# Small RNA sequencing of cryopreserved semen from single bull revealed altered miRNAs and piRNAs expression between High- and Low-motile sperm populations

**DOI:** 10.1186/s12864-016-3394-7

**Published:** 2017-01-04

**Authors:** E. Capra, F. Turri, B. Lazzari, P. Cremonesi, T. M. Gliozzi, I. Fojadelli, A. Stella, F. Pizzi

**Affiliations:** 1 Istituto di Biologia e Biotecnologia Agraria, Consiglio Nazionale delle Ricerche, via Einstein, 26900 Lodi, Italy; 2 Parco Tecnologico Padano, via Einstein, 26900 Lodi, Italy

**Keywords:** Sperm, Cryopreserved, Sequencing, miRNA, piRNA

## Abstract

**Background:**

Small RNAs present in bovine ejaculate can be linked to sperm abnormalities and fertility disorders. At present, quality parameters routinely used in semen evaluation are not fully reliable to predict bull fertility. In order to provide additional quality measurements for cryopreserved semen used for breeding, a method based on deep sequencing of sperm microRNA (miRNA) and Piwi-interacting RNA (piRNA) from individual bulls was developed.

To validate our method, two populations of spermatozoa isolated from high and low motile fractions separated by Percoll were sequenced, and their small RNAs content characterized.

**Results:**

Sperm cells from frozen thawed semen samples of 4 bulls were successfully separated in two fractions. We identified 83 miRNAs and 79 putative piRNAs clusters that were differentially expressed in both fractions. Gene pathways targeted by 40 known differentially expressed miRNAs were related to apoptosis. Dysregulation of miR-17-5p, miR-26a-5p, miR-486-5p, miR-122-5p, miR-184 and miR-20a-5p was found to target three pathways (PTEN, PI3K/AKT and STAT).

**Conclusions:**

Small RNAs sequencing data obtained from single bulls are consistent with previous findings. Specific miRNAs are differentially represented in low versus high motile sperm, suggesting an alteration of cell functions and increased germ cell apoptosis in the low motile fraction.

**Electronic supplementary material:**

The online version of this article (doi:10.1186/s12864-016-3394-7) contains supplementary material, which is available to authorized users.

## Background

Reproductive success is crucial for species’ survival. Infertility is a disorder affecting humans as well as other animals. Concerning these, latter infertility is a major cause of economic losses and a major limitation to the achievement of optimum efficiency in the livestock production system. The causes of infertility can be numerous and complexes. In human, infertility is prevalently due to anatomical problems and endocrine disorders causing low sperm counts and poor sperm quality, and in part to genetic disorders [1]. In cattle, a number of bulls considered of high-merit based on their spermatozoa motility and morphology were reported to be unable to produce successful full-term pregnancies, according to extensive fertility data and progeny records [2, 3], suggesting that molecular defects affect the ability of spermatozoa to fertilize and contribute to normal embryo development [4–6]. Individual bulls differ in their ability to fertilize oocytes in vitro depending on different sperm traits, like motility, membrane and acrosome integrity, and the ability to penetrate oocytes [7]. Cryopreserved semen is used worldwide in farm animal husbandry and for animal genetic resources conservation. Several advanced technologies can be used to examine quality of spermatozoa - as Computer-Assisted Semen Analysis (CASA) and flow cytometry (FCM) - which can provide accurate and unbiased evaluation of sperm functions. It is generally accepted that sperm motility is a determining factor in normal male fertility because of its essential role in reaching the site of fertilization [8], as a consequence, the evaluation of sperm motility is useful for the diagnosis and treatment of low fertility and infertility [9]. Despite their relevance, the molecular mechanisms controlling sperm motility are still partially unknown. The integration of several tests, from standard procedures for the evaluation of sperm motility and viability, to sperm molecular investigation, is a promising approach to achieve a better understanding of sperm functions as well as to evaluate semen quality and predict bull fertility.

During fertilization, besides the paternal genome, spermatozoa transport coding and non coding RNAs into the oocyte. Mammalian sperm contains an array of RNAs including messenger RNAs (mRNAs), ribosomal RNAs (rRNAs) and small RNAs (sRNAs), largely representing remnant transcripts produced during spermatogenesis [10–12]. RNA-Seq characterization of bovine spermatozoa revealed the presence of degraded and full-length nuclear-encoded transcripts involved in capacitation and fertilization, suggesting that RNA could be translated after spermatogenesis and potentially contribute to capacitation and early embryogenesis [13]. Furthermore, sperm transcripts retain information of the past events of spermatogenesis and probably contribute to egg fertilization and development. Comparisons between sperm from fertile and infertile males in different species indicate that sperm transcripts may have diagnostic value, and suggest a relationship between sperm transcripts composition and proper sperm functions [8, 14–17].

sRNAs are a class of short non-coding RNAs including different types of RNAs (i.e. microRNA (miRNA) and Piwi-interacting RNA (piRNA)), that play an essential regulatory role in spermatogenesis, such as maintenance and transposon silencing. piRNAs are known to be important to maintain fertility, as confirmed by the defects in fertility observed in mutants lacking Piwi in *C. elegans* [18], *Danio rerio* [19] and *Mus musculus* [20]. miRNAs were found to regulate spermatogonial stem cell (SSCs) renewal at the post-transcriptional level via targeting specific genes [21]. The testicular expressed miRNAs were reported to change depending on the stage of spermatogenesis [22, 23]. miRNAs participate in the control of many functions, such as maintenance of spermatogonial stem cells (SSCs) status, regulation of SSCs differentiation, meitoic and post-meiotic processing and spermiogenesis [24]. Dysregulation in miRNAs’ expression patterns is severely affected in different types of reproduction abnormalities [25–27]. Sperm miRNA profiling alteration was detected in bulls with high vs low fertility level, indicating a possible role of miRNAs in male infertility [28].

Since the first genome-wide miRNA and piRNA profiling in human testis was reported [29], the Next Generation Sequencing (NGS) technology was adopted to detect sRNAs dysregulation associated to sperm characteristic alterations. Recently, the bull sperm microRNAome was found to be altered in the “fescue toxicosis” syndrome, a disease related to consumption of alkaloids contaminated feed, which has negative effects on growth and reproduction in animals [30]. However, due to the low yields in miRNA recovery from frozen semen, analyses were conducted on RNAs from several pooled individuals.

Here, we propose the first integrated approach to compare miRNA and piRNA expression between high and low motility sperm populations isolated after Percoll gradient from cryopreserved spermatozoa collected from single bulls. Deep sequencing information from single animal was achieved to explore how miRNA and piRNA expression variations can potentially affect bovine sperm characteristics, such as motility and kinetic parameters. The development of a reliable method for small RNA profiling in bovine sperm isolated from frozen thawed sperm through NGS could be an important step in deciphering the contents of miRNA and piRNA sequences in animals that are well characterized for different traits such as fertility.

## Methods

### Isolation of spermatozoa through Percoll gradient

Frozen semen straws from four mature progeny tested Holstein bulls with satisfactory semen quality were obtained from an ﻿Artificial Insemination ﻿﻿AI center (INSEME,﻿ Zorlesco, Lodi, Italy).

For each bull 12 frozen semen doses (0.5 mL, 20x10^6^ cells per dose) were simultaneously thawed in a water bath at 37 °C for 20 seconds and pooled. The pool (6 mL) was split in 3 aliquots of 2 mL that were overlaid on a dual-layer (90–45%) discontinuous Percoll gradient (Sigma-Aldrich, St. Louis, USA) in three 15 ml conical tubes and centrifuged at 700 × *g* for 30 min at 20 °C. The Percoll layers were prepared by diluting Percoll solution as previously described [31]. The Percoll gradient is a colloidal suspension of silica particles coated with polyvinylpyrrolidone (PVP). By using two discontinuous layers (45% and 90%) by centrifugation it is possible to obtain a different sedimentation according to sperm motion. The two fractions obtained (High Motile = HM and Low Motile = LM) from each of the three tubes (replicates) were washed in Tyrode’s albumin lactate pyruvate (TALP) buffer at 700 × *g* for 10 min at 20 °C; the obtained pellets were re-suspended in 150 μl of TALP. For each bull an aliquot of semen of the High Motile and Low Motile fractions was evaluated immediately after Percoll density gradient centrifugation. Three technical replicates per bull were evaluated for sperm kinetic parameters by CASA, and sperm viability and acrosomal status by flow cytometer in both fractions. Aliquots from each replicate were kept at −80 °C until RNA extraction (approximately 1 month later).

### Evaluation of sperm characteristics

#### Motility

Sperm kinetics parameters were assessed using a CASA (Computer-Assisted Semen Analysis) system (ISAS^®^ v1, Spain). A 10 μl drop of semen was placed on a pre-warmed (37 °C) Makler chamber. During the analysis, the microscope heating stage was maintained at 37 °C. Using a 10× objective in phase contrast, the image was relayed, digitized and analyzed by the ISAS^®^ software with user-defined settings as follows: frames acquired, 25; frame rate, 20Hz; minimum particles area 20 μm^﻿2﻿^; maximum particles areas 70 μm^2^; progressivity of the straightness 70%. Spermatozoa speed was assigned to 3 broad categories: rapid (50 μm/s), medium (25 μm/s) and slow (10 μm/s). CASA kinetics parameters were: total motility (MOT TOT, %), progressive motility (PRG, %), curvilinear velocity (VCL, μm⁄s), straight-line velocity (VSL, μm⁄s), average path velocity (VAP, μm⁄s), linearity coefficient (LIN, %= VSL/VCL × 100), amplitude of lateral head displacement (ALH, μm), straightness coefficient (STR, % = VSL/VAP × 100), wobble coefficient (WOB, % = VAP/VCL × 100) and beat cross frequency (BCF, Hz).

#### Flow cytometry analysis

Measurements were performed on a Guava Easycyte^TM^ 5HT microcapillary flow cytometer (Merck KGaA Darmstadt) with the CytoSoft™﻿ and IMV EasySoft software for semen analysis (IMV Technologies, France). The fluorescent probes were excited by an Argon ion blue laser (488 nm). A forward and side-scatter gate were used to separate sperm cells from debris. Non sperm events were excluded from further analysis. Detection of fluorescence was set with three photomultiplier tubes (green: 525/30 nm, orange/yellow: 586/26 nm, and red: 690/50 nm). Compensation for spectra overlap between fluorochromes was set (http://www.drmr.com/compensation). Calibration was carried out using standard beads with the Guava Easy Check Kit (Guava Technologies, Inc., Millipore). Acquisitions were performed using the CytoSoft™ software. A total of 5000 events per sample were analyzed with a flow rate of 200 cells/s. The assessment of sperm viability and acrosome integrity was performed by using EasyKit 5 (IMV Technologies, France). The percentage of cells with disrupted acrosome within viable or dead sperm fractions was measured. Each well of the ready-to-use 96-well plate was filled with 200 μL of Embryo Holding solution (IMV Technologies, France), 40.000 sperm cells were added and incubated for 45 min at 37 °C in the dark. Spermatozoa with disrupted acrosomes were labeled with a green probe, dead spermatozoa with damaged plasma membrane were labeled with a red fluorochrome, consequently the percentages of alive and dead sperm fractions with intact or damaged acrosomal membrane were computed.

### RNA extraction

For each bull, HM and LM sperm fractions obtained from three technical replicates (equivalent to approximately four frozen semen doses each) were used for RNA isolation. RNA was extracted using TRIzol^®^ (Invitrogen, Carlsbad, CA) according to Govindaraju et al. [28], with some modifications. Briefly, 400 μl of TRIzol were added into each sperm cell pellet and then homogenized at high speed for 30 s. Glycogen (3 μl of 20 mg/ml) was added to the tubes and another 400 μl of TRIzol^®^ were then added, mixed and incubated for 15 min at 65 °C. Total RNA was then purified with the NucleoSpin^®^miRNA kit (Macherey-Nagel, Germany), following the protocol in combination with TRIzol^® ^lysis with small and large RNA in one fraction (total RNA). RNA concentration and quality were determined by Agilent 2100 Bioanalyzer (Santa Clara, CA). The isolated RNAs were stored at −80 °C until use.

### Library preparation and sequencing

Six sperm RNA samples, representing three technical replicates for both HM and LM fractions, were obtained from each single bull. RNA extraction from semen straws typically resulted in few picograms of RNA: a quantity not compatible for single small RNA library sequencing. Therefore pool of sample has been usually used for semen small RNA sequencing. In order to avoid pooling samples, our approach provide a library preparation from each single RNA sample with proper index. Libraries from single samples were then combined, approximately fifteen-fold concentrated in volume and isolated. Small RNA libraries were generated using the Illumina Truseq Small RNA Preparation kit according to manufacturer’s instructions with the following modifications: before size selection, libraries were pooled together and added with Agencourt^®^AMPure^®^ XP (Beckman, Coulter, Brea, CA) (1 Vol. sample: 1.8 Vol. beads). Libraries were eluted in 1/15 volume of the initial pool solution (15X libraries pool). The libraries pool was purified on a Pippin Prep system (Sage Science, MA, USA) to recover the 125 to 167 nt fraction containing mature miRNAs (Additional file 1). The quality and yield after sample preparation was measured with an Agilent 2200 Tape Station, High Sensitivity D1000. Libraries were sequenced on a single lane of Illumina Hiseq 2000 (San Diego, CA).

### piRNA analysis

Preliminary quality control of raw reads was carried out with FastQC (http://www.bioinformatics.babraham.ac.uk/projects/fastqc/). Illumina raw sequences were then trimmed with Trimmomatic [32] to remove primers, Illumina adapters and low quality regions and sequences. A minimum average base quality of 15 over a 4 bases sliding window and a minimum length of 12 bases of the trimmed sequence were used as thresholds.

Small RNA sequences ranging from 26 to 33 nt in length after trimming were selected for piRNA detection. Sequences were collapsed to remove identical sequences but retain information on read counts using the collapse tool from the NGS toolbox [33]. Furthermore, low-complexity reads were removed using the duster tool from the NGS toolbox. The resulting sequences were mapped to the *Bos taurus* 3.1 (Bt3.1) genome assembly and to chromosome Y from the 4.6.1 assembly with sRNA mapper. Only the best-scoring alignments were taken into account, and up to two non-templated 3′ nucleotides were allowed in order to successfully map sequences that were subject to post-transcriptional 3′ editing [34]. After mapping, the program reallocate (http://www.smallrnagroup-mainz.de/software.html) was used to assign read counts of multiple mapping sequences according to estimated local transcription rates based on uniquely mapping sequences.

piRNA cluster detection was performed with proTRAC version 2.1 [35, 36], imposing a piRNA length of 26 to 33 bp and a minimum cluster length of 5000 bp. Genes falling within the detected clusters were retrieved according to Bt3.1 NCBI annotation, repeats and transposable elements were also retrieved, according to the Repeat Masker annotation available at the NCBI. Overlaps among HM and LM clusters were assessed with BedTools Intersect (http://bedtools.readthedocs.org).

### miRNA detection and analysis

miRNA detection and discovery was carried out with Mirdeep2 on Illumina high quality trimmed sequences. *Bos taurus* miRNAs available at MirBase (http://www.mirbase.org/) were used to accomplish known miRNA detection on the trimmed sequences. Known miRNAs from related species (sheep, goat and horse) available at MirBase were also input into Mirdeep2 to support the individuation of novel miRNAs.

The Mirdeep2 quantifier module was used to quantify expression and retrieve counts for the detected known and novel miRNAs. Differential expression analyses between the HM and LM fractions were run with the Bioconductor edgeR package [37]. miRNA cluster analysis was performed with Genesis [38]. Box-plot graphic was generated with BoxPlotR [39]. miRNA target prediction and functional analysis were performed by Ingenuity Pathway Analysis (IPA, Ingenuity System, www.ingenuity.com). Human homologous miRNAs were analyzed with microRNA Target filter (IPA) to attribute (experimentally observed) target genes. Finally miRNA target mRNA and the corresponding experimental Log Ratios were used for pathway analysis.

### Statistical analysis

Data obtained from CASA and flow cytometry measurements were analyzed using the SAS™ package v 9.4 (SAS Institute Inc., Cary, NC, USA). The General Linear Model procedure (PROC GLM) was used to analyze the effect of technical replicates on semen quality parameters in the two fractions. The model included as fixed effects the bull and the replicate nested in the sperm fractions (HM and LM).

A mixed model procedure (PROC MIXED) was used to perform analysis on sperm quality parameters in order to evaluate the efficiency of the sperm separation into the HM and LM sperm fractions. The mixed model included the fixed effect of the sperm fraction (HM and LM), and bull as random. Results are given as adjusted least squares means ± standard error means (LSM ± SEM).

## Results

### Isolation of spermatozoa and evaluation of sperm characteristics

Concerning semen quality parameters in the two fractions (HM and LM) any statistical significant difference was detected among technical replicates. Sperm cells were successfully fractionated in HM and LM populations after Percoll centrifugation considering both sperm kinetics parameters and sperm acrosomal status as shown in Table 1. Considering MOT TOT, PRG, VSL, VCL, VAP, ALH, BCF, VIA, DIA and DDA variables, a significant (*P* < 0.05) improvement of the sperm quality occurred in HM fraction. The improvement occurred, although to a less significant extent, also for LIN, STR and WOB kinetics parameters.

**Table 1:** Sperm quality variables assessed in High Motile and Low Motile sperm fractions

Variables	High Motile	Low Motile
MOT TOT (%)	48.44 ± 4.65^a^	3.78 ± 4.65^b^
PRG (%)	39.94 ± 4.64^a^	1.86 ± 4.64^b^
VSL (μm/s)	66.65 ± 5.66^a^	25.25 ± 5.66^b^
VCL (μm/s)	102.47 ± 6.90^a^	50.79 ± 6.90^b^
VAP (μm/s)	72.00 ± 5.43^a^	31.60 ± 5.43^b^
LIN (%)	63.94 ± 5.57	48.59 ± 5.57
STR (%)	92.02 ± 4.07	82.03 ± 4.07
WOB (%)	69.15 ± 4.25	57.60 ± 4.25
ALH (μm)	3.04 ± 0.23^a^	2.14 ± 0.23^b^
BCF (Hz)	9.34 ± 0.68^a^	4.12 ± 0.68^b^
VIA (%)	68.75 ± 3.90^a^	10.39 ± 3.90^b^
DIA (%)	23.72 ± 3.25^a^	36.27 ± 3.25^b^
VDA (%)	1.24 ± 0.54	0.78 ± 0.54
DDA (%)	6.39 ± 2.56^a^	52.54 ± 2.56^b^

*MOT TOT* total motility, *PRG* cells progressive motility, *VSL* straight-line velocity, *VCL* curvilinear velocity, *VAP* average path velocity, *LIN* linear coefficient, *STR* straightness coefficient, *WOB* wobble coefficient, *ALH* amplitude of lateral head displacement, *BCF* beat cross-frequency, VIA viable with intact acrosome, *DIA* dead with intact acrosome, *VDA* viable with disrupted acrosome, *DDA* dead with disrupted acrosome

^a, b^values within a row with different superscripts differ significantly at *P* <0.05

### Small RNA sequencing

Hiseq sequencing resulted in 110,394,322 reads after trimming, with an average production of 4,599,763 reads per sample.

#### piRNA

A total of 99 and 51 putative piRNA clusters were assigned by proTRAC to the HM and LM fractions, respectively (Additional file 2). Among these, only 36 clusters were shared between the two fractions, indicating a significant diversity of piRNA content. Unique sequences in putative piRNA clusters represent the 3.77% and the 3.29% of the unique sequences of size 26 to 33 bp in the HM and LM fractions, respectively. Apart from two clusters (Cluster14 and Cluster24) of the HM fraction, all the other clusters overlap clusters reported to be expressed in bull testis libraries at the piRNA cluster database (http://www.smallrnagroup.uni-mainz.de/piRNAclusterDB.html, [36]). 74.5% and 85.0% of the unique putative piRNA sequences mapping within clusters in the HM and LM fractions, respectively, are identical to piRNA sequences falling within the piRNA cluster database testis clusters. piRNA clusters details, including genes, repeats, transposable elements and transcription factors binding sites falling within the cluster regions, are given in Additional file 3 (HM fraction) and Additional file 4 (LM fraction).

#### miRNA

In total, 813 unique miRNAs were detected by Mirdeep2. Among these, 478 were known *Bos taurus* miRNAs, 103 were homologous of known miRNAs from other species and 232 were new candidate miRNAs (Additional file 5).

### Differentially expressed miRNAs and pathway analysis of predicted miRNA targets

**Fig. 1:**
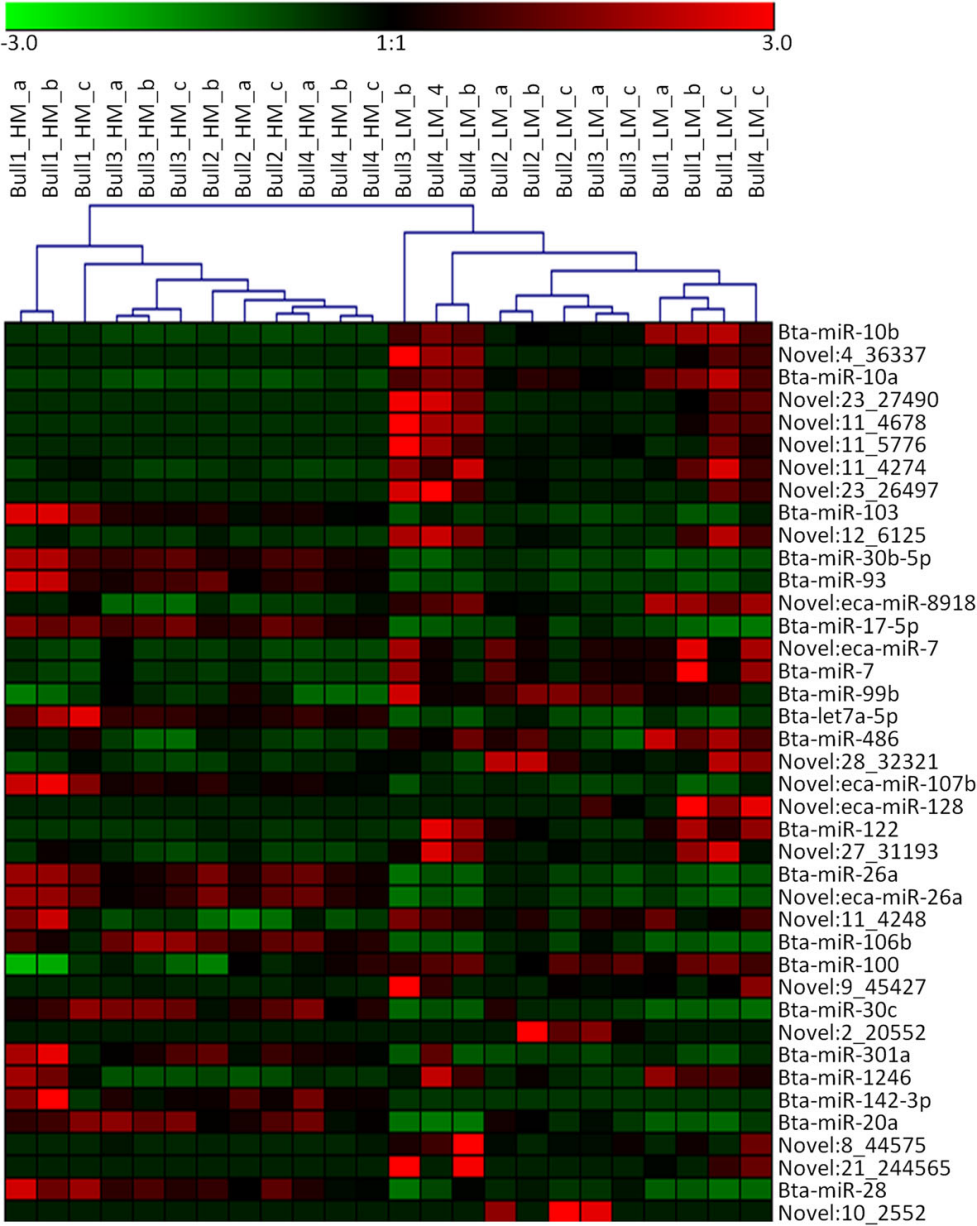
Cluster analysis of the 83 differentially expressed DEmiRNAs (FDR <0.01) in High Motile (HM) and Low Motile (LM) fraction. In figure are shown the first 40 DEmiRNAs

**Fig. 2:**
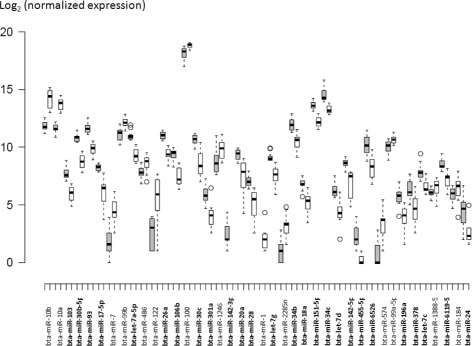
Box plot showing the differentially expressed (DE) *Bos taurus* known miRNAs in high (gray bar) and low (white bar) motile fractions isolated from cryopreserved bovine semen. Central lines inside the boxes indicate median values; box width indicates 25 and 75% quartile ranges around the median; “T” indicates the maximum and minimum values, and black dots represent outliers. *N* = 12 for each treatment. In bold miRNA highly expressed in HM fraction

After applying a stringent filter approach to compare high and low motile sperm (FDR < 0.01), we identified 83 differentially expressed miRNAs (DEmiRNAs), 40 of which were known and the remaining were novel (Additional file 6). A tree with a clear distinction between the two separated fractions was generated by cluster analysis (Fig. 1). Among all the known DEmiRNAs, 26 miRNAs showed greater expression in HM sperm (Fig. 2). It is interesting to note that many known miRNAs found in our study (19/40) were previously reported to be differentially expressed in sperm with abnormalities in human, sheep and cattle (Table 2).

**Table 2:** Comparison between known DEmiRNAs found in our study and in other studies. miRNAs were obtained from A) high and low motile fractions isolated﻿﻿ from bovine cryopreserved semen, B) adult testis tissue from sheep well or under fed, C) human spermatozoa isolated from patients with normal or abnormal semen, D) human spermatozoa isolated from patients with normal semen or spermatogenic impairments, E) human spermatozoa isolated from patients with normal or vasectomized epididymis

	This study		Guan et al., [55]		Liu et al., [26]		Abu-Halima et al., [27]		Belleanée et al., [56]		
Specie	*Bos taurus*		*Ovis* *aries*		*Homo sapiens*		*Homo* *sapiens*		*Homo* *sapiens*		
Study type	A		B		C		D		E		
miRNA	High motile	Low motile	Well-fed	Underfed	Normal semen	Abnormal semen	Normal semen	Abnormal semen	Normal epididymis	Vasectomized epididymis	Agreement with previous study
**bta-miR-103**	+				+						Yes
**bta-miR-30b-5p**	+		+		+						Yes
bta-miR-93	+										
**bta-miR-17-5p**	+		+								Yes
bta-let-7a-5p	+										
**bta-miR-26a**	+			+				+			No
**bta-miR-106b**	+		+								Yes
bta-miR-30c	+										
bta-miR-301a	+										
**bta-miR-142-3p**	+		+								Yes
bta-miR-20a	+										
bta-miR-28	+										
bta-let-7 g	+										
**bta-miR-34b**	+						+				Yes
**bta-miR-18a**	+				+				+		Yes
bta-miR-151-5p	+										
**bta-miR-34c**	+						+				Yes
bta-let-7d	+										
bta-miR-142-5p	+										
**bta-miR-455-5p**	+				+						Yes
bta-miR-6526	+										
bta-miR-196a	+										
bta-miR-378	+										
bta-let-7c	+										
bta-miR-6119-5p	+										
**bta-miR-24**	+							+			No
**bta-miR-10b**		+		+							Yes
bta-miR-10a		+									
bta-miR-7		+									
**bta-miR-99b**		+						+			Yes
bta-miR-486		+									
**bta-miR-122**		+				+	+				Yes/No
**bta-miR-100**		+	+		+						No
**bta-miR-1246**		+								+	Yes
bta-miR-1		+									
bta-miR-2285n		+									
**bta-miR-574**		+				+			+		Yes/No
**bta-miR-99a-5p**		+		+				+			Yes
**bta-miR-1388-5p**		+									Yes
bta-miR-184		+									

﻿In bold miRNAs have been previous﻿ reported in sperm or testis tissue﻿

**Fig. 3:**
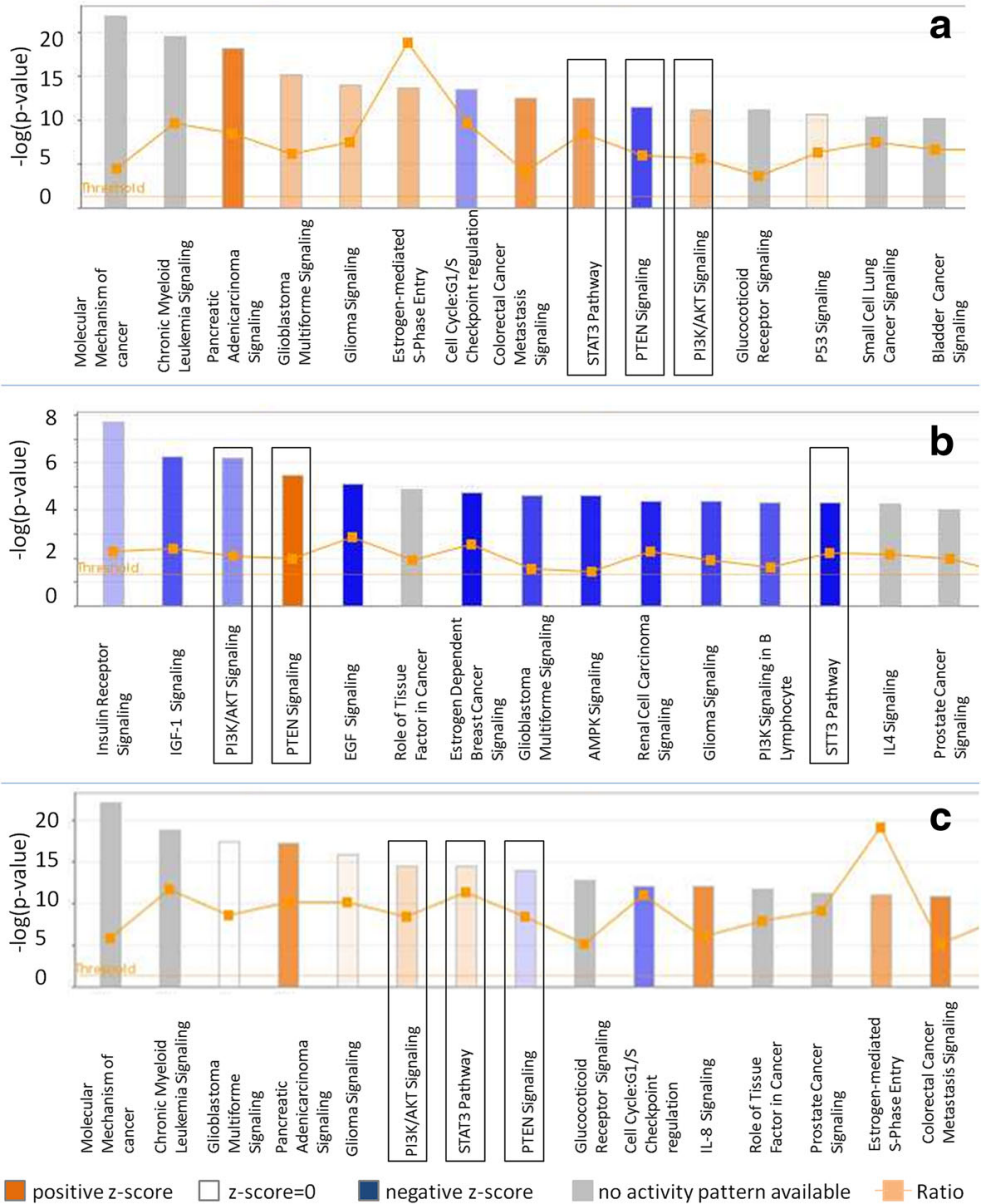
Canonical Pathway Chart of the (experimentally observed) genes targeted by 20 differentially expressed miRNA that found correspondence with human miRNA. Pathways analyses were calculated from: **a**) miRNAs up-regulated in the High Motile (HM) fraction; **b**) miRNAs up-regulated in the Low Motile (LM) fraction and **c**) Total of miRNAs differentially expressed between the (HM) and (LM) fractions. In squares, pathways that showed a positive or negative score and were shared between miRNAs up-regulated in the HM and LM fractions. The first 15 pathways are shown in the figure

**Fig. 4:**
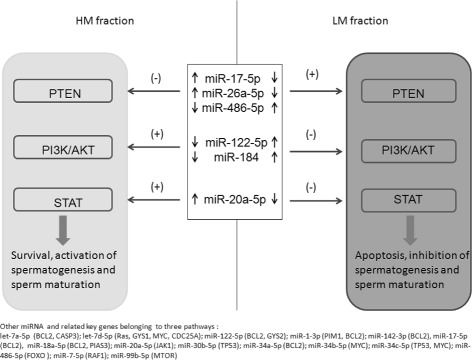
Working hypothesis of the mechanism through which different miRNAs regulate the PTEN pathway, PI3K/AKT and STAT signalling in sperm. Arrows indicate miRNA expression and (+) activation or (−) inhibition of the related pathway. At the bottom of the figure other miRNAs and related target genes involved in the pathway regulation are reported

Target genes of the 40 known miRNAs found in this study were predicted, and pathways potentially affecting sperm motility were identified. 14/26 miRNAs highly expressed in the HM fraction (let-7d-5p, miR-103a-3p, miR-142-3p, miR-17-5p, miR-18a-5p, miR-196a-5p, miR-20a-5p, miR-24-1-5p, miR-26a-5p, miR-301a-3p, miR-30b-5p, miR-34b-5p, miR-34c-5p, miR-378a-3p) and 7/14 miRNAs highly expressed in the LM fraction (miR-10b-5p, miR-122-5p, miR-1-3p, miR-184, miR-486-5p, miR-7-5p, miR-99b-5p) were predicted to target 327 and 281 experimentally observed genes, respectively. The canonical pathway analysis revealed that these genes are involved in different biological pathways. Interestingly, we observed that in some instances, pathway regulation by miRNAs highly expressed in HM and LM fractions turned out to have opposite effects (positive or negative z-score, Fig. 3). Among these pathways, “PTEN Signaling” was regulated by miR-17-5p, miR-26a-5p and miR-486-5p, “PI3K/AKT Signaling” by 122-5p and miR-184 and “STAT3 Pathway” by miR-20a-5p (Fig. 4).

## Discussion

Due to limitations in ﻿sRNA recovery from frozen thawed sperm, usually miRNA sperm profiling was achieved exclusively by microarray experiments or Real Time PCR [28, 30–40]. sRNAs profiling through NGS sequencing provides different advantages vs microarray experiments, such as discrimination of miRNAs that are very similar in sequence (isomiRs), detection of novel miRNAs [41] and the simultaneous piRNAs detection. This work firstly provides a comprehensive description of small RNAs isolated from HM and LM fractions from individual bull cryopreserved spermatozoa obtained by NGS sequencing and analysis.

HM and LM fractions obtained after Percoll showed a good reproducibility between technical replicates, confirming the efficiency of the isolation of sperm fraction by Percoll gradient.

This procedure attenuated samples variability [42, 43]. The increase of the proportion of living spermatozoa with intact acrosome (VIA) in HM fraction reported in this study was in agreement with previously results [44–46] in frozen-thawed bull and buffalo semen, indicating that the major part of dead spermatozoa were retained in the upper layers of the gradient. Moreover, the HM fraction obtained in this study was characterized by sperm with fast motion characteristics and membranes integrity, two aspects strictly related to the fertilizing capacity [47–49].

Since piRNAs were firstly observed to have a putative role in gametogenesis in developing mouse male germ cells, they have been thought to be absent from mature spermatozoa [50]. Later, a survey of small RNAs in human sperm revealed sequence reads aligned to piRNA clusters located on several chromosomes and speculated their possible role in early embryo development in sperm [51]. A recent study identified a panel of piRNAs presents in seminal plasma that can serve as markers to distinguish fertile from infertile males [52]. Here we present the first characterization of piRNAs in two sperm fractions obtained by Percoll fractionation, showing a high diversity of piRNA content between the HM an LM fractions and the presence of a higher number of piRNA clusters in the HM fraction. To our knowledge, this is the first study able to compare piRNA expression in different sperm samples. Because of the low level of piRNAs conservation between even closely related species [50–53], the functional role of piRNAs dysregulation in HM and LM fraction remain to be further understood. On the contrary, bovine miRNA content in sperm was explored in different studies. In this study the total number of known bovine miRNAs isolated from semen cryopreserved in straws was in agreement with previously reported data obtained from NGS miRNA profiling of sperm isolated from caudal epididymis [54] or frozen sperm pellet [30] in *Bos taurus*. Finally, several of the top expressed miRNAs in this study have been previously reported as the most abundant in bovine sperm [30]. Moreover, miRNA expression comparison between the two fractions showed that about 10% of the miRNA are differentailly expressed. A similar percentage of expressed miRNA variation was observed in the semen of infertile men with semen abnormalities analyzed by microarray [25]. The high level of miRNA conservation among species supports a direct comparison of our data with data presented in previous studies on different species. About half of the known miRNAs found in this study have been previously reported in sperm or testis tissue from other species: *Ovis aries* [55] and *Homo sapiens* [26, 27, 56]. The relative expression in HM and LM fractions of several of these known miRNAs, including bta-miR-103, bta-miR-30b-5p, bta-miR-17-5p, bta-miR-106b, bta-miR-142-3p, bta-miR-34b, bta-miR-18a, bta-miR-34c, bta-miR-455-5p, bta-miR-10b, bta-miR-99b, bta-miR-1246, bta-miR-99a-5p, and bta-miR-1388-5p, was consistent with the relative abundance of their homologous miRNAs, observed in the normal vs abnormal sperm. Conversely, bta-miR-26a, bta-miR-24 and bta-miR-100 showed an opposite expression in our samples with respect to what previously described in literature [26, 27, 55]. bta-miR-122 and bta-miR-574 expression in the HM or LM fraction was only partially in agreement with what reported in previous studies [26, 27, 56]. Different miRNAs, including miR10b, miR-26, miR-34c and miR-99a, were also seen to change their expression level in underfed animals, and these variations were postulated to cause reduction in spermatozoa quality by disruption to Sertoli cell function and to increase germ cell apoptosis [55].

Functional analysis of the known DEmiRNAs showed targeting to mRNAs involved in different pathways, in particular “STAT3 Pathway”, “PI3K/AKT Signaling” and “PTEN Signaling”. The PTEN pathway is a crucial mediator of mitochondria-dependent apoptosis [57]. The role of PTEN in mammalian spermatogenesis under normal physiological conditions, consists in suppressing AKT activity to maintain activation of the RAF1/ERK signaling, which in turn maintains the normal function of the initial segment and, therefore, normal sperm maturation [58]. PTEN function is linked to its capacity of antagonizing the PI3K/AKT signaling. *Akt1* and *Akt2* knockout was seen to increase PTEN activity, probably inducing sperm apoptosis, decreasing spermatogenesis, sperm maturation and fertilization in male mice [59]. The inhibition of the STAT pathway in spermatozoa was reported to increase ROS production and calcium levels, and to decrease cellular ATP levels and mitochondrial membrane potential, that is consistent with cells undergoing apoptosis [60].

We postulate that the simultaneous low expression and up-regulation of different miRNAs could dysregulate PTEN, PI3K/AKT and STAT signaling and influence the apoptosis, vitality and motility in spermatozoa (Fig. 4). PTEN could be targeted by the simultaneous action of miR-17-5p, miR-26a-5p, miR-486-5p. According to previous results, miR-17-5p, miR-26a-5p up-regulation enhances AKT pathway activation by PTEN suppression and promotes cancer [61, 62]. On the contrary, miR-486 plays a pro-apoptotic tumor-suppressor role [63], and its high expression was associated with a good prognosis in gastric adenocarcinoma [64]; however, miR-486 over expression in dystrophin-deficient mice was also observed to reduce PTEN expression [65]. In agreement with our results, two miRNAs found to be highly expressed in the LM fraction were reported to target the AKT pathway and promote apoptosis. miR-122 was reported to play a pivotal role as tumor suppressor by decreasing AKT3 levels, inhibiting cell migration and proliferation and inducing apoptosis [66], whereas miR-184 was found to be involved in suppressing cell survival and growth by targeting AKT2 in neuroblastoma cells [67]. Finally, miR-17-5p and miR-20a-5p, that we found to be under-expressed in the LM fraction and potentially target PTEN and STAT signaling, if down-regulated were proved to trigger cell apoptosis [68].

## Conclusion

In conclusion we provide a protocol, based on small RNA sequencing, enabling to characterize miRNA and piRNA contents in cryopreserved bovine spermatozoa from single animals. We also provide a dataset of novel bovine miRNAs and a first description of piRNA genomic clusters expressed in bovine spermatozoa in high and low motile sperm population. Small RNAs were seen to differ between HM and LM sperm fractions. Furthermore, some miRNAs differentially expressed in HM and LM fraction targeted genes associated with cell apoptosis, mitochondrial membrane potential and spermatogenesis alteration, indicating a functional redundancy, which might influence sperm motility and thus bull fertility.

## Additional files

Additional file 1: Agilent Tape station profile of small RNA library (152 bp) obtained from pool of 24 samples concentrated with magnetic beads and size selected with Pippin Prep. 128 bp size peak represents primer dimmers co-purified with the library. (DOCX 40 kb)
Additional file 2: Putative piRNA clusters that were assigned by proTRAC to the High Motile (HM) and Low Motile (LM) sperm fractions. (XLSX 21 kb)
Additional file 3: Details for each piRNA clusters found in High Motile (HM) sperm fraction. Genes, repeats, transposable elements and transcription factors binding sites falling within the cluster regions were reported. (ZIP 1896 kb)
Additional file 4: Details for each piRNA clusters found in Low Motile (LM) sperm fraction. Genes, repeats, transposable elements and transcription factors binding sites falling within the cluster regions were reported. (ZIP 1034 kb)
Additional file 5: miRNAs detected in High Motile (HM) and Low Motile (LM) sperm fraction by Mirdeep2. Normalized read counts were reported for each bull (Bull1, Bull2, Bull3, Bull4) and replicate (a, b, c). (XLSX 140 kb)
Additional file 6: Differentially expressed miRNAs between the High Motile (HM) and Low Motile (LM) spermfractions calculated with the Bioconductor edgeR package. (XLSX 70 kb)
Additional file 7: Novel miRNA precursors sequences identified in High Motile (HM) and Low Motile (LM) sperm fractions. (FA 25 kb)
Additional file 8: Novel miRNA mature sequences identified in High Motile (HM) and Low Motile (LM) sperm fractions. (FA 12 kb)
Additional file 9: piRNA clusters identify in High Motile (HM) and Low Motile (LM) sperm fractions. (FASTA 1073 kb)

## Abbreviations

sRNA: Small RNA
miRNA: MicroRNA
piRNA: Piwi-interacting RNA
CASA: Computer-assisted sperm analysis
FCM: Flow cytometry
NGS: Next generation sequencing
HM: High motile
LM: Low motile
TALP: Tyrode’s albumin lactate pyruvate
MOT TOT: Total motility
PRG: Cell progressive motility
VCL: Curvilinear velocity
VSL: Straight-line velocity
VAP: Average path velocity
LIN: Linearity coefficient
ALH: Amplitude of lateral head displacement
STR: Straightness coefficient
WOB: Wobble coefficient
BCF: Beat cross frequency
VIA: Viable with intact acrosome
DIA: Dead with intact acrosome
VDA: Viable with disrupted acrosome
DDA: Dead with disrupted acrosome
IPA: Ingenuity pathway analysis
GLM: General linear model
LSM: Least squares means
SEM: Standard error means
DE: Differentially expressed
