# Supplementary material for: Small RNA sequencing of cryopreserved semen from single bull revealed altered miRNAs and piRNAs expression between High- and Low-motile sperm populations
Source: BMC Genomics. 2017 Jan 4;18:14. doi: 10.1186/s12864-016-3394-7 (PMC5209821; doi:10.1186/s12864-016-3394-7)
Supplement: Additional file 1: — Agilent Tape station profile of small RNA library (152 bp) obtained from pool of 24 samples concentrated with magnetic beads and size selected with Pippin Prep. 128 bp size peak represents primer dimmers co-purified with the library. (DOCX 40 kb) [file 12864_2016_3394_MOESM1_ESM.docx]

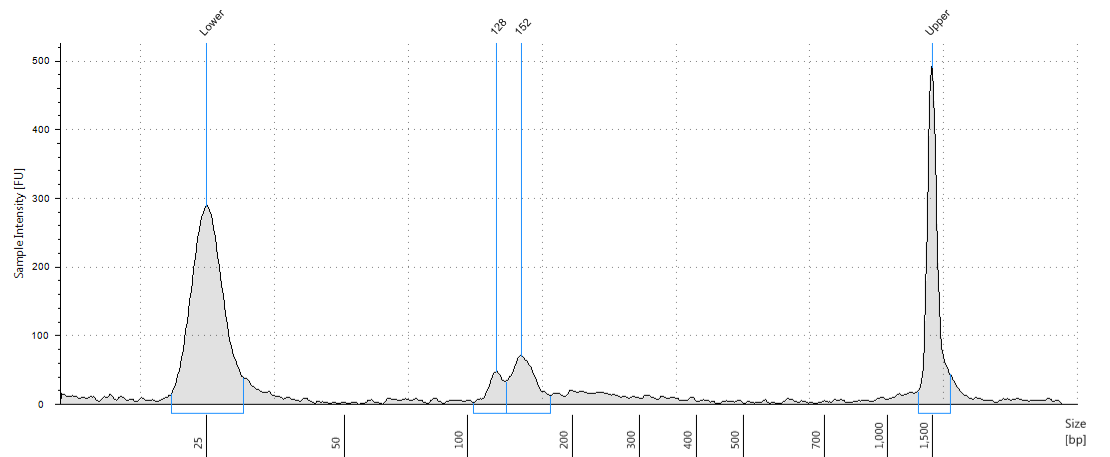


## Peak Table

| **Size [bp]** | **Calibrated Conc. [pg/µl]** | **Assigned Conc. [pg/µl]** | **Peak Molarity [pmol/l]** | **% Integrated Area** | **Peak Comment** | **Observations** |
| --- | --- | --- | --- | --- | --- | --- |
| 25 | 453 | - | 27900 | - |  | Lower Marker |
| 128 | 33.4 | - | 401 | 29.68 |  |  |
| 152 | 79.2 | - | 804 | 70.32 |  |  |
| 1,500 | 250 | 250 | 256 | - |  | Upper Marker |

Supplementary material S1. Agilent Tape station profile of small RNA library (152 bp) obtained from pool of 24 samples concentrated with magnetic beads and size selected with Pippin Prep. 128 bp size peak represent primer dimmers co-purified with the library.
