# Supplementary material for: Small RNA sequencing of cryopreserved semen from single bull revealed altered miRNAs and piRNAs expression between High- and Low-motile sperm populations
Source: BMC Genomics. 2017 Jan 4;18:14. doi: 10.1186/s12864-016-3394-7 (PMC5209821; doi:10.1186/s12864-016-3394-7)
Supplement: Additional file 3: — Details for each piRNA clusters found in High Motile (HM) sperm fraction. Genes, repeats, transposable elements and transcription factors binding sites falling within the cluster regions were reported. (ZIP 1896 kb) [file 12864_2016_3394_MOESM3_ESM.zip › 1.html]

piRNA cluster 1


Predicted piRNA cluster no. 1     previous   next
  

Show proTRAC run info
Hide proTRAC run info

================================= proTRAC ====================================  
VERSION: 2.1                                    LAST MODIFIED: 06. October 2015  
  
Please cite:  
Rosenkranz D, Zischler H. proTRAC - a software for probabilistic piRNA cluster  
detection, visualization and analysis. 2012. BMC Bioinformatics 13:5.  
  
and (for proTRAC 2.0 and later):  
Rosenkranz D, Rudloff S, Bastuck K, Ketting RF, Zischler H. Tupaia small RNAs  
provide insights into function and evolution of RNAi-based transposon defense  
in mammals. 2015. RNA 21(5):911-922.  
  
Contact:  
David Rosenkranz  
Institute of Anthropology, small RNA group  
Johannes Gutenberg University Mainz  
email: rosenkranz@uni-mainz.de  
  
You can find the latest proTRAC version at:  
http://sourceforge.net/projects/protrac/files  
http://www.smallRNAgroup-mainz.de/software  
==============================================================================  
  
PARAMETERS:  
Map file: .............../storage/core/barbara/genhome/smallRNA/fertility/Sample\_motile/pirna/Sample\_motile\_26-33\_collapsed.fa.no-dust.map.weighted-10000-1000-b-0  
Genome file: ............/storage/core/barbara/genhome/smallRNA/fertility/Sample\_all/pirna/bt\_311\_chrY.fa  
RepeatMasker annotation: /storage/genomes/bt\_umd31/GCF\_000003055.6\_Bos\_taurus\_UMD\_3.1.1\_repeatMasker\_chr.out  
GeneSet:................./storage/core/barbara/genhome/smallRNA/fertility/Sample\_all/pirna/full.gtf  
  
Significant (p<=0.01) hit density will be calculated based  
on observed hit distribution.  
  
Sliding window size: ........................................ 5000 bp  
Sliding window increament: .................................. 1000 bp  
Normalize each hit by number of genomic hits: ............... 1 [0=no/1=yes]  
Normalize each hit by number of sequence reads: ............. 1 [0=no/1=yes]  
Normalize values (-> per million mapped reads): ............. 1 [0=no/1=yes]  
Min. fraction of hits with 1T(U) or 10A: .................... 0.75  
Alternatively: Min. fraction of hits with 1T(U) and 10A: .... 0.5  
Min. fraction of hits with typical piRNA length: ............ 0.75  
Typical piRNA length: ....................................... 26-33 nt  
Min. size of a piRNA cluster: ............................... 5000 bp.  
Min. number of hits (absolute): ............................. 0  
Min. number of hits (normalized): ........................... 0  
Min. fraction of hits on the mainstrand: .................... 0.75  
Top fraction of mapped sequences (in terms of read counts): . 1%  
Top fraction accounts for max. n% of sequence reads: ........ 90%  
Min. fraction of hits on each arm of a bidirectional cluster: 0.1  
Output image file for each cluster: ......................... 0 [0=no/1=yes]  
Output html file for each cluster: .......................... 1 [0=no/1=yes]  
Output a summary table: ..................................... 1 [0=no/1=yes]  
Output a FASTA file for each cluster (piRNA sequences): ..... 1 [0=no/1=yes]  
Output a FASTA file comprising cluster sequences: ........... 1 [0=no/1=yes]  
Search DNA motifs in clusters: .............................. 1 [0=no/1=yes]  
Output flanking sequences: +/- .............................. 0 bp  
Output ~.pTi file: .......................................... 1 [0=no/1=yes]  
==============================================================================  
  
  
Genome size (without gaps): ............ 2678902517 bp  
Gaps (N/X/-): .......................... 53837044 bp  
Mapped reads: .......................... 658825247023  
Non-identical sequences: ............... 514171  
Genomic hits: .......................... 764233  
Significant densitiy of mapped reads: .. 12867599.5173724 reads/kb

Show proTRAC cluster info
Hide proTRAC cluster info

|  |  |
| --- | --- |
| Location | chr1 |
| Coordinates | 80730804-80736822 |
| Size [bp] | 6019 |
| Sequence hit loci | 483 |
| Mapped reads (normalized) | 566227345 |
| Mapped reads (normalized) per kb | 94073325.3 |
| Normalized reads with 1T (1U) | 86.1% |
| Normalized reads with 10A | 27.7% |
| Normalized reads with length 26-33 nt | 100% |
| Normalized reads on the main strand(s) | 100% |
| Predicted directionality | mono:plus |

100%

0%

1T (1U)  
reads

10A reads

26-33 nt  
reads

reads on mainstrand

**Either the amount of reads with 1T (1U) OR 10A has to exceed 75% (set with option: -1Tor10A)  
Alternatively the amount of reads with 1T (1U) AND 10A has to exceed 50% (set with option: -1Tand10A)  
Minimum amount of reads with preferred size is 75% (set with option: -pisize)  
Minimum amount of reads on the main strand(s) is 75% (set with option: -clstrand)**

Show read coverage
Hide read coverage

WHAT DO I SEE HERE?  
This chart shows the location of mapped sequence reads within a predicted piRNA cluster. The color refers to the number of genomic hits produced by the sequence read in question. A dark red bar indicates that this sequence read produces many other hits elsewhere in the genome. Many adjacent red or yellow bars can indicate the presence of a multi-copy element such as transposons or rRNA genes. A dark green bar indicates that this sequence read maps uniquely to this locus.

1 hit

2-5 hits

6-10 hits

11-20 hits

21-50 hits

51-100 hits

> 100 hits

chr1

80730804

80736822

Gene Set

RepeatMasker

Mapped  
Reads

67.35

plus strand

minus strand

67.35

Region: chr1 1-80730810. Max. coverage (+): 1.02. Max coverage (-): 0

Region: chr1 80730811-80730822. Max. coverage (+): 1.02. Max coverage (-): 0

Region: chr1 80730823-80730834. Max. coverage (+): 0. Max coverage (-): 0

Region: chr1 80730835-80730846. Max. coverage (+): 0. Max coverage (-): 0

Region: chr1 80730847-80730858. Max. coverage (+): 0. Max coverage (-): 0

Region: chr1 80730859-80730870. Max. coverage (+): 1.92. Max coverage (-): 0

Region: chr1 80730871-80730882. Max. coverage (+): 1.92. Max coverage (-): 0

Region: chr1 80730883-80730894. Max. coverage (+): 1.48. Max coverage (-): 0

Region: chr1 80730895-80730906. Max. coverage (+): 1.48. Max coverage (-): 0

Region: chr1 80730907-80730918. Max. coverage (+): 0. Max coverage (-): 0

Region: chr1 80730919-80730930. Max. coverage (+): 1.92. Max coverage (-): 0

Region: chr1 80730931-80730942. Max. coverage (+): 1.92. Max coverage (-): 0

Region: chr1 80730943-80730954. Max. coverage (+): 0. Max coverage (-): 0

Region: chr1 80730955-80730966. Max. coverage (+): 0. Max coverage (-): 0

Region: chr1 80730967-80730978. Max. coverage (+): 0. Max coverage (-): 0

Region: chr1 80730979-80730990. Max. coverage (+): 0. Max coverage (-): 0

Region: chr1 80730991-80731002. Max. coverage (+): 0. Max coverage (-): 0

Region: chr1 80731003-80731014. Max. coverage (+): 0. Max coverage (-): 0

Region: chr1 80731015-80731026. Max. coverage (+): 0. Max coverage (-): 0

Region: chr1 80731027-80731038. Max. coverage (+): 0. Max coverage (-): 0

Region: chr1 80731039-80731050. Max. coverage (+): 0. Max coverage (-): 0

Region: chr1 80731051-80731062. Max. coverage (+): 0. Max coverage (-): 0

Region: chr1 80731063-80731074. Max. coverage (+): 0. Max coverage (-): 0

Region: chr1 80731075-80731086. Max. coverage (+): 0. Max coverage (-): 0

Region: chr1 80731087-80731098. Max. coverage (+): 0. Max coverage (-): 0

Region: chr1 80731099-80731110. Max. coverage (+): 0. Max coverage (-): 0

Region: chr1 80731111-80731123. Max. coverage (+): 0. Max coverage (-): 0

Region: chr1 80731124-80731135. Max. coverage (+): 0. Max coverage (-): 0

Region: chr1 80731136-80731147. Max. coverage (+): 0. Max coverage (-): 0

Region: chr1 80731148-80731159. Max. coverage (+): 0. Max coverage (-): 0

Region: chr1 80731160-80731171. Max. coverage (+): 1.68. Max coverage (-): 0

Region: chr1 80731172-80731183. Max. coverage (+): 1.68. Max coverage (-): 0

Region: chr1 80731184-80731195. Max. coverage (+): 0. Max coverage (-): 0

Region: chr1 80731196-80731207. Max. coverage (+): 0. Max coverage (-): 0

Region: chr1 80731208-80731219. Max. coverage (+): 0. Max coverage (-): 0

Region: chr1 80731220-80731231. Max. coverage (+): 0. Max coverage (-): 0

Region: chr1 80731232-80731243. Max. coverage (+): 2.69. Max coverage (-): 0

Region: chr1 80731244-80731255. Max. coverage (+): 1.91. Max coverage (-): 0

Region: chr1 80731256-80731267. Max. coverage (+): 0. Max coverage (-): 0

Region: chr1 80731268-80731279. Max. coverage (+): 0. Max coverage (-): 0

Region: chr1 80731280-80731291. Max. coverage (+): 0. Max coverage (-): 0

Region: chr1 80731292-80731303. Max. coverage (+): 0. Max coverage (-): 0

Region: chr1 80731304-80731315. Max. coverage (+): 0. Max coverage (-): 0

Region: chr1 80731316-80731327. Max. coverage (+): 0. Max coverage (-): 0

Region: chr1 80731328-80731339. Max. coverage (+): 0. Max coverage (-): 0

Region: chr1 80731340-80731351. Max. coverage (+): 0. Max coverage (-): 0

Region: chr1 80731352-80731363. Max. coverage (+): 0. Max coverage (-): 0

Region: chr1 80731364-80731375. Max. coverage (+): 0. Max coverage (-): 0

Region: chr1 80731376-80731387. Max. coverage (+): 0. Max coverage (-): 0

Region: chr1 80731388-80731399. Max. coverage (+): 0. Max coverage (-): 0

Region: chr1 80731400-80731411. Max. coverage (+): 0. Max coverage (-): 0

Region: chr1 80731412-80731423. Max. coverage (+): 0. Max coverage (-): 0

Region: chr1 80731424-80731435. Max. coverage (+): 0. Max coverage (-): 0

Region: chr1 80731436-80731448. Max. coverage (+): 0. Max coverage (-): 0

Region: chr1 80731449-80731460. Max. coverage (+): 0. Max coverage (-): 0

Region: chr1 80731461-80731472. Max. coverage (+): 0. Max coverage (-): 0

Region: chr1 80731473-80731484. Max. coverage (+): 0. Max coverage (-): 0

Region: chr1 80731485-80731496. Max. coverage (+): 0. Max coverage (-): 0

Region: chr1 80731497-80731508. Max. coverage (+): 0. Max coverage (-): 0

Region: chr1 80731509-80731520. Max. coverage (+): 6.35. Max coverage (-): 0

Region: chr1 80731521-80731532. Max. coverage (+): 6.35. Max coverage (-): 0

Region: chr1 80731533-80731544. Max. coverage (+): 6.39. Max coverage (-): 0

Region: chr1 80731545-80731556. Max. coverage (+): 2.51. Max coverage (-): 0

Region: chr1 80731557-80731568. Max. coverage (+): 0.46. Max coverage (-): 0

Region: chr1 80731569-80731580. Max. coverage (+): 0. Max coverage (-): 0

Region: chr1 80731581-80731592. Max. coverage (+): 0. Max coverage (-): 0

Region: chr1 80731593-80731604. Max. coverage (+): 0. Max coverage (-): 0

Region: chr1 80731605-80731616. Max. coverage (+): 0. Max coverage (-): 0

Region: chr1 80731617-80731628. Max. coverage (+): 0. Max coverage (-): 0

Region: chr1 80731629-80731640. Max. coverage (+): 0. Max coverage (-): 0

Region: chr1 80731641-80731652. Max. coverage (+): 0. Max coverage (-): 0

Region: chr1 80731653-80731664. Max. coverage (+): 0. Max coverage (-): 0

Region: chr1 80731665-80731676. Max. coverage (+): 0. Max coverage (-): 0

Region: chr1 80731677-80731688. Max. coverage (+): 0. Max coverage (-): 0

Region: chr1 80731689-80731700. Max. coverage (+): 0. Max coverage (-): 0

Region: chr1 80731701-80731712. Max. coverage (+): 0. Max coverage (-): 0

Region: chr1 80731713-80731724. Max. coverage (+): 0. Max coverage (-): 0

Region: chr1 80731725-80731736. Max. coverage (+): 1.5. Max coverage (-): 0

Region: chr1 80731737-80731748. Max. coverage (+): 0. Max coverage (-): 0

Region: chr1 80731749-80731761. Max. coverage (+): 0.26. Max coverage (-): 0

Region: chr1 80731762-80731773. Max. coverage (+): 0.26. Max coverage (-): 0

Region: chr1 80731774-80731785. Max. coverage (+): 0. Max coverage (-): 0

Region: chr1 80731786-80731797. Max. coverage (+): 0. Max coverage (-): 0

Region: chr1 80731798-80731809. Max. coverage (+): 0. Max coverage (-): 0

Region: chr1 80731810-80731821. Max. coverage (+): 0. Max coverage (-): 0

Region: chr1 80731822-80731833. Max. coverage (+): 0. Max coverage (-): 0

Region: chr1 80731834-80731845. Max. coverage (+): 0. Max coverage (-): 0

Region: chr1 80731846-80731857. Max. coverage (+): 0. Max coverage (-): 0

Region: chr1 80731858-80731869. Max. coverage (+): 0. Max coverage (-): 0

Region: chr1 80731870-80731881. Max. coverage (+): 0. Max coverage (-): 0

Region: chr1 80731882-80731893. Max. coverage (+): 0. Max coverage (-): 0

Region: chr1 80731894-80731905. Max. coverage (+): 0. Max coverage (-): 0

Region: chr1 80731906-80731917. Max. coverage (+): 0. Max coverage (-): 0

Region: chr1 80731918-80731929. Max. coverage (+): 0. Max coverage (-): 0

Region: chr1 80731930-80731941. Max. coverage (+): 0. Max coverage (-): 0

Region: chr1 80731942-80731953. Max. coverage (+): 0. Max coverage (-): 0

Region: chr1 80731954-80731965. Max. coverage (+): 0. Max coverage (-): 0

Region: chr1 80731966-80731977. Max. coverage (+): 0. Max coverage (-): 0

Region: chr1 80731978-80731989. Max. coverage (+): 0. Max coverage (-): 0

Region: chr1 80731990-80732001. Max. coverage (+): 0. Max coverage (-): 0

Region: chr1 80732002-80732013. Max. coverage (+): 0. Max coverage (-): 0

Region: chr1 80732014-80732025. Max. coverage (+): 0. Max coverage (-): 0

Region: chr1 80732026-80732037. Max. coverage (+): 0. Max coverage (-): 0

Region: chr1 80732038-80732049. Max. coverage (+): 0. Max coverage (-): 0

Region: chr1 80732050-80732061. Max. coverage (+): 0. Max coverage (-): 0

Region: chr1 80732062-80732074. Max. coverage (+): 0. Max coverage (-): 0

Region: chr1 80732075-80732086. Max. coverage (+): 0. Max coverage (-): 0

Region: chr1 80732087-80732098. Max. coverage (+): 0. Max coverage (-): 0

Region: chr1 80732099-80732110. Max. coverage (+): 0. Max coverage (-): 0

Region: chr1 80732111-80732122. Max. coverage (+): 0. Max coverage (-): 0

Region: chr1 80732123-80732134. Max. coverage (+): 0. Max coverage (-): 0

Region: chr1 80732135-80732146. Max. coverage (+): 0. Max coverage (-): 0

Region: chr1 80732147-80732158. Max. coverage (+): 0. Max coverage (-): 0

Region: chr1 80732159-80732170. Max. coverage (+): 0. Max coverage (-): 0

Region: chr1 80732171-80732182. Max. coverage (+): 0. Max coverage (-): 0

Region: chr1 80732183-80732194. Max. coverage (+): 0. Max coverage (-): 0

Region: chr1 80732195-80732206. Max. coverage (+): 0. Max coverage (-): 0

Region: chr1 80732207-80732218. Max. coverage (+): 0. Max coverage (-): 0

Region: chr1 80732219-80732230. Max. coverage (+): 0. Max coverage (-): 0

Region: chr1 80732231-80732242. Max. coverage (+): 0. Max coverage (-): 0

Region: chr1 80732243-80732254. Max. coverage (+): 0. Max coverage (-): 0

Region: chr1 80732255-80732266. Max. coverage (+): 8.06. Max coverage (-): 0

Region: chr1 80732267-80732278. Max. coverage (+): 0. Max coverage (-): 0

Region: chr1 80732279-80732290. Max. coverage (+): 0. Max coverage (-): 0

Region: chr1 80732291-80732302. Max. coverage (+): 0. Max coverage (-): 0

Region: chr1 80732303-80732314. Max. coverage (+): 0. Max coverage (-): 0

Region: chr1 80732315-80732326. Max. coverage (+): 0. Max coverage (-): 0

Region: chr1 80732327-80732338. Max. coverage (+): 0. Max coverage (-): 0

Region: chr1 80732339-80732350. Max. coverage (+): 0. Max coverage (-): 0

Region: chr1 80732351-80732362. Max. coverage (+): 0. Max coverage (-): 0

Region: chr1 80732363-80732374. Max. coverage (+): 0. Max coverage (-): 0

Region: chr1 80732375-80732386. Max. coverage (+): 0. Max coverage (-): 0

Region: chr1 80732387-80732399. Max. coverage (+): 0. Max coverage (-): 0

Region: chr1 80732400-80732411. Max. coverage (+): 0. Max coverage (-): 0

Region: chr1 80732412-80732423. Max. coverage (+): 0. Max coverage (-): 0

Region: chr1 80732424-80732435. Max. coverage (+): 0. Max coverage (-): 0

Region: chr1 80732436-80732447. Max. coverage (+): 0. Max coverage (-): 0

Region: chr1 80732448-80732459. Max. coverage (+): 0. Max coverage (-): 0

Region: chr1 80732460-80732471. Max. coverage (+): 0. Max coverage (-): 0

Region: chr1 80732472-80732483. Max. coverage (+): 0. Max coverage (-): 0

Region: chr1 80732484-80732495. Max. coverage (+): 0. Max coverage (-): 0

Region: chr1 80732496-80732507. Max. coverage (+): 0. Max coverage (-): 0

Region: chr1 80732508-80732519. Max. coverage (+): 4.39. Max coverage (-): 0

Region: chr1 80732520-80732531. Max. coverage (+): 4.39. Max coverage (-): 0

Region: chr1 80732532-80732543. Max. coverage (+): 6.2. Max coverage (-): 0

Region: chr1 80732544-80732555. Max. coverage (+): 4.24. Max coverage (-): 0

Region: chr1 80732556-80732567. Max. coverage (+): 4.24. Max coverage (-): 0

Region: chr1 80732568-80732579. Max. coverage (+): 3.3. Max coverage (-): 0

Region: chr1 80732580-80732591. Max. coverage (+): 9.32. Max coverage (-): 0

Region: chr1 80732592-80732603. Max. coverage (+): 8.6. Max coverage (-): 0

Region: chr1 80732604-80732615. Max. coverage (+): 1.91. Max coverage (-): 0

Region: chr1 80732616-80732627. Max. coverage (+): 1.91. Max coverage (-): 0

Region: chr1 80732628-80732639. Max. coverage (+): 0. Max coverage (-): 0

Region: chr1 80732640-80732651. Max. coverage (+): 0. Max coverage (-): 0

Region: chr1 80732652-80732663. Max. coverage (+): 0. Max coverage (-): 0

Region: chr1 80732664-80732675. Max. coverage (+): 0. Max coverage (-): 0

Region: chr1 80732676-80732687. Max. coverage (+): 0. Max coverage (-): 0

Region: chr1 80732688-80732699. Max. coverage (+): 0. Max coverage (-): 0

Region: chr1 80732700-80732712. Max. coverage (+): 0. Max coverage (-): 0

Region: chr1 80732713-80732724. Max. coverage (+): 0. Max coverage (-): 0

Region: chr1 80732725-80732736. Max. coverage (+): 0. Max coverage (-): 0

Region: chr1 80732737-80732748. Max. coverage (+): 0. Max coverage (-): 0

Region: chr1 80732749-80732760. Max. coverage (+): 2.49. Max coverage (-): 0

Region: chr1 80732761-80732772. Max. coverage (+): 5.2. Max coverage (-): 0

Region: chr1 80732773-80732784. Max. coverage (+): 45.83. Max coverage (-): 0

Region: chr1 80732785-80732796. Max. coverage (+): 3.5. Max coverage (-): 0

Region: chr1 80732797-80732808. Max. coverage (+): 0.9. Max coverage (-): 0

Region: chr1 80732809-80732820. Max. coverage (+): 0. Max coverage (-): 0

Region: chr1 80732821-80732832. Max. coverage (+): 7.04. Max coverage (-): 0

Region: chr1 80732833-80732844. Max. coverage (+): 28.82. Max coverage (-): 0

Region: chr1 80732845-80732856. Max. coverage (+): 14.32. Max coverage (-): 0

Region: chr1 80732857-80732868. Max. coverage (+): 7.52. Max coverage (-): 0

Region: chr1 80732869-80732880. Max. coverage (+): 5.42. Max coverage (-): 0

Region: chr1 80732881-80732892. Max. coverage (+): 5.42. Max coverage (-): 0

Region: chr1 80732893-80732904. Max. coverage (+): 4.53. Max coverage (-): 0

Region: chr1 80732905-80732916. Max. coverage (+): 1.18. Max coverage (-): 0

Region: chr1 80732917-80732928. Max. coverage (+): 0. Max coverage (-): 0

Region: chr1 80732929-80732940. Max. coverage (+): 0. Max coverage (-): 0

Region: chr1 80732941-80732952. Max. coverage (+): 0. Max coverage (-): 0

Region: chr1 80732953-80732964. Max. coverage (+): 0. Max coverage (-): 0

Region: chr1 80732965-80732976. Max. coverage (+): 0. Max coverage (-): 0

Region: chr1 80732977-80732988. Max. coverage (+): 0. Max coverage (-): 0

Region: chr1 80732989-80733000. Max. coverage (+): 0. Max coverage (-): 0

Region: chr1 80733001-80733012. Max. coverage (+): 0. Max coverage (-): 0

Region: chr1 80733013-80733025. Max. coverage (+): 0. Max coverage (-): 0

Region: chr1 80733026-80733037. Max. coverage (+): 0. Max coverage (-): 0

Region: chr1 80733038-80733049. Max. coverage (+): 0. Max coverage (-): 0

Region: chr1 80733050-80733061. Max. coverage (+): 0. Max coverage (-): 0

Region: chr1 80733062-80733073. Max. coverage (+): 0. Max coverage (-): 0

Region: chr1 80733074-80733085. Max. coverage (+): 0. Max coverage (-): 0

Region: chr1 80733086-80733097. Max. coverage (+): 0. Max coverage (-): 0

Region: chr1 80733098-80733109. Max. coverage (+): 0. Max coverage (-): 0

Region: chr1 80733110-80733121. Max. coverage (+): 0. Max coverage (-): 0

Region: chr1 80733122-80733133. Max. coverage (+): 0. Max coverage (-): 0

Region: chr1 80733134-80733145. Max. coverage (+): 1.42. Max coverage (-): 0

Region: chr1 80733146-80733157. Max. coverage (+): 7.32. Max coverage (-): 0

Region: chr1 80733158-80733169. Max. coverage (+): 1.07. Max coverage (-): 0

Region: chr1 80733170-80733181. Max. coverage (+): 13.74. Max coverage (-): 0

Region: chr1 80733182-80733193. Max. coverage (+): 13.39. Max coverage (-): 0

Region: chr1 80733194-80733205. Max. coverage (+): 0. Max coverage (-): 0

Region: chr1 80733206-80733217. Max. coverage (+): 0. Max coverage (-): 0

Region: chr1 80733218-80733229. Max. coverage (+): 5.48. Max coverage (-): 0

Region: chr1 80733230-80733241. Max. coverage (+): 5.48. Max coverage (-): 0

Region: chr1 80733242-80733253. Max. coverage (+): 6.3. Max coverage (-): 0

Region: chr1 80733254-80733265. Max. coverage (+): 11.89. Max coverage (-): 0

Region: chr1 80733266-80733277. Max. coverage (+): 13.68. Max coverage (-): 0

Region: chr1 80733278-80733289. Max. coverage (+): 3.08. Max coverage (-): 0

Region: chr1 80733290-80733301. Max. coverage (+): 2.04. Max coverage (-): 0

Region: chr1 80733302-80733313. Max. coverage (+): 13.39. Max coverage (-): 0

Region: chr1 80733314-80733325. Max. coverage (+): 14.38. Max coverage (-): 0

Region: chr1 80733326-80733337. Max. coverage (+): 0.77. Max coverage (-): 0

Region: chr1 80733338-80733350. Max. coverage (+): 2.22. Max coverage (-): 0

Region: chr1 80733351-80733362. Max. coverage (+): 0. Max coverage (-): 0

Region: chr1 80733363-80733374. Max. coverage (+): 0. Max coverage (-): 0

Region: chr1 80733375-80733386. Max. coverage (+): 0. Max coverage (-): 0

Region: chr1 80733387-80733398. Max. coverage (+): 0.49. Max coverage (-): 0

Region: chr1 80733399-80733410. Max. coverage (+): 0.49. Max coverage (-): 0

Region: chr1 80733411-80733422. Max. coverage (+): 8.9. Max coverage (-): 0

Region: chr1 80733423-80733434. Max. coverage (+): 4.45. Max coverage (-): 0

Region: chr1 80733435-80733446. Max. coverage (+): 0.94. Max coverage (-): 0

Region: chr1 80733447-80733458. Max. coverage (+): 0.94. Max coverage (-): 0

Region: chr1 80733459-80733470. Max. coverage (+): 1.77. Max coverage (-): 0

Region: chr1 80733471-80733482. Max. coverage (+): 7.05. Max coverage (-): 0

Region: chr1 80733483-80733494. Max. coverage (+): 0. Max coverage (-): 0

Region: chr1 80733495-80733506. Max. coverage (+): 2.99. Max coverage (-): 0

Region: chr1 80733507-80733518. Max. coverage (+): 11.3. Max coverage (-): 0

Region: chr1 80733519-80733530. Max. coverage (+): 10.36. Max coverage (-): 0

Region: chr1 80733531-80733542. Max. coverage (+): 8.68. Max coverage (-): 0

Region: chr1 80733543-80733554. Max. coverage (+): 0. Max coverage (-): 0

Region: chr1 80733555-80733566. Max. coverage (+): 4.58. Max coverage (-): 0

Region: chr1 80733567-80733578. Max. coverage (+): 4.13. Max coverage (-): 0

Region: chr1 80733579-80733590. Max. coverage (+): 4.13. Max coverage (-): 0

Region: chr1 80733591-80733602. Max. coverage (+): 4.33. Max coverage (-): 0

Region: chr1 80733603-80733614. Max. coverage (+): 2.43. Max coverage (-): 0

Region: chr1 80733615-80733626. Max. coverage (+): 0. Max coverage (-): 0

Region: chr1 80733627-80733638. Max. coverage (+): 0. Max coverage (-): 0

Region: chr1 80733639-80733650. Max. coverage (+): 6.51. Max coverage (-): 0

Region: chr1 80733651-80733663. Max. coverage (+): 13.96. Max coverage (-): 0

Region: chr1 80733664-80733675. Max. coverage (+): 13.96. Max coverage (-): 0

Region: chr1 80733676-80733687. Max. coverage (+): 1.86. Max coverage (-): 0

Region: chr1 80733688-80733699. Max. coverage (+): 2.05. Max coverage (-): 0

Region: chr1 80733700-80733711. Max. coverage (+): 2.05. Max coverage (-): 0

Region: chr1 80733712-80733723. Max. coverage (+): 21.1. Max coverage (-): 0

Region: chr1 80733724-80733735. Max. coverage (+): 17.83. Max coverage (-): 0

Region: chr1 80733736-80733747. Max. coverage (+): 7.21. Max coverage (-): 0

Region: chr1 80733748-80733759. Max. coverage (+): 5.76. Max coverage (-): 0

Region: chr1 80733760-80733771. Max. coverage (+): 5.76. Max coverage (-): 0

Region: chr1 80733772-80733783. Max. coverage (+): 3.71. Max coverage (-): 0

Region: chr1 80733784-80733795. Max. coverage (+): 0. Max coverage (-): 0

Region: chr1 80733796-80733807. Max. coverage (+): 0. Max coverage (-): 0

Region: chr1 80733808-80733819. Max. coverage (+): 0. Max coverage (-): 0

Region: chr1 80733820-80733831. Max. coverage (+): 2.75. Max coverage (-): 0

Region: chr1 80733832-80733843. Max. coverage (+): 4.71. Max coverage (-): 0

Region: chr1 80733844-80733855. Max. coverage (+): 0.62. Max coverage (-): 0

Region: chr1 80733856-80733867. Max. coverage (+): 0. Max coverage (-): 0

Region: chr1 80733868-80733879. Max. coverage (+): 34.67. Max coverage (-): 0

Region: chr1 80733880-80733891. Max. coverage (+): 22.83. Max coverage (-): 0

Region: chr1 80733892-80733903. Max. coverage (+): 0.69. Max coverage (-): 0

Region: chr1 80733904-80733915. Max. coverage (+): 0. Max coverage (-): 0

Region: chr1 80733916-80733927. Max. coverage (+): 0. Max coverage (-): 0

Region: chr1 80733928-80733939. Max. coverage (+): 0. Max coverage (-): 0

Region: chr1 80733940-80733951. Max. coverage (+): 0. Max coverage (-): 0

Region: chr1 80733952-80733963. Max. coverage (+): 2.2. Max coverage (-): 0

Region: chr1 80733964-80733976. Max. coverage (+): 6.19. Max coverage (-): 0

Region: chr1 80733977-80733988. Max. coverage (+): 11.08. Max coverage (-): 0

Region: chr1 80733989-80734000. Max. coverage (+): 11.08. Max coverage (-): 0

Region: chr1 80734001-80734012. Max. coverage (+): 10.5. Max coverage (-): 0

Region: chr1 80734013-80734024. Max. coverage (+): 0.86. Max coverage (-): 0

Region: chr1 80734025-80734036. Max. coverage (+): 2.29. Max coverage (-): 0

Region: chr1 80734037-80734048. Max. coverage (+): 25.92. Max coverage (-): 0

Region: chr1 80734049-80734060. Max. coverage (+): 24.52. Max coverage (-): 0

Region: chr1 80734061-80734072. Max. coverage (+): 3.84. Max coverage (-): 0

Region: chr1 80734073-80734084. Max. coverage (+): 6.77. Max coverage (-): 0

Region: chr1 80734085-80734096. Max. coverage (+): 14.87. Max coverage (-): 0

Region: chr1 80734097-80734108. Max. coverage (+): 14.87. Max coverage (-): 0

Region: chr1 80734109-80734120. Max. coverage (+): 2.04. Max coverage (-): 0

Region: chr1 80734121-80734132. Max. coverage (+): 1.29. Max coverage (-): 0

Region: chr1 80734133-80734144. Max. coverage (+): 7.68. Max coverage (-): 0

Region: chr1 80734145-80734156. Max. coverage (+): 0. Max coverage (-): 0

Region: chr1 80734157-80734168. Max. coverage (+): 0. Max coverage (-): 0

Region: chr1 80734169-80734180. Max. coverage (+): 0. Max coverage (-): 0

Region: chr1 80734181-80734192. Max. coverage (+): 7.16. Max coverage (-): 0

Region: chr1 80734193-80734204. Max. coverage (+): 10.1. Max coverage (-): 0

Region: chr1 80734205-80734216. Max. coverage (+): 3.78. Max coverage (-): 0

Region: chr1 80734217-80734228. Max. coverage (+): 3.68. Max coverage (-): 0

Region: chr1 80734229-80734240. Max. coverage (+): 5.79. Max coverage (-): 0

Region: chr1 80734241-80734252. Max. coverage (+): 6.96. Max coverage (-): 0

Region: chr1 80734253-80734264. Max. coverage (+): 4.2. Max coverage (-): 0

Region: chr1 80734265-80734276. Max. coverage (+): 12.49. Max coverage (-): 0

Region: chr1 80734277-80734289. Max. coverage (+): 4.89. Max coverage (-): 0

Region: chr1 80734290-80734301. Max. coverage (+): 0. Max coverage (-): 0

Region: chr1 80734302-80734313. Max. coverage (+): 10.6. Max coverage (-): 0

Region: chr1 80734314-80734325. Max. coverage (+): 4.78. Max coverage (-): 0

Region: chr1 80734326-80734337. Max. coverage (+): 0. Max coverage (-): 0

Region: chr1 80734338-80734349. Max. coverage (+): 30.97. Max coverage (-): 0

Region: chr1 80734350-80734361. Max. coverage (+): 67.35. Max coverage (-): 0

Region: chr1 80734362-80734373. Max. coverage (+): 25.56. Max coverage (-): 0

Region: chr1 80734374-80734385. Max. coverage (+): 0. Max coverage (-): 0

Region: chr1 80734386-80734397. Max. coverage (+): 0. Max coverage (-): 0

Region: chr1 80734398-80734409. Max. coverage (+): 5.05. Max coverage (-): 0

Region: chr1 80734410-80734421. Max. coverage (+): 3.78. Max coverage (-): 0

Region: chr1 80734422-80734433. Max. coverage (+): 9.5. Max coverage (-): 0

Region: chr1 80734434-80734445. Max. coverage (+): 0.78. Max coverage (-): 0

Region: chr1 80734446-80734457. Max. coverage (+): 0. Max coverage (-): 0

Region: chr1 80734458-80734469. Max. coverage (+): 0. Max coverage (-): 0

Region: chr1 80734470-80734481. Max. coverage (+): 0. Max coverage (-): 0

Region: chr1 80734482-80734493. Max. coverage (+): 0. Max coverage (-): 0

Region: chr1 80734494-80734505. Max. coverage (+): 0. Max coverage (-): 0

Region: chr1 80734506-80734517. Max. coverage (+): 1.61. Max coverage (-): 0

Region: chr1 80734518-80734529. Max. coverage (+): 0. Max coverage (-): 0

Region: chr1 80734530-80734541. Max. coverage (+): 2.2. Max coverage (-): 0

Region: chr1 80734542-80734553. Max. coverage (+): 2.2. Max coverage (-): 0

Region: chr1 80734554-80734565. Max. coverage (+): 0.82. Max coverage (-): 0

Region: chr1 80734566-80734577. Max. coverage (+): 4.08. Max coverage (-): 0

Region: chr1 80734578-80734589. Max. coverage (+): 20.61. Max coverage (-): 0

Region: chr1 80734590-80734601. Max. coverage (+): 5.77. Max coverage (-): 0

Region: chr1 80734602-80734614. Max. coverage (+): 0. Max coverage (-): 0

Region: chr1 80734615-80734626. Max. coverage (+): 0. Max coverage (-): 0

Region: chr1 80734627-80734638. Max. coverage (+): 5.37. Max coverage (-): 0

Region: chr1 80734639-80734650. Max. coverage (+): 4.44. Max coverage (-): 0

Region: chr1 80734651-80734662. Max. coverage (+): 0. Max coverage (-): 0

Region: chr1 80734663-80734674. Max. coverage (+): 0. Max coverage (-): 0

Region: chr1 80734675-80734686. Max. coverage (+): 0. Max coverage (-): 0

Region: chr1 80734687-80734698. Max. coverage (+): 0.97. Max coverage (-): 0

Region: chr1 80734699-80734710. Max. coverage (+): 5.87. Max coverage (-): 0

Region: chr1 80734711-80734722. Max. coverage (+): 8.48. Max coverage (-): 0

Region: chr1 80734723-80734734. Max. coverage (+): 5.6. Max coverage (-): 0

Region: chr1 80734735-80734746. Max. coverage (+): 0. Max coverage (-): 0

Region: chr1 80734747-80734758. Max. coverage (+): 2.75. Max coverage (-): 0

Region: chr1 80734759-80734770. Max. coverage (+): 2.75. Max coverage (-): 0

Region: chr1 80734771-80734782. Max. coverage (+): 1.2. Max coverage (-): 0

Region: chr1 80734783-80734794. Max. coverage (+): 0. Max coverage (-): 0

Region: chr1 80734795-80734806. Max. coverage (+): 0. Max coverage (-): 0

Region: chr1 80734807-80734818. Max. coverage (+): 0. Max coverage (-): 0

Region: chr1 80734819-80734830. Max. coverage (+): 0. Max coverage (-): 0

Region: chr1 80734831-80734842. Max. coverage (+): 0. Max coverage (-): 0

Region: chr1 80734843-80734854. Max. coverage (+): 0.95. Max coverage (-): 0

Region: chr1 80734855-80734866. Max. coverage (+): 4.94. Max coverage (-): 0

Region: chr1 80734867-80734878. Max. coverage (+): 0. Max coverage (-): 0

Region: chr1 80734879-80734890. Max. coverage (+): 0. Max coverage (-): 0

Region: chr1 80734891-80734902. Max. coverage (+): 0. Max coverage (-): 0

Region: chr1 80734903-80734914. Max. coverage (+): 0. Max coverage (-): 0

Region: chr1 80734915-80734927. Max. coverage (+): 0. Max coverage (-): 0

Region: chr1 80734928-80734939. Max. coverage (+): 0. Max coverage (-): 0

Region: chr1 80734940-80734951. Max. coverage (+): 0. Max coverage (-): 0

Region: chr1 80734952-80734963. Max. coverage (+): 0. Max coverage (-): 0

Region: chr1 80734964-80734975. Max. coverage (+): 0. Max coverage (-): 0

Region: chr1 80734976-80734987. Max. coverage (+): 0. Max coverage (-): 0

Region: chr1 80734988-80734999. Max. coverage (+): 0. Max coverage (-): 0

Region: chr1 80735000-80735011. Max. coverage (+): 0. Max coverage (-): 0

Region: chr1 80735012-80735023. Max. coverage (+): 0. Max coverage (-): 0

Region: chr1 80735024-80735035. Max. coverage (+): 0. Max coverage (-): 0

Region: chr1 80735036-80735047. Max. coverage (+): 0. Max coverage (-): 0

Region: chr1 80735048-80735059. Max. coverage (+): 0. Max coverage (-): 0

Region: chr1 80735060-80735071. Max. coverage (+): 0. Max coverage (-): 0

Region: chr1 80735072-80735083. Max. coverage (+): 0. Max coverage (-): 0

Region: chr1 80735084-80735095. Max. coverage (+): 0. Max coverage (-): 0

Region: chr1 80735096-80735107. Max. coverage (+): 0. Max coverage (-): 0

Region: chr1 80735108-80735119. Max. coverage (+): 0. Max coverage (-): 0

Region: chr1 80735120-80735131. Max. coverage (+): 0. Max coverage (-): 0

Region: chr1 80735132-80735143. Max. coverage (+): 0. Max coverage (-): 0

Region: chr1 80735144-80735155. Max. coverage (+): 0. Max coverage (-): 0

Region: chr1 80735156-80735167. Max. coverage (+): 0. Max coverage (-): 0

Region: chr1 80735168-80735179. Max. coverage (+): 0. Max coverage (-): 0

Region: chr1 80735180-80735191. Max. coverage (+): 0. Max coverage (-): 0

Region: chr1 80735192-80735203. Max. coverage (+): 0. Max coverage (-): 0

Region: chr1 80735204-80735215. Max. coverage (+): 0. Max coverage (-): 0

Region: chr1 80735216-80735227. Max. coverage (+): 0. Max coverage (-): 0

Region: chr1 80735228-80735240. Max. coverage (+): 0. Max coverage (-): 0

Region: chr1 80735241-80735252. Max. coverage (+): 0. Max coverage (-): 0

Region: chr1 80735253-80735264. Max. coverage (+): 0. Max coverage (-): 0

Region: chr1 80735265-80735276. Max. coverage (+): 0. Max coverage (-): 0

Region: chr1 80735277-80735288. Max. coverage (+): 0. Max coverage (-): 0

Region: chr1 80735289-80735300. Max. coverage (+): 0. Max coverage (-): 0

Region: chr1 80735301-80735312. Max. coverage (+): 0. Max coverage (-): 0

Region: chr1 80735313-80735324. Max. coverage (+): 0. Max coverage (-): 0

Region: chr1 80735325-80735336. Max. coverage (+): 0. Max coverage (-): 0

Region: chr1 80735337-80735348. Max. coverage (+): 0. Max coverage (-): 0

Region: chr1 80735349-80735360. Max. coverage (+): 0. Max coverage (-): 0

Region: chr1 80735361-80735372. Max. coverage (+): 2.53. Max coverage (-): 0

Region: chr1 80735373-80735384. Max. coverage (+): 0.44. Max coverage (-): 0

Region: chr1 80735385-80735396. Max. coverage (+): 4.88. Max coverage (-): 0

Region: chr1 80735397-80735408. Max. coverage (+): 4.88. Max coverage (-): 0

Region: chr1 80735409-80735420. Max. coverage (+): 0. Max coverage (-): 0

Region: chr1 80735421-80735432. Max. coverage (+): 0.84. Max coverage (-): 0

Region: chr1 80735433-80735444. Max. coverage (+): 0.84. Max coverage (-): 0

Region: chr1 80735445-80735456. Max. coverage (+): 1.11. Max coverage (-): 0

Region: chr1 80735457-80735468. Max. coverage (+): 0.64. Max coverage (-): 0

Region: chr1 80735469-80735480. Max. coverage (+): 0. Max coverage (-): 0

Region: chr1 80735481-80735492. Max. coverage (+): 0. Max coverage (-): 0

Region: chr1 80735493-80735504. Max. coverage (+): 1.29. Max coverage (-): 0

Region: chr1 80735505-80735516. Max. coverage (+): 0. Max coverage (-): 0

Region: chr1 80735517-80735528. Max. coverage (+): 0. Max coverage (-): 0

Region: chr1 80735529-80735540. Max. coverage (+): 1.65. Max coverage (-): 0

Region: chr1 80735541-80735552. Max. coverage (+): 0.55. Max coverage (-): 0

Region: chr1 80735553-80735565. Max. coverage (+): 0. Max coverage (-): 0

Region: chr1 80735566-80735577. Max. coverage (+): 0. Max coverage (-): 0

Region: chr1 80735578-80735589. Max. coverage (+): 0. Max coverage (-): 0

Region: chr1 80735590-80735601. Max. coverage (+): 0. Max coverage (-): 0

Region: chr1 80735602-80735613. Max. coverage (+): 0. Max coverage (-): 0

Region: chr1 80735614-80735625. Max. coverage (+): 0. Max coverage (-): 0

Region: chr1 80735626-80735637. Max. coverage (+): 0. Max coverage (-): 0

Region: chr1 80735638-80735649. Max. coverage (+): 4.59. Max coverage (-): 0

Region: chr1 80735650-80735661. Max. coverage (+): 0. Max coverage (-): 0

Region: chr1 80735662-80735673. Max. coverage (+): 2.87. Max coverage (-): 0

Region: chr1 80735674-80735685. Max. coverage (+): 2.87. Max coverage (-): 0

Region: chr1 80735686-80735697. Max. coverage (+): 0. Max coverage (-): 0

Region: chr1 80735698-80735709. Max. coverage (+): 0. Max coverage (-): 0

Region: chr1 80735710-80735721. Max. coverage (+): 0. Max coverage (-): 0

Region: chr1 80735722-80735733. Max. coverage (+): 0. Max coverage (-): 0

Region: chr1 80735734-80735745. Max. coverage (+): 0. Max coverage (-): 0

Region: chr1 80735746-80735757. Max. coverage (+): 0. Max coverage (-): 0

Region: chr1 80735758-80735769. Max. coverage (+): 1.78. Max coverage (-): 0

Region: chr1 80735770-80735781. Max. coverage (+): 2.87. Max coverage (-): 0

Region: chr1 80735782-80735793. Max. coverage (+): 0. Max coverage (-): 0

Region: chr1 80735794-80735805. Max. coverage (+): 0. Max coverage (-): 0

Region: chr1 80735806-80735817. Max. coverage (+): 22.66. Max coverage (-): 0

Region: chr1 80735818-80735829. Max. coverage (+): 5.75. Max coverage (-): 0

Region: chr1 80735830-80735841. Max. coverage (+): 5.75. Max coverage (-): 0

Region: chr1 80735842-80735853. Max. coverage (+): 0. Max coverage (-): 0

Region: chr1 80735854-80735865. Max. coverage (+): 1.46. Max coverage (-): 0

Region: chr1 80735866-80735878. Max. coverage (+): 1.8. Max coverage (-): 0

Region: chr1 80735879-80735890. Max. coverage (+): 2.03. Max coverage (-): 0

Region: chr1 80735891-80735902. Max. coverage (+): 5.17. Max coverage (-): 0

Region: chr1 80735903-80735914. Max. coverage (+): 5.17. Max coverage (-): 0

Region: chr1 80735915-80735926. Max. coverage (+): 1.94. Max coverage (-): 0

Region: chr1 80735927-80735938. Max. coverage (+): 0. Max coverage (-): 0

Region: chr1 80735939-80735950. Max. coverage (+): 0. Max coverage (-): 0

Region: chr1 80735951-80735962. Max. coverage (+): 0.97. Max coverage (-): 0

Region: chr1 80735963-80735974. Max. coverage (+): 0.97. Max coverage (-): 0

Region: chr1 80735975-80735986. Max. coverage (+): 2.37. Max coverage (-): 0

Region: chr1 80735987-80735998. Max. coverage (+): 0. Max coverage (-): 0

Region: chr1 80735999-80736010. Max. coverage (+): 1.69. Max coverage (-): 0

Region: chr1 80736011-80736022. Max. coverage (+): 1.69. Max coverage (-): 0

Region: chr1 80736023-80736034. Max. coverage (+): 0. Max coverage (-): 0

Region: chr1 80736035-80736046. Max. coverage (+): 6.57. Max coverage (-): 0

Region: chr1 80736047-80736058. Max. coverage (+): 5.14. Max coverage (-): 0

Region: chr1 80736059-80736070. Max. coverage (+): 13.26. Max coverage (-): 0

Region: chr1 80736071-80736082. Max. coverage (+): 0. Max coverage (-): 0

Region: chr1 80736083-80736094. Max. coverage (+): 0. Max coverage (-): 0

Region: chr1 80736095-80736106. Max. coverage (+): 0. Max coverage (-): 0

Region: chr1 80736107-80736118. Max. coverage (+): 0. Max coverage (-): 0

Region: chr1 80736119-80736130. Max. coverage (+): 0. Max coverage (-): 0

Region: chr1 80736131-80736142. Max. coverage (+): 0. Max coverage (-): 0

Region: chr1 80736143-80736154. Max. coverage (+): 0. Max coverage (-): 0

Region: chr1 80736155-80736166. Max. coverage (+): 0. Max coverage (-): 0

Region: chr1 80736167-80736178. Max. coverage (+): 0.9. Max coverage (-): 0

Region: chr1 80736179-80736191. Max. coverage (+): 0. Max coverage (-): 0

Region: chr1 80736192-80736203. Max. coverage (+): 1.34. Max coverage (-): 0

Region: chr1 80736204-80736215. Max. coverage (+): 4.01. Max coverage (-): 0

Region: chr1 80736216-80736227. Max. coverage (+): 4.01. Max coverage (-): 0

Region: chr1 80736228-80736239. Max. coverage (+): 0. Max coverage (-): 0

Region: chr1 80736240-80736251. Max. coverage (+): 0. Max coverage (-): 0

Region: chr1 80736252-80736263. Max. coverage (+): 0.66. Max coverage (-): 0

Region: chr1 80736264-80736275. Max. coverage (+): 0.66. Max coverage (-): 0

Region: chr1 80736276-80736287. Max. coverage (+): 0. Max coverage (-): 0

Region: chr1 80736288-80736299. Max. coverage (+): 0. Max coverage (-): 0

Region: chr1 80736300-80736311. Max. coverage (+): 0. Max coverage (-): 0

Region: chr1 80736312-80736323. Max. coverage (+): 0. Max coverage (-): 0

Region: chr1 80736324-80736335. Max. coverage (+): 0. Max coverage (-): 0

Region: chr1 80736336-80736347. Max. coverage (+): 0. Max coverage (-): 0

Region: chr1 80736348-80736359. Max. coverage (+): 0. Max coverage (-): 0

Region: chr1 80736360-80736371. Max. coverage (+): 0. Max coverage (-): 0

Region: chr1 80736372-80736383. Max. coverage (+): 1.01. Max coverage (-): 0

Region: chr1 80736384-80736395. Max. coverage (+): 0. Max coverage (-): 0

Region: chr1 80736396-80736407. Max. coverage (+): 0. Max coverage (-): 0

Region: chr1 80736408-80736419. Max. coverage (+): 0. Max coverage (-): 0

Region: chr1 80736420-80736431. Max. coverage (+): 0. Max coverage (-): 0

Region: chr1 80736432-80736443. Max. coverage (+): 0. Max coverage (-): 0

Region: chr1 80736444-80736455. Max. coverage (+): 0. Max coverage (-): 0

Region: chr1 80736456-80736467. Max. coverage (+): 0. Max coverage (-): 0

Region: chr1 80736468-80736479. Max. coverage (+): 0. Max coverage (-): 0

Region: chr1 80736480-80736491. Max. coverage (+): 0. Max coverage (-): 0

Region: chr1 80736492-80736503. Max. coverage (+): 0. Max coverage (-): 0

Region: chr1 80736504-80736516. Max. coverage (+): 0. Max coverage (-): 0

Region: chr1 80736517-80736528. Max. coverage (+): 0. Max coverage (-): 0

Region: chr1 80736529-80736540. Max. coverage (+): 0. Max coverage (-): 0

Region: chr1 80736541-80736552. Max. coverage (+): 0. Max coverage (-): 0

Region: chr1 80736553-80736564. Max. coverage (+): 0. Max coverage (-): 0

Region: chr1 80736565-80736576. Max. coverage (+): 0. Max coverage (-): 0

Region: chr1 80736577-80736588. Max. coverage (+): 0. Max coverage (-): 0

Region: chr1 80736589-80736600. Max. coverage (+): 0. Max coverage (-): 0

Region: chr1 80736601-80736612. Max. coverage (+): 0. Max coverage (-): 0

Region: chr1 80736613-80736624. Max. coverage (+): 0. Max coverage (-): 0

Region: chr1 80736625-80736636. Max. coverage (+): 0. Max coverage (-): 0

Region: chr1 80736637-80736648. Max. coverage (+): 0. Max coverage (-): 0

Region: chr1 80736649-80736660. Max. coverage (+): 0. Max coverage (-): 0

Region: chr1 80736661-80736672. Max. coverage (+): 0. Max coverage (-): 0

Region: chr1 80736673-80736684. Max. coverage (+): 0. Max coverage (-): 0

Region: chr1 80736685-80736696. Max. coverage (+): 0. Max coverage (-): 0

Region: chr1 80736697-80736708. Max. coverage (+): 0. Max coverage (-): 0

Region: chr1 80736709-80736720. Max. coverage (+): 0. Max coverage (-): 0

Region: chr1 80736721-80736732. Max. coverage (+): 0. Max coverage (-): 0

Region: chr1 80736733-80736744. Max. coverage (+): 0. Max coverage (-): 0

Region: chr1 80736745-80736756. Max. coverage (+): 0.74. Max coverage (-): 0

Region: chr1 80736757-80736768. Max. coverage (+): 0. Max coverage (-): 0

Region: chr1 80736769-80736780. Max. coverage (+): 0. Max coverage (-): 0

Region: chr1 80736781-80736792. Max. coverage (+): 11.1. Max coverage (-): 0

Region: chr1 80736793-80736804. Max. coverage (+): 11.1. Max coverage (-): 0

Region: chr1 80736805-80736816. Max. coverage (+): 0. Max coverage (-): 0

Region: chr1 80736817-. Max. coverage (+): 0. Max coverage (-): 0

RepeatMasker Color Code

**+**

100-98% Identity

<98-95% Identity

<95-90% Identity

<90-85% Identity

<85-80% Identity

<80-75% Identity

<75-70% Identity

<70% Identity

**-**

Gene Set Color Code

**+**

Gene

Pseudogene

**-**

Topology/Coverage Color Code

Coverage Plus Strand

Coverage Minus Strand

Mainstrand: Plus

Mainstrand: Minus

Complementary Strand

Flanking Region  
(if option -flank >0)

Gene Set Annotation  
  
RepeatMasker Annotation  

**1. L1ME3B**: 80731990-80732139 (-), Divergence to consensus: 42.1%  
**2. MER5A**: 80732154-80732234 (+), Divergence to consensus: 29.6%  
**3. MER5A**: 80732292-80732330 (-), Divergence to consensus: 17.9%  
**4. BOV-A2**: 80732328-80732448 (-), Divergence to consensus: 8.2%  
**5. MER5A**: 80732449-80732499 (-), Divergence to consensus: 29.4%  
**6. MERX**: 80732645-80732691 (+), Divergence to consensus: 19.1%  
**7. L1MEg**: 80732938-80733114 (-), Divergence to consensus: 39.6%  
**8. ART2A**: 80735087-80735226 (-), Divergence to consensus: 19.4%  
**9. (TATG)n**: 80735253-80735352 (+), Divergence to consensus: 26.5%

  
Transcription Factor Binding Sites  

**Gata4** (Sequence: AGATAAC (-): 80730957)  
**Gata4** (Sequence: AGATAAC (-): 80736067)  
**Gata4** (Sequence: CTTATCT (+): 80734328)
