# Supplementary material for: Small RNA sequencing of cryopreserved semen from single bull revealed altered miRNAs and piRNAs expression between High- and Low-motile sperm populations
Source: BMC Genomics. 2017 Jan 4;18:14. doi: 10.1186/s12864-016-3394-7 (PMC5209821; doi:10.1186/s12864-016-3394-7)
Supplement: Additional file 3: — Details for each piRNA clusters found in High Motile (HM) sperm fraction. Genes, repeats, transposable elements and transcription factors binding sites falling within the cluster regions were reported. (ZIP 1896 kb) [file 12864_2016_3394_MOESM3_ESM.zip › 10.html]

piRNA cluster 10


Predicted piRNA cluster no. 10     previous   next
  

Show proTRAC run info
Hide proTRAC run info

================================= proTRAC ====================================  
VERSION: 2.1                                    LAST MODIFIED: 06. October 2015  
  
Please cite:  
Rosenkranz D, Zischler H. proTRAC - a software for probabilistic piRNA cluster  
detection, visualization and analysis. 2012. BMC Bioinformatics 13:5.  
  
and (for proTRAC 2.0 and later):  
Rosenkranz D, Rudloff S, Bastuck K, Ketting RF, Zischler H. Tupaia small RNAs  
provide insights into function and evolution of RNAi-based transposon defense  
in mammals. 2015. RNA 21(5):911-922.  
  
Contact:  
David Rosenkranz  
Institute of Anthropology, small RNA group  
Johannes Gutenberg University Mainz  
email: rosenkranz@uni-mainz.de  
  
You can find the latest proTRAC version at:  
http://sourceforge.net/projects/protrac/files  
http://www.smallRNAgroup-mainz.de/software  
==============================================================================  
  
PARAMETERS:  
Map file: .............../storage/core/barbara/genhome/smallRNA/fertility/Sample\_motile/pirna/Sample\_motile\_26-33\_collapsed.fa.no-dust.map.weighted-10000-1000-b-0  
Genome file: ............/storage/core/barbara/genhome/smallRNA/fertility/Sample\_all/pirna/bt\_311\_chrY.fa  
RepeatMasker annotation: /storage/genomes/bt\_umd31/GCF\_000003055.6\_Bos\_taurus\_UMD\_3.1.1\_repeatMasker\_chr.out  
GeneSet:................./storage/core/barbara/genhome/smallRNA/fertility/Sample\_all/pirna/full.gtf  
  
Significant (p<=0.01) hit density will be calculated based  
on observed hit distribution.  
  
Sliding window size: ........................................ 5000 bp  
Sliding window increament: .................................. 1000 bp  
Normalize each hit by number of genomic hits: ............... 1 [0=no/1=yes]  
Normalize each hit by number of sequence reads: ............. 1 [0=no/1=yes]  
Normalize values (-> per million mapped reads): ............. 1 [0=no/1=yes]  
Min. fraction of hits with 1T(U) or 10A: .................... 0.75  
Alternatively: Min. fraction of hits with 1T(U) and 10A: .... 0.5  
Min. fraction of hits with typical piRNA length: ............ 0.75  
Typical piRNA length: ....................................... 26-33 nt  
Min. size of a piRNA cluster: ............................... 5000 bp.  
Min. number of hits (absolute): ............................. 0  
Min. number of hits (normalized): ........................... 0  
Min. fraction of hits on the mainstrand: .................... 0.75  
Top fraction of mapped sequences (in terms of read counts): . 1%  
Top fraction accounts for max. n% of sequence reads: ........ 90%  
Min. fraction of hits on each arm of a bidirectional cluster: 0.1  
Output image file for each cluster: ......................... 0 [0=no/1=yes]  
Output html file for each cluster: .......................... 1 [0=no/1=yes]  
Output a summary table: ..................................... 1 [0=no/1=yes]  
Output a FASTA file for each cluster (piRNA sequences): ..... 1 [0=no/1=yes]  
Output a FASTA file comprising cluster sequences: ........... 1 [0=no/1=yes]  
Search DNA motifs in clusters: .............................. 1 [0=no/1=yes]  
Output flanking sequences: +/- .............................. 0 bp  
Output ~.pTi file: .......................................... 1 [0=no/1=yes]  
==============================================================================  
  
  
Genome size (without gaps): ............ 2678902517 bp  
Gaps (N/X/-): .......................... 53837044 bp  
Mapped reads: .......................... 658825247023  
Non-identical sequences: ............... 514171  
Genomic hits: .......................... 764233  
Significant densitiy of mapped reads: .. 12867599.5173724 reads/kb

Show proTRAC cluster info
Hide proTRAC cluster info

|  |  |
| --- | --- |
| Location | chr11 |
| Coordinates | 49231047-49236490 |
| Size [bp] | 5444 |
| Sequence hit loci | 65 |
| Mapped reads (normalized) | 93432972 |
| Mapped reads (normalized) per kb | 17162559.1 |
| Normalized reads with 1T (1U) | 91.2% |
| Normalized reads with 10A | 21.8% |
| Normalized reads with length 26-33 nt | 100% |
| Normalized reads on the main strand(s) | 100% |
| Predicted directionality | mono:plus |

100%

0%

1T (1U)  
reads

10A reads

26-33 nt  
reads

reads on mainstrand

**Either the amount of reads with 1T (1U) OR 10A has to exceed 75% (set with option: -1Tor10A)  
Alternatively the amount of reads with 1T (1U) AND 10A has to exceed 50% (set with option: -1Tand10A)  
Minimum amount of reads with preferred size is 75% (set with option: -pisize)  
Minimum amount of reads on the main strand(s) is 75% (set with option: -clstrand)**

Show read coverage
Hide read coverage

WHAT DO I SEE HERE?  
This chart shows the location of mapped sequence reads within a predicted piRNA cluster. The color refers to the number of genomic hits produced by the sequence read in question. A dark red bar indicates that this sequence read produces many other hits elsewhere in the genome. Many adjacent red or yellow bars can indicate the presence of a multi-copy element such as transposons or rRNA genes. A dark green bar indicates that this sequence read maps uniquely to this locus.

1 hit

2-5 hits

6-10 hits

11-20 hits

21-50 hits

51-100 hits

> 100 hits

chr11

49231047

49236490

Gene Set

RepeatMasker

Mapped  
Reads

15.93

plus strand

minus strand

15.93

Region: chr11 100991968-49231052. Max. coverage (+): 4.04. Max coverage (-): 0

Region: chr11 49231053-49231063. Max. coverage (+): 4.04. Max coverage (-): 0

Region: chr11 49231064-49231074. Max. coverage (+): 0. Max coverage (-): 0

Region: chr11 49231075-49231085. Max. coverage (+): 0. Max coverage (-): 0

Region: chr11 49231086-49231095. Max. coverage (+): 0. Max coverage (-): 0

Region: chr11 49231096-49231106. Max. coverage (+): 0. Max coverage (-): 0

Region: chr11 49231107-49231117. Max. coverage (+): 0. Max coverage (-): 0

Region: chr11 49231118-49231128. Max. coverage (+): 0. Max coverage (-): 0

Region: chr11 49231129-49231139. Max. coverage (+): 0. Max coverage (-): 0

Region: chr11 49231140-49231150. Max. coverage (+): 0. Max coverage (-): 0

Region: chr11 49231151-49231161. Max. coverage (+): 0. Max coverage (-): 0

Region: chr11 49231162-49231172. Max. coverage (+): 0. Max coverage (-): 0

Region: chr11 49231173-49231183. Max. coverage (+): 0. Max coverage (-): 0

Region: chr11 49231184-49231193. Max. coverage (+): 0. Max coverage (-): 0

Region: chr11 49231194-49231204. Max. coverage (+): 0. Max coverage (-): 0

Region: chr11 49231205-49231215. Max. coverage (+): 0. Max coverage (-): 0

Region: chr11 49231216-49231226. Max. coverage (+): 0. Max coverage (-): 0

Region: chr11 49231227-49231237. Max. coverage (+): 0. Max coverage (-): 0

Region: chr11 49231238-49231248. Max. coverage (+): 0. Max coverage (-): 0

Region: chr11 49231249-49231259. Max. coverage (+): 0. Max coverage (-): 0

Region: chr11 49231260-49231270. Max. coverage (+): 0. Max coverage (-): 0

Region: chr11 49231271-49231281. Max. coverage (+): 0. Max coverage (-): 0

Region: chr11 49231282-49231291. Max. coverage (+): 0. Max coverage (-): 0

Region: chr11 49231292-49231302. Max. coverage (+): 0. Max coverage (-): 0

Region: chr11 49231303-49231313. Max. coverage (+): 0. Max coverage (-): 0

Region: chr11 49231314-49231324. Max. coverage (+): 0. Max coverage (-): 0

Region: chr11 49231325-49231335. Max. coverage (+): 0. Max coverage (-): 0

Region: chr11 49231336-49231346. Max. coverage (+): 0. Max coverage (-): 0

Region: chr11 49231347-49231357. Max. coverage (+): 0. Max coverage (-): 0

Region: chr11 49231358-49231368. Max. coverage (+): 0. Max coverage (-): 0

Region: chr11 49231369-49231379. Max. coverage (+): 0. Max coverage (-): 0

Region: chr11 49231380-49231389. Max. coverage (+): 0. Max coverage (-): 0

Region: chr11 49231390-49231400. Max. coverage (+): 0. Max coverage (-): 0

Region: chr11 49231401-49231411. Max. coverage (+): 0. Max coverage (-): 0

Region: chr11 49231412-49231422. Max. coverage (+): 0. Max coverage (-): 0

Region: chr11 49231423-49231433. Max. coverage (+): 0. Max coverage (-): 0

Region: chr11 49231434-49231444. Max. coverage (+): 0. Max coverage (-): 0

Region: chr11 49231445-49231455. Max. coverage (+): 1.71. Max coverage (-): 0

Region: chr11 49231456-49231466. Max. coverage (+): 1.71. Max coverage (-): 0

Region: chr11 49231467-49231477. Max. coverage (+): 0. Max coverage (-): 0

Region: chr11 49231478-49231487. Max. coverage (+): 0. Max coverage (-): 0

Region: chr11 49231488-49231498. Max. coverage (+): 0. Max coverage (-): 0

Region: chr11 49231499-49231509. Max. coverage (+): 3.47. Max coverage (-): 0

Region: chr11 49231510-49231520. Max. coverage (+): 3.47. Max coverage (-): 0

Region: chr11 49231521-49231531. Max. coverage (+): 0. Max coverage (-): 0

Region: chr11 49231532-49231542. Max. coverage (+): 0. Max coverage (-): 0

Region: chr11 49231543-49231553. Max. coverage (+): 0. Max coverage (-): 0

Region: chr11 49231554-49231564. Max. coverage (+): 0. Max coverage (-): 0

Region: chr11 49231565-49231575. Max. coverage (+): 0. Max coverage (-): 0

Region: chr11 49231576-49231585. Max. coverage (+): 0. Max coverage (-): 0

Region: chr11 49231586-49231596. Max. coverage (+): 0. Max coverage (-): 0

Region: chr11 49231597-49231607. Max. coverage (+): 0. Max coverage (-): 0

Region: chr11 49231608-49231618. Max. coverage (+): 0. Max coverage (-): 0

Region: chr11 49231619-49231629. Max. coverage (+): 0. Max coverage (-): 0

Region: chr11 49231630-49231640. Max. coverage (+): 0. Max coverage (-): 0

Region: chr11 49231641-49231651. Max. coverage (+): 0. Max coverage (-): 0

Region: chr11 49231652-49231662. Max. coverage (+): 0. Max coverage (-): 0

Region: chr11 49231663-49231673. Max. coverage (+): 0. Max coverage (-): 0

Region: chr11 49231674-49231683. Max. coverage (+): 0. Max coverage (-): 0

Region: chr11 49231684-49231694. Max. coverage (+): 0. Max coverage (-): 0

Region: chr11 49231695-49231705. Max. coverage (+): 0. Max coverage (-): 0

Region: chr11 49231706-49231716. Max. coverage (+): 0. Max coverage (-): 0

Region: chr11 49231717-49231727. Max. coverage (+): 0. Max coverage (-): 0

Region: chr11 49231728-49231738. Max. coverage (+): 0. Max coverage (-): 0

Region: chr11 49231739-49231749. Max. coverage (+): 0. Max coverage (-): 0

Region: chr11 49231750-49231760. Max. coverage (+): 0. Max coverage (-): 0

Region: chr11 49231761-49231771. Max. coverage (+): 0. Max coverage (-): 0

Region: chr11 49231772-49231781. Max. coverage (+): 0. Max coverage (-): 0

Region: chr11 49231782-49231792. Max. coverage (+): 0. Max coverage (-): 0

Region: chr11 49231793-49231803. Max. coverage (+): 0. Max coverage (-): 0

Region: chr11 49231804-49231814. Max. coverage (+): 0. Max coverage (-): 0

Region: chr11 49231815-49231825. Max. coverage (+): 0. Max coverage (-): 0

Region: chr11 49231826-49231836. Max. coverage (+): 0. Max coverage (-): 0

Region: chr11 49231837-49231847. Max. coverage (+): 0. Max coverage (-): 0

Region: chr11 49231848-49231858. Max. coverage (+): 0. Max coverage (-): 0

Region: chr11 49231859-49231869. Max. coverage (+): 0. Max coverage (-): 0

Region: chr11 49231870-49231879. Max. coverage (+): 0. Max coverage (-): 0

Region: chr11 49231880-49231890. Max. coverage (+): 0. Max coverage (-): 0

Region: chr11 49231891-49231901. Max. coverage (+): 0. Max coverage (-): 0

Region: chr11 49231902-49231912. Max. coverage (+): 0. Max coverage (-): 0

Region: chr11 49231913-49231923. Max. coverage (+): 0. Max coverage (-): 0

Region: chr11 49231924-49231934. Max. coverage (+): 0. Max coverage (-): 0

Region: chr11 49231935-49231945. Max. coverage (+): 0. Max coverage (-): 0

Region: chr11 49231946-49231956. Max. coverage (+): 0. Max coverage (-): 0

Region: chr11 49231957-49231967. Max. coverage (+): 0. Max coverage (-): 0

Region: chr11 49231968-49231977. Max. coverage (+): 0. Max coverage (-): 0

Region: chr11 49231978-49231988. Max. coverage (+): 0. Max coverage (-): 0

Region: chr11 49231989-49231999. Max. coverage (+): 0. Max coverage (-): 0

Region: chr11 49232000-49232010. Max. coverage (+): 0. Max coverage (-): 0

Region: chr11 49232011-49232021. Max. coverage (+): 0. Max coverage (-): 0

Region: chr11 49232022-49232032. Max. coverage (+): 0. Max coverage (-): 0

Region: chr11 49232033-49232043. Max. coverage (+): 0. Max coverage (-): 0

Region: chr11 49232044-49232054. Max. coverage (+): 0. Max coverage (-): 0

Region: chr11 49232055-49232065. Max. coverage (+): 0. Max coverage (-): 0

Region: chr11 49232066-49232075. Max. coverage (+): 0. Max coverage (-): 0

Region: chr11 49232076-49232086. Max. coverage (+): 0. Max coverage (-): 0

Region: chr11 49232087-49232097. Max. coverage (+): 0. Max coverage (-): 0

Region: chr11 49232098-49232108. Max. coverage (+): 0. Max coverage (-): 0

Region: chr11 49232109-49232119. Max. coverage (+): 0. Max coverage (-): 0

Region: chr11 49232120-49232130. Max. coverage (+): 0. Max coverage (-): 0

Region: chr11 49232131-49232141. Max. coverage (+): 0. Max coverage (-): 0

Region: chr11 49232142-49232152. Max. coverage (+): 0. Max coverage (-): 0

Region: chr11 49232153-49232163. Max. coverage (+): 0. Max coverage (-): 0

Region: chr11 49232164-49232173. Max. coverage (+): 0. Max coverage (-): 0

Region: chr11 49232174-49232184. Max. coverage (+): 0. Max coverage (-): 0

Region: chr11 49232185-49232195. Max. coverage (+): 0. Max coverage (-): 0

Region: chr11 49232196-49232206. Max. coverage (+): 0. Max coverage (-): 0

Region: chr11 49232207-49232217. Max. coverage (+): 0. Max coverage (-): 0

Region: chr11 49232218-49232228. Max. coverage (+): 0. Max coverage (-): 0

Region: chr11 49232229-49232239. Max. coverage (+): 0. Max coverage (-): 0

Region: chr11 49232240-49232250. Max. coverage (+): 0. Max coverage (-): 0

Region: chr11 49232251-49232261. Max. coverage (+): 0. Max coverage (-): 0

Region: chr11 49232262-49232271. Max. coverage (+): 0. Max coverage (-): 0

Region: chr11 49232272-49232282. Max. coverage (+): 0. Max coverage (-): 0

Region: chr11 49232283-49232293. Max. coverage (+): 0. Max coverage (-): 0

Region: chr11 49232294-49232304. Max. coverage (+): 0. Max coverage (-): 0

Region: chr11 49232305-49232315. Max. coverage (+): 0. Max coverage (-): 0

Region: chr11 49232316-49232326. Max. coverage (+): 0. Max coverage (-): 0

Region: chr11 49232327-49232337. Max. coverage (+): 0. Max coverage (-): 0

Region: chr11 49232338-49232348. Max. coverage (+): 0. Max coverage (-): 0

Region: chr11 49232349-49232359. Max. coverage (+): 0. Max coverage (-): 0

Region: chr11 49232360-49232369. Max. coverage (+): 0. Max coverage (-): 0

Region: chr11 49232370-49232380. Max. coverage (+): 0. Max coverage (-): 0

Region: chr11 49232381-49232391. Max. coverage (+): 0. Max coverage (-): 0

Region: chr11 49232392-49232402. Max. coverage (+): 0. Max coverage (-): 0

Region: chr11 49232403-49232413. Max. coverage (+): 0. Max coverage (-): 0

Region: chr11 49232414-49232424. Max. coverage (+): 0. Max coverage (-): 0

Region: chr11 49232425-49232435. Max. coverage (+): 0. Max coverage (-): 0

Region: chr11 49232436-49232446. Max. coverage (+): 0. Max coverage (-): 0

Region: chr11 49232447-49232456. Max. coverage (+): 0. Max coverage (-): 0

Region: chr11 49232457-49232467. Max. coverage (+): 0. Max coverage (-): 0

Region: chr11 49232468-49232478. Max. coverage (+): 0. Max coverage (-): 0

Region: chr11 49232479-49232489. Max. coverage (+): 3.99. Max coverage (-): 0

Region: chr11 49232490-49232500. Max. coverage (+): 0. Max coverage (-): 0

Region: chr11 49232501-49232511. Max. coverage (+): 0. Max coverage (-): 0

Region: chr11 49232512-49232522. Max. coverage (+): 0. Max coverage (-): 0

Region: chr11 49232523-49232533. Max. coverage (+): 0. Max coverage (-): 0

Region: chr11 49232534-49232544. Max. coverage (+): 0. Max coverage (-): 0

Region: chr11 49232545-49232554. Max. coverage (+): 0. Max coverage (-): 0

Region: chr11 49232555-49232565. Max. coverage (+): 0. Max coverage (-): 0

Region: chr11 49232566-49232576. Max. coverage (+): 0. Max coverage (-): 0

Region: chr11 49232577-49232587. Max. coverage (+): 0. Max coverage (-): 0

Region: chr11 49232588-49232598. Max. coverage (+): 0. Max coverage (-): 0

Region: chr11 49232599-49232609. Max. coverage (+): 0. Max coverage (-): 0

Region: chr11 49232610-49232620. Max. coverage (+): 0. Max coverage (-): 0

Region: chr11 49232621-49232631. Max. coverage (+): 0. Max coverage (-): 0

Region: chr11 49232632-49232642. Max. coverage (+): 0. Max coverage (-): 0

Region: chr11 49232643-49232652. Max. coverage (+): 0. Max coverage (-): 0

Region: chr11 49232653-49232663. Max. coverage (+): 0. Max coverage (-): 0

Region: chr11 49232664-49232674. Max. coverage (+): 0. Max coverage (-): 0

Region: chr11 49232675-49232685. Max. coverage (+): 0. Max coverage (-): 0

Region: chr11 49232686-49232696. Max. coverage (+): 0. Max coverage (-): 0

Region: chr11 49232697-49232707. Max. coverage (+): 0. Max coverage (-): 0

Region: chr11 49232708-49232718. Max. coverage (+): 0. Max coverage (-): 0

Region: chr11 49232719-49232729. Max. coverage (+): 0. Max coverage (-): 0

Region: chr11 49232730-49232740. Max. coverage (+): 0. Max coverage (-): 0

Region: chr11 49232741-49232750. Max. coverage (+): 0. Max coverage (-): 0

Region: chr11 49232751-49232761. Max. coverage (+): 0. Max coverage (-): 0

Region: chr11 49232762-49232772. Max. coverage (+): 0. Max coverage (-): 0

Region: chr11 49232773-49232783. Max. coverage (+): 0. Max coverage (-): 0

Region: chr11 49232784-49232794. Max. coverage (+): 0. Max coverage (-): 0

Region: chr11 49232795-49232805. Max. coverage (+): 0. Max coverage (-): 0

Region: chr11 49232806-49232816. Max. coverage (+): 0. Max coverage (-): 0

Region: chr11 49232817-49232827. Max. coverage (+): 0. Max coverage (-): 0

Region: chr11 49232828-49232838. Max. coverage (+): 0. Max coverage (-): 0

Region: chr11 49232839-49232848. Max. coverage (+): 0. Max coverage (-): 0

Region: chr11 49232849-49232859. Max. coverage (+): 0. Max coverage (-): 0

Region: chr11 49232860-49232870. Max. coverage (+): 0. Max coverage (-): 0

Region: chr11 49232871-49232881. Max. coverage (+): 0. Max coverage (-): 0

Region: chr11 49232882-49232892. Max. coverage (+): 0. Max coverage (-): 0

Region: chr11 49232893-49232903. Max. coverage (+): 0. Max coverage (-): 0

Region: chr11 49232904-49232914. Max. coverage (+): 0. Max coverage (-): 0

Region: chr11 49232915-49232925. Max. coverage (+): 0. Max coverage (-): 0

Region: chr11 49232926-49232936. Max. coverage (+): 0. Max coverage (-): 0

Region: chr11 49232937-49232946. Max. coverage (+): 0. Max coverage (-): 0

Region: chr11 49232947-49232957. Max. coverage (+): 0. Max coverage (-): 0

Region: chr11 49232958-49232968. Max. coverage (+): 0. Max coverage (-): 0

Region: chr11 49232969-49232979. Max. coverage (+): 0. Max coverage (-): 0

Region: chr11 49232980-49232990. Max. coverage (+): 0. Max coverage (-): 0

Region: chr11 49232991-49233001. Max. coverage (+): 0. Max coverage (-): 0

Region: chr11 49233002-49233012. Max. coverage (+): 0. Max coverage (-): 0

Region: chr11 49233013-49233023. Max. coverage (+): 0. Max coverage (-): 0

Region: chr11 49233024-49233034. Max. coverage (+): 0. Max coverage (-): 0

Region: chr11 49233035-49233044. Max. coverage (+): 0. Max coverage (-): 0

Region: chr11 49233045-49233055. Max. coverage (+): 0. Max coverage (-): 0

Region: chr11 49233056-49233066. Max. coverage (+): 0. Max coverage (-): 0

Region: chr11 49233067-49233077. Max. coverage (+): 0. Max coverage (-): 0

Region: chr11 49233078-49233088. Max. coverage (+): 0. Max coverage (-): 0

Region: chr11 49233089-49233099. Max. coverage (+): 0. Max coverage (-): 0

Region: chr11 49233100-49233110. Max. coverage (+): 0. Max coverage (-): 0

Region: chr11 49233111-49233121. Max. coverage (+): 0. Max coverage (-): 0

Region: chr11 49233122-49233132. Max. coverage (+): 0. Max coverage (-): 0

Region: chr11 49233133-49233142. Max. coverage (+): 0. Max coverage (-): 0

Region: chr11 49233143-49233153. Max. coverage (+): 0. Max coverage (-): 0

Region: chr11 49233154-49233164. Max. coverage (+): 0. Max coverage (-): 0

Region: chr11 49233165-49233175. Max. coverage (+): 0. Max coverage (-): 0

Region: chr11 49233176-49233186. Max. coverage (+): 0. Max coverage (-): 0

Region: chr11 49233187-49233197. Max. coverage (+): 0. Max coverage (-): 0

Region: chr11 49233198-49233208. Max. coverage (+): 0. Max coverage (-): 0

Region: chr11 49233209-49233219. Max. coverage (+): 0. Max coverage (-): 0

Region: chr11 49233220-49233230. Max. coverage (+): 0. Max coverage (-): 0

Region: chr11 49233231-49233240. Max. coverage (+): 0. Max coverage (-): 0

Region: chr11 49233241-49233251. Max. coverage (+): 0. Max coverage (-): 0

Region: chr11 49233252-49233262. Max. coverage (+): 0. Max coverage (-): 0

Region: chr11 49233263-49233273. Max. coverage (+): 0. Max coverage (-): 0

Region: chr11 49233274-49233284. Max. coverage (+): 0. Max coverage (-): 0

Region: chr11 49233285-49233295. Max. coverage (+): 0. Max coverage (-): 0

Region: chr11 49233296-49233306. Max. coverage (+): 0. Max coverage (-): 0

Region: chr11 49233307-49233317. Max. coverage (+): 0. Max coverage (-): 0

Region: chr11 49233318-49233328. Max. coverage (+): 0.9. Max coverage (-): 0

Region: chr11 49233329-49233338. Max. coverage (+): 0. Max coverage (-): 0

Region: chr11 49233339-49233349. Max. coverage (+): 1.33. Max coverage (-): 0

Region: chr11 49233350-49233360. Max. coverage (+): 0. Max coverage (-): 0

Region: chr11 49233361-49233371. Max. coverage (+): 0. Max coverage (-): 0

Region: chr11 49233372-49233382. Max. coverage (+): 0. Max coverage (-): 0

Region: chr11 49233383-49233393. Max. coverage (+): 0. Max coverage (-): 0

Region: chr11 49233394-49233404. Max. coverage (+): 0. Max coverage (-): 0

Region: chr11 49233405-49233415. Max. coverage (+): 0. Max coverage (-): 0

Region: chr11 49233416-49233426. Max. coverage (+): 0. Max coverage (-): 0

Region: chr11 49233427-49233436. Max. coverage (+): 0. Max coverage (-): 0

Region: chr11 49233437-49233447. Max. coverage (+): 0. Max coverage (-): 0

Region: chr11 49233448-49233458. Max. coverage (+): 0. Max coverage (-): 0

Region: chr11 49233459-49233469. Max. coverage (+): 2.18. Max coverage (-): 0

Region: chr11 49233470-49233480. Max. coverage (+): 2.18. Max coverage (-): 0

Region: chr11 49233481-49233491. Max. coverage (+): 1.1. Max coverage (-): 0

Region: chr11 49233492-49233502. Max. coverage (+): 0. Max coverage (-): 0

Region: chr11 49233503-49233513. Max. coverage (+): 0. Max coverage (-): 0

Region: chr11 49233514-49233524. Max. coverage (+): 0.58. Max coverage (-): 0

Region: chr11 49233525-49233534. Max. coverage (+): 0.58. Max coverage (-): 0

Region: chr11 49233535-49233545. Max. coverage (+): 0. Max coverage (-): 0

Region: chr11 49233546-49233556. Max. coverage (+): 0. Max coverage (-): 0

Region: chr11 49233557-49233567. Max. coverage (+): 0. Max coverage (-): 0

Region: chr11 49233568-49233578. Max. coverage (+): 0. Max coverage (-): 0

Region: chr11 49233579-49233589. Max. coverage (+): 0. Max coverage (-): 0

Region: chr11 49233590-49233600. Max. coverage (+): 0. Max coverage (-): 0

Region: chr11 49233601-49233611. Max. coverage (+): 0. Max coverage (-): 0

Region: chr11 49233612-49233622. Max. coverage (+): 0. Max coverage (-): 0

Region: chr11 49233623-49233632. Max. coverage (+): 0. Max coverage (-): 0

Region: chr11 49233633-49233643. Max. coverage (+): 3.16. Max coverage (-): 0

Region: chr11 49233644-49233654. Max. coverage (+): 0. Max coverage (-): 0

Region: chr11 49233655-49233665. Max. coverage (+): 0. Max coverage (-): 0

Region: chr11 49233666-49233676. Max. coverage (+): 0. Max coverage (-): 0

Region: chr11 49233677-49233687. Max. coverage (+): 0. Max coverage (-): 0

Region: chr11 49233688-49233698. Max. coverage (+): 0. Max coverage (-): 0

Region: chr11 49233699-49233709. Max. coverage (+): 0. Max coverage (-): 0

Region: chr11 49233710-49233720. Max. coverage (+): 0. Max coverage (-): 0

Region: chr11 49233721-49233730. Max. coverage (+): 0. Max coverage (-): 0

Region: chr11 49233731-49233741. Max. coverage (+): 0. Max coverage (-): 0

Region: chr11 49233742-49233752. Max. coverage (+): 0. Max coverage (-): 0

Region: chr11 49233753-49233763. Max. coverage (+): 0. Max coverage (-): 0

Region: chr11 49233764-49233774. Max. coverage (+): 0. Max coverage (-): 0

Region: chr11 49233775-49233785. Max. coverage (+): 0. Max coverage (-): 0

Region: chr11 49233786-49233796. Max. coverage (+): 0. Max coverage (-): 0

Region: chr11 49233797-49233807. Max. coverage (+): 0. Max coverage (-): 0

Region: chr11 49233808-49233817. Max. coverage (+): 0. Max coverage (-): 0

Region: chr11 49233818-49233828. Max. coverage (+): 0. Max coverage (-): 0

Region: chr11 49233829-49233839. Max. coverage (+): 0. Max coverage (-): 0

Region: chr11 49233840-49233850. Max. coverage (+): 0. Max coverage (-): 0

Region: chr11 49233851-49233861. Max. coverage (+): 0. Max coverage (-): 0

Region: chr11 49233862-49233872. Max. coverage (+): 0.95. Max coverage (-): 0

Region: chr11 49233873-49233883. Max. coverage (+): 0.95. Max coverage (-): 0

Region: chr11 49233884-49233894. Max. coverage (+): 0. Max coverage (-): 0

Region: chr11 49233895-49233905. Max. coverage (+): 0. Max coverage (-): 0

Region: chr11 49233906-49233915. Max. coverage (+): 0. Max coverage (-): 0

Region: chr11 49233916-49233926. Max. coverage (+): 0. Max coverage (-): 0

Region: chr11 49233927-49233937. Max. coverage (+): 0. Max coverage (-): 0

Region: chr11 49233938-49233948. Max. coverage (+): 0. Max coverage (-): 0

Region: chr11 49233949-49233959. Max. coverage (+): 0. Max coverage (-): 0

Region: chr11 49233960-49233970. Max. coverage (+): 0. Max coverage (-): 0

Region: chr11 49233971-49233981. Max. coverage (+): 0. Max coverage (-): 0

Region: chr11 49233982-49233992. Max. coverage (+): 0. Max coverage (-): 0

Region: chr11 49233993-49234003. Max. coverage (+): 0. Max coverage (-): 0

Region: chr11 49234004-49234013. Max. coverage (+): 0. Max coverage (-): 0

Region: chr11 49234014-49234024. Max. coverage (+): 0. Max coverage (-): 0

Region: chr11 49234025-49234035. Max. coverage (+): 0. Max coverage (-): 0

Region: chr11 49234036-49234046. Max. coverage (+): 0. Max coverage (-): 0

Region: chr11 49234047-49234057. Max. coverage (+): 0. Max coverage (-): 0

Region: chr11 49234058-49234068. Max. coverage (+): 0. Max coverage (-): 0

Region: chr11 49234069-49234079. Max. coverage (+): 0. Max coverage (-): 0

Region: chr11 49234080-49234090. Max. coverage (+): 0. Max coverage (-): 0

Region: chr11 49234091-49234101. Max. coverage (+): 0. Max coverage (-): 0

Region: chr11 49234102-49234111. Max. coverage (+): 0. Max coverage (-): 0

Region: chr11 49234112-49234122. Max. coverage (+): 0. Max coverage (-): 0

Region: chr11 49234123-49234133. Max. coverage (+): 0. Max coverage (-): 0

Region: chr11 49234134-49234144. Max. coverage (+): 0. Max coverage (-): 0

Region: chr11 49234145-49234155. Max. coverage (+): 0. Max coverage (-): 0

Region: chr11 49234156-49234166. Max. coverage (+): 0. Max coverage (-): 0

Region: chr11 49234167-49234177. Max. coverage (+): 0. Max coverage (-): 0

Region: chr11 49234178-49234188. Max. coverage (+): 0. Max coverage (-): 0

Region: chr11 49234189-49234199. Max. coverage (+): 0. Max coverage (-): 0

Region: chr11 49234200-49234209. Max. coverage (+): 0. Max coverage (-): 0

Region: chr11 49234210-49234220. Max. coverage (+): 0. Max coverage (-): 0

Region: chr11 49234221-49234231. Max. coverage (+): 0. Max coverage (-): 0

Region: chr11 49234232-49234242. Max. coverage (+): 0. Max coverage (-): 0

Region: chr11 49234243-49234253. Max. coverage (+): 0. Max coverage (-): 0

Region: chr11 49234254-49234264. Max. coverage (+): 0. Max coverage (-): 0

Region: chr11 49234265-49234275. Max. coverage (+): 0. Max coverage (-): 0

Region: chr11 49234276-49234286. Max. coverage (+): 0. Max coverage (-): 0

Region: chr11 49234287-49234297. Max. coverage (+): 0. Max coverage (-): 0

Region: chr11 49234298-49234307. Max. coverage (+): 0. Max coverage (-): 0

Region: chr11 49234308-49234318. Max. coverage (+): 0. Max coverage (-): 0

Region: chr11 49234319-49234329. Max. coverage (+): 0. Max coverage (-): 0

Region: chr11 49234330-49234340. Max. coverage (+): 0. Max coverage (-): 0

Region: chr11 49234341-49234351. Max. coverage (+): 2.1. Max coverage (-): 0

Region: chr11 49234352-49234362. Max. coverage (+): 3. Max coverage (-): 0

Region: chr11 49234363-49234373. Max. coverage (+): 0. Max coverage (-): 0

Region: chr11 49234374-49234384. Max. coverage (+): 15.93. Max coverage (-): 0

Region: chr11 49234385-49234395. Max. coverage (+): 14.11. Max coverage (-): 0

Region: chr11 49234396-49234405. Max. coverage (+): 4.99. Max coverage (-): 0

Region: chr11 49234406-49234416. Max. coverage (+): 8.3. Max coverage (-): 0

Region: chr11 49234417-49234427. Max. coverage (+): 0. Max coverage (-): 0

Region: chr11 49234428-49234438. Max. coverage (+): 0. Max coverage (-): 0

Region: chr11 49234439-49234449. Max. coverage (+): 3.36. Max coverage (-): 0

Region: chr11 49234450-49234460. Max. coverage (+): 6.19. Max coverage (-): 0

Region: chr11 49234461-49234471. Max. coverage (+): 0.7. Max coverage (-): 0

Region: chr11 49234472-49234482. Max. coverage (+): 2.36. Max coverage (-): 0

Region: chr11 49234483-49234493. Max. coverage (+): 0. Max coverage (-): 0

Region: chr11 49234494-49234503. Max. coverage (+): 2.64. Max coverage (-): 0

Region: chr11 49234504-49234514. Max. coverage (+): 2.64. Max coverage (-): 0

Region: chr11 49234515-49234525. Max. coverage (+): 0. Max coverage (-): 0

Region: chr11 49234526-49234536. Max. coverage (+): 1.95. Max coverage (-): 0

Region: chr11 49234537-49234547. Max. coverage (+): 1.95. Max coverage (-): 0

Region: chr11 49234548-49234558. Max. coverage (+): 0. Max coverage (-): 0

Region: chr11 49234559-49234569. Max. coverage (+): 1.42. Max coverage (-): 0

Region: chr11 49234570-49234580. Max. coverage (+): 2.65. Max coverage (-): 0

Region: chr11 49234581-49234591. Max. coverage (+): 2.65. Max coverage (-): 0

Region: chr11 49234592-49234601. Max. coverage (+): 0. Max coverage (-): 0

Region: chr11 49234602-49234612. Max. coverage (+): 0. Max coverage (-): 0

Region: chr11 49234613-49234623. Max. coverage (+): 0. Max coverage (-): 0

Region: chr11 49234624-49234634. Max. coverage (+): 0. Max coverage (-): 0

Region: chr11 49234635-49234645. Max. coverage (+): 0. Max coverage (-): 0

Region: chr11 49234646-49234656. Max. coverage (+): 0. Max coverage (-): 0

Region: chr11 49234657-49234667. Max. coverage (+): 6.05. Max coverage (-): 0

Region: chr11 49234668-49234678. Max. coverage (+): 6.05. Max coverage (-): 0

Region: chr11 49234679-49234689. Max. coverage (+): 0. Max coverage (-): 0

Region: chr11 49234690-49234699. Max. coverage (+): 0. Max coverage (-): 0

Region: chr11 49234700-49234710. Max. coverage (+): 0. Max coverage (-): 0

Region: chr11 49234711-49234721. Max. coverage (+): 0. Max coverage (-): 0

Region: chr11 49234722-49234732. Max. coverage (+): 0. Max coverage (-): 0

Region: chr11 49234733-49234743. Max. coverage (+): 5.14. Max coverage (-): 0

Region: chr11 49234744-49234754. Max. coverage (+): 5.14. Max coverage (-): 0

Region: chr11 49234755-49234765. Max. coverage (+): 0. Max coverage (-): 0

Region: chr11 49234766-49234776. Max. coverage (+): 0. Max coverage (-): 0

Region: chr11 49234777-49234787. Max. coverage (+): 0. Max coverage (-): 0

Region: chr11 49234788-49234797. Max. coverage (+): 0. Max coverage (-): 0

Region: chr11 49234798-49234808. Max. coverage (+): 0. Max coverage (-): 0

Region: chr11 49234809-49234819. Max. coverage (+): 0. Max coverage (-): 0

Region: chr11 49234820-49234830. Max. coverage (+): 0. Max coverage (-): 0

Region: chr11 49234831-49234841. Max. coverage (+): 0. Max coverage (-): 0

Region: chr11 49234842-49234852. Max. coverage (+): 0. Max coverage (-): 0

Region: chr11 49234853-49234863. Max. coverage (+): 0. Max coverage (-): 0

Region: chr11 49234864-49234874. Max. coverage (+): 0. Max coverage (-): 0

Region: chr11 49234875-49234885. Max. coverage (+): 0. Max coverage (-): 0

Region: chr11 49234886-49234895. Max. coverage (+): 0. Max coverage (-): 0

Region: chr11 49234896-49234906. Max. coverage (+): 0. Max coverage (-): 0

Region: chr11 49234907-49234917. Max. coverage (+): 0. Max coverage (-): 0

Region: chr11 49234918-49234928. Max. coverage (+): 0. Max coverage (-): 0

Region: chr11 49234929-49234939. Max. coverage (+): 1.08. Max coverage (-): 0

Region: chr11 49234940-49234950. Max. coverage (+): 0. Max coverage (-): 0

Region: chr11 49234951-49234961. Max. coverage (+): 0. Max coverage (-): 0

Region: chr11 49234962-49234972. Max. coverage (+): 0. Max coverage (-): 0

Region: chr11 49234973-49234983. Max. coverage (+): 0. Max coverage (-): 0

Region: chr11 49234984-49234993. Max. coverage (+): 0. Max coverage (-): 0

Region: chr11 49234994-49235004. Max. coverage (+): 0. Max coverage (-): 0

Region: chr11 49235005-49235015. Max. coverage (+): 0. Max coverage (-): 0

Region: chr11 49235016-49235026. Max. coverage (+): 0. Max coverage (-): 0

Region: chr11 49235027-49235037. Max. coverage (+): 0. Max coverage (-): 0

Region: chr11 49235038-49235048. Max. coverage (+): 0. Max coverage (-): 0

Region: chr11 49235049-49235059. Max. coverage (+): 0. Max coverage (-): 0

Region: chr11 49235060-49235070. Max. coverage (+): 0. Max coverage (-): 0

Region: chr11 49235071-49235081. Max. coverage (+): 0. Max coverage (-): 0

Region: chr11 49235082-49235091. Max. coverage (+): 6.97. Max coverage (-): 0

Region: chr11 49235092-49235102. Max. coverage (+): 12. Max coverage (-): 0

Region: chr11 49235103-49235113. Max. coverage (+): 10.41. Max coverage (-): 0

Region: chr11 49235114-49235124. Max. coverage (+): 0. Max coverage (-): 0

Region: chr11 49235125-49235135. Max. coverage (+): 0. Max coverage (-): 0

Region: chr11 49235136-49235146. Max. coverage (+): 3.98. Max coverage (-): 0

Region: chr11 49235147-49235157. Max. coverage (+): 3.98. Max coverage (-): 0

Region: chr11 49235158-49235168. Max. coverage (+): 0.82. Max coverage (-): 0

Region: chr11 49235169-49235178. Max. coverage (+): 0.82. Max coverage (-): 0

Region: chr11 49235179-49235189. Max. coverage (+): 0. Max coverage (-): 0

Region: chr11 49235190-49235200. Max. coverage (+): 0. Max coverage (-): 0

Region: chr11 49235201-49235211. Max. coverage (+): 4.32. Max coverage (-): 0

Region: chr11 49235212-49235222. Max. coverage (+): 0. Max coverage (-): 0

Region: chr11 49235223-49235233. Max. coverage (+): 0. Max coverage (-): 0

Region: chr11 49235234-49235244. Max. coverage (+): 0. Max coverage (-): 0

Region: chr11 49235245-49235255. Max. coverage (+): 0. Max coverage (-): 0

Region: chr11 49235256-49235266. Max. coverage (+): 0. Max coverage (-): 0

Region: chr11 49235267-49235276. Max. coverage (+): 9.73. Max coverage (-): 0

Region: chr11 49235277-49235287. Max. coverage (+): 5.26. Max coverage (-): 0

Region: chr11 49235288-49235298. Max. coverage (+): 4.9. Max coverage (-): 0

Region: chr11 49235299-49235309. Max. coverage (+): 2.04. Max coverage (-): 0

Region: chr11 49235310-49235320. Max. coverage (+): 0. Max coverage (-): 0

Region: chr11 49235321-49235331. Max. coverage (+): 0. Max coverage (-): 0

Region: chr11 49235332-49235342. Max. coverage (+): 2.02. Max coverage (-): 0

Region: chr11 49235343-49235353. Max. coverage (+): 3.84. Max coverage (-): 0

Region: chr11 49235354-49235364. Max. coverage (+): 3.84. Max coverage (-): 0

Region: chr11 49235365-49235374. Max. coverage (+): 0.57. Max coverage (-): 0

Region: chr11 49235375-49235385. Max. coverage (+): 0. Max coverage (-): 0

Region: chr11 49235386-49235396. Max. coverage (+): 0. Max coverage (-): 0

Region: chr11 49235397-49235407. Max. coverage (+): 0. Max coverage (-): 0

Region: chr11 49235408-49235418. Max. coverage (+): 0. Max coverage (-): 0

Region: chr11 49235419-49235429. Max. coverage (+): 0. Max coverage (-): 0

Region: chr11 49235430-49235440. Max. coverage (+): 0. Max coverage (-): 0

Region: chr11 49235441-49235451. Max. coverage (+): 0. Max coverage (-): 0

Region: chr11 49235452-49235462. Max. coverage (+): 0. Max coverage (-): 0

Region: chr11 49235463-49235472. Max. coverage (+): 0. Max coverage (-): 0

Region: chr11 49235473-49235483. Max. coverage (+): 0. Max coverage (-): 0

Region: chr11 49235484-49235494. Max. coverage (+): 0. Max coverage (-): 0

Region: chr11 49235495-49235505. Max. coverage (+): 0. Max coverage (-): 0

Region: chr11 49235506-49235516. Max. coverage (+): 0. Max coverage (-): 0

Region: chr11 49235517-49235527. Max. coverage (+): 0. Max coverage (-): 0

Region: chr11 49235528-49235538. Max. coverage (+): 0. Max coverage (-): 0

Region: chr11 49235539-49235549. Max. coverage (+): 0. Max coverage (-): 0

Region: chr11 49235550-49235560. Max. coverage (+): 0. Max coverage (-): 0

Region: chr11 49235561-49235570. Max. coverage (+): 0. Max coverage (-): 0

Region: chr11 49235571-49235581. Max. coverage (+): 0. Max coverage (-): 0

Region: chr11 49235582-49235592. Max. coverage (+): 0. Max coverage (-): 0

Region: chr11 49235593-49235603. Max. coverage (+): 0. Max coverage (-): 0

Region: chr11 49235604-49235614. Max. coverage (+): 0. Max coverage (-): 0

Region: chr11 49235615-49235625. Max. coverage (+): 0. Max coverage (-): 0

Region: chr11 49235626-49235636. Max. coverage (+): 0. Max coverage (-): 0

Region: chr11 49235637-49235647. Max. coverage (+): 1.01. Max coverage (-): 0

Region: chr11 49235648-49235658. Max. coverage (+): 1.01. Max coverage (-): 0

Region: chr11 49235659-49235668. Max. coverage (+): 0. Max coverage (-): 0

Region: chr11 49235669-49235679. Max. coverage (+): 0. Max coverage (-): 0

Region: chr11 49235680-49235690. Max. coverage (+): 0. Max coverage (-): 0

Region: chr11 49235691-49235701. Max. coverage (+): 0. Max coverage (-): 0

Region: chr11 49235702-49235712. Max. coverage (+): 0. Max coverage (-): 0

Region: chr11 49235713-49235723. Max. coverage (+): 0. Max coverage (-): 0

Region: chr11 49235724-49235734. Max. coverage (+): 0. Max coverage (-): 0

Region: chr11 49235735-49235745. Max. coverage (+): 0. Max coverage (-): 0

Region: chr11 49235746-49235756. Max. coverage (+): 0. Max coverage (-): 0

Region: chr11 49235757-49235766. Max. coverage (+): 0. Max coverage (-): 0

Region: chr11 49235767-49235777. Max. coverage (+): 0. Max coverage (-): 0

Region: chr11 49235778-49235788. Max. coverage (+): 0. Max coverage (-): 0

Region: chr11 49235789-49235799. Max. coverage (+): 3.89. Max coverage (-): 0

Region: chr11 49235800-49235810. Max. coverage (+): 1.22. Max coverage (-): 0

Region: chr11 49235811-49235821. Max. coverage (+): 1.22. Max coverage (-): 0

Region: chr11 49235822-49235832. Max. coverage (+): 0. Max coverage (-): 0

Region: chr11 49235833-49235843. Max. coverage (+): 0. Max coverage (-): 0

Region: chr11 49235844-49235854. Max. coverage (+): 0. Max coverage (-): 0

Region: chr11 49235855-49235864. Max. coverage (+): 0. Max coverage (-): 0

Region: chr11 49235865-49235875. Max. coverage (+): 0. Max coverage (-): 0

Region: chr11 49235876-49235886. Max. coverage (+): 0. Max coverage (-): 0

Region: chr11 49235887-49235897. Max. coverage (+): 0. Max coverage (-): 0

Region: chr11 49235898-49235908. Max. coverage (+): 0. Max coverage (-): 0

Region: chr11 49235909-49235919. Max. coverage (+): 0. Max coverage (-): 0

Region: chr11 49235920-49235930. Max. coverage (+): 0. Max coverage (-): 0

Region: chr11 49235931-49235941. Max. coverage (+): 0. Max coverage (-): 0

Region: chr11 49235942-49235952. Max. coverage (+): 0. Max coverage (-): 0

Region: chr11 49235953-49235962. Max. coverage (+): 0. Max coverage (-): 0

Region: chr11 49235963-49235973. Max. coverage (+): 0. Max coverage (-): 0

Region: chr11 49235974-49235984. Max. coverage (+): 0. Max coverage (-): 0

Region: chr11 49235985-49235995. Max. coverage (+): 0. Max coverage (-): 0

Region: chr11 49235996-49236006. Max. coverage (+): 0. Max coverage (-): 0

Region: chr11 49236007-49236017. Max. coverage (+): 0. Max coverage (-): 0

Region: chr11 49236018-49236028. Max. coverage (+): 0. Max coverage (-): 0

Region: chr11 49236029-49236039. Max. coverage (+): 0. Max coverage (-): 0

Region: chr11 49236040-49236050. Max. coverage (+): 0. Max coverage (-): 0

Region: chr11 49236051-49236060. Max. coverage (+): 0. Max coverage (-): 0

Region: chr11 49236061-49236071. Max. coverage (+): 0. Max coverage (-): 0

Region: chr11 49236072-49236082. Max. coverage (+): 0. Max coverage (-): 0

Region: chr11 49236083-49236093. Max. coverage (+): 0. Max coverage (-): 0

Region: chr11 49236094-49236104. Max. coverage (+): 0. Max coverage (-): 0

Region: chr11 49236105-49236115. Max. coverage (+): 0. Max coverage (-): 0

Region: chr11 49236116-49236126. Max. coverage (+): 0. Max coverage (-): 0

Region: chr11 49236127-49236137. Max. coverage (+): 0. Max coverage (-): 0

Region: chr11 49236138-49236148. Max. coverage (+): 0. Max coverage (-): 0

Region: chr11 49236149-49236158. Max. coverage (+): 0. Max coverage (-): 0

Region: chr11 49236159-49236169. Max. coverage (+): 0. Max coverage (-): 0

Region: chr11 49236170-49236180. Max. coverage (+): 0. Max coverage (-): 0

Region: chr11 49236181-49236191. Max. coverage (+): 0. Max coverage (-): 0

Region: chr11 49236192-49236202. Max. coverage (+): 0. Max coverage (-): 0

Region: chr11 49236203-49236213. Max. coverage (+): 0. Max coverage (-): 0

Region: chr11 49236214-49236224. Max. coverage (+): 0. Max coverage (-): 0

Region: chr11 49236225-49236235. Max. coverage (+): 0. Max coverage (-): 0

Region: chr11 49236236-49236246. Max. coverage (+): 0. Max coverage (-): 0

Region: chr11 49236247-49236256. Max. coverage (+): 0. Max coverage (-): 0

Region: chr11 49236257-49236267. Max. coverage (+): 0. Max coverage (-): 0

Region: chr11 49236268-49236278. Max. coverage (+): 0. Max coverage (-): 0

Region: chr11 49236279-49236289. Max. coverage (+): 0. Max coverage (-): 0

Region: chr11 49236290-49236300. Max. coverage (+): 0. Max coverage (-): 0

Region: chr11 49236301-49236311. Max. coverage (+): 0. Max coverage (-): 0

Region: chr11 49236312-49236322. Max. coverage (+): 0. Max coverage (-): 0

Region: chr11 49236323-49236333. Max. coverage (+): 0. Max coverage (-): 0

Region: chr11 49236334-49236344. Max. coverage (+): 0. Max coverage (-): 0

Region: chr11 49236345-49236354. Max. coverage (+): 0. Max coverage (-): 0

Region: chr11 49236355-49236365. Max. coverage (+): 0. Max coverage (-): 0

Region: chr11 49236366-49236376. Max. coverage (+): 0. Max coverage (-): 0

Region: chr11 49236377-49236387. Max. coverage (+): 0. Max coverage (-): 0

Region: chr11 49236388-49236398. Max. coverage (+): 1.9. Max coverage (-): 0

Region: chr11 49236399-49236409. Max. coverage (+): 1.9. Max coverage (-): 0

Region: chr11 49236410-49236420. Max. coverage (+): 0. Max coverage (-): 0

Region: chr11 49236421-49236431. Max. coverage (+): 0. Max coverage (-): 0

Region: chr11 49236432-49236442. Max. coverage (+): 0. Max coverage (-): 0

Region: chr11 49236443-49236452. Max. coverage (+): 0. Max coverage (-): 0

Region: chr11 49236453-49236463. Max. coverage (+): 0.73. Max coverage (-): 0

Region: chr11 49236464-49236474. Max. coverage (+): 0.73. Max coverage (-): 0

Region: chr11 49236475-49236485. Max. coverage (+): 0. Max coverage (-): 0

Region: chr11 49236486-. Max. coverage (+): 0. Max coverage (-): 0

RepeatMasker Color Code

**+**

100-98% Identity

<98-95% Identity

<95-90% Identity

<90-85% Identity

<85-80% Identity

<80-75% Identity

<75-70% Identity

<70% Identity

**-**

Gene Set Color Code

**+**

Gene

Pseudogene

**-**

Topology/Coverage Color Code

Coverage Plus Strand

Coverage Minus Strand

Mainstrand: Plus

Mainstrand: Minus

Complementary Strand

Flanking Region  
(if option -flank >0)

Gene Set Annotation  

**1. C2orf68 (protein coding, ENSBTAG00000018291) Tr:00000024344 Ex:1**: 49230913-49231081 (+)  
**2. C2orf68 (protein coding, ENSBTAG00000018291) Tr:00000024344 Ex:2**: 49231165-49231283 (+)  
**3. C2orf68 (protein coding, ENSBTAG00000018291) Tr:00000024344 Ex:3**: 49232486-49232637 (+)  
**4. C2orf68 (protein coding, ENSBTAG00000018291) Tr:00000024344 Ex:4**: 49235689-49236832 (+)  
**5. C2orf68 (protein coding, ENSBTAG00000018291) Tr:00000064372 Ex:1**: 49230913-49231081 (+)  
**6. C2orf68 (protein coding, ENSBTAG00000018291) Tr:00000064372 Ex:2**: 49231165-49231283 (+)  
**7. C2orf68 (protein coding, ENSBTAG00000018291) Tr:00000064372 Ex:3**: 49232486-49232637 (+)  
**8. C2orf68 (protein coding, ENSBTAG00000018291) Tr:00000064372 Ex:4**: 49233004-49233126 (+)

  
RepeatMasker Annotation  

**1. (CCCCG)n**: 49231074-49231158 (+), Divergence to consensus: 31.3%  
**2. MIR**: 49231742-49231944 (-), Divergence to consensus: 36.9%  
**3. CHR-2B**: 49232097-49232389 (-), Divergence to consensus: 29.8%  
**4. Bov-tA2**: 49234046-49234259 (+), Divergence to consensus: 14%

  
Transcription Factor Binding Sites  

**Gata4** (Sequence: AGATAAC (-): 49231697)  
**Gata4** (Sequence: GTTATCT (+): 49236479)
