# Supplementary material for: Small RNA sequencing of cryopreserved semen from single bull revealed altered miRNAs and piRNAs expression between High- and Low-motile sperm populations
Source: BMC Genomics. 2017 Jan 4;18:14. doi: 10.1186/s12864-016-3394-7 (PMC5209821; doi:10.1186/s12864-016-3394-7)
Supplement: Additional file 3: — Details for each piRNA clusters found in High Motile (HM) sperm fraction. Genes, repeats, transposable elements and transcription factors binding sites falling within the cluster regions were reported. (ZIP 1896 kb) [file 12864_2016_3394_MOESM3_ESM.zip › 11.html]

piRNA cluster 11


Predicted piRNA cluster no. 11     previous   next
  

Show proTRAC run info
Hide proTRAC run info

================================= proTRAC ====================================  
VERSION: 2.1                                    LAST MODIFIED: 06. October 2015  
  
Please cite:  
Rosenkranz D, Zischler H. proTRAC - a software for probabilistic piRNA cluster  
detection, visualization and analysis. 2012. BMC Bioinformatics 13:5.  
  
and (for proTRAC 2.0 and later):  
Rosenkranz D, Rudloff S, Bastuck K, Ketting RF, Zischler H. Tupaia small RNAs  
provide insights into function and evolution of RNAi-based transposon defense  
in mammals. 2015. RNA 21(5):911-922.  
  
Contact:  
David Rosenkranz  
Institute of Anthropology, small RNA group  
Johannes Gutenberg University Mainz  
email: rosenkranz@uni-mainz.de  
  
You can find the latest proTRAC version at:  
http://sourceforge.net/projects/protrac/files  
http://www.smallRNAgroup-mainz.de/software  
==============================================================================  
  
PARAMETERS:  
Map file: .............../storage/core/barbara/genhome/smallRNA/fertility/Sample\_motile/pirna/Sample\_motile\_26-33\_collapsed.fa.no-dust.map.weighted-10000-1000-b-0  
Genome file: ............/storage/core/barbara/genhome/smallRNA/fertility/Sample\_all/pirna/bt\_311\_chrY.fa  
RepeatMasker annotation: /storage/genomes/bt\_umd31/GCF\_000003055.6\_Bos\_taurus\_UMD\_3.1.1\_repeatMasker\_chr.out  
GeneSet:................./storage/core/barbara/genhome/smallRNA/fertility/Sample\_all/pirna/full.gtf  
  
Significant (p<=0.01) hit density will be calculated based  
on observed hit distribution.  
  
Sliding window size: ........................................ 5000 bp  
Sliding window increament: .................................. 1000 bp  
Normalize each hit by number of genomic hits: ............... 1 [0=no/1=yes]  
Normalize each hit by number of sequence reads: ............. 1 [0=no/1=yes]  
Normalize values (-> per million mapped reads): ............. 1 [0=no/1=yes]  
Min. fraction of hits with 1T(U) or 10A: .................... 0.75  
Alternatively: Min. fraction of hits with 1T(U) and 10A: .... 0.5  
Min. fraction of hits with typical piRNA length: ............ 0.75  
Typical piRNA length: ....................................... 26-33 nt  
Min. size of a piRNA cluster: ............................... 5000 bp.  
Min. number of hits (absolute): ............................. 0  
Min. number of hits (normalized): ........................... 0  
Min. fraction of hits on the mainstrand: .................... 0.75  
Top fraction of mapped sequences (in terms of read counts): . 1%  
Top fraction accounts for max. n% of sequence reads: ........ 90%  
Min. fraction of hits on each arm of a bidirectional cluster: 0.1  
Output image file for each cluster: ......................... 0 [0=no/1=yes]  
Output html file for each cluster: .......................... 1 [0=no/1=yes]  
Output a summary table: ..................................... 1 [0=no/1=yes]  
Output a FASTA file for each cluster (piRNA sequences): ..... 1 [0=no/1=yes]  
Output a FASTA file comprising cluster sequences: ........... 1 [0=no/1=yes]  
Search DNA motifs in clusters: .............................. 1 [0=no/1=yes]  
Output flanking sequences: +/- .............................. 0 bp  
Output ~.pTi file: .......................................... 1 [0=no/1=yes]  
==============================================================================  
  
  
Genome size (without gaps): ............ 2678902517 bp  
Gaps (N/X/-): .......................... 53837044 bp  
Mapped reads: .......................... 658825247023  
Non-identical sequences: ............... 514171  
Genomic hits: .......................... 764233  
Significant densitiy of mapped reads: .. 12867599.5173724 reads/kb

Show proTRAC cluster info
Hide proTRAC cluster info

|  |  |
| --- | --- |
| Location | chr11 |
| Coordinates | 94267448-94276893 |
| Size [bp] | 9446 |
| Sequence hit loci | 113 |
| Mapped reads (normalized) | 166466872 |
| Mapped reads (normalized) per kb | 17623001.5 |
| Normalized reads with 1T (1U) | 84.1% |
| Normalized reads with 10A | 32.4% |
| Normalized reads with length 26-33 nt | 100% |
| Normalized reads on the main strand(s) | 97.1% |
| Predicted directionality | mono:minus |

100%

0%

1T (1U)  
reads

10A reads

26-33 nt  
reads

reads on mainstrand

**Either the amount of reads with 1T (1U) OR 10A has to exceed 75% (set with option: -1Tor10A)  
Alternatively the amount of reads with 1T (1U) AND 10A has to exceed 50% (set with option: -1Tand10A)  
Minimum amount of reads with preferred size is 75% (set with option: -pisize)  
Minimum amount of reads on the main strand(s) is 75% (set with option: -clstrand)**

Show read coverage
Hide read coverage

WHAT DO I SEE HERE?  
This chart shows the location of mapped sequence reads within a predicted piRNA cluster. The color refers to the number of genomic hits produced by the sequence read in question. A dark red bar indicates that this sequence read produces many other hits elsewhere in the genome. Many adjacent red or yellow bars can indicate the presence of a multi-copy element such as transposons or rRNA genes. A dark green bar indicates that this sequence read maps uniquely to this locus.

1 hit

2-5 hits

6-10 hits

11-20 hits

21-50 hits

51-100 hits

> 100 hits

chr11

94267448

94276893

Gene Set

RepeatMasker

Mapped  
Reads

19.07

plus strand

minus strand

19.07

Region: chr11 49236487-94267457. Max. coverage (+): 0.58. Max coverage (-): 0

Region: chr11 94267458-94267476. Max. coverage (+): 0. Max coverage (-): 0

Region: chr11 94267477-94267495. Max. coverage (+): 0. Max coverage (-): 0

Region: chr11 94267496-94267514. Max. coverage (+): 0. Max coverage (-): 0

Region: chr11 94267515-94267533. Max. coverage (+): 0. Max coverage (-): 0

Region: chr11 94267534-94267551. Max. coverage (+): 0. Max coverage (-): 0

Region: chr11 94267552-94267570. Max. coverage (+): 0. Max coverage (-): 0

Region: chr11 94267571-94267589. Max. coverage (+): 0. Max coverage (-): 0

Region: chr11 94267590-94267608. Max. coverage (+): 0. Max coverage (-): 0

Region: chr11 94267609-94267627. Max. coverage (+): 0. Max coverage (-): 0

Region: chr11 94267628-94267646. Max. coverage (+): 0. Max coverage (-): 0

Region: chr11 94267647-94267665. Max. coverage (+): 0. Max coverage (-): 0

Region: chr11 94267666-94267684. Max. coverage (+): 0. Max coverage (-): 0

Region: chr11 94267685-94267703. Max. coverage (+): 0. Max coverage (-): 0

Region: chr11 94267704-94267721. Max. coverage (+): 0. Max coverage (-): 1.73

Region: chr11 94267722-94267740. Max. coverage (+): 0. Max coverage (-): 1.06

Region: chr11 94267741-94267759. Max. coverage (+): 0. Max coverage (-): 0

Region: chr11 94267760-94267778. Max. coverage (+): 0. Max coverage (-): 0

Region: chr11 94267779-94267797. Max. coverage (+): 0. Max coverage (-): 0

Region: chr11 94267798-94267816. Max. coverage (+): 0. Max coverage (-): 0

Region: chr11 94267817-94267835. Max. coverage (+): 0. Max coverage (-): 0

Region: chr11 94267836-94267854. Max. coverage (+): 0. Max coverage (-): 0

Region: chr11 94267855-94267873. Max. coverage (+): 0. Max coverage (-): 0

Region: chr11 94267874-94267891. Max. coverage (+): 0. Max coverage (-): 0

Region: chr11 94267892-94267910. Max. coverage (+): 0. Max coverage (-): 0

Region: chr11 94267911-94267929. Max. coverage (+): 0. Max coverage (-): 0

Region: chr11 94267930-94267948. Max. coverage (+): 0. Max coverage (-): 0

Region: chr11 94267949-94267967. Max. coverage (+): 0. Max coverage (-): 0

Region: chr11 94267968-94267986. Max. coverage (+): 0. Max coverage (-): 0

Region: chr11 94267987-94268005. Max. coverage (+): 0. Max coverage (-): 0

Region: chr11 94268006-94268024. Max. coverage (+): 0. Max coverage (-): 0

Region: chr11 94268025-94268043. Max. coverage (+): 0. Max coverage (-): 0

Region: chr11 94268044-94268061. Max. coverage (+): 0. Max coverage (-): 0

Region: chr11 94268062-94268080. Max. coverage (+): 0. Max coverage (-): 0

Region: chr11 94268081-94268099. Max. coverage (+): 0. Max coverage (-): 0

Region: chr11 94268100-94268118. Max. coverage (+): 0. Max coverage (-): 0

Region: chr11 94268119-94268137. Max. coverage (+): 0. Max coverage (-): 0

Region: chr11 94268138-94268156. Max. coverage (+): 0. Max coverage (-): 0

Region: chr11 94268157-94268175. Max. coverage (+): 0. Max coverage (-): 0

Region: chr11 94268176-94268194. Max. coverage (+): 0. Max coverage (-): 0

Region: chr11 94268195-94268213. Max. coverage (+): 0. Max coverage (-): 0

Region: chr11 94268214-94268232. Max. coverage (+): 0. Max coverage (-): 0

Region: chr11 94268233-94268250. Max. coverage (+): 0. Max coverage (-): 0

Region: chr11 94268251-94268269. Max. coverage (+): 0. Max coverage (-): 0

Region: chr11 94268270-94268288. Max. coverage (+): 0. Max coverage (-): 0

Region: chr11 94268289-94268307. Max. coverage (+): 0. Max coverage (-): 0

Region: chr11 94268308-94268326. Max. coverage (+): 0. Max coverage (-): 0

Region: chr11 94268327-94268345. Max. coverage (+): 0. Max coverage (-): 0

Region: chr11 94268346-94268364. Max. coverage (+): 0. Max coverage (-): 1.39

Region: chr11 94268365-94268383. Max. coverage (+): 0. Max coverage (-): 1.14

Region: chr11 94268384-94268402. Max. coverage (+): 0. Max coverage (-): 1.14

Region: chr11 94268403-94268420. Max. coverage (+): 0. Max coverage (-): 0

Region: chr11 94268421-94268439. Max. coverage (+): 0. Max coverage (-): 0

Region: chr11 94268440-94268458. Max. coverage (+): 0. Max coverage (-): 0

Region: chr11 94268459-94268477. Max. coverage (+): 0. Max coverage (-): 0

Region: chr11 94268478-94268496. Max. coverage (+): 0. Max coverage (-): 10.02

Region: chr11 94268497-94268515. Max. coverage (+): 0. Max coverage (-): 0

Region: chr11 94268516-94268534. Max. coverage (+): 0. Max coverage (-): 0

Region: chr11 94268535-94268553. Max. coverage (+): 0. Max coverage (-): 0

Region: chr11 94268554-94268572. Max. coverage (+): 0. Max coverage (-): 0

Region: chr11 94268573-94268590. Max. coverage (+): 0. Max coverage (-): 0

Region: chr11 94268591-94268609. Max. coverage (+): 0. Max coverage (-): 0

Region: chr11 94268610-94268628. Max. coverage (+): 0. Max coverage (-): 0

Region: chr11 94268629-94268647. Max. coverage (+): 0. Max coverage (-): 0

Region: chr11 94268648-94268666. Max. coverage (+): 0. Max coverage (-): 0

Region: chr11 94268667-94268685. Max. coverage (+): 0. Max coverage (-): 1.04

Region: chr11 94268686-94268704. Max. coverage (+): 0. Max coverage (-): 1.04

Region: chr11 94268705-94268723. Max. coverage (+): 0. Max coverage (-): 0

Region: chr11 94268724-94268742. Max. coverage (+): 0. Max coverage (-): 0

Region: chr11 94268743-94268760. Max. coverage (+): 0. Max coverage (-): 0

Region: chr11 94268761-94268779. Max. coverage (+): 0. Max coverage (-): 3.32

Region: chr11 94268780-94268798. Max. coverage (+): 0. Max coverage (-): 3.32

Region: chr11 94268799-94268817. Max. coverage (+): 0. Max coverage (-): 0

Region: chr11 94268818-94268836. Max. coverage (+): 0. Max coverage (-): 0

Region: chr11 94268837-94268855. Max. coverage (+): 0. Max coverage (-): 0

Region: chr11 94268856-94268874. Max. coverage (+): 0. Max coverage (-): 0

Region: chr11 94268875-94268893. Max. coverage (+): 0. Max coverage (-): 0

Region: chr11 94268894-94268912. Max. coverage (+): 0. Max coverage (-): 6.23

Region: chr11 94268913-94268931. Max. coverage (+): 0. Max coverage (-): 0

Region: chr11 94268932-94268949. Max. coverage (+): 0. Max coverage (-): 0

Region: chr11 94268950-94268968. Max. coverage (+): 0. Max coverage (-): 0

Region: chr11 94268969-94268987. Max. coverage (+): 0. Max coverage (-): 0

Region: chr11 94268988-94269006. Max. coverage (+): 0. Max coverage (-): 9.37

Region: chr11 94269007-94269025. Max. coverage (+): 0. Max coverage (-): 0

Region: chr11 94269026-94269044. Max. coverage (+): 0. Max coverage (-): 0

Region: chr11 94269045-94269063. Max. coverage (+): 0. Max coverage (-): 1.36

Region: chr11 94269064-94269082. Max. coverage (+): 0. Max coverage (-): 1.36

Region: chr11 94269083-94269101. Max. coverage (+): 0. Max coverage (-): 0

Region: chr11 94269102-94269119. Max. coverage (+): 0. Max coverage (-): 0

Region: chr11 94269120-94269138. Max. coverage (+): 0. Max coverage (-): 0

Region: chr11 94269139-94269157. Max. coverage (+): 0. Max coverage (-): 0

Region: chr11 94269158-94269176. Max. coverage (+): 0. Max coverage (-): 0

Region: chr11 94269177-94269195. Max. coverage (+): 0. Max coverage (-): 0

Region: chr11 94269196-94269214. Max. coverage (+): 0. Max coverage (-): 0

Region: chr11 94269215-94269233. Max. coverage (+): 0. Max coverage (-): 3.02

Region: chr11 94269234-94269252. Max. coverage (+): 0. Max coverage (-): 3.02

Region: chr11 94269253-94269271. Max. coverage (+): 0. Max coverage (-): 0

Region: chr11 94269272-94269289. Max. coverage (+): 0. Max coverage (-): 0

Region: chr11 94269290-94269308. Max. coverage (+): 0. Max coverage (-): 0.9

Region: chr11 94269309-94269327. Max. coverage (+): 0. Max coverage (-): 0.9

Region: chr11 94269328-94269346. Max. coverage (+): 0. Max coverage (-): 0

Region: chr11 94269347-94269365. Max. coverage (+): 0. Max coverage (-): 0

Region: chr11 94269366-94269384. Max. coverage (+): 0. Max coverage (-): 0

Region: chr11 94269385-94269403. Max. coverage (+): 0. Max coverage (-): 0

Region: chr11 94269404-94269422. Max. coverage (+): 0. Max coverage (-): 0

Region: chr11 94269423-94269441. Max. coverage (+): 0. Max coverage (-): 0

Region: chr11 94269442-94269459. Max. coverage (+): 0. Max coverage (-): 0

Region: chr11 94269460-94269478. Max. coverage (+): 0. Max coverage (-): 0

Region: chr11 94269479-94269497. Max. coverage (+): 0. Max coverage (-): 0

Region: chr11 94269498-94269516. Max. coverage (+): 0. Max coverage (-): 0

Region: chr11 94269517-94269535. Max. coverage (+): 0. Max coverage (-): 0

Region: chr11 94269536-94269554. Max. coverage (+): 0. Max coverage (-): 0

Region: chr11 94269555-94269573. Max. coverage (+): 0. Max coverage (-): 0

Region: chr11 94269574-94269592. Max. coverage (+): 0. Max coverage (-): 0

Region: chr11 94269593-94269611. Max. coverage (+): 0. Max coverage (-): 0

Region: chr11 94269612-94269630. Max. coverage (+): 0. Max coverage (-): 2.04

Region: chr11 94269631-94269648. Max. coverage (+): 0. Max coverage (-): 7.12

Region: chr11 94269649-94269667. Max. coverage (+): 0. Max coverage (-): 7.12

Region: chr11 94269668-94269686. Max. coverage (+): 0. Max coverage (-): 6.14

Region: chr11 94269687-94269705. Max. coverage (+): 0. Max coverage (-): 0

Region: chr11 94269706-94269724. Max. coverage (+): 0. Max coverage (-): 0

Region: chr11 94269725-94269743. Max. coverage (+): 0. Max coverage (-): 0

Region: chr11 94269744-94269762. Max. coverage (+): 0. Max coverage (-): 0

Region: chr11 94269763-94269781. Max. coverage (+): 0. Max coverage (-): 8.36

Region: chr11 94269782-94269800. Max. coverage (+): 0. Max coverage (-): 8.36

Region: chr11 94269801-94269818. Max. coverage (+): 0. Max coverage (-): 0

Region: chr11 94269819-94269837. Max. coverage (+): 0. Max coverage (-): 0

Region: chr11 94269838-94269856. Max. coverage (+): 0. Max coverage (-): 0

Region: chr11 94269857-94269875. Max. coverage (+): 0. Max coverage (-): 0

Region: chr11 94269876-94269894. Max. coverage (+): 0. Max coverage (-): 0

Region: chr11 94269895-94269913. Max. coverage (+): 0. Max coverage (-): 9.45

Region: chr11 94269914-94269932. Max. coverage (+): 0. Max coverage (-): 4.98

Region: chr11 94269933-94269951. Max. coverage (+): 0. Max coverage (-): 0

Region: chr11 94269952-94269970. Max. coverage (+): 0. Max coverage (-): 0

Region: chr11 94269971-94269988. Max. coverage (+): 0. Max coverage (-): 1.57

Region: chr11 94269989-94270007. Max. coverage (+): 0. Max coverage (-): 0

Region: chr11 94270008-94270026. Max. coverage (+): 0. Max coverage (-): 0

Region: chr11 94270027-94270045. Max. coverage (+): 0. Max coverage (-): 0

Region: chr11 94270046-94270064. Max. coverage (+): 0. Max coverage (-): 0

Region: chr11 94270065-94270083. Max. coverage (+): 0. Max coverage (-): 0

Region: chr11 94270084-94270102. Max. coverage (+): 0. Max coverage (-): 0

Region: chr11 94270103-94270121. Max. coverage (+): 0. Max coverage (-): 0

Region: chr11 94270122-94270140. Max. coverage (+): 0. Max coverage (-): 0

Region: chr11 94270141-94270159. Max. coverage (+): 0. Max coverage (-): 0

Region: chr11 94270160-94270177. Max. coverage (+): 0. Max coverage (-): 0

Region: chr11 94270178-94270196. Max. coverage (+): 0. Max coverage (-): 0.7

Region: chr11 94270197-94270215. Max. coverage (+): 0. Max coverage (-): 0

Region: chr11 94270216-94270234. Max. coverage (+): 0. Max coverage (-): 0

Region: chr11 94270235-94270253. Max. coverage (+): 0. Max coverage (-): 0

Region: chr11 94270254-94270272. Max. coverage (+): 0. Max coverage (-): 0

Region: chr11 94270273-94270291. Max. coverage (+): 0. Max coverage (-): 0

Region: chr11 94270292-94270310. Max. coverage (+): 0. Max coverage (-): 0

Region: chr11 94270311-94270329. Max. coverage (+): 0. Max coverage (-): 4.21

Region: chr11 94270330-94270347. Max. coverage (+): 0. Max coverage (-): 4.21

Region: chr11 94270348-94270366. Max. coverage (+): 0. Max coverage (-): 0

Region: chr11 94270367-94270385. Max. coverage (+): 0. Max coverage (-): 1.54

Region: chr11 94270386-94270404. Max. coverage (+): 0. Max coverage (-): 1.54

Region: chr11 94270405-94270423. Max. coverage (+): 0. Max coverage (-): 0

Region: chr11 94270424-94270442. Max. coverage (+): 0. Max coverage (-): 0

Region: chr11 94270443-94270461. Max. coverage (+): 0. Max coverage (-): 0

Region: chr11 94270462-94270480. Max. coverage (+): 0. Max coverage (-): 3.49

Region: chr11 94270481-94270499. Max. coverage (+): 0. Max coverage (-): 0

Region: chr11 94270500-94270517. Max. coverage (+): 0. Max coverage (-): 0

Region: chr11 94270518-94270536. Max. coverage (+): 0. Max coverage (-): 0

Region: chr11 94270537-94270555. Max. coverage (+): 0. Max coverage (-): 0

Region: chr11 94270556-94270574. Max. coverage (+): 0. Max coverage (-): 0

Region: chr11 94270575-94270593. Max. coverage (+): 0. Max coverage (-): 0

Region: chr11 94270594-94270612. Max. coverage (+): 0. Max coverage (-): 0

Region: chr11 94270613-94270631. Max. coverage (+): 0. Max coverage (-): 3.19

Region: chr11 94270632-94270650. Max. coverage (+): 0. Max coverage (-): 0

Region: chr11 94270651-94270669. Max. coverage (+): 0. Max coverage (-): 0

Region: chr11 94270670-94270687. Max. coverage (+): 0. Max coverage (-): 0

Region: chr11 94270688-94270706. Max. coverage (+): 0. Max coverage (-): 0

Region: chr11 94270707-94270725. Max. coverage (+): 0. Max coverage (-): 0

Region: chr11 94270726-94270744. Max. coverage (+): 0. Max coverage (-): 0.7

Region: chr11 94270745-94270763. Max. coverage (+): 0. Max coverage (-): 2.37

Region: chr11 94270764-94270782. Max. coverage (+): 0. Max coverage (-): 0

Region: chr11 94270783-94270801. Max. coverage (+): 0. Max coverage (-): 0

Region: chr11 94270802-94270820. Max. coverage (+): 3.5. Max coverage (-): 0

Region: chr11 94270821-94270839. Max. coverage (+): 0. Max coverage (-): 0

Region: chr11 94270840-94270858. Max. coverage (+): 0. Max coverage (-): 0

Region: chr11 94270859-94270876. Max. coverage (+): 0. Max coverage (-): 4.86

Region: chr11 94270877-94270895. Max. coverage (+): 0. Max coverage (-): 4.86

Region: chr11 94270896-94270914. Max. coverage (+): 0. Max coverage (-): 7.2

Region: chr11 94270915-94270933. Max. coverage (+): 0. Max coverage (-): 7.2

Region: chr11 94270934-94270952. Max. coverage (+): 0. Max coverage (-): 0

Region: chr11 94270953-94270971. Max. coverage (+): 0. Max coverage (-): 0

Region: chr11 94270972-94270990. Max. coverage (+): 0. Max coverage (-): 0

Region: chr11 94270991-94271009. Max. coverage (+): 0. Max coverage (-): 19.07

Region: chr11 94271010-94271028. Max. coverage (+): 0. Max coverage (-): 0

Region: chr11 94271029-94271046. Max. coverage (+): 0. Max coverage (-): 1.64

Region: chr11 94271047-94271065. Max. coverage (+): 0. Max coverage (-): 3.46

Region: chr11 94271066-94271084. Max. coverage (+): 0. Max coverage (-): 0

Region: chr11 94271085-94271103. Max. coverage (+): 0. Max coverage (-): 0

Region: chr11 94271104-94271122. Max. coverage (+): 0. Max coverage (-): 0

Region: chr11 94271123-94271141. Max. coverage (+): 0. Max coverage (-): 0

Region: chr11 94271142-94271160. Max. coverage (+): 0. Max coverage (-): 0

Region: chr11 94271161-94271179. Max. coverage (+): 0. Max coverage (-): 0

Region: chr11 94271180-94271198. Max. coverage (+): 0. Max coverage (-): 0

Region: chr11 94271199-94271216. Max. coverage (+): 0. Max coverage (-): 0

Region: chr11 94271217-94271235. Max. coverage (+): 0. Max coverage (-): 0

Region: chr11 94271236-94271254. Max. coverage (+): 0. Max coverage (-): 0

Region: chr11 94271255-94271273. Max. coverage (+): 0. Max coverage (-): 0

Region: chr11 94271274-94271292. Max. coverage (+): 0. Max coverage (-): 0

Region: chr11 94271293-94271311. Max. coverage (+): 0. Max coverage (-): 0

Region: chr11 94271312-94271330. Max. coverage (+): 0. Max coverage (-): 0

Region: chr11 94271331-94271349. Max. coverage (+): 0. Max coverage (-): 0

Region: chr11 94271350-94271368. Max. coverage (+): 0. Max coverage (-): 0

Region: chr11 94271369-94271386. Max. coverage (+): 0. Max coverage (-): 0

Region: chr11 94271387-94271405. Max. coverage (+): 0. Max coverage (-): 0

Region: chr11 94271406-94271424. Max. coverage (+): 0. Max coverage (-): 0

Region: chr11 94271425-94271443. Max. coverage (+): 0. Max coverage (-): 0

Region: chr11 94271444-94271462. Max. coverage (+): 0. Max coverage (-): 0

Region: chr11 94271463-94271481. Max. coverage (+): 0. Max coverage (-): 0

Region: chr11 94271482-94271500. Max. coverage (+): 0. Max coverage (-): 1.93

Region: chr11 94271501-94271519. Max. coverage (+): 0. Max coverage (-): 0

Region: chr11 94271520-94271538. Max. coverage (+): 0. Max coverage (-): 0

Region: chr11 94271539-94271557. Max. coverage (+): 0. Max coverage (-): 0

Region: chr11 94271558-94271575. Max. coverage (+): 0. Max coverage (-): 0

Region: chr11 94271576-94271594. Max. coverage (+): 0. Max coverage (-): 0

Region: chr11 94271595-94271613. Max. coverage (+): 0. Max coverage (-): 0

Region: chr11 94271614-94271632. Max. coverage (+): 0. Max coverage (-): 0

Region: chr11 94271633-94271651. Max. coverage (+): 0. Max coverage (-): 0

Region: chr11 94271652-94271670. Max. coverage (+): 0. Max coverage (-): 0

Region: chr11 94271671-94271689. Max. coverage (+): 0. Max coverage (-): 0

Region: chr11 94271690-94271708. Max. coverage (+): 0. Max coverage (-): 0

Region: chr11 94271709-94271727. Max. coverage (+): 0. Max coverage (-): 0

Region: chr11 94271728-94271745. Max. coverage (+): 0. Max coverage (-): 0

Region: chr11 94271746-94271764. Max. coverage (+): 0. Max coverage (-): 0

Region: chr11 94271765-94271783. Max. coverage (+): 0. Max coverage (-): 0

Region: chr11 94271784-94271802. Max. coverage (+): 0. Max coverage (-): 0

Region: chr11 94271803-94271821. Max. coverage (+): 0. Max coverage (-): 0

Region: chr11 94271822-94271840. Max. coverage (+): 0. Max coverage (-): 0

Region: chr11 94271841-94271859. Max. coverage (+): 0. Max coverage (-): 0

Region: chr11 94271860-94271878. Max. coverage (+): 0. Max coverage (-): 1.86

Region: chr11 94271879-94271897. Max. coverage (+): 0. Max coverage (-): 0.98

Region: chr11 94271898-94271915. Max. coverage (+): 0. Max coverage (-): 0

Region: chr11 94271916-94271934. Max. coverage (+): 0. Max coverage (-): 0

Region: chr11 94271935-94271953. Max. coverage (+): 0. Max coverage (-): 0

Region: chr11 94271954-94271972. Max. coverage (+): 0. Max coverage (-): 0

Region: chr11 94271973-94271991. Max. coverage (+): 0. Max coverage (-): 0

Region: chr11 94271992-94272010. Max. coverage (+): 0. Max coverage (-): 0

Region: chr11 94272011-94272029. Max. coverage (+): 0. Max coverage (-): 0

Region: chr11 94272030-94272048. Max. coverage (+): 0. Max coverage (-): 0

Region: chr11 94272049-94272067. Max. coverage (+): 0. Max coverage (-): 0

Region: chr11 94272068-94272085. Max. coverage (+): 0. Max coverage (-): 0

Region: chr11 94272086-94272104. Max. coverage (+): 0. Max coverage (-): 0

Region: chr11 94272105-94272123. Max. coverage (+): 0. Max coverage (-): 7.47

Region: chr11 94272124-94272142. Max. coverage (+): 1.15. Max coverage (-): 0.64

Region: chr11 94272143-94272161. Max. coverage (+): 0. Max coverage (-): 0

Region: chr11 94272162-94272180. Max. coverage (+): 0. Max coverage (-): 0

Region: chr11 94272181-94272199. Max. coverage (+): 0. Max coverage (-): 0

Region: chr11 94272200-94272218. Max. coverage (+): 0. Max coverage (-): 0

Region: chr11 94272219-94272237. Max. coverage (+): 0. Max coverage (-): 1.7

Region: chr11 94272238-94272256. Max. coverage (+): 0. Max coverage (-): 0

Region: chr11 94272257-94272274. Max. coverage (+): 0. Max coverage (-): 3.36

Region: chr11 94272275-94272293. Max. coverage (+): 0. Max coverage (-): 4.68

Region: chr11 94272294-94272312. Max. coverage (+): 0. Max coverage (-): 0

Region: chr11 94272313-94272331. Max. coverage (+): 0. Max coverage (-): 0

Region: chr11 94272332-94272350. Max. coverage (+): 0. Max coverage (-): 0

Region: chr11 94272351-94272369. Max. coverage (+): 0. Max coverage (-): 1.77

Region: chr11 94272370-94272388. Max. coverage (+): 0. Max coverage (-): 0

Region: chr11 94272389-94272407. Max. coverage (+): 0. Max coverage (-): 0

Region: chr11 94272408-94272426. Max. coverage (+): 0. Max coverage (-): 0

Region: chr11 94272427-94272444. Max. coverage (+): 0. Max coverage (-): 0.99

Region: chr11 94272445-94272463. Max. coverage (+): 0. Max coverage (-): 1.98

Region: chr11 94272464-94272482. Max. coverage (+): 0. Max coverage (-): 1.98

Region: chr11 94272483-94272501. Max. coverage (+): 0. Max coverage (-): 10.03

Region: chr11 94272502-94272520. Max. coverage (+): 0. Max coverage (-): 4.13

Region: chr11 94272521-94272539. Max. coverage (+): 0. Max coverage (-): 0

Region: chr11 94272540-94272558. Max. coverage (+): 0. Max coverage (-): 0

Region: chr11 94272559-94272577. Max. coverage (+): 0. Max coverage (-): 0

Region: chr11 94272578-94272596. Max. coverage (+): 0. Max coverage (-): 0

Region: chr11 94272597-94272614. Max. coverage (+): 0. Max coverage (-): 0

Region: chr11 94272615-94272633. Max. coverage (+): 0. Max coverage (-): 1.37

Region: chr11 94272634-94272652. Max. coverage (+): 0. Max coverage (-): 0

Region: chr11 94272653-94272671. Max. coverage (+): 0. Max coverage (-): 0

Region: chr11 94272672-94272690. Max. coverage (+): 0. Max coverage (-): 0

Region: chr11 94272691-94272709. Max. coverage (+): 0. Max coverage (-): 0

Region: chr11 94272710-94272728. Max. coverage (+): 0. Max coverage (-): 0

Region: chr11 94272729-94272747. Max. coverage (+): 0. Max coverage (-): 3.42

Region: chr11 94272748-94272766. Max. coverage (+): 0. Max coverage (-): 0

Region: chr11 94272767-94272784. Max. coverage (+): 0. Max coverage (-): 5.36

Region: chr11 94272785-94272803. Max. coverage (+): 0. Max coverage (-): 0

Region: chr11 94272804-94272822. Max. coverage (+): 0. Max coverage (-): 0

Region: chr11 94272823-94272841. Max. coverage (+): 0. Max coverage (-): 0

Region: chr11 94272842-94272860. Max. coverage (+): 0. Max coverage (-): 0

Region: chr11 94272861-94272879. Max. coverage (+): 0. Max coverage (-): 0

Region: chr11 94272880-94272898. Max. coverage (+): 0. Max coverage (-): 0

Region: chr11 94272899-94272917. Max. coverage (+): 0. Max coverage (-): 0

Region: chr11 94272918-94272936. Max. coverage (+): 0. Max coverage (-): 0

Region: chr11 94272937-94272955. Max. coverage (+): 0. Max coverage (-): 4

Region: chr11 94272956-94272973. Max. coverage (+): 0. Max coverage (-): 0

Region: chr11 94272974-94272992. Max. coverage (+): 0. Max coverage (-): 0

Region: chr11 94272993-94273011. Max. coverage (+): 0. Max coverage (-): 0

Region: chr11 94273012-94273030. Max. coverage (+): 0. Max coverage (-): 0

Region: chr11 94273031-94273049. Max. coverage (+): 0. Max coverage (-): 0

Region: chr11 94273050-94273068. Max. coverage (+): 0. Max coverage (-): 0

Region: chr11 94273069-94273087. Max. coverage (+): 0. Max coverage (-): 0.53

Region: chr11 94273088-94273106. Max. coverage (+): 0. Max coverage (-): 2.22

Region: chr11 94273107-94273125. Max. coverage (+): 0. Max coverage (-): 0

Region: chr11 94273126-94273143. Max. coverage (+): 0. Max coverage (-): 0

Region: chr11 94273144-94273162. Max. coverage (+): 0. Max coverage (-): 0

Region: chr11 94273163-94273181. Max. coverage (+): 0. Max coverage (-): 0

Region: chr11 94273182-94273200. Max. coverage (+): 0. Max coverage (-): 0

Region: chr11 94273201-94273219. Max. coverage (+): 0. Max coverage (-): 0

Region: chr11 94273220-94273238. Max. coverage (+): 0. Max coverage (-): 0

Region: chr11 94273239-94273257. Max. coverage (+): 0. Max coverage (-): 0

Region: chr11 94273258-94273276. Max. coverage (+): 0. Max coverage (-): 0

Region: chr11 94273277-94273295. Max. coverage (+): 0. Max coverage (-): 0

Region: chr11 94273296-94273313. Max. coverage (+): 0. Max coverage (-): 0

Region: chr11 94273314-94273332. Max. coverage (+): 0. Max coverage (-): 0

Region: chr11 94273333-94273351. Max. coverage (+): 0. Max coverage (-): 0

Region: chr11 94273352-94273370. Max. coverage (+): 0. Max coverage (-): 0

Region: chr11 94273371-94273389. Max. coverage (+): 0. Max coverage (-): 0

Region: chr11 94273390-94273408. Max. coverage (+): 0. Max coverage (-): 0

Region: chr11 94273409-94273427. Max. coverage (+): 0. Max coverage (-): 0

Region: chr11 94273428-94273446. Max. coverage (+): 0. Max coverage (-): 0

Region: chr11 94273447-94273465. Max. coverage (+): 0. Max coverage (-): 0

Region: chr11 94273466-94273483. Max. coverage (+): 0. Max coverage (-): 0

Region: chr11 94273484-94273502. Max. coverage (+): 0. Max coverage (-): 0

Region: chr11 94273503-94273521. Max. coverage (+): 0. Max coverage (-): 3.83

Region: chr11 94273522-94273540. Max. coverage (+): 0. Max coverage (-): 3.83

Region: chr11 94273541-94273559. Max. coverage (+): 0. Max coverage (-): 0

Region: chr11 94273560-94273578. Max. coverage (+): 0. Max coverage (-): 0.61

Region: chr11 94273579-94273597. Max. coverage (+): 0. Max coverage (-): 0.61

Region: chr11 94273598-94273616. Max. coverage (+): 0. Max coverage (-): 0

Region: chr11 94273617-94273635. Max. coverage (+): 0. Max coverage (-): 0

Region: chr11 94273636-94273654. Max. coverage (+): 0. Max coverage (-): 0

Region: chr11 94273655-94273672. Max. coverage (+): 0. Max coverage (-): 0

Region: chr11 94273673-94273691. Max. coverage (+): 0. Max coverage (-): 0

Region: chr11 94273692-94273710. Max. coverage (+): 0. Max coverage (-): 0

Region: chr11 94273711-94273729. Max. coverage (+): 0. Max coverage (-): 0

Region: chr11 94273730-94273748. Max. coverage (+): 0. Max coverage (-): 0

Region: chr11 94273749-94273767. Max. coverage (+): 0. Max coverage (-): 0

Region: chr11 94273768-94273786. Max. coverage (+): 0. Max coverage (-): 0

Region: chr11 94273787-94273805. Max. coverage (+): 0. Max coverage (-): 0

Region: chr11 94273806-94273824. Max. coverage (+): 0. Max coverage (-): 0

Region: chr11 94273825-94273842. Max. coverage (+): 0. Max coverage (-): 0

Region: chr11 94273843-94273861. Max. coverage (+): 0. Max coverage (-): 0

Region: chr11 94273862-94273880. Max. coverage (+): 0. Max coverage (-): 0

Region: chr11 94273881-94273899. Max. coverage (+): 0. Max coverage (-): 0

Region: chr11 94273900-94273918. Max. coverage (+): 0. Max coverage (-): 0

Region: chr11 94273919-94273937. Max. coverage (+): 0. Max coverage (-): 4.85

Region: chr11 94273938-94273956. Max. coverage (+): 0. Max coverage (-): 0

Region: chr11 94273957-94273975. Max. coverage (+): 0. Max coverage (-): 0

Region: chr11 94273976-94273994. Max. coverage (+): 0. Max coverage (-): 0

Region: chr11 94273995-94274012. Max. coverage (+): 0. Max coverage (-): 0

Region: chr11 94274013-94274031. Max. coverage (+): 0. Max coverage (-): 0

Region: chr11 94274032-94274050. Max. coverage (+): 0. Max coverage (-): 0

Region: chr11 94274051-94274069. Max. coverage (+): 0. Max coverage (-): 5.06

Region: chr11 94274070-94274088. Max. coverage (+): 0. Max coverage (-): 0

Region: chr11 94274089-94274107. Max. coverage (+): 0. Max coverage (-): 1.9

Region: chr11 94274108-94274126. Max. coverage (+): 0. Max coverage (-): 0

Region: chr11 94274127-94274145. Max. coverage (+): 0. Max coverage (-): 0

Region: chr11 94274146-94274164. Max. coverage (+): 0. Max coverage (-): 0

Region: chr11 94274165-94274182. Max. coverage (+): 0. Max coverage (-): 0

Region: chr11 94274183-94274201. Max. coverage (+): 0. Max coverage (-): 0

Region: chr11 94274202-94274220. Max. coverage (+): 0. Max coverage (-): 0

Region: chr11 94274221-94274239. Max. coverage (+): 0. Max coverage (-): 0

Region: chr11 94274240-94274258. Max. coverage (+): 0. Max coverage (-): 0

Region: chr11 94274259-94274277. Max. coverage (+): 0. Max coverage (-): 0

Region: chr11 94274278-94274296. Max. coverage (+): 0. Max coverage (-): 3.25

Region: chr11 94274297-94274315. Max. coverage (+): 0. Max coverage (-): 0

Region: chr11 94274316-94274334. Max. coverage (+): 0. Max coverage (-): 0

Region: chr11 94274335-94274353. Max. coverage (+): 0. Max coverage (-): 0

Region: chr11 94274354-94274371. Max. coverage (+): 0. Max coverage (-): 0

Region: chr11 94274372-94274390. Max. coverage (+): 0. Max coverage (-): 0

Region: chr11 94274391-94274409. Max. coverage (+): 0. Max coverage (-): 0

Region: chr11 94274410-94274428. Max. coverage (+): 0. Max coverage (-): 0

Region: chr11 94274429-94274447. Max. coverage (+): 0. Max coverage (-): 0

Region: chr11 94274448-94274466. Max. coverage (+): 0. Max coverage (-): 0

Region: chr11 94274467-94274485. Max. coverage (+): 0. Max coverage (-): 0

Region: chr11 94274486-94274504. Max. coverage (+): 0. Max coverage (-): 0

Region: chr11 94274505-94274523. Max. coverage (+): 0. Max coverage (-): 0

Region: chr11 94274524-94274541. Max. coverage (+): 0. Max coverage (-): 0

Region: chr11 94274542-94274560. Max. coverage (+): 0. Max coverage (-): 3.93

Region: chr11 94274561-94274579. Max. coverage (+): 0. Max coverage (-): 0.76

Region: chr11 94274580-94274598. Max. coverage (+): 0. Max coverage (-): 9.8

Region: chr11 94274599-94274617. Max. coverage (+): 0. Max coverage (-): 11.65

Region: chr11 94274618-94274636. Max. coverage (+): 0. Max coverage (-): 0

Region: chr11 94274637-94274655. Max. coverage (+): 0. Max coverage (-): 0

Region: chr11 94274656-94274674. Max. coverage (+): 0. Max coverage (-): 0

Region: chr11 94274675-94274693. Max. coverage (+): 0. Max coverage (-): 0

Region: chr11 94274694-94274711. Max. coverage (+): 0. Max coverage (-): 0

Region: chr11 94274712-94274730. Max. coverage (+): 0. Max coverage (-): 0

Region: chr11 94274731-94274749. Max. coverage (+): 0. Max coverage (-): 0

Region: chr11 94274750-94274768. Max. coverage (+): 0. Max coverage (-): 2.62

Region: chr11 94274769-94274787. Max. coverage (+): 0. Max coverage (-): 0

Region: chr11 94274788-94274806. Max. coverage (+): 0. Max coverage (-): 0

Region: chr11 94274807-94274825. Max. coverage (+): 0. Max coverage (-): 0

Region: chr11 94274826-94274844. Max. coverage (+): 0. Max coverage (-): 0

Region: chr11 94274845-94274863. Max. coverage (+): 0. Max coverage (-): 3.41

Region: chr11 94274864-94274882. Max. coverage (+): 0. Max coverage (-): 0

Region: chr11 94274883-94274900. Max. coverage (+): 0. Max coverage (-): 0

Region: chr11 94274901-94274919. Max. coverage (+): 0. Max coverage (-): 0

Region: chr11 94274920-94274938. Max. coverage (+): 0. Max coverage (-): 0

Region: chr11 94274939-94274957. Max. coverage (+): 0. Max coverage (-): 0

Region: chr11 94274958-94274976. Max. coverage (+): 0. Max coverage (-): 0

Region: chr11 94274977-94274995. Max. coverage (+): 0. Max coverage (-): 0

Region: chr11 94274996-94275014. Max. coverage (+): 0. Max coverage (-): 1.11

Region: chr11 94275015-94275033. Max. coverage (+): 0. Max coverage (-): 3.6

Region: chr11 94275034-94275052. Max. coverage (+): 0. Max coverage (-): 0

Region: chr11 94275053-94275070. Max. coverage (+): 0. Max coverage (-): 0

Region: chr11 94275071-94275089. Max. coverage (+): 0. Max coverage (-): 0

Region: chr11 94275090-94275108. Max. coverage (+): 0. Max coverage (-): 0

Region: chr11 94275109-94275127. Max. coverage (+): 0. Max coverage (-): 1.58

Region: chr11 94275128-94275146. Max. coverage (+): 0. Max coverage (-): 0

Region: chr11 94275147-94275165. Max. coverage (+): 0. Max coverage (-): 0

Region: chr11 94275166-94275184. Max. coverage (+): 0. Max coverage (-): 0

Region: chr11 94275185-94275203. Max. coverage (+): 0. Max coverage (-): 0

Region: chr11 94275204-94275222. Max. coverage (+): 0. Max coverage (-): 1.19

Region: chr11 94275223-94275240. Max. coverage (+): 2.09. Max coverage (-): 1.19

Region: chr11 94275241-94275259. Max. coverage (+): 0. Max coverage (-): 0

Region: chr11 94275260-94275278. Max. coverage (+): 0. Max coverage (-): 0

Region: chr11 94275279-94275297. Max. coverage (+): 0. Max coverage (-): 0

Region: chr11 94275298-94275316. Max. coverage (+): 0. Max coverage (-): 0

Region: chr11 94275317-94275335. Max. coverage (+): 0. Max coverage (-): 0

Region: chr11 94275336-94275354. Max. coverage (+): 0. Max coverage (-): 0

Region: chr11 94275355-94275373. Max. coverage (+): 0. Max coverage (-): 0

Region: chr11 94275374-94275392. Max. coverage (+): 0. Max coverage (-): 0

Region: chr11 94275393-94275410. Max. coverage (+): 0. Max coverage (-): 0

Region: chr11 94275411-94275429. Max. coverage (+): 0. Max coverage (-): 0

Region: chr11 94275430-94275448. Max. coverage (+): 0. Max coverage (-): 0

Region: chr11 94275449-94275467. Max. coverage (+): 0. Max coverage (-): 0

Region: chr11 94275468-94275486. Max. coverage (+): 0. Max coverage (-): 0

Region: chr11 94275487-94275505. Max. coverage (+): 0. Max coverage (-): 0

Region: chr11 94275506-94275524. Max. coverage (+): 0. Max coverage (-): 0

Region: chr11 94275525-94275543. Max. coverage (+): 0. Max coverage (-): 0

Region: chr11 94275544-94275562. Max. coverage (+): 0. Max coverage (-): 0

Region: chr11 94275563-94275581. Max. coverage (+): 0. Max coverage (-): 0

Region: chr11 94275582-94275599. Max. coverage (+): 0. Max coverage (-): 0

Region: chr11 94275600-94275618. Max. coverage (+): 0. Max coverage (-): 0

Region: chr11 94275619-94275637. Max. coverage (+): 0. Max coverage (-): 0

Region: chr11 94275638-94275656. Max. coverage (+): 0. Max coverage (-): 0

Region: chr11 94275657-94275675. Max. coverage (+): 0. Max coverage (-): 0

Region: chr11 94275676-94275694. Max. coverage (+): 0. Max coverage (-): 0

Region: chr11 94275695-94275713. Max. coverage (+): 0. Max coverage (-): 5.11

Region: chr11 94275714-94275732. Max. coverage (+): 0. Max coverage (-): 0

Region: chr11 94275733-94275751. Max. coverage (+): 0. Max coverage (-): 0

Region: chr11 94275752-94275769. Max. coverage (+): 0. Max coverage (-): 0

Region: chr11 94275770-94275788. Max. coverage (+): 0. Max coverage (-): 0

Region: chr11 94275789-94275807. Max. coverage (+): 0. Max coverage (-): 0

Region: chr11 94275808-94275826. Max. coverage (+): 0. Max coverage (-): 0

Region: chr11 94275827-94275845. Max. coverage (+): 0. Max coverage (-): 0

Region: chr11 94275846-94275864. Max. coverage (+): 0. Max coverage (-): 0

Region: chr11 94275865-94275883. Max. coverage (+): 0. Max coverage (-): 0

Region: chr11 94275884-94275902. Max. coverage (+): 0. Max coverage (-): 0

Region: chr11 94275903-94275921. Max. coverage (+): 0. Max coverage (-): 0

Region: chr11 94275922-94275939. Max. coverage (+): 0. Max coverage (-): 0

Region: chr11 94275940-94275958. Max. coverage (+): 0. Max coverage (-): 0

Region: chr11 94275959-94275977. Max. coverage (+): 0. Max coverage (-): 0

Region: chr11 94275978-94275996. Max. coverage (+): 0. Max coverage (-): 0

Region: chr11 94275997-94276015. Max. coverage (+): 0. Max coverage (-): 0

Region: chr11 94276016-94276034. Max. coverage (+): 0. Max coverage (-): 0

Region: chr11 94276035-94276053. Max. coverage (+): 0. Max coverage (-): 0

Region: chr11 94276054-94276072. Max. coverage (+): 0. Max coverage (-): 0

Region: chr11 94276073-94276091. Max. coverage (+): 0. Max coverage (-): 0

Region: chr11 94276092-94276109. Max. coverage (+): 0. Max coverage (-): 0

Region: chr11 94276110-94276128. Max. coverage (+): 0. Max coverage (-): 0

Region: chr11 94276129-94276147. Max. coverage (+): 0. Max coverage (-): 0

Region: chr11 94276148-94276166. Max. coverage (+): 0. Max coverage (-): 0

Region: chr11 94276167-94276185. Max. coverage (+): 0. Max coverage (-): 0

Region: chr11 94276186-94276204. Max. coverage (+): 0. Max coverage (-): 0

Region: chr11 94276205-94276223. Max. coverage (+): 0. Max coverage (-): 2.86

Region: chr11 94276224-94276242. Max. coverage (+): 0. Max coverage (-): 0

Region: chr11 94276243-94276261. Max. coverage (+): 0. Max coverage (-): 0

Region: chr11 94276262-94276280. Max. coverage (+): 0. Max coverage (-): 0

Region: chr11 94276281-94276298. Max. coverage (+): 0. Max coverage (-): 0

Region: chr11 94276299-94276317. Max. coverage (+): 0. Max coverage (-): 0

Region: chr11 94276318-94276336. Max. coverage (+): 0. Max coverage (-): 0

Region: chr11 94276337-94276355. Max. coverage (+): 0. Max coverage (-): 0

Region: chr11 94276356-94276374. Max. coverage (+): 0. Max coverage (-): 0

Region: chr11 94276375-94276393. Max. coverage (+): 0. Max coverage (-): 0

Region: chr11 94276394-94276412. Max. coverage (+): 0. Max coverage (-): 0

Region: chr11 94276413-94276431. Max. coverage (+): 0. Max coverage (-): 0

Region: chr11 94276432-94276450. Max. coverage (+): 0. Max coverage (-): 0

Region: chr11 94276451-94276468. Max. coverage (+): 0. Max coverage (-): 0

Region: chr11 94276469-94276487. Max. coverage (+): 0. Max coverage (-): 0

Region: chr11 94276488-94276506. Max. coverage (+): 0. Max coverage (-): 0

Region: chr11 94276507-94276525. Max. coverage (+): 0. Max coverage (-): 0

Region: chr11 94276526-94276544. Max. coverage (+): 0. Max coverage (-): 0

Region: chr11 94276545-94276563. Max. coverage (+): 0. Max coverage (-): 0

Region: chr11 94276564-94276582. Max. coverage (+): 0. Max coverage (-): 0

Region: chr11 94276583-94276601. Max. coverage (+): 0. Max coverage (-): 0

Region: chr11 94276602-94276620. Max. coverage (+): 0. Max coverage (-): 0

Region: chr11 94276621-94276638. Max. coverage (+): 0. Max coverage (-): 0

Region: chr11 94276639-94276657. Max. coverage (+): 0. Max coverage (-): 0

Region: chr11 94276658-94276676. Max. coverage (+): 0. Max coverage (-): 0

Region: chr11 94276677-94276695. Max. coverage (+): 0. Max coverage (-): 0

Region: chr11 94276696-94276714. Max. coverage (+): 0. Max coverage (-): 0

Region: chr11 94276715-94276733. Max. coverage (+): 0. Max coverage (-): 0

Region: chr11 94276734-94276752. Max. coverage (+): 0. Max coverage (-): 0

Region: chr11 94276753-94276771. Max. coverage (+): 0. Max coverage (-): 0

Region: chr11 94276772-94276790. Max. coverage (+): 0. Max coverage (-): 0

Region: chr11 94276791-94276808. Max. coverage (+): 0. Max coverage (-): 0

Region: chr11 94276809-94276827. Max. coverage (+): 0. Max coverage (-): 0

Region: chr11 94276828-94276846. Max. coverage (+): 0. Max coverage (-): 0

Region: chr11 94276847-94276865. Max. coverage (+): 0. Max coverage (-): 4.54

Region: chr11 94276866-94276884. Max. coverage (+): 0. Max coverage (-): 4.54

Region: chr11 94276885-. Max. coverage (+): 0. Max coverage (-): 0

RepeatMasker Color Code

**+**

100-98% Identity

<98-95% Identity

<95-90% Identity

<90-85% Identity

<85-80% Identity

<80-75% Identity

<75-70% Identity

<70% Identity

**-**

Gene Set Color Code

**+**

Gene

Pseudogene

**-**

Topology/Coverage Color Code

Coverage Plus Strand

Coverage Minus Strand

Mainstrand: Plus

Mainstrand: Minus

Complementary Strand

Flanking Region  
(if option -flank >0)

Gene Set Annotation  
  
RepeatMasker Annotation  

**1. L2**: 94270225-94270308 (+), Divergence to consensus: 35.4%  
**2. MIRb**: 94273131-94273272 (+), Divergence to consensus: 40.9%  
**3. MIRc**: 94273331-94273418 (+), Divergence to consensus: 45.5%  
**4. MIRc**: 94274315-94274386 (+), Divergence to consensus: 40.8%  
**5. MIRb**: 94274385-94274449 (+), Divergence to consensus: 36.9%  
**6. BOV-A2**: 94275323-94275594 (-), Divergence to consensus: 5.2%  
**7. (TA)n**: 94275596-94275633 (+), Divergence to consensus: 10.5%  
**8. (T)n**: 94275634-94275655 (+), Divergence to consensus: 0%  
**9. MIR**: 94276346-94276426 (-), Divergence to consensus: 23.5%

  
Transcription Factor Binding Sites  

**Gata4** (Sequence: AGATAAC (-): 94267783)  
**Gata4** (Sequence: AGATAAG (-): 94272992)
