# Supplementary material for: Small RNA sequencing of cryopreserved semen from single bull revealed altered miRNAs and piRNAs expression between High- and Low-motile sperm populations
Source: BMC Genomics. 2017 Jan 4;18:14. doi: 10.1186/s12864-016-3394-7 (PMC5209821; doi:10.1186/s12864-016-3394-7)
Supplement: Additional file 3: — Details for each piRNA clusters found in High Motile (HM) sperm fraction. Genes, repeats, transposable elements and transcription factors binding sites falling within the cluster regions were reported. (ZIP 1896 kb) [file 12864_2016_3394_MOESM3_ESM.zip › 12.html]

piRNA cluster 12


Predicted piRNA cluster no. 12     previous   next
  

Show proTRAC run info
Hide proTRAC run info

================================= proTRAC ====================================  
VERSION: 2.1                                    LAST MODIFIED: 06. October 2015  
  
Please cite:  
Rosenkranz D, Zischler H. proTRAC - a software for probabilistic piRNA cluster  
detection, visualization and analysis. 2012. BMC Bioinformatics 13:5.  
  
and (for proTRAC 2.0 and later):  
Rosenkranz D, Rudloff S, Bastuck K, Ketting RF, Zischler H. Tupaia small RNAs  
provide insights into function and evolution of RNAi-based transposon defense  
in mammals. 2015. RNA 21(5):911-922.  
  
Contact:  
David Rosenkranz  
Institute of Anthropology, small RNA group  
Johannes Gutenberg University Mainz  
email: rosenkranz@uni-mainz.de  
  
You can find the latest proTRAC version at:  
http://sourceforge.net/projects/protrac/files  
http://www.smallRNAgroup-mainz.de/software  
==============================================================================  
  
PARAMETERS:  
Map file: .............../storage/core/barbara/genhome/smallRNA/fertility/Sample\_motile/pirna/Sample\_motile\_26-33\_collapsed.fa.no-dust.map.weighted-10000-1000-b-0  
Genome file: ............/storage/core/barbara/genhome/smallRNA/fertility/Sample\_all/pirna/bt\_311\_chrY.fa  
RepeatMasker annotation: /storage/genomes/bt\_umd31/GCF\_000003055.6\_Bos\_taurus\_UMD\_3.1.1\_repeatMasker\_chr.out  
GeneSet:................./storage/core/barbara/genhome/smallRNA/fertility/Sample\_all/pirna/full.gtf  
  
Significant (p<=0.01) hit density will be calculated based  
on observed hit distribution.  
  
Sliding window size: ........................................ 5000 bp  
Sliding window increament: .................................. 1000 bp  
Normalize each hit by number of genomic hits: ............... 1 [0=no/1=yes]  
Normalize each hit by number of sequence reads: ............. 1 [0=no/1=yes]  
Normalize values (-> per million mapped reads): ............. 1 [0=no/1=yes]  
Min. fraction of hits with 1T(U) or 10A: .................... 0.75  
Alternatively: Min. fraction of hits with 1T(U) and 10A: .... 0.5  
Min. fraction of hits with typical piRNA length: ............ 0.75  
Typical piRNA length: ....................................... 26-33 nt  
Min. size of a piRNA cluster: ............................... 5000 bp.  
Min. number of hits (absolute): ............................. 0  
Min. number of hits (normalized): ........................... 0  
Min. fraction of hits on the mainstrand: .................... 0.75  
Top fraction of mapped sequences (in terms of read counts): . 1%  
Top fraction accounts for max. n% of sequence reads: ........ 90%  
Min. fraction of hits on each arm of a bidirectional cluster: 0.1  
Output image file for each cluster: ......................... 0 [0=no/1=yes]  
Output html file for each cluster: .......................... 1 [0=no/1=yes]  
Output a summary table: ..................................... 1 [0=no/1=yes]  
Output a FASTA file for each cluster (piRNA sequences): ..... 1 [0=no/1=yes]  
Output a FASTA file comprising cluster sequences: ........... 1 [0=no/1=yes]  
Search DNA motifs in clusters: .............................. 1 [0=no/1=yes]  
Output flanking sequences: +/- .............................. 0 bp  
Output ~.pTi file: .......................................... 1 [0=no/1=yes]  
==============================================================================  
  
  
Genome size (without gaps): ............ 2678902517 bp  
Gaps (N/X/-): .......................... 53837044 bp  
Mapped reads: .......................... 658825247023  
Non-identical sequences: ............... 514171  
Genomic hits: .......................... 764233  
Significant densitiy of mapped reads: .. 12867599.5173724 reads/kb

Show proTRAC cluster info
Hide proTRAC cluster info

|  |  |
| --- | --- |
| Location | chr11 |
| Coordinates | 97605349-97610629 |
| Size [bp] | 5281 |
| Sequence hit loci | 367 |
| Mapped reads (normalized) | 403560166 |
| Mapped reads (normalized) per kb | 76417376.6 |
| Normalized reads with 1T (1U) | 78.4% |
| Normalized reads with 10A | 25.5% |
| Normalized reads with length 26-33 nt | 100% |
| Normalized reads on the main strand(s) | 100% |
| Predicted directionality | mono:plus |

100%

0%

1T (1U)  
reads

10A reads

26-33 nt  
reads

reads on mainstrand

**Either the amount of reads with 1T (1U) OR 10A has to exceed 75% (set with option: -1Tor10A)  
Alternatively the amount of reads with 1T (1U) AND 10A has to exceed 50% (set with option: -1Tand10A)  
Minimum amount of reads with preferred size is 75% (set with option: -pisize)  
Minimum amount of reads on the main strand(s) is 75% (set with option: -clstrand)**

Show read coverage
Hide read coverage

WHAT DO I SEE HERE?  
This chart shows the location of mapped sequence reads within a predicted piRNA cluster. The color refers to the number of genomic hits produced by the sequence read in question. A dark red bar indicates that this sequence read produces many other hits elsewhere in the genome. Many adjacent red or yellow bars can indicate the presence of a multi-copy element such as transposons or rRNA genes. A dark green bar indicates that this sequence read maps uniquely to this locus.

1 hit

2-5 hits

6-10 hits

11-20 hits

21-50 hits

51-100 hits

> 100 hits

chr11

97605349

97610629

Gene Set

RepeatMasker

Mapped  
Reads

29.67

plus strand

minus strand

29.67

Region: chr11 94276886-97605354. Max. coverage (+): 9.29. Max coverage (-): 0

Region: chr11 97605355-97605364. Max. coverage (+): 9.29. Max coverage (-): 0

Region: chr11 97605365-97605375. Max. coverage (+): 0. Max coverage (-): 0

Region: chr11 97605376-97605385. Max. coverage (+): 0. Max coverage (-): 0

Region: chr11 97605386-97605396. Max. coverage (+): 11.96. Max coverage (-): 0

Region: chr11 97605397-97605407. Max. coverage (+): 7.83. Max coverage (-): 0

Region: chr11 97605408-97605417. Max. coverage (+): 0. Max coverage (-): 0

Region: chr11 97605418-97605428. Max. coverage (+): 1.63. Max coverage (-): 0

Region: chr11 97605429-97605438. Max. coverage (+): 0. Max coverage (-): 0

Region: chr11 97605439-97605449. Max. coverage (+): 0. Max coverage (-): 0

Region: chr11 97605450-97605459. Max. coverage (+): 0. Max coverage (-): 0

Region: chr11 97605460-97605470. Max. coverage (+): 3.27. Max coverage (-): 0

Region: chr11 97605471-97605481. Max. coverage (+): 3.27. Max coverage (-): 0

Region: chr11 97605482-97605491. Max. coverage (+): 0. Max coverage (-): 0

Region: chr11 97605492-97605502. Max. coverage (+): 7.04. Max coverage (-): 0

Region: chr11 97605503-97605512. Max. coverage (+): 7.04. Max coverage (-): 0

Region: chr11 97605513-97605523. Max. coverage (+): 3.38. Max coverage (-): 0

Region: chr11 97605524-97605533. Max. coverage (+): 3.38. Max coverage (-): 0

Region: chr11 97605534-97605544. Max. coverage (+): 4.09. Max coverage (-): 0

Region: chr11 97605545-97605554. Max. coverage (+): 0.7. Max coverage (-): 0

Region: chr11 97605555-97605565. Max. coverage (+): 4.72. Max coverage (-): 0

Region: chr11 97605566-97605576. Max. coverage (+): 4.72. Max coverage (-): 0

Region: chr11 97605577-97605586. Max. coverage (+): 10.14. Max coverage (-): 0

Region: chr11 97605587-97605597. Max. coverage (+): 2.4. Max coverage (-): 0

Region: chr11 97605598-97605607. Max. coverage (+): 2.4. Max coverage (-): 0

Region: chr11 97605608-97605618. Max. coverage (+): 9.83. Max coverage (-): 0

Region: chr11 97605619-97605628. Max. coverage (+): 0. Max coverage (-): 0

Region: chr11 97605629-97605639. Max. coverage (+): 0. Max coverage (-): 0

Region: chr11 97605640-97605650. Max. coverage (+): 0. Max coverage (-): 0

Region: chr11 97605651-97605660. Max. coverage (+): 3.79. Max coverage (-): 0

Region: chr11 97605661-97605671. Max. coverage (+): 4.11. Max coverage (-): 0

Region: chr11 97605672-97605681. Max. coverage (+): 4.11. Max coverage (-): 0

Region: chr11 97605682-97605692. Max. coverage (+): 0. Max coverage (-): 0

Region: chr11 97605693-97605702. Max. coverage (+): 0. Max coverage (-): 0

Region: chr11 97605703-97605713. Max. coverage (+): 0. Max coverage (-): 0

Region: chr11 97605714-97605723. Max. coverage (+): 2.51. Max coverage (-): 0

Region: chr11 97605724-97605734. Max. coverage (+): 2.51. Max coverage (-): 0

Region: chr11 97605735-97605745. Max. coverage (+): 3.53. Max coverage (-): 0

Region: chr11 97605746-97605755. Max. coverage (+): 3.67. Max coverage (-): 0

Region: chr11 97605756-97605766. Max. coverage (+): 2.4. Max coverage (-): 0

Region: chr11 97605767-97605776. Max. coverage (+): 0. Max coverage (-): 0

Region: chr11 97605777-97605787. Max. coverage (+): 0. Max coverage (-): 0

Region: chr11 97605788-97605797. Max. coverage (+): 0. Max coverage (-): 0

Region: chr11 97605798-97605808. Max. coverage (+): 4.6. Max coverage (-): 0

Region: chr11 97605809-97605819. Max. coverage (+): 27.06. Max coverage (-): 0

Region: chr11 97605820-97605829. Max. coverage (+): 1.21. Max coverage (-): 0

Region: chr11 97605830-97605840. Max. coverage (+): 1.21. Max coverage (-): 0

Region: chr11 97605841-97605850. Max. coverage (+): 0. Max coverage (-): 0

Region: chr11 97605851-97605861. Max. coverage (+): 0. Max coverage (-): 0

Region: chr11 97605862-97605871. Max. coverage (+): 0. Max coverage (-): 0

Region: chr11 97605872-97605882. Max. coverage (+): 1.24. Max coverage (-): 0

Region: chr11 97605883-97605892. Max. coverage (+): 0.6. Max coverage (-): 0

Region: chr11 97605893-97605903. Max. coverage (+): 0.6. Max coverage (-): 0

Region: chr11 97605904-97605914. Max. coverage (+): 0. Max coverage (-): 0

Region: chr11 97605915-97605924. Max. coverage (+): 0. Max coverage (-): 0

Region: chr11 97605925-97605935. Max. coverage (+): 5.03. Max coverage (-): 0

Region: chr11 97605936-97605945. Max. coverage (+): 0. Max coverage (-): 0

Region: chr11 97605946-97605956. Max. coverage (+): 0. Max coverage (-): 0

Region: chr11 97605957-97605966. Max. coverage (+): 4.98. Max coverage (-): 0

Region: chr11 97605967-97605977. Max. coverage (+): 4.98. Max coverage (-): 0

Region: chr11 97605978-97605988. Max. coverage (+): 0. Max coverage (-): 0

Region: chr11 97605989-97605998. Max. coverage (+): 0. Max coverage (-): 0

Region: chr11 97605999-97606009. Max. coverage (+): 0. Max coverage (-): 0

Region: chr11 97606010-97606019. Max. coverage (+): 0. Max coverage (-): 0

Region: chr11 97606020-97606030. Max. coverage (+): 0. Max coverage (-): 0

Region: chr11 97606031-97606040. Max. coverage (+): 0. Max coverage (-): 0

Region: chr11 97606041-97606051. Max. coverage (+): 0. Max coverage (-): 0

Region: chr11 97606052-97606061. Max. coverage (+): 0. Max coverage (-): 0

Region: chr11 97606062-97606072. Max. coverage (+): 0. Max coverage (-): 0

Region: chr11 97606073-97606083. Max. coverage (+): 0. Max coverage (-): 0

Region: chr11 97606084-97606093. Max. coverage (+): 3.88. Max coverage (-): 0

Region: chr11 97606094-97606104. Max. coverage (+): 1.61. Max coverage (-): 0

Region: chr11 97606105-97606114. Max. coverage (+): 0. Max coverage (-): 0

Region: chr11 97606115-97606125. Max. coverage (+): 0. Max coverage (-): 0

Region: chr11 97606126-97606135. Max. coverage (+): 0. Max coverage (-): 0

Region: chr11 97606136-97606146. Max. coverage (+): 0. Max coverage (-): 0

Region: chr11 97606147-97606156. Max. coverage (+): 0. Max coverage (-): 0

Region: chr11 97606157-97606167. Max. coverage (+): 0. Max coverage (-): 0

Region: chr11 97606168-97606178. Max. coverage (+): 0. Max coverage (-): 0

Region: chr11 97606179-97606188. Max. coverage (+): 9.13. Max coverage (-): 0

Region: chr11 97606189-97606199. Max. coverage (+): 9.13. Max coverage (-): 0

Region: chr11 97606200-97606209. Max. coverage (+): 0. Max coverage (-): 0

Region: chr11 97606210-97606220. Max. coverage (+): 0. Max coverage (-): 0

Region: chr11 97606221-97606230. Max. coverage (+): 1.32. Max coverage (-): 0

Region: chr11 97606231-97606241. Max. coverage (+): 1.32. Max coverage (-): 0

Region: chr11 97606242-97606252. Max. coverage (+): 0. Max coverage (-): 0

Region: chr11 97606253-97606262. Max. coverage (+): 1.69. Max coverage (-): 0

Region: chr11 97606263-97606273. Max. coverage (+): 0. Max coverage (-): 0

Region: chr11 97606274-97606283. Max. coverage (+): 4.72. Max coverage (-): 0

Region: chr11 97606284-97606294. Max. coverage (+): 4.72. Max coverage (-): 0

Region: chr11 97606295-97606304. Max. coverage (+): 0. Max coverage (-): 0

Region: chr11 97606305-97606315. Max. coverage (+): 0. Max coverage (-): 0

Region: chr11 97606316-97606325. Max. coverage (+): 0. Max coverage (-): 0

Region: chr11 97606326-97606336. Max. coverage (+): 0. Max coverage (-): 0

Region: chr11 97606337-97606347. Max. coverage (+): 0. Max coverage (-): 0

Region: chr11 97606348-97606357. Max. coverage (+): 0. Max coverage (-): 0

Region: chr11 97606358-97606368. Max. coverage (+): 0. Max coverage (-): 0

Region: chr11 97606369-97606378. Max. coverage (+): 0. Max coverage (-): 0

Region: chr11 97606379-97606389. Max. coverage (+): 0. Max coverage (-): 0

Region: chr11 97606390-97606399. Max. coverage (+): 0. Max coverage (-): 0

Region: chr11 97606400-97606410. Max. coverage (+): 1.77. Max coverage (-): 0

Region: chr11 97606411-97606421. Max. coverage (+): 4.08. Max coverage (-): 0

Region: chr11 97606422-97606431. Max. coverage (+): 0. Max coverage (-): 0

Region: chr11 97606432-97606442. Max. coverage (+): 0. Max coverage (-): 0

Region: chr11 97606443-97606452. Max. coverage (+): 3.46. Max coverage (-): 0

Region: chr11 97606453-97606463. Max. coverage (+): 3.46. Max coverage (-): 0

Region: chr11 97606464-97606473. Max. coverage (+): 0. Max coverage (-): 0

Region: chr11 97606474-97606484. Max. coverage (+): 0. Max coverage (-): 0

Region: chr11 97606485-97606494. Max. coverage (+): 0. Max coverage (-): 0

Region: chr11 97606495-97606505. Max. coverage (+): 0. Max coverage (-): 0

Region: chr11 97606506-97606516. Max. coverage (+): 0. Max coverage (-): 0

Region: chr11 97606517-97606526. Max. coverage (+): 1.61. Max coverage (-): 0

Region: chr11 97606527-97606537. Max. coverage (+): 1.61. Max coverage (-): 0

Region: chr11 97606538-97606547. Max. coverage (+): 0. Max coverage (-): 0

Region: chr11 97606548-97606558. Max. coverage (+): 0. Max coverage (-): 0

Region: chr11 97606559-97606568. Max. coverage (+): 0. Max coverage (-): 0

Region: chr11 97606569-97606579. Max. coverage (+): 0. Max coverage (-): 0

Region: chr11 97606580-97606590. Max. coverage (+): 2.32. Max coverage (-): 0

Region: chr11 97606591-97606600. Max. coverage (+): 2.32. Max coverage (-): 0

Region: chr11 97606601-97606611. Max. coverage (+): 0. Max coverage (-): 0

Region: chr11 97606612-97606621. Max. coverage (+): 0. Max coverage (-): 0

Region: chr11 97606622-97606632. Max. coverage (+): 0. Max coverage (-): 0

Region: chr11 97606633-97606642. Max. coverage (+): 0. Max coverage (-): 0

Region: chr11 97606643-97606653. Max. coverage (+): 1.86. Max coverage (-): 0

Region: chr11 97606654-97606663. Max. coverage (+): 3.68. Max coverage (-): 0

Region: chr11 97606664-97606674. Max. coverage (+): 3.68. Max coverage (-): 0

Region: chr11 97606675-97606685. Max. coverage (+): 0. Max coverage (-): 0

Region: chr11 97606686-97606695. Max. coverage (+): 0.88. Max coverage (-): 0

Region: chr11 97606696-97606706. Max. coverage (+): 0.88. Max coverage (-): 0

Region: chr11 97606707-97606716. Max. coverage (+): 0. Max coverage (-): 0

Region: chr11 97606717-97606727. Max. coverage (+): 2.69. Max coverage (-): 0

Region: chr11 97606728-97606737. Max. coverage (+): 8.98. Max coverage (-): 0

Region: chr11 97606738-97606748. Max. coverage (+): 9.35. Max coverage (-): 0

Region: chr11 97606749-97606759. Max. coverage (+): 4.48. Max coverage (-): 0

Region: chr11 97606760-97606769. Max. coverage (+): 2.69. Max coverage (-): 0

Region: chr11 97606770-97606780. Max. coverage (+): 2.69. Max coverage (-): 0

Region: chr11 97606781-97606790. Max. coverage (+): 3.84. Max coverage (-): 0

Region: chr11 97606791-97606801. Max. coverage (+): 10.29. Max coverage (-): 0

Region: chr11 97606802-97606811. Max. coverage (+): 3.93. Max coverage (-): 0

Region: chr11 97606812-97606822. Max. coverage (+): 2.69. Max coverage (-): 0

Region: chr11 97606823-97606832. Max. coverage (+): 2.69. Max coverage (-): 0

Region: chr11 97606833-97606843. Max. coverage (+): 12.57. Max coverage (-): 0

Region: chr11 97606844-97606854. Max. coverage (+): 12.57. Max coverage (-): 0

Region: chr11 97606855-97606864. Max. coverage (+): 0. Max coverage (-): 0

Region: chr11 97606865-97606875. Max. coverage (+): 0. Max coverage (-): 0

Region: chr11 97606876-97606885. Max. coverage (+): 3.58. Max coverage (-): 0

Region: chr11 97606886-97606896. Max. coverage (+): 3.58. Max coverage (-): 0

Region: chr11 97606897-97606906. Max. coverage (+): 0. Max coverage (-): 0

Region: chr11 97606907-97606917. Max. coverage (+): 0. Max coverage (-): 0

Region: chr11 97606918-97606928. Max. coverage (+): 0. Max coverage (-): 0

Region: chr11 97606929-97606938. Max. coverage (+): 1.99. Max coverage (-): 0

Region: chr11 97606939-97606949. Max. coverage (+): 1.99. Max coverage (-): 0

Region: chr11 97606950-97606959. Max. coverage (+): 0. Max coverage (-): 0

Region: chr11 97606960-97606970. Max. coverage (+): 0. Max coverage (-): 0

Region: chr11 97606971-97606980. Max. coverage (+): 12.81. Max coverage (-): 0

Region: chr11 97606981-97606991. Max. coverage (+): 12.81. Max coverage (-): 0

Region: chr11 97606992-97607001. Max. coverage (+): 0. Max coverage (-): 0

Region: chr11 97607002-97607012. Max. coverage (+): 21.61. Max coverage (-): 0

Region: chr11 97607013-97607023. Max. coverage (+): 21.61. Max coverage (-): 0

Region: chr11 97607024-97607033. Max. coverage (+): 0. Max coverage (-): 0

Region: chr11 97607034-97607044. Max. coverage (+): 0. Max coverage (-): 0

Region: chr11 97607045-97607054. Max. coverage (+): 0. Max coverage (-): 0

Region: chr11 97607055-97607065. Max. coverage (+): 0. Max coverage (-): 0

Region: chr11 97607066-97607075. Max. coverage (+): 0. Max coverage (-): 0

Region: chr11 97607076-97607086. Max. coverage (+): 0. Max coverage (-): 0

Region: chr11 97607087-97607097. Max. coverage (+): 0. Max coverage (-): 0

Region: chr11 97607098-97607107. Max. coverage (+): 0.49. Max coverage (-): 0

Region: chr11 97607108-97607118. Max. coverage (+): 0. Max coverage (-): 0

Region: chr11 97607119-97607128. Max. coverage (+): 0. Max coverage (-): 0

Region: chr11 97607129-97607139. Max. coverage (+): 11.11. Max coverage (-): 0

Region: chr11 97607140-97607149. Max. coverage (+): 3.89. Max coverage (-): 0

Region: chr11 97607150-97607160. Max. coverage (+): 0. Max coverage (-): 0

Region: chr11 97607161-97607170. Max. coverage (+): 0. Max coverage (-): 0

Region: chr11 97607171-97607181. Max. coverage (+): 0. Max coverage (-): 0

Region: chr11 97607182-97607192. Max. coverage (+): 0. Max coverage (-): 0

Region: chr11 97607193-97607202. Max. coverage (+): 0. Max coverage (-): 0

Region: chr11 97607203-97607213. Max. coverage (+): 0. Max coverage (-): 0

Region: chr11 97607214-97607223. Max. coverage (+): 4.93. Max coverage (-): 0

Region: chr11 97607224-97607234. Max. coverage (+): 4.93. Max coverage (-): 0

Region: chr11 97607235-97607244. Max. coverage (+): 0. Max coverage (-): 0

Region: chr11 97607245-97607255. Max. coverage (+): 0. Max coverage (-): 0

Region: chr11 97607256-97607266. Max. coverage (+): 0. Max coverage (-): 0

Region: chr11 97607267-97607276. Max. coverage (+): 0. Max coverage (-): 0

Region: chr11 97607277-97607287. Max. coverage (+): 0. Max coverage (-): 0

Region: chr11 97607288-97607297. Max. coverage (+): 0. Max coverage (-): 0

Region: chr11 97607298-97607308. Max. coverage (+): 0. Max coverage (-): 0

Region: chr11 97607309-97607318. Max. coverage (+): 0. Max coverage (-): 0

Region: chr11 97607319-97607329. Max. coverage (+): 11.77. Max coverage (-): 0

Region: chr11 97607330-97607339. Max. coverage (+): 8.21. Max coverage (-): 0

Region: chr11 97607340-97607350. Max. coverage (+): 5.25. Max coverage (-): 0

Region: chr11 97607351-97607361. Max. coverage (+): 4.98. Max coverage (-): 0

Region: chr11 97607362-97607371. Max. coverage (+): 4.98. Max coverage (-): 0

Region: chr11 97607372-97607382. Max. coverage (+): 0. Max coverage (-): 0

Region: chr11 97607383-97607392. Max. coverage (+): 0. Max coverage (-): 0

Region: chr11 97607393-97607403. Max. coverage (+): 0. Max coverage (-): 0

Region: chr11 97607404-97607413. Max. coverage (+): 0. Max coverage (-): 0

Region: chr11 97607414-97607424. Max. coverage (+): 0. Max coverage (-): 0

Region: chr11 97607425-97607434. Max. coverage (+): 0. Max coverage (-): 0

Region: chr11 97607435-97607445. Max. coverage (+): 0. Max coverage (-): 0

Region: chr11 97607446-97607456. Max. coverage (+): 0. Max coverage (-): 0

Region: chr11 97607457-97607466. Max. coverage (+): 8.49. Max coverage (-): 0

Region: chr11 97607467-97607477. Max. coverage (+): 13.28. Max coverage (-): 0

Region: chr11 97607478-97607487. Max. coverage (+): 7.53. Max coverage (-): 0

Region: chr11 97607488-97607498. Max. coverage (+): 3.51. Max coverage (-): 0

Region: chr11 97607499-97607508. Max. coverage (+): 0. Max coverage (-): 0

Region: chr11 97607509-97607519. Max. coverage (+): 1.07. Max coverage (-): 0

Region: chr11 97607520-97607530. Max. coverage (+): 0. Max coverage (-): 0

Region: chr11 97607531-97607540. Max. coverage (+): 0. Max coverage (-): 0

Region: chr11 97607541-97607551. Max. coverage (+): 0. Max coverage (-): 0

Region: chr11 97607552-97607561. Max. coverage (+): 11.73. Max coverage (-): 0

Region: chr11 97607562-97607572. Max. coverage (+): 20.24. Max coverage (-): 0

Region: chr11 97607573-97607582. Max. coverage (+): 0. Max coverage (-): 0

Region: chr11 97607583-97607593. Max. coverage (+): 0. Max coverage (-): 0

Region: chr11 97607594-97607603. Max. coverage (+): 0. Max coverage (-): 0

Region: chr11 97607604-97607614. Max. coverage (+): 0. Max coverage (-): 0

Region: chr11 97607615-97607625. Max. coverage (+): 7.33. Max coverage (-): 0

Region: chr11 97607626-97607635. Max. coverage (+): 0. Max coverage (-): 0

Region: chr11 97607636-97607646. Max. coverage (+): 0. Max coverage (-): 0

Region: chr11 97607647-97607656. Max. coverage (+): 0. Max coverage (-): 0

Region: chr11 97607657-97607667. Max. coverage (+): 0. Max coverage (-): 0

Region: chr11 97607668-97607677. Max. coverage (+): 5.92. Max coverage (-): 0

Region: chr11 97607678-97607688. Max. coverage (+): 2.41. Max coverage (-): 0

Region: chr11 97607689-97607699. Max. coverage (+): 2.91. Max coverage (-): 0

Region: chr11 97607700-97607709. Max. coverage (+): 2.15. Max coverage (-): 0

Region: chr11 97607710-97607720. Max. coverage (+): 0. Max coverage (-): 0

Region: chr11 97607721-97607730. Max. coverage (+): 0.14. Max coverage (-): 0

Region: chr11 97607731-97607741. Max. coverage (+): 2.45. Max coverage (-): 0

Region: chr11 97607742-97607751. Max. coverage (+): 2.45. Max coverage (-): 0

Region: chr11 97607752-97607762. Max. coverage (+): 0. Max coverage (-): 0

Region: chr11 97607763-97607772. Max. coverage (+): 0. Max coverage (-): 0

Region: chr11 97607773-97607783. Max. coverage (+): 0. Max coverage (-): 0

Region: chr11 97607784-97607794. Max. coverage (+): 0. Max coverage (-): 0

Region: chr11 97607795-97607804. Max. coverage (+): 1.64. Max coverage (-): 0

Region: chr11 97607805-97607815. Max. coverage (+): 10.24. Max coverage (-): 0

Region: chr11 97607816-97607825. Max. coverage (+): 1.96. Max coverage (-): 0

Region: chr11 97607826-97607836. Max. coverage (+): 1.96. Max coverage (-): 0

Region: chr11 97607837-97607846. Max. coverage (+): 0. Max coverage (-): 0

Region: chr11 97607847-97607857. Max. coverage (+): 6.08. Max coverage (-): 0

Region: chr11 97607858-97607868. Max. coverage (+): 29.67. Max coverage (-): 0

Region: chr11 97607869-97607878. Max. coverage (+): 21.79. Max coverage (-): 0

Region: chr11 97607879-97607889. Max. coverage (+): 6.06. Max coverage (-): 0

Region: chr11 97607890-97607899. Max. coverage (+): 6.06. Max coverage (-): 0

Region: chr11 97607900-97607910. Max. coverage (+): 0. Max coverage (-): 0

Region: chr11 97607911-97607920. Max. coverage (+): 0. Max coverage (-): 0

Region: chr11 97607921-97607931. Max. coverage (+): 0. Max coverage (-): 0

Region: chr11 97607932-97607941. Max. coverage (+): 0. Max coverage (-): 0

Region: chr11 97607942-97607952. Max. coverage (+): 0.21. Max coverage (-): 0

Region: chr11 97607953-97607963. Max. coverage (+): 2.72. Max coverage (-): 0

Region: chr11 97607964-97607973. Max. coverage (+): 2.51. Max coverage (-): 0

Region: chr11 97607974-97607984. Max. coverage (+): 0. Max coverage (-): 0

Region: chr11 97607985-97607994. Max. coverage (+): 0. Max coverage (-): 0

Region: chr11 97607995-97608005. Max. coverage (+): 0. Max coverage (-): 0

Region: chr11 97608006-97608015. Max. coverage (+): 2.95. Max coverage (-): 0

Region: chr11 97608016-97608026. Max. coverage (+): 2.95. Max coverage (-): 0

Region: chr11 97608027-97608037. Max. coverage (+): 0. Max coverage (-): 0

Region: chr11 97608038-97608047. Max. coverage (+): 0. Max coverage (-): 0

Region: chr11 97608048-97608058. Max. coverage (+): 0. Max coverage (-): 0

Region: chr11 97608059-97608068. Max. coverage (+): 1.04. Max coverage (-): 0

Region: chr11 97608069-97608079. Max. coverage (+): 0. Max coverage (-): 0

Region: chr11 97608080-97608089. Max. coverage (+): 0.84. Max coverage (-): 0

Region: chr11 97608090-97608100. Max. coverage (+): 0. Max coverage (-): 0

Region: chr11 97608101-97608110. Max. coverage (+): 2.24. Max coverage (-): 0

Region: chr11 97608111-97608121. Max. coverage (+): 0. Max coverage (-): 0

Region: chr11 97608122-97608132. Max. coverage (+): 0. Max coverage (-): 0

Region: chr11 97608133-97608142. Max. coverage (+): 0. Max coverage (-): 0

Region: chr11 97608143-97608153. Max. coverage (+): 0. Max coverage (-): 0

Region: chr11 97608154-97608163. Max. coverage (+): 3.66. Max coverage (-): 0

Region: chr11 97608164-97608174. Max. coverage (+): 2.96. Max coverage (-): 0

Region: chr11 97608175-97608184. Max. coverage (+): 1.26. Max coverage (-): 0

Region: chr11 97608185-97608195. Max. coverage (+): 1.07. Max coverage (-): 0

Region: chr11 97608196-97608206. Max. coverage (+): 0. Max coverage (-): 0

Region: chr11 97608207-97608216. Max. coverage (+): 1.38. Max coverage (-): 0

Region: chr11 97608217-97608227. Max. coverage (+): 1.87. Max coverage (-): 0

Region: chr11 97608228-97608237. Max. coverage (+): 11.8. Max coverage (-): 0

Region: chr11 97608238-97608248. Max. coverage (+): 19.34. Max coverage (-): 0

Region: chr11 97608249-97608258. Max. coverage (+): 0. Max coverage (-): 0

Region: chr11 97608259-97608269. Max. coverage (+): 0. Max coverage (-): 0

Region: chr11 97608270-97608279. Max. coverage (+): 0. Max coverage (-): 0

Region: chr11 97608280-97608290. Max. coverage (+): 0. Max coverage (-): 0

Region: chr11 97608291-97608301. Max. coverage (+): 0. Max coverage (-): 0

Region: chr11 97608302-97608311. Max. coverage (+): 0. Max coverage (-): 0

Region: chr11 97608312-97608322. Max. coverage (+): 0. Max coverage (-): 0

Region: chr11 97608323-97608332. Max. coverage (+): 0. Max coverage (-): 0

Region: chr11 97608333-97608343. Max. coverage (+): 0. Max coverage (-): 0

Region: chr11 97608344-97608353. Max. coverage (+): 0. Max coverage (-): 0

Region: chr11 97608354-97608364. Max. coverage (+): 0. Max coverage (-): 0

Region: chr11 97608365-97608375. Max. coverage (+): 0. Max coverage (-): 0

Region: chr11 97608376-97608385. Max. coverage (+): 0. Max coverage (-): 0

Region: chr11 97608386-97608396. Max. coverage (+): 1.5. Max coverage (-): 0

Region: chr11 97608397-97608406. Max. coverage (+): 0.35. Max coverage (-): 0

Region: chr11 97608407-97608417. Max. coverage (+): 3.93. Max coverage (-): 0

Region: chr11 97608418-97608427. Max. coverage (+): 12.65. Max coverage (-): 0

Region: chr11 97608428-97608438. Max. coverage (+): 0. Max coverage (-): 0

Region: chr11 97608439-97608448. Max. coverage (+): 0. Max coverage (-): 0

Region: chr11 97608449-97608459. Max. coverage (+): 9.36. Max coverage (-): 0

Region: chr11 97608460-97608470. Max. coverage (+): 9.36. Max coverage (-): 0

Region: chr11 97608471-97608480. Max. coverage (+): 0. Max coverage (-): 0

Region: chr11 97608481-97608491. Max. coverage (+): 0. Max coverage (-): 0

Region: chr11 97608492-97608501. Max. coverage (+): 0. Max coverage (-): 0

Region: chr11 97608502-97608512. Max. coverage (+): 0. Max coverage (-): 0

Region: chr11 97608513-97608522. Max. coverage (+): 0. Max coverage (-): 0

Region: chr11 97608523-97608533. Max. coverage (+): 0. Max coverage (-): 0

Region: chr11 97608534-97608544. Max. coverage (+): 0. Max coverage (-): 0

Region: chr11 97608545-97608554. Max. coverage (+): 0. Max coverage (-): 0

Region: chr11 97608555-97608565. Max. coverage (+): 0.9. Max coverage (-): 0

Region: chr11 97608566-97608575. Max. coverage (+): 0. Max coverage (-): 0

Region: chr11 97608576-97608586. Max. coverage (+): 0. Max coverage (-): 0

Region: chr11 97608587-97608596. Max. coverage (+): 0. Max coverage (-): 0

Region: chr11 97608597-97608607. Max. coverage (+): 0. Max coverage (-): 0

Region: chr11 97608608-97608617. Max. coverage (+): 0. Max coverage (-): 0

Region: chr11 97608618-97608628. Max. coverage (+): 0. Max coverage (-): 0

Region: chr11 97608629-97608639. Max. coverage (+): 0. Max coverage (-): 0

Region: chr11 97608640-97608649. Max. coverage (+): 0. Max coverage (-): 0

Region: chr11 97608650-97608660. Max. coverage (+): 0. Max coverage (-): 0

Region: chr11 97608661-97608670. Max. coverage (+): 0. Max coverage (-): 0

Region: chr11 97608671-97608681. Max. coverage (+): 0. Max coverage (-): 0

Region: chr11 97608682-97608691. Max. coverage (+): 1.83. Max coverage (-): 0

Region: chr11 97608692-97608702. Max. coverage (+): 1.83. Max coverage (-): 0

Region: chr11 97608703-97608712. Max. coverage (+): 4.05. Max coverage (-): 0

Region: chr11 97608713-97608723. Max. coverage (+): 4.05. Max coverage (-): 0

Region: chr11 97608724-97608734. Max. coverage (+): 5.95. Max coverage (-): 0

Region: chr11 97608735-97608744. Max. coverage (+): 10.01. Max coverage (-): 0

Region: chr11 97608745-97608755. Max. coverage (+): 4.65. Max coverage (-): 0

Region: chr11 97608756-97608765. Max. coverage (+): 0. Max coverage (-): 0

Region: chr11 97608766-97608776. Max. coverage (+): 0. Max coverage (-): 0

Region: chr11 97608777-97608786. Max. coverage (+): 0. Max coverage (-): 0

Region: chr11 97608787-97608797. Max. coverage (+): 0.72. Max coverage (-): 0

Region: chr11 97608798-97608808. Max. coverage (+): 0.72. Max coverage (-): 0

Region: chr11 97608809-97608818. Max. coverage (+): 0. Max coverage (-): 0

Region: chr11 97608819-97608829. Max. coverage (+): 5.51. Max coverage (-): 0

Region: chr11 97608830-97608839. Max. coverage (+): 5.51. Max coverage (-): 0

Region: chr11 97608840-97608850. Max. coverage (+): 0. Max coverage (-): 0

Region: chr11 97608851-97608860. Max. coverage (+): 0. Max coverage (-): 0

Region: chr11 97608861-97608871. Max. coverage (+): 0.61. Max coverage (-): 0

Region: chr11 97608872-97608881. Max. coverage (+): 3.24. Max coverage (-): 0

Region: chr11 97608882-97608892. Max. coverage (+): 5.53. Max coverage (-): 0

Region: chr11 97608893-97608903. Max. coverage (+): 5.53. Max coverage (-): 0

Region: chr11 97608904-97608913. Max. coverage (+): 0. Max coverage (-): 0

Region: chr11 97608914-97608924. Max. coverage (+): 0.8. Max coverage (-): 0

Region: chr11 97608925-97608934. Max. coverage (+): 0.8. Max coverage (-): 0

Region: chr11 97608935-97608945. Max. coverage (+): 0. Max coverage (-): 0

Region: chr11 97608946-97608955. Max. coverage (+): 0. Max coverage (-): 0

Region: chr11 97608956-97608966. Max. coverage (+): 0. Max coverage (-): 0

Region: chr11 97608967-97608977. Max. coverage (+): 1.64. Max coverage (-): 0

Region: chr11 97608978-97608987. Max. coverage (+): 4.15. Max coverage (-): 0

Region: chr11 97608988-97608998. Max. coverage (+): 0. Max coverage (-): 0

Region: chr11 97608999-97609008. Max. coverage (+): 0.65. Max coverage (-): 0

Region: chr11 97609009-97609019. Max. coverage (+): 0.65. Max coverage (-): 0

Region: chr11 97609020-97609029. Max. coverage (+): 7.03. Max coverage (-): 0

Region: chr11 97609030-97609040. Max. coverage (+): 7.03. Max coverage (-): 0

Region: chr11 97609041-97609050. Max. coverage (+): 0.14. Max coverage (-): 0

Region: chr11 97609051-97609061. Max. coverage (+): 0. Max coverage (-): 0

Region: chr11 97609062-97609072. Max. coverage (+): 1.91. Max coverage (-): 0

Region: chr11 97609073-97609082. Max. coverage (+): 6.32. Max coverage (-): 0

Region: chr11 97609083-97609093. Max. coverage (+): 6.32. Max coverage (-): 0

Region: chr11 97609094-97609103. Max. coverage (+): 0. Max coverage (-): 0

Region: chr11 97609104-97609114. Max. coverage (+): 0. Max coverage (-): 0

Region: chr11 97609115-97609124. Max. coverage (+): 0. Max coverage (-): 0

Region: chr11 97609125-97609135. Max. coverage (+): 0. Max coverage (-): 0

Region: chr11 97609136-97609146. Max. coverage (+): 0.42. Max coverage (-): 0

Region: chr11 97609147-97609156. Max. coverage (+): 0.42. Max coverage (-): 0

Region: chr11 97609157-97609167. Max. coverage (+): 0. Max coverage (-): 0

Region: chr11 97609168-97609177. Max. coverage (+): 0. Max coverage (-): 0

Region: chr11 97609178-97609188. Max. coverage (+): 0. Max coverage (-): 0

Region: chr11 97609189-97609198. Max. coverage (+): 0. Max coverage (-): 0

Region: chr11 97609199-97609209. Max. coverage (+): 0. Max coverage (-): 0

Region: chr11 97609210-97609219. Max. coverage (+): 0.46. Max coverage (-): 0

Region: chr11 97609220-97609230. Max. coverage (+): 0.46. Max coverage (-): 0

Region: chr11 97609231-97609241. Max. coverage (+): 4.38. Max coverage (-): 0

Region: chr11 97609242-97609251. Max. coverage (+): 7.58. Max coverage (-): 0

Region: chr11 97609252-97609262. Max. coverage (+): 3.21. Max coverage (-): 0

Region: chr11 97609263-97609272. Max. coverage (+): 0. Max coverage (-): 0

Region: chr11 97609273-97609283. Max. coverage (+): 0. Max coverage (-): 0

Region: chr11 97609284-97609293. Max. coverage (+): 0. Max coverage (-): 0

Region: chr11 97609294-97609304. Max. coverage (+): 0. Max coverage (-): 0

Region: chr11 97609305-97609315. Max. coverage (+): 0.53. Max coverage (-): 0

Region: chr11 97609316-97609325. Max. coverage (+): 0.53. Max coverage (-): 0

Region: chr11 97609326-97609336. Max. coverage (+): 0. Max coverage (-): 0

Region: chr11 97609337-97609346. Max. coverage (+): 0. Max coverage (-): 0

Region: chr11 97609347-97609357. Max. coverage (+): 3.1. Max coverage (-): 0

Region: chr11 97609358-97609367. Max. coverage (+): 0. Max coverage (-): 0

Region: chr11 97609368-97609378. Max. coverage (+): 0. Max coverage (-): 0

Region: chr11 97609379-97609388. Max. coverage (+): 0. Max coverage (-): 0

Region: chr11 97609389-97609399. Max. coverage (+): 0. Max coverage (-): 0

Region: chr11 97609400-97609410. Max. coverage (+): 0. Max coverage (-): 0

Region: chr11 97609411-97609420. Max. coverage (+): 0. Max coverage (-): 0

Region: chr11 97609421-97609431. Max. coverage (+): 0. Max coverage (-): 0

Region: chr11 97609432-97609441. Max. coverage (+): 0. Max coverage (-): 0

Region: chr11 97609442-97609452. Max. coverage (+): 3.87. Max coverage (-): 0

Region: chr11 97609453-97609462. Max. coverage (+): 16.43. Max coverage (-): 0

Region: chr11 97609463-97609473. Max. coverage (+): 0.82. Max coverage (-): 0

Region: chr11 97609474-97609484. Max. coverage (+): 3.53. Max coverage (-): 0

Region: chr11 97609485-97609494. Max. coverage (+): 3.53. Max coverage (-): 0

Region: chr11 97609495-97609505. Max. coverage (+): 0. Max coverage (-): 0

Region: chr11 97609506-97609515. Max. coverage (+): 0. Max coverage (-): 0

Region: chr11 97609516-97609526. Max. coverage (+): 0. Max coverage (-): 0

Region: chr11 97609527-97609536. Max. coverage (+): 0. Max coverage (-): 0

Region: chr11 97609537-97609547. Max. coverage (+): 5.85. Max coverage (-): 0

Region: chr11 97609548-97609557. Max. coverage (+): 1.92. Max coverage (-): 0

Region: chr11 97609558-97609568. Max. coverage (+): 0. Max coverage (-): 0

Region: chr11 97609569-97609579. Max. coverage (+): 0. Max coverage (-): 0

Region: chr11 97609580-97609589. Max. coverage (+): 0. Max coverage (-): 0

Region: chr11 97609590-97609600. Max. coverage (+): 0. Max coverage (-): 0

Region: chr11 97609601-97609610. Max. coverage (+): 3.14. Max coverage (-): 0

Region: chr11 97609611-97609621. Max. coverage (+): 0. Max coverage (-): 0

Region: chr11 97609622-97609631. Max. coverage (+): 0. Max coverage (-): 0

Region: chr11 97609632-97609642. Max. coverage (+): 0. Max coverage (-): 0

Region: chr11 97609643-97609653. Max. coverage (+): 0. Max coverage (-): 0

Region: chr11 97609654-97609663. Max. coverage (+): 0.33. Max coverage (-): 0

Region: chr11 97609664-97609674. Max. coverage (+): 3.26. Max coverage (-): 0

Region: chr11 97609675-97609684. Max. coverage (+): 0. Max coverage (-): 0

Region: chr11 97609685-97609695. Max. coverage (+): 0. Max coverage (-): 0

Region: chr11 97609696-97609705. Max. coverage (+): 0. Max coverage (-): 0

Region: chr11 97609706-97609716. Max. coverage (+): 0. Max coverage (-): 0

Region: chr11 97609717-97609726. Max. coverage (+): 0. Max coverage (-): 0

Region: chr11 97609727-97609737. Max. coverage (+): 3.44. Max coverage (-): 0

Region: chr11 97609738-97609748. Max. coverage (+): 0. Max coverage (-): 0

Region: chr11 97609749-97609758. Max. coverage (+): 0. Max coverage (-): 0

Region: chr11 97609759-97609769. Max. coverage (+): 0. Max coverage (-): 0

Region: chr11 97609770-97609779. Max. coverage (+): 0.73. Max coverage (-): 0

Region: chr11 97609780-97609790. Max. coverage (+): 0.73. Max coverage (-): 0

Region: chr11 97609791-97609800. Max. coverage (+): 0. Max coverage (-): 0

Region: chr11 97609801-97609811. Max. coverage (+): 0. Max coverage (-): 0

Region: chr11 97609812-97609822. Max. coverage (+): 0. Max coverage (-): 0

Region: chr11 97609823-97609832. Max. coverage (+): 0. Max coverage (-): 0

Region: chr11 97609833-97609843. Max. coverage (+): 0.87. Max coverage (-): 0

Region: chr11 97609844-97609853. Max. coverage (+): 0. Max coverage (-): 0

Region: chr11 97609854-97609864. Max. coverage (+): 0. Max coverage (-): 0

Region: chr11 97609865-97609874. Max. coverage (+): 0. Max coverage (-): 0

Region: chr11 97609875-97609885. Max. coverage (+): 0. Max coverage (-): 0

Region: chr11 97609886-97609895. Max. coverage (+): 0. Max coverage (-): 0

Region: chr11 97609896-97609906. Max. coverage (+): 0. Max coverage (-): 0

Region: chr11 97609907-97609917. Max. coverage (+): 0. Max coverage (-): 0

Region: chr11 97609918-97609927. Max. coverage (+): 0. Max coverage (-): 0

Region: chr11 97609928-97609938. Max. coverage (+): 1.21. Max coverage (-): 0

Region: chr11 97609939-97609948. Max. coverage (+): 1.21. Max coverage (-): 0

Region: chr11 97609949-97609959. Max. coverage (+): 0. Max coverage (-): 0

Region: chr11 97609960-97609969. Max. coverage (+): 0. Max coverage (-): 0

Region: chr11 97609970-97609980. Max. coverage (+): 0. Max coverage (-): 0

Region: chr11 97609981-97609990. Max. coverage (+): 0. Max coverage (-): 0

Region: chr11 97609991-97610001. Max. coverage (+): 4.32. Max coverage (-): 0

Region: chr11 97610002-97610012. Max. coverage (+): 4.32. Max coverage (-): 0

Region: chr11 97610013-97610022. Max. coverage (+): 0. Max coverage (-): 0

Region: chr11 97610023-97610033. Max. coverage (+): 0. Max coverage (-): 0

Region: chr11 97610034-97610043. Max. coverage (+): 0. Max coverage (-): 0

Region: chr11 97610044-97610054. Max. coverage (+): 0. Max coverage (-): 0

Region: chr11 97610055-97610064. Max. coverage (+): 0. Max coverage (-): 0

Region: chr11 97610065-97610075. Max. coverage (+): 0. Max coverage (-): 0

Region: chr11 97610076-97610086. Max. coverage (+): 0. Max coverage (-): 0

Region: chr11 97610087-97610096. Max. coverage (+): 0. Max coverage (-): 0

Region: chr11 97610097-97610107. Max. coverage (+): 0. Max coverage (-): 0

Region: chr11 97610108-97610117. Max. coverage (+): 0.8. Max coverage (-): 0

Region: chr11 97610118-97610128. Max. coverage (+): 0.8. Max coverage (-): 0

Region: chr11 97610129-97610138. Max. coverage (+): 0. Max coverage (-): 0

Region: chr11 97610139-97610149. Max. coverage (+): 0. Max coverage (-): 0

Region: chr11 97610150-97610159. Max. coverage (+): 0. Max coverage (-): 0

Region: chr11 97610160-97610170. Max. coverage (+): 0. Max coverage (-): 0

Region: chr11 97610171-97610181. Max. coverage (+): 0. Max coverage (-): 0

Region: chr11 97610182-97610191. Max. coverage (+): 0. Max coverage (-): 0

Region: chr11 97610192-97610202. Max. coverage (+): 0. Max coverage (-): 0

Region: chr11 97610203-97610212. Max. coverage (+): 0. Max coverage (-): 0

Region: chr11 97610213-97610223. Max. coverage (+): 0. Max coverage (-): 0

Region: chr11 97610224-97610233. Max. coverage (+): 0. Max coverage (-): 0

Region: chr11 97610234-97610244. Max. coverage (+): 0. Max coverage (-): 0

Region: chr11 97610245-97610255. Max. coverage (+): 0. Max coverage (-): 0

Region: chr11 97610256-97610265. Max. coverage (+): 0.52. Max coverage (-): 0

Region: chr11 97610266-97610276. Max. coverage (+): 0.52. Max coverage (-): 0

Region: chr11 97610277-97610286. Max. coverage (+): 0. Max coverage (-): 0

Region: chr11 97610287-97610297. Max. coverage (+): 0. Max coverage (-): 0

Region: chr11 97610298-97610307. Max. coverage (+): 0. Max coverage (-): 0

Region: chr11 97610308-97610318. Max. coverage (+): 0. Max coverage (-): 0

Region: chr11 97610319-97610328. Max. coverage (+): 0. Max coverage (-): 0

Region: chr11 97610329-97610339. Max. coverage (+): 0.68. Max coverage (-): 0

Region: chr11 97610340-97610350. Max. coverage (+): 0.68. Max coverage (-): 0

Region: chr11 97610351-97610360. Max. coverage (+): 0. Max coverage (-): 0

Region: chr11 97610361-97610371. Max. coverage (+): 0. Max coverage (-): 0

Region: chr11 97610372-97610381. Max. coverage (+): 0. Max coverage (-): 0

Region: chr11 97610382-97610392. Max. coverage (+): 0. Max coverage (-): 0

Region: chr11 97610393-97610402. Max. coverage (+): 0. Max coverage (-): 0

Region: chr11 97610403-97610413. Max. coverage (+): 0. Max coverage (-): 0

Region: chr11 97610414-97610424. Max. coverage (+): 0. Max coverage (-): 0

Region: chr11 97610425-97610434. Max. coverage (+): 0. Max coverage (-): 0

Region: chr11 97610435-97610445. Max. coverage (+): 0. Max coverage (-): 0

Region: chr11 97610446-97610455. Max. coverage (+): 0. Max coverage (-): 0

Region: chr11 97610456-97610466. Max. coverage (+): 0. Max coverage (-): 0

Region: chr11 97610467-97610476. Max. coverage (+): 0. Max coverage (-): 0

Region: chr11 97610477-97610487. Max. coverage (+): 0. Max coverage (-): 0

Region: chr11 97610488-97610497. Max. coverage (+): 0. Max coverage (-): 0

Region: chr11 97610498-97610508. Max. coverage (+): 0. Max coverage (-): 0

Region: chr11 97610509-97610519. Max. coverage (+): 0. Max coverage (-): 0

Region: chr11 97610520-97610529. Max. coverage (+): 0. Max coverage (-): 0

Region: chr11 97610530-97610540. Max. coverage (+): 0. Max coverage (-): 0

Region: chr11 97610541-97610550. Max. coverage (+): 0. Max coverage (-): 0

Region: chr11 97610551-97610561. Max. coverage (+): 0. Max coverage (-): 0

Region: chr11 97610562-97610571. Max. coverage (+): 0. Max coverage (-): 0

Region: chr11 97610572-97610582. Max. coverage (+): 0. Max coverage (-): 0

Region: chr11 97610583-97610593. Max. coverage (+): 0. Max coverage (-): 0

Region: chr11 97610594-97610603. Max. coverage (+): 4.89. Max coverage (-): 0

Region: chr11 97610604-97610614. Max. coverage (+): 4.89. Max coverage (-): 0

Region: chr11 97610615-97610624. Max. coverage (+): 0. Max coverage (-): 0

Region: chr11 97610625-. Max. coverage (+): 0. Max coverage (-): 0

RepeatMasker Color Code

**+**

100-98% Identity

<98-95% Identity

<95-90% Identity

<90-85% Identity

<85-80% Identity

<80-75% Identity

<75-70% Identity

<70% Identity

**-**

Gene Set Color Code

**+**

Gene

Pseudogene

**-**

Topology/Coverage Color Code

Coverage Plus Strand

Coverage Minus Strand

Mainstrand: Plus

Mainstrand: Minus

Complementary Strand

Flanking Region  
(if option -flank >0)

Gene Set Annotation  

**1. ZBTB43 (protein coding, ENSBTAG00000003438) Tr:00000004465 Ex:2**: 97605347-97608142 (+)

  
RepeatMasker Annotation  

**1. AT\_rich**: 97608595-97608634 (+), Divergence to consensus: 65%  
**2. GC\_rich**: 97610100-97610122 (+), Divergence to consensus: 47.8%

  
Transcription Factor Binding Sites  

**SOX9** (Sequence: CCATTGTT (+): 97605475)  
**A-MYB** (Sequence: CCAACTGTCA (-): 97607858)
