# Supplementary material for: Small RNA sequencing of cryopreserved semen from single bull revealed altered miRNAs and piRNAs expression between High- and Low-motile sperm populations
Source: BMC Genomics. 2017 Jan 4;18:14. doi: 10.1186/s12864-016-3394-7 (PMC5209821; doi:10.1186/s12864-016-3394-7)
Supplement: Additional file 3: — Details for each piRNA clusters found in High Motile (HM) sperm fraction. Genes, repeats, transposable elements and transcription factors binding sites falling within the cluster regions were reported. (ZIP 1896 kb) [file 12864_2016_3394_MOESM3_ESM.zip › 13.html]

piRNA cluster 13


Predicted piRNA cluster no. 13     previous   next
  

Show proTRAC run info
Hide proTRAC run info

================================= proTRAC ====================================  
VERSION: 2.1                                    LAST MODIFIED: 06. October 2015  
  
Please cite:  
Rosenkranz D, Zischler H. proTRAC - a software for probabilistic piRNA cluster  
detection, visualization and analysis. 2012. BMC Bioinformatics 13:5.  
  
and (for proTRAC 2.0 and later):  
Rosenkranz D, Rudloff S, Bastuck K, Ketting RF, Zischler H. Tupaia small RNAs  
provide insights into function and evolution of RNAi-based transposon defense  
in mammals. 2015. RNA 21(5):911-922.  
  
Contact:  
David Rosenkranz  
Institute of Anthropology, small RNA group  
Johannes Gutenberg University Mainz  
email: rosenkranz@uni-mainz.de  
  
You can find the latest proTRAC version at:  
http://sourceforge.net/projects/protrac/files  
http://www.smallRNAgroup-mainz.de/software  
==============================================================================  
  
PARAMETERS:  
Map file: .............../storage/core/barbara/genhome/smallRNA/fertility/Sample\_motile/pirna/Sample\_motile\_26-33\_collapsed.fa.no-dust.map.weighted-10000-1000-b-0  
Genome file: ............/storage/core/barbara/genhome/smallRNA/fertility/Sample\_all/pirna/bt\_311\_chrY.fa  
RepeatMasker annotation: /storage/genomes/bt\_umd31/GCF\_000003055.6\_Bos\_taurus\_UMD\_3.1.1\_repeatMasker\_chr.out  
GeneSet:................./storage/core/barbara/genhome/smallRNA/fertility/Sample\_all/pirna/full.gtf  
  
Significant (p<=0.01) hit density will be calculated based  
on observed hit distribution.  
  
Sliding window size: ........................................ 5000 bp  
Sliding window increament: .................................. 1000 bp  
Normalize each hit by number of genomic hits: ............... 1 [0=no/1=yes]  
Normalize each hit by number of sequence reads: ............. 1 [0=no/1=yes]  
Normalize values (-> per million mapped reads): ............. 1 [0=no/1=yes]  
Min. fraction of hits with 1T(U) or 10A: .................... 0.75  
Alternatively: Min. fraction of hits with 1T(U) and 10A: .... 0.5  
Min. fraction of hits with typical piRNA length: ............ 0.75  
Typical piRNA length: ....................................... 26-33 nt  
Min. size of a piRNA cluster: ............................... 5000 bp.  
Min. number of hits (absolute): ............................. 0  
Min. number of hits (normalized): ........................... 0  
Min. fraction of hits on the mainstrand: .................... 0.75  
Top fraction of mapped sequences (in terms of read counts): . 1%  
Top fraction accounts for max. n% of sequence reads: ........ 90%  
Min. fraction of hits on each arm of a bidirectional cluster: 0.1  
Output image file for each cluster: ......................... 0 [0=no/1=yes]  
Output html file for each cluster: .......................... 1 [0=no/1=yes]  
Output a summary table: ..................................... 1 [0=no/1=yes]  
Output a FASTA file for each cluster (piRNA sequences): ..... 1 [0=no/1=yes]  
Output a FASTA file comprising cluster sequences: ........... 1 [0=no/1=yes]  
Search DNA motifs in clusters: .............................. 1 [0=no/1=yes]  
Output flanking sequences: +/- .............................. 0 bp  
Output ~.pTi file: .......................................... 1 [0=no/1=yes]  
==============================================================================  
  
  
Genome size (without gaps): ............ 2678902517 bp  
Gaps (N/X/-): .......................... 53837044 bp  
Mapped reads: .......................... 658825247023  
Non-identical sequences: ............... 514171  
Genomic hits: .......................... 764233  
Significant densitiy of mapped reads: .. 12867599.5173724 reads/kb

Show proTRAC cluster info
Hide proTRAC cluster info

|  |  |
| --- | --- |
| Location | chr12 |
| Coordinates | 29588446-29652271 |
| Size [bp] | 63826 |
| Sequence hit loci | 10010 |
| Mapped reads (normalized) | 11447139861.1 |
| Mapped reads (normalized) per kb | 179349165.9 |
| Normalized reads with 1T (1U) | 76.1% |
| Normalized reads with 10A | 29.2% |
| Normalized reads with length 26-33 nt | 100% |
| Normalized reads on the main strand(s) | 99.7% |
| Predicted directionality | mono:plus |

100%

0%

1T (1U)  
reads

10A reads

26-33 nt  
reads

reads on mainstrand

**Either the amount of reads with 1T (1U) OR 10A has to exceed 75% (set with option: -1Tor10A)  
Alternatively the amount of reads with 1T (1U) AND 10A has to exceed 50% (set with option: -1Tand10A)  
Minimum amount of reads with preferred size is 75% (set with option: -pisize)  
Minimum amount of reads on the main strand(s) is 75% (set with option: -clstrand)**

Show read coverage
Hide read coverage

WHAT DO I SEE HERE?  
This chart shows the location of mapped sequence reads within a predicted piRNA cluster. The color refers to the number of genomic hits produced by the sequence read in question. A dark red bar indicates that this sequence read produces many other hits elsewhere in the genome. Many adjacent red or yellow bars can indicate the presence of a multi-copy element such as transposons or rRNA genes. A dark green bar indicates that this sequence read maps uniquely to this locus.

1 hit

2-5 hits

6-10 hits

11-20 hits

21-50 hits

51-100 hits

> 100 hits

chr12

29588446

29652271

Gene Set

RepeatMasker

Mapped  
Reads

284.29

plus strand

minus strand

284.29

Region: chr12 97610626-29588509. Max. coverage (+): 3.97. Max coverage (-): 2.52

Region: chr12 29588510-29588637. Max. coverage (+): 0. Max coverage (-): 0

Region: chr12 29588638-29588765. Max. coverage (+): 0. Max coverage (-): 0

Region: chr12 29588766-29588892. Max. coverage (+): 0. Max coverage (-): 0

Region: chr12 29588893-29589020. Max. coverage (+): 0. Max coverage (-): 0.52

Region: chr12 29589021-29589148. Max. coverage (+): 0. Max coverage (-): 0

Region: chr12 29589149-29589275. Max. coverage (+): 12.11. Max coverage (-): 3.4

Region: chr12 29589276-29589403. Max. coverage (+): 34.16. Max coverage (-): 0

Region: chr12 29589404-29589531. Max. coverage (+): 16.16. Max coverage (-): 0

Region: chr12 29589532-29589658. Max. coverage (+): 6.36. Max coverage (-): 0

Region: chr12 29589659-29589786. Max. coverage (+): 28.06. Max coverage (-): 0

Region: chr12 29589787-29589913. Max. coverage (+): 33.77. Max coverage (-): 1.58

Region: chr12 29589914-29590041. Max. coverage (+): 29.06. Max coverage (-): 0

Region: chr12 29590042-29590169. Max. coverage (+): 47.12. Max coverage (-): 0

Region: chr12 29590170-29590296. Max. coverage (+): 32.52. Max coverage (-): 0

Region: chr12 29590297-29590424. Max. coverage (+): 33.84. Max coverage (-): 0

Region: chr12 29590425-29590552. Max. coverage (+): 13.15. Max coverage (-): 0

Region: chr12 29590553-29590679. Max. coverage (+): 11. Max coverage (-): 0

Region: chr12 29590680-29590807. Max. coverage (+): 21.94. Max coverage (-): 0

Region: chr12 29590808-29590935. Max. coverage (+): 65.87. Max coverage (-): 7.69

Region: chr12 29590936-29591062. Max. coverage (+): 26. Max coverage (-): 3.45

Region: chr12 29591063-29591190. Max. coverage (+): 9.42. Max coverage (-): 0

Region: chr12 29591191-29591318. Max. coverage (+): 11.85. Max coverage (-): 0

Region: chr12 29591319-29591445. Max. coverage (+): 8.25. Max coverage (-): 0

Region: chr12 29591446-29591573. Max. coverage (+): 13.93. Max coverage (-): 0

Region: chr12 29591574-29591701. Max. coverage (+): 22.05. Max coverage (-): 0

Region: chr12 29591702-29591828. Max. coverage (+): 36.32. Max coverage (-): 0

Region: chr12 29591829-29591956. Max. coverage (+): 0. Max coverage (-): 0

Region: chr12 29591957-29592084. Max. coverage (+): 10.01. Max coverage (-): 0

Region: chr12 29592085-29592211. Max. coverage (+): 8.78. Max coverage (-): 0

Region: chr12 29592212-29592339. Max. coverage (+): 1.67. Max coverage (-): 0

Region: chr12 29592340-29592467. Max. coverage (+): 2.09. Max coverage (-): 0

Region: chr12 29592468-29592594. Max. coverage (+): 14.54. Max coverage (-): 0

Region: chr12 29592595-29592722. Max. coverage (+): 2.05. Max coverage (-): 0

Region: chr12 29592723-29592849. Max. coverage (+): 5.88. Max coverage (-): 0

Region: chr12 29592850-29592977. Max. coverage (+): 0.55. Max coverage (-): 0

Region: chr12 29592978-29593105. Max. coverage (+): 1.12. Max coverage (-): 0

Region: chr12 29593106-29593232. Max. coverage (+): 0. Max coverage (-): 0

Region: chr12 29593233-29593360. Max. coverage (+): 7.49. Max coverage (-): 0

Region: chr12 29593361-29593488. Max. coverage (+): 5.25. Max coverage (-): 0

Region: chr12 29593489-29593615. Max. coverage (+): 8.85. Max coverage (-): 0

Region: chr12 29593616-29593743. Max. coverage (+): 27.75. Max coverage (-): 0

Region: chr12 29593744-29593871. Max. coverage (+): 9.98. Max coverage (-): 0

Region: chr12 29593872-29593998. Max. coverage (+): 26.22. Max coverage (-): 0

Region: chr12 29593999-29594126. Max. coverage (+): 7.22. Max coverage (-): 0

Region: chr12 29594127-29594254. Max. coverage (+): 65.31. Max coverage (-): 0

Region: chr12 29594255-29594381. Max. coverage (+): 9.24. Max coverage (-): 0

Region: chr12 29594382-29594509. Max. coverage (+): 23.05. Max coverage (-): 0

Region: chr12 29594510-29594637. Max. coverage (+): 10.61. Max coverage (-): 0

Region: chr12 29594638-29594764. Max. coverage (+): 13.74. Max coverage (-): 0

Region: chr12 29594765-29594892. Max. coverage (+): 9.7. Max coverage (-): 0

Region: chr12 29594893-29595020. Max. coverage (+): 16.31. Max coverage (-): 0

Region: chr12 29595021-29595147. Max. coverage (+): 3.23. Max coverage (-): 0

Region: chr12 29595148-29595275. Max. coverage (+): 0. Max coverage (-): 0

Region: chr12 29595276-29595403. Max. coverage (+): 18.76. Max coverage (-): 0

Region: chr12 29595404-29595530. Max. coverage (+): 99.5. Max coverage (-): 0

Region: chr12 29595531-29595658. Max. coverage (+): 57.71. Max coverage (-): 0

Region: chr12 29595659-29595785. Max. coverage (+): 0. Max coverage (-): 0

Region: chr12 29595786-29595913. Max. coverage (+): 26.53. Max coverage (-): 0

Region: chr12 29595914-29596041. Max. coverage (+): 16.52. Max coverage (-): 0

Region: chr12 29596042-29596168. Max. coverage (+): 115. Max coverage (-): 0

Region: chr12 29596169-29596296. Max. coverage (+): 49.92. Max coverage (-): 0

Region: chr12 29596297-29596424. Max. coverage (+): 47.27. Max coverage (-): 0

Region: chr12 29596425-29596551. Max. coverage (+): 23.5. Max coverage (-): 0

Region: chr12 29596552-29596679. Max. coverage (+): 86.68. Max coverage (-): 0

Region: chr12 29596680-29596807. Max. coverage (+): 28.35. Max coverage (-): 0

Region: chr12 29596808-29596934. Max. coverage (+): 121.66. Max coverage (-): 0

Region: chr12 29596935-29597062. Max. coverage (+): 72.98. Max coverage (-): 0

Region: chr12 29597063-29597190. Max. coverage (+): 11.4. Max coverage (-): 2.6

Region: chr12 29597191-29597317. Max. coverage (+): 8.16. Max coverage (-): 0

Region: chr12 29597318-29597445. Max. coverage (+): 6.4. Max coverage (-): 0

Region: chr12 29597446-29597573. Max. coverage (+): 3.72. Max coverage (-): 0

Region: chr12 29597574-29597700. Max. coverage (+): 3.02. Max coverage (-): 0

Region: chr12 29597701-29597828. Max. coverage (+): 0. Max coverage (-): 0

Region: chr12 29597829-29597956. Max. coverage (+): 39.81. Max coverage (-): 0

Region: chr12 29597957-29598083. Max. coverage (+): 34.5. Max coverage (-): 0

Region: chr12 29598084-29598211. Max. coverage (+): 122.14. Max coverage (-): 1.1

Region: chr12 29598212-29598339. Max. coverage (+): 25.36. Max coverage (-): 0

Region: chr12 29598340-29598466. Max. coverage (+): 35.85. Max coverage (-): 0

Region: chr12 29598467-29598594. Max. coverage (+): 29.61. Max coverage (-): 0

Region: chr12 29598595-29598721. Max. coverage (+): 23.96. Max coverage (-): 0

Region: chr12 29598722-29598849. Max. coverage (+): 9.79. Max coverage (-): 0

Region: chr12 29598850-29598977. Max. coverage (+): 61.15. Max coverage (-): 0

Region: chr12 29598978-29599104. Max. coverage (+): 79.19. Max coverage (-): 2.33

Region: chr12 29599105-29599232. Max. coverage (+): 155.48. Max coverage (-): 0

Region: chr12 29599233-29599360. Max. coverage (+): 29.11. Max coverage (-): 0

Region: chr12 29599361-29599487. Max. coverage (+): 36.52. Max coverage (-): 3.98

Region: chr12 29599488-29599615. Max. coverage (+): 35.06. Max coverage (-): 0

Region: chr12 29599616-29599743. Max. coverage (+): 45. Max coverage (-): 0

Region: chr12 29599744-29599870. Max. coverage (+): 18.65. Max coverage (-): 0

Region: chr12 29599871-29599998. Max. coverage (+): 41. Max coverage (-): 0

Region: chr12 29599999-29600126. Max. coverage (+): 16.61. Max coverage (-): 0

Region: chr12 29600127-29600253. Max. coverage (+): 28.17. Max coverage (-): 0

Region: chr12 29600254-29600381. Max. coverage (+): 13.49. Max coverage (-): 0

Region: chr12 29600382-29600509. Max. coverage (+): 74.11. Max coverage (-): 1.94

Region: chr12 29600510-29600636. Max. coverage (+): 40.55. Max coverage (-): 0

Region: chr12 29600637-29600764. Max. coverage (+): 53.7. Max coverage (-): 0

Region: chr12 29600765-29600892. Max. coverage (+): 45.52. Max coverage (-): 0

Region: chr12 29600893-29601019. Max. coverage (+): 27.58. Max coverage (-): 0

Region: chr12 29601020-29601147. Max. coverage (+): 15.63. Max coverage (-): 0

Region: chr12 29601148-29601275. Max. coverage (+): 19.37. Max coverage (-): 0

Region: chr12 29601276-29601402. Max. coverage (+): 15.93. Max coverage (-): 0

Region: chr12 29601403-29601530. Max. coverage (+): 10.71. Max coverage (-): 0

Region: chr12 29601531-29601657. Max. coverage (+): 16.23. Max coverage (-): 0

Region: chr12 29601658-29601785. Max. coverage (+): 10.95. Max coverage (-): 0

Region: chr12 29601786-29601913. Max. coverage (+): 3.66. Max coverage (-): 0

Region: chr12 29601914-29602040. Max. coverage (+): 1.67. Max coverage (-): 0

Region: chr12 29602041-29602168. Max. coverage (+): 0. Max coverage (-): 0

Region: chr12 29602169-29602296. Max. coverage (+): 0. Max coverage (-): 0

Region: chr12 29602297-29602423. Max. coverage (+): 0. Max coverage (-): 0

Region: chr12 29602424-29602551. Max. coverage (+): 4.31. Max coverage (-): 0

Region: chr12 29602552-29602679. Max. coverage (+): 11.52. Max coverage (-): 0

Region: chr12 29602680-29602806. Max. coverage (+): 1.23. Max coverage (-): 0

Region: chr12 29602807-29602934. Max. coverage (+): 1.92. Max coverage (-): 0

Region: chr12 29602935-29603062. Max. coverage (+): 0. Max coverage (-): 0

Region: chr12 29603063-29603189. Max. coverage (+): 2.38. Max coverage (-): 0

Region: chr12 29603190-29603317. Max. coverage (+): 6.37. Max coverage (-): 0

Region: chr12 29603318-29603445. Max. coverage (+): 12.28. Max coverage (-): 0

Region: chr12 29603446-29603572. Max. coverage (+): 2.69. Max coverage (-): 0

Region: chr12 29603573-29603700. Max. coverage (+): 0. Max coverage (-): 0

Region: chr12 29603701-29603828. Max. coverage (+): 20.98. Max coverage (-): 0

Region: chr12 29603829-29603955. Max. coverage (+): 12.35. Max coverage (-): 0

Region: chr12 29603956-29604083. Max. coverage (+): 3.08. Max coverage (-): 0

Region: chr12 29604084-29604211. Max. coverage (+): 3.83. Max coverage (-): 0

Region: chr12 29604212-29604338. Max. coverage (+): 3.83. Max coverage (-): 0

Region: chr12 29604339-29604466. Max. coverage (+): 12.68. Max coverage (-): 0

Region: chr12 29604467-29604593. Max. coverage (+): 3.07. Max coverage (-): 0

Region: chr12 29604594-29604721. Max. coverage (+): 9.45. Max coverage (-): 0

Region: chr12 29604722-29604849. Max. coverage (+): 2.57. Max coverage (-): 0

Region: chr12 29604850-29604976. Max. coverage (+): 9.07. Max coverage (-): 0

Region: chr12 29604977-29605104. Max. coverage (+): 0. Max coverage (-): 0

Region: chr12 29605105-29605232. Max. coverage (+): 0. Max coverage (-): 0

Region: chr12 29605233-29605359. Max. coverage (+): 0. Max coverage (-): 0

Region: chr12 29605360-29605487. Max. coverage (+): 0. Max coverage (-): 0

Region: chr12 29605488-29605615. Max. coverage (+): 0. Max coverage (-): 0

Region: chr12 29605616-29605742. Max. coverage (+): 0. Max coverage (-): 0

Region: chr12 29605743-29605870. Max. coverage (+): 0. Max coverage (-): 0

Region: chr12 29605871-29605998. Max. coverage (+): 0. Max coverage (-): 0

Region: chr12 29605999-29606125. Max. coverage (+): 0. Max coverage (-): 0

Region: chr12 29606126-29606253. Max. coverage (+): 0. Max coverage (-): 0

Region: chr12 29606254-29606381. Max. coverage (+): 0. Max coverage (-): 0

Region: chr12 29606382-29606508. Max. coverage (+): 0. Max coverage (-): 0

Region: chr12 29606509-29606636. Max. coverage (+): 0. Max coverage (-): 0

Region: chr12 29606637-29606764. Max. coverage (+): 3.29. Max coverage (-): 0

Region: chr12 29606765-29606891. Max. coverage (+): 6.51. Max coverage (-): 0

Region: chr12 29606892-29607019. Max. coverage (+): 0. Max coverage (-): 0

Region: chr12 29607020-29607147. Max. coverage (+): 4.87. Max coverage (-): 0

Region: chr12 29607148-29607274. Max. coverage (+): 1.23. Max coverage (-): 0

Region: chr12 29607275-29607402. Max. coverage (+): 43.69. Max coverage (-): 0

Region: chr12 29607403-29607529. Max. coverage (+): 4.76. Max coverage (-): 0

Region: chr12 29607530-29607657. Max. coverage (+): 6.57. Max coverage (-): 0

Region: chr12 29607658-29607785. Max. coverage (+): 0.57. Max coverage (-): 0

Region: chr12 29607786-29607912. Max. coverage (+): 0. Max coverage (-): 0

Region: chr12 29607913-29608040. Max. coverage (+): 0. Max coverage (-): 0

Region: chr12 29608041-29608168. Max. coverage (+): 0. Max coverage (-): 0

Region: chr12 29608169-29608295. Max. coverage (+): 0. Max coverage (-): 0

Region: chr12 29608296-29608423. Max. coverage (+): 0. Max coverage (-): 0

Region: chr12 29608424-29608551. Max. coverage (+): 0. Max coverage (-): 0

Region: chr12 29608552-29608678. Max. coverage (+): 0. Max coverage (-): 0

Region: chr12 29608679-29608806. Max. coverage (+): 8.42. Max coverage (-): 0

Region: chr12 29608807-29608934. Max. coverage (+): 0. Max coverage (-): 0

Region: chr12 29608935-29609061. Max. coverage (+): 5.7. Max coverage (-): 0

Region: chr12 29609062-29609189. Max. coverage (+): 3.71. Max coverage (-): 0

Region: chr12 29609190-29609317. Max. coverage (+): 7.36. Max coverage (-): 0

Region: chr12 29609318-29609444. Max. coverage (+): 1.44. Max coverage (-): 0

Region: chr12 29609445-29609572. Max. coverage (+): 0. Max coverage (-): 0

Region: chr12 29609573-29609700. Max. coverage (+): 5.5. Max coverage (-): 0

Region: chr12 29609701-29609827. Max. coverage (+): 0.47. Max coverage (-): 0

Region: chr12 29609828-29609955. Max. coverage (+): 7.67. Max coverage (-): 0

Region: chr12 29609956-29610083. Max. coverage (+): 4.21. Max coverage (-): 0

Region: chr12 29610084-29610210. Max. coverage (+): 11.88. Max coverage (-): 0

Region: chr12 29610211-29610338. Max. coverage (+): 16.76. Max coverage (-): 0

Region: chr12 29610339-29610465. Max. coverage (+): 8.34. Max coverage (-): 0

Region: chr12 29610466-29610593. Max. coverage (+): 6.42. Max coverage (-): 0

Region: chr12 29610594-29610721. Max. coverage (+): 8.42. Max coverage (-): 0

Region: chr12 29610722-29610848. Max. coverage (+): 18.6. Max coverage (-): 0

Region: chr12 29610849-29610976. Max. coverage (+): 179.22. Max coverage (-): 0

Region: chr12 29610977-29611104. Max. coverage (+): 105.69. Max coverage (-): 0

Region: chr12 29611105-29611231. Max. coverage (+): 97.41. Max coverage (-): 0

Region: chr12 29611232-29611359. Max. coverage (+): 21.72. Max coverage (-): 0

Region: chr12 29611360-29611487. Max. coverage (+): 59.76. Max coverage (-): 0

Region: chr12 29611488-29611614. Max. coverage (+): 29.46. Max coverage (-): 0

Region: chr12 29611615-29611742. Max. coverage (+): 94.56. Max coverage (-): 0

Region: chr12 29611743-29611870. Max. coverage (+): 39.88. Max coverage (-): 0

Region: chr12 29611871-29611997. Max. coverage (+): 26.07. Max coverage (-): 0

Region: chr12 29611998-29612125. Max. coverage (+): 70.78. Max coverage (-): 0

Region: chr12 29612126-29612253. Max. coverage (+): 55.75. Max coverage (-): 0

Region: chr12 29612254-29612380. Max. coverage (+): 39.15. Max coverage (-): 0

Region: chr12 29612381-29612508. Max. coverage (+): 86.91. Max coverage (-): 0

Region: chr12 29612509-29612636. Max. coverage (+): 22.16. Max coverage (-): 3.03

Region: chr12 29612637-29612763. Max. coverage (+): 24.91. Max coverage (-): 0

Region: chr12 29612764-29612891. Max. coverage (+): 14.08. Max coverage (-): 0

Region: chr12 29612892-29613019. Max. coverage (+): 0. Max coverage (-): 0

Region: chr12 29613020-29613146. Max. coverage (+): 0. Max coverage (-): 0

Region: chr12 29613147-29613274. Max. coverage (+): 0. Max coverage (-): 0

Region: chr12 29613275-29613401. Max. coverage (+): 1.9. Max coverage (-): 0

Region: chr12 29613402-29613529. Max. coverage (+): 18.03. Max coverage (-): 0

Region: chr12 29613530-29613657. Max. coverage (+): 110.25. Max coverage (-): 0

Region: chr12 29613658-29613784. Max. coverage (+): 11.86. Max coverage (-): 0

Region: chr12 29613785-29613912. Max. coverage (+): 0. Max coverage (-): 0

Region: chr12 29613913-29614040. Max. coverage (+): 32.29. Max coverage (-): 0

Region: chr12 29614041-29614167. Max. coverage (+): 17.26. Max coverage (-): 0

Region: chr12 29614168-29614295. Max. coverage (+): 45.72. Max coverage (-): 0

Region: chr12 29614296-29614423. Max. coverage (+): 53.2. Max coverage (-): 0

Region: chr12 29614424-29614550. Max. coverage (+): 7.3. Max coverage (-): 0

Region: chr12 29614551-29614678. Max. coverage (+): 41.08. Max coverage (-): 0

Region: chr12 29614679-29614806. Max. coverage (+): 5.06. Max coverage (-): 0

Region: chr12 29614807-29614933. Max. coverage (+): 8.11. Max coverage (-): 0

Region: chr12 29614934-29615061. Max. coverage (+): 13.65. Max coverage (-): 0

Region: chr12 29615062-29615189. Max. coverage (+): 18.77. Max coverage (-): 0

Region: chr12 29615190-29615316. Max. coverage (+): 13.73. Max coverage (-): 0

Region: chr12 29615317-29615444. Max. coverage (+): 0. Max coverage (-): 0

Region: chr12 29615445-29615572. Max. coverage (+): 13.06. Max coverage (-): 0

Region: chr12 29615573-29615699. Max. coverage (+): 117.42. Max coverage (-): 0

Region: chr12 29615700-29615827. Max. coverage (+): 127.51. Max coverage (-): 0

Region: chr12 29615828-29615955. Max. coverage (+): 40.47. Max coverage (-): 0

Region: chr12 29615956-29616082. Max. coverage (+): 13.14. Max coverage (-): 0

Region: chr12 29616083-29616210. Max. coverage (+): 11.36. Max coverage (-): 0

Region: chr12 29616211-29616337. Max. coverage (+): 89.55. Max coverage (-): 0

Region: chr12 29616338-29616465. Max. coverage (+): 77.06. Max coverage (-): 0

Region: chr12 29616466-29616593. Max. coverage (+): 0. Max coverage (-): 0

Region: chr12 29616594-29616720. Max. coverage (+): 0. Max coverage (-): 0

Region: chr12 29616721-29616848. Max. coverage (+): 25.7. Max coverage (-): 0

Region: chr12 29616849-29616976. Max. coverage (+): 70.12. Max coverage (-): 0

Region: chr12 29616977-29617103. Max. coverage (+): 36.46. Max coverage (-): 0

Region: chr12 29617104-29617231. Max. coverage (+): 14.03. Max coverage (-): 0

Region: chr12 29617232-29617359. Max. coverage (+): 1.51. Max coverage (-): 0

Region: chr12 29617360-29617486. Max. coverage (+): 7.57. Max coverage (-): 0

Region: chr12 29617487-29617614. Max. coverage (+): 24.26. Max coverage (-): 0

Region: chr12 29617615-29617742. Max. coverage (+): 20.24. Max coverage (-): 0

Region: chr12 29617743-29617869. Max. coverage (+): 0. Max coverage (-): 0

Region: chr12 29617870-29617997. Max. coverage (+): 0. Max coverage (-): 0

Region: chr12 29617998-29618125. Max. coverage (+): 0. Max coverage (-): 0

Region: chr12 29618126-29618252. Max. coverage (+): 0. Max coverage (-): 0

Region: chr12 29618253-29618380. Max. coverage (+): 0. Max coverage (-): 0

Region: chr12 29618381-29618508. Max. coverage (+): 0. Max coverage (-): 0

Region: chr12 29618509-29618635. Max. coverage (+): 0. Max coverage (-): 0

Region: chr12 29618636-29618763. Max. coverage (+): 0. Max coverage (-): 0

Region: chr12 29618764-29618891. Max. coverage (+): 0. Max coverage (-): 0

Region: chr12 29618892-29619018. Max. coverage (+): 4.24. Max coverage (-): 0

Region: chr12 29619019-29619146. Max. coverage (+): 58.08. Max coverage (-): 0

Region: chr12 29619147-29619273. Max. coverage (+): 31.76. Max coverage (-): 0

Region: chr12 29619274-29619401. Max. coverage (+): 0. Max coverage (-): 0

Region: chr12 29619402-29619529. Max. coverage (+): 0. Max coverage (-): 0

Region: chr12 29619530-29619656. Max. coverage (+): 0. Max coverage (-): 0

Region: chr12 29619657-29619784. Max. coverage (+): 0. Max coverage (-): 0

Region: chr12 29619785-29619912. Max. coverage (+): 30.99. Max coverage (-): 0

Region: chr12 29619913-29620039. Max. coverage (+): 8.54. Max coverage (-): 0

Region: chr12 29620040-29620167. Max. coverage (+): 15.96. Max coverage (-): 0

Region: chr12 29620168-29620295. Max. coverage (+): 25.52. Max coverage (-): 0

Region: chr12 29620296-29620422. Max. coverage (+): 0. Max coverage (-): 0

Region: chr12 29620423-29620550. Max. coverage (+): 0. Max coverage (-): 0

Region: chr12 29620551-29620678. Max. coverage (+): 40.55. Max coverage (-): 0

Region: chr12 29620679-29620805. Max. coverage (+): 49.61. Max coverage (-): 0

Region: chr12 29620806-29620933. Max. coverage (+): 55.1. Max coverage (-): 0

Region: chr12 29620934-29621061. Max. coverage (+): 13.75. Max coverage (-): 0

Region: chr12 29621062-29621188. Max. coverage (+): 7.76. Max coverage (-): 0

Region: chr12 29621189-29621316. Max. coverage (+): 9.32. Max coverage (-): 0

Region: chr12 29621317-29621444. Max. coverage (+): 0. Max coverage (-): 0

Region: chr12 29621445-29621571. Max. coverage (+): 0. Max coverage (-): 0

Region: chr12 29621572-29621699. Max. coverage (+): 102.56. Max coverage (-): 0

Region: chr12 29621700-29621826. Max. coverage (+): 129.07. Max coverage (-): 0

Region: chr12 29621827-29621954. Max. coverage (+): 68.97. Max coverage (-): 0

Region: chr12 29621955-29622082. Max. coverage (+): 0. Max coverage (-): 0

Region: chr12 29622083-29622209. Max. coverage (+): 30.84. Max coverage (-): 0

Region: chr12 29622210-29622337. Max. coverage (+): 32.18. Max coverage (-): 0

Region: chr12 29622338-29622465. Max. coverage (+): 34.64. Max coverage (-): 0

Region: chr12 29622466-29622592. Max. coverage (+): 284.29. Max coverage (-): 2.98

Region: chr12 29622593-29622720. Max. coverage (+): 55.2. Max coverage (-): 0

Region: chr12 29622721-29622848. Max. coverage (+): 0. Max coverage (-): 0

Region: chr12 29622849-29622975. Max. coverage (+): 94.3. Max coverage (-): 0

Region: chr12 29622976-29623103. Max. coverage (+): 1.68. Max coverage (-): 0

Region: chr12 29623104-29623231. Max. coverage (+): 39.01. Max coverage (-): 0

Region: chr12 29623232-29623358. Max. coverage (+): 0. Max coverage (-): 0

Region: chr12 29623359-29623486. Max. coverage (+): 0. Max coverage (-): 0

Region: chr12 29623487-29623614. Max. coverage (+): 0. Max coverage (-): 0

Region: chr12 29623615-29623741. Max. coverage (+): 0. Max coverage (-): 0

Region: chr12 29623742-29623869. Max. coverage (+): 0. Max coverage (-): 0

Region: chr12 29623870-29623997. Max. coverage (+): 0. Max coverage (-): 0

Region: chr12 29623998-29624124. Max. coverage (+): 0. Max coverage (-): 0

Region: chr12 29624125-29624252. Max. coverage (+): 0. Max coverage (-): 0

Region: chr12 29624253-29624380. Max. coverage (+): 0. Max coverage (-): 0

Region: chr12 29624381-29624507. Max. coverage (+): 0. Max coverage (-): 0

Region: chr12 29624508-29624635. Max. coverage (+): 0. Max coverage (-): 0

Region: chr12 29624636-29624762. Max. coverage (+): 0. Max coverage (-): 0

Region: chr12 29624763-29624890. Max. coverage (+): 0. Max coverage (-): 0

Region: chr12 29624891-29625018. Max. coverage (+): 0. Max coverage (-): 0

Region: chr12 29625019-29625145. Max. coverage (+): 0. Max coverage (-): 0

Region: chr12 29625146-29625273. Max. coverage (+): 0. Max coverage (-): 0

Region: chr12 29625274-29625401. Max. coverage (+): 0. Max coverage (-): 0

Region: chr12 29625402-29625528. Max. coverage (+): 0. Max coverage (-): 0

Region: chr12 29625529-29625656. Max. coverage (+): 0. Max coverage (-): 0

Region: chr12 29625657-29625784. Max. coverage (+): 0. Max coverage (-): 0

Region: chr12 29625785-29625911. Max. coverage (+): 0. Max coverage (-): 0

Region: chr12 29625912-29626039. Max. coverage (+): 0. Max coverage (-): 0

Region: chr12 29626040-29626167. Max. coverage (+): 0. Max coverage (-): 0

Region: chr12 29626168-29626294. Max. coverage (+): 2.21. Max coverage (-): 0

Region: chr12 29626295-29626422. Max. coverage (+): 5.9. Max coverage (-): 0

Region: chr12 29626423-29626550. Max. coverage (+): 0. Max coverage (-): 0

Region: chr12 29626551-29626677. Max. coverage (+): 0. Max coverage (-): 0

Region: chr12 29626678-29626805. Max. coverage (+): 0. Max coverage (-): 0

Region: chr12 29626806-29626933. Max. coverage (+): 48.4. Max coverage (-): 0

Region: chr12 29626934-29627060. Max. coverage (+): 21.1. Max coverage (-): 0

Region: chr12 29627061-29627188. Max. coverage (+): 22.13. Max coverage (-): 0

Region: chr12 29627189-29627316. Max. coverage (+): 13.13. Max coverage (-): 0

Region: chr12 29627317-29627443. Max. coverage (+): 0. Max coverage (-): 0

Region: chr12 29627444-29627571. Max. coverage (+): 8.17. Max coverage (-): 0

Region: chr12 29627572-29627698. Max. coverage (+): 31.48. Max coverage (-): 0

Region: chr12 29627699-29627826. Max. coverage (+): 26.36. Max coverage (-): 0

Region: chr12 29627827-29627954. Max. coverage (+): 14.49. Max coverage (-): 0

Region: chr12 29627955-29628081. Max. coverage (+): 12.64. Max coverage (-): 0

Region: chr12 29628082-29628209. Max. coverage (+): 0. Max coverage (-): 0

Region: chr12 29628210-29628337. Max. coverage (+): 0. Max coverage (-): 0

Region: chr12 29628338-29628464. Max. coverage (+): 6.07. Max coverage (-): 0

Region: chr12 29628465-29628592. Max. coverage (+): 7.19. Max coverage (-): 0

Region: chr12 29628593-29628720. Max. coverage (+): 2.3. Max coverage (-): 0

Region: chr12 29628721-29628847. Max. coverage (+): 0. Max coverage (-): 0

Region: chr12 29628848-29628975. Max. coverage (+): 0. Max coverage (-): 0

Region: chr12 29628976-29629103. Max. coverage (+): 0. Max coverage (-): 0

Region: chr12 29629104-29629230. Max. coverage (+): 0. Max coverage (-): 0

Region: chr12 29629231-29629358. Max. coverage (+): 7.59. Max coverage (-): 0

Region: chr12 29629359-29629486. Max. coverage (+): 16.38. Max coverage (-): 0

Region: chr12 29629487-29629613. Max. coverage (+): 38.04. Max coverage (-): 0

Region: chr12 29629614-29629741. Max. coverage (+): 29.25. Max coverage (-): 0

Region: chr12 29629742-29629869. Max. coverage (+): 3.15. Max coverage (-): 0

Region: chr12 29629870-29629996. Max. coverage (+): 0. Max coverage (-): 0

Region: chr12 29629997-29630124. Max. coverage (+): 5.03. Max coverage (-): 0

Region: chr12 29630125-29630252. Max. coverage (+): 0. Max coverage (-): 0

Region: chr12 29630253-29630379. Max. coverage (+): 0. Max coverage (-): 0

Region: chr12 29630380-29630507. Max. coverage (+): 0. Max coverage (-): 0

Region: chr12 29630508-29630634. Max. coverage (+): 0. Max coverage (-): 0

Region: chr12 29630635-29630762. Max. coverage (+): 0. Max coverage (-): 0

Region: chr12 29630763-29630890. Max. coverage (+): 23.1. Max coverage (-): 0

Region: chr12 29630891-29631017. Max. coverage (+): 0. Max coverage (-): 0

Region: chr12 29631018-29631145. Max. coverage (+): 58.88. Max coverage (-): 0

Region: chr12 29631146-29631273. Max. coverage (+): 4.28. Max coverage (-): 0

Region: chr12 29631274-29631400. Max. coverage (+): 16.21. Max coverage (-): 0

Region: chr12 29631401-29631528. Max. coverage (+): 19.69. Max coverage (-): 0

Region: chr12 29631529-29631656. Max. coverage (+): 41.84. Max coverage (-): 0

Region: chr12 29631657-29631783. Max. coverage (+): 17.04. Max coverage (-): 0

Region: chr12 29631784-29631911. Max. coverage (+): 0. Max coverage (-): 0

Region: chr12 29631912-29632039. Max. coverage (+): 3.53. Max coverage (-): 0

Region: chr12 29632040-29632166. Max. coverage (+): 20.82. Max coverage (-): 0

Region: chr12 29632167-29632294. Max. coverage (+): 0. Max coverage (-): 0

Region: chr12 29632295-29632422. Max. coverage (+): 0. Max coverage (-): 0

Region: chr12 29632423-29632549. Max. coverage (+): 0. Max coverage (-): 0

Region: chr12 29632550-29632677. Max. coverage (+): 0. Max coverage (-): 0

Region: chr12 29632678-29632805. Max. coverage (+): 0. Max coverage (-): 0

Region: chr12 29632806-29632932. Max. coverage (+): 0. Max coverage (-): 0

Region: chr12 29632933-29633060. Max. coverage (+): 3.64. Max coverage (-): 0

Region: chr12 29633061-29633188. Max. coverage (+): 0. Max coverage (-): 0

Region: chr12 29633189-29633315. Max. coverage (+): 0. Max coverage (-): 0

Region: chr12 29633316-29633443. Max. coverage (+): 0. Max coverage (-): 0

Region: chr12 29633444-29633570. Max. coverage (+): 33.13. Max coverage (-): 0.8

Region: chr12 29633571-29633698. Max. coverage (+): 52.15. Max coverage (-): 0

Region: chr12 29633699-29633826. Max. coverage (+): 58.58. Max coverage (-): 5.05

Region: chr12 29633827-29633953. Max. coverage (+): 10.66. Max coverage (-): 0

Region: chr12 29633954-29634081. Max. coverage (+): 9.49. Max coverage (-): 0

Region: chr12 29634082-29634209. Max. coverage (+): 0. Max coverage (-): 0

Region: chr12 29634210-29634336. Max. coverage (+): 0. Max coverage (-): 0

Region: chr12 29634337-29634464. Max. coverage (+): 12.9. Max coverage (-): 0

Region: chr12 29634465-29634592. Max. coverage (+): 8.79. Max coverage (-): 0

Region: chr12 29634593-29634719. Max. coverage (+): 9.52. Max coverage (-): 0

Region: chr12 29634720-29634847. Max. coverage (+): 11.7. Max coverage (-): 0

Region: chr12 29634848-29634975. Max. coverage (+): 33.59. Max coverage (-): 0

Region: chr12 29634976-29635102. Max. coverage (+): 8.07. Max coverage (-): 0

Region: chr12 29635103-29635230. Max. coverage (+): 8.77. Max coverage (-): 0

Region: chr12 29635231-29635358. Max. coverage (+): 17.12. Max coverage (-): 0

Region: chr12 29635359-29635485. Max. coverage (+): 31.82. Max coverage (-): 0

Region: chr12 29635486-29635613. Max. coverage (+): 37.55. Max coverage (-): 0

Region: chr12 29635614-29635741. Max. coverage (+): 19.9. Max coverage (-): 0

Region: chr12 29635742-29635868. Max. coverage (+): 18.09. Max coverage (-): 0

Region: chr12 29635869-29635996. Max. coverage (+): 0. Max coverage (-): 0

Region: chr12 29635997-29636124. Max. coverage (+): 0. Max coverage (-): 0

Region: chr12 29636125-29636251. Max. coverage (+): 0. Max coverage (-): 0

Region: chr12 29636252-29636379. Max. coverage (+): 3.85. Max coverage (-): 0

Region: chr12 29636380-29636506. Max. coverage (+): 3.43. Max coverage (-): 0

Region: chr12 29636507-29636634. Max. coverage (+): 4.96. Max coverage (-): 0

Region: chr12 29636635-29636762. Max. coverage (+): 0. Max coverage (-): 0

Region: chr12 29636763-29636889. Max. coverage (+): 0. Max coverage (-): 0

Region: chr12 29636890-29637017. Max. coverage (+): 12.7. Max coverage (-): 0

Region: chr12 29637018-29637145. Max. coverage (+): 5.17. Max coverage (-): 0

Region: chr12 29637146-29637272. Max. coverage (+): 1.34. Max coverage (-): 0

Region: chr12 29637273-29637400. Max. coverage (+): 0. Max coverage (-): 0

Region: chr12 29637401-29637528. Max. coverage (+): 0.52. Max coverage (-): 0

Region: chr12 29637529-29637655. Max. coverage (+): 1.97. Max coverage (-): 0

Region: chr12 29637656-29637783. Max. coverage (+): 0. Max coverage (-): 0

Region: chr12 29637784-29637911. Max. coverage (+): 0.21. Max coverage (-): 0

Region: chr12 29637912-29638038. Max. coverage (+): 0. Max coverage (-): 0

Region: chr12 29638039-29638166. Max. coverage (+): 2.27. Max coverage (-): 0

Region: chr12 29638167-29638294. Max. coverage (+): 2.12. Max coverage (-): 0

Region: chr12 29638295-29638421. Max. coverage (+): 2.28. Max coverage (-): 0

Region: chr12 29638422-29638549. Max. coverage (+): 1.93. Max coverage (-): 0

Region: chr12 29638550-29638677. Max. coverage (+): 1.35. Max coverage (-): 0

Region: chr12 29638678-29638804. Max. coverage (+): 1.35. Max coverage (-): 0

Region: chr12 29638805-29638932. Max. coverage (+): 1.44. Max coverage (-): 0

Region: chr12 29638933-29639060. Max. coverage (+): 7.29. Max coverage (-): 0

Region: chr12 29639061-29639187. Max. coverage (+): 0. Max coverage (-): 0

Region: chr12 29639188-29639315. Max. coverage (+): 3.39. Max coverage (-): 0

Region: chr12 29639316-29639442. Max. coverage (+): 4.83. Max coverage (-): 0

Region: chr12 29639443-29639570. Max. coverage (+): 0. Max coverage (-): 0

Region: chr12 29639571-29639698. Max. coverage (+): 0. Max coverage (-): 0

Region: chr12 29639699-29639825. Max. coverage (+): 0. Max coverage (-): 0

Region: chr12 29639826-29639953. Max. coverage (+): 0. Max coverage (-): 0

Region: chr12 29639954-29640081. Max. coverage (+): 7.68. Max coverage (-): 0

Region: chr12 29640082-29640208. Max. coverage (+): 3.6. Max coverage (-): 0

Region: chr12 29640209-29640336. Max. coverage (+): 2.03. Max coverage (-): 0

Region: chr12 29640337-29640464. Max. coverage (+): 1.72. Max coverage (-): 0

Region: chr12 29640465-29640591. Max. coverage (+): 2.76. Max coverage (-): 0

Region: chr12 29640592-29640719. Max. coverage (+): 5.08. Max coverage (-): 0

Region: chr12 29640720-29640847. Max. coverage (+): 0. Max coverage (-): 0

Region: chr12 29640848-29640974. Max. coverage (+): 0. Max coverage (-): 0

Region: chr12 29640975-29641102. Max. coverage (+): 0. Max coverage (-): 0

Region: chr12 29641103-29641230. Max. coverage (+): 0. Max coverage (-): 0

Region: chr12 29641231-29641357. Max. coverage (+): 0. Max coverage (-): 0

Region: chr12 29641358-29641485. Max. coverage (+): 0. Max coverage (-): 0

Region: chr12 29641486-29641613. Max. coverage (+): 4.62. Max coverage (-): 0

Region: chr12 29641614-29641740. Max. coverage (+): 0. Max coverage (-): 0

Region: chr12 29641741-29641868. Max. coverage (+): 0. Max coverage (-): 0

Region: chr12 29641869-29641996. Max. coverage (+): 0. Max coverage (-): 0

Region: chr12 29641997-29642123. Max. coverage (+): 10.6. Max coverage (-): 0

Region: chr12 29642124-29642251. Max. coverage (+): 0. Max coverage (-): 0

Region: chr12 29642252-29642378. Max. coverage (+): 0. Max coverage (-): 0

Region: chr12 29642379-29642506. Max. coverage (+): 0. Max coverage (-): 0

Region: chr12 29642507-29642634. Max. coverage (+): 0. Max coverage (-): 0

Region: chr12 29642635-29642761. Max. coverage (+): 1.64. Max coverage (-): 0

Region: chr12 29642762-29642889. Max. coverage (+): 13.36. Max coverage (-): 0

Region: chr12 29642890-29643017. Max. coverage (+): 0. Max coverage (-): 0

Region: chr12 29643018-29643144. Max. coverage (+): 0. Max coverage (-): 0

Region: chr12 29643145-29643272. Max. coverage (+): 0. Max coverage (-): 0

Region: chr12 29643273-29643400. Max. coverage (+): 8.11. Max coverage (-): 0

Region: chr12 29643401-29643527. Max. coverage (+): 11.77. Max coverage (-): 0

Region: chr12 29643528-29643655. Max. coverage (+): 3.56. Max coverage (-): 0

Region: chr12 29643656-29643783. Max. coverage (+): 8.3. Max coverage (-): 0

Region: chr12 29643784-29643910. Max. coverage (+): 3.91. Max coverage (-): 0

Region: chr12 29643911-29644038. Max. coverage (+): 4.84. Max coverage (-): 0

Region: chr12 29644039-29644166. Max. coverage (+): 12.04. Max coverage (-): 0

Region: chr12 29644167-29644293. Max. coverage (+): 16.42. Max coverage (-): 0

Region: chr12 29644294-29644421. Max. coverage (+): 1.31. Max coverage (-): 0

Region: chr12 29644422-29644549. Max. coverage (+): 3.37. Max coverage (-): 0

Region: chr12 29644550-29644676. Max. coverage (+): 0. Max coverage (-): 0

Region: chr12 29644677-29644804. Max. coverage (+): 1.38. Max coverage (-): 0

Region: chr12 29644805-29644932. Max. coverage (+): 1.6. Max coverage (-): 0

Region: chr12 29644933-29645059. Max. coverage (+): 0. Max coverage (-): 0

Region: chr12 29645060-29645187. Max. coverage (+): 0. Max coverage (-): 0

Region: chr12 29645188-29645314. Max. coverage (+): 0. Max coverage (-): 0

Region: chr12 29645315-29645442. Max. coverage (+): 0. Max coverage (-): 0

Region: chr12 29645443-29645570. Max. coverage (+): 14.28. Max coverage (-): 0

Region: chr12 29645571-29645697. Max. coverage (+): 3.25. Max coverage (-): 0

Region: chr12 29645698-29645825. Max. coverage (+): 1.3. Max coverage (-): 0

Region: chr12 29645826-29645953. Max. coverage (+): 4.39. Max coverage (-): 0

Region: chr12 29645954-29646080. Max. coverage (+): 2.12. Max coverage (-): 0

Region: chr12 29646081-29646208. Max. coverage (+): 0. Max coverage (-): 0

Region: chr12 29646209-29646336. Max. coverage (+): 0. Max coverage (-): 0

Region: chr12 29646337-29646463. Max. coverage (+): 0. Max coverage (-): 0

Region: chr12 29646464-29646591. Max. coverage (+): 0. Max coverage (-): 0

Region: chr12 29646592-29646719. Max. coverage (+): 0. Max coverage (-): 0

Region: chr12 29646720-29646846. Max. coverage (+): 0. Max coverage (-): 0

Region: chr12 29646847-29646974. Max. coverage (+): 0. Max coverage (-): 0

Region: chr12 29646975-29647102. Max. coverage (+): 0. Max coverage (-): 0

Region: chr12 29647103-29647229. Max. coverage (+): 0. Max coverage (-): 0

Region: chr12 29647230-29647357. Max. coverage (+): 0. Max coverage (-): 0

Region: chr12 29647358-29647485. Max. coverage (+): 0. Max coverage (-): 0

Region: chr12 29647486-29647612. Max. coverage (+): 0. Max coverage (-): 0

Region: chr12 29647613-29647740. Max. coverage (+): 0. Max coverage (-): 0

Region: chr12 29647741-29647868. Max. coverage (+): 0. Max coverage (-): 0

Region: chr12 29647869-29647995. Max. coverage (+): 0. Max coverage (-): 0

Region: chr12 29647996-29648123. Max. coverage (+): 0. Max coverage (-): 0

Region: chr12 29648124-29648250. Max. coverage (+): 0. Max coverage (-): 0

Region: chr12 29648251-29648378. Max. coverage (+): 4.23. Max coverage (-): 0

Region: chr12 29648379-29648506. Max. coverage (+): 15.37. Max coverage (-): 0

Region: chr12 29648507-29648633. Max. coverage (+): 0. Max coverage (-): 0

Region: chr12 29648634-29648761. Max. coverage (+): 8.67. Max coverage (-): 0

Region: chr12 29648762-29648889. Max. coverage (+): 2.65. Max coverage (-): 0

Region: chr12 29648890-29649016. Max. coverage (+): 1.34. Max coverage (-): 0

Region: chr12 29649017-29649144. Max. coverage (+): 5.78. Max coverage (-): 0

Region: chr12 29649145-29649272. Max. coverage (+): 11.59. Max coverage (-): 0

Region: chr12 29649273-29649399. Max. coverage (+): 0. Max coverage (-): 0

Region: chr12 29649400-29649527. Max. coverage (+): 0. Max coverage (-): 0

Region: chr12 29649528-29649655. Max. coverage (+): 0.98. Max coverage (-): 0

Region: chr12 29649656-29649782. Max. coverage (+): 4.83. Max coverage (-): 0

Region: chr12 29649783-29649910. Max. coverage (+): 8.94. Max coverage (-): 0

Region: chr12 29649911-29650038. Max. coverage (+): 3.14. Max coverage (-): 0

Region: chr12 29650039-29650165. Max. coverage (+): 6.63. Max coverage (-): 0

Region: chr12 29650166-29650293. Max. coverage (+): 2.12. Max coverage (-): 0

Region: chr12 29650294-29650421. Max. coverage (+): 0. Max coverage (-): 0

Region: chr12 29650422-29650548. Max. coverage (+): 0. Max coverage (-): 0

Region: chr12 29650549-29650676. Max. coverage (+): 13.84. Max coverage (-): 0

Region: chr12 29650677-29650804. Max. coverage (+): 18.05. Max coverage (-): 0

Region: chr12 29650805-29650931. Max. coverage (+): 3.01. Max coverage (-): 0

Region: chr12 29650932-29651059. Max. coverage (+): 6.97. Max coverage (-): 0

Region: chr12 29651060-29651186. Max. coverage (+): 4.74. Max coverage (-): 0

Region: chr12 29651187-29651314. Max. coverage (+): 4.32. Max coverage (-): 0

Region: chr12 29651315-29651442. Max. coverage (+): 5.36. Max coverage (-): 0

Region: chr12 29651443-29651569. Max. coverage (+): 0. Max coverage (-): 0

Region: chr12 29651570-29651697. Max. coverage (+): 0. Max coverage (-): 0

Region: chr12 29651698-29651825. Max. coverage (+): 0. Max coverage (-): 0

Region: chr12 29651826-29651952. Max. coverage (+): 0. Max coverage (-): 0

Region: chr12 29651953-29652080. Max. coverage (+): 2.74. Max coverage (-): 0

Region: chr12 29652081-29652208. Max. coverage (+): 0. Max coverage (-): 0

Region: chr12 29652209-. Max. coverage (+): 4.1. Max coverage (-): 0

RepeatMasker Color Code

**+**

100-98% Identity

<98-95% Identity

<95-90% Identity

<90-85% Identity

<85-80% Identity

<80-75% Identity

<75-70% Identity

<70% Identity

**-**

Gene Set Color Code

**+**

Gene

Pseudogene

**-**

Topology/Coverage Color Code

Coverage Plus Strand

Coverage Minus Strand

Mainstrand: Plus

Mainstrand: Minus

Complementary Strand

Flanking Region  
(if option -flank >0)

Gene Set Annotation  
  
RepeatMasker Annotation  

**1. L1MA7**: 29588335-29588516 (+), Divergence to consensus: 35.8%  
**2. L1MD**: 29588525-29588820 (+), Divergence to consensus: 25.2%  
**3. AT\_rich**: 29591399-29591426 (+), Divergence to consensus: 42.9%  
**4. L2**: 29593323-29593623 (+), Divergence to consensus: 45.6%  
**5. BOV-A2**: 29595205-29595321 (-), Divergence to consensus: 5.2%  
**6. L1ME3B**: 29595732-29595847 (+), Divergence to consensus: 34.5%  
**7. MIRb**: 29595973-29596043 (-), Divergence to consensus: 35.2%  
**8. Charlie16a**: 29597433-29597494 (+), Divergence to consensus: 27.6%  
**9. MIR**: 29597704-29597806 (+), Divergence to consensus: 30%  
**10. L2**: 29600239-29600500 (-), Divergence to consensus: 43.6%  
**11. L1\_Art**: 29602038-29602414 (-), Divergence to consensus: 34.7%  
**12. AT\_rich**: 29603464-29603506 (+), Divergence to consensus: 74.4%  
**13. L2b**: 29604402-29604454 (-), Divergence to consensus: 26.4%  
**14. L2c**: 29605051-29605122 (-), Divergence to consensus: 36.3%  
**15. MIR**: 29605128-29605265 (+), Divergence to consensus: 36.8%  
**16. BTLTR1C**: 29605280-29606538 (-), Divergence to consensus: 5.4%  
**17. G-rich**: 29606569-29606659 (+), Divergence to consensus: 32.2%  
**18. MIRb**: 29606678-29606753 (+), Divergence to consensus: 38.6%  
**19. MIR**: 29606818-29607015 (-), Divergence to consensus: 36.6%  
**20. L1MC5a**: 29607860-29608341 (-), Divergence to consensus: 42.2%  
**21. L1MC5a**: 29608393-29608641 (+), Divergence to consensus: 37.4%  
**22. (CCA)n**: 29608839-29608861 (+), Divergence to consensus: 4.3%  
**23. UCON2**: 29609779-29609906 (-), Divergence to consensus: 37.5%  
**24. MamGypLTR1a**: 29612503-29612545 (+), Divergence to consensus: 18.6%  
**25. MLT2F**: 29612835-29613389 (-), Divergence to consensus: 54.1%  
**26. Bov-tA2**: 29613494-29613623 (-), Divergence to consensus: 17.7%  
**27. MER102c**: 29613692-29613761 (-), Divergence to consensus: 31.4%  
**28. MER102b**: 29613796-29613967 (-), Divergence to consensus: 34.6%  
**29. L2b**: 29614502-29614592 (+), Divergence to consensus: 46%  
**30. BOV-A2**: 29615281-29615529 (-), Divergence to consensus: 10.4%  
**31. MIRb**: 29616025-29616055 (-), Divergence to consensus: 12.9%  
**32. L2**: 29616102-29616201 (-), Divergence to consensus: 39%  
**33. MLT1J2**: 29616397-29616791 (-), Divergence to consensus: 42.6%  
**34. MER102c**: 29617211-29617321 (-), Divergence to consensus: 28.4%  
**35. ERV2-1C-LTR\_BT**: 29617669-29618930 (-), Divergence to consensus: 21.3%  
**36. BTLTR1F**: 29618931-29618986 (-), Divergence to consensus: 8.9%  
**37. L1MC4**: 29619284-29619778 (+), Divergence to consensus: 35.2%  
**38. BOV-A2**: 29620282-29620359 (-), Divergence to consensus: 24.4%  
**39. ART2A**: 29620291-29620576 (-), Divergence to consensus: 18.2%  
**40. L1ME3**: 29621373-29621616 (-), Divergence to consensus: 37.4%  
**41. MER58A**: 29621737-29621800 (-), Divergence to consensus: 25.5%  
**42. MER58A**: 29621801-29621870 (-), Divergence to consensus: 25.7%  
**43. L1ME3**: 29621873-29622091 (-), Divergence to consensus: 36.2%  
**44. MLT1F2**: 29622293-29622489 (-), Divergence to consensus: 39.6%  
**45. L1ME3**: 29622718-29622922 (-), Divergence to consensus: 46.8%  
**46. MER20**: 29622924-29623067 (-), Divergence to consensus: 34.4%  
**47. L1ME3**: 29623208-29624074 (-), Divergence to consensus: 38.5%  
**48. L1ME3**: 29624069-29624332 (-), Divergence to consensus: 34.2%  
**49. SINE2-2\_BT**: 29624333-29624439 (-), Divergence to consensus: 31.8%  
**50. L1ME3**: 29624440-29624644 (-), Divergence to consensus: 34.2%  
**51. L1ME3**: 29624672-29625650 (-), Divergence to consensus: 42.7%  
**52. ART2A**: 29625685-29625806 (-), Divergence to consensus: 18%  
**53. L1ME3**: 29625824-29626260 (-), Divergence to consensus: 42.7%  
**54. L1ME3**: 29626479-29626603 (-), Divergence to consensus: 46.4%  
**55. ART2A**: 29626640-29626651 (-), Divergence to consensus: 28.1%  
**56. (CTG)n**: 29626652-29626677 (+), Divergence to consensus: 0%  
**57. ART2A**: 29626678-29626744 (-), Divergence to consensus: 28.1%  
**58. BOV-A2**: 29626745-29626853 (-), Divergence to consensus: 5.1%  
**59. MLT1L**: 29627282-29627501 (+), Divergence to consensus: 44.2%  
**60. MLT1A**: 29628042-29628431 (-), Divergence to consensus: 34.5%  
**61. FordPrefect**: 29628665-29629116 (+), Divergence to consensus: 34.2%  
**62. FordPrefect**: 29629116-29629301 (+), Divergence to consensus: 31.2%  
**63. MIRb**: 29629886-29630009 (+), Divergence to consensus: 37.6%  
**64. L1\_BT**: 29630082-29630783 (-), Divergence to consensus: 17.6%  
**65. LTR41B**: 29631165-29631360 (+), Divergence to consensus: 27.2%  
**66. MIR**: 29631807-29632014 (-), Divergence to consensus: 47.9%  
**67. L2**: 29632172-29632947 (+), Divergence to consensus: 47.8%  
**68. MER58C**: 29633038-29633168 (-), Divergence to consensus: 31.4%  
**69. (CAGTT)n**: 29633169-29633189 (+), Divergence to consensus: 0%  
**70. ART2A**: 29633190-29633493 (-), Divergence to consensus: 16.1%  
**71. MER58C**: 29633613-29633690 (-), Divergence to consensus: 29.9%  
**72. MLT1I**: 29633691-29633834 (-), Divergence to consensus: 36.2%  
**73. LTR40a**: 29633991-29634401 (+), Divergence to consensus: 34.5%  
**74. tRNA-Gly-GGG**: 29635874-29635909 (-), Divergence to consensus: 5.6%  
**75. CHR-2A**: 29636581-29636898 (-), Divergence to consensus: 22.5%  
**76. Charlie22a**: 29637211-29637383 (+), Divergence to consensus: 35.3%  
**77. Bov-tA1**: 29637856-29638053 (-), Divergence to consensus: 14.1%  
**78. HAL1ME**: 29640636-29640703 (-), Divergence to consensus: 26.5%  
**79. HAL1ME**: 29640750-29641272 (-), Divergence to consensus: 45.1%  
**80. L1ME1**: 29641271-29641486 (+), Divergence to consensus: 30.5%  
**81. L1MB5**: 29641599-29641982 (+), Divergence to consensus: 29.6%  
**82. MLT1E1A**: 29642122-29642642 (+), Divergence to consensus: 39.2%  
**83. L1MEg**: 29642813-29643246 (+), Divergence to consensus: 50.3%  
**84. L1ME4a**: 29643499-29643626 (+), Divergence to consensus: 41.5%  
**85. MER5B**: 29643937-29644021 (+), Divergence to consensus: 44.7%  
**86. (TG)n**: 29644803-29644829 (+), Divergence to consensus: 0%  
**87. Bov-tA2**: 29644970-29645155 (-), Divergence to consensus: 24.2%  
**88. BOV-A2**: 29645188-29645435 (+), Divergence to consensus: 8.9%  
**89. Charlie2a**: 29646136-29646225 (+), Divergence to consensus: 42.7%  
**90. BOV-A2**: 29646226-29646497 (-), Divergence to consensus: 5.5%  
**91. Charlie2a**: 29646498-29646547 (+), Divergence to consensus: 42.7%  
**92. Charlie2a**: 29646559-29646786 (+), Divergence to consensus: 24.1%  
**93. Charlie2a**: 29646815-29647105 (+), Divergence to consensus: 36.4%  
**94. Charlie2a**: 29647116-29647309 (+), Divergence to consensus: 32.3%  
**95. LTR37B**: 29647310-29647404 (-), Divergence to consensus: 34.8%  
**96. SINE2-2\_BT**: 29647449-29647539 (-), Divergence to consensus: 31.9%  
**97. LTR37B**: 29647573-29647875 (-), Divergence to consensus: 48.2%  
**98. AT\_rich**: 29647922-29647943 (+), Divergence to consensus: 40.9%  
**99. Charlie2a**: 29647980-29648079 (+), Divergence to consensus: 30%  
**100. BOV-A2**: 29648117-29648351 (+), Divergence to consensus: 21.3%  
**101. ERVL-B4-int**: 29648523-29648880 (+), Divergence to consensus: 38%  
**102. ERV1-2C-LTR\_BT**: 29648905-29648953 (-), Divergence to consensus: 26.5%  
**103. ERV1-2C-LTR\_BT**: 29649315-29649446 (-), Divergence to consensus: 43.4%  
**104. ERVL-B4-int**: 29649447-29649634 (+), Divergence to consensus: 37.1%  
**105. LTR71\_BT**: 29650265-29650627 (+), Divergence to consensus: 20.9%  
**106. MLT1D**: 29651462-29651967 (+), Divergence to consensus: 37.7%

  
Transcription Factor Binding Sites  

**RFX4\_2** (Sequence: GTAACTAAG (-): 29602838)  
**RFX4\_1** (Sequence: GTTGCCATG (-): 29597368)  
**RFX4\_1** (Sequence: CTTGGCAAC (+): 29611581)  
**SPZ1** (Sequence: CTCATACCCT (-): 29615076)  
**RFX4\_2** (Sequence: CATGGATAC (+): 29594476)  
**RFX4\_2** (Sequence: CCTGGATAC (+): 29627649)  
**Gata4** (Sequence: AGATAAG (-): 29604722)  
**Gata4** (Sequence: AGATAAC (-): 29629485)  
**SOX9** (Sequence: AACAATGA (-): 29629686)  
**SOX9** (Sequence: CCATTGTT (+): 29602421)  
**SOX9** (Sequence: TTATTGTT (+): 29612147)  
**SOX9** (Sequence: CCATTGTT (+): 29623101)  
**SOX9** (Sequence: TCATTGTT (+): 29635498)  
**SOX9** (Sequence: TTATTGTT (+): 29647711)  
**A-MYB** (Sequence: CCAACTGTCT (-): 29623077)  
**SPZ1** (Sequence: AGGGTTACAG (+): 29603177)  
**SPZ1** (Sequence: GGGGTTAGAG (+): 29622679)  
**Mybl1\_1** (Sequence: AACCGTTA (+): 29621170)  
**Gata4** (Sequence: CTTATCT (+): 29593447)  
**Gata4** (Sequence: GTTATCT (+): 29612152)  
**Gata4** (Sequence: GTTATCT (+): 29640467)
