# Supplementary material for: Small RNA sequencing of cryopreserved semen from single bull revealed altered miRNAs and piRNAs expression between High- and Low-motile sperm populations
Source: BMC Genomics. 2017 Jan 4;18:14. doi: 10.1186/s12864-016-3394-7 (PMC5209821; doi:10.1186/s12864-016-3394-7)
Supplement: Additional file 3: — Details for each piRNA clusters found in High Motile (HM) sperm fraction. Genes, repeats, transposable elements and transcription factors binding sites falling within the cluster regions were reported. (ZIP 1896 kb) [file 12864_2016_3394_MOESM3_ESM.zip › 14.html]

piRNA cluster 14


Predicted piRNA cluster no. 14     previous   next
  

Show proTRAC run info
Hide proTRAC run info

================================= proTRAC ====================================  
VERSION: 2.1                                    LAST MODIFIED: 06. October 2015  
  
Please cite:  
Rosenkranz D, Zischler H. proTRAC - a software for probabilistic piRNA cluster  
detection, visualization and analysis. 2012. BMC Bioinformatics 13:5.  
  
and (for proTRAC 2.0 and later):  
Rosenkranz D, Rudloff S, Bastuck K, Ketting RF, Zischler H. Tupaia small RNAs  
provide insights into function and evolution of RNAi-based transposon defense  
in mammals. 2015. RNA 21(5):911-922.  
  
Contact:  
David Rosenkranz  
Institute of Anthropology, small RNA group  
Johannes Gutenberg University Mainz  
email: rosenkranz@uni-mainz.de  
  
You can find the latest proTRAC version at:  
http://sourceforge.net/projects/protrac/files  
http://www.smallRNAgroup-mainz.de/software  
==============================================================================  
  
PARAMETERS:  
Map file: .............../storage/core/barbara/genhome/smallRNA/fertility/Sample\_motile/pirna/Sample\_motile\_26-33\_collapsed.fa.no-dust.map.weighted-10000-1000-b-0  
Genome file: ............/storage/core/barbara/genhome/smallRNA/fertility/Sample\_all/pirna/bt\_311\_chrY.fa  
RepeatMasker annotation: /storage/genomes/bt\_umd31/GCF\_000003055.6\_Bos\_taurus\_UMD\_3.1.1\_repeatMasker\_chr.out  
GeneSet:................./storage/core/barbara/genhome/smallRNA/fertility/Sample\_all/pirna/full.gtf  
  
Significant (p<=0.01) hit density will be calculated based  
on observed hit distribution.  
  
Sliding window size: ........................................ 5000 bp  
Sliding window increament: .................................. 1000 bp  
Normalize each hit by number of genomic hits: ............... 1 [0=no/1=yes]  
Normalize each hit by number of sequence reads: ............. 1 [0=no/1=yes]  
Normalize values (-> per million mapped reads): ............. 1 [0=no/1=yes]  
Min. fraction of hits with 1T(U) or 10A: .................... 0.75  
Alternatively: Min. fraction of hits with 1T(U) and 10A: .... 0.5  
Min. fraction of hits with typical piRNA length: ............ 0.75  
Typical piRNA length: ....................................... 26-33 nt  
Min. size of a piRNA cluster: ............................... 5000 bp.  
Min. number of hits (absolute): ............................. 0  
Min. number of hits (normalized): ........................... 0  
Min. fraction of hits on the mainstrand: .................... 0.75  
Top fraction of mapped sequences (in terms of read counts): . 1%  
Top fraction accounts for max. n% of sequence reads: ........ 90%  
Min. fraction of hits on each arm of a bidirectional cluster: 0.1  
Output image file for each cluster: ......................... 0 [0=no/1=yes]  
Output html file for each cluster: .......................... 1 [0=no/1=yes]  
Output a summary table: ..................................... 1 [0=no/1=yes]  
Output a FASTA file for each cluster (piRNA sequences): ..... 1 [0=no/1=yes]  
Output a FASTA file comprising cluster sequences: ........... 1 [0=no/1=yes]  
Search DNA motifs in clusters: .............................. 1 [0=no/1=yes]  
Output flanking sequences: +/- .............................. 0 bp  
Output ~.pTi file: .......................................... 1 [0=no/1=yes]  
==============================================================================  
  
  
Genome size (without gaps): ............ 2678902517 bp  
Gaps (N/X/-): .......................... 53837044 bp  
Mapped reads: .......................... 658825247023  
Non-identical sequences: ............... 514171  
Genomic hits: .......................... 764233  
Significant densitiy of mapped reads: .. 12867599.5173724 reads/kb

Show proTRAC cluster info
Hide proTRAC cluster info

|  |  |
| --- | --- |
| Location | chr12 |
| Coordinates | 34906127-34911948 |
| Size [bp] | 5822 |
| Sequence hit loci | 53 |
| Mapped reads (normalized) | 71590736 |
| Mapped reads (normalized) per kb | 12296588.1 |
| Normalized reads with 1T (1U) | 84.8% |
| Normalized reads with 10A | 30.7% |
| Normalized reads with length 26-33 nt | 100% |
| Normalized reads on the main strand(s) | 100% |
| Predicted directionality | mono:plus |

100%

0%

1T (1U)  
reads

10A reads

26-33 nt  
reads

reads on mainstrand

**Either the amount of reads with 1T (1U) OR 10A has to exceed 75% (set with option: -1Tor10A)  
Alternatively the amount of reads with 1T (1U) AND 10A has to exceed 50% (set with option: -1Tand10A)  
Minimum amount of reads with preferred size is 75% (set with option: -pisize)  
Minimum amount of reads on the main strand(s) is 75% (set with option: -clstrand)**

Show read coverage
Hide read coverage

WHAT DO I SEE HERE?  
This chart shows the location of mapped sequence reads within a predicted piRNA cluster. The color refers to the number of genomic hits produced by the sequence read in question. A dark red bar indicates that this sequence read produces many other hits elsewhere in the genome. Many adjacent red or yellow bars can indicate the presence of a multi-copy element such as transposons or rRNA genes. A dark green bar indicates that this sequence read maps uniquely to this locus.

1 hit

2-5 hits

6-10 hits

11-20 hits

21-50 hits

51-100 hits

> 100 hits

chr12

34906127

34911948

Gene Set

RepeatMasker

Mapped  
Reads

7.95

plus strand

minus strand

7.95

Region: chr12 29652210-34906132. Max. coverage (+): 0.42. Max coverage (-): 0

Region: chr12 34906133-34906144. Max. coverage (+): 0.42. Max coverage (-): 0

Region: chr12 34906145-34906156. Max. coverage (+): 0. Max coverage (-): 0

Region: chr12 34906157-34906167. Max. coverage (+): 0. Max coverage (-): 0

Region: chr12 34906168-34906179. Max. coverage (+): 0. Max coverage (-): 0

Region: chr12 34906180-34906191. Max. coverage (+): 0. Max coverage (-): 0

Region: chr12 34906192-34906202. Max. coverage (+): 0. Max coverage (-): 0

Region: chr12 34906203-34906214. Max. coverage (+): 0. Max coverage (-): 0

Region: chr12 34906215-34906225. Max. coverage (+): 0. Max coverage (-): 0

Region: chr12 34906226-34906237. Max. coverage (+): 0. Max coverage (-): 0

Region: chr12 34906238-34906249. Max. coverage (+): 0. Max coverage (-): 0

Region: chr12 34906250-34906260. Max. coverage (+): 0. Max coverage (-): 0

Region: chr12 34906261-34906272. Max. coverage (+): 0. Max coverage (-): 0

Region: chr12 34906273-34906284. Max. coverage (+): 0. Max coverage (-): 0

Region: chr12 34906285-34906295. Max. coverage (+): 0. Max coverage (-): 0

Region: chr12 34906296-34906307. Max. coverage (+): 0. Max coverage (-): 0

Region: chr12 34906308-34906319. Max. coverage (+): 0. Max coverage (-): 0

Region: chr12 34906320-34906330. Max. coverage (+): 0. Max coverage (-): 0

Region: chr12 34906331-34906342. Max. coverage (+): 4.36. Max coverage (-): 0

Region: chr12 34906343-34906354. Max. coverage (+): 4.36. Max coverage (-): 0

Region: chr12 34906355-34906365. Max. coverage (+): 0. Max coverage (-): 0

Region: chr12 34906366-34906377. Max. coverage (+): 0. Max coverage (-): 0

Region: chr12 34906378-34906388. Max. coverage (+): 0. Max coverage (-): 0

Region: chr12 34906389-34906400. Max. coverage (+): 0. Max coverage (-): 0

Region: chr12 34906401-34906412. Max. coverage (+): 0. Max coverage (-): 0

Region: chr12 34906413-34906423. Max. coverage (+): 0. Max coverage (-): 0

Region: chr12 34906424-34906435. Max. coverage (+): 0. Max coverage (-): 0

Region: chr12 34906436-34906447. Max. coverage (+): 0. Max coverage (-): 0

Region: chr12 34906448-34906458. Max. coverage (+): 0. Max coverage (-): 0

Region: chr12 34906459-34906470. Max. coverage (+): 0. Max coverage (-): 0

Region: chr12 34906471-34906482. Max. coverage (+): 0. Max coverage (-): 0

Region: chr12 34906483-34906493. Max. coverage (+): 0. Max coverage (-): 0

Region: chr12 34906494-34906505. Max. coverage (+): 0. Max coverage (-): 0

Region: chr12 34906506-34906517. Max. coverage (+): 0. Max coverage (-): 0

Region: chr12 34906518-34906528. Max. coverage (+): 0. Max coverage (-): 0

Region: chr12 34906529-34906540. Max. coverage (+): 0. Max coverage (-): 0

Region: chr12 34906541-34906552. Max. coverage (+): 0. Max coverage (-): 0

Region: chr12 34906553-34906563. Max. coverage (+): 0. Max coverage (-): 0

Region: chr12 34906564-34906575. Max. coverage (+): 0. Max coverage (-): 0

Region: chr12 34906576-34906586. Max. coverage (+): 0. Max coverage (-): 0

Region: chr12 34906587-34906598. Max. coverage (+): 0. Max coverage (-): 0

Region: chr12 34906599-34906610. Max. coverage (+): 1.65. Max coverage (-): 0

Region: chr12 34906611-34906621. Max. coverage (+): 1.65. Max coverage (-): 0

Region: chr12 34906622-34906633. Max. coverage (+): 0. Max coverage (-): 0

Region: chr12 34906634-34906645. Max. coverage (+): 0. Max coverage (-): 0

Region: chr12 34906646-34906656. Max. coverage (+): 0. Max coverage (-): 0

Region: chr12 34906657-34906668. Max. coverage (+): 0. Max coverage (-): 0

Region: chr12 34906669-34906680. Max. coverage (+): 0. Max coverage (-): 0

Region: chr12 34906681-34906691. Max. coverage (+): 0. Max coverage (-): 0

Region: chr12 34906692-34906703. Max. coverage (+): 0. Max coverage (-): 0

Region: chr12 34906704-34906715. Max. coverage (+): 0. Max coverage (-): 0

Region: chr12 34906716-34906726. Max. coverage (+): 0. Max coverage (-): 0

Region: chr12 34906727-34906738. Max. coverage (+): 0. Max coverage (-): 0

Region: chr12 34906739-34906749. Max. coverage (+): 0. Max coverage (-): 0

Region: chr12 34906750-34906761. Max. coverage (+): 0. Max coverage (-): 0

Region: chr12 34906762-34906773. Max. coverage (+): 0. Max coverage (-): 0

Region: chr12 34906774-34906784. Max. coverage (+): 0. Max coverage (-): 0

Region: chr12 34906785-34906796. Max. coverage (+): 0. Max coverage (-): 0

Region: chr12 34906797-34906808. Max. coverage (+): 0. Max coverage (-): 0

Region: chr12 34906809-34906819. Max. coverage (+): 0. Max coverage (-): 0

Region: chr12 34906820-34906831. Max. coverage (+): 0. Max coverage (-): 0

Region: chr12 34906832-34906843. Max. coverage (+): 0. Max coverage (-): 0

Region: chr12 34906844-34906854. Max. coverage (+): 0. Max coverage (-): 0

Region: chr12 34906855-34906866. Max. coverage (+): 0. Max coverage (-): 0

Region: chr12 34906867-34906878. Max. coverage (+): 0. Max coverage (-): 0

Region: chr12 34906879-34906889. Max. coverage (+): 0. Max coverage (-): 0

Region: chr12 34906890-34906901. Max. coverage (+): 0. Max coverage (-): 0

Region: chr12 34906902-34906912. Max. coverage (+): 0. Max coverage (-): 0

Region: chr12 34906913-34906924. Max. coverage (+): 0. Max coverage (-): 0

Region: chr12 34906925-34906936. Max. coverage (+): 0. Max coverage (-): 0

Region: chr12 34906937-34906947. Max. coverage (+): 0. Max coverage (-): 0

Region: chr12 34906948-34906959. Max. coverage (+): 0. Max coverage (-): 0

Region: chr12 34906960-34906971. Max. coverage (+): 0. Max coverage (-): 0

Region: chr12 34906972-34906982. Max. coverage (+): 0. Max coverage (-): 0

Region: chr12 34906983-34906994. Max. coverage (+): 0. Max coverage (-): 0

Region: chr12 34906995-34907006. Max. coverage (+): 0. Max coverage (-): 0

Region: chr12 34907007-34907017. Max. coverage (+): 0. Max coverage (-): 0

Region: chr12 34907018-34907029. Max. coverage (+): 0. Max coverage (-): 0

Region: chr12 34907030-34907041. Max. coverage (+): 0. Max coverage (-): 0

Region: chr12 34907042-34907052. Max. coverage (+): 0. Max coverage (-): 0

Region: chr12 34907053-34907064. Max. coverage (+): 0. Max coverage (-): 0

Region: chr12 34907065-34907075. Max. coverage (+): 0. Max coverage (-): 0

Region: chr12 34907076-34907087. Max. coverage (+): 0. Max coverage (-): 0

Region: chr12 34907088-34907099. Max. coverage (+): 0. Max coverage (-): 0

Region: chr12 34907100-34907110. Max. coverage (+): 0. Max coverage (-): 0

Region: chr12 34907111-34907122. Max. coverage (+): 0. Max coverage (-): 0

Region: chr12 34907123-34907134. Max. coverage (+): 0. Max coverage (-): 0

Region: chr12 34907135-34907145. Max. coverage (+): 0. Max coverage (-): 0

Region: chr12 34907146-34907157. Max. coverage (+): 0. Max coverage (-): 0

Region: chr12 34907158-34907169. Max. coverage (+): 0. Max coverage (-): 0

Region: chr12 34907170-34907180. Max. coverage (+): 0. Max coverage (-): 0

Region: chr12 34907181-34907192. Max. coverage (+): 0. Max coverage (-): 0

Region: chr12 34907193-34907204. Max. coverage (+): 0. Max coverage (-): 0

Region: chr12 34907205-34907215. Max. coverage (+): 0. Max coverage (-): 0

Region: chr12 34907216-34907227. Max. coverage (+): 0. Max coverage (-): 0

Region: chr12 34907228-34907239. Max. coverage (+): 2.12. Max coverage (-): 0

Region: chr12 34907240-34907250. Max. coverage (+): 2.12. Max coverage (-): 0

Region: chr12 34907251-34907262. Max. coverage (+): 0. Max coverage (-): 0

Region: chr12 34907263-34907273. Max. coverage (+): 0. Max coverage (-): 0

Region: chr12 34907274-34907285. Max. coverage (+): 2.45. Max coverage (-): 0

Region: chr12 34907286-34907297. Max. coverage (+): 2.45. Max coverage (-): 0

Region: chr12 34907298-34907308. Max. coverage (+): 0. Max coverage (-): 0

Region: chr12 34907309-34907320. Max. coverage (+): 0. Max coverage (-): 0

Region: chr12 34907321-34907332. Max. coverage (+): 0. Max coverage (-): 0

Region: chr12 34907333-34907343. Max. coverage (+): 0. Max coverage (-): 0

Region: chr12 34907344-34907355. Max. coverage (+): 0. Max coverage (-): 0

Region: chr12 34907356-34907367. Max. coverage (+): 0. Max coverage (-): 0

Region: chr12 34907368-34907378. Max. coverage (+): 0. Max coverage (-): 0

Region: chr12 34907379-34907390. Max. coverage (+): 0. Max coverage (-): 0

Region: chr12 34907391-34907402. Max. coverage (+): 0. Max coverage (-): 0

Region: chr12 34907403-34907413. Max. coverage (+): 0. Max coverage (-): 0

Region: chr12 34907414-34907425. Max. coverage (+): 0. Max coverage (-): 0

Region: chr12 34907426-34907436. Max. coverage (+): 0. Max coverage (-): 0

Region: chr12 34907437-34907448. Max. coverage (+): 0. Max coverage (-): 0

Region: chr12 34907449-34907460. Max. coverage (+): 0. Max coverage (-): 0

Region: chr12 34907461-34907471. Max. coverage (+): 0. Max coverage (-): 0

Region: chr12 34907472-34907483. Max. coverage (+): 0. Max coverage (-): 0

Region: chr12 34907484-34907495. Max. coverage (+): 0. Max coverage (-): 0

Region: chr12 34907496-34907506. Max. coverage (+): 0. Max coverage (-): 0

Region: chr12 34907507-34907518. Max. coverage (+): 0. Max coverage (-): 0

Region: chr12 34907519-34907530. Max. coverage (+): 0. Max coverage (-): 0

Region: chr12 34907531-34907541. Max. coverage (+): 0. Max coverage (-): 0

Region: chr12 34907542-34907553. Max. coverage (+): 0. Max coverage (-): 0

Region: chr12 34907554-34907565. Max. coverage (+): 0. Max coverage (-): 0

Region: chr12 34907566-34907576. Max. coverage (+): 0. Max coverage (-): 0

Region: chr12 34907577-34907588. Max. coverage (+): 0. Max coverage (-): 0

Region: chr12 34907589-34907599. Max. coverage (+): 0. Max coverage (-): 0

Region: chr12 34907600-34907611. Max. coverage (+): 0. Max coverage (-): 0

Region: chr12 34907612-34907623. Max. coverage (+): 0. Max coverage (-): 0

Region: chr12 34907624-34907634. Max. coverage (+): 0. Max coverage (-): 0

Region: chr12 34907635-34907646. Max. coverage (+): 0. Max coverage (-): 0

Region: chr12 34907647-34907658. Max. coverage (+): 0. Max coverage (-): 0

Region: chr12 34907659-34907669. Max. coverage (+): 0. Max coverage (-): 0

Region: chr12 34907670-34907681. Max. coverage (+): 0. Max coverage (-): 0

Region: chr12 34907682-34907693. Max. coverage (+): 0. Max coverage (-): 0

Region: chr12 34907694-34907704. Max. coverage (+): 0. Max coverage (-): 0

Region: chr12 34907705-34907716. Max. coverage (+): 0. Max coverage (-): 0

Region: chr12 34907717-34907728. Max. coverage (+): 0. Max coverage (-): 0

Region: chr12 34907729-34907739. Max. coverage (+): 0. Max coverage (-): 0

Region: chr12 34907740-34907751. Max. coverage (+): 0. Max coverage (-): 0

Region: chr12 34907752-34907762. Max. coverage (+): 0. Max coverage (-): 0

Region: chr12 34907763-34907774. Max. coverage (+): 0. Max coverage (-): 0

Region: chr12 34907775-34907786. Max. coverage (+): 0. Max coverage (-): 0

Region: chr12 34907787-34907797. Max. coverage (+): 0. Max coverage (-): 0

Region: chr12 34907798-34907809. Max. coverage (+): 0. Max coverage (-): 0

Region: chr12 34907810-34907821. Max. coverage (+): 0. Max coverage (-): 0

Region: chr12 34907822-34907832. Max. coverage (+): 0. Max coverage (-): 0

Region: chr12 34907833-34907844. Max. coverage (+): 0. Max coverage (-): 0

Region: chr12 34907845-34907856. Max. coverage (+): 0. Max coverage (-): 0

Region: chr12 34907857-34907867. Max. coverage (+): 0. Max coverage (-): 0

Region: chr12 34907868-34907879. Max. coverage (+): 0. Max coverage (-): 0

Region: chr12 34907880-34907891. Max. coverage (+): 0. Max coverage (-): 0

Region: chr12 34907892-34907902. Max. coverage (+): 0. Max coverage (-): 0

Region: chr12 34907903-34907914. Max. coverage (+): 0. Max coverage (-): 0

Region: chr12 34907915-34907925. Max. coverage (+): 0. Max coverage (-): 0

Region: chr12 34907926-34907937. Max. coverage (+): 0. Max coverage (-): 0

Region: chr12 34907938-34907949. Max. coverage (+): 0. Max coverage (-): 0

Region: chr12 34907950-34907960. Max. coverage (+): 0. Max coverage (-): 0

Region: chr12 34907961-34907972. Max. coverage (+): 0. Max coverage (-): 0

Region: chr12 34907973-34907984. Max. coverage (+): 0. Max coverage (-): 0

Region: chr12 34907985-34907995. Max. coverage (+): 0. Max coverage (-): 0

Region: chr12 34907996-34908007. Max. coverage (+): 0. Max coverage (-): 0

Region: chr12 34908008-34908019. Max. coverage (+): 0. Max coverage (-): 0

Region: chr12 34908020-34908030. Max. coverage (+): 0. Max coverage (-): 0

Region: chr12 34908031-34908042. Max. coverage (+): 0. Max coverage (-): 0

Region: chr12 34908043-34908054. Max. coverage (+): 0. Max coverage (-): 0

Region: chr12 34908055-34908065. Max. coverage (+): 0. Max coverage (-): 0

Region: chr12 34908066-34908077. Max. coverage (+): 0. Max coverage (-): 0

Region: chr12 34908078-34908089. Max. coverage (+): 0. Max coverage (-): 0

Region: chr12 34908090-34908100. Max. coverage (+): 0. Max coverage (-): 0

Region: chr12 34908101-34908112. Max. coverage (+): 2.07. Max coverage (-): 0

Region: chr12 34908113-34908123. Max. coverage (+): 2.07. Max coverage (-): 0

Region: chr12 34908124-34908135. Max. coverage (+): 0. Max coverage (-): 0

Region: chr12 34908136-34908147. Max. coverage (+): 0. Max coverage (-): 0

Region: chr12 34908148-34908158. Max. coverage (+): 0. Max coverage (-): 0

Region: chr12 34908159-34908170. Max. coverage (+): 0. Max coverage (-): 0

Region: chr12 34908171-34908182. Max. coverage (+): 0. Max coverage (-): 0

Region: chr12 34908183-34908193. Max. coverage (+): 0. Max coverage (-): 0

Region: chr12 34908194-34908205. Max. coverage (+): 0. Max coverage (-): 0

Region: chr12 34908206-34908217. Max. coverage (+): 0. Max coverage (-): 0

Region: chr12 34908218-34908228. Max. coverage (+): 0. Max coverage (-): 0

Region: chr12 34908229-34908240. Max. coverage (+): 0. Max coverage (-): 0

Region: chr12 34908241-34908252. Max. coverage (+): 0. Max coverage (-): 0

Region: chr12 34908253-34908263. Max. coverage (+): 0. Max coverage (-): 0

Region: chr12 34908264-34908275. Max. coverage (+): 7.89. Max coverage (-): 0

Region: chr12 34908276-34908286. Max. coverage (+): 7.89. Max coverage (-): 0

Region: chr12 34908287-34908298. Max. coverage (+): 0. Max coverage (-): 0

Region: chr12 34908299-34908310. Max. coverage (+): 0.58. Max coverage (-): 0

Region: chr12 34908311-34908321. Max. coverage (+): 0. Max coverage (-): 0

Region: chr12 34908322-34908333. Max. coverage (+): 0. Max coverage (-): 0

Region: chr12 34908334-34908345. Max. coverage (+): 0. Max coverage (-): 0

Region: chr12 34908346-34908356. Max. coverage (+): 0. Max coverage (-): 0

Region: chr12 34908357-34908368. Max. coverage (+): 0. Max coverage (-): 0

Region: chr12 34908369-34908380. Max. coverage (+): 7.95. Max coverage (-): 0

Region: chr12 34908381-34908391. Max. coverage (+): 7.95. Max coverage (-): 0

Region: chr12 34908392-34908403. Max. coverage (+): 0. Max coverage (-): 0

Region: chr12 34908404-34908415. Max. coverage (+): 0. Max coverage (-): 0

Region: chr12 34908416-34908426. Max. coverage (+): 0. Max coverage (-): 0

Region: chr12 34908427-34908438. Max. coverage (+): 0. Max coverage (-): 0

Region: chr12 34908439-34908449. Max. coverage (+): 0. Max coverage (-): 0

Region: chr12 34908450-34908461. Max. coverage (+): 0. Max coverage (-): 0

Region: chr12 34908462-34908473. Max. coverage (+): 0. Max coverage (-): 0

Region: chr12 34908474-34908484. Max. coverage (+): 0. Max coverage (-): 0

Region: chr12 34908485-34908496. Max. coverage (+): 0. Max coverage (-): 0

Region: chr12 34908497-34908508. Max. coverage (+): 0. Max coverage (-): 0

Region: chr12 34908509-34908519. Max. coverage (+): 0. Max coverage (-): 0

Region: chr12 34908520-34908531. Max. coverage (+): 0. Max coverage (-): 0

Region: chr12 34908532-34908543. Max. coverage (+): 0. Max coverage (-): 0

Region: chr12 34908544-34908554. Max. coverage (+): 0. Max coverage (-): 0

Region: chr12 34908555-34908566. Max. coverage (+): 0. Max coverage (-): 0

Region: chr12 34908567-34908578. Max. coverage (+): 0. Max coverage (-): 0

Region: chr12 34908579-34908589. Max. coverage (+): 0. Max coverage (-): 0

Region: chr12 34908590-34908601. Max. coverage (+): 0. Max coverage (-): 0

Region: chr12 34908602-34908612. Max. coverage (+): 0. Max coverage (-): 0

Region: chr12 34908613-34908624. Max. coverage (+): 0. Max coverage (-): 0

Region: chr12 34908625-34908636. Max. coverage (+): 1.75. Max coverage (-): 0

Region: chr12 34908637-34908647. Max. coverage (+): 1.75. Max coverage (-): 0

Region: chr12 34908648-34908659. Max. coverage (+): 0. Max coverage (-): 0

Region: chr12 34908660-34908671. Max. coverage (+): 0. Max coverage (-): 0

Region: chr12 34908672-34908682. Max. coverage (+): 0. Max coverage (-): 0

Region: chr12 34908683-34908694. Max. coverage (+): 0. Max coverage (-): 0

Region: chr12 34908695-34908706. Max. coverage (+): 0. Max coverage (-): 0

Region: chr12 34908707-34908717. Max. coverage (+): 0. Max coverage (-): 0

Region: chr12 34908718-34908729. Max. coverage (+): 0. Max coverage (-): 0

Region: chr12 34908730-34908741. Max. coverage (+): 0. Max coverage (-): 0

Region: chr12 34908742-34908752. Max. coverage (+): 0. Max coverage (-): 0

Region: chr12 34908753-34908764. Max. coverage (+): 0. Max coverage (-): 0

Region: chr12 34908765-34908776. Max. coverage (+): 0. Max coverage (-): 0

Region: chr12 34908777-34908787. Max. coverage (+): 0. Max coverage (-): 0

Region: chr12 34908788-34908799. Max. coverage (+): 0.7. Max coverage (-): 0

Region: chr12 34908800-34908810. Max. coverage (+): 0.7. Max coverage (-): 0

Region: chr12 34908811-34908822. Max. coverage (+): 0. Max coverage (-): 0

Region: chr12 34908823-34908834. Max. coverage (+): 0. Max coverage (-): 0

Region: chr12 34908835-34908845. Max. coverage (+): 0. Max coverage (-): 0

Region: chr12 34908846-34908857. Max. coverage (+): 0. Max coverage (-): 0

Region: chr12 34908858-34908869. Max. coverage (+): 0. Max coverage (-): 0

Region: chr12 34908870-34908880. Max. coverage (+): 0. Max coverage (-): 0

Region: chr12 34908881-34908892. Max. coverage (+): 0. Max coverage (-): 0

Region: chr12 34908893-34908904. Max. coverage (+): 0. Max coverage (-): 0

Region: chr12 34908905-34908915. Max. coverage (+): 0. Max coverage (-): 0

Region: chr12 34908916-34908927. Max. coverage (+): 0. Max coverage (-): 0

Region: chr12 34908928-34908939. Max. coverage (+): 0. Max coverage (-): 0

Region: chr12 34908940-34908950. Max. coverage (+): 7.03. Max coverage (-): 0

Region: chr12 34908951-34908962. Max. coverage (+): 2.01. Max coverage (-): 0

Region: chr12 34908963-34908973. Max. coverage (+): 0.97. Max coverage (-): 0

Region: chr12 34908974-34908985. Max. coverage (+): 0. Max coverage (-): 0

Region: chr12 34908986-34908997. Max. coverage (+): 0. Max coverage (-): 0

Region: chr12 34908998-34909008. Max. coverage (+): 0. Max coverage (-): 0

Region: chr12 34909009-34909020. Max. coverage (+): 0. Max coverage (-): 0

Region: chr12 34909021-34909032. Max. coverage (+): 0. Max coverage (-): 0

Region: chr12 34909033-34909043. Max. coverage (+): 0. Max coverage (-): 0

Region: chr12 34909044-34909055. Max. coverage (+): 0. Max coverage (-): 0

Region: chr12 34909056-34909067. Max. coverage (+): 0. Max coverage (-): 0

Region: chr12 34909068-34909078. Max. coverage (+): 0. Max coverage (-): 0

Region: chr12 34909079-34909090. Max. coverage (+): 0. Max coverage (-): 0

Region: chr12 34909091-34909102. Max. coverage (+): 5.18. Max coverage (-): 0

Region: chr12 34909103-34909113. Max. coverage (+): 5.18. Max coverage (-): 0

Region: chr12 34909114-34909125. Max. coverage (+): 0. Max coverage (-): 0

Region: chr12 34909126-34909136. Max. coverage (+): 0. Max coverage (-): 0

Region: chr12 34909137-34909148. Max. coverage (+): 0. Max coverage (-): 0

Region: chr12 34909149-34909160. Max. coverage (+): 0. Max coverage (-): 0

Region: chr12 34909161-34909171. Max. coverage (+): 0. Max coverage (-): 0

Region: chr12 34909172-34909183. Max. coverage (+): 0. Max coverage (-): 0

Region: chr12 34909184-34909195. Max. coverage (+): 4.91. Max coverage (-): 0

Region: chr12 34909196-34909206. Max. coverage (+): 7.17. Max coverage (-): 0

Region: chr12 34909207-34909218. Max. coverage (+): 0. Max coverage (-): 0

Region: chr12 34909219-34909230. Max. coverage (+): 0. Max coverage (-): 0

Region: chr12 34909231-34909241. Max. coverage (+): 0. Max coverage (-): 0

Region: chr12 34909242-34909253. Max. coverage (+): 0. Max coverage (-): 0

Region: chr12 34909254-34909265. Max. coverage (+): 0. Max coverage (-): 0

Region: chr12 34909266-34909276. Max. coverage (+): 0. Max coverage (-): 0

Region: chr12 34909277-34909288. Max. coverage (+): 0. Max coverage (-): 0

Region: chr12 34909289-34909299. Max. coverage (+): 0. Max coverage (-): 0

Region: chr12 34909300-34909311. Max. coverage (+): 0. Max coverage (-): 0

Region: chr12 34909312-34909323. Max. coverage (+): 0. Max coverage (-): 0

Region: chr12 34909324-34909334. Max. coverage (+): 0. Max coverage (-): 0

Region: chr12 34909335-34909346. Max. coverage (+): 0. Max coverage (-): 0

Region: chr12 34909347-34909358. Max. coverage (+): 0. Max coverage (-): 0

Region: chr12 34909359-34909369. Max. coverage (+): 0. Max coverage (-): 0

Region: chr12 34909370-34909381. Max. coverage (+): 0. Max coverage (-): 0

Region: chr12 34909382-34909393. Max. coverage (+): 0. Max coverage (-): 0

Region: chr12 34909394-34909404. Max. coverage (+): 0. Max coverage (-): 0

Region: chr12 34909405-34909416. Max. coverage (+): 0. Max coverage (-): 0

Region: chr12 34909417-34909428. Max. coverage (+): 0. Max coverage (-): 0

Region: chr12 34909429-34909439. Max. coverage (+): 0. Max coverage (-): 0

Region: chr12 34909440-34909451. Max. coverage (+): 0. Max coverage (-): 0

Region: chr12 34909452-34909463. Max. coverage (+): 0. Max coverage (-): 0

Region: chr12 34909464-34909474. Max. coverage (+): 0. Max coverage (-): 0

Region: chr12 34909475-34909486. Max. coverage (+): 0. Max coverage (-): 0

Region: chr12 34909487-34909497. Max. coverage (+): 0. Max coverage (-): 0

Region: chr12 34909498-34909509. Max. coverage (+): 0. Max coverage (-): 0

Region: chr12 34909510-34909521. Max. coverage (+): 0. Max coverage (-): 0

Region: chr12 34909522-34909532. Max. coverage (+): 0. Max coverage (-): 0

Region: chr12 34909533-34909544. Max. coverage (+): 0. Max coverage (-): 0

Region: chr12 34909545-34909556. Max. coverage (+): 0. Max coverage (-): 0

Region: chr12 34909557-34909567. Max. coverage (+): 5.06. Max coverage (-): 0

Region: chr12 34909568-34909579. Max. coverage (+): 5.06. Max coverage (-): 0

Region: chr12 34909580-34909591. Max. coverage (+): 0. Max coverage (-): 0

Region: chr12 34909592-34909602. Max. coverage (+): 0. Max coverage (-): 0

Region: chr12 34909603-34909614. Max. coverage (+): 0. Max coverage (-): 0

Region: chr12 34909615-34909626. Max. coverage (+): 0. Max coverage (-): 0

Region: chr12 34909627-34909637. Max. coverage (+): 0. Max coverage (-): 0

Region: chr12 34909638-34909649. Max. coverage (+): 0. Max coverage (-): 0

Region: chr12 34909650-34909660. Max. coverage (+): 0. Max coverage (-): 0

Region: chr12 34909661-34909672. Max. coverage (+): 0. Max coverage (-): 0

Region: chr12 34909673-34909684. Max. coverage (+): 0. Max coverage (-): 0

Region: chr12 34909685-34909695. Max. coverage (+): 0. Max coverage (-): 0

Region: chr12 34909696-34909707. Max. coverage (+): 0.62. Max coverage (-): 0

Region: chr12 34909708-34909719. Max. coverage (+): 7.42. Max coverage (-): 0

Region: chr12 34909720-34909730. Max. coverage (+): 0. Max coverage (-): 0

Region: chr12 34909731-34909742. Max. coverage (+): 0. Max coverage (-): 0

Region: chr12 34909743-34909754. Max. coverage (+): 0. Max coverage (-): 0

Region: chr12 34909755-34909765. Max. coverage (+): 4.04. Max coverage (-): 0

Region: chr12 34909766-34909777. Max. coverage (+): 4.04. Max coverage (-): 0

Region: chr12 34909778-34909789. Max. coverage (+): 0. Max coverage (-): 0

Region: chr12 34909790-34909800. Max. coverage (+): 0. Max coverage (-): 0

Region: chr12 34909801-34909812. Max. coverage (+): 0. Max coverage (-): 0

Region: chr12 34909813-34909823. Max. coverage (+): 0. Max coverage (-): 0

Region: chr12 34909824-34909835. Max. coverage (+): 0. Max coverage (-): 0

Region: chr12 34909836-34909847. Max. coverage (+): 0. Max coverage (-): 0

Region: chr12 34909848-34909858. Max. coverage (+): 2.17. Max coverage (-): 0

Region: chr12 34909859-34909870. Max. coverage (+): 0. Max coverage (-): 0

Region: chr12 34909871-34909882. Max. coverage (+): 0. Max coverage (-): 0

Region: chr12 34909883-34909893. Max. coverage (+): 3.77. Max coverage (-): 0

Region: chr12 34909894-34909905. Max. coverage (+): 0. Max coverage (-): 0

Region: chr12 34909906-34909917. Max. coverage (+): 0. Max coverage (-): 0

Region: chr12 34909918-34909928. Max. coverage (+): 0. Max coverage (-): 0

Region: chr12 34909929-34909940. Max. coverage (+): 0. Max coverage (-): 0

Region: chr12 34909941-34909952. Max. coverage (+): 0. Max coverage (-): 0

Region: chr12 34909953-34909963. Max. coverage (+): 0. Max coverage (-): 0

Region: chr12 34909964-34909975. Max. coverage (+): 0. Max coverage (-): 0

Region: chr12 34909976-34909986. Max. coverage (+): 0. Max coverage (-): 0

Region: chr12 34909987-34909998. Max. coverage (+): 0. Max coverage (-): 0

Region: chr12 34909999-34910010. Max. coverage (+): 0. Max coverage (-): 0

Region: chr12 34910011-34910021. Max. coverage (+): 0. Max coverage (-): 0

Region: chr12 34910022-34910033. Max. coverage (+): 0. Max coverage (-): 0

Region: chr12 34910034-34910045. Max. coverage (+): 0. Max coverage (-): 0

Region: chr12 34910046-34910056. Max. coverage (+): 0. Max coverage (-): 0

Region: chr12 34910057-34910068. Max. coverage (+): 0. Max coverage (-): 0

Region: chr12 34910069-34910080. Max. coverage (+): 1.43. Max coverage (-): 0

Region: chr12 34910081-34910091. Max. coverage (+): 3.38. Max coverage (-): 0

Region: chr12 34910092-34910103. Max. coverage (+): 6.41. Max coverage (-): 0

Region: chr12 34910104-34910115. Max. coverage (+): 0. Max coverage (-): 0

Region: chr12 34910116-34910126. Max. coverage (+): 0. Max coverage (-): 0

Region: chr12 34910127-34910138. Max. coverage (+): 0. Max coverage (-): 0

Region: chr12 34910139-34910150. Max. coverage (+): 0. Max coverage (-): 0

Region: chr12 34910151-34910161. Max. coverage (+): 0. Max coverage (-): 0

Region: chr12 34910162-34910173. Max. coverage (+): 0. Max coverage (-): 0

Region: chr12 34910174-34910184. Max. coverage (+): 0. Max coverage (-): 0

Region: chr12 34910185-34910196. Max. coverage (+): 0. Max coverage (-): 0

Region: chr12 34910197-34910208. Max. coverage (+): 0. Max coverage (-): 0

Region: chr12 34910209-34910219. Max. coverage (+): 0. Max coverage (-): 0

Region: chr12 34910220-34910231. Max. coverage (+): 0. Max coverage (-): 0

Region: chr12 34910232-34910243. Max. coverage (+): 0. Max coverage (-): 0

Region: chr12 34910244-34910254. Max. coverage (+): 0. Max coverage (-): 0

Region: chr12 34910255-34910266. Max. coverage (+): 0. Max coverage (-): 0

Region: chr12 34910267-34910278. Max. coverage (+): 0. Max coverage (-): 0

Region: chr12 34910279-34910289. Max. coverage (+): 0. Max coverage (-): 0

Region: chr12 34910290-34910301. Max. coverage (+): 0. Max coverage (-): 0

Region: chr12 34910302-34910313. Max. coverage (+): 0. Max coverage (-): 0

Region: chr12 34910314-34910324. Max. coverage (+): 0. Max coverage (-): 0

Region: chr12 34910325-34910336. Max. coverage (+): 0. Max coverage (-): 0

Region: chr12 34910337-34910347. Max. coverage (+): 3.16. Max coverage (-): 0

Region: chr12 34910348-34910359. Max. coverage (+): 0. Max coverage (-): 0

Region: chr12 34910360-34910371. Max. coverage (+): 0. Max coverage (-): 0

Region: chr12 34910372-34910382. Max. coverage (+): 0. Max coverage (-): 0

Region: chr12 34910383-34910394. Max. coverage (+): 0. Max coverage (-): 0

Region: chr12 34910395-34910406. Max. coverage (+): 2.3. Max coverage (-): 0

Region: chr12 34910407-34910417. Max. coverage (+): 2.3. Max coverage (-): 0

Region: chr12 34910418-34910429. Max. coverage (+): 0. Max coverage (-): 0

Region: chr12 34910430-34910441. Max. coverage (+): 0. Max coverage (-): 0

Region: chr12 34910442-34910452. Max. coverage (+): 0. Max coverage (-): 0

Region: chr12 34910453-34910464. Max. coverage (+): 0. Max coverage (-): 0

Region: chr12 34910465-34910476. Max. coverage (+): 0.84. Max coverage (-): 0

Region: chr12 34910477-34910487. Max. coverage (+): 0. Max coverage (-): 0

Region: chr12 34910488-34910499. Max. coverage (+): 0. Max coverage (-): 0

Region: chr12 34910500-34910510. Max. coverage (+): 0. Max coverage (-): 0

Region: chr12 34910511-34910522. Max. coverage (+): 0. Max coverage (-): 0

Region: chr12 34910523-34910534. Max. coverage (+): 0. Max coverage (-): 0

Region: chr12 34910535-34910545. Max. coverage (+): 0. Max coverage (-): 0

Region: chr12 34910546-34910557. Max. coverage (+): 0. Max coverage (-): 0

Region: chr12 34910558-34910569. Max. coverage (+): 2.66. Max coverage (-): 0

Region: chr12 34910570-34910580. Max. coverage (+): 0. Max coverage (-): 0

Region: chr12 34910581-34910592. Max. coverage (+): 0. Max coverage (-): 0

Region: chr12 34910593-34910604. Max. coverage (+): 0. Max coverage (-): 0

Region: chr12 34910605-34910615. Max. coverage (+): 0. Max coverage (-): 0

Region: chr12 34910616-34910627. Max. coverage (+): 0. Max coverage (-): 0

Region: chr12 34910628-34910639. Max. coverage (+): 0. Max coverage (-): 0

Region: chr12 34910640-34910650. Max. coverage (+): 0. Max coverage (-): 0

Region: chr12 34910651-34910662. Max. coverage (+): 0. Max coverage (-): 0

Region: chr12 34910663-34910673. Max. coverage (+): 0. Max coverage (-): 0

Region: chr12 34910674-34910685. Max. coverage (+): 0. Max coverage (-): 0

Region: chr12 34910686-34910697. Max. coverage (+): 0. Max coverage (-): 0

Region: chr12 34910698-34910708. Max. coverage (+): 0. Max coverage (-): 0

Region: chr12 34910709-34910720. Max. coverage (+): 0. Max coverage (-): 0

Region: chr12 34910721-34910732. Max. coverage (+): 0. Max coverage (-): 0

Region: chr12 34910733-34910743. Max. coverage (+): 0. Max coverage (-): 0

Region: chr12 34910744-34910755. Max. coverage (+): 0. Max coverage (-): 0

Region: chr12 34910756-34910767. Max. coverage (+): 0. Max coverage (-): 0

Region: chr12 34910768-34910778. Max. coverage (+): 1.02. Max coverage (-): 0

Region: chr12 34910779-34910790. Max. coverage (+): 1.02. Max coverage (-): 0

Region: chr12 34910791-34910802. Max. coverage (+): 0. Max coverage (-): 0

Region: chr12 34910803-34910813. Max. coverage (+): 0. Max coverage (-): 0

Region: chr12 34910814-34910825. Max. coverage (+): 0. Max coverage (-): 0

Region: chr12 34910826-34910836. Max. coverage (+): 0. Max coverage (-): 0

Region: chr12 34910837-34910848. Max. coverage (+): 0. Max coverage (-): 0

Region: chr12 34910849-34910860. Max. coverage (+): 0. Max coverage (-): 0

Region: chr12 34910861-34910871. Max. coverage (+): 0. Max coverage (-): 0

Region: chr12 34910872-34910883. Max. coverage (+): 0. Max coverage (-): 0

Region: chr12 34910884-34910895. Max. coverage (+): 0. Max coverage (-): 0

Region: chr12 34910896-34910906. Max. coverage (+): 0. Max coverage (-): 0

Region: chr12 34910907-34910918. Max. coverage (+): 0. Max coverage (-): 0

Region: chr12 34910919-34910930. Max. coverage (+): 0. Max coverage (-): 0

Region: chr12 34910931-34910941. Max. coverage (+): 0. Max coverage (-): 0

Region: chr12 34910942-34910953. Max. coverage (+): 0. Max coverage (-): 0

Region: chr12 34910954-34910965. Max. coverage (+): 0. Max coverage (-): 0

Region: chr12 34910966-34910976. Max. coverage (+): 0. Max coverage (-): 0

Region: chr12 34910977-34910988. Max. coverage (+): 0. Max coverage (-): 0

Region: chr12 34910989-34911000. Max. coverage (+): 0. Max coverage (-): 0

Region: chr12 34911001-34911011. Max. coverage (+): 0. Max coverage (-): 0

Region: chr12 34911012-34911023. Max. coverage (+): 0. Max coverage (-): 0

Region: chr12 34911024-34911034. Max. coverage (+): 0. Max coverage (-): 0

Region: chr12 34911035-34911046. Max. coverage (+): 0. Max coverage (-): 0

Region: chr12 34911047-34911058. Max. coverage (+): 0. Max coverage (-): 0

Region: chr12 34911059-34911069. Max. coverage (+): 2.29. Max coverage (-): 0

Region: chr12 34911070-34911081. Max. coverage (+): 2.29. Max coverage (-): 0

Region: chr12 34911082-34911093. Max. coverage (+): 0. Max coverage (-): 0

Region: chr12 34911094-34911104. Max. coverage (+): 0. Max coverage (-): 0

Region: chr12 34911105-34911116. Max. coverage (+): 0. Max coverage (-): 0

Region: chr12 34911117-34911128. Max. coverage (+): 0. Max coverage (-): 0

Region: chr12 34911129-34911139. Max. coverage (+): 0. Max coverage (-): 0

Region: chr12 34911140-34911151. Max. coverage (+): 0. Max coverage (-): 0

Region: chr12 34911152-34911163. Max. coverage (+): 0. Max coverage (-): 0

Region: chr12 34911164-34911174. Max. coverage (+): 5.04. Max coverage (-): 0

Region: chr12 34911175-34911186. Max. coverage (+): 0. Max coverage (-): 0

Region: chr12 34911187-34911197. Max. coverage (+): 0. Max coverage (-): 0

Region: chr12 34911198-34911209. Max. coverage (+): 0. Max coverage (-): 0

Region: chr12 34911210-34911221. Max. coverage (+): 0. Max coverage (-): 0

Region: chr12 34911222-34911232. Max. coverage (+): 0. Max coverage (-): 0

Region: chr12 34911233-34911244. Max. coverage (+): 0. Max coverage (-): 0

Region: chr12 34911245-34911256. Max. coverage (+): 0. Max coverage (-): 0

Region: chr12 34911257-34911267. Max. coverage (+): 0. Max coverage (-): 0

Region: chr12 34911268-34911279. Max. coverage (+): 0. Max coverage (-): 0

Region: chr12 34911280-34911291. Max. coverage (+): 0. Max coverage (-): 0

Region: chr12 34911292-34911302. Max. coverage (+): 0. Max coverage (-): 0

Region: chr12 34911303-34911314. Max. coverage (+): 0. Max coverage (-): 0

Region: chr12 34911315-34911326. Max. coverage (+): 0. Max coverage (-): 0

Region: chr12 34911327-34911337. Max. coverage (+): 0. Max coverage (-): 0

Region: chr12 34911338-34911349. Max. coverage (+): 0. Max coverage (-): 0

Region: chr12 34911350-34911360. Max. coverage (+): 0. Max coverage (-): 0

Region: chr12 34911361-34911372. Max. coverage (+): 0. Max coverage (-): 0

Region: chr12 34911373-34911384. Max. coverage (+): 0. Max coverage (-): 0

Region: chr12 34911385-34911395. Max. coverage (+): 0. Max coverage (-): 0

Region: chr12 34911396-34911407. Max. coverage (+): 0. Max coverage (-): 0

Region: chr12 34911408-34911419. Max. coverage (+): 0. Max coverage (-): 0

Region: chr12 34911420-34911430. Max. coverage (+): 0. Max coverage (-): 0

Region: chr12 34911431-34911442. Max. coverage (+): 0. Max coverage (-): 0

Region: chr12 34911443-34911454. Max. coverage (+): 0. Max coverage (-): 0

Region: chr12 34911455-34911465. Max. coverage (+): 0. Max coverage (-): 0

Region: chr12 34911466-34911477. Max. coverage (+): 0. Max coverage (-): 0

Region: chr12 34911478-34911489. Max. coverage (+): 0. Max coverage (-): 0

Region: chr12 34911490-34911500. Max. coverage (+): 0. Max coverage (-): 0

Region: chr12 34911501-34911512. Max. coverage (+): 0. Max coverage (-): 0

Region: chr12 34911513-34911523. Max. coverage (+): 0. Max coverage (-): 0

Region: chr12 34911524-34911535. Max. coverage (+): 0. Max coverage (-): 0

Region: chr12 34911536-34911547. Max. coverage (+): 0. Max coverage (-): 0

Region: chr12 34911548-34911558. Max. coverage (+): 0. Max coverage (-): 0

Region: chr12 34911559-34911570. Max. coverage (+): 0. Max coverage (-): 0

Region: chr12 34911571-34911582. Max. coverage (+): 0. Max coverage (-): 0

Region: chr12 34911583-34911593. Max. coverage (+): 0. Max coverage (-): 0

Region: chr12 34911594-34911605. Max. coverage (+): 0. Max coverage (-): 0

Region: chr12 34911606-34911617. Max. coverage (+): 0. Max coverage (-): 0

Region: chr12 34911618-34911628. Max. coverage (+): 0. Max coverage (-): 0

Region: chr12 34911629-34911640. Max. coverage (+): 0. Max coverage (-): 0

Region: chr12 34911641-34911652. Max. coverage (+): 0. Max coverage (-): 0

Region: chr12 34911653-34911663. Max. coverage (+): 0. Max coverage (-): 0

Region: chr12 34911664-34911675. Max. coverage (+): 0. Max coverage (-): 0

Region: chr12 34911676-34911687. Max. coverage (+): 0. Max coverage (-): 0

Region: chr12 34911688-34911698. Max. coverage (+): 0. Max coverage (-): 0

Region: chr12 34911699-34911710. Max. coverage (+): 0. Max coverage (-): 0

Region: chr12 34911711-34911721. Max. coverage (+): 0. Max coverage (-): 0

Region: chr12 34911722-34911733. Max. coverage (+): 0. Max coverage (-): 0

Region: chr12 34911734-34911745. Max. coverage (+): 0. Max coverage (-): 0

Region: chr12 34911746-34911756. Max. coverage (+): 0. Max coverage (-): 0

Region: chr12 34911757-34911768. Max. coverage (+): 0. Max coverage (-): 0

Region: chr12 34911769-34911780. Max. coverage (+): 0. Max coverage (-): 0

Region: chr12 34911781-34911791. Max. coverage (+): 0. Max coverage (-): 0

Region: chr12 34911792-34911803. Max. coverage (+): 1. Max coverage (-): 0

Region: chr12 34911804-34911815. Max. coverage (+): 1. Max coverage (-): 0

Region: chr12 34911816-34911826. Max. coverage (+): 0. Max coverage (-): 0

Region: chr12 34911827-34911838. Max. coverage (+): 0. Max coverage (-): 0

Region: chr12 34911839-34911850. Max. coverage (+): 0. Max coverage (-): 0

Region: chr12 34911851-34911861. Max. coverage (+): 0. Max coverage (-): 0

Region: chr12 34911862-34911873. Max. coverage (+): 0. Max coverage (-): 0

Region: chr12 34911874-34911884. Max. coverage (+): 0. Max coverage (-): 0

Region: chr12 34911885-34911896. Max. coverage (+): 0. Max coverage (-): 0

Region: chr12 34911897-34911908. Max. coverage (+): 0. Max coverage (-): 0

Region: chr12 34911909-34911919. Max. coverage (+): 0.44. Max coverage (-): 0

Region: chr12 34911920-34911931. Max. coverage (+): 0.44. Max coverage (-): 0

Region: chr12 34911932-34911943. Max. coverage (+): 0. Max coverage (-): 0

Region: chr12 34911944-. Max. coverage (+): 0. Max coverage (-): 0

RepeatMasker Color Code

**+**

100-98% Identity

<98-95% Identity

<95-90% Identity

<90-85% Identity

<85-80% Identity

<80-75% Identity

<75-70% Identity

<70% Identity

**-**

Gene Set Color Code

**+**

Gene

Pseudogene

**-**

Topology/Coverage Color Code

Coverage Plus Strand

Coverage Minus Strand

Mainstrand: Plus

Mainstrand: Minus

Complementary Strand

Flanking Region  
(if option -flank >0)

Gene Set Annotation  

**1. SACS (protein coding, ENSBTAG00000001867) Tr:00000002433 Ex:8**: 34906073-34917630 (+)

  
RepeatMasker Annotation  

**1. AT\_rich**: 34911012-34911032 (+), Divergence to consensus: 66.7%

  
Transcription Factor Binding Sites  

**RFX4\_2** (Sequence: GTAACCAGG (-): 34906553)
