# Supplementary material for: Small RNA sequencing of cryopreserved semen from single bull revealed altered miRNAs and piRNAs expression between High- and Low-motile sperm populations
Source: BMC Genomics. 2017 Jan 4;18:14. doi: 10.1186/s12864-016-3394-7 (PMC5209821; doi:10.1186/s12864-016-3394-7)
Supplement: Additional file 3: — Details for each piRNA clusters found in High Motile (HM) sperm fraction. Genes, repeats, transposable elements and transcription factors binding sites falling within the cluster regions were reported. (ZIP 1896 kb) [file 12864_2016_3394_MOESM3_ESM.zip › 15.html]

piRNA cluster 15


Predicted piRNA cluster no. 15     previous   next
  

Show proTRAC run info
Hide proTRAC run info

================================= proTRAC ====================================  
VERSION: 2.1                                    LAST MODIFIED: 06. October 2015  
  
Please cite:  
Rosenkranz D, Zischler H. proTRAC - a software for probabilistic piRNA cluster  
detection, visualization and analysis. 2012. BMC Bioinformatics 13:5.  
  
and (for proTRAC 2.0 and later):  
Rosenkranz D, Rudloff S, Bastuck K, Ketting RF, Zischler H. Tupaia small RNAs  
provide insights into function and evolution of RNAi-based transposon defense  
in mammals. 2015. RNA 21(5):911-922.  
  
Contact:  
David Rosenkranz  
Institute of Anthropology, small RNA group  
Johannes Gutenberg University Mainz  
email: rosenkranz@uni-mainz.de  
  
You can find the latest proTRAC version at:  
http://sourceforge.net/projects/protrac/files  
http://www.smallRNAgroup-mainz.de/software  
==============================================================================  
  
PARAMETERS:  
Map file: .............../storage/core/barbara/genhome/smallRNA/fertility/Sample\_motile/pirna/Sample\_motile\_26-33\_collapsed.fa.no-dust.map.weighted-10000-1000-b-0  
Genome file: ............/storage/core/barbara/genhome/smallRNA/fertility/Sample\_all/pirna/bt\_311\_chrY.fa  
RepeatMasker annotation: /storage/genomes/bt\_umd31/GCF\_000003055.6\_Bos\_taurus\_UMD\_3.1.1\_repeatMasker\_chr.out  
GeneSet:................./storage/core/barbara/genhome/smallRNA/fertility/Sample\_all/pirna/full.gtf  
  
Significant (p<=0.01) hit density will be calculated based  
on observed hit distribution.  
  
Sliding window size: ........................................ 5000 bp  
Sliding window increament: .................................. 1000 bp  
Normalize each hit by number of genomic hits: ............... 1 [0=no/1=yes]  
Normalize each hit by number of sequence reads: ............. 1 [0=no/1=yes]  
Normalize values (-> per million mapped reads): ............. 1 [0=no/1=yes]  
Min. fraction of hits with 1T(U) or 10A: .................... 0.75  
Alternatively: Min. fraction of hits with 1T(U) and 10A: .... 0.5  
Min. fraction of hits with typical piRNA length: ............ 0.75  
Typical piRNA length: ....................................... 26-33 nt  
Min. size of a piRNA cluster: ............................... 5000 bp.  
Min. number of hits (absolute): ............................. 0  
Min. number of hits (normalized): ........................... 0  
Min. fraction of hits on the mainstrand: .................... 0.75  
Top fraction of mapped sequences (in terms of read counts): . 1%  
Top fraction accounts for max. n% of sequence reads: ........ 90%  
Min. fraction of hits on each arm of a bidirectional cluster: 0.1  
Output image file for each cluster: ......................... 0 [0=no/1=yes]  
Output html file for each cluster: .......................... 1 [0=no/1=yes]  
Output a summary table: ..................................... 1 [0=no/1=yes]  
Output a FASTA file for each cluster (piRNA sequences): ..... 1 [0=no/1=yes]  
Output a FASTA file comprising cluster sequences: ........... 1 [0=no/1=yes]  
Search DNA motifs in clusters: .............................. 1 [0=no/1=yes]  
Output flanking sequences: +/- .............................. 0 bp  
Output ~.pTi file: .......................................... 1 [0=no/1=yes]  
==============================================================================  
  
  
Genome size (without gaps): ............ 2678902517 bp  
Gaps (N/X/-): .......................... 53837044 bp  
Mapped reads: .......................... 658825247023  
Non-identical sequences: ............... 514171  
Genomic hits: .......................... 764233  
Significant densitiy of mapped reads: .. 12867599.5173724 reads/kb

Show proTRAC cluster info
Hide proTRAC cluster info

|  |  |
| --- | --- |
| Location | chr13 |
| Coordinates | 11232074-11238759 |
| Size [bp] | 6686 |
| Sequence hit loci | 68 |
| Mapped reads (normalized) | 80817873.6 |
| Mapped reads (normalized) per kb | 12087626.9 |
| Normalized reads with 1T (1U) | 77.9% |
| Normalized reads with 10A | 43% |
| Normalized reads with length 26-33 nt | 100% |
| Normalized reads on the main strand(s) | 82.9% |
| Predicted directionality | mono:plus |

100%

0%

1T (1U)  
reads

10A reads

26-33 nt  
reads

reads on mainstrand

**Either the amount of reads with 1T (1U) OR 10A has to exceed 75% (set with option: -1Tor10A)  
Alternatively the amount of reads with 1T (1U) AND 10A has to exceed 50% (set with option: -1Tand10A)  
Minimum amount of reads with preferred size is 75% (set with option: -pisize)  
Minimum amount of reads on the main strand(s) is 75% (set with option: -clstrand)**

Show read coverage
Hide read coverage

WHAT DO I SEE HERE?  
This chart shows the location of mapped sequence reads within a predicted piRNA cluster. The color refers to the number of genomic hits produced by the sequence read in question. A dark red bar indicates that this sequence read produces many other hits elsewhere in the genome. Many adjacent red or yellow bars can indicate the presence of a multi-copy element such as transposons or rRNA genes. A dark green bar indicates that this sequence read maps uniquely to this locus.

1 hit

2-5 hits

6-10 hits

11-20 hits

21-50 hits

51-100 hits

> 100 hits

chr13

11232074

11238759

Gene Set

RepeatMasker

Mapped  
Reads

11.99

plus strand

minus strand

11.99

Region: chr13 34911945-11232080. Max. coverage (+): 3.97. Max coverage (-): 0

Region: chr13 11232081-11232094. Max. coverage (+): 3.97. Max coverage (-): 0

Region: chr13 11232095-11232107. Max. coverage (+): 4.55. Max coverage (-): 0

Region: chr13 11232108-11232120. Max. coverage (+): 4.55. Max coverage (-): 0

Region: chr13 11232121-11232134. Max. coverage (+): 0. Max coverage (-): 0

Region: chr13 11232135-11232147. Max. coverage (+): 0. Max coverage (-): 0

Region: chr13 11232148-11232160. Max. coverage (+): 0. Max coverage (-): 0

Region: chr13 11232161-11232174. Max. coverage (+): 0. Max coverage (-): 0

Region: chr13 11232175-11232187. Max. coverage (+): 0. Max coverage (-): 0

Region: chr13 11232188-11232201. Max. coverage (+): 0. Max coverage (-): 0

Region: chr13 11232202-11232214. Max. coverage (+): 0. Max coverage (-): 0

Region: chr13 11232215-11232227. Max. coverage (+): 0. Max coverage (-): 0

Region: chr13 11232228-11232241. Max. coverage (+): 0. Max coverage (-): 0

Region: chr13 11232242-11232254. Max. coverage (+): 0. Max coverage (-): 0

Region: chr13 11232255-11232267. Max. coverage (+): 0. Max coverage (-): 0

Region: chr13 11232268-11232281. Max. coverage (+): 0. Max coverage (-): 0

Region: chr13 11232282-11232294. Max. coverage (+): 0. Max coverage (-): 0

Region: chr13 11232295-11232308. Max. coverage (+): 0. Max coverage (-): 0

Region: chr13 11232309-11232321. Max. coverage (+): 0. Max coverage (-): 0

Region: chr13 11232322-11232334. Max. coverage (+): 0. Max coverage (-): 0

Region: chr13 11232335-11232348. Max. coverage (+): 0. Max coverage (-): 0

Region: chr13 11232349-11232361. Max. coverage (+): 0. Max coverage (-): 0

Region: chr13 11232362-11232374. Max. coverage (+): 0. Max coverage (-): 0

Region: chr13 11232375-11232388. Max. coverage (+): 0. Max coverage (-): 0

Region: chr13 11232389-11232401. Max. coverage (+): 0. Max coverage (-): 0

Region: chr13 11232402-11232414. Max. coverage (+): 2.51. Max coverage (-): 0

Region: chr13 11232415-11232428. Max. coverage (+): 2.51. Max coverage (-): 0

Region: chr13 11232429-11232441. Max. coverage (+): 0. Max coverage (-): 0

Region: chr13 11232442-11232455. Max. coverage (+): 0. Max coverage (-): 0

Region: chr13 11232456-11232468. Max. coverage (+): 0. Max coverage (-): 0

Region: chr13 11232469-11232481. Max. coverage (+): 0. Max coverage (-): 0

Region: chr13 11232482-11232495. Max. coverage (+): 0. Max coverage (-): 0

Region: chr13 11232496-11232508. Max. coverage (+): 0. Max coverage (-): 0

Region: chr13 11232509-11232521. Max. coverage (+): 0. Max coverage (-): 0

Region: chr13 11232522-11232535. Max. coverage (+): 0. Max coverage (-): 0

Region: chr13 11232536-11232548. Max. coverage (+): 0. Max coverage (-): 0

Region: chr13 11232549-11232562. Max. coverage (+): 0. Max coverage (-): 0

Region: chr13 11232563-11232575. Max. coverage (+): 0. Max coverage (-): 0

Region: chr13 11232576-11232588. Max. coverage (+): 0. Max coverage (-): 0

Region: chr13 11232589-11232602. Max. coverage (+): 0. Max coverage (-): 0

Region: chr13 11232603-11232615. Max. coverage (+): 0. Max coverage (-): 0

Region: chr13 11232616-11232628. Max. coverage (+): 0. Max coverage (-): 0

Region: chr13 11232629-11232642. Max. coverage (+): 0. Max coverage (-): 0

Region: chr13 11232643-11232655. Max. coverage (+): 0. Max coverage (-): 0

Region: chr13 11232656-11232669. Max. coverage (+): 0. Max coverage (-): 0

Region: chr13 11232670-11232682. Max. coverage (+): 0. Max coverage (-): 0

Region: chr13 11232683-11232695. Max. coverage (+): 0. Max coverage (-): 0

Region: chr13 11232696-11232709. Max. coverage (+): 0. Max coverage (-): 0

Region: chr13 11232710-11232722. Max. coverage (+): 0. Max coverage (-): 0

Region: chr13 11232723-11232735. Max. coverage (+): 0. Max coverage (-): 0

Region: chr13 11232736-11232749. Max. coverage (+): 0. Max coverage (-): 0

Region: chr13 11232750-11232762. Max. coverage (+): 0. Max coverage (-): 0

Region: chr13 11232763-11232776. Max. coverage (+): 0. Max coverage (-): 0

Region: chr13 11232777-11232789. Max. coverage (+): 0. Max coverage (-): 0

Region: chr13 11232790-11232802. Max. coverage (+): 0. Max coverage (-): 0

Region: chr13 11232803-11232816. Max. coverage (+): 0. Max coverage (-): 0

Region: chr13 11232817-11232829. Max. coverage (+): 0. Max coverage (-): 0

Region: chr13 11232830-11232842. Max. coverage (+): 0. Max coverage (-): 0

Region: chr13 11232843-11232856. Max. coverage (+): 0. Max coverage (-): 0

Region: chr13 11232857-11232869. Max. coverage (+): 0. Max coverage (-): 0

Region: chr13 11232870-11232883. Max. coverage (+): 0. Max coverage (-): 0

Region: chr13 11232884-11232896. Max. coverage (+): 0. Max coverage (-): 0

Region: chr13 11232897-11232909. Max. coverage (+): 0. Max coverage (-): 0

Region: chr13 11232910-11232923. Max. coverage (+): 0. Max coverage (-): 0

Region: chr13 11232924-11232936. Max. coverage (+): 0. Max coverage (-): 0

Region: chr13 11232937-11232949. Max. coverage (+): 0. Max coverage (-): 0

Region: chr13 11232950-11232963. Max. coverage (+): 0. Max coverage (-): 0

Region: chr13 11232964-11232976. Max. coverage (+): 0. Max coverage (-): 0

Region: chr13 11232977-11232989. Max. coverage (+): 0. Max coverage (-): 0

Region: chr13 11232990-11233003. Max. coverage (+): 0. Max coverage (-): 0

Region: chr13 11233004-11233016. Max. coverage (+): 0. Max coverage (-): 0

Region: chr13 11233017-11233030. Max. coverage (+): 0. Max coverage (-): 0

Region: chr13 11233031-11233043. Max. coverage (+): 0. Max coverage (-): 0

Region: chr13 11233044-11233056. Max. coverage (+): 0. Max coverage (-): 0

Region: chr13 11233057-11233070. Max. coverage (+): 0. Max coverage (-): 0

Region: chr13 11233071-11233083. Max. coverage (+): 0. Max coverage (-): 0

Region: chr13 11233084-11233096. Max. coverage (+): 0. Max coverage (-): 0

Region: chr13 11233097-11233110. Max. coverage (+): 0. Max coverage (-): 0

Region: chr13 11233111-11233123. Max. coverage (+): 0. Max coverage (-): 0

Region: chr13 11233124-11233137. Max. coverage (+): 0. Max coverage (-): 0

Region: chr13 11233138-11233150. Max. coverage (+): 0. Max coverage (-): 0

Region: chr13 11233151-11233163. Max. coverage (+): 0. Max coverage (-): 0

Region: chr13 11233164-11233177. Max. coverage (+): 0. Max coverage (-): 0

Region: chr13 11233178-11233190. Max. coverage (+): 0. Max coverage (-): 0

Region: chr13 11233191-11233203. Max. coverage (+): 0. Max coverage (-): 0

Region: chr13 11233204-11233217. Max. coverage (+): 0. Max coverage (-): 0

Region: chr13 11233218-11233230. Max. coverage (+): 0. Max coverage (-): 0

Region: chr13 11233231-11233244. Max. coverage (+): 0. Max coverage (-): 0

Region: chr13 11233245-11233257. Max. coverage (+): 3.08. Max coverage (-): 0

Region: chr13 11233258-11233270. Max. coverage (+): 2.36. Max coverage (-): 0

Region: chr13 11233271-11233284. Max. coverage (+): 0. Max coverage (-): 0

Region: chr13 11233285-11233297. Max. coverage (+): 0. Max coverage (-): 0

Region: chr13 11233298-11233310. Max. coverage (+): 0. Max coverage (-): 0

Region: chr13 11233311-11233324. Max. coverage (+): 0. Max coverage (-): 0

Region: chr13 11233325-11233337. Max. coverage (+): 0. Max coverage (-): 0

Region: chr13 11233338-11233351. Max. coverage (+): 0. Max coverage (-): 0

Region: chr13 11233352-11233364. Max. coverage (+): 0. Max coverage (-): 0

Region: chr13 11233365-11233377. Max. coverage (+): 0. Max coverage (-): 0

Region: chr13 11233378-11233391. Max. coverage (+): 0. Max coverage (-): 0

Region: chr13 11233392-11233404. Max. coverage (+): 0. Max coverage (-): 0

Region: chr13 11233405-11233417. Max. coverage (+): 0. Max coverage (-): 0

Region: chr13 11233418-11233431. Max. coverage (+): 0. Max coverage (-): 0

Region: chr13 11233432-11233444. Max. coverage (+): 0. Max coverage (-): 0

Region: chr13 11233445-11233458. Max. coverage (+): 0. Max coverage (-): 0

Region: chr13 11233459-11233471. Max. coverage (+): 0. Max coverage (-): 0

Region: chr13 11233472-11233484. Max. coverage (+): 0. Max coverage (-): 0

Region: chr13 11233485-11233498. Max. coverage (+): 0. Max coverage (-): 0

Region: chr13 11233499-11233511. Max. coverage (+): 0. Max coverage (-): 0

Region: chr13 11233512-11233524. Max. coverage (+): 0. Max coverage (-): 0

Region: chr13 11233525-11233538. Max. coverage (+): 0. Max coverage (-): 0

Region: chr13 11233539-11233551. Max. coverage (+): 0. Max coverage (-): 0

Region: chr13 11233552-11233564. Max. coverage (+): 0. Max coverage (-): 0

Region: chr13 11233565-11233578. Max. coverage (+): 0. Max coverage (-): 0

Region: chr13 11233579-11233591. Max. coverage (+): 0. Max coverage (-): 0

Region: chr13 11233592-11233605. Max. coverage (+): 0. Max coverage (-): 0

Region: chr13 11233606-11233618. Max. coverage (+): 0. Max coverage (-): 0

Region: chr13 11233619-11233631. Max. coverage (+): 0. Max coverage (-): 0

Region: chr13 11233632-11233645. Max. coverage (+): 0. Max coverage (-): 0

Region: chr13 11233646-11233658. Max. coverage (+): 0. Max coverage (-): 0

Region: chr13 11233659-11233671. Max. coverage (+): 0. Max coverage (-): 0

Region: chr13 11233672-11233685. Max. coverage (+): 0. Max coverage (-): 0

Region: chr13 11233686-11233698. Max. coverage (+): 0. Max coverage (-): 0

Region: chr13 11233699-11233712. Max. coverage (+): 0. Max coverage (-): 0

Region: chr13 11233713-11233725. Max. coverage (+): 0. Max coverage (-): 0

Region: chr13 11233726-11233738. Max. coverage (+): 0. Max coverage (-): 0

Region: chr13 11233739-11233752. Max. coverage (+): 0. Max coverage (-): 0

Region: chr13 11233753-11233765. Max. coverage (+): 0. Max coverage (-): 0

Region: chr13 11233766-11233778. Max. coverage (+): 0. Max coverage (-): 4.92

Region: chr13 11233779-11233792. Max. coverage (+): 0. Max coverage (-): 0

Region: chr13 11233793-11233805. Max. coverage (+): 0. Max coverage (-): 0

Region: chr13 11233806-11233819. Max. coverage (+): 0. Max coverage (-): 0

Region: chr13 11233820-11233832. Max. coverage (+): 0. Max coverage (-): 0

Region: chr13 11233833-11233845. Max. coverage (+): 0. Max coverage (-): 0

Region: chr13 11233846-11233859. Max. coverage (+): 0. Max coverage (-): 0

Region: chr13 11233860-11233872. Max. coverage (+): 0. Max coverage (-): 0

Region: chr13 11233873-11233885. Max. coverage (+): 0. Max coverage (-): 0

Region: chr13 11233886-11233899. Max. coverage (+): 0. Max coverage (-): 0

Region: chr13 11233900-11233912. Max. coverage (+): 0. Max coverage (-): 0

Region: chr13 11233913-11233926. Max. coverage (+): 0. Max coverage (-): 0

Region: chr13 11233927-11233939. Max. coverage (+): 0. Max coverage (-): 0

Region: chr13 11233940-11233952. Max. coverage (+): 0. Max coverage (-): 0

Region: chr13 11233953-11233966. Max. coverage (+): 0. Max coverage (-): 0

Region: chr13 11233967-11233979. Max. coverage (+): 0.93. Max coverage (-): 0

Region: chr13 11233980-11233992. Max. coverage (+): 0. Max coverage (-): 0

Region: chr13 11233993-11234006. Max. coverage (+): 1.3. Max coverage (-): 0

Region: chr13 11234007-11234019. Max. coverage (+): 0. Max coverage (-): 0

Region: chr13 11234020-11234032. Max. coverage (+): 0. Max coverage (-): 0

Region: chr13 11234033-11234046. Max. coverage (+): 0. Max coverage (-): 0

Region: chr13 11234047-11234059. Max. coverage (+): 0. Max coverage (-): 0

Region: chr13 11234060-11234073. Max. coverage (+): 0. Max coverage (-): 0

Region: chr13 11234074-11234086. Max. coverage (+): 0. Max coverage (-): 0

Region: chr13 11234087-11234099. Max. coverage (+): 0. Max coverage (-): 0

Region: chr13 11234100-11234113. Max. coverage (+): 0. Max coverage (-): 0

Region: chr13 11234114-11234126. Max. coverage (+): 0. Max coverage (-): 0

Region: chr13 11234127-11234139. Max. coverage (+): 0. Max coverage (-): 0

Region: chr13 11234140-11234153. Max. coverage (+): 0. Max coverage (-): 0

Region: chr13 11234154-11234166. Max. coverage (+): 0. Max coverage (-): 0

Region: chr13 11234167-11234180. Max. coverage (+): 0. Max coverage (-): 0

Region: chr13 11234181-11234193. Max. coverage (+): 0. Max coverage (-): 0

Region: chr13 11234194-11234206. Max. coverage (+): 0. Max coverage (-): 0

Region: chr13 11234207-11234220. Max. coverage (+): 0. Max coverage (-): 0

Region: chr13 11234221-11234233. Max. coverage (+): 0. Max coverage (-): 0

Region: chr13 11234234-11234246. Max. coverage (+): 0. Max coverage (-): 0

Region: chr13 11234247-11234260. Max. coverage (+): 0. Max coverage (-): 0

Region: chr13 11234261-11234273. Max. coverage (+): 0. Max coverage (-): 0

Region: chr13 11234274-11234287. Max. coverage (+): 1.67. Max coverage (-): 0

Region: chr13 11234288-11234300. Max. coverage (+): 1.67. Max coverage (-): 0

Region: chr13 11234301-11234313. Max. coverage (+): 3.41. Max coverage (-): 0

Region: chr13 11234314-11234327. Max. coverage (+): 0. Max coverage (-): 0

Region: chr13 11234328-11234340. Max. coverage (+): 0. Max coverage (-): 2.65

Region: chr13 11234341-11234353. Max. coverage (+): 11.99. Max coverage (-): 2.65

Region: chr13 11234354-11234367. Max. coverage (+): 0. Max coverage (-): 0

Region: chr13 11234368-11234380. Max. coverage (+): 0. Max coverage (-): 0

Region: chr13 11234381-11234394. Max. coverage (+): 2.63. Max coverage (-): 0

Region: chr13 11234395-11234407. Max. coverage (+): 5.45. Max coverage (-): 0

Region: chr13 11234408-11234420. Max. coverage (+): 1.99. Max coverage (-): 0

Region: chr13 11234421-11234434. Max. coverage (+): 1.99. Max coverage (-): 0

Region: chr13 11234435-11234447. Max. coverage (+): 0. Max coverage (-): 0.92

Region: chr13 11234448-11234460. Max. coverage (+): 2.41. Max coverage (-): 0.92

Region: chr13 11234461-11234474. Max. coverage (+): 1.55. Max coverage (-): 0

Region: chr13 11234475-11234487. Max. coverage (+): 0. Max coverage (-): 0

Region: chr13 11234488-11234501. Max. coverage (+): 0. Max coverage (-): 0

Region: chr13 11234502-11234514. Max. coverage (+): 0. Max coverage (-): 0

Region: chr13 11234515-11234527. Max. coverage (+): 0. Max coverage (-): 0

Region: chr13 11234528-11234541. Max. coverage (+): 0. Max coverage (-): 0

Region: chr13 11234542-11234554. Max. coverage (+): 0. Max coverage (-): 0

Region: chr13 11234555-11234567. Max. coverage (+): 0. Max coverage (-): 0

Region: chr13 11234568-11234581. Max. coverage (+): 0. Max coverage (-): 0

Region: chr13 11234582-11234594. Max. coverage (+): 0. Max coverage (-): 0

Region: chr13 11234595-11234607. Max. coverage (+): 0. Max coverage (-): 0

Region: chr13 11234608-11234621. Max. coverage (+): 1.75. Max coverage (-): 0

Region: chr13 11234622-11234634. Max. coverage (+): 1.75. Max coverage (-): 0

Region: chr13 11234635-11234648. Max. coverage (+): 0. Max coverage (-): 0

Region: chr13 11234649-11234661. Max. coverage (+): 0. Max coverage (-): 0

Region: chr13 11234662-11234674. Max. coverage (+): 0. Max coverage (-): 0

Region: chr13 11234675-11234688. Max. coverage (+): 0. Max coverage (-): 0.35

Region: chr13 11234689-11234701. Max. coverage (+): 0. Max coverage (-): 0.35

Region: chr13 11234702-11234714. Max. coverage (+): 2.91. Max coverage (-): 0

Region: chr13 11234715-11234728. Max. coverage (+): 0. Max coverage (-): 0

Region: chr13 11234729-11234741. Max. coverage (+): 0. Max coverage (-): 3.12

Region: chr13 11234742-11234755. Max. coverage (+): 0. Max coverage (-): 3.12

Region: chr13 11234756-11234768. Max. coverage (+): 0. Max coverage (-): 0

Region: chr13 11234769-11234781. Max. coverage (+): 0. Max coverage (-): 0

Region: chr13 11234782-11234795. Max. coverage (+): 0.23. Max coverage (-): 0

Region: chr13 11234796-11234808. Max. coverage (+): 0. Max coverage (-): 0

Region: chr13 11234809-11234821. Max. coverage (+): 0. Max coverage (-): 0

Region: chr13 11234822-11234835. Max. coverage (+): 0. Max coverage (-): 0

Region: chr13 11234836-11234848. Max. coverage (+): 0. Max coverage (-): 0

Region: chr13 11234849-11234862. Max. coverage (+): 4.73. Max coverage (-): 0

Region: chr13 11234863-11234875. Max. coverage (+): 9.94. Max coverage (-): 0

Region: chr13 11234876-11234888. Max. coverage (+): 2.38. Max coverage (-): 0

Region: chr13 11234889-11234902. Max. coverage (+): 0. Max coverage (-): 0

Region: chr13 11234903-11234915. Max. coverage (+): 0. Max coverage (-): 0

Region: chr13 11234916-11234928. Max. coverage (+): 0. Max coverage (-): 0

Region: chr13 11234929-11234942. Max. coverage (+): 0. Max coverage (-): 3.94

Region: chr13 11234943-11234955. Max. coverage (+): 0. Max coverage (-): 3.94

Region: chr13 11234956-11234969. Max. coverage (+): 1.43. Max coverage (-): 0

Region: chr13 11234970-11234982. Max. coverage (+): 0.61. Max coverage (-): 0

Region: chr13 11234983-11234995. Max. coverage (+): 0. Max coverage (-): 0

Region: chr13 11234996-11235009. Max. coverage (+): 0. Max coverage (-): 0

Region: chr13 11235010-11235022. Max. coverage (+): 0.95. Max coverage (-): 2.56

Region: chr13 11235023-11235035. Max. coverage (+): 0.95. Max coverage (-): 0

Region: chr13 11235036-11235049. Max. coverage (+): 0. Max coverage (-): 0

Region: chr13 11235050-11235062. Max. coverage (+): 2.88. Max coverage (-): 0

Region: chr13 11235063-11235076. Max. coverage (+): 0. Max coverage (-): 0

Region: chr13 11235077-11235089. Max. coverage (+): 3.63. Max coverage (-): 0

Region: chr13 11235090-11235102. Max. coverage (+): 0. Max coverage (-): 0

Region: chr13 11235103-11235116. Max. coverage (+): 0. Max coverage (-): 0

Region: chr13 11235117-11235129. Max. coverage (+): 0. Max coverage (-): 0

Region: chr13 11235130-11235142. Max. coverage (+): 1.88. Max coverage (-): 1.13

Region: chr13 11235143-11235156. Max. coverage (+): 1.88. Max coverage (-): 0

Region: chr13 11235157-11235169. Max. coverage (+): 0. Max coverage (-): 0

Region: chr13 11235170-11235182. Max. coverage (+): 0. Max coverage (-): 0

Region: chr13 11235183-11235196. Max. coverage (+): 0. Max coverage (-): 0

Region: chr13 11235197-11235209. Max. coverage (+): 0. Max coverage (-): 0

Region: chr13 11235210-11235223. Max. coverage (+): 0. Max coverage (-): 0

Region: chr13 11235224-11235236. Max. coverage (+): 0. Max coverage (-): 0

Region: chr13 11235237-11235249. Max. coverage (+): 0. Max coverage (-): 0

Region: chr13 11235250-11235263. Max. coverage (+): 0. Max coverage (-): 0

Region: chr13 11235264-11235276. Max. coverage (+): 0. Max coverage (-): 0

Region: chr13 11235277-11235289. Max. coverage (+): 0. Max coverage (-): 0

Region: chr13 11235290-11235303. Max. coverage (+): 3.21. Max coverage (-): 0

Region: chr13 11235304-11235316. Max. coverage (+): 8.07. Max coverage (-): 0

Region: chr13 11235317-11235330. Max. coverage (+): 3.75. Max coverage (-): 0

Region: chr13 11235331-11235343. Max. coverage (+): 1.77. Max coverage (-): 0

Region: chr13 11235344-11235356. Max. coverage (+): 0. Max coverage (-): 0

Region: chr13 11235357-11235370. Max. coverage (+): 0. Max coverage (-): 0

Region: chr13 11235371-11235383. Max. coverage (+): 0. Max coverage (-): 0

Region: chr13 11235384-11235396. Max. coverage (+): 0. Max coverage (-): 0

Region: chr13 11235397-11235410. Max. coverage (+): 0. Max coverage (-): 0

Region: chr13 11235411-11235423. Max. coverage (+): 0. Max coverage (-): 0

Region: chr13 11235424-11235437. Max. coverage (+): 0. Max coverage (-): 0

Region: chr13 11235438-11235450. Max. coverage (+): 0. Max coverage (-): 0

Region: chr13 11235451-11235463. Max. coverage (+): 0. Max coverage (-): 0

Region: chr13 11235464-11235477. Max. coverage (+): 0. Max coverage (-): 0

Region: chr13 11235478-11235490. Max. coverage (+): 0. Max coverage (-): 0

Region: chr13 11235491-11235503. Max. coverage (+): 0. Max coverage (-): 0

Region: chr13 11235504-11235517. Max. coverage (+): 0. Max coverage (-): 0

Region: chr13 11235518-11235530. Max. coverage (+): 0. Max coverage (-): 0

Region: chr13 11235531-11235544. Max. coverage (+): 0. Max coverage (-): 0

Region: chr13 11235545-11235557. Max. coverage (+): 0.56. Max coverage (-): 0

Region: chr13 11235558-11235570. Max. coverage (+): 0. Max coverage (-): 0

Region: chr13 11235571-11235584. Max. coverage (+): 0. Max coverage (-): 0

Region: chr13 11235585-11235597. Max. coverage (+): 0. Max coverage (-): 0

Region: chr13 11235598-11235610. Max. coverage (+): 0. Max coverage (-): 1.38

Region: chr13 11235611-11235624. Max. coverage (+): 0. Max coverage (-): 0

Region: chr13 11235625-11235637. Max. coverage (+): 0. Max coverage (-): 0

Region: chr13 11235638-11235651. Max. coverage (+): 1.59. Max coverage (-): 0

Region: chr13 11235652-11235664. Max. coverage (+): 0. Max coverage (-): 0

Region: chr13 11235665-11235677. Max. coverage (+): 0. Max coverage (-): 0

Region: chr13 11235678-11235691. Max. coverage (+): 0. Max coverage (-): 0

Region: chr13 11235692-11235704. Max. coverage (+): 0. Max coverage (-): 0

Region: chr13 11235705-11235717. Max. coverage (+): 3.7. Max coverage (-): 0

Region: chr13 11235718-11235731. Max. coverage (+): 3.7. Max coverage (-): 0

Region: chr13 11235732-11235744. Max. coverage (+): 0. Max coverage (-): 0

Region: chr13 11235745-11235757. Max. coverage (+): 0. Max coverage (-): 0

Region: chr13 11235758-11235771. Max. coverage (+): 0. Max coverage (-): 0

Region: chr13 11235772-11235784. Max. coverage (+): 0. Max coverage (-): 0

Region: chr13 11235785-11235798. Max. coverage (+): 0. Max coverage (-): 0

Region: chr13 11235799-11235811. Max. coverage (+): 0. Max coverage (-): 0

Region: chr13 11235812-11235824. Max. coverage (+): 0. Max coverage (-): 0

Region: chr13 11235825-11235838. Max. coverage (+): 0. Max coverage (-): 0

Region: chr13 11235839-11235851. Max. coverage (+): 0. Max coverage (-): 0

Region: chr13 11235852-11235864. Max. coverage (+): 0. Max coverage (-): 0

Region: chr13 11235865-11235878. Max. coverage (+): 0. Max coverage (-): 0

Region: chr13 11235879-11235891. Max. coverage (+): 0. Max coverage (-): 0

Region: chr13 11235892-11235905. Max. coverage (+): 0. Max coverage (-): 0

Region: chr13 11235906-11235918. Max. coverage (+): 0. Max coverage (-): 0

Region: chr13 11235919-11235931. Max. coverage (+): 0. Max coverage (-): 0

Region: chr13 11235932-11235945. Max. coverage (+): 0. Max coverage (-): 0

Region: chr13 11235946-11235958. Max. coverage (+): 0. Max coverage (-): 0

Region: chr13 11235959-11235971. Max. coverage (+): 0. Max coverage (-): 0

Region: chr13 11235972-11235985. Max. coverage (+): 0. Max coverage (-): 0

Region: chr13 11235986-11235998. Max. coverage (+): 0. Max coverage (-): 0

Region: chr13 11235999-11236012. Max. coverage (+): 0. Max coverage (-): 0

Region: chr13 11236013-11236025. Max. coverage (+): 0. Max coverage (-): 0

Region: chr13 11236026-11236038. Max. coverage (+): 0. Max coverage (-): 0

Region: chr13 11236039-11236052. Max. coverage (+): 0. Max coverage (-): 0

Region: chr13 11236053-11236065. Max. coverage (+): 0.23. Max coverage (-): 0

Region: chr13 11236066-11236078. Max. coverage (+): 0.23. Max coverage (-): 0

Region: chr13 11236079-11236092. Max. coverage (+): 0. Max coverage (-): 0

Region: chr13 11236093-11236105. Max. coverage (+): 0. Max coverage (-): 0

Region: chr13 11236106-11236119. Max. coverage (+): 0. Max coverage (-): 0

Region: chr13 11236120-11236132. Max. coverage (+): 0. Max coverage (-): 0

Region: chr13 11236133-11236145. Max. coverage (+): 0. Max coverage (-): 0

Region: chr13 11236146-11236159. Max. coverage (+): 0. Max coverage (-): 0

Region: chr13 11236160-11236172. Max. coverage (+): 0. Max coverage (-): 0

Region: chr13 11236173-11236185. Max. coverage (+): 0. Max coverage (-): 0

Region: chr13 11236186-11236199. Max. coverage (+): 0. Max coverage (-): 0

Region: chr13 11236200-11236212. Max. coverage (+): 0. Max coverage (-): 0

Region: chr13 11236213-11236226. Max. coverage (+): 0. Max coverage (-): 0

Region: chr13 11236227-11236239. Max. coverage (+): 0. Max coverage (-): 0

Region: chr13 11236240-11236252. Max. coverage (+): 0. Max coverage (-): 0

Region: chr13 11236253-11236266. Max. coverage (+): 0. Max coverage (-): 0

Region: chr13 11236267-11236279. Max. coverage (+): 0. Max coverage (-): 0

Region: chr13 11236280-11236292. Max. coverage (+): 0. Max coverage (-): 0

Region: chr13 11236293-11236306. Max. coverage (+): 0. Max coverage (-): 0

Region: chr13 11236307-11236319. Max. coverage (+): 0. Max coverage (-): 0

Region: chr13 11236320-11236332. Max. coverage (+): 0. Max coverage (-): 0

Region: chr13 11236333-11236346. Max. coverage (+): 0. Max coverage (-): 0

Region: chr13 11236347-11236359. Max. coverage (+): 0. Max coverage (-): 0

Region: chr13 11236360-11236373. Max. coverage (+): 0. Max coverage (-): 0

Region: chr13 11236374-11236386. Max. coverage (+): 0. Max coverage (-): 0

Region: chr13 11236387-11236399. Max. coverage (+): 0. Max coverage (-): 0

Region: chr13 11236400-11236413. Max. coverage (+): 0. Max coverage (-): 0

Region: chr13 11236414-11236426. Max. coverage (+): 0. Max coverage (-): 0

Region: chr13 11236427-11236439. Max. coverage (+): 0. Max coverage (-): 0

Region: chr13 11236440-11236453. Max. coverage (+): 0. Max coverage (-): 0

Region: chr13 11236454-11236466. Max. coverage (+): 0. Max coverage (-): 0

Region: chr13 11236467-11236480. Max. coverage (+): 0. Max coverage (-): 0

Region: chr13 11236481-11236493. Max. coverage (+): 0. Max coverage (-): 0

Region: chr13 11236494-11236506. Max. coverage (+): 0. Max coverage (-): 0

Region: chr13 11236507-11236520. Max. coverage (+): 0. Max coverage (-): 0

Region: chr13 11236521-11236533. Max. coverage (+): 0. Max coverage (-): 0

Region: chr13 11236534-11236546. Max. coverage (+): 0. Max coverage (-): 0

Region: chr13 11236547-11236560. Max. coverage (+): 0. Max coverage (-): 0

Region: chr13 11236561-11236573. Max. coverage (+): 0. Max coverage (-): 0

Region: chr13 11236574-11236587. Max. coverage (+): 0. Max coverage (-): 0

Region: chr13 11236588-11236600. Max. coverage (+): 0. Max coverage (-): 0

Region: chr13 11236601-11236613. Max. coverage (+): 0. Max coverage (-): 0

Region: chr13 11236614-11236627. Max. coverage (+): 0. Max coverage (-): 0

Region: chr13 11236628-11236640. Max. coverage (+): 0. Max coverage (-): 0

Region: chr13 11236641-11236653. Max. coverage (+): 0. Max coverage (-): 0

Region: chr13 11236654-11236667. Max. coverage (+): 0. Max coverage (-): 0

Region: chr13 11236668-11236680. Max. coverage (+): 0. Max coverage (-): 0

Region: chr13 11236681-11236694. Max. coverage (+): 0. Max coverage (-): 0

Region: chr13 11236695-11236707. Max. coverage (+): 0. Max coverage (-): 0

Region: chr13 11236708-11236720. Max. coverage (+): 0. Max coverage (-): 0

Region: chr13 11236721-11236734. Max. coverage (+): 0. Max coverage (-): 0

Region: chr13 11236735-11236747. Max. coverage (+): 0. Max coverage (-): 0

Region: chr13 11236748-11236760. Max. coverage (+): 0. Max coverage (-): 0

Region: chr13 11236761-11236774. Max. coverage (+): 0. Max coverage (-): 0

Region: chr13 11236775-11236787. Max. coverage (+): 0. Max coverage (-): 0

Region: chr13 11236788-11236801. Max. coverage (+): 0. Max coverage (-): 0

Region: chr13 11236802-11236814. Max. coverage (+): 0. Max coverage (-): 0

Region: chr13 11236815-11236827. Max. coverage (+): 0. Max coverage (-): 0

Region: chr13 11236828-11236841. Max. coverage (+): 0. Max coverage (-): 0

Region: chr13 11236842-11236854. Max. coverage (+): 0. Max coverage (-): 0

Region: chr13 11236855-11236867. Max. coverage (+): 0. Max coverage (-): 0

Region: chr13 11236868-11236881. Max. coverage (+): 0. Max coverage (-): 0

Region: chr13 11236882-11236894. Max. coverage (+): 0. Max coverage (-): 0

Region: chr13 11236895-11236907. Max. coverage (+): 0. Max coverage (-): 0

Region: chr13 11236908-11236921. Max. coverage (+): 0. Max coverage (-): 0

Region: chr13 11236922-11236934. Max. coverage (+): 0. Max coverage (-): 0

Region: chr13 11236935-11236948. Max. coverage (+): 0. Max coverage (-): 0

Region: chr13 11236949-11236961. Max. coverage (+): 0. Max coverage (-): 0

Region: chr13 11236962-11236974. Max. coverage (+): 0. Max coverage (-): 0

Region: chr13 11236975-11236988. Max. coverage (+): 0. Max coverage (-): 0

Region: chr13 11236989-11237001. Max. coverage (+): 0. Max coverage (-): 0

Region: chr13 11237002-11237014. Max. coverage (+): 0. Max coverage (-): 0

Region: chr13 11237015-11237028. Max. coverage (+): 0. Max coverage (-): 0

Region: chr13 11237029-11237041. Max. coverage (+): 0. Max coverage (-): 0

Region: chr13 11237042-11237055. Max. coverage (+): 0. Max coverage (-): 0

Region: chr13 11237056-11237068. Max. coverage (+): 0. Max coverage (-): 0

Region: chr13 11237069-11237081. Max. coverage (+): 0. Max coverage (-): 0

Region: chr13 11237082-11237095. Max. coverage (+): 0. Max coverage (-): 0

Region: chr13 11237096-11237108. Max. coverage (+): 0. Max coverage (-): 0

Region: chr13 11237109-11237121. Max. coverage (+): 0. Max coverage (-): 0

Region: chr13 11237122-11237135. Max. coverage (+): 0. Max coverage (-): 0

Region: chr13 11237136-11237148. Max. coverage (+): 0. Max coverage (-): 0

Region: chr13 11237149-11237162. Max. coverage (+): 0. Max coverage (-): 0

Region: chr13 11237163-11237175. Max. coverage (+): 0. Max coverage (-): 0

Region: chr13 11237176-11237188. Max. coverage (+): 0. Max coverage (-): 0

Region: chr13 11237189-11237202. Max. coverage (+): 0. Max coverage (-): 0

Region: chr13 11237203-11237215. Max. coverage (+): 0. Max coverage (-): 0

Region: chr13 11237216-11237228. Max. coverage (+): 0. Max coverage (-): 0

Region: chr13 11237229-11237242. Max. coverage (+): 0. Max coverage (-): 0

Region: chr13 11237243-11237255. Max. coverage (+): 0. Max coverage (-): 0

Region: chr13 11237256-11237269. Max. coverage (+): 0. Max coverage (-): 0

Region: chr13 11237270-11237282. Max. coverage (+): 0. Max coverage (-): 0

Region: chr13 11237283-11237295. Max. coverage (+): 0. Max coverage (-): 0

Region: chr13 11237296-11237309. Max. coverage (+): 0. Max coverage (-): 0

Region: chr13 11237310-11237322. Max. coverage (+): 0. Max coverage (-): 0

Region: chr13 11237323-11237335. Max. coverage (+): 0. Max coverage (-): 0

Region: chr13 11237336-11237349. Max. coverage (+): 0. Max coverage (-): 0

Region: chr13 11237350-11237362. Max. coverage (+): 0. Max coverage (-): 0

Region: chr13 11237363-11237375. Max. coverage (+): 0. Max coverage (-): 0

Region: chr13 11237376-11237389. Max. coverage (+): 0. Max coverage (-): 0

Region: chr13 11237390-11237402. Max. coverage (+): 0. Max coverage (-): 0

Region: chr13 11237403-11237416. Max. coverage (+): 0. Max coverage (-): 0

Region: chr13 11237417-11237429. Max. coverage (+): 0. Max coverage (-): 0

Region: chr13 11237430-11237442. Max. coverage (+): 0. Max coverage (-): 0

Region: chr13 11237443-11237456. Max. coverage (+): 0. Max coverage (-): 0

Region: chr13 11237457-11237469. Max. coverage (+): 0. Max coverage (-): 0

Region: chr13 11237470-11237482. Max. coverage (+): 0. Max coverage (-): 0

Region: chr13 11237483-11237496. Max. coverage (+): 0. Max coverage (-): 0

Region: chr13 11237497-11237509. Max. coverage (+): 0. Max coverage (-): 0

Region: chr13 11237510-11237523. Max. coverage (+): 0. Max coverage (-): 0

Region: chr13 11237524-11237536. Max. coverage (+): 0. Max coverage (-): 0

Region: chr13 11237537-11237549. Max. coverage (+): 0. Max coverage (-): 0

Region: chr13 11237550-11237563. Max. coverage (+): 0. Max coverage (-): 0

Region: chr13 11237564-11237576. Max. coverage (+): 0. Max coverage (-): 0

Region: chr13 11237577-11237589. Max. coverage (+): 0. Max coverage (-): 0

Region: chr13 11237590-11237603. Max. coverage (+): 0. Max coverage (-): 0

Region: chr13 11237604-11237616. Max. coverage (+): 0. Max coverage (-): 0

Region: chr13 11237617-11237630. Max. coverage (+): 0. Max coverage (-): 0

Region: chr13 11237631-11237643. Max. coverage (+): 0. Max coverage (-): 0

Region: chr13 11237644-11237656. Max. coverage (+): 0. Max coverage (-): 0

Region: chr13 11237657-11237670. Max. coverage (+): 0. Max coverage (-): 0

Region: chr13 11237671-11237683. Max. coverage (+): 0. Max coverage (-): 0

Region: chr13 11237684-11237696. Max. coverage (+): 0. Max coverage (-): 0

Region: chr13 11237697-11237710. Max. coverage (+): 0. Max coverage (-): 0

Region: chr13 11237711-11237723. Max. coverage (+): 2.28. Max coverage (-): 0

Region: chr13 11237724-11237737. Max. coverage (+): 2.28. Max coverage (-): 0

Region: chr13 11237738-11237750. Max. coverage (+): 0. Max coverage (-): 0

Region: chr13 11237751-11237763. Max. coverage (+): 0. Max coverage (-): 0

Region: chr13 11237764-11237777. Max. coverage (+): 0. Max coverage (-): 0

Region: chr13 11237778-11237790. Max. coverage (+): 0. Max coverage (-): 0

Region: chr13 11237791-11237803. Max. coverage (+): 0. Max coverage (-): 0

Region: chr13 11237804-11237817. Max. coverage (+): 0. Max coverage (-): 0

Region: chr13 11237818-11237830. Max. coverage (+): 0. Max coverage (-): 0

Region: chr13 11237831-11237844. Max. coverage (+): 0. Max coverage (-): 0

Region: chr13 11237845-11237857. Max. coverage (+): 0. Max coverage (-): 0

Region: chr13 11237858-11237870. Max. coverage (+): 0. Max coverage (-): 0

Region: chr13 11237871-11237884. Max. coverage (+): 0. Max coverage (-): 0

Region: chr13 11237885-11237897. Max. coverage (+): 0. Max coverage (-): 0

Region: chr13 11237898-11237910. Max. coverage (+): 0. Max coverage (-): 0

Region: chr13 11237911-11237924. Max. coverage (+): 0. Max coverage (-): 0

Region: chr13 11237925-11237937. Max. coverage (+): 0. Max coverage (-): 0

Region: chr13 11237938-11237950. Max. coverage (+): 0. Max coverage (-): 0

Region: chr13 11237951-11237964. Max. coverage (+): 0. Max coverage (-): 0

Region: chr13 11237965-11237977. Max. coverage (+): 0. Max coverage (-): 0

Region: chr13 11237978-11237991. Max. coverage (+): 0. Max coverage (-): 0

Region: chr13 11237992-11238004. Max. coverage (+): 0. Max coverage (-): 0

Region: chr13 11238005-11238017. Max. coverage (+): 0. Max coverage (-): 0

Region: chr13 11238018-11238031. Max. coverage (+): 0. Max coverage (-): 0

Region: chr13 11238032-11238044. Max. coverage (+): 0. Max coverage (-): 0

Region: chr13 11238045-11238057. Max. coverage (+): 0. Max coverage (-): 0

Region: chr13 11238058-11238071. Max. coverage (+): 0. Max coverage (-): 0

Region: chr13 11238072-11238084. Max. coverage (+): 0. Max coverage (-): 0

Region: chr13 11238085-11238098. Max. coverage (+): 0. Max coverage (-): 0

Region: chr13 11238099-11238111. Max. coverage (+): 0. Max coverage (-): 0

Region: chr13 11238112-11238124. Max. coverage (+): 0. Max coverage (-): 0

Region: chr13 11238125-11238138. Max. coverage (+): 0. Max coverage (-): 0

Region: chr13 11238139-11238151. Max. coverage (+): 0. Max coverage (-): 0

Region: chr13 11238152-11238164. Max. coverage (+): 0. Max coverage (-): 0

Region: chr13 11238165-11238178. Max. coverage (+): 0. Max coverage (-): 0

Region: chr13 11238179-11238191. Max. coverage (+): 0. Max coverage (-): 0

Region: chr13 11238192-11238205. Max. coverage (+): 0. Max coverage (-): 0

Region: chr13 11238206-11238218. Max. coverage (+): 0. Max coverage (-): 0

Region: chr13 11238219-11238231. Max. coverage (+): 0. Max coverage (-): 0

Region: chr13 11238232-11238245. Max. coverage (+): 0. Max coverage (-): 0

Region: chr13 11238246-11238258. Max. coverage (+): 0. Max coverage (-): 0

Region: chr13 11238259-11238271. Max. coverage (+): 0. Max coverage (-): 0

Region: chr13 11238272-11238285. Max. coverage (+): 0. Max coverage (-): 0

Region: chr13 11238286-11238298. Max. coverage (+): 0. Max coverage (-): 0

Region: chr13 11238299-11238312. Max. coverage (+): 0. Max coverage (-): 0

Region: chr13 11238313-11238325. Max. coverage (+): 0. Max coverage (-): 0

Region: chr13 11238326-11238338. Max. coverage (+): 0. Max coverage (-): 0

Region: chr13 11238339-11238352. Max. coverage (+): 0. Max coverage (-): 0

Region: chr13 11238353-11238365. Max. coverage (+): 0. Max coverage (-): 0

Region: chr13 11238366-11238378. Max. coverage (+): 0. Max coverage (-): 0

Region: chr13 11238379-11238392. Max. coverage (+): 0. Max coverage (-): 0

Region: chr13 11238393-11238405. Max. coverage (+): 0. Max coverage (-): 0

Region: chr13 11238406-11238419. Max. coverage (+): 0. Max coverage (-): 0

Region: chr13 11238420-11238432. Max. coverage (+): 0. Max coverage (-): 0

Region: chr13 11238433-11238445. Max. coverage (+): 0. Max coverage (-): 0

Region: chr13 11238446-11238459. Max. coverage (+): 0. Max coverage (-): 0

Region: chr13 11238460-11238472. Max. coverage (+): 0. Max coverage (-): 0

Region: chr13 11238473-11238485. Max. coverage (+): 0. Max coverage (-): 0

Region: chr13 11238486-11238499. Max. coverage (+): 0. Max coverage (-): 0

Region: chr13 11238500-11238512. Max. coverage (+): 0. Max coverage (-): 0

Region: chr13 11238513-11238525. Max. coverage (+): 0. Max coverage (-): 0

Region: chr13 11238526-11238539. Max. coverage (+): 0. Max coverage (-): 0

Region: chr13 11238540-11238552. Max. coverage (+): 0. Max coverage (-): 0

Region: chr13 11238553-11238566. Max. coverage (+): 0. Max coverage (-): 0

Region: chr13 11238567-11238579. Max. coverage (+): 0. Max coverage (-): 0

Region: chr13 11238580-11238592. Max. coverage (+): 0. Max coverage (-): 0

Region: chr13 11238593-11238606. Max. coverage (+): 0. Max coverage (-): 0

Region: chr13 11238607-11238619. Max. coverage (+): 1.3. Max coverage (-): 0

Region: chr13 11238620-11238632. Max. coverage (+): 0. Max coverage (-): 0

Region: chr13 11238633-11238646. Max. coverage (+): 0. Max coverage (-): 0

Region: chr13 11238647-11238659. Max. coverage (+): 0. Max coverage (-): 0

Region: chr13 11238660-11238673. Max. coverage (+): 0. Max coverage (-): 0

Region: chr13 11238674-11238686. Max. coverage (+): 0. Max coverage (-): 0

Region: chr13 11238687-11238699. Max. coverage (+): 0. Max coverage (-): 0

Region: chr13 11238700-11238713. Max. coverage (+): 0. Max coverage (-): 0

Region: chr13 11238714-11238726. Max. coverage (+): 0. Max coverage (-): 0

Region: chr13 11238727-11238739. Max. coverage (+): 0.98. Max coverage (-): 0

Region: chr13 11238740-11238753. Max. coverage (+): 0.98. Max coverage (-): 0

Region: chr13 11238754-. Max. coverage (+): 0. Max coverage (-): 0

RepeatMasker Color Code

**+**

100-98% Identity

<98-95% Identity

<95-90% Identity

<90-85% Identity

<85-80% Identity

<80-75% Identity

<75-70% Identity

<70% Identity

**-**

Gene Set Color Code

**+**

Gene

Pseudogene

**-**

Topology/Coverage Color Code

Coverage Plus Strand

Coverage Minus Strand

Mainstrand: Plus

Mainstrand: Minus

Complementary Strand

Flanking Region  
(if option -flank >0)

Gene Set Annotation  
  
RepeatMasker Annotation  

**1. Bov-tA3**: 11234057-11234260 (+), Divergence to consensus: 8.8%  
**2. BTLTR1**: 11236375-11236443 (-), Divergence to consensus: 11.5%  
**3. BovB**: 11236445-11236575 (+), Divergence to consensus: 4.6%  
**4. BOV-A2**: 11236577-11236691 (+), Divergence to consensus: 8.7%  
**5. (CAG)n**: 11236692-11236718 (+), Divergence to consensus: 0%  
**6. BovB**: 11236719-11237436 (+), Divergence to consensus: 8.3%  
**7. ART2A**: 11237437-11237582 (+), Divergence to consensus: 13%  
**8. L1-3\_BT**: 11237586-11237681 (+), Divergence to consensus: 9.4%  
**9. L2c**: 11237949-11238356 (+), Divergence to consensus: 51.7%

  
Transcription Factor Binding Sites  

**Gata4** (Sequence: AGATAAC (-): 11232629)  
**SOX9** (Sequence: AACAATAA (-): 11232772)  
**SOX9** (Sequence: TCATTGTT (+): 11233611)
