# Supplementary material for: Small RNA sequencing of cryopreserved semen from single bull revealed altered miRNAs and piRNAs expression between High- and Low-motile sperm populations
Source: BMC Genomics. 2017 Jan 4;18:14. doi: 10.1186/s12864-016-3394-7 (PMC5209821; doi:10.1186/s12864-016-3394-7)
Supplement: Additional file 3: — Details for each piRNA clusters found in High Motile (HM) sperm fraction. Genes, repeats, transposable elements and transcription factors binding sites falling within the cluster regions were reported. (ZIP 1896 kb) [file 12864_2016_3394_MOESM3_ESM.zip › 16.html]

piRNA cluster 16


Predicted piRNA cluster no. 16     previous   next
  

Show proTRAC run info
Hide proTRAC run info

================================= proTRAC ====================================  
VERSION: 2.1                                    LAST MODIFIED: 06. October 2015  
  
Please cite:  
Rosenkranz D, Zischler H. proTRAC - a software for probabilistic piRNA cluster  
detection, visualization and analysis. 2012. BMC Bioinformatics 13:5.  
  
and (for proTRAC 2.0 and later):  
Rosenkranz D, Rudloff S, Bastuck K, Ketting RF, Zischler H. Tupaia small RNAs  
provide insights into function and evolution of RNAi-based transposon defense  
in mammals. 2015. RNA 21(5):911-922.  
  
Contact:  
David Rosenkranz  
Institute of Anthropology, small RNA group  
Johannes Gutenberg University Mainz  
email: rosenkranz@uni-mainz.de  
  
You can find the latest proTRAC version at:  
http://sourceforge.net/projects/protrac/files  
http://www.smallRNAgroup-mainz.de/software  
==============================================================================  
  
PARAMETERS:  
Map file: .............../storage/core/barbara/genhome/smallRNA/fertility/Sample\_motile/pirna/Sample\_motile\_26-33\_collapsed.fa.no-dust.map.weighted-10000-1000-b-0  
Genome file: ............/storage/core/barbara/genhome/smallRNA/fertility/Sample\_all/pirna/bt\_311\_chrY.fa  
RepeatMasker annotation: /storage/genomes/bt\_umd31/GCF\_000003055.6\_Bos\_taurus\_UMD\_3.1.1\_repeatMasker\_chr.out  
GeneSet:................./storage/core/barbara/genhome/smallRNA/fertility/Sample\_all/pirna/full.gtf  
  
Significant (p<=0.01) hit density will be calculated based  
on observed hit distribution.  
  
Sliding window size: ........................................ 5000 bp  
Sliding window increament: .................................. 1000 bp  
Normalize each hit by number of genomic hits: ............... 1 [0=no/1=yes]  
Normalize each hit by number of sequence reads: ............. 1 [0=no/1=yes]  
Normalize values (-> per million mapped reads): ............. 1 [0=no/1=yes]  
Min. fraction of hits with 1T(U) or 10A: .................... 0.75  
Alternatively: Min. fraction of hits with 1T(U) and 10A: .... 0.5  
Min. fraction of hits with typical piRNA length: ............ 0.75  
Typical piRNA length: ....................................... 26-33 nt  
Min. size of a piRNA cluster: ............................... 5000 bp.  
Min. number of hits (absolute): ............................. 0  
Min. number of hits (normalized): ........................... 0  
Min. fraction of hits on the mainstrand: .................... 0.75  
Top fraction of mapped sequences (in terms of read counts): . 1%  
Top fraction accounts for max. n% of sequence reads: ........ 90%  
Min. fraction of hits on each arm of a bidirectional cluster: 0.1  
Output image file for each cluster: ......................... 0 [0=no/1=yes]  
Output html file for each cluster: .......................... 1 [0=no/1=yes]  
Output a summary table: ..................................... 1 [0=no/1=yes]  
Output a FASTA file for each cluster (piRNA sequences): ..... 1 [0=no/1=yes]  
Output a FASTA file comprising cluster sequences: ........... 1 [0=no/1=yes]  
Search DNA motifs in clusters: .............................. 1 [0=no/1=yes]  
Output flanking sequences: +/- .............................. 0 bp  
Output ~.pTi file: .......................................... 1 [0=no/1=yes]  
==============================================================================  
  
  
Genome size (without gaps): ............ 2678902517 bp  
Gaps (N/X/-): .......................... 53837044 bp  
Mapped reads: .......................... 658825247023  
Non-identical sequences: ............... 514171  
Genomic hits: .......................... 764233  
Significant densitiy of mapped reads: .. 12867599.5173724 reads/kb

Show proTRAC cluster info
Hide proTRAC cluster info

|  |  |
| --- | --- |
| Location | chr13 |
| Coordinates | 66729482-66736949 |
| Size [bp] | 7468 |
| Sequence hit loci | 136 |
| Mapped reads (normalized) | 163208628 |
| Mapped reads (normalized) per kb | 21854395.8 |
| Normalized reads with 1T (1U) | 92.7% |
| Normalized reads with 10A | 26.1% |
| Normalized reads with length 26-33 nt | 100% |
| Normalized reads on the main strand(s) | 97.4% |
| Predicted directionality | bi:minus-plus (split between 66735298 and 66736361) |

100%

0%

1T (1U)  
reads

10A reads

26-33 nt  
reads

reads on mainstrand

**Either the amount of reads with 1T (1U) OR 10A has to exceed 75% (set with option: -1Tor10A)  
Alternatively the amount of reads with 1T (1U) AND 10A has to exceed 50% (set with option: -1Tand10A)  
Minimum amount of reads with preferred size is 75% (set with option: -pisize)  
Minimum amount of reads on the main strand(s) is 75% (set with option: -clstrand)**

Show read coverage
Hide read coverage

WHAT DO I SEE HERE?  
This chart shows the location of mapped sequence reads within a predicted piRNA cluster. The color refers to the number of genomic hits produced by the sequence read in question. A dark red bar indicates that this sequence read produces many other hits elsewhere in the genome. Many adjacent red or yellow bars can indicate the presence of a multi-copy element such as transposons or rRNA genes. A dark green bar indicates that this sequence read maps uniquely to this locus.

1 hit

2-5 hits

6-10 hits

11-20 hits

21-50 hits

51-100 hits

> 100 hits

chr13

66729482

66736949

Gene Set

RepeatMasker

Mapped  
Reads

18.34

plus strand

minus strand

18.34

Region: chr13 11238755-66729489. Max. coverage (+): 0. Max coverage (-): 1.06

Region: chr13 66729490-66729504. Max. coverage (+): 0. Max coverage (-): 0

Region: chr13 66729505-66729519. Max. coverage (+): 0. Max coverage (-): 0

Region: chr13 66729520-66729534. Max. coverage (+): 0. Max coverage (-): 0

Region: chr13 66729535-66729549. Max. coverage (+): 0. Max coverage (-): 0

Region: chr13 66729550-66729564. Max. coverage (+): 0. Max coverage (-): 0

Region: chr13 66729565-66729579. Max. coverage (+): 0. Max coverage (-): 0

Region: chr13 66729580-66729594. Max. coverage (+): 0. Max coverage (-): 0

Region: chr13 66729595-66729608. Max. coverage (+): 0. Max coverage (-): 0

Region: chr13 66729609-66729623. Max. coverage (+): 0. Max coverage (-): 0

Region: chr13 66729624-66729638. Max. coverage (+): 0. Max coverage (-): 0

Region: chr13 66729639-66729653. Max. coverage (+): 0. Max coverage (-): 0

Region: chr13 66729654-66729668. Max. coverage (+): 0. Max coverage (-): 0

Region: chr13 66729669-66729683. Max. coverage (+): 0. Max coverage (-): 0

Region: chr13 66729684-66729698. Max. coverage (+): 0. Max coverage (-): 0

Region: chr13 66729699-66729713. Max. coverage (+): 0. Max coverage (-): 0

Region: chr13 66729714-66729728. Max. coverage (+): 0. Max coverage (-): 0

Region: chr13 66729729-66729743. Max. coverage (+): 0. Max coverage (-): 0

Region: chr13 66729744-66729758. Max. coverage (+): 0. Max coverage (-): 0

Region: chr13 66729759-66729773. Max. coverage (+): 0. Max coverage (-): 0

Region: chr13 66729774-66729788. Max. coverage (+): 0. Max coverage (-): 0

Region: chr13 66729789-66729803. Max. coverage (+): 0. Max coverage (-): 0

Region: chr13 66729804-66729818. Max. coverage (+): 0. Max coverage (-): 0

Region: chr13 66729819-66729832. Max. coverage (+): 0. Max coverage (-): 0

Region: chr13 66729833-66729847. Max. coverage (+): 0. Max coverage (-): 0

Region: chr13 66729848-66729862. Max. coverage (+): 0. Max coverage (-): 0

Region: chr13 66729863-66729877. Max. coverage (+): 0. Max coverage (-): 0

Region: chr13 66729878-66729892. Max. coverage (+): 0. Max coverage (-): 0

Region: chr13 66729893-66729907. Max. coverage (+): 0. Max coverage (-): 0

Region: chr13 66729908-66729922. Max. coverage (+): 0. Max coverage (-): 0

Region: chr13 66729923-66729937. Max. coverage (+): 0. Max coverage (-): 0

Region: chr13 66729938-66729952. Max. coverage (+): 0. Max coverage (-): 0

Region: chr13 66729953-66729967. Max. coverage (+): 0. Max coverage (-): 0

Region: chr13 66729968-66729982. Max. coverage (+): 0. Max coverage (-): 0

Region: chr13 66729983-66729997. Max. coverage (+): 0. Max coverage (-): 0

Region: chr13 66729998-66730012. Max. coverage (+): 0. Max coverage (-): 0

Region: chr13 66730013-66730027. Max. coverage (+): 0. Max coverage (-): 0

Region: chr13 66730028-66730042. Max. coverage (+): 0. Max coverage (-): 0

Region: chr13 66730043-66730057. Max. coverage (+): 0. Max coverage (-): 0

Region: chr13 66730058-66730071. Max. coverage (+): 0. Max coverage (-): 0

Region: chr13 66730072-66730086. Max. coverage (+): 0. Max coverage (-): 0

Region: chr13 66730087-66730101. Max. coverage (+): 0. Max coverage (-): 0

Region: chr13 66730102-66730116. Max. coverage (+): 0. Max coverage (-): 0

Region: chr13 66730117-66730131. Max. coverage (+): 0. Max coverage (-): 0

Region: chr13 66730132-66730146. Max. coverage (+): 0. Max coverage (-): 0

Region: chr13 66730147-66730161. Max. coverage (+): 0. Max coverage (-): 0

Region: chr13 66730162-66730176. Max. coverage (+): 0. Max coverage (-): 0

Region: chr13 66730177-66730191. Max. coverage (+): 0. Max coverage (-): 0

Region: chr13 66730192-66730206. Max. coverage (+): 0. Max coverage (-): 0

Region: chr13 66730207-66730221. Max. coverage (+): 0. Max coverage (-): 0

Region: chr13 66730222-66730236. Max. coverage (+): 0. Max coverage (-): 0

Region: chr13 66730237-66730251. Max. coverage (+): 0. Max coverage (-): 0

Region: chr13 66730252-66730266. Max. coverage (+): 0. Max coverage (-): 0

Region: chr13 66730267-66730281. Max. coverage (+): 0. Max coverage (-): 0

Region: chr13 66730282-66730296. Max. coverage (+): 0. Max coverage (-): 0

Region: chr13 66730297-66730310. Max. coverage (+): 0. Max coverage (-): 0

Region: chr13 66730311-66730325. Max. coverage (+): 0. Max coverage (-): 0

Region: chr13 66730326-66730340. Max. coverage (+): 0. Max coverage (-): 0

Region: chr13 66730341-66730355. Max. coverage (+): 0. Max coverage (-): 0

Region: chr13 66730356-66730370. Max. coverage (+): 0. Max coverage (-): 0

Region: chr13 66730371-66730385. Max. coverage (+): 0. Max coverage (-): 0

Region: chr13 66730386-66730400. Max. coverage (+): 0. Max coverage (-): 0

Region: chr13 66730401-66730415. Max. coverage (+): 0. Max coverage (-): 0

Region: chr13 66730416-66730430. Max. coverage (+): 0. Max coverage (-): 0

Region: chr13 66730431-66730445. Max. coverage (+): 0. Max coverage (-): 0

Region: chr13 66730446-66730460. Max. coverage (+): 0. Max coverage (-): 0

Region: chr13 66730461-66730475. Max. coverage (+): 0. Max coverage (-): 0

Region: chr13 66730476-66730490. Max. coverage (+): 0. Max coverage (-): 0

Region: chr13 66730491-66730505. Max. coverage (+): 0. Max coverage (-): 0

Region: chr13 66730506-66730520. Max. coverage (+): 0. Max coverage (-): 0

Region: chr13 66730521-66730534. Max. coverage (+): 0. Max coverage (-): 0

Region: chr13 66730535-66730549. Max. coverage (+): 0. Max coverage (-): 0

Region: chr13 66730550-66730564. Max. coverage (+): 0. Max coverage (-): 0

Region: chr13 66730565-66730579. Max. coverage (+): 0. Max coverage (-): 0

Region: chr13 66730580-66730594. Max. coverage (+): 0. Max coverage (-): 0

Region: chr13 66730595-66730609. Max. coverage (+): 0. Max coverage (-): 1.82

Region: chr13 66730610-66730624. Max. coverage (+): 0. Max coverage (-): 0

Region: chr13 66730625-66730639. Max. coverage (+): 0. Max coverage (-): 0

Region: chr13 66730640-66730654. Max. coverage (+): 0. Max coverage (-): 0

Region: chr13 66730655-66730669. Max. coverage (+): 0. Max coverage (-): 0

Region: chr13 66730670-66730684. Max. coverage (+): 0. Max coverage (-): 0

Region: chr13 66730685-66730699. Max. coverage (+): 0. Max coverage (-): 0

Region: chr13 66730700-66730714. Max. coverage (+): 0. Max coverage (-): 0

Region: chr13 66730715-66730729. Max. coverage (+): 0. Max coverage (-): 1.9

Region: chr13 66730730-66730744. Max. coverage (+): 0. Max coverage (-): 0

Region: chr13 66730745-66730759. Max. coverage (+): 0. Max coverage (-): 0

Region: chr13 66730760-66730773. Max. coverage (+): 0. Max coverage (-): 0

Region: chr13 66730774-66730788. Max. coverage (+): 0. Max coverage (-): 0

Region: chr13 66730789-66730803. Max. coverage (+): 0. Max coverage (-): 0

Region: chr13 66730804-66730818. Max. coverage (+): 0. Max coverage (-): 0

Region: chr13 66730819-66730833. Max. coverage (+): 0. Max coverage (-): 0

Region: chr13 66730834-66730848. Max. coverage (+): 0. Max coverage (-): 0

Region: chr13 66730849-66730863. Max. coverage (+): 0. Max coverage (-): 0

Region: chr13 66730864-66730878. Max. coverage (+): 0. Max coverage (-): 0

Region: chr13 66730879-66730893. Max. coverage (+): 0. Max coverage (-): 0

Region: chr13 66730894-66730908. Max. coverage (+): 0. Max coverage (-): 0

Region: chr13 66730909-66730923. Max. coverage (+): 0. Max coverage (-): 0

Region: chr13 66730924-66730938. Max. coverage (+): 0. Max coverage (-): 0

Region: chr13 66730939-66730953. Max. coverage (+): 0. Max coverage (-): 0

Region: chr13 66730954-66730968. Max. coverage (+): 0. Max coverage (-): 0

Region: chr13 66730969-66730983. Max. coverage (+): 0. Max coverage (-): 0

Region: chr13 66730984-66730998. Max. coverage (+): 0. Max coverage (-): 0

Region: chr13 66730999-66731012. Max. coverage (+): 0. Max coverage (-): 0

Region: chr13 66731013-66731027. Max. coverage (+): 0. Max coverage (-): 0

Region: chr13 66731028-66731042. Max. coverage (+): 0. Max coverage (-): 0

Region: chr13 66731043-66731057. Max. coverage (+): 0. Max coverage (-): 0

Region: chr13 66731058-66731072. Max. coverage (+): 0. Max coverage (-): 2.06

Region: chr13 66731073-66731087. Max. coverage (+): 0. Max coverage (-): 0.99

Region: chr13 66731088-66731102. Max. coverage (+): 0. Max coverage (-): 0

Region: chr13 66731103-66731117. Max. coverage (+): 0. Max coverage (-): 0

Region: chr13 66731118-66731132. Max. coverage (+): 0. Max coverage (-): 1.09

Region: chr13 66731133-66731147. Max. coverage (+): 0. Max coverage (-): 1.09

Region: chr13 66731148-66731162. Max. coverage (+): 0. Max coverage (-): 0

Region: chr13 66731163-66731177. Max. coverage (+): 0. Max coverage (-): 0

Region: chr13 66731178-66731192. Max. coverage (+): 0. Max coverage (-): 0

Region: chr13 66731193-66731207. Max. coverage (+): 0. Max coverage (-): 1.87

Region: chr13 66731208-66731222. Max. coverage (+): 0. Max coverage (-): 0

Region: chr13 66731223-66731236. Max. coverage (+): 0. Max coverage (-): 0

Region: chr13 66731237-66731251. Max. coverage (+): 0. Max coverage (-): 2.3

Region: chr13 66731252-66731266. Max. coverage (+): 0. Max coverage (-): 2.3

Region: chr13 66731267-66731281. Max. coverage (+): 0. Max coverage (-): 0

Region: chr13 66731282-66731296. Max. coverage (+): 0. Max coverage (-): 0

Region: chr13 66731297-66731311. Max. coverage (+): 0. Max coverage (-): 0

Region: chr13 66731312-66731326. Max. coverage (+): 0. Max coverage (-): 0

Region: chr13 66731327-66731341. Max. coverage (+): 0. Max coverage (-): 0

Region: chr13 66731342-66731356. Max. coverage (+): 0. Max coverage (-): 0

Region: chr13 66731357-66731371. Max. coverage (+): 0. Max coverage (-): 3.06

Region: chr13 66731372-66731386. Max. coverage (+): 0. Max coverage (-): 3.06

Region: chr13 66731387-66731401. Max. coverage (+): 0. Max coverage (-): 0

Region: chr13 66731402-66731416. Max. coverage (+): 0. Max coverage (-): 0

Region: chr13 66731417-66731431. Max. coverage (+): 0. Max coverage (-): 0

Region: chr13 66731432-66731446. Max. coverage (+): 0. Max coverage (-): 0

Region: chr13 66731447-66731461. Max. coverage (+): 0. Max coverage (-): 0

Region: chr13 66731462-66731475. Max. coverage (+): 0. Max coverage (-): 0

Region: chr13 66731476-66731490. Max. coverage (+): 0. Max coverage (-): 0

Region: chr13 66731491-66731505. Max. coverage (+): 0. Max coverage (-): 0

Region: chr13 66731506-66731520. Max. coverage (+): 0. Max coverage (-): 0

Region: chr13 66731521-66731535. Max. coverage (+): 0. Max coverage (-): 0

Region: chr13 66731536-66731550. Max. coverage (+): 0. Max coverage (-): 0

Region: chr13 66731551-66731565. Max. coverage (+): 0. Max coverage (-): 0

Region: chr13 66731566-66731580. Max. coverage (+): 0. Max coverage (-): 0

Region: chr13 66731581-66731595. Max. coverage (+): 0. Max coverage (-): 0

Region: chr13 66731596-66731610. Max. coverage (+): 0. Max coverage (-): 0

Region: chr13 66731611-66731625. Max. coverage (+): 0. Max coverage (-): 0

Region: chr13 66731626-66731640. Max. coverage (+): 0. Max coverage (-): 0

Region: chr13 66731641-66731655. Max. coverage (+): 0. Max coverage (-): 0

Region: chr13 66731656-66731670. Max. coverage (+): 0. Max coverage (-): 0

Region: chr13 66731671-66731685. Max. coverage (+): 0. Max coverage (-): 0

Region: chr13 66731686-66731699. Max. coverage (+): 0. Max coverage (-): 0

Region: chr13 66731700-66731714. Max. coverage (+): 0. Max coverage (-): 0

Region: chr13 66731715-66731729. Max. coverage (+): 0. Max coverage (-): 0

Region: chr13 66731730-66731744. Max. coverage (+): 0. Max coverage (-): 0

Region: chr13 66731745-66731759. Max. coverage (+): 0. Max coverage (-): 0

Region: chr13 66731760-66731774. Max. coverage (+): 0. Max coverage (-): 0

Region: chr13 66731775-66731789. Max. coverage (+): 0. Max coverage (-): 0

Region: chr13 66731790-66731804. Max. coverage (+): 0. Max coverage (-): 0

Region: chr13 66731805-66731819. Max. coverage (+): 0. Max coverage (-): 0

Region: chr13 66731820-66731834. Max. coverage (+): 0. Max coverage (-): 0

Region: chr13 66731835-66731849. Max. coverage (+): 0. Max coverage (-): 0

Region: chr13 66731850-66731864. Max. coverage (+): 0. Max coverage (-): 0

Region: chr13 66731865-66731879. Max. coverage (+): 0. Max coverage (-): 0

Region: chr13 66731880-66731894. Max. coverage (+): 0. Max coverage (-): 0

Region: chr13 66731895-66731909. Max. coverage (+): 0. Max coverage (-): 0

Region: chr13 66731910-66731924. Max. coverage (+): 0. Max coverage (-): 0

Region: chr13 66731925-66731938. Max. coverage (+): 0. Max coverage (-): 0

Region: chr13 66731939-66731953. Max. coverage (+): 0. Max coverage (-): 0

Region: chr13 66731954-66731968. Max. coverage (+): 0. Max coverage (-): 0

Region: chr13 66731969-66731983. Max. coverage (+): 0. Max coverage (-): 0

Region: chr13 66731984-66731998. Max. coverage (+): 0. Max coverage (-): 0

Region: chr13 66731999-66732013. Max. coverage (+): 0. Max coverage (-): 0

Region: chr13 66732014-66732028. Max. coverage (+): 0. Max coverage (-): 0

Region: chr13 66732029-66732043. Max. coverage (+): 0. Max coverage (-): 0

Region: chr13 66732044-66732058. Max. coverage (+): 0. Max coverage (-): 0

Region: chr13 66732059-66732073. Max. coverage (+): 0. Max coverage (-): 0

Region: chr13 66732074-66732088. Max. coverage (+): 0. Max coverage (-): 0

Region: chr13 66732089-66732103. Max. coverage (+): 0. Max coverage (-): 0

Region: chr13 66732104-66732118. Max. coverage (+): 0. Max coverage (-): 0

Region: chr13 66732119-66732133. Max. coverage (+): 0. Max coverage (-): 0

Region: chr13 66732134-66732148. Max. coverage (+): 0. Max coverage (-): 0

Region: chr13 66732149-66732163. Max. coverage (+): 0. Max coverage (-): 0

Region: chr13 66732164-66732177. Max. coverage (+): 0. Max coverage (-): 0

Region: chr13 66732178-66732192. Max. coverage (+): 0. Max coverage (-): 0

Region: chr13 66732193-66732207. Max. coverage (+): 0. Max coverage (-): 0

Region: chr13 66732208-66732222. Max. coverage (+): 0. Max coverage (-): 18.34

Region: chr13 66732223-66732237. Max. coverage (+): 0. Max coverage (-): 16.66

Region: chr13 66732238-66732252. Max. coverage (+): 0. Max coverage (-): 0

Region: chr13 66732253-66732267. Max. coverage (+): 0. Max coverage (-): 0

Region: chr13 66732268-66732282. Max. coverage (+): 0. Max coverage (-): 0

Region: chr13 66732283-66732297. Max. coverage (+): 0. Max coverage (-): 0

Region: chr13 66732298-66732312. Max. coverage (+): 0. Max coverage (-): 0

Region: chr13 66732313-66732327. Max. coverage (+): 0. Max coverage (-): 0

Region: chr13 66732328-66732342. Max. coverage (+): 0. Max coverage (-): 0

Region: chr13 66732343-66732357. Max. coverage (+): 0. Max coverage (-): 0

Region: chr13 66732358-66732372. Max. coverage (+): 0. Max coverage (-): 0

Region: chr13 66732373-66732387. Max. coverage (+): 0. Max coverage (-): 1.82

Region: chr13 66732388-66732401. Max. coverage (+): 0. Max coverage (-): 0

Region: chr13 66732402-66732416. Max. coverage (+): 0. Max coverage (-): 0

Region: chr13 66732417-66732431. Max. coverage (+): 0. Max coverage (-): 0

Region: chr13 66732432-66732446. Max. coverage (+): 0. Max coverage (-): 0

Region: chr13 66732447-66732461. Max. coverage (+): 0. Max coverage (-): 0

Region: chr13 66732462-66732476. Max. coverage (+): 0. Max coverage (-): 1.9

Region: chr13 66732477-66732491. Max. coverage (+): 0. Max coverage (-): 1.9

Region: chr13 66732492-66732506. Max. coverage (+): 0. Max coverage (-): 1.49

Region: chr13 66732507-66732521. Max. coverage (+): 0. Max coverage (-): 0

Region: chr13 66732522-66732536. Max. coverage (+): 0. Max coverage (-): 1.58

Region: chr13 66732537-66732551. Max. coverage (+): 0. Max coverage (-): 0

Region: chr13 66732552-66732566. Max. coverage (+): 0. Max coverage (-): 0

Region: chr13 66732567-66732581. Max. coverage (+): 0. Max coverage (-): 9.39

Region: chr13 66732582-66732596. Max. coverage (+): 0. Max coverage (-): 4.48

Region: chr13 66732597-66732611. Max. coverage (+): 0. Max coverage (-): 0

Region: chr13 66732612-66732626. Max. coverage (+): 0. Max coverage (-): 0

Region: chr13 66732627-66732640. Max. coverage (+): 0. Max coverage (-): 0

Region: chr13 66732641-66732655. Max. coverage (+): 0. Max coverage (-): 0

Region: chr13 66732656-66732670. Max. coverage (+): 0. Max coverage (-): 0

Region: chr13 66732671-66732685. Max. coverage (+): 0. Max coverage (-): 0

Region: chr13 66732686-66732700. Max. coverage (+): 0. Max coverage (-): 2.16

Region: chr13 66732701-66732715. Max. coverage (+): 0. Max coverage (-): 0

Region: chr13 66732716-66732730. Max. coverage (+): 0. Max coverage (-): 7.26

Region: chr13 66732731-66732745. Max. coverage (+): 0. Max coverage (-): 0

Region: chr13 66732746-66732760. Max. coverage (+): 0. Max coverage (-): 0

Region: chr13 66732761-66732775. Max. coverage (+): 0. Max coverage (-): 0

Region: chr13 66732776-66732790. Max. coverage (+): 0. Max coverage (-): 4.06

Region: chr13 66732791-66732805. Max. coverage (+): 0. Max coverage (-): 2.21

Region: chr13 66732806-66732820. Max. coverage (+): 0. Max coverage (-): 14.47

Region: chr13 66732821-66732835. Max. coverage (+): 0. Max coverage (-): 10.07

Region: chr13 66732836-66732850. Max. coverage (+): 0. Max coverage (-): 0

Region: chr13 66732851-66732865. Max. coverage (+): 0. Max coverage (-): 0

Region: chr13 66732866-66732879. Max. coverage (+): 0. Max coverage (-): 0

Region: chr13 66732880-66732894. Max. coverage (+): 0. Max coverage (-): 0

Region: chr13 66732895-66732909. Max. coverage (+): 0. Max coverage (-): 0

Region: chr13 66732910-66732924. Max. coverage (+): 0. Max coverage (-): 0

Region: chr13 66732925-66732939. Max. coverage (+): 0. Max coverage (-): 0

Region: chr13 66732940-66732954. Max. coverage (+): 0. Max coverage (-): 1.71

Region: chr13 66732955-66732969. Max. coverage (+): 0. Max coverage (-): 0

Region: chr13 66732970-66732984. Max. coverage (+): 0. Max coverage (-): 0

Region: chr13 66732985-66732999. Max. coverage (+): 0. Max coverage (-): 0

Region: chr13 66733000-66733014. Max. coverage (+): 0. Max coverage (-): 0

Region: chr13 66733015-66733029. Max. coverage (+): 0. Max coverage (-): 0

Region: chr13 66733030-66733044. Max. coverage (+): 0. Max coverage (-): 0

Region: chr13 66733045-66733059. Max. coverage (+): 0. Max coverage (-): 0

Region: chr13 66733060-66733074. Max. coverage (+): 0. Max coverage (-): 0

Region: chr13 66733075-66733089. Max. coverage (+): 0. Max coverage (-): 0

Region: chr13 66733090-66733103. Max. coverage (+): 0. Max coverage (-): 0

Region: chr13 66733104-66733118. Max. coverage (+): 0. Max coverage (-): 0

Region: chr13 66733119-66733133. Max. coverage (+): 0. Max coverage (-): 0

Region: chr13 66733134-66733148. Max. coverage (+): 0. Max coverage (-): 0

Region: chr13 66733149-66733163. Max. coverage (+): 0. Max coverage (-): 0

Region: chr13 66733164-66733178. Max. coverage (+): 0. Max coverage (-): 0

Region: chr13 66733179-66733193. Max. coverage (+): 0. Max coverage (-): 0

Region: chr13 66733194-66733208. Max. coverage (+): 0. Max coverage (-): 0

Region: chr13 66733209-66733223. Max. coverage (+): 0. Max coverage (-): 0

Region: chr13 66733224-66733238. Max. coverage (+): 0. Max coverage (-): 0

Region: chr13 66733239-66733253. Max. coverage (+): 0. Max coverage (-): 0

Region: chr13 66733254-66733268. Max. coverage (+): 0. Max coverage (-): 0

Region: chr13 66733269-66733283. Max. coverage (+): 0. Max coverage (-): 0

Region: chr13 66733284-66733298. Max. coverage (+): 0. Max coverage (-): 0

Region: chr13 66733299-66733313. Max. coverage (+): 0. Max coverage (-): 0

Region: chr13 66733314-66733328. Max. coverage (+): 0. Max coverage (-): 0

Region: chr13 66733329-66733342. Max. coverage (+): 0. Max coverage (-): 0

Region: chr13 66733343-66733357. Max. coverage (+): 0. Max coverage (-): 0

Region: chr13 66733358-66733372. Max. coverage (+): 0. Max coverage (-): 0

Region: chr13 66733373-66733387. Max. coverage (+): 0. Max coverage (-): 0

Region: chr13 66733388-66733402. Max. coverage (+): 0. Max coverage (-): 0

Region: chr13 66733403-66733417. Max. coverage (+): 0. Max coverage (-): 0

Region: chr13 66733418-66733432. Max. coverage (+): 0. Max coverage (-): 0

Region: chr13 66733433-66733447. Max. coverage (+): 0. Max coverage (-): 0

Region: chr13 66733448-66733462. Max. coverage (+): 0. Max coverage (-): 0

Region: chr13 66733463-66733477. Max. coverage (+): 0. Max coverage (-): 0

Region: chr13 66733478-66733492. Max. coverage (+): 0. Max coverage (-): 0

Region: chr13 66733493-66733507. Max. coverage (+): 0. Max coverage (-): 0

Region: chr13 66733508-66733522. Max. coverage (+): 0. Max coverage (-): 0

Region: chr13 66733523-66733537. Max. coverage (+): 0. Max coverage (-): 0

Region: chr13 66733538-66733552. Max. coverage (+): 0. Max coverage (-): 0

Region: chr13 66733553-66733566. Max. coverage (+): 0. Max coverage (-): 0

Region: chr13 66733567-66733581. Max. coverage (+): 0. Max coverage (-): 0

Region: chr13 66733582-66733596. Max. coverage (+): 0. Max coverage (-): 0

Region: chr13 66733597-66733611. Max. coverage (+): 0. Max coverage (-): 0

Region: chr13 66733612-66733626. Max. coverage (+): 0. Max coverage (-): 3.15

Region: chr13 66733627-66733641. Max. coverage (+): 0. Max coverage (-): 0

Region: chr13 66733642-66733656. Max. coverage (+): 0. Max coverage (-): 0

Region: chr13 66733657-66733671. Max. coverage (+): 0. Max coverage (-): 3.54

Region: chr13 66733672-66733686. Max. coverage (+): 0. Max coverage (-): 3.54

Region: chr13 66733687-66733701. Max. coverage (+): 0. Max coverage (-): 2.26

Region: chr13 66733702-66733716. Max. coverage (+): 0. Max coverage (-): 2.4

Region: chr13 66733717-66733731. Max. coverage (+): 0. Max coverage (-): 2.4

Region: chr13 66733732-66733746. Max. coverage (+): 0. Max coverage (-): 0

Region: chr13 66733747-66733761. Max. coverage (+): 0. Max coverage (-): 4.9

Region: chr13 66733762-66733776. Max. coverage (+): 0. Max coverage (-): 0.48

Region: chr13 66733777-66733791. Max. coverage (+): 0. Max coverage (-): 2.5

Region: chr13 66733792-66733805. Max. coverage (+): 0. Max coverage (-): 8.94

Region: chr13 66733806-66733820. Max. coverage (+): 0. Max coverage (-): 0

Region: chr13 66733821-66733835. Max. coverage (+): 0. Max coverage (-): 5.09

Region: chr13 66733836-66733850. Max. coverage (+): 0. Max coverage (-): 12.95

Region: chr13 66733851-66733865. Max. coverage (+): 0. Max coverage (-): 12.95

Region: chr13 66733866-66733880. Max. coverage (+): 0. Max coverage (-): 2.1

Region: chr13 66733881-66733895. Max. coverage (+): 0. Max coverage (-): 2.06

Region: chr13 66733896-66733910. Max. coverage (+): 0. Max coverage (-): 14.19

Region: chr13 66733911-66733925. Max. coverage (+): 0. Max coverage (-): 14.19

Region: chr13 66733926-66733940. Max. coverage (+): 0. Max coverage (-): 1.45

Region: chr13 66733941-66733955. Max. coverage (+): 0. Max coverage (-): 2.78

Region: chr13 66733956-66733970. Max. coverage (+): 0. Max coverage (-): 0

Region: chr13 66733971-66733985. Max. coverage (+): 0. Max coverage (-): 1.99

Region: chr13 66733986-66734000. Max. coverage (+): 0. Max coverage (-): 2.78

Region: chr13 66734001-66734015. Max. coverage (+): 0. Max coverage (-): 2.29

Region: chr13 66734016-66734030. Max. coverage (+): 0. Max coverage (-): 3.41

Region: chr13 66734031-66734044. Max. coverage (+): 0. Max coverage (-): 17.77

Region: chr13 66734045-66734059. Max. coverage (+): 0. Max coverage (-): 5.53

Region: chr13 66734060-66734074. Max. coverage (+): 0. Max coverage (-): 0.65

Region: chr13 66734075-66734089. Max. coverage (+): 0. Max coverage (-): 1.84

Region: chr13 66734090-66734104. Max. coverage (+): 0. Max coverage (-): 1.84

Region: chr13 66734105-66734119. Max. coverage (+): 0. Max coverage (-): 0.68

Region: chr13 66734120-66734134. Max. coverage (+): 0. Max coverage (-): 5.66

Region: chr13 66734135-66734149. Max. coverage (+): 0. Max coverage (-): 0

Region: chr13 66734150-66734164. Max. coverage (+): 0. Max coverage (-): 0

Region: chr13 66734165-66734179. Max. coverage (+): 0. Max coverage (-): 0

Region: chr13 66734180-66734194. Max. coverage (+): 0. Max coverage (-): 0

Region: chr13 66734195-66734209. Max. coverage (+): 0. Max coverage (-): 2.87

Region: chr13 66734210-66734224. Max. coverage (+): 0. Max coverage (-): 0

Region: chr13 66734225-66734239. Max. coverage (+): 0. Max coverage (-): 0

Region: chr13 66734240-66734254. Max. coverage (+): 0. Max coverage (-): 0

Region: chr13 66734255-66734268. Max. coverage (+): 0. Max coverage (-): 0

Region: chr13 66734269-66734283. Max. coverage (+): 0. Max coverage (-): 0

Region: chr13 66734284-66734298. Max. coverage (+): 0. Max coverage (-): 3.29

Region: chr13 66734299-66734313. Max. coverage (+): 0. Max coverage (-): 3.29

Region: chr13 66734314-66734328. Max. coverage (+): 0. Max coverage (-): 0

Region: chr13 66734329-66734343. Max. coverage (+): 0. Max coverage (-): 0

Region: chr13 66734344-66734358. Max. coverage (+): 0. Max coverage (-): 0

Region: chr13 66734359-66734373. Max. coverage (+): 0. Max coverage (-): 0

Region: chr13 66734374-66734388. Max. coverage (+): 0. Max coverage (-): 0

Region: chr13 66734389-66734403. Max. coverage (+): 0. Max coverage (-): 0

Region: chr13 66734404-66734418. Max. coverage (+): 0. Max coverage (-): 1.92

Region: chr13 66734419-66734433. Max. coverage (+): 0. Max coverage (-): 1.92

Region: chr13 66734434-66734448. Max. coverage (+): 0. Max coverage (-): 0

Region: chr13 66734449-66734463. Max. coverage (+): 0. Max coverage (-): 0

Region: chr13 66734464-66734478. Max. coverage (+): 0. Max coverage (-): 0

Region: chr13 66734479-66734493. Max. coverage (+): 0. Max coverage (-): 0

Region: chr13 66734494-66734507. Max. coverage (+): 0. Max coverage (-): 0

Region: chr13 66734508-66734522. Max. coverage (+): 0. Max coverage (-): 0

Region: chr13 66734523-66734537. Max. coverage (+): 0. Max coverage (-): 0

Region: chr13 66734538-66734552. Max. coverage (+): 0. Max coverage (-): 0

Region: chr13 66734553-66734567. Max. coverage (+): 0. Max coverage (-): 0

Region: chr13 66734568-66734582. Max. coverage (+): 0. Max coverage (-): 0

Region: chr13 66734583-66734597. Max. coverage (+): 0. Max coverage (-): 0

Region: chr13 66734598-66734612. Max. coverage (+): 0. Max coverage (-): 0

Region: chr13 66734613-66734627. Max. coverage (+): 0. Max coverage (-): 0

Region: chr13 66734628-66734642. Max. coverage (+): 0. Max coverage (-): 0

Region: chr13 66734643-66734657. Max. coverage (+): 0. Max coverage (-): 0

Region: chr13 66734658-66734672. Max. coverage (+): 1.84. Max coverage (-): 0

Region: chr13 66734673-66734687. Max. coverage (+): 0. Max coverage (-): 0

Region: chr13 66734688-66734702. Max. coverage (+): 0. Max coverage (-): 0

Region: chr13 66734703-66734717. Max. coverage (+): 0. Max coverage (-): 0

Region: chr13 66734718-66734732. Max. coverage (+): 0. Max coverage (-): 0

Region: chr13 66734733-66734746. Max. coverage (+): 0. Max coverage (-): 0

Region: chr13 66734747-66734761. Max. coverage (+): 0. Max coverage (-): 0

Region: chr13 66734762-66734776. Max. coverage (+): 0. Max coverage (-): 0

Region: chr13 66734777-66734791. Max. coverage (+): 0. Max coverage (-): 0

Region: chr13 66734792-66734806. Max. coverage (+): 0. Max coverage (-): 0

Region: chr13 66734807-66734821. Max. coverage (+): 0. Max coverage (-): 0

Region: chr13 66734822-66734836. Max. coverage (+): 4.56. Max coverage (-): 0

Region: chr13 66734837-66734851. Max. coverage (+): 4.56. Max coverage (-): 0

Region: chr13 66734852-66734866. Max. coverage (+): 0. Max coverage (-): 0

Region: chr13 66734867-66734881. Max. coverage (+): 0. Max coverage (-): 0

Region: chr13 66734882-66734896. Max. coverage (+): 0. Max coverage (-): 0

Region: chr13 66734897-66734911. Max. coverage (+): 0. Max coverage (-): 0

Region: chr13 66734912-66734926. Max. coverage (+): 0. Max coverage (-): 0

Region: chr13 66734927-66734941. Max. coverage (+): 0. Max coverage (-): 0

Region: chr13 66734942-66734956. Max. coverage (+): 0. Max coverage (-): 0

Region: chr13 66734957-66734970. Max. coverage (+): 0. Max coverage (-): 5.55

Region: chr13 66734971-66734985. Max. coverage (+): 0. Max coverage (-): 1.59

Region: chr13 66734986-66735000. Max. coverage (+): 0. Max coverage (-): 0

Region: chr13 66735001-66735015. Max. coverage (+): 0. Max coverage (-): 0

Region: chr13 66735016-66735030. Max. coverage (+): 0. Max coverage (-): 0

Region: chr13 66735031-66735045. Max. coverage (+): 0. Max coverage (-): 0

Region: chr13 66735046-66735060. Max. coverage (+): 0. Max coverage (-): 0

Region: chr13 66735061-66735075. Max. coverage (+): 0. Max coverage (-): 0

Region: chr13 66735076-66735090. Max. coverage (+): 0. Max coverage (-): 0

Region: chr13 66735091-66735105. Max. coverage (+): 0. Max coverage (-): 0

Region: chr13 66735106-66735120. Max. coverage (+): 0. Max coverage (-): 0

Region: chr13 66735121-66735135. Max. coverage (+): 0. Max coverage (-): 0

Region: chr13 66735136-66735150. Max. coverage (+): 0. Max coverage (-): 0

Region: chr13 66735151-66735165. Max. coverage (+): 0. Max coverage (-): 0.77

Region: chr13 66735166-66735180. Max. coverage (+): 0. Max coverage (-): 0.72

Region: chr13 66735181-66735195. Max. coverage (+): 0. Max coverage (-): 2.24

Region: chr13 66735196-66735209. Max. coverage (+): 0. Max coverage (-): 0

Region: chr13 66735210-66735224. Max. coverage (+): 0. Max coverage (-): 0

Region: chr13 66735225-66735239. Max. coverage (+): 0. Max coverage (-): 0

Region: chr13 66735240-66735254. Max. coverage (+): 0. Max coverage (-): 0.91

Region: chr13 66735255-66735269. Max. coverage (+): 0. Max coverage (-): 2.05

Region: chr13 66735270-66735284. Max. coverage (+): 0. Max coverage (-): 0

Region: chr13 66735285-66735299. Max. coverage (+): 0. Max coverage (-): 3.33

Region: chr13 66735300-66735314. Max. coverage (+): 0. Max coverage (-): 3.33

Region: chr13 66735315-66735329. Max. coverage (+): 0. Max coverage (-): 0

Region: chr13 66735330-66735344. Max. coverage (+): 0. Max coverage (-): 0

Region: chr13 66735345-66735359. Max. coverage (+): 0. Max coverage (-): 0

Region: chr13 66735360-66735374. Max. coverage (+): 0. Max coverage (-): 0

Region: chr13 66735375-66735389. Max. coverage (+): 0. Max coverage (-): 0

Region: chr13 66735390-66735404. Max. coverage (+): 0. Max coverage (-): 0

Region: chr13 66735405-66735419. Max. coverage (+): 0. Max coverage (-): 0

Region: chr13 66735420-66735433. Max. coverage (+): 0. Max coverage (-): 0

Region: chr13 66735434-66735448. Max. coverage (+): 0. Max coverage (-): 0

Region: chr13 66735449-66735463. Max. coverage (+): 0. Max coverage (-): 0

Region: chr13 66735464-66735478. Max. coverage (+): 0. Max coverage (-): 0

Region: chr13 66735479-66735493. Max. coverage (+): 0. Max coverage (-): 0

Region: chr13 66735494-66735508. Max. coverage (+): 0. Max coverage (-): 0

Region: chr13 66735509-66735523. Max. coverage (+): 0. Max coverage (-): 0

Region: chr13 66735524-66735538. Max. coverage (+): 0. Max coverage (-): 0

Region: chr13 66735539-66735553. Max. coverage (+): 0. Max coverage (-): 0

Region: chr13 66735554-66735568. Max. coverage (+): 0. Max coverage (-): 0

Region: chr13 66735569-66735583. Max. coverage (+): 0. Max coverage (-): 0

Region: chr13 66735584-66735598. Max. coverage (+): 0. Max coverage (-): 0

Region: chr13 66735599-66735613. Max. coverage (+): 0. Max coverage (-): 0

Region: chr13 66735614-66735628. Max. coverage (+): 0. Max coverage (-): 0

Region: chr13 66735629-66735643. Max. coverage (+): 0. Max coverage (-): 0

Region: chr13 66735644-66735658. Max. coverage (+): 0. Max coverage (-): 0

Region: chr13 66735659-66735672. Max. coverage (+): 0. Max coverage (-): 0

Region: chr13 66735673-66735687. Max. coverage (+): 0. Max coverage (-): 0

Region: chr13 66735688-66735702. Max. coverage (+): 0. Max coverage (-): 0

Region: chr13 66735703-66735717. Max. coverage (+): 0. Max coverage (-): 0

Region: chr13 66735718-66735732. Max. coverage (+): 0. Max coverage (-): 0

Region: chr13 66735733-66735747. Max. coverage (+): 0. Max coverage (-): 0

Region: chr13 66735748-66735762. Max. coverage (+): 0. Max coverage (-): 0

Region: chr13 66735763-66735777. Max. coverage (+): 0. Max coverage (-): 0

Region: chr13 66735778-66735792. Max. coverage (+): 0. Max coverage (-): 0

Region: chr13 66735793-66735807. Max. coverage (+): 0. Max coverage (-): 0

Region: chr13 66735808-66735822. Max. coverage (+): 0. Max coverage (-): 0

Region: chr13 66735823-66735837. Max. coverage (+): 0. Max coverage (-): 0

Region: chr13 66735838-66735852. Max. coverage (+): 0. Max coverage (-): 0

Region: chr13 66735853-66735867. Max. coverage (+): 0. Max coverage (-): 0

Region: chr13 66735868-66735882. Max. coverage (+): 0. Max coverage (-): 0

Region: chr13 66735883-66735897. Max. coverage (+): 0. Max coverage (-): 0

Region: chr13 66735898-66735911. Max. coverage (+): 0. Max coverage (-): 0

Region: chr13 66735912-66735926. Max. coverage (+): 0. Max coverage (-): 0

Region: chr13 66735927-66735941. Max. coverage (+): 0. Max coverage (-): 0

Region: chr13 66735942-66735956. Max. coverage (+): 0. Max coverage (-): 0

Region: chr13 66735957-66735971. Max. coverage (+): 0. Max coverage (-): 0

Region: chr13 66735972-66735986. Max. coverage (+): 0. Max coverage (-): 0

Region: chr13 66735987-66736001. Max. coverage (+): 0. Max coverage (-): 0

Region: chr13 66736002-66736016. Max. coverage (+): 0. Max coverage (-): 0

Region: chr13 66736017-66736031. Max. coverage (+): 0. Max coverage (-): 0

Region: chr13 66736032-66736046. Max. coverage (+): 0. Max coverage (-): 0

Region: chr13 66736047-66736061. Max. coverage (+): 0. Max coverage (-): 0

Region: chr13 66736062-66736076. Max. coverage (+): 0. Max coverage (-): 0

Region: chr13 66736077-66736091. Max. coverage (+): 0. Max coverage (-): 0

Region: chr13 66736092-66736106. Max. coverage (+): 0. Max coverage (-): 0

Region: chr13 66736107-66736121. Max. coverage (+): 0. Max coverage (-): 0

Region: chr13 66736122-66736135. Max. coverage (+): 0. Max coverage (-): 0

Region: chr13 66736136-66736150. Max. coverage (+): 0. Max coverage (-): 0

Region: chr13 66736151-66736165. Max. coverage (+): 0. Max coverage (-): 0

Region: chr13 66736166-66736180. Max. coverage (+): 0. Max coverage (-): 0

Region: chr13 66736181-66736195. Max. coverage (+): 0. Max coverage (-): 0

Region: chr13 66736196-66736210. Max. coverage (+): 0. Max coverage (-): 0

Region: chr13 66736211-66736225. Max. coverage (+): 0. Max coverage (-): 0

Region: chr13 66736226-66736240. Max. coverage (+): 0. Max coverage (-): 0

Region: chr13 66736241-66736255. Max. coverage (+): 0. Max coverage (-): 0

Region: chr13 66736256-66736270. Max. coverage (+): 0. Max coverage (-): 0

Region: chr13 66736271-66736285. Max. coverage (+): 0. Max coverage (-): 0

Region: chr13 66736286-66736300. Max. coverage (+): 0. Max coverage (-): 0

Region: chr13 66736301-66736315. Max. coverage (+): 0. Max coverage (-): 0

Region: chr13 66736316-66736330. Max. coverage (+): 0. Max coverage (-): 0

Region: chr13 66736331-66736345. Max. coverage (+): 0. Max coverage (-): 0

Region: chr13 66736346-66736360. Max. coverage (+): 0. Max coverage (-): 0

Region: chr13 66736361-66736374. Max. coverage (+): 1.73. Max coverage (-): 0

Region: chr13 66736375-66736389. Max. coverage (+): 0.9. Max coverage (-): 0

Region: chr13 66736390-66736404. Max. coverage (+): 0. Max coverage (-): 0

Region: chr13 66736405-66736419. Max. coverage (+): 2.26. Max coverage (-): 0

Region: chr13 66736420-66736434. Max. coverage (+): 0. Max coverage (-): 0

Region: chr13 66736435-66736449. Max. coverage (+): 12.98. Max coverage (-): 0

Region: chr13 66736450-66736464. Max. coverage (+): 9.85. Max coverage (-): 0

Region: chr13 66736465-66736479. Max. coverage (+): 0. Max coverage (-): 0

Region: chr13 66736480-66736494. Max. coverage (+): 0. Max coverage (-): 0

Region: chr13 66736495-66736509. Max. coverage (+): 0.93. Max coverage (-): 0

Region: chr13 66736510-66736524. Max. coverage (+): 0. Max coverage (-): 0

Region: chr13 66736525-66736539. Max. coverage (+): 0. Max coverage (-): 0

Region: chr13 66736540-66736554. Max. coverage (+): 0. Max coverage (-): 0

Region: chr13 66736555-66736569. Max. coverage (+): 0. Max coverage (-): 0

Region: chr13 66736570-66736584. Max. coverage (+): 0. Max coverage (-): 0

Region: chr13 66736585-66736599. Max. coverage (+): 0. Max coverage (-): 0

Region: chr13 66736600-66736613. Max. coverage (+): 0. Max coverage (-): 0

Region: chr13 66736614-66736628. Max. coverage (+): 0. Max coverage (-): 0

Region: chr13 66736629-66736643. Max. coverage (+): 0. Max coverage (-): 0

Region: chr13 66736644-66736658. Max. coverage (+): 0. Max coverage (-): 0

Region: chr13 66736659-66736673. Max. coverage (+): 0. Max coverage (-): 0

Region: chr13 66736674-66736688. Max. coverage (+): 0. Max coverage (-): 0

Region: chr13 66736689-66736703. Max. coverage (+): 0. Max coverage (-): 0

Region: chr13 66736704-66736718. Max. coverage (+): 0. Max coverage (-): 0

Region: chr13 66736719-66736733. Max. coverage (+): 0. Max coverage (-): 0

Region: chr13 66736734-66736748. Max. coverage (+): 0. Max coverage (-): 0

Region: chr13 66736749-66736763. Max. coverage (+): 0. Max coverage (-): 0

Region: chr13 66736764-66736778. Max. coverage (+): 0. Max coverage (-): 0

Region: chr13 66736779-66736793. Max. coverage (+): 0. Max coverage (-): 0

Region: chr13 66736794-66736808. Max. coverage (+): 0. Max coverage (-): 0

Region: chr13 66736809-66736823. Max. coverage (+): 1.33. Max coverage (-): 0

Region: chr13 66736824-66736837. Max. coverage (+): 1.33. Max coverage (-): 0

Region: chr13 66736838-66736852. Max. coverage (+): 0. Max coverage (-): 0

Region: chr13 66736853-66736867. Max. coverage (+): 3.63. Max coverage (-): 0

Region: chr13 66736868-66736882. Max. coverage (+): 1.41. Max coverage (-): 0

Region: chr13 66736883-66736897. Max. coverage (+): 0. Max coverage (-): 0

Region: chr13 66736898-66736912. Max. coverage (+): 0. Max coverage (-): 0

Region: chr13 66736913-66736927. Max. coverage (+): 4.31. Max coverage (-): 0

Region: chr13 66736928-66736942. Max. coverage (+): 4.31. Max coverage (-): 0

Region: chr13 66736943-. Max. coverage (+): 0. Max coverage (-): 0

RepeatMasker Color Code

**+**

100-98% Identity

<98-95% Identity

<95-90% Identity

<90-85% Identity

<85-80% Identity

<80-75% Identity

<75-70% Identity

<70% Identity

**-**

Gene Set Color Code

**+**

Gene

Pseudogene

**-**

Topology/Coverage Color Code

Coverage Plus Strand

Coverage Minus Strand

Mainstrand: Plus

Mainstrand: Minus

Complementary Strand

Flanking Region  
(if option -flank >0)

Gene Set Annotation  

**1. RBL1 (protein coding, ENSBTAG00000011541) Tr:00000015333 Ex:1**: 66730434-66730589 (-)

  
RepeatMasker Annotation  

**1. MIRc**: 66729581-66729706 (+), Divergence to consensus: 42.1%  
**2. MIRc**: 66729773-66729823 (-), Divergence to consensus: 21.4%  
**3. MIRb**: 66729899-66730096 (+), Divergence to consensus: 34%  
**4. MIR3**: 66730207-66730304 (+), Divergence to consensus: 37.7%  
**5. L2a**: 66731428-66731625 (+), Divergence to consensus: 35.5%  
**6. L2a**: 66731683-66731871 (+), Divergence to consensus: 47%  
**7. (CACG)n**: 66731875-66731962 (+), Divergence to consensus: 17.1%  
**8. L2a**: 66731963-66732128 (+), Divergence to consensus: 41.8%  
**9. SINE2-2\_BT**: 66732865-66732930 (-), Divergence to consensus: 18.2%  
**10. L2a**: 66734238-66734293 (-), Divergence to consensus: 23.6%  
**11. MamTip1**: 66735387-66735613 (-), Divergence to consensus: 30.1%  
**12. BOV-A2**: 66736036-66736223 (-), Divergence to consensus: 12.7%  
**13. MIRc**: 66736594-66736788 (+), Divergence to consensus: 33.7%

  
Transcription Factor Binding Sites  

**Gata4** (Sequence: AGATAAG (-): 66735861)  
**SOX9** (Sequence: CCATTGTT (+): 66732162)
