# Supplementary material for: Small RNA sequencing of cryopreserved semen from single bull revealed altered miRNAs and piRNAs expression between High- and Low-motile sperm populations
Source: BMC Genomics. 2017 Jan 4;18:14. doi: 10.1186/s12864-016-3394-7 (PMC5209821; doi:10.1186/s12864-016-3394-7)
Supplement: Additional file 3: — Details for each piRNA clusters found in High Motile (HM) sperm fraction. Genes, repeats, transposable elements and transcription factors binding sites falling within the cluster regions were reported. (ZIP 1896 kb) [file 12864_2016_3394_MOESM3_ESM.zip › 17.html]

piRNA cluster 17


Predicted piRNA cluster no. 17     previous   next
  

Show proTRAC run info
Hide proTRAC run info

================================= proTRAC ====================================  
VERSION: 2.1                                    LAST MODIFIED: 06. October 2015  
  
Please cite:  
Rosenkranz D, Zischler H. proTRAC - a software for probabilistic piRNA cluster  
detection, visualization and analysis. 2012. BMC Bioinformatics 13:5.  
  
and (for proTRAC 2.0 and later):  
Rosenkranz D, Rudloff S, Bastuck K, Ketting RF, Zischler H. Tupaia small RNAs  
provide insights into function and evolution of RNAi-based transposon defense  
in mammals. 2015. RNA 21(5):911-922.  
  
Contact:  
David Rosenkranz  
Institute of Anthropology, small RNA group  
Johannes Gutenberg University Mainz  
email: rosenkranz@uni-mainz.de  
  
You can find the latest proTRAC version at:  
http://sourceforge.net/projects/protrac/files  
http://www.smallRNAgroup-mainz.de/software  
==============================================================================  
  
PARAMETERS:  
Map file: .............../storage/core/barbara/genhome/smallRNA/fertility/Sample\_motile/pirna/Sample\_motile\_26-33\_collapsed.fa.no-dust.map.weighted-10000-1000-b-0  
Genome file: ............/storage/core/barbara/genhome/smallRNA/fertility/Sample\_all/pirna/bt\_311\_chrY.fa  
RepeatMasker annotation: /storage/genomes/bt\_umd31/GCF\_000003055.6\_Bos\_taurus\_UMD\_3.1.1\_repeatMasker\_chr.out  
GeneSet:................./storage/core/barbara/genhome/smallRNA/fertility/Sample\_all/pirna/full.gtf  
  
Significant (p<=0.01) hit density will be calculated based  
on observed hit distribution.  
  
Sliding window size: ........................................ 5000 bp  
Sliding window increament: .................................. 1000 bp  
Normalize each hit by number of genomic hits: ............... 1 [0=no/1=yes]  
Normalize each hit by number of sequence reads: ............. 1 [0=no/1=yes]  
Normalize values (-> per million mapped reads): ............. 1 [0=no/1=yes]  
Min. fraction of hits with 1T(U) or 10A: .................... 0.75  
Alternatively: Min. fraction of hits with 1T(U) and 10A: .... 0.5  
Min. fraction of hits with typical piRNA length: ............ 0.75  
Typical piRNA length: ....................................... 26-33 nt  
Min. size of a piRNA cluster: ............................... 5000 bp.  
Min. number of hits (absolute): ............................. 0  
Min. number of hits (normalized): ........................... 0  
Min. fraction of hits on the mainstrand: .................... 0.75  
Top fraction of mapped sequences (in terms of read counts): . 1%  
Top fraction accounts for max. n% of sequence reads: ........ 90%  
Min. fraction of hits on each arm of a bidirectional cluster: 0.1  
Output image file for each cluster: ......................... 0 [0=no/1=yes]  
Output html file for each cluster: .......................... 1 [0=no/1=yes]  
Output a summary table: ..................................... 1 [0=no/1=yes]  
Output a FASTA file for each cluster (piRNA sequences): ..... 1 [0=no/1=yes]  
Output a FASTA file comprising cluster sequences: ........... 1 [0=no/1=yes]  
Search DNA motifs in clusters: .............................. 1 [0=no/1=yes]  
Output flanking sequences: +/- .............................. 0 bp  
Output ~.pTi file: .......................................... 1 [0=no/1=yes]  
==============================================================================  
  
  
Genome size (without gaps): ............ 2678902517 bp  
Gaps (N/X/-): .......................... 53837044 bp  
Mapped reads: .......................... 658825247023  
Non-identical sequences: ............... 514171  
Genomic hits: .......................... 764233  
Significant densitiy of mapped reads: .. 12867599.5173724 reads/kb

Show proTRAC cluster info
Hide proTRAC cluster info

|  |  |
| --- | --- |
| Location | chr13 |
| Coordinates | 66749019-66755808 |
| Size [bp] | 6790 |
| Sequence hit loci | 86 |
| Mapped reads (normalized) | 111486532 |
| Mapped reads (normalized) per kb | 16419224.2 |
| Normalized reads with 1T (1U) | 80.6% |
| Normalized reads with 10A | 40.8% |
| Normalized reads with length 26-33 nt | 100% |
| Normalized reads on the main strand(s) | 89.4% |
| Predicted directionality | mono:plus |

100%

0%

1T (1U)  
reads

10A reads

26-33 nt  
reads

reads on mainstrand

**Either the amount of reads with 1T (1U) OR 10A has to exceed 75% (set with option: -1Tor10A)  
Alternatively the amount of reads with 1T (1U) AND 10A has to exceed 50% (set with option: -1Tand10A)  
Minimum amount of reads with preferred size is 75% (set with option: -pisize)  
Minimum amount of reads on the main strand(s) is 75% (set with option: -clstrand)**

Show read coverage
Hide read coverage

WHAT DO I SEE HERE?  
This chart shows the location of mapped sequence reads within a predicted piRNA cluster. The color refers to the number of genomic hits produced by the sequence read in question. A dark red bar indicates that this sequence read produces many other hits elsewhere in the genome. Many adjacent red or yellow bars can indicate the presence of a multi-copy element such as transposons or rRNA genes. A dark green bar indicates that this sequence read maps uniquely to this locus.

1 hit

2-5 hits

6-10 hits

11-20 hits

21-50 hits

51-100 hits

> 100 hits

chr13

66749019

66755808

Gene Set

RepeatMasker

Mapped  
Reads

12.29

plus strand

minus strand

12.29

Region: chr13 66736944-66749025. Max. coverage (+): 4.19. Max coverage (-): 0

Region: chr13 66749026-66749039. Max. coverage (+): 4.19. Max coverage (-): 0

Region: chr13 66749040-66749052. Max. coverage (+): 0. Max coverage (-): 0

Region: chr13 66749053-66749066. Max. coverage (+): 0. Max coverage (-): 0

Region: chr13 66749067-66749080. Max. coverage (+): 0. Max coverage (-): 0

Region: chr13 66749081-66749093. Max. coverage (+): 0. Max coverage (-): 0

Region: chr13 66749094-66749107. Max. coverage (+): 0. Max coverage (-): 0

Region: chr13 66749108-66749120. Max. coverage (+): 0. Max coverage (-): 0

Region: chr13 66749121-66749134. Max. coverage (+): 0. Max coverage (-): 0

Region: chr13 66749135-66749148. Max. coverage (+): 0. Max coverage (-): 0

Region: chr13 66749149-66749161. Max. coverage (+): 0. Max coverage (-): 0

Region: chr13 66749162-66749175. Max. coverage (+): 0. Max coverage (-): 0

Region: chr13 66749176-66749188. Max. coverage (+): 0. Max coverage (-): 0

Region: chr13 66749189-66749202. Max. coverage (+): 0. Max coverage (-): 0

Region: chr13 66749203-66749215. Max. coverage (+): 0. Max coverage (-): 0

Region: chr13 66749216-66749229. Max. coverage (+): 0. Max coverage (-): 0

Region: chr13 66749230-66749243. Max. coverage (+): 0. Max coverage (-): 0

Region: chr13 66749244-66749256. Max. coverage (+): 0. Max coverage (-): 0

Region: chr13 66749257-66749270. Max. coverage (+): 0. Max coverage (-): 0

Region: chr13 66749271-66749283. Max. coverage (+): 0. Max coverage (-): 0

Region: chr13 66749284-66749297. Max. coverage (+): 0. Max coverage (-): 0

Region: chr13 66749298-66749310. Max. coverage (+): 0. Max coverage (-): 0

Region: chr13 66749311-66749324. Max. coverage (+): 0. Max coverage (-): 0

Region: chr13 66749325-66749338. Max. coverage (+): 0. Max coverage (-): 0

Region: chr13 66749339-66749351. Max. coverage (+): 0. Max coverage (-): 0

Region: chr13 66749352-66749365. Max. coverage (+): 2.32. Max coverage (-): 0

Region: chr13 66749366-66749378. Max. coverage (+): 0. Max coverage (-): 0

Region: chr13 66749379-66749392. Max. coverage (+): 0. Max coverage (-): 0

Region: chr13 66749393-66749406. Max. coverage (+): 0. Max coverage (-): 0

Region: chr13 66749407-66749419. Max. coverage (+): 0. Max coverage (-): 0

Region: chr13 66749420-66749433. Max. coverage (+): 0. Max coverage (-): 0

Region: chr13 66749434-66749446. Max. coverage (+): 0. Max coverage (-): 0

Region: chr13 66749447-66749460. Max. coverage (+): 0. Max coverage (-): 0

Region: chr13 66749461-66749473. Max. coverage (+): 0. Max coverage (-): 0

Region: chr13 66749474-66749487. Max. coverage (+): 0. Max coverage (-): 0

Region: chr13 66749488-66749501. Max. coverage (+): 0. Max coverage (-): 0

Region: chr13 66749502-66749514. Max. coverage (+): 0. Max coverage (-): 0

Region: chr13 66749515-66749528. Max. coverage (+): 0. Max coverage (-): 0

Region: chr13 66749529-66749541. Max. coverage (+): 0. Max coverage (-): 0

Region: chr13 66749542-66749555. Max. coverage (+): 0. Max coverage (-): 0

Region: chr13 66749556-66749568. Max. coverage (+): 0. Max coverage (-): 0

Region: chr13 66749569-66749582. Max. coverage (+): 0. Max coverage (-): 0

Region: chr13 66749583-66749596. Max. coverage (+): 0. Max coverage (-): 0

Region: chr13 66749597-66749609. Max. coverage (+): 0. Max coverage (-): 0

Region: chr13 66749610-66749623. Max. coverage (+): 0. Max coverage (-): 0

Region: chr13 66749624-66749636. Max. coverage (+): 0. Max coverage (-): 0

Region: chr13 66749637-66749650. Max. coverage (+): 0. Max coverage (-): 0

Region: chr13 66749651-66749664. Max. coverage (+): 1.55. Max coverage (-): 0

Region: chr13 66749665-66749677. Max. coverage (+): 1.55. Max coverage (-): 0

Region: chr13 66749678-66749691. Max. coverage (+): 0. Max coverage (-): 0

Region: chr13 66749692-66749704. Max. coverage (+): 0. Max coverage (-): 0

Region: chr13 66749705-66749718. Max. coverage (+): 0. Max coverage (-): 0

Region: chr13 66749719-66749731. Max. coverage (+): 0. Max coverage (-): 0

Region: chr13 66749732-66749745. Max. coverage (+): 0. Max coverage (-): 0

Region: chr13 66749746-66749759. Max. coverage (+): 0. Max coverage (-): 0

Region: chr13 66749760-66749772. Max. coverage (+): 0. Max coverage (-): 0

Region: chr13 66749773-66749786. Max. coverage (+): 0. Max coverage (-): 0

Region: chr13 66749787-66749799. Max. coverage (+): 0. Max coverage (-): 0

Region: chr13 66749800-66749813. Max. coverage (+): 0. Max coverage (-): 0

Region: chr13 66749814-66749827. Max. coverage (+): 0. Max coverage (-): 0

Region: chr13 66749828-66749840. Max. coverage (+): 0. Max coverage (-): 0

Region: chr13 66749841-66749854. Max. coverage (+): 0. Max coverage (-): 0

Region: chr13 66749855-66749867. Max. coverage (+): 0. Max coverage (-): 0

Region: chr13 66749868-66749881. Max. coverage (+): 0. Max coverage (-): 0

Region: chr13 66749882-66749894. Max. coverage (+): 0. Max coverage (-): 0

Region: chr13 66749895-66749908. Max. coverage (+): 0. Max coverage (-): 0

Region: chr13 66749909-66749922. Max. coverage (+): 0. Max coverage (-): 0

Region: chr13 66749923-66749935. Max. coverage (+): 0. Max coverage (-): 0

Region: chr13 66749936-66749949. Max. coverage (+): 0. Max coverage (-): 0

Region: chr13 66749950-66749962. Max. coverage (+): 0. Max coverage (-): 0

Region: chr13 66749963-66749976. Max. coverage (+): 0. Max coverage (-): 0

Region: chr13 66749977-66749989. Max. coverage (+): 0. Max coverage (-): 0

Region: chr13 66749990-66750003. Max. coverage (+): 0. Max coverage (-): 0

Region: chr13 66750004-66750017. Max. coverage (+): 0. Max coverage (-): 0

Region: chr13 66750018-66750030. Max. coverage (+): 0. Max coverage (-): 0

Region: chr13 66750031-66750044. Max. coverage (+): 0. Max coverage (-): 0

Region: chr13 66750045-66750057. Max. coverage (+): 2.61. Max coverage (-): 0

Region: chr13 66750058-66750071. Max. coverage (+): 2.61. Max coverage (-): 0

Region: chr13 66750072-66750085. Max. coverage (+): 2.61. Max coverage (-): 0

Region: chr13 66750086-66750098. Max. coverage (+): 2.61. Max coverage (-): 0

Region: chr13 66750099-66750112. Max. coverage (+): 0. Max coverage (-): 0

Region: chr13 66750113-66750125. Max. coverage (+): 0. Max coverage (-): 0

Region: chr13 66750126-66750139. Max. coverage (+): 0. Max coverage (-): 0

Region: chr13 66750140-66750152. Max. coverage (+): 0. Max coverage (-): 0

Region: chr13 66750153-66750166. Max. coverage (+): 0. Max coverage (-): 0

Region: chr13 66750167-66750180. Max. coverage (+): 0. Max coverage (-): 0

Region: chr13 66750181-66750193. Max. coverage (+): 0. Max coverage (-): 0

Region: chr13 66750194-66750207. Max. coverage (+): 0. Max coverage (-): 0

Region: chr13 66750208-66750220. Max. coverage (+): 0. Max coverage (-): 0

Region: chr13 66750221-66750234. Max. coverage (+): 0. Max coverage (-): 0

Region: chr13 66750235-66750247. Max. coverage (+): 0. Max coverage (-): 0

Region: chr13 66750248-66750261. Max. coverage (+): 0. Max coverage (-): 0

Region: chr13 66750262-66750275. Max. coverage (+): 0. Max coverage (-): 0

Region: chr13 66750276-66750288. Max. coverage (+): 0. Max coverage (-): 0

Region: chr13 66750289-66750302. Max. coverage (+): 0. Max coverage (-): 0

Region: chr13 66750303-66750315. Max. coverage (+): 0. Max coverage (-): 0

Region: chr13 66750316-66750329. Max. coverage (+): 0. Max coverage (-): 0

Region: chr13 66750330-66750343. Max. coverage (+): 0. Max coverage (-): 0

Region: chr13 66750344-66750356. Max. coverage (+): 0. Max coverage (-): 0

Region: chr13 66750357-66750370. Max. coverage (+): 0. Max coverage (-): 0

Region: chr13 66750371-66750383. Max. coverage (+): 0. Max coverage (-): 0

Region: chr13 66750384-66750397. Max. coverage (+): 0. Max coverage (-): 0

Region: chr13 66750398-66750410. Max. coverage (+): 0. Max coverage (-): 0

Region: chr13 66750411-66750424. Max. coverage (+): 0. Max coverage (-): 0

Region: chr13 66750425-66750438. Max. coverage (+): 0. Max coverage (-): 0

Region: chr13 66750439-66750451. Max. coverage (+): 0. Max coverage (-): 0

Region: chr13 66750452-66750465. Max. coverage (+): 0. Max coverage (-): 0

Region: chr13 66750466-66750478. Max. coverage (+): 0. Max coverage (-): 0

Region: chr13 66750479-66750492. Max. coverage (+): 0. Max coverage (-): 0

Region: chr13 66750493-66750506. Max. coverage (+): 0. Max coverage (-): 0

Region: chr13 66750507-66750519. Max. coverage (+): 0. Max coverage (-): 0

Region: chr13 66750520-66750533. Max. coverage (+): 0. Max coverage (-): 0

Region: chr13 66750534-66750546. Max. coverage (+): 0. Max coverage (-): 0

Region: chr13 66750547-66750560. Max. coverage (+): 0. Max coverage (-): 0

Region: chr13 66750561-66750573. Max. coverage (+): 0. Max coverage (-): 0

Region: chr13 66750574-66750587. Max. coverage (+): 0. Max coverage (-): 0

Region: chr13 66750588-66750601. Max. coverage (+): 0. Max coverage (-): 0

Region: chr13 66750602-66750614. Max. coverage (+): 0. Max coverage (-): 0

Region: chr13 66750615-66750628. Max. coverage (+): 0. Max coverage (-): 0

Region: chr13 66750629-66750641. Max. coverage (+): 0. Max coverage (-): 0

Region: chr13 66750642-66750655. Max. coverage (+): 0. Max coverage (-): 0

Region: chr13 66750656-66750668. Max. coverage (+): 0. Max coverage (-): 0

Region: chr13 66750669-66750682. Max. coverage (+): 0. Max coverage (-): 0

Region: chr13 66750683-66750696. Max. coverage (+): 0. Max coverage (-): 1.51

Region: chr13 66750697-66750709. Max. coverage (+): 0. Max coverage (-): 0

Region: chr13 66750710-66750723. Max. coverage (+): 0. Max coverage (-): 0

Region: chr13 66750724-66750736. Max. coverage (+): 1.28. Max coverage (-): 0

Region: chr13 66750737-66750750. Max. coverage (+): 1.28. Max coverage (-): 0

Region: chr13 66750751-66750764. Max. coverage (+): 0. Max coverage (-): 0

Region: chr13 66750765-66750777. Max. coverage (+): 0. Max coverage (-): 0

Region: chr13 66750778-66750791. Max. coverage (+): 0. Max coverage (-): 0

Region: chr13 66750792-66750804. Max. coverage (+): 0. Max coverage (-): 0

Region: chr13 66750805-66750818. Max. coverage (+): 0. Max coverage (-): 0

Region: chr13 66750819-66750831. Max. coverage (+): 0. Max coverage (-): 0

Region: chr13 66750832-66750845. Max. coverage (+): 0. Max coverage (-): 0

Region: chr13 66750846-66750859. Max. coverage (+): 2.42. Max coverage (-): 0

Region: chr13 66750860-66750872. Max. coverage (+): 2.42. Max coverage (-): 0

Region: chr13 66750873-66750886. Max. coverage (+): 1.44. Max coverage (-): 0

Region: chr13 66750887-66750899. Max. coverage (+): 0. Max coverage (-): 0

Region: chr13 66750900-66750913. Max. coverage (+): 0. Max coverage (-): 0

Region: chr13 66750914-66750926. Max. coverage (+): 0. Max coverage (-): 0

Region: chr13 66750927-66750940. Max. coverage (+): 0. Max coverage (-): 0

Region: chr13 66750941-66750954. Max. coverage (+): 0. Max coverage (-): 0

Region: chr13 66750955-66750967. Max. coverage (+): 0. Max coverage (-): 0

Region: chr13 66750968-66750981. Max. coverage (+): 0. Max coverage (-): 0

Region: chr13 66750982-66750994. Max. coverage (+): 0. Max coverage (-): 0

Region: chr13 66750995-66751008. Max. coverage (+): 0. Max coverage (-): 0

Region: chr13 66751009-66751022. Max. coverage (+): 0. Max coverage (-): 0

Region: chr13 66751023-66751035. Max. coverage (+): 0. Max coverage (-): 0

Region: chr13 66751036-66751049. Max. coverage (+): 0. Max coverage (-): 0

Region: chr13 66751050-66751062. Max. coverage (+): 0. Max coverage (-): 0

Region: chr13 66751063-66751076. Max. coverage (+): 0. Max coverage (-): 0

Region: chr13 66751077-66751089. Max. coverage (+): 5.56. Max coverage (-): 0

Region: chr13 66751090-66751103. Max. coverage (+): 5.56. Max coverage (-): 0

Region: chr13 66751104-66751117. Max. coverage (+): 2.16. Max coverage (-): 0

Region: chr13 66751118-66751130. Max. coverage (+): 0. Max coverage (-): 0

Region: chr13 66751131-66751144. Max. coverage (+): 0. Max coverage (-): 0

Region: chr13 66751145-66751157. Max. coverage (+): 0. Max coverage (-): 0

Region: chr13 66751158-66751171. Max. coverage (+): 0. Max coverage (-): 0

Region: chr13 66751172-66751185. Max. coverage (+): 0. Max coverage (-): 0

Region: chr13 66751186-66751198. Max. coverage (+): 0. Max coverage (-): 0

Region: chr13 66751199-66751212. Max. coverage (+): 0. Max coverage (-): 0

Region: chr13 66751213-66751225. Max. coverage (+): 0. Max coverage (-): 0

Region: chr13 66751226-66751239. Max. coverage (+): 0. Max coverage (-): 0

Region: chr13 66751240-66751252. Max. coverage (+): 0. Max coverage (-): 0

Region: chr13 66751253-66751266. Max. coverage (+): 0. Max coverage (-): 0

Region: chr13 66751267-66751280. Max. coverage (+): 0. Max coverage (-): 0

Region: chr13 66751281-66751293. Max. coverage (+): 0. Max coverage (-): 0

Region: chr13 66751294-66751307. Max. coverage (+): 0. Max coverage (-): 0

Region: chr13 66751308-66751320. Max. coverage (+): 0. Max coverage (-): 0

Region: chr13 66751321-66751334. Max. coverage (+): 0. Max coverage (-): 0

Region: chr13 66751335-66751347. Max. coverage (+): 0. Max coverage (-): 0

Region: chr13 66751348-66751361. Max. coverage (+): 4.16. Max coverage (-): 0

Region: chr13 66751362-66751375. Max. coverage (+): 0. Max coverage (-): 0

Region: chr13 66751376-66751388. Max. coverage (+): 0. Max coverage (-): 0

Region: chr13 66751389-66751402. Max. coverage (+): 0. Max coverage (-): 0

Region: chr13 66751403-66751415. Max. coverage (+): 0. Max coverage (-): 0

Region: chr13 66751416-66751429. Max. coverage (+): 0. Max coverage (-): 0

Region: chr13 66751430-66751443. Max. coverage (+): 3.06. Max coverage (-): 0

Region: chr13 66751444-66751456. Max. coverage (+): 0. Max coverage (-): 0

Region: chr13 66751457-66751470. Max. coverage (+): 0. Max coverage (-): 0

Region: chr13 66751471-66751483. Max. coverage (+): 0. Max coverage (-): 0

Region: chr13 66751484-66751497. Max. coverage (+): 0. Max coverage (-): 0

Region: chr13 66751498-66751510. Max. coverage (+): 0. Max coverage (-): 0

Region: chr13 66751511-66751524. Max. coverage (+): 0. Max coverage (-): 0

Region: chr13 66751525-66751538. Max. coverage (+): 3.03. Max coverage (-): 0

Region: chr13 66751539-66751551. Max. coverage (+): 0. Max coverage (-): 0

Region: chr13 66751552-66751565. Max. coverage (+): 0. Max coverage (-): 0

Region: chr13 66751566-66751578. Max. coverage (+): 0. Max coverage (-): 0

Region: chr13 66751579-66751592. Max. coverage (+): 0. Max coverage (-): 0

Region: chr13 66751593-66751605. Max. coverage (+): 0. Max coverage (-): 0

Region: chr13 66751606-66751619. Max. coverage (+): 0. Max coverage (-): 0

Region: chr13 66751620-66751633. Max. coverage (+): 0. Max coverage (-): 0

Region: chr13 66751634-66751646. Max. coverage (+): 0. Max coverage (-): 0

Region: chr13 66751647-66751660. Max. coverage (+): 0. Max coverage (-): 0

Region: chr13 66751661-66751673. Max. coverage (+): 0. Max coverage (-): 0

Region: chr13 66751674-66751687. Max. coverage (+): 0. Max coverage (-): 0

Region: chr13 66751688-66751701. Max. coverage (+): 0. Max coverage (-): 0

Region: chr13 66751702-66751714. Max. coverage (+): 0. Max coverage (-): 0

Region: chr13 66751715-66751728. Max. coverage (+): 0. Max coverage (-): 0

Region: chr13 66751729-66751741. Max. coverage (+): 0. Max coverage (-): 0

Region: chr13 66751742-66751755. Max. coverage (+): 3.64. Max coverage (-): 0

Region: chr13 66751756-66751768. Max. coverage (+): 3.64. Max coverage (-): 2.08

Region: chr13 66751769-66751782. Max. coverage (+): 0. Max coverage (-): 2.08

Region: chr13 66751783-66751796. Max. coverage (+): 0. Max coverage (-): 0

Region: chr13 66751797-66751809. Max. coverage (+): 0. Max coverage (-): 0

Region: chr13 66751810-66751823. Max. coverage (+): 0. Max coverage (-): 0

Region: chr13 66751824-66751836. Max. coverage (+): 0. Max coverage (-): 0

Region: chr13 66751837-66751850. Max. coverage (+): 0. Max coverage (-): 0

Region: chr13 66751851-66751864. Max. coverage (+): 0. Max coverage (-): 0

Region: chr13 66751865-66751877. Max. coverage (+): 0. Max coverage (-): 0

Region: chr13 66751878-66751891. Max. coverage (+): 0. Max coverage (-): 0

Region: chr13 66751892-66751904. Max. coverage (+): 0. Max coverage (-): 0

Region: chr13 66751905-66751918. Max. coverage (+): 0. Max coverage (-): 0

Region: chr13 66751919-66751931. Max. coverage (+): 0. Max coverage (-): 0

Region: chr13 66751932-66751945. Max. coverage (+): 0. Max coverage (-): 0

Region: chr13 66751946-66751959. Max. coverage (+): 0. Max coverage (-): 0

Region: chr13 66751960-66751972. Max. coverage (+): 0. Max coverage (-): 0

Region: chr13 66751973-66751986. Max. coverage (+): 0. Max coverage (-): 0

Region: chr13 66751987-66751999. Max. coverage (+): 0. Max coverage (-): 0

Region: chr13 66752000-66752013. Max. coverage (+): 1.01. Max coverage (-): 0

Region: chr13 66752014-66752026. Max. coverage (+): 1.01. Max coverage (-): 0

Region: chr13 66752027-66752040. Max. coverage (+): 0. Max coverage (-): 0

Region: chr13 66752041-66752054. Max. coverage (+): 0. Max coverage (-): 0

Region: chr13 66752055-66752067. Max. coverage (+): 0. Max coverage (-): 0

Region: chr13 66752068-66752081. Max. coverage (+): 0. Max coverage (-): 0

Region: chr13 66752082-66752094. Max. coverage (+): 0. Max coverage (-): 0

Region: chr13 66752095-66752108. Max. coverage (+): 0. Max coverage (-): 0

Region: chr13 66752109-66752122. Max. coverage (+): 0. Max coverage (-): 0

Region: chr13 66752123-66752135. Max. coverage (+): 0. Max coverage (-): 0

Region: chr13 66752136-66752149. Max. coverage (+): 0. Max coverage (-): 0

Region: chr13 66752150-66752162. Max. coverage (+): 0. Max coverage (-): 0

Region: chr13 66752163-66752176. Max. coverage (+): 0. Max coverage (-): 0

Region: chr13 66752177-66752189. Max. coverage (+): 0. Max coverage (-): 0

Region: chr13 66752190-66752203. Max. coverage (+): 0. Max coverage (-): 0

Region: chr13 66752204-66752217. Max. coverage (+): 0. Max coverage (-): 0

Region: chr13 66752218-66752230. Max. coverage (+): 0. Max coverage (-): 0

Region: chr13 66752231-66752244. Max. coverage (+): 0. Max coverage (-): 0

Region: chr13 66752245-66752257. Max. coverage (+): 0. Max coverage (-): 0

Region: chr13 66752258-66752271. Max. coverage (+): 0. Max coverage (-): 0

Region: chr13 66752272-66752284. Max. coverage (+): 0. Max coverage (-): 0

Region: chr13 66752285-66752298. Max. coverage (+): 0. Max coverage (-): 0

Region: chr13 66752299-66752312. Max. coverage (+): 0. Max coverage (-): 0

Region: chr13 66752313-66752325. Max. coverage (+): 0. Max coverage (-): 0

Region: chr13 66752326-66752339. Max. coverage (+): 0. Max coverage (-): 0

Region: chr13 66752340-66752352. Max. coverage (+): 0. Max coverage (-): 0

Region: chr13 66752353-66752366. Max. coverage (+): 0. Max coverage (-): 0

Region: chr13 66752367-66752380. Max. coverage (+): 0. Max coverage (-): 0

Region: chr13 66752381-66752393. Max. coverage (+): 0. Max coverage (-): 0

Region: chr13 66752394-66752407. Max. coverage (+): 3.13. Max coverage (-): 0

Region: chr13 66752408-66752420. Max. coverage (+): 10.67. Max coverage (-): 0

Region: chr13 66752421-66752434. Max. coverage (+): 9.53. Max coverage (-): 0

Region: chr13 66752435-66752447. Max. coverage (+): 0. Max coverage (-): 0

Region: chr13 66752448-66752461. Max. coverage (+): 0. Max coverage (-): 0

Region: chr13 66752462-66752475. Max. coverage (+): 0. Max coverage (-): 0

Region: chr13 66752476-66752488. Max. coverage (+): 0. Max coverage (-): 0

Region: chr13 66752489-66752502. Max. coverage (+): 0. Max coverage (-): 0

Region: chr13 66752503-66752515. Max. coverage (+): 0. Max coverage (-): 0

Region: chr13 66752516-66752529. Max. coverage (+): 0. Max coverage (-): 0

Region: chr13 66752530-66752543. Max. coverage (+): 0. Max coverage (-): 0

Region: chr13 66752544-66752556. Max. coverage (+): 0. Max coverage (-): 0

Region: chr13 66752557-66752570. Max. coverage (+): 0. Max coverage (-): 0

Region: chr13 66752571-66752583. Max. coverage (+): 0. Max coverage (-): 0

Region: chr13 66752584-66752597. Max. coverage (+): 0. Max coverage (-): 0

Region: chr13 66752598-66752610. Max. coverage (+): 0. Max coverage (-): 0

Region: chr13 66752611-66752624. Max. coverage (+): 0. Max coverage (-): 0

Region: chr13 66752625-66752638. Max. coverage (+): 0. Max coverage (-): 0

Region: chr13 66752639-66752651. Max. coverage (+): 2.52. Max coverage (-): 0

Region: chr13 66752652-66752665. Max. coverage (+): 10.31. Max coverage (-): 0

Region: chr13 66752666-66752678. Max. coverage (+): 10.31. Max coverage (-): 0

Region: chr13 66752679-66752692. Max. coverage (+): 0. Max coverage (-): 0

Region: chr13 66752693-66752705. Max. coverage (+): 1.52. Max coverage (-): 0

Region: chr13 66752706-66752719. Max. coverage (+): 2.97. Max coverage (-): 0

Region: chr13 66752720-66752733. Max. coverage (+): 0. Max coverage (-): 0

Region: chr13 66752734-66752746. Max. coverage (+): 0. Max coverage (-): 0

Region: chr13 66752747-66752760. Max. coverage (+): 0. Max coverage (-): 0

Region: chr13 66752761-66752773. Max. coverage (+): 0. Max coverage (-): 0

Region: chr13 66752774-66752787. Max. coverage (+): 0. Max coverage (-): 0

Region: chr13 66752788-66752801. Max. coverage (+): 0. Max coverage (-): 0

Region: chr13 66752802-66752814. Max. coverage (+): 0. Max coverage (-): 0

Region: chr13 66752815-66752828. Max. coverage (+): 0. Max coverage (-): 0

Region: chr13 66752829-66752841. Max. coverage (+): 0. Max coverage (-): 0

Region: chr13 66752842-66752855. Max. coverage (+): 0. Max coverage (-): 0

Region: chr13 66752856-66752868. Max. coverage (+): 0. Max coverage (-): 0

Region: chr13 66752869-66752882. Max. coverage (+): 0. Max coverage (-): 0

Region: chr13 66752883-66752896. Max. coverage (+): 0. Max coverage (-): 0

Region: chr13 66752897-66752909. Max. coverage (+): 0. Max coverage (-): 0

Region: chr13 66752910-66752923. Max. coverage (+): 0. Max coverage (-): 0

Region: chr13 66752924-66752936. Max. coverage (+): 0. Max coverage (-): 0

Region: chr13 66752937-66752950. Max. coverage (+): 0. Max coverage (-): 0

Region: chr13 66752951-66752963. Max. coverage (+): 0. Max coverage (-): 0

Region: chr13 66752964-66752977. Max. coverage (+): 0. Max coverage (-): 0

Region: chr13 66752978-66752991. Max. coverage (+): 0. Max coverage (-): 0

Region: chr13 66752992-66753004. Max. coverage (+): 0. Max coverage (-): 0

Region: chr13 66753005-66753018. Max. coverage (+): 0. Max coverage (-): 0

Region: chr13 66753019-66753031. Max. coverage (+): 0. Max coverage (-): 0

Region: chr13 66753032-66753045. Max. coverage (+): 0.63. Max coverage (-): 0

Region: chr13 66753046-66753059. Max. coverage (+): 0.63. Max coverage (-): 0

Region: chr13 66753060-66753072. Max. coverage (+): 1.64. Max coverage (-): 5.92

Region: chr13 66753073-66753086. Max. coverage (+): 0. Max coverage (-): 5.92

Region: chr13 66753087-66753099. Max. coverage (+): 0. Max coverage (-): 0

Region: chr13 66753100-66753113. Max. coverage (+): 0. Max coverage (-): 0

Region: chr13 66753114-66753126. Max. coverage (+): 0. Max coverage (-): 0

Region: chr13 66753127-66753140. Max. coverage (+): 0. Max coverage (-): 0

Region: chr13 66753141-66753154. Max. coverage (+): 0. Max coverage (-): 0

Region: chr13 66753155-66753167. Max. coverage (+): 0. Max coverage (-): 0

Region: chr13 66753168-66753181. Max. coverage (+): 0. Max coverage (-): 0

Region: chr13 66753182-66753194. Max. coverage (+): 0. Max coverage (-): 0

Region: chr13 66753195-66753208. Max. coverage (+): 0. Max coverage (-): 0

Region: chr13 66753209-66753222. Max. coverage (+): 0. Max coverage (-): 0

Region: chr13 66753223-66753235. Max. coverage (+): 0. Max coverage (-): 0

Region: chr13 66753236-66753249. Max. coverage (+): 0. Max coverage (-): 0

Region: chr13 66753250-66753262. Max. coverage (+): 0. Max coverage (-): 0

Region: chr13 66753263-66753276. Max. coverage (+): 0. Max coverage (-): 0

Region: chr13 66753277-66753289. Max. coverage (+): 0. Max coverage (-): 0

Region: chr13 66753290-66753303. Max. coverage (+): 0. Max coverage (-): 0

Region: chr13 66753304-66753317. Max. coverage (+): 0. Max coverage (-): 0

Region: chr13 66753318-66753330. Max. coverage (+): 0. Max coverage (-): 0

Region: chr13 66753331-66753344. Max. coverage (+): 0. Max coverage (-): 0

Region: chr13 66753345-66753357. Max. coverage (+): 0. Max coverage (-): 0

Region: chr13 66753358-66753371. Max. coverage (+): 0. Max coverage (-): 0

Region: chr13 66753372-66753384. Max. coverage (+): 0. Max coverage (-): 0

Region: chr13 66753385-66753398. Max. coverage (+): 0. Max coverage (-): 0

Region: chr13 66753399-66753412. Max. coverage (+): 0. Max coverage (-): 0

Region: chr13 66753413-66753425. Max. coverage (+): 0. Max coverage (-): 0

Region: chr13 66753426-66753439. Max. coverage (+): 0. Max coverage (-): 0

Region: chr13 66753440-66753452. Max. coverage (+): 0. Max coverage (-): 0

Region: chr13 66753453-66753466. Max. coverage (+): 0. Max coverage (-): 0

Region: chr13 66753467-66753480. Max. coverage (+): 0. Max coverage (-): 0

Region: chr13 66753481-66753493. Max. coverage (+): 0. Max coverage (-): 0

Region: chr13 66753494-66753507. Max. coverage (+): 0. Max coverage (-): 1.96

Region: chr13 66753508-66753520. Max. coverage (+): 0.91. Max coverage (-): 1.96

Region: chr13 66753521-66753534. Max. coverage (+): 0.91. Max coverage (-): 6.5

Region: chr13 66753535-66753547. Max. coverage (+): 0. Max coverage (-): 6.5

Region: chr13 66753548-66753561. Max. coverage (+): 1.93. Max coverage (-): 0

Region: chr13 66753562-66753575. Max. coverage (+): 0. Max coverage (-): 0

Region: chr13 66753576-66753588. Max. coverage (+): 0. Max coverage (-): 0

Region: chr13 66753589-66753602. Max. coverage (+): 7.08. Max coverage (-): 0

Region: chr13 66753603-66753615. Max. coverage (+): 7.08. Max coverage (-): 0

Region: chr13 66753616-66753629. Max. coverage (+): 1.85. Max coverage (-): 0

Region: chr13 66753630-66753642. Max. coverage (+): 0. Max coverage (-): 0

Region: chr13 66753643-66753656. Max. coverage (+): 0. Max coverage (-): 0

Region: chr13 66753657-66753670. Max. coverage (+): 0. Max coverage (-): 0

Region: chr13 66753671-66753683. Max. coverage (+): 5.55. Max coverage (-): 0

Region: chr13 66753684-66753697. Max. coverage (+): 5.06. Max coverage (-): 0

Region: chr13 66753698-66753710. Max. coverage (+): 5.06. Max coverage (-): 0

Region: chr13 66753711-66753724. Max. coverage (+): 3.26. Max coverage (-): 0

Region: chr13 66753725-66753738. Max. coverage (+): 1.46. Max coverage (-): 0

Region: chr13 66753739-66753751. Max. coverage (+): 1.46. Max coverage (-): 0

Region: chr13 66753752-66753765. Max. coverage (+): 0. Max coverage (-): 0

Region: chr13 66753766-66753778. Max. coverage (+): 4.5. Max coverage (-): 0

Region: chr13 66753779-66753792. Max. coverage (+): 4.84. Max coverage (-): 0

Region: chr13 66753793-66753805. Max. coverage (+): 0. Max coverage (-): 0

Region: chr13 66753806-66753819. Max. coverage (+): 0. Max coverage (-): 0

Region: chr13 66753820-66753833. Max. coverage (+): 3.53. Max coverage (-): 0

Region: chr13 66753834-66753846. Max. coverage (+): 0. Max coverage (-): 0

Region: chr13 66753847-66753860. Max. coverage (+): 0. Max coverage (-): 0

Region: chr13 66753861-66753873. Max. coverage (+): 0. Max coverage (-): 0

Region: chr13 66753874-66753887. Max. coverage (+): 0. Max coverage (-): 0

Region: chr13 66753888-66753901. Max. coverage (+): 1.37. Max coverage (-): 0

Region: chr13 66753902-66753914. Max. coverage (+): 3.44. Max coverage (-): 0

Region: chr13 66753915-66753928. Max. coverage (+): 2.07. Max coverage (-): 0

Region: chr13 66753929-66753941. Max. coverage (+): 0. Max coverage (-): 0

Region: chr13 66753942-66753955. Max. coverage (+): 0. Max coverage (-): 0

Region: chr13 66753956-66753968. Max. coverage (+): 0. Max coverage (-): 0

Region: chr13 66753969-66753982. Max. coverage (+): 0. Max coverage (-): 0

Region: chr13 66753983-66753996. Max. coverage (+): 0. Max coverage (-): 0

Region: chr13 66753997-66754009. Max. coverage (+): 0. Max coverage (-): 0

Region: chr13 66754010-66754023. Max. coverage (+): 0. Max coverage (-): 0

Region: chr13 66754024-66754036. Max. coverage (+): 0. Max coverage (-): 0

Region: chr13 66754037-66754050. Max. coverage (+): 9.07. Max coverage (-): 0

Region: chr13 66754051-66754063. Max. coverage (+): 9.07. Max coverage (-): 0

Region: chr13 66754064-66754077. Max. coverage (+): 0. Max coverage (-): 0

Region: chr13 66754078-66754091. Max. coverage (+): 1.07. Max coverage (-): 0

Region: chr13 66754092-66754104. Max. coverage (+): 1.07. Max coverage (-): 0

Region: chr13 66754105-66754118. Max. coverage (+): 0. Max coverage (-): 0

Region: chr13 66754119-66754131. Max. coverage (+): 0. Max coverage (-): 0

Region: chr13 66754132-66754145. Max. coverage (+): 0. Max coverage (-): 0

Region: chr13 66754146-66754159. Max. coverage (+): 0. Max coverage (-): 0

Region: chr13 66754160-66754172. Max. coverage (+): 0. Max coverage (-): 0

Region: chr13 66754173-66754186. Max. coverage (+): 0. Max coverage (-): 0

Region: chr13 66754187-66754199. Max. coverage (+): 0. Max coverage (-): 0

Region: chr13 66754200-66754213. Max. coverage (+): 0. Max coverage (-): 0

Region: chr13 66754214-66754226. Max. coverage (+): 0. Max coverage (-): 0

Region: chr13 66754227-66754240. Max. coverage (+): 0. Max coverage (-): 0

Region: chr13 66754241-66754254. Max. coverage (+): 0. Max coverage (-): 0

Region: chr13 66754255-66754267. Max. coverage (+): 0. Max coverage (-): 0

Region: chr13 66754268-66754281. Max. coverage (+): 0. Max coverage (-): 0

Region: chr13 66754282-66754294. Max. coverage (+): 0. Max coverage (-): 0

Region: chr13 66754295-66754308. Max. coverage (+): 0. Max coverage (-): 0

Region: chr13 66754309-66754321. Max. coverage (+): 0. Max coverage (-): 0

Region: chr13 66754322-66754335. Max. coverage (+): 0. Max coverage (-): 0

Region: chr13 66754336-66754349. Max. coverage (+): 0. Max coverage (-): 0

Region: chr13 66754350-66754362. Max. coverage (+): 0. Max coverage (-): 0

Region: chr13 66754363-66754376. Max. coverage (+): 0. Max coverage (-): 0

Region: chr13 66754377-66754389. Max. coverage (+): 0. Max coverage (-): 0

Region: chr13 66754390-66754403. Max. coverage (+): 0. Max coverage (-): 0

Region: chr13 66754404-66754417. Max. coverage (+): 0. Max coverage (-): 0

Region: chr13 66754418-66754430. Max. coverage (+): 0. Max coverage (-): 0

Region: chr13 66754431-66754444. Max. coverage (+): 0. Max coverage (-): 0

Region: chr13 66754445-66754457. Max. coverage (+): 0. Max coverage (-): 0

Region: chr13 66754458-66754471. Max. coverage (+): 0. Max coverage (-): 0

Region: chr13 66754472-66754484. Max. coverage (+): 0. Max coverage (-): 0

Region: chr13 66754485-66754498. Max. coverage (+): 0. Max coverage (-): 0

Region: chr13 66754499-66754512. Max. coverage (+): 0. Max coverage (-): 0

Region: chr13 66754513-66754525. Max. coverage (+): 0. Max coverage (-): 0

Region: chr13 66754526-66754539. Max. coverage (+): 0. Max coverage (-): 0

Region: chr13 66754540-66754552. Max. coverage (+): 0. Max coverage (-): 0

Region: chr13 66754553-66754566. Max. coverage (+): 0. Max coverage (-): 0

Region: chr13 66754567-66754580. Max. coverage (+): 0. Max coverage (-): 0

Region: chr13 66754581-66754593. Max. coverage (+): 0. Max coverage (-): 0

Region: chr13 66754594-66754607. Max. coverage (+): 0. Max coverage (-): 0

Region: chr13 66754608-66754620. Max. coverage (+): 0. Max coverage (-): 0

Region: chr13 66754621-66754634. Max. coverage (+): 0. Max coverage (-): 0

Region: chr13 66754635-66754647. Max. coverage (+): 0. Max coverage (-): 0

Region: chr13 66754648-66754661. Max. coverage (+): 0. Max coverage (-): 0

Region: chr13 66754662-66754675. Max. coverage (+): 0. Max coverage (-): 0

Region: chr13 66754676-66754688. Max. coverage (+): 0. Max coverage (-): 0

Region: chr13 66754689-66754702. Max. coverage (+): 0. Max coverage (-): 0

Region: chr13 66754703-66754715. Max. coverage (+): 0. Max coverage (-): 0

Region: chr13 66754716-66754729. Max. coverage (+): 0. Max coverage (-): 0

Region: chr13 66754730-66754742. Max. coverage (+): 0. Max coverage (-): 0

Region: chr13 66754743-66754756. Max. coverage (+): 0. Max coverage (-): 0

Region: chr13 66754757-66754770. Max. coverage (+): 0. Max coverage (-): 0

Region: chr13 66754771-66754783. Max. coverage (+): 1.63. Max coverage (-): 0

Region: chr13 66754784-66754797. Max. coverage (+): 1.63. Max coverage (-): 0

Region: chr13 66754798-66754810. Max. coverage (+): 0. Max coverage (-): 0

Region: chr13 66754811-66754824. Max. coverage (+): 0. Max coverage (-): 0

Region: chr13 66754825-66754838. Max. coverage (+): 0. Max coverage (-): 0

Region: chr13 66754839-66754851. Max. coverage (+): 0. Max coverage (-): 0

Region: chr13 66754852-66754865. Max. coverage (+): 0. Max coverage (-): 0

Region: chr13 66754866-66754878. Max. coverage (+): 0. Max coverage (-): 0

Region: chr13 66754879-66754892. Max. coverage (+): 0. Max coverage (-): 0

Region: chr13 66754893-66754905. Max. coverage (+): 0. Max coverage (-): 0

Region: chr13 66754906-66754919. Max. coverage (+): 0.55. Max coverage (-): 0

Region: chr13 66754920-66754933. Max. coverage (+): 0. Max coverage (-): 0

Region: chr13 66754934-66754946. Max. coverage (+): 0. Max coverage (-): 0

Region: chr13 66754947-66754960. Max. coverage (+): 0. Max coverage (-): 0

Region: chr13 66754961-66754973. Max. coverage (+): 0. Max coverage (-): 0

Region: chr13 66754974-66754987. Max. coverage (+): 0. Max coverage (-): 0

Region: chr13 66754988-66755000. Max. coverage (+): 0. Max coverage (-): 0

Region: chr13 66755001-66755014. Max. coverage (+): 0. Max coverage (-): 0

Region: chr13 66755015-66755028. Max. coverage (+): 0. Max coverage (-): 0

Region: chr13 66755029-66755041. Max. coverage (+): 0. Max coverage (-): 0

Region: chr13 66755042-66755055. Max. coverage (+): 0. Max coverage (-): 0

Region: chr13 66755056-66755068. Max. coverage (+): 0. Max coverage (-): 0

Region: chr13 66755069-66755082. Max. coverage (+): 0. Max coverage (-): 0

Region: chr13 66755083-66755096. Max. coverage (+): 0. Max coverage (-): 0

Region: chr13 66755097-66755109. Max. coverage (+): 0. Max coverage (-): 0

Region: chr13 66755110-66755123. Max. coverage (+): 0. Max coverage (-): 0

Region: chr13 66755124-66755136. Max. coverage (+): 0. Max coverage (-): 0

Region: chr13 66755137-66755150. Max. coverage (+): 0. Max coverage (-): 0

Region: chr13 66755151-66755163. Max. coverage (+): 0. Max coverage (-): 0

Region: chr13 66755164-66755177. Max. coverage (+): 0. Max coverage (-): 0

Region: chr13 66755178-66755191. Max. coverage (+): 0. Max coverage (-): 0

Region: chr13 66755192-66755204. Max. coverage (+): 0. Max coverage (-): 0

Region: chr13 66755205-66755218. Max. coverage (+): 0. Max coverage (-): 0

Region: chr13 66755219-66755231. Max. coverage (+): 0. Max coverage (-): 0

Region: chr13 66755232-66755245. Max. coverage (+): 0. Max coverage (-): 0

Region: chr13 66755246-66755259. Max. coverage (+): 0. Max coverage (-): 0

Region: chr13 66755260-66755272. Max. coverage (+): 0. Max coverage (-): 0

Region: chr13 66755273-66755286. Max. coverage (+): 0. Max coverage (-): 0

Region: chr13 66755287-66755299. Max. coverage (+): 0. Max coverage (-): 0

Region: chr13 66755300-66755313. Max. coverage (+): 0. Max coverage (-): 0

Region: chr13 66755314-66755326. Max. coverage (+): 0. Max coverage (-): 0

Region: chr13 66755327-66755340. Max. coverage (+): 0. Max coverage (-): 0

Region: chr13 66755341-66755354. Max. coverage (+): 0. Max coverage (-): 0

Region: chr13 66755355-66755367. Max. coverage (+): 0. Max coverage (-): 0

Region: chr13 66755368-66755381. Max. coverage (+): 0. Max coverage (-): 0

Region: chr13 66755382-66755394. Max. coverage (+): 0. Max coverage (-): 0

Region: chr13 66755395-66755408. Max. coverage (+): 0. Max coverage (-): 0

Region: chr13 66755409-66755421. Max. coverage (+): 0. Max coverage (-): 0

Region: chr13 66755422-66755435. Max. coverage (+): 0. Max coverage (-): 0

Region: chr13 66755436-66755449. Max. coverage (+): 0. Max coverage (-): 0

Region: chr13 66755450-66755462. Max. coverage (+): 0. Max coverage (-): 0

Region: chr13 66755463-66755476. Max. coverage (+): 0. Max coverage (-): 0

Region: chr13 66755477-66755489. Max. coverage (+): 0. Max coverage (-): 0

Region: chr13 66755490-66755503. Max. coverage (+): 0. Max coverage (-): 0

Region: chr13 66755504-66755517. Max. coverage (+): 0. Max coverage (-): 0

Region: chr13 66755518-66755530. Max. coverage (+): 0. Max coverage (-): 0

Region: chr13 66755531-66755544. Max. coverage (+): 0. Max coverage (-): 0

Region: chr13 66755545-66755557. Max. coverage (+): 0. Max coverage (-): 0

Region: chr13 66755558-66755571. Max. coverage (+): 11.75. Max coverage (-): 0

Region: chr13 66755572-66755584. Max. coverage (+): 12.29. Max coverage (-): 0

Region: chr13 66755585-66755598. Max. coverage (+): 0. Max coverage (-): 0

Region: chr13 66755599-66755612. Max. coverage (+): 0. Max coverage (-): 0

Region: chr13 66755613-66755625. Max. coverage (+): 0. Max coverage (-): 0

Region: chr13 66755626-66755639. Max. coverage (+): 0. Max coverage (-): 0

Region: chr13 66755640-66755652. Max. coverage (+): 0. Max coverage (-): 0

Region: chr13 66755653-66755666. Max. coverage (+): 0. Max coverage (-): 0

Region: chr13 66755667-66755679. Max. coverage (+): 0. Max coverage (-): 0

Region: chr13 66755680-66755693. Max. coverage (+): 0. Max coverage (-): 0

Region: chr13 66755694-66755707. Max. coverage (+): 5.15. Max coverage (-): 0

Region: chr13 66755708-66755720. Max. coverage (+): 5.15. Max coverage (-): 0

Region: chr13 66755721-66755734. Max. coverage (+): 0. Max coverage (-): 0

Region: chr13 66755735-66755747. Max. coverage (+): 0. Max coverage (-): 0

Region: chr13 66755748-66755761. Max. coverage (+): 2.08. Max coverage (-): 0

Region: chr13 66755762-66755775. Max. coverage (+): 3.5. Max coverage (-): 0

Region: chr13 66755776-66755788. Max. coverage (+): 3.89. Max coverage (-): 0

Region: chr13 66755789-66755802. Max. coverage (+): 0. Max coverage (-): 0

Region: chr13 66755803-. Max. coverage (+): 0. Max coverage (-): 0

RepeatMasker Color Code

**+**

100-98% Identity

<98-95% Identity

<95-90% Identity

<90-85% Identity

<85-80% Identity

<80-75% Identity

<75-70% Identity

<70% Identity

**-**

Gene Set Color Code

**+**

Gene

Pseudogene

**-**

Topology/Coverage Color Code

Coverage Plus Strand

Coverage Minus Strand

Mainstrand: Plus

Mainstrand: Minus

Complementary Strand

Flanking Region  
(if option -flank >0)

Gene Set Annotation  

**1. MROH8 (protein coding, ENSBTAG00000013737) Tr:00000018251 Ex:14**: 66754510-66754647 (-)  
**2. MROH8 (protein coding, ENSBTAG00000013737) Tr:00000018251 Ex:15**: 66753935-66754049 (-)  
**3. MROH8 (protein coding, ENSBTAG00000013737) Tr:00000018251 Ex:16**: 66753467-66753607 (-)  
**4. MROH8 (protein coding, ENSBTAG00000013737) Tr:00000018251 Ex:17**: 66752964-66753128 (-)  
**5. MROH8 (protein coding, ENSBTAG00000013737) Tr:00000018251 Ex:18**: 66751767-66751922 (-)  
**6. MROH8 (protein coding, ENSBTAG00000013737) Tr:00000018251 Ex:19**: 66749537-66749659 (-)  
**7. MROH8 (protein coding, ENSBTAG00000013737) Tr:00000063909 Ex:8**: 66754510-66754647 (-)  
**8. MROH8 (protein coding, ENSBTAG00000013737) Tr:00000063909 Ex:9**: 66753935-66754049 (-)  
**9. MROH8 (protein coding, ENSBTAG00000013737) Tr:00000063909 Ex:10**: 66753467-66753607 (-)  
**10. MROH8 (protein coding, ENSBTAG00000013737) Tr:00000063909 Ex:11**: 66752964-66753128 (-)  
**11. MROH8 (protein coding, ENSBTAG00000013737) Tr:00000063909 Ex:12**: 66751767-66751922 (-)  
**12. MROH8 (protein coding, ENSBTAG00000013737) Tr:00000063909 Ex:13**: 66749537-66749659 (-)

  
RepeatMasker Annotation  

**1. Charlie15a**: 66749688-66749790 (-), Divergence to consensus: 43.7%  
**2. L1ME3C**: 66750255-66750653 (+), Divergence to consensus: 43.1%  
**3. L2c**: 66751636-66751690 (-), Divergence to consensus: 26.1%  
**4. Bov-tA1**: 66752166-66752390 (+), Divergence to consensus: 15.1%  
**5. SINE2-2\_BT**: 66752473-66752596 (-), Divergence to consensus: 19.4%  
**6. MIRc**: 66752758-66752893 (+), Divergence to consensus: 39.9%  
**7. L1\_Art**: 66753335-66753454 (+), Divergence to consensus: 17.3%  
**8. ART2A**: 66754178-66754347 (-), Divergence to consensus: 11.8%  
**9. MIR3**: 66755026-66755163 (-), Divergence to consensus: 44.4%  
**10. CHR-2B**: 66755164-66755465 (+), Divergence to consensus: 26.6%  
**11. MIR3**: 66755466-66755508 (-), Divergence to consensus: 44.4%  
**12. Bov-tA2**: 66755808-66755929 (+), Divergence to consensus: 17.9%

  
Transcription Factor Binding Sites  

**Gata4** (Sequence: CTTATCT (+): 66751848)  
**Gata4** (Sequence: CTTATCT (+): 66752954)
