# Supplementary material for: Small RNA sequencing of cryopreserved semen from single bull revealed altered miRNAs and piRNAs expression between High- and Low-motile sperm populations
Source: BMC Genomics. 2017 Jan 4;18:14. doi: 10.1186/s12864-016-3394-7 (PMC5209821; doi:10.1186/s12864-016-3394-7)
Supplement: Additional file 3: — Details for each piRNA clusters found in High Motile (HM) sperm fraction. Genes, repeats, transposable elements and transcription factors binding sites falling within the cluster regions were reported. (ZIP 1896 kb) [file 12864_2016_3394_MOESM3_ESM.zip › 18.html]

piRNA cluster 18


Predicted piRNA cluster no. 18     previous   next
  

Show proTRAC run info
Hide proTRAC run info

================================= proTRAC ====================================  
VERSION: 2.1                                    LAST MODIFIED: 06. October 2015  
  
Please cite:  
Rosenkranz D, Zischler H. proTRAC - a software for probabilistic piRNA cluster  
detection, visualization and analysis. 2012. BMC Bioinformatics 13:5.  
  
and (for proTRAC 2.0 and later):  
Rosenkranz D, Rudloff S, Bastuck K, Ketting RF, Zischler H. Tupaia small RNAs  
provide insights into function and evolution of RNAi-based transposon defense  
in mammals. 2015. RNA 21(5):911-922.  
  
Contact:  
David Rosenkranz  
Institute of Anthropology, small RNA group  
Johannes Gutenberg University Mainz  
email: rosenkranz@uni-mainz.de  
  
You can find the latest proTRAC version at:  
http://sourceforge.net/projects/protrac/files  
http://www.smallRNAgroup-mainz.de/software  
==============================================================================  
  
PARAMETERS:  
Map file: .............../storage/core/barbara/genhome/smallRNA/fertility/Sample\_motile/pirna/Sample\_motile\_26-33\_collapsed.fa.no-dust.map.weighted-10000-1000-b-0  
Genome file: ............/storage/core/barbara/genhome/smallRNA/fertility/Sample\_all/pirna/bt\_311\_chrY.fa  
RepeatMasker annotation: /storage/genomes/bt\_umd31/GCF\_000003055.6\_Bos\_taurus\_UMD\_3.1.1\_repeatMasker\_chr.out  
GeneSet:................./storage/core/barbara/genhome/smallRNA/fertility/Sample\_all/pirna/full.gtf  
  
Significant (p<=0.01) hit density will be calculated based  
on observed hit distribution.  
  
Sliding window size: ........................................ 5000 bp  
Sliding window increament: .................................. 1000 bp  
Normalize each hit by number of genomic hits: ............... 1 [0=no/1=yes]  
Normalize each hit by number of sequence reads: ............. 1 [0=no/1=yes]  
Normalize values (-> per million mapped reads): ............. 1 [0=no/1=yes]  
Min. fraction of hits with 1T(U) or 10A: .................... 0.75  
Alternatively: Min. fraction of hits with 1T(U) and 10A: .... 0.5  
Min. fraction of hits with typical piRNA length: ............ 0.75  
Typical piRNA length: ....................................... 26-33 nt  
Min. size of a piRNA cluster: ............................... 5000 bp.  
Min. number of hits (absolute): ............................. 0  
Min. number of hits (normalized): ........................... 0  
Min. fraction of hits on the mainstrand: .................... 0.75  
Top fraction of mapped sequences (in terms of read counts): . 1%  
Top fraction accounts for max. n% of sequence reads: ........ 90%  
Min. fraction of hits on each arm of a bidirectional cluster: 0.1  
Output image file for each cluster: ......................... 0 [0=no/1=yes]  
Output html file for each cluster: .......................... 1 [0=no/1=yes]  
Output a summary table: ..................................... 1 [0=no/1=yes]  
Output a FASTA file for each cluster (piRNA sequences): ..... 1 [0=no/1=yes]  
Output a FASTA file comprising cluster sequences: ........... 1 [0=no/1=yes]  
Search DNA motifs in clusters: .............................. 1 [0=no/1=yes]  
Output flanking sequences: +/- .............................. 0 bp  
Output ~.pTi file: .......................................... 1 [0=no/1=yes]  
==============================================================================  
  
  
Genome size (without gaps): ............ 2678902517 bp  
Gaps (N/X/-): .......................... 53837044 bp  
Mapped reads: .......................... 658825247023  
Non-identical sequences: ............... 514171  
Genomic hits: .......................... 764233  
Significant densitiy of mapped reads: .. 12867599.5173724 reads/kb

Show proTRAC cluster info
Hide proTRAC cluster info

|  |  |
| --- | --- |
| Location | chr13 |
| Coordinates | 70529295-70535032 |
| Size [bp] | 5738 |
| Sequence hit loci | 116 |
| Mapped reads (normalized) | 154156597 |
| Mapped reads (normalized) per kb | 26865910.9 |
| Normalized reads with 1T (1U) | 82.8% |
| Normalized reads with 10A | 46.5% |
| Normalized reads with length 26-33 nt | 100% |
| Normalized reads on the main strand(s) | 91.6% |
| Predicted directionality | mono:minus |

100%

0%

1T (1U)  
reads

10A reads

26-33 nt  
reads

reads on mainstrand

**Either the amount of reads with 1T (1U) OR 10A has to exceed 75% (set with option: -1Tor10A)  
Alternatively the amount of reads with 1T (1U) AND 10A has to exceed 50% (set with option: -1Tand10A)  
Minimum amount of reads with preferred size is 75% (set with option: -pisize)  
Minimum amount of reads on the main strand(s) is 75% (set with option: -clstrand)**

Show read coverage
Hide read coverage

WHAT DO I SEE HERE?  
This chart shows the location of mapped sequence reads within a predicted piRNA cluster. The color refers to the number of genomic hits produced by the sequence read in question. A dark red bar indicates that this sequence read produces many other hits elsewhere in the genome. Many adjacent red or yellow bars can indicate the presence of a multi-copy element such as transposons or rRNA genes. A dark green bar indicates that this sequence read maps uniquely to this locus.

1 hit

2-5 hits

6-10 hits

11-20 hits

21-50 hits

51-100 hits

> 100 hits

chr13

70529295

70535032

Gene Set

RepeatMasker

Mapped  
Reads

16.42

plus strand

minus strand

16.42

Region: chr13 66755804-70529300. Max. coverage (+): 0. Max coverage (-): 5.53

Region: chr13 70529301-70529312. Max. coverage (+): 0. Max coverage (-): 5.53

Region: chr13 70529313-70529323. Max. coverage (+): 0. Max coverage (-): 0

Region: chr13 70529324-70529335. Max. coverage (+): 0. Max coverage (-): 0

Region: chr13 70529336-70529346. Max. coverage (+): 0. Max coverage (-): 0

Region: chr13 70529347-70529358. Max. coverage (+): 0. Max coverage (-): 0

Region: chr13 70529359-70529369. Max. coverage (+): 0. Max coverage (-): 0

Region: chr13 70529370-70529381. Max. coverage (+): 0. Max coverage (-): 0

Region: chr13 70529382-70529392. Max. coverage (+): 0. Max coverage (-): 0

Region: chr13 70529393-70529404. Max. coverage (+): 0. Max coverage (-): 0

Region: chr13 70529405-70529415. Max. coverage (+): 0. Max coverage (-): 0

Region: chr13 70529416-70529426. Max. coverage (+): 0. Max coverage (-): 0

Region: chr13 70529427-70529438. Max. coverage (+): 0. Max coverage (-): 0

Region: chr13 70529439-70529449. Max. coverage (+): 0. Max coverage (-): 0

Region: chr13 70529450-70529461. Max. coverage (+): 0. Max coverage (-): 0

Region: chr13 70529462-70529472. Max. coverage (+): 0. Max coverage (-): 0

Region: chr13 70529473-70529484. Max. coverage (+): 0. Max coverage (-): 0

Region: chr13 70529485-70529495. Max. coverage (+): 0. Max coverage (-): 0

Region: chr13 70529496-70529507. Max. coverage (+): 0. Max coverage (-): 0

Region: chr13 70529508-70529518. Max. coverage (+): 0. Max coverage (-): 0

Region: chr13 70529519-70529530. Max. coverage (+): 0. Max coverage (-): 0

Region: chr13 70529531-70529541. Max. coverage (+): 0. Max coverage (-): 0

Region: chr13 70529542-70529553. Max. coverage (+): 0. Max coverage (-): 0

Region: chr13 70529554-70529564. Max. coverage (+): 0. Max coverage (-): 0

Region: chr13 70529565-70529576. Max. coverage (+): 0. Max coverage (-): 0

Region: chr13 70529577-70529587. Max. coverage (+): 0. Max coverage (-): 0

Region: chr13 70529588-70529599. Max. coverage (+): 0. Max coverage (-): 0

Region: chr13 70529600-70529610. Max. coverage (+): 0. Max coverage (-): 0

Region: chr13 70529611-70529622. Max. coverage (+): 0. Max coverage (-): 0

Region: chr13 70529623-70529633. Max. coverage (+): 0. Max coverage (-): 0

Region: chr13 70529634-70529645. Max. coverage (+): 0. Max coverage (-): 0

Region: chr13 70529646-70529656. Max. coverage (+): 0. Max coverage (-): 0

Region: chr13 70529657-70529667. Max. coverage (+): 0. Max coverage (-): 0

Region: chr13 70529668-70529679. Max. coverage (+): 0. Max coverage (-): 0

Region: chr13 70529680-70529690. Max. coverage (+): 0. Max coverage (-): 0

Region: chr13 70529691-70529702. Max. coverage (+): 0. Max coverage (-): 0

Region: chr13 70529703-70529713. Max. coverage (+): 0. Max coverage (-): 0

Region: chr13 70529714-70529725. Max. coverage (+): 0. Max coverage (-): 0

Region: chr13 70529726-70529736. Max. coverage (+): 0. Max coverage (-): 0

Region: chr13 70529737-70529748. Max. coverage (+): 0. Max coverage (-): 0

Region: chr13 70529749-70529759. Max. coverage (+): 0. Max coverage (-): 0

Region: chr13 70529760-70529771. Max. coverage (+): 0. Max coverage (-): 0

Region: chr13 70529772-70529782. Max. coverage (+): 0. Max coverage (-): 0

Region: chr13 70529783-70529794. Max. coverage (+): 0. Max coverage (-): 0

Region: chr13 70529795-70529805. Max. coverage (+): 0. Max coverage (-): 0

Region: chr13 70529806-70529817. Max. coverage (+): 0. Max coverage (-): 0

Region: chr13 70529818-70529828. Max. coverage (+): 0. Max coverage (-): 0

Region: chr13 70529829-70529840. Max. coverage (+): 0. Max coverage (-): 0

Region: chr13 70529841-70529851. Max. coverage (+): 0. Max coverage (-): 0

Region: chr13 70529852-70529863. Max. coverage (+): 0. Max coverage (-): 0

Region: chr13 70529864-70529874. Max. coverage (+): 0. Max coverage (-): 0

Region: chr13 70529875-70529886. Max. coverage (+): 0. Max coverage (-): 0

Region: chr13 70529887-70529897. Max. coverage (+): 0. Max coverage (-): 0

Region: chr13 70529898-70529908. Max. coverage (+): 0. Max coverage (-): 0

Region: chr13 70529909-70529920. Max. coverage (+): 0. Max coverage (-): 0

Region: chr13 70529921-70529931. Max. coverage (+): 0. Max coverage (-): 0

Region: chr13 70529932-70529943. Max. coverage (+): 0. Max coverage (-): 0

Region: chr13 70529944-70529954. Max. coverage (+): 0. Max coverage (-): 0

Region: chr13 70529955-70529966. Max. coverage (+): 0. Max coverage (-): 0

Region: chr13 70529967-70529977. Max. coverage (+): 0. Max coverage (-): 0

Region: chr13 70529978-70529989. Max. coverage (+): 0. Max coverage (-): 0

Region: chr13 70529990-70530000. Max. coverage (+): 0. Max coverage (-): 0

Region: chr13 70530001-70530012. Max. coverage (+): 0. Max coverage (-): 0

Region: chr13 70530013-70530023. Max. coverage (+): 0. Max coverage (-): 0

Region: chr13 70530024-70530035. Max. coverage (+): 0. Max coverage (-): 0

Region: chr13 70530036-70530046. Max. coverage (+): 0. Max coverage (-): 0

Region: chr13 70530047-70530058. Max. coverage (+): 0. Max coverage (-): 0

Region: chr13 70530059-70530069. Max. coverage (+): 0. Max coverage (-): 0

Region: chr13 70530070-70530081. Max. coverage (+): 0. Max coverage (-): 0

Region: chr13 70530082-70530092. Max. coverage (+): 3.87. Max coverage (-): 0

Region: chr13 70530093-70530104. Max. coverage (+): 3.87. Max coverage (-): 0

Region: chr13 70530105-70530115. Max. coverage (+): 0. Max coverage (-): 0

Region: chr13 70530116-70530127. Max. coverage (+): 0. Max coverage (-): 0

Region: chr13 70530128-70530138. Max. coverage (+): 0. Max coverage (-): 0

Region: chr13 70530139-70530149. Max. coverage (+): 0. Max coverage (-): 0

Region: chr13 70530150-70530161. Max. coverage (+): 0. Max coverage (-): 0

Region: chr13 70530162-70530172. Max. coverage (+): 0. Max coverage (-): 0

Region: chr13 70530173-70530184. Max. coverage (+): 0. Max coverage (-): 0

Region: chr13 70530185-70530195. Max. coverage (+): 1.35. Max coverage (-): 0

Region: chr13 70530196-70530207. Max. coverage (+): 1.55. Max coverage (-): 6.66

Region: chr13 70530208-70530218. Max. coverage (+): 1.55. Max coverage (-): 6.66

Region: chr13 70530219-70530230. Max. coverage (+): 0. Max coverage (-): 0

Region: chr13 70530231-70530241. Max. coverage (+): 0. Max coverage (-): 1.33

Region: chr13 70530242-70530253. Max. coverage (+): 0. Max coverage (-): 4.63

Region: chr13 70530254-70530264. Max. coverage (+): 0. Max coverage (-): 0

Region: chr13 70530265-70530276. Max. coverage (+): 2.93. Max coverage (-): 0

Region: chr13 70530277-70530287. Max. coverage (+): 0. Max coverage (-): 0

Region: chr13 70530288-70530299. Max. coverage (+): 0. Max coverage (-): 0

Region: chr13 70530300-70530310. Max. coverage (+): 0. Max coverage (-): 0

Region: chr13 70530311-70530322. Max. coverage (+): 0. Max coverage (-): 0

Region: chr13 70530323-70530333. Max. coverage (+): 0. Max coverage (-): 0

Region: chr13 70530334-70530345. Max. coverage (+): 0. Max coverage (-): 0

Region: chr13 70530346-70530356. Max. coverage (+): 0. Max coverage (-): 0

Region: chr13 70530357-70530368. Max. coverage (+): 0. Max coverage (-): 0

Region: chr13 70530369-70530379. Max. coverage (+): 0. Max coverage (-): 0

Region: chr13 70530380-70530390. Max. coverage (+): 0. Max coverage (-): 0

Region: chr13 70530391-70530402. Max. coverage (+): 0. Max coverage (-): 3.58

Region: chr13 70530403-70530413. Max. coverage (+): 0. Max coverage (-): 3.58

Region: chr13 70530414-70530425. Max. coverage (+): 0. Max coverage (-): 2.27

Region: chr13 70530426-70530436. Max. coverage (+): 0. Max coverage (-): 2.27

Region: chr13 70530437-70530448. Max. coverage (+): 0. Max coverage (-): 0

Region: chr13 70530449-70530459. Max. coverage (+): 0. Max coverage (-): 0

Region: chr13 70530460-70530471. Max. coverage (+): 0. Max coverage (-): 0

Region: chr13 70530472-70530482. Max. coverage (+): 0. Max coverage (-): 0

Region: chr13 70530483-70530494. Max. coverage (+): 0. Max coverage (-): 0

Region: chr13 70530495-70530505. Max. coverage (+): 0. Max coverage (-): 0

Region: chr13 70530506-70530517. Max. coverage (+): 0. Max coverage (-): 4.59

Region: chr13 70530518-70530528. Max. coverage (+): 0. Max coverage (-): 0

Region: chr13 70530529-70530540. Max. coverage (+): 0. Max coverage (-): 0

Region: chr13 70530541-70530551. Max. coverage (+): 0. Max coverage (-): 0

Region: chr13 70530552-70530563. Max. coverage (+): 0. Max coverage (-): 0

Region: chr13 70530564-70530574. Max. coverage (+): 0. Max coverage (-): 0

Region: chr13 70530575-70530586. Max. coverage (+): 0. Max coverage (-): 0

Region: chr13 70530587-70530597. Max. coverage (+): 0. Max coverage (-): 0

Region: chr13 70530598-70530609. Max. coverage (+): 0. Max coverage (-): 0

Region: chr13 70530610-70530620. Max. coverage (+): 0. Max coverage (-): 0

Region: chr13 70530621-70530631. Max. coverage (+): 0. Max coverage (-): 0

Region: chr13 70530632-70530643. Max. coverage (+): 0. Max coverage (-): 0

Region: chr13 70530644-70530654. Max. coverage (+): 0. Max coverage (-): 0

Region: chr13 70530655-70530666. Max. coverage (+): 0. Max coverage (-): 0

Region: chr13 70530667-70530677. Max. coverage (+): 0. Max coverage (-): 0

Region: chr13 70530678-70530689. Max. coverage (+): 0. Max coverage (-): 0

Region: chr13 70530690-70530700. Max. coverage (+): 0. Max coverage (-): 0

Region: chr13 70530701-70530712. Max. coverage (+): 0. Max coverage (-): 0

Region: chr13 70530713-70530723. Max. coverage (+): 0. Max coverage (-): 0

Region: chr13 70530724-70530735. Max. coverage (+): 0. Max coverage (-): 0

Region: chr13 70530736-70530746. Max. coverage (+): 0. Max coverage (-): 0

Region: chr13 70530747-70530758. Max. coverage (+): 0. Max coverage (-): 0

Region: chr13 70530759-70530769. Max. coverage (+): 0. Max coverage (-): 0

Region: chr13 70530770-70530781. Max. coverage (+): 0. Max coverage (-): 0

Region: chr13 70530782-70530792. Max. coverage (+): 0. Max coverage (-): 0

Region: chr13 70530793-70530804. Max. coverage (+): 0. Max coverage (-): 0

Region: chr13 70530805-70530815. Max. coverage (+): 0. Max coverage (-): 0

Region: chr13 70530816-70530827. Max. coverage (+): 0. Max coverage (-): 0

Region: chr13 70530828-70530838. Max. coverage (+): 0. Max coverage (-): 0

Region: chr13 70530839-70530849. Max. coverage (+): 0. Max coverage (-): 0

Region: chr13 70530850-70530861. Max. coverage (+): 0. Max coverage (-): 0

Region: chr13 70530862-70530872. Max. coverage (+): 0. Max coverage (-): 0

Region: chr13 70530873-70530884. Max. coverage (+): 0. Max coverage (-): 1.46

Region: chr13 70530885-70530895. Max. coverage (+): 0. Max coverage (-): 1.46

Region: chr13 70530896-70530907. Max. coverage (+): 0. Max coverage (-): 0

Region: chr13 70530908-70530918. Max. coverage (+): 0. Max coverage (-): 0

Region: chr13 70530919-70530930. Max. coverage (+): 0. Max coverage (-): 4.41

Region: chr13 70530931-70530941. Max. coverage (+): 0. Max coverage (-): 0

Region: chr13 70530942-70530953. Max. coverage (+): 0. Max coverage (-): 0

Region: chr13 70530954-70530964. Max. coverage (+): 0. Max coverage (-): 0

Region: chr13 70530965-70530976. Max. coverage (+): 0. Max coverage (-): 4.7

Region: chr13 70530977-70530987. Max. coverage (+): 0. Max coverage (-): 4.7

Region: chr13 70530988-70530999. Max. coverage (+): 0. Max coverage (-): 0

Region: chr13 70531000-70531010. Max. coverage (+): 0. Max coverage (-): 0

Region: chr13 70531011-70531022. Max. coverage (+): 0. Max coverage (-): 0

Region: chr13 70531023-70531033. Max. coverage (+): 0. Max coverage (-): 3.67

Region: chr13 70531034-70531045. Max. coverage (+): 0. Max coverage (-): 0

Region: chr13 70531046-70531056. Max. coverage (+): 0. Max coverage (-): 0

Region: chr13 70531057-70531068. Max. coverage (+): 0. Max coverage (-): 1.6

Region: chr13 70531069-70531079. Max. coverage (+): 0. Max coverage (-): 1.6

Region: chr13 70531080-70531090. Max. coverage (+): 0. Max coverage (-): 8.42

Region: chr13 70531091-70531102. Max. coverage (+): 0. Max coverage (-): 9.94

Region: chr13 70531103-70531113. Max. coverage (+): 0. Max coverage (-): 0

Region: chr13 70531114-70531125. Max. coverage (+): 0. Max coverage (-): 16.42

Region: chr13 70531126-70531136. Max. coverage (+): 0. Max coverage (-): 16.42

Region: chr13 70531137-70531148. Max. coverage (+): 0. Max coverage (-): 1.3

Region: chr13 70531149-70531159. Max. coverage (+): 0. Max coverage (-): 0

Region: chr13 70531160-70531171. Max. coverage (+): 0. Max coverage (-): 0

Region: chr13 70531172-70531182. Max. coverage (+): 0. Max coverage (-): 0

Region: chr13 70531183-70531194. Max. coverage (+): 0. Max coverage (-): 0

Region: chr13 70531195-70531205. Max. coverage (+): 0. Max coverage (-): 0

Region: chr13 70531206-70531217. Max. coverage (+): 0. Max coverage (-): 2.29

Region: chr13 70531218-70531228. Max. coverage (+): 0. Max coverage (-): 2.29

Region: chr13 70531229-70531240. Max. coverage (+): 0. Max coverage (-): 0

Region: chr13 70531241-70531251. Max. coverage (+): 0. Max coverage (-): 0

Region: chr13 70531252-70531263. Max. coverage (+): 0. Max coverage (-): 0

Region: chr13 70531264-70531274. Max. coverage (+): 0. Max coverage (-): 0

Region: chr13 70531275-70531286. Max. coverage (+): 0. Max coverage (-): 0

Region: chr13 70531287-70531297. Max. coverage (+): 0. Max coverage (-): 1.59

Region: chr13 70531298-70531309. Max. coverage (+): 0. Max coverage (-): 1.59

Region: chr13 70531310-70531320. Max. coverage (+): 0. Max coverage (-): 0

Region: chr13 70531321-70531331. Max. coverage (+): 0. Max coverage (-): 0

Region: chr13 70531332-70531343. Max. coverage (+): 0. Max coverage (-): 0

Region: chr13 70531344-70531354. Max. coverage (+): 1.01. Max coverage (-): 13.92

Region: chr13 70531355-70531366. Max. coverage (+): 1.01. Max coverage (-): 10.32

Region: chr13 70531367-70531377. Max. coverage (+): 0. Max coverage (-): 0

Region: chr13 70531378-70531389. Max. coverage (+): 0. Max coverage (-): 0

Region: chr13 70531390-70531400. Max. coverage (+): 0. Max coverage (-): 0

Region: chr13 70531401-70531412. Max. coverage (+): 0. Max coverage (-): 0

Region: chr13 70531413-70531423. Max. coverage (+): 0. Max coverage (-): 0

Region: chr13 70531424-70531435. Max. coverage (+): 0. Max coverage (-): 0

Region: chr13 70531436-70531446. Max. coverage (+): 0. Max coverage (-): 4.33

Region: chr13 70531447-70531458. Max. coverage (+): 0. Max coverage (-): 4.33

Region: chr13 70531459-70531469. Max. coverage (+): 0. Max coverage (-): 3.75

Region: chr13 70531470-70531481. Max. coverage (+): 0. Max coverage (-): 0

Region: chr13 70531482-70531492. Max. coverage (+): 0. Max coverage (-): 0

Region: chr13 70531493-70531504. Max. coverage (+): 0. Max coverage (-): 0

Region: chr13 70531505-70531515. Max. coverage (+): 0. Max coverage (-): 0

Region: chr13 70531516-70531527. Max. coverage (+): 0. Max coverage (-): 0

Region: chr13 70531528-70531538. Max. coverage (+): 0. Max coverage (-): 0

Region: chr13 70531539-70531550. Max. coverage (+): 0. Max coverage (-): 0

Region: chr13 70531551-70531561. Max. coverage (+): 0. Max coverage (-): 0.87

Region: chr13 70531562-70531572. Max. coverage (+): 0. Max coverage (-): 2.52

Region: chr13 70531573-70531584. Max. coverage (+): 0. Max coverage (-): 2.52

Region: chr13 70531585-70531595. Max. coverage (+): 0. Max coverage (-): 0

Region: chr13 70531596-70531607. Max. coverage (+): 0. Max coverage (-): 0.6

Region: chr13 70531608-70531618. Max. coverage (+): 0. Max coverage (-): 0.6

Region: chr13 70531619-70531630. Max. coverage (+): 0. Max coverage (-): 0

Region: chr13 70531631-70531641. Max. coverage (+): 0. Max coverage (-): 5.16

Region: chr13 70531642-70531653. Max. coverage (+): 0. Max coverage (-): 6.94

Region: chr13 70531654-70531664. Max. coverage (+): 0. Max coverage (-): 0

Region: chr13 70531665-70531676. Max. coverage (+): 0. Max coverage (-): 0

Region: chr13 70531677-70531687. Max. coverage (+): 0. Max coverage (-): 0

Region: chr13 70531688-70531699. Max. coverage (+): 0. Max coverage (-): 0

Region: chr13 70531700-70531710. Max. coverage (+): 0. Max coverage (-): 2.82

Region: chr13 70531711-70531722. Max. coverage (+): 0. Max coverage (-): 4.56

Region: chr13 70531723-70531733. Max. coverage (+): 0. Max coverage (-): 0

Region: chr13 70531734-70531745. Max. coverage (+): 0. Max coverage (-): 0

Region: chr13 70531746-70531756. Max. coverage (+): 0. Max coverage (-): 0

Region: chr13 70531757-70531768. Max. coverage (+): 0. Max coverage (-): 3.94

Region: chr13 70531769-70531779. Max. coverage (+): 0. Max coverage (-): 5.17

Region: chr13 70531780-70531791. Max. coverage (+): 0. Max coverage (-): 7.56

Region: chr13 70531792-70531802. Max. coverage (+): 0. Max coverage (-): 0.77

Region: chr13 70531803-70531813. Max. coverage (+): 0. Max coverage (-): 0

Region: chr13 70531814-70531825. Max. coverage (+): 0. Max coverage (-): 0

Region: chr13 70531826-70531836. Max. coverage (+): 0. Max coverage (-): 0

Region: chr13 70531837-70531848. Max. coverage (+): 0. Max coverage (-): 0

Region: chr13 70531849-70531859. Max. coverage (+): 0. Max coverage (-): 0

Region: chr13 70531860-70531871. Max. coverage (+): 0. Max coverage (-): 3.05

Region: chr13 70531872-70531882. Max. coverage (+): 0. Max coverage (-): 0

Region: chr13 70531883-70531894. Max. coverage (+): 0. Max coverage (-): 0

Region: chr13 70531895-70531905. Max. coverage (+): 0. Max coverage (-): 0

Region: chr13 70531906-70531917. Max. coverage (+): 0. Max coverage (-): 0

Region: chr13 70531918-70531928. Max. coverage (+): 0. Max coverage (-): 0

Region: chr13 70531929-70531940. Max. coverage (+): 0. Max coverage (-): 0

Region: chr13 70531941-70531951. Max. coverage (+): 0. Max coverage (-): 0

Region: chr13 70531952-70531963. Max. coverage (+): 0. Max coverage (-): 0

Region: chr13 70531964-70531974. Max. coverage (+): 0. Max coverage (-): 0

Region: chr13 70531975-70531986. Max. coverage (+): 0. Max coverage (-): 0

Region: chr13 70531987-70531997. Max. coverage (+): 0. Max coverage (-): 0

Region: chr13 70531998-70532009. Max. coverage (+): 0. Max coverage (-): 0

Region: chr13 70532010-70532020. Max. coverage (+): 0. Max coverage (-): 0

Region: chr13 70532021-70532032. Max. coverage (+): 0. Max coverage (-): 0

Region: chr13 70532033-70532043. Max. coverage (+): 0. Max coverage (-): 2.28

Region: chr13 70532044-70532054. Max. coverage (+): 0. Max coverage (-): 2.28

Region: chr13 70532055-70532066. Max. coverage (+): 1.88. Max coverage (-): 0

Region: chr13 70532067-70532077. Max. coverage (+): 0. Max coverage (-): 0

Region: chr13 70532078-70532089. Max. coverage (+): 0. Max coverage (-): 0

Region: chr13 70532090-70532100. Max. coverage (+): 0. Max coverage (-): 0

Region: chr13 70532101-70532112. Max. coverage (+): 0. Max coverage (-): 0

Region: chr13 70532113-70532123. Max. coverage (+): 0. Max coverage (-): 6.81

Region: chr13 70532124-70532135. Max. coverage (+): 0. Max coverage (-): 10.94

Region: chr13 70532136-70532146. Max. coverage (+): 0. Max coverage (-): 0

Region: chr13 70532147-70532158. Max. coverage (+): 0. Max coverage (-): 0

Region: chr13 70532159-70532169. Max. coverage (+): 0. Max coverage (-): 0

Region: chr13 70532170-70532181. Max. coverage (+): 0. Max coverage (-): 0

Region: chr13 70532182-70532192. Max. coverage (+): 0. Max coverage (-): 0

Region: chr13 70532193-70532204. Max. coverage (+): 0. Max coverage (-): 0

Region: chr13 70532205-70532215. Max. coverage (+): 0. Max coverage (-): 0

Region: chr13 70532216-70532227. Max. coverage (+): 0. Max coverage (-): 0

Region: chr13 70532228-70532238. Max. coverage (+): 0. Max coverage (-): 0

Region: chr13 70532239-70532250. Max. coverage (+): 0. Max coverage (-): 0

Region: chr13 70532251-70532261. Max. coverage (+): 0. Max coverage (-): 0

Region: chr13 70532262-70532273. Max. coverage (+): 0. Max coverage (-): 3.06

Region: chr13 70532274-70532284. Max. coverage (+): 0. Max coverage (-): 3.06

Region: chr13 70532285-70532295. Max. coverage (+): 0. Max coverage (-): 0

Region: chr13 70532296-70532307. Max. coverage (+): 0. Max coverage (-): 0

Region: chr13 70532308-70532318. Max. coverage (+): 0. Max coverage (-): 0

Region: chr13 70532319-70532330. Max. coverage (+): 0. Max coverage (-): 0

Region: chr13 70532331-70532341. Max. coverage (+): 0. Max coverage (-): 0

Region: chr13 70532342-70532353. Max. coverage (+): 0. Max coverage (-): 0

Region: chr13 70532354-70532364. Max. coverage (+): 0. Max coverage (-): 0

Region: chr13 70532365-70532376. Max. coverage (+): 0. Max coverage (-): 0

Region: chr13 70532377-70532387. Max. coverage (+): 0. Max coverage (-): 0

Region: chr13 70532388-70532399. Max. coverage (+): 0. Max coverage (-): 0

Region: chr13 70532400-70532410. Max. coverage (+): 0. Max coverage (-): 0

Region: chr13 70532411-70532422. Max. coverage (+): 0. Max coverage (-): 0

Region: chr13 70532423-70532433. Max. coverage (+): 0. Max coverage (-): 0

Region: chr13 70532434-70532445. Max. coverage (+): 0. Max coverage (-): 0

Region: chr13 70532446-70532456. Max. coverage (+): 0. Max coverage (-): 0

Region: chr13 70532457-70532468. Max. coverage (+): 0. Max coverage (-): 0

Region: chr13 70532469-70532479. Max. coverage (+): 0. Max coverage (-): 0

Region: chr13 70532480-70532491. Max. coverage (+): 0. Max coverage (-): 0

Region: chr13 70532492-70532502. Max. coverage (+): 0. Max coverage (-): 0

Region: chr13 70532503-70532514. Max. coverage (+): 0. Max coverage (-): 0

Region: chr13 70532515-70532525. Max. coverage (+): 0. Max coverage (-): 0

Region: chr13 70532526-70532536. Max. coverage (+): 0. Max coverage (-): 0

Region: chr13 70532537-70532548. Max. coverage (+): 0. Max coverage (-): 0

Region: chr13 70532549-70532559. Max. coverage (+): 0. Max coverage (-): 0

Region: chr13 70532560-70532571. Max. coverage (+): 0. Max coverage (-): 0.79

Region: chr13 70532572-70532582. Max. coverage (+): 0. Max coverage (-): 3.65

Region: chr13 70532583-70532594. Max. coverage (+): 0. Max coverage (-): 2.21

Region: chr13 70532595-70532605. Max. coverage (+): 0. Max coverage (-): 0

Region: chr13 70532606-70532617. Max. coverage (+): 0. Max coverage (-): 1.2

Region: chr13 70532618-70532628. Max. coverage (+): 2.28. Max coverage (-): 1.2

Region: chr13 70532629-70532640. Max. coverage (+): 2.28. Max coverage (-): 8.66

Region: chr13 70532641-70532651. Max. coverage (+): 0. Max coverage (-): 4.31

Region: chr13 70532652-70532663. Max. coverage (+): 0. Max coverage (-): 0

Region: chr13 70532664-70532674. Max. coverage (+): 0. Max coverage (-): 0

Region: chr13 70532675-70532686. Max. coverage (+): 2.25. Max coverage (-): 0

Region: chr13 70532687-70532697. Max. coverage (+): 2.25. Max coverage (-): 1.14

Region: chr13 70532698-70532709. Max. coverage (+): 0. Max coverage (-): 1.14

Region: chr13 70532710-70532720. Max. coverage (+): 0. Max coverage (-): 1.02

Region: chr13 70532721-70532732. Max. coverage (+): 0. Max coverage (-): 1.02

Region: chr13 70532733-70532743. Max. coverage (+): 0. Max coverage (-): 0

Region: chr13 70532744-70532755. Max. coverage (+): 0. Max coverage (-): 0

Region: chr13 70532756-70532766. Max. coverage (+): 0. Max coverage (-): 0

Region: chr13 70532767-70532777. Max. coverage (+): 0. Max coverage (-): 0

Region: chr13 70532778-70532789. Max. coverage (+): 0. Max coverage (-): 0

Region: chr13 70532790-70532800. Max. coverage (+): 0. Max coverage (-): 0

Region: chr13 70532801-70532812. Max. coverage (+): 0. Max coverage (-): 0

Region: chr13 70532813-70532823. Max. coverage (+): 0. Max coverage (-): 0

Region: chr13 70532824-70532835. Max. coverage (+): 0. Max coverage (-): 0

Region: chr13 70532836-70532846. Max. coverage (+): 0. Max coverage (-): 0

Region: chr13 70532847-70532858. Max. coverage (+): 0. Max coverage (-): 0

Region: chr13 70532859-70532869. Max. coverage (+): 0. Max coverage (-): 0

Region: chr13 70532870-70532881. Max. coverage (+): 0. Max coverage (-): 0

Region: chr13 70532882-70532892. Max. coverage (+): 0. Max coverage (-): 0

Region: chr13 70532893-70532904. Max. coverage (+): 0. Max coverage (-): 0

Region: chr13 70532905-70532915. Max. coverage (+): 0. Max coverage (-): 0

Region: chr13 70532916-70532927. Max. coverage (+): 0. Max coverage (-): 0

Region: chr13 70532928-70532938. Max. coverage (+): 0. Max coverage (-): 0

Region: chr13 70532939-70532950. Max. coverage (+): 0. Max coverage (-): 0

Region: chr13 70532951-70532961. Max. coverage (+): 0. Max coverage (-): 0

Region: chr13 70532962-70532973. Max. coverage (+): 0. Max coverage (-): 0

Region: chr13 70532974-70532984. Max. coverage (+): 0. Max coverage (-): 0

Region: chr13 70532985-70532996. Max. coverage (+): 0. Max coverage (-): 0

Region: chr13 70532997-70533007. Max. coverage (+): 0. Max coverage (-): 0

Region: chr13 70533008-70533018. Max. coverage (+): 0. Max coverage (-): 0

Region: chr13 70533019-70533030. Max. coverage (+): 0. Max coverage (-): 0

Region: chr13 70533031-70533041. Max. coverage (+): 0. Max coverage (-): 0

Region: chr13 70533042-70533053. Max. coverage (+): 0. Max coverage (-): 0

Region: chr13 70533054-70533064. Max. coverage (+): 0. Max coverage (-): 0

Region: chr13 70533065-70533076. Max. coverage (+): 0. Max coverage (-): 0

Region: chr13 70533077-70533087. Max. coverage (+): 0. Max coverage (-): 0

Region: chr13 70533088-70533099. Max. coverage (+): 0. Max coverage (-): 0

Region: chr13 70533100-70533110. Max. coverage (+): 0. Max coverage (-): 0

Region: chr13 70533111-70533122. Max. coverage (+): 0. Max coverage (-): 0

Region: chr13 70533123-70533133. Max. coverage (+): 0. Max coverage (-): 0

Region: chr13 70533134-70533145. Max. coverage (+): 0. Max coverage (-): 0

Region: chr13 70533146-70533156. Max. coverage (+): 0. Max coverage (-): 0

Region: chr13 70533157-70533168. Max. coverage (+): 0. Max coverage (-): 0

Region: chr13 70533169-70533179. Max. coverage (+): 0. Max coverage (-): 0

Region: chr13 70533180-70533191. Max. coverage (+): 0. Max coverage (-): 0

Region: chr13 70533192-70533202. Max. coverage (+): 0. Max coverage (-): 7.33

Region: chr13 70533203-70533214. Max. coverage (+): 0. Max coverage (-): 5.18

Region: chr13 70533215-70533225. Max. coverage (+): 0. Max coverage (-): 0

Region: chr13 70533226-70533237. Max. coverage (+): 0. Max coverage (-): 0

Region: chr13 70533238-70533248. Max. coverage (+): 0. Max coverage (-): 0

Region: chr13 70533249-70533259. Max. coverage (+): 0. Max coverage (-): 0

Region: chr13 70533260-70533271. Max. coverage (+): 0. Max coverage (-): 0

Region: chr13 70533272-70533282. Max. coverage (+): 0. Max coverage (-): 0

Region: chr13 70533283-70533294. Max. coverage (+): 0. Max coverage (-): 6.77

Region: chr13 70533295-70533305. Max. coverage (+): 0. Max coverage (-): 5.81

Region: chr13 70533306-70533317. Max. coverage (+): 1. Max coverage (-): 3.39

Region: chr13 70533318-70533328. Max. coverage (+): 0. Max coverage (-): 0

Region: chr13 70533329-70533340. Max. coverage (+): 0. Max coverage (-): 0

Region: chr13 70533341-70533351. Max. coverage (+): 0. Max coverage (-): 0

Region: chr13 70533352-70533363. Max. coverage (+): 0. Max coverage (-): 0

Region: chr13 70533364-70533374. Max. coverage (+): 0. Max coverage (-): 0

Region: chr13 70533375-70533386. Max. coverage (+): 0. Max coverage (-): 0

Region: chr13 70533387-70533397. Max. coverage (+): 0. Max coverage (-): 0

Region: chr13 70533398-70533409. Max. coverage (+): 0. Max coverage (-): 0

Region: chr13 70533410-70533420. Max. coverage (+): 0. Max coverage (-): 0

Region: chr13 70533421-70533432. Max. coverage (+): 0. Max coverage (-): 0

Region: chr13 70533433-70533443. Max. coverage (+): 0. Max coverage (-): 0

Region: chr13 70533444-70533455. Max. coverage (+): 0. Max coverage (-): 0

Region: chr13 70533456-70533466. Max. coverage (+): 0. Max coverage (-): 0

Region: chr13 70533467-70533478. Max. coverage (+): 0. Max coverage (-): 0

Region: chr13 70533479-70533489. Max. coverage (+): 0. Max coverage (-): 0

Region: chr13 70533490-70533500. Max. coverage (+): 0. Max coverage (-): 0

Region: chr13 70533501-70533512. Max. coverage (+): 0. Max coverage (-): 0

Region: chr13 70533513-70533523. Max. coverage (+): 0. Max coverage (-): 0

Region: chr13 70533524-70533535. Max. coverage (+): 0. Max coverage (-): 0

Region: chr13 70533536-70533546. Max. coverage (+): 0. Max coverage (-): 0

Region: chr13 70533547-70533558. Max. coverage (+): 0. Max coverage (-): 0

Region: chr13 70533559-70533569. Max. coverage (+): 0. Max coverage (-): 0

Region: chr13 70533570-70533581. Max. coverage (+): 0. Max coverage (-): 0

Region: chr13 70533582-70533592. Max. coverage (+): 0. Max coverage (-): 0

Region: chr13 70533593-70533604. Max. coverage (+): 0. Max coverage (-): 0

Region: chr13 70533605-70533615. Max. coverage (+): 0. Max coverage (-): 6.61

Region: chr13 70533616-70533627. Max. coverage (+): 0. Max coverage (-): 7.74

Region: chr13 70533628-70533638. Max. coverage (+): 0. Max coverage (-): 4.02

Region: chr13 70533639-70533650. Max. coverage (+): 0. Max coverage (-): 0

Region: chr13 70533651-70533661. Max. coverage (+): 0. Max coverage (-): 0

Region: chr13 70533662-70533673. Max. coverage (+): 0. Max coverage (-): 0

Region: chr13 70533674-70533684. Max. coverage (+): 0. Max coverage (-): 0

Region: chr13 70533685-70533696. Max. coverage (+): 0. Max coverage (-): 0

Region: chr13 70533697-70533707. Max. coverage (+): 0. Max coverage (-): 1.76

Region: chr13 70533708-70533718. Max. coverage (+): 0. Max coverage (-): 1.76

Region: chr13 70533719-70533730. Max. coverage (+): 0. Max coverage (-): 1.33

Region: chr13 70533731-70533741. Max. coverage (+): 0. Max coverage (-): 0

Region: chr13 70533742-70533753. Max. coverage (+): 0. Max coverage (-): 0

Region: chr13 70533754-70533764. Max. coverage (+): 0. Max coverage (-): 0

Region: chr13 70533765-70533776. Max. coverage (+): 0. Max coverage (-): 0

Region: chr13 70533777-70533787. Max. coverage (+): 0. Max coverage (-): 0

Region: chr13 70533788-70533799. Max. coverage (+): 0. Max coverage (-): 0

Region: chr13 70533800-70533810. Max. coverage (+): 0. Max coverage (-): 0

Region: chr13 70533811-70533822. Max. coverage (+): 0. Max coverage (-): 0

Region: chr13 70533823-70533833. Max. coverage (+): 0. Max coverage (-): 0

Region: chr13 70533834-70533845. Max. coverage (+): 0. Max coverage (-): 0

Region: chr13 70533846-70533856. Max. coverage (+): 0. Max coverage (-): 0

Region: chr13 70533857-70533868. Max. coverage (+): 0. Max coverage (-): 0

Region: chr13 70533869-70533879. Max. coverage (+): 0. Max coverage (-): 0

Region: chr13 70533880-70533891. Max. coverage (+): 0. Max coverage (-): 0

Region: chr13 70533892-70533902. Max. coverage (+): 0. Max coverage (-): 0

Region: chr13 70533903-70533914. Max. coverage (+): 0. Max coverage (-): 0

Region: chr13 70533915-70533925. Max. coverage (+): 0. Max coverage (-): 0

Region: chr13 70533926-70533937. Max. coverage (+): 0. Max coverage (-): 0

Region: chr13 70533938-70533948. Max. coverage (+): 0. Max coverage (-): 0

Region: chr13 70533949-70533959. Max. coverage (+): 0. Max coverage (-): 0

Region: chr13 70533960-70533971. Max. coverage (+): 0. Max coverage (-): 0

Region: chr13 70533972-70533982. Max. coverage (+): 0. Max coverage (-): 0

Region: chr13 70533983-70533994. Max. coverage (+): 0. Max coverage (-): 0

Region: chr13 70533995-70534005. Max. coverage (+): 0. Max coverage (-): 0

Region: chr13 70534006-70534017. Max. coverage (+): 0. Max coverage (-): 0

Region: chr13 70534018-70534028. Max. coverage (+): 0. Max coverage (-): 0

Region: chr13 70534029-70534040. Max. coverage (+): 0. Max coverage (-): 0

Region: chr13 70534041-70534051. Max. coverage (+): 0. Max coverage (-): 0.9

Region: chr13 70534052-70534063. Max. coverage (+): 0. Max coverage (-): 0.9

Region: chr13 70534064-70534074. Max. coverage (+): 0. Max coverage (-): 0

Region: chr13 70534075-70534086. Max. coverage (+): 1.59. Max coverage (-): 0

Region: chr13 70534087-70534097. Max. coverage (+): 0. Max coverage (-): 0

Region: chr13 70534098-70534109. Max. coverage (+): 0. Max coverage (-): 0

Region: chr13 70534110-70534120. Max. coverage (+): 0. Max coverage (-): 1.09

Region: chr13 70534121-70534132. Max. coverage (+): 0. Max coverage (-): 1.09

Region: chr13 70534133-70534143. Max. coverage (+): 0. Max coverage (-): 0

Region: chr13 70534144-70534155. Max. coverage (+): 0. Max coverage (-): 0

Region: chr13 70534156-70534166. Max. coverage (+): 0. Max coverage (-): 0

Region: chr13 70534167-70534178. Max. coverage (+): 0. Max coverage (-): 0

Region: chr13 70534179-70534189. Max. coverage (+): 0. Max coverage (-): 0

Region: chr13 70534190-70534200. Max. coverage (+): 0. Max coverage (-): 0

Region: chr13 70534201-70534212. Max. coverage (+): 0. Max coverage (-): 0

Region: chr13 70534213-70534223. Max. coverage (+): 0. Max coverage (-): 4.29

Region: chr13 70534224-70534235. Max. coverage (+): 0. Max coverage (-): 0

Region: chr13 70534236-70534246. Max. coverage (+): 0. Max coverage (-): 0

Region: chr13 70534247-70534258. Max. coverage (+): 0. Max coverage (-): 0

Region: chr13 70534259-70534269. Max. coverage (+): 0. Max coverage (-): 0

Region: chr13 70534270-70534281. Max. coverage (+): 0. Max coverage (-): 0

Region: chr13 70534282-70534292. Max. coverage (+): 0. Max coverage (-): 0

Region: chr13 70534293-70534304. Max. coverage (+): 0. Max coverage (-): 0

Region: chr13 70534305-70534315. Max. coverage (+): 0. Max coverage (-): 0

Region: chr13 70534316-70534327. Max. coverage (+): 0. Max coverage (-): 0

Region: chr13 70534328-70534338. Max. coverage (+): 0. Max coverage (-): 0

Region: chr13 70534339-70534350. Max. coverage (+): 0. Max coverage (-): 0

Region: chr13 70534351-70534361. Max. coverage (+): 0. Max coverage (-): 0

Region: chr13 70534362-70534373. Max. coverage (+): 0. Max coverage (-): 0

Region: chr13 70534374-70534384. Max. coverage (+): 0. Max coverage (-): 0

Region: chr13 70534385-70534396. Max. coverage (+): 0. Max coverage (-): 0

Region: chr13 70534397-70534407. Max. coverage (+): 0. Max coverage (-): 0

Region: chr13 70534408-70534419. Max. coverage (+): 0. Max coverage (-): 0

Region: chr13 70534420-70534430. Max. coverage (+): 0. Max coverage (-): 0

Region: chr13 70534431-70534441. Max. coverage (+): 0. Max coverage (-): 0

Region: chr13 70534442-70534453. Max. coverage (+): 0. Max coverage (-): 0

Region: chr13 70534454-70534464. Max. coverage (+): 0. Max coverage (-): 0

Region: chr13 70534465-70534476. Max. coverage (+): 0. Max coverage (-): 0

Region: chr13 70534477-70534487. Max. coverage (+): 0. Max coverage (-): 0

Region: chr13 70534488-70534499. Max. coverage (+): 0. Max coverage (-): 0

Region: chr13 70534500-70534510. Max. coverage (+): 0. Max coverage (-): 0

Region: chr13 70534511-70534522. Max. coverage (+): 0. Max coverage (-): 0

Region: chr13 70534523-70534533. Max. coverage (+): 0. Max coverage (-): 0

Region: chr13 70534534-70534545. Max. coverage (+): 0. Max coverage (-): 0

Region: chr13 70534546-70534556. Max. coverage (+): 0. Max coverage (-): 0

Region: chr13 70534557-70534568. Max. coverage (+): 0. Max coverage (-): 0

Region: chr13 70534569-70534579. Max. coverage (+): 0. Max coverage (-): 0

Region: chr13 70534580-70534591. Max. coverage (+): 0. Max coverage (-): 0

Region: chr13 70534592-70534602. Max. coverage (+): 0. Max coverage (-): 0

Region: chr13 70534603-70534614. Max. coverage (+): 0. Max coverage (-): 0

Region: chr13 70534615-70534625. Max. coverage (+): 0. Max coverage (-): 0

Region: chr13 70534626-70534637. Max. coverage (+): 0. Max coverage (-): 0

Region: chr13 70534638-70534648. Max. coverage (+): 0. Max coverage (-): 0

Region: chr13 70534649-70534660. Max. coverage (+): 0. Max coverage (-): 0

Region: chr13 70534661-70534671. Max. coverage (+): 0. Max coverage (-): 0

Region: chr13 70534672-70534682. Max. coverage (+): 0. Max coverage (-): 0

Region: chr13 70534683-70534694. Max. coverage (+): 0. Max coverage (-): 0

Region: chr13 70534695-70534705. Max. coverage (+): 0. Max coverage (-): 0

Region: chr13 70534706-70534717. Max. coverage (+): 0. Max coverage (-): 0

Region: chr13 70534718-70534728. Max. coverage (+): 0. Max coverage (-): 0

Region: chr13 70534729-70534740. Max. coverage (+): 0. Max coverage (-): 0

Region: chr13 70534741-70534751. Max. coverage (+): 0. Max coverage (-): 0

Region: chr13 70534752-70534763. Max. coverage (+): 0. Max coverage (-): 0

Region: chr13 70534764-70534774. Max. coverage (+): 0. Max coverage (-): 0

Region: chr13 70534775-70534786. Max. coverage (+): 0. Max coverage (-): 0

Region: chr13 70534787-70534797. Max. coverage (+): 0. Max coverage (-): 0

Region: chr13 70534798-70534809. Max. coverage (+): 0. Max coverage (-): 0

Region: chr13 70534810-70534820. Max. coverage (+): 0. Max coverage (-): 0

Region: chr13 70534821-70534832. Max. coverage (+): 0. Max coverage (-): 0

Region: chr13 70534833-70534843. Max. coverage (+): 0. Max coverage (-): 0

Region: chr13 70534844-70534855. Max. coverage (+): 0. Max coverage (-): 0

Region: chr13 70534856-70534866. Max. coverage (+): 0. Max coverage (-): 0

Region: chr13 70534867-70534878. Max. coverage (+): 0. Max coverage (-): 0

Region: chr13 70534879-70534889. Max. coverage (+): 0. Max coverage (-): 0

Region: chr13 70534890-70534901. Max. coverage (+): 0. Max coverage (-): 0

Region: chr13 70534902-70534912. Max. coverage (+): 0. Max coverage (-): 0

Region: chr13 70534913-70534923. Max. coverage (+): 0. Max coverage (-): 0

Region: chr13 70534924-70534935. Max. coverage (+): 0. Max coverage (-): 0

Region: chr13 70534936-70534946. Max. coverage (+): 0. Max coverage (-): 1.95

Region: chr13 70534947-70534958. Max. coverage (+): 0. Max coverage (-): 0

Region: chr13 70534959-70534969. Max. coverage (+): 0. Max coverage (-): 0

Region: chr13 70534970-70534981. Max. coverage (+): 0. Max coverage (-): 0

Region: chr13 70534982-70534992. Max. coverage (+): 0. Max coverage (-): 0

Region: chr13 70534993-70535004. Max. coverage (+): 0. Max coverage (-): 0.69

Region: chr13 70535005-70535015. Max. coverage (+): 0. Max coverage (-): 0.69

Region: chr13 70535016-70535027. Max. coverage (+): 0. Max coverage (-): 0

Region: chr13 70535028-. Max. coverage (+): 0. Max coverage (-): 0

RepeatMasker Color Code

**+**

100-98% Identity

<98-95% Identity

<95-90% Identity

<90-85% Identity

<85-80% Identity

<80-75% Identity

<75-70% Identity

<70% Identity

**-**

Gene Set Color Code

**+**

Gene

Pseudogene

**-**

Topology/Coverage Color Code

Coverage Plus Strand

Coverage Minus Strand

Mainstrand: Plus

Mainstrand: Minus

Complementary Strand

Flanking Region  
(if option -flank >0)

Gene Set Annotation  
  
RepeatMasker Annotation  
  
Transcription Factor Binding Sites  

**SPZ1** (Sequence: CTGAAACCCT (-): 70530629)  
**RFX4\_2** (Sequence: CCTGGATAC (+): 70534925)
