# Supplementary material for: Small RNA sequencing of cryopreserved semen from single bull revealed altered miRNAs and piRNAs expression between High- and Low-motile sperm populations
Source: BMC Genomics. 2017 Jan 4;18:14. doi: 10.1186/s12864-016-3394-7 (PMC5209821; doi:10.1186/s12864-016-3394-7)
Supplement: Additional file 3: — Details for each piRNA clusters found in High Motile (HM) sperm fraction. Genes, repeats, transposable elements and transcription factors binding sites falling within the cluster regions were reported. (ZIP 1896 kb) [file 12864_2016_3394_MOESM3_ESM.zip › 19.html]

piRNA cluster 19


Predicted piRNA cluster no. 19     previous   next
  

Show proTRAC run info
Hide proTRAC run info

================================= proTRAC ====================================  
VERSION: 2.1                                    LAST MODIFIED: 06. October 2015  
  
Please cite:  
Rosenkranz D, Zischler H. proTRAC - a software for probabilistic piRNA cluster  
detection, visualization and analysis. 2012. BMC Bioinformatics 13:5.  
  
and (for proTRAC 2.0 and later):  
Rosenkranz D, Rudloff S, Bastuck K, Ketting RF, Zischler H. Tupaia small RNAs  
provide insights into function and evolution of RNAi-based transposon defense  
in mammals. 2015. RNA 21(5):911-922.  
  
Contact:  
David Rosenkranz  
Institute of Anthropology, small RNA group  
Johannes Gutenberg University Mainz  
email: rosenkranz@uni-mainz.de  
  
You can find the latest proTRAC version at:  
http://sourceforge.net/projects/protrac/files  
http://www.smallRNAgroup-mainz.de/software  
==============================================================================  
  
PARAMETERS:  
Map file: .............../storage/core/barbara/genhome/smallRNA/fertility/Sample\_motile/pirna/Sample\_motile\_26-33\_collapsed.fa.no-dust.map.weighted-10000-1000-b-0  
Genome file: ............/storage/core/barbara/genhome/smallRNA/fertility/Sample\_all/pirna/bt\_311\_chrY.fa  
RepeatMasker annotation: /storage/genomes/bt\_umd31/GCF\_000003055.6\_Bos\_taurus\_UMD\_3.1.1\_repeatMasker\_chr.out  
GeneSet:................./storage/core/barbara/genhome/smallRNA/fertility/Sample\_all/pirna/full.gtf  
  
Significant (p<=0.01) hit density will be calculated based  
on observed hit distribution.  
  
Sliding window size: ........................................ 5000 bp  
Sliding window increament: .................................. 1000 bp  
Normalize each hit by number of genomic hits: ............... 1 [0=no/1=yes]  
Normalize each hit by number of sequence reads: ............. 1 [0=no/1=yes]  
Normalize values (-> per million mapped reads): ............. 1 [0=no/1=yes]  
Min. fraction of hits with 1T(U) or 10A: .................... 0.75  
Alternatively: Min. fraction of hits with 1T(U) and 10A: .... 0.5  
Min. fraction of hits with typical piRNA length: ............ 0.75  
Typical piRNA length: ....................................... 26-33 nt  
Min. size of a piRNA cluster: ............................... 5000 bp.  
Min. number of hits (absolute): ............................. 0  
Min. number of hits (normalized): ........................... 0  
Min. fraction of hits on the mainstrand: .................... 0.75  
Top fraction of mapped sequences (in terms of read counts): . 1%  
Top fraction accounts for max. n% of sequence reads: ........ 90%  
Min. fraction of hits on each arm of a bidirectional cluster: 0.1  
Output image file for each cluster: ......................... 0 [0=no/1=yes]  
Output html file for each cluster: .......................... 1 [0=no/1=yes]  
Output a summary table: ..................................... 1 [0=no/1=yes]  
Output a FASTA file for each cluster (piRNA sequences): ..... 1 [0=no/1=yes]  
Output a FASTA file comprising cluster sequences: ........... 1 [0=no/1=yes]  
Search DNA motifs in clusters: .............................. 1 [0=no/1=yes]  
Output flanking sequences: +/- .............................. 0 bp  
Output ~.pTi file: .......................................... 1 [0=no/1=yes]  
==============================================================================  
  
  
Genome size (without gaps): ............ 2678902517 bp  
Gaps (N/X/-): .......................... 53837044 bp  
Mapped reads: .......................... 658825247023  
Non-identical sequences: ............... 514171  
Genomic hits: .......................... 764233  
Significant densitiy of mapped reads: .. 12867599.5173724 reads/kb

Show proTRAC cluster info
Hide proTRAC cluster info

|  |  |
| --- | --- |
| Location | chr14 |
| Coordinates | 204367-213582 |
| Size [bp] | 9216 |
| Sequence hit loci | 386 |
| Mapped reads (normalized) | 171695427.8 |
| Mapped reads (normalized) per kb | 18630146.2 |
| Normalized reads with 1T (1U) | 77.5% |
| Normalized reads with 10A | 53.1% |
| Normalized reads with length 26-33 nt | 100% |
| Normalized reads on the main strand(s) | 80.4% |
| Predicted directionality | mono:plus |

100%

0%

1T (1U)  
reads

10A reads

26-33 nt  
reads

reads on mainstrand

**Either the amount of reads with 1T (1U) OR 10A has to exceed 75% (set with option: -1Tor10A)  
Alternatively the amount of reads with 1T (1U) AND 10A has to exceed 50% (set with option: -1Tand10A)  
Minimum amount of reads with preferred size is 75% (set with option: -pisize)  
Minimum amount of reads on the main strand(s) is 75% (set with option: -clstrand)**

Show read coverage
Hide read coverage

WHAT DO I SEE HERE?  
This chart shows the location of mapped sequence reads within a predicted piRNA cluster. The color refers to the number of genomic hits produced by the sequence read in question. A dark red bar indicates that this sequence read produces many other hits elsewhere in the genome. Many adjacent red or yellow bars can indicate the presence of a multi-copy element such as transposons or rRNA genes. A dark green bar indicates that this sequence read maps uniquely to this locus.

1 hit

2-5 hits

6-10 hits

11-20 hits

21-50 hits

51-100 hits

> 100 hits

chr14

204367

213582

Gene Set

RepeatMasker

Mapped  
Reads

27.12

plus strand

minus strand

27.12

Region: chr14 70535029-204376. Max. coverage (+): 0.4. Max coverage (-): 0

Region: chr14 204377-204394. Max. coverage (+): 0. Max coverage (-): 0

Region: chr14 204395-204413. Max. coverage (+): 0.79. Max coverage (-): 0

Region: chr14 204414-204431. Max. coverage (+): 1.87. Max coverage (-): 0

Region: chr14 204432-204449. Max. coverage (+): 0.24. Max coverage (-): 0

Region: chr14 204450-204468. Max. coverage (+): 2.34. Max coverage (-): 0

Region: chr14 204469-204486. Max. coverage (+): 0. Max coverage (-): 0

Region: chr14 204487-204505. Max. coverage (+): 1.38. Max coverage (-): 0

Region: chr14 204506-204523. Max. coverage (+): 0. Max coverage (-): 0

Region: chr14 204524-204542. Max. coverage (+): 0. Max coverage (-): 0

Region: chr14 204543-204560. Max. coverage (+): 0.34. Max coverage (-): 0

Region: chr14 204561-204578. Max. coverage (+): 0. Max coverage (-): 0

Region: chr14 204579-204597. Max. coverage (+): 0. Max coverage (-): 0

Region: chr14 204598-204615. Max. coverage (+): 0. Max coverage (-): 0

Region: chr14 204616-204634. Max. coverage (+): 0. Max coverage (-): 0

Region: chr14 204635-204652. Max. coverage (+): 0. Max coverage (-): 0

Region: chr14 204653-204671. Max. coverage (+): 0. Max coverage (-): 0

Region: chr14 204672-204689. Max. coverage (+): 0. Max coverage (-): 0

Region: chr14 204690-204707. Max. coverage (+): 0. Max coverage (-): 0

Region: chr14 204708-204726. Max. coverage (+): 22.32. Max coverage (-): 0

Region: chr14 204727-204744. Max. coverage (+): 0. Max coverage (-): 0

Region: chr14 204745-204763. Max. coverage (+): 0. Max coverage (-): 0

Region: chr14 204764-204781. Max. coverage (+): 0.07. Max coverage (-): 0

Region: chr14 204782-204800. Max. coverage (+): 0. Max coverage (-): 0

Region: chr14 204801-204818. Max. coverage (+): 0.53. Max coverage (-): 0

Region: chr14 204819-204837. Max. coverage (+): 1.87. Max coverage (-): 0

Region: chr14 204838-204855. Max. coverage (+): 0. Max coverage (-): 0

Region: chr14 204856-204873. Max. coverage (+): 0. Max coverage (-): 0

Region: chr14 204874-204892. Max. coverage (+): 0. Max coverage (-): 0

Region: chr14 204893-204910. Max. coverage (+): 1.59. Max coverage (-): 0

Region: chr14 204911-204929. Max. coverage (+): 0. Max coverage (-): 0

Region: chr14 204930-204947. Max. coverage (+): 1.69. Max coverage (-): 0

Region: chr14 204948-204966. Max. coverage (+): 3.68. Max coverage (-): 0

Region: chr14 204967-204984. Max. coverage (+): 1.8. Max coverage (-): 0

Region: chr14 204985-205002. Max. coverage (+): 4.98. Max coverage (-): 0

Region: chr14 205003-205021. Max. coverage (+): 4.32. Max coverage (-): 0

Region: chr14 205022-205039. Max. coverage (+): 0. Max coverage (-): 0.31

Region: chr14 205040-205058. Max. coverage (+): 0. Max coverage (-): 0

Region: chr14 205059-205076. Max. coverage (+): 0. Max coverage (-): 0

Region: chr14 205077-205095. Max. coverage (+): 0. Max coverage (-): 0

Region: chr14 205096-205113. Max. coverage (+): 0. Max coverage (-): 0

Region: chr14 205114-205131. Max. coverage (+): 0. Max coverage (-): 0

Region: chr14 205132-205150. Max. coverage (+): 0. Max coverage (-): 0

Region: chr14 205151-205168. Max. coverage (+): 0. Max coverage (-): 0.32

Region: chr14 205169-205187. Max. coverage (+): 0. Max coverage (-): 0

Region: chr14 205188-205205. Max. coverage (+): 0. Max coverage (-): 0

Region: chr14 205206-205224. Max. coverage (+): 0. Max coverage (-): 0

Region: chr14 205225-205242. Max. coverage (+): 0. Max coverage (-): 0

Region: chr14 205243-205260. Max. coverage (+): 0. Max coverage (-): 0

Region: chr14 205261-205279. Max. coverage (+): 0. Max coverage (-): 0

Region: chr14 205280-205297. Max. coverage (+): 0. Max coverage (-): 0

Region: chr14 205298-205316. Max. coverage (+): 0. Max coverage (-): 0

Region: chr14 205317-205334. Max. coverage (+): 0. Max coverage (-): 0

Region: chr14 205335-205353. Max. coverage (+): 0. Max coverage (-): 0

Region: chr14 205354-205371. Max. coverage (+): 0. Max coverage (-): 0

Region: chr14 205372-205389. Max. coverage (+): 0. Max coverage (-): 0

Region: chr14 205390-205408. Max. coverage (+): 0. Max coverage (-): 0

Region: chr14 205409-205426. Max. coverage (+): 0. Max coverage (-): 0

Region: chr14 205427-205445. Max. coverage (+): 0. Max coverage (-): 0

Region: chr14 205446-205463. Max. coverage (+): 0. Max coverage (-): 0

Region: chr14 205464-205482. Max. coverage (+): 0. Max coverage (-): 0

Region: chr14 205483-205500. Max. coverage (+): 0. Max coverage (-): 0

Region: chr14 205501-205518. Max. coverage (+): 0. Max coverage (-): 0

Region: chr14 205519-205537. Max. coverage (+): 0. Max coverage (-): 0

Region: chr14 205538-205555. Max. coverage (+): 0.52. Max coverage (-): 0

Region: chr14 205556-205574. Max. coverage (+): 0.52. Max coverage (-): 0

Region: chr14 205575-205592. Max. coverage (+): 0. Max coverage (-): 0

Region: chr14 205593-205611. Max. coverage (+): 0. Max coverage (-): 0

Region: chr14 205612-205629. Max. coverage (+): 1.87. Max coverage (-): 0

Region: chr14 205630-205648. Max. coverage (+): 1.93. Max coverage (-): 0

Region: chr14 205649-205666. Max. coverage (+): 1.93. Max coverage (-): 0

Region: chr14 205667-205684. Max. coverage (+): 2.28. Max coverage (-): 0

Region: chr14 205685-205703. Max. coverage (+): 1.57. Max coverage (-): 0

Region: chr14 205704-205721. Max. coverage (+): 2.85. Max coverage (-): 0

Region: chr14 205722-205740. Max. coverage (+): 0. Max coverage (-): 0

Region: chr14 205741-205758. Max. coverage (+): 0. Max coverage (-): 0

Region: chr14 205759-205777. Max. coverage (+): 0. Max coverage (-): 0

Region: chr14 205778-205795. Max. coverage (+): 0.82. Max coverage (-): 0

Region: chr14 205796-205813. Max. coverage (+): 0.41. Max coverage (-): 0

Region: chr14 205814-205832. Max. coverage (+): 0. Max coverage (-): 0

Region: chr14 205833-205850. Max. coverage (+): 0. Max coverage (-): 0

Region: chr14 205851-205869. Max. coverage (+): 0. Max coverage (-): 0

Region: chr14 205870-205887. Max. coverage (+): 0. Max coverage (-): 0

Region: chr14 205888-205906. Max. coverage (+): 0. Max coverage (-): 0

Region: chr14 205907-205924. Max. coverage (+): 0. Max coverage (-): 0

Region: chr14 205925-205942. Max. coverage (+): 0. Max coverage (-): 0

Region: chr14 205943-205961. Max. coverage (+): 0. Max coverage (-): 0

Region: chr14 205962-205979. Max. coverage (+): 27.12. Max coverage (-): 0

Region: chr14 205980-205998. Max. coverage (+): 0. Max coverage (-): 0

Region: chr14 205999-206016. Max. coverage (+): 0. Max coverage (-): 0

Region: chr14 206017-206035. Max. coverage (+): 0.09. Max coverage (-): 0

Region: chr14 206036-206053. Max. coverage (+): 0. Max coverage (-): 0

Region: chr14 206054-206071. Max. coverage (+): 0.64. Max coverage (-): 0

Region: chr14 206072-206090. Max. coverage (+): 2.28. Max coverage (-): 0

Region: chr14 206091-206108. Max. coverage (+): 0. Max coverage (-): 0

Region: chr14 206109-206127. Max. coverage (+): 0. Max coverage (-): 0

Region: chr14 206128-206145. Max. coverage (+): 0.8. Max coverage (-): 0

Region: chr14 206146-206164. Max. coverage (+): 1.93. Max coverage (-): 0

Region: chr14 206165-206182. Max. coverage (+): 0. Max coverage (-): 0

Region: chr14 206183-206200. Max. coverage (+): 2.05. Max coverage (-): 0

Region: chr14 206201-206219. Max. coverage (+): 4.47. Max coverage (-): 0

Region: chr14 206220-206237. Max. coverage (+): 2.18. Max coverage (-): 0

Region: chr14 206238-206256. Max. coverage (+): 6.05. Max coverage (-): 0

Region: chr14 206257-206274. Max. coverage (+): 0.34. Max coverage (-): 0

Region: chr14 206275-206293. Max. coverage (+): 0. Max coverage (-): 0.38

Region: chr14 206294-206311. Max. coverage (+): 0. Max coverage (-): 0

Region: chr14 206312-206330. Max. coverage (+): 0. Max coverage (-): 0

Region: chr14 206331-206348. Max. coverage (+): 0. Max coverage (-): 0

Region: chr14 206349-206366. Max. coverage (+): 0. Max coverage (-): 0

Region: chr14 206367-206385. Max. coverage (+): 0. Max coverage (-): 0

Region: chr14 206386-206403. Max. coverage (+): 0. Max coverage (-): 0

Region: chr14 206404-206422. Max. coverage (+): 0. Max coverage (-): 0

Region: chr14 206423-206440. Max. coverage (+): 0. Max coverage (-): 0

Region: chr14 206441-206459. Max. coverage (+): 0. Max coverage (-): 0

Region: chr14 206460-206477. Max. coverage (+): 0. Max coverage (-): 0

Region: chr14 206478-206495. Max. coverage (+): 0. Max coverage (-): 0

Region: chr14 206496-206514. Max. coverage (+): 0. Max coverage (-): 0

Region: chr14 206515-206532. Max. coverage (+): 0. Max coverage (-): 0

Region: chr14 206533-206551. Max. coverage (+): 0. Max coverage (-): 0

Region: chr14 206552-206569. Max. coverage (+): 0. Max coverage (-): 0

Region: chr14 206570-206588. Max. coverage (+): 0. Max coverage (-): 0

Region: chr14 206589-206606. Max. coverage (+): 0. Max coverage (-): 0

Region: chr14 206607-206624. Max. coverage (+): 0. Max coverage (-): 0

Region: chr14 206625-206643. Max. coverage (+): 0. Max coverage (-): 0

Region: chr14 206644-206661. Max. coverage (+): 0. Max coverage (-): 0

Region: chr14 206662-206680. Max. coverage (+): 0. Max coverage (-): 0

Region: chr14 206681-206698. Max. coverage (+): 0. Max coverage (-): 0

Region: chr14 206699-206717. Max. coverage (+): 0. Max coverage (-): 0

Region: chr14 206718-206735. Max. coverage (+): 0. Max coverage (-): 0

Region: chr14 206736-206753. Max. coverage (+): 0. Max coverage (-): 0

Region: chr14 206754-206772. Max. coverage (+): 0. Max coverage (-): 0

Region: chr14 206773-206790. Max. coverage (+): 0. Max coverage (-): 0

Region: chr14 206791-206809. Max. coverage (+): 0. Max coverage (-): 0

Region: chr14 206810-206827. Max. coverage (+): 0. Max coverage (-): 0

Region: chr14 206828-206846. Max. coverage (+): 0. Max coverage (-): 0

Region: chr14 206847-206864. Max. coverage (+): 0. Max coverage (-): 0

Region: chr14 206865-206882. Max. coverage (+): 0. Max coverage (-): 0

Region: chr14 206883-206901. Max. coverage (+): 0. Max coverage (-): 0

Region: chr14 206902-206919. Max. coverage (+): 0. Max coverage (-): 0

Region: chr14 206920-206938. Max. coverage (+): 0. Max coverage (-): 0

Region: chr14 206939-206956. Max. coverage (+): 0. Max coverage (-): 0

Region: chr14 206957-206975. Max. coverage (+): 0. Max coverage (-): 0

Region: chr14 206976-206993. Max. coverage (+): 0. Max coverage (-): 0

Region: chr14 206994-207011. Max. coverage (+): 0. Max coverage (-): 0

Region: chr14 207012-207030. Max. coverage (+): 0. Max coverage (-): 0

Region: chr14 207031-207048. Max. coverage (+): 0. Max coverage (-): 0

Region: chr14 207049-207067. Max. coverage (+): 0. Max coverage (-): 0

Region: chr14 207068-207085. Max. coverage (+): 0. Max coverage (-): 0

Region: chr14 207086-207104. Max. coverage (+): 0. Max coverage (-): 0

Region: chr14 207105-207122. Max. coverage (+): 0. Max coverage (-): 0

Region: chr14 207123-207141. Max. coverage (+): 0. Max coverage (-): 0

Region: chr14 207142-207159. Max. coverage (+): 0. Max coverage (-): 0

Region: chr14 207160-207177. Max. coverage (+): 0. Max coverage (-): 0

Region: chr14 207178-207196. Max. coverage (+): 0. Max coverage (-): 0

Region: chr14 207197-207214. Max. coverage (+): 0. Max coverage (-): 0

Region: chr14 207215-207233. Max. coverage (+): 0. Max coverage (-): 0

Region: chr14 207234-207251. Max. coverage (+): 0. Max coverage (-): 0

Region: chr14 207252-207270. Max. coverage (+): 0. Max coverage (-): 0

Region: chr14 207271-207288. Max. coverage (+): 0. Max coverage (-): 0

Region: chr14 207289-207306. Max. coverage (+): 0. Max coverage (-): 0

Region: chr14 207307-207325. Max. coverage (+): 0. Max coverage (-): 0

Region: chr14 207326-207343. Max. coverage (+): 0. Max coverage (-): 0

Region: chr14 207344-207362. Max. coverage (+): 0. Max coverage (-): 0

Region: chr14 207363-207380. Max. coverage (+): 0. Max coverage (-): 0

Region: chr14 207381-207399. Max. coverage (+): 0. Max coverage (-): 0

Region: chr14 207400-207417. Max. coverage (+): 0. Max coverage (-): 0

Region: chr14 207418-207435. Max. coverage (+): 0. Max coverage (-): 0

Region: chr14 207436-207454. Max. coverage (+): 0. Max coverage (-): 0

Region: chr14 207455-207472. Max. coverage (+): 0. Max coverage (-): 0

Region: chr14 207473-207491. Max. coverage (+): 0. Max coverage (-): 0

Region: chr14 207492-207509. Max. coverage (+): 0. Max coverage (-): 0

Region: chr14 207510-207528. Max. coverage (+): 0. Max coverage (-): 0

Region: chr14 207529-207546. Max. coverage (+): 0. Max coverage (-): 0

Region: chr14 207547-207564. Max. coverage (+): 0. Max coverage (-): 0

Region: chr14 207565-207583. Max. coverage (+): 0. Max coverage (-): 0

Region: chr14 207584-207601. Max. coverage (+): 0. Max coverage (-): 0

Region: chr14 207602-207620. Max. coverage (+): 0. Max coverage (-): 0

Region: chr14 207621-207638. Max. coverage (+): 0. Max coverage (-): 0

Region: chr14 207639-207657. Max. coverage (+): 0. Max coverage (-): 0

Region: chr14 207658-207675. Max. coverage (+): 0. Max coverage (-): 0

Region: chr14 207676-207693. Max. coverage (+): 0. Max coverage (-): 0

Region: chr14 207694-207712. Max. coverage (+): 0. Max coverage (-): 0

Region: chr14 207713-207730. Max. coverage (+): 0. Max coverage (-): 0

Region: chr14 207731-207749. Max. coverage (+): 0. Max coverage (-): 0

Region: chr14 207750-207767. Max. coverage (+): 0. Max coverage (-): 0

Region: chr14 207768-207786. Max. coverage (+): 0. Max coverage (-): 0

Region: chr14 207787-207804. Max. coverage (+): 0. Max coverage (-): 0

Region: chr14 207805-207822. Max. coverage (+): 0. Max coverage (-): 0

Region: chr14 207823-207841. Max. coverage (+): 0. Max coverage (-): 0

Region: chr14 207842-207859. Max. coverage (+): 0. Max coverage (-): 0

Region: chr14 207860-207878. Max. coverage (+): 0. Max coverage (-): 0

Region: chr14 207879-207896. Max. coverage (+): 0. Max coverage (-): 0

Region: chr14 207897-207915. Max. coverage (+): 0. Max coverage (-): 0

Region: chr14 207916-207933. Max. coverage (+): 0. Max coverage (-): 0

Region: chr14 207934-207952. Max. coverage (+): 0. Max coverage (-): 0

Region: chr14 207953-207970. Max. coverage (+): 0. Max coverage (-): 0

Region: chr14 207971-207988. Max. coverage (+): 0. Max coverage (-): 0

Region: chr14 207989-208007. Max. coverage (+): 0. Max coverage (-): 0

Region: chr14 208008-208025. Max. coverage (+): 0. Max coverage (-): 0

Region: chr14 208026-208044. Max. coverage (+): 0. Max coverage (-): 0

Region: chr14 208045-208062. Max. coverage (+): 0. Max coverage (-): 0

Region: chr14 208063-208081. Max. coverage (+): 0. Max coverage (-): 0

Region: chr14 208082-208099. Max. coverage (+): 0. Max coverage (-): 0

Region: chr14 208100-208117. Max. coverage (+): 0. Max coverage (-): 0

Region: chr14 208118-208136. Max. coverage (+): 0. Max coverage (-): 0

Region: chr14 208137-208154. Max. coverage (+): 0. Max coverage (-): 0

Region: chr14 208155-208173. Max. coverage (+): 0. Max coverage (-): 0

Region: chr14 208174-208191. Max. coverage (+): 0. Max coverage (-): 0

Region: chr14 208192-208210. Max. coverage (+): 0. Max coverage (-): 0

Region: chr14 208211-208228. Max. coverage (+): 0. Max coverage (-): 0

Region: chr14 208229-208246. Max. coverage (+): 0. Max coverage (-): 0

Region: chr14 208247-208265. Max. coverage (+): 0. Max coverage (-): 0

Region: chr14 208266-208283. Max. coverage (+): 0. Max coverage (-): 0

Region: chr14 208284-208302. Max. coverage (+): 0. Max coverage (-): 0

Region: chr14 208303-208320. Max. coverage (+): 0. Max coverage (-): 0

Region: chr14 208321-208339. Max. coverage (+): 0. Max coverage (-): 0

Region: chr14 208340-208357. Max. coverage (+): 0. Max coverage (-): 0

Region: chr14 208358-208375. Max. coverage (+): 0. Max coverage (-): 0

Region: chr14 208376-208394. Max. coverage (+): 0. Max coverage (-): 0

Region: chr14 208395-208412. Max. coverage (+): 0. Max coverage (-): 0

Region: chr14 208413-208431. Max. coverage (+): 0. Max coverage (-): 0

Region: chr14 208432-208449. Max. coverage (+): 0. Max coverage (-): 0

Region: chr14 208450-208468. Max. coverage (+): 0. Max coverage (-): 0

Region: chr14 208469-208486. Max. coverage (+): 1.53. Max coverage (-): 0

Region: chr14 208487-208504. Max. coverage (+): 0. Max coverage (-): 0

Region: chr14 208505-208523. Max. coverage (+): 0. Max coverage (-): 0

Region: chr14 208524-208541. Max. coverage (+): 0. Max coverage (-): 0

Region: chr14 208542-208560. Max. coverage (+): 0. Max coverage (-): 0

Region: chr14 208561-208578. Max. coverage (+): 1.02. Max coverage (-): 0

Region: chr14 208579-208597. Max. coverage (+): 0. Max coverage (-): 0

Region: chr14 208598-208615. Max. coverage (+): 0. Max coverage (-): 0

Region: chr14 208616-208634. Max. coverage (+): 0. Max coverage (-): 0

Region: chr14 208635-208652. Max. coverage (+): 0. Max coverage (-): 0

Region: chr14 208653-208670. Max. coverage (+): 0. Max coverage (-): 0

Region: chr14 208671-208689. Max. coverage (+): 0. Max coverage (-): 0

Region: chr14 208690-208707. Max. coverage (+): 0. Max coverage (-): 0

Region: chr14 208708-208726. Max. coverage (+): 0. Max coverage (-): 0

Region: chr14 208727-208744. Max. coverage (+): 0. Max coverage (-): 0

Region: chr14 208745-208763. Max. coverage (+): 0. Max coverage (-): 0

Region: chr14 208764-208781. Max. coverage (+): 0. Max coverage (-): 0

Region: chr14 208782-208799. Max. coverage (+): 0. Max coverage (-): 0

Region: chr14 208800-208818. Max. coverage (+): 0. Max coverage (-): 0

Region: chr14 208819-208836. Max. coverage (+): 0. Max coverage (-): 0

Region: chr14 208837-208855. Max. coverage (+): 0. Max coverage (-): 0

Region: chr14 208856-208873. Max. coverage (+): 0. Max coverage (-): 0

Region: chr14 208874-208892. Max. coverage (+): 0. Max coverage (-): 0

Region: chr14 208893-208910. Max. coverage (+): 0. Max coverage (-): 0

Region: chr14 208911-208928. Max. coverage (+): 0. Max coverage (-): 0

Region: chr14 208929-208947. Max. coverage (+): 0. Max coverage (-): 0

Region: chr14 208948-208965. Max. coverage (+): 0. Max coverage (-): 1.21

Region: chr14 208966-208984. Max. coverage (+): 0. Max coverage (-): 4.29

Region: chr14 208985-209002. Max. coverage (+): 0. Max coverage (-): 0

Region: chr14 209003-209021. Max. coverage (+): 0. Max coverage (-): 0

Region: chr14 209022-209039. Max. coverage (+): 0. Max coverage (-): 1.87

Region: chr14 209040-209057. Max. coverage (+): 12.83. Max coverage (-): 1.87

Region: chr14 209058-209076. Max. coverage (+): 1.11. Max coverage (-): 0

Region: chr14 209077-209094. Max. coverage (+): 20.42. Max coverage (-): 0

Region: chr14 209095-209113. Max. coverage (+): 1.31. Max coverage (-): 0

Region: chr14 209114-209131. Max. coverage (+): 0. Max coverage (-): 0

Region: chr14 209132-209150. Max. coverage (+): 0. Max coverage (-): 0

Region: chr14 209151-209168. Max. coverage (+): 0. Max coverage (-): 0

Region: chr14 209169-209186. Max. coverage (+): 0. Max coverage (-): 0

Region: chr14 209187-209205. Max. coverage (+): 0. Max coverage (-): 0

Region: chr14 209206-209223. Max. coverage (+): 0. Max coverage (-): 0

Region: chr14 209224-209242. Max. coverage (+): 0. Max coverage (-): 0

Region: chr14 209243-209260. Max. coverage (+): 0. Max coverage (-): 16.14

Region: chr14 209261-209279. Max. coverage (+): 0. Max coverage (-): 2.41

Region: chr14 209280-209297. Max. coverage (+): 0. Max coverage (-): 0.33

Region: chr14 209298-209315. Max. coverage (+): 0. Max coverage (-): 0.11

Region: chr14 209316-209334. Max. coverage (+): 0. Max coverage (-): 0

Region: chr14 209335-209352. Max. coverage (+): 0. Max coverage (-): 0

Region: chr14 209353-209371. Max. coverage (+): 0. Max coverage (-): 0

Region: chr14 209372-209389. Max. coverage (+): 0. Max coverage (-): 0

Region: chr14 209390-209408. Max. coverage (+): 0. Max coverage (-): 0

Region: chr14 209409-209426. Max. coverage (+): 0. Max coverage (-): 0

Region: chr14 209427-209445. Max. coverage (+): 0. Max coverage (-): 0

Region: chr14 209446-209463. Max. coverage (+): 0. Max coverage (-): 0

Region: chr14 209464-209481. Max. coverage (+): 0. Max coverage (-): 0

Region: chr14 209482-209500. Max. coverage (+): 0. Max coverage (-): 0

Region: chr14 209501-209518. Max. coverage (+): 0. Max coverage (-): 0

Region: chr14 209519-209537. Max. coverage (+): 0. Max coverage (-): 0

Region: chr14 209538-209555. Max. coverage (+): 0. Max coverage (-): 0

Region: chr14 209556-209574. Max. coverage (+): 0. Max coverage (-): 0

Region: chr14 209575-209592. Max. coverage (+): 0.2. Max coverage (-): 0

Region: chr14 209593-209610. Max. coverage (+): 0.2. Max coverage (-): 0

Region: chr14 209611-209629. Max. coverage (+): 0. Max coverage (-): 0

Region: chr14 209630-209647. Max. coverage (+): 11.06. Max coverage (-): 0

Region: chr14 209648-209666. Max. coverage (+): 0. Max coverage (-): 0

Region: chr14 209667-209684. Max. coverage (+): 0. Max coverage (-): 0

Region: chr14 209685-209703. Max. coverage (+): 0. Max coverage (-): 0

Region: chr14 209704-209721. Max. coverage (+): 0. Max coverage (-): 0

Region: chr14 209722-209739. Max. coverage (+): 6.32. Max coverage (-): 0

Region: chr14 209740-209758. Max. coverage (+): 1.34. Max coverage (-): 0

Region: chr14 209759-209776. Max. coverage (+): 2.33. Max coverage (-): 0

Region: chr14 209777-209795. Max. coverage (+): 0. Max coverage (-): 0

Region: chr14 209796-209813. Max. coverage (+): 0. Max coverage (-): 0

Region: chr14 209814-209832. Max. coverage (+): 0. Max coverage (-): 0

Region: chr14 209833-209850. Max. coverage (+): 0. Max coverage (-): 0

Region: chr14 209851-209868. Max. coverage (+): 0. Max coverage (-): 0

Region: chr14 209869-209887. Max. coverage (+): 0. Max coverage (-): 0

Region: chr14 209888-209905. Max. coverage (+): 0. Max coverage (-): 0

Region: chr14 209906-209924. Max. coverage (+): 0. Max coverage (-): 0

Region: chr14 209925-209942. Max. coverage (+): 0. Max coverage (-): 0

Region: chr14 209943-209961. Max. coverage (+): 0. Max coverage (-): 0

Region: chr14 209962-209979. Max. coverage (+): 0. Max coverage (-): 0

Region: chr14 209980-209997. Max. coverage (+): 0. Max coverage (-): 0

Region: chr14 209998-210016. Max. coverage (+): 0. Max coverage (-): 0

Region: chr14 210017-210034. Max. coverage (+): 0. Max coverage (-): 0

Region: chr14 210035-210053. Max. coverage (+): 0. Max coverage (-): 0

Region: chr14 210054-210071. Max. coverage (+): 0. Max coverage (-): 0

Region: chr14 210072-210090. Max. coverage (+): 0. Max coverage (-): 0

Region: chr14 210091-210108. Max. coverage (+): 0. Max coverage (-): 0

Region: chr14 210109-210126. Max. coverage (+): 0. Max coverage (-): 0

Region: chr14 210127-210145. Max. coverage (+): 0. Max coverage (-): 0

Region: chr14 210146-210163. Max. coverage (+): 0. Max coverage (-): 0

Region: chr14 210164-210182. Max. coverage (+): 0. Max coverage (-): 0

Region: chr14 210183-210200. Max. coverage (+): 0. Max coverage (-): 0

Region: chr14 210201-210219. Max. coverage (+): 0. Max coverage (-): 0

Region: chr14 210220-210237. Max. coverage (+): 0. Max coverage (-): 0

Region: chr14 210238-210256. Max. coverage (+): 0. Max coverage (-): 0

Region: chr14 210257-210274. Max. coverage (+): 0. Max coverage (-): 0

Region: chr14 210275-210292. Max. coverage (+): 0.55. Max coverage (-): 0

Region: chr14 210293-210311. Max. coverage (+): 0.55. Max coverage (-): 0

Region: chr14 210312-210329. Max. coverage (+): 3.8. Max coverage (-): 0

Region: chr14 210330-210348. Max. coverage (+): 0. Max coverage (-): 0

Region: chr14 210349-210366. Max. coverage (+): 0. Max coverage (-): 0

Region: chr14 210367-210385. Max. coverage (+): 0. Max coverage (-): 0

Region: chr14 210386-210403. Max. coverage (+): 0. Max coverage (-): 0

Region: chr14 210404-210421. Max. coverage (+): 0. Max coverage (-): 0

Region: chr14 210422-210440. Max. coverage (+): 0. Max coverage (-): 0

Region: chr14 210441-210458. Max. coverage (+): 0. Max coverage (-): 0

Region: chr14 210459-210477. Max. coverage (+): 0. Max coverage (-): 0

Region: chr14 210478-210495. Max. coverage (+): 0. Max coverage (-): 0

Region: chr14 210496-210514. Max. coverage (+): 0. Max coverage (-): 0

Region: chr14 210515-210532. Max. coverage (+): 0. Max coverage (-): 0

Region: chr14 210533-210550. Max. coverage (+): 0. Max coverage (-): 0

Region: chr14 210551-210569. Max. coverage (+): 0. Max coverage (-): 0

Region: chr14 210570-210587. Max. coverage (+): 0. Max coverage (-): 0

Region: chr14 210588-210606. Max. coverage (+): 0. Max coverage (-): 0

Region: chr14 210607-210624. Max. coverage (+): 0. Max coverage (-): 0

Region: chr14 210625-210643. Max. coverage (+): 0. Max coverage (-): 0

Region: chr14 210644-210661. Max. coverage (+): 0. Max coverage (-): 0

Region: chr14 210662-210679. Max. coverage (+): 0. Max coverage (-): 0

Region: chr14 210680-210698. Max. coverage (+): 0. Max coverage (-): 0

Region: chr14 210699-210716. Max. coverage (+): 0. Max coverage (-): 0

Region: chr14 210717-210735. Max. coverage (+): 0. Max coverage (-): 0.34

Region: chr14 210736-210753. Max. coverage (+): 0. Max coverage (-): 0.34

Region: chr14 210754-210772. Max. coverage (+): 0. Max coverage (-): 0

Region: chr14 210773-210790. Max. coverage (+): 0. Max coverage (-): 0

Region: chr14 210791-210808. Max. coverage (+): 0. Max coverage (-): 0

Region: chr14 210809-210827. Max. coverage (+): 0. Max coverage (-): 0

Region: chr14 210828-210845. Max. coverage (+): 0. Max coverage (-): 0

Region: chr14 210846-210864. Max. coverage (+): 0. Max coverage (-): 0

Region: chr14 210865-210882. Max. coverage (+): 0. Max coverage (-): 0

Region: chr14 210883-210901. Max. coverage (+): 0. Max coverage (-): 0

Region: chr14 210902-210919. Max. coverage (+): 0. Max coverage (-): 0

Region: chr14 210920-210938. Max. coverage (+): 0. Max coverage (-): 0.36

Region: chr14 210939-210956. Max. coverage (+): 0. Max coverage (-): 0

Region: chr14 210957-210974. Max. coverage (+): 0. Max coverage (-): 0

Region: chr14 210975-210993. Max. coverage (+): 0.09. Max coverage (-): 0

Region: chr14 210994-211011. Max. coverage (+): 0. Max coverage (-): 0

Region: chr14 211012-211030. Max. coverage (+): 0. Max coverage (-): 0

Region: chr14 211031-211048. Max. coverage (+): 0. Max coverage (-): 0

Region: chr14 211049-211067. Max. coverage (+): 0. Max coverage (-): 0

Region: chr14 211068-211085. Max. coverage (+): 0. Max coverage (-): 0

Region: chr14 211086-211103. Max. coverage (+): 0. Max coverage (-): 0

Region: chr14 211104-211122. Max. coverage (+): 0. Max coverage (-): 0

Region: chr14 211123-211140. Max. coverage (+): 0. Max coverage (-): 0

Region: chr14 211141-211159. Max. coverage (+): 0. Max coverage (-): 0

Region: chr14 211160-211177. Max. coverage (+): 0. Max coverage (-): 0

Region: chr14 211178-211196. Max. coverage (+): 0. Max coverage (-): 0

Region: chr14 211197-211214. Max. coverage (+): 0. Max coverage (-): 0

Region: chr14 211215-211232. Max. coverage (+): 0. Max coverage (-): 0

Region: chr14 211233-211251. Max. coverage (+): 0. Max coverage (-): 0

Region: chr14 211252-211269. Max. coverage (+): 0. Max coverage (-): 0

Region: chr14 211270-211288. Max. coverage (+): 0. Max coverage (-): 0

Region: chr14 211289-211306. Max. coverage (+): 0. Max coverage (-): 0

Region: chr14 211307-211325. Max. coverage (+): 0. Max coverage (-): 0

Region: chr14 211326-211343. Max. coverage (+): 0. Max coverage (-): 0.35

Region: chr14 211344-211361. Max. coverage (+): 0.4. Max coverage (-): 0

Region: chr14 211362-211380. Max. coverage (+): 0.22. Max coverage (-): 0

Region: chr14 211381-211398. Max. coverage (+): 0.1. Max coverage (-): 0

Region: chr14 211399-211417. Max. coverage (+): 0. Max coverage (-): 0

Region: chr14 211418-211435. Max. coverage (+): 0. Max coverage (-): 0

Region: chr14 211436-211454. Max. coverage (+): 0. Max coverage (-): 0

Region: chr14 211455-211472. Max. coverage (+): 0. Max coverage (-): 0

Region: chr14 211473-211490. Max. coverage (+): 0. Max coverage (-): 0

Region: chr14 211491-211509. Max. coverage (+): 0. Max coverage (-): 0

Region: chr14 211510-211527. Max. coverage (+): 0. Max coverage (-): 0

Region: chr14 211528-211546. Max. coverage (+): 0. Max coverage (-): 0

Region: chr14 211547-211564. Max. coverage (+): 0. Max coverage (-): 0

Region: chr14 211565-211583. Max. coverage (+): 0. Max coverage (-): 0

Region: chr14 211584-211601. Max. coverage (+): 0. Max coverage (-): 0

Region: chr14 211602-211619. Max. coverage (+): 0. Max coverage (-): 0

Region: chr14 211620-211638. Max. coverage (+): 0. Max coverage (-): 0

Region: chr14 211639-211656. Max. coverage (+): 0. Max coverage (-): 0

Region: chr14 211657-211675. Max. coverage (+): 0. Max coverage (-): 0

Region: chr14 211676-211693. Max. coverage (+): 0. Max coverage (-): 0

Region: chr14 211694-211712. Max. coverage (+): 0. Max coverage (-): 0

Region: chr14 211713-211730. Max. coverage (+): 0. Max coverage (-): 0

Region: chr14 211731-211749. Max. coverage (+): 0. Max coverage (-): 0

Region: chr14 211750-211767. Max. coverage (+): 0. Max coverage (-): 0

Region: chr14 211768-211785. Max. coverage (+): 0. Max coverage (-): 0

Region: chr14 211786-211804. Max. coverage (+): 0. Max coverage (-): 0

Region: chr14 211805-211822. Max. coverage (+): 0. Max coverage (-): 0

Region: chr14 211823-211841. Max. coverage (+): 0. Max coverage (-): 0

Region: chr14 211842-211859. Max. coverage (+): 0. Max coverage (-): 0

Region: chr14 211860-211878. Max. coverage (+): 0. Max coverage (-): 0

Region: chr14 211879-211896. Max. coverage (+): 0. Max coverage (-): 0

Region: chr14 211897-211914. Max. coverage (+): 0. Max coverage (-): 0

Region: chr14 211915-211933. Max. coverage (+): 0. Max coverage (-): 0

Region: chr14 211934-211951. Max. coverage (+): 0. Max coverage (-): 0

Region: chr14 211952-211970. Max. coverage (+): 0. Max coverage (-): 0

Region: chr14 211971-211988. Max. coverage (+): 0. Max coverage (-): 0

Region: chr14 211989-212007. Max. coverage (+): 0. Max coverage (-): 0

Region: chr14 212008-212025. Max. coverage (+): 0. Max coverage (-): 0

Region: chr14 212026-212043. Max. coverage (+): 0. Max coverage (-): 0

Region: chr14 212044-212062. Max. coverage (+): 0. Max coverage (-): 0

Region: chr14 212063-212080. Max. coverage (+): 0. Max coverage (-): 0

Region: chr14 212081-212099. Max. coverage (+): 0. Max coverage (-): 0

Region: chr14 212100-212117. Max. coverage (+): 0. Max coverage (-): 0

Region: chr14 212118-212136. Max. coverage (+): 0. Max coverage (-): 0

Region: chr14 212137-212154. Max. coverage (+): 0. Max coverage (-): 0

Region: chr14 212155-212172. Max. coverage (+): 0. Max coverage (-): 0

Region: chr14 212173-212191. Max. coverage (+): 0. Max coverage (-): 0

Region: chr14 212192-212209. Max. coverage (+): 0. Max coverage (-): 0

Region: chr14 212210-212228. Max. coverage (+): 0. Max coverage (-): 0

Region: chr14 212229-212246. Max. coverage (+): 0. Max coverage (-): 0

Region: chr14 212247-212265. Max. coverage (+): 0. Max coverage (-): 0

Region: chr14 212266-212283. Max. coverage (+): 1. Max coverage (-): 0

Region: chr14 212284-212301. Max. coverage (+): 0. Max coverage (-): 0

Region: chr14 212302-212320. Max. coverage (+): 0. Max coverage (-): 0

Region: chr14 212321-212338. Max. coverage (+): 0.76. Max coverage (-): 0

Region: chr14 212339-212357. Max. coverage (+): 4.95. Max coverage (-): 0

Region: chr14 212358-212375. Max. coverage (+): 3.26. Max coverage (-): 0

Region: chr14 212376-212394. Max. coverage (+): 0. Max coverage (-): 0

Region: chr14 212395-212412. Max. coverage (+): 0. Max coverage (-): 0

Region: chr14 212413-212430. Max. coverage (+): 4.41. Max coverage (-): 0

Region: chr14 212431-212449. Max. coverage (+): 0. Max coverage (-): 0

Region: chr14 212450-212467. Max. coverage (+): 0. Max coverage (-): 0

Region: chr14 212468-212486. Max. coverage (+): 0. Max coverage (-): 0

Region: chr14 212487-212504. Max. coverage (+): 0. Max coverage (-): 0

Region: chr14 212505-212523. Max. coverage (+): 0. Max coverage (-): 0

Region: chr14 212524-212541. Max. coverage (+): 0. Max coverage (-): 0.95

Region: chr14 212542-212560. Max. coverage (+): 0. Max coverage (-): 0

Region: chr14 212561-212578. Max. coverage (+): 0.63. Max coverage (-): 0

Region: chr14 212579-212596. Max. coverage (+): 0.63. Max coverage (-): 0

Region: chr14 212597-212615. Max. coverage (+): 0. Max coverage (-): 0.66

Region: chr14 212616-212633. Max. coverage (+): 0. Max coverage (-): 0

Region: chr14 212634-212652. Max. coverage (+): 0. Max coverage (-): 0

Region: chr14 212653-212670. Max. coverage (+): 0. Max coverage (-): 0

Region: chr14 212671-212689. Max. coverage (+): 0. Max coverage (-): 0.27

Region: chr14 212690-212707. Max. coverage (+): 1.17. Max coverage (-): 0

Region: chr14 212708-212725. Max. coverage (+): 1.17. Max coverage (-): 0

Region: chr14 212726-212744. Max. coverage (+): 0. Max coverage (-): 0

Region: chr14 212745-212762. Max. coverage (+): 0. Max coverage (-): 0

Region: chr14 212763-212781. Max. coverage (+): 0. Max coverage (-): 0.25

Region: chr14 212782-212799. Max. coverage (+): 1.21. Max coverage (-): 0

Region: chr14 212800-212818. Max. coverage (+): 0. Max coverage (-): 0

Region: chr14 212819-212836. Max. coverage (+): 0. Max coverage (-): 0

Region: chr14 212837-212854. Max. coverage (+): 0. Max coverage (-): 0

Region: chr14 212855-212873. Max. coverage (+): 0. Max coverage (-): 0

Region: chr14 212874-212891. Max. coverage (+): 0. Max coverage (-): 0

Region: chr14 212892-212910. Max. coverage (+): 0. Max coverage (-): 0

Region: chr14 212911-212928. Max. coverage (+): 0. Max coverage (-): 1.76

Region: chr14 212929-212947. Max. coverage (+): 0. Max coverage (-): 2.26

Region: chr14 212948-212965. Max. coverage (+): 0. Max coverage (-): 2.26

Region: chr14 212966-212983. Max. coverage (+): 0. Max coverage (-): 0

Region: chr14 212984-213002. Max. coverage (+): 0. Max coverage (-): 0

Region: chr14 213003-213020. Max. coverage (+): 0. Max coverage (-): 0

Region: chr14 213021-213039. Max. coverage (+): 0. Max coverage (-): 0

Region: chr14 213040-213057. Max. coverage (+): 0. Max coverage (-): 0

Region: chr14 213058-213076. Max. coverage (+): 0. Max coverage (-): 0

Region: chr14 213077-213094. Max. coverage (+): 0. Max coverage (-): 6.63

Region: chr14 213095-213112. Max. coverage (+): 0. Max coverage (-): 12.79

Region: chr14 213113-213131. Max. coverage (+): 0. Max coverage (-): 0

Region: chr14 213132-213149. Max. coverage (+): 0.33. Max coverage (-): 0

Region: chr14 213150-213168. Max. coverage (+): 0. Max coverage (-): 0

Region: chr14 213169-213186. Max. coverage (+): 0. Max coverage (-): 0

Region: chr14 213187-213205. Max. coverage (+): 0. Max coverage (-): 0

Region: chr14 213206-213223. Max. coverage (+): 0. Max coverage (-): 0

Region: chr14 213224-213242. Max. coverage (+): 0. Max coverage (-): 0

Region: chr14 213243-213260. Max. coverage (+): 0. Max coverage (-): 0

Region: chr14 213261-213278. Max. coverage (+): 0. Max coverage (-): 0

Region: chr14 213279-213297. Max. coverage (+): 7.54. Max coverage (-): 0

Region: chr14 213298-213315. Max. coverage (+): 0. Max coverage (-): 0

Region: chr14 213316-213334. Max. coverage (+): 0. Max coverage (-): 0

Region: chr14 213335-213352. Max. coverage (+): 4.29. Max coverage (-): 0

Region: chr14 213353-213371. Max. coverage (+): 0. Max coverage (-): 0

Region: chr14 213372-213389. Max. coverage (+): 0.94. Max coverage (-): 0

Region: chr14 213390-213407. Max. coverage (+): 0. Max coverage (-): 0

Region: chr14 213408-213426. Max. coverage (+): 6.87. Max coverage (-): 0

Region: chr14 213427-213444. Max. coverage (+): 0. Max coverage (-): 0

Region: chr14 213445-213463. Max. coverage (+): 0. Max coverage (-): 0

Region: chr14 213464-213481. Max. coverage (+): 0. Max coverage (-): 0

Region: chr14 213482-213500. Max. coverage (+): 0. Max coverage (-): 0

Region: chr14 213501-213518. Max. coverage (+): 0. Max coverage (-): 0

Region: chr14 213519-213536. Max. coverage (+): 0. Max coverage (-): 0

Region: chr14 213537-213555. Max. coverage (+): 0. Max coverage (-): 0

Region: chr14 213556-213573. Max. coverage (+): 0.31. Max coverage (-): 0.73

Region: chr14 213574-. Max. coverage (+): 0. Max coverage (-): 0

RepeatMasker Color Code

**+**

100-98% Identity

<98-95% Identity

<95-90% Identity

<90-85% Identity

<85-80% Identity

<80-75% Identity

<75-70% Identity

<70% Identity

**-**

Gene Set Color Code

**+**

Gene

Pseudogene

**-**

Topology/Coverage Color Code

Coverage Plus Strand

Coverage Minus Strand

Mainstrand: Plus

Mainstrand: Minus

Complementary Strand

Flanking Region  
(if option -flank >0)

Gene Set Annotation  

**1. 5S\_rRNA (protein coding, ENSBTAG00000046736) Tr:00000064110 Ex:1**: 208693-208812 (-)

  
RepeatMasker Annotation  

**1. G-rich**: 205578-205641 (+), Divergence to consensus: 28.1%  
**2. L1-3\_BT**: 206520-206577 (-), Divergence to consensus: 20.7%  
**3. LTR32\_BT**: 207529-207609 (-), Divergence to consensus: 23.1%  
**4. CHR-2A**: 207610-207696 (+), Divergence to consensus: 25.5%  
**5. L1-3\_BT**: 208044-208303 (+), Divergence to consensus: 31.9%  
**6. 5S**: 208691-208810 (-), Divergence to consensus: 10%  
**7. SINE2-2\_BT**: 209357-209467 (-), Divergence to consensus: 30.9%  
**8. Bov-tA2**: 209938-210132 (-), Divergence to consensus: 20.1%  
**9. (CGTG)n**: 210801-210823 (+), Divergence to consensus: 4.3%  
**10. (CA)n**: 211410-211449 (+), Divergence to consensus: 0%  
**11. Bov-tA3**: 211483-211666 (-), Divergence to consensus: 20.9%

  
Transcription Factor Binding Sites  

**RFX4\_2** (Sequence: GTATCTATG (-): 210329)  
**RFX4\_2** (Sequence: GTATCCATG (-): 213490)  
**SOX9** (Sequence: AACAATGA (-): 204975)  
**SOX9** (Sequence: AACAATGA (-): 206225)  
**SOX9** (Sequence: AACAATGA (-): 213387)  
**SPZ1** (Sequence: AGGGTTTGAG (+): 204368)  
**SPZ1** (Sequence: AGGGTTTGAG (+): 205620)
