# Supplementary material for: Small RNA sequencing of cryopreserved semen from single bull revealed altered miRNAs and piRNAs expression between High- and Low-motile sperm populations
Source: BMC Genomics. 2017 Jan 4;18:14. doi: 10.1186/s12864-016-3394-7 (PMC5209821; doi:10.1186/s12864-016-3394-7)
Supplement: Additional file 3: — Details for each piRNA clusters found in High Motile (HM) sperm fraction. Genes, repeats, transposable elements and transcription factors binding sites falling within the cluster regions were reported. (ZIP 1896 kb) [file 12864_2016_3394_MOESM3_ESM.zip › 2.html]

piRNA cluster 2


Predicted piRNA cluster no. 2     previous   next
  

Show proTRAC run info
Hide proTRAC run info

================================= proTRAC ====================================  
VERSION: 2.1                                    LAST MODIFIED: 06. October 2015  
  
Please cite:  
Rosenkranz D, Zischler H. proTRAC - a software for probabilistic piRNA cluster  
detection, visualization and analysis. 2012. BMC Bioinformatics 13:5.  
  
and (for proTRAC 2.0 and later):  
Rosenkranz D, Rudloff S, Bastuck K, Ketting RF, Zischler H. Tupaia small RNAs  
provide insights into function and evolution of RNAi-based transposon defense  
in mammals. 2015. RNA 21(5):911-922.  
  
Contact:  
David Rosenkranz  
Institute of Anthropology, small RNA group  
Johannes Gutenberg University Mainz  
email: rosenkranz@uni-mainz.de  
  
You can find the latest proTRAC version at:  
http://sourceforge.net/projects/protrac/files  
http://www.smallRNAgroup-mainz.de/software  
==============================================================================  
  
PARAMETERS:  
Map file: .............../storage/core/barbara/genhome/smallRNA/fertility/Sample\_motile/pirna/Sample\_motile\_26-33\_collapsed.fa.no-dust.map.weighted-10000-1000-b-0  
Genome file: ............/storage/core/barbara/genhome/smallRNA/fertility/Sample\_all/pirna/bt\_311\_chrY.fa  
RepeatMasker annotation: /storage/genomes/bt\_umd31/GCF\_000003055.6\_Bos\_taurus\_UMD\_3.1.1\_repeatMasker\_chr.out  
GeneSet:................./storage/core/barbara/genhome/smallRNA/fertility/Sample\_all/pirna/full.gtf  
  
Significant (p<=0.01) hit density will be calculated based  
on observed hit distribution.  
  
Sliding window size: ........................................ 5000 bp  
Sliding window increament: .................................. 1000 bp  
Normalize each hit by number of genomic hits: ............... 1 [0=no/1=yes]  
Normalize each hit by number of sequence reads: ............. 1 [0=no/1=yes]  
Normalize values (-> per million mapped reads): ............. 1 [0=no/1=yes]  
Min. fraction of hits with 1T(U) or 10A: .................... 0.75  
Alternatively: Min. fraction of hits with 1T(U) and 10A: .... 0.5  
Min. fraction of hits with typical piRNA length: ............ 0.75  
Typical piRNA length: ....................................... 26-33 nt  
Min. size of a piRNA cluster: ............................... 5000 bp.  
Min. number of hits (absolute): ............................. 0  
Min. number of hits (normalized): ........................... 0  
Min. fraction of hits on the mainstrand: .................... 0.75  
Top fraction of mapped sequences (in terms of read counts): . 1%  
Top fraction accounts for max. n% of sequence reads: ........ 90%  
Min. fraction of hits on each arm of a bidirectional cluster: 0.1  
Output image file for each cluster: ......................... 0 [0=no/1=yes]  
Output html file for each cluster: .......................... 1 [0=no/1=yes]  
Output a summary table: ..................................... 1 [0=no/1=yes]  
Output a FASTA file for each cluster (piRNA sequences): ..... 1 [0=no/1=yes]  
Output a FASTA file comprising cluster sequences: ........... 1 [0=no/1=yes]  
Search DNA motifs in clusters: .............................. 1 [0=no/1=yes]  
Output flanking sequences: +/- .............................. 0 bp  
Output ~.pTi file: .......................................... 1 [0=no/1=yes]  
==============================================================================  
  
  
Genome size (without gaps): ............ 2678902517 bp  
Gaps (N/X/-): .......................... 53837044 bp  
Mapped reads: .......................... 658825247023  
Non-identical sequences: ............... 514171  
Genomic hits: .......................... 764233  
Significant densitiy of mapped reads: .. 12867599.5173724 reads/kb

Show proTRAC cluster info
Hide proTRAC cluster info

|  |  |
| --- | --- |
| Location | chr10 |
| Coordinates | 36886496-36892767 |
| Size [bp] | 6272 |
| Sequence hit loci | 188 |
| Mapped reads (normalized) | 231435071.5 |
| Mapped reads (normalized) per kb | 36899724.4 |
| Normalized reads with 1T (1U) | 79.4% |
| Normalized reads with 10A | 34.4% |
| Normalized reads with length 26-33 nt | 100% |
| Normalized reads on the main strand(s) | 100% |
| Predicted directionality | mono:plus |

100%

0%

1T (1U)  
reads

10A reads

26-33 nt  
reads

reads on mainstrand

**Either the amount of reads with 1T (1U) OR 10A has to exceed 75% (set with option: -1Tor10A)  
Alternatively the amount of reads with 1T (1U) AND 10A has to exceed 50% (set with option: -1Tand10A)  
Minimum amount of reads with preferred size is 75% (set with option: -pisize)  
Minimum amount of reads on the main strand(s) is 75% (set with option: -clstrand)**

Show read coverage
Hide read coverage

WHAT DO I SEE HERE?  
This chart shows the location of mapped sequence reads within a predicted piRNA cluster. The color refers to the number of genomic hits produced by the sequence read in question. A dark red bar indicates that this sequence read produces many other hits elsewhere in the genome. Many adjacent red or yellow bars can indicate the presence of a multi-copy element such as transposons or rRNA genes. A dark green bar indicates that this sequence read maps uniquely to this locus.

1 hit

2-5 hits

6-10 hits

11-20 hits

21-50 hits

51-100 hits

> 100 hits

chr10

36886496

36892767

Gene Set

RepeatMasker

Mapped  
Reads

22.34

plus strand

minus strand

22.34

Region: chr10 80736818-36886502. Max. coverage (+): 1.91. Max coverage (-): 0

Region: chr10 36886503-36886514. Max. coverage (+): 1.91. Max coverage (-): 0

Region: chr10 36886515-36886527. Max. coverage (+): 0. Max coverage (-): 0

Region: chr10 36886528-36886539. Max. coverage (+): 0. Max coverage (-): 0

Region: chr10 36886540-36886552. Max. coverage (+): 0. Max coverage (-): 0

Region: chr10 36886553-36886564. Max. coverage (+): 0. Max coverage (-): 0

Region: chr10 36886565-36886577. Max. coverage (+): 0. Max coverage (-): 0

Region: chr10 36886578-36886590. Max. coverage (+): 0. Max coverage (-): 0

Region: chr10 36886591-36886602. Max. coverage (+): 0. Max coverage (-): 0

Region: chr10 36886603-36886615. Max. coverage (+): 0. Max coverage (-): 0

Region: chr10 36886616-36886627. Max. coverage (+): 0. Max coverage (-): 0

Region: chr10 36886628-36886640. Max. coverage (+): 0. Max coverage (-): 0

Region: chr10 36886641-36886652. Max. coverage (+): 2.2. Max coverage (-): 0

Region: chr10 36886653-36886665. Max. coverage (+): 2.2. Max coverage (-): 0

Region: chr10 36886666-36886677. Max. coverage (+): 4.6. Max coverage (-): 0

Region: chr10 36886678-36886690. Max. coverage (+): 0. Max coverage (-): 0

Region: chr10 36886691-36886702. Max. coverage (+): 0. Max coverage (-): 0

Region: chr10 36886703-36886715. Max. coverage (+): 0. Max coverage (-): 0

Region: chr10 36886716-36886728. Max. coverage (+): 0. Max coverage (-): 0

Region: chr10 36886729-36886740. Max. coverage (+): 0. Max coverage (-): 0

Region: chr10 36886741-36886753. Max. coverage (+): 0. Max coverage (-): 0

Region: chr10 36886754-36886765. Max. coverage (+): 0. Max coverage (-): 0

Region: chr10 36886766-36886778. Max. coverage (+): 0. Max coverage (-): 0

Region: chr10 36886779-36886790. Max. coverage (+): 0. Max coverage (-): 0

Region: chr10 36886791-36886803. Max. coverage (+): 0. Max coverage (-): 0

Region: chr10 36886804-36886815. Max. coverage (+): 0. Max coverage (-): 0

Region: chr10 36886816-36886828. Max. coverage (+): 0. Max coverage (-): 0

Region: chr10 36886829-36886840. Max. coverage (+): 0. Max coverage (-): 0

Region: chr10 36886841-36886853. Max. coverage (+): 0. Max coverage (-): 0

Region: chr10 36886854-36886866. Max. coverage (+): 0. Max coverage (-): 0

Region: chr10 36886867-36886878. Max. coverage (+): 0. Max coverage (-): 0

Region: chr10 36886879-36886891. Max. coverage (+): 0. Max coverage (-): 0

Region: chr10 36886892-36886903. Max. coverage (+): 0. Max coverage (-): 0

Region: chr10 36886904-36886916. Max. coverage (+): 0. Max coverage (-): 0

Region: chr10 36886917-36886928. Max. coverage (+): 0. Max coverage (-): 0

Region: chr10 36886929-36886941. Max. coverage (+): 0. Max coverage (-): 0

Region: chr10 36886942-36886953. Max. coverage (+): 0. Max coverage (-): 0

Region: chr10 36886954-36886966. Max. coverage (+): 0. Max coverage (-): 0

Region: chr10 36886967-36886978. Max. coverage (+): 0. Max coverage (-): 0

Region: chr10 36886979-36886991. Max. coverage (+): 0. Max coverage (-): 0

Region: chr10 36886992-36887004. Max. coverage (+): 0. Max coverage (-): 0

Region: chr10 36887005-36887016. Max. coverage (+): 0. Max coverage (-): 0

Region: chr10 36887017-36887029. Max. coverage (+): 0. Max coverage (-): 0

Region: chr10 36887030-36887041. Max. coverage (+): 0. Max coverage (-): 0

Region: chr10 36887042-36887054. Max. coverage (+): 0. Max coverage (-): 0

Region: chr10 36887055-36887066. Max. coverage (+): 0. Max coverage (-): 0

Region: chr10 36887067-36887079. Max. coverage (+): 0. Max coverage (-): 0

Region: chr10 36887080-36887091. Max. coverage (+): 0. Max coverage (-): 0

Region: chr10 36887092-36887104. Max. coverage (+): 0. Max coverage (-): 0

Region: chr10 36887105-36887116. Max. coverage (+): 0. Max coverage (-): 0

Region: chr10 36887117-36887129. Max. coverage (+): 0. Max coverage (-): 0

Region: chr10 36887130-36887142. Max. coverage (+): 0. Max coverage (-): 0

Region: chr10 36887143-36887154. Max. coverage (+): 4.67. Max coverage (-): 0

Region: chr10 36887155-36887167. Max. coverage (+): 4.67. Max coverage (-): 0

Region: chr10 36887168-36887179. Max. coverage (+): 0. Max coverage (-): 0

Region: chr10 36887180-36887192. Max. coverage (+): 0. Max coverage (-): 0

Region: chr10 36887193-36887204. Max. coverage (+): 0. Max coverage (-): 0

Region: chr10 36887205-36887217. Max. coverage (+): 0. Max coverage (-): 0

Region: chr10 36887218-36887229. Max. coverage (+): 0. Max coverage (-): 0

Region: chr10 36887230-36887242. Max. coverage (+): 1.19. Max coverage (-): 0

Region: chr10 36887243-36887254. Max. coverage (+): 1.19. Max coverage (-): 0

Region: chr10 36887255-36887267. Max. coverage (+): 0. Max coverage (-): 0

Region: chr10 36887268-36887279. Max. coverage (+): 0. Max coverage (-): 0

Region: chr10 36887280-36887292. Max. coverage (+): 0. Max coverage (-): 0

Region: chr10 36887293-36887305. Max. coverage (+): 0. Max coverage (-): 0

Region: chr10 36887306-36887317. Max. coverage (+): 0. Max coverage (-): 0

Region: chr10 36887318-36887330. Max. coverage (+): 0. Max coverage (-): 0

Region: chr10 36887331-36887342. Max. coverage (+): 0. Max coverage (-): 0

Region: chr10 36887343-36887355. Max. coverage (+): 0. Max coverage (-): 0

Region: chr10 36887356-36887367. Max. coverage (+): 0. Max coverage (-): 0

Region: chr10 36887368-36887380. Max. coverage (+): 0. Max coverage (-): 0

Region: chr10 36887381-36887392. Max. coverage (+): 0. Max coverage (-): 0

Region: chr10 36887393-36887405. Max. coverage (+): 0. Max coverage (-): 0

Region: chr10 36887406-36887417. Max. coverage (+): 2.9. Max coverage (-): 0

Region: chr10 36887418-36887430. Max. coverage (+): 2.9. Max coverage (-): 0

Region: chr10 36887431-36887443. Max. coverage (+): 2.35. Max coverage (-): 0

Region: chr10 36887444-36887455. Max. coverage (+): 0. Max coverage (-): 0

Region: chr10 36887456-36887468. Max. coverage (+): 0. Max coverage (-): 0

Region: chr10 36887469-36887480. Max. coverage (+): 0. Max coverage (-): 0

Region: chr10 36887481-36887493. Max. coverage (+): 2.03. Max coverage (-): 0

Region: chr10 36887494-36887505. Max. coverage (+): 2.03. Max coverage (-): 0

Region: chr10 36887506-36887518. Max. coverage (+): 4.86. Max coverage (-): 0

Region: chr10 36887519-36887530. Max. coverage (+): 4.86. Max coverage (-): 0

Region: chr10 36887531-36887543. Max. coverage (+): 0. Max coverage (-): 0

Region: chr10 36887544-36887555. Max. coverage (+): 0. Max coverage (-): 0

Region: chr10 36887556-36887568. Max. coverage (+): 0. Max coverage (-): 0

Region: chr10 36887569-36887581. Max. coverage (+): 0. Max coverage (-): 0

Region: chr10 36887582-36887593. Max. coverage (+): 0. Max coverage (-): 0

Region: chr10 36887594-36887606. Max. coverage (+): 0. Max coverage (-): 0

Region: chr10 36887607-36887618. Max. coverage (+): 0. Max coverage (-): 0

Region: chr10 36887619-36887631. Max. coverage (+): 0. Max coverage (-): 0

Region: chr10 36887632-36887643. Max. coverage (+): 0. Max coverage (-): 0

Region: chr10 36887644-36887656. Max. coverage (+): 0. Max coverage (-): 0

Region: chr10 36887657-36887668. Max. coverage (+): 0. Max coverage (-): 0

Region: chr10 36887669-36887681. Max. coverage (+): 0. Max coverage (-): 0

Region: chr10 36887682-36887693. Max. coverage (+): 0. Max coverage (-): 0

Region: chr10 36887694-36887706. Max. coverage (+): 0. Max coverage (-): 0

Region: chr10 36887707-36887719. Max. coverage (+): 0. Max coverage (-): 0

Region: chr10 36887720-36887731. Max. coverage (+): 0. Max coverage (-): 0

Region: chr10 36887732-36887744. Max. coverage (+): 0. Max coverage (-): 0

Region: chr10 36887745-36887756. Max. coverage (+): 2.12. Max coverage (-): 0

Region: chr10 36887757-36887769. Max. coverage (+): 9.83. Max coverage (-): 0

Region: chr10 36887770-36887781. Max. coverage (+): 9.83. Max coverage (-): 0

Region: chr10 36887782-36887794. Max. coverage (+): 0. Max coverage (-): 0

Region: chr10 36887795-36887806. Max. coverage (+): 3.84. Max coverage (-): 0

Region: chr10 36887807-36887819. Max. coverage (+): 0. Max coverage (-): 0

Region: chr10 36887820-36887831. Max. coverage (+): 0. Max coverage (-): 0

Region: chr10 36887832-36887844. Max. coverage (+): 0. Max coverage (-): 0

Region: chr10 36887845-36887857. Max. coverage (+): 0. Max coverage (-): 0

Region: chr10 36887858-36887869. Max. coverage (+): 2.29. Max coverage (-): 0

Region: chr10 36887870-36887882. Max. coverage (+): 1.13. Max coverage (-): 0

Region: chr10 36887883-36887894. Max. coverage (+): 0. Max coverage (-): 0

Region: chr10 36887895-36887907. Max. coverage (+): 0. Max coverage (-): 0

Region: chr10 36887908-36887919. Max. coverage (+): 0. Max coverage (-): 0

Region: chr10 36887920-36887932. Max. coverage (+): 0. Max coverage (-): 0

Region: chr10 36887933-36887944. Max. coverage (+): 0. Max coverage (-): 0

Region: chr10 36887945-36887957. Max. coverage (+): 0. Max coverage (-): 0

Region: chr10 36887958-36887969. Max. coverage (+): 0. Max coverage (-): 0

Region: chr10 36887970-36887982. Max. coverage (+): 0. Max coverage (-): 0

Region: chr10 36887983-36887995. Max. coverage (+): 0. Max coverage (-): 0

Region: chr10 36887996-36888007. Max. coverage (+): 0. Max coverage (-): 0

Region: chr10 36888008-36888020. Max. coverage (+): 6.41. Max coverage (-): 0

Region: chr10 36888021-36888032. Max. coverage (+): 6.41. Max coverage (-): 0

Region: chr10 36888033-36888045. Max. coverage (+): 0. Max coverage (-): 0

Region: chr10 36888046-36888057. Max. coverage (+): 0. Max coverage (-): 0

Region: chr10 36888058-36888070. Max. coverage (+): 0. Max coverage (-): 0

Region: chr10 36888071-36888082. Max. coverage (+): 0. Max coverage (-): 0

Region: chr10 36888083-36888095. Max. coverage (+): 0. Max coverage (-): 0

Region: chr10 36888096-36888107. Max. coverage (+): 0. Max coverage (-): 0

Region: chr10 36888108-36888120. Max. coverage (+): 0. Max coverage (-): 0

Region: chr10 36888121-36888132. Max. coverage (+): 0. Max coverage (-): 0

Region: chr10 36888133-36888145. Max. coverage (+): 0. Max coverage (-): 0

Region: chr10 36888146-36888158. Max. coverage (+): 0. Max coverage (-): 0

Region: chr10 36888159-36888170. Max. coverage (+): 0. Max coverage (-): 0

Region: chr10 36888171-36888183. Max. coverage (+): 0. Max coverage (-): 0

Region: chr10 36888184-36888195. Max. coverage (+): 0. Max coverage (-): 0

Region: chr10 36888196-36888208. Max. coverage (+): 0. Max coverage (-): 0

Region: chr10 36888209-36888220. Max. coverage (+): 0. Max coverage (-): 0

Region: chr10 36888221-36888233. Max. coverage (+): 0. Max coverage (-): 0

Region: chr10 36888234-36888245. Max. coverage (+): 0. Max coverage (-): 0

Region: chr10 36888246-36888258. Max. coverage (+): 0. Max coverage (-): 0

Region: chr10 36888259-36888270. Max. coverage (+): 0. Max coverage (-): 0

Region: chr10 36888271-36888283. Max. coverage (+): 0. Max coverage (-): 0

Region: chr10 36888284-36888296. Max. coverage (+): 0. Max coverage (-): 0

Region: chr10 36888297-36888308. Max. coverage (+): 0. Max coverage (-): 0

Region: chr10 36888309-36888321. Max. coverage (+): 0. Max coverage (-): 0

Region: chr10 36888322-36888333. Max. coverage (+): 0. Max coverage (-): 0

Region: chr10 36888334-36888346. Max. coverage (+): 0. Max coverage (-): 0

Region: chr10 36888347-36888358. Max. coverage (+): 0. Max coverage (-): 0

Region: chr10 36888359-36888371. Max. coverage (+): 0. Max coverage (-): 0

Region: chr10 36888372-36888383. Max. coverage (+): 0. Max coverage (-): 0

Region: chr10 36888384-36888396. Max. coverage (+): 0. Max coverage (-): 0

Region: chr10 36888397-36888408. Max. coverage (+): 0. Max coverage (-): 0

Region: chr10 36888409-36888421. Max. coverage (+): 0. Max coverage (-): 0

Region: chr10 36888422-36888434. Max. coverage (+): 0. Max coverage (-): 0

Region: chr10 36888435-36888446. Max. coverage (+): 0. Max coverage (-): 0

Region: chr10 36888447-36888459. Max. coverage (+): 0. Max coverage (-): 0

Region: chr10 36888460-36888471. Max. coverage (+): 0. Max coverage (-): 0

Region: chr10 36888472-36888484. Max. coverage (+): 0. Max coverage (-): 0

Region: chr10 36888485-36888496. Max. coverage (+): 0. Max coverage (-): 0

Region: chr10 36888497-36888509. Max. coverage (+): 6.65. Max coverage (-): 0

Region: chr10 36888510-36888521. Max. coverage (+): 6.65. Max coverage (-): 0

Region: chr10 36888522-36888534. Max. coverage (+): 0.45. Max coverage (-): 0

Region: chr10 36888535-36888546. Max. coverage (+): 0. Max coverage (-): 0

Region: chr10 36888547-36888559. Max. coverage (+): 0. Max coverage (-): 0

Region: chr10 36888560-36888572. Max. coverage (+): 0. Max coverage (-): 0

Region: chr10 36888573-36888584. Max. coverage (+): 0. Max coverage (-): 0

Region: chr10 36888585-36888597. Max. coverage (+): 0. Max coverage (-): 0

Region: chr10 36888598-36888609. Max. coverage (+): 0. Max coverage (-): 0

Region: chr10 36888610-36888622. Max. coverage (+): 0. Max coverage (-): 0

Region: chr10 36888623-36888634. Max. coverage (+): 0. Max coverage (-): 0

Region: chr10 36888635-36888647. Max. coverage (+): 0. Max coverage (-): 0

Region: chr10 36888648-36888659. Max. coverage (+): 0. Max coverage (-): 0

Region: chr10 36888660-36888672. Max. coverage (+): 0. Max coverage (-): 0

Region: chr10 36888673-36888684. Max. coverage (+): 0. Max coverage (-): 0

Region: chr10 36888685-36888697. Max. coverage (+): 0. Max coverage (-): 0

Region: chr10 36888698-36888710. Max. coverage (+): 0. Max coverage (-): 0

Region: chr10 36888711-36888722. Max. coverage (+): 0. Max coverage (-): 0

Region: chr10 36888723-36888735. Max. coverage (+): 0.53. Max coverage (-): 0

Region: chr10 36888736-36888747. Max. coverage (+): 0. Max coverage (-): 0

Region: chr10 36888748-36888760. Max. coverage (+): 0. Max coverage (-): 0

Region: chr10 36888761-36888772. Max. coverage (+): 0. Max coverage (-): 0

Region: chr10 36888773-36888785. Max. coverage (+): 0. Max coverage (-): 0

Region: chr10 36888786-36888797. Max. coverage (+): 0. Max coverage (-): 0

Region: chr10 36888798-36888810. Max. coverage (+): 0. Max coverage (-): 0

Region: chr10 36888811-36888822. Max. coverage (+): 0. Max coverage (-): 0

Region: chr10 36888823-36888835. Max. coverage (+): 4.8. Max coverage (-): 0

Region: chr10 36888836-36888847. Max. coverage (+): 0. Max coverage (-): 0

Region: chr10 36888848-36888860. Max. coverage (+): 0. Max coverage (-): 0

Region: chr10 36888861-36888873. Max. coverage (+): 0. Max coverage (-): 0

Region: chr10 36888874-36888885. Max. coverage (+): 0.86. Max coverage (-): 0

Region: chr10 36888886-36888898. Max. coverage (+): 0.86. Max coverage (-): 0

Region: chr10 36888899-36888910. Max. coverage (+): 0. Max coverage (-): 0

Region: chr10 36888911-36888923. Max. coverage (+): 0. Max coverage (-): 0

Region: chr10 36888924-36888935. Max. coverage (+): 3.24. Max coverage (-): 0

Region: chr10 36888936-36888948. Max. coverage (+): 0. Max coverage (-): 0

Region: chr10 36888949-36888960. Max. coverage (+): 0. Max coverage (-): 0

Region: chr10 36888961-36888973. Max. coverage (+): 0.57. Max coverage (-): 0

Region: chr10 36888974-36888985. Max. coverage (+): 0.57. Max coverage (-): 0

Region: chr10 36888986-36888998. Max. coverage (+): 1.93. Max coverage (-): 0

Region: chr10 36888999-36889011. Max. coverage (+): 0. Max coverage (-): 0

Region: chr10 36889012-36889023. Max. coverage (+): 0. Max coverage (-): 0

Region: chr10 36889024-36889036. Max. coverage (+): 0. Max coverage (-): 0

Region: chr10 36889037-36889048. Max. coverage (+): 0. Max coverage (-): 0

Region: chr10 36889049-36889061. Max. coverage (+): 0. Max coverage (-): 0

Region: chr10 36889062-36889073. Max. coverage (+): 0. Max coverage (-): 0

Region: chr10 36889074-36889086. Max. coverage (+): 0. Max coverage (-): 0

Region: chr10 36889087-36889098. Max. coverage (+): 0.65. Max coverage (-): 0

Region: chr10 36889099-36889111. Max. coverage (+): 0. Max coverage (-): 0

Region: chr10 36889112-36889123. Max. coverage (+): 2.25. Max coverage (-): 0

Region: chr10 36889124-36889136. Max. coverage (+): 2.25. Max coverage (-): 0

Region: chr10 36889137-36889149. Max. coverage (+): 5.89. Max coverage (-): 0

Region: chr10 36889150-36889161. Max. coverage (+): 8.72. Max coverage (-): 0

Region: chr10 36889162-36889174. Max. coverage (+): 8.72. Max coverage (-): 0

Region: chr10 36889175-36889186. Max. coverage (+): 0. Max coverage (-): 0

Region: chr10 36889187-36889199. Max. coverage (+): 0. Max coverage (-): 0

Region: chr10 36889200-36889211. Max. coverage (+): 0. Max coverage (-): 0

Region: chr10 36889212-36889224. Max. coverage (+): 0. Max coverage (-): 0

Region: chr10 36889225-36889236. Max. coverage (+): 2.01. Max coverage (-): 0

Region: chr10 36889237-36889249. Max. coverage (+): 5.14. Max coverage (-): 0

Region: chr10 36889250-36889261. Max. coverage (+): 2.13. Max coverage (-): 0

Region: chr10 36889262-36889274. Max. coverage (+): 0. Max coverage (-): 0

Region: chr10 36889275-36889287. Max. coverage (+): 12.63. Max coverage (-): 0

Region: chr10 36889288-36889299. Max. coverage (+): 5.9. Max coverage (-): 0

Region: chr10 36889300-36889312. Max. coverage (+): 0. Max coverage (-): 0

Region: chr10 36889313-36889324. Max. coverage (+): 0. Max coverage (-): 0

Region: chr10 36889325-36889337. Max. coverage (+): 0. Max coverage (-): 0

Region: chr10 36889338-36889349. Max. coverage (+): 0.32. Max coverage (-): 0

Region: chr10 36889350-36889362. Max. coverage (+): 0.32. Max coverage (-): 0

Region: chr10 36889363-36889374. Max. coverage (+): 4.66. Max coverage (-): 0

Region: chr10 36889375-36889387. Max. coverage (+): 4.66. Max coverage (-): 0

Region: chr10 36889388-36889399. Max. coverage (+): 1.72. Max coverage (-): 0

Region: chr10 36889400-36889412. Max. coverage (+): 0. Max coverage (-): 0

Region: chr10 36889413-36889425. Max. coverage (+): 4.18. Max coverage (-): 0

Region: chr10 36889426-36889437. Max. coverage (+): 4.18. Max coverage (-): 0

Region: chr10 36889438-36889450. Max. coverage (+): 0. Max coverage (-): 0

Region: chr10 36889451-36889462. Max. coverage (+): 0. Max coverage (-): 0

Region: chr10 36889463-36889475. Max. coverage (+): 4.85. Max coverage (-): 0

Region: chr10 36889476-36889487. Max. coverage (+): 4.85. Max coverage (-): 0

Region: chr10 36889488-36889500. Max. coverage (+): 0. Max coverage (-): 0

Region: chr10 36889501-36889512. Max. coverage (+): 0. Max coverage (-): 0

Region: chr10 36889513-36889525. Max. coverage (+): 1.67. Max coverage (-): 0

Region: chr10 36889526-36889537. Max. coverage (+): 1.67. Max coverage (-): 0

Region: chr10 36889538-36889550. Max. coverage (+): 0. Max coverage (-): 0

Region: chr10 36889551-36889563. Max. coverage (+): 0. Max coverage (-): 0

Region: chr10 36889564-36889575. Max. coverage (+): 0. Max coverage (-): 0

Region: chr10 36889576-36889588. Max. coverage (+): 0. Max coverage (-): 0

Region: chr10 36889589-36889600. Max. coverage (+): 0. Max coverage (-): 0

Region: chr10 36889601-36889613. Max. coverage (+): 0. Max coverage (-): 0

Region: chr10 36889614-36889625. Max. coverage (+): 0. Max coverage (-): 0

Region: chr10 36889626-36889638. Max. coverage (+): 0. Max coverage (-): 0

Region: chr10 36889639-36889650. Max. coverage (+): 0. Max coverage (-): 0

Region: chr10 36889651-36889663. Max. coverage (+): 0. Max coverage (-): 0

Region: chr10 36889664-36889675. Max. coverage (+): 4.85. Max coverage (-): 0

Region: chr10 36889676-36889688. Max. coverage (+): 0. Max coverage (-): 0

Region: chr10 36889689-36889700. Max. coverage (+): 1.84. Max coverage (-): 0

Region: chr10 36889701-36889713. Max. coverage (+): 1.84. Max coverage (-): 0

Region: chr10 36889714-36889726. Max. coverage (+): 0. Max coverage (-): 0

Region: chr10 36889727-36889738. Max. coverage (+): 0. Max coverage (-): 0

Region: chr10 36889739-36889751. Max. coverage (+): 3.86. Max coverage (-): 0

Region: chr10 36889752-36889763. Max. coverage (+): 3.86. Max coverage (-): 0

Region: chr10 36889764-36889776. Max. coverage (+): 0. Max coverage (-): 0

Region: chr10 36889777-36889788. Max. coverage (+): 0. Max coverage (-): 0

Region: chr10 36889789-36889801. Max. coverage (+): 22.34. Max coverage (-): 0

Region: chr10 36889802-36889813. Max. coverage (+): 12.32. Max coverage (-): 0

Region: chr10 36889814-36889826. Max. coverage (+): 0. Max coverage (-): 0

Region: chr10 36889827-36889838. Max. coverage (+): 0. Max coverage (-): 0

Region: chr10 36889839-36889851. Max. coverage (+): 2.61. Max coverage (-): 0

Region: chr10 36889852-36889864. Max. coverage (+): 2.61. Max coverage (-): 0

Region: chr10 36889865-36889876. Max. coverage (+): 0. Max coverage (-): 0

Region: chr10 36889877-36889889. Max. coverage (+): 3.1. Max coverage (-): 0

Region: chr10 36889890-36889901. Max. coverage (+): 2.33. Max coverage (-): 0

Region: chr10 36889902-36889914. Max. coverage (+): 0. Max coverage (-): 0

Region: chr10 36889915-36889926. Max. coverage (+): 0. Max coverage (-): 0

Region: chr10 36889927-36889939. Max. coverage (+): 1.11. Max coverage (-): 0

Region: chr10 36889940-36889951. Max. coverage (+): 1.11. Max coverage (-): 0

Region: chr10 36889952-36889964. Max. coverage (+): 0. Max coverage (-): 0

Region: chr10 36889965-36889976. Max. coverage (+): 0. Max coverage (-): 0

Region: chr10 36889977-36889989. Max. coverage (+): 4.09. Max coverage (-): 0

Region: chr10 36889990-36890002. Max. coverage (+): 4.72. Max coverage (-): 0

Region: chr10 36890003-36890014. Max. coverage (+): 0. Max coverage (-): 0

Region: chr10 36890015-36890027. Max. coverage (+): 0.81. Max coverage (-): 0

Region: chr10 36890028-36890039. Max. coverage (+): 5.09. Max coverage (-): 0

Region: chr10 36890040-36890052. Max. coverage (+): 0. Max coverage (-): 0

Region: chr10 36890053-36890064. Max. coverage (+): 0. Max coverage (-): 0

Region: chr10 36890065-36890077. Max. coverage (+): 0. Max coverage (-): 0

Region: chr10 36890078-36890089. Max. coverage (+): 0. Max coverage (-): 0

Region: chr10 36890090-36890102. Max. coverage (+): 0. Max coverage (-): 0

Region: chr10 36890103-36890114. Max. coverage (+): 0. Max coverage (-): 0

Region: chr10 36890115-36890127. Max. coverage (+): 0. Max coverage (-): 0

Region: chr10 36890128-36890140. Max. coverage (+): 0. Max coverage (-): 0

Region: chr10 36890141-36890152. Max. coverage (+): 4.81. Max coverage (-): 0

Region: chr10 36890153-36890165. Max. coverage (+): 4.81. Max coverage (-): 0

Region: chr10 36890166-36890177. Max. coverage (+): 0.51. Max coverage (-): 0

Region: chr10 36890178-36890190. Max. coverage (+): 0.51. Max coverage (-): 0

Region: chr10 36890191-36890202. Max. coverage (+): 0. Max coverage (-): 0

Region: chr10 36890203-36890215. Max. coverage (+): 0. Max coverage (-): 0

Region: chr10 36890216-36890227. Max. coverage (+): 1.55. Max coverage (-): 0

Region: chr10 36890228-36890240. Max. coverage (+): 1.55. Max coverage (-): 0

Region: chr10 36890241-36890252. Max. coverage (+): 5.23. Max coverage (-): 0

Region: chr10 36890253-36890265. Max. coverage (+): 0. Max coverage (-): 0

Region: chr10 36890266-36890278. Max. coverage (+): 0. Max coverage (-): 0

Region: chr10 36890279-36890290. Max. coverage (+): 0.49. Max coverage (-): 0

Region: chr10 36890291-36890303. Max. coverage (+): 0. Max coverage (-): 0

Region: chr10 36890304-36890315. Max. coverage (+): 0. Max coverage (-): 0

Region: chr10 36890316-36890328. Max. coverage (+): 0. Max coverage (-): 0

Region: chr10 36890329-36890340. Max. coverage (+): 0.53. Max coverage (-): 0

Region: chr10 36890341-36890353. Max. coverage (+): 0.53. Max coverage (-): 0

Region: chr10 36890354-36890365. Max. coverage (+): 8.67. Max coverage (-): 0

Region: chr10 36890366-36890378. Max. coverage (+): 5.17. Max coverage (-): 0

Region: chr10 36890379-36890390. Max. coverage (+): 3.35. Max coverage (-): 0

Region: chr10 36890391-36890403. Max. coverage (+): 3.35. Max coverage (-): 0

Region: chr10 36890404-36890415. Max. coverage (+): 0. Max coverage (-): 0

Region: chr10 36890416-36890428. Max. coverage (+): 1.21. Max coverage (-): 0

Region: chr10 36890429-36890441. Max. coverage (+): 1.21. Max coverage (-): 0

Region: chr10 36890442-36890453. Max. coverage (+): 0.57. Max coverage (-): 0

Region: chr10 36890454-36890466. Max. coverage (+): 0. Max coverage (-): 0

Region: chr10 36890467-36890478. Max. coverage (+): 0.99. Max coverage (-): 0

Region: chr10 36890479-36890491. Max. coverage (+): 12.61. Max coverage (-): 0

Region: chr10 36890492-36890503. Max. coverage (+): 0. Max coverage (-): 0

Region: chr10 36890504-36890516. Max. coverage (+): 0. Max coverage (-): 0

Region: chr10 36890517-36890528. Max. coverage (+): 5.13. Max coverage (-): 0

Region: chr10 36890529-36890541. Max. coverage (+): 3.29. Max coverage (-): 0

Region: chr10 36890542-36890553. Max. coverage (+): 3.62. Max coverage (-): 0

Region: chr10 36890554-36890566. Max. coverage (+): 1.39. Max coverage (-): 0

Region: chr10 36890567-36890579. Max. coverage (+): 0. Max coverage (-): 0

Region: chr10 36890580-36890591. Max. coverage (+): 0. Max coverage (-): 0

Region: chr10 36890592-36890604. Max. coverage (+): 0. Max coverage (-): 0

Region: chr10 36890605-36890616. Max. coverage (+): 3.09. Max coverage (-): 0

Region: chr10 36890617-36890629. Max. coverage (+): 5.06. Max coverage (-): 0

Region: chr10 36890630-36890641. Max. coverage (+): 10.27. Max coverage (-): 0

Region: chr10 36890642-36890654. Max. coverage (+): 3.95. Max coverage (-): 0

Region: chr10 36890655-36890666. Max. coverage (+): 0. Max coverage (-): 0

Region: chr10 36890667-36890679. Max. coverage (+): 0. Max coverage (-): 0

Region: chr10 36890680-36890691. Max. coverage (+): 0. Max coverage (-): 0

Region: chr10 36890692-36890704. Max. coverage (+): 0. Max coverage (-): 0

Region: chr10 36890705-36890717. Max. coverage (+): 0. Max coverage (-): 0

Region: chr10 36890718-36890729. Max. coverage (+): 0. Max coverage (-): 0

Region: chr10 36890730-36890742. Max. coverage (+): 0. Max coverage (-): 0

Region: chr10 36890743-36890754. Max. coverage (+): 1.92. Max coverage (-): 0

Region: chr10 36890755-36890767. Max. coverage (+): 0. Max coverage (-): 0

Region: chr10 36890768-36890779. Max. coverage (+): 0. Max coverage (-): 0

Region: chr10 36890780-36890792. Max. coverage (+): 0. Max coverage (-): 0

Region: chr10 36890793-36890804. Max. coverage (+): 0. Max coverage (-): 0

Region: chr10 36890805-36890817. Max. coverage (+): 5.54. Max coverage (-): 0

Region: chr10 36890818-36890829. Max. coverage (+): 0.86. Max coverage (-): 0

Region: chr10 36890830-36890842. Max. coverage (+): 0. Max coverage (-): 0

Region: chr10 36890843-36890855. Max. coverage (+): 0. Max coverage (-): 0

Region: chr10 36890856-36890867. Max. coverage (+): 0. Max coverage (-): 0

Region: chr10 36890868-36890880. Max. coverage (+): 0. Max coverage (-): 0

Region: chr10 36890881-36890892. Max. coverage (+): 0. Max coverage (-): 0

Region: chr10 36890893-36890905. Max. coverage (+): 0.99. Max coverage (-): 0

Region: chr10 36890906-36890917. Max. coverage (+): 0. Max coverage (-): 0

Region: chr10 36890918-36890930. Max. coverage (+): 0. Max coverage (-): 0

Region: chr10 36890931-36890942. Max. coverage (+): 2.29. Max coverage (-): 0

Region: chr10 36890943-36890955. Max. coverage (+): 7.37. Max coverage (-): 0

Region: chr10 36890956-36890967. Max. coverage (+): 7.37. Max coverage (-): 0

Region: chr10 36890968-36890980. Max. coverage (+): 7.52. Max coverage (-): 0

Region: chr10 36890981-36890993. Max. coverage (+): 0. Max coverage (-): 0

Region: chr10 36890994-36891005. Max. coverage (+): 0. Max coverage (-): 0

Region: chr10 36891006-36891018. Max. coverage (+): 0. Max coverage (-): 0

Region: chr10 36891019-36891030. Max. coverage (+): 0. Max coverage (-): 0

Region: chr10 36891031-36891043. Max. coverage (+): 1.78. Max coverage (-): 0

Region: chr10 36891044-36891055. Max. coverage (+): 0. Max coverage (-): 0

Region: chr10 36891056-36891068. Max. coverage (+): 0. Max coverage (-): 0

Region: chr10 36891069-36891080. Max. coverage (+): 0. Max coverage (-): 0

Region: chr10 36891081-36891093. Max. coverage (+): 0. Max coverage (-): 0

Region: chr10 36891094-36891105. Max. coverage (+): 0. Max coverage (-): 0

Region: chr10 36891106-36891118. Max. coverage (+): 0. Max coverage (-): 0

Region: chr10 36891119-36891131. Max. coverage (+): 4.36. Max coverage (-): 0

Region: chr10 36891132-36891143. Max. coverage (+): 0. Max coverage (-): 0

Region: chr10 36891144-36891156. Max. coverage (+): 2.94. Max coverage (-): 0

Region: chr10 36891157-36891168. Max. coverage (+): 0. Max coverage (-): 0

Region: chr10 36891169-36891181. Max. coverage (+): 0. Max coverage (-): 0

Region: chr10 36891182-36891193. Max. coverage (+): 0. Max coverage (-): 0

Region: chr10 36891194-36891206. Max. coverage (+): 0. Max coverage (-): 0

Region: chr10 36891207-36891218. Max. coverage (+): 0. Max coverage (-): 0

Region: chr10 36891219-36891231. Max. coverage (+): 0. Max coverage (-): 0

Region: chr10 36891232-36891243. Max. coverage (+): 0. Max coverage (-): 0

Region: chr10 36891244-36891256. Max. coverage (+): 0. Max coverage (-): 0

Region: chr10 36891257-36891268. Max. coverage (+): 0. Max coverage (-): 0

Region: chr10 36891269-36891281. Max. coverage (+): 0. Max coverage (-): 0

Region: chr10 36891282-36891294. Max. coverage (+): 0. Max coverage (-): 0

Region: chr10 36891295-36891306. Max. coverage (+): 0. Max coverage (-): 0

Region: chr10 36891307-36891319. Max. coverage (+): 0. Max coverage (-): 0

Region: chr10 36891320-36891331. Max. coverage (+): 0. Max coverage (-): 0

Region: chr10 36891332-36891344. Max. coverage (+): 0. Max coverage (-): 0

Region: chr10 36891345-36891356. Max. coverage (+): 2.12. Max coverage (-): 0

Region: chr10 36891357-36891369. Max. coverage (+): 2.75. Max coverage (-): 0

Region: chr10 36891370-36891381. Max. coverage (+): 0. Max coverage (-): 0

Region: chr10 36891382-36891394. Max. coverage (+): 0. Max coverage (-): 0

Region: chr10 36891395-36891406. Max. coverage (+): 0. Max coverage (-): 0

Region: chr10 36891407-36891419. Max. coverage (+): 1.04. Max coverage (-): 0

Region: chr10 36891420-36891432. Max. coverage (+): 0. Max coverage (-): 0

Region: chr10 36891433-36891444. Max. coverage (+): 0. Max coverage (-): 0

Region: chr10 36891445-36891457. Max. coverage (+): 0. Max coverage (-): 0

Region: chr10 36891458-36891469. Max. coverage (+): 0. Max coverage (-): 0

Region: chr10 36891470-36891482. Max. coverage (+): 0. Max coverage (-): 0

Region: chr10 36891483-36891494. Max. coverage (+): 0. Max coverage (-): 0

Region: chr10 36891495-36891507. Max. coverage (+): 0. Max coverage (-): 0

Region: chr10 36891508-36891519. Max. coverage (+): 0. Max coverage (-): 0

Region: chr10 36891520-36891532. Max. coverage (+): 0. Max coverage (-): 0

Region: chr10 36891533-36891544. Max. coverage (+): 0.76. Max coverage (-): 0

Region: chr10 36891545-36891557. Max. coverage (+): 0. Max coverage (-): 0

Region: chr10 36891558-36891570. Max. coverage (+): 0. Max coverage (-): 0

Region: chr10 36891571-36891582. Max. coverage (+): 0. Max coverage (-): 0

Region: chr10 36891583-36891595. Max. coverage (+): 0. Max coverage (-): 0

Region: chr10 36891596-36891607. Max. coverage (+): 0. Max coverage (-): 0

Region: chr10 36891608-36891620. Max. coverage (+): 0. Max coverage (-): 0

Region: chr10 36891621-36891632. Max. coverage (+): 0. Max coverage (-): 0

Region: chr10 36891633-36891645. Max. coverage (+): 11.88. Max coverage (-): 0

Region: chr10 36891646-36891657. Max. coverage (+): 0. Max coverage (-): 0

Region: chr10 36891658-36891670. Max. coverage (+): 14.15. Max coverage (-): 0

Region: chr10 36891671-36891682. Max. coverage (+): 14.15. Max coverage (-): 0

Region: chr10 36891683-36891695. Max. coverage (+): 0. Max coverage (-): 0

Region: chr10 36891696-36891708. Max. coverage (+): 0. Max coverage (-): 0

Region: chr10 36891709-36891720. Max. coverage (+): 0. Max coverage (-): 0

Region: chr10 36891721-36891733. Max. coverage (+): 4.77. Max coverage (-): 0

Region: chr10 36891734-36891745. Max. coverage (+): 1.06. Max coverage (-): 0

Region: chr10 36891746-36891758. Max. coverage (+): 2.95. Max coverage (-): 0

Region: chr10 36891759-36891770. Max. coverage (+): 6.08. Max coverage (-): 0

Region: chr10 36891771-36891783. Max. coverage (+): 0. Max coverage (-): 0

Region: chr10 36891784-36891795. Max. coverage (+): 0. Max coverage (-): 0

Region: chr10 36891796-36891808. Max. coverage (+): 0. Max coverage (-): 0

Region: chr10 36891809-36891820. Max. coverage (+): 7.23. Max coverage (-): 0

Region: chr10 36891821-36891833. Max. coverage (+): 3.4. Max coverage (-): 0

Region: chr10 36891834-36891846. Max. coverage (+): 0. Max coverage (-): 0

Region: chr10 36891847-36891858. Max. coverage (+): 0. Max coverage (-): 0

Region: chr10 36891859-36891871. Max. coverage (+): 0. Max coverage (-): 0

Region: chr10 36891872-36891883. Max. coverage (+): 0. Max coverage (-): 0

Region: chr10 36891884-36891896. Max. coverage (+): 0. Max coverage (-): 0

Region: chr10 36891897-36891908. Max. coverage (+): 0. Max coverage (-): 0

Region: chr10 36891909-36891921. Max. coverage (+): 0. Max coverage (-): 0

Region: chr10 36891922-36891933. Max. coverage (+): 0. Max coverage (-): 0

Region: chr10 36891934-36891946. Max. coverage (+): 3.99. Max coverage (-): 0

Region: chr10 36891947-36891958. Max. coverage (+): 1.05. Max coverage (-): 0

Region: chr10 36891959-36891971. Max. coverage (+): 0. Max coverage (-): 0

Region: chr10 36891972-36891983. Max. coverage (+): 3.22. Max coverage (-): 0

Region: chr10 36891984-36891996. Max. coverage (+): 0. Max coverage (-): 0

Region: chr10 36891997-36892009. Max. coverage (+): 0. Max coverage (-): 0

Region: chr10 36892010-36892021. Max. coverage (+): 0. Max coverage (-): 0

Region: chr10 36892022-36892034. Max. coverage (+): 0. Max coverage (-): 0

Region: chr10 36892035-36892046. Max. coverage (+): 5.36. Max coverage (-): 0

Region: chr10 36892047-36892059. Max. coverage (+): 0. Max coverage (-): 0

Region: chr10 36892060-36892071. Max. coverage (+): 3.09. Max coverage (-): 0

Region: chr10 36892072-36892084. Max. coverage (+): 0. Max coverage (-): 0

Region: chr10 36892085-36892096. Max. coverage (+): 0. Max coverage (-): 0

Region: chr10 36892097-36892109. Max. coverage (+): 0. Max coverage (-): 0

Region: chr10 36892110-36892121. Max. coverage (+): 0. Max coverage (-): 0

Region: chr10 36892122-36892134. Max. coverage (+): 0. Max coverage (-): 0

Region: chr10 36892135-36892147. Max. coverage (+): 0. Max coverage (-): 0

Region: chr10 36892148-36892159. Max. coverage (+): 0. Max coverage (-): 0

Region: chr10 36892160-36892172. Max. coverage (+): 0. Max coverage (-): 0

Region: chr10 36892173-36892184. Max. coverage (+): 0. Max coverage (-): 0

Region: chr10 36892185-36892197. Max. coverage (+): 0. Max coverage (-): 0

Region: chr10 36892198-36892209. Max. coverage (+): 0. Max coverage (-): 0

Region: chr10 36892210-36892222. Max. coverage (+): 0. Max coverage (-): 0

Region: chr10 36892223-36892234. Max. coverage (+): 0. Max coverage (-): 0

Region: chr10 36892235-36892247. Max. coverage (+): 0. Max coverage (-): 0

Region: chr10 36892248-36892259. Max. coverage (+): 0. Max coverage (-): 0

Region: chr10 36892260-36892272. Max. coverage (+): 0. Max coverage (-): 0

Region: chr10 36892273-36892285. Max. coverage (+): 0. Max coverage (-): 0

Region: chr10 36892286-36892297. Max. coverage (+): 0. Max coverage (-): 0

Region: chr10 36892298-36892310. Max. coverage (+): 0. Max coverage (-): 0

Region: chr10 36892311-36892322. Max. coverage (+): 0. Max coverage (-): 0

Region: chr10 36892323-36892335. Max. coverage (+): 0. Max coverage (-): 0

Region: chr10 36892336-36892347. Max. coverage (+): 0. Max coverage (-): 0

Region: chr10 36892348-36892360. Max. coverage (+): 0. Max coverage (-): 0

Region: chr10 36892361-36892372. Max. coverage (+): 0. Max coverage (-): 0

Region: chr10 36892373-36892385. Max. coverage (+): 0. Max coverage (-): 0

Region: chr10 36892386-36892397. Max. coverage (+): 0. Max coverage (-): 0

Region: chr10 36892398-36892410. Max. coverage (+): 0. Max coverage (-): 0

Region: chr10 36892411-36892423. Max. coverage (+): 0. Max coverage (-): 0

Region: chr10 36892424-36892435. Max. coverage (+): 0. Max coverage (-): 0

Region: chr10 36892436-36892448. Max. coverage (+): 0. Max coverage (-): 0

Region: chr10 36892449-36892460. Max. coverage (+): 0. Max coverage (-): 0

Region: chr10 36892461-36892473. Max. coverage (+): 0. Max coverage (-): 0

Region: chr10 36892474-36892485. Max. coverage (+): 0. Max coverage (-): 0

Region: chr10 36892486-36892498. Max. coverage (+): 0. Max coverage (-): 0

Region: chr10 36892499-36892510. Max. coverage (+): 0. Max coverage (-): 0

Region: chr10 36892511-36892523. Max. coverage (+): 0. Max coverage (-): 0

Region: chr10 36892524-36892535. Max. coverage (+): 0. Max coverage (-): 0

Region: chr10 36892536-36892548. Max. coverage (+): 0. Max coverage (-): 0

Region: chr10 36892549-36892561. Max. coverage (+): 0. Max coverage (-): 0

Region: chr10 36892562-36892573. Max. coverage (+): 0. Max coverage (-): 0

Region: chr10 36892574-36892586. Max. coverage (+): 0. Max coverage (-): 0

Region: chr10 36892587-36892598. Max. coverage (+): 0. Max coverage (-): 0

Region: chr10 36892599-36892611. Max. coverage (+): 0. Max coverage (-): 0

Region: chr10 36892612-36892623. Max. coverage (+): 0. Max coverage (-): 0

Region: chr10 36892624-36892636. Max. coverage (+): 0. Max coverage (-): 0

Region: chr10 36892637-36892648. Max. coverage (+): 0. Max coverage (-): 0

Region: chr10 36892649-36892661. Max. coverage (+): 0. Max coverage (-): 0

Region: chr10 36892662-36892673. Max. coverage (+): 0. Max coverage (-): 0

Region: chr10 36892674-36892686. Max. coverage (+): 0. Max coverage (-): 0

Region: chr10 36892687-36892699. Max. coverage (+): 0. Max coverage (-): 0

Region: chr10 36892700-36892711. Max. coverage (+): 0. Max coverage (-): 0

Region: chr10 36892712-36892724. Max. coverage (+): 0. Max coverage (-): 0

Region: chr10 36892725-36892736. Max. coverage (+): 0. Max coverage (-): 0

Region: chr10 36892737-36892749. Max. coverage (+): 1.71. Max coverage (-): 0

Region: chr10 36892750-36892761. Max. coverage (+): 0. Max coverage (-): 0

Region: chr10 36892762-. Max. coverage (+): 0. Max coverage (-): 0

RepeatMasker Color Code

**+**

100-98% Identity

<98-95% Identity

<95-90% Identity

<90-85% Identity

<85-80% Identity

<80-75% Identity

<75-70% Identity

<70% Identity

**-**

Gene Set Color Code

**+**

Gene

Pseudogene

**-**

Topology/Coverage Color Code

Coverage Plus Strand

Coverage Minus Strand

Mainstrand: Plus

Mainstrand: Minus

Complementary Strand

Flanking Region  
(if option -flank >0)

Gene Set Annotation  
  
RepeatMasker Annotation  

**1. L2a**: 36891562-36891608 (-), Divergence to consensus: 29.8%

  
Transcription Factor Binding Sites  

**RFX4\_2** (Sequence: GTAACCAGG (-): 36892656)  
**Gata4** (Sequence: AGATAAG (-): 36887009)  
**SOX9** (Sequence: CCATTGTT (+): 36888844)  
**SOX9** (Sequence: TTATTGTT (+): 36889552)  
**SOX9** (Sequence: TTATTGTT (+): 36892383)  
**Gata4** (Sequence: CTTATCT (+): 36886503)  
**Gata4** (Sequence: CTTATCT (+): 36890639)
