# Supplementary material for: Small RNA sequencing of cryopreserved semen from single bull revealed altered miRNAs and piRNAs expression between High- and Low-motile sperm populations
Source: BMC Genomics. 2017 Jan 4;18:14. doi: 10.1186/s12864-016-3394-7 (PMC5209821; doi:10.1186/s12864-016-3394-7)
Supplement: Additional file 3: — Details for each piRNA clusters found in High Motile (HM) sperm fraction. Genes, repeats, transposable elements and transcription factors binding sites falling within the cluster regions were reported. (ZIP 1896 kb) [file 12864_2016_3394_MOESM3_ESM.zip › 20.html]

piRNA cluster 20


Predicted piRNA cluster no. 20     previous   next
  

Show proTRAC run info
Hide proTRAC run info

================================= proTRAC ====================================  
VERSION: 2.1                                    LAST MODIFIED: 06. October 2015  
  
Please cite:  
Rosenkranz D, Zischler H. proTRAC - a software for probabilistic piRNA cluster  
detection, visualization and analysis. 2012. BMC Bioinformatics 13:5.  
  
and (for proTRAC 2.0 and later):  
Rosenkranz D, Rudloff S, Bastuck K, Ketting RF, Zischler H. Tupaia small RNAs  
provide insights into function and evolution of RNAi-based transposon defense  
in mammals. 2015. RNA 21(5):911-922.  
  
Contact:  
David Rosenkranz  
Institute of Anthropology, small RNA group  
Johannes Gutenberg University Mainz  
email: rosenkranz@uni-mainz.de  
  
You can find the latest proTRAC version at:  
http://sourceforge.net/projects/protrac/files  
http://www.smallRNAgroup-mainz.de/software  
==============================================================================  
  
PARAMETERS:  
Map file: .............../storage/core/barbara/genhome/smallRNA/fertility/Sample\_motile/pirna/Sample\_motile\_26-33\_collapsed.fa.no-dust.map.weighted-10000-1000-b-0  
Genome file: ............/storage/core/barbara/genhome/smallRNA/fertility/Sample\_all/pirna/bt\_311\_chrY.fa  
RepeatMasker annotation: /storage/genomes/bt\_umd31/GCF\_000003055.6\_Bos\_taurus\_UMD\_3.1.1\_repeatMasker\_chr.out  
GeneSet:................./storage/core/barbara/genhome/smallRNA/fertility/Sample\_all/pirna/full.gtf  
  
Significant (p<=0.01) hit density will be calculated based  
on observed hit distribution.  
  
Sliding window size: ........................................ 5000 bp  
Sliding window increament: .................................. 1000 bp  
Normalize each hit by number of genomic hits: ............... 1 [0=no/1=yes]  
Normalize each hit by number of sequence reads: ............. 1 [0=no/1=yes]  
Normalize values (-> per million mapped reads): ............. 1 [0=no/1=yes]  
Min. fraction of hits with 1T(U) or 10A: .................... 0.75  
Alternatively: Min. fraction of hits with 1T(U) and 10A: .... 0.5  
Min. fraction of hits with typical piRNA length: ............ 0.75  
Typical piRNA length: ....................................... 26-33 nt  
Min. size of a piRNA cluster: ............................... 5000 bp.  
Min. number of hits (absolute): ............................. 0  
Min. number of hits (normalized): ........................... 0  
Min. fraction of hits on the mainstrand: .................... 0.75  
Top fraction of mapped sequences (in terms of read counts): . 1%  
Top fraction accounts for max. n% of sequence reads: ........ 90%  
Min. fraction of hits on each arm of a bidirectional cluster: 0.1  
Output image file for each cluster: ......................... 0 [0=no/1=yes]  
Output html file for each cluster: .......................... 1 [0=no/1=yes]  
Output a summary table: ..................................... 1 [0=no/1=yes]  
Output a FASTA file for each cluster (piRNA sequences): ..... 1 [0=no/1=yes]  
Output a FASTA file comprising cluster sequences: ........... 1 [0=no/1=yes]  
Search DNA motifs in clusters: .............................. 1 [0=no/1=yes]  
Output flanking sequences: +/- .............................. 0 bp  
Output ~.pTi file: .......................................... 1 [0=no/1=yes]  
==============================================================================  
  
  
Genome size (without gaps): ............ 2678902517 bp  
Gaps (N/X/-): .......................... 53837044 bp  
Mapped reads: .......................... 658825247023  
Non-identical sequences: ............... 514171  
Genomic hits: .......................... 764233  
Significant densitiy of mapped reads: .. 12867599.5173724 reads/kb

Show proTRAC cluster info
Hide proTRAC cluster info

|  |  |
| --- | --- |
| Location | chr14 |
| Coordinates | 4167520-4175452 |
| Size [bp] | 7933 |
| Sequence hit loci | 95 |
| Mapped reads (normalized) | 131922267 |
| Mapped reads (normalized) per kb | 16629555.9 |
| Normalized reads with 1T (1U) | 81.1% |
| Normalized reads with 10A | 16.9% |
| Normalized reads with length 26-33 nt | 100% |
| Normalized reads on the main strand(s) | 96.8% |
| Predicted directionality | mono:plus |

100%

0%

1T (1U)  
reads

10A reads

26-33 nt  
reads

reads on mainstrand

**Either the amount of reads with 1T (1U) OR 10A has to exceed 75% (set with option: -1Tor10A)  
Alternatively the amount of reads with 1T (1U) AND 10A has to exceed 50% (set with option: -1Tand10A)  
Minimum amount of reads with preferred size is 75% (set with option: -pisize)  
Minimum amount of reads on the main strand(s) is 75% (set with option: -clstrand)**

Show read coverage
Hide read coverage

WHAT DO I SEE HERE?  
This chart shows the location of mapped sequence reads within a predicted piRNA cluster. The color refers to the number of genomic hits produced by the sequence read in question. A dark red bar indicates that this sequence read produces many other hits elsewhere in the genome. Many adjacent red or yellow bars can indicate the presence of a multi-copy element such as transposons or rRNA genes. A dark green bar indicates that this sequence read maps uniquely to this locus.

1 hit

2-5 hits

6-10 hits

11-20 hits

21-50 hits

51-100 hits

> 100 hits

chr14

4167520

4175452

Gene Set

RepeatMasker

Mapped  
Reads

13.06

plus strand

minus strand

13.06

Region: chr14 213575-4167527. Max. coverage (+): 1.24. Max coverage (-): 0

Region: chr14 4167528-4167543. Max. coverage (+): 0. Max coverage (-): 0

Region: chr14 4167544-4167559. Max. coverage (+): 0. Max coverage (-): 0

Region: chr14 4167560-4167575. Max. coverage (+): 0. Max coverage (-): 0

Region: chr14 4167576-4167591. Max. coverage (+): 0. Max coverage (-): 0

Region: chr14 4167592-4167607. Max. coverage (+): 0. Max coverage (-): 0

Region: chr14 4167608-4167623. Max. coverage (+): 0. Max coverage (-): 0

Region: chr14 4167624-4167638. Max. coverage (+): 0. Max coverage (-): 0

Region: chr14 4167639-4167654. Max. coverage (+): 0. Max coverage (-): 0

Region: chr14 4167655-4167670. Max. coverage (+): 0. Max coverage (-): 0

Region: chr14 4167671-4167686. Max. coverage (+): 0. Max coverage (-): 0

Region: chr14 4167687-4167702. Max. coverage (+): 0. Max coverage (-): 0

Region: chr14 4167703-4167718. Max. coverage (+): 0. Max coverage (-): 0

Region: chr14 4167719-4167734. Max. coverage (+): 0. Max coverage (-): 0

Region: chr14 4167735-4167750. Max. coverage (+): 0. Max coverage (-): 0

Region: chr14 4167751-4167765. Max. coverage (+): 0. Max coverage (-): 0

Region: chr14 4167766-4167781. Max. coverage (+): 0. Max coverage (-): 0

Region: chr14 4167782-4167797. Max. coverage (+): 0. Max coverage (-): 0

Region: chr14 4167798-4167813. Max. coverage (+): 0. Max coverage (-): 0

Region: chr14 4167814-4167829. Max. coverage (+): 0. Max coverage (-): 0

Region: chr14 4167830-4167845. Max. coverage (+): 0. Max coverage (-): 0

Region: chr14 4167846-4167861. Max. coverage (+): 0. Max coverage (-): 0

Region: chr14 4167862-4167876. Max. coverage (+): 0. Max coverage (-): 0

Region: chr14 4167877-4167892. Max. coverage (+): 0. Max coverage (-): 0

Region: chr14 4167893-4167908. Max. coverage (+): 1.19. Max coverage (-): 0

Region: chr14 4167909-4167924. Max. coverage (+): 0. Max coverage (-): 0

Region: chr14 4167925-4167940. Max. coverage (+): 0. Max coverage (-): 0

Region: chr14 4167941-4167956. Max. coverage (+): 0. Max coverage (-): 0

Region: chr14 4167957-4167972. Max. coverage (+): 0. Max coverage (-): 0

Region: chr14 4167973-4167988. Max. coverage (+): 0. Max coverage (-): 0

Region: chr14 4167989-4168003. Max. coverage (+): 0. Max coverage (-): 0

Region: chr14 4168004-4168019. Max. coverage (+): 0. Max coverage (-): 0

Region: chr14 4168020-4168035. Max. coverage (+): 0. Max coverage (-): 0

Region: chr14 4168036-4168051. Max. coverage (+): 0. Max coverage (-): 0

Region: chr14 4168052-4168067. Max. coverage (+): 0. Max coverage (-): 0

Region: chr14 4168068-4168083. Max. coverage (+): 0. Max coverage (-): 0

Region: chr14 4168084-4168099. Max. coverage (+): 0. Max coverage (-): 0

Region: chr14 4168100-4168114. Max. coverage (+): 0. Max coverage (-): 0

Region: chr14 4168115-4168130. Max. coverage (+): 0. Max coverage (-): 0

Region: chr14 4168131-4168146. Max. coverage (+): 0. Max coverage (-): 0

Region: chr14 4168147-4168162. Max. coverage (+): 0. Max coverage (-): 0

Region: chr14 4168163-4168178. Max. coverage (+): 0. Max coverage (-): 0

Region: chr14 4168179-4168194. Max. coverage (+): 0. Max coverage (-): 0

Region: chr14 4168195-4168210. Max. coverage (+): 0. Max coverage (-): 0

Region: chr14 4168211-4168226. Max. coverage (+): 0. Max coverage (-): 0

Region: chr14 4168227-4168241. Max. coverage (+): 0. Max coverage (-): 0

Region: chr14 4168242-4168257. Max. coverage (+): 0. Max coverage (-): 0

Region: chr14 4168258-4168273. Max. coverage (+): 0. Max coverage (-): 0

Region: chr14 4168274-4168289. Max. coverage (+): 0. Max coverage (-): 0

Region: chr14 4168290-4168305. Max. coverage (+): 0. Max coverage (-): 0

Region: chr14 4168306-4168321. Max. coverage (+): 0. Max coverage (-): 0

Region: chr14 4168322-4168337. Max. coverage (+): 0. Max coverage (-): 0

Region: chr14 4168338-4168352. Max. coverage (+): 0. Max coverage (-): 0

Region: chr14 4168353-4168368. Max. coverage (+): 0. Max coverage (-): 0

Region: chr14 4168369-4168384. Max. coverage (+): 0. Max coverage (-): 0

Region: chr14 4168385-4168400. Max. coverage (+): 0. Max coverage (-): 0

Region: chr14 4168401-4168416. Max. coverage (+): 0. Max coverage (-): 0

Region: chr14 4168417-4168432. Max. coverage (+): 0. Max coverage (-): 0

Region: chr14 4168433-4168448. Max. coverage (+): 0. Max coverage (-): 0

Region: chr14 4168449-4168464. Max. coverage (+): 0. Max coverage (-): 0

Region: chr14 4168465-4168479. Max. coverage (+): 0. Max coverage (-): 0

Region: chr14 4168480-4168495. Max. coverage (+): 0. Max coverage (-): 0

Region: chr14 4168496-4168511. Max. coverage (+): 0. Max coverage (-): 0

Region: chr14 4168512-4168527. Max. coverage (+): 0. Max coverage (-): 0

Region: chr14 4168528-4168543. Max. coverage (+): 0. Max coverage (-): 0

Region: chr14 4168544-4168559. Max. coverage (+): 0.68. Max coverage (-): 0

Region: chr14 4168560-4168575. Max. coverage (+): 0.68. Max coverage (-): 0

Region: chr14 4168576-4168590. Max. coverage (+): 0. Max coverage (-): 0

Region: chr14 4168591-4168606. Max. coverage (+): 0. Max coverage (-): 0

Region: chr14 4168607-4168622. Max. coverage (+): 0. Max coverage (-): 0

Region: chr14 4168623-4168638. Max. coverage (+): 0. Max coverage (-): 0

Region: chr14 4168639-4168654. Max. coverage (+): 0. Max coverage (-): 0

Region: chr14 4168655-4168670. Max. coverage (+): 0. Max coverage (-): 0

Region: chr14 4168671-4168686. Max. coverage (+): 0. Max coverage (-): 0

Region: chr14 4168687-4168702. Max. coverage (+): 0. Max coverage (-): 0

Region: chr14 4168703-4168717. Max. coverage (+): 0. Max coverage (-): 0

Region: chr14 4168718-4168733. Max. coverage (+): 0. Max coverage (-): 0

Region: chr14 4168734-4168749. Max. coverage (+): 0. Max coverage (-): 0

Region: chr14 4168750-4168765. Max. coverage (+): 0. Max coverage (-): 0

Region: chr14 4168766-4168781. Max. coverage (+): 2.15. Max coverage (-): 0

Region: chr14 4168782-4168797. Max. coverage (+): 0. Max coverage (-): 0

Region: chr14 4168798-4168813. Max. coverage (+): 0. Max coverage (-): 0

Region: chr14 4168814-4168828. Max. coverage (+): 0. Max coverage (-): 0

Region: chr14 4168829-4168844. Max. coverage (+): 3.07. Max coverage (-): 0

Region: chr14 4168845-4168860. Max. coverage (+): 3.07. Max coverage (-): 0

Region: chr14 4168861-4168876. Max. coverage (+): 3.34. Max coverage (-): 0

Region: chr14 4168877-4168892. Max. coverage (+): 0. Max coverage (-): 0

Region: chr14 4168893-4168908. Max. coverage (+): 0. Max coverage (-): 0

Region: chr14 4168909-4168924. Max. coverage (+): 0. Max coverage (-): 0

Region: chr14 4168925-4168940. Max. coverage (+): 0. Max coverage (-): 0

Region: chr14 4168941-4168955. Max. coverage (+): 0. Max coverage (-): 0

Region: chr14 4168956-4168971. Max. coverage (+): 0. Max coverage (-): 0

Region: chr14 4168972-4168987. Max. coverage (+): 0. Max coverage (-): 0

Region: chr14 4168988-4169003. Max. coverage (+): 0. Max coverage (-): 0

Region: chr14 4169004-4169019. Max. coverage (+): 0. Max coverage (-): 0

Region: chr14 4169020-4169035. Max. coverage (+): 0. Max coverage (-): 0

Region: chr14 4169036-4169051. Max. coverage (+): 0. Max coverage (-): 0

Region: chr14 4169052-4169066. Max. coverage (+): 0. Max coverage (-): 0

Region: chr14 4169067-4169082. Max. coverage (+): 0. Max coverage (-): 0

Region: chr14 4169083-4169098. Max. coverage (+): 0. Max coverage (-): 0

Region: chr14 4169099-4169114. Max. coverage (+): 0. Max coverage (-): 0

Region: chr14 4169115-4169130. Max. coverage (+): 0. Max coverage (-): 0

Region: chr14 4169131-4169146. Max. coverage (+): 0. Max coverage (-): 0

Region: chr14 4169147-4169162. Max. coverage (+): 0. Max coverage (-): 0

Region: chr14 4169163-4169177. Max. coverage (+): 0. Max coverage (-): 0

Region: chr14 4169178-4169193. Max. coverage (+): 0. Max coverage (-): 0

Region: chr14 4169194-4169209. Max. coverage (+): 0. Max coverage (-): 0

Region: chr14 4169210-4169225. Max. coverage (+): 0. Max coverage (-): 0

Region: chr14 4169226-4169241. Max. coverage (+): 4.91. Max coverage (-): 0

Region: chr14 4169242-4169257. Max. coverage (+): 0. Max coverage (-): 0

Region: chr14 4169258-4169273. Max. coverage (+): 0. Max coverage (-): 0

Region: chr14 4169274-4169289. Max. coverage (+): 0. Max coverage (-): 0

Region: chr14 4169290-4169304. Max. coverage (+): 0. Max coverage (-): 0

Region: chr14 4169305-4169320. Max. coverage (+): 0. Max coverage (-): 0

Region: chr14 4169321-4169336. Max. coverage (+): 0. Max coverage (-): 0

Region: chr14 4169337-4169352. Max. coverage (+): 0. Max coverage (-): 0

Region: chr14 4169353-4169368. Max. coverage (+): 0. Max coverage (-): 0

Region: chr14 4169369-4169384. Max. coverage (+): 0. Max coverage (-): 0

Region: chr14 4169385-4169400. Max. coverage (+): 0. Max coverage (-): 0

Region: chr14 4169401-4169415. Max. coverage (+): 0. Max coverage (-): 0

Region: chr14 4169416-4169431. Max. coverage (+): 0. Max coverage (-): 0

Region: chr14 4169432-4169447. Max. coverage (+): 0. Max coverage (-): 0

Region: chr14 4169448-4169463. Max. coverage (+): 0. Max coverage (-): 0

Region: chr14 4169464-4169479. Max. coverage (+): 0. Max coverage (-): 0

Region: chr14 4169480-4169495. Max. coverage (+): 0. Max coverage (-): 0

Region: chr14 4169496-4169511. Max. coverage (+): 0. Max coverage (-): 0

Region: chr14 4169512-4169527. Max. coverage (+): 0. Max coverage (-): 0

Region: chr14 4169528-4169542. Max. coverage (+): 8.86. Max coverage (-): 0

Region: chr14 4169543-4169558. Max. coverage (+): 5.59. Max coverage (-): 0

Region: chr14 4169559-4169574. Max. coverage (+): 0. Max coverage (-): 0

Region: chr14 4169575-4169590. Max. coverage (+): 0.74. Max coverage (-): 0

Region: chr14 4169591-4169606. Max. coverage (+): 0. Max coverage (-): 0

Region: chr14 4169607-4169622. Max. coverage (+): 0. Max coverage (-): 0

Region: chr14 4169623-4169638. Max. coverage (+): 0. Max coverage (-): 0

Region: chr14 4169639-4169653. Max. coverage (+): 0. Max coverage (-): 0

Region: chr14 4169654-4169669. Max. coverage (+): 0. Max coverage (-): 0

Region: chr14 4169670-4169685. Max. coverage (+): 0. Max coverage (-): 0

Region: chr14 4169686-4169701. Max. coverage (+): 0. Max coverage (-): 0

Region: chr14 4169702-4169717. Max. coverage (+): 0. Max coverage (-): 0

Region: chr14 4169718-4169733. Max. coverage (+): 0. Max coverage (-): 0

Region: chr14 4169734-4169749. Max. coverage (+): 0. Max coverage (-): 0

Region: chr14 4169750-4169765. Max. coverage (+): 0. Max coverage (-): 0

Region: chr14 4169766-4169780. Max. coverage (+): 0. Max coverage (-): 0

Region: chr14 4169781-4169796. Max. coverage (+): 0. Max coverage (-): 0

Region: chr14 4169797-4169812. Max. coverage (+): 0. Max coverage (-): 0

Region: chr14 4169813-4169828. Max. coverage (+): 4.81. Max coverage (-): 0

Region: chr14 4169829-4169844. Max. coverage (+): 4.81. Max coverage (-): 0

Region: chr14 4169845-4169860. Max. coverage (+): 0. Max coverage (-): 0

Region: chr14 4169861-4169876. Max. coverage (+): 0.58. Max coverage (-): 0

Region: chr14 4169877-4169891. Max. coverage (+): 0.58. Max coverage (-): 0

Region: chr14 4169892-4169907. Max. coverage (+): 0. Max coverage (-): 0

Region: chr14 4169908-4169923. Max. coverage (+): 0. Max coverage (-): 0

Region: chr14 4169924-4169939. Max. coverage (+): 2.04. Max coverage (-): 0

Region: chr14 4169940-4169955. Max. coverage (+): 0. Max coverage (-): 0

Region: chr14 4169956-4169971. Max. coverage (+): 0. Max coverage (-): 0

Region: chr14 4169972-4169987. Max. coverage (+): 0. Max coverage (-): 0

Region: chr14 4169988-4170003. Max. coverage (+): 0. Max coverage (-): 0

Region: chr14 4170004-4170018. Max. coverage (+): 0. Max coverage (-): 0

Region: chr14 4170019-4170034. Max. coverage (+): 0. Max coverage (-): 0

Region: chr14 4170035-4170050. Max. coverage (+): 1.75. Max coverage (-): 0

Region: chr14 4170051-4170066. Max. coverage (+): 0. Max coverage (-): 0

Region: chr14 4170067-4170082. Max. coverage (+): 0. Max coverage (-): 0

Region: chr14 4170083-4170098. Max. coverage (+): 0. Max coverage (-): 0

Region: chr14 4170099-4170114. Max. coverage (+): 0. Max coverage (-): 0

Region: chr14 4170115-4170129. Max. coverage (+): 0. Max coverage (-): 0

Region: chr14 4170130-4170145. Max. coverage (+): 0. Max coverage (-): 0

Region: chr14 4170146-4170161. Max. coverage (+): 13.06. Max coverage (-): 0

Region: chr14 4170162-4170177. Max. coverage (+): 11.63. Max coverage (-): 0

Region: chr14 4170178-4170193. Max. coverage (+): 4.08. Max coverage (-): 0

Region: chr14 4170194-4170209. Max. coverage (+): 0. Max coverage (-): 0

Region: chr14 4170210-4170225. Max. coverage (+): 0. Max coverage (-): 0

Region: chr14 4170226-4170241. Max. coverage (+): 0. Max coverage (-): 0

Region: chr14 4170242-4170256. Max. coverage (+): 0. Max coverage (-): 0

Region: chr14 4170257-4170272. Max. coverage (+): 0. Max coverage (-): 0

Region: chr14 4170273-4170288. Max. coverage (+): 1.42. Max coverage (-): 0

Region: chr14 4170289-4170304. Max. coverage (+): 1.42. Max coverage (-): 0

Region: chr14 4170305-4170320. Max. coverage (+): 0. Max coverage (-): 0

Region: chr14 4170321-4170336. Max. coverage (+): 0. Max coverage (-): 0

Region: chr14 4170337-4170352. Max. coverage (+): 0. Max coverage (-): 0

Region: chr14 4170353-4170367. Max. coverage (+): 0. Max coverage (-): 0

Region: chr14 4170368-4170383. Max. coverage (+): 0. Max coverage (-): 0

Region: chr14 4170384-4170399. Max. coverage (+): 0. Max coverage (-): 0

Region: chr14 4170400-4170415. Max. coverage (+): 0. Max coverage (-): 0

Region: chr14 4170416-4170431. Max. coverage (+): 0. Max coverage (-): 0

Region: chr14 4170432-4170447. Max. coverage (+): 0. Max coverage (-): 0

Region: chr14 4170448-4170463. Max. coverage (+): 0. Max coverage (-): 0

Region: chr14 4170464-4170479. Max. coverage (+): 0. Max coverage (-): 0

Region: chr14 4170480-4170494. Max. coverage (+): 0. Max coverage (-): 0

Region: chr14 4170495-4170510. Max. coverage (+): 0. Max coverage (-): 0

Region: chr14 4170511-4170526. Max. coverage (+): 0. Max coverage (-): 0

Region: chr14 4170527-4170542. Max. coverage (+): 0. Max coverage (-): 0

Region: chr14 4170543-4170558. Max. coverage (+): 2.07. Max coverage (-): 0

Region: chr14 4170559-4170574. Max. coverage (+): 0. Max coverage (-): 0

Region: chr14 4170575-4170590. Max. coverage (+): 0. Max coverage (-): 0

Region: chr14 4170591-4170605. Max. coverage (+): 0. Max coverage (-): 0

Region: chr14 4170606-4170621. Max. coverage (+): 0.52. Max coverage (-): 0

Region: chr14 4170622-4170637. Max. coverage (+): 0.52. Max coverage (-): 0

Region: chr14 4170638-4170653. Max. coverage (+): 0. Max coverage (-): 0

Region: chr14 4170654-4170669. Max. coverage (+): 4.74. Max coverage (-): 0

Region: chr14 4170670-4170685. Max. coverage (+): 7.42. Max coverage (-): 0

Region: chr14 4170686-4170701. Max. coverage (+): 0.92. Max coverage (-): 0

Region: chr14 4170702-4170716. Max. coverage (+): 3.27. Max coverage (-): 0

Region: chr14 4170717-4170732. Max. coverage (+): 2.87. Max coverage (-): 0

Region: chr14 4170733-4170748. Max. coverage (+): 3.92. Max coverage (-): 0

Region: chr14 4170749-4170764. Max. coverage (+): 0. Max coverage (-): 0

Region: chr14 4170765-4170780. Max. coverage (+): 0. Max coverage (-): 0

Region: chr14 4170781-4170796. Max. coverage (+): 0. Max coverage (-): 0

Region: chr14 4170797-4170812. Max. coverage (+): 0. Max coverage (-): 0

Region: chr14 4170813-4170828. Max. coverage (+): 0. Max coverage (-): 0

Region: chr14 4170829-4170843. Max. coverage (+): 4.17. Max coverage (-): 0

Region: chr14 4170844-4170859. Max. coverage (+): 6.81. Max coverage (-): 0

Region: chr14 4170860-4170875. Max. coverage (+): 6.81. Max coverage (-): 0

Region: chr14 4170876-4170891. Max. coverage (+): 0. Max coverage (-): 0

Region: chr14 4170892-4170907. Max. coverage (+): 0. Max coverage (-): 0

Region: chr14 4170908-4170923. Max. coverage (+): 0. Max coverage (-): 0

Region: chr14 4170924-4170939. Max. coverage (+): 0. Max coverage (-): 0

Region: chr14 4170940-4170954. Max. coverage (+): 0. Max coverage (-): 0

Region: chr14 4170955-4170970. Max. coverage (+): 0. Max coverage (-): 0

Region: chr14 4170971-4170986. Max. coverage (+): 0. Max coverage (-): 0

Region: chr14 4170987-4171002. Max. coverage (+): 1.82. Max coverage (-): 0

Region: chr14 4171003-4171018. Max. coverage (+): 0. Max coverage (-): 0

Region: chr14 4171019-4171034. Max. coverage (+): 0. Max coverage (-): 0

Region: chr14 4171035-4171050. Max. coverage (+): 0. Max coverage (-): 0

Region: chr14 4171051-4171066. Max. coverage (+): 0. Max coverage (-): 0

Region: chr14 4171067-4171081. Max. coverage (+): 0. Max coverage (-): 0

Region: chr14 4171082-4171097. Max. coverage (+): 0. Max coverage (-): 0

Region: chr14 4171098-4171113. Max. coverage (+): 0. Max coverage (-): 0

Region: chr14 4171114-4171129. Max. coverage (+): 0. Max coverage (-): 0

Region: chr14 4171130-4171145. Max. coverage (+): 1.31. Max coverage (-): 0

Region: chr14 4171146-4171161. Max. coverage (+): 0. Max coverage (-): 0

Region: chr14 4171162-4171177. Max. coverage (+): 0. Max coverage (-): 0

Region: chr14 4171178-4171192. Max. coverage (+): 0. Max coverage (-): 0

Region: chr14 4171193-4171208. Max. coverage (+): 0. Max coverage (-): 0

Region: chr14 4171209-4171224. Max. coverage (+): 0. Max coverage (-): 0

Region: chr14 4171225-4171240. Max. coverage (+): 0.9. Max coverage (-): 0

Region: chr14 4171241-4171256. Max. coverage (+): 0.9. Max coverage (-): 0

Region: chr14 4171257-4171272. Max. coverage (+): 0. Max coverage (-): 0

Region: chr14 4171273-4171288. Max. coverage (+): 0. Max coverage (-): 0

Region: chr14 4171289-4171304. Max. coverage (+): 0. Max coverage (-): 0

Region: chr14 4171305-4171319. Max. coverage (+): 0. Max coverage (-): 0

Region: chr14 4171320-4171335. Max. coverage (+): 0. Max coverage (-): 0

Region: chr14 4171336-4171351. Max. coverage (+): 0. Max coverage (-): 0

Region: chr14 4171352-4171367. Max. coverage (+): 2.96. Max coverage (-): 0

Region: chr14 4171368-4171383. Max. coverage (+): 0. Max coverage (-): 0

Region: chr14 4171384-4171399. Max. coverage (+): 0. Max coverage (-): 0

Region: chr14 4171400-4171415. Max. coverage (+): 0. Max coverage (-): 0

Region: chr14 4171416-4171430. Max. coverage (+): 0. Max coverage (-): 0

Region: chr14 4171431-4171446. Max. coverage (+): 0. Max coverage (-): 0

Region: chr14 4171447-4171462. Max. coverage (+): 0. Max coverage (-): 0

Region: chr14 4171463-4171478. Max. coverage (+): 0. Max coverage (-): 0

Region: chr14 4171479-4171494. Max. coverage (+): 0. Max coverage (-): 0

Region: chr14 4171495-4171510. Max. coverage (+): 0.93. Max coverage (-): 0

Region: chr14 4171511-4171526. Max. coverage (+): 0. Max coverage (-): 0

Region: chr14 4171527-4171542. Max. coverage (+): 0. Max coverage (-): 0

Region: chr14 4171543-4171557. Max. coverage (+): 0. Max coverage (-): 0

Region: chr14 4171558-4171573. Max. coverage (+): 0.77. Max coverage (-): 0

Region: chr14 4171574-4171589. Max. coverage (+): 0.77. Max coverage (-): 0

Region: chr14 4171590-4171605. Max. coverage (+): 0. Max coverage (-): 0

Region: chr14 4171606-4171621. Max. coverage (+): 0. Max coverage (-): 0

Region: chr14 4171622-4171637. Max. coverage (+): 0. Max coverage (-): 0

Region: chr14 4171638-4171653. Max. coverage (+): 0. Max coverage (-): 0

Region: chr14 4171654-4171668. Max. coverage (+): 0. Max coverage (-): 0

Region: chr14 4171669-4171684. Max. coverage (+): 0. Max coverage (-): 0

Region: chr14 4171685-4171700. Max. coverage (+): 0. Max coverage (-): 0

Region: chr14 4171701-4171716. Max. coverage (+): 0. Max coverage (-): 0

Region: chr14 4171717-4171732. Max. coverage (+): 0. Max coverage (-): 0

Region: chr14 4171733-4171748. Max. coverage (+): 0. Max coverage (-): 0

Region: chr14 4171749-4171764. Max. coverage (+): 9.89. Max coverage (-): 0

Region: chr14 4171765-4171780. Max. coverage (+): 9.89. Max coverage (-): 0

Region: chr14 4171781-4171795. Max. coverage (+): 0. Max coverage (-): 0

Region: chr14 4171796-4171811. Max. coverage (+): 0. Max coverage (-): 0

Region: chr14 4171812-4171827. Max. coverage (+): 0. Max coverage (-): 0

Region: chr14 4171828-4171843. Max. coverage (+): 0. Max coverage (-): 0

Region: chr14 4171844-4171859. Max. coverage (+): 0. Max coverage (-): 0

Region: chr14 4171860-4171875. Max. coverage (+): 2. Max coverage (-): 0

Region: chr14 4171876-4171891. Max. coverage (+): 2. Max coverage (-): 0

Region: chr14 4171892-4171906. Max. coverage (+): 0. Max coverage (-): 0

Region: chr14 4171907-4171922. Max. coverage (+): 0. Max coverage (-): 0

Region: chr14 4171923-4171938. Max. coverage (+): 0. Max coverage (-): 0

Region: chr14 4171939-4171954. Max. coverage (+): 0. Max coverage (-): 0

Region: chr14 4171955-4171970. Max. coverage (+): 0. Max coverage (-): 0

Region: chr14 4171971-4171986. Max. coverage (+): 4.02. Max coverage (-): 0

Region: chr14 4171987-4172002. Max. coverage (+): 0. Max coverage (-): 0

Region: chr14 4172003-4172018. Max. coverage (+): 0. Max coverage (-): 0

Region: chr14 4172019-4172033. Max. coverage (+): 0. Max coverage (-): 0

Region: chr14 4172034-4172049. Max. coverage (+): 0. Max coverage (-): 0

Region: chr14 4172050-4172065. Max. coverage (+): 0. Max coverage (-): 0

Region: chr14 4172066-4172081. Max. coverage (+): 0. Max coverage (-): 0

Region: chr14 4172082-4172097. Max. coverage (+): 0. Max coverage (-): 0

Region: chr14 4172098-4172113. Max. coverage (+): 0. Max coverage (-): 0

Region: chr14 4172114-4172129. Max. coverage (+): 0. Max coverage (-): 0

Region: chr14 4172130-4172144. Max. coverage (+): 0. Max coverage (-): 0

Region: chr14 4172145-4172160. Max. coverage (+): 0. Max coverage (-): 0

Region: chr14 4172161-4172176. Max. coverage (+): 0. Max coverage (-): 0

Region: chr14 4172177-4172192. Max. coverage (+): 0. Max coverage (-): 0

Region: chr14 4172193-4172208. Max. coverage (+): 0. Max coverage (-): 0

Region: chr14 4172209-4172224. Max. coverage (+): 7.68. Max coverage (-): 0

Region: chr14 4172225-4172240. Max. coverage (+): 0. Max coverage (-): 0

Region: chr14 4172241-4172256. Max. coverage (+): 0. Max coverage (-): 0

Region: chr14 4172257-4172271. Max. coverage (+): 0. Max coverage (-): 0

Region: chr14 4172272-4172287. Max. coverage (+): 0. Max coverage (-): 0

Region: chr14 4172288-4172303. Max. coverage (+): 0. Max coverage (-): 0

Region: chr14 4172304-4172319. Max. coverage (+): 0. Max coverage (-): 0

Region: chr14 4172320-4172335. Max. coverage (+): 0. Max coverage (-): 0

Region: chr14 4172336-4172351. Max. coverage (+): 0. Max coverage (-): 0

Region: chr14 4172352-4172367. Max. coverage (+): 0. Max coverage (-): 0

Region: chr14 4172368-4172382. Max. coverage (+): 0. Max coverage (-): 0

Region: chr14 4172383-4172398. Max. coverage (+): 0. Max coverage (-): 0

Region: chr14 4172399-4172414. Max. coverage (+): 0. Max coverage (-): 0

Region: chr14 4172415-4172430. Max. coverage (+): 0. Max coverage (-): 0

Region: chr14 4172431-4172446. Max. coverage (+): 0. Max coverage (-): 0

Region: chr14 4172447-4172462. Max. coverage (+): 5. Max coverage (-): 0

Region: chr14 4172463-4172478. Max. coverage (+): 5. Max coverage (-): 0

Region: chr14 4172479-4172493. Max. coverage (+): 0. Max coverage (-): 0

Region: chr14 4172494-4172509. Max. coverage (+): 0. Max coverage (-): 0

Region: chr14 4172510-4172525. Max. coverage (+): 3.77. Max coverage (-): 0

Region: chr14 4172526-4172541. Max. coverage (+): 3.77. Max coverage (-): 0

Region: chr14 4172542-4172557. Max. coverage (+): 0. Max coverage (-): 0

Region: chr14 4172558-4172573. Max. coverage (+): 3.67. Max coverage (-): 0

Region: chr14 4172574-4172589. Max. coverage (+): 4.97. Max coverage (-): 0

Region: chr14 4172590-4172605. Max. coverage (+): 0. Max coverage (-): 0

Region: chr14 4172606-4172620. Max. coverage (+): 0. Max coverage (-): 0

Region: chr14 4172621-4172636. Max. coverage (+): 0. Max coverage (-): 0

Region: chr14 4172637-4172652. Max. coverage (+): 0. Max coverage (-): 0

Region: chr14 4172653-4172668. Max. coverage (+): 4.64. Max coverage (-): 0

Region: chr14 4172669-4172684. Max. coverage (+): 0. Max coverage (-): 0

Region: chr14 4172685-4172700. Max. coverage (+): 0. Max coverage (-): 0

Region: chr14 4172701-4172716. Max. coverage (+): 0. Max coverage (-): 0

Region: chr14 4172717-4172731. Max. coverage (+): 0. Max coverage (-): 0

Region: chr14 4172732-4172747. Max. coverage (+): 0. Max coverage (-): 0

Region: chr14 4172748-4172763. Max. coverage (+): 0. Max coverage (-): 0

Region: chr14 4172764-4172779. Max. coverage (+): 0. Max coverage (-): 0

Region: chr14 4172780-4172795. Max. coverage (+): 7.23. Max coverage (-): 0

Region: chr14 4172796-4172811. Max. coverage (+): 0. Max coverage (-): 0

Region: chr14 4172812-4172827. Max. coverage (+): 0. Max coverage (-): 0

Region: chr14 4172828-4172843. Max. coverage (+): 0. Max coverage (-): 0

Region: chr14 4172844-4172858. Max. coverage (+): 0. Max coverage (-): 0

Region: chr14 4172859-4172874. Max. coverage (+): 0. Max coverage (-): 0

Region: chr14 4172875-4172890. Max. coverage (+): 0. Max coverage (-): 0

Region: chr14 4172891-4172906. Max. coverage (+): 0. Max coverage (-): 0

Region: chr14 4172907-4172922. Max. coverage (+): 0. Max coverage (-): 0

Region: chr14 4172923-4172938. Max. coverage (+): 0. Max coverage (-): 0

Region: chr14 4172939-4172954. Max. coverage (+): 0. Max coverage (-): 0

Region: chr14 4172955-4172969. Max. coverage (+): 0. Max coverage (-): 0

Region: chr14 4172970-4172985. Max. coverage (+): 0. Max coverage (-): 0

Region: chr14 4172986-4173001. Max. coverage (+): 0. Max coverage (-): 0

Region: chr14 4173002-4173017. Max. coverage (+): 0. Max coverage (-): 0

Region: chr14 4173018-4173033. Max. coverage (+): 0. Max coverage (-): 0

Region: chr14 4173034-4173049. Max. coverage (+): 0. Max coverage (-): 0

Region: chr14 4173050-4173065. Max. coverage (+): 0. Max coverage (-): 0

Region: chr14 4173066-4173081. Max. coverage (+): 0. Max coverage (-): 0

Region: chr14 4173082-4173096. Max. coverage (+): 0. Max coverage (-): 0

Region: chr14 4173097-4173112. Max. coverage (+): 0. Max coverage (-): 0

Region: chr14 4173113-4173128. Max. coverage (+): 1.61. Max coverage (-): 0

Region: chr14 4173129-4173144. Max. coverage (+): 1.61. Max coverage (-): 0

Region: chr14 4173145-4173160. Max. coverage (+): 0. Max coverage (-): 0

Region: chr14 4173161-4173176. Max. coverage (+): 0. Max coverage (-): 0

Region: chr14 4173177-4173192. Max. coverage (+): 0.32. Max coverage (-): 0

Region: chr14 4173193-4173207. Max. coverage (+): 1.41. Max coverage (-): 0

Region: chr14 4173208-4173223. Max. coverage (+): 1.41. Max coverage (-): 0

Region: chr14 4173224-4173239. Max. coverage (+): 0. Max coverage (-): 0

Region: chr14 4173240-4173255. Max. coverage (+): 3.41. Max coverage (-): 0

Region: chr14 4173256-4173271. Max. coverage (+): 3.41. Max coverage (-): 0

Region: chr14 4173272-4173287. Max. coverage (+): 0. Max coverage (-): 0

Region: chr14 4173288-4173303. Max. coverage (+): 0. Max coverage (-): 0

Region: chr14 4173304-4173319. Max. coverage (+): 0. Max coverage (-): 0

Region: chr14 4173320-4173334. Max. coverage (+): 0. Max coverage (-): 0

Region: chr14 4173335-4173350. Max. coverage (+): 0. Max coverage (-): 0

Region: chr14 4173351-4173366. Max. coverage (+): 0. Max coverage (-): 0

Region: chr14 4173367-4173382. Max. coverage (+): 0. Max coverage (-): 0

Region: chr14 4173383-4173398. Max. coverage (+): 0. Max coverage (-): 0

Region: chr14 4173399-4173414. Max. coverage (+): 0. Max coverage (-): 0

Region: chr14 4173415-4173430. Max. coverage (+): 0. Max coverage (-): 0

Region: chr14 4173431-4173445. Max. coverage (+): 0. Max coverage (-): 0

Region: chr14 4173446-4173461. Max. coverage (+): 0. Max coverage (-): 0

Region: chr14 4173462-4173477. Max. coverage (+): 0. Max coverage (-): 0

Region: chr14 4173478-4173493. Max. coverage (+): 0. Max coverage (-): 0

Region: chr14 4173494-4173509. Max. coverage (+): 0. Max coverage (-): 0

Region: chr14 4173510-4173525. Max. coverage (+): 0. Max coverage (-): 0

Region: chr14 4173526-4173541. Max. coverage (+): 0. Max coverage (-): 0

Region: chr14 4173542-4173557. Max. coverage (+): 6.18. Max coverage (-): 0

Region: chr14 4173558-4173572. Max. coverage (+): 0. Max coverage (-): 0

Region: chr14 4173573-4173588. Max. coverage (+): 0. Max coverage (-): 0

Region: chr14 4173589-4173604. Max. coverage (+): 0. Max coverage (-): 0

Region: chr14 4173605-4173620. Max. coverage (+): 2.21. Max coverage (-): 0

Region: chr14 4173621-4173636. Max. coverage (+): 0. Max coverage (-): 0

Region: chr14 4173637-4173652. Max. coverage (+): 0. Max coverage (-): 0

Region: chr14 4173653-4173668. Max. coverage (+): 0. Max coverage (-): 0

Region: chr14 4173669-4173683. Max. coverage (+): 0. Max coverage (-): 0

Region: chr14 4173684-4173699. Max. coverage (+): 0. Max coverage (-): 0

Region: chr14 4173700-4173715. Max. coverage (+): 0. Max coverage (-): 1.19

Region: chr14 4173716-4173731. Max. coverage (+): 0. Max coverage (-): 0

Region: chr14 4173732-4173747. Max. coverage (+): 0. Max coverage (-): 0

Region: chr14 4173748-4173763. Max. coverage (+): 0. Max coverage (-): 0

Region: chr14 4173764-4173779. Max. coverage (+): 0. Max coverage (-): 0

Region: chr14 4173780-4173795. Max. coverage (+): 0. Max coverage (-): 0

Region: chr14 4173796-4173810. Max. coverage (+): 0. Max coverage (-): 0

Region: chr14 4173811-4173826. Max. coverage (+): 0. Max coverage (-): 0

Region: chr14 4173827-4173842. Max. coverage (+): 0. Max coverage (-): 0

Region: chr14 4173843-4173858. Max. coverage (+): 0. Max coverage (-): 0

Region: chr14 4173859-4173874. Max. coverage (+): 0. Max coverage (-): 0

Region: chr14 4173875-4173890. Max. coverage (+): 3.39. Max coverage (-): 0

Region: chr14 4173891-4173906. Max. coverage (+): 0. Max coverage (-): 0

Region: chr14 4173907-4173921. Max. coverage (+): 0. Max coverage (-): 0

Region: chr14 4173922-4173937. Max. coverage (+): 0.84. Max coverage (-): 0

Region: chr14 4173938-4173953. Max. coverage (+): 0. Max coverage (-): 0

Region: chr14 4173954-4173969. Max. coverage (+): 4.94. Max coverage (-): 0

Region: chr14 4173970-4173985. Max. coverage (+): 0.87. Max coverage (-): 0

Region: chr14 4173986-4174001. Max. coverage (+): 0. Max coverage (-): 0

Region: chr14 4174002-4174017. Max. coverage (+): 0. Max coverage (-): 0

Region: chr14 4174018-4174032. Max. coverage (+): 0. Max coverage (-): 0

Region: chr14 4174033-4174048. Max. coverage (+): 0. Max coverage (-): 0

Region: chr14 4174049-4174064. Max. coverage (+): 3.37. Max coverage (-): 0

Region: chr14 4174065-4174080. Max. coverage (+): 3.37. Max coverage (-): 0

Region: chr14 4174081-4174096. Max. coverage (+): 0. Max coverage (-): 0

Region: chr14 4174097-4174112. Max. coverage (+): 0. Max coverage (-): 0

Region: chr14 4174113-4174128. Max. coverage (+): 0. Max coverage (-): 0

Region: chr14 4174129-4174144. Max. coverage (+): 0. Max coverage (-): 0

Region: chr14 4174145-4174159. Max. coverage (+): 0. Max coverage (-): 0

Region: chr14 4174160-4174175. Max. coverage (+): 0. Max coverage (-): 5.24

Region: chr14 4174176-4174191. Max. coverage (+): 0. Max coverage (-): 0

Region: chr14 4174192-4174207. Max. coverage (+): 0. Max coverage (-): 0

Region: chr14 4174208-4174223. Max. coverage (+): 0. Max coverage (-): 0

Region: chr14 4174224-4174239. Max. coverage (+): 0. Max coverage (-): 0

Region: chr14 4174240-4174255. Max. coverage (+): 0. Max coverage (-): 0

Region: chr14 4174256-4174270. Max. coverage (+): 0. Max coverage (-): 0

Region: chr14 4174271-4174286. Max. coverage (+): 0. Max coverage (-): 0

Region: chr14 4174287-4174302. Max. coverage (+): 0. Max coverage (-): 0

Region: chr14 4174303-4174318. Max. coverage (+): 0. Max coverage (-): 0

Region: chr14 4174319-4174334. Max. coverage (+): 0.49. Max coverage (-): 0

Region: chr14 4174335-4174350. Max. coverage (+): 0. Max coverage (-): 0

Region: chr14 4174351-4174366. Max. coverage (+): 0. Max coverage (-): 0

Region: chr14 4174367-4174382. Max. coverage (+): 0. Max coverage (-): 0

Region: chr14 4174383-4174397. Max. coverage (+): 0. Max coverage (-): 0

Region: chr14 4174398-4174413. Max. coverage (+): 5.66. Max coverage (-): 0

Region: chr14 4174414-4174429. Max. coverage (+): 5.66. Max coverage (-): 0

Region: chr14 4174430-4174445. Max. coverage (+): 0. Max coverage (-): 0

Region: chr14 4174446-4174461. Max. coverage (+): 7.17. Max coverage (-): 0

Region: chr14 4174462-4174477. Max. coverage (+): 7.17. Max coverage (-): 0

Region: chr14 4174478-4174493. Max. coverage (+): 0. Max coverage (-): 0

Region: chr14 4174494-4174508. Max. coverage (+): 0. Max coverage (-): 0

Region: chr14 4174509-4174524. Max. coverage (+): 0. Max coverage (-): 0

Region: chr14 4174525-4174540. Max. coverage (+): 0. Max coverage (-): 0

Region: chr14 4174541-4174556. Max. coverage (+): 0. Max coverage (-): 0

Region: chr14 4174557-4174572. Max. coverage (+): 0. Max coverage (-): 0

Region: chr14 4174573-4174588. Max. coverage (+): 0. Max coverage (-): 0

Region: chr14 4174589-4174604. Max. coverage (+): 0. Max coverage (-): 0

Region: chr14 4174605-4174620. Max. coverage (+): 0. Max coverage (-): 0

Region: chr14 4174621-4174635. Max. coverage (+): 0. Max coverage (-): 0

Region: chr14 4174636-4174651. Max. coverage (+): 0. Max coverage (-): 0

Region: chr14 4174652-4174667. Max. coverage (+): 0. Max coverage (-): 0

Region: chr14 4174668-4174683. Max. coverage (+): 0. Max coverage (-): 0

Region: chr14 4174684-4174699. Max. coverage (+): 0. Max coverage (-): 0

Region: chr14 4174700-4174715. Max. coverage (+): 1.28. Max coverage (-): 0

Region: chr14 4174716-4174731. Max. coverage (+): 1.28. Max coverage (-): 0

Region: chr14 4174732-4174746. Max. coverage (+): 0. Max coverage (-): 0

Region: chr14 4174747-4174762. Max. coverage (+): 0. Max coverage (-): 0

Region: chr14 4174763-4174778. Max. coverage (+): 0. Max coverage (-): 0

Region: chr14 4174779-4174794. Max. coverage (+): 0. Max coverage (-): 0

Region: chr14 4174795-4174810. Max. coverage (+): 0. Max coverage (-): 0

Region: chr14 4174811-4174826. Max. coverage (+): 0. Max coverage (-): 0

Region: chr14 4174827-4174842. Max. coverage (+): 0. Max coverage (-): 0

Region: chr14 4174843-4174858. Max. coverage (+): 0. Max coverage (-): 0

Region: chr14 4174859-4174873. Max. coverage (+): 0. Max coverage (-): 0

Region: chr14 4174874-4174889. Max. coverage (+): 0. Max coverage (-): 0

Region: chr14 4174890-4174905. Max. coverage (+): 0. Max coverage (-): 0

Region: chr14 4174906-4174921. Max. coverage (+): 0. Max coverage (-): 0

Region: chr14 4174922-4174937. Max. coverage (+): 0. Max coverage (-): 0

Region: chr14 4174938-4174953. Max. coverage (+): 0. Max coverage (-): 0

Region: chr14 4174954-4174969. Max. coverage (+): 0. Max coverage (-): 0

Region: chr14 4174970-4174984. Max. coverage (+): 0. Max coverage (-): 0

Region: chr14 4174985-4175000. Max. coverage (+): 0. Max coverage (-): 0

Region: chr14 4175001-4175016. Max. coverage (+): 0. Max coverage (-): 0

Region: chr14 4175017-4175032. Max. coverage (+): 0. Max coverage (-): 0

Region: chr14 4175033-4175048. Max. coverage (+): 0. Max coverage (-): 0

Region: chr14 4175049-4175064. Max. coverage (+): 0. Max coverage (-): 0

Region: chr14 4175065-4175080. Max. coverage (+): 0. Max coverage (-): 0

Region: chr14 4175081-4175096. Max. coverage (+): 0. Max coverage (-): 0

Region: chr14 4175097-4175111. Max. coverage (+): 0. Max coverage (-): 0

Region: chr14 4175112-4175127. Max. coverage (+): 0. Max coverage (-): 0

Region: chr14 4175128-4175143. Max. coverage (+): 0. Max coverage (-): 0

Region: chr14 4175144-4175159. Max. coverage (+): 0. Max coverage (-): 0

Region: chr14 4175160-4175175. Max. coverage (+): 0. Max coverage (-): 0

Region: chr14 4175176-4175191. Max. coverage (+): 0. Max coverage (-): 0

Region: chr14 4175192-4175207. Max. coverage (+): 0. Max coverage (-): 0

Region: chr14 4175208-4175222. Max. coverage (+): 0. Max coverage (-): 0

Region: chr14 4175223-4175238. Max. coverage (+): 0. Max coverage (-): 0

Region: chr14 4175239-4175254. Max. coverage (+): 0. Max coverage (-): 0

Region: chr14 4175255-4175270. Max. coverage (+): 0. Max coverage (-): 0

Region: chr14 4175271-4175286. Max. coverage (+): 0. Max coverage (-): 0

Region: chr14 4175287-4175302. Max. coverage (+): 0.7. Max coverage (-): 0

Region: chr14 4175303-4175318. Max. coverage (+): 0. Max coverage (-): 0

Region: chr14 4175319-4175334. Max. coverage (+): 0. Max coverage (-): 0

Region: chr14 4175335-4175349. Max. coverage (+): 0. Max coverage (-): 0

Region: chr14 4175350-4175365. Max. coverage (+): 0. Max coverage (-): 0

Region: chr14 4175366-4175381. Max. coverage (+): 0. Max coverage (-): 0

Region: chr14 4175382-4175397. Max. coverage (+): 0. Max coverage (-): 0

Region: chr14 4175398-4175413. Max. coverage (+): 0. Max coverage (-): 0

Region: chr14 4175414-4175429. Max. coverage (+): 1.02. Max coverage (-): 0

Region: chr14 4175430-4175445. Max. coverage (+): 1.02. Max coverage (-): 0

Region: chr14 4175446-. Max. coverage (+): 0. Max coverage (-): 0

RepeatMasker Color Code

**+**

100-98% Identity

<98-95% Identity

<95-90% Identity

<90-85% Identity

<85-80% Identity

<80-75% Identity

<75-70% Identity

<70% Identity

**-**

Gene Set Color Code

**+**

Gene

Pseudogene

**-**

Topology/Coverage Color Code

Coverage Plus Strand

Coverage Minus Strand

Mainstrand: Plus

Mainstrand: Minus

Complementary Strand

Flanking Region  
(if option -flank >0)

Gene Set Annotation  

**1. AGO2 (protein coding, ENSBTAG00000001579) Tr:00000002068 Ex:19**: 4167492-4168483 (+)

  
RepeatMasker Annotation  

**1. AT\_rich**: 4168283-4168310 (+), Divergence to consensus: 71.4%  
**2. (CCCCCT)n**: 4168708-4168738 (+), Divergence to consensus: 6.5%  
**3. (TG)n**: 4169389-4169436 (+), Divergence to consensus: 29.2%  
**4. (TG)n**: 4172390-4172425 (+), Divergence to consensus: 5.6%

  
Transcription Factor Binding Sites  

**Gata4** (Sequence: AGATAAG (-): 4170261)  
**SOX9** (Sequence: TTATTGTT (+): 4171265)
