# Supplementary material for: Small RNA sequencing of cryopreserved semen from single bull revealed altered miRNAs and piRNAs expression between High- and Low-motile sperm populations
Source: BMC Genomics. 2017 Jan 4;18:14. doi: 10.1186/s12864-016-3394-7 (PMC5209821; doi:10.1186/s12864-016-3394-7)
Supplement: Additional file 3: — Details for each piRNA clusters found in High Motile (HM) sperm fraction. Genes, repeats, transposable elements and transcription factors binding sites falling within the cluster regions were reported. (ZIP 1896 kb) [file 12864_2016_3394_MOESM3_ESM.zip › 21.html]

piRNA cluster 21


Predicted piRNA cluster no. 21     previous   next
  

Show proTRAC run info
Hide proTRAC run info

================================= proTRAC ====================================  
VERSION: 2.1                                    LAST MODIFIED: 06. October 2015  
  
Please cite:  
Rosenkranz D, Zischler H. proTRAC - a software for probabilistic piRNA cluster  
detection, visualization and analysis. 2012. BMC Bioinformatics 13:5.  
  
and (for proTRAC 2.0 and later):  
Rosenkranz D, Rudloff S, Bastuck K, Ketting RF, Zischler H. Tupaia small RNAs  
provide insights into function and evolution of RNAi-based transposon defense  
in mammals. 2015. RNA 21(5):911-922.  
  
Contact:  
David Rosenkranz  
Institute of Anthropology, small RNA group  
Johannes Gutenberg University Mainz  
email: rosenkranz@uni-mainz.de  
  
You can find the latest proTRAC version at:  
http://sourceforge.net/projects/protrac/files  
http://www.smallRNAgroup-mainz.de/software  
==============================================================================  
  
PARAMETERS:  
Map file: .............../storage/core/barbara/genhome/smallRNA/fertility/Sample\_motile/pirna/Sample\_motile\_26-33\_collapsed.fa.no-dust.map.weighted-10000-1000-b-0  
Genome file: ............/storage/core/barbara/genhome/smallRNA/fertility/Sample\_all/pirna/bt\_311\_chrY.fa  
RepeatMasker annotation: /storage/genomes/bt\_umd31/GCF\_000003055.6\_Bos\_taurus\_UMD\_3.1.1\_repeatMasker\_chr.out  
GeneSet:................./storage/core/barbara/genhome/smallRNA/fertility/Sample\_all/pirna/full.gtf  
  
Significant (p<=0.01) hit density will be calculated based  
on observed hit distribution.  
  
Sliding window size: ........................................ 5000 bp  
Sliding window increament: .................................. 1000 bp  
Normalize each hit by number of genomic hits: ............... 1 [0=no/1=yes]  
Normalize each hit by number of sequence reads: ............. 1 [0=no/1=yes]  
Normalize values (-> per million mapped reads): ............. 1 [0=no/1=yes]  
Min. fraction of hits with 1T(U) or 10A: .................... 0.75  
Alternatively: Min. fraction of hits with 1T(U) and 10A: .... 0.5  
Min. fraction of hits with typical piRNA length: ............ 0.75  
Typical piRNA length: ....................................... 26-33 nt  
Min. size of a piRNA cluster: ............................... 5000 bp.  
Min. number of hits (absolute): ............................. 0  
Min. number of hits (normalized): ........................... 0  
Min. fraction of hits on the mainstrand: .................... 0.75  
Top fraction of mapped sequences (in terms of read counts): . 1%  
Top fraction accounts for max. n% of sequence reads: ........ 90%  
Min. fraction of hits on each arm of a bidirectional cluster: 0.1  
Output image file for each cluster: ......................... 0 [0=no/1=yes]  
Output html file for each cluster: .......................... 1 [0=no/1=yes]  
Output a summary table: ..................................... 1 [0=no/1=yes]  
Output a FASTA file for each cluster (piRNA sequences): ..... 1 [0=no/1=yes]  
Output a FASTA file comprising cluster sequences: ........... 1 [0=no/1=yes]  
Search DNA motifs in clusters: .............................. 1 [0=no/1=yes]  
Output flanking sequences: +/- .............................. 0 bp  
Output ~.pTi file: .......................................... 1 [0=no/1=yes]  
==============================================================================  
  
  
Genome size (without gaps): ............ 2678902517 bp  
Gaps (N/X/-): .......................... 53837044 bp  
Mapped reads: .......................... 658825247023  
Non-identical sequences: ............... 514171  
Genomic hits: .......................... 764233  
Significant densitiy of mapped reads: .. 12867599.5173724 reads/kb

Show proTRAC cluster info
Hide proTRAC cluster info

|  |  |
| --- | --- |
| Location | chr14 |
| Coordinates | 15168079-15194870 |
| Size [bp] | 26792 |
| Sequence hit loci | 5183 |
| Mapped reads (normalized) | 1994345633.3 |
| Mapped reads (normalized) per kb | 74438102.2 |
| Normalized reads with 1T (1U) | 76.9% |
| Normalized reads with 10A | 34.1% |
| Normalized reads with length 26-33 nt | 100% |
| Normalized reads on the main strand(s) | 95% |
| Predicted directionality | mono:minus |

100%

0%

1T (1U)  
reads

10A reads

26-33 nt  
reads

reads on mainstrand

**Either the amount of reads with 1T (1U) OR 10A has to exceed 75% (set with option: -1Tor10A)  
Alternatively the amount of reads with 1T (1U) AND 10A has to exceed 50% (set with option: -1Tand10A)  
Minimum amount of reads with preferred size is 75% (set with option: -pisize)  
Minimum amount of reads on the main strand(s) is 75% (set with option: -clstrand)**

Show read coverage
Hide read coverage

WHAT DO I SEE HERE?  
This chart shows the location of mapped sequence reads within a predicted piRNA cluster. The color refers to the number of genomic hits produced by the sequence read in question. A dark red bar indicates that this sequence read produces many other hits elsewhere in the genome. Many adjacent red or yellow bars can indicate the presence of a multi-copy element such as transposons or rRNA genes. A dark green bar indicates that this sequence read maps uniquely to this locus.

1 hit

2-5 hits

6-10 hits

11-20 hits

21-50 hits

51-100 hits

> 100 hits

chr14

15168079

15194870

Gene Set

RepeatMasker

Mapped  
Reads

65.65

plus strand

minus strand

65.65

Region: chr14 4175447-15168105. Max. coverage (+): 0. Max coverage (-): 1.21

Region: chr14 15168106-15168159. Max. coverage (+): 0. Max coverage (-): 1.04

Region: chr14 15168160-15168212. Max. coverage (+): 0. Max coverage (-): 0

Region: chr14 15168213-15168266. Max. coverage (+): 0. Max coverage (-): 1.11

Region: chr14 15168267-15168320. Max. coverage (+): 0. Max coverage (-): 0.13

Region: chr14 15168321-15168373. Max. coverage (+): 0. Max coverage (-): 0.41

Region: chr14 15168374-15168427. Max. coverage (+): 0. Max coverage (-): 0.2

Region: chr14 15168428-15168480. Max. coverage (+): 0.23. Max coverage (-): 0.2

Region: chr14 15168481-15168534. Max. coverage (+): 0. Max coverage (-): 0.78

Region: chr14 15168535-15168588. Max. coverage (+): 0. Max coverage (-): 1.84

Region: chr14 15168589-15168641. Max. coverage (+): 0. Max coverage (-): 3.78

Region: chr14 15168642-15168695. Max. coverage (+): 0. Max coverage (-): 0

Region: chr14 15168696-15168748. Max. coverage (+): 0. Max coverage (-): 0

Region: chr14 15168749-15168802. Max. coverage (+): 0. Max coverage (-): 0

Region: chr14 15168803-15168855. Max. coverage (+): 0. Max coverage (-): 0

Region: chr14 15168856-15168909. Max. coverage (+): 1.28. Max coverage (-): 0.99

Region: chr14 15168910-15168963. Max. coverage (+): 0.3. Max coverage (-): 0

Region: chr14 15168964-15169016. Max. coverage (+): 1.04. Max coverage (-): 0.74

Region: chr14 15169017-15169070. Max. coverage (+): 0.73. Max coverage (-): 2.25

Region: chr14 15169071-15169123. Max. coverage (+): 0. Max coverage (-): 0

Region: chr14 15169124-15169177. Max. coverage (+): 0. Max coverage (-): 2.7

Region: chr14 15169178-15169231. Max. coverage (+): 0. Max coverage (-): 0

Region: chr14 15169232-15169284. Max. coverage (+): 0. Max coverage (-): 0

Region: chr14 15169285-15169338. Max. coverage (+): 0. Max coverage (-): 0

Region: chr14 15169339-15169391. Max. coverage (+): 0. Max coverage (-): 0

Region: chr14 15169392-15169445. Max. coverage (+): 0. Max coverage (-): 0

Region: chr14 15169446-15169498. Max. coverage (+): 0.93. Max coverage (-): 0

Region: chr14 15169499-15169552. Max. coverage (+): 0. Max coverage (-): 2.32

Region: chr14 15169553-15169606. Max. coverage (+): 0. Max coverage (-): 0

Region: chr14 15169607-15169659. Max. coverage (+): 0. Max coverage (-): 2.98

Region: chr14 15169660-15169713. Max. coverage (+): 0. Max coverage (-): 2.24

Region: chr14 15169714-15169766. Max. coverage (+): 0. Max coverage (-): 0

Region: chr14 15169767-15169820. Max. coverage (+): 0. Max coverage (-): 0

Region: chr14 15169821-15169874. Max. coverage (+): 0. Max coverage (-): 0

Region: chr14 15169875-15169927. Max. coverage (+): 0.32. Max coverage (-): 0

Region: chr14 15169928-15169981. Max. coverage (+): 1.12. Max coverage (-): 2.43

Region: chr14 15169982-15170034. Max. coverage (+): 0. Max coverage (-): 0

Region: chr14 15170035-15170088. Max. coverage (+): 0. Max coverage (-): 2.92

Region: chr14 15170089-15170141. Max. coverage (+): 0. Max coverage (-): 0

Region: chr14 15170142-15170195. Max. coverage (+): 0. Max coverage (-): 0

Region: chr14 15170196-15170249. Max. coverage (+): 0. Max coverage (-): 0

Region: chr14 15170250-15170302. Max. coverage (+): 0. Max coverage (-): 0

Region: chr14 15170303-15170356. Max. coverage (+): 0. Max coverage (-): 0

Region: chr14 15170357-15170409. Max. coverage (+): 1. Max coverage (-): 0

Region: chr14 15170410-15170463. Max. coverage (+): 0. Max coverage (-): 2.32

Region: chr14 15170464-15170517. Max. coverage (+): 0. Max coverage (-): 2.32

Region: chr14 15170518-15170570. Max. coverage (+): 0. Max coverage (-): 5.99

Region: chr14 15170571-15170624. Max. coverage (+): 0. Max coverage (-): 2.52

Region: chr14 15170625-15170677. Max. coverage (+): 0. Max coverage (-): 0

Region: chr14 15170678-15170731. Max. coverage (+): 0. Max coverage (-): 0

Region: chr14 15170732-15170784. Max. coverage (+): 0. Max coverage (-): 0

Region: chr14 15170785-15170838. Max. coverage (+): 0. Max coverage (-): 0

Region: chr14 15170839-15170892. Max. coverage (+): 0. Max coverage (-): 0

Region: chr14 15170893-15170945. Max. coverage (+): 0. Max coverage (-): 0

Region: chr14 15170946-15170999. Max. coverage (+): 0. Max coverage (-): 0

Region: chr14 15171000-15171052. Max. coverage (+): 0. Max coverage (-): 0

Region: chr14 15171053-15171106. Max. coverage (+): 0. Max coverage (-): 0

Region: chr14 15171107-15171160. Max. coverage (+): 1.13. Max coverage (-): 0

Region: chr14 15171161-15171213. Max. coverage (+): 1.13. Max coverage (-): 0

Region: chr14 15171214-15171267. Max. coverage (+): 0. Max coverage (-): 0

Region: chr14 15171268-15171320. Max. coverage (+): 0. Max coverage (-): 5.99

Region: chr14 15171321-15171374. Max. coverage (+): 0. Max coverage (-): 0.88

Region: chr14 15171375-15171427. Max. coverage (+): 0. Max coverage (-): 2.52

Region: chr14 15171428-15171481. Max. coverage (+): 0. Max coverage (-): 0

Region: chr14 15171482-15171535. Max. coverage (+): 0. Max coverage (-): 0

Region: chr14 15171536-15171588. Max. coverage (+): 0. Max coverage (-): 0

Region: chr14 15171589-15171642. Max. coverage (+): 0. Max coverage (-): 0

Region: chr14 15171643-15171695. Max. coverage (+): 0. Max coverage (-): 0

Region: chr14 15171696-15171749. Max. coverage (+): 0. Max coverage (-): 0

Region: chr14 15171750-15171803. Max. coverage (+): 0.31. Max coverage (-): 0.9

Region: chr14 15171804-15171856. Max. coverage (+): 0. Max coverage (-): 2.84

Region: chr14 15171857-15171910. Max. coverage (+): 0. Max coverage (-): 0

Region: chr14 15171911-15171963. Max. coverage (+): 0. Max coverage (-): 0

Region: chr14 15171964-15172017. Max. coverage (+): 0. Max coverage (-): 0

Region: chr14 15172018-15172071. Max. coverage (+): 0.31. Max coverage (-): 0.9

Region: chr14 15172072-15172124. Max. coverage (+): 0. Max coverage (-): 4.44

Region: chr14 15172125-15172178. Max. coverage (+): 0. Max coverage (-): 2.16

Region: chr14 15172179-15172231. Max. coverage (+): 0. Max coverage (-): 1.7

Region: chr14 15172232-15172285. Max. coverage (+): 0. Max coverage (-): 0

Region: chr14 15172286-15172338. Max. coverage (+): 0. Max coverage (-): 0

Region: chr14 15172339-15172392. Max. coverage (+): 0. Max coverage (-): 6.52

Region: chr14 15172393-15172446. Max. coverage (+): 0. Max coverage (-): 6.52

Region: chr14 15172447-15172499. Max. coverage (+): 0. Max coverage (-): 3.14

Region: chr14 15172500-15172553. Max. coverage (+): 0. Max coverage (-): 2.04

Region: chr14 15172554-15172606. Max. coverage (+): 2.21. Max coverage (-): 0

Region: chr14 15172607-15172660. Max. coverage (+): 0. Max coverage (-): 0

Region: chr14 15172661-15172714. Max. coverage (+): 0. Max coverage (-): 1.3

Region: chr14 15172715-15172767. Max. coverage (+): 0. Max coverage (-): 0

Region: chr14 15172768-15172821. Max. coverage (+): 0. Max coverage (-): 0

Region: chr14 15172822-15172874. Max. coverage (+): 8.92. Max coverage (-): 0.63

Region: chr14 15172875-15172928. Max. coverage (+): 0. Max coverage (-): 0

Region: chr14 15172929-15172981. Max. coverage (+): 0. Max coverage (-): 0

Region: chr14 15172982-15173035. Max. coverage (+): 0. Max coverage (-): 0

Region: chr14 15173036-15173089. Max. coverage (+): 0. Max coverage (-): 1.64

Region: chr14 15173090-15173142. Max. coverage (+): 0. Max coverage (-): 1.64

Region: chr14 15173143-15173196. Max. coverage (+): 0. Max coverage (-): 0.6

Region: chr14 15173197-15173249. Max. coverage (+): 0. Max coverage (-): 0

Region: chr14 15173250-15173303. Max. coverage (+): 0. Max coverage (-): 0

Region: chr14 15173304-15173357. Max. coverage (+): 0. Max coverage (-): 0

Region: chr14 15173358-15173410. Max. coverage (+): 0. Max coverage (-): 0

Region: chr14 15173411-15173464. Max. coverage (+): 0. Max coverage (-): 0

Region: chr14 15173465-15173517. Max. coverage (+): 0.36. Max coverage (-): 1.04

Region: chr14 15173518-15173571. Max. coverage (+): 0. Max coverage (-): 5.17

Region: chr14 15173572-15173624. Max. coverage (+): 0. Max coverage (-): 0.53

Region: chr14 15173625-15173678. Max. coverage (+): 0. Max coverage (-): 2.51

Region: chr14 15173679-15173732. Max. coverage (+): 0. Max coverage (-): 0

Region: chr14 15173733-15173785. Max. coverage (+): 0. Max coverage (-): 0

Region: chr14 15173786-15173839. Max. coverage (+): 0. Max coverage (-): 6.38

Region: chr14 15173840-15173892. Max. coverage (+): 0. Max coverage (-): 8.7

Region: chr14 15173893-15173946. Max. coverage (+): 0. Max coverage (-): 4.19

Region: chr14 15173947-15174000. Max. coverage (+): 0. Max coverage (-): 2.72

Region: chr14 15174001-15174053. Max. coverage (+): 2.94. Max coverage (-): 0.23

Region: chr14 15174054-15174107. Max. coverage (+): 0. Max coverage (-): 5.05

Region: chr14 15174108-15174160. Max. coverage (+): 0. Max coverage (-): 1.74

Region: chr14 15174161-15174214. Max. coverage (+): 0. Max coverage (-): 0.72

Region: chr14 15174215-15174267. Max. coverage (+): 10.22. Max coverage (-): 0

Region: chr14 15174268-15174321. Max. coverage (+): 0. Max coverage (-): 0

Region: chr14 15174322-15174375. Max. coverage (+): 0. Max coverage (-): 0

Region: chr14 15174376-15174428. Max. coverage (+): 0. Max coverage (-): 0

Region: chr14 15174429-15174482. Max. coverage (+): 0. Max coverage (-): 1.88

Region: chr14 15174483-15174535. Max. coverage (+): 0. Max coverage (-): 3.26

Region: chr14 15174536-15174589. Max. coverage (+): 0. Max coverage (-): 1.5

Region: chr14 15174590-15174643. Max. coverage (+): 0. Max coverage (-): 2.28

Region: chr14 15174644-15174696. Max. coverage (+): 0. Max coverage (-): 0

Region: chr14 15174697-15174750. Max. coverage (+): 1.2. Max coverage (-): 0

Region: chr14 15174751-15174803. Max. coverage (+): 0. Max coverage (-): 0

Region: chr14 15174804-15174857. Max. coverage (+): 0. Max coverage (-): 0

Region: chr14 15174858-15174910. Max. coverage (+): 0. Max coverage (-): 3.27

Region: chr14 15174911-15174964. Max. coverage (+): 0. Max coverage (-): 2.32

Region: chr14 15174965-15175018. Max. coverage (+): 0. Max coverage (-): 0

Region: chr14 15175019-15175071. Max. coverage (+): 0. Max coverage (-): 0.55

Region: chr14 15175072-15175125. Max. coverage (+): 0. Max coverage (-): 0

Region: chr14 15175126-15175178. Max. coverage (+): 0. Max coverage (-): 0

Region: chr14 15175179-15175232. Max. coverage (+): 0. Max coverage (-): 0

Region: chr14 15175233-15175286. Max. coverage (+): 0. Max coverage (-): 0

Region: chr14 15175287-15175339. Max. coverage (+): 0. Max coverage (-): 0

Region: chr14 15175340-15175393. Max. coverage (+): 0. Max coverage (-): 3.72

Region: chr14 15175394-15175446. Max. coverage (+): 0. Max coverage (-): 1.71

Region: chr14 15175447-15175500. Max. coverage (+): 0. Max coverage (-): 1.19

Region: chr14 15175501-15175553. Max. coverage (+): 0. Max coverage (-): 2.6

Region: chr14 15175554-15175607. Max. coverage (+): 0.75. Max coverage (-): 0

Region: chr14 15175608-15175661. Max. coverage (+): 1.36. Max coverage (-): 0

Region: chr14 15175662-15175714. Max. coverage (+): 0. Max coverage (-): 0

Region: chr14 15175715-15175768. Max. coverage (+): 0. Max coverage (-): 0

Region: chr14 15175769-15175821. Max. coverage (+): 0. Max coverage (-): 3.27

Region: chr14 15175822-15175875. Max. coverage (+): 0. Max coverage (-): 2.32

Region: chr14 15175876-15175929. Max. coverage (+): 0. Max coverage (-): 0.61

Region: chr14 15175930-15175982. Max. coverage (+): 0. Max coverage (-): 0.61

Region: chr14 15175983-15176036. Max. coverage (+): 0. Max coverage (-): 6.06

Region: chr14 15176037-15176089. Max. coverage (+): 0. Max coverage (-): 6.06

Region: chr14 15176090-15176143. Max. coverage (+): 0. Max coverage (-): 0

Region: chr14 15176144-15176196. Max. coverage (+): 0. Max coverage (-): 1.38

Region: chr14 15176197-15176250. Max. coverage (+): 0. Max coverage (-): 0

Region: chr14 15176251-15176304. Max. coverage (+): 0. Max coverage (-): 6.26

Region: chr14 15176305-15176357. Max. coverage (+): 0. Max coverage (-): 0

Region: chr14 15176358-15176411. Max. coverage (+): 0. Max coverage (-): 0

Region: chr14 15176412-15176464. Max. coverage (+): 0. Max coverage (-): 0

Region: chr14 15176465-15176518. Max. coverage (+): 0. Max coverage (-): 0

Region: chr14 15176519-15176572. Max. coverage (+): 0. Max coverage (-): 0

Region: chr14 15176573-15176625. Max. coverage (+): 0. Max coverage (-): 0

Region: chr14 15176626-15176679. Max. coverage (+): 0. Max coverage (-): 0

Region: chr14 15176680-15176732. Max. coverage (+): 0. Max coverage (-): 0

Region: chr14 15176733-15176786. Max. coverage (+): 0. Max coverage (-): 0

Region: chr14 15176787-15176839. Max. coverage (+): 0. Max coverage (-): 0

Region: chr14 15176840-15176893. Max. coverage (+): 0. Max coverage (-): 0

Region: chr14 15176894-15176947. Max. coverage (+): 0. Max coverage (-): 0

Region: chr14 15176948-15177000. Max. coverage (+): 0. Max coverage (-): 0

Region: chr14 15177001-15177054. Max. coverage (+): 0. Max coverage (-): 0

Region: chr14 15177055-15177107. Max. coverage (+): 0. Max coverage (-): 0

Region: chr14 15177108-15177161. Max. coverage (+): 0. Max coverage (-): 0

Region: chr14 15177162-15177215. Max. coverage (+): 0. Max coverage (-): 0

Region: chr14 15177216-15177268. Max. coverage (+): 0. Max coverage (-): 0

Region: chr14 15177269-15177322. Max. coverage (+): 0. Max coverage (-): 0

Region: chr14 15177323-15177375. Max. coverage (+): 0. Max coverage (-): 9.51

Region: chr14 15177376-15177429. Max. coverage (+): 0. Max coverage (-): 9.51

Region: chr14 15177430-15177482. Max. coverage (+): 0.64. Max coverage (-): 9.06

Region: chr14 15177483-15177536. Max. coverage (+): 0. Max coverage (-): 14.31

Region: chr14 15177537-15177590. Max. coverage (+): 2.14. Max coverage (-): 20.04

Region: chr14 15177591-15177643. Max. coverage (+): 0. Max coverage (-): 26.96

Region: chr14 15177644-15177697. Max. coverage (+): 0.66. Max coverage (-): 47.09

Region: chr14 15177698-15177750. Max. coverage (+): 0. Max coverage (-): 65.65

Region: chr14 15177751-15177804. Max. coverage (+): 0. Max coverage (-): 22.09

Region: chr14 15177805-15177858. Max. coverage (+): 0. Max coverage (-): 0

Region: chr14 15177859-15177911. Max. coverage (+): 0. Max coverage (-): 0

Region: chr14 15177912-15177965. Max. coverage (+): 0. Max coverage (-): 0

Region: chr14 15177966-15178018. Max. coverage (+): 0. Max coverage (-): 0

Region: chr14 15178019-15178072. Max. coverage (+): 0. Max coverage (-): 0

Region: chr14 15178073-15178125. Max. coverage (+): 0. Max coverage (-): 0

Region: chr14 15178126-15178179. Max. coverage (+): 0. Max coverage (-): 0

Region: chr14 15178180-15178233. Max. coverage (+): 3.55. Max coverage (-): 18.51

Region: chr14 15178234-15178286. Max. coverage (+): 0. Max coverage (-): 11.23

Region: chr14 15178287-15178340. Max. coverage (+): 0. Max coverage (-): 2.75

Region: chr14 15178341-15178393. Max. coverage (+): 2.64. Max coverage (-): 1.06

Region: chr14 15178394-15178447. Max. coverage (+): 0. Max coverage (-): 17.4

Region: chr14 15178448-15178501. Max. coverage (+): 0. Max coverage (-): 0

Region: chr14 15178502-15178554. Max. coverage (+): 0. Max coverage (-): 0

Region: chr14 15178555-15178608. Max. coverage (+): 0. Max coverage (-): 0

Region: chr14 15178609-15178661. Max. coverage (+): 0. Max coverage (-): 0

Region: chr14 15178662-15178715. Max. coverage (+): 0. Max coverage (-): 13.74

Region: chr14 15178716-15178769. Max. coverage (+): 0. Max coverage (-): 34.75

Region: chr14 15178770-15178822. Max. coverage (+): 0. Max coverage (-): 0

Region: chr14 15178823-15178876. Max. coverage (+): 0. Max coverage (-): 0

Region: chr14 15178877-15178929. Max. coverage (+): 0. Max coverage (-): 0

Region: chr14 15178930-15178983. Max. coverage (+): 0. Max coverage (-): 0

Region: chr14 15178984-15179036. Max. coverage (+): 0. Max coverage (-): 0

Region: chr14 15179037-15179090. Max. coverage (+): 0. Max coverage (-): 0

Region: chr14 15179091-15179144. Max. coverage (+): 0. Max coverage (-): 0

Region: chr14 15179145-15179197. Max. coverage (+): 0. Max coverage (-): 0

Region: chr14 15179198-15179251. Max. coverage (+): 0. Max coverage (-): 0

Region: chr14 15179252-15179304. Max. coverage (+): 0. Max coverage (-): 0

Region: chr14 15179305-15179358. Max. coverage (+): 0. Max coverage (-): 0

Region: chr14 15179359-15179412. Max. coverage (+): 0. Max coverage (-): 0

Region: chr14 15179413-15179465. Max. coverage (+): 0. Max coverage (-): 0

Region: chr14 15179466-15179519. Max. coverage (+): 0. Max coverage (-): 0

Region: chr14 15179520-15179572. Max. coverage (+): 0. Max coverage (-): 2.29

Region: chr14 15179573-15179626. Max. coverage (+): 0. Max coverage (-): 1.32

Region: chr14 15179627-15179679. Max. coverage (+): 1.76. Max coverage (-): 2.11

Region: chr14 15179680-15179733. Max. coverage (+): 0. Max coverage (-): 6.69

Region: chr14 15179734-15179787. Max. coverage (+): 0. Max coverage (-): 20.44

Region: chr14 15179788-15179840. Max. coverage (+): 0.52. Max coverage (-): 37.58

Region: chr14 15179841-15179894. Max. coverage (+): 0. Max coverage (-): 5.96

Region: chr14 15179895-15179947. Max. coverage (+): 0.58. Max coverage (-): 6.94

Region: chr14 15179948-15180001. Max. coverage (+): 0. Max coverage (-): 0.91

Region: chr14 15180002-15180055. Max. coverage (+): 0. Max coverage (-): 0

Region: chr14 15180056-15180108. Max. coverage (+): 0. Max coverage (-): 0

Region: chr14 15180109-15180162. Max. coverage (+): 0.1. Max coverage (-): 19.17

Region: chr14 15180163-15180215. Max. coverage (+): 0. Max coverage (-): 3.23

Region: chr14 15180216-15180269. Max. coverage (+): 0. Max coverage (-): 3.91

Region: chr14 15180270-15180322. Max. coverage (+): 0. Max coverage (-): 2.29

Region: chr14 15180323-15180376. Max. coverage (+): 0. Max coverage (-): 0.29

Region: chr14 15180377-15180430. Max. coverage (+): 1.76. Max coverage (-): 1.34

Region: chr14 15180431-15180483. Max. coverage (+): 0. Max coverage (-): 13.5

Region: chr14 15180484-15180537. Max. coverage (+): 0. Max coverage (-): 5.99

Region: chr14 15180538-15180590. Max. coverage (+): 0.52. Max coverage (-): 1.52

Region: chr14 15180591-15180644. Max. coverage (+): 0. Max coverage (-): 0

Region: chr14 15180645-15180698. Max. coverage (+): 0. Max coverage (-): 0

Region: chr14 15180699-15180751. Max. coverage (+): 0. Max coverage (-): 0

Region: chr14 15180752-15180805. Max. coverage (+): 0. Max coverage (-): 0

Region: chr14 15180806-15180858. Max. coverage (+): 0. Max coverage (-): 0

Region: chr14 15180859-15180912. Max. coverage (+): 0. Max coverage (-): 0

Region: chr14 15180913-15180965. Max. coverage (+): 0. Max coverage (-): 0

Region: chr14 15180966-15181019. Max. coverage (+): 0. Max coverage (-): 0

Region: chr14 15181020-15181073. Max. coverage (+): 0. Max coverage (-): 0

Region: chr14 15181074-15181126. Max. coverage (+): 0. Max coverage (-): 0

Region: chr14 15181127-15181180. Max. coverage (+): 0. Max coverage (-): 0

Region: chr14 15181181-15181233. Max. coverage (+): 0. Max coverage (-): 0

Region: chr14 15181234-15181287. Max. coverage (+): 0. Max coverage (-): 0

Region: chr14 15181288-15181341. Max. coverage (+): 0. Max coverage (-): 0

Region: chr14 15181342-15181394. Max. coverage (+): 0. Max coverage (-): 0

Region: chr14 15181395-15181448. Max. coverage (+): 0. Max coverage (-): 0

Region: chr14 15181449-15181501. Max. coverage (+): 0. Max coverage (-): 0

Region: chr14 15181502-15181555. Max. coverage (+): 0. Max coverage (-): 0

Region: chr14 15181556-15181608. Max. coverage (+): 0. Max coverage (-): 0

Region: chr14 15181609-15181662. Max. coverage (+): 0. Max coverage (-): 0

Region: chr14 15181663-15181716. Max. coverage (+): 0. Max coverage (-): 0

Region: chr14 15181717-15181769. Max. coverage (+): 0. Max coverage (-): 0

Region: chr14 15181770-15181823. Max. coverage (+): 0. Max coverage (-): 10.03

Region: chr14 15181824-15181876. Max. coverage (+): 2.36. Max coverage (-): 2.45

Region: chr14 15181877-15181930. Max. coverage (+): 0.09. Max coverage (-): 12.72

Region: chr14 15181931-15181984. Max. coverage (+): 0. Max coverage (-): 22.02

Region: chr14 15181985-15182037. Max. coverage (+): 0. Max coverage (-): 3.42

Region: chr14 15182038-15182091. Max. coverage (+): 0. Max coverage (-): 1.48

Region: chr14 15182092-15182144. Max. coverage (+): 0. Max coverage (-): 2.01

Region: chr14 15182145-15182198. Max. coverage (+): 1.54. Max coverage (-): 1.85

Region: chr14 15182199-15182251. Max. coverage (+): 0. Max coverage (-): 5.86

Region: chr14 15182252-15182305. Max. coverage (+): 0. Max coverage (-): 11.83

Region: chr14 15182306-15182359. Max. coverage (+): 0.45. Max coverage (-): 32.94

Region: chr14 15182360-15182412. Max. coverage (+): 0. Max coverage (-): 17.69

Region: chr14 15182413-15182466. Max. coverage (+): 0.51. Max coverage (-): 6.09

Region: chr14 15182467-15182519. Max. coverage (+): 0. Max coverage (-): 3.67

Region: chr14 15182520-15182573. Max. coverage (+): 0. Max coverage (-): 20.89

Region: chr14 15182574-15182627. Max. coverage (+): 4.69. Max coverage (-): 36.14

Region: chr14 15182628-15182680. Max. coverage (+): 0.71. Max coverage (-): 6.7

Region: chr14 15182681-15182734. Max. coverage (+): 4.69. Max coverage (-): 36.14

Region: chr14 15182735-15182787. Max. coverage (+): 1.48. Max coverage (-): 6.7

Region: chr14 15182788-15182841. Max. coverage (+): 4.28. Max coverage (-): 33.03

Region: chr14 15182842-15182894. Max. coverage (+): 4.28. Max coverage (-): 21.07

Region: chr14 15182895-15182948. Max. coverage (+): 2.94. Max coverage (-): 33.03

Region: chr14 15182949-15183002. Max. coverage (+): 4.28. Max coverage (-): 32.34

Region: chr14 15183003-15183055. Max. coverage (+): 2.95. Max coverage (-): 23.22

Region: chr14 15183056-15183109. Max. coverage (+): 0.26. Max coverage (-): 5.47

Region: chr14 15183110-15183162. Max. coverage (+): 0. Max coverage (-): 1.29

Region: chr14 15183163-15183216. Max. coverage (+): 0.67. Max coverage (-): 0.38

Region: chr14 15183217-15183270. Max. coverage (+): 0.89. Max coverage (-): 3.86

Region: chr14 15183271-15183323. Max. coverage (+): 0. Max coverage (-): 3.86

Region: chr14 15183324-15183377. Max. coverage (+): 0. Max coverage (-): 1.48

Region: chr14 15183378-15183430. Max. coverage (+): 1.69. Max coverage (-): 6.19

Region: chr14 15183431-15183484. Max. coverage (+): 0. Max coverage (-): 1.24

Region: chr14 15183485-15183537. Max. coverage (+): 0. Max coverage (-): 9.64

Region: chr14 15183538-15183591. Max. coverage (+): 0. Max coverage (-): 3.05

Region: chr14 15183592-15183645. Max. coverage (+): 0. Max coverage (-): 2.63

Region: chr14 15183646-15183698. Max. coverage (+): 0. Max coverage (-): 1.63

Region: chr14 15183699-15183752. Max. coverage (+): 0.25. Max coverage (-): 12.2

Region: chr14 15183753-15183805. Max. coverage (+): 0. Max coverage (-): 1.33

Region: chr14 15183806-15183859. Max. coverage (+): 0.07. Max coverage (-): 0.86

Region: chr14 15183860-15183913. Max. coverage (+): 0. Max coverage (-): 0

Region: chr14 15183914-15183966. Max. coverage (+): 0. Max coverage (-): 12.88

Region: chr14 15183967-15184020. Max. coverage (+): 0. Max coverage (-): 18.25

Region: chr14 15184021-15184073. Max. coverage (+): 0. Max coverage (-): 2.84

Region: chr14 15184074-15184127. Max. coverage (+): 0. Max coverage (-): 2.21

Region: chr14 15184128-15184180. Max. coverage (+): 0. Max coverage (-): 0.91

Region: chr14 15184181-15184234. Max. coverage (+): 1.28. Max coverage (-): 1.53

Region: chr14 15184235-15184288. Max. coverage (+): 0. Max coverage (-): 9.81

Region: chr14 15184289-15184341. Max. coverage (+): 0. Max coverage (-): 19.31

Region: chr14 15184342-15184395. Max. coverage (+): 0.37. Max coverage (-): 23.4

Region: chr14 15184396-15184448. Max. coverage (+): 0.42. Max coverage (-): 1.93

Region: chr14 15184449-15184502. Max. coverage (+): 0.42. Max coverage (-): 5.04

Region: chr14 15184503-15184556. Max. coverage (+): 0. Max coverage (-): 9.02

Region: chr14 15184557-15184609. Max. coverage (+): 0.59. Max coverage (-): 6.26

Region: chr14 15184610-15184663. Max. coverage (+): 0. Max coverage (-): 9.39

Region: chr14 15184664-15184716. Max. coverage (+): 0.59. Max coverage (-): 9.39

Region: chr14 15184717-15184770. Max. coverage (+): 2.68. Max coverage (-): 4.84

Region: chr14 15184771-15184823. Max. coverage (+): 0.21. Max coverage (-): 4.96

Region: chr14 15184824-15184877. Max. coverage (+): 0. Max coverage (-): 1.06

Region: chr14 15184878-15184931. Max. coverage (+): 0.55. Max coverage (-): 0

Region: chr14 15184932-15184984. Max. coverage (+): 0.73. Max coverage (-): 3.18

Region: chr14 15184985-15185038. Max. coverage (+): 0. Max coverage (-): 3.18

Region: chr14 15185039-15185091. Max. coverage (+): 0. Max coverage (-): 1.22

Region: chr14 15185092-15185145. Max. coverage (+): 1.39. Max coverage (-): 5.09

Region: chr14 15185146-15185199. Max. coverage (+): 0. Max coverage (-): 0

Region: chr14 15185200-15185252. Max. coverage (+): 0. Max coverage (-): 7.92

Region: chr14 15185253-15185306. Max. coverage (+): 0. Max coverage (-): 2.16

Region: chr14 15185307-15185359. Max. coverage (+): 0. Max coverage (-): 2.16

Region: chr14 15185360-15185413. Max. coverage (+): 0. Max coverage (-): 1.34

Region: chr14 15185414-15185467. Max. coverage (+): 0.21. Max coverage (-): 10.03

Region: chr14 15185468-15185520. Max. coverage (+): 0. Max coverage (-): 0

Region: chr14 15185521-15185574. Max. coverage (+): 0. Max coverage (-): 0

Region: chr14 15185575-15185627. Max. coverage (+): 2.43. Max coverage (-): 2.12

Region: chr14 15185628-15185681. Max. coverage (+): 0.21. Max coverage (-): 4.49

Region: chr14 15185682-15185734. Max. coverage (+): 0. Max coverage (-): 1.06

Region: chr14 15185735-15185788. Max. coverage (+): 0.55. Max coverage (-): 0.44

Region: chr14 15185789-15185842. Max. coverage (+): 0.73. Max coverage (-): 2.9

Region: chr14 15185843-15185895. Max. coverage (+): 0. Max coverage (-): 1.56

Region: chr14 15185896-15185949. Max. coverage (+): 0.21. Max coverage (-): 1.11

Region: chr14 15185950-15186002. Max. coverage (+): 1.27. Max coverage (-): 4.65

Region: chr14 15186003-15186056. Max. coverage (+): 0. Max coverage (-): 0.93

Region: chr14 15186057-15186110. Max. coverage (+): 0. Max coverage (-): 7.25

Region: chr14 15186111-15186163. Max. coverage (+): 0. Max coverage (-): 2.29

Region: chr14 15186164-15186217. Max. coverage (+): 0. Max coverage (-): 0.65

Region: chr14 15186218-15186270. Max. coverage (+): 0.19. Max coverage (-): 9.17

Region: chr14 15186271-15186324. Max. coverage (+): 0.82. Max coverage (-): 0.73

Region: chr14 15186325-15186377. Max. coverage (+): 0.05. Max coverage (-): 1.32

Region: chr14 15186378-15186431. Max. coverage (+): 1.14. Max coverage (-): 1.51

Region: chr14 15186432-15186485. Max. coverage (+): 0.52. Max coverage (-): 12.24

Region: chr14 15186486-15186538. Max. coverage (+): 0. Max coverage (-): 2.55

Region: chr14 15186539-15186592. Max. coverage (+): 1.65. Max coverage (-): 2.11

Region: chr14 15186593-15186645. Max. coverage (+): 0. Max coverage (-): 0.6

Region: chr14 15186646-15186699. Max. coverage (+): 0. Max coverage (-): 0

Region: chr14 15186700-15186753. Max. coverage (+): 0. Max coverage (-): 3.01

Region: chr14 15186754-15186806. Max. coverage (+): 0.23. Max coverage (-): 14.47

Region: chr14 15186807-15186860. Max. coverage (+): 0.23. Max coverage (-): 1.19

Region: chr14 15186861-15186913. Max. coverage (+): 0. Max coverage (-): 0

Region: chr14 15186914-15186967. Max. coverage (+): 0. Max coverage (-): 0

Region: chr14 15186968-15187020. Max. coverage (+): 0. Max coverage (-): 6.57

Region: chr14 15187021-15187074. Max. coverage (+): 0. Max coverage (-): 2.11

Region: chr14 15187075-15187128. Max. coverage (+): 0. Max coverage (-): 0

Region: chr14 15187129-15187181. Max. coverage (+): 0.17. Max coverage (-): 7.32

Region: chr14 15187182-15187235. Max. coverage (+): 0.76. Max coverage (-): 8.45

Region: chr14 15187236-15187288. Max. coverage (+): 0.05. Max coverage (-): 1.01

Region: chr14 15187289-15187342. Max. coverage (+): 1.05. Max coverage (-): 1.21

Region: chr14 15187343-15187396. Max. coverage (+): 0.48. Max coverage (-): 11.27

Region: chr14 15187397-15187449. Max. coverage (+): 0. Max coverage (-): 2.35

Region: chr14 15187450-15187503. Max. coverage (+): 0.77. Max coverage (-): 1.94

Region: chr14 15187504-15187556. Max. coverage (+): 1.52. Max coverage (-): 0.56

Region: chr14 15187557-15187610. Max. coverage (+): 0. Max coverage (-): 0

Region: chr14 15187611-15187663. Max. coverage (+): 0. Max coverage (-): 0

Region: chr14 15187664-15187717. Max. coverage (+): 0. Max coverage (-): 13.34

Region: chr14 15187718-15187771. Max. coverage (+): 0.21. Max coverage (-): 4.24

Region: chr14 15187772-15187824. Max. coverage (+): 0. Max coverage (-): 0

Region: chr14 15187825-15187878. Max. coverage (+): 0.64. Max coverage (-): 0.45

Region: chr14 15187879-15187931. Max. coverage (+): 1.81. Max coverage (-): 0.84

Region: chr14 15187932-15187985. Max. coverage (+): 0. Max coverage (-): 0

Region: chr14 15187986-15188039. Max. coverage (+): 0. Max coverage (-): 0

Region: chr14 15188040-15188092. Max. coverage (+): 0. Max coverage (-): 0

Region: chr14 15188093-15188146. Max. coverage (+): 0. Max coverage (-): 6.27

Region: chr14 15188147-15188199. Max. coverage (+): 0. Max coverage (-): 1.71

Region: chr14 15188200-15188253. Max. coverage (+): 0. Max coverage (-): 0.56

Region: chr14 15188254-15188306. Max. coverage (+): 0.16. Max coverage (-): 7.94

Region: chr14 15188307-15188360. Max. coverage (+): 0.71. Max coverage (-): 0.93

Region: chr14 15188361-15188414. Max. coverage (+): 0.05. Max coverage (-): 1.14

Region: chr14 15188415-15188467. Max. coverage (+): 0.99. Max coverage (-): 1.31

Region: chr14 15188468-15188521. Max. coverage (+): 5.82. Max coverage (-): 3.24

Region: chr14 15188522-15188574. Max. coverage (+): 0. Max coverage (-): 2.74

Region: chr14 15188575-15188628. Max. coverage (+): 0. Max coverage (-): 3.9

Region: chr14 15188629-15188682. Max. coverage (+): 0. Max coverage (-): 0

Region: chr14 15188683-15188735. Max. coverage (+): 0. Max coverage (-): 0

Region: chr14 15188736-15188789. Max. coverage (+): 0. Max coverage (-): 2.61

Region: chr14 15188790-15188842. Max. coverage (+): 0.2. Max coverage (-): 12.52

Region: chr14 15188843-15188896. Max. coverage (+): 0.2. Max coverage (-): 1.23

Region: chr14 15188897-15188949. Max. coverage (+): 0. Max coverage (-): 0.42

Region: chr14 15188950-15189003. Max. coverage (+): 1.7. Max coverage (-): 0.42

Region: chr14 15189004-15189057. Max. coverage (+): 1.52. Max coverage (-): 0.79

Region: chr14 15189058-15189110. Max. coverage (+): 0. Max coverage (-): 4.65

Region: chr14 15189111-15189164. Max. coverage (+): 0. Max coverage (-): 1.9

Region: chr14 15189165-15189217. Max. coverage (+): 0. Max coverage (-): 6.76

Region: chr14 15189218-15189271. Max. coverage (+): 0. Max coverage (-): 30.79

Region: chr14 15189272-15189325. Max. coverage (+): 0. Max coverage (-): 0.23

Region: chr14 15189326-15189378. Max. coverage (+): 0. Max coverage (-): 8.42

Region: chr14 15189379-15189432. Max. coverage (+): 2.17. Max coverage (-): 0

Region: chr14 15189433-15189485. Max. coverage (+): 0. Max coverage (-): 1.28

Region: chr14 15189486-15189539. Max. coverage (+): 0. Max coverage (-): 0.88

Region: chr14 15189540-15189592. Max. coverage (+): 0. Max coverage (-): 0.8

Region: chr14 15189593-15189646. Max. coverage (+): 0. Max coverage (-): 0

Region: chr14 15189647-15189700. Max. coverage (+): 0. Max coverage (-): 0.47

Region: chr14 15189701-15189753. Max. coverage (+): 0.15. Max coverage (-): 7.42

Region: chr14 15189754-15189807. Max. coverage (+): 0. Max coverage (-): 0

Region: chr14 15189808-15189860. Max. coverage (+): 0.04. Max coverage (-): 1.07

Region: chr14 15189861-15189914. Max. coverage (+): 0.93. Max coverage (-): 8.77

Region: chr14 15189915-15189968. Max. coverage (+): 0. Max coverage (-): 9.9

Region: chr14 15189969-15190021. Max. coverage (+): 0. Max coverage (-): 1.7

Region: chr14 15190022-15190075. Max. coverage (+): 1.33. Max coverage (-): 0.49

Region: chr14 15190076-15190128. Max. coverage (+): 0. Max coverage (-): 0

Region: chr14 15190129-15190182. Max. coverage (+): 0. Max coverage (-): 0

Region: chr14 15190183-15190235. Max. coverage (+): 0. Max coverage (-): 2.21

Region: chr14 15190236-15190289. Max. coverage (+): 0.17. Max coverage (-): 10.62

Region: chr14 15190290-15190343. Max. coverage (+): 0. Max coverage (-): 0

Region: chr14 15190344-15190396. Max. coverage (+): 0. Max coverage (-): 0.36

Region: chr14 15190397-15190450. Max. coverage (+): 1.44. Max coverage (-): 0.25

Region: chr14 15190451-15190503. Max. coverage (+): 1.29. Max coverage (-): 0.67

Region: chr14 15190504-15190557. Max. coverage (+): 0. Max coverage (-): 3.61

Region: chr14 15190558-15190611. Max. coverage (+): 0.19. Max coverage (-): 19.55

Region: chr14 15190612-15190664. Max. coverage (+): 0. Max coverage (-): 6.13

Region: chr14 15190665-15190718. Max. coverage (+): 0. Max coverage (-): 27.94

Region: chr14 15190719-15190771. Max. coverage (+): 0. Max coverage (-): 0.21

Region: chr14 15190772-15190825. Max. coverage (+): 0. Max coverage (-): 7.64

Region: chr14 15190826-15190878. Max. coverage (+): 1.97. Max coverage (-): 0

Region: chr14 15190879-15190932. Max. coverage (+): 0. Max coverage (-): 1.16

Region: chr14 15190933-15190986. Max. coverage (+): 0. Max coverage (-): 0.8

Region: chr14 15190987-15191039. Max. coverage (+): 0.36. Max coverage (-): 0.99

Region: chr14 15191040-15191093. Max. coverage (+): 0. Max coverage (-): 62.84

Region: chr14 15191094-15191146. Max. coverage (+): 0.28. Max coverage (-): 4.64

Region: chr14 15191147-15191200. Max. coverage (+): 0. Max coverage (-): 0

Region: chr14 15191201-15191254. Max. coverage (+): 0. Max coverage (-): 2.58

Region: chr14 15191255-15191307. Max. coverage (+): 0. Max coverage (-): 24.97

Region: chr14 15191308-15191361. Max. coverage (+): 0. Max coverage (-): 0.18

Region: chr14 15191362-15191414. Max. coverage (+): 0. Max coverage (-): 5.07

Region: chr14 15191415-15191468. Max. coverage (+): 0. Max coverage (-): 6.83

Region: chr14 15191469-15191521. Max. coverage (+): 1.76. Max coverage (-): 1.04

Region: chr14 15191522-15191575. Max. coverage (+): 0. Max coverage (-): 0

Region: chr14 15191576-15191629. Max. coverage (+): 0.29. Max coverage (-): 0.81

Region: chr14 15191630-15191682. Max. coverage (+): 0.32. Max coverage (-): 56.17

Region: chr14 15191683-15191736. Max. coverage (+): 0.25. Max coverage (-): 49.86

Region: chr14 15191737-15191789. Max. coverage (+): 0.47. Max coverage (-): 2.52

Region: chr14 15191790-15191843. Max. coverage (+): 0.54. Max coverage (-): 9.79

Region: chr14 15191844-15191897. Max. coverage (+): 0.33. Max coverage (-): 2.94

Region: chr14 15191898-15191950. Max. coverage (+): 0.33. Max coverage (-): 14.53

Region: chr14 15191951-15192004. Max. coverage (+): 0. Max coverage (-): 3.61

Region: chr14 15192005-15192057. Max. coverage (+): 0. Max coverage (-): 0

Region: chr14 15192058-15192111. Max. coverage (+): 0. Max coverage (-): 0

Region: chr14 15192112-15192165. Max. coverage (+): 0. Max coverage (-): 0

Region: chr14 15192166-15192218. Max. coverage (+): 0. Max coverage (-): 0

Region: chr14 15192219-15192272. Max. coverage (+): 0. Max coverage (-): 9.62

Region: chr14 15192273-15192325. Max. coverage (+): 0. Max coverage (-): 1.34

Region: chr14 15192326-15192379. Max. coverage (+): 0. Max coverage (-): 0

Region: chr14 15192380-15192432. Max. coverage (+): 0. Max coverage (-): 11.69

Region: chr14 15192433-15192486. Max. coverage (+): 0. Max coverage (-): 5.9

Region: chr14 15192487-15192540. Max. coverage (+): 0. Max coverage (-): 34.05

Region: chr14 15192541-15192593. Max. coverage (+): 0. Max coverage (-): 21.84

Region: chr14 15192594-15192647. Max. coverage (+): 0. Max coverage (-): 2.48

Region: chr14 15192648-15192700. Max. coverage (+): 0. Max coverage (-): 5.06

Region: chr14 15192701-15192754. Max. coverage (+): 0. Max coverage (-): 8.39

Region: chr14 15192755-15192808. Max. coverage (+): 0. Max coverage (-): 0

Region: chr14 15192809-15192861. Max. coverage (+): 0. Max coverage (-): 0

Region: chr14 15192862-15192915. Max. coverage (+): 0. Max coverage (-): 3.19

Region: chr14 15192916-15192968. Max. coverage (+): 0. Max coverage (-): 0

Region: chr14 15192969-15193022. Max. coverage (+): 0. Max coverage (-): 0

Region: chr14 15193023-15193075. Max. coverage (+): 0. Max coverage (-): 0

Region: chr14 15193076-15193129. Max. coverage (+): 0. Max coverage (-): 0.73

Region: chr14 15193130-15193183. Max. coverage (+): 0. Max coverage (-): 9.62

Region: chr14 15193184-15193236. Max. coverage (+): 0. Max coverage (-): 1.34

Region: chr14 15193237-15193290. Max. coverage (+): 0. Max coverage (-): 0

Region: chr14 15193291-15193343. Max. coverage (+): 0. Max coverage (-): 9.98

Region: chr14 15193344-15193397. Max. coverage (+): 0. Max coverage (-): 2.69

Region: chr14 15193398-15193451. Max. coverage (+): 0. Max coverage (-): 29.07

Region: chr14 15193452-15193504. Max. coverage (+): 0. Max coverage (-): 18.65

Region: chr14 15193505-15193558. Max. coverage (+): 0. Max coverage (-): 4.32

Region: chr14 15193559-15193611. Max. coverage (+): 0. Max coverage (-): 4.32

Region: chr14 15193612-15193665. Max. coverage (+): 0. Max coverage (-): 0

Region: chr14 15193666-15193718. Max. coverage (+): 0. Max coverage (-): 0

Region: chr14 15193719-15193772. Max. coverage (+): 0. Max coverage (-): 0

Region: chr14 15193773-15193826. Max. coverage (+): 0. Max coverage (-): 0

Region: chr14 15193827-15193879. Max. coverage (+): 0. Max coverage (-): 0.77

Region: chr14 15193880-15193933. Max. coverage (+): 0.24. Max coverage (-): 0

Region: chr14 15193934-15193986. Max. coverage (+): 0.24. Max coverage (-): 1.32

Region: chr14 15193987-15194040. Max. coverage (+): 0.43. Max coverage (-): 2.49

Region: chr14 15194041-15194094. Max. coverage (+): 0. Max coverage (-): 0

Region: chr14 15194095-15194147. Max. coverage (+): 0. Max coverage (-): 0

Region: chr14 15194148-15194201. Max. coverage (+): 0. Max coverage (-): 0

Region: chr14 15194202-15194254. Max. coverage (+): 0. Max coverage (-): 0

Region: chr14 15194255-15194308. Max. coverage (+): 0. Max coverage (-): 13.71

Region: chr14 15194309-15194361. Max. coverage (+): 0. Max coverage (-): 0

Region: chr14 15194362-15194415. Max. coverage (+): 0. Max coverage (-): 3.6

Region: chr14 15194416-15194469. Max. coverage (+): 0. Max coverage (-): 0

Region: chr14 15194470-15194522. Max. coverage (+): 0. Max coverage (-): 0

Region: chr14 15194523-15194576. Max. coverage (+): 0. Max coverage (-): 0

Region: chr14 15194577-15194629. Max. coverage (+): 0. Max coverage (-): 0

Region: chr14 15194630-15194683. Max. coverage (+): 0. Max coverage (-): 0.64

Region: chr14 15194684-15194737. Max. coverage (+): 0. Max coverage (-): 0

Region: chr14 15194738-15194790. Max. coverage (+): 0.2. Max coverage (-): 0

Region: chr14 15194791-15194844. Max. coverage (+): 0.36. Max coverage (-): 2.07

Region: chr14 15194845-. Max. coverage (+): 0. Max coverage (-): 2.07

RepeatMasker Color Code

**+**

100-98% Identity

<98-95% Identity

<95-90% Identity

<90-85% Identity

<85-80% Identity

<80-75% Identity

<75-70% Identity

<70% Identity

**-**

Gene Set Color Code

**+**

Gene

Pseudogene

**-**

Topology/Coverage Color Code

Coverage Plus Strand

Coverage Minus Strand

Mainstrand: Plus

Mainstrand: Minus

Complementary Strand

Flanking Region  
(if option -flank >0)

Gene Set Annotation  
  
RepeatMasker Annotation  

**1. Bov-tA1**: 15169171-15169382 (+), Divergence to consensus: 27.1%  
**2. Bov-tA1**: 15170108-15170319 (+), Divergence to consensus: 27.1%  
**3. Bov-tA2**: 15170722-15170810 (-), Divergence to consensus: 13.6%  
**4. Bov-tA3**: 15170926-15171082 (+), Divergence to consensus: 24.8%  
**5. Bov-tA2**: 15171491-15171694 (-), Divergence to consensus: 13.7%  
**6. (TTTTA)n**: 15172758-15172802 (+), Divergence to consensus: 6.8%  
**7. BOV-A2**: 15172903-15172955 (+), Divergence to consensus: 13.2%  
**8. L2b**: 15172988-15173087 (+), Divergence to consensus: 33.3%  
**9. BOV-A2**: 15174270-15174322 (+), Divergence to consensus: 13.2%  
**10. L2a**: 15174355-15174458 (+), Divergence to consensus: 34%  
**11. L2a**: 15175243-15175346 (+), Divergence to consensus: 34%  
**12. MIR**: 15176364-15176484 (-), Divergence to consensus: 34.1%  
**13. ERV1-2-I\_BT-int**: 15176485-15177020 (+), Divergence to consensus: 24.7%  
**14. ERV1-2-I\_BT-int**: 15177121-15177371 (+), Divergence to consensus: 26.3%  
**15. BOV-A2**: 15177798-15177930 (+), Divergence to consensus: 9%  
**16. ERV1-2-I\_BT-int**: 15178056-15178177 (+), Divergence to consensus: 26.7%  
**17. BOV-A2**: 15178801-15178933 (+), Divergence to consensus: 7.5%  
**18. ERV1-2-I\_BT-int**: 15179059-15179180 (+), Divergence to consensus: 25.9%  
**19. SINE2-3\_BT**: 15192024-15192202 (-), Divergence to consensus: 18%  
**20. (T)n**: 15192616-15192645 (+), Divergence to consensus: 13.3%  
**21. SINE2-3\_BT**: 15192928-15193106 (-), Divergence to consensus: 18%  
**22. T-rich**: 15193519-15193549 (+), Divergence to consensus: 16.1%  
**23. LTR103\_Mam**: 15193611-15193779 (-), Divergence to consensus: 45.4%  
**24. L1MC4**: 15194094-15194171 (+), Divergence to consensus: 24.7%  
**25. LTR103\_Mam**: 15194443-15194611 (-), Divergence to consensus: 45.4%

  
Transcription Factor Binding Sites  

**RFX4\_1** (Sequence: CTTAGCAAC (+): 15181078)  
**RFX4\_1** (Sequence: CTTAGCAAC (+): 15183303)  
**RFX4\_1** (Sequence: CTTAGCAAC (+): 15185016)  
**RFX4\_1** (Sequence: CTTAGCAAC (+): 15185854)  
**RFX4\_2** (Sequence: CCTAGATAC (+): 15174780)  
**RFX4\_2** (Sequence: CCTAGATAC (+): 15175668)  
**Gata4** (Sequence: AGATAAC (-): 15176138)  
**Gata4** (Sequence: AGATAAG (-): 15176253)  
**Gata4** (Sequence: AGATAAC (-): 15181319)  
**Gata4** (Sequence: AGATAAC (-): 15183544)  
**Gata4** (Sequence: AGATAAC (-): 15185258)  
**Gata4** (Sequence: AGATAAC (-): 15186095)  
**Gata4** (Sequence: AGATAAC (-): 15187018)  
**Gata4** (Sequence: AGATAAC (-): 15188133)  
**SOX9** (Sequence: AACAATAA (-): 15169152)  
**SOX9** (Sequence: AACAATAA (-): 15170089)  
**SOX9** (Sequence: AACAATAA (-): 15180807)  
**SOX9** (Sequence: AACAATAA (-): 15182590)  
**SOX9** (Sequence: AACAATAA (-): 15183032)  
**SOX9** (Sequence: AACAATAA (-): 15184745)  
**SOX9** (Sequence: AACAATAA (-): 15185583)  
**Gata4** (Sequence: GTTATCT (+): 15169049)  
**Gata4** (Sequence: GTTATCT (+): 15169986)  
**Gata4** (Sequence: CTTATCT (+): 15171777)  
**Gata4** (Sequence: CTTATCT (+): 15172043)  
**Gata4** (Sequence: CTTATCT (+): 15173492)
