# Supplementary material for: Small RNA sequencing of cryopreserved semen from single bull revealed altered miRNAs and piRNAs expression between High- and Low-motile sperm populations
Source: BMC Genomics. 2017 Jan 4;18:14. doi: 10.1186/s12864-016-3394-7 (PMC5209821; doi:10.1186/s12864-016-3394-7)
Supplement: Additional file 3: — Details for each piRNA clusters found in High Motile (HM) sperm fraction. Genes, repeats, transposable elements and transcription factors binding sites falling within the cluster regions were reported. (ZIP 1896 kb) [file 12864_2016_3394_MOESM3_ESM.zip › 22.html]

piRNA cluster 22


Predicted piRNA cluster no. 22     previous   next
  

Show proTRAC run info
Hide proTRAC run info

================================= proTRAC ====================================  
VERSION: 2.1                                    LAST MODIFIED: 06. October 2015  
  
Please cite:  
Rosenkranz D, Zischler H. proTRAC - a software for probabilistic piRNA cluster  
detection, visualization and analysis. 2012. BMC Bioinformatics 13:5.  
  
and (for proTRAC 2.0 and later):  
Rosenkranz D, Rudloff S, Bastuck K, Ketting RF, Zischler H. Tupaia small RNAs  
provide insights into function and evolution of RNAi-based transposon defense  
in mammals. 2015. RNA 21(5):911-922.  
  
Contact:  
David Rosenkranz  
Institute of Anthropology, small RNA group  
Johannes Gutenberg University Mainz  
email: rosenkranz@uni-mainz.de  
  
You can find the latest proTRAC version at:  
http://sourceforge.net/projects/protrac/files  
http://www.smallRNAgroup-mainz.de/software  
==============================================================================  
  
PARAMETERS:  
Map file: .............../storage/core/barbara/genhome/smallRNA/fertility/Sample\_motile/pirna/Sample\_motile\_26-33\_collapsed.fa.no-dust.map.weighted-10000-1000-b-0  
Genome file: ............/storage/core/barbara/genhome/smallRNA/fertility/Sample\_all/pirna/bt\_311\_chrY.fa  
RepeatMasker annotation: /storage/genomes/bt\_umd31/GCF\_000003055.6\_Bos\_taurus\_UMD\_3.1.1\_repeatMasker\_chr.out  
GeneSet:................./storage/core/barbara/genhome/smallRNA/fertility/Sample\_all/pirna/full.gtf  
  
Significant (p<=0.01) hit density will be calculated based  
on observed hit distribution.  
  
Sliding window size: ........................................ 5000 bp  
Sliding window increament: .................................. 1000 bp  
Normalize each hit by number of genomic hits: ............... 1 [0=no/1=yes]  
Normalize each hit by number of sequence reads: ............. 1 [0=no/1=yes]  
Normalize values (-> per million mapped reads): ............. 1 [0=no/1=yes]  
Min. fraction of hits with 1T(U) or 10A: .................... 0.75  
Alternatively: Min. fraction of hits with 1T(U) and 10A: .... 0.5  
Min. fraction of hits with typical piRNA length: ............ 0.75  
Typical piRNA length: ....................................... 26-33 nt  
Min. size of a piRNA cluster: ............................... 5000 bp.  
Min. number of hits (absolute): ............................. 0  
Min. number of hits (normalized): ........................... 0  
Min. fraction of hits on the mainstrand: .................... 0.75  
Top fraction of mapped sequences (in terms of read counts): . 1%  
Top fraction accounts for max. n% of sequence reads: ........ 90%  
Min. fraction of hits on each arm of a bidirectional cluster: 0.1  
Output image file for each cluster: ......................... 0 [0=no/1=yes]  
Output html file for each cluster: .......................... 1 [0=no/1=yes]  
Output a summary table: ..................................... 1 [0=no/1=yes]  
Output a FASTA file for each cluster (piRNA sequences): ..... 1 [0=no/1=yes]  
Output a FASTA file comprising cluster sequences: ........... 1 [0=no/1=yes]  
Search DNA motifs in clusters: .............................. 1 [0=no/1=yes]  
Output flanking sequences: +/- .............................. 0 bp  
Output ~.pTi file: .......................................... 1 [0=no/1=yes]  
==============================================================================  
  
  
Genome size (without gaps): ............ 2678902517 bp  
Gaps (N/X/-): .......................... 53837044 bp  
Mapped reads: .......................... 658825247023  
Non-identical sequences: ............... 514171  
Genomic hits: .......................... 764233  
Significant densitiy of mapped reads: .. 12867599.5173724 reads/kb

Show proTRAC cluster info
Hide proTRAC cluster info

|  |  |
| --- | --- |
| Location | chr14 |
| Coordinates | 15241069-15251040 |
| Size [bp] | 9972 |
| Sequence hit loci | 332 |
| Mapped reads (normalized) | 199685263.4 |
| Mapped reads (normalized) per kb | 20024595.2 |
| Normalized reads with 1T (1U) | 76.7% |
| Normalized reads with 10A | 40.7% |
| Normalized reads with length 26-33 nt | 100% |
| Normalized reads on the main strand(s) | 90.9% |
| Predicted directionality | mono:plus |

100%

0%

1T (1U)  
reads

10A reads

26-33 nt  
reads

reads on mainstrand

**Either the amount of reads with 1T (1U) OR 10A has to exceed 75% (set with option: -1Tor10A)  
Alternatively the amount of reads with 1T (1U) AND 10A has to exceed 50% (set with option: -1Tand10A)  
Minimum amount of reads with preferred size is 75% (set with option: -pisize)  
Minimum amount of reads on the main strand(s) is 75% (set with option: -clstrand)**

Show read coverage
Hide read coverage

WHAT DO I SEE HERE?  
This chart shows the location of mapped sequence reads within a predicted piRNA cluster. The color refers to the number of genomic hits produced by the sequence read in question. A dark red bar indicates that this sequence read produces many other hits elsewhere in the genome. Many adjacent red or yellow bars can indicate the presence of a multi-copy element such as transposons or rRNA genes. A dark green bar indicates that this sequence read maps uniquely to this locus.

1 hit

2-5 hits

6-10 hits

11-20 hits

21-50 hits

51-100 hits

> 100 hits

chr14

15241069

15251040

Gene Set

RepeatMasker

Mapped  
Reads

17.16

plus strand

minus strand

17.16

Region: chr14 15194846-15241078. Max. coverage (+): 0. Max coverage (-): 3.9

Region: chr14 15241079-15241098. Max. coverage (+): 0. Max coverage (-): 1.7

Region: chr14 15241099-15241118. Max. coverage (+): 0. Max coverage (-): 0

Region: chr14 15241119-15241138. Max. coverage (+): 0. Max coverage (-): 0

Region: chr14 15241139-15241158. Max. coverage (+): 0. Max coverage (-): 0

Region: chr14 15241159-15241178. Max. coverage (+): 0. Max coverage (-): 0

Region: chr14 15241179-15241198. Max. coverage (+): 0. Max coverage (-): 0

Region: chr14 15241199-15241218. Max. coverage (+): 0. Max coverage (-): 0

Region: chr14 15241219-15241238. Max. coverage (+): 0. Max coverage (-): 0

Region: chr14 15241239-15241258. Max. coverage (+): 0. Max coverage (-): 0

Region: chr14 15241259-15241278. Max. coverage (+): 0. Max coverage (-): 0

Region: chr14 15241279-15241298. Max. coverage (+): 0. Max coverage (-): 0

Region: chr14 15241299-15241318. Max. coverage (+): 0. Max coverage (-): 0

Region: chr14 15241319-15241338. Max. coverage (+): 0. Max coverage (-): 0

Region: chr14 15241339-15241358. Max. coverage (+): 0. Max coverage (-): 0

Region: chr14 15241359-15241378. Max. coverage (+): 0. Max coverage (-): 0

Region: chr14 15241379-15241398. Max. coverage (+): 0. Max coverage (-): 0

Region: chr14 15241399-15241418. Max. coverage (+): 0. Max coverage (-): 0

Region: chr14 15241419-15241437. Max. coverage (+): 0. Max coverage (-): 0

Region: chr14 15241438-15241457. Max. coverage (+): 0. Max coverage (-): 0

Region: chr14 15241458-15241477. Max. coverage (+): 0. Max coverage (-): 0

Region: chr14 15241478-15241497. Max. coverage (+): 0. Max coverage (-): 0

Region: chr14 15241498-15241517. Max. coverage (+): 0. Max coverage (-): 0

Region: chr14 15241518-15241537. Max. coverage (+): 0. Max coverage (-): 0

Region: chr14 15241538-15241557. Max. coverage (+): 0. Max coverage (-): 0

Region: chr14 15241558-15241577. Max. coverage (+): 0. Max coverage (-): 0

Region: chr14 15241578-15241597. Max. coverage (+): 0. Max coverage (-): 0

Region: chr14 15241598-15241617. Max. coverage (+): 0. Max coverage (-): 0

Region: chr14 15241618-15241637. Max. coverage (+): 0. Max coverage (-): 0

Region: chr14 15241638-15241657. Max. coverage (+): 0. Max coverage (-): 0

Region: chr14 15241658-15241677. Max. coverage (+): 0. Max coverage (-): 0

Region: chr14 15241678-15241697. Max. coverage (+): 0. Max coverage (-): 0

Region: chr14 15241698-15241717. Max. coverage (+): 0. Max coverage (-): 0

Region: chr14 15241718-15241737. Max. coverage (+): 0. Max coverage (-): 0

Region: chr14 15241738-15241757. Max. coverage (+): 0. Max coverage (-): 0

Region: chr14 15241758-15241777. Max. coverage (+): 0. Max coverage (-): 0

Region: chr14 15241778-15241796. Max. coverage (+): 0.56. Max coverage (-): 0

Region: chr14 15241797-15241816. Max. coverage (+): 2.57. Max coverage (-): 0.68

Region: chr14 15241817-15241836. Max. coverage (+): 1.21. Max coverage (-): 0

Region: chr14 15241837-15241856. Max. coverage (+): 2.3. Max coverage (-): 0

Region: chr14 15241857-15241876. Max. coverage (+): 4.73. Max coverage (-): 0

Region: chr14 15241877-15241896. Max. coverage (+): 4.56. Max coverage (-): 0

Region: chr14 15241897-15241916. Max. coverage (+): 0. Max coverage (-): 0

Region: chr14 15241917-15241936. Max. coverage (+): 0. Max coverage (-): 0

Region: chr14 15241937-15241956. Max. coverage (+): 17.16. Max coverage (-): 0

Region: chr14 15241957-15241976. Max. coverage (+): 0. Max coverage (-): 0

Region: chr14 15241977-15241996. Max. coverage (+): 0. Max coverage (-): 0

Region: chr14 15241997-15242016. Max. coverage (+): 0. Max coverage (-): 0

Region: chr14 15242017-15242036. Max. coverage (+): 0. Max coverage (-): 0

Region: chr14 15242037-15242056. Max. coverage (+): 0. Max coverage (-): 0

Region: chr14 15242057-15242076. Max. coverage (+): 0. Max coverage (-): 0

Region: chr14 15242077-15242096. Max. coverage (+): 0. Max coverage (-): 0

Region: chr14 15242097-15242116. Max. coverage (+): 0. Max coverage (-): 0

Region: chr14 15242117-15242136. Max. coverage (+): 0. Max coverage (-): 0

Region: chr14 15242137-15242155. Max. coverage (+): 0. Max coverage (-): 0

Region: chr14 15242156-15242175. Max. coverage (+): 0. Max coverage (-): 0

Region: chr14 15242176-15242195. Max. coverage (+): 0. Max coverage (-): 0

Region: chr14 15242196-15242215. Max. coverage (+): 0. Max coverage (-): 0

Region: chr14 15242216-15242235. Max. coverage (+): 0. Max coverage (-): 0

Region: chr14 15242236-15242255. Max. coverage (+): 0. Max coverage (-): 0

Region: chr14 15242256-15242275. Max. coverage (+): 0. Max coverage (-): 0

Region: chr14 15242276-15242295. Max. coverage (+): 0. Max coverage (-): 0

Region: chr14 15242296-15242315. Max. coverage (+): 0. Max coverage (-): 0

Region: chr14 15242316-15242335. Max. coverage (+): 0. Max coverage (-): 0

Region: chr14 15242336-15242355. Max. coverage (+): 0. Max coverage (-): 0

Region: chr14 15242356-15242375. Max. coverage (+): 0. Max coverage (-): 0

Region: chr14 15242376-15242395. Max. coverage (+): 0. Max coverage (-): 0

Region: chr14 15242396-15242415. Max. coverage (+): 0. Max coverage (-): 0

Region: chr14 15242416-15242435. Max. coverage (+): 0. Max coverage (-): 0

Region: chr14 15242436-15242455. Max. coverage (+): 0. Max coverage (-): 0

Region: chr14 15242456-15242475. Max. coverage (+): 0. Max coverage (-): 0

Region: chr14 15242476-15242494. Max. coverage (+): 0. Max coverage (-): 0

Region: chr14 15242495-15242514. Max. coverage (+): 0. Max coverage (-): 0.51

Region: chr14 15242515-15242534. Max. coverage (+): 0. Max coverage (-): 1.78

Region: chr14 15242535-15242554. Max. coverage (+): 0. Max coverage (-): 3.43

Region: chr14 15242555-15242574. Max. coverage (+): 0. Max coverage (-): 0

Region: chr14 15242575-15242594. Max. coverage (+): 0. Max coverage (-): 0

Region: chr14 15242595-15242614. Max. coverage (+): 0. Max coverage (-): 0

Region: chr14 15242615-15242634. Max. coverage (+): 0. Max coverage (-): 0

Region: chr14 15242635-15242654. Max. coverage (+): 0. Max coverage (-): 0

Region: chr14 15242655-15242674. Max. coverage (+): 0. Max coverage (-): 0

Region: chr14 15242675-15242694. Max. coverage (+): 0. Max coverage (-): 0

Region: chr14 15242695-15242714. Max. coverage (+): 0. Max coverage (-): 0

Region: chr14 15242715-15242734. Max. coverage (+): 0. Max coverage (-): 2.48

Region: chr14 15242735-15242754. Max. coverage (+): 0. Max coverage (-): 0

Region: chr14 15242755-15242774. Max. coverage (+): 0. Max coverage (-): 0

Region: chr14 15242775-15242794. Max. coverage (+): 0. Max coverage (-): 0

Region: chr14 15242795-15242814. Max. coverage (+): 0. Max coverage (-): 0

Region: chr14 15242815-15242834. Max. coverage (+): 0. Max coverage (-): 0

Region: chr14 15242835-15242853. Max. coverage (+): 0. Max coverage (-): 0

Region: chr14 15242854-15242873. Max. coverage (+): 0. Max coverage (-): 0

Region: chr14 15242874-15242893. Max. coverage (+): 0. Max coverage (-): 0

Region: chr14 15242894-15242913. Max. coverage (+): 0. Max coverage (-): 0

Region: chr14 15242914-15242933. Max. coverage (+): 0. Max coverage (-): 0

Region: chr14 15242934-15242953. Max. coverage (+): 0. Max coverage (-): 0

Region: chr14 15242954-15242973. Max. coverage (+): 0. Max coverage (-): 0

Region: chr14 15242974-15242993. Max. coverage (+): 0. Max coverage (-): 0

Region: chr14 15242994-15243013. Max. coverage (+): 0. Max coverage (-): 0

Region: chr14 15243014-15243033. Max. coverage (+): 0. Max coverage (-): 0

Region: chr14 15243034-15243053. Max. coverage (+): 0. Max coverage (-): 0

Region: chr14 15243054-15243073. Max. coverage (+): 0. Max coverage (-): 0

Region: chr14 15243074-15243093. Max. coverage (+): 0. Max coverage (-): 0

Region: chr14 15243094-15243113. Max. coverage (+): 0. Max coverage (-): 0

Region: chr14 15243114-15243133. Max. coverage (+): 0. Max coverage (-): 0.7

Region: chr14 15243134-15243153. Max. coverage (+): 0. Max coverage (-): 0.31

Region: chr14 15243154-15243173. Max. coverage (+): 0. Max coverage (-): 0

Region: chr14 15243174-15243193. Max. coverage (+): 0. Max coverage (-): 0

Region: chr14 15243194-15243212. Max. coverage (+): 0. Max coverage (-): 0

Region: chr14 15243213-15243232. Max. coverage (+): 0. Max coverage (-): 0

Region: chr14 15243233-15243252. Max. coverage (+): 0. Max coverage (-): 0

Region: chr14 15243253-15243272. Max. coverage (+): 0. Max coverage (-): 0

Region: chr14 15243273-15243292. Max. coverage (+): 0. Max coverage (-): 0

Region: chr14 15243293-15243312. Max. coverage (+): 0. Max coverage (-): 0

Region: chr14 15243313-15243332. Max. coverage (+): 0. Max coverage (-): 0

Region: chr14 15243333-15243352. Max. coverage (+): 0. Max coverage (-): 0

Region: chr14 15243353-15243372. Max. coverage (+): 0. Max coverage (-): 0

Region: chr14 15243373-15243392. Max. coverage (+): 0. Max coverage (-): 0

Region: chr14 15243393-15243412. Max. coverage (+): 0. Max coverage (-): 0

Region: chr14 15243413-15243432. Max. coverage (+): 0. Max coverage (-): 0

Region: chr14 15243433-15243452. Max. coverage (+): 0. Max coverage (-): 0

Region: chr14 15243453-15243472. Max. coverage (+): 0. Max coverage (-): 0

Region: chr14 15243473-15243492. Max. coverage (+): 0. Max coverage (-): 3.68

Region: chr14 15243493-15243512. Max. coverage (+): 0. Max coverage (-): 0

Region: chr14 15243513-15243532. Max. coverage (+): 0. Max coverage (-): 0

Region: chr14 15243533-15243552. Max. coverage (+): 0. Max coverage (-): 0

Region: chr14 15243553-15243571. Max. coverage (+): 0. Max coverage (-): 0

Region: chr14 15243572-15243591. Max. coverage (+): 0. Max coverage (-): 0

Region: chr14 15243592-15243611. Max. coverage (+): 0. Max coverage (-): 0

Region: chr14 15243612-15243631. Max. coverage (+): 0. Max coverage (-): 0

Region: chr14 15243632-15243651. Max. coverage (+): 0. Max coverage (-): 0

Region: chr14 15243652-15243671. Max. coverage (+): 0. Max coverage (-): 0

Region: chr14 15243672-15243691. Max. coverage (+): 0. Max coverage (-): 0

Region: chr14 15243692-15243711. Max. coverage (+): 0. Max coverage (-): 0

Region: chr14 15243712-15243731. Max. coverage (+): 0. Max coverage (-): 0

Region: chr14 15243732-15243751. Max. coverage (+): 0.41. Max coverage (-): 0

Region: chr14 15243752-15243771. Max. coverage (+): 0.22. Max coverage (-): 0

Region: chr14 15243772-15243791. Max. coverage (+): 3.29. Max coverage (-): 0.08

Region: chr14 15243792-15243811. Max. coverage (+): 3.29. Max coverage (-): 0

Region: chr14 15243812-15243831. Max. coverage (+): 0. Max coverage (-): 0

Region: chr14 15243832-15243851. Max. coverage (+): 1.02. Max coverage (-): 0

Region: chr14 15243852-15243871. Max. coverage (+): 1.13. Max coverage (-): 0

Region: chr14 15243872-15243891. Max. coverage (+): 0. Max coverage (-): 0

Region: chr14 15243892-15243911. Max. coverage (+): 1.57. Max coverage (-): 0

Region: chr14 15243912-15243930. Max. coverage (+): 6.69. Max coverage (-): 0

Region: chr14 15243931-15243950. Max. coverage (+): 0. Max coverage (-): 0

Region: chr14 15243951-15243970. Max. coverage (+): 0. Max coverage (-): 0

Region: chr14 15243971-15243990. Max. coverage (+): 0. Max coverage (-): 0

Region: chr14 15243991-15244010. Max. coverage (+): 0. Max coverage (-): 0

Region: chr14 15244011-15244030. Max. coverage (+): 0. Max coverage (-): 0

Region: chr14 15244031-15244050. Max. coverage (+): 0. Max coverage (-): 0

Region: chr14 15244051-15244070. Max. coverage (+): 1.05. Max coverage (-): 0

Region: chr14 15244071-15244090. Max. coverage (+): 1.13. Max coverage (-): 0

Region: chr14 15244091-15244110. Max. coverage (+): 1.1. Max coverage (-): 0.15

Region: chr14 15244111-15244130. Max. coverage (+): 0.15. Max coverage (-): 0.15

Region: chr14 15244131-15244150. Max. coverage (+): 0.15. Max coverage (-): 0

Region: chr14 15244151-15244170. Max. coverage (+): 0. Max coverage (-): 0

Region: chr14 15244171-15244190. Max. coverage (+): 0. Max coverage (-): 0

Region: chr14 15244191-15244210. Max. coverage (+): 0. Max coverage (-): 0

Region: chr14 15244211-15244230. Max. coverage (+): 0. Max coverage (-): 0

Region: chr14 15244231-15244250. Max. coverage (+): 0. Max coverage (-): 0

Region: chr14 15244251-15244270. Max. coverage (+): 0. Max coverage (-): 0

Region: chr14 15244271-15244289. Max. coverage (+): 0. Max coverage (-): 0

Region: chr14 15244290-15244309. Max. coverage (+): 0. Max coverage (-): 0

Region: chr14 15244310-15244329. Max. coverage (+): 0. Max coverage (-): 0

Region: chr14 15244330-15244349. Max. coverage (+): 0. Max coverage (-): 0

Region: chr14 15244350-15244369. Max. coverage (+): 0. Max coverage (-): 0

Region: chr14 15244370-15244389. Max. coverage (+): 0. Max coverage (-): 0

Region: chr14 15244390-15244409. Max. coverage (+): 0. Max coverage (-): 0

Region: chr14 15244410-15244429. Max. coverage (+): 0. Max coverage (-): 0

Region: chr14 15244430-15244449. Max. coverage (+): 0. Max coverage (-): 0

Region: chr14 15244450-15244469. Max. coverage (+): 0.22. Max coverage (-): 0

Region: chr14 15244470-15244489. Max. coverage (+): 2.21. Max coverage (-): 0

Region: chr14 15244490-15244509. Max. coverage (+): 0. Max coverage (-): 0

Region: chr14 15244510-15244529. Max. coverage (+): 0. Max coverage (-): 0

Region: chr14 15244530-15244549. Max. coverage (+): 0. Max coverage (-): 0

Region: chr14 15244550-15244569. Max. coverage (+): 0. Max coverage (-): 0

Region: chr14 15244570-15244589. Max. coverage (+): 0. Max coverage (-): 0

Region: chr14 15244590-15244609. Max. coverage (+): 0. Max coverage (-): 0

Region: chr14 15244610-15244629. Max. coverage (+): 0. Max coverage (-): 0

Region: chr14 15244630-15244648. Max. coverage (+): 0. Max coverage (-): 0

Region: chr14 15244649-15244668. Max. coverage (+): 0. Max coverage (-): 0

Region: chr14 15244669-15244688. Max. coverage (+): 0. Max coverage (-): 0

Region: chr14 15244689-15244708. Max. coverage (+): 0. Max coverage (-): 0

Region: chr14 15244709-15244728. Max. coverage (+): 0. Max coverage (-): 0

Region: chr14 15244729-15244748. Max. coverage (+): 0. Max coverage (-): 0

Region: chr14 15244749-15244768. Max. coverage (+): 0. Max coverage (-): 0

Region: chr14 15244769-15244788. Max. coverage (+): 0. Max coverage (-): 0

Region: chr14 15244789-15244808. Max. coverage (+): 0. Max coverage (-): 0

Region: chr14 15244809-15244828. Max. coverage (+): 0. Max coverage (-): 0

Region: chr14 15244829-15244848. Max. coverage (+): 0. Max coverage (-): 0

Region: chr14 15244849-15244868. Max. coverage (+): 0. Max coverage (-): 0

Region: chr14 15244869-15244888. Max. coverage (+): 0. Max coverage (-): 0

Region: chr14 15244889-15244908. Max. coverage (+): 0. Max coverage (-): 0

Region: chr14 15244909-15244928. Max. coverage (+): 0. Max coverage (-): 0

Region: chr14 15244929-15244948. Max. coverage (+): 0. Max coverage (-): 0

Region: chr14 15244949-15244968. Max. coverage (+): 0. Max coverage (-): 0

Region: chr14 15244969-15244987. Max. coverage (+): 0. Max coverage (-): 0

Region: chr14 15244988-15245007. Max. coverage (+): 0. Max coverage (-): 0

Region: chr14 15245008-15245027. Max. coverage (+): 4.98. Max coverage (-): 0

Region: chr14 15245028-15245047. Max. coverage (+): 4.98. Max coverage (-): 0

Region: chr14 15245048-15245067. Max. coverage (+): 0. Max coverage (-): 0

Region: chr14 15245068-15245087. Max. coverage (+): 1.09. Max coverage (-): 0

Region: chr14 15245088-15245107. Max. coverage (+): 0. Max coverage (-): 0

Region: chr14 15245108-15245127. Max. coverage (+): 1.91. Max coverage (-): 0

Region: chr14 15245128-15245147. Max. coverage (+): 2.29. Max coverage (-): 0

Region: chr14 15245148-15245167. Max. coverage (+): 1.47. Max coverage (-): 0

Region: chr14 15245168-15245187. Max. coverage (+): 1.87. Max coverage (-): 3.27

Region: chr14 15245188-15245207. Max. coverage (+): 0.68. Max coverage (-): 0

Region: chr14 15245208-15245227. Max. coverage (+): 1.55. Max coverage (-): 0

Region: chr14 15245228-15245247. Max. coverage (+): 1.55. Max coverage (-): 0

Region: chr14 15245248-15245267. Max. coverage (+): 0.39. Max coverage (-): 0

Region: chr14 15245268-15245287. Max. coverage (+): 0.69. Max coverage (-): 0

Region: chr14 15245288-15245307. Max. coverage (+): 0.42. Max coverage (-): 0

Region: chr14 15245308-15245327. Max. coverage (+): 0.38. Max coverage (-): 0

Region: chr14 15245328-15245346. Max. coverage (+): 0. Max coverage (-): 0

Region: chr14 15245347-15245366. Max. coverage (+): 0. Max coverage (-): 0

Region: chr14 15245367-15245386. Max. coverage (+): 0. Max coverage (-): 0

Region: chr14 15245387-15245406. Max. coverage (+): 0. Max coverage (-): 0

Region: chr14 15245407-15245426. Max. coverage (+): 2.39. Max coverage (-): 0

Region: chr14 15245427-15245446. Max. coverage (+): 0. Max coverage (-): 0

Region: chr14 15245447-15245466. Max. coverage (+): 0. Max coverage (-): 0

Region: chr14 15245467-15245486. Max. coverage (+): 0. Max coverage (-): 0

Region: chr14 15245487-15245506. Max. coverage (+): 0. Max coverage (-): 0

Region: chr14 15245507-15245526. Max. coverage (+): 0. Max coverage (-): 0

Region: chr14 15245527-15245546. Max. coverage (+): 0. Max coverage (-): 0

Region: chr14 15245547-15245566. Max. coverage (+): 0. Max coverage (-): 0

Region: chr14 15245567-15245586. Max. coverage (+): 0. Max coverage (-): 0

Region: chr14 15245587-15245606. Max. coverage (+): 0. Max coverage (-): 0

Region: chr14 15245607-15245626. Max. coverage (+): 0. Max coverage (-): 0

Region: chr14 15245627-15245646. Max. coverage (+): 0. Max coverage (-): 0

Region: chr14 15245647-15245666. Max. coverage (+): 0. Max coverage (-): 0

Region: chr14 15245667-15245686. Max. coverage (+): 0. Max coverage (-): 0

Region: chr14 15245687-15245705. Max. coverage (+): 0. Max coverage (-): 0

Region: chr14 15245706-15245725. Max. coverage (+): 0. Max coverage (-): 0

Region: chr14 15245726-15245745. Max. coverage (+): 0. Max coverage (-): 0

Region: chr14 15245746-15245765. Max. coverage (+): 0. Max coverage (-): 0

Region: chr14 15245766-15245785. Max. coverage (+): 0. Max coverage (-): 0

Region: chr14 15245786-15245805. Max. coverage (+): 0. Max coverage (-): 0

Region: chr14 15245806-15245825. Max. coverage (+): 0. Max coverage (-): 0

Region: chr14 15245826-15245845. Max. coverage (+): 5.58. Max coverage (-): 0

Region: chr14 15245846-15245865. Max. coverage (+): 5.58. Max coverage (-): 0

Region: chr14 15245866-15245885. Max. coverage (+): 4.08. Max coverage (-): 1.08

Region: chr14 15245886-15245905. Max. coverage (+): 1.93. Max coverage (-): 0

Region: chr14 15245906-15245925. Max. coverage (+): 3.66. Max coverage (-): 0

Region: chr14 15245926-15245945. Max. coverage (+): 7.53. Max coverage (-): 0

Region: chr14 15245946-15245965. Max. coverage (+): 3.92. Max coverage (-): 0

Region: chr14 15245966-15245985. Max. coverage (+): 0. Max coverage (-): 0

Region: chr14 15245986-15246005. Max. coverage (+): 0. Max coverage (-): 0

Region: chr14 15246006-15246025. Max. coverage (+): 0. Max coverage (-): 0

Region: chr14 15246026-15246045. Max. coverage (+): 0. Max coverage (-): 0

Region: chr14 15246046-15246064. Max. coverage (+): 0. Max coverage (-): 0

Region: chr14 15246065-15246084. Max. coverage (+): 0. Max coverage (-): 0

Region: chr14 15246085-15246104. Max. coverage (+): 0. Max coverage (-): 0

Region: chr14 15246105-15246124. Max. coverage (+): 3.05. Max coverage (-): 0

Region: chr14 15246125-15246144. Max. coverage (+): 3.05. Max coverage (-): 0

Region: chr14 15246145-15246164. Max. coverage (+): 14.12. Max coverage (-): 0

Region: chr14 15246165-15246184. Max. coverage (+): 14.12. Max coverage (-): 0

Region: chr14 15246185-15246204. Max. coverage (+): 11.95. Max coverage (-): 0

Region: chr14 15246205-15246224. Max. coverage (+): 3.07. Max coverage (-): 2

Region: chr14 15246225-15246244. Max. coverage (+): 2.09. Max coverage (-): 0

Region: chr14 15246245-15246264. Max. coverage (+): 0.45. Max coverage (-): 0

Region: chr14 15246265-15246284. Max. coverage (+): 0. Max coverage (-): 0

Region: chr14 15246285-15246304. Max. coverage (+): 0. Max coverage (-): 0

Region: chr14 15246305-15246324. Max. coverage (+): 0. Max coverage (-): 0

Region: chr14 15246325-15246344. Max. coverage (+): 0. Max coverage (-): 0

Region: chr14 15246345-15246364. Max. coverage (+): 0. Max coverage (-): 0

Region: chr14 15246365-15246384. Max. coverage (+): 0. Max coverage (-): 0

Region: chr14 15246385-15246404. Max. coverage (+): 0. Max coverage (-): 0

Region: chr14 15246405-15246423. Max. coverage (+): 0. Max coverage (-): 0

Region: chr14 15246424-15246443. Max. coverage (+): 0. Max coverage (-): 0

Region: chr14 15246444-15246463. Max. coverage (+): 0. Max coverage (-): 0

Region: chr14 15246464-15246483. Max. coverage (+): 0. Max coverage (-): 0

Region: chr14 15246484-15246503. Max. coverage (+): 0. Max coverage (-): 0

Region: chr14 15246504-15246523. Max. coverage (+): 11.73. Max coverage (-): 0

Region: chr14 15246524-15246543. Max. coverage (+): 11.73. Max coverage (-): 0

Region: chr14 15246544-15246563. Max. coverage (+): 0.57. Max coverage (-): 0

Region: chr14 15246564-15246583. Max. coverage (+): 0.57. Max coverage (-): 0

Region: chr14 15246584-15246603. Max. coverage (+): 0. Max coverage (-): 0

Region: chr14 15246604-15246623. Max. coverage (+): 0. Max coverage (-): 0

Region: chr14 15246624-15246643. Max. coverage (+): 0. Max coverage (-): 0

Region: chr14 15246644-15246663. Max. coverage (+): 0. Max coverage (-): 0

Region: chr14 15246664-15246683. Max. coverage (+): 0. Max coverage (-): 0

Region: chr14 15246684-15246703. Max. coverage (+): 0. Max coverage (-): 0

Region: chr14 15246704-15246723. Max. coverage (+): 0. Max coverage (-): 0

Region: chr14 15246724-15246743. Max. coverage (+): 0. Max coverage (-): 0

Region: chr14 15246744-15246763. Max. coverage (+): 0. Max coverage (-): 0

Region: chr14 15246764-15246782. Max. coverage (+): 0. Max coverage (-): 0

Region: chr14 15246783-15246802. Max. coverage (+): 0. Max coverage (-): 0

Region: chr14 15246803-15246822. Max. coverage (+): 0. Max coverage (-): 0

Region: chr14 15246823-15246842. Max. coverage (+): 0. Max coverage (-): 0

Region: chr14 15246843-15246862. Max. coverage (+): 0. Max coverage (-): 0

Region: chr14 15246863-15246882. Max. coverage (+): 0. Max coverage (-): 0

Region: chr14 15246883-15246902. Max. coverage (+): 0. Max coverage (-): 0

Region: chr14 15246903-15246922. Max. coverage (+): 0. Max coverage (-): 0

Region: chr14 15246923-15246942. Max. coverage (+): 0. Max coverage (-): 0

Region: chr14 15246943-15246962. Max. coverage (+): 0. Max coverage (-): 0

Region: chr14 15246963-15246982. Max. coverage (+): 2.09. Max coverage (-): 0

Region: chr14 15246983-15247002. Max. coverage (+): 2.09. Max coverage (-): 0

Region: chr14 15247003-15247022. Max. coverage (+): 0.46. Max coverage (-): 0

Region: chr14 15247023-15247042. Max. coverage (+): 10.55. Max coverage (-): 0

Region: chr14 15247043-15247062. Max. coverage (+): 0. Max coverage (-): 0

Region: chr14 15247063-15247082. Max. coverage (+): 0. Max coverage (-): 0

Region: chr14 15247083-15247102. Max. coverage (+): 0. Max coverage (-): 0

Region: chr14 15247103-15247122. Max. coverage (+): 0. Max coverage (-): 0

Region: chr14 15247123-15247141. Max. coverage (+): 0. Max coverage (-): 0

Region: chr14 15247142-15247161. Max. coverage (+): 0. Max coverage (-): 0

Region: chr14 15247162-15247181. Max. coverage (+): 0. Max coverage (-): 0

Region: chr14 15247182-15247201. Max. coverage (+): 0. Max coverage (-): 0

Region: chr14 15247202-15247221. Max. coverage (+): 0. Max coverage (-): 0

Region: chr14 15247222-15247241. Max. coverage (+): 0. Max coverage (-): 0

Region: chr14 15247242-15247261. Max. coverage (+): 0. Max coverage (-): 0

Region: chr14 15247262-15247281. Max. coverage (+): 0. Max coverage (-): 0

Region: chr14 15247282-15247301. Max. coverage (+): 0. Max coverage (-): 0

Region: chr14 15247302-15247321. Max. coverage (+): 0. Max coverage (-): 0

Region: chr14 15247322-15247341. Max. coverage (+): 0. Max coverage (-): 0

Region: chr14 15247342-15247361. Max. coverage (+): 0. Max coverage (-): 0

Region: chr14 15247362-15247381. Max. coverage (+): 0. Max coverage (-): 0

Region: chr14 15247382-15247401. Max. coverage (+): 0. Max coverage (-): 0

Region: chr14 15247402-15247421. Max. coverage (+): 0. Max coverage (-): 0

Region: chr14 15247422-15247441. Max. coverage (+): 0. Max coverage (-): 0

Region: chr14 15247442-15247461. Max. coverage (+): 0. Max coverage (-): 0

Region: chr14 15247462-15247480. Max. coverage (+): 0. Max coverage (-): 0

Region: chr14 15247481-15247500. Max. coverage (+): 0. Max coverage (-): 0

Region: chr14 15247501-15247520. Max. coverage (+): 0. Max coverage (-): 0

Region: chr14 15247521-15247540. Max. coverage (+): 0. Max coverage (-): 0

Region: chr14 15247541-15247560. Max. coverage (+): 0. Max coverage (-): 0

Region: chr14 15247561-15247580. Max. coverage (+): 0. Max coverage (-): 0

Region: chr14 15247581-15247600. Max. coverage (+): 0. Max coverage (-): 0

Region: chr14 15247601-15247620. Max. coverage (+): 0. Max coverage (-): 0

Region: chr14 15247621-15247640. Max. coverage (+): 0. Max coverage (-): 0

Region: chr14 15247641-15247660. Max. coverage (+): 0. Max coverage (-): 0

Region: chr14 15247661-15247680. Max. coverage (+): 0. Max coverage (-): 0

Region: chr14 15247681-15247700. Max. coverage (+): 0. Max coverage (-): 0

Region: chr14 15247701-15247720. Max. coverage (+): 0. Max coverage (-): 0

Region: chr14 15247721-15247740. Max. coverage (+): 0. Max coverage (-): 0

Region: chr14 15247741-15247760. Max. coverage (+): 0. Max coverage (-): 0

Region: chr14 15247761-15247780. Max. coverage (+): 0. Max coverage (-): 0

Region: chr14 15247781-15247800. Max. coverage (+): 0. Max coverage (-): 0

Region: chr14 15247801-15247820. Max. coverage (+): 4.31. Max coverage (-): 0

Region: chr14 15247821-15247839. Max. coverage (+): 2.17. Max coverage (-): 0

Region: chr14 15247840-15247859. Max. coverage (+): 0. Max coverage (-): 0

Region: chr14 15247860-15247879. Max. coverage (+): 0. Max coverage (-): 0

Region: chr14 15247880-15247899. Max. coverage (+): 0. Max coverage (-): 0

Region: chr14 15247900-15247919. Max. coverage (+): 1.17. Max coverage (-): 0

Region: chr14 15247920-15247939. Max. coverage (+): 0.15. Max coverage (-): 0

Region: chr14 15247940-15247959. Max. coverage (+): 0. Max coverage (-): 0

Region: chr14 15247960-15247979. Max. coverage (+): 2.46. Max coverage (-): 0

Region: chr14 15247980-15247999. Max. coverage (+): 4.84. Max coverage (-): 0

Region: chr14 15248000-15248019. Max. coverage (+): 0.52. Max coverage (-): 0

Region: chr14 15248020-15248039. Max. coverage (+): 1.34. Max coverage (-): 0

Region: chr14 15248040-15248059. Max. coverage (+): 4.28. Max coverage (-): 0

Region: chr14 15248060-15248079. Max. coverage (+): 0. Max coverage (-): 0

Region: chr14 15248080-15248099. Max. coverage (+): 0. Max coverage (-): 0

Region: chr14 15248100-15248119. Max. coverage (+): 0. Max coverage (-): 0

Region: chr14 15248120-15248139. Max. coverage (+): 0. Max coverage (-): 0

Region: chr14 15248140-15248159. Max. coverage (+): 0. Max coverage (-): 0

Region: chr14 15248160-15248179. Max. coverage (+): 0. Max coverage (-): 0

Region: chr14 15248180-15248198. Max. coverage (+): 0. Max coverage (-): 0

Region: chr14 15248199-15248218. Max. coverage (+): 0. Max coverage (-): 0

Region: chr14 15248219-15248238. Max. coverage (+): 0. Max coverage (-): 0

Region: chr14 15248239-15248258. Max. coverage (+): 2.29. Max coverage (-): 0

Region: chr14 15248259-15248278. Max. coverage (+): 2.29. Max coverage (-): 0

Region: chr14 15248279-15248298. Max. coverage (+): 2.29. Max coverage (-): 0

Region: chr14 15248299-15248318. Max. coverage (+): 0. Max coverage (-): 0

Region: chr14 15248319-15248338. Max. coverage (+): 0. Max coverage (-): 0

Region: chr14 15248339-15248358. Max. coverage (+): 0. Max coverage (-): 0

Region: chr14 15248359-15248378. Max. coverage (+): 0. Max coverage (-): 0

Region: chr14 15248379-15248398. Max. coverage (+): 0. Max coverage (-): 0

Region: chr14 15248399-15248418. Max. coverage (+): 0. Max coverage (-): 0

Region: chr14 15248419-15248438. Max. coverage (+): 0. Max coverage (-): 0

Region: chr14 15248439-15248458. Max. coverage (+): 0. Max coverage (-): 0

Region: chr14 15248459-15248478. Max. coverage (+): 0. Max coverage (-): 0

Region: chr14 15248479-15248498. Max. coverage (+): 0. Max coverage (-): 0

Region: chr14 15248499-15248518. Max. coverage (+): 0. Max coverage (-): 0

Region: chr14 15248519-15248538. Max. coverage (+): 0. Max coverage (-): 0

Region: chr14 15248539-15248557. Max. coverage (+): 0. Max coverage (-): 0

Region: chr14 15248558-15248577. Max. coverage (+): 0. Max coverage (-): 0

Region: chr14 15248578-15248597. Max. coverage (+): 0. Max coverage (-): 0

Region: chr14 15248598-15248617. Max. coverage (+): 0. Max coverage (-): 0

Region: chr14 15248618-15248637. Max. coverage (+): 0. Max coverage (-): 0

Region: chr14 15248638-15248657. Max. coverage (+): 0. Max coverage (-): 0

Region: chr14 15248658-15248677. Max. coverage (+): 0. Max coverage (-): 0

Region: chr14 15248678-15248697. Max. coverage (+): 0. Max coverage (-): 0

Region: chr14 15248698-15248717. Max. coverage (+): 0. Max coverage (-): 0

Region: chr14 15248718-15248737. Max. coverage (+): 0. Max coverage (-): 0

Region: chr14 15248738-15248757. Max. coverage (+): 0. Max coverage (-): 0

Region: chr14 15248758-15248777. Max. coverage (+): 4.14. Max coverage (-): 0

Region: chr14 15248778-15248797. Max. coverage (+): 3.98. Max coverage (-): 0

Region: chr14 15248798-15248817. Max. coverage (+): 0. Max coverage (-): 0

Region: chr14 15248818-15248837. Max. coverage (+): 0. Max coverage (-): 0

Region: chr14 15248838-15248857. Max. coverage (+): 0. Max coverage (-): 0

Region: chr14 15248858-15248877. Max. coverage (+): 1.13. Max coverage (-): 0

Region: chr14 15248878-15248897. Max. coverage (+): 1.13. Max coverage (-): 0

Region: chr14 15248898-15248916. Max. coverage (+): 0. Max coverage (-): 0

Region: chr14 15248917-15248936. Max. coverage (+): 1.98. Max coverage (-): 0

Region: chr14 15248937-15248956. Max. coverage (+): 4.84. Max coverage (-): 0

Region: chr14 15248957-15248976. Max. coverage (+): 9.43. Max coverage (-): 0

Region: chr14 15248977-15248996. Max. coverage (+): 0.83. Max coverage (-): 0

Region: chr14 15248997-15249016. Max. coverage (+): 15.5. Max coverage (-): 0

Region: chr14 15249017-15249036. Max. coverage (+): 3.48. Max coverage (-): 0

Region: chr14 15249037-15249056. Max. coverage (+): 1.04. Max coverage (-): 0

Region: chr14 15249057-15249076. Max. coverage (+): 0.66. Max coverage (-): 0

Region: chr14 15249077-15249096. Max. coverage (+): 0.66. Max coverage (-): 0

Region: chr14 15249097-15249116. Max. coverage (+): 0. Max coverage (-): 0

Region: chr14 15249117-15249136. Max. coverage (+): 0.37. Max coverage (-): 0

Region: chr14 15249137-15249156. Max. coverage (+): 0. Max coverage (-): 0

Region: chr14 15249157-15249176. Max. coverage (+): 0. Max coverage (-): 0

Region: chr14 15249177-15249196. Max. coverage (+): 2.09. Max coverage (-): 0

Region: chr14 15249197-15249216. Max. coverage (+): 0. Max coverage (-): 0

Region: chr14 15249217-15249236. Max. coverage (+): 0. Max coverage (-): 0

Region: chr14 15249237-15249256. Max. coverage (+): 0. Max coverage (-): 0

Region: chr14 15249257-15249275. Max. coverage (+): 0. Max coverage (-): 0

Region: chr14 15249276-15249295. Max. coverage (+): 0. Max coverage (-): 0

Region: chr14 15249296-15249315. Max. coverage (+): 0. Max coverage (-): 0

Region: chr14 15249316-15249335. Max. coverage (+): 0. Max coverage (-): 0

Region: chr14 15249336-15249355. Max. coverage (+): 0. Max coverage (-): 0

Region: chr14 15249356-15249375. Max. coverage (+): 0. Max coverage (-): 0

Region: chr14 15249376-15249395. Max. coverage (+): 0. Max coverage (-): 0

Region: chr14 15249396-15249415. Max. coverage (+): 0. Max coverage (-): 0

Region: chr14 15249416-15249435. Max. coverage (+): 0. Max coverage (-): 0

Region: chr14 15249436-15249455. Max. coverage (+): 0. Max coverage (-): 0

Region: chr14 15249456-15249475. Max. coverage (+): 0. Max coverage (-): 0

Region: chr14 15249476-15249495. Max. coverage (+): 0. Max coverage (-): 0

Region: chr14 15249496-15249515. Max. coverage (+): 0. Max coverage (-): 0

Region: chr14 15249516-15249535. Max. coverage (+): 0.81. Max coverage (-): 0

Region: chr14 15249536-15249555. Max. coverage (+): 0.81. Max coverage (-): 0

Region: chr14 15249556-15249575. Max. coverage (+): 0. Max coverage (-): 0

Region: chr14 15249576-15249595. Max. coverage (+): 0. Max coverage (-): 0

Region: chr14 15249596-15249615. Max. coverage (+): 0. Max coverage (-): 0

Region: chr14 15249616-15249634. Max. coverage (+): 0. Max coverage (-): 0

Region: chr14 15249635-15249654. Max. coverage (+): 0. Max coverage (-): 0

Region: chr14 15249655-15249674. Max. coverage (+): 0. Max coverage (-): 0

Region: chr14 15249675-15249694. Max. coverage (+): 0. Max coverage (-): 0

Region: chr14 15249695-15249714. Max. coverage (+): 0. Max coverage (-): 0

Region: chr14 15249715-15249734. Max. coverage (+): 4.77. Max coverage (-): 0

Region: chr14 15249735-15249754. Max. coverage (+): 0. Max coverage (-): 0

Region: chr14 15249755-15249774. Max. coverage (+): 1.04. Max coverage (-): 0

Region: chr14 15249775-15249794. Max. coverage (+): 1.04. Max coverage (-): 0

Region: chr14 15249795-15249814. Max. coverage (+): 0. Max coverage (-): 0

Region: chr14 15249815-15249834. Max. coverage (+): 1.83. Max coverage (-): 0

Region: chr14 15249835-15249854. Max. coverage (+): 2.19. Max coverage (-): 0

Region: chr14 15249855-15249874. Max. coverage (+): 1.79. Max coverage (-): 3.13

Region: chr14 15249875-15249894. Max. coverage (+): 0.97. Max coverage (-): 3.13

Region: chr14 15249895-15249914. Max. coverage (+): 0. Max coverage (-): 0

Region: chr14 15249915-15249934. Max. coverage (+): 1.49. Max coverage (-): 0

Region: chr14 15249935-15249954. Max. coverage (+): 0.6. Max coverage (-): 0

Region: chr14 15249955-15249973. Max. coverage (+): 0.66. Max coverage (-): 0

Region: chr14 15249974-15249993. Max. coverage (+): 0.66. Max coverage (-): 0

Region: chr14 15249994-15250013. Max. coverage (+): 0. Max coverage (-): 0

Region: chr14 15250014-15250033. Max. coverage (+): 0.33. Max coverage (-): 0

Region: chr14 15250034-15250053. Max. coverage (+): 0. Max coverage (-): 0

Region: chr14 15250054-15250073. Max. coverage (+): 0. Max coverage (-): 0

Region: chr14 15250074-15250093. Max. coverage (+): 0. Max coverage (-): 0

Region: chr14 15250094-15250113. Max. coverage (+): 0. Max coverage (-): 0

Region: chr14 15250114-15250133. Max. coverage (+): 2.04. Max coverage (-): 0

Region: chr14 15250134-15250153. Max. coverage (+): 0. Max coverage (-): 0

Region: chr14 15250154-15250173. Max. coverage (+): 0. Max coverage (-): 0

Region: chr14 15250174-15250193. Max. coverage (+): 0. Max coverage (-): 0

Region: chr14 15250194-15250213. Max. coverage (+): 0. Max coverage (-): 0

Region: chr14 15250214-15250233. Max. coverage (+): 0. Max coverage (-): 0

Region: chr14 15250234-15250253. Max. coverage (+): 0. Max coverage (-): 0

Region: chr14 15250254-15250273. Max. coverage (+): 0. Max coverage (-): 0

Region: chr14 15250274-15250293. Max. coverage (+): 0. Max coverage (-): 0

Region: chr14 15250294-15250313. Max. coverage (+): 0. Max coverage (-): 0

Region: chr14 15250314-15250332. Max. coverage (+): 0. Max coverage (-): 0

Region: chr14 15250333-15250352. Max. coverage (+): 0. Max coverage (-): 0

Region: chr14 15250353-15250372. Max. coverage (+): 0. Max coverage (-): 0

Region: chr14 15250373-15250392. Max. coverage (+): 0. Max coverage (-): 0

Region: chr14 15250393-15250412. Max. coverage (+): 0. Max coverage (-): 0

Region: chr14 15250413-15250432. Max. coverage (+): 0. Max coverage (-): 0

Region: chr14 15250433-15250452. Max. coverage (+): 0. Max coverage (-): 0

Region: chr14 15250453-15250472. Max. coverage (+): 0. Max coverage (-): 0

Region: chr14 15250473-15250492. Max. coverage (+): 0. Max coverage (-): 0

Region: chr14 15250493-15250512. Max. coverage (+): 0. Max coverage (-): 0

Region: chr14 15250513-15250532. Max. coverage (+): 0. Max coverage (-): 0

Region: chr14 15250533-15250552. Max. coverage (+): 0. Max coverage (-): 0

Region: chr14 15250553-15250572. Max. coverage (+): 0. Max coverage (-): 0

Region: chr14 15250573-15250592. Max. coverage (+): 0. Max coverage (-): 0

Region: chr14 15250593-15250612. Max. coverage (+): 0. Max coverage (-): 0

Region: chr14 15250613-15250632. Max. coverage (+): 0. Max coverage (-): 0

Region: chr14 15250633-15250652. Max. coverage (+): 0. Max coverage (-): 0

Region: chr14 15250653-15250672. Max. coverage (+): 0. Max coverage (-): 0

Region: chr14 15250673-15250691. Max. coverage (+): 0. Max coverage (-): 0

Region: chr14 15250692-15250711. Max. coverage (+): 0. Max coverage (-): 0

Region: chr14 15250712-15250731. Max. coverage (+): 0. Max coverage (-): 0

Region: chr14 15250732-15250751. Max. coverage (+): 0. Max coverage (-): 0

Region: chr14 15250752-15250771. Max. coverage (+): 0. Max coverage (-): 0

Region: chr14 15250772-15250791. Max. coverage (+): 0. Max coverage (-): 0

Region: chr14 15250792-15250811. Max. coverage (+): 0. Max coverage (-): 0

Region: chr14 15250812-15250831. Max. coverage (+): 0. Max coverage (-): 0

Region: chr14 15250832-15250851. Max. coverage (+): 0. Max coverage (-): 0

Region: chr14 15250852-15250871. Max. coverage (+): 0. Max coverage (-): 0

Region: chr14 15250872-15250891. Max. coverage (+): 0. Max coverage (-): 0

Region: chr14 15250892-15250911. Max. coverage (+): 0. Max coverage (-): 0

Region: chr14 15250912-15250931. Max. coverage (+): 0. Max coverage (-): 0

Region: chr14 15250932-15250951. Max. coverage (+): 0. Max coverage (-): 0

Region: chr14 15250952-15250971. Max. coverage (+): 0. Max coverage (-): 0

Region: chr14 15250972-15250991. Max. coverage (+): 0. Max coverage (-): 0

Region: chr14 15250992-15251011. Max. coverage (+): 3.56. Max coverage (-): 0

Region: chr14 15251012-15251031. Max. coverage (+): 3.56. Max coverage (-): 0

Region: chr14 15251032-. Max. coverage (+): 0. Max coverage (-): 0

RepeatMasker Color Code

**+**

100-98% Identity

<98-95% Identity

<95-90% Identity

<90-85% Identity

<85-80% Identity

<80-75% Identity

<75-70% Identity

<70% Identity

**-**

Gene Set Color Code

**+**

Gene

Pseudogene

**-**

Topology/Coverage Color Code

Coverage Plus Strand

Coverage Minus Strand

Mainstrand: Plus

Mainstrand: Minus

Complementary Strand

Flanking Region  
(if option -flank >0)

Gene Set Annotation  
  
RepeatMasker Annotation  

**1. ART2A**: 15241150-15241391 (-), Divergence to consensus: 28.6%  
**2. L1\_BT**: 15242140-15242419 (+), Divergence to consensus: 19.7%  
**3. Bov-tA3**: 15242782-15242995 (+), Divergence to consensus: 15.8%  
**4. L2c**: 15243017-15243117 (-), Divergence to consensus: 31.1%  
**5. BOV-A2**: 15243181-15243202 (-), Divergence to consensus: 27%  
**6. (CAGTT)n**: 15243203-15243225 (+), Divergence to consensus: 0%  
**7. BOV-A2**: 15243226-15243286 (-), Divergence to consensus: 27%  
**8. ART2A**: 15243287-15243447 (-), Divergence to consensus: 30.2%  
**9. T-rich**: 15243958-15243995 (+), Divergence to consensus: 15.8%  
**10. L3**: 15244189-15244258 (-), Divergence to consensus: 35.8%  
**11. Bov-tA1**: 15244504-15244723 (+), Divergence to consensus: 25.2%  
**12. L3**: 15244769-15244954 (-), Divergence to consensus: 44.1%  
**13. L2c**: 15245543-15245699 (-), Divergence to consensus: 45.3%  
**14. T-rich**: 15246056-15246096 (+), Divergence to consensus: 17.5%  
**15. L3**: 15246304-15246354 (-), Divergence to consensus: 31.4%  
**16. Bov-tA1**: 15246591-15246808 (+), Divergence to consensus: 22.5%  
**17. L3**: 15247057-15247107 (-), Divergence to consensus: 33.3%  
**18. Bov-tA1**: 15247344-15247561 (+), Divergence to consensus: 22.5%  
**19. L3**: 15247609-15247794 (-), Divergence to consensus: 44.1%  
**20. Bov-tA1**: 15248308-15248525 (+), Divergence to consensus: 22.5%  
**21. L3**: 15248573-15248758 (-), Divergence to consensus: 43.6%  
**22. L2c**: 15249341-15249521 (-), Divergence to consensus: 47.5%  
**23. L2c**: 15250244-15250678 (-), Divergence to consensus: 48.1%

  
Transcription Factor Binding Sites  

**Gata4** (Sequence: CTTATCT (+): 15244958)  
**Gata4** (Sequence: CTTATCT (+): 15247798)  
**Gata4** (Sequence: CTTATCT (+): 15248762)
