# Supplementary material for: Small RNA sequencing of cryopreserved semen from single bull revealed altered miRNAs and piRNAs expression between High- and Low-motile sperm populations
Source: BMC Genomics. 2017 Jan 4;18:14. doi: 10.1186/s12864-016-3394-7 (PMC5209821; doi:10.1186/s12864-016-3394-7)
Supplement: Additional file 3: — Details for each piRNA clusters found in High Motile (HM) sperm fraction. Genes, repeats, transposable elements and transcription factors binding sites falling within the cluster regions were reported. (ZIP 1896 kb) [file 12864_2016_3394_MOESM3_ESM.zip › 23.html]

piRNA cluster 23


Predicted piRNA cluster no. 23     previous   next
  

Show proTRAC run info
Hide proTRAC run info

================================= proTRAC ====================================  
VERSION: 2.1                                    LAST MODIFIED: 06. October 2015  
  
Please cite:  
Rosenkranz D, Zischler H. proTRAC - a software for probabilistic piRNA cluster  
detection, visualization and analysis. 2012. BMC Bioinformatics 13:5.  
  
and (for proTRAC 2.0 and later):  
Rosenkranz D, Rudloff S, Bastuck K, Ketting RF, Zischler H. Tupaia small RNAs  
provide insights into function and evolution of RNAi-based transposon defense  
in mammals. 2015. RNA 21(5):911-922.  
  
Contact:  
David Rosenkranz  
Institute of Anthropology, small RNA group  
Johannes Gutenberg University Mainz  
email: rosenkranz@uni-mainz.de  
  
You can find the latest proTRAC version at:  
http://sourceforge.net/projects/protrac/files  
http://www.smallRNAgroup-mainz.de/software  
==============================================================================  
  
PARAMETERS:  
Map file: .............../storage/core/barbara/genhome/smallRNA/fertility/Sample\_motile/pirna/Sample\_motile\_26-33\_collapsed.fa.no-dust.map.weighted-10000-1000-b-0  
Genome file: ............/storage/core/barbara/genhome/smallRNA/fertility/Sample\_all/pirna/bt\_311\_chrY.fa  
RepeatMasker annotation: /storage/genomes/bt\_umd31/GCF\_000003055.6\_Bos\_taurus\_UMD\_3.1.1\_repeatMasker\_chr.out  
GeneSet:................./storage/core/barbara/genhome/smallRNA/fertility/Sample\_all/pirna/full.gtf  
  
Significant (p<=0.01) hit density will be calculated based  
on observed hit distribution.  
  
Sliding window size: ........................................ 5000 bp  
Sliding window increament: .................................. 1000 bp  
Normalize each hit by number of genomic hits: ............... 1 [0=no/1=yes]  
Normalize each hit by number of sequence reads: ............. 1 [0=no/1=yes]  
Normalize values (-> per million mapped reads): ............. 1 [0=no/1=yes]  
Min. fraction of hits with 1T(U) or 10A: .................... 0.75  
Alternatively: Min. fraction of hits with 1T(U) and 10A: .... 0.5  
Min. fraction of hits with typical piRNA length: ............ 0.75  
Typical piRNA length: ....................................... 26-33 nt  
Min. size of a piRNA cluster: ............................... 5000 bp.  
Min. number of hits (absolute): ............................. 0  
Min. number of hits (normalized): ........................... 0  
Min. fraction of hits on the mainstrand: .................... 0.75  
Top fraction of mapped sequences (in terms of read counts): . 1%  
Top fraction accounts for max. n% of sequence reads: ........ 90%  
Min. fraction of hits on each arm of a bidirectional cluster: 0.1  
Output image file for each cluster: ......................... 0 [0=no/1=yes]  
Output html file for each cluster: .......................... 1 [0=no/1=yes]  
Output a summary table: ..................................... 1 [0=no/1=yes]  
Output a FASTA file for each cluster (piRNA sequences): ..... 1 [0=no/1=yes]  
Output a FASTA file comprising cluster sequences: ........... 1 [0=no/1=yes]  
Search DNA motifs in clusters: .............................. 1 [0=no/1=yes]  
Output flanking sequences: +/- .............................. 0 bp  
Output ~.pTi file: .......................................... 1 [0=no/1=yes]  
==============================================================================  
  
  
Genome size (without gaps): ............ 2678902517 bp  
Gaps (N/X/-): .......................... 53837044 bp  
Mapped reads: .......................... 658825247023  
Non-identical sequences: ............... 514171  
Genomic hits: .......................... 764233  
Significant densitiy of mapped reads: .. 12867599.5173724 reads/kb

Show proTRAC cluster info
Hide proTRAC cluster info

|  |  |
| --- | --- |
| Location | chr14 |
| Coordinates | 15258224-15267594 |
| Size [bp] | 9371 |
| Sequence hit loci | 663 |
| Mapped reads (normalized) | 659403273.9 |
| Mapped reads (normalized) per kb | 70366372.2 |
| Normalized reads with 1T (1U) | 64% |
| Normalized reads with 10A | 50.9% |
| Normalized reads with length 26-33 nt | 100% |
| Normalized reads on the main strand(s) | 87.4% |
| Predicted directionality | mono:plus |

100%

0%

1T (1U)  
reads

10A reads

26-33 nt  
reads

reads on mainstrand

**Either the amount of reads with 1T (1U) OR 10A has to exceed 75% (set with option: -1Tor10A)  
Alternatively the amount of reads with 1T (1U) AND 10A has to exceed 50% (set with option: -1Tand10A)  
Minimum amount of reads with preferred size is 75% (set with option: -pisize)  
Minimum amount of reads on the main strand(s) is 75% (set with option: -clstrand)**

Show read coverage
Hide read coverage

WHAT DO I SEE HERE?  
This chart shows the location of mapped sequence reads within a predicted piRNA cluster. The color refers to the number of genomic hits produced by the sequence read in question. A dark red bar indicates that this sequence read produces many other hits elsewhere in the genome. Many adjacent red or yellow bars can indicate the presence of a multi-copy element such as transposons or rRNA genes. A dark green bar indicates that this sequence read maps uniquely to this locus.

1 hit

2-5 hits

6-10 hits

11-20 hits

21-50 hits

51-100 hits

> 100 hits

chr14

15258224

15267594

Gene Set

RepeatMasker

Mapped  
Reads

63.78

plus strand

minus strand

63.78

Region: chr14 15251033-15258233. Max. coverage (+): 4.55. Max coverage (-): 0

Region: chr14 15258234-15258252. Max. coverage (+): 4. Max coverage (-): 0

Region: chr14 15258253-15258270. Max. coverage (+): 0. Max coverage (-): 0

Region: chr14 15258271-15258289. Max. coverage (+): 0. Max coverage (-): 0

Region: chr14 15258290-15258308. Max. coverage (+): 0. Max coverage (-): 0

Region: chr14 15258309-15258327. Max. coverage (+): 0. Max coverage (-): 0

Region: chr14 15258328-15258345. Max. coverage (+): 0. Max coverage (-): 0

Region: chr14 15258346-15258364. Max. coverage (+): 0. Max coverage (-): 0

Region: chr14 15258365-15258383. Max. coverage (+): 0. Max coverage (-): 0

Region: chr14 15258384-15258402. Max. coverage (+): 0. Max coverage (-): 0

Region: chr14 15258403-15258420. Max. coverage (+): 0. Max coverage (-): 0

Region: chr14 15258421-15258439. Max. coverage (+): 0. Max coverage (-): 0

Region: chr14 15258440-15258458. Max. coverage (+): 0. Max coverage (-): 0

Region: chr14 15258459-15258477. Max. coverage (+): 0. Max coverage (-): 0

Region: chr14 15258478-15258495. Max. coverage (+): 2.82. Max coverage (-): 0

Region: chr14 15258496-15258514. Max. coverage (+): 2.82. Max coverage (-): 0

Region: chr14 15258515-15258533. Max. coverage (+): 0. Max coverage (-): 0

Region: chr14 15258534-15258551. Max. coverage (+): 0. Max coverage (-): 0

Region: chr14 15258552-15258570. Max. coverage (+): 0. Max coverage (-): 0

Region: chr14 15258571-15258589. Max. coverage (+): 0. Max coverage (-): 0

Region: chr14 15258590-15258608. Max. coverage (+): 0. Max coverage (-): 0

Region: chr14 15258609-15258626. Max. coverage (+): 0. Max coverage (-): 0

Region: chr14 15258627-15258645. Max. coverage (+): 0. Max coverage (-): 0

Region: chr14 15258646-15258664. Max. coverage (+): 0. Max coverage (-): 0

Region: chr14 15258665-15258683. Max. coverage (+): 0. Max coverage (-): 0

Region: chr14 15258684-15258701. Max. coverage (+): 1.38. Max coverage (-): 0

Region: chr14 15258702-15258720. Max. coverage (+): 3.22. Max coverage (-): 0

Region: chr14 15258721-15258739. Max. coverage (+): 0. Max coverage (-): 0

Region: chr14 15258740-15258758. Max. coverage (+): 0. Max coverage (-): 0

Region: chr14 15258759-15258776. Max. coverage (+): 0. Max coverage (-): 0

Region: chr14 15258777-15258795. Max. coverage (+): 0. Max coverage (-): 0

Region: chr14 15258796-15258814. Max. coverage (+): 0. Max coverage (-): 0

Region: chr14 15258815-15258833. Max. coverage (+): 0. Max coverage (-): 0

Region: chr14 15258834-15258851. Max. coverage (+): 0. Max coverage (-): 0

Region: chr14 15258852-15258870. Max. coverage (+): 0. Max coverage (-): 0

Region: chr14 15258871-15258889. Max. coverage (+): 0. Max coverage (-): 0

Region: chr14 15258890-15258908. Max. coverage (+): 0. Max coverage (-): 1.89

Region: chr14 15258909-15258926. Max. coverage (+): 0. Max coverage (-): 1

Region: chr14 15258927-15258945. Max. coverage (+): 0. Max coverage (-): 0

Region: chr14 15258946-15258964. Max. coverage (+): 5.44. Max coverage (-): 0

Region: chr14 15258965-15258983. Max. coverage (+): 5.44. Max coverage (-): 0

Region: chr14 15258984-15259001. Max. coverage (+): 0. Max coverage (-): 0

Region: chr14 15259002-15259020. Max. coverage (+): 10.36. Max coverage (-): 0

Region: chr14 15259021-15259039. Max. coverage (+): 0. Max coverage (-): 0

Region: chr14 15259040-15259058. Max. coverage (+): 6.32. Max coverage (-): 4.21

Region: chr14 15259059-15259076. Max. coverage (+): 1. Max coverage (-): 0

Region: chr14 15259077-15259095. Max. coverage (+): 31.69. Max coverage (-): 0

Region: chr14 15259096-15259114. Max. coverage (+): 0. Max coverage (-): 0

Region: chr14 15259115-15259132. Max. coverage (+): 0. Max coverage (-): 0

Region: chr14 15259133-15259151. Max. coverage (+): 0.76. Max coverage (-): 0

Region: chr14 15259152-15259170. Max. coverage (+): 0.76. Max coverage (-): 0

Region: chr14 15259171-15259189. Max. coverage (+): 5.03. Max coverage (-): 0

Region: chr14 15259190-15259207. Max. coverage (+): 5.03. Max coverage (-): 0

Region: chr14 15259208-15259226. Max. coverage (+): 0. Max coverage (-): 0

Region: chr14 15259227-15259245. Max. coverage (+): 0. Max coverage (-): 0

Region: chr14 15259246-15259264. Max. coverage (+): 9.7. Max coverage (-): 0

Region: chr14 15259265-15259282. Max. coverage (+): 9.7. Max coverage (-): 0

Region: chr14 15259283-15259301. Max. coverage (+): 10.02. Max coverage (-): 0

Region: chr14 15259302-15259320. Max. coverage (+): 0. Max coverage (-): 0

Region: chr14 15259321-15259339. Max. coverage (+): 0. Max coverage (-): 0

Region: chr14 15259340-15259357. Max. coverage (+): 0. Max coverage (-): 0

Region: chr14 15259358-15259376. Max. coverage (+): 0. Max coverage (-): 0

Region: chr14 15259377-15259395. Max. coverage (+): 0. Max coverage (-): 0

Region: chr14 15259396-15259414. Max. coverage (+): 0. Max coverage (-): 0

Region: chr14 15259415-15259432. Max. coverage (+): 0. Max coverage (-): 0

Region: chr14 15259433-15259451. Max. coverage (+): 0. Max coverage (-): 0

Region: chr14 15259452-15259470. Max. coverage (+): 0. Max coverage (-): 0

Region: chr14 15259471-15259489. Max. coverage (+): 0. Max coverage (-): 0

Region: chr14 15259490-15259507. Max. coverage (+): 0. Max coverage (-): 0

Region: chr14 15259508-15259526. Max. coverage (+): 3.45. Max coverage (-): 0

Region: chr14 15259527-15259545. Max. coverage (+): 3.61. Max coverage (-): 0

Region: chr14 15259546-15259564. Max. coverage (+): 0. Max coverage (-): 0

Region: chr14 15259565-15259582. Max. coverage (+): 10.27. Max coverage (-): 0

Region: chr14 15259583-15259601. Max. coverage (+): 10.27. Max coverage (-): 0

Region: chr14 15259602-15259620. Max. coverage (+): 0. Max coverage (-): 0

Region: chr14 15259621-15259639. Max. coverage (+): 0. Max coverage (-): 0

Region: chr14 15259640-15259657. Max. coverage (+): 3.28. Max coverage (-): 0

Region: chr14 15259658-15259676. Max. coverage (+): 1.79. Max coverage (-): 0

Region: chr14 15259677-15259695. Max. coverage (+): 0. Max coverage (-): 0

Region: chr14 15259696-15259713. Max. coverage (+): 0. Max coverage (-): 0

Region: chr14 15259714-15259732. Max. coverage (+): 3.36. Max coverage (-): 0.16

Region: chr14 15259733-15259751. Max. coverage (+): 0.52. Max coverage (-): 0.16

Region: chr14 15259752-15259770. Max. coverage (+): 0. Max coverage (-): 0

Region: chr14 15259771-15259788. Max. coverage (+): 2.24. Max coverage (-): 2.91

Region: chr14 15259789-15259807. Max. coverage (+): 4.88. Max coverage (-): 2.91

Region: chr14 15259808-15259826. Max. coverage (+): 4.88. Max coverage (-): 0

Region: chr14 15259827-15259845. Max. coverage (+): 4.68. Max coverage (-): 0

Region: chr14 15259846-15259863. Max. coverage (+): 0. Max coverage (-): 6.18

Region: chr14 15259864-15259882. Max. coverage (+): 0. Max coverage (-): 0

Region: chr14 15259883-15259901. Max. coverage (+): 0. Max coverage (-): 0

Region: chr14 15259902-15259920. Max. coverage (+): 1.08. Max coverage (-): 0

Region: chr14 15259921-15259938. Max. coverage (+): 0.59. Max coverage (-): 0

Region: chr14 15259939-15259957. Max. coverage (+): 0.59. Max coverage (-): 0

Region: chr14 15259958-15259976. Max. coverage (+): 0. Max coverage (-): 3.34

Region: chr14 15259977-15259995. Max. coverage (+): 0. Max coverage (-): 3.34

Region: chr14 15259996-15260013. Max. coverage (+): 1.25. Max coverage (-): 0

Region: chr14 15260014-15260032. Max. coverage (+): 1.25. Max coverage (-): 0

Region: chr14 15260033-15260051. Max. coverage (+): 5. Max coverage (-): 0

Region: chr14 15260052-15260070. Max. coverage (+): 0. Max coverage (-): 0

Region: chr14 15260071-15260088. Max. coverage (+): 0. Max coverage (-): 0

Region: chr14 15260089-15260107. Max. coverage (+): 0. Max coverage (-): 0

Region: chr14 15260108-15260126. Max. coverage (+): 0. Max coverage (-): 0

Region: chr14 15260127-15260145. Max. coverage (+): 0. Max coverage (-): 0

Region: chr14 15260146-15260163. Max. coverage (+): 0.17. Max coverage (-): 0

Region: chr14 15260164-15260182. Max. coverage (+): 0.33. Max coverage (-): 0

Region: chr14 15260183-15260201. Max. coverage (+): 0.33. Max coverage (-): 0

Region: chr14 15260202-15260220. Max. coverage (+): 0. Max coverage (-): 1.09

Region: chr14 15260221-15260238. Max. coverage (+): 0. Max coverage (-): 0

Region: chr14 15260239-15260257. Max. coverage (+): 0. Max coverage (-): 0

Region: chr14 15260258-15260276. Max. coverage (+): 0. Max coverage (-): 0

Region: chr14 15260277-15260294. Max. coverage (+): 7.67. Max coverage (-): 0

Region: chr14 15260295-15260313. Max. coverage (+): 0. Max coverage (-): 0

Region: chr14 15260314-15260332. Max. coverage (+): 6.77. Max coverage (-): 0

Region: chr14 15260333-15260351. Max. coverage (+): 12.11. Max coverage (-): 0

Region: chr14 15260352-15260369. Max. coverage (+): 17.09. Max coverage (-): 0

Region: chr14 15260370-15260388. Max. coverage (+): 9.23. Max coverage (-): 0

Region: chr14 15260389-15260407. Max. coverage (+): 0. Max coverage (-): 0

Region: chr14 15260408-15260426. Max. coverage (+): 0. Max coverage (-): 0

Region: chr14 15260427-15260444. Max. coverage (+): 0. Max coverage (-): 0

Region: chr14 15260445-15260463. Max. coverage (+): 0. Max coverage (-): 0

Region: chr14 15260464-15260482. Max. coverage (+): 2.81. Max coverage (-): 0

Region: chr14 15260483-15260501. Max. coverage (+): 4.68. Max coverage (-): 0

Region: chr14 15260502-15260519. Max. coverage (+): 0. Max coverage (-): 0

Region: chr14 15260520-15260538. Max. coverage (+): 2.86. Max coverage (-): 0

Region: chr14 15260539-15260557. Max. coverage (+): 6.92. Max coverage (-): 0

Region: chr14 15260558-15260576. Max. coverage (+): 0. Max coverage (-): 0

Region: chr14 15260577-15260594. Max. coverage (+): 0. Max coverage (-): 0

Region: chr14 15260595-15260613. Max. coverage (+): 0. Max coverage (-): 0

Region: chr14 15260614-15260632. Max. coverage (+): 0. Max coverage (-): 0

Region: chr14 15260633-15260651. Max. coverage (+): 0. Max coverage (-): 0

Region: chr14 15260652-15260669. Max. coverage (+): 0. Max coverage (-): 0

Region: chr14 15260670-15260688. Max. coverage (+): 0. Max coverage (-): 0

Region: chr14 15260689-15260707. Max. coverage (+): 0. Max coverage (-): 0

Region: chr14 15260708-15260726. Max. coverage (+): 0. Max coverage (-): 0

Region: chr14 15260727-15260744. Max. coverage (+): 2.87. Max coverage (-): 1.64

Region: chr14 15260745-15260763. Max. coverage (+): 3.32. Max coverage (-): 1.64

Region: chr14 15260764-15260782. Max. coverage (+): 8.14. Max coverage (-): 0

Region: chr14 15260783-15260801. Max. coverage (+): 9.35. Max coverage (-): 0

Region: chr14 15260802-15260819. Max. coverage (+): 1.59. Max coverage (-): 0

Region: chr14 15260820-15260838. Max. coverage (+): 0. Max coverage (-): 0

Region: chr14 15260839-15260857. Max. coverage (+): 7.44. Max coverage (-): 0

Region: chr14 15260858-15260875. Max. coverage (+): 0. Max coverage (-): 0

Region: chr14 15260876-15260894. Max. coverage (+): 1.39. Max coverage (-): 0

Region: chr14 15260895-15260913. Max. coverage (+): 1.87. Max coverage (-): 0

Region: chr14 15260914-15260932. Max. coverage (+): 0. Max coverage (-): 0

Region: chr14 15260933-15260950. Max. coverage (+): 4.1. Max coverage (-): 0

Region: chr14 15260951-15260969. Max. coverage (+): 3.21. Max coverage (-): 0

Region: chr14 15260970-15260988. Max. coverage (+): 1.49. Max coverage (-): 0

Region: chr14 15260989-15261007. Max. coverage (+): 0. Max coverage (-): 0

Region: chr14 15261008-15261025. Max. coverage (+): 5.65. Max coverage (-): 0

Region: chr14 15261026-15261044. Max. coverage (+): 4.89. Max coverage (-): 0

Region: chr14 15261045-15261063. Max. coverage (+): 0. Max coverage (-): 0

Region: chr14 15261064-15261082. Max. coverage (+): 0. Max coverage (-): 0

Region: chr14 15261083-15261100. Max. coverage (+): 0. Max coverage (-): 0

Region: chr14 15261101-15261119. Max. coverage (+): 0. Max coverage (-): 1.32

Region: chr14 15261120-15261138. Max. coverage (+): 0. Max coverage (-): 0.81

Region: chr14 15261139-15261157. Max. coverage (+): 0. Max coverage (-): 0

Region: chr14 15261158-15261175. Max. coverage (+): 0. Max coverage (-): 0

Region: chr14 15261176-15261194. Max. coverage (+): 1.21. Max coverage (-): 0

Region: chr14 15261195-15261213. Max. coverage (+): 0. Max coverage (-): 0

Region: chr14 15261214-15261232. Max. coverage (+): 0. Max coverage (-): 7.51

Region: chr14 15261233-15261250. Max. coverage (+): 0. Max coverage (-): 1.04

Region: chr14 15261251-15261269. Max. coverage (+): 0. Max coverage (-): 0

Region: chr14 15261270-15261288. Max. coverage (+): 0. Max coverage (-): 0

Region: chr14 15261289-15261307. Max. coverage (+): 0. Max coverage (-): 0

Region: chr14 15261308-15261325. Max. coverage (+): 0. Max coverage (-): 0

Region: chr14 15261326-15261344. Max. coverage (+): 0. Max coverage (-): 0

Region: chr14 15261345-15261363. Max. coverage (+): 0. Max coverage (-): 0

Region: chr14 15261364-15261382. Max. coverage (+): 0. Max coverage (-): 0

Region: chr14 15261383-15261400. Max. coverage (+): 0. Max coverage (-): 0

Region: chr14 15261401-15261419. Max. coverage (+): 0.32. Max coverage (-): 0

Region: chr14 15261420-15261438. Max. coverage (+): 0.32. Max coverage (-): 0

Region: chr14 15261439-15261456. Max. coverage (+): 3.04. Max coverage (-): 0

Region: chr14 15261457-15261475. Max. coverage (+): 4.08. Max coverage (-): 0

Region: chr14 15261476-15261494. Max. coverage (+): 4.08. Max coverage (-): 0

Region: chr14 15261495-15261513. Max. coverage (+): 0. Max coverage (-): 0

Region: chr14 15261514-15261531. Max. coverage (+): 0. Max coverage (-): 0

Region: chr14 15261532-15261550. Max. coverage (+): 0. Max coverage (-): 0

Region: chr14 15261551-15261569. Max. coverage (+): 0. Max coverage (-): 0

Region: chr14 15261570-15261588. Max. coverage (+): 0. Max coverage (-): 0

Region: chr14 15261589-15261606. Max. coverage (+): 4.93. Max coverage (-): 0

Region: chr14 15261607-15261625. Max. coverage (+): 0. Max coverage (-): 0

Region: chr14 15261626-15261644. Max. coverage (+): 0. Max coverage (-): 0

Region: chr14 15261645-15261663. Max. coverage (+): 0. Max coverage (-): 0

Region: chr14 15261664-15261681. Max. coverage (+): 0. Max coverage (-): 0

Region: chr14 15261682-15261700. Max. coverage (+): 4.38. Max coverage (-): 0

Region: chr14 15261701-15261719. Max. coverage (+): 4.38. Max coverage (-): 0

Region: chr14 15261720-15261738. Max. coverage (+): 1.11. Max coverage (-): 0

Region: chr14 15261739-15261756. Max. coverage (+): 0. Max coverage (-): 0

Region: chr14 15261757-15261775. Max. coverage (+): 7.05. Max coverage (-): 0

Region: chr14 15261776-15261794. Max. coverage (+): 2.14. Max coverage (-): 0

Region: chr14 15261795-15261813. Max. coverage (+): 63.78. Max coverage (-): 0

Region: chr14 15261814-15261831. Max. coverage (+): 4.99. Max coverage (-): 0

Region: chr14 15261832-15261850. Max. coverage (+): 1.5. Max coverage (-): 0

Region: chr14 15261851-15261869. Max. coverage (+): 13.85. Max coverage (-): 0

Region: chr14 15261870-15261888. Max. coverage (+): 0. Max coverage (-): 0

Region: chr14 15261889-15261906. Max. coverage (+): 0. Max coverage (-): 0

Region: chr14 15261907-15261925. Max. coverage (+): 2.59. Max coverage (-): 0

Region: chr14 15261926-15261944. Max. coverage (+): 0. Max coverage (-): 0

Region: chr14 15261945-15261963. Max. coverage (+): 2.53. Max coverage (-): 0

Region: chr14 15261964-15261981. Max. coverage (+): 2.53. Max coverage (-): 0

Region: chr14 15261982-15262000. Max. coverage (+): 0. Max coverage (-): 0

Region: chr14 15262001-15262019. Max. coverage (+): 0. Max coverage (-): 0

Region: chr14 15262020-15262037. Max. coverage (+): 51.73. Max coverage (-): 0

Region: chr14 15262038-15262056. Max. coverage (+): 18.53. Max coverage (-): 0

Region: chr14 15262057-15262075. Max. coverage (+): 10.55. Max coverage (-): 0

Region: chr14 15262076-15262094. Max. coverage (+): 0. Max coverage (-): 0

Region: chr14 15262095-15262112. Max. coverage (+): 22.04. Max coverage (-): 0

Region: chr14 15262113-15262131. Max. coverage (+): 11.17. Max coverage (-): 0

Region: chr14 15262132-15262150. Max. coverage (+): 0. Max coverage (-): 0

Region: chr14 15262151-15262169. Max. coverage (+): 1.15. Max coverage (-): 0

Region: chr14 15262170-15262187. Max. coverage (+): 1.15. Max coverage (-): 0

Region: chr14 15262188-15262206. Max. coverage (+): 0. Max coverage (-): 0

Region: chr14 15262207-15262225. Max. coverage (+): 0. Max coverage (-): 0

Region: chr14 15262226-15262244. Max. coverage (+): 0. Max coverage (-): 0

Region: chr14 15262245-15262262. Max. coverage (+): 0. Max coverage (-): 0

Region: chr14 15262263-15262281. Max. coverage (+): 0. Max coverage (-): 0

Region: chr14 15262282-15262300. Max. coverage (+): 4.12. Max coverage (-): 0

Region: chr14 15262301-15262319. Max. coverage (+): 3.81. Max coverage (-): 0

Region: chr14 15262320-15262337. Max. coverage (+): 0. Max coverage (-): 0

Region: chr14 15262338-15262356. Max. coverage (+): 12.51. Max coverage (-): 0

Region: chr14 15262357-15262375. Max. coverage (+): 0. Max coverage (-): 0

Region: chr14 15262376-15262394. Max. coverage (+): 0. Max coverage (-): 0

Region: chr14 15262395-15262412. Max. coverage (+): 0. Max coverage (-): 0

Region: chr14 15262413-15262431. Max. coverage (+): 0. Max coverage (-): 1.08

Region: chr14 15262432-15262450. Max. coverage (+): 0. Max coverage (-): 7.95

Region: chr14 15262451-15262469. Max. coverage (+): 0. Max coverage (-): 0

Region: chr14 15262470-15262487. Max. coverage (+): 0. Max coverage (-): 0

Region: chr14 15262488-15262506. Max. coverage (+): 2.22. Max coverage (-): 0.19

Region: chr14 15262507-15262525. Max. coverage (+): 0.63. Max coverage (-): 0.19

Region: chr14 15262526-15262544. Max. coverage (+): 4.7. Max coverage (-): 0

Region: chr14 15262545-15262562. Max. coverage (+): 4.7. Max coverage (-): 0

Region: chr14 15262563-15262581. Max. coverage (+): 6.45. Max coverage (-): 0

Region: chr14 15262582-15262600. Max. coverage (+): 6.45. Max coverage (-): 0

Region: chr14 15262601-15262618. Max. coverage (+): 0. Max coverage (-): 0

Region: chr14 15262619-15262637. Max. coverage (+): 0. Max coverage (-): 0

Region: chr14 15262638-15262656. Max. coverage (+): 2.12. Max coverage (-): 0

Region: chr14 15262657-15262675. Max. coverage (+): 9.61. Max coverage (-): 0

Region: chr14 15262676-15262693. Max. coverage (+): 9.61. Max coverage (-): 0

Region: chr14 15262694-15262712. Max. coverage (+): 0. Max coverage (-): 0

Region: chr14 15262713-15262731. Max. coverage (+): 0. Max coverage (-): 0

Region: chr14 15262732-15262750. Max. coverage (+): 0. Max coverage (-): 0

Region: chr14 15262751-15262768. Max. coverage (+): 0. Max coverage (-): 0

Region: chr14 15262769-15262787. Max. coverage (+): 0. Max coverage (-): 0

Region: chr14 15262788-15262806. Max. coverage (+): 1.95. Max coverage (-): 0

Region: chr14 15262807-15262825. Max. coverage (+): 1.95. Max coverage (-): 0

Region: chr14 15262826-15262843. Max. coverage (+): 0. Max coverage (-): 0

Region: chr14 15262844-15262862. Max. coverage (+): 0. Max coverage (-): 0

Region: chr14 15262863-15262881. Max. coverage (+): 2.95. Max coverage (-): 0

Region: chr14 15262882-15262900. Max. coverage (+): 1.09. Max coverage (-): 0

Region: chr14 15262901-15262918. Max. coverage (+): 1.09. Max coverage (-): 0

Region: chr14 15262919-15262937. Max. coverage (+): 0.21. Max coverage (-): 0

Region: chr14 15262938-15262956. Max. coverage (+): 4.81. Max coverage (-): 0

Region: chr14 15262957-15262975. Max. coverage (+): 2.44. Max coverage (-): 0

Region: chr14 15262976-15262993. Max. coverage (+): 0. Max coverage (-): 0.01

Region: chr14 15262994-15263012. Max. coverage (+): 0. Max coverage (-): 0

Region: chr14 15263013-15263031. Max. coverage (+): 0. Max coverage (-): 0

Region: chr14 15263032-15263050. Max. coverage (+): 0. Max coverage (-): 0

Region: chr14 15263051-15263068. Max. coverage (+): 0. Max coverage (-): 0

Region: chr14 15263069-15263087. Max. coverage (+): 0. Max coverage (-): 0

Region: chr14 15263088-15263106. Max. coverage (+): 0. Max coverage (-): 0

Region: chr14 15263107-15263125. Max. coverage (+): 7.77. Max coverage (-): 0

Region: chr14 15263126-15263143. Max. coverage (+): 30.06. Max coverage (-): 0

Region: chr14 15263144-15263162. Max. coverage (+): 22.8. Max coverage (-): 0

Region: chr14 15263163-15263181. Max. coverage (+): 8.12. Max coverage (-): 0

Region: chr14 15263182-15263200. Max. coverage (+): 4.35. Max coverage (-): 0

Region: chr14 15263201-15263218. Max. coverage (+): 6.27. Max coverage (-): 0

Region: chr14 15263219-15263237. Max. coverage (+): 0. Max coverage (-): 0

Region: chr14 15263238-15263256. Max. coverage (+): 0. Max coverage (-): 0

Region: chr14 15263257-15263274. Max. coverage (+): 0. Max coverage (-): 0

Region: chr14 15263275-15263293. Max. coverage (+): 0. Max coverage (-): 0.89

Region: chr14 15263294-15263312. Max. coverage (+): 0. Max coverage (-): 0

Region: chr14 15263313-15263331. Max. coverage (+): 4.22. Max coverage (-): 0

Region: chr14 15263332-15263349. Max. coverage (+): 4.73. Max coverage (-): 0

Region: chr14 15263350-15263368. Max. coverage (+): 5.7. Max coverage (-): 0

Region: chr14 15263369-15263387. Max. coverage (+): 4.72. Max coverage (-): 0

Region: chr14 15263388-15263406. Max. coverage (+): 0. Max coverage (-): 0

Region: chr14 15263407-15263424. Max. coverage (+): 9.94. Max coverage (-): 0

Region: chr14 15263425-15263443. Max. coverage (+): 0.12. Max coverage (-): 0

Region: chr14 15263444-15263462. Max. coverage (+): 0. Max coverage (-): 0

Region: chr14 15263463-15263481. Max. coverage (+): 5.04. Max coverage (-): 0

Region: chr14 15263482-15263499. Max. coverage (+): 5.04. Max coverage (-): 0

Region: chr14 15263500-15263518. Max. coverage (+): 0. Max coverage (-): 0

Region: chr14 15263519-15263537. Max. coverage (+): 0.01. Max coverage (-): 0

Region: chr14 15263538-15263556. Max. coverage (+): 0. Max coverage (-): 0

Region: chr14 15263557-15263574. Max. coverage (+): 0. Max coverage (-): 0

Region: chr14 15263575-15263593. Max. coverage (+): 0. Max coverage (-): 0

Region: chr14 15263594-15263612. Max. coverage (+): 0. Max coverage (-): 0

Region: chr14 15263613-15263631. Max. coverage (+): 12.18. Max coverage (-): 0

Region: chr14 15263632-15263649. Max. coverage (+): 0. Max coverage (-): 0

Region: chr14 15263650-15263668. Max. coverage (+): 0. Max coverage (-): 0

Region: chr14 15263669-15263687. Max. coverage (+): 0. Max coverage (-): 0

Region: chr14 15263688-15263706. Max. coverage (+): 0. Max coverage (-): 0

Region: chr14 15263707-15263724. Max. coverage (+): 0. Max coverage (-): 0

Region: chr14 15263725-15263743. Max. coverage (+): 0. Max coverage (-): 0

Region: chr14 15263744-15263762. Max. coverage (+): 4.56. Max coverage (-): 0

Region: chr14 15263763-15263781. Max. coverage (+): 12.51. Max coverage (-): 0

Region: chr14 15263782-15263799. Max. coverage (+): 4.39. Max coverage (-): 0

Region: chr14 15263800-15263818. Max. coverage (+): 4.39. Max coverage (-): 0

Region: chr14 15263819-15263837. Max. coverage (+): 0. Max coverage (-): 0

Region: chr14 15263838-15263855. Max. coverage (+): 0. Max coverage (-): 0

Region: chr14 15263856-15263874. Max. coverage (+): 20.25. Max coverage (-): 0

Region: chr14 15263875-15263893. Max. coverage (+): 0. Max coverage (-): 0

Region: chr14 15263894-15263912. Max. coverage (+): 2.37. Max coverage (-): 0

Region: chr14 15263913-15263930. Max. coverage (+): 2.37. Max coverage (-): 0

Region: chr14 15263931-15263949. Max. coverage (+): 0. Max coverage (-): 0

Region: chr14 15263950-15263968. Max. coverage (+): 0. Max coverage (-): 0

Region: chr14 15263969-15263987. Max. coverage (+): 0. Max coverage (-): 0

Region: chr14 15263988-15264005. Max. coverage (+): 0. Max coverage (-): 0

Region: chr14 15264006-15264024. Max. coverage (+): 0. Max coverage (-): 0

Region: chr14 15264025-15264043. Max. coverage (+): 6.95. Max coverage (-): 0

Region: chr14 15264044-15264062. Max. coverage (+): 2.76. Max coverage (-): 0

Region: chr14 15264063-15264080. Max. coverage (+): 0. Max coverage (-): 0

Region: chr14 15264081-15264099. Max. coverage (+): 5.83. Max coverage (-): 0

Region: chr14 15264100-15264118. Max. coverage (+): 5.83. Max coverage (-): 0

Region: chr14 15264119-15264137. Max. coverage (+): 0. Max coverage (-): 0

Region: chr14 15264138-15264155. Max. coverage (+): 0. Max coverage (-): 0

Region: chr14 15264156-15264174. Max. coverage (+): 1.37. Max coverage (-): 0

Region: chr14 15264175-15264193. Max. coverage (+): 1.37. Max coverage (-): 0

Region: chr14 15264194-15264212. Max. coverage (+): 0. Max coverage (-): 0

Region: chr14 15264213-15264230. Max. coverage (+): 0. Max coverage (-): 0

Region: chr14 15264231-15264249. Max. coverage (+): 1.57. Max coverage (-): 0

Region: chr14 15264250-15264268. Max. coverage (+): 1.92. Max coverage (-): 0

Region: chr14 15264269-15264287. Max. coverage (+): 4.04. Max coverage (-): 0

Region: chr14 15264288-15264305. Max. coverage (+): 4.04. Max coverage (-): 0

Region: chr14 15264306-15264324. Max. coverage (+): 0. Max coverage (-): 0

Region: chr14 15264325-15264343. Max. coverage (+): 2.21. Max coverage (-): 0

Region: chr14 15264344-15264362. Max. coverage (+): 18.95. Max coverage (-): 0

Region: chr14 15264363-15264380. Max. coverage (+): 0. Max coverage (-): 0

Region: chr14 15264381-15264399. Max. coverage (+): 0. Max coverage (-): 0

Region: chr14 15264400-15264418. Max. coverage (+): 5.24. Max coverage (-): 0

Region: chr14 15264419-15264436. Max. coverage (+): 5.24. Max coverage (-): 0

Region: chr14 15264437-15264455. Max. coverage (+): 0. Max coverage (-): 0

Region: chr14 15264456-15264474. Max. coverage (+): 3.39. Max coverage (-): 0

Region: chr14 15264475-15264493. Max. coverage (+): 3.39. Max coverage (-): 0

Region: chr14 15264494-15264511. Max. coverage (+): 0. Max coverage (-): 0

Region: chr14 15264512-15264530. Max. coverage (+): 0. Max coverage (-): 0

Region: chr14 15264531-15264549. Max. coverage (+): 0. Max coverage (-): 0

Region: chr14 15264550-15264568. Max. coverage (+): 0.76. Max coverage (-): 0

Region: chr14 15264569-15264586. Max. coverage (+): 0.98. Max coverage (-): 0

Region: chr14 15264587-15264605. Max. coverage (+): 2.03. Max coverage (-): 0

Region: chr14 15264606-15264624. Max. coverage (+): 3.26. Max coverage (-): 0

Region: chr14 15264625-15264643. Max. coverage (+): 3.74. Max coverage (-): 0

Region: chr14 15264644-15264661. Max. coverage (+): 0. Max coverage (-): 0

Region: chr14 15264662-15264680. Max. coverage (+): 3.86. Max coverage (-): 0

Region: chr14 15264681-15264699. Max. coverage (+): 3.86. Max coverage (-): 0

Region: chr14 15264700-15264718. Max. coverage (+): 0. Max coverage (-): 0

Region: chr14 15264719-15264736. Max. coverage (+): 0. Max coverage (-): 0

Region: chr14 15264737-15264755. Max. coverage (+): 4.83. Max coverage (-): 0

Region: chr14 15264756-15264774. Max. coverage (+): 4.83. Max coverage (-): 0

Region: chr14 15264775-15264793. Max. coverage (+): 0. Max coverage (-): 0

Region: chr14 15264794-15264811. Max. coverage (+): 0. Max coverage (-): 0

Region: chr14 15264812-15264830. Max. coverage (+): 0. Max coverage (-): 0

Region: chr14 15264831-15264849. Max. coverage (+): 0. Max coverage (-): 0

Region: chr14 15264850-15264868. Max. coverage (+): 0. Max coverage (-): 0

Region: chr14 15264869-15264886. Max. coverage (+): 0. Max coverage (-): 0

Region: chr14 15264887-15264905. Max. coverage (+): 0. Max coverage (-): 0

Region: chr14 15264906-15264924. Max. coverage (+): 0. Max coverage (-): 0

Region: chr14 15264925-15264943. Max. coverage (+): 0. Max coverage (-): 0

Region: chr14 15264944-15264961. Max. coverage (+): 0. Max coverage (-): 0

Region: chr14 15264962-15264980. Max. coverage (+): 5.92. Max coverage (-): 0

Region: chr14 15264981-15264999. Max. coverage (+): 0. Max coverage (-): 0

Region: chr14 15265000-15265017. Max. coverage (+): 0. Max coverage (-): 0

Region: chr14 15265018-15265036. Max. coverage (+): 0. Max coverage (-): 0

Region: chr14 15265037-15265055. Max. coverage (+): 0. Max coverage (-): 0

Region: chr14 15265056-15265074. Max. coverage (+): 11.84. Max coverage (-): 0

Region: chr14 15265075-15265092. Max. coverage (+): 9.73. Max coverage (-): 0

Region: chr14 15265093-15265111. Max. coverage (+): 0. Max coverage (-): 0

Region: chr14 15265112-15265130. Max. coverage (+): 0. Max coverage (-): 0

Region: chr14 15265131-15265149. Max. coverage (+): 0. Max coverage (-): 0

Region: chr14 15265150-15265167. Max. coverage (+): 0. Max coverage (-): 0

Region: chr14 15265168-15265186. Max. coverage (+): 0. Max coverage (-): 0

Region: chr14 15265187-15265205. Max. coverage (+): 0. Max coverage (-): 0

Region: chr14 15265206-15265224. Max. coverage (+): 1.46. Max coverage (-): 0

Region: chr14 15265225-15265242. Max. coverage (+): 1.38. Max coverage (-): 0

Region: chr14 15265243-15265261. Max. coverage (+): 0. Max coverage (-): 0

Region: chr14 15265262-15265280. Max. coverage (+): 0. Max coverage (-): 0

Region: chr14 15265281-15265299. Max. coverage (+): 0. Max coverage (-): 0

Region: chr14 15265300-15265317. Max. coverage (+): 0. Max coverage (-): 0

Region: chr14 15265318-15265336. Max. coverage (+): 0. Max coverage (-): 0

Region: chr14 15265337-15265355. Max. coverage (+): 0. Max coverage (-): 0

Region: chr14 15265356-15265374. Max. coverage (+): 10.92. Max coverage (-): 0

Region: chr14 15265375-15265392. Max. coverage (+): 7.95. Max coverage (-): 0

Region: chr14 15265393-15265411. Max. coverage (+): 0. Max coverage (-): 0

Region: chr14 15265412-15265430. Max. coverage (+): 0. Max coverage (-): 0

Region: chr14 15265431-15265449. Max. coverage (+): 0. Max coverage (-): 0

Region: chr14 15265450-15265467. Max. coverage (+): 0. Max coverage (-): 0

Region: chr14 15265468-15265486. Max. coverage (+): 0. Max coverage (-): 0

Region: chr14 15265487-15265505. Max. coverage (+): 2.34. Max coverage (-): 0

Region: chr14 15265506-15265524. Max. coverage (+): 2.7. Max coverage (-): 1.33

Region: chr14 15265525-15265542. Max. coverage (+): 0. Max coverage (-): 0

Region: chr14 15265543-15265561. Max. coverage (+): 0. Max coverage (-): 0

Region: chr14 15265562-15265580. Max. coverage (+): 1.3. Max coverage (-): 0

Region: chr14 15265581-15265598. Max. coverage (+): 3.82. Max coverage (-): 0

Region: chr14 15265599-15265617. Max. coverage (+): 0. Max coverage (-): 0

Region: chr14 15265618-15265636. Max. coverage (+): 0. Max coverage (-): 0

Region: chr14 15265637-15265655. Max. coverage (+): 0. Max coverage (-): 0

Region: chr14 15265656-15265673. Max. coverage (+): 4.19. Max coverage (-): 0

Region: chr14 15265674-15265692. Max. coverage (+): 1. Max coverage (-): 0

Region: chr14 15265693-15265711. Max. coverage (+): 0. Max coverage (-): 0

Region: chr14 15265712-15265730. Max. coverage (+): 2.83. Max coverage (-): 0

Region: chr14 15265731-15265748. Max. coverage (+): 4.42. Max coverage (-): 0

Region: chr14 15265749-15265767. Max. coverage (+): 0. Max coverage (-): 0

Region: chr14 15265768-15265786. Max. coverage (+): 0. Max coverage (-): 22.99

Region: chr14 15265787-15265805. Max. coverage (+): 1.93. Max coverage (-): 0

Region: chr14 15265806-15265823. Max. coverage (+): 0.91. Max coverage (-): 0

Region: chr14 15265824-15265842. Max. coverage (+): 0. Max coverage (-): 0

Region: chr14 15265843-15265861. Max. coverage (+): 0. Max coverage (-): 0

Region: chr14 15265862-15265880. Max. coverage (+): 0. Max coverage (-): 1.07

Region: chr14 15265881-15265898. Max. coverage (+): 0. Max coverage (-): 1.07

Region: chr14 15265899-15265917. Max. coverage (+): 0. Max coverage (-): 0

Region: chr14 15265918-15265936. Max. coverage (+): 3.96. Max coverage (-): 0

Region: chr14 15265937-15265955. Max. coverage (+): 0. Max coverage (-): 0

Region: chr14 15265956-15265973. Max. coverage (+): 0. Max coverage (-): 0

Region: chr14 15265974-15265992. Max. coverage (+): 0. Max coverage (-): 0

Region: chr14 15265993-15266011. Max. coverage (+): 0. Max coverage (-): 0.39

Region: chr14 15266012-15266030. Max. coverage (+): 0. Max coverage (-): 0

Region: chr14 15266031-15266048. Max. coverage (+): 0. Max coverage (-): 0

Region: chr14 15266049-15266067. Max. coverage (+): 0. Max coverage (-): 0

Region: chr14 15266068-15266086. Max. coverage (+): 0. Max coverage (-): 3.58

Region: chr14 15266087-15266105. Max. coverage (+): 1.69. Max coverage (-): 4.76

Region: chr14 15266106-15266123. Max. coverage (+): 0. Max coverage (-): 0

Region: chr14 15266124-15266142. Max. coverage (+): 1.88. Max coverage (-): 5.24

Region: chr14 15266143-15266161. Max. coverage (+): 0. Max coverage (-): 20.94

Region: chr14 15266162-15266179. Max. coverage (+): 0. Max coverage (-): 0

Region: chr14 15266180-15266198. Max. coverage (+): 4.44. Max coverage (-): 0

Region: chr14 15266199-15266217. Max. coverage (+): 0. Max coverage (-): 0

Region: chr14 15266218-15266236. Max. coverage (+): 0. Max coverage (-): 0

Region: chr14 15266237-15266254. Max. coverage (+): 0. Max coverage (-): 0

Region: chr14 15266255-15266273. Max. coverage (+): 0. Max coverage (-): 0

Region: chr14 15266274-15266292. Max. coverage (+): 3.21. Max coverage (-): 5.31

Region: chr14 15266293-15266311. Max. coverage (+): 0.76. Max coverage (-): 3.04

Region: chr14 15266312-15266329. Max. coverage (+): 0.76. Max coverage (-): 1.56

Region: chr14 15266330-15266348. Max. coverage (+): 0. Max coverage (-): 7.03

Region: chr14 15266349-15266367. Max. coverage (+): 0. Max coverage (-): 0

Region: chr14 15266368-15266386. Max. coverage (+): 0. Max coverage (-): 6.83

Region: chr14 15266387-15266404. Max. coverage (+): 1.96. Max coverage (-): 6.6

Region: chr14 15266405-15266423. Max. coverage (+): 0. Max coverage (-): 0

Region: chr14 15266424-15266442. Max. coverage (+): 0. Max coverage (-): 0

Region: chr14 15266443-15266461. Max. coverage (+): 0. Max coverage (-): 0

Region: chr14 15266462-15266479. Max. coverage (+): 0. Max coverage (-): 0

Region: chr14 15266480-15266498. Max. coverage (+): 0. Max coverage (-): 0

Region: chr14 15266499-15266517. Max. coverage (+): 0. Max coverage (-): 0

Region: chr14 15266518-15266536. Max. coverage (+): 0. Max coverage (-): 0

Region: chr14 15266537-15266554. Max. coverage (+): 0. Max coverage (-): 0

Region: chr14 15266555-15266573. Max. coverage (+): 0. Max coverage (-): 0

Region: chr14 15266574-15266592. Max. coverage (+): 0. Max coverage (-): 0

Region: chr14 15266593-15266611. Max. coverage (+): 0. Max coverage (-): 0

Region: chr14 15266612-15266629. Max. coverage (+): 0. Max coverage (-): 0

Region: chr14 15266630-15266648. Max. coverage (+): 0. Max coverage (-): 0

Region: chr14 15266649-15266667. Max. coverage (+): 0. Max coverage (-): 0

Region: chr14 15266668-15266686. Max. coverage (+): 0. Max coverage (-): 0

Region: chr14 15266687-15266704. Max. coverage (+): 0. Max coverage (-): 0

Region: chr14 15266705-15266723. Max. coverage (+): 0. Max coverage (-): 0

Region: chr14 15266724-15266742. Max. coverage (+): 2.84. Max coverage (-): 0

Region: chr14 15266743-15266760. Max. coverage (+): 0. Max coverage (-): 0

Region: chr14 15266761-15266779. Max. coverage (+): 0. Max coverage (-): 0

Region: chr14 15266780-15266798. Max. coverage (+): 0. Max coverage (-): 0

Region: chr14 15266799-15266817. Max. coverage (+): 0. Max coverage (-): 0.03

Region: chr14 15266818-15266835. Max. coverage (+): 0. Max coverage (-): 0

Region: chr14 15266836-15266854. Max. coverage (+): 0. Max coverage (-): 0

Region: chr14 15266855-15266873. Max. coverage (+): 0. Max coverage (-): 0

Region: chr14 15266874-15266892. Max. coverage (+): 0. Max coverage (-): 0

Region: chr14 15266893-15266910. Max. coverage (+): 0. Max coverage (-): 0.03

Region: chr14 15266911-15266929. Max. coverage (+): 0. Max coverage (-): 0.43

Region: chr14 15266930-15266948. Max. coverage (+): 0. Max coverage (-): 0.43

Region: chr14 15266949-15266967. Max. coverage (+): 0. Max coverage (-): 0

Region: chr14 15266968-15266985. Max. coverage (+): 0. Max coverage (-): 0

Region: chr14 15266986-15267004. Max. coverage (+): 0. Max coverage (-): 0

Region: chr14 15267005-15267023. Max. coverage (+): 0. Max coverage (-): 0

Region: chr14 15267024-15267042. Max. coverage (+): 0. Max coverage (-): 0

Region: chr14 15267043-15267060. Max. coverage (+): 0. Max coverage (-): 0.02

Region: chr14 15267061-15267079. Max. coverage (+): 0. Max coverage (-): 0

Region: chr14 15267080-15267098. Max. coverage (+): 4.41. Max coverage (-): 0

Region: chr14 15267099-15267117. Max. coverage (+): 0. Max coverage (-): 0.47

Region: chr14 15267118-15267135. Max. coverage (+): 0.62. Max coverage (-): 0.62

Region: chr14 15267136-15267154. Max. coverage (+): 0. Max coverage (-): 0

Region: chr14 15267155-15267173. Max. coverage (+): 0. Max coverage (-): 0

Region: chr14 15267174-15267192. Max. coverage (+): 0. Max coverage (-): 0.25

Region: chr14 15267193-15267210. Max. coverage (+): 0.6. Max coverage (-): 0.07

Region: chr14 15267211-15267229. Max. coverage (+): 0.38. Max coverage (-): 0

Region: chr14 15267230-15267248. Max. coverage (+): 0. Max coverage (-): 0

Region: chr14 15267249-15267267. Max. coverage (+): 0. Max coverage (-): 0

Region: chr14 15267268-15267285. Max. coverage (+): 0. Max coverage (-): 0

Region: chr14 15267286-15267304. Max. coverage (+): 0. Max coverage (-): 0

Region: chr14 15267305-15267323. Max. coverage (+): 0.29. Max coverage (-): 0

Region: chr14 15267324-15267341. Max. coverage (+): 0.29. Max coverage (-): 0

Region: chr14 15267342-15267360. Max. coverage (+): 0. Max coverage (-): 0

Region: chr14 15267361-15267379. Max. coverage (+): 0. Max coverage (-): 0

Region: chr14 15267380-15267398. Max. coverage (+): 0. Max coverage (-): 0

Region: chr14 15267399-15267416. Max. coverage (+): 0. Max coverage (-): 0.02

Region: chr14 15267417-15267435. Max. coverage (+): 0. Max coverage (-): 0

Region: chr14 15267436-15267454. Max. coverage (+): 0. Max coverage (-): 0

Region: chr14 15267455-15267473. Max. coverage (+): 0. Max coverage (-): 0

Region: chr14 15267474-15267491. Max. coverage (+): 0. Max coverage (-): 0

Region: chr14 15267492-15267510. Max. coverage (+): 0.01. Max coverage (-): 0

Region: chr14 15267511-15267529. Max. coverage (+): 0. Max coverage (-): 0

Region: chr14 15267530-15267548. Max. coverage (+): 0. Max coverage (-): 0

Region: chr14 15267549-15267566. Max. coverage (+): 0.01. Max coverage (-): 0

Region: chr14 15267567-15267585. Max. coverage (+): 0.01. Max coverage (-): 0

Region: chr14 15267586-. Max. coverage (+): 0. Max coverage (-): 0

RepeatMasker Color Code

**+**

100-98% Identity

<98-95% Identity

<95-90% Identity

<90-85% Identity

<85-80% Identity

<80-75% Identity

<75-70% Identity

<70% Identity

**-**

Gene Set Color Code

**+**

Gene

Pseudogene

**-**

Topology/Coverage Color Code

Coverage Plus Strand

Coverage Minus Strand

Mainstrand: Plus

Mainstrand: Minus

Complementary Strand

Flanking Region  
(if option -flank >0)

Gene Set Annotation  
  
RepeatMasker Annotation  

**1. T-rich**: 15258299-15258359 (+), Divergence to consensus: 24.6%  
**2. T-rich**: 15258377-15258466 (+), Divergence to consensus: 30%  
**3. SINE2-3\_BT**: 15266518-15266698 (-), Divergence to consensus: 17.3%

  
Transcription Factor Binding Sites  

**RFX4\_2** (Sequence: GTATCCAAG (-): 15264247)  
**RFX4\_1** (Sequence: CATGGCAAC (+): 15260553)  
**RFX4\_1** (Sequence: CATGGCAAC (+): 15265427)  
**SOX9** (Sequence: AACAATGA (-): 15262036)  
**SOX9** (Sequence: AACAATGA (-): 15264724)  
**SOX9** (Sequence: AACAATGG (-): 15265559)
