# Supplementary material for: Small RNA sequencing of cryopreserved semen from single bull revealed altered miRNAs and piRNAs expression between High- and Low-motile sperm populations
Source: BMC Genomics. 2017 Jan 4;18:14. doi: 10.1186/s12864-016-3394-7 (PMC5209821; doi:10.1186/s12864-016-3394-7)
Supplement: Additional file 3: — Details for each piRNA clusters found in High Motile (HM) sperm fraction. Genes, repeats, transposable elements and transcription factors binding sites falling within the cluster regions were reported. (ZIP 1896 kb) [file 12864_2016_3394_MOESM3_ESM.zip › 24.html]

piRNA cluster 24


Predicted piRNA cluster no. 24     previous   next
  

Show proTRAC run info
Hide proTRAC run info

================================= proTRAC ====================================  
VERSION: 2.1                                    LAST MODIFIED: 06. October 2015  
  
Please cite:  
Rosenkranz D, Zischler H. proTRAC - a software for probabilistic piRNA cluster  
detection, visualization and analysis. 2012. BMC Bioinformatics 13:5.  
  
and (for proTRAC 2.0 and later):  
Rosenkranz D, Rudloff S, Bastuck K, Ketting RF, Zischler H. Tupaia small RNAs  
provide insights into function and evolution of RNAi-based transposon defense  
in mammals. 2015. RNA 21(5):911-922.  
  
Contact:  
David Rosenkranz  
Institute of Anthropology, small RNA group  
Johannes Gutenberg University Mainz  
email: rosenkranz@uni-mainz.de  
  
You can find the latest proTRAC version at:  
http://sourceforge.net/projects/protrac/files  
http://www.smallRNAgroup-mainz.de/software  
==============================================================================  
  
PARAMETERS:  
Map file: .............../storage/core/barbara/genhome/smallRNA/fertility/Sample\_motile/pirna/Sample\_motile\_26-33\_collapsed.fa.no-dust.map.weighted-10000-1000-b-0  
Genome file: ............/storage/core/barbara/genhome/smallRNA/fertility/Sample\_all/pirna/bt\_311\_chrY.fa  
RepeatMasker annotation: /storage/genomes/bt\_umd31/GCF\_000003055.6\_Bos\_taurus\_UMD\_3.1.1\_repeatMasker\_chr.out  
GeneSet:................./storage/core/barbara/genhome/smallRNA/fertility/Sample\_all/pirna/full.gtf  
  
Significant (p<=0.01) hit density will be calculated based  
on observed hit distribution.  
  
Sliding window size: ........................................ 5000 bp  
Sliding window increament: .................................. 1000 bp  
Normalize each hit by number of genomic hits: ............... 1 [0=no/1=yes]  
Normalize each hit by number of sequence reads: ............. 1 [0=no/1=yes]  
Normalize values (-> per million mapped reads): ............. 1 [0=no/1=yes]  
Min. fraction of hits with 1T(U) or 10A: .................... 0.75  
Alternatively: Min. fraction of hits with 1T(U) and 10A: .... 0.5  
Min. fraction of hits with typical piRNA length: ............ 0.75  
Typical piRNA length: ....................................... 26-33 nt  
Min. size of a piRNA cluster: ............................... 5000 bp.  
Min. number of hits (absolute): ............................. 0  
Min. number of hits (normalized): ........................... 0  
Min. fraction of hits on the mainstrand: .................... 0.75  
Top fraction of mapped sequences (in terms of read counts): . 1%  
Top fraction accounts for max. n% of sequence reads: ........ 90%  
Min. fraction of hits on each arm of a bidirectional cluster: 0.1  
Output image file for each cluster: ......................... 0 [0=no/1=yes]  
Output html file for each cluster: .......................... 1 [0=no/1=yes]  
Output a summary table: ..................................... 1 [0=no/1=yes]  
Output a FASTA file for each cluster (piRNA sequences): ..... 1 [0=no/1=yes]  
Output a FASTA file comprising cluster sequences: ........... 1 [0=no/1=yes]  
Search DNA motifs in clusters: .............................. 1 [0=no/1=yes]  
Output flanking sequences: +/- .............................. 0 bp  
Output ~.pTi file: .......................................... 1 [0=no/1=yes]  
==============================================================================  
  
  
Genome size (without gaps): ............ 2678902517 bp  
Gaps (N/X/-): .......................... 53837044 bp  
Mapped reads: .......................... 658825247023  
Non-identical sequences: ............... 514171  
Genomic hits: .......................... 764233  
Significant densitiy of mapped reads: .. 12867599.5173724 reads/kb

Show proTRAC cluster info
Hide proTRAC cluster info

|  |  |
| --- | --- |
| Location | chr14 |
| Coordinates | 15345069-15350915 |
| Size [bp] | 5847 |
| Sequence hit loci | 204 |
| Mapped reads (normalized) | 102973117.4 |
| Mapped reads (normalized) per kb | 17611273.7 |
| Normalized reads with 1T (1U) | 65.9% |
| Normalized reads with 10A | 50.4% |
| Normalized reads with length 26-33 nt | 100% |
| Normalized reads on the main strand(s) | 78.5% |
| Predicted directionality | mono:plus |

100%

0%

1T (1U)  
reads

10A reads

26-33 nt  
reads

reads on mainstrand

**Either the amount of reads with 1T (1U) OR 10A has to exceed 75% (set with option: -1Tor10A)  
Alternatively the amount of reads with 1T (1U) AND 10A has to exceed 50% (set with option: -1Tand10A)  
Minimum amount of reads with preferred size is 75% (set with option: -pisize)  
Minimum amount of reads on the main strand(s) is 75% (set with option: -clstrand)**

Show read coverage
Hide read coverage

WHAT DO I SEE HERE?  
This chart shows the location of mapped sequence reads within a predicted piRNA cluster. The color refers to the number of genomic hits produced by the sequence read in question. A dark red bar indicates that this sequence read produces many other hits elsewhere in the genome. Many adjacent red or yellow bars can indicate the presence of a multi-copy element such as transposons or rRNA genes. A dark green bar indicates that this sequence read maps uniquely to this locus.

1 hit

2-5 hits

6-10 hits

11-20 hits

21-50 hits

51-100 hits

> 100 hits

chr14

15345069

15350915

Gene Set

RepeatMasker

Mapped  
Reads

11

plus strand

minus strand

11

Region: chr14 15267587-15345074. Max. coverage (+): 1.88. Max coverage (-): 0

Region: chr14 15345075-15345086. Max. coverage (+): 1.88. Max coverage (-): 0

Region: chr14 15345087-15345098. Max. coverage (+): 0. Max coverage (-): 0

Region: chr14 15345099-15345109. Max. coverage (+): 0. Max coverage (-): 0

Region: chr14 15345110-15345121. Max. coverage (+): 0. Max coverage (-): 0

Region: chr14 15345122-15345133. Max. coverage (+): 3.48. Max coverage (-): 0

Region: chr14 15345134-15345145. Max. coverage (+): 2.89. Max coverage (-): 0

Region: chr14 15345146-15345156. Max. coverage (+): 7.43. Max coverage (-): 0

Region: chr14 15345157-15345168. Max. coverage (+): 1.57. Max coverage (-): 0

Region: chr14 15345169-15345180. Max. coverage (+): 0.52. Max coverage (-): 0.27

Region: chr14 15345181-15345191. Max. coverage (+): 0. Max coverage (-): 1.16

Region: chr14 15345192-15345203. Max. coverage (+): 0.08. Max coverage (-): 0.66

Region: chr14 15345204-15345215. Max. coverage (+): 0.08. Max coverage (-): 0.25

Region: chr14 15345216-15345226. Max. coverage (+): 0. Max coverage (-): 0

Region: chr14 15345227-15345238. Max. coverage (+): 0. Max coverage (-): 0.1

Region: chr14 15345239-15345250. Max. coverage (+): 0. Max coverage (-): 0.1

Region: chr14 15345251-15345261. Max. coverage (+): 0. Max coverage (-): 0

Region: chr14 15345262-15345273. Max. coverage (+): 0. Max coverage (-): 0

Region: chr14 15345274-15345285. Max. coverage (+): 0. Max coverage (-): 0

Region: chr14 15345286-15345297. Max. coverage (+): 0. Max coverage (-): 0

Region: chr14 15345298-15345308. Max. coverage (+): 0. Max coverage (-): 0

Region: chr14 15345309-15345320. Max. coverage (+): 0. Max coverage (-): 0

Region: chr14 15345321-15345332. Max. coverage (+): 0. Max coverage (-): 0.16

Region: chr14 15345333-15345343. Max. coverage (+): 0.26. Max coverage (-): 0

Region: chr14 15345344-15345355. Max. coverage (+): 0.26. Max coverage (-): 0

Region: chr14 15345356-15345367. Max. coverage (+): 0. Max coverage (-): 0

Region: chr14 15345368-15345378. Max. coverage (+): 0. Max coverage (-): 0

Region: chr14 15345379-15345390. Max. coverage (+): 0. Max coverage (-): 0

Region: chr14 15345391-15345402. Max. coverage (+): 0. Max coverage (-): 0

Region: chr14 15345403-15345413. Max. coverage (+): 0. Max coverage (-): 0

Region: chr14 15345414-15345425. Max. coverage (+): 0. Max coverage (-): 0

Region: chr14 15345426-15345437. Max. coverage (+): 0. Max coverage (-): 0

Region: chr14 15345438-15345449. Max. coverage (+): 0. Max coverage (-): 0

Region: chr14 15345450-15345460. Max. coverage (+): 0. Max coverage (-): 0

Region: chr14 15345461-15345472. Max. coverage (+): 0. Max coverage (-): 0

Region: chr14 15345473-15345484. Max. coverage (+): 0. Max coverage (-): 0

Region: chr14 15345485-15345495. Max. coverage (+): 0.09. Max coverage (-): 0

Region: chr14 15345496-15345507. Max. coverage (+): 0.57. Max coverage (-): 0.31

Region: chr14 15345508-15345519. Max. coverage (+): 0.57. Max coverage (-): 0

Region: chr14 15345520-15345530. Max. coverage (+): 0. Max coverage (-): 5.05

Region: chr14 15345531-15345542. Max. coverage (+): 0. Max coverage (-): 0

Region: chr14 15345543-15345554. Max. coverage (+): 0. Max coverage (-): 0

Region: chr14 15345555-15345565. Max. coverage (+): 0. Max coverage (-): 0

Region: chr14 15345566-15345577. Max. coverage (+): 3.34. Max coverage (-): 0

Region: chr14 15345578-15345589. Max. coverage (+): 0. Max coverage (-): 0

Region: chr14 15345590-15345601. Max. coverage (+): 0. Max coverage (-): 0

Region: chr14 15345602-15345612. Max. coverage (+): 0. Max coverage (-): 0

Region: chr14 15345613-15345624. Max. coverage (+): 0. Max coverage (-): 0

Region: chr14 15345625-15345636. Max. coverage (+): 0. Max coverage (-): 0

Region: chr14 15345637-15345647. Max. coverage (+): 4.96. Max coverage (-): 0

Region: chr14 15345648-15345659. Max. coverage (+): 6.64. Max coverage (-): 0

Region: chr14 15345660-15345671. Max. coverage (+): 9.1. Max coverage (-): 0

Region: chr14 15345672-15345682. Max. coverage (+): 0. Max coverage (-): 0

Region: chr14 15345683-15345694. Max. coverage (+): 0. Max coverage (-): 0

Region: chr14 15345695-15345706. Max. coverage (+): 0. Max coverage (-): 0

Region: chr14 15345707-15345718. Max. coverage (+): 0. Max coverage (-): 0

Region: chr14 15345719-15345729. Max. coverage (+): 0. Max coverage (-): 0

Region: chr14 15345730-15345741. Max. coverage (+): 0. Max coverage (-): 0

Region: chr14 15345742-15345753. Max. coverage (+): 0. Max coverage (-): 0

Region: chr14 15345754-15345764. Max. coverage (+): 0. Max coverage (-): 0

Region: chr14 15345765-15345776. Max. coverage (+): 0. Max coverage (-): 0

Region: chr14 15345777-15345788. Max. coverage (+): 0. Max coverage (-): 0

Region: chr14 15345789-15345799. Max. coverage (+): 0. Max coverage (-): 0

Region: chr14 15345800-15345811. Max. coverage (+): 0. Max coverage (-): 0

Region: chr14 15345812-15345823. Max. coverage (+): 0. Max coverage (-): 0

Region: chr14 15345824-15345834. Max. coverage (+): 0. Max coverage (-): 0

Region: chr14 15345835-15345846. Max. coverage (+): 0. Max coverage (-): 0

Region: chr14 15345847-15345858. Max. coverage (+): 0. Max coverage (-): 0

Region: chr14 15345859-15345870. Max. coverage (+): 0. Max coverage (-): 0

Region: chr14 15345871-15345881. Max. coverage (+): 0. Max coverage (-): 0

Region: chr14 15345882-15345893. Max. coverage (+): 0. Max coverage (-): 0

Region: chr14 15345894-15345905. Max. coverage (+): 0. Max coverage (-): 0

Region: chr14 15345906-15345916. Max. coverage (+): 0. Max coverage (-): 0

Region: chr14 15345917-15345928. Max. coverage (+): 0. Max coverage (-): 0

Region: chr14 15345929-15345940. Max. coverage (+): 0. Max coverage (-): 0

Region: chr14 15345941-15345951. Max. coverage (+): 0. Max coverage (-): 0

Region: chr14 15345952-15345963. Max. coverage (+): 0. Max coverage (-): 0

Region: chr14 15345964-15345975. Max. coverage (+): 0. Max coverage (-): 0

Region: chr14 15345976-15345986. Max. coverage (+): 0. Max coverage (-): 0

Region: chr14 15345987-15345998. Max. coverage (+): 0. Max coverage (-): 0

Region: chr14 15345999-15346010. Max. coverage (+): 0. Max coverage (-): 0

Region: chr14 15346011-15346022. Max. coverage (+): 0. Max coverage (-): 0

Region: chr14 15346023-15346033. Max. coverage (+): 0. Max coverage (-): 0

Region: chr14 15346034-15346045. Max. coverage (+): 0.73. Max coverage (-): 0

Region: chr14 15346046-15346057. Max. coverage (+): 0. Max coverage (-): 0

Region: chr14 15346058-15346068. Max. coverage (+): 0. Max coverage (-): 0

Region: chr14 15346069-15346080. Max. coverage (+): 0. Max coverage (-): 0

Region: chr14 15346081-15346092. Max. coverage (+): 0. Max coverage (-): 0

Region: chr14 15346093-15346103. Max. coverage (+): 0. Max coverage (-): 0

Region: chr14 15346104-15346115. Max. coverage (+): 0. Max coverage (-): 0

Region: chr14 15346116-15346127. Max. coverage (+): 0. Max coverage (-): 0

Region: chr14 15346128-15346139. Max. coverage (+): 0. Max coverage (-): 0

Region: chr14 15346140-15346150. Max. coverage (+): 0. Max coverage (-): 0

Region: chr14 15346151-15346162. Max. coverage (+): 0. Max coverage (-): 0

Region: chr14 15346163-15346174. Max. coverage (+): 0. Max coverage (-): 0

Region: chr14 15346175-15346185. Max. coverage (+): 0. Max coverage (-): 0

Region: chr14 15346186-15346197. Max. coverage (+): 0. Max coverage (-): 0

Region: chr14 15346198-15346209. Max. coverage (+): 0. Max coverage (-): 0

Region: chr14 15346210-15346220. Max. coverage (+): 0. Max coverage (-): 0

Region: chr14 15346221-15346232. Max. coverage (+): 0. Max coverage (-): 0

Region: chr14 15346233-15346244. Max. coverage (+): 0. Max coverage (-): 0

Region: chr14 15346245-15346255. Max. coverage (+): 0. Max coverage (-): 0

Region: chr14 15346256-15346267. Max. coverage (+): 0. Max coverage (-): 0

Region: chr14 15346268-15346279. Max. coverage (+): 0. Max coverage (-): 0

Region: chr14 15346280-15346291. Max. coverage (+): 0. Max coverage (-): 0

Region: chr14 15346292-15346302. Max. coverage (+): 0. Max coverage (-): 0

Region: chr14 15346303-15346314. Max. coverage (+): 0. Max coverage (-): 0

Region: chr14 15346315-15346326. Max. coverage (+): 0. Max coverage (-): 0

Region: chr14 15346327-15346337. Max. coverage (+): 2.2. Max coverage (-): 0

Region: chr14 15346338-15346349. Max. coverage (+): 0.65. Max coverage (-): 0

Region: chr14 15346350-15346361. Max. coverage (+): 0. Max coverage (-): 0

Region: chr14 15346362-15346372. Max. coverage (+): 0. Max coverage (-): 0

Region: chr14 15346373-15346384. Max. coverage (+): 4.07. Max coverage (-): 0

Region: chr14 15346385-15346396. Max. coverage (+): 4.07. Max coverage (-): 0

Region: chr14 15346397-15346407. Max. coverage (+): 4.3. Max coverage (-): 0

Region: chr14 15346408-15346419. Max. coverage (+): 8.68. Max coverage (-): 0

Region: chr14 15346420-15346431. Max. coverage (+): 0.61. Max coverage (-): 0

Region: chr14 15346432-15346443. Max. coverage (+): 0.61. Max coverage (-): 1.33

Region: chr14 15346444-15346454. Max. coverage (+): 0. Max coverage (-): 1.36

Region: chr14 15346455-15346466. Max. coverage (+): 0.09. Max coverage (-): 0.77

Region: chr14 15346467-15346478. Max. coverage (+): 0.09. Max coverage (-): 0.13

Region: chr14 15346479-15346489. Max. coverage (+): 0. Max coverage (-): 0

Region: chr14 15346490-15346501. Max. coverage (+): 0. Max coverage (-): 0

Region: chr14 15346502-15346513. Max. coverage (+): 0. Max coverage (-): 0

Region: chr14 15346514-15346524. Max. coverage (+): 0. Max coverage (-): 0

Region: chr14 15346525-15346536. Max. coverage (+): 0. Max coverage (-): 0

Region: chr14 15346537-15346548. Max. coverage (+): 0. Max coverage (-): 0

Region: chr14 15346549-15346559. Max. coverage (+): 0. Max coverage (-): 0

Region: chr14 15346560-15346571. Max. coverage (+): 0. Max coverage (-): 0

Region: chr14 15346572-15346583. Max. coverage (+): 0. Max coverage (-): 0

Region: chr14 15346584-15346595. Max. coverage (+): 0. Max coverage (-): 0

Region: chr14 15346596-15346606. Max. coverage (+): 0. Max coverage (-): 0

Region: chr14 15346607-15346618. Max. coverage (+): 0. Max coverage (-): 0

Region: chr14 15346619-15346630. Max. coverage (+): 0. Max coverage (-): 0

Region: chr14 15346631-15346641. Max. coverage (+): 0. Max coverage (-): 0

Region: chr14 15346642-15346653. Max. coverage (+): 0. Max coverage (-): 0

Region: chr14 15346654-15346665. Max. coverage (+): 0. Max coverage (-): 0

Region: chr14 15346666-15346676. Max. coverage (+): 0. Max coverage (-): 0

Region: chr14 15346677-15346688. Max. coverage (+): 0. Max coverage (-): 0

Region: chr14 15346689-15346700. Max. coverage (+): 0. Max coverage (-): 0

Region: chr14 15346701-15346712. Max. coverage (+): 0. Max coverage (-): 0

Region: chr14 15346713-15346723. Max. coverage (+): 0. Max coverage (-): 0

Region: chr14 15346724-15346735. Max. coverage (+): 0. Max coverage (-): 0

Region: chr14 15346736-15346747. Max. coverage (+): 0. Max coverage (-): 0

Region: chr14 15346748-15346758. Max. coverage (+): 0. Max coverage (-): 0

Region: chr14 15346759-15346770. Max. coverage (+): 0. Max coverage (-): 0

Region: chr14 15346771-15346782. Max. coverage (+): 0. Max coverage (-): 0

Region: chr14 15346783-15346793. Max. coverage (+): 0. Max coverage (-): 0

Region: chr14 15346794-15346805. Max. coverage (+): 0. Max coverage (-): 0

Region: chr14 15346806-15346817. Max. coverage (+): 0. Max coverage (-): 0

Region: chr14 15346818-15346828. Max. coverage (+): 0. Max coverage (-): 0

Region: chr14 15346829-15346840. Max. coverage (+): 0. Max coverage (-): 0

Region: chr14 15346841-15346852. Max. coverage (+): 0. Max coverage (-): 0

Region: chr14 15346853-15346864. Max. coverage (+): 0. Max coverage (-): 0

Region: chr14 15346865-15346875. Max. coverage (+): 0. Max coverage (-): 0

Region: chr14 15346876-15346887. Max. coverage (+): 0. Max coverage (-): 0

Region: chr14 15346888-15346899. Max. coverage (+): 0. Max coverage (-): 0

Region: chr14 15346900-15346910. Max. coverage (+): 0. Max coverage (-): 0

Region: chr14 15346911-15346922. Max. coverage (+): 0. Max coverage (-): 0

Region: chr14 15346923-15346934. Max. coverage (+): 0. Max coverage (-): 0

Region: chr14 15346935-15346945. Max. coverage (+): 0. Max coverage (-): 0

Region: chr14 15346946-15346957. Max. coverage (+): 0. Max coverage (-): 0

Region: chr14 15346958-15346969. Max. coverage (+): 0. Max coverage (-): 0

Region: chr14 15346970-15346980. Max. coverage (+): 0. Max coverage (-): 0

Region: chr14 15346981-15346992. Max. coverage (+): 0. Max coverage (-): 0

Region: chr14 15346993-15347004. Max. coverage (+): 0. Max coverage (-): 0

Region: chr14 15347005-15347016. Max. coverage (+): 0. Max coverage (-): 0

Region: chr14 15347017-15347027. Max. coverage (+): 0.22. Max coverage (-): 0

Region: chr14 15347028-15347039. Max. coverage (+): 0. Max coverage (-): 0

Region: chr14 15347040-15347051. Max. coverage (+): 0. Max coverage (-): 0

Region: chr14 15347052-15347062. Max. coverage (+): 0. Max coverage (-): 0

Region: chr14 15347063-15347074. Max. coverage (+): 0. Max coverage (-): 0

Region: chr14 15347075-15347086. Max. coverage (+): 0. Max coverage (-): 0

Region: chr14 15347087-15347097. Max. coverage (+): 0. Max coverage (-): 0

Region: chr14 15347098-15347109. Max. coverage (+): 0. Max coverage (-): 0

Region: chr14 15347110-15347121. Max. coverage (+): 0. Max coverage (-): 0

Region: chr14 15347122-15347132. Max. coverage (+): 0. Max coverage (-): 0

Region: chr14 15347133-15347144. Max. coverage (+): 0. Max coverage (-): 0

Region: chr14 15347145-15347156. Max. coverage (+): 0. Max coverage (-): 0

Region: chr14 15347157-15347168. Max. coverage (+): 0. Max coverage (-): 0

Region: chr14 15347169-15347179. Max. coverage (+): 0. Max coverage (-): 0

Region: chr14 15347180-15347191. Max. coverage (+): 0. Max coverage (-): 0

Region: chr14 15347192-15347203. Max. coverage (+): 0. Max coverage (-): 0

Region: chr14 15347204-15347214. Max. coverage (+): 0. Max coverage (-): 0

Region: chr14 15347215-15347226. Max. coverage (+): 0. Max coverage (-): 0

Region: chr14 15347227-15347238. Max. coverage (+): 0. Max coverage (-): 0

Region: chr14 15347239-15347249. Max. coverage (+): 0. Max coverage (-): 0

Region: chr14 15347250-15347261. Max. coverage (+): 0. Max coverage (-): 0

Region: chr14 15347262-15347273. Max. coverage (+): 0. Max coverage (-): 0

Region: chr14 15347274-15347285. Max. coverage (+): 0. Max coverage (-): 0

Region: chr14 15347286-15347296. Max. coverage (+): 0. Max coverage (-): 0

Region: chr14 15347297-15347308. Max. coverage (+): 0. Max coverage (-): 0

Region: chr14 15347309-15347320. Max. coverage (+): 0. Max coverage (-): 0

Region: chr14 15347321-15347331. Max. coverage (+): 0. Max coverage (-): 0

Region: chr14 15347332-15347343. Max. coverage (+): 0. Max coverage (-): 0

Region: chr14 15347344-15347355. Max. coverage (+): 0. Max coverage (-): 0

Region: chr14 15347356-15347366. Max. coverage (+): 0. Max coverage (-): 0

Region: chr14 15347367-15347378. Max. coverage (+): 0. Max coverage (-): 0

Region: chr14 15347379-15347390. Max. coverage (+): 0. Max coverage (-): 0

Region: chr14 15347391-15347401. Max. coverage (+): 0. Max coverage (-): 0

Region: chr14 15347402-15347413. Max. coverage (+): 0. Max coverage (-): 0

Region: chr14 15347414-15347425. Max. coverage (+): 0. Max coverage (-): 0

Region: chr14 15347426-15347437. Max. coverage (+): 0. Max coverage (-): 0

Region: chr14 15347438-15347448. Max. coverage (+): 0. Max coverage (-): 0

Region: chr14 15347449-15347460. Max. coverage (+): 0. Max coverage (-): 0

Region: chr14 15347461-15347472. Max. coverage (+): 0. Max coverage (-): 0

Region: chr14 15347473-15347483. Max. coverage (+): 0. Max coverage (-): 0

Region: chr14 15347484-15347495. Max. coverage (+): 0. Max coverage (-): 0

Region: chr14 15347496-15347507. Max. coverage (+): 0. Max coverage (-): 0

Region: chr14 15347508-15347518. Max. coverage (+): 0. Max coverage (-): 0

Region: chr14 15347519-15347530. Max. coverage (+): 0. Max coverage (-): 0

Region: chr14 15347531-15347542. Max. coverage (+): 0. Max coverage (-): 0

Region: chr14 15347543-15347553. Max. coverage (+): 0. Max coverage (-): 0

Region: chr14 15347554-15347565. Max. coverage (+): 0. Max coverage (-): 0

Region: chr14 15347566-15347577. Max. coverage (+): 0. Max coverage (-): 0

Region: chr14 15347578-15347589. Max. coverage (+): 0. Max coverage (-): 0

Region: chr14 15347590-15347600. Max. coverage (+): 0. Max coverage (-): 0

Region: chr14 15347601-15347612. Max. coverage (+): 0. Max coverage (-): 0

Region: chr14 15347613-15347624. Max. coverage (+): 0. Max coverage (-): 0

Region: chr14 15347625-15347635. Max. coverage (+): 0. Max coverage (-): 0

Region: chr14 15347636-15347647. Max. coverage (+): 0. Max coverage (-): 0

Region: chr14 15347648-15347659. Max. coverage (+): 0. Max coverage (-): 0

Region: chr14 15347660-15347670. Max. coverage (+): 0. Max coverage (-): 0

Region: chr14 15347671-15347682. Max. coverage (+): 0. Max coverage (-): 0

Region: chr14 15347683-15347694. Max. coverage (+): 0. Max coverage (-): 0

Region: chr14 15347695-15347705. Max. coverage (+): 0. Max coverage (-): 0

Region: chr14 15347706-15347717. Max. coverage (+): 0. Max coverage (-): 0

Region: chr14 15347718-15347729. Max. coverage (+): 0. Max coverage (-): 0

Region: chr14 15347730-15347741. Max. coverage (+): 0. Max coverage (-): 0

Region: chr14 15347742-15347752. Max. coverage (+): 0. Max coverage (-): 0

Region: chr14 15347753-15347764. Max. coverage (+): 0. Max coverage (-): 0

Region: chr14 15347765-15347776. Max. coverage (+): 0. Max coverage (-): 0

Region: chr14 15347777-15347787. Max. coverage (+): 0. Max coverage (-): 0

Region: chr14 15347788-15347799. Max. coverage (+): 0. Max coverage (-): 0

Region: chr14 15347800-15347811. Max. coverage (+): 0. Max coverage (-): 0

Region: chr14 15347812-15347822. Max. coverage (+): 0. Max coverage (-): 0

Region: chr14 15347823-15347834. Max. coverage (+): 0. Max coverage (-): 0

Region: chr14 15347835-15347846. Max. coverage (+): 0. Max coverage (-): 0

Region: chr14 15347847-15347858. Max. coverage (+): 0. Max coverage (-): 0

Region: chr14 15347859-15347869. Max. coverage (+): 0. Max coverage (-): 0

Region: chr14 15347870-15347881. Max. coverage (+): 0. Max coverage (-): 0

Region: chr14 15347882-15347893. Max. coverage (+): 0. Max coverage (-): 0

Region: chr14 15347894-15347904. Max. coverage (+): 0. Max coverage (-): 0

Region: chr14 15347905-15347916. Max. coverage (+): 0. Max coverage (-): 0

Region: chr14 15347917-15347928. Max. coverage (+): 0. Max coverage (-): 0

Region: chr14 15347929-15347939. Max. coverage (+): 0. Max coverage (-): 0

Region: chr14 15347940-15347951. Max. coverage (+): 0. Max coverage (-): 0

Region: chr14 15347952-15347963. Max. coverage (+): 0. Max coverage (-): 0

Region: chr14 15347964-15347974. Max. coverage (+): 0. Max coverage (-): 0

Region: chr14 15347975-15347986. Max. coverage (+): 0. Max coverage (-): 0

Region: chr14 15347987-15347998. Max. coverage (+): 0. Max coverage (-): 0

Region: chr14 15347999-15348010. Max. coverage (+): 0. Max coverage (-): 0

Region: chr14 15348011-15348021. Max. coverage (+): 0. Max coverage (-): 0

Region: chr14 15348022-15348033. Max. coverage (+): 0. Max coverage (-): 0

Region: chr14 15348034-15348045. Max. coverage (+): 0. Max coverage (-): 0

Region: chr14 15348046-15348056. Max. coverage (+): 0.22. Max coverage (-): 0

Region: chr14 15348057-15348068. Max. coverage (+): 0.22. Max coverage (-): 0

Region: chr14 15348069-15348080. Max. coverage (+): 0. Max coverage (-): 0

Region: chr14 15348081-15348091. Max. coverage (+): 0. Max coverage (-): 0

Region: chr14 15348092-15348103. Max. coverage (+): 0. Max coverage (-): 0

Region: chr14 15348104-15348115. Max. coverage (+): 0. Max coverage (-): 0

Region: chr14 15348116-15348126. Max. coverage (+): 0. Max coverage (-): 0

Region: chr14 15348127-15348138. Max. coverage (+): 0. Max coverage (-): 0

Region: chr14 15348139-15348150. Max. coverage (+): 0. Max coverage (-): 0

Region: chr14 15348151-15348162. Max. coverage (+): 0. Max coverage (-): 0

Region: chr14 15348163-15348173. Max. coverage (+): 0. Max coverage (-): 0

Region: chr14 15348174-15348185. Max. coverage (+): 0. Max coverage (-): 0

Region: chr14 15348186-15348197. Max. coverage (+): 0. Max coverage (-): 0

Region: chr14 15348198-15348208. Max. coverage (+): 0.55. Max coverage (-): 0

Region: chr14 15348209-15348220. Max. coverage (+): 0.55. Max coverage (-): 0

Region: chr14 15348221-15348232. Max. coverage (+): 0. Max coverage (-): 0

Region: chr14 15348233-15348243. Max. coverage (+): 0. Max coverage (-): 0

Region: chr14 15348244-15348255. Max. coverage (+): 0. Max coverage (-): 0

Region: chr14 15348256-15348267. Max. coverage (+): 0. Max coverage (-): 0

Region: chr14 15348268-15348279. Max. coverage (+): 0. Max coverage (-): 0

Region: chr14 15348280-15348290. Max. coverage (+): 0. Max coverage (-): 0

Region: chr14 15348291-15348302. Max. coverage (+): 0. Max coverage (-): 0

Region: chr14 15348303-15348314. Max. coverage (+): 0. Max coverage (-): 0

Region: chr14 15348315-15348325. Max. coverage (+): 0. Max coverage (-): 0

Region: chr14 15348326-15348337. Max. coverage (+): 0. Max coverage (-): 0

Region: chr14 15348338-15348349. Max. coverage (+): 0. Max coverage (-): 0

Region: chr14 15348350-15348360. Max. coverage (+): 3.69. Max coverage (-): 0

Region: chr14 15348361-15348372. Max. coverage (+): 0. Max coverage (-): 0

Region: chr14 15348373-15348384. Max. coverage (+): 0. Max coverage (-): 0

Region: chr14 15348385-15348395. Max. coverage (+): 0. Max coverage (-): 0

Region: chr14 15348396-15348407. Max. coverage (+): 0. Max coverage (-): 0

Region: chr14 15348408-15348419. Max. coverage (+): 0. Max coverage (-): 0

Region: chr14 15348420-15348431. Max. coverage (+): 0. Max coverage (-): 0

Region: chr14 15348432-15348442. Max. coverage (+): 0. Max coverage (-): 0

Region: chr14 15348443-15348454. Max. coverage (+): 0. Max coverage (-): 0

Region: chr14 15348455-15348466. Max. coverage (+): 0. Max coverage (-): 0

Region: chr14 15348467-15348477. Max. coverage (+): 0. Max coverage (-): 0

Region: chr14 15348478-15348489. Max. coverage (+): 0. Max coverage (-): 0

Region: chr14 15348490-15348501. Max. coverage (+): 0. Max coverage (-): 0

Region: chr14 15348502-15348512. Max. coverage (+): 0. Max coverage (-): 0

Region: chr14 15348513-15348524. Max. coverage (+): 0. Max coverage (-): 0

Region: chr14 15348525-15348536. Max. coverage (+): 0. Max coverage (-): 0

Region: chr14 15348537-15348547. Max. coverage (+): 0. Max coverage (-): 0

Region: chr14 15348548-15348559. Max. coverage (+): 0. Max coverage (-): 0

Region: chr14 15348560-15348571. Max. coverage (+): 0. Max coverage (-): 0

Region: chr14 15348572-15348583. Max. coverage (+): 0.02. Max coverage (-): 0

Region: chr14 15348584-15348594. Max. coverage (+): 0. Max coverage (-): 0

Region: chr14 15348595-15348606. Max. coverage (+): 11. Max coverage (-): 0

Region: chr14 15348607-15348618. Max. coverage (+): 0. Max coverage (-): 0

Region: chr14 15348619-15348629. Max. coverage (+): 0. Max coverage (-): 0

Region: chr14 15348630-15348641. Max. coverage (+): 0. Max coverage (-): 0

Region: chr14 15348642-15348653. Max. coverage (+): 0. Max coverage (-): 0

Region: chr14 15348654-15348664. Max. coverage (+): 0. Max coverage (-): 0

Region: chr14 15348665-15348676. Max. coverage (+): 0. Max coverage (-): 0

Region: chr14 15348677-15348688. Max. coverage (+): 1.26. Max coverage (-): 0

Region: chr14 15348689-15348699. Max. coverage (+): 1.26. Max coverage (-): 0

Region: chr14 15348700-15348711. Max. coverage (+): 0. Max coverage (-): 0

Region: chr14 15348712-15348723. Max. coverage (+): 0. Max coverage (-): 0

Region: chr14 15348724-15348735. Max. coverage (+): 0. Max coverage (-): 0

Region: chr14 15348736-15348746. Max. coverage (+): 0. Max coverage (-): 0

Region: chr14 15348747-15348758. Max. coverage (+): 0. Max coverage (-): 0

Region: chr14 15348759-15348770. Max. coverage (+): 0. Max coverage (-): 0

Region: chr14 15348771-15348781. Max. coverage (+): 0. Max coverage (-): 0

Region: chr14 15348782-15348793. Max. coverage (+): 0. Max coverage (-): 0

Region: chr14 15348794-15348805. Max. coverage (+): 0. Max coverage (-): 0

Region: chr14 15348806-15348816. Max. coverage (+): 0. Max coverage (-): 0

Region: chr14 15348817-15348828. Max. coverage (+): 2.16. Max coverage (-): 0

Region: chr14 15348829-15348840. Max. coverage (+): 0. Max coverage (-): 0

Region: chr14 15348841-15348852. Max. coverage (+): 0.09. Max coverage (-): 0

Region: chr14 15348853-15348863. Max. coverage (+): 1. Max coverage (-): 0

Region: chr14 15348864-15348875. Max. coverage (+): 1.15. Max coverage (-): 0

Region: chr14 15348876-15348887. Max. coverage (+): 0.12. Max coverage (-): 0

Region: chr14 15348888-15348898. Max. coverage (+): 0. Max coverage (-): 0

Region: chr14 15348899-15348910. Max. coverage (+): 0. Max coverage (-): 0

Region: chr14 15348911-15348922. Max. coverage (+): 0. Max coverage (-): 0

Region: chr14 15348923-15348933. Max. coverage (+): 0.68. Max coverage (-): 0

Region: chr14 15348934-15348945. Max. coverage (+): 0.68. Max coverage (-): 0

Region: chr14 15348946-15348957. Max. coverage (+): 0.48. Max coverage (-): 0

Region: chr14 15348958-15348968. Max. coverage (+): 0.48. Max coverage (-): 0

Region: chr14 15348969-15348980. Max. coverage (+): 0.17. Max coverage (-): 0

Region: chr14 15348981-15348992. Max. coverage (+): 0.23. Max coverage (-): 0

Region: chr14 15348993-15349004. Max. coverage (+): 0.15. Max coverage (-): 0

Region: chr14 15349005-15349015. Max. coverage (+): 0. Max coverage (-): 0

Region: chr14 15349016-15349027. Max. coverage (+): 0. Max coverage (-): 0

Region: chr14 15349028-15349039. Max. coverage (+): 0. Max coverage (-): 0

Region: chr14 15349040-15349050. Max. coverage (+): 0. Max coverage (-): 0

Region: chr14 15349051-15349062. Max. coverage (+): 6. Max coverage (-): 0

Region: chr14 15349063-15349074. Max. coverage (+): 1.1. Max coverage (-): 0

Region: chr14 15349075-15349085. Max. coverage (+): 1.1. Max coverage (-): 0

Region: chr14 15349086-15349097. Max. coverage (+): 4.61. Max coverage (-): 0

Region: chr14 15349098-15349109. Max. coverage (+): 7.93. Max coverage (-): 0

Region: chr14 15349110-15349120. Max. coverage (+): 2.78. Max coverage (-): 0

Region: chr14 15349121-15349132. Max. coverage (+): 0. Max coverage (-): 0

Region: chr14 15349133-15349144. Max. coverage (+): 0. Max coverage (-): 0

Region: chr14 15349145-15349156. Max. coverage (+): 0. Max coverage (-): 0

Region: chr14 15349157-15349167. Max. coverage (+): 0. Max coverage (-): 0

Region: chr14 15349168-15349179. Max. coverage (+): 0. Max coverage (-): 0

Region: chr14 15349180-15349191. Max. coverage (+): 0. Max coverage (-): 0

Region: chr14 15349192-15349202. Max. coverage (+): 0. Max coverage (-): 0

Region: chr14 15349203-15349214. Max. coverage (+): 0. Max coverage (-): 0

Region: chr14 15349215-15349226. Max. coverage (+): 0. Max coverage (-): 0

Region: chr14 15349227-15349237. Max. coverage (+): 0. Max coverage (-): 0

Region: chr14 15349238-15349249. Max. coverage (+): 0. Max coverage (-): 0

Region: chr14 15349250-15349261. Max. coverage (+): 0. Max coverage (-): 0

Region: chr14 15349262-15349272. Max. coverage (+): 0. Max coverage (-): 0

Region: chr14 15349273-15349284. Max. coverage (+): 0. Max coverage (-): 0

Region: chr14 15349285-15349296. Max. coverage (+): 1.58. Max coverage (-): 0

Region: chr14 15349297-15349308. Max. coverage (+): 0. Max coverage (-): 0.93

Region: chr14 15349309-15349319. Max. coverage (+): 0. Max coverage (-): 0.93

Region: chr14 15349320-15349331. Max. coverage (+): 0. Max coverage (-): 0

Region: chr14 15349332-15349343. Max. coverage (+): 0. Max coverage (-): 0

Region: chr14 15349344-15349354. Max. coverage (+): 1.9. Max coverage (-): 0

Region: chr14 15349355-15349366. Max. coverage (+): 1.9. Max coverage (-): 0

Region: chr14 15349367-15349378. Max. coverage (+): 0. Max coverage (-): 0

Region: chr14 15349379-15349389. Max. coverage (+): 0. Max coverage (-): 0

Region: chr14 15349390-15349401. Max. coverage (+): 0. Max coverage (-): 0.54

Region: chr14 15349402-15349413. Max. coverage (+): 0. Max coverage (-): 0.72

Region: chr14 15349414-15349425. Max. coverage (+): 0.26. Max coverage (-): 0.33

Region: chr14 15349426-15349436. Max. coverage (+): 0. Max coverage (-): 0

Region: chr14 15349437-15349448. Max. coverage (+): 0. Max coverage (-): 0

Region: chr14 15349449-15349460. Max. coverage (+): 0. Max coverage (-): 0

Region: chr14 15349461-15349471. Max. coverage (+): 0. Max coverage (-): 0

Region: chr14 15349472-15349483. Max. coverage (+): 0. Max coverage (-): 0

Region: chr14 15349484-15349495. Max. coverage (+): 0. Max coverage (-): 0

Region: chr14 15349496-15349506. Max. coverage (+): 0. Max coverage (-): 0

Region: chr14 15349507-15349518. Max. coverage (+): 0. Max coverage (-): 0

Region: chr14 15349519-15349530. Max. coverage (+): 0. Max coverage (-): 1.77

Region: chr14 15349531-15349541. Max. coverage (+): 0. Max coverage (-): 0.96

Region: chr14 15349542-15349553. Max. coverage (+): 0. Max coverage (-): 0

Region: chr14 15349554-15349565. Max. coverage (+): 0. Max coverage (-): 0

Region: chr14 15349566-15349577. Max. coverage (+): 0. Max coverage (-): 0

Region: chr14 15349578-15349588. Max. coverage (+): 0. Max coverage (-): 0

Region: chr14 15349589-15349600. Max. coverage (+): 0.41. Max coverage (-): 0.39

Region: chr14 15349601-15349612. Max. coverage (+): 0.48. Max coverage (-): 0.79

Region: chr14 15349613-15349623. Max. coverage (+): 0. Max coverage (-): 0.38

Region: chr14 15349624-15349635. Max. coverage (+): 0. Max coverage (-): 0

Region: chr14 15349636-15349647. Max. coverage (+): 0. Max coverage (-): 0

Region: chr14 15349648-15349658. Max. coverage (+): 5.05. Max coverage (-): 0

Region: chr14 15349659-15349670. Max. coverage (+): 0. Max coverage (-): 0

Region: chr14 15349671-15349682. Max. coverage (+): 0. Max coverage (-): 0

Region: chr14 15349683-15349693. Max. coverage (+): 0. Max coverage (-): 2.59

Region: chr14 15349694-15349705. Max. coverage (+): 0.29. Max coverage (-): 1.02

Region: chr14 15349706-15349717. Max. coverage (+): 0.29. Max coverage (-): 0.99

Region: chr14 15349718-15349729. Max. coverage (+): 0. Max coverage (-): 0

Region: chr14 15349730-15349740. Max. coverage (+): 0. Max coverage (-): 0

Region: chr14 15349741-15349752. Max. coverage (+): 0. Max coverage (-): 0

Region: chr14 15349753-15349764. Max. coverage (+): 0. Max coverage (-): 0

Region: chr14 15349765-15349775. Max. coverage (+): 0. Max coverage (-): 0

Region: chr14 15349776-15349787. Max. coverage (+): 0. Max coverage (-): 0

Region: chr14 15349788-15349799. Max. coverage (+): 0. Max coverage (-): 0.61

Region: chr14 15349800-15349810. Max. coverage (+): 0. Max coverage (-): 3.19

Region: chr14 15349811-15349822. Max. coverage (+): 0. Max coverage (-): 2.56

Region: chr14 15349823-15349834. Max. coverage (+): 0. Max coverage (-): 0

Region: chr14 15349835-15349845. Max. coverage (+): 0. Max coverage (-): 0

Region: chr14 15349846-15349857. Max. coverage (+): 0. Max coverage (-): 0

Region: chr14 15349858-15349869. Max. coverage (+): 0. Max coverage (-): 0

Region: chr14 15349870-15349881. Max. coverage (+): 0. Max coverage (-): 0

Region: chr14 15349882-15349892. Max. coverage (+): 0. Max coverage (-): 0

Region: chr14 15349893-15349904. Max. coverage (+): 0. Max coverage (-): 0

Region: chr14 15349905-15349916. Max. coverage (+): 0. Max coverage (-): 0

Region: chr14 15349917-15349927. Max. coverage (+): 0. Max coverage (-): 0

Region: chr14 15349928-15349939. Max. coverage (+): 0. Max coverage (-): 0

Region: chr14 15349940-15349951. Max. coverage (+): 0. Max coverage (-): 0

Region: chr14 15349952-15349962. Max. coverage (+): 0. Max coverage (-): 0

Region: chr14 15349963-15349974. Max. coverage (+): 0. Max coverage (-): 0

Region: chr14 15349975-15349986. Max. coverage (+): 0. Max coverage (-): 0

Region: chr14 15349987-15349998. Max. coverage (+): 0. Max coverage (-): 0

Region: chr14 15349999-15350009. Max. coverage (+): 0. Max coverage (-): 0

Region: chr14 15350010-15350021. Max. coverage (+): 0. Max coverage (-): 0

Region: chr14 15350022-15350033. Max. coverage (+): 0. Max coverage (-): 0

Region: chr14 15350034-15350044. Max. coverage (+): 0. Max coverage (-): 0

Region: chr14 15350045-15350056. Max. coverage (+): 0. Max coverage (-): 0

Region: chr14 15350057-15350068. Max. coverage (+): 5.81. Max coverage (-): 0

Region: chr14 15350069-15350079. Max. coverage (+): 5.81. Max coverage (-): 0

Region: chr14 15350080-15350091. Max. coverage (+): 0. Max coverage (-): 0

Region: chr14 15350092-15350103. Max. coverage (+): 0. Max coverage (-): 0.17

Region: chr14 15350104-15350114. Max. coverage (+): 0. Max coverage (-): 1.19

Region: chr14 15350115-15350126. Max. coverage (+): 0. Max coverage (-): 1.34

Region: chr14 15350127-15350138. Max. coverage (+): 0. Max coverage (-): 0

Region: chr14 15350139-15350150. Max. coverage (+): 0. Max coverage (-): 0

Region: chr14 15350151-15350161. Max. coverage (+): 0. Max coverage (-): 0

Region: chr14 15350162-15350173. Max. coverage (+): 0. Max coverage (-): 0

Region: chr14 15350174-15350185. Max. coverage (+): 0. Max coverage (-): 0

Region: chr14 15350186-15350196. Max. coverage (+): 0. Max coverage (-): 0

Region: chr14 15350197-15350208. Max. coverage (+): 0. Max coverage (-): 0

Region: chr14 15350209-15350220. Max. coverage (+): 0. Max coverage (-): 0

Region: chr14 15350221-15350231. Max. coverage (+): 0. Max coverage (-): 0

Region: chr14 15350232-15350243. Max. coverage (+): 0. Max coverage (-): 0

Region: chr14 15350244-15350255. Max. coverage (+): 0. Max coverage (-): 0

Region: chr14 15350256-15350266. Max. coverage (+): 0. Max coverage (-): 0

Region: chr14 15350267-15350278. Max. coverage (+): 0. Max coverage (-): 0

Region: chr14 15350279-15350290. Max. coverage (+): 0. Max coverage (-): 0

Region: chr14 15350291-15350302. Max. coverage (+): 0. Max coverage (-): 0.21

Region: chr14 15350303-15350313. Max. coverage (+): 0. Max coverage (-): 1.53

Region: chr14 15350314-15350325. Max. coverage (+): 0. Max coverage (-): 0

Region: chr14 15350326-15350337. Max. coverage (+): 0. Max coverage (-): 0

Region: chr14 15350338-15350348. Max. coverage (+): 0. Max coverage (-): 0.84

Region: chr14 15350349-15350360. Max. coverage (+): 0. Max coverage (-): 0

Region: chr14 15350361-15350372. Max. coverage (+): 0. Max coverage (-): 0

Region: chr14 15350373-15350383. Max. coverage (+): 0. Max coverage (-): 0

Region: chr14 15350384-15350395. Max. coverage (+): 0. Max coverage (-): 0

Region: chr14 15350396-15350407. Max. coverage (+): 0. Max coverage (-): 0

Region: chr14 15350408-15350419. Max. coverage (+): 0. Max coverage (-): 0

Region: chr14 15350420-15350430. Max. coverage (+): 0. Max coverage (-): 0

Region: chr14 15350431-15350442. Max. coverage (+): 8.13. Max coverage (-): 0

Region: chr14 15350443-15350454. Max. coverage (+): 0. Max coverage (-): 0

Region: chr14 15350455-15350465. Max. coverage (+): 0. Max coverage (-): 0

Region: chr14 15350466-15350477. Max. coverage (+): 0. Max coverage (-): 0

Region: chr14 15350478-15350489. Max. coverage (+): 0. Max coverage (-): 2.48

Region: chr14 15350490-15350500. Max. coverage (+): 0. Max coverage (-): 6.48

Region: chr14 15350501-15350512. Max. coverage (+): 0. Max coverage (-): 0

Region: chr14 15350513-15350524. Max. coverage (+): 0. Max coverage (-): 0

Region: chr14 15350525-15350535. Max. coverage (+): 0. Max coverage (-): 0

Region: chr14 15350536-15350547. Max. coverage (+): 0. Max coverage (-): 0

Region: chr14 15350548-15350559. Max. coverage (+): 0. Max coverage (-): 0

Region: chr14 15350560-15350571. Max. coverage (+): 0. Max coverage (-): 0

Region: chr14 15350572-15350582. Max. coverage (+): 0. Max coverage (-): 0

Region: chr14 15350583-15350594. Max. coverage (+): 0. Max coverage (-): 0

Region: chr14 15350595-15350606. Max. coverage (+): 0. Max coverage (-): 0

Region: chr14 15350607-15350617. Max. coverage (+): 0. Max coverage (-): 0

Region: chr14 15350618-15350629. Max. coverage (+): 0. Max coverage (-): 0

Region: chr14 15350630-15350641. Max. coverage (+): 0. Max coverage (-): 0

Region: chr14 15350642-15350652. Max. coverage (+): 0. Max coverage (-): 0

Region: chr14 15350653-15350664. Max. coverage (+): 0. Max coverage (-): 0

Region: chr14 15350665-15350676. Max. coverage (+): 0. Max coverage (-): 0

Region: chr14 15350677-15350687. Max. coverage (+): 0. Max coverage (-): 0

Region: chr14 15350688-15350699. Max. coverage (+): 0. Max coverage (-): 0

Region: chr14 15350700-15350711. Max. coverage (+): 0. Max coverage (-): 0

Region: chr14 15350712-15350723. Max. coverage (+): 0. Max coverage (-): 0

Region: chr14 15350724-15350734. Max. coverage (+): 0. Max coverage (-): 0

Region: chr14 15350735-15350746. Max. coverage (+): 0. Max coverage (-): 0

Region: chr14 15350747-15350758. Max. coverage (+): 0. Max coverage (-): 0

Region: chr14 15350759-15350769. Max. coverage (+): 0. Max coverage (-): 0

Region: chr14 15350770-15350781. Max. coverage (+): 0. Max coverage (-): 0

Region: chr14 15350782-15350793. Max. coverage (+): 0. Max coverage (-): 0

Region: chr14 15350794-15350804. Max. coverage (+): 0. Max coverage (-): 0

Region: chr14 15350805-15350816. Max. coverage (+): 0. Max coverage (-): 0

Region: chr14 15350817-15350828. Max. coverage (+): 0.26. Max coverage (-): 0

Region: chr14 15350829-15350839. Max. coverage (+): 0. Max coverage (-): 0

Region: chr14 15350840-15350851. Max. coverage (+): 0. Max coverage (-): 0

Region: chr14 15350852-15350863. Max. coverage (+): 0. Max coverage (-): 0

Region: chr14 15350864-15350875. Max. coverage (+): 0. Max coverage (-): 0

Region: chr14 15350876-15350886. Max. coverage (+): 7.37. Max coverage (-): 0

Region: chr14 15350887-15350898. Max. coverage (+): 8.9. Max coverage (-): 0

Region: chr14 15350899-15350910. Max. coverage (+): 0. Max coverage (-): 0

Region: chr14 15350911-. Max. coverage (+): 0. Max coverage (-): 0

RepeatMasker Color Code

**+**

100-98% Identity

<98-95% Identity

<95-90% Identity

<90-85% Identity

<85-80% Identity

<80-75% Identity

<75-70% Identity

<70% Identity

**-**

Gene Set Color Code

**+**

Gene

Pseudogene

**-**

Topology/Coverage Color Code

Coverage Plus Strand

Coverage Minus Strand

Mainstrand: Plus

Mainstrand: Minus

Complementary Strand

Flanking Region  
(if option -flank >0)

Gene Set Annotation  
  
RepeatMasker Annotation  

**1. SINE2-3\_BT**: 15349835-15350014 (-), Divergence to consensus: 16.8%  
**2. (TTTTC)n**: 15350406-15350435 (+), Divergence to consensus: 13.3%

  
Transcription Factor Binding Sites  

**RFX4\_1** (Sequence: CTTAGCAAC (+): 15345407)  
**RFX4\_1** (Sequence: CTTAGCAAC (+): 15346666)  
**RFX4\_1** (Sequence: CTTAGCAAC (+): 15347705)  
**RFX4\_1** (Sequence: CATGGCAAC (+): 15348710)  
**Gata4** (Sequence: AGATAAC (-): 15350882)
