# Supplementary material for: Small RNA sequencing of cryopreserved semen from single bull revealed altered miRNAs and piRNAs expression between High- and Low-motile sperm populations
Source: BMC Genomics. 2017 Jan 4;18:14. doi: 10.1186/s12864-016-3394-7 (PMC5209821; doi:10.1186/s12864-016-3394-7)
Supplement: Additional file 3: — Details for each piRNA clusters found in High Motile (HM) sperm fraction. Genes, repeats, transposable elements and transcription factors binding sites falling within the cluster regions were reported. (ZIP 1896 kb) [file 12864_2016_3394_MOESM3_ESM.zip › 25.html]

piRNA cluster 25


Predicted piRNA cluster no. 25     previous   next
  

Show proTRAC run info
Hide proTRAC run info

================================= proTRAC ====================================  
VERSION: 2.1                                    LAST MODIFIED: 06. October 2015  
  
Please cite:  
Rosenkranz D, Zischler H. proTRAC - a software for probabilistic piRNA cluster  
detection, visualization and analysis. 2012. BMC Bioinformatics 13:5.  
  
and (for proTRAC 2.0 and later):  
Rosenkranz D, Rudloff S, Bastuck K, Ketting RF, Zischler H. Tupaia small RNAs  
provide insights into function and evolution of RNAi-based transposon defense  
in mammals. 2015. RNA 21(5):911-922.  
  
Contact:  
David Rosenkranz  
Institute of Anthropology, small RNA group  
Johannes Gutenberg University Mainz  
email: rosenkranz@uni-mainz.de  
  
You can find the latest proTRAC version at:  
http://sourceforge.net/projects/protrac/files  
http://www.smallRNAgroup-mainz.de/software  
==============================================================================  
  
PARAMETERS:  
Map file: .............../storage/core/barbara/genhome/smallRNA/fertility/Sample\_motile/pirna/Sample\_motile\_26-33\_collapsed.fa.no-dust.map.weighted-10000-1000-b-0  
Genome file: ............/storage/core/barbara/genhome/smallRNA/fertility/Sample\_all/pirna/bt\_311\_chrY.fa  
RepeatMasker annotation: /storage/genomes/bt\_umd31/GCF\_000003055.6\_Bos\_taurus\_UMD\_3.1.1\_repeatMasker\_chr.out  
GeneSet:................./storage/core/barbara/genhome/smallRNA/fertility/Sample\_all/pirna/full.gtf  
  
Significant (p<=0.01) hit density will be calculated based  
on observed hit distribution.  
  
Sliding window size: ........................................ 5000 bp  
Sliding window increament: .................................. 1000 bp  
Normalize each hit by number of genomic hits: ............... 1 [0=no/1=yes]  
Normalize each hit by number of sequence reads: ............. 1 [0=no/1=yes]  
Normalize values (-> per million mapped reads): ............. 1 [0=no/1=yes]  
Min. fraction of hits with 1T(U) or 10A: .................... 0.75  
Alternatively: Min. fraction of hits with 1T(U) and 10A: .... 0.5  
Min. fraction of hits with typical piRNA length: ............ 0.75  
Typical piRNA length: ....................................... 26-33 nt  
Min. size of a piRNA cluster: ............................... 5000 bp.  
Min. number of hits (absolute): ............................. 0  
Min. number of hits (normalized): ........................... 0  
Min. fraction of hits on the mainstrand: .................... 0.75  
Top fraction of mapped sequences (in terms of read counts): . 1%  
Top fraction accounts for max. n% of sequence reads: ........ 90%  
Min. fraction of hits on each arm of a bidirectional cluster: 0.1  
Output image file for each cluster: ......................... 0 [0=no/1=yes]  
Output html file for each cluster: .......................... 1 [0=no/1=yes]  
Output a summary table: ..................................... 1 [0=no/1=yes]  
Output a FASTA file for each cluster (piRNA sequences): ..... 1 [0=no/1=yes]  
Output a FASTA file comprising cluster sequences: ........... 1 [0=no/1=yes]  
Search DNA motifs in clusters: .............................. 1 [0=no/1=yes]  
Output flanking sequences: +/- .............................. 0 bp  
Output ~.pTi file: .......................................... 1 [0=no/1=yes]  
==============================================================================  
  
  
Genome size (without gaps): ............ 2678902517 bp  
Gaps (N/X/-): .......................... 53837044 bp  
Mapped reads: .......................... 658825247023  
Non-identical sequences: ............... 514171  
Genomic hits: .......................... 764233  
Significant densitiy of mapped reads: .. 12867599.5173724 reads/kb

Show proTRAC cluster info
Hide proTRAC cluster info

|  |  |
| --- | --- |
| Location | chr14 |
| Coordinates | 16776112-16810189 |
| Size [bp] | 34078 |
| Sequence hit loci | 2116 |
| Mapped reads (normalized) | 2478407994.1 |
| Mapped reads (normalized) per kb | 72727507.3 |
| Normalized reads with 1T (1U) | 81.6% |
| Normalized reads with 10A | 32.7% |
| Normalized reads with length 26-33 nt | 100% |
| Normalized reads on the main strand(s) | 99.3% |
| Predicted directionality | bi:minus-plus (split between 16783949 and 16783952) |

100%

0%

1T (1U)  
reads

10A reads

26-33 nt  
reads

reads on mainstrand

**Either the amount of reads with 1T (1U) OR 10A has to exceed 75% (set with option: -1Tor10A)  
Alternatively the amount of reads with 1T (1U) AND 10A has to exceed 50% (set with option: -1Tand10A)  
Minimum amount of reads with preferred size is 75% (set with option: -pisize)  
Minimum amount of reads on the main strand(s) is 75% (set with option: -clstrand)**

Show read coverage
Hide read coverage

WHAT DO I SEE HERE?  
This chart shows the location of mapped sequence reads within a predicted piRNA cluster. The color refers to the number of genomic hits produced by the sequence read in question. A dark red bar indicates that this sequence read produces many other hits elsewhere in the genome. Many adjacent red or yellow bars can indicate the presence of a multi-copy element such as transposons or rRNA genes. A dark green bar indicates that this sequence read maps uniquely to this locus.

1 hit

2-5 hits

6-10 hits

11-20 hits

21-50 hits

51-100 hits

> 100 hits

chr14

16776112

16810189

Gene Set

RepeatMasker

Mapped  
Reads

75.79

plus strand

minus strand

75.79

Region: chr14 15350912-16776146. Max. coverage (+): 0. Max coverage (-): 5.01

Region: chr14 16776147-16776214. Max. coverage (+): 0. Max coverage (-): 7.47

Region: chr14 16776215-16776282. Max. coverage (+): 0. Max coverage (-): 0

Region: chr14 16776283-16776350. Max. coverage (+): 0. Max coverage (-): 0

Region: chr14 16776351-16776418. Max. coverage (+): 0. Max coverage (-): 0

Region: chr14 16776419-16776486. Max. coverage (+): 0. Max coverage (-): 4.7

Region: chr14 16776487-16776555. Max. coverage (+): 0. Max coverage (-): 0

Region: chr14 16776556-16776623. Max. coverage (+): 0. Max coverage (-): 0

Region: chr14 16776624-16776691. Max. coverage (+): 0. Max coverage (-): 22.45

Region: chr14 16776692-16776759. Max. coverage (+): 0. Max coverage (-): 0

Region: chr14 16776760-16776827. Max. coverage (+): 0. Max coverage (-): 0

Region: chr14 16776828-16776895. Max. coverage (+): 0. Max coverage (-): 0

Region: chr14 16776896-16776963. Max. coverage (+): 0. Max coverage (-): 0

Region: chr14 16776964-16777032. Max. coverage (+): 0. Max coverage (-): 0

Region: chr14 16777033-16777100. Max. coverage (+): 0. Max coverage (-): 0

Region: chr14 16777101-16777168. Max. coverage (+): 0. Max coverage (-): 0

Region: chr14 16777169-16777236. Max. coverage (+): 0. Max coverage (-): 0

Region: chr14 16777237-16777304. Max. coverage (+): 0. Max coverage (-): 0

Region: chr14 16777305-16777372. Max. coverage (+): 0. Max coverage (-): 0

Region: chr14 16777373-16777441. Max. coverage (+): 0. Max coverage (-): 0

Region: chr14 16777442-16777509. Max. coverage (+): 0. Max coverage (-): 0

Region: chr14 16777510-16777577. Max. coverage (+): 0. Max coverage (-): 13.22

Region: chr14 16777578-16777645. Max. coverage (+): 0. Max coverage (-): 4.31

Region: chr14 16777646-16777713. Max. coverage (+): 0. Max coverage (-): 0

Region: chr14 16777714-16777781. Max. coverage (+): 0. Max coverage (-): 0

Region: chr14 16777782-16777849. Max. coverage (+): 0. Max coverage (-): 0

Region: chr14 16777850-16777918. Max. coverage (+): 0. Max coverage (-): 4.17

Region: chr14 16777919-16777986. Max. coverage (+): 0. Max coverage (-): 4.07

Region: chr14 16777987-16778054. Max. coverage (+): 0. Max coverage (-): 0

Region: chr14 16778055-16778122. Max. coverage (+): 0. Max coverage (-): 0

Region: chr14 16778123-16778190. Max. coverage (+): 0. Max coverage (-): 0

Region: chr14 16778191-16778258. Max. coverage (+): 0. Max coverage (-): 0

Region: chr14 16778259-16778327. Max. coverage (+): 0. Max coverage (-): 0

Region: chr14 16778328-16778395. Max. coverage (+): 0. Max coverage (-): 0

Region: chr14 16778396-16778463. Max. coverage (+): 0. Max coverage (-): 2.35

Region: chr14 16778464-16778531. Max. coverage (+): 0. Max coverage (-): 4.17

Region: chr14 16778532-16778599. Max. coverage (+): 0. Max coverage (-): 0

Region: chr14 16778600-16778667. Max. coverage (+): 0. Max coverage (-): 0

Region: chr14 16778668-16778736. Max. coverage (+): 0. Max coverage (-): 0

Region: chr14 16778737-16778804. Max. coverage (+): 0. Max coverage (-): 0

Region: chr14 16778805-16778872. Max. coverage (+): 0. Max coverage (-): 0

Region: chr14 16778873-16778940. Max. coverage (+): 0. Max coverage (-): 0

Region: chr14 16778941-16779008. Max. coverage (+): 0. Max coverage (-): 0

Region: chr14 16779009-16779076. Max. coverage (+): 0. Max coverage (-): 0

Region: chr14 16779077-16779144. Max. coverage (+): 0. Max coverage (-): 0

Region: chr14 16779145-16779213. Max. coverage (+): 0. Max coverage (-): 0

Region: chr14 16779214-16779281. Max. coverage (+): 0. Max coverage (-): 0

Region: chr14 16779282-16779349. Max. coverage (+): 0. Max coverage (-): 0

Region: chr14 16779350-16779417. Max. coverage (+): 0. Max coverage (-): 3.45

Region: chr14 16779418-16779485. Max. coverage (+): 0. Max coverage (-): 3.13

Region: chr14 16779486-16779553. Max. coverage (+): 0. Max coverage (-): 1.75

Region: chr14 16779554-16779622. Max. coverage (+): 0. Max coverage (-): 0

Region: chr14 16779623-16779690. Max. coverage (+): 0. Max coverage (-): 7.65

Region: chr14 16779691-16779758. Max. coverage (+): 0. Max coverage (-): 0

Region: chr14 16779759-16779826. Max. coverage (+): 0. Max coverage (-): 1.33

Region: chr14 16779827-16779894. Max. coverage (+): 0. Max coverage (-): 10.33

Region: chr14 16779895-16779962. Max. coverage (+): 0. Max coverage (-): 0.91

Region: chr14 16779963-16780030. Max. coverage (+): 0. Max coverage (-): 0.43

Region: chr14 16780031-16780099. Max. coverage (+): 0. Max coverage (-): 1.49

Region: chr14 16780100-16780167. Max. coverage (+): 0. Max coverage (-): 5.09

Region: chr14 16780168-16780235. Max. coverage (+): 0. Max coverage (-): 3.91

Region: chr14 16780236-16780303. Max. coverage (+): 0. Max coverage (-): 1.48

Region: chr14 16780304-16780371. Max. coverage (+): 0. Max coverage (-): 0

Region: chr14 16780372-16780439. Max. coverage (+): 0. Max coverage (-): 3.16

Region: chr14 16780440-16780508. Max. coverage (+): 0. Max coverage (-): 1.38

Region: chr14 16780509-16780576. Max. coverage (+): 0. Max coverage (-): 0

Region: chr14 16780577-16780644. Max. coverage (+): 0. Max coverage (-): 0

Region: chr14 16780645-16780712. Max. coverage (+): 0. Max coverage (-): 0

Region: chr14 16780713-16780780. Max. coverage (+): 0. Max coverage (-): 0

Region: chr14 16780781-16780848. Max. coverage (+): 0. Max coverage (-): 0

Region: chr14 16780849-16780916. Max. coverage (+): 0. Max coverage (-): 0

Region: chr14 16780917-16780985. Max. coverage (+): 0. Max coverage (-): 6.88

Region: chr14 16780986-16781053. Max. coverage (+): 0. Max coverage (-): 1.89

Region: chr14 16781054-16781121. Max. coverage (+): 0. Max coverage (-): 2.17

Region: chr14 16781122-16781189. Max. coverage (+): 0. Max coverage (-): 0

Region: chr14 16781190-16781257. Max. coverage (+): 0. Max coverage (-): 0

Region: chr14 16781258-16781325. Max. coverage (+): 0. Max coverage (-): 0

Region: chr14 16781326-16781394. Max. coverage (+): 0. Max coverage (-): 0

Region: chr14 16781395-16781462. Max. coverage (+): 0. Max coverage (-): 0

Region: chr14 16781463-16781530. Max. coverage (+): 0. Max coverage (-): 0

Region: chr14 16781531-16781598. Max. coverage (+): 0. Max coverage (-): 0

Region: chr14 16781599-16781666. Max. coverage (+): 0. Max coverage (-): 2.4

Region: chr14 16781667-16781734. Max. coverage (+): 0. Max coverage (-): 0

Region: chr14 16781735-16781803. Max. coverage (+): 0. Max coverage (-): 0

Region: chr14 16781804-16781871. Max. coverage (+): 0. Max coverage (-): 6.13

Region: chr14 16781872-16781939. Max. coverage (+): 0. Max coverage (-): 1.1

Region: chr14 16781940-16782007. Max. coverage (+): 0. Max coverage (-): 8.85

Region: chr14 16782008-16782075. Max. coverage (+): 0. Max coverage (-): 8.25

Region: chr14 16782076-16782143. Max. coverage (+): 0. Max coverage (-): 8.77

Region: chr14 16782144-16782211. Max. coverage (+): 0. Max coverage (-): 9.07

Region: chr14 16782212-16782280. Max. coverage (+): 0. Max coverage (-): 12.05

Region: chr14 16782281-16782348. Max. coverage (+): 2.49. Max coverage (-): 4.43

Region: chr14 16782349-16782416. Max. coverage (+): 0. Max coverage (-): 2.94

Region: chr14 16782417-16782484. Max. coverage (+): 0. Max coverage (-): 0

Region: chr14 16782485-16782552. Max. coverage (+): 0. Max coverage (-): 0

Region: chr14 16782553-16782620. Max. coverage (+): 0. Max coverage (-): 8.41

Region: chr14 16782621-16782689. Max. coverage (+): 0. Max coverage (-): 10.73

Region: chr14 16782690-16782757. Max. coverage (+): 2.13. Max coverage (-): 8.2

Region: chr14 16782758-16782825. Max. coverage (+): 0. Max coverage (-): 5.39

Region: chr14 16782826-16782893. Max. coverage (+): 0. Max coverage (-): 2.91

Region: chr14 16782894-16782961. Max. coverage (+): 0. Max coverage (-): 10.77

Region: chr14 16782962-16783029. Max. coverage (+): 0.78. Max coverage (-): 8.93

Region: chr14 16783030-16783097. Max. coverage (+): 1.81. Max coverage (-): 8.99

Region: chr14 16783098-16783166. Max. coverage (+): 0. Max coverage (-): 2.81

Region: chr14 16783167-16783234. Max. coverage (+): 0. Max coverage (-): 0

Region: chr14 16783235-16783302. Max. coverage (+): 0. Max coverage (-): 0

Region: chr14 16783303-16783370. Max. coverage (+): 0. Max coverage (-): 0

Region: chr14 16783371-16783438. Max. coverage (+): 0. Max coverage (-): 0

Region: chr14 16783439-16783506. Max. coverage (+): 0. Max coverage (-): 0

Region: chr14 16783507-16783575. Max. coverage (+): 0. Max coverage (-): 0

Region: chr14 16783576-16783643. Max. coverage (+): 0. Max coverage (-): 0

Region: chr14 16783644-16783711. Max. coverage (+): 0. Max coverage (-): 0

Region: chr14 16783712-16783779. Max. coverage (+): 1.56. Max coverage (-): 1.27

Region: chr14 16783780-16783847. Max. coverage (+): 3.29. Max coverage (-): 0

Region: chr14 16783848-16783915. Max. coverage (+): 6.16. Max coverage (-): 0

Region: chr14 16783916-16783984. Max. coverage (+): 13.08. Max coverage (-): 0

Region: chr14 16783985-16784052. Max. coverage (+): 0. Max coverage (-): 0

Region: chr14 16784053-16784120. Max. coverage (+): 0. Max coverage (-): 0

Region: chr14 16784121-16784188. Max. coverage (+): 13.93. Max coverage (-): 0

Region: chr14 16784189-16784256. Max. coverage (+): 4.24. Max coverage (-): 0

Region: chr14 16784257-16784324. Max. coverage (+): 8.92. Max coverage (-): 0

Region: chr14 16784325-16784392. Max. coverage (+): 11.45. Max coverage (-): 0

Region: chr14 16784393-16784461. Max. coverage (+): 7.94. Max coverage (-): 0

Region: chr14 16784462-16784529. Max. coverage (+): 26.12. Max coverage (-): 0

Region: chr14 16784530-16784597. Max. coverage (+): 1.39. Max coverage (-): 0

Region: chr14 16784598-16784665. Max. coverage (+): 0. Max coverage (-): 0

Region: chr14 16784666-16784733. Max. coverage (+): 0. Max coverage (-): 0

Region: chr14 16784734-16784801. Max. coverage (+): 0. Max coverage (-): 0

Region: chr14 16784802-16784870. Max. coverage (+): 8.16. Max coverage (-): 0

Region: chr14 16784871-16784938. Max. coverage (+): 0. Max coverage (-): 0

Region: chr14 16784939-16785006. Max. coverage (+): 2.57. Max coverage (-): 0

Region: chr14 16785007-16785074. Max. coverage (+): 2.57. Max coverage (-): 0

Region: chr14 16785075-16785142. Max. coverage (+): 0. Max coverage (-): 0

Region: chr14 16785143-16785210. Max. coverage (+): 0. Max coverage (-): 0

Region: chr14 16785211-16785278. Max. coverage (+): 0. Max coverage (-): 0

Region: chr14 16785279-16785347. Max. coverage (+): 0.97. Max coverage (-): 0

Region: chr14 16785348-16785415. Max. coverage (+): 0.97. Max coverage (-): 0

Region: chr14 16785416-16785483. Max. coverage (+): 0. Max coverage (-): 0

Region: chr14 16785484-16785551. Max. coverage (+): 0. Max coverage (-): 0

Region: chr14 16785552-16785619. Max. coverage (+): 0. Max coverage (-): 0

Region: chr14 16785620-16785687. Max. coverage (+): 0. Max coverage (-): 0

Region: chr14 16785688-16785756. Max. coverage (+): 0. Max coverage (-): 0

Region: chr14 16785757-16785824. Max. coverage (+): 0. Max coverage (-): 0

Region: chr14 16785825-16785892. Max. coverage (+): 0. Max coverage (-): 0

Region: chr14 16785893-16785960. Max. coverage (+): 0. Max coverage (-): 0

Region: chr14 16785961-16786028. Max. coverage (+): 0. Max coverage (-): 0

Region: chr14 16786029-16786096. Max. coverage (+): 0. Max coverage (-): 0

Region: chr14 16786097-16786165. Max. coverage (+): 1.65. Max coverage (-): 0

Region: chr14 16786166-16786233. Max. coverage (+): 4.5. Max coverage (-): 0

Region: chr14 16786234-16786301. Max. coverage (+): 4.5. Max coverage (-): 0

Region: chr14 16786302-16786369. Max. coverage (+): 0. Max coverage (-): 0

Region: chr14 16786370-16786437. Max. coverage (+): 0. Max coverage (-): 0

Region: chr14 16786438-16786505. Max. coverage (+): 12.53. Max coverage (-): 0

Region: chr14 16786506-16786573. Max. coverage (+): 8.97. Max coverage (-): 0

Region: chr14 16786574-16786642. Max. coverage (+): 0. Max coverage (-): 0

Region: chr14 16786643-16786710. Max. coverage (+): 0. Max coverage (-): 0

Region: chr14 16786711-16786778. Max. coverage (+): 0. Max coverage (-): 0

Region: chr14 16786779-16786846. Max. coverage (+): 4.46. Max coverage (-): 0

Region: chr14 16786847-16786914. Max. coverage (+): 1.77. Max coverage (-): 0

Region: chr14 16786915-16786982. Max. coverage (+): 0. Max coverage (-): 0

Region: chr14 16786983-16787051. Max. coverage (+): 0. Max coverage (-): 0

Region: chr14 16787052-16787119. Max. coverage (+): 0. Max coverage (-): 0

Region: chr14 16787120-16787187. Max. coverage (+): 0. Max coverage (-): 0

Region: chr14 16787188-16787255. Max. coverage (+): 0. Max coverage (-): 0

Region: chr14 16787256-16787323. Max. coverage (+): 0. Max coverage (-): 0

Region: chr14 16787324-16787391. Max. coverage (+): 0. Max coverage (-): 0

Region: chr14 16787392-16787459. Max. coverage (+): 0. Max coverage (-): 0

Region: chr14 16787460-16787528. Max. coverage (+): 0. Max coverage (-): 0

Region: chr14 16787529-16787596. Max. coverage (+): 0. Max coverage (-): 0

Region: chr14 16787597-16787664. Max. coverage (+): 4.57. Max coverage (-): 0

Region: chr14 16787665-16787732. Max. coverage (+): 5.03. Max coverage (-): 0

Region: chr14 16787733-16787800. Max. coverage (+): 0. Max coverage (-): 0

Region: chr14 16787801-16787868. Max. coverage (+): 0. Max coverage (-): 0

Region: chr14 16787869-16787937. Max. coverage (+): 0. Max coverage (-): 0

Region: chr14 16787938-16788005. Max. coverage (+): 0. Max coverage (-): 0

Region: chr14 16788006-16788073. Max. coverage (+): 3.39. Max coverage (-): 0

Region: chr14 16788074-16788141. Max. coverage (+): 0. Max coverage (-): 0

Region: chr14 16788142-16788209. Max. coverage (+): 16.96. Max coverage (-): 0

Region: chr14 16788210-16788277. Max. coverage (+): 0.74. Max coverage (-): 0

Region: chr14 16788278-16788346. Max. coverage (+): 0. Max coverage (-): 0

Region: chr14 16788347-16788414. Max. coverage (+): 0. Max coverage (-): 0

Region: chr14 16788415-16788482. Max. coverage (+): 0. Max coverage (-): 0

Region: chr14 16788483-16788550. Max. coverage (+): 0. Max coverage (-): 0

Region: chr14 16788551-16788618. Max. coverage (+): 0. Max coverage (-): 0

Region: chr14 16788619-16788686. Max. coverage (+): 0. Max coverage (-): 0

Region: chr14 16788687-16788754. Max. coverage (+): 2.14. Max coverage (-): 0

Region: chr14 16788755-16788823. Max. coverage (+): 0. Max coverage (-): 0

Region: chr14 16788824-16788891. Max. coverage (+): 0. Max coverage (-): 0

Region: chr14 16788892-16788959. Max. coverage (+): 0. Max coverage (-): 0

Region: chr14 16788960-16789027. Max. coverage (+): 0. Max coverage (-): 0

Region: chr14 16789028-16789095. Max. coverage (+): 0. Max coverage (-): 0

Region: chr14 16789096-16789163. Max. coverage (+): 0. Max coverage (-): 0

Region: chr14 16789164-16789232. Max. coverage (+): 0. Max coverage (-): 0

Region: chr14 16789233-16789300. Max. coverage (+): 0. Max coverage (-): 0

Region: chr14 16789301-16789368. Max. coverage (+): 0. Max coverage (-): 0

Region: chr14 16789369-16789436. Max. coverage (+): 0. Max coverage (-): 0

Region: chr14 16789437-16789504. Max. coverage (+): 0. Max coverage (-): 0

Region: chr14 16789505-16789572. Max. coverage (+): 0. Max coverage (-): 0

Region: chr14 16789573-16789640. Max. coverage (+): 0. Max coverage (-): 0

Region: chr14 16789641-16789709. Max. coverage (+): 0. Max coverage (-): 0

Region: chr14 16789710-16789777. Max. coverage (+): 0. Max coverage (-): 0

Region: chr14 16789778-16789845. Max. coverage (+): 0. Max coverage (-): 0

Region: chr14 16789846-16789913. Max. coverage (+): 0. Max coverage (-): 0

Region: chr14 16789914-16789981. Max. coverage (+): 0. Max coverage (-): 0

Region: chr14 16789982-16790049. Max. coverage (+): 0. Max coverage (-): 0

Region: chr14 16790050-16790118. Max. coverage (+): 0.9. Max coverage (-): 0

Region: chr14 16790119-16790186. Max. coverage (+): 5.14. Max coverage (-): 0

Region: chr14 16790187-16790254. Max. coverage (+): 0. Max coverage (-): 0

Region: chr14 16790255-16790322. Max. coverage (+): 0. Max coverage (-): 0

Region: chr14 16790323-16790390. Max. coverage (+): 0. Max coverage (-): 0

Region: chr14 16790391-16790458. Max. coverage (+): 0. Max coverage (-): 0

Region: chr14 16790459-16790526. Max. coverage (+): 0. Max coverage (-): 0

Region: chr14 16790527-16790595. Max. coverage (+): 0. Max coverage (-): 0

Region: chr14 16790596-16790663. Max. coverage (+): 0. Max coverage (-): 0

Region: chr14 16790664-16790731. Max. coverage (+): 0. Max coverage (-): 0

Region: chr14 16790732-16790799. Max. coverage (+): 0. Max coverage (-): 0

Region: chr14 16790800-16790867. Max. coverage (+): 0. Max coverage (-): 0

Region: chr14 16790868-16790935. Max. coverage (+): 0. Max coverage (-): 0

Region: chr14 16790936-16791004. Max. coverage (+): 0. Max coverage (-): 0

Region: chr14 16791005-16791072. Max. coverage (+): 3.69. Max coverage (-): 0

Region: chr14 16791073-16791140. Max. coverage (+): 1.1. Max coverage (-): 0

Region: chr14 16791141-16791208. Max. coverage (+): 2.59. Max coverage (-): 0

Region: chr14 16791209-16791276. Max. coverage (+): 0. Max coverage (-): 0

Region: chr14 16791277-16791344. Max. coverage (+): 0.46. Max coverage (-): 0

Region: chr14 16791345-16791413. Max. coverage (+): 0. Max coverage (-): 0

Region: chr14 16791414-16791481. Max. coverage (+): 1.61. Max coverage (-): 0

Region: chr14 16791482-16791549. Max. coverage (+): 0. Max coverage (-): 0

Region: chr14 16791550-16791617. Max. coverage (+): 0. Max coverage (-): 0

Region: chr14 16791618-16791685. Max. coverage (+): 0. Max coverage (-): 0

Region: chr14 16791686-16791753. Max. coverage (+): 0. Max coverage (-): 0

Region: chr14 16791754-16791821. Max. coverage (+): 0. Max coverage (-): 0

Region: chr14 16791822-16791890. Max. coverage (+): 0. Max coverage (-): 0

Region: chr14 16791891-16791958. Max. coverage (+): 2.2. Max coverage (-): 0

Region: chr14 16791959-16792026. Max. coverage (+): 1.25. Max coverage (-): 0

Region: chr14 16792027-16792094. Max. coverage (+): 0. Max coverage (-): 0

Region: chr14 16792095-16792162. Max. coverage (+): 0. Max coverage (-): 0

Region: chr14 16792163-16792230. Max. coverage (+): 2.74. Max coverage (-): 0

Region: chr14 16792231-16792299. Max. coverage (+): 0. Max coverage (-): 0

Region: chr14 16792300-16792367. Max. coverage (+): 0. Max coverage (-): 0

Region: chr14 16792368-16792435. Max. coverage (+): 1.76. Max coverage (-): 0

Region: chr14 16792436-16792503. Max. coverage (+): 0. Max coverage (-): 0

Region: chr14 16792504-16792571. Max. coverage (+): 0. Max coverage (-): 0

Region: chr14 16792572-16792639. Max. coverage (+): 0. Max coverage (-): 0

Region: chr14 16792640-16792707. Max. coverage (+): 0. Max coverage (-): 0

Region: chr14 16792708-16792776. Max. coverage (+): 0. Max coverage (-): 0

Region: chr14 16792777-16792844. Max. coverage (+): 0. Max coverage (-): 0

Region: chr14 16792845-16792912. Max. coverage (+): 3.01. Max coverage (-): 0

Region: chr14 16792913-16792980. Max. coverage (+): 12.79. Max coverage (-): 0

Region: chr14 16792981-16793048. Max. coverage (+): 52.28. Max coverage (-): 0

Region: chr14 16793049-16793116. Max. coverage (+): 48.3. Max coverage (-): 0

Region: chr14 16793117-16793185. Max. coverage (+): 26.07. Max coverage (-): 0

Region: chr14 16793186-16793253. Max. coverage (+): 6.84. Max coverage (-): 0

Region: chr14 16793254-16793321. Max. coverage (+): 0. Max coverage (-): 0

Region: chr14 16793322-16793389. Max. coverage (+): 0. Max coverage (-): 0

Region: chr14 16793390-16793457. Max. coverage (+): 0. Max coverage (-): 0

Region: chr14 16793458-16793525. Max. coverage (+): 0. Max coverage (-): 0

Region: chr14 16793526-16793594. Max. coverage (+): 0. Max coverage (-): 0

Region: chr14 16793595-16793662. Max. coverage (+): 0. Max coverage (-): 0

Region: chr14 16793663-16793730. Max. coverage (+): 0. Max coverage (-): 0

Region: chr14 16793731-16793798. Max. coverage (+): 0. Max coverage (-): 0

Region: chr14 16793799-16793866. Max. coverage (+): 0. Max coverage (-): 0

Region: chr14 16793867-16793934. Max. coverage (+): 0. Max coverage (-): 0

Region: chr14 16793935-16794002. Max. coverage (+): 0. Max coverage (-): 0

Region: chr14 16794003-16794071. Max. coverage (+): 0. Max coverage (-): 0

Region: chr14 16794072-16794139. Max. coverage (+): 0. Max coverage (-): 0

Region: chr14 16794140-16794207. Max. coverage (+): 0. Max coverage (-): 0

Region: chr14 16794208-16794275. Max. coverage (+): 0. Max coverage (-): 0

Region: chr14 16794276-16794343. Max. coverage (+): 11.07. Max coverage (-): 0

Region: chr14 16794344-16794411. Max. coverage (+): 10.56. Max coverage (-): 0

Region: chr14 16794412-16794480. Max. coverage (+): 0. Max coverage (-): 0

Region: chr14 16794481-16794548. Max. coverage (+): 0. Max coverage (-): 0

Region: chr14 16794549-16794616. Max. coverage (+): 0. Max coverage (-): 0

Region: chr14 16794617-16794684. Max. coverage (+): 75.79. Max coverage (-): 0

Region: chr14 16794685-16794752. Max. coverage (+): 4.18. Max coverage (-): 0

Region: chr14 16794753-16794820. Max. coverage (+): 20.62. Max coverage (-): 0

Region: chr14 16794821-16794888. Max. coverage (+): 13.75. Max coverage (-): 0

Region: chr14 16794889-16794957. Max. coverage (+): 0. Max coverage (-): 0

Region: chr14 16794958-16795025. Max. coverage (+): 0. Max coverage (-): 0

Region: chr14 16795026-16795093. Max. coverage (+): 0. Max coverage (-): 0

Region: chr14 16795094-16795161. Max. coverage (+): 0. Max coverage (-): 0

Region: chr14 16795162-16795229. Max. coverage (+): 6.05. Max coverage (-): 0

Region: chr14 16795230-16795297. Max. coverage (+): 6.92. Max coverage (-): 0

Region: chr14 16795298-16795366. Max. coverage (+): 13.14. Max coverage (-): 0

Region: chr14 16795367-16795434. Max. coverage (+): 3.97. Max coverage (-): 0

Region: chr14 16795435-16795502. Max. coverage (+): 0. Max coverage (-): 0

Region: chr14 16795503-16795570. Max. coverage (+): 0. Max coverage (-): 0

Region: chr14 16795571-16795638. Max. coverage (+): 0. Max coverage (-): 0

Region: chr14 16795639-16795706. Max. coverage (+): 0. Max coverage (-): 0

Region: chr14 16795707-16795775. Max. coverage (+): 54.52. Max coverage (-): 0

Region: chr14 16795776-16795843. Max. coverage (+): 2.84. Max coverage (-): 0

Region: chr14 16795844-16795911. Max. coverage (+): 0. Max coverage (-): 0

Region: chr14 16795912-16795979. Max. coverage (+): 0. Max coverage (-): 0

Region: chr14 16795980-16796047. Max. coverage (+): 0. Max coverage (-): 0

Region: chr14 16796048-16796115. Max. coverage (+): 1.69. Max coverage (-): 0

Region: chr14 16796116-16796183. Max. coverage (+): 7.64. Max coverage (-): 0

Region: chr14 16796184-16796252. Max. coverage (+): 4.82. Max coverage (-): 0

Region: chr14 16796253-16796320. Max. coverage (+): 14.03. Max coverage (-): 0

Region: chr14 16796321-16796388. Max. coverage (+): 0.49. Max coverage (-): 0

Region: chr14 16796389-16796456. Max. coverage (+): 0.15. Max coverage (-): 0

Region: chr14 16796457-16796524. Max. coverage (+): 0. Max coverage (-): 0

Region: chr14 16796525-16796592. Max. coverage (+): 0. Max coverage (-): 0

Region: chr14 16796593-16796661. Max. coverage (+): 34.85. Max coverage (-): 0

Region: chr14 16796662-16796729. Max. coverage (+): 12.64. Max coverage (-): 0

Region: chr14 16796730-16796797. Max. coverage (+): 0. Max coverage (-): 0

Region: chr14 16796798-16796865. Max. coverage (+): 0. Max coverage (-): 0

Region: chr14 16796866-16796933. Max. coverage (+): 0. Max coverage (-): 0

Region: chr14 16796934-16797001. Max. coverage (+): 9.34. Max coverage (-): 0

Region: chr14 16797002-16797069. Max. coverage (+): 10.6. Max coverage (-): 0

Region: chr14 16797070-16797138. Max. coverage (+): 13. Max coverage (-): 0

Region: chr14 16797139-16797206. Max. coverage (+): 21.13. Max coverage (-): 0

Region: chr14 16797207-16797274. Max. coverage (+): 17.87. Max coverage (-): 0

Region: chr14 16797275-16797342. Max. coverage (+): 38.92. Max coverage (-): 0

Region: chr14 16797343-16797410. Max. coverage (+): 5.64. Max coverage (-): 0

Region: chr14 16797411-16797478. Max. coverage (+): 12.92. Max coverage (-): 0

Region: chr14 16797479-16797547. Max. coverage (+): 10.43. Max coverage (-): 0

Region: chr14 16797548-16797615. Max. coverage (+): 0. Max coverage (-): 0

Region: chr14 16797616-16797683. Max. coverage (+): 0. Max coverage (-): 0

Region: chr14 16797684-16797751. Max. coverage (+): 0. Max coverage (-): 0

Region: chr14 16797752-16797819. Max. coverage (+): 25.1. Max coverage (-): 0

Region: chr14 16797820-16797887. Max. coverage (+): 14.85. Max coverage (-): 0

Region: chr14 16797888-16797955. Max. coverage (+): 0. Max coverage (-): 0

Region: chr14 16797956-16798024. Max. coverage (+): 0. Max coverage (-): 0

Region: chr14 16798025-16798092. Max. coverage (+): 0. Max coverage (-): 0

Region: chr14 16798093-16798160. Max. coverage (+): 0. Max coverage (-): 0

Region: chr14 16798161-16798228. Max. coverage (+): 0. Max coverage (-): 0

Region: chr14 16798229-16798296. Max. coverage (+): 0. Max coverage (-): 0

Region: chr14 16798297-16798364. Max. coverage (+): 0. Max coverage (-): 0

Region: chr14 16798365-16798433. Max. coverage (+): 1.32. Max coverage (-): 0

Region: chr14 16798434-16798501. Max. coverage (+): 0. Max coverage (-): 0

Region: chr14 16798502-16798569. Max. coverage (+): 0. Max coverage (-): 0

Region: chr14 16798570-16798637. Max. coverage (+): 0. Max coverage (-): 0

Region: chr14 16798638-16798705. Max. coverage (+): 23.23. Max coverage (-): 0

Region: chr14 16798706-16798773. Max. coverage (+): 0. Max coverage (-): 0

Region: chr14 16798774-16798842. Max. coverage (+): 0. Max coverage (-): 0

Region: chr14 16798843-16798910. Max. coverage (+): 0. Max coverage (-): 0

Region: chr14 16798911-16798978. Max. coverage (+): 0. Max coverage (-): 0

Region: chr14 16798979-16799046. Max. coverage (+): 0. Max coverage (-): 0

Region: chr14 16799047-16799114. Max. coverage (+): 0. Max coverage (-): 0

Region: chr14 16799115-16799182. Max. coverage (+): 10.53. Max coverage (-): 0

Region: chr14 16799183-16799250. Max. coverage (+): 17.21. Max coverage (-): 0

Region: chr14 16799251-16799319. Max. coverage (+): 17.21. Max coverage (-): 0

Region: chr14 16799320-16799387. Max. coverage (+): 5.07. Max coverage (-): 0

Region: chr14 16799388-16799455. Max. coverage (+): 9.01. Max coverage (-): 0

Region: chr14 16799456-16799523. Max. coverage (+): 5.56. Max coverage (-): 0

Region: chr14 16799524-16799591. Max. coverage (+): 2.08. Max coverage (-): 0

Region: chr14 16799592-16799659. Max. coverage (+): 0. Max coverage (-): 0

Region: chr14 16799660-16799728. Max. coverage (+): 0. Max coverage (-): 0

Region: chr14 16799729-16799796. Max. coverage (+): 9.44. Max coverage (-): 0

Region: chr14 16799797-16799864. Max. coverage (+): 5.87. Max coverage (-): 0

Region: chr14 16799865-16799932. Max. coverage (+): 2.84. Max coverage (-): 0

Region: chr14 16799933-16800000. Max. coverage (+): 1.04. Max coverage (-): 0

Region: chr14 16800001-16800068. Max. coverage (+): 0. Max coverage (-): 0

Region: chr14 16800069-16800136. Max. coverage (+): 0. Max coverage (-): 0

Region: chr14 16800137-16800205. Max. coverage (+): 0. Max coverage (-): 0

Region: chr14 16800206-16800273. Max. coverage (+): 0. Max coverage (-): 0

Region: chr14 16800274-16800341. Max. coverage (+): 0. Max coverage (-): 0

Region: chr14 16800342-16800409. Max. coverage (+): 0. Max coverage (-): 0

Region: chr14 16800410-16800477. Max. coverage (+): 0. Max coverage (-): 0

Region: chr14 16800478-16800545. Max. coverage (+): 0. Max coverage (-): 0

Region: chr14 16800546-16800614. Max. coverage (+): 0. Max coverage (-): 0

Region: chr14 16800615-16800682. Max. coverage (+): 0. Max coverage (-): 0

Region: chr14 16800683-16800750. Max. coverage (+): 2.18. Max coverage (-): 0

Region: chr14 16800751-16800818. Max. coverage (+): 0. Max coverage (-): 0

Region: chr14 16800819-16800886. Max. coverage (+): 0. Max coverage (-): 0

Region: chr14 16800887-16800954. Max. coverage (+): 0. Max coverage (-): 0

Region: chr14 16800955-16801023. Max. coverage (+): 0. Max coverage (-): 0

Region: chr14 16801024-16801091. Max. coverage (+): 0. Max coverage (-): 0

Region: chr14 16801092-16801159. Max. coverage (+): 0. Max coverage (-): 0

Region: chr14 16801160-16801227. Max. coverage (+): 0. Max coverage (-): 0

Region: chr14 16801228-16801295. Max. coverage (+): 0. Max coverage (-): 0

Region: chr14 16801296-16801363. Max. coverage (+): 0. Max coverage (-): 0

Region: chr14 16801364-16801431. Max. coverage (+): 0. Max coverage (-): 0

Region: chr14 16801432-16801500. Max. coverage (+): 0. Max coverage (-): 0

Region: chr14 16801501-16801568. Max. coverage (+): 0. Max coverage (-): 0

Region: chr14 16801569-16801636. Max. coverage (+): 0. Max coverage (-): 0

Region: chr14 16801637-16801704. Max. coverage (+): 3.57. Max coverage (-): 0

Region: chr14 16801705-16801772. Max. coverage (+): 0. Max coverage (-): 0

Region: chr14 16801773-16801840. Max. coverage (+): 0. Max coverage (-): 0

Region: chr14 16801841-16801909. Max. coverage (+): 0. Max coverage (-): 0

Region: chr14 16801910-16801977. Max. coverage (+): 0. Max coverage (-): 0

Region: chr14 16801978-16802045. Max. coverage (+): 0. Max coverage (-): 0

Region: chr14 16802046-16802113. Max. coverage (+): 0. Max coverage (-): 0

Region: chr14 16802114-16802181. Max. coverage (+): 0. Max coverage (-): 0

Region: chr14 16802182-16802249. Max. coverage (+): 0. Max coverage (-): 0

Region: chr14 16802250-16802317. Max. coverage (+): 0. Max coverage (-): 0

Region: chr14 16802318-16802386. Max. coverage (+): 1.03. Max coverage (-): 0

Region: chr14 16802387-16802454. Max. coverage (+): 4.35. Max coverage (-): 0

Region: chr14 16802455-16802522. Max. coverage (+): 68.01. Max coverage (-): 0

Region: chr14 16802523-16802590. Max. coverage (+): 40.01. Max coverage (-): 0

Region: chr14 16802591-16802658. Max. coverage (+): 0. Max coverage (-): 0

Region: chr14 16802659-16802726. Max. coverage (+): 5.08. Max coverage (-): 0

Region: chr14 16802727-16802795. Max. coverage (+): 16.19. Max coverage (-): 0

Region: chr14 16802796-16802863. Max. coverage (+): 0. Max coverage (-): 0

Region: chr14 16802864-16802931. Max. coverage (+): 0. Max coverage (-): 0

Region: chr14 16802932-16802999. Max. coverage (+): 0. Max coverage (-): 0

Region: chr14 16803000-16803067. Max. coverage (+): 0. Max coverage (-): 0

Region: chr14 16803068-16803135. Max. coverage (+): 0. Max coverage (-): 0

Region: chr14 16803136-16803204. Max. coverage (+): 0. Max coverage (-): 0

Region: chr14 16803205-16803272. Max. coverage (+): 0. Max coverage (-): 0

Region: chr14 16803273-16803340. Max. coverage (+): 0. Max coverage (-): 0

Region: chr14 16803341-16803408. Max. coverage (+): 0. Max coverage (-): 0

Region: chr14 16803409-16803476. Max. coverage (+): 0. Max coverage (-): 0

Region: chr14 16803477-16803544. Max. coverage (+): 0. Max coverage (-): 0

Region: chr14 16803545-16803612. Max. coverage (+): 0. Max coverage (-): 0

Region: chr14 16803613-16803681. Max. coverage (+): 0. Max coverage (-): 0

Region: chr14 16803682-16803749. Max. coverage (+): 0. Max coverage (-): 0

Region: chr14 16803750-16803817. Max. coverage (+): 0. Max coverage (-): 0

Region: chr14 16803818-16803885. Max. coverage (+): 0. Max coverage (-): 0

Region: chr14 16803886-16803953. Max. coverage (+): 10.4. Max coverage (-): 0

Region: chr14 16803954-16804021. Max. coverage (+): 12.99. Max coverage (-): 0

Region: chr14 16804022-16804090. Max. coverage (+): 18.36. Max coverage (-): 0

Region: chr14 16804091-16804158. Max. coverage (+): 21.6. Max coverage (-): 0

Region: chr14 16804159-16804226. Max. coverage (+): 0. Max coverage (-): 0

Region: chr14 16804227-16804294. Max. coverage (+): 9.66. Max coverage (-): 0

Region: chr14 16804295-16804362. Max. coverage (+): 29.18. Max coverage (-): 0

Region: chr14 16804363-16804430. Max. coverage (+): 7.12. Max coverage (-): 0

Region: chr14 16804431-16804498. Max. coverage (+): 0. Max coverage (-): 0

Region: chr14 16804499-16804567. Max. coverage (+): 0. Max coverage (-): 0

Region: chr14 16804568-16804635. Max. coverage (+): 2.46. Max coverage (-): 0

Region: chr14 16804636-16804703. Max. coverage (+): 67.48. Max coverage (-): 0

Region: chr14 16804704-16804771. Max. coverage (+): 16.76. Max coverage (-): 0

Region: chr14 16804772-16804839. Max. coverage (+): 60.23. Max coverage (-): 0

Region: chr14 16804840-16804907. Max. coverage (+): 0. Max coverage (-): 0

Region: chr14 16804908-16804976. Max. coverage (+): 0. Max coverage (-): 0

Region: chr14 16804977-16805044. Max. coverage (+): 0. Max coverage (-): 0

Region: chr14 16805045-16805112. Max. coverage (+): 15.35. Max coverage (-): 0

Region: chr14 16805113-16805180. Max. coverage (+): 29.23. Max coverage (-): 0

Region: chr14 16805181-16805248. Max. coverage (+): 48.82. Max coverage (-): 0

Region: chr14 16805249-16805316. Max. coverage (+): 38.57. Max coverage (-): 0

Region: chr14 16805317-16805385. Max. coverage (+): 19.78. Max coverage (-): 0

Region: chr14 16805386-16805453. Max. coverage (+): 12.35. Max coverage (-): 0

Region: chr14 16805454-16805521. Max. coverage (+): 12.85. Max coverage (-): 0

Region: chr14 16805522-16805589. Max. coverage (+): 52.15. Max coverage (-): 0

Region: chr14 16805590-16805657. Max. coverage (+): 29.3. Max coverage (-): 0

Region: chr14 16805658-16805725. Max. coverage (+): 39.88. Max coverage (-): 0

Region: chr14 16805726-16805793. Max. coverage (+): 73.22. Max coverage (-): 0

Region: chr14 16805794-16805862. Max. coverage (+): 28.91. Max coverage (-): 0

Region: chr14 16805863-16805930. Max. coverage (+): 0. Max coverage (-): 0

Region: chr14 16805931-16805998. Max. coverage (+): 0. Max coverage (-): 0

Region: chr14 16805999-16806066. Max. coverage (+): 0. Max coverage (-): 0

Region: chr14 16806067-16806134. Max. coverage (+): 4.29. Max coverage (-): 0

Region: chr14 16806135-16806202. Max. coverage (+): 9.04. Max coverage (-): 0

Region: chr14 16806203-16806271. Max. coverage (+): 18.48. Max coverage (-): 0

Region: chr14 16806272-16806339. Max. coverage (+): 0. Max coverage (-): 0

Region: chr14 16806340-16806407. Max. coverage (+): 33.52. Max coverage (-): 0

Region: chr14 16806408-16806475. Max. coverage (+): 41.95. Max coverage (-): 0

Region: chr14 16806476-16806543. Max. coverage (+): 19.04. Max coverage (-): 0

Region: chr14 16806544-16806611. Max. coverage (+): 15.3. Max coverage (-): 0

Region: chr14 16806612-16806679. Max. coverage (+): 12.25. Max coverage (-): 0

Region: chr14 16806680-16806748. Max. coverage (+): 0. Max coverage (-): 0

Region: chr14 16806749-16806816. Max. coverage (+): 1.99. Max coverage (-): 0

Region: chr14 16806817-16806884. Max. coverage (+): 0. Max coverage (-): 0

Region: chr14 16806885-16806952. Max. coverage (+): 10.96. Max coverage (-): 0

Region: chr14 16806953-16807020. Max. coverage (+): 11.57. Max coverage (-): 0

Region: chr14 16807021-16807088. Max. coverage (+): 11.57. Max coverage (-): 0

Region: chr14 16807089-16807157. Max. coverage (+): 0. Max coverage (-): 0

Region: chr14 16807158-16807225. Max. coverage (+): 0. Max coverage (-): 0

Region: chr14 16807226-16807293. Max. coverage (+): 0. Max coverage (-): 0

Region: chr14 16807294-16807361. Max. coverage (+): 0. Max coverage (-): 0

Region: chr14 16807362-16807429. Max. coverage (+): 0. Max coverage (-): 0

Region: chr14 16807430-16807497. Max. coverage (+): 0. Max coverage (-): 0

Region: chr14 16807498-16807565. Max. coverage (+): 0. Max coverage (-): 0

Region: chr14 16807566-16807634. Max. coverage (+): 4.66. Max coverage (-): 0

Region: chr14 16807635-16807702. Max. coverage (+): 13.67. Max coverage (-): 0

Region: chr14 16807703-16807770. Max. coverage (+): 6.04. Max coverage (-): 0

Region: chr14 16807771-16807838. Max. coverage (+): 9.36. Max coverage (-): 0

Region: chr14 16807839-16807906. Max. coverage (+): 9.36. Max coverage (-): 0

Region: chr14 16807907-16807974. Max. coverage (+): 0. Max coverage (-): 0

Region: chr14 16807975-16808043. Max. coverage (+): 3.01. Max coverage (-): 0

Region: chr14 16808044-16808111. Max. coverage (+): 9.51. Max coverage (-): 0

Region: chr14 16808112-16808179. Max. coverage (+): 0. Max coverage (-): 0

Region: chr14 16808180-16808247. Max. coverage (+): 0. Max coverage (-): 0

Region: chr14 16808248-16808315. Max. coverage (+): 0. Max coverage (-): 0

Region: chr14 16808316-16808383. Max. coverage (+): 0. Max coverage (-): 0

Region: chr14 16808384-16808452. Max. coverage (+): 8.52. Max coverage (-): 0

Region: chr14 16808453-16808520. Max. coverage (+): 11.01. Max coverage (-): 0

Region: chr14 16808521-16808588. Max. coverage (+): 0. Max coverage (-): 0

Region: chr14 16808589-16808656. Max. coverage (+): 0. Max coverage (-): 0

Region: chr14 16808657-16808724. Max. coverage (+): 14.42. Max coverage (-): 0

Region: chr14 16808725-16808792. Max. coverage (+): 8.17. Max coverage (-): 0

Region: chr14 16808793-16808860. Max. coverage (+): 28.42. Max coverage (-): 0

Region: chr14 16808861-16808929. Max. coverage (+): 7.22. Max coverage (-): 0

Region: chr14 16808930-16808997. Max. coverage (+): 14.62. Max coverage (-): 0

Region: chr14 16808998-16809065. Max. coverage (+): 23.69. Max coverage (-): 0

Region: chr14 16809066-16809133. Max. coverage (+): 22.21. Max coverage (-): 0

Region: chr14 16809134-16809201. Max. coverage (+): 36.9. Max coverage (-): 0

Region: chr14 16809202-16809269. Max. coverage (+): 11.94. Max coverage (-): 0

Region: chr14 16809270-16809338. Max. coverage (+): 19.43. Max coverage (-): 0

Region: chr14 16809339-16809406. Max. coverage (+): 5.16. Max coverage (-): 0

Region: chr14 16809407-16809474. Max. coverage (+): 11.68. Max coverage (-): 0

Region: chr14 16809475-16809542. Max. coverage (+): 4.1. Max coverage (-): 0

Region: chr14 16809543-16809610. Max. coverage (+): 10.95. Max coverage (-): 0

Region: chr14 16809611-16809678. Max. coverage (+): 4.03. Max coverage (-): 0

Region: chr14 16809679-16809746. Max. coverage (+): 8.59. Max coverage (-): 0

Region: chr14 16809747-16809815. Max. coverage (+): 3.51. Max coverage (-): 0

Region: chr14 16809816-16809883. Max. coverage (+): 3.16. Max coverage (-): 0

Region: chr14 16809884-16809951. Max. coverage (+): 2.07. Max coverage (-): 0

Region: chr14 16809952-16810019. Max. coverage (+): 2.07. Max coverage (-): 0

Region: chr14 16810020-16810087. Max. coverage (+): 0. Max coverage (-): 0

Region: chr14 16810088-16810155. Max. coverage (+): 1.48. Max coverage (-): 0

Region: chr14 16810156-. Max. coverage (+): 3.99. Max coverage (-): 0

RepeatMasker Color Code

**+**

100-98% Identity

<98-95% Identity

<95-90% Identity

<90-85% Identity

<85-80% Identity

<80-75% Identity

<75-70% Identity

<70% Identity

**-**

Gene Set Color Code

**+**

Gene

Pseudogene

**-**

Topology/Coverage Color Code

Coverage Plus Strand

Coverage Minus Strand

Mainstrand: Plus

Mainstrand: Minus

Complementary Strand

Flanking Region  
(if option -flank >0)

Gene Set Annotation  
  
RepeatMasker Annotation  

**1. Bov-tA2**: 16776223-16776430 (-), Divergence to consensus: 15.3%  
**2. MER20**: 16776509-16776640 (+), Divergence to consensus: 29.6%  
**3. ART2A**: 16776704-16776823 (-), Divergence to consensus: 15.9%  
**4. L1ME4c**: 16776920-16777096 (-), Divergence to consensus: 37.4%  
**5. L1M5**: 16777308-16777528 (-), Divergence to consensus: 45.8%  
**6. CHR-2A**: 16778140-16778399 (+), Divergence to consensus: 38.1%  
**7. MLT1G**: 16778545-16778785 (-), Divergence to consensus: 34%  
**8. L1MC4**: 16778881-16779027 (-), Divergence to consensus: 48.3%  
**9. L2b**: 16779036-16779303 (-), Divergence to consensus: 40%  
**10. MIRb**: 16780536-16780657 (+), Divergence to consensus: 39%  
**11. MIR**: 16780715-16780882 (+), Divergence to consensus: 39.8%  
**12. CHR-2\_BT**: 16781121-16781310 (+), Divergence to consensus: 33.2%  
**13. Bov-tA1**: 16781311-16781530 (-), Divergence to consensus: 20.6%  
**14. CHR-2\_BT**: 16781531-16781582 (+), Divergence to consensus: 33.2%  
**15. LTR16**: 16782532-16782584 (+), Divergence to consensus: 22.6%  
**16. L2b**: 16783145-16783210 (-), Divergence to consensus: 33.4%  
**17. MLT1I**: 16783315-16783628 (-), Divergence to consensus: 39.1%  
**18. MIRc**: 16783986-16784119 (+), Divergence to consensus: 31.5%  
**19. MIR3**: 16784568-16784654 (-), Divergence to consensus: 32.6%  
**20. LTR86A1**: 16785042-16785154 (+), Divergence to consensus: 36.1%  
**21. Bov-tA2**: 16785191-16785333 (+), Divergence to consensus: 14.1%  
**22. Bov-tA1**: 16785376-16785594 (+), Divergence to consensus: 14.8%  
**23. LTR86A1**: 16785614-16785880 (+), Divergence to consensus: 46.3%  
**24. Bov-tA2**: 16785923-16786117 (+), Divergence to consensus: 18%  
**25. L1-2\_BT**: 16786331-16786423 (-), Divergence to consensus: 16.2%  
**26. MLT1J**: 16786603-16786827 (-), Divergence to consensus: 49.9%  
**27. MLT1J-int**: 16786922-16787355 (-), Divergence to consensus: 45.4%  
**28. BOV-A2**: 16787356-16787616 (-), Divergence to consensus: 5%  
**29. MLT1J-int**: 16787736-16788010 (-), Divergence to consensus: 42.2%  
**30. MLT1G**: 16788367-16788681 (+), Divergence to consensus: 37.4%  
**31. CHRL1\_BT**: 16788762-16788878 (+), Divergence to consensus: 36.9%  
**32. L1MC5a**: 16788903-16788979 (-), Divergence to consensus: 22.1%  
**33. L1\_BT**: 16788976-16789385 (-), Divergence to consensus: 17.7%  
**34. BTLTR1J**: 16789386-16789437 (+), Divergence to consensus: 23.1%  
**35. L1\_BT**: 16789438-16790080 (-), Divergence to consensus: 17.7%  
**36. MER20**: 16790234-16790392 (-), Divergence to consensus: 42.6%  
**37. L1MC5a**: 16790398-16790562 (-), Divergence to consensus: 28.2%  
**38. ART2A**: 16790563-16790680 (+), Divergence to consensus: 20.7%  
**39. L1MC5a**: 16790684-16790930 (-), Divergence to consensus: 38.6%  
**40. L1MC5a**: 16791499-16791816 (-), Divergence to consensus: 46.6%  
**41. L3**: 16792035-16792152 (-), Divergence to consensus: 41.5%  
**42. Bov-tA2**: 16792227-16792400 (-), Divergence to consensus: 34.2%  
**43. Bov-tA2**: 16792444-16792576 (-), Divergence to consensus: 18.8%  
**44. BOV-A2**: 16792593-16792863 (-), Divergence to consensus: 5.2%  
**45. MIR**: 16793275-16793504 (+), Divergence to consensus: 41.4%  
**46. MIR**: 16793512-16793645 (+), Divergence to consensus: 43.8%  
**47. Bov-tA1**: 16793646-16793869 (-), Divergence to consensus: 13.8%  
**48. MIR**: 16793870-16793955 (+), Divergence to consensus: 43.8%  
**49. MamGypLTR1c**: 16793990-16794253 (-), Divergence to consensus: 48.9%  
**50. CHR-2\_BT**: 16794397-16794627 (+), Divergence to consensus: 33.4%  
**51. L2c**: 16794864-16794975 (-), Divergence to consensus: 39.7%  
**52. MER20**: 16794980-16795151 (+), Divergence to consensus: 18.6%  
**53. MIR**: 16795638-16795715 (-), Divergence to consensus: 28.2%  
**54. L2b**: 16795826-16796034 (+), Divergence to consensus: 42.3%  
**55. CHRL**: 16796433-16796588 (+), Divergence to consensus: 30.5%  
**56. Bov-tA1**: 16796691-16796936 (-), Divergence to consensus: 30.7%  
**57. L2b**: 16797321-16797356 (+), Divergence to consensus: 22.2%  
**58. L2b**: 16797507-16797771 (+), Divergence to consensus: 47.2%  
**59. MLT1F**: 16797860-16798411 (+), Divergence to consensus: 43.1%  
**60. Bov-tA2**: 16798449-16798655 (-), Divergence to consensus: 15.5%  
**61. L2a**: 16798714-16798897 (-), Divergence to consensus: 36.7%  
**62. MIRb**: 16798915-16799112 (-), Divergence to consensus: 36.9%  
**63. MIR**: 16799546-16799738 (-), Divergence to consensus: 47.9%  
**64. L2b**: 16799842-16799899 (-), Divergence to consensus: 25.9%  
**65. L2a**: 16800265-16800519 (-), Divergence to consensus: 45.8%  
**66. MIRc**: 16800555-16800655 (-), Divergence to consensus: 36.3%  
**67. L2b**: 16800779-16801342 (-), Divergence to consensus: 46.2%  
**68. L1-3\_BT**: 16801387-16801541 (-), Divergence to consensus: 7.2%  
**69. L1MD1**: 16801543-16801664 (-), Divergence to consensus: 27.2%  
**70. Bov-tA1**: 16801704-16801924 (-), Divergence to consensus: 14.4%  
**71. L1MD1**: 16801926-16802276 (-), Divergence to consensus: 24.1%  
**72. L2b**: 16802278-16802357 (-), Divergence to consensus: 32.6%  
**73. MIRb**: 16802571-16802689 (-), Divergence to consensus: 34.8%  
**74. Charlie1a**: 16802803-16803060 (+), Divergence to consensus: 23.1%  
**75. Charlie1**: 16803066-16803453 (+), Divergence to consensus: 32.6%  
**76. Bov-tA3**: 16803452-16803549 (-), Divergence to consensus: 13.4%  
**77. Charlie1**: 16803541-16803947 (+), Divergence to consensus: 33.2%  
**78. Charlie1**: 16804429-16804617 (+), Divergence to consensus: 31%  
**79. MIRc**: 16804839-16805045 (+), Divergence to consensus: 49.8%  
**80. CHR-2B**: 16805853-16806086 (-), Divergence to consensus: 49.1%  
**81. L2b**: 16806311-16806355 (-), Divergence to consensus: 17.8%  
**82. MIRb**: 16806647-16806682 (-), Divergence to consensus: 25%  
**83. MLT1J**: 16806687-16806751 (+), Divergence to consensus: 27.7%  
**84. MLT1J**: 16806817-16806889 (+), Divergence to consensus: 43.8%  
**85. MLT1A0**: 16807094-16807229 (-), Divergence to consensus: 25.8%  
**86. Bov-tA1**: 16807241-16807458 (-), Divergence to consensus: 17.5%  
**87. MLT1A0**: 16807461-16807584 (-), Divergence to consensus: 22.4%  
**88. MIRb**: 16808144-16808284 (-), Divergence to consensus: 36.6%  
**89. CHRL1\_BT**: 16808324-16808394 (+), Divergence to consensus: 20.3%  
**90. L4\_C\_Mam**: 16808516-16808581 (+), Divergence to consensus: 28.8%  
**91. AT\_rich**: 16809270-16809301 (+), Divergence to consensus: 50%  
**92. L2c**: 16809978-16810104 (-), Divergence to consensus: 37.3%

  
Transcription Factor Binding Sites  

**RFX4\_2** (Sequence: GTATCCATG (-): 16783033)  
**RFX4\_2** (Sequence: GTATCCAGG (-): 16797823)  
**RFX4\_1** (Sequence: CTTGGCAAC (+): 16802485)  
**SPZ1** (Sequence: CTCAAACCCT (-): 16794665)  
**RFX4\_2** (Sequence: CATAGATAC (+): 16792987)  
**Gata4** (Sequence: AGATAAG (-): 16779551)  
**SOX9** (Sequence: AACAATAA (-): 16805139)  
**SOX9** (Sequence: AACAATAG (-): 16805203)  
**SOX9** (Sequence: AACAATAA (-): 16809579)  
**Gata4** (Sequence: GTTATCT (+): 16791145)
