# Supplementary material for: Small RNA sequencing of cryopreserved semen from single bull revealed altered miRNAs and piRNAs expression between High- and Low-motile sperm populations
Source: BMC Genomics. 2017 Jan 4;18:14. doi: 10.1186/s12864-016-3394-7 (PMC5209821; doi:10.1186/s12864-016-3394-7)
Supplement: Additional file 3: — Details for each piRNA clusters found in High Motile (HM) sperm fraction. Genes, repeats, transposable elements and transcription factors binding sites falling within the cluster regions were reported. (ZIP 1896 kb) [file 12864_2016_3394_MOESM3_ESM.zip › 26.html]

piRNA cluster 26


Predicted piRNA cluster no. 26     previous   next
  

Show proTRAC run info
Hide proTRAC run info

================================= proTRAC ====================================  
VERSION: 2.1                                    LAST MODIFIED: 06. October 2015  
  
Please cite:  
Rosenkranz D, Zischler H. proTRAC - a software for probabilistic piRNA cluster  
detection, visualization and analysis. 2012. BMC Bioinformatics 13:5.  
  
and (for proTRAC 2.0 and later):  
Rosenkranz D, Rudloff S, Bastuck K, Ketting RF, Zischler H. Tupaia small RNAs  
provide insights into function and evolution of RNAi-based transposon defense  
in mammals. 2015. RNA 21(5):911-922.  
  
Contact:  
David Rosenkranz  
Institute of Anthropology, small RNA group  
Johannes Gutenberg University Mainz  
email: rosenkranz@uni-mainz.de  
  
You can find the latest proTRAC version at:  
http://sourceforge.net/projects/protrac/files  
http://www.smallRNAgroup-mainz.de/software  
==============================================================================  
  
PARAMETERS:  
Map file: .............../storage/core/barbara/genhome/smallRNA/fertility/Sample\_motile/pirna/Sample\_motile\_26-33\_collapsed.fa.no-dust.map.weighted-10000-1000-b-0  
Genome file: ............/storage/core/barbara/genhome/smallRNA/fertility/Sample\_all/pirna/bt\_311\_chrY.fa  
RepeatMasker annotation: /storage/genomes/bt\_umd31/GCF\_000003055.6\_Bos\_taurus\_UMD\_3.1.1\_repeatMasker\_chr.out  
GeneSet:................./storage/core/barbara/genhome/smallRNA/fertility/Sample\_all/pirna/full.gtf  
  
Significant (p<=0.01) hit density will be calculated based  
on observed hit distribution.  
  
Sliding window size: ........................................ 5000 bp  
Sliding window increament: .................................. 1000 bp  
Normalize each hit by number of genomic hits: ............... 1 [0=no/1=yes]  
Normalize each hit by number of sequence reads: ............. 1 [0=no/1=yes]  
Normalize values (-> per million mapped reads): ............. 1 [0=no/1=yes]  
Min. fraction of hits with 1T(U) or 10A: .................... 0.75  
Alternatively: Min. fraction of hits with 1T(U) and 10A: .... 0.5  
Min. fraction of hits with typical piRNA length: ............ 0.75  
Typical piRNA length: ....................................... 26-33 nt  
Min. size of a piRNA cluster: ............................... 5000 bp.  
Min. number of hits (absolute): ............................. 0  
Min. number of hits (normalized): ........................... 0  
Min. fraction of hits on the mainstrand: .................... 0.75  
Top fraction of mapped sequences (in terms of read counts): . 1%  
Top fraction accounts for max. n% of sequence reads: ........ 90%  
Min. fraction of hits on each arm of a bidirectional cluster: 0.1  
Output image file for each cluster: ......................... 0 [0=no/1=yes]  
Output html file for each cluster: .......................... 1 [0=no/1=yes]  
Output a summary table: ..................................... 1 [0=no/1=yes]  
Output a FASTA file for each cluster (piRNA sequences): ..... 1 [0=no/1=yes]  
Output a FASTA file comprising cluster sequences: ........... 1 [0=no/1=yes]  
Search DNA motifs in clusters: .............................. 1 [0=no/1=yes]  
Output flanking sequences: +/- .............................. 0 bp  
Output ~.pTi file: .......................................... 1 [0=no/1=yes]  
==============================================================================  
  
  
Genome size (without gaps): ............ 2678902517 bp  
Gaps (N/X/-): .......................... 53837044 bp  
Mapped reads: .......................... 658825247023  
Non-identical sequences: ............... 514171  
Genomic hits: .......................... 764233  
Significant densitiy of mapped reads: .. 12867599.5173724 reads/kb

Show proTRAC cluster info
Hide proTRAC cluster info

|  |  |
| --- | --- |
| Location | chr15 |
| Coordinates | 28396729-28403015 |
| Size [bp] | 6287 |
| Sequence hit loci | 67 |
| Mapped reads (normalized) | 95811493 |
| Mapped reads (normalized) per kb | 15239620.3 |
| Normalized reads with 1T (1U) | 86.6% |
| Normalized reads with 10A | 22.4% |
| Normalized reads with length 26-33 nt | 100% |
| Normalized reads on the main strand(s) | 100% |
| Predicted directionality | mono:plus |

100%

0%

1T (1U)  
reads

10A reads

26-33 nt  
reads

reads on mainstrand

**Either the amount of reads with 1T (1U) OR 10A has to exceed 75% (set with option: -1Tor10A)  
Alternatively the amount of reads with 1T (1U) AND 10A has to exceed 50% (set with option: -1Tand10A)  
Minimum amount of reads with preferred size is 75% (set with option: -pisize)  
Minimum amount of reads on the main strand(s) is 75% (set with option: -clstrand)**

Show read coverage
Hide read coverage

WHAT DO I SEE HERE?  
This chart shows the location of mapped sequence reads within a predicted piRNA cluster. The color refers to the number of genomic hits produced by the sequence read in question. A dark red bar indicates that this sequence read produces many other hits elsewhere in the genome. Many adjacent red or yellow bars can indicate the presence of a multi-copy element such as transposons or rRNA genes. A dark green bar indicates that this sequence read maps uniquely to this locus.

1 hit

2-5 hits

6-10 hits

11-20 hits

21-50 hits

51-100 hits

> 100 hits

chr15

28396729

28403015

Gene Set

RepeatMasker

Mapped  
Reads

24.17

plus strand

minus strand

24.17

Region: chr15 16810157-28396735. Max. coverage (+): 2. Max coverage (-): 0

Region: chr15 28396736-28396747. Max. coverage (+): 2. Max coverage (-): 0

Region: chr15 28396748-28396760. Max. coverage (+): 0. Max coverage (-): 0

Region: chr15 28396761-28396773. Max. coverage (+): 0. Max coverage (-): 0

Region: chr15 28396774-28396785. Max. coverage (+): 0. Max coverage (-): 0

Region: chr15 28396786-28396798. Max. coverage (+): 0. Max coverage (-): 0

Region: chr15 28396799-28396810. Max. coverage (+): 0. Max coverage (-): 0

Region: chr15 28396811-28396823. Max. coverage (+): 0. Max coverage (-): 0

Region: chr15 28396824-28396835. Max. coverage (+): 0. Max coverage (-): 0

Region: chr15 28396836-28396848. Max. coverage (+): 0. Max coverage (-): 0

Region: chr15 28396849-28396861. Max. coverage (+): 0. Max coverage (-): 0

Region: chr15 28396862-28396873. Max. coverage (+): 0. Max coverage (-): 0

Region: chr15 28396874-28396886. Max. coverage (+): 0. Max coverage (-): 0

Region: chr15 28396887-28396898. Max. coverage (+): 0. Max coverage (-): 0

Region: chr15 28396899-28396911. Max. coverage (+): 0. Max coverage (-): 0

Region: chr15 28396912-28396923. Max. coverage (+): 0. Max coverage (-): 0

Region: chr15 28396924-28396936. Max. coverage (+): 0. Max coverage (-): 0

Region: chr15 28396937-28396949. Max. coverage (+): 0. Max coverage (-): 0

Region: chr15 28396950-28396961. Max. coverage (+): 0. Max coverage (-): 0

Region: chr15 28396962-28396974. Max. coverage (+): 0. Max coverage (-): 0

Region: chr15 28396975-28396986. Max. coverage (+): 0. Max coverage (-): 0

Region: chr15 28396987-28396999. Max. coverage (+): 0. Max coverage (-): 0

Region: chr15 28397000-28397011. Max. coverage (+): 0. Max coverage (-): 0

Region: chr15 28397012-28397024. Max. coverage (+): 0. Max coverage (-): 0

Region: chr15 28397025-28397037. Max. coverage (+): 0. Max coverage (-): 0

Region: chr15 28397038-28397049. Max. coverage (+): 0. Max coverage (-): 0

Region: chr15 28397050-28397062. Max. coverage (+): 0. Max coverage (-): 0

Region: chr15 28397063-28397074. Max. coverage (+): 0. Max coverage (-): 0

Region: chr15 28397075-28397087. Max. coverage (+): 0. Max coverage (-): 0

Region: chr15 28397088-28397099. Max. coverage (+): 0. Max coverage (-): 0

Region: chr15 28397100-28397112. Max. coverage (+): 0. Max coverage (-): 0

Region: chr15 28397113-28397125. Max. coverage (+): 0. Max coverage (-): 0

Region: chr15 28397126-28397137. Max. coverage (+): 0. Max coverage (-): 0

Region: chr15 28397138-28397150. Max. coverage (+): 0. Max coverage (-): 0

Region: chr15 28397151-28397162. Max. coverage (+): 0. Max coverage (-): 0

Region: chr15 28397163-28397175. Max. coverage (+): 0. Max coverage (-): 0

Region: chr15 28397176-28397187. Max. coverage (+): 0. Max coverage (-): 0

Region: chr15 28397188-28397200. Max. coverage (+): 0. Max coverage (-): 0

Region: chr15 28397201-28397213. Max. coverage (+): 0. Max coverage (-): 0

Region: chr15 28397214-28397225. Max. coverage (+): 0. Max coverage (-): 0

Region: chr15 28397226-28397238. Max. coverage (+): 0. Max coverage (-): 0

Region: chr15 28397239-28397250. Max. coverage (+): 0. Max coverage (-): 0

Region: chr15 28397251-28397263. Max. coverage (+): 0. Max coverage (-): 0

Region: chr15 28397264-28397275. Max. coverage (+): 0. Max coverage (-): 0

Region: chr15 28397276-28397288. Max. coverage (+): 0. Max coverage (-): 0

Region: chr15 28397289-28397301. Max. coverage (+): 0. Max coverage (-): 0

Region: chr15 28397302-28397313. Max. coverage (+): 0. Max coverage (-): 0

Region: chr15 28397314-28397326. Max. coverage (+): 0. Max coverage (-): 0

Region: chr15 28397327-28397338. Max. coverage (+): 0. Max coverage (-): 0

Region: chr15 28397339-28397351. Max. coverage (+): 0. Max coverage (-): 0

Region: chr15 28397352-28397363. Max. coverage (+): 0. Max coverage (-): 0

Region: chr15 28397364-28397376. Max. coverage (+): 0. Max coverage (-): 0

Region: chr15 28397377-28397389. Max. coverage (+): 0. Max coverage (-): 0

Region: chr15 28397390-28397401. Max. coverage (+): 0. Max coverage (-): 0

Region: chr15 28397402-28397414. Max. coverage (+): 0. Max coverage (-): 0

Region: chr15 28397415-28397426. Max. coverage (+): 0. Max coverage (-): 0

Region: chr15 28397427-28397439. Max. coverage (+): 0. Max coverage (-): 0

Region: chr15 28397440-28397452. Max. coverage (+): 0. Max coverage (-): 0

Region: chr15 28397453-28397464. Max. coverage (+): 0. Max coverage (-): 0

Region: chr15 28397465-28397477. Max. coverage (+): 0. Max coverage (-): 0

Region: chr15 28397478-28397489. Max. coverage (+): 0. Max coverage (-): 0

Region: chr15 28397490-28397502. Max. coverage (+): 0. Max coverage (-): 0

Region: chr15 28397503-28397514. Max. coverage (+): 0. Max coverage (-): 0

Region: chr15 28397515-28397527. Max. coverage (+): 0. Max coverage (-): 0

Region: chr15 28397528-28397540. Max. coverage (+): 0. Max coverage (-): 0

Region: chr15 28397541-28397552. Max. coverage (+): 0. Max coverage (-): 0

Region: chr15 28397553-28397565. Max. coverage (+): 0. Max coverage (-): 0

Region: chr15 28397566-28397577. Max. coverage (+): 0. Max coverage (-): 0

Region: chr15 28397578-28397590. Max. coverage (+): 0. Max coverage (-): 0

Region: chr15 28397591-28397602. Max. coverage (+): 0. Max coverage (-): 0

Region: chr15 28397603-28397615. Max. coverage (+): 0. Max coverage (-): 0

Region: chr15 28397616-28397628. Max. coverage (+): 0. Max coverage (-): 0

Region: chr15 28397629-28397640. Max. coverage (+): 0. Max coverage (-): 0

Region: chr15 28397641-28397653. Max. coverage (+): 0. Max coverage (-): 0

Region: chr15 28397654-28397665. Max. coverage (+): 0. Max coverage (-): 0

Region: chr15 28397666-28397678. Max. coverage (+): 0. Max coverage (-): 0

Region: chr15 28397679-28397690. Max. coverage (+): 0. Max coverage (-): 0

Region: chr15 28397691-28397703. Max. coverage (+): 0. Max coverage (-): 0

Region: chr15 28397704-28397716. Max. coverage (+): 0. Max coverage (-): 0

Region: chr15 28397717-28397728. Max. coverage (+): 0. Max coverage (-): 0

Region: chr15 28397729-28397741. Max. coverage (+): 0. Max coverage (-): 0

Region: chr15 28397742-28397753. Max. coverage (+): 0. Max coverage (-): 0

Region: chr15 28397754-28397766. Max. coverage (+): 0. Max coverage (-): 0

Region: chr15 28397767-28397778. Max. coverage (+): 0. Max coverage (-): 0

Region: chr15 28397779-28397791. Max. coverage (+): 0. Max coverage (-): 0

Region: chr15 28397792-28397804. Max. coverage (+): 0. Max coverage (-): 0

Region: chr15 28397805-28397816. Max. coverage (+): 0. Max coverage (-): 0

Region: chr15 28397817-28397829. Max. coverage (+): 0. Max coverage (-): 0

Region: chr15 28397830-28397841. Max. coverage (+): 0. Max coverage (-): 0

Region: chr15 28397842-28397854. Max. coverage (+): 1.7. Max coverage (-): 0

Region: chr15 28397855-28397866. Max. coverage (+): 0. Max coverage (-): 0

Region: chr15 28397867-28397879. Max. coverage (+): 0. Max coverage (-): 0

Region: chr15 28397880-28397892. Max. coverage (+): 0. Max coverage (-): 0

Region: chr15 28397893-28397904. Max. coverage (+): 0. Max coverage (-): 0

Region: chr15 28397905-28397917. Max. coverage (+): 0. Max coverage (-): 0

Region: chr15 28397918-28397929. Max. coverage (+): 0. Max coverage (-): 0

Region: chr15 28397930-28397942. Max. coverage (+): 0. Max coverage (-): 0

Region: chr15 28397943-28397954. Max. coverage (+): 0. Max coverage (-): 0

Region: chr15 28397955-28397967. Max. coverage (+): 0. Max coverage (-): 0

Region: chr15 28397968-28397980. Max. coverage (+): 0. Max coverage (-): 0

Region: chr15 28397981-28397992. Max. coverage (+): 0. Max coverage (-): 0

Region: chr15 28397993-28398005. Max. coverage (+): 0. Max coverage (-): 0

Region: chr15 28398006-28398017. Max. coverage (+): 0. Max coverage (-): 0

Region: chr15 28398018-28398030. Max. coverage (+): 0. Max coverage (-): 0

Region: chr15 28398031-28398042. Max. coverage (+): 0. Max coverage (-): 0

Region: chr15 28398043-28398055. Max. coverage (+): 0. Max coverage (-): 0

Region: chr15 28398056-28398068. Max. coverage (+): 0. Max coverage (-): 0

Region: chr15 28398069-28398080. Max. coverage (+): 0. Max coverage (-): 0

Region: chr15 28398081-28398093. Max. coverage (+): 0. Max coverage (-): 0

Region: chr15 28398094-28398105. Max. coverage (+): 0. Max coverage (-): 0

Region: chr15 28398106-28398118. Max. coverage (+): 0. Max coverage (-): 0

Region: chr15 28398119-28398131. Max. coverage (+): 0. Max coverage (-): 0

Region: chr15 28398132-28398143. Max. coverage (+): 0. Max coverage (-): 0

Region: chr15 28398144-28398156. Max. coverage (+): 0. Max coverage (-): 0

Region: chr15 28398157-28398168. Max. coverage (+): 2.03. Max coverage (-): 0

Region: chr15 28398169-28398181. Max. coverage (+): 1.54. Max coverage (-): 0

Region: chr15 28398182-28398193. Max. coverage (+): 24.17. Max coverage (-): 0

Region: chr15 28398194-28398206. Max. coverage (+): 3.61. Max coverage (-): 0

Region: chr15 28398207-28398219. Max. coverage (+): 0. Max coverage (-): 0

Region: chr15 28398220-28398231. Max. coverage (+): 0. Max coverage (-): 0

Region: chr15 28398232-28398244. Max. coverage (+): 0. Max coverage (-): 0

Region: chr15 28398245-28398256. Max. coverage (+): 0. Max coverage (-): 0

Region: chr15 28398257-28398269. Max. coverage (+): 0. Max coverage (-): 0

Region: chr15 28398270-28398281. Max. coverage (+): 10.08. Max coverage (-): 0

Region: chr15 28398282-28398294. Max. coverage (+): 10.08. Max coverage (-): 0

Region: chr15 28398295-28398307. Max. coverage (+): 5.49. Max coverage (-): 0

Region: chr15 28398308-28398319. Max. coverage (+): 3.37. Max coverage (-): 0

Region: chr15 28398320-28398332. Max. coverage (+): 0.91. Max coverage (-): 0

Region: chr15 28398333-28398344. Max. coverage (+): 0.91. Max coverage (-): 0

Region: chr15 28398345-28398357. Max. coverage (+): 0. Max coverage (-): 0

Region: chr15 28398358-28398369. Max. coverage (+): 0. Max coverage (-): 0

Region: chr15 28398370-28398382. Max. coverage (+): 8.76. Max coverage (-): 0

Region: chr15 28398383-28398395. Max. coverage (+): 8.76. Max coverage (-): 0

Region: chr15 28398396-28398407. Max. coverage (+): 0. Max coverage (-): 0

Region: chr15 28398408-28398420. Max. coverage (+): 0. Max coverage (-): 0

Region: chr15 28398421-28398432. Max. coverage (+): 4.91. Max coverage (-): 0

Region: chr15 28398433-28398445. Max. coverage (+): 4.91. Max coverage (-): 0

Region: chr15 28398446-28398457. Max. coverage (+): 4.04. Max coverage (-): 0

Region: chr15 28398458-28398470. Max. coverage (+): 0. Max coverage (-): 0

Region: chr15 28398471-28398483. Max. coverage (+): 0. Max coverage (-): 0

Region: chr15 28398484-28398495. Max. coverage (+): 0. Max coverage (-): 0

Region: chr15 28398496-28398508. Max. coverage (+): 0. Max coverage (-): 0

Region: chr15 28398509-28398520. Max. coverage (+): 0. Max coverage (-): 0

Region: chr15 28398521-28398533. Max. coverage (+): 0. Max coverage (-): 0

Region: chr15 28398534-28398545. Max. coverage (+): 0. Max coverage (-): 0

Region: chr15 28398546-28398558. Max. coverage (+): 0. Max coverage (-): 0

Region: chr15 28398559-28398571. Max. coverage (+): 0. Max coverage (-): 0

Region: chr15 28398572-28398583. Max. coverage (+): 0. Max coverage (-): 0

Region: chr15 28398584-28398596. Max. coverage (+): 0. Max coverage (-): 0

Region: chr15 28398597-28398608. Max. coverage (+): 0. Max coverage (-): 0

Region: chr15 28398609-28398621. Max. coverage (+): 0. Max coverage (-): 0

Region: chr15 28398622-28398633. Max. coverage (+): 0. Max coverage (-): 0

Region: chr15 28398634-28398646. Max. coverage (+): 0. Max coverage (-): 0

Region: chr15 28398647-28398659. Max. coverage (+): 0. Max coverage (-): 0

Region: chr15 28398660-28398671. Max. coverage (+): 0. Max coverage (-): 0

Region: chr15 28398672-28398684. Max. coverage (+): 0. Max coverage (-): 0

Region: chr15 28398685-28398696. Max. coverage (+): 0. Max coverage (-): 0

Region: chr15 28398697-28398709. Max. coverage (+): 1.1. Max coverage (-): 0

Region: chr15 28398710-28398721. Max. coverage (+): 1.1. Max coverage (-): 0

Region: chr15 28398722-28398734. Max. coverage (+): 0. Max coverage (-): 0

Region: chr15 28398735-28398747. Max. coverage (+): 0. Max coverage (-): 0

Region: chr15 28398748-28398759. Max. coverage (+): 0. Max coverage (-): 0

Region: chr15 28398760-28398772. Max. coverage (+): 0. Max coverage (-): 0

Region: chr15 28398773-28398784. Max. coverage (+): 0. Max coverage (-): 0

Region: chr15 28398785-28398797. Max. coverage (+): 0. Max coverage (-): 0

Region: chr15 28398798-28398809. Max. coverage (+): 0. Max coverage (-): 0

Region: chr15 28398810-28398822. Max. coverage (+): 0. Max coverage (-): 0

Region: chr15 28398823-28398835. Max. coverage (+): 0. Max coverage (-): 0

Region: chr15 28398836-28398847. Max. coverage (+): 0. Max coverage (-): 0

Region: chr15 28398848-28398860. Max. coverage (+): 0. Max coverage (-): 0

Region: chr15 28398861-28398872. Max. coverage (+): 0. Max coverage (-): 0

Region: chr15 28398873-28398885. Max. coverage (+): 0. Max coverage (-): 0

Region: chr15 28398886-28398898. Max. coverage (+): 0. Max coverage (-): 0

Region: chr15 28398899-28398910. Max. coverage (+): 3.48. Max coverage (-): 0

Region: chr15 28398911-28398923. Max. coverage (+): 0. Max coverage (-): 0

Region: chr15 28398924-28398935. Max. coverage (+): 0. Max coverage (-): 0

Region: chr15 28398936-28398948. Max. coverage (+): 0. Max coverage (-): 0

Region: chr15 28398949-28398960. Max. coverage (+): 0. Max coverage (-): 0

Region: chr15 28398961-28398973. Max. coverage (+): 0. Max coverage (-): 0

Region: chr15 28398974-28398986. Max. coverage (+): 0. Max coverage (-): 0

Region: chr15 28398987-28398998. Max. coverage (+): 0. Max coverage (-): 0

Region: chr15 28398999-28399011. Max. coverage (+): 0. Max coverage (-): 0

Region: chr15 28399012-28399023. Max. coverage (+): 0. Max coverage (-): 0

Region: chr15 28399024-28399036. Max. coverage (+): 0. Max coverage (-): 0

Region: chr15 28399037-28399048. Max. coverage (+): 0. Max coverage (-): 0

Region: chr15 28399049-28399061. Max. coverage (+): 0. Max coverage (-): 0

Region: chr15 28399062-28399074. Max. coverage (+): 0. Max coverage (-): 0

Region: chr15 28399075-28399086. Max. coverage (+): 0. Max coverage (-): 0

Region: chr15 28399087-28399099. Max. coverage (+): 0. Max coverage (-): 0

Region: chr15 28399100-28399111. Max. coverage (+): 3.69. Max coverage (-): 0

Region: chr15 28399112-28399124. Max. coverage (+): 0. Max coverage (-): 0

Region: chr15 28399125-28399136. Max. coverage (+): 0. Max coverage (-): 0

Region: chr15 28399137-28399149. Max. coverage (+): 0. Max coverage (-): 0

Region: chr15 28399150-28399162. Max. coverage (+): 0. Max coverage (-): 0

Region: chr15 28399163-28399174. Max. coverage (+): 0. Max coverage (-): 0

Region: chr15 28399175-28399187. Max. coverage (+): 0. Max coverage (-): 0

Region: chr15 28399188-28399199. Max. coverage (+): 0. Max coverage (-): 0

Region: chr15 28399200-28399212. Max. coverage (+): 0. Max coverage (-): 0

Region: chr15 28399213-28399224. Max. coverage (+): 0. Max coverage (-): 0

Region: chr15 28399225-28399237. Max. coverage (+): 0. Max coverage (-): 0

Region: chr15 28399238-28399250. Max. coverage (+): 0. Max coverage (-): 0

Region: chr15 28399251-28399262. Max. coverage (+): 0. Max coverage (-): 0

Region: chr15 28399263-28399275. Max. coverage (+): 0. Max coverage (-): 0

Region: chr15 28399276-28399287. Max. coverage (+): 0. Max coverage (-): 0

Region: chr15 28399288-28399300. Max. coverage (+): 0. Max coverage (-): 0

Region: chr15 28399301-28399312. Max. coverage (+): 0. Max coverage (-): 0

Region: chr15 28399313-28399325. Max. coverage (+): 0. Max coverage (-): 0

Region: chr15 28399326-28399338. Max. coverage (+): 0. Max coverage (-): 0

Region: chr15 28399339-28399350. Max. coverage (+): 1.89. Max coverage (-): 0

Region: chr15 28399351-28399363. Max. coverage (+): 1.89. Max coverage (-): 0

Region: chr15 28399364-28399375. Max. coverage (+): 0. Max coverage (-): 0

Region: chr15 28399376-28399388. Max. coverage (+): 0. Max coverage (-): 0

Region: chr15 28399389-28399400. Max. coverage (+): 0. Max coverage (-): 0

Region: chr15 28399401-28399413. Max. coverage (+): 0. Max coverage (-): 0

Region: chr15 28399414-28399426. Max. coverage (+): 0. Max coverage (-): 0

Region: chr15 28399427-28399438. Max. coverage (+): 0. Max coverage (-): 0

Region: chr15 28399439-28399451. Max. coverage (+): 0. Max coverage (-): 0

Region: chr15 28399452-28399463. Max. coverage (+): 0. Max coverage (-): 0

Region: chr15 28399464-28399476. Max. coverage (+): 0. Max coverage (-): 0

Region: chr15 28399477-28399488. Max. coverage (+): 0. Max coverage (-): 0

Region: chr15 28399489-28399501. Max. coverage (+): 0. Max coverage (-): 0

Region: chr15 28399502-28399514. Max. coverage (+): 0. Max coverage (-): 0

Region: chr15 28399515-28399526. Max. coverage (+): 0. Max coverage (-): 0

Region: chr15 28399527-28399539. Max. coverage (+): 0. Max coverage (-): 0

Region: chr15 28399540-28399551. Max. coverage (+): 0. Max coverage (-): 0

Region: chr15 28399552-28399564. Max. coverage (+): 0. Max coverage (-): 0

Region: chr15 28399565-28399577. Max. coverage (+): 0. Max coverage (-): 0

Region: chr15 28399578-28399589. Max. coverage (+): 0. Max coverage (-): 0

Region: chr15 28399590-28399602. Max. coverage (+): 0. Max coverage (-): 0

Region: chr15 28399603-28399614. Max. coverage (+): 0. Max coverage (-): 0

Region: chr15 28399615-28399627. Max. coverage (+): 0. Max coverage (-): 0

Region: chr15 28399628-28399639. Max. coverage (+): 0. Max coverage (-): 0

Region: chr15 28399640-28399652. Max. coverage (+): 0. Max coverage (-): 0

Region: chr15 28399653-28399665. Max. coverage (+): 0. Max coverage (-): 0

Region: chr15 28399666-28399677. Max. coverage (+): 0. Max coverage (-): 0

Region: chr15 28399678-28399690. Max. coverage (+): 0. Max coverage (-): 0

Region: chr15 28399691-28399702. Max. coverage (+): 0. Max coverage (-): 0

Region: chr15 28399703-28399715. Max. coverage (+): 0. Max coverage (-): 0

Region: chr15 28399716-28399727. Max. coverage (+): 0. Max coverage (-): 0

Region: chr15 28399728-28399740. Max. coverage (+): 0. Max coverage (-): 0

Region: chr15 28399741-28399753. Max. coverage (+): 0. Max coverage (-): 0

Region: chr15 28399754-28399765. Max. coverage (+): 0. Max coverage (-): 0

Region: chr15 28399766-28399778. Max. coverage (+): 0. Max coverage (-): 0

Region: chr15 28399779-28399790. Max. coverage (+): 0. Max coverage (-): 0

Region: chr15 28399791-28399803. Max. coverage (+): 0. Max coverage (-): 0

Region: chr15 28399804-28399815. Max. coverage (+): 0. Max coverage (-): 0

Region: chr15 28399816-28399828. Max. coverage (+): 0. Max coverage (-): 0

Region: chr15 28399829-28399841. Max. coverage (+): 0. Max coverage (-): 0

Region: chr15 28399842-28399853. Max. coverage (+): 0. Max coverage (-): 0

Region: chr15 28399854-28399866. Max. coverage (+): 0. Max coverage (-): 0

Region: chr15 28399867-28399878. Max. coverage (+): 0. Max coverage (-): 0

Region: chr15 28399879-28399891. Max. coverage (+): 0. Max coverage (-): 0

Region: chr15 28399892-28399903. Max. coverage (+): 0. Max coverage (-): 0

Region: chr15 28399904-28399916. Max. coverage (+): 0. Max coverage (-): 0

Region: chr15 28399917-28399929. Max. coverage (+): 0. Max coverage (-): 0

Region: chr15 28399930-28399941. Max. coverage (+): 0. Max coverage (-): 0

Region: chr15 28399942-28399954. Max. coverage (+): 0. Max coverage (-): 0

Region: chr15 28399955-28399966. Max. coverage (+): 0. Max coverage (-): 0

Region: chr15 28399967-28399979. Max. coverage (+): 0. Max coverage (-): 0

Region: chr15 28399980-28399991. Max. coverage (+): 0. Max coverage (-): 0

Region: chr15 28399992-28400004. Max. coverage (+): 0. Max coverage (-): 0

Region: chr15 28400005-28400017. Max. coverage (+): 0. Max coverage (-): 0

Region: chr15 28400018-28400029. Max. coverage (+): 0. Max coverage (-): 0

Region: chr15 28400030-28400042. Max. coverage (+): 3.32. Max coverage (-): 0

Region: chr15 28400043-28400054. Max. coverage (+): 3.32. Max coverage (-): 0

Region: chr15 28400055-28400067. Max. coverage (+): 0. Max coverage (-): 0

Region: chr15 28400068-28400079. Max. coverage (+): 0. Max coverage (-): 0

Region: chr15 28400080-28400092. Max. coverage (+): 0. Max coverage (-): 0

Region: chr15 28400093-28400105. Max. coverage (+): 2.79. Max coverage (-): 0

Region: chr15 28400106-28400117. Max. coverage (+): 0. Max coverage (-): 0

Region: chr15 28400118-28400130. Max. coverage (+): 6.72. Max coverage (-): 0

Region: chr15 28400131-28400142. Max. coverage (+): 6.72. Max coverage (-): 0

Region: chr15 28400143-28400155. Max. coverage (+): 0. Max coverage (-): 0

Region: chr15 28400156-28400167. Max. coverage (+): 0. Max coverage (-): 0

Region: chr15 28400168-28400180. Max. coverage (+): 0. Max coverage (-): 0

Region: chr15 28400181-28400193. Max. coverage (+): 0. Max coverage (-): 0

Region: chr15 28400194-28400205. Max. coverage (+): 0. Max coverage (-): 0

Region: chr15 28400206-28400218. Max. coverage (+): 0. Max coverage (-): 0

Region: chr15 28400219-28400230. Max. coverage (+): 0. Max coverage (-): 0

Region: chr15 28400231-28400243. Max. coverage (+): 0. Max coverage (-): 0

Region: chr15 28400244-28400256. Max. coverage (+): 1.74. Max coverage (-): 0

Region: chr15 28400257-28400268. Max. coverage (+): 1.74. Max coverage (-): 0

Region: chr15 28400269-28400281. Max. coverage (+): 0. Max coverage (-): 0

Region: chr15 28400282-28400293. Max. coverage (+): 0. Max coverage (-): 0

Region: chr15 28400294-28400306. Max. coverage (+): 0. Max coverage (-): 0

Region: chr15 28400307-28400318. Max. coverage (+): 0. Max coverage (-): 0

Region: chr15 28400319-28400331. Max. coverage (+): 0. Max coverage (-): 0

Region: chr15 28400332-28400344. Max. coverage (+): 0. Max coverage (-): 0

Region: chr15 28400345-28400356. Max. coverage (+): 0. Max coverage (-): 0

Region: chr15 28400357-28400369. Max. coverage (+): 0. Max coverage (-): 0

Region: chr15 28400370-28400381. Max. coverage (+): 0. Max coverage (-): 0

Region: chr15 28400382-28400394. Max. coverage (+): 0. Max coverage (-): 0

Region: chr15 28400395-28400406. Max. coverage (+): 0. Max coverage (-): 0

Region: chr15 28400407-28400419. Max. coverage (+): 0. Max coverage (-): 0

Region: chr15 28400420-28400432. Max. coverage (+): 0. Max coverage (-): 0

Region: chr15 28400433-28400444. Max. coverage (+): 0. Max coverage (-): 0

Region: chr15 28400445-28400457. Max. coverage (+): 0. Max coverage (-): 0

Region: chr15 28400458-28400469. Max. coverage (+): 0. Max coverage (-): 0

Region: chr15 28400470-28400482. Max. coverage (+): 0. Max coverage (-): 0

Region: chr15 28400483-28400494. Max. coverage (+): 0. Max coverage (-): 0

Region: chr15 28400495-28400507. Max. coverage (+): 9.15. Max coverage (-): 0

Region: chr15 28400508-28400520. Max. coverage (+): 0. Max coverage (-): 0

Region: chr15 28400521-28400532. Max. coverage (+): 0. Max coverage (-): 0

Region: chr15 28400533-28400545. Max. coverage (+): 0. Max coverage (-): 0

Region: chr15 28400546-28400557. Max. coverage (+): 0. Max coverage (-): 0

Region: chr15 28400558-28400570. Max. coverage (+): 0. Max coverage (-): 0

Region: chr15 28400571-28400582. Max. coverage (+): 0. Max coverage (-): 0

Region: chr15 28400583-28400595. Max. coverage (+): 0. Max coverage (-): 0

Region: chr15 28400596-28400608. Max. coverage (+): 4.32. Max coverage (-): 0

Region: chr15 28400609-28400620. Max. coverage (+): 0. Max coverage (-): 0

Region: chr15 28400621-28400633. Max. coverage (+): 0. Max coverage (-): 0

Region: chr15 28400634-28400645. Max. coverage (+): 0. Max coverage (-): 0

Region: chr15 28400646-28400658. Max. coverage (+): 0. Max coverage (-): 0

Region: chr15 28400659-28400670. Max. coverage (+): 0. Max coverage (-): 0

Region: chr15 28400671-28400683. Max. coverage (+): 0. Max coverage (-): 0

Region: chr15 28400684-28400696. Max. coverage (+): 0. Max coverage (-): 0

Region: chr15 28400697-28400708. Max. coverage (+): 0. Max coverage (-): 0

Region: chr15 28400709-28400721. Max. coverage (+): 0. Max coverage (-): 0

Region: chr15 28400722-28400733. Max. coverage (+): 0. Max coverage (-): 0

Region: chr15 28400734-28400746. Max. coverage (+): 0. Max coverage (-): 0

Region: chr15 28400747-28400758. Max. coverage (+): 0. Max coverage (-): 0

Region: chr15 28400759-28400771. Max. coverage (+): 1.45. Max coverage (-): 0

Region: chr15 28400772-28400784. Max. coverage (+): 7.6. Max coverage (-): 0

Region: chr15 28400785-28400796. Max. coverage (+): 7.6. Max coverage (-): 0

Region: chr15 28400797-28400809. Max. coverage (+): 0. Max coverage (-): 0

Region: chr15 28400810-28400821. Max. coverage (+): 0. Max coverage (-): 0

Region: chr15 28400822-28400834. Max. coverage (+): 0. Max coverage (-): 0

Region: chr15 28400835-28400846. Max. coverage (+): 0. Max coverage (-): 0

Region: chr15 28400847-28400859. Max. coverage (+): 0. Max coverage (-): 0

Region: chr15 28400860-28400872. Max. coverage (+): 0. Max coverage (-): 0

Region: chr15 28400873-28400884. Max. coverage (+): 0. Max coverage (-): 0

Region: chr15 28400885-28400897. Max. coverage (+): 0. Max coverage (-): 0

Region: chr15 28400898-28400909. Max. coverage (+): 0. Max coverage (-): 0

Region: chr15 28400910-28400922. Max. coverage (+): 0. Max coverage (-): 0

Region: chr15 28400923-28400935. Max. coverage (+): 0. Max coverage (-): 0

Region: chr15 28400936-28400947. Max. coverage (+): 0. Max coverage (-): 0

Region: chr15 28400948-28400960. Max. coverage (+): 0. Max coverage (-): 0

Region: chr15 28400961-28400972. Max. coverage (+): 0. Max coverage (-): 0

Region: chr15 28400973-28400985. Max. coverage (+): 0. Max coverage (-): 0

Region: chr15 28400986-28400997. Max. coverage (+): 0. Max coverage (-): 0

Region: chr15 28400998-28401010. Max. coverage (+): 4.51. Max coverage (-): 0

Region: chr15 28401011-28401023. Max. coverage (+): 5.75. Max coverage (-): 0

Region: chr15 28401024-28401035. Max. coverage (+): 0. Max coverage (-): 0

Region: chr15 28401036-28401048. Max. coverage (+): 0. Max coverage (-): 0

Region: chr15 28401049-28401060. Max. coverage (+): 0. Max coverage (-): 0

Region: chr15 28401061-28401073. Max. coverage (+): 0. Max coverage (-): 0

Region: chr15 28401074-28401085. Max. coverage (+): 0. Max coverage (-): 0

Region: chr15 28401086-28401098. Max. coverage (+): 0. Max coverage (-): 0

Region: chr15 28401099-28401111. Max. coverage (+): 0. Max coverage (-): 0

Region: chr15 28401112-28401123. Max. coverage (+): 0. Max coverage (-): 0

Region: chr15 28401124-28401136. Max. coverage (+): 0. Max coverage (-): 0

Region: chr15 28401137-28401148. Max. coverage (+): 0. Max coverage (-): 0

Region: chr15 28401149-28401161. Max. coverage (+): 0. Max coverage (-): 0

Region: chr15 28401162-28401173. Max. coverage (+): 0. Max coverage (-): 0

Region: chr15 28401174-28401186. Max. coverage (+): 0. Max coverage (-): 0

Region: chr15 28401187-28401199. Max. coverage (+): 0. Max coverage (-): 0

Region: chr15 28401200-28401211. Max. coverage (+): 0. Max coverage (-): 0

Region: chr15 28401212-28401224. Max. coverage (+): 0. Max coverage (-): 0

Region: chr15 28401225-28401236. Max. coverage (+): 0. Max coverage (-): 0

Region: chr15 28401237-28401249. Max. coverage (+): 0. Max coverage (-): 0

Region: chr15 28401250-28401261. Max. coverage (+): 0. Max coverage (-): 0

Region: chr15 28401262-28401274. Max. coverage (+): 0. Max coverage (-): 0

Region: chr15 28401275-28401287. Max. coverage (+): 0. Max coverage (-): 0

Region: chr15 28401288-28401299. Max. coverage (+): 0. Max coverage (-): 0

Region: chr15 28401300-28401312. Max. coverage (+): 0. Max coverage (-): 0

Region: chr15 28401313-28401324. Max. coverage (+): 0. Max coverage (-): 0

Region: chr15 28401325-28401337. Max. coverage (+): 0. Max coverage (-): 0

Region: chr15 28401338-28401349. Max. coverage (+): 0. Max coverage (-): 0

Region: chr15 28401350-28401362. Max. coverage (+): 0. Max coverage (-): 0

Region: chr15 28401363-28401375. Max. coverage (+): 0. Max coverage (-): 0

Region: chr15 28401376-28401387. Max. coverage (+): 0. Max coverage (-): 0

Region: chr15 28401388-28401400. Max. coverage (+): 0. Max coverage (-): 0

Region: chr15 28401401-28401412. Max. coverage (+): 0. Max coverage (-): 0

Region: chr15 28401413-28401425. Max. coverage (+): 0. Max coverage (-): 0

Region: chr15 28401426-28401437. Max. coverage (+): 0. Max coverage (-): 0

Region: chr15 28401438-28401450. Max. coverage (+): 0. Max coverage (-): 0

Region: chr15 28401451-28401463. Max. coverage (+): 0. Max coverage (-): 0

Region: chr15 28401464-28401475. Max. coverage (+): 0. Max coverage (-): 0

Region: chr15 28401476-28401488. Max. coverage (+): 0. Max coverage (-): 0

Region: chr15 28401489-28401500. Max. coverage (+): 0. Max coverage (-): 0

Region: chr15 28401501-28401513. Max. coverage (+): 0. Max coverage (-): 0

Region: chr15 28401514-28401525. Max. coverage (+): 0. Max coverage (-): 0

Region: chr15 28401526-28401538. Max. coverage (+): 0. Max coverage (-): 0

Region: chr15 28401539-28401551. Max. coverage (+): 0. Max coverage (-): 0

Region: chr15 28401552-28401563. Max. coverage (+): 0. Max coverage (-): 0

Region: chr15 28401564-28401576. Max. coverage (+): 0. Max coverage (-): 0

Region: chr15 28401577-28401588. Max. coverage (+): 0. Max coverage (-): 0

Region: chr15 28401589-28401601. Max. coverage (+): 0. Max coverage (-): 0

Region: chr15 28401602-28401613. Max. coverage (+): 0. Max coverage (-): 0

Region: chr15 28401614-28401626. Max. coverage (+): 0. Max coverage (-): 0

Region: chr15 28401627-28401639. Max. coverage (+): 0. Max coverage (-): 0

Region: chr15 28401640-28401651. Max. coverage (+): 0. Max coverage (-): 0

Region: chr15 28401652-28401664. Max. coverage (+): 0. Max coverage (-): 0

Region: chr15 28401665-28401676. Max. coverage (+): 0. Max coverage (-): 0

Region: chr15 28401677-28401689. Max. coverage (+): 0. Max coverage (-): 0

Region: chr15 28401690-28401702. Max. coverage (+): 0. Max coverage (-): 0

Region: chr15 28401703-28401714. Max. coverage (+): 0. Max coverage (-): 0

Region: chr15 28401715-28401727. Max. coverage (+): 0. Max coverage (-): 0

Region: chr15 28401728-28401739. Max. coverage (+): 0. Max coverage (-): 0

Region: chr15 28401740-28401752. Max. coverage (+): 0. Max coverage (-): 0

Region: chr15 28401753-28401764. Max. coverage (+): 10.92. Max coverage (-): 0

Region: chr15 28401765-28401777. Max. coverage (+): 0. Max coverage (-): 0

Region: chr15 28401778-28401790. Max. coverage (+): 0. Max coverage (-): 0

Region: chr15 28401791-28401802. Max. coverage (+): 0. Max coverage (-): 0

Region: chr15 28401803-28401815. Max. coverage (+): 0. Max coverage (-): 0

Region: chr15 28401816-28401827. Max. coverage (+): 0. Max coverage (-): 0

Region: chr15 28401828-28401840. Max. coverage (+): 0. Max coverage (-): 0

Region: chr15 28401841-28401852. Max. coverage (+): 0. Max coverage (-): 0

Region: chr15 28401853-28401865. Max. coverage (+): 0. Max coverage (-): 0

Region: chr15 28401866-28401878. Max. coverage (+): 0. Max coverage (-): 0

Region: chr15 28401879-28401890. Max. coverage (+): 0. Max coverage (-): 0

Region: chr15 28401891-28401903. Max. coverage (+): 0. Max coverage (-): 0

Region: chr15 28401904-28401915. Max. coverage (+): 0.87. Max coverage (-): 0

Region: chr15 28401916-28401928. Max. coverage (+): 0. Max coverage (-): 0

Region: chr15 28401929-28401940. Max. coverage (+): 0. Max coverage (-): 0

Region: chr15 28401941-28401953. Max. coverage (+): 0. Max coverage (-): 0

Region: chr15 28401954-28401966. Max. coverage (+): 0. Max coverage (-): 0

Region: chr15 28401967-28401978. Max. coverage (+): 0. Max coverage (-): 0

Region: chr15 28401979-28401991. Max. coverage (+): 0. Max coverage (-): 0

Region: chr15 28401992-28402003. Max. coverage (+): 0. Max coverage (-): 0

Region: chr15 28402004-28402016. Max. coverage (+): 0. Max coverage (-): 0

Region: chr15 28402017-28402028. Max. coverage (+): 0. Max coverage (-): 0

Region: chr15 28402029-28402041. Max. coverage (+): 0. Max coverage (-): 0

Region: chr15 28402042-28402054. Max. coverage (+): 0. Max coverage (-): 0

Region: chr15 28402055-28402066. Max. coverage (+): 0. Max coverage (-): 0

Region: chr15 28402067-28402079. Max. coverage (+): 0. Max coverage (-): 0

Region: chr15 28402080-28402091. Max. coverage (+): 0. Max coverage (-): 0

Region: chr15 28402092-28402104. Max. coverage (+): 0. Max coverage (-): 0

Region: chr15 28402105-28402116. Max. coverage (+): 0. Max coverage (-): 0

Region: chr15 28402117-28402129. Max. coverage (+): 0. Max coverage (-): 0

Region: chr15 28402130-28402142. Max. coverage (+): 0. Max coverage (-): 0

Region: chr15 28402143-28402154. Max. coverage (+): 0. Max coverage (-): 0

Region: chr15 28402155-28402167. Max. coverage (+): 1.18. Max coverage (-): 0

Region: chr15 28402168-28402179. Max. coverage (+): 0. Max coverage (-): 0

Region: chr15 28402180-28402192. Max. coverage (+): 0. Max coverage (-): 0

Region: chr15 28402193-28402204. Max. coverage (+): 0. Max coverage (-): 0

Region: chr15 28402205-28402217. Max. coverage (+): 0. Max coverage (-): 0

Region: chr15 28402218-28402230. Max. coverage (+): 0. Max coverage (-): 0

Region: chr15 28402231-28402242. Max. coverage (+): 0. Max coverage (-): 0

Region: chr15 28402243-28402255. Max. coverage (+): 0. Max coverage (-): 0

Region: chr15 28402256-28402267. Max. coverage (+): 0. Max coverage (-): 0

Region: chr15 28402268-28402280. Max. coverage (+): 0. Max coverage (-): 0

Region: chr15 28402281-28402292. Max. coverage (+): 0. Max coverage (-): 0

Region: chr15 28402293-28402305. Max. coverage (+): 0. Max coverage (-): 0

Region: chr15 28402306-28402318. Max. coverage (+): 0. Max coverage (-): 0

Region: chr15 28402319-28402330. Max. coverage (+): 0. Max coverage (-): 0

Region: chr15 28402331-28402343. Max. coverage (+): 0. Max coverage (-): 0

Region: chr15 28402344-28402355. Max. coverage (+): 0. Max coverage (-): 0

Region: chr15 28402356-28402368. Max. coverage (+): 0. Max coverage (-): 0

Region: chr15 28402369-28402381. Max. coverage (+): 0. Max coverage (-): 0

Region: chr15 28402382-28402393. Max. coverage (+): 0. Max coverage (-): 0

Region: chr15 28402394-28402406. Max. coverage (+): 0. Max coverage (-): 0

Region: chr15 28402407-28402418. Max. coverage (+): 0. Max coverage (-): 0

Region: chr15 28402419-28402431. Max. coverage (+): 0. Max coverage (-): 0

Region: chr15 28402432-28402443. Max. coverage (+): 0. Max coverage (-): 0

Region: chr15 28402444-28402456. Max. coverage (+): 0. Max coverage (-): 0

Region: chr15 28402457-28402469. Max. coverage (+): 0. Max coverage (-): 0

Region: chr15 28402470-28402481. Max. coverage (+): 0. Max coverage (-): 0

Region: chr15 28402482-28402494. Max. coverage (+): 6.01. Max coverage (-): 0

Region: chr15 28402495-28402506. Max. coverage (+): 9.87. Max coverage (-): 0

Region: chr15 28402507-28402519. Max. coverage (+): 0. Max coverage (-): 0

Region: chr15 28402520-28402531. Max. coverage (+): 0. Max coverage (-): 0

Region: chr15 28402532-28402544. Max. coverage (+): 0. Max coverage (-): 0

Region: chr15 28402545-28402557. Max. coverage (+): 0. Max coverage (-): 0

Region: chr15 28402558-28402569. Max. coverage (+): 0. Max coverage (-): 0

Region: chr15 28402570-28402582. Max. coverage (+): 0. Max coverage (-): 0

Region: chr15 28402583-28402594. Max. coverage (+): 0. Max coverage (-): 0

Region: chr15 28402595-28402607. Max. coverage (+): 0.4. Max coverage (-): 0

Region: chr15 28402608-28402619. Max. coverage (+): 0.4. Max coverage (-): 0

Region: chr15 28402620-28402632. Max. coverage (+): 0. Max coverage (-): 0

Region: chr15 28402633-28402645. Max. coverage (+): 0. Max coverage (-): 0

Region: chr15 28402646-28402657. Max. coverage (+): 0. Max coverage (-): 0

Region: chr15 28402658-28402670. Max. coverage (+): 0. Max coverage (-): 0

Region: chr15 28402671-28402682. Max. coverage (+): 0. Max coverage (-): 0

Region: chr15 28402683-28402695. Max. coverage (+): 0. Max coverage (-): 0

Region: chr15 28402696-28402707. Max. coverage (+): 0. Max coverage (-): 0

Region: chr15 28402708-28402720. Max. coverage (+): 0. Max coverage (-): 0

Region: chr15 28402721-28402733. Max. coverage (+): 0. Max coverage (-): 0

Region: chr15 28402734-28402745. Max. coverage (+): 0. Max coverage (-): 0

Region: chr15 28402746-28402758. Max. coverage (+): 0. Max coverage (-): 0

Region: chr15 28402759-28402770. Max. coverage (+): 0. Max coverage (-): 0

Region: chr15 28402771-28402783. Max. coverage (+): 0. Max coverage (-): 0

Region: chr15 28402784-28402795. Max. coverage (+): 0. Max coverage (-): 0

Region: chr15 28402796-28402808. Max. coverage (+): 0. Max coverage (-): 0

Region: chr15 28402809-28402821. Max. coverage (+): 0. Max coverage (-): 0

Region: chr15 28402822-28402833. Max. coverage (+): 0. Max coverage (-): 0

Region: chr15 28402834-28402846. Max. coverage (+): 0. Max coverage (-): 0

Region: chr15 28402847-28402858. Max. coverage (+): 0. Max coverage (-): 0

Region: chr15 28402859-28402871. Max. coverage (+): 0. Max coverage (-): 0

Region: chr15 28402872-28402883. Max. coverage (+): 0. Max coverage (-): 0

Region: chr15 28402884-28402896. Max. coverage (+): 0. Max coverage (-): 0

Region: chr15 28402897-28402909. Max. coverage (+): 0. Max coverage (-): 0

Region: chr15 28402910-28402921. Max. coverage (+): 0. Max coverage (-): 0

Region: chr15 28402922-28402934. Max. coverage (+): 0. Max coverage (-): 0

Region: chr15 28402935-28402946. Max. coverage (+): 0. Max coverage (-): 0

Region: chr15 28402947-28402959. Max. coverage (+): 0. Max coverage (-): 0

Region: chr15 28402960-28402971. Max. coverage (+): 0. Max coverage (-): 0

Region: chr15 28402972-28402984. Max. coverage (+): 0. Max coverage (-): 0

Region: chr15 28402985-28402997. Max. coverage (+): 2.27. Max coverage (-): 0

Region: chr15 28402998-28403009. Max. coverage (+): 0. Max coverage (-): 0

Region: chr15 28403010-. Max. coverage (+): 0. Max coverage (-): 0

RepeatMasker Color Code

**+**

100-98% Identity

<98-95% Identity

<95-90% Identity

<90-85% Identity

<85-80% Identity

<80-75% Identity

<75-70% Identity

<70% Identity

**-**

Gene Set Color Code

**+**

Gene

Pseudogene

**-**

Topology/Coverage Color Code

Coverage Plus Strand

Coverage Minus Strand

Mainstrand: Plus

Mainstrand: Minus

Complementary Strand

Flanking Region  
(if option -flank >0)

Gene Set Annotation  

**1. RNF214 (protein coding, ENSBTAG00000019358) Tr:00000025789 Ex:15**: 28396609-28397142 (+)  
**2. BACE1 (protein coding, ENSBTAG00000019365) Tr:00000040056 Ex:7**: 28402549-28402698 (-)  
**3. BACE1 (protein coding, ENSBTAG00000019365) Tr:00000040056 Ex:8**: 28402115-28402286 (-)  
**4. BACE1 (protein coding, ENSBTAG00000019365) Tr:00000040056 Ex:9**: 28400714-28401481 (-)

  
RepeatMasker Annotation  

**1. CHR-2A**: 28397931-28397984 (-), Divergence to consensus: 16.1%  
**2. Bov-tA2**: 28397985-28398103 (-), Divergence to consensus: 10.8%  
**3. AT\_rich**: 28400697-28400717 (+), Divergence to consensus: 47.6%

  
Transcription Factor Binding Sites  

**RFX4\_2** (Sequence: GTAACCAGG (-): 28397333)  
**SOX9** (Sequence: AACAATGA (-): 28402720)  
**SOX9** (Sequence: CCATTGTT (+): 28398921)  
**Gata4** (Sequence: GTTATCT (+): 28402988)
