# Supplementary material for: Small RNA sequencing of cryopreserved semen from single bull revealed altered miRNAs and piRNAs expression between High- and Low-motile sperm populations
Source: BMC Genomics. 2017 Jan 4;18:14. doi: 10.1186/s12864-016-3394-7 (PMC5209821; doi:10.1186/s12864-016-3394-7)
Supplement: Additional file 3: — Details for each piRNA clusters found in High Motile (HM) sperm fraction. Genes, repeats, transposable elements and transcription factors binding sites falling within the cluster regions were reported. (ZIP 1896 kb) [file 12864_2016_3394_MOESM3_ESM.zip › 27.html]

piRNA cluster 27


Predicted piRNA cluster no. 27     previous   next
  

Show proTRAC run info
Hide proTRAC run info

================================= proTRAC ====================================  
VERSION: 2.1                                    LAST MODIFIED: 06. October 2015  
  
Please cite:  
Rosenkranz D, Zischler H. proTRAC - a software for probabilistic piRNA cluster  
detection, visualization and analysis. 2012. BMC Bioinformatics 13:5.  
  
and (for proTRAC 2.0 and later):  
Rosenkranz D, Rudloff S, Bastuck K, Ketting RF, Zischler H. Tupaia small RNAs  
provide insights into function and evolution of RNAi-based transposon defense  
in mammals. 2015. RNA 21(5):911-922.  
  
Contact:  
David Rosenkranz  
Institute of Anthropology, small RNA group  
Johannes Gutenberg University Mainz  
email: rosenkranz@uni-mainz.de  
  
You can find the latest proTRAC version at:  
http://sourceforge.net/projects/protrac/files  
http://www.smallRNAgroup-mainz.de/software  
==============================================================================  
  
PARAMETERS:  
Map file: .............../storage/core/barbara/genhome/smallRNA/fertility/Sample\_motile/pirna/Sample\_motile\_26-33\_collapsed.fa.no-dust.map.weighted-10000-1000-b-0  
Genome file: ............/storage/core/barbara/genhome/smallRNA/fertility/Sample\_all/pirna/bt\_311\_chrY.fa  
RepeatMasker annotation: /storage/genomes/bt\_umd31/GCF\_000003055.6\_Bos\_taurus\_UMD\_3.1.1\_repeatMasker\_chr.out  
GeneSet:................./storage/core/barbara/genhome/smallRNA/fertility/Sample\_all/pirna/full.gtf  
  
Significant (p<=0.01) hit density will be calculated based  
on observed hit distribution.  
  
Sliding window size: ........................................ 5000 bp  
Sliding window increament: .................................. 1000 bp  
Normalize each hit by number of genomic hits: ............... 1 [0=no/1=yes]  
Normalize each hit by number of sequence reads: ............. 1 [0=no/1=yes]  
Normalize values (-> per million mapped reads): ............. 1 [0=no/1=yes]  
Min. fraction of hits with 1T(U) or 10A: .................... 0.75  
Alternatively: Min. fraction of hits with 1T(U) and 10A: .... 0.5  
Min. fraction of hits with typical piRNA length: ............ 0.75  
Typical piRNA length: ....................................... 26-33 nt  
Min. size of a piRNA cluster: ............................... 5000 bp.  
Min. number of hits (absolute): ............................. 0  
Min. number of hits (normalized): ........................... 0  
Min. fraction of hits on the mainstrand: .................... 0.75  
Top fraction of mapped sequences (in terms of read counts): . 1%  
Top fraction accounts for max. n% of sequence reads: ........ 90%  
Min. fraction of hits on each arm of a bidirectional cluster: 0.1  
Output image file for each cluster: ......................... 0 [0=no/1=yes]  
Output html file for each cluster: .......................... 1 [0=no/1=yes]  
Output a summary table: ..................................... 1 [0=no/1=yes]  
Output a FASTA file for each cluster (piRNA sequences): ..... 1 [0=no/1=yes]  
Output a FASTA file comprising cluster sequences: ........... 1 [0=no/1=yes]  
Search DNA motifs in clusters: .............................. 1 [0=no/1=yes]  
Output flanking sequences: +/- .............................. 0 bp  
Output ~.pTi file: .......................................... 1 [0=no/1=yes]  
==============================================================================  
  
  
Genome size (without gaps): ............ 2678902517 bp  
Gaps (N/X/-): .......................... 53837044 bp  
Mapped reads: .......................... 658825247023  
Non-identical sequences: ............... 514171  
Genomic hits: .......................... 764233  
Significant densitiy of mapped reads: .. 12867599.5173724 reads/kb

Show proTRAC cluster info
Hide proTRAC cluster info

|  |  |
| --- | --- |
| Location | chr15 |
| Coordinates | 30403169-30413850 |
| Size [bp] | 10682 |
| Sequence hit loci | 895 |
| Mapped reads (normalized) | 1068715455 |
| Mapped reads (normalized) per kb | 100048254.5 |
| Normalized reads with 1T (1U) | 85.4% |
| Normalized reads with 10A | 27.8% |
| Normalized reads with length 26-33 nt | 100% |
| Normalized reads on the main strand(s) | 100% |
| Predicted directionality | mono:plus |

100%

0%

1T (1U)  
reads

10A reads

26-33 nt  
reads

reads on mainstrand

**Either the amount of reads with 1T (1U) OR 10A has to exceed 75% (set with option: -1Tor10A)  
Alternatively the amount of reads with 1T (1U) AND 10A has to exceed 50% (set with option: -1Tand10A)  
Minimum amount of reads with preferred size is 75% (set with option: -pisize)  
Minimum amount of reads on the main strand(s) is 75% (set with option: -clstrand)**

Show read coverage
Hide read coverage

WHAT DO I SEE HERE?  
This chart shows the location of mapped sequence reads within a predicted piRNA cluster. The color refers to the number of genomic hits produced by the sequence read in question. A dark red bar indicates that this sequence read produces many other hits elsewhere in the genome. Many adjacent red or yellow bars can indicate the presence of a multi-copy element such as transposons or rRNA genes. A dark green bar indicates that this sequence read maps uniquely to this locus.

1 hit

2-5 hits

6-10 hits

11-20 hits

21-50 hits

51-100 hits

> 100 hits

chr15

30403169

30413850

Gene Set

RepeatMasker

Mapped  
Reads

47.06

plus strand

minus strand

47.06

Region: chr15 28403011-30403179. Max. coverage (+): 1. Max coverage (-): 0

Region: chr15 30403180-30403201. Max. coverage (+): 1.31. Max coverage (-): 0

Region: chr15 30403202-30403222. Max. coverage (+): 0. Max coverage (-): 0

Region: chr15 30403223-30403243. Max. coverage (+): 0. Max coverage (-): 0

Region: chr15 30403244-30403265. Max. coverage (+): 0. Max coverage (-): 0

Region: chr15 30403266-30403286. Max. coverage (+): 0. Max coverage (-): 0

Region: chr15 30403287-30403307. Max. coverage (+): 0. Max coverage (-): 0

Region: chr15 30403308-30403329. Max. coverage (+): 0. Max coverage (-): 0

Region: chr15 30403330-30403350. Max. coverage (+): 0. Max coverage (-): 0

Region: chr15 30403351-30403371. Max. coverage (+): 0. Max coverage (-): 0

Region: chr15 30403372-30403393. Max. coverage (+): 0. Max coverage (-): 0

Region: chr15 30403394-30403414. Max. coverage (+): 0. Max coverage (-): 0

Region: chr15 30403415-30403436. Max. coverage (+): 0. Max coverage (-): 0

Region: chr15 30403437-30403457. Max. coverage (+): 0. Max coverage (-): 0

Region: chr15 30403458-30403478. Max. coverage (+): 0. Max coverage (-): 0

Region: chr15 30403479-30403500. Max. coverage (+): 0. Max coverage (-): 0

Region: chr15 30403501-30403521. Max. coverage (+): 0. Max coverage (-): 0

Region: chr15 30403522-30403542. Max. coverage (+): 0. Max coverage (-): 0

Region: chr15 30403543-30403564. Max. coverage (+): 0. Max coverage (-): 0

Region: chr15 30403565-30403585. Max. coverage (+): 0. Max coverage (-): 0

Region: chr15 30403586-30403606. Max. coverage (+): 1.94. Max coverage (-): 0

Region: chr15 30403607-30403628. Max. coverage (+): 1.94. Max coverage (-): 0

Region: chr15 30403629-30403649. Max. coverage (+): 0. Max coverage (-): 0

Region: chr15 30403650-30403671. Max. coverage (+): 0. Max coverage (-): 0

Region: chr15 30403672-30403692. Max. coverage (+): 0. Max coverage (-): 0

Region: chr15 30403693-30403713. Max. coverage (+): 0. Max coverage (-): 0

Region: chr15 30403714-30403735. Max. coverage (+): 0. Max coverage (-): 0

Region: chr15 30403736-30403756. Max. coverage (+): 0. Max coverage (-): 0

Region: chr15 30403757-30403777. Max. coverage (+): 0. Max coverage (-): 0

Region: chr15 30403778-30403799. Max. coverage (+): 0. Max coverage (-): 0

Region: chr15 30403800-30403820. Max. coverage (+): 0. Max coverage (-): 0

Region: chr15 30403821-30403841. Max. coverage (+): 0. Max coverage (-): 0

Region: chr15 30403842-30403863. Max. coverage (+): 0. Max coverage (-): 0

Region: chr15 30403864-30403884. Max. coverage (+): 0. Max coverage (-): 0

Region: chr15 30403885-30403906. Max. coverage (+): 0. Max coverage (-): 0

Region: chr15 30403907-30403927. Max. coverage (+): 0. Max coverage (-): 0

Region: chr15 30403928-30403948. Max. coverage (+): 0. Max coverage (-): 0

Region: chr15 30403949-30403970. Max. coverage (+): 0. Max coverage (-): 0

Region: chr15 30403971-30403991. Max. coverage (+): 0. Max coverage (-): 0

Region: chr15 30403992-30404012. Max. coverage (+): 0. Max coverage (-): 0

Region: chr15 30404013-30404034. Max. coverage (+): 0. Max coverage (-): 0

Region: chr15 30404035-30404055. Max. coverage (+): 0. Max coverage (-): 0

Region: chr15 30404056-30404076. Max. coverage (+): 0. Max coverage (-): 0

Region: chr15 30404077-30404098. Max. coverage (+): 0. Max coverage (-): 0

Region: chr15 30404099-30404119. Max. coverage (+): 0. Max coverage (-): 0

Region: chr15 30404120-30404141. Max. coverage (+): 0. Max coverage (-): 0

Region: chr15 30404142-30404162. Max. coverage (+): 0. Max coverage (-): 0

Region: chr15 30404163-30404183. Max. coverage (+): 0. Max coverage (-): 0

Region: chr15 30404184-30404205. Max. coverage (+): 0. Max coverage (-): 0

Region: chr15 30404206-30404226. Max. coverage (+): 0. Max coverage (-): 0

Region: chr15 30404227-30404247. Max. coverage (+): 0. Max coverage (-): 0

Region: chr15 30404248-30404269. Max. coverage (+): 0. Max coverage (-): 0

Region: chr15 30404270-30404290. Max. coverage (+): 0. Max coverage (-): 0

Region: chr15 30404291-30404311. Max. coverage (+): 0. Max coverage (-): 0

Region: chr15 30404312-30404333. Max. coverage (+): 0. Max coverage (-): 0

Region: chr15 30404334-30404354. Max. coverage (+): 0. Max coverage (-): 0

Region: chr15 30404355-30404376. Max. coverage (+): 0. Max coverage (-): 0

Region: chr15 30404377-30404397. Max. coverage (+): 0. Max coverage (-): 0

Region: chr15 30404398-30404418. Max. coverage (+): 0. Max coverage (-): 0

Region: chr15 30404419-30404440. Max. coverage (+): 0. Max coverage (-): 0

Region: chr15 30404441-30404461. Max. coverage (+): 0. Max coverage (-): 0

Region: chr15 30404462-30404482. Max. coverage (+): 0. Max coverage (-): 0

Region: chr15 30404483-30404504. Max. coverage (+): 0. Max coverage (-): 0

Region: chr15 30404505-30404525. Max. coverage (+): 0. Max coverage (-): 0

Region: chr15 30404526-30404546. Max. coverage (+): 0. Max coverage (-): 0

Region: chr15 30404547-30404568. Max. coverage (+): 0. Max coverage (-): 0

Region: chr15 30404569-30404589. Max. coverage (+): 0. Max coverage (-): 0

Region: chr15 30404590-30404611. Max. coverage (+): 0. Max coverage (-): 0

Region: chr15 30404612-30404632. Max. coverage (+): 0. Max coverage (-): 0

Region: chr15 30404633-30404653. Max. coverage (+): 0. Max coverage (-): 0

Region: chr15 30404654-30404675. Max. coverage (+): 0. Max coverage (-): 0

Region: chr15 30404676-30404696. Max. coverage (+): 0. Max coverage (-): 0

Region: chr15 30404697-30404717. Max. coverage (+): 0. Max coverage (-): 0

Region: chr15 30404718-30404739. Max. coverage (+): 0. Max coverage (-): 0

Region: chr15 30404740-30404760. Max. coverage (+): 0.4. Max coverage (-): 0

Region: chr15 30404761-30404781. Max. coverage (+): 0. Max coverage (-): 0

Region: chr15 30404782-30404803. Max. coverage (+): 0. Max coverage (-): 0

Region: chr15 30404804-30404824. Max. coverage (+): 0. Max coverage (-): 0

Region: chr15 30404825-30404846. Max. coverage (+): 0. Max coverage (-): 0

Region: chr15 30404847-30404867. Max. coverage (+): 0. Max coverage (-): 0

Region: chr15 30404868-30404888. Max. coverage (+): 0. Max coverage (-): 0

Region: chr15 30404889-30404910. Max. coverage (+): 0. Max coverage (-): 0

Region: chr15 30404911-30404931. Max. coverage (+): 0. Max coverage (-): 0

Region: chr15 30404932-30404952. Max. coverage (+): 0. Max coverage (-): 0

Region: chr15 30404953-30404974. Max. coverage (+): 0. Max coverage (-): 0

Region: chr15 30404975-30404995. Max. coverage (+): 0. Max coverage (-): 0

Region: chr15 30404996-30405016. Max. coverage (+): 0. Max coverage (-): 0

Region: chr15 30405017-30405038. Max. coverage (+): 0. Max coverage (-): 0

Region: chr15 30405039-30405059. Max. coverage (+): 0. Max coverage (-): 0

Region: chr15 30405060-30405081. Max. coverage (+): 0. Max coverage (-): 0

Region: chr15 30405082-30405102. Max. coverage (+): 0. Max coverage (-): 0

Region: chr15 30405103-30405123. Max. coverage (+): 0. Max coverage (-): 0

Region: chr15 30405124-30405145. Max. coverage (+): 0. Max coverage (-): 0

Region: chr15 30405146-30405166. Max. coverage (+): 0. Max coverage (-): 0

Region: chr15 30405167-30405187. Max. coverage (+): 0. Max coverage (-): 0

Region: chr15 30405188-30405209. Max. coverage (+): 0. Max coverage (-): 0

Region: chr15 30405210-30405230. Max. coverage (+): 0. Max coverage (-): 0

Region: chr15 30405231-30405251. Max. coverage (+): 0. Max coverage (-): 0

Region: chr15 30405252-30405273. Max. coverage (+): 0. Max coverage (-): 0

Region: chr15 30405274-30405294. Max. coverage (+): 0. Max coverage (-): 0

Region: chr15 30405295-30405316. Max. coverage (+): 0. Max coverage (-): 0

Region: chr15 30405317-30405337. Max. coverage (+): 0. Max coverage (-): 0

Region: chr15 30405338-30405358. Max. coverage (+): 0. Max coverage (-): 0

Region: chr15 30405359-30405380. Max. coverage (+): 0. Max coverage (-): 0

Region: chr15 30405381-30405401. Max. coverage (+): 0. Max coverage (-): 0

Region: chr15 30405402-30405422. Max. coverage (+): 0. Max coverage (-): 0

Region: chr15 30405423-30405444. Max. coverage (+): 0. Max coverage (-): 0

Region: chr15 30405445-30405465. Max. coverage (+): 0. Max coverage (-): 0

Region: chr15 30405466-30405486. Max. coverage (+): 0. Max coverage (-): 0

Region: chr15 30405487-30405508. Max. coverage (+): 0. Max coverage (-): 0

Region: chr15 30405509-30405529. Max. coverage (+): 0. Max coverage (-): 0

Region: chr15 30405530-30405551. Max. coverage (+): 0. Max coverage (-): 0

Region: chr15 30405552-30405572. Max. coverage (+): 0. Max coverage (-): 0

Region: chr15 30405573-30405593. Max. coverage (+): 0. Max coverage (-): 0

Region: chr15 30405594-30405615. Max. coverage (+): 0. Max coverage (-): 0

Region: chr15 30405616-30405636. Max. coverage (+): 0. Max coverage (-): 0

Region: chr15 30405637-30405657. Max. coverage (+): 0. Max coverage (-): 0

Region: chr15 30405658-30405679. Max. coverage (+): 0. Max coverage (-): 0

Region: chr15 30405680-30405700. Max. coverage (+): 0. Max coverage (-): 0

Region: chr15 30405701-30405721. Max. coverage (+): 0. Max coverage (-): 0

Region: chr15 30405722-30405743. Max. coverage (+): 0. Max coverage (-): 0

Region: chr15 30405744-30405764. Max. coverage (+): 0. Max coverage (-): 0

Region: chr15 30405765-30405786. Max. coverage (+): 0. Max coverage (-): 0

Region: chr15 30405787-30405807. Max. coverage (+): 3.05. Max coverage (-): 0

Region: chr15 30405808-30405828. Max. coverage (+): 0. Max coverage (-): 0

Region: chr15 30405829-30405850. Max. coverage (+): 2.95. Max coverage (-): 0

Region: chr15 30405851-30405871. Max. coverage (+): 3.1. Max coverage (-): 0

Region: chr15 30405872-30405892. Max. coverage (+): 0. Max coverage (-): 0

Region: chr15 30405893-30405914. Max. coverage (+): 0. Max coverage (-): 0

Region: chr15 30405915-30405935. Max. coverage (+): 0.85. Max coverage (-): 0

Region: chr15 30405936-30405957. Max. coverage (+): 0. Max coverage (-): 0

Region: chr15 30405958-30405978. Max. coverage (+): 1.87. Max coverage (-): 0

Region: chr15 30405979-30405999. Max. coverage (+): 0. Max coverage (-): 0

Region: chr15 30406000-30406021. Max. coverage (+): 0. Max coverage (-): 0

Region: chr15 30406022-30406042. Max. coverage (+): 0. Max coverage (-): 0

Region: chr15 30406043-30406063. Max. coverage (+): 0. Max coverage (-): 0

Region: chr15 30406064-30406085. Max. coverage (+): 0. Max coverage (-): 0

Region: chr15 30406086-30406106. Max. coverage (+): 0. Max coverage (-): 0

Region: chr15 30406107-30406127. Max. coverage (+): 0. Max coverage (-): 0

Region: chr15 30406128-30406149. Max. coverage (+): 0. Max coverage (-): 0

Region: chr15 30406150-30406170. Max. coverage (+): 0. Max coverage (-): 0

Region: chr15 30406171-30406192. Max. coverage (+): 0. Max coverage (-): 0

Region: chr15 30406193-30406213. Max. coverage (+): 0. Max coverage (-): 0

Region: chr15 30406214-30406234. Max. coverage (+): 0. Max coverage (-): 0

Region: chr15 30406235-30406256. Max. coverage (+): 0. Max coverage (-): 0

Region: chr15 30406257-30406277. Max. coverage (+): 0. Max coverage (-): 0

Region: chr15 30406278-30406298. Max. coverage (+): 0. Max coverage (-): 0

Region: chr15 30406299-30406320. Max. coverage (+): 5.47. Max coverage (-): 0

Region: chr15 30406321-30406341. Max. coverage (+): 5.47. Max coverage (-): 0

Region: chr15 30406342-30406362. Max. coverage (+): 0. Max coverage (-): 0

Region: chr15 30406363-30406384. Max. coverage (+): 1.35. Max coverage (-): 0

Region: chr15 30406385-30406405. Max. coverage (+): 1.35. Max coverage (-): 0

Region: chr15 30406406-30406427. Max. coverage (+): 0. Max coverage (-): 0

Region: chr15 30406428-30406448. Max. coverage (+): 0. Max coverage (-): 0

Region: chr15 30406449-30406469. Max. coverage (+): 0. Max coverage (-): 0

Region: chr15 30406470-30406491. Max. coverage (+): 0. Max coverage (-): 0

Region: chr15 30406492-30406512. Max. coverage (+): 0. Max coverage (-): 0

Region: chr15 30406513-30406533. Max. coverage (+): 0. Max coverage (-): 0

Region: chr15 30406534-30406555. Max. coverage (+): 0. Max coverage (-): 0

Region: chr15 30406556-30406576. Max. coverage (+): 0. Max coverage (-): 0

Region: chr15 30406577-30406597. Max. coverage (+): 0. Max coverage (-): 0

Region: chr15 30406598-30406619. Max. coverage (+): 0. Max coverage (-): 0

Region: chr15 30406620-30406640. Max. coverage (+): 0. Max coverage (-): 0

Region: chr15 30406641-30406662. Max. coverage (+): 4.22. Max coverage (-): 0

Region: chr15 30406663-30406683. Max. coverage (+): 0. Max coverage (-): 0

Region: chr15 30406684-30406704. Max. coverage (+): 0. Max coverage (-): 0

Region: chr15 30406705-30406726. Max. coverage (+): 0. Max coverage (-): 0

Region: chr15 30406727-30406747. Max. coverage (+): 4.99. Max coverage (-): 0

Region: chr15 30406748-30406768. Max. coverage (+): 0. Max coverage (-): 0

Region: chr15 30406769-30406790. Max. coverage (+): 4.64. Max coverage (-): 0

Region: chr15 30406791-30406811. Max. coverage (+): 8.53. Max coverage (-): 0

Region: chr15 30406812-30406832. Max. coverage (+): 0.61. Max coverage (-): 0

Region: chr15 30406833-30406854. Max. coverage (+): 3.12. Max coverage (-): 0

Region: chr15 30406855-30406875. Max. coverage (+): 8.7. Max coverage (-): 0

Region: chr15 30406876-30406897. Max. coverage (+): 1.18. Max coverage (-): 0

Region: chr15 30406898-30406918. Max. coverage (+): 0. Max coverage (-): 0

Region: chr15 30406919-30406939. Max. coverage (+): 0. Max coverage (-): 0

Region: chr15 30406940-30406961. Max. coverage (+): 0. Max coverage (-): 0

Region: chr15 30406962-30406982. Max. coverage (+): 1.59. Max coverage (-): 0

Region: chr15 30406983-30407003. Max. coverage (+): 0. Max coverage (-): 0

Region: chr15 30407004-30407025. Max. coverage (+): 0. Max coverage (-): 0

Region: chr15 30407026-30407046. Max. coverage (+): 0. Max coverage (-): 0

Region: chr15 30407047-30407067. Max. coverage (+): 6.94. Max coverage (-): 0

Region: chr15 30407068-30407089. Max. coverage (+): 0. Max coverage (-): 0

Region: chr15 30407090-30407110. Max. coverage (+): 0. Max coverage (-): 0

Region: chr15 30407111-30407132. Max. coverage (+): 0. Max coverage (-): 0

Region: chr15 30407133-30407153. Max. coverage (+): 0. Max coverage (-): 0

Region: chr15 30407154-30407174. Max. coverage (+): 0. Max coverage (-): 0

Region: chr15 30407175-30407196. Max. coverage (+): 0. Max coverage (-): 0

Region: chr15 30407197-30407217. Max. coverage (+): 0. Max coverage (-): 0

Region: chr15 30407218-30407238. Max. coverage (+): 6.43. Max coverage (-): 0

Region: chr15 30407239-30407260. Max. coverage (+): 0.81. Max coverage (-): 0

Region: chr15 30407261-30407281. Max. coverage (+): 2.85. Max coverage (-): 0

Region: chr15 30407282-30407302. Max. coverage (+): 0.43. Max coverage (-): 0

Region: chr15 30407303-30407324. Max. coverage (+): 1.34. Max coverage (-): 0

Region: chr15 30407325-30407345. Max. coverage (+): 1.34. Max coverage (-): 0

Region: chr15 30407346-30407367. Max. coverage (+): 5.28. Max coverage (-): 0

Region: chr15 30407368-30407388. Max. coverage (+): 7.53. Max coverage (-): 0

Region: chr15 30407389-30407409. Max. coverage (+): 2.62. Max coverage (-): 0

Region: chr15 30407410-30407431. Max. coverage (+): 6.24. Max coverage (-): 0

Region: chr15 30407432-30407452. Max. coverage (+): 8.34. Max coverage (-): 0

Region: chr15 30407453-30407473. Max. coverage (+): 2.92. Max coverage (-): 0

Region: chr15 30407474-30407495. Max. coverage (+): 4.6. Max coverage (-): 0

Region: chr15 30407496-30407516. Max. coverage (+): 0. Max coverage (-): 0

Region: chr15 30407517-30407537. Max. coverage (+): 3.76. Max coverage (-): 0

Region: chr15 30407538-30407559. Max. coverage (+): 6.86. Max coverage (-): 0

Region: chr15 30407560-30407580. Max. coverage (+): 6.86. Max coverage (-): 0

Region: chr15 30407581-30407602. Max. coverage (+): 0. Max coverage (-): 0

Region: chr15 30407603-30407623. Max. coverage (+): 11.1. Max coverage (-): 0

Region: chr15 30407624-30407644. Max. coverage (+): 4.88. Max coverage (-): 0

Region: chr15 30407645-30407666. Max. coverage (+): 10.98. Max coverage (-): 0

Region: chr15 30407667-30407687. Max. coverage (+): 9.87. Max coverage (-): 0

Region: chr15 30407688-30407708. Max. coverage (+): 0. Max coverage (-): 0

Region: chr15 30407709-30407730. Max. coverage (+): 1.89. Max coverage (-): 0

Region: chr15 30407731-30407751. Max. coverage (+): 0. Max coverage (-): 0

Region: chr15 30407752-30407772. Max. coverage (+): 0. Max coverage (-): 0

Region: chr15 30407773-30407794. Max. coverage (+): 0. Max coverage (-): 0

Region: chr15 30407795-30407815. Max. coverage (+): 3.14. Max coverage (-): 0

Region: chr15 30407816-30407837. Max. coverage (+): 3.14. Max coverage (-): 0

Region: chr15 30407838-30407858. Max. coverage (+): 2.37. Max coverage (-): 0

Region: chr15 30407859-30407879. Max. coverage (+): 1.26. Max coverage (-): 0

Region: chr15 30407880-30407901. Max. coverage (+): 16.54. Max coverage (-): 0

Region: chr15 30407902-30407922. Max. coverage (+): 16.22. Max coverage (-): 0

Region: chr15 30407923-30407943. Max. coverage (+): 1.05. Max coverage (-): 0

Region: chr15 30407944-30407965. Max. coverage (+): 2.53. Max coverage (-): 0

Region: chr15 30407966-30407986. Max. coverage (+): 3.32. Max coverage (-): 0

Region: chr15 30407987-30408007. Max. coverage (+): 7.84. Max coverage (-): 0

Region: chr15 30408008-30408029. Max. coverage (+): 1.24. Max coverage (-): 0

Region: chr15 30408030-30408050. Max. coverage (+): 4.59. Max coverage (-): 0

Region: chr15 30408051-30408072. Max. coverage (+): 3.08. Max coverage (-): 0

Region: chr15 30408073-30408093. Max. coverage (+): 3.08. Max coverage (-): 0

Region: chr15 30408094-30408114. Max. coverage (+): 9.67. Max coverage (-): 0

Region: chr15 30408115-30408136. Max. coverage (+): 9.67. Max coverage (-): 0

Region: chr15 30408137-30408157. Max. coverage (+): 34.47. Max coverage (-): 0

Region: chr15 30408158-30408178. Max. coverage (+): 34.47. Max coverage (-): 0

Region: chr15 30408179-30408200. Max. coverage (+): 10.76. Max coverage (-): 0

Region: chr15 30408201-30408221. Max. coverage (+): 0. Max coverage (-): 0

Region: chr15 30408222-30408242. Max. coverage (+): 2.07. Max coverage (-): 0

Region: chr15 30408243-30408264. Max. coverage (+): 24.28. Max coverage (-): 0

Region: chr15 30408265-30408285. Max. coverage (+): 11.81. Max coverage (-): 0

Region: chr15 30408286-30408307. Max. coverage (+): 0. Max coverage (-): 0

Region: chr15 30408308-30408328. Max. coverage (+): 0. Max coverage (-): 0

Region: chr15 30408329-30408349. Max. coverage (+): 1.8. Max coverage (-): 0

Region: chr15 30408350-30408371. Max. coverage (+): 0. Max coverage (-): 0

Region: chr15 30408372-30408392. Max. coverage (+): 1.65. Max coverage (-): 0

Region: chr15 30408393-30408413. Max. coverage (+): 0. Max coverage (-): 0

Region: chr15 30408414-30408435. Max. coverage (+): 2.88. Max coverage (-): 0

Region: chr15 30408436-30408456. Max. coverage (+): 2.88. Max coverage (-): 0

Region: chr15 30408457-30408477. Max. coverage (+): 1.84. Max coverage (-): 0

Region: chr15 30408478-30408499. Max. coverage (+): 30.55. Max coverage (-): 0

Region: chr15 30408500-30408520. Max. coverage (+): 6.26. Max coverage (-): 0

Region: chr15 30408521-30408542. Max. coverage (+): 0. Max coverage (-): 0

Region: chr15 30408543-30408563. Max. coverage (+): 1.95. Max coverage (-): 0

Region: chr15 30408564-30408584. Max. coverage (+): 1.95. Max coverage (-): 0

Region: chr15 30408585-30408606. Max. coverage (+): 4.98. Max coverage (-): 0

Region: chr15 30408607-30408627. Max. coverage (+): 4.98. Max coverage (-): 0

Region: chr15 30408628-30408648. Max. coverage (+): 0. Max coverage (-): 0

Region: chr15 30408649-30408670. Max. coverage (+): 0. Max coverage (-): 0

Region: chr15 30408671-30408691. Max. coverage (+): 11.4. Max coverage (-): 0

Region: chr15 30408692-30408712. Max. coverage (+): 0. Max coverage (-): 0

Region: chr15 30408713-30408734. Max. coverage (+): 3.62. Max coverage (-): 0

Region: chr15 30408735-30408755. Max. coverage (+): 11.95. Max coverage (-): 0

Region: chr15 30408756-30408777. Max. coverage (+): 16.06. Max coverage (-): 0

Region: chr15 30408778-30408798. Max. coverage (+): 8.92. Max coverage (-): 0

Region: chr15 30408799-30408819. Max. coverage (+): 0. Max coverage (-): 0

Region: chr15 30408820-30408841. Max. coverage (+): 3.79. Max coverage (-): 0

Region: chr15 30408842-30408862. Max. coverage (+): 7.6. Max coverage (-): 0

Region: chr15 30408863-30408883. Max. coverage (+): 0. Max coverage (-): 0

Region: chr15 30408884-30408905. Max. coverage (+): 2.2. Max coverage (-): 0

Region: chr15 30408906-30408926. Max. coverage (+): 16.58. Max coverage (-): 0

Region: chr15 30408927-30408947. Max. coverage (+): 6.27. Max coverage (-): 0

Region: chr15 30408948-30408969. Max. coverage (+): 3.32. Max coverage (-): 0

Region: chr15 30408970-30408990. Max. coverage (+): 3.3. Max coverage (-): 0

Region: chr15 30408991-30409012. Max. coverage (+): 0. Max coverage (-): 0

Region: chr15 30409013-30409033. Max. coverage (+): 1.73. Max coverage (-): 0

Region: chr15 30409034-30409054. Max. coverage (+): 7.14. Max coverage (-): 0

Region: chr15 30409055-30409076. Max. coverage (+): 7.14. Max coverage (-): 0

Region: chr15 30409077-30409097. Max. coverage (+): 4.99. Max coverage (-): 0

Region: chr15 30409098-30409118. Max. coverage (+): 4.99. Max coverage (-): 0

Region: chr15 30409119-30409140. Max. coverage (+): 2.3. Max coverage (-): 0

Region: chr15 30409141-30409161. Max. coverage (+): 2.3. Max coverage (-): 0

Region: chr15 30409162-30409182. Max. coverage (+): 0. Max coverage (-): 0

Region: chr15 30409183-30409204. Max. coverage (+): 0. Max coverage (-): 0

Region: chr15 30409205-30409225. Max. coverage (+): 0. Max coverage (-): 0

Region: chr15 30409226-30409247. Max. coverage (+): 24.25. Max coverage (-): 0

Region: chr15 30409248-30409268. Max. coverage (+): 3.22. Max coverage (-): 0

Region: chr15 30409269-30409289. Max. coverage (+): 0. Max coverage (-): 0

Region: chr15 30409290-30409311. Max. coverage (+): 0. Max coverage (-): 0

Region: chr15 30409312-30409332. Max. coverage (+): 5.61. Max coverage (-): 0

Region: chr15 30409333-30409353. Max. coverage (+): 3.25. Max coverage (-): 0

Region: chr15 30409354-30409375. Max. coverage (+): 4.34. Max coverage (-): 0

Region: chr15 30409376-30409396. Max. coverage (+): 1.87. Max coverage (-): 0

Region: chr15 30409397-30409417. Max. coverage (+): 0. Max coverage (-): 0

Region: chr15 30409418-30409439. Max. coverage (+): 1.43. Max coverage (-): 0

Region: chr15 30409440-30409460. Max. coverage (+): 2.25. Max coverage (-): 0

Region: chr15 30409461-30409482. Max. coverage (+): 8.17. Max coverage (-): 0

Region: chr15 30409483-30409503. Max. coverage (+): 0.85. Max coverage (-): 0

Region: chr15 30409504-30409524. Max. coverage (+): 0. Max coverage (-): 0

Region: chr15 30409525-30409546. Max. coverage (+): 0. Max coverage (-): 0

Region: chr15 30409547-30409567. Max. coverage (+): 0. Max coverage (-): 0

Region: chr15 30409568-30409588. Max. coverage (+): 7.1. Max coverage (-): 0

Region: chr15 30409589-30409610. Max. coverage (+): 1.47. Max coverage (-): 0

Region: chr15 30409611-30409631. Max. coverage (+): 19.37. Max coverage (-): 0

Region: chr15 30409632-30409652. Max. coverage (+): 18.83. Max coverage (-): 0

Region: chr15 30409653-30409674. Max. coverage (+): 3.25. Max coverage (-): 0

Region: chr15 30409675-30409695. Max. coverage (+): 0.25. Max coverage (-): 0

Region: chr15 30409696-30409717. Max. coverage (+): 42.86. Max coverage (-): 0

Region: chr15 30409718-30409738. Max. coverage (+): 0. Max coverage (-): 0

Region: chr15 30409739-30409759. Max. coverage (+): 0. Max coverage (-): 0

Region: chr15 30409760-30409781. Max. coverage (+): 18.72. Max coverage (-): 0

Region: chr15 30409782-30409802. Max. coverage (+): 10.56. Max coverage (-): 0

Region: chr15 30409803-30409823. Max. coverage (+): 2.59. Max coverage (-): 0

Region: chr15 30409824-30409845. Max. coverage (+): 2.59. Max coverage (-): 0

Region: chr15 30409846-30409866. Max. coverage (+): 4.79. Max coverage (-): 0

Region: chr15 30409867-30409887. Max. coverage (+): 22.89. Max coverage (-): 0

Region: chr15 30409888-30409909. Max. coverage (+): 9.79. Max coverage (-): 0

Region: chr15 30409910-30409930. Max. coverage (+): 4.3. Max coverage (-): 0

Region: chr15 30409931-30409952. Max. coverage (+): 2.99. Max coverage (-): 0

Region: chr15 30409953-30409973. Max. coverage (+): 2.99. Max coverage (-): 0

Region: chr15 30409974-30409994. Max. coverage (+): 0. Max coverage (-): 0

Region: chr15 30409995-30410016. Max. coverage (+): 5.21. Max coverage (-): 0

Region: chr15 30410017-30410037. Max. coverage (+): 11.05. Max coverage (-): 0

Region: chr15 30410038-30410058. Max. coverage (+): 11.89. Max coverage (-): 0

Region: chr15 30410059-30410080. Max. coverage (+): 7.24. Max coverage (-): 0

Region: chr15 30410081-30410101. Max. coverage (+): 1.99. Max coverage (-): 0

Region: chr15 30410102-30410122. Max. coverage (+): 9.07. Max coverage (-): 0

Region: chr15 30410123-30410144. Max. coverage (+): 12.64. Max coverage (-): 0

Region: chr15 30410145-30410165. Max. coverage (+): 4.12. Max coverage (-): 0

Region: chr15 30410166-30410187. Max. coverage (+): 4.33. Max coverage (-): 0

Region: chr15 30410188-30410208. Max. coverage (+): 4.77. Max coverage (-): 0

Region: chr15 30410209-30410229. Max. coverage (+): 4.77. Max coverage (-): 0

Region: chr15 30410230-30410251. Max. coverage (+): 3.31. Max coverage (-): 0

Region: chr15 30410252-30410272. Max. coverage (+): 0. Max coverage (-): 0

Region: chr15 30410273-30410293. Max. coverage (+): 0. Max coverage (-): 0

Region: chr15 30410294-30410315. Max. coverage (+): 1.74. Max coverage (-): 0

Region: chr15 30410316-30410336. Max. coverage (+): 12.13. Max coverage (-): 0

Region: chr15 30410337-30410357. Max. coverage (+): 12.87. Max coverage (-): 0

Region: chr15 30410358-30410379. Max. coverage (+): 11.25. Max coverage (-): 0

Region: chr15 30410380-30410400. Max. coverage (+): 12.74. Max coverage (-): 0

Region: chr15 30410401-30410422. Max. coverage (+): 4.44. Max coverage (-): 0

Region: chr15 30410423-30410443. Max. coverage (+): 3.52. Max coverage (-): 0

Region: chr15 30410444-30410464. Max. coverage (+): 11.77. Max coverage (-): 0

Region: chr15 30410465-30410486. Max. coverage (+): 4.87. Max coverage (-): 0

Region: chr15 30410487-30410507. Max. coverage (+): 12.23. Max coverage (-): 0

Region: chr15 30410508-30410528. Max. coverage (+): 3.18. Max coverage (-): 0

Region: chr15 30410529-30410550. Max. coverage (+): 3.18. Max coverage (-): 0

Region: chr15 30410551-30410571. Max. coverage (+): 0. Max coverage (-): 0

Region: chr15 30410572-30410592. Max. coverage (+): 6.13. Max coverage (-): 0

Region: chr15 30410593-30410614. Max. coverage (+): 9.89. Max coverage (-): 0

Region: chr15 30410615-30410635. Max. coverage (+): 19.3. Max coverage (-): 0

Region: chr15 30410636-30410657. Max. coverage (+): 13.66. Max coverage (-): 0

Region: chr15 30410658-30410678. Max. coverage (+): 0. Max coverage (-): 0

Region: chr15 30410679-30410699. Max. coverage (+): 0. Max coverage (-): 0

Region: chr15 30410700-30410721. Max. coverage (+): 0. Max coverage (-): 0

Region: chr15 30410722-30410742. Max. coverage (+): 2.97. Max coverage (-): 0

Region: chr15 30410743-30410763. Max. coverage (+): 7.25. Max coverage (-): 0

Region: chr15 30410764-30410785. Max. coverage (+): 15.41. Max coverage (-): 0

Region: chr15 30410786-30410806. Max. coverage (+): 5.03. Max coverage (-): 0

Region: chr15 30410807-30410827. Max. coverage (+): 0.8. Max coverage (-): 0

Region: chr15 30410828-30410849. Max. coverage (+): 2.11. Max coverage (-): 0

Region: chr15 30410850-30410870. Max. coverage (+): 0.75. Max coverage (-): 0

Region: chr15 30410871-30410892. Max. coverage (+): 3.85. Max coverage (-): 0

Region: chr15 30410893-30410913. Max. coverage (+): 6.79. Max coverage (-): 0

Region: chr15 30410914-30410934. Max. coverage (+): 4.97. Max coverage (-): 0

Region: chr15 30410935-30410956. Max. coverage (+): 0. Max coverage (-): 0

Region: chr15 30410957-30410977. Max. coverage (+): 0. Max coverage (-): 0

Region: chr15 30410978-30410998. Max. coverage (+): 0. Max coverage (-): 0

Region: chr15 30410999-30411020. Max. coverage (+): 0. Max coverage (-): 0

Region: chr15 30411021-30411041. Max. coverage (+): 0. Max coverage (-): 0

Region: chr15 30411042-30411062. Max. coverage (+): 0. Max coverage (-): 0

Region: chr15 30411063-30411084. Max. coverage (+): 1.67. Max coverage (-): 0

Region: chr15 30411085-30411105. Max. coverage (+): 0. Max coverage (-): 0

Region: chr15 30411106-30411127. Max. coverage (+): 0. Max coverage (-): 0

Region: chr15 30411128-30411148. Max. coverage (+): 0. Max coverage (-): 0

Region: chr15 30411149-30411169. Max. coverage (+): 1.02. Max coverage (-): 0

Region: chr15 30411170-30411191. Max. coverage (+): 4.18. Max coverage (-): 0

Region: chr15 30411192-30411212. Max. coverage (+): 4.18. Max coverage (-): 0

Region: chr15 30411213-30411233. Max. coverage (+): 3.3. Max coverage (-): 0

Region: chr15 30411234-30411255. Max. coverage (+): 22.01. Max coverage (-): 0

Region: chr15 30411256-30411276. Max. coverage (+): 4.91. Max coverage (-): 0

Region: chr15 30411277-30411298. Max. coverage (+): 7.49. Max coverage (-): 0

Region: chr15 30411299-30411319. Max. coverage (+): 16.82. Max coverage (-): 0

Region: chr15 30411320-30411340. Max. coverage (+): 16.82. Max coverage (-): 0

Region: chr15 30411341-30411362. Max. coverage (+): 9.37. Max coverage (-): 0

Region: chr15 30411363-30411383. Max. coverage (+): 16.68. Max coverage (-): 0

Region: chr15 30411384-30411404. Max. coverage (+): 10.19. Max coverage (-): 0

Region: chr15 30411405-30411426. Max. coverage (+): 0. Max coverage (-): 0

Region: chr15 30411427-30411447. Max. coverage (+): 5.99. Max coverage (-): 0

Region: chr15 30411448-30411468. Max. coverage (+): 7.91. Max coverage (-): 0

Region: chr15 30411469-30411490. Max. coverage (+): 0. Max coverage (-): 0

Region: chr15 30411491-30411511. Max. coverage (+): 0. Max coverage (-): 0

Region: chr15 30411512-30411533. Max. coverage (+): 4.38. Max coverage (-): 0

Region: chr15 30411534-30411554. Max. coverage (+): 4.06. Max coverage (-): 0

Region: chr15 30411555-30411575. Max. coverage (+): 8.31. Max coverage (-): 0

Region: chr15 30411576-30411597. Max. coverage (+): 14.83. Max coverage (-): 0

Region: chr15 30411598-30411618. Max. coverage (+): 6.64. Max coverage (-): 0

Region: chr15 30411619-30411639. Max. coverage (+): 5.39. Max coverage (-): 0

Region: chr15 30411640-30411661. Max. coverage (+): 7.26. Max coverage (-): 0

Region: chr15 30411662-30411682. Max. coverage (+): 10.53. Max coverage (-): 0

Region: chr15 30411683-30411703. Max. coverage (+): 0. Max coverage (-): 0

Region: chr15 30411704-30411725. Max. coverage (+): 47.06. Max coverage (-): 0

Region: chr15 30411726-30411746. Max. coverage (+): 5.02. Max coverage (-): 0

Region: chr15 30411747-30411768. Max. coverage (+): 3.17. Max coverage (-): 0

Region: chr15 30411769-30411789. Max. coverage (+): 16.01. Max coverage (-): 0

Region: chr15 30411790-30411810. Max. coverage (+): 7.57. Max coverage (-): 0

Region: chr15 30411811-30411832. Max. coverage (+): 0. Max coverage (-): 0

Region: chr15 30411833-30411853. Max. coverage (+): 0. Max coverage (-): 0

Region: chr15 30411854-30411874. Max. coverage (+): 6.57. Max coverage (-): 0

Region: chr15 30411875-30411896. Max. coverage (+): 9.51. Max coverage (-): 0

Region: chr15 30411897-30411917. Max. coverage (+): 9.1. Max coverage (-): 0

Region: chr15 30411918-30411938. Max. coverage (+): 0. Max coverage (-): 0

Region: chr15 30411939-30411960. Max. coverage (+): 1.63. Max coverage (-): 0

Region: chr15 30411961-30411981. Max. coverage (+): 0. Max coverage (-): 0

Region: chr15 30411982-30412003. Max. coverage (+): 0. Max coverage (-): 0

Region: chr15 30412004-30412024. Max. coverage (+): 0.38. Max coverage (-): 0

Region: chr15 30412025-30412045. Max. coverage (+): 0.38. Max coverage (-): 0

Region: chr15 30412046-30412067. Max. coverage (+): 2.68. Max coverage (-): 0

Region: chr15 30412068-30412088. Max. coverage (+): 2.68. Max coverage (-): 0

Region: chr15 30412089-30412109. Max. coverage (+): 0. Max coverage (-): 0

Region: chr15 30412110-30412131. Max. coverage (+): 0. Max coverage (-): 0

Region: chr15 30412132-30412152. Max. coverage (+): 44.51. Max coverage (-): 0

Region: chr15 30412153-30412173. Max. coverage (+): 0.19. Max coverage (-): 0

Region: chr15 30412174-30412195. Max. coverage (+): 23.28. Max coverage (-): 0

Region: chr15 30412196-30412216. Max. coverage (+): 10.77. Max coverage (-): 0

Region: chr15 30412217-30412238. Max. coverage (+): 42.16. Max coverage (-): 0

Region: chr15 30412239-30412259. Max. coverage (+): 27.47. Max coverage (-): 0

Region: chr15 30412260-30412280. Max. coverage (+): 25.38. Max coverage (-): 0

Region: chr15 30412281-30412302. Max. coverage (+): 18.99. Max coverage (-): 0

Region: chr15 30412303-30412323. Max. coverage (+): 18.93. Max coverage (-): 0

Region: chr15 30412324-30412344. Max. coverage (+): 5.3. Max coverage (-): 0

Region: chr15 30412345-30412366. Max. coverage (+): 4.98. Max coverage (-): 0

Region: chr15 30412367-30412387. Max. coverage (+): 2.36. Max coverage (-): 0

Region: chr15 30412388-30412408. Max. coverage (+): 0. Max coverage (-): 0

Region: chr15 30412409-30412430. Max. coverage (+): 0. Max coverage (-): 0

Region: chr15 30412431-30412451. Max. coverage (+): 0. Max coverage (-): 0

Region: chr15 30412452-30412473. Max. coverage (+): 0. Max coverage (-): 0

Region: chr15 30412474-30412494. Max. coverage (+): 0. Max coverage (-): 0

Region: chr15 30412495-30412515. Max. coverage (+): 10.62. Max coverage (-): 0

Region: chr15 30412516-30412537. Max. coverage (+): 11.51. Max coverage (-): 0

Region: chr15 30412538-30412558. Max. coverage (+): 2.62. Max coverage (-): 0

Region: chr15 30412559-30412579. Max. coverage (+): 5.75. Max coverage (-): 0

Region: chr15 30412580-30412601. Max. coverage (+): 5.75. Max coverage (-): 0

Region: chr15 30412602-30412622. Max. coverage (+): 0. Max coverage (-): 0

Region: chr15 30412623-30412643. Max. coverage (+): 1. Max coverage (-): 0

Region: chr15 30412644-30412665. Max. coverage (+): 1. Max coverage (-): 0

Region: chr15 30412666-30412686. Max. coverage (+): 0. Max coverage (-): 0

Region: chr15 30412687-30412708. Max. coverage (+): 0. Max coverage (-): 0

Region: chr15 30412709-30412729. Max. coverage (+): 0.88. Max coverage (-): 0

Region: chr15 30412730-30412750. Max. coverage (+): 0.88. Max coverage (-): 0

Region: chr15 30412751-30412772. Max. coverage (+): 21.62. Max coverage (-): 0

Region: chr15 30412773-30412793. Max. coverage (+): 2.96. Max coverage (-): 0

Region: chr15 30412794-30412814. Max. coverage (+): 0. Max coverage (-): 0

Region: chr15 30412815-30412836. Max. coverage (+): 0. Max coverage (-): 0

Region: chr15 30412837-30412857. Max. coverage (+): 10.87. Max coverage (-): 0

Region: chr15 30412858-30412878. Max. coverage (+): 0. Max coverage (-): 0

Region: chr15 30412879-30412900. Max. coverage (+): 0. Max coverage (-): 0

Region: chr15 30412901-30412921. Max. coverage (+): 0. Max coverage (-): 0

Region: chr15 30412922-30412943. Max. coverage (+): 0. Max coverage (-): 0

Region: chr15 30412944-30412964. Max. coverage (+): 0. Max coverage (-): 0

Region: chr15 30412965-30412985. Max. coverage (+): 12.88. Max coverage (-): 0

Region: chr15 30412986-30413007. Max. coverage (+): 9.81. Max coverage (-): 0

Region: chr15 30413008-30413028. Max. coverage (+): 0. Max coverage (-): 0

Region: chr15 30413029-30413049. Max. coverage (+): 15.32. Max coverage (-): 0

Region: chr15 30413050-30413071. Max. coverage (+): 0. Max coverage (-): 0

Region: chr15 30413072-30413092. Max. coverage (+): 0. Max coverage (-): 0

Region: chr15 30413093-30413113. Max. coverage (+): 0. Max coverage (-): 0

Region: chr15 30413114-30413135. Max. coverage (+): 0. Max coverage (-): 0

Region: chr15 30413136-30413156. Max. coverage (+): 5.47. Max coverage (-): 0

Region: chr15 30413157-30413178. Max. coverage (+): 8.38. Max coverage (-): 0

Region: chr15 30413179-30413199. Max. coverage (+): 0. Max coverage (-): 0

Region: chr15 30413200-30413220. Max. coverage (+): 2.24. Max coverage (-): 0

Region: chr15 30413221-30413242. Max. coverage (+): 0. Max coverage (-): 0

Region: chr15 30413243-30413263. Max. coverage (+): 0. Max coverage (-): 0

Region: chr15 30413264-30413284. Max. coverage (+): 0. Max coverage (-): 0

Region: chr15 30413285-30413306. Max. coverage (+): 2.03. Max coverage (-): 0

Region: chr15 30413307-30413327. Max. coverage (+): 0. Max coverage (-): 0

Region: chr15 30413328-30413348. Max. coverage (+): 0. Max coverage (-): 0

Region: chr15 30413349-30413370. Max. coverage (+): 0. Max coverage (-): 0

Region: chr15 30413371-30413391. Max. coverage (+): 0. Max coverage (-): 0

Region: chr15 30413392-30413413. Max. coverage (+): 0. Max coverage (-): 0

Region: chr15 30413414-30413434. Max. coverage (+): 0.71. Max coverage (-): 0

Region: chr15 30413435-30413455. Max. coverage (+): 0. Max coverage (-): 0

Region: chr15 30413456-30413477. Max. coverage (+): 0. Max coverage (-): 0

Region: chr15 30413478-30413498. Max. coverage (+): 0. Max coverage (-): 0

Region: chr15 30413499-30413519. Max. coverage (+): 0. Max coverage (-): 0

Region: chr15 30413520-30413541. Max. coverage (+): 0. Max coverage (-): 0

Region: chr15 30413542-30413562. Max. coverage (+): 0. Max coverage (-): 0

Region: chr15 30413563-30413583. Max. coverage (+): 0. Max coverage (-): 0

Region: chr15 30413584-30413605. Max. coverage (+): 0. Max coverage (-): 0

Region: chr15 30413606-30413626. Max. coverage (+): 0. Max coverage (-): 0

Region: chr15 30413627-30413648. Max. coverage (+): 0. Max coverage (-): 0

Region: chr15 30413649-30413669. Max. coverage (+): 0. Max coverage (-): 0

Region: chr15 30413670-30413690. Max. coverage (+): 0. Max coverage (-): 0

Region: chr15 30413691-30413712. Max. coverage (+): 0.48. Max coverage (-): 0

Region: chr15 30413713-30413733. Max. coverage (+): 0. Max coverage (-): 0

Region: chr15 30413734-30413754. Max. coverage (+): 0. Max coverage (-): 0

Region: chr15 30413755-30413776. Max. coverage (+): 0. Max coverage (-): 0

Region: chr15 30413777-30413797. Max. coverage (+): 0. Max coverage (-): 0

Region: chr15 30413798-30413818. Max. coverage (+): 0. Max coverage (-): 0

Region: chr15 30413819-30413840. Max. coverage (+): 5.05. Max coverage (-): 0

Region: chr15 30413841-. Max. coverage (+): 0. Max coverage (-): 0

RepeatMasker Color Code

**+**

100-98% Identity

<98-95% Identity

<95-90% Identity

<90-85% Identity

<85-80% Identity

<80-75% Identity

<75-70% Identity

<70% Identity

**-**

Gene Set Color Code

**+**

Gene

Pseudogene

**-**

Topology/Coverage Color Code

Coverage Plus Strand

Coverage Minus Strand

Mainstrand: Plus

Mainstrand: Minus

Complementary Strand

Flanking Region  
(if option -flank >0)

Gene Set Annotation  

**1. CBL (protein coding, ENSBTAG00000006817) Tr:00000008961 Ex:13**: 30403076-30403216 (+)  
**2. CBL (protein coding, ENSBTAG00000006817) Tr:00000008961 Ex:14**: 30403594-30403700 (+)  
**3. CBL (protein coding, ENSBTAG00000006817) Tr:00000008961 Ex:15**: 30404623-30404805 (+)  
**4. CBL (protein coding, ENSBTAG00000006817) Tr:00000008961 Ex:16**: 30405793-30406073 (+)

  
RepeatMasker Annotation  

**1. CHRL**: 30403749-30403926 (-), Divergence to consensus: 18.6%  
**2. L2a**: 30404465-30404555 (+), Divergence to consensus: 45.1%  
**3. MIRc**: 30405389-30405536 (+), Divergence to consensus: 43.6%

  
Transcription Factor Binding Sites  

**RFX4\_1** (Sequence: GTTGCCAGG (-): 30411619)  
**RFX4\_2** (Sequence: CGTGGTTAC (+): 30407980)  
**Gata4** (Sequence: AGATAAC (-): 30403546)  
**Gata4** (Sequence: GTTATCT (+): 30403485)  
**Gata4** (Sequence: GTTATCT (+): 30409936)  
**Gata4** (Sequence: CTTATCT (+): 30411526)
