# Supplementary material for: Small RNA sequencing of cryopreserved semen from single bull revealed altered miRNAs and piRNAs expression between High- and Low-motile sperm populations
Source: BMC Genomics. 2017 Jan 4;18:14. doi: 10.1186/s12864-016-3394-7 (PMC5209821; doi:10.1186/s12864-016-3394-7)
Supplement: Additional file 3: — Details for each piRNA clusters found in High Motile (HM) sperm fraction. Genes, repeats, transposable elements and transcription factors binding sites falling within the cluster regions were reported. (ZIP 1896 kb) [file 12864_2016_3394_MOESM3_ESM.zip › 28.html]

piRNA cluster 28


Predicted piRNA cluster no. 28     previous   next
  

Show proTRAC run info
Hide proTRAC run info

================================= proTRAC ====================================  
VERSION: 2.1                                    LAST MODIFIED: 06. October 2015  
  
Please cite:  
Rosenkranz D, Zischler H. proTRAC - a software for probabilistic piRNA cluster  
detection, visualization and analysis. 2012. BMC Bioinformatics 13:5.  
  
and (for proTRAC 2.0 and later):  
Rosenkranz D, Rudloff S, Bastuck K, Ketting RF, Zischler H. Tupaia small RNAs  
provide insights into function and evolution of RNAi-based transposon defense  
in mammals. 2015. RNA 21(5):911-922.  
  
Contact:  
David Rosenkranz  
Institute of Anthropology, small RNA group  
Johannes Gutenberg University Mainz  
email: rosenkranz@uni-mainz.de  
  
You can find the latest proTRAC version at:  
http://sourceforge.net/projects/protrac/files  
http://www.smallRNAgroup-mainz.de/software  
==============================================================================  
  
PARAMETERS:  
Map file: .............../storage/core/barbara/genhome/smallRNA/fertility/Sample\_motile/pirna/Sample\_motile\_26-33\_collapsed.fa.no-dust.map.weighted-10000-1000-b-0  
Genome file: ............/storage/core/barbara/genhome/smallRNA/fertility/Sample\_all/pirna/bt\_311\_chrY.fa  
RepeatMasker annotation: /storage/genomes/bt\_umd31/GCF\_000003055.6\_Bos\_taurus\_UMD\_3.1.1\_repeatMasker\_chr.out  
GeneSet:................./storage/core/barbara/genhome/smallRNA/fertility/Sample\_all/pirna/full.gtf  
  
Significant (p<=0.01) hit density will be calculated based  
on observed hit distribution.  
  
Sliding window size: ........................................ 5000 bp  
Sliding window increament: .................................. 1000 bp  
Normalize each hit by number of genomic hits: ............... 1 [0=no/1=yes]  
Normalize each hit by number of sequence reads: ............. 1 [0=no/1=yes]  
Normalize values (-> per million mapped reads): ............. 1 [0=no/1=yes]  
Min. fraction of hits with 1T(U) or 10A: .................... 0.75  
Alternatively: Min. fraction of hits with 1T(U) and 10A: .... 0.5  
Min. fraction of hits with typical piRNA length: ............ 0.75  
Typical piRNA length: ....................................... 26-33 nt  
Min. size of a piRNA cluster: ............................... 5000 bp.  
Min. number of hits (absolute): ............................. 0  
Min. number of hits (normalized): ........................... 0  
Min. fraction of hits on the mainstrand: .................... 0.75  
Top fraction of mapped sequences (in terms of read counts): . 1%  
Top fraction accounts for max. n% of sequence reads: ........ 90%  
Min. fraction of hits on each arm of a bidirectional cluster: 0.1  
Output image file for each cluster: ......................... 0 [0=no/1=yes]  
Output html file for each cluster: .......................... 1 [0=no/1=yes]  
Output a summary table: ..................................... 1 [0=no/1=yes]  
Output a FASTA file for each cluster (piRNA sequences): ..... 1 [0=no/1=yes]  
Output a FASTA file comprising cluster sequences: ........... 1 [0=no/1=yes]  
Search DNA motifs in clusters: .............................. 1 [0=no/1=yes]  
Output flanking sequences: +/- .............................. 0 bp  
Output ~.pTi file: .......................................... 1 [0=no/1=yes]  
==============================================================================  
  
  
Genome size (without gaps): ............ 2678902517 bp  
Gaps (N/X/-): .......................... 53837044 bp  
Mapped reads: .......................... 658825247023  
Non-identical sequences: ............... 514171  
Genomic hits: .......................... 764233  
Significant densitiy of mapped reads: .. 12867599.5173724 reads/kb

Show proTRAC cluster info
Hide proTRAC cluster info

|  |  |
| --- | --- |
| Location | chr15 |
| Coordinates | 55041103-55058419 |
| Size [bp] | 17317 |
| Sequence hit loci | 1546 |
| Mapped reads (normalized) | 1852269189.6 |
| Mapped reads (normalized) per kb | 106962475.6 |
| Normalized reads with 1T (1U) | 82.4% |
| Normalized reads with 10A | 31% |
| Normalized reads with length 26-33 nt | 100% |
| Normalized reads on the main strand(s) | 100% |
| Predicted directionality | mono:plus |

100%

0%

1T (1U)  
reads

10A reads

26-33 nt  
reads

reads on mainstrand

**Either the amount of reads with 1T (1U) OR 10A has to exceed 75% (set with option: -1Tor10A)  
Alternatively the amount of reads with 1T (1U) AND 10A has to exceed 50% (set with option: -1Tand10A)  
Minimum amount of reads with preferred size is 75% (set with option: -pisize)  
Minimum amount of reads on the main strand(s) is 75% (set with option: -clstrand)**

Show read coverage
Hide read coverage

WHAT DO I SEE HERE?  
This chart shows the location of mapped sequence reads within a predicted piRNA cluster. The color refers to the number of genomic hits produced by the sequence read in question. A dark red bar indicates that this sequence read produces many other hits elsewhere in the genome. Many adjacent red or yellow bars can indicate the presence of a multi-copy element such as transposons or rRNA genes. A dark green bar indicates that this sequence read maps uniquely to this locus.

1 hit

2-5 hits

6-10 hits

11-20 hits

21-50 hits

51-100 hits

> 100 hits

chr15

55041103

55058419

Gene Set

RepeatMasker

Mapped  
Reads

47.02

plus strand

minus strand

47.02

Region: chr15 30413842-55041120. Max. coverage (+): 9.1. Max coverage (-): 0

Region: chr15 55041121-55041154. Max. coverage (+): 7.25. Max coverage (-): 0

Region: chr15 55041155-55041189. Max. coverage (+): 6.05. Max coverage (-): 0

Region: chr15 55041190-55041224. Max. coverage (+): 6.05. Max coverage (-): 0

Region: chr15 55041225-55041258. Max. coverage (+): 0. Max coverage (-): 0

Region: chr15 55041259-55041293. Max. coverage (+): 0. Max coverage (-): 0

Region: chr15 55041294-55041328. Max. coverage (+): 0. Max coverage (-): 0

Region: chr15 55041329-55041362. Max. coverage (+): 0. Max coverage (-): 0

Region: chr15 55041363-55041397. Max. coverage (+): 0. Max coverage (-): 0

Region: chr15 55041398-55041432. Max. coverage (+): 2.95. Max coverage (-): 0

Region: chr15 55041433-55041466. Max. coverage (+): 2.99. Max coverage (-): 0

Region: chr15 55041467-55041501. Max. coverage (+): 2.42. Max coverage (-): 0

Region: chr15 55041502-55041535. Max. coverage (+): 0. Max coverage (-): 0

Region: chr15 55041536-55041570. Max. coverage (+): 0. Max coverage (-): 0

Region: chr15 55041571-55041605. Max. coverage (+): 2.55. Max coverage (-): 0

Region: chr15 55041606-55041639. Max. coverage (+): 0. Max coverage (-): 0

Region: chr15 55041640-55041674. Max. coverage (+): 6.02. Max coverage (-): 0

Region: chr15 55041675-55041709. Max. coverage (+): 0. Max coverage (-): 0

Region: chr15 55041710-55041743. Max. coverage (+): 0. Max coverage (-): 0

Region: chr15 55041744-55041778. Max. coverage (+): 0. Max coverage (-): 0

Region: chr15 55041779-55041812. Max. coverage (+): 0. Max coverage (-): 0

Region: chr15 55041813-55041847. Max. coverage (+): 2.14. Max coverage (-): 0

Region: chr15 55041848-55041882. Max. coverage (+): 9.78. Max coverage (-): 0

Region: chr15 55041883-55041916. Max. coverage (+): 4.32. Max coverage (-): 0

Region: chr15 55041917-55041951. Max. coverage (+): 0. Max coverage (-): 0

Region: chr15 55041952-55041986. Max. coverage (+): 1.59. Max coverage (-): 0

Region: chr15 55041987-55042020. Max. coverage (+): 3.61. Max coverage (-): 0

Region: chr15 55042021-55042055. Max. coverage (+): 4.5. Max coverage (-): 0

Region: chr15 55042056-55042090. Max. coverage (+): 34.42. Max coverage (-): 0

Region: chr15 55042091-55042124. Max. coverage (+): 13.62. Max coverage (-): 0

Region: chr15 55042125-55042159. Max. coverage (+): 10.67. Max coverage (-): 0

Region: chr15 55042160-55042193. Max. coverage (+): 13.69. Max coverage (-): 0

Region: chr15 55042194-55042228. Max. coverage (+): 4.54. Max coverage (-): 0

Region: chr15 55042229-55042263. Max. coverage (+): 5.67. Max coverage (-): 0

Region: chr15 55042264-55042297. Max. coverage (+): 7.24. Max coverage (-): 0

Region: chr15 55042298-55042332. Max. coverage (+): 15.67. Max coverage (-): 0

Region: chr15 55042333-55042367. Max. coverage (+): 0. Max coverage (-): 0

Region: chr15 55042368-55042401. Max. coverage (+): 0. Max coverage (-): 0

Region: chr15 55042402-55042436. Max. coverage (+): 0. Max coverage (-): 0

Region: chr15 55042437-55042471. Max. coverage (+): 0. Max coverage (-): 0

Region: chr15 55042472-55042505. Max. coverage (+): 0. Max coverage (-): 0

Region: chr15 55042506-55042540. Max. coverage (+): 1.84. Max coverage (-): 0

Region: chr15 55042541-55042574. Max. coverage (+): 31.56. Max coverage (-): 0

Region: chr15 55042575-55042609. Max. coverage (+): 3.16. Max coverage (-): 0

Region: chr15 55042610-55042644. Max. coverage (+): 4.6. Max coverage (-): 0

Region: chr15 55042645-55042678. Max. coverage (+): 4.91. Max coverage (-): 0

Region: chr15 55042679-55042713. Max. coverage (+): 14.64. Max coverage (-): 0

Region: chr15 55042714-55042748. Max. coverage (+): 16.3. Max coverage (-): 0

Region: chr15 55042749-55042782. Max. coverage (+): 11.63. Max coverage (-): 0

Region: chr15 55042783-55042817. Max. coverage (+): 10.69. Max coverage (-): 0

Region: chr15 55042818-55042852. Max. coverage (+): 36.73. Max coverage (-): 0

Region: chr15 55042853-55042886. Max. coverage (+): 12.45. Max coverage (-): 0

Region: chr15 55042887-55042921. Max. coverage (+): 14.2. Max coverage (-): 0

Region: chr15 55042922-55042955. Max. coverage (+): 3.55. Max coverage (-): 0

Region: chr15 55042956-55042990. Max. coverage (+): 21.05. Max coverage (-): 0

Region: chr15 55042991-55043025. Max. coverage (+): 1.15. Max coverage (-): 0

Region: chr15 55043026-55043059. Max. coverage (+): 0. Max coverage (-): 0

Region: chr15 55043060-55043094. Max. coverage (+): 5.94. Max coverage (-): 0

Region: chr15 55043095-55043129. Max. coverage (+): 8.49. Max coverage (-): 0

Region: chr15 55043130-55043163. Max. coverage (+): 24.18. Max coverage (-): 0

Region: chr15 55043164-55043198. Max. coverage (+): 24.18. Max coverage (-): 0

Region: chr15 55043199-55043232. Max. coverage (+): 6.41. Max coverage (-): 0

Region: chr15 55043233-55043267. Max. coverage (+): 23.27. Max coverage (-): 0

Region: chr15 55043268-55043302. Max. coverage (+): 26.45. Max coverage (-): 0

Region: chr15 55043303-55043336. Max. coverage (+): 43.39. Max coverage (-): 0

Region: chr15 55043337-55043371. Max. coverage (+): 6.03. Max coverage (-): 0

Region: chr15 55043372-55043406. Max. coverage (+): 24.53. Max coverage (-): 0

Region: chr15 55043407-55043440. Max. coverage (+): 9.47. Max coverage (-): 0

Region: chr15 55043441-55043475. Max. coverage (+): 29.13. Max coverage (-): 0

Region: chr15 55043476-55043510. Max. coverage (+): 14.33. Max coverage (-): 0

Region: chr15 55043511-55043544. Max. coverage (+): 37.42. Max coverage (-): 0

Region: chr15 55043545-55043579. Max. coverage (+): 47.02. Max coverage (-): 0

Region: chr15 55043580-55043613. Max. coverage (+): 18.04. Max coverage (-): 0

Region: chr15 55043614-55043648. Max. coverage (+): 11.87. Max coverage (-): 0

Region: chr15 55043649-55043683. Max. coverage (+): 7.68. Max coverage (-): 0

Region: chr15 55043684-55043717. Max. coverage (+): 5.14. Max coverage (-): 0

Region: chr15 55043718-55043752. Max. coverage (+): 4.53. Max coverage (-): 0

Region: chr15 55043753-55043787. Max. coverage (+): 4.06. Max coverage (-): 0

Region: chr15 55043788-55043821. Max. coverage (+): 3.09. Max coverage (-): 0

Region: chr15 55043822-55043856. Max. coverage (+): 1.52. Max coverage (-): 0

Region: chr15 55043857-55043891. Max. coverage (+): 13.2. Max coverage (-): 0

Region: chr15 55043892-55043925. Max. coverage (+): 15.76. Max coverage (-): 0

Region: chr15 55043926-55043960. Max. coverage (+): 20.48. Max coverage (-): 0

Region: chr15 55043961-55043994. Max. coverage (+): 14.75. Max coverage (-): 0

Region: chr15 55043995-55044029. Max. coverage (+): 1.55. Max coverage (-): 0

Region: chr15 55044030-55044064. Max. coverage (+): 0. Max coverage (-): 0

Region: chr15 55044065-55044098. Max. coverage (+): 0. Max coverage (-): 0

Region: chr15 55044099-55044133. Max. coverage (+): 7.52. Max coverage (-): 0

Region: chr15 55044134-55044168. Max. coverage (+): 1.88. Max coverage (-): 0

Region: chr15 55044169-55044202. Max. coverage (+): 5.18. Max coverage (-): 0

Region: chr15 55044203-55044237. Max. coverage (+): 13.39. Max coverage (-): 0

Region: chr15 55044238-55044272. Max. coverage (+): 5.01. Max coverage (-): 0

Region: chr15 55044273-55044306. Max. coverage (+): 0. Max coverage (-): 0

Region: chr15 55044307-55044341. Max. coverage (+): 2.47. Max coverage (-): 0

Region: chr15 55044342-55044375. Max. coverage (+): 4.65. Max coverage (-): 0

Region: chr15 55044376-55044410. Max. coverage (+): 25.32. Max coverage (-): 0

Region: chr15 55044411-55044445. Max. coverage (+): 3.7. Max coverage (-): 0

Region: chr15 55044446-55044479. Max. coverage (+): 19.42. Max coverage (-): 0

Region: chr15 55044480-55044514. Max. coverage (+): 8.3. Max coverage (-): 0

Region: chr15 55044515-55044549. Max. coverage (+): 0. Max coverage (-): 0

Region: chr15 55044550-55044583. Max. coverage (+): 0. Max coverage (-): 0

Region: chr15 55044584-55044618. Max. coverage (+): 4.55. Max coverage (-): 0

Region: chr15 55044619-55044652. Max. coverage (+): 6.37. Max coverage (-): 0

Region: chr15 55044653-55044687. Max. coverage (+): 0. Max coverage (-): 0

Region: chr15 55044688-55044722. Max. coverage (+): 13.31. Max coverage (-): 0

Region: chr15 55044723-55044756. Max. coverage (+): 13.31. Max coverage (-): 0

Region: chr15 55044757-55044791. Max. coverage (+): 6.28. Max coverage (-): 0

Region: chr15 55044792-55044826. Max. coverage (+): 6.9. Max coverage (-): 0

Region: chr15 55044827-55044860. Max. coverage (+): 2.88. Max coverage (-): 0

Region: chr15 55044861-55044895. Max. coverage (+): 1.31. Max coverage (-): 0

Region: chr15 55044896-55044930. Max. coverage (+): 3.88. Max coverage (-): 0

Region: chr15 55044931-55044964. Max. coverage (+): 4.36. Max coverage (-): 0

Region: chr15 55044965-55044999. Max. coverage (+): 4.36. Max coverage (-): 0

Region: chr15 55045000-55045033. Max. coverage (+): 0. Max coverage (-): 0

Region: chr15 55045034-55045068. Max. coverage (+): 0. Max coverage (-): 0

Region: chr15 55045069-55045103. Max. coverage (+): 2.9. Max coverage (-): 0

Region: chr15 55045104-55045137. Max. coverage (+): 3.11. Max coverage (-): 0

Region: chr15 55045138-55045172. Max. coverage (+): 3.02. Max coverage (-): 0

Region: chr15 55045173-55045207. Max. coverage (+): 0. Max coverage (-): 0

Region: chr15 55045208-55045241. Max. coverage (+): 0. Max coverage (-): 0

Region: chr15 55045242-55045276. Max. coverage (+): 0. Max coverage (-): 0

Region: chr15 55045277-55045311. Max. coverage (+): 0. Max coverage (-): 0

Region: chr15 55045312-55045345. Max. coverage (+): 0. Max coverage (-): 0

Region: chr15 55045346-55045380. Max. coverage (+): 0. Max coverage (-): 0

Region: chr15 55045381-55045414. Max. coverage (+): 24.63. Max coverage (-): 0

Region: chr15 55045415-55045449. Max. coverage (+): 24.63. Max coverage (-): 0

Region: chr15 55045450-55045484. Max. coverage (+): 7.69. Max coverage (-): 0

Region: chr15 55045485-55045518. Max. coverage (+): 6.28. Max coverage (-): 0

Region: chr15 55045519-55045553. Max. coverage (+): 0. Max coverage (-): 0

Region: chr15 55045554-55045588. Max. coverage (+): 7.73. Max coverage (-): 0

Region: chr15 55045589-55045622. Max. coverage (+): 7.73. Max coverage (-): 0

Region: chr15 55045623-55045657. Max. coverage (+): 6.84. Max coverage (-): 0

Region: chr15 55045658-55045692. Max. coverage (+): 0. Max coverage (-): 0

Region: chr15 55045693-55045726. Max. coverage (+): 3.82. Max coverage (-): 0

Region: chr15 55045727-55045761. Max. coverage (+): 4.88. Max coverage (-): 0

Region: chr15 55045762-55045795. Max. coverage (+): 21.35. Max coverage (-): 0

Region: chr15 55045796-55045830. Max. coverage (+): 32.22. Max coverage (-): 0

Region: chr15 55045831-55045865. Max. coverage (+): 0. Max coverage (-): 0

Region: chr15 55045866-55045899. Max. coverage (+): 0. Max coverage (-): 0

Region: chr15 55045900-55045934. Max. coverage (+): 0. Max coverage (-): 0

Region: chr15 55045935-55045969. Max. coverage (+): 0. Max coverage (-): 0

Region: chr15 55045970-55046003. Max. coverage (+): 8.66. Max coverage (-): 0

Region: chr15 55046004-55046038. Max. coverage (+): 0. Max coverage (-): 0

Region: chr15 55046039-55046072. Max. coverage (+): 8.72. Max coverage (-): 0

Region: chr15 55046073-55046107. Max. coverage (+): 39.95. Max coverage (-): 0

Region: chr15 55046108-55046142. Max. coverage (+): 21.64. Max coverage (-): 0

Region: chr15 55046143-55046176. Max. coverage (+): 3.14. Max coverage (-): 0

Region: chr15 55046177-55046211. Max. coverage (+): 1.31. Max coverage (-): 0

Region: chr15 55046212-55046246. Max. coverage (+): 24.37. Max coverage (-): 0

Region: chr15 55046247-55046280. Max. coverage (+): 11.03. Max coverage (-): 0

Region: chr15 55046281-55046315. Max. coverage (+): 2.2. Max coverage (-): 0

Region: chr15 55046316-55046350. Max. coverage (+): 10.53. Max coverage (-): 0

Region: chr15 55046351-55046384. Max. coverage (+): 33.15. Max coverage (-): 0

Region: chr15 55046385-55046419. Max. coverage (+): 3.41. Max coverage (-): 0

Region: chr15 55046420-55046453. Max. coverage (+): 2.63. Max coverage (-): 0

Region: chr15 55046454-55046488. Max. coverage (+): 3.06. Max coverage (-): 0

Region: chr15 55046489-55046523. Max. coverage (+): 3.41. Max coverage (-): 0

Region: chr15 55046524-55046557. Max. coverage (+): 7.56. Max coverage (-): 0

Region: chr15 55046558-55046592. Max. coverage (+): 8.17. Max coverage (-): 0

Region: chr15 55046593-55046627. Max. coverage (+): 3.26. Max coverage (-): 0

Region: chr15 55046628-55046661. Max. coverage (+): 7.05. Max coverage (-): 0

Region: chr15 55046662-55046696. Max. coverage (+): 5.2. Max coverage (-): 0

Region: chr15 55046697-55046731. Max. coverage (+): 2.04. Max coverage (-): 0

Region: chr15 55046732-55046765. Max. coverage (+): 1.42. Max coverage (-): 0

Region: chr15 55046766-55046800. Max. coverage (+): 6. Max coverage (-): 0

Region: chr15 55046801-55046834. Max. coverage (+): 14.88. Max coverage (-): 0

Region: chr15 55046835-55046869. Max. coverage (+): 14.61. Max coverage (-): 0

Region: chr15 55046870-55046904. Max. coverage (+): 1.79. Max coverage (-): 0

Region: chr15 55046905-55046938. Max. coverage (+): 2.11. Max coverage (-): 0

Region: chr15 55046939-55046973. Max. coverage (+): 2.63. Max coverage (-): 0

Region: chr15 55046974-55047008. Max. coverage (+): 2.63. Max coverage (-): 0

Region: chr15 55047009-55047042. Max. coverage (+): 0. Max coverage (-): 0

Region: chr15 55047043-55047077. Max. coverage (+): 2.1. Max coverage (-): 0

Region: chr15 55047078-55047111. Max. coverage (+): 8.41. Max coverage (-): 0

Region: chr15 55047112-55047146. Max. coverage (+): 4.71. Max coverage (-): 0

Region: chr15 55047147-55047181. Max. coverage (+): 4.78. Max coverage (-): 0

Region: chr15 55047182-55047215. Max. coverage (+): 0. Max coverage (-): 0

Region: chr15 55047216-55047250. Max. coverage (+): 0. Max coverage (-): 0

Region: chr15 55047251-55047285. Max. coverage (+): 0. Max coverage (-): 0

Region: chr15 55047286-55047319. Max. coverage (+): 4.75. Max coverage (-): 0

Region: chr15 55047320-55047354. Max. coverage (+): 6.39. Max coverage (-): 0

Region: chr15 55047355-55047389. Max. coverage (+): 4.98. Max coverage (-): 0

Region: chr15 55047390-55047423. Max. coverage (+): 12.58. Max coverage (-): 0

Region: chr15 55047424-55047458. Max. coverage (+): 11.7. Max coverage (-): 0

Region: chr15 55047459-55047492. Max. coverage (+): 17.42. Max coverage (-): 0

Region: chr15 55047493-55047527. Max. coverage (+): 7.37. Max coverage (-): 0

Region: chr15 55047528-55047562. Max. coverage (+): 6.19. Max coverage (-): 0

Region: chr15 55047563-55047596. Max. coverage (+): 0. Max coverage (-): 0

Region: chr15 55047597-55047631. Max. coverage (+): 13.29. Max coverage (-): 0

Region: chr15 55047632-55047666. Max. coverage (+): 13.29. Max coverage (-): 0

Region: chr15 55047667-55047700. Max. coverage (+): 3.49. Max coverage (-): 0

Region: chr15 55047701-55047735. Max. coverage (+): 0. Max coverage (-): 0

Region: chr15 55047736-55047770. Max. coverage (+): 9.18. Max coverage (-): 0

Region: chr15 55047771-55047804. Max. coverage (+): 9.18. Max coverage (-): 0

Region: chr15 55047805-55047839. Max. coverage (+): 5.65. Max coverage (-): 0

Region: chr15 55047840-55047873. Max. coverage (+): 5.54. Max coverage (-): 0

Region: chr15 55047874-55047908. Max. coverage (+): 8.72. Max coverage (-): 0

Region: chr15 55047909-55047943. Max. coverage (+): 0. Max coverage (-): 0

Region: chr15 55047944-55047977. Max. coverage (+): 0. Max coverage (-): 0

Region: chr15 55047978-55048012. Max. coverage (+): 0. Max coverage (-): 0

Region: chr15 55048013-55048047. Max. coverage (+): 0. Max coverage (-): 0

Region: chr15 55048048-55048081. Max. coverage (+): 0. Max coverage (-): 0

Region: chr15 55048082-55048116. Max. coverage (+): 0. Max coverage (-): 0

Region: chr15 55048117-55048151. Max. coverage (+): 0. Max coverage (-): 0

Region: chr15 55048152-55048185. Max. coverage (+): 7.25. Max coverage (-): 0

Region: chr15 55048186-55048220. Max. coverage (+): 15.38. Max coverage (-): 0

Region: chr15 55048221-55048254. Max. coverage (+): 0. Max coverage (-): 0

Region: chr15 55048255-55048289. Max. coverage (+): 0. Max coverage (-): 0

Region: chr15 55048290-55048324. Max. coverage (+): 0. Max coverage (-): 0

Region: chr15 55048325-55048358. Max. coverage (+): 5.78. Max coverage (-): 0

Region: chr15 55048359-55048393. Max. coverage (+): 16.82. Max coverage (-): 0

Region: chr15 55048394-55048428. Max. coverage (+): 9.26. Max coverage (-): 0

Region: chr15 55048429-55048462. Max. coverage (+): 3.19. Max coverage (-): 0

Region: chr15 55048463-55048497. Max. coverage (+): 3.99. Max coverage (-): 0

Region: chr15 55048498-55048531. Max. coverage (+): 5.61. Max coverage (-): 0

Region: chr15 55048532-55048566. Max. coverage (+): 0. Max coverage (-): 0

Region: chr15 55048567-55048601. Max. coverage (+): 0. Max coverage (-): 0

Region: chr15 55048602-55048635. Max. coverage (+): 5.76. Max coverage (-): 0

Region: chr15 55048636-55048670. Max. coverage (+): 0. Max coverage (-): 0

Region: chr15 55048671-55048705. Max. coverage (+): 7.19. Max coverage (-): 0

Region: chr15 55048706-55048739. Max. coverage (+): 0.76. Max coverage (-): 0

Region: chr15 55048740-55048774. Max. coverage (+): 1.1. Max coverage (-): 0

Region: chr15 55048775-55048809. Max. coverage (+): 10. Max coverage (-): 0

Region: chr15 55048810-55048843. Max. coverage (+): 4.73. Max coverage (-): 0

Region: chr15 55048844-55048878. Max. coverage (+): 0. Max coverage (-): 0

Region: chr15 55048879-55048912. Max. coverage (+): 7.41. Max coverage (-): 0

Region: chr15 55048913-55048947. Max. coverage (+): 7.72. Max coverage (-): 0

Region: chr15 55048948-55048982. Max. coverage (+): 0. Max coverage (-): 0

Region: chr15 55048983-55049016. Max. coverage (+): 3.25. Max coverage (-): 0

Region: chr15 55049017-55049051. Max. coverage (+): 3.25. Max coverage (-): 0

Region: chr15 55049052-55049086. Max. coverage (+): 0. Max coverage (-): 0

Region: chr15 55049087-55049120. Max. coverage (+): 3.91. Max coverage (-): 0

Region: chr15 55049121-55049155. Max. coverage (+): 0. Max coverage (-): 0

Region: chr15 55049156-55049190. Max. coverage (+): 0. Max coverage (-): 0

Region: chr15 55049191-55049224. Max. coverage (+): 0. Max coverage (-): 0

Region: chr15 55049225-55049259. Max. coverage (+): 0. Max coverage (-): 0

Region: chr15 55049260-55049293. Max. coverage (+): 0. Max coverage (-): 0

Region: chr15 55049294-55049328. Max. coverage (+): 0. Max coverage (-): 0

Region: chr15 55049329-55049363. Max. coverage (+): 0. Max coverage (-): 0

Region: chr15 55049364-55049397. Max. coverage (+): 0.79. Max coverage (-): 0

Region: chr15 55049398-55049432. Max. coverage (+): 0. Max coverage (-): 0

Region: chr15 55049433-55049467. Max. coverage (+): 0. Max coverage (-): 0

Region: chr15 55049468-55049501. Max. coverage (+): 0. Max coverage (-): 0

Region: chr15 55049502-55049536. Max. coverage (+): 0. Max coverage (-): 0

Region: chr15 55049537-55049571. Max. coverage (+): 0. Max coverage (-): 0

Region: chr15 55049572-55049605. Max. coverage (+): 0. Max coverage (-): 0

Region: chr15 55049606-55049640. Max. coverage (+): 0. Max coverage (-): 0

Region: chr15 55049641-55049674. Max. coverage (+): 0. Max coverage (-): 0

Region: chr15 55049675-55049709. Max. coverage (+): 0. Max coverage (-): 0

Region: chr15 55049710-55049744. Max. coverage (+): 0. Max coverage (-): 0

Region: chr15 55049745-55049778. Max. coverage (+): 0. Max coverage (-): 0

Region: chr15 55049779-55049813. Max. coverage (+): 0. Max coverage (-): 0

Region: chr15 55049814-55049848. Max. coverage (+): 0. Max coverage (-): 0

Region: chr15 55049849-55049882. Max. coverage (+): 0. Max coverage (-): 0

Region: chr15 55049883-55049917. Max. coverage (+): 0. Max coverage (-): 0

Region: chr15 55049918-55049951. Max. coverage (+): 0. Max coverage (-): 0

Region: chr15 55049952-55049986. Max. coverage (+): 0. Max coverage (-): 0

Region: chr15 55049987-55050021. Max. coverage (+): 0. Max coverage (-): 0

Region: chr15 55050022-55050055. Max. coverage (+): 0. Max coverage (-): 0

Region: chr15 55050056-55050090. Max. coverage (+): 0. Max coverage (-): 0

Region: chr15 55050091-55050125. Max. coverage (+): 0. Max coverage (-): 0

Region: chr15 55050126-55050159. Max. coverage (+): 0. Max coverage (-): 0

Region: chr15 55050160-55050194. Max. coverage (+): 0. Max coverage (-): 0

Region: chr15 55050195-55050229. Max. coverage (+): 0. Max coverage (-): 0

Region: chr15 55050230-55050263. Max. coverage (+): 0. Max coverage (-): 0

Region: chr15 55050264-55050298. Max. coverage (+): 0. Max coverage (-): 0

Region: chr15 55050299-55050332. Max. coverage (+): 0. Max coverage (-): 0

Region: chr15 55050333-55050367. Max. coverage (+): 0. Max coverage (-): 0

Region: chr15 55050368-55050402. Max. coverage (+): 0. Max coverage (-): 0

Region: chr15 55050403-55050436. Max. coverage (+): 0. Max coverage (-): 0

Region: chr15 55050437-55050471. Max. coverage (+): 0. Max coverage (-): 0

Region: chr15 55050472-55050506. Max. coverage (+): 0. Max coverage (-): 0

Region: chr15 55050507-55050540. Max. coverage (+): 0. Max coverage (-): 0

Region: chr15 55050541-55050575. Max. coverage (+): 0. Max coverage (-): 0

Region: chr15 55050576-55050610. Max. coverage (+): 0. Max coverage (-): 0

Region: chr15 55050611-55050644. Max. coverage (+): 0. Max coverage (-): 0

Region: chr15 55050645-55050679. Max. coverage (+): 0. Max coverage (-): 0

Region: chr15 55050680-55050713. Max. coverage (+): 0. Max coverage (-): 0

Region: chr15 55050714-55050748. Max. coverage (+): 14.6. Max coverage (-): 0

Region: chr15 55050749-55050783. Max. coverage (+): 10.25. Max coverage (-): 0

Region: chr15 55050784-55050817. Max. coverage (+): 0. Max coverage (-): 0

Region: chr15 55050818-55050852. Max. coverage (+): 3.92. Max coverage (-): 0

Region: chr15 55050853-55050887. Max. coverage (+): 11. Max coverage (-): 0

Region: chr15 55050888-55050921. Max. coverage (+): 0.63. Max coverage (-): 0

Region: chr15 55050922-55050956. Max. coverage (+): 5.29. Max coverage (-): 0

Region: chr15 55050957-55050991. Max. coverage (+): 2.45. Max coverage (-): 0

Region: chr15 55050992-55051025. Max. coverage (+): 2.45. Max coverage (-): 0

Region: chr15 55051026-55051060. Max. coverage (+): 0. Max coverage (-): 0

Region: chr15 55051061-55051094. Max. coverage (+): 0. Max coverage (-): 0

Region: chr15 55051095-55051129. Max. coverage (+): 0. Max coverage (-): 0

Region: chr15 55051130-55051164. Max. coverage (+): 2.39. Max coverage (-): 0

Region: chr15 55051165-55051198. Max. coverage (+): 16. Max coverage (-): 0

Region: chr15 55051199-55051233. Max. coverage (+): 16. Max coverage (-): 0

Region: chr15 55051234-55051268. Max. coverage (+): 17.14. Max coverage (-): 0

Region: chr15 55051269-55051302. Max. coverage (+): 0. Max coverage (-): 0

Region: chr15 55051303-55051337. Max. coverage (+): 11.24. Max coverage (-): 0

Region: chr15 55051338-55051371. Max. coverage (+): 4.08. Max coverage (-): 0

Region: chr15 55051372-55051406. Max. coverage (+): 4.08. Max coverage (-): 0

Region: chr15 55051407-55051441. Max. coverage (+): 10.45. Max coverage (-): 0

Region: chr15 55051442-55051475. Max. coverage (+): 16.7. Max coverage (-): 0

Region: chr15 55051476-55051510. Max. coverage (+): 31.37. Max coverage (-): 0

Region: chr15 55051511-55051545. Max. coverage (+): 29.96. Max coverage (-): 0

Region: chr15 55051546-55051579. Max. coverage (+): 0. Max coverage (-): 0

Region: chr15 55051580-55051614. Max. coverage (+): 10.39. Max coverage (-): 0

Region: chr15 55051615-55051649. Max. coverage (+): 12.06. Max coverage (-): 0

Region: chr15 55051650-55051683. Max. coverage (+): 5.86. Max coverage (-): 0

Region: chr15 55051684-55051718. Max. coverage (+): 0. Max coverage (-): 0

Region: chr15 55051719-55051752. Max. coverage (+): 0. Max coverage (-): 0

Region: chr15 55051753-55051787. Max. coverage (+): 0. Max coverage (-): 0

Region: chr15 55051788-55051822. Max. coverage (+): 0. Max coverage (-): 0

Region: chr15 55051823-55051856. Max. coverage (+): 0. Max coverage (-): 0

Region: chr15 55051857-55051891. Max. coverage (+): 0. Max coverage (-): 0

Region: chr15 55051892-55051926. Max. coverage (+): 0. Max coverage (-): 0

Region: chr15 55051927-55051960. Max. coverage (+): 0. Max coverage (-): 0

Region: chr15 55051961-55051995. Max. coverage (+): 0. Max coverage (-): 0

Region: chr15 55051996-55052030. Max. coverage (+): 1.28. Max coverage (-): 0

Region: chr15 55052031-55052064. Max. coverage (+): 33.66. Max coverage (-): 0

Region: chr15 55052065-55052099. Max. coverage (+): 13.21. Max coverage (-): 0

Region: chr15 55052100-55052133. Max. coverage (+): 8.52. Max coverage (-): 0

Region: chr15 55052134-55052168. Max. coverage (+): 25.48. Max coverage (-): 0

Region: chr15 55052169-55052203. Max. coverage (+): 14.13. Max coverage (-): 0

Region: chr15 55052204-55052237. Max. coverage (+): 11.85. Max coverage (-): 0

Region: chr15 55052238-55052272. Max. coverage (+): 1.45. Max coverage (-): 0

Region: chr15 55052273-55052307. Max. coverage (+): 3.62. Max coverage (-): 0

Region: chr15 55052308-55052341. Max. coverage (+): 19.58. Max coverage (-): 0

Region: chr15 55052342-55052376. Max. coverage (+): 4.05. Max coverage (-): 0

Region: chr15 55052377-55052411. Max. coverage (+): 3.24. Max coverage (-): 0

Region: chr15 55052412-55052445. Max. coverage (+): 32.64. Max coverage (-): 0

Region: chr15 55052446-55052480. Max. coverage (+): 4.65. Max coverage (-): 0

Region: chr15 55052481-55052514. Max. coverage (+): 3.6. Max coverage (-): 0

Region: chr15 55052515-55052549. Max. coverage (+): 27.79. Max coverage (-): 0

Region: chr15 55052550-55052584. Max. coverage (+): 18.98. Max coverage (-): 0

Region: chr15 55052585-55052618. Max. coverage (+): 9.71. Max coverage (-): 0

Region: chr15 55052619-55052653. Max. coverage (+): 10.58. Max coverage (-): 0

Region: chr15 55052654-55052688. Max. coverage (+): 8.68. Max coverage (-): 0

Region: chr15 55052689-55052722. Max. coverage (+): 6.82. Max coverage (-): 0

Region: chr15 55052723-55052757. Max. coverage (+): 3.67. Max coverage (-): 0

Region: chr15 55052758-55052791. Max. coverage (+): 0. Max coverage (-): 0

Region: chr15 55052792-55052826. Max. coverage (+): 2.25. Max coverage (-): 0

Region: chr15 55052827-55052861. Max. coverage (+): 5.03. Max coverage (-): 0

Region: chr15 55052862-55052895. Max. coverage (+): 19.63. Max coverage (-): 0

Region: chr15 55052896-55052930. Max. coverage (+): 19.63. Max coverage (-): 0

Region: chr15 55052931-55052965. Max. coverage (+): 23.71. Max coverage (-): 0

Region: chr15 55052966-55052999. Max. coverage (+): 0. Max coverage (-): 0

Region: chr15 55053000-55053034. Max. coverage (+): 10.93. Max coverage (-): 0

Region: chr15 55053035-55053069. Max. coverage (+): 13.06. Max coverage (-): 0

Region: chr15 55053070-55053103. Max. coverage (+): 4.15. Max coverage (-): 0

Region: chr15 55053104-55053138. Max. coverage (+): 1.2. Max coverage (-): 0

Region: chr15 55053139-55053172. Max. coverage (+): 6.29. Max coverage (-): 0

Region: chr15 55053173-55053207. Max. coverage (+): 15.47. Max coverage (-): 0

Region: chr15 55053208-55053242. Max. coverage (+): 37. Max coverage (-): 0

Region: chr15 55053243-55053276. Max. coverage (+): 0. Max coverage (-): 0

Region: chr15 55053277-55053311. Max. coverage (+): 5.32. Max coverage (-): 0

Region: chr15 55053312-55053346. Max. coverage (+): 5.32. Max coverage (-): 0

Region: chr15 55053347-55053380. Max. coverage (+): 2.59. Max coverage (-): 0

Region: chr15 55053381-55053415. Max. coverage (+): 0. Max coverage (-): 0

Region: chr15 55053416-55053450. Max. coverage (+): 2.1. Max coverage (-): 0

Region: chr15 55053451-55053484. Max. coverage (+): 10.16. Max coverage (-): 0

Region: chr15 55053485-55053519. Max. coverage (+): 1.92. Max coverage (-): 0

Region: chr15 55053520-55053553. Max. coverage (+): 9.96. Max coverage (-): 0

Region: chr15 55053554-55053588. Max. coverage (+): 13.97. Max coverage (-): 0

Region: chr15 55053589-55053623. Max. coverage (+): 6.38. Max coverage (-): 0

Region: chr15 55053624-55053657. Max. coverage (+): 6.38. Max coverage (-): 0

Region: chr15 55053658-55053692. Max. coverage (+): 0. Max coverage (-): 0

Region: chr15 55053693-55053727. Max. coverage (+): 1.45. Max coverage (-): 0

Region: chr15 55053728-55053761. Max. coverage (+): 0. Max coverage (-): 0

Region: chr15 55053762-55053796. Max. coverage (+): 0. Max coverage (-): 0

Region: chr15 55053797-55053830. Max. coverage (+): 0. Max coverage (-): 0

Region: chr15 55053831-55053865. Max. coverage (+): 0. Max coverage (-): 0

Region: chr15 55053866-55053900. Max. coverage (+): 0. Max coverage (-): 0

Region: chr15 55053901-55053934. Max. coverage (+): 0. Max coverage (-): 0

Region: chr15 55053935-55053969. Max. coverage (+): 0. Max coverage (-): 0

Region: chr15 55053970-55054004. Max. coverage (+): 0. Max coverage (-): 0

Region: chr15 55054005-55054038. Max. coverage (+): 0. Max coverage (-): 0

Region: chr15 55054039-55054073. Max. coverage (+): 9.56. Max coverage (-): 0

Region: chr15 55054074-55054108. Max. coverage (+): 1.45. Max coverage (-): 0

Region: chr15 55054109-55054142. Max. coverage (+): 10.83. Max coverage (-): 0

Region: chr15 55054143-55054177. Max. coverage (+): 12.78. Max coverage (-): 0

Region: chr15 55054178-55054211. Max. coverage (+): 17.96. Max coverage (-): 0

Region: chr15 55054212-55054246. Max. coverage (+): 11.06. Max coverage (-): 0

Region: chr15 55054247-55054281. Max. coverage (+): 0.45. Max coverage (-): 0

Region: chr15 55054282-55054315. Max. coverage (+): 0. Max coverage (-): 0

Region: chr15 55054316-55054350. Max. coverage (+): 0. Max coverage (-): 0

Region: chr15 55054351-55054385. Max. coverage (+): 4.13. Max coverage (-): 0

Region: chr15 55054386-55054419. Max. coverage (+): 0. Max coverage (-): 0

Region: chr15 55054420-55054454. Max. coverage (+): 0. Max coverage (-): 0

Region: chr15 55054455-55054489. Max. coverage (+): 0.57. Max coverage (-): 0

Region: chr15 55054490-55054523. Max. coverage (+): 1.26. Max coverage (-): 0

Region: chr15 55054524-55054558. Max. coverage (+): 0. Max coverage (-): 0

Region: chr15 55054559-55054592. Max. coverage (+): 0. Max coverage (-): 0

Region: chr15 55054593-55054627. Max. coverage (+): 1.98. Max coverage (-): 0

Region: chr15 55054628-55054662. Max. coverage (+): 1.87. Max coverage (-): 0

Region: chr15 55054663-55054696. Max. coverage (+): 0. Max coverage (-): 0

Region: chr15 55054697-55054731. Max. coverage (+): 2.49. Max coverage (-): 0

Region: chr15 55054732-55054766. Max. coverage (+): 0. Max coverage (-): 0

Region: chr15 55054767-55054800. Max. coverage (+): 0. Max coverage (-): 0

Region: chr15 55054801-55054835. Max. coverage (+): 0. Max coverage (-): 0

Region: chr15 55054836-55054870. Max. coverage (+): 0. Max coverage (-): 0

Region: chr15 55054871-55054904. Max. coverage (+): 0. Max coverage (-): 0

Region: chr15 55054905-55054939. Max. coverage (+): 0. Max coverage (-): 0

Region: chr15 55054940-55054973. Max. coverage (+): 0. Max coverage (-): 0

Region: chr15 55054974-55055008. Max. coverage (+): 0. Max coverage (-): 0

Region: chr15 55055009-55055043. Max. coverage (+): 0. Max coverage (-): 0

Region: chr15 55055044-55055077. Max. coverage (+): 0. Max coverage (-): 0

Region: chr15 55055078-55055112. Max. coverage (+): 0. Max coverage (-): 0

Region: chr15 55055113-55055147. Max. coverage (+): 0. Max coverage (-): 0

Region: chr15 55055148-55055181. Max. coverage (+): 0. Max coverage (-): 0

Region: chr15 55055182-55055216. Max. coverage (+): 0. Max coverage (-): 0

Region: chr15 55055217-55055250. Max. coverage (+): 1.98. Max coverage (-): 0

Region: chr15 55055251-55055285. Max. coverage (+): 9.75. Max coverage (-): 0

Region: chr15 55055286-55055320. Max. coverage (+): 2.59. Max coverage (-): 0

Region: chr15 55055321-55055354. Max. coverage (+): 0.77. Max coverage (-): 0

Region: chr15 55055355-55055389. Max. coverage (+): 0.77. Max coverage (-): 0

Region: chr15 55055390-55055424. Max. coverage (+): 2.29. Max coverage (-): 0

Region: chr15 55055425-55055458. Max. coverage (+): 6.42. Max coverage (-): 0

Region: chr15 55055459-55055493. Max. coverage (+): 0. Max coverage (-): 0

Region: chr15 55055494-55055528. Max. coverage (+): 3.97. Max coverage (-): 0

Region: chr15 55055529-55055562. Max. coverage (+): 14.9. Max coverage (-): 0

Region: chr15 55055563-55055597. Max. coverage (+): 0. Max coverage (-): 0

Region: chr15 55055598-55055631. Max. coverage (+): 0. Max coverage (-): 0

Region: chr15 55055632-55055666. Max. coverage (+): 0. Max coverage (-): 0

Region: chr15 55055667-55055701. Max. coverage (+): 5.07. Max coverage (-): 0

Region: chr15 55055702-55055735. Max. coverage (+): 5.07. Max coverage (-): 0

Region: chr15 55055736-55055770. Max. coverage (+): 2.49. Max coverage (-): 0

Region: chr15 55055771-55055805. Max. coverage (+): 2.49. Max coverage (-): 0

Region: chr15 55055806-55055839. Max. coverage (+): 0. Max coverage (-): 0

Region: chr15 55055840-55055874. Max. coverage (+): 3.89. Max coverage (-): 0

Region: chr15 55055875-55055909. Max. coverage (+): 0. Max coverage (-): 0

Region: chr15 55055910-55055943. Max. coverage (+): 0. Max coverage (-): 0

Region: chr15 55055944-55055978. Max. coverage (+): 0. Max coverage (-): 0

Region: chr15 55055979-55056012. Max. coverage (+): 4.01. Max coverage (-): 0

Region: chr15 55056013-55056047. Max. coverage (+): 0. Max coverage (-): 0

Region: chr15 55056048-55056082. Max. coverage (+): 0. Max coverage (-): 0

Region: chr15 55056083-55056116. Max. coverage (+): 0. Max coverage (-): 0

Region: chr15 55056117-55056151. Max. coverage (+): 0. Max coverage (-): 0

Region: chr15 55056152-55056186. Max. coverage (+): 0. Max coverage (-): 0

Region: chr15 55056187-55056220. Max. coverage (+): 0. Max coverage (-): 0

Region: chr15 55056221-55056255. Max. coverage (+): 0. Max coverage (-): 0

Region: chr15 55056256-55056290. Max. coverage (+): 0. Max coverage (-): 0

Region: chr15 55056291-55056324. Max. coverage (+): 0. Max coverage (-): 0

Region: chr15 55056325-55056359. Max. coverage (+): 0. Max coverage (-): 0

Region: chr15 55056360-55056393. Max. coverage (+): 0. Max coverage (-): 0

Region: chr15 55056394-55056428. Max. coverage (+): 0. Max coverage (-): 0

Region: chr15 55056429-55056463. Max. coverage (+): 0. Max coverage (-): 0

Region: chr15 55056464-55056497. Max. coverage (+): 0. Max coverage (-): 0

Region: chr15 55056498-55056532. Max. coverage (+): 0. Max coverage (-): 0

Region: chr15 55056533-55056567. Max. coverage (+): 0. Max coverage (-): 0

Region: chr15 55056568-55056601. Max. coverage (+): 0. Max coverage (-): 0

Region: chr15 55056602-55056636. Max. coverage (+): 0. Max coverage (-): 0

Region: chr15 55056637-55056670. Max. coverage (+): 0. Max coverage (-): 0

Region: chr15 55056671-55056705. Max. coverage (+): 0. Max coverage (-): 0

Region: chr15 55056706-55056740. Max. coverage (+): 0. Max coverage (-): 0

Region: chr15 55056741-55056774. Max. coverage (+): 2.24. Max coverage (-): 0

Region: chr15 55056775-55056809. Max. coverage (+): 2.24. Max coverage (-): 0

Region: chr15 55056810-55056844. Max. coverage (+): 0. Max coverage (-): 0

Region: chr15 55056845-55056878. Max. coverage (+): 0. Max coverage (-): 0

Region: chr15 55056879-55056913. Max. coverage (+): 0. Max coverage (-): 0

Region: chr15 55056914-55056948. Max. coverage (+): 0. Max coverage (-): 0

Region: chr15 55056949-55056982. Max. coverage (+): 0. Max coverage (-): 0

Region: chr15 55056983-55057017. Max. coverage (+): 0. Max coverage (-): 0

Region: chr15 55057018-55057051. Max. coverage (+): 0. Max coverage (-): 0

Region: chr15 55057052-55057086. Max. coverage (+): 0. Max coverage (-): 0

Region: chr15 55057087-55057121. Max. coverage (+): 0. Max coverage (-): 0

Region: chr15 55057122-55057155. Max. coverage (+): 0. Max coverage (-): 0

Region: chr15 55057156-55057190. Max. coverage (+): 0. Max coverage (-): 0

Region: chr15 55057191-55057225. Max. coverage (+): 0. Max coverage (-): 0

Region: chr15 55057226-55057259. Max. coverage (+): 0. Max coverage (-): 0

Region: chr15 55057260-55057294. Max. coverage (+): 0. Max coverage (-): 0

Region: chr15 55057295-55057329. Max. coverage (+): 0. Max coverage (-): 0

Region: chr15 55057330-55057363. Max. coverage (+): 0. Max coverage (-): 0

Region: chr15 55057364-55057398. Max. coverage (+): 0. Max coverage (-): 0

Region: chr15 55057399-55057432. Max. coverage (+): 0. Max coverage (-): 0

Region: chr15 55057433-55057467. Max. coverage (+): 0. Max coverage (-): 0

Region: chr15 55057468-55057502. Max. coverage (+): 0. Max coverage (-): 0

Region: chr15 55057503-55057536. Max. coverage (+): 0. Max coverage (-): 0

Region: chr15 55057537-55057571. Max. coverage (+): 0. Max coverage (-): 0

Region: chr15 55057572-55057606. Max. coverage (+): 0. Max coverage (-): 0

Region: chr15 55057607-55057640. Max. coverage (+): 0. Max coverage (-): 0

Region: chr15 55057641-55057675. Max. coverage (+): 0. Max coverage (-): 0

Region: chr15 55057676-55057710. Max. coverage (+): 0.81. Max coverage (-): 0

Region: chr15 55057711-55057744. Max. coverage (+): 0. Max coverage (-): 0

Region: chr15 55057745-55057779. Max. coverage (+): 0. Max coverage (-): 0

Region: chr15 55057780-55057813. Max. coverage (+): 1.04. Max coverage (-): 0

Region: chr15 55057814-55057848. Max. coverage (+): 0. Max coverage (-): 0

Region: chr15 55057849-55057883. Max. coverage (+): 0. Max coverage (-): 0

Region: chr15 55057884-55057917. Max. coverage (+): 0. Max coverage (-): 0

Region: chr15 55057918-55057952. Max. coverage (+): 0. Max coverage (-): 0

Region: chr15 55057953-55057987. Max. coverage (+): 0. Max coverage (-): 0

Region: chr15 55057988-55058021. Max. coverage (+): 0. Max coverage (-): 0

Region: chr15 55058022-55058056. Max. coverage (+): 0. Max coverage (-): 0

Region: chr15 55058057-55058090. Max. coverage (+): 0. Max coverage (-): 0

Region: chr15 55058091-55058125. Max. coverage (+): 0. Max coverage (-): 0

Region: chr15 55058126-55058160. Max. coverage (+): 0. Max coverage (-): 0

Region: chr15 55058161-55058194. Max. coverage (+): 0. Max coverage (-): 0

Region: chr15 55058195-55058229. Max. coverage (+): 0. Max coverage (-): 0

Region: chr15 55058230-55058264. Max. coverage (+): 0. Max coverage (-): 0

Region: chr15 55058265-55058298. Max. coverage (+): 0. Max coverage (-): 0

Region: chr15 55058299-55058333. Max. coverage (+): 0. Max coverage (-): 0

Region: chr15 55058334-55058368. Max. coverage (+): 0. Max coverage (-): 0

Region: chr15 55058369-55058402. Max. coverage (+): 8.54. Max coverage (-): 0

Region: chr15 55058403-. Max. coverage (+): 0. Max coverage (-): 0

RepeatMasker Color Code

**+**

100-98% Identity

<98-95% Identity

<95-90% Identity

<90-85% Identity

<85-80% Identity

<80-75% Identity

<75-70% Identity

<70% Identity

**-**

Gene Set Color Code

**+**

Gene

Pseudogene

**-**

Topology/Coverage Color Code

Coverage Plus Strand

Coverage Minus Strand

Mainstrand: Plus

Mainstrand: Minus

Complementary Strand

Flanking Region  
(if option -flank >0)

Gene Set Annotation  

**1. NEU3 (protein coding, ENSBTAG00000025931) Tr:00000036738 Ex:3**: 55041084-55043623 (+)

  
RepeatMasker Annotation  

**1. MIR3**: 55042356-55042515 (+), Divergence to consensus: 32.8%  
**2. L2c**: 55043020-55043063 (-), Divergence to consensus: 26.9%  
**3. Bov-tA2**: 55045183-55045390 (-), Divergence to consensus: 16.4%  
**4. L2c**: 55045741-55045807 (+), Divergence to consensus: 35.8%  
**5. L2b**: 55045845-55045957 (+), Divergence to consensus: 28.4%  
**6. MamRep1151**: 55047220-55047295 (-), Divergence to consensus: 30.3%  
**7. AT\_rich**: 55047718-55047739 (+), Divergence to consensus: 40.9%  
**8. MIRc**: 55047941-55048125 (+), Divergence to consensus: 39.7%  
**9. MIRc**: 55049215-55049312 (-), Divergence to consensus: 44.9%  
**10. BovB**: 55049446-55050184 (+), Divergence to consensus: 10.9%  
**11. ART2A**: 55050186-55050718 (+), Divergence to consensus: 15.3%  
**12. L2c**: 55051053-55051151 (+), Divergence to consensus: 34.3%  
**13. LTR18C\_BT**: 55051688-55051913 (+), Divergence to consensus: 6.2%  
**14. ERVL-B4-int**: 55053707-55054045 (+), Divergence to consensus: 34.8%  
**15. ART2A**: 55054746-55055229 (+), Divergence to consensus: 17%  
**16. LTR18C\_BT**: 55056019-55056290 (+), Divergence to consensus: 6.6%  
**17. MER91A**: 55056428-55056485 (+), Divergence to consensus: 34.5%  
**18. MIRb**: 55056801-55057028 (-), Divergence to consensus: 40.6%  
**19. MIRb**: 55058192-55058379 (+), Divergence to consensus: 37.8%

  
Transcription Factor Binding Sites  

**RFX4\_1** (Sequence: GTTGCTAAG (-): 55057364)  
**SPZ1** (Sequence: CTGAAACCCT (-): 55053629)  
**SOX9** (Sequence: AACAATGG (-): 55047212)  
**SOX9** (Sequence: CCATTGTT (+): 55048183)  
**Gata4** (Sequence: CTTATCT (+): 55042575)  
**Gata4** (Sequence: CTTATCT (+): 55045617)
