# Supplementary material for: Small RNA sequencing of cryopreserved semen from single bull revealed altered miRNAs and piRNAs expression between High- and Low-motile sperm populations
Source: BMC Genomics. 2017 Jan 4;18:14. doi: 10.1186/s12864-016-3394-7 (PMC5209821; doi:10.1186/s12864-016-3394-7)
Supplement: Additional file 3: — Details for each piRNA clusters found in High Motile (HM) sperm fraction. Genes, repeats, transposable elements and transcription factors binding sites falling within the cluster regions were reported. (ZIP 1896 kb) [file 12864_2016_3394_MOESM3_ESM.zip › 29.html]

piRNA cluster 29


Predicted piRNA cluster no. 29     previous   next
  

Show proTRAC run info
Hide proTRAC run info

================================= proTRAC ====================================  
VERSION: 2.1                                    LAST MODIFIED: 06. October 2015  
  
Please cite:  
Rosenkranz D, Zischler H. proTRAC - a software for probabilistic piRNA cluster  
detection, visualization and analysis. 2012. BMC Bioinformatics 13:5.  
  
and (for proTRAC 2.0 and later):  
Rosenkranz D, Rudloff S, Bastuck K, Ketting RF, Zischler H. Tupaia small RNAs  
provide insights into function and evolution of RNAi-based transposon defense  
in mammals. 2015. RNA 21(5):911-922.  
  
Contact:  
David Rosenkranz  
Institute of Anthropology, small RNA group  
Johannes Gutenberg University Mainz  
email: rosenkranz@uni-mainz.de  
  
You can find the latest proTRAC version at:  
http://sourceforge.net/projects/protrac/files  
http://www.smallRNAgroup-mainz.de/software  
==============================================================================  
  
PARAMETERS:  
Map file: .............../storage/core/barbara/genhome/smallRNA/fertility/Sample\_motile/pirna/Sample\_motile\_26-33\_collapsed.fa.no-dust.map.weighted-10000-1000-b-0  
Genome file: ............/storage/core/barbara/genhome/smallRNA/fertility/Sample\_all/pirna/bt\_311\_chrY.fa  
RepeatMasker annotation: /storage/genomes/bt\_umd31/GCF\_000003055.6\_Bos\_taurus\_UMD\_3.1.1\_repeatMasker\_chr.out  
GeneSet:................./storage/core/barbara/genhome/smallRNA/fertility/Sample\_all/pirna/full.gtf  
  
Significant (p<=0.01) hit density will be calculated based  
on observed hit distribution.  
  
Sliding window size: ........................................ 5000 bp  
Sliding window increament: .................................. 1000 bp  
Normalize each hit by number of genomic hits: ............... 1 [0=no/1=yes]  
Normalize each hit by number of sequence reads: ............. 1 [0=no/1=yes]  
Normalize values (-> per million mapped reads): ............. 1 [0=no/1=yes]  
Min. fraction of hits with 1T(U) or 10A: .................... 0.75  
Alternatively: Min. fraction of hits with 1T(U) and 10A: .... 0.5  
Min. fraction of hits with typical piRNA length: ............ 0.75  
Typical piRNA length: ....................................... 26-33 nt  
Min. size of a piRNA cluster: ............................... 5000 bp.  
Min. number of hits (absolute): ............................. 0  
Min. number of hits (normalized): ........................... 0  
Min. fraction of hits on the mainstrand: .................... 0.75  
Top fraction of mapped sequences (in terms of read counts): . 1%  
Top fraction accounts for max. n% of sequence reads: ........ 90%  
Min. fraction of hits on each arm of a bidirectional cluster: 0.1  
Output image file for each cluster: ......................... 0 [0=no/1=yes]  
Output html file for each cluster: .......................... 1 [0=no/1=yes]  
Output a summary table: ..................................... 1 [0=no/1=yes]  
Output a FASTA file for each cluster (piRNA sequences): ..... 1 [0=no/1=yes]  
Output a FASTA file comprising cluster sequences: ........... 1 [0=no/1=yes]  
Search DNA motifs in clusters: .............................. 1 [0=no/1=yes]  
Output flanking sequences: +/- .............................. 0 bp  
Output ~.pTi file: .......................................... 1 [0=no/1=yes]  
==============================================================================  
  
  
Genome size (without gaps): ............ 2678902517 bp  
Gaps (N/X/-): .......................... 53837044 bp  
Mapped reads: .......................... 658825247023  
Non-identical sequences: ............... 514171  
Genomic hits: .......................... 764233  
Significant densitiy of mapped reads: .. 12867599.5173724 reads/kb

Show proTRAC cluster info
Hide proTRAC cluster info

|  |  |
| --- | --- |
| Location | chr15 |
| Coordinates | 76585907-76654714 |
| Size [bp] | 68808 |
| Sequence hit loci | 18804 |
| Mapped reads (normalized) | 20991853910 |
| Mapped reads (normalized) per kb | 305078681.4 |
| Normalized reads with 1T (1U) | 77.1% |
| Normalized reads with 10A | 30.2% |
| Normalized reads with length 26-33 nt | 100% |
| Normalized reads on the main strand(s) | 97.7% |
| Predicted directionality | mono:minus |

100%

0%

1T (1U)  
reads

10A reads

26-33 nt  
reads

reads on mainstrand

**Either the amount of reads with 1T (1U) OR 10A has to exceed 75% (set with option: -1Tor10A)  
Alternatively the amount of reads with 1T (1U) AND 10A has to exceed 50% (set with option: -1Tand10A)  
Minimum amount of reads with preferred size is 75% (set with option: -pisize)  
Minimum amount of reads on the main strand(s) is 75% (set with option: -clstrand)**

Show read coverage
Hide read coverage

WHAT DO I SEE HERE?  
This chart shows the location of mapped sequence reads within a predicted piRNA cluster. The color refers to the number of genomic hits produced by the sequence read in question. A dark red bar indicates that this sequence read produces many other hits elsewhere in the genome. Many adjacent red or yellow bars can indicate the presence of a multi-copy element such as transposons or rRNA genes. A dark green bar indicates that this sequence read maps uniquely to this locus.

1 hit

2-5 hits

6-10 hits

11-20 hits

21-50 hits

51-100 hits

> 100 hits

chr15

76585907

76654714

Gene Set

RepeatMasker

Mapped  
Reads

186.12

plus strand

minus strand

186.12

Region: chr15 55058404-76585975. Max. coverage (+): 0. Max coverage (-): 3.6

Region: chr15 76585976-76586113. Max. coverage (+): 0. Max coverage (-): 2.16

Region: chr15 76586114-76586251. Max. coverage (+): 0. Max coverage (-): 0

Region: chr15 76586252-76586388. Max. coverage (+): 0. Max coverage (-): 0

Region: chr15 76586389-76586526. Max. coverage (+): 0. Max coverage (-): 3.94

Region: chr15 76586527-76586663. Max. coverage (+): 0. Max coverage (-): 1.35

Region: chr15 76586664-76586801. Max. coverage (+): 0. Max coverage (-): 0

Region: chr15 76586802-76586939. Max. coverage (+): 0. Max coverage (-): 0.8

Region: chr15 76586940-76587076. Max. coverage (+): 0. Max coverage (-): 4.95

Region: chr15 76587077-76587214. Max. coverage (+): 0. Max coverage (-): 13.6

Region: chr15 76587215-76587351. Max. coverage (+): 0. Max coverage (-): 6.75

Region: chr15 76587352-76587489. Max. coverage (+): 0. Max coverage (-): 4.46

Region: chr15 76587490-76587627. Max. coverage (+): 0. Max coverage (-): 2.69

Region: chr15 76587628-76587764. Max. coverage (+): 0. Max coverage (-): 13.2

Region: chr15 76587765-76587902. Max. coverage (+): 0. Max coverage (-): 5.27

Region: chr15 76587903-76588040. Max. coverage (+): 0. Max coverage (-): 0

Region: chr15 76588041-76588177. Max. coverage (+): 0. Max coverage (-): 0

Region: chr15 76588178-76588315. Max. coverage (+): 0. Max coverage (-): 0

Region: chr15 76588316-76588452. Max. coverage (+): 0. Max coverage (-): 0

Region: chr15 76588453-76588590. Max. coverage (+): 0. Max coverage (-): 0

Region: chr15 76588591-76588728. Max. coverage (+): 0. Max coverage (-): 0

Region: chr15 76588729-76588865. Max. coverage (+): 0. Max coverage (-): 1.82

Region: chr15 76588866-76589003. Max. coverage (+): 0. Max coverage (-): 0

Region: chr15 76589004-76589140. Max. coverage (+): 0. Max coverage (-): 15.96

Region: chr15 76589141-76589278. Max. coverage (+): 0. Max coverage (-): 7.23

Region: chr15 76589279-76589416. Max. coverage (+): 0. Max coverage (-): 0

Region: chr15 76589417-76589553. Max. coverage (+): 0. Max coverage (-): 1.47

Region: chr15 76589554-76589691. Max. coverage (+): 0. Max coverage (-): 6.76

Region: chr15 76589692-76589829. Max. coverage (+): 0. Max coverage (-): 8.15

Region: chr15 76589830-76589966. Max. coverage (+): 0. Max coverage (-): 0

Region: chr15 76589967-76590104. Max. coverage (+): 0. Max coverage (-): 0

Region: chr15 76590105-76590241. Max. coverage (+): 0. Max coverage (-): 4.95

Region: chr15 76590242-76590379. Max. coverage (+): 0. Max coverage (-): 0

Region: chr15 76590380-76590517. Max. coverage (+): 0. Max coverage (-): 4.77

Region: chr15 76590518-76590654. Max. coverage (+): 0. Max coverage (-): 1.35

Region: chr15 76590655-76590792. Max. coverage (+): 0. Max coverage (-): 0

Region: chr15 76590793-76590929. Max. coverage (+): 0. Max coverage (-): 0

Region: chr15 76590930-76591067. Max. coverage (+): 0. Max coverage (-): 0

Region: chr15 76591068-76591205. Max. coverage (+): 0. Max coverage (-): 0

Region: chr15 76591206-76591342. Max. coverage (+): 0. Max coverage (-): 3.69

Region: chr15 76591343-76591480. Max. coverage (+): 0. Max coverage (-): 0

Region: chr15 76591481-76591618. Max. coverage (+): 0. Max coverage (-): 0

Region: chr15 76591619-76591755. Max. coverage (+): 0. Max coverage (-): 0

Region: chr15 76591756-76591893. Max. coverage (+): 0. Max coverage (-): 0

Region: chr15 76591894-76592030. Max. coverage (+): 0. Max coverage (-): 0

Region: chr15 76592031-76592168. Max. coverage (+): 0. Max coverage (-): 0

Region: chr15 76592169-76592306. Max. coverage (+): 0. Max coverage (-): 0

Region: chr15 76592307-76592443. Max. coverage (+): 0. Max coverage (-): 0

Region: chr15 76592444-76592581. Max. coverage (+): 0. Max coverage (-): 0

Region: chr15 76592582-76592718. Max. coverage (+): 0. Max coverage (-): 0

Region: chr15 76592719-76592856. Max. coverage (+): 0. Max coverage (-): 0

Region: chr15 76592857-76592994. Max. coverage (+): 0. Max coverage (-): 0

Region: chr15 76592995-76593131. Max. coverage (+): 0. Max coverage (-): 1.62

Region: chr15 76593132-76593269. Max. coverage (+): 0. Max coverage (-): 2.2

Region: chr15 76593270-76593407. Max. coverage (+): 0. Max coverage (-): 2.2

Region: chr15 76593408-76593544. Max. coverage (+): 0. Max coverage (-): 0

Region: chr15 76593545-76593682. Max. coverage (+): 0. Max coverage (-): 0

Region: chr15 76593683-76593819. Max. coverage (+): 0. Max coverage (-): 1.8

Region: chr15 76593820-76593957. Max. coverage (+): 0. Max coverage (-): 4.29

Region: chr15 76593958-76594095. Max. coverage (+): 0. Max coverage (-): 0

Region: chr15 76594096-76594232. Max. coverage (+): 0. Max coverage (-): 0

Region: chr15 76594233-76594370. Max. coverage (+): 0. Max coverage (-): 5.76

Region: chr15 76594371-76594507. Max. coverage (+): 0. Max coverage (-): 4.77

Region: chr15 76594508-76594645. Max. coverage (+): 0. Max coverage (-): 0.9

Region: chr15 76594646-76594783. Max. coverage (+): 0. Max coverage (-): 9.82

Region: chr15 76594784-76594920. Max. coverage (+): 0. Max coverage (-): 4.86

Region: chr15 76594921-76595058. Max. coverage (+): 0. Max coverage (-): 0

Region: chr15 76595059-76595196. Max. coverage (+): 0. Max coverage (-): 0

Region: chr15 76595197-76595333. Max. coverage (+): 0. Max coverage (-): 6.89

Region: chr15 76595334-76595471. Max. coverage (+): 0. Max coverage (-): 0

Region: chr15 76595472-76595608. Max. coverage (+): 2.17. Max coverage (-): 0

Region: chr15 76595609-76595746. Max. coverage (+): 0. Max coverage (-): 1.98

Region: chr15 76595747-76595884. Max. coverage (+): 0. Max coverage (-): 3.48

Region: chr15 76595885-76596021. Max. coverage (+): 0. Max coverage (-): 2.96

Region: chr15 76596022-76596159. Max. coverage (+): 0. Max coverage (-): 0

Region: chr15 76596160-76596297. Max. coverage (+): 0. Max coverage (-): 0

Region: chr15 76596298-76596434. Max. coverage (+): 0. Max coverage (-): 0

Region: chr15 76596435-76596572. Max. coverage (+): 0. Max coverage (-): 7.12

Region: chr15 76596573-76596709. Max. coverage (+): 0. Max coverage (-): 7.05

Region: chr15 76596710-76596847. Max. coverage (+): 0. Max coverage (-): 0

Region: chr15 76596848-76596985. Max. coverage (+): 0. Max coverage (-): 0

Region: chr15 76596986-76597122. Max. coverage (+): 0. Max coverage (-): 13.05

Region: chr15 76597123-76597260. Max. coverage (+): 0. Max coverage (-): 1.92

Region: chr15 76597261-76597397. Max. coverage (+): 0. Max coverage (-): 0

Region: chr15 76597398-76597535. Max. coverage (+): 0. Max coverage (-): 10.52

Region: chr15 76597536-76597673. Max. coverage (+): 0. Max coverage (-): 1.5

Region: chr15 76597674-76597810. Max. coverage (+): 0. Max coverage (-): 8.13

Region: chr15 76597811-76597948. Max. coverage (+): 0. Max coverage (-): 6.83

Region: chr15 76597949-76598086. Max. coverage (+): 0. Max coverage (-): 4.37

Region: chr15 76598087-76598223. Max. coverage (+): 0. Max coverage (-): 3.67

Region: chr15 76598224-76598361. Max. coverage (+): 0. Max coverage (-): 0

Region: chr15 76598362-76598498. Max. coverage (+): 0. Max coverage (-): 2.43

Region: chr15 76598499-76598636. Max. coverage (+): 0. Max coverage (-): 5.5

Region: chr15 76598637-76598774. Max. coverage (+): 0. Max coverage (-): 0.86

Region: chr15 76598775-76598911. Max. coverage (+): 0. Max coverage (-): 4.64

Region: chr15 76598912-76599049. Max. coverage (+): 0. Max coverage (-): 0

Region: chr15 76599050-76599186. Max. coverage (+): 0. Max coverage (-): 10.34

Region: chr15 76599187-76599324. Max. coverage (+): 0. Max coverage (-): 95.72

Region: chr15 76599325-76599462. Max. coverage (+): 0. Max coverage (-): 64.83

Region: chr15 76599463-76599599. Max. coverage (+): 0. Max coverage (-): 0

Region: chr15 76599600-76599737. Max. coverage (+): 0. Max coverage (-): 8.55

Region: chr15 76599738-76599875. Max. coverage (+): 0. Max coverage (-): 11.4

Region: chr15 76599876-76600012. Max. coverage (+): 0. Max coverage (-): 10.74

Region: chr15 76600013-76600150. Max. coverage (+): 0. Max coverage (-): 9.44

Region: chr15 76600151-76600287. Max. coverage (+): 0. Max coverage (-): 6.74

Region: chr15 76600288-76600425. Max. coverage (+): 0. Max coverage (-): 6.55

Region: chr15 76600426-76600563. Max. coverage (+): 0. Max coverage (-): 15.14

Region: chr15 76600564-76600700. Max. coverage (+): 0. Max coverage (-): 0

Region: chr15 76600701-76600838. Max. coverage (+): 0. Max coverage (-): 53.6

Region: chr15 76600839-76600975. Max. coverage (+): 0. Max coverage (-): 16.59

Region: chr15 76600976-76601113. Max. coverage (+): 0. Max coverage (-): 47.06

Region: chr15 76601114-76601251. Max. coverage (+): 0. Max coverage (-): 8.9

Region: chr15 76601252-76601388. Max. coverage (+): 0. Max coverage (-): 18.67

Region: chr15 76601389-76601526. Max. coverage (+): 0. Max coverage (-): 15.61

Region: chr15 76601527-76601664. Max. coverage (+): 0. Max coverage (-): 29.53

Region: chr15 76601665-76601801. Max. coverage (+): 0. Max coverage (-): 7.34

Region: chr15 76601802-76601939. Max. coverage (+): 0. Max coverage (-): 81.59

Region: chr15 76601940-76602076. Max. coverage (+): 0. Max coverage (-): 77.19

Region: chr15 76602077-76602214. Max. coverage (+): 0. Max coverage (-): 0

Region: chr15 76602215-76602352. Max. coverage (+): 0. Max coverage (-): 19.55

Region: chr15 76602353-76602489. Max. coverage (+): 0. Max coverage (-): 25.83

Region: chr15 76602490-76602627. Max. coverage (+): 0. Max coverage (-): 43

Region: chr15 76602628-76602764. Max. coverage (+): 0. Max coverage (-): 27.03

Region: chr15 76602765-76602902. Max. coverage (+): 0. Max coverage (-): 57.02

Region: chr15 76602903-76603040. Max. coverage (+): 0. Max coverage (-): 128.89

Region: chr15 76603041-76603177. Max. coverage (+): 0. Max coverage (-): 40.93

Region: chr15 76603178-76603315. Max. coverage (+): 0. Max coverage (-): 0

Region: chr15 76603316-76603453. Max. coverage (+): 0. Max coverage (-): 18.24

Region: chr15 76603454-76603590. Max. coverage (+): 0. Max coverage (-): 28.84

Region: chr15 76603591-76603728. Max. coverage (+): 0. Max coverage (-): 90.72

Region: chr15 76603729-76603865. Max. coverage (+): 0. Max coverage (-): 14.53

Region: chr15 76603866-76604003. Max. coverage (+): 0. Max coverage (-): 3

Region: chr15 76604004-76604141. Max. coverage (+): 0. Max coverage (-): 19.03

Region: chr15 76604142-76604278. Max. coverage (+): 0. Max coverage (-): 46.09

Region: chr15 76604279-76604416. Max. coverage (+): 0. Max coverage (-): 9.72

Region: chr15 76604417-76604553. Max. coverage (+): 0. Max coverage (-): 59.81

Region: chr15 76604554-76604691. Max. coverage (+): 0. Max coverage (-): 0

Region: chr15 76604692-76604829. Max. coverage (+): 0. Max coverage (-): 21.19

Region: chr15 76604830-76604966. Max. coverage (+): 0. Max coverage (-): 33.95

Region: chr15 76604967-76605104. Max. coverage (+): 0. Max coverage (-): 59.78

Region: chr15 76605105-76605242. Max. coverage (+): 0. Max coverage (-): 143.24

Region: chr15 76605243-76605379. Max. coverage (+): 0. Max coverage (-): 23.59

Region: chr15 76605380-76605517. Max. coverage (+): 0. Max coverage (-): 49.06

Region: chr15 76605518-76605654. Max. coverage (+): 0. Max coverage (-): 8.9

Region: chr15 76605655-76605792. Max. coverage (+): 0. Max coverage (-): 34.61

Region: chr15 76605793-76605930. Max. coverage (+): 0. Max coverage (-): 0

Region: chr15 76605931-76606067. Max. coverage (+): 0. Max coverage (-): 79.86

Region: chr15 76606068-76606205. Max. coverage (+): 0. Max coverage (-): 28.66

Region: chr15 76606206-76606342. Max. coverage (+): 0. Max coverage (-): 1.05

Region: chr15 76606343-76606480. Max. coverage (+): 0. Max coverage (-): 32.01

Region: chr15 76606481-76606618. Max. coverage (+): 0. Max coverage (-): 16.9

Region: chr15 76606619-76606755. Max. coverage (+): 0. Max coverage (-): 33.97

Region: chr15 76606756-76606893. Max. coverage (+): 0. Max coverage (-): 37.56

Region: chr15 76606894-76607031. Max. coverage (+): 0. Max coverage (-): 25.81

Region: chr15 76607032-76607168. Max. coverage (+): 0. Max coverage (-): 12.72

Region: chr15 76607169-76607306. Max. coverage (+): 0. Max coverage (-): 17.79

Region: chr15 76607307-76607443. Max. coverage (+): 0. Max coverage (-): 52.53

Region: chr15 76607444-76607581. Max. coverage (+): 0. Max coverage (-): 11.58

Region: chr15 76607582-76607719. Max. coverage (+): 0. Max coverage (-): 60

Region: chr15 76607720-76607856. Max. coverage (+): 0. Max coverage (-): 14.15

Region: chr15 76607857-76607994. Max. coverage (+): 0. Max coverage (-): 22.55

Region: chr15 76607995-76608131. Max. coverage (+): 0. Max coverage (-): 29.63

Region: chr15 76608132-76608269. Max. coverage (+): 0. Max coverage (-): 49.21

Region: chr15 76608270-76608407. Max. coverage (+): 0. Max coverage (-): 50.85

Region: chr15 76608408-76608544. Max. coverage (+): 0. Max coverage (-): 70.5

Region: chr15 76608545-76608682. Max. coverage (+): 0. Max coverage (-): 91.81

Region: chr15 76608683-76608820. Max. coverage (+): 0. Max coverage (-): 22.45

Region: chr15 76608821-76608957. Max. coverage (+): 0. Max coverage (-): 12.45

Region: chr15 76608958-76609095. Max. coverage (+): 0. Max coverage (-): 47.48

Region: chr15 76609096-76609232. Max. coverage (+): 0. Max coverage (-): 18.19

Region: chr15 76609233-76609370. Max. coverage (+): 0. Max coverage (-): 30.04

Region: chr15 76609371-76609508. Max. coverage (+): 0. Max coverage (-): 107.96

Region: chr15 76609509-76609645. Max. coverage (+): 0. Max coverage (-): 32.26

Region: chr15 76609646-76609783. Max. coverage (+): 0. Max coverage (-): 29.54

Region: chr15 76609784-76609920. Max. coverage (+): 0. Max coverage (-): 30.57

Region: chr15 76609921-76610058. Max. coverage (+): 0. Max coverage (-): 59.1

Region: chr15 76610059-76610196. Max. coverage (+): 0. Max coverage (-): 96.79

Region: chr15 76610197-76610333. Max. coverage (+): 0. Max coverage (-): 0

Region: chr15 76610334-76610471. Max. coverage (+): 0. Max coverage (-): 0

Region: chr15 76610472-76610609. Max. coverage (+): 0. Max coverage (-): 26.35

Region: chr15 76610610-76610746. Max. coverage (+): 0. Max coverage (-): 0

Region: chr15 76610747-76610884. Max. coverage (+): 0. Max coverage (-): 13.19

Region: chr15 76610885-76611021. Max. coverage (+): 0. Max coverage (-): 6.95

Region: chr15 76611022-76611159. Max. coverage (+): 0. Max coverage (-): 14.37

Region: chr15 76611160-76611297. Max. coverage (+): 0. Max coverage (-): 21.57

Region: chr15 76611298-76611434. Max. coverage (+): 0. Max coverage (-): 41.55

Region: chr15 76611435-76611572. Max. coverage (+): 0. Max coverage (-): 24.19

Region: chr15 76611573-76611709. Max. coverage (+): 0. Max coverage (-): 27.43

Region: chr15 76611710-76611847. Max. coverage (+): 0. Max coverage (-): 89.9

Region: chr15 76611848-76611985. Max. coverage (+): 0. Max coverage (-): 49.92

Region: chr15 76611986-76612122. Max. coverage (+): 0. Max coverage (-): 88.13

Region: chr15 76612123-76612260. Max. coverage (+): 0. Max coverage (-): 79.8

Region: chr15 76612261-76612398. Max. coverage (+): 0. Max coverage (-): 0

Region: chr15 76612399-76612535. Max. coverage (+): 0. Max coverage (-): 0

Region: chr15 76612536-76612673. Max. coverage (+): 0. Max coverage (-): 0

Region: chr15 76612674-76612810. Max. coverage (+): 0. Max coverage (-): 6.54

Region: chr15 76612811-76612948. Max. coverage (+): 0. Max coverage (-): 45.76

Region: chr15 76612949-76613086. Max. coverage (+): 0. Max coverage (-): 0

Region: chr15 76613087-76613223. Max. coverage (+): 0. Max coverage (-): 81.84

Region: chr15 76613224-76613361. Max. coverage (+): 0. Max coverage (-): 21.21

Region: chr15 76613362-76613499. Max. coverage (+): 0. Max coverage (-): 10.04

Region: chr15 76613500-76613636. Max. coverage (+): 0. Max coverage (-): 64.49

Region: chr15 76613637-76613774. Max. coverage (+): 0. Max coverage (-): 70.54

Region: chr15 76613775-76613911. Max. coverage (+): 0. Max coverage (-): 35.22

Region: chr15 76613912-76614049. Max. coverage (+): 0. Max coverage (-): 9.61

Region: chr15 76614050-76614187. Max. coverage (+): 0. Max coverage (-): 55.53

Region: chr15 76614188-76614324. Max. coverage (+): 0. Max coverage (-): 20.93

Region: chr15 76614325-76614462. Max. coverage (+): 0. Max coverage (-): 42.5

Region: chr15 76614463-76614599. Max. coverage (+): 0. Max coverage (-): 30.27

Region: chr15 76614600-76614737. Max. coverage (+): 0. Max coverage (-): 67.74

Region: chr15 76614738-76614875. Max. coverage (+): 0. Max coverage (-): 11.69

Region: chr15 76614876-76615012. Max. coverage (+): 0. Max coverage (-): 30.13

Region: chr15 76615013-76615150. Max. coverage (+): 0. Max coverage (-): 13.09

Region: chr15 76615151-76615288. Max. coverage (+): 0. Max coverage (-): 23.86

Region: chr15 76615289-76615425. Max. coverage (+): 0. Max coverage (-): 158.15

Region: chr15 76615426-76615563. Max. coverage (+): 0. Max coverage (-): 46.63

Region: chr15 76615564-76615700. Max. coverage (+): 0. Max coverage (-): 36.5

Region: chr15 76615701-76615838. Max. coverage (+): 0. Max coverage (-): 73.96

Region: chr15 76615839-76615976. Max. coverage (+): 0. Max coverage (-): 116

Region: chr15 76615977-76616113. Max. coverage (+): 0. Max coverage (-): 115.06

Region: chr15 76616114-76616251. Max. coverage (+): 0. Max coverage (-): 63.89

Region: chr15 76616252-76616388. Max. coverage (+): 0. Max coverage (-): 54.36

Region: chr15 76616389-76616526. Max. coverage (+): 1.35. Max coverage (-): 25.65

Region: chr15 76616527-76616664. Max. coverage (+): 0. Max coverage (-): 23.92

Region: chr15 76616665-76616801. Max. coverage (+): 0. Max coverage (-): 6.99

Region: chr15 76616802-76616939. Max. coverage (+): 0. Max coverage (-): 0

Region: chr15 76616940-76617077. Max. coverage (+): 0. Max coverage (-): 6

Region: chr15 76617078-76617214. Max. coverage (+): 0. Max coverage (-): 0

Region: chr15 76617215-76617352. Max. coverage (+): 0. Max coverage (-): 11.45

Region: chr15 76617353-76617489. Max. coverage (+): 0. Max coverage (-): 4.11

Region: chr15 76617490-76617627. Max. coverage (+): 0. Max coverage (-): 27.82

Region: chr15 76617628-76617765. Max. coverage (+): 0. Max coverage (-): 100.36

Region: chr15 76617766-76617902. Max. coverage (+): 0. Max coverage (-): 48.01

Region: chr15 76617903-76618040. Max. coverage (+): 0. Max coverage (-): 39.07

Region: chr15 76618041-76618177. Max. coverage (+): 0. Max coverage (-): 25.92

Region: chr15 76618178-76618315. Max. coverage (+): 0. Max coverage (-): 34.08

Region: chr15 76618316-76618453. Max. coverage (+): 0. Max coverage (-): 24.76

Region: chr15 76618454-76618590. Max. coverage (+): 0. Max coverage (-): 13.89

Region: chr15 76618591-76618728. Max. coverage (+): 0. Max coverage (-): 82.71

Region: chr15 76618729-76618866. Max. coverage (+): 0. Max coverage (-): 65.3

Region: chr15 76618867-76619003. Max. coverage (+): 0. Max coverage (-): 56.55

Region: chr15 76619004-76619141. Max. coverage (+): 0. Max coverage (-): 62.63

Region: chr15 76619142-76619278. Max. coverage (+): 0. Max coverage (-): 58.12

Region: chr15 76619279-76619416. Max. coverage (+): 0. Max coverage (-): 82.33

Region: chr15 76619417-76619554. Max. coverage (+): 3.55. Max coverage (-): 76.01

Region: chr15 76619555-76619691. Max. coverage (+): 0. Max coverage (-): 4.54

Region: chr15 76619692-76619829. Max. coverage (+): 0. Max coverage (-): 6.49

Region: chr15 76619830-76619966. Max. coverage (+): 0. Max coverage (-): 6.49

Region: chr15 76619967-76620104. Max. coverage (+): 0. Max coverage (-): 26.8

Region: chr15 76620105-76620242. Max. coverage (+): 0. Max coverage (-): 76.72

Region: chr15 76620243-76620379. Max. coverage (+): 0. Max coverage (-): 96.97

Region: chr15 76620380-76620517. Max. coverage (+): 0. Max coverage (-): 49.01

Region: chr15 76620518-76620655. Max. coverage (+): 0. Max coverage (-): 39.53

Region: chr15 76620656-76620792. Max. coverage (+): 0. Max coverage (-): 54.23

Region: chr15 76620793-76620930. Max. coverage (+): 0. Max coverage (-): 156.64

Region: chr15 76620931-76621067. Max. coverage (+): 0. Max coverage (-): 0

Region: chr15 76621068-76621205. Max. coverage (+): 1.26. Max coverage (-): 79.64

Region: chr15 76621206-76621343. Max. coverage (+): 1.46. Max coverage (-): 13.1

Region: chr15 76621344-76621480. Max. coverage (+): 0. Max coverage (-): 148.78

Region: chr15 76621481-76621618. Max. coverage (+): 0. Max coverage (-): 31.41

Region: chr15 76621619-76621755. Max. coverage (+): 0. Max coverage (-): 39.99

Region: chr15 76621756-76621893. Max. coverage (+): 3.51. Max coverage (-): 38.6

Region: chr15 76621894-76622031. Max. coverage (+): 0. Max coverage (-): 25.26

Region: chr15 76622032-76622168. Max. coverage (+): 0. Max coverage (-): 38.89

Region: chr15 76622169-76622306. Max. coverage (+): 0. Max coverage (-): 37.74

Region: chr15 76622307-76622444. Max. coverage (+): 0. Max coverage (-): 26.86

Region: chr15 76622445-76622581. Max. coverage (+): 0. Max coverage (-): 27.36

Region: chr15 76622582-76622719. Max. coverage (+): 0. Max coverage (-): 79.18

Region: chr15 76622720-76622856. Max. coverage (+): 0. Max coverage (-): 5.26

Region: chr15 76622857-76622994. Max. coverage (+): 0. Max coverage (-): 1.5

Region: chr15 76622995-76623132. Max. coverage (+): 0. Max coverage (-): 46.46

Region: chr15 76623133-76623269. Max. coverage (+): 0. Max coverage (-): 23.2

Region: chr15 76623270-76623407. Max. coverage (+): 2.04. Max coverage (-): 15.26

Region: chr15 76623408-76623544. Max. coverage (+): 0. Max coverage (-): 20.52

Region: chr15 76623545-76623682. Max. coverage (+): 1.27. Max coverage (-): 82.38

Region: chr15 76623683-76623820. Max. coverage (+): 0. Max coverage (-): 68.02

Region: chr15 76623821-76623957. Max. coverage (+): 1.21. Max coverage (-): 27.12

Region: chr15 76623958-76624095. Max. coverage (+): 0.63. Max coverage (-): 10.58

Region: chr15 76624096-76624233. Max. coverage (+): 2.01. Max coverage (-): 53.64

Region: chr15 76624234-76624370. Max. coverage (+): 0. Max coverage (-): 79.28

Region: chr15 76624371-76624508. Max. coverage (+): 0. Max coverage (-): 0

Region: chr15 76624509-76624645. Max. coverage (+): 1.63. Max coverage (-): 27.83

Region: chr15 76624646-76624783. Max. coverage (+): 0. Max coverage (-): 102.25

Region: chr15 76624784-76624921. Max. coverage (+): 0. Max coverage (-): 0

Region: chr15 76624922-76625058. Max. coverage (+): 0. Max coverage (-): 0

Region: chr15 76625059-76625196. Max. coverage (+): 0. Max coverage (-): 30.95

Region: chr15 76625197-76625333. Max. coverage (+): 0. Max coverage (-): 58

Region: chr15 76625334-76625471. Max. coverage (+): 0. Max coverage (-): 30.04

Region: chr15 76625472-76625609. Max. coverage (+): 0. Max coverage (-): 4.8

Region: chr15 76625610-76625746. Max. coverage (+): 0. Max coverage (-): 29.56

Region: chr15 76625747-76625884. Max. coverage (+): 0. Max coverage (-): 18.46

Region: chr15 76625885-76626022. Max. coverage (+): 0. Max coverage (-): 45.68

Region: chr15 76626023-76626159. Max. coverage (+): 0. Max coverage (-): 13.91

Region: chr15 76626160-76626297. Max. coverage (+): 0. Max coverage (-): 0

Region: chr15 76626298-76626434. Max. coverage (+): 0. Max coverage (-): 0

Region: chr15 76626435-76626572. Max. coverage (+): 0. Max coverage (-): 60.56

Region: chr15 76626573-76626710. Max. coverage (+): 0. Max coverage (-): 24.13

Region: chr15 76626711-76626847. Max. coverage (+): 0. Max coverage (-): 57.16

Region: chr15 76626848-76626985. Max. coverage (+): 0. Max coverage (-): 5.66

Region: chr15 76626986-76627122. Max. coverage (+): 0. Max coverage (-): 16.9

Region: chr15 76627123-76627260. Max. coverage (+): 0. Max coverage (-): 39.3

Region: chr15 76627261-76627398. Max. coverage (+): 0. Max coverage (-): 18.99

Region: chr15 76627399-76627535. Max. coverage (+): 1.14. Max coverage (-): 36.11

Region: chr15 76627536-76627673. Max. coverage (+): 1.24. Max coverage (-): 30.57

Region: chr15 76627674-76627811. Max. coverage (+): 3.5. Max coverage (-): 48.26

Region: chr15 76627812-76627948. Max. coverage (+): 0. Max coverage (-): 0.44

Region: chr15 76627949-76628086. Max. coverage (+): 1.36. Max coverage (-): 42.31

Region: chr15 76628087-76628223. Max. coverage (+): 0. Max coverage (-): 21.8

Region: chr15 76628224-76628361. Max. coverage (+): 0. Max coverage (-): 6.23

Region: chr15 76628362-76628499. Max. coverage (+): 0. Max coverage (-): 47.49

Region: chr15 76628500-76628636. Max. coverage (+): 1.24. Max coverage (-): 38.2

Region: chr15 76628637-76628774. Max. coverage (+): 1.24. Max coverage (-): 10.55

Region: chr15 76628775-76628911. Max. coverage (+): 0. Max coverage (-): 7.42

Region: chr15 76628912-76629049. Max. coverage (+): 0. Max coverage (-): 38.97

Region: chr15 76629050-76629187. Max. coverage (+): 2.01. Max coverage (-): 35.97

Region: chr15 76629188-76629324. Max. coverage (+): 0. Max coverage (-): 0

Region: chr15 76629325-76629462. Max. coverage (+): 0. Max coverage (-): 2.91

Region: chr15 76629463-76629600. Max. coverage (+): 0. Max coverage (-): 21.37

Region: chr15 76629601-76629737. Max. coverage (+): 0. Max coverage (-): 70.25

Region: chr15 76629738-76629875. Max. coverage (+): 2.82. Max coverage (-): 14.93

Region: chr15 76629876-76630012. Max. coverage (+): 0. Max coverage (-): 66.73

Region: chr15 76630013-76630150. Max. coverage (+): 0. Max coverage (-): 24.74

Region: chr15 76630151-76630288. Max. coverage (+): 0. Max coverage (-): 21.34

Region: chr15 76630289-76630425. Max. coverage (+): 0. Max coverage (-): 39.66

Region: chr15 76630426-76630563. Max. coverage (+): 0. Max coverage (-): 57.21

Region: chr15 76630564-76630701. Max. coverage (+): 0. Max coverage (-): 20.03

Region: chr15 76630702-76630838. Max. coverage (+): 0. Max coverage (-): 24.48

Region: chr15 76630839-76630976. Max. coverage (+): 0. Max coverage (-): 23.2

Region: chr15 76630977-76631113. Max. coverage (+): 0. Max coverage (-): 52.07

Region: chr15 76631114-76631251. Max. coverage (+): 0. Max coverage (-): 46.28

Region: chr15 76631252-76631389. Max. coverage (+): 0. Max coverage (-): 0

Region: chr15 76631390-76631526. Max. coverage (+): 0. Max coverage (-): 55.25

Region: chr15 76631527-76631664. Max. coverage (+): 0. Max coverage (-): 0.65

Region: chr15 76631665-76631801. Max. coverage (+): 0. Max coverage (-): 35.42

Region: chr15 76631802-76631939. Max. coverage (+): 0. Max coverage (-): 16

Region: chr15 76631940-76632077. Max. coverage (+): 0. Max coverage (-): 18.48

Region: chr15 76632078-76632214. Max. coverage (+): 0. Max coverage (-): 3.45

Region: chr15 76632215-76632352. Max. coverage (+): 0. Max coverage (-): 9.48

Region: chr15 76632353-76632490. Max. coverage (+): 0. Max coverage (-): 5.02

Region: chr15 76632491-76632627. Max. coverage (+): 0. Max coverage (-): 11.39

Region: chr15 76632628-76632765. Max. coverage (+): 0. Max coverage (-): 19.41

Region: chr15 76632766-76632902. Max. coverage (+): 0. Max coverage (-): 8.68

Region: chr15 76632903-76633040. Max. coverage (+): 0. Max coverage (-): 14.39

Region: chr15 76633041-76633178. Max. coverage (+): 0. Max coverage (-): 22.61

Region: chr15 76633179-76633315. Max. coverage (+): 0. Max coverage (-): 53.05

Region: chr15 76633316-76633453. Max. coverage (+): 4.21. Max coverage (-): 79.36

Region: chr15 76633454-76633590. Max. coverage (+): 0. Max coverage (-): 0

Region: chr15 76633591-76633728. Max. coverage (+): 0. Max coverage (-): 0

Region: chr15 76633729-76633866. Max. coverage (+): 0. Max coverage (-): 0

Region: chr15 76633867-76634003. Max. coverage (+): 0. Max coverage (-): 0

Region: chr15 76634004-76634141. Max. coverage (+): 0. Max coverage (-): 0

Region: chr15 76634142-76634279. Max. coverage (+): 0. Max coverage (-): 0

Region: chr15 76634280-76634416. Max. coverage (+): 0. Max coverage (-): 0

Region: chr15 76634417-76634554. Max. coverage (+): 0. Max coverage (-): 19.16

Region: chr15 76634555-76634691. Max. coverage (+): 0. Max coverage (-): 18.79

Region: chr15 76634692-76634829. Max. coverage (+): 0. Max coverage (-): 0

Region: chr15 76634830-76634967. Max. coverage (+): 0. Max coverage (-): 8.23

Region: chr15 76634968-76635104. Max. coverage (+): 0. Max coverage (-): 20.67

Region: chr15 76635105-76635242. Max. coverage (+): 0. Max coverage (-): 45.19

Region: chr15 76635243-76635379. Max. coverage (+): 0. Max coverage (-): 11.76

Region: chr15 76635380-76635517. Max. coverage (+): 0. Max coverage (-): 17.35

Region: chr15 76635518-76635655. Max. coverage (+): 0. Max coverage (-): 36.21

Region: chr15 76635656-76635792. Max. coverage (+): 0. Max coverage (-): 33.51

Region: chr15 76635793-76635930. Max. coverage (+): 0. Max coverage (-): 36.9

Region: chr15 76635931-76636068. Max. coverage (+): 0. Max coverage (-): 5.7

Region: chr15 76636069-76636205. Max. coverage (+): 0. Max coverage (-): 76.13

Region: chr15 76636206-76636343. Max. coverage (+): 2.94. Max coverage (-): 26.4

Region: chr15 76636344-76636480. Max. coverage (+): 2.94. Max coverage (-): 35.37

Region: chr15 76636481-76636618. Max. coverage (+): 0. Max coverage (-): 22.5

Region: chr15 76636619-76636756. Max. coverage (+): 0. Max coverage (-): 54.99

Region: chr15 76636757-76636893. Max. coverage (+): 0. Max coverage (-): 41.09

Region: chr15 76636894-76637031. Max. coverage (+): 0. Max coverage (-): 49.93

Region: chr15 76637032-76637168. Max. coverage (+): 0. Max coverage (-): 22.11

Region: chr15 76637169-76637306. Max. coverage (+): 0. Max coverage (-): 0

Region: chr15 76637307-76637444. Max. coverage (+): 0. Max coverage (-): 0

Region: chr15 76637445-76637581. Max. coverage (+): 0. Max coverage (-): 0

Region: chr15 76637582-76637719. Max. coverage (+): 0. Max coverage (-): 0

Region: chr15 76637720-76637857. Max. coverage (+): 0. Max coverage (-): 0

Region: chr15 76637858-76637994. Max. coverage (+): 0. Max coverage (-): 0

Region: chr15 76637995-76638132. Max. coverage (+): 0. Max coverage (-): 34.13

Region: chr15 76638133-76638269. Max. coverage (+): 0. Max coverage (-): 44.19

Region: chr15 76638270-76638407. Max. coverage (+): 0. Max coverage (-): 78.86

Region: chr15 76638408-76638545. Max. coverage (+): 0. Max coverage (-): 52.06

Region: chr15 76638546-76638682. Max. coverage (+): 0. Max coverage (-): 0

Region: chr15 76638683-76638820. Max. coverage (+): 0. Max coverage (-): 0

Region: chr15 76638821-76638957. Max. coverage (+): 0. Max coverage (-): 0

Region: chr15 76638958-76639095. Max. coverage (+): 0. Max coverage (-): 0

Region: chr15 76639096-76639233. Max. coverage (+): 0. Max coverage (-): 0

Region: chr15 76639234-76639370. Max. coverage (+): 0. Max coverage (-): 22.87

Region: chr15 76639371-76639508. Max. coverage (+): 0. Max coverage (-): 20.47

Region: chr15 76639509-76639646. Max. coverage (+): 0. Max coverage (-): 12.2

Region: chr15 76639647-76639783. Max. coverage (+): 0. Max coverage (-): 3.33

Region: chr15 76639784-76639921. Max. coverage (+): 0. Max coverage (-): 20.73

Region: chr15 76639922-76640058. Max. coverage (+): 0. Max coverage (-): 27.61

Region: chr15 76640059-76640196. Max. coverage (+): 0. Max coverage (-): 1.66

Region: chr15 76640197-76640334. Max. coverage (+): 0. Max coverage (-): 13.19

Region: chr15 76640335-76640471. Max. coverage (+): 0. Max coverage (-): 17.95

Region: chr15 76640472-76640609. Max. coverage (+): 0. Max coverage (-): 22.96

Region: chr15 76640610-76640746. Max. coverage (+): 0. Max coverage (-): 44.98

Region: chr15 76640747-76640884. Max. coverage (+): 0. Max coverage (-): 14.94

Region: chr15 76640885-76641022. Max. coverage (+): 0. Max coverage (-): 18.63

Region: chr15 76641023-76641159. Max. coverage (+): 0. Max coverage (-): 74.15

Region: chr15 76641160-76641297. Max. coverage (+): 0. Max coverage (-): 43.36

Region: chr15 76641298-76641435. Max. coverage (+): 0. Max coverage (-): 7.5

Region: chr15 76641436-76641572. Max. coverage (+): 0. Max coverage (-): 22.63

Region: chr15 76641573-76641710. Max. coverage (+): 0. Max coverage (-): 12.87

Region: chr15 76641711-76641847. Max. coverage (+): 5.09. Max coverage (-): 17.12

Region: chr15 76641848-76641985. Max. coverage (+): 0. Max coverage (-): 14.66

Region: chr15 76641986-76642123. Max. coverage (+): 0. Max coverage (-): 13.43

Region: chr15 76642124-76642260. Max. coverage (+): 0. Max coverage (-): 0

Region: chr15 76642261-76642398. Max. coverage (+): 0. Max coverage (-): 39.39

Region: chr15 76642399-76642535. Max. coverage (+): 0. Max coverage (-): 23.62

Region: chr15 76642536-76642673. Max. coverage (+): 0. Max coverage (-): 9.13

Region: chr15 76642674-76642811. Max. coverage (+): 0. Max coverage (-): 16.93

Region: chr15 76642812-76642948. Max. coverage (+): 0. Max coverage (-): 31.61

Region: chr15 76642949-76643086. Max. coverage (+): 0. Max coverage (-): 46.41

Region: chr15 76643087-76643224. Max. coverage (+): 0. Max coverage (-): 50.52

Region: chr15 76643225-76643361. Max. coverage (+): 0. Max coverage (-): 24.52

Region: chr15 76643362-76643499. Max. coverage (+): 0. Max coverage (-): 51.14

Region: chr15 76643500-76643636. Max. coverage (+): 0. Max coverage (-): 23.98

Region: chr15 76643637-76643774. Max. coverage (+): 0. Max coverage (-): 14

Region: chr15 76643775-76643912. Max. coverage (+): 0. Max coverage (-): 16.77

Region: chr15 76643913-76644049. Max. coverage (+): 0. Max coverage (-): 17

Region: chr15 76644050-76644187. Max. coverage (+): 0. Max coverage (-): 13.76

Region: chr15 76644188-76644324. Max. coverage (+): 0. Max coverage (-): 18.76

Region: chr15 76644325-76644462. Max. coverage (+): 0. Max coverage (-): 49.82

Region: chr15 76644463-76644600. Max. coverage (+): 0. Max coverage (-): 37.97

Region: chr15 76644601-76644737. Max. coverage (+): 0. Max coverage (-): 63.74

Region: chr15 76644738-76644875. Max. coverage (+): 0. Max coverage (-): 37.11

Region: chr15 76644876-76645013. Max. coverage (+): 0. Max coverage (-): 0

Region: chr15 76645014-76645150. Max. coverage (+): 0. Max coverage (-): 30.92

Region: chr15 76645151-76645288. Max. coverage (+): 0. Max coverage (-): 44.33

Region: chr15 76645289-76645425. Max. coverage (+): 0. Max coverage (-): 60.26

Region: chr15 76645426-76645563. Max. coverage (+): 0. Max coverage (-): 11.1

Region: chr15 76645564-76645701. Max. coverage (+): 0. Max coverage (-): 15.49

Region: chr15 76645702-76645838. Max. coverage (+): 0. Max coverage (-): 9.55

Region: chr15 76645839-76645976. Max. coverage (+): 0. Max coverage (-): 11.49

Region: chr15 76645977-76646113. Max. coverage (+): 0. Max coverage (-): 52.39

Region: chr15 76646114-76646251. Max. coverage (+): 3.76. Max coverage (-): 81.7

Region: chr15 76646252-76646389. Max. coverage (+): 0. Max coverage (-): 9.62

Region: chr15 76646390-76646526. Max. coverage (+): 0. Max coverage (-): 30.09

Region: chr15 76646527-76646664. Max. coverage (+): 1.88. Max coverage (-): 10.66

Region: chr15 76646665-76646802. Max. coverage (+): 2.13. Max coverage (-): 28.21

Region: chr15 76646803-76646939. Max. coverage (+): 0. Max coverage (-): 33.58

Region: chr15 76646940-76647077. Max. coverage (+): 0. Max coverage (-): 50.73

Region: chr15 76647078-76647214. Max. coverage (+): 0. Max coverage (-): 147.2

Region: chr15 76647215-76647352. Max. coverage (+): 0. Max coverage (-): 85.08

Region: chr15 76647353-76647490. Max. coverage (+): 0. Max coverage (-): 22.95

Region: chr15 76647491-76647627. Max. coverage (+): 0. Max coverage (-): 45.32

Region: chr15 76647628-76647765. Max. coverage (+): 0. Max coverage (-): 12.58

Region: chr15 76647766-76647903. Max. coverage (+): 0. Max coverage (-): 102.73

Region: chr15 76647904-76648040. Max. coverage (+): 0. Max coverage (-): 20.64

Region: chr15 76648041-76648178. Max. coverage (+): 1.56. Max coverage (-): 26.58

Region: chr15 76648179-76648315. Max. coverage (+): 8.99. Max coverage (-): 27.13

Region: chr15 76648316-76648453. Max. coverage (+): 1.05. Max coverage (-): 16.48

Region: chr15 76648454-76648591. Max. coverage (+): 5.07. Max coverage (-): 54.98

Region: chr15 76648592-76648728. Max. coverage (+): 5.07. Max coverage (-): 17.69

Region: chr15 76648729-76648866. Max. coverage (+): 0. Max coverage (-): 20.47

Region: chr15 76648867-76649003. Max. coverage (+): 0. Max coverage (-): 36.24

Region: chr15 76649004-76649141. Max. coverage (+): 1.35. Max coverage (-): 62.89

Region: chr15 76649142-76649279. Max. coverage (+): 0. Max coverage (-): 11.01

Region: chr15 76649280-76649416. Max. coverage (+): 0. Max coverage (-): 34.92

Region: chr15 76649417-76649554. Max. coverage (+): 0. Max coverage (-): 3.82

Region: chr15 76649555-76649692. Max. coverage (+): 0. Max coverage (-): 25.23

Region: chr15 76649693-76649829. Max. coverage (+): 20.16. Max coverage (-): 13.73

Region: chr15 76649830-76649967. Max. coverage (+): 36.33. Max coverage (-): 2.77

Region: chr15 76649968-76650104. Max. coverage (+): 0. Max coverage (-): 0

Region: chr15 76650105-76650242. Max. coverage (+): 15.97. Max coverage (-): 2.18

Region: chr15 76650243-76650380. Max. coverage (+): 15.97. Max coverage (-): 0

Region: chr15 76650381-76650517. Max. coverage (+): 1.66. Max coverage (-): 0

Region: chr15 76650518-76650655. Max. coverage (+): 12.38. Max coverage (-): 0

Region: chr15 76650656-76650792. Max. coverage (+): 2.68. Max coverage (-): 0

Region: chr15 76650793-76650930. Max. coverage (+): 3.4. Max coverage (-): 0

Region: chr15 76650931-76651068. Max. coverage (+): 3.48. Max coverage (-): 0

Region: chr15 76651069-76651205. Max. coverage (+): 0. Max coverage (-): 0

Region: chr15 76651206-76651343. Max. coverage (+): 0.08. Max coverage (-): 0

Region: chr15 76651344-76651481. Max. coverage (+): 0. Max coverage (-): 0

Region: chr15 76651482-76651618. Max. coverage (+): 91.95. Max coverage (-): 0

Region: chr15 76651619-76651756. Max. coverage (+): 15. Max coverage (-): 0

Region: chr15 76651757-76651893. Max. coverage (+): 186.12. Max coverage (-): 0

Region: chr15 76651894-76652031. Max. coverage (+): 50.62. Max coverage (-): 0

Region: chr15 76652032-76652169. Max. coverage (+): 4.36. Max coverage (-): 0

Region: chr15 76652170-76652306. Max. coverage (+): 0. Max coverage (-): 0

Region: chr15 76652307-76652444. Max. coverage (+): 4.21. Max coverage (-): 0

Region: chr15 76652445-76652581. Max. coverage (+): 4.16. Max coverage (-): 0

Region: chr15 76652582-76652719. Max. coverage (+): 1.24. Max coverage (-): 0

Region: chr15 76652720-76652857. Max. coverage (+): 3.79. Max coverage (-): 0

Region: chr15 76652858-76652994. Max. coverage (+): 0. Max coverage (-): 0

Region: chr15 76652995-76653132. Max. coverage (+): 0. Max coverage (-): 0

Region: chr15 76653133-76653270. Max. coverage (+): 0. Max coverage (-): 0

Region: chr15 76653271-76653407. Max. coverage (+): 0. Max coverage (-): 0

Region: chr15 76653408-76653545. Max. coverage (+): 7.75. Max coverage (-): 0.94

Region: chr15 76653546-76653682. Max. coverage (+): 6.72. Max coverage (-): 3.4

Region: chr15 76653683-76653820. Max. coverage (+): 5.49. Max coverage (-): 0

Region: chr15 76653821-76653958. Max. coverage (+): 3.95. Max coverage (-): 1.72

Region: chr15 76653959-76654095. Max. coverage (+): 6.47. Max coverage (-): 1.58

Region: chr15 76654096-76654233. Max. coverage (+): 5.02. Max coverage (-): 4.42

Region: chr15 76654234-76654370. Max. coverage (+): 2.24. Max coverage (-): 0

Region: chr15 76654371-76654508. Max. coverage (+): 2.26. Max coverage (-): 0

Region: chr15 76654509-76654646. Max. coverage (+): 2.95. Max coverage (-): 1.57

Region: chr15 76654647-. Max. coverage (+): 4.39. Max coverage (-): 0

RepeatMasker Color Code

**+**

100-98% Identity

<98-95% Identity

<95-90% Identity

<90-85% Identity

<85-80% Identity

<80-75% Identity

<75-70% Identity

<70% Identity

**-**

Gene Set Color Code

**+**

Gene

Pseudogene

**-**

Topology/Coverage Color Code

Coverage Plus Strand

Coverage Minus Strand

Mainstrand: Plus

Mainstrand: Minus

Complementary Strand

Flanking Region  
(if option -flank >0)

Gene Set Annotation  
  
RepeatMasker Annotation  

**1. ERV1-1C-LTR\_BT**: 76586112-76586445 (-), Divergence to consensus: 9.4%  
**2. MIRb**: 76586505-76586601 (-), Divergence to consensus: 39.5%  
**3. Plat\_L3**: 76588202-76588324 (+), Divergence to consensus: 31.1%  
**4. Plat\_L3**: 76588508-76588613 (+), Divergence to consensus: 33.2%  
**5. MIR**: 76589716-76589914 (-), Divergence to consensus: 40.5%  
**6. GC\_rich**: 76592554-76592581 (+), Divergence to consensus: 53.6%  
**7. L2b**: 76593458-76593646 (-), Divergence to consensus: 43%  
**8. MIRb**: 76593957-76594090 (+), Divergence to consensus: 33.5%  
**9. L2b**: 76594964-76595266 (-), Divergence to consensus: 49.8%  
**10. MIR**: 76595316-76595570 (-), Divergence to consensus: 32.3%  
**11. MIR3**: 76596102-76596245 (-), Divergence to consensus: 34%  
**12. MIR**: 76599311-76599442 (-), Divergence to consensus: 50%  
**13. L1ME3**: 76599450-76599647 (+), Divergence to consensus: 39.9%  
**14. L1ME4b**: 76599642-76600004 (+), Divergence to consensus: 47.1%  
**15. MIR**: 76600597-76600725 (+), Divergence to consensus: 30.6%  
**16. MIRc**: 76601214-76601271 (+), Divergence to consensus: 29.4%  
**17. CHRL**: 76602077-76602284 (+), Divergence to consensus: 25.3%  
**18. MIR**: 76603204-76603329 (+), Divergence to consensus: 32.8%  
**19. L2a**: 76604589-76604775 (+), Divergence to consensus: 40.7%  
**20. ART2A**: 76605179-76605315 (-), Divergence to consensus: 11.7%  
**21. MER103C**: 76605797-76606024 (+), Divergence to consensus: 46.4%  
**22. CHRL1\_BT**: 76606240-76606352 (-), Divergence to consensus: 29.3%  
**23. MIR3**: 76606516-76606667 (+), Divergence to consensus: 45.4%  
**24. AT\_rich**: 76608370-76608392 (+), Divergence to consensus: 43.5%  
**25. MER90a**: 76609904-76609983 (+), Divergence to consensus: 28.1%  
**26. MER110-int**: 76610191-76610505 (+), Divergence to consensus: 42.3%  
**27. Bov-tA3**: 76610552-76610760 (+), Divergence to consensus: 14.1%  
**28. AT\_rich**: 76611025-76611046 (+), Divergence to consensus: 45.5%  
**29. Bov-tA3**: 76612224-76612377 (-), Divergence to consensus: 24.3%  
**30. MER90a**: 76612393-76612769 (+), Divergence to consensus: 21.2%  
**31. BOV-A2**: 76612972-76613085 (-), Divergence to consensus: 7.9%  
**32. MIR3**: 76613931-76613970 (-), Divergence to consensus: 22.5%  
**33. MIR3**: 76615021-76615125 (+), Divergence to consensus: 37.2%  
**34. MIR3**: 76616563-76616655 (-), Divergence to consensus: 33.3%  
**35. Bov-tA1**: 76617043-76617269 (+), Divergence to consensus: 13.2%  
**36. MIRb**: 76617988-76618059 (+), Divergence to consensus: 19.4%  
**37. MER5A**: 76618435-76618549 (+), Divergence to consensus: 30.1%  
**38. MIR**: 76619592-76619824 (-), Divergence to consensus: 33.4%  
**39. L1ME4a**: 76621881-76622121 (+), Divergence to consensus: 48.4%  
**40. L1ME4a**: 76622187-76622287 (+), Divergence to consensus: 41.8%  
**41. A-rich**: 76622304-76622381 (+), Divergence to consensus: 33.8%  
**42. T-rich**: 76622868-76622931 (+), Divergence to consensus: 28.6%  
**43. MIRb**: 76624409-76624597 (+), Divergence to consensus: 37.1%  
**44. (TTCA)n**: 76624692-76624723 (+), Divergence to consensus: 9.4%  
**45. L2a**: 76624762-76625084 (-), Divergence to consensus: 48.4%  
**46. CHRL**: 76625260-76625413 (-), Divergence to consensus: 20%  
**47. MIR3**: 76625489-76625609 (-), Divergence to consensus: 41.6%  
**48. L2c**: 76626061-76626437 (-), Divergence to consensus: 50.1%  
**49. L2c**: 76626862-76626959 (-), Divergence to consensus: 36%  
**50. MIRb**: 76627821-76627945 (-), Divergence to consensus: 36.9%  
**51. MIRc**: 76628826-76628978 (+), Divergence to consensus: 37.2%  
**52. MIRb**: 76629223-76629445 (+), Divergence to consensus: 48.6%  
**53. A-rich**: 76630071-76630107 (+), Divergence to consensus: 18.9%  
**54. MIRb**: 76630255-76630393 (-), Divergence to consensus: 37.7%  
**55. L2a**: 76631297-76631394 (+), Divergence to consensus: 38.3%  
**56. MER41\_BT**: 76631493-76631605 (+), Divergence to consensus: 23%  
**57. L2a**: 76632128-76632250 (+), Divergence to consensus: 40.1%  
**58. L2a**: 76633466-76634521 (-), Divergence to consensus: 48.4%  
**59. L2b**: 76634699-76634774 (-), Divergence to consensus: 29%  
**60. MamGypLTR1a**: 76635941-76636049 (+), Divergence to consensus: 38.5%  
**61. L3**: 76637175-76637393 (+), Divergence to consensus: 40.3%  
**62. L3**: 76637410-76637662 (+), Divergence to consensus: 43.9%  
**63. L3**: 76637686-76638067 (+), Divergence to consensus: 43.1%  
**64. ART2A**: 76638549-76639033 (+), Divergence to consensus: 18.3%  
**65. L2c**: 76639058-76639096 (-), Divergence to consensus: 25.6%  
**66. Tigger7**: 76639228-76639566 (+), Divergence to consensus: 26.5%  
**67. L2c**: 76639607-76639750 (-), Divergence to consensus: 40.2%  
**68. Charlie18a**: 76639998-76640118 (+), Divergence to consensus: 31.6%  
**69. L2c**: 76641033-76641134 (-), Divergence to consensus: 38.4%  
**70. CHRL**: 76642127-76642288 (+), Divergence to consensus: 18.5%  
**71. CT-rich**: 76642530-76642574 (+), Divergence to consensus: 24.4%  
**72. Bov-tA2**: 76644874-76645023 (-), Divergence to consensus: 13.5%  
**73. MIR**: 76648870-76648933 (-), Divergence to consensus: 24%  
**74. L2a**: 76648886-76648960 (-), Divergence to consensus: 48.2%  
**75. MIR3**: 76650019-76650156 (+), Divergence to consensus: 41.9%  
**76. C-rich**: 76650738-76650814 (+), Divergence to consensus: 37.8%  
**77. Bov-tA2**: 76651040-76651244 (+), Divergence to consensus: 19.3%  
**78. L1\_BT**: 76651935-76652009 (-), Divergence to consensus: 13.4%  
**79. MIRc**: 76652764-76652844 (+), Divergence to consensus: 39.5%  
**80. L2a**: 76652914-76653216 (+), Divergence to consensus: 50.4%  
**81. MIRb**: 76653232-76653407 (-), Divergence to consensus: 57.3%

  
Transcription Factor Binding Sites  

**RFX4\_2** (Sequence: GTAACCATG (-): 76633360)  
**RFX4\_1** (Sequence: CTTGGCAAC (+): 76628792)  
**RFX4\_1** (Sequence: CTTGGCAAC (+): 76647746)  
**SPZ1** (Sequence: CTGTTACCCC (-): 76619880)  
**SPZ1** (Sequence: CTCAAACCCC (-): 76620842)  
**RFX4\_2** (Sequence: CCTAGATAC (+): 76599356)  
**Gata4** (Sequence: AGATAAG (-): 76603600)  
**Gata4** (Sequence: AGATAAG (-): 76615741)  
**Gata4** (Sequence: AGATAAC (-): 76630015)  
**Gata4** (Sequence: AGATAAC (-): 76637146)  
**Gata4** (Sequence: AGATAAC (-): 76644843)  
**SOX9** (Sequence: AACAATGG (-): 76599117)  
**SOX9** (Sequence: AACAATGA (-): 76600014)  
**SOX9** (Sequence: AACAATAG (-): 76605104)  
**SOX9** (Sequence: AACAATGA (-): 76623990)  
**SOX9** (Sequence: AACAATGA (-): 76625150)  
**SOX9** (Sequence: AACAATAA (-): 76635602)  
**SOX9** (Sequence: AACAATGG (-): 76649595)  
**SOX9** (Sequence: CCATTGTT (+): 76614018)  
**SOX9** (Sequence: CCATTGTT (+): 76648066)  
**SPZ1** (Sequence: AGGGTTTCAG (+): 76602941)  
**SPZ1** (Sequence: GGGGTAAGAG (+): 76641570)  
**Mybl1\_1** (Sequence: AACCGTTA (+): 76649621)  
**Gata4** (Sequence: GTTATCT (+): 76589262)  
**Gata4** (Sequence: CTTATCT (+): 76591228)  
**Gata4** (Sequence: GTTATCT (+): 76593747)  
**Gata4** (Sequence: CTTATCT (+): 76596262)  
**Gata4** (Sequence: GTTATCT (+): 76602434)  
**Gata4** (Sequence: CTTATCT (+): 76606419)  
**Gata4** (Sequence: GTTATCT (+): 76622187)  
**Gata4** (Sequence: CTTATCT (+): 76632401)  
**Gata4** (Sequence: CTTATCT (+): 76638370)  
**Gata4** (Sequence: GTTATCT (+): 76640520)  
**Gata4** (Sequence: CTTATCT (+): 76643108)  
**Gata4** (Sequence: GTTATCT (+): 76643567)  
**Gata4** (Sequence: CTTATCT (+): 76647901)  
**Gata4** (Sequence: CTTATCT (+): 76648710)
