# Supplementary material for: Small RNA sequencing of cryopreserved semen from single bull revealed altered miRNAs and piRNAs expression between High- and Low-motile sperm populations
Source: BMC Genomics. 2017 Jan 4;18:14. doi: 10.1186/s12864-016-3394-7 (PMC5209821; doi:10.1186/s12864-016-3394-7)
Supplement: Additional file 3: — Details for each piRNA clusters found in High Motile (HM) sperm fraction. Genes, repeats, transposable elements and transcription factors binding sites falling within the cluster regions were reported. (ZIP 1896 kb) [file 12864_2016_3394_MOESM3_ESM.zip › 3.html]

piRNA cluster 3


Predicted piRNA cluster no. 3     previous   next
  

Show proTRAC run info
Hide proTRAC run info

================================= proTRAC ====================================  
VERSION: 2.1                                    LAST MODIFIED: 06. October 2015  
  
Please cite:  
Rosenkranz D, Zischler H. proTRAC - a software for probabilistic piRNA cluster  
detection, visualization and analysis. 2012. BMC Bioinformatics 13:5.  
  
and (for proTRAC 2.0 and later):  
Rosenkranz D, Rudloff S, Bastuck K, Ketting RF, Zischler H. Tupaia small RNAs  
provide insights into function and evolution of RNAi-based transposon defense  
in mammals. 2015. RNA 21(5):911-922.  
  
Contact:  
David Rosenkranz  
Institute of Anthropology, small RNA group  
Johannes Gutenberg University Mainz  
email: rosenkranz@uni-mainz.de  
  
You can find the latest proTRAC version at:  
http://sourceforge.net/projects/protrac/files  
http://www.smallRNAgroup-mainz.de/software  
==============================================================================  
  
PARAMETERS:  
Map file: .............../storage/core/barbara/genhome/smallRNA/fertility/Sample\_motile/pirna/Sample\_motile\_26-33\_collapsed.fa.no-dust.map.weighted-10000-1000-b-0  
Genome file: ............/storage/core/barbara/genhome/smallRNA/fertility/Sample\_all/pirna/bt\_311\_chrY.fa  
RepeatMasker annotation: /storage/genomes/bt\_umd31/GCF\_000003055.6\_Bos\_taurus\_UMD\_3.1.1\_repeatMasker\_chr.out  
GeneSet:................./storage/core/barbara/genhome/smallRNA/fertility/Sample\_all/pirna/full.gtf  
  
Significant (p<=0.01) hit density will be calculated based  
on observed hit distribution.  
  
Sliding window size: ........................................ 5000 bp  
Sliding window increament: .................................. 1000 bp  
Normalize each hit by number of genomic hits: ............... 1 [0=no/1=yes]  
Normalize each hit by number of sequence reads: ............. 1 [0=no/1=yes]  
Normalize values (-> per million mapped reads): ............. 1 [0=no/1=yes]  
Min. fraction of hits with 1T(U) or 10A: .................... 0.75  
Alternatively: Min. fraction of hits with 1T(U) and 10A: .... 0.5  
Min. fraction of hits with typical piRNA length: ............ 0.75  
Typical piRNA length: ....................................... 26-33 nt  
Min. size of a piRNA cluster: ............................... 5000 bp.  
Min. number of hits (absolute): ............................. 0  
Min. number of hits (normalized): ........................... 0  
Min. fraction of hits on the mainstrand: .................... 0.75  
Top fraction of mapped sequences (in terms of read counts): . 1%  
Top fraction accounts for max. n% of sequence reads: ........ 90%  
Min. fraction of hits on each arm of a bidirectional cluster: 0.1  
Output image file for each cluster: ......................... 0 [0=no/1=yes]  
Output html file for each cluster: .......................... 1 [0=no/1=yes]  
Output a summary table: ..................................... 1 [0=no/1=yes]  
Output a FASTA file for each cluster (piRNA sequences): ..... 1 [0=no/1=yes]  
Output a FASTA file comprising cluster sequences: ........... 1 [0=no/1=yes]  
Search DNA motifs in clusters: .............................. 1 [0=no/1=yes]  
Output flanking sequences: +/- .............................. 0 bp  
Output ~.pTi file: .......................................... 1 [0=no/1=yes]  
==============================================================================  
  
  
Genome size (without gaps): ............ 2678902517 bp  
Gaps (N/X/-): .......................... 53837044 bp  
Mapped reads: .......................... 658825247023  
Non-identical sequences: ............... 514171  
Genomic hits: .......................... 764233  
Significant densitiy of mapped reads: .. 12867599.5173724 reads/kb

Show proTRAC cluster info
Hide proTRAC cluster info

|  |  |
| --- | --- |
| Location | chr10 |
| Coordinates | 45866880-45871993 |
| Size [bp] | 5114 |
| Sequence hit loci | 71 |
| Mapped reads (normalized) | 112239540 |
| Mapped reads (normalized) per kb | 21947504.9 |
| Normalized reads with 1T (1U) | 81% |
| Normalized reads with 10A | 33.3% |
| Normalized reads with length 26-33 nt | 100% |
| Normalized reads on the main strand(s) | 100% |
| Predicted directionality | mono:plus |

100%

0%

1T (1U)  
reads

10A reads

26-33 nt  
reads

reads on mainstrand

**Either the amount of reads with 1T (1U) OR 10A has to exceed 75% (set with option: -1Tor10A)  
Alternatively the amount of reads with 1T (1U) AND 10A has to exceed 50% (set with option: -1Tand10A)  
Minimum amount of reads with preferred size is 75% (set with option: -pisize)  
Minimum amount of reads on the main strand(s) is 75% (set with option: -clstrand)**

Show read coverage
Hide read coverage

WHAT DO I SEE HERE?  
This chart shows the location of mapped sequence reads within a predicted piRNA cluster. The color refers to the number of genomic hits produced by the sequence read in question. A dark red bar indicates that this sequence read produces many other hits elsewhere in the genome. Many adjacent red or yellow bars can indicate the presence of a multi-copy element such as transposons or rRNA genes. A dark green bar indicates that this sequence read maps uniquely to this locus.

1 hit

2-5 hits

6-10 hits

11-20 hits

21-50 hits

51-100 hits

> 100 hits

chr10

45866880

45871993

Gene Set

RepeatMasker

Mapped  
Reads

14.23

plus strand

minus strand

14.23

Region: chr10 36892763-45866885. Max. coverage (+): 4.31. Max coverage (-): 0

Region: chr10 45866886-45866895. Max. coverage (+): 4.31. Max coverage (-): 0

Region: chr10 45866896-45866905. Max. coverage (+): 0. Max coverage (-): 0

Region: chr10 45866906-45866915. Max. coverage (+): 0. Max coverage (-): 0

Region: chr10 45866916-45866926. Max. coverage (+): 0. Max coverage (-): 0

Region: chr10 45866927-45866936. Max. coverage (+): 0. Max coverage (-): 0

Region: chr10 45866937-45866946. Max. coverage (+): 0. Max coverage (-): 0

Region: chr10 45866947-45866956. Max. coverage (+): 0. Max coverage (-): 0

Region: chr10 45866957-45866966. Max. coverage (+): 0. Max coverage (-): 0

Region: chr10 45866967-45866977. Max. coverage (+): 0. Max coverage (-): 0

Region: chr10 45866978-45866987. Max. coverage (+): 0. Max coverage (-): 0

Region: chr10 45866988-45866997. Max. coverage (+): 0. Max coverage (-): 0

Region: chr10 45866998-45867007. Max. coverage (+): 0. Max coverage (-): 0

Region: chr10 45867008-45867018. Max. coverage (+): 0. Max coverage (-): 0

Region: chr10 45867019-45867028. Max. coverage (+): 0. Max coverage (-): 0

Region: chr10 45867029-45867038. Max. coverage (+): 0. Max coverage (-): 0

Region: chr10 45867039-45867048. Max. coverage (+): 0. Max coverage (-): 0

Region: chr10 45867049-45867058. Max. coverage (+): 0. Max coverage (-): 0

Region: chr10 45867059-45867069. Max. coverage (+): 0. Max coverage (-): 0

Region: chr10 45867070-45867079. Max. coverage (+): 0. Max coverage (-): 0

Region: chr10 45867080-45867089. Max. coverage (+): 0. Max coverage (-): 0

Region: chr10 45867090-45867099. Max. coverage (+): 0. Max coverage (-): 0

Region: chr10 45867100-45867110. Max. coverage (+): 0. Max coverage (-): 0

Region: chr10 45867111-45867120. Max. coverage (+): 0. Max coverage (-): 0

Region: chr10 45867121-45867130. Max. coverage (+): 0. Max coverage (-): 0

Region: chr10 45867131-45867140. Max. coverage (+): 0. Max coverage (-): 0

Region: chr10 45867141-45867151. Max. coverage (+): 0. Max coverage (-): 0

Region: chr10 45867152-45867161. Max. coverage (+): 0. Max coverage (-): 0

Region: chr10 45867162-45867171. Max. coverage (+): 0. Max coverage (-): 0

Region: chr10 45867172-45867181. Max. coverage (+): 0. Max coverage (-): 0

Region: chr10 45867182-45867191. Max. coverage (+): 0. Max coverage (-): 0

Region: chr10 45867192-45867202. Max. coverage (+): 0. Max coverage (-): 0

Region: chr10 45867203-45867212. Max. coverage (+): 0. Max coverage (-): 0

Region: chr10 45867213-45867222. Max. coverage (+): 0. Max coverage (-): 0

Region: chr10 45867223-45867232. Max. coverage (+): 0. Max coverage (-): 0

Region: chr10 45867233-45867243. Max. coverage (+): 0. Max coverage (-): 0

Region: chr10 45867244-45867253. Max. coverage (+): 3.19. Max coverage (-): 0

Region: chr10 45867254-45867263. Max. coverage (+): 3.19. Max coverage (-): 0

Region: chr10 45867264-45867273. Max. coverage (+): 0. Max coverage (-): 0

Region: chr10 45867274-45867284. Max. coverage (+): 0. Max coverage (-): 0

Region: chr10 45867285-45867294. Max. coverage (+): 0. Max coverage (-): 0

Region: chr10 45867295-45867304. Max. coverage (+): 0. Max coverage (-): 0

Region: chr10 45867305-45867314. Max. coverage (+): 0. Max coverage (-): 0

Region: chr10 45867315-45867324. Max. coverage (+): 0. Max coverage (-): 0

Region: chr10 45867325-45867335. Max. coverage (+): 0. Max coverage (-): 0

Region: chr10 45867336-45867345. Max. coverage (+): 0. Max coverage (-): 0

Region: chr10 45867346-45867355. Max. coverage (+): 0. Max coverage (-): 0

Region: chr10 45867356-45867365. Max. coverage (+): 0. Max coverage (-): 0

Region: chr10 45867366-45867376. Max. coverage (+): 0. Max coverage (-): 0

Region: chr10 45867377-45867386. Max. coverage (+): 0. Max coverage (-): 0

Region: chr10 45867387-45867396. Max. coverage (+): 0. Max coverage (-): 0

Region: chr10 45867397-45867406. Max. coverage (+): 0. Max coverage (-): 0

Region: chr10 45867407-45867416. Max. coverage (+): 0. Max coverage (-): 0

Region: chr10 45867417-45867427. Max. coverage (+): 0. Max coverage (-): 0

Region: chr10 45867428-45867437. Max. coverage (+): 0. Max coverage (-): 0

Region: chr10 45867438-45867447. Max. coverage (+): 0. Max coverage (-): 0

Region: chr10 45867448-45867457. Max. coverage (+): 0. Max coverage (-): 0

Region: chr10 45867458-45867468. Max. coverage (+): 0. Max coverage (-): 0

Region: chr10 45867469-45867478. Max. coverage (+): 0. Max coverage (-): 0

Region: chr10 45867479-45867488. Max. coverage (+): 0. Max coverage (-): 0

Region: chr10 45867489-45867498. Max. coverage (+): 0. Max coverage (-): 0

Region: chr10 45867499-45867509. Max. coverage (+): 0. Max coverage (-): 0

Region: chr10 45867510-45867519. Max. coverage (+): 0. Max coverage (-): 0

Region: chr10 45867520-45867529. Max. coverage (+): 1.24. Max coverage (-): 0

Region: chr10 45867530-45867539. Max. coverage (+): 1.24. Max coverage (-): 0

Region: chr10 45867540-45867549. Max. coverage (+): 0. Max coverage (-): 0

Region: chr10 45867550-45867560. Max. coverage (+): 0. Max coverage (-): 0

Region: chr10 45867561-45867570. Max. coverage (+): 0. Max coverage (-): 0

Region: chr10 45867571-45867580. Max. coverage (+): 0. Max coverage (-): 0

Region: chr10 45867581-45867590. Max. coverage (+): 0. Max coverage (-): 0

Region: chr10 45867591-45867601. Max. coverage (+): 0. Max coverage (-): 0

Region: chr10 45867602-45867611. Max. coverage (+): 0. Max coverage (-): 0

Region: chr10 45867612-45867621. Max. coverage (+): 0. Max coverage (-): 0

Region: chr10 45867622-45867631. Max. coverage (+): 3.72. Max coverage (-): 0

Region: chr10 45867632-45867641. Max. coverage (+): 3.72. Max coverage (-): 0

Region: chr10 45867642-45867652. Max. coverage (+): 0. Max coverage (-): 0

Region: chr10 45867653-45867662. Max. coverage (+): 0. Max coverage (-): 0

Region: chr10 45867663-45867672. Max. coverage (+): 0. Max coverage (-): 0

Region: chr10 45867673-45867682. Max. coverage (+): 0. Max coverage (-): 0

Region: chr10 45867683-45867693. Max. coverage (+): 0.35. Max coverage (-): 0

Region: chr10 45867694-45867703. Max. coverage (+): 0.49. Max coverage (-): 0

Region: chr10 45867704-45867713. Max. coverage (+): 0.49. Max coverage (-): 0

Region: chr10 45867714-45867723. Max. coverage (+): 0. Max coverage (-): 0

Region: chr10 45867724-45867734. Max. coverage (+): 0. Max coverage (-): 0

Region: chr10 45867735-45867744. Max. coverage (+): 0. Max coverage (-): 0

Region: chr10 45867745-45867754. Max. coverage (+): 0. Max coverage (-): 0

Region: chr10 45867755-45867764. Max. coverage (+): 0. Max coverage (-): 0

Region: chr10 45867765-45867774. Max. coverage (+): 0. Max coverage (-): 0

Region: chr10 45867775-45867785. Max. coverage (+): 0. Max coverage (-): 0

Region: chr10 45867786-45867795. Max. coverage (+): 0. Max coverage (-): 0

Region: chr10 45867796-45867805. Max. coverage (+): 4.12. Max coverage (-): 0

Region: chr10 45867806-45867815. Max. coverage (+): 4.12. Max coverage (-): 0

Region: chr10 45867816-45867826. Max. coverage (+): 0. Max coverage (-): 0

Region: chr10 45867827-45867836. Max. coverage (+): 0. Max coverage (-): 0

Region: chr10 45867837-45867846. Max. coverage (+): 0. Max coverage (-): 0

Region: chr10 45867847-45867856. Max. coverage (+): 0. Max coverage (-): 0

Region: chr10 45867857-45867867. Max. coverage (+): 0. Max coverage (-): 0

Region: chr10 45867868-45867877. Max. coverage (+): 0. Max coverage (-): 0

Region: chr10 45867878-45867887. Max. coverage (+): 0. Max coverage (-): 0

Region: chr10 45867888-45867897. Max. coverage (+): 0. Max coverage (-): 0

Region: chr10 45867898-45867907. Max. coverage (+): 0. Max coverage (-): 0

Region: chr10 45867908-45867918. Max. coverage (+): 0. Max coverage (-): 0

Region: chr10 45867919-45867928. Max. coverage (+): 2.4. Max coverage (-): 0

Region: chr10 45867929-45867938. Max. coverage (+): 2.4. Max coverage (-): 0

Region: chr10 45867939-45867948. Max. coverage (+): 1.03. Max coverage (-): 0

Region: chr10 45867949-45867959. Max. coverage (+): 1.03. Max coverage (-): 0

Region: chr10 45867960-45867969. Max. coverage (+): 0. Max coverage (-): 0

Region: chr10 45867970-45867979. Max. coverage (+): 0.99. Max coverage (-): 0

Region: chr10 45867980-45867989. Max. coverage (+): 0.99. Max coverage (-): 0

Region: chr10 45867990-45867999. Max. coverage (+): 0. Max coverage (-): 0

Region: chr10 45868000-45868010. Max. coverage (+): 0. Max coverage (-): 0

Region: chr10 45868011-45868020. Max. coverage (+): 0. Max coverage (-): 0

Region: chr10 45868021-45868030. Max. coverage (+): 0. Max coverage (-): 0

Region: chr10 45868031-45868040. Max. coverage (+): 0. Max coverage (-): 0

Region: chr10 45868041-45868051. Max. coverage (+): 0. Max coverage (-): 0

Region: chr10 45868052-45868061. Max. coverage (+): 0. Max coverage (-): 0

Region: chr10 45868062-45868071. Max. coverage (+): 0. Max coverage (-): 0

Region: chr10 45868072-45868081. Max. coverage (+): 0.86. Max coverage (-): 0

Region: chr10 45868082-45868092. Max. coverage (+): 0.86. Max coverage (-): 0

Region: chr10 45868093-45868102. Max. coverage (+): 0. Max coverage (-): 0

Region: chr10 45868103-45868112. Max. coverage (+): 0. Max coverage (-): 0

Region: chr10 45868113-45868122. Max. coverage (+): 0. Max coverage (-): 0

Region: chr10 45868123-45868132. Max. coverage (+): 1.19. Max coverage (-): 0

Region: chr10 45868133-45868143. Max. coverage (+): 2.05. Max coverage (-): 0

Region: chr10 45868144-45868153. Max. coverage (+): 0. Max coverage (-): 0

Region: chr10 45868154-45868163. Max. coverage (+): 0. Max coverage (-): 0

Region: chr10 45868164-45868173. Max. coverage (+): 0. Max coverage (-): 0

Region: chr10 45868174-45868184. Max. coverage (+): 0. Max coverage (-): 0

Region: chr10 45868185-45868194. Max. coverage (+): 0. Max coverage (-): 0

Region: chr10 45868195-45868204. Max. coverage (+): 0. Max coverage (-): 0

Region: chr10 45868205-45868214. Max. coverage (+): 1.41. Max coverage (-): 0

Region: chr10 45868215-45868224. Max. coverage (+): 1.41. Max coverage (-): 0

Region: chr10 45868225-45868235. Max. coverage (+): 0. Max coverage (-): 0

Region: chr10 45868236-45868245. Max. coverage (+): 0. Max coverage (-): 0

Region: chr10 45868246-45868255. Max. coverage (+): 0. Max coverage (-): 0

Region: chr10 45868256-45868265. Max. coverage (+): 0. Max coverage (-): 0

Region: chr10 45868266-45868276. Max. coverage (+): 0. Max coverage (-): 0

Region: chr10 45868277-45868286. Max. coverage (+): 0. Max coverage (-): 0

Region: chr10 45868287-45868296. Max. coverage (+): 1.93. Max coverage (-): 0

Region: chr10 45868297-45868306. Max. coverage (+): 1.93. Max coverage (-): 0

Region: chr10 45868307-45868317. Max. coverage (+): 0. Max coverage (-): 0

Region: chr10 45868318-45868327. Max. coverage (+): 0. Max coverage (-): 0

Region: chr10 45868328-45868337. Max. coverage (+): 0. Max coverage (-): 0

Region: chr10 45868338-45868347. Max. coverage (+): 0. Max coverage (-): 0

Region: chr10 45868348-45868357. Max. coverage (+): 0. Max coverage (-): 0

Region: chr10 45868358-45868368. Max. coverage (+): 6. Max coverage (-): 0

Region: chr10 45868369-45868378. Max. coverage (+): 0. Max coverage (-): 0

Region: chr10 45868379-45868388. Max. coverage (+): 0. Max coverage (-): 0

Region: chr10 45868389-45868398. Max. coverage (+): 0. Max coverage (-): 0

Region: chr10 45868399-45868409. Max. coverage (+): 0. Max coverage (-): 0

Region: chr10 45868410-45868419. Max. coverage (+): 0. Max coverage (-): 0

Region: chr10 45868420-45868429. Max. coverage (+): 0. Max coverage (-): 0

Region: chr10 45868430-45868439. Max. coverage (+): 0. Max coverage (-): 0

Region: chr10 45868440-45868449. Max. coverage (+): 0. Max coverage (-): 0

Region: chr10 45868450-45868460. Max. coverage (+): 0. Max coverage (-): 0

Region: chr10 45868461-45868470. Max. coverage (+): 0. Max coverage (-): 0

Region: chr10 45868471-45868480. Max. coverage (+): 0. Max coverage (-): 0

Region: chr10 45868481-45868490. Max. coverage (+): 0. Max coverage (-): 0

Region: chr10 45868491-45868501. Max. coverage (+): 0. Max coverage (-): 0

Region: chr10 45868502-45868511. Max. coverage (+): 0. Max coverage (-): 0

Region: chr10 45868512-45868521. Max. coverage (+): 0. Max coverage (-): 0

Region: chr10 45868522-45868531. Max. coverage (+): 0. Max coverage (-): 0

Region: chr10 45868532-45868542. Max. coverage (+): 0. Max coverage (-): 0

Region: chr10 45868543-45868552. Max. coverage (+): 0. Max coverage (-): 0

Region: chr10 45868553-45868562. Max. coverage (+): 0. Max coverage (-): 0

Region: chr10 45868563-45868572. Max. coverage (+): 2.86. Max coverage (-): 0

Region: chr10 45868573-45868582. Max. coverage (+): 9.45. Max coverage (-): 0

Region: chr10 45868583-45868593. Max. coverage (+): 8.23. Max coverage (-): 0

Region: chr10 45868594-45868603. Max. coverage (+): 0. Max coverage (-): 0

Region: chr10 45868604-45868613. Max. coverage (+): 0. Max coverage (-): 0

Region: chr10 45868614-45868623. Max. coverage (+): 0. Max coverage (-): 0

Region: chr10 45868624-45868634. Max. coverage (+): 0. Max coverage (-): 0

Region: chr10 45868635-45868644. Max. coverage (+): 0. Max coverage (-): 0

Region: chr10 45868645-45868654. Max. coverage (+): 0. Max coverage (-): 0

Region: chr10 45868655-45868664. Max. coverage (+): 0. Max coverage (-): 0

Region: chr10 45868665-45868675. Max. coverage (+): 0. Max coverage (-): 0

Region: chr10 45868676-45868685. Max. coverage (+): 0. Max coverage (-): 0

Region: chr10 45868686-45868695. Max. coverage (+): 0. Max coverage (-): 0

Region: chr10 45868696-45868705. Max. coverage (+): 0. Max coverage (-): 0

Region: chr10 45868706-45868715. Max. coverage (+): 0. Max coverage (-): 0

Region: chr10 45868716-45868726. Max. coverage (+): 4.91. Max coverage (-): 0

Region: chr10 45868727-45868736. Max. coverage (+): 4.5. Max coverage (-): 0

Region: chr10 45868737-45868746. Max. coverage (+): 0. Max coverage (-): 0

Region: chr10 45868747-45868756. Max. coverage (+): 3.43. Max coverage (-): 0

Region: chr10 45868757-45868767. Max. coverage (+): 3.43. Max coverage (-): 0

Region: chr10 45868768-45868777. Max. coverage (+): 0. Max coverage (-): 0

Region: chr10 45868778-45868787. Max. coverage (+): 0. Max coverage (-): 0

Region: chr10 45868788-45868797. Max. coverage (+): 0. Max coverage (-): 0

Region: chr10 45868798-45868807. Max. coverage (+): 0. Max coverage (-): 0

Region: chr10 45868808-45868818. Max. coverage (+): 0. Max coverage (-): 0

Region: chr10 45868819-45868828. Max. coverage (+): 0. Max coverage (-): 0

Region: chr10 45868829-45868838. Max. coverage (+): 0. Max coverage (-): 0

Region: chr10 45868839-45868848. Max. coverage (+): 0. Max coverage (-): 0

Region: chr10 45868849-45868859. Max. coverage (+): 0. Max coverage (-): 0

Region: chr10 45868860-45868869. Max. coverage (+): 0. Max coverage (-): 0

Region: chr10 45868870-45868879. Max. coverage (+): 0. Max coverage (-): 0

Region: chr10 45868880-45868889. Max. coverage (+): 0. Max coverage (-): 0

Region: chr10 45868890-45868900. Max. coverage (+): 0. Max coverage (-): 0

Region: chr10 45868901-45868910. Max. coverage (+): 0. Max coverage (-): 0

Region: chr10 45868911-45868920. Max. coverage (+): 0. Max coverage (-): 0

Region: chr10 45868921-45868930. Max. coverage (+): 0. Max coverage (-): 0

Region: chr10 45868931-45868940. Max. coverage (+): 0. Max coverage (-): 0

Region: chr10 45868941-45868951. Max. coverage (+): 0. Max coverage (-): 0

Region: chr10 45868952-45868961. Max. coverage (+): 0. Max coverage (-): 0

Region: chr10 45868962-45868971. Max. coverage (+): 0. Max coverage (-): 0

Region: chr10 45868972-45868981. Max. coverage (+): 0. Max coverage (-): 0

Region: chr10 45868982-45868992. Max. coverage (+): 0. Max coverage (-): 0

Region: chr10 45868993-45869002. Max. coverage (+): 0. Max coverage (-): 0

Region: chr10 45869003-45869012. Max. coverage (+): 0. Max coverage (-): 0

Region: chr10 45869013-45869022. Max. coverage (+): 3.28. Max coverage (-): 0

Region: chr10 45869023-45869032. Max. coverage (+): 3.28. Max coverage (-): 0

Region: chr10 45869033-45869043. Max. coverage (+): 0. Max coverage (-): 0

Region: chr10 45869044-45869053. Max. coverage (+): 0. Max coverage (-): 0

Region: chr10 45869054-45869063. Max. coverage (+): 0. Max coverage (-): 0

Region: chr10 45869064-45869073. Max. coverage (+): 0. Max coverage (-): 0

Region: chr10 45869074-45869084. Max. coverage (+): 0.83. Max coverage (-): 0

Region: chr10 45869085-45869094. Max. coverage (+): 0. Max coverage (-): 0

Region: chr10 45869095-45869104. Max. coverage (+): 0. Max coverage (-): 0

Region: chr10 45869105-45869114. Max. coverage (+): 0. Max coverage (-): 0

Region: chr10 45869115-45869125. Max. coverage (+): 0. Max coverage (-): 0

Region: chr10 45869126-45869135. Max. coverage (+): 0. Max coverage (-): 0

Region: chr10 45869136-45869145. Max. coverage (+): 0. Max coverage (-): 0

Region: chr10 45869146-45869155. Max. coverage (+): 0. Max coverage (-): 0

Region: chr10 45869156-45869165. Max. coverage (+): 0. Max coverage (-): 0

Region: chr10 45869166-45869176. Max. coverage (+): 0. Max coverage (-): 0

Region: chr10 45869177-45869186. Max. coverage (+): 0. Max coverage (-): 0

Region: chr10 45869187-45869196. Max. coverage (+): 4.44. Max coverage (-): 0

Region: chr10 45869197-45869206. Max. coverage (+): 4.44. Max coverage (-): 0

Region: chr10 45869207-45869217. Max. coverage (+): 0. Max coverage (-): 0

Region: chr10 45869218-45869227. Max. coverage (+): 5.19. Max coverage (-): 0

Region: chr10 45869228-45869237. Max. coverage (+): 5.19. Max coverage (-): 0

Region: chr10 45869238-45869247. Max. coverage (+): 14.23. Max coverage (-): 0

Region: chr10 45869248-45869258. Max. coverage (+): 12.23. Max coverage (-): 0

Region: chr10 45869259-45869268. Max. coverage (+): 0. Max coverage (-): 0

Region: chr10 45869269-45869278. Max. coverage (+): 4.34. Max coverage (-): 0

Region: chr10 45869279-45869288. Max. coverage (+): 4.34. Max coverage (-): 0

Region: chr10 45869289-45869298. Max. coverage (+): 0. Max coverage (-): 0

Region: chr10 45869299-45869309. Max. coverage (+): 0. Max coverage (-): 0

Region: chr10 45869310-45869319. Max. coverage (+): 0. Max coverage (-): 0

Region: chr10 45869320-45869329. Max. coverage (+): 0. Max coverage (-): 0

Region: chr10 45869330-45869339. Max. coverage (+): 0. Max coverage (-): 0

Region: chr10 45869340-45869350. Max. coverage (+): 0. Max coverage (-): 0

Region: chr10 45869351-45869360. Max. coverage (+): 0. Max coverage (-): 0

Region: chr10 45869361-45869370. Max. coverage (+): 0. Max coverage (-): 0

Region: chr10 45869371-45869380. Max. coverage (+): 0. Max coverage (-): 0

Region: chr10 45869381-45869390. Max. coverage (+): 0. Max coverage (-): 0

Region: chr10 45869391-45869401. Max. coverage (+): 0. Max coverage (-): 0

Region: chr10 45869402-45869411. Max. coverage (+): 0. Max coverage (-): 0

Region: chr10 45869412-45869421. Max. coverage (+): 3.69. Max coverage (-): 0

Region: chr10 45869422-45869431. Max. coverage (+): 10.67. Max coverage (-): 0

Region: chr10 45869432-45869442. Max. coverage (+): 6.98. Max coverage (-): 0

Region: chr10 45869443-45869452. Max. coverage (+): 0. Max coverage (-): 0

Region: chr10 45869453-45869462. Max. coverage (+): 6.56. Max coverage (-): 0

Region: chr10 45869463-45869472. Max. coverage (+): 10.71. Max coverage (-): 0

Region: chr10 45869473-45869483. Max. coverage (+): 0. Max coverage (-): 0

Region: chr10 45869484-45869493. Max. coverage (+): 0. Max coverage (-): 0

Region: chr10 45869494-45869503. Max. coverage (+): 0. Max coverage (-): 0

Region: chr10 45869504-45869513. Max. coverage (+): 0. Max coverage (-): 0

Region: chr10 45869514-45869523. Max. coverage (+): 0. Max coverage (-): 0

Region: chr10 45869524-45869534. Max. coverage (+): 2.11. Max coverage (-): 0

Region: chr10 45869535-45869544. Max. coverage (+): 2.11. Max coverage (-): 0

Region: chr10 45869545-45869554. Max. coverage (+): 0. Max coverage (-): 0

Region: chr10 45869555-45869564. Max. coverage (+): 0. Max coverage (-): 0

Region: chr10 45869565-45869575. Max. coverage (+): 0. Max coverage (-): 0

Region: chr10 45869576-45869585. Max. coverage (+): 0. Max coverage (-): 0

Region: chr10 45869586-45869595. Max. coverage (+): 0. Max coverage (-): 0

Region: chr10 45869596-45869605. Max. coverage (+): 0. Max coverage (-): 0

Region: chr10 45869606-45869615. Max. coverage (+): 0. Max coverage (-): 0

Region: chr10 45869616-45869626. Max. coverage (+): 2.57. Max coverage (-): 0

Region: chr10 45869627-45869636. Max. coverage (+): 0. Max coverage (-): 0

Region: chr10 45869637-45869646. Max. coverage (+): 0. Max coverage (-): 0

Region: chr10 45869647-45869656. Max. coverage (+): 0. Max coverage (-): 0

Region: chr10 45869657-45869667. Max. coverage (+): 0. Max coverage (-): 0

Region: chr10 45869668-45869677. Max. coverage (+): 0. Max coverage (-): 0

Region: chr10 45869678-45869687. Max. coverage (+): 0. Max coverage (-): 0

Region: chr10 45869688-45869697. Max. coverage (+): 0. Max coverage (-): 0

Region: chr10 45869698-45869708. Max. coverage (+): 0. Max coverage (-): 0

Region: chr10 45869709-45869718. Max. coverage (+): 0. Max coverage (-): 0

Region: chr10 45869719-45869728. Max. coverage (+): 0. Max coverage (-): 0

Region: chr10 45869729-45869738. Max. coverage (+): 0. Max coverage (-): 0

Region: chr10 45869739-45869748. Max. coverage (+): 0. Max coverage (-): 0

Region: chr10 45869749-45869759. Max. coverage (+): 0. Max coverage (-): 0

Region: chr10 45869760-45869769. Max. coverage (+): 0. Max coverage (-): 0

Region: chr10 45869770-45869779. Max. coverage (+): 2.04. Max coverage (-): 0

Region: chr10 45869780-45869789. Max. coverage (+): 4.13. Max coverage (-): 0

Region: chr10 45869790-45869800. Max. coverage (+): 1.96. Max coverage (-): 0

Region: chr10 45869801-45869810. Max. coverage (+): 0. Max coverage (-): 0

Region: chr10 45869811-45869820. Max. coverage (+): 0. Max coverage (-): 0

Region: chr10 45869821-45869830. Max. coverage (+): 0. Max coverage (-): 0

Region: chr10 45869831-45869841. Max. coverage (+): 0. Max coverage (-): 0

Region: chr10 45869842-45869851. Max. coverage (+): 0. Max coverage (-): 0

Region: chr10 45869852-45869861. Max. coverage (+): 0. Max coverage (-): 0

Region: chr10 45869862-45869871. Max. coverage (+): 0. Max coverage (-): 0

Region: chr10 45869872-45869881. Max. coverage (+): 0. Max coverage (-): 0

Region: chr10 45869882-45869892. Max. coverage (+): 0. Max coverage (-): 0

Region: chr10 45869893-45869902. Max. coverage (+): 0. Max coverage (-): 0

Region: chr10 45869903-45869912. Max. coverage (+): 0. Max coverage (-): 0

Region: chr10 45869913-45869922. Max. coverage (+): 0. Max coverage (-): 0

Region: chr10 45869923-45869933. Max. coverage (+): 0. Max coverage (-): 0

Region: chr10 45869934-45869943. Max. coverage (+): 0. Max coverage (-): 0

Region: chr10 45869944-45869953. Max. coverage (+): 0. Max coverage (-): 0

Region: chr10 45869954-45869963. Max. coverage (+): 0. Max coverage (-): 0

Region: chr10 45869964-45869973. Max. coverage (+): 0. Max coverage (-): 0

Region: chr10 45869974-45869984. Max. coverage (+): 0. Max coverage (-): 0

Region: chr10 45869985-45869994. Max. coverage (+): 0. Max coverage (-): 0

Region: chr10 45869995-45870004. Max. coverage (+): 0. Max coverage (-): 0

Region: chr10 45870005-45870014. Max. coverage (+): 2.82. Max coverage (-): 0

Region: chr10 45870015-45870025. Max. coverage (+): 2.82. Max coverage (-): 0

Region: chr10 45870026-45870035. Max. coverage (+): 0. Max coverage (-): 0

Region: chr10 45870036-45870045. Max. coverage (+): 0. Max coverage (-): 0

Region: chr10 45870046-45870055. Max. coverage (+): 0. Max coverage (-): 0

Region: chr10 45870056-45870066. Max. coverage (+): 0. Max coverage (-): 0

Region: chr10 45870067-45870076. Max. coverage (+): 0. Max coverage (-): 0

Region: chr10 45870077-45870086. Max. coverage (+): 0. Max coverage (-): 0

Region: chr10 45870087-45870096. Max. coverage (+): 0. Max coverage (-): 0

Region: chr10 45870097-45870106. Max. coverage (+): 4.41. Max coverage (-): 0

Region: chr10 45870107-45870117. Max. coverage (+): 0. Max coverage (-): 0

Region: chr10 45870118-45870127. Max. coverage (+): 0. Max coverage (-): 0

Region: chr10 45870128-45870137. Max. coverage (+): 0. Max coverage (-): 0

Region: chr10 45870138-45870147. Max. coverage (+): 0. Max coverage (-): 0

Region: chr10 45870148-45870158. Max. coverage (+): 0. Max coverage (-): 0

Region: chr10 45870159-45870168. Max. coverage (+): 0. Max coverage (-): 0

Region: chr10 45870169-45870178. Max. coverage (+): 0. Max coverage (-): 0

Region: chr10 45870179-45870188. Max. coverage (+): 1.17. Max coverage (-): 0

Region: chr10 45870189-45870198. Max. coverage (+): 1.17. Max coverage (-): 0

Region: chr10 45870199-45870209. Max. coverage (+): 0. Max coverage (-): 0

Region: chr10 45870210-45870219. Max. coverage (+): 0. Max coverage (-): 0

Region: chr10 45870220-45870229. Max. coverage (+): 0. Max coverage (-): 0

Region: chr10 45870230-45870239. Max. coverage (+): 0. Max coverage (-): 0

Region: chr10 45870240-45870250. Max. coverage (+): 0. Max coverage (-): 0

Region: chr10 45870251-45870260. Max. coverage (+): 0. Max coverage (-): 0

Region: chr10 45870261-45870270. Max. coverage (+): 0. Max coverage (-): 0

Region: chr10 45870271-45870280. Max. coverage (+): 0. Max coverage (-): 0

Region: chr10 45870281-45870291. Max. coverage (+): 0. Max coverage (-): 0

Region: chr10 45870292-45870301. Max. coverage (+): 0. Max coverage (-): 0

Region: chr10 45870302-45870311. Max. coverage (+): 0. Max coverage (-): 0

Region: chr10 45870312-45870321. Max. coverage (+): 0. Max coverage (-): 0

Region: chr10 45870322-45870331. Max. coverage (+): 0. Max coverage (-): 0

Region: chr10 45870332-45870342. Max. coverage (+): 0. Max coverage (-): 0

Region: chr10 45870343-45870352. Max. coverage (+): 3.63. Max coverage (-): 0

Region: chr10 45870353-45870362. Max. coverage (+): 2.29. Max coverage (-): 0

Region: chr10 45870363-45870372. Max. coverage (+): 3.34. Max coverage (-): 0

Region: chr10 45870373-45870383. Max. coverage (+): 0. Max coverage (-): 0

Region: chr10 45870384-45870393. Max. coverage (+): 0. Max coverage (-): 0

Region: chr10 45870394-45870403. Max. coverage (+): 0. Max coverage (-): 0

Region: chr10 45870404-45870413. Max. coverage (+): 4.4. Max coverage (-): 0

Region: chr10 45870414-45870424. Max. coverage (+): 4.4. Max coverage (-): 0

Region: chr10 45870425-45870434. Max. coverage (+): 0. Max coverage (-): 0

Region: chr10 45870435-45870444. Max. coverage (+): 0. Max coverage (-): 0

Region: chr10 45870445-45870454. Max. coverage (+): 0. Max coverage (-): 0

Region: chr10 45870455-45870464. Max. coverage (+): 0. Max coverage (-): 0

Region: chr10 45870465-45870475. Max. coverage (+): 0. Max coverage (-): 0

Region: chr10 45870476-45870485. Max. coverage (+): 0. Max coverage (-): 0

Region: chr10 45870486-45870495. Max. coverage (+): 0. Max coverage (-): 0

Region: chr10 45870496-45870505. Max. coverage (+): 0. Max coverage (-): 0

Region: chr10 45870506-45870516. Max. coverage (+): 0. Max coverage (-): 0

Region: chr10 45870517-45870526. Max. coverage (+): 0. Max coverage (-): 0

Region: chr10 45870527-45870536. Max. coverage (+): 3.25. Max coverage (-): 0

Region: chr10 45870537-45870546. Max. coverage (+): 0. Max coverage (-): 0

Region: chr10 45870547-45870556. Max. coverage (+): 0. Max coverage (-): 0

Region: chr10 45870557-45870567. Max. coverage (+): 1.85. Max coverage (-): 0

Region: chr10 45870568-45870577. Max. coverage (+): 3.45. Max coverage (-): 0

Region: chr10 45870578-45870587. Max. coverage (+): 0. Max coverage (-): 0

Region: chr10 45870588-45870597. Max. coverage (+): 0. Max coverage (-): 0

Region: chr10 45870598-45870608. Max. coverage (+): 0. Max coverage (-): 0

Region: chr10 45870609-45870618. Max. coverage (+): 0. Max coverage (-): 0

Region: chr10 45870619-45870628. Max. coverage (+): 0. Max coverage (-): 0

Region: chr10 45870629-45870638. Max. coverage (+): 0. Max coverage (-): 0

Region: chr10 45870639-45870649. Max. coverage (+): 0. Max coverage (-): 0

Region: chr10 45870650-45870659. Max. coverage (+): 0. Max coverage (-): 0

Region: chr10 45870660-45870669. Max. coverage (+): 0. Max coverage (-): 0

Region: chr10 45870670-45870679. Max. coverage (+): 0. Max coverage (-): 0

Region: chr10 45870680-45870689. Max. coverage (+): 0. Max coverage (-): 0

Region: chr10 45870690-45870700. Max. coverage (+): 0. Max coverage (-): 0

Region: chr10 45870701-45870710. Max. coverage (+): 0. Max coverage (-): 0

Region: chr10 45870711-45870720. Max. coverage (+): 0. Max coverage (-): 0

Region: chr10 45870721-45870730. Max. coverage (+): 0. Max coverage (-): 0

Region: chr10 45870731-45870741. Max. coverage (+): 0. Max coverage (-): 0

Region: chr10 45870742-45870751. Max. coverage (+): 0. Max coverage (-): 0

Region: chr10 45870752-45870761. Max. coverage (+): 0. Max coverage (-): 0

Region: chr10 45870762-45870771. Max. coverage (+): 1.38. Max coverage (-): 0

Region: chr10 45870772-45870781. Max. coverage (+): 3.93. Max coverage (-): 0

Region: chr10 45870782-45870792. Max. coverage (+): 3.93. Max coverage (-): 0

Region: chr10 45870793-45870802. Max. coverage (+): 0. Max coverage (-): 0

Region: chr10 45870803-45870812. Max. coverage (+): 0. Max coverage (-): 0

Region: chr10 45870813-45870822. Max. coverage (+): 0. Max coverage (-): 0

Region: chr10 45870823-45870833. Max. coverage (+): 0. Max coverage (-): 0

Region: chr10 45870834-45870843. Max. coverage (+): 0. Max coverage (-): 0

Region: chr10 45870844-45870853. Max. coverage (+): 0. Max coverage (-): 0

Region: chr10 45870854-45870863. Max. coverage (+): 0. Max coverage (-): 0

Region: chr10 45870864-45870874. Max. coverage (+): 0. Max coverage (-): 0

Region: chr10 45870875-45870884. Max. coverage (+): 0. Max coverage (-): 0

Region: chr10 45870885-45870894. Max. coverage (+): 0. Max coverage (-): 0

Region: chr10 45870895-45870904. Max. coverage (+): 0. Max coverage (-): 0

Region: chr10 45870905-45870914. Max. coverage (+): 0. Max coverage (-): 0

Region: chr10 45870915-45870925. Max. coverage (+): 0. Max coverage (-): 0

Region: chr10 45870926-45870935. Max. coverage (+): 0. Max coverage (-): 0

Region: chr10 45870936-45870945. Max. coverage (+): 0. Max coverage (-): 0

Region: chr10 45870946-45870955. Max. coverage (+): 0. Max coverage (-): 0

Region: chr10 45870956-45870966. Max. coverage (+): 0. Max coverage (-): 0

Region: chr10 45870967-45870976. Max. coverage (+): 0. Max coverage (-): 0

Region: chr10 45870977-45870986. Max. coverage (+): 0. Max coverage (-): 0

Region: chr10 45870987-45870996. Max. coverage (+): 0. Max coverage (-): 0

Region: chr10 45870997-45871006. Max. coverage (+): 0. Max coverage (-): 0

Region: chr10 45871007-45871017. Max. coverage (+): 0. Max coverage (-): 0

Region: chr10 45871018-45871027. Max. coverage (+): 0. Max coverage (-): 0

Region: chr10 45871028-45871037. Max. coverage (+): 0. Max coverage (-): 0

Region: chr10 45871038-45871047. Max. coverage (+): 0. Max coverage (-): 0

Region: chr10 45871048-45871058. Max. coverage (+): 0. Max coverage (-): 0

Region: chr10 45871059-45871068. Max. coverage (+): 0. Max coverage (-): 0

Region: chr10 45871069-45871078. Max. coverage (+): 0. Max coverage (-): 0

Region: chr10 45871079-45871088. Max. coverage (+): 0. Max coverage (-): 0

Region: chr10 45871089-45871099. Max. coverage (+): 0. Max coverage (-): 0

Region: chr10 45871100-45871109. Max. coverage (+): 0. Max coverage (-): 0

Region: chr10 45871110-45871119. Max. coverage (+): 0. Max coverage (-): 0

Region: chr10 45871120-45871129. Max. coverage (+): 0. Max coverage (-): 0

Region: chr10 45871130-45871139. Max. coverage (+): 0. Max coverage (-): 0

Region: chr10 45871140-45871150. Max. coverage (+): 0. Max coverage (-): 0

Region: chr10 45871151-45871160. Max. coverage (+): 0. Max coverage (-): 0

Region: chr10 45871161-45871170. Max. coverage (+): 0. Max coverage (-): 0

Region: chr10 45871171-45871180. Max. coverage (+): 0. Max coverage (-): 0

Region: chr10 45871181-45871191. Max. coverage (+): 0. Max coverage (-): 0

Region: chr10 45871192-45871201. Max. coverage (+): 0. Max coverage (-): 0

Region: chr10 45871202-45871211. Max. coverage (+): 0. Max coverage (-): 0

Region: chr10 45871212-45871221. Max. coverage (+): 0. Max coverage (-): 0

Region: chr10 45871222-45871232. Max. coverage (+): 0. Max coverage (-): 0

Region: chr10 45871233-45871242. Max. coverage (+): 0. Max coverage (-): 0

Region: chr10 45871243-45871252. Max. coverage (+): 0. Max coverage (-): 0

Region: chr10 45871253-45871262. Max. coverage (+): 0. Max coverage (-): 0

Region: chr10 45871263-45871272. Max. coverage (+): 0. Max coverage (-): 0

Region: chr10 45871273-45871283. Max. coverage (+): 0. Max coverage (-): 0

Region: chr10 45871284-45871293. Max. coverage (+): 0. Max coverage (-): 0

Region: chr10 45871294-45871303. Max. coverage (+): 0. Max coverage (-): 0

Region: chr10 45871304-45871313. Max. coverage (+): 0. Max coverage (-): 0

Region: chr10 45871314-45871324. Max. coverage (+): 0. Max coverage (-): 0

Region: chr10 45871325-45871334. Max. coverage (+): 0. Max coverage (-): 0

Region: chr10 45871335-45871344. Max. coverage (+): 0. Max coverage (-): 0

Region: chr10 45871345-45871354. Max. coverage (+): 0. Max coverage (-): 0

Region: chr10 45871355-45871364. Max. coverage (+): 2.27. Max coverage (-): 0

Region: chr10 45871365-45871375. Max. coverage (+): 1.55. Max coverage (-): 0

Region: chr10 45871376-45871385. Max. coverage (+): 0. Max coverage (-): 0

Region: chr10 45871386-45871395. Max. coverage (+): 0. Max coverage (-): 0

Region: chr10 45871396-45871405. Max. coverage (+): 0. Max coverage (-): 0

Region: chr10 45871406-45871416. Max. coverage (+): 0. Max coverage (-): 0

Region: chr10 45871417-45871426. Max. coverage (+): 0. Max coverage (-): 0

Region: chr10 45871427-45871436. Max. coverage (+): 0. Max coverage (-): 0

Region: chr10 45871437-45871446. Max. coverage (+): 0. Max coverage (-): 0

Region: chr10 45871447-45871457. Max. coverage (+): 0. Max coverage (-): 0

Region: chr10 45871458-45871467. Max. coverage (+): 0. Max coverage (-): 0

Region: chr10 45871468-45871477. Max. coverage (+): 0. Max coverage (-): 0

Region: chr10 45871478-45871487. Max. coverage (+): 0. Max coverage (-): 0

Region: chr10 45871488-45871497. Max. coverage (+): 0. Max coverage (-): 0

Region: chr10 45871498-45871508. Max. coverage (+): 0. Max coverage (-): 0

Region: chr10 45871509-45871518. Max. coverage (+): 0. Max coverage (-): 0

Region: chr10 45871519-45871528. Max. coverage (+): 0. Max coverage (-): 0

Region: chr10 45871529-45871538. Max. coverage (+): 0. Max coverage (-): 0

Region: chr10 45871539-45871549. Max. coverage (+): 0. Max coverage (-): 0

Region: chr10 45871550-45871559. Max. coverage (+): 0. Max coverage (-): 0

Region: chr10 45871560-45871569. Max. coverage (+): 0. Max coverage (-): 0

Region: chr10 45871570-45871579. Max. coverage (+): 0. Max coverage (-): 0

Region: chr10 45871580-45871589. Max. coverage (+): 0. Max coverage (-): 0

Region: chr10 45871590-45871600. Max. coverage (+): 0. Max coverage (-): 0

Region: chr10 45871601-45871610. Max. coverage (+): 0. Max coverage (-): 0

Region: chr10 45871611-45871620. Max. coverage (+): 0. Max coverage (-): 0

Region: chr10 45871621-45871630. Max. coverage (+): 0. Max coverage (-): 0

Region: chr10 45871631-45871641. Max. coverage (+): 0. Max coverage (-): 0

Region: chr10 45871642-45871651. Max. coverage (+): 0. Max coverage (-): 0

Region: chr10 45871652-45871661. Max. coverage (+): 0. Max coverage (-): 0

Region: chr10 45871662-45871671. Max. coverage (+): 0. Max coverage (-): 0

Region: chr10 45871672-45871682. Max. coverage (+): 0. Max coverage (-): 0

Region: chr10 45871683-45871692. Max. coverage (+): 0. Max coverage (-): 0

Region: chr10 45871693-45871702. Max. coverage (+): 0. Max coverage (-): 0

Region: chr10 45871703-45871712. Max. coverage (+): 0. Max coverage (-): 0

Region: chr10 45871713-45871722. Max. coverage (+): 0. Max coverage (-): 0

Region: chr10 45871723-45871733. Max. coverage (+): 0. Max coverage (-): 0

Region: chr10 45871734-45871743. Max. coverage (+): 0. Max coverage (-): 0

Region: chr10 45871744-45871753. Max. coverage (+): 0. Max coverage (-): 0

Region: chr10 45871754-45871763. Max. coverage (+): 0. Max coverage (-): 0

Region: chr10 45871764-45871774. Max. coverage (+): 0. Max coverage (-): 0

Region: chr10 45871775-45871784. Max. coverage (+): 0. Max coverage (-): 0

Region: chr10 45871785-45871794. Max. coverage (+): 0. Max coverage (-): 0

Region: chr10 45871795-45871804. Max. coverage (+): 0. Max coverage (-): 0

Region: chr10 45871805-45871815. Max. coverage (+): 0. Max coverage (-): 0

Region: chr10 45871816-45871825. Max. coverage (+): 0. Max coverage (-): 0

Region: chr10 45871826-45871835. Max. coverage (+): 0. Max coverage (-): 0

Region: chr10 45871836-45871845. Max. coverage (+): 0. Max coverage (-): 0

Region: chr10 45871846-45871855. Max. coverage (+): 0. Max coverage (-): 0

Region: chr10 45871856-45871866. Max. coverage (+): 0. Max coverage (-): 0

Region: chr10 45871867-45871876. Max. coverage (+): 0. Max coverage (-): 0

Region: chr10 45871877-45871886. Max. coverage (+): 0. Max coverage (-): 0

Region: chr10 45871887-45871896. Max. coverage (+): 0. Max coverage (-): 0

Region: chr10 45871897-45871907. Max. coverage (+): 0. Max coverage (-): 0

Region: chr10 45871908-45871917. Max. coverage (+): 0. Max coverage (-): 0

Region: chr10 45871918-45871927. Max. coverage (+): 0. Max coverage (-): 0

Region: chr10 45871928-45871937. Max. coverage (+): 0. Max coverage (-): 0

Region: chr10 45871938-45871947. Max. coverage (+): 0. Max coverage (-): 0

Region: chr10 45871948-45871958. Max. coverage (+): 0. Max coverage (-): 0

Region: chr10 45871959-45871968. Max. coverage (+): 4.72. Max coverage (-): 0

Region: chr10 45871969-45871978. Max. coverage (+): 4.72. Max coverage (-): 0

Region: chr10 45871979-45871988. Max. coverage (+): 0. Max coverage (-): 0

Region: chr10 45871989-. Max. coverage (+): 0. Max coverage (-): 0

RepeatMasker Color Code

**+**

100-98% Identity

<98-95% Identity

<95-90% Identity

<90-85% Identity

<85-80% Identity

<80-75% Identity

<75-70% Identity

<70% Identity

**-**

Gene Set Color Code

**+**

Gene

Pseudogene

**-**

Topology/Coverage Color Code

Coverage Plus Strand

Coverage Minus Strand

Mainstrand: Plus

Mainstrand: Minus

Complementary Strand

Flanking Region  
(if option -flank >0)

Gene Set Annotation  

**1. CSNK1G1 (protein coding, ENSBTAG00000016823) Tr:00000022379 Ex:13**: 45866862-45867730 (+)

  
RepeatMasker Annotation  

**1. (TG)n**: 45871889-45871940 (+), Divergence to consensus: 25%

  
Transcription Factor Binding Sites  

**Gata4** (Sequence: AGATAAG (-): 45868519)  
**SOX9** (Sequence: CTATTGTT (+): 45867190)  
**A-MYB** (Sequence: CCAACTGCCA (-): 45868245)
