# Supplementary material for: Small RNA sequencing of cryopreserved semen from single bull revealed altered miRNAs and piRNAs expression between High- and Low-motile sperm populations
Source: BMC Genomics. 2017 Jan 4;18:14. doi: 10.1186/s12864-016-3394-7 (PMC5209821; doi:10.1186/s12864-016-3394-7)
Supplement: Additional file 3: — Details for each piRNA clusters found in High Motile (HM) sperm fraction. Genes, repeats, transposable elements and transcription factors binding sites falling within the cluster regions were reported. (ZIP 1896 kb) [file 12864_2016_3394_MOESM3_ESM.zip › 30.html]

piRNA cluster 30


Predicted piRNA cluster no. 30     previous   next
  

Show proTRAC run info
Hide proTRAC run info

================================= proTRAC ====================================  
VERSION: 2.1                                    LAST MODIFIED: 06. October 2015  
  
Please cite:  
Rosenkranz D, Zischler H. proTRAC - a software for probabilistic piRNA cluster  
detection, visualization and analysis. 2012. BMC Bioinformatics 13:5.  
  
and (for proTRAC 2.0 and later):  
Rosenkranz D, Rudloff S, Bastuck K, Ketting RF, Zischler H. Tupaia small RNAs  
provide insights into function and evolution of RNAi-based transposon defense  
in mammals. 2015. RNA 21(5):911-922.  
  
Contact:  
David Rosenkranz  
Institute of Anthropology, small RNA group  
Johannes Gutenberg University Mainz  
email: rosenkranz@uni-mainz.de  
  
You can find the latest proTRAC version at:  
http://sourceforge.net/projects/protrac/files  
http://www.smallRNAgroup-mainz.de/software  
==============================================================================  
  
PARAMETERS:  
Map file: .............../storage/core/barbara/genhome/smallRNA/fertility/Sample\_motile/pirna/Sample\_motile\_26-33\_collapsed.fa.no-dust.map.weighted-10000-1000-b-0  
Genome file: ............/storage/core/barbara/genhome/smallRNA/fertility/Sample\_all/pirna/bt\_311\_chrY.fa  
RepeatMasker annotation: /storage/genomes/bt\_umd31/GCF\_000003055.6\_Bos\_taurus\_UMD\_3.1.1\_repeatMasker\_chr.out  
GeneSet:................./storage/core/barbara/genhome/smallRNA/fertility/Sample\_all/pirna/full.gtf  
  
Significant (p<=0.01) hit density will be calculated based  
on observed hit distribution.  
  
Sliding window size: ........................................ 5000 bp  
Sliding window increament: .................................. 1000 bp  
Normalize each hit by number of genomic hits: ............... 1 [0=no/1=yes]  
Normalize each hit by number of sequence reads: ............. 1 [0=no/1=yes]  
Normalize values (-> per million mapped reads): ............. 1 [0=no/1=yes]  
Min. fraction of hits with 1T(U) or 10A: .................... 0.75  
Alternatively: Min. fraction of hits with 1T(U) and 10A: .... 0.5  
Min. fraction of hits with typical piRNA length: ............ 0.75  
Typical piRNA length: ....................................... 26-33 nt  
Min. size of a piRNA cluster: ............................... 5000 bp.  
Min. number of hits (absolute): ............................. 0  
Min. number of hits (normalized): ........................... 0  
Min. fraction of hits on the mainstrand: .................... 0.75  
Top fraction of mapped sequences (in terms of read counts): . 1%  
Top fraction accounts for max. n% of sequence reads: ........ 90%  
Min. fraction of hits on each arm of a bidirectional cluster: 0.1  
Output image file for each cluster: ......................... 0 [0=no/1=yes]  
Output html file for each cluster: .......................... 1 [0=no/1=yes]  
Output a summary table: ..................................... 1 [0=no/1=yes]  
Output a FASTA file for each cluster (piRNA sequences): ..... 1 [0=no/1=yes]  
Output a FASTA file comprising cluster sequences: ........... 1 [0=no/1=yes]  
Search DNA motifs in clusters: .............................. 1 [0=no/1=yes]  
Output flanking sequences: +/- .............................. 0 bp  
Output ~.pTi file: .......................................... 1 [0=no/1=yes]  
==============================================================================  
  
  
Genome size (without gaps): ............ 2678902517 bp  
Gaps (N/X/-): .......................... 53837044 bp  
Mapped reads: .......................... 658825247023  
Non-identical sequences: ............... 514171  
Genomic hits: .......................... 764233  
Significant densitiy of mapped reads: .. 12867599.5173724 reads/kb

Show proTRAC cluster info
Hide proTRAC cluster info

|  |  |
| --- | --- |
| Location | chr16 |
| Coordinates | 61892258-61897730 |
| Size [bp] | 5473 |
| Sequence hit loci | 65 |
| Mapped reads (normalized) | 83276977.2 |
| Mapped reads (normalized) per kb | 15215965.1 |
| Normalized reads with 1T (1U) | 88.4% |
| Normalized reads with 10A | 45.8% |
| Normalized reads with length 26-33 nt | 100% |
| Normalized reads on the main strand(s) | 100% |
| Predicted directionality | mono:minus |

100%

0%

1T (1U)  
reads

10A reads

26-33 nt  
reads

reads on mainstrand

**Either the amount of reads with 1T (1U) OR 10A has to exceed 75% (set with option: -1Tor10A)  
Alternatively the amount of reads with 1T (1U) AND 10A has to exceed 50% (set with option: -1Tand10A)  
Minimum amount of reads with preferred size is 75% (set with option: -pisize)  
Minimum amount of reads on the main strand(s) is 75% (set with option: -clstrand)**

Show read coverage
Hide read coverage

WHAT DO I SEE HERE?  
This chart shows the location of mapped sequence reads within a predicted piRNA cluster. The color refers to the number of genomic hits produced by the sequence read in question. A dark red bar indicates that this sequence read produces many other hits elsewhere in the genome. Many adjacent red or yellow bars can indicate the presence of a multi-copy element such as transposons or rRNA genes. A dark green bar indicates that this sequence read maps uniquely to this locus.

1 hit

2-5 hits

6-10 hits

11-20 hits

21-50 hits

51-100 hits

> 100 hits

chr16

61892258

61897730

Gene Set

RepeatMasker

Mapped  
Reads

16.59

plus strand

minus strand

16.59

Region: chr16 76654648-61892263. Max. coverage (+): 0. Max coverage (-): 3.25

Region: chr16 61892264-61892274. Max. coverage (+): 0. Max coverage (-): 3.25

Region: chr16 61892275-61892285. Max. coverage (+): 0. Max coverage (-): 0

Region: chr16 61892286-61892296. Max. coverage (+): 0. Max coverage (-): 0

Region: chr16 61892297-61892307. Max. coverage (+): 0. Max coverage (-): 0

Region: chr16 61892308-61892318. Max. coverage (+): 0. Max coverage (-): 0

Region: chr16 61892319-61892329. Max. coverage (+): 0. Max coverage (-): 0

Region: chr16 61892330-61892340. Max. coverage (+): 0. Max coverage (-): 0

Region: chr16 61892341-61892351. Max. coverage (+): 0. Max coverage (-): 0

Region: chr16 61892352-61892361. Max. coverage (+): 0. Max coverage (-): 0

Region: chr16 61892362-61892372. Max. coverage (+): 0. Max coverage (-): 0

Region: chr16 61892373-61892383. Max. coverage (+): 0. Max coverage (-): 0

Region: chr16 61892384-61892394. Max. coverage (+): 0. Max coverage (-): 0

Region: chr16 61892395-61892405. Max. coverage (+): 0. Max coverage (-): 0

Region: chr16 61892406-61892416. Max. coverage (+): 0. Max coverage (-): 0

Region: chr16 61892417-61892427. Max. coverage (+): 0. Max coverage (-): 0

Region: chr16 61892428-61892438. Max. coverage (+): 0. Max coverage (-): 0

Region: chr16 61892439-61892449. Max. coverage (+): 0. Max coverage (-): 0

Region: chr16 61892450-61892460. Max. coverage (+): 0. Max coverage (-): 0

Region: chr16 61892461-61892471. Max. coverage (+): 0. Max coverage (-): 0

Region: chr16 61892472-61892482. Max. coverage (+): 0. Max coverage (-): 0

Region: chr16 61892483-61892493. Max. coverage (+): 0. Max coverage (-): 0

Region: chr16 61892494-61892504. Max. coverage (+): 0. Max coverage (-): 0

Region: chr16 61892505-61892515. Max. coverage (+): 0. Max coverage (-): 0

Region: chr16 61892516-61892526. Max. coverage (+): 0. Max coverage (-): 0

Region: chr16 61892527-61892537. Max. coverage (+): 0. Max coverage (-): 0

Region: chr16 61892538-61892548. Max. coverage (+): 0. Max coverage (-): 0

Region: chr16 61892549-61892559. Max. coverage (+): 0. Max coverage (-): 0

Region: chr16 61892560-61892569. Max. coverage (+): 0. Max coverage (-): 2.26

Region: chr16 61892570-61892580. Max. coverage (+): 0. Max coverage (-): 2.26

Region: chr16 61892581-61892591. Max. coverage (+): 0. Max coverage (-): 0

Region: chr16 61892592-61892602. Max. coverage (+): 0. Max coverage (-): 0

Region: chr16 61892603-61892613. Max. coverage (+): 0. Max coverage (-): 0

Region: chr16 61892614-61892624. Max. coverage (+): 0. Max coverage (-): 2.15

Region: chr16 61892625-61892635. Max. coverage (+): 0. Max coverage (-): 0

Region: chr16 61892636-61892646. Max. coverage (+): 0. Max coverage (-): 0

Region: chr16 61892647-61892657. Max. coverage (+): 0. Max coverage (-): 0

Region: chr16 61892658-61892668. Max. coverage (+): 0. Max coverage (-): 2.51

Region: chr16 61892669-61892679. Max. coverage (+): 0. Max coverage (-): 2.51

Region: chr16 61892680-61892690. Max. coverage (+): 0. Max coverage (-): 0

Region: chr16 61892691-61892701. Max. coverage (+): 0. Max coverage (-): 0

Region: chr16 61892702-61892712. Max. coverage (+): 0. Max coverage (-): 0

Region: chr16 61892713-61892723. Max. coverage (+): 0. Max coverage (-): 0

Region: chr16 61892724-61892734. Max. coverage (+): 0. Max coverage (-): 0

Region: chr16 61892735-61892745. Max. coverage (+): 0. Max coverage (-): 0

Region: chr16 61892746-61892756. Max. coverage (+): 0. Max coverage (-): 0

Region: chr16 61892757-61892766. Max. coverage (+): 0. Max coverage (-): 0

Region: chr16 61892767-61892777. Max. coverage (+): 0. Max coverage (-): 0

Region: chr16 61892778-61892788. Max. coverage (+): 0. Max coverage (-): 0

Region: chr16 61892789-61892799. Max. coverage (+): 0. Max coverage (-): 2.48

Region: chr16 61892800-61892810. Max. coverage (+): 0. Max coverage (-): 2.48

Region: chr16 61892811-61892821. Max. coverage (+): 0. Max coverage (-): 0

Region: chr16 61892822-61892832. Max. coverage (+): 0. Max coverage (-): 0

Region: chr16 61892833-61892843. Max. coverage (+): 0. Max coverage (-): 2.06

Region: chr16 61892844-61892854. Max. coverage (+): 0. Max coverage (-): 0

Region: chr16 61892855-61892865. Max. coverage (+): 0. Max coverage (-): 0

Region: chr16 61892866-61892876. Max. coverage (+): 0. Max coverage (-): 0

Region: chr16 61892877-61892887. Max. coverage (+): 0. Max coverage (-): 0

Region: chr16 61892888-61892898. Max. coverage (+): 0. Max coverage (-): 0

Region: chr16 61892899-61892909. Max. coverage (+): 0. Max coverage (-): 0

Region: chr16 61892910-61892920. Max. coverage (+): 0. Max coverage (-): 0

Region: chr16 61892921-61892931. Max. coverage (+): 0. Max coverage (-): 0

Region: chr16 61892932-61892942. Max. coverage (+): 0. Max coverage (-): 0

Region: chr16 61892943-61892953. Max. coverage (+): 0. Max coverage (-): 0

Region: chr16 61892954-61892964. Max. coverage (+): 0. Max coverage (-): 0

Region: chr16 61892965-61892974. Max. coverage (+): 0. Max coverage (-): 0

Region: chr16 61892975-61892985. Max. coverage (+): 0. Max coverage (-): 0

Region: chr16 61892986-61892996. Max. coverage (+): 0. Max coverage (-): 0

Region: chr16 61892997-61893007. Max. coverage (+): 0. Max coverage (-): 0

Region: chr16 61893008-61893018. Max. coverage (+): 0. Max coverage (-): 0

Region: chr16 61893019-61893029. Max. coverage (+): 0. Max coverage (-): 0

Region: chr16 61893030-61893040. Max. coverage (+): 0. Max coverage (-): 0

Region: chr16 61893041-61893051. Max. coverage (+): 0. Max coverage (-): 0

Region: chr16 61893052-61893062. Max. coverage (+): 0. Max coverage (-): 0

Region: chr16 61893063-61893073. Max. coverage (+): 0. Max coverage (-): 1.91

Region: chr16 61893074-61893084. Max. coverage (+): 0. Max coverage (-): 1.91

Region: chr16 61893085-61893095. Max. coverage (+): 0. Max coverage (-): 0

Region: chr16 61893096-61893106. Max. coverage (+): 0. Max coverage (-): 0

Region: chr16 61893107-61893117. Max. coverage (+): 0. Max coverage (-): 0

Region: chr16 61893118-61893128. Max. coverage (+): 0. Max coverage (-): 0

Region: chr16 61893129-61893139. Max. coverage (+): 0. Max coverage (-): 0

Region: chr16 61893140-61893150. Max. coverage (+): 0. Max coverage (-): 0

Region: chr16 61893151-61893161. Max. coverage (+): 0. Max coverage (-): 0

Region: chr16 61893162-61893171. Max. coverage (+): 0. Max coverage (-): 0

Region: chr16 61893172-61893182. Max. coverage (+): 0. Max coverage (-): 0

Region: chr16 61893183-61893193. Max. coverage (+): 0. Max coverage (-): 1.9

Region: chr16 61893194-61893204. Max. coverage (+): 0. Max coverage (-): 2.55

Region: chr16 61893205-61893215. Max. coverage (+): 0. Max coverage (-): 2.55

Region: chr16 61893216-61893226. Max. coverage (+): 0. Max coverage (-): 1.24

Region: chr16 61893227-61893237. Max. coverage (+): 0. Max coverage (-): 1.24

Region: chr16 61893238-61893248. Max. coverage (+): 0. Max coverage (-): 0

Region: chr16 61893249-61893259. Max. coverage (+): 0. Max coverage (-): 0

Region: chr16 61893260-61893270. Max. coverage (+): 0. Max coverage (-): 0

Region: chr16 61893271-61893281. Max. coverage (+): 0. Max coverage (-): 0

Region: chr16 61893282-61893292. Max. coverage (+): 0. Max coverage (-): 0

Region: chr16 61893293-61893303. Max. coverage (+): 0. Max coverage (-): 0

Region: chr16 61893304-61893314. Max. coverage (+): 0. Max coverage (-): 0

Region: chr16 61893315-61893325. Max. coverage (+): 0. Max coverage (-): 0

Region: chr16 61893326-61893336. Max. coverage (+): 0. Max coverage (-): 0

Region: chr16 61893337-61893347. Max. coverage (+): 0. Max coverage (-): 0

Region: chr16 61893348-61893358. Max. coverage (+): 0. Max coverage (-): 0

Region: chr16 61893359-61893369. Max. coverage (+): 0. Max coverage (-): 0

Region: chr16 61893370-61893379. Max. coverage (+): 0. Max coverage (-): 0

Region: chr16 61893380-61893390. Max. coverage (+): 0. Max coverage (-): 0

Region: chr16 61893391-61893401. Max. coverage (+): 0. Max coverage (-): 0

Region: chr16 61893402-61893412. Max. coverage (+): 0. Max coverage (-): 0

Region: chr16 61893413-61893423. Max. coverage (+): 0. Max coverage (-): 0

Region: chr16 61893424-61893434. Max. coverage (+): 0. Max coverage (-): 0

Region: chr16 61893435-61893445. Max. coverage (+): 0. Max coverage (-): 5.35

Region: chr16 61893446-61893456. Max. coverage (+): 0. Max coverage (-): 0.8

Region: chr16 61893457-61893467. Max. coverage (+): 0. Max coverage (-): 0

Region: chr16 61893468-61893478. Max. coverage (+): 0. Max coverage (-): 0

Region: chr16 61893479-61893489. Max. coverage (+): 0. Max coverage (-): 0

Region: chr16 61893490-61893500. Max. coverage (+): 0. Max coverage (-): 0

Region: chr16 61893501-61893511. Max. coverage (+): 0. Max coverage (-): 0

Region: chr16 61893512-61893522. Max. coverage (+): 0. Max coverage (-): 0

Region: chr16 61893523-61893533. Max. coverage (+): 0. Max coverage (-): 0

Region: chr16 61893534-61893544. Max. coverage (+): 0. Max coverage (-): 0

Region: chr16 61893545-61893555. Max. coverage (+): 0. Max coverage (-): 0

Region: chr16 61893556-61893566. Max. coverage (+): 0. Max coverage (-): 0

Region: chr16 61893567-61893576. Max. coverage (+): 0. Max coverage (-): 5.64

Region: chr16 61893577-61893587. Max. coverage (+): 0. Max coverage (-): 1.2

Region: chr16 61893588-61893598. Max. coverage (+): 0. Max coverage (-): 0

Region: chr16 61893599-61893609. Max. coverage (+): 0. Max coverage (-): 0

Region: chr16 61893610-61893620. Max. coverage (+): 0. Max coverage (-): 0

Region: chr16 61893621-61893631. Max. coverage (+): 0. Max coverage (-): 0

Region: chr16 61893632-61893642. Max. coverage (+): 0. Max coverage (-): 0

Region: chr16 61893643-61893653. Max. coverage (+): 0. Max coverage (-): 0

Region: chr16 61893654-61893664. Max. coverage (+): 0. Max coverage (-): 0

Region: chr16 61893665-61893675. Max. coverage (+): 0. Max coverage (-): 0

Region: chr16 61893676-61893686. Max. coverage (+): 0. Max coverage (-): 0

Region: chr16 61893687-61893697. Max. coverage (+): 0. Max coverage (-): 0

Region: chr16 61893698-61893708. Max. coverage (+): 0. Max coverage (-): 0

Region: chr16 61893709-61893719. Max. coverage (+): 0. Max coverage (-): 0.33

Region: chr16 61893720-61893730. Max. coverage (+): 0. Max coverage (-): 0

Region: chr16 61893731-61893741. Max. coverage (+): 0. Max coverage (-): 0

Region: chr16 61893742-61893752. Max. coverage (+): 0. Max coverage (-): 0

Region: chr16 61893753-61893763. Max. coverage (+): 0. Max coverage (-): 0

Region: chr16 61893764-61893774. Max. coverage (+): 0. Max coverage (-): 0

Region: chr16 61893775-61893784. Max. coverage (+): 0. Max coverage (-): 0

Region: chr16 61893785-61893795. Max. coverage (+): 0. Max coverage (-): 0

Region: chr16 61893796-61893806. Max. coverage (+): 0. Max coverage (-): 0

Region: chr16 61893807-61893817. Max. coverage (+): 0. Max coverage (-): 0

Region: chr16 61893818-61893828. Max. coverage (+): 0. Max coverage (-): 0

Region: chr16 61893829-61893839. Max. coverage (+): 0. Max coverage (-): 2.17

Region: chr16 61893840-61893850. Max. coverage (+): 0. Max coverage (-): 2.17

Region: chr16 61893851-61893861. Max. coverage (+): 0. Max coverage (-): 0

Region: chr16 61893862-61893872. Max. coverage (+): 0. Max coverage (-): 0

Region: chr16 61893873-61893883. Max. coverage (+): 0. Max coverage (-): 0

Region: chr16 61893884-61893894. Max. coverage (+): 0. Max coverage (-): 0

Region: chr16 61893895-61893905. Max. coverage (+): 0. Max coverage (-): 0

Region: chr16 61893906-61893916. Max. coverage (+): 0. Max coverage (-): 0

Region: chr16 61893917-61893927. Max. coverage (+): 0. Max coverage (-): 0

Region: chr16 61893928-61893938. Max. coverage (+): 0. Max coverage (-): 2.76

Region: chr16 61893939-61893949. Max. coverage (+): 0. Max coverage (-): 2.76

Region: chr16 61893950-61893960. Max. coverage (+): 0. Max coverage (-): 0

Region: chr16 61893961-61893971. Max. coverage (+): 0. Max coverage (-): 0

Region: chr16 61893972-61893981. Max. coverage (+): 0. Max coverage (-): 0

Region: chr16 61893982-61893992. Max. coverage (+): 0. Max coverage (-): 0

Region: chr16 61893993-61894003. Max. coverage (+): 0. Max coverage (-): 0

Region: chr16 61894004-61894014. Max. coverage (+): 0. Max coverage (-): 0

Region: chr16 61894015-61894025. Max. coverage (+): 0. Max coverage (-): 0

Region: chr16 61894026-61894036. Max. coverage (+): 0. Max coverage (-): 0

Region: chr16 61894037-61894047. Max. coverage (+): 0. Max coverage (-): 0

Region: chr16 61894048-61894058. Max. coverage (+): 0. Max coverage (-): 0

Region: chr16 61894059-61894069. Max. coverage (+): 0. Max coverage (-): 0

Region: chr16 61894070-61894080. Max. coverage (+): 0. Max coverage (-): 1.14

Region: chr16 61894081-61894091. Max. coverage (+): 0. Max coverage (-): 1.14

Region: chr16 61894092-61894102. Max. coverage (+): 0. Max coverage (-): 0.83

Region: chr16 61894103-61894113. Max. coverage (+): 0. Max coverage (-): 2.49

Region: chr16 61894114-61894124. Max. coverage (+): 0. Max coverage (-): 0

Region: chr16 61894125-61894135. Max. coverage (+): 0. Max coverage (-): 0

Region: chr16 61894136-61894146. Max. coverage (+): 0. Max coverage (-): 0

Region: chr16 61894147-61894157. Max. coverage (+): 0. Max coverage (-): 0

Region: chr16 61894158-61894168. Max. coverage (+): 0. Max coverage (-): 0

Region: chr16 61894169-61894179. Max. coverage (+): 0. Max coverage (-): 0

Region: chr16 61894180-61894189. Max. coverage (+): 0. Max coverage (-): 0

Region: chr16 61894190-61894200. Max. coverage (+): 0. Max coverage (-): 0

Region: chr16 61894201-61894211. Max. coverage (+): 0. Max coverage (-): 0

Region: chr16 61894212-61894222. Max. coverage (+): 0. Max coverage (-): 0

Region: chr16 61894223-61894233. Max. coverage (+): 0. Max coverage (-): 16.59

Region: chr16 61894234-61894244. Max. coverage (+): 0. Max coverage (-): 16.59

Region: chr16 61894245-61894255. Max. coverage (+): 0. Max coverage (-): 0

Region: chr16 61894256-61894266. Max. coverage (+): 0. Max coverage (-): 0

Region: chr16 61894267-61894277. Max. coverage (+): 0. Max coverage (-): 0

Region: chr16 61894278-61894288. Max. coverage (+): 0. Max coverage (-): 0

Region: chr16 61894289-61894299. Max. coverage (+): 0. Max coverage (-): 0

Region: chr16 61894300-61894310. Max. coverage (+): 0. Max coverage (-): 0

Region: chr16 61894311-61894321. Max. coverage (+): 0. Max coverage (-): 0

Region: chr16 61894322-61894332. Max. coverage (+): 0. Max coverage (-): 0

Region: chr16 61894333-61894343. Max. coverage (+): 0. Max coverage (-): 0

Region: chr16 61894344-61894354. Max. coverage (+): 0. Max coverage (-): 0

Region: chr16 61894355-61894365. Max. coverage (+): 0. Max coverage (-): 0

Region: chr16 61894366-61894376. Max. coverage (+): 0. Max coverage (-): 0

Region: chr16 61894377-61894386. Max. coverage (+): 0. Max coverage (-): 0

Region: chr16 61894387-61894397. Max. coverage (+): 0. Max coverage (-): 0

Region: chr16 61894398-61894408. Max. coverage (+): 0. Max coverage (-): 0

Region: chr16 61894409-61894419. Max. coverage (+): 0. Max coverage (-): 0

Region: chr16 61894420-61894430. Max. coverage (+): 0. Max coverage (-): 0

Region: chr16 61894431-61894441. Max. coverage (+): 0. Max coverage (-): 0

Region: chr16 61894442-61894452. Max. coverage (+): 0. Max coverage (-): 0

Region: chr16 61894453-61894463. Max. coverage (+): 0. Max coverage (-): 0

Region: chr16 61894464-61894474. Max. coverage (+): 0. Max coverage (-): 0

Region: chr16 61894475-61894485. Max. coverage (+): 0. Max coverage (-): 0

Region: chr16 61894486-61894496. Max. coverage (+): 0. Max coverage (-): 0

Region: chr16 61894497-61894507. Max. coverage (+): 0. Max coverage (-): 0

Region: chr16 61894508-61894518. Max. coverage (+): 0. Max coverage (-): 0

Region: chr16 61894519-61894529. Max. coverage (+): 0. Max coverage (-): 0

Region: chr16 61894530-61894540. Max. coverage (+): 0. Max coverage (-): 0

Region: chr16 61894541-61894551. Max. coverage (+): 0. Max coverage (-): 0

Region: chr16 61894552-61894562. Max. coverage (+): 0. Max coverage (-): 0

Region: chr16 61894563-61894573. Max. coverage (+): 0. Max coverage (-): 0

Region: chr16 61894574-61894584. Max. coverage (+): 0. Max coverage (-): 0

Region: chr16 61894585-61894594. Max. coverage (+): 0. Max coverage (-): 0

Region: chr16 61894595-61894605. Max. coverage (+): 0. Max coverage (-): 0

Region: chr16 61894606-61894616. Max. coverage (+): 0. Max coverage (-): 0

Region: chr16 61894617-61894627. Max. coverage (+): 0. Max coverage (-): 0

Region: chr16 61894628-61894638. Max. coverage (+): 0. Max coverage (-): 0

Region: chr16 61894639-61894649. Max. coverage (+): 0. Max coverage (-): 0

Region: chr16 61894650-61894660. Max. coverage (+): 0. Max coverage (-): 0

Region: chr16 61894661-61894671. Max. coverage (+): 0. Max coverage (-): 0

Region: chr16 61894672-61894682. Max. coverage (+): 0. Max coverage (-): 0

Region: chr16 61894683-61894693. Max. coverage (+): 0. Max coverage (-): 0

Region: chr16 61894694-61894704. Max. coverage (+): 0. Max coverage (-): 0

Region: chr16 61894705-61894715. Max. coverage (+): 0. Max coverage (-): 0

Region: chr16 61894716-61894726. Max. coverage (+): 0. Max coverage (-): 0

Region: chr16 61894727-61894737. Max. coverage (+): 0. Max coverage (-): 0

Region: chr16 61894738-61894748. Max. coverage (+): 0. Max coverage (-): 0

Region: chr16 61894749-61894759. Max. coverage (+): 0. Max coverage (-): 0

Region: chr16 61894760-61894770. Max. coverage (+): 0. Max coverage (-): 0

Region: chr16 61894771-61894781. Max. coverage (+): 0. Max coverage (-): 0

Region: chr16 61894782-61894791. Max. coverage (+): 0. Max coverage (-): 0

Region: chr16 61894792-61894802. Max. coverage (+): 0. Max coverage (-): 0

Region: chr16 61894803-61894813. Max. coverage (+): 0. Max coverage (-): 0

Region: chr16 61894814-61894824. Max. coverage (+): 0. Max coverage (-): 0

Region: chr16 61894825-61894835. Max. coverage (+): 0. Max coverage (-): 0

Region: chr16 61894836-61894846. Max. coverage (+): 0. Max coverage (-): 0

Region: chr16 61894847-61894857. Max. coverage (+): 0. Max coverage (-): 0

Region: chr16 61894858-61894868. Max. coverage (+): 0. Max coverage (-): 0

Region: chr16 61894869-61894879. Max. coverage (+): 0. Max coverage (-): 0

Region: chr16 61894880-61894890. Max. coverage (+): 0. Max coverage (-): 0

Region: chr16 61894891-61894901. Max. coverage (+): 0. Max coverage (-): 0

Region: chr16 61894902-61894912. Max. coverage (+): 0. Max coverage (-): 0

Region: chr16 61894913-61894923. Max. coverage (+): 0. Max coverage (-): 0

Region: chr16 61894924-61894934. Max. coverage (+): 0. Max coverage (-): 0

Region: chr16 61894935-61894945. Max. coverage (+): 0. Max coverage (-): 0

Region: chr16 61894946-61894956. Max. coverage (+): 0. Max coverage (-): 0

Region: chr16 61894957-61894967. Max. coverage (+): 0. Max coverage (-): 0

Region: chr16 61894968-61894978. Max. coverage (+): 0. Max coverage (-): 0

Region: chr16 61894979-61894989. Max. coverage (+): 0. Max coverage (-): 0

Region: chr16 61894990-61894999. Max. coverage (+): 0. Max coverage (-): 0

Region: chr16 61895000-61895010. Max. coverage (+): 0. Max coverage (-): 5.23

Region: chr16 61895011-61895021. Max. coverage (+): 0. Max coverage (-): 5.23

Region: chr16 61895022-61895032. Max. coverage (+): 0. Max coverage (-): 0

Region: chr16 61895033-61895043. Max. coverage (+): 0. Max coverage (-): 0

Region: chr16 61895044-61895054. Max. coverage (+): 0. Max coverage (-): 0

Region: chr16 61895055-61895065. Max. coverage (+): 0. Max coverage (-): 0

Region: chr16 61895066-61895076. Max. coverage (+): 0. Max coverage (-): 0

Region: chr16 61895077-61895087. Max. coverage (+): 0. Max coverage (-): 0

Region: chr16 61895088-61895098. Max. coverage (+): 0. Max coverage (-): 0

Region: chr16 61895099-61895109. Max. coverage (+): 0. Max coverage (-): 0

Region: chr16 61895110-61895120. Max. coverage (+): 0. Max coverage (-): 0

Region: chr16 61895121-61895131. Max. coverage (+): 0. Max coverage (-): 0

Region: chr16 61895132-61895142. Max. coverage (+): 0. Max coverage (-): 0

Region: chr16 61895143-61895153. Max. coverage (+): 0. Max coverage (-): 0

Region: chr16 61895154-61895164. Max. coverage (+): 0. Max coverage (-): 0

Region: chr16 61895165-61895175. Max. coverage (+): 0. Max coverage (-): 0

Region: chr16 61895176-61895186. Max. coverage (+): 0. Max coverage (-): 0

Region: chr16 61895187-61895197. Max. coverage (+): 0. Max coverage (-): 0

Region: chr16 61895198-61895207. Max. coverage (+): 0. Max coverage (-): 0

Region: chr16 61895208-61895218. Max. coverage (+): 0. Max coverage (-): 0

Region: chr16 61895219-61895229. Max. coverage (+): 0. Max coverage (-): 0

Region: chr16 61895230-61895240. Max. coverage (+): 0. Max coverage (-): 0

Region: chr16 61895241-61895251. Max. coverage (+): 0. Max coverage (-): 0

Region: chr16 61895252-61895262. Max. coverage (+): 0. Max coverage (-): 0

Region: chr16 61895263-61895273. Max. coverage (+): 0. Max coverage (-): 0

Region: chr16 61895274-61895284. Max. coverage (+): 0. Max coverage (-): 0

Region: chr16 61895285-61895295. Max. coverage (+): 0. Max coverage (-): 0

Region: chr16 61895296-61895306. Max. coverage (+): 0. Max coverage (-): 0

Region: chr16 61895307-61895317. Max. coverage (+): 0. Max coverage (-): 0

Region: chr16 61895318-61895328. Max. coverage (+): 0. Max coverage (-): 0

Region: chr16 61895329-61895339. Max. coverage (+): 0. Max coverage (-): 0

Region: chr16 61895340-61895350. Max. coverage (+): 0. Max coverage (-): 0

Region: chr16 61895351-61895361. Max. coverage (+): 0. Max coverage (-): 0

Region: chr16 61895362-61895372. Max. coverage (+): 0. Max coverage (-): 0

Region: chr16 61895373-61895383. Max. coverage (+): 0. Max coverage (-): 0

Region: chr16 61895384-61895394. Max. coverage (+): 0. Max coverage (-): 0

Region: chr16 61895395-61895404. Max. coverage (+): 0. Max coverage (-): 0

Region: chr16 61895405-61895415. Max. coverage (+): 0. Max coverage (-): 0

Region: chr16 61895416-61895426. Max. coverage (+): 0. Max coverage (-): 0

Region: chr16 61895427-61895437. Max. coverage (+): 0. Max coverage (-): 0

Region: chr16 61895438-61895448. Max. coverage (+): 0. Max coverage (-): 0

Region: chr16 61895449-61895459. Max. coverage (+): 0. Max coverage (-): 0

Region: chr16 61895460-61895470. Max. coverage (+): 0. Max coverage (-): 0

Region: chr16 61895471-61895481. Max. coverage (+): 0. Max coverage (-): 0

Region: chr16 61895482-61895492. Max. coverage (+): 0. Max coverage (-): 0

Region: chr16 61895493-61895503. Max. coverage (+): 0. Max coverage (-): 0

Region: chr16 61895504-61895514. Max. coverage (+): 0. Max coverage (-): 0

Region: chr16 61895515-61895525. Max. coverage (+): 0. Max coverage (-): 0

Region: chr16 61895526-61895536. Max. coverage (+): 0. Max coverage (-): 0

Region: chr16 61895537-61895547. Max. coverage (+): 0. Max coverage (-): 0

Region: chr16 61895548-61895558. Max. coverage (+): 0. Max coverage (-): 0

Region: chr16 61895559-61895569. Max. coverage (+): 0. Max coverage (-): 0.73

Region: chr16 61895570-61895580. Max. coverage (+): 0. Max coverage (-): 0.73

Region: chr16 61895581-61895591. Max. coverage (+): 0. Max coverage (-): 3.8

Region: chr16 61895592-61895602. Max. coverage (+): 0. Max coverage (-): 2.52

Region: chr16 61895603-61895612. Max. coverage (+): 0. Max coverage (-): 0

Region: chr16 61895613-61895623. Max. coverage (+): 0. Max coverage (-): 0

Region: chr16 61895624-61895634. Max. coverage (+): 0. Max coverage (-): 0

Region: chr16 61895635-61895645. Max. coverage (+): 0. Max coverage (-): 0

Region: chr16 61895646-61895656. Max. coverage (+): 0. Max coverage (-): 0

Region: chr16 61895657-61895667. Max. coverage (+): 0. Max coverage (-): 2.39

Region: chr16 61895668-61895678. Max. coverage (+): 0. Max coverage (-): 2.39

Region: chr16 61895679-61895689. Max. coverage (+): 0. Max coverage (-): 0

Region: chr16 61895690-61895700. Max. coverage (+): 0. Max coverage (-): 0

Region: chr16 61895701-61895711. Max. coverage (+): 0. Max coverage (-): 0

Region: chr16 61895712-61895722. Max. coverage (+): 0. Max coverage (-): 0

Region: chr16 61895723-61895733. Max. coverage (+): 0. Max coverage (-): 0

Region: chr16 61895734-61895744. Max. coverage (+): 0. Max coverage (-): 0

Region: chr16 61895745-61895755. Max. coverage (+): 0. Max coverage (-): 0

Region: chr16 61895756-61895766. Max. coverage (+): 0. Max coverage (-): 0

Region: chr16 61895767-61895777. Max. coverage (+): 0. Max coverage (-): 0

Region: chr16 61895778-61895788. Max. coverage (+): 0. Max coverage (-): 0

Region: chr16 61895789-61895799. Max. coverage (+): 0. Max coverage (-): 0

Region: chr16 61895800-61895809. Max. coverage (+): 0. Max coverage (-): 9.95

Region: chr16 61895810-61895820. Max. coverage (+): 0. Max coverage (-): 0

Region: chr16 61895821-61895831. Max. coverage (+): 0. Max coverage (-): 0

Region: chr16 61895832-61895842. Max. coverage (+): 0. Max coverage (-): 4.87

Region: chr16 61895843-61895853. Max. coverage (+): 0. Max coverage (-): 4.8

Region: chr16 61895854-61895864. Max. coverage (+): 0. Max coverage (-): 1.09

Region: chr16 61895865-61895875. Max. coverage (+): 0. Max coverage (-): 1.09

Region: chr16 61895876-61895886. Max. coverage (+): 0. Max coverage (-): 0

Region: chr16 61895887-61895897. Max. coverage (+): 0. Max coverage (-): 4.43

Region: chr16 61895898-61895908. Max. coverage (+): 0. Max coverage (-): 4.43

Region: chr16 61895909-61895919. Max. coverage (+): 0. Max coverage (-): 0

Region: chr16 61895920-61895930. Max. coverage (+): 0. Max coverage (-): 0

Region: chr16 61895931-61895941. Max. coverage (+): 0. Max coverage (-): 0

Region: chr16 61895942-61895952. Max. coverage (+): 0. Max coverage (-): 0

Region: chr16 61895953-61895963. Max. coverage (+): 0. Max coverage (-): 0

Region: chr16 61895964-61895974. Max. coverage (+): 0. Max coverage (-): 0

Region: chr16 61895975-61895985. Max. coverage (+): 0. Max coverage (-): 0

Region: chr16 61895986-61895996. Max. coverage (+): 0. Max coverage (-): 0

Region: chr16 61895997-61896007. Max. coverage (+): 0. Max coverage (-): 0

Region: chr16 61896008-61896017. Max. coverage (+): 0. Max coverage (-): 0

Region: chr16 61896018-61896028. Max. coverage (+): 0. Max coverage (-): 0

Region: chr16 61896029-61896039. Max. coverage (+): 0. Max coverage (-): 0

Region: chr16 61896040-61896050. Max. coverage (+): 0. Max coverage (-): 0

Region: chr16 61896051-61896061. Max. coverage (+): 0. Max coverage (-): 0

Region: chr16 61896062-61896072. Max. coverage (+): 0. Max coverage (-): 0

Region: chr16 61896073-61896083. Max. coverage (+): 0. Max coverage (-): 0

Region: chr16 61896084-61896094. Max. coverage (+): 0. Max coverage (-): 0

Region: chr16 61896095-61896105. Max. coverage (+): 0. Max coverage (-): 0

Region: chr16 61896106-61896116. Max. coverage (+): 0. Max coverage (-): 0

Region: chr16 61896117-61896127. Max. coverage (+): 0. Max coverage (-): 0

Region: chr16 61896128-61896138. Max. coverage (+): 0. Max coverage (-): 0

Region: chr16 61896139-61896149. Max. coverage (+): 0. Max coverage (-): 0

Region: chr16 61896150-61896160. Max. coverage (+): 0. Max coverage (-): 0

Region: chr16 61896161-61896171. Max. coverage (+): 0. Max coverage (-): 0

Region: chr16 61896172-61896182. Max. coverage (+): 0. Max coverage (-): 0

Region: chr16 61896183-61896193. Max. coverage (+): 0. Max coverage (-): 0

Region: chr16 61896194-61896204. Max. coverage (+): 0. Max coverage (-): 0

Region: chr16 61896205-61896214. Max. coverage (+): 0. Max coverage (-): 0

Region: chr16 61896215-61896225. Max. coverage (+): 0. Max coverage (-): 0

Region: chr16 61896226-61896236. Max. coverage (+): 0. Max coverage (-): 0

Region: chr16 61896237-61896247. Max. coverage (+): 0. Max coverage (-): 2.25

Region: chr16 61896248-61896258. Max. coverage (+): 0. Max coverage (-): 2.25

Region: chr16 61896259-61896269. Max. coverage (+): 0. Max coverage (-): 0

Region: chr16 61896270-61896280. Max. coverage (+): 0. Max coverage (-): 0

Region: chr16 61896281-61896291. Max. coverage (+): 0. Max coverage (-): 0

Region: chr16 61896292-61896302. Max. coverage (+): 0. Max coverage (-): 0

Region: chr16 61896303-61896313. Max. coverage (+): 0. Max coverage (-): 0

Region: chr16 61896314-61896324. Max. coverage (+): 0. Max coverage (-): 0

Region: chr16 61896325-61896335. Max. coverage (+): 0. Max coverage (-): 1.61

Region: chr16 61896336-61896346. Max. coverage (+): 0. Max coverage (-): 1.61

Region: chr16 61896347-61896357. Max. coverage (+): 0. Max coverage (-): 0

Region: chr16 61896358-61896368. Max. coverage (+): 0. Max coverage (-): 0

Region: chr16 61896369-61896379. Max. coverage (+): 0. Max coverage (-): 0

Region: chr16 61896380-61896390. Max. coverage (+): 0. Max coverage (-): 3.79

Region: chr16 61896391-61896401. Max. coverage (+): 0. Max coverage (-): 0

Region: chr16 61896402-61896412. Max. coverage (+): 0. Max coverage (-): 0

Region: chr16 61896413-61896422. Max. coverage (+): 0. Max coverage (-): 0

Region: chr16 61896423-61896433. Max. coverage (+): 0. Max coverage (-): 0

Region: chr16 61896434-61896444. Max. coverage (+): 0. Max coverage (-): 0

Region: chr16 61896445-61896455. Max. coverage (+): 0. Max coverage (-): 0

Region: chr16 61896456-61896466. Max. coverage (+): 0. Max coverage (-): 0

Region: chr16 61896467-61896477. Max. coverage (+): 0. Max coverage (-): 0

Region: chr16 61896478-61896488. Max. coverage (+): 0. Max coverage (-): 0

Region: chr16 61896489-61896499. Max. coverage (+): 0. Max coverage (-): 0

Region: chr16 61896500-61896510. Max. coverage (+): 0. Max coverage (-): 0

Region: chr16 61896511-61896521. Max. coverage (+): 0. Max coverage (-): 0

Region: chr16 61896522-61896532. Max. coverage (+): 0. Max coverage (-): 0

Region: chr16 61896533-61896543. Max. coverage (+): 0. Max coverage (-): 0

Region: chr16 61896544-61896554. Max. coverage (+): 0. Max coverage (-): 0

Region: chr16 61896555-61896565. Max. coverage (+): 0. Max coverage (-): 0

Region: chr16 61896566-61896576. Max. coverage (+): 0. Max coverage (-): 0

Region: chr16 61896577-61896587. Max. coverage (+): 0. Max coverage (-): 0

Region: chr16 61896588-61896598. Max. coverage (+): 0. Max coverage (-): 0

Region: chr16 61896599-61896609. Max. coverage (+): 0. Max coverage (-): 0

Region: chr16 61896610-61896619. Max. coverage (+): 0. Max coverage (-): 0

Region: chr16 61896620-61896630. Max. coverage (+): 0. Max coverage (-): 0

Region: chr16 61896631-61896641. Max. coverage (+): 0. Max coverage (-): 0

Region: chr16 61896642-61896652. Max. coverage (+): 0. Max coverage (-): 0

Region: chr16 61896653-61896663. Max. coverage (+): 0. Max coverage (-): 0

Region: chr16 61896664-61896674. Max. coverage (+): 0. Max coverage (-): 0

Region: chr16 61896675-61896685. Max. coverage (+): 0. Max coverage (-): 0

Region: chr16 61896686-61896696. Max. coverage (+): 0. Max coverage (-): 0

Region: chr16 61896697-61896707. Max. coverage (+): 0. Max coverage (-): 0

Region: chr16 61896708-61896718. Max. coverage (+): 0. Max coverage (-): 0

Region: chr16 61896719-61896729. Max. coverage (+): 0. Max coverage (-): 0

Region: chr16 61896730-61896740. Max. coverage (+): 0. Max coverage (-): 0

Region: chr16 61896741-61896751. Max. coverage (+): 0. Max coverage (-): 0

Region: chr16 61896752-61896762. Max. coverage (+): 0. Max coverage (-): 0

Region: chr16 61896763-61896773. Max. coverage (+): 0. Max coverage (-): 0

Region: chr16 61896774-61896784. Max. coverage (+): 0. Max coverage (-): 0

Region: chr16 61896785-61896795. Max. coverage (+): 0. Max coverage (-): 0

Region: chr16 61896796-61896806. Max. coverage (+): 0. Max coverage (-): 0

Region: chr16 61896807-61896817. Max. coverage (+): 0. Max coverage (-): 0

Region: chr16 61896818-61896827. Max. coverage (+): 0. Max coverage (-): 0

Region: chr16 61896828-61896838. Max. coverage (+): 0. Max coverage (-): 0

Region: chr16 61896839-61896849. Max. coverage (+): 0. Max coverage (-): 0

Region: chr16 61896850-61896860. Max. coverage (+): 0. Max coverage (-): 0

Region: chr16 61896861-61896871. Max. coverage (+): 0. Max coverage (-): 0

Region: chr16 61896872-61896882. Max. coverage (+): 0. Max coverage (-): 0

Region: chr16 61896883-61896893. Max. coverage (+): 0. Max coverage (-): 0

Region: chr16 61896894-61896904. Max. coverage (+): 0. Max coverage (-): 0.87

Region: chr16 61896905-61896915. Max. coverage (+): 0. Max coverage (-): 2.79

Region: chr16 61896916-61896926. Max. coverage (+): 0. Max coverage (-): 1.91

Region: chr16 61896927-61896937. Max. coverage (+): 0. Max coverage (-): 0

Region: chr16 61896938-61896948. Max. coverage (+): 0. Max coverage (-): 0

Region: chr16 61896949-61896959. Max. coverage (+): 0. Max coverage (-): 0

Region: chr16 61896960-61896970. Max. coverage (+): 0. Max coverage (-): 1.26

Region: chr16 61896971-61896981. Max. coverage (+): 0. Max coverage (-): 1.26

Region: chr16 61896982-61896992. Max. coverage (+): 0. Max coverage (-): 0

Region: chr16 61896993-61897003. Max. coverage (+): 0. Max coverage (-): 0

Region: chr16 61897004-61897014. Max. coverage (+): 0. Max coverage (-): 0

Region: chr16 61897015-61897024. Max. coverage (+): 0. Max coverage (-): 0

Region: chr16 61897025-61897035. Max. coverage (+): 0. Max coverage (-): 0

Region: chr16 61897036-61897046. Max. coverage (+): 0. Max coverage (-): 0

Region: chr16 61897047-61897057. Max. coverage (+): 0. Max coverage (-): 1.41

Region: chr16 61897058-61897068. Max. coverage (+): 0. Max coverage (-): 0

Region: chr16 61897069-61897079. Max. coverage (+): 0. Max coverage (-): 0

Region: chr16 61897080-61897090. Max. coverage (+): 0. Max coverage (-): 0

Region: chr16 61897091-61897101. Max. coverage (+): 0. Max coverage (-): 1.16

Region: chr16 61897102-61897112. Max. coverage (+): 0. Max coverage (-): 1.16

Region: chr16 61897113-61897123. Max. coverage (+): 0. Max coverage (-): 0

Region: chr16 61897124-61897134. Max. coverage (+): 0. Max coverage (-): 0

Region: chr16 61897135-61897145. Max. coverage (+): 0. Max coverage (-): 0

Region: chr16 61897146-61897156. Max. coverage (+): 0. Max coverage (-): 0

Region: chr16 61897157-61897167. Max. coverage (+): 0. Max coverage (-): 0

Region: chr16 61897168-61897178. Max. coverage (+): 0. Max coverage (-): 0

Region: chr16 61897179-61897189. Max. coverage (+): 0. Max coverage (-): 0

Region: chr16 61897190-61897200. Max. coverage (+): 0. Max coverage (-): 0

Region: chr16 61897201-61897211. Max. coverage (+): 0. Max coverage (-): 0

Region: chr16 61897212-61897222. Max. coverage (+): 0. Max coverage (-): 0

Region: chr16 61897223-61897232. Max. coverage (+): 0. Max coverage (-): 0

Region: chr16 61897233-61897243. Max. coverage (+): 0. Max coverage (-): 0

Region: chr16 61897244-61897254. Max. coverage (+): 0. Max coverage (-): 1.24

Region: chr16 61897255-61897265. Max. coverage (+): 0. Max coverage (-): 1.95

Region: chr16 61897266-61897276. Max. coverage (+): 0. Max coverage (-): 5.39

Region: chr16 61897277-61897287. Max. coverage (+): 0. Max coverage (-): 0

Region: chr16 61897288-61897298. Max. coverage (+): 0. Max coverage (-): 0

Region: chr16 61897299-61897309. Max. coverage (+): 0. Max coverage (-): 5.01

Region: chr16 61897310-61897320. Max. coverage (+): 0. Max coverage (-): 0

Region: chr16 61897321-61897331. Max. coverage (+): 0. Max coverage (-): 0

Region: chr16 61897332-61897342. Max. coverage (+): 0. Max coverage (-): 0

Region: chr16 61897343-61897353. Max. coverage (+): 0. Max coverage (-): 3.37

Region: chr16 61897354-61897364. Max. coverage (+): 0. Max coverage (-): 3.37

Region: chr16 61897365-61897375. Max. coverage (+): 0. Max coverage (-): 0.91

Region: chr16 61897376-61897386. Max. coverage (+): 0. Max coverage (-): 0

Region: chr16 61897387-61897397. Max. coverage (+): 0. Max coverage (-): 0

Region: chr16 61897398-61897408. Max. coverage (+): 0. Max coverage (-): 1.86

Region: chr16 61897409-61897419. Max. coverage (+): 0. Max coverage (-): 1.86

Region: chr16 61897420-61897429. Max. coverage (+): 0. Max coverage (-): 0

Region: chr16 61897430-61897440. Max. coverage (+): 0. Max coverage (-): 0

Region: chr16 61897441-61897451. Max. coverage (+): 0. Max coverage (-): 0

Region: chr16 61897452-61897462. Max. coverage (+): 0. Max coverage (-): 0

Region: chr16 61897463-61897473. Max. coverage (+): 0. Max coverage (-): 0

Region: chr16 61897474-61897484. Max. coverage (+): 0. Max coverage (-): 0

Region: chr16 61897485-61897495. Max. coverage (+): 0. Max coverage (-): 0

Region: chr16 61897496-61897506. Max. coverage (+): 0. Max coverage (-): 0

Region: chr16 61897507-61897517. Max. coverage (+): 0. Max coverage (-): 0

Region: chr16 61897518-61897528. Max. coverage (+): 0. Max coverage (-): 0

Region: chr16 61897529-61897539. Max. coverage (+): 0. Max coverage (-): 0

Region: chr16 61897540-61897550. Max. coverage (+): 0. Max coverage (-): 0

Region: chr16 61897551-61897561. Max. coverage (+): 0. Max coverage (-): 0

Region: chr16 61897562-61897572. Max. coverage (+): 0. Max coverage (-): 0

Region: chr16 61897573-61897583. Max. coverage (+): 0. Max coverage (-): 0

Region: chr16 61897584-61897594. Max. coverage (+): 0. Max coverage (-): 0

Region: chr16 61897595-61897605. Max. coverage (+): 0. Max coverage (-): 0

Region: chr16 61897606-61897616. Max. coverage (+): 0. Max coverage (-): 0

Region: chr16 61897617-61897627. Max. coverage (+): 0. Max coverage (-): 0

Region: chr16 61897628-61897637. Max. coverage (+): 0. Max coverage (-): 0

Region: chr16 61897638-61897648. Max. coverage (+): 0. Max coverage (-): 0

Region: chr16 61897649-61897659. Max. coverage (+): 0. Max coverage (-): 0

Region: chr16 61897660-61897670. Max. coverage (+): 0. Max coverage (-): 0

Region: chr16 61897671-61897681. Max. coverage (+): 0. Max coverage (-): 0

Region: chr16 61897682-61897692. Max. coverage (+): 0. Max coverage (-): 0

Region: chr16 61897693-61897703. Max. coverage (+): 0. Max coverage (-): 0

Region: chr16 61897704-61897714. Max. coverage (+): 0. Max coverage (-): 0.74

Region: chr16 61897715-61897725. Max. coverage (+): 0. Max coverage (-): 0

Region: chr16 61897726-. Max. coverage (+): 0. Max coverage (-): 0

RepeatMasker Color Code

**+**

100-98% Identity

<98-95% Identity

<95-90% Identity

<90-85% Identity

<85-80% Identity

<80-75% Identity

<75-70% Identity

<70% Identity

**-**

Gene Set Color Code

**+**

Gene

Pseudogene

**-**

Topology/Coverage Color Code

Coverage Plus Strand

Coverage Minus Strand

Mainstrand: Plus

Mainstrand: Minus

Complementary Strand

Flanking Region  
(if option -flank >0)

Gene Set Annotation  
  
RepeatMasker Annotation  

**1. MIR3**: 61893978-61894079 (+), Divergence to consensus: 39.3%  
**2. MIRb**: 61894263-61894434 (-), Divergence to consensus: 26.5%  
**3. BOV-A2**: 61894658-61894920 (-), Divergence to consensus: 3.4%

  
Transcription Factor Binding Sites  

**RFX4\_1** (Sequence: GTTGCTAAG (-): 61893721)  
**SOX9** (Sequence: AACAATAA (-): 61893615)  
**SOX9** (Sequence: AACAATAA (-): 61896611)
