# Supplementary material for: Small RNA sequencing of cryopreserved semen from single bull revealed altered miRNAs and piRNAs expression between High- and Low-motile sperm populations
Source: BMC Genomics. 2017 Jan 4;18:14. doi: 10.1186/s12864-016-3394-7 (PMC5209821; doi:10.1186/s12864-016-3394-7)
Supplement: Additional file 3: — Details for each piRNA clusters found in High Motile (HM) sperm fraction. Genes, repeats, transposable elements and transcription factors binding sites falling within the cluster regions were reported. (ZIP 1896 kb) [file 12864_2016_3394_MOESM3_ESM.zip › 31.html]

piRNA cluster 31


Predicted piRNA cluster no. 31     previous   next
  

Show proTRAC run info
Hide proTRAC run info

================================= proTRAC ====================================  
VERSION: 2.1                                    LAST MODIFIED: 06. October 2015  
  
Please cite:  
Rosenkranz D, Zischler H. proTRAC - a software for probabilistic piRNA cluster  
detection, visualization and analysis. 2012. BMC Bioinformatics 13:5.  
  
and (for proTRAC 2.0 and later):  
Rosenkranz D, Rudloff S, Bastuck K, Ketting RF, Zischler H. Tupaia small RNAs  
provide insights into function and evolution of RNAi-based transposon defense  
in mammals. 2015. RNA 21(5):911-922.  
  
Contact:  
David Rosenkranz  
Institute of Anthropology, small RNA group  
Johannes Gutenberg University Mainz  
email: rosenkranz@uni-mainz.de  
  
You can find the latest proTRAC version at:  
http://sourceforge.net/projects/protrac/files  
http://www.smallRNAgroup-mainz.de/software  
==============================================================================  
  
PARAMETERS:  
Map file: .............../storage/core/barbara/genhome/smallRNA/fertility/Sample\_motile/pirna/Sample\_motile\_26-33\_collapsed.fa.no-dust.map.weighted-10000-1000-b-0  
Genome file: ............/storage/core/barbara/genhome/smallRNA/fertility/Sample\_all/pirna/bt\_311\_chrY.fa  
RepeatMasker annotation: /storage/genomes/bt\_umd31/GCF\_000003055.6\_Bos\_taurus\_UMD\_3.1.1\_repeatMasker\_chr.out  
GeneSet:................./storage/core/barbara/genhome/smallRNA/fertility/Sample\_all/pirna/full.gtf  
  
Significant (p<=0.01) hit density will be calculated based  
on observed hit distribution.  
  
Sliding window size: ........................................ 5000 bp  
Sliding window increament: .................................. 1000 bp  
Normalize each hit by number of genomic hits: ............... 1 [0=no/1=yes]  
Normalize each hit by number of sequence reads: ............. 1 [0=no/1=yes]  
Normalize values (-> per million mapped reads): ............. 1 [0=no/1=yes]  
Min. fraction of hits with 1T(U) or 10A: .................... 0.75  
Alternatively: Min. fraction of hits with 1T(U) and 10A: .... 0.5  
Min. fraction of hits with typical piRNA length: ............ 0.75  
Typical piRNA length: ....................................... 26-33 nt  
Min. size of a piRNA cluster: ............................... 5000 bp.  
Min. number of hits (absolute): ............................. 0  
Min. number of hits (normalized): ........................... 0  
Min. fraction of hits on the mainstrand: .................... 0.75  
Top fraction of mapped sequences (in terms of read counts): . 1%  
Top fraction accounts for max. n% of sequence reads: ........ 90%  
Min. fraction of hits on each arm of a bidirectional cluster: 0.1  
Output image file for each cluster: ......................... 0 [0=no/1=yes]  
Output html file for each cluster: .......................... 1 [0=no/1=yes]  
Output a summary table: ..................................... 1 [0=no/1=yes]  
Output a FASTA file for each cluster (piRNA sequences): ..... 1 [0=no/1=yes]  
Output a FASTA file comprising cluster sequences: ........... 1 [0=no/1=yes]  
Search DNA motifs in clusters: .............................. 1 [0=no/1=yes]  
Output flanking sequences: +/- .............................. 0 bp  
Output ~.pTi file: .......................................... 1 [0=no/1=yes]  
==============================================================================  
  
  
Genome size (without gaps): ............ 2678902517 bp  
Gaps (N/X/-): .......................... 53837044 bp  
Mapped reads: .......................... 658825247023  
Non-identical sequences: ............... 514171  
Genomic hits: .......................... 764233  
Significant densitiy of mapped reads: .. 12867599.5173724 reads/kb

Show proTRAC cluster info
Hide proTRAC cluster info

|  |  |
| --- | --- |
| Location | chr17 |
| Coordinates | 65194575-65201120 |
| Size [bp] | 6546 |
| Sequence hit loci | 144 |
| Mapped reads (normalized) | 189471915 |
| Mapped reads (normalized) per kb | 28944686.1 |
| Normalized reads with 1T (1U) | 87% |
| Normalized reads with 10A | 23.6% |
| Normalized reads with length 26-33 nt | 100% |
| Normalized reads on the main strand(s) | 99.3% |
| Predicted directionality | mono:plus |

100%

0%

1T (1U)  
reads

10A reads

26-33 nt  
reads

reads on mainstrand

**Either the amount of reads with 1T (1U) OR 10A has to exceed 75% (set with option: -1Tor10A)  
Alternatively the amount of reads with 1T (1U) AND 10A has to exceed 50% (set with option: -1Tand10A)  
Minimum amount of reads with preferred size is 75% (set with option: -pisize)  
Minimum amount of reads on the main strand(s) is 75% (set with option: -clstrand)**

Show read coverage
Hide read coverage

WHAT DO I SEE HERE?  
This chart shows the location of mapped sequence reads within a predicted piRNA cluster. The color refers to the number of genomic hits produced by the sequence read in question. A dark red bar indicates that this sequence read produces many other hits elsewhere in the genome. Many adjacent red or yellow bars can indicate the presence of a multi-copy element such as transposons or rRNA genes. A dark green bar indicates that this sequence read maps uniquely to this locus.

1 hit

2-5 hits

6-10 hits

11-20 hits

21-50 hits

51-100 hits

> 100 hits

chr17

65194575

65201120

Gene Set

RepeatMasker

Mapped  
Reads

24.88

plus strand

minus strand

24.88

Region: chr17 61897727-65194581. Max. coverage (+): 8.25. Max coverage (-): 0

Region: chr17 65194582-65194594. Max. coverage (+): 8.25. Max coverage (-): 0

Region: chr17 65194595-65194607. Max. coverage (+): 0. Max coverage (-): 0

Region: chr17 65194608-65194620. Max. coverage (+): 0. Max coverage (-): 0

Region: chr17 65194621-65194633. Max. coverage (+): 2.34. Max coverage (-): 0

Region: chr17 65194634-65194647. Max. coverage (+): 2.34. Max coverage (-): 0

Region: chr17 65194648-65194660. Max. coverage (+): 0. Max coverage (-): 0

Region: chr17 65194661-65194673. Max. coverage (+): 0. Max coverage (-): 0

Region: chr17 65194674-65194686. Max. coverage (+): 2.09. Max coverage (-): 0

Region: chr17 65194687-65194699. Max. coverage (+): 0. Max coverage (-): 0

Region: chr17 65194700-65194712. Max. coverage (+): 0. Max coverage (-): 0

Region: chr17 65194713-65194725. Max. coverage (+): 3.24. Max coverage (-): 0

Region: chr17 65194726-65194738. Max. coverage (+): 3.24. Max coverage (-): 0

Region: chr17 65194739-65194751. Max. coverage (+): 0. Max coverage (-): 0

Region: chr17 65194752-65194764. Max. coverage (+): 1.43. Max coverage (-): 0

Region: chr17 65194765-65194777. Max. coverage (+): 0. Max coverage (-): 0

Region: chr17 65194778-65194791. Max. coverage (+): 0. Max coverage (-): 0

Region: chr17 65194792-65194804. Max. coverage (+): 0. Max coverage (-): 0

Region: chr17 65194805-65194817. Max. coverage (+): 0. Max coverage (-): 0

Region: chr17 65194818-65194830. Max. coverage (+): 0. Max coverage (-): 0

Region: chr17 65194831-65194843. Max. coverage (+): 0. Max coverage (-): 0

Region: chr17 65194844-65194856. Max. coverage (+): 0. Max coverage (-): 0

Region: chr17 65194857-65194869. Max. coverage (+): 0. Max coverage (-): 0

Region: chr17 65194870-65194882. Max. coverage (+): 0. Max coverage (-): 0

Region: chr17 65194883-65194895. Max. coverage (+): 0. Max coverage (-): 0

Region: chr17 65194896-65194908. Max. coverage (+): 0. Max coverage (-): 0

Region: chr17 65194909-65194921. Max. coverage (+): 0. Max coverage (-): 0

Region: chr17 65194922-65194935. Max. coverage (+): 0. Max coverage (-): 0

Region: chr17 65194936-65194948. Max. coverage (+): 0. Max coverage (-): 0

Region: chr17 65194949-65194961. Max. coverage (+): 0. Max coverage (-): 0

Region: chr17 65194962-65194974. Max. coverage (+): 0. Max coverage (-): 0

Region: chr17 65194975-65194987. Max. coverage (+): 0. Max coverage (-): 0

Region: chr17 65194988-65195000. Max. coverage (+): 0. Max coverage (-): 0

Region: chr17 65195001-65195013. Max. coverage (+): 0. Max coverage (-): 0

Region: chr17 65195014-65195026. Max. coverage (+): 0. Max coverage (-): 0

Region: chr17 65195027-65195039. Max. coverage (+): 0. Max coverage (-): 0

Region: chr17 65195040-65195052. Max. coverage (+): 0. Max coverage (-): 0

Region: chr17 65195053-65195065. Max. coverage (+): 0. Max coverage (-): 0

Region: chr17 65195066-65195079. Max. coverage (+): 0. Max coverage (-): 0

Region: chr17 65195080-65195092. Max. coverage (+): 0. Max coverage (-): 0

Region: chr17 65195093-65195105. Max. coverage (+): 0. Max coverage (-): 0

Region: chr17 65195106-65195118. Max. coverage (+): 0. Max coverage (-): 0

Region: chr17 65195119-65195131. Max. coverage (+): 0.87. Max coverage (-): 0

Region: chr17 65195132-65195144. Max. coverage (+): 0.87. Max coverage (-): 0

Region: chr17 65195145-65195157. Max. coverage (+): 0. Max coverage (-): 0

Region: chr17 65195158-65195170. Max. coverage (+): 0. Max coverage (-): 0

Region: chr17 65195171-65195183. Max. coverage (+): 0. Max coverage (-): 0

Region: chr17 65195184-65195196. Max. coverage (+): 0. Max coverage (-): 0

Region: chr17 65195197-65195209. Max. coverage (+): 0. Max coverage (-): 0

Region: chr17 65195210-65195223. Max. coverage (+): 0. Max coverage (-): 0

Region: chr17 65195224-65195236. Max. coverage (+): 2.24. Max coverage (-): 0

Region: chr17 65195237-65195249. Max. coverage (+): 0. Max coverage (-): 0

Region: chr17 65195250-65195262. Max. coverage (+): 0. Max coverage (-): 0

Region: chr17 65195263-65195275. Max. coverage (+): 0. Max coverage (-): 0

Region: chr17 65195276-65195288. Max. coverage (+): 0. Max coverage (-): 0

Region: chr17 65195289-65195301. Max. coverage (+): 0. Max coverage (-): 0

Region: chr17 65195302-65195314. Max. coverage (+): 0. Max coverage (-): 0

Region: chr17 65195315-65195327. Max. coverage (+): 1.28. Max coverage (-): 0

Region: chr17 65195328-65195340. Max. coverage (+): 1.28. Max coverage (-): 0

Region: chr17 65195341-65195353. Max. coverage (+): 0. Max coverage (-): 0

Region: chr17 65195354-65195367. Max. coverage (+): 0. Max coverage (-): 0

Region: chr17 65195368-65195380. Max. coverage (+): 7.55. Max coverage (-): 0

Region: chr17 65195381-65195393. Max. coverage (+): 9.62. Max coverage (-): 0

Region: chr17 65195394-65195406. Max. coverage (+): 2.84. Max coverage (-): 0

Region: chr17 65195407-65195419. Max. coverage (+): 0.97. Max coverage (-): 0

Region: chr17 65195420-65195432. Max. coverage (+): 0.97. Max coverage (-): 0

Region: chr17 65195433-65195445. Max. coverage (+): 0. Max coverage (-): 0

Region: chr17 65195446-65195458. Max. coverage (+): 0. Max coverage (-): 0

Region: chr17 65195459-65195471. Max. coverage (+): 0. Max coverage (-): 0

Region: chr17 65195472-65195484. Max. coverage (+): 0. Max coverage (-): 0

Region: chr17 65195485-65195497. Max. coverage (+): 11.97. Max coverage (-): 0

Region: chr17 65195498-65195511. Max. coverage (+): 23.02. Max coverage (-): 0

Region: chr17 65195512-65195524. Max. coverage (+): 3.81. Max coverage (-): 0

Region: chr17 65195525-65195537. Max. coverage (+): 0. Max coverage (-): 0

Region: chr17 65195538-65195550. Max. coverage (+): 0. Max coverage (-): 0

Region: chr17 65195551-65195563. Max. coverage (+): 0. Max coverage (-): 0

Region: chr17 65195564-65195576. Max. coverage (+): 0. Max coverage (-): 0

Region: chr17 65195577-65195589. Max. coverage (+): 0. Max coverage (-): 0

Region: chr17 65195590-65195602. Max. coverage (+): 0. Max coverage (-): 0

Region: chr17 65195603-65195615. Max. coverage (+): 7.7. Max coverage (-): 0

Region: chr17 65195616-65195628. Max. coverage (+): 7.7. Max coverage (-): 0

Region: chr17 65195629-65195641. Max. coverage (+): 0. Max coverage (-): 0

Region: chr17 65195642-65195655. Max. coverage (+): 0. Max coverage (-): 0

Region: chr17 65195656-65195668. Max. coverage (+): 1.15. Max coverage (-): 0

Region: chr17 65195669-65195681. Max. coverage (+): 0. Max coverage (-): 0

Region: chr17 65195682-65195694. Max. coverage (+): 0. Max coverage (-): 0

Region: chr17 65195695-65195707. Max. coverage (+): 2.17. Max coverage (-): 0

Region: chr17 65195708-65195720. Max. coverage (+): 0. Max coverage (-): 0

Region: chr17 65195721-65195733. Max. coverage (+): 0. Max coverage (-): 0

Region: chr17 65195734-65195746. Max. coverage (+): 0. Max coverage (-): 0

Region: chr17 65195747-65195759. Max. coverage (+): 2.21. Max coverage (-): 0

Region: chr17 65195760-65195772. Max. coverage (+): 3.97. Max coverage (-): 0

Region: chr17 65195773-65195786. Max. coverage (+): 1.91. Max coverage (-): 0

Region: chr17 65195787-65195799. Max. coverage (+): 5.27. Max coverage (-): 0

Region: chr17 65195800-65195812. Max. coverage (+): 5.27. Max coverage (-): 0

Region: chr17 65195813-65195825. Max. coverage (+): 0. Max coverage (-): 0

Region: chr17 65195826-65195838. Max. coverage (+): 5.29. Max coverage (-): 0

Region: chr17 65195839-65195851. Max. coverage (+): 0.74. Max coverage (-): 0

Region: chr17 65195852-65195864. Max. coverage (+): 2.15. Max coverage (-): 0

Region: chr17 65195865-65195877. Max. coverage (+): 0. Max coverage (-): 0

Region: chr17 65195878-65195890. Max. coverage (+): 0. Max coverage (-): 0

Region: chr17 65195891-65195903. Max. coverage (+): 0. Max coverage (-): 0

Region: chr17 65195904-65195916. Max. coverage (+): 0. Max coverage (-): 0

Region: chr17 65195917-65195930. Max. coverage (+): 0. Max coverage (-): 0

Region: chr17 65195931-65195943. Max. coverage (+): 0.95. Max coverage (-): 0

Region: chr17 65195944-65195956. Max. coverage (+): 0. Max coverage (-): 0

Region: chr17 65195957-65195969. Max. coverage (+): 0. Max coverage (-): 0

Region: chr17 65195970-65195982. Max. coverage (+): 0. Max coverage (-): 0

Region: chr17 65195983-65195995. Max. coverage (+): 4.42. Max coverage (-): 0

Region: chr17 65195996-65196008. Max. coverage (+): 0. Max coverage (-): 0

Region: chr17 65196009-65196021. Max. coverage (+): 0. Max coverage (-): 0

Region: chr17 65196022-65196034. Max. coverage (+): 0. Max coverage (-): 0

Region: chr17 65196035-65196047. Max. coverage (+): 0. Max coverage (-): 0

Region: chr17 65196048-65196060. Max. coverage (+): 0. Max coverage (-): 1.94

Region: chr17 65196061-65196074. Max. coverage (+): 0. Max coverage (-): 0

Region: chr17 65196075-65196087. Max. coverage (+): 0. Max coverage (-): 0

Region: chr17 65196088-65196100. Max. coverage (+): 0. Max coverage (-): 0

Region: chr17 65196101-65196113. Max. coverage (+): 0. Max coverage (-): 0

Region: chr17 65196114-65196126. Max. coverage (+): 0. Max coverage (-): 0

Region: chr17 65196127-65196139. Max. coverage (+): 1.77. Max coverage (-): 0

Region: chr17 65196140-65196152. Max. coverage (+): 1.77. Max coverage (-): 0

Region: chr17 65196153-65196165. Max. coverage (+): 0. Max coverage (-): 0

Region: chr17 65196166-65196178. Max. coverage (+): 0. Max coverage (-): 0

Region: chr17 65196179-65196191. Max. coverage (+): 0. Max coverage (-): 0

Region: chr17 65196192-65196204. Max. coverage (+): 0. Max coverage (-): 0

Region: chr17 65196205-65196218. Max. coverage (+): 0. Max coverage (-): 0

Region: chr17 65196219-65196231. Max. coverage (+): 0. Max coverage (-): 0

Region: chr17 65196232-65196244. Max. coverage (+): 0. Max coverage (-): 0

Region: chr17 65196245-65196257. Max. coverage (+): 0. Max coverage (-): 0

Region: chr17 65196258-65196270. Max. coverage (+): 0. Max coverage (-): 0

Region: chr17 65196271-65196283. Max. coverage (+): 0. Max coverage (-): 0

Region: chr17 65196284-65196296. Max. coverage (+): 0. Max coverage (-): 0

Region: chr17 65196297-65196309. Max. coverage (+): 0. Max coverage (-): 0

Region: chr17 65196310-65196322. Max. coverage (+): 0. Max coverage (-): 0

Region: chr17 65196323-65196335. Max. coverage (+): 1.29. Max coverage (-): 0

Region: chr17 65196336-65196348. Max. coverage (+): 7.55. Max coverage (-): 0

Region: chr17 65196349-65196362. Max. coverage (+): 0. Max coverage (-): 0

Region: chr17 65196363-65196375. Max. coverage (+): 0. Max coverage (-): 0

Region: chr17 65196376-65196388. Max. coverage (+): 0. Max coverage (-): 0

Region: chr17 65196389-65196401. Max. coverage (+): 0. Max coverage (-): 0

Region: chr17 65196402-65196414. Max. coverage (+): 5.09. Max coverage (-): 0

Region: chr17 65196415-65196427. Max. coverage (+): 0. Max coverage (-): 0

Region: chr17 65196428-65196440. Max. coverage (+): 0. Max coverage (-): 0

Region: chr17 65196441-65196453. Max. coverage (+): 0. Max coverage (-): 0

Region: chr17 65196454-65196466. Max. coverage (+): 0. Max coverage (-): 0

Region: chr17 65196467-65196479. Max. coverage (+): 0. Max coverage (-): 0

Region: chr17 65196480-65196492. Max. coverage (+): 1.05. Max coverage (-): 0

Region: chr17 65196493-65196506. Max. coverage (+): 3.26. Max coverage (-): 0

Region: chr17 65196507-65196519. Max. coverage (+): 1.85. Max coverage (-): 0

Region: chr17 65196520-65196532. Max. coverage (+): 5.53. Max coverage (-): 0

Region: chr17 65196533-65196545. Max. coverage (+): 3.56. Max coverage (-): 0

Region: chr17 65196546-65196558. Max. coverage (+): 0. Max coverage (-): 0

Region: chr17 65196559-65196571. Max. coverage (+): 0. Max coverage (-): 0

Region: chr17 65196572-65196584. Max. coverage (+): 0. Max coverage (-): 0

Region: chr17 65196585-65196597. Max. coverage (+): 0. Max coverage (-): 0

Region: chr17 65196598-65196610. Max. coverage (+): 0. Max coverage (-): 0

Region: chr17 65196611-65196623. Max. coverage (+): 0. Max coverage (-): 0

Region: chr17 65196624-65196636. Max. coverage (+): 0. Max coverage (-): 0

Region: chr17 65196637-65196650. Max. coverage (+): 0. Max coverage (-): 0

Region: chr17 65196651-65196663. Max. coverage (+): 0. Max coverage (-): 0

Region: chr17 65196664-65196676. Max. coverage (+): 0. Max coverage (-): 0

Region: chr17 65196677-65196689. Max. coverage (+): 0. Max coverage (-): 0

Region: chr17 65196690-65196702. Max. coverage (+): 0. Max coverage (-): 0

Region: chr17 65196703-65196715. Max. coverage (+): 0. Max coverage (-): 0

Region: chr17 65196716-65196728. Max. coverage (+): 0. Max coverage (-): 0

Region: chr17 65196729-65196741. Max. coverage (+): 0. Max coverage (-): 0

Region: chr17 65196742-65196754. Max. coverage (+): 0. Max coverage (-): 0

Region: chr17 65196755-65196767. Max. coverage (+): 0. Max coverage (-): 0

Region: chr17 65196768-65196781. Max. coverage (+): 0. Max coverage (-): 0

Region: chr17 65196782-65196794. Max. coverage (+): 0. Max coverage (-): 0

Region: chr17 65196795-65196807. Max. coverage (+): 0. Max coverage (-): 0

Region: chr17 65196808-65196820. Max. coverage (+): 0. Max coverage (-): 0

Region: chr17 65196821-65196833. Max. coverage (+): 0. Max coverage (-): 0

Region: chr17 65196834-65196846. Max. coverage (+): 0. Max coverage (-): 0

Region: chr17 65196847-65196859. Max. coverage (+): 0. Max coverage (-): 0

Region: chr17 65196860-65196872. Max. coverage (+): 0. Max coverage (-): 0

Region: chr17 65196873-65196885. Max. coverage (+): 0. Max coverage (-): 0

Region: chr17 65196886-65196898. Max. coverage (+): 0. Max coverage (-): 0

Region: chr17 65196899-65196911. Max. coverage (+): 0. Max coverage (-): 0

Region: chr17 65196912-65196925. Max. coverage (+): 0. Max coverage (-): 0

Region: chr17 65196926-65196938. Max. coverage (+): 4.04. Max coverage (-): 0

Region: chr17 65196939-65196951. Max. coverage (+): 4.04. Max coverage (-): 0

Region: chr17 65196952-65196964. Max. coverage (+): 0. Max coverage (-): 0

Region: chr17 65196965-65196977. Max. coverage (+): 3.58. Max coverage (-): 0

Region: chr17 65196978-65196990. Max. coverage (+): 0. Max coverage (-): 0

Region: chr17 65196991-65197003. Max. coverage (+): 0. Max coverage (-): 0

Region: chr17 65197004-65197016. Max. coverage (+): 0. Max coverage (-): 0

Region: chr17 65197017-65197029. Max. coverage (+): 0. Max coverage (-): 0

Region: chr17 65197030-65197042. Max. coverage (+): 0. Max coverage (-): 0

Region: chr17 65197043-65197055. Max. coverage (+): 0. Max coverage (-): 0

Region: chr17 65197056-65197069. Max. coverage (+): 0. Max coverage (-): 0

Region: chr17 65197070-65197082. Max. coverage (+): 1.3. Max coverage (-): 0

Region: chr17 65197083-65197095. Max. coverage (+): 0. Max coverage (-): 0

Region: chr17 65197096-65197108. Max. coverage (+): 0. Max coverage (-): 0

Region: chr17 65197109-65197121. Max. coverage (+): 0. Max coverage (-): 0

Region: chr17 65197122-65197134. Max. coverage (+): 1.23. Max coverage (-): 0

Region: chr17 65197135-65197147. Max. coverage (+): 0. Max coverage (-): 0

Region: chr17 65197148-65197160. Max. coverage (+): 0. Max coverage (-): 0

Region: chr17 65197161-65197173. Max. coverage (+): 0. Max coverage (-): 0

Region: chr17 65197174-65197186. Max. coverage (+): 1.45. Max coverage (-): 0

Region: chr17 65197187-65197199. Max. coverage (+): 1.45. Max coverage (-): 0

Region: chr17 65197200-65197213. Max. coverage (+): 0. Max coverage (-): 0

Region: chr17 65197214-65197226. Max. coverage (+): 0. Max coverage (-): 0

Region: chr17 65197227-65197239. Max. coverage (+): 0. Max coverage (-): 0

Region: chr17 65197240-65197252. Max. coverage (+): 0. Max coverage (-): 0

Region: chr17 65197253-65197265. Max. coverage (+): 0. Max coverage (-): 0

Region: chr17 65197266-65197278. Max. coverage (+): 0. Max coverage (-): 0

Region: chr17 65197279-65197291. Max. coverage (+): 0. Max coverage (-): 0

Region: chr17 65197292-65197304. Max. coverage (+): 0. Max coverage (-): 0

Region: chr17 65197305-65197317. Max. coverage (+): 0. Max coverage (-): 0

Region: chr17 65197318-65197330. Max. coverage (+): 0. Max coverage (-): 0

Region: chr17 65197331-65197343. Max. coverage (+): 0. Max coverage (-): 0

Region: chr17 65197344-65197357. Max. coverage (+): 0. Max coverage (-): 0

Region: chr17 65197358-65197370. Max. coverage (+): 0. Max coverage (-): 0

Region: chr17 65197371-65197383. Max. coverage (+): 0. Max coverage (-): 0

Region: chr17 65197384-65197396. Max. coverage (+): 0. Max coverage (-): 0

Region: chr17 65197397-65197409. Max. coverage (+): 0. Max coverage (-): 0

Region: chr17 65197410-65197422. Max. coverage (+): 0. Max coverage (-): 0

Region: chr17 65197423-65197435. Max. coverage (+): 0. Max coverage (-): 0

Region: chr17 65197436-65197448. Max. coverage (+): 0. Max coverage (-): 0

Region: chr17 65197449-65197461. Max. coverage (+): 0. Max coverage (-): 0

Region: chr17 65197462-65197474. Max. coverage (+): 0. Max coverage (-): 0

Region: chr17 65197475-65197487. Max. coverage (+): 1.86. Max coverage (-): 0

Region: chr17 65197488-65197501. Max. coverage (+): 0. Max coverage (-): 0

Region: chr17 65197502-65197514. Max. coverage (+): 4.08. Max coverage (-): 0

Region: chr17 65197515-65197527. Max. coverage (+): 4.2. Max coverage (-): 0

Region: chr17 65197528-65197540. Max. coverage (+): 0. Max coverage (-): 0

Region: chr17 65197541-65197553. Max. coverage (+): 0. Max coverage (-): 0

Region: chr17 65197554-65197566. Max. coverage (+): 0. Max coverage (-): 0

Region: chr17 65197567-65197579. Max. coverage (+): 24.88. Max coverage (-): 0

Region: chr17 65197580-65197592. Max. coverage (+): 1.31. Max coverage (-): 0

Region: chr17 65197593-65197605. Max. coverage (+): 0. Max coverage (-): 0

Region: chr17 65197606-65197618. Max. coverage (+): 0. Max coverage (-): 0

Region: chr17 65197619-65197631. Max. coverage (+): 5.23. Max coverage (-): 0

Region: chr17 65197632-65197645. Max. coverage (+): 0. Max coverage (-): 0

Region: chr17 65197646-65197658. Max. coverage (+): 0. Max coverage (-): 0

Region: chr17 65197659-65197671. Max. coverage (+): 0.74. Max coverage (-): 0

Region: chr17 65197672-65197684. Max. coverage (+): 0.74. Max coverage (-): 0

Region: chr17 65197685-65197697. Max. coverage (+): 0. Max coverage (-): 0

Region: chr17 65197698-65197710. Max. coverage (+): 0. Max coverage (-): 0

Region: chr17 65197711-65197723. Max. coverage (+): 0. Max coverage (-): 0

Region: chr17 65197724-65197736. Max. coverage (+): 0. Max coverage (-): 0

Region: chr17 65197737-65197749. Max. coverage (+): 0. Max coverage (-): 0

Region: chr17 65197750-65197762. Max. coverage (+): 0. Max coverage (-): 0

Region: chr17 65197763-65197775. Max. coverage (+): 0. Max coverage (-): 0

Region: chr17 65197776-65197789. Max. coverage (+): 0. Max coverage (-): 0

Region: chr17 65197790-65197802. Max. coverage (+): 0. Max coverage (-): 0

Region: chr17 65197803-65197815. Max. coverage (+): 0. Max coverage (-): 0

Region: chr17 65197816-65197828. Max. coverage (+): 7.88. Max coverage (-): 0

Region: chr17 65197829-65197841. Max. coverage (+): 12.02. Max coverage (-): 0

Region: chr17 65197842-65197854. Max. coverage (+): 10.87. Max coverage (-): 0

Region: chr17 65197855-65197867. Max. coverage (+): 0. Max coverage (-): 0

Region: chr17 65197868-65197880. Max. coverage (+): 0. Max coverage (-): 0

Region: chr17 65197881-65197893. Max. coverage (+): 0. Max coverage (-): 0

Region: chr17 65197894-65197906. Max. coverage (+): 0. Max coverage (-): 0

Region: chr17 65197907-65197920. Max. coverage (+): 0. Max coverage (-): 0

Region: chr17 65197921-65197933. Max. coverage (+): 0. Max coverage (-): 0

Region: chr17 65197934-65197946. Max. coverage (+): 0. Max coverage (-): 0

Region: chr17 65197947-65197959. Max. coverage (+): 2.61. Max coverage (-): 0

Region: chr17 65197960-65197972. Max. coverage (+): 2.61. Max coverage (-): 0

Region: chr17 65197973-65197985. Max. coverage (+): 0. Max coverage (-): 0

Region: chr17 65197986-65197998. Max. coverage (+): 3.01. Max coverage (-): 0

Region: chr17 65197999-65198011. Max. coverage (+): 0. Max coverage (-): 0

Region: chr17 65198012-65198024. Max. coverage (+): 1.21. Max coverage (-): 0

Region: chr17 65198025-65198037. Max. coverage (+): 1.21. Max coverage (-): 0

Region: chr17 65198038-65198050. Max. coverage (+): 0. Max coverage (-): 0

Region: chr17 65198051-65198064. Max. coverage (+): 0. Max coverage (-): 0

Region: chr17 65198065-65198077. Max. coverage (+): 0. Max coverage (-): 0

Region: chr17 65198078-65198090. Max. coverage (+): 0. Max coverage (-): 0

Region: chr17 65198091-65198103. Max. coverage (+): 0. Max coverage (-): 0

Region: chr17 65198104-65198116. Max. coverage (+): 8.61. Max coverage (-): 0

Region: chr17 65198117-65198129. Max. coverage (+): 8.61. Max coverage (-): 0

Region: chr17 65198130-65198142. Max. coverage (+): 1.51. Max coverage (-): 0

Region: chr17 65198143-65198155. Max. coverage (+): 2.75. Max coverage (-): 0

Region: chr17 65198156-65198168. Max. coverage (+): 4.69. Max coverage (-): 0

Region: chr17 65198169-65198181. Max. coverage (+): 3.61. Max coverage (-): 0

Region: chr17 65198182-65198194. Max. coverage (+): 0. Max coverage (-): 0

Region: chr17 65198195-65198208. Max. coverage (+): 0. Max coverage (-): 0

Region: chr17 65198209-65198221. Max. coverage (+): 0. Max coverage (-): 0

Region: chr17 65198222-65198234. Max. coverage (+): 4.85. Max coverage (-): 0

Region: chr17 65198235-65198247. Max. coverage (+): 8.83. Max coverage (-): 0

Region: chr17 65198248-65198260. Max. coverage (+): 0. Max coverage (-): 0

Region: chr17 65198261-65198273. Max. coverage (+): 0. Max coverage (-): 0

Region: chr17 65198274-65198286. Max. coverage (+): 0. Max coverage (-): 0

Region: chr17 65198287-65198299. Max. coverage (+): 0. Max coverage (-): 0

Region: chr17 65198300-65198312. Max. coverage (+): 0. Max coverage (-): 0

Region: chr17 65198313-65198325. Max. coverage (+): 0. Max coverage (-): 0

Region: chr17 65198326-65198338. Max. coverage (+): 0. Max coverage (-): 0

Region: chr17 65198339-65198352. Max. coverage (+): 0. Max coverage (-): 0

Region: chr17 65198353-65198365. Max. coverage (+): 0. Max coverage (-): 0

Region: chr17 65198366-65198378. Max. coverage (+): 0. Max coverage (-): 0

Region: chr17 65198379-65198391. Max. coverage (+): 0. Max coverage (-): 0

Region: chr17 65198392-65198404. Max. coverage (+): 0. Max coverage (-): 0

Region: chr17 65198405-65198417. Max. coverage (+): 0. Max coverage (-): 0

Region: chr17 65198418-65198430. Max. coverage (+): 1.5. Max coverage (-): 0

Region: chr17 65198431-65198443. Max. coverage (+): 0. Max coverage (-): 0

Region: chr17 65198444-65198456. Max. coverage (+): 0. Max coverage (-): 0

Region: chr17 65198457-65198469. Max. coverage (+): 0. Max coverage (-): 0

Region: chr17 65198470-65198482. Max. coverage (+): 0. Max coverage (-): 0

Region: chr17 65198483-65198496. Max. coverage (+): 0. Max coverage (-): 0

Region: chr17 65198497-65198509. Max. coverage (+): 0. Max coverage (-): 0

Region: chr17 65198510-65198522. Max. coverage (+): 1.24. Max coverage (-): 0

Region: chr17 65198523-65198535. Max. coverage (+): 0. Max coverage (-): 0

Region: chr17 65198536-65198548. Max. coverage (+): 0. Max coverage (-): 0

Region: chr17 65198549-65198561. Max. coverage (+): 0. Max coverage (-): 0

Region: chr17 65198562-65198574. Max. coverage (+): 0. Max coverage (-): 0

Region: chr17 65198575-65198587. Max. coverage (+): 0. Max coverage (-): 0

Region: chr17 65198588-65198600. Max. coverage (+): 0. Max coverage (-): 0

Region: chr17 65198601-65198613. Max. coverage (+): 1.22. Max coverage (-): 0

Region: chr17 65198614-65198626. Max. coverage (+): 1.22. Max coverage (-): 0

Region: chr17 65198627-65198640. Max. coverage (+): 0. Max coverage (-): 0

Region: chr17 65198641-65198653. Max. coverage (+): 11.13. Max coverage (-): 0

Region: chr17 65198654-65198666. Max. coverage (+): 0. Max coverage (-): 0

Region: chr17 65198667-65198679. Max. coverage (+): 0. Max coverage (-): 0

Region: chr17 65198680-65198692. Max. coverage (+): 0. Max coverage (-): 0

Region: chr17 65198693-65198705. Max. coverage (+): 0. Max coverage (-): 0

Region: chr17 65198706-65198718. Max. coverage (+): 0. Max coverage (-): 0

Region: chr17 65198719-65198731. Max. coverage (+): 0. Max coverage (-): 0

Region: chr17 65198732-65198744. Max. coverage (+): 0. Max coverage (-): 0

Region: chr17 65198745-65198757. Max. coverage (+): 0. Max coverage (-): 0

Region: chr17 65198758-65198770. Max. coverage (+): 0. Max coverage (-): 0

Region: chr17 65198771-65198784. Max. coverage (+): 0. Max coverage (-): 0

Region: chr17 65198785-65198797. Max. coverage (+): 0. Max coverage (-): 0

Region: chr17 65198798-65198810. Max. coverage (+): 0. Max coverage (-): 0

Region: chr17 65198811-65198823. Max. coverage (+): 0. Max coverage (-): 0

Region: chr17 65198824-65198836. Max. coverage (+): 0. Max coverage (-): 0

Region: chr17 65198837-65198849. Max. coverage (+): 0. Max coverage (-): 0

Region: chr17 65198850-65198862. Max. coverage (+): 0. Max coverage (-): 0

Region: chr17 65198863-65198875. Max. coverage (+): 0. Max coverage (-): 0

Region: chr17 65198876-65198888. Max. coverage (+): 0. Max coverage (-): 0

Region: chr17 65198889-65198901. Max. coverage (+): 0.46. Max coverage (-): 0

Region: chr17 65198902-65198914. Max. coverage (+): 0.46. Max coverage (-): 0

Region: chr17 65198915-65198928. Max. coverage (+): 0. Max coverage (-): 0

Region: chr17 65198929-65198941. Max. coverage (+): 0. Max coverage (-): 0

Region: chr17 65198942-65198954. Max. coverage (+): 0. Max coverage (-): 0

Region: chr17 65198955-65198967. Max. coverage (+): 0. Max coverage (-): 0

Region: chr17 65198968-65198980. Max. coverage (+): 0. Max coverage (-): 0

Region: chr17 65198981-65198993. Max. coverage (+): 8.34. Max coverage (-): 0

Region: chr17 65198994-65199006. Max. coverage (+): 8.34. Max coverage (-): 0

Region: chr17 65199007-65199019. Max. coverage (+): 0. Max coverage (-): 0

Region: chr17 65199020-65199032. Max. coverage (+): 0. Max coverage (-): 0

Region: chr17 65199033-65199045. Max. coverage (+): 0. Max coverage (-): 0

Region: chr17 65199046-65199059. Max. coverage (+): 0. Max coverage (-): 0

Region: chr17 65199060-65199072. Max. coverage (+): 0. Max coverage (-): 0

Region: chr17 65199073-65199085. Max. coverage (+): 0. Max coverage (-): 0

Region: chr17 65199086-65199098. Max. coverage (+): 0. Max coverage (-): 0

Region: chr17 65199099-65199111. Max. coverage (+): 0. Max coverage (-): 0

Region: chr17 65199112-65199124. Max. coverage (+): 0. Max coverage (-): 0

Region: chr17 65199125-65199137. Max. coverage (+): 0. Max coverage (-): 0

Region: chr17 65199138-65199150. Max. coverage (+): 0. Max coverage (-): 0

Region: chr17 65199151-65199163. Max. coverage (+): 0. Max coverage (-): 0

Region: chr17 65199164-65199176. Max. coverage (+): 0. Max coverage (-): 0

Region: chr17 65199177-65199189. Max. coverage (+): 0. Max coverage (-): 0

Region: chr17 65199190-65199203. Max. coverage (+): 0. Max coverage (-): 0

Region: chr17 65199204-65199216. Max. coverage (+): 0. Max coverage (-): 0

Region: chr17 65199217-65199229. Max. coverage (+): 0. Max coverage (-): 0

Region: chr17 65199230-65199242. Max. coverage (+): 0. Max coverage (-): 0

Region: chr17 65199243-65199255. Max. coverage (+): 0. Max coverage (-): 0

Region: chr17 65199256-65199268. Max. coverage (+): 0. Max coverage (-): 0

Region: chr17 65199269-65199281. Max. coverage (+): 0. Max coverage (-): 0

Region: chr17 65199282-65199294. Max. coverage (+): 0. Max coverage (-): 0

Region: chr17 65199295-65199307. Max. coverage (+): 0. Max coverage (-): 0

Region: chr17 65199308-65199320. Max. coverage (+): 0. Max coverage (-): 0

Region: chr17 65199321-65199333. Max. coverage (+): 0. Max coverage (-): 0

Region: chr17 65199334-65199347. Max. coverage (+): 0. Max coverage (-): 0

Region: chr17 65199348-65199360. Max. coverage (+): 0. Max coverage (-): 0

Region: chr17 65199361-65199373. Max. coverage (+): 0. Max coverage (-): 0

Region: chr17 65199374-65199386. Max. coverage (+): 1.59. Max coverage (-): 0

Region: chr17 65199387-65199399. Max. coverage (+): 1.59. Max coverage (-): 0

Region: chr17 65199400-65199412. Max. coverage (+): 0. Max coverage (-): 0

Region: chr17 65199413-65199425. Max. coverage (+): 0. Max coverage (-): 0

Region: chr17 65199426-65199438. Max. coverage (+): 0. Max coverage (-): 0

Region: chr17 65199439-65199451. Max. coverage (+): 0. Max coverage (-): 0

Region: chr17 65199452-65199464. Max. coverage (+): 5.24. Max coverage (-): 0

Region: chr17 65199465-65199477. Max. coverage (+): 2.97. Max coverage (-): 0

Region: chr17 65199478-65199491. Max. coverage (+): 2.97. Max coverage (-): 0

Region: chr17 65199492-65199504. Max. coverage (+): 0. Max coverage (-): 0

Region: chr17 65199505-65199517. Max. coverage (+): 0. Max coverage (-): 0

Region: chr17 65199518-65199530. Max. coverage (+): 0. Max coverage (-): 0

Region: chr17 65199531-65199543. Max. coverage (+): 0. Max coverage (-): 0

Region: chr17 65199544-65199556. Max. coverage (+): 0. Max coverage (-): 0

Region: chr17 65199557-65199569. Max. coverage (+): 0. Max coverage (-): 0

Region: chr17 65199570-65199582. Max. coverage (+): 0. Max coverage (-): 0

Region: chr17 65199583-65199595. Max. coverage (+): 0. Max coverage (-): 0

Region: chr17 65199596-65199608. Max. coverage (+): 0. Max coverage (-): 0

Region: chr17 65199609-65199621. Max. coverage (+): 0. Max coverage (-): 0

Region: chr17 65199622-65199635. Max. coverage (+): 0. Max coverage (-): 0

Region: chr17 65199636-65199648. Max. coverage (+): 0. Max coverage (-): 0

Region: chr17 65199649-65199661. Max. coverage (+): 0. Max coverage (-): 0

Region: chr17 65199662-65199674. Max. coverage (+): 0. Max coverage (-): 0

Region: chr17 65199675-65199687. Max. coverage (+): 0. Max coverage (-): 0

Region: chr17 65199688-65199700. Max. coverage (+): 0. Max coverage (-): 0

Region: chr17 65199701-65199713. Max. coverage (+): 0. Max coverage (-): 0

Region: chr17 65199714-65199726. Max. coverage (+): 6.55. Max coverage (-): 0

Region: chr17 65199727-65199739. Max. coverage (+): 0. Max coverage (-): 0

Region: chr17 65199740-65199752. Max. coverage (+): 0. Max coverage (-): 0

Region: chr17 65199753-65199765. Max. coverage (+): 0. Max coverage (-): 0

Region: chr17 65199766-65199779. Max. coverage (+): 0. Max coverage (-): 0

Region: chr17 65199780-65199792. Max. coverage (+): 0. Max coverage (-): 0

Region: chr17 65199793-65199805. Max. coverage (+): 0. Max coverage (-): 0

Region: chr17 65199806-65199818. Max. coverage (+): 0. Max coverage (-): 0

Region: chr17 65199819-65199831. Max. coverage (+): 0. Max coverage (-): 0

Region: chr17 65199832-65199844. Max. coverage (+): 0. Max coverage (-): 0

Region: chr17 65199845-65199857. Max. coverage (+): 0. Max coverage (-): 0

Region: chr17 65199858-65199870. Max. coverage (+): 0. Max coverage (-): 0

Region: chr17 65199871-65199883. Max. coverage (+): 0. Max coverage (-): 0

Region: chr17 65199884-65199896. Max. coverage (+): 0. Max coverage (-): 0

Region: chr17 65199897-65199909. Max. coverage (+): 0. Max coverage (-): 0

Region: chr17 65199910-65199923. Max. coverage (+): 0. Max coverage (-): 0

Region: chr17 65199924-65199936. Max. coverage (+): 0. Max coverage (-): 0

Region: chr17 65199937-65199949. Max. coverage (+): 0. Max coverage (-): 0

Region: chr17 65199950-65199962. Max. coverage (+): 0. Max coverage (-): 0

Region: chr17 65199963-65199975. Max. coverage (+): 0. Max coverage (-): 0

Region: chr17 65199976-65199988. Max. coverage (+): 0. Max coverage (-): 0

Region: chr17 65199989-65200001. Max. coverage (+): 0. Max coverage (-): 0

Region: chr17 65200002-65200014. Max. coverage (+): 0. Max coverage (-): 0

Region: chr17 65200015-65200027. Max. coverage (+): 0. Max coverage (-): 0

Region: chr17 65200028-65200040. Max. coverage (+): 0. Max coverage (-): 0

Region: chr17 65200041-65200054. Max. coverage (+): 0. Max coverage (-): 0

Region: chr17 65200055-65200067. Max. coverage (+): 0. Max coverage (-): 0

Region: chr17 65200068-65200080. Max. coverage (+): 0. Max coverage (-): 0

Region: chr17 65200081-65200093. Max. coverage (+): 0. Max coverage (-): 0

Region: chr17 65200094-65200106. Max. coverage (+): 0. Max coverage (-): 0

Region: chr17 65200107-65200119. Max. coverage (+): 0. Max coverage (-): 0

Region: chr17 65200120-65200132. Max. coverage (+): 0. Max coverage (-): 0

Region: chr17 65200133-65200145. Max. coverage (+): 0. Max coverage (-): 0

Region: chr17 65200146-65200158. Max. coverage (+): 0. Max coverage (-): 0

Region: chr17 65200159-65200171. Max. coverage (+): 0. Max coverage (-): 0

Region: chr17 65200172-65200184. Max. coverage (+): 0. Max coverage (-): 0

Region: chr17 65200185-65200198. Max. coverage (+): 0. Max coverage (-): 0

Region: chr17 65200199-65200211. Max. coverage (+): 0. Max coverage (-): 0

Region: chr17 65200212-65200224. Max. coverage (+): 0. Max coverage (-): 0

Region: chr17 65200225-65200237. Max. coverage (+): 0. Max coverage (-): 0

Region: chr17 65200238-65200250. Max. coverage (+): 0. Max coverage (-): 0

Region: chr17 65200251-65200263. Max. coverage (+): 0. Max coverage (-): 0

Region: chr17 65200264-65200276. Max. coverage (+): 0. Max coverage (-): 0

Region: chr17 65200277-65200289. Max. coverage (+): 3.66. Max coverage (-): 0

Region: chr17 65200290-65200302. Max. coverage (+): 3.66. Max coverage (-): 0

Region: chr17 65200303-65200315. Max. coverage (+): 0. Max coverage (-): 0

Region: chr17 65200316-65200328. Max. coverage (+): 0. Max coverage (-): 0

Region: chr17 65200329-65200342. Max. coverage (+): 0. Max coverage (-): 0

Region: chr17 65200343-65200355. Max. coverage (+): 0. Max coverage (-): 0

Region: chr17 65200356-65200368. Max. coverage (+): 0. Max coverage (-): 0

Region: chr17 65200369-65200381. Max. coverage (+): 0.8. Max coverage (-): 0

Region: chr17 65200382-65200394. Max. coverage (+): 0.8. Max coverage (-): 0

Region: chr17 65200395-65200407. Max. coverage (+): 0. Max coverage (-): 0

Region: chr17 65200408-65200420. Max. coverage (+): 0. Max coverage (-): 0

Region: chr17 65200421-65200433. Max. coverage (+): 2.17. Max coverage (-): 0

Region: chr17 65200434-65200446. Max. coverage (+): 2.17. Max coverage (-): 0

Region: chr17 65200447-65200459. Max. coverage (+): 0. Max coverage (-): 0

Region: chr17 65200460-65200472. Max. coverage (+): 0. Max coverage (-): 0

Region: chr17 65200473-65200486. Max. coverage (+): 0. Max coverage (-): 0

Region: chr17 65200487-65200499. Max. coverage (+): 0. Max coverage (-): 0

Region: chr17 65200500-65200512. Max. coverage (+): 0. Max coverage (-): 0

Region: chr17 65200513-65200525. Max. coverage (+): 0. Max coverage (-): 0

Region: chr17 65200526-65200538. Max. coverage (+): 0. Max coverage (-): 0

Region: chr17 65200539-65200551. Max. coverage (+): 0. Max coverage (-): 0

Region: chr17 65200552-65200564. Max. coverage (+): 0. Max coverage (-): 0

Region: chr17 65200565-65200577. Max. coverage (+): 0. Max coverage (-): 0

Region: chr17 65200578-65200590. Max. coverage (+): 0. Max coverage (-): 0

Region: chr17 65200591-65200603. Max. coverage (+): 0. Max coverage (-): 0

Region: chr17 65200604-65200616. Max. coverage (+): 0. Max coverage (-): 0

Region: chr17 65200617-65200630. Max. coverage (+): 0. Max coverage (-): 0

Region: chr17 65200631-65200643. Max. coverage (+): 0. Max coverage (-): 0

Region: chr17 65200644-65200656. Max. coverage (+): 0. Max coverage (-): 0

Region: chr17 65200657-65200669. Max. coverage (+): 0. Max coverage (-): 0

Region: chr17 65200670-65200682. Max. coverage (+): 0. Max coverage (-): 0

Region: chr17 65200683-65200695. Max. coverage (+): 0. Max coverage (-): 0

Region: chr17 65200696-65200708. Max. coverage (+): 0. Max coverage (-): 0

Region: chr17 65200709-65200721. Max. coverage (+): 0. Max coverage (-): 0

Region: chr17 65200722-65200734. Max. coverage (+): 0. Max coverage (-): 0

Region: chr17 65200735-65200747. Max. coverage (+): 0. Max coverage (-): 0

Region: chr17 65200748-65200760. Max. coverage (+): 0. Max coverage (-): 0

Region: chr17 65200761-65200774. Max. coverage (+): 0. Max coverage (-): 0

Region: chr17 65200775-65200787. Max. coverage (+): 0. Max coverage (-): 0

Region: chr17 65200788-65200800. Max. coverage (+): 0. Max coverage (-): 0

Region: chr17 65200801-65200813. Max. coverage (+): 0. Max coverage (-): 0

Region: chr17 65200814-65200826. Max. coverage (+): 0. Max coverage (-): 0

Region: chr17 65200827-65200839. Max. coverage (+): 0. Max coverage (-): 0

Region: chr17 65200840-65200852. Max. coverage (+): 0. Max coverage (-): 0

Region: chr17 65200853-65200865. Max. coverage (+): 0. Max coverage (-): 0

Region: chr17 65200866-65200878. Max. coverage (+): 0. Max coverage (-): 0

Region: chr17 65200879-65200891. Max. coverage (+): 0. Max coverage (-): 0

Region: chr17 65200892-65200904. Max. coverage (+): 0. Max coverage (-): 0

Region: chr17 65200905-65200918. Max. coverage (+): 0. Max coverage (-): 0

Region: chr17 65200919-65200931. Max. coverage (+): 0. Max coverage (-): 0

Region: chr17 65200932-65200944. Max. coverage (+): 0. Max coverage (-): 0

Region: chr17 65200945-65200957. Max. coverage (+): 0. Max coverage (-): 0

Region: chr17 65200958-65200970. Max. coverage (+): 0. Max coverage (-): 0

Region: chr17 65200971-65200983. Max. coverage (+): 0. Max coverage (-): 0

Region: chr17 65200984-65200996. Max. coverage (+): 0. Max coverage (-): 0

Region: chr17 65200997-65201009. Max. coverage (+): 0. Max coverage (-): 0

Region: chr17 65201010-65201022. Max. coverage (+): 0. Max coverage (-): 0

Region: chr17 65201023-65201035. Max. coverage (+): 0. Max coverage (-): 0

Region: chr17 65201036-65201048. Max. coverage (+): 0. Max coverage (-): 0

Region: chr17 65201049-65201062. Max. coverage (+): 0. Max coverage (-): 0

Region: chr17 65201063-65201075. Max. coverage (+): 0. Max coverage (-): 0

Region: chr17 65201076-65201088. Max. coverage (+): 0. Max coverage (-): 0

Region: chr17 65201089-65201101. Max. coverage (+): 2.09. Max coverage (-): 0

Region: chr17 65201102-65201114. Max. coverage (+): 0. Max coverage (-): 0

Region: chr17 65201115-. Max. coverage (+): 0. Max coverage (-): 0

RepeatMasker Color Code

**+**

100-98% Identity

<98-95% Identity

<95-90% Identity

<90-85% Identity

<85-80% Identity

<80-75% Identity

<75-70% Identity

<70% Identity

**-**

Gene Set Color Code

**+**

Gene

Pseudogene

**-**

Topology/Coverage Color Code

Coverage Plus Strand

Coverage Minus Strand

Mainstrand: Plus

Mainstrand: Minus

Complementary Strand

Flanking Region  
(if option -flank >0)

Gene Set Annotation  

**1. MLEC (protein coding, ENSBTAG00000020050) Tr:00000026715 Ex:5**: 65194493-65194879 (+)

  
RepeatMasker Annotation  

**1. (CA)n**: 65194827-65194857 (+), Divergence to consensus: 0%  
**2. (C)n**: 65199222-65199245 (+), Divergence to consensus: 4.2%  
**3. MIRb**: 65200517-65200625 (-), Divergence to consensus: 29.6%  
**4. MIR3**: 65200754-65200848 (-), Divergence to consensus: 29.2%  
**5. GC\_rich**: 65200851-65200871 (+), Divergence to consensus: 33.3%

  
Transcription Factor Binding Sites  

**RFX4\_2** (Sequence: GTATCCAAG (-): 65195393)
