# Supplementary material for: Small RNA sequencing of cryopreserved semen from single bull revealed altered miRNAs and piRNAs expression between High- and Low-motile sperm populations
Source: BMC Genomics. 2017 Jan 4;18:14. doi: 10.1186/s12864-016-3394-7 (PMC5209821; doi:10.1186/s12864-016-3394-7)
Supplement: Additional file 3: — Details for each piRNA clusters found in High Motile (HM) sperm fraction. Genes, repeats, transposable elements and transcription factors binding sites falling within the cluster regions were reported. (ZIP 1896 kb) [file 12864_2016_3394_MOESM3_ESM.zip › 32.html]

piRNA cluster 32


Predicted piRNA cluster no. 32     previous   next
  

Show proTRAC run info
Hide proTRAC run info

================================= proTRAC ====================================  
VERSION: 2.1                                    LAST MODIFIED: 06. October 2015  
  
Please cite:  
Rosenkranz D, Zischler H. proTRAC - a software for probabilistic piRNA cluster  
detection, visualization and analysis. 2012. BMC Bioinformatics 13:5.  
  
and (for proTRAC 2.0 and later):  
Rosenkranz D, Rudloff S, Bastuck K, Ketting RF, Zischler H. Tupaia small RNAs  
provide insights into function and evolution of RNAi-based transposon defense  
in mammals. 2015. RNA 21(5):911-922.  
  
Contact:  
David Rosenkranz  
Institute of Anthropology, small RNA group  
Johannes Gutenberg University Mainz  
email: rosenkranz@uni-mainz.de  
  
You can find the latest proTRAC version at:  
http://sourceforge.net/projects/protrac/files  
http://www.smallRNAgroup-mainz.de/software  
==============================================================================  
  
PARAMETERS:  
Map file: .............../storage/core/barbara/genhome/smallRNA/fertility/Sample\_motile/pirna/Sample\_motile\_26-33\_collapsed.fa.no-dust.map.weighted-10000-1000-b-0  
Genome file: ............/storage/core/barbara/genhome/smallRNA/fertility/Sample\_all/pirna/bt\_311\_chrY.fa  
RepeatMasker annotation: /storage/genomes/bt\_umd31/GCF\_000003055.6\_Bos\_taurus\_UMD\_3.1.1\_repeatMasker\_chr.out  
GeneSet:................./storage/core/barbara/genhome/smallRNA/fertility/Sample\_all/pirna/full.gtf  
  
Significant (p<=0.01) hit density will be calculated based  
on observed hit distribution.  
  
Sliding window size: ........................................ 5000 bp  
Sliding window increament: .................................. 1000 bp  
Normalize each hit by number of genomic hits: ............... 1 [0=no/1=yes]  
Normalize each hit by number of sequence reads: ............. 1 [0=no/1=yes]  
Normalize values (-> per million mapped reads): ............. 1 [0=no/1=yes]  
Min. fraction of hits with 1T(U) or 10A: .................... 0.75  
Alternatively: Min. fraction of hits with 1T(U) and 10A: .... 0.5  
Min. fraction of hits with typical piRNA length: ............ 0.75  
Typical piRNA length: ....................................... 26-33 nt  
Min. size of a piRNA cluster: ............................... 5000 bp.  
Min. number of hits (absolute): ............................. 0  
Min. number of hits (normalized): ........................... 0  
Min. fraction of hits on the mainstrand: .................... 0.75  
Top fraction of mapped sequences (in terms of read counts): . 1%  
Top fraction accounts for max. n% of sequence reads: ........ 90%  
Min. fraction of hits on each arm of a bidirectional cluster: 0.1  
Output image file for each cluster: ......................... 0 [0=no/1=yes]  
Output html file for each cluster: .......................... 1 [0=no/1=yes]  
Output a summary table: ..................................... 1 [0=no/1=yes]  
Output a FASTA file for each cluster (piRNA sequences): ..... 1 [0=no/1=yes]  
Output a FASTA file comprising cluster sequences: ........... 1 [0=no/1=yes]  
Search DNA motifs in clusters: .............................. 1 [0=no/1=yes]  
Output flanking sequences: +/- .............................. 0 bp  
Output ~.pTi file: .......................................... 1 [0=no/1=yes]  
==============================================================================  
  
  
Genome size (without gaps): ............ 2678902517 bp  
Gaps (N/X/-): .......................... 53837044 bp  
Mapped reads: .......................... 658825247023  
Non-identical sequences: ............... 514171  
Genomic hits: .......................... 764233  
Significant densitiy of mapped reads: .. 12867599.5173724 reads/kb

Show proTRAC cluster info
Hide proTRAC cluster info

|  |  |
| --- | --- |
| Location | chr17 |
| Coordinates | 67223543-67230676 |
| Size [bp] | 7134 |
| Sequence hit loci | 181 |
| Mapped reads (normalized) | 225172220 |
| Mapped reads (normalized) per kb | 31563249.2 |
| Normalized reads with 1T (1U) | 79.1% |
| Normalized reads with 10A | 29.6% |
| Normalized reads with length 26-33 nt | 100% |
| Normalized reads on the main strand(s) | 100% |
| Predicted directionality | mono:plus |

100%

0%

1T (1U)  
reads

10A reads

26-33 nt  
reads

reads on mainstrand

**Either the amount of reads with 1T (1U) OR 10A has to exceed 75% (set with option: -1Tor10A)  
Alternatively the amount of reads with 1T (1U) AND 10A has to exceed 50% (set with option: -1Tand10A)  
Minimum amount of reads with preferred size is 75% (set with option: -pisize)  
Minimum amount of reads on the main strand(s) is 75% (set with option: -clstrand)**

Show read coverage
Hide read coverage

WHAT DO I SEE HERE?  
This chart shows the location of mapped sequence reads within a predicted piRNA cluster. The color refers to the number of genomic hits produced by the sequence read in question. A dark red bar indicates that this sequence read produces many other hits elsewhere in the genome. Many adjacent red or yellow bars can indicate the presence of a multi-copy element such as transposons or rRNA genes. A dark green bar indicates that this sequence read maps uniquely to this locus.

1 hit

2-5 hits

6-10 hits

11-20 hits

21-50 hits

51-100 hits

> 100 hits

chr17

67223543

67230676

Gene Set

RepeatMasker

Mapped  
Reads

44.7

plus strand

minus strand

44.7

Region: chr17 65201116-67223550. Max. coverage (+): 1.11. Max coverage (-): 0

Region: chr17 67223551-67223564. Max. coverage (+): 0. Max coverage (-): 0

Region: chr17 67223565-67223578. Max. coverage (+): 0. Max coverage (-): 0

Region: chr17 67223579-67223592. Max. coverage (+): 0. Max coverage (-): 0

Region: chr17 67223593-67223607. Max. coverage (+): 0. Max coverage (-): 0

Region: chr17 67223608-67223621. Max. coverage (+): 0. Max coverage (-): 0

Region: chr17 67223622-67223635. Max. coverage (+): 0. Max coverage (-): 0

Region: chr17 67223636-67223650. Max. coverage (+): 0. Max coverage (-): 0

Region: chr17 67223651-67223664. Max. coverage (+): 0. Max coverage (-): 0

Region: chr17 67223665-67223678. Max. coverage (+): 0. Max coverage (-): 0

Region: chr17 67223679-67223692. Max. coverage (+): 0. Max coverage (-): 0

Region: chr17 67223693-67223707. Max. coverage (+): 0. Max coverage (-): 0

Region: chr17 67223708-67223721. Max. coverage (+): 0. Max coverage (-): 0

Region: chr17 67223722-67223735. Max. coverage (+): 0. Max coverage (-): 0

Region: chr17 67223736-67223749. Max. coverage (+): 0. Max coverage (-): 0

Region: chr17 67223750-67223764. Max. coverage (+): 0. Max coverage (-): 0

Region: chr17 67223765-67223778. Max. coverage (+): 0. Max coverage (-): 0

Region: chr17 67223779-67223792. Max. coverage (+): 0. Max coverage (-): 0

Region: chr17 67223793-67223806. Max. coverage (+): 0. Max coverage (-): 0

Region: chr17 67223807-67223821. Max. coverage (+): 0. Max coverage (-): 0

Region: chr17 67223822-67223835. Max. coverage (+): 0. Max coverage (-): 0

Region: chr17 67223836-67223849. Max. coverage (+): 0. Max coverage (-): 0

Region: chr17 67223850-67223864. Max. coverage (+): 0. Max coverage (-): 0

Region: chr17 67223865-67223878. Max. coverage (+): 0. Max coverage (-): 0

Region: chr17 67223879-67223892. Max. coverage (+): 0. Max coverage (-): 0

Region: chr17 67223893-67223906. Max. coverage (+): 0. Max coverage (-): 0

Region: chr17 67223907-67223921. Max. coverage (+): 0. Max coverage (-): 0

Region: chr17 67223922-67223935. Max. coverage (+): 0. Max coverage (-): 0

Region: chr17 67223936-67223949. Max. coverage (+): 0. Max coverage (-): 0

Region: chr17 67223950-67223963. Max. coverage (+): 0. Max coverage (-): 0

Region: chr17 67223964-67223978. Max. coverage (+): 0. Max coverage (-): 0

Region: chr17 67223979-67223992. Max. coverage (+): 0. Max coverage (-): 0

Region: chr17 67223993-67224006. Max. coverage (+): 0. Max coverage (-): 0

Region: chr17 67224007-67224020. Max. coverage (+): 0. Max coverage (-): 0

Region: chr17 67224021-67224035. Max. coverage (+): 0. Max coverage (-): 0

Region: chr17 67224036-67224049. Max. coverage (+): 0. Max coverage (-): 0

Region: chr17 67224050-67224063. Max. coverage (+): 0. Max coverage (-): 0

Region: chr17 67224064-67224078. Max. coverage (+): 0. Max coverage (-): 0

Region: chr17 67224079-67224092. Max. coverage (+): 0. Max coverage (-): 0

Region: chr17 67224093-67224106. Max. coverage (+): 0. Max coverage (-): 0

Region: chr17 67224107-67224120. Max. coverage (+): 0. Max coverage (-): 0

Region: chr17 67224121-67224135. Max. coverage (+): 0. Max coverage (-): 0

Region: chr17 67224136-67224149. Max. coverage (+): 0. Max coverage (-): 0

Region: chr17 67224150-67224163. Max. coverage (+): 0. Max coverage (-): 0

Region: chr17 67224164-67224177. Max. coverage (+): 0. Max coverage (-): 0

Region: chr17 67224178-67224192. Max. coverage (+): 0. Max coverage (-): 0

Region: chr17 67224193-67224206. Max. coverage (+): 0. Max coverage (-): 0

Region: chr17 67224207-67224220. Max. coverage (+): 0. Max coverage (-): 0

Region: chr17 67224221-67224234. Max. coverage (+): 0. Max coverage (-): 0

Region: chr17 67224235-67224249. Max. coverage (+): 0. Max coverage (-): 0

Region: chr17 67224250-67224263. Max. coverage (+): 0. Max coverage (-): 0

Region: chr17 67224264-67224277. Max. coverage (+): 0. Max coverage (-): 0

Region: chr17 67224278-67224292. Max. coverage (+): 0. Max coverage (-): 0

Region: chr17 67224293-67224306. Max. coverage (+): 0. Max coverage (-): 0

Region: chr17 67224307-67224320. Max. coverage (+): 0. Max coverage (-): 0

Region: chr17 67224321-67224334. Max. coverage (+): 0. Max coverage (-): 0

Region: chr17 67224335-67224349. Max. coverage (+): 0. Max coverage (-): 0

Region: chr17 67224350-67224363. Max. coverage (+): 0. Max coverage (-): 0

Region: chr17 67224364-67224377. Max. coverage (+): 0. Max coverage (-): 0

Region: chr17 67224378-67224391. Max. coverage (+): 0. Max coverage (-): 0

Region: chr17 67224392-67224406. Max. coverage (+): 0. Max coverage (-): 0

Region: chr17 67224407-67224420. Max. coverage (+): 0. Max coverage (-): 0

Region: chr17 67224421-67224434. Max. coverage (+): 0. Max coverage (-): 0

Region: chr17 67224435-67224449. Max. coverage (+): 0. Max coverage (-): 0

Region: chr17 67224450-67224463. Max. coverage (+): 0. Max coverage (-): 0

Region: chr17 67224464-67224477. Max. coverage (+): 0. Max coverage (-): 0

Region: chr17 67224478-67224491. Max. coverage (+): 0. Max coverage (-): 0

Region: chr17 67224492-67224506. Max. coverage (+): 0. Max coverage (-): 0

Region: chr17 67224507-67224520. Max. coverage (+): 0. Max coverage (-): 0

Region: chr17 67224521-67224534. Max. coverage (+): 0. Max coverage (-): 0

Region: chr17 67224535-67224548. Max. coverage (+): 0. Max coverage (-): 0

Region: chr17 67224549-67224563. Max. coverage (+): 0. Max coverage (-): 0

Region: chr17 67224564-67224577. Max. coverage (+): 0. Max coverage (-): 0

Region: chr17 67224578-67224591. Max. coverage (+): 0. Max coverage (-): 0

Region: chr17 67224592-67224605. Max. coverage (+): 0. Max coverage (-): 0

Region: chr17 67224606-67224620. Max. coverage (+): 0. Max coverage (-): 0

Region: chr17 67224621-67224634. Max. coverage (+): 0. Max coverage (-): 0

Region: chr17 67224635-67224648. Max. coverage (+): 0. Max coverage (-): 0

Region: chr17 67224649-67224663. Max. coverage (+): 0. Max coverage (-): 0

Region: chr17 67224664-67224677. Max. coverage (+): 0. Max coverage (-): 0

Region: chr17 67224678-67224691. Max. coverage (+): 0. Max coverage (-): 0

Region: chr17 67224692-67224705. Max. coverage (+): 0. Max coverage (-): 0

Region: chr17 67224706-67224720. Max. coverage (+): 0. Max coverage (-): 0

Region: chr17 67224721-67224734. Max. coverage (+): 11.12. Max coverage (-): 0

Region: chr17 67224735-67224748. Max. coverage (+): 44.7. Max coverage (-): 0

Region: chr17 67224749-67224762. Max. coverage (+): 9.16. Max coverage (-): 0

Region: chr17 67224763-67224777. Max. coverage (+): 1.13. Max coverage (-): 0

Region: chr17 67224778-67224791. Max. coverage (+): 8.45. Max coverage (-): 0

Region: chr17 67224792-67224805. Max. coverage (+): 3.7. Max coverage (-): 0

Region: chr17 67224806-67224819. Max. coverage (+): 0. Max coverage (-): 0

Region: chr17 67224820-67224834. Max. coverage (+): 2.32. Max coverage (-): 0

Region: chr17 67224835-67224848. Max. coverage (+): 8.58. Max coverage (-): 0

Region: chr17 67224849-67224862. Max. coverage (+): 12.74. Max coverage (-): 0

Region: chr17 67224863-67224877. Max. coverage (+): 0.97. Max coverage (-): 0

Region: chr17 67224878-67224891. Max. coverage (+): 0. Max coverage (-): 0

Region: chr17 67224892-67224905. Max. coverage (+): 0.71. Max coverage (-): 0

Region: chr17 67224906-67224919. Max. coverage (+): 14.93. Max coverage (-): 0

Region: chr17 67224920-67224934. Max. coverage (+): 9.16. Max coverage (-): 0

Region: chr17 67224935-67224948. Max. coverage (+): 15.6. Max coverage (-): 0

Region: chr17 67224949-67224962. Max. coverage (+): 12.82. Max coverage (-): 0

Region: chr17 67224963-67224976. Max. coverage (+): 7.79. Max coverage (-): 0

Region: chr17 67224977-67224991. Max. coverage (+): 0. Max coverage (-): 0

Region: chr17 67224992-67225005. Max. coverage (+): 0. Max coverage (-): 0

Region: chr17 67225006-67225019. Max. coverage (+): 0. Max coverage (-): 0

Region: chr17 67225020-67225034. Max. coverage (+): 0. Max coverage (-): 0

Region: chr17 67225035-67225048. Max. coverage (+): 8.76. Max coverage (-): 0

Region: chr17 67225049-67225062. Max. coverage (+): 8.76. Max coverage (-): 0

Region: chr17 67225063-67225076. Max. coverage (+): 4.31. Max coverage (-): 0

Region: chr17 67225077-67225091. Max. coverage (+): 0. Max coverage (-): 0

Region: chr17 67225092-67225105. Max. coverage (+): 0. Max coverage (-): 0

Region: chr17 67225106-67225119. Max. coverage (+): 2.99. Max coverage (-): 0

Region: chr17 67225120-67225133. Max. coverage (+): 2.99. Max coverage (-): 0

Region: chr17 67225134-67225148. Max. coverage (+): 0. Max coverage (-): 0

Region: chr17 67225149-67225162. Max. coverage (+): 0. Max coverage (-): 0

Region: chr17 67225163-67225176. Max. coverage (+): 0. Max coverage (-): 0

Region: chr17 67225177-67225190. Max. coverage (+): 0. Max coverage (-): 0

Region: chr17 67225191-67225205. Max. coverage (+): 0. Max coverage (-): 0

Region: chr17 67225206-67225219. Max. coverage (+): 0. Max coverage (-): 0

Region: chr17 67225220-67225233. Max. coverage (+): 0. Max coverage (-): 0

Region: chr17 67225234-67225248. Max. coverage (+): 0. Max coverage (-): 0

Region: chr17 67225249-67225262. Max. coverage (+): 0. Max coverage (-): 0

Region: chr17 67225263-67225276. Max. coverage (+): 6.29. Max coverage (-): 0

Region: chr17 67225277-67225290. Max. coverage (+): 8.37. Max coverage (-): 0

Region: chr17 67225291-67225305. Max. coverage (+): 0.71. Max coverage (-): 0

Region: chr17 67225306-67225319. Max. coverage (+): 0.43. Max coverage (-): 0

Region: chr17 67225320-67225333. Max. coverage (+): 0.43. Max coverage (-): 0

Region: chr17 67225334-67225347. Max. coverage (+): 0. Max coverage (-): 0

Region: chr17 67225348-67225362. Max. coverage (+): 0. Max coverage (-): 0

Region: chr17 67225363-67225376. Max. coverage (+): 0. Max coverage (-): 0

Region: chr17 67225377-67225390. Max. coverage (+): 7.44. Max coverage (-): 0

Region: chr17 67225391-67225404. Max. coverage (+): 7.44. Max coverage (-): 0

Region: chr17 67225405-67225419. Max. coverage (+): 6.25. Max coverage (-): 0

Region: chr17 67225420-67225433. Max. coverage (+): 1.7. Max coverage (-): 0

Region: chr17 67225434-67225447. Max. coverage (+): 4.66. Max coverage (-): 0

Region: chr17 67225448-67225462. Max. coverage (+): 4.89. Max coverage (-): 0

Region: chr17 67225463-67225476. Max. coverage (+): 1.73. Max coverage (-): 0

Region: chr17 67225477-67225490. Max. coverage (+): 5.04. Max coverage (-): 0

Region: chr17 67225491-67225504. Max. coverage (+): 3.88. Max coverage (-): 0

Region: chr17 67225505-67225519. Max. coverage (+): 0.97. Max coverage (-): 0

Region: chr17 67225520-67225533. Max. coverage (+): 2.17. Max coverage (-): 0

Region: chr17 67225534-67225547. Max. coverage (+): 0. Max coverage (-): 0

Region: chr17 67225548-67225561. Max. coverage (+): 4.65. Max coverage (-): 0

Region: chr17 67225562-67225576. Max. coverage (+): 2.1. Max coverage (-): 0

Region: chr17 67225577-67225590. Max. coverage (+): 0. Max coverage (-): 0

Region: chr17 67225591-67225604. Max. coverage (+): 0. Max coverage (-): 0

Region: chr17 67225605-67225618. Max. coverage (+): 0. Max coverage (-): 0

Region: chr17 67225619-67225633. Max. coverage (+): 0. Max coverage (-): 0

Region: chr17 67225634-67225647. Max. coverage (+): 0. Max coverage (-): 0

Region: chr17 67225648-67225661. Max. coverage (+): 0. Max coverage (-): 0

Region: chr17 67225662-67225676. Max. coverage (+): 0. Max coverage (-): 0

Region: chr17 67225677-67225690. Max. coverage (+): 0. Max coverage (-): 0

Region: chr17 67225691-67225704. Max. coverage (+): 0. Max coverage (-): 0

Region: chr17 67225705-67225718. Max. coverage (+): 0. Max coverage (-): 0

Region: chr17 67225719-67225733. Max. coverage (+): 0. Max coverage (-): 0

Region: chr17 67225734-67225747. Max. coverage (+): 0. Max coverage (-): 0

Region: chr17 67225748-67225761. Max. coverage (+): 0. Max coverage (-): 0

Region: chr17 67225762-67225775. Max. coverage (+): 0. Max coverage (-): 0

Region: chr17 67225776-67225790. Max. coverage (+): 0. Max coverage (-): 0

Region: chr17 67225791-67225804. Max. coverage (+): 0. Max coverage (-): 0

Region: chr17 67225805-67225818. Max. coverage (+): 0. Max coverage (-): 0

Region: chr17 67225819-67225833. Max. coverage (+): 0. Max coverage (-): 0

Region: chr17 67225834-67225847. Max. coverage (+): 0. Max coverage (-): 0

Region: chr17 67225848-67225861. Max. coverage (+): 5.9. Max coverage (-): 0

Region: chr17 67225862-67225875. Max. coverage (+): 0. Max coverage (-): 0

Region: chr17 67225876-67225890. Max. coverage (+): 0. Max coverage (-): 0

Region: chr17 67225891-67225904. Max. coverage (+): 0. Max coverage (-): 0

Region: chr17 67225905-67225918. Max. coverage (+): 1.62. Max coverage (-): 0

Region: chr17 67225919-67225932. Max. coverage (+): 22.31. Max coverage (-): 0

Region: chr17 67225933-67225947. Max. coverage (+): 8.21. Max coverage (-): 0

Region: chr17 67225948-67225961. Max. coverage (+): 6.98. Max coverage (-): 0

Region: chr17 67225962-67225975. Max. coverage (+): 2.1. Max coverage (-): 0

Region: chr17 67225976-67225989. Max. coverage (+): 0. Max coverage (-): 0

Region: chr17 67225990-67226004. Max. coverage (+): 1.44. Max coverage (-): 0

Region: chr17 67226005-67226018. Max. coverage (+): 1.44. Max coverage (-): 0

Region: chr17 67226019-67226032. Max. coverage (+): 0. Max coverage (-): 0

Region: chr17 67226033-67226047. Max. coverage (+): 17.76. Max coverage (-): 0

Region: chr17 67226048-67226061. Max. coverage (+): 17.76. Max coverage (-): 0

Region: chr17 67226062-67226075. Max. coverage (+): 6.74. Max coverage (-): 0

Region: chr17 67226076-67226089. Max. coverage (+): 4.29. Max coverage (-): 0

Region: chr17 67226090-67226104. Max. coverage (+): 0. Max coverage (-): 0

Region: chr17 67226105-67226118. Max. coverage (+): 0. Max coverage (-): 0

Region: chr17 67226119-67226132. Max. coverage (+): 4.92. Max coverage (-): 0

Region: chr17 67226133-67226146. Max. coverage (+): 0. Max coverage (-): 0

Region: chr17 67226147-67226161. Max. coverage (+): 0. Max coverage (-): 0

Region: chr17 67226162-67226175. Max. coverage (+): 0. Max coverage (-): 0

Region: chr17 67226176-67226189. Max. coverage (+): 0. Max coverage (-): 0

Region: chr17 67226190-67226203. Max. coverage (+): 0. Max coverage (-): 0

Region: chr17 67226204-67226218. Max. coverage (+): 5.79. Max coverage (-): 0

Region: chr17 67226219-67226232. Max. coverage (+): 1.96. Max coverage (-): 0

Region: chr17 67226233-67226246. Max. coverage (+): 1.09. Max coverage (-): 0

Region: chr17 67226247-67226261. Max. coverage (+): 0. Max coverage (-): 0

Region: chr17 67226262-67226275. Max. coverage (+): 0. Max coverage (-): 0

Region: chr17 67226276-67226289. Max. coverage (+): 0. Max coverage (-): 0

Region: chr17 67226290-67226303. Max. coverage (+): 0. Max coverage (-): 0

Region: chr17 67226304-67226318. Max. coverage (+): 1.63. Max coverage (-): 0

Region: chr17 67226319-67226332. Max. coverage (+): 0. Max coverage (-): 0

Region: chr17 67226333-67226346. Max. coverage (+): 0. Max coverage (-): 0

Region: chr17 67226347-67226360. Max. coverage (+): 0. Max coverage (-): 0

Region: chr17 67226361-67226375. Max. coverage (+): 0. Max coverage (-): 0

Region: chr17 67226376-67226389. Max. coverage (+): 1.14. Max coverage (-): 0

Region: chr17 67226390-67226403. Max. coverage (+): 1.14. Max coverage (-): 0

Region: chr17 67226404-67226418. Max. coverage (+): 0. Max coverage (-): 0

Region: chr17 67226419-67226432. Max. coverage (+): 0. Max coverage (-): 0

Region: chr17 67226433-67226446. Max. coverage (+): 3.15. Max coverage (-): 0

Region: chr17 67226447-67226460. Max. coverage (+): 3.15. Max coverage (-): 0

Region: chr17 67226461-67226475. Max. coverage (+): 0. Max coverage (-): 0

Region: chr17 67226476-67226489. Max. coverage (+): 0. Max coverage (-): 0

Region: chr17 67226490-67226503. Max. coverage (+): 0. Max coverage (-): 0

Region: chr17 67226504-67226517. Max. coverage (+): 0. Max coverage (-): 0

Region: chr17 67226518-67226532. Max. coverage (+): 0. Max coverage (-): 0

Region: chr17 67226533-67226546. Max. coverage (+): 0. Max coverage (-): 0

Region: chr17 67226547-67226560. Max. coverage (+): 0. Max coverage (-): 0

Region: chr17 67226561-67226574. Max. coverage (+): 1.05. Max coverage (-): 0

Region: chr17 67226575-67226589. Max. coverage (+): 1.05. Max coverage (-): 0

Region: chr17 67226590-67226603. Max. coverage (+): 0. Max coverage (-): 0

Region: chr17 67226604-67226617. Max. coverage (+): 0. Max coverage (-): 0

Region: chr17 67226618-67226632. Max. coverage (+): 0. Max coverage (-): 0

Region: chr17 67226633-67226646. Max. coverage (+): 0. Max coverage (-): 0

Region: chr17 67226647-67226660. Max. coverage (+): 0. Max coverage (-): 0

Region: chr17 67226661-67226674. Max. coverage (+): 0. Max coverage (-): 0

Region: chr17 67226675-67226689. Max. coverage (+): 0. Max coverage (-): 0

Region: chr17 67226690-67226703. Max. coverage (+): 0. Max coverage (-): 0

Region: chr17 67226704-67226717. Max. coverage (+): 0. Max coverage (-): 0

Region: chr17 67226718-67226731. Max. coverage (+): 0. Max coverage (-): 0

Region: chr17 67226732-67226746. Max. coverage (+): 11.47. Max coverage (-): 0

Region: chr17 67226747-67226760. Max. coverage (+): 0. Max coverage (-): 0

Region: chr17 67226761-67226774. Max. coverage (+): 0. Max coverage (-): 0

Region: chr17 67226775-67226788. Max. coverage (+): 1.36. Max coverage (-): 0

Region: chr17 67226789-67226803. Max. coverage (+): 1.98. Max coverage (-): 0

Region: chr17 67226804-67226817. Max. coverage (+): 0. Max coverage (-): 0

Region: chr17 67226818-67226831. Max. coverage (+): 0. Max coverage (-): 0

Region: chr17 67226832-67226846. Max. coverage (+): 0. Max coverage (-): 0

Region: chr17 67226847-67226860. Max. coverage (+): 2.36. Max coverage (-): 0

Region: chr17 67226861-67226874. Max. coverage (+): 2.8. Max coverage (-): 0

Region: chr17 67226875-67226888. Max. coverage (+): 3.65. Max coverage (-): 0

Region: chr17 67226889-67226903. Max. coverage (+): 3.65. Max coverage (-): 0

Region: chr17 67226904-67226917. Max. coverage (+): 0. Max coverage (-): 0

Region: chr17 67226918-67226931. Max. coverage (+): 1.1. Max coverage (-): 0

Region: chr17 67226932-67226945. Max. coverage (+): 0. Max coverage (-): 0

Region: chr17 67226946-67226960. Max. coverage (+): 2.88. Max coverage (-): 0

Region: chr17 67226961-67226974. Max. coverage (+): 2.88. Max coverage (-): 0

Region: chr17 67226975-67226988. Max. coverage (+): 0. Max coverage (-): 0

Region: chr17 67226989-67227002. Max. coverage (+): 0. Max coverage (-): 0

Region: chr17 67227003-67227017. Max. coverage (+): 0. Max coverage (-): 0

Region: chr17 67227018-67227031. Max. coverage (+): 0. Max coverage (-): 0

Region: chr17 67227032-67227045. Max. coverage (+): 0. Max coverage (-): 0

Region: chr17 67227046-67227060. Max. coverage (+): 2.25. Max coverage (-): 0

Region: chr17 67227061-67227074. Max. coverage (+): 0. Max coverage (-): 0

Region: chr17 67227075-67227088. Max. coverage (+): 0. Max coverage (-): 0

Region: chr17 67227089-67227102. Max. coverage (+): 0. Max coverage (-): 0

Region: chr17 67227103-67227117. Max. coverage (+): 0. Max coverage (-): 0

Region: chr17 67227118-67227131. Max. coverage (+): 0. Max coverage (-): 0

Region: chr17 67227132-67227145. Max. coverage (+): 0. Max coverage (-): 0

Region: chr17 67227146-67227159. Max. coverage (+): 0. Max coverage (-): 0

Region: chr17 67227160-67227174. Max. coverage (+): 0. Max coverage (-): 0

Region: chr17 67227175-67227188. Max. coverage (+): 0. Max coverage (-): 0

Region: chr17 67227189-67227202. Max. coverage (+): 0. Max coverage (-): 0

Region: chr17 67227203-67227217. Max. coverage (+): 0. Max coverage (-): 0

Region: chr17 67227218-67227231. Max. coverage (+): 0. Max coverage (-): 0

Region: chr17 67227232-67227245. Max. coverage (+): 0. Max coverage (-): 0

Region: chr17 67227246-67227259. Max. coverage (+): 0. Max coverage (-): 0

Region: chr17 67227260-67227274. Max. coverage (+): 0. Max coverage (-): 0

Region: chr17 67227275-67227288. Max. coverage (+): 0. Max coverage (-): 0

Region: chr17 67227289-67227302. Max. coverage (+): 0. Max coverage (-): 0

Region: chr17 67227303-67227316. Max. coverage (+): 0. Max coverage (-): 0

Region: chr17 67227317-67227331. Max. coverage (+): 0. Max coverage (-): 0

Region: chr17 67227332-67227345. Max. coverage (+): 0. Max coverage (-): 0

Region: chr17 67227346-67227359. Max. coverage (+): 1.24. Max coverage (-): 0

Region: chr17 67227360-67227373. Max. coverage (+): 0. Max coverage (-): 0

Region: chr17 67227374-67227388. Max. coverage (+): 0. Max coverage (-): 0

Region: chr17 67227389-67227402. Max. coverage (+): 0. Max coverage (-): 0

Region: chr17 67227403-67227416. Max. coverage (+): 0. Max coverage (-): 0

Region: chr17 67227417-67227431. Max. coverage (+): 0. Max coverage (-): 0

Region: chr17 67227432-67227445. Max. coverage (+): 0. Max coverage (-): 0

Region: chr17 67227446-67227459. Max. coverage (+): 0. Max coverage (-): 0

Region: chr17 67227460-67227473. Max. coverage (+): 0. Max coverage (-): 0

Region: chr17 67227474-67227488. Max. coverage (+): 0. Max coverage (-): 0

Region: chr17 67227489-67227502. Max. coverage (+): 0. Max coverage (-): 0

Region: chr17 67227503-67227516. Max. coverage (+): 0. Max coverage (-): 0

Region: chr17 67227517-67227530. Max. coverage (+): 0. Max coverage (-): 0

Region: chr17 67227531-67227545. Max. coverage (+): 0. Max coverage (-): 0

Region: chr17 67227546-67227559. Max. coverage (+): 0. Max coverage (-): 0

Region: chr17 67227560-67227573. Max. coverage (+): 0. Max coverage (-): 0

Region: chr17 67227574-67227587. Max. coverage (+): 0. Max coverage (-): 0

Region: chr17 67227588-67227602. Max. coverage (+): 0. Max coverage (-): 0

Region: chr17 67227603-67227616. Max. coverage (+): 0. Max coverage (-): 0

Region: chr17 67227617-67227630. Max. coverage (+): 0. Max coverage (-): 0

Region: chr17 67227631-67227645. Max. coverage (+): 0. Max coverage (-): 0

Region: chr17 67227646-67227659. Max. coverage (+): 0. Max coverage (-): 0

Region: chr17 67227660-67227673. Max. coverage (+): 0. Max coverage (-): 0

Region: chr17 67227674-67227687. Max. coverage (+): 0. Max coverage (-): 0

Region: chr17 67227688-67227702. Max. coverage (+): 0. Max coverage (-): 0

Region: chr17 67227703-67227716. Max. coverage (+): 0. Max coverage (-): 0

Region: chr17 67227717-67227730. Max. coverage (+): 0. Max coverage (-): 0

Region: chr17 67227731-67227744. Max. coverage (+): 0. Max coverage (-): 0

Region: chr17 67227745-67227759. Max. coverage (+): 0. Max coverage (-): 0

Region: chr17 67227760-67227773. Max. coverage (+): 0. Max coverage (-): 0

Region: chr17 67227774-67227787. Max. coverage (+): 0. Max coverage (-): 0

Region: chr17 67227788-67227801. Max. coverage (+): 0. Max coverage (-): 0

Region: chr17 67227802-67227816. Max. coverage (+): 0. Max coverage (-): 0

Region: chr17 67227817-67227830. Max. coverage (+): 0. Max coverage (-): 0

Region: chr17 67227831-67227844. Max. coverage (+): 0. Max coverage (-): 0

Region: chr17 67227845-67227859. Max. coverage (+): 0. Max coverage (-): 0

Region: chr17 67227860-67227873. Max. coverage (+): 0. Max coverage (-): 0

Region: chr17 67227874-67227887. Max. coverage (+): 0. Max coverage (-): 0

Region: chr17 67227888-67227901. Max. coverage (+): 0. Max coverage (-): 0

Region: chr17 67227902-67227916. Max. coverage (+): 0. Max coverage (-): 0

Region: chr17 67227917-67227930. Max. coverage (+): 0. Max coverage (-): 0

Region: chr17 67227931-67227944. Max. coverage (+): 0. Max coverage (-): 0

Region: chr17 67227945-67227958. Max. coverage (+): 0. Max coverage (-): 0

Region: chr17 67227959-67227973. Max. coverage (+): 0. Max coverage (-): 0

Region: chr17 67227974-67227987. Max. coverage (+): 0. Max coverage (-): 0

Region: chr17 67227988-67228001. Max. coverage (+): 0. Max coverage (-): 0

Region: chr17 67228002-67228016. Max. coverage (+): 0. Max coverage (-): 0

Region: chr17 67228017-67228030. Max. coverage (+): 0. Max coverage (-): 0

Region: chr17 67228031-67228044. Max. coverage (+): 0. Max coverage (-): 0

Region: chr17 67228045-67228058. Max. coverage (+): 0. Max coverage (-): 0

Region: chr17 67228059-67228073. Max. coverage (+): 0. Max coverage (-): 0

Region: chr17 67228074-67228087. Max. coverage (+): 0. Max coverage (-): 0

Region: chr17 67228088-67228101. Max. coverage (+): 0. Max coverage (-): 0

Region: chr17 67228102-67228115. Max. coverage (+): 0. Max coverage (-): 0

Region: chr17 67228116-67228130. Max. coverage (+): 0. Max coverage (-): 0

Region: chr17 67228131-67228144. Max. coverage (+): 0. Max coverage (-): 0

Region: chr17 67228145-67228158. Max. coverage (+): 0. Max coverage (-): 0

Region: chr17 67228159-67228172. Max. coverage (+): 0. Max coverage (-): 0

Region: chr17 67228173-67228187. Max. coverage (+): 0. Max coverage (-): 0

Region: chr17 67228188-67228201. Max. coverage (+): 0. Max coverage (-): 0

Region: chr17 67228202-67228215. Max. coverage (+): 0. Max coverage (-): 0

Region: chr17 67228216-67228230. Max. coverage (+): 12. Max coverage (-): 0

Region: chr17 67228231-67228244. Max. coverage (+): 12. Max coverage (-): 0

Region: chr17 67228245-67228258. Max. coverage (+): 0. Max coverage (-): 0

Region: chr17 67228259-67228272. Max. coverage (+): 0. Max coverage (-): 0

Region: chr17 67228273-67228287. Max. coverage (+): 0. Max coverage (-): 0

Region: chr17 67228288-67228301. Max. coverage (+): 0. Max coverage (-): 0

Region: chr17 67228302-67228315. Max. coverage (+): 0. Max coverage (-): 0

Region: chr17 67228316-67228329. Max. coverage (+): 0. Max coverage (-): 0

Region: chr17 67228330-67228344. Max. coverage (+): 0. Max coverage (-): 0

Region: chr17 67228345-67228358. Max. coverage (+): 0. Max coverage (-): 0

Region: chr17 67228359-67228372. Max. coverage (+): 0. Max coverage (-): 0

Region: chr17 67228373-67228386. Max. coverage (+): 0. Max coverage (-): 0

Region: chr17 67228387-67228401. Max. coverage (+): 0. Max coverage (-): 0

Region: chr17 67228402-67228415. Max. coverage (+): 0. Max coverage (-): 0

Region: chr17 67228416-67228429. Max. coverage (+): 0. Max coverage (-): 0

Region: chr17 67228430-67228444. Max. coverage (+): 0. Max coverage (-): 0

Region: chr17 67228445-67228458. Max. coverage (+): 0. Max coverage (-): 0

Region: chr17 67228459-67228472. Max. coverage (+): 0. Max coverage (-): 0

Region: chr17 67228473-67228486. Max. coverage (+): 0. Max coverage (-): 0

Region: chr17 67228487-67228501. Max. coverage (+): 0. Max coverage (-): 0

Region: chr17 67228502-67228515. Max. coverage (+): 0. Max coverage (-): 0

Region: chr17 67228516-67228529. Max. coverage (+): 0. Max coverage (-): 0

Region: chr17 67228530-67228543. Max. coverage (+): 0. Max coverage (-): 0

Region: chr17 67228544-67228558. Max. coverage (+): 0. Max coverage (-): 0

Region: chr17 67228559-67228572. Max. coverage (+): 0. Max coverage (-): 0

Region: chr17 67228573-67228586. Max. coverage (+): 0. Max coverage (-): 0

Region: chr17 67228587-67228601. Max. coverage (+): 0. Max coverage (-): 0

Region: chr17 67228602-67228615. Max. coverage (+): 0. Max coverage (-): 0

Region: chr17 67228616-67228629. Max. coverage (+): 0. Max coverage (-): 0

Region: chr17 67228630-67228643. Max. coverage (+): 0. Max coverage (-): 0

Region: chr17 67228644-67228658. Max. coverage (+): 0. Max coverage (-): 0

Region: chr17 67228659-67228672. Max. coverage (+): 0. Max coverage (-): 0

Region: chr17 67228673-67228686. Max. coverage (+): 0. Max coverage (-): 0

Region: chr17 67228687-67228700. Max. coverage (+): 0. Max coverage (-): 0

Region: chr17 67228701-67228715. Max. coverage (+): 0. Max coverage (-): 0

Region: chr17 67228716-67228729. Max. coverage (+): 0. Max coverage (-): 0

Region: chr17 67228730-67228743. Max. coverage (+): 0. Max coverage (-): 0

Region: chr17 67228744-67228757. Max. coverage (+): 0. Max coverage (-): 0

Region: chr17 67228758-67228772. Max. coverage (+): 0. Max coverage (-): 0

Region: chr17 67228773-67228786. Max. coverage (+): 0. Max coverage (-): 0

Region: chr17 67228787-67228800. Max. coverage (+): 0. Max coverage (-): 0

Region: chr17 67228801-67228815. Max. coverage (+): 0. Max coverage (-): 0

Region: chr17 67228816-67228829. Max. coverage (+): 0. Max coverage (-): 0

Region: chr17 67228830-67228843. Max. coverage (+): 0. Max coverage (-): 0

Region: chr17 67228844-67228857. Max. coverage (+): 0. Max coverage (-): 0

Region: chr17 67228858-67228872. Max. coverage (+): 0. Max coverage (-): 0

Region: chr17 67228873-67228886. Max. coverage (+): 0. Max coverage (-): 0

Region: chr17 67228887-67228900. Max. coverage (+): 0. Max coverage (-): 0

Region: chr17 67228901-67228914. Max. coverage (+): 0. Max coverage (-): 0

Region: chr17 67228915-67228929. Max. coverage (+): 0. Max coverage (-): 0

Region: chr17 67228930-67228943. Max. coverage (+): 0. Max coverage (-): 0

Region: chr17 67228944-67228957. Max. coverage (+): 0. Max coverage (-): 0

Region: chr17 67228958-67228971. Max. coverage (+): 0. Max coverage (-): 0

Region: chr17 67228972-67228986. Max. coverage (+): 0. Max coverage (-): 0

Region: chr17 67228987-67229000. Max. coverage (+): 0. Max coverage (-): 0

Region: chr17 67229001-67229014. Max. coverage (+): 0. Max coverage (-): 0

Region: chr17 67229015-67229029. Max. coverage (+): 0. Max coverage (-): 0

Region: chr17 67229030-67229043. Max. coverage (+): 0. Max coverage (-): 0

Region: chr17 67229044-67229057. Max. coverage (+): 0. Max coverage (-): 0

Region: chr17 67229058-67229071. Max. coverage (+): 0. Max coverage (-): 0

Region: chr17 67229072-67229086. Max. coverage (+): 0. Max coverage (-): 0

Region: chr17 67229087-67229100. Max. coverage (+): 4.84. Max coverage (-): 0

Region: chr17 67229101-67229114. Max. coverage (+): 4.84. Max coverage (-): 0

Region: chr17 67229115-67229128. Max. coverage (+): 0. Max coverage (-): 0

Region: chr17 67229129-67229143. Max. coverage (+): 0. Max coverage (-): 0

Region: chr17 67229144-67229157. Max. coverage (+): 0. Max coverage (-): 0

Region: chr17 67229158-67229171. Max. coverage (+): 0. Max coverage (-): 0

Region: chr17 67229172-67229185. Max. coverage (+): 0. Max coverage (-): 0

Region: chr17 67229186-67229200. Max. coverage (+): 0. Max coverage (-): 0

Region: chr17 67229201-67229214. Max. coverage (+): 0. Max coverage (-): 0

Region: chr17 67229215-67229228. Max. coverage (+): 0. Max coverage (-): 0

Region: chr17 67229229-67229243. Max. coverage (+): 0. Max coverage (-): 0

Region: chr17 67229244-67229257. Max. coverage (+): 0. Max coverage (-): 0

Region: chr17 67229258-67229271. Max. coverage (+): 0. Max coverage (-): 0

Region: chr17 67229272-67229285. Max. coverage (+): 0. Max coverage (-): 0

Region: chr17 67229286-67229300. Max. coverage (+): 0. Max coverage (-): 0

Region: chr17 67229301-67229314. Max. coverage (+): 0. Max coverage (-): 0

Region: chr17 67229315-67229328. Max. coverage (+): 0. Max coverage (-): 0

Region: chr17 67229329-67229342. Max. coverage (+): 0. Max coverage (-): 0

Region: chr17 67229343-67229357. Max. coverage (+): 0. Max coverage (-): 0

Region: chr17 67229358-67229371. Max. coverage (+): 0. Max coverage (-): 0

Region: chr17 67229372-67229385. Max. coverage (+): 0. Max coverage (-): 0

Region: chr17 67229386-67229400. Max. coverage (+): 0. Max coverage (-): 0

Region: chr17 67229401-67229414. Max. coverage (+): 0. Max coverage (-): 0

Region: chr17 67229415-67229428. Max. coverage (+): 0. Max coverage (-): 0

Region: chr17 67229429-67229442. Max. coverage (+): 0. Max coverage (-): 0

Region: chr17 67229443-67229457. Max. coverage (+): 0. Max coverage (-): 0

Region: chr17 67229458-67229471. Max. coverage (+): 0. Max coverage (-): 0

Region: chr17 67229472-67229485. Max. coverage (+): 0. Max coverage (-): 0

Region: chr17 67229486-67229499. Max. coverage (+): 4.91. Max coverage (-): 0

Region: chr17 67229500-67229514. Max. coverage (+): 4.91. Max coverage (-): 0

Region: chr17 67229515-67229528. Max. coverage (+): 0. Max coverage (-): 0

Region: chr17 67229529-67229542. Max. coverage (+): 0. Max coverage (-): 0

Region: chr17 67229543-67229556. Max. coverage (+): 0. Max coverage (-): 0

Region: chr17 67229557-67229571. Max. coverage (+): 0. Max coverage (-): 0

Region: chr17 67229572-67229585. Max. coverage (+): 0. Max coverage (-): 0

Region: chr17 67229586-67229599. Max. coverage (+): 0. Max coverage (-): 0

Region: chr17 67229600-67229614. Max. coverage (+): 0. Max coverage (-): 0

Region: chr17 67229615-67229628. Max. coverage (+): 0. Max coverage (-): 0

Region: chr17 67229629-67229642. Max. coverage (+): 0. Max coverage (-): 0

Region: chr17 67229643-67229656. Max. coverage (+): 0. Max coverage (-): 0

Region: chr17 67229657-67229671. Max. coverage (+): 0. Max coverage (-): 0

Region: chr17 67229672-67229685. Max. coverage (+): 0. Max coverage (-): 0

Region: chr17 67229686-67229699. Max. coverage (+): 0. Max coverage (-): 0

Region: chr17 67229700-67229713. Max. coverage (+): 0. Max coverage (-): 0

Region: chr17 67229714-67229728. Max. coverage (+): 0. Max coverage (-): 0

Region: chr17 67229729-67229742. Max. coverage (+): 0. Max coverage (-): 0

Region: chr17 67229743-67229756. Max. coverage (+): 0. Max coverage (-): 0

Region: chr17 67229757-67229770. Max. coverage (+): 0. Max coverage (-): 0

Region: chr17 67229771-67229785. Max. coverage (+): 0. Max coverage (-): 0

Region: chr17 67229786-67229799. Max. coverage (+): 0. Max coverage (-): 0

Region: chr17 67229800-67229813. Max. coverage (+): 0. Max coverage (-): 0

Region: chr17 67229814-67229828. Max. coverage (+): 0. Max coverage (-): 0

Region: chr17 67229829-67229842. Max. coverage (+): 0. Max coverage (-): 0

Region: chr17 67229843-67229856. Max. coverage (+): 0. Max coverage (-): 0

Region: chr17 67229857-67229870. Max. coverage (+): 0. Max coverage (-): 0

Region: chr17 67229871-67229885. Max. coverage (+): 0. Max coverage (-): 0

Region: chr17 67229886-67229899. Max. coverage (+): 0. Max coverage (-): 0

Region: chr17 67229900-67229913. Max. coverage (+): 0. Max coverage (-): 0

Region: chr17 67229914-67229927. Max. coverage (+): 0. Max coverage (-): 0

Region: chr17 67229928-67229942. Max. coverage (+): 0. Max coverage (-): 0

Region: chr17 67229943-67229956. Max. coverage (+): 0. Max coverage (-): 0

Region: chr17 67229957-67229970. Max. coverage (+): 0. Max coverage (-): 0

Region: chr17 67229971-67229985. Max. coverage (+): 0. Max coverage (-): 0

Region: chr17 67229986-67229999. Max. coverage (+): 0. Max coverage (-): 0

Region: chr17 67230000-67230013. Max. coverage (+): 0. Max coverage (-): 0

Region: chr17 67230014-67230027. Max. coverage (+): 0. Max coverage (-): 0

Region: chr17 67230028-67230042. Max. coverage (+): 0. Max coverage (-): 0

Region: chr17 67230043-67230056. Max. coverage (+): 0. Max coverage (-): 0

Region: chr17 67230057-67230070. Max. coverage (+): 0. Max coverage (-): 0

Region: chr17 67230071-67230084. Max. coverage (+): 0. Max coverage (-): 0

Region: chr17 67230085-67230099. Max. coverage (+): 0. Max coverage (-): 0

Region: chr17 67230100-67230113. Max. coverage (+): 0. Max coverage (-): 0

Region: chr17 67230114-67230127. Max. coverage (+): 0. Max coverage (-): 0

Region: chr17 67230128-67230141. Max. coverage (+): 0. Max coverage (-): 0

Region: chr17 67230142-67230156. Max. coverage (+): 0. Max coverage (-): 0

Region: chr17 67230157-67230170. Max. coverage (+): 0. Max coverage (-): 0

Region: chr17 67230171-67230184. Max. coverage (+): 0. Max coverage (-): 0

Region: chr17 67230185-67230199. Max. coverage (+): 0. Max coverage (-): 0

Region: chr17 67230200-67230213. Max. coverage (+): 0. Max coverage (-): 0

Region: chr17 67230214-67230227. Max. coverage (+): 0. Max coverage (-): 0

Region: chr17 67230228-67230241. Max. coverage (+): 1.39. Max coverage (-): 0

Region: chr17 67230242-67230256. Max. coverage (+): 0. Max coverage (-): 0

Region: chr17 67230257-67230270. Max. coverage (+): 0. Max coverage (-): 0

Region: chr17 67230271-67230284. Max. coverage (+): 0. Max coverage (-): 0

Region: chr17 67230285-67230298. Max. coverage (+): 0. Max coverage (-): 0

Region: chr17 67230299-67230313. Max. coverage (+): 0. Max coverage (-): 0

Region: chr17 67230314-67230327. Max. coverage (+): 0. Max coverage (-): 0

Region: chr17 67230328-67230341. Max. coverage (+): 0. Max coverage (-): 0

Region: chr17 67230342-67230355. Max. coverage (+): 0. Max coverage (-): 0

Region: chr17 67230356-67230370. Max. coverage (+): 0. Max coverage (-): 0

Region: chr17 67230371-67230384. Max. coverage (+): 0. Max coverage (-): 0

Region: chr17 67230385-67230398. Max. coverage (+): 0. Max coverage (-): 0

Region: chr17 67230399-67230413. Max. coverage (+): 0. Max coverage (-): 0

Region: chr17 67230414-67230427. Max. coverage (+): 0. Max coverage (-): 0

Region: chr17 67230428-67230441. Max. coverage (+): 0. Max coverage (-): 0

Region: chr17 67230442-67230455. Max. coverage (+): 0. Max coverage (-): 0

Region: chr17 67230456-67230470. Max. coverage (+): 0. Max coverage (-): 0

Region: chr17 67230471-67230484. Max. coverage (+): 0. Max coverage (-): 0

Region: chr17 67230485-67230498. Max. coverage (+): 0. Max coverage (-): 0

Region: chr17 67230499-67230512. Max. coverage (+): 0. Max coverage (-): 0

Region: chr17 67230513-67230527. Max. coverage (+): 0. Max coverage (-): 0

Region: chr17 67230528-67230541. Max. coverage (+): 0. Max coverage (-): 0

Region: chr17 67230542-67230555. Max. coverage (+): 0. Max coverage (-): 0

Region: chr17 67230556-67230569. Max. coverage (+): 0. Max coverage (-): 0

Region: chr17 67230570-67230584. Max. coverage (+): 0. Max coverage (-): 0

Region: chr17 67230585-67230598. Max. coverage (+): 0. Max coverage (-): 0

Region: chr17 67230599-67230612. Max. coverage (+): 0. Max coverage (-): 0

Region: chr17 67230613-67230627. Max. coverage (+): 0. Max coverage (-): 0

Region: chr17 67230628-67230641. Max. coverage (+): 0. Max coverage (-): 0

Region: chr17 67230642-67230655. Max. coverage (+): 2.27. Max coverage (-): 0

Region: chr17 67230656-67230669. Max. coverage (+): 0. Max coverage (-): 0

Region: chr17 67230670-. Max. coverage (+): 0. Max coverage (-): 0

RepeatMasker Color Code

**+**

100-98% Identity

<98-95% Identity

<95-90% Identity

<90-85% Identity

<85-80% Identity

<80-75% Identity

<75-70% Identity

<70% Identity

**-**

Gene Set Color Code

**+**

Gene

Pseudogene

**-**

Topology/Coverage Color Code

Coverage Plus Strand

Coverage Minus Strand

Mainstrand: Plus

Mainstrand: Minus

Complementary Strand

Flanking Region  
(if option -flank >0)

Gene Set Annotation  

**1. PIWIL3 (protein coding, ENSBTAG00000015526) Tr:00000020633 Ex:15**: 67227117-67227317 (-)  
**2. PIWIL3 (protein coding, ENSBTAG00000015526) Tr:00000020633 Ex:16**: 67226882-67226985 (-)  
**3. PIWIL3 (protein coding, ENSBTAG00000015526) Tr:00000020633 Ex:17**: 67225872-67225942 (-)  
**4. PIWIL3 (protein coding, ENSBTAG00000015526) Tr:00000020633 Ex:18**: 67225285-67225438 (-)  
**5. PIWIL3 (protein coding, ENSBTAG00000015526) Tr:00000020633 Ex:19**: 67224435-67224487 (-)  
**6. PIWIL3 (protein coding, ENSBTAG00000015526) Tr:00000020633 Ex:20**: 67224314-67224367 (-)  
**7. PIWIL3 (protein coding, ENSBTAG00000015526) Tr:00000020633 Ex:21**: 67224135-67224138 (-)

  
RepeatMasker Annotation  

**1. BOV-A2**: 67224597-67224718 (+), Divergence to consensus: 4.9%  
**2. MER20**: 67225590-67225772 (-), Divergence to consensus: 38%  
**3. MLT1E3**: 67226598-67226716 (+), Divergence to consensus: 38.9%  
**4. MIRb**: 67227660-67227766 (-), Divergence to consensus: 38.3%  
**5. Bov-tA2**: 67228018-67228220 (-), Divergence to consensus: 26.2%  
**6. L1MC4**: 67228343-67228512 (+), Divergence to consensus: 39%  
**7. Bov-tA1**: 67228548-67228757 (-), Divergence to consensus: 24.4%  
**8. Bov-tA1**: 67228863-67229079 (+), Divergence to consensus: 15.8%  
**9. BOV-A2**: 67229901-67230169 (-), Divergence to consensus: 3.8%  
**10. L3**: 67230313-67230423 (+), Divergence to consensus: 41.4%

  
Transcription Factor Binding Sites  

**SOX9** (Sequence: AACAATGG (-): 67229314)  
**Gata4** (Sequence: GTTATCT (+): 67224526)
