# Supplementary material for: Small RNA sequencing of cryopreserved semen from single bull revealed altered miRNAs and piRNAs expression between High- and Low-motile sperm populations
Source: BMC Genomics. 2017 Jan 4;18:14. doi: 10.1186/s12864-016-3394-7 (PMC5209821; doi:10.1186/s12864-016-3394-7)
Supplement: Additional file 3: — Details for each piRNA clusters found in High Motile (HM) sperm fraction. Genes, repeats, transposable elements and transcription factors binding sites falling within the cluster regions were reported. (ZIP 1896 kb) [file 12864_2016_3394_MOESM3_ESM.zip › 33.html]

piRNA cluster 33


Predicted piRNA cluster no. 33     previous   next
  

Show proTRAC run info
Hide proTRAC run info

================================= proTRAC ====================================  
VERSION: 2.1                                    LAST MODIFIED: 06. October 2015  
  
Please cite:  
Rosenkranz D, Zischler H. proTRAC - a software for probabilistic piRNA cluster  
detection, visualization and analysis. 2012. BMC Bioinformatics 13:5.  
  
and (for proTRAC 2.0 and later):  
Rosenkranz D, Rudloff S, Bastuck K, Ketting RF, Zischler H. Tupaia small RNAs  
provide insights into function and evolution of RNAi-based transposon defense  
in mammals. 2015. RNA 21(5):911-922.  
  
Contact:  
David Rosenkranz  
Institute of Anthropology, small RNA group  
Johannes Gutenberg University Mainz  
email: rosenkranz@uni-mainz.de  
  
You can find the latest proTRAC version at:  
http://sourceforge.net/projects/protrac/files  
http://www.smallRNAgroup-mainz.de/software  
==============================================================================  
  
PARAMETERS:  
Map file: .............../storage/core/barbara/genhome/smallRNA/fertility/Sample\_motile/pirna/Sample\_motile\_26-33\_collapsed.fa.no-dust.map.weighted-10000-1000-b-0  
Genome file: ............/storage/core/barbara/genhome/smallRNA/fertility/Sample\_all/pirna/bt\_311\_chrY.fa  
RepeatMasker annotation: /storage/genomes/bt\_umd31/GCF\_000003055.6\_Bos\_taurus\_UMD\_3.1.1\_repeatMasker\_chr.out  
GeneSet:................./storage/core/barbara/genhome/smallRNA/fertility/Sample\_all/pirna/full.gtf  
  
Significant (p<=0.01) hit density will be calculated based  
on observed hit distribution.  
  
Sliding window size: ........................................ 5000 bp  
Sliding window increament: .................................. 1000 bp  
Normalize each hit by number of genomic hits: ............... 1 [0=no/1=yes]  
Normalize each hit by number of sequence reads: ............. 1 [0=no/1=yes]  
Normalize values (-> per million mapped reads): ............. 1 [0=no/1=yes]  
Min. fraction of hits with 1T(U) or 10A: .................... 0.75  
Alternatively: Min. fraction of hits with 1T(U) and 10A: .... 0.5  
Min. fraction of hits with typical piRNA length: ............ 0.75  
Typical piRNA length: ....................................... 26-33 nt  
Min. size of a piRNA cluster: ............................... 5000 bp.  
Min. number of hits (absolute): ............................. 0  
Min. number of hits (normalized): ........................... 0  
Min. fraction of hits on the mainstrand: .................... 0.75  
Top fraction of mapped sequences (in terms of read counts): . 1%  
Top fraction accounts for max. n% of sequence reads: ........ 90%  
Min. fraction of hits on each arm of a bidirectional cluster: 0.1  
Output image file for each cluster: ......................... 0 [0=no/1=yes]  
Output html file for each cluster: .......................... 1 [0=no/1=yes]  
Output a summary table: ..................................... 1 [0=no/1=yes]  
Output a FASTA file for each cluster (piRNA sequences): ..... 1 [0=no/1=yes]  
Output a FASTA file comprising cluster sequences: ........... 1 [0=no/1=yes]  
Search DNA motifs in clusters: .............................. 1 [0=no/1=yes]  
Output flanking sequences: +/- .............................. 0 bp  
Output ~.pTi file: .......................................... 1 [0=no/1=yes]  
==============================================================================  
  
  
Genome size (without gaps): ............ 2678902517 bp  
Gaps (N/X/-): .......................... 53837044 bp  
Mapped reads: .......................... 658825247023  
Non-identical sequences: ............... 514171  
Genomic hits: .......................... 764233  
Significant densitiy of mapped reads: .. 12867599.5173724 reads/kb

Show proTRAC cluster info
Hide proTRAC cluster info

|  |  |
| --- | --- |
| Location | chr17 |
| Coordinates | 72230402-72243797 |
| Size [bp] | 13396 |
| Sequence hit loci | 359 |
| Mapped reads (normalized) | 469654472 |
| Mapped reads (normalized) per kb | 35059306.7 |
| Normalized reads with 1T (1U) | 83.4% |
| Normalized reads with 10A | 35.1% |
| Normalized reads with length 26-33 nt | 100% |
| Normalized reads on the main strand(s) | 97.9% |
| Predicted directionality | mono:plus |

100%

0%

1T (1U)  
reads

10A reads

26-33 nt  
reads

reads on mainstrand

**Either the amount of reads with 1T (1U) OR 10A has to exceed 75% (set with option: -1Tor10A)  
Alternatively the amount of reads with 1T (1U) AND 10A has to exceed 50% (set with option: -1Tand10A)  
Minimum amount of reads with preferred size is 75% (set with option: -pisize)  
Minimum amount of reads on the main strand(s) is 75% (set with option: -clstrand)**

Show read coverage
Hide read coverage

WHAT DO I SEE HERE?  
This chart shows the location of mapped sequence reads within a predicted piRNA cluster. The color refers to the number of genomic hits produced by the sequence read in question. A dark red bar indicates that this sequence read produces many other hits elsewhere in the genome. Many adjacent red or yellow bars can indicate the presence of a multi-copy element such as transposons or rRNA genes. A dark green bar indicates that this sequence read maps uniquely to this locus.

1 hit

2-5 hits

6-10 hits

11-20 hits

21-50 hits

51-100 hits

> 100 hits

chr17

72230402

72243797

Gene Set

RepeatMasker

Mapped  
Reads

30.11

plus strand

minus strand

30.11

Region: chr17 67230671-72230415. Max. coverage (+): 0. Max coverage (-): 1.32

Region: chr17 72230416-72230442. Max. coverage (+): 0. Max coverage (-): 1.12

Region: chr17 72230443-72230468. Max. coverage (+): 0. Max coverage (-): 0

Region: chr17 72230469-72230495. Max. coverage (+): 0. Max coverage (-): 0

Region: chr17 72230496-72230522. Max. coverage (+): 1.12. Max coverage (-): 0

Region: chr17 72230523-72230549. Max. coverage (+): 1.12. Max coverage (-): 0

Region: chr17 72230550-72230576. Max. coverage (+): 0. Max coverage (-): 0

Region: chr17 72230577-72230602. Max. coverage (+): 0. Max coverage (-): 0

Region: chr17 72230603-72230629. Max. coverage (+): 0. Max coverage (-): 0

Region: chr17 72230630-72230656. Max. coverage (+): 0. Max coverage (-): 0

Region: chr17 72230657-72230683. Max. coverage (+): 0. Max coverage (-): 0

Region: chr17 72230684-72230710. Max. coverage (+): 0. Max coverage (-): 0

Region: chr17 72230711-72230736. Max. coverage (+): 3.92. Max coverage (-): 0

Region: chr17 72230737-72230763. Max. coverage (+): 0. Max coverage (-): 0

Region: chr17 72230764-72230790. Max. coverage (+): 0. Max coverage (-): 0

Region: chr17 72230791-72230817. Max. coverage (+): 0. Max coverage (-): 0

Region: chr17 72230818-72230844. Max. coverage (+): 4.31. Max coverage (-): 0

Region: chr17 72230845-72230870. Max. coverage (+): 0. Max coverage (-): 0

Region: chr17 72230871-72230897. Max. coverage (+): 11.37. Max coverage (-): 7.16

Region: chr17 72230898-72230924. Max. coverage (+): 0.77. Max coverage (-): 0

Region: chr17 72230925-72230951. Max. coverage (+): 0.77. Max coverage (-): 0

Region: chr17 72230952-72230978. Max. coverage (+): 0. Max coverage (-): 0

Region: chr17 72230979-72231004. Max. coverage (+): 0. Max coverage (-): 0

Region: chr17 72231005-72231031. Max. coverage (+): 2. Max coverage (-): 0

Region: chr17 72231032-72231058. Max. coverage (+): 6.76. Max coverage (-): 0

Region: chr17 72231059-72231085. Max. coverage (+): 0. Max coverage (-): 0

Region: chr17 72231086-72231111. Max. coverage (+): 0. Max coverage (-): 0

Region: chr17 72231112-72231138. Max. coverage (+): 2.53. Max coverage (-): 0

Region: chr17 72231139-72231165. Max. coverage (+): 0. Max coverage (-): 0

Region: chr17 72231166-72231192. Max. coverage (+): 0. Max coverage (-): 0

Region: chr17 72231193-72231219. Max. coverage (+): 0. Max coverage (-): 0

Region: chr17 72231220-72231245. Max. coverage (+): 0. Max coverage (-): 0

Region: chr17 72231246-72231272. Max. coverage (+): 1.05. Max coverage (-): 0

Region: chr17 72231273-72231299. Max. coverage (+): 1.05. Max coverage (-): 0

Region: chr17 72231300-72231326. Max. coverage (+): 0. Max coverage (-): 0

Region: chr17 72231327-72231353. Max. coverage (+): 5.62. Max coverage (-): 0

Region: chr17 72231354-72231379. Max. coverage (+): 5.62. Max coverage (-): 0

Region: chr17 72231380-72231406. Max. coverage (+): 2.18. Max coverage (-): 0

Region: chr17 72231407-72231433. Max. coverage (+): 5.15. Max coverage (-): 0

Region: chr17 72231434-72231460. Max. coverage (+): 1.39. Max coverage (-): 0

Region: chr17 72231461-72231487. Max. coverage (+): 0. Max coverage (-): 0

Region: chr17 72231488-72231513. Max. coverage (+): 0. Max coverage (-): 0

Region: chr17 72231514-72231540. Max. coverage (+): 0. Max coverage (-): 0

Region: chr17 72231541-72231567. Max. coverage (+): 7.25. Max coverage (-): 0

Region: chr17 72231568-72231594. Max. coverage (+): 0. Max coverage (-): 0

Region: chr17 72231595-72231621. Max. coverage (+): 0. Max coverage (-): 1.8

Region: chr17 72231622-72231647. Max. coverage (+): 15.92. Max coverage (-): 1.8

Region: chr17 72231648-72231674. Max. coverage (+): 0. Max coverage (-): 0

Region: chr17 72231675-72231701. Max. coverage (+): 1.47. Max coverage (-): 0

Region: chr17 72231702-72231728. Max. coverage (+): 1.47. Max coverage (-): 0

Region: chr17 72231729-72231754. Max. coverage (+): 0. Max coverage (-): 0

Region: chr17 72231755-72231781. Max. coverage (+): 1.82. Max coverage (-): 3.3

Region: chr17 72231782-72231808. Max. coverage (+): 0. Max coverage (-): 0

Region: chr17 72231809-72231835. Max. coverage (+): 0. Max coverage (-): 0

Region: chr17 72231836-72231862. Max. coverage (+): 0. Max coverage (-): 0

Region: chr17 72231863-72231888. Max. coverage (+): 0. Max coverage (-): 0

Region: chr17 72231889-72231915. Max. coverage (+): 0. Max coverage (-): 0

Region: chr17 72231916-72231942. Max. coverage (+): 5.22. Max coverage (-): 0

Region: chr17 72231943-72231969. Max. coverage (+): 3.39. Max coverage (-): 0

Region: chr17 72231970-72231996. Max. coverage (+): 0.7. Max coverage (-): 0

Region: chr17 72231997-72232022. Max. coverage (+): 0. Max coverage (-): 0

Region: chr17 72232023-72232049. Max. coverage (+): 0. Max coverage (-): 0

Region: chr17 72232050-72232076. Max. coverage (+): 0. Max coverage (-): 0

Region: chr17 72232077-72232103. Max. coverage (+): 0. Max coverage (-): 0

Region: chr17 72232104-72232130. Max. coverage (+): 0. Max coverage (-): 0

Region: chr17 72232131-72232156. Max. coverage (+): 0. Max coverage (-): 0

Region: chr17 72232157-72232183. Max. coverage (+): 0. Max coverage (-): 0

Region: chr17 72232184-72232210. Max. coverage (+): 0. Max coverage (-): 0

Region: chr17 72232211-72232237. Max. coverage (+): 0. Max coverage (-): 0

Region: chr17 72232238-72232264. Max. coverage (+): 0. Max coverage (-): 0

Region: chr17 72232265-72232290. Max. coverage (+): 1.68. Max coverage (-): 0

Region: chr17 72232291-72232317. Max. coverage (+): 0. Max coverage (-): 0

Region: chr17 72232318-72232344. Max. coverage (+): 0. Max coverage (-): 0

Region: chr17 72232345-72232371. Max. coverage (+): 0. Max coverage (-): 0

Region: chr17 72232372-72232398. Max. coverage (+): 0. Max coverage (-): 0

Region: chr17 72232399-72232424. Max. coverage (+): 0. Max coverage (-): 0

Region: chr17 72232425-72232451. Max. coverage (+): 0. Max coverage (-): 0

Region: chr17 72232452-72232478. Max. coverage (+): 0. Max coverage (-): 0

Region: chr17 72232479-72232505. Max. coverage (+): 0. Max coverage (-): 0

Region: chr17 72232506-72232531. Max. coverage (+): 0. Max coverage (-): 0

Region: chr17 72232532-72232558. Max. coverage (+): 0.92. Max coverage (-): 0

Region: chr17 72232559-72232585. Max. coverage (+): 0. Max coverage (-): 0

Region: chr17 72232586-72232612. Max. coverage (+): 6.51. Max coverage (-): 0

Region: chr17 72232613-72232639. Max. coverage (+): 0. Max coverage (-): 0

Region: chr17 72232640-72232665. Max. coverage (+): 0. Max coverage (-): 0

Region: chr17 72232666-72232692. Max. coverage (+): 0. Max coverage (-): 0

Region: chr17 72232693-72232719. Max. coverage (+): 0. Max coverage (-): 0

Region: chr17 72232720-72232746. Max. coverage (+): 0. Max coverage (-): 0

Region: chr17 72232747-72232773. Max. coverage (+): 4.29. Max coverage (-): 0

Region: chr17 72232774-72232799. Max. coverage (+): 1.53. Max coverage (-): 0

Region: chr17 72232800-72232826. Max. coverage (+): 0. Max coverage (-): 0

Region: chr17 72232827-72232853. Max. coverage (+): 0. Max coverage (-): 0

Region: chr17 72232854-72232880. Max. coverage (+): 0. Max coverage (-): 0

Region: chr17 72232881-72232907. Max. coverage (+): 0. Max coverage (-): 0

Region: chr17 72232908-72232933. Max. coverage (+): 0. Max coverage (-): 0

Region: chr17 72232934-72232960. Max. coverage (+): 3.71. Max coverage (-): 0

Region: chr17 72232961-72232987. Max. coverage (+): 0. Max coverage (-): 0

Region: chr17 72232988-72233014. Max. coverage (+): 0. Max coverage (-): 0

Region: chr17 72233015-72233041. Max. coverage (+): 5.1. Max coverage (-): 0

Region: chr17 72233042-72233067. Max. coverage (+): 0. Max coverage (-): 0

Region: chr17 72233068-72233094. Max. coverage (+): 0. Max coverage (-): 0

Region: chr17 72233095-72233121. Max. coverage (+): 0. Max coverage (-): 0

Region: chr17 72233122-72233148. Max. coverage (+): 0.42. Max coverage (-): 0

Region: chr17 72233149-72233174. Max. coverage (+): 5.05. Max coverage (-): 0

Region: chr17 72233175-72233201. Max. coverage (+): 5.05. Max coverage (-): 0

Region: chr17 72233202-72233228. Max. coverage (+): 5.08. Max coverage (-): 0

Region: chr17 72233229-72233255. Max. coverage (+): 0. Max coverage (-): 0

Region: chr17 72233256-72233282. Max. coverage (+): 0. Max coverage (-): 0

Region: chr17 72233283-72233308. Max. coverage (+): 0. Max coverage (-): 0

Region: chr17 72233309-72233335. Max. coverage (+): 0. Max coverage (-): 0

Region: chr17 72233336-72233362. Max. coverage (+): 0. Max coverage (-): 0

Region: chr17 72233363-72233389. Max. coverage (+): 0. Max coverage (-): 0

Region: chr17 72233390-72233416. Max. coverage (+): 0. Max coverage (-): 0

Region: chr17 72233417-72233442. Max. coverage (+): 0. Max coverage (-): 0

Region: chr17 72233443-72233469. Max. coverage (+): 0. Max coverage (-): 0

Region: chr17 72233470-72233496. Max. coverage (+): 0. Max coverage (-): 0

Region: chr17 72233497-72233523. Max. coverage (+): 0. Max coverage (-): 0

Region: chr17 72233524-72233550. Max. coverage (+): 0. Max coverage (-): 0

Region: chr17 72233551-72233576. Max. coverage (+): 0. Max coverage (-): 0

Region: chr17 72233577-72233603. Max. coverage (+): 7.06. Max coverage (-): 0

Region: chr17 72233604-72233630. Max. coverage (+): 2.14. Max coverage (-): 0

Region: chr17 72233631-72233657. Max. coverage (+): 0. Max coverage (-): 0

Region: chr17 72233658-72233684. Max. coverage (+): 0. Max coverage (-): 0

Region: chr17 72233685-72233710. Max. coverage (+): 0. Max coverage (-): 0

Region: chr17 72233711-72233737. Max. coverage (+): 0. Max coverage (-): 0

Region: chr17 72233738-72233764. Max. coverage (+): 0. Max coverage (-): 0

Region: chr17 72233765-72233791. Max. coverage (+): 0. Max coverage (-): 0

Region: chr17 72233792-72233817. Max. coverage (+): 0. Max coverage (-): 0

Region: chr17 72233818-72233844. Max. coverage (+): 0. Max coverage (-): 0

Region: chr17 72233845-72233871. Max. coverage (+): 14.58. Max coverage (-): 0

Region: chr17 72233872-72233898. Max. coverage (+): 4.8. Max coverage (-): 0

Region: chr17 72233899-72233925. Max. coverage (+): 4.49. Max coverage (-): 0

Region: chr17 72233926-72233951. Max. coverage (+): 2.08. Max coverage (-): 0

Region: chr17 72233952-72233978. Max. coverage (+): 0. Max coverage (-): 0

Region: chr17 72233979-72234005. Max. coverage (+): 0. Max coverage (-): 0

Region: chr17 72234006-72234032. Max. coverage (+): 0. Max coverage (-): 0

Region: chr17 72234033-72234059. Max. coverage (+): 0.16. Max coverage (-): 0

Region: chr17 72234060-72234085. Max. coverage (+): 0. Max coverage (-): 0

Region: chr17 72234086-72234112. Max. coverage (+): 0. Max coverage (-): 0

Region: chr17 72234113-72234139. Max. coverage (+): 0. Max coverage (-): 0

Region: chr17 72234140-72234166. Max. coverage (+): 0. Max coverage (-): 0

Region: chr17 72234167-72234193. Max. coverage (+): 0. Max coverage (-): 0

Region: chr17 72234194-72234219. Max. coverage (+): 0. Max coverage (-): 0

Region: chr17 72234220-72234246. Max. coverage (+): 0. Max coverage (-): 0

Region: chr17 72234247-72234273. Max. coverage (+): 0. Max coverage (-): 0

Region: chr17 72234274-72234300. Max. coverage (+): 0. Max coverage (-): 0

Region: chr17 72234301-72234327. Max. coverage (+): 0. Max coverage (-): 0

Region: chr17 72234328-72234353. Max. coverage (+): 9.33. Max coverage (-): 0

Region: chr17 72234354-72234380. Max. coverage (+): 9.33. Max coverage (-): 0

Region: chr17 72234381-72234407. Max. coverage (+): 2.76. Max coverage (-): 0

Region: chr17 72234408-72234434. Max. coverage (+): 3.43. Max coverage (-): 0

Region: chr17 72234435-72234460. Max. coverage (+): 3.43. Max coverage (-): 0

Region: chr17 72234461-72234487. Max. coverage (+): 0. Max coverage (-): 0

Region: chr17 72234488-72234514. Max. coverage (+): 0. Max coverage (-): 0

Region: chr17 72234515-72234541. Max. coverage (+): 0. Max coverage (-): 0

Region: chr17 72234542-72234568. Max. coverage (+): 0. Max coverage (-): 0

Region: chr17 72234569-72234594. Max. coverage (+): 0. Max coverage (-): 0

Region: chr17 72234595-72234621. Max. coverage (+): 0. Max coverage (-): 0

Region: chr17 72234622-72234648. Max. coverage (+): 0. Max coverage (-): 0

Region: chr17 72234649-72234675. Max. coverage (+): 1.97. Max coverage (-): 0

Region: chr17 72234676-72234702. Max. coverage (+): 0. Max coverage (-): 0

Region: chr17 72234703-72234728. Max. coverage (+): 0. Max coverage (-): 0

Region: chr17 72234729-72234755. Max. coverage (+): 0. Max coverage (-): 0

Region: chr17 72234756-72234782. Max. coverage (+): 0. Max coverage (-): 0

Region: chr17 72234783-72234809. Max. coverage (+): 0. Max coverage (-): 0

Region: chr17 72234810-72234836. Max. coverage (+): 0. Max coverage (-): 0

Region: chr17 72234837-72234862. Max. coverage (+): 0. Max coverage (-): 0

Region: chr17 72234863-72234889. Max. coverage (+): 5.1. Max coverage (-): 0

Region: chr17 72234890-72234916. Max. coverage (+): 0. Max coverage (-): 0

Region: chr17 72234917-72234943. Max. coverage (+): 0. Max coverage (-): 0

Region: chr17 72234944-72234970. Max. coverage (+): 0. Max coverage (-): 0

Region: chr17 72234971-72234996. Max. coverage (+): 0. Max coverage (-): 0

Region: chr17 72234997-72235023. Max. coverage (+): 4.43. Max coverage (-): 0

Region: chr17 72235024-72235050. Max. coverage (+): 0. Max coverage (-): 0

Region: chr17 72235051-72235077. Max. coverage (+): 4.51. Max coverage (-): 0

Region: chr17 72235078-72235103. Max. coverage (+): 2.69. Max coverage (-): 0

Region: chr17 72235104-72235130. Max. coverage (+): 5. Max coverage (-): 0

Region: chr17 72235131-72235157. Max. coverage (+): 10.32. Max coverage (-): 0

Region: chr17 72235158-72235184. Max. coverage (+): 1.32. Max coverage (-): 0

Region: chr17 72235185-72235211. Max. coverage (+): 0. Max coverage (-): 0

Region: chr17 72235212-72235237. Max. coverage (+): 0. Max coverage (-): 0

Region: chr17 72235238-72235264. Max. coverage (+): 0. Max coverage (-): 0

Region: chr17 72235265-72235291. Max. coverage (+): 0. Max coverage (-): 0

Region: chr17 72235292-72235318. Max. coverage (+): 0. Max coverage (-): 0

Region: chr17 72235319-72235345. Max. coverage (+): 0. Max coverage (-): 0

Region: chr17 72235346-72235371. Max. coverage (+): 0. Max coverage (-): 0

Region: chr17 72235372-72235398. Max. coverage (+): 0. Max coverage (-): 0

Region: chr17 72235399-72235425. Max. coverage (+): 0. Max coverage (-): 0

Region: chr17 72235426-72235452. Max. coverage (+): 0. Max coverage (-): 0

Region: chr17 72235453-72235479. Max. coverage (+): 0. Max coverage (-): 0

Region: chr17 72235480-72235505. Max. coverage (+): 1.76. Max coverage (-): 0

Region: chr17 72235506-72235532. Max. coverage (+): 0. Max coverage (-): 0

Region: chr17 72235533-72235559. Max. coverage (+): 0. Max coverage (-): 0

Region: chr17 72235560-72235586. Max. coverage (+): 1.36. Max coverage (-): 0

Region: chr17 72235587-72235613. Max. coverage (+): 6.74. Max coverage (-): 0

Region: chr17 72235614-72235639. Max. coverage (+): 11.08. Max coverage (-): 0

Region: chr17 72235640-72235666. Max. coverage (+): 11.08. Max coverage (-): 0

Region: chr17 72235667-72235693. Max. coverage (+): 1.13. Max coverage (-): 0

Region: chr17 72235694-72235720. Max. coverage (+): 13.97. Max coverage (-): 0

Region: chr17 72235721-72235747. Max. coverage (+): 2.08. Max coverage (-): 0

Region: chr17 72235748-72235773. Max. coverage (+): 0. Max coverage (-): 0

Region: chr17 72235774-72235800. Max. coverage (+): 0. Max coverage (-): 0

Region: chr17 72235801-72235827. Max. coverage (+): 4.68. Max coverage (-): 0

Region: chr17 72235828-72235854. Max. coverage (+): 5.26. Max coverage (-): 0

Region: chr17 72235855-72235880. Max. coverage (+): 0. Max coverage (-): 0

Region: chr17 72235881-72235907. Max. coverage (+): 0. Max coverage (-): 0

Region: chr17 72235908-72235934. Max. coverage (+): 4.63. Max coverage (-): 0

Region: chr17 72235935-72235961. Max. coverage (+): 4.63. Max coverage (-): 0

Region: chr17 72235962-72235988. Max. coverage (+): 0.41. Max coverage (-): 0

Region: chr17 72235989-72236014. Max. coverage (+): 0. Max coverage (-): 0

Region: chr17 72236015-72236041. Max. coverage (+): 0. Max coverage (-): 0

Region: chr17 72236042-72236068. Max. coverage (+): 8.85. Max coverage (-): 0

Region: chr17 72236069-72236095. Max. coverage (+): 0. Max coverage (-): 0

Region: chr17 72236096-72236122. Max. coverage (+): 0. Max coverage (-): 0

Region: chr17 72236123-72236148. Max. coverage (+): 0. Max coverage (-): 0

Region: chr17 72236149-72236175. Max. coverage (+): 0. Max coverage (-): 0

Region: chr17 72236176-72236202. Max. coverage (+): 0. Max coverage (-): 0

Region: chr17 72236203-72236229. Max. coverage (+): 8.27. Max coverage (-): 0

Region: chr17 72236230-72236256. Max. coverage (+): 2.13. Max coverage (-): 0

Region: chr17 72236257-72236282. Max. coverage (+): 5.43. Max coverage (-): 0

Region: chr17 72236283-72236309. Max. coverage (+): 6.53. Max coverage (-): 0

Region: chr17 72236310-72236336. Max. coverage (+): 7.13. Max coverage (-): 0

Region: chr17 72236337-72236363. Max. coverage (+): 5.73. Max coverage (-): 0

Region: chr17 72236364-72236390. Max. coverage (+): 5.37. Max coverage (-): 0

Region: chr17 72236391-72236416. Max. coverage (+): 0. Max coverage (-): 0

Region: chr17 72236417-72236443. Max. coverage (+): 2.95. Max coverage (-): 0

Region: chr17 72236444-72236470. Max. coverage (+): 0. Max coverage (-): 0

Region: chr17 72236471-72236497. Max. coverage (+): 7.37. Max coverage (-): 0

Region: chr17 72236498-72236523. Max. coverage (+): 0. Max coverage (-): 0

Region: chr17 72236524-72236550. Max. coverage (+): 0. Max coverage (-): 0

Region: chr17 72236551-72236577. Max. coverage (+): 0. Max coverage (-): 0

Region: chr17 72236578-72236604. Max. coverage (+): 0. Max coverage (-): 0

Region: chr17 72236605-72236631. Max. coverage (+): 0. Max coverage (-): 0

Region: chr17 72236632-72236657. Max. coverage (+): 0. Max coverage (-): 0

Region: chr17 72236658-72236684. Max. coverage (+): 7.1. Max coverage (-): 0

Region: chr17 72236685-72236711. Max. coverage (+): 0. Max coverage (-): 0

Region: chr17 72236712-72236738. Max. coverage (+): 3.92. Max coverage (-): 0

Region: chr17 72236739-72236765. Max. coverage (+): 3.63. Max coverage (-): 0

Region: chr17 72236766-72236791. Max. coverage (+): 4.54. Max coverage (-): 0

Region: chr17 72236792-72236818. Max. coverage (+): 0. Max coverage (-): 0

Region: chr17 72236819-72236845. Max. coverage (+): 0. Max coverage (-): 0

Region: chr17 72236846-72236872. Max. coverage (+): 0. Max coverage (-): 0

Region: chr17 72236873-72236899. Max. coverage (+): 2.2. Max coverage (-): 0

Region: chr17 72236900-72236925. Max. coverage (+): 3.67. Max coverage (-): 0

Region: chr17 72236926-72236952. Max. coverage (+): 3.67. Max coverage (-): 0

Region: chr17 72236953-72236979. Max. coverage (+): 6.29. Max coverage (-): 0

Region: chr17 72236980-72237006. Max. coverage (+): 6.29. Max coverage (-): 0

Region: chr17 72237007-72237033. Max. coverage (+): 0. Max coverage (-): 0

Region: chr17 72237034-72237059. Max. coverage (+): 0. Max coverage (-): 0

Region: chr17 72237060-72237086. Max. coverage (+): 9.88. Max coverage (-): 0

Region: chr17 72237087-72237113. Max. coverage (+): 9.88. Max coverage (-): 0

Region: chr17 72237114-72237140. Max. coverage (+): 0. Max coverage (-): 0

Region: chr17 72237141-72237166. Max. coverage (+): 0. Max coverage (-): 0

Region: chr17 72237167-72237193. Max. coverage (+): 0. Max coverage (-): 0

Region: chr17 72237194-72237220. Max. coverage (+): 0. Max coverage (-): 0

Region: chr17 72237221-72237247. Max. coverage (+): 0. Max coverage (-): 0

Region: chr17 72237248-72237274. Max. coverage (+): 0. Max coverage (-): 0

Region: chr17 72237275-72237300. Max. coverage (+): 0. Max coverage (-): 0

Region: chr17 72237301-72237327. Max. coverage (+): 0. Max coverage (-): 0

Region: chr17 72237328-72237354. Max. coverage (+): 0. Max coverage (-): 0

Region: chr17 72237355-72237381. Max. coverage (+): 0. Max coverage (-): 0

Region: chr17 72237382-72237408. Max. coverage (+): 0. Max coverage (-): 0

Region: chr17 72237409-72237434. Max. coverage (+): 0. Max coverage (-): 0

Region: chr17 72237435-72237461. Max. coverage (+): 0. Max coverage (-): 0

Region: chr17 72237462-72237488. Max. coverage (+): 4.71. Max coverage (-): 0

Region: chr17 72237489-72237515. Max. coverage (+): 7.19. Max coverage (-): 0

Region: chr17 72237516-72237542. Max. coverage (+): 0. Max coverage (-): 0

Region: chr17 72237543-72237568. Max. coverage (+): 0. Max coverage (-): 0

Region: chr17 72237569-72237595. Max. coverage (+): 6.34. Max coverage (-): 0

Region: chr17 72237596-72237622. Max. coverage (+): 14.51. Max coverage (-): 0

Region: chr17 72237623-72237649. Max. coverage (+): 0. Max coverage (-): 0

Region: chr17 72237650-72237676. Max. coverage (+): 2.46. Max coverage (-): 0

Region: chr17 72237677-72237702. Max. coverage (+): 0. Max coverage (-): 0

Region: chr17 72237703-72237729. Max. coverage (+): 0. Max coverage (-): 0

Region: chr17 72237730-72237756. Max. coverage (+): 3.97. Max coverage (-): 0

Region: chr17 72237757-72237783. Max. coverage (+): 14.84. Max coverage (-): 0

Region: chr17 72237784-72237809. Max. coverage (+): 15. Max coverage (-): 0

Region: chr17 72237810-72237836. Max. coverage (+): 14.99. Max coverage (-): 0

Region: chr17 72237837-72237863. Max. coverage (+): 30.11. Max coverage (-): 0

Region: chr17 72237864-72237890. Max. coverage (+): 2.03. Max coverage (-): 0

Region: chr17 72237891-72237917. Max. coverage (+): 0. Max coverage (-): 0

Region: chr17 72237918-72237943. Max. coverage (+): 0. Max coverage (-): 0

Region: chr17 72237944-72237970. Max. coverage (+): 0. Max coverage (-): 0

Region: chr17 72237971-72237997. Max. coverage (+): 0. Max coverage (-): 0

Region: chr17 72237998-72238024. Max. coverage (+): 0. Max coverage (-): 0

Region: chr17 72238025-72238051. Max. coverage (+): 1.32. Max coverage (-): 0

Region: chr17 72238052-72238077. Max. coverage (+): 7.96. Max coverage (-): 0

Region: chr17 72238078-72238104. Max. coverage (+): 0. Max coverage (-): 0

Region: chr17 72238105-72238131. Max. coverage (+): 0. Max coverage (-): 0

Region: chr17 72238132-72238158. Max. coverage (+): 0. Max coverage (-): 0

Region: chr17 72238159-72238185. Max. coverage (+): 0. Max coverage (-): 0

Region: chr17 72238186-72238211. Max. coverage (+): 1.7. Max coverage (-): 0

Region: chr17 72238212-72238238. Max. coverage (+): 4.63. Max coverage (-): 0

Region: chr17 72238239-72238265. Max. coverage (+): 0. Max coverage (-): 0

Region: chr17 72238266-72238292. Max. coverage (+): 0. Max coverage (-): 0

Region: chr17 72238293-72238319. Max. coverage (+): 0. Max coverage (-): 0

Region: chr17 72238320-72238345. Max. coverage (+): 0. Max coverage (-): 0

Region: chr17 72238346-72238372. Max. coverage (+): 0. Max coverage (-): 0

Region: chr17 72238373-72238399. Max. coverage (+): 0. Max coverage (-): 0

Region: chr17 72238400-72238426. Max. coverage (+): 0. Max coverage (-): 0

Region: chr17 72238427-72238452. Max. coverage (+): 0. Max coverage (-): 0

Region: chr17 72238453-72238479. Max. coverage (+): 0. Max coverage (-): 0

Region: chr17 72238480-72238506. Max. coverage (+): 13.3. Max coverage (-): 0

Region: chr17 72238507-72238533. Max. coverage (+): 0. Max coverage (-): 0

Region: chr17 72238534-72238560. Max. coverage (+): 0. Max coverage (-): 0

Region: chr17 72238561-72238586. Max. coverage (+): 0. Max coverage (-): 0

Region: chr17 72238587-72238613. Max. coverage (+): 0. Max coverage (-): 0

Region: chr17 72238614-72238640. Max. coverage (+): 2.88. Max coverage (-): 0

Region: chr17 72238641-72238667. Max. coverage (+): 7.05. Max coverage (-): 0

Region: chr17 72238668-72238694. Max. coverage (+): 6.7. Max coverage (-): 0

Region: chr17 72238695-72238720. Max. coverage (+): 1.62. Max coverage (-): 0

Region: chr17 72238721-72238747. Max. coverage (+): 0. Max coverage (-): 0

Region: chr17 72238748-72238774. Max. coverage (+): 0. Max coverage (-): 0

Region: chr17 72238775-72238801. Max. coverage (+): 4.29. Max coverage (-): 0

Region: chr17 72238802-72238828. Max. coverage (+): 10.13. Max coverage (-): 0

Region: chr17 72238829-72238854. Max. coverage (+): 0. Max coverage (-): 0

Region: chr17 72238855-72238881. Max. coverage (+): 0. Max coverage (-): 0

Region: chr17 72238882-72238908. Max. coverage (+): 0. Max coverage (-): 0

Region: chr17 72238909-72238935. Max. coverage (+): 0. Max coverage (-): 0

Region: chr17 72238936-72238962. Max. coverage (+): 0. Max coverage (-): 0

Region: chr17 72238963-72238988. Max. coverage (+): 0. Max coverage (-): 0

Region: chr17 72238989-72239015. Max. coverage (+): 0. Max coverage (-): 0

Region: chr17 72239016-72239042. Max. coverage (+): 0. Max coverage (-): 0

Region: chr17 72239043-72239069. Max. coverage (+): 0. Max coverage (-): 0

Region: chr17 72239070-72239096. Max. coverage (+): 3.67. Max coverage (-): 0

Region: chr17 72239097-72239122. Max. coverage (+): 3.67. Max coverage (-): 0

Region: chr17 72239123-72239149. Max. coverage (+): 0. Max coverage (-): 0

Region: chr17 72239150-72239176. Max. coverage (+): 0. Max coverage (-): 0

Region: chr17 72239177-72239203. Max. coverage (+): 0. Max coverage (-): 0

Region: chr17 72239204-72239229. Max. coverage (+): 0. Max coverage (-): 0

Region: chr17 72239230-72239256. Max. coverage (+): 0. Max coverage (-): 0

Region: chr17 72239257-72239283. Max. coverage (+): 0. Max coverage (-): 0

Region: chr17 72239284-72239310. Max. coverage (+): 0. Max coverage (-): 0

Region: chr17 72239311-72239337. Max. coverage (+): 0. Max coverage (-): 0

Region: chr17 72239338-72239363. Max. coverage (+): 3.36. Max coverage (-): 0

Region: chr17 72239364-72239390. Max. coverage (+): 12.69. Max coverage (-): 0

Region: chr17 72239391-72239417. Max. coverage (+): 12.69. Max coverage (-): 0

Region: chr17 72239418-72239444. Max. coverage (+): 5.54. Max coverage (-): 0

Region: chr17 72239445-72239471. Max. coverage (+): 6.22. Max coverage (-): 0

Region: chr17 72239472-72239497. Max. coverage (+): 6.22. Max coverage (-): 0

Region: chr17 72239498-72239524. Max. coverage (+): 11.8. Max coverage (-): 0

Region: chr17 72239525-72239551. Max. coverage (+): 0. Max coverage (-): 0

Region: chr17 72239552-72239578. Max. coverage (+): 0. Max coverage (-): 0

Region: chr17 72239579-72239605. Max. coverage (+): 0. Max coverage (-): 0

Region: chr17 72239606-72239631. Max. coverage (+): 0. Max coverage (-): 0

Region: chr17 72239632-72239658. Max. coverage (+): 0. Max coverage (-): 0

Region: chr17 72239659-72239685. Max. coverage (+): 0. Max coverage (-): 0

Region: chr17 72239686-72239712. Max. coverage (+): 0. Max coverage (-): 0

Region: chr17 72239713-72239739. Max. coverage (+): 0. Max coverage (-): 0

Region: chr17 72239740-72239765. Max. coverage (+): 0. Max coverage (-): 0

Region: chr17 72239766-72239792. Max. coverage (+): 0. Max coverage (-): 0

Region: chr17 72239793-72239819. Max. coverage (+): 0. Max coverage (-): 0

Region: chr17 72239820-72239846. Max. coverage (+): 0. Max coverage (-): 0

Region: chr17 72239847-72239872. Max. coverage (+): 0. Max coverage (-): 0

Region: chr17 72239873-72239899. Max. coverage (+): 0. Max coverage (-): 0

Region: chr17 72239900-72239926. Max. coverage (+): 0. Max coverage (-): 0

Region: chr17 72239927-72239953. Max. coverage (+): 0. Max coverage (-): 0

Region: chr17 72239954-72239980. Max. coverage (+): 0. Max coverage (-): 0

Region: chr17 72239981-72240006. Max. coverage (+): 0.31. Max coverage (-): 0

Region: chr17 72240007-72240033. Max. coverage (+): 4.6. Max coverage (-): 0

Region: chr17 72240034-72240060. Max. coverage (+): 7.58. Max coverage (-): 0

Region: chr17 72240061-72240087. Max. coverage (+): 4.49. Max coverage (-): 0

Region: chr17 72240088-72240114. Max. coverage (+): 0. Max coverage (-): 0

Region: chr17 72240115-72240140. Max. coverage (+): 0. Max coverage (-): 0

Region: chr17 72240141-72240167. Max. coverage (+): 0. Max coverage (-): 0

Region: chr17 72240168-72240194. Max. coverage (+): 0. Max coverage (-): 0

Region: chr17 72240195-72240221. Max. coverage (+): 0. Max coverage (-): 0

Region: chr17 72240222-72240248. Max. coverage (+): 0. Max coverage (-): 0

Region: chr17 72240249-72240274. Max. coverage (+): 2.04. Max coverage (-): 0

Region: chr17 72240275-72240301. Max. coverage (+): 2.04. Max coverage (-): 0

Region: chr17 72240302-72240328. Max. coverage (+): 0. Max coverage (-): 0

Region: chr17 72240329-72240355. Max. coverage (+): 0. Max coverage (-): 0

Region: chr17 72240356-72240382. Max. coverage (+): 0. Max coverage (-): 0

Region: chr17 72240383-72240408. Max. coverage (+): 0. Max coverage (-): 0

Region: chr17 72240409-72240435. Max. coverage (+): 0. Max coverage (-): 0

Region: chr17 72240436-72240462. Max. coverage (+): 0.51. Max coverage (-): 0

Region: chr17 72240463-72240489. Max. coverage (+): 0. Max coverage (-): 0

Region: chr17 72240490-72240515. Max. coverage (+): 9.65. Max coverage (-): 0

Region: chr17 72240516-72240542. Max. coverage (+): 0. Max coverage (-): 0

Region: chr17 72240543-72240569. Max. coverage (+): 1.41. Max coverage (-): 0

Region: chr17 72240570-72240596. Max. coverage (+): 0. Max coverage (-): 0

Region: chr17 72240597-72240623. Max. coverage (+): 3.3. Max coverage (-): 0

Region: chr17 72240624-72240649. Max. coverage (+): 0. Max coverage (-): 0

Region: chr17 72240650-72240676. Max. coverage (+): 0. Max coverage (-): 0

Region: chr17 72240677-72240703. Max. coverage (+): 0. Max coverage (-): 0

Region: chr17 72240704-72240730. Max. coverage (+): 3.78. Max coverage (-): 0

Region: chr17 72240731-72240757. Max. coverage (+): 0.61. Max coverage (-): 0

Region: chr17 72240758-72240783. Max. coverage (+): 0. Max coverage (-): 0

Region: chr17 72240784-72240810. Max. coverage (+): 0. Max coverage (-): 0

Region: chr17 72240811-72240837. Max. coverage (+): 0. Max coverage (-): 0

Region: chr17 72240838-72240864. Max. coverage (+): 0. Max coverage (-): 0

Region: chr17 72240865-72240891. Max. coverage (+): 0. Max coverage (-): 0

Region: chr17 72240892-72240917. Max. coverage (+): 0. Max coverage (-): 0

Region: chr17 72240918-72240944. Max. coverage (+): 0. Max coverage (-): 0

Region: chr17 72240945-72240971. Max. coverage (+): 0. Max coverage (-): 0

Region: chr17 72240972-72240998. Max. coverage (+): 0. Max coverage (-): 0

Region: chr17 72240999-72241025. Max. coverage (+): 0. Max coverage (-): 0

Region: chr17 72241026-72241051. Max. coverage (+): 0. Max coverage (-): 0

Region: chr17 72241052-72241078. Max. coverage (+): 0. Max coverage (-): 0

Region: chr17 72241079-72241105. Max. coverage (+): 1.75. Max coverage (-): 0

Region: chr17 72241106-72241132. Max. coverage (+): 0. Max coverage (-): 0

Region: chr17 72241133-72241158. Max. coverage (+): 0. Max coverage (-): 0

Region: chr17 72241159-72241185. Max. coverage (+): 0. Max coverage (-): 0

Region: chr17 72241186-72241212. Max. coverage (+): 0. Max coverage (-): 0

Region: chr17 72241213-72241239. Max. coverage (+): 0. Max coverage (-): 0

Region: chr17 72241240-72241266. Max. coverage (+): 0. Max coverage (-): 0

Region: chr17 72241267-72241292. Max. coverage (+): 0. Max coverage (-): 0

Region: chr17 72241293-72241319. Max. coverage (+): 0. Max coverage (-): 0

Region: chr17 72241320-72241346. Max. coverage (+): 0. Max coverage (-): 0

Region: chr17 72241347-72241373. Max. coverage (+): 0. Max coverage (-): 0

Region: chr17 72241374-72241400. Max. coverage (+): 0. Max coverage (-): 0

Region: chr17 72241401-72241426. Max. coverage (+): 0. Max coverage (-): 0

Region: chr17 72241427-72241453. Max. coverage (+): 0. Max coverage (-): 0

Region: chr17 72241454-72241480. Max. coverage (+): 0. Max coverage (-): 0

Region: chr17 72241481-72241507. Max. coverage (+): 0. Max coverage (-): 0

Region: chr17 72241508-72241534. Max. coverage (+): 0. Max coverage (-): 0

Region: chr17 72241535-72241560. Max. coverage (+): 0. Max coverage (-): 0

Region: chr17 72241561-72241587. Max. coverage (+): 0. Max coverage (-): 0

Region: chr17 72241588-72241614. Max. coverage (+): 0. Max coverage (-): 0

Region: chr17 72241615-72241641. Max. coverage (+): 0. Max coverage (-): 0

Region: chr17 72241642-72241668. Max. coverage (+): 0. Max coverage (-): 0

Region: chr17 72241669-72241694. Max. coverage (+): 0. Max coverage (-): 0

Region: chr17 72241695-72241721. Max. coverage (+): 0. Max coverage (-): 0

Region: chr17 72241722-72241748. Max. coverage (+): 0. Max coverage (-): 0

Region: chr17 72241749-72241775. Max. coverage (+): 0. Max coverage (-): 0

Region: chr17 72241776-72241801. Max. coverage (+): 0. Max coverage (-): 0

Region: chr17 72241802-72241828. Max. coverage (+): 0. Max coverage (-): 0

Region: chr17 72241829-72241855. Max. coverage (+): 0. Max coverage (-): 0

Region: chr17 72241856-72241882. Max. coverage (+): 0. Max coverage (-): 0

Region: chr17 72241883-72241909. Max. coverage (+): 0. Max coverage (-): 0

Region: chr17 72241910-72241935. Max. coverage (+): 0. Max coverage (-): 0

Region: chr17 72241936-72241962. Max. coverage (+): 0. Max coverage (-): 0

Region: chr17 72241963-72241989. Max. coverage (+): 0. Max coverage (-): 0

Region: chr17 72241990-72242016. Max. coverage (+): 0. Max coverage (-): 0

Region: chr17 72242017-72242043. Max. coverage (+): 0. Max coverage (-): 0

Region: chr17 72242044-72242069. Max. coverage (+): 0. Max coverage (-): 0

Region: chr17 72242070-72242096. Max. coverage (+): 0. Max coverage (-): 0

Region: chr17 72242097-72242123. Max. coverage (+): 0. Max coverage (-): 0

Region: chr17 72242124-72242150. Max. coverage (+): 0. Max coverage (-): 0

Region: chr17 72242151-72242177. Max. coverage (+): 0. Max coverage (-): 0

Region: chr17 72242178-72242203. Max. coverage (+): 0. Max coverage (-): 0

Region: chr17 72242204-72242230. Max. coverage (+): 0. Max coverage (-): 0

Region: chr17 72242231-72242257. Max. coverage (+): 14.11. Max coverage (-): 0

Region: chr17 72242258-72242284. Max. coverage (+): 14.11. Max coverage (-): 0

Region: chr17 72242285-72242311. Max. coverage (+): 0. Max coverage (-): 0

Region: chr17 72242312-72242337. Max. coverage (+): 0. Max coverage (-): 0

Region: chr17 72242338-72242364. Max. coverage (+): 0. Max coverage (-): 0

Region: chr17 72242365-72242391. Max. coverage (+): 0. Max coverage (-): 0

Region: chr17 72242392-72242418. Max. coverage (+): 0. Max coverage (-): 0

Region: chr17 72242419-72242445. Max. coverage (+): 4.3. Max coverage (-): 0

Region: chr17 72242446-72242471. Max. coverage (+): 4.3. Max coverage (-): 0

Region: chr17 72242472-72242498. Max. coverage (+): 0. Max coverage (-): 0

Region: chr17 72242499-72242525. Max. coverage (+): 1.99. Max coverage (-): 0

Region: chr17 72242526-72242552. Max. coverage (+): 0. Max coverage (-): 0

Region: chr17 72242553-72242578. Max. coverage (+): 0. Max coverage (-): 0

Region: chr17 72242579-72242605. Max. coverage (+): 0. Max coverage (-): 0

Region: chr17 72242606-72242632. Max. coverage (+): 0. Max coverage (-): 0

Region: chr17 72242633-72242659. Max. coverage (+): 0. Max coverage (-): 0

Region: chr17 72242660-72242686. Max. coverage (+): 0. Max coverage (-): 0

Region: chr17 72242687-72242712. Max. coverage (+): 0. Max coverage (-): 0

Region: chr17 72242713-72242739. Max. coverage (+): 0. Max coverage (-): 0

Region: chr17 72242740-72242766. Max. coverage (+): 0. Max coverage (-): 0

Region: chr17 72242767-72242793. Max. coverage (+): 0. Max coverage (-): 0

Region: chr17 72242794-72242820. Max. coverage (+): 0. Max coverage (-): 0

Region: chr17 72242821-72242846. Max. coverage (+): 0. Max coverage (-): 0

Region: chr17 72242847-72242873. Max. coverage (+): 0. Max coverage (-): 0

Region: chr17 72242874-72242900. Max. coverage (+): 0. Max coverage (-): 0

Region: chr17 72242901-72242927. Max. coverage (+): 0. Max coverage (-): 0

Region: chr17 72242928-72242954. Max. coverage (+): 0. Max coverage (-): 0

Region: chr17 72242955-72242980. Max. coverage (+): 0. Max coverage (-): 0

Region: chr17 72242981-72243007. Max. coverage (+): 0. Max coverage (-): 0

Region: chr17 72243008-72243034. Max. coverage (+): 0. Max coverage (-): 0

Region: chr17 72243035-72243061. Max. coverage (+): 3.79. Max coverage (-): 0

Region: chr17 72243062-72243088. Max. coverage (+): 0. Max coverage (-): 0

Region: chr17 72243089-72243114. Max. coverage (+): 0. Max coverage (-): 0

Region: chr17 72243115-72243141. Max. coverage (+): 0. Max coverage (-): 0

Region: chr17 72243142-72243168. Max. coverage (+): 0. Max coverage (-): 0

Region: chr17 72243169-72243195. Max. coverage (+): 0. Max coverage (-): 0

Region: chr17 72243196-72243221. Max. coverage (+): 0. Max coverage (-): 0

Region: chr17 72243222-72243248. Max. coverage (+): 0. Max coverage (-): 0

Region: chr17 72243249-72243275. Max. coverage (+): 0. Max coverage (-): 0

Region: chr17 72243276-72243302. Max. coverage (+): 0. Max coverage (-): 0

Region: chr17 72243303-72243329. Max. coverage (+): 0. Max coverage (-): 0

Region: chr17 72243330-72243355. Max. coverage (+): 0. Max coverage (-): 0

Region: chr17 72243356-72243382. Max. coverage (+): 0. Max coverage (-): 0

Region: chr17 72243383-72243409. Max. coverage (+): 0. Max coverage (-): 0

Region: chr17 72243410-72243436. Max. coverage (+): 0. Max coverage (-): 0

Region: chr17 72243437-72243463. Max. coverage (+): 0. Max coverage (-): 0

Region: chr17 72243464-72243489. Max. coverage (+): 0.52. Max coverage (-): 0

Region: chr17 72243490-72243516. Max. coverage (+): 0.52. Max coverage (-): 0

Region: chr17 72243517-72243543. Max. coverage (+): 1.42. Max coverage (-): 0

Region: chr17 72243544-72243570. Max. coverage (+): 0. Max coverage (-): 0

Region: chr17 72243571-72243597. Max. coverage (+): 0. Max coverage (-): 0

Region: chr17 72243598-72243623. Max. coverage (+): 0. Max coverage (-): 0

Region: chr17 72243624-72243650. Max. coverage (+): 0. Max coverage (-): 0

Region: chr17 72243651-72243677. Max. coverage (+): 0. Max coverage (-): 0

Region: chr17 72243678-72243704. Max. coverage (+): 0. Max coverage (-): 0

Region: chr17 72243705-72243731. Max. coverage (+): 3.88. Max coverage (-): 0

Region: chr17 72243732-72243757. Max. coverage (+): 0. Max coverage (-): 0

Region: chr17 72243758-72243784. Max. coverage (+): 12.24. Max coverage (-): 0

Region: chr17 72243785-. Max. coverage (+): 0. Max coverage (-): 0

RepeatMasker Color Code

**+**

100-98% Identity

<98-95% Identity

<95-90% Identity

<90-85% Identity

<85-80% Identity

<80-75% Identity

<75-70% Identity

<70% Identity

**-**

Gene Set Color Code

**+**

Gene

Pseudogene

**-**

Topology/Coverage Color Code

Coverage Plus Strand

Coverage Minus Strand

Mainstrand: Plus

Mainstrand: Minus

Complementary Strand

Flanking Region  
(if option -flank >0)

Gene Set Annotation  

**1. PATZ1 (protein coding, ENSBTAG00000005478) Tr:00000029432 Ex:3**: 72238208-72238379 (-)  
**2. PATZ1 (protein coding, ENSBTAG00000005478) Tr:00000029432 Ex:4**: 72232450-72232587 (-)  
**3. PATZ1 (protein coding, ENSBTAG00000005478) Tr:00000029432 Ex:5**: 72229523-72231042 (-)

  
RepeatMasker Annotation  

**1. CHRL1\_BT**: 72233704-72233853 (-), Divergence to consensus: 31.4%  
**2. Bov-tA1**: 72234161-72234345 (-), Divergence to consensus: 22.3%  
**3. MIRc**: 72237211-72237437 (-), Divergence to consensus: 38.6%  
**4. Charlie15b**: 72239591-72239762 (+), Divergence to consensus: 43.3%  
**5. Tigger2a\_Art**: 72239763-72239997 (+), Divergence to consensus: 38%  
**6. Tigger19a**: 72240763-72241003 (+), Divergence to consensus: 49.9%

  
Transcription Factor Binding Sites  

**SPZ1** (Sequence: CTGAAACCCT (-): 72236765)  
**SOX9** (Sequence: AACAATGA (-): 72233174)  
**SOX9** (Sequence: CTATTGTT (+): 72233908)
