# Supplementary material for: Small RNA sequencing of cryopreserved semen from single bull revealed altered miRNAs and piRNAs expression between High- and Low-motile sperm populations
Source: BMC Genomics. 2017 Jan 4;18:14. doi: 10.1186/s12864-016-3394-7 (PMC5209821; doi:10.1186/s12864-016-3394-7)
Supplement: Additional file 3: — Details for each piRNA clusters found in High Motile (HM) sperm fraction. Genes, repeats, transposable elements and transcription factors binding sites falling within the cluster regions were reported. (ZIP 1896 kb) [file 12864_2016_3394_MOESM3_ESM.zip › 34.html]

piRNA cluster 34


Predicted piRNA cluster no. 34     previous   next
  

Show proTRAC run info
Hide proTRAC run info

================================= proTRAC ====================================  
VERSION: 2.1                                    LAST MODIFIED: 06. October 2015  
  
Please cite:  
Rosenkranz D, Zischler H. proTRAC - a software for probabilistic piRNA cluster  
detection, visualization and analysis. 2012. BMC Bioinformatics 13:5.  
  
and (for proTRAC 2.0 and later):  
Rosenkranz D, Rudloff S, Bastuck K, Ketting RF, Zischler H. Tupaia small RNAs  
provide insights into function and evolution of RNAi-based transposon defense  
in mammals. 2015. RNA 21(5):911-922.  
  
Contact:  
David Rosenkranz  
Institute of Anthropology, small RNA group  
Johannes Gutenberg University Mainz  
email: rosenkranz@uni-mainz.de  
  
You can find the latest proTRAC version at:  
http://sourceforge.net/projects/protrac/files  
http://www.smallRNAgroup-mainz.de/software  
==============================================================================  
  
PARAMETERS:  
Map file: .............../storage/core/barbara/genhome/smallRNA/fertility/Sample\_motile/pirna/Sample\_motile\_26-33\_collapsed.fa.no-dust.map.weighted-10000-1000-b-0  
Genome file: ............/storage/core/barbara/genhome/smallRNA/fertility/Sample\_all/pirna/bt\_311\_chrY.fa  
RepeatMasker annotation: /storage/genomes/bt\_umd31/GCF\_000003055.6\_Bos\_taurus\_UMD\_3.1.1\_repeatMasker\_chr.out  
GeneSet:................./storage/core/barbara/genhome/smallRNA/fertility/Sample\_all/pirna/full.gtf  
  
Significant (p<=0.01) hit density will be calculated based  
on observed hit distribution.  
  
Sliding window size: ........................................ 5000 bp  
Sliding window increament: .................................. 1000 bp  
Normalize each hit by number of genomic hits: ............... 1 [0=no/1=yes]  
Normalize each hit by number of sequence reads: ............. 1 [0=no/1=yes]  
Normalize values (-> per million mapped reads): ............. 1 [0=no/1=yes]  
Min. fraction of hits with 1T(U) or 10A: .................... 0.75  
Alternatively: Min. fraction of hits with 1T(U) and 10A: .... 0.5  
Min. fraction of hits with typical piRNA length: ............ 0.75  
Typical piRNA length: ....................................... 26-33 nt  
Min. size of a piRNA cluster: ............................... 5000 bp.  
Min. number of hits (absolute): ............................. 0  
Min. number of hits (normalized): ........................... 0  
Min. fraction of hits on the mainstrand: .................... 0.75  
Top fraction of mapped sequences (in terms of read counts): . 1%  
Top fraction accounts for max. n% of sequence reads: ........ 90%  
Min. fraction of hits on each arm of a bidirectional cluster: 0.1  
Output image file for each cluster: ......................... 0 [0=no/1=yes]  
Output html file for each cluster: .......................... 1 [0=no/1=yes]  
Output a summary table: ..................................... 1 [0=no/1=yes]  
Output a FASTA file for each cluster (piRNA sequences): ..... 1 [0=no/1=yes]  
Output a FASTA file comprising cluster sequences: ........... 1 [0=no/1=yes]  
Search DNA motifs in clusters: .............................. 1 [0=no/1=yes]  
Output flanking sequences: +/- .............................. 0 bp  
Output ~.pTi file: .......................................... 1 [0=no/1=yes]  
==============================================================================  
  
  
Genome size (without gaps): ............ 2678902517 bp  
Gaps (N/X/-): .......................... 53837044 bp  
Mapped reads: .......................... 658825247023  
Non-identical sequences: ............... 514171  
Genomic hits: .......................... 764233  
Significant densitiy of mapped reads: .. 12867599.5173724 reads/kb

Show proTRAC cluster info
Hide proTRAC cluster info

|  |  |
| --- | --- |
| Location | chr17 |
| Coordinates | 73754017-73778011 |
| Size [bp] | 23995 |
| Sequence hit loci | 678 |
| Mapped reads (normalized) | 898239336.2 |
| Mapped reads (normalized) per kb | 37434437.8 |
| Normalized reads with 1T (1U) | 84% |
| Normalized reads with 10A | 26% |
| Normalized reads with length 26-33 nt | 100% |
| Normalized reads on the main strand(s) | 100% |
| Predicted directionality | mono:minus |

100%

0%

1T (1U)  
reads

10A reads

26-33 nt  
reads

reads on mainstrand

**Either the amount of reads with 1T (1U) OR 10A has to exceed 75% (set with option: -1Tor10A)  
Alternatively the amount of reads with 1T (1U) AND 10A has to exceed 50% (set with option: -1Tand10A)  
Minimum amount of reads with preferred size is 75% (set with option: -pisize)  
Minimum amount of reads on the main strand(s) is 75% (set with option: -clstrand)**

Show read coverage
Hide read coverage

WHAT DO I SEE HERE?  
This chart shows the location of mapped sequence reads within a predicted piRNA cluster. The color refers to the number of genomic hits produced by the sequence read in question. A dark red bar indicates that this sequence read produces many other hits elsewhere in the genome. Many adjacent red or yellow bars can indicate the presence of a multi-copy element such as transposons or rRNA genes. A dark green bar indicates that this sequence read maps uniquely to this locus.

1 hit

2-5 hits

6-10 hits

11-20 hits

21-50 hits

51-100 hits

> 100 hits

chr17

73754017

73778011

Gene Set

RepeatMasker

Mapped  
Reads

46.7

plus strand

minus strand

46.7

Region: chr17 72243786-73754040. Max. coverage (+): 0. Max coverage (-): 1.68

Region: chr17 73754041-73754088. Max. coverage (+): 0. Max coverage (-): 0

Region: chr17 73754089-73754136. Max. coverage (+): 0. Max coverage (-): 0

Region: chr17 73754137-73754184. Max. coverage (+): 0. Max coverage (-): 0

Region: chr17 73754185-73754232. Max. coverage (+): 0. Max coverage (-): 0

Region: chr17 73754233-73754280. Max. coverage (+): 0. Max coverage (-): 0

Region: chr17 73754281-73754328. Max. coverage (+): 0. Max coverage (-): 0

Region: chr17 73754329-73754376. Max. coverage (+): 0. Max coverage (-): 0

Region: chr17 73754377-73754424. Max. coverage (+): 0. Max coverage (-): 0

Region: chr17 73754425-73754472. Max. coverage (+): 0. Max coverage (-): 4.64

Region: chr17 73754473-73754520. Max. coverage (+): 0. Max coverage (-): 4.64

Region: chr17 73754521-73754568. Max. coverage (+): 0. Max coverage (-): 0

Region: chr17 73754569-73754616. Max. coverage (+): 0. Max coverage (-): 0

Region: chr17 73754617-73754664. Max. coverage (+): 0. Max coverage (-): 0

Region: chr17 73754665-73754712. Max. coverage (+): 0. Max coverage (-): 0

Region: chr17 73754713-73754760. Max. coverage (+): 0. Max coverage (-): 0

Region: chr17 73754761-73754808. Max. coverage (+): 0. Max coverage (-): 0

Region: chr17 73754809-73754856. Max. coverage (+): 0. Max coverage (-): 4.57

Region: chr17 73754857-73754904. Max. coverage (+): 0. Max coverage (-): 0

Region: chr17 73754905-73754952. Max. coverage (+): 0. Max coverage (-): 0

Region: chr17 73754953-73755000. Max. coverage (+): 0. Max coverage (-): 0

Region: chr17 73755001-73755048. Max. coverage (+): 0. Max coverage (-): 0.43

Region: chr17 73755049-73755096. Max. coverage (+): 0. Max coverage (-): 0

Region: chr17 73755097-73755144. Max. coverage (+): 0. Max coverage (-): 0

Region: chr17 73755145-73755192. Max. coverage (+): 0. Max coverage (-): 0

Region: chr17 73755193-73755240. Max. coverage (+): 0. Max coverage (-): 0

Region: chr17 73755241-73755288. Max. coverage (+): 0. Max coverage (-): 0

Region: chr17 73755289-73755336. Max. coverage (+): 0. Max coverage (-): 0

Region: chr17 73755337-73755384. Max. coverage (+): 0. Max coverage (-): 0

Region: chr17 73755385-73755432. Max. coverage (+): 0. Max coverage (-): 0

Region: chr17 73755433-73755480. Max. coverage (+): 0. Max coverage (-): 2.23

Region: chr17 73755481-73755528. Max. coverage (+): 0. Max coverage (-): 0

Region: chr17 73755529-73755576. Max. coverage (+): 0. Max coverage (-): 0

Region: chr17 73755577-73755624. Max. coverage (+): 0. Max coverage (-): 0

Region: chr17 73755625-73755672. Max. coverage (+): 0. Max coverage (-): 0

Region: chr17 73755673-73755720. Max. coverage (+): 0. Max coverage (-): 0

Region: chr17 73755721-73755768. Max. coverage (+): 0. Max coverage (-): 0

Region: chr17 73755769-73755816. Max. coverage (+): 0. Max coverage (-): 2.84

Region: chr17 73755817-73755864. Max. coverage (+): 0. Max coverage (-): 2.84

Region: chr17 73755865-73755912. Max. coverage (+): 0. Max coverage (-): 0

Region: chr17 73755913-73755960. Max. coverage (+): 0. Max coverage (-): 0

Region: chr17 73755961-73756008. Max. coverage (+): 0. Max coverage (-): 0

Region: chr17 73756009-73756056. Max. coverage (+): 0. Max coverage (-): 0

Region: chr17 73756057-73756104. Max. coverage (+): 0. Max coverage (-): 1.85

Region: chr17 73756105-73756152. Max. coverage (+): 0. Max coverage (-): 0

Region: chr17 73756153-73756200. Max. coverage (+): 0. Max coverage (-): 0

Region: chr17 73756201-73756248. Max. coverage (+): 0. Max coverage (-): 0

Region: chr17 73756249-73756296. Max. coverage (+): 0. Max coverage (-): 1.2

Region: chr17 73756297-73756344. Max. coverage (+): 0. Max coverage (-): 1.2

Region: chr17 73756345-73756392. Max. coverage (+): 0. Max coverage (-): 7.56

Region: chr17 73756393-73756440. Max. coverage (+): 0. Max coverage (-): 1.41

Region: chr17 73756441-73756488. Max. coverage (+): 0. Max coverage (-): 0

Region: chr17 73756489-73756536. Max. coverage (+): 0. Max coverage (-): 0

Region: chr17 73756537-73756584. Max. coverage (+): 0. Max coverage (-): 0

Region: chr17 73756585-73756632. Max. coverage (+): 0. Max coverage (-): 0

Region: chr17 73756633-73756680. Max. coverage (+): 0. Max coverage (-): 0

Region: chr17 73756681-73756728. Max. coverage (+): 0. Max coverage (-): 0

Region: chr17 73756729-73756776. Max. coverage (+): 0. Max coverage (-): 0.94

Region: chr17 73756777-73756824. Max. coverage (+): 0. Max coverage (-): 3.4

Region: chr17 73756825-73756872. Max. coverage (+): 0. Max coverage (-): 0

Region: chr17 73756873-73756920. Max. coverage (+): 0. Max coverage (-): 0

Region: chr17 73756921-73756968. Max. coverage (+): 0. Max coverage (-): 3.98

Region: chr17 73756969-73757016. Max. coverage (+): 0. Max coverage (-): 0

Region: chr17 73757017-73757064. Max. coverage (+): 0. Max coverage (-): 0

Region: chr17 73757065-73757112. Max. coverage (+): 0. Max coverage (-): 0

Region: chr17 73757113-73757160. Max. coverage (+): 0. Max coverage (-): 10.51

Region: chr17 73757161-73757208. Max. coverage (+): 0. Max coverage (-): 6.3

Region: chr17 73757209-73757256. Max. coverage (+): 0. Max coverage (-): 0

Region: chr17 73757257-73757304. Max. coverage (+): 0. Max coverage (-): 0

Region: chr17 73757305-73757352. Max. coverage (+): 0. Max coverage (-): 0

Region: chr17 73757353-73757400. Max. coverage (+): 0. Max coverage (-): 2.66

Region: chr17 73757401-73757448. Max. coverage (+): 0. Max coverage (-): 0

Region: chr17 73757449-73757496. Max. coverage (+): 0. Max coverage (-): 0

Region: chr17 73757497-73757544. Max. coverage (+): 0. Max coverage (-): 0.57

Region: chr17 73757545-73757592. Max. coverage (+): 0. Max coverage (-): 0

Region: chr17 73757593-73757640. Max. coverage (+): 0. Max coverage (-): 0

Region: chr17 73757641-73757688. Max. coverage (+): 0. Max coverage (-): 1.14

Region: chr17 73757689-73757736. Max. coverage (+): 0. Max coverage (-): 9.95

Region: chr17 73757737-73757784. Max. coverage (+): 0. Max coverage (-): 0

Region: chr17 73757785-73757832. Max. coverage (+): 0. Max coverage (-): 0

Region: chr17 73757833-73757880. Max. coverage (+): 0. Max coverage (-): 0

Region: chr17 73757881-73757928. Max. coverage (+): 0. Max coverage (-): 3.23

Region: chr17 73757929-73757976. Max. coverage (+): 0. Max coverage (-): 0

Region: chr17 73757977-73758024. Max. coverage (+): 0. Max coverage (-): 0

Region: chr17 73758025-73758072. Max. coverage (+): 0. Max coverage (-): 5.85

Region: chr17 73758073-73758120. Max. coverage (+): 0. Max coverage (-): 15.59

Region: chr17 73758121-73758168. Max. coverage (+): 0. Max coverage (-): 2.9

Region: chr17 73758169-73758216. Max. coverage (+): 0. Max coverage (-): 3.07

Region: chr17 73758217-73758264. Max. coverage (+): 0. Max coverage (-): 1.83

Region: chr17 73758265-73758312. Max. coverage (+): 0. Max coverage (-): 4.23

Region: chr17 73758313-73758360. Max. coverage (+): 0. Max coverage (-): 20.05

Region: chr17 73758361-73758408. Max. coverage (+): 0. Max coverage (-): 3.62

Region: chr17 73758409-73758456. Max. coverage (+): 0. Max coverage (-): 0

Region: chr17 73758457-73758504. Max. coverage (+): 0. Max coverage (-): 0

Region: chr17 73758505-73758552. Max. coverage (+): 0. Max coverage (-): 1.19

Region: chr17 73758553-73758600. Max. coverage (+): 0. Max coverage (-): 0

Region: chr17 73758601-73758648. Max. coverage (+): 0. Max coverage (-): 4.88

Region: chr17 73758649-73758696. Max. coverage (+): 0. Max coverage (-): 11.87

Region: chr17 73758697-73758744. Max. coverage (+): 0. Max coverage (-): 2.2

Region: chr17 73758745-73758792. Max. coverage (+): 0. Max coverage (-): 0

Region: chr17 73758793-73758839. Max. coverage (+): 0. Max coverage (-): 0

Region: chr17 73758840-73758887. Max. coverage (+): 0. Max coverage (-): 0

Region: chr17 73758888-73758935. Max. coverage (+): 0. Max coverage (-): 0

Region: chr17 73758936-73758983. Max. coverage (+): 0. Max coverage (-): 6.41

Region: chr17 73758984-73759031. Max. coverage (+): 0. Max coverage (-): 0

Region: chr17 73759032-73759079. Max. coverage (+): 0. Max coverage (-): 0

Region: chr17 73759080-73759127. Max. coverage (+): 0. Max coverage (-): 0

Region: chr17 73759128-73759175. Max. coverage (+): 0. Max coverage (-): 0

Region: chr17 73759176-73759223. Max. coverage (+): 0. Max coverage (-): 0

Region: chr17 73759224-73759271. Max. coverage (+): 0. Max coverage (-): 0

Region: chr17 73759272-73759319. Max. coverage (+): 0. Max coverage (-): 0.85

Region: chr17 73759320-73759367. Max. coverage (+): 0. Max coverage (-): 4.99

Region: chr17 73759368-73759415. Max. coverage (+): 0. Max coverage (-): 0

Region: chr17 73759416-73759463. Max. coverage (+): 0. Max coverage (-): 0

Region: chr17 73759464-73759511. Max. coverage (+): 0. Max coverage (-): 0

Region: chr17 73759512-73759559. Max. coverage (+): 0. Max coverage (-): 0

Region: chr17 73759560-73759607. Max. coverage (+): 0. Max coverage (-): 0

Region: chr17 73759608-73759655. Max. coverage (+): 0. Max coverage (-): 10.68

Region: chr17 73759656-73759703. Max. coverage (+): 0. Max coverage (-): 20.48

Region: chr17 73759704-73759751. Max. coverage (+): 0. Max coverage (-): 20.48

Region: chr17 73759752-73759799. Max. coverage (+): 0. Max coverage (-): 0

Region: chr17 73759800-73759847. Max. coverage (+): 0. Max coverage (-): 0

Region: chr17 73759848-73759895. Max. coverage (+): 0. Max coverage (-): 0

Region: chr17 73759896-73759943. Max. coverage (+): 0. Max coverage (-): 0

Region: chr17 73759944-73759991. Max. coverage (+): 0. Max coverage (-): 0

Region: chr17 73759992-73760039. Max. coverage (+): 0. Max coverage (-): 0

Region: chr17 73760040-73760087. Max. coverage (+): 0. Max coverage (-): 0

Region: chr17 73760088-73760135. Max. coverage (+): 0. Max coverage (-): 0

Region: chr17 73760136-73760183. Max. coverage (+): 0. Max coverage (-): 0

Region: chr17 73760184-73760231. Max. coverage (+): 0. Max coverage (-): 0

Region: chr17 73760232-73760279. Max. coverage (+): 0. Max coverage (-): 0

Region: chr17 73760280-73760327. Max. coverage (+): 0. Max coverage (-): 0

Region: chr17 73760328-73760375. Max. coverage (+): 0. Max coverage (-): 2.28

Region: chr17 73760376-73760423. Max. coverage (+): 0. Max coverage (-): 0

Region: chr17 73760424-73760471. Max. coverage (+): 0. Max coverage (-): 8.11

Region: chr17 73760472-73760519. Max. coverage (+): 0. Max coverage (-): 0

Region: chr17 73760520-73760567. Max. coverage (+): 0. Max coverage (-): 0

Region: chr17 73760568-73760615. Max. coverage (+): 0. Max coverage (-): 0

Region: chr17 73760616-73760663. Max. coverage (+): 0. Max coverage (-): 5.76

Region: chr17 73760664-73760711. Max. coverage (+): 0. Max coverage (-): 6.18

Region: chr17 73760712-73760759. Max. coverage (+): 0. Max coverage (-): 4.58

Region: chr17 73760760-73760807. Max. coverage (+): 0. Max coverage (-): 6.98

Region: chr17 73760808-73760855. Max. coverage (+): 0. Max coverage (-): 5.83

Region: chr17 73760856-73760903. Max. coverage (+): 0. Max coverage (-): 7.18

Region: chr17 73760904-73760951. Max. coverage (+): 0. Max coverage (-): 4.9

Region: chr17 73760952-73760999. Max. coverage (+): 0. Max coverage (-): 2.18

Region: chr17 73761000-73761047. Max. coverage (+): 0. Max coverage (-): 2.61

Region: chr17 73761048-73761095. Max. coverage (+): 0. Max coverage (-): 0

Region: chr17 73761096-73761143. Max. coverage (+): 0. Max coverage (-): 0

Region: chr17 73761144-73761191. Max. coverage (+): 0. Max coverage (-): 0

Region: chr17 73761192-73761239. Max. coverage (+): 0. Max coverage (-): 9.62

Region: chr17 73761240-73761287. Max. coverage (+): 0. Max coverage (-): 6.56

Region: chr17 73761288-73761335. Max. coverage (+): 0. Max coverage (-): 5.59

Region: chr17 73761336-73761383. Max. coverage (+): 0. Max coverage (-): 0

Region: chr17 73761384-73761431. Max. coverage (+): 0. Max coverage (-): 0

Region: chr17 73761432-73761479. Max. coverage (+): 0. Max coverage (-): 0

Region: chr17 73761480-73761527. Max. coverage (+): 0. Max coverage (-): 9.99

Region: chr17 73761528-73761575. Max. coverage (+): 0. Max coverage (-): 0

Region: chr17 73761576-73761623. Max. coverage (+): 0. Max coverage (-): 10.96

Region: chr17 73761624-73761671. Max. coverage (+): 0. Max coverage (-): 5.15

Region: chr17 73761672-73761719. Max. coverage (+): 0. Max coverage (-): 0

Region: chr17 73761720-73761767. Max. coverage (+): 0. Max coverage (-): 9.25

Region: chr17 73761768-73761815. Max. coverage (+): 0. Max coverage (-): 0

Region: chr17 73761816-73761863. Max. coverage (+): 0. Max coverage (-): 2.54

Region: chr17 73761864-73761911. Max. coverage (+): 0. Max coverage (-): 5.11

Region: chr17 73761912-73761959. Max. coverage (+): 0. Max coverage (-): 4.48

Region: chr17 73761960-73762007. Max. coverage (+): 0. Max coverage (-): 0

Region: chr17 73762008-73762055. Max. coverage (+): 0. Max coverage (-): 0

Region: chr17 73762056-73762103. Max. coverage (+): 0. Max coverage (-): 3.55

Region: chr17 73762104-73762151. Max. coverage (+): 0. Max coverage (-): 3.55

Region: chr17 73762152-73762199. Max. coverage (+): 0. Max coverage (-): 6.36

Region: chr17 73762200-73762247. Max. coverage (+): 0. Max coverage (-): 6.06

Region: chr17 73762248-73762295. Max. coverage (+): 0. Max coverage (-): 1.53

Region: chr17 73762296-73762343. Max. coverage (+): 0. Max coverage (-): 0

Region: chr17 73762344-73762391. Max. coverage (+): 0. Max coverage (-): 0

Region: chr17 73762392-73762439. Max. coverage (+): 0. Max coverage (-): 3.54

Region: chr17 73762440-73762487. Max. coverage (+): 0. Max coverage (-): 9.86

Region: chr17 73762488-73762535. Max. coverage (+): 0. Max coverage (-): 0

Region: chr17 73762536-73762583. Max. coverage (+): 0. Max coverage (-): 0

Region: chr17 73762584-73762631. Max. coverage (+): 0. Max coverage (-): 0

Region: chr17 73762632-73762679. Max. coverage (+): 0. Max coverage (-): 0

Region: chr17 73762680-73762727. Max. coverage (+): 0. Max coverage (-): 1.12

Region: chr17 73762728-73762775. Max. coverage (+): 0. Max coverage (-): 31.88

Region: chr17 73762776-73762823. Max. coverage (+): 0. Max coverage (-): 30.92

Region: chr17 73762824-73762871. Max. coverage (+): 0. Max coverage (-): 0

Region: chr17 73762872-73762919. Max. coverage (+): 0. Max coverage (-): 0

Region: chr17 73762920-73762967. Max. coverage (+): 0. Max coverage (-): 0

Region: chr17 73762968-73763015. Max. coverage (+): 0. Max coverage (-): 3.56

Region: chr17 73763016-73763063. Max. coverage (+): 0. Max coverage (-): 8.43

Region: chr17 73763064-73763111. Max. coverage (+): 0. Max coverage (-): 0

Region: chr17 73763112-73763159. Max. coverage (+): 0. Max coverage (-): 4.58

Region: chr17 73763160-73763207. Max. coverage (+): 0. Max coverage (-): 0

Region: chr17 73763208-73763255. Max. coverage (+): 0. Max coverage (-): 24.13

Region: chr17 73763256-73763303. Max. coverage (+): 0. Max coverage (-): 9.31

Region: chr17 73763304-73763351. Max. coverage (+): 0. Max coverage (-): 0

Region: chr17 73763352-73763399. Max. coverage (+): 0. Max coverage (-): 0

Region: chr17 73763400-73763447. Max. coverage (+): 0. Max coverage (-): 0

Region: chr17 73763448-73763495. Max. coverage (+): 0. Max coverage (-): 0

Region: chr17 73763496-73763543. Max. coverage (+): 0. Max coverage (-): 0.83

Region: chr17 73763544-73763591. Max. coverage (+): 0. Max coverage (-): 0

Region: chr17 73763592-73763638. Max. coverage (+): 0. Max coverage (-): 0

Region: chr17 73763639-73763686. Max. coverage (+): 0. Max coverage (-): 0

Region: chr17 73763687-73763734. Max. coverage (+): 0. Max coverage (-): 0

Region: chr17 73763735-73763782. Max. coverage (+): 0. Max coverage (-): 0

Region: chr17 73763783-73763830. Max. coverage (+): 0. Max coverage (-): 3.39

Region: chr17 73763831-73763878. Max. coverage (+): 0. Max coverage (-): 3.39

Region: chr17 73763879-73763926. Max. coverage (+): 0. Max coverage (-): 4.3

Region: chr17 73763927-73763974. Max. coverage (+): 0. Max coverage (-): 0

Region: chr17 73763975-73764022. Max. coverage (+): 0. Max coverage (-): 0

Region: chr17 73764023-73764070. Max. coverage (+): 0. Max coverage (-): 0

Region: chr17 73764071-73764118. Max. coverage (+): 0. Max coverage (-): 0

Region: chr17 73764119-73764166. Max. coverage (+): 0. Max coverage (-): 0

Region: chr17 73764167-73764214. Max. coverage (+): 0. Max coverage (-): 0

Region: chr17 73764215-73764262. Max. coverage (+): 0. Max coverage (-): 1.03

Region: chr17 73764263-73764310. Max. coverage (+): 0. Max coverage (-): 5.65

Region: chr17 73764311-73764358. Max. coverage (+): 0. Max coverage (-): 0

Region: chr17 73764359-73764406. Max. coverage (+): 0. Max coverage (-): 0

Region: chr17 73764407-73764454. Max. coverage (+): 0. Max coverage (-): 4.27

Region: chr17 73764455-73764502. Max. coverage (+): 0. Max coverage (-): 4.62

Region: chr17 73764503-73764550. Max. coverage (+): 0. Max coverage (-): 2.04

Region: chr17 73764551-73764598. Max. coverage (+): 0. Max coverage (-): 0

Region: chr17 73764599-73764646. Max. coverage (+): 0. Max coverage (-): 0

Region: chr17 73764647-73764694. Max. coverage (+): 0. Max coverage (-): 0

Region: chr17 73764695-73764742. Max. coverage (+): 0. Max coverage (-): 0

Region: chr17 73764743-73764790. Max. coverage (+): 0. Max coverage (-): 0

Region: chr17 73764791-73764838. Max. coverage (+): 0. Max coverage (-): 0

Region: chr17 73764839-73764886. Max. coverage (+): 0. Max coverage (-): 0

Region: chr17 73764887-73764934. Max. coverage (+): 0. Max coverage (-): 0

Region: chr17 73764935-73764982. Max. coverage (+): 0. Max coverage (-): 0.79

Region: chr17 73764983-73765030. Max. coverage (+): 0. Max coverage (-): 0

Region: chr17 73765031-73765078. Max. coverage (+): 0. Max coverage (-): 0

Region: chr17 73765079-73765126. Max. coverage (+): 0. Max coverage (-): 2.01

Region: chr17 73765127-73765174. Max. coverage (+): 0. Max coverage (-): 0

Region: chr17 73765175-73765222. Max. coverage (+): 0. Max coverage (-): 2.24

Region: chr17 73765223-73765270. Max. coverage (+): 0. Max coverage (-): 1.64

Region: chr17 73765271-73765318. Max. coverage (+): 0. Max coverage (-): 0

Region: chr17 73765319-73765366. Max. coverage (+): 0. Max coverage (-): 0

Region: chr17 73765367-73765414. Max. coverage (+): 0. Max coverage (-): 0

Region: chr17 73765415-73765462. Max. coverage (+): 0. Max coverage (-): 0

Region: chr17 73765463-73765510. Max. coverage (+): 0. Max coverage (-): 0

Region: chr17 73765511-73765558. Max. coverage (+): 0. Max coverage (-): 0

Region: chr17 73765559-73765606. Max. coverage (+): 0. Max coverage (-): 0

Region: chr17 73765607-73765654. Max. coverage (+): 0. Max coverage (-): 0

Region: chr17 73765655-73765702. Max. coverage (+): 0. Max coverage (-): 0

Region: chr17 73765703-73765750. Max. coverage (+): 0. Max coverage (-): 3.72

Region: chr17 73765751-73765798. Max. coverage (+): 0. Max coverage (-): 0

Region: chr17 73765799-73765846. Max. coverage (+): 0. Max coverage (-): 4.49

Region: chr17 73765847-73765894. Max. coverage (+): 0. Max coverage (-): 2.13

Region: chr17 73765895-73765942. Max. coverage (+): 0. Max coverage (-): 5.24

Region: chr17 73765943-73765990. Max. coverage (+): 0. Max coverage (-): 6.6

Region: chr17 73765991-73766038. Max. coverage (+): 0. Max coverage (-): 9.68

Region: chr17 73766039-73766086. Max. coverage (+): 0. Max coverage (-): 4.63

Region: chr17 73766087-73766134. Max. coverage (+): 0. Max coverage (-): 12.09

Region: chr17 73766135-73766182. Max. coverage (+): 0. Max coverage (-): 0

Region: chr17 73766183-73766230. Max. coverage (+): 0. Max coverage (-): 0

Region: chr17 73766231-73766278. Max. coverage (+): 0. Max coverage (-): 0

Region: chr17 73766279-73766326. Max. coverage (+): 0. Max coverage (-): 0

Region: chr17 73766327-73766374. Max. coverage (+): 0. Max coverage (-): 0

Region: chr17 73766375-73766422. Max. coverage (+): 0. Max coverage (-): 0

Region: chr17 73766423-73766470. Max. coverage (+): 0. Max coverage (-): 0

Region: chr17 73766471-73766518. Max. coverage (+): 0. Max coverage (-): 6.76

Region: chr17 73766519-73766566. Max. coverage (+): 0. Max coverage (-): 0

Region: chr17 73766567-73766614. Max. coverage (+): 0. Max coverage (-): 0

Region: chr17 73766615-73766662. Max. coverage (+): 0. Max coverage (-): 0

Region: chr17 73766663-73766710. Max. coverage (+): 0. Max coverage (-): 0

Region: chr17 73766711-73766758. Max. coverage (+): 0. Max coverage (-): 0

Region: chr17 73766759-73766806. Max. coverage (+): 0. Max coverage (-): 0

Region: chr17 73766807-73766854. Max. coverage (+): 0. Max coverage (-): 0

Region: chr17 73766855-73766902. Max. coverage (+): 0. Max coverage (-): 0

Region: chr17 73766903-73766950. Max. coverage (+): 0. Max coverage (-): 0

Region: chr17 73766951-73766998. Max. coverage (+): 0. Max coverage (-): 0

Region: chr17 73766999-73767046. Max. coverage (+): 0. Max coverage (-): 0

Region: chr17 73767047-73767094. Max. coverage (+): 0. Max coverage (-): 0

Region: chr17 73767095-73767142. Max. coverage (+): 0. Max coverage (-): 0

Region: chr17 73767143-73767190. Max. coverage (+): 0. Max coverage (-): 1.42

Region: chr17 73767191-73767238. Max. coverage (+): 0. Max coverage (-): 1.42

Region: chr17 73767239-73767286. Max. coverage (+): 0. Max coverage (-): 0

Region: chr17 73767287-73767334. Max. coverage (+): 0. Max coverage (-): 0

Region: chr17 73767335-73767382. Max. coverage (+): 0. Max coverage (-): 3.3

Region: chr17 73767383-73767430. Max. coverage (+): 0. Max coverage (-): 0

Region: chr17 73767431-73767478. Max. coverage (+): 0. Max coverage (-): 0

Region: chr17 73767479-73767526. Max. coverage (+): 0. Max coverage (-): 1.22

Region: chr17 73767527-73767574. Max. coverage (+): 0. Max coverage (-): 0

Region: chr17 73767575-73767622. Max. coverage (+): 0. Max coverage (-): 14.6

Region: chr17 73767623-73767670. Max. coverage (+): 0. Max coverage (-): 0

Region: chr17 73767671-73767718. Max. coverage (+): 0. Max coverage (-): 1.51

Region: chr17 73767719-73767766. Max. coverage (+): 0. Max coverage (-): 7.45

Region: chr17 73767767-73767814. Max. coverage (+): 0. Max coverage (-): 16.02

Region: chr17 73767815-73767862. Max. coverage (+): 0. Max coverage (-): 14.46

Region: chr17 73767863-73767910. Max. coverage (+): 0. Max coverage (-): 0

Region: chr17 73767911-73767958. Max. coverage (+): 0. Max coverage (-): 0

Region: chr17 73767959-73768006. Max. coverage (+): 0. Max coverage (-): 0

Region: chr17 73768007-73768054. Max. coverage (+): 0. Max coverage (-): 0.58

Region: chr17 73768055-73768102. Max. coverage (+): 0. Max coverage (-): 9.21

Region: chr17 73768103-73768150. Max. coverage (+): 0. Max coverage (-): 16.28

Region: chr17 73768151-73768198. Max. coverage (+): 0. Max coverage (-): 0

Region: chr17 73768199-73768246. Max. coverage (+): 0. Max coverage (-): 0.77

Region: chr17 73768247-73768294. Max. coverage (+): 0. Max coverage (-): 0

Region: chr17 73768295-73768342. Max. coverage (+): 0. Max coverage (-): 0

Region: chr17 73768343-73768390. Max. coverage (+): 0. Max coverage (-): 0

Region: chr17 73768391-73768437. Max. coverage (+): 0. Max coverage (-): 4.33

Region: chr17 73768438-73768485. Max. coverage (+): 0. Max coverage (-): 5.42

Region: chr17 73768486-73768533. Max. coverage (+): 0. Max coverage (-): 0

Region: chr17 73768534-73768581. Max. coverage (+): 0. Max coverage (-): 4.58

Region: chr17 73768582-73768629. Max. coverage (+): 0. Max coverage (-): 19.87

Region: chr17 73768630-73768677. Max. coverage (+): 0. Max coverage (-): 3.86

Region: chr17 73768678-73768725. Max. coverage (+): 0. Max coverage (-): 3.18

Region: chr17 73768726-73768773. Max. coverage (+): 0. Max coverage (-): 3.22

Region: chr17 73768774-73768821. Max. coverage (+): 0. Max coverage (-): 0

Region: chr17 73768822-73768869. Max. coverage (+): 0. Max coverage (-): 0

Region: chr17 73768870-73768917. Max. coverage (+): 0. Max coverage (-): 0

Region: chr17 73768918-73768965. Max. coverage (+): 0. Max coverage (-): 0

Region: chr17 73768966-73769013. Max. coverage (+): 0. Max coverage (-): 0

Region: chr17 73769014-73769061. Max. coverage (+): 0. Max coverage (-): 0

Region: chr17 73769062-73769109. Max. coverage (+): 0. Max coverage (-): 1.39

Region: chr17 73769110-73769157. Max. coverage (+): 0. Max coverage (-): 1.27

Region: chr17 73769158-73769205. Max. coverage (+): 0. Max coverage (-): 4.79

Region: chr17 73769206-73769253. Max. coverage (+): 0. Max coverage (-): 0

Region: chr17 73769254-73769301. Max. coverage (+): 0. Max coverage (-): 8.66

Region: chr17 73769302-73769349. Max. coverage (+): 0. Max coverage (-): 0

Region: chr17 73769350-73769397. Max. coverage (+): 0. Max coverage (-): 0

Region: chr17 73769398-73769445. Max. coverage (+): 0. Max coverage (-): 0

Region: chr17 73769446-73769493. Max. coverage (+): 0. Max coverage (-): 0

Region: chr17 73769494-73769541. Max. coverage (+): 0. Max coverage (-): 1.19

Region: chr17 73769542-73769589. Max. coverage (+): 0. Max coverage (-): 3.26

Region: chr17 73769590-73769637. Max. coverage (+): 0. Max coverage (-): 0

Region: chr17 73769638-73769685. Max. coverage (+): 0. Max coverage (-): 16.6

Region: chr17 73769686-73769733. Max. coverage (+): 0. Max coverage (-): 0

Region: chr17 73769734-73769781. Max. coverage (+): 0. Max coverage (-): 0

Region: chr17 73769782-73769829. Max. coverage (+): 0. Max coverage (-): 9.57

Region: chr17 73769830-73769877. Max. coverage (+): 0. Max coverage (-): 0

Region: chr17 73769878-73769925. Max. coverage (+): 0. Max coverage (-): 2.07

Region: chr17 73769926-73769973. Max. coverage (+): 0. Max coverage (-): 0

Region: chr17 73769974-73770021. Max. coverage (+): 0. Max coverage (-): 0

Region: chr17 73770022-73770069. Max. coverage (+): 0. Max coverage (-): 7.55

Region: chr17 73770070-73770117. Max. coverage (+): 0. Max coverage (-): 0

Region: chr17 73770118-73770165. Max. coverage (+): 0. Max coverage (-): 6.95

Region: chr17 73770166-73770213. Max. coverage (+): 0. Max coverage (-): 0

Region: chr17 73770214-73770261. Max. coverage (+): 0. Max coverage (-): 0

Region: chr17 73770262-73770309. Max. coverage (+): 0. Max coverage (-): 0

Region: chr17 73770310-73770357. Max. coverage (+): 0. Max coverage (-): 0

Region: chr17 73770358-73770405. Max. coverage (+): 0. Max coverage (-): 0

Region: chr17 73770406-73770453. Max. coverage (+): 0. Max coverage (-): 0

Region: chr17 73770454-73770501. Max. coverage (+): 0. Max coverage (-): 0

Region: chr17 73770502-73770549. Max. coverage (+): 0. Max coverage (-): 0

Region: chr17 73770550-73770597. Max. coverage (+): 0. Max coverage (-): 0

Region: chr17 73770598-73770645. Max. coverage (+): 0. Max coverage (-): 0

Region: chr17 73770646-73770693. Max. coverage (+): 0. Max coverage (-): 0

Region: chr17 73770694-73770741. Max. coverage (+): 0. Max coverage (-): 0

Region: chr17 73770742-73770789. Max. coverage (+): 0. Max coverage (-): 0

Region: chr17 73770790-73770837. Max. coverage (+): 0. Max coverage (-): 28.95

Region: chr17 73770838-73770885. Max. coverage (+): 0. Max coverage (-): 46.7

Region: chr17 73770886-73770933. Max. coverage (+): 0. Max coverage (-): 0

Region: chr17 73770934-73770981. Max. coverage (+): 0. Max coverage (-): 4.46

Region: chr17 73770982-73771029. Max. coverage (+): 0. Max coverage (-): 0

Region: chr17 73771030-73771077. Max. coverage (+): 0. Max coverage (-): 0

Region: chr17 73771078-73771125. Max. coverage (+): 0. Max coverage (-): 0

Region: chr17 73771126-73771173. Max. coverage (+): 0. Max coverage (-): 0

Region: chr17 73771174-73771221. Max. coverage (+): 0. Max coverage (-): 0

Region: chr17 73771222-73771269. Max. coverage (+): 0. Max coverage (-): 1.39

Region: chr17 73771270-73771317. Max. coverage (+): 0. Max coverage (-): 13.78

Region: chr17 73771318-73771365. Max. coverage (+): 0. Max coverage (-): 4.78

Region: chr17 73771366-73771413. Max. coverage (+): 0. Max coverage (-): 0

Region: chr17 73771414-73771461. Max. coverage (+): 0. Max coverage (-): 0

Region: chr17 73771462-73771509. Max. coverage (+): 0. Max coverage (-): 0

Region: chr17 73771510-73771557. Max. coverage (+): 0. Max coverage (-): 1.41

Region: chr17 73771558-73771605. Max. coverage (+): 0. Max coverage (-): 1.45

Region: chr17 73771606-73771653. Max. coverage (+): 0. Max coverage (-): 7.42

Region: chr17 73771654-73771701. Max. coverage (+): 0. Max coverage (-): 1.08

Region: chr17 73771702-73771749. Max. coverage (+): 0. Max coverage (-): 1.28

Region: chr17 73771750-73771797. Max. coverage (+): 0. Max coverage (-): 0.22

Region: chr17 73771798-73771845. Max. coverage (+): 0. Max coverage (-): 7.6

Region: chr17 73771846-73771893. Max. coverage (+): 0. Max coverage (-): 0

Region: chr17 73771894-73771941. Max. coverage (+): 0. Max coverage (-): 0

Region: chr17 73771942-73771989. Max. coverage (+): 0. Max coverage (-): 0

Region: chr17 73771990-73772037. Max. coverage (+): 0. Max coverage (-): 0

Region: chr17 73772038-73772085. Max. coverage (+): 0. Max coverage (-): 2.04

Region: chr17 73772086-73772133. Max. coverage (+): 0. Max coverage (-): 0

Region: chr17 73772134-73772181. Max. coverage (+): 0. Max coverage (-): 0

Region: chr17 73772182-73772229. Max. coverage (+): 0. Max coverage (-): 21.99

Region: chr17 73772230-73772277. Max. coverage (+): 0. Max coverage (-): 0

Region: chr17 73772278-73772325. Max. coverage (+): 0. Max coverage (-): 1.07

Region: chr17 73772326-73772373. Max. coverage (+): 0. Max coverage (-): 1.43

Region: chr17 73772374-73772421. Max. coverage (+): 0. Max coverage (-): 22.13

Region: chr17 73772422-73772469. Max. coverage (+): 0. Max coverage (-): 14.14

Region: chr17 73772470-73772517. Max. coverage (+): 0. Max coverage (-): 3.2

Region: chr17 73772518-73772565. Max. coverage (+): 0. Max coverage (-): 4.59

Region: chr17 73772566-73772613. Max. coverage (+): 0. Max coverage (-): 0

Region: chr17 73772614-73772661. Max. coverage (+): 0. Max coverage (-): 4.01

Region: chr17 73772662-73772709. Max. coverage (+): 0. Max coverage (-): 16.26

Region: chr17 73772710-73772757. Max. coverage (+): 0. Max coverage (-): 16.26

Region: chr17 73772758-73772805. Max. coverage (+): 0. Max coverage (-): 10.65

Region: chr17 73772806-73772853. Max. coverage (+): 0. Max coverage (-): 0

Region: chr17 73772854-73772901. Max. coverage (+): 0. Max coverage (-): 4.8

Region: chr17 73772902-73772949. Max. coverage (+): 0. Max coverage (-): 0

Region: chr17 73772950-73772997. Max. coverage (+): 0. Max coverage (-): 0.68

Region: chr17 73772998-73773045. Max. coverage (+): 0. Max coverage (-): 3.58

Region: chr17 73773046-73773093. Max. coverage (+): 0. Max coverage (-): 3.58

Region: chr17 73773094-73773141. Max. coverage (+): 0. Max coverage (-): 4.19

Region: chr17 73773142-73773189. Max. coverage (+): 0. Max coverage (-): 16.97

Region: chr17 73773190-73773236. Max. coverage (+): 0. Max coverage (-): 12.99

Region: chr17 73773237-73773284. Max. coverage (+): 0. Max coverage (-): 0

Region: chr17 73773285-73773332. Max. coverage (+): 0. Max coverage (-): 0

Region: chr17 73773333-73773380. Max. coverage (+): 0. Max coverage (-): 0

Region: chr17 73773381-73773428. Max. coverage (+): 0. Max coverage (-): 3.72

Region: chr17 73773429-73773476. Max. coverage (+): 0. Max coverage (-): 0

Region: chr17 73773477-73773524. Max. coverage (+): 0. Max coverage (-): 0

Region: chr17 73773525-73773572. Max. coverage (+): 0. Max coverage (-): 0

Region: chr17 73773573-73773620. Max. coverage (+): 0. Max coverage (-): 0

Region: chr17 73773621-73773668. Max. coverage (+): 0. Max coverage (-): 0

Region: chr17 73773669-73773716. Max. coverage (+): 0. Max coverage (-): 0

Region: chr17 73773717-73773764. Max. coverage (+): 0. Max coverage (-): 0

Region: chr17 73773765-73773812. Max. coverage (+): 0. Max coverage (-): 0

Region: chr17 73773813-73773860. Max. coverage (+): 0. Max coverage (-): 0

Region: chr17 73773861-73773908. Max. coverage (+): 0. Max coverage (-): 0

Region: chr17 73773909-73773956. Max. coverage (+): 0. Max coverage (-): 0

Region: chr17 73773957-73774004. Max. coverage (+): 0. Max coverage (-): 0

Region: chr17 73774005-73774052. Max. coverage (+): 0. Max coverage (-): 0

Region: chr17 73774053-73774100. Max. coverage (+): 0. Max coverage (-): 0

Region: chr17 73774101-73774148. Max. coverage (+): 0. Max coverage (-): 6.85

Region: chr17 73774149-73774196. Max. coverage (+): 0. Max coverage (-): 5.77

Region: chr17 73774197-73774244. Max. coverage (+): 0. Max coverage (-): 0

Region: chr17 73774245-73774292. Max. coverage (+): 0. Max coverage (-): 0

Region: chr17 73774293-73774340. Max. coverage (+): 0. Max coverage (-): 13.52

Region: chr17 73774341-73774388. Max. coverage (+): 0. Max coverage (-): 3.31

Region: chr17 73774389-73774436. Max. coverage (+): 0. Max coverage (-): 0

Region: chr17 73774437-73774484. Max. coverage (+): 0. Max coverage (-): 0

Region: chr17 73774485-73774532. Max. coverage (+): 0. Max coverage (-): 0

Region: chr17 73774533-73774580. Max. coverage (+): 0. Max coverage (-): 0

Region: chr17 73774581-73774628. Max. coverage (+): 0. Max coverage (-): 0

Region: chr17 73774629-73774676. Max. coverage (+): 0. Max coverage (-): 1.4

Region: chr17 73774677-73774724. Max. coverage (+): 0. Max coverage (-): 4.98

Region: chr17 73774725-73774772. Max. coverage (+): 0. Max coverage (-): 0

Region: chr17 73774773-73774820. Max. coverage (+): 0. Max coverage (-): 9.87

Region: chr17 73774821-73774868. Max. coverage (+): 0. Max coverage (-): 1.71

Region: chr17 73774869-73774916. Max. coverage (+): 0. Max coverage (-): 0

Region: chr17 73774917-73774964. Max. coverage (+): 0. Max coverage (-): 0

Region: chr17 73774965-73775012. Max. coverage (+): 0. Max coverage (-): 0

Region: chr17 73775013-73775060. Max. coverage (+): 0. Max coverage (-): 4.86

Region: chr17 73775061-73775108. Max. coverage (+): 0. Max coverage (-): 3.16

Region: chr17 73775109-73775156. Max. coverage (+): 0. Max coverage (-): 15.66

Region: chr17 73775157-73775204. Max. coverage (+): 0. Max coverage (-): 3.32

Region: chr17 73775205-73775252. Max. coverage (+): 0. Max coverage (-): 5

Region: chr17 73775253-73775300. Max. coverage (+): 0. Max coverage (-): 8.35

Region: chr17 73775301-73775348. Max. coverage (+): 0. Max coverage (-): 2.85

Region: chr17 73775349-73775396. Max. coverage (+): 0. Max coverage (-): 5.85

Region: chr17 73775397-73775444. Max. coverage (+): 0. Max coverage (-): 0

Region: chr17 73775445-73775492. Max. coverage (+): 0. Max coverage (-): 0

Region: chr17 73775493-73775540. Max. coverage (+): 0. Max coverage (-): 2.06

Region: chr17 73775541-73775588. Max. coverage (+): 0. Max coverage (-): 0

Region: chr17 73775589-73775636. Max. coverage (+): 0. Max coverage (-): 0

Region: chr17 73775637-73775684. Max. coverage (+): 0. Max coverage (-): 0

Region: chr17 73775685-73775732. Max. coverage (+): 0. Max coverage (-): 0

Region: chr17 73775733-73775780. Max. coverage (+): 0. Max coverage (-): 0

Region: chr17 73775781-73775828. Max. coverage (+): 0. Max coverage (-): 4.54

Region: chr17 73775829-73775876. Max. coverage (+): 0. Max coverage (-): 0

Region: chr17 73775877-73775924. Max. coverage (+): 0. Max coverage (-): 0

Region: chr17 73775925-73775972. Max. coverage (+): 0. Max coverage (-): 0

Region: chr17 73775973-73776020. Max. coverage (+): 0. Max coverage (-): 0

Region: chr17 73776021-73776068. Max. coverage (+): 0. Max coverage (-): 0

Region: chr17 73776069-73776116. Max. coverage (+): 0. Max coverage (-): 0

Region: chr17 73776117-73776164. Max. coverage (+): 0. Max coverage (-): 0

Region: chr17 73776165-73776212. Max. coverage (+): 0. Max coverage (-): 0

Region: chr17 73776213-73776260. Max. coverage (+): 0. Max coverage (-): 0

Region: chr17 73776261-73776308. Max. coverage (+): 0. Max coverage (-): 0

Region: chr17 73776309-73776356. Max. coverage (+): 0. Max coverage (-): 0

Region: chr17 73776357-73776404. Max. coverage (+): 0. Max coverage (-): 0

Region: chr17 73776405-73776452. Max. coverage (+): 0. Max coverage (-): 0.97

Region: chr17 73776453-73776500. Max. coverage (+): 0. Max coverage (-): 6.79

Region: chr17 73776501-73776548. Max. coverage (+): 0. Max coverage (-): 0

Region: chr17 73776549-73776596. Max. coverage (+): 0. Max coverage (-): 0

Region: chr17 73776597-73776644. Max. coverage (+): 0. Max coverage (-): 0

Region: chr17 73776645-73776692. Max. coverage (+): 0. Max coverage (-): 0

Region: chr17 73776693-73776740. Max. coverage (+): 0. Max coverage (-): 4.59

Region: chr17 73776741-73776788. Max. coverage (+): 0. Max coverage (-): 1.99

Region: chr17 73776789-73776836. Max. coverage (+): 0. Max coverage (-): 4.82

Region: chr17 73776837-73776884. Max. coverage (+): 0. Max coverage (-): 7.33

Region: chr17 73776885-73776932. Max. coverage (+): 0. Max coverage (-): 0

Region: chr17 73776933-73776980. Max. coverage (+): 0. Max coverage (-): 2.77

Region: chr17 73776981-73777028. Max. coverage (+): 0. Max coverage (-): 4.3

Region: chr17 73777029-73777076. Max. coverage (+): 0. Max coverage (-): 1.12

Region: chr17 73777077-73777124. Max. coverage (+): 0. Max coverage (-): 0

Region: chr17 73777125-73777172. Max. coverage (+): 0. Max coverage (-): 1.76

Region: chr17 73777173-73777220. Max. coverage (+): 0. Max coverage (-): 1.76

Region: chr17 73777221-73777268. Max. coverage (+): 0. Max coverage (-): 2.11

Region: chr17 73777269-73777316. Max. coverage (+): 0. Max coverage (-): 4.46

Region: chr17 73777317-73777364. Max. coverage (+): 0. Max coverage (-): 0

Region: chr17 73777365-73777412. Max. coverage (+): 0. Max coverage (-): 0

Region: chr17 73777413-73777460. Max. coverage (+): 0. Max coverage (-): 5.9

Region: chr17 73777461-73777508. Max. coverage (+): 0. Max coverage (-): 4.66

Region: chr17 73777509-73777556. Max. coverage (+): 0. Max coverage (-): 3.87

Region: chr17 73777557-73777604. Max. coverage (+): 0. Max coverage (-): 0

Region: chr17 73777605-73777652. Max. coverage (+): 0. Max coverage (-): 0

Region: chr17 73777653-73777700. Max. coverage (+): 0. Max coverage (-): 4.89

Region: chr17 73777701-73777748. Max. coverage (+): 0. Max coverage (-): 0

Region: chr17 73777749-73777796. Max. coverage (+): 0. Max coverage (-): 0

Region: chr17 73777797-73777844. Max. coverage (+): 0. Max coverage (-): 0

Region: chr17 73777845-73777892. Max. coverage (+): 0. Max coverage (-): 0

Region: chr17 73777893-73777940. Max. coverage (+): 0. Max coverage (-): 0

Region: chr17 73777941-73777988. Max. coverage (+): 0. Max coverage (-): 4.08

Region: chr17 73777989-. Max. coverage (+): 0. Max coverage (-): 4.08

RepeatMasker Color Code

**+**

100-98% Identity

<98-95% Identity

<95-90% Identity

<90-85% Identity

<85-80% Identity

<80-75% Identity

<75-70% Identity

<70% Identity

**-**

Gene Set Color Code

**+**

Gene

Pseudogene

**-**

Topology/Coverage Color Code

Coverage Plus Strand

Coverage Minus Strand

Mainstrand: Plus

Mainstrand: Minus

Complementary Strand

Flanking Region  
(if option -flank >0)

Gene Set Annotation  

**1. BCR (protein coding, ENSBTAG00000020566) Tr:00000029392 Ex:1**: 73777868-73778104 (-)  
**2. BCR (protein coding, ENSBTAG00000020566) Tr:00000029392 Ex:2**: 73777816-73777840 (-)

  
RepeatMasker Annotation  

**1. MIR3**: 73755251-73755394 (-), Divergence to consensus: 40.7%  
**2. L2a**: 73759432-73759552 (-), Divergence to consensus: 38.6%  
**3. CHR-2\_BT**: 73759779-73759877 (+), Divergence to consensus: 19.2%  
**4. GC\_rich**: 73760267-73760288 (+), Divergence to consensus: 54.5%  
**5. L1MC4a**: 73760364-73760430 (-), Divergence to consensus: 27.6%  
**6. L1ME4c**: 73761666-73761734 (-), Divergence to consensus: 24.8%  
**7. L1MB3**: 73762465-73762707 (+), Divergence to consensus: 42.8%  
**8. SINE2-3\_BT**: 73763321-73763482 (+), Divergence to consensus: 36.5%  
**9. GC\_rich**: 73764944-73764965 (+), Divergence to consensus: 45.5%  
**10. GC\_rich**: 73765277-73765315 (+), Divergence to consensus: 69.2%  
**11. Bov-tA2**: 73766139-73766345 (-), Divergence to consensus: 12.8%  
**12. Bov-tA2**: 73766379-73766428 (+), Divergence to consensus: 22%  
**13. L1MB2**: 73766544-73766956 (+), Divergence to consensus: 45.6%  
**14. (TGGA)n**: 73767927-73767996 (+), Divergence to consensus: 12.9%  
**15. L1MC5a**: 73768164-73768207 (-), Divergence to consensus: 18.2%  
**16. Bov-tA2**: 73768793-73768969 (-), Divergence to consensus: 27.6%  
**17. Bov-tA2**: 73768970-73769090 (-), Divergence to consensus: 14.9%  
**18. MLT1E1**: 73769314-73769504 (+), Divergence to consensus: 36.6%  
**19. SINE2-1\_BT**: 73773246-73773367 (+), Divergence to consensus: 28.6%  
**20. MLT1B**: 73773599-73773966 (+), Divergence to consensus: 41.8%  
**21. Bov-tA2**: 73774368-73774570 (+), Divergence to consensus: 12%

  
Transcription Factor Binding Sites  

**RFX4\_1** (Sequence: CGTGGCAAC (+): 73774071)  
**RFX4\_2** (Sequence: CCTGGATAC (+): 73754336)  
**Gata4** (Sequence: AGATAAG (-): 73764094)  
**Gata4** (Sequence: CTTATCT (+): 73775840)
