# Supplementary material for: Small RNA sequencing of cryopreserved semen from single bull revealed altered miRNAs and piRNAs expression between High- and Low-motile sperm populations
Source: BMC Genomics. 2017 Jan 4;18:14. doi: 10.1186/s12864-016-3394-7 (PMC5209821; doi:10.1186/s12864-016-3394-7)
Supplement: Additional file 3: — Details for each piRNA clusters found in High Motile (HM) sperm fraction. Genes, repeats, transposable elements and transcription factors binding sites falling within the cluster regions were reported. (ZIP 1896 kb) [file 12864_2016_3394_MOESM3_ESM.zip › 35.html]

piRNA cluster 35


Predicted piRNA cluster no. 35     previous   next
  

Show proTRAC run info
Hide proTRAC run info

================================= proTRAC ====================================  
VERSION: 2.1                                    LAST MODIFIED: 06. October 2015  
  
Please cite:  
Rosenkranz D, Zischler H. proTRAC - a software for probabilistic piRNA cluster  
detection, visualization and analysis. 2012. BMC Bioinformatics 13:5.  
  
and (for proTRAC 2.0 and later):  
Rosenkranz D, Rudloff S, Bastuck K, Ketting RF, Zischler H. Tupaia small RNAs  
provide insights into function and evolution of RNAi-based transposon defense  
in mammals. 2015. RNA 21(5):911-922.  
  
Contact:  
David Rosenkranz  
Institute of Anthropology, small RNA group  
Johannes Gutenberg University Mainz  
email: rosenkranz@uni-mainz.de  
  
You can find the latest proTRAC version at:  
http://sourceforge.net/projects/protrac/files  
http://www.smallRNAgroup-mainz.de/software  
==============================================================================  
  
PARAMETERS:  
Map file: .............../storage/core/barbara/genhome/smallRNA/fertility/Sample\_motile/pirna/Sample\_motile\_26-33\_collapsed.fa.no-dust.map.weighted-10000-1000-b-0  
Genome file: ............/storage/core/barbara/genhome/smallRNA/fertility/Sample\_all/pirna/bt\_311\_chrY.fa  
RepeatMasker annotation: /storage/genomes/bt\_umd31/GCF\_000003055.6\_Bos\_taurus\_UMD\_3.1.1\_repeatMasker\_chr.out  
GeneSet:................./storage/core/barbara/genhome/smallRNA/fertility/Sample\_all/pirna/full.gtf  
  
Significant (p<=0.01) hit density will be calculated based  
on observed hit distribution.  
  
Sliding window size: ........................................ 5000 bp  
Sliding window increament: .................................. 1000 bp  
Normalize each hit by number of genomic hits: ............... 1 [0=no/1=yes]  
Normalize each hit by number of sequence reads: ............. 1 [0=no/1=yes]  
Normalize values (-> per million mapped reads): ............. 1 [0=no/1=yes]  
Min. fraction of hits with 1T(U) or 10A: .................... 0.75  
Alternatively: Min. fraction of hits with 1T(U) and 10A: .... 0.5  
Min. fraction of hits with typical piRNA length: ............ 0.75  
Typical piRNA length: ....................................... 26-33 nt  
Min. size of a piRNA cluster: ............................... 5000 bp.  
Min. number of hits (absolute): ............................. 0  
Min. number of hits (normalized): ........................... 0  
Min. fraction of hits on the mainstrand: .................... 0.75  
Top fraction of mapped sequences (in terms of read counts): . 1%  
Top fraction accounts for max. n% of sequence reads: ........ 90%  
Min. fraction of hits on each arm of a bidirectional cluster: 0.1  
Output image file for each cluster: ......................... 0 [0=no/1=yes]  
Output html file for each cluster: .......................... 1 [0=no/1=yes]  
Output a summary table: ..................................... 1 [0=no/1=yes]  
Output a FASTA file for each cluster (piRNA sequences): ..... 1 [0=no/1=yes]  
Output a FASTA file comprising cluster sequences: ........... 1 [0=no/1=yes]  
Search DNA motifs in clusters: .............................. 1 [0=no/1=yes]  
Output flanking sequences: +/- .............................. 0 bp  
Output ~.pTi file: .......................................... 1 [0=no/1=yes]  
==============================================================================  
  
  
Genome size (without gaps): ............ 2678902517 bp  
Gaps (N/X/-): .......................... 53837044 bp  
Mapped reads: .......................... 658825247023  
Non-identical sequences: ............... 514171  
Genomic hits: .......................... 764233  
Significant densitiy of mapped reads: .. 12867599.5173724 reads/kb

Show proTRAC cluster info
Hide proTRAC cluster info

|  |  |
| --- | --- |
| Location | chr18 |
| Coordinates | 8029412-8036551 |
| Size [bp] | 7140 |
| Sequence hit loci | 110 |
| Mapped reads (normalized) | 155795018 |
| Mapped reads (normalized) per kb | 21820030.5 |
| Normalized reads with 1T (1U) | 93.2% |
| Normalized reads with 10A | 29.5% |
| Normalized reads with length 26-33 nt | 100% |
| Normalized reads on the main strand(s) | 100% |
| Predicted directionality | mono:plus |

100%

0%

1T (1U)  
reads

10A reads

26-33 nt  
reads

reads on mainstrand

**Either the amount of reads with 1T (1U) OR 10A has to exceed 75% (set with option: -1Tor10A)  
Alternatively the amount of reads with 1T (1U) AND 10A has to exceed 50% (set with option: -1Tand10A)  
Minimum amount of reads with preferred size is 75% (set with option: -pisize)  
Minimum amount of reads on the main strand(s) is 75% (set with option: -clstrand)**

Show read coverage
Hide read coverage

WHAT DO I SEE HERE?  
This chart shows the location of mapped sequence reads within a predicted piRNA cluster. The color refers to the number of genomic hits produced by the sequence read in question. A dark red bar indicates that this sequence read produces many other hits elsewhere in the genome. Many adjacent red or yellow bars can indicate the presence of a multi-copy element such as transposons or rRNA genes. A dark green bar indicates that this sequence read maps uniquely to this locus.

1 hit

2-5 hits

6-10 hits

11-20 hits

21-50 hits

51-100 hits

> 100 hits

chr18

8029412

8036551

Gene Set

RepeatMasker

Mapped  
Reads

17.92

plus strand

minus strand

17.92

Region: chr18 73777990-8029419. Max. coverage (+): 4.61. Max coverage (-): 0

Region: chr18 8029420-8029433. Max. coverage (+): 0. Max coverage (-): 0

Region: chr18 8029434-8029447. Max. coverage (+): 0. Max coverage (-): 0

Region: chr18 8029448-8029461. Max. coverage (+): 3.68. Max coverage (-): 0

Region: chr18 8029462-8029476. Max. coverage (+): 0. Max coverage (-): 0

Region: chr18 8029477-8029490. Max. coverage (+): 0. Max coverage (-): 0

Region: chr18 8029491-8029504. Max. coverage (+): 0. Max coverage (-): 0

Region: chr18 8029505-8029519. Max. coverage (+): 0. Max coverage (-): 0

Region: chr18 8029520-8029533. Max. coverage (+): 1.41. Max coverage (-): 0

Region: chr18 8029534-8029547. Max. coverage (+): 0. Max coverage (-): 0

Region: chr18 8029548-8029561. Max. coverage (+): 0. Max coverage (-): 0

Region: chr18 8029562-8029576. Max. coverage (+): 0.87. Max coverage (-): 0

Region: chr18 8029577-8029590. Max. coverage (+): 2.39. Max coverage (-): 0

Region: chr18 8029591-8029604. Max. coverage (+): 0. Max coverage (-): 0

Region: chr18 8029605-8029619. Max. coverage (+): 0. Max coverage (-): 0

Region: chr18 8029620-8029633. Max. coverage (+): 0. Max coverage (-): 0

Region: chr18 8029634-8029647. Max. coverage (+): 0. Max coverage (-): 0

Region: chr18 8029648-8029661. Max. coverage (+): 0. Max coverage (-): 0

Region: chr18 8029662-8029676. Max. coverage (+): 0. Max coverage (-): 0

Region: chr18 8029677-8029690. Max. coverage (+): 0. Max coverage (-): 0

Region: chr18 8029691-8029704. Max. coverage (+): 0. Max coverage (-): 0

Region: chr18 8029705-8029719. Max. coverage (+): 0. Max coverage (-): 0

Region: chr18 8029720-8029733. Max. coverage (+): 0. Max coverage (-): 0

Region: chr18 8029734-8029747. Max. coverage (+): 0. Max coverage (-): 0

Region: chr18 8029748-8029761. Max. coverage (+): 0. Max coverage (-): 0

Region: chr18 8029762-8029776. Max. coverage (+): 0. Max coverage (-): 0

Region: chr18 8029777-8029790. Max. coverage (+): 8.76. Max coverage (-): 0

Region: chr18 8029791-8029804. Max. coverage (+): 8.25. Max coverage (-): 0

Region: chr18 8029805-8029818. Max. coverage (+): 0. Max coverage (-): 0

Region: chr18 8029819-8029833. Max. coverage (+): 0. Max coverage (-): 0

Region: chr18 8029834-8029847. Max. coverage (+): 0. Max coverage (-): 0

Region: chr18 8029848-8029861. Max. coverage (+): 0. Max coverage (-): 0

Region: chr18 8029862-8029876. Max. coverage (+): 1.13. Max coverage (-): 0

Region: chr18 8029877-8029890. Max. coverage (+): 1.13. Max coverage (-): 0

Region: chr18 8029891-8029904. Max. coverage (+): 0. Max coverage (-): 0

Region: chr18 8029905-8029918. Max. coverage (+): 1.37. Max coverage (-): 0

Region: chr18 8029919-8029933. Max. coverage (+): 0. Max coverage (-): 0

Region: chr18 8029934-8029947. Max. coverage (+): 0. Max coverage (-): 0

Region: chr18 8029948-8029961. Max. coverage (+): 5.06. Max coverage (-): 0

Region: chr18 8029962-8029976. Max. coverage (+): 0. Max coverage (-): 0

Region: chr18 8029977-8029990. Max. coverage (+): 0. Max coverage (-): 0

Region: chr18 8029991-8030004. Max. coverage (+): 0. Max coverage (-): 0

Region: chr18 8030005-8030018. Max. coverage (+): 0. Max coverage (-): 0

Region: chr18 8030019-8030033. Max. coverage (+): 0. Max coverage (-): 0

Region: chr18 8030034-8030047. Max. coverage (+): 0. Max coverage (-): 0

Region: chr18 8030048-8030061. Max. coverage (+): 0. Max coverage (-): 0

Region: chr18 8030062-8030076. Max. coverage (+): 0. Max coverage (-): 0

Region: chr18 8030077-8030090. Max. coverage (+): 0. Max coverage (-): 0

Region: chr18 8030091-8030104. Max. coverage (+): 6.01. Max coverage (-): 0

Region: chr18 8030105-8030118. Max. coverage (+): 10.09. Max coverage (-): 0

Region: chr18 8030119-8030133. Max. coverage (+): 0. Max coverage (-): 0

Region: chr18 8030134-8030147. Max. coverage (+): 0. Max coverage (-): 0

Region: chr18 8030148-8030161. Max. coverage (+): 1.77. Max coverage (-): 0

Region: chr18 8030162-8030175. Max. coverage (+): 0. Max coverage (-): 0

Region: chr18 8030176-8030190. Max. coverage (+): 0. Max coverage (-): 0

Region: chr18 8030191-8030204. Max. coverage (+): 0. Max coverage (-): 0

Region: chr18 8030205-8030218. Max. coverage (+): 0. Max coverage (-): 0

Region: chr18 8030219-8030233. Max. coverage (+): 0. Max coverage (-): 0

Region: chr18 8030234-8030247. Max. coverage (+): 0. Max coverage (-): 0

Region: chr18 8030248-8030261. Max. coverage (+): 0. Max coverage (-): 0

Region: chr18 8030262-8030275. Max. coverage (+): 0. Max coverage (-): 0

Region: chr18 8030276-8030290. Max. coverage (+): 0. Max coverage (-): 0

Region: chr18 8030291-8030304. Max. coverage (+): 0. Max coverage (-): 0

Region: chr18 8030305-8030318. Max. coverage (+): 0. Max coverage (-): 0

Region: chr18 8030319-8030333. Max. coverage (+): 0. Max coverage (-): 0

Region: chr18 8030334-8030347. Max. coverage (+): 0. Max coverage (-): 0

Region: chr18 8030348-8030361. Max. coverage (+): 0. Max coverage (-): 0

Region: chr18 8030362-8030375. Max. coverage (+): 3.07. Max coverage (-): 0

Region: chr18 8030376-8030390. Max. coverage (+): 0. Max coverage (-): 0

Region: chr18 8030391-8030404. Max. coverage (+): 0. Max coverage (-): 0

Region: chr18 8030405-8030418. Max. coverage (+): 6.28. Max coverage (-): 0

Region: chr18 8030419-8030433. Max. coverage (+): 0. Max coverage (-): 0

Region: chr18 8030434-8030447. Max. coverage (+): 6.84. Max coverage (-): 0

Region: chr18 8030448-8030461. Max. coverage (+): 11.29. Max coverage (-): 0

Region: chr18 8030462-8030475. Max. coverage (+): 1.08. Max coverage (-): 0

Region: chr18 8030476-8030490. Max. coverage (+): 0. Max coverage (-): 0

Region: chr18 8030491-8030504. Max. coverage (+): 0. Max coverage (-): 0

Region: chr18 8030505-8030518. Max. coverage (+): 0. Max coverage (-): 0

Region: chr18 8030519-8030532. Max. coverage (+): 0. Max coverage (-): 0

Region: chr18 8030533-8030547. Max. coverage (+): 0. Max coverage (-): 0

Region: chr18 8030548-8030561. Max. coverage (+): 0. Max coverage (-): 0

Region: chr18 8030562-8030575. Max. coverage (+): 0. Max coverage (-): 0

Region: chr18 8030576-8030590. Max. coverage (+): 1.25. Max coverage (-): 0

Region: chr18 8030591-8030604. Max. coverage (+): 0.87. Max coverage (-): 0

Region: chr18 8030605-8030618. Max. coverage (+): 3.84. Max coverage (-): 0

Region: chr18 8030619-8030632. Max. coverage (+): 7.95. Max coverage (-): 0

Region: chr18 8030633-8030647. Max. coverage (+): 5.04. Max coverage (-): 0

Region: chr18 8030648-8030661. Max. coverage (+): 0. Max coverage (-): 0

Region: chr18 8030662-8030675. Max. coverage (+): 0. Max coverage (-): 0

Region: chr18 8030676-8030690. Max. coverage (+): 2.86. Max coverage (-): 0

Region: chr18 8030691-8030704. Max. coverage (+): 3.28. Max coverage (-): 0

Region: chr18 8030705-8030718. Max. coverage (+): 0. Max coverage (-): 0

Region: chr18 8030719-8030732. Max. coverage (+): 0. Max coverage (-): 0

Region: chr18 8030733-8030747. Max. coverage (+): 0. Max coverage (-): 0

Region: chr18 8030748-8030761. Max. coverage (+): 0. Max coverage (-): 0

Region: chr18 8030762-8030775. Max. coverage (+): 3.85. Max coverage (-): 0

Region: chr18 8030776-8030790. Max. coverage (+): 3.85. Max coverage (-): 0

Region: chr18 8030791-8030804. Max. coverage (+): 0. Max coverage (-): 0

Region: chr18 8030805-8030818. Max. coverage (+): 0. Max coverage (-): 0

Region: chr18 8030819-8030832. Max. coverage (+): 0. Max coverage (-): 0

Region: chr18 8030833-8030847. Max. coverage (+): 0. Max coverage (-): 0

Region: chr18 8030848-8030861. Max. coverage (+): 7.26. Max coverage (-): 0

Region: chr18 8030862-8030875. Max. coverage (+): 0. Max coverage (-): 0

Region: chr18 8030876-8030889. Max. coverage (+): 0. Max coverage (-): 0

Region: chr18 8030890-8030904. Max. coverage (+): 0. Max coverage (-): 0

Region: chr18 8030905-8030918. Max. coverage (+): 0. Max coverage (-): 0

Region: chr18 8030919-8030932. Max. coverage (+): 0. Max coverage (-): 0

Region: chr18 8030933-8030947. Max. coverage (+): 2.5. Max coverage (-): 0

Region: chr18 8030948-8030961. Max. coverage (+): 0. Max coverage (-): 0

Region: chr18 8030962-8030975. Max. coverage (+): 0. Max coverage (-): 0

Region: chr18 8030976-8030989. Max. coverage (+): 0. Max coverage (-): 0

Region: chr18 8030990-8031004. Max. coverage (+): 6.93. Max coverage (-): 0

Region: chr18 8031005-8031018. Max. coverage (+): 6.93. Max coverage (-): 0

Region: chr18 8031019-8031032. Max. coverage (+): 0. Max coverage (-): 0

Region: chr18 8031033-8031047. Max. coverage (+): 0. Max coverage (-): 0

Region: chr18 8031048-8031061. Max. coverage (+): 0. Max coverage (-): 0

Region: chr18 8031062-8031075. Max. coverage (+): 0.91. Max coverage (-): 0

Region: chr18 8031076-8031089. Max. coverage (+): 0. Max coverage (-): 0

Region: chr18 8031090-8031104. Max. coverage (+): 0. Max coverage (-): 0

Region: chr18 8031105-8031118. Max. coverage (+): 0. Max coverage (-): 0

Region: chr18 8031119-8031132. Max. coverage (+): 0. Max coverage (-): 0

Region: chr18 8031133-8031147. Max. coverage (+): 0. Max coverage (-): 0

Region: chr18 8031148-8031161. Max. coverage (+): 0. Max coverage (-): 0

Region: chr18 8031162-8031175. Max. coverage (+): 0. Max coverage (-): 0

Region: chr18 8031176-8031189. Max. coverage (+): 0. Max coverage (-): 0

Region: chr18 8031190-8031204. Max. coverage (+): 2.29. Max coverage (-): 0

Region: chr18 8031205-8031218. Max. coverage (+): 0. Max coverage (-): 0

Region: chr18 8031219-8031232. Max. coverage (+): 0. Max coverage (-): 0

Region: chr18 8031233-8031246. Max. coverage (+): 0. Max coverage (-): 0

Region: chr18 8031247-8031261. Max. coverage (+): 0. Max coverage (-): 0

Region: chr18 8031262-8031275. Max. coverage (+): 0. Max coverage (-): 0

Region: chr18 8031276-8031289. Max. coverage (+): 0. Max coverage (-): 0

Region: chr18 8031290-8031304. Max. coverage (+): 0. Max coverage (-): 0

Region: chr18 8031305-8031318. Max. coverage (+): 0. Max coverage (-): 0

Region: chr18 8031319-8031332. Max. coverage (+): 0. Max coverage (-): 0

Region: chr18 8031333-8031346. Max. coverage (+): 0. Max coverage (-): 0

Region: chr18 8031347-8031361. Max. coverage (+): 0. Max coverage (-): 0

Region: chr18 8031362-8031375. Max. coverage (+): 0. Max coverage (-): 0

Region: chr18 8031376-8031389. Max. coverage (+): 0. Max coverage (-): 0

Region: chr18 8031390-8031404. Max. coverage (+): 0. Max coverage (-): 0

Region: chr18 8031405-8031418. Max. coverage (+): 0. Max coverage (-): 0

Region: chr18 8031419-8031432. Max. coverage (+): 0. Max coverage (-): 0

Region: chr18 8031433-8031446. Max. coverage (+): 1.04. Max coverage (-): 0

Region: chr18 8031447-8031461. Max. coverage (+): 1.04. Max coverage (-): 0

Region: chr18 8031462-8031475. Max. coverage (+): 0. Max coverage (-): 0

Region: chr18 8031476-8031489. Max. coverage (+): 0. Max coverage (-): 0

Region: chr18 8031490-8031504. Max. coverage (+): 0. Max coverage (-): 0

Region: chr18 8031505-8031518. Max. coverage (+): 0. Max coverage (-): 0

Region: chr18 8031519-8031532. Max. coverage (+): 0. Max coverage (-): 0

Region: chr18 8031533-8031546. Max. coverage (+): 0. Max coverage (-): 0

Region: chr18 8031547-8031561. Max. coverage (+): 0. Max coverage (-): 0

Region: chr18 8031562-8031575. Max. coverage (+): 0. Max coverage (-): 0

Region: chr18 8031576-8031589. Max. coverage (+): 0. Max coverage (-): 0

Region: chr18 8031590-8031603. Max. coverage (+): 0. Max coverage (-): 0

Region: chr18 8031604-8031618. Max. coverage (+): 0. Max coverage (-): 0

Region: chr18 8031619-8031632. Max. coverage (+): 0. Max coverage (-): 0

Region: chr18 8031633-8031646. Max. coverage (+): 0. Max coverage (-): 0

Region: chr18 8031647-8031661. Max. coverage (+): 0. Max coverage (-): 0

Region: chr18 8031662-8031675. Max. coverage (+): 0. Max coverage (-): 0

Region: chr18 8031676-8031689. Max. coverage (+): 0. Max coverage (-): 0

Region: chr18 8031690-8031703. Max. coverage (+): 0. Max coverage (-): 0

Region: chr18 8031704-8031718. Max. coverage (+): 0. Max coverage (-): 0

Region: chr18 8031719-8031732. Max. coverage (+): 0. Max coverage (-): 0

Region: chr18 8031733-8031746. Max. coverage (+): 0. Max coverage (-): 0

Region: chr18 8031747-8031761. Max. coverage (+): 0. Max coverage (-): 0

Region: chr18 8031762-8031775. Max. coverage (+): 0. Max coverage (-): 0

Region: chr18 8031776-8031789. Max. coverage (+): 0. Max coverage (-): 0

Region: chr18 8031790-8031803. Max. coverage (+): 0. Max coverage (-): 0

Region: chr18 8031804-8031818. Max. coverage (+): 0. Max coverage (-): 0

Region: chr18 8031819-8031832. Max. coverage (+): 0. Max coverage (-): 0

Region: chr18 8031833-8031846. Max. coverage (+): 0. Max coverage (-): 0

Region: chr18 8031847-8031861. Max. coverage (+): 0. Max coverage (-): 0

Region: chr18 8031862-8031875. Max. coverage (+): 0. Max coverage (-): 0

Region: chr18 8031876-8031889. Max. coverage (+): 0. Max coverage (-): 0

Region: chr18 8031890-8031903. Max. coverage (+): 0. Max coverage (-): 0

Region: chr18 8031904-8031918. Max. coverage (+): 0. Max coverage (-): 0

Region: chr18 8031919-8031932. Max. coverage (+): 0. Max coverage (-): 0

Region: chr18 8031933-8031946. Max. coverage (+): 0. Max coverage (-): 0

Region: chr18 8031947-8031960. Max. coverage (+): 0. Max coverage (-): 0

Region: chr18 8031961-8031975. Max. coverage (+): 0. Max coverage (-): 0

Region: chr18 8031976-8031989. Max. coverage (+): 0. Max coverage (-): 0

Region: chr18 8031990-8032003. Max. coverage (+): 0. Max coverage (-): 0

Region: chr18 8032004-8032018. Max. coverage (+): 0. Max coverage (-): 0

Region: chr18 8032019-8032032. Max. coverage (+): 0. Max coverage (-): 0

Region: chr18 8032033-8032046. Max. coverage (+): 0. Max coverage (-): 0

Region: chr18 8032047-8032060. Max. coverage (+): 0. Max coverage (-): 0

Region: chr18 8032061-8032075. Max. coverage (+): 0. Max coverage (-): 0

Region: chr18 8032076-8032089. Max. coverage (+): 0. Max coverage (-): 0

Region: chr18 8032090-8032103. Max. coverage (+): 0. Max coverage (-): 0

Region: chr18 8032104-8032118. Max. coverage (+): 0. Max coverage (-): 0

Region: chr18 8032119-8032132. Max. coverage (+): 0. Max coverage (-): 0

Region: chr18 8032133-8032146. Max. coverage (+): 2.96. Max coverage (-): 0

Region: chr18 8032147-8032160. Max. coverage (+): 2.96. Max coverage (-): 0

Region: chr18 8032161-8032175. Max. coverage (+): 1.21. Max coverage (-): 0

Region: chr18 8032176-8032189. Max. coverage (+): 1.74. Max coverage (-): 0

Region: chr18 8032190-8032203. Max. coverage (+): 0. Max coverage (-): 0

Region: chr18 8032204-8032218. Max. coverage (+): 0. Max coverage (-): 0

Region: chr18 8032219-8032232. Max. coverage (+): 0. Max coverage (-): 0

Region: chr18 8032233-8032246. Max. coverage (+): 0. Max coverage (-): 0

Region: chr18 8032247-8032260. Max. coverage (+): 1.68. Max coverage (-): 0

Region: chr18 8032261-8032275. Max. coverage (+): 5.7. Max coverage (-): 0

Region: chr18 8032276-8032289. Max. coverage (+): 0.94. Max coverage (-): 0

Region: chr18 8032290-8032303. Max. coverage (+): 0. Max coverage (-): 0

Region: chr18 8032304-8032317. Max. coverage (+): 0. Max coverage (-): 0

Region: chr18 8032318-8032332. Max. coverage (+): 0. Max coverage (-): 0

Region: chr18 8032333-8032346. Max. coverage (+): 0. Max coverage (-): 0

Region: chr18 8032347-8032360. Max. coverage (+): 0. Max coverage (-): 0

Region: chr18 8032361-8032375. Max. coverage (+): 0. Max coverage (-): 0

Region: chr18 8032376-8032389. Max. coverage (+): 0. Max coverage (-): 0

Region: chr18 8032390-8032403. Max. coverage (+): 0. Max coverage (-): 0

Region: chr18 8032404-8032417. Max. coverage (+): 0. Max coverage (-): 0

Region: chr18 8032418-8032432. Max. coverage (+): 0. Max coverage (-): 0

Region: chr18 8032433-8032446. Max. coverage (+): 0. Max coverage (-): 0

Region: chr18 8032447-8032460. Max. coverage (+): 0. Max coverage (-): 0

Region: chr18 8032461-8032475. Max. coverage (+): 0.48. Max coverage (-): 0

Region: chr18 8032476-8032489. Max. coverage (+): 5.24. Max coverage (-): 0

Region: chr18 8032490-8032503. Max. coverage (+): 6. Max coverage (-): 0

Region: chr18 8032504-8032517. Max. coverage (+): 0. Max coverage (-): 0

Region: chr18 8032518-8032532. Max. coverage (+): 0. Max coverage (-): 0

Region: chr18 8032533-8032546. Max. coverage (+): 0. Max coverage (-): 0

Region: chr18 8032547-8032560. Max. coverage (+): 0. Max coverage (-): 0

Region: chr18 8032561-8032575. Max. coverage (+): 0. Max coverage (-): 0

Region: chr18 8032576-8032589. Max. coverage (+): 0. Max coverage (-): 0

Region: chr18 8032590-8032603. Max. coverage (+): 0. Max coverage (-): 0

Region: chr18 8032604-8032617. Max. coverage (+): 0. Max coverage (-): 0

Region: chr18 8032618-8032632. Max. coverage (+): 4.34. Max coverage (-): 0

Region: chr18 8032633-8032646. Max. coverage (+): 0. Max coverage (-): 0

Region: chr18 8032647-8032660. Max. coverage (+): 0. Max coverage (-): 0

Region: chr18 8032661-8032674. Max. coverage (+): 0. Max coverage (-): 0

Region: chr18 8032675-8032689. Max. coverage (+): 0. Max coverage (-): 0

Region: chr18 8032690-8032703. Max. coverage (+): 1.19. Max coverage (-): 0

Region: chr18 8032704-8032717. Max. coverage (+): 0. Max coverage (-): 0

Region: chr18 8032718-8032732. Max. coverage (+): 0.41. Max coverage (-): 0

Region: chr18 8032733-8032746. Max. coverage (+): 4.34. Max coverage (-): 0

Region: chr18 8032747-8032760. Max. coverage (+): 0. Max coverage (-): 0

Region: chr18 8032761-8032774. Max. coverage (+): 0. Max coverage (-): 0

Region: chr18 8032775-8032789. Max. coverage (+): 0. Max coverage (-): 0

Region: chr18 8032790-8032803. Max. coverage (+): 11.47. Max coverage (-): 0

Region: chr18 8032804-8032817. Max. coverage (+): 8.72. Max coverage (-): 0

Region: chr18 8032818-8032832. Max. coverage (+): 0. Max coverage (-): 0

Region: chr18 8032833-8032846. Max. coverage (+): 0. Max coverage (-): 0

Region: chr18 8032847-8032860. Max. coverage (+): 4.66. Max coverage (-): 0

Region: chr18 8032861-8032874. Max. coverage (+): 1.89. Max coverage (-): 0

Region: chr18 8032875-8032889. Max. coverage (+): 1.89. Max coverage (-): 0

Region: chr18 8032890-8032903. Max. coverage (+): 0. Max coverage (-): 0

Region: chr18 8032904-8032917. Max. coverage (+): 0. Max coverage (-): 0

Region: chr18 8032918-8032932. Max. coverage (+): 3.99. Max coverage (-): 0

Region: chr18 8032933-8032946. Max. coverage (+): 1.56. Max coverage (-): 0

Region: chr18 8032947-8032960. Max. coverage (+): 0. Max coverage (-): 0

Region: chr18 8032961-8032974. Max. coverage (+): 0. Max coverage (-): 0

Region: chr18 8032975-8032989. Max. coverage (+): 0. Max coverage (-): 0

Region: chr18 8032990-8033003. Max. coverage (+): 0. Max coverage (-): 0

Region: chr18 8033004-8033017. Max. coverage (+): 0. Max coverage (-): 0

Region: chr18 8033018-8033031. Max. coverage (+): 0. Max coverage (-): 0

Region: chr18 8033032-8033046. Max. coverage (+): 0. Max coverage (-): 0

Region: chr18 8033047-8033060. Max. coverage (+): 0. Max coverage (-): 0

Region: chr18 8033061-8033074. Max. coverage (+): 0. Max coverage (-): 0

Region: chr18 8033075-8033089. Max. coverage (+): 0. Max coverage (-): 0

Region: chr18 8033090-8033103. Max. coverage (+): 0. Max coverage (-): 0

Region: chr18 8033104-8033117. Max. coverage (+): 0.84. Max coverage (-): 0

Region: chr18 8033118-8033131. Max. coverage (+): 0. Max coverage (-): 0

Region: chr18 8033132-8033146. Max. coverage (+): 1.42. Max coverage (-): 0

Region: chr18 8033147-8033160. Max. coverage (+): 0. Max coverage (-): 0

Region: chr18 8033161-8033174. Max. coverage (+): 0. Max coverage (-): 0

Region: chr18 8033175-8033189. Max. coverage (+): 0. Max coverage (-): 0

Region: chr18 8033190-8033203. Max. coverage (+): 0. Max coverage (-): 0

Region: chr18 8033204-8033217. Max. coverage (+): 0. Max coverage (-): 0

Region: chr18 8033218-8033231. Max. coverage (+): 0. Max coverage (-): 0

Region: chr18 8033232-8033246. Max. coverage (+): 0. Max coverage (-): 0

Region: chr18 8033247-8033260. Max. coverage (+): 0. Max coverage (-): 0

Region: chr18 8033261-8033274. Max. coverage (+): 0. Max coverage (-): 0

Region: chr18 8033275-8033289. Max. coverage (+): 0. Max coverage (-): 0

Region: chr18 8033290-8033303. Max. coverage (+): 0. Max coverage (-): 0

Region: chr18 8033304-8033317. Max. coverage (+): 0. Max coverage (-): 0

Region: chr18 8033318-8033331. Max. coverage (+): 1.3. Max coverage (-): 0

Region: chr18 8033332-8033346. Max. coverage (+): 0. Max coverage (-): 0

Region: chr18 8033347-8033360. Max. coverage (+): 0. Max coverage (-): 0

Region: chr18 8033361-8033374. Max. coverage (+): 0. Max coverage (-): 0

Region: chr18 8033375-8033388. Max. coverage (+): 0. Max coverage (-): 0

Region: chr18 8033389-8033403. Max. coverage (+): 0. Max coverage (-): 0

Region: chr18 8033404-8033417. Max. coverage (+): 0. Max coverage (-): 0

Region: chr18 8033418-8033431. Max. coverage (+): 5.89. Max coverage (-): 0

Region: chr18 8033432-8033446. Max. coverage (+): 0. Max coverage (-): 0

Region: chr18 8033447-8033460. Max. coverage (+): 0. Max coverage (-): 0

Region: chr18 8033461-8033474. Max. coverage (+): 0. Max coverage (-): 0

Region: chr18 8033475-8033488. Max. coverage (+): 0. Max coverage (-): 0

Region: chr18 8033489-8033503. Max. coverage (+): 0. Max coverage (-): 0

Region: chr18 8033504-8033517. Max. coverage (+): 0. Max coverage (-): 0

Region: chr18 8033518-8033531. Max. coverage (+): 0. Max coverage (-): 0

Region: chr18 8033532-8033546. Max. coverage (+): 1.14. Max coverage (-): 0

Region: chr18 8033547-8033560. Max. coverage (+): 0. Max coverage (-): 0

Region: chr18 8033561-8033574. Max. coverage (+): 0. Max coverage (-): 0

Region: chr18 8033575-8033588. Max. coverage (+): 0. Max coverage (-): 0

Region: chr18 8033589-8033603. Max. coverage (+): 0. Max coverage (-): 0

Region: chr18 8033604-8033617. Max. coverage (+): 0. Max coverage (-): 0

Region: chr18 8033618-8033631. Max. coverage (+): 1.96. Max coverage (-): 0

Region: chr18 8033632-8033646. Max. coverage (+): 0. Max coverage (-): 0

Region: chr18 8033647-8033660. Max. coverage (+): 0. Max coverage (-): 0

Region: chr18 8033661-8033674. Max. coverage (+): 0. Max coverage (-): 0

Region: chr18 8033675-8033688. Max. coverage (+): 0. Max coverage (-): 0

Region: chr18 8033689-8033703. Max. coverage (+): 0.91. Max coverage (-): 0

Region: chr18 8033704-8033717. Max. coverage (+): 9.12. Max coverage (-): 0

Region: chr18 8033718-8033731. Max. coverage (+): 17.92. Max coverage (-): 0

Region: chr18 8033732-8033745. Max. coverage (+): 17.92. Max coverage (-): 0

Region: chr18 8033746-8033760. Max. coverage (+): 0. Max coverage (-): 0

Region: chr18 8033761-8033774. Max. coverage (+): 0. Max coverage (-): 0

Region: chr18 8033775-8033788. Max. coverage (+): 0. Max coverage (-): 0

Region: chr18 8033789-8033803. Max. coverage (+): 0. Max coverage (-): 0

Region: chr18 8033804-8033817. Max. coverage (+): 0. Max coverage (-): 0

Region: chr18 8033818-8033831. Max. coverage (+): 0. Max coverage (-): 0

Region: chr18 8033832-8033845. Max. coverage (+): 0. Max coverage (-): 0

Region: chr18 8033846-8033860. Max. coverage (+): 0. Max coverage (-): 0

Region: chr18 8033861-8033874. Max. coverage (+): 0. Max coverage (-): 0

Region: chr18 8033875-8033888. Max. coverage (+): 0. Max coverage (-): 0

Region: chr18 8033889-8033903. Max. coverage (+): 0. Max coverage (-): 0

Region: chr18 8033904-8033917. Max. coverage (+): 0. Max coverage (-): 0

Region: chr18 8033918-8033931. Max. coverage (+): 0. Max coverage (-): 0

Region: chr18 8033932-8033945. Max. coverage (+): 0. Max coverage (-): 0

Region: chr18 8033946-8033960. Max. coverage (+): 0. Max coverage (-): 0

Region: chr18 8033961-8033974. Max. coverage (+): 0. Max coverage (-): 0

Region: chr18 8033975-8033988. Max. coverage (+): 0. Max coverage (-): 0

Region: chr18 8033989-8034003. Max. coverage (+): 0. Max coverage (-): 0

Region: chr18 8034004-8034017. Max. coverage (+): 0. Max coverage (-): 0

Region: chr18 8034018-8034031. Max. coverage (+): 0. Max coverage (-): 0

Region: chr18 8034032-8034045. Max. coverage (+): 0. Max coverage (-): 0

Region: chr18 8034046-8034060. Max. coverage (+): 0. Max coverage (-): 0

Region: chr18 8034061-8034074. Max. coverage (+): 0. Max coverage (-): 0

Region: chr18 8034075-8034088. Max. coverage (+): 0. Max coverage (-): 0

Region: chr18 8034089-8034102. Max. coverage (+): 0. Max coverage (-): 0

Region: chr18 8034103-8034117. Max. coverage (+): 0. Max coverage (-): 0

Region: chr18 8034118-8034131. Max. coverage (+): 0. Max coverage (-): 0

Region: chr18 8034132-8034145. Max. coverage (+): 0. Max coverage (-): 0

Region: chr18 8034146-8034160. Max. coverage (+): 0. Max coverage (-): 0

Region: chr18 8034161-8034174. Max. coverage (+): 0. Max coverage (-): 0

Region: chr18 8034175-8034188. Max. coverage (+): 0. Max coverage (-): 0

Region: chr18 8034189-8034202. Max. coverage (+): 0. Max coverage (-): 0

Region: chr18 8034203-8034217. Max. coverage (+): 0. Max coverage (-): 0

Region: chr18 8034218-8034231. Max. coverage (+): 0. Max coverage (-): 0

Region: chr18 8034232-8034245. Max. coverage (+): 0. Max coverage (-): 0

Region: chr18 8034246-8034260. Max. coverage (+): 0. Max coverage (-): 0

Region: chr18 8034261-8034274. Max. coverage (+): 0. Max coverage (-): 0

Region: chr18 8034275-8034288. Max. coverage (+): 0. Max coverage (-): 0

Region: chr18 8034289-8034302. Max. coverage (+): 0. Max coverage (-): 0

Region: chr18 8034303-8034317. Max. coverage (+): 0. Max coverage (-): 0

Region: chr18 8034318-8034331. Max. coverage (+): 0. Max coverage (-): 0

Region: chr18 8034332-8034345. Max. coverage (+): 0. Max coverage (-): 0

Region: chr18 8034346-8034360. Max. coverage (+): 0. Max coverage (-): 0

Region: chr18 8034361-8034374. Max. coverage (+): 0. Max coverage (-): 0

Region: chr18 8034375-8034388. Max. coverage (+): 0. Max coverage (-): 0

Region: chr18 8034389-8034402. Max. coverage (+): 0. Max coverage (-): 0

Region: chr18 8034403-8034417. Max. coverage (+): 0. Max coverage (-): 0

Region: chr18 8034418-8034431. Max. coverage (+): 0. Max coverage (-): 0

Region: chr18 8034432-8034445. Max. coverage (+): 0. Max coverage (-): 0

Region: chr18 8034446-8034459. Max. coverage (+): 0. Max coverage (-): 0

Region: chr18 8034460-8034474. Max. coverage (+): 0. Max coverage (-): 0

Region: chr18 8034475-8034488. Max. coverage (+): 0. Max coverage (-): 0

Region: chr18 8034489-8034502. Max. coverage (+): 0. Max coverage (-): 0

Region: chr18 8034503-8034517. Max. coverage (+): 0. Max coverage (-): 0

Region: chr18 8034518-8034531. Max. coverage (+): 0. Max coverage (-): 0

Region: chr18 8034532-8034545. Max. coverage (+): 0. Max coverage (-): 0

Region: chr18 8034546-8034559. Max. coverage (+): 0. Max coverage (-): 0

Region: chr18 8034560-8034574. Max. coverage (+): 0. Max coverage (-): 0

Region: chr18 8034575-8034588. Max. coverage (+): 0. Max coverage (-): 0

Region: chr18 8034589-8034602. Max. coverage (+): 0. Max coverage (-): 0

Region: chr18 8034603-8034617. Max. coverage (+): 0. Max coverage (-): 0

Region: chr18 8034618-8034631. Max. coverage (+): 3.39. Max coverage (-): 0

Region: chr18 8034632-8034645. Max. coverage (+): 3.39. Max coverage (-): 0

Region: chr18 8034646-8034659. Max. coverage (+): 0. Max coverage (-): 0

Region: chr18 8034660-8034674. Max. coverage (+): 0. Max coverage (-): 0

Region: chr18 8034675-8034688. Max. coverage (+): 0. Max coverage (-): 0

Region: chr18 8034689-8034702. Max. coverage (+): 0. Max coverage (-): 0

Region: chr18 8034703-8034717. Max. coverage (+): 2.62. Max coverage (-): 0

Region: chr18 8034718-8034731. Max. coverage (+): 2.62. Max coverage (-): 0

Region: chr18 8034732-8034745. Max. coverage (+): 0. Max coverage (-): 0

Region: chr18 8034746-8034759. Max. coverage (+): 0. Max coverage (-): 0

Region: chr18 8034760-8034774. Max. coverage (+): 0. Max coverage (-): 0

Region: chr18 8034775-8034788. Max. coverage (+): 0. Max coverage (-): 0

Region: chr18 8034789-8034802. Max. coverage (+): 0. Max coverage (-): 0

Region: chr18 8034803-8034816. Max. coverage (+): 0. Max coverage (-): 0

Region: chr18 8034817-8034831. Max. coverage (+): 0. Max coverage (-): 0

Region: chr18 8034832-8034845. Max. coverage (+): 0. Max coverage (-): 0

Region: chr18 8034846-8034859. Max. coverage (+): 0. Max coverage (-): 0

Region: chr18 8034860-8034874. Max. coverage (+): 0. Max coverage (-): 0

Region: chr18 8034875-8034888. Max. coverage (+): 0. Max coverage (-): 0

Region: chr18 8034889-8034902. Max. coverage (+): 0. Max coverage (-): 0

Region: chr18 8034903-8034916. Max. coverage (+): 0. Max coverage (-): 0

Region: chr18 8034917-8034931. Max. coverage (+): 0. Max coverage (-): 0

Region: chr18 8034932-8034945. Max. coverage (+): 0. Max coverage (-): 0

Region: chr18 8034946-8034959. Max. coverage (+): 0. Max coverage (-): 0

Region: chr18 8034960-8034974. Max. coverage (+): 0. Max coverage (-): 0

Region: chr18 8034975-8034988. Max. coverage (+): 0. Max coverage (-): 0

Region: chr18 8034989-8035002. Max. coverage (+): 0. Max coverage (-): 0

Region: chr18 8035003-8035016. Max. coverage (+): 0. Max coverage (-): 0

Region: chr18 8035017-8035031. Max. coverage (+): 4.38. Max coverage (-): 0

Region: chr18 8035032-8035045. Max. coverage (+): 4.38. Max coverage (-): 0

Region: chr18 8035046-8035059. Max. coverage (+): 0. Max coverage (-): 0

Region: chr18 8035060-8035074. Max. coverage (+): 0. Max coverage (-): 0

Region: chr18 8035075-8035088. Max. coverage (+): 0. Max coverage (-): 0

Region: chr18 8035089-8035102. Max. coverage (+): 0. Max coverage (-): 0

Region: chr18 8035103-8035116. Max. coverage (+): 0. Max coverage (-): 0

Region: chr18 8035117-8035131. Max. coverage (+): 0. Max coverage (-): 0

Region: chr18 8035132-8035145. Max. coverage (+): 0. Max coverage (-): 0

Region: chr18 8035146-8035159. Max. coverage (+): 0. Max coverage (-): 0

Region: chr18 8035160-8035173. Max. coverage (+): 0. Max coverage (-): 0

Region: chr18 8035174-8035188. Max. coverage (+): 0. Max coverage (-): 0

Region: chr18 8035189-8035202. Max. coverage (+): 0. Max coverage (-): 0

Region: chr18 8035203-8035216. Max. coverage (+): 0. Max coverage (-): 0

Region: chr18 8035217-8035231. Max. coverage (+): 0. Max coverage (-): 0

Region: chr18 8035232-8035245. Max. coverage (+): 0. Max coverage (-): 0

Region: chr18 8035246-8035259. Max. coverage (+): 0. Max coverage (-): 0

Region: chr18 8035260-8035273. Max. coverage (+): 0. Max coverage (-): 0

Region: chr18 8035274-8035288. Max. coverage (+): 0. Max coverage (-): 0

Region: chr18 8035289-8035302. Max. coverage (+): 0. Max coverage (-): 0

Region: chr18 8035303-8035316. Max. coverage (+): 0. Max coverage (-): 0

Region: chr18 8035317-8035331. Max. coverage (+): 0. Max coverage (-): 0

Region: chr18 8035332-8035345. Max. coverage (+): 0. Max coverage (-): 0

Region: chr18 8035346-8035359. Max. coverage (+): 0. Max coverage (-): 0

Region: chr18 8035360-8035373. Max. coverage (+): 0. Max coverage (-): 0

Region: chr18 8035374-8035388. Max. coverage (+): 0. Max coverage (-): 0

Region: chr18 8035389-8035402. Max. coverage (+): 0. Max coverage (-): 0

Region: chr18 8035403-8035416. Max. coverage (+): 0. Max coverage (-): 0

Region: chr18 8035417-8035431. Max. coverage (+): 0. Max coverage (-): 0

Region: chr18 8035432-8035445. Max. coverage (+): 0. Max coverage (-): 0

Region: chr18 8035446-8035459. Max. coverage (+): 0. Max coverage (-): 0

Region: chr18 8035460-8035473. Max. coverage (+): 0. Max coverage (-): 0

Region: chr18 8035474-8035488. Max. coverage (+): 0. Max coverage (-): 0

Region: chr18 8035489-8035502. Max. coverage (+): 0. Max coverage (-): 0

Region: chr18 8035503-8035516. Max. coverage (+): 7.51. Max coverage (-): 0

Region: chr18 8035517-8035530. Max. coverage (+): 0. Max coverage (-): 0

Region: chr18 8035531-8035545. Max. coverage (+): 0. Max coverage (-): 0

Region: chr18 8035546-8035559. Max. coverage (+): 0. Max coverage (-): 0

Region: chr18 8035560-8035573. Max. coverage (+): 0. Max coverage (-): 0

Region: chr18 8035574-8035588. Max. coverage (+): 0. Max coverage (-): 0

Region: chr18 8035589-8035602. Max. coverage (+): 0. Max coverage (-): 0

Region: chr18 8035603-8035616. Max. coverage (+): 2.83. Max coverage (-): 0

Region: chr18 8035617-8035630. Max. coverage (+): 0. Max coverage (-): 0

Region: chr18 8035631-8035645. Max. coverage (+): 0. Max coverage (-): 0

Region: chr18 8035646-8035659. Max. coverage (+): 0. Max coverage (-): 0

Region: chr18 8035660-8035673. Max. coverage (+): 1.26. Max coverage (-): 0

Region: chr18 8035674-8035688. Max. coverage (+): 0. Max coverage (-): 0

Region: chr18 8035689-8035702. Max. coverage (+): 0. Max coverage (-): 0

Region: chr18 8035703-8035716. Max. coverage (+): 0. Max coverage (-): 0

Region: chr18 8035717-8035730. Max. coverage (+): 0. Max coverage (-): 0

Region: chr18 8035731-8035745. Max. coverage (+): 0. Max coverage (-): 0

Region: chr18 8035746-8035759. Max. coverage (+): 0. Max coverage (-): 0

Region: chr18 8035760-8035773. Max. coverage (+): 0. Max coverage (-): 0

Region: chr18 8035774-8035788. Max. coverage (+): 0. Max coverage (-): 0

Region: chr18 8035789-8035802. Max. coverage (+): 0. Max coverage (-): 0

Region: chr18 8035803-8035816. Max. coverage (+): 0. Max coverage (-): 0

Region: chr18 8035817-8035830. Max. coverage (+): 0. Max coverage (-): 0

Region: chr18 8035831-8035845. Max. coverage (+): 0. Max coverage (-): 0

Region: chr18 8035846-8035859. Max. coverage (+): 0. Max coverage (-): 0

Region: chr18 8035860-8035873. Max. coverage (+): 0. Max coverage (-): 0

Region: chr18 8035874-8035887. Max. coverage (+): 0. Max coverage (-): 0

Region: chr18 8035888-8035902. Max. coverage (+): 0. Max coverage (-): 0

Region: chr18 8035903-8035916. Max. coverage (+): 2.47. Max coverage (-): 0

Region: chr18 8035917-8035930. Max. coverage (+): 2.47. Max coverage (-): 0

Region: chr18 8035931-8035945. Max. coverage (+): 0. Max coverage (-): 0

Region: chr18 8035946-8035959. Max. coverage (+): 0. Max coverage (-): 0

Region: chr18 8035960-8035973. Max. coverage (+): 0. Max coverage (-): 0

Region: chr18 8035974-8035987. Max. coverage (+): 0. Max coverage (-): 0

Region: chr18 8035988-8036002. Max. coverage (+): 0. Max coverage (-): 0

Region: chr18 8036003-8036016. Max. coverage (+): 0. Max coverage (-): 0

Region: chr18 8036017-8036030. Max. coverage (+): 0. Max coverage (-): 0

Region: chr18 8036031-8036045. Max. coverage (+): 0. Max coverage (-): 0

Region: chr18 8036046-8036059. Max. coverage (+): 0. Max coverage (-): 0

Region: chr18 8036060-8036073. Max. coverage (+): 0. Max coverage (-): 0

Region: chr18 8036074-8036087. Max. coverage (+): 0. Max coverage (-): 0

Region: chr18 8036088-8036102. Max. coverage (+): 0. Max coverage (-): 0

Region: chr18 8036103-8036116. Max. coverage (+): 0. Max coverage (-): 0

Region: chr18 8036117-8036130. Max. coverage (+): 0. Max coverage (-): 0

Region: chr18 8036131-8036145. Max. coverage (+): 0. Max coverage (-): 0

Region: chr18 8036146-8036159. Max. coverage (+): 0. Max coverage (-): 0

Region: chr18 8036160-8036173. Max. coverage (+): 0. Max coverage (-): 0

Region: chr18 8036174-8036187. Max. coverage (+): 0. Max coverage (-): 0

Region: chr18 8036188-8036202. Max. coverage (+): 0. Max coverage (-): 0

Region: chr18 8036203-8036216. Max. coverage (+): 0. Max coverage (-): 0

Region: chr18 8036217-8036230. Max. coverage (+): 0. Max coverage (-): 0

Region: chr18 8036231-8036244. Max. coverage (+): 0. Max coverage (-): 0

Region: chr18 8036245-8036259. Max. coverage (+): 0. Max coverage (-): 0

Region: chr18 8036260-8036273. Max. coverage (+): 0. Max coverage (-): 0

Region: chr18 8036274-8036287. Max. coverage (+): 0. Max coverage (-): 0

Region: chr18 8036288-8036302. Max. coverage (+): 0. Max coverage (-): 0

Region: chr18 8036303-8036316. Max. coverage (+): 0. Max coverage (-): 0

Region: chr18 8036317-8036330. Max. coverage (+): 0. Max coverage (-): 0

Region: chr18 8036331-8036344. Max. coverage (+): 0. Max coverage (-): 0

Region: chr18 8036345-8036359. Max. coverage (+): 0. Max coverage (-): 0

Region: chr18 8036360-8036373. Max. coverage (+): 0. Max coverage (-): 0

Region: chr18 8036374-8036387. Max. coverage (+): 0. Max coverage (-): 0

Region: chr18 8036388-8036402. Max. coverage (+): 0. Max coverage (-): 0

Region: chr18 8036403-8036416. Max. coverage (+): 0. Max coverage (-): 0

Region: chr18 8036417-8036430. Max. coverage (+): 0. Max coverage (-): 0

Region: chr18 8036431-8036444. Max. coverage (+): 0. Max coverage (-): 0

Region: chr18 8036445-8036459. Max. coverage (+): 0. Max coverage (-): 0

Region: chr18 8036460-8036473. Max. coverage (+): 0. Max coverage (-): 0

Region: chr18 8036474-8036487. Max. coverage (+): 0. Max coverage (-): 0

Region: chr18 8036488-8036502. Max. coverage (+): 0. Max coverage (-): 0

Region: chr18 8036503-8036516. Max. coverage (+): 0. Max coverage (-): 0

Region: chr18 8036517-8036530. Max. coverage (+): 5.24. Max coverage (-): 0

Region: chr18 8036531-8036544. Max. coverage (+): 0. Max coverage (-): 0

Region: chr18 8036545-. Max. coverage (+): 0. Max coverage (-): 0

RepeatMasker Color Code

**+**

100-98% Identity

<98-95% Identity

<95-90% Identity

<90-85% Identity

<85-80% Identity

<80-75% Identity

<75-70% Identity

<70% Identity

**-**

Gene Set Color Code

**+**

Gene

Pseudogene

**-**

Topology/Coverage Color Code

Coverage Plus Strand

Coverage Minus Strand

Mainstrand: Plus

Mainstrand: Minus

Complementary Strand

Flanking Region  
(if option -flank >0)

Gene Set Annotation  
  
RepeatMasker Annotation  

**1. L2c**: 8030234-8030339 (+), Divergence to consensus: 31.5%  
**2. ART2A**: 8031549-8032071 (-), Divergence to consensus: 19.6%  
**3. BOV-A2**: 8034252-8034520 (-), Divergence to consensus: 3.7%  
**4. L3**: 8034818-8034952 (+), Divergence to consensus: 39.5%  
**5. L3**: 8036032-8036136 (+), Divergence to consensus: 40.3%

  
Transcription Factor Binding Sites  

**RFX4\_2** (Sequence: CGTAGATAC (+): 8030692)  
**SOX9** (Sequence: AACAATAA (-): 8032843)  
**SOX9** (Sequence: CTATTGTT (+): 8031343)
