# Supplementary material for: Small RNA sequencing of cryopreserved semen from single bull revealed altered miRNAs and piRNAs expression between High- and Low-motile sperm populations
Source: BMC Genomics. 2017 Jan 4;18:14. doi: 10.1186/s12864-016-3394-7 (PMC5209821; doi:10.1186/s12864-016-3394-7)
Supplement: Additional file 3: — Details for each piRNA clusters found in High Motile (HM) sperm fraction. Genes, repeats, transposable elements and transcription factors binding sites falling within the cluster regions were reported. (ZIP 1896 kb) [file 12864_2016_3394_MOESM3_ESM.zip › 36.html]

piRNA cluster 36


Predicted piRNA cluster no. 36     previous   next
  

Show proTRAC run info
Hide proTRAC run info

================================= proTRAC ====================================  
VERSION: 2.1                                    LAST MODIFIED: 06. October 2015  
  
Please cite:  
Rosenkranz D, Zischler H. proTRAC - a software for probabilistic piRNA cluster  
detection, visualization and analysis. 2012. BMC Bioinformatics 13:5.  
  
and (for proTRAC 2.0 and later):  
Rosenkranz D, Rudloff S, Bastuck K, Ketting RF, Zischler H. Tupaia small RNAs  
provide insights into function and evolution of RNAi-based transposon defense  
in mammals. 2015. RNA 21(5):911-922.  
  
Contact:  
David Rosenkranz  
Institute of Anthropology, small RNA group  
Johannes Gutenberg University Mainz  
email: rosenkranz@uni-mainz.de  
  
You can find the latest proTRAC version at:  
http://sourceforge.net/projects/protrac/files  
http://www.smallRNAgroup-mainz.de/software  
==============================================================================  
  
PARAMETERS:  
Map file: .............../storage/core/barbara/genhome/smallRNA/fertility/Sample\_motile/pirna/Sample\_motile\_26-33\_collapsed.fa.no-dust.map.weighted-10000-1000-b-0  
Genome file: ............/storage/core/barbara/genhome/smallRNA/fertility/Sample\_all/pirna/bt\_311\_chrY.fa  
RepeatMasker annotation: /storage/genomes/bt\_umd31/GCF\_000003055.6\_Bos\_taurus\_UMD\_3.1.1\_repeatMasker\_chr.out  
GeneSet:................./storage/core/barbara/genhome/smallRNA/fertility/Sample\_all/pirna/full.gtf  
  
Significant (p<=0.01) hit density will be calculated based  
on observed hit distribution.  
  
Sliding window size: ........................................ 5000 bp  
Sliding window increament: .................................. 1000 bp  
Normalize each hit by number of genomic hits: ............... 1 [0=no/1=yes]  
Normalize each hit by number of sequence reads: ............. 1 [0=no/1=yes]  
Normalize values (-> per million mapped reads): ............. 1 [0=no/1=yes]  
Min. fraction of hits with 1T(U) or 10A: .................... 0.75  
Alternatively: Min. fraction of hits with 1T(U) and 10A: .... 0.5  
Min. fraction of hits with typical piRNA length: ............ 0.75  
Typical piRNA length: ....................................... 26-33 nt  
Min. size of a piRNA cluster: ............................... 5000 bp.  
Min. number of hits (absolute): ............................. 0  
Min. number of hits (normalized): ........................... 0  
Min. fraction of hits on the mainstrand: .................... 0.75  
Top fraction of mapped sequences (in terms of read counts): . 1%  
Top fraction accounts for max. n% of sequence reads: ........ 90%  
Min. fraction of hits on each arm of a bidirectional cluster: 0.1  
Output image file for each cluster: ......................... 0 [0=no/1=yes]  
Output html file for each cluster: .......................... 1 [0=no/1=yes]  
Output a summary table: ..................................... 1 [0=no/1=yes]  
Output a FASTA file for each cluster (piRNA sequences): ..... 1 [0=no/1=yes]  
Output a FASTA file comprising cluster sequences: ........... 1 [0=no/1=yes]  
Search DNA motifs in clusters: .............................. 1 [0=no/1=yes]  
Output flanking sequences: +/- .............................. 0 bp  
Output ~.pTi file: .......................................... 1 [0=no/1=yes]  
==============================================================================  
  
  
Genome size (without gaps): ............ 2678902517 bp  
Gaps (N/X/-): .......................... 53837044 bp  
Mapped reads: .......................... 658825247023  
Non-identical sequences: ............... 514171  
Genomic hits: .......................... 764233  
Significant densitiy of mapped reads: .. 12867599.5173724 reads/kb

Show proTRAC cluster info
Hide proTRAC cluster info

|  |  |
| --- | --- |
| Location | chr18 |
| Coordinates | 39370744-39378390 |
| Size [bp] | 7647 |
| Sequence hit loci | 136 |
| Mapped reads (normalized) | 198057290 |
| Mapped reads (normalized) per kb | 25899998.7 |
| Normalized reads with 1T (1U) | 81% |
| Normalized reads with 10A | 35.6% |
| Normalized reads with length 26-33 nt | 100% |
| Normalized reads on the main strand(s) | 100% |
| Predicted directionality | mono:minus |

100%

0%

1T (1U)  
reads

10A reads

26-33 nt  
reads

reads on mainstrand

**Either the amount of reads with 1T (1U) OR 10A has to exceed 75% (set with option: -1Tor10A)  
Alternatively the amount of reads with 1T (1U) AND 10A has to exceed 50% (set with option: -1Tand10A)  
Minimum amount of reads with preferred size is 75% (set with option: -pisize)  
Minimum amount of reads on the main strand(s) is 75% (set with option: -clstrand)**

Show read coverage
Hide read coverage

WHAT DO I SEE HERE?  
This chart shows the location of mapped sequence reads within a predicted piRNA cluster. The color refers to the number of genomic hits produced by the sequence read in question. A dark red bar indicates that this sequence read produces many other hits elsewhere in the genome. Many adjacent red or yellow bars can indicate the presence of a multi-copy element such as transposons or rRNA genes. A dark green bar indicates that this sequence read maps uniquely to this locus.

1 hit

2-5 hits

6-10 hits

11-20 hits

21-50 hits

51-100 hits

> 100 hits

chr18

39370744

39378390

Gene Set

RepeatMasker

Mapped  
Reads

23.68

plus strand

minus strand

23.68

Region: chr18 8036546-39370751. Max. coverage (+): 0. Max coverage (-): 1.04

Region: chr18 39370752-39370766. Max. coverage (+): 0. Max coverage (-): 0

Region: chr18 39370767-39370782. Max. coverage (+): 0. Max coverage (-): 0

Region: chr18 39370783-39370797. Max. coverage (+): 0. Max coverage (-): 0

Region: chr18 39370798-39370812. Max. coverage (+): 0. Max coverage (-): 0

Region: chr18 39370813-39370828. Max. coverage (+): 0. Max coverage (-): 0

Region: chr18 39370829-39370843. Max. coverage (+): 0. Max coverage (-): 0

Region: chr18 39370844-39370858. Max. coverage (+): 0. Max coverage (-): 0

Region: chr18 39370859-39370873. Max. coverage (+): 0. Max coverage (-): 0

Region: chr18 39370874-39370889. Max. coverage (+): 0. Max coverage (-): 0

Region: chr18 39370890-39370904. Max. coverage (+): 0. Max coverage (-): 0

Region: chr18 39370905-39370919. Max. coverage (+): 0. Max coverage (-): 0

Region: chr18 39370920-39370935. Max. coverage (+): 0. Max coverage (-): 0

Region: chr18 39370936-39370950. Max. coverage (+): 0. Max coverage (-): 0

Region: chr18 39370951-39370965. Max. coverage (+): 0. Max coverage (-): 0

Region: chr18 39370966-39370981. Max. coverage (+): 0. Max coverage (-): 0

Region: chr18 39370982-39370996. Max. coverage (+): 0. Max coverage (-): 0

Region: chr18 39370997-39371011. Max. coverage (+): 0. Max coverage (-): 0

Region: chr18 39371012-39371026. Max. coverage (+): 0. Max coverage (-): 0

Region: chr18 39371027-39371042. Max. coverage (+): 0. Max coverage (-): 0

Region: chr18 39371043-39371057. Max. coverage (+): 0. Max coverage (-): 0

Region: chr18 39371058-39371072. Max. coverage (+): 0. Max coverage (-): 0

Region: chr18 39371073-39371088. Max. coverage (+): 0. Max coverage (-): 0

Region: chr18 39371089-39371103. Max. coverage (+): 0. Max coverage (-): 0

Region: chr18 39371104-39371118. Max. coverage (+): 0. Max coverage (-): 0

Region: chr18 39371119-39371133. Max. coverage (+): 0. Max coverage (-): 0

Region: chr18 39371134-39371149. Max. coverage (+): 0. Max coverage (-): 0

Region: chr18 39371150-39371164. Max. coverage (+): 0. Max coverage (-): 0

Region: chr18 39371165-39371179. Max. coverage (+): 0. Max coverage (-): 0

Region: chr18 39371180-39371195. Max. coverage (+): 0. Max coverage (-): 0

Region: chr18 39371196-39371210. Max. coverage (+): 0. Max coverage (-): 0

Region: chr18 39371211-39371225. Max. coverage (+): 0. Max coverage (-): 0

Region: chr18 39371226-39371241. Max. coverage (+): 0. Max coverage (-): 0

Region: chr18 39371242-39371256. Max. coverage (+): 0. Max coverage (-): 0

Region: chr18 39371257-39371271. Max. coverage (+): 0. Max coverage (-): 0

Region: chr18 39371272-39371286. Max. coverage (+): 0. Max coverage (-): 4.18

Region: chr18 39371287-39371302. Max. coverage (+): 0. Max coverage (-): 0

Region: chr18 39371303-39371317. Max. coverage (+): 0. Max coverage (-): 0

Region: chr18 39371318-39371332. Max. coverage (+): 0. Max coverage (-): 0

Region: chr18 39371333-39371348. Max. coverage (+): 0. Max coverage (-): 0

Region: chr18 39371349-39371363. Max. coverage (+): 0. Max coverage (-): 0

Region: chr18 39371364-39371378. Max. coverage (+): 0. Max coverage (-): 0

Region: chr18 39371379-39371393. Max. coverage (+): 0. Max coverage (-): 0

Region: chr18 39371394-39371409. Max. coverage (+): 0. Max coverage (-): 0

Region: chr18 39371410-39371424. Max. coverage (+): 0. Max coverage (-): 0

Region: chr18 39371425-39371439. Max. coverage (+): 0. Max coverage (-): 0

Region: chr18 39371440-39371455. Max. coverage (+): 0. Max coverage (-): 0

Region: chr18 39371456-39371470. Max. coverage (+): 0. Max coverage (-): 0

Region: chr18 39371471-39371485. Max. coverage (+): 0. Max coverage (-): 0

Region: chr18 39371486-39371501. Max. coverage (+): 0. Max coverage (-): 0

Region: chr18 39371502-39371516. Max. coverage (+): 0. Max coverage (-): 0

Region: chr18 39371517-39371531. Max. coverage (+): 0. Max coverage (-): 0

Region: chr18 39371532-39371546. Max. coverage (+): 0. Max coverage (-): 0

Region: chr18 39371547-39371562. Max. coverage (+): 0. Max coverage (-): 0

Region: chr18 39371563-39371577. Max. coverage (+): 0. Max coverage (-): 0

Region: chr18 39371578-39371592. Max. coverage (+): 0. Max coverage (-): 0

Region: chr18 39371593-39371608. Max. coverage (+): 0. Max coverage (-): 0

Region: chr18 39371609-39371623. Max. coverage (+): 0. Max coverage (-): 0

Region: chr18 39371624-39371638. Max. coverage (+): 0. Max coverage (-): 0

Region: chr18 39371639-39371653. Max. coverage (+): 0. Max coverage (-): 0

Region: chr18 39371654-39371669. Max. coverage (+): 0. Max coverage (-): 0

Region: chr18 39371670-39371684. Max. coverage (+): 0. Max coverage (-): 0

Region: chr18 39371685-39371699. Max. coverage (+): 0. Max coverage (-): 0

Region: chr18 39371700-39371715. Max. coverage (+): 0. Max coverage (-): 0

Region: chr18 39371716-39371730. Max. coverage (+): 0. Max coverage (-): 0

Region: chr18 39371731-39371745. Max. coverage (+): 0. Max coverage (-): 0

Region: chr18 39371746-39371761. Max. coverage (+): 0. Max coverage (-): 0

Region: chr18 39371762-39371776. Max. coverage (+): 0. Max coverage (-): 0

Region: chr18 39371777-39371791. Max. coverage (+): 0. Max coverage (-): 1.93

Region: chr18 39371792-39371806. Max. coverage (+): 0. Max coverage (-): 1.93

Region: chr18 39371807-39371822. Max. coverage (+): 0. Max coverage (-): 0

Region: chr18 39371823-39371837. Max. coverage (+): 0. Max coverage (-): 4.85

Region: chr18 39371838-39371852. Max. coverage (+): 0. Max coverage (-): 4.85

Region: chr18 39371853-39371868. Max. coverage (+): 0. Max coverage (-): 0

Region: chr18 39371869-39371883. Max. coverage (+): 0. Max coverage (-): 4.4

Region: chr18 39371884-39371898. Max. coverage (+): 0. Max coverage (-): 0

Region: chr18 39371899-39371913. Max. coverage (+): 0. Max coverage (-): 0

Region: chr18 39371914-39371929. Max. coverage (+): 0. Max coverage (-): 2.66

Region: chr18 39371930-39371944. Max. coverage (+): 0. Max coverage (-): 2.66

Region: chr18 39371945-39371959. Max. coverage (+): 0. Max coverage (-): 0

Region: chr18 39371960-39371975. Max. coverage (+): 0. Max coverage (-): 0

Region: chr18 39371976-39371990. Max. coverage (+): 0. Max coverage (-): 5.63

Region: chr18 39371991-39372005. Max. coverage (+): 0. Max coverage (-): 0

Region: chr18 39372006-39372021. Max. coverage (+): 0. Max coverage (-): 1.96

Region: chr18 39372022-39372036. Max. coverage (+): 0. Max coverage (-): 1.39

Region: chr18 39372037-39372051. Max. coverage (+): 0. Max coverage (-): 0

Region: chr18 39372052-39372066. Max. coverage (+): 0. Max coverage (-): 0

Region: chr18 39372067-39372082. Max. coverage (+): 0. Max coverage (-): 0

Region: chr18 39372083-39372097. Max. coverage (+): 0. Max coverage (-): 2.57

Region: chr18 39372098-39372112. Max. coverage (+): 0. Max coverage (-): 2.57

Region: chr18 39372113-39372128. Max. coverage (+): 0. Max coverage (-): 0

Region: chr18 39372129-39372143. Max. coverage (+): 0. Max coverage (-): 0.83

Region: chr18 39372144-39372158. Max. coverage (+): 0. Max coverage (-): 0

Region: chr18 39372159-39372173. Max. coverage (+): 0. Max coverage (-): 0

Region: chr18 39372174-39372189. Max. coverage (+): 0. Max coverage (-): 0.64

Region: chr18 39372190-39372204. Max. coverage (+): 0. Max coverage (-): 0.64

Region: chr18 39372205-39372219. Max. coverage (+): 0. Max coverage (-): 1.72

Region: chr18 39372220-39372235. Max. coverage (+): 0. Max coverage (-): 1.72

Region: chr18 39372236-39372250. Max. coverage (+): 0. Max coverage (-): 0

Region: chr18 39372251-39372265. Max. coverage (+): 0. Max coverage (-): 0

Region: chr18 39372266-39372281. Max. coverage (+): 0. Max coverage (-): 0

Region: chr18 39372282-39372296. Max. coverage (+): 0. Max coverage (-): 0

Region: chr18 39372297-39372311. Max. coverage (+): 0. Max coverage (-): 0

Region: chr18 39372312-39372326. Max. coverage (+): 0. Max coverage (-): 0

Region: chr18 39372327-39372342. Max. coverage (+): 0. Max coverage (-): 0

Region: chr18 39372343-39372357. Max. coverage (+): 0. Max coverage (-): 0

Region: chr18 39372358-39372372. Max. coverage (+): 0. Max coverage (-): 0

Region: chr18 39372373-39372388. Max. coverage (+): 0. Max coverage (-): 0

Region: chr18 39372389-39372403. Max. coverage (+): 0. Max coverage (-): 0.7

Region: chr18 39372404-39372418. Max. coverage (+): 0. Max coverage (-): 0.7

Region: chr18 39372419-39372433. Max. coverage (+): 0. Max coverage (-): 0

Region: chr18 39372434-39372449. Max. coverage (+): 0. Max coverage (-): 0

Region: chr18 39372450-39372464. Max. coverage (+): 0. Max coverage (-): 3.72

Region: chr18 39372465-39372479. Max. coverage (+): 0. Max coverage (-): 0

Region: chr18 39372480-39372495. Max. coverage (+): 0. Max coverage (-): 0

Region: chr18 39372496-39372510. Max. coverage (+): 0. Max coverage (-): 0

Region: chr18 39372511-39372525. Max. coverage (+): 0. Max coverage (-): 0

Region: chr18 39372526-39372541. Max. coverage (+): 0. Max coverage (-): 0

Region: chr18 39372542-39372556. Max. coverage (+): 0. Max coverage (-): 0

Region: chr18 39372557-39372571. Max. coverage (+): 0. Max coverage (-): 0

Region: chr18 39372572-39372586. Max. coverage (+): 0. Max coverage (-): 0

Region: chr18 39372587-39372602. Max. coverage (+): 0. Max coverage (-): 0

Region: chr18 39372603-39372617. Max. coverage (+): 0. Max coverage (-): 7.26

Region: chr18 39372618-39372632. Max. coverage (+): 0. Max coverage (-): 0

Region: chr18 39372633-39372648. Max. coverage (+): 0. Max coverage (-): 0

Region: chr18 39372649-39372663. Max. coverage (+): 0. Max coverage (-): 0.69

Region: chr18 39372664-39372678. Max. coverage (+): 0. Max coverage (-): 2.2

Region: chr18 39372679-39372693. Max. coverage (+): 0. Max coverage (-): 4.79

Region: chr18 39372694-39372709. Max. coverage (+): 0. Max coverage (-): 0

Region: chr18 39372710-39372724. Max. coverage (+): 0. Max coverage (-): 0

Region: chr18 39372725-39372739. Max. coverage (+): 0. Max coverage (-): 0

Region: chr18 39372740-39372755. Max. coverage (+): 0. Max coverage (-): 0

Region: chr18 39372756-39372770. Max. coverage (+): 0. Max coverage (-): 0

Region: chr18 39372771-39372785. Max. coverage (+): 0. Max coverage (-): 2.16

Region: chr18 39372786-39372801. Max. coverage (+): 0. Max coverage (-): 0

Region: chr18 39372802-39372816. Max. coverage (+): 0. Max coverage (-): 0

Region: chr18 39372817-39372831. Max. coverage (+): 0. Max coverage (-): 0

Region: chr18 39372832-39372846. Max. coverage (+): 0. Max coverage (-): 1.12

Region: chr18 39372847-39372862. Max. coverage (+): 0. Max coverage (-): 1.12

Region: chr18 39372863-39372877. Max. coverage (+): 0. Max coverage (-): 0

Region: chr18 39372878-39372892. Max. coverage (+): 0. Max coverage (-): 0.99

Region: chr18 39372893-39372908. Max. coverage (+): 0. Max coverage (-): 2.03

Region: chr18 39372909-39372923. Max. coverage (+): 0. Max coverage (-): 0

Region: chr18 39372924-39372938. Max. coverage (+): 0. Max coverage (-): 0

Region: chr18 39372939-39372953. Max. coverage (+): 0. Max coverage (-): 20.53

Region: chr18 39372954-39372969. Max. coverage (+): 0. Max coverage (-): 20.53

Region: chr18 39372970-39372984. Max. coverage (+): 0. Max coverage (-): 0

Region: chr18 39372985-39372999. Max. coverage (+): 0. Max coverage (-): 0

Region: chr18 39373000-39373015. Max. coverage (+): 0. Max coverage (-): 0

Region: chr18 39373016-39373030. Max. coverage (+): 0. Max coverage (-): 0

Region: chr18 39373031-39373045. Max. coverage (+): 0. Max coverage (-): 0

Region: chr18 39373046-39373061. Max. coverage (+): 0. Max coverage (-): 0

Region: chr18 39373062-39373076. Max. coverage (+): 0. Max coverage (-): 4.88

Region: chr18 39373077-39373091. Max. coverage (+): 0. Max coverage (-): 4.88

Region: chr18 39373092-39373106. Max. coverage (+): 0. Max coverage (-): 2.16

Region: chr18 39373107-39373122. Max. coverage (+): 0. Max coverage (-): 2.16

Region: chr18 39373123-39373137. Max. coverage (+): 0. Max coverage (-): 0

Region: chr18 39373138-39373152. Max. coverage (+): 0. Max coverage (-): 4.86

Region: chr18 39373153-39373168. Max. coverage (+): 0. Max coverage (-): 0

Region: chr18 39373169-39373183. Max. coverage (+): 0. Max coverage (-): 2.23

Region: chr18 39373184-39373198. Max. coverage (+): 0. Max coverage (-): 1.64

Region: chr18 39373199-39373213. Max. coverage (+): 0. Max coverage (-): 0

Region: chr18 39373214-39373229. Max. coverage (+): 0. Max coverage (-): 0

Region: chr18 39373230-39373244. Max. coverage (+): 0. Max coverage (-): 0

Region: chr18 39373245-39373259. Max. coverage (+): 0. Max coverage (-): 0

Region: chr18 39373260-39373275. Max. coverage (+): 0. Max coverage (-): 0

Region: chr18 39373276-39373290. Max. coverage (+): 0. Max coverage (-): 0

Region: chr18 39373291-39373305. Max. coverage (+): 0. Max coverage (-): 22.35

Region: chr18 39373306-39373321. Max. coverage (+): 0. Max coverage (-): 6.87

Region: chr18 39373322-39373336. Max. coverage (+): 0. Max coverage (-): 0

Region: chr18 39373337-39373351. Max. coverage (+): 0. Max coverage (-): 0

Region: chr18 39373352-39373366. Max. coverage (+): 0. Max coverage (-): 3.64

Region: chr18 39373367-39373382. Max. coverage (+): 0. Max coverage (-): 7.48

Region: chr18 39373383-39373397. Max. coverage (+): 0. Max coverage (-): 0

Region: chr18 39373398-39373412. Max. coverage (+): 0. Max coverage (-): 0

Region: chr18 39373413-39373428. Max. coverage (+): 0. Max coverage (-): 1.45

Region: chr18 39373429-39373443. Max. coverage (+): 0. Max coverage (-): 2.97

Region: chr18 39373444-39373458. Max. coverage (+): 0. Max coverage (-): 4.5

Region: chr18 39373459-39373473. Max. coverage (+): 0. Max coverage (-): 4.5

Region: chr18 39373474-39373489. Max. coverage (+): 0. Max coverage (-): 1.04

Region: chr18 39373490-39373504. Max. coverage (+): 0. Max coverage (-): 0

Region: chr18 39373505-39373519. Max. coverage (+): 0. Max coverage (-): 4.39

Region: chr18 39373520-39373535. Max. coverage (+): 0. Max coverage (-): 3.19

Region: chr18 39373536-39373550. Max. coverage (+): 0. Max coverage (-): 0

Region: chr18 39373551-39373565. Max. coverage (+): 0. Max coverage (-): 0

Region: chr18 39373566-39373581. Max. coverage (+): 0. Max coverage (-): 0

Region: chr18 39373582-39373596. Max. coverage (+): 0. Max coverage (-): 0

Region: chr18 39373597-39373611. Max. coverage (+): 0. Max coverage (-): 0

Region: chr18 39373612-39373626. Max. coverage (+): 0. Max coverage (-): 0

Region: chr18 39373627-39373642. Max. coverage (+): 0. Max coverage (-): 18.83

Region: chr18 39373643-39373657. Max. coverage (+): 0. Max coverage (-): 17.15

Region: chr18 39373658-39373672. Max. coverage (+): 0. Max coverage (-): 5.16

Region: chr18 39373673-39373688. Max. coverage (+): 0. Max coverage (-): 6.5

Region: chr18 39373689-39373703. Max. coverage (+): 0. Max coverage (-): 6.5

Region: chr18 39373704-39373718. Max. coverage (+): 0. Max coverage (-): 0

Region: chr18 39373719-39373733. Max. coverage (+): 0. Max coverage (-): 10.98

Region: chr18 39373734-39373749. Max. coverage (+): 0. Max coverage (-): 10.98

Region: chr18 39373750-39373764. Max. coverage (+): 0. Max coverage (-): 3.68

Region: chr18 39373765-39373779. Max. coverage (+): 0. Max coverage (-): 0

Region: chr18 39373780-39373795. Max. coverage (+): 0. Max coverage (-): 0

Region: chr18 39373796-39373810. Max. coverage (+): 0. Max coverage (-): 0

Region: chr18 39373811-39373825. Max. coverage (+): 0. Max coverage (-): 0

Region: chr18 39373826-39373841. Max. coverage (+): 0. Max coverage (-): 0

Region: chr18 39373842-39373856. Max. coverage (+): 0. Max coverage (-): 0

Region: chr18 39373857-39373871. Max. coverage (+): 0. Max coverage (-): 0

Region: chr18 39373872-39373886. Max. coverage (+): 0. Max coverage (-): 0

Region: chr18 39373887-39373902. Max. coverage (+): 0. Max coverage (-): 0

Region: chr18 39373903-39373917. Max. coverage (+): 0. Max coverage (-): 0

Region: chr18 39373918-39373932. Max. coverage (+): 0. Max coverage (-): 0

Region: chr18 39373933-39373948. Max. coverage (+): 0. Max coverage (-): 0

Region: chr18 39373949-39373963. Max. coverage (+): 0. Max coverage (-): 0

Region: chr18 39373964-39373978. Max. coverage (+): 0. Max coverage (-): 0

Region: chr18 39373979-39373993. Max. coverage (+): 0. Max coverage (-): 0

Region: chr18 39373994-39374009. Max. coverage (+): 0. Max coverage (-): 0

Region: chr18 39374010-39374024. Max. coverage (+): 0. Max coverage (-): 0

Region: chr18 39374025-39374039. Max. coverage (+): 0. Max coverage (-): 0

Region: chr18 39374040-39374055. Max. coverage (+): 0. Max coverage (-): 0

Region: chr18 39374056-39374070. Max. coverage (+): 0. Max coverage (-): 0

Region: chr18 39374071-39374085. Max. coverage (+): 0. Max coverage (-): 0

Region: chr18 39374086-39374101. Max. coverage (+): 0. Max coverage (-): 0

Region: chr18 39374102-39374116. Max. coverage (+): 0. Max coverage (-): 0

Region: chr18 39374117-39374131. Max. coverage (+): 0. Max coverage (-): 0

Region: chr18 39374132-39374146. Max. coverage (+): 0. Max coverage (-): 0

Region: chr18 39374147-39374162. Max. coverage (+): 0. Max coverage (-): 0

Region: chr18 39374163-39374177. Max. coverage (+): 0. Max coverage (-): 0

Region: chr18 39374178-39374192. Max. coverage (+): 0. Max coverage (-): 0

Region: chr18 39374193-39374208. Max. coverage (+): 0. Max coverage (-): 2.75

Region: chr18 39374209-39374223. Max. coverage (+): 0. Max coverage (-): 2.75

Region: chr18 39374224-39374238. Max. coverage (+): 0. Max coverage (-): 0

Region: chr18 39374239-39374253. Max. coverage (+): 0. Max coverage (-): 0.59

Region: chr18 39374254-39374269. Max. coverage (+): 0. Max coverage (-): 0.59

Region: chr18 39374270-39374284. Max. coverage (+): 0. Max coverage (-): 0

Region: chr18 39374285-39374299. Max. coverage (+): 0. Max coverage (-): 3.9

Region: chr18 39374300-39374315. Max. coverage (+): 0. Max coverage (-): 3.9

Region: chr18 39374316-39374330. Max. coverage (+): 0. Max coverage (-): 0

Region: chr18 39374331-39374345. Max. coverage (+): 0. Max coverage (-): 0

Region: chr18 39374346-39374361. Max. coverage (+): 0. Max coverage (-): 0

Region: chr18 39374362-39374376. Max. coverage (+): 0. Max coverage (-): 0

Region: chr18 39374377-39374391. Max. coverage (+): 0. Max coverage (-): 1.89

Region: chr18 39374392-39374406. Max. coverage (+): 0. Max coverage (-): 1.89

Region: chr18 39374407-39374422. Max. coverage (+): 0. Max coverage (-): 0

Region: chr18 39374423-39374437. Max. coverage (+): 0. Max coverage (-): 0

Region: chr18 39374438-39374452. Max. coverage (+): 0. Max coverage (-): 0

Region: chr18 39374453-39374468. Max. coverage (+): 0. Max coverage (-): 0

Region: chr18 39374469-39374483. Max. coverage (+): 0. Max coverage (-): 0

Region: chr18 39374484-39374498. Max. coverage (+): 0. Max coverage (-): 0

Region: chr18 39374499-39374513. Max. coverage (+): 0. Max coverage (-): 0

Region: chr18 39374514-39374529. Max. coverage (+): 0. Max coverage (-): 0

Region: chr18 39374530-39374544. Max. coverage (+): 0. Max coverage (-): 0

Region: chr18 39374545-39374559. Max. coverage (+): 0. Max coverage (-): 0

Region: chr18 39374560-39374575. Max. coverage (+): 0. Max coverage (-): 0

Region: chr18 39374576-39374590. Max. coverage (+): 0. Max coverage (-): 0

Region: chr18 39374591-39374605. Max. coverage (+): 0. Max coverage (-): 0

Region: chr18 39374606-39374621. Max. coverage (+): 0. Max coverage (-): 0

Region: chr18 39374622-39374636. Max. coverage (+): 0. Max coverage (-): 2.09

Region: chr18 39374637-39374651. Max. coverage (+): 0. Max coverage (-): 1.85

Region: chr18 39374652-39374666. Max. coverage (+): 0. Max coverage (-): 1.85

Region: chr18 39374667-39374682. Max. coverage (+): 0. Max coverage (-): 0

Region: chr18 39374683-39374697. Max. coverage (+): 0. Max coverage (-): 0

Region: chr18 39374698-39374712. Max. coverage (+): 0. Max coverage (-): 0

Region: chr18 39374713-39374728. Max. coverage (+): 0. Max coverage (-): 0

Region: chr18 39374729-39374743. Max. coverage (+): 0. Max coverage (-): 1.88

Region: chr18 39374744-39374758. Max. coverage (+): 0. Max coverage (-): 20.98

Region: chr18 39374759-39374773. Max. coverage (+): 0. Max coverage (-): 23.68

Region: chr18 39374774-39374789. Max. coverage (+): 0. Max coverage (-): 3.78

Region: chr18 39374790-39374804. Max. coverage (+): 0. Max coverage (-): 0.97

Region: chr18 39374805-39374819. Max. coverage (+): 0. Max coverage (-): 0

Region: chr18 39374820-39374835. Max. coverage (+): 0. Max coverage (-): 9.17

Region: chr18 39374836-39374850. Max. coverage (+): 0. Max coverage (-): 2.54

Region: chr18 39374851-39374865. Max. coverage (+): 0. Max coverage (-): 0

Region: chr18 39374866-39374881. Max. coverage (+): 0. Max coverage (-): 4.7

Region: chr18 39374882-39374896. Max. coverage (+): 0. Max coverage (-): 0

Region: chr18 39374897-39374911. Max. coverage (+): 0. Max coverage (-): 0

Region: chr18 39374912-39374926. Max. coverage (+): 0. Max coverage (-): 0

Region: chr18 39374927-39374942. Max. coverage (+): 0. Max coverage (-): 0

Region: chr18 39374943-39374957. Max. coverage (+): 0. Max coverage (-): 0

Region: chr18 39374958-39374972. Max. coverage (+): 0. Max coverage (-): 0

Region: chr18 39374973-39374988. Max. coverage (+): 0. Max coverage (-): 0

Region: chr18 39374989-39375003. Max. coverage (+): 0. Max coverage (-): 0

Region: chr18 39375004-39375018. Max. coverage (+): 0. Max coverage (-): 0

Region: chr18 39375019-39375033. Max. coverage (+): 0. Max coverage (-): 0

Region: chr18 39375034-39375049. Max. coverage (+): 0. Max coverage (-): 0

Region: chr18 39375050-39375064. Max. coverage (+): 0. Max coverage (-): 0

Region: chr18 39375065-39375079. Max. coverage (+): 0. Max coverage (-): 0

Region: chr18 39375080-39375095. Max. coverage (+): 0. Max coverage (-): 0

Region: chr18 39375096-39375110. Max. coverage (+): 0. Max coverage (-): 0

Region: chr18 39375111-39375125. Max. coverage (+): 0. Max coverage (-): 0

Region: chr18 39375126-39375141. Max. coverage (+): 0. Max coverage (-): 0

Region: chr18 39375142-39375156. Max. coverage (+): 0. Max coverage (-): 0

Region: chr18 39375157-39375171. Max. coverage (+): 0. Max coverage (-): 0

Region: chr18 39375172-39375186. Max. coverage (+): 0. Max coverage (-): 4.77

Region: chr18 39375187-39375202. Max. coverage (+): 0. Max coverage (-): 4.77

Region: chr18 39375203-39375217. Max. coverage (+): 0. Max coverage (-): 0

Region: chr18 39375218-39375232. Max. coverage (+): 0. Max coverage (-): 3.54

Region: chr18 39375233-39375248. Max. coverage (+): 0. Max coverage (-): 0

Region: chr18 39375249-39375263. Max. coverage (+): 0. Max coverage (-): 0

Region: chr18 39375264-39375278. Max. coverage (+): 0. Max coverage (-): 0

Region: chr18 39375279-39375293. Max. coverage (+): 0. Max coverage (-): 0

Region: chr18 39375294-39375309. Max. coverage (+): 0. Max coverage (-): 0

Region: chr18 39375310-39375324. Max. coverage (+): 0. Max coverage (-): 0

Region: chr18 39375325-39375339. Max. coverage (+): 0. Max coverage (-): 0

Region: chr18 39375340-39375355. Max. coverage (+): 0. Max coverage (-): 1.09

Region: chr18 39375356-39375370. Max. coverage (+): 0. Max coverage (-): 0

Region: chr18 39375371-39375385. Max. coverage (+): 0. Max coverage (-): 0

Region: chr18 39375386-39375401. Max. coverage (+): 0. Max coverage (-): 0

Region: chr18 39375402-39375416. Max. coverage (+): 0. Max coverage (-): 0

Region: chr18 39375417-39375431. Max. coverage (+): 0. Max coverage (-): 0

Region: chr18 39375432-39375446. Max. coverage (+): 0. Max coverage (-): 0

Region: chr18 39375447-39375462. Max. coverage (+): 0. Max coverage (-): 0

Region: chr18 39375463-39375477. Max. coverage (+): 0. Max coverage (-): 0

Region: chr18 39375478-39375492. Max. coverage (+): 0. Max coverage (-): 0

Region: chr18 39375493-39375508. Max. coverage (+): 0. Max coverage (-): 0.86

Region: chr18 39375509-39375523. Max. coverage (+): 0. Max coverage (-): 0

Region: chr18 39375524-39375538. Max. coverage (+): 0. Max coverage (-): 1.5

Region: chr18 39375539-39375553. Max. coverage (+): 0. Max coverage (-): 3.03

Region: chr18 39375554-39375569. Max. coverage (+): 0. Max coverage (-): 0

Region: chr18 39375570-39375584. Max. coverage (+): 0. Max coverage (-): 0

Region: chr18 39375585-39375599. Max. coverage (+): 0. Max coverage (-): 0

Region: chr18 39375600-39375615. Max. coverage (+): 0. Max coverage (-): 0

Region: chr18 39375616-39375630. Max. coverage (+): 0. Max coverage (-): 0

Region: chr18 39375631-39375645. Max. coverage (+): 0. Max coverage (-): 0

Region: chr18 39375646-39375661. Max. coverage (+): 0. Max coverage (-): 0

Region: chr18 39375662-39375676. Max. coverage (+): 0. Max coverage (-): 0

Region: chr18 39375677-39375691. Max. coverage (+): 0. Max coverage (-): 0

Region: chr18 39375692-39375706. Max. coverage (+): 0. Max coverage (-): 0

Region: chr18 39375707-39375722. Max. coverage (+): 0. Max coverage (-): 0

Region: chr18 39375723-39375737. Max. coverage (+): 0. Max coverage (-): 0

Region: chr18 39375738-39375752. Max. coverage (+): 0. Max coverage (-): 0

Region: chr18 39375753-39375768. Max. coverage (+): 0. Max coverage (-): 0

Region: chr18 39375769-39375783. Max. coverage (+): 0. Max coverage (-): 0

Region: chr18 39375784-39375798. Max. coverage (+): 0. Max coverage (-): 0

Region: chr18 39375799-39375813. Max. coverage (+): 0. Max coverage (-): 0

Region: chr18 39375814-39375829. Max. coverage (+): 0. Max coverage (-): 0

Region: chr18 39375830-39375844. Max. coverage (+): 0. Max coverage (-): 0

Region: chr18 39375845-39375859. Max. coverage (+): 0. Max coverage (-): 1.96

Region: chr18 39375860-39375875. Max. coverage (+): 0. Max coverage (-): 0

Region: chr18 39375876-39375890. Max. coverage (+): 0. Max coverage (-): 0

Region: chr18 39375891-39375905. Max. coverage (+): 0. Max coverage (-): 0

Region: chr18 39375906-39375921. Max. coverage (+): 0. Max coverage (-): 0

Region: chr18 39375922-39375936. Max. coverage (+): 0. Max coverage (-): 0

Region: chr18 39375937-39375951. Max. coverage (+): 0. Max coverage (-): 0

Region: chr18 39375952-39375966. Max. coverage (+): 0. Max coverage (-): 0

Region: chr18 39375967-39375982. Max. coverage (+): 0. Max coverage (-): 0

Region: chr18 39375983-39375997. Max. coverage (+): 0. Max coverage (-): 0

Region: chr18 39375998-39376012. Max. coverage (+): 0. Max coverage (-): 0

Region: chr18 39376013-39376028. Max. coverage (+): 0. Max coverage (-): 0

Region: chr18 39376029-39376043. Max. coverage (+): 0. Max coverage (-): 0

Region: chr18 39376044-39376058. Max. coverage (+): 0. Max coverage (-): 0

Region: chr18 39376059-39376073. Max. coverage (+): 0. Max coverage (-): 0

Region: chr18 39376074-39376089. Max. coverage (+): 0. Max coverage (-): 0

Region: chr18 39376090-39376104. Max. coverage (+): 0. Max coverage (-): 0

Region: chr18 39376105-39376119. Max. coverage (+): 0. Max coverage (-): 0

Region: chr18 39376120-39376135. Max. coverage (+): 0. Max coverage (-): 0

Region: chr18 39376136-39376150. Max. coverage (+): 0. Max coverage (-): 0

Region: chr18 39376151-39376165. Max. coverage (+): 0. Max coverage (-): 0

Region: chr18 39376166-39376181. Max. coverage (+): 0. Max coverage (-): 0

Region: chr18 39376182-39376196. Max. coverage (+): 0. Max coverage (-): 3.35

Region: chr18 39376197-39376211. Max. coverage (+): 0. Max coverage (-): 3.35

Region: chr18 39376212-39376226. Max. coverage (+): 0. Max coverage (-): 0

Region: chr18 39376227-39376242. Max. coverage (+): 0. Max coverage (-): 0

Region: chr18 39376243-39376257. Max. coverage (+): 0. Max coverage (-): 0

Region: chr18 39376258-39376272. Max. coverage (+): 0. Max coverage (-): 0

Region: chr18 39376273-39376288. Max. coverage (+): 0. Max coverage (-): 0

Region: chr18 39376289-39376303. Max. coverage (+): 0. Max coverage (-): 0

Region: chr18 39376304-39376318. Max. coverage (+): 0. Max coverage (-): 0

Region: chr18 39376319-39376333. Max. coverage (+): 0. Max coverage (-): 0

Region: chr18 39376334-39376349. Max. coverage (+): 0. Max coverage (-): 0

Region: chr18 39376350-39376364. Max. coverage (+): 0. Max coverage (-): 0

Region: chr18 39376365-39376379. Max. coverage (+): 0. Max coverage (-): 0

Region: chr18 39376380-39376395. Max. coverage (+): 0. Max coverage (-): 0

Region: chr18 39376396-39376410. Max. coverage (+): 0. Max coverage (-): 0

Region: chr18 39376411-39376425. Max. coverage (+): 0. Max coverage (-): 0

Region: chr18 39376426-39376441. Max. coverage (+): 0. Max coverage (-): 0

Region: chr18 39376442-39376456. Max. coverage (+): 0. Max coverage (-): 0

Region: chr18 39376457-39376471. Max. coverage (+): 0. Max coverage (-): 0

Region: chr18 39376472-39376486. Max. coverage (+): 0. Max coverage (-): 0

Region: chr18 39376487-39376502. Max. coverage (+): 0. Max coverage (-): 0

Region: chr18 39376503-39376517. Max. coverage (+): 0. Max coverage (-): 0

Region: chr18 39376518-39376532. Max. coverage (+): 0. Max coverage (-): 0

Region: chr18 39376533-39376548. Max. coverage (+): 0. Max coverage (-): 0

Region: chr18 39376549-39376563. Max. coverage (+): 0. Max coverage (-): 0

Region: chr18 39376564-39376578. Max. coverage (+): 0. Max coverage (-): 0

Region: chr18 39376579-39376593. Max. coverage (+): 0. Max coverage (-): 0

Region: chr18 39376594-39376609. Max. coverage (+): 0. Max coverage (-): 2.11

Region: chr18 39376610-39376624. Max. coverage (+): 0. Max coverage (-): 0.69

Region: chr18 39376625-39376639. Max. coverage (+): 0. Max coverage (-): 0

Region: chr18 39376640-39376655. Max. coverage (+): 0. Max coverage (-): 0

Region: chr18 39376656-39376670. Max. coverage (+): 0. Max coverage (-): 0

Region: chr18 39376671-39376685. Max. coverage (+): 0. Max coverage (-): 0

Region: chr18 39376686-39376701. Max. coverage (+): 0. Max coverage (-): 0

Region: chr18 39376702-39376716. Max. coverage (+): 0. Max coverage (-): 0

Region: chr18 39376717-39376731. Max. coverage (+): 0. Max coverage (-): 0

Region: chr18 39376732-39376746. Max. coverage (+): 0. Max coverage (-): 0

Region: chr18 39376747-39376762. Max. coverage (+): 0. Max coverage (-): 0

Region: chr18 39376763-39376777. Max. coverage (+): 0. Max coverage (-): 0

Region: chr18 39376778-39376792. Max. coverage (+): 0. Max coverage (-): 0

Region: chr18 39376793-39376808. Max. coverage (+): 0. Max coverage (-): 0

Region: chr18 39376809-39376823. Max. coverage (+): 0. Max coverage (-): 0

Region: chr18 39376824-39376838. Max. coverage (+): 0. Max coverage (-): 0

Region: chr18 39376839-39376853. Max. coverage (+): 0. Max coverage (-): 0

Region: chr18 39376854-39376869. Max. coverage (+): 0. Max coverage (-): 0

Region: chr18 39376870-39376884. Max. coverage (+): 0. Max coverage (-): 0

Region: chr18 39376885-39376899. Max. coverage (+): 0. Max coverage (-): 0

Region: chr18 39376900-39376915. Max. coverage (+): 0. Max coverage (-): 0

Region: chr18 39376916-39376930. Max. coverage (+): 0. Max coverage (-): 0

Region: chr18 39376931-39376945. Max. coverage (+): 0. Max coverage (-): 0

Region: chr18 39376946-39376961. Max. coverage (+): 0. Max coverage (-): 0

Region: chr18 39376962-39376976. Max. coverage (+): 0. Max coverage (-): 0

Region: chr18 39376977-39376991. Max. coverage (+): 0. Max coverage (-): 4.35

Region: chr18 39376992-39377006. Max. coverage (+): 0. Max coverage (-): 4.35

Region: chr18 39377007-39377022. Max. coverage (+): 0. Max coverage (-): 0

Region: chr18 39377023-39377037. Max. coverage (+): 0. Max coverage (-): 0

Region: chr18 39377038-39377052. Max. coverage (+): 0. Max coverage (-): 0

Region: chr18 39377053-39377068. Max. coverage (+): 0. Max coverage (-): 0

Region: chr18 39377069-39377083. Max. coverage (+): 0. Max coverage (-): 0

Region: chr18 39377084-39377098. Max. coverage (+): 0. Max coverage (-): 0

Region: chr18 39377099-39377113. Max. coverage (+): 0. Max coverage (-): 0

Region: chr18 39377114-39377129. Max. coverage (+): 0. Max coverage (-): 0

Region: chr18 39377130-39377144. Max. coverage (+): 0. Max coverage (-): 0

Region: chr18 39377145-39377159. Max. coverage (+): 0. Max coverage (-): 0

Region: chr18 39377160-39377175. Max. coverage (+): 0. Max coverage (-): 0

Region: chr18 39377176-39377190. Max. coverage (+): 0. Max coverage (-): 2.24

Region: chr18 39377191-39377205. Max. coverage (+): 0. Max coverage (-): 0

Region: chr18 39377206-39377221. Max. coverage (+): 0. Max coverage (-): 0

Region: chr18 39377222-39377236. Max. coverage (+): 0. Max coverage (-): 0

Region: chr18 39377237-39377251. Max. coverage (+): 0. Max coverage (-): 0

Region: chr18 39377252-39377266. Max. coverage (+): 0. Max coverage (-): 0

Region: chr18 39377267-39377282. Max. coverage (+): 0. Max coverage (-): 0

Region: chr18 39377283-39377297. Max. coverage (+): 0. Max coverage (-): 0

Region: chr18 39377298-39377312. Max. coverage (+): 0. Max coverage (-): 0

Region: chr18 39377313-39377328. Max. coverage (+): 0. Max coverage (-): 0

Region: chr18 39377329-39377343. Max. coverage (+): 0. Max coverage (-): 0

Region: chr18 39377344-39377358. Max. coverage (+): 0. Max coverage (-): 0

Region: chr18 39377359-39377373. Max. coverage (+): 0. Max coverage (-): 0

Region: chr18 39377374-39377389. Max. coverage (+): 0. Max coverage (-): 0

Region: chr18 39377390-39377404. Max. coverage (+): 0. Max coverage (-): 0

Region: chr18 39377405-39377419. Max. coverage (+): 0. Max coverage (-): 0

Region: chr18 39377420-39377435. Max. coverage (+): 0. Max coverage (-): 0

Region: chr18 39377436-39377450. Max. coverage (+): 0. Max coverage (-): 0

Region: chr18 39377451-39377465. Max. coverage (+): 0. Max coverage (-): 0

Region: chr18 39377466-39377481. Max. coverage (+): 0. Max coverage (-): 0

Region: chr18 39377482-39377496. Max. coverage (+): 0. Max coverage (-): 0

Region: chr18 39377497-39377511. Max. coverage (+): 0. Max coverage (-): 0

Region: chr18 39377512-39377526. Max. coverage (+): 0. Max coverage (-): 0

Region: chr18 39377527-39377542. Max. coverage (+): 0. Max coverage (-): 0

Region: chr18 39377543-39377557. Max. coverage (+): 0. Max coverage (-): 0

Region: chr18 39377558-39377572. Max. coverage (+): 0. Max coverage (-): 0

Region: chr18 39377573-39377588. Max. coverage (+): 0. Max coverage (-): 0

Region: chr18 39377589-39377603. Max. coverage (+): 0. Max coverage (-): 0

Region: chr18 39377604-39377618. Max. coverage (+): 0. Max coverage (-): 0

Region: chr18 39377619-39377633. Max. coverage (+): 0. Max coverage (-): 0

Region: chr18 39377634-39377649. Max. coverage (+): 0. Max coverage (-): 0

Region: chr18 39377650-39377664. Max. coverage (+): 0. Max coverage (-): 0

Region: chr18 39377665-39377679. Max. coverage (+): 0. Max coverage (-): 0

Region: chr18 39377680-39377695. Max. coverage (+): 0. Max coverage (-): 0

Region: chr18 39377696-39377710. Max. coverage (+): 0. Max coverage (-): 0

Region: chr18 39377711-39377725. Max. coverage (+): 0. Max coverage (-): 0

Region: chr18 39377726-39377741. Max. coverage (+): 0. Max coverage (-): 0

Region: chr18 39377742-39377756. Max. coverage (+): 0. Max coverage (-): 0

Region: chr18 39377757-39377771. Max. coverage (+): 0. Max coverage (-): 0

Region: chr18 39377772-39377786. Max. coverage (+): 0. Max coverage (-): 0

Region: chr18 39377787-39377802. Max. coverage (+): 0. Max coverage (-): 0

Region: chr18 39377803-39377817. Max. coverage (+): 0. Max coverage (-): 0

Region: chr18 39377818-39377832. Max. coverage (+): 0. Max coverage (-): 0

Region: chr18 39377833-39377848. Max. coverage (+): 0. Max coverage (-): 0

Region: chr18 39377849-39377863. Max. coverage (+): 0. Max coverage (-): 0

Region: chr18 39377864-39377878. Max. coverage (+): 0. Max coverage (-): 0

Region: chr18 39377879-39377893. Max. coverage (+): 0. Max coverage (-): 0

Region: chr18 39377894-39377909. Max. coverage (+): 0. Max coverage (-): 0

Region: chr18 39377910-39377924. Max. coverage (+): 0. Max coverage (-): 0

Region: chr18 39377925-39377939. Max. coverage (+): 0. Max coverage (-): 0

Region: chr18 39377940-39377955. Max. coverage (+): 0. Max coverage (-): 0

Region: chr18 39377956-39377970. Max. coverage (+): 0. Max coverage (-): 0

Region: chr18 39377971-39377985. Max. coverage (+): 0. Max coverage (-): 0

Region: chr18 39377986-39378001. Max. coverage (+): 0. Max coverage (-): 0

Region: chr18 39378002-39378016. Max. coverage (+): 0. Max coverage (-): 0

Region: chr18 39378017-39378031. Max. coverage (+): 0. Max coverage (-): 0

Region: chr18 39378032-39378046. Max. coverage (+): 0. Max coverage (-): 0

Region: chr18 39378047-39378062. Max. coverage (+): 0. Max coverage (-): 0

Region: chr18 39378063-39378077. Max. coverage (+): 0. Max coverage (-): 0

Region: chr18 39378078-39378092. Max. coverage (+): 0. Max coverage (-): 0

Region: chr18 39378093-39378108. Max. coverage (+): 0. Max coverage (-): 0

Region: chr18 39378109-39378123. Max. coverage (+): 0. Max coverage (-): 0

Region: chr18 39378124-39378138. Max. coverage (+): 0. Max coverage (-): 5.13

Region: chr18 39378139-39378153. Max. coverage (+): 0. Max coverage (-): 5.13

Region: chr18 39378154-39378169. Max. coverage (+): 0. Max coverage (-): 0

Region: chr18 39378170-39378184. Max. coverage (+): 0. Max coverage (-): 0

Region: chr18 39378185-39378199. Max. coverage (+): 0. Max coverage (-): 0

Region: chr18 39378200-39378215. Max. coverage (+): 0. Max coverage (-): 0

Region: chr18 39378216-39378230. Max. coverage (+): 0. Max coverage (-): 0

Region: chr18 39378231-39378245. Max. coverage (+): 0. Max coverage (-): 0

Region: chr18 39378246-39378261. Max. coverage (+): 0. Max coverage (-): 0

Region: chr18 39378262-39378276. Max. coverage (+): 0. Max coverage (-): 0

Region: chr18 39378277-39378291. Max. coverage (+): 0. Max coverage (-): 0

Region: chr18 39378292-39378306. Max. coverage (+): 0. Max coverage (-): 0

Region: chr18 39378307-39378322. Max. coverage (+): 0. Max coverage (-): 2.06

Region: chr18 39378323-39378337. Max. coverage (+): 0. Max coverage (-): 0

Region: chr18 39378338-39378352. Max. coverage (+): 0. Max coverage (-): 0

Region: chr18 39378353-39378368. Max. coverage (+): 0. Max coverage (-): 4.29

Region: chr18 39378369-39378383. Max. coverage (+): 0. Max coverage (-): 4.29

Region: chr18 39378384-. Max. coverage (+): 0. Max coverage (-): 0

RepeatMasker Color Code

**+**

100-98% Identity

<98-95% Identity

<95-90% Identity

<90-85% Identity

<85-80% Identity

<80-75% Identity

<75-70% Identity

<70% Identity

**-**

Gene Set Color Code

**+**

Gene

Pseudogene

**-**

Topology/Coverage Color Code

Coverage Plus Strand

Coverage Minus Strand

Mainstrand: Plus

Mainstrand: Minus

Complementary Strand

Flanking Region  
(if option -flank >0)

Gene Set Annotation  

**1. U6 (protein coding, ENSBTAG00000042970) Tr:00000059962 Ex:1**: 39373803-39373909 (-)  
**2. ATXN1L (protein coding, ENSBTAG00000046255) Tr:00000063114 Ex:1**: 39376391-39378454 (-)

  
RepeatMasker Annotation  

**1. U6**: 39373805-39373909 (-), Divergence to consensus: 0%  
**2. MIRb**: 39373921-39373962 (-), Divergence to consensus: 21.4%  
**3. MIR**: 39374424-39374575 (-), Divergence to consensus: 44.2%  
**4. SINE2-1\_BT**: 39374939-39375050 (+), Divergence to consensus: 20.4%

  
Transcription Factor Binding Sites  

**Gata4** (Sequence: AGATAAG (-): 39373420)  
**SOX9** (Sequence: AACAATAA (-): 39370749)
