# Supplementary material for: Small RNA sequencing of cryopreserved semen from single bull revealed altered miRNAs and piRNAs expression between High- and Low-motile sperm populations
Source: BMC Genomics. 2017 Jan 4;18:14. doi: 10.1186/s12864-016-3394-7 (PMC5209821; doi:10.1186/s12864-016-3394-7)
Supplement: Additional file 3: — Details for each piRNA clusters found in High Motile (HM) sperm fraction. Genes, repeats, transposable elements and transcription factors binding sites falling within the cluster regions were reported. (ZIP 1896 kb) [file 12864_2016_3394_MOESM3_ESM.zip › 37.html]

piRNA cluster 37


Predicted piRNA cluster no. 37     previous   next
  

Show proTRAC run info
Hide proTRAC run info

================================= proTRAC ====================================  
VERSION: 2.1                                    LAST MODIFIED: 06. October 2015  
  
Please cite:  
Rosenkranz D, Zischler H. proTRAC - a software for probabilistic piRNA cluster  
detection, visualization and analysis. 2012. BMC Bioinformatics 13:5.  
  
and (for proTRAC 2.0 and later):  
Rosenkranz D, Rudloff S, Bastuck K, Ketting RF, Zischler H. Tupaia small RNAs  
provide insights into function and evolution of RNAi-based transposon defense  
in mammals. 2015. RNA 21(5):911-922.  
  
Contact:  
David Rosenkranz  
Institute of Anthropology, small RNA group  
Johannes Gutenberg University Mainz  
email: rosenkranz@uni-mainz.de  
  
You can find the latest proTRAC version at:  
http://sourceforge.net/projects/protrac/files  
http://www.smallRNAgroup-mainz.de/software  
==============================================================================  
  
PARAMETERS:  
Map file: .............../storage/core/barbara/genhome/smallRNA/fertility/Sample\_motile/pirna/Sample\_motile\_26-33\_collapsed.fa.no-dust.map.weighted-10000-1000-b-0  
Genome file: ............/storage/core/barbara/genhome/smallRNA/fertility/Sample\_all/pirna/bt\_311\_chrY.fa  
RepeatMasker annotation: /storage/genomes/bt\_umd31/GCF\_000003055.6\_Bos\_taurus\_UMD\_3.1.1\_repeatMasker\_chr.out  
GeneSet:................./storage/core/barbara/genhome/smallRNA/fertility/Sample\_all/pirna/full.gtf  
  
Significant (p<=0.01) hit density will be calculated based  
on observed hit distribution.  
  
Sliding window size: ........................................ 5000 bp  
Sliding window increament: .................................. 1000 bp  
Normalize each hit by number of genomic hits: ............... 1 [0=no/1=yes]  
Normalize each hit by number of sequence reads: ............. 1 [0=no/1=yes]  
Normalize values (-> per million mapped reads): ............. 1 [0=no/1=yes]  
Min. fraction of hits with 1T(U) or 10A: .................... 0.75  
Alternatively: Min. fraction of hits with 1T(U) and 10A: .... 0.5  
Min. fraction of hits with typical piRNA length: ............ 0.75  
Typical piRNA length: ....................................... 26-33 nt  
Min. size of a piRNA cluster: ............................... 5000 bp.  
Min. number of hits (absolute): ............................. 0  
Min. number of hits (normalized): ........................... 0  
Min. fraction of hits on the mainstrand: .................... 0.75  
Top fraction of mapped sequences (in terms of read counts): . 1%  
Top fraction accounts for max. n% of sequence reads: ........ 90%  
Min. fraction of hits on each arm of a bidirectional cluster: 0.1  
Output image file for each cluster: ......................... 0 [0=no/1=yes]  
Output html file for each cluster: .......................... 1 [0=no/1=yes]  
Output a summary table: ..................................... 1 [0=no/1=yes]  
Output a FASTA file for each cluster (piRNA sequences): ..... 1 [0=no/1=yes]  
Output a FASTA file comprising cluster sequences: ........... 1 [0=no/1=yes]  
Search DNA motifs in clusters: .............................. 1 [0=no/1=yes]  
Output flanking sequences: +/- .............................. 0 bp  
Output ~.pTi file: .......................................... 1 [0=no/1=yes]  
==============================================================================  
  
  
Genome size (without gaps): ............ 2678902517 bp  
Gaps (N/X/-): .......................... 53837044 bp  
Mapped reads: .......................... 658825247023  
Non-identical sequences: ............... 514171  
Genomic hits: .......................... 764233  
Significant densitiy of mapped reads: .. 12867599.5173724 reads/kb

Show proTRAC cluster info
Hide proTRAC cluster info

|  |  |
| --- | --- |
| Location | chr18 |
| Coordinates | 39784573-39800577 |
| Size [bp] | 16005 |
| Sequence hit loci | 3036 |
| Mapped reads (normalized) | 3417582384 |
| Mapped reads (normalized) per kb | 213532170.2 |
| Normalized reads with 1T (1U) | 78.2% |
| Normalized reads with 10A | 22.1% |
| Normalized reads with length 26-33 nt | 100% |
| Normalized reads on the main strand(s) | 99.8% |
| Predicted directionality | mono:plus |

100%

0%

1T (1U)  
reads

10A reads

26-33 nt  
reads

reads on mainstrand

**Either the amount of reads with 1T (1U) OR 10A has to exceed 75% (set with option: -1Tor10A)  
Alternatively the amount of reads with 1T (1U) AND 10A has to exceed 50% (set with option: -1Tand10A)  
Minimum amount of reads with preferred size is 75% (set with option: -pisize)  
Minimum amount of reads on the main strand(s) is 75% (set with option: -clstrand)**

Show read coverage
Hide read coverage

WHAT DO I SEE HERE?  
This chart shows the location of mapped sequence reads within a predicted piRNA cluster. The color refers to the number of genomic hits produced by the sequence read in question. A dark red bar indicates that this sequence read produces many other hits elsewhere in the genome. Many adjacent red or yellow bars can indicate the presence of a multi-copy element such as transposons or rRNA genes. A dark green bar indicates that this sequence read maps uniquely to this locus.

1 hit

2-5 hits

6-10 hits

11-20 hits

21-50 hits

51-100 hits

> 100 hits

chr18

39784573

39800577

Gene Set

RepeatMasker

Mapped  
Reads

80.88

plus strand

minus strand

80.88

Region: chr18 39378385-39784589. Max. coverage (+): 0. Max coverage (-): 3.57

Region: chr18 39784590-39784621. Max. coverage (+): 0. Max coverage (-): 0

Region: chr18 39784622-39784653. Max. coverage (+): 0. Max coverage (-): 0

Region: chr18 39784654-39784685. Max. coverage (+): 0. Max coverage (-): 0

Region: chr18 39784686-39784717. Max. coverage (+): 0. Max coverage (-): 0

Region: chr18 39784718-39784749. Max. coverage (+): 0. Max coverage (-): 0

Region: chr18 39784750-39784781. Max. coverage (+): 0. Max coverage (-): 0

Region: chr18 39784782-39784813. Max. coverage (+): 0. Max coverage (-): 0

Region: chr18 39784814-39784845. Max. coverage (+): 0. Max coverage (-): 0

Region: chr18 39784846-39784877. Max. coverage (+): 0. Max coverage (-): 0

Region: chr18 39784878-39784909. Max. coverage (+): 0. Max coverage (-): 0

Region: chr18 39784910-39784941. Max. coverage (+): 0. Max coverage (-): 0

Region: chr18 39784942-39784973. Max. coverage (+): 0. Max coverage (-): 0

Region: chr18 39784974-39785005. Max. coverage (+): 0. Max coverage (-): 0

Region: chr18 39785006-39785037. Max. coverage (+): 0. Max coverage (-): 0

Region: chr18 39785038-39785069. Max. coverage (+): 0. Max coverage (-): 0

Region: chr18 39785070-39785101. Max. coverage (+): 0. Max coverage (-): 0

Region: chr18 39785102-39785133. Max. coverage (+): 0. Max coverage (-): 0

Region: chr18 39785134-39785165. Max. coverage (+): 0. Max coverage (-): 0

Region: chr18 39785166-39785197. Max. coverage (+): 0. Max coverage (-): 0

Region: chr18 39785198-39785229. Max. coverage (+): 0. Max coverage (-): 0

Region: chr18 39785230-39785261. Max. coverage (+): 0. Max coverage (-): 0

Region: chr18 39785262-39785293. Max. coverage (+): 0. Max coverage (-): 0

Region: chr18 39785294-39785325. Max. coverage (+): 0. Max coverage (-): 0

Region: chr18 39785326-39785357. Max. coverage (+): 0. Max coverage (-): 0

Region: chr18 39785358-39785389. Max. coverage (+): 0. Max coverage (-): 0

Region: chr18 39785390-39785421. Max. coverage (+): 0. Max coverage (-): 0

Region: chr18 39785422-39785453. Max. coverage (+): 0. Max coverage (-): 0

Region: chr18 39785454-39785485. Max. coverage (+): 0. Max coverage (-): 0

Region: chr18 39785486-39785517. Max. coverage (+): 0. Max coverage (-): 0

Region: chr18 39785518-39785549. Max. coverage (+): 0. Max coverage (-): 0

Region: chr18 39785550-39785581. Max. coverage (+): 0. Max coverage (-): 0

Region: chr18 39785582-39785613. Max. coverage (+): 0. Max coverage (-): 0

Region: chr18 39785614-39785645. Max. coverage (+): 0. Max coverage (-): 0

Region: chr18 39785646-39785677. Max. coverage (+): 0. Max coverage (-): 0

Region: chr18 39785678-39785709. Max. coverage (+): 0. Max coverage (-): 0

Region: chr18 39785710-39785741. Max. coverage (+): 0. Max coverage (-): 0

Region: chr18 39785742-39785773. Max. coverage (+): 0. Max coverage (-): 1.34

Region: chr18 39785774-39785805. Max. coverage (+): 0. Max coverage (-): 0

Region: chr18 39785806-39785837. Max. coverage (+): 0. Max coverage (-): 0

Region: chr18 39785838-39785869. Max. coverage (+): 0. Max coverage (-): 0.77

Region: chr18 39785870-39785901. Max. coverage (+): 0. Max coverage (-): 0

Region: chr18 39785902-39785933. Max. coverage (+): 0. Max coverage (-): 0

Region: chr18 39785934-39785965. Max. coverage (+): 0. Max coverage (-): 0

Region: chr18 39785966-39785997. Max. coverage (+): 0. Max coverage (-): 0

Region: chr18 39785998-39786029. Max. coverage (+): 0. Max coverage (-): 0

Region: chr18 39786030-39786061. Max. coverage (+): 0. Max coverage (-): 0

Region: chr18 39786062-39786093. Max. coverage (+): 0. Max coverage (-): 2.41

Region: chr18 39786094-39786125. Max. coverage (+): 0. Max coverage (-): 0

Region: chr18 39786126-39786157. Max. coverage (+): 1.65. Max coverage (-): 0

Region: chr18 39786158-39786189. Max. coverage (+): 0. Max coverage (-): 0

Region: chr18 39786190-39786221. Max. coverage (+): 0. Max coverage (-): 0

Region: chr18 39786222-39786253. Max. coverage (+): 0. Max coverage (-): 0

Region: chr18 39786254-39786285. Max. coverage (+): 0. Max coverage (-): 0

Region: chr18 39786286-39786317. Max. coverage (+): 0. Max coverage (-): 0

Region: chr18 39786318-39786349. Max. coverage (+): 0. Max coverage (-): 0

Region: chr18 39786350-39786381. Max. coverage (+): 0. Max coverage (-): 0

Region: chr18 39786382-39786413. Max. coverage (+): 0. Max coverage (-): 0

Region: chr18 39786414-39786445. Max. coverage (+): 0. Max coverage (-): 0

Region: chr18 39786446-39786477. Max. coverage (+): 0. Max coverage (-): 0

Region: chr18 39786478-39786509. Max. coverage (+): 0. Max coverage (-): 0

Region: chr18 39786510-39786541. Max. coverage (+): 0. Max coverage (-): 0

Region: chr18 39786542-39786573. Max. coverage (+): 0. Max coverage (-): 0

Region: chr18 39786574-39786605. Max. coverage (+): 0. Max coverage (-): 0

Region: chr18 39786606-39786637. Max. coverage (+): 0. Max coverage (-): 0

Region: chr18 39786638-39786669. Max. coverage (+): 0. Max coverage (-): 0

Region: chr18 39786670-39786701. Max. coverage (+): 0. Max coverage (-): 0

Region: chr18 39786702-39786733. Max. coverage (+): 0. Max coverage (-): 0

Region: chr18 39786734-39786765. Max. coverage (+): 0. Max coverage (-): 0

Region: chr18 39786766-39786797. Max. coverage (+): 0. Max coverage (-): 0

Region: chr18 39786798-39786829. Max. coverage (+): 0. Max coverage (-): 0

Region: chr18 39786830-39786861. Max. coverage (+): 0. Max coverage (-): 0

Region: chr18 39786862-39786893. Max. coverage (+): 0. Max coverage (-): 0

Region: chr18 39786894-39786925. Max. coverage (+): 0. Max coverage (-): 0

Region: chr18 39786926-39786957. Max. coverage (+): 0. Max coverage (-): 0

Region: chr18 39786958-39786989. Max. coverage (+): 0. Max coverage (-): 0

Region: chr18 39786990-39787021. Max. coverage (+): 0. Max coverage (-): 0

Region: chr18 39787022-39787053. Max. coverage (+): 0. Max coverage (-): 0

Region: chr18 39787054-39787085. Max. coverage (+): 0. Max coverage (-): 0

Region: chr18 39787086-39787117. Max. coverage (+): 0. Max coverage (-): 0

Region: chr18 39787118-39787149. Max. coverage (+): 0. Max coverage (-): 0

Region: chr18 39787150-39787181. Max. coverage (+): 0. Max coverage (-): 0

Region: chr18 39787182-39787213. Max. coverage (+): 0. Max coverage (-): 0

Region: chr18 39787214-39787245. Max. coverage (+): 0. Max coverage (-): 0

Region: chr18 39787246-39787277. Max. coverage (+): 0. Max coverage (-): 0

Region: chr18 39787278-39787309. Max. coverage (+): 0. Max coverage (-): 0

Region: chr18 39787310-39787341. Max. coverage (+): 0. Max coverage (-): 0

Region: chr18 39787342-39787373. Max. coverage (+): 0. Max coverage (-): 0

Region: chr18 39787374-39787405. Max. coverage (+): 0. Max coverage (-): 0

Region: chr18 39787406-39787437. Max. coverage (+): 0. Max coverage (-): 0

Region: chr18 39787438-39787469. Max. coverage (+): 0. Max coverage (-): 0

Region: chr18 39787470-39787501. Max. coverage (+): 0. Max coverage (-): 0

Region: chr18 39787502-39787533. Max. coverage (+): 0. Max coverage (-): 0

Region: chr18 39787534-39787565. Max. coverage (+): 0. Max coverage (-): 0

Region: chr18 39787566-39787597. Max. coverage (+): 0. Max coverage (-): 0

Region: chr18 39787598-39787629. Max. coverage (+): 0. Max coverage (-): 0

Region: chr18 39787630-39787661. Max. coverage (+): 0. Max coverage (-): 0

Region: chr18 39787662-39787693. Max. coverage (+): 0. Max coverage (-): 0

Region: chr18 39787694-39787725. Max. coverage (+): 0. Max coverage (-): 0

Region: chr18 39787726-39787757. Max. coverage (+): 0. Max coverage (-): 0

Region: chr18 39787758-39787790. Max. coverage (+): 0. Max coverage (-): 0

Region: chr18 39787791-39787822. Max. coverage (+): 0. Max coverage (-): 0

Region: chr18 39787823-39787854. Max. coverage (+): 0. Max coverage (-): 0

Region: chr18 39787855-39787886. Max. coverage (+): 1.95. Max coverage (-): 0

Region: chr18 39787887-39787918. Max. coverage (+): 2.35. Max coverage (-): 0

Region: chr18 39787919-39787950. Max. coverage (+): 4.79. Max coverage (-): 0

Region: chr18 39787951-39787982. Max. coverage (+): 0.13. Max coverage (-): 0

Region: chr18 39787983-39788014. Max. coverage (+): 3.84. Max coverage (-): 0

Region: chr18 39788015-39788046. Max. coverage (+): 1.17. Max coverage (-): 0

Region: chr18 39788047-39788078. Max. coverage (+): 0. Max coverage (-): 0

Region: chr18 39788079-39788110. Max. coverage (+): 9.84. Max coverage (-): 0

Region: chr18 39788111-39788142. Max. coverage (+): 2.64. Max coverage (-): 0

Region: chr18 39788143-39788174. Max. coverage (+): 0. Max coverage (-): 0

Region: chr18 39788175-39788206. Max. coverage (+): 0. Max coverage (-): 0

Region: chr18 39788207-39788238. Max. coverage (+): 3.58. Max coverage (-): 0

Region: chr18 39788239-39788270. Max. coverage (+): 4.24. Max coverage (-): 0

Region: chr18 39788271-39788302. Max. coverage (+): 1.03. Max coverage (-): 0

Region: chr18 39788303-39788334. Max. coverage (+): 0. Max coverage (-): 0

Region: chr18 39788335-39788366. Max. coverage (+): 0. Max coverage (-): 0

Region: chr18 39788367-39788398. Max. coverage (+): 0. Max coverage (-): 0

Region: chr18 39788399-39788430. Max. coverage (+): 0. Max coverage (-): 0

Region: chr18 39788431-39788462. Max. coverage (+): 0. Max coverage (-): 0

Region: chr18 39788463-39788494. Max. coverage (+): 0. Max coverage (-): 0

Region: chr18 39788495-39788526. Max. coverage (+): 5.18. Max coverage (-): 0

Region: chr18 39788527-39788558. Max. coverage (+): 0. Max coverage (-): 0

Region: chr18 39788559-39788590. Max. coverage (+): 3.04. Max coverage (-): 0

Region: chr18 39788591-39788622. Max. coverage (+): 3.04. Max coverage (-): 0

Region: chr18 39788623-39788654. Max. coverage (+): 0. Max coverage (-): 0

Region: chr18 39788655-39788686. Max. coverage (+): 8.33. Max coverage (-): 0

Region: chr18 39788687-39788718. Max. coverage (+): 0. Max coverage (-): 0

Region: chr18 39788719-39788750. Max. coverage (+): 0. Max coverage (-): 0

Region: chr18 39788751-39788782. Max. coverage (+): 0. Max coverage (-): 0

Region: chr18 39788783-39788814. Max. coverage (+): 0. Max coverage (-): 0

Region: chr18 39788815-39788846. Max. coverage (+): 3.91. Max coverage (-): 0

Region: chr18 39788847-39788878. Max. coverage (+): 0.65. Max coverage (-): 0

Region: chr18 39788879-39788910. Max. coverage (+): 4.03. Max coverage (-): 0

Region: chr18 39788911-39788942. Max. coverage (+): 10.81. Max coverage (-): 0

Region: chr18 39788943-39788974. Max. coverage (+): 54.66. Max coverage (-): 0

Region: chr18 39788975-39789006. Max. coverage (+): 0. Max coverage (-): 0

Region: chr18 39789007-39789038. Max. coverage (+): 0. Max coverage (-): 0

Region: chr18 39789039-39789070. Max. coverage (+): 0. Max coverage (-): 0

Region: chr18 39789071-39789102. Max. coverage (+): 0. Max coverage (-): 0

Region: chr18 39789103-39789134. Max. coverage (+): 0. Max coverage (-): 0

Region: chr18 39789135-39789166. Max. coverage (+): 0. Max coverage (-): 0

Region: chr18 39789167-39789198. Max. coverage (+): 0. Max coverage (-): 0

Region: chr18 39789199-39789230. Max. coverage (+): 0. Max coverage (-): 0

Region: chr18 39789231-39789262. Max. coverage (+): 0. Max coverage (-): 0

Region: chr18 39789263-39789294. Max. coverage (+): 0. Max coverage (-): 0

Region: chr18 39789295-39789326. Max. coverage (+): 0. Max coverage (-): 0

Region: chr18 39789327-39789358. Max. coverage (+): 0. Max coverage (-): 0

Region: chr18 39789359-39789390. Max. coverage (+): 0. Max coverage (-): 0

Region: chr18 39789391-39789422. Max. coverage (+): 0. Max coverage (-): 0

Region: chr18 39789423-39789454. Max. coverage (+): 0. Max coverage (-): 0

Region: chr18 39789455-39789486. Max. coverage (+): 0. Max coverage (-): 0

Region: chr18 39789487-39789518. Max. coverage (+): 0. Max coverage (-): 0

Region: chr18 39789519-39789550. Max. coverage (+): 0. Max coverage (-): 0

Region: chr18 39789551-39789582. Max. coverage (+): 0. Max coverage (-): 0

Region: chr18 39789583-39789614. Max. coverage (+): 0. Max coverage (-): 0

Region: chr18 39789615-39789646. Max. coverage (+): 0. Max coverage (-): 0

Region: chr18 39789647-39789678. Max. coverage (+): 0. Max coverage (-): 0

Region: chr18 39789679-39789710. Max. coverage (+): 0. Max coverage (-): 0

Region: chr18 39789711-39789742. Max. coverage (+): 0. Max coverage (-): 0

Region: chr18 39789743-39789774. Max. coverage (+): 0. Max coverage (-): 0

Region: chr18 39789775-39789806. Max. coverage (+): 0. Max coverage (-): 0

Region: chr18 39789807-39789838. Max. coverage (+): 0. Max coverage (-): 0

Region: chr18 39789839-39789870. Max. coverage (+): 0. Max coverage (-): 0

Region: chr18 39789871-39789902. Max. coverage (+): 0. Max coverage (-): 0

Region: chr18 39789903-39789934. Max. coverage (+): 0. Max coverage (-): 0

Region: chr18 39789935-39789966. Max. coverage (+): 0. Max coverage (-): 0

Region: chr18 39789967-39789998. Max. coverage (+): 0. Max coverage (-): 0

Region: chr18 39789999-39790030. Max. coverage (+): 0. Max coverage (-): 0

Region: chr18 39790031-39790062. Max. coverage (+): 0. Max coverage (-): 0

Region: chr18 39790063-39790094. Max. coverage (+): 0. Max coverage (-): 0

Region: chr18 39790095-39790126. Max. coverage (+): 0. Max coverage (-): 0

Region: chr18 39790127-39790158. Max. coverage (+): 0. Max coverage (-): 0

Region: chr18 39790159-39790190. Max. coverage (+): 0. Max coverage (-): 0

Region: chr18 39790191-39790222. Max. coverage (+): 0. Max coverage (-): 0

Region: chr18 39790223-39790254. Max. coverage (+): 0. Max coverage (-): 0

Region: chr18 39790255-39790286. Max. coverage (+): 0. Max coverage (-): 0

Region: chr18 39790287-39790318. Max. coverage (+): 0. Max coverage (-): 0

Region: chr18 39790319-39790350. Max. coverage (+): 0. Max coverage (-): 0

Region: chr18 39790351-39790382. Max. coverage (+): 0. Max coverage (-): 0

Region: chr18 39790383-39790414. Max. coverage (+): 0. Max coverage (-): 0

Region: chr18 39790415-39790446. Max. coverage (+): 0. Max coverage (-): 0

Region: chr18 39790447-39790478. Max. coverage (+): 0. Max coverage (-): 0

Region: chr18 39790479-39790510. Max. coverage (+): 0. Max coverage (-): 0

Region: chr18 39790511-39790542. Max. coverage (+): 0. Max coverage (-): 0

Region: chr18 39790543-39790574. Max. coverage (+): 0. Max coverage (-): 0

Region: chr18 39790575-39790606. Max. coverage (+): 0. Max coverage (-): 0

Region: chr18 39790607-39790638. Max. coverage (+): 0. Max coverage (-): 0

Region: chr18 39790639-39790670. Max. coverage (+): 0. Max coverage (-): 0

Region: chr18 39790671-39790702. Max. coverage (+): 0. Max coverage (-): 0

Region: chr18 39790703-39790734. Max. coverage (+): 0. Max coverage (-): 0

Region: chr18 39790735-39790766. Max. coverage (+): 0. Max coverage (-): 0

Region: chr18 39790767-39790798. Max. coverage (+): 0. Max coverage (-): 0

Region: chr18 39790799-39790830. Max. coverage (+): 0. Max coverage (-): 0

Region: chr18 39790831-39790862. Max. coverage (+): 0. Max coverage (-): 0

Region: chr18 39790863-39790894. Max. coverage (+): 0. Max coverage (-): 0

Region: chr18 39790895-39790926. Max. coverage (+): 0. Max coverage (-): 0

Region: chr18 39790927-39790958. Max. coverage (+): 0. Max coverage (-): 0

Region: chr18 39790959-39790991. Max. coverage (+): 0. Max coverage (-): 0

Region: chr18 39790992-39791023. Max. coverage (+): 0. Max coverage (-): 0

Region: chr18 39791024-39791055. Max. coverage (+): 0. Max coverage (-): 0

Region: chr18 39791056-39791087. Max. coverage (+): 0. Max coverage (-): 0

Region: chr18 39791088-39791119. Max. coverage (+): 0. Max coverage (-): 0

Region: chr18 39791120-39791151. Max. coverage (+): 0. Max coverage (-): 0

Region: chr18 39791152-39791183. Max. coverage (+): 0. Max coverage (-): 0

Region: chr18 39791184-39791215. Max. coverage (+): 0. Max coverage (-): 0

Region: chr18 39791216-39791247. Max. coverage (+): 0. Max coverage (-): 0

Region: chr18 39791248-39791279. Max. coverage (+): 0. Max coverage (-): 0

Region: chr18 39791280-39791311. Max. coverage (+): 0. Max coverage (-): 0

Region: chr18 39791312-39791343. Max. coverage (+): 0. Max coverage (-): 0

Region: chr18 39791344-39791375. Max. coverage (+): 0. Max coverage (-): 0

Region: chr18 39791376-39791407. Max. coverage (+): 0. Max coverage (-): 0

Region: chr18 39791408-39791439. Max. coverage (+): 0. Max coverage (-): 0

Region: chr18 39791440-39791471. Max. coverage (+): 0. Max coverage (-): 0

Region: chr18 39791472-39791503. Max. coverage (+): 0. Max coverage (-): 0

Region: chr18 39791504-39791535. Max. coverage (+): 0. Max coverage (-): 0

Region: chr18 39791536-39791567. Max. coverage (+): 0. Max coverage (-): 0

Region: chr18 39791568-39791599. Max. coverage (+): 0. Max coverage (-): 0

Region: chr18 39791600-39791631. Max. coverage (+): 0. Max coverage (-): 0

Region: chr18 39791632-39791663. Max. coverage (+): 0. Max coverage (-): 0

Region: chr18 39791664-39791695. Max. coverage (+): 0. Max coverage (-): 0

Region: chr18 39791696-39791727. Max. coverage (+): 0. Max coverage (-): 0

Region: chr18 39791728-39791759. Max. coverage (+): 0. Max coverage (-): 0

Region: chr18 39791760-39791791. Max. coverage (+): 0. Max coverage (-): 0

Region: chr18 39791792-39791823. Max. coverage (+): 0. Max coverage (-): 0

Region: chr18 39791824-39791855. Max. coverage (+): 0. Max coverage (-): 0

Region: chr18 39791856-39791887. Max. coverage (+): 0. Max coverage (-): 0

Region: chr18 39791888-39791919. Max. coverage (+): 0. Max coverage (-): 0

Region: chr18 39791920-39791951. Max. coverage (+): 0. Max coverage (-): 0

Region: chr18 39791952-39791983. Max. coverage (+): 0. Max coverage (-): 0

Region: chr18 39791984-39792015. Max. coverage (+): 0. Max coverage (-): 0

Region: chr18 39792016-39792047. Max. coverage (+): 0. Max coverage (-): 0

Region: chr18 39792048-39792079. Max. coverage (+): 0. Max coverage (-): 0

Region: chr18 39792080-39792111. Max. coverage (+): 0. Max coverage (-): 0

Region: chr18 39792112-39792143. Max. coverage (+): 0. Max coverage (-): 0

Region: chr18 39792144-39792175. Max. coverage (+): 0. Max coverage (-): 0

Region: chr18 39792176-39792207. Max. coverage (+): 0. Max coverage (-): 0

Region: chr18 39792208-39792239. Max. coverage (+): 0. Max coverage (-): 0

Region: chr18 39792240-39792271. Max. coverage (+): 0. Max coverage (-): 0

Region: chr18 39792272-39792303. Max. coverage (+): 0. Max coverage (-): 0

Region: chr18 39792304-39792335. Max. coverage (+): 0. Max coverage (-): 0

Region: chr18 39792336-39792367. Max. coverage (+): 0. Max coverage (-): 0

Region: chr18 39792368-39792399. Max. coverage (+): 0. Max coverage (-): 0

Region: chr18 39792400-39792431. Max. coverage (+): 0. Max coverage (-): 0

Region: chr18 39792432-39792463. Max. coverage (+): 0. Max coverage (-): 0

Region: chr18 39792464-39792495. Max. coverage (+): 0. Max coverage (-): 0

Region: chr18 39792496-39792527. Max. coverage (+): 0. Max coverage (-): 0

Region: chr18 39792528-39792559. Max. coverage (+): 0. Max coverage (-): 0

Region: chr18 39792560-39792591. Max. coverage (+): 0. Max coverage (-): 0

Region: chr18 39792592-39792623. Max. coverage (+): 0. Max coverage (-): 0

Region: chr18 39792624-39792655. Max. coverage (+): 0. Max coverage (-): 0

Region: chr18 39792656-39792687. Max. coverage (+): 0. Max coverage (-): 0

Region: chr18 39792688-39792719. Max. coverage (+): 0. Max coverage (-): 0

Region: chr18 39792720-39792751. Max. coverage (+): 0. Max coverage (-): 0

Region: chr18 39792752-39792783. Max. coverage (+): 4.57. Max coverage (-): 0

Region: chr18 39792784-39792815. Max. coverage (+): 0. Max coverage (-): 0

Region: chr18 39792816-39792847. Max. coverage (+): 0. Max coverage (-): 0

Region: chr18 39792848-39792879. Max. coverage (+): 1.1. Max coverage (-): 0

Region: chr18 39792880-39792911. Max. coverage (+): 0. Max coverage (-): 0

Region: chr18 39792912-39792943. Max. coverage (+): 0. Max coverage (-): 0

Region: chr18 39792944-39792975. Max. coverage (+): 0. Max coverage (-): 0

Region: chr18 39792976-39793007. Max. coverage (+): 0. Max coverage (-): 0

Region: chr18 39793008-39793039. Max. coverage (+): 0. Max coverage (-): 0

Region: chr18 39793040-39793071. Max. coverage (+): 0. Max coverage (-): 0

Region: chr18 39793072-39793103. Max. coverage (+): 0. Max coverage (-): 0

Region: chr18 39793104-39793135. Max. coverage (+): 0.41. Max coverage (-): 0

Region: chr18 39793136-39793167. Max. coverage (+): 37.01. Max coverage (-): 0

Region: chr18 39793168-39793199. Max. coverage (+): 7.58. Max coverage (-): 0

Region: chr18 39793200-39793231. Max. coverage (+): 2.47. Max coverage (-): 0

Region: chr18 39793232-39793263. Max. coverage (+): 1.79. Max coverage (-): 0

Region: chr18 39793264-39793295. Max. coverage (+): 12.03. Max coverage (-): 0

Region: chr18 39793296-39793327. Max. coverage (+): 6.71. Max coverage (-): 0

Region: chr18 39793328-39793359. Max. coverage (+): 4.69. Max coverage (-): 0

Region: chr18 39793360-39793391. Max. coverage (+): 18.66. Max coverage (-): 0

Region: chr18 39793392-39793423. Max. coverage (+): 26.02. Max coverage (-): 0

Region: chr18 39793424-39793455. Max. coverage (+): 0. Max coverage (-): 0

Region: chr18 39793456-39793487. Max. coverage (+): 0. Max coverage (-): 0

Region: chr18 39793488-39793519. Max. coverage (+): 14.51. Max coverage (-): 0

Region: chr18 39793520-39793551. Max. coverage (+): 10.73. Max coverage (-): 0

Region: chr18 39793552-39793583. Max. coverage (+): 1.05. Max coverage (-): 0

Region: chr18 39793584-39793615. Max. coverage (+): 6.75. Max coverage (-): 0

Region: chr18 39793616-39793647. Max. coverage (+): 7.24. Max coverage (-): 0

Region: chr18 39793648-39793679. Max. coverage (+): 9.32. Max coverage (-): 0

Region: chr18 39793680-39793711. Max. coverage (+): 0. Max coverage (-): 0

Region: chr18 39793712-39793743. Max. coverage (+): 5.14. Max coverage (-): 0

Region: chr18 39793744-39793775. Max. coverage (+): 4.25. Max coverage (-): 0

Region: chr18 39793776-39793807. Max. coverage (+): 0. Max coverage (-): 0

Region: chr18 39793808-39793839. Max. coverage (+): 0. Max coverage (-): 0

Region: chr18 39793840-39793871. Max. coverage (+): 6.56. Max coverage (-): 0

Region: chr18 39793872-39793903. Max. coverage (+): 0. Max coverage (-): 0

Region: chr18 39793904-39793935. Max. coverage (+): 0. Max coverage (-): 0

Region: chr18 39793936-39793967. Max. coverage (+): 4.75. Max coverage (-): 0

Region: chr18 39793968-39793999. Max. coverage (+): 5.04. Max coverage (-): 0

Region: chr18 39794000-39794031. Max. coverage (+): 0. Max coverage (-): 0

Region: chr18 39794032-39794063. Max. coverage (+): 0. Max coverage (-): 0

Region: chr18 39794064-39794095. Max. coverage (+): 0. Max coverage (-): 0

Region: chr18 39794096-39794127. Max. coverage (+): 0. Max coverage (-): 0

Region: chr18 39794128-39794159. Max. coverage (+): 0. Max coverage (-): 0

Region: chr18 39794160-39794192. Max. coverage (+): 0. Max coverage (-): 0

Region: chr18 39794193-39794224. Max. coverage (+): 11.98. Max coverage (-): 0

Region: chr18 39794225-39794256. Max. coverage (+): 23.62. Max coverage (-): 0

Region: chr18 39794257-39794288. Max. coverage (+): 0. Max coverage (-): 0

Region: chr18 39794289-39794320. Max. coverage (+): 0. Max coverage (-): 0

Region: chr18 39794321-39794352. Max. coverage (+): 15.38. Max coverage (-): 0

Region: chr18 39794353-39794384. Max. coverage (+): 7.86. Max coverage (-): 0

Region: chr18 39794385-39794416. Max. coverage (+): 3.72. Max coverage (-): 0

Region: chr18 39794417-39794448. Max. coverage (+): 13.38. Max coverage (-): 0

Region: chr18 39794449-39794480. Max. coverage (+): 0. Max coverage (-): 0

Region: chr18 39794481-39794512. Max. coverage (+): 0. Max coverage (-): 0

Region: chr18 39794513-39794544. Max. coverage (+): 0. Max coverage (-): 0

Region: chr18 39794545-39794576. Max. coverage (+): 0. Max coverage (-): 0

Region: chr18 39794577-39794608. Max. coverage (+): 0. Max coverage (-): 0

Region: chr18 39794609-39794640. Max. coverage (+): 0. Max coverage (-): 0

Region: chr18 39794641-39794672. Max. coverage (+): 0. Max coverage (-): 0

Region: chr18 39794673-39794704. Max. coverage (+): 0. Max coverage (-): 0

Region: chr18 39794705-39794736. Max. coverage (+): 3.51. Max coverage (-): 0

Region: chr18 39794737-39794768. Max. coverage (+): 76.65. Max coverage (-): 0

Region: chr18 39794769-39794800. Max. coverage (+): 30.09. Max coverage (-): 0

Region: chr18 39794801-39794832. Max. coverage (+): 11.76. Max coverage (-): 0

Region: chr18 39794833-39794864. Max. coverage (+): 27.12. Max coverage (-): 0

Region: chr18 39794865-39794896. Max. coverage (+): 23.22. Max coverage (-): 0

Region: chr18 39794897-39794928. Max. coverage (+): 43.8. Max coverage (-): 0

Region: chr18 39794929-39794960. Max. coverage (+): 60.06. Max coverage (-): 0

Region: chr18 39794961-39794992. Max. coverage (+): 14.71. Max coverage (-): 0

Region: chr18 39794993-39795024. Max. coverage (+): 40.1. Max coverage (-): 0

Region: chr18 39795025-39795056. Max. coverage (+): 18.09. Max coverage (-): 0

Region: chr18 39795057-39795088. Max. coverage (+): 13.71. Max coverage (-): 0

Region: chr18 39795089-39795120. Max. coverage (+): 5.34. Max coverage (-): 0

Region: chr18 39795121-39795152. Max. coverage (+): 3.57. Max coverage (-): 0

Region: chr18 39795153-39795184. Max. coverage (+): 7.69. Max coverage (-): 0

Region: chr18 39795185-39795216. Max. coverage (+): 4.29. Max coverage (-): 0

Region: chr18 39795217-39795248. Max. coverage (+): 9.11. Max coverage (-): 0

Region: chr18 39795249-39795280. Max. coverage (+): 10.57. Max coverage (-): 0

Region: chr18 39795281-39795312. Max. coverage (+): 15.99. Max coverage (-): 0

Region: chr18 39795313-39795344. Max. coverage (+): 12.95. Max coverage (-): 0

Region: chr18 39795345-39795376. Max. coverage (+): 21.88. Max coverage (-): 0

Region: chr18 39795377-39795408. Max. coverage (+): 51.84. Max coverage (-): 0

Region: chr18 39795409-39795440. Max. coverage (+): 2.07. Max coverage (-): 0

Region: chr18 39795441-39795472. Max. coverage (+): 41.69. Max coverage (-): 0

Region: chr18 39795473-39795504. Max. coverage (+): 2.29. Max coverage (-): 0

Region: chr18 39795505-39795536. Max. coverage (+): 20.3. Max coverage (-): 0

Region: chr18 39795537-39795568. Max. coverage (+): 9.74. Max coverage (-): 0

Region: chr18 39795569-39795600. Max. coverage (+): 0. Max coverage (-): 0

Region: chr18 39795601-39795632. Max. coverage (+): 7.26. Max coverage (-): 0

Region: chr18 39795633-39795664. Max. coverage (+): 8.81. Max coverage (-): 0

Region: chr18 39795665-39795696. Max. coverage (+): 0.7. Max coverage (-): 0

Region: chr18 39795697-39795728. Max. coverage (+): 0. Max coverage (-): 0

Region: chr18 39795729-39795760. Max. coverage (+): 3.26. Max coverage (-): 0

Region: chr18 39795761-39795792. Max. coverage (+): 2.84. Max coverage (-): 0

Region: chr18 39795793-39795824. Max. coverage (+): 34.31. Max coverage (-): 0

Region: chr18 39795825-39795856. Max. coverage (+): 3.31. Max coverage (-): 0

Region: chr18 39795857-39795888. Max. coverage (+): 14.54. Max coverage (-): 0

Region: chr18 39795889-39795920. Max. coverage (+): 20. Max coverage (-): 0

Region: chr18 39795921-39795952. Max. coverage (+): 16.5. Max coverage (-): 0

Region: chr18 39795953-39795984. Max. coverage (+): 23.89. Max coverage (-): 0

Region: chr18 39795985-39796016. Max. coverage (+): 16.16. Max coverage (-): 0

Region: chr18 39796017-39796048. Max. coverage (+): 29.15. Max coverage (-): 0

Region: chr18 39796049-39796080. Max. coverage (+): 34.05. Max coverage (-): 0

Region: chr18 39796081-39796112. Max. coverage (+): 7.6. Max coverage (-): 0

Region: chr18 39796113-39796144. Max. coverage (+): 16.49. Max coverage (-): 0

Region: chr18 39796145-39796176. Max. coverage (+): 73.19. Max coverage (-): 0

Region: chr18 39796177-39796208. Max. coverage (+): 29.43. Max coverage (-): 0

Region: chr18 39796209-39796240. Max. coverage (+): 5.4. Max coverage (-): 0

Region: chr18 39796241-39796272. Max. coverage (+): 0. Max coverage (-): 0

Region: chr18 39796273-39796304. Max. coverage (+): 80.88. Max coverage (-): 0

Region: chr18 39796305-39796336. Max. coverage (+): 1.6. Max coverage (-): 0

Region: chr18 39796337-39796368. Max. coverage (+): 13.48. Max coverage (-): 0

Region: chr18 39796369-39796400. Max. coverage (+): 29.56. Max coverage (-): 0

Region: chr18 39796401-39796432. Max. coverage (+): 26.55. Max coverage (-): 0

Region: chr18 39796433-39796464. Max. coverage (+): 79.53. Max coverage (-): 0

Region: chr18 39796465-39796496. Max. coverage (+): 3.59. Max coverage (-): 0

Region: chr18 39796497-39796528. Max. coverage (+): 1.62. Max coverage (-): 0

Region: chr18 39796529-39796560. Max. coverage (+): 5.02. Max coverage (-): 0

Region: chr18 39796561-39796592. Max. coverage (+): 14.44. Max coverage (-): 0

Region: chr18 39796593-39796624. Max. coverage (+): 9.71. Max coverage (-): 0

Region: chr18 39796625-39796656. Max. coverage (+): 58.22. Max coverage (-): 0

Region: chr18 39796657-39796688. Max. coverage (+): 36. Max coverage (-): 0

Region: chr18 39796689-39796720. Max. coverage (+): 26.21. Max coverage (-): 0

Region: chr18 39796721-39796752. Max. coverage (+): 37.42. Max coverage (-): 0

Region: chr18 39796753-39796784. Max. coverage (+): 0. Max coverage (-): 0

Region: chr18 39796785-39796816. Max. coverage (+): 18.18. Max coverage (-): 0

Region: chr18 39796817-39796848. Max. coverage (+): 16.14. Max coverage (-): 0

Region: chr18 39796849-39796880. Max. coverage (+): 15.81. Max coverage (-): 0

Region: chr18 39796881-39796912. Max. coverage (+): 12.53. Max coverage (-): 0

Region: chr18 39796913-39796944. Max. coverage (+): 2.34. Max coverage (-): 0

Region: chr18 39796945-39796976. Max. coverage (+): 2.34. Max coverage (-): 0

Region: chr18 39796977-39797008. Max. coverage (+): 4.66. Max coverage (-): 0

Region: chr18 39797009-39797040. Max. coverage (+): 0. Max coverage (-): 0

Region: chr18 39797041-39797072. Max. coverage (+): 33.87. Max coverage (-): 0

Region: chr18 39797073-39797104. Max. coverage (+): 15.21. Max coverage (-): 0

Region: chr18 39797105-39797136. Max. coverage (+): 61.71. Max coverage (-): 0

Region: chr18 39797137-39797168. Max. coverage (+): 21.42. Max coverage (-): 0

Region: chr18 39797169-39797200. Max. coverage (+): 6.32. Max coverage (-): 0

Region: chr18 39797201-39797232. Max. coverage (+): 25.46. Max coverage (-): 0

Region: chr18 39797233-39797264. Max. coverage (+): 36.31. Max coverage (-): 0

Region: chr18 39797265-39797296. Max. coverage (+): 31.65. Max coverage (-): 0

Region: chr18 39797297-39797328. Max. coverage (+): 30.05. Max coverage (-): 0

Region: chr18 39797329-39797360. Max. coverage (+): 28.6. Max coverage (-): 0

Region: chr18 39797361-39797393. Max. coverage (+): 35.05. Max coverage (-): 0

Region: chr18 39797394-39797425. Max. coverage (+): 15.73. Max coverage (-): 0

Region: chr18 39797426-39797457. Max. coverage (+): 11.4. Max coverage (-): 0

Region: chr18 39797458-39797489. Max. coverage (+): 15.09. Max coverage (-): 0

Region: chr18 39797490-39797521. Max. coverage (+): 0. Max coverage (-): 0

Region: chr18 39797522-39797553. Max. coverage (+): 38.95. Max coverage (-): 0

Region: chr18 39797554-39797585. Max. coverage (+): 47.97. Max coverage (-): 0

Region: chr18 39797586-39797617. Max. coverage (+): 17.81. Max coverage (-): 0

Region: chr18 39797618-39797649. Max. coverage (+): 36.72. Max coverage (-): 0

Region: chr18 39797650-39797681. Max. coverage (+): 12.98. Max coverage (-): 0

Region: chr18 39797682-39797713. Max. coverage (+): 39.13. Max coverage (-): 0

Region: chr18 39797714-39797745. Max. coverage (+): 12.45. Max coverage (-): 0

Region: chr18 39797746-39797777. Max. coverage (+): 12.45. Max coverage (-): 0

Region: chr18 39797778-39797809. Max. coverage (+): 38.75. Max coverage (-): 0

Region: chr18 39797810-39797841. Max. coverage (+): 49.66. Max coverage (-): 0

Region: chr18 39797842-39797873. Max. coverage (+): 34.5. Max coverage (-): 0

Region: chr18 39797874-39797905. Max. coverage (+): 36.9. Max coverage (-): 0

Region: chr18 39797906-39797937. Max. coverage (+): 3.53. Max coverage (-): 0

Region: chr18 39797938-39797969. Max. coverage (+): 3.53. Max coverage (-): 0

Region: chr18 39797970-39798001. Max. coverage (+): 0. Max coverage (-): 0

Region: chr18 39798002-39798033. Max. coverage (+): 70.03. Max coverage (-): 0

Region: chr18 39798034-39798065. Max. coverage (+): 70.03. Max coverage (-): 0

Region: chr18 39798066-39798097. Max. coverage (+): 46.79. Max coverage (-): 0

Region: chr18 39798098-39798129. Max. coverage (+): 11.25. Max coverage (-): 0

Region: chr18 39798130-39798161. Max. coverage (+): 25.08. Max coverage (-): 0

Region: chr18 39798162-39798193. Max. coverage (+): 41.38. Max coverage (-): 0

Region: chr18 39798194-39798225. Max. coverage (+): 34.45. Max coverage (-): 0

Region: chr18 39798226-39798257. Max. coverage (+): 67.07. Max coverage (-): 0

Region: chr18 39798258-39798289. Max. coverage (+): 2.25. Max coverage (-): 0

Region: chr18 39798290-39798321. Max. coverage (+): 61.09. Max coverage (-): 0

Region: chr18 39798322-39798353. Max. coverage (+): 12.03. Max coverage (-): 0

Region: chr18 39798354-39798385. Max. coverage (+): 12.03. Max coverage (-): 0

Region: chr18 39798386-39798417. Max. coverage (+): 41.49. Max coverage (-): 0

Region: chr18 39798418-39798449. Max. coverage (+): 2.1. Max coverage (-): 0

Region: chr18 39798450-39798481. Max. coverage (+): 30.84. Max coverage (-): 0

Region: chr18 39798482-39798513. Max. coverage (+): 18.87. Max coverage (-): 0

Region: chr18 39798514-39798545. Max. coverage (+): 15.36. Max coverage (-): 0

Region: chr18 39798546-39798577. Max. coverage (+): 9.47. Max coverage (-): 0

Region: chr18 39798578-39798609. Max. coverage (+): 9.14. Max coverage (-): 0

Region: chr18 39798610-39798641. Max. coverage (+): 15.06. Max coverage (-): 0

Region: chr18 39798642-39798673. Max. coverage (+): 13.47. Max coverage (-): 0

Region: chr18 39798674-39798705. Max. coverage (+): 13.47. Max coverage (-): 0

Region: chr18 39798706-39798737. Max. coverage (+): 0. Max coverage (-): 0

Region: chr18 39798738-39798769. Max. coverage (+): 0. Max coverage (-): 0

Region: chr18 39798770-39798801. Max. coverage (+): 0. Max coverage (-): 0

Region: chr18 39798802-39798833. Max. coverage (+): 0. Max coverage (-): 0

Region: chr18 39798834-39798865. Max. coverage (+): 0. Max coverage (-): 0

Region: chr18 39798866-39798897. Max. coverage (+): 7.91. Max coverage (-): 0

Region: chr18 39798898-39798929. Max. coverage (+): 4.42. Max coverage (-): 0

Region: chr18 39798930-39798961. Max. coverage (+): 15.62. Max coverage (-): 0

Region: chr18 39798962-39798993. Max. coverage (+): 0. Max coverage (-): 0

Region: chr18 39798994-39799025. Max. coverage (+): 28.8. Max coverage (-): 0

Region: chr18 39799026-39799057. Max. coverage (+): 26.33. Max coverage (-): 0

Region: chr18 39799058-39799089. Max. coverage (+): 15.77. Max coverage (-): 0

Region: chr18 39799090-39799121. Max. coverage (+): 4.77. Max coverage (-): 0

Region: chr18 39799122-39799153. Max. coverage (+): 9.78. Max coverage (-): 0

Region: chr18 39799154-39799185. Max. coverage (+): 66.6. Max coverage (-): 0

Region: chr18 39799186-39799217. Max. coverage (+): 19.59. Max coverage (-): 0

Region: chr18 39799218-39799249. Max. coverage (+): 39.27. Max coverage (-): 0

Region: chr18 39799250-39799281. Max. coverage (+): 40.87. Max coverage (-): 0

Region: chr18 39799282-39799313. Max. coverage (+): 14.23. Max coverage (-): 0

Region: chr18 39799314-39799345. Max. coverage (+): 45.36. Max coverage (-): 0

Region: chr18 39799346-39799377. Max. coverage (+): 13.98. Max coverage (-): 0

Region: chr18 39799378-39799409. Max. coverage (+): 21.84. Max coverage (-): 0

Region: chr18 39799410-39799441. Max. coverage (+): 42.75. Max coverage (-): 0

Region: chr18 39799442-39799473. Max. coverage (+): 69.56. Max coverage (-): 0

Region: chr18 39799474-39799505. Max. coverage (+): 22.36. Max coverage (-): 0

Region: chr18 39799506-39799537. Max. coverage (+): 11.92. Max coverage (-): 0

Region: chr18 39799538-39799569. Max. coverage (+): 19.23. Max coverage (-): 0

Region: chr18 39799570-39799601. Max. coverage (+): 38.7. Max coverage (-): 0

Region: chr18 39799602-39799633. Max. coverage (+): 6.01. Max coverage (-): 0

Region: chr18 39799634-39799665. Max. coverage (+): 6.01. Max coverage (-): 0

Region: chr18 39799666-39799697. Max. coverage (+): 29.65. Max coverage (-): 0

Region: chr18 39799698-39799729. Max. coverage (+): 29.65. Max coverage (-): 0

Region: chr18 39799730-39799761. Max. coverage (+): 3.34. Max coverage (-): 0

Region: chr18 39799762-39799793. Max. coverage (+): 0. Max coverage (-): 0

Region: chr18 39799794-39799825. Max. coverage (+): 0. Max coverage (-): 0

Region: chr18 39799826-39799857. Max. coverage (+): 9.97. Max coverage (-): 0

Region: chr18 39799858-39799889. Max. coverage (+): 27.52. Max coverage (-): 0

Region: chr18 39799890-39799921. Max. coverage (+): 1.9. Max coverage (-): 0

Region: chr18 39799922-39799953. Max. coverage (+): 1.76. Max coverage (-): 0

Region: chr18 39799954-39799985. Max. coverage (+): 8.14. Max coverage (-): 0

Region: chr18 39799986-39800017. Max. coverage (+): 8.14. Max coverage (-): 0

Region: chr18 39800018-39800049. Max. coverage (+): 5.27. Max coverage (-): 0

Region: chr18 39800050-39800081. Max. coverage (+): 0. Max coverage (-): 0

Region: chr18 39800082-39800113. Max. coverage (+): 0. Max coverage (-): 0

Region: chr18 39800114-39800145. Max. coverage (+): 0. Max coverage (-): 0

Region: chr18 39800146-39800177. Max. coverage (+): 0. Max coverage (-): 0

Region: chr18 39800178-39800209. Max. coverage (+): 0. Max coverage (-): 0

Region: chr18 39800210-39800241. Max. coverage (+): 0. Max coverage (-): 0

Region: chr18 39800242-39800273. Max. coverage (+): 0. Max coverage (-): 0

Region: chr18 39800274-39800305. Max. coverage (+): 2.84. Max coverage (-): 0

Region: chr18 39800306-39800337. Max. coverage (+): 27.95. Max coverage (-): 0

Region: chr18 39800338-39800369. Max. coverage (+): 7.05. Max coverage (-): 0

Region: chr18 39800370-39800401. Max. coverage (+): 19.91. Max coverage (-): 0

Region: chr18 39800402-39800433. Max. coverage (+): 12.21. Max coverage (-): 0

Region: chr18 39800434-39800465. Max. coverage (+): 0. Max coverage (-): 0

Region: chr18 39800466-39800497. Max. coverage (+): 1.85. Max coverage (-): 0

Region: chr18 39800498-39800529. Max. coverage (+): 8.78. Max coverage (-): 0

Region: chr18 39800530-39800561. Max. coverage (+): 27.23. Max coverage (-): 0

Region: chr18 39800562-. Max. coverage (+): 0. Max coverage (-): 0

RepeatMasker Color Code

**+**

100-98% Identity

<98-95% Identity

<95-90% Identity

<90-85% Identity

<85-80% Identity

<80-75% Identity

<75-70% Identity

<70% Identity

**-**

Gene Set Color Code

**+**

Gene

Pseudogene

**-**

Topology/Coverage Color Code

Coverage Plus Strand

Coverage Minus Strand

Mainstrand: Plus

Mainstrand: Minus

Complementary Strand

Flanking Region  
(if option -flank >0)

Gene Set Annotation  
  
RepeatMasker Annotation  

**1. (CAGAT)n**: 39784625-39784644 (+), Divergence to consensus: 0%  
**2. ART2A**: 39784645-39784993 (-), Divergence to consensus: 10.1%  
**3. ART2A**: 39784991-39785026 (-), Divergence to consensus: 2.8%  
**4. BovB**: 39785027-39785361 (-), Divergence to consensus: 3.6%  
**5. L2b**: 39785846-39785930 (+), Divergence to consensus: 37.6%  
**6. C-rich**: 39786259-39786316 (+), Divergence to consensus: 18.3%  
**7. L1MC4a**: 39786934-39787164 (+), Divergence to consensus: 27.3%  
**8. MER5B**: 39787194-39787292 (-), Divergence to consensus: 36%  
**9. AT\_rich**: 39787511-39787536 (+), Divergence to consensus: 76.9%  
**10. L2c**: 39787582-39787797 (-), Divergence to consensus: 46.4%  
**11. MIR**: 39788468-39788549 (+), Divergence to consensus: 31.7%  
**12. Bov-tA2**: 39789205-39789385 (-), Divergence to consensus: 31%  
**13. Bov-tA2**: 39789386-39789506 (-), Divergence to consensus: 13.2%  
**14. MIR3**: 39789990-39790157 (+), Divergence to consensus: 43.5%  
**15. MER5A1**: 39790166-39790315 (-), Divergence to consensus: 19.3%  
**16. MER5B**: 39790450-39790565 (+), Divergence to consensus: 28.6%  
**17. BOV-A2**: 39790712-39790940 (+), Divergence to consensus: 2.6%  
**18. BOV-A2**: 39791946-39792205 (+), Divergence to consensus: 6.2%  
**19. (CAG)n**: 39792206-39792234 (+), Divergence to consensus: 3.5%  
**20. MIR3**: 39792528-39792624 (+), Divergence to consensus: 37.2%  
**21. MIR**: 39794006-39794167 (+), Divergence to consensus: 32.6%  
**22. AT\_rich**: 39794169-39794194 (+), Divergence to consensus: 61.5%  
**23. MER20**: 39794466-39794697 (-), Divergence to consensus: 28.8%  
**24. LTR16C**: 39797962-39798018 (-), Divergence to consensus: 19.3%  
**25. MER45A**: 39798701-39798859 (+), Divergence to consensus: 42.7%  
**26. Charlie1b**: 39800057-39800250 (-), Divergence to consensus: 28.3%  
**27. Charlie1b**: 39800223-39800275 (-), Divergence to consensus: 15.1%  
**28. LTR37-int**: 39800466-39800522 (+), Divergence to consensus: 17.6%

  
Transcription Factor Binding Sites  

**RFX4\_1** (Sequence: CCTGGCAAC (+): 39795910)  
**Gata4** (Sequence: AGATAAC (-): 39788627)  
**Gata4** (Sequence: AGATAAC (-): 39795059)  
**SOX9** (Sequence: TTATTGTT (+): 39785434)  
**SOX9** (Sequence: TTATTGTT (+): 39785668)  
**SOX9** (Sequence: TCATTGTT (+): 39796574)  
**SOX9** (Sequence: CTATTGTT (+): 39799355)  
**A-MYB** (Sequence: AGGCAGTTGG (+): 39787839)  
**SPZ1** (Sequence: AGGGTTTCAG (+): 39796430)
