# Supplementary material for: Small RNA sequencing of cryopreserved semen from single bull revealed altered miRNAs and piRNAs expression between High- and Low-motile sperm populations
Source: BMC Genomics. 2017 Jan 4;18:14. doi: 10.1186/s12864-016-3394-7 (PMC5209821; doi:10.1186/s12864-016-3394-7)
Supplement: Additional file 3: — Details for each piRNA clusters found in High Motile (HM) sperm fraction. Genes, repeats, transposable elements and transcription factors binding sites falling within the cluster regions were reported. (ZIP 1896 kb) [file 12864_2016_3394_MOESM3_ESM.zip › 38.html]

piRNA cluster 38


Predicted piRNA cluster no. 38     previous   next
  

Show proTRAC run info
Hide proTRAC run info

================================= proTRAC ====================================  
VERSION: 2.1                                    LAST MODIFIED: 06. October 2015  
  
Please cite:  
Rosenkranz D, Zischler H. proTRAC - a software for probabilistic piRNA cluster  
detection, visualization and analysis. 2012. BMC Bioinformatics 13:5.  
  
and (for proTRAC 2.0 and later):  
Rosenkranz D, Rudloff S, Bastuck K, Ketting RF, Zischler H. Tupaia small RNAs  
provide insights into function and evolution of RNAi-based transposon defense  
in mammals. 2015. RNA 21(5):911-922.  
  
Contact:  
David Rosenkranz  
Institute of Anthropology, small RNA group  
Johannes Gutenberg University Mainz  
email: rosenkranz@uni-mainz.de  
  
You can find the latest proTRAC version at:  
http://sourceforge.net/projects/protrac/files  
http://www.smallRNAgroup-mainz.de/software  
==============================================================================  
  
PARAMETERS:  
Map file: .............../storage/core/barbara/genhome/smallRNA/fertility/Sample\_motile/pirna/Sample\_motile\_26-33\_collapsed.fa.no-dust.map.weighted-10000-1000-b-0  
Genome file: ............/storage/core/barbara/genhome/smallRNA/fertility/Sample\_all/pirna/bt\_311\_chrY.fa  
RepeatMasker annotation: /storage/genomes/bt\_umd31/GCF\_000003055.6\_Bos\_taurus\_UMD\_3.1.1\_repeatMasker\_chr.out  
GeneSet:................./storage/core/barbara/genhome/smallRNA/fertility/Sample\_all/pirna/full.gtf  
  
Significant (p<=0.01) hit density will be calculated based  
on observed hit distribution.  
  
Sliding window size: ........................................ 5000 bp  
Sliding window increament: .................................. 1000 bp  
Normalize each hit by number of genomic hits: ............... 1 [0=no/1=yes]  
Normalize each hit by number of sequence reads: ............. 1 [0=no/1=yes]  
Normalize values (-> per million mapped reads): ............. 1 [0=no/1=yes]  
Min. fraction of hits with 1T(U) or 10A: .................... 0.75  
Alternatively: Min. fraction of hits with 1T(U) and 10A: .... 0.5  
Min. fraction of hits with typical piRNA length: ............ 0.75  
Typical piRNA length: ....................................... 26-33 nt  
Min. size of a piRNA cluster: ............................... 5000 bp.  
Min. number of hits (absolute): ............................. 0  
Min. number of hits (normalized): ........................... 0  
Min. fraction of hits on the mainstrand: .................... 0.75  
Top fraction of mapped sequences (in terms of read counts): . 1%  
Top fraction accounts for max. n% of sequence reads: ........ 90%  
Min. fraction of hits on each arm of a bidirectional cluster: 0.1  
Output image file for each cluster: ......................... 0 [0=no/1=yes]  
Output html file for each cluster: .......................... 1 [0=no/1=yes]  
Output a summary table: ..................................... 1 [0=no/1=yes]  
Output a FASTA file for each cluster (piRNA sequences): ..... 1 [0=no/1=yes]  
Output a FASTA file comprising cluster sequences: ........... 1 [0=no/1=yes]  
Search DNA motifs in clusters: .............................. 1 [0=no/1=yes]  
Output flanking sequences: +/- .............................. 0 bp  
Output ~.pTi file: .......................................... 1 [0=no/1=yes]  
==============================================================================  
  
  
Genome size (without gaps): ............ 2678902517 bp  
Gaps (N/X/-): .......................... 53837044 bp  
Mapped reads: .......................... 658825247023  
Non-identical sequences: ............... 514171  
Genomic hits: .......................... 764233  
Significant densitiy of mapped reads: .. 12867599.5173724 reads/kb

Show proTRAC cluster info
Hide proTRAC cluster info

|  |  |
| --- | --- |
| Location | chr18 |
| Coordinates | 45799158-45807914 |
| Size [bp] | 8757 |
| Sequence hit loci | 777 |
| Mapped reads (normalized) | 866407250.2 |
| Mapped reads (normalized) per kb | 98938820.4 |
| Normalized reads with 1T (1U) | 83.8% |
| Normalized reads with 10A | 35.2% |
| Normalized reads with length 26-33 nt | 100% |
| Normalized reads on the main strand(s) | 99.1% |
| Predicted directionality | mono:minus |

100%

0%

1T (1U)  
reads

10A reads

26-33 nt  
reads

reads on mainstrand

**Either the amount of reads with 1T (1U) OR 10A has to exceed 75% (set with option: -1Tor10A)  
Alternatively the amount of reads with 1T (1U) AND 10A has to exceed 50% (set with option: -1Tand10A)  
Minimum amount of reads with preferred size is 75% (set with option: -pisize)  
Minimum amount of reads on the main strand(s) is 75% (set with option: -clstrand)**

Show read coverage
Hide read coverage

WHAT DO I SEE HERE?  
This chart shows the location of mapped sequence reads within a predicted piRNA cluster. The color refers to the number of genomic hits produced by the sequence read in question. A dark red bar indicates that this sequence read produces many other hits elsewhere in the genome. Many adjacent red or yellow bars can indicate the presence of a multi-copy element such as transposons or rRNA genes. A dark green bar indicates that this sequence read maps uniquely to this locus.

1 hit

2-5 hits

6-10 hits

11-20 hits

21-50 hits

51-100 hits

> 100 hits

chr18

45799158

45807914

Gene Set

RepeatMasker

Mapped  
Reads

61.39

plus strand

minus strand

61.39

Region: chr18 39800563-45799166. Max. coverage (+): 0. Max coverage (-): 5.03

Region: chr18 45799167-45799184. Max. coverage (+): 0. Max coverage (-): 1.38

Region: chr18 45799185-45799201. Max. coverage (+): 0. Max coverage (-): 0

Region: chr18 45799202-45799219. Max. coverage (+): 0. Max coverage (-): 0

Region: chr18 45799220-45799236. Max. coverage (+): 0. Max coverage (-): 0

Region: chr18 45799237-45799254. Max. coverage (+): 0. Max coverage (-): 0

Region: chr18 45799255-45799271. Max. coverage (+): 0. Max coverage (-): 0

Region: chr18 45799272-45799289. Max. coverage (+): 0. Max coverage (-): 0

Region: chr18 45799290-45799306. Max. coverage (+): 0. Max coverage (-): 0

Region: chr18 45799307-45799324. Max. coverage (+): 0. Max coverage (-): 0

Region: chr18 45799325-45799341. Max. coverage (+): 0. Max coverage (-): 0

Region: chr18 45799342-45799359. Max. coverage (+): 0. Max coverage (-): 0

Region: chr18 45799360-45799376. Max. coverage (+): 0. Max coverage (-): 0

Region: chr18 45799377-45799394. Max. coverage (+): 0. Max coverage (-): 0

Region: chr18 45799395-45799411. Max. coverage (+): 0. Max coverage (-): 0

Region: chr18 45799412-45799429. Max. coverage (+): 0. Max coverage (-): 0

Region: chr18 45799430-45799446. Max. coverage (+): 0. Max coverage (-): 0

Region: chr18 45799447-45799464. Max. coverage (+): 0. Max coverage (-): 0

Region: chr18 45799465-45799482. Max. coverage (+): 0. Max coverage (-): 0

Region: chr18 45799483-45799499. Max. coverage (+): 0. Max coverage (-): 0

Region: chr18 45799500-45799517. Max. coverage (+): 0. Max coverage (-): 0

Region: chr18 45799518-45799534. Max. coverage (+): 0. Max coverage (-): 0

Region: chr18 45799535-45799552. Max. coverage (+): 0. Max coverage (-): 0

Region: chr18 45799553-45799569. Max. coverage (+): 0. Max coverage (-): 0

Region: chr18 45799570-45799587. Max. coverage (+): 0. Max coverage (-): 0

Region: chr18 45799588-45799604. Max. coverage (+): 0. Max coverage (-): 0

Region: chr18 45799605-45799622. Max. coverage (+): 0. Max coverage (-): 0

Region: chr18 45799623-45799639. Max. coverage (+): 0. Max coverage (-): 0

Region: chr18 45799640-45799657. Max. coverage (+): 0. Max coverage (-): 0

Region: chr18 45799658-45799674. Max. coverage (+): 0. Max coverage (-): 0

Region: chr18 45799675-45799692. Max. coverage (+): 0. Max coverage (-): 0

Region: chr18 45799693-45799709. Max. coverage (+): 0. Max coverage (-): 0

Region: chr18 45799710-45799727. Max. coverage (+): 0. Max coverage (-): 0

Region: chr18 45799728-45799744. Max. coverage (+): 0. Max coverage (-): 0

Region: chr18 45799745-45799762. Max. coverage (+): 0. Max coverage (-): 0

Region: chr18 45799763-45799779. Max. coverage (+): 0. Max coverage (-): 0

Region: chr18 45799780-45799797. Max. coverage (+): 0. Max coverage (-): 0

Region: chr18 45799798-45799814. Max. coverage (+): 0. Max coverage (-): 0

Region: chr18 45799815-45799832. Max. coverage (+): 0. Max coverage (-): 0

Region: chr18 45799833-45799849. Max. coverage (+): 0. Max coverage (-): 0

Region: chr18 45799850-45799867. Max. coverage (+): 0. Max coverage (-): 0

Region: chr18 45799868-45799884. Max. coverage (+): 0. Max coverage (-): 0

Region: chr18 45799885-45799902. Max. coverage (+): 0. Max coverage (-): 0

Region: chr18 45799903-45799919. Max. coverage (+): 0. Max coverage (-): 0

Region: chr18 45799920-45799937. Max. coverage (+): 0. Max coverage (-): 0

Region: chr18 45799938-45799954. Max. coverage (+): 0. Max coverage (-): 0

Region: chr18 45799955-45799972. Max. coverage (+): 0. Max coverage (-): 0

Region: chr18 45799973-45799989. Max. coverage (+): 0. Max coverage (-): 0

Region: chr18 45799990-45800007. Max. coverage (+): 0. Max coverage (-): 0

Region: chr18 45800008-45800024. Max. coverage (+): 0. Max coverage (-): 0

Region: chr18 45800025-45800042. Max. coverage (+): 0. Max coverage (-): 0

Region: chr18 45800043-45800059. Max. coverage (+): 0. Max coverage (-): 0

Region: chr18 45800060-45800077. Max. coverage (+): 0. Max coverage (-): 0

Region: chr18 45800078-45800094. Max. coverage (+): 0. Max coverage (-): 0

Region: chr18 45800095-45800112. Max. coverage (+): 0. Max coverage (-): 0

Region: chr18 45800113-45800130. Max. coverage (+): 0. Max coverage (-): 0

Region: chr18 45800131-45800147. Max. coverage (+): 0. Max coverage (-): 0

Region: chr18 45800148-45800165. Max. coverage (+): 0. Max coverage (-): 0

Region: chr18 45800166-45800182. Max. coverage (+): 0. Max coverage (-): 0

Region: chr18 45800183-45800200. Max. coverage (+): 0. Max coverage (-): 0

Region: chr18 45800201-45800217. Max. coverage (+): 0. Max coverage (-): 0

Region: chr18 45800218-45800235. Max. coverage (+): 0. Max coverage (-): 0

Region: chr18 45800236-45800252. Max. coverage (+): 0. Max coverage (-): 0

Region: chr18 45800253-45800270. Max. coverage (+): 0. Max coverage (-): 0

Region: chr18 45800271-45800287. Max. coverage (+): 0. Max coverage (-): 0

Region: chr18 45800288-45800305. Max. coverage (+): 0. Max coverage (-): 0

Region: chr18 45800306-45800322. Max. coverage (+): 0. Max coverage (-): 0

Region: chr18 45800323-45800340. Max. coverage (+): 0. Max coverage (-): 0

Region: chr18 45800341-45800357. Max. coverage (+): 0. Max coverage (-): 0

Region: chr18 45800358-45800375. Max. coverage (+): 0. Max coverage (-): 0

Region: chr18 45800376-45800392. Max. coverage (+): 0. Max coverage (-): 0

Region: chr18 45800393-45800410. Max. coverage (+): 0. Max coverage (-): 0

Region: chr18 45800411-45800427. Max. coverage (+): 0. Max coverage (-): 0

Region: chr18 45800428-45800445. Max. coverage (+): 0. Max coverage (-): 0

Region: chr18 45800446-45800462. Max. coverage (+): 0. Max coverage (-): 0

Region: chr18 45800463-45800480. Max. coverage (+): 0. Max coverage (-): 0

Region: chr18 45800481-45800497. Max. coverage (+): 0. Max coverage (-): 0

Region: chr18 45800498-45800515. Max. coverage (+): 0. Max coverage (-): 3.39

Region: chr18 45800516-45800532. Max. coverage (+): 0. Max coverage (-): 0

Region: chr18 45800533-45800550. Max. coverage (+): 0. Max coverage (-): 0

Region: chr18 45800551-45800567. Max. coverage (+): 0. Max coverage (-): 0

Region: chr18 45800568-45800585. Max. coverage (+): 0. Max coverage (-): 0

Region: chr18 45800586-45800602. Max. coverage (+): 0. Max coverage (-): 0

Region: chr18 45800603-45800620. Max. coverage (+): 0. Max coverage (-): 0

Region: chr18 45800621-45800637. Max. coverage (+): 0. Max coverage (-): 0

Region: chr18 45800638-45800655. Max. coverage (+): 0. Max coverage (-): 0

Region: chr18 45800656-45800672. Max. coverage (+): 0. Max coverage (-): 0

Region: chr18 45800673-45800690. Max. coverage (+): 0. Max coverage (-): 0

Region: chr18 45800691-45800707. Max. coverage (+): 0. Max coverage (-): 0

Region: chr18 45800708-45800725. Max. coverage (+): 0. Max coverage (-): 0

Region: chr18 45800726-45800743. Max. coverage (+): 0. Max coverage (-): 0

Region: chr18 45800744-45800760. Max. coverage (+): 0. Max coverage (-): 0

Region: chr18 45800761-45800778. Max. coverage (+): 0. Max coverage (-): 1.35

Region: chr18 45800779-45800795. Max. coverage (+): 0. Max coverage (-): 0

Region: chr18 45800796-45800813. Max. coverage (+): 0. Max coverage (-): 0

Region: chr18 45800814-45800830. Max. coverage (+): 0. Max coverage (-): 0

Region: chr18 45800831-45800848. Max. coverage (+): 0. Max coverage (-): 0

Region: chr18 45800849-45800865. Max. coverage (+): 0. Max coverage (-): 0

Region: chr18 45800866-45800883. Max. coverage (+): 0. Max coverage (-): 0

Region: chr18 45800884-45800900. Max. coverage (+): 0. Max coverage (-): 0

Region: chr18 45800901-45800918. Max. coverage (+): 0. Max coverage (-): 0

Region: chr18 45800919-45800935. Max. coverage (+): 0. Max coverage (-): 0

Region: chr18 45800936-45800953. Max. coverage (+): 0. Max coverage (-): 0

Region: chr18 45800954-45800970. Max. coverage (+): 0. Max coverage (-): 5.25

Region: chr18 45800971-45800988. Max. coverage (+): 0. Max coverage (-): 5.25

Region: chr18 45800989-45801005. Max. coverage (+): 0. Max coverage (-): 0

Region: chr18 45801006-45801023. Max. coverage (+): 0. Max coverage (-): 0

Region: chr18 45801024-45801040. Max. coverage (+): 0. Max coverage (-): 0

Region: chr18 45801041-45801058. Max. coverage (+): 0. Max coverage (-): 0

Region: chr18 45801059-45801075. Max. coverage (+): 0. Max coverage (-): 0

Region: chr18 45801076-45801093. Max. coverage (+): 0. Max coverage (-): 0

Region: chr18 45801094-45801110. Max. coverage (+): 0. Max coverage (-): 0

Region: chr18 45801111-45801128. Max. coverage (+): 0. Max coverage (-): 0

Region: chr18 45801129-45801145. Max. coverage (+): 0. Max coverage (-): 0

Region: chr18 45801146-45801163. Max. coverage (+): 0. Max coverage (-): 2.81

Region: chr18 45801164-45801180. Max. coverage (+): 0. Max coverage (-): 0

Region: chr18 45801181-45801198. Max. coverage (+): 0. Max coverage (-): 0

Region: chr18 45801199-45801215. Max. coverage (+): 0. Max coverage (-): 0

Region: chr18 45801216-45801233. Max. coverage (+): 0. Max coverage (-): 0

Region: chr18 45801234-45801250. Max. coverage (+): 0. Max coverage (-): 0

Region: chr18 45801251-45801268. Max. coverage (+): 0. Max coverage (-): 0

Region: chr18 45801269-45801285. Max. coverage (+): 0. Max coverage (-): 0

Region: chr18 45801286-45801303. Max. coverage (+): 0. Max coverage (-): 3.25

Region: chr18 45801304-45801320. Max. coverage (+): 0. Max coverage (-): 1.56

Region: chr18 45801321-45801338. Max. coverage (+): 0. Max coverage (-): 1.56

Region: chr18 45801339-45801356. Max. coverage (+): 0. Max coverage (-): 9.86

Region: chr18 45801357-45801373. Max. coverage (+): 0. Max coverage (-): 19.69

Region: chr18 45801374-45801391. Max. coverage (+): 0. Max coverage (-): 1.77

Region: chr18 45801392-45801408. Max. coverage (+): 0. Max coverage (-): 0

Region: chr18 45801409-45801426. Max. coverage (+): 0. Max coverage (-): 0

Region: chr18 45801427-45801443. Max. coverage (+): 0. Max coverage (-): 5.65

Region: chr18 45801444-45801461. Max. coverage (+): 0. Max coverage (-): 5.65

Region: chr18 45801462-45801478. Max. coverage (+): 0. Max coverage (-): 2.19

Region: chr18 45801479-45801496. Max. coverage (+): 0. Max coverage (-): 0

Region: chr18 45801497-45801513. Max. coverage (+): 0. Max coverage (-): 2.15

Region: chr18 45801514-45801531. Max. coverage (+): 0. Max coverage (-): 0

Region: chr18 45801532-45801548. Max. coverage (+): 0. Max coverage (-): 0

Region: chr18 45801549-45801566. Max. coverage (+): 0. Max coverage (-): 12.3

Region: chr18 45801567-45801583. Max. coverage (+): 0. Max coverage (-): 3.36

Region: chr18 45801584-45801601. Max. coverage (+): 0. Max coverage (-): 0

Region: chr18 45801602-45801618. Max. coverage (+): 0. Max coverage (-): 0

Region: chr18 45801619-45801636. Max. coverage (+): 0. Max coverage (-): 0

Region: chr18 45801637-45801653. Max. coverage (+): 0. Max coverage (-): 3.05

Region: chr18 45801654-45801671. Max. coverage (+): 0. Max coverage (-): 0

Region: chr18 45801672-45801688. Max. coverage (+): 0. Max coverage (-): 0

Region: chr18 45801689-45801706. Max. coverage (+): 0. Max coverage (-): 0

Region: chr18 45801707-45801723. Max. coverage (+): 0. Max coverage (-): 0

Region: chr18 45801724-45801741. Max. coverage (+): 0. Max coverage (-): 1.33

Region: chr18 45801742-45801758. Max. coverage (+): 0. Max coverage (-): 0

Region: chr18 45801759-45801776. Max. coverage (+): 0. Max coverage (-): 7.2

Region: chr18 45801777-45801793. Max. coverage (+): 0. Max coverage (-): 5.88

Region: chr18 45801794-45801811. Max. coverage (+): 0. Max coverage (-): 8.2

Region: chr18 45801812-45801828. Max. coverage (+): 0. Max coverage (-): 6.55

Region: chr18 45801829-45801846. Max. coverage (+): 0. Max coverage (-): 0

Region: chr18 45801847-45801863. Max. coverage (+): 0. Max coverage (-): 0.55

Region: chr18 45801864-45801881. Max. coverage (+): 0. Max coverage (-): 41.29

Region: chr18 45801882-45801898. Max. coverage (+): 0. Max coverage (-): 1.38

Region: chr18 45801899-45801916. Max. coverage (+): 0. Max coverage (-): 3.4

Region: chr18 45801917-45801933. Max. coverage (+): 0. Max coverage (-): 6.01

Region: chr18 45801934-45801951. Max. coverage (+): 0. Max coverage (-): 0

Region: chr18 45801952-45801968. Max. coverage (+): 0. Max coverage (-): 0

Region: chr18 45801969-45801986. Max. coverage (+): 0. Max coverage (-): 7.65

Region: chr18 45801987-45802004. Max. coverage (+): 0. Max coverage (-): 5.52

Region: chr18 45802005-45802021. Max. coverage (+): 0. Max coverage (-): 2.11

Region: chr18 45802022-45802039. Max. coverage (+): 0. Max coverage (-): 17.88

Region: chr18 45802040-45802056. Max. coverage (+): 0. Max coverage (-): 9.07

Region: chr18 45802057-45802074. Max. coverage (+): 0. Max coverage (-): 5

Region: chr18 45802075-45802091. Max. coverage (+): 0. Max coverage (-): 8.79

Region: chr18 45802092-45802109. Max. coverage (+): 0. Max coverage (-): 20.7

Region: chr18 45802110-45802126. Max. coverage (+): 0. Max coverage (-): 0

Region: chr18 45802127-45802144. Max. coverage (+): 0. Max coverage (-): 11.38

Region: chr18 45802145-45802161. Max. coverage (+): 0. Max coverage (-): 26.71

Region: chr18 45802162-45802179. Max. coverage (+): 0. Max coverage (-): 4.84

Region: chr18 45802180-45802196. Max. coverage (+): 0. Max coverage (-): 0

Region: chr18 45802197-45802214. Max. coverage (+): 0. Max coverage (-): 11.58

Region: chr18 45802215-45802231. Max. coverage (+): 0. Max coverage (-): 25.36

Region: chr18 45802232-45802249. Max. coverage (+): 0. Max coverage (-): 13.74

Region: chr18 45802250-45802266. Max. coverage (+): 0. Max coverage (-): 1.48

Region: chr18 45802267-45802284. Max. coverage (+): 0. Max coverage (-): 0

Region: chr18 45802285-45802301. Max. coverage (+): 0. Max coverage (-): 19.04

Region: chr18 45802302-45802319. Max. coverage (+): 0. Max coverage (-): 3.08

Region: chr18 45802320-45802336. Max. coverage (+): 0. Max coverage (-): 34.38

Region: chr18 45802337-45802354. Max. coverage (+): 0. Max coverage (-): 3.06

Region: chr18 45802355-45802371. Max. coverage (+): 0. Max coverage (-): 12.68

Region: chr18 45802372-45802389. Max. coverage (+): 0. Max coverage (-): 22.84

Region: chr18 45802390-45802406. Max. coverage (+): 0. Max coverage (-): 0

Region: chr18 45802407-45802424. Max. coverage (+): 0. Max coverage (-): 0

Region: chr18 45802425-45802441. Max. coverage (+): 0. Max coverage (-): 0

Region: chr18 45802442-45802459. Max. coverage (+): 0. Max coverage (-): 0

Region: chr18 45802460-45802476. Max. coverage (+): 0. Max coverage (-): 0

Region: chr18 45802477-45802494. Max. coverage (+): 0. Max coverage (-): 0.63

Region: chr18 45802495-45802511. Max. coverage (+): 0. Max coverage (-): 10.15

Region: chr18 45802512-45802529. Max. coverage (+): 0. Max coverage (-): 0.77

Region: chr18 45802530-45802546. Max. coverage (+): 0. Max coverage (-): 0

Region: chr18 45802547-45802564. Max. coverage (+): 0. Max coverage (-): 7.7

Region: chr18 45802565-45802581. Max. coverage (+): 0. Max coverage (-): 4.48

Region: chr18 45802582-45802599. Max. coverage (+): 0. Max coverage (-): 6.79

Region: chr18 45802600-45802617. Max. coverage (+): 0. Max coverage (-): 0

Region: chr18 45802618-45802634. Max. coverage (+): 0. Max coverage (-): 0

Region: chr18 45802635-45802652. Max. coverage (+): 0. Max coverage (-): 0

Region: chr18 45802653-45802669. Max. coverage (+): 0. Max coverage (-): 0

Region: chr18 45802670-45802687. Max. coverage (+): 0. Max coverage (-): 0

Region: chr18 45802688-45802704. Max. coverage (+): 0. Max coverage (-): 1.68

Region: chr18 45802705-45802722. Max. coverage (+): 0. Max coverage (-): 1.68

Region: chr18 45802723-45802739. Max. coverage (+): 0. Max coverage (-): 4.88

Region: chr18 45802740-45802757. Max. coverage (+): 0. Max coverage (-): 9.03

Region: chr18 45802758-45802774. Max. coverage (+): 0. Max coverage (-): 12.53

Region: chr18 45802775-45802792. Max. coverage (+): 0. Max coverage (-): 0

Region: chr18 45802793-45802809. Max. coverage (+): 0. Max coverage (-): 0

Region: chr18 45802810-45802827. Max. coverage (+): 0. Max coverage (-): 5.83

Region: chr18 45802828-45802844. Max. coverage (+): 0. Max coverage (-): 4.37

Region: chr18 45802845-45802862. Max. coverage (+): 0. Max coverage (-): 0

Region: chr18 45802863-45802879. Max. coverage (+): 0. Max coverage (-): 0

Region: chr18 45802880-45802897. Max. coverage (+): 0. Max coverage (-): 2.16

Region: chr18 45802898-45802914. Max. coverage (+): 0. Max coverage (-): 18.39

Region: chr18 45802915-45802932. Max. coverage (+): 0. Max coverage (-): 22.59

Region: chr18 45802933-45802949. Max. coverage (+): 0. Max coverage (-): 16.14

Region: chr18 45802950-45802967. Max. coverage (+): 0. Max coverage (-): 14.42

Region: chr18 45802968-45802984. Max. coverage (+): 0. Max coverage (-): 13.24

Region: chr18 45802985-45803002. Max. coverage (+): 0. Max coverage (-): 7.23

Region: chr18 45803003-45803019. Max. coverage (+): 0. Max coverage (-): 0.94

Region: chr18 45803020-45803037. Max. coverage (+): 0. Max coverage (-): 5.91

Region: chr18 45803038-45803054. Max. coverage (+): 0. Max coverage (-): 13.95

Region: chr18 45803055-45803072. Max. coverage (+): 0. Max coverage (-): 0

Region: chr18 45803073-45803089. Max. coverage (+): 0. Max coverage (-): 10.78

Region: chr18 45803090-45803107. Max. coverage (+): 0. Max coverage (-): 18.07

Region: chr18 45803108-45803124. Max. coverage (+): 0. Max coverage (-): 15.5

Region: chr18 45803125-45803142. Max. coverage (+): 0. Max coverage (-): 4.16

Region: chr18 45803143-45803159. Max. coverage (+): 0. Max coverage (-): 0

Region: chr18 45803160-45803177. Max. coverage (+): 0. Max coverage (-): 15.13

Region: chr18 45803178-45803194. Max. coverage (+): 0. Max coverage (-): 51.64

Region: chr18 45803195-45803212. Max. coverage (+): 0. Max coverage (-): 48.14

Region: chr18 45803213-45803230. Max. coverage (+): 0. Max coverage (-): 14.18

Region: chr18 45803231-45803247. Max. coverage (+): 0. Max coverage (-): 5.34

Region: chr18 45803248-45803265. Max. coverage (+): 0. Max coverage (-): 12.24

Region: chr18 45803266-45803282. Max. coverage (+): 0. Max coverage (-): 6.38

Region: chr18 45803283-45803300. Max. coverage (+): 0. Max coverage (-): 4.92

Region: chr18 45803301-45803317. Max. coverage (+): 0. Max coverage (-): 0.97

Region: chr18 45803318-45803335. Max. coverage (+): 0. Max coverage (-): 0.97

Region: chr18 45803336-45803352. Max. coverage (+): 0. Max coverage (-): 0

Region: chr18 45803353-45803370. Max. coverage (+): 0. Max coverage (-): 18.92

Region: chr18 45803371-45803387. Max. coverage (+): 0. Max coverage (-): 6.26

Region: chr18 45803388-45803405. Max. coverage (+): 0. Max coverage (-): 20.93

Region: chr18 45803406-45803422. Max. coverage (+): 0. Max coverage (-): 2.95

Region: chr18 45803423-45803440. Max. coverage (+): 0. Max coverage (-): 0.56

Region: chr18 45803441-45803457. Max. coverage (+): 0. Max coverage (-): 18.81

Region: chr18 45803458-45803475. Max. coverage (+): 0. Max coverage (-): 9.12

Region: chr18 45803476-45803492. Max. coverage (+): 0. Max coverage (-): 19.69

Region: chr18 45803493-45803510. Max. coverage (+): 0. Max coverage (-): 14.78

Region: chr18 45803511-45803527. Max. coverage (+): 0. Max coverage (-): 9.46

Region: chr18 45803528-45803545. Max. coverage (+): 0. Max coverage (-): 9.46

Region: chr18 45803546-45803562. Max. coverage (+): 0. Max coverage (-): 23.93

Region: chr18 45803563-45803580. Max. coverage (+): 0. Max coverage (-): 4.51

Region: chr18 45803581-45803597. Max. coverage (+): 0. Max coverage (-): 0

Region: chr18 45803598-45803615. Max. coverage (+): 0. Max coverage (-): 9.39

Region: chr18 45803616-45803632. Max. coverage (+): 0. Max coverage (-): 9.39

Region: chr18 45803633-45803650. Max. coverage (+): 0. Max coverage (-): 12.54

Region: chr18 45803651-45803667. Max. coverage (+): 0. Max coverage (-): 12.39

Region: chr18 45803668-45803685. Max. coverage (+): 0. Max coverage (-): 19.27

Region: chr18 45803686-45803702. Max. coverage (+): 0. Max coverage (-): 4.6

Region: chr18 45803703-45803720. Max. coverage (+): 0. Max coverage (-): 4.83

Region: chr18 45803721-45803737. Max. coverage (+): 0. Max coverage (-): 4.55

Region: chr18 45803738-45803755. Max. coverage (+): 0. Max coverage (-): 6.8

Region: chr18 45803756-45803772. Max. coverage (+): 0. Max coverage (-): 5.04

Region: chr18 45803773-45803790. Max. coverage (+): 0. Max coverage (-): 4.49

Region: chr18 45803791-45803807. Max. coverage (+): 0. Max coverage (-): 10.41

Region: chr18 45803808-45803825. Max. coverage (+): 0. Max coverage (-): 14.11

Region: chr18 45803826-45803842. Max. coverage (+): 0. Max coverage (-): 4.2

Region: chr18 45803843-45803860. Max. coverage (+): 0. Max coverage (-): 4.2

Region: chr18 45803861-45803878. Max. coverage (+): 0. Max coverage (-): 1.09

Region: chr18 45803879-45803895. Max. coverage (+): 0. Max coverage (-): 5.13

Region: chr18 45803896-45803913. Max. coverage (+): 0. Max coverage (-): 21.68

Region: chr18 45803914-45803930. Max. coverage (+): 0. Max coverage (-): 28.51

Region: chr18 45803931-45803948. Max. coverage (+): 0. Max coverage (-): 14.24

Region: chr18 45803949-45803965. Max. coverage (+): 0. Max coverage (-): 4.99

Region: chr18 45803966-45803983. Max. coverage (+): 0. Max coverage (-): 0

Region: chr18 45803984-45804000. Max. coverage (+): 0. Max coverage (-): 0.85

Region: chr18 45804001-45804018. Max. coverage (+): 0. Max coverage (-): 19.43

Region: chr18 45804019-45804035. Max. coverage (+): 0. Max coverage (-): 24.35

Region: chr18 45804036-45804053. Max. coverage (+): 0. Max coverage (-): 12.6

Region: chr18 45804054-45804070. Max. coverage (+): 0. Max coverage (-): 12.6

Region: chr18 45804071-45804088. Max. coverage (+): 0. Max coverage (-): 3.67

Region: chr18 45804089-45804105. Max. coverage (+): 0. Max coverage (-): 61.39

Region: chr18 45804106-45804123. Max. coverage (+): 0. Max coverage (-): 44.72

Region: chr18 45804124-45804140. Max. coverage (+): 0. Max coverage (-): 23.04

Region: chr18 45804141-45804158. Max. coverage (+): 0. Max coverage (-): 12.59

Region: chr18 45804159-45804175. Max. coverage (+): 0. Max coverage (-): 0

Region: chr18 45804176-45804193. Max. coverage (+): 0. Max coverage (-): 0

Region: chr18 45804194-45804210. Max. coverage (+): 0. Max coverage (-): 0

Region: chr18 45804211-45804228. Max. coverage (+): 0. Max coverage (-): 0

Region: chr18 45804229-45804245. Max. coverage (+): 0. Max coverage (-): 0

Region: chr18 45804246-45804263. Max. coverage (+): 0. Max coverage (-): 0

Region: chr18 45804264-45804280. Max. coverage (+): 0. Max coverage (-): 0

Region: chr18 45804281-45804298. Max. coverage (+): 0. Max coverage (-): 0

Region: chr18 45804299-45804315. Max. coverage (+): 0. Max coverage (-): 0

Region: chr18 45804316-45804333. Max. coverage (+): 0. Max coverage (-): 0

Region: chr18 45804334-45804350. Max. coverage (+): 0. Max coverage (-): 0

Region: chr18 45804351-45804368. Max. coverage (+): 0. Max coverage (-): 0

Region: chr18 45804369-45804385. Max. coverage (+): 0. Max coverage (-): 0

Region: chr18 45804386-45804403. Max. coverage (+): 0. Max coverage (-): 8.28

Region: chr18 45804404-45804420. Max. coverage (+): 0. Max coverage (-): 0

Region: chr18 45804421-45804438. Max. coverage (+): 0. Max coverage (-): 0

Region: chr18 45804439-45804455. Max. coverage (+): 0. Max coverage (-): 0

Region: chr18 45804456-45804473. Max. coverage (+): 0. Max coverage (-): 0

Region: chr18 45804474-45804491. Max. coverage (+): 0. Max coverage (-): 0

Region: chr18 45804492-45804508. Max. coverage (+): 0. Max coverage (-): 0

Region: chr18 45804509-45804526. Max. coverage (+): 0. Max coverage (-): 0

Region: chr18 45804527-45804543. Max. coverage (+): 0. Max coverage (-): 0

Region: chr18 45804544-45804561. Max. coverage (+): 0. Max coverage (-): 0

Region: chr18 45804562-45804578. Max. coverage (+): 0. Max coverage (-): 0

Region: chr18 45804579-45804596. Max. coverage (+): 0. Max coverage (-): 0

Region: chr18 45804597-45804613. Max. coverage (+): 0. Max coverage (-): 0

Region: chr18 45804614-45804631. Max. coverage (+): 0. Max coverage (-): 0

Region: chr18 45804632-45804648. Max. coverage (+): 0. Max coverage (-): 1.04

Region: chr18 45804649-45804666. Max. coverage (+): 0. Max coverage (-): 1.78

Region: chr18 45804667-45804683. Max. coverage (+): 0. Max coverage (-): 0

Region: chr18 45804684-45804701. Max. coverage (+): 0. Max coverage (-): 0

Region: chr18 45804702-45804718. Max. coverage (+): 0. Max coverage (-): 0

Region: chr18 45804719-45804736. Max. coverage (+): 0. Max coverage (-): 0

Region: chr18 45804737-45804753. Max. coverage (+): 0. Max coverage (-): 0

Region: chr18 45804754-45804771. Max. coverage (+): 0. Max coverage (-): 0

Region: chr18 45804772-45804788. Max. coverage (+): 0. Max coverage (-): 0

Region: chr18 45804789-45804806. Max. coverage (+): 0. Max coverage (-): 0

Region: chr18 45804807-45804823. Max. coverage (+): 0. Max coverage (-): 0

Region: chr18 45804824-45804841. Max. coverage (+): 0. Max coverage (-): 0

Region: chr18 45804842-45804858. Max. coverage (+): 0. Max coverage (-): 0

Region: chr18 45804859-45804876. Max. coverage (+): 0. Max coverage (-): 0

Region: chr18 45804877-45804893. Max. coverage (+): 0. Max coverage (-): 0

Region: chr18 45804894-45804911. Max. coverage (+): 0. Max coverage (-): 0

Region: chr18 45804912-45804928. Max. coverage (+): 0. Max coverage (-): 0

Region: chr18 45804929-45804946. Max. coverage (+): 0. Max coverage (-): 0

Region: chr18 45804947-45804963. Max. coverage (+): 0. Max coverage (-): 0

Region: chr18 45804964-45804981. Max. coverage (+): 0. Max coverage (-): 0

Region: chr18 45804982-45804998. Max. coverage (+): 0. Max coverage (-): 0

Region: chr18 45804999-45805016. Max. coverage (+): 0. Max coverage (-): 0

Region: chr18 45805017-45805033. Max. coverage (+): 0. Max coverage (-): 0

Region: chr18 45805034-45805051. Max. coverage (+): 0. Max coverage (-): 0

Region: chr18 45805052-45805068. Max. coverage (+): 0. Max coverage (-): 0

Region: chr18 45805069-45805086. Max. coverage (+): 0. Max coverage (-): 0

Region: chr18 45805087-45805104. Max. coverage (+): 0. Max coverage (-): 0

Region: chr18 45805105-45805121. Max. coverage (+): 0. Max coverage (-): 0

Region: chr18 45805122-45805139. Max. coverage (+): 0. Max coverage (-): 0

Region: chr18 45805140-45805156. Max. coverage (+): 0. Max coverage (-): 0

Region: chr18 45805157-45805174. Max. coverage (+): 0. Max coverage (-): 0

Region: chr18 45805175-45805191. Max. coverage (+): 0. Max coverage (-): 0

Region: chr18 45805192-45805209. Max. coverage (+): 0. Max coverage (-): 0

Region: chr18 45805210-45805226. Max. coverage (+): 0. Max coverage (-): 0

Region: chr18 45805227-45805244. Max. coverage (+): 0. Max coverage (-): 0

Region: chr18 45805245-45805261. Max. coverage (+): 0. Max coverage (-): 0

Region: chr18 45805262-45805279. Max. coverage (+): 0. Max coverage (-): 0

Region: chr18 45805280-45805296. Max. coverage (+): 0. Max coverage (-): 0

Region: chr18 45805297-45805314. Max. coverage (+): 0. Max coverage (-): 0

Region: chr18 45805315-45805331. Max. coverage (+): 0. Max coverage (-): 0

Region: chr18 45805332-45805349. Max. coverage (+): 0. Max coverage (-): 0

Region: chr18 45805350-45805366. Max. coverage (+): 0. Max coverage (-): 0

Region: chr18 45805367-45805384. Max. coverage (+): 0. Max coverage (-): 0

Region: chr18 45805385-45805401. Max. coverage (+): 0. Max coverage (-): 0

Region: chr18 45805402-45805419. Max. coverage (+): 0. Max coverage (-): 0

Region: chr18 45805420-45805436. Max. coverage (+): 0. Max coverage (-): 0

Region: chr18 45805437-45805454. Max. coverage (+): 0. Max coverage (-): 0

Region: chr18 45805455-45805471. Max. coverage (+): 0. Max coverage (-): 0

Region: chr18 45805472-45805489. Max. coverage (+): 0. Max coverage (-): 0

Region: chr18 45805490-45805506. Max. coverage (+): 0. Max coverage (-): 0

Region: chr18 45805507-45805524. Max. coverage (+): 0. Max coverage (-): 0

Region: chr18 45805525-45805541. Max. coverage (+): 0. Max coverage (-): 4.9

Region: chr18 45805542-45805559. Max. coverage (+): 0. Max coverage (-): 4.9

Region: chr18 45805560-45805576. Max. coverage (+): 0. Max coverage (-): 0

Region: chr18 45805577-45805594. Max. coverage (+): 0. Max coverage (-): 0

Region: chr18 45805595-45805611. Max. coverage (+): 0. Max coverage (-): 0

Region: chr18 45805612-45805629. Max. coverage (+): 0. Max coverage (-): 0

Region: chr18 45805630-45805646. Max. coverage (+): 0. Max coverage (-): 0

Region: chr18 45805647-45805664. Max. coverage (+): 0. Max coverage (-): 0

Region: chr18 45805665-45805681. Max. coverage (+): 0. Max coverage (-): 0

Region: chr18 45805682-45805699. Max. coverage (+): 0. Max coverage (-): 0

Region: chr18 45805700-45805716. Max. coverage (+): 0. Max coverage (-): 0

Region: chr18 45805717-45805734. Max. coverage (+): 0. Max coverage (-): 0

Region: chr18 45805735-45805752. Max. coverage (+): 0. Max coverage (-): 0

Region: chr18 45805753-45805769. Max. coverage (+): 0. Max coverage (-): 0

Region: chr18 45805770-45805787. Max. coverage (+): 0. Max coverage (-): 0

Region: chr18 45805788-45805804. Max. coverage (+): 0. Max coverage (-): 0

Region: chr18 45805805-45805822. Max. coverage (+): 0. Max coverage (-): 0

Region: chr18 45805823-45805839. Max. coverage (+): 0. Max coverage (-): 0

Region: chr18 45805840-45805857. Max. coverage (+): 0. Max coverage (-): 0

Region: chr18 45805858-45805874. Max. coverage (+): 0. Max coverage (-): 0

Region: chr18 45805875-45805892. Max. coverage (+): 0. Max coverage (-): 0

Region: chr18 45805893-45805909. Max. coverage (+): 0. Max coverage (-): 0

Region: chr18 45805910-45805927. Max. coverage (+): 0. Max coverage (-): 0

Region: chr18 45805928-45805944. Max. coverage (+): 0. Max coverage (-): 0

Region: chr18 45805945-45805962. Max. coverage (+): 0. Max coverage (-): 0

Region: chr18 45805963-45805979. Max. coverage (+): 0. Max coverage (-): 0

Region: chr18 45805980-45805997. Max. coverage (+): 0. Max coverage (-): 0

Region: chr18 45805998-45806014. Max. coverage (+): 0. Max coverage (-): 0

Region: chr18 45806015-45806032. Max. coverage (+): 0. Max coverage (-): 0

Region: chr18 45806033-45806049. Max. coverage (+): 0. Max coverage (-): 0

Region: chr18 45806050-45806067. Max. coverage (+): 0. Max coverage (-): 0

Region: chr18 45806068-45806084. Max. coverage (+): 0. Max coverage (-): 0

Region: chr18 45806085-45806102. Max. coverage (+): 0. Max coverage (-): 0

Region: chr18 45806103-45806119. Max. coverage (+): 0. Max coverage (-): 0

Region: chr18 45806120-45806137. Max. coverage (+): 0. Max coverage (-): 0

Region: chr18 45806138-45806154. Max. coverage (+): 0. Max coverage (-): 0

Region: chr18 45806155-45806172. Max. coverage (+): 0. Max coverage (-): 0

Region: chr18 45806173-45806189. Max. coverage (+): 0. Max coverage (-): 0

Region: chr18 45806190-45806207. Max. coverage (+): 0. Max coverage (-): 0

Region: chr18 45806208-45806224. Max. coverage (+): 0. Max coverage (-): 0

Region: chr18 45806225-45806242. Max. coverage (+): 0. Max coverage (-): 0

Region: chr18 45806243-45806259. Max. coverage (+): 0. Max coverage (-): 0

Region: chr18 45806260-45806277. Max. coverage (+): 0. Max coverage (-): 0

Region: chr18 45806278-45806294. Max. coverage (+): 0. Max coverage (-): 0

Region: chr18 45806295-45806312. Max. coverage (+): 0. Max coverage (-): 0

Region: chr18 45806313-45806329. Max. coverage (+): 0. Max coverage (-): 0

Region: chr18 45806330-45806347. Max. coverage (+): 0. Max coverage (-): 0

Region: chr18 45806348-45806365. Max. coverage (+): 0. Max coverage (-): 0

Region: chr18 45806366-45806382. Max. coverage (+): 0. Max coverage (-): 0

Region: chr18 45806383-45806400. Max. coverage (+): 0. Max coverage (-): 0

Region: chr18 45806401-45806417. Max. coverage (+): 0. Max coverage (-): 0

Region: chr18 45806418-45806435. Max. coverage (+): 0. Max coverage (-): 0

Region: chr18 45806436-45806452. Max. coverage (+): 0. Max coverage (-): 0

Region: chr18 45806453-45806470. Max. coverage (+): 0. Max coverage (-): 0

Region: chr18 45806471-45806487. Max. coverage (+): 0. Max coverage (-): 0

Region: chr18 45806488-45806505. Max. coverage (+): 0. Max coverage (-): 0

Region: chr18 45806506-45806522. Max. coverage (+): 0. Max coverage (-): 0

Region: chr18 45806523-45806540. Max. coverage (+): 0. Max coverage (-): 0

Region: chr18 45806541-45806557. Max. coverage (+): 0. Max coverage (-): 0

Region: chr18 45806558-45806575. Max. coverage (+): 0. Max coverage (-): 0

Region: chr18 45806576-45806592. Max. coverage (+): 0. Max coverage (-): 10.03

Region: chr18 45806593-45806610. Max. coverage (+): 0. Max coverage (-): 0

Region: chr18 45806611-45806627. Max. coverage (+): 0. Max coverage (-): 1.95

Region: chr18 45806628-45806645. Max. coverage (+): 0. Max coverage (-): 0

Region: chr18 45806646-45806662. Max. coverage (+): 0. Max coverage (-): 0

Region: chr18 45806663-45806680. Max. coverage (+): 0. Max coverage (-): 0

Region: chr18 45806681-45806697. Max. coverage (+): 0. Max coverage (-): 0

Region: chr18 45806698-45806715. Max. coverage (+): 0. Max coverage (-): 0

Region: chr18 45806716-45806732. Max. coverage (+): 0. Max coverage (-): 0

Region: chr18 45806733-45806750. Max. coverage (+): 0. Max coverage (-): 0

Region: chr18 45806751-45806767. Max. coverage (+): 0. Max coverage (-): 0

Region: chr18 45806768-45806785. Max. coverage (+): 0. Max coverage (-): 0

Region: chr18 45806786-45806802. Max. coverage (+): 0. Max coverage (-): 0

Region: chr18 45806803-45806820. Max. coverage (+): 0. Max coverage (-): 0

Region: chr18 45806821-45806837. Max. coverage (+): 0. Max coverage (-): 0

Region: chr18 45806838-45806855. Max. coverage (+): 0. Max coverage (-): 0

Region: chr18 45806856-45806872. Max. coverage (+): 0. Max coverage (-): 0

Region: chr18 45806873-45806890. Max. coverage (+): 0. Max coverage (-): 0

Region: chr18 45806891-45806907. Max. coverage (+): 0. Max coverage (-): 0

Region: chr18 45806908-45806925. Max. coverage (+): 0. Max coverage (-): 0

Region: chr18 45806926-45806942. Max. coverage (+): 0. Max coverage (-): 0

Region: chr18 45806943-45806960. Max. coverage (+): 0. Max coverage (-): 2.75

Region: chr18 45806961-45806978. Max. coverage (+): 0. Max coverage (-): 5.72

Region: chr18 45806979-45806995. Max. coverage (+): 0. Max coverage (-): 4.49

Region: chr18 45806996-45807013. Max. coverage (+): 0. Max coverage (-): 0

Region: chr18 45807014-45807030. Max. coverage (+): 0. Max coverage (-): 0

Region: chr18 45807031-45807048. Max. coverage (+): 0. Max coverage (-): 1.84

Region: chr18 45807049-45807065. Max. coverage (+): 0. Max coverage (-): 0

Region: chr18 45807066-45807083. Max. coverage (+): 0. Max coverage (-): 0

Region: chr18 45807084-45807100. Max. coverage (+): 0. Max coverage (-): 0

Region: chr18 45807101-45807118. Max. coverage (+): 0. Max coverage (-): 0

Region: chr18 45807119-45807135. Max. coverage (+): 0. Max coverage (-): 0

Region: chr18 45807136-45807153. Max. coverage (+): 0. Max coverage (-): 0

Region: chr18 45807154-45807170. Max. coverage (+): 0. Max coverage (-): 1.77

Region: chr18 45807171-45807188. Max. coverage (+): 0. Max coverage (-): 1.77

Region: chr18 45807189-45807205. Max. coverage (+): 0. Max coverage (-): 0

Region: chr18 45807206-45807223. Max. coverage (+): 0. Max coverage (-): 0

Region: chr18 45807224-45807240. Max. coverage (+): 0. Max coverage (-): 0

Region: chr18 45807241-45807258. Max. coverage (+): 2.77. Max coverage (-): 0

Region: chr18 45807259-45807275. Max. coverage (+): 2.77. Max coverage (-): 0

Region: chr18 45807276-45807293. Max. coverage (+): 0. Max coverage (-): 0

Region: chr18 45807294-45807310. Max. coverage (+): 0. Max coverage (-): 0

Region: chr18 45807311-45807328. Max. coverage (+): 0. Max coverage (-): 0

Region: chr18 45807329-45807345. Max. coverage (+): 0. Max coverage (-): 0

Region: chr18 45807346-45807363. Max. coverage (+): 1.11. Max coverage (-): 0

Region: chr18 45807364-45807380. Max. coverage (+): 0. Max coverage (-): 0

Region: chr18 45807381-45807398. Max. coverage (+): 0. Max coverage (-): 0

Region: chr18 45807399-45807415. Max. coverage (+): 0. Max coverage (-): 3.04

Region: chr18 45807416-45807433. Max. coverage (+): 0. Max coverage (-): 3.04

Region: chr18 45807434-45807450. Max. coverage (+): 0. Max coverage (-): 0

Region: chr18 45807451-45807468. Max. coverage (+): 0. Max coverage (-): 0

Region: chr18 45807469-45807485. Max. coverage (+): 0. Max coverage (-): 0

Region: chr18 45807486-45807503. Max. coverage (+): 0. Max coverage (-): 0

Region: chr18 45807504-45807520. Max. coverage (+): 0. Max coverage (-): 0

Region: chr18 45807521-45807538. Max. coverage (+): 0. Max coverage (-): 0

Region: chr18 45807539-45807555. Max. coverage (+): 0. Max coverage (-): 0

Region: chr18 45807556-45807573. Max. coverage (+): 0. Max coverage (-): 0

Region: chr18 45807574-45807590. Max. coverage (+): 0. Max coverage (-): 0

Region: chr18 45807591-45807608. Max. coverage (+): 0. Max coverage (-): 0

Region: chr18 45807609-45807626. Max. coverage (+): 0. Max coverage (-): 0

Region: chr18 45807627-45807643. Max. coverage (+): 0. Max coverage (-): 1.05

Region: chr18 45807644-45807661. Max. coverage (+): 0. Max coverage (-): 1.05

Region: chr18 45807662-45807678. Max. coverage (+): 0. Max coverage (-): 0

Region: chr18 45807679-45807696. Max. coverage (+): 0. Max coverage (-): 0

Region: chr18 45807697-45807713. Max. coverage (+): 0. Max coverage (-): 0

Region: chr18 45807714-45807731. Max. coverage (+): 0. Max coverage (-): 0

Region: chr18 45807732-45807748. Max. coverage (+): 2.03. Max coverage (-): 0

Region: chr18 45807749-45807766. Max. coverage (+): 0.79. Max coverage (-): 4.73

Region: chr18 45807767-45807783. Max. coverage (+): 0. Max coverage (-): 0

Region: chr18 45807784-45807801. Max. coverage (+): 0. Max coverage (-): 0

Region: chr18 45807802-45807818. Max. coverage (+): 0. Max coverage (-): 0

Region: chr18 45807819-45807836. Max. coverage (+): 0.66. Max coverage (-): 0

Region: chr18 45807837-45807853. Max. coverage (+): 0. Max coverage (-): 0

Region: chr18 45807854-45807871. Max. coverage (+): 0. Max coverage (-): 0

Region: chr18 45807872-45807888. Max. coverage (+): 5.04. Max coverage (-): 2.63

Region: chr18 45807889-45807906. Max. coverage (+): 5.04. Max coverage (-): 0

Region: chr18 45807907-. Max. coverage (+): 0. Max coverage (-): 0

RepeatMasker Color Code

**+**

100-98% Identity

<98-95% Identity

<95-90% Identity

<90-85% Identity

<85-80% Identity

<80-75% Identity

<75-70% Identity

<70% Identity

**-**

Gene Set Color Code

**+**

Gene

Pseudogene

**-**

Topology/Coverage Color Code

Coverage Plus Strand

Coverage Minus Strand

Mainstrand: Plus

Mainstrand: Minus

Complementary Strand

Flanking Region  
(if option -flank >0)

Gene Set Annotation  
  
RepeatMasker Annotation  

**1. L1M5**: 45799261-45799589 (+), Divergence to consensus: 38.5%  
**2. SINE2-2\_BT**: 45799590-45799704 (-), Divergence to consensus: 23.5%  
**3. L1M5**: 45799705-45800017 (+), Divergence to consensus: 36.7%  
**4. L1M5**: 45800019-45800486 (+), Divergence to consensus: 37.5%  
**5. MIRb**: 45800865-45801011 (+), Divergence to consensus: 44.4%  
**6. L1\_Art**: 45804437-45804534 (-), Divergence to consensus: 23.7%  
**7. Bov-tA2**: 45804535-45804578 (+), Divergence to consensus: 4.5%  
**8. L1ME1**: 45804732-45804894 (+), Divergence to consensus: 28.8%  
**9. Bov-tA2**: 45804899-45805088 (+), Divergence to consensus: 11.2%  
**10. MER58C**: 45805129-45805316 (-), Divergence to consensus: 39.3%  
**11. L1ME1**: 45805319-45805419 (+), Divergence to consensus: 24.8%  
**12. LTR87**: 45805426-45805718 (-), Divergence to consensus: 46.6%  
**13. SINE2-3\_BT**: 45805782-45805923 (+), Divergence to consensus: 23.4%  
**14. Bov-tA2**: 45806276-45806472 (+), Divergence to consensus: 20.9%

  
Transcription Factor Binding Sites  

**Gata4** (Sequence: AGATAAC (-): 45800606)  
**Gata4** (Sequence: AGATAAC (-): 45805539)  
**Gata4** (Sequence: GTTATCT (+): 45804359)
