# Supplementary material for: Small RNA sequencing of cryopreserved semen from single bull revealed altered miRNAs and piRNAs expression between High- and Low-motile sperm populations
Source: BMC Genomics. 2017 Jan 4;18:14. doi: 10.1186/s12864-016-3394-7 (PMC5209821; doi:10.1186/s12864-016-3394-7)
Supplement: Additional file 3: — Details for each piRNA clusters found in High Motile (HM) sperm fraction. Genes, repeats, transposable elements and transcription factors binding sites falling within the cluster regions were reported. (ZIP 1896 kb) [file 12864_2016_3394_MOESM3_ESM.zip › 39.html]

piRNA cluster 39


Predicted piRNA cluster no. 39     previous   next
  

Show proTRAC run info
Hide proTRAC run info

================================= proTRAC ====================================  
VERSION: 2.1                                    LAST MODIFIED: 06. October 2015  
  
Please cite:  
Rosenkranz D, Zischler H. proTRAC - a software for probabilistic piRNA cluster  
detection, visualization and analysis. 2012. BMC Bioinformatics 13:5.  
  
and (for proTRAC 2.0 and later):  
Rosenkranz D, Rudloff S, Bastuck K, Ketting RF, Zischler H. Tupaia small RNAs  
provide insights into function and evolution of RNAi-based transposon defense  
in mammals. 2015. RNA 21(5):911-922.  
  
Contact:  
David Rosenkranz  
Institute of Anthropology, small RNA group  
Johannes Gutenberg University Mainz  
email: rosenkranz@uni-mainz.de  
  
You can find the latest proTRAC version at:  
http://sourceforge.net/projects/protrac/files  
http://www.smallRNAgroup-mainz.de/software  
==============================================================================  
  
PARAMETERS:  
Map file: .............../storage/core/barbara/genhome/smallRNA/fertility/Sample\_motile/pirna/Sample\_motile\_26-33\_collapsed.fa.no-dust.map.weighted-10000-1000-b-0  
Genome file: ............/storage/core/barbara/genhome/smallRNA/fertility/Sample\_all/pirna/bt\_311\_chrY.fa  
RepeatMasker annotation: /storage/genomes/bt\_umd31/GCF\_000003055.6\_Bos\_taurus\_UMD\_3.1.1\_repeatMasker\_chr.out  
GeneSet:................./storage/core/barbara/genhome/smallRNA/fertility/Sample\_all/pirna/full.gtf  
  
Significant (p<=0.01) hit density will be calculated based  
on observed hit distribution.  
  
Sliding window size: ........................................ 5000 bp  
Sliding window increament: .................................. 1000 bp  
Normalize each hit by number of genomic hits: ............... 1 [0=no/1=yes]  
Normalize each hit by number of sequence reads: ............. 1 [0=no/1=yes]  
Normalize values (-> per million mapped reads): ............. 1 [0=no/1=yes]  
Min. fraction of hits with 1T(U) or 10A: .................... 0.75  
Alternatively: Min. fraction of hits with 1T(U) and 10A: .... 0.5  
Min. fraction of hits with typical piRNA length: ............ 0.75  
Typical piRNA length: ....................................... 26-33 nt  
Min. size of a piRNA cluster: ............................... 5000 bp.  
Min. number of hits (absolute): ............................. 0  
Min. number of hits (normalized): ........................... 0  
Min. fraction of hits on the mainstrand: .................... 0.75  
Top fraction of mapped sequences (in terms of read counts): . 1%  
Top fraction accounts for max. n% of sequence reads: ........ 90%  
Min. fraction of hits on each arm of a bidirectional cluster: 0.1  
Output image file for each cluster: ......................... 0 [0=no/1=yes]  
Output html file for each cluster: .......................... 1 [0=no/1=yes]  
Output a summary table: ..................................... 1 [0=no/1=yes]  
Output a FASTA file for each cluster (piRNA sequences): ..... 1 [0=no/1=yes]  
Output a FASTA file comprising cluster sequences: ........... 1 [0=no/1=yes]  
Search DNA motifs in clusters: .............................. 1 [0=no/1=yes]  
Output flanking sequences: +/- .............................. 0 bp  
Output ~.pTi file: .......................................... 1 [0=no/1=yes]  
==============================================================================  
  
  
Genome size (without gaps): ............ 2678902517 bp  
Gaps (N/X/-): .......................... 53837044 bp  
Mapped reads: .......................... 658825247023  
Non-identical sequences: ............... 514171  
Genomic hits: .......................... 764233  
Significant densitiy of mapped reads: .. 12867599.5173724 reads/kb

Show proTRAC cluster info
Hide proTRAC cluster info

|  |  |
| --- | --- |
| Location | chr18 |
| Coordinates | 64809406-64814538 |
| Size [bp] | 5133 |
| Sequence hit loci | 66 |
| Mapped reads (normalized) | 96686357.3 |
| Mapped reads (normalized) per kb | 18836227.8 |
| Normalized reads with 1T (1U) | 88.7% |
| Normalized reads with 10A | 28% |
| Normalized reads with length 26-33 nt | 100% |
| Normalized reads on the main strand(s) | 99.7% |
| Predicted directionality | mono:plus |

100%

0%

1T (1U)  
reads

10A reads

26-33 nt  
reads

reads on mainstrand

**Either the amount of reads with 1T (1U) OR 10A has to exceed 75% (set with option: -1Tor10A)  
Alternatively the amount of reads with 1T (1U) AND 10A has to exceed 50% (set with option: -1Tand10A)  
Minimum amount of reads with preferred size is 75% (set with option: -pisize)  
Minimum amount of reads on the main strand(s) is 75% (set with option: -clstrand)**

Show read coverage
Hide read coverage

WHAT DO I SEE HERE?  
This chart shows the location of mapped sequence reads within a predicted piRNA cluster. The color refers to the number of genomic hits produced by the sequence read in question. A dark red bar indicates that this sequence read produces many other hits elsewhere in the genome. Many adjacent red or yellow bars can indicate the presence of a multi-copy element such as transposons or rRNA genes. A dark green bar indicates that this sequence read maps uniquely to this locus.

1 hit

2-5 hits

6-10 hits

11-20 hits

21-50 hits

51-100 hits

> 100 hits

chr18

64809406

64814538

Gene Set

RepeatMasker

Mapped  
Reads

17.04

plus strand

minus strand

17.04

Region: chr18 45807908-64809411. Max. coverage (+): 4.97. Max coverage (-): 0

Region: chr18 64809412-64809421. Max. coverage (+): 4.97. Max coverage (-): 0

Region: chr18 64809422-64809431. Max. coverage (+): 0. Max coverage (-): 0

Region: chr18 64809432-64809441. Max. coverage (+): 0. Max coverage (-): 0

Region: chr18 64809442-64809452. Max. coverage (+): 0. Max coverage (-): 0

Region: chr18 64809453-64809462. Max. coverage (+): 0. Max coverage (-): 0

Region: chr18 64809463-64809472. Max. coverage (+): 0. Max coverage (-): 0

Region: chr18 64809473-64809482. Max. coverage (+): 0. Max coverage (-): 0

Region: chr18 64809483-64809493. Max. coverage (+): 0. Max coverage (-): 0

Region: chr18 64809494-64809503. Max. coverage (+): 0. Max coverage (-): 0

Region: chr18 64809504-64809513. Max. coverage (+): 0. Max coverage (-): 0

Region: chr18 64809514-64809524. Max. coverage (+): 0. Max coverage (-): 0

Region: chr18 64809525-64809534. Max. coverage (+): 0. Max coverage (-): 0

Region: chr18 64809535-64809544. Max. coverage (+): 0. Max coverage (-): 0

Region: chr18 64809545-64809554. Max. coverage (+): 4.64. Max coverage (-): 0

Region: chr18 64809555-64809565. Max. coverage (+): 0. Max coverage (-): 0

Region: chr18 64809566-64809575. Max. coverage (+): 0. Max coverage (-): 0

Region: chr18 64809576-64809585. Max. coverage (+): 0. Max coverage (-): 0

Region: chr18 64809586-64809595. Max. coverage (+): 0. Max coverage (-): 0

Region: chr18 64809596-64809606. Max. coverage (+): 0. Max coverage (-): 0

Region: chr18 64809607-64809616. Max. coverage (+): 0. Max coverage (-): 0

Region: chr18 64809617-64809626. Max. coverage (+): 0. Max coverage (-): 0

Region: chr18 64809627-64809636. Max. coverage (+): 0. Max coverage (-): 0

Region: chr18 64809637-64809647. Max. coverage (+): 0. Max coverage (-): 0

Region: chr18 64809648-64809657. Max. coverage (+): 0. Max coverage (-): 0

Region: chr18 64809658-64809667. Max. coverage (+): 0. Max coverage (-): 0

Region: chr18 64809668-64809678. Max. coverage (+): 0. Max coverage (-): 0

Region: chr18 64809679-64809688. Max. coverage (+): 0. Max coverage (-): 0

Region: chr18 64809689-64809698. Max. coverage (+): 0. Max coverage (-): 0

Region: chr18 64809699-64809708. Max. coverage (+): 0. Max coverage (-): 0

Region: chr18 64809709-64809719. Max. coverage (+): 0. Max coverage (-): 0

Region: chr18 64809720-64809729. Max. coverage (+): 0. Max coverage (-): 0

Region: chr18 64809730-64809739. Max. coverage (+): 0. Max coverage (-): 0

Region: chr18 64809740-64809749. Max. coverage (+): 0. Max coverage (-): 0

Region: chr18 64809750-64809760. Max. coverage (+): 0. Max coverage (-): 0

Region: chr18 64809761-64809770. Max. coverage (+): 0. Max coverage (-): 0

Region: chr18 64809771-64809780. Max. coverage (+): 0. Max coverage (-): 0

Region: chr18 64809781-64809790. Max. coverage (+): 0. Max coverage (-): 0

Region: chr18 64809791-64809801. Max. coverage (+): 0. Max coverage (-): 0

Region: chr18 64809802-64809811. Max. coverage (+): 0. Max coverage (-): 0

Region: chr18 64809812-64809821. Max. coverage (+): 0. Max coverage (-): 0

Region: chr18 64809822-64809832. Max. coverage (+): 0. Max coverage (-): 0

Region: chr18 64809833-64809842. Max. coverage (+): 0. Max coverage (-): 0

Region: chr18 64809843-64809852. Max. coverage (+): 0. Max coverage (-): 0

Region: chr18 64809853-64809862. Max. coverage (+): 0. Max coverage (-): 0

Region: chr18 64809863-64809873. Max. coverage (+): 0. Max coverage (-): 0

Region: chr18 64809874-64809883. Max. coverage (+): 0. Max coverage (-): 0

Region: chr18 64809884-64809893. Max. coverage (+): 0. Max coverage (-): 0

Region: chr18 64809894-64809903. Max. coverage (+): 0. Max coverage (-): 0

Region: chr18 64809904-64809914. Max. coverage (+): 0. Max coverage (-): 0

Region: chr18 64809915-64809924. Max. coverage (+): 0. Max coverage (-): 0

Region: chr18 64809925-64809934. Max. coverage (+): 0. Max coverage (-): 0

Region: chr18 64809935-64809944. Max. coverage (+): 0. Max coverage (-): 0

Region: chr18 64809945-64809955. Max. coverage (+): 0. Max coverage (-): 0

Region: chr18 64809956-64809965. Max. coverage (+): 0. Max coverage (-): 0

Region: chr18 64809966-64809975. Max. coverage (+): 0. Max coverage (-): 0

Region: chr18 64809976-64809986. Max. coverage (+): 0. Max coverage (-): 0

Region: chr18 64809987-64809996. Max. coverage (+): 0. Max coverage (-): 0

Region: chr18 64809997-64810006. Max. coverage (+): 0. Max coverage (-): 0

Region: chr18 64810007-64810016. Max. coverage (+): 0. Max coverage (-): 0

Region: chr18 64810017-64810027. Max. coverage (+): 0. Max coverage (-): 0

Region: chr18 64810028-64810037. Max. coverage (+): 0. Max coverage (-): 0

Region: chr18 64810038-64810047. Max. coverage (+): 0. Max coverage (-): 0

Region: chr18 64810048-64810057. Max. coverage (+): 0. Max coverage (-): 0

Region: chr18 64810058-64810068. Max. coverage (+): 0. Max coverage (-): 0

Region: chr18 64810069-64810078. Max. coverage (+): 0. Max coverage (-): 0

Region: chr18 64810079-64810088. Max. coverage (+): 0. Max coverage (-): 0

Region: chr18 64810089-64810098. Max. coverage (+): 0. Max coverage (-): 0

Region: chr18 64810099-64810109. Max. coverage (+): 0. Max coverage (-): 0

Region: chr18 64810110-64810119. Max. coverage (+): 0. Max coverage (-): 0

Region: chr18 64810120-64810129. Max. coverage (+): 0. Max coverage (-): 0

Region: chr18 64810130-64810140. Max. coverage (+): 0. Max coverage (-): 0

Region: chr18 64810141-64810150. Max. coverage (+): 0. Max coverage (-): 0

Region: chr18 64810151-64810160. Max. coverage (+): 7.18. Max coverage (-): 0

Region: chr18 64810161-64810170. Max. coverage (+): 7.18. Max coverage (-): 0

Region: chr18 64810171-64810181. Max. coverage (+): 0. Max coverage (-): 0

Region: chr18 64810182-64810191. Max. coverage (+): 0. Max coverage (-): 0

Region: chr18 64810192-64810201. Max. coverage (+): 0. Max coverage (-): 0

Region: chr18 64810202-64810211. Max. coverage (+): 0. Max coverage (-): 0

Region: chr18 64810212-64810222. Max. coverage (+): 0. Max coverage (-): 0

Region: chr18 64810223-64810232. Max. coverage (+): 0. Max coverage (-): 0

Region: chr18 64810233-64810242. Max. coverage (+): 0. Max coverage (-): 0

Region: chr18 64810243-64810252. Max. coverage (+): 0. Max coverage (-): 0

Region: chr18 64810253-64810263. Max. coverage (+): 0. Max coverage (-): 0

Region: chr18 64810264-64810273. Max. coverage (+): 0. Max coverage (-): 0

Region: chr18 64810274-64810283. Max. coverage (+): 0. Max coverage (-): 0

Region: chr18 64810284-64810294. Max. coverage (+): 0. Max coverage (-): 0

Region: chr18 64810295-64810304. Max. coverage (+): 0. Max coverage (-): 0

Region: chr18 64810305-64810314. Max. coverage (+): 0. Max coverage (-): 0

Region: chr18 64810315-64810324. Max. coverage (+): 0. Max coverage (-): 0

Region: chr18 64810325-64810335. Max. coverage (+): 0. Max coverage (-): 0

Region: chr18 64810336-64810345. Max. coverage (+): 0. Max coverage (-): 0

Region: chr18 64810346-64810355. Max. coverage (+): 0. Max coverage (-): 0

Region: chr18 64810356-64810365. Max. coverage (+): 0. Max coverage (-): 0

Region: chr18 64810366-64810376. Max. coverage (+): 0. Max coverage (-): 0

Region: chr18 64810377-64810386. Max. coverage (+): 0. Max coverage (-): 0

Region: chr18 64810387-64810396. Max. coverage (+): 0. Max coverage (-): 0

Region: chr18 64810397-64810406. Max. coverage (+): 0. Max coverage (-): 0

Region: chr18 64810407-64810417. Max. coverage (+): 4.41. Max coverage (-): 0

Region: chr18 64810418-64810427. Max. coverage (+): 4.41. Max coverage (-): 0

Region: chr18 64810428-64810437. Max. coverage (+): 0. Max coverage (-): 0

Region: chr18 64810438-64810447. Max. coverage (+): 0. Max coverage (-): 0

Region: chr18 64810448-64810458. Max. coverage (+): 0. Max coverage (-): 0

Region: chr18 64810459-64810468. Max. coverage (+): 0. Max coverage (-): 0

Region: chr18 64810469-64810478. Max. coverage (+): 0. Max coverage (-): 0

Region: chr18 64810479-64810489. Max. coverage (+): 0. Max coverage (-): 0

Region: chr18 64810490-64810499. Max. coverage (+): 2.68. Max coverage (-): 0

Region: chr18 64810500-64810509. Max. coverage (+): 2.68. Max coverage (-): 0

Region: chr18 64810510-64810519. Max. coverage (+): 0. Max coverage (-): 0

Region: chr18 64810520-64810530. Max. coverage (+): 0. Max coverage (-): 0

Region: chr18 64810531-64810540. Max. coverage (+): 0. Max coverage (-): 0

Region: chr18 64810541-64810550. Max. coverage (+): 0. Max coverage (-): 0

Region: chr18 64810551-64810560. Max. coverage (+): 0. Max coverage (-): 0

Region: chr18 64810561-64810571. Max. coverage (+): 0. Max coverage (-): 0

Region: chr18 64810572-64810581. Max. coverage (+): 0. Max coverage (-): 0

Region: chr18 64810582-64810591. Max. coverage (+): 0. Max coverage (-): 0

Region: chr18 64810592-64810601. Max. coverage (+): 0. Max coverage (-): 0

Region: chr18 64810602-64810612. Max. coverage (+): 0. Max coverage (-): 0

Region: chr18 64810613-64810622. Max. coverage (+): 0. Max coverage (-): 0

Region: chr18 64810623-64810632. Max. coverage (+): 0. Max coverage (-): 0

Region: chr18 64810633-64810643. Max. coverage (+): 0. Max coverage (-): 0

Region: chr18 64810644-64810653. Max. coverage (+): 0. Max coverage (-): 0

Region: chr18 64810654-64810663. Max. coverage (+): 0. Max coverage (-): 0

Region: chr18 64810664-64810673. Max. coverage (+): 0. Max coverage (-): 0

Region: chr18 64810674-64810684. Max. coverage (+): 0. Max coverage (-): 0

Region: chr18 64810685-64810694. Max. coverage (+): 0. Max coverage (-): 0

Region: chr18 64810695-64810704. Max. coverage (+): 0. Max coverage (-): 0

Region: chr18 64810705-64810714. Max. coverage (+): 0. Max coverage (-): 0

Region: chr18 64810715-64810725. Max. coverage (+): 0. Max coverage (-): 0

Region: chr18 64810726-64810735. Max. coverage (+): 0. Max coverage (-): 0

Region: chr18 64810736-64810745. Max. coverage (+): 0. Max coverage (-): 0

Region: chr18 64810746-64810755. Max. coverage (+): 0. Max coverage (-): 0

Region: chr18 64810756-64810766. Max. coverage (+): 0. Max coverage (-): 0

Region: chr18 64810767-64810776. Max. coverage (+): 0. Max coverage (-): 0

Region: chr18 64810777-64810786. Max. coverage (+): 0. Max coverage (-): 0

Region: chr18 64810787-64810797. Max. coverage (+): 0. Max coverage (-): 0

Region: chr18 64810798-64810807. Max. coverage (+): 0. Max coverage (-): 0

Region: chr18 64810808-64810817. Max. coverage (+): 0. Max coverage (-): 0

Region: chr18 64810818-64810827. Max. coverage (+): 0. Max coverage (-): 0

Region: chr18 64810828-64810838. Max. coverage (+): 0. Max coverage (-): 0

Region: chr18 64810839-64810848. Max. coverage (+): 0. Max coverage (-): 0

Region: chr18 64810849-64810858. Max. coverage (+): 0. Max coverage (-): 0

Region: chr18 64810859-64810868. Max. coverage (+): 0. Max coverage (-): 0

Region: chr18 64810869-64810879. Max. coverage (+): 0. Max coverage (-): 0

Region: chr18 64810880-64810889. Max. coverage (+): 0. Max coverage (-): 0

Region: chr18 64810890-64810899. Max. coverage (+): 0. Max coverage (-): 0

Region: chr18 64810900-64810909. Max. coverage (+): 0. Max coverage (-): 0

Region: chr18 64810910-64810920. Max. coverage (+): 0. Max coverage (-): 0

Region: chr18 64810921-64810930. Max. coverage (+): 0. Max coverage (-): 0

Region: chr18 64810931-64810940. Max. coverage (+): 0. Max coverage (-): 0

Region: chr18 64810941-64810951. Max. coverage (+): 0. Max coverage (-): 0

Region: chr18 64810952-64810961. Max. coverage (+): 0. Max coverage (-): 0

Region: chr18 64810962-64810971. Max. coverage (+): 0. Max coverage (-): 0

Region: chr18 64810972-64810981. Max. coverage (+): 2.68. Max coverage (-): 0

Region: chr18 64810982-64810992. Max. coverage (+): 0. Max coverage (-): 0

Region: chr18 64810993-64811002. Max. coverage (+): 0. Max coverage (-): 0

Region: chr18 64811003-64811012. Max. coverage (+): 0. Max coverage (-): 0

Region: chr18 64811013-64811022. Max. coverage (+): 0. Max coverage (-): 0

Region: chr18 64811023-64811033. Max. coverage (+): 0. Max coverage (-): 0

Region: chr18 64811034-64811043. Max. coverage (+): 0. Max coverage (-): 0

Region: chr18 64811044-64811053. Max. coverage (+): 0. Max coverage (-): 0

Region: chr18 64811054-64811063. Max. coverage (+): 0. Max coverage (-): 0

Region: chr18 64811064-64811074. Max. coverage (+): 0. Max coverage (-): 0

Region: chr18 64811075-64811084. Max. coverage (+): 2.66. Max coverage (-): 0

Region: chr18 64811085-64811094. Max. coverage (+): 5.78. Max coverage (-): 0

Region: chr18 64811095-64811105. Max. coverage (+): 0. Max coverage (-): 0

Region: chr18 64811106-64811115. Max. coverage (+): 0. Max coverage (-): 0

Region: chr18 64811116-64811125. Max. coverage (+): 0. Max coverage (-): 0

Region: chr18 64811126-64811135. Max. coverage (+): 0. Max coverage (-): 0

Region: chr18 64811136-64811146. Max. coverage (+): 0. Max coverage (-): 0

Region: chr18 64811147-64811156. Max. coverage (+): 0. Max coverage (-): 0

Region: chr18 64811157-64811166. Max. coverage (+): 0. Max coverage (-): 0

Region: chr18 64811167-64811176. Max. coverage (+): 0. Max coverage (-): 0

Region: chr18 64811177-64811187. Max. coverage (+): 0. Max coverage (-): 0

Region: chr18 64811188-64811197. Max. coverage (+): 0. Max coverage (-): 0

Region: chr18 64811198-64811207. Max. coverage (+): 0. Max coverage (-): 0

Region: chr18 64811208-64811217. Max. coverage (+): 1.96. Max coverage (-): 0

Region: chr18 64811218-64811228. Max. coverage (+): 1.96. Max coverage (-): 0

Region: chr18 64811229-64811238. Max. coverage (+): 0. Max coverage (-): 0

Region: chr18 64811239-64811248. Max. coverage (+): 0. Max coverage (-): 0

Region: chr18 64811249-64811259. Max. coverage (+): 0. Max coverage (-): 0

Region: chr18 64811260-64811269. Max. coverage (+): 0. Max coverage (-): 0

Region: chr18 64811270-64811279. Max. coverage (+): 0. Max coverage (-): 0

Region: chr18 64811280-64811289. Max. coverage (+): 0. Max coverage (-): 0

Region: chr18 64811290-64811300. Max. coverage (+): 0. Max coverage (-): 0

Region: chr18 64811301-64811310. Max. coverage (+): 0. Max coverage (-): 0

Region: chr18 64811311-64811320. Max. coverage (+): 0. Max coverage (-): 0

Region: chr18 64811321-64811330. Max. coverage (+): 0. Max coverage (-): 0

Region: chr18 64811331-64811341. Max. coverage (+): 0. Max coverage (-): 0

Region: chr18 64811342-64811351. Max. coverage (+): 0. Max coverage (-): 0

Region: chr18 64811352-64811361. Max. coverage (+): 0. Max coverage (-): 0

Region: chr18 64811362-64811371. Max. coverage (+): 0. Max coverage (-): 0

Region: chr18 64811372-64811382. Max. coverage (+): 0. Max coverage (-): 0

Region: chr18 64811383-64811392. Max. coverage (+): 0. Max coverage (-): 0

Region: chr18 64811393-64811402. Max. coverage (+): 0. Max coverage (-): 0

Region: chr18 64811403-64811413. Max. coverage (+): 5.02. Max coverage (-): 0

Region: chr18 64811414-64811423. Max. coverage (+): 5.02. Max coverage (-): 0

Region: chr18 64811424-64811433. Max. coverage (+): 0. Max coverage (-): 0

Region: chr18 64811434-64811443. Max. coverage (+): 0. Max coverage (-): 0

Region: chr18 64811444-64811454. Max. coverage (+): 0. Max coverage (-): 0

Region: chr18 64811455-64811464. Max. coverage (+): 0. Max coverage (-): 0

Region: chr18 64811465-64811474. Max. coverage (+): 0.56. Max coverage (-): 0

Region: chr18 64811475-64811484. Max. coverage (+): 0. Max coverage (-): 0

Region: chr18 64811485-64811495. Max. coverage (+): 0. Max coverage (-): 0

Region: chr18 64811496-64811505. Max. coverage (+): 0. Max coverage (-): 0

Region: chr18 64811506-64811515. Max. coverage (+): 0. Max coverage (-): 0

Region: chr18 64811516-64811525. Max. coverage (+): 0. Max coverage (-): 0

Region: chr18 64811526-64811536. Max. coverage (+): 0. Max coverage (-): 0

Region: chr18 64811537-64811546. Max. coverage (+): 0. Max coverage (-): 0

Region: chr18 64811547-64811556. Max. coverage (+): 0. Max coverage (-): 0

Region: chr18 64811557-64811566. Max. coverage (+): 0. Max coverage (-): 0

Region: chr18 64811567-64811577. Max. coverage (+): 0. Max coverage (-): 0

Region: chr18 64811578-64811587. Max. coverage (+): 0. Max coverage (-): 0.43

Region: chr18 64811588-64811597. Max. coverage (+): 0. Max coverage (-): 0.43

Region: chr18 64811598-64811608. Max. coverage (+): 0.48. Max coverage (-): 0

Region: chr18 64811609-64811618. Max. coverage (+): 0. Max coverage (-): 0

Region: chr18 64811619-64811628. Max. coverage (+): 0. Max coverage (-): 0

Region: chr18 64811629-64811638. Max. coverage (+): 0. Max coverage (-): 0

Region: chr18 64811639-64811649. Max. coverage (+): 0. Max coverage (-): 0

Region: chr18 64811650-64811659. Max. coverage (+): 10.46. Max coverage (-): 0

Region: chr18 64811660-64811669. Max. coverage (+): 17.04. Max coverage (-): 0

Region: chr18 64811670-64811679. Max. coverage (+): 17.04. Max coverage (-): 0

Region: chr18 64811680-64811690. Max. coverage (+): 0. Max coverage (-): 0

Region: chr18 64811691-64811700. Max. coverage (+): 0. Max coverage (-): 0

Region: chr18 64811701-64811710. Max. coverage (+): 0. Max coverage (-): 0

Region: chr18 64811711-64811720. Max. coverage (+): 0. Max coverage (-): 0

Region: chr18 64811721-64811731. Max. coverage (+): 0. Max coverage (-): 0

Region: chr18 64811732-64811741. Max. coverage (+): 0. Max coverage (-): 0

Region: chr18 64811742-64811751. Max. coverage (+): 0. Max coverage (-): 0

Region: chr18 64811752-64811762. Max. coverage (+): 0. Max coverage (-): 0

Region: chr18 64811763-64811772. Max. coverage (+): 0. Max coverage (-): 0

Region: chr18 64811773-64811782. Max. coverage (+): 0. Max coverage (-): 0

Region: chr18 64811783-64811792. Max. coverage (+): 0. Max coverage (-): 0

Region: chr18 64811793-64811803. Max. coverage (+): 0. Max coverage (-): 0

Region: chr18 64811804-64811813. Max. coverage (+): 0. Max coverage (-): 0

Region: chr18 64811814-64811823. Max. coverage (+): 0. Max coverage (-): 0

Region: chr18 64811824-64811833. Max. coverage (+): 0. Max coverage (-): 0

Region: chr18 64811834-64811844. Max. coverage (+): 0. Max coverage (-): 0

Region: chr18 64811845-64811854. Max. coverage (+): 0. Max coverage (-): 0

Region: chr18 64811855-64811864. Max. coverage (+): 0. Max coverage (-): 0

Region: chr18 64811865-64811874. Max. coverage (+): 0. Max coverage (-): 0

Region: chr18 64811875-64811885. Max. coverage (+): 0.82. Max coverage (-): 0

Region: chr18 64811886-64811895. Max. coverage (+): 0.82. Max coverage (-): 0

Region: chr18 64811896-64811905. Max. coverage (+): 0. Max coverage (-): 0

Region: chr18 64811906-64811916. Max. coverage (+): 0. Max coverage (-): 0

Region: chr18 64811917-64811926. Max. coverage (+): 0. Max coverage (-): 0

Region: chr18 64811927-64811936. Max. coverage (+): 0. Max coverage (-): 0

Region: chr18 64811937-64811946. Max. coverage (+): 0. Max coverage (-): 0

Region: chr18 64811947-64811957. Max. coverage (+): 0. Max coverage (-): 0

Region: chr18 64811958-64811967. Max. coverage (+): 0. Max coverage (-): 0

Region: chr18 64811968-64811977. Max. coverage (+): 4.32. Max coverage (-): 0

Region: chr18 64811978-64811987. Max. coverage (+): 4.32. Max coverage (-): 0

Region: chr18 64811988-64811998. Max. coverage (+): 0. Max coverage (-): 0

Region: chr18 64811999-64812008. Max. coverage (+): 4.29. Max coverage (-): 0

Region: chr18 64812009-64812018. Max. coverage (+): 4.29. Max coverage (-): 0

Region: chr18 64812019-64812028. Max. coverage (+): 0. Max coverage (-): 0

Region: chr18 64812029-64812039. Max. coverage (+): 1.22. Max coverage (-): 0

Region: chr18 64812040-64812049. Max. coverage (+): 0. Max coverage (-): 0

Region: chr18 64812050-64812059. Max. coverage (+): 0. Max coverage (-): 0

Region: chr18 64812060-64812070. Max. coverage (+): 4.63. Max coverage (-): 0

Region: chr18 64812071-64812080. Max. coverage (+): 4.63. Max coverage (-): 0

Region: chr18 64812081-64812090. Max. coverage (+): 0. Max coverage (-): 0

Region: chr18 64812091-64812100. Max. coverage (+): 0. Max coverage (-): 0

Region: chr18 64812101-64812111. Max. coverage (+): 0. Max coverage (-): 0

Region: chr18 64812112-64812121. Max. coverage (+): 0. Max coverage (-): 0

Region: chr18 64812122-64812131. Max. coverage (+): 0. Max coverage (-): 0

Region: chr18 64812132-64812141. Max. coverage (+): 0.18. Max coverage (-): 0

Region: chr18 64812142-64812152. Max. coverage (+): 0. Max coverage (-): 0

Region: chr18 64812153-64812162. Max. coverage (+): 0. Max coverage (-): 0

Region: chr18 64812163-64812172. Max. coverage (+): 0. Max coverage (-): 0

Region: chr18 64812173-64812182. Max. coverage (+): 0. Max coverage (-): 0

Region: chr18 64812183-64812193. Max. coverage (+): 0.87. Max coverage (-): 0

Region: chr18 64812194-64812203. Max. coverage (+): 2.27. Max coverage (-): 0

Region: chr18 64812204-64812213. Max. coverage (+): 2.27. Max coverage (-): 0

Region: chr18 64812214-64812224. Max. coverage (+): 0. Max coverage (-): 0

Region: chr18 64812225-64812234. Max. coverage (+): 0. Max coverage (-): 0

Region: chr18 64812235-64812244. Max. coverage (+): 0. Max coverage (-): 0

Region: chr18 64812245-64812254. Max. coverage (+): 0. Max coverage (-): 0

Region: chr18 64812255-64812265. Max. coverage (+): 0. Max coverage (-): 0

Region: chr18 64812266-64812275. Max. coverage (+): 0. Max coverage (-): 0

Region: chr18 64812276-64812285. Max. coverage (+): 0. Max coverage (-): 0

Region: chr18 64812286-64812295. Max. coverage (+): 0. Max coverage (-): 0

Region: chr18 64812296-64812306. Max. coverage (+): 0. Max coverage (-): 0

Region: chr18 64812307-64812316. Max. coverage (+): 0. Max coverage (-): 0

Region: chr18 64812317-64812326. Max. coverage (+): 0. Max coverage (-): 0

Region: chr18 64812327-64812336. Max. coverage (+): 0. Max coverage (-): 0

Region: chr18 64812337-64812347. Max. coverage (+): 0. Max coverage (-): 0

Region: chr18 64812348-64812357. Max. coverage (+): 0. Max coverage (-): 0

Region: chr18 64812358-64812367. Max. coverage (+): 0. Max coverage (-): 0

Region: chr18 64812368-64812378. Max. coverage (+): 0. Max coverage (-): 0

Region: chr18 64812379-64812388. Max. coverage (+): 0. Max coverage (-): 0

Region: chr18 64812389-64812398. Max. coverage (+): 0. Max coverage (-): 0

Region: chr18 64812399-64812408. Max. coverage (+): 0. Max coverage (-): 0

Region: chr18 64812409-64812419. Max. coverage (+): 0. Max coverage (-): 0

Region: chr18 64812420-64812429. Max. coverage (+): 0. Max coverage (-): 0

Region: chr18 64812430-64812439. Max. coverage (+): 0.86. Max coverage (-): 0

Region: chr18 64812440-64812449. Max. coverage (+): 0. Max coverage (-): 0

Region: chr18 64812450-64812460. Max. coverage (+): 0. Max coverage (-): 0

Region: chr18 64812461-64812470. Max. coverage (+): 4.12. Max coverage (-): 0

Region: chr18 64812471-64812480. Max. coverage (+): 10.95. Max coverage (-): 0

Region: chr18 64812481-64812490. Max. coverage (+): 1.72. Max coverage (-): 0

Region: chr18 64812491-64812501. Max. coverage (+): 0. Max coverage (-): 0

Region: chr18 64812502-64812511. Max. coverage (+): 0. Max coverage (-): 0

Region: chr18 64812512-64812521. Max. coverage (+): 0. Max coverage (-): 0

Region: chr18 64812522-64812531. Max. coverage (+): 0. Max coverage (-): 0

Region: chr18 64812532-64812542. Max. coverage (+): 0. Max coverage (-): 0

Region: chr18 64812543-64812552. Max. coverage (+): 0. Max coverage (-): 0

Region: chr18 64812553-64812562. Max. coverage (+): 0. Max coverage (-): 0

Region: chr18 64812563-64812573. Max. coverage (+): 0. Max coverage (-): 0

Region: chr18 64812574-64812583. Max. coverage (+): 1.58. Max coverage (-): 0

Region: chr18 64812584-64812593. Max. coverage (+): 1.58. Max coverage (-): 0

Region: chr18 64812594-64812603. Max. coverage (+): 0. Max coverage (-): 0

Region: chr18 64812604-64812614. Max. coverage (+): 0. Max coverage (-): 0

Region: chr18 64812615-64812624. Max. coverage (+): 0. Max coverage (-): 0

Region: chr18 64812625-64812634. Max. coverage (+): 0. Max coverage (-): 0

Region: chr18 64812635-64812644. Max. coverage (+): 0. Max coverage (-): 0

Region: chr18 64812645-64812655. Max. coverage (+): 0. Max coverage (-): 0

Region: chr18 64812656-64812665. Max. coverage (+): 2.2. Max coverage (-): 0

Region: chr18 64812666-64812675. Max. coverage (+): 2.2. Max coverage (-): 0

Region: chr18 64812676-64812685. Max. coverage (+): 0. Max coverage (-): 0

Region: chr18 64812686-64812696. Max. coverage (+): 0. Max coverage (-): 0

Region: chr18 64812697-64812706. Max. coverage (+): 0. Max coverage (-): 0

Region: chr18 64812707-64812716. Max. coverage (+): 0. Max coverage (-): 0

Region: chr18 64812717-64812727. Max. coverage (+): 0. Max coverage (-): 0

Region: chr18 64812728-64812737. Max. coverage (+): 0. Max coverage (-): 0

Region: chr18 64812738-64812747. Max. coverage (+): 0. Max coverage (-): 0

Region: chr18 64812748-64812757. Max. coverage (+): 0. Max coverage (-): 0

Region: chr18 64812758-64812768. Max. coverage (+): 0. Max coverage (-): 0

Region: chr18 64812769-64812778. Max. coverage (+): 0. Max coverage (-): 0

Region: chr18 64812779-64812788. Max. coverage (+): 0. Max coverage (-): 0

Region: chr18 64812789-64812798. Max. coverage (+): 2.59. Max coverage (-): 0

Region: chr18 64812799-64812809. Max. coverage (+): 2.59. Max coverage (-): 0

Region: chr18 64812810-64812819. Max. coverage (+): 0. Max coverage (-): 0

Region: chr18 64812820-64812829. Max. coverage (+): 0. Max coverage (-): 0

Region: chr18 64812830-64812839. Max. coverage (+): 0. Max coverage (-): 0

Region: chr18 64812840-64812850. Max. coverage (+): 0. Max coverage (-): 0

Region: chr18 64812851-64812860. Max. coverage (+): 0. Max coverage (-): 0

Region: chr18 64812861-64812870. Max. coverage (+): 0. Max coverage (-): 0

Region: chr18 64812871-64812881. Max. coverage (+): 0. Max coverage (-): 0

Region: chr18 64812882-64812891. Max. coverage (+): 0. Max coverage (-): 0

Region: chr18 64812892-64812901. Max. coverage (+): 0. Max coverage (-): 0

Region: chr18 64812902-64812911. Max. coverage (+): 0. Max coverage (-): 0

Region: chr18 64812912-64812922. Max. coverage (+): 0. Max coverage (-): 0

Region: chr18 64812923-64812932. Max. coverage (+): 0. Max coverage (-): 0

Region: chr18 64812933-64812942. Max. coverage (+): 0. Max coverage (-): 0

Region: chr18 64812943-64812952. Max. coverage (+): 0. Max coverage (-): 0

Region: chr18 64812953-64812963. Max. coverage (+): 0. Max coverage (-): 0

Region: chr18 64812964-64812973. Max. coverage (+): 0. Max coverage (-): 0

Region: chr18 64812974-64812983. Max. coverage (+): 8.88. Max coverage (-): 0

Region: chr18 64812984-64812993. Max. coverage (+): 8.88. Max coverage (-): 0

Region: chr18 64812994-64813004. Max. coverage (+): 0. Max coverage (-): 0

Region: chr18 64813005-64813014. Max. coverage (+): 4.42. Max coverage (-): 0

Region: chr18 64813015-64813024. Max. coverage (+): 4.42. Max coverage (-): 0

Region: chr18 64813025-64813035. Max. coverage (+): 0.59. Max coverage (-): 0

Region: chr18 64813036-64813045. Max. coverage (+): 0. Max coverage (-): 0

Region: chr18 64813046-64813055. Max. coverage (+): 0. Max coverage (-): 0

Region: chr18 64813056-64813065. Max. coverage (+): 4.08. Max coverage (-): 0

Region: chr18 64813066-64813076. Max. coverage (+): 0. Max coverage (-): 0

Region: chr18 64813077-64813086. Max. coverage (+): 0. Max coverage (-): 0

Region: chr18 64813087-64813096. Max. coverage (+): 0. Max coverage (-): 0

Region: chr18 64813097-64813106. Max. coverage (+): 0. Max coverage (-): 0

Region: chr18 64813107-64813117. Max. coverage (+): 0. Max coverage (-): 0

Region: chr18 64813118-64813127. Max. coverage (+): 0. Max coverage (-): 0

Region: chr18 64813128-64813137. Max. coverage (+): 0. Max coverage (-): 0

Region: chr18 64813138-64813147. Max. coverage (+): 0. Max coverage (-): 0

Region: chr18 64813148-64813158. Max. coverage (+): 1.1. Max coverage (-): 0

Region: chr18 64813159-64813168. Max. coverage (+): 0. Max coverage (-): 0

Region: chr18 64813169-64813178. Max. coverage (+): 5.73. Max coverage (-): 0

Region: chr18 64813179-64813189. Max. coverage (+): 5.73. Max coverage (-): 0

Region: chr18 64813190-64813199. Max. coverage (+): 0. Max coverage (-): 0

Region: chr18 64813200-64813209. Max. coverage (+): 0. Max coverage (-): 0

Region: chr18 64813210-64813219. Max. coverage (+): 2.14. Max coverage (-): 0

Region: chr18 64813220-64813230. Max. coverage (+): 0. Max coverage (-): 0

Region: chr18 64813231-64813240. Max. coverage (+): 0. Max coverage (-): 0

Region: chr18 64813241-64813250. Max. coverage (+): 0. Max coverage (-): 0

Region: chr18 64813251-64813260. Max. coverage (+): 0. Max coverage (-): 0

Region: chr18 64813261-64813271. Max. coverage (+): 0. Max coverage (-): 0

Region: chr18 64813272-64813281. Max. coverage (+): 0. Max coverage (-): 0

Region: chr18 64813282-64813291. Max. coverage (+): 2.6. Max coverage (-): 0

Region: chr18 64813292-64813301. Max. coverage (+): 2.6. Max coverage (-): 0

Region: chr18 64813302-64813312. Max. coverage (+): 0. Max coverage (-): 0

Region: chr18 64813313-64813322. Max. coverage (+): 0. Max coverage (-): 0

Region: chr18 64813323-64813332. Max. coverage (+): 0. Max coverage (-): 0

Region: chr18 64813333-64813343. Max. coverage (+): 0. Max coverage (-): 0

Region: chr18 64813344-64813353. Max. coverage (+): 0. Max coverage (-): 0

Region: chr18 64813354-64813363. Max. coverage (+): 0. Max coverage (-): 0

Region: chr18 64813364-64813373. Max. coverage (+): 0.72. Max coverage (-): 0

Region: chr18 64813374-64813384. Max. coverage (+): 0.72. Max coverage (-): 0

Region: chr18 64813385-64813394. Max. coverage (+): 0. Max coverage (-): 0

Region: chr18 64813395-64813404. Max. coverage (+): 0. Max coverage (-): 0

Region: chr18 64813405-64813414. Max. coverage (+): 0. Max coverage (-): 0

Region: chr18 64813415-64813425. Max. coverage (+): 0. Max coverage (-): 0

Region: chr18 64813426-64813435. Max. coverage (+): 0. Max coverage (-): 0

Region: chr18 64813436-64813445. Max. coverage (+): 0. Max coverage (-): 0

Region: chr18 64813446-64813455. Max. coverage (+): 0. Max coverage (-): 0

Region: chr18 64813456-64813466. Max. coverage (+): 3.72. Max coverage (-): 0

Region: chr18 64813467-64813476. Max. coverage (+): 3.72. Max coverage (-): 0

Region: chr18 64813477-64813486. Max. coverage (+): 0. Max coverage (-): 0

Region: chr18 64813487-64813497. Max. coverage (+): 0. Max coverage (-): 0

Region: chr18 64813498-64813507. Max. coverage (+): 0. Max coverage (-): 0

Region: chr18 64813508-64813517. Max. coverage (+): 0. Max coverage (-): 0

Region: chr18 64813518-64813527. Max. coverage (+): 0. Max coverage (-): 0

Region: chr18 64813528-64813538. Max. coverage (+): 0. Max coverage (-): 0

Region: chr18 64813539-64813548. Max. coverage (+): 0. Max coverage (-): 0

Region: chr18 64813549-64813558. Max. coverage (+): 0. Max coverage (-): 0

Region: chr18 64813559-64813568. Max. coverage (+): 0. Max coverage (-): 0

Region: chr18 64813569-64813579. Max. coverage (+): 0. Max coverage (-): 0

Region: chr18 64813580-64813589. Max. coverage (+): 0. Max coverage (-): 0

Region: chr18 64813590-64813599. Max. coverage (+): 0. Max coverage (-): 0

Region: chr18 64813600-64813609. Max. coverage (+): 0. Max coverage (-): 0

Region: chr18 64813610-64813620. Max. coverage (+): 0. Max coverage (-): 0

Region: chr18 64813621-64813630. Max. coverage (+): 0. Max coverage (-): 0

Region: chr18 64813631-64813640. Max. coverage (+): 0. Max coverage (-): 0

Region: chr18 64813641-64813650. Max. coverage (+): 0. Max coverage (-): 0

Region: chr18 64813651-64813661. Max. coverage (+): 0. Max coverage (-): 0

Region: chr18 64813662-64813671. Max. coverage (+): 0. Max coverage (-): 0

Region: chr18 64813672-64813681. Max. coverage (+): 0. Max coverage (-): 0

Region: chr18 64813682-64813692. Max. coverage (+): 0. Max coverage (-): 0

Region: chr18 64813693-64813702. Max. coverage (+): 0. Max coverage (-): 0

Region: chr18 64813703-64813712. Max. coverage (+): 0. Max coverage (-): 0

Region: chr18 64813713-64813722. Max. coverage (+): 0. Max coverage (-): 0

Region: chr18 64813723-64813733. Max. coverage (+): 0. Max coverage (-): 0

Region: chr18 64813734-64813743. Max. coverage (+): 0. Max coverage (-): 0

Region: chr18 64813744-64813753. Max. coverage (+): 0. Max coverage (-): 0

Region: chr18 64813754-64813763. Max. coverage (+): 0. Max coverage (-): 0

Region: chr18 64813764-64813774. Max. coverage (+): 0. Max coverage (-): 0

Region: chr18 64813775-64813784. Max. coverage (+): 0. Max coverage (-): 0

Region: chr18 64813785-64813794. Max. coverage (+): 0. Max coverage (-): 0

Region: chr18 64813795-64813804. Max. coverage (+): 0. Max coverage (-): 0

Region: chr18 64813805-64813815. Max. coverage (+): 0. Max coverage (-): 0

Region: chr18 64813816-64813825. Max. coverage (+): 0. Max coverage (-): 0

Region: chr18 64813826-64813835. Max. coverage (+): 0. Max coverage (-): 0

Region: chr18 64813836-64813846. Max. coverage (+): 0. Max coverage (-): 0

Region: chr18 64813847-64813856. Max. coverage (+): 0. Max coverage (-): 0

Region: chr18 64813857-64813866. Max. coverage (+): 0. Max coverage (-): 0

Region: chr18 64813867-64813876. Max. coverage (+): 0. Max coverage (-): 0

Region: chr18 64813877-64813887. Max. coverage (+): 0. Max coverage (-): 0

Region: chr18 64813888-64813897. Max. coverage (+): 0. Max coverage (-): 0

Region: chr18 64813898-64813907. Max. coverage (+): 0. Max coverage (-): 0

Region: chr18 64813908-64813917. Max. coverage (+): 0. Max coverage (-): 0

Region: chr18 64813918-64813928. Max. coverage (+): 0. Max coverage (-): 0

Region: chr18 64813929-64813938. Max. coverage (+): 0. Max coverage (-): 0

Region: chr18 64813939-64813948. Max. coverage (+): 0. Max coverage (-): 0

Region: chr18 64813949-64813958. Max. coverage (+): 0. Max coverage (-): 0

Region: chr18 64813959-64813969. Max. coverage (+): 0. Max coverage (-): 0

Region: chr18 64813970-64813979. Max. coverage (+): 0. Max coverage (-): 0

Region: chr18 64813980-64813989. Max. coverage (+): 0. Max coverage (-): 0

Region: chr18 64813990-64814000. Max. coverage (+): 0. Max coverage (-): 0

Region: chr18 64814001-64814010. Max. coverage (+): 0. Max coverage (-): 0

Region: chr18 64814011-64814020. Max. coverage (+): 0. Max coverage (-): 0

Region: chr18 64814021-64814030. Max. coverage (+): 0. Max coverage (-): 0

Region: chr18 64814031-64814041. Max. coverage (+): 0. Max coverage (-): 0

Region: chr18 64814042-64814051. Max. coverage (+): 0. Max coverage (-): 0

Region: chr18 64814052-64814061. Max. coverage (+): 0. Max coverage (-): 0

Region: chr18 64814062-64814071. Max. coverage (+): 0. Max coverage (-): 0

Region: chr18 64814072-64814082. Max. coverage (+): 0. Max coverage (-): 0

Region: chr18 64814083-64814092. Max. coverage (+): 0. Max coverage (-): 0

Region: chr18 64814093-64814102. Max. coverage (+): 0. Max coverage (-): 0

Region: chr18 64814103-64814112. Max. coverage (+): 0. Max coverage (-): 0

Region: chr18 64814113-64814123. Max. coverage (+): 0. Max coverage (-): 0

Region: chr18 64814124-64814133. Max. coverage (+): 0. Max coverage (-): 0

Region: chr18 64814134-64814143. Max. coverage (+): 0. Max coverage (-): 0

Region: chr18 64814144-64814154. Max. coverage (+): 0. Max coverage (-): 0

Region: chr18 64814155-64814164. Max. coverage (+): 0. Max coverage (-): 0

Region: chr18 64814165-64814174. Max. coverage (+): 0. Max coverage (-): 0

Region: chr18 64814175-64814184. Max. coverage (+): 0. Max coverage (-): 0

Region: chr18 64814185-64814195. Max. coverage (+): 0. Max coverage (-): 0

Region: chr18 64814196-64814205. Max. coverage (+): 0. Max coverage (-): 0

Region: chr18 64814206-64814215. Max. coverage (+): 0. Max coverage (-): 0

Region: chr18 64814216-64814225. Max. coverage (+): 0. Max coverage (-): 0

Region: chr18 64814226-64814236. Max. coverage (+): 0. Max coverage (-): 0

Region: chr18 64814237-64814246. Max. coverage (+): 0. Max coverage (-): 0

Region: chr18 64814247-64814256. Max. coverage (+): 0. Max coverage (-): 0

Region: chr18 64814257-64814266. Max. coverage (+): 0. Max coverage (-): 0

Region: chr18 64814267-64814277. Max. coverage (+): 0. Max coverage (-): 0

Region: chr18 64814278-64814287. Max. coverage (+): 0. Max coverage (-): 0

Region: chr18 64814288-64814297. Max. coverage (+): 0. Max coverage (-): 0

Region: chr18 64814298-64814308. Max. coverage (+): 0. Max coverage (-): 0

Region: chr18 64814309-64814318. Max. coverage (+): 0. Max coverage (-): 0

Region: chr18 64814319-64814328. Max. coverage (+): 0. Max coverage (-): 0

Region: chr18 64814329-64814338. Max. coverage (+): 0. Max coverage (-): 0

Region: chr18 64814339-64814349. Max. coverage (+): 0. Max coverage (-): 0

Region: chr18 64814350-64814359. Max. coverage (+): 0. Max coverage (-): 0

Region: chr18 64814360-64814369. Max. coverage (+): 0. Max coverage (-): 0

Region: chr18 64814370-64814379. Max. coverage (+): 0. Max coverage (-): 0

Region: chr18 64814380-64814390. Max. coverage (+): 0. Max coverage (-): 0

Region: chr18 64814391-64814400. Max. coverage (+): 0. Max coverage (-): 0

Region: chr18 64814401-64814410. Max. coverage (+): 0. Max coverage (-): 0

Region: chr18 64814411-64814420. Max. coverage (+): 0. Max coverage (-): 0

Region: chr18 64814421-64814431. Max. coverage (+): 0. Max coverage (-): 0

Region: chr18 64814432-64814441. Max. coverage (+): 0. Max coverage (-): 0

Region: chr18 64814442-64814451. Max. coverage (+): 0. Max coverage (-): 0

Region: chr18 64814452-64814462. Max. coverage (+): 0. Max coverage (-): 0

Region: chr18 64814463-64814472. Max. coverage (+): 0. Max coverage (-): 0

Region: chr18 64814473-64814482. Max. coverage (+): 0. Max coverage (-): 0

Region: chr18 64814483-64814492. Max. coverage (+): 0. Max coverage (-): 0

Region: chr18 64814493-64814503. Max. coverage (+): 0. Max coverage (-): 0

Region: chr18 64814504-64814513. Max. coverage (+): 7.73. Max coverage (-): 0

Region: chr18 64814514-64814523. Max. coverage (+): 7.73. Max coverage (-): 0

Region: chr18 64814524-64814533. Max. coverage (+): 0. Max coverage (-): 0

Region: chr18 64814534-. Max. coverage (+): 0. Max coverage (-): 0

RepeatMasker Color Code

**+**

100-98% Identity

<98-95% Identity

<95-90% Identity

<90-85% Identity

<85-80% Identity

<80-75% Identity

<75-70% Identity

<70% Identity

**-**

Gene Set Color Code

**+**

Gene

Pseudogene

**-**

Topology/Coverage Color Code

Coverage Plus Strand

Coverage Minus Strand

Mainstrand: Plus

Mainstrand: Minus

Complementary Strand

Flanking Region  
(if option -flank >0)

Gene Set Annotation  

**1. (protein coding, ENSBTAG00000000195) Tr:00000021095 Ex:2**: 64809488-64809614 (+)  
**2. (protein coding, ENSBTAG00000000195) Tr:00000021095 Ex:3**: 64810144-64810266 (+)  
**3. (protein coding, ENSBTAG00000000195) Tr:00000021095 Ex:4**: 64810933-64811000 (+)  
**4. (protein coding, ENSBTAG00000000195) Tr:00000021095 Ex:5**: 64811202-64812263 (+)

  
RepeatMasker Annotation  

**1. L1ME1**: 64813662-64813972 (-), Divergence to consensus: 46.2%  
**2. SINE2-1\_BT**: 64813973-64814077 (-), Divergence to consensus: 31.4%  
**3. L1ME1**: 64814078-64814264 (-), Divergence to consensus: 46.2%  
**4. L1MD**: 64814261-64814480 (-), Divergence to consensus: 43.5%

  
Transcription Factor Binding Sites
