# Supplementary material for: Small RNA sequencing of cryopreserved semen from single bull revealed altered miRNAs and piRNAs expression between High- and Low-motile sperm populations
Source: BMC Genomics. 2017 Jan 4;18:14. doi: 10.1186/s12864-016-3394-7 (PMC5209821; doi:10.1186/s12864-016-3394-7)
Supplement: Additional file 3: — Details for each piRNA clusters found in High Motile (HM) sperm fraction. Genes, repeats, transposable elements and transcription factors binding sites falling within the cluster regions were reported. (ZIP 1896 kb) [file 12864_2016_3394_MOESM3_ESM.zip › 4.html]

piRNA cluster 4


Predicted piRNA cluster no. 4     previous   next
  

Show proTRAC run info
Hide proTRAC run info

================================= proTRAC ====================================  
VERSION: 2.1                                    LAST MODIFIED: 06. October 2015  
  
Please cite:  
Rosenkranz D, Zischler H. proTRAC - a software for probabilistic piRNA cluster  
detection, visualization and analysis. 2012. BMC Bioinformatics 13:5.  
  
and (for proTRAC 2.0 and later):  
Rosenkranz D, Rudloff S, Bastuck K, Ketting RF, Zischler H. Tupaia small RNAs  
provide insights into function and evolution of RNAi-based transposon defense  
in mammals. 2015. RNA 21(5):911-922.  
  
Contact:  
David Rosenkranz  
Institute of Anthropology, small RNA group  
Johannes Gutenberg University Mainz  
email: rosenkranz@uni-mainz.de  
  
You can find the latest proTRAC version at:  
http://sourceforge.net/projects/protrac/files  
http://www.smallRNAgroup-mainz.de/software  
==============================================================================  
  
PARAMETERS:  
Map file: .............../storage/core/barbara/genhome/smallRNA/fertility/Sample\_motile/pirna/Sample\_motile\_26-33\_collapsed.fa.no-dust.map.weighted-10000-1000-b-0  
Genome file: ............/storage/core/barbara/genhome/smallRNA/fertility/Sample\_all/pirna/bt\_311\_chrY.fa  
RepeatMasker annotation: /storage/genomes/bt\_umd31/GCF\_000003055.6\_Bos\_taurus\_UMD\_3.1.1\_repeatMasker\_chr.out  
GeneSet:................./storage/core/barbara/genhome/smallRNA/fertility/Sample\_all/pirna/full.gtf  
  
Significant (p<=0.01) hit density will be calculated based  
on observed hit distribution.  
  
Sliding window size: ........................................ 5000 bp  
Sliding window increament: .................................. 1000 bp  
Normalize each hit by number of genomic hits: ............... 1 [0=no/1=yes]  
Normalize each hit by number of sequence reads: ............. 1 [0=no/1=yes]  
Normalize values (-> per million mapped reads): ............. 1 [0=no/1=yes]  
Min. fraction of hits with 1T(U) or 10A: .................... 0.75  
Alternatively: Min. fraction of hits with 1T(U) and 10A: .... 0.5  
Min. fraction of hits with typical piRNA length: ............ 0.75  
Typical piRNA length: ....................................... 26-33 nt  
Min. size of a piRNA cluster: ............................... 5000 bp.  
Min. number of hits (absolute): ............................. 0  
Min. number of hits (normalized): ........................... 0  
Min. fraction of hits on the mainstrand: .................... 0.75  
Top fraction of mapped sequences (in terms of read counts): . 1%  
Top fraction accounts for max. n% of sequence reads: ........ 90%  
Min. fraction of hits on each arm of a bidirectional cluster: 0.1  
Output image file for each cluster: ......................... 0 [0=no/1=yes]  
Output html file for each cluster: .......................... 1 [0=no/1=yes]  
Output a summary table: ..................................... 1 [0=no/1=yes]  
Output a FASTA file for each cluster (piRNA sequences): ..... 1 [0=no/1=yes]  
Output a FASTA file comprising cluster sequences: ........... 1 [0=no/1=yes]  
Search DNA motifs in clusters: .............................. 1 [0=no/1=yes]  
Output flanking sequences: +/- .............................. 0 bp  
Output ~.pTi file: .......................................... 1 [0=no/1=yes]  
==============================================================================  
  
  
Genome size (without gaps): ............ 2678902517 bp  
Gaps (N/X/-): .......................... 53837044 bp  
Mapped reads: .......................... 658825247023  
Non-identical sequences: ............... 514171  
Genomic hits: .......................... 764233  
Significant densitiy of mapped reads: .. 12867599.5173724 reads/kb

Show proTRAC cluster info
Hide proTRAC cluster info

|  |  |
| --- | --- |
| Location | chr10 |
| Coordinates | 47898839-47913229 |
| Size [bp] | 14391 |
| Sequence hit loci | 4515 |
| Mapped reads (normalized) | 5028440445 |
| Mapped reads (normalized) per kb | 349415637.9 |
| Normalized reads with 1T (1U) | 80% |
| Normalized reads with 10A | 31.6% |
| Normalized reads with length 26-33 nt | 100% |
| Normalized reads on the main strand(s) | 100% |
| Predicted directionality | mono:minus |

100%

0%

1T (1U)  
reads

10A reads

26-33 nt  
reads

reads on mainstrand

**Either the amount of reads with 1T (1U) OR 10A has to exceed 75% (set with option: -1Tor10A)  
Alternatively the amount of reads with 1T (1U) AND 10A has to exceed 50% (set with option: -1Tand10A)  
Minimum amount of reads with preferred size is 75% (set with option: -pisize)  
Minimum amount of reads on the main strand(s) is 75% (set with option: -clstrand)**

Show read coverage
Hide read coverage

WHAT DO I SEE HERE?  
This chart shows the location of mapped sequence reads within a predicted piRNA cluster. The color refers to the number of genomic hits produced by the sequence read in question. A dark red bar indicates that this sequence read produces many other hits elsewhere in the genome. Many adjacent red or yellow bars can indicate the presence of a multi-copy element such as transposons or rRNA genes. A dark green bar indicates that this sequence read maps uniquely to this locus.

1 hit

2-5 hits

6-10 hits

11-20 hits

21-50 hits

51-100 hits

> 100 hits

chr10

47898839

47913229

Gene Set

RepeatMasker

Mapped  
Reads

166.02

plus strand

minus strand

166.02

Region: chr10 45871990-47898853. Max. coverage (+): 0. Max coverage (-): 1.15

Region: chr10 47898854-47898882. Max. coverage (+): 0. Max coverage (-): 0

Region: chr10 47898883-47898910. Max. coverage (+): 0. Max coverage (-): 0

Region: chr10 47898911-47898939. Max. coverage (+): 0. Max coverage (-): 0

Region: chr10 47898940-47898968. Max. coverage (+): 0. Max coverage (-): 0

Region: chr10 47898969-47898997. Max. coverage (+): 0. Max coverage (-): 0

Region: chr10 47898998-47899026. Max. coverage (+): 0. Max coverage (-): 0

Region: chr10 47899027-47899054. Max. coverage (+): 0. Max coverage (-): 0.9

Region: chr10 47899055-47899083. Max. coverage (+): 0. Max coverage (-): 0

Region: chr10 47899084-47899112. Max. coverage (+): 0. Max coverage (-): 0

Region: chr10 47899113-47899141. Max. coverage (+): 0. Max coverage (-): 0

Region: chr10 47899142-47899169. Max. coverage (+): 0. Max coverage (-): 0

Region: chr10 47899170-47899198. Max. coverage (+): 0. Max coverage (-): 16.88

Region: chr10 47899199-47899227. Max. coverage (+): 0. Max coverage (-): 16.88

Region: chr10 47899228-47899256. Max. coverage (+): 0. Max coverage (-): 0

Region: chr10 47899257-47899285. Max. coverage (+): 0. Max coverage (-): 11.88

Region: chr10 47899286-47899313. Max. coverage (+): 0. Max coverage (-): 1.56

Region: chr10 47899314-47899342. Max. coverage (+): 0. Max coverage (-): 0

Region: chr10 47899343-47899371. Max. coverage (+): 0. Max coverage (-): 0

Region: chr10 47899372-47899400. Max. coverage (+): 0. Max coverage (-): 0

Region: chr10 47899401-47899429. Max. coverage (+): 0. Max coverage (-): 0

Region: chr10 47899430-47899457. Max. coverage (+): 0. Max coverage (-): 0

Region: chr10 47899458-47899486. Max. coverage (+): 0. Max coverage (-): 0

Region: chr10 47899487-47899515. Max. coverage (+): 0. Max coverage (-): 0

Region: chr10 47899516-47899544. Max. coverage (+): 0. Max coverage (-): 0

Region: chr10 47899545-47899572. Max. coverage (+): 0. Max coverage (-): 0

Region: chr10 47899573-47899601. Max. coverage (+): 0. Max coverage (-): 1.34

Region: chr10 47899602-47899630. Max. coverage (+): 0. Max coverage (-): 0

Region: chr10 47899631-47899659. Max. coverage (+): 0. Max coverage (-): 5.15

Region: chr10 47899660-47899688. Max. coverage (+): 0. Max coverage (-): 4.62

Region: chr10 47899689-47899716. Max. coverage (+): 0. Max coverage (-): 1.87

Region: chr10 47899717-47899745. Max. coverage (+): 0. Max coverage (-): 0

Region: chr10 47899746-47899774. Max. coverage (+): 0. Max coverage (-): 0

Region: chr10 47899775-47899803. Max. coverage (+): 0. Max coverage (-): 0

Region: chr10 47899804-47899831. Max. coverage (+): 0. Max coverage (-): 0

Region: chr10 47899832-47899860. Max. coverage (+): 0. Max coverage (-): 0

Region: chr10 47899861-47899889. Max. coverage (+): 0. Max coverage (-): 0

Region: chr10 47899890-47899918. Max. coverage (+): 0. Max coverage (-): 4.04

Region: chr10 47899919-47899947. Max. coverage (+): 0. Max coverage (-): 4.06

Region: chr10 47899948-47899975. Max. coverage (+): 0. Max coverage (-): 3.58

Region: chr10 47899976-47900004. Max. coverage (+): 0. Max coverage (-): 0.52

Region: chr10 47900005-47900033. Max. coverage (+): 0. Max coverage (-): 34.37

Region: chr10 47900034-47900062. Max. coverage (+): 0. Max coverage (-): 11.46

Region: chr10 47900063-47900091. Max. coverage (+): 0. Max coverage (-): 0

Region: chr10 47900092-47900119. Max. coverage (+): 0. Max coverage (-): 0

Region: chr10 47900120-47900148. Max. coverage (+): 0. Max coverage (-): 0

Region: chr10 47900149-47900177. Max. coverage (+): 0. Max coverage (-): 0

Region: chr10 47900178-47900206. Max. coverage (+): 0. Max coverage (-): 0

Region: chr10 47900207-47900234. Max. coverage (+): 0. Max coverage (-): 0

Region: chr10 47900235-47900263. Max. coverage (+): 0. Max coverage (-): 0

Region: chr10 47900264-47900292. Max. coverage (+): 0. Max coverage (-): 0

Region: chr10 47900293-47900321. Max. coverage (+): 0. Max coverage (-): 0

Region: chr10 47900322-47900350. Max. coverage (+): 0. Max coverage (-): 0

Region: chr10 47900351-47900378. Max. coverage (+): 0. Max coverage (-): 0

Region: chr10 47900379-47900407. Max. coverage (+): 0. Max coverage (-): 0

Region: chr10 47900408-47900436. Max. coverage (+): 0. Max coverage (-): 0

Region: chr10 47900437-47900465. Max. coverage (+): 0. Max coverage (-): 0

Region: chr10 47900466-47900493. Max. coverage (+): 0. Max coverage (-): 0

Region: chr10 47900494-47900522. Max. coverage (+): 0. Max coverage (-): 0

Region: chr10 47900523-47900551. Max. coverage (+): 0. Max coverage (-): 0

Region: chr10 47900552-47900580. Max. coverage (+): 0. Max coverage (-): 0

Region: chr10 47900581-47900609. Max. coverage (+): 0. Max coverage (-): 0

Region: chr10 47900610-47900637. Max. coverage (+): 0. Max coverage (-): 0

Region: chr10 47900638-47900666. Max. coverage (+): 0. Max coverage (-): 0

Region: chr10 47900667-47900695. Max. coverage (+): 0. Max coverage (-): 0

Region: chr10 47900696-47900724. Max. coverage (+): 0. Max coverage (-): 0

Region: chr10 47900725-47900753. Max. coverage (+): 0. Max coverage (-): 0

Region: chr10 47900754-47900781. Max. coverage (+): 0. Max coverage (-): 0

Region: chr10 47900782-47900810. Max. coverage (+): 0. Max coverage (-): 0

Region: chr10 47900811-47900839. Max. coverage (+): 0. Max coverage (-): 0

Region: chr10 47900840-47900868. Max. coverage (+): 0. Max coverage (-): 0

Region: chr10 47900869-47900896. Max. coverage (+): 0. Max coverage (-): 0

Region: chr10 47900897-47900925. Max. coverage (+): 0. Max coverage (-): 0

Region: chr10 47900926-47900954. Max. coverage (+): 0. Max coverage (-): 0

Region: chr10 47900955-47900983. Max. coverage (+): 0. Max coverage (-): 0

Region: chr10 47900984-47901012. Max. coverage (+): 0. Max coverage (-): 0

Region: chr10 47901013-47901040. Max. coverage (+): 0. Max coverage (-): 0

Region: chr10 47901041-47901069. Max. coverage (+): 0. Max coverage (-): 0

Region: chr10 47901070-47901098. Max. coverage (+): 0. Max coverage (-): 0

Region: chr10 47901099-47901127. Max. coverage (+): 0. Max coverage (-): 0

Region: chr10 47901128-47901155. Max. coverage (+): 0. Max coverage (-): 0

Region: chr10 47901156-47901184. Max. coverage (+): 0. Max coverage (-): 0

Region: chr10 47901185-47901213. Max. coverage (+): 0. Max coverage (-): 0

Region: chr10 47901214-47901242. Max. coverage (+): 0. Max coverage (-): 0

Region: chr10 47901243-47901271. Max. coverage (+): 0. Max coverage (-): 0

Region: chr10 47901272-47901299. Max. coverage (+): 0. Max coverage (-): 0

Region: chr10 47901300-47901328. Max. coverage (+): 0. Max coverage (-): 0

Region: chr10 47901329-47901357. Max. coverage (+): 0. Max coverage (-): 0

Region: chr10 47901358-47901386. Max. coverage (+): 0. Max coverage (-): 0

Region: chr10 47901387-47901414. Max. coverage (+): 0. Max coverage (-): 0

Region: chr10 47901415-47901443. Max. coverage (+): 0. Max coverage (-): 0

Region: chr10 47901444-47901472. Max. coverage (+): 0. Max coverage (-): 0

Region: chr10 47901473-47901501. Max. coverage (+): 0. Max coverage (-): 0

Region: chr10 47901502-47901530. Max. coverage (+): 0. Max coverage (-): 0

Region: chr10 47901531-47901558. Max. coverage (+): 0. Max coverage (-): 0

Region: chr10 47901559-47901587. Max. coverage (+): 0. Max coverage (-): 0

Region: chr10 47901588-47901616. Max. coverage (+): 0. Max coverage (-): 0

Region: chr10 47901617-47901645. Max. coverage (+): 0. Max coverage (-): 0

Region: chr10 47901646-47901674. Max. coverage (+): 0. Max coverage (-): 0

Region: chr10 47901675-47901702. Max. coverage (+): 0. Max coverage (-): 0

Region: chr10 47901703-47901731. Max. coverage (+): 0. Max coverage (-): 2.85

Region: chr10 47901732-47901760. Max. coverage (+): 0. Max coverage (-): 16.89

Region: chr10 47901761-47901789. Max. coverage (+): 0. Max coverage (-): 8.25

Region: chr10 47901790-47901817. Max. coverage (+): 0. Max coverage (-): 17.31

Region: chr10 47901818-47901846. Max. coverage (+): 0. Max coverage (-): 6.36

Region: chr10 47901847-47901875. Max. coverage (+): 0. Max coverage (-): 9.56

Region: chr10 47901876-47901904. Max. coverage (+): 0. Max coverage (-): 4.1

Region: chr10 47901905-47901933. Max. coverage (+): 0. Max coverage (-): 6.74

Region: chr10 47901934-47901961. Max. coverage (+): 0. Max coverage (-): 2.77

Region: chr10 47901962-47901990. Max. coverage (+): 0. Max coverage (-): 3.08

Region: chr10 47901991-47902019. Max. coverage (+): 0. Max coverage (-): 3.08

Region: chr10 47902020-47902048. Max. coverage (+): 0. Max coverage (-): 20.53

Region: chr10 47902049-47902076. Max. coverage (+): 0. Max coverage (-): 15.1

Region: chr10 47902077-47902105. Max. coverage (+): 0. Max coverage (-): 15.1

Region: chr10 47902106-47902134. Max. coverage (+): 0. Max coverage (-): 22.83

Region: chr10 47902135-47902163. Max. coverage (+): 0. Max coverage (-): 24.85

Region: chr10 47902164-47902192. Max. coverage (+): 0. Max coverage (-): 5.15

Region: chr10 47902193-47902220. Max. coverage (+): 0. Max coverage (-): 6.69

Region: chr10 47902221-47902249. Max. coverage (+): 0. Max coverage (-): 9.35

Region: chr10 47902250-47902278. Max. coverage (+): 0. Max coverage (-): 6.92

Region: chr10 47902279-47902307. Max. coverage (+): 0. Max coverage (-): 10.9

Region: chr10 47902308-47902336. Max. coverage (+): 0. Max coverage (-): 4.53

Region: chr10 47902337-47902364. Max. coverage (+): 0. Max coverage (-): 1.55

Region: chr10 47902365-47902393. Max. coverage (+): 0. Max coverage (-): 86.34

Region: chr10 47902394-47902422. Max. coverage (+): 0. Max coverage (-): 75.68

Region: chr10 47902423-47902451. Max. coverage (+): 0. Max coverage (-): 5.75

Region: chr10 47902452-47902479. Max. coverage (+): 0. Max coverage (-): 4.29

Region: chr10 47902480-47902508. Max. coverage (+): 0. Max coverage (-): 1.74

Region: chr10 47902509-47902537. Max. coverage (+): 0. Max coverage (-): 10.07

Region: chr10 47902538-47902566. Max. coverage (+): 0. Max coverage (-): 11.07

Region: chr10 47902567-47902595. Max. coverage (+): 0. Max coverage (-): 14.94

Region: chr10 47902596-47902623. Max. coverage (+): 0. Max coverage (-): 6.83

Region: chr10 47902624-47902652. Max. coverage (+): 0. Max coverage (-): 21.97

Region: chr10 47902653-47902681. Max. coverage (+): 0. Max coverage (-): 166.02

Region: chr10 47902682-47902710. Max. coverage (+): 0. Max coverage (-): 9.35

Region: chr10 47902711-47902738. Max. coverage (+): 0. Max coverage (-): 13.69

Region: chr10 47902739-47902767. Max. coverage (+): 0. Max coverage (-): 34.44

Region: chr10 47902768-47902796. Max. coverage (+): 0. Max coverage (-): 12.95

Region: chr10 47902797-47902825. Max. coverage (+): 0. Max coverage (-): 33.41

Region: chr10 47902826-47902854. Max. coverage (+): 0. Max coverage (-): 33.17

Region: chr10 47902855-47902882. Max. coverage (+): 0. Max coverage (-): 28.2

Region: chr10 47902883-47902911. Max. coverage (+): 0. Max coverage (-): 49.9

Region: chr10 47902912-47902940. Max. coverage (+): 0. Max coverage (-): 71.69

Region: chr10 47902941-47902969. Max. coverage (+): 0. Max coverage (-): 19.94

Region: chr10 47902970-47902997. Max. coverage (+): 0. Max coverage (-): 6.11

Region: chr10 47902998-47903026. Max. coverage (+): 0. Max coverage (-): 24.92

Region: chr10 47903027-47903055. Max. coverage (+): 0. Max coverage (-): 10.79

Region: chr10 47903056-47903084. Max. coverage (+): 0. Max coverage (-): 58.26

Region: chr10 47903085-47903113. Max. coverage (+): 0. Max coverage (-): 24.11

Region: chr10 47903114-47903141. Max. coverage (+): 0. Max coverage (-): 12.49

Region: chr10 47903142-47903170. Max. coverage (+): 0. Max coverage (-): 37.26

Region: chr10 47903171-47903199. Max. coverage (+): 0. Max coverage (-): 34.78

Region: chr10 47903200-47903228. Max. coverage (+): 0. Max coverage (-): 15.8

Region: chr10 47903229-47903257. Max. coverage (+): 0. Max coverage (-): 14.83

Region: chr10 47903258-47903285. Max. coverage (+): 0. Max coverage (-): 40.91

Region: chr10 47903286-47903314. Max. coverage (+): 0. Max coverage (-): 56.41

Region: chr10 47903315-47903343. Max. coverage (+): 0. Max coverage (-): 17.78

Region: chr10 47903344-47903372. Max. coverage (+): 0. Max coverage (-): 11.67

Region: chr10 47903373-47903400. Max. coverage (+): 0. Max coverage (-): 32.64

Region: chr10 47903401-47903429. Max. coverage (+): 0. Max coverage (-): 0.81

Region: chr10 47903430-47903458. Max. coverage (+): 0. Max coverage (-): 0

Region: chr10 47903459-47903487. Max. coverage (+): 0. Max coverage (-): 0

Region: chr10 47903488-47903516. Max. coverage (+): 0. Max coverage (-): 0

Region: chr10 47903517-47903544. Max. coverage (+): 0. Max coverage (-): 0

Region: chr10 47903545-47903573. Max. coverage (+): 0. Max coverage (-): 0

Region: chr10 47903574-47903602. Max. coverage (+): 0. Max coverage (-): 0

Region: chr10 47903603-47903631. Max. coverage (+): 0. Max coverage (-): 0

Region: chr10 47903632-47903659. Max. coverage (+): 0. Max coverage (-): 0

Region: chr10 47903660-47903688. Max. coverage (+): 0. Max coverage (-): 0

Region: chr10 47903689-47903717. Max. coverage (+): 0. Max coverage (-): 0

Region: chr10 47903718-47903746. Max. coverage (+): 0. Max coverage (-): 7.1

Region: chr10 47903747-47903775. Max. coverage (+): 0. Max coverage (-): 7.1

Region: chr10 47903776-47903803. Max. coverage (+): 0. Max coverage (-): 21.9

Region: chr10 47903804-47903832. Max. coverage (+): 0. Max coverage (-): 5.08

Region: chr10 47903833-47903861. Max. coverage (+): 0. Max coverage (-): 15.23

Region: chr10 47903862-47903890. Max. coverage (+): 0. Max coverage (-): 5

Region: chr10 47903891-47903919. Max. coverage (+): 0. Max coverage (-): 13.09

Region: chr10 47903920-47903947. Max. coverage (+): 0. Max coverage (-): 20.98

Region: chr10 47903948-47903976. Max. coverage (+): 0. Max coverage (-): 3.85

Region: chr10 47903977-47904005. Max. coverage (+): 0. Max coverage (-): 25.94

Region: chr10 47904006-47904034. Max. coverage (+): 0. Max coverage (-): 26.39

Region: chr10 47904035-47904062. Max. coverage (+): 0. Max coverage (-): 28.04

Region: chr10 47904063-47904091. Max. coverage (+): 0. Max coverage (-): 4.53

Region: chr10 47904092-47904120. Max. coverage (+): 0. Max coverage (-): 29.32

Region: chr10 47904121-47904149. Max. coverage (+): 0. Max coverage (-): 2.55

Region: chr10 47904150-47904178. Max. coverage (+): 0. Max coverage (-): 0

Region: chr10 47904179-47904206. Max. coverage (+): 0. Max coverage (-): 9.58

Region: chr10 47904207-47904235. Max. coverage (+): 0. Max coverage (-): 11.47

Region: chr10 47904236-47904264. Max. coverage (+): 0. Max coverage (-): 11.47

Region: chr10 47904265-47904293. Max. coverage (+): 0. Max coverage (-): 35.61

Region: chr10 47904294-47904321. Max. coverage (+): 0. Max coverage (-): 12.03

Region: chr10 47904322-47904350. Max. coverage (+): 0. Max coverage (-): 21.88

Region: chr10 47904351-47904379. Max. coverage (+): 0. Max coverage (-): 15.38

Region: chr10 47904380-47904408. Max. coverage (+): 0. Max coverage (-): 14.16

Region: chr10 47904409-47904437. Max. coverage (+): 0. Max coverage (-): 25.32

Region: chr10 47904438-47904465. Max. coverage (+): 0. Max coverage (-): 0

Region: chr10 47904466-47904494. Max. coverage (+): 0. Max coverage (-): 0

Region: chr10 47904495-47904523. Max. coverage (+): 0. Max coverage (-): 0

Region: chr10 47904524-47904552. Max. coverage (+): 0. Max coverage (-): 0

Region: chr10 47904553-47904581. Max. coverage (+): 0. Max coverage (-): 0

Region: chr10 47904582-47904609. Max. coverage (+): 0. Max coverage (-): 0

Region: chr10 47904610-47904638. Max. coverage (+): 0. Max coverage (-): 0

Region: chr10 47904639-47904667. Max. coverage (+): 0. Max coverage (-): 0

Region: chr10 47904668-47904696. Max. coverage (+): 0. Max coverage (-): 0

Region: chr10 47904697-47904724. Max. coverage (+): 0. Max coverage (-): 29.82

Region: chr10 47904725-47904753. Max. coverage (+): 0. Max coverage (-): 13.09

Region: chr10 47904754-47904782. Max. coverage (+): 0. Max coverage (-): 11.28

Region: chr10 47904783-47904811. Max. coverage (+): 0. Max coverage (-): 16.3

Region: chr10 47904812-47904840. Max. coverage (+): 0. Max coverage (-): 4.67

Region: chr10 47904841-47904868. Max. coverage (+): 0. Max coverage (-): 0

Region: chr10 47904869-47904897. Max. coverage (+): 0. Max coverage (-): 1.57

Region: chr10 47904898-47904926. Max. coverage (+): 0. Max coverage (-): 5.9

Region: chr10 47904927-47904955. Max. coverage (+): 0. Max coverage (-): 15.2

Region: chr10 47904956-47904983. Max. coverage (+): 0. Max coverage (-): 10.3

Region: chr10 47904984-47905012. Max. coverage (+): 0. Max coverage (-): 2.08

Region: chr10 47905013-47905041. Max. coverage (+): 0. Max coverage (-): 2.18

Region: chr10 47905042-47905070. Max. coverage (+): 0. Max coverage (-): 0

Region: chr10 47905071-47905099. Max. coverage (+): 0. Max coverage (-): 0.51

Region: chr10 47905100-47905127. Max. coverage (+): 0. Max coverage (-): 0.51

Region: chr10 47905128-47905156. Max. coverage (+): 0. Max coverage (-): 5.02

Region: chr10 47905157-47905185. Max. coverage (+): 0. Max coverage (-): 9.88

Region: chr10 47905186-47905214. Max. coverage (+): 0. Max coverage (-): 8.06

Region: chr10 47905215-47905242. Max. coverage (+): 0. Max coverage (-): 25.35

Region: chr10 47905243-47905271. Max. coverage (+): 0. Max coverage (-): 5.33

Region: chr10 47905272-47905300. Max. coverage (+): 0. Max coverage (-): 0

Region: chr10 47905301-47905329. Max. coverage (+): 0. Max coverage (-): 7.49

Region: chr10 47905330-47905358. Max. coverage (+): 0. Max coverage (-): 22.65

Region: chr10 47905359-47905386. Max. coverage (+): 0. Max coverage (-): 44.62

Region: chr10 47905387-47905415. Max. coverage (+): 0. Max coverage (-): 22.89

Region: chr10 47905416-47905444. Max. coverage (+): 0. Max coverage (-): 16.29

Region: chr10 47905445-47905473. Max. coverage (+): 0. Max coverage (-): 26.22

Region: chr10 47905474-47905502. Max. coverage (+): 0. Max coverage (-): 0

Region: chr10 47905503-47905530. Max. coverage (+): 0. Max coverage (-): 46.09

Region: chr10 47905531-47905559. Max. coverage (+): 0. Max coverage (-): 31.43

Region: chr10 47905560-47905588. Max. coverage (+): 0. Max coverage (-): 8.27

Region: chr10 47905589-47905617. Max. coverage (+): 0. Max coverage (-): 106.13

Region: chr10 47905618-47905645. Max. coverage (+): 0. Max coverage (-): 0

Region: chr10 47905646-47905674. Max. coverage (+): 0. Max coverage (-): 3.58

Region: chr10 47905675-47905703. Max. coverage (+): 0. Max coverage (-): 13.56

Region: chr10 47905704-47905732. Max. coverage (+): 0. Max coverage (-): 28.15

Region: chr10 47905733-47905761. Max. coverage (+): 0. Max coverage (-): 32.65

Region: chr10 47905762-47905789. Max. coverage (+): 0. Max coverage (-): 35.65

Region: chr10 47905790-47905818. Max. coverage (+): 0. Max coverage (-): 19.77

Region: chr10 47905819-47905847. Max. coverage (+): 0. Max coverage (-): 15.04

Region: chr10 47905848-47905876. Max. coverage (+): 0. Max coverage (-): 0

Region: chr10 47905877-47905904. Max. coverage (+): 0. Max coverage (-): 14.85

Region: chr10 47905905-47905933. Max. coverage (+): 0. Max coverage (-): 40.32

Region: chr10 47905934-47905962. Max. coverage (+): 0. Max coverage (-): 8.81

Region: chr10 47905963-47905991. Max. coverage (+): 0. Max coverage (-): 8.46

Region: chr10 47905992-47906020. Max. coverage (+): 0. Max coverage (-): 22.24

Region: chr10 47906021-47906048. Max. coverage (+): 0. Max coverage (-): 7.59

Region: chr10 47906049-47906077. Max. coverage (+): 0. Max coverage (-): 43.71

Region: chr10 47906078-47906106. Max. coverage (+): 0. Max coverage (-): 3.24

Region: chr10 47906107-47906135. Max. coverage (+): 0. Max coverage (-): 9.01

Region: chr10 47906136-47906164. Max. coverage (+): 0. Max coverage (-): 0

Region: chr10 47906165-47906192. Max. coverage (+): 0. Max coverage (-): 5.23

Region: chr10 47906193-47906221. Max. coverage (+): 0. Max coverage (-): 8.67

Region: chr10 47906222-47906250. Max. coverage (+): 0. Max coverage (-): 0

Region: chr10 47906251-47906279. Max. coverage (+): 0. Max coverage (-): 2.99

Region: chr10 47906280-47906307. Max. coverage (+): 0. Max coverage (-): 9.73

Region: chr10 47906308-47906336. Max. coverage (+): 0. Max coverage (-): 0

Region: chr10 47906337-47906365. Max. coverage (+): 0. Max coverage (-): 76.16

Region: chr10 47906366-47906394. Max. coverage (+): 0. Max coverage (-): 79.59

Region: chr10 47906395-47906423. Max. coverage (+): 0. Max coverage (-): 5.28

Region: chr10 47906424-47906451. Max. coverage (+): 0. Max coverage (-): 1.68

Region: chr10 47906452-47906480. Max. coverage (+): 0. Max coverage (-): 1.68

Region: chr10 47906481-47906509. Max. coverage (+): 0. Max coverage (-): 4.83

Region: chr10 47906510-47906538. Max. coverage (+): 0. Max coverage (-): 9.87

Region: chr10 47906539-47906566. Max. coverage (+): 0. Max coverage (-): 33.31

Region: chr10 47906567-47906595. Max. coverage (+): 0. Max coverage (-): 20.39

Region: chr10 47906596-47906624. Max. coverage (+): 0. Max coverage (-): 13.4

Region: chr10 47906625-47906653. Max. coverage (+): 0. Max coverage (-): 2.25

Region: chr10 47906654-47906682. Max. coverage (+): 0. Max coverage (-): 16.38

Region: chr10 47906683-47906710. Max. coverage (+): 0. Max coverage (-): 10.06

Region: chr10 47906711-47906739. Max. coverage (+): 0. Max coverage (-): 12.4

Region: chr10 47906740-47906768. Max. coverage (+): 0. Max coverage (-): 11.08

Region: chr10 47906769-47906797. Max. coverage (+): 0. Max coverage (-): 6.9

Region: chr10 47906798-47906826. Max. coverage (+): 0. Max coverage (-): 2.83

Region: chr10 47906827-47906854. Max. coverage (+): 0. Max coverage (-): 0

Region: chr10 47906855-47906883. Max. coverage (+): 0. Max coverage (-): 1.55

Region: chr10 47906884-47906912. Max. coverage (+): 0. Max coverage (-): 9.81

Region: chr10 47906913-47906941. Max. coverage (+): 0. Max coverage (-): 41.44

Region: chr10 47906942-47906969. Max. coverage (+): 0. Max coverage (-): 12.15

Region: chr10 47906970-47906998. Max. coverage (+): 0. Max coverage (-): 34.84

Region: chr10 47906999-47907027. Max. coverage (+): 0. Max coverage (-): 41.94

Region: chr10 47907028-47907056. Max. coverage (+): 0. Max coverage (-): 29.42

Region: chr10 47907057-47907085. Max. coverage (+): 0. Max coverage (-): 4.67

Region: chr10 47907086-47907113. Max. coverage (+): 0. Max coverage (-): 0

Region: chr10 47907114-47907142. Max. coverage (+): 0. Max coverage (-): 19.96

Region: chr10 47907143-47907171. Max. coverage (+): 0. Max coverage (-): 18.99

Region: chr10 47907172-47907200. Max. coverage (+): 0. Max coverage (-): 16.18

Region: chr10 47907201-47907228. Max. coverage (+): 0. Max coverage (-): 14.14

Region: chr10 47907229-47907257. Max. coverage (+): 0. Max coverage (-): 35.72

Region: chr10 47907258-47907286. Max. coverage (+): 0. Max coverage (-): 28.14

Region: chr10 47907287-47907315. Max. coverage (+): 0. Max coverage (-): 19.4

Region: chr10 47907316-47907344. Max. coverage (+): 0. Max coverage (-): 30.52

Region: chr10 47907345-47907372. Max. coverage (+): 0. Max coverage (-): 48.84

Region: chr10 47907373-47907401. Max. coverage (+): 0. Max coverage (-): 9.57

Region: chr10 47907402-47907430. Max. coverage (+): 0. Max coverage (-): 22.73

Region: chr10 47907431-47907459. Max. coverage (+): 0. Max coverage (-): 25.78

Region: chr10 47907460-47907487. Max. coverage (+): 0. Max coverage (-): 30.87

Region: chr10 47907488-47907516. Max. coverage (+): 0. Max coverage (-): 3.09

Region: chr10 47907517-47907545. Max. coverage (+): 0. Max coverage (-): 4.6

Region: chr10 47907546-47907574. Max. coverage (+): 0. Max coverage (-): 6.09

Region: chr10 47907575-47907603. Max. coverage (+): 0. Max coverage (-): 7.03

Region: chr10 47907604-47907631. Max. coverage (+): 0. Max coverage (-): 23.89

Region: chr10 47907632-47907660. Max. coverage (+): 0. Max coverage (-): 17.42

Region: chr10 47907661-47907689. Max. coverage (+): 0. Max coverage (-): 15.61

Region: chr10 47907690-47907718. Max. coverage (+): 0. Max coverage (-): 8.07

Region: chr10 47907719-47907747. Max. coverage (+): 0. Max coverage (-): 18.92

Region: chr10 47907748-47907775. Max. coverage (+): 0. Max coverage (-): 84.69

Region: chr10 47907776-47907804. Max. coverage (+): 0. Max coverage (-): 27.24

Region: chr10 47907805-47907833. Max. coverage (+): 0. Max coverage (-): 20.43

Region: chr10 47907834-47907862. Max. coverage (+): 0. Max coverage (-): 19.39

Region: chr10 47907863-47907890. Max. coverage (+): 0. Max coverage (-): 10.03

Region: chr10 47907891-47907919. Max. coverage (+): 0. Max coverage (-): 11.84

Region: chr10 47907920-47907948. Max. coverage (+): 0. Max coverage (-): 43.48

Region: chr10 47907949-47907977. Max. coverage (+): 0. Max coverage (-): 15.23

Region: chr10 47907978-47908006. Max. coverage (+): 0. Max coverage (-): 18.8

Region: chr10 47908007-47908034. Max. coverage (+): 0. Max coverage (-): 26.97

Region: chr10 47908035-47908063. Max. coverage (+): 0. Max coverage (-): 87.28

Region: chr10 47908064-47908092. Max. coverage (+): 0. Max coverage (-): 5.44

Region: chr10 47908093-47908121. Max. coverage (+): 0. Max coverage (-): 10.73

Region: chr10 47908122-47908149. Max. coverage (+): 0. Max coverage (-): 39.89

Region: chr10 47908150-47908178. Max. coverage (+): 0. Max coverage (-): 12.06

Region: chr10 47908179-47908207. Max. coverage (+): 0. Max coverage (-): 20.49

Region: chr10 47908208-47908236. Max. coverage (+): 0. Max coverage (-): 8.17

Region: chr10 47908237-47908265. Max. coverage (+): 0. Max coverage (-): 24.25

Region: chr10 47908266-47908293. Max. coverage (+): 0. Max coverage (-): 11.15

Region: chr10 47908294-47908322. Max. coverage (+): 0. Max coverage (-): 94.03

Region: chr10 47908323-47908351. Max. coverage (+): 0. Max coverage (-): 23.26

Region: chr10 47908352-47908380. Max. coverage (+): 0. Max coverage (-): 27.02

Region: chr10 47908381-47908409. Max. coverage (+): 0. Max coverage (-): 27.02

Region: chr10 47908410-47908437. Max. coverage (+): 0. Max coverage (-): 43.03

Region: chr10 47908438-47908466. Max. coverage (+): 0. Max coverage (-): 17.57

Region: chr10 47908467-47908495. Max. coverage (+): 0. Max coverage (-): 25.33

Region: chr10 47908496-47908524. Max. coverage (+): 0. Max coverage (-): 39.07

Region: chr10 47908525-47908552. Max. coverage (+): 0. Max coverage (-): 50.98

Region: chr10 47908553-47908581. Max. coverage (+): 0. Max coverage (-): 22.61

Region: chr10 47908582-47908610. Max. coverage (+): 0. Max coverage (-): 25.6

Region: chr10 47908611-47908639. Max. coverage (+): 0. Max coverage (-): 72.09

Region: chr10 47908640-47908668. Max. coverage (+): 0. Max coverage (-): 73.36

Region: chr10 47908669-47908696. Max. coverage (+): 0. Max coverage (-): 106.11

Region: chr10 47908697-47908725. Max. coverage (+): 0. Max coverage (-): 90.79

Region: chr10 47908726-47908754. Max. coverage (+): 0. Max coverage (-): 56.9

Region: chr10 47908755-47908783. Max. coverage (+): 0. Max coverage (-): 8.23

Region: chr10 47908784-47908811. Max. coverage (+): 0. Max coverage (-): 80.48

Region: chr10 47908812-47908840. Max. coverage (+): 0. Max coverage (-): 111.84

Region: chr10 47908841-47908869. Max. coverage (+): 0. Max coverage (-): 3.84

Region: chr10 47908870-47908898. Max. coverage (+): 0. Max coverage (-): 109.71

Region: chr10 47908899-47908927. Max. coverage (+): 0. Max coverage (-): 0

Region: chr10 47908928-47908955. Max. coverage (+): 0. Max coverage (-): 2.32

Region: chr10 47908956-47908984. Max. coverage (+): 0. Max coverage (-): 5.65

Region: chr10 47908985-47909013. Max. coverage (+): 0. Max coverage (-): 5.96

Region: chr10 47909014-47909042. Max. coverage (+): 0. Max coverage (-): 3.93

Region: chr10 47909043-47909071. Max. coverage (+): 0. Max coverage (-): 8.61

Region: chr10 47909072-47909099. Max. coverage (+): 0. Max coverage (-): 5.52

Region: chr10 47909100-47909128. Max. coverage (+): 0. Max coverage (-): 18.53

Region: chr10 47909129-47909157. Max. coverage (+): 0. Max coverage (-): 5.3

Region: chr10 47909158-47909186. Max. coverage (+): 0. Max coverage (-): 19.51

Region: chr10 47909187-47909214. Max. coverage (+): 0. Max coverage (-): 19.51

Region: chr10 47909215-47909243. Max. coverage (+): 0. Max coverage (-): 8.7

Region: chr10 47909244-47909272. Max. coverage (+): 0. Max coverage (-): 16.3

Region: chr10 47909273-47909301. Max. coverage (+): 0. Max coverage (-): 17.11

Region: chr10 47909302-47909330. Max. coverage (+): 0. Max coverage (-): 3.73

Region: chr10 47909331-47909358. Max. coverage (+): 0. Max coverage (-): 0

Region: chr10 47909359-47909387. Max. coverage (+): 0. Max coverage (-): 0

Region: chr10 47909388-47909416. Max. coverage (+): 0. Max coverage (-): 0

Region: chr10 47909417-47909445. Max. coverage (+): 0. Max coverage (-): 0

Region: chr10 47909446-47909473. Max. coverage (+): 0. Max coverage (-): 0

Region: chr10 47909474-47909502. Max. coverage (+): 0. Max coverage (-): 0

Region: chr10 47909503-47909531. Max. coverage (+): 0. Max coverage (-): 0

Region: chr10 47909532-47909560. Max. coverage (+): 0. Max coverage (-): 0

Region: chr10 47909561-47909589. Max. coverage (+): 0. Max coverage (-): 0

Region: chr10 47909590-47909617. Max. coverage (+): 0. Max coverage (-): 0

Region: chr10 47909618-47909646. Max. coverage (+): 0. Max coverage (-): 0

Region: chr10 47909647-47909675. Max. coverage (+): 0. Max coverage (-): 0

Region: chr10 47909676-47909704. Max. coverage (+): 0. Max coverage (-): 0

Region: chr10 47909705-47909732. Max. coverage (+): 0. Max coverage (-): 0.82

Region: chr10 47909733-47909761. Max. coverage (+): 0. Max coverage (-): 6.45

Region: chr10 47909762-47909790. Max. coverage (+): 0. Max coverage (-): 7.97

Region: chr10 47909791-47909819. Max. coverage (+): 0. Max coverage (-): 7.97

Region: chr10 47909820-47909848. Max. coverage (+): 0. Max coverage (-): 21.63

Region: chr10 47909849-47909876. Max. coverage (+): 0. Max coverage (-): 19.46

Region: chr10 47909877-47909905. Max. coverage (+): 0. Max coverage (-): 0

Region: chr10 47909906-47909934. Max. coverage (+): 0. Max coverage (-): 6.21

Region: chr10 47909935-47909963. Max. coverage (+): 0. Max coverage (-): 0

Region: chr10 47909964-47909992. Max. coverage (+): 0. Max coverage (-): 2.3

Region: chr10 47909993-47910020. Max. coverage (+): 0. Max coverage (-): 5.72

Region: chr10 47910021-47910049. Max. coverage (+): 0. Max coverage (-): 0

Region: chr10 47910050-47910078. Max. coverage (+): 0. Max coverage (-): 1.47

Region: chr10 47910079-47910107. Max. coverage (+): 0. Max coverage (-): 0

Region: chr10 47910108-47910135. Max. coverage (+): 0. Max coverage (-): 0

Region: chr10 47910136-47910164. Max. coverage (+): 0. Max coverage (-): 6.55

Region: chr10 47910165-47910193. Max. coverage (+): 0. Max coverage (-): 8.58

Region: chr10 47910194-47910222. Max. coverage (+): 0. Max coverage (-): 4.99

Region: chr10 47910223-47910251. Max. coverage (+): 0. Max coverage (-): 4.85

Region: chr10 47910252-47910279. Max. coverage (+): 0. Max coverage (-): 4.53

Region: chr10 47910280-47910308. Max. coverage (+): 0. Max coverage (-): 3.36

Region: chr10 47910309-47910337. Max. coverage (+): 0. Max coverage (-): 2.17

Region: chr10 47910338-47910366. Max. coverage (+): 0. Max coverage (-): 0

Region: chr10 47910367-47910394. Max. coverage (+): 0. Max coverage (-): 0.47

Region: chr10 47910395-47910423. Max. coverage (+): 0. Max coverage (-): 6.7

Region: chr10 47910424-47910452. Max. coverage (+): 0. Max coverage (-): 16.18

Region: chr10 47910453-47910481. Max. coverage (+): 0. Max coverage (-): 11.69

Region: chr10 47910482-47910510. Max. coverage (+): 0. Max coverage (-): 18.93

Region: chr10 47910511-47910538. Max. coverage (+): 0. Max coverage (-): 15.83

Region: chr10 47910539-47910567. Max. coverage (+): 0. Max coverage (-): 0

Region: chr10 47910568-47910596. Max. coverage (+): 0. Max coverage (-): 3.08

Region: chr10 47910597-47910625. Max. coverage (+): 0. Max coverage (-): 14.77

Region: chr10 47910626-47910654. Max. coverage (+): 0. Max coverage (-): 15.43

Region: chr10 47910655-47910682. Max. coverage (+): 0. Max coverage (-): 10.35

Region: chr10 47910683-47910711. Max. coverage (+): 0. Max coverage (-): 50.55

Region: chr10 47910712-47910740. Max. coverage (+): 0. Max coverage (-): 31.46

Region: chr10 47910741-47910769. Max. coverage (+): 0. Max coverage (-): 29.78

Region: chr10 47910770-47910797. Max. coverage (+): 0. Max coverage (-): 11.23

Region: chr10 47910798-47910826. Max. coverage (+): 0. Max coverage (-): 0

Region: chr10 47910827-47910855. Max. coverage (+): 0. Max coverage (-): 4.61

Region: chr10 47910856-47910884. Max. coverage (+): 0. Max coverage (-): 15.64

Region: chr10 47910885-47910913. Max. coverage (+): 0. Max coverage (-): 17.5

Region: chr10 47910914-47910941. Max. coverage (+): 0. Max coverage (-): 13.21

Region: chr10 47910942-47910970. Max. coverage (+): 0. Max coverage (-): 8.89

Region: chr10 47910971-47910999. Max. coverage (+): 0. Max coverage (-): 64.06

Region: chr10 47911000-47911028. Max. coverage (+): 0. Max coverage (-): 7.1

Region: chr10 47911029-47911056. Max. coverage (+): 0. Max coverage (-): 4.63

Region: chr10 47911057-47911085. Max. coverage (+): 0. Max coverage (-): 6.42

Region: chr10 47911086-47911114. Max. coverage (+): 0. Max coverage (-): 14.33

Region: chr10 47911115-47911143. Max. coverage (+): 0. Max coverage (-): 0.98

Region: chr10 47911144-47911172. Max. coverage (+): 0. Max coverage (-): 5.62

Region: chr10 47911173-47911200. Max. coverage (+): 0. Max coverage (-): 0.72

Region: chr10 47911201-47911229. Max. coverage (+): 0. Max coverage (-): 0

Region: chr10 47911230-47911258. Max. coverage (+): 0. Max coverage (-): 4

Region: chr10 47911259-47911287. Max. coverage (+): 0. Max coverage (-): 5.98

Region: chr10 47911288-47911315. Max. coverage (+): 0. Max coverage (-): 4.89

Region: chr10 47911316-47911344. Max. coverage (+): 0. Max coverage (-): 9.88

Region: chr10 47911345-47911373. Max. coverage (+): 0. Max coverage (-): 15.25

Region: chr10 47911374-47911402. Max. coverage (+): 0. Max coverage (-): 8.12

Region: chr10 47911403-47911431. Max. coverage (+): 0. Max coverage (-): 4.99

Region: chr10 47911432-47911459. Max. coverage (+): 0. Max coverage (-): 2.76

Region: chr10 47911460-47911488. Max. coverage (+): 0. Max coverage (-): 4.21

Region: chr10 47911489-47911517. Max. coverage (+): 0. Max coverage (-): 5.09

Region: chr10 47911518-47911546. Max. coverage (+): 0. Max coverage (-): 0.41

Region: chr10 47911547-47911575. Max. coverage (+): 0. Max coverage (-): 3.74

Region: chr10 47911576-47911603. Max. coverage (+): 0. Max coverage (-): 3.74

Region: chr10 47911604-47911632. Max. coverage (+): 0. Max coverage (-): 10.69

Region: chr10 47911633-47911661. Max. coverage (+): 0. Max coverage (-): 2.16

Region: chr10 47911662-47911690. Max. coverage (+): 0. Max coverage (-): 1.3

Region: chr10 47911691-47911718. Max. coverage (+): 0. Max coverage (-): 2.44

Region: chr10 47911719-47911747. Max. coverage (+): 0. Max coverage (-): 0.78

Region: chr10 47911748-47911776. Max. coverage (+): 0. Max coverage (-): 1.64

Region: chr10 47911777-47911805. Max. coverage (+): 0. Max coverage (-): 0

Region: chr10 47911806-47911834. Max. coverage (+): 0. Max coverage (-): 0

Region: chr10 47911835-47911862. Max. coverage (+): 0. Max coverage (-): 6.03

Region: chr10 47911863-47911891. Max. coverage (+): 0. Max coverage (-): 6.84

Region: chr10 47911892-47911920. Max. coverage (+): 0. Max coverage (-): 12.89

Region: chr10 47911921-47911949. Max. coverage (+): 0. Max coverage (-): 0

Region: chr10 47911950-47911977. Max. coverage (+): 0. Max coverage (-): 0

Region: chr10 47911978-47912006. Max. coverage (+): 0. Max coverage (-): 0

Region: chr10 47912007-47912035. Max. coverage (+): 0. Max coverage (-): 0

Region: chr10 47912036-47912064. Max. coverage (+): 0. Max coverage (-): 20.72

Region: chr10 47912065-47912093. Max. coverage (+): 0. Max coverage (-): 15.59

Region: chr10 47912094-47912121. Max. coverage (+): 0. Max coverage (-): 15.18

Region: chr10 47912122-47912150. Max. coverage (+): 0. Max coverage (-): 4.01

Region: chr10 47912151-47912179. Max. coverage (+): 0. Max coverage (-): 4.4

Region: chr10 47912180-47912208. Max. coverage (+): 0. Max coverage (-): 3.91

Region: chr10 47912209-47912237. Max. coverage (+): 0. Max coverage (-): 5.16

Region: chr10 47912238-47912265. Max. coverage (+): 0. Max coverage (-): 10.55

Region: chr10 47912266-47912294. Max. coverage (+): 0. Max coverage (-): 13.4

Region: chr10 47912295-47912323. Max. coverage (+): 0. Max coverage (-): 0

Region: chr10 47912324-47912352. Max. coverage (+): 0. Max coverage (-): 10.67

Region: chr10 47912353-47912380. Max. coverage (+): 0. Max coverage (-): 1.72

Region: chr10 47912381-47912409. Max. coverage (+): 0. Max coverage (-): 1.72

Region: chr10 47912410-47912438. Max. coverage (+): 0. Max coverage (-): 16.32

Region: chr10 47912439-47912467. Max. coverage (+): 0. Max coverage (-): 8.63

Region: chr10 47912468-47912496. Max. coverage (+): 0. Max coverage (-): 15.56

Region: chr10 47912497-47912524. Max. coverage (+): 0. Max coverage (-): 7.34

Region: chr10 47912525-47912553. Max. coverage (+): 0. Max coverage (-): 5.96

Region: chr10 47912554-47912582. Max. coverage (+): 0. Max coverage (-): 4.19

Region: chr10 47912583-47912611. Max. coverage (+): 0. Max coverage (-): 35.03

Region: chr10 47912612-47912639. Max. coverage (+): 0. Max coverage (-): 10.43

Region: chr10 47912640-47912668. Max. coverage (+): 0. Max coverage (-): 28.75

Region: chr10 47912669-47912697. Max. coverage (+): 0. Max coverage (-): 6.74

Region: chr10 47912698-47912726. Max. coverage (+): 0. Max coverage (-): 10.58

Region: chr10 47912727-47912755. Max. coverage (+): 0. Max coverage (-): 6.95

Region: chr10 47912756-47912783. Max. coverage (+): 0. Max coverage (-): 11.28

Region: chr10 47912784-47912812. Max. coverage (+): 0. Max coverage (-): 18.43

Region: chr10 47912813-47912841. Max. coverage (+): 0. Max coverage (-): 21.69

Region: chr10 47912842-47912870. Max. coverage (+): 0. Max coverage (-): 16.79

Region: chr10 47912871-47912899. Max. coverage (+): 0. Max coverage (-): 24.11

Region: chr10 47912900-47912927. Max. coverage (+): 0. Max coverage (-): 6.7

Region: chr10 47912928-47912956. Max. coverage (+): 0. Max coverage (-): 15.33

Region: chr10 47912957-47912985. Max. coverage (+): 0. Max coverage (-): 1.01

Region: chr10 47912986-47913014. Max. coverage (+): 0. Max coverage (-): 21.22

Region: chr10 47913015-47913042. Max. coverage (+): 0. Max coverage (-): 3.3

Region: chr10 47913043-47913071. Max. coverage (+): 0. Max coverage (-): 14.4

Region: chr10 47913072-47913100. Max. coverage (+): 0. Max coverage (-): 12.34

Region: chr10 47913101-47913129. Max. coverage (+): 0. Max coverage (-): 3.68

Region: chr10 47913130-47913158. Max. coverage (+): 0. Max coverage (-): 10.06

Region: chr10 47913159-47913186. Max. coverage (+): 0. Max coverage (-): 22.83

Region: chr10 47913187-47913215. Max. coverage (+): 0. Max coverage (-): 20.18

Region: chr10 47913216-. Max. coverage (+): 0. Max coverage (-): 0

RepeatMasker Color Code

**+**

100-98% Identity

<98-95% Identity

<95-90% Identity

<90-85% Identity

<85-80% Identity

<80-75% Identity

<75-70% Identity

<70% Identity

**-**

Gene Set Color Code

**+**

Gene

Pseudogene

**-**

Topology/Coverage Color Code

Coverage Plus Strand

Coverage Minus Strand

Mainstrand: Plus

Mainstrand: Minus

Complementary Strand

Flanking Region  
(if option -flank >0)

Gene Set Annotation  
  
RepeatMasker Annotation  

**1. MIR3**: 47899410-47899530 (-), Divergence to consensus: 31.8%  
**2. Charlie4z**: 47899774-47899895 (-), Divergence to consensus: 37.4%  
**3. BTLTR1**: 47900092-47900123 (+), Divergence to consensus: 12.5%  
**4. BovB**: 47900124-47900384 (-), Divergence to consensus: 4.2%  
**5. BovB**: 47900377-47901218 (+), Divergence to consensus: 3%  
**6. ART2A**: 47901219-47901689 (+), Divergence to consensus: 19.1%  
**7. (AACTG)n**: 47901690-47901713 (+), Divergence to consensus: 4.2%  
**8. ART2A**: 47903422-47903721 (+), Divergence to consensus: 18.4%  
**9. CHR-2B**: 47904464-47904703 (-), Divergence to consensus: 47.8%  
**10. AT\_rich**: 47904721-47904746 (+), Divergence to consensus: 38.5%  
**11. AT\_rich**: 47904724-47904749 (+), Divergence to consensus: 69.2%  
**12. CHR-2B**: 47909351-47909591 (-), Divergence to consensus: 51.3%  
**13. AT\_rich**: 47911793-47911835 (+), Divergence to consensus: 72.1%  
**14. L2c**: 47911939-47912035 (+), Divergence to consensus: 46.4%

  
Transcription Factor Binding Sites  

**SPZ1** (Sequence: CTGAAACCCT (-): 47899754)  
**SPZ1** (Sequence: CTCTAACCCC (-): 47908460)  
**Gata4** (Sequence: AGATAAG (-): 47905236)  
**Mybl1\_1** (Sequence: AACCGTTA (+): 47911834)
