# Supplementary material for: Small RNA sequencing of cryopreserved semen from single bull revealed altered miRNAs and piRNAs expression between High- and Low-motile sperm populations
Source: BMC Genomics. 2017 Jan 4;18:14. doi: 10.1186/s12864-016-3394-7 (PMC5209821; doi:10.1186/s12864-016-3394-7)
Supplement: Additional file 3: — Details for each piRNA clusters found in High Motile (HM) sperm fraction. Genes, repeats, transposable elements and transcription factors binding sites falling within the cluster regions were reported. (ZIP 1896 kb) [file 12864_2016_3394_MOESM3_ESM.zip › 40.html]

piRNA cluster 40


Predicted piRNA cluster no. 40     previous   next
  

Show proTRAC run info
Hide proTRAC run info

================================= proTRAC ====================================  
VERSION: 2.1                                    LAST MODIFIED: 06. October 2015  
  
Please cite:  
Rosenkranz D, Zischler H. proTRAC - a software for probabilistic piRNA cluster  
detection, visualization and analysis. 2012. BMC Bioinformatics 13:5.  
  
and (for proTRAC 2.0 and later):  
Rosenkranz D, Rudloff S, Bastuck K, Ketting RF, Zischler H. Tupaia small RNAs  
provide insights into function and evolution of RNAi-based transposon defense  
in mammals. 2015. RNA 21(5):911-922.  
  
Contact:  
David Rosenkranz  
Institute of Anthropology, small RNA group  
Johannes Gutenberg University Mainz  
email: rosenkranz@uni-mainz.de  
  
You can find the latest proTRAC version at:  
http://sourceforge.net/projects/protrac/files  
http://www.smallRNAgroup-mainz.de/software  
==============================================================================  
  
PARAMETERS:  
Map file: .............../storage/core/barbara/genhome/smallRNA/fertility/Sample\_motile/pirna/Sample\_motile\_26-33\_collapsed.fa.no-dust.map.weighted-10000-1000-b-0  
Genome file: ............/storage/core/barbara/genhome/smallRNA/fertility/Sample\_all/pirna/bt\_311\_chrY.fa  
RepeatMasker annotation: /storage/genomes/bt\_umd31/GCF\_000003055.6\_Bos\_taurus\_UMD\_3.1.1\_repeatMasker\_chr.out  
GeneSet:................./storage/core/barbara/genhome/smallRNA/fertility/Sample\_all/pirna/full.gtf  
  
Significant (p<=0.01) hit density will be calculated based  
on observed hit distribution.  
  
Sliding window size: ........................................ 5000 bp  
Sliding window increament: .................................. 1000 bp  
Normalize each hit by number of genomic hits: ............... 1 [0=no/1=yes]  
Normalize each hit by number of sequence reads: ............. 1 [0=no/1=yes]  
Normalize values (-> per million mapped reads): ............. 1 [0=no/1=yes]  
Min. fraction of hits with 1T(U) or 10A: .................... 0.75  
Alternatively: Min. fraction of hits with 1T(U) and 10A: .... 0.5  
Min. fraction of hits with typical piRNA length: ............ 0.75  
Typical piRNA length: ....................................... 26-33 nt  
Min. size of a piRNA cluster: ............................... 5000 bp.  
Min. number of hits (absolute): ............................. 0  
Min. number of hits (normalized): ........................... 0  
Min. fraction of hits on the mainstrand: .................... 0.75  
Top fraction of mapped sequences (in terms of read counts): . 1%  
Top fraction accounts for max. n% of sequence reads: ........ 90%  
Min. fraction of hits on each arm of a bidirectional cluster: 0.1  
Output image file for each cluster: ......................... 0 [0=no/1=yes]  
Output html file for each cluster: .......................... 1 [0=no/1=yes]  
Output a summary table: ..................................... 1 [0=no/1=yes]  
Output a FASTA file for each cluster (piRNA sequences): ..... 1 [0=no/1=yes]  
Output a FASTA file comprising cluster sequences: ........... 1 [0=no/1=yes]  
Search DNA motifs in clusters: .............................. 1 [0=no/1=yes]  
Output flanking sequences: +/- .............................. 0 bp  
Output ~.pTi file: .......................................... 1 [0=no/1=yes]  
==============================================================================  
  
  
Genome size (without gaps): ............ 2678902517 bp  
Gaps (N/X/-): .......................... 53837044 bp  
Mapped reads: .......................... 658825247023  
Non-identical sequences: ............... 514171  
Genomic hits: .......................... 764233  
Significant densitiy of mapped reads: .. 12867599.5173724 reads/kb

Show proTRAC cluster info
Hide proTRAC cluster info

|  |  |
| --- | --- |
| Location | chr19 |
| Coordinates | 23956210-23961423 |
| Size [bp] | 5214 |
| Sequence hit loci | 58 |
| Mapped reads (normalized) | 72251806 |
| Mapped reads (normalized) per kb | 13857270 |
| Normalized reads with 1T (1U) | 86.5% |
| Normalized reads with 10A | 25.4% |
| Normalized reads with length 26-33 nt | 100% |
| Normalized reads on the main strand(s) | 100% |
| Predicted directionality | mono:minus |

100%

0%

1T (1U)  
reads

10A reads

26-33 nt  
reads

reads on mainstrand

**Either the amount of reads with 1T (1U) OR 10A has to exceed 75% (set with option: -1Tor10A)  
Alternatively the amount of reads with 1T (1U) AND 10A has to exceed 50% (set with option: -1Tand10A)  
Minimum amount of reads with preferred size is 75% (set with option: -pisize)  
Minimum amount of reads on the main strand(s) is 75% (set with option: -clstrand)**

Show read coverage
Hide read coverage

WHAT DO I SEE HERE?  
This chart shows the location of mapped sequence reads within a predicted piRNA cluster. The color refers to the number of genomic hits produced by the sequence read in question. A dark red bar indicates that this sequence read produces many other hits elsewhere in the genome. Many adjacent red or yellow bars can indicate the presence of a multi-copy element such as transposons or rRNA genes. A dark green bar indicates that this sequence read maps uniquely to this locus.

1 hit

2-5 hits

6-10 hits

11-20 hits

21-50 hits

51-100 hits

> 100 hits

chr19

23956210

23961423

Gene Set

RepeatMasker

Mapped  
Reads

19.84

plus strand

minus strand

19.84

Region: chr19 64814535-23956215. Max. coverage (+): 0. Max coverage (-): 0.62

Region: chr19 23956216-23956225. Max. coverage (+): 0. Max coverage (-): 1.39

Region: chr19 23956226-23956236. Max. coverage (+): 0. Max coverage (-): 0

Region: chr19 23956237-23956246. Max. coverage (+): 0. Max coverage (-): 0

Region: chr19 23956247-23956256. Max. coverage (+): 0. Max coverage (-): 0

Region: chr19 23956257-23956267. Max. coverage (+): 0. Max coverage (-): 0

Region: chr19 23956268-23956277. Max. coverage (+): 0. Max coverage (-): 0

Region: chr19 23956278-23956288. Max. coverage (+): 0. Max coverage (-): 0

Region: chr19 23956289-23956298. Max. coverage (+): 0. Max coverage (-): 0

Region: chr19 23956299-23956309. Max. coverage (+): 0. Max coverage (-): 0

Region: chr19 23956310-23956319. Max. coverage (+): 0. Max coverage (-): 0

Region: chr19 23956320-23956329. Max. coverage (+): 0. Max coverage (-): 0

Region: chr19 23956330-23956340. Max. coverage (+): 0. Max coverage (-): 1.06

Region: chr19 23956341-23956350. Max. coverage (+): 0. Max coverage (-): 0

Region: chr19 23956351-23956361. Max. coverage (+): 0. Max coverage (-): 0

Region: chr19 23956362-23956371. Max. coverage (+): 0. Max coverage (-): 0

Region: chr19 23956372-23956382. Max. coverage (+): 0. Max coverage (-): 0

Region: chr19 23956383-23956392. Max. coverage (+): 0. Max coverage (-): 0

Region: chr19 23956393-23956402. Max. coverage (+): 0. Max coverage (-): 0

Region: chr19 23956403-23956413. Max. coverage (+): 0. Max coverage (-): 0

Region: chr19 23956414-23956423. Max. coverage (+): 0. Max coverage (-): 0

Region: chr19 23956424-23956434. Max. coverage (+): 0. Max coverage (-): 0

Region: chr19 23956435-23956444. Max. coverage (+): 0. Max coverage (-): 0

Region: chr19 23956445-23956455. Max. coverage (+): 0. Max coverage (-): 4.17

Region: chr19 23956456-23956465. Max. coverage (+): 0. Max coverage (-): 0

Region: chr19 23956466-23956475. Max. coverage (+): 0. Max coverage (-): 0

Region: chr19 23956476-23956486. Max. coverage (+): 0. Max coverage (-): 0

Region: chr19 23956487-23956496. Max. coverage (+): 0. Max coverage (-): 0

Region: chr19 23956497-23956507. Max. coverage (+): 0. Max coverage (-): 0

Region: chr19 23956508-23956517. Max. coverage (+): 0. Max coverage (-): 0

Region: chr19 23956518-23956528. Max. coverage (+): 0. Max coverage (-): 0

Region: chr19 23956529-23956538. Max. coverage (+): 0. Max coverage (-): 0

Region: chr19 23956539-23956548. Max. coverage (+): 0. Max coverage (-): 0

Region: chr19 23956549-23956559. Max. coverage (+): 0. Max coverage (-): 0.91

Region: chr19 23956560-23956569. Max. coverage (+): 0. Max coverage (-): 0.91

Region: chr19 23956570-23956580. Max. coverage (+): 0. Max coverage (-): 0

Region: chr19 23956581-23956590. Max. coverage (+): 0. Max coverage (-): 0

Region: chr19 23956591-23956601. Max. coverage (+): 0. Max coverage (-): 0

Region: chr19 23956602-23956611. Max. coverage (+): 0. Max coverage (-): 0

Region: chr19 23956612-23956621. Max. coverage (+): 0. Max coverage (-): 0

Region: chr19 23956622-23956632. Max. coverage (+): 0. Max coverage (-): 0

Region: chr19 23956633-23956642. Max. coverage (+): 0. Max coverage (-): 2.08

Region: chr19 23956643-23956653. Max. coverage (+): 0. Max coverage (-): 2.08

Region: chr19 23956654-23956663. Max. coverage (+): 0. Max coverage (-): 0

Region: chr19 23956664-23956674. Max. coverage (+): 0. Max coverage (-): 0

Region: chr19 23956675-23956684. Max. coverage (+): 0. Max coverage (-): 0

Region: chr19 23956685-23956694. Max. coverage (+): 0. Max coverage (-): 0

Region: chr19 23956695-23956705. Max. coverage (+): 0. Max coverage (-): 0

Region: chr19 23956706-23956715. Max. coverage (+): 0. Max coverage (-): 0

Region: chr19 23956716-23956726. Max. coverage (+): 0. Max coverage (-): 0

Region: chr19 23956727-23956736. Max. coverage (+): 0. Max coverage (-): 0

Region: chr19 23956737-23956747. Max. coverage (+): 0. Max coverage (-): 0

Region: chr19 23956748-23956757. Max. coverage (+): 0. Max coverage (-): 0

Region: chr19 23956758-23956767. Max. coverage (+): 0. Max coverage (-): 0

Region: chr19 23956768-23956778. Max. coverage (+): 0. Max coverage (-): 0

Region: chr19 23956779-23956788. Max. coverage (+): 0. Max coverage (-): 0

Region: chr19 23956789-23956799. Max. coverage (+): 0. Max coverage (-): 0

Region: chr19 23956800-23956809. Max. coverage (+): 0. Max coverage (-): 0

Region: chr19 23956810-23956820. Max. coverage (+): 0. Max coverage (-): 0

Region: chr19 23956821-23956830. Max. coverage (+): 0. Max coverage (-): 0

Region: chr19 23956831-23956840. Max. coverage (+): 0. Max coverage (-): 0

Region: chr19 23956841-23956851. Max. coverage (+): 0. Max coverage (-): 0

Region: chr19 23956852-23956861. Max. coverage (+): 0. Max coverage (-): 0

Region: chr19 23956862-23956872. Max. coverage (+): 0. Max coverage (-): 0

Region: chr19 23956873-23956882. Max. coverage (+): 0. Max coverage (-): 0

Region: chr19 23956883-23956893. Max. coverage (+): 0. Max coverage (-): 0

Region: chr19 23956894-23956903. Max. coverage (+): 0. Max coverage (-): 0

Region: chr19 23956904-23956913. Max. coverage (+): 0. Max coverage (-): 0

Region: chr19 23956914-23956924. Max. coverage (+): 0. Max coverage (-): 0

Region: chr19 23956925-23956934. Max. coverage (+): 0. Max coverage (-): 0

Region: chr19 23956935-23956945. Max. coverage (+): 0. Max coverage (-): 0

Region: chr19 23956946-23956955. Max. coverage (+): 0. Max coverage (-): 0

Region: chr19 23956956-23956966. Max. coverage (+): 0. Max coverage (-): 0

Region: chr19 23956967-23956976. Max. coverage (+): 0. Max coverage (-): 0

Region: chr19 23956977-23956986. Max. coverage (+): 0. Max coverage (-): 1.49

Region: chr19 23956987-23956997. Max. coverage (+): 0. Max coverage (-): 1.49

Region: chr19 23956998-23957007. Max. coverage (+): 0. Max coverage (-): 0

Region: chr19 23957008-23957018. Max. coverage (+): 0. Max coverage (-): 0

Region: chr19 23957019-23957028. Max. coverage (+): 0. Max coverage (-): 0

Region: chr19 23957029-23957039. Max. coverage (+): 0. Max coverage (-): 0

Region: chr19 23957040-23957049. Max. coverage (+): 0. Max coverage (-): 0

Region: chr19 23957050-23957059. Max. coverage (+): 0. Max coverage (-): 0

Region: chr19 23957060-23957070. Max. coverage (+): 0. Max coverage (-): 0

Region: chr19 23957071-23957080. Max. coverage (+): 0. Max coverage (-): 0

Region: chr19 23957081-23957091. Max. coverage (+): 0. Max coverage (-): 0

Region: chr19 23957092-23957101. Max. coverage (+): 0. Max coverage (-): 0

Region: chr19 23957102-23957112. Max. coverage (+): 0. Max coverage (-): 0

Region: chr19 23957113-23957122. Max. coverage (+): 0. Max coverage (-): 0

Region: chr19 23957123-23957132. Max. coverage (+): 0. Max coverage (-): 0

Region: chr19 23957133-23957143. Max. coverage (+): 0. Max coverage (-): 0

Region: chr19 23957144-23957153. Max. coverage (+): 0. Max coverage (-): 0

Region: chr19 23957154-23957164. Max. coverage (+): 0. Max coverage (-): 0

Region: chr19 23957165-23957174. Max. coverage (+): 0. Max coverage (-): 0

Region: chr19 23957175-23957185. Max. coverage (+): 0. Max coverage (-): 0

Region: chr19 23957186-23957195. Max. coverage (+): 0. Max coverage (-): 0

Region: chr19 23957196-23957205. Max. coverage (+): 0. Max coverage (-): 0

Region: chr19 23957206-23957216. Max. coverage (+): 0. Max coverage (-): 0

Region: chr19 23957217-23957226. Max. coverage (+): 0. Max coverage (-): 0

Region: chr19 23957227-23957237. Max. coverage (+): 0. Max coverage (-): 0

Region: chr19 23957238-23957247. Max. coverage (+): 0. Max coverage (-): 0

Region: chr19 23957248-23957258. Max. coverage (+): 0. Max coverage (-): 3.77

Region: chr19 23957259-23957268. Max. coverage (+): 0. Max coverage (-): 0

Region: chr19 23957269-23957278. Max. coverage (+): 0. Max coverage (-): 0

Region: chr19 23957279-23957289. Max. coverage (+): 0. Max coverage (-): 0

Region: chr19 23957290-23957299. Max. coverage (+): 0. Max coverage (-): 0

Region: chr19 23957300-23957310. Max. coverage (+): 0. Max coverage (-): 0

Region: chr19 23957311-23957320. Max. coverage (+): 0. Max coverage (-): 5.92

Region: chr19 23957321-23957331. Max. coverage (+): 0. Max coverage (-): 5.92

Region: chr19 23957332-23957341. Max. coverage (+): 0. Max coverage (-): 0

Region: chr19 23957342-23957351. Max. coverage (+): 0. Max coverage (-): 0

Region: chr19 23957352-23957362. Max. coverage (+): 0. Max coverage (-): 0

Region: chr19 23957363-23957372. Max. coverage (+): 0. Max coverage (-): 0

Region: chr19 23957373-23957383. Max. coverage (+): 0. Max coverage (-): 0

Region: chr19 23957384-23957393. Max. coverage (+): 0. Max coverage (-): 0

Region: chr19 23957394-23957404. Max. coverage (+): 0. Max coverage (-): 0

Region: chr19 23957405-23957414. Max. coverage (+): 0. Max coverage (-): 0

Region: chr19 23957415-23957424. Max. coverage (+): 0. Max coverage (-): 0

Region: chr19 23957425-23957435. Max. coverage (+): 0. Max coverage (-): 0

Region: chr19 23957436-23957445. Max. coverage (+): 0. Max coverage (-): 0

Region: chr19 23957446-23957456. Max. coverage (+): 0. Max coverage (-): 0

Region: chr19 23957457-23957466. Max. coverage (+): 0. Max coverage (-): 0

Region: chr19 23957467-23957477. Max. coverage (+): 0. Max coverage (-): 0

Region: chr19 23957478-23957487. Max. coverage (+): 0. Max coverage (-): 0

Region: chr19 23957488-23957497. Max. coverage (+): 0. Max coverage (-): 3.85

Region: chr19 23957498-23957508. Max. coverage (+): 0. Max coverage (-): 3.85

Region: chr19 23957509-23957518. Max. coverage (+): 0. Max coverage (-): 0

Region: chr19 23957519-23957529. Max. coverage (+): 0. Max coverage (-): 0

Region: chr19 23957530-23957539. Max. coverage (+): 0. Max coverage (-): 0

Region: chr19 23957540-23957549. Max. coverage (+): 0. Max coverage (-): 0

Region: chr19 23957550-23957560. Max. coverage (+): 0. Max coverage (-): 0

Region: chr19 23957561-23957570. Max. coverage (+): 0. Max coverage (-): 0

Region: chr19 23957571-23957581. Max. coverage (+): 0. Max coverage (-): 0

Region: chr19 23957582-23957591. Max. coverage (+): 0. Max coverage (-): 0

Region: chr19 23957592-23957602. Max. coverage (+): 0. Max coverage (-): 0

Region: chr19 23957603-23957612. Max. coverage (+): 0. Max coverage (-): 0

Region: chr19 23957613-23957622. Max. coverage (+): 0. Max coverage (-): 0

Region: chr19 23957623-23957633. Max. coverage (+): 0. Max coverage (-): 0

Region: chr19 23957634-23957643. Max. coverage (+): 0. Max coverage (-): 0

Region: chr19 23957644-23957654. Max. coverage (+): 0. Max coverage (-): 0

Region: chr19 23957655-23957664. Max. coverage (+): 0. Max coverage (-): 0

Region: chr19 23957665-23957675. Max. coverage (+): 0. Max coverage (-): 0

Region: chr19 23957676-23957685. Max. coverage (+): 0. Max coverage (-): 0

Region: chr19 23957686-23957695. Max. coverage (+): 0. Max coverage (-): 0

Region: chr19 23957696-23957706. Max. coverage (+): 0. Max coverage (-): 0

Region: chr19 23957707-23957716. Max. coverage (+): 0. Max coverage (-): 0

Region: chr19 23957717-23957727. Max. coverage (+): 0. Max coverage (-): 0

Region: chr19 23957728-23957737. Max. coverage (+): 0. Max coverage (-): 0

Region: chr19 23957738-23957748. Max. coverage (+): 0. Max coverage (-): 0

Region: chr19 23957749-23957758. Max. coverage (+): 0. Max coverage (-): 0

Region: chr19 23957759-23957768. Max. coverage (+): 0. Max coverage (-): 0

Region: chr19 23957769-23957779. Max. coverage (+): 0. Max coverage (-): 0

Region: chr19 23957780-23957789. Max. coverage (+): 0. Max coverage (-): 0

Region: chr19 23957790-23957800. Max. coverage (+): 0. Max coverage (-): 0

Region: chr19 23957801-23957810. Max. coverage (+): 0. Max coverage (-): 0

Region: chr19 23957811-23957821. Max. coverage (+): 0. Max coverage (-): 0

Region: chr19 23957822-23957831. Max. coverage (+): 0. Max coverage (-): 0

Region: chr19 23957832-23957841. Max. coverage (+): 0. Max coverage (-): 0

Region: chr19 23957842-23957852. Max. coverage (+): 0. Max coverage (-): 0

Region: chr19 23957853-23957862. Max. coverage (+): 0. Max coverage (-): 0

Region: chr19 23957863-23957873. Max. coverage (+): 0. Max coverage (-): 0

Region: chr19 23957874-23957883. Max. coverage (+): 0. Max coverage (-): 0

Region: chr19 23957884-23957894. Max. coverage (+): 0. Max coverage (-): 0

Region: chr19 23957895-23957904. Max. coverage (+): 0. Max coverage (-): 0

Region: chr19 23957905-23957914. Max. coverage (+): 0. Max coverage (-): 0

Region: chr19 23957915-23957925. Max. coverage (+): 0. Max coverage (-): 0.75

Region: chr19 23957926-23957935. Max. coverage (+): 0. Max coverage (-): 0.75

Region: chr19 23957936-23957946. Max. coverage (+): 0. Max coverage (-): 2.13

Region: chr19 23957947-23957956. Max. coverage (+): 0. Max coverage (-): 0

Region: chr19 23957957-23957967. Max. coverage (+): 0. Max coverage (-): 0

Region: chr19 23957968-23957977. Max. coverage (+): 0. Max coverage (-): 0

Region: chr19 23957978-23957987. Max. coverage (+): 0. Max coverage (-): 0

Region: chr19 23957988-23957998. Max. coverage (+): 0. Max coverage (-): 0

Region: chr19 23957999-23958008. Max. coverage (+): 0. Max coverage (-): 0

Region: chr19 23958009-23958019. Max. coverage (+): 0. Max coverage (-): 0

Region: chr19 23958020-23958029. Max. coverage (+): 0. Max coverage (-): 0

Region: chr19 23958030-23958040. Max. coverage (+): 0. Max coverage (-): 0

Region: chr19 23958041-23958050. Max. coverage (+): 0. Max coverage (-): 0

Region: chr19 23958051-23958060. Max. coverage (+): 0. Max coverage (-): 0

Region: chr19 23958061-23958071. Max. coverage (+): 0. Max coverage (-): 0

Region: chr19 23958072-23958081. Max. coverage (+): 0. Max coverage (-): 0

Region: chr19 23958082-23958092. Max. coverage (+): 0. Max coverage (-): 0

Region: chr19 23958093-23958102. Max. coverage (+): 0. Max coverage (-): 0

Region: chr19 23958103-23958113. Max. coverage (+): 0. Max coverage (-): 0

Region: chr19 23958114-23958123. Max. coverage (+): 0. Max coverage (-): 0

Region: chr19 23958124-23958133. Max. coverage (+): 0. Max coverage (-): 0

Region: chr19 23958134-23958144. Max. coverage (+): 0. Max coverage (-): 0

Region: chr19 23958145-23958154. Max. coverage (+): 0. Max coverage (-): 0

Region: chr19 23958155-23958165. Max. coverage (+): 0. Max coverage (-): 0

Region: chr19 23958166-23958175. Max. coverage (+): 0. Max coverage (-): 0

Region: chr19 23958176-23958186. Max. coverage (+): 0. Max coverage (-): 0

Region: chr19 23958187-23958196. Max. coverage (+): 0. Max coverage (-): 0

Region: chr19 23958197-23958206. Max. coverage (+): 0. Max coverage (-): 0

Region: chr19 23958207-23958217. Max. coverage (+): 0. Max coverage (-): 4.2

Region: chr19 23958218-23958227. Max. coverage (+): 0. Max coverage (-): 0

Region: chr19 23958228-23958238. Max. coverage (+): 0. Max coverage (-): 0

Region: chr19 23958239-23958248. Max. coverage (+): 0. Max coverage (-): 0

Region: chr19 23958249-23958259. Max. coverage (+): 0. Max coverage (-): 0

Region: chr19 23958260-23958269. Max. coverage (+): 0. Max coverage (-): 0

Region: chr19 23958270-23958279. Max. coverage (+): 0. Max coverage (-): 0

Region: chr19 23958280-23958290. Max. coverage (+): 0. Max coverage (-): 0

Region: chr19 23958291-23958300. Max. coverage (+): 0. Max coverage (-): 0

Region: chr19 23958301-23958311. Max. coverage (+): 0. Max coverage (-): 0

Region: chr19 23958312-23958321. Max. coverage (+): 0. Max coverage (-): 0

Region: chr19 23958322-23958332. Max. coverage (+): 0. Max coverage (-): 0

Region: chr19 23958333-23958342. Max. coverage (+): 0. Max coverage (-): 0

Region: chr19 23958343-23958352. Max. coverage (+): 0. Max coverage (-): 0

Region: chr19 23958353-23958363. Max. coverage (+): 0. Max coverage (-): 0

Region: chr19 23958364-23958373. Max. coverage (+): 0. Max coverage (-): 0

Region: chr19 23958374-23958384. Max. coverage (+): 0. Max coverage (-): 0

Region: chr19 23958385-23958394. Max. coverage (+): 0. Max coverage (-): 0

Region: chr19 23958395-23958405. Max. coverage (+): 0. Max coverage (-): 0

Region: chr19 23958406-23958415. Max. coverage (+): 0. Max coverage (-): 0

Region: chr19 23958416-23958425. Max. coverage (+): 0. Max coverage (-): 0

Region: chr19 23958426-23958436. Max. coverage (+): 0. Max coverage (-): 0

Region: chr19 23958437-23958446. Max. coverage (+): 0. Max coverage (-): 0

Region: chr19 23958447-23958457. Max. coverage (+): 0. Max coverage (-): 0

Region: chr19 23958458-23958467. Max. coverage (+): 0. Max coverage (-): 0

Region: chr19 23958468-23958478. Max. coverage (+): 0. Max coverage (-): 0

Region: chr19 23958479-23958488. Max. coverage (+): 0. Max coverage (-): 0

Region: chr19 23958489-23958498. Max. coverage (+): 0. Max coverage (-): 0

Region: chr19 23958499-23958509. Max. coverage (+): 0. Max coverage (-): 0

Region: chr19 23958510-23958519. Max. coverage (+): 0. Max coverage (-): 0.27

Region: chr19 23958520-23958530. Max. coverage (+): 0. Max coverage (-): 0.27

Region: chr19 23958531-23958540. Max. coverage (+): 0. Max coverage (-): 0

Region: chr19 23958541-23958551. Max. coverage (+): 0. Max coverage (-): 0

Region: chr19 23958552-23958561. Max. coverage (+): 0. Max coverage (-): 0

Region: chr19 23958562-23958571. Max. coverage (+): 0. Max coverage (-): 0

Region: chr19 23958572-23958582. Max. coverage (+): 0. Max coverage (-): 0

Region: chr19 23958583-23958592. Max. coverage (+): 0. Max coverage (-): 0

Region: chr19 23958593-23958603. Max. coverage (+): 0. Max coverage (-): 0

Region: chr19 23958604-23958613. Max. coverage (+): 0. Max coverage (-): 0

Region: chr19 23958614-23958624. Max. coverage (+): 0. Max coverage (-): 0

Region: chr19 23958625-23958634. Max. coverage (+): 0. Max coverage (-): 0

Region: chr19 23958635-23958644. Max. coverage (+): 0. Max coverage (-): 0

Region: chr19 23958645-23958655. Max. coverage (+): 0. Max coverage (-): 0

Region: chr19 23958656-23958665. Max. coverage (+): 0. Max coverage (-): 0

Region: chr19 23958666-23958676. Max. coverage (+): 0. Max coverage (-): 0

Region: chr19 23958677-23958686. Max. coverage (+): 0. Max coverage (-): 0

Region: chr19 23958687-23958697. Max. coverage (+): 0. Max coverage (-): 0

Region: chr19 23958698-23958707. Max. coverage (+): 0. Max coverage (-): 0

Region: chr19 23958708-23958717. Max. coverage (+): 0. Max coverage (-): 0

Region: chr19 23958718-23958728. Max. coverage (+): 0. Max coverage (-): 0

Region: chr19 23958729-23958738. Max. coverage (+): 0. Max coverage (-): 0

Region: chr19 23958739-23958749. Max. coverage (+): 0. Max coverage (-): 0

Region: chr19 23958750-23958759. Max. coverage (+): 0. Max coverage (-): 0

Region: chr19 23958760-23958770. Max. coverage (+): 0. Max coverage (-): 0.86

Region: chr19 23958771-23958780. Max. coverage (+): 0. Max coverage (-): 0

Region: chr19 23958781-23958790. Max. coverage (+): 0. Max coverage (-): 0

Region: chr19 23958791-23958801. Max. coverage (+): 0. Max coverage (-): 0

Region: chr19 23958802-23958811. Max. coverage (+): 0. Max coverage (-): 0

Region: chr19 23958812-23958822. Max. coverage (+): 0. Max coverage (-): 0

Region: chr19 23958823-23958832. Max. coverage (+): 0. Max coverage (-): 0

Region: chr19 23958833-23958843. Max. coverage (+): 0. Max coverage (-): 0

Region: chr19 23958844-23958853. Max. coverage (+): 0. Max coverage (-): 0

Region: chr19 23958854-23958863. Max. coverage (+): 0. Max coverage (-): 0

Region: chr19 23958864-23958874. Max. coverage (+): 0. Max coverage (-): 0

Region: chr19 23958875-23958884. Max. coverage (+): 0. Max coverage (-): 0

Region: chr19 23958885-23958895. Max. coverage (+): 0. Max coverage (-): 0

Region: chr19 23958896-23958905. Max. coverage (+): 0. Max coverage (-): 5.26

Region: chr19 23958906-23958916. Max. coverage (+): 0. Max coverage (-): 0

Region: chr19 23958917-23958926. Max. coverage (+): 0. Max coverage (-): 0

Region: chr19 23958927-23958936. Max. coverage (+): 0. Max coverage (-): 0

Region: chr19 23958937-23958947. Max. coverage (+): 0. Max coverage (-): 0

Region: chr19 23958948-23958957. Max. coverage (+): 0. Max coverage (-): 0

Region: chr19 23958958-23958968. Max. coverage (+): 0. Max coverage (-): 2.75

Region: chr19 23958969-23958978. Max. coverage (+): 0. Max coverage (-): 0

Region: chr19 23958979-23958989. Max. coverage (+): 0. Max coverage (-): 0

Region: chr19 23958990-23958999. Max. coverage (+): 0. Max coverage (-): 0

Region: chr19 23959000-23959009. Max. coverage (+): 0. Max coverage (-): 0

Region: chr19 23959010-23959020. Max. coverage (+): 0. Max coverage (-): 1.55

Region: chr19 23959021-23959030. Max. coverage (+): 0. Max coverage (-): 1.55

Region: chr19 23959031-23959041. Max. coverage (+): 0. Max coverage (-): 0

Region: chr19 23959042-23959051. Max. coverage (+): 0. Max coverage (-): 0

Region: chr19 23959052-23959062. Max. coverage (+): 0. Max coverage (-): 0

Region: chr19 23959063-23959072. Max. coverage (+): 0. Max coverage (-): 0

Region: chr19 23959073-23959082. Max. coverage (+): 0. Max coverage (-): 0

Region: chr19 23959083-23959093. Max. coverage (+): 0. Max coverage (-): 0

Region: chr19 23959094-23959103. Max. coverage (+): 0. Max coverage (-): 0

Region: chr19 23959104-23959114. Max. coverage (+): 0. Max coverage (-): 0

Region: chr19 23959115-23959124. Max. coverage (+): 0. Max coverage (-): 0

Region: chr19 23959125-23959135. Max. coverage (+): 0. Max coverage (-): 0

Region: chr19 23959136-23959145. Max. coverage (+): 0. Max coverage (-): 0

Region: chr19 23959146-23959155. Max. coverage (+): 0. Max coverage (-): 0

Region: chr19 23959156-23959166. Max. coverage (+): 0. Max coverage (-): 0

Region: chr19 23959167-23959176. Max. coverage (+): 0. Max coverage (-): 0

Region: chr19 23959177-23959187. Max. coverage (+): 0. Max coverage (-): 0

Region: chr19 23959188-23959197. Max. coverage (+): 0. Max coverage (-): 0

Region: chr19 23959198-23959208. Max. coverage (+): 0. Max coverage (-): 0

Region: chr19 23959209-23959218. Max. coverage (+): 0. Max coverage (-): 0

Region: chr19 23959219-23959228. Max. coverage (+): 0. Max coverage (-): 0

Region: chr19 23959229-23959239. Max. coverage (+): 0. Max coverage (-): 0

Region: chr19 23959240-23959249. Max. coverage (+): 0. Max coverage (-): 0

Region: chr19 23959250-23959260. Max. coverage (+): 0. Max coverage (-): 0

Region: chr19 23959261-23959270. Max. coverage (+): 0. Max coverage (-): 0

Region: chr19 23959271-23959281. Max. coverage (+): 0. Max coverage (-): 0

Region: chr19 23959282-23959291. Max. coverage (+): 0. Max coverage (-): 0

Region: chr19 23959292-23959301. Max. coverage (+): 0. Max coverage (-): 0

Region: chr19 23959302-23959312. Max. coverage (+): 0. Max coverage (-): 0

Region: chr19 23959313-23959322. Max. coverage (+): 0. Max coverage (-): 0

Region: chr19 23959323-23959333. Max. coverage (+): 0. Max coverage (-): 0

Region: chr19 23959334-23959343. Max. coverage (+): 0. Max coverage (-): 0

Region: chr19 23959344-23959354. Max. coverage (+): 0. Max coverage (-): 5.16

Region: chr19 23959355-23959364. Max. coverage (+): 0. Max coverage (-): 5.16

Region: chr19 23959365-23959374. Max. coverage (+): 0. Max coverage (-): 0

Region: chr19 23959375-23959385. Max. coverage (+): 0. Max coverage (-): 0

Region: chr19 23959386-23959395. Max. coverage (+): 0. Max coverage (-): 4.07

Region: chr19 23959396-23959406. Max. coverage (+): 0. Max coverage (-): 5.04

Region: chr19 23959407-23959416. Max. coverage (+): 0. Max coverage (-): 3.65

Region: chr19 23959417-23959427. Max. coverage (+): 0. Max coverage (-): 0

Region: chr19 23959428-23959437. Max. coverage (+): 0. Max coverage (-): 2.26

Region: chr19 23959438-23959447. Max. coverage (+): 0. Max coverage (-): 2.26

Region: chr19 23959448-23959458. Max. coverage (+): 0. Max coverage (-): 0

Region: chr19 23959459-23959468. Max. coverage (+): 0. Max coverage (-): 0

Region: chr19 23959469-23959479. Max. coverage (+): 0. Max coverage (-): 0

Region: chr19 23959480-23959489. Max. coverage (+): 0. Max coverage (-): 0

Region: chr19 23959490-23959500. Max. coverage (+): 0. Max coverage (-): 0

Region: chr19 23959501-23959510. Max. coverage (+): 0. Max coverage (-): 0

Region: chr19 23959511-23959520. Max. coverage (+): 0. Max coverage (-): 0

Region: chr19 23959521-23959531. Max. coverage (+): 0. Max coverage (-): 0

Region: chr19 23959532-23959541. Max. coverage (+): 0. Max coverage (-): 0

Region: chr19 23959542-23959552. Max. coverage (+): 0. Max coverage (-): 0

Region: chr19 23959553-23959562. Max. coverage (+): 0. Max coverage (-): 0

Region: chr19 23959563-23959573. Max. coverage (+): 0. Max coverage (-): 0

Region: chr19 23959574-23959583. Max. coverage (+): 0. Max coverage (-): 0

Region: chr19 23959584-23959593. Max. coverage (+): 0. Max coverage (-): 0

Region: chr19 23959594-23959604. Max. coverage (+): 0. Max coverage (-): 0

Region: chr19 23959605-23959614. Max. coverage (+): 0. Max coverage (-): 0

Region: chr19 23959615-23959625. Max. coverage (+): 0. Max coverage (-): 0

Region: chr19 23959626-23959635. Max. coverage (+): 0. Max coverage (-): 0

Region: chr19 23959636-23959646. Max. coverage (+): 0. Max coverage (-): 0

Region: chr19 23959647-23959656. Max. coverage (+): 0. Max coverage (-): 0

Region: chr19 23959657-23959666. Max. coverage (+): 0. Max coverage (-): 0

Region: chr19 23959667-23959677. Max. coverage (+): 0. Max coverage (-): 0

Region: chr19 23959678-23959687. Max. coverage (+): 0. Max coverage (-): 0

Region: chr19 23959688-23959698. Max. coverage (+): 0. Max coverage (-): 0

Region: chr19 23959699-23959708. Max. coverage (+): 0. Max coverage (-): 0

Region: chr19 23959709-23959719. Max. coverage (+): 0. Max coverage (-): 0

Region: chr19 23959720-23959729. Max. coverage (+): 0. Max coverage (-): 0

Region: chr19 23959730-23959739. Max. coverage (+): 0. Max coverage (-): 0

Region: chr19 23959740-23959750. Max. coverage (+): 0. Max coverage (-): 0

Region: chr19 23959751-23959760. Max. coverage (+): 0. Max coverage (-): 0

Region: chr19 23959761-23959771. Max. coverage (+): 0. Max coverage (-): 0

Region: chr19 23959772-23959781. Max. coverage (+): 0. Max coverage (-): 0

Region: chr19 23959782-23959792. Max. coverage (+): 0. Max coverage (-): 0

Region: chr19 23959793-23959802. Max. coverage (+): 0. Max coverage (-): 0

Region: chr19 23959803-23959812. Max. coverage (+): 0. Max coverage (-): 0

Region: chr19 23959813-23959823. Max. coverage (+): 0. Max coverage (-): 0

Region: chr19 23959824-23959833. Max. coverage (+): 0. Max coverage (-): 0

Region: chr19 23959834-23959844. Max. coverage (+): 0. Max coverage (-): 0

Region: chr19 23959845-23959854. Max. coverage (+): 0. Max coverage (-): 0

Region: chr19 23959855-23959865. Max. coverage (+): 0. Max coverage (-): 1.16

Region: chr19 23959866-23959875. Max. coverage (+): 0. Max coverage (-): 1.16

Region: chr19 23959876-23959885. Max. coverage (+): 0. Max coverage (-): 0

Region: chr19 23959886-23959896. Max. coverage (+): 0. Max coverage (-): 0

Region: chr19 23959897-23959906. Max. coverage (+): 0. Max coverage (-): 0

Region: chr19 23959907-23959917. Max. coverage (+): 0. Max coverage (-): 0

Region: chr19 23959918-23959927. Max. coverage (+): 0. Max coverage (-): 0

Region: chr19 23959928-23959938. Max. coverage (+): 0. Max coverage (-): 0

Region: chr19 23959939-23959948. Max. coverage (+): 0. Max coverage (-): 0

Region: chr19 23959949-23959958. Max. coverage (+): 0. Max coverage (-): 0

Region: chr19 23959959-23959969. Max. coverage (+): 0. Max coverage (-): 0

Region: chr19 23959970-23959979. Max. coverage (+): 0. Max coverage (-): 0

Region: chr19 23959980-23959990. Max. coverage (+): 0. Max coverage (-): 0

Region: chr19 23959991-23960000. Max. coverage (+): 0. Max coverage (-): 0

Region: chr19 23960001-23960011. Max. coverage (+): 0. Max coverage (-): 0

Region: chr19 23960012-23960021. Max. coverage (+): 0. Max coverage (-): 0

Region: chr19 23960022-23960031. Max. coverage (+): 0. Max coverage (-): 0

Region: chr19 23960032-23960042. Max. coverage (+): 0. Max coverage (-): 0

Region: chr19 23960043-23960052. Max. coverage (+): 0. Max coverage (-): 0

Region: chr19 23960053-23960063. Max. coverage (+): 0. Max coverage (-): 0

Region: chr19 23960064-23960073. Max. coverage (+): 0. Max coverage (-): 0

Region: chr19 23960074-23960084. Max. coverage (+): 0. Max coverage (-): 0

Region: chr19 23960085-23960094. Max. coverage (+): 0. Max coverage (-): 1.94

Region: chr19 23960095-23960104. Max. coverage (+): 0. Max coverage (-): 1.94

Region: chr19 23960105-23960115. Max. coverage (+): 0. Max coverage (-): 0

Region: chr19 23960116-23960125. Max. coverage (+): 0. Max coverage (-): 0

Region: chr19 23960126-23960136. Max. coverage (+): 0. Max coverage (-): 0

Region: chr19 23960137-23960146. Max. coverage (+): 0. Max coverage (-): 0

Region: chr19 23960147-23960156. Max. coverage (+): 0. Max coverage (-): 0

Region: chr19 23960157-23960167. Max. coverage (+): 0. Max coverage (-): 7.79

Region: chr19 23960168-23960177. Max. coverage (+): 0. Max coverage (-): 2.53

Region: chr19 23960178-23960188. Max. coverage (+): 0. Max coverage (-): 0

Region: chr19 23960189-23960198. Max. coverage (+): 0. Max coverage (-): 0

Region: chr19 23960199-23960209. Max. coverage (+): 0. Max coverage (-): 0

Region: chr19 23960210-23960219. Max. coverage (+): 0. Max coverage (-): 0

Region: chr19 23960220-23960229. Max. coverage (+): 0. Max coverage (-): 0

Region: chr19 23960230-23960240. Max. coverage (+): 0. Max coverage (-): 0

Region: chr19 23960241-23960250. Max. coverage (+): 0. Max coverage (-): 0

Region: chr19 23960251-23960261. Max. coverage (+): 0. Max coverage (-): 0

Region: chr19 23960262-23960271. Max. coverage (+): 0. Max coverage (-): 0

Region: chr19 23960272-23960282. Max. coverage (+): 0. Max coverage (-): 0

Region: chr19 23960283-23960292. Max. coverage (+): 0. Max coverage (-): 0

Region: chr19 23960293-23960302. Max. coverage (+): 0. Max coverage (-): 0

Region: chr19 23960303-23960313. Max. coverage (+): 0. Max coverage (-): 0

Region: chr19 23960314-23960323. Max. coverage (+): 0. Max coverage (-): 0

Region: chr19 23960324-23960334. Max. coverage (+): 0. Max coverage (-): 0

Region: chr19 23960335-23960344. Max. coverage (+): 0. Max coverage (-): 0

Region: chr19 23960345-23960355. Max. coverage (+): 0. Max coverage (-): 0

Region: chr19 23960356-23960365. Max. coverage (+): 0. Max coverage (-): 0

Region: chr19 23960366-23960375. Max. coverage (+): 0. Max coverage (-): 0

Region: chr19 23960376-23960386. Max. coverage (+): 0. Max coverage (-): 0

Region: chr19 23960387-23960396. Max. coverage (+): 0. Max coverage (-): 0.93

Region: chr19 23960397-23960407. Max. coverage (+): 0. Max coverage (-): 0.93

Region: chr19 23960408-23960417. Max. coverage (+): 0. Max coverage (-): 0

Region: chr19 23960418-23960428. Max. coverage (+): 0. Max coverage (-): 0

Region: chr19 23960429-23960438. Max. coverage (+): 0. Max coverage (-): 0

Region: chr19 23960439-23960448. Max. coverage (+): 0. Max coverage (-): 0

Region: chr19 23960449-23960459. Max. coverage (+): 0. Max coverage (-): 0

Region: chr19 23960460-23960469. Max. coverage (+): 0. Max coverage (-): 0

Region: chr19 23960470-23960480. Max. coverage (+): 0. Max coverage (-): 0

Region: chr19 23960481-23960490. Max. coverage (+): 0. Max coverage (-): 0

Region: chr19 23960491-23960501. Max. coverage (+): 0. Max coverage (-): 0

Region: chr19 23960502-23960511. Max. coverage (+): 0. Max coverage (-): 1.35

Region: chr19 23960512-23960521. Max. coverage (+): 0. Max coverage (-): 1.35

Region: chr19 23960522-23960532. Max. coverage (+): 0. Max coverage (-): 0

Region: chr19 23960533-23960542. Max. coverage (+): 0. Max coverage (-): 0

Region: chr19 23960543-23960553. Max. coverage (+): 0. Max coverage (-): 0

Region: chr19 23960554-23960563. Max. coverage (+): 0. Max coverage (-): 4.14

Region: chr19 23960564-23960574. Max. coverage (+): 0. Max coverage (-): 4.14

Region: chr19 23960575-23960584. Max. coverage (+): 0. Max coverage (-): 0

Region: chr19 23960585-23960594. Max. coverage (+): 0. Max coverage (-): 1.86

Region: chr19 23960595-23960605. Max. coverage (+): 0. Max coverage (-): 1.86

Region: chr19 23960606-23960615. Max. coverage (+): 0. Max coverage (-): 1.01

Region: chr19 23960616-23960626. Max. coverage (+): 0. Max coverage (-): 0

Region: chr19 23960627-23960636. Max. coverage (+): 0. Max coverage (-): 0

Region: chr19 23960637-23960647. Max. coverage (+): 0. Max coverage (-): 0

Region: chr19 23960648-23960657. Max. coverage (+): 0. Max coverage (-): 0

Region: chr19 23960658-23960667. Max. coverage (+): 0. Max coverage (-): 0

Region: chr19 23960668-23960678. Max. coverage (+): 0. Max coverage (-): 0

Region: chr19 23960679-23960688. Max. coverage (+): 0. Max coverage (-): 0

Region: chr19 23960689-23960699. Max. coverage (+): 0. Max coverage (-): 0

Region: chr19 23960700-23960709. Max. coverage (+): 0. Max coverage (-): 0

Region: chr19 23960710-23960720. Max. coverage (+): 0. Max coverage (-): 0

Region: chr19 23960721-23960730. Max. coverage (+): 0. Max coverage (-): 1.92

Region: chr19 23960731-23960740. Max. coverage (+): 0. Max coverage (-): 1.55

Region: chr19 23960741-23960751. Max. coverage (+): 0. Max coverage (-): 3.8

Region: chr19 23960752-23960761. Max. coverage (+): 0. Max coverage (-): 3.8

Region: chr19 23960762-23960772. Max. coverage (+): 0. Max coverage (-): 0

Region: chr19 23960773-23960782. Max. coverage (+): 0. Max coverage (-): 0

Region: chr19 23960783-23960793. Max. coverage (+): 0. Max coverage (-): 0

Region: chr19 23960794-23960803. Max. coverage (+): 0. Max coverage (-): 0

Region: chr19 23960804-23960813. Max. coverage (+): 0. Max coverage (-): 0

Region: chr19 23960814-23960824. Max. coverage (+): 0. Max coverage (-): 0

Region: chr19 23960825-23960834. Max. coverage (+): 0. Max coverage (-): 0

Region: chr19 23960835-23960845. Max. coverage (+): 0. Max coverage (-): 0

Region: chr19 23960846-23960855. Max. coverage (+): 0. Max coverage (-): 1

Region: chr19 23960856-23960866. Max. coverage (+): 0. Max coverage (-): 0

Region: chr19 23960867-23960876. Max. coverage (+): 0. Max coverage (-): 1.82

Region: chr19 23960877-23960886. Max. coverage (+): 0. Max coverage (-): 19.84

Region: chr19 23960887-23960897. Max. coverage (+): 0. Max coverage (-): 2.71

Region: chr19 23960898-23960907. Max. coverage (+): 0. Max coverage (-): 0

Region: chr19 23960908-23960918. Max. coverage (+): 0. Max coverage (-): 0

Region: chr19 23960919-23960928. Max. coverage (+): 0. Max coverage (-): 1.51

Region: chr19 23960929-23960939. Max. coverage (+): 0. Max coverage (-): 1.51

Region: chr19 23960940-23960949. Max. coverage (+): 0. Max coverage (-): 0

Region: chr19 23960950-23960959. Max. coverage (+): 0. Max coverage (-): 0

Region: chr19 23960960-23960970. Max. coverage (+): 0. Max coverage (-): 0

Region: chr19 23960971-23960980. Max. coverage (+): 0. Max coverage (-): 0

Region: chr19 23960981-23960991. Max. coverage (+): 0. Max coverage (-): 0

Region: chr19 23960992-23961001. Max. coverage (+): 0. Max coverage (-): 0

Region: chr19 23961002-23961012. Max. coverage (+): 0. Max coverage (-): 0

Region: chr19 23961013-23961022. Max. coverage (+): 0. Max coverage (-): 0

Region: chr19 23961023-23961032. Max. coverage (+): 0. Max coverage (-): 0

Region: chr19 23961033-23961043. Max. coverage (+): 0. Max coverage (-): 0

Region: chr19 23961044-23961053. Max. coverage (+): 0. Max coverage (-): 0

Region: chr19 23961054-23961064. Max. coverage (+): 0. Max coverage (-): 0

Region: chr19 23961065-23961074. Max. coverage (+): 0. Max coverage (-): 0

Region: chr19 23961075-23961085. Max. coverage (+): 0. Max coverage (-): 0

Region: chr19 23961086-23961095. Max. coverage (+): 0. Max coverage (-): 0

Region: chr19 23961096-23961105. Max. coverage (+): 0. Max coverage (-): 0

Region: chr19 23961106-23961116. Max. coverage (+): 0. Max coverage (-): 0

Region: chr19 23961117-23961126. Max. coverage (+): 0. Max coverage (-): 0

Region: chr19 23961127-23961137. Max. coverage (+): 0. Max coverage (-): 0

Region: chr19 23961138-23961147. Max. coverage (+): 0. Max coverage (-): 0

Region: chr19 23961148-23961158. Max. coverage (+): 0. Max coverage (-): 0

Region: chr19 23961159-23961168. Max. coverage (+): 0. Max coverage (-): 0

Region: chr19 23961169-23961178. Max. coverage (+): 0. Max coverage (-): 0

Region: chr19 23961179-23961189. Max. coverage (+): 0. Max coverage (-): 0

Region: chr19 23961190-23961199. Max. coverage (+): 0. Max coverage (-): 0

Region: chr19 23961200-23961210. Max. coverage (+): 0. Max coverage (-): 0

Region: chr19 23961211-23961220. Max. coverage (+): 0. Max coverage (-): 0

Region: chr19 23961221-23961231. Max. coverage (+): 0. Max coverage (-): 0

Region: chr19 23961232-23961241. Max. coverage (+): 0. Max coverage (-): 0

Region: chr19 23961242-23961251. Max. coverage (+): 0. Max coverage (-): 0

Region: chr19 23961252-23961262. Max. coverage (+): 0. Max coverage (-): 0

Region: chr19 23961263-23961272. Max. coverage (+): 0. Max coverage (-): 0

Region: chr19 23961273-23961283. Max. coverage (+): 0. Max coverage (-): 0

Region: chr19 23961284-23961293. Max. coverage (+): 0. Max coverage (-): 0

Region: chr19 23961294-23961304. Max. coverage (+): 0. Max coverage (-): 0

Region: chr19 23961305-23961314. Max. coverage (+): 0. Max coverage (-): 0

Region: chr19 23961315-23961324. Max. coverage (+): 0. Max coverage (-): 0

Region: chr19 23961325-23961335. Max. coverage (+): 0. Max coverage (-): 0

Region: chr19 23961336-23961345. Max. coverage (+): 0. Max coverage (-): 0

Region: chr19 23961346-23961356. Max. coverage (+): 0. Max coverage (-): 0

Region: chr19 23961357-23961366. Max. coverage (+): 0. Max coverage (-): 0

Region: chr19 23961367-23961377. Max. coverage (+): 0. Max coverage (-): 0

Region: chr19 23961378-23961387. Max. coverage (+): 0. Max coverage (-): 0

Region: chr19 23961388-23961397. Max. coverage (+): 0. Max coverage (-): 1.72

Region: chr19 23961398-23961408. Max. coverage (+): 0. Max coverage (-): 1.72

Region: chr19 23961409-23961418. Max. coverage (+): 0. Max coverage (-): 0

Region: chr19 23961419-. Max. coverage (+): 0. Max coverage (-): 0

RepeatMasker Color Code

**+**

100-98% Identity

<98-95% Identity

<95-90% Identity

<90-85% Identity

<85-80% Identity

<80-75% Identity

<75-70% Identity

<70% Identity

**-**

Gene Set Color Code

**+**

Gene

Pseudogene

**-**

Topology/Coverage Color Code

Coverage Plus Strand

Coverage Minus Strand

Mainstrand: Plus

Mainstrand: Minus

Complementary Strand

Flanking Region  
(if option -flank >0)

Gene Set Annotation  

**1. (protein coding, ENSBTAG00000017440) Tr:00000035153 Ex:10**: 23961074-23962022 (-)

  
RepeatMasker Annotation  

**1. L1ME4a**: 23959213-23959348 (+), Divergence to consensus: 43.3%  
**2. Bov-tA2**: 23960972-23961104 (-), Divergence to consensus: 9.5%

  
Transcription Factor Binding Sites  

**Gata4** (Sequence: CTTATCT (+): 23960231)
