# Supplementary material for: Small RNA sequencing of cryopreserved semen from single bull revealed altered miRNAs and piRNAs expression between High- and Low-motile sperm populations
Source: BMC Genomics. 2017 Jan 4;18:14. doi: 10.1186/s12864-016-3394-7 (PMC5209821; doi:10.1186/s12864-016-3394-7)
Supplement: Additional file 3: — Details for each piRNA clusters found in High Motile (HM) sperm fraction. Genes, repeats, transposable elements and transcription factors binding sites falling within the cluster regions were reported. (ZIP 1896 kb) [file 12864_2016_3394_MOESM3_ESM.zip › 41.html]

piRNA cluster 41


Predicted piRNA cluster no. 41     previous   next
  

Show proTRAC run info
Hide proTRAC run info

================================= proTRAC ====================================  
VERSION: 2.1                                    LAST MODIFIED: 06. October 2015  
  
Please cite:  
Rosenkranz D, Zischler H. proTRAC - a software for probabilistic piRNA cluster  
detection, visualization and analysis. 2012. BMC Bioinformatics 13:5.  
  
and (for proTRAC 2.0 and later):  
Rosenkranz D, Rudloff S, Bastuck K, Ketting RF, Zischler H. Tupaia small RNAs  
provide insights into function and evolution of RNAi-based transposon defense  
in mammals. 2015. RNA 21(5):911-922.  
  
Contact:  
David Rosenkranz  
Institute of Anthropology, small RNA group  
Johannes Gutenberg University Mainz  
email: rosenkranz@uni-mainz.de  
  
You can find the latest proTRAC version at:  
http://sourceforge.net/projects/protrac/files  
http://www.smallRNAgroup-mainz.de/software  
==============================================================================  
  
PARAMETERS:  
Map file: .............../storage/core/barbara/genhome/smallRNA/fertility/Sample\_motile/pirna/Sample\_motile\_26-33\_collapsed.fa.no-dust.map.weighted-10000-1000-b-0  
Genome file: ............/storage/core/barbara/genhome/smallRNA/fertility/Sample\_all/pirna/bt\_311\_chrY.fa  
RepeatMasker annotation: /storage/genomes/bt\_umd31/GCF\_000003055.6\_Bos\_taurus\_UMD\_3.1.1\_repeatMasker\_chr.out  
GeneSet:................./storage/core/barbara/genhome/smallRNA/fertility/Sample\_all/pirna/full.gtf  
  
Significant (p<=0.01) hit density will be calculated based  
on observed hit distribution.  
  
Sliding window size: ........................................ 5000 bp  
Sliding window increament: .................................. 1000 bp  
Normalize each hit by number of genomic hits: ............... 1 [0=no/1=yes]  
Normalize each hit by number of sequence reads: ............. 1 [0=no/1=yes]  
Normalize values (-> per million mapped reads): ............. 1 [0=no/1=yes]  
Min. fraction of hits with 1T(U) or 10A: .................... 0.75  
Alternatively: Min. fraction of hits with 1T(U) and 10A: .... 0.5  
Min. fraction of hits with typical piRNA length: ............ 0.75  
Typical piRNA length: ....................................... 26-33 nt  
Min. size of a piRNA cluster: ............................... 5000 bp.  
Min. number of hits (absolute): ............................. 0  
Min. number of hits (normalized): ........................... 0  
Min. fraction of hits on the mainstrand: .................... 0.75  
Top fraction of mapped sequences (in terms of read counts): . 1%  
Top fraction accounts for max. n% of sequence reads: ........ 90%  
Min. fraction of hits on each arm of a bidirectional cluster: 0.1  
Output image file for each cluster: ......................... 0 [0=no/1=yes]  
Output html file for each cluster: .......................... 1 [0=no/1=yes]  
Output a summary table: ..................................... 1 [0=no/1=yes]  
Output a FASTA file for each cluster (piRNA sequences): ..... 1 [0=no/1=yes]  
Output a FASTA file comprising cluster sequences: ........... 1 [0=no/1=yes]  
Search DNA motifs in clusters: .............................. 1 [0=no/1=yes]  
Output flanking sequences: +/- .............................. 0 bp  
Output ~.pTi file: .......................................... 1 [0=no/1=yes]  
==============================================================================  
  
  
Genome size (without gaps): ............ 2678902517 bp  
Gaps (N/X/-): .......................... 53837044 bp  
Mapped reads: .......................... 658825247023  
Non-identical sequences: ............... 514171  
Genomic hits: .......................... 764233  
Significant densitiy of mapped reads: .. 12867599.5173724 reads/kb

Show proTRAC cluster info
Hide proTRAC cluster info

|  |  |
| --- | --- |
| Location | chr19 |
| Coordinates | 34930297-34935881 |
| Size [bp] | 5585 |
| Sequence hit loci | 54 |
| Mapped reads (normalized) | 84678330 |
| Mapped reads (normalized) per kb | 15161742.2 |
| Normalized reads with 1T (1U) | 83.7% |
| Normalized reads with 10A | 37.4% |
| Normalized reads with length 26-33 nt | 100% |
| Normalized reads on the main strand(s) | 100% |
| Predicted directionality | mono:minus |

100%

0%

1T (1U)  
reads

10A reads

26-33 nt  
reads

reads on mainstrand

**Either the amount of reads with 1T (1U) OR 10A has to exceed 75% (set with option: -1Tor10A)  
Alternatively the amount of reads with 1T (1U) AND 10A has to exceed 50% (set with option: -1Tand10A)  
Minimum amount of reads with preferred size is 75% (set with option: -pisize)  
Minimum amount of reads on the main strand(s) is 75% (set with option: -clstrand)**

Show read coverage
Hide read coverage

WHAT DO I SEE HERE?  
This chart shows the location of mapped sequence reads within a predicted piRNA cluster. The color refers to the number of genomic hits produced by the sequence read in question. A dark red bar indicates that this sequence read produces many other hits elsewhere in the genome. Many adjacent red or yellow bars can indicate the presence of a multi-copy element such as transposons or rRNA genes. A dark green bar indicates that this sequence read maps uniquely to this locus.

1 hit

2-5 hits

6-10 hits

11-20 hits

21-50 hits

51-100 hits

> 100 hits

chr19

34930297

34935881

Gene Set

RepeatMasker

Mapped  
Reads

14.79

plus strand

minus strand

14.79

Region: chr19 23961420-34930302. Max. coverage (+): 0. Max coverage (-): 1.39

Region: chr19 34930303-34930313. Max. coverage (+): 0. Max coverage (-): 1.39

Region: chr19 34930314-34930324. Max. coverage (+): 0. Max coverage (-): 0

Region: chr19 34930325-34930336. Max. coverage (+): 0. Max coverage (-): 0

Region: chr19 34930337-34930347. Max. coverage (+): 0. Max coverage (-): 0

Region: chr19 34930348-34930358. Max. coverage (+): 0. Max coverage (-): 0

Region: chr19 34930359-34930369. Max. coverage (+): 0. Max coverage (-): 0

Region: chr19 34930370-34930380. Max. coverage (+): 0. Max coverage (-): 0

Region: chr19 34930381-34930391. Max. coverage (+): 0. Max coverage (-): 0

Region: chr19 34930392-34930403. Max. coverage (+): 0. Max coverage (-): 0

Region: chr19 34930404-34930414. Max. coverage (+): 0. Max coverage (-): 0

Region: chr19 34930415-34930425. Max. coverage (+): 0. Max coverage (-): 0

Region: chr19 34930426-34930436. Max. coverage (+): 0. Max coverage (-): 0

Region: chr19 34930437-34930447. Max. coverage (+): 0. Max coverage (-): 0

Region: chr19 34930448-34930458. Max. coverage (+): 0. Max coverage (-): 0

Region: chr19 34930459-34930470. Max. coverage (+): 0. Max coverage (-): 12.88

Region: chr19 34930471-34930481. Max. coverage (+): 0. Max coverage (-): 12.88

Region: chr19 34930482-34930492. Max. coverage (+): 0. Max coverage (-): 0

Region: chr19 34930493-34930503. Max. coverage (+): 0. Max coverage (-): 0

Region: chr19 34930504-34930514. Max. coverage (+): 0. Max coverage (-): 0

Region: chr19 34930515-34930525. Max. coverage (+): 0. Max coverage (-): 0

Region: chr19 34930526-34930537. Max. coverage (+): 0. Max coverage (-): 1.27

Region: chr19 34930538-34930548. Max. coverage (+): 0. Max coverage (-): 1.27

Region: chr19 34930549-34930559. Max. coverage (+): 0. Max coverage (-): 0

Region: chr19 34930560-34930570. Max. coverage (+): 0. Max coverage (-): 0

Region: chr19 34930571-34930581. Max. coverage (+): 0. Max coverage (-): 0

Region: chr19 34930582-34930593. Max. coverage (+): 0. Max coverage (-): 4.66

Region: chr19 34930594-34930604. Max. coverage (+): 0. Max coverage (-): 4.84

Region: chr19 34930605-34930615. Max. coverage (+): 0. Max coverage (-): 8.74

Region: chr19 34930616-34930626. Max. coverage (+): 0. Max coverage (-): 0

Region: chr19 34930627-34930637. Max. coverage (+): 0. Max coverage (-): 0

Region: chr19 34930638-34930648. Max. coverage (+): 0. Max coverage (-): 0

Region: chr19 34930649-34930660. Max. coverage (+): 0. Max coverage (-): 0

Region: chr19 34930661-34930671. Max. coverage (+): 0. Max coverage (-): 0

Region: chr19 34930672-34930682. Max. coverage (+): 0. Max coverage (-): 0

Region: chr19 34930683-34930693. Max. coverage (+): 0. Max coverage (-): 0

Region: chr19 34930694-34930704. Max. coverage (+): 0. Max coverage (-): 0

Region: chr19 34930705-34930715. Max. coverage (+): 0. Max coverage (-): 0

Region: chr19 34930716-34930727. Max. coverage (+): 0. Max coverage (-): 0

Region: chr19 34930728-34930738. Max. coverage (+): 0. Max coverage (-): 0

Region: chr19 34930739-34930749. Max. coverage (+): 0. Max coverage (-): 0

Region: chr19 34930750-34930760. Max. coverage (+): 0. Max coverage (-): 0

Region: chr19 34930761-34930771. Max. coverage (+): 0. Max coverage (-): 0

Region: chr19 34930772-34930782. Max. coverage (+): 0. Max coverage (-): 0

Region: chr19 34930783-34930794. Max. coverage (+): 0. Max coverage (-): 0

Region: chr19 34930795-34930805. Max. coverage (+): 0. Max coverage (-): 0

Region: chr19 34930806-34930816. Max. coverage (+): 0. Max coverage (-): 0

Region: chr19 34930817-34930827. Max. coverage (+): 0. Max coverage (-): 0

Region: chr19 34930828-34930838. Max. coverage (+): 0. Max coverage (-): 0

Region: chr19 34930839-34930849. Max. coverage (+): 0. Max coverage (-): 0

Region: chr19 34930850-34930861. Max. coverage (+): 0. Max coverage (-): 0

Region: chr19 34930862-34930872. Max. coverage (+): 0. Max coverage (-): 0

Region: chr19 34930873-34930883. Max. coverage (+): 0. Max coverage (-): 0

Region: chr19 34930884-34930894. Max. coverage (+): 0. Max coverage (-): 0

Region: chr19 34930895-34930905. Max. coverage (+): 0. Max coverage (-): 0

Region: chr19 34930906-34930916. Max. coverage (+): 0. Max coverage (-): 0

Region: chr19 34930917-34930928. Max. coverage (+): 0. Max coverage (-): 0

Region: chr19 34930929-34930939. Max. coverage (+): 0. Max coverage (-): 1.14

Region: chr19 34930940-34930950. Max. coverage (+): 0. Max coverage (-): 1.14

Region: chr19 34930951-34930961. Max. coverage (+): 0. Max coverage (-): 0

Region: chr19 34930962-34930972. Max. coverage (+): 0. Max coverage (-): 0

Region: chr19 34930973-34930983. Max. coverage (+): 0. Max coverage (-): 0

Region: chr19 34930984-34930995. Max. coverage (+): 0. Max coverage (-): 0

Region: chr19 34930996-34931006. Max. coverage (+): 0. Max coverage (-): 0

Region: chr19 34931007-34931017. Max. coverage (+): 0. Max coverage (-): 0

Region: chr19 34931018-34931028. Max. coverage (+): 0. Max coverage (-): 0

Region: chr19 34931029-34931039. Max. coverage (+): 0. Max coverage (-): 2.26

Region: chr19 34931040-34931050. Max. coverage (+): 0. Max coverage (-): 3.72

Region: chr19 34931051-34931062. Max. coverage (+): 0. Max coverage (-): 3.72

Region: chr19 34931063-34931073. Max. coverage (+): 0. Max coverage (-): 0

Region: chr19 34931074-34931084. Max. coverage (+): 0. Max coverage (-): 0

Region: chr19 34931085-34931095. Max. coverage (+): 0. Max coverage (-): 0

Region: chr19 34931096-34931106. Max. coverage (+): 0. Max coverage (-): 0

Region: chr19 34931107-34931117. Max. coverage (+): 0. Max coverage (-): 0

Region: chr19 34931118-34931129. Max. coverage (+): 0. Max coverage (-): 0

Region: chr19 34931130-34931140. Max. coverage (+): 0. Max coverage (-): 0

Region: chr19 34931141-34931151. Max. coverage (+): 0. Max coverage (-): 0

Region: chr19 34931152-34931162. Max. coverage (+): 0. Max coverage (-): 0

Region: chr19 34931163-34931173. Max. coverage (+): 0. Max coverage (-): 0

Region: chr19 34931174-34931185. Max. coverage (+): 0. Max coverage (-): 0

Region: chr19 34931186-34931196. Max. coverage (+): 0. Max coverage (-): 0

Region: chr19 34931197-34931207. Max. coverage (+): 0. Max coverage (-): 0

Region: chr19 34931208-34931218. Max. coverage (+): 0. Max coverage (-): 0

Region: chr19 34931219-34931229. Max. coverage (+): 0. Max coverage (-): 0

Region: chr19 34931230-34931240. Max. coverage (+): 0. Max coverage (-): 0

Region: chr19 34931241-34931252. Max. coverage (+): 0. Max coverage (-): 2.3

Region: chr19 34931253-34931263. Max. coverage (+): 0. Max coverage (-): 2.3

Region: chr19 34931264-34931274. Max. coverage (+): 0. Max coverage (-): 0

Region: chr19 34931275-34931285. Max. coverage (+): 0. Max coverage (-): 0

Region: chr19 34931286-34931296. Max. coverage (+): 0. Max coverage (-): 0

Region: chr19 34931297-34931307. Max. coverage (+): 0. Max coverage (-): 0

Region: chr19 34931308-34931319. Max. coverage (+): 0. Max coverage (-): 0

Region: chr19 34931320-34931330. Max. coverage (+): 0. Max coverage (-): 0

Region: chr19 34931331-34931341. Max. coverage (+): 0. Max coverage (-): 0

Region: chr19 34931342-34931352. Max. coverage (+): 0. Max coverage (-): 0

Region: chr19 34931353-34931363. Max. coverage (+): 0. Max coverage (-): 0

Region: chr19 34931364-34931374. Max. coverage (+): 0. Max coverage (-): 0

Region: chr19 34931375-34931386. Max. coverage (+): 0. Max coverage (-): 0

Region: chr19 34931387-34931397. Max. coverage (+): 0. Max coverage (-): 0.35

Region: chr19 34931398-34931408. Max. coverage (+): 0. Max coverage (-): 0.35

Region: chr19 34931409-34931419. Max. coverage (+): 0. Max coverage (-): 0

Region: chr19 34931420-34931430. Max. coverage (+): 0. Max coverage (-): 0

Region: chr19 34931431-34931441. Max. coverage (+): 0. Max coverage (-): 0

Region: chr19 34931442-34931453. Max. coverage (+): 0. Max coverage (-): 0

Region: chr19 34931454-34931464. Max. coverage (+): 0. Max coverage (-): 1.35

Region: chr19 34931465-34931475. Max. coverage (+): 0. Max coverage (-): 7.92

Region: chr19 34931476-34931486. Max. coverage (+): 0. Max coverage (-): 6.56

Region: chr19 34931487-34931497. Max. coverage (+): 0. Max coverage (-): 0

Region: chr19 34931498-34931508. Max. coverage (+): 0. Max coverage (-): 0

Region: chr19 34931509-34931520. Max. coverage (+): 0. Max coverage (-): 14.79

Region: chr19 34931521-34931531. Max. coverage (+): 0. Max coverage (-): 14.79

Region: chr19 34931532-34931542. Max. coverage (+): 0. Max coverage (-): 1.63

Region: chr19 34931543-34931553. Max. coverage (+): 0. Max coverage (-): 0

Region: chr19 34931554-34931564. Max. coverage (+): 0. Max coverage (-): 3.87

Region: chr19 34931565-34931575. Max. coverage (+): 0. Max coverage (-): 3.87

Region: chr19 34931576-34931587. Max. coverage (+): 0. Max coverage (-): 0

Region: chr19 34931588-34931598. Max. coverage (+): 0. Max coverage (-): 0

Region: chr19 34931599-34931609. Max. coverage (+): 0. Max coverage (-): 0

Region: chr19 34931610-34931620. Max. coverage (+): 0. Max coverage (-): 0

Region: chr19 34931621-34931631. Max. coverage (+): 0. Max coverage (-): 0

Region: chr19 34931632-34931642. Max. coverage (+): 0. Max coverage (-): 0

Region: chr19 34931643-34931654. Max. coverage (+): 0. Max coverage (-): 0

Region: chr19 34931655-34931665. Max. coverage (+): 0. Max coverage (-): 0

Region: chr19 34931666-34931676. Max. coverage (+): 0. Max coverage (-): 0

Region: chr19 34931677-34931687. Max. coverage (+): 0. Max coverage (-): 0

Region: chr19 34931688-34931698. Max. coverage (+): 0. Max coverage (-): 0

Region: chr19 34931699-34931710. Max. coverage (+): 0. Max coverage (-): 0

Region: chr19 34931711-34931721. Max. coverage (+): 0. Max coverage (-): 0

Region: chr19 34931722-34931732. Max. coverage (+): 0. Max coverage (-): 0

Region: chr19 34931733-34931743. Max. coverage (+): 0. Max coverage (-): 0

Region: chr19 34931744-34931754. Max. coverage (+): 0. Max coverage (-): 0

Region: chr19 34931755-34931765. Max. coverage (+): 0. Max coverage (-): 0

Region: chr19 34931766-34931777. Max. coverage (+): 0. Max coverage (-): 0

Region: chr19 34931778-34931788. Max. coverage (+): 0. Max coverage (-): 0

Region: chr19 34931789-34931799. Max. coverage (+): 0. Max coverage (-): 0

Region: chr19 34931800-34931810. Max. coverage (+): 0. Max coverage (-): 0

Region: chr19 34931811-34931821. Max. coverage (+): 0. Max coverage (-): 0

Region: chr19 34931822-34931832. Max. coverage (+): 0. Max coverage (-): 4.93

Region: chr19 34931833-34931844. Max. coverage (+): 0. Max coverage (-): 6.54

Region: chr19 34931845-34931855. Max. coverage (+): 0. Max coverage (-): 0

Region: chr19 34931856-34931866. Max. coverage (+): 0. Max coverage (-): 0

Region: chr19 34931867-34931877. Max. coverage (+): 0. Max coverage (-): 0

Region: chr19 34931878-34931888. Max. coverage (+): 0. Max coverage (-): 0

Region: chr19 34931889-34931899. Max. coverage (+): 0. Max coverage (-): 0

Region: chr19 34931900-34931911. Max. coverage (+): 0. Max coverage (-): 0

Region: chr19 34931912-34931922. Max. coverage (+): 0. Max coverage (-): 0

Region: chr19 34931923-34931933. Max. coverage (+): 0. Max coverage (-): 0

Region: chr19 34931934-34931944. Max. coverage (+): 0. Max coverage (-): 0

Region: chr19 34931945-34931955. Max. coverage (+): 0. Max coverage (-): 0

Region: chr19 34931956-34931966. Max. coverage (+): 0. Max coverage (-): 0

Region: chr19 34931967-34931978. Max. coverage (+): 0. Max coverage (-): 0

Region: chr19 34931979-34931989. Max. coverage (+): 0. Max coverage (-): 0

Region: chr19 34931990-34932000. Max. coverage (+): 0. Max coverage (-): 0

Region: chr19 34932001-34932011. Max. coverage (+): 0. Max coverage (-): 0

Region: chr19 34932012-34932022. Max. coverage (+): 0. Max coverage (-): 0

Region: chr19 34932023-34932033. Max. coverage (+): 0. Max coverage (-): 0

Region: chr19 34932034-34932045. Max. coverage (+): 0. Max coverage (-): 0

Region: chr19 34932046-34932056. Max. coverage (+): 0. Max coverage (-): 0

Region: chr19 34932057-34932067. Max. coverage (+): 0. Max coverage (-): 0

Region: chr19 34932068-34932078. Max. coverage (+): 0. Max coverage (-): 0

Region: chr19 34932079-34932089. Max. coverage (+): 0. Max coverage (-): 0

Region: chr19 34932090-34932100. Max. coverage (+): 0. Max coverage (-): 0

Region: chr19 34932101-34932112. Max. coverage (+): 0. Max coverage (-): 0

Region: chr19 34932113-34932123. Max. coverage (+): 0. Max coverage (-): 0

Region: chr19 34932124-34932134. Max. coverage (+): 0. Max coverage (-): 0

Region: chr19 34932135-34932145. Max. coverage (+): 0. Max coverage (-): 0

Region: chr19 34932146-34932156. Max. coverage (+): 0. Max coverage (-): 0

Region: chr19 34932157-34932167. Max. coverage (+): 0. Max coverage (-): 0

Region: chr19 34932168-34932179. Max. coverage (+): 0. Max coverage (-): 0

Region: chr19 34932180-34932190. Max. coverage (+): 0. Max coverage (-): 0

Region: chr19 34932191-34932201. Max. coverage (+): 0. Max coverage (-): 0

Region: chr19 34932202-34932212. Max. coverage (+): 0. Max coverage (-): 0

Region: chr19 34932213-34932223. Max. coverage (+): 0. Max coverage (-): 0

Region: chr19 34932224-34932234. Max. coverage (+): 0. Max coverage (-): 0

Region: chr19 34932235-34932246. Max. coverage (+): 0. Max coverage (-): 0

Region: chr19 34932247-34932257. Max. coverage (+): 0. Max coverage (-): 0

Region: chr19 34932258-34932268. Max. coverage (+): 0. Max coverage (-): 0

Region: chr19 34932269-34932279. Max. coverage (+): 0. Max coverage (-): 0

Region: chr19 34932280-34932290. Max. coverage (+): 0. Max coverage (-): 0

Region: chr19 34932291-34932302. Max. coverage (+): 0. Max coverage (-): 0

Region: chr19 34932303-34932313. Max. coverage (+): 0. Max coverage (-): 0

Region: chr19 34932314-34932324. Max. coverage (+): 0. Max coverage (-): 0

Region: chr19 34932325-34932335. Max. coverage (+): 0. Max coverage (-): 0

Region: chr19 34932336-34932346. Max. coverage (+): 0. Max coverage (-): 0

Region: chr19 34932347-34932357. Max. coverage (+): 0. Max coverage (-): 0

Region: chr19 34932358-34932369. Max. coverage (+): 0. Max coverage (-): 0

Region: chr19 34932370-34932380. Max. coverage (+): 0. Max coverage (-): 0

Region: chr19 34932381-34932391. Max. coverage (+): 0. Max coverage (-): 0

Region: chr19 34932392-34932402. Max. coverage (+): 0. Max coverage (-): 0

Region: chr19 34932403-34932413. Max. coverage (+): 0. Max coverage (-): 0

Region: chr19 34932414-34932424. Max. coverage (+): 0. Max coverage (-): 0

Region: chr19 34932425-34932436. Max. coverage (+): 0. Max coverage (-): 0

Region: chr19 34932437-34932447. Max. coverage (+): 0. Max coverage (-): 0

Region: chr19 34932448-34932458. Max. coverage (+): 0. Max coverage (-): 5.03

Region: chr19 34932459-34932469. Max. coverage (+): 0. Max coverage (-): 1.56

Region: chr19 34932470-34932480. Max. coverage (+): 0. Max coverage (-): 0

Region: chr19 34932481-34932491. Max. coverage (+): 0. Max coverage (-): 0

Region: chr19 34932492-34932503. Max. coverage (+): 0. Max coverage (-): 0

Region: chr19 34932504-34932514. Max. coverage (+): 0. Max coverage (-): 0

Region: chr19 34932515-34932525. Max. coverage (+): 0. Max coverage (-): 0

Region: chr19 34932526-34932536. Max. coverage (+): 0. Max coverage (-): 1.7

Region: chr19 34932537-34932547. Max. coverage (+): 0. Max coverage (-): 1.7

Region: chr19 34932548-34932558. Max. coverage (+): 0. Max coverage (-): 0

Region: chr19 34932559-34932570. Max. coverage (+): 0. Max coverage (-): 0

Region: chr19 34932571-34932581. Max. coverage (+): 0. Max coverage (-): 0

Region: chr19 34932582-34932592. Max. coverage (+): 0. Max coverage (-): 0

Region: chr19 34932593-34932603. Max. coverage (+): 0. Max coverage (-): 0

Region: chr19 34932604-34932614. Max. coverage (+): 0. Max coverage (-): 0

Region: chr19 34932615-34932625. Max. coverage (+): 0. Max coverage (-): 0

Region: chr19 34932626-34932637. Max. coverage (+): 0. Max coverage (-): 0

Region: chr19 34932638-34932648. Max. coverage (+): 0. Max coverage (-): 0

Region: chr19 34932649-34932659. Max. coverage (+): 0. Max coverage (-): 0

Region: chr19 34932660-34932670. Max. coverage (+): 0. Max coverage (-): 0

Region: chr19 34932671-34932681. Max. coverage (+): 0. Max coverage (-): 0

Region: chr19 34932682-34932692. Max. coverage (+): 0. Max coverage (-): 0

Region: chr19 34932693-34932704. Max. coverage (+): 0. Max coverage (-): 0

Region: chr19 34932705-34932715. Max. coverage (+): 0. Max coverage (-): 0

Region: chr19 34932716-34932726. Max. coverage (+): 0. Max coverage (-): 0

Region: chr19 34932727-34932737. Max. coverage (+): 0. Max coverage (-): 0

Region: chr19 34932738-34932748. Max. coverage (+): 0. Max coverage (-): 0

Region: chr19 34932749-34932759. Max. coverage (+): 0. Max coverage (-): 0

Region: chr19 34932760-34932771. Max. coverage (+): 0. Max coverage (-): 0

Region: chr19 34932772-34932782. Max. coverage (+): 0. Max coverage (-): 0

Region: chr19 34932783-34932793. Max. coverage (+): 0. Max coverage (-): 0

Region: chr19 34932794-34932804. Max. coverage (+): 0. Max coverage (-): 0

Region: chr19 34932805-34932815. Max. coverage (+): 0. Max coverage (-): 0

Region: chr19 34932816-34932827. Max. coverage (+): 0. Max coverage (-): 0

Region: chr19 34932828-34932838. Max. coverage (+): 0. Max coverage (-): 0

Region: chr19 34932839-34932849. Max. coverage (+): 0. Max coverage (-): 0

Region: chr19 34932850-34932860. Max. coverage (+): 0. Max coverage (-): 0

Region: chr19 34932861-34932871. Max. coverage (+): 0. Max coverage (-): 0

Region: chr19 34932872-34932882. Max. coverage (+): 0. Max coverage (-): 0

Region: chr19 34932883-34932894. Max. coverage (+): 0. Max coverage (-): 0

Region: chr19 34932895-34932905. Max. coverage (+): 0. Max coverage (-): 0.98

Region: chr19 34932906-34932916. Max. coverage (+): 0. Max coverage (-): 1.46

Region: chr19 34932917-34932927. Max. coverage (+): 0. Max coverage (-): 0.48

Region: chr19 34932928-34932938. Max. coverage (+): 0. Max coverage (-): 0

Region: chr19 34932939-34932949. Max. coverage (+): 0. Max coverage (-): 0

Region: chr19 34932950-34932961. Max. coverage (+): 0. Max coverage (-): 0

Region: chr19 34932962-34932972. Max. coverage (+): 0. Max coverage (-): 0

Region: chr19 34932973-34932983. Max. coverage (+): 0. Max coverage (-): 0

Region: chr19 34932984-34932994. Max. coverage (+): 0. Max coverage (-): 0

Region: chr19 34932995-34933005. Max. coverage (+): 0. Max coverage (-): 0

Region: chr19 34933006-34933016. Max. coverage (+): 0. Max coverage (-): 0

Region: chr19 34933017-34933028. Max. coverage (+): 0. Max coverage (-): 0

Region: chr19 34933029-34933039. Max. coverage (+): 0. Max coverage (-): 0

Region: chr19 34933040-34933050. Max. coverage (+): 0. Max coverage (-): 0

Region: chr19 34933051-34933061. Max. coverage (+): 0. Max coverage (-): 0

Region: chr19 34933062-34933072. Max. coverage (+): 0. Max coverage (-): 0

Region: chr19 34933073-34933083. Max. coverage (+): 0. Max coverage (-): 0

Region: chr19 34933084-34933095. Max. coverage (+): 0. Max coverage (-): 0.37

Region: chr19 34933096-34933106. Max. coverage (+): 0. Max coverage (-): 0.37

Region: chr19 34933107-34933117. Max. coverage (+): 0. Max coverage (-): 0

Region: chr19 34933118-34933128. Max. coverage (+): 0. Max coverage (-): 0

Region: chr19 34933129-34933139. Max. coverage (+): 0. Max coverage (-): 0

Region: chr19 34933140-34933150. Max. coverage (+): 0. Max coverage (-): 0

Region: chr19 34933151-34933162. Max. coverage (+): 0. Max coverage (-): 0

Region: chr19 34933163-34933173. Max. coverage (+): 0. Max coverage (-): 0

Region: chr19 34933174-34933184. Max. coverage (+): 0. Max coverage (-): 0

Region: chr19 34933185-34933195. Max. coverage (+): 0. Max coverage (-): 0

Region: chr19 34933196-34933206. Max. coverage (+): 0. Max coverage (-): 0

Region: chr19 34933207-34933217. Max. coverage (+): 0. Max coverage (-): 0

Region: chr19 34933218-34933229. Max. coverage (+): 0. Max coverage (-): 0

Region: chr19 34933230-34933240. Max. coverage (+): 0. Max coverage (-): 0

Region: chr19 34933241-34933251. Max. coverage (+): 0. Max coverage (-): 0

Region: chr19 34933252-34933262. Max. coverage (+): 0. Max coverage (-): 0

Region: chr19 34933263-34933273. Max. coverage (+): 0. Max coverage (-): 0

Region: chr19 34933274-34933284. Max. coverage (+): 0. Max coverage (-): 0

Region: chr19 34933285-34933296. Max. coverage (+): 0. Max coverage (-): 0

Region: chr19 34933297-34933307. Max. coverage (+): 0. Max coverage (-): 0

Region: chr19 34933308-34933318. Max. coverage (+): 0. Max coverage (-): 0

Region: chr19 34933319-34933329. Max. coverage (+): 0. Max coverage (-): 0

Region: chr19 34933330-34933340. Max. coverage (+): 0. Max coverage (-): 0

Region: chr19 34933341-34933351. Max. coverage (+): 0. Max coverage (-): 0

Region: chr19 34933352-34933363. Max. coverage (+): 0. Max coverage (-): 0

Region: chr19 34933364-34933374. Max. coverage (+): 0. Max coverage (-): 0

Region: chr19 34933375-34933385. Max. coverage (+): 0. Max coverage (-): 0

Region: chr19 34933386-34933396. Max. coverage (+): 0. Max coverage (-): 0

Region: chr19 34933397-34933407. Max. coverage (+): 0. Max coverage (-): 0

Region: chr19 34933408-34933419. Max. coverage (+): 0. Max coverage (-): 4.84

Region: chr19 34933420-34933430. Max. coverage (+): 0. Max coverage (-): 4.84

Region: chr19 34933431-34933441. Max. coverage (+): 0. Max coverage (-): 0

Region: chr19 34933442-34933452. Max. coverage (+): 0. Max coverage (-): 0

Region: chr19 34933453-34933463. Max. coverage (+): 0. Max coverage (-): 0

Region: chr19 34933464-34933474. Max. coverage (+): 0. Max coverage (-): 0

Region: chr19 34933475-34933486. Max. coverage (+): 0. Max coverage (-): 0

Region: chr19 34933487-34933497. Max. coverage (+): 0. Max coverage (-): 0

Region: chr19 34933498-34933508. Max. coverage (+): 0. Max coverage (-): 0

Region: chr19 34933509-34933519. Max. coverage (+): 0. Max coverage (-): 0.48

Region: chr19 34933520-34933530. Max. coverage (+): 0. Max coverage (-): 0.97

Region: chr19 34933531-34933541. Max. coverage (+): 0. Max coverage (-): 0

Region: chr19 34933542-34933553. Max. coverage (+): 0. Max coverage (-): 0

Region: chr19 34933554-34933564. Max. coverage (+): 0. Max coverage (-): 0

Region: chr19 34933565-34933575. Max. coverage (+): 0. Max coverage (-): 0

Region: chr19 34933576-34933586. Max. coverage (+): 0. Max coverage (-): 3.89

Region: chr19 34933587-34933597. Max. coverage (+): 0. Max coverage (-): 3.89

Region: chr19 34933598-34933608. Max. coverage (+): 0. Max coverage (-): 0

Region: chr19 34933609-34933620. Max. coverage (+): 0. Max coverage (-): 0

Region: chr19 34933621-34933631. Max. coverage (+): 0. Max coverage (-): 0

Region: chr19 34933632-34933642. Max. coverage (+): 0. Max coverage (-): 0

Region: chr19 34933643-34933653. Max. coverage (+): 0. Max coverage (-): 0

Region: chr19 34933654-34933664. Max. coverage (+): 0. Max coverage (-): 5.37

Region: chr19 34933665-34933675. Max. coverage (+): 0. Max coverage (-): 0

Region: chr19 34933676-34933687. Max. coverage (+): 0. Max coverage (-): 0

Region: chr19 34933688-34933698. Max. coverage (+): 0. Max coverage (-): 0

Region: chr19 34933699-34933709. Max. coverage (+): 0. Max coverage (-): 0

Region: chr19 34933710-34933720. Max. coverage (+): 0. Max coverage (-): 0

Region: chr19 34933721-34933731. Max. coverage (+): 0. Max coverage (-): 0

Region: chr19 34933732-34933742. Max. coverage (+): 0. Max coverage (-): 0

Region: chr19 34933743-34933754. Max. coverage (+): 0. Max coverage (-): 0

Region: chr19 34933755-34933765. Max. coverage (+): 0. Max coverage (-): 1.32

Region: chr19 34933766-34933776. Max. coverage (+): 0. Max coverage (-): 1.32

Region: chr19 34933777-34933787. Max. coverage (+): 0. Max coverage (-): 0

Region: chr19 34933788-34933798. Max. coverage (+): 0. Max coverage (-): 0

Region: chr19 34933799-34933809. Max. coverage (+): 0. Max coverage (-): 0

Region: chr19 34933810-34933821. Max. coverage (+): 0. Max coverage (-): 0

Region: chr19 34933822-34933832. Max. coverage (+): 0. Max coverage (-): 0

Region: chr19 34933833-34933843. Max. coverage (+): 0. Max coverage (-): 0

Region: chr19 34933844-34933854. Max. coverage (+): 0. Max coverage (-): 0

Region: chr19 34933855-34933865. Max. coverage (+): 0. Max coverage (-): 0

Region: chr19 34933866-34933876. Max. coverage (+): 0. Max coverage (-): 0

Region: chr19 34933877-34933888. Max. coverage (+): 0. Max coverage (-): 0

Region: chr19 34933889-34933899. Max. coverage (+): 0. Max coverage (-): 0

Region: chr19 34933900-34933910. Max. coverage (+): 0. Max coverage (-): 0

Region: chr19 34933911-34933921. Max. coverage (+): 0. Max coverage (-): 0

Region: chr19 34933922-34933932. Max. coverage (+): 0. Max coverage (-): 0

Region: chr19 34933933-34933944. Max. coverage (+): 0. Max coverage (-): 0

Region: chr19 34933945-34933955. Max. coverage (+): 0. Max coverage (-): 0

Region: chr19 34933956-34933966. Max. coverage (+): 0. Max coverage (-): 0

Region: chr19 34933967-34933977. Max. coverage (+): 0. Max coverage (-): 0

Region: chr19 34933978-34933988. Max. coverage (+): 0. Max coverage (-): 0

Region: chr19 34933989-34933999. Max. coverage (+): 0. Max coverage (-): 0

Region: chr19 34934000-34934011. Max. coverage (+): 0. Max coverage (-): 0

Region: chr19 34934012-34934022. Max. coverage (+): 0. Max coverage (-): 0

Region: chr19 34934023-34934033. Max. coverage (+): 0. Max coverage (-): 0

Region: chr19 34934034-34934044. Max. coverage (+): 0. Max coverage (-): 0

Region: chr19 34934045-34934055. Max. coverage (+): 0. Max coverage (-): 0

Region: chr19 34934056-34934066. Max. coverage (+): 0. Max coverage (-): 0

Region: chr19 34934067-34934078. Max. coverage (+): 0. Max coverage (-): 0

Region: chr19 34934079-34934089. Max. coverage (+): 0. Max coverage (-): 0

Region: chr19 34934090-34934100. Max. coverage (+): 0. Max coverage (-): 0

Region: chr19 34934101-34934111. Max. coverage (+): 0. Max coverage (-): 0

Region: chr19 34934112-34934122. Max. coverage (+): 0. Max coverage (-): 0

Region: chr19 34934123-34934133. Max. coverage (+): 0. Max coverage (-): 0

Region: chr19 34934134-34934145. Max. coverage (+): 0. Max coverage (-): 0

Region: chr19 34934146-34934156. Max. coverage (+): 0. Max coverage (-): 0

Region: chr19 34934157-34934167. Max. coverage (+): 0. Max coverage (-): 0

Region: chr19 34934168-34934178. Max. coverage (+): 0. Max coverage (-): 0

Region: chr19 34934179-34934189. Max. coverage (+): 0. Max coverage (-): 0

Region: chr19 34934190-34934200. Max. coverage (+): 0. Max coverage (-): 0

Region: chr19 34934201-34934212. Max. coverage (+): 0. Max coverage (-): 0

Region: chr19 34934213-34934223. Max. coverage (+): 0. Max coverage (-): 0

Region: chr19 34934224-34934234. Max. coverage (+): 0. Max coverage (-): 0

Region: chr19 34934235-34934245. Max. coverage (+): 0. Max coverage (-): 0

Region: chr19 34934246-34934256. Max. coverage (+): 0. Max coverage (-): 0

Region: chr19 34934257-34934267. Max. coverage (+): 0. Max coverage (-): 0

Region: chr19 34934268-34934279. Max. coverage (+): 0. Max coverage (-): 0

Region: chr19 34934280-34934290. Max. coverage (+): 0. Max coverage (-): 0

Region: chr19 34934291-34934301. Max. coverage (+): 0. Max coverage (-): 0

Region: chr19 34934302-34934312. Max. coverage (+): 0. Max coverage (-): 0

Region: chr19 34934313-34934323. Max. coverage (+): 0. Max coverage (-): 0

Region: chr19 34934324-34934334. Max. coverage (+): 0. Max coverage (-): 0

Region: chr19 34934335-34934346. Max. coverage (+): 0. Max coverage (-): 0

Region: chr19 34934347-34934357. Max. coverage (+): 0. Max coverage (-): 0

Region: chr19 34934358-34934368. Max. coverage (+): 0. Max coverage (-): 0

Region: chr19 34934369-34934379. Max. coverage (+): 0. Max coverage (-): 0

Region: chr19 34934380-34934390. Max. coverage (+): 0. Max coverage (-): 0

Region: chr19 34934391-34934401. Max. coverage (+): 0. Max coverage (-): 0

Region: chr19 34934402-34934413. Max. coverage (+): 0. Max coverage (-): 0

Region: chr19 34934414-34934424. Max. coverage (+): 0. Max coverage (-): 0

Region: chr19 34934425-34934435. Max. coverage (+): 0. Max coverage (-): 0

Region: chr19 34934436-34934446. Max. coverage (+): 0. Max coverage (-): 0

Region: chr19 34934447-34934457. Max. coverage (+): 0. Max coverage (-): 0

Region: chr19 34934458-34934468. Max. coverage (+): 0. Max coverage (-): 0

Region: chr19 34934469-34934480. Max. coverage (+): 0. Max coverage (-): 0

Region: chr19 34934481-34934491. Max. coverage (+): 0. Max coverage (-): 0

Region: chr19 34934492-34934502. Max. coverage (+): 0. Max coverage (-): 0

Region: chr19 34934503-34934513. Max. coverage (+): 0. Max coverage (-): 0

Region: chr19 34934514-34934524. Max. coverage (+): 0. Max coverage (-): 0

Region: chr19 34934525-34934536. Max. coverage (+): 0. Max coverage (-): 0

Region: chr19 34934537-34934547. Max. coverage (+): 0. Max coverage (-): 0

Region: chr19 34934548-34934558. Max. coverage (+): 0. Max coverage (-): 0

Region: chr19 34934559-34934569. Max. coverage (+): 0. Max coverage (-): 0

Region: chr19 34934570-34934580. Max. coverage (+): 0. Max coverage (-): 0

Region: chr19 34934581-34934591. Max. coverage (+): 0. Max coverage (-): 0

Region: chr19 34934592-34934603. Max. coverage (+): 0. Max coverage (-): 0

Region: chr19 34934604-34934614. Max. coverage (+): 0. Max coverage (-): 0

Region: chr19 34934615-34934625. Max. coverage (+): 0. Max coverage (-): 0

Region: chr19 34934626-34934636. Max. coverage (+): 0. Max coverage (-): 0

Region: chr19 34934637-34934647. Max. coverage (+): 0. Max coverage (-): 0

Region: chr19 34934648-34934658. Max. coverage (+): 0. Max coverage (-): 0

Region: chr19 34934659-34934670. Max. coverage (+): 0. Max coverage (-): 0

Region: chr19 34934671-34934681. Max. coverage (+): 0. Max coverage (-): 0

Region: chr19 34934682-34934692. Max. coverage (+): 0. Max coverage (-): 0

Region: chr19 34934693-34934703. Max. coverage (+): 0. Max coverage (-): 0

Region: chr19 34934704-34934714. Max. coverage (+): 0. Max coverage (-): 4.21

Region: chr19 34934715-34934725. Max. coverage (+): 0. Max coverage (-): 4.21

Region: chr19 34934726-34934737. Max. coverage (+): 0. Max coverage (-): 0

Region: chr19 34934738-34934748. Max. coverage (+): 0. Max coverage (-): 0

Region: chr19 34934749-34934759. Max. coverage (+): 0. Max coverage (-): 0

Region: chr19 34934760-34934770. Max. coverage (+): 0. Max coverage (-): 0

Region: chr19 34934771-34934781. Max. coverage (+): 0. Max coverage (-): 9.36

Region: chr19 34934782-34934792. Max. coverage (+): 0. Max coverage (-): 7.69

Region: chr19 34934793-34934804. Max. coverage (+): 0. Max coverage (-): 0

Region: chr19 34934805-34934815. Max. coverage (+): 0. Max coverage (-): 0

Region: chr19 34934816-34934826. Max. coverage (+): 0. Max coverage (-): 0

Region: chr19 34934827-34934837. Max. coverage (+): 0. Max coverage (-): 0

Region: chr19 34934838-34934848. Max. coverage (+): 0. Max coverage (-): 0

Region: chr19 34934849-34934859. Max. coverage (+): 0. Max coverage (-): 0

Region: chr19 34934860-34934871. Max. coverage (+): 0. Max coverage (-): 0

Region: chr19 34934872-34934882. Max. coverage (+): 0. Max coverage (-): 0

Region: chr19 34934883-34934893. Max. coverage (+): 0. Max coverage (-): 0

Region: chr19 34934894-34934904. Max. coverage (+): 0. Max coverage (-): 0

Region: chr19 34934905-34934915. Max. coverage (+): 0. Max coverage (-): 3.74

Region: chr19 34934916-34934926. Max. coverage (+): 0. Max coverage (-): 3.74

Region: chr19 34934927-34934938. Max. coverage (+): 0. Max coverage (-): 0

Region: chr19 34934939-34934949. Max. coverage (+): 0. Max coverage (-): 0

Region: chr19 34934950-34934960. Max. coverage (+): 0. Max coverage (-): 3.03

Region: chr19 34934961-34934971. Max. coverage (+): 0. Max coverage (-): 0

Region: chr19 34934972-34934982. Max. coverage (+): 0. Max coverage (-): 0

Region: chr19 34934983-34934993. Max. coverage (+): 0. Max coverage (-): 0

Region: chr19 34934994-34935005. Max. coverage (+): 0. Max coverage (-): 0

Region: chr19 34935006-34935016. Max. coverage (+): 0. Max coverage (-): 0

Region: chr19 34935017-34935027. Max. coverage (+): 0. Max coverage (-): 0

Region: chr19 34935028-34935038. Max. coverage (+): 0. Max coverage (-): 0

Region: chr19 34935039-34935049. Max. coverage (+): 0. Max coverage (-): 0

Region: chr19 34935050-34935061. Max. coverage (+): 0. Max coverage (-): 0

Region: chr19 34935062-34935072. Max. coverage (+): 0. Max coverage (-): 0

Region: chr19 34935073-34935083. Max. coverage (+): 0. Max coverage (-): 0

Region: chr19 34935084-34935094. Max. coverage (+): 0. Max coverage (-): 0

Region: chr19 34935095-34935105. Max. coverage (+): 0. Max coverage (-): 0

Region: chr19 34935106-34935116. Max. coverage (+): 0. Max coverage (-): 0

Region: chr19 34935117-34935128. Max. coverage (+): 0. Max coverage (-): 0

Region: chr19 34935129-34935139. Max. coverage (+): 0. Max coverage (-): 0

Region: chr19 34935140-34935150. Max. coverage (+): 0. Max coverage (-): 0

Region: chr19 34935151-34935161. Max. coverage (+): 0. Max coverage (-): 0

Region: chr19 34935162-34935172. Max. coverage (+): 0. Max coverage (-): 0

Region: chr19 34935173-34935183. Max. coverage (+): 0. Max coverage (-): 0

Region: chr19 34935184-34935195. Max. coverage (+): 0. Max coverage (-): 0

Region: chr19 34935196-34935206. Max. coverage (+): 0. Max coverage (-): 0

Region: chr19 34935207-34935217. Max. coverage (+): 0. Max coverage (-): 0

Region: chr19 34935218-34935228. Max. coverage (+): 0. Max coverage (-): 1.1

Region: chr19 34935229-34935239. Max. coverage (+): 0. Max coverage (-): 1.1

Region: chr19 34935240-34935250. Max. coverage (+): 0. Max coverage (-): 0

Region: chr19 34935251-34935262. Max. coverage (+): 0. Max coverage (-): 0

Region: chr19 34935263-34935273. Max. coverage (+): 0. Max coverage (-): 0

Region: chr19 34935274-34935284. Max. coverage (+): 0. Max coverage (-): 0

Region: chr19 34935285-34935295. Max. coverage (+): 0. Max coverage (-): 0

Region: chr19 34935296-34935306. Max. coverage (+): 0. Max coverage (-): 0

Region: chr19 34935307-34935317. Max. coverage (+): 0. Max coverage (-): 0

Region: chr19 34935318-34935329. Max. coverage (+): 0. Max coverage (-): 0

Region: chr19 34935330-34935340. Max. coverage (+): 0. Max coverage (-): 0

Region: chr19 34935341-34935351. Max. coverage (+): 0. Max coverage (-): 0

Region: chr19 34935352-34935362. Max. coverage (+): 0. Max coverage (-): 0

Region: chr19 34935363-34935373. Max. coverage (+): 0. Max coverage (-): 0

Region: chr19 34935374-34935384. Max. coverage (+): 0. Max coverage (-): 0

Region: chr19 34935385-34935396. Max. coverage (+): 0. Max coverage (-): 0

Region: chr19 34935397-34935407. Max. coverage (+): 0. Max coverage (-): 0

Region: chr19 34935408-34935418. Max. coverage (+): 0. Max coverage (-): 0

Region: chr19 34935419-34935429. Max. coverage (+): 0. Max coverage (-): 0

Region: chr19 34935430-34935440. Max. coverage (+): 0. Max coverage (-): 0

Region: chr19 34935441-34935451. Max. coverage (+): 0. Max coverage (-): 0

Region: chr19 34935452-34935463. Max. coverage (+): 0. Max coverage (-): 0

Region: chr19 34935464-34935474. Max. coverage (+): 0. Max coverage (-): 0

Region: chr19 34935475-34935485. Max. coverage (+): 0. Max coverage (-): 0

Region: chr19 34935486-34935496. Max. coverage (+): 0. Max coverage (-): 0

Region: chr19 34935497-34935507. Max. coverage (+): 0. Max coverage (-): 0

Region: chr19 34935508-34935518. Max. coverage (+): 0. Max coverage (-): 0

Region: chr19 34935519-34935530. Max. coverage (+): 0. Max coverage (-): 0

Region: chr19 34935531-34935541. Max. coverage (+): 0. Max coverage (-): 0

Region: chr19 34935542-34935552. Max. coverage (+): 0. Max coverage (-): 0

Region: chr19 34935553-34935563. Max. coverage (+): 0. Max coverage (-): 0

Region: chr19 34935564-34935574. Max. coverage (+): 0. Max coverage (-): 1.59

Region: chr19 34935575-34935585. Max. coverage (+): 0. Max coverage (-): 4.99

Region: chr19 34935586-34935597. Max. coverage (+): 0. Max coverage (-): 0

Region: chr19 34935598-34935608. Max. coverage (+): 0. Max coverage (-): 0

Region: chr19 34935609-34935619. Max. coverage (+): 0. Max coverage (-): 0

Region: chr19 34935620-34935630. Max. coverage (+): 0. Max coverage (-): 0

Region: chr19 34935631-34935641. Max. coverage (+): 0. Max coverage (-): 0

Region: chr19 34935642-34935653. Max. coverage (+): 0. Max coverage (-): 0

Region: chr19 34935654-34935664. Max. coverage (+): 0. Max coverage (-): 0

Region: chr19 34935665-34935675. Max. coverage (+): 0. Max coverage (-): 0.51

Region: chr19 34935676-34935686. Max. coverage (+): 0. Max coverage (-): 0.51

Region: chr19 34935687-34935697. Max. coverage (+): 0. Max coverage (-): 0

Region: chr19 34935698-34935708. Max. coverage (+): 0. Max coverage (-): 0

Region: chr19 34935709-34935720. Max. coverage (+): 0. Max coverage (-): 0

Region: chr19 34935721-34935731. Max. coverage (+): 0. Max coverage (-): 0

Region: chr19 34935732-34935742. Max. coverage (+): 0. Max coverage (-): 0

Region: chr19 34935743-34935753. Max. coverage (+): 0. Max coverage (-): 0

Region: chr19 34935754-34935764. Max. coverage (+): 0. Max coverage (-): 0

Region: chr19 34935765-34935775. Max. coverage (+): 0. Max coverage (-): 0

Region: chr19 34935776-34935787. Max. coverage (+): 0. Max coverage (-): 0

Region: chr19 34935788-34935798. Max. coverage (+): 0. Max coverage (-): 0

Region: chr19 34935799-34935809. Max. coverage (+): 0. Max coverage (-): 0

Region: chr19 34935810-34935820. Max. coverage (+): 0. Max coverage (-): 0

Region: chr19 34935821-34935831. Max. coverage (+): 0. Max coverage (-): 0

Region: chr19 34935832-34935842. Max. coverage (+): 0. Max coverage (-): 0

Region: chr19 34935843-34935854. Max. coverage (+): 0. Max coverage (-): 0

Region: chr19 34935855-34935865. Max. coverage (+): 0. Max coverage (-): 3.19

Region: chr19 34935866-34935876. Max. coverage (+): 0. Max coverage (-): 0

Region: chr19 34935877-. Max. coverage (+): 0. Max coverage (-): 0

RepeatMasker Color Code

**+**

100-98% Identity

<98-95% Identity

<95-90% Identity

<90-85% Identity

<85-80% Identity

<80-75% Identity

<75-70% Identity

<70% Identity

**-**

Gene Set Color Code

**+**

Gene

Pseudogene

**-**

Topology/Coverage Color Code

Coverage Plus Strand

Coverage Minus Strand

Mainstrand: Plus

Mainstrand: Minus

Complementary Strand

Flanking Region  
(if option -flank >0)

Gene Set Annotation  

**1. SMCR8 (protein coding, ENSBTAG00000017090) Tr:00000022723 Ex:7**: 34935284-34936451 (-)  
**2. SMCR8 (protein coding, ENSBTAG00000017090) Tr:00000022723 Ex:8**: 34932550-34933003 (-)

  
RepeatMasker Annotation  

**1. L2a**: 34934436-34934507 (+), Divergence to consensus: 34.8%  
**2. MER5B**: 34935010-34935128 (+), Divergence to consensus: 35.5%  
**3. MIR3**: 34935149-34935184 (-), Divergence to consensus: 25%

  
Transcription Factor Binding Sites  

**Gata4** (Sequence: GTTATCT (+): 34933768)
