# Supplementary material for: Small RNA sequencing of cryopreserved semen from single bull revealed altered miRNAs and piRNAs expression between High- and Low-motile sperm populations
Source: BMC Genomics. 2017 Jan 4;18:14. doi: 10.1186/s12864-016-3394-7 (PMC5209821; doi:10.1186/s12864-016-3394-7)
Supplement: Additional file 3: — Details for each piRNA clusters found in High Motile (HM) sperm fraction. Genes, repeats, transposable elements and transcription factors binding sites falling within the cluster regions were reported. (ZIP 1896 kb) [file 12864_2016_3394_MOESM3_ESM.zip › 42.html]

piRNA cluster 42


Predicted piRNA cluster no. 42     previous   next
  

Show proTRAC run info
Hide proTRAC run info

================================= proTRAC ====================================  
VERSION: 2.1                                    LAST MODIFIED: 06. October 2015  
  
Please cite:  
Rosenkranz D, Zischler H. proTRAC - a software for probabilistic piRNA cluster  
detection, visualization and analysis. 2012. BMC Bioinformatics 13:5.  
  
and (for proTRAC 2.0 and later):  
Rosenkranz D, Rudloff S, Bastuck K, Ketting RF, Zischler H. Tupaia small RNAs  
provide insights into function and evolution of RNAi-based transposon defense  
in mammals. 2015. RNA 21(5):911-922.  
  
Contact:  
David Rosenkranz  
Institute of Anthropology, small RNA group  
Johannes Gutenberg University Mainz  
email: rosenkranz@uni-mainz.de  
  
You can find the latest proTRAC version at:  
http://sourceforge.net/projects/protrac/files  
http://www.smallRNAgroup-mainz.de/software  
==============================================================================  
  
PARAMETERS:  
Map file: .............../storage/core/barbara/genhome/smallRNA/fertility/Sample\_motile/pirna/Sample\_motile\_26-33\_collapsed.fa.no-dust.map.weighted-10000-1000-b-0  
Genome file: ............/storage/core/barbara/genhome/smallRNA/fertility/Sample\_all/pirna/bt\_311\_chrY.fa  
RepeatMasker annotation: /storage/genomes/bt\_umd31/GCF\_000003055.6\_Bos\_taurus\_UMD\_3.1.1\_repeatMasker\_chr.out  
GeneSet:................./storage/core/barbara/genhome/smallRNA/fertility/Sample\_all/pirna/full.gtf  
  
Significant (p<=0.01) hit density will be calculated based  
on observed hit distribution.  
  
Sliding window size: ........................................ 5000 bp  
Sliding window increament: .................................. 1000 bp  
Normalize each hit by number of genomic hits: ............... 1 [0=no/1=yes]  
Normalize each hit by number of sequence reads: ............. 1 [0=no/1=yes]  
Normalize values (-> per million mapped reads): ............. 1 [0=no/1=yes]  
Min. fraction of hits with 1T(U) or 10A: .................... 0.75  
Alternatively: Min. fraction of hits with 1T(U) and 10A: .... 0.5  
Min. fraction of hits with typical piRNA length: ............ 0.75  
Typical piRNA length: ....................................... 26-33 nt  
Min. size of a piRNA cluster: ............................... 5000 bp.  
Min. number of hits (absolute): ............................. 0  
Min. number of hits (normalized): ........................... 0  
Min. fraction of hits on the mainstrand: .................... 0.75  
Top fraction of mapped sequences (in terms of read counts): . 1%  
Top fraction accounts for max. n% of sequence reads: ........ 90%  
Min. fraction of hits on each arm of a bidirectional cluster: 0.1  
Output image file for each cluster: ......................... 0 [0=no/1=yes]  
Output html file for each cluster: .......................... 1 [0=no/1=yes]  
Output a summary table: ..................................... 1 [0=no/1=yes]  
Output a FASTA file for each cluster (piRNA sequences): ..... 1 [0=no/1=yes]  
Output a FASTA file comprising cluster sequences: ........... 1 [0=no/1=yes]  
Search DNA motifs in clusters: .............................. 1 [0=no/1=yes]  
Output flanking sequences: +/- .............................. 0 bp  
Output ~.pTi file: .......................................... 1 [0=no/1=yes]  
==============================================================================  
  
  
Genome size (without gaps): ............ 2678902517 bp  
Gaps (N/X/-): .......................... 53837044 bp  
Mapped reads: .......................... 658825247023  
Non-identical sequences: ............... 514171  
Genomic hits: .......................... 764233  
Significant densitiy of mapped reads: .. 12867599.5173724 reads/kb

Show proTRAC cluster info
Hide proTRAC cluster info

|  |  |
| --- | --- |
| Location | chr19 |
| Coordinates | 35559126-35571566 |
| Size [bp] | 12441 |
| Sequence hit loci | 118 |
| Mapped reads (normalized) | 171281191 |
| Mapped reads (normalized) per kb | 13767477.8 |
| Normalized reads with 1T (1U) | 80.8% |
| Normalized reads with 10A | 30.6% |
| Normalized reads with length 26-33 nt | 100% |
| Normalized reads on the main strand(s) | 100% |
| Predicted directionality | mono:plus |

100%

0%

1T (1U)  
reads

10A reads

26-33 nt  
reads

reads on mainstrand

**Either the amount of reads with 1T (1U) OR 10A has to exceed 75% (set with option: -1Tor10A)  
Alternatively the amount of reads with 1T (1U) AND 10A has to exceed 50% (set with option: -1Tand10A)  
Minimum amount of reads with preferred size is 75% (set with option: -pisize)  
Minimum amount of reads on the main strand(s) is 75% (set with option: -clstrand)**

Show read coverage
Hide read coverage

WHAT DO I SEE HERE?  
This chart shows the location of mapped sequence reads within a predicted piRNA cluster. The color refers to the number of genomic hits produced by the sequence read in question. A dark red bar indicates that this sequence read produces many other hits elsewhere in the genome. Many adjacent red or yellow bars can indicate the presence of a multi-copy element such as transposons or rRNA genes. A dark green bar indicates that this sequence read maps uniquely to this locus.

1 hit

2-5 hits

6-10 hits

11-20 hits

21-50 hits

51-100 hits

> 100 hits

chr19

35559126

35571566

Gene Set

RepeatMasker

Mapped  
Reads

12.99

plus strand

minus strand

12.99

Region: chr19 34935878-35559138. Max. coverage (+): 5.13. Max coverage (-): 0

Region: chr19 35559139-35559163. Max. coverage (+): 0.94. Max coverage (-): 0

Region: chr19 35559164-35559188. Max. coverage (+): 0.94. Max coverage (-): 0

Region: chr19 35559189-35559213. Max. coverage (+): 0. Max coverage (-): 0

Region: chr19 35559214-35559237. Max. coverage (+): 0. Max coverage (-): 0

Region: chr19 35559238-35559262. Max. coverage (+): 0. Max coverage (-): 0

Region: chr19 35559263-35559287. Max. coverage (+): 4.68. Max coverage (-): 0

Region: chr19 35559288-35559312. Max. coverage (+): 0. Max coverage (-): 0

Region: chr19 35559313-35559337. Max. coverage (+): 0. Max coverage (-): 0

Region: chr19 35559338-35559362. Max. coverage (+): 0. Max coverage (-): 0

Region: chr19 35559363-35559387. Max. coverage (+): 0. Max coverage (-): 0

Region: chr19 35559388-35559412. Max. coverage (+): 0. Max coverage (-): 0

Region: chr19 35559413-35559437. Max. coverage (+): 0. Max coverage (-): 0

Region: chr19 35559438-35559461. Max. coverage (+): 0.96. Max coverage (-): 0

Region: chr19 35559462-35559486. Max. coverage (+): 2.3. Max coverage (-): 0

Region: chr19 35559487-35559511. Max. coverage (+): 0. Max coverage (-): 0

Region: chr19 35559512-35559536. Max. coverage (+): 0. Max coverage (-): 0

Region: chr19 35559537-35559561. Max. coverage (+): 0. Max coverage (-): 0

Region: chr19 35559562-35559586. Max. coverage (+): 0. Max coverage (-): 0

Region: chr19 35559587-35559611. Max. coverage (+): 0. Max coverage (-): 0

Region: chr19 35559612-35559636. Max. coverage (+): 7.14. Max coverage (-): 0

Region: chr19 35559637-35559660. Max. coverage (+): 7.14. Max coverage (-): 0

Region: chr19 35559661-35559685. Max. coverage (+): 0. Max coverage (-): 0

Region: chr19 35559686-35559710. Max. coverage (+): 2.83. Max coverage (-): 0

Region: chr19 35559711-35559735. Max. coverage (+): 3.99. Max coverage (-): 0

Region: chr19 35559736-35559760. Max. coverage (+): 1.18. Max coverage (-): 0

Region: chr19 35559761-35559785. Max. coverage (+): 1.18. Max coverage (-): 0

Region: chr19 35559786-35559810. Max. coverage (+): 0. Max coverage (-): 0

Region: chr19 35559811-35559835. Max. coverage (+): 0. Max coverage (-): 0

Region: chr19 35559836-35559860. Max. coverage (+): 0. Max coverage (-): 0

Region: chr19 35559861-35559884. Max. coverage (+): 0. Max coverage (-): 0

Region: chr19 35559885-35559909. Max. coverage (+): 0. Max coverage (-): 0

Region: chr19 35559910-35559934. Max. coverage (+): 0. Max coverage (-): 0

Region: chr19 35559935-35559959. Max. coverage (+): 0. Max coverage (-): 0

Region: chr19 35559960-35559984. Max. coverage (+): 0. Max coverage (-): 0

Region: chr19 35559985-35560009. Max. coverage (+): 0. Max coverage (-): 0

Region: chr19 35560010-35560034. Max. coverage (+): 0. Max coverage (-): 0

Region: chr19 35560035-35560059. Max. coverage (+): 0. Max coverage (-): 0

Region: chr19 35560060-35560083. Max. coverage (+): 0. Max coverage (-): 0

Region: chr19 35560084-35560108. Max. coverage (+): 0. Max coverage (-): 0

Region: chr19 35560109-35560133. Max. coverage (+): 0. Max coverage (-): 0

Region: chr19 35560134-35560158. Max. coverage (+): 0. Max coverage (-): 0

Region: chr19 35560159-35560183. Max. coverage (+): 0. Max coverage (-): 0

Region: chr19 35560184-35560208. Max. coverage (+): 0. Max coverage (-): 0

Region: chr19 35560209-35560233. Max. coverage (+): 0. Max coverage (-): 0

Region: chr19 35560234-35560258. Max. coverage (+): 0. Max coverage (-): 0

Region: chr19 35560259-35560283. Max. coverage (+): 0. Max coverage (-): 0

Region: chr19 35560284-35560307. Max. coverage (+): 0. Max coverage (-): 0

Region: chr19 35560308-35560332. Max. coverage (+): 0. Max coverage (-): 0

Region: chr19 35560333-35560357. Max. coverage (+): 0. Max coverage (-): 0

Region: chr19 35560358-35560382. Max. coverage (+): 0. Max coverage (-): 0

Region: chr19 35560383-35560407. Max. coverage (+): 1.16. Max coverage (-): 0

Region: chr19 35560408-35560432. Max. coverage (+): 1.03. Max coverage (-): 0

Region: chr19 35560433-35560457. Max. coverage (+): 1.03. Max coverage (-): 0

Region: chr19 35560458-35560482. Max. coverage (+): 0. Max coverage (-): 0

Region: chr19 35560483-35560506. Max. coverage (+): 0. Max coverage (-): 0

Region: chr19 35560507-35560531. Max. coverage (+): 0. Max coverage (-): 0

Region: chr19 35560532-35560556. Max. coverage (+): 0. Max coverage (-): 0

Region: chr19 35560557-35560581. Max. coverage (+): 0. Max coverage (-): 0

Region: chr19 35560582-35560606. Max. coverage (+): 5.18. Max coverage (-): 0

Region: chr19 35560607-35560631. Max. coverage (+): 0. Max coverage (-): 0

Region: chr19 35560632-35560656. Max. coverage (+): 1.2. Max coverage (-): 0

Region: chr19 35560657-35560681. Max. coverage (+): 0. Max coverage (-): 0

Region: chr19 35560682-35560706. Max. coverage (+): 0. Max coverage (-): 0

Region: chr19 35560707-35560730. Max. coverage (+): 0. Max coverage (-): 0

Region: chr19 35560731-35560755. Max. coverage (+): 0. Max coverage (-): 0

Region: chr19 35560756-35560780. Max. coverage (+): 0. Max coverage (-): 0

Region: chr19 35560781-35560805. Max. coverage (+): 0. Max coverage (-): 0

Region: chr19 35560806-35560830. Max. coverage (+): 0. Max coverage (-): 0

Region: chr19 35560831-35560855. Max. coverage (+): 0. Max coverage (-): 0

Region: chr19 35560856-35560880. Max. coverage (+): 0. Max coverage (-): 0

Region: chr19 35560881-35560905. Max. coverage (+): 0. Max coverage (-): 0

Region: chr19 35560906-35560929. Max. coverage (+): 0. Max coverage (-): 0

Region: chr19 35560930-35560954. Max. coverage (+): 1.66. Max coverage (-): 0

Region: chr19 35560955-35560979. Max. coverage (+): 1.66. Max coverage (-): 0

Region: chr19 35560980-35561004. Max. coverage (+): 0. Max coverage (-): 0

Region: chr19 35561005-35561029. Max. coverage (+): 0. Max coverage (-): 0

Region: chr19 35561030-35561054. Max. coverage (+): 3.15. Max coverage (-): 0

Region: chr19 35561055-35561079. Max. coverage (+): 0. Max coverage (-): 0

Region: chr19 35561080-35561104. Max. coverage (+): 3.1. Max coverage (-): 0

Region: chr19 35561105-35561129. Max. coverage (+): 3.1. Max coverage (-): 0

Region: chr19 35561130-35561153. Max. coverage (+): 0. Max coverage (-): 0

Region: chr19 35561154-35561178. Max. coverage (+): 0.47. Max coverage (-): 0

Region: chr19 35561179-35561203. Max. coverage (+): 0.78. Max coverage (-): 0

Region: chr19 35561204-35561228. Max. coverage (+): 0. Max coverage (-): 0

Region: chr19 35561229-35561253. Max. coverage (+): 0. Max coverage (-): 0

Region: chr19 35561254-35561278. Max. coverage (+): 0. Max coverage (-): 0

Region: chr19 35561279-35561303. Max. coverage (+): 6.26. Max coverage (-): 0

Region: chr19 35561304-35561328. Max. coverage (+): 0. Max coverage (-): 0

Region: chr19 35561329-35561352. Max. coverage (+): 0. Max coverage (-): 0

Region: chr19 35561353-35561377. Max. coverage (+): 0.41. Max coverage (-): 0

Region: chr19 35561378-35561402. Max. coverage (+): 0. Max coverage (-): 0

Region: chr19 35561403-35561427. Max. coverage (+): 0. Max coverage (-): 0

Region: chr19 35561428-35561452. Max. coverage (+): 0. Max coverage (-): 0

Region: chr19 35561453-35561477. Max. coverage (+): 1.38. Max coverage (-): 0

Region: chr19 35561478-35561502. Max. coverage (+): 6.97. Max coverage (-): 0

Region: chr19 35561503-35561527. Max. coverage (+): 0. Max coverage (-): 0

Region: chr19 35561528-35561551. Max. coverage (+): 2.37. Max coverage (-): 0

Region: chr19 35561552-35561576. Max. coverage (+): 0. Max coverage (-): 0

Region: chr19 35561577-35561601. Max. coverage (+): 0. Max coverage (-): 0

Region: chr19 35561602-35561626. Max. coverage (+): 0. Max coverage (-): 0

Region: chr19 35561627-35561651. Max. coverage (+): 0. Max coverage (-): 0

Region: chr19 35561652-35561676. Max. coverage (+): 0. Max coverage (-): 0

Region: chr19 35561677-35561701. Max. coverage (+): 0. Max coverage (-): 0

Region: chr19 35561702-35561726. Max. coverage (+): 0. Max coverage (-): 0

Region: chr19 35561727-35561751. Max. coverage (+): 0. Max coverage (-): 0

Region: chr19 35561752-35561775. Max. coverage (+): 0. Max coverage (-): 0

Region: chr19 35561776-35561800. Max. coverage (+): 0. Max coverage (-): 0

Region: chr19 35561801-35561825. Max. coverage (+): 0. Max coverage (-): 0

Region: chr19 35561826-35561850. Max. coverage (+): 0. Max coverage (-): 0

Region: chr19 35561851-35561875. Max. coverage (+): 0. Max coverage (-): 0

Region: chr19 35561876-35561900. Max. coverage (+): 1.37. Max coverage (-): 0

Region: chr19 35561901-35561925. Max. coverage (+): 1.47. Max coverage (-): 0

Region: chr19 35561926-35561950. Max. coverage (+): 0. Max coverage (-): 0

Region: chr19 35561951-35561974. Max. coverage (+): 0. Max coverage (-): 0

Region: chr19 35561975-35561999. Max. coverage (+): 0. Max coverage (-): 0

Region: chr19 35562000-35562024. Max. coverage (+): 0. Max coverage (-): 0

Region: chr19 35562025-35562049. Max. coverage (+): 0. Max coverage (-): 0

Region: chr19 35562050-35562074. Max. coverage (+): 0. Max coverage (-): 0

Region: chr19 35562075-35562099. Max. coverage (+): 0. Max coverage (-): 0

Region: chr19 35562100-35562124. Max. coverage (+): 0. Max coverage (-): 0

Region: chr19 35562125-35562149. Max. coverage (+): 0. Max coverage (-): 0

Region: chr19 35562150-35562174. Max. coverage (+): 0. Max coverage (-): 0

Region: chr19 35562175-35562198. Max. coverage (+): 0. Max coverage (-): 0

Region: chr19 35562199-35562223. Max. coverage (+): 0. Max coverage (-): 0

Region: chr19 35562224-35562248. Max. coverage (+): 0. Max coverage (-): 0

Region: chr19 35562249-35562273. Max. coverage (+): 4.21. Max coverage (-): 0

Region: chr19 35562274-35562298. Max. coverage (+): 0. Max coverage (-): 0

Region: chr19 35562299-35562323. Max. coverage (+): 0. Max coverage (-): 0

Region: chr19 35562324-35562348. Max. coverage (+): 0. Max coverage (-): 0

Region: chr19 35562349-35562373. Max. coverage (+): 1.89. Max coverage (-): 0

Region: chr19 35562374-35562397. Max. coverage (+): 0. Max coverage (-): 0

Region: chr19 35562398-35562422. Max. coverage (+): 6.92. Max coverage (-): 0

Region: chr19 35562423-35562447. Max. coverage (+): 6.92. Max coverage (-): 0

Region: chr19 35562448-35562472. Max. coverage (+): 0. Max coverage (-): 0

Region: chr19 35562473-35562497. Max. coverage (+): 0. Max coverage (-): 0

Region: chr19 35562498-35562522. Max. coverage (+): 0. Max coverage (-): 0

Region: chr19 35562523-35562547. Max. coverage (+): 0. Max coverage (-): 0

Region: chr19 35562548-35562572. Max. coverage (+): 0. Max coverage (-): 0

Region: chr19 35562573-35562597. Max. coverage (+): 0. Max coverage (-): 0

Region: chr19 35562598-35562621. Max. coverage (+): 0. Max coverage (-): 0

Region: chr19 35562622-35562646. Max. coverage (+): 0. Max coverage (-): 0

Region: chr19 35562647-35562671. Max. coverage (+): 0. Max coverage (-): 0

Region: chr19 35562672-35562696. Max. coverage (+): 0. Max coverage (-): 0

Region: chr19 35562697-35562721. Max. coverage (+): 0.93. Max coverage (-): 0

Region: chr19 35562722-35562746. Max. coverage (+): 0. Max coverage (-): 0

Region: chr19 35562747-35562771. Max. coverage (+): 0. Max coverage (-): 0

Region: chr19 35562772-35562796. Max. coverage (+): 0. Max coverage (-): 0

Region: chr19 35562797-35562820. Max. coverage (+): 0. Max coverage (-): 0

Region: chr19 35562821-35562845. Max. coverage (+): 0. Max coverage (-): 0

Region: chr19 35562846-35562870. Max. coverage (+): 0. Max coverage (-): 0

Region: chr19 35562871-35562895. Max. coverage (+): 0. Max coverage (-): 0

Region: chr19 35562896-35562920. Max. coverage (+): 0. Max coverage (-): 0

Region: chr19 35562921-35562945. Max. coverage (+): 0. Max coverage (-): 0

Region: chr19 35562946-35562970. Max. coverage (+): 0. Max coverage (-): 0

Region: chr19 35562971-35562995. Max. coverage (+): 0. Max coverage (-): 0

Region: chr19 35562996-35563020. Max. coverage (+): 0. Max coverage (-): 0

Region: chr19 35563021-35563044. Max. coverage (+): 0. Max coverage (-): 0

Region: chr19 35563045-35563069. Max. coverage (+): 0. Max coverage (-): 0

Region: chr19 35563070-35563094. Max. coverage (+): 0. Max coverage (-): 0

Region: chr19 35563095-35563119. Max. coverage (+): 0. Max coverage (-): 0

Region: chr19 35563120-35563144. Max. coverage (+): 0. Max coverage (-): 0

Region: chr19 35563145-35563169. Max. coverage (+): 0. Max coverage (-): 0

Region: chr19 35563170-35563194. Max. coverage (+): 0. Max coverage (-): 0

Region: chr19 35563195-35563219. Max. coverage (+): 0. Max coverage (-): 0

Region: chr19 35563220-35563243. Max. coverage (+): 0. Max coverage (-): 0

Region: chr19 35563244-35563268. Max. coverage (+): 0. Max coverage (-): 0

Region: chr19 35563269-35563293. Max. coverage (+): 0.44. Max coverage (-): 0

Region: chr19 35563294-35563318. Max. coverage (+): 12.76. Max coverage (-): 0

Region: chr19 35563319-35563343. Max. coverage (+): 8.95. Max coverage (-): 0

Region: chr19 35563344-35563368. Max. coverage (+): 0. Max coverage (-): 0

Region: chr19 35563369-35563393. Max. coverage (+): 0. Max coverage (-): 0

Region: chr19 35563394-35563418. Max. coverage (+): 1.51. Max coverage (-): 0

Region: chr19 35563419-35563443. Max. coverage (+): 4.12. Max coverage (-): 0

Region: chr19 35563444-35563467. Max. coverage (+): 0. Max coverage (-): 0

Region: chr19 35563468-35563492. Max. coverage (+): 0. Max coverage (-): 0

Region: chr19 35563493-35563517. Max. coverage (+): 0. Max coverage (-): 0

Region: chr19 35563518-35563542. Max. coverage (+): 0. Max coverage (-): 0

Region: chr19 35563543-35563567. Max. coverage (+): 0. Max coverage (-): 0

Region: chr19 35563568-35563592. Max. coverage (+): 0. Max coverage (-): 0

Region: chr19 35563593-35563617. Max. coverage (+): 0. Max coverage (-): 0

Region: chr19 35563618-35563642. Max. coverage (+): 0. Max coverage (-): 0

Region: chr19 35563643-35563666. Max. coverage (+): 0. Max coverage (-): 0

Region: chr19 35563667-35563691. Max. coverage (+): 0. Max coverage (-): 0

Region: chr19 35563692-35563716. Max. coverage (+): 0. Max coverage (-): 0

Region: chr19 35563717-35563741. Max. coverage (+): 0. Max coverage (-): 0

Region: chr19 35563742-35563766. Max. coverage (+): 0. Max coverage (-): 0

Region: chr19 35563767-35563791. Max. coverage (+): 0. Max coverage (-): 0

Region: chr19 35563792-35563816. Max. coverage (+): 0. Max coverage (-): 0

Region: chr19 35563817-35563841. Max. coverage (+): 0. Max coverage (-): 0

Region: chr19 35563842-35563866. Max. coverage (+): 0.76. Max coverage (-): 0

Region: chr19 35563867-35563890. Max. coverage (+): 0. Max coverage (-): 0

Region: chr19 35563891-35563915. Max. coverage (+): 0. Max coverage (-): 0

Region: chr19 35563916-35563940. Max. coverage (+): 0. Max coverage (-): 0

Region: chr19 35563941-35563965. Max. coverage (+): 0. Max coverage (-): 0

Region: chr19 35563966-35563990. Max. coverage (+): 0. Max coverage (-): 0

Region: chr19 35563991-35564015. Max. coverage (+): 4.91. Max coverage (-): 0

Region: chr19 35564016-35564040. Max. coverage (+): 0. Max coverage (-): 0

Region: chr19 35564041-35564065. Max. coverage (+): 0. Max coverage (-): 0

Region: chr19 35564066-35564089. Max. coverage (+): 0. Max coverage (-): 0

Region: chr19 35564090-35564114. Max. coverage (+): 0. Max coverage (-): 0

Region: chr19 35564115-35564139. Max. coverage (+): 0. Max coverage (-): 0

Region: chr19 35564140-35564164. Max. coverage (+): 0. Max coverage (-): 0

Region: chr19 35564165-35564189. Max. coverage (+): 0. Max coverage (-): 0

Region: chr19 35564190-35564214. Max. coverage (+): 0. Max coverage (-): 0

Region: chr19 35564215-35564239. Max. coverage (+): 0. Max coverage (-): 0

Region: chr19 35564240-35564264. Max. coverage (+): 0. Max coverage (-): 0

Region: chr19 35564265-35564289. Max. coverage (+): 0. Max coverage (-): 0

Region: chr19 35564290-35564313. Max. coverage (+): 0. Max coverage (-): 0

Region: chr19 35564314-35564338. Max. coverage (+): 0. Max coverage (-): 0

Region: chr19 35564339-35564363. Max. coverage (+): 0. Max coverage (-): 0

Region: chr19 35564364-35564388. Max. coverage (+): 4.13. Max coverage (-): 0

Region: chr19 35564389-35564413. Max. coverage (+): 4.23. Max coverage (-): 0

Region: chr19 35564414-35564438. Max. coverage (+): 1.74. Max coverage (-): 0

Region: chr19 35564439-35564463. Max. coverage (+): 1.74. Max coverage (-): 0

Region: chr19 35564464-35564488. Max. coverage (+): 0. Max coverage (-): 0

Region: chr19 35564489-35564512. Max. coverage (+): 0. Max coverage (-): 0

Region: chr19 35564513-35564537. Max. coverage (+): 0. Max coverage (-): 0

Region: chr19 35564538-35564562. Max. coverage (+): 0. Max coverage (-): 0

Region: chr19 35564563-35564587. Max. coverage (+): 0. Max coverage (-): 0

Region: chr19 35564588-35564612. Max. coverage (+): 0. Max coverage (-): 0

Region: chr19 35564613-35564637. Max. coverage (+): 0.55. Max coverage (-): 0

Region: chr19 35564638-35564662. Max. coverage (+): 0.55. Max coverage (-): 0

Region: chr19 35564663-35564687. Max. coverage (+): 0. Max coverage (-): 0

Region: chr19 35564688-35564712. Max. coverage (+): 0. Max coverage (-): 0

Region: chr19 35564713-35564736. Max. coverage (+): 0. Max coverage (-): 0

Region: chr19 35564737-35564761. Max. coverage (+): 2.59. Max coverage (-): 0

Region: chr19 35564762-35564786. Max. coverage (+): 0. Max coverage (-): 0

Region: chr19 35564787-35564811. Max. coverage (+): 0. Max coverage (-): 0

Region: chr19 35564812-35564836. Max. coverage (+): 0. Max coverage (-): 0

Region: chr19 35564837-35564861. Max. coverage (+): 0. Max coverage (-): 0

Region: chr19 35564862-35564886. Max. coverage (+): 0. Max coverage (-): 0

Region: chr19 35564887-35564911. Max. coverage (+): 0. Max coverage (-): 0

Region: chr19 35564912-35564935. Max. coverage (+): 0.73. Max coverage (-): 0

Region: chr19 35564936-35564960. Max. coverage (+): 0. Max coverage (-): 0

Region: chr19 35564961-35564985. Max. coverage (+): 0. Max coverage (-): 0

Region: chr19 35564986-35565010. Max. coverage (+): 0. Max coverage (-): 0

Region: chr19 35565011-35565035. Max. coverage (+): 0. Max coverage (-): 0

Region: chr19 35565036-35565060. Max. coverage (+): 0. Max coverage (-): 0

Region: chr19 35565061-35565085. Max. coverage (+): 0. Max coverage (-): 0

Region: chr19 35565086-35565110. Max. coverage (+): 0. Max coverage (-): 0

Region: chr19 35565111-35565135. Max. coverage (+): 2.7. Max coverage (-): 0

Region: chr19 35565136-35565159. Max. coverage (+): 0. Max coverage (-): 0

Region: chr19 35565160-35565184. Max. coverage (+): 0. Max coverage (-): 0

Region: chr19 35565185-35565209. Max. coverage (+): 0. Max coverage (-): 0

Region: chr19 35565210-35565234. Max. coverage (+): 0. Max coverage (-): 0

Region: chr19 35565235-35565259. Max. coverage (+): 0. Max coverage (-): 0

Region: chr19 35565260-35565284. Max. coverage (+): 0. Max coverage (-): 0

Region: chr19 35565285-35565309. Max. coverage (+): 0. Max coverage (-): 0

Region: chr19 35565310-35565334. Max. coverage (+): 0. Max coverage (-): 0

Region: chr19 35565335-35565358. Max. coverage (+): 0. Max coverage (-): 0

Region: chr19 35565359-35565383. Max. coverage (+): 0. Max coverage (-): 0

Region: chr19 35565384-35565408. Max. coverage (+): 0. Max coverage (-): 0

Region: chr19 35565409-35565433. Max. coverage (+): 0. Max coverage (-): 0

Region: chr19 35565434-35565458. Max. coverage (+): 0. Max coverage (-): 0

Region: chr19 35565459-35565483. Max. coverage (+): 0. Max coverage (-): 0

Region: chr19 35565484-35565508. Max. coverage (+): 0. Max coverage (-): 0

Region: chr19 35565509-35565533. Max. coverage (+): 0. Max coverage (-): 0

Region: chr19 35565534-35565557. Max. coverage (+): 0. Max coverage (-): 0

Region: chr19 35565558-35565582. Max. coverage (+): 0. Max coverage (-): 0

Region: chr19 35565583-35565607. Max. coverage (+): 0. Max coverage (-): 0

Region: chr19 35565608-35565632. Max. coverage (+): 0. Max coverage (-): 0

Region: chr19 35565633-35565657. Max. coverage (+): 0. Max coverage (-): 0

Region: chr19 35565658-35565682. Max. coverage (+): 0. Max coverage (-): 0

Region: chr19 35565683-35565707. Max. coverage (+): 0. Max coverage (-): 0

Region: chr19 35565708-35565732. Max. coverage (+): 0. Max coverage (-): 0

Region: chr19 35565733-35565757. Max. coverage (+): 0. Max coverage (-): 0

Region: chr19 35565758-35565781. Max. coverage (+): 0. Max coverage (-): 0

Region: chr19 35565782-35565806. Max. coverage (+): 0. Max coverage (-): 0

Region: chr19 35565807-35565831. Max. coverage (+): 0. Max coverage (-): 0

Region: chr19 35565832-35565856. Max. coverage (+): 0. Max coverage (-): 0

Region: chr19 35565857-35565881. Max. coverage (+): 0. Max coverage (-): 0

Region: chr19 35565882-35565906. Max. coverage (+): 0. Max coverage (-): 0

Region: chr19 35565907-35565931. Max. coverage (+): 0. Max coverage (-): 0

Region: chr19 35565932-35565956. Max. coverage (+): 0.62. Max coverage (-): 0

Region: chr19 35565957-35565980. Max. coverage (+): 0. Max coverage (-): 0

Region: chr19 35565981-35566005. Max. coverage (+): 0. Max coverage (-): 0

Region: chr19 35566006-35566030. Max. coverage (+): 6.25. Max coverage (-): 0

Region: chr19 35566031-35566055. Max. coverage (+): 6.25. Max coverage (-): 0

Region: chr19 35566056-35566080. Max. coverage (+): 0. Max coverage (-): 0

Region: chr19 35566081-35566105. Max. coverage (+): 0. Max coverage (-): 0

Region: chr19 35566106-35566130. Max. coverage (+): 0. Max coverage (-): 0

Region: chr19 35566131-35566155. Max. coverage (+): 0. Max coverage (-): 0

Region: chr19 35566156-35566180. Max. coverage (+): 0. Max coverage (-): 0

Region: chr19 35566181-35566204. Max. coverage (+): 0. Max coverage (-): 0

Region: chr19 35566205-35566229. Max. coverage (+): 0. Max coverage (-): 0

Region: chr19 35566230-35566254. Max. coverage (+): 0. Max coverage (-): 0

Region: chr19 35566255-35566279. Max. coverage (+): 1.42. Max coverage (-): 0

Region: chr19 35566280-35566304. Max. coverage (+): 1.42. Max coverage (-): 0

Region: chr19 35566305-35566329. Max. coverage (+): 0. Max coverage (-): 0

Region: chr19 35566330-35566354. Max. coverage (+): 0. Max coverage (-): 0

Region: chr19 35566355-35566379. Max. coverage (+): 0. Max coverage (-): 0

Region: chr19 35566380-35566403. Max. coverage (+): 3.84. Max coverage (-): 0

Region: chr19 35566404-35566428. Max. coverage (+): 0. Max coverage (-): 0

Region: chr19 35566429-35566453. Max. coverage (+): 0. Max coverage (-): 0

Region: chr19 35566454-35566478. Max. coverage (+): 0. Max coverage (-): 0

Region: chr19 35566479-35566503. Max. coverage (+): 0. Max coverage (-): 0

Region: chr19 35566504-35566528. Max. coverage (+): 0. Max coverage (-): 0

Region: chr19 35566529-35566553. Max. coverage (+): 2.25. Max coverage (-): 0

Region: chr19 35566554-35566578. Max. coverage (+): 0. Max coverage (-): 0

Region: chr19 35566579-35566603. Max. coverage (+): 0. Max coverage (-): 0

Region: chr19 35566604-35566627. Max. coverage (+): 0. Max coverage (-): 0

Region: chr19 35566628-35566652. Max. coverage (+): 0. Max coverage (-): 0

Region: chr19 35566653-35566677. Max. coverage (+): 2.06. Max coverage (-): 0

Region: chr19 35566678-35566702. Max. coverage (+): 0. Max coverage (-): 0

Region: chr19 35566703-35566727. Max. coverage (+): 0. Max coverage (-): 0

Region: chr19 35566728-35566752. Max. coverage (+): 6.1. Max coverage (-): 0

Region: chr19 35566753-35566777. Max. coverage (+): 6.1. Max coverage (-): 0

Region: chr19 35566778-35566802. Max. coverage (+): 0. Max coverage (-): 0

Region: chr19 35566803-35566826. Max. coverage (+): 0. Max coverage (-): 0

Region: chr19 35566827-35566851. Max. coverage (+): 0. Max coverage (-): 0

Region: chr19 35566852-35566876. Max. coverage (+): 3.64. Max coverage (-): 0

Region: chr19 35566877-35566901. Max. coverage (+): 0. Max coverage (-): 0

Region: chr19 35566902-35566926. Max. coverage (+): 0.54. Max coverage (-): 0

Region: chr19 35566927-35566951. Max. coverage (+): 4.55. Max coverage (-): 0

Region: chr19 35566952-35566976. Max. coverage (+): 0. Max coverage (-): 0

Region: chr19 35566977-35567001. Max. coverage (+): 0. Max coverage (-): 0

Region: chr19 35567002-35567026. Max. coverage (+): 0. Max coverage (-): 0

Region: chr19 35567027-35567050. Max. coverage (+): 2.58. Max coverage (-): 0

Region: chr19 35567051-35567075. Max. coverage (+): 2.58. Max coverage (-): 0

Region: chr19 35567076-35567100. Max. coverage (+): 0. Max coverage (-): 0

Region: chr19 35567101-35567125. Max. coverage (+): 0. Max coverage (-): 0

Region: chr19 35567126-35567150. Max. coverage (+): 0. Max coverage (-): 0

Region: chr19 35567151-35567175. Max. coverage (+): 0. Max coverage (-): 0

Region: chr19 35567176-35567200. Max. coverage (+): 0. Max coverage (-): 0

Region: chr19 35567201-35567225. Max. coverage (+): 0. Max coverage (-): 0

Region: chr19 35567226-35567249. Max. coverage (+): 0. Max coverage (-): 0

Region: chr19 35567250-35567274. Max. coverage (+): 0. Max coverage (-): 0

Region: chr19 35567275-35567299. Max. coverage (+): 0. Max coverage (-): 0

Region: chr19 35567300-35567324. Max. coverage (+): 0. Max coverage (-): 0

Region: chr19 35567325-35567349. Max. coverage (+): 4.5. Max coverage (-): 0

Region: chr19 35567350-35567374. Max. coverage (+): 4.38. Max coverage (-): 0

Region: chr19 35567375-35567399. Max. coverage (+): 0. Max coverage (-): 0

Region: chr19 35567400-35567424. Max. coverage (+): 0. Max coverage (-): 0

Region: chr19 35567425-35567449. Max. coverage (+): 0. Max coverage (-): 0

Region: chr19 35567450-35567473. Max. coverage (+): 0. Max coverage (-): 0

Region: chr19 35567474-35567498. Max. coverage (+): 0. Max coverage (-): 0

Region: chr19 35567499-35567523. Max. coverage (+): 0.37. Max coverage (-): 0

Region: chr19 35567524-35567548. Max. coverage (+): 3.74. Max coverage (-): 0

Region: chr19 35567549-35567573. Max. coverage (+): 2.02. Max coverage (-): 0

Region: chr19 35567574-35567598. Max. coverage (+): 0. Max coverage (-): 0

Region: chr19 35567599-35567623. Max. coverage (+): 0. Max coverage (-): 0

Region: chr19 35567624-35567648. Max. coverage (+): 1.61. Max coverage (-): 0

Region: chr19 35567649-35567672. Max. coverage (+): 0.94. Max coverage (-): 0

Region: chr19 35567673-35567697. Max. coverage (+): 0. Max coverage (-): 0

Region: chr19 35567698-35567722. Max. coverage (+): 0. Max coverage (-): 0

Region: chr19 35567723-35567747. Max. coverage (+): 0. Max coverage (-): 0

Region: chr19 35567748-35567772. Max. coverage (+): 0. Max coverage (-): 0

Region: chr19 35567773-35567797. Max. coverage (+): 0. Max coverage (-): 0

Region: chr19 35567798-35567822. Max. coverage (+): 0. Max coverage (-): 0

Region: chr19 35567823-35567847. Max. coverage (+): 3.48. Max coverage (-): 0

Region: chr19 35567848-35567872. Max. coverage (+): 0. Max coverage (-): 0

Region: chr19 35567873-35567896. Max. coverage (+): 0. Max coverage (-): 0

Region: chr19 35567897-35567921. Max. coverage (+): 0. Max coverage (-): 0

Region: chr19 35567922-35567946. Max. coverage (+): 0. Max coverage (-): 0

Region: chr19 35567947-35567971. Max. coverage (+): 0. Max coverage (-): 0

Region: chr19 35567972-35567996. Max. coverage (+): 0. Max coverage (-): 0

Region: chr19 35567997-35568021. Max. coverage (+): 0. Max coverage (-): 0

Region: chr19 35568022-35568046. Max. coverage (+): 0. Max coverage (-): 0

Region: chr19 35568047-35568071. Max. coverage (+): 0. Max coverage (-): 0

Region: chr19 35568072-35568095. Max. coverage (+): 0. Max coverage (-): 0

Region: chr19 35568096-35568120. Max. coverage (+): 0. Max coverage (-): 0

Region: chr19 35568121-35568145. Max. coverage (+): 0. Max coverage (-): 0

Region: chr19 35568146-35568170. Max. coverage (+): 0. Max coverage (-): 0

Region: chr19 35568171-35568195. Max. coverage (+): 0. Max coverage (-): 0

Region: chr19 35568196-35568220. Max. coverage (+): 0. Max coverage (-): 0

Region: chr19 35568221-35568245. Max. coverage (+): 0. Max coverage (-): 0

Region: chr19 35568246-35568270. Max. coverage (+): 0. Max coverage (-): 0

Region: chr19 35568271-35568295. Max. coverage (+): 0. Max coverage (-): 0

Region: chr19 35568296-35568319. Max. coverage (+): 0. Max coverage (-): 0

Region: chr19 35568320-35568344. Max. coverage (+): 0. Max coverage (-): 0

Region: chr19 35568345-35568369. Max. coverage (+): 0. Max coverage (-): 0

Region: chr19 35568370-35568394. Max. coverage (+): 0. Max coverage (-): 0

Region: chr19 35568395-35568419. Max. coverage (+): 1.55. Max coverage (-): 0

Region: chr19 35568420-35568444. Max. coverage (+): 1.92. Max coverage (-): 0

Region: chr19 35568445-35568469. Max. coverage (+): 0. Max coverage (-): 0

Region: chr19 35568470-35568494. Max. coverage (+): 0. Max coverage (-): 0

Region: chr19 35568495-35568518. Max. coverage (+): 0. Max coverage (-): 0

Region: chr19 35568519-35568543. Max. coverage (+): 1.85. Max coverage (-): 0

Region: chr19 35568544-35568568. Max. coverage (+): 0. Max coverage (-): 0

Region: chr19 35568569-35568593. Max. coverage (+): 12.99. Max coverage (-): 0

Region: chr19 35568594-35568618. Max. coverage (+): 4. Max coverage (-): 0

Region: chr19 35568619-35568643. Max. coverage (+): 0. Max coverage (-): 0

Region: chr19 35568644-35568668. Max. coverage (+): 0. Max coverage (-): 0

Region: chr19 35568669-35568693. Max. coverage (+): 0. Max coverage (-): 0

Region: chr19 35568694-35568718. Max. coverage (+): 0. Max coverage (-): 0

Region: chr19 35568719-35568742. Max. coverage (+): 0. Max coverage (-): 0

Region: chr19 35568743-35568767. Max. coverage (+): 0. Max coverage (-): 0

Region: chr19 35568768-35568792. Max. coverage (+): 1.81. Max coverage (-): 0

Region: chr19 35568793-35568817. Max. coverage (+): 1.81. Max coverage (-): 0

Region: chr19 35568818-35568842. Max. coverage (+): 0. Max coverage (-): 0

Region: chr19 35568843-35568867. Max. coverage (+): 0. Max coverage (-): 0

Region: chr19 35568868-35568892. Max. coverage (+): 0. Max coverage (-): 0

Region: chr19 35568893-35568917. Max. coverage (+): 0. Max coverage (-): 0

Region: chr19 35568918-35568941. Max. coverage (+): 0. Max coverage (-): 0

Region: chr19 35568942-35568966. Max. coverage (+): 0. Max coverage (-): 0

Region: chr19 35568967-35568991. Max. coverage (+): 0. Max coverage (-): 0

Region: chr19 35568992-35569016. Max. coverage (+): 0. Max coverage (-): 0

Region: chr19 35569017-35569041. Max. coverage (+): 0. Max coverage (-): 0

Region: chr19 35569042-35569066. Max. coverage (+): 0. Max coverage (-): 0

Region: chr19 35569067-35569091. Max. coverage (+): 10.22. Max coverage (-): 0

Region: chr19 35569092-35569116. Max. coverage (+): 2.1. Max coverage (-): 0

Region: chr19 35569117-35569141. Max. coverage (+): 0. Max coverage (-): 0

Region: chr19 35569142-35569165. Max. coverage (+): 0. Max coverage (-): 0

Region: chr19 35569166-35569190. Max. coverage (+): 0. Max coverage (-): 0

Region: chr19 35569191-35569215. Max. coverage (+): 0. Max coverage (-): 0

Region: chr19 35569216-35569240. Max. coverage (+): 4.17. Max coverage (-): 0

Region: chr19 35569241-35569265. Max. coverage (+): 0. Max coverage (-): 0

Region: chr19 35569266-35569290. Max. coverage (+): 0. Max coverage (-): 0

Region: chr19 35569291-35569315. Max. coverage (+): 2.11. Max coverage (-): 0

Region: chr19 35569316-35569340. Max. coverage (+): 0. Max coverage (-): 0

Region: chr19 35569341-35569364. Max. coverage (+): 0. Max coverage (-): 0

Region: chr19 35569365-35569389. Max. coverage (+): 3.21. Max coverage (-): 0

Region: chr19 35569390-35569414. Max. coverage (+): 0. Max coverage (-): 0

Region: chr19 35569415-35569439. Max. coverage (+): 0. Max coverage (-): 0

Region: chr19 35569440-35569464. Max. coverage (+): 0. Max coverage (-): 0

Region: chr19 35569465-35569489. Max. coverage (+): 0. Max coverage (-): 0

Region: chr19 35569490-35569514. Max. coverage (+): 0. Max coverage (-): 0

Region: chr19 35569515-35569539. Max. coverage (+): 0. Max coverage (-): 0

Region: chr19 35569540-35569563. Max. coverage (+): 0. Max coverage (-): 0

Region: chr19 35569564-35569588. Max. coverage (+): 0. Max coverage (-): 0

Region: chr19 35569589-35569613. Max. coverage (+): 0. Max coverage (-): 0

Region: chr19 35569614-35569638. Max. coverage (+): 0. Max coverage (-): 0

Region: chr19 35569639-35569663. Max. coverage (+): 0. Max coverage (-): 0

Region: chr19 35569664-35569688. Max. coverage (+): 0. Max coverage (-): 0

Region: chr19 35569689-35569713. Max. coverage (+): 0. Max coverage (-): 0

Region: chr19 35569714-35569738. Max. coverage (+): 0. Max coverage (-): 0

Region: chr19 35569739-35569763. Max. coverage (+): 0. Max coverage (-): 0

Region: chr19 35569764-35569787. Max. coverage (+): 0. Max coverage (-): 0

Region: chr19 35569788-35569812. Max. coverage (+): 0. Max coverage (-): 0

Region: chr19 35569813-35569837. Max. coverage (+): 0. Max coverage (-): 0

Region: chr19 35569838-35569862. Max. coverage (+): 0. Max coverage (-): 0

Region: chr19 35569863-35569887. Max. coverage (+): 0. Max coverage (-): 0

Region: chr19 35569888-35569912. Max. coverage (+): 6.54. Max coverage (-): 0

Region: chr19 35569913-35569937. Max. coverage (+): 0. Max coverage (-): 0

Region: chr19 35569938-35569962. Max. coverage (+): 0. Max coverage (-): 0

Region: chr19 35569963-35569986. Max. coverage (+): 0. Max coverage (-): 0

Region: chr19 35569987-35570011. Max. coverage (+): 6.87. Max coverage (-): 0

Region: chr19 35570012-35570036. Max. coverage (+): 3.58. Max coverage (-): 0

Region: chr19 35570037-35570061. Max. coverage (+): 0. Max coverage (-): 0

Region: chr19 35570062-35570086. Max. coverage (+): 0. Max coverage (-): 0

Region: chr19 35570087-35570111. Max. coverage (+): 0. Max coverage (-): 0

Region: chr19 35570112-35570136. Max. coverage (+): 5.23. Max coverage (-): 0

Region: chr19 35570137-35570161. Max. coverage (+): 0. Max coverage (-): 0

Region: chr19 35570162-35570186. Max. coverage (+): 0. Max coverage (-): 0

Region: chr19 35570187-35570210. Max. coverage (+): 0. Max coverage (-): 0

Region: chr19 35570211-35570235. Max. coverage (+): 0. Max coverage (-): 0

Region: chr19 35570236-35570260. Max. coverage (+): 0. Max coverage (-): 0

Region: chr19 35570261-35570285. Max. coverage (+): 0. Max coverage (-): 0

Region: chr19 35570286-35570310. Max. coverage (+): 0. Max coverage (-): 0

Region: chr19 35570311-35570335. Max. coverage (+): 0. Max coverage (-): 0

Region: chr19 35570336-35570360. Max. coverage (+): 0. Max coverage (-): 0

Region: chr19 35570361-35570385. Max. coverage (+): 0. Max coverage (-): 0

Region: chr19 35570386-35570409. Max. coverage (+): 0. Max coverage (-): 0

Region: chr19 35570410-35570434. Max. coverage (+): 0. Max coverage (-): 0

Region: chr19 35570435-35570459. Max. coverage (+): 0. Max coverage (-): 0

Region: chr19 35570460-35570484. Max. coverage (+): 0. Max coverage (-): 0

Region: chr19 35570485-35570509. Max. coverage (+): 0. Max coverage (-): 0

Region: chr19 35570510-35570534. Max. coverage (+): 0. Max coverage (-): 0

Region: chr19 35570535-35570559. Max. coverage (+): 0. Max coverage (-): 0

Region: chr19 35570560-35570584. Max. coverage (+): 0. Max coverage (-): 0

Region: chr19 35570585-35570609. Max. coverage (+): 0. Max coverage (-): 0

Region: chr19 35570610-35570633. Max. coverage (+): 0. Max coverage (-): 0

Region: chr19 35570634-35570658. Max. coverage (+): 0. Max coverage (-): 0

Region: chr19 35570659-35570683. Max. coverage (+): 0. Max coverage (-): 0

Region: chr19 35570684-35570708. Max. coverage (+): 0. Max coverage (-): 0

Region: chr19 35570709-35570733. Max. coverage (+): 0. Max coverage (-): 0

Region: chr19 35570734-35570758. Max. coverage (+): 0. Max coverage (-): 0

Region: chr19 35570759-35570783. Max. coverage (+): 0. Max coverage (-): 0

Region: chr19 35570784-35570808. Max. coverage (+): 0. Max coverage (-): 0

Region: chr19 35570809-35570832. Max. coverage (+): 0. Max coverage (-): 0

Region: chr19 35570833-35570857. Max. coverage (+): 0. Max coverage (-): 0

Region: chr19 35570858-35570882. Max. coverage (+): 0. Max coverage (-): 0

Region: chr19 35570883-35570907. Max. coverage (+): 0. Max coverage (-): 0

Region: chr19 35570908-35570932. Max. coverage (+): 0. Max coverage (-): 0

Region: chr19 35570933-35570957. Max. coverage (+): 0. Max coverage (-): 0

Region: chr19 35570958-35570982. Max. coverage (+): 0. Max coverage (-): 0

Region: chr19 35570983-35571007. Max. coverage (+): 0. Max coverage (-): 0

Region: chr19 35571008-35571032. Max. coverage (+): 0. Max coverage (-): 0

Region: chr19 35571033-35571056. Max. coverage (+): 0. Max coverage (-): 0

Region: chr19 35571057-35571081. Max. coverage (+): 0. Max coverage (-): 0

Region: chr19 35571082-35571106. Max. coverage (+): 0. Max coverage (-): 0

Region: chr19 35571107-35571131. Max. coverage (+): 0. Max coverage (-): 0

Region: chr19 35571132-35571156. Max. coverage (+): 0. Max coverage (-): 0

Region: chr19 35571157-35571181. Max. coverage (+): 0. Max coverage (-): 0

Region: chr19 35571182-35571206. Max. coverage (+): 0. Max coverage (-): 0

Region: chr19 35571207-35571231. Max. coverage (+): 0. Max coverage (-): 0

Region: chr19 35571232-35571255. Max. coverage (+): 0. Max coverage (-): 0

Region: chr19 35571256-35571280. Max. coverage (+): 0. Max coverage (-): 0

Region: chr19 35571281-35571305. Max. coverage (+): 0. Max coverage (-): 0

Region: chr19 35571306-35571330. Max. coverage (+): 0. Max coverage (-): 0

Region: chr19 35571331-35571355. Max. coverage (+): 0. Max coverage (-): 0

Region: chr19 35571356-35571380. Max. coverage (+): 0. Max coverage (-): 0

Region: chr19 35571381-35571405. Max. coverage (+): 0. Max coverage (-): 0

Region: chr19 35571406-35571430. Max. coverage (+): 0. Max coverage (-): 0

Region: chr19 35571431-35571455. Max. coverage (+): 0. Max coverage (-): 0

Region: chr19 35571456-35571479. Max. coverage (+): 0. Max coverage (-): 0

Region: chr19 35571480-35571504. Max. coverage (+): 0. Max coverage (-): 0

Region: chr19 35571505-35571529. Max. coverage (+): 5.08. Max coverage (-): 0

Region: chr19 35571530-35571554. Max. coverage (+): 0.31. Max coverage (-): 0

Region: chr19 35571555-. Max. coverage (+): 0. Max coverage (-): 0

RepeatMasker Color Code

**+**

100-98% Identity

<98-95% Identity

<95-90% Identity

<90-85% Identity

<85-80% Identity

<80-75% Identity

<75-70% Identity

<70% Identity

**-**

Gene Set Color Code

**+**

Gene

Pseudogene

**-**

Topology/Coverage Color Code

Coverage Plus Strand

Coverage Minus Strand

Mainstrand: Plus

Mainstrand: Minus

Complementary Strand

Flanking Region  
(if option -flank >0)

Gene Set Annotation  

**1. MPRIP (protein coding, ENSBTAG00000010534) Tr:00000013929 Ex:18**: 35565250-35565397 (-)  
**2. MPRIP (protein coding, ENSBTAG00000010534) Tr:00000013929 Ex:19**: 35564610-35564727 (-)  
**3. MPRIP (protein coding, ENSBTAG00000010534) Tr:00000013929 Ex:20**: 35563538-35563697 (-)  
**4. MPRIP (protein coding, ENSBTAG00000010534) Tr:00000013929 Ex:21**: 35562706-35562825 (-)  
**5. MPRIP (protein coding, ENSBTAG00000010534) Tr:00000013929 Ex:22**: 35562339-35562425 (-)  
**6. MPRIP (protein coding, ENSBTAG00000010534) Tr:00000013929 Ex:23**: 35561743-35561905 (-)  
**7. MPRIP (protein coding, ENSBTAG00000010534) Tr:00000013929 Ex:24**: 35560958-35561043 (-)  
**8. MPRIP (protein coding, ENSBTAG00000010534) Tr:00000013929 Ex:25**: 35560537-35560598 (-)

  
RepeatMasker Annotation  

**1. SINE2-1\_BT**: 35559360-35559459 (-), Divergence to consensus: 26%  
**2. AT\_rich**: 35564878-35564902 (+), Divergence to consensus: 56%  
**3. AT\_rich**: 35564880-35564904 (+), Divergence to consensus: 48%  
**4. L2b**: 35565738-35565851 (-), Divergence to consensus: 38.2%  
**5. MIR3**: 35565826-35565931 (+), Divergence to consensus: 40.4%

  
Transcription Factor Binding Sites  

**Gata4** (Sequence: AGATAAG (-): 35566670)  
**Gata4** (Sequence: AGATAAG (-): 35567551)  
**A-MYB** (Sequence: TGGCAGTTGG (+): 35564817)  
**Gata4** (Sequence: CTTATCT (+): 35568755)
