# Supplementary material for: Small RNA sequencing of cryopreserved semen from single bull revealed altered miRNAs and piRNAs expression between High- and Low-motile sperm populations
Source: BMC Genomics. 2017 Jan 4;18:14. doi: 10.1186/s12864-016-3394-7 (PMC5209821; doi:10.1186/s12864-016-3394-7)
Supplement: Additional file 3: — Details for each piRNA clusters found in High Motile (HM) sperm fraction. Genes, repeats, transposable elements and transcription factors binding sites falling within the cluster regions were reported. (ZIP 1896 kb) [file 12864_2016_3394_MOESM3_ESM.zip › 43.html]

piRNA cluster 43


Predicted piRNA cluster no. 43     previous   next
  

Show proTRAC run info
Hide proTRAC run info

================================= proTRAC ====================================  
VERSION: 2.1                                    LAST MODIFIED: 06. October 2015  
  
Please cite:  
Rosenkranz D, Zischler H. proTRAC - a software for probabilistic piRNA cluster  
detection, visualization and analysis. 2012. BMC Bioinformatics 13:5.  
  
and (for proTRAC 2.0 and later):  
Rosenkranz D, Rudloff S, Bastuck K, Ketting RF, Zischler H. Tupaia small RNAs  
provide insights into function and evolution of RNAi-based transposon defense  
in mammals. 2015. RNA 21(5):911-922.  
  
Contact:  
David Rosenkranz  
Institute of Anthropology, small RNA group  
Johannes Gutenberg University Mainz  
email: rosenkranz@uni-mainz.de  
  
You can find the latest proTRAC version at:  
http://sourceforge.net/projects/protrac/files  
http://www.smallRNAgroup-mainz.de/software  
==============================================================================  
  
PARAMETERS:  
Map file: .............../storage/core/barbara/genhome/smallRNA/fertility/Sample\_motile/pirna/Sample\_motile\_26-33\_collapsed.fa.no-dust.map.weighted-10000-1000-b-0  
Genome file: ............/storage/core/barbara/genhome/smallRNA/fertility/Sample\_all/pirna/bt\_311\_chrY.fa  
RepeatMasker annotation: /storage/genomes/bt\_umd31/GCF\_000003055.6\_Bos\_taurus\_UMD\_3.1.1\_repeatMasker\_chr.out  
GeneSet:................./storage/core/barbara/genhome/smallRNA/fertility/Sample\_all/pirna/full.gtf  
  
Significant (p<=0.01) hit density will be calculated based  
on observed hit distribution.  
  
Sliding window size: ........................................ 5000 bp  
Sliding window increament: .................................. 1000 bp  
Normalize each hit by number of genomic hits: ............... 1 [0=no/1=yes]  
Normalize each hit by number of sequence reads: ............. 1 [0=no/1=yes]  
Normalize values (-> per million mapped reads): ............. 1 [0=no/1=yes]  
Min. fraction of hits with 1T(U) or 10A: .................... 0.75  
Alternatively: Min. fraction of hits with 1T(U) and 10A: .... 0.5  
Min. fraction of hits with typical piRNA length: ............ 0.75  
Typical piRNA length: ....................................... 26-33 nt  
Min. size of a piRNA cluster: ............................... 5000 bp.  
Min. number of hits (absolute): ............................. 0  
Min. number of hits (normalized): ........................... 0  
Min. fraction of hits on the mainstrand: .................... 0.75  
Top fraction of mapped sequences (in terms of read counts): . 1%  
Top fraction accounts for max. n% of sequence reads: ........ 90%  
Min. fraction of hits on each arm of a bidirectional cluster: 0.1  
Output image file for each cluster: ......................... 0 [0=no/1=yes]  
Output html file for each cluster: .......................... 1 [0=no/1=yes]  
Output a summary table: ..................................... 1 [0=no/1=yes]  
Output a FASTA file for each cluster (piRNA sequences): ..... 1 [0=no/1=yes]  
Output a FASTA file comprising cluster sequences: ........... 1 [0=no/1=yes]  
Search DNA motifs in clusters: .............................. 1 [0=no/1=yes]  
Output flanking sequences: +/- .............................. 0 bp  
Output ~.pTi file: .......................................... 1 [0=no/1=yes]  
==============================================================================  
  
  
Genome size (without gaps): ............ 2678902517 bp  
Gaps (N/X/-): .......................... 53837044 bp  
Mapped reads: .......................... 658825247023  
Non-identical sequences: ............... 514171  
Genomic hits: .......................... 764233  
Significant densitiy of mapped reads: .. 12867599.5173724 reads/kb

Show proTRAC cluster info
Hide proTRAC cluster info

|  |  |
| --- | --- |
| Location | chr19 |
| Coordinates | 41177285-41183423 |
| Size [bp] | 6139 |
| Sequence hit loci | 124 |
| Mapped reads (normalized) | 157820654 |
| Mapped reads (normalized) per kb | 25707876.5 |
| Normalized reads with 1T (1U) | 79.6% |
| Normalized reads with 10A | 23.7% |
| Normalized reads with length 26-33 nt | 100% |
| Normalized reads on the main strand(s) | 100% |
| Predicted directionality | mono:plus |

100%

0%

1T (1U)  
reads

10A reads

26-33 nt  
reads

reads on mainstrand

**Either the amount of reads with 1T (1U) OR 10A has to exceed 75% (set with option: -1Tor10A)  
Alternatively the amount of reads with 1T (1U) AND 10A has to exceed 50% (set with option: -1Tand10A)  
Minimum amount of reads with preferred size is 75% (set with option: -pisize)  
Minimum amount of reads on the main strand(s) is 75% (set with option: -clstrand)**

Show read coverage
Hide read coverage

WHAT DO I SEE HERE?  
This chart shows the location of mapped sequence reads within a predicted piRNA cluster. The color refers to the number of genomic hits produced by the sequence read in question. A dark red bar indicates that this sequence read produces many other hits elsewhere in the genome. Many adjacent red or yellow bars can indicate the presence of a multi-copy element such as transposons or rRNA genes. A dark green bar indicates that this sequence read maps uniquely to this locus.

1 hit

2-5 hits

6-10 hits

11-20 hits

21-50 hits

51-100 hits

> 100 hits

chr19

41177285

41183423

Gene Set

RepeatMasker

Mapped  
Reads

13.57

plus strand

minus strand

13.57

Region: chr19 35571556-41177291. Max. coverage (+): 6.97. Max coverage (-): 0

Region: chr19 41177292-41177303. Max. coverage (+): 6.97. Max coverage (-): 0

Region: chr19 41177304-41177315. Max. coverage (+): 0. Max coverage (-): 0

Region: chr19 41177316-41177327. Max. coverage (+): 0. Max coverage (-): 0

Region: chr19 41177328-41177340. Max. coverage (+): 0. Max coverage (-): 0

Region: chr19 41177341-41177352. Max. coverage (+): 0. Max coverage (-): 0

Region: chr19 41177353-41177364. Max. coverage (+): 0. Max coverage (-): 0

Region: chr19 41177365-41177377. Max. coverage (+): 0. Max coverage (-): 0

Region: chr19 41177378-41177389. Max. coverage (+): 0. Max coverage (-): 0

Region: chr19 41177390-41177401. Max. coverage (+): 0. Max coverage (-): 0

Region: chr19 41177402-41177413. Max. coverage (+): 0. Max coverage (-): 0

Region: chr19 41177414-41177426. Max. coverage (+): 0. Max coverage (-): 0

Region: chr19 41177427-41177438. Max. coverage (+): 0. Max coverage (-): 0

Region: chr19 41177439-41177450. Max. coverage (+): 0. Max coverage (-): 0

Region: chr19 41177451-41177463. Max. coverage (+): 0. Max coverage (-): 0

Region: chr19 41177464-41177475. Max. coverage (+): 0. Max coverage (-): 0

Region: chr19 41177476-41177487. Max. coverage (+): 0. Max coverage (-): 0

Region: chr19 41177488-41177499. Max. coverage (+): 0. Max coverage (-): 0

Region: chr19 41177500-41177512. Max. coverage (+): 0. Max coverage (-): 0

Region: chr19 41177513-41177524. Max. coverage (+): 0. Max coverage (-): 0

Region: chr19 41177525-41177536. Max. coverage (+): 0. Max coverage (-): 0

Region: chr19 41177537-41177548. Max. coverage (+): 0. Max coverage (-): 0

Region: chr19 41177549-41177561. Max. coverage (+): 0. Max coverage (-): 0

Region: chr19 41177562-41177573. Max. coverage (+): 0. Max coverage (-): 0

Region: chr19 41177574-41177585. Max. coverage (+): 0. Max coverage (-): 0

Region: chr19 41177586-41177598. Max. coverage (+): 0. Max coverage (-): 0

Region: chr19 41177599-41177610. Max. coverage (+): 0. Max coverage (-): 0

Region: chr19 41177611-41177622. Max. coverage (+): 0. Max coverage (-): 0

Region: chr19 41177623-41177634. Max. coverage (+): 0. Max coverage (-): 0

Region: chr19 41177635-41177647. Max. coverage (+): 0. Max coverage (-): 0

Region: chr19 41177648-41177659. Max. coverage (+): 0. Max coverage (-): 0

Region: chr19 41177660-41177671. Max. coverage (+): 0. Max coverage (-): 0

Region: chr19 41177672-41177684. Max. coverage (+): 0. Max coverage (-): 0

Region: chr19 41177685-41177696. Max. coverage (+): 0. Max coverage (-): 0

Region: chr19 41177697-41177708. Max. coverage (+): 0. Max coverage (-): 0

Region: chr19 41177709-41177720. Max. coverage (+): 0. Max coverage (-): 0

Region: chr19 41177721-41177733. Max. coverage (+): 0. Max coverage (-): 0

Region: chr19 41177734-41177745. Max. coverage (+): 0. Max coverage (-): 0

Region: chr19 41177746-41177757. Max. coverage (+): 0. Max coverage (-): 0

Region: chr19 41177758-41177769. Max. coverage (+): 2.66. Max coverage (-): 0

Region: chr19 41177770-41177782. Max. coverage (+): 2.66. Max coverage (-): 0

Region: chr19 41177783-41177794. Max. coverage (+): 0. Max coverage (-): 0

Region: chr19 41177795-41177806. Max. coverage (+): 4.21. Max coverage (-): 0

Region: chr19 41177807-41177819. Max. coverage (+): 7.16. Max coverage (-): 0

Region: chr19 41177820-41177831. Max. coverage (+): 7.16. Max coverage (-): 0

Region: chr19 41177832-41177843. Max. coverage (+): 0. Max coverage (-): 0

Region: chr19 41177844-41177855. Max. coverage (+): 0. Max coverage (-): 0

Region: chr19 41177856-41177868. Max. coverage (+): 0. Max coverage (-): 0

Region: chr19 41177869-41177880. Max. coverage (+): 0. Max coverage (-): 0

Region: chr19 41177881-41177892. Max. coverage (+): 0. Max coverage (-): 0

Region: chr19 41177893-41177905. Max. coverage (+): 0. Max coverage (-): 0

Region: chr19 41177906-41177917. Max. coverage (+): 0.84. Max coverage (-): 0

Region: chr19 41177918-41177929. Max. coverage (+): 0. Max coverage (-): 0

Region: chr19 41177930-41177941. Max. coverage (+): 0. Max coverage (-): 0

Region: chr19 41177942-41177954. Max. coverage (+): 13.57. Max coverage (-): 0

Region: chr19 41177955-41177966. Max. coverage (+): 5.15. Max coverage (-): 0

Region: chr19 41177967-41177978. Max. coverage (+): 0. Max coverage (-): 0

Region: chr19 41177979-41177990. Max. coverage (+): 0. Max coverage (-): 0

Region: chr19 41177991-41178003. Max. coverage (+): 0. Max coverage (-): 0

Region: chr19 41178004-41178015. Max. coverage (+): 0. Max coverage (-): 0

Region: chr19 41178016-41178027. Max. coverage (+): 0. Max coverage (-): 0

Region: chr19 41178028-41178040. Max. coverage (+): 0. Max coverage (-): 0

Region: chr19 41178041-41178052. Max. coverage (+): 0. Max coverage (-): 0

Region: chr19 41178053-41178064. Max. coverage (+): 5.47. Max coverage (-): 0

Region: chr19 41178065-41178076. Max. coverage (+): 0. Max coverage (-): 0

Region: chr19 41178077-41178089. Max. coverage (+): 0. Max coverage (-): 0

Region: chr19 41178090-41178101. Max. coverage (+): 0. Max coverage (-): 0

Region: chr19 41178102-41178113. Max. coverage (+): 0. Max coverage (-): 0

Region: chr19 41178114-41178126. Max. coverage (+): 0. Max coverage (-): 0

Region: chr19 41178127-41178138. Max. coverage (+): 0. Max coverage (-): 0

Region: chr19 41178139-41178150. Max. coverage (+): 0. Max coverage (-): 0

Region: chr19 41178151-41178162. Max. coverage (+): 0. Max coverage (-): 0

Region: chr19 41178163-41178175. Max. coverage (+): 0.77. Max coverage (-): 0

Region: chr19 41178176-41178187. Max. coverage (+): 0.77. Max coverage (-): 0

Region: chr19 41178188-41178199. Max. coverage (+): 0. Max coverage (-): 0

Region: chr19 41178200-41178211. Max. coverage (+): 0. Max coverage (-): 0

Region: chr19 41178212-41178224. Max. coverage (+): 0. Max coverage (-): 0

Region: chr19 41178225-41178236. Max. coverage (+): 0. Max coverage (-): 0

Region: chr19 41178237-41178248. Max. coverage (+): 0. Max coverage (-): 0

Region: chr19 41178249-41178261. Max. coverage (+): 1.4. Max coverage (-): 0

Region: chr19 41178262-41178273. Max. coverage (+): 0. Max coverage (-): 0

Region: chr19 41178274-41178285. Max. coverage (+): 1.4. Max coverage (-): 0

Region: chr19 41178286-41178297. Max. coverage (+): 0. Max coverage (-): 0

Region: chr19 41178298-41178310. Max. coverage (+): 0. Max coverage (-): 0

Region: chr19 41178311-41178322. Max. coverage (+): 0. Max coverage (-): 0

Region: chr19 41178323-41178334. Max. coverage (+): 0. Max coverage (-): 0

Region: chr19 41178335-41178347. Max. coverage (+): 0. Max coverage (-): 0

Region: chr19 41178348-41178359. Max. coverage (+): 0. Max coverage (-): 0

Region: chr19 41178360-41178371. Max. coverage (+): 0. Max coverage (-): 0

Region: chr19 41178372-41178383. Max. coverage (+): 0. Max coverage (-): 0

Region: chr19 41178384-41178396. Max. coverage (+): 0. Max coverage (-): 0

Region: chr19 41178397-41178408. Max. coverage (+): 0. Max coverage (-): 0

Region: chr19 41178409-41178420. Max. coverage (+): 0. Max coverage (-): 0

Region: chr19 41178421-41178432. Max. coverage (+): 0. Max coverage (-): 0

Region: chr19 41178433-41178445. Max. coverage (+): 0. Max coverage (-): 0

Region: chr19 41178446-41178457. Max. coverage (+): 0. Max coverage (-): 0

Region: chr19 41178458-41178469. Max. coverage (+): 0. Max coverage (-): 0

Region: chr19 41178470-41178482. Max. coverage (+): 0. Max coverage (-): 0

Region: chr19 41178483-41178494. Max. coverage (+): 0. Max coverage (-): 0

Region: chr19 41178495-41178506. Max. coverage (+): 0. Max coverage (-): 0

Region: chr19 41178507-41178518. Max. coverage (+): 0. Max coverage (-): 0

Region: chr19 41178519-41178531. Max. coverage (+): 0. Max coverage (-): 0

Region: chr19 41178532-41178543. Max. coverage (+): 0. Max coverage (-): 0

Region: chr19 41178544-41178555. Max. coverage (+): 0. Max coverage (-): 0

Region: chr19 41178556-41178568. Max. coverage (+): 0. Max coverage (-): 0

Region: chr19 41178569-41178580. Max. coverage (+): 0. Max coverage (-): 0

Region: chr19 41178581-41178592. Max. coverage (+): 0. Max coverage (-): 0

Region: chr19 41178593-41178604. Max. coverage (+): 0. Max coverage (-): 0

Region: chr19 41178605-41178617. Max. coverage (+): 0. Max coverage (-): 0

Region: chr19 41178618-41178629. Max. coverage (+): 0. Max coverage (-): 0

Region: chr19 41178630-41178641. Max. coverage (+): 0. Max coverage (-): 0

Region: chr19 41178642-41178653. Max. coverage (+): 0. Max coverage (-): 0

Region: chr19 41178654-41178666. Max. coverage (+): 0. Max coverage (-): 0

Region: chr19 41178667-41178678. Max. coverage (+): 0. Max coverage (-): 0

Region: chr19 41178679-41178690. Max. coverage (+): 0. Max coverage (-): 0

Region: chr19 41178691-41178703. Max. coverage (+): 0. Max coverage (-): 0

Region: chr19 41178704-41178715. Max. coverage (+): 2.3. Max coverage (-): 0

Region: chr19 41178716-41178727. Max. coverage (+): 0. Max coverage (-): 0

Region: chr19 41178728-41178739. Max. coverage (+): 0.5. Max coverage (-): 0

Region: chr19 41178740-41178752. Max. coverage (+): 0. Max coverage (-): 0

Region: chr19 41178753-41178764. Max. coverage (+): 0. Max coverage (-): 0

Region: chr19 41178765-41178776. Max. coverage (+): 0. Max coverage (-): 0

Region: chr19 41178777-41178789. Max. coverage (+): 0. Max coverage (-): 0

Region: chr19 41178790-41178801. Max. coverage (+): 4.4. Max coverage (-): 0

Region: chr19 41178802-41178813. Max. coverage (+): 5.09. Max coverage (-): 0

Region: chr19 41178814-41178825. Max. coverage (+): 0. Max coverage (-): 0

Region: chr19 41178826-41178838. Max. coverage (+): 0. Max coverage (-): 0

Region: chr19 41178839-41178850. Max. coverage (+): 0.23. Max coverage (-): 0

Region: chr19 41178851-41178862. Max. coverage (+): 1.66. Max coverage (-): 0

Region: chr19 41178863-41178875. Max. coverage (+): 0. Max coverage (-): 0

Region: chr19 41178876-41178887. Max. coverage (+): 2.55. Max coverage (-): 0

Region: chr19 41178888-41178899. Max. coverage (+): 2.55. Max coverage (-): 0

Region: chr19 41178900-41178911. Max. coverage (+): 3.19. Max coverage (-): 0

Region: chr19 41178912-41178924. Max. coverage (+): 0. Max coverage (-): 0

Region: chr19 41178925-41178936. Max. coverage (+): 0. Max coverage (-): 0

Region: chr19 41178937-41178948. Max. coverage (+): 0. Max coverage (-): 0

Region: chr19 41178949-41178960. Max. coverage (+): 0. Max coverage (-): 0

Region: chr19 41178961-41178973. Max. coverage (+): 0. Max coverage (-): 0

Region: chr19 41178974-41178985. Max. coverage (+): 0. Max coverage (-): 0

Region: chr19 41178986-41178997. Max. coverage (+): 0. Max coverage (-): 0

Region: chr19 41178998-41179010. Max. coverage (+): 0. Max coverage (-): 0

Region: chr19 41179011-41179022. Max. coverage (+): 0. Max coverage (-): 0

Region: chr19 41179023-41179034. Max. coverage (+): 0. Max coverage (-): 0

Region: chr19 41179035-41179046. Max. coverage (+): 0. Max coverage (-): 0

Region: chr19 41179047-41179059. Max. coverage (+): 0. Max coverage (-): 0

Region: chr19 41179060-41179071. Max. coverage (+): 0. Max coverage (-): 0

Region: chr19 41179072-41179083. Max. coverage (+): 0. Max coverage (-): 0

Region: chr19 41179084-41179096. Max. coverage (+): 0. Max coverage (-): 0

Region: chr19 41179097-41179108. Max. coverage (+): 0. Max coverage (-): 0

Region: chr19 41179109-41179120. Max. coverage (+): 0. Max coverage (-): 0

Region: chr19 41179121-41179132. Max. coverage (+): 0. Max coverage (-): 0

Region: chr19 41179133-41179145. Max. coverage (+): 0. Max coverage (-): 0

Region: chr19 41179146-41179157. Max. coverage (+): 0. Max coverage (-): 0

Region: chr19 41179158-41179169. Max. coverage (+): 0. Max coverage (-): 0

Region: chr19 41179170-41179181. Max. coverage (+): 9.53. Max coverage (-): 0

Region: chr19 41179182-41179194. Max. coverage (+): 9.53. Max coverage (-): 0

Region: chr19 41179195-41179206. Max. coverage (+): 0. Max coverage (-): 0

Region: chr19 41179207-41179218. Max. coverage (+): 0. Max coverage (-): 0

Region: chr19 41179219-41179231. Max. coverage (+): 6.73. Max coverage (-): 0

Region: chr19 41179232-41179243. Max. coverage (+): 3.3. Max coverage (-): 0

Region: chr19 41179244-41179255. Max. coverage (+): 0. Max coverage (-): 0

Region: chr19 41179256-41179267. Max. coverage (+): 0. Max coverage (-): 0

Region: chr19 41179268-41179280. Max. coverage (+): 0. Max coverage (-): 0

Region: chr19 41179281-41179292. Max. coverage (+): 4.6. Max coverage (-): 0

Region: chr19 41179293-41179304. Max. coverage (+): 4.6. Max coverage (-): 0

Region: chr19 41179305-41179317. Max. coverage (+): 0. Max coverage (-): 0

Region: chr19 41179318-41179329. Max. coverage (+): 0. Max coverage (-): 0

Region: chr19 41179330-41179341. Max. coverage (+): 0. Max coverage (-): 0

Region: chr19 41179342-41179353. Max. coverage (+): 0. Max coverage (-): 0

Region: chr19 41179354-41179366. Max. coverage (+): 0. Max coverage (-): 0

Region: chr19 41179367-41179378. Max. coverage (+): 0. Max coverage (-): 0

Region: chr19 41179379-41179390. Max. coverage (+): 0. Max coverage (-): 0

Region: chr19 41179391-41179402. Max. coverage (+): 0. Max coverage (-): 0

Region: chr19 41179403-41179415. Max. coverage (+): 0. Max coverage (-): 0

Region: chr19 41179416-41179427. Max. coverage (+): 0. Max coverage (-): 0

Region: chr19 41179428-41179439. Max. coverage (+): 0. Max coverage (-): 0

Region: chr19 41179440-41179452. Max. coverage (+): 0. Max coverage (-): 0

Region: chr19 41179453-41179464. Max. coverage (+): 0. Max coverage (-): 0

Region: chr19 41179465-41179476. Max. coverage (+): 0. Max coverage (-): 0

Region: chr19 41179477-41179488. Max. coverage (+): 0. Max coverage (-): 0

Region: chr19 41179489-41179501. Max. coverage (+): 0. Max coverage (-): 0

Region: chr19 41179502-41179513. Max. coverage (+): 0. Max coverage (-): 0

Region: chr19 41179514-41179525. Max. coverage (+): 0. Max coverage (-): 0

Region: chr19 41179526-41179538. Max. coverage (+): 0. Max coverage (-): 0

Region: chr19 41179539-41179550. Max. coverage (+): 0. Max coverage (-): 0

Region: chr19 41179551-41179562. Max. coverage (+): 4.08. Max coverage (-): 0

Region: chr19 41179563-41179574. Max. coverage (+): 12.66. Max coverage (-): 0

Region: chr19 41179575-41179587. Max. coverage (+): 4.66. Max coverage (-): 0

Region: chr19 41179588-41179599. Max. coverage (+): 0. Max coverage (-): 0

Region: chr19 41179600-41179611. Max. coverage (+): 0. Max coverage (-): 0

Region: chr19 41179612-41179623. Max. coverage (+): 0. Max coverage (-): 0

Region: chr19 41179624-41179636. Max. coverage (+): 0. Max coverage (-): 0

Region: chr19 41179637-41179648. Max. coverage (+): 0. Max coverage (-): 0

Region: chr19 41179649-41179660. Max. coverage (+): 0. Max coverage (-): 0

Region: chr19 41179661-41179673. Max. coverage (+): 0. Max coverage (-): 0

Region: chr19 41179674-41179685. Max. coverage (+): 0. Max coverage (-): 0

Region: chr19 41179686-41179697. Max. coverage (+): 0. Max coverage (-): 0

Region: chr19 41179698-41179709. Max. coverage (+): 0. Max coverage (-): 0

Region: chr19 41179710-41179722. Max. coverage (+): 1.41. Max coverage (-): 0

Region: chr19 41179723-41179734. Max. coverage (+): 1.41. Max coverage (-): 0

Region: chr19 41179735-41179746. Max. coverage (+): 0. Max coverage (-): 0

Region: chr19 41179747-41179759. Max. coverage (+): 0. Max coverage (-): 0

Region: chr19 41179760-41179771. Max. coverage (+): 1.32. Max coverage (-): 0

Region: chr19 41179772-41179783. Max. coverage (+): 1.32. Max coverage (-): 0

Region: chr19 41179784-41179795. Max. coverage (+): 0. Max coverage (-): 0

Region: chr19 41179796-41179808. Max. coverage (+): 8.72. Max coverage (-): 0

Region: chr19 41179809-41179820. Max. coverage (+): 2.49. Max coverage (-): 0

Region: chr19 41179821-41179832. Max. coverage (+): 0. Max coverage (-): 0

Region: chr19 41179833-41179844. Max. coverage (+): 0. Max coverage (-): 0

Region: chr19 41179845-41179857. Max. coverage (+): 0. Max coverage (-): 0

Region: chr19 41179858-41179869. Max. coverage (+): 0. Max coverage (-): 0

Region: chr19 41179870-41179881. Max. coverage (+): 0. Max coverage (-): 0

Region: chr19 41179882-41179894. Max. coverage (+): 0. Max coverage (-): 0

Region: chr19 41179895-41179906. Max. coverage (+): 0. Max coverage (-): 0

Region: chr19 41179907-41179918. Max. coverage (+): 0. Max coverage (-): 0

Region: chr19 41179919-41179930. Max. coverage (+): 0. Max coverage (-): 0

Region: chr19 41179931-41179943. Max. coverage (+): 0. Max coverage (-): 0

Region: chr19 41179944-41179955. Max. coverage (+): 0. Max coverage (-): 0

Region: chr19 41179956-41179967. Max. coverage (+): 0. Max coverage (-): 0

Region: chr19 41179968-41179980. Max. coverage (+): 4.63. Max coverage (-): 0

Region: chr19 41179981-41179992. Max. coverage (+): 0. Max coverage (-): 0

Region: chr19 41179993-41180004. Max. coverage (+): 1.15. Max coverage (-): 0

Region: chr19 41180005-41180016. Max. coverage (+): 1.15. Max coverage (-): 0

Region: chr19 41180017-41180029. Max. coverage (+): 0. Max coverage (-): 0

Region: chr19 41180030-41180041. Max. coverage (+): 0. Max coverage (-): 0

Region: chr19 41180042-41180053. Max. coverage (+): 0. Max coverage (-): 0

Region: chr19 41180054-41180065. Max. coverage (+): 0. Max coverage (-): 0

Region: chr19 41180066-41180078. Max. coverage (+): 0. Max coverage (-): 0

Region: chr19 41180079-41180090. Max. coverage (+): 0. Max coverage (-): 0

Region: chr19 41180091-41180102. Max. coverage (+): 0. Max coverage (-): 0

Region: chr19 41180103-41180115. Max. coverage (+): 1.98. Max coverage (-): 0

Region: chr19 41180116-41180127. Max. coverage (+): 2.88. Max coverage (-): 0

Region: chr19 41180128-41180139. Max. coverage (+): 1.18. Max coverage (-): 0

Region: chr19 41180140-41180151. Max. coverage (+): 0. Max coverage (-): 0

Region: chr19 41180152-41180164. Max. coverage (+): 0. Max coverage (-): 0

Region: chr19 41180165-41180176. Max. coverage (+): 5.24. Max coverage (-): 0

Region: chr19 41180177-41180188. Max. coverage (+): 0. Max coverage (-): 0

Region: chr19 41180189-41180201. Max. coverage (+): 0. Max coverage (-): 0

Region: chr19 41180202-41180213. Max. coverage (+): 0. Max coverage (-): 0

Region: chr19 41180214-41180225. Max. coverage (+): 5.02. Max coverage (-): 0

Region: chr19 41180226-41180237. Max. coverage (+): 5.02. Max coverage (-): 0

Region: chr19 41180238-41180250. Max. coverage (+): 0. Max coverage (-): 0

Region: chr19 41180251-41180262. Max. coverage (+): 0. Max coverage (-): 0

Region: chr19 41180263-41180274. Max. coverage (+): 0. Max coverage (-): 0

Region: chr19 41180275-41180286. Max. coverage (+): 0. Max coverage (-): 0

Region: chr19 41180287-41180299. Max. coverage (+): 0. Max coverage (-): 0

Region: chr19 41180300-41180311. Max. coverage (+): 8.12. Max coverage (-): 0

Region: chr19 41180312-41180323. Max. coverage (+): 0. Max coverage (-): 0

Region: chr19 41180324-41180336. Max. coverage (+): 0. Max coverage (-): 0

Region: chr19 41180337-41180348. Max. coverage (+): 0. Max coverage (-): 0

Region: chr19 41180349-41180360. Max. coverage (+): 9.45. Max coverage (-): 0

Region: chr19 41180361-41180372. Max. coverage (+): 2.59. Max coverage (-): 0

Region: chr19 41180373-41180385. Max. coverage (+): 0. Max coverage (-): 0

Region: chr19 41180386-41180397. Max. coverage (+): 0. Max coverage (-): 0

Region: chr19 41180398-41180409. Max. coverage (+): 0. Max coverage (-): 0

Region: chr19 41180410-41180422. Max. coverage (+): 0. Max coverage (-): 0

Region: chr19 41180423-41180434. Max. coverage (+): 0. Max coverage (-): 0

Region: chr19 41180435-41180446. Max. coverage (+): 0. Max coverage (-): 0

Region: chr19 41180447-41180458. Max. coverage (+): 0. Max coverage (-): 0

Region: chr19 41180459-41180471. Max. coverage (+): 0. Max coverage (-): 0

Region: chr19 41180472-41180483. Max. coverage (+): 0. Max coverage (-): 0

Region: chr19 41180484-41180495. Max. coverage (+): 0. Max coverage (-): 0

Region: chr19 41180496-41180507. Max. coverage (+): 0. Max coverage (-): 0

Region: chr19 41180508-41180520. Max. coverage (+): 0. Max coverage (-): 0

Region: chr19 41180521-41180532. Max. coverage (+): 0. Max coverage (-): 0

Region: chr19 41180533-41180544. Max. coverage (+): 0. Max coverage (-): 0

Region: chr19 41180545-41180557. Max. coverage (+): 0. Max coverage (-): 0

Region: chr19 41180558-41180569. Max. coverage (+): 0. Max coverage (-): 0

Region: chr19 41180570-41180581. Max. coverage (+): 0. Max coverage (-): 0

Region: chr19 41180582-41180593. Max. coverage (+): 0. Max coverage (-): 0

Region: chr19 41180594-41180606. Max. coverage (+): 0. Max coverage (-): 0

Region: chr19 41180607-41180618. Max. coverage (+): 0. Max coverage (-): 0

Region: chr19 41180619-41180630. Max. coverage (+): 0. Max coverage (-): 0

Region: chr19 41180631-41180643. Max. coverage (+): 0. Max coverage (-): 0

Region: chr19 41180644-41180655. Max. coverage (+): 0. Max coverage (-): 0

Region: chr19 41180656-41180667. Max. coverage (+): 0. Max coverage (-): 0

Region: chr19 41180668-41180679. Max. coverage (+): 0. Max coverage (-): 0

Region: chr19 41180680-41180692. Max. coverage (+): 0.81. Max coverage (-): 0

Region: chr19 41180693-41180704. Max. coverage (+): 0. Max coverage (-): 0

Region: chr19 41180705-41180716. Max. coverage (+): 4.45. Max coverage (-): 0

Region: chr19 41180717-41180728. Max. coverage (+): 2.11. Max coverage (-): 0

Region: chr19 41180729-41180741. Max. coverage (+): 1.05. Max coverage (-): 0

Region: chr19 41180742-41180753. Max. coverage (+): 0. Max coverage (-): 0

Region: chr19 41180754-41180765. Max. coverage (+): 0. Max coverage (-): 0

Region: chr19 41180766-41180778. Max. coverage (+): 0. Max coverage (-): 0

Region: chr19 41180779-41180790. Max. coverage (+): 0. Max coverage (-): 0

Region: chr19 41180791-41180802. Max. coverage (+): 0. Max coverage (-): 0

Region: chr19 41180803-41180814. Max. coverage (+): 0. Max coverage (-): 0

Region: chr19 41180815-41180827. Max. coverage (+): 0. Max coverage (-): 0

Region: chr19 41180828-41180839. Max. coverage (+): 0. Max coverage (-): 0

Region: chr19 41180840-41180851. Max. coverage (+): 0. Max coverage (-): 0

Region: chr19 41180852-41180864. Max. coverage (+): 6.06. Max coverage (-): 0

Region: chr19 41180865-41180876. Max. coverage (+): 4.2. Max coverage (-): 0

Region: chr19 41180877-41180888. Max. coverage (+): 11.68. Max coverage (-): 0

Region: chr19 41180889-41180900. Max. coverage (+): 11.68. Max coverage (-): 0

Region: chr19 41180901-41180913. Max. coverage (+): 0. Max coverage (-): 0

Region: chr19 41180914-41180925. Max. coverage (+): 0. Max coverage (-): 0

Region: chr19 41180926-41180937. Max. coverage (+): 0. Max coverage (-): 0

Region: chr19 41180938-41180949. Max. coverage (+): 0. Max coverage (-): 0

Region: chr19 41180950-41180962. Max. coverage (+): 0. Max coverage (-): 0

Region: chr19 41180963-41180974. Max. coverage (+): 0. Max coverage (-): 0

Region: chr19 41180975-41180986. Max. coverage (+): 0. Max coverage (-): 0

Region: chr19 41180987-41180999. Max. coverage (+): 0. Max coverage (-): 0

Region: chr19 41181000-41181011. Max. coverage (+): 0. Max coverage (-): 0

Region: chr19 41181012-41181023. Max. coverage (+): 0. Max coverage (-): 0

Region: chr19 41181024-41181035. Max. coverage (+): 0. Max coverage (-): 0

Region: chr19 41181036-41181048. Max. coverage (+): 0. Max coverage (-): 0

Region: chr19 41181049-41181060. Max. coverage (+): 0. Max coverage (-): 0

Region: chr19 41181061-41181072. Max. coverage (+): 0. Max coverage (-): 0

Region: chr19 41181073-41181085. Max. coverage (+): 0. Max coverage (-): 0

Region: chr19 41181086-41181097. Max. coverage (+): 0. Max coverage (-): 0

Region: chr19 41181098-41181109. Max. coverage (+): 0. Max coverage (-): 0

Region: chr19 41181110-41181121. Max. coverage (+): 0. Max coverage (-): 0

Region: chr19 41181122-41181134. Max. coverage (+): 0. Max coverage (-): 0

Region: chr19 41181135-41181146. Max. coverage (+): 3.32. Max coverage (-): 0

Region: chr19 41181147-41181158. Max. coverage (+): 0. Max coverage (-): 0

Region: chr19 41181159-41181170. Max. coverage (+): 0. Max coverage (-): 0

Region: chr19 41181171-41181183. Max. coverage (+): 0. Max coverage (-): 0

Region: chr19 41181184-41181195. Max. coverage (+): 0. Max coverage (-): 0

Region: chr19 41181196-41181207. Max. coverage (+): 0. Max coverage (-): 0

Region: chr19 41181208-41181220. Max. coverage (+): 0. Max coverage (-): 0

Region: chr19 41181221-41181232. Max. coverage (+): 0.83. Max coverage (-): 0

Region: chr19 41181233-41181244. Max. coverage (+): 0.83. Max coverage (-): 0

Region: chr19 41181245-41181256. Max. coverage (+): 0. Max coverage (-): 0

Region: chr19 41181257-41181269. Max. coverage (+): 0. Max coverage (-): 0

Region: chr19 41181270-41181281. Max. coverage (+): 0. Max coverage (-): 0

Region: chr19 41181282-41181293. Max. coverage (+): 10.5. Max coverage (-): 0

Region: chr19 41181294-41181306. Max. coverage (+): 10.5. Max coverage (-): 0

Region: chr19 41181307-41181318. Max. coverage (+): 0. Max coverage (-): 0

Region: chr19 41181319-41181330. Max. coverage (+): 0. Max coverage (-): 0

Region: chr19 41181331-41181342. Max. coverage (+): 0. Max coverage (-): 0

Region: chr19 41181343-41181355. Max. coverage (+): 0. Max coverage (-): 0

Region: chr19 41181356-41181367. Max. coverage (+): 0. Max coverage (-): 0

Region: chr19 41181368-41181379. Max. coverage (+): 0. Max coverage (-): 0

Region: chr19 41181380-41181391. Max. coverage (+): 0. Max coverage (-): 0

Region: chr19 41181392-41181404. Max. coverage (+): 0. Max coverage (-): 0

Region: chr19 41181405-41181416. Max. coverage (+): 0. Max coverage (-): 0

Region: chr19 41181417-41181428. Max. coverage (+): 0. Max coverage (-): 0

Region: chr19 41181429-41181441. Max. coverage (+): 0. Max coverage (-): 0

Region: chr19 41181442-41181453. Max. coverage (+): 0. Max coverage (-): 0

Region: chr19 41181454-41181465. Max. coverage (+): 0. Max coverage (-): 0

Region: chr19 41181466-41181477. Max. coverage (+): 0. Max coverage (-): 0

Region: chr19 41181478-41181490. Max. coverage (+): 0. Max coverage (-): 0

Region: chr19 41181491-41181502. Max. coverage (+): 0.88. Max coverage (-): 0

Region: chr19 41181503-41181514. Max. coverage (+): 0.88. Max coverage (-): 0

Region: chr19 41181515-41181527. Max. coverage (+): 0. Max coverage (-): 0

Region: chr19 41181528-41181539. Max. coverage (+): 0. Max coverage (-): 0

Region: chr19 41181540-41181551. Max. coverage (+): 0. Max coverage (-): 0

Region: chr19 41181552-41181563. Max. coverage (+): 0. Max coverage (-): 0

Region: chr19 41181564-41181576. Max. coverage (+): 0. Max coverage (-): 0

Region: chr19 41181577-41181588. Max. coverage (+): 0. Max coverage (-): 0

Region: chr19 41181589-41181600. Max. coverage (+): 0. Max coverage (-): 0

Region: chr19 41181601-41181612. Max. coverage (+): 0. Max coverage (-): 0

Region: chr19 41181613-41181625. Max. coverage (+): 0. Max coverage (-): 0

Region: chr19 41181626-41181637. Max. coverage (+): 2.05. Max coverage (-): 0

Region: chr19 41181638-41181649. Max. coverage (+): 0. Max coverage (-): 0

Region: chr19 41181650-41181662. Max. coverage (+): 0. Max coverage (-): 0

Region: chr19 41181663-41181674. Max. coverage (+): 0. Max coverage (-): 0

Region: chr19 41181675-41181686. Max. coverage (+): 0. Max coverage (-): 0

Region: chr19 41181687-41181698. Max. coverage (+): 0. Max coverage (-): 0

Region: chr19 41181699-41181711. Max. coverage (+): 0. Max coverage (-): 0

Region: chr19 41181712-41181723. Max. coverage (+): 0. Max coverage (-): 0

Region: chr19 41181724-41181735. Max. coverage (+): 0. Max coverage (-): 0

Region: chr19 41181736-41181748. Max. coverage (+): 0. Max coverage (-): 0

Region: chr19 41181749-41181760. Max. coverage (+): 0. Max coverage (-): 0

Region: chr19 41181761-41181772. Max. coverage (+): 0. Max coverage (-): 0

Region: chr19 41181773-41181784. Max. coverage (+): 0. Max coverage (-): 0

Region: chr19 41181785-41181797. Max. coverage (+): 3.78. Max coverage (-): 0

Region: chr19 41181798-41181809. Max. coverage (+): 2.3. Max coverage (-): 0

Region: chr19 41181810-41181821. Max. coverage (+): 1.12. Max coverage (-): 0

Region: chr19 41181822-41181833. Max. coverage (+): 0. Max coverage (-): 0

Region: chr19 41181834-41181846. Max. coverage (+): 0. Max coverage (-): 0

Region: chr19 41181847-41181858. Max. coverage (+): 0. Max coverage (-): 0

Region: chr19 41181859-41181870. Max. coverage (+): 0. Max coverage (-): 0

Region: chr19 41181871-41181883. Max. coverage (+): 0. Max coverage (-): 0

Region: chr19 41181884-41181895. Max. coverage (+): 0. Max coverage (-): 0

Region: chr19 41181896-41181907. Max. coverage (+): 5.98. Max coverage (-): 0

Region: chr19 41181908-41181919. Max. coverage (+): 4.1. Max coverage (-): 0

Region: chr19 41181920-41181932. Max. coverage (+): 0. Max coverage (-): 0

Region: chr19 41181933-41181944. Max. coverage (+): 0. Max coverage (-): 0

Region: chr19 41181945-41181956. Max. coverage (+): 0. Max coverage (-): 0

Region: chr19 41181957-41181969. Max. coverage (+): 0. Max coverage (-): 0

Region: chr19 41181970-41181981. Max. coverage (+): 0. Max coverage (-): 0

Region: chr19 41181982-41181993. Max. coverage (+): 0. Max coverage (-): 0

Region: chr19 41181994-41182005. Max. coverage (+): 0. Max coverage (-): 0

Region: chr19 41182006-41182018. Max. coverage (+): 0. Max coverage (-): 0

Region: chr19 41182019-41182030. Max. coverage (+): 0. Max coverage (-): 0

Region: chr19 41182031-41182042. Max. coverage (+): 0. Max coverage (-): 0

Region: chr19 41182043-41182055. Max. coverage (+): 0. Max coverage (-): 0

Region: chr19 41182056-41182067. Max. coverage (+): 0. Max coverage (-): 0

Region: chr19 41182068-41182079. Max. coverage (+): 0. Max coverage (-): 0

Region: chr19 41182080-41182091. Max. coverage (+): 0. Max coverage (-): 0

Region: chr19 41182092-41182104. Max. coverage (+): 0. Max coverage (-): 0

Region: chr19 41182105-41182116. Max. coverage (+): 0. Max coverage (-): 0

Region: chr19 41182117-41182128. Max. coverage (+): 0. Max coverage (-): 0

Region: chr19 41182129-41182140. Max. coverage (+): 0. Max coverage (-): 0

Region: chr19 41182141-41182153. Max. coverage (+): 0. Max coverage (-): 0

Region: chr19 41182154-41182165. Max. coverage (+): 0. Max coverage (-): 0

Region: chr19 41182166-41182177. Max. coverage (+): 0. Max coverage (-): 0

Region: chr19 41182178-41182190. Max. coverage (+): 2.12. Max coverage (-): 0

Region: chr19 41182191-41182202. Max. coverage (+): 2.12. Max coverage (-): 0

Region: chr19 41182203-41182214. Max. coverage (+): 0. Max coverage (-): 0

Region: chr19 41182215-41182226. Max. coverage (+): 0. Max coverage (-): 0

Region: chr19 41182227-41182239. Max. coverage (+): 0. Max coverage (-): 0

Region: chr19 41182240-41182251. Max. coverage (+): 0. Max coverage (-): 0

Region: chr19 41182252-41182263. Max. coverage (+): 0. Max coverage (-): 0

Region: chr19 41182264-41182276. Max. coverage (+): 0. Max coverage (-): 0

Region: chr19 41182277-41182288. Max. coverage (+): 0. Max coverage (-): 0

Region: chr19 41182289-41182300. Max. coverage (+): 0. Max coverage (-): 0

Region: chr19 41182301-41182312. Max. coverage (+): 0. Max coverage (-): 0

Region: chr19 41182313-41182325. Max. coverage (+): 0. Max coverage (-): 0

Region: chr19 41182326-41182337. Max. coverage (+): 0. Max coverage (-): 0

Region: chr19 41182338-41182349. Max. coverage (+): 0. Max coverage (-): 0

Region: chr19 41182350-41182361. Max. coverage (+): 0. Max coverage (-): 0

Region: chr19 41182362-41182374. Max. coverage (+): 0. Max coverage (-): 0

Region: chr19 41182375-41182386. Max. coverage (+): 0. Max coverage (-): 0

Region: chr19 41182387-41182398. Max. coverage (+): 4.24. Max coverage (-): 0

Region: chr19 41182399-41182411. Max. coverage (+): 3.69. Max coverage (-): 0

Region: chr19 41182412-41182423. Max. coverage (+): 3.69. Max coverage (-): 0

Region: chr19 41182424-41182435. Max. coverage (+): 0. Max coverage (-): 0

Region: chr19 41182436-41182447. Max. coverage (+): 0. Max coverage (-): 0

Region: chr19 41182448-41182460. Max. coverage (+): 0. Max coverage (-): 0

Region: chr19 41182461-41182472. Max. coverage (+): 0. Max coverage (-): 0

Region: chr19 41182473-41182484. Max. coverage (+): 1.91. Max coverage (-): 0

Region: chr19 41182485-41182497. Max. coverage (+): 0. Max coverage (-): 0

Region: chr19 41182498-41182509. Max. coverage (+): 0. Max coverage (-): 0

Region: chr19 41182510-41182521. Max. coverage (+): 0. Max coverage (-): 0

Region: chr19 41182522-41182533. Max. coverage (+): 0. Max coverage (-): 0

Region: chr19 41182534-41182546. Max. coverage (+): 5.04. Max coverage (-): 0

Region: chr19 41182547-41182558. Max. coverage (+): 5.04. Max coverage (-): 0

Region: chr19 41182559-41182570. Max. coverage (+): 0. Max coverage (-): 0

Region: chr19 41182571-41182582. Max. coverage (+): 0. Max coverage (-): 0

Region: chr19 41182583-41182595. Max. coverage (+): 0. Max coverage (-): 0

Region: chr19 41182596-41182607. Max. coverage (+): 0. Max coverage (-): 0

Region: chr19 41182608-41182619. Max. coverage (+): 0. Max coverage (-): 0

Region: chr19 41182620-41182632. Max. coverage (+): 0. Max coverage (-): 0

Region: chr19 41182633-41182644. Max. coverage (+): 0. Max coverage (-): 0

Region: chr19 41182645-41182656. Max. coverage (+): 0. Max coverage (-): 0

Region: chr19 41182657-41182668. Max. coverage (+): 0. Max coverage (-): 0

Region: chr19 41182669-41182681. Max. coverage (+): 0. Max coverage (-): 0

Region: chr19 41182682-41182693. Max. coverage (+): 0. Max coverage (-): 0

Region: chr19 41182694-41182705. Max. coverage (+): 0. Max coverage (-): 0

Region: chr19 41182706-41182718. Max. coverage (+): 0. Max coverage (-): 0

Region: chr19 41182719-41182730. Max. coverage (+): 0. Max coverage (-): 0

Region: chr19 41182731-41182742. Max. coverage (+): 0. Max coverage (-): 0

Region: chr19 41182743-41182754. Max. coverage (+): 0. Max coverage (-): 0

Region: chr19 41182755-41182767. Max. coverage (+): 0. Max coverage (-): 0

Region: chr19 41182768-41182779. Max. coverage (+): 0. Max coverage (-): 0

Region: chr19 41182780-41182791. Max. coverage (+): 0. Max coverage (-): 0

Region: chr19 41182792-41182803. Max. coverage (+): 0. Max coverage (-): 0

Region: chr19 41182804-41182816. Max. coverage (+): 0. Max coverage (-): 0

Region: chr19 41182817-41182828. Max. coverage (+): 0. Max coverage (-): 0

Region: chr19 41182829-41182840. Max. coverage (+): 0. Max coverage (-): 0

Region: chr19 41182841-41182853. Max. coverage (+): 0. Max coverage (-): 0

Region: chr19 41182854-41182865. Max. coverage (+): 0. Max coverage (-): 0

Region: chr19 41182866-41182877. Max. coverage (+): 0. Max coverage (-): 0

Region: chr19 41182878-41182889. Max. coverage (+): 0. Max coverage (-): 0

Region: chr19 41182890-41182902. Max. coverage (+): 0. Max coverage (-): 0

Region: chr19 41182903-41182914. Max. coverage (+): 0. Max coverage (-): 0

Region: chr19 41182915-41182926. Max. coverage (+): 0. Max coverage (-): 0

Region: chr19 41182927-41182939. Max. coverage (+): 0. Max coverage (-): 0

Region: chr19 41182940-41182951. Max. coverage (+): 0. Max coverage (-): 0

Region: chr19 41182952-41182963. Max. coverage (+): 0. Max coverage (-): 0

Region: chr19 41182964-41182975. Max. coverage (+): 0. Max coverage (-): 0

Region: chr19 41182976-41182988. Max. coverage (+): 0. Max coverage (-): 0

Region: chr19 41182989-41183000. Max. coverage (+): 0. Max coverage (-): 0

Region: chr19 41183001-41183012. Max. coverage (+): 0. Max coverage (-): 0

Region: chr19 41183013-41183024. Max. coverage (+): 0. Max coverage (-): 0

Region: chr19 41183025-41183037. Max. coverage (+): 0. Max coverage (-): 0

Region: chr19 41183038-41183049. Max. coverage (+): 0. Max coverage (-): 0

Region: chr19 41183050-41183061. Max. coverage (+): 0. Max coverage (-): 0

Region: chr19 41183062-41183074. Max. coverage (+): 0. Max coverage (-): 0

Region: chr19 41183075-41183086. Max. coverage (+): 0. Max coverage (-): 0

Region: chr19 41183087-41183098. Max. coverage (+): 0. Max coverage (-): 0

Region: chr19 41183099-41183110. Max. coverage (+): 0. Max coverage (-): 0

Region: chr19 41183111-41183123. Max. coverage (+): 0. Max coverage (-): 0

Region: chr19 41183124-41183135. Max. coverage (+): 0. Max coverage (-): 0

Region: chr19 41183136-41183147. Max. coverage (+): 0. Max coverage (-): 0

Region: chr19 41183148-41183160. Max. coverage (+): 0. Max coverage (-): 0

Region: chr19 41183161-41183172. Max. coverage (+): 0. Max coverage (-): 0

Region: chr19 41183173-41183184. Max. coverage (+): 0. Max coverage (-): 0

Region: chr19 41183185-41183196. Max. coverage (+): 0. Max coverage (-): 0

Region: chr19 41183197-41183209. Max. coverage (+): 0. Max coverage (-): 0

Region: chr19 41183210-41183221. Max. coverage (+): 0. Max coverage (-): 0

Region: chr19 41183222-41183233. Max. coverage (+): 0. Max coverage (-): 0

Region: chr19 41183234-41183245. Max. coverage (+): 0. Max coverage (-): 0

Region: chr19 41183246-41183258. Max. coverage (+): 0. Max coverage (-): 0

Region: chr19 41183259-41183270. Max. coverage (+): 0. Max coverage (-): 0

Region: chr19 41183271-41183282. Max. coverage (+): 0. Max coverage (-): 0

Region: chr19 41183283-41183295. Max. coverage (+): 0.71. Max coverage (-): 0

Region: chr19 41183296-41183307. Max. coverage (+): 0.71. Max coverage (-): 0

Region: chr19 41183308-41183319. Max. coverage (+): 0. Max coverage (-): 0

Region: chr19 41183320-41183331. Max. coverage (+): 0. Max coverage (-): 0

Region: chr19 41183332-41183344. Max. coverage (+): 0. Max coverage (-): 0

Region: chr19 41183345-41183356. Max. coverage (+): 0. Max coverage (-): 0

Region: chr19 41183357-41183368. Max. coverage (+): 0. Max coverage (-): 0

Region: chr19 41183369-41183381. Max. coverage (+): 6.16. Max coverage (-): 0

Region: chr19 41183382-41183393. Max. coverage (+): 0.78. Max coverage (-): 0

Region: chr19 41183394-41183405. Max. coverage (+): 0.78. Max coverage (-): 0

Region: chr19 41183406-41183417. Max. coverage (+): 0. Max coverage (-): 0

Region: chr19 41183418-. Max. coverage (+): 0. Max coverage (-): 0

RepeatMasker Color Code

**+**

100-98% Identity

<98-95% Identity

<95-90% Identity

<90-85% Identity

<85-80% Identity

<80-75% Identity

<75-70% Identity

<70% Identity

**-**

Gene Set Color Code

**+**

Gene

Pseudogene

**-**

Topology/Coverage Color Code

Coverage Plus Strand

Coverage Minus Strand

Mainstrand: Plus

Mainstrand: Minus

Complementary Strand

Flanking Region  
(if option -flank >0)

Gene Set Annotation  

**1. WIPF2 (protein coding, ENSBTAG00000038126) Tr:00000013695 Ex:6**: 41177259-41177360 (+)  
**2. WIPF2 (protein coding, ENSBTAG00000038126) Tr:00000013695 Ex:7**: 41177599-41177893 (+)

  
RepeatMasker Annotation  

**1. L3**: 41183050-41183091 (+), Divergence to consensus: 26.2%  
**2. L2c**: 41183096-41183218 (-), Divergence to consensus: 32.2%

  
Transcription Factor Binding Sites  

**RFX4\_1** (Sequence: CATGGCAAC (+): 41181370)  
**SPZ1** (Sequence: CTCAAACCCT (-): 41177854)  
**SPZ1** (Sequence: CTGTAACCCC (-): 41178217)  
**RFX4\_2** (Sequence: CATAGTTAC (+): 41180001)  
**SOX9** (Sequence: TCATTGTT (+): 41177964)  
**SOX9** (Sequence: CTATTGTT (+): 41179139)  
**SOX9** (Sequence: TCATTGTT (+): 41182440)
