# Supplementary material for: Small RNA sequencing of cryopreserved semen from single bull revealed altered miRNAs and piRNAs expression between High- and Low-motile sperm populations
Source: BMC Genomics. 2017 Jan 4;18:14. doi: 10.1186/s12864-016-3394-7 (PMC5209821; doi:10.1186/s12864-016-3394-7)
Supplement: Additional file 3: — Details for each piRNA clusters found in High Motile (HM) sperm fraction. Genes, repeats, transposable elements and transcription factors binding sites falling within the cluster regions were reported. (ZIP 1896 kb) [file 12864_2016_3394_MOESM3_ESM.zip › 44.html]

piRNA cluster 44


Predicted piRNA cluster no. 44     previous   next
  

Show proTRAC run info
Hide proTRAC run info

================================= proTRAC ====================================  
VERSION: 2.1                                    LAST MODIFIED: 06. October 2015  
  
Please cite:  
Rosenkranz D, Zischler H. proTRAC - a software for probabilistic piRNA cluster  
detection, visualization and analysis. 2012. BMC Bioinformatics 13:5.  
  
and (for proTRAC 2.0 and later):  
Rosenkranz D, Rudloff S, Bastuck K, Ketting RF, Zischler H. Tupaia small RNAs  
provide insights into function and evolution of RNAi-based transposon defense  
in mammals. 2015. RNA 21(5):911-922.  
  
Contact:  
David Rosenkranz  
Institute of Anthropology, small RNA group  
Johannes Gutenberg University Mainz  
email: rosenkranz@uni-mainz.de  
  
You can find the latest proTRAC version at:  
http://sourceforge.net/projects/protrac/files  
http://www.smallRNAgroup-mainz.de/software  
==============================================================================  
  
PARAMETERS:  
Map file: .............../storage/core/barbara/genhome/smallRNA/fertility/Sample\_motile/pirna/Sample\_motile\_26-33\_collapsed.fa.no-dust.map.weighted-10000-1000-b-0  
Genome file: ............/storage/core/barbara/genhome/smallRNA/fertility/Sample\_all/pirna/bt\_311\_chrY.fa  
RepeatMasker annotation: /storage/genomes/bt\_umd31/GCF\_000003055.6\_Bos\_taurus\_UMD\_3.1.1\_repeatMasker\_chr.out  
GeneSet:................./storage/core/barbara/genhome/smallRNA/fertility/Sample\_all/pirna/full.gtf  
  
Significant (p<=0.01) hit density will be calculated based  
on observed hit distribution.  
  
Sliding window size: ........................................ 5000 bp  
Sliding window increament: .................................. 1000 bp  
Normalize each hit by number of genomic hits: ............... 1 [0=no/1=yes]  
Normalize each hit by number of sequence reads: ............. 1 [0=no/1=yes]  
Normalize values (-> per million mapped reads): ............. 1 [0=no/1=yes]  
Min. fraction of hits with 1T(U) or 10A: .................... 0.75  
Alternatively: Min. fraction of hits with 1T(U) and 10A: .... 0.5  
Min. fraction of hits with typical piRNA length: ............ 0.75  
Typical piRNA length: ....................................... 26-33 nt  
Min. size of a piRNA cluster: ............................... 5000 bp.  
Min. number of hits (absolute): ............................. 0  
Min. number of hits (normalized): ........................... 0  
Min. fraction of hits on the mainstrand: .................... 0.75  
Top fraction of mapped sequences (in terms of read counts): . 1%  
Top fraction accounts for max. n% of sequence reads: ........ 90%  
Min. fraction of hits on each arm of a bidirectional cluster: 0.1  
Output image file for each cluster: ......................... 0 [0=no/1=yes]  
Output html file for each cluster: .......................... 1 [0=no/1=yes]  
Output a summary table: ..................................... 1 [0=no/1=yes]  
Output a FASTA file for each cluster (piRNA sequences): ..... 1 [0=no/1=yes]  
Output a FASTA file comprising cluster sequences: ........... 1 [0=no/1=yes]  
Search DNA motifs in clusters: .............................. 1 [0=no/1=yes]  
Output flanking sequences: +/- .............................. 0 bp  
Output ~.pTi file: .......................................... 1 [0=no/1=yes]  
==============================================================================  
  
  
Genome size (without gaps): ............ 2678902517 bp  
Gaps (N/X/-): .......................... 53837044 bp  
Mapped reads: .......................... 658825247023  
Non-identical sequences: ............... 514171  
Genomic hits: .......................... 764233  
Significant densitiy of mapped reads: .. 12867599.5173724 reads/kb

Show proTRAC cluster info
Hide proTRAC cluster info

|  |  |
| --- | --- |
| Location | chr19 |
| Coordinates | 45404027-45409119 |
| Size [bp] | 5093 |
| Sequence hit loci | 51 |
| Mapped reads (normalized) | 78561009 |
| Mapped reads (normalized) per kb | 15425291.4 |
| Normalized reads with 1T (1U) | 83% |
| Normalized reads with 10A | 29.4% |
| Normalized reads with length 26-33 nt | 100% |
| Normalized reads on the main strand(s) | 94% |
| Predicted directionality | mono:plus |

100%

0%

1T (1U)  
reads

10A reads

26-33 nt  
reads

reads on mainstrand

**Either the amount of reads with 1T (1U) OR 10A has to exceed 75% (set with option: -1Tor10A)  
Alternatively the amount of reads with 1T (1U) AND 10A has to exceed 50% (set with option: -1Tand10A)  
Minimum amount of reads with preferred size is 75% (set with option: -pisize)  
Minimum amount of reads on the main strand(s) is 75% (set with option: -clstrand)**

Show read coverage
Hide read coverage

WHAT DO I SEE HERE?  
This chart shows the location of mapped sequence reads within a predicted piRNA cluster. The color refers to the number of genomic hits produced by the sequence read in question. A dark red bar indicates that this sequence read produces many other hits elsewhere in the genome. Many adjacent red or yellow bars can indicate the presence of a multi-copy element such as transposons or rRNA genes. A dark green bar indicates that this sequence read maps uniquely to this locus.

1 hit

2-5 hits

6-10 hits

11-20 hits

21-50 hits

51-100 hits

> 100 hits

chr19

45404027

45409119

Gene Set

RepeatMasker

Mapped  
Reads

14.15

plus strand

minus strand

14.15

Region: chr19 41183419-45404032. Max. coverage (+): 2.26. Max coverage (-): 0

Region: chr19 45404033-45404042. Max. coverage (+): 2.26. Max coverage (-): 0

Region: chr19 45404043-45404052. Max. coverage (+): 0. Max coverage (-): 0

Region: chr19 45404053-45404062. Max. coverage (+): 0. Max coverage (-): 0

Region: chr19 45404063-45404072. Max. coverage (+): 0. Max coverage (-): 0

Region: chr19 45404073-45404083. Max. coverage (+): 0. Max coverage (-): 0

Region: chr19 45404084-45404093. Max. coverage (+): 0. Max coverage (-): 0

Region: chr19 45404094-45404103. Max. coverage (+): 4.14. Max coverage (-): 0

Region: chr19 45404104-45404113. Max. coverage (+): 4.14. Max coverage (-): 0

Region: chr19 45404114-45404123. Max. coverage (+): 0. Max coverage (-): 0

Region: chr19 45404124-45404133. Max. coverage (+): 0. Max coverage (-): 0

Region: chr19 45404134-45404144. Max. coverage (+): 0. Max coverage (-): 0

Region: chr19 45404145-45404154. Max. coverage (+): 0. Max coverage (-): 0

Region: chr19 45404155-45404164. Max. coverage (+): 0. Max coverage (-): 0

Region: chr19 45404165-45404174. Max. coverage (+): 0. Max coverage (-): 0

Region: chr19 45404175-45404184. Max. coverage (+): 0. Max coverage (-): 0

Region: chr19 45404185-45404195. Max. coverage (+): 0. Max coverage (-): 0

Region: chr19 45404196-45404205. Max. coverage (+): 0. Max coverage (-): 0

Region: chr19 45404206-45404215. Max. coverage (+): 0. Max coverage (-): 0

Region: chr19 45404216-45404225. Max. coverage (+): 0. Max coverage (-): 0

Region: chr19 45404226-45404235. Max. coverage (+): 0. Max coverage (-): 0

Region: chr19 45404236-45404245. Max. coverage (+): 0. Max coverage (-): 0

Region: chr19 45404246-45404256. Max. coverage (+): 0. Max coverage (-): 0

Region: chr19 45404257-45404266. Max. coverage (+): 0. Max coverage (-): 0

Region: chr19 45404267-45404276. Max. coverage (+): 0. Max coverage (-): 0

Region: chr19 45404277-45404286. Max. coverage (+): 0. Max coverage (-): 0

Region: chr19 45404287-45404296. Max. coverage (+): 0. Max coverage (-): 0

Region: chr19 45404297-45404307. Max. coverage (+): 0. Max coverage (-): 0

Region: chr19 45404308-45404317. Max. coverage (+): 0. Max coverage (-): 0

Region: chr19 45404318-45404327. Max. coverage (+): 0. Max coverage (-): 0

Region: chr19 45404328-45404337. Max. coverage (+): 0. Max coverage (-): 0

Region: chr19 45404338-45404347. Max. coverage (+): 0. Max coverage (-): 0

Region: chr19 45404348-45404358. Max. coverage (+): 0. Max coverage (-): 0

Region: chr19 45404359-45404368. Max. coverage (+): 0. Max coverage (-): 0

Region: chr19 45404369-45404378. Max. coverage (+): 0. Max coverage (-): 0

Region: chr19 45404379-45404388. Max. coverage (+): 0. Max coverage (-): 0

Region: chr19 45404389-45404398. Max. coverage (+): 0. Max coverage (-): 0

Region: chr19 45404399-45404408. Max. coverage (+): 0. Max coverage (-): 0

Region: chr19 45404409-45404419. Max. coverage (+): 0. Max coverage (-): 0

Region: chr19 45404420-45404429. Max. coverage (+): 0. Max coverage (-): 0

Region: chr19 45404430-45404439. Max. coverage (+): 0. Max coverage (-): 0

Region: chr19 45404440-45404449. Max. coverage (+): 0. Max coverage (-): 0

Region: chr19 45404450-45404459. Max. coverage (+): 0. Max coverage (-): 0

Region: chr19 45404460-45404470. Max. coverage (+): 0. Max coverage (-): 0

Region: chr19 45404471-45404480. Max. coverage (+): 0. Max coverage (-): 0

Region: chr19 45404481-45404490. Max. coverage (+): 0. Max coverage (-): 0

Region: chr19 45404491-45404500. Max. coverage (+): 0. Max coverage (-): 0

Region: chr19 45404501-45404510. Max. coverage (+): 0. Max coverage (-): 0

Region: chr19 45404511-45404521. Max. coverage (+): 0. Max coverage (-): 0

Region: chr19 45404522-45404531. Max. coverage (+): 0. Max coverage (-): 0

Region: chr19 45404532-45404541. Max. coverage (+): 0. Max coverage (-): 0

Region: chr19 45404542-45404551. Max. coverage (+): 0. Max coverage (-): 0

Region: chr19 45404552-45404561. Max. coverage (+): 0. Max coverage (-): 0

Region: chr19 45404562-45404571. Max. coverage (+): 0. Max coverage (-): 0

Region: chr19 45404572-45404582. Max. coverage (+): 0. Max coverage (-): 0

Region: chr19 45404583-45404592. Max. coverage (+): 0. Max coverage (-): 0

Region: chr19 45404593-45404602. Max. coverage (+): 0. Max coverage (-): 0

Region: chr19 45404603-45404612. Max. coverage (+): 0. Max coverage (-): 0

Region: chr19 45404613-45404622. Max. coverage (+): 0. Max coverage (-): 0

Region: chr19 45404623-45404633. Max. coverage (+): 0. Max coverage (-): 0

Region: chr19 45404634-45404643. Max. coverage (+): 0. Max coverage (-): 0

Region: chr19 45404644-45404653. Max. coverage (+): 0. Max coverage (-): 0

Region: chr19 45404654-45404663. Max. coverage (+): 0. Max coverage (-): 0

Region: chr19 45404664-45404673. Max. coverage (+): 0. Max coverage (-): 0

Region: chr19 45404674-45404683. Max. coverage (+): 0. Max coverage (-): 0

Region: chr19 45404684-45404694. Max. coverage (+): 0. Max coverage (-): 0

Region: chr19 45404695-45404704. Max. coverage (+): 0. Max coverage (-): 0

Region: chr19 45404705-45404714. Max. coverage (+): 0. Max coverage (-): 0

Region: chr19 45404715-45404724. Max. coverage (+): 0. Max coverage (-): 0

Region: chr19 45404725-45404734. Max. coverage (+): 0. Max coverage (-): 0

Region: chr19 45404735-45404745. Max. coverage (+): 0. Max coverage (-): 0

Region: chr19 45404746-45404755. Max. coverage (+): 0. Max coverage (-): 0

Region: chr19 45404756-45404765. Max. coverage (+): 0. Max coverage (-): 0

Region: chr19 45404766-45404775. Max. coverage (+): 0. Max coverage (-): 0

Region: chr19 45404776-45404785. Max. coverage (+): 0. Max coverage (-): 0

Region: chr19 45404786-45404796. Max. coverage (+): 0. Max coverage (-): 0

Region: chr19 45404797-45404806. Max. coverage (+): 0. Max coverage (-): 0

Region: chr19 45404807-45404816. Max. coverage (+): 0. Max coverage (-): 0

Region: chr19 45404817-45404826. Max. coverage (+): 0. Max coverage (-): 0

Region: chr19 45404827-45404836. Max. coverage (+): 0. Max coverage (-): 0

Region: chr19 45404837-45404846. Max. coverage (+): 0. Max coverage (-): 0

Region: chr19 45404847-45404857. Max. coverage (+): 0. Max coverage (-): 0

Region: chr19 45404858-45404867. Max. coverage (+): 0. Max coverage (-): 0

Region: chr19 45404868-45404877. Max. coverage (+): 0. Max coverage (-): 0

Region: chr19 45404878-45404887. Max. coverage (+): 0. Max coverage (-): 0

Region: chr19 45404888-45404897. Max. coverage (+): 0. Max coverage (-): 0

Region: chr19 45404898-45404908. Max. coverage (+): 0. Max coverage (-): 0

Region: chr19 45404909-45404918. Max. coverage (+): 0. Max coverage (-): 0

Region: chr19 45404919-45404928. Max. coverage (+): 0. Max coverage (-): 0

Region: chr19 45404929-45404938. Max. coverage (+): 0. Max coverage (-): 0

Region: chr19 45404939-45404948. Max. coverage (+): 0. Max coverage (-): 0

Region: chr19 45404949-45404959. Max. coverage (+): 0. Max coverage (-): 0

Region: chr19 45404960-45404969. Max. coverage (+): 0. Max coverage (-): 0

Region: chr19 45404970-45404979. Max. coverage (+): 0. Max coverage (-): 0

Region: chr19 45404980-45404989. Max. coverage (+): 0. Max coverage (-): 0

Region: chr19 45404990-45404999. Max. coverage (+): 0. Max coverage (-): 0

Region: chr19 45405000-45405009. Max. coverage (+): 0. Max coverage (-): 0

Region: chr19 45405010-45405020. Max. coverage (+): 0. Max coverage (-): 0

Region: chr19 45405021-45405030. Max. coverage (+): 0. Max coverage (-): 0

Region: chr19 45405031-45405040. Max. coverage (+): 0. Max coverage (-): 0

Region: chr19 45405041-45405050. Max. coverage (+): 0. Max coverage (-): 0

Region: chr19 45405051-45405060. Max. coverage (+): 0. Max coverage (-): 0

Region: chr19 45405061-45405071. Max. coverage (+): 0. Max coverage (-): 0

Region: chr19 45405072-45405081. Max. coverage (+): 0. Max coverage (-): 0

Region: chr19 45405082-45405091. Max. coverage (+): 0. Max coverage (-): 0

Region: chr19 45405092-45405101. Max. coverage (+): 0. Max coverage (-): 0

Region: chr19 45405102-45405111. Max. coverage (+): 0. Max coverage (-): 0

Region: chr19 45405112-45405121. Max. coverage (+): 0. Max coverage (-): 0

Region: chr19 45405122-45405132. Max. coverage (+): 0. Max coverage (-): 1.07

Region: chr19 45405133-45405142. Max. coverage (+): 0. Max coverage (-): 1.07

Region: chr19 45405143-45405152. Max. coverage (+): 0. Max coverage (-): 0

Region: chr19 45405153-45405162. Max. coverage (+): 0. Max coverage (-): 0

Region: chr19 45405163-45405172. Max. coverage (+): 0. Max coverage (-): 0

Region: chr19 45405173-45405183. Max. coverage (+): 0. Max coverage (-): 0

Region: chr19 45405184-45405193. Max. coverage (+): 0. Max coverage (-): 0

Region: chr19 45405194-45405203. Max. coverage (+): 0. Max coverage (-): 0

Region: chr19 45405204-45405213. Max. coverage (+): 0. Max coverage (-): 0

Region: chr19 45405214-45405223. Max. coverage (+): 0. Max coverage (-): 0

Region: chr19 45405224-45405234. Max. coverage (+): 0. Max coverage (-): 0

Region: chr19 45405235-45405244. Max. coverage (+): 0. Max coverage (-): 0

Region: chr19 45405245-45405254. Max. coverage (+): 0. Max coverage (-): 0

Region: chr19 45405255-45405264. Max. coverage (+): 0. Max coverage (-): 0

Region: chr19 45405265-45405274. Max. coverage (+): 0. Max coverage (-): 0

Region: chr19 45405275-45405284. Max. coverage (+): 0. Max coverage (-): 0

Region: chr19 45405285-45405295. Max. coverage (+): 0. Max coverage (-): 0

Region: chr19 45405296-45405305. Max. coverage (+): 0. Max coverage (-): 0

Region: chr19 45405306-45405315. Max. coverage (+): 0. Max coverage (-): 0

Region: chr19 45405316-45405325. Max. coverage (+): 0. Max coverage (-): 0

Region: chr19 45405326-45405335. Max. coverage (+): 0. Max coverage (-): 0

Region: chr19 45405336-45405346. Max. coverage (+): 0. Max coverage (-): 0

Region: chr19 45405347-45405356. Max. coverage (+): 0. Max coverage (-): 0

Region: chr19 45405357-45405366. Max. coverage (+): 0. Max coverage (-): 0

Region: chr19 45405367-45405376. Max. coverage (+): 0. Max coverage (-): 0

Region: chr19 45405377-45405386. Max. coverage (+): 0. Max coverage (-): 0

Region: chr19 45405387-45405397. Max. coverage (+): 0. Max coverage (-): 0

Region: chr19 45405398-45405407. Max. coverage (+): 0. Max coverage (-): 0

Region: chr19 45405408-45405417. Max. coverage (+): 0. Max coverage (-): 0

Region: chr19 45405418-45405427. Max. coverage (+): 0. Max coverage (-): 0

Region: chr19 45405428-45405437. Max. coverage (+): 0. Max coverage (-): 0

Region: chr19 45405438-45405447. Max. coverage (+): 0. Max coverage (-): 0

Region: chr19 45405448-45405458. Max. coverage (+): 0. Max coverage (-): 0

Region: chr19 45405459-45405468. Max. coverage (+): 0. Max coverage (-): 0

Region: chr19 45405469-45405478. Max. coverage (+): 0. Max coverage (-): 0

Region: chr19 45405479-45405488. Max. coverage (+): 0. Max coverage (-): 0

Region: chr19 45405489-45405498. Max. coverage (+): 0. Max coverage (-): 0

Region: chr19 45405499-45405509. Max. coverage (+): 0. Max coverage (-): 0

Region: chr19 45405510-45405519. Max. coverage (+): 0. Max coverage (-): 0

Region: chr19 45405520-45405529. Max. coverage (+): 0. Max coverage (-): 0

Region: chr19 45405530-45405539. Max. coverage (+): 0. Max coverage (-): 0

Region: chr19 45405540-45405549. Max. coverage (+): 0. Max coverage (-): 0

Region: chr19 45405550-45405559. Max. coverage (+): 0. Max coverage (-): 0

Region: chr19 45405560-45405570. Max. coverage (+): 0. Max coverage (-): 0

Region: chr19 45405571-45405580. Max. coverage (+): 0. Max coverage (-): 0

Region: chr19 45405581-45405590. Max. coverage (+): 0. Max coverage (-): 0

Region: chr19 45405591-45405600. Max. coverage (+): 0. Max coverage (-): 0

Region: chr19 45405601-45405610. Max. coverage (+): 0. Max coverage (-): 0

Region: chr19 45405611-45405621. Max. coverage (+): 0. Max coverage (-): 0

Region: chr19 45405622-45405631. Max. coverage (+): 0. Max coverage (-): 0

Region: chr19 45405632-45405641. Max. coverage (+): 0. Max coverage (-): 0

Region: chr19 45405642-45405651. Max. coverage (+): 0. Max coverage (-): 0

Region: chr19 45405652-45405661. Max. coverage (+): 0. Max coverage (-): 0

Region: chr19 45405662-45405672. Max. coverage (+): 0. Max coverage (-): 0

Region: chr19 45405673-45405682. Max. coverage (+): 0. Max coverage (-): 0

Region: chr19 45405683-45405692. Max. coverage (+): 0. Max coverage (-): 0

Region: chr19 45405693-45405702. Max. coverage (+): 0. Max coverage (-): 0

Region: chr19 45405703-45405712. Max. coverage (+): 0. Max coverage (-): 0

Region: chr19 45405713-45405722. Max. coverage (+): 0. Max coverage (-): 0

Region: chr19 45405723-45405733. Max. coverage (+): 0. Max coverage (-): 0

Region: chr19 45405734-45405743. Max. coverage (+): 0. Max coverage (-): 0

Region: chr19 45405744-45405753. Max. coverage (+): 0. Max coverage (-): 0

Region: chr19 45405754-45405763. Max. coverage (+): 0. Max coverage (-): 0

Region: chr19 45405764-45405773. Max. coverage (+): 0. Max coverage (-): 0

Region: chr19 45405774-45405784. Max. coverage (+): 0. Max coverage (-): 0

Region: chr19 45405785-45405794. Max. coverage (+): 0. Max coverage (-): 0

Region: chr19 45405795-45405804. Max. coverage (+): 0. Max coverage (-): 0

Region: chr19 45405805-45405814. Max. coverage (+): 0. Max coverage (-): 0

Region: chr19 45405815-45405824. Max. coverage (+): 0. Max coverage (-): 0

Region: chr19 45405825-45405835. Max. coverage (+): 0. Max coverage (-): 0

Region: chr19 45405836-45405845. Max. coverage (+): 0. Max coverage (-): 0

Region: chr19 45405846-45405855. Max. coverage (+): 0. Max coverage (-): 0

Region: chr19 45405856-45405865. Max. coverage (+): 0. Max coverage (-): 0

Region: chr19 45405866-45405875. Max. coverage (+): 0. Max coverage (-): 0

Region: chr19 45405876-45405885. Max. coverage (+): 0. Max coverage (-): 0

Region: chr19 45405886-45405896. Max. coverage (+): 0. Max coverage (-): 0

Region: chr19 45405897-45405906. Max. coverage (+): 0. Max coverage (-): 0

Region: chr19 45405907-45405916. Max. coverage (+): 0. Max coverage (-): 0

Region: chr19 45405917-45405926. Max. coverage (+): 0. Max coverage (-): 0

Region: chr19 45405927-45405936. Max. coverage (+): 0. Max coverage (-): 0

Region: chr19 45405937-45405947. Max. coverage (+): 0. Max coverage (-): 0

Region: chr19 45405948-45405957. Max. coverage (+): 0. Max coverage (-): 0

Region: chr19 45405958-45405967. Max. coverage (+): 0. Max coverage (-): 0

Region: chr19 45405968-45405977. Max. coverage (+): 0. Max coverage (-): 0

Region: chr19 45405978-45405987. Max. coverage (+): 0. Max coverage (-): 0

Region: chr19 45405988-45405997. Max. coverage (+): 6.39. Max coverage (-): 0

Region: chr19 45405998-45406008. Max. coverage (+): 6.39. Max coverage (-): 0

Region: chr19 45406009-45406018. Max. coverage (+): 0. Max coverage (-): 0

Region: chr19 45406019-45406028. Max. coverage (+): 0. Max coverage (-): 0

Region: chr19 45406029-45406038. Max. coverage (+): 0. Max coverage (-): 0

Region: chr19 45406039-45406048. Max. coverage (+): 0. Max coverage (-): 0

Region: chr19 45406049-45406059. Max. coverage (+): 0. Max coverage (-): 0

Region: chr19 45406060-45406069. Max. coverage (+): 0. Max coverage (-): 0

Region: chr19 45406070-45406079. Max. coverage (+): 0. Max coverage (-): 0

Region: chr19 45406080-45406089. Max. coverage (+): 1.96. Max coverage (-): 0

Region: chr19 45406090-45406099. Max. coverage (+): 1.96. Max coverage (-): 0

Region: chr19 45406100-45406110. Max. coverage (+): 0. Max coverage (-): 0

Region: chr19 45406111-45406120. Max. coverage (+): 0. Max coverage (-): 0

Region: chr19 45406121-45406130. Max. coverage (+): 0. Max coverage (-): 0

Region: chr19 45406131-45406140. Max. coverage (+): 0. Max coverage (-): 0

Region: chr19 45406141-45406150. Max. coverage (+): 0. Max coverage (-): 0

Region: chr19 45406151-45406160. Max. coverage (+): 0. Max coverage (-): 0

Region: chr19 45406161-45406171. Max. coverage (+): 0. Max coverage (-): 0

Region: chr19 45406172-45406181. Max. coverage (+): 0. Max coverage (-): 0

Region: chr19 45406182-45406191. Max. coverage (+): 0. Max coverage (-): 0

Region: chr19 45406192-45406201. Max. coverage (+): 0. Max coverage (-): 0

Region: chr19 45406202-45406211. Max. coverage (+): 1.03. Max coverage (-): 0

Region: chr19 45406212-45406222. Max. coverage (+): 1.03. Max coverage (-): 0

Region: chr19 45406223-45406232. Max. coverage (+): 0. Max coverage (-): 0

Region: chr19 45406233-45406242. Max. coverage (+): 0. Max coverage (-): 0

Region: chr19 45406243-45406252. Max. coverage (+): 0. Max coverage (-): 0

Region: chr19 45406253-45406262. Max. coverage (+): 0. Max coverage (-): 0

Region: chr19 45406263-45406273. Max. coverage (+): 0. Max coverage (-): 0

Region: chr19 45406274-45406283. Max. coverage (+): 0. Max coverage (-): 0

Region: chr19 45406284-45406293. Max. coverage (+): 0. Max coverage (-): 0

Region: chr19 45406294-45406303. Max. coverage (+): 0. Max coverage (-): 0

Region: chr19 45406304-45406313. Max. coverage (+): 0. Max coverage (-): 0

Region: chr19 45406314-45406323. Max. coverage (+): 0. Max coverage (-): 0

Region: chr19 45406324-45406334. Max. coverage (+): 0. Max coverage (-): 0

Region: chr19 45406335-45406344. Max. coverage (+): 0. Max coverage (-): 0

Region: chr19 45406345-45406354. Max. coverage (+): 0. Max coverage (-): 0

Region: chr19 45406355-45406364. Max. coverage (+): 0. Max coverage (-): 0

Region: chr19 45406365-45406374. Max. coverage (+): 0. Max coverage (-): 0

Region: chr19 45406375-45406385. Max. coverage (+): 0. Max coverage (-): 0

Region: chr19 45406386-45406395. Max. coverage (+): 2.12. Max coverage (-): 0

Region: chr19 45406396-45406405. Max. coverage (+): 0. Max coverage (-): 0

Region: chr19 45406406-45406415. Max. coverage (+): 0. Max coverage (-): 0

Region: chr19 45406416-45406425. Max. coverage (+): 0. Max coverage (-): 0

Region: chr19 45406426-45406435. Max. coverage (+): 0.53. Max coverage (-): 0

Region: chr19 45406436-45406446. Max. coverage (+): 0.53. Max coverage (-): 0

Region: chr19 45406447-45406456. Max. coverage (+): 0. Max coverage (-): 0

Region: chr19 45406457-45406466. Max. coverage (+): 0. Max coverage (-): 0

Region: chr19 45406467-45406476. Max. coverage (+): 0. Max coverage (-): 0

Region: chr19 45406477-45406486. Max. coverage (+): 0. Max coverage (-): 0

Region: chr19 45406487-45406497. Max. coverage (+): 0. Max coverage (-): 0

Region: chr19 45406498-45406507. Max. coverage (+): 0. Max coverage (-): 0

Region: chr19 45406508-45406517. Max. coverage (+): 0. Max coverage (-): 0

Region: chr19 45406518-45406527. Max. coverage (+): 0. Max coverage (-): 0

Region: chr19 45406528-45406537. Max. coverage (+): 0. Max coverage (-): 0

Region: chr19 45406538-45406548. Max. coverage (+): 0. Max coverage (-): 0

Region: chr19 45406549-45406558. Max. coverage (+): 0. Max coverage (-): 0

Region: chr19 45406559-45406568. Max. coverage (+): 0. Max coverage (-): 0

Region: chr19 45406569-45406578. Max. coverage (+): 0. Max coverage (-): 0

Region: chr19 45406579-45406588. Max. coverage (+): 0. Max coverage (-): 0

Region: chr19 45406589-45406598. Max. coverage (+): 0. Max coverage (-): 0

Region: chr19 45406599-45406609. Max. coverage (+): 0. Max coverage (-): 0

Region: chr19 45406610-45406619. Max. coverage (+): 0. Max coverage (-): 0

Region: chr19 45406620-45406629. Max. coverage (+): 0. Max coverage (-): 0

Region: chr19 45406630-45406639. Max. coverage (+): 0. Max coverage (-): 0

Region: chr19 45406640-45406649. Max. coverage (+): 0. Max coverage (-): 0

Region: chr19 45406650-45406660. Max. coverage (+): 0. Max coverage (-): 0

Region: chr19 45406661-45406670. Max. coverage (+): 0. Max coverage (-): 0

Region: chr19 45406671-45406680. Max. coverage (+): 0. Max coverage (-): 0

Region: chr19 45406681-45406690. Max. coverage (+): 0. Max coverage (-): 0

Region: chr19 45406691-45406700. Max. coverage (+): 0. Max coverage (-): 0

Region: chr19 45406701-45406711. Max. coverage (+): 0. Max coverage (-): 0

Region: chr19 45406712-45406721. Max. coverage (+): 2.04. Max coverage (-): 0

Region: chr19 45406722-45406731. Max. coverage (+): 0. Max coverage (-): 0

Region: chr19 45406732-45406741. Max. coverage (+): 0. Max coverage (-): 0

Region: chr19 45406742-45406751. Max. coverage (+): 0. Max coverage (-): 0

Region: chr19 45406752-45406761. Max. coverage (+): 0. Max coverage (-): 0

Region: chr19 45406762-45406772. Max. coverage (+): 0. Max coverage (-): 0

Region: chr19 45406773-45406782. Max. coverage (+): 2.13. Max coverage (-): 0

Region: chr19 45406783-45406792. Max. coverage (+): 2.13. Max coverage (-): 0

Region: chr19 45406793-45406802. Max. coverage (+): 0. Max coverage (-): 0

Region: chr19 45406803-45406812. Max. coverage (+): 0. Max coverage (-): 0

Region: chr19 45406813-45406823. Max. coverage (+): 2.76. Max coverage (-): 0

Region: chr19 45406824-45406833. Max. coverage (+): 0. Max coverage (-): 0

Region: chr19 45406834-45406843. Max. coverage (+): 0. Max coverage (-): 0

Region: chr19 45406844-45406853. Max. coverage (+): 0. Max coverage (-): 0

Region: chr19 45406854-45406863. Max. coverage (+): 1.26. Max coverage (-): 0

Region: chr19 45406864-45406873. Max. coverage (+): 0. Max coverage (-): 0

Region: chr19 45406874-45406884. Max. coverage (+): 0. Max coverage (-): 0

Region: chr19 45406885-45406894. Max. coverage (+): 0. Max coverage (-): 0

Region: chr19 45406895-45406904. Max. coverage (+): 0. Max coverage (-): 0

Region: chr19 45406905-45406914. Max. coverage (+): 1.94. Max coverage (-): 0

Region: chr19 45406915-45406924. Max. coverage (+): 2.74. Max coverage (-): 0

Region: chr19 45406925-45406935. Max. coverage (+): 0. Max coverage (-): 0

Region: chr19 45406936-45406945. Max. coverage (+): 0. Max coverage (-): 0

Region: chr19 45406946-45406955. Max. coverage (+): 0. Max coverage (-): 0

Region: chr19 45406956-45406965. Max. coverage (+): 0. Max coverage (-): 0

Region: chr19 45406966-45406975. Max. coverage (+): 4.62. Max coverage (-): 0

Region: chr19 45406976-45406986. Max. coverage (+): 4.62. Max coverage (-): 0

Region: chr19 45406987-45406996. Max. coverage (+): 0. Max coverage (-): 0

Region: chr19 45406997-45407006. Max. coverage (+): 0. Max coverage (-): 0

Region: chr19 45407007-45407016. Max. coverage (+): 0. Max coverage (-): 0

Region: chr19 45407017-45407026. Max. coverage (+): 0. Max coverage (-): 0

Region: chr19 45407027-45407036. Max. coverage (+): 0. Max coverage (-): 0

Region: chr19 45407037-45407047. Max. coverage (+): 0. Max coverage (-): 0

Region: chr19 45407048-45407057. Max. coverage (+): 0. Max coverage (-): 0

Region: chr19 45407058-45407067. Max. coverage (+): 0. Max coverage (-): 0

Region: chr19 45407068-45407077. Max. coverage (+): 0. Max coverage (-): 0

Region: chr19 45407078-45407087. Max. coverage (+): 0. Max coverage (-): 0

Region: chr19 45407088-45407098. Max. coverage (+): 0. Max coverage (-): 0

Region: chr19 45407099-45407108. Max. coverage (+): 0. Max coverage (-): 0

Region: chr19 45407109-45407118. Max. coverage (+): 0. Max coverage (-): 0

Region: chr19 45407119-45407128. Max. coverage (+): 0. Max coverage (-): 0

Region: chr19 45407129-45407138. Max. coverage (+): 0. Max coverage (-): 0

Region: chr19 45407139-45407149. Max. coverage (+): 0. Max coverage (-): 0

Region: chr19 45407150-45407159. Max. coverage (+): 0. Max coverage (-): 0

Region: chr19 45407160-45407169. Max. coverage (+): 0. Max coverage (-): 0

Region: chr19 45407170-45407179. Max. coverage (+): 0. Max coverage (-): 0

Region: chr19 45407180-45407189. Max. coverage (+): 7.31. Max coverage (-): 0

Region: chr19 45407190-45407199. Max. coverage (+): 5.02. Max coverage (-): 0

Region: chr19 45407200-45407210. Max. coverage (+): 0. Max coverage (-): 0

Region: chr19 45407211-45407220. Max. coverage (+): 2.94. Max coverage (-): 0

Region: chr19 45407221-45407230. Max. coverage (+): 2.94. Max coverage (-): 0

Region: chr19 45407231-45407240. Max. coverage (+): 0. Max coverage (-): 0

Region: chr19 45407241-45407250. Max. coverage (+): 4.53. Max coverage (-): 0

Region: chr19 45407251-45407261. Max. coverage (+): 4.53. Max coverage (-): 0

Region: chr19 45407262-45407271. Max. coverage (+): 0. Max coverage (-): 0

Region: chr19 45407272-45407281. Max. coverage (+): 0. Max coverage (-): 0

Region: chr19 45407282-45407291. Max. coverage (+): 0. Max coverage (-): 0

Region: chr19 45407292-45407301. Max. coverage (+): 0. Max coverage (-): 0

Region: chr19 45407302-45407311. Max. coverage (+): 0. Max coverage (-): 0

Region: chr19 45407312-45407322. Max. coverage (+): 0. Max coverage (-): 2.96

Region: chr19 45407323-45407332. Max. coverage (+): 0. Max coverage (-): 2.96

Region: chr19 45407333-45407342. Max. coverage (+): 0. Max coverage (-): 0

Region: chr19 45407343-45407352. Max. coverage (+): 0. Max coverage (-): 0

Region: chr19 45407353-45407362. Max. coverage (+): 0. Max coverage (-): 0

Region: chr19 45407363-45407373. Max. coverage (+): 0. Max coverage (-): 0

Region: chr19 45407374-45407383. Max. coverage (+): 7.99. Max coverage (-): 0

Region: chr19 45407384-45407393. Max. coverage (+): 8.94. Max coverage (-): 0

Region: chr19 45407394-45407403. Max. coverage (+): 0. Max coverage (-): 0

Region: chr19 45407404-45407413. Max. coverage (+): 0. Max coverage (-): 0

Region: chr19 45407414-45407424. Max. coverage (+): 0. Max coverage (-): 0

Region: chr19 45407425-45407434. Max. coverage (+): 0. Max coverage (-): 0

Region: chr19 45407435-45407444. Max. coverage (+): 0. Max coverage (-): 0

Region: chr19 45407445-45407454. Max. coverage (+): 0. Max coverage (-): 0

Region: chr19 45407455-45407464. Max. coverage (+): 1.78. Max coverage (-): 0

Region: chr19 45407465-45407474. Max. coverage (+): 1.78. Max coverage (-): 0

Region: chr19 45407475-45407485. Max. coverage (+): 1.66. Max coverage (-): 0

Region: chr19 45407486-45407495. Max. coverage (+): 0. Max coverage (-): 0

Region: chr19 45407496-45407505. Max. coverage (+): 0. Max coverage (-): 0

Region: chr19 45407506-45407515. Max. coverage (+): 0. Max coverage (-): 0

Region: chr19 45407516-45407525. Max. coverage (+): 0. Max coverage (-): 0

Region: chr19 45407526-45407536. Max. coverage (+): 5.25. Max coverage (-): 0

Region: chr19 45407537-45407546. Max. coverage (+): 0. Max coverage (-): 0

Region: chr19 45407547-45407556. Max. coverage (+): 0. Max coverage (-): 0

Region: chr19 45407557-45407566. Max. coverage (+): 0. Max coverage (-): 0

Region: chr19 45407567-45407576. Max. coverage (+): 0. Max coverage (-): 0

Region: chr19 45407577-45407587. Max. coverage (+): 0. Max coverage (-): 0

Region: chr19 45407588-45407597. Max. coverage (+): 0. Max coverage (-): 0

Region: chr19 45407598-45407607. Max. coverage (+): 0. Max coverage (-): 0

Region: chr19 45407608-45407617. Max. coverage (+): 0. Max coverage (-): 0

Region: chr19 45407618-45407627. Max. coverage (+): 0. Max coverage (-): 0

Region: chr19 45407628-45407637. Max. coverage (+): 0. Max coverage (-): 0

Region: chr19 45407638-45407648. Max. coverage (+): 0. Max coverage (-): 0

Region: chr19 45407649-45407658. Max. coverage (+): 0. Max coverage (-): 0

Region: chr19 45407659-45407668. Max. coverage (+): 0. Max coverage (-): 0

Region: chr19 45407669-45407678. Max. coverage (+): 0. Max coverage (-): 0

Region: chr19 45407679-45407688. Max. coverage (+): 0. Max coverage (-): 0

Region: chr19 45407689-45407699. Max. coverage (+): 0. Max coverage (-): 0

Region: chr19 45407700-45407709. Max. coverage (+): 0. Max coverage (-): 0

Region: chr19 45407710-45407719. Max. coverage (+): 0. Max coverage (-): 0

Region: chr19 45407720-45407729. Max. coverage (+): 0. Max coverage (-): 0

Region: chr19 45407730-45407739. Max. coverage (+): 0. Max coverage (-): 0

Region: chr19 45407740-45407749. Max. coverage (+): 0. Max coverage (-): 0

Region: chr19 45407750-45407760. Max. coverage (+): 0. Max coverage (-): 0

Region: chr19 45407761-45407770. Max. coverage (+): 0. Max coverage (-): 0

Region: chr19 45407771-45407780. Max. coverage (+): 0. Max coverage (-): 0

Region: chr19 45407781-45407790. Max. coverage (+): 0. Max coverage (-): 0

Region: chr19 45407791-45407800. Max. coverage (+): 0. Max coverage (-): 0

Region: chr19 45407801-45407811. Max. coverage (+): 0. Max coverage (-): 0

Region: chr19 45407812-45407821. Max. coverage (+): 2.26. Max coverage (-): 0

Region: chr19 45407822-45407831. Max. coverage (+): 2.26. Max coverage (-): 0

Region: chr19 45407832-45407841. Max. coverage (+): 0. Max coverage (-): 0

Region: chr19 45407842-45407851. Max. coverage (+): 0. Max coverage (-): 0

Region: chr19 45407852-45407862. Max. coverage (+): 0. Max coverage (-): 0

Region: chr19 45407863-45407872. Max. coverage (+): 0. Max coverage (-): 0

Region: chr19 45407873-45407882. Max. coverage (+): 0. Max coverage (-): 0

Region: chr19 45407883-45407892. Max. coverage (+): 0. Max coverage (-): 0

Region: chr19 45407893-45407902. Max. coverage (+): 0. Max coverage (-): 0

Region: chr19 45407903-45407912. Max. coverage (+): 0. Max coverage (-): 0

Region: chr19 45407913-45407923. Max. coverage (+): 0. Max coverage (-): 0

Region: chr19 45407924-45407933. Max. coverage (+): 0. Max coverage (-): 0

Region: chr19 45407934-45407943. Max. coverage (+): 0. Max coverage (-): 0

Region: chr19 45407944-45407953. Max. coverage (+): 0. Max coverage (-): 0

Region: chr19 45407954-45407963. Max. coverage (+): 0. Max coverage (-): 0

Region: chr19 45407964-45407974. Max. coverage (+): 0. Max coverage (-): 0

Region: chr19 45407975-45407984. Max. coverage (+): 0. Max coverage (-): 0

Region: chr19 45407985-45407994. Max. coverage (+): 0. Max coverage (-): 0

Region: chr19 45407995-45408004. Max. coverage (+): 0. Max coverage (-): 0

Region: chr19 45408005-45408014. Max. coverage (+): 0. Max coverage (-): 0

Region: chr19 45408015-45408025. Max. coverage (+): 0. Max coverage (-): 0

Region: chr19 45408026-45408035. Max. coverage (+): 0. Max coverage (-): 0

Region: chr19 45408036-45408045. Max. coverage (+): 0. Max coverage (-): 0

Region: chr19 45408046-45408055. Max. coverage (+): 0. Max coverage (-): 0

Region: chr19 45408056-45408065. Max. coverage (+): 0. Max coverage (-): 3.16

Region: chr19 45408066-45408075. Max. coverage (+): 0. Max coverage (-): 3.16

Region: chr19 45408076-45408086. Max. coverage (+): 0. Max coverage (-): 0

Region: chr19 45408087-45408096. Max. coverage (+): 0. Max coverage (-): 0

Region: chr19 45408097-45408106. Max. coverage (+): 0. Max coverage (-): 0

Region: chr19 45408107-45408116. Max. coverage (+): 3.41. Max coverage (-): 0

Region: chr19 45408117-45408126. Max. coverage (+): 0. Max coverage (-): 0

Region: chr19 45408127-45408137. Max. coverage (+): 0. Max coverage (-): 0

Region: chr19 45408138-45408147. Max. coverage (+): 0. Max coverage (-): 0

Region: chr19 45408148-45408157. Max. coverage (+): 0. Max coverage (-): 0

Region: chr19 45408158-45408167. Max. coverage (+): 0. Max coverage (-): 0

Region: chr19 45408168-45408177. Max. coverage (+): 0. Max coverage (-): 0

Region: chr19 45408178-45408187. Max. coverage (+): 0. Max coverage (-): 0

Region: chr19 45408188-45408198. Max. coverage (+): 1.34. Max coverage (-): 0

Region: chr19 45408199-45408208. Max. coverage (+): 0. Max coverage (-): 0

Region: chr19 45408209-45408218. Max. coverage (+): 0. Max coverage (-): 0

Region: chr19 45408219-45408228. Max. coverage (+): 0. Max coverage (-): 0

Region: chr19 45408229-45408238. Max. coverage (+): 0. Max coverage (-): 0

Region: chr19 45408239-45408249. Max. coverage (+): 0. Max coverage (-): 0

Region: chr19 45408250-45408259. Max. coverage (+): 0. Max coverage (-): 0

Region: chr19 45408260-45408269. Max. coverage (+): 0. Max coverage (-): 0

Region: chr19 45408270-45408279. Max. coverage (+): 0. Max coverage (-): 0

Region: chr19 45408280-45408289. Max. coverage (+): 0. Max coverage (-): 0

Region: chr19 45408290-45408300. Max. coverage (+): 0. Max coverage (-): 0

Region: chr19 45408301-45408310. Max. coverage (+): 0. Max coverage (-): 0

Region: chr19 45408311-45408320. Max. coverage (+): 0. Max coverage (-): 0

Region: chr19 45408321-45408330. Max. coverage (+): 0. Max coverage (-): 0

Region: chr19 45408331-45408340. Max. coverage (+): 4.19. Max coverage (-): 0

Region: chr19 45408341-45408350. Max. coverage (+): 4.19. Max coverage (-): 0

Region: chr19 45408351-45408361. Max. coverage (+): 0. Max coverage (-): 0

Region: chr19 45408362-45408371. Max. coverage (+): 10.4. Max coverage (-): 0

Region: chr19 45408372-45408381. Max. coverage (+): 14.15. Max coverage (-): 0

Region: chr19 45408382-45408391. Max. coverage (+): 0. Max coverage (-): 0

Region: chr19 45408392-45408401. Max. coverage (+): 1.34. Max coverage (-): 0

Region: chr19 45408402-45408412. Max. coverage (+): 1.34. Max coverage (-): 0

Region: chr19 45408413-45408422. Max. coverage (+): 0. Max coverage (-): 0

Region: chr19 45408423-45408432. Max. coverage (+): 0. Max coverage (-): 0

Region: chr19 45408433-45408442. Max. coverage (+): 0. Max coverage (-): 0

Region: chr19 45408443-45408452. Max. coverage (+): 0. Max coverage (-): 0

Region: chr19 45408453-45408463. Max. coverage (+): 0. Max coverage (-): 0

Region: chr19 45408464-45408473. Max. coverage (+): 0. Max coverage (-): 0

Region: chr19 45408474-45408483. Max. coverage (+): 0. Max coverage (-): 0

Region: chr19 45408484-45408493. Max. coverage (+): 0. Max coverage (-): 0

Region: chr19 45408494-45408503. Max. coverage (+): 6.51. Max coverage (-): 0

Region: chr19 45408504-45408513. Max. coverage (+): 6.51. Max coverage (-): 0

Region: chr19 45408514-45408524. Max. coverage (+): 0. Max coverage (-): 0

Region: chr19 45408525-45408534. Max. coverage (+): 0. Max coverage (-): 0

Region: chr19 45408535-45408544. Max. coverage (+): 0. Max coverage (-): 0

Region: chr19 45408545-45408554. Max. coverage (+): 0. Max coverage (-): 0

Region: chr19 45408555-45408564. Max. coverage (+): 0. Max coverage (-): 0

Region: chr19 45408565-45408575. Max. coverage (+): 0. Max coverage (-): 0

Region: chr19 45408576-45408585. Max. coverage (+): 0.75. Max coverage (-): 0

Region: chr19 45408586-45408595. Max. coverage (+): 0.75. Max coverage (-): 0

Region: chr19 45408596-45408605. Max. coverage (+): 0. Max coverage (-): 0

Region: chr19 45408606-45408615. Max. coverage (+): 0. Max coverage (-): 0

Region: chr19 45408616-45408625. Max. coverage (+): 0. Max coverage (-): 0

Region: chr19 45408626-45408636. Max. coverage (+): 0. Max coverage (-): 0

Region: chr19 45408637-45408646. Max. coverage (+): 0. Max coverage (-): 0

Region: chr19 45408647-45408656. Max. coverage (+): 0. Max coverage (-): 0

Region: chr19 45408657-45408666. Max. coverage (+): 0. Max coverage (-): 0

Region: chr19 45408667-45408676. Max. coverage (+): 0. Max coverage (-): 0

Region: chr19 45408677-45408687. Max. coverage (+): 0. Max coverage (-): 0

Region: chr19 45408688-45408697. Max. coverage (+): 0. Max coverage (-): 0

Region: chr19 45408698-45408707. Max. coverage (+): 0. Max coverage (-): 0

Region: chr19 45408708-45408717. Max. coverage (+): 0. Max coverage (-): 0

Region: chr19 45408718-45408727. Max. coverage (+): 0. Max coverage (-): 0

Region: chr19 45408728-45408738. Max. coverage (+): 0. Max coverage (-): 0

Region: chr19 45408739-45408748. Max. coverage (+): 0. Max coverage (-): 0

Region: chr19 45408749-45408758. Max. coverage (+): 0. Max coverage (-): 0

Region: chr19 45408759-45408768. Max. coverage (+): 0. Max coverage (-): 0

Region: chr19 45408769-45408778. Max. coverage (+): 0. Max coverage (-): 0

Region: chr19 45408779-45408788. Max. coverage (+): 0. Max coverage (-): 0

Region: chr19 45408789-45408799. Max. coverage (+): 0. Max coverage (-): 0

Region: chr19 45408800-45408809. Max. coverage (+): 0. Max coverage (-): 0

Region: chr19 45408810-45408819. Max. coverage (+): 0. Max coverage (-): 0

Region: chr19 45408820-45408829. Max. coverage (+): 0. Max coverage (-): 0

Region: chr19 45408830-45408839. Max. coverage (+): 0.81. Max coverage (-): 0

Region: chr19 45408840-45408850. Max. coverage (+): 0. Max coverage (-): 0

Region: chr19 45408851-45408860. Max. coverage (+): 0. Max coverage (-): 0

Region: chr19 45408861-45408870. Max. coverage (+): 0. Max coverage (-): 0

Region: chr19 45408871-45408880. Max. coverage (+): 0. Max coverage (-): 0

Region: chr19 45408881-45408890. Max. coverage (+): 7.76. Max coverage (-): 0

Region: chr19 45408891-45408901. Max. coverage (+): 7.76. Max coverage (-): 0

Region: chr19 45408902-45408911. Max. coverage (+): 0. Max coverage (-): 0

Region: chr19 45408912-45408921. Max. coverage (+): 0. Max coverage (-): 0

Region: chr19 45408922-45408931. Max. coverage (+): 0. Max coverage (-): 0

Region: chr19 45408932-45408941. Max. coverage (+): 0. Max coverage (-): 0

Region: chr19 45408942-45408951. Max. coverage (+): 0. Max coverage (-): 0

Region: chr19 45408952-45408962. Max. coverage (+): 0. Max coverage (-): 0

Region: chr19 45408963-45408972. Max. coverage (+): 0. Max coverage (-): 0

Region: chr19 45408973-45408982. Max. coverage (+): 0. Max coverage (-): 0

Region: chr19 45408983-45408992. Max. coverage (+): 0. Max coverage (-): 0

Region: chr19 45408993-45409002. Max. coverage (+): 0. Max coverage (-): 0

Region: chr19 45409003-45409013. Max. coverage (+): 0. Max coverage (-): 0

Region: chr19 45409014-45409023. Max. coverage (+): 0. Max coverage (-): 0

Region: chr19 45409024-45409033. Max. coverage (+): 0. Max coverage (-): 0

Region: chr19 45409034-45409043. Max. coverage (+): 0. Max coverage (-): 0

Region: chr19 45409044-45409053. Max. coverage (+): 0. Max coverage (-): 0

Region: chr19 45409054-45409063. Max. coverage (+): 0. Max coverage (-): 0

Region: chr19 45409064-45409074. Max. coverage (+): 0. Max coverage (-): 0

Region: chr19 45409075-45409084. Max. coverage (+): 0. Max coverage (-): 0

Region: chr19 45409085-45409094. Max. coverage (+): 1.19. Max coverage (-): 0

Region: chr19 45409095-45409104. Max. coverage (+): 1.19. Max coverage (-): 0

Region: chr19 45409105-45409114. Max. coverage (+): 0. Max coverage (-): 0

Region: chr19 45409115-. Max. coverage (+): 0. Max coverage (-): 0

RepeatMasker Color Code

**+**

100-98% Identity

<98-95% Identity

<95-90% Identity

<90-85% Identity

<85-80% Identity

<80-75% Identity

<75-70% Identity

<70% Identity

**-**

Gene Set Color Code

**+**

Gene

Pseudogene

**-**

Topology/Coverage Color Code

Coverage Plus Strand

Coverage Minus Strand

Mainstrand: Plus

Mainstrand: Minus

Complementary Strand

Flanking Region  
(if option -flank >0)

Gene Set Annotation  

**1. NMT1 (protein coding, ENSBTAG00000006051) Tr:00000007950 Ex:10**: 45403972-45404139 (+)  
**2. NMT1 (protein coding, ENSBTAG00000006051) Tr:00000007950 Ex:11**: 45405126-45405263 (+)  
**3. NMT1 (protein coding, ENSBTAG00000006051) Tr:00000007950 Ex:12**: 45405947-45406315 (+)

  
RepeatMasker Annotation  

**1. AT\_rich**: 45406272-45406321 (+), Divergence to consensus: 86%

  
Transcription Factor Binding Sites  

**RFX4\_1** (Sequence: CTTGGCAAC (+): 45407163)
