# Supplementary material for: Small RNA sequencing of cryopreserved semen from single bull revealed altered miRNAs and piRNAs expression between High- and Low-motile sperm populations
Source: BMC Genomics. 2017 Jan 4;18:14. doi: 10.1186/s12864-016-3394-7 (PMC5209821; doi:10.1186/s12864-016-3394-7)
Supplement: Additional file 3: — Details for each piRNA clusters found in High Motile (HM) sperm fraction. Genes, repeats, transposable elements and transcription factors binding sites falling within the cluster regions were reported. (ZIP 1896 kb) [file 12864_2016_3394_MOESM3_ESM.zip › 45.html]

piRNA cluster 45


Predicted piRNA cluster no. 45     previous   next
  

Show proTRAC run info
Hide proTRAC run info

================================= proTRAC ====================================  
VERSION: 2.1                                    LAST MODIFIED: 06. October 2015  
  
Please cite:  
Rosenkranz D, Zischler H. proTRAC - a software for probabilistic piRNA cluster  
detection, visualization and analysis. 2012. BMC Bioinformatics 13:5.  
  
and (for proTRAC 2.0 and later):  
Rosenkranz D, Rudloff S, Bastuck K, Ketting RF, Zischler H. Tupaia small RNAs  
provide insights into function and evolution of RNAi-based transposon defense  
in mammals. 2015. RNA 21(5):911-922.  
  
Contact:  
David Rosenkranz  
Institute of Anthropology, small RNA group  
Johannes Gutenberg University Mainz  
email: rosenkranz@uni-mainz.de  
  
You can find the latest proTRAC version at:  
http://sourceforge.net/projects/protrac/files  
http://www.smallRNAgroup-mainz.de/software  
==============================================================================  
  
PARAMETERS:  
Map file: .............../storage/core/barbara/genhome/smallRNA/fertility/Sample\_motile/pirna/Sample\_motile\_26-33\_collapsed.fa.no-dust.map.weighted-10000-1000-b-0  
Genome file: ............/storage/core/barbara/genhome/smallRNA/fertility/Sample\_all/pirna/bt\_311\_chrY.fa  
RepeatMasker annotation: /storage/genomes/bt\_umd31/GCF\_000003055.6\_Bos\_taurus\_UMD\_3.1.1\_repeatMasker\_chr.out  
GeneSet:................./storage/core/barbara/genhome/smallRNA/fertility/Sample\_all/pirna/full.gtf  
  
Significant (p<=0.01) hit density will be calculated based  
on observed hit distribution.  
  
Sliding window size: ........................................ 5000 bp  
Sliding window increament: .................................. 1000 bp  
Normalize each hit by number of genomic hits: ............... 1 [0=no/1=yes]  
Normalize each hit by number of sequence reads: ............. 1 [0=no/1=yes]  
Normalize values (-> per million mapped reads): ............. 1 [0=no/1=yes]  
Min. fraction of hits with 1T(U) or 10A: .................... 0.75  
Alternatively: Min. fraction of hits with 1T(U) and 10A: .... 0.5  
Min. fraction of hits with typical piRNA length: ............ 0.75  
Typical piRNA length: ....................................... 26-33 nt  
Min. size of a piRNA cluster: ............................... 5000 bp.  
Min. number of hits (absolute): ............................. 0  
Min. number of hits (normalized): ........................... 0  
Min. fraction of hits on the mainstrand: .................... 0.75  
Top fraction of mapped sequences (in terms of read counts): . 1%  
Top fraction accounts for max. n% of sequence reads: ........ 90%  
Min. fraction of hits on each arm of a bidirectional cluster: 0.1  
Output image file for each cluster: ......................... 0 [0=no/1=yes]  
Output html file for each cluster: .......................... 1 [0=no/1=yes]  
Output a summary table: ..................................... 1 [0=no/1=yes]  
Output a FASTA file for each cluster (piRNA sequences): ..... 1 [0=no/1=yes]  
Output a FASTA file comprising cluster sequences: ........... 1 [0=no/1=yes]  
Search DNA motifs in clusters: .............................. 1 [0=no/1=yes]  
Output flanking sequences: +/- .............................. 0 bp  
Output ~.pTi file: .......................................... 1 [0=no/1=yes]  
==============================================================================  
  
  
Genome size (without gaps): ............ 2678902517 bp  
Gaps (N/X/-): .......................... 53837044 bp  
Mapped reads: .......................... 658825247023  
Non-identical sequences: ............... 514171  
Genomic hits: .......................... 764233  
Significant densitiy of mapped reads: .. 12867599.5173724 reads/kb

Show proTRAC cluster info
Hide proTRAC cluster info

|  |  |
| --- | --- |
| Location | chr21 |
| Coordinates | 21106863-21111954 |
| Size [bp] | 5092 |
| Sequence hit loci | 137 |
| Mapped reads (normalized) | 168711916 |
| Mapped reads (normalized) per kb | 33132740.8 |
| Normalized reads with 1T (1U) | 82.7% |
| Normalized reads with 10A | 24.1% |
| Normalized reads with length 26-33 nt | 100% |
| Normalized reads on the main strand(s) | 100% |
| Predicted directionality | mono:plus |

100%

0%

1T (1U)  
reads

10A reads

26-33 nt  
reads

reads on mainstrand

**Either the amount of reads with 1T (1U) OR 10A has to exceed 75% (set with option: -1Tor10A)  
Alternatively the amount of reads with 1T (1U) AND 10A has to exceed 50% (set with option: -1Tand10A)  
Minimum amount of reads with preferred size is 75% (set with option: -pisize)  
Minimum amount of reads on the main strand(s) is 75% (set with option: -clstrand)**

Show read coverage
Hide read coverage

WHAT DO I SEE HERE?  
This chart shows the location of mapped sequence reads within a predicted piRNA cluster. The color refers to the number of genomic hits produced by the sequence read in question. A dark red bar indicates that this sequence read produces many other hits elsewhere in the genome. Many adjacent red or yellow bars can indicate the presence of a multi-copy element such as transposons or rRNA genes. A dark green bar indicates that this sequence read maps uniquely to this locus.

1 hit

2-5 hits

6-10 hits

11-20 hits

21-50 hits

51-100 hits

> 100 hits

chr21

21106863

21111954

Gene Set

RepeatMasker

Mapped  
Reads

22.29

plus strand

minus strand

22.29

Region: chr21 45409116-21106868. Max. coverage (+): 4.92. Max coverage (-): 0

Region: chr21 21106869-21106878. Max. coverage (+): 4.92. Max coverage (-): 0

Region: chr21 21106879-21106888. Max. coverage (+): 0. Max coverage (-): 0

Region: chr21 21106889-21106898. Max. coverage (+): 0. Max coverage (-): 0

Region: chr21 21106899-21106908. Max. coverage (+): 0. Max coverage (-): 0

Region: chr21 21106909-21106919. Max. coverage (+): 0. Max coverage (-): 0

Region: chr21 21106920-21106929. Max. coverage (+): 0. Max coverage (-): 0

Region: chr21 21106930-21106939. Max. coverage (+): 0. Max coverage (-): 0

Region: chr21 21106940-21106949. Max. coverage (+): 0. Max coverage (-): 0

Region: chr21 21106950-21106959. Max. coverage (+): 0. Max coverage (-): 0

Region: chr21 21106960-21106969. Max. coverage (+): 0. Max coverage (-): 0

Region: chr21 21106970-21106980. Max. coverage (+): 0. Max coverage (-): 0

Region: chr21 21106981-21106990. Max. coverage (+): 0. Max coverage (-): 0

Region: chr21 21106991-21107000. Max. coverage (+): 0. Max coverage (-): 0

Region: chr21 21107001-21107010. Max. coverage (+): 0. Max coverage (-): 0

Region: chr21 21107011-21107020. Max. coverage (+): 0. Max coverage (-): 0

Region: chr21 21107021-21107031. Max. coverage (+): 0. Max coverage (-): 0

Region: chr21 21107032-21107041. Max. coverage (+): 0. Max coverage (-): 0

Region: chr21 21107042-21107051. Max. coverage (+): 0. Max coverage (-): 0

Region: chr21 21107052-21107061. Max. coverage (+): 0. Max coverage (-): 0

Region: chr21 21107062-21107071. Max. coverage (+): 0. Max coverage (-): 0

Region: chr21 21107072-21107081. Max. coverage (+): 0. Max coverage (-): 0

Region: chr21 21107082-21107092. Max. coverage (+): 0. Max coverage (-): 0

Region: chr21 21107093-21107102. Max. coverage (+): 0. Max coverage (-): 0

Region: chr21 21107103-21107112. Max. coverage (+): 0. Max coverage (-): 0

Region: chr21 21107113-21107122. Max. coverage (+): 0. Max coverage (-): 0

Region: chr21 21107123-21107132. Max. coverage (+): 0. Max coverage (-): 0

Region: chr21 21107133-21107143. Max. coverage (+): 0. Max coverage (-): 0

Region: chr21 21107144-21107153. Max. coverage (+): 0. Max coverage (-): 0

Region: chr21 21107154-21107163. Max. coverage (+): 0. Max coverage (-): 0

Region: chr21 21107164-21107173. Max. coverage (+): 0. Max coverage (-): 0

Region: chr21 21107174-21107183. Max. coverage (+): 0. Max coverage (-): 0

Region: chr21 21107184-21107193. Max. coverage (+): 0. Max coverage (-): 0

Region: chr21 21107194-21107204. Max. coverage (+): 0. Max coverage (-): 0

Region: chr21 21107205-21107214. Max. coverage (+): 0. Max coverage (-): 0

Region: chr21 21107215-21107224. Max. coverage (+): 0. Max coverage (-): 0

Region: chr21 21107225-21107234. Max. coverage (+): 0. Max coverage (-): 0

Region: chr21 21107235-21107244. Max. coverage (+): 0. Max coverage (-): 0

Region: chr21 21107245-21107255. Max. coverage (+): 0. Max coverage (-): 0

Region: chr21 21107256-21107265. Max. coverage (+): 0. Max coverage (-): 0

Region: chr21 21107266-21107275. Max. coverage (+): 0. Max coverage (-): 0

Region: chr21 21107276-21107285. Max. coverage (+): 0. Max coverage (-): 0

Region: chr21 21107286-21107295. Max. coverage (+): 0. Max coverage (-): 0

Region: chr21 21107296-21107306. Max. coverage (+): 0. Max coverage (-): 0

Region: chr21 21107307-21107316. Max. coverage (+): 0. Max coverage (-): 0

Region: chr21 21107317-21107326. Max. coverage (+): 0. Max coverage (-): 0

Region: chr21 21107327-21107336. Max. coverage (+): 0. Max coverage (-): 0

Region: chr21 21107337-21107346. Max. coverage (+): 0. Max coverage (-): 0

Region: chr21 21107347-21107356. Max. coverage (+): 0. Max coverage (-): 0

Region: chr21 21107357-21107367. Max. coverage (+): 0. Max coverage (-): 0

Region: chr21 21107368-21107377. Max. coverage (+): 0. Max coverage (-): 0

Region: chr21 21107378-21107387. Max. coverage (+): 0. Max coverage (-): 0

Region: chr21 21107388-21107397. Max. coverage (+): 0. Max coverage (-): 0

Region: chr21 21107398-21107407. Max. coverage (+): 0. Max coverage (-): 0

Region: chr21 21107408-21107418. Max. coverage (+): 0. Max coverage (-): 0

Region: chr21 21107419-21107428. Max. coverage (+): 0. Max coverage (-): 0

Region: chr21 21107429-21107438. Max. coverage (+): 0. Max coverage (-): 0

Region: chr21 21107439-21107448. Max. coverage (+): 0. Max coverage (-): 0

Region: chr21 21107449-21107458. Max. coverage (+): 0. Max coverage (-): 0

Region: chr21 21107459-21107468. Max. coverage (+): 0. Max coverage (-): 0

Region: chr21 21107469-21107479. Max. coverage (+): 0. Max coverage (-): 0

Region: chr21 21107480-21107489. Max. coverage (+): 0. Max coverage (-): 0

Region: chr21 21107490-21107499. Max. coverage (+): 0. Max coverage (-): 0

Region: chr21 21107500-21107509. Max. coverage (+): 0. Max coverage (-): 0

Region: chr21 21107510-21107519. Max. coverage (+): 0. Max coverage (-): 0

Region: chr21 21107520-21107530. Max. coverage (+): 0. Max coverage (-): 0

Region: chr21 21107531-21107540. Max. coverage (+): 0. Max coverage (-): 0

Region: chr21 21107541-21107550. Max. coverage (+): 0. Max coverage (-): 0

Region: chr21 21107551-21107560. Max. coverage (+): 0. Max coverage (-): 0

Region: chr21 21107561-21107570. Max. coverage (+): 0. Max coverage (-): 0

Region: chr21 21107571-21107580. Max. coverage (+): 0. Max coverage (-): 0

Region: chr21 21107581-21107591. Max. coverage (+): 0. Max coverage (-): 0

Region: chr21 21107592-21107601. Max. coverage (+): 0. Max coverage (-): 0

Region: chr21 21107602-21107611. Max. coverage (+): 0. Max coverage (-): 0

Region: chr21 21107612-21107621. Max. coverage (+): 0. Max coverage (-): 0

Region: chr21 21107622-21107631. Max. coverage (+): 0. Max coverage (-): 0

Region: chr21 21107632-21107642. Max. coverage (+): 0. Max coverage (-): 0

Region: chr21 21107643-21107652. Max. coverage (+): 0. Max coverage (-): 0

Region: chr21 21107653-21107662. Max. coverage (+): 0. Max coverage (-): 0

Region: chr21 21107663-21107672. Max. coverage (+): 0. Max coverage (-): 0

Region: chr21 21107673-21107682. Max. coverage (+): 0. Max coverage (-): 0

Region: chr21 21107683-21107692. Max. coverage (+): 0. Max coverage (-): 0

Region: chr21 21107693-21107703. Max. coverage (+): 0. Max coverage (-): 0

Region: chr21 21107704-21107713. Max. coverage (+): 0. Max coverage (-): 0

Region: chr21 21107714-21107723. Max. coverage (+): 0. Max coverage (-): 0

Region: chr21 21107724-21107733. Max. coverage (+): 0. Max coverage (-): 0

Region: chr21 21107734-21107743. Max. coverage (+): 0. Max coverage (-): 0

Region: chr21 21107744-21107754. Max. coverage (+): 0.95. Max coverage (-): 0

Region: chr21 21107755-21107764. Max. coverage (+): 6.29. Max coverage (-): 0

Region: chr21 21107765-21107774. Max. coverage (+): 4.16. Max coverage (-): 0

Region: chr21 21107775-21107784. Max. coverage (+): 5.88. Max coverage (-): 0

Region: chr21 21107785-21107794. Max. coverage (+): 0. Max coverage (-): 0

Region: chr21 21107795-21107805. Max. coverage (+): 2.85. Max coverage (-): 0

Region: chr21 21107806-21107815. Max. coverage (+): 2.85. Max coverage (-): 0

Region: chr21 21107816-21107825. Max. coverage (+): 7.8. Max coverage (-): 0

Region: chr21 21107826-21107835. Max. coverage (+): 7.8. Max coverage (-): 0

Region: chr21 21107836-21107845. Max. coverage (+): 22.29. Max coverage (-): 0

Region: chr21 21107846-21107855. Max. coverage (+): 20.4. Max coverage (-): 0

Region: chr21 21107856-21107866. Max. coverage (+): 0. Max coverage (-): 0

Region: chr21 21107867-21107876. Max. coverage (+): 0. Max coverage (-): 0

Region: chr21 21107877-21107886. Max. coverage (+): 0. Max coverage (-): 0

Region: chr21 21107887-21107896. Max. coverage (+): 0. Max coverage (-): 0

Region: chr21 21107897-21107906. Max. coverage (+): 0. Max coverage (-): 0

Region: chr21 21107907-21107917. Max. coverage (+): 0. Max coverage (-): 0

Region: chr21 21107918-21107927. Max. coverage (+): 0. Max coverage (-): 0

Region: chr21 21107928-21107937. Max. coverage (+): 0. Max coverage (-): 0

Region: chr21 21107938-21107947. Max. coverage (+): 0. Max coverage (-): 0

Region: chr21 21107948-21107957. Max. coverage (+): 6.05. Max coverage (-): 0

Region: chr21 21107958-21107967. Max. coverage (+): 7.64. Max coverage (-): 0

Region: chr21 21107968-21107978. Max. coverage (+): 14.7. Max coverage (-): 0

Region: chr21 21107979-21107988. Max. coverage (+): 11.05. Max coverage (-): 0

Region: chr21 21107989-21107998. Max. coverage (+): 0. Max coverage (-): 0

Region: chr21 21107999-21108008. Max. coverage (+): 0. Max coverage (-): 0

Region: chr21 21108009-21108018. Max. coverage (+): 1.6. Max coverage (-): 0

Region: chr21 21108019-21108029. Max. coverage (+): 2.27. Max coverage (-): 0

Region: chr21 21108030-21108039. Max. coverage (+): 0.74. Max coverage (-): 0

Region: chr21 21108040-21108049. Max. coverage (+): 5.21. Max coverage (-): 0

Region: chr21 21108050-21108059. Max. coverage (+): 0.38. Max coverage (-): 0

Region: chr21 21108060-21108069. Max. coverage (+): 0. Max coverage (-): 0

Region: chr21 21108070-21108079. Max. coverage (+): 0. Max coverage (-): 0

Region: chr21 21108080-21108090. Max. coverage (+): 0.82. Max coverage (-): 0

Region: chr21 21108091-21108100. Max. coverage (+): 0. Max coverage (-): 0

Region: chr21 21108101-21108110. Max. coverage (+): 0. Max coverage (-): 0

Region: chr21 21108111-21108120. Max. coverage (+): 0. Max coverage (-): 0

Region: chr21 21108121-21108130. Max. coverage (+): 0. Max coverage (-): 0

Region: chr21 21108131-21108141. Max. coverage (+): 0. Max coverage (-): 0

Region: chr21 21108142-21108151. Max. coverage (+): 0. Max coverage (-): 0

Region: chr21 21108152-21108161. Max. coverage (+): 0. Max coverage (-): 0

Region: chr21 21108162-21108171. Max. coverage (+): 0. Max coverage (-): 0

Region: chr21 21108172-21108181. Max. coverage (+): 0. Max coverage (-): 0

Region: chr21 21108182-21108192. Max. coverage (+): 1.76. Max coverage (-): 0

Region: chr21 21108193-21108202. Max. coverage (+): 0. Max coverage (-): 0

Region: chr21 21108203-21108212. Max. coverage (+): 0. Max coverage (-): 0

Region: chr21 21108213-21108222. Max. coverage (+): 2.68. Max coverage (-): 0

Region: chr21 21108223-21108232. Max. coverage (+): 3.77. Max coverage (-): 0

Region: chr21 21108233-21108242. Max. coverage (+): 0. Max coverage (-): 0

Region: chr21 21108243-21108253. Max. coverage (+): 0.85. Max coverage (-): 0

Region: chr21 21108254-21108263. Max. coverage (+): 0. Max coverage (-): 0

Region: chr21 21108264-21108273. Max. coverage (+): 0. Max coverage (-): 0

Region: chr21 21108274-21108283. Max. coverage (+): 3.72. Max coverage (-): 0

Region: chr21 21108284-21108293. Max. coverage (+): 0.77. Max coverage (-): 0

Region: chr21 21108294-21108304. Max. coverage (+): 0. Max coverage (-): 0

Region: chr21 21108305-21108314. Max. coverage (+): 0. Max coverage (-): 0

Region: chr21 21108315-21108324. Max. coverage (+): 1.96. Max coverage (-): 0

Region: chr21 21108325-21108334. Max. coverage (+): 1.96. Max coverage (-): 0

Region: chr21 21108335-21108344. Max. coverage (+): 0. Max coverage (-): 0

Region: chr21 21108345-21108354. Max. coverage (+): 0. Max coverage (-): 0

Region: chr21 21108355-21108365. Max. coverage (+): 0. Max coverage (-): 0

Region: chr21 21108366-21108375. Max. coverage (+): 0. Max coverage (-): 0

Region: chr21 21108376-21108385. Max. coverage (+): 0. Max coverage (-): 0

Region: chr21 21108386-21108395. Max. coverage (+): 0. Max coverage (-): 0

Region: chr21 21108396-21108405. Max. coverage (+): 0. Max coverage (-): 0

Region: chr21 21108406-21108416. Max. coverage (+): 0. Max coverage (-): 0

Region: chr21 21108417-21108426. Max. coverage (+): 0. Max coverage (-): 0

Region: chr21 21108427-21108436. Max. coverage (+): 9.03. Max coverage (-): 0

Region: chr21 21108437-21108446. Max. coverage (+): 9.03. Max coverage (-): 0

Region: chr21 21108447-21108456. Max. coverage (+): 0. Max coverage (-): 0

Region: chr21 21108457-21108466. Max. coverage (+): 0. Max coverage (-): 0

Region: chr21 21108467-21108477. Max. coverage (+): 0. Max coverage (-): 0

Region: chr21 21108478-21108487. Max. coverage (+): 4.07. Max coverage (-): 0

Region: chr21 21108488-21108497. Max. coverage (+): 6.13. Max coverage (-): 0

Region: chr21 21108498-21108507. Max. coverage (+): 1.21. Max coverage (-): 0

Region: chr21 21108508-21108517. Max. coverage (+): 1.21. Max coverage (-): 0

Region: chr21 21108518-21108528. Max. coverage (+): 0. Max coverage (-): 0

Region: chr21 21108529-21108538. Max. coverage (+): 0. Max coverage (-): 0

Region: chr21 21108539-21108548. Max. coverage (+): 0. Max coverage (-): 0

Region: chr21 21108549-21108558. Max. coverage (+): 2.31. Max coverage (-): 0

Region: chr21 21108559-21108568. Max. coverage (+): 2.31. Max coverage (-): 0

Region: chr21 21108569-21108579. Max. coverage (+): 0. Max coverage (-): 0

Region: chr21 21108580-21108589. Max. coverage (+): 4.35. Max coverage (-): 0

Region: chr21 21108590-21108599. Max. coverage (+): 4.35. Max coverage (-): 0

Region: chr21 21108600-21108609. Max. coverage (+): 0. Max coverage (-): 0

Region: chr21 21108610-21108619. Max. coverage (+): 0. Max coverage (-): 0

Region: chr21 21108620-21108629. Max. coverage (+): 0. Max coverage (-): 0

Region: chr21 21108630-21108640. Max. coverage (+): 0. Max coverage (-): 0

Region: chr21 21108641-21108650. Max. coverage (+): 0. Max coverage (-): 0

Region: chr21 21108651-21108660. Max. coverage (+): 0. Max coverage (-): 0

Region: chr21 21108661-21108670. Max. coverage (+): 0. Max coverage (-): 0

Region: chr21 21108671-21108680. Max. coverage (+): 0. Max coverage (-): 0

Region: chr21 21108681-21108691. Max. coverage (+): 0. Max coverage (-): 0

Region: chr21 21108692-21108701. Max. coverage (+): 0. Max coverage (-): 0

Region: chr21 21108702-21108711. Max. coverage (+): 0. Max coverage (-): 0

Region: chr21 21108712-21108721. Max. coverage (+): 0. Max coverage (-): 0

Region: chr21 21108722-21108731. Max. coverage (+): 0. Max coverage (-): 0

Region: chr21 21108732-21108741. Max. coverage (+): 0. Max coverage (-): 0

Region: chr21 21108742-21108752. Max. coverage (+): 0. Max coverage (-): 0

Region: chr21 21108753-21108762. Max. coverage (+): 2.08. Max coverage (-): 0

Region: chr21 21108763-21108772. Max. coverage (+): 0. Max coverage (-): 0

Region: chr21 21108773-21108782. Max. coverage (+): 0. Max coverage (-): 0

Region: chr21 21108783-21108792. Max. coverage (+): 0. Max coverage (-): 0

Region: chr21 21108793-21108803. Max. coverage (+): 0. Max coverage (-): 0

Region: chr21 21108804-21108813. Max. coverage (+): 0. Max coverage (-): 0

Region: chr21 21108814-21108823. Max. coverage (+): 0. Max coverage (-): 0

Region: chr21 21108824-21108833. Max. coverage (+): 0. Max coverage (-): 0

Region: chr21 21108834-21108843. Max. coverage (+): 0. Max coverage (-): 0

Region: chr21 21108844-21108853. Max. coverage (+): 4.66. Max coverage (-): 0

Region: chr21 21108854-21108864. Max. coverage (+): 4.66. Max coverage (-): 0

Region: chr21 21108865-21108874. Max. coverage (+): 0.35. Max coverage (-): 0

Region: chr21 21108875-21108884. Max. coverage (+): 0. Max coverage (-): 0

Region: chr21 21108885-21108894. Max. coverage (+): 0. Max coverage (-): 0

Region: chr21 21108895-21108904. Max. coverage (+): 0. Max coverage (-): 0

Region: chr21 21108905-21108915. Max. coverage (+): 3.29. Max coverage (-): 0

Region: chr21 21108916-21108925. Max. coverage (+): 3.29. Max coverage (-): 0

Region: chr21 21108926-21108935. Max. coverage (+): 3.26. Max coverage (-): 0

Region: chr21 21108936-21108945. Max. coverage (+): 0. Max coverage (-): 0

Region: chr21 21108946-21108955. Max. coverage (+): 2.14. Max coverage (-): 0

Region: chr21 21108956-21108965. Max. coverage (+): 2.14. Max coverage (-): 0

Region: chr21 21108966-21108976. Max. coverage (+): 0. Max coverage (-): 0

Region: chr21 21108977-21108986. Max. coverage (+): 0. Max coverage (-): 0

Region: chr21 21108987-21108996. Max. coverage (+): 0.55. Max coverage (-): 0

Region: chr21 21108997-21109006. Max. coverage (+): 2.01. Max coverage (-): 0

Region: chr21 21109007-21109016. Max. coverage (+): 0. Max coverage (-): 0

Region: chr21 21109017-21109027. Max. coverage (+): 0. Max coverage (-): 0

Region: chr21 21109028-21109037. Max. coverage (+): 0. Max coverage (-): 0

Region: chr21 21109038-21109047. Max. coverage (+): 0. Max coverage (-): 0

Region: chr21 21109048-21109057. Max. coverage (+): 0. Max coverage (-): 0

Region: chr21 21109058-21109067. Max. coverage (+): 0. Max coverage (-): 0

Region: chr21 21109068-21109078. Max. coverage (+): 0. Max coverage (-): 0

Region: chr21 21109079-21109088. Max. coverage (+): 0. Max coverage (-): 0

Region: chr21 21109089-21109098. Max. coverage (+): 0. Max coverage (-): 0

Region: chr21 21109099-21109108. Max. coverage (+): 0. Max coverage (-): 0

Region: chr21 21109109-21109118. Max. coverage (+): 0. Max coverage (-): 0

Region: chr21 21109119-21109128. Max. coverage (+): 0. Max coverage (-): 0

Region: chr21 21109129-21109139. Max. coverage (+): 7.35. Max coverage (-): 0

Region: chr21 21109140-21109149. Max. coverage (+): 7.35. Max coverage (-): 0

Region: chr21 21109150-21109159. Max. coverage (+): 3.58. Max coverage (-): 0

Region: chr21 21109160-21109169. Max. coverage (+): 0. Max coverage (-): 0

Region: chr21 21109170-21109179. Max. coverage (+): 0. Max coverage (-): 0

Region: chr21 21109180-21109190. Max. coverage (+): 0. Max coverage (-): 0

Region: chr21 21109191-21109200. Max. coverage (+): 0. Max coverage (-): 0

Region: chr21 21109201-21109210. Max. coverage (+): 0. Max coverage (-): 0

Region: chr21 21109211-21109220. Max. coverage (+): 0. Max coverage (-): 0

Region: chr21 21109221-21109230. Max. coverage (+): 0. Max coverage (-): 0

Region: chr21 21109231-21109240. Max. coverage (+): 0. Max coverage (-): 0

Region: chr21 21109241-21109251. Max. coverage (+): 0. Max coverage (-): 0

Region: chr21 21109252-21109261. Max. coverage (+): 0. Max coverage (-): 0

Region: chr21 21109262-21109271. Max. coverage (+): 0. Max coverage (-): 0

Region: chr21 21109272-21109281. Max. coverage (+): 0. Max coverage (-): 0

Region: chr21 21109282-21109291. Max. coverage (+): 0. Max coverage (-): 0

Region: chr21 21109292-21109302. Max. coverage (+): 0. Max coverage (-): 0

Region: chr21 21109303-21109312. Max. coverage (+): 0. Max coverage (-): 0

Region: chr21 21109313-21109322. Max. coverage (+): 0. Max coverage (-): 0

Region: chr21 21109323-21109332. Max. coverage (+): 0. Max coverage (-): 0

Region: chr21 21109333-21109342. Max. coverage (+): 0. Max coverage (-): 0

Region: chr21 21109343-21109352. Max. coverage (+): 0. Max coverage (-): 0

Region: chr21 21109353-21109363. Max. coverage (+): 0. Max coverage (-): 0

Region: chr21 21109364-21109373. Max. coverage (+): 0. Max coverage (-): 0

Region: chr21 21109374-21109383. Max. coverage (+): 0. Max coverage (-): 0

Region: chr21 21109384-21109393. Max. coverage (+): 0. Max coverage (-): 0

Region: chr21 21109394-21109403. Max. coverage (+): 0. Max coverage (-): 0

Region: chr21 21109404-21109414. Max. coverage (+): 0. Max coverage (-): 0

Region: chr21 21109415-21109424. Max. coverage (+): 0. Max coverage (-): 0

Region: chr21 21109425-21109434. Max. coverage (+): 0. Max coverage (-): 0

Region: chr21 21109435-21109444. Max. coverage (+): 0. Max coverage (-): 0

Region: chr21 21109445-21109454. Max. coverage (+): 0. Max coverage (-): 0

Region: chr21 21109455-21109465. Max. coverage (+): 0. Max coverage (-): 0

Region: chr21 21109466-21109475. Max. coverage (+): 0. Max coverage (-): 0

Region: chr21 21109476-21109485. Max. coverage (+): 0. Max coverage (-): 0

Region: chr21 21109486-21109495. Max. coverage (+): 0. Max coverage (-): 0

Region: chr21 21109496-21109505. Max. coverage (+): 0. Max coverage (-): 0

Region: chr21 21109506-21109515. Max. coverage (+): 0. Max coverage (-): 0

Region: chr21 21109516-21109526. Max. coverage (+): 0. Max coverage (-): 0

Region: chr21 21109527-21109536. Max. coverage (+): 0. Max coverage (-): 0

Region: chr21 21109537-21109546. Max. coverage (+): 0. Max coverage (-): 0

Region: chr21 21109547-21109556. Max. coverage (+): 0. Max coverage (-): 0

Region: chr21 21109557-21109566. Max. coverage (+): 0. Max coverage (-): 0

Region: chr21 21109567-21109577. Max. coverage (+): 0. Max coverage (-): 0

Region: chr21 21109578-21109587. Max. coverage (+): 0. Max coverage (-): 0

Region: chr21 21109588-21109597. Max. coverage (+): 0. Max coverage (-): 0

Region: chr21 21109598-21109607. Max. coverage (+): 0. Max coverage (-): 0

Region: chr21 21109608-21109617. Max. coverage (+): 0. Max coverage (-): 0

Region: chr21 21109618-21109627. Max. coverage (+): 0. Max coverage (-): 0

Region: chr21 21109628-21109638. Max. coverage (+): 1.59. Max coverage (-): 0

Region: chr21 21109639-21109648. Max. coverage (+): 1.59. Max coverage (-): 0

Region: chr21 21109649-21109658. Max. coverage (+): 0. Max coverage (-): 0

Region: chr21 21109659-21109668. Max. coverage (+): 0. Max coverage (-): 0

Region: chr21 21109669-21109678. Max. coverage (+): 0. Max coverage (-): 0

Region: chr21 21109679-21109689. Max. coverage (+): 0. Max coverage (-): 0

Region: chr21 21109690-21109699. Max. coverage (+): 0. Max coverage (-): 0

Region: chr21 21109700-21109709. Max. coverage (+): 0. Max coverage (-): 0

Region: chr21 21109710-21109719. Max. coverage (+): 0. Max coverage (-): 0

Region: chr21 21109720-21109729. Max. coverage (+): 1.09. Max coverage (-): 0

Region: chr21 21109730-21109739. Max. coverage (+): 1.09. Max coverage (-): 0

Region: chr21 21109740-21109750. Max. coverage (+): 0. Max coverage (-): 0

Region: chr21 21109751-21109760. Max. coverage (+): 5.08. Max coverage (-): 0

Region: chr21 21109761-21109770. Max. coverage (+): 0. Max coverage (-): 0

Region: chr21 21109771-21109780. Max. coverage (+): 0.35. Max coverage (-): 0

Region: chr21 21109781-21109790. Max. coverage (+): 7.45. Max coverage (-): 0

Region: chr21 21109791-21109801. Max. coverage (+): 4.46. Max coverage (-): 0

Region: chr21 21109802-21109811. Max. coverage (+): 1.56. Max coverage (-): 0

Region: chr21 21109812-21109821. Max. coverage (+): 0. Max coverage (-): 0

Region: chr21 21109822-21109831. Max. coverage (+): 0. Max coverage (-): 0

Region: chr21 21109832-21109841. Max. coverage (+): 1.67. Max coverage (-): 0

Region: chr21 21109842-21109852. Max. coverage (+): 1.67. Max coverage (-): 0

Region: chr21 21109853-21109862. Max. coverage (+): 0. Max coverage (-): 0

Region: chr21 21109863-21109872. Max. coverage (+): 0. Max coverage (-): 0

Region: chr21 21109873-21109882. Max. coverage (+): 0. Max coverage (-): 0

Region: chr21 21109883-21109892. Max. coverage (+): 0. Max coverage (-): 0

Region: chr21 21109893-21109902. Max. coverage (+): 0. Max coverage (-): 0

Region: chr21 21109903-21109913. Max. coverage (+): 0. Max coverage (-): 0

Region: chr21 21109914-21109923. Max. coverage (+): 0. Max coverage (-): 0

Region: chr21 21109924-21109933. Max. coverage (+): 0. Max coverage (-): 0

Region: chr21 21109934-21109943. Max. coverage (+): 0. Max coverage (-): 0

Region: chr21 21109944-21109953. Max. coverage (+): 0. Max coverage (-): 0

Region: chr21 21109954-21109964. Max. coverage (+): 0. Max coverage (-): 0

Region: chr21 21109965-21109974. Max. coverage (+): 0. Max coverage (-): 0

Region: chr21 21109975-21109984. Max. coverage (+): 3.06. Max coverage (-): 0

Region: chr21 21109985-21109994. Max. coverage (+): 3.06. Max coverage (-): 0

Region: chr21 21109995-21110004. Max. coverage (+): 3.07. Max coverage (-): 0

Region: chr21 21110005-21110014. Max. coverage (+): 9.33. Max coverage (-): 0

Region: chr21 21110015-21110025. Max. coverage (+): 9.33. Max coverage (-): 0

Region: chr21 21110026-21110035. Max. coverage (+): 0. Max coverage (-): 0

Region: chr21 21110036-21110045. Max. coverage (+): 0. Max coverage (-): 0

Region: chr21 21110046-21110055. Max. coverage (+): 0. Max coverage (-): 0

Region: chr21 21110056-21110065. Max. coverage (+): 3.49. Max coverage (-): 0

Region: chr21 21110066-21110076. Max. coverage (+): 11.86. Max coverage (-): 0

Region: chr21 21110077-21110086. Max. coverage (+): 0. Max coverage (-): 0

Region: chr21 21110087-21110096. Max. coverage (+): 0. Max coverage (-): 0

Region: chr21 21110097-21110106. Max. coverage (+): 0. Max coverage (-): 0

Region: chr21 21110107-21110116. Max. coverage (+): 0. Max coverage (-): 0

Region: chr21 21110117-21110126. Max. coverage (+): 0. Max coverage (-): 0

Region: chr21 21110127-21110137. Max. coverage (+): 0. Max coverage (-): 0

Region: chr21 21110138-21110147. Max. coverage (+): 1.12. Max coverage (-): 0

Region: chr21 21110148-21110157. Max. coverage (+): 1.12. Max coverage (-): 0

Region: chr21 21110158-21110167. Max. coverage (+): 0. Max coverage (-): 0

Region: chr21 21110168-21110177. Max. coverage (+): 0. Max coverage (-): 0

Region: chr21 21110178-21110188. Max. coverage (+): 0. Max coverage (-): 0

Region: chr21 21110189-21110198. Max. coverage (+): 0. Max coverage (-): 0

Region: chr21 21110199-21110208. Max. coverage (+): 0. Max coverage (-): 0

Region: chr21 21110209-21110218. Max. coverage (+): 0. Max coverage (-): 0

Region: chr21 21110219-21110228. Max. coverage (+): 0. Max coverage (-): 0

Region: chr21 21110229-21110238. Max. coverage (+): 0. Max coverage (-): 0

Region: chr21 21110239-21110249. Max. coverage (+): 0. Max coverage (-): 0

Region: chr21 21110250-21110259. Max. coverage (+): 0. Max coverage (-): 0

Region: chr21 21110260-21110269. Max. coverage (+): 1.13. Max coverage (-): 0

Region: chr21 21110270-21110279. Max. coverage (+): 1.13. Max coverage (-): 0

Region: chr21 21110280-21110289. Max. coverage (+): 0. Max coverage (-): 0

Region: chr21 21110290-21110300. Max. coverage (+): 0. Max coverage (-): 0

Region: chr21 21110301-21110310. Max. coverage (+): 0. Max coverage (-): 0

Region: chr21 21110311-21110320. Max. coverage (+): 0. Max coverage (-): 0

Region: chr21 21110321-21110330. Max. coverage (+): 0. Max coverage (-): 0

Region: chr21 21110331-21110340. Max. coverage (+): 0. Max coverage (-): 0

Region: chr21 21110341-21110351. Max. coverage (+): 0. Max coverage (-): 0

Region: chr21 21110352-21110361. Max. coverage (+): 5.24. Max coverage (-): 0

Region: chr21 21110362-21110371. Max. coverage (+): 5.24. Max coverage (-): 0

Region: chr21 21110372-21110381. Max. coverage (+): 0. Max coverage (-): 0

Region: chr21 21110382-21110391. Max. coverage (+): 0. Max coverage (-): 0

Region: chr21 21110392-21110401. Max. coverage (+): 1.08. Max coverage (-): 0

Region: chr21 21110402-21110412. Max. coverage (+): 1.08. Max coverage (-): 0

Region: chr21 21110413-21110422. Max. coverage (+): 1.41. Max coverage (-): 0

Region: chr21 21110423-21110432. Max. coverage (+): 1.41. Max coverage (-): 0

Region: chr21 21110433-21110442. Max. coverage (+): 0. Max coverage (-): 0

Region: chr21 21110443-21110452. Max. coverage (+): 0. Max coverage (-): 0

Region: chr21 21110453-21110463. Max. coverage (+): 0. Max coverage (-): 0

Region: chr21 21110464-21110473. Max. coverage (+): 0. Max coverage (-): 0

Region: chr21 21110474-21110483. Max. coverage (+): 0. Max coverage (-): 0

Region: chr21 21110484-21110493. Max. coverage (+): 0. Max coverage (-): 0

Region: chr21 21110494-21110503. Max. coverage (+): 0. Max coverage (-): 0

Region: chr21 21110504-21110513. Max. coverage (+): 0. Max coverage (-): 0

Region: chr21 21110514-21110524. Max. coverage (+): 0. Max coverage (-): 0

Region: chr21 21110525-21110534. Max. coverage (+): 0. Max coverage (-): 0

Region: chr21 21110535-21110544. Max. coverage (+): 0. Max coverage (-): 0

Region: chr21 21110545-21110554. Max. coverage (+): 0. Max coverage (-): 0

Region: chr21 21110555-21110564. Max. coverage (+): 9.52. Max coverage (-): 0

Region: chr21 21110565-21110575. Max. coverage (+): 4.56. Max coverage (-): 0

Region: chr21 21110576-21110585. Max. coverage (+): 0. Max coverage (-): 0

Region: chr21 21110586-21110595. Max. coverage (+): 1.64. Max coverage (-): 0

Region: chr21 21110596-21110605. Max. coverage (+): 0. Max coverage (-): 0

Region: chr21 21110606-21110615. Max. coverage (+): 0. Max coverage (-): 0

Region: chr21 21110616-21110625. Max. coverage (+): 0. Max coverage (-): 0

Region: chr21 21110626-21110636. Max. coverage (+): 0. Max coverage (-): 0

Region: chr21 21110637-21110646. Max. coverage (+): 1.07. Max coverage (-): 0

Region: chr21 21110647-21110656. Max. coverage (+): 0. Max coverage (-): 0

Region: chr21 21110657-21110666. Max. coverage (+): 1.25. Max coverage (-): 0

Region: chr21 21110667-21110676. Max. coverage (+): 1.25. Max coverage (-): 0

Region: chr21 21110677-21110687. Max. coverage (+): 0. Max coverage (-): 0

Region: chr21 21110688-21110697. Max. coverage (+): 0. Max coverage (-): 0

Region: chr21 21110698-21110707. Max. coverage (+): 0. Max coverage (-): 0

Region: chr21 21110708-21110717. Max. coverage (+): 0. Max coverage (-): 0

Region: chr21 21110718-21110727. Max. coverage (+): 0. Max coverage (-): 0

Region: chr21 21110728-21110738. Max. coverage (+): 0. Max coverage (-): 0

Region: chr21 21110739-21110748. Max. coverage (+): 0. Max coverage (-): 0

Region: chr21 21110749-21110758. Max. coverage (+): 0. Max coverage (-): 0

Region: chr21 21110759-21110768. Max. coverage (+): 5.27. Max coverage (-): 0

Region: chr21 21110769-21110778. Max. coverage (+): 5.27. Max coverage (-): 0

Region: chr21 21110779-21110788. Max. coverage (+): 1.48. Max coverage (-): 0

Region: chr21 21110789-21110799. Max. coverage (+): 1.48. Max coverage (-): 0

Region: chr21 21110800-21110809. Max. coverage (+): 0. Max coverage (-): 0

Region: chr21 21110810-21110819. Max. coverage (+): 0. Max coverage (-): 0

Region: chr21 21110820-21110829. Max. coverage (+): 0. Max coverage (-): 0

Region: chr21 21110830-21110839. Max. coverage (+): 0. Max coverage (-): 0

Region: chr21 21110840-21110850. Max. coverage (+): 0. Max coverage (-): 0

Region: chr21 21110851-21110860. Max. coverage (+): 0. Max coverage (-): 0

Region: chr21 21110861-21110870. Max. coverage (+): 0. Max coverage (-): 0

Region: chr21 21110871-21110880. Max. coverage (+): 0. Max coverage (-): 0

Region: chr21 21110881-21110890. Max. coverage (+): 0. Max coverage (-): 0

Region: chr21 21110891-21110900. Max. coverage (+): 0. Max coverage (-): 0

Region: chr21 21110901-21110911. Max. coverage (+): 0. Max coverage (-): 0

Region: chr21 21110912-21110921. Max. coverage (+): 8.77. Max coverage (-): 0

Region: chr21 21110922-21110931. Max. coverage (+): 15.59. Max coverage (-): 0

Region: chr21 21110932-21110941. Max. coverage (+): 0. Max coverage (-): 0

Region: chr21 21110942-21110951. Max. coverage (+): 0. Max coverage (-): 0

Region: chr21 21110952-21110962. Max. coverage (+): 0. Max coverage (-): 0

Region: chr21 21110963-21110972. Max. coverage (+): 0. Max coverage (-): 0

Region: chr21 21110973-21110982. Max. coverage (+): 0. Max coverage (-): 0

Region: chr21 21110983-21110992. Max. coverage (+): 0. Max coverage (-): 0

Region: chr21 21110993-21111002. Max. coverage (+): 0. Max coverage (-): 0

Region: chr21 21111003-21111012. Max. coverage (+): 0. Max coverage (-): 0

Region: chr21 21111013-21111023. Max. coverage (+): 0. Max coverage (-): 0

Region: chr21 21111024-21111033. Max. coverage (+): 0. Max coverage (-): 0

Region: chr21 21111034-21111043. Max. coverage (+): 0. Max coverage (-): 0

Region: chr21 21111044-21111053. Max. coverage (+): 0. Max coverage (-): 0

Region: chr21 21111054-21111063. Max. coverage (+): 0. Max coverage (-): 0

Region: chr21 21111064-21111074. Max. coverage (+): 0. Max coverage (-): 0

Region: chr21 21111075-21111084. Max. coverage (+): 0. Max coverage (-): 0

Region: chr21 21111085-21111094. Max. coverage (+): 0. Max coverage (-): 0

Region: chr21 21111095-21111104. Max. coverage (+): 0. Max coverage (-): 0

Region: chr21 21111105-21111114. Max. coverage (+): 0. Max coverage (-): 0

Region: chr21 21111115-21111125. Max. coverage (+): 0. Max coverage (-): 0

Region: chr21 21111126-21111135. Max. coverage (+): 0. Max coverage (-): 0

Region: chr21 21111136-21111145. Max. coverage (+): 0. Max coverage (-): 0

Region: chr21 21111146-21111155. Max. coverage (+): 0. Max coverage (-): 0

Region: chr21 21111156-21111165. Max. coverage (+): 0. Max coverage (-): 0

Region: chr21 21111166-21111175. Max. coverage (+): 0. Max coverage (-): 0

Region: chr21 21111176-21111186. Max. coverage (+): 0. Max coverage (-): 0

Region: chr21 21111187-21111196. Max. coverage (+): 0. Max coverage (-): 0

Region: chr21 21111197-21111206. Max. coverage (+): 0. Max coverage (-): 0

Region: chr21 21111207-21111216. Max. coverage (+): 0. Max coverage (-): 0

Region: chr21 21111217-21111226. Max. coverage (+): 0. Max coverage (-): 0

Region: chr21 21111227-21111237. Max. coverage (+): 0. Max coverage (-): 0

Region: chr21 21111238-21111247. Max. coverage (+): 0. Max coverage (-): 0

Region: chr21 21111248-21111257. Max. coverage (+): 0. Max coverage (-): 0

Region: chr21 21111258-21111267. Max. coverage (+): 0. Max coverage (-): 0

Region: chr21 21111268-21111277. Max. coverage (+): 0. Max coverage (-): 0

Region: chr21 21111278-21111287. Max. coverage (+): 3.71. Max coverage (-): 0

Region: chr21 21111288-21111298. Max. coverage (+): 3.71. Max coverage (-): 0

Region: chr21 21111299-21111308. Max. coverage (+): 0. Max coverage (-): 0

Region: chr21 21111309-21111318. Max. coverage (+): 0. Max coverage (-): 0

Region: chr21 21111319-21111328. Max. coverage (+): 0. Max coverage (-): 0

Region: chr21 21111329-21111338. Max. coverage (+): 0. Max coverage (-): 0

Region: chr21 21111339-21111349. Max. coverage (+): 0. Max coverage (-): 0

Region: chr21 21111350-21111359. Max. coverage (+): 0. Max coverage (-): 0

Region: chr21 21111360-21111369. Max. coverage (+): 0. Max coverage (-): 0

Region: chr21 21111370-21111379. Max. coverage (+): 0. Max coverage (-): 0

Region: chr21 21111380-21111389. Max. coverage (+): 0. Max coverage (-): 0

Region: chr21 21111390-21111399. Max. coverage (+): 0. Max coverage (-): 0

Region: chr21 21111400-21111410. Max. coverage (+): 0. Max coverage (-): 0

Region: chr21 21111411-21111420. Max. coverage (+): 0. Max coverage (-): 0

Region: chr21 21111421-21111430. Max. coverage (+): 0. Max coverage (-): 0

Region: chr21 21111431-21111440. Max. coverage (+): 0. Max coverage (-): 0

Region: chr21 21111441-21111450. Max. coverage (+): 0. Max coverage (-): 0

Region: chr21 21111451-21111461. Max. coverage (+): 0. Max coverage (-): 0

Region: chr21 21111462-21111471. Max. coverage (+): 0. Max coverage (-): 0

Region: chr21 21111472-21111481. Max. coverage (+): 0. Max coverage (-): 0

Region: chr21 21111482-21111491. Max. coverage (+): 0. Max coverage (-): 0

Region: chr21 21111492-21111501. Max. coverage (+): 0. Max coverage (-): 0

Region: chr21 21111502-21111511. Max. coverage (+): 0. Max coverage (-): 0

Region: chr21 21111512-21111522. Max. coverage (+): 0. Max coverage (-): 0

Region: chr21 21111523-21111532. Max. coverage (+): 0. Max coverage (-): 0

Region: chr21 21111533-21111542. Max. coverage (+): 0. Max coverage (-): 0

Region: chr21 21111543-21111552. Max. coverage (+): 0. Max coverage (-): 0

Region: chr21 21111553-21111562. Max. coverage (+): 0. Max coverage (-): 0

Region: chr21 21111563-21111573. Max. coverage (+): 0. Max coverage (-): 0

Region: chr21 21111574-21111583. Max. coverage (+): 4.76. Max coverage (-): 0

Region: chr21 21111584-21111593. Max. coverage (+): 4.76. Max coverage (-): 0

Region: chr21 21111594-21111603. Max. coverage (+): 0. Max coverage (-): 0

Region: chr21 21111604-21111613. Max. coverage (+): 0. Max coverage (-): 0

Region: chr21 21111614-21111624. Max. coverage (+): 0. Max coverage (-): 0

Region: chr21 21111625-21111634. Max. coverage (+): 0. Max coverage (-): 0

Region: chr21 21111635-21111644. Max. coverage (+): 0. Max coverage (-): 0

Region: chr21 21111645-21111654. Max. coverage (+): 0. Max coverage (-): 0

Region: chr21 21111655-21111664. Max. coverage (+): 0. Max coverage (-): 0

Region: chr21 21111665-21111674. Max. coverage (+): 0. Max coverage (-): 0

Region: chr21 21111675-21111685. Max. coverage (+): 0. Max coverage (-): 0

Region: chr21 21111686-21111695. Max. coverage (+): 0. Max coverage (-): 0

Region: chr21 21111696-21111705. Max. coverage (+): 0. Max coverage (-): 0

Region: chr21 21111706-21111715. Max. coverage (+): 0. Max coverage (-): 0

Region: chr21 21111716-21111725. Max. coverage (+): 0. Max coverage (-): 0

Region: chr21 21111726-21111736. Max. coverage (+): 0. Max coverage (-): 0

Region: chr21 21111737-21111746. Max. coverage (+): 0. Max coverage (-): 0

Region: chr21 21111747-21111756. Max. coverage (+): 0. Max coverage (-): 0

Region: chr21 21111757-21111766. Max. coverage (+): 0. Max coverage (-): 0

Region: chr21 21111767-21111776. Max. coverage (+): 0. Max coverage (-): 0

Region: chr21 21111777-21111786. Max. coverage (+): 0. Max coverage (-): 0

Region: chr21 21111787-21111797. Max. coverage (+): 0. Max coverage (-): 0

Region: chr21 21111798-21111807. Max. coverage (+): 0. Max coverage (-): 0

Region: chr21 21111808-21111817. Max. coverage (+): 0. Max coverage (-): 0

Region: chr21 21111818-21111827. Max. coverage (+): 0. Max coverage (-): 0

Region: chr21 21111828-21111837. Max. coverage (+): 0. Max coverage (-): 0

Region: chr21 21111838-21111848. Max. coverage (+): 0. Max coverage (-): 0

Region: chr21 21111849-21111858. Max. coverage (+): 0. Max coverage (-): 0

Region: chr21 21111859-21111868. Max. coverage (+): 0. Max coverage (-): 0

Region: chr21 21111869-21111878. Max. coverage (+): 0. Max coverage (-): 0

Region: chr21 21111879-21111888. Max. coverage (+): 0. Max coverage (-): 0

Region: chr21 21111889-21111898. Max. coverage (+): 0. Max coverage (-): 0

Region: chr21 21111899-21111909. Max. coverage (+): 0. Max coverage (-): 0

Region: chr21 21111910-21111919. Max. coverage (+): 0. Max coverage (-): 0

Region: chr21 21111920-21111929. Max. coverage (+): 2.08. Max coverage (-): 0

Region: chr21 21111930-21111939. Max. coverage (+): 2.08. Max coverage (-): 0

Region: chr21 21111940-21111949. Max. coverage (+): 0. Max coverage (-): 0

Region: chr21 21111950-. Max. coverage (+): 0. Max coverage (-): 0

RepeatMasker Color Code

**+**

100-98% Identity

<98-95% Identity

<95-90% Identity

<90-85% Identity

<85-80% Identity

<80-75% Identity

<75-70% Identity

<70% Identity

**-**

Gene Set Color Code

**+**

Gene

Pseudogene

**-**

Topology/Coverage Color Code

Coverage Plus Strand

Coverage Minus Strand

Mainstrand: Plus

Mainstrand: Minus

Complementary Strand

Flanking Region  
(if option -flank >0)

Gene Set Annotation  
  
RepeatMasker Annotation  

**1. MER5A**: 21107008-21107134 (-), Divergence to consensus: 33.6%  
**2. MIR3**: 21107274-21107451 (+), Divergence to consensus: 39.2%  
**3. Bov-tA3**: 21107631-21107743 (-), Divergence to consensus: 7.1%  
**4. (CA)n**: 21108350-21108369 (+), Divergence to consensus: 0%  
**5. Bov-tA1**: 21109416-21109632 (-), Divergence to consensus: 17.1%  
**6. MIR3**: 21109901-21109983 (+), Divergence to consensus: 36.2%  
**7. SINE2-2\_BT**: 21111096-21111205 (+), Divergence to consensus: 26.3%

  
Transcription Factor Binding Sites  

**RFX4\_2** (Sequence: CCTAGATAC (+): 21108050)  
**RFX4\_2** (Sequence: CTTGGATAC (+): 21110957)  
**Gata4** (Sequence: AGATAAG (-): 21110073)  
**Gata4** (Sequence: GTTATCT (+): 21108291)
