# Supplementary material for: Small RNA sequencing of cryopreserved semen from single bull revealed altered miRNAs and piRNAs expression between High- and Low-motile sperm populations
Source: BMC Genomics. 2017 Jan 4;18:14. doi: 10.1186/s12864-016-3394-7 (PMC5209821; doi:10.1186/s12864-016-3394-7)
Supplement: Additional file 3: — Details for each piRNA clusters found in High Motile (HM) sperm fraction. Genes, repeats, transposable elements and transcription factors binding sites falling within the cluster regions were reported. (ZIP 1896 kb) [file 12864_2016_3394_MOESM3_ESM.zip › 46.html]

piRNA cluster 46


Predicted piRNA cluster no. 46     previous   next
  

Show proTRAC run info
Hide proTRAC run info

================================= proTRAC ====================================  
VERSION: 2.1                                    LAST MODIFIED: 06. October 2015  
  
Please cite:  
Rosenkranz D, Zischler H. proTRAC - a software for probabilistic piRNA cluster  
detection, visualization and analysis. 2012. BMC Bioinformatics 13:5.  
  
and (for proTRAC 2.0 and later):  
Rosenkranz D, Rudloff S, Bastuck K, Ketting RF, Zischler H. Tupaia small RNAs  
provide insights into function and evolution of RNAi-based transposon defense  
in mammals. 2015. RNA 21(5):911-922.  
  
Contact:  
David Rosenkranz  
Institute of Anthropology, small RNA group  
Johannes Gutenberg University Mainz  
email: rosenkranz@uni-mainz.de  
  
You can find the latest proTRAC version at:  
http://sourceforge.net/projects/protrac/files  
http://www.smallRNAgroup-mainz.de/software  
==============================================================================  
  
PARAMETERS:  
Map file: .............../storage/core/barbara/genhome/smallRNA/fertility/Sample\_motile/pirna/Sample\_motile\_26-33\_collapsed.fa.no-dust.map.weighted-10000-1000-b-0  
Genome file: ............/storage/core/barbara/genhome/smallRNA/fertility/Sample\_all/pirna/bt\_311\_chrY.fa  
RepeatMasker annotation: /storage/genomes/bt\_umd31/GCF\_000003055.6\_Bos\_taurus\_UMD\_3.1.1\_repeatMasker\_chr.out  
GeneSet:................./storage/core/barbara/genhome/smallRNA/fertility/Sample\_all/pirna/full.gtf  
  
Significant (p<=0.01) hit density will be calculated based  
on observed hit distribution.  
  
Sliding window size: ........................................ 5000 bp  
Sliding window increament: .................................. 1000 bp  
Normalize each hit by number of genomic hits: ............... 1 [0=no/1=yes]  
Normalize each hit by number of sequence reads: ............. 1 [0=no/1=yes]  
Normalize values (-> per million mapped reads): ............. 1 [0=no/1=yes]  
Min. fraction of hits with 1T(U) or 10A: .................... 0.75  
Alternatively: Min. fraction of hits with 1T(U) and 10A: .... 0.5  
Min. fraction of hits with typical piRNA length: ............ 0.75  
Typical piRNA length: ....................................... 26-33 nt  
Min. size of a piRNA cluster: ............................... 5000 bp.  
Min. number of hits (absolute): ............................. 0  
Min. number of hits (normalized): ........................... 0  
Min. fraction of hits on the mainstrand: .................... 0.75  
Top fraction of mapped sequences (in terms of read counts): . 1%  
Top fraction accounts for max. n% of sequence reads: ........ 90%  
Min. fraction of hits on each arm of a bidirectional cluster: 0.1  
Output image file for each cluster: ......................... 0 [0=no/1=yes]  
Output html file for each cluster: .......................... 1 [0=no/1=yes]  
Output a summary table: ..................................... 1 [0=no/1=yes]  
Output a FASTA file for each cluster (piRNA sequences): ..... 1 [0=no/1=yes]  
Output a FASTA file comprising cluster sequences: ........... 1 [0=no/1=yes]  
Search DNA motifs in clusters: .............................. 1 [0=no/1=yes]  
Output flanking sequences: +/- .............................. 0 bp  
Output ~.pTi file: .......................................... 1 [0=no/1=yes]  
==============================================================================  
  
  
Genome size (without gaps): ............ 2678902517 bp  
Gaps (N/X/-): .......................... 53837044 bp  
Mapped reads: .......................... 658825247023  
Non-identical sequences: ............... 514171  
Genomic hits: .......................... 764233  
Significant densitiy of mapped reads: .. 12867599.5173724 reads/kb

Show proTRAC cluster info
Hide proTRAC cluster info

|  |  |
| --- | --- |
| Location | chr21 |
| Coordinates | 23752077-23764946 |
| Size [bp] | 12870 |
| Sequence hit loci | 181 |
| Mapped reads (normalized) | 252257280.7 |
| Mapped reads (normalized) per kb | 19600410.3 |
| Normalized reads with 1T (1U) | 93.7% |
| Normalized reads with 10A | 32.1% |
| Normalized reads with length 26-33 nt | 100% |
| Normalized reads on the main strand(s) | 100% |
| Predicted directionality | mono:plus |

100%

0%

1T (1U)  
reads

10A reads

26-33 nt  
reads

reads on mainstrand

**Either the amount of reads with 1T (1U) OR 10A has to exceed 75% (set with option: -1Tor10A)  
Alternatively the amount of reads with 1T (1U) AND 10A has to exceed 50% (set with option: -1Tand10A)  
Minimum amount of reads with preferred size is 75% (set with option: -pisize)  
Minimum amount of reads on the main strand(s) is 75% (set with option: -clstrand)**

Show read coverage
Hide read coverage

WHAT DO I SEE HERE?  
This chart shows the location of mapped sequence reads within a predicted piRNA cluster. The color refers to the number of genomic hits produced by the sequence read in question. A dark red bar indicates that this sequence read produces many other hits elsewhere in the genome. Many adjacent red or yellow bars can indicate the presence of a multi-copy element such as transposons or rRNA genes. A dark green bar indicates that this sequence read maps uniquely to this locus.

1 hit

2-5 hits

6-10 hits

11-20 hits

21-50 hits

51-100 hits

> 100 hits

chr21

23752077

23764946

Gene Set

RepeatMasker

Mapped  
Reads

16.18

plus strand

minus strand

16.18

Region: chr21 21111951-23752089. Max. coverage (+): 0.36. Max coverage (-): 0

Region: chr21 23752090-23752115. Max. coverage (+): 12. Max coverage (-): 0

Region: chr21 23752116-23752141. Max. coverage (+): 0. Max coverage (-): 0

Region: chr21 23752142-23752167. Max. coverage (+): 0. Max coverage (-): 0

Region: chr21 23752168-23752192. Max. coverage (+): 0. Max coverage (-): 0

Region: chr21 23752193-23752218. Max. coverage (+): 1.46. Max coverage (-): 0

Region: chr21 23752219-23752244. Max. coverage (+): 0. Max coverage (-): 0

Region: chr21 23752245-23752270. Max. coverage (+): 0. Max coverage (-): 0

Region: chr21 23752271-23752295. Max. coverage (+): 4.12. Max coverage (-): 0

Region: chr21 23752296-23752321. Max. coverage (+): 0. Max coverage (-): 0

Region: chr21 23752322-23752347. Max. coverage (+): 0. Max coverage (-): 0

Region: chr21 23752348-23752373. Max. coverage (+): 0. Max coverage (-): 0

Region: chr21 23752374-23752398. Max. coverage (+): 0. Max coverage (-): 0

Region: chr21 23752399-23752424. Max. coverage (+): 0. Max coverage (-): 0

Region: chr21 23752425-23752450. Max. coverage (+): 0. Max coverage (-): 0

Region: chr21 23752451-23752475. Max. coverage (+): 0. Max coverage (-): 0

Region: chr21 23752476-23752501. Max. coverage (+): 0. Max coverage (-): 0

Region: chr21 23752502-23752527. Max. coverage (+): 0. Max coverage (-): 0

Region: chr21 23752528-23752553. Max. coverage (+): 0. Max coverage (-): 0

Region: chr21 23752554-23752578. Max. coverage (+): 0. Max coverage (-): 0

Region: chr21 23752579-23752604. Max. coverage (+): 0. Max coverage (-): 0

Region: chr21 23752605-23752630. Max. coverage (+): 0. Max coverage (-): 0

Region: chr21 23752631-23752656. Max. coverage (+): 0. Max coverage (-): 0

Region: chr21 23752657-23752681. Max. coverage (+): 0. Max coverage (-): 0

Region: chr21 23752682-23752707. Max. coverage (+): 0. Max coverage (-): 0

Region: chr21 23752708-23752733. Max. coverage (+): 0. Max coverage (-): 0

Region: chr21 23752734-23752759. Max. coverage (+): 0. Max coverage (-): 0

Region: chr21 23752760-23752784. Max. coverage (+): 0. Max coverage (-): 0

Region: chr21 23752785-23752810. Max. coverage (+): 0. Max coverage (-): 0

Region: chr21 23752811-23752836. Max. coverage (+): 0. Max coverage (-): 0

Region: chr21 23752837-23752862. Max. coverage (+): 0. Max coverage (-): 0

Region: chr21 23752863-23752887. Max. coverage (+): 0. Max coverage (-): 0

Region: chr21 23752888-23752913. Max. coverage (+): 0. Max coverage (-): 0

Region: chr21 23752914-23752939. Max. coverage (+): 0. Max coverage (-): 0

Region: chr21 23752940-23752965. Max. coverage (+): 0. Max coverage (-): 0

Region: chr21 23752966-23752990. Max. coverage (+): 0. Max coverage (-): 0

Region: chr21 23752991-23753016. Max. coverage (+): 0. Max coverage (-): 0

Region: chr21 23753017-23753042. Max. coverage (+): 1.06. Max coverage (-): 0

Region: chr21 23753043-23753067. Max. coverage (+): 0. Max coverage (-): 0

Region: chr21 23753068-23753093. Max. coverage (+): 0. Max coverage (-): 0

Region: chr21 23753094-23753119. Max. coverage (+): 0. Max coverage (-): 0

Region: chr21 23753120-23753145. Max. coverage (+): 0. Max coverage (-): 0

Region: chr21 23753146-23753170. Max. coverage (+): 0. Max coverage (-): 0

Region: chr21 23753171-23753196. Max. coverage (+): 0. Max coverage (-): 0

Region: chr21 23753197-23753222. Max. coverage (+): 0. Max coverage (-): 0

Region: chr21 23753223-23753248. Max. coverage (+): 0. Max coverage (-): 0

Region: chr21 23753249-23753273. Max. coverage (+): 0. Max coverage (-): 0

Region: chr21 23753274-23753299. Max. coverage (+): 0. Max coverage (-): 0

Region: chr21 23753300-23753325. Max. coverage (+): 0. Max coverage (-): 0

Region: chr21 23753326-23753351. Max. coverage (+): 0. Max coverage (-): 0

Region: chr21 23753352-23753376. Max. coverage (+): 0. Max coverage (-): 0

Region: chr21 23753377-23753402. Max. coverage (+): 0. Max coverage (-): 0

Region: chr21 23753403-23753428. Max. coverage (+): 0. Max coverage (-): 0

Region: chr21 23753429-23753454. Max. coverage (+): 0. Max coverage (-): 0

Region: chr21 23753455-23753479. Max. coverage (+): 0. Max coverage (-): 0

Region: chr21 23753480-23753505. Max. coverage (+): 0. Max coverage (-): 0

Region: chr21 23753506-23753531. Max. coverage (+): 0. Max coverage (-): 0

Region: chr21 23753532-23753557. Max. coverage (+): 0. Max coverage (-): 0

Region: chr21 23753558-23753582. Max. coverage (+): 0. Max coverage (-): 0

Region: chr21 23753583-23753608. Max. coverage (+): 0. Max coverage (-): 0

Region: chr21 23753609-23753634. Max. coverage (+): 0. Max coverage (-): 0

Region: chr21 23753635-23753660. Max. coverage (+): 0. Max coverage (-): 0

Region: chr21 23753661-23753685. Max. coverage (+): 0. Max coverage (-): 0

Region: chr21 23753686-23753711. Max. coverage (+): 0. Max coverage (-): 0

Region: chr21 23753712-23753737. Max. coverage (+): 0. Max coverage (-): 0

Region: chr21 23753738-23753762. Max. coverage (+): 0. Max coverage (-): 0

Region: chr21 23753763-23753788. Max. coverage (+): 0. Max coverage (-): 0

Region: chr21 23753789-23753814. Max. coverage (+): 0. Max coverage (-): 0

Region: chr21 23753815-23753840. Max. coverage (+): 0. Max coverage (-): 0

Region: chr21 23753841-23753865. Max. coverage (+): 0. Max coverage (-): 0

Region: chr21 23753866-23753891. Max. coverage (+): 0. Max coverage (-): 0

Region: chr21 23753892-23753917. Max. coverage (+): 0. Max coverage (-): 0

Region: chr21 23753918-23753943. Max. coverage (+): 0. Max coverage (-): 0

Region: chr21 23753944-23753968. Max. coverage (+): 0. Max coverage (-): 0

Region: chr21 23753969-23753994. Max. coverage (+): 0. Max coverage (-): 0

Region: chr21 23753995-23754020. Max. coverage (+): 0. Max coverage (-): 0

Region: chr21 23754021-23754046. Max. coverage (+): 0. Max coverage (-): 0

Region: chr21 23754047-23754071. Max. coverage (+): 0. Max coverage (-): 0

Region: chr21 23754072-23754097. Max. coverage (+): 0. Max coverage (-): 0

Region: chr21 23754098-23754123. Max. coverage (+): 0. Max coverage (-): 0

Region: chr21 23754124-23754149. Max. coverage (+): 0. Max coverage (-): 0

Region: chr21 23754150-23754174. Max. coverage (+): 0. Max coverage (-): 0

Region: chr21 23754175-23754200. Max. coverage (+): 0. Max coverage (-): 0

Region: chr21 23754201-23754226. Max. coverage (+): 0. Max coverage (-): 0

Region: chr21 23754227-23754252. Max. coverage (+): 0. Max coverage (-): 0

Region: chr21 23754253-23754277. Max. coverage (+): 0. Max coverage (-): 0

Region: chr21 23754278-23754303. Max. coverage (+): 0. Max coverage (-): 0

Region: chr21 23754304-23754329. Max. coverage (+): 0. Max coverage (-): 0

Region: chr21 23754330-23754354. Max. coverage (+): 0. Max coverage (-): 0

Region: chr21 23754355-23754380. Max. coverage (+): 0. Max coverage (-): 0

Region: chr21 23754381-23754406. Max. coverage (+): 0. Max coverage (-): 0

Region: chr21 23754407-23754432. Max. coverage (+): 0. Max coverage (-): 0

Region: chr21 23754433-23754457. Max. coverage (+): 0. Max coverage (-): 0

Region: chr21 23754458-23754483. Max. coverage (+): 0. Max coverage (-): 0

Region: chr21 23754484-23754509. Max. coverage (+): 0. Max coverage (-): 0

Region: chr21 23754510-23754535. Max. coverage (+): 4.35. Max coverage (-): 0

Region: chr21 23754536-23754560. Max. coverage (+): 4.35. Max coverage (-): 0

Region: chr21 23754561-23754586. Max. coverage (+): 1.74. Max coverage (-): 0

Region: chr21 23754587-23754612. Max. coverage (+): 0. Max coverage (-): 0

Region: chr21 23754613-23754638. Max. coverage (+): 0. Max coverage (-): 0

Region: chr21 23754639-23754663. Max. coverage (+): 0. Max coverage (-): 0

Region: chr21 23754664-23754689. Max. coverage (+): 0. Max coverage (-): 0

Region: chr21 23754690-23754715. Max. coverage (+): 1.37. Max coverage (-): 0

Region: chr21 23754716-23754741. Max. coverage (+): 0. Max coverage (-): 0

Region: chr21 23754742-23754766. Max. coverage (+): 0. Max coverage (-): 0

Region: chr21 23754767-23754792. Max. coverage (+): 0. Max coverage (-): 0

Region: chr21 23754793-23754818. Max. coverage (+): 0. Max coverage (-): 0

Region: chr21 23754819-23754844. Max. coverage (+): 0. Max coverage (-): 0

Region: chr21 23754845-23754869. Max. coverage (+): 0. Max coverage (-): 0

Region: chr21 23754870-23754895. Max. coverage (+): 0. Max coverage (-): 0

Region: chr21 23754896-23754921. Max. coverage (+): 0. Max coverage (-): 0

Region: chr21 23754922-23754947. Max. coverage (+): 0. Max coverage (-): 0

Region: chr21 23754948-23754972. Max. coverage (+): 0. Max coverage (-): 0

Region: chr21 23754973-23754998. Max. coverage (+): 0. Max coverage (-): 0

Region: chr21 23754999-23755024. Max. coverage (+): 0. Max coverage (-): 0

Region: chr21 23755025-23755049. Max. coverage (+): 0. Max coverage (-): 0

Region: chr21 23755050-23755075. Max. coverage (+): 0. Max coverage (-): 0

Region: chr21 23755076-23755101. Max. coverage (+): 0. Max coverage (-): 0

Region: chr21 23755102-23755127. Max. coverage (+): 0. Max coverage (-): 0

Region: chr21 23755128-23755152. Max. coverage (+): 0. Max coverage (-): 0

Region: chr21 23755153-23755178. Max. coverage (+): 0. Max coverage (-): 0

Region: chr21 23755179-23755204. Max. coverage (+): 0. Max coverage (-): 0

Region: chr21 23755205-23755230. Max. coverage (+): 1.31. Max coverage (-): 0

Region: chr21 23755231-23755255. Max. coverage (+): 0. Max coverage (-): 0

Region: chr21 23755256-23755281. Max. coverage (+): 0. Max coverage (-): 0

Region: chr21 23755282-23755307. Max. coverage (+): 0. Max coverage (-): 0

Region: chr21 23755308-23755333. Max. coverage (+): 0. Max coverage (-): 0

Region: chr21 23755334-23755358. Max. coverage (+): 0. Max coverage (-): 0

Region: chr21 23755359-23755384. Max. coverage (+): 5.13. Max coverage (-): 0

Region: chr21 23755385-23755410. Max. coverage (+): 9.59. Max coverage (-): 0

Region: chr21 23755411-23755436. Max. coverage (+): 0. Max coverage (-): 0

Region: chr21 23755437-23755461. Max. coverage (+): 2.28. Max coverage (-): 0

Region: chr21 23755462-23755487. Max. coverage (+): 1.47. Max coverage (-): 0

Region: chr21 23755488-23755513. Max. coverage (+): 0. Max coverage (-): 0

Region: chr21 23755514-23755539. Max. coverage (+): 5.74. Max coverage (-): 0

Region: chr21 23755540-23755564. Max. coverage (+): 0.53. Max coverage (-): 0

Region: chr21 23755565-23755590. Max. coverage (+): 0. Max coverage (-): 0

Region: chr21 23755591-23755616. Max. coverage (+): 2.07. Max coverage (-): 0

Region: chr21 23755617-23755641. Max. coverage (+): 2.07. Max coverage (-): 0

Region: chr21 23755642-23755667. Max. coverage (+): 0. Max coverage (-): 0

Region: chr21 23755668-23755693. Max. coverage (+): 0.37. Max coverage (-): 0

Region: chr21 23755694-23755719. Max. coverage (+): 0. Max coverage (-): 0

Region: chr21 23755720-23755744. Max. coverage (+): 2.12. Max coverage (-): 0

Region: chr21 23755745-23755770. Max. coverage (+): 0. Max coverage (-): 0

Region: chr21 23755771-23755796. Max. coverage (+): 0. Max coverage (-): 0

Region: chr21 23755797-23755822. Max. coverage (+): 0.57. Max coverage (-): 0

Region: chr21 23755823-23755847. Max. coverage (+): 0.93. Max coverage (-): 0

Region: chr21 23755848-23755873. Max. coverage (+): 3.27. Max coverage (-): 0

Region: chr21 23755874-23755899. Max. coverage (+): 4.74. Max coverage (-): 0

Region: chr21 23755900-23755925. Max. coverage (+): 5.09. Max coverage (-): 0

Region: chr21 23755926-23755950. Max. coverage (+): 0. Max coverage (-): 0

Region: chr21 23755951-23755976. Max. coverage (+): 0. Max coverage (-): 0

Region: chr21 23755977-23756002. Max. coverage (+): 0. Max coverage (-): 0

Region: chr21 23756003-23756028. Max. coverage (+): 0. Max coverage (-): 0

Region: chr21 23756029-23756053. Max. coverage (+): 0. Max coverage (-): 0

Region: chr21 23756054-23756079. Max. coverage (+): 0. Max coverage (-): 0

Region: chr21 23756080-23756105. Max. coverage (+): 0. Max coverage (-): 0

Region: chr21 23756106-23756131. Max. coverage (+): 0. Max coverage (-): 0

Region: chr21 23756132-23756156. Max. coverage (+): 0. Max coverage (-): 0

Region: chr21 23756157-23756182. Max. coverage (+): 0. Max coverage (-): 0

Region: chr21 23756183-23756208. Max. coverage (+): 0. Max coverage (-): 0

Region: chr21 23756209-23756234. Max. coverage (+): 0. Max coverage (-): 0

Region: chr21 23756235-23756259. Max. coverage (+): 6.71. Max coverage (-): 0

Region: chr21 23756260-23756285. Max. coverage (+): 1.66. Max coverage (-): 0

Region: chr21 23756286-23756311. Max. coverage (+): 0. Max coverage (-): 0

Region: chr21 23756312-23756336. Max. coverage (+): 1.71. Max coverage (-): 0

Region: chr21 23756337-23756362. Max. coverage (+): 9.41. Max coverage (-): 0

Region: chr21 23756363-23756388. Max. coverage (+): 0. Max coverage (-): 0

Region: chr21 23756389-23756414. Max. coverage (+): 0. Max coverage (-): 0

Region: chr21 23756415-23756439. Max. coverage (+): 0. Max coverage (-): 0

Region: chr21 23756440-23756465. Max. coverage (+): 0. Max coverage (-): 0

Region: chr21 23756466-23756491. Max. coverage (+): 0. Max coverage (-): 0

Region: chr21 23756492-23756517. Max. coverage (+): 0. Max coverage (-): 0

Region: chr21 23756518-23756542. Max. coverage (+): 0. Max coverage (-): 0

Region: chr21 23756543-23756568. Max. coverage (+): 8.9. Max coverage (-): 0

Region: chr21 23756569-23756594. Max. coverage (+): 8.9. Max coverage (-): 0

Region: chr21 23756595-23756620. Max. coverage (+): 0. Max coverage (-): 0

Region: chr21 23756621-23756645. Max. coverage (+): 0. Max coverage (-): 0

Region: chr21 23756646-23756671. Max. coverage (+): 0. Max coverage (-): 0

Region: chr21 23756672-23756697. Max. coverage (+): 0. Max coverage (-): 0

Region: chr21 23756698-23756723. Max. coverage (+): 0. Max coverage (-): 0

Region: chr21 23756724-23756748. Max. coverage (+): 0. Max coverage (-): 0

Region: chr21 23756749-23756774. Max. coverage (+): 0. Max coverage (-): 0

Region: chr21 23756775-23756800. Max. coverage (+): 0. Max coverage (-): 0

Region: chr21 23756801-23756826. Max. coverage (+): 0. Max coverage (-): 0

Region: chr21 23756827-23756851. Max. coverage (+): 0. Max coverage (-): 0

Region: chr21 23756852-23756877. Max. coverage (+): 0. Max coverage (-): 0

Region: chr21 23756878-23756903. Max. coverage (+): 0. Max coverage (-): 0

Region: chr21 23756904-23756928. Max. coverage (+): 0. Max coverage (-): 0

Region: chr21 23756929-23756954. Max. coverage (+): 0. Max coverage (-): 0

Region: chr21 23756955-23756980. Max. coverage (+): 0. Max coverage (-): 0

Region: chr21 23756981-23757006. Max. coverage (+): 0. Max coverage (-): 0

Region: chr21 23757007-23757031. Max. coverage (+): 0. Max coverage (-): 0

Region: chr21 23757032-23757057. Max. coverage (+): 3.93. Max coverage (-): 0

Region: chr21 23757058-23757083. Max. coverage (+): 3.93. Max coverage (-): 0

Region: chr21 23757084-23757109. Max. coverage (+): 6.94. Max coverage (-): 0

Region: chr21 23757110-23757134. Max. coverage (+): 7.35. Max coverage (-): 0

Region: chr21 23757135-23757160. Max. coverage (+): 0. Max coverage (-): 0

Region: chr21 23757161-23757186. Max. coverage (+): 3.94. Max coverage (-): 0

Region: chr21 23757187-23757212. Max. coverage (+): 3.94. Max coverage (-): 0

Region: chr21 23757213-23757237. Max. coverage (+): 1.62. Max coverage (-): 0

Region: chr21 23757238-23757263. Max. coverage (+): 0. Max coverage (-): 0

Region: chr21 23757264-23757289. Max. coverage (+): 0. Max coverage (-): 0

Region: chr21 23757290-23757315. Max. coverage (+): 5.24. Max coverage (-): 0

Region: chr21 23757316-23757340. Max. coverage (+): 5.24. Max coverage (-): 0

Region: chr21 23757341-23757366. Max. coverage (+): 0. Max coverage (-): 0

Region: chr21 23757367-23757392. Max. coverage (+): 0. Max coverage (-): 0

Region: chr21 23757393-23757418. Max. coverage (+): 0.88. Max coverage (-): 0

Region: chr21 23757419-23757443. Max. coverage (+): 0. Max coverage (-): 0

Region: chr21 23757444-23757469. Max. coverage (+): 0. Max coverage (-): 0

Region: chr21 23757470-23757495. Max. coverage (+): 0. Max coverage (-): 0

Region: chr21 23757496-23757521. Max. coverage (+): 0. Max coverage (-): 0

Region: chr21 23757522-23757546. Max. coverage (+): 2.02. Max coverage (-): 0

Region: chr21 23757547-23757572. Max. coverage (+): 3.59. Max coverage (-): 0

Region: chr21 23757573-23757598. Max. coverage (+): 0. Max coverage (-): 0

Region: chr21 23757599-23757623. Max. coverage (+): 0. Max coverage (-): 0

Region: chr21 23757624-23757649. Max. coverage (+): 0. Max coverage (-): 0

Region: chr21 23757650-23757675. Max. coverage (+): 0. Max coverage (-): 0

Region: chr21 23757676-23757701. Max. coverage (+): 7.66. Max coverage (-): 0

Region: chr21 23757702-23757726. Max. coverage (+): 0. Max coverage (-): 0

Region: chr21 23757727-23757752. Max. coverage (+): 0. Max coverage (-): 0

Region: chr21 23757753-23757778. Max. coverage (+): 0. Max coverage (-): 0

Region: chr21 23757779-23757804. Max. coverage (+): 2.84. Max coverage (-): 0

Region: chr21 23757805-23757829. Max. coverage (+): 2.84. Max coverage (-): 0

Region: chr21 23757830-23757855. Max. coverage (+): 4.28. Max coverage (-): 0

Region: chr21 23757856-23757881. Max. coverage (+): 0. Max coverage (-): 0

Region: chr21 23757882-23757907. Max. coverage (+): 0. Max coverage (-): 0

Region: chr21 23757908-23757932. Max. coverage (+): 5.27. Max coverage (-): 0

Region: chr21 23757933-23757958. Max. coverage (+): 2.2. Max coverage (-): 0

Region: chr21 23757959-23757984. Max. coverage (+): 0. Max coverage (-): 0

Region: chr21 23757985-23758010. Max. coverage (+): 0. Max coverage (-): 0

Region: chr21 23758011-23758035. Max. coverage (+): 0. Max coverage (-): 0

Region: chr21 23758036-23758061. Max. coverage (+): 2.07. Max coverage (-): 0

Region: chr21 23758062-23758087. Max. coverage (+): 7.3. Max coverage (-): 0

Region: chr21 23758088-23758113. Max. coverage (+): 0. Max coverage (-): 0

Region: chr21 23758114-23758138. Max. coverage (+): 1.02. Max coverage (-): 0

Region: chr21 23758139-23758164. Max. coverage (+): 0. Max coverage (-): 0

Region: chr21 23758165-23758190. Max. coverage (+): 5.07. Max coverage (-): 0

Region: chr21 23758191-23758215. Max. coverage (+): 5.07. Max coverage (-): 0

Region: chr21 23758216-23758241. Max. coverage (+): 1.99. Max coverage (-): 0

Region: chr21 23758242-23758267. Max. coverage (+): 0.46. Max coverage (-): 0

Region: chr21 23758268-23758293. Max. coverage (+): 0. Max coverage (-): 0

Region: chr21 23758294-23758318. Max. coverage (+): 0.74. Max coverage (-): 0

Region: chr21 23758319-23758344. Max. coverage (+): 0.74. Max coverage (-): 0

Region: chr21 23758345-23758370. Max. coverage (+): 0. Max coverage (-): 0

Region: chr21 23758371-23758396. Max. coverage (+): 0. Max coverage (-): 0

Region: chr21 23758397-23758421. Max. coverage (+): 6.71. Max coverage (-): 0

Region: chr21 23758422-23758447. Max. coverage (+): 7.33. Max coverage (-): 0

Region: chr21 23758448-23758473. Max. coverage (+): 0. Max coverage (-): 0

Region: chr21 23758474-23758499. Max. coverage (+): 0. Max coverage (-): 0

Region: chr21 23758500-23758524. Max. coverage (+): 3.31. Max coverage (-): 0

Region: chr21 23758525-23758550. Max. coverage (+): 0. Max coverage (-): 0

Region: chr21 23758551-23758576. Max. coverage (+): 0. Max coverage (-): 0

Region: chr21 23758577-23758602. Max. coverage (+): 0. Max coverage (-): 0

Region: chr21 23758603-23758627. Max. coverage (+): 0. Max coverage (-): 0

Region: chr21 23758628-23758653. Max. coverage (+): 0. Max coverage (-): 0

Region: chr21 23758654-23758679. Max. coverage (+): 0. Max coverage (-): 0

Region: chr21 23758680-23758705. Max. coverage (+): 0. Max coverage (-): 0

Region: chr21 23758706-23758730. Max. coverage (+): 0. Max coverage (-): 0

Region: chr21 23758731-23758756. Max. coverage (+): 0. Max coverage (-): 0

Region: chr21 23758757-23758782. Max. coverage (+): 0. Max coverage (-): 0

Region: chr21 23758783-23758808. Max. coverage (+): 0. Max coverage (-): 0

Region: chr21 23758809-23758833. Max. coverage (+): 0. Max coverage (-): 0

Region: chr21 23758834-23758859. Max. coverage (+): 0. Max coverage (-): 0

Region: chr21 23758860-23758885. Max. coverage (+): 0. Max coverage (-): 0

Region: chr21 23758886-23758910. Max. coverage (+): 0. Max coverage (-): 0

Region: chr21 23758911-23758936. Max. coverage (+): 0. Max coverage (-): 0

Region: chr21 23758937-23758962. Max. coverage (+): 0. Max coverage (-): 0

Region: chr21 23758963-23758988. Max. coverage (+): 0. Max coverage (-): 0

Region: chr21 23758989-23759013. Max. coverage (+): 0. Max coverage (-): 0

Region: chr21 23759014-23759039. Max. coverage (+): 0. Max coverage (-): 0

Region: chr21 23759040-23759065. Max. coverage (+): 0. Max coverage (-): 0

Region: chr21 23759066-23759091. Max. coverage (+): 0. Max coverage (-): 0

Region: chr21 23759092-23759116. Max. coverage (+): 0. Max coverage (-): 0

Region: chr21 23759117-23759142. Max. coverage (+): 0. Max coverage (-): 0

Region: chr21 23759143-23759168. Max. coverage (+): 0. Max coverage (-): 0

Region: chr21 23759169-23759194. Max. coverage (+): 0. Max coverage (-): 0

Region: chr21 23759195-23759219. Max. coverage (+): 0. Max coverage (-): 0

Region: chr21 23759220-23759245. Max. coverage (+): 0. Max coverage (-): 0

Region: chr21 23759246-23759271. Max. coverage (+): 0. Max coverage (-): 0

Region: chr21 23759272-23759297. Max. coverage (+): 0. Max coverage (-): 0

Region: chr21 23759298-23759322. Max. coverage (+): 0. Max coverage (-): 0

Region: chr21 23759323-23759348. Max. coverage (+): 0. Max coverage (-): 0

Region: chr21 23759349-23759374. Max. coverage (+): 0. Max coverage (-): 0

Region: chr21 23759375-23759400. Max. coverage (+): 0. Max coverage (-): 0

Region: chr21 23759401-23759425. Max. coverage (+): 0. Max coverage (-): 0

Region: chr21 23759426-23759451. Max. coverage (+): 0. Max coverage (-): 0

Region: chr21 23759452-23759477. Max. coverage (+): 0. Max coverage (-): 0

Region: chr21 23759478-23759502. Max. coverage (+): 0. Max coverage (-): 0

Region: chr21 23759503-23759528. Max. coverage (+): 0. Max coverage (-): 0

Region: chr21 23759529-23759554. Max. coverage (+): 0. Max coverage (-): 0

Region: chr21 23759555-23759580. Max. coverage (+): 0. Max coverage (-): 0

Region: chr21 23759581-23759605. Max. coverage (+): 0. Max coverage (-): 0

Region: chr21 23759606-23759631. Max. coverage (+): 0. Max coverage (-): 0

Region: chr21 23759632-23759657. Max. coverage (+): 0. Max coverage (-): 0

Region: chr21 23759658-23759683. Max. coverage (+): 0. Max coverage (-): 0

Region: chr21 23759684-23759708. Max. coverage (+): 0. Max coverage (-): 0

Region: chr21 23759709-23759734. Max. coverage (+): 0. Max coverage (-): 0

Region: chr21 23759735-23759760. Max. coverage (+): 0. Max coverage (-): 0

Region: chr21 23759761-23759786. Max. coverage (+): 0. Max coverage (-): 0

Region: chr21 23759787-23759811. Max. coverage (+): 0. Max coverage (-): 0

Region: chr21 23759812-23759837. Max. coverage (+): 0. Max coverage (-): 0

Region: chr21 23759838-23759863. Max. coverage (+): 0. Max coverage (-): 0

Region: chr21 23759864-23759889. Max. coverage (+): 0. Max coverage (-): 0

Region: chr21 23759890-23759914. Max. coverage (+): 0. Max coverage (-): 0

Region: chr21 23759915-23759940. Max. coverage (+): 0. Max coverage (-): 0

Region: chr21 23759941-23759966. Max. coverage (+): 0. Max coverage (-): 0

Region: chr21 23759967-23759992. Max. coverage (+): 0. Max coverage (-): 0

Region: chr21 23759993-23760017. Max. coverage (+): 0. Max coverage (-): 0

Region: chr21 23760018-23760043. Max. coverage (+): 0. Max coverage (-): 0

Region: chr21 23760044-23760069. Max. coverage (+): 0. Max coverage (-): 0

Region: chr21 23760070-23760095. Max. coverage (+): 0. Max coverage (-): 0

Region: chr21 23760096-23760120. Max. coverage (+): 0. Max coverage (-): 0

Region: chr21 23760121-23760146. Max. coverage (+): 0. Max coverage (-): 0

Region: chr21 23760147-23760172. Max. coverage (+): 0. Max coverage (-): 0

Region: chr21 23760173-23760197. Max. coverage (+): 0. Max coverage (-): 0

Region: chr21 23760198-23760223. Max. coverage (+): 0. Max coverage (-): 0

Region: chr21 23760224-23760249. Max. coverage (+): 0. Max coverage (-): 0

Region: chr21 23760250-23760275. Max. coverage (+): 0. Max coverage (-): 0

Region: chr21 23760276-23760300. Max. coverage (+): 0. Max coverage (-): 0

Region: chr21 23760301-23760326. Max. coverage (+): 0. Max coverage (-): 0

Region: chr21 23760327-23760352. Max. coverage (+): 0. Max coverage (-): 0

Region: chr21 23760353-23760378. Max. coverage (+): 0. Max coverage (-): 0

Region: chr21 23760379-23760403. Max. coverage (+): 0. Max coverage (-): 0

Region: chr21 23760404-23760429. Max. coverage (+): 0. Max coverage (-): 0

Region: chr21 23760430-23760455. Max. coverage (+): 0. Max coverage (-): 0

Region: chr21 23760456-23760481. Max. coverage (+): 0. Max coverage (-): 0

Region: chr21 23760482-23760506. Max. coverage (+): 0. Max coverage (-): 0

Region: chr21 23760507-23760532. Max. coverage (+): 0. Max coverage (-): 0

Region: chr21 23760533-23760558. Max. coverage (+): 0. Max coverage (-): 0

Region: chr21 23760559-23760584. Max. coverage (+): 0. Max coverage (-): 0

Region: chr21 23760585-23760609. Max. coverage (+): 0. Max coverage (-): 0

Region: chr21 23760610-23760635. Max. coverage (+): 0. Max coverage (-): 0

Region: chr21 23760636-23760661. Max. coverage (+): 0. Max coverage (-): 0

Region: chr21 23760662-23760687. Max. coverage (+): 0. Max coverage (-): 0

Region: chr21 23760688-23760712. Max. coverage (+): 0. Max coverage (-): 0

Region: chr21 23760713-23760738. Max. coverage (+): 0. Max coverage (-): 0

Region: chr21 23760739-23760764. Max. coverage (+): 0. Max coverage (-): 0

Region: chr21 23760765-23760789. Max. coverage (+): 0. Max coverage (-): 0

Region: chr21 23760790-23760815. Max. coverage (+): 0. Max coverage (-): 0

Region: chr21 23760816-23760841. Max. coverage (+): 0. Max coverage (-): 0

Region: chr21 23760842-23760867. Max. coverage (+): 0. Max coverage (-): 0

Region: chr21 23760868-23760892. Max. coverage (+): 0. Max coverage (-): 0

Region: chr21 23760893-23760918. Max. coverage (+): 0. Max coverage (-): 0

Region: chr21 23760919-23760944. Max. coverage (+): 0. Max coverage (-): 0

Region: chr21 23760945-23760970. Max. coverage (+): 0. Max coverage (-): 0

Region: chr21 23760971-23760995. Max. coverage (+): 0. Max coverage (-): 0

Region: chr21 23760996-23761021. Max. coverage (+): 0. Max coverage (-): 0

Region: chr21 23761022-23761047. Max. coverage (+): 0. Max coverage (-): 0

Region: chr21 23761048-23761073. Max. coverage (+): 0. Max coverage (-): 0

Region: chr21 23761074-23761098. Max. coverage (+): 0. Max coverage (-): 0

Region: chr21 23761099-23761124. Max. coverage (+): 0. Max coverage (-): 0

Region: chr21 23761125-23761150. Max. coverage (+): 0. Max coverage (-): 0

Region: chr21 23761151-23761176. Max. coverage (+): 0. Max coverage (-): 0

Region: chr21 23761177-23761201. Max. coverage (+): 0. Max coverage (-): 0

Region: chr21 23761202-23761227. Max. coverage (+): 0. Max coverage (-): 0

Region: chr21 23761228-23761253. Max. coverage (+): 0. Max coverage (-): 0

Region: chr21 23761254-23761279. Max. coverage (+): 0. Max coverage (-): 0

Region: chr21 23761280-23761304. Max. coverage (+): 0. Max coverage (-): 0

Region: chr21 23761305-23761330. Max. coverage (+): 0. Max coverage (-): 0

Region: chr21 23761331-23761356. Max. coverage (+): 0. Max coverage (-): 0

Region: chr21 23761357-23761382. Max. coverage (+): 0. Max coverage (-): 0

Region: chr21 23761383-23761407. Max. coverage (+): 0. Max coverage (-): 0

Region: chr21 23761408-23761433. Max. coverage (+): 0. Max coverage (-): 0

Region: chr21 23761434-23761459. Max. coverage (+): 0. Max coverage (-): 0

Region: chr21 23761460-23761484. Max. coverage (+): 0. Max coverage (-): 0

Region: chr21 23761485-23761510. Max. coverage (+): 0. Max coverage (-): 0

Region: chr21 23761511-23761536. Max. coverage (+): 0. Max coverage (-): 0

Region: chr21 23761537-23761562. Max. coverage (+): 0. Max coverage (-): 0

Region: chr21 23761563-23761587. Max. coverage (+): 0. Max coverage (-): 0

Region: chr21 23761588-23761613. Max. coverage (+): 0. Max coverage (-): 0

Region: chr21 23761614-23761639. Max. coverage (+): 0. Max coverage (-): 0

Region: chr21 23761640-23761665. Max. coverage (+): 0. Max coverage (-): 0

Region: chr21 23761666-23761690. Max. coverage (+): 0. Max coverage (-): 0

Region: chr21 23761691-23761716. Max. coverage (+): 0. Max coverage (-): 0

Region: chr21 23761717-23761742. Max. coverage (+): 0. Max coverage (-): 0

Region: chr21 23761743-23761768. Max. coverage (+): 0. Max coverage (-): 0

Region: chr21 23761769-23761793. Max. coverage (+): 0. Max coverage (-): 0

Region: chr21 23761794-23761819. Max. coverage (+): 2.1. Max coverage (-): 0

Region: chr21 23761820-23761845. Max. coverage (+): 6.72. Max coverage (-): 0

Region: chr21 23761846-23761871. Max. coverage (+): 0. Max coverage (-): 0

Region: chr21 23761872-23761896. Max. coverage (+): 3.01. Max coverage (-): 0

Region: chr21 23761897-23761922. Max. coverage (+): 0. Max coverage (-): 0

Region: chr21 23761923-23761948. Max. coverage (+): 11.1. Max coverage (-): 0

Region: chr21 23761949-23761974. Max. coverage (+): 16.18. Max coverage (-): 0

Region: chr21 23761975-23761999. Max. coverage (+): 1.58. Max coverage (-): 0

Region: chr21 23762000-23762025. Max. coverage (+): 0. Max coverage (-): 0

Region: chr21 23762026-23762051. Max. coverage (+): 5.66. Max coverage (-): 0

Region: chr21 23762052-23762076. Max. coverage (+): 4.58. Max coverage (-): 0

Region: chr21 23762077-23762102. Max. coverage (+): 4.58. Max coverage (-): 0

Region: chr21 23762103-23762128. Max. coverage (+): 0. Max coverage (-): 0

Region: chr21 23762129-23762154. Max. coverage (+): 4.61. Max coverage (-): 0

Region: chr21 23762155-23762179. Max. coverage (+): 4.61. Max coverage (-): 0

Region: chr21 23762180-23762205. Max. coverage (+): 9.75. Max coverage (-): 0

Region: chr21 23762206-23762231. Max. coverage (+): 0. Max coverage (-): 0

Region: chr21 23762232-23762257. Max. coverage (+): 2.24. Max coverage (-): 0

Region: chr21 23762258-23762282. Max. coverage (+): 5.18. Max coverage (-): 0

Region: chr21 23762283-23762308. Max. coverage (+): 3.41. Max coverage (-): 0

Region: chr21 23762309-23762334. Max. coverage (+): 0. Max coverage (-): 0

Region: chr21 23762335-23762360. Max. coverage (+): 14.9. Max coverage (-): 0

Region: chr21 23762361-23762385. Max. coverage (+): 1.97. Max coverage (-): 0

Region: chr21 23762386-23762411. Max. coverage (+): 0. Max coverage (-): 0

Region: chr21 23762412-23762437. Max. coverage (+): 0. Max coverage (-): 0

Region: chr21 23762438-23762463. Max. coverage (+): 0. Max coverage (-): 0

Region: chr21 23762464-23762488. Max. coverage (+): 2.26. Max coverage (-): 0

Region: chr21 23762489-23762514. Max. coverage (+): 0. Max coverage (-): 0

Region: chr21 23762515-23762540. Max. coverage (+): 15.79. Max coverage (-): 0

Region: chr21 23762541-23762566. Max. coverage (+): 0. Max coverage (-): 0

Region: chr21 23762567-23762591. Max. coverage (+): 0. Max coverage (-): 0

Region: chr21 23762592-23762617. Max. coverage (+): 10.75. Max coverage (-): 0

Region: chr21 23762618-23762643. Max. coverage (+): 0.54. Max coverage (-): 0

Region: chr21 23762644-23762669. Max. coverage (+): 9.09. Max coverage (-): 0

Region: chr21 23762670-23762694. Max. coverage (+): 4.41. Max coverage (-): 0

Region: chr21 23762695-23762720. Max. coverage (+): 1.43. Max coverage (-): 0

Region: chr21 23762721-23762746. Max. coverage (+): 0. Max coverage (-): 0

Region: chr21 23762747-23762771. Max. coverage (+): 0. Max coverage (-): 0

Region: chr21 23762772-23762797. Max. coverage (+): 0. Max coverage (-): 0

Region: chr21 23762798-23762823. Max. coverage (+): 0. Max coverage (-): 0

Region: chr21 23762824-23762849. Max. coverage (+): 0. Max coverage (-): 0

Region: chr21 23762850-23762874. Max. coverage (+): 0. Max coverage (-): 0

Region: chr21 23762875-23762900. Max. coverage (+): 0. Max coverage (-): 0

Region: chr21 23762901-23762926. Max. coverage (+): 0. Max coverage (-): 0

Region: chr21 23762927-23762952. Max. coverage (+): 6.92. Max coverage (-): 0

Region: chr21 23762953-23762977. Max. coverage (+): 10.59. Max coverage (-): 0

Region: chr21 23762978-23763003. Max. coverage (+): 8.19. Max coverage (-): 0

Region: chr21 23763004-23763029. Max. coverage (+): 0. Max coverage (-): 0

Region: chr21 23763030-23763055. Max. coverage (+): 4.54. Max coverage (-): 0

Region: chr21 23763056-23763080. Max. coverage (+): 0. Max coverage (-): 0

Region: chr21 23763081-23763106. Max. coverage (+): 0. Max coverage (-): 0

Region: chr21 23763107-23763132. Max. coverage (+): 4.35. Max coverage (-): 0

Region: chr21 23763133-23763158. Max. coverage (+): 0. Max coverage (-): 0

Region: chr21 23763159-23763183. Max. coverage (+): 6.01. Max coverage (-): 0

Region: chr21 23763184-23763209. Max. coverage (+): 2.36. Max coverage (-): 0

Region: chr21 23763210-23763235. Max. coverage (+): 2.36. Max coverage (-): 0

Region: chr21 23763236-23763261. Max. coverage (+): 0. Max coverage (-): 0

Region: chr21 23763262-23763286. Max. coverage (+): 0. Max coverage (-): 0

Region: chr21 23763287-23763312. Max. coverage (+): 0. Max coverage (-): 0

Region: chr21 23763313-23763338. Max. coverage (+): 0. Max coverage (-): 0

Region: chr21 23763339-23763363. Max. coverage (+): 0. Max coverage (-): 0

Region: chr21 23763364-23763389. Max. coverage (+): 0. Max coverage (-): 0

Region: chr21 23763390-23763415. Max. coverage (+): 0. Max coverage (-): 0

Region: chr21 23763416-23763441. Max. coverage (+): 0. Max coverage (-): 0

Region: chr21 23763442-23763466. Max. coverage (+): 0. Max coverage (-): 0

Region: chr21 23763467-23763492. Max. coverage (+): 0. Max coverage (-): 0

Region: chr21 23763493-23763518. Max. coverage (+): 0. Max coverage (-): 0

Region: chr21 23763519-23763544. Max. coverage (+): 0. Max coverage (-): 0

Region: chr21 23763545-23763569. Max. coverage (+): 0. Max coverage (-): 0

Region: chr21 23763570-23763595. Max. coverage (+): 0. Max coverage (-): 0

Region: chr21 23763596-23763621. Max. coverage (+): 0. Max coverage (-): 0

Region: chr21 23763622-23763647. Max. coverage (+): 0. Max coverage (-): 0

Region: chr21 23763648-23763672. Max. coverage (+): 0. Max coverage (-): 0

Region: chr21 23763673-23763698. Max. coverage (+): 0. Max coverage (-): 0

Region: chr21 23763699-23763724. Max. coverage (+): 0. Max coverage (-): 0

Region: chr21 23763725-23763750. Max. coverage (+): 0. Max coverage (-): 0

Region: chr21 23763751-23763775. Max. coverage (+): 0. Max coverage (-): 0

Region: chr21 23763776-23763801. Max. coverage (+): 0. Max coverage (-): 0

Region: chr21 23763802-23763827. Max. coverage (+): 0. Max coverage (-): 0

Region: chr21 23763828-23763853. Max. coverage (+): 0. Max coverage (-): 0

Region: chr21 23763854-23763878. Max. coverage (+): 0. Max coverage (-): 0

Region: chr21 23763879-23763904. Max. coverage (+): 0. Max coverage (-): 0

Region: chr21 23763905-23763930. Max. coverage (+): 0. Max coverage (-): 0

Region: chr21 23763931-23763956. Max. coverage (+): 0. Max coverage (-): 0

Region: chr21 23763957-23763981. Max. coverage (+): 0. Max coverage (-): 0

Region: chr21 23763982-23764007. Max. coverage (+): 0. Max coverage (-): 0

Region: chr21 23764008-23764033. Max. coverage (+): 0. Max coverage (-): 0

Region: chr21 23764034-23764058. Max. coverage (+): 0. Max coverage (-): 0

Region: chr21 23764059-23764084. Max. coverage (+): 0. Max coverage (-): 0

Region: chr21 23764085-23764110. Max. coverage (+): 0. Max coverage (-): 0

Region: chr21 23764111-23764136. Max. coverage (+): 0. Max coverage (-): 0

Region: chr21 23764137-23764161. Max. coverage (+): 0. Max coverage (-): 0

Region: chr21 23764162-23764187. Max. coverage (+): 0. Max coverage (-): 0

Region: chr21 23764188-23764213. Max. coverage (+): 3.26. Max coverage (-): 0

Region: chr21 23764214-23764239. Max. coverage (+): 0. Max coverage (-): 0

Region: chr21 23764240-23764264. Max. coverage (+): 4.1. Max coverage (-): 0

Region: chr21 23764265-23764290. Max. coverage (+): 0. Max coverage (-): 0

Region: chr21 23764291-23764316. Max. coverage (+): 1.1. Max coverage (-): 0

Region: chr21 23764317-23764342. Max. coverage (+): 1.28. Max coverage (-): 0

Region: chr21 23764343-23764367. Max. coverage (+): 0. Max coverage (-): 0

Region: chr21 23764368-23764393. Max. coverage (+): 0. Max coverage (-): 0

Region: chr21 23764394-23764419. Max. coverage (+): 0. Max coverage (-): 0

Region: chr21 23764420-23764445. Max. coverage (+): 0. Max coverage (-): 0

Region: chr21 23764446-23764470. Max. coverage (+): 0. Max coverage (-): 0

Region: chr21 23764471-23764496. Max. coverage (+): 0. Max coverage (-): 0

Region: chr21 23764497-23764522. Max. coverage (+): 0. Max coverage (-): 0

Region: chr21 23764523-23764548. Max. coverage (+): 0. Max coverage (-): 0

Region: chr21 23764549-23764573. Max. coverage (+): 0. Max coverage (-): 0

Region: chr21 23764574-23764599. Max. coverage (+): 0. Max coverage (-): 0

Region: chr21 23764600-23764625. Max. coverage (+): 0. Max coverage (-): 0

Region: chr21 23764626-23764650. Max. coverage (+): 0. Max coverage (-): 0

Region: chr21 23764651-23764676. Max. coverage (+): 0. Max coverage (-): 0

Region: chr21 23764677-23764702. Max. coverage (+): 0. Max coverage (-): 0

Region: chr21 23764703-23764728. Max. coverage (+): 0. Max coverage (-): 0

Region: chr21 23764729-23764753. Max. coverage (+): 0. Max coverage (-): 0

Region: chr21 23764754-23764779. Max. coverage (+): 0. Max coverage (-): 0

Region: chr21 23764780-23764805. Max. coverage (+): 0. Max coverage (-): 0

Region: chr21 23764806-23764831. Max. coverage (+): 4.75. Max coverage (-): 0

Region: chr21 23764832-23764856. Max. coverage (+): 0. Max coverage (-): 0

Region: chr21 23764857-23764882. Max. coverage (+): 0. Max coverage (-): 0

Region: chr21 23764883-23764908. Max. coverage (+): 0. Max coverage (-): 0

Region: chr21 23764909-23764934. Max. coverage (+): 4.65. Max coverage (-): 0

Region: chr21 23764935-. Max. coverage (+): 0. Max coverage (-): 0

RepeatMasker Color Code

**+**

100-98% Identity

<98-95% Identity

<95-90% Identity

<90-85% Identity

<85-80% Identity

<80-75% Identity

<75-70% Identity

<70% Identity

**-**

Gene Set Color Code

**+**

Gene

Pseudogene

**-**

Topology/Coverage Color Code

Coverage Plus Strand

Coverage Minus Strand

Mainstrand: Plus

Mainstrand: Minus

Complementary Strand

Flanking Region  
(if option -flank >0)

Gene Set Annotation  
  
RepeatMasker Annotation  

**1. MER5A**: 23752648-23752834 (-), Divergence to consensus: 21.4%  
**2. L2**: 23753226-23753366 (+), Divergence to consensus: 41.5%  
**3. L1-2\_BT**: 23753373-23753684 (-), Divergence to consensus: 33.1%  
**4. L1MCa**: 23753752-23753819 (-), Divergence to consensus: 20.7%  
**5. L2b**: 23754605-23754822 (-), Divergence to consensus: 49.7%  
**6. Bov-tA2**: 23754983-23755187 (+), Divergence to consensus: 17%  
**7. SINE2-2\_BT**: 23755254-23755346 (+), Divergence to consensus: 26.2%  
**8. MIR**: 23755984-23756221 (-), Divergence to consensus: 37.8%  
**9. Charlie8**: 23756486-23756559 (-), Divergence to consensus: 27.3%  
**10. MLT1I**: 23756615-23756994 (-), Divergence to consensus: 36.7%  
**11. AT\_rich**: 23757287-23757309 (+), Divergence to consensus: 47.8%  
**12. L2**: 23758030-23758541 (+), Divergence to consensus: 51%  
**13. L1MC5**: 23758560-23758812 (-), Divergence to consensus: 33.2%  
**14. SINE2-2\_BT**: 23758820-23758936 (-), Divergence to consensus: 21.3%  
**15. BovB**: 23758990-23759656 (+), Divergence to consensus: 3%  
**16. BTLTR1**: 23759657-23759727 (-), Divergence to consensus: 8.4%  
**17. BovB**: 23759728-23760571 (+), Divergence to consensus: 3.3%  
**18. ART2A**: 23760572-23761091 (+), Divergence to consensus: 7.6%  
**19. BOV-A2**: 23761025-23761220 (+), Divergence to consensus: 22%  
**20. MER5A**: 23761209-23761285 (+), Divergence to consensus: 45.3%  
**21. L1MB4**: 23761286-23761520 (-), Divergence to consensus: 22.1%  
**22. MER5A**: 23761521-23761553 (+), Divergence to consensus: 45.3%  
**23. Bov-tA3**: 23761664-23761816 (-), Divergence to consensus: 18.3%  
**24. MIR**: 23762768-23762935 (-), Divergence to consensus: 35.9%  
**25. L2c**: 23763070-23763145 (-), Divergence to consensus: 37.3%  
**26. L2c**: 23763231-23763408 (-), Divergence to consensus: 40.8%  
**27. L1M5**: 23763417-23763579 (-), Divergence to consensus: 40.8%  
**28. HAL1ME**: 23763686-23764220 (-), Divergence to consensus: 52.1%  
**29. CHR-2A**: 23764404-23764646 (-), Divergence to consensus: 37.9%

  
Transcription Factor Binding Sites  

**Gata4** (Sequence: AGATAAC (-): 23752195)  
**SOX9** (Sequence: AACAATAG (-): 23752335)  
**SOX9** (Sequence: TCATTGTT (+): 23762518)  
**SOX9** (Sequence: TCATTGTT (+): 23764927)  
**Gata4** (Sequence: CTTATCT (+): 23753902)
