# Supplementary material for: Small RNA sequencing of cryopreserved semen from single bull revealed altered miRNAs and piRNAs expression between High- and Low-motile sperm populations
Source: BMC Genomics. 2017 Jan 4;18:14. doi: 10.1186/s12864-016-3394-7 (PMC5209821; doi:10.1186/s12864-016-3394-7)
Supplement: Additional file 3: — Details for each piRNA clusters found in High Motile (HM) sperm fraction. Genes, repeats, transposable elements and transcription factors binding sites falling within the cluster regions were reported. (ZIP 1896 kb) [file 12864_2016_3394_MOESM3_ESM.zip › 47.html]

piRNA cluster 47


Predicted piRNA cluster no. 47     previous   next
  

Show proTRAC run info
Hide proTRAC run info

================================= proTRAC ====================================  
VERSION: 2.1                                    LAST MODIFIED: 06. October 2015  
  
Please cite:  
Rosenkranz D, Zischler H. proTRAC - a software for probabilistic piRNA cluster  
detection, visualization and analysis. 2012. BMC Bioinformatics 13:5.  
  
and (for proTRAC 2.0 and later):  
Rosenkranz D, Rudloff S, Bastuck K, Ketting RF, Zischler H. Tupaia small RNAs  
provide insights into function and evolution of RNAi-based transposon defense  
in mammals. 2015. RNA 21(5):911-922.  
  
Contact:  
David Rosenkranz  
Institute of Anthropology, small RNA group  
Johannes Gutenberg University Mainz  
email: rosenkranz@uni-mainz.de  
  
You can find the latest proTRAC version at:  
http://sourceforge.net/projects/protrac/files  
http://www.smallRNAgroup-mainz.de/software  
==============================================================================  
  
PARAMETERS:  
Map file: .............../storage/core/barbara/genhome/smallRNA/fertility/Sample\_motile/pirna/Sample\_motile\_26-33\_collapsed.fa.no-dust.map.weighted-10000-1000-b-0  
Genome file: ............/storage/core/barbara/genhome/smallRNA/fertility/Sample\_all/pirna/bt\_311\_chrY.fa  
RepeatMasker annotation: /storage/genomes/bt\_umd31/GCF\_000003055.6\_Bos\_taurus\_UMD\_3.1.1\_repeatMasker\_chr.out  
GeneSet:................./storage/core/barbara/genhome/smallRNA/fertility/Sample\_all/pirna/full.gtf  
  
Significant (p<=0.01) hit density will be calculated based  
on observed hit distribution.  
  
Sliding window size: ........................................ 5000 bp  
Sliding window increament: .................................. 1000 bp  
Normalize each hit by number of genomic hits: ............... 1 [0=no/1=yes]  
Normalize each hit by number of sequence reads: ............. 1 [0=no/1=yes]  
Normalize values (-> per million mapped reads): ............. 1 [0=no/1=yes]  
Min. fraction of hits with 1T(U) or 10A: .................... 0.75  
Alternatively: Min. fraction of hits with 1T(U) and 10A: .... 0.5  
Min. fraction of hits with typical piRNA length: ............ 0.75  
Typical piRNA length: ....................................... 26-33 nt  
Min. size of a piRNA cluster: ............................... 5000 bp.  
Min. number of hits (absolute): ............................. 0  
Min. number of hits (normalized): ........................... 0  
Min. fraction of hits on the mainstrand: .................... 0.75  
Top fraction of mapped sequences (in terms of read counts): . 1%  
Top fraction accounts for max. n% of sequence reads: ........ 90%  
Min. fraction of hits on each arm of a bidirectional cluster: 0.1  
Output image file for each cluster: ......................... 0 [0=no/1=yes]  
Output html file for each cluster: .......................... 1 [0=no/1=yes]  
Output a summary table: ..................................... 1 [0=no/1=yes]  
Output a FASTA file for each cluster (piRNA sequences): ..... 1 [0=no/1=yes]  
Output a FASTA file comprising cluster sequences: ........... 1 [0=no/1=yes]  
Search DNA motifs in clusters: .............................. 1 [0=no/1=yes]  
Output flanking sequences: +/- .............................. 0 bp  
Output ~.pTi file: .......................................... 1 [0=no/1=yes]  
==============================================================================  
  
  
Genome size (without gaps): ............ 2678902517 bp  
Gaps (N/X/-): .......................... 53837044 bp  
Mapped reads: .......................... 658825247023  
Non-identical sequences: ............... 514171  
Genomic hits: .......................... 764233  
Significant densitiy of mapped reads: .. 12867599.5173724 reads/kb

Show proTRAC cluster info
Hide proTRAC cluster info

|  |  |
| --- | --- |
| Location | chr21 |
| Coordinates | 23768046-23775670 |
| Size [bp] | 7625 |
| Sequence hit loci | 79 |
| Mapped reads (normalized) | 99989712 |
| Mapped reads (normalized) per kb | 13113404.9 |
| Normalized reads with 1T (1U) | 91.6% |
| Normalized reads with 10A | 43.8% |
| Normalized reads with length 26-33 nt | 100% |
| Normalized reads on the main strand(s) | 100% |
| Predicted directionality | mono:plus |

100%

0%

1T (1U)  
reads

10A reads

26-33 nt  
reads

reads on mainstrand

**Either the amount of reads with 1T (1U) OR 10A has to exceed 75% (set with option: -1Tor10A)  
Alternatively the amount of reads with 1T (1U) AND 10A has to exceed 50% (set with option: -1Tand10A)  
Minimum amount of reads with preferred size is 75% (set with option: -pisize)  
Minimum amount of reads on the main strand(s) is 75% (set with option: -clstrand)**

Show read coverage
Hide read coverage

WHAT DO I SEE HERE?  
This chart shows the location of mapped sequence reads within a predicted piRNA cluster. The color refers to the number of genomic hits produced by the sequence read in question. A dark red bar indicates that this sequence read produces many other hits elsewhere in the genome. Many adjacent red or yellow bars can indicate the presence of a multi-copy element such as transposons or rRNA genes. A dark green bar indicates that this sequence read maps uniquely to this locus.

1 hit

2-5 hits

6-10 hits

11-20 hits

21-50 hits

51-100 hits

> 100 hits

chr21

23768046

23775670

Gene Set

RepeatMasker

Mapped  
Reads

17.34

plus strand

minus strand

17.34

Region: chr21 23764936-23768053. Max. coverage (+): 1.67. Max coverage (-): 0

Region: chr21 23768054-23768068. Max. coverage (+): 0. Max coverage (-): 0

Region: chr21 23768069-23768084. Max. coverage (+): 0. Max coverage (-): 0

Region: chr21 23768085-23768099. Max. coverage (+): 0. Max coverage (-): 0

Region: chr21 23768100-23768114. Max. coverage (+): 0. Max coverage (-): 0

Region: chr21 23768115-23768129. Max. coverage (+): 0. Max coverage (-): 0

Region: chr21 23768130-23768145. Max. coverage (+): 0. Max coverage (-): 0

Region: chr21 23768146-23768160. Max. coverage (+): 0. Max coverage (-): 0

Region: chr21 23768161-23768175. Max. coverage (+): 0. Max coverage (-): 0

Region: chr21 23768176-23768190. Max. coverage (+): 0. Max coverage (-): 0

Region: chr21 23768191-23768206. Max. coverage (+): 0. Max coverage (-): 0

Region: chr21 23768207-23768221. Max. coverage (+): 0. Max coverage (-): 0

Region: chr21 23768222-23768236. Max. coverage (+): 0. Max coverage (-): 0

Region: chr21 23768237-23768251. Max. coverage (+): 0. Max coverage (-): 0

Region: chr21 23768252-23768267. Max. coverage (+): 0. Max coverage (-): 0

Region: chr21 23768268-23768282. Max. coverage (+): 0. Max coverage (-): 0

Region: chr21 23768283-23768297. Max. coverage (+): 1.75. Max coverage (-): 0

Region: chr21 23768298-23768312. Max. coverage (+): 1.75. Max coverage (-): 0

Region: chr21 23768313-23768328. Max. coverage (+): 4.04. Max coverage (-): 0

Region: chr21 23768329-23768343. Max. coverage (+): 0. Max coverage (-): 0

Region: chr21 23768344-23768358. Max. coverage (+): 0. Max coverage (-): 0

Region: chr21 23768359-23768373. Max. coverage (+): 0. Max coverage (-): 0

Region: chr21 23768374-23768389. Max. coverage (+): 0. Max coverage (-): 0

Region: chr21 23768390-23768404. Max. coverage (+): 0. Max coverage (-): 0

Region: chr21 23768405-23768419. Max. coverage (+): 0. Max coverage (-): 0

Region: chr21 23768420-23768434. Max. coverage (+): 0. Max coverage (-): 0

Region: chr21 23768435-23768450. Max. coverage (+): 0. Max coverage (-): 0

Region: chr21 23768451-23768465. Max. coverage (+): 0. Max coverage (-): 0

Region: chr21 23768466-23768480. Max. coverage (+): 0. Max coverage (-): 0

Region: chr21 23768481-23768495. Max. coverage (+): 0. Max coverage (-): 0

Region: chr21 23768496-23768511. Max. coverage (+): 0. Max coverage (-): 0

Region: chr21 23768512-23768526. Max. coverage (+): 0. Max coverage (-): 0

Region: chr21 23768527-23768541. Max. coverage (+): 0. Max coverage (-): 0

Region: chr21 23768542-23768556. Max. coverage (+): 0. Max coverage (-): 0

Region: chr21 23768557-23768572. Max. coverage (+): 0. Max coverage (-): 0

Region: chr21 23768573-23768587. Max. coverage (+): 0. Max coverage (-): 0

Region: chr21 23768588-23768602. Max. coverage (+): 0. Max coverage (-): 0

Region: chr21 23768603-23768617. Max. coverage (+): 0. Max coverage (-): 0

Region: chr21 23768618-23768633. Max. coverage (+): 0. Max coverage (-): 0

Region: chr21 23768634-23768648. Max. coverage (+): 0. Max coverage (-): 0

Region: chr21 23768649-23768663. Max. coverage (+): 0. Max coverage (-): 0

Region: chr21 23768664-23768678. Max. coverage (+): 0. Max coverage (-): 0

Region: chr21 23768679-23768694. Max. coverage (+): 0. Max coverage (-): 0

Region: chr21 23768695-23768709. Max. coverage (+): 0. Max coverage (-): 0

Region: chr21 23768710-23768724. Max. coverage (+): 0. Max coverage (-): 0

Region: chr21 23768725-23768739. Max. coverage (+): 0. Max coverage (-): 0

Region: chr21 23768740-23768755. Max. coverage (+): 0. Max coverage (-): 0

Region: chr21 23768756-23768770. Max. coverage (+): 0. Max coverage (-): 0

Region: chr21 23768771-23768785. Max. coverage (+): 0. Max coverage (-): 0

Region: chr21 23768786-23768800. Max. coverage (+): 0. Max coverage (-): 0

Region: chr21 23768801-23768816. Max. coverage (+): 0. Max coverage (-): 0

Region: chr21 23768817-23768831. Max. coverage (+): 0. Max coverage (-): 0

Region: chr21 23768832-23768846. Max. coverage (+): 0. Max coverage (-): 0

Region: chr21 23768847-23768861. Max. coverage (+): 0. Max coverage (-): 0

Region: chr21 23768862-23768877. Max. coverage (+): 0. Max coverage (-): 0

Region: chr21 23768878-23768892. Max. coverage (+): 0. Max coverage (-): 0

Region: chr21 23768893-23768907. Max. coverage (+): 0. Max coverage (-): 0

Region: chr21 23768908-23768922. Max. coverage (+): 0. Max coverage (-): 0

Region: chr21 23768923-23768938. Max. coverage (+): 0. Max coverage (-): 0

Region: chr21 23768939-23768953. Max. coverage (+): 0. Max coverage (-): 0

Region: chr21 23768954-23768968. Max. coverage (+): 0. Max coverage (-): 0

Region: chr21 23768969-23768983. Max. coverage (+): 3.34. Max coverage (-): 0

Region: chr21 23768984-23768999. Max. coverage (+): 0. Max coverage (-): 0

Region: chr21 23769000-23769014. Max. coverage (+): 0. Max coverage (-): 0

Region: chr21 23769015-23769029. Max. coverage (+): 0. Max coverage (-): 0

Region: chr21 23769030-23769044. Max. coverage (+): 0. Max coverage (-): 0

Region: chr21 23769045-23769060. Max. coverage (+): 0. Max coverage (-): 0

Region: chr21 23769061-23769075. Max. coverage (+): 0. Max coverage (-): 0

Region: chr21 23769076-23769090. Max. coverage (+): 1.18. Max coverage (-): 0

Region: chr21 23769091-23769105. Max. coverage (+): 2.01. Max coverage (-): 0

Region: chr21 23769106-23769121. Max. coverage (+): 0. Max coverage (-): 0

Region: chr21 23769122-23769136. Max. coverage (+): 0. Max coverage (-): 0

Region: chr21 23769137-23769151. Max. coverage (+): 0. Max coverage (-): 0

Region: chr21 23769152-23769166. Max. coverage (+): 0. Max coverage (-): 0

Region: chr21 23769167-23769182. Max. coverage (+): 1.41. Max coverage (-): 0

Region: chr21 23769183-23769197. Max. coverage (+): 0. Max coverage (-): 0

Region: chr21 23769198-23769212. Max. coverage (+): 0. Max coverage (-): 0

Region: chr21 23769213-23769227. Max. coverage (+): 0. Max coverage (-): 0

Region: chr21 23769228-23769243. Max. coverage (+): 0. Max coverage (-): 0

Region: chr21 23769244-23769258. Max. coverage (+): 0. Max coverage (-): 0

Region: chr21 23769259-23769273. Max. coverage (+): 0. Max coverage (-): 0

Region: chr21 23769274-23769288. Max. coverage (+): 0. Max coverage (-): 0

Region: chr21 23769289-23769304. Max. coverage (+): 1.82. Max coverage (-): 0

Region: chr21 23769305-23769319. Max. coverage (+): 0. Max coverage (-): 0

Region: chr21 23769320-23769334. Max. coverage (+): 0. Max coverage (-): 0

Region: chr21 23769335-23769349. Max. coverage (+): 0. Max coverage (-): 0

Region: chr21 23769350-23769365. Max. coverage (+): 0. Max coverage (-): 0

Region: chr21 23769366-23769380. Max. coverage (+): 0. Max coverage (-): 0

Region: chr21 23769381-23769395. Max. coverage (+): 0.86. Max coverage (-): 0

Region: chr21 23769396-23769410. Max. coverage (+): 0.86. Max coverage (-): 0

Region: chr21 23769411-23769426. Max. coverage (+): 0. Max coverage (-): 0

Region: chr21 23769427-23769441. Max. coverage (+): 0. Max coverage (-): 0

Region: chr21 23769442-23769456. Max. coverage (+): 0. Max coverage (-): 0

Region: chr21 23769457-23769471. Max. coverage (+): 0. Max coverage (-): 0

Region: chr21 23769472-23769487. Max. coverage (+): 0. Max coverage (-): 0

Region: chr21 23769488-23769502. Max. coverage (+): 0. Max coverage (-): 0

Region: chr21 23769503-23769517. Max. coverage (+): 0. Max coverage (-): 0

Region: chr21 23769518-23769532. Max. coverage (+): 0. Max coverage (-): 0

Region: chr21 23769533-23769548. Max. coverage (+): 0. Max coverage (-): 0

Region: chr21 23769549-23769563. Max. coverage (+): 0. Max coverage (-): 0

Region: chr21 23769564-23769578. Max. coverage (+): 0. Max coverage (-): 0

Region: chr21 23769579-23769593. Max. coverage (+): 0. Max coverage (-): 0

Region: chr21 23769594-23769609. Max. coverage (+): 0. Max coverage (-): 0

Region: chr21 23769610-23769624. Max. coverage (+): 7.22. Max coverage (-): 0

Region: chr21 23769625-23769639. Max. coverage (+): 7.22. Max coverage (-): 0

Region: chr21 23769640-23769654. Max. coverage (+): 2.3. Max coverage (-): 0

Region: chr21 23769655-23769670. Max. coverage (+): 2.3. Max coverage (-): 0

Region: chr21 23769671-23769685. Max. coverage (+): 4.71. Max coverage (-): 0

Region: chr21 23769686-23769700. Max. coverage (+): 0. Max coverage (-): 0

Region: chr21 23769701-23769715. Max. coverage (+): 0. Max coverage (-): 0

Region: chr21 23769716-23769731. Max. coverage (+): 0. Max coverage (-): 0

Region: chr21 23769732-23769746. Max. coverage (+): 0. Max coverage (-): 0

Region: chr21 23769747-23769761. Max. coverage (+): 0. Max coverage (-): 0

Region: chr21 23769762-23769776. Max. coverage (+): 0. Max coverage (-): 0

Region: chr21 23769777-23769792. Max. coverage (+): 0. Max coverage (-): 0

Region: chr21 23769793-23769807. Max. coverage (+): 0. Max coverage (-): 0

Region: chr21 23769808-23769822. Max. coverage (+): 0. Max coverage (-): 0

Region: chr21 23769823-23769837. Max. coverage (+): 0. Max coverage (-): 0

Region: chr21 23769838-23769853. Max. coverage (+): 0. Max coverage (-): 0

Region: chr21 23769854-23769868. Max. coverage (+): 0. Max coverage (-): 0

Region: chr21 23769869-23769883. Max. coverage (+): 0. Max coverage (-): 0

Region: chr21 23769884-23769898. Max. coverage (+): 0. Max coverage (-): 0

Region: chr21 23769899-23769914. Max. coverage (+): 0. Max coverage (-): 0

Region: chr21 23769915-23769929. Max. coverage (+): 0. Max coverage (-): 0

Region: chr21 23769930-23769944. Max. coverage (+): 0. Max coverage (-): 0

Region: chr21 23769945-23769959. Max. coverage (+): 0. Max coverage (-): 0

Region: chr21 23769960-23769975. Max. coverage (+): 0. Max coverage (-): 0

Region: chr21 23769976-23769990. Max. coverage (+): 0. Max coverage (-): 0

Region: chr21 23769991-23770005. Max. coverage (+): 0. Max coverage (-): 0

Region: chr21 23770006-23770020. Max. coverage (+): 0. Max coverage (-): 0

Region: chr21 23770021-23770036. Max. coverage (+): 0. Max coverage (-): 0

Region: chr21 23770037-23770051. Max. coverage (+): 0. Max coverage (-): 0

Region: chr21 23770052-23770066. Max. coverage (+): 0. Max coverage (-): 0

Region: chr21 23770067-23770081. Max. coverage (+): 0. Max coverage (-): 0

Region: chr21 23770082-23770097. Max. coverage (+): 0. Max coverage (-): 0

Region: chr21 23770098-23770112. Max. coverage (+): 0. Max coverage (-): 0

Region: chr21 23770113-23770127. Max. coverage (+): 0. Max coverage (-): 0

Region: chr21 23770128-23770142. Max. coverage (+): 0. Max coverage (-): 0

Region: chr21 23770143-23770158. Max. coverage (+): 0. Max coverage (-): 0

Region: chr21 23770159-23770173. Max. coverage (+): 0. Max coverage (-): 0

Region: chr21 23770174-23770188. Max. coverage (+): 0. Max coverage (-): 0

Region: chr21 23770189-23770203. Max. coverage (+): 0. Max coverage (-): 0

Region: chr21 23770204-23770219. Max. coverage (+): 0. Max coverage (-): 0

Region: chr21 23770220-23770234. Max. coverage (+): 0. Max coverage (-): 0

Region: chr21 23770235-23770249. Max. coverage (+): 0. Max coverage (-): 0

Region: chr21 23770250-23770264. Max. coverage (+): 0. Max coverage (-): 0

Region: chr21 23770265-23770280. Max. coverage (+): 0. Max coverage (-): 0

Region: chr21 23770281-23770295. Max. coverage (+): 0. Max coverage (-): 0

Region: chr21 23770296-23770310. Max. coverage (+): 0. Max coverage (-): 0

Region: chr21 23770311-23770325. Max. coverage (+): 0. Max coverage (-): 0

Region: chr21 23770326-23770341. Max. coverage (+): 0. Max coverage (-): 0

Region: chr21 23770342-23770356. Max. coverage (+): 0. Max coverage (-): 0

Region: chr21 23770357-23770371. Max. coverage (+): 0. Max coverage (-): 0

Region: chr21 23770372-23770386. Max. coverage (+): 0. Max coverage (-): 0

Region: chr21 23770387-23770402. Max. coverage (+): 0. Max coverage (-): 0

Region: chr21 23770403-23770417. Max. coverage (+): 0. Max coverage (-): 0

Region: chr21 23770418-23770432. Max. coverage (+): 0. Max coverage (-): 0

Region: chr21 23770433-23770447. Max. coverage (+): 0. Max coverage (-): 0

Region: chr21 23770448-23770463. Max. coverage (+): 0. Max coverage (-): 0

Region: chr21 23770464-23770478. Max. coverage (+): 0. Max coverage (-): 0

Region: chr21 23770479-23770493. Max. coverage (+): 0. Max coverage (-): 0

Region: chr21 23770494-23770508. Max. coverage (+): 0. Max coverage (-): 0

Region: chr21 23770509-23770524. Max. coverage (+): 0. Max coverage (-): 0

Region: chr21 23770525-23770539. Max. coverage (+): 0. Max coverage (-): 0

Region: chr21 23770540-23770554. Max. coverage (+): 0. Max coverage (-): 0

Region: chr21 23770555-23770569. Max. coverage (+): 0. Max coverage (-): 0

Region: chr21 23770570-23770585. Max. coverage (+): 0. Max coverage (-): 0

Region: chr21 23770586-23770600. Max. coverage (+): 0. Max coverage (-): 0

Region: chr21 23770601-23770615. Max. coverage (+): 0. Max coverage (-): 0

Region: chr21 23770616-23770630. Max. coverage (+): 0. Max coverage (-): 0

Region: chr21 23770631-23770646. Max. coverage (+): 0. Max coverage (-): 0

Region: chr21 23770647-23770661. Max. coverage (+): 0. Max coverage (-): 0

Region: chr21 23770662-23770676. Max. coverage (+): 0. Max coverage (-): 0

Region: chr21 23770677-23770691. Max. coverage (+): 0. Max coverage (-): 0

Region: chr21 23770692-23770707. Max. coverage (+): 0. Max coverage (-): 0

Region: chr21 23770708-23770722. Max. coverage (+): 0. Max coverage (-): 0

Region: chr21 23770723-23770737. Max. coverage (+): 0. Max coverage (-): 0

Region: chr21 23770738-23770752. Max. coverage (+): 0. Max coverage (-): 0

Region: chr21 23770753-23770768. Max. coverage (+): 0. Max coverage (-): 0

Region: chr21 23770769-23770783. Max. coverage (+): 0. Max coverage (-): 0

Region: chr21 23770784-23770798. Max. coverage (+): 0. Max coverage (-): 0

Region: chr21 23770799-23770813. Max. coverage (+): 0. Max coverage (-): 0

Region: chr21 23770814-23770829. Max. coverage (+): 0. Max coverage (-): 0

Region: chr21 23770830-23770844. Max. coverage (+): 0. Max coverage (-): 0

Region: chr21 23770845-23770859. Max. coverage (+): 0. Max coverage (-): 0

Region: chr21 23770860-23770874. Max. coverage (+): 5.16. Max coverage (-): 0

Region: chr21 23770875-23770890. Max. coverage (+): 0. Max coverage (-): 0

Region: chr21 23770891-23770905. Max. coverage (+): 0. Max coverage (-): 0

Region: chr21 23770906-23770920. Max. coverage (+): 0. Max coverage (-): 0

Region: chr21 23770921-23770935. Max. coverage (+): 0. Max coverage (-): 0

Region: chr21 23770936-23770951. Max. coverage (+): 0. Max coverage (-): 0

Region: chr21 23770952-23770966. Max. coverage (+): 0. Max coverage (-): 0

Region: chr21 23770967-23770981. Max. coverage (+): 0. Max coverage (-): 0

Region: chr21 23770982-23770996. Max. coverage (+): 0. Max coverage (-): 0

Region: chr21 23770997-23771012. Max. coverage (+): 0. Max coverage (-): 0

Region: chr21 23771013-23771027. Max. coverage (+): 0. Max coverage (-): 0

Region: chr21 23771028-23771042. Max. coverage (+): 0. Max coverage (-): 0

Region: chr21 23771043-23771057. Max. coverage (+): 0. Max coverage (-): 0

Region: chr21 23771058-23771073. Max. coverage (+): 0. Max coverage (-): 0

Region: chr21 23771074-23771088. Max. coverage (+): 0. Max coverage (-): 0

Region: chr21 23771089-23771103. Max. coverage (+): 0. Max coverage (-): 0

Region: chr21 23771104-23771118. Max. coverage (+): 0. Max coverage (-): 0

Region: chr21 23771119-23771134. Max. coverage (+): 0. Max coverage (-): 0

Region: chr21 23771135-23771149. Max. coverage (+): 0. Max coverage (-): 0

Region: chr21 23771150-23771164. Max. coverage (+): 0. Max coverage (-): 0

Region: chr21 23771165-23771179. Max. coverage (+): 0. Max coverage (-): 0

Region: chr21 23771180-23771195. Max. coverage (+): 0. Max coverage (-): 0

Region: chr21 23771196-23771210. Max. coverage (+): 0. Max coverage (-): 0

Region: chr21 23771211-23771225. Max. coverage (+): 0. Max coverage (-): 0

Region: chr21 23771226-23771240. Max. coverage (+): 0. Max coverage (-): 0

Region: chr21 23771241-23771256. Max. coverage (+): 0. Max coverage (-): 0

Region: chr21 23771257-23771271. Max. coverage (+): 0. Max coverage (-): 0

Region: chr21 23771272-23771286. Max. coverage (+): 0. Max coverage (-): 0

Region: chr21 23771287-23771301. Max. coverage (+): 0. Max coverage (-): 0

Region: chr21 23771302-23771317. Max. coverage (+): 0. Max coverage (-): 0

Region: chr21 23771318-23771332. Max. coverage (+): 0. Max coverage (-): 0

Region: chr21 23771333-23771347. Max. coverage (+): 0. Max coverage (-): 0

Region: chr21 23771348-23771362. Max. coverage (+): 0. Max coverage (-): 0

Region: chr21 23771363-23771378. Max. coverage (+): 0. Max coverage (-): 0

Region: chr21 23771379-23771393. Max. coverage (+): 0. Max coverage (-): 0

Region: chr21 23771394-23771408. Max. coverage (+): 0. Max coverage (-): 0

Region: chr21 23771409-23771423. Max. coverage (+): 0. Max coverage (-): 0

Region: chr21 23771424-23771439. Max. coverage (+): 0. Max coverage (-): 0

Region: chr21 23771440-23771454. Max. coverage (+): 0. Max coverage (-): 0

Region: chr21 23771455-23771469. Max. coverage (+): 0. Max coverage (-): 0

Region: chr21 23771470-23771484. Max. coverage (+): 0. Max coverage (-): 0

Region: chr21 23771485-23771500. Max. coverage (+): 0. Max coverage (-): 0

Region: chr21 23771501-23771515. Max. coverage (+): 0. Max coverage (-): 0

Region: chr21 23771516-23771530. Max. coverage (+): 0. Max coverage (-): 0

Region: chr21 23771531-23771545. Max. coverage (+): 0. Max coverage (-): 0

Region: chr21 23771546-23771561. Max. coverage (+): 0. Max coverage (-): 0

Region: chr21 23771562-23771576. Max. coverage (+): 0. Max coverage (-): 0

Region: chr21 23771577-23771591. Max. coverage (+): 0. Max coverage (-): 0

Region: chr21 23771592-23771606. Max. coverage (+): 0. Max coverage (-): 0

Region: chr21 23771607-23771622. Max. coverage (+): 0. Max coverage (-): 0

Region: chr21 23771623-23771637. Max. coverage (+): 0. Max coverage (-): 0

Region: chr21 23771638-23771652. Max. coverage (+): 0. Max coverage (-): 0

Region: chr21 23771653-23771667. Max. coverage (+): 0. Max coverage (-): 0

Region: chr21 23771668-23771683. Max. coverage (+): 0. Max coverage (-): 0

Region: chr21 23771684-23771698. Max. coverage (+): 0. Max coverage (-): 0

Region: chr21 23771699-23771713. Max. coverage (+): 0. Max coverage (-): 0

Region: chr21 23771714-23771728. Max. coverage (+): 0. Max coverage (-): 0

Region: chr21 23771729-23771744. Max. coverage (+): 0. Max coverage (-): 0

Region: chr21 23771745-23771759. Max. coverage (+): 0. Max coverage (-): 0

Region: chr21 23771760-23771774. Max. coverage (+): 0. Max coverage (-): 0

Region: chr21 23771775-23771789. Max. coverage (+): 0. Max coverage (-): 0

Region: chr21 23771790-23771805. Max. coverage (+): 0. Max coverage (-): 0

Region: chr21 23771806-23771820. Max. coverage (+): 0. Max coverage (-): 0

Region: chr21 23771821-23771835. Max. coverage (+): 0. Max coverage (-): 0

Region: chr21 23771836-23771850. Max. coverage (+): 0. Max coverage (-): 0

Region: chr21 23771851-23771866. Max. coverage (+): 0. Max coverage (-): 0

Region: chr21 23771867-23771881. Max. coverage (+): 0. Max coverage (-): 0

Region: chr21 23771882-23771896. Max. coverage (+): 0. Max coverage (-): 0

Region: chr21 23771897-23771911. Max. coverage (+): 0. Max coverage (-): 0

Region: chr21 23771912-23771927. Max. coverage (+): 0. Max coverage (-): 0

Region: chr21 23771928-23771942. Max. coverage (+): 0. Max coverage (-): 0

Region: chr21 23771943-23771957. Max. coverage (+): 0. Max coverage (-): 0

Region: chr21 23771958-23771972. Max. coverage (+): 17.34. Max coverage (-): 0

Region: chr21 23771973-23771988. Max. coverage (+): 9.05. Max coverage (-): 0

Region: chr21 23771989-23772003. Max. coverage (+): 0. Max coverage (-): 0

Region: chr21 23772004-23772018. Max. coverage (+): 0. Max coverage (-): 0

Region: chr21 23772019-23772033. Max. coverage (+): 11.42. Max coverage (-): 0

Region: chr21 23772034-23772049. Max. coverage (+): 13.65. Max coverage (-): 0

Region: chr21 23772050-23772064. Max. coverage (+): 0. Max coverage (-): 0

Region: chr21 23772065-23772079. Max. coverage (+): 0. Max coverage (-): 0

Region: chr21 23772080-23772094. Max. coverage (+): 2.17. Max coverage (-): 0

Region: chr21 23772095-23772110. Max. coverage (+): 4.7. Max coverage (-): 0

Region: chr21 23772111-23772125. Max. coverage (+): 0. Max coverage (-): 0

Region: chr21 23772126-23772140. Max. coverage (+): 0. Max coverage (-): 0

Region: chr21 23772141-23772155. Max. coverage (+): 0. Max coverage (-): 0

Region: chr21 23772156-23772171. Max. coverage (+): 0. Max coverage (-): 0

Region: chr21 23772172-23772186. Max. coverage (+): 0. Max coverage (-): 0

Region: chr21 23772187-23772201. Max. coverage (+): 0. Max coverage (-): 0

Region: chr21 23772202-23772216. Max. coverage (+): 0. Max coverage (-): 0

Region: chr21 23772217-23772232. Max. coverage (+): 0. Max coverage (-): 0

Region: chr21 23772233-23772247. Max. coverage (+): 1.23. Max coverage (-): 0

Region: chr21 23772248-23772262. Max. coverage (+): 1.23. Max coverage (-): 0

Region: chr21 23772263-23772277. Max. coverage (+): 1.15. Max coverage (-): 0

Region: chr21 23772278-23772293. Max. coverage (+): 0. Max coverage (-): 0

Region: chr21 23772294-23772308. Max. coverage (+): 0. Max coverage (-): 0

Region: chr21 23772309-23772323. Max. coverage (+): 0. Max coverage (-): 0

Region: chr21 23772324-23772338. Max. coverage (+): 0. Max coverage (-): 0

Region: chr21 23772339-23772354. Max. coverage (+): 0. Max coverage (-): 0

Region: chr21 23772355-23772369. Max. coverage (+): 0. Max coverage (-): 0

Region: chr21 23772370-23772384. Max. coverage (+): 0. Max coverage (-): 0

Region: chr21 23772385-23772399. Max. coverage (+): 0. Max coverage (-): 0

Region: chr21 23772400-23772415. Max. coverage (+): 0. Max coverage (-): 0

Region: chr21 23772416-23772430. Max. coverage (+): 0. Max coverage (-): 0

Region: chr21 23772431-23772445. Max. coverage (+): 0. Max coverage (-): 0

Region: chr21 23772446-23772460. Max. coverage (+): 0. Max coverage (-): 0

Region: chr21 23772461-23772476. Max. coverage (+): 0. Max coverage (-): 0

Region: chr21 23772477-23772491. Max. coverage (+): 0. Max coverage (-): 0

Region: chr21 23772492-23772506. Max. coverage (+): 0. Max coverage (-): 0

Region: chr21 23772507-23772521. Max. coverage (+): 0. Max coverage (-): 0

Region: chr21 23772522-23772537. Max. coverage (+): 0. Max coverage (-): 0

Region: chr21 23772538-23772552. Max. coverage (+): 0. Max coverage (-): 0

Region: chr21 23772553-23772567. Max. coverage (+): 0. Max coverage (-): 0

Region: chr21 23772568-23772582. Max. coverage (+): 0. Max coverage (-): 0

Region: chr21 23772583-23772598. Max. coverage (+): 0. Max coverage (-): 0

Region: chr21 23772599-23772613. Max. coverage (+): 0. Max coverage (-): 0

Region: chr21 23772614-23772628. Max. coverage (+): 0. Max coverage (-): 0

Region: chr21 23772629-23772643. Max. coverage (+): 0. Max coverage (-): 0

Region: chr21 23772644-23772659. Max. coverage (+): 0. Max coverage (-): 0

Region: chr21 23772660-23772674. Max. coverage (+): 1.68. Max coverage (-): 0

Region: chr21 23772675-23772689. Max. coverage (+): 3.28. Max coverage (-): 0

Region: chr21 23772690-23772704. Max. coverage (+): 3.28. Max coverage (-): 0

Region: chr21 23772705-23772720. Max. coverage (+): 0. Max coverage (-): 0

Region: chr21 23772721-23772735. Max. coverage (+): 0. Max coverage (-): 0

Region: chr21 23772736-23772750. Max. coverage (+): 0. Max coverage (-): 0

Region: chr21 23772751-23772765. Max. coverage (+): 6.62. Max coverage (-): 0

Region: chr21 23772766-23772781. Max. coverage (+): 6.62. Max coverage (-): 0

Region: chr21 23772782-23772796. Max. coverage (+): 0. Max coverage (-): 0

Region: chr21 23772797-23772811. Max. coverage (+): 0. Max coverage (-): 0

Region: chr21 23772812-23772826. Max. coverage (+): 0. Max coverage (-): 0

Region: chr21 23772827-23772842. Max. coverage (+): 0. Max coverage (-): 0

Region: chr21 23772843-23772857. Max. coverage (+): 0. Max coverage (-): 0

Region: chr21 23772858-23772872. Max. coverage (+): 0. Max coverage (-): 0

Region: chr21 23772873-23772887. Max. coverage (+): 3.61. Max coverage (-): 0

Region: chr21 23772888-23772903. Max. coverage (+): 9.94. Max coverage (-): 0

Region: chr21 23772904-23772918. Max. coverage (+): 14.61. Max coverage (-): 0

Region: chr21 23772919-23772933. Max. coverage (+): 1.77. Max coverage (-): 0

Region: chr21 23772934-23772948. Max. coverage (+): 0. Max coverage (-): 0

Region: chr21 23772949-23772964. Max. coverage (+): 0. Max coverage (-): 0

Region: chr21 23772965-23772979. Max. coverage (+): 1.24. Max coverage (-): 0

Region: chr21 23772980-23772994. Max. coverage (+): 0. Max coverage (-): 0

Region: chr21 23772995-23773009. Max. coverage (+): 0. Max coverage (-): 0

Region: chr21 23773010-23773025. Max. coverage (+): 0. Max coverage (-): 0

Region: chr21 23773026-23773040. Max. coverage (+): 0. Max coverage (-): 0

Region: chr21 23773041-23773055. Max. coverage (+): 0. Max coverage (-): 0

Region: chr21 23773056-23773070. Max. coverage (+): 0. Max coverage (-): 0

Region: chr21 23773071-23773086. Max. coverage (+): 1.54. Max coverage (-): 0

Region: chr21 23773087-23773101. Max. coverage (+): 1.54. Max coverage (-): 0

Region: chr21 23773102-23773116. Max. coverage (+): 0. Max coverage (-): 0

Region: chr21 23773117-23773131. Max. coverage (+): 0. Max coverage (-): 0

Region: chr21 23773132-23773147. Max. coverage (+): 0. Max coverage (-): 0

Region: chr21 23773148-23773162. Max. coverage (+): 0. Max coverage (-): 0

Region: chr21 23773163-23773177. Max. coverage (+): 0. Max coverage (-): 0

Region: chr21 23773178-23773192. Max. coverage (+): 0. Max coverage (-): 0

Region: chr21 23773193-23773208. Max. coverage (+): 2.28. Max coverage (-): 0

Region: chr21 23773209-23773223. Max. coverage (+): 0. Max coverage (-): 0

Region: chr21 23773224-23773238. Max. coverage (+): 0. Max coverage (-): 0

Region: chr21 23773239-23773253. Max. coverage (+): 0. Max coverage (-): 0

Region: chr21 23773254-23773269. Max. coverage (+): 2.17. Max coverage (-): 0

Region: chr21 23773270-23773284. Max. coverage (+): 3.25. Max coverage (-): 0

Region: chr21 23773285-23773299. Max. coverage (+): 3.25. Max coverage (-): 0

Region: chr21 23773300-23773314. Max. coverage (+): 0. Max coverage (-): 0

Region: chr21 23773315-23773330. Max. coverage (+): 0. Max coverage (-): 0

Region: chr21 23773331-23773345. Max. coverage (+): 0. Max coverage (-): 0

Region: chr21 23773346-23773360. Max. coverage (+): 0. Max coverage (-): 0

Region: chr21 23773361-23773375. Max. coverage (+): 0. Max coverage (-): 0

Region: chr21 23773376-23773391. Max. coverage (+): 0. Max coverage (-): 0

Region: chr21 23773392-23773406. Max. coverage (+): 0. Max coverage (-): 0

Region: chr21 23773407-23773421. Max. coverage (+): 0. Max coverage (-): 0

Region: chr21 23773422-23773436. Max. coverage (+): 0. Max coverage (-): 0

Region: chr21 23773437-23773452. Max. coverage (+): 0. Max coverage (-): 0

Region: chr21 23773453-23773467. Max. coverage (+): 0. Max coverage (-): 0

Region: chr21 23773468-23773482. Max. coverage (+): 0. Max coverage (-): 0

Region: chr21 23773483-23773497. Max. coverage (+): 0. Max coverage (-): 0

Region: chr21 23773498-23773513. Max. coverage (+): 0. Max coverage (-): 0

Region: chr21 23773514-23773528. Max. coverage (+): 0. Max coverage (-): 0

Region: chr21 23773529-23773543. Max. coverage (+): 0. Max coverage (-): 0

Region: chr21 23773544-23773558. Max. coverage (+): 0. Max coverage (-): 0

Region: chr21 23773559-23773574. Max. coverage (+): 0. Max coverage (-): 0

Region: chr21 23773575-23773589. Max. coverage (+): 0. Max coverage (-): 0

Region: chr21 23773590-23773604. Max. coverage (+): 0. Max coverage (-): 0

Region: chr21 23773605-23773619. Max. coverage (+): 0. Max coverage (-): 0

Region: chr21 23773620-23773635. Max. coverage (+): 0. Max coverage (-): 0

Region: chr21 23773636-23773650. Max. coverage (+): 0. Max coverage (-): 0

Region: chr21 23773651-23773665. Max. coverage (+): 1.96. Max coverage (-): 0

Region: chr21 23773666-23773680. Max. coverage (+): 7.84. Max coverage (-): 0

Region: chr21 23773681-23773696. Max. coverage (+): 1.86. Max coverage (-): 0

Region: chr21 23773697-23773711. Max. coverage (+): 1.86. Max coverage (-): 0

Region: chr21 23773712-23773726. Max. coverage (+): 0. Max coverage (-): 0

Region: chr21 23773727-23773741. Max. coverage (+): 0. Max coverage (-): 0

Region: chr21 23773742-23773757. Max. coverage (+): 0. Max coverage (-): 0

Region: chr21 23773758-23773772. Max. coverage (+): 0. Max coverage (-): 0

Region: chr21 23773773-23773787. Max. coverage (+): 0. Max coverage (-): 0

Region: chr21 23773788-23773802. Max. coverage (+): 0. Max coverage (-): 0

Region: chr21 23773803-23773818. Max. coverage (+): 0. Max coverage (-): 0

Region: chr21 23773819-23773833. Max. coverage (+): 0. Max coverage (-): 0

Region: chr21 23773834-23773848. Max. coverage (+): 0. Max coverage (-): 0

Region: chr21 23773849-23773863. Max. coverage (+): 0. Max coverage (-): 0

Region: chr21 23773864-23773879. Max. coverage (+): 0. Max coverage (-): 0

Region: chr21 23773880-23773894. Max. coverage (+): 0. Max coverage (-): 0

Region: chr21 23773895-23773909. Max. coverage (+): 0. Max coverage (-): 0

Region: chr21 23773910-23773924. Max. coverage (+): 0. Max coverage (-): 0

Region: chr21 23773925-23773940. Max. coverage (+): 0. Max coverage (-): 0

Region: chr21 23773941-23773955. Max. coverage (+): 0. Max coverage (-): 0

Region: chr21 23773956-23773970. Max. coverage (+): 3.78. Max coverage (-): 0

Region: chr21 23773971-23773985. Max. coverage (+): 3.78. Max coverage (-): 0

Region: chr21 23773986-23774001. Max. coverage (+): 0. Max coverage (-): 0

Region: chr21 23774002-23774016. Max. coverage (+): 3.84. Max coverage (-): 0

Region: chr21 23774017-23774031. Max. coverage (+): 3.84. Max coverage (-): 0

Region: chr21 23774032-23774046. Max. coverage (+): 0. Max coverage (-): 0

Region: chr21 23774047-23774062. Max. coverage (+): 0. Max coverage (-): 0

Region: chr21 23774063-23774077. Max. coverage (+): 0. Max coverage (-): 0

Region: chr21 23774078-23774092. Max. coverage (+): 0. Max coverage (-): 0

Region: chr21 23774093-23774107. Max. coverage (+): 0. Max coverage (-): 0

Region: chr21 23774108-23774123. Max. coverage (+): 0. Max coverage (-): 0

Region: chr21 23774124-23774138. Max. coverage (+): 0. Max coverage (-): 0

Region: chr21 23774139-23774153. Max. coverage (+): 0. Max coverage (-): 0

Region: chr21 23774154-23774168. Max. coverage (+): 0. Max coverage (-): 0

Region: chr21 23774169-23774184. Max. coverage (+): 2.49. Max coverage (-): 0

Region: chr21 23774185-23774199. Max. coverage (+): 2.49. Max coverage (-): 0

Region: chr21 23774200-23774214. Max. coverage (+): 1.05. Max coverage (-): 0

Region: chr21 23774215-23774229. Max. coverage (+): 0. Max coverage (-): 0

Region: chr21 23774230-23774245. Max. coverage (+): 0. Max coverage (-): 0

Region: chr21 23774246-23774260. Max. coverage (+): 0. Max coverage (-): 0

Region: chr21 23774261-23774275. Max. coverage (+): 0. Max coverage (-): 0

Region: chr21 23774276-23774290. Max. coverage (+): 0. Max coverage (-): 0

Region: chr21 23774291-23774306. Max. coverage (+): 0. Max coverage (-): 0

Region: chr21 23774307-23774321. Max. coverage (+): 0. Max coverage (-): 0

Region: chr21 23774322-23774336. Max. coverage (+): 0. Max coverage (-): 0

Region: chr21 23774337-23774351. Max. coverage (+): 0. Max coverage (-): 0

Region: chr21 23774352-23774367. Max. coverage (+): 0. Max coverage (-): 0

Region: chr21 23774368-23774382. Max. coverage (+): 0. Max coverage (-): 0

Region: chr21 23774383-23774397. Max. coverage (+): 0. Max coverage (-): 0

Region: chr21 23774398-23774412. Max. coverage (+): 0. Max coverage (-): 0

Region: chr21 23774413-23774428. Max. coverage (+): 0. Max coverage (-): 0

Region: chr21 23774429-23774443. Max. coverage (+): 0. Max coverage (-): 0

Region: chr21 23774444-23774458. Max. coverage (+): 0. Max coverage (-): 0

Region: chr21 23774459-23774473. Max. coverage (+): 0. Max coverage (-): 0

Region: chr21 23774474-23774489. Max. coverage (+): 0. Max coverage (-): 0

Region: chr21 23774490-23774504. Max. coverage (+): 0. Max coverage (-): 0

Region: chr21 23774505-23774519. Max. coverage (+): 0. Max coverage (-): 0

Region: chr21 23774520-23774534. Max. coverage (+): 0. Max coverage (-): 0

Region: chr21 23774535-23774550. Max. coverage (+): 0. Max coverage (-): 0

Region: chr21 23774551-23774565. Max. coverage (+): 0. Max coverage (-): 0

Region: chr21 23774566-23774580. Max. coverage (+): 0. Max coverage (-): 0

Region: chr21 23774581-23774595. Max. coverage (+): 0. Max coverage (-): 0

Region: chr21 23774596-23774611. Max. coverage (+): 0. Max coverage (-): 0

Region: chr21 23774612-23774626. Max. coverage (+): 0. Max coverage (-): 0

Region: chr21 23774627-23774641. Max. coverage (+): 0. Max coverage (-): 0

Region: chr21 23774642-23774656. Max. coverage (+): 0. Max coverage (-): 0

Region: chr21 23774657-23774672. Max. coverage (+): 0. Max coverage (-): 0

Region: chr21 23774673-23774687. Max. coverage (+): 0. Max coverage (-): 0

Region: chr21 23774688-23774702. Max. coverage (+): 0. Max coverage (-): 0

Region: chr21 23774703-23774717. Max. coverage (+): 0. Max coverage (-): 0

Region: chr21 23774718-23774733. Max. coverage (+): 0. Max coverage (-): 0

Region: chr21 23774734-23774748. Max. coverage (+): 0. Max coverage (-): 0

Region: chr21 23774749-23774763. Max. coverage (+): 0. Max coverage (-): 0

Region: chr21 23774764-23774778. Max. coverage (+): 0. Max coverage (-): 0

Region: chr21 23774779-23774794. Max. coverage (+): 0. Max coverage (-): 0

Region: chr21 23774795-23774809. Max. coverage (+): 0. Max coverage (-): 0

Region: chr21 23774810-23774824. Max. coverage (+): 0. Max coverage (-): 0

Region: chr21 23774825-23774839. Max. coverage (+): 0. Max coverage (-): 0

Region: chr21 23774840-23774855. Max. coverage (+): 0.36. Max coverage (-): 0

Region: chr21 23774856-23774870. Max. coverage (+): 0. Max coverage (-): 0

Region: chr21 23774871-23774885. Max. coverage (+): 0. Max coverage (-): 0

Region: chr21 23774886-23774900. Max. coverage (+): 0. Max coverage (-): 0

Region: chr21 23774901-23774916. Max. coverage (+): 0. Max coverage (-): 0

Region: chr21 23774917-23774931. Max. coverage (+): 0. Max coverage (-): 0

Region: chr21 23774932-23774946. Max. coverage (+): 0. Max coverage (-): 0

Region: chr21 23774947-23774961. Max. coverage (+): 0. Max coverage (-): 0

Region: chr21 23774962-23774977. Max. coverage (+): 0.74. Max coverage (-): 0

Region: chr21 23774978-23774992. Max. coverage (+): 0.74. Max coverage (-): 0

Region: chr21 23774993-23775007. Max. coverage (+): 0. Max coverage (-): 0

Region: chr21 23775008-23775022. Max. coverage (+): 0. Max coverage (-): 0

Region: chr21 23775023-23775038. Max. coverage (+): 0. Max coverage (-): 0

Region: chr21 23775039-23775053. Max. coverage (+): 0. Max coverage (-): 0

Region: chr21 23775054-23775068. Max. coverage (+): 0. Max coverage (-): 0

Region: chr21 23775069-23775083. Max. coverage (+): 0. Max coverage (-): 0

Region: chr21 23775084-23775099. Max. coverage (+): 0. Max coverage (-): 0

Region: chr21 23775100-23775114. Max. coverage (+): 0. Max coverage (-): 0

Region: chr21 23775115-23775129. Max. coverage (+): 0. Max coverage (-): 0

Region: chr21 23775130-23775144. Max. coverage (+): 0. Max coverage (-): 0

Region: chr21 23775145-23775160. Max. coverage (+): 0. Max coverage (-): 0

Region: chr21 23775161-23775175. Max. coverage (+): 0. Max coverage (-): 0

Region: chr21 23775176-23775190. Max. coverage (+): 0. Max coverage (-): 0

Region: chr21 23775191-23775205. Max. coverage (+): 0. Max coverage (-): 0

Region: chr21 23775206-23775221. Max. coverage (+): 0. Max coverage (-): 0

Region: chr21 23775222-23775236. Max. coverage (+): 0. Max coverage (-): 0

Region: chr21 23775237-23775251. Max. coverage (+): 0. Max coverage (-): 0

Region: chr21 23775252-23775266. Max. coverage (+): 0. Max coverage (-): 0

Region: chr21 23775267-23775282. Max. coverage (+): 0. Max coverage (-): 0

Region: chr21 23775283-23775297. Max. coverage (+): 0. Max coverage (-): 0

Region: chr21 23775298-23775312. Max. coverage (+): 0. Max coverage (-): 0

Region: chr21 23775313-23775327. Max. coverage (+): 0. Max coverage (-): 0

Region: chr21 23775328-23775343. Max. coverage (+): 0. Max coverage (-): 0

Region: chr21 23775344-23775358. Max. coverage (+): 0. Max coverage (-): 0

Region: chr21 23775359-23775373. Max. coverage (+): 0. Max coverage (-): 0

Region: chr21 23775374-23775388. Max. coverage (+): 0. Max coverage (-): 0

Region: chr21 23775389-23775404. Max. coverage (+): 0. Max coverage (-): 0

Region: chr21 23775405-23775419. Max. coverage (+): 0. Max coverage (-): 0

Region: chr21 23775420-23775434. Max. coverage (+): 0. Max coverage (-): 0

Region: chr21 23775435-23775449. Max. coverage (+): 0. Max coverage (-): 0

Region: chr21 23775450-23775465. Max. coverage (+): 0. Max coverage (-): 0

Region: chr21 23775466-23775480. Max. coverage (+): 2.04. Max coverage (-): 0

Region: chr21 23775481-23775495. Max. coverage (+): 2.04. Max coverage (-): 0

Region: chr21 23775496-23775510. Max. coverage (+): 0. Max coverage (-): 0

Region: chr21 23775511-23775526. Max. coverage (+): 0. Max coverage (-): 0

Region: chr21 23775527-23775541. Max. coverage (+): 0. Max coverage (-): 0

Region: chr21 23775542-23775556. Max. coverage (+): 0. Max coverage (-): 0

Region: chr21 23775557-23775571. Max. coverage (+): 0. Max coverage (-): 0

Region: chr21 23775572-23775587. Max. coverage (+): 0. Max coverage (-): 0

Region: chr21 23775588-23775602. Max. coverage (+): 0. Max coverage (-): 0

Region: chr21 23775603-23775617. Max. coverage (+): 1.91. Max coverage (-): 0

Region: chr21 23775618-23775632. Max. coverage (+): 0. Max coverage (-): 0

Region: chr21 23775633-23775648. Max. coverage (+): 1.09. Max coverage (-): 0

Region: chr21 23775649-23775663. Max. coverage (+): 0. Max coverage (-): 0

Region: chr21 23775664-. Max. coverage (+): 0. Max coverage (-): 0

RepeatMasker Color Code

**+**

100-98% Identity

<98-95% Identity

<95-90% Identity

<90-85% Identity

<85-80% Identity

<80-75% Identity

<75-70% Identity

<70% Identity

**-**

Gene Set Color Code

**+**

Gene

Pseudogene

**-**

Topology/Coverage Color Code

Coverage Plus Strand

Coverage Minus Strand

Mainstrand: Plus

Mainstrand: Minus

Complementary Strand

Flanking Region  
(if option -flank >0)

Gene Set Annotation  
  
RepeatMasker Annotation  

**1. BOV-A2**: 23768689-23768946 (+), Divergence to consensus: 8.1%  
**2. AT\_rich**: 23769485-23769505 (+), Divergence to consensus: 52.4%  
**3. SINE2-2\_BT**: 23769506-23769609 (-), Divergence to consensus: 32.7%  
**4. BOV-A2**: 23769712-23769841 (+), Divergence to consensus: 10%  
**5. ART2A**: 23770017-23770534 (-), Divergence to consensus: 23.1%  
**6. BovB**: 23770536-23770773 (-), Divergence to consensus: 25.6%  
**7. Bov-tA2**: 23771049-23771163 (+), Divergence to consensus: 23.5%  
**8. Bov-tA2**: 23771164-23771345 (+), Divergence to consensus: 23.1%  
**9. L1\_Art**: 23771383-23771621 (-), Divergence to consensus: 34%  
**10. Bov-tA1**: 23771622-23771839 (+), Divergence to consensus: 17.3%  
**11. L3**: 23771885-23771955 (-), Divergence to consensus: 26.1%  
**12. SINE2-3\_BT**: 23772424-23772566 (+), Divergence to consensus: 14.8%  
**13. Bov-tA3**: 23774403-23774456 (-), Divergence to consensus: 25.2%  
**14. MLT1B**: 23774450-23774676 (-), Divergence to consensus: 30.7%  
**15. MLT1B-int**: 23774678-23774826 (-), Divergence to consensus: 22.2%  
**16. ART2A**: 23775106-23775256 (+), Divergence to consensus: 16.7%  
**17. L1M2**: 23775257-23775409 (-), Divergence to consensus: 23.5%

  
Transcription Factor Binding Sites  

**RFX4\_2** (Sequence: CCTGGTTAC (+): 23774177)  
**SOX9** (Sequence: TCATTGTT (+): 23769132)
