# Supplementary material for: Small RNA sequencing of cryopreserved semen from single bull revealed altered miRNAs and piRNAs expression between High- and Low-motile sperm populations
Source: BMC Genomics. 2017 Jan 4;18:14. doi: 10.1186/s12864-016-3394-7 (PMC5209821; doi:10.1186/s12864-016-3394-7)
Supplement: Additional file 3: — Details for each piRNA clusters found in High Motile (HM) sperm fraction. Genes, repeats, transposable elements and transcription factors binding sites falling within the cluster regions were reported. (ZIP 1896 kb) [file 12864_2016_3394_MOESM3_ESM.zip › 48.html]

piRNA cluster 48


Predicted piRNA cluster no. 48     previous   next
  

Show proTRAC run info
Hide proTRAC run info

================================= proTRAC ====================================  
VERSION: 2.1                                    LAST MODIFIED: 06. October 2015  
  
Please cite:  
Rosenkranz D, Zischler H. proTRAC - a software for probabilistic piRNA cluster  
detection, visualization and analysis. 2012. BMC Bioinformatics 13:5.  
  
and (for proTRAC 2.0 and later):  
Rosenkranz D, Rudloff S, Bastuck K, Ketting RF, Zischler H. Tupaia small RNAs  
provide insights into function and evolution of RNAi-based transposon defense  
in mammals. 2015. RNA 21(5):911-922.  
  
Contact:  
David Rosenkranz  
Institute of Anthropology, small RNA group  
Johannes Gutenberg University Mainz  
email: rosenkranz@uni-mainz.de  
  
You can find the latest proTRAC version at:  
http://sourceforge.net/projects/protrac/files  
http://www.smallRNAgroup-mainz.de/software  
==============================================================================  
  
PARAMETERS:  
Map file: .............../storage/core/barbara/genhome/smallRNA/fertility/Sample\_motile/pirna/Sample\_motile\_26-33\_collapsed.fa.no-dust.map.weighted-10000-1000-b-0  
Genome file: ............/storage/core/barbara/genhome/smallRNA/fertility/Sample\_all/pirna/bt\_311\_chrY.fa  
RepeatMasker annotation: /storage/genomes/bt\_umd31/GCF\_000003055.6\_Bos\_taurus\_UMD\_3.1.1\_repeatMasker\_chr.out  
GeneSet:................./storage/core/barbara/genhome/smallRNA/fertility/Sample\_all/pirna/full.gtf  
  
Significant (p<=0.01) hit density will be calculated based  
on observed hit distribution.  
  
Sliding window size: ........................................ 5000 bp  
Sliding window increament: .................................. 1000 bp  
Normalize each hit by number of genomic hits: ............... 1 [0=no/1=yes]  
Normalize each hit by number of sequence reads: ............. 1 [0=no/1=yes]  
Normalize values (-> per million mapped reads): ............. 1 [0=no/1=yes]  
Min. fraction of hits with 1T(U) or 10A: .................... 0.75  
Alternatively: Min. fraction of hits with 1T(U) and 10A: .... 0.5  
Min. fraction of hits with typical piRNA length: ............ 0.75  
Typical piRNA length: ....................................... 26-33 nt  
Min. size of a piRNA cluster: ............................... 5000 bp.  
Min. number of hits (absolute): ............................. 0  
Min. number of hits (normalized): ........................... 0  
Min. fraction of hits on the mainstrand: .................... 0.75  
Top fraction of mapped sequences (in terms of read counts): . 1%  
Top fraction accounts for max. n% of sequence reads: ........ 90%  
Min. fraction of hits on each arm of a bidirectional cluster: 0.1  
Output image file for each cluster: ......................... 0 [0=no/1=yes]  
Output html file for each cluster: .......................... 1 [0=no/1=yes]  
Output a summary table: ..................................... 1 [0=no/1=yes]  
Output a FASTA file for each cluster (piRNA sequences): ..... 1 [0=no/1=yes]  
Output a FASTA file comprising cluster sequences: ........... 1 [0=no/1=yes]  
Search DNA motifs in clusters: .............................. 1 [0=no/1=yes]  
Output flanking sequences: +/- .............................. 0 bp  
Output ~.pTi file: .......................................... 1 [0=no/1=yes]  
==============================================================================  
  
  
Genome size (without gaps): ............ 2678902517 bp  
Gaps (N/X/-): .......................... 53837044 bp  
Mapped reads: .......................... 658825247023  
Non-identical sequences: ............... 514171  
Genomic hits: .......................... 764233  
Significant densitiy of mapped reads: .. 12867599.5173724 reads/kb

Show proTRAC cluster info
Hide proTRAC cluster info

|  |  |
| --- | --- |
| Location | chr21 |
| Coordinates | 29761174-29770759 |
| Size [bp] | 9586 |
| Sequence hit loci | 495 |
| Mapped reads (normalized) | 596855336.5 |
| Mapped reads (normalized) per kb | 62263231.4 |
| Normalized reads with 1T (1U) | 86.3% |
| Normalized reads with 10A | 30.5% |
| Normalized reads with length 26-33 nt | 100% |
| Normalized reads on the main strand(s) | 100% |
| Predicted directionality | mono:plus |

100%

0%

1T (1U)  
reads

10A reads

26-33 nt  
reads

reads on mainstrand

**Either the amount of reads with 1T (1U) OR 10A has to exceed 75% (set with option: -1Tor10A)  
Alternatively the amount of reads with 1T (1U) AND 10A has to exceed 50% (set with option: -1Tand10A)  
Minimum amount of reads with preferred size is 75% (set with option: -pisize)  
Minimum amount of reads on the main strand(s) is 75% (set with option: -clstrand)**

Show read coverage
Hide read coverage

WHAT DO I SEE HERE?  
This chart shows the location of mapped sequence reads within a predicted piRNA cluster. The color refers to the number of genomic hits produced by the sequence read in question. A dark red bar indicates that this sequence read produces many other hits elsewhere in the genome. Many adjacent red or yellow bars can indicate the presence of a multi-copy element such as transposons or rRNA genes. A dark green bar indicates that this sequence read maps uniquely to this locus.

1 hit

2-5 hits

6-10 hits

11-20 hits

21-50 hits

51-100 hits

> 100 hits

chr21

29761174

29770759

Gene Set

RepeatMasker

Mapped  
Reads

36.07

plus strand

minus strand

36.07

Region: chr21 23775665-29761183. Max. coverage (+): 0.61. Max coverage (-): 0

Region: chr21 29761184-29761202. Max. coverage (+): 6.83. Max coverage (-): 0

Region: chr21 29761203-29761221. Max. coverage (+): 0. Max coverage (-): 0

Region: chr21 29761222-29761241. Max. coverage (+): 0. Max coverage (-): 0

Region: chr21 29761242-29761260. Max. coverage (+): 0. Max coverage (-): 0

Region: chr21 29761261-29761279. Max. coverage (+): 0.3. Max coverage (-): 0

Region: chr21 29761280-29761298. Max. coverage (+): 9.83. Max coverage (-): 0

Region: chr21 29761299-29761317. Max. coverage (+): 0. Max coverage (-): 0

Region: chr21 29761318-29761336. Max. coverage (+): 0. Max coverage (-): 0

Region: chr21 29761337-29761356. Max. coverage (+): 0. Max coverage (-): 0

Region: chr21 29761357-29761375. Max. coverage (+): 0. Max coverage (-): 0

Region: chr21 29761376-29761394. Max. coverage (+): 0. Max coverage (-): 0

Region: chr21 29761395-29761413. Max. coverage (+): 0. Max coverage (-): 0

Region: chr21 29761414-29761432. Max. coverage (+): 0. Max coverage (-): 0

Region: chr21 29761433-29761451. Max. coverage (+): 0. Max coverage (-): 0

Region: chr21 29761452-29761471. Max. coverage (+): 0. Max coverage (-): 0

Region: chr21 29761472-29761490. Max. coverage (+): 0. Max coverage (-): 0

Region: chr21 29761491-29761509. Max. coverage (+): 0. Max coverage (-): 0

Region: chr21 29761510-29761528. Max. coverage (+): 0. Max coverage (-): 0

Region: chr21 29761529-29761547. Max. coverage (+): 0. Max coverage (-): 0

Region: chr21 29761548-29761567. Max. coverage (+): 4.41. Max coverage (-): 0

Region: chr21 29761568-29761586. Max. coverage (+): 0. Max coverage (-): 0

Region: chr21 29761587-29761605. Max. coverage (+): 2.21. Max coverage (-): 0

Region: chr21 29761606-29761624. Max. coverage (+): 1.47. Max coverage (-): 0

Region: chr21 29761625-29761643. Max. coverage (+): 1.47. Max coverage (-): 0

Region: chr21 29761644-29761662. Max. coverage (+): 0. Max coverage (-): 0

Region: chr21 29761663-29761682. Max. coverage (+): 0. Max coverage (-): 0

Region: chr21 29761683-29761701. Max. coverage (+): 5.52. Max coverage (-): 0

Region: chr21 29761702-29761720. Max. coverage (+): 5.52. Max coverage (-): 0

Region: chr21 29761721-29761739. Max. coverage (+): 4.52. Max coverage (-): 0

Region: chr21 29761740-29761758. Max. coverage (+): 0. Max coverage (-): 0

Region: chr21 29761759-29761777. Max. coverage (+): 0. Max coverage (-): 0

Region: chr21 29761778-29761797. Max. coverage (+): 4.53. Max coverage (-): 0

Region: chr21 29761798-29761816. Max. coverage (+): 0. Max coverage (-): 0

Region: chr21 29761817-29761835. Max. coverage (+): 0. Max coverage (-): 0

Region: chr21 29761836-29761854. Max. coverage (+): 0. Max coverage (-): 0

Region: chr21 29761855-29761873. Max. coverage (+): 0. Max coverage (-): 0

Region: chr21 29761874-29761892. Max. coverage (+): 1.94. Max coverage (-): 0

Region: chr21 29761893-29761912. Max. coverage (+): 0. Max coverage (-): 0

Region: chr21 29761913-29761931. Max. coverage (+): 0. Max coverage (-): 0

Region: chr21 29761932-29761950. Max. coverage (+): 12.54. Max coverage (-): 0

Region: chr21 29761951-29761969. Max. coverage (+): 4.5. Max coverage (-): 0

Region: chr21 29761970-29761988. Max. coverage (+): 15.18. Max coverage (-): 0

Region: chr21 29761989-29762007. Max. coverage (+): 15.18. Max coverage (-): 0

Region: chr21 29762008-29762027. Max. coverage (+): 6.92. Max coverage (-): 0

Region: chr21 29762028-29762046. Max. coverage (+): 8.63. Max coverage (-): 0

Region: chr21 29762047-29762065. Max. coverage (+): 6.69. Max coverage (-): 0

Region: chr21 29762066-29762084. Max. coverage (+): 10.62. Max coverage (-): 0

Region: chr21 29762085-29762103. Max. coverage (+): 0. Max coverage (-): 0

Region: chr21 29762104-29762123. Max. coverage (+): 1.6. Max coverage (-): 0

Region: chr21 29762124-29762142. Max. coverage (+): 7.92. Max coverage (-): 0

Region: chr21 29762143-29762161. Max. coverage (+): 3.45. Max coverage (-): 0

Region: chr21 29762162-29762180. Max. coverage (+): 2.11. Max coverage (-): 0

Region: chr21 29762181-29762199. Max. coverage (+): 2.51. Max coverage (-): 0

Region: chr21 29762200-29762218. Max. coverage (+): 0. Max coverage (-): 0

Region: chr21 29762219-29762238. Max. coverage (+): 5.69. Max coverage (-): 0

Region: chr21 29762239-29762257. Max. coverage (+): 1.63. Max coverage (-): 0

Region: chr21 29762258-29762276. Max. coverage (+): 2.18. Max coverage (-): 0

Region: chr21 29762277-29762295. Max. coverage (+): 0. Max coverage (-): 0

Region: chr21 29762296-29762314. Max. coverage (+): 0. Max coverage (-): 0

Region: chr21 29762315-29762333. Max. coverage (+): 1.34. Max coverage (-): 0

Region: chr21 29762334-29762353. Max. coverage (+): 6.4. Max coverage (-): 0

Region: chr21 29762354-29762372. Max. coverage (+): 0. Max coverage (-): 0

Region: chr21 29762373-29762391. Max. coverage (+): 0. Max coverage (-): 0

Region: chr21 29762392-29762410. Max. coverage (+): 0. Max coverage (-): 0

Region: chr21 29762411-29762429. Max. coverage (+): 0. Max coverage (-): 0

Region: chr21 29762430-29762448. Max. coverage (+): 0. Max coverage (-): 0

Region: chr21 29762449-29762468. Max. coverage (+): 0. Max coverage (-): 0

Region: chr21 29762469-29762487. Max. coverage (+): 0. Max coverage (-): 0

Region: chr21 29762488-29762506. Max. coverage (+): 0. Max coverage (-): 0

Region: chr21 29762507-29762525. Max. coverage (+): 0. Max coverage (-): 0

Region: chr21 29762526-29762544. Max. coverage (+): 0. Max coverage (-): 0

Region: chr21 29762545-29762563. Max. coverage (+): 0. Max coverage (-): 0

Region: chr21 29762564-29762583. Max. coverage (+): 0. Max coverage (-): 0

Region: chr21 29762584-29762602. Max. coverage (+): 0. Max coverage (-): 0

Region: chr21 29762603-29762621. Max. coverage (+): 0. Max coverage (-): 0

Region: chr21 29762622-29762640. Max. coverage (+): 0. Max coverage (-): 0

Region: chr21 29762641-29762659. Max. coverage (+): 0. Max coverage (-): 0

Region: chr21 29762660-29762679. Max. coverage (+): 0. Max coverage (-): 0

Region: chr21 29762680-29762698. Max. coverage (+): 0. Max coverage (-): 0

Region: chr21 29762699-29762717. Max. coverage (+): 0. Max coverage (-): 0

Region: chr21 29762718-29762736. Max. coverage (+): 0. Max coverage (-): 0

Region: chr21 29762737-29762755. Max. coverage (+): 0. Max coverage (-): 0

Region: chr21 29762756-29762774. Max. coverage (+): 0. Max coverage (-): 0

Region: chr21 29762775-29762794. Max. coverage (+): 0. Max coverage (-): 0

Region: chr21 29762795-29762813. Max. coverage (+): 0.74. Max coverage (-): 0

Region: chr21 29762814-29762832. Max. coverage (+): 0. Max coverage (-): 0

Region: chr21 29762833-29762851. Max. coverage (+): 7.22. Max coverage (-): 0

Region: chr21 29762852-29762870. Max. coverage (+): 9.46. Max coverage (-): 0

Region: chr21 29762871-29762889. Max. coverage (+): 0. Max coverage (-): 0

Region: chr21 29762890-29762909. Max. coverage (+): 36.07. Max coverage (-): 0

Region: chr21 29762910-29762928. Max. coverage (+): 12.08. Max coverage (-): 0

Region: chr21 29762929-29762947. Max. coverage (+): 2.12. Max coverage (-): 0

Region: chr21 29762948-29762966. Max. coverage (+): 4.29. Max coverage (-): 0

Region: chr21 29762967-29762985. Max. coverage (+): 4.29. Max coverage (-): 0

Region: chr21 29762986-29763004. Max. coverage (+): 0. Max coverage (-): 0

Region: chr21 29763005-29763024. Max. coverage (+): 0. Max coverage (-): 0

Region: chr21 29763025-29763043. Max. coverage (+): 0. Max coverage (-): 0

Region: chr21 29763044-29763062. Max. coverage (+): 0. Max coverage (-): 0

Region: chr21 29763063-29763081. Max. coverage (+): 4.82. Max coverage (-): 0

Region: chr21 29763082-29763100. Max. coverage (+): 0. Max coverage (-): 0

Region: chr21 29763101-29763119. Max. coverage (+): 0. Max coverage (-): 0

Region: chr21 29763120-29763139. Max. coverage (+): 0.75. Max coverage (-): 0

Region: chr21 29763140-29763158. Max. coverage (+): 0. Max coverage (-): 0

Region: chr21 29763159-29763177. Max. coverage (+): 0. Max coverage (-): 0

Region: chr21 29763178-29763196. Max. coverage (+): 0. Max coverage (-): 0

Region: chr21 29763197-29763215. Max. coverage (+): 0. Max coverage (-): 0

Region: chr21 29763216-29763234. Max. coverage (+): 12.18. Max coverage (-): 0

Region: chr21 29763235-29763254. Max. coverage (+): 1.96. Max coverage (-): 0

Region: chr21 29763255-29763273. Max. coverage (+): 0. Max coverage (-): 0

Region: chr21 29763274-29763292. Max. coverage (+): 0. Max coverage (-): 0

Region: chr21 29763293-29763311. Max. coverage (+): 5.85. Max coverage (-): 0

Region: chr21 29763312-29763330. Max. coverage (+): 27.11. Max coverage (-): 0

Region: chr21 29763331-29763350. Max. coverage (+): 13.36. Max coverage (-): 0

Region: chr21 29763351-29763369. Max. coverage (+): 0.77. Max coverage (-): 0

Region: chr21 29763370-29763388. Max. coverage (+): 2.21. Max coverage (-): 0

Region: chr21 29763389-29763407. Max. coverage (+): 2.21. Max coverage (-): 0

Region: chr21 29763408-29763426. Max. coverage (+): 0. Max coverage (-): 0

Region: chr21 29763427-29763445. Max. coverage (+): 9.32. Max coverage (-): 0

Region: chr21 29763446-29763465. Max. coverage (+): 1.96. Max coverage (-): 0

Region: chr21 29763466-29763484. Max. coverage (+): 0. Max coverage (-): 0

Region: chr21 29763485-29763503. Max. coverage (+): 0. Max coverage (-): 0

Region: chr21 29763504-29763522. Max. coverage (+): 0.79. Max coverage (-): 0

Region: chr21 29763523-29763541. Max. coverage (+): 0.79. Max coverage (-): 0

Region: chr21 29763542-29763560. Max. coverage (+): 0. Max coverage (-): 0

Region: chr21 29763561-29763580. Max. coverage (+): 0. Max coverage (-): 0

Region: chr21 29763581-29763599. Max. coverage (+): 2.66. Max coverage (-): 0

Region: chr21 29763600-29763618. Max. coverage (+): 4.44. Max coverage (-): 0

Region: chr21 29763619-29763637. Max. coverage (+): 9.25. Max coverage (-): 0

Region: chr21 29763638-29763656. Max. coverage (+): 0. Max coverage (-): 0

Region: chr21 29763657-29763675. Max. coverage (+): 0. Max coverage (-): 0

Region: chr21 29763676-29763695. Max. coverage (+): 0. Max coverage (-): 0

Region: chr21 29763696-29763714. Max. coverage (+): 0. Max coverage (-): 0

Region: chr21 29763715-29763733. Max. coverage (+): 0. Max coverage (-): 0

Region: chr21 29763734-29763752. Max. coverage (+): 1.65. Max coverage (-): 0

Region: chr21 29763753-29763771. Max. coverage (+): 6.28. Max coverage (-): 0

Region: chr21 29763772-29763790. Max. coverage (+): 0. Max coverage (-): 0

Region: chr21 29763791-29763810. Max. coverage (+): 0. Max coverage (-): 0

Region: chr21 29763811-29763829. Max. coverage (+): 2.17. Max coverage (-): 0

Region: chr21 29763830-29763848. Max. coverage (+): 2.17. Max coverage (-): 0

Region: chr21 29763849-29763867. Max. coverage (+): 1.56. Max coverage (-): 0

Region: chr21 29763868-29763886. Max. coverage (+): 1.56. Max coverage (-): 0

Region: chr21 29763887-29763906. Max. coverage (+): 0. Max coverage (-): 0

Region: chr21 29763907-29763925. Max. coverage (+): 0. Max coverage (-): 0

Region: chr21 29763926-29763944. Max. coverage (+): 6.72. Max coverage (-): 0

Region: chr21 29763945-29763963. Max. coverage (+): 6.72. Max coverage (-): 0

Region: chr21 29763964-29763982. Max. coverage (+): 0. Max coverage (-): 0

Region: chr21 29763983-29764001. Max. coverage (+): 2.54. Max coverage (-): 0

Region: chr21 29764002-29764021. Max. coverage (+): 0. Max coverage (-): 0

Region: chr21 29764022-29764040. Max. coverage (+): 0. Max coverage (-): 0

Region: chr21 29764041-29764059. Max. coverage (+): 0. Max coverage (-): 0

Region: chr21 29764060-29764078. Max. coverage (+): 0. Max coverage (-): 0

Region: chr21 29764079-29764097. Max. coverage (+): 0. Max coverage (-): 0

Region: chr21 29764098-29764116. Max. coverage (+): 0. Max coverage (-): 0

Region: chr21 29764117-29764136. Max. coverage (+): 0. Max coverage (-): 0

Region: chr21 29764137-29764155. Max. coverage (+): 0. Max coverage (-): 0

Region: chr21 29764156-29764174. Max. coverage (+): 0. Max coverage (-): 0

Region: chr21 29764175-29764193. Max. coverage (+): 0. Max coverage (-): 0

Region: chr21 29764194-29764212. Max. coverage (+): 2.18. Max coverage (-): 0

Region: chr21 29764213-29764231. Max. coverage (+): 9.14. Max coverage (-): 0

Region: chr21 29764232-29764251. Max. coverage (+): 12.71. Max coverage (-): 0

Region: chr21 29764252-29764270. Max. coverage (+): 0. Max coverage (-): 0

Region: chr21 29764271-29764289. Max. coverage (+): 0. Max coverage (-): 0

Region: chr21 29764290-29764308. Max. coverage (+): 0. Max coverage (-): 0

Region: chr21 29764309-29764327. Max. coverage (+): 0. Max coverage (-): 0

Region: chr21 29764328-29764346. Max. coverage (+): 1.43. Max coverage (-): 0

Region: chr21 29764347-29764366. Max. coverage (+): 1.17. Max coverage (-): 0

Region: chr21 29764367-29764385. Max. coverage (+): 20.07. Max coverage (-): 0

Region: chr21 29764386-29764404. Max. coverage (+): 1.18. Max coverage (-): 0

Region: chr21 29764405-29764423. Max. coverage (+): 0. Max coverage (-): 0

Region: chr21 29764424-29764442. Max. coverage (+): 0. Max coverage (-): 0

Region: chr21 29764443-29764461. Max. coverage (+): 0. Max coverage (-): 0

Region: chr21 29764462-29764481. Max. coverage (+): 0. Max coverage (-): 0

Region: chr21 29764482-29764500. Max. coverage (+): 0. Max coverage (-): 0

Region: chr21 29764501-29764519. Max. coverage (+): 0. Max coverage (-): 0

Region: chr21 29764520-29764538. Max. coverage (+): 0.07. Max coverage (-): 0

Region: chr21 29764539-29764557. Max. coverage (+): 7.91. Max coverage (-): 0

Region: chr21 29764558-29764577. Max. coverage (+): 0. Max coverage (-): 0

Region: chr21 29764578-29764596. Max. coverage (+): 0. Max coverage (-): 0

Region: chr21 29764597-29764615. Max. coverage (+): 0. Max coverage (-): 0

Region: chr21 29764616-29764634. Max. coverage (+): 0.54. Max coverage (-): 0

Region: chr21 29764635-29764653. Max. coverage (+): 4.37. Max coverage (-): 0

Region: chr21 29764654-29764672. Max. coverage (+): 4.37. Max coverage (-): 0

Region: chr21 29764673-29764692. Max. coverage (+): 2.59. Max coverage (-): 0

Region: chr21 29764693-29764711. Max. coverage (+): 0. Max coverage (-): 0

Region: chr21 29764712-29764730. Max. coverage (+): 2.73. Max coverage (-): 0

Region: chr21 29764731-29764749. Max. coverage (+): 6.69. Max coverage (-): 0

Region: chr21 29764750-29764768. Max. coverage (+): 0. Max coverage (-): 0

Region: chr21 29764769-29764787. Max. coverage (+): 3.35. Max coverage (-): 0

Region: chr21 29764788-29764807. Max. coverage (+): 2.13. Max coverage (-): 0

Region: chr21 29764808-29764826. Max. coverage (+): 1.69. Max coverage (-): 0

Region: chr21 29764827-29764845. Max. coverage (+): 0. Max coverage (-): 0

Region: chr21 29764846-29764864. Max. coverage (+): 0. Max coverage (-): 0

Region: chr21 29764865-29764883. Max. coverage (+): 5.99. Max coverage (-): 0

Region: chr21 29764884-29764902. Max. coverage (+): 5.99. Max coverage (-): 0

Region: chr21 29764903-29764922. Max. coverage (+): 0. Max coverage (-): 0

Region: chr21 29764923-29764941. Max. coverage (+): 0. Max coverage (-): 0

Region: chr21 29764942-29764960. Max. coverage (+): 5.01. Max coverage (-): 0

Region: chr21 29764961-29764979. Max. coverage (+): 9.82. Max coverage (-): 0

Region: chr21 29764980-29764998. Max. coverage (+): 5.08. Max coverage (-): 0

Region: chr21 29764999-29765017. Max. coverage (+): 1.51. Max coverage (-): 0

Region: chr21 29765018-29765037. Max. coverage (+): 0. Max coverage (-): 0

Region: chr21 29765038-29765056. Max. coverage (+): 3.2. Max coverage (-): 0

Region: chr21 29765057-29765075. Max. coverage (+): 3.2. Max coverage (-): 0

Region: chr21 29765076-29765094. Max. coverage (+): 0. Max coverage (-): 0

Region: chr21 29765095-29765113. Max. coverage (+): 0. Max coverage (-): 0

Region: chr21 29765114-29765133. Max. coverage (+): 2.17. Max coverage (-): 0

Region: chr21 29765134-29765152. Max. coverage (+): 2.04. Max coverage (-): 0

Region: chr21 29765153-29765171. Max. coverage (+): 18.66. Max coverage (-): 0

Region: chr21 29765172-29765190. Max. coverage (+): 6.84. Max coverage (-): 0

Region: chr21 29765191-29765209. Max. coverage (+): 3.67. Max coverage (-): 0

Region: chr21 29765210-29765228. Max. coverage (+): 3.67. Max coverage (-): 0

Region: chr21 29765229-29765248. Max. coverage (+): 0. Max coverage (-): 0

Region: chr21 29765249-29765267. Max. coverage (+): 0. Max coverage (-): 0

Region: chr21 29765268-29765286. Max. coverage (+): 2.53. Max coverage (-): 0

Region: chr21 29765287-29765305. Max. coverage (+): 8.27. Max coverage (-): 0

Region: chr21 29765306-29765324. Max. coverage (+): 6.52. Max coverage (-): 0

Region: chr21 29765325-29765343. Max. coverage (+): 0. Max coverage (-): 0

Region: chr21 29765344-29765363. Max. coverage (+): 9.33. Max coverage (-): 0

Region: chr21 29765364-29765382. Max. coverage (+): 9.33. Max coverage (-): 0

Region: chr21 29765383-29765401. Max. coverage (+): 0.46. Max coverage (-): 0

Region: chr21 29765402-29765420. Max. coverage (+): 0. Max coverage (-): 0

Region: chr21 29765421-29765439. Max. coverage (+): 0. Max coverage (-): 0

Region: chr21 29765440-29765458. Max. coverage (+): 0. Max coverage (-): 0

Region: chr21 29765459-29765478. Max. coverage (+): 0. Max coverage (-): 0

Region: chr21 29765479-29765497. Max. coverage (+): 10.31. Max coverage (-): 0

Region: chr21 29765498-29765516. Max. coverage (+): 3.56. Max coverage (-): 0

Region: chr21 29765517-29765535. Max. coverage (+): 0. Max coverage (-): 0

Region: chr21 29765536-29765554. Max. coverage (+): 1.58. Max coverage (-): 0

Region: chr21 29765555-29765573. Max. coverage (+): 0. Max coverage (-): 0

Region: chr21 29765574-29765593. Max. coverage (+): 0. Max coverage (-): 0

Region: chr21 29765594-29765612. Max. coverage (+): 4.17. Max coverage (-): 0

Region: chr21 29765613-29765631. Max. coverage (+): 4.33. Max coverage (-): 0

Region: chr21 29765632-29765650. Max. coverage (+): 4.33. Max coverage (-): 0

Region: chr21 29765651-29765669. Max. coverage (+): 12.94. Max coverage (-): 0

Region: chr21 29765670-29765689. Max. coverage (+): 12.94. Max coverage (-): 0

Region: chr21 29765690-29765708. Max. coverage (+): 0. Max coverage (-): 0

Region: chr21 29765709-29765727. Max. coverage (+): 1.25. Max coverage (-): 0

Region: chr21 29765728-29765746. Max. coverage (+): 1.25. Max coverage (-): 0

Region: chr21 29765747-29765765. Max. coverage (+): 4.94. Max coverage (-): 0

Region: chr21 29765766-29765784. Max. coverage (+): 4.94. Max coverage (-): 0

Region: chr21 29765785-29765804. Max. coverage (+): 4.03. Max coverage (-): 0

Region: chr21 29765805-29765823. Max. coverage (+): 5.03. Max coverage (-): 0

Region: chr21 29765824-29765842. Max. coverage (+): 5.03. Max coverage (-): 0

Region: chr21 29765843-29765861. Max. coverage (+): 0. Max coverage (-): 0

Region: chr21 29765862-29765880. Max. coverage (+): 0. Max coverage (-): 0

Region: chr21 29765881-29765899. Max. coverage (+): 0.22. Max coverage (-): 0

Region: chr21 29765900-29765919. Max. coverage (+): 4.81. Max coverage (-): 0

Region: chr21 29765920-29765938. Max. coverage (+): 2.65. Max coverage (-): 0

Region: chr21 29765939-29765957. Max. coverage (+): 0. Max coverage (-): 0

Region: chr21 29765958-29765976. Max. coverage (+): 0. Max coverage (-): 0

Region: chr21 29765977-29765995. Max. coverage (+): 1.57. Max coverage (-): 0

Region: chr21 29765996-29766014. Max. coverage (+): 5.57. Max coverage (-): 0

Region: chr21 29766015-29766034. Max. coverage (+): 0.99. Max coverage (-): 0

Region: chr21 29766035-29766053. Max. coverage (+): 0. Max coverage (-): 0

Region: chr21 29766054-29766072. Max. coverage (+): 1.07. Max coverage (-): 0

Region: chr21 29766073-29766091. Max. coverage (+): 1.67. Max coverage (-): 0

Region: chr21 29766092-29766110. Max. coverage (+): 1.67. Max coverage (-): 0

Region: chr21 29766111-29766129. Max. coverage (+): 9.54. Max coverage (-): 0

Region: chr21 29766130-29766149. Max. coverage (+): 0. Max coverage (-): 0

Region: chr21 29766150-29766168. Max. coverage (+): 0. Max coverage (-): 0

Region: chr21 29766169-29766187. Max. coverage (+): 1.07. Max coverage (-): 0

Region: chr21 29766188-29766206. Max. coverage (+): 1.16. Max coverage (-): 0

Region: chr21 29766207-29766225. Max. coverage (+): 7.95. Max coverage (-): 0

Region: chr21 29766226-29766244. Max. coverage (+): 12.56. Max coverage (-): 0

Region: chr21 29766245-29766264. Max. coverage (+): 0.87. Max coverage (-): 0

Region: chr21 29766265-29766283. Max. coverage (+): 0. Max coverage (-): 0

Region: chr21 29766284-29766302. Max. coverage (+): 0. Max coverage (-): 0

Region: chr21 29766303-29766321. Max. coverage (+): 0. Max coverage (-): 0

Region: chr21 29766322-29766340. Max. coverage (+): 22.47. Max coverage (-): 0

Region: chr21 29766341-29766360. Max. coverage (+): 0. Max coverage (-): 0

Region: chr21 29766361-29766379. Max. coverage (+): 0. Max coverage (-): 0

Region: chr21 29766380-29766398. Max. coverage (+): 8.45. Max coverage (-): 0

Region: chr21 29766399-29766417. Max. coverage (+): 18.25. Max coverage (-): 0

Region: chr21 29766418-29766436. Max. coverage (+): 0. Max coverage (-): 0

Region: chr21 29766437-29766455. Max. coverage (+): 7.2. Max coverage (-): 0

Region: chr21 29766456-29766475. Max. coverage (+): 12.69. Max coverage (-): 0

Region: chr21 29766476-29766494. Max. coverage (+): 22.4. Max coverage (-): 0

Region: chr21 29766495-29766513. Max. coverage (+): 17.52. Max coverage (-): 0

Region: chr21 29766514-29766532. Max. coverage (+): 0. Max coverage (-): 0

Region: chr21 29766533-29766551. Max. coverage (+): 0. Max coverage (-): 0

Region: chr21 29766552-29766570. Max. coverage (+): 0. Max coverage (-): 0

Region: chr21 29766571-29766590. Max. coverage (+): 0. Max coverage (-): 0

Region: chr21 29766591-29766609. Max. coverage (+): 0. Max coverage (-): 0

Region: chr21 29766610-29766628. Max. coverage (+): 0. Max coverage (-): 0

Region: chr21 29766629-29766647. Max. coverage (+): 0. Max coverage (-): 0

Region: chr21 29766648-29766666. Max. coverage (+): 0. Max coverage (-): 0

Region: chr21 29766667-29766685. Max. coverage (+): 0. Max coverage (-): 0

Region: chr21 29766686-29766705. Max. coverage (+): 3.18. Max coverage (-): 0

Region: chr21 29766706-29766724. Max. coverage (+): 7.04. Max coverage (-): 0

Region: chr21 29766725-29766743. Max. coverage (+): 7.04. Max coverage (-): 0

Region: chr21 29766744-29766762. Max. coverage (+): 8.93. Max coverage (-): 0

Region: chr21 29766763-29766781. Max. coverage (+): 11.38. Max coverage (-): 0

Region: chr21 29766782-29766800. Max. coverage (+): 17.64. Max coverage (-): 0

Region: chr21 29766801-29766820. Max. coverage (+): 12.72. Max coverage (-): 0

Region: chr21 29766821-29766839. Max. coverage (+): 0. Max coverage (-): 0

Region: chr21 29766840-29766858. Max. coverage (+): 0. Max coverage (-): 0

Region: chr21 29766859-29766877. Max. coverage (+): 0. Max coverage (-): 0

Region: chr21 29766878-29766896. Max. coverage (+): 0. Max coverage (-): 0

Region: chr21 29766897-29766916. Max. coverage (+): 0. Max coverage (-): 0

Region: chr21 29766917-29766935. Max. coverage (+): 0. Max coverage (-): 0

Region: chr21 29766936-29766954. Max. coverage (+): 0. Max coverage (-): 0

Region: chr21 29766955-29766973. Max. coverage (+): 4.53. Max coverage (-): 0

Region: chr21 29766974-29766992. Max. coverage (+): 4.53. Max coverage (-): 0

Region: chr21 29766993-29767011. Max. coverage (+): 0. Max coverage (-): 0

Region: chr21 29767012-29767031. Max. coverage (+): 0. Max coverage (-): 0

Region: chr21 29767032-29767050. Max. coverage (+): 0. Max coverage (-): 0

Region: chr21 29767051-29767069. Max. coverage (+): 0. Max coverage (-): 0

Region: chr21 29767070-29767088. Max. coverage (+): 0. Max coverage (-): 0

Region: chr21 29767089-29767107. Max. coverage (+): 0. Max coverage (-): 0

Region: chr21 29767108-29767126. Max. coverage (+): 0. Max coverage (-): 0

Region: chr21 29767127-29767146. Max. coverage (+): 0. Max coverage (-): 0

Region: chr21 29767147-29767165. Max. coverage (+): 0. Max coverage (-): 0

Region: chr21 29767166-29767184. Max. coverage (+): 0. Max coverage (-): 0

Region: chr21 29767185-29767203. Max. coverage (+): 2.69. Max coverage (-): 0

Region: chr21 29767204-29767222. Max. coverage (+): 0. Max coverage (-): 0

Region: chr21 29767223-29767241. Max. coverage (+): 9.24. Max coverage (-): 0

Region: chr21 29767242-29767261. Max. coverage (+): 0. Max coverage (-): 0

Region: chr21 29767262-29767280. Max. coverage (+): 0. Max coverage (-): 0

Region: chr21 29767281-29767299. Max. coverage (+): 2.59. Max coverage (-): 0

Region: chr21 29767300-29767318. Max. coverage (+): 0. Max coverage (-): 0

Region: chr21 29767319-29767337. Max. coverage (+): 0. Max coverage (-): 0

Region: chr21 29767338-29767356. Max. coverage (+): 0. Max coverage (-): 0

Region: chr21 29767357-29767376. Max. coverage (+): 1.73. Max coverage (-): 0

Region: chr21 29767377-29767395. Max. coverage (+): 2.98. Max coverage (-): 0

Region: chr21 29767396-29767414. Max. coverage (+): 0. Max coverage (-): 0

Region: chr21 29767415-29767433. Max. coverage (+): 0. Max coverage (-): 0

Region: chr21 29767434-29767452. Max. coverage (+): 9.44. Max coverage (-): 0

Region: chr21 29767453-29767472. Max. coverage (+): 9.44. Max coverage (-): 0

Region: chr21 29767473-29767491. Max. coverage (+): 3.47. Max coverage (-): 0

Region: chr21 29767492-29767510. Max. coverage (+): 0.86. Max coverage (-): 0

Region: chr21 29767511-29767529. Max. coverage (+): 0. Max coverage (-): 0

Region: chr21 29767530-29767548. Max. coverage (+): 0. Max coverage (-): 0

Region: chr21 29767549-29767567. Max. coverage (+): 4.96. Max coverage (-): 0

Region: chr21 29767568-29767587. Max. coverage (+): 0. Max coverage (-): 0

Region: chr21 29767588-29767606. Max. coverage (+): 0.82. Max coverage (-): 0

Region: chr21 29767607-29767625. Max. coverage (+): 0. Max coverage (-): 0

Region: chr21 29767626-29767644. Max. coverage (+): 0. Max coverage (-): 0

Region: chr21 29767645-29767663. Max. coverage (+): 0. Max coverage (-): 0

Region: chr21 29767664-29767682. Max. coverage (+): 0.62. Max coverage (-): 0

Region: chr21 29767683-29767702. Max. coverage (+): 0.62. Max coverage (-): 0

Region: chr21 29767703-29767721. Max. coverage (+): 0. Max coverage (-): 0

Region: chr21 29767722-29767740. Max. coverage (+): 0. Max coverage (-): 0

Region: chr21 29767741-29767759. Max. coverage (+): 8.59. Max coverage (-): 0

Region: chr21 29767760-29767778. Max. coverage (+): 0. Max coverage (-): 0

Region: chr21 29767779-29767797. Max. coverage (+): 3.55. Max coverage (-): 0

Region: chr21 29767798-29767817. Max. coverage (+): 4.78. Max coverage (-): 0

Region: chr21 29767818-29767836. Max. coverage (+): 4.78. Max coverage (-): 0

Region: chr21 29767837-29767855. Max. coverage (+): 0. Max coverage (-): 0

Region: chr21 29767856-29767874. Max. coverage (+): 11.52. Max coverage (-): 0

Region: chr21 29767875-29767893. Max. coverage (+): 2.89. Max coverage (-): 0

Region: chr21 29767894-29767912. Max. coverage (+): 5.33. Max coverage (-): 0

Region: chr21 29767913-29767932. Max. coverage (+): 5.33. Max coverage (-): 0

Region: chr21 29767933-29767951. Max. coverage (+): 0. Max coverage (-): 0

Region: chr21 29767952-29767970. Max. coverage (+): 1.45. Max coverage (-): 0

Region: chr21 29767971-29767989. Max. coverage (+): 0. Max coverage (-): 0

Region: chr21 29767990-29768008. Max. coverage (+): 0. Max coverage (-): 0

Region: chr21 29768009-29768027. Max. coverage (+): 0. Max coverage (-): 0

Region: chr21 29768028-29768047. Max. coverage (+): 0. Max coverage (-): 0

Region: chr21 29768048-29768066. Max. coverage (+): 4.11. Max coverage (-): 0

Region: chr21 29768067-29768085. Max. coverage (+): 0. Max coverage (-): 0

Region: chr21 29768086-29768104. Max. coverage (+): 4.75. Max coverage (-): 0

Region: chr21 29768105-29768123. Max. coverage (+): 4.3. Max coverage (-): 0

Region: chr21 29768124-29768143. Max. coverage (+): 0. Max coverage (-): 0

Region: chr21 29768144-29768162. Max. coverage (+): 0. Max coverage (-): 0

Region: chr21 29768163-29768181. Max. coverage (+): 0. Max coverage (-): 0

Region: chr21 29768182-29768200. Max. coverage (+): 0. Max coverage (-): 0

Region: chr21 29768201-29768219. Max. coverage (+): 0. Max coverage (-): 0

Region: chr21 29768220-29768238. Max. coverage (+): 0. Max coverage (-): 0

Region: chr21 29768239-29768258. Max. coverage (+): 0. Max coverage (-): 0

Region: chr21 29768259-29768277. Max. coverage (+): 0. Max coverage (-): 0

Region: chr21 29768278-29768296. Max. coverage (+): 7.01. Max coverage (-): 0

Region: chr21 29768297-29768315. Max. coverage (+): 1.27. Max coverage (-): 0

Region: chr21 29768316-29768334. Max. coverage (+): 0. Max coverage (-): 0

Region: chr21 29768335-29768353. Max. coverage (+): 0. Max coverage (-): 0

Region: chr21 29768354-29768373. Max. coverage (+): 0. Max coverage (-): 0

Region: chr21 29768374-29768392. Max. coverage (+): 0. Max coverage (-): 0

Region: chr21 29768393-29768411. Max. coverage (+): 0. Max coverage (-): 0

Region: chr21 29768412-29768430. Max. coverage (+): 0. Max coverage (-): 0

Region: chr21 29768431-29768449. Max. coverage (+): 0. Max coverage (-): 0

Region: chr21 29768450-29768468. Max. coverage (+): 0. Max coverage (-): 0

Region: chr21 29768469-29768488. Max. coverage (+): 0. Max coverage (-): 0

Region: chr21 29768489-29768507. Max. coverage (+): 0. Max coverage (-): 0

Region: chr21 29768508-29768526. Max. coverage (+): 0. Max coverage (-): 0

Region: chr21 29768527-29768545. Max. coverage (+): 0. Max coverage (-): 0

Region: chr21 29768546-29768564. Max. coverage (+): 0. Max coverage (-): 0

Region: chr21 29768565-29768583. Max. coverage (+): 0. Max coverage (-): 0

Region: chr21 29768584-29768603. Max. coverage (+): 0. Max coverage (-): 0

Region: chr21 29768604-29768622. Max. coverage (+): 0. Max coverage (-): 0

Region: chr21 29768623-29768641. Max. coverage (+): 0. Max coverage (-): 0

Region: chr21 29768642-29768660. Max. coverage (+): 0. Max coverage (-): 0

Region: chr21 29768661-29768679. Max. coverage (+): 0. Max coverage (-): 0

Region: chr21 29768680-29768699. Max. coverage (+): 0. Max coverage (-): 0

Region: chr21 29768700-29768718. Max. coverage (+): 0. Max coverage (-): 0

Region: chr21 29768719-29768737. Max. coverage (+): 0. Max coverage (-): 0

Region: chr21 29768738-29768756. Max. coverage (+): 0. Max coverage (-): 0

Region: chr21 29768757-29768775. Max. coverage (+): 0. Max coverage (-): 0

Region: chr21 29768776-29768794. Max. coverage (+): 0. Max coverage (-): 0

Region: chr21 29768795-29768814. Max. coverage (+): 0. Max coverage (-): 0

Region: chr21 29768815-29768833. Max. coverage (+): 0. Max coverage (-): 0

Region: chr21 29768834-29768852. Max. coverage (+): 0. Max coverage (-): 0

Region: chr21 29768853-29768871. Max. coverage (+): 0. Max coverage (-): 0

Region: chr21 29768872-29768890. Max. coverage (+): 0. Max coverage (-): 0

Region: chr21 29768891-29768909. Max. coverage (+): 0. Max coverage (-): 0

Region: chr21 29768910-29768929. Max. coverage (+): 0. Max coverage (-): 0

Region: chr21 29768930-29768948. Max. coverage (+): 0. Max coverage (-): 0

Region: chr21 29768949-29768967. Max. coverage (+): 0. Max coverage (-): 0

Region: chr21 29768968-29768986. Max. coverage (+): 0. Max coverage (-): 0

Region: chr21 29768987-29769005. Max. coverage (+): 0. Max coverage (-): 0

Region: chr21 29769006-29769024. Max. coverage (+): 0. Max coverage (-): 0

Region: chr21 29769025-29769044. Max. coverage (+): 0. Max coverage (-): 0

Region: chr21 29769045-29769063. Max. coverage (+): 0. Max coverage (-): 0

Region: chr21 29769064-29769082. Max. coverage (+): 0. Max coverage (-): 0

Region: chr21 29769083-29769101. Max. coverage (+): 0. Max coverage (-): 0

Region: chr21 29769102-29769120. Max. coverage (+): 0. Max coverage (-): 0

Region: chr21 29769121-29769139. Max. coverage (+): 0. Max coverage (-): 0

Region: chr21 29769140-29769159. Max. coverage (+): 7.26. Max coverage (-): 0

Region: chr21 29769160-29769178. Max. coverage (+): 7.26. Max coverage (-): 0

Region: chr21 29769179-29769197. Max. coverage (+): 6.06. Max coverage (-): 0

Region: chr21 29769198-29769216. Max. coverage (+): 0. Max coverage (-): 0

Region: chr21 29769217-29769235. Max. coverage (+): 0. Max coverage (-): 0

Region: chr21 29769236-29769254. Max. coverage (+): 0. Max coverage (-): 0

Region: chr21 29769255-29769274. Max. coverage (+): 0. Max coverage (-): 0

Region: chr21 29769275-29769293. Max. coverage (+): 0. Max coverage (-): 0

Region: chr21 29769294-29769312. Max. coverage (+): 0. Max coverage (-): 0

Region: chr21 29769313-29769331. Max. coverage (+): 0. Max coverage (-): 0

Region: chr21 29769332-29769350. Max. coverage (+): 0. Max coverage (-): 0

Region: chr21 29769351-29769370. Max. coverage (+): 0. Max coverage (-): 0

Region: chr21 29769371-29769389. Max. coverage (+): 0. Max coverage (-): 0

Region: chr21 29769390-29769408. Max. coverage (+): 0. Max coverage (-): 0

Region: chr21 29769409-29769427. Max. coverage (+): 0. Max coverage (-): 0

Region: chr21 29769428-29769446. Max. coverage (+): 0. Max coverage (-): 0

Region: chr21 29769447-29769465. Max. coverage (+): 0. Max coverage (-): 0

Region: chr21 29769466-29769485. Max. coverage (+): 0. Max coverage (-): 0

Region: chr21 29769486-29769504. Max. coverage (+): 0. Max coverage (-): 0

Region: chr21 29769505-29769523. Max. coverage (+): 0. Max coverage (-): 0

Region: chr21 29769524-29769542. Max. coverage (+): 0. Max coverage (-): 0

Region: chr21 29769543-29769561. Max. coverage (+): 0. Max coverage (-): 0

Region: chr21 29769562-29769580. Max. coverage (+): 0. Max coverage (-): 0

Region: chr21 29769581-29769600. Max. coverage (+): 0. Max coverage (-): 0

Region: chr21 29769601-29769619. Max. coverage (+): 0. Max coverage (-): 0

Region: chr21 29769620-29769638. Max. coverage (+): 0. Max coverage (-): 0

Region: chr21 29769639-29769657. Max. coverage (+): 0. Max coverage (-): 0

Region: chr21 29769658-29769676. Max. coverage (+): 0. Max coverage (-): 0

Region: chr21 29769677-29769695. Max. coverage (+): 0. Max coverage (-): 0

Region: chr21 29769696-29769715. Max. coverage (+): 0. Max coverage (-): 0

Region: chr21 29769716-29769734. Max. coverage (+): 0. Max coverage (-): 0

Region: chr21 29769735-29769753. Max. coverage (+): 0. Max coverage (-): 0

Region: chr21 29769754-29769772. Max. coverage (+): 0. Max coverage (-): 0

Region: chr21 29769773-29769791. Max. coverage (+): 0. Max coverage (-): 0

Region: chr21 29769792-29769810. Max. coverage (+): 0. Max coverage (-): 0

Region: chr21 29769811-29769830. Max. coverage (+): 0. Max coverage (-): 0

Region: chr21 29769831-29769849. Max. coverage (+): 0. Max coverage (-): 0

Region: chr21 29769850-29769868. Max. coverage (+): 0. Max coverage (-): 0

Region: chr21 29769869-29769887. Max. coverage (+): 0. Max coverage (-): 0

Region: chr21 29769888-29769906. Max. coverage (+): 0. Max coverage (-): 0

Region: chr21 29769907-29769926. Max. coverage (+): 0. Max coverage (-): 0

Region: chr21 29769927-29769945. Max. coverage (+): 0. Max coverage (-): 0

Region: chr21 29769946-29769964. Max. coverage (+): 0. Max coverage (-): 0

Region: chr21 29769965-29769983. Max. coverage (+): 0. Max coverage (-): 0

Region: chr21 29769984-29770002. Max. coverage (+): 0. Max coverage (-): 0

Region: chr21 29770003-29770021. Max. coverage (+): 0. Max coverage (-): 0

Region: chr21 29770022-29770041. Max. coverage (+): 0. Max coverage (-): 0

Region: chr21 29770042-29770060. Max. coverage (+): 0. Max coverage (-): 0

Region: chr21 29770061-29770079. Max. coverage (+): 0. Max coverage (-): 0

Region: chr21 29770080-29770098. Max. coverage (+): 0. Max coverage (-): 0

Region: chr21 29770099-29770117. Max. coverage (+): 0. Max coverage (-): 0

Region: chr21 29770118-29770136. Max. coverage (+): 1.61. Max coverage (-): 0

Region: chr21 29770137-29770156. Max. coverage (+): 2.59. Max coverage (-): 0

Region: chr21 29770157-29770175. Max. coverage (+): 0. Max coverage (-): 0

Region: chr21 29770176-29770194. Max. coverage (+): 0. Max coverage (-): 0

Region: chr21 29770195-29770213. Max. coverage (+): 0. Max coverage (-): 0

Region: chr21 29770214-29770232. Max. coverage (+): 0. Max coverage (-): 0

Region: chr21 29770233-29770251. Max. coverage (+): 0. Max coverage (-): 0

Region: chr21 29770252-29770271. Max. coverage (+): 0. Max coverage (-): 0

Region: chr21 29770272-29770290. Max. coverage (+): 0. Max coverage (-): 0

Region: chr21 29770291-29770309. Max. coverage (+): 0. Max coverage (-): 0

Region: chr21 29770310-29770328. Max. coverage (+): 5.03. Max coverage (-): 0

Region: chr21 29770329-29770347. Max. coverage (+): 3.9. Max coverage (-): 0

Region: chr21 29770348-29770366. Max. coverage (+): 3.9. Max coverage (-): 0

Region: chr21 29770367-29770386. Max. coverage (+): 0. Max coverage (-): 0

Region: chr21 29770387-29770405. Max. coverage (+): 0.66. Max coverage (-): 0

Region: chr21 29770406-29770424. Max. coverage (+): 0. Max coverage (-): 0

Region: chr21 29770425-29770443. Max. coverage (+): 2.62. Max coverage (-): 0

Region: chr21 29770444-29770462. Max. coverage (+): 0. Max coverage (-): 0

Region: chr21 29770463-29770482. Max. coverage (+): 0. Max coverage (-): 0

Region: chr21 29770483-29770501. Max. coverage (+): 0. Max coverage (-): 0

Region: chr21 29770502-29770520. Max. coverage (+): 4.7. Max coverage (-): 0

Region: chr21 29770521-29770539. Max. coverage (+): 0. Max coverage (-): 0

Region: chr21 29770540-29770558. Max. coverage (+): 0.62. Max coverage (-): 0

Region: chr21 29770559-29770577. Max. coverage (+): 0.62. Max coverage (-): 0

Region: chr21 29770578-29770597. Max. coverage (+): 0. Max coverage (-): 0

Region: chr21 29770598-29770616. Max. coverage (+): 0.89. Max coverage (-): 0

Region: chr21 29770617-29770635. Max. coverage (+): 0. Max coverage (-): 0

Region: chr21 29770636-29770654. Max. coverage (+): 2.76. Max coverage (-): 0

Region: chr21 29770655-29770673. Max. coverage (+): 2.31. Max coverage (-): 0

Region: chr21 29770674-29770692. Max. coverage (+): 0.98. Max coverage (-): 0

Region: chr21 29770693-29770712. Max. coverage (+): 1.65. Max coverage (-): 0

Region: chr21 29770713-29770731. Max. coverage (+): 4.55. Max coverage (-): 0

Region: chr21 29770732-29770750. Max. coverage (+): 4.12. Max coverage (-): 0

Region: chr21 29770751-. Max. coverage (+): 0. Max coverage (-): 0

RepeatMasker Color Code

**+**

100-98% Identity

<98-95% Identity

<95-90% Identity

<90-85% Identity

<85-80% Identity

<80-75% Identity

<75-70% Identity

<70% Identity

**-**

Gene Set Color Code

**+**

Gene

Pseudogene

**-**

Topology/Coverage Color Code

Coverage Plus Strand

Coverage Minus Strand

Mainstrand: Plus

Mainstrand: Minus

Complementary Strand

Flanking Region  
(if option -flank >0)

Gene Set Annotation  

**1. (protein coding, ENSBTAG00000003957) Tr:00000005175 Ex:1**: 29766181-29766253 (-)  
**2. (protein coding, ENSBTAG00000003957) Tr:00000005175 Ex:2**: 29765462-29765730 (-)  
**3. (protein coding, ENSBTAG00000003957) Tr:00000005175 Ex:3**: 29764983-29765050 (-)  
**4. (protein coding, ENSBTAG00000003957) Tr:00000005175 Ex:4**: 29763705-29763898 (-)  
**5. (protein coding, ENSBTAG00000003957) Tr:00000005175 Ex:5**: 29762148-29762413 (-)

  
RepeatMasker Annotation  

**1. CHRL1\_BT**: 29761379-29761527 (-), Divergence to consensus: 23.5%  
**2. BOV-A2**: 29762591-29762793 (-), Divergence to consensus: 11.8%  
**3. AT\_rich**: 29766505-29766541 (+), Divergence to consensus: 70.3%  
**4. SINE2-2\_BT**: 29766565-29766677 (-), Divergence to consensus: 22.1%  
**5. L2a**: 29766835-29766916 (-), Divergence to consensus: 34.5%  
**6. MIRc**: 29767032-29767159 (-), Divergence to consensus: 44.5%  
**7. ERV1-2C-LTR\_BT**: 29768355-29768836 (+), Divergence to consensus: 18.9%  
**8. ERV1-2-I\_BT-int**: 29768873-29769533 (+), Divergence to consensus: 21.5%  
**9. MER41\_BT**: 29769512-29769978 (-), Divergence to consensus: 38.5%  
**10. ERV1-2-I\_BT-int**: 29769979-29770093 (+), Divergence to consensus: 17.1%  
**11. ERV1-2-I\_BT-int**: 29770758-29772583 (+), Divergence to consensus: 13.1%

  
Transcription Factor Binding Sites  

**Gata4** (Sequence: AGATAAC (-): 29770509)
