# Supplementary material for: Small RNA sequencing of cryopreserved semen from single bull revealed altered miRNAs and piRNAs expression between High- and Low-motile sperm populations
Source: BMC Genomics. 2017 Jan 4;18:14. doi: 10.1186/s12864-016-3394-7 (PMC5209821; doi:10.1186/s12864-016-3394-7)
Supplement: Additional file 3: — Details for each piRNA clusters found in High Motile (HM) sperm fraction. Genes, repeats, transposable elements and transcription factors binding sites falling within the cluster regions were reported. (ZIP 1896 kb) [file 12864_2016_3394_MOESM3_ESM.zip › 49.html]

piRNA cluster 49


Predicted piRNA cluster no. 49     previous   next
  

Show proTRAC run info
Hide proTRAC run info

================================= proTRAC ====================================  
VERSION: 2.1                                    LAST MODIFIED: 06. October 2015  
  
Please cite:  
Rosenkranz D, Zischler H. proTRAC - a software for probabilistic piRNA cluster  
detection, visualization and analysis. 2012. BMC Bioinformatics 13:5.  
  
and (for proTRAC 2.0 and later):  
Rosenkranz D, Rudloff S, Bastuck K, Ketting RF, Zischler H. Tupaia small RNAs  
provide insights into function and evolution of RNAi-based transposon defense  
in mammals. 2015. RNA 21(5):911-922.  
  
Contact:  
David Rosenkranz  
Institute of Anthropology, small RNA group  
Johannes Gutenberg University Mainz  
email: rosenkranz@uni-mainz.de  
  
You can find the latest proTRAC version at:  
http://sourceforge.net/projects/protrac/files  
http://www.smallRNAgroup-mainz.de/software  
==============================================================================  
  
PARAMETERS:  
Map file: .............../storage/core/barbara/genhome/smallRNA/fertility/Sample\_motile/pirna/Sample\_motile\_26-33\_collapsed.fa.no-dust.map.weighted-10000-1000-b-0  
Genome file: ............/storage/core/barbara/genhome/smallRNA/fertility/Sample\_all/pirna/bt\_311\_chrY.fa  
RepeatMasker annotation: /storage/genomes/bt\_umd31/GCF\_000003055.6\_Bos\_taurus\_UMD\_3.1.1\_repeatMasker\_chr.out  
GeneSet:................./storage/core/barbara/genhome/smallRNA/fertility/Sample\_all/pirna/full.gtf  
  
Significant (p<=0.01) hit density will be calculated based  
on observed hit distribution.  
  
Sliding window size: ........................................ 5000 bp  
Sliding window increament: .................................. 1000 bp  
Normalize each hit by number of genomic hits: ............... 1 [0=no/1=yes]  
Normalize each hit by number of sequence reads: ............. 1 [0=no/1=yes]  
Normalize values (-> per million mapped reads): ............. 1 [0=no/1=yes]  
Min. fraction of hits with 1T(U) or 10A: .................... 0.75  
Alternatively: Min. fraction of hits with 1T(U) and 10A: .... 0.5  
Min. fraction of hits with typical piRNA length: ............ 0.75  
Typical piRNA length: ....................................... 26-33 nt  
Min. size of a piRNA cluster: ............................... 5000 bp.  
Min. number of hits (absolute): ............................. 0  
Min. number of hits (normalized): ........................... 0  
Min. fraction of hits on the mainstrand: .................... 0.75  
Top fraction of mapped sequences (in terms of read counts): . 1%  
Top fraction accounts for max. n% of sequence reads: ........ 90%  
Min. fraction of hits on each arm of a bidirectional cluster: 0.1  
Output image file for each cluster: ......................... 0 [0=no/1=yes]  
Output html file for each cluster: .......................... 1 [0=no/1=yes]  
Output a summary table: ..................................... 1 [0=no/1=yes]  
Output a FASTA file for each cluster (piRNA sequences): ..... 1 [0=no/1=yes]  
Output a FASTA file comprising cluster sequences: ........... 1 [0=no/1=yes]  
Search DNA motifs in clusters: .............................. 1 [0=no/1=yes]  
Output flanking sequences: +/- .............................. 0 bp  
Output ~.pTi file: .......................................... 1 [0=no/1=yes]  
==============================================================================  
  
  
Genome size (without gaps): ............ 2678902517 bp  
Gaps (N/X/-): .......................... 53837044 bp  
Mapped reads: .......................... 658825247023  
Non-identical sequences: ............... 514171  
Genomic hits: .......................... 764233  
Significant densitiy of mapped reads: .. 12867599.5173724 reads/kb

Show proTRAC cluster info
Hide proTRAC cluster info

|  |  |
| --- | --- |
| Location | chr22 |
| Coordinates | 12739964-12753743 |
| Size [bp] | 13780 |
| Sequence hit loci | 260 |
| Mapped reads (normalized) | 336959908.6 |
| Mapped reads (normalized) per kb | 24452823.6 |
| Normalized reads with 1T (1U) | 83.7% |
| Normalized reads with 10A | 34% |
| Normalized reads with length 26-33 nt | 100% |
| Normalized reads on the main strand(s) | 95.6% |
| Predicted directionality | bi:minus-plus (split between 12750181 and 12750924) |

100%

0%

1T (1U)  
reads

10A reads

26-33 nt  
reads

reads on mainstrand

**Either the amount of reads with 1T (1U) OR 10A has to exceed 75% (set with option: -1Tor10A)  
Alternatively the amount of reads with 1T (1U) AND 10A has to exceed 50% (set with option: -1Tand10A)  
Minimum amount of reads with preferred size is 75% (set with option: -pisize)  
Minimum amount of reads on the main strand(s) is 75% (set with option: -clstrand)**

Show read coverage
Hide read coverage

WHAT DO I SEE HERE?  
This chart shows the location of mapped sequence reads within a predicted piRNA cluster. The color refers to the number of genomic hits produced by the sequence read in question. A dark red bar indicates that this sequence read produces many other hits elsewhere in the genome. Many adjacent red or yellow bars can indicate the presence of a multi-copy element such as transposons or rRNA genes. A dark green bar indicates that this sequence read maps uniquely to this locus.

1 hit

2-5 hits

6-10 hits

11-20 hits

21-50 hits

51-100 hits

> 100 hits

chr22

12739964

12753743

Gene Set

RepeatMasker

Mapped  
Reads

41.96

plus strand

minus strand

41.96

Region: chr22 29770752-12739977. Max. coverage (+): 1.22. Max coverage (-): 0

Region: chr22 12739978-12740005. Max. coverage (+): 6.86. Max coverage (-): 0

Region: chr22 12740006-12740032. Max. coverage (+): 4.94. Max coverage (-): 0

Region: chr22 12740033-12740060. Max. coverage (+): 0. Max coverage (-): 0

Region: chr22 12740061-12740088. Max. coverage (+): 0. Max coverage (-): 0

Region: chr22 12740089-12740115. Max. coverage (+): 0. Max coverage (-): 0

Region: chr22 12740116-12740143. Max. coverage (+): 10.34. Max coverage (-): 0

Region: chr22 12740144-12740170. Max. coverage (+): 0. Max coverage (-): 0

Region: chr22 12740171-12740198. Max. coverage (+): 0. Max coverage (-): 0

Region: chr22 12740199-12740225. Max. coverage (+): 0. Max coverage (-): 0

Region: chr22 12740226-12740253. Max. coverage (+): 0. Max coverage (-): 0

Region: chr22 12740254-12740280. Max. coverage (+): 0. Max coverage (-): 0

Region: chr22 12740281-12740308. Max. coverage (+): 0. Max coverage (-): 0

Region: chr22 12740309-12740336. Max. coverage (+): 0. Max coverage (-): 0

Region: chr22 12740337-12740363. Max. coverage (+): 0. Max coverage (-): 0

Region: chr22 12740364-12740391. Max. coverage (+): 3.15. Max coverage (-): 0

Region: chr22 12740392-12740418. Max. coverage (+): 0. Max coverage (-): 0

Region: chr22 12740419-12740446. Max. coverage (+): 0. Max coverage (-): 0

Region: chr22 12740447-12740473. Max. coverage (+): 0. Max coverage (-): 0

Region: chr22 12740474-12740501. Max. coverage (+): 0. Max coverage (-): 0

Region: chr22 12740502-12740528. Max. coverage (+): 0. Max coverage (-): 0

Region: chr22 12740529-12740556. Max. coverage (+): 0. Max coverage (-): 0

Region: chr22 12740557-12740584. Max. coverage (+): 0. Max coverage (-): 0

Region: chr22 12740585-12740611. Max. coverage (+): 0. Max coverage (-): 0

Region: chr22 12740612-12740639. Max. coverage (+): 0. Max coverage (-): 0

Region: chr22 12740640-12740666. Max. coverage (+): 0. Max coverage (-): 0

Region: chr22 12740667-12740694. Max. coverage (+): 0. Max coverage (-): 0

Region: chr22 12740695-12740721. Max. coverage (+): 0. Max coverage (-): 0

Region: chr22 12740722-12740749. Max. coverage (+): 0. Max coverage (-): 0

Region: chr22 12740750-12740777. Max. coverage (+): 0. Max coverage (-): 9.21

Region: chr22 12740778-12740804. Max. coverage (+): 0. Max coverage (-): 9.21

Region: chr22 12740805-12740832. Max. coverage (+): 0. Max coverage (-): 0

Region: chr22 12740833-12740859. Max. coverage (+): 0. Max coverage (-): 2.3

Region: chr22 12740860-12740887. Max. coverage (+): 0. Max coverage (-): 1.48

Region: chr22 12740888-12740914. Max. coverage (+): 0. Max coverage (-): 11.34

Region: chr22 12740915-12740942. Max. coverage (+): 0. Max coverage (-): 5.19

Region: chr22 12740943-12740969. Max. coverage (+): 0. Max coverage (-): 0

Region: chr22 12740970-12740997. Max. coverage (+): 0. Max coverage (-): 0

Region: chr22 12740998-12741025. Max. coverage (+): 0. Max coverage (-): 0

Region: chr22 12741026-12741052. Max. coverage (+): 0. Max coverage (-): 22.66

Region: chr22 12741053-12741080. Max. coverage (+): 0. Max coverage (-): 10.46

Region: chr22 12741081-12741107. Max. coverage (+): 0. Max coverage (-): 5.52

Region: chr22 12741108-12741135. Max. coverage (+): 0. Max coverage (-): 3.65

Region: chr22 12741136-12741162. Max. coverage (+): 0. Max coverage (-): 11.28

Region: chr22 12741163-12741190. Max. coverage (+): 0. Max coverage (-): 9.98

Region: chr22 12741191-12741217. Max. coverage (+): 0. Max coverage (-): 0

Region: chr22 12741218-12741245. Max. coverage (+): 0. Max coverage (-): 23.61

Region: chr22 12741246-12741273. Max. coverage (+): 0. Max coverage (-): 20.3

Region: chr22 12741274-12741300. Max. coverage (+): 0. Max coverage (-): 2.14

Region: chr22 12741301-12741328. Max. coverage (+): 0.81. Max coverage (-): 2.63

Region: chr22 12741329-12741355. Max. coverage (+): 0. Max coverage (-): 12.86

Region: chr22 12741356-12741383. Max. coverage (+): 0. Max coverage (-): 10.73

Region: chr22 12741384-12741410. Max. coverage (+): 0. Max coverage (-): 0

Region: chr22 12741411-12741438. Max. coverage (+): 0. Max coverage (-): 0

Region: chr22 12741439-12741466. Max. coverage (+): 0. Max coverage (-): 0

Region: chr22 12741467-12741493. Max. coverage (+): 0. Max coverage (-): 0

Region: chr22 12741494-12741521. Max. coverage (+): 0. Max coverage (-): 0

Region: chr22 12741522-12741548. Max. coverage (+): 0. Max coverage (-): 0

Region: chr22 12741549-12741576. Max. coverage (+): 0. Max coverage (-): 0

Region: chr22 12741577-12741603. Max. coverage (+): 0. Max coverage (-): 0

Region: chr22 12741604-12741631. Max. coverage (+): 0. Max coverage (-): 0

Region: chr22 12741632-12741658. Max. coverage (+): 0. Max coverage (-): 0

Region: chr22 12741659-12741686. Max. coverage (+): 0. Max coverage (-): 0

Region: chr22 12741687-12741714. Max. coverage (+): 0. Max coverage (-): 0

Region: chr22 12741715-12741741. Max. coverage (+): 0. Max coverage (-): 0

Region: chr22 12741742-12741769. Max. coverage (+): 0. Max coverage (-): 0

Region: chr22 12741770-12741796. Max. coverage (+): 0. Max coverage (-): 0

Region: chr22 12741797-12741824. Max. coverage (+): 0. Max coverage (-): 0

Region: chr22 12741825-12741851. Max. coverage (+): 0. Max coverage (-): 0

Region: chr22 12741852-12741879. Max. coverage (+): 0. Max coverage (-): 0

Region: chr22 12741880-12741906. Max. coverage (+): 0. Max coverage (-): 0

Region: chr22 12741907-12741934. Max. coverage (+): 0. Max coverage (-): 0

Region: chr22 12741935-12741962. Max. coverage (+): 0. Max coverage (-): 0

Region: chr22 12741963-12741989. Max. coverage (+): 0. Max coverage (-): 0

Region: chr22 12741990-12742017. Max. coverage (+): 0. Max coverage (-): 3.18

Region: chr22 12742018-12742044. Max. coverage (+): 0. Max coverage (-): 0

Region: chr22 12742045-12742072. Max. coverage (+): 0. Max coverage (-): 0

Region: chr22 12742073-12742099. Max. coverage (+): 0. Max coverage (-): 0

Region: chr22 12742100-12742127. Max. coverage (+): 0. Max coverage (-): 0

Region: chr22 12742128-12742155. Max. coverage (+): 0. Max coverage (-): 0

Region: chr22 12742156-12742182. Max. coverage (+): 0. Max coverage (-): 0

Region: chr22 12742183-12742210. Max. coverage (+): 0. Max coverage (-): 0

Region: chr22 12742211-12742237. Max. coverage (+): 0. Max coverage (-): 0

Region: chr22 12742238-12742265. Max. coverage (+): 0. Max coverage (-): 0

Region: chr22 12742266-12742292. Max. coverage (+): 0. Max coverage (-): 0

Region: chr22 12742293-12742320. Max. coverage (+): 0. Max coverage (-): 0

Region: chr22 12742321-12742347. Max. coverage (+): 0. Max coverage (-): 0

Region: chr22 12742348-12742375. Max. coverage (+): 0. Max coverage (-): 0

Region: chr22 12742376-12742403. Max. coverage (+): 0. Max coverage (-): 0

Region: chr22 12742404-12742430. Max. coverage (+): 0. Max coverage (-): 0

Region: chr22 12742431-12742458. Max. coverage (+): 0. Max coverage (-): 0

Region: chr22 12742459-12742485. Max. coverage (+): 0. Max coverage (-): 0

Region: chr22 12742486-12742513. Max. coverage (+): 0. Max coverage (-): 0

Region: chr22 12742514-12742540. Max. coverage (+): 0. Max coverage (-): 0

Region: chr22 12742541-12742568. Max. coverage (+): 0. Max coverage (-): 0

Region: chr22 12742569-12742595. Max. coverage (+): 0. Max coverage (-): 0

Region: chr22 12742596-12742623. Max. coverage (+): 0. Max coverage (-): 0

Region: chr22 12742624-12742651. Max. coverage (+): 0. Max coverage (-): 0

Region: chr22 12742652-12742678. Max. coverage (+): 0. Max coverage (-): 0

Region: chr22 12742679-12742706. Max. coverage (+): 0. Max coverage (-): 0

Region: chr22 12742707-12742733. Max. coverage (+): 0. Max coverage (-): 0

Region: chr22 12742734-12742761. Max. coverage (+): 0. Max coverage (-): 0

Region: chr22 12742762-12742788. Max. coverage (+): 0. Max coverage (-): 0

Region: chr22 12742789-12742816. Max. coverage (+): 0. Max coverage (-): 0

Region: chr22 12742817-12742844. Max. coverage (+): 0. Max coverage (-): 0

Region: chr22 12742845-12742871. Max. coverage (+): 0. Max coverage (-): 0

Region: chr22 12742872-12742899. Max. coverage (+): 0. Max coverage (-): 0

Region: chr22 12742900-12742926. Max. coverage (+): 0. Max coverage (-): 0

Region: chr22 12742927-12742954. Max. coverage (+): 0. Max coverage (-): 0

Region: chr22 12742955-12742981. Max. coverage (+): 0. Max coverage (-): 0

Region: chr22 12742982-12743009. Max. coverage (+): 0. Max coverage (-): 0

Region: chr22 12743010-12743036. Max. coverage (+): 0. Max coverage (-): 0

Region: chr22 12743037-12743064. Max. coverage (+): 0. Max coverage (-): 0

Region: chr22 12743065-12743092. Max. coverage (+): 0. Max coverage (-): 0

Region: chr22 12743093-12743119. Max. coverage (+): 0. Max coverage (-): 0

Region: chr22 12743120-12743147. Max. coverage (+): 0. Max coverage (-): 0

Region: chr22 12743148-12743174. Max. coverage (+): 0. Max coverage (-): 0

Region: chr22 12743175-12743202. Max. coverage (+): 0. Max coverage (-): 0

Region: chr22 12743203-12743229. Max. coverage (+): 0. Max coverage (-): 0

Region: chr22 12743230-12743257. Max. coverage (+): 0. Max coverage (-): 0

Region: chr22 12743258-12743284. Max. coverage (+): 0. Max coverage (-): 0

Region: chr22 12743285-12743312. Max. coverage (+): 0. Max coverage (-): 0

Region: chr22 12743313-12743340. Max. coverage (+): 0. Max coverage (-): 0

Region: chr22 12743341-12743367. Max. coverage (+): 0. Max coverage (-): 0

Region: chr22 12743368-12743395. Max. coverage (+): 0. Max coverage (-): 0

Region: chr22 12743396-12743422. Max. coverage (+): 0. Max coverage (-): 0

Region: chr22 12743423-12743450. Max. coverage (+): 0. Max coverage (-): 0

Region: chr22 12743451-12743477. Max. coverage (+): 0. Max coverage (-): 0

Region: chr22 12743478-12743505. Max. coverage (+): 0. Max coverage (-): 0

Region: chr22 12743506-12743533. Max. coverage (+): 0. Max coverage (-): 0

Region: chr22 12743534-12743560. Max. coverage (+): 0. Max coverage (-): 0

Region: chr22 12743561-12743588. Max. coverage (+): 0. Max coverage (-): 0

Region: chr22 12743589-12743615. Max. coverage (+): 0. Max coverage (-): 0

Region: chr22 12743616-12743643. Max. coverage (+): 0. Max coverage (-): 0

Region: chr22 12743644-12743670. Max. coverage (+): 0. Max coverage (-): 0

Region: chr22 12743671-12743698. Max. coverage (+): 0. Max coverage (-): 0

Region: chr22 12743699-12743725. Max. coverage (+): 0. Max coverage (-): 0

Region: chr22 12743726-12743753. Max. coverage (+): 0. Max coverage (-): 0

Region: chr22 12743754-12743781. Max. coverage (+): 0. Max coverage (-): 0

Region: chr22 12743782-12743808. Max. coverage (+): 0. Max coverage (-): 0

Region: chr22 12743809-12743836. Max. coverage (+): 0. Max coverage (-): 0

Region: chr22 12743837-12743863. Max. coverage (+): 0. Max coverage (-): 0

Region: chr22 12743864-12743891. Max. coverage (+): 0. Max coverage (-): 0

Region: chr22 12743892-12743918. Max. coverage (+): 0. Max coverage (-): 0

Region: chr22 12743919-12743946. Max. coverage (+): 0. Max coverage (-): 0

Region: chr22 12743947-12743973. Max. coverage (+): 0. Max coverage (-): 0

Region: chr22 12743974-12744001. Max. coverage (+): 0. Max coverage (-): 0

Region: chr22 12744002-12744029. Max. coverage (+): 0. Max coverage (-): 0

Region: chr22 12744030-12744056. Max. coverage (+): 0. Max coverage (-): 0

Region: chr22 12744057-12744084. Max. coverage (+): 0. Max coverage (-): 0

Region: chr22 12744085-12744111. Max. coverage (+): 0. Max coverage (-): 0

Region: chr22 12744112-12744139. Max. coverage (+): 0. Max coverage (-): 0

Region: chr22 12744140-12744166. Max. coverage (+): 0. Max coverage (-): 0

Region: chr22 12744167-12744194. Max. coverage (+): 0. Max coverage (-): 0

Region: chr22 12744195-12744222. Max. coverage (+): 0. Max coverage (-): 0

Region: chr22 12744223-12744249. Max. coverage (+): 0. Max coverage (-): 0

Region: chr22 12744250-12744277. Max. coverage (+): 0. Max coverage (-): 0

Region: chr22 12744278-12744304. Max. coverage (+): 0. Max coverage (-): 0

Region: chr22 12744305-12744332. Max. coverage (+): 0. Max coverage (-): 0

Region: chr22 12744333-12744359. Max. coverage (+): 0. Max coverage (-): 0

Region: chr22 12744360-12744387. Max. coverage (+): 0. Max coverage (-): 0

Region: chr22 12744388-12744414. Max. coverage (+): 0. Max coverage (-): 0

Region: chr22 12744415-12744442. Max. coverage (+): 0. Max coverage (-): 0

Region: chr22 12744443-12744470. Max. coverage (+): 0. Max coverage (-): 0

Region: chr22 12744471-12744497. Max. coverage (+): 0. Max coverage (-): 0

Region: chr22 12744498-12744525. Max. coverage (+): 0. Max coverage (-): 0

Region: chr22 12744526-12744552. Max. coverage (+): 0. Max coverage (-): 0

Region: chr22 12744553-12744580. Max. coverage (+): 0. Max coverage (-): 0

Region: chr22 12744581-12744607. Max. coverage (+): 0. Max coverage (-): 0

Region: chr22 12744608-12744635. Max. coverage (+): 0. Max coverage (-): 0

Region: chr22 12744636-12744662. Max. coverage (+): 0. Max coverage (-): 0

Region: chr22 12744663-12744690. Max. coverage (+): 0. Max coverage (-): 0

Region: chr22 12744691-12744718. Max. coverage (+): 0. Max coverage (-): 0

Region: chr22 12744719-12744745. Max. coverage (+): 0. Max coverage (-): 0

Region: chr22 12744746-12744773. Max. coverage (+): 0. Max coverage (-): 0

Region: chr22 12744774-12744800. Max. coverage (+): 0. Max coverage (-): 0

Region: chr22 12744801-12744828. Max. coverage (+): 0. Max coverage (-): 0

Region: chr22 12744829-12744855. Max. coverage (+): 0. Max coverage (-): 0

Region: chr22 12744856-12744883. Max. coverage (+): 0. Max coverage (-): 0

Region: chr22 12744884-12744911. Max. coverage (+): 0. Max coverage (-): 0

Region: chr22 12744912-12744938. Max. coverage (+): 0. Max coverage (-): 0

Region: chr22 12744939-12744966. Max. coverage (+): 0. Max coverage (-): 0

Region: chr22 12744967-12744993. Max. coverage (+): 0. Max coverage (-): 0

Region: chr22 12744994-12745021. Max. coverage (+): 0. Max coverage (-): 0

Region: chr22 12745022-12745048. Max. coverage (+): 0. Max coverage (-): 0

Region: chr22 12745049-12745076. Max. coverage (+): 0. Max coverage (-): 0

Region: chr22 12745077-12745103. Max. coverage (+): 0. Max coverage (-): 0

Region: chr22 12745104-12745131. Max. coverage (+): 0. Max coverage (-): 0

Region: chr22 12745132-12745159. Max. coverage (+): 0. Max coverage (-): 0

Region: chr22 12745160-12745186. Max. coverage (+): 0. Max coverage (-): 0

Region: chr22 12745187-12745214. Max. coverage (+): 0. Max coverage (-): 0

Region: chr22 12745215-12745241. Max. coverage (+): 0. Max coverage (-): 0

Region: chr22 12745242-12745269. Max. coverage (+): 0. Max coverage (-): 0

Region: chr22 12745270-12745296. Max. coverage (+): 0. Max coverage (-): 0

Region: chr22 12745297-12745324. Max. coverage (+): 0. Max coverage (-): 0

Region: chr22 12745325-12745351. Max. coverage (+): 0. Max coverage (-): 0

Region: chr22 12745352-12745379. Max. coverage (+): 0. Max coverage (-): 0

Region: chr22 12745380-12745407. Max. coverage (+): 0. Max coverage (-): 0

Region: chr22 12745408-12745434. Max. coverage (+): 0. Max coverage (-): 0

Region: chr22 12745435-12745462. Max. coverage (+): 0. Max coverage (-): 0

Region: chr22 12745463-12745489. Max. coverage (+): 0. Max coverage (-): 0

Region: chr22 12745490-12745517. Max. coverage (+): 0. Max coverage (-): 0

Region: chr22 12745518-12745544. Max. coverage (+): 0. Max coverage (-): 0

Region: chr22 12745545-12745572. Max. coverage (+): 0. Max coverage (-): 0

Region: chr22 12745573-12745600. Max. coverage (+): 0. Max coverage (-): 0

Region: chr22 12745601-12745627. Max. coverage (+): 0. Max coverage (-): 0

Region: chr22 12745628-12745655. Max. coverage (+): 0. Max coverage (-): 0

Region: chr22 12745656-12745682. Max. coverage (+): 0. Max coverage (-): 0

Region: chr22 12745683-12745710. Max. coverage (+): 0. Max coverage (-): 0

Region: chr22 12745711-12745737. Max. coverage (+): 0. Max coverage (-): 0

Region: chr22 12745738-12745765. Max. coverage (+): 0. Max coverage (-): 0

Region: chr22 12745766-12745792. Max. coverage (+): 0. Max coverage (-): 0

Region: chr22 12745793-12745820. Max. coverage (+): 0. Max coverage (-): 0

Region: chr22 12745821-12745848. Max. coverage (+): 0. Max coverage (-): 0

Region: chr22 12745849-12745875. Max. coverage (+): 0. Max coverage (-): 0

Region: chr22 12745876-12745903. Max. coverage (+): 0. Max coverage (-): 0.69

Region: chr22 12745904-12745930. Max. coverage (+): 0. Max coverage (-): 0

Region: chr22 12745931-12745958. Max. coverage (+): 0. Max coverage (-): 0

Region: chr22 12745959-12745985. Max. coverage (+): 0. Max coverage (-): 0

Region: chr22 12745986-12746013. Max. coverage (+): 0. Max coverage (-): 0

Region: chr22 12746014-12746040. Max. coverage (+): 0. Max coverage (-): 0

Region: chr22 12746041-12746068. Max. coverage (+): 0. Max coverage (-): 0

Region: chr22 12746069-12746096. Max. coverage (+): 0. Max coverage (-): 4.16

Region: chr22 12746097-12746123. Max. coverage (+): 0. Max coverage (-): 4.16

Region: chr22 12746124-12746151. Max. coverage (+): 0. Max coverage (-): 0

Region: chr22 12746152-12746178. Max. coverage (+): 0. Max coverage (-): 0

Region: chr22 12746179-12746206. Max. coverage (+): 0. Max coverage (-): 0

Region: chr22 12746207-12746233. Max. coverage (+): 0. Max coverage (-): 0

Region: chr22 12746234-12746261. Max. coverage (+): 0. Max coverage (-): 0

Region: chr22 12746262-12746289. Max. coverage (+): 0. Max coverage (-): 0

Region: chr22 12746290-12746316. Max. coverage (+): 0. Max coverage (-): 0

Region: chr22 12746317-12746344. Max. coverage (+): 0. Max coverage (-): 0

Region: chr22 12746345-12746371. Max. coverage (+): 0. Max coverage (-): 0

Region: chr22 12746372-12746399. Max. coverage (+): 0. Max coverage (-): 0

Region: chr22 12746400-12746426. Max. coverage (+): 0. Max coverage (-): 4.39

Region: chr22 12746427-12746454. Max. coverage (+): 0. Max coverage (-): 0

Region: chr22 12746455-12746481. Max. coverage (+): 0. Max coverage (-): 0

Region: chr22 12746482-12746509. Max. coverage (+): 0. Max coverage (-): 0

Region: chr22 12746510-12746537. Max. coverage (+): 0. Max coverage (-): 0

Region: chr22 12746538-12746564. Max. coverage (+): 0. Max coverage (-): 0

Region: chr22 12746565-12746592. Max. coverage (+): 0. Max coverage (-): 0

Region: chr22 12746593-12746619. Max. coverage (+): 0. Max coverage (-): 0

Region: chr22 12746620-12746647. Max. coverage (+): 0. Max coverage (-): 0

Region: chr22 12746648-12746674. Max. coverage (+): 0. Max coverage (-): 0

Region: chr22 12746675-12746702. Max. coverage (+): 0. Max coverage (-): 0

Region: chr22 12746703-12746729. Max. coverage (+): 0. Max coverage (-): 0

Region: chr22 12746730-12746757. Max. coverage (+): 0. Max coverage (-): 0

Region: chr22 12746758-12746785. Max. coverage (+): 0. Max coverage (-): 0

Region: chr22 12746786-12746812. Max. coverage (+): 0. Max coverage (-): 0

Region: chr22 12746813-12746840. Max. coverage (+): 0. Max coverage (-): 0

Region: chr22 12746841-12746867. Max. coverage (+): 0. Max coverage (-): 0

Region: chr22 12746868-12746895. Max. coverage (+): 0. Max coverage (-): 0

Region: chr22 12746896-12746922. Max. coverage (+): 0. Max coverage (-): 0

Region: chr22 12746923-12746950. Max. coverage (+): 0. Max coverage (-): 0

Region: chr22 12746951-12746978. Max. coverage (+): 0. Max coverage (-): 0

Region: chr22 12746979-12747005. Max. coverage (+): 0. Max coverage (-): 0

Region: chr22 12747006-12747033. Max. coverage (+): 0. Max coverage (-): 0

Region: chr22 12747034-12747060. Max. coverage (+): 0. Max coverage (-): 0

Region: chr22 12747061-12747088. Max. coverage (+): 0. Max coverage (-): 0

Region: chr22 12747089-12747115. Max. coverage (+): 0. Max coverage (-): 0

Region: chr22 12747116-12747143. Max. coverage (+): 0. Max coverage (-): 0

Region: chr22 12747144-12747170. Max. coverage (+): 0. Max coverage (-): 5.11

Region: chr22 12747171-12747198. Max. coverage (+): 0. Max coverage (-): 0

Region: chr22 12747199-12747226. Max. coverage (+): 0. Max coverage (-): 0

Region: chr22 12747227-12747253. Max. coverage (+): 0. Max coverage (-): 0

Region: chr22 12747254-12747281. Max. coverage (+): 0. Max coverage (-): 0

Region: chr22 12747282-12747308. Max. coverage (+): 0. Max coverage (-): 0

Region: chr22 12747309-12747336. Max. coverage (+): 0. Max coverage (-): 0

Region: chr22 12747337-12747363. Max. coverage (+): 0. Max coverage (-): 0

Region: chr22 12747364-12747391. Max. coverage (+): 0. Max coverage (-): 0

Region: chr22 12747392-12747418. Max. coverage (+): 0. Max coverage (-): 0

Region: chr22 12747419-12747446. Max. coverage (+): 0. Max coverage (-): 0

Region: chr22 12747447-12747474. Max. coverage (+): 0. Max coverage (-): 0

Region: chr22 12747475-12747501. Max. coverage (+): 0. Max coverage (-): 0

Region: chr22 12747502-12747529. Max. coverage (+): 0. Max coverage (-): 0

Region: chr22 12747530-12747556. Max. coverage (+): 0. Max coverage (-): 0

Region: chr22 12747557-12747584. Max. coverage (+): 0. Max coverage (-): 0

Region: chr22 12747585-12747611. Max. coverage (+): 0. Max coverage (-): 0

Region: chr22 12747612-12747639. Max. coverage (+): 0. Max coverage (-): 0

Region: chr22 12747640-12747667. Max. coverage (+): 0. Max coverage (-): 0

Region: chr22 12747668-12747694. Max. coverage (+): 0. Max coverage (-): 0

Region: chr22 12747695-12747722. Max. coverage (+): 0. Max coverage (-): 0

Region: chr22 12747723-12747749. Max. coverage (+): 0. Max coverage (-): 0

Region: chr22 12747750-12747777. Max. coverage (+): 0. Max coverage (-): 0

Region: chr22 12747778-12747804. Max. coverage (+): 0. Max coverage (-): 0

Region: chr22 12747805-12747832. Max. coverage (+): 0. Max coverage (-): 0

Region: chr22 12747833-12747859. Max. coverage (+): 0. Max coverage (-): 0

Region: chr22 12747860-12747887. Max. coverage (+): 0. Max coverage (-): 0

Region: chr22 12747888-12747915. Max. coverage (+): 0. Max coverage (-): 0

Region: chr22 12747916-12747942. Max. coverage (+): 0. Max coverage (-): 0

Region: chr22 12747943-12747970. Max. coverage (+): 0. Max coverage (-): 0

Region: chr22 12747971-12747997. Max. coverage (+): 0. Max coverage (-): 0

Region: chr22 12747998-12748025. Max. coverage (+): 0. Max coverage (-): 0

Region: chr22 12748026-12748052. Max. coverage (+): 0. Max coverage (-): 0

Region: chr22 12748053-12748080. Max. coverage (+): 0. Max coverage (-): 0

Region: chr22 12748081-12748107. Max. coverage (+): 0. Max coverage (-): 0

Region: chr22 12748108-12748135. Max. coverage (+): 0. Max coverage (-): 0

Region: chr22 12748136-12748163. Max. coverage (+): 0. Max coverage (-): 0

Region: chr22 12748164-12748190. Max. coverage (+): 0. Max coverage (-): 0

Region: chr22 12748191-12748218. Max. coverage (+): 0. Max coverage (-): 0

Region: chr22 12748219-12748245. Max. coverage (+): 0. Max coverage (-): 0

Region: chr22 12748246-12748273. Max. coverage (+): 0. Max coverage (-): 20.54

Region: chr22 12748274-12748300. Max. coverage (+): 0. Max coverage (-): 15.72

Region: chr22 12748301-12748328. Max. coverage (+): 0. Max coverage (-): 12.36

Region: chr22 12748329-12748356. Max. coverage (+): 0. Max coverage (-): 9.63

Region: chr22 12748357-12748383. Max. coverage (+): 0. Max coverage (-): 0

Region: chr22 12748384-12748411. Max. coverage (+): 0. Max coverage (-): 0

Region: chr22 12748412-12748438. Max. coverage (+): 0. Max coverage (-): 0

Region: chr22 12748439-12748466. Max. coverage (+): 0. Max coverage (-): 0

Region: chr22 12748467-12748493. Max. coverage (+): 0. Max coverage (-): 0

Region: chr22 12748494-12748521. Max. coverage (+): 0. Max coverage (-): 0

Region: chr22 12748522-12748548. Max. coverage (+): 0. Max coverage (-): 0

Region: chr22 12748549-12748576. Max. coverage (+): 0. Max coverage (-): 0

Region: chr22 12748577-12748604. Max. coverage (+): 0. Max coverage (-): 0

Region: chr22 12748605-12748631. Max. coverage (+): 0. Max coverage (-): 0

Region: chr22 12748632-12748659. Max. coverage (+): 0. Max coverage (-): 0

Region: chr22 12748660-12748686. Max. coverage (+): 0. Max coverage (-): 0

Region: chr22 12748687-12748714. Max. coverage (+): 0. Max coverage (-): 0

Region: chr22 12748715-12748741. Max. coverage (+): 0. Max coverage (-): 0

Region: chr22 12748742-12748769. Max. coverage (+): 0. Max coverage (-): 0

Region: chr22 12748770-12748796. Max. coverage (+): 0. Max coverage (-): 0.83

Region: chr22 12748797-12748824. Max. coverage (+): 0. Max coverage (-): 0

Region: chr22 12748825-12748852. Max. coverage (+): 0. Max coverage (-): 0

Region: chr22 12748853-12748879. Max. coverage (+): 0. Max coverage (-): 0

Region: chr22 12748880-12748907. Max. coverage (+): 0. Max coverage (-): 0

Region: chr22 12748908-12748934. Max. coverage (+): 0. Max coverage (-): 0

Region: chr22 12748935-12748962. Max. coverage (+): 0. Max coverage (-): 0

Region: chr22 12748963-12748989. Max. coverage (+): 0. Max coverage (-): 0

Region: chr22 12748990-12749017. Max. coverage (+): 0. Max coverage (-): 0

Region: chr22 12749018-12749045. Max. coverage (+): 0. Max coverage (-): 0

Region: chr22 12749046-12749072. Max. coverage (+): 0. Max coverage (-): 0

Region: chr22 12749073-12749100. Max. coverage (+): 0. Max coverage (-): 0

Region: chr22 12749101-12749127. Max. coverage (+): 0. Max coverage (-): 0

Region: chr22 12749128-12749155. Max. coverage (+): 0. Max coverage (-): 0

Region: chr22 12749156-12749182. Max. coverage (+): 0. Max coverage (-): 0

Region: chr22 12749183-12749210. Max. coverage (+): 0. Max coverage (-): 0

Region: chr22 12749211-12749237. Max. coverage (+): 0. Max coverage (-): 0

Region: chr22 12749238-12749265. Max. coverage (+): 0. Max coverage (-): 2

Region: chr22 12749266-12749293. Max. coverage (+): 0. Max coverage (-): 2.81

Region: chr22 12749294-12749320. Max. coverage (+): 0. Max coverage (-): 0

Region: chr22 12749321-12749348. Max. coverage (+): 0. Max coverage (-): 0

Region: chr22 12749349-12749375. Max. coverage (+): 0. Max coverage (-): 6.15

Region: chr22 12749376-12749403. Max. coverage (+): 0. Max coverage (-): 22.06

Region: chr22 12749404-12749430. Max. coverage (+): 0. Max coverage (-): 34.97

Region: chr22 12749431-12749458. Max. coverage (+): 0. Max coverage (-): 6.9

Region: chr22 12749459-12749485. Max. coverage (+): 0. Max coverage (-): 0

Region: chr22 12749486-12749513. Max. coverage (+): 0. Max coverage (-): 0

Region: chr22 12749514-12749541. Max. coverage (+): 0. Max coverage (-): 0

Region: chr22 12749542-12749568. Max. coverage (+): 0. Max coverage (-): 0

Region: chr22 12749569-12749596. Max. coverage (+): 0. Max coverage (-): 0

Region: chr22 12749597-12749623. Max. coverage (+): 0. Max coverage (-): 9.7

Region: chr22 12749624-12749651. Max. coverage (+): 0. Max coverage (-): 5.84

Region: chr22 12749652-12749678. Max. coverage (+): 0. Max coverage (-): 4.52

Region: chr22 12749679-12749706. Max. coverage (+): 0. Max coverage (-): 2.8

Region: chr22 12749707-12749734. Max. coverage (+): 0. Max coverage (-): 0.57

Region: chr22 12749735-12749761. Max. coverage (+): 0. Max coverage (-): 1.22

Region: chr22 12749762-12749789. Max. coverage (+): 0. Max coverage (-): 15.28

Region: chr22 12749790-12749816. Max. coverage (+): 0. Max coverage (-): 0

Region: chr22 12749817-12749844. Max. coverage (+): 0. Max coverage (-): 41.96

Region: chr22 12749845-12749871. Max. coverage (+): 0. Max coverage (-): 6.42

Region: chr22 12749872-12749899. Max. coverage (+): 0. Max coverage (-): 1.85

Region: chr22 12749900-12749926. Max. coverage (+): 0. Max coverage (-): 0

Region: chr22 12749927-12749954. Max. coverage (+): 0. Max coverage (-): 0

Region: chr22 12749955-12749982. Max. coverage (+): 0. Max coverage (-): 0

Region: chr22 12749983-12750009. Max. coverage (+): 0. Max coverage (-): 3.76

Region: chr22 12750010-12750037. Max. coverage (+): 0. Max coverage (-): 3.76

Region: chr22 12750038-12750064. Max. coverage (+): 0. Max coverage (-): 0

Region: chr22 12750065-12750092. Max. coverage (+): 0. Max coverage (-): 2.71

Region: chr22 12750093-12750119. Max. coverage (+): 0. Max coverage (-): 0

Region: chr22 12750120-12750147. Max. coverage (+): 0. Max coverage (-): 0

Region: chr22 12750148-12750174. Max. coverage (+): 0. Max coverage (-): 1.73

Region: chr22 12750175-12750202. Max. coverage (+): 0. Max coverage (-): 1.73

Region: chr22 12750203-12750230. Max. coverage (+): 0. Max coverage (-): 0

Region: chr22 12750231-12750257. Max. coverage (+): 0. Max coverage (-): 0

Region: chr22 12750258-12750285. Max. coverage (+): 0. Max coverage (-): 0

Region: chr22 12750286-12750312. Max. coverage (+): 0. Max coverage (-): 0

Region: chr22 12750313-12750340. Max. coverage (+): 0. Max coverage (-): 0

Region: chr22 12750341-12750367. Max. coverage (+): 0. Max coverage (-): 0

Region: chr22 12750368-12750395. Max. coverage (+): 0. Max coverage (-): 0

Region: chr22 12750396-12750423. Max. coverage (+): 0. Max coverage (-): 0

Region: chr22 12750424-12750450. Max. coverage (+): 0. Max coverage (-): 0

Region: chr22 12750451-12750478. Max. coverage (+): 0. Max coverage (-): 0

Region: chr22 12750479-12750505. Max. coverage (+): 0. Max coverage (-): 0

Region: chr22 12750506-12750533. Max. coverage (+): 0. Max coverage (-): 0

Region: chr22 12750534-12750560. Max. coverage (+): 0. Max coverage (-): 0

Region: chr22 12750561-12750588. Max. coverage (+): 0. Max coverage (-): 0

Region: chr22 12750589-12750615. Max. coverage (+): 0. Max coverage (-): 0

Region: chr22 12750616-12750643. Max. coverage (+): 0. Max coverage (-): 0

Region: chr22 12750644-12750671. Max. coverage (+): 0. Max coverage (-): 0

Region: chr22 12750672-12750698. Max. coverage (+): 0. Max coverage (-): 0

Region: chr22 12750699-12750726. Max. coverage (+): 0. Max coverage (-): 0

Region: chr22 12750727-12750753. Max. coverage (+): 0. Max coverage (-): 0

Region: chr22 12750754-12750781. Max. coverage (+): 0. Max coverage (-): 0

Region: chr22 12750782-12750808. Max. coverage (+): 0. Max coverage (-): 0

Region: chr22 12750809-12750836. Max. coverage (+): 0. Max coverage (-): 0

Region: chr22 12750837-12750863. Max. coverage (+): 0. Max coverage (-): 0

Region: chr22 12750864-12750891. Max. coverage (+): 0. Max coverage (-): 0

Region: chr22 12750892-12750919. Max. coverage (+): 0. Max coverage (-): 0

Region: chr22 12750920-12750946. Max. coverage (+): 1.38. Max coverage (-): 0

Region: chr22 12750947-12750974. Max. coverage (+): 0. Max coverage (-): 0

Region: chr22 12750975-12751001. Max. coverage (+): 0. Max coverage (-): 0

Region: chr22 12751002-12751029. Max. coverage (+): 0. Max coverage (-): 0

Region: chr22 12751030-12751056. Max. coverage (+): 0. Max coverage (-): 0

Region: chr22 12751057-12751084. Max. coverage (+): 1.79. Max coverage (-): 0

Region: chr22 12751085-12751112. Max. coverage (+): 3.88. Max coverage (-): 0

Region: chr22 12751113-12751139. Max. coverage (+): 0. Max coverage (-): 0

Region: chr22 12751140-12751167. Max. coverage (+): 0. Max coverage (-): 0

Region: chr22 12751168-12751194. Max. coverage (+): 0. Max coverage (-): 0

Region: chr22 12751195-12751222. Max. coverage (+): 0. Max coverage (-): 0

Region: chr22 12751223-12751249. Max. coverage (+): 0. Max coverage (-): 0

Region: chr22 12751250-12751277. Max. coverage (+): 0. Max coverage (-): 0

Region: chr22 12751278-12751304. Max. coverage (+): 0. Max coverage (-): 0

Region: chr22 12751305-12751332. Max. coverage (+): 0. Max coverage (-): 0

Region: chr22 12751333-12751360. Max. coverage (+): 0. Max coverage (-): 0

Region: chr22 12751361-12751387. Max. coverage (+): 0. Max coverage (-): 0

Region: chr22 12751388-12751415. Max. coverage (+): 0. Max coverage (-): 0

Region: chr22 12751416-12751442. Max. coverage (+): 0. Max coverage (-): 0

Region: chr22 12751443-12751470. Max. coverage (+): 0. Max coverage (-): 0

Region: chr22 12751471-12751497. Max. coverage (+): 0. Max coverage (-): 0

Region: chr22 12751498-12751525. Max. coverage (+): 0. Max coverage (-): 0

Region: chr22 12751526-12751552. Max. coverage (+): 0. Max coverage (-): 0

Region: chr22 12751553-12751580. Max. coverage (+): 0. Max coverage (-): 0

Region: chr22 12751581-12751608. Max. coverage (+): 0. Max coverage (-): 0

Region: chr22 12751609-12751635. Max. coverage (+): 0. Max coverage (-): 0

Region: chr22 12751636-12751663. Max. coverage (+): 0. Max coverage (-): 0

Region: chr22 12751664-12751690. Max. coverage (+): 0. Max coverage (-): 0

Region: chr22 12751691-12751718. Max. coverage (+): 0. Max coverage (-): 0

Region: chr22 12751719-12751745. Max. coverage (+): 0. Max coverage (-): 0

Region: chr22 12751746-12751773. Max. coverage (+): 0. Max coverage (-): 0

Region: chr22 12751774-12751801. Max. coverage (+): 0. Max coverage (-): 0

Region: chr22 12751802-12751828. Max. coverage (+): 0. Max coverage (-): 0

Region: chr22 12751829-12751856. Max. coverage (+): 0. Max coverage (-): 0

Region: chr22 12751857-12751883. Max. coverage (+): 0. Max coverage (-): 0

Region: chr22 12751884-12751911. Max. coverage (+): 0. Max coverage (-): 0

Region: chr22 12751912-12751938. Max. coverage (+): 0. Max coverage (-): 0

Region: chr22 12751939-12751966. Max. coverage (+): 0. Max coverage (-): 0

Region: chr22 12751967-12751993. Max. coverage (+): 0. Max coverage (-): 0

Region: chr22 12751994-12752021. Max. coverage (+): 0. Max coverage (-): 0

Region: chr22 12752022-12752049. Max. coverage (+): 0. Max coverage (-): 0

Region: chr22 12752050-12752076. Max. coverage (+): 0. Max coverage (-): 0

Region: chr22 12752077-12752104. Max. coverage (+): 0. Max coverage (-): 0

Region: chr22 12752105-12752131. Max. coverage (+): 0. Max coverage (-): 0

Region: chr22 12752132-12752159. Max. coverage (+): 0. Max coverage (-): 0

Region: chr22 12752160-12752186. Max. coverage (+): 0. Max coverage (-): 0

Region: chr22 12752187-12752214. Max. coverage (+): 0. Max coverage (-): 0

Region: chr22 12752215-12752241. Max. coverage (+): 0. Max coverage (-): 0

Region: chr22 12752242-12752269. Max. coverage (+): 0. Max coverage (-): 0

Region: chr22 12752270-12752297. Max. coverage (+): 7.12. Max coverage (-): 0

Region: chr22 12752298-12752324. Max. coverage (+): 0. Max coverage (-): 0

Region: chr22 12752325-12752352. Max. coverage (+): 0. Max coverage (-): 0

Region: chr22 12752353-12752379. Max. coverage (+): 0. Max coverage (-): 0

Region: chr22 12752380-12752407. Max. coverage (+): 2.28. Max coverage (-): 0

Region: chr22 12752408-12752434. Max. coverage (+): 2.28. Max coverage (-): 0

Region: chr22 12752435-12752462. Max. coverage (+): 7.7. Max coverage (-): 0

Region: chr22 12752463-12752490. Max. coverage (+): 0. Max coverage (-): 0

Region: chr22 12752491-12752517. Max. coverage (+): 4.73. Max coverage (-): 0

Region: chr22 12752518-12752545. Max. coverage (+): 9.67. Max coverage (-): 0

Region: chr22 12752546-12752572. Max. coverage (+): 0. Max coverage (-): 0

Region: chr22 12752573-12752600. Max. coverage (+): 0. Max coverage (-): 0

Region: chr22 12752601-12752627. Max. coverage (+): 0. Max coverage (-): 0

Region: chr22 12752628-12752655. Max. coverage (+): 1.97. Max coverage (-): 0

Region: chr22 12752656-12752682. Max. coverage (+): 3.88. Max coverage (-): 0

Region: chr22 12752683-12752710. Max. coverage (+): 3.88. Max coverage (-): 0

Region: chr22 12752711-12752738. Max. coverage (+): 0. Max coverage (-): 0

Region: chr22 12752739-12752765. Max. coverage (+): 0. Max coverage (-): 0

Region: chr22 12752766-12752793. Max. coverage (+): 0. Max coverage (-): 0

Region: chr22 12752794-12752820. Max. coverage (+): 2.65. Max coverage (-): 0

Region: chr22 12752821-12752848. Max. coverage (+): 1.73. Max coverage (-): 0

Region: chr22 12752849-12752875. Max. coverage (+): 0. Max coverage (-): 0

Region: chr22 12752876-12752903. Max. coverage (+): 2.59. Max coverage (-): 0

Region: chr22 12752904-12752930. Max. coverage (+): 2.05. Max coverage (-): 0

Region: chr22 12752931-12752958. Max. coverage (+): 0. Max coverage (-): 0

Region: chr22 12752959-12752986. Max. coverage (+): 0. Max coverage (-): 0

Region: chr22 12752987-12753013. Max. coverage (+): 0. Max coverage (-): 0

Region: chr22 12753014-12753041. Max. coverage (+): 0. Max coverage (-): 0

Region: chr22 12753042-12753068. Max. coverage (+): 0. Max coverage (-): 0

Region: chr22 12753069-12753096. Max. coverage (+): 0. Max coverage (-): 0

Region: chr22 12753097-12753123. Max. coverage (+): 0. Max coverage (-): 0

Region: chr22 12753124-12753151. Max. coverage (+): 0. Max coverage (-): 0

Region: chr22 12753152-12753179. Max. coverage (+): 0. Max coverage (-): 0

Region: chr22 12753180-12753206. Max. coverage (+): 0. Max coverage (-): 0

Region: chr22 12753207-12753234. Max. coverage (+): 0. Max coverage (-): 0

Region: chr22 12753235-12753261. Max. coverage (+): 0. Max coverage (-): 0

Region: chr22 12753262-12753289. Max. coverage (+): 0. Max coverage (-): 0

Region: chr22 12753290-12753316. Max. coverage (+): 0. Max coverage (-): 0

Region: chr22 12753317-12753344. Max. coverage (+): 0. Max coverage (-): 0

Region: chr22 12753345-12753371. Max. coverage (+): 0. Max coverage (-): 0

Region: chr22 12753372-12753399. Max. coverage (+): 0. Max coverage (-): 0

Region: chr22 12753400-12753427. Max. coverage (+): 0. Max coverage (-): 0

Region: chr22 12753428-12753454. Max. coverage (+): 0. Max coverage (-): 0

Region: chr22 12753455-12753482. Max. coverage (+): 0. Max coverage (-): 0

Region: chr22 12753483-12753509. Max. coverage (+): 0. Max coverage (-): 0

Region: chr22 12753510-12753537. Max. coverage (+): 1.77. Max coverage (-): 0

Region: chr22 12753538-12753564. Max. coverage (+): 0. Max coverage (-): 0

Region: chr22 12753565-12753592. Max. coverage (+): 0. Max coverage (-): 0

Region: chr22 12753593-12753619. Max. coverage (+): 0. Max coverage (-): 0

Region: chr22 12753620-12753647. Max. coverage (+): 0. Max coverage (-): 0

Region: chr22 12753648-12753675. Max. coverage (+): 0. Max coverage (-): 0

Region: chr22 12753676-12753702. Max. coverage (+): 0. Max coverage (-): 0

Region: chr22 12753703-12753730. Max. coverage (+): 11.87. Max coverage (-): 0

Region: chr22 12753731-. Max. coverage (+): 0. Max coverage (-): 0

RepeatMasker Color Code

**+**

100-98% Identity

<98-95% Identity

<95-90% Identity

<90-85% Identity

<85-80% Identity

<80-75% Identity

<75-70% Identity

<70% Identity

**-**

Gene Set Color Code

**+**

Gene

Pseudogene

**-**

Topology/Coverage Color Code

Coverage Plus Strand

Coverage Minus Strand

Mainstrand: Plus

Mainstrand: Minus

Complementary Strand

Flanking Region  
(if option -flank >0)

Gene Set Annotation  

**1. RPSA (protein coding, ENSBTAG00000009757) Tr:00000012866 Ex:3**: 12739961-12740206 (+)  
**2. RPSA (protein coding, ENSBTAG00000009757) Tr:00000012866 Ex:4**: 12740956-12741084 (+)  
**3. RPSA (protein coding, ENSBTAG00000009757) Tr:00000012866 Ex:5**: 12741204-12741369 (+)  
**4. RPSA (protein coding, ENSBTAG00000009757) Tr:00000012866 Ex:6**: 12741478-12741653 (+)  
**5. SNORA62 (protein coding, ENSBTAG00000042875) Tr:00000059867 Ex:1**: 12740261-12740409 (+)

  
RepeatMasker Annotation  

**1. MER5B**: 12740676-12740729 (+), Divergence to consensus: 29.7%  
**2. Bov-tA1**: 12742488-12742677 (-), Divergence to consensus: 24.2%  
**3. Bov-tA2**: 12742738-12742907 (-), Divergence to consensus: 29.4%  
**4. L1-2\_BT**: 12743038-12743246 (+), Divergence to consensus: 42.1%  
**5. Bov-tA2**: 12743385-12743444 (+), Divergence to consensus: 20.3%  
**6. Bov-tA2**: 12743565-12743642 (-), Divergence to consensus: 24.4%  
**7. L1-2\_BT**: 12744066-12744328 (-), Divergence to consensus: 22.9%  
**8. L1-2\_BT**: 12744343-12744462 (+), Divergence to consensus: 42%  
**9. CHR-2B**: 12744463-12744760 (+), Divergence to consensus: 24%  
**10. L1-2\_BT**: 12744761-12745333 (+), Divergence to consensus: 42%  
**11. SINE2-1\_BT**: 12746941-12747038 (+), Divergence to consensus: 22.8%  
**12. MER87\_BT**: 12748407-12748733 (-), Divergence to consensus: 55%  
**13. Bov-tA3**: 12748886-12748993 (-), Divergence to consensus: 16.7%  
**14. MLT1H2**: 12749478-12749599 (-), Divergence to consensus: 33.2%  
**15. GC\_rich**: 12750528-12750552 (+), Divergence to consensus: 48%  
**16. BOV-A2**: 12751369-12751423 (+), Divergence to consensus: 7.3%  
**17. BOV-A2**: 12751424-12751695 (+), Divergence to consensus: 5.2%

  
Transcription Factor Binding Sites  

**RFX4\_1** (Sequence: GTTGCCAAG (-): 12748274)  
**RFX4\_2** (Sequence: CATGGATAC (+): 12741127)  
**Gata4** (Sequence: AGATAAC (-): 12746924)  
**Gata4** (Sequence: AGATAAC (-): 12751858)  
**SOX9** (Sequence: TTATTGTT (+): 12740907)  
**A-MYB** (Sequence: AGGCAGTTGG (+): 12746216)
