# Supplementary material for: Small RNA sequencing of cryopreserved semen from single bull revealed altered miRNAs and piRNAs expression between High- and Low-motile sperm populations
Source: BMC Genomics. 2017 Jan 4;18:14. doi: 10.1186/s12864-016-3394-7 (PMC5209821; doi:10.1186/s12864-016-3394-7)
Supplement: Additional file 3: — Details for each piRNA clusters found in High Motile (HM) sperm fraction. Genes, repeats, transposable elements and transcription factors binding sites falling within the cluster regions were reported. (ZIP 1896 kb) [file 12864_2016_3394_MOESM3_ESM.zip › 5.html]

piRNA cluster 5


Predicted piRNA cluster no. 5     previous   next
  

Show proTRAC run info
Hide proTRAC run info

================================= proTRAC ====================================  
VERSION: 2.1                                    LAST MODIFIED: 06. October 2015  
  
Please cite:  
Rosenkranz D, Zischler H. proTRAC - a software for probabilistic piRNA cluster  
detection, visualization and analysis. 2012. BMC Bioinformatics 13:5.  
  
and (for proTRAC 2.0 and later):  
Rosenkranz D, Rudloff S, Bastuck K, Ketting RF, Zischler H. Tupaia small RNAs  
provide insights into function and evolution of RNAi-based transposon defense  
in mammals. 2015. RNA 21(5):911-922.  
  
Contact:  
David Rosenkranz  
Institute of Anthropology, small RNA group  
Johannes Gutenberg University Mainz  
email: rosenkranz@uni-mainz.de  
  
You can find the latest proTRAC version at:  
http://sourceforge.net/projects/protrac/files  
http://www.smallRNAgroup-mainz.de/software  
==============================================================================  
  
PARAMETERS:  
Map file: .............../storage/core/barbara/genhome/smallRNA/fertility/Sample\_motile/pirna/Sample\_motile\_26-33\_collapsed.fa.no-dust.map.weighted-10000-1000-b-0  
Genome file: ............/storage/core/barbara/genhome/smallRNA/fertility/Sample\_all/pirna/bt\_311\_chrY.fa  
RepeatMasker annotation: /storage/genomes/bt\_umd31/GCF\_000003055.6\_Bos\_taurus\_UMD\_3.1.1\_repeatMasker\_chr.out  
GeneSet:................./storage/core/barbara/genhome/smallRNA/fertility/Sample\_all/pirna/full.gtf  
  
Significant (p<=0.01) hit density will be calculated based  
on observed hit distribution.  
  
Sliding window size: ........................................ 5000 bp  
Sliding window increament: .................................. 1000 bp  
Normalize each hit by number of genomic hits: ............... 1 [0=no/1=yes]  
Normalize each hit by number of sequence reads: ............. 1 [0=no/1=yes]  
Normalize values (-> per million mapped reads): ............. 1 [0=no/1=yes]  
Min. fraction of hits with 1T(U) or 10A: .................... 0.75  
Alternatively: Min. fraction of hits with 1T(U) and 10A: .... 0.5  
Min. fraction of hits with typical piRNA length: ............ 0.75  
Typical piRNA length: ....................................... 26-33 nt  
Min. size of a piRNA cluster: ............................... 5000 bp.  
Min. number of hits (absolute): ............................. 0  
Min. number of hits (normalized): ........................... 0  
Min. fraction of hits on the mainstrand: .................... 0.75  
Top fraction of mapped sequences (in terms of read counts): . 1%  
Top fraction accounts for max. n% of sequence reads: ........ 90%  
Min. fraction of hits on each arm of a bidirectional cluster: 0.1  
Output image file for each cluster: ......................... 0 [0=no/1=yes]  
Output html file for each cluster: .......................... 1 [0=no/1=yes]  
Output a summary table: ..................................... 1 [0=no/1=yes]  
Output a FASTA file for each cluster (piRNA sequences): ..... 1 [0=no/1=yes]  
Output a FASTA file comprising cluster sequences: ........... 1 [0=no/1=yes]  
Search DNA motifs in clusters: .............................. 1 [0=no/1=yes]  
Output flanking sequences: +/- .............................. 0 bp  
Output ~.pTi file: .......................................... 1 [0=no/1=yes]  
==============================================================================  
  
  
Genome size (without gaps): ............ 2678902517 bp  
Gaps (N/X/-): .......................... 53837044 bp  
Mapped reads: .......................... 658825247023  
Non-identical sequences: ............... 514171  
Genomic hits: .......................... 764233  
Significant densitiy of mapped reads: .. 12867599.5173724 reads/kb

Show proTRAC cluster info
Hide proTRAC cluster info

|  |  |
| --- | --- |
| Location | chr10 |
| Coordinates | 47924021-47947874 |
| Size [bp] | 23854 |
| Sequence hit loci | 4009 |
| Mapped reads (normalized) | 4494651833 |
| Mapped reads (normalized) per kb | 188423402.1 |
| Normalized reads with 1T (1U) | 77.8% |
| Normalized reads with 10A | 31.5% |
| Normalized reads with length 26-33 nt | 100% |
| Normalized reads on the main strand(s) | 99.3% |
| Predicted directionality | bi:minus-plus (split between 47930773 and 47930775) |

100%

0%

1T (1U)  
reads

10A reads

26-33 nt  
reads

reads on mainstrand

**Either the amount of reads with 1T (1U) OR 10A has to exceed 75% (set with option: -1Tor10A)  
Alternatively the amount of reads with 1T (1U) AND 10A has to exceed 50% (set with option: -1Tand10A)  
Minimum amount of reads with preferred size is 75% (set with option: -pisize)  
Minimum amount of reads on the main strand(s) is 75% (set with option: -clstrand)**

Show read coverage
Hide read coverage

WHAT DO I SEE HERE?  
This chart shows the location of mapped sequence reads within a predicted piRNA cluster. The color refers to the number of genomic hits produced by the sequence read in question. A dark red bar indicates that this sequence read produces many other hits elsewhere in the genome. Many adjacent red or yellow bars can indicate the presence of a multi-copy element such as transposons or rRNA genes. A dark green bar indicates that this sequence read maps uniquely to this locus.

1 hit

2-5 hits

6-10 hits

11-20 hits

21-50 hits

51-100 hits

> 100 hits

chr10

47924021

47947874

Gene Set

RepeatMasker

Mapped  
Reads

223.68

plus strand

minus strand

223.68

Region: chr10 47913217-47924044. Max. coverage (+): 0. Max coverage (-): 2.62

Region: chr10 47924045-47924092. Max. coverage (+): 0. Max coverage (-): 0

Region: chr10 47924093-47924140. Max. coverage (+): 0. Max coverage (-): 0

Region: chr10 47924141-47924187. Max. coverage (+): 0. Max coverage (-): 0

Region: chr10 47924188-47924235. Max. coverage (+): 0. Max coverage (-): 1.48

Region: chr10 47924236-47924283. Max. coverage (+): 0. Max coverage (-): 0

Region: chr10 47924284-47924331. Max. coverage (+): 0. Max coverage (-): 0

Region: chr10 47924332-47924378. Max. coverage (+): 0. Max coverage (-): 0

Region: chr10 47924379-47924426. Max. coverage (+): 0. Max coverage (-): 0

Region: chr10 47924427-47924474. Max. coverage (+): 0. Max coverage (-): 0

Region: chr10 47924475-47924521. Max. coverage (+): 0. Max coverage (-): 0

Region: chr10 47924522-47924569. Max. coverage (+): 0. Max coverage (-): 0

Region: chr10 47924570-47924617. Max. coverage (+): 0. Max coverage (-): 0

Region: chr10 47924618-47924665. Max. coverage (+): 0. Max coverage (-): 1.59

Region: chr10 47924666-47924712. Max. coverage (+): 0. Max coverage (-): 1.59

Region: chr10 47924713-47924760. Max. coverage (+): 0. Max coverage (-): 0

Region: chr10 47924761-47924808. Max. coverage (+): 0. Max coverage (-): 0

Region: chr10 47924809-47924855. Max. coverage (+): 0. Max coverage (-): 0

Region: chr10 47924856-47924903. Max. coverage (+): 0. Max coverage (-): 0

Region: chr10 47924904-47924951. Max. coverage (+): 0. Max coverage (-): 0

Region: chr10 47924952-47924999. Max. coverage (+): 0. Max coverage (-): 0

Region: chr10 47925000-47925046. Max. coverage (+): 0. Max coverage (-): 0

Region: chr10 47925047-47925094. Max. coverage (+): 0. Max coverage (-): 1.28

Region: chr10 47925095-47925142. Max. coverage (+): 0. Max coverage (-): 0

Region: chr10 47925143-47925189. Max. coverage (+): 0. Max coverage (-): 2.94

Region: chr10 47925190-47925237. Max. coverage (+): 0. Max coverage (-): 0

Region: chr10 47925238-47925285. Max. coverage (+): 0. Max coverage (-): 0

Region: chr10 47925286-47925332. Max. coverage (+): 0. Max coverage (-): 0

Region: chr10 47925333-47925380. Max. coverage (+): 0. Max coverage (-): 0

Region: chr10 47925381-47925428. Max. coverage (+): 0. Max coverage (-): 0

Region: chr10 47925429-47925476. Max. coverage (+): 0. Max coverage (-): 4.63

Region: chr10 47925477-47925523. Max. coverage (+): 0. Max coverage (-): 0

Region: chr10 47925524-47925571. Max. coverage (+): 0. Max coverage (-): 0

Region: chr10 47925572-47925619. Max. coverage (+): 0. Max coverage (-): 0

Region: chr10 47925620-47925666. Max. coverage (+): 0. Max coverage (-): 0

Region: chr10 47925667-47925714. Max. coverage (+): 0. Max coverage (-): 0

Region: chr10 47925715-47925762. Max. coverage (+): 0. Max coverage (-): 0

Region: chr10 47925763-47925810. Max. coverage (+): 0. Max coverage (-): 0

Region: chr10 47925811-47925857. Max. coverage (+): 0. Max coverage (-): 0

Region: chr10 47925858-47925905. Max. coverage (+): 0. Max coverage (-): 0

Region: chr10 47925906-47925953. Max. coverage (+): 0. Max coverage (-): 0

Region: chr10 47925954-47926000. Max. coverage (+): 0. Max coverage (-): 0

Region: chr10 47926001-47926048. Max. coverage (+): 0. Max coverage (-): 0

Region: chr10 47926049-47926096. Max. coverage (+): 0. Max coverage (-): 0

Region: chr10 47926097-47926144. Max. coverage (+): 0. Max coverage (-): 1.56

Region: chr10 47926145-47926191. Max. coverage (+): 0. Max coverage (-): 0

Region: chr10 47926192-47926239. Max. coverage (+): 0. Max coverage (-): 0

Region: chr10 47926240-47926287. Max. coverage (+): 0. Max coverage (-): 0

Region: chr10 47926288-47926334. Max. coverage (+): 0. Max coverage (-): 0

Region: chr10 47926335-47926382. Max. coverage (+): 0. Max coverage (-): 0

Region: chr10 47926383-47926430. Max. coverage (+): 0. Max coverage (-): 12.83

Region: chr10 47926431-47926477. Max. coverage (+): 0. Max coverage (-): 15.5

Region: chr10 47926478-47926525. Max. coverage (+): 0. Max coverage (-): 16.85

Region: chr10 47926526-47926573. Max. coverage (+): 0. Max coverage (-): 0.72

Region: chr10 47926574-47926621. Max. coverage (+): 0. Max coverage (-): 4.99

Region: chr10 47926622-47926668. Max. coverage (+): 0. Max coverage (-): 0

Region: chr10 47926669-47926716. Max. coverage (+): 0. Max coverage (-): 4.58

Region: chr10 47926717-47926764. Max. coverage (+): 0. Max coverage (-): 0

Region: chr10 47926765-47926811. Max. coverage (+): 0. Max coverage (-): 0

Region: chr10 47926812-47926859. Max. coverage (+): 0. Max coverage (-): 0

Region: chr10 47926860-47926907. Max. coverage (+): 0. Max coverage (-): 0

Region: chr10 47926908-47926955. Max. coverage (+): 0. Max coverage (-): 2.77

Region: chr10 47926956-47927002. Max. coverage (+): 0. Max coverage (-): 4.76

Region: chr10 47927003-47927050. Max. coverage (+): 0. Max coverage (-): 6.48

Region: chr10 47927051-47927098. Max. coverage (+): 0. Max coverage (-): 4.8

Region: chr10 47927099-47927145. Max. coverage (+): 0. Max coverage (-): 5.01

Region: chr10 47927146-47927193. Max. coverage (+): 0. Max coverage (-): 3.94

Region: chr10 47927194-47927241. Max. coverage (+): 0. Max coverage (-): 0

Region: chr10 47927242-47927288. Max. coverage (+): 0. Max coverage (-): 0

Region: chr10 47927289-47927336. Max. coverage (+): 0. Max coverage (-): 0

Region: chr10 47927337-47927384. Max. coverage (+): 0. Max coverage (-): 1.2

Region: chr10 47927385-47927432. Max. coverage (+): 0. Max coverage (-): 2.51

Region: chr10 47927433-47927479. Max. coverage (+): 0. Max coverage (-): 1.52

Region: chr10 47927480-47927527. Max. coverage (+): 0. Max coverage (-): 0

Region: chr10 47927528-47927575. Max. coverage (+): 0. Max coverage (-): 7.91

Region: chr10 47927576-47927622. Max. coverage (+): 0. Max coverage (-): 1.42

Region: chr10 47927623-47927670. Max. coverage (+): 0. Max coverage (-): 0

Region: chr10 47927671-47927718. Max. coverage (+): 0. Max coverage (-): 0

Region: chr10 47927719-47927766. Max. coverage (+): 0. Max coverage (-): 0

Region: chr10 47927767-47927813. Max. coverage (+): 0. Max coverage (-): 0

Region: chr10 47927814-47927861. Max. coverage (+): 0. Max coverage (-): 0

Region: chr10 47927862-47927909. Max. coverage (+): 0. Max coverage (-): 0

Region: chr10 47927910-47927956. Max. coverage (+): 0. Max coverage (-): 0

Region: chr10 47927957-47928004. Max. coverage (+): 0. Max coverage (-): 0

Region: chr10 47928005-47928052. Max. coverage (+): 0. Max coverage (-): 0

Region: chr10 47928053-47928100. Max. coverage (+): 0. Max coverage (-): 0

Region: chr10 47928101-47928147. Max. coverage (+): 0. Max coverage (-): 0

Region: chr10 47928148-47928195. Max. coverage (+): 0. Max coverage (-): 1.02

Region: chr10 47928196-47928243. Max. coverage (+): 0. Max coverage (-): 4.08

Region: chr10 47928244-47928290. Max. coverage (+): 0. Max coverage (-): 2.26

Region: chr10 47928291-47928338. Max. coverage (+): 0. Max coverage (-): 0

Region: chr10 47928339-47928386. Max. coverage (+): 0. Max coverage (-): 1.62

Region: chr10 47928387-47928433. Max. coverage (+): 0. Max coverage (-): 0

Region: chr10 47928434-47928481. Max. coverage (+): 0. Max coverage (-): 0

Region: chr10 47928482-47928529. Max. coverage (+): 0. Max coverage (-): 1.82

Region: chr10 47928530-47928577. Max. coverage (+): 0. Max coverage (-): 6.18

Region: chr10 47928578-47928624. Max. coverage (+): 0. Max coverage (-): 9.41

Region: chr10 47928625-47928672. Max. coverage (+): 0. Max coverage (-): 0

Region: chr10 47928673-47928720. Max. coverage (+): 0. Max coverage (-): 22.93

Region: chr10 47928721-47928767. Max. coverage (+): 1.13. Max coverage (-): 25.6

Region: chr10 47928768-47928815. Max. coverage (+): 1.13. Max coverage (-): 9.82

Region: chr10 47928816-47928863. Max. coverage (+): 0. Max coverage (-): 17.46

Region: chr10 47928864-47928911. Max. coverage (+): 0. Max coverage (-): 8.97

Region: chr10 47928912-47928958. Max. coverage (+): 0. Max coverage (-): 69.21

Region: chr10 47928959-47929006. Max. coverage (+): 3.64. Max coverage (-): 26.99

Region: chr10 47929007-47929054. Max. coverage (+): 3.64. Max coverage (-): 18.61

Region: chr10 47929055-47929101. Max. coverage (+): 0.61. Max coverage (-): 28.44

Region: chr10 47929102-47929149. Max. coverage (+): 3.48. Max coverage (-): 7.26

Region: chr10 47929150-47929197. Max. coverage (+): 0. Max coverage (-): 0

Region: chr10 47929198-47929245. Max. coverage (+): 0. Max coverage (-): 0

Region: chr10 47929246-47929292. Max. coverage (+): 0. Max coverage (-): 28.62

Region: chr10 47929293-47929340. Max. coverage (+): 0. Max coverage (-): 23.01

Region: chr10 47929341-47929388. Max. coverage (+): 0. Max coverage (-): 12.43

Region: chr10 47929389-47929435. Max. coverage (+): 0. Max coverage (-): 29.17

Region: chr10 47929436-47929483. Max. coverage (+): 0. Max coverage (-): 52.06

Region: chr10 47929484-47929531. Max. coverage (+): 0. Max coverage (-): 4.4

Region: chr10 47929532-47929578. Max. coverage (+): 2.54. Max coverage (-): 8.99

Region: chr10 47929579-47929626. Max. coverage (+): 2.54. Max coverage (-): 15.56

Region: chr10 47929627-47929674. Max. coverage (+): 0. Max coverage (-): 45.57

Region: chr10 47929675-47929722. Max. coverage (+): 0. Max coverage (-): 93.89

Region: chr10 47929723-47929769. Max. coverage (+): 0. Max coverage (-): 24.18

Region: chr10 47929770-47929817. Max. coverage (+): 0. Max coverage (-): 10.16

Region: chr10 47929818-47929865. Max. coverage (+): 0. Max coverage (-): 19.21

Region: chr10 47929866-47929912. Max. coverage (+): 0. Max coverage (-): 27.87

Region: chr10 47929913-47929960. Max. coverage (+): 0. Max coverage (-): 13.58

Region: chr10 47929961-47930008. Max. coverage (+): 0. Max coverage (-): 9.7

Region: chr10 47930009-47930056. Max. coverage (+): 0. Max coverage (-): 6.96

Region: chr10 47930057-47930103. Max. coverage (+): 0. Max coverage (-): 18.82

Region: chr10 47930104-47930151. Max. coverage (+): 0. Max coverage (-): 14.76

Region: chr10 47930152-47930199. Max. coverage (+): 0. Max coverage (-): 16.61

Region: chr10 47930200-47930246. Max. coverage (+): 0. Max coverage (-): 14.39

Region: chr10 47930247-47930294. Max. coverage (+): 0. Max coverage (-): 21.64

Region: chr10 47930295-47930342. Max. coverage (+): 0.99. Max coverage (-): 10.1

Region: chr10 47930343-47930390. Max. coverage (+): 0. Max coverage (-): 0

Region: chr10 47930391-47930437. Max. coverage (+): 0. Max coverage (-): 7.62

Region: chr10 47930438-47930485. Max. coverage (+): 0. Max coverage (-): 10.73

Region: chr10 47930486-47930533. Max. coverage (+): 0. Max coverage (-): 16.59

Region: chr10 47930534-47930580. Max. coverage (+): 3.03. Max coverage (-): 0

Region: chr10 47930581-47930628. Max. coverage (+): 2.03. Max coverage (-): 2.73

Region: chr10 47930629-47930676. Max. coverage (+): 0. Max coverage (-): 4.01

Region: chr10 47930677-47930723. Max. coverage (+): 0. Max coverage (-): 0

Region: chr10 47930724-47930771. Max. coverage (+): 0. Max coverage (-): 2.62

Region: chr10 47930772-47930819. Max. coverage (+): 4.52. Max coverage (-): 5.38

Region: chr10 47930820-47930867. Max. coverage (+): 0. Max coverage (-): 0

Region: chr10 47930868-47930914. Max. coverage (+): 0. Max coverage (-): 0

Region: chr10 47930915-47930962. Max. coverage (+): 0. Max coverage (-): 0

Region: chr10 47930963-47931010. Max. coverage (+): 5.13. Max coverage (-): 0

Region: chr10 47931011-47931057. Max. coverage (+): 8.93. Max coverage (-): 0

Region: chr10 47931058-47931105. Max. coverage (+): 0. Max coverage (-): 0

Region: chr10 47931106-47931153. Max. coverage (+): 30.41. Max coverage (-): 2.08

Region: chr10 47931154-47931201. Max. coverage (+): 16.33. Max coverage (-): 2.08

Region: chr10 47931202-47931248. Max. coverage (+): 40.09. Max coverage (-): 0

Region: chr10 47931249-47931296. Max. coverage (+): 6.9. Max coverage (-): 0

Region: chr10 47931297-47931344. Max. coverage (+): 4.31. Max coverage (-): 0

Region: chr10 47931345-47931391. Max. coverage (+): 15.5. Max coverage (-): 0

Region: chr10 47931392-47931439. Max. coverage (+): 15.26. Max coverage (-): 0

Region: chr10 47931440-47931487. Max. coverage (+): 43.96. Max coverage (-): 0

Region: chr10 47931488-47931535. Max. coverage (+): 31.43. Max coverage (-): 0

Region: chr10 47931536-47931582. Max. coverage (+): 6.36. Max coverage (-): 1.89

Region: chr10 47931583-47931630. Max. coverage (+): 37.57. Max coverage (-): 0

Region: chr10 47931631-47931678. Max. coverage (+): 33.44. Max coverage (-): 0

Region: chr10 47931679-47931725. Max. coverage (+): 6.87. Max coverage (-): 0

Region: chr10 47931726-47931773. Max. coverage (+): 9.14. Max coverage (-): 0

Region: chr10 47931774-47931821. Max. coverage (+): 16.28. Max coverage (-): 3.31

Region: chr10 47931822-47931868. Max. coverage (+): 1.29. Max coverage (-): 2.21

Region: chr10 47931869-47931916. Max. coverage (+): 2.7. Max coverage (-): 0

Region: chr10 47931917-47931964. Max. coverage (+): 5.93. Max coverage (-): 2.59

Region: chr10 47931965-47932012. Max. coverage (+): 65.68. Max coverage (-): 0

Region: chr10 47932013-47932059. Max. coverage (+): 13.92. Max coverage (-): 0

Region: chr10 47932060-47932107. Max. coverage (+): 4.76. Max coverage (-): 0

Region: chr10 47932108-47932155. Max. coverage (+): 29.54. Max coverage (-): 0

Region: chr10 47932156-47932202. Max. coverage (+): 31.18. Max coverage (-): 5.74

Region: chr10 47932203-47932250. Max. coverage (+): 30.92. Max coverage (-): 0

Region: chr10 47932251-47932298. Max. coverage (+): 85.86. Max coverage (-): 0

Region: chr10 47932299-47932346. Max. coverage (+): 58.5. Max coverage (-): 0

Region: chr10 47932347-47932393. Max. coverage (+): 82.69. Max coverage (-): 0

Region: chr10 47932394-47932441. Max. coverage (+): 37.7. Max coverage (-): 0

Region: chr10 47932442-47932489. Max. coverage (+): 39.94. Max coverage (-): 0

Region: chr10 47932490-47932536. Max. coverage (+): 6.41. Max coverage (-): 1.11

Region: chr10 47932537-47932584. Max. coverage (+): 15.11. Max coverage (-): 1.11

Region: chr10 47932585-47932632. Max. coverage (+): 5.67. Max coverage (-): 0

Region: chr10 47932633-47932680. Max. coverage (+): 27.09. Max coverage (-): 0

Region: chr10 47932681-47932727. Max. coverage (+): 11.08. Max coverage (-): 0

Region: chr10 47932728-47932775. Max. coverage (+): 24.77. Max coverage (-): 0

Region: chr10 47932776-47932823. Max. coverage (+): 17.36. Max coverage (-): 0

Region: chr10 47932824-47932870. Max. coverage (+): 19.3. Max coverage (-): 0

Region: chr10 47932871-47932918. Max. coverage (+): 9.24. Max coverage (-): 0

Region: chr10 47932919-47932966. Max. coverage (+): 8.11. Max coverage (-): 0

Region: chr10 47932967-47933013. Max. coverage (+): 7.19. Max coverage (-): 0

Region: chr10 47933014-47933061. Max. coverage (+): 4.34. Max coverage (-): 0

Region: chr10 47933062-47933109. Max. coverage (+): 0. Max coverage (-): 0

Region: chr10 47933110-47933157. Max. coverage (+): 10.55. Max coverage (-): 0

Region: chr10 47933158-47933204. Max. coverage (+): 27.48. Max coverage (-): 0

Region: chr10 47933205-47933252. Max. coverage (+): 26.17. Max coverage (-): 0

Region: chr10 47933253-47933300. Max. coverage (+): 189.38. Max coverage (-): 0

Region: chr10 47933301-47933347. Max. coverage (+): 11.32. Max coverage (-): 0

Region: chr10 47933348-47933395. Max. coverage (+): 25.94. Max coverage (-): 0

Region: chr10 47933396-47933443. Max. coverage (+): 43.31. Max coverage (-): 0

Region: chr10 47933444-47933491. Max. coverage (+): 32.37. Max coverage (-): 0

Region: chr10 47933492-47933538. Max. coverage (+): 43.29. Max coverage (-): 0

Region: chr10 47933539-47933586. Max. coverage (+): 27.09. Max coverage (-): 0

Region: chr10 47933587-47933634. Max. coverage (+): 24.12. Max coverage (-): 0

Region: chr10 47933635-47933681. Max. coverage (+): 10.58. Max coverage (-): 0

Region: chr10 47933682-47933729. Max. coverage (+): 29.59. Max coverage (-): 4.94

Region: chr10 47933730-47933777. Max. coverage (+): 0. Max coverage (-): 0

Region: chr10 47933778-47933824. Max. coverage (+): 0. Max coverage (-): 0

Region: chr10 47933825-47933872. Max. coverage (+): 17.33. Max coverage (-): 0

Region: chr10 47933873-47933920. Max. coverage (+): 68.56. Max coverage (-): 0

Region: chr10 47933921-47933968. Max. coverage (+): 86.69. Max coverage (-): 0

Region: chr10 47933969-47934015. Max. coverage (+): 44.04. Max coverage (-): 0

Region: chr10 47934016-47934063. Max. coverage (+): 31.23. Max coverage (-): 0

Region: chr10 47934064-47934111. Max. coverage (+): 14.69. Max coverage (-): 0

Region: chr10 47934112-47934158. Max. coverage (+): 31.74. Max coverage (-): 0

Region: chr10 47934159-47934206. Max. coverage (+): 5.95. Max coverage (-): 0

Region: chr10 47934207-47934254. Max. coverage (+): 8.34. Max coverage (-): 0

Region: chr10 47934255-47934302. Max. coverage (+): 18.58. Max coverage (-): 0

Region: chr10 47934303-47934349. Max. coverage (+): 7.2. Max coverage (-): 0

Region: chr10 47934350-47934397. Max. coverage (+): 4.03. Max coverage (-): 0

Region: chr10 47934398-47934445. Max. coverage (+): 9.71. Max coverage (-): 0

Region: chr10 47934446-47934492. Max. coverage (+): 223.68. Max coverage (-): 0

Region: chr10 47934493-47934540. Max. coverage (+): 40.02. Max coverage (-): 0

Region: chr10 47934541-47934588. Max. coverage (+): 30.44. Max coverage (-): 0

Region: chr10 47934589-47934636. Max. coverage (+): 31.22. Max coverage (-): 0

Region: chr10 47934637-47934683. Max. coverage (+): 42.66. Max coverage (-): 0

Region: chr10 47934684-47934731. Max. coverage (+): 48.19. Max coverage (-): 0

Region: chr10 47934732-47934779. Max. coverage (+): 42. Max coverage (-): 0

Region: chr10 47934780-47934826. Max. coverage (+): 16.3. Max coverage (-): 0

Region: chr10 47934827-47934874. Max. coverage (+): 56.72. Max coverage (-): 0

Region: chr10 47934875-47934922. Max. coverage (+): 45.15. Max coverage (-): 0

Region: chr10 47934923-47934969. Max. coverage (+): 17.19. Max coverage (-): 0

Region: chr10 47934970-47935017. Max. coverage (+): 18.04. Max coverage (-): 0

Region: chr10 47935018-47935065. Max. coverage (+): 10.11. Max coverage (-): 0

Region: chr10 47935066-47935113. Max. coverage (+): 23.46. Max coverage (-): 0

Region: chr10 47935114-47935160. Max. coverage (+): 43.96. Max coverage (-): 0

Region: chr10 47935161-47935208. Max. coverage (+): 43.24. Max coverage (-): 0

Region: chr10 47935209-47935256. Max. coverage (+): 20.69. Max coverage (-): 0

Region: chr10 47935257-47935303. Max. coverage (+): 37.72. Max coverage (-): 0

Region: chr10 47935304-47935351. Max. coverage (+): 34.14. Max coverage (-): 0

Region: chr10 47935352-47935399. Max. coverage (+): 51.68. Max coverage (-): 0

Region: chr10 47935400-47935447. Max. coverage (+): 32.79. Max coverage (-): 1.15

Region: chr10 47935448-47935494. Max. coverage (+): 50.43. Max coverage (-): 0

Region: chr10 47935495-47935542. Max. coverage (+): 13.67. Max coverage (-): 0

Region: chr10 47935543-47935590. Max. coverage (+): 89. Max coverage (-): 0

Region: chr10 47935591-47935637. Max. coverage (+): 85.98. Max coverage (-): 0

Region: chr10 47935638-47935685. Max. coverage (+): 0. Max coverage (-): 0

Region: chr10 47935686-47935733. Max. coverage (+): 0. Max coverage (-): 0

Region: chr10 47935734-47935781. Max. coverage (+): 0. Max coverage (-): 0

Region: chr10 47935782-47935828. Max. coverage (+): 0. Max coverage (-): 0

Region: chr10 47935829-47935876. Max. coverage (+): 0. Max coverage (-): 0

Region: chr10 47935877-47935924. Max. coverage (+): 0. Max coverage (-): 0

Region: chr10 47935925-47935971. Max. coverage (+): 0. Max coverage (-): 0

Region: chr10 47935972-47936019. Max. coverage (+): 0. Max coverage (-): 0

Region: chr10 47936020-47936067. Max. coverage (+): 0. Max coverage (-): 0

Region: chr10 47936068-47936114. Max. coverage (+): 5.93. Max coverage (-): 0

Region: chr10 47936115-47936162. Max. coverage (+): 13.15. Max coverage (-): 0

Region: chr10 47936163-47936210. Max. coverage (+): 8.81. Max coverage (-): 0

Region: chr10 47936211-47936258. Max. coverage (+): 0. Max coverage (-): 0

Region: chr10 47936259-47936305. Max. coverage (+): 0. Max coverage (-): 0

Region: chr10 47936306-47936353. Max. coverage (+): 0. Max coverage (-): 0

Region: chr10 47936354-47936401. Max. coverage (+): 0. Max coverage (-): 0

Region: chr10 47936402-47936448. Max. coverage (+): 15.55. Max coverage (-): 0

Region: chr10 47936449-47936496. Max. coverage (+): 12.53. Max coverage (-): 0

Region: chr10 47936497-47936544. Max. coverage (+): 10.58. Max coverage (-): 0

Region: chr10 47936545-47936592. Max. coverage (+): 0. Max coverage (-): 0

Region: chr10 47936593-47936639. Max. coverage (+): 0. Max coverage (-): 0

Region: chr10 47936640-47936687. Max. coverage (+): 6.47. Max coverage (-): 0

Region: chr10 47936688-47936735. Max. coverage (+): 0. Max coverage (-): 0

Region: chr10 47936736-47936782. Max. coverage (+): 12.64. Max coverage (-): 0

Region: chr10 47936783-47936830. Max. coverage (+): 10.94. Max coverage (-): 0

Region: chr10 47936831-47936878. Max. coverage (+): 10.85. Max coverage (-): 0

Region: chr10 47936879-47936926. Max. coverage (+): 5.3. Max coverage (-): 0

Region: chr10 47936927-47936973. Max. coverage (+): 0. Max coverage (-): 0

Region: chr10 47936974-47937021. Max. coverage (+): 21.7. Max coverage (-): 0

Region: chr10 47937022-47937069. Max. coverage (+): 23.3. Max coverage (-): 0

Region: chr10 47937070-47937116. Max. coverage (+): 2.52. Max coverage (-): 0

Region: chr10 47937117-47937164. Max. coverage (+): 0. Max coverage (-): 0

Region: chr10 47937165-47937212. Max. coverage (+): 13.76. Max coverage (-): 0

Region: chr10 47937213-47937259. Max. coverage (+): 7.33. Max coverage (-): 0

Region: chr10 47937260-47937307. Max. coverage (+): 5.27. Max coverage (-): 0

Region: chr10 47937308-47937355. Max. coverage (+): 0. Max coverage (-): 0

Region: chr10 47937356-47937403. Max. coverage (+): 15.13. Max coverage (-): 0

Region: chr10 47937404-47937450. Max. coverage (+): 2.3. Max coverage (-): 0

Region: chr10 47937451-47937498. Max. coverage (+): 5.41. Max coverage (-): 0

Region: chr10 47937499-47937546. Max. coverage (+): 0. Max coverage (-): 0

Region: chr10 47937547-47937593. Max. coverage (+): 0. Max coverage (-): 0

Region: chr10 47937594-47937641. Max. coverage (+): 1.17. Max coverage (-): 0

Region: chr10 47937642-47937689. Max. coverage (+): 0.15. Max coverage (-): 0

Region: chr10 47937690-47937737. Max. coverage (+): 0. Max coverage (-): 0

Region: chr10 47937738-47937784. Max. coverage (+): 0. Max coverage (-): 0

Region: chr10 47937785-47937832. Max. coverage (+): 0. Max coverage (-): 0

Region: chr10 47937833-47937880. Max. coverage (+): 0. Max coverage (-): 0

Region: chr10 47937881-47937927. Max. coverage (+): 0. Max coverage (-): 0

Region: chr10 47937928-47937975. Max. coverage (+): 4.31. Max coverage (-): 0

Region: chr10 47937976-47938023. Max. coverage (+): 22.85. Max coverage (-): 0

Region: chr10 47938024-47938071. Max. coverage (+): 16.72. Max coverage (-): 0

Region: chr10 47938072-47938118. Max. coverage (+): 12.89. Max coverage (-): 0

Region: chr10 47938119-47938166. Max. coverage (+): 5. Max coverage (-): 0

Region: chr10 47938167-47938214. Max. coverage (+): 5. Max coverage (-): 0

Region: chr10 47938215-47938261. Max. coverage (+): 0. Max coverage (-): 0

Region: chr10 47938262-47938309. Max. coverage (+): 0. Max coverage (-): 0

Region: chr10 47938310-47938357. Max. coverage (+): 0. Max coverage (-): 0

Region: chr10 47938358-47938404. Max. coverage (+): 0. Max coverage (-): 0

Region: chr10 47938405-47938452. Max. coverage (+): 0. Max coverage (-): 0

Region: chr10 47938453-47938500. Max. coverage (+): 0. Max coverage (-): 0

Region: chr10 47938501-47938548. Max. coverage (+): 0. Max coverage (-): 0

Region: chr10 47938549-47938595. Max. coverage (+): 0. Max coverage (-): 0

Region: chr10 47938596-47938643. Max. coverage (+): 0. Max coverage (-): 0

Region: chr10 47938644-47938691. Max. coverage (+): 0. Max coverage (-): 0

Region: chr10 47938692-47938738. Max. coverage (+): 0. Max coverage (-): 0

Region: chr10 47938739-47938786. Max. coverage (+): 0. Max coverage (-): 0

Region: chr10 47938787-47938834. Max. coverage (+): 0. Max coverage (-): 0

Region: chr10 47938835-47938882. Max. coverage (+): 0. Max coverage (-): 0

Region: chr10 47938883-47938929. Max. coverage (+): 0. Max coverage (-): 0

Region: chr10 47938930-47938977. Max. coverage (+): 0. Max coverage (-): 0

Region: chr10 47938978-47939025. Max. coverage (+): 0. Max coverage (-): 0

Region: chr10 47939026-47939072. Max. coverage (+): 0. Max coverage (-): 0

Region: chr10 47939073-47939120. Max. coverage (+): 0. Max coverage (-): 0

Region: chr10 47939121-47939168. Max. coverage (+): 0. Max coverage (-): 0

Region: chr10 47939169-47939215. Max. coverage (+): 0. Max coverage (-): 0

Region: chr10 47939216-47939263. Max. coverage (+): 0. Max coverage (-): 0

Region: chr10 47939264-47939311. Max. coverage (+): 0. Max coverage (-): 0

Region: chr10 47939312-47939359. Max. coverage (+): 0. Max coverage (-): 0

Region: chr10 47939360-47939406. Max. coverage (+): 0. Max coverage (-): 0

Region: chr10 47939407-47939454. Max. coverage (+): 0. Max coverage (-): 0

Region: chr10 47939455-47939502. Max. coverage (+): 0. Max coverage (-): 0

Region: chr10 47939503-47939549. Max. coverage (+): 0. Max coverage (-): 0

Region: chr10 47939550-47939597. Max. coverage (+): 0. Max coverage (-): 0

Region: chr10 47939598-47939645. Max. coverage (+): 0. Max coverage (-): 0

Region: chr10 47939646-47939693. Max. coverage (+): 0. Max coverage (-): 0

Region: chr10 47939694-47939740. Max. coverage (+): 0. Max coverage (-): 0

Region: chr10 47939741-47939788. Max. coverage (+): 0. Max coverage (-): 0

Region: chr10 47939789-47939836. Max. coverage (+): 0. Max coverage (-): 0

Region: chr10 47939837-47939883. Max. coverage (+): 0. Max coverage (-): 0

Region: chr10 47939884-47939931. Max. coverage (+): 0. Max coverage (-): 0

Region: chr10 47939932-47939979. Max. coverage (+): 0. Max coverage (-): 0

Region: chr10 47939980-47940027. Max. coverage (+): 0. Max coverage (-): 0

Region: chr10 47940028-47940074. Max. coverage (+): 0. Max coverage (-): 0

Region: chr10 47940075-47940122. Max. coverage (+): 0. Max coverage (-): 0

Region: chr10 47940123-47940170. Max. coverage (+): 0. Max coverage (-): 0

Region: chr10 47940171-47940217. Max. coverage (+): 0. Max coverage (-): 0

Region: chr10 47940218-47940265. Max. coverage (+): 0. Max coverage (-): 0

Region: chr10 47940266-47940313. Max. coverage (+): 0.37. Max coverage (-): 0

Region: chr10 47940314-47940360. Max. coverage (+): 0. Max coverage (-): 0

Region: chr10 47940361-47940408. Max. coverage (+): 0. Max coverage (-): 0

Region: chr10 47940409-47940456. Max. coverage (+): 0. Max coverage (-): 0

Region: chr10 47940457-47940504. Max. coverage (+): 2.91. Max coverage (-): 0

Region: chr10 47940505-47940551. Max. coverage (+): 0. Max coverage (-): 0

Region: chr10 47940552-47940599. Max. coverage (+): 1.47. Max coverage (-): 0

Region: chr10 47940600-47940647. Max. coverage (+): 0. Max coverage (-): 0

Region: chr10 47940648-47940694. Max. coverage (+): 4.79. Max coverage (-): 0

Region: chr10 47940695-47940742. Max. coverage (+): 4.24. Max coverage (-): 0

Region: chr10 47940743-47940790. Max. coverage (+): 0. Max coverage (-): 0

Region: chr10 47940791-47940838. Max. coverage (+): 0. Max coverage (-): 0

Region: chr10 47940839-47940885. Max. coverage (+): 0. Max coverage (-): 0

Region: chr10 47940886-47940933. Max. coverage (+): 0. Max coverage (-): 0

Region: chr10 47940934-47940981. Max. coverage (+): 0. Max coverage (-): 0

Region: chr10 47940982-47941028. Max. coverage (+): 0. Max coverage (-): 0

Region: chr10 47941029-47941076. Max. coverage (+): 0.76. Max coverage (-): 0

Region: chr10 47941077-47941124. Max. coverage (+): 0. Max coverage (-): 0

Region: chr10 47941125-47941172. Max. coverage (+): 0. Max coverage (-): 0

Region: chr10 47941173-47941219. Max. coverage (+): 0. Max coverage (-): 0

Region: chr10 47941220-47941267. Max. coverage (+): 0. Max coverage (-): 0

Region: chr10 47941268-47941315. Max. coverage (+): 0. Max coverage (-): 0

Region: chr10 47941316-47941362. Max. coverage (+): 0. Max coverage (-): 0

Region: chr10 47941363-47941410. Max. coverage (+): 0. Max coverage (-): 0

Region: chr10 47941411-47941458. Max. coverage (+): 0. Max coverage (-): 0

Region: chr10 47941459-47941505. Max. coverage (+): 0. Max coverage (-): 0

Region: chr10 47941506-47941553. Max. coverage (+): 0. Max coverage (-): 0

Region: chr10 47941554-47941601. Max. coverage (+): 0. Max coverage (-): 0

Region: chr10 47941602-47941649. Max. coverage (+): 0. Max coverage (-): 0

Region: chr10 47941650-47941696. Max. coverage (+): 0. Max coverage (-): 0

Region: chr10 47941697-47941744. Max. coverage (+): 0. Max coverage (-): 0

Region: chr10 47941745-47941792. Max. coverage (+): 0. Max coverage (-): 0

Region: chr10 47941793-47941839. Max. coverage (+): 0. Max coverage (-): 0

Region: chr10 47941840-47941887. Max. coverage (+): 0. Max coverage (-): 0

Region: chr10 47941888-47941935. Max. coverage (+): 0. Max coverage (-): 0

Region: chr10 47941936-47941983. Max. coverage (+): 0.95. Max coverage (-): 0

Region: chr10 47941984-47942030. Max. coverage (+): 0. Max coverage (-): 0

Region: chr10 47942031-47942078. Max. coverage (+): 0. Max coverage (-): 0

Region: chr10 47942079-47942126. Max. coverage (+): 0. Max coverage (-): 0

Region: chr10 47942127-47942173. Max. coverage (+): 0. Max coverage (-): 0

Region: chr10 47942174-47942221. Max. coverage (+): 0. Max coverage (-): 0

Region: chr10 47942222-47942269. Max. coverage (+): 0. Max coverage (-): 0

Region: chr10 47942270-47942317. Max. coverage (+): 0. Max coverage (-): 0

Region: chr10 47942318-47942364. Max. coverage (+): 0. Max coverage (-): 0

Region: chr10 47942365-47942412. Max. coverage (+): 0. Max coverage (-): 0

Region: chr10 47942413-47942460. Max. coverage (+): 0. Max coverage (-): 0

Region: chr10 47942461-47942507. Max. coverage (+): 2.17. Max coverage (-): 0

Region: chr10 47942508-47942555. Max. coverage (+): 0. Max coverage (-): 0

Region: chr10 47942556-47942603. Max. coverage (+): 1.81. Max coverage (-): 0

Region: chr10 47942604-47942650. Max. coverage (+): 1.19. Max coverage (-): 0

Region: chr10 47942651-47942698. Max. coverage (+): 6.85. Max coverage (-): 0

Region: chr10 47942699-47942746. Max. coverage (+): 0. Max coverage (-): 0

Region: chr10 47942747-47942794. Max. coverage (+): 0. Max coverage (-): 0

Region: chr10 47942795-47942841. Max. coverage (+): 0. Max coverage (-): 0

Region: chr10 47942842-47942889. Max. coverage (+): 1.01. Max coverage (-): 0

Region: chr10 47942890-47942937. Max. coverage (+): 0. Max coverage (-): 0

Region: chr10 47942938-47942984. Max. coverage (+): 6.67. Max coverage (-): 0

Region: chr10 47942985-47943032. Max. coverage (+): 4.46. Max coverage (-): 0

Region: chr10 47943033-47943080. Max. coverage (+): 4.46. Max coverage (-): 0

Region: chr10 47943081-47943128. Max. coverage (+): 5.71. Max coverage (-): 0

Region: chr10 47943129-47943175. Max. coverage (+): 0.88. Max coverage (-): 0

Region: chr10 47943176-47943223. Max. coverage (+): 4.1. Max coverage (-): 0

Region: chr10 47943224-47943271. Max. coverage (+): 0. Max coverage (-): 0

Region: chr10 47943272-47943318. Max. coverage (+): 0. Max coverage (-): 0

Region: chr10 47943319-47943366. Max. coverage (+): 0. Max coverage (-): 0

Region: chr10 47943367-47943414. Max. coverage (+): 10.88. Max coverage (-): 0

Region: chr10 47943415-47943462. Max. coverage (+): 6.63. Max coverage (-): 0

Region: chr10 47943463-47943509. Max. coverage (+): 0.94. Max coverage (-): 0

Region: chr10 47943510-47943557. Max. coverage (+): 4.83. Max coverage (-): 0

Region: chr10 47943558-47943605. Max. coverage (+): 0. Max coverage (-): 0

Region: chr10 47943606-47943652. Max. coverage (+): 0.8. Max coverage (-): 0

Region: chr10 47943653-47943700. Max. coverage (+): 0.8. Max coverage (-): 0

Region: chr10 47943701-47943748. Max. coverage (+): 0. Max coverage (-): 0

Region: chr10 47943749-47943795. Max. coverage (+): 0.7. Max coverage (-): 0

Region: chr10 47943796-47943843. Max. coverage (+): 1.92. Max coverage (-): 0

Region: chr10 47943844-47943891. Max. coverage (+): 2.22. Max coverage (-): 0

Region: chr10 47943892-47943939. Max. coverage (+): 0. Max coverage (-): 0

Region: chr10 47943940-47943986. Max. coverage (+): 8.46. Max coverage (-): 0

Region: chr10 47943987-47944034. Max. coverage (+): 0. Max coverage (-): 0

Region: chr10 47944035-47944082. Max. coverage (+): 0. Max coverage (-): 0

Region: chr10 47944083-47944129. Max. coverage (+): 0. Max coverage (-): 0

Region: chr10 47944130-47944177. Max. coverage (+): 0. Max coverage (-): 0

Region: chr10 47944178-47944225. Max. coverage (+): 0. Max coverage (-): 0

Region: chr10 47944226-47944273. Max. coverage (+): 0.81. Max coverage (-): 0

Region: chr10 47944274-47944320. Max. coverage (+): 0. Max coverage (-): 0

Region: chr10 47944321-47944368. Max. coverage (+): 0. Max coverage (-): 0

Region: chr10 47944369-47944416. Max. coverage (+): 0. Max coverage (-): 0

Region: chr10 47944417-47944463. Max. coverage (+): 0. Max coverage (-): 0

Region: chr10 47944464-47944511. Max. coverage (+): 0. Max coverage (-): 0

Region: chr10 47944512-47944559. Max. coverage (+): 1.13. Max coverage (-): 0

Region: chr10 47944560-47944607. Max. coverage (+): 0. Max coverage (-): 0

Region: chr10 47944608-47944654. Max. coverage (+): 2.21. Max coverage (-): 0

Region: chr10 47944655-47944702. Max. coverage (+): 0. Max coverage (-): 0

Region: chr10 47944703-47944750. Max. coverage (+): 0. Max coverage (-): 0

Region: chr10 47944751-47944797. Max. coverage (+): 0. Max coverage (-): 0

Region: chr10 47944798-47944845. Max. coverage (+): 0. Max coverage (-): 0

Region: chr10 47944846-47944893. Max. coverage (+): 0. Max coverage (-): 0

Region: chr10 47944894-47944940. Max. coverage (+): 0. Max coverage (-): 0

Region: chr10 47944941-47944988. Max. coverage (+): 0. Max coverage (-): 0

Region: chr10 47944989-47945036. Max. coverage (+): 3.4. Max coverage (-): 0

Region: chr10 47945037-47945084. Max. coverage (+): 6.59. Max coverage (-): 0

Region: chr10 47945085-47945131. Max. coverage (+): 0. Max coverage (-): 0

Region: chr10 47945132-47945179. Max. coverage (+): 0. Max coverage (-): 0

Region: chr10 47945180-47945227. Max. coverage (+): 0. Max coverage (-): 0

Region: chr10 47945228-47945274. Max. coverage (+): 0. Max coverage (-): 0

Region: chr10 47945275-47945322. Max. coverage (+): 0. Max coverage (-): 0

Region: chr10 47945323-47945370. Max. coverage (+): 0. Max coverage (-): 0

Region: chr10 47945371-47945418. Max. coverage (+): 0. Max coverage (-): 0

Region: chr10 47945419-47945465. Max. coverage (+): 0. Max coverage (-): 0

Region: chr10 47945466-47945513. Max. coverage (+): 0. Max coverage (-): 0

Region: chr10 47945514-47945561. Max. coverage (+): 1.46. Max coverage (-): 0

Region: chr10 47945562-47945608. Max. coverage (+): 0. Max coverage (-): 0

Region: chr10 47945609-47945656. Max. coverage (+): 0. Max coverage (-): 0

Region: chr10 47945657-47945704. Max. coverage (+): 0. Max coverage (-): 0

Region: chr10 47945705-47945751. Max. coverage (+): 0. Max coverage (-): 0

Region: chr10 47945752-47945799. Max. coverage (+): 0. Max coverage (-): 0

Region: chr10 47945800-47945847. Max. coverage (+): 0. Max coverage (-): 0

Region: chr10 47945848-47945895. Max. coverage (+): 0. Max coverage (-): 0

Region: chr10 47945896-47945942. Max. coverage (+): 0. Max coverage (-): 0

Region: chr10 47945943-47945990. Max. coverage (+): 0. Max coverage (-): 0

Region: chr10 47945991-47946038. Max. coverage (+): 0. Max coverage (-): 0

Region: chr10 47946039-47946085. Max. coverage (+): 0. Max coverage (-): 0

Region: chr10 47946086-47946133. Max. coverage (+): 0. Max coverage (-): 0

Region: chr10 47946134-47946181. Max. coverage (+): 0.88. Max coverage (-): 0

Region: chr10 47946182-47946229. Max. coverage (+): 5.19. Max coverage (-): 0

Region: chr10 47946230-47946276. Max. coverage (+): 4.06. Max coverage (-): 0

Region: chr10 47946277-47946324. Max. coverage (+): 4.23. Max coverage (-): 0

Region: chr10 47946325-47946372. Max. coverage (+): 17.49. Max coverage (-): 0

Region: chr10 47946373-47946419. Max. coverage (+): 3.83. Max coverage (-): 0

Region: chr10 47946420-47946467. Max. coverage (+): 0.45. Max coverage (-): 0

Region: chr10 47946468-47946515. Max. coverage (+): 0. Max coverage (-): 0

Region: chr10 47946516-47946563. Max. coverage (+): 0. Max coverage (-): 0

Region: chr10 47946564-47946610. Max. coverage (+): 0. Max coverage (-): 0

Region: chr10 47946611-47946658. Max. coverage (+): 0. Max coverage (-): 0

Region: chr10 47946659-47946706. Max. coverage (+): 0. Max coverage (-): 0

Region: chr10 47946707-47946753. Max. coverage (+): 0. Max coverage (-): 0

Region: chr10 47946754-47946801. Max. coverage (+): 0. Max coverage (-): 0

Region: chr10 47946802-47946849. Max. coverage (+): 0. Max coverage (-): 0

Region: chr10 47946850-47946896. Max. coverage (+): 0. Max coverage (-): 0

Region: chr10 47946897-47946944. Max. coverage (+): 0. Max coverage (-): 0

Region: chr10 47946945-47946992. Max. coverage (+): 0. Max coverage (-): 0

Region: chr10 47946993-47947040. Max. coverage (+): 0. Max coverage (-): 0

Region: chr10 47947041-47947087. Max. coverage (+): 0.92. Max coverage (-): 0

Region: chr10 47947088-47947135. Max. coverage (+): 0. Max coverage (-): 0

Region: chr10 47947136-47947183. Max. coverage (+): 0. Max coverage (-): 0

Region: chr10 47947184-47947230. Max. coverage (+): 0. Max coverage (-): 0

Region: chr10 47947231-47947278. Max. coverage (+): 0. Max coverage (-): 0

Region: chr10 47947279-47947326. Max. coverage (+): 0. Max coverage (-): 0

Region: chr10 47947327-47947374. Max. coverage (+): 5.03. Max coverage (-): 0

Region: chr10 47947375-47947421. Max. coverage (+): 0. Max coverage (-): 0

Region: chr10 47947422-47947469. Max. coverage (+): 0. Max coverage (-): 0

Region: chr10 47947470-47947517. Max. coverage (+): 2.1. Max coverage (-): 0

Region: chr10 47947518-47947564. Max. coverage (+): 0. Max coverage (-): 0

Region: chr10 47947565-47947612. Max. coverage (+): 0. Max coverage (-): 0

Region: chr10 47947613-47947660. Max. coverage (+): 6.23. Max coverage (-): 0

Region: chr10 47947661-47947708. Max. coverage (+): 0. Max coverage (-): 0

Region: chr10 47947709-47947755. Max. coverage (+): 0. Max coverage (-): 0

Region: chr10 47947756-47947803. Max. coverage (+): 0. Max coverage (-): 0

Region: chr10 47947804-47947851. Max. coverage (+): 18.44. Max coverage (-): 0

Region: chr10 47947852-. Max. coverage (+): 0. Max coverage (-): 0

RepeatMasker Color Code

**+**

100-98% Identity

<98-95% Identity

<95-90% Identity

<90-85% Identity

<85-80% Identity

<80-75% Identity

<75-70% Identity

<70% Identity

**-**

Gene Set Color Code

**+**

Gene

Pseudogene

**-**

Topology/Coverage Color Code

Coverage Plus Strand

Coverage Minus Strand

Mainstrand: Plus

Mainstrand: Minus

Complementary Strand

Flanking Region  
(if option -flank >0)

Gene Set Annotation  
  
RepeatMasker Annotation  

**1. MIR**: 47924840-47925051 (-), Divergence to consensus: 34.5%  
**2. (TG)n**: 47925640-47925668 (+), Divergence to consensus: 6.9%  
**3. AT\_rich**: 47925681-47925701 (+), Divergence to consensus: 38.1%  
**4. (TTTA)n**: 47925738-47925761 (+), Divergence to consensus: 0%  
**5. L2c**: 47925837-47926108 (+), Divergence to consensus: 44.7%  
**6. Bov-tA2**: 47926163-47926347 (+), Divergence to consensus: 20%  
**7. (TA)n**: 47926792-47926910 (+), Divergence to consensus: 31.3%  
**8. L1MEj**: 47927693-47928080 (-), Divergence to consensus: 40.6%  
**9. MIRb**: 47929138-47929286 (-), Divergence to consensus: 41.7%  
**10. GC\_rich**: 47930581-47930602 (+), Divergence to consensus: 36.4%  
**11. L2b**: 47933757-47933858 (-), Divergence to consensus: 37.4%  
**12. LTR5\_BT**: 47935638-47936062 (+), Divergence to consensus: 14.4%  
**13. MIRb**: 47936233-47936389 (+), Divergence to consensus: 28.3%  
**14. Charlie4z**: 47936546-47936643 (+), Divergence to consensus: 32.7%  
**15. MER94B**: 47936777-47936819 (-), Divergence to consensus: 27.8%  
**16. MIR3**: 47936905-47936979 (+), Divergence to consensus: 35.8%  
**17. L1MC2**: 47937560-47937624 (-), Divergence to consensus: 22.9%  
**18. L1M5**: 47937677-47937924 (-), Divergence to consensus: 32.3%  
**19. Bov-tA2**: 47938344-47938530 (+), Divergence to consensus: 22.5%  
**20. L1M5**: 47938540-47939399 (-), Divergence to consensus: 40.6%  
**21. Bov-tA2**: 47939400-47939450 (-), Divergence to consensus: 21.6%  
**22. L1M5**: 47939451-47940062 (-), Divergence to consensus: 40.6%  
**23. MER58C**: 47940154-47940250 (-), Divergence to consensus: 30.8%  
**24. MIRb**: 47940857-47940935 (+), Divergence to consensus: 31.2%  
**25. SINE2-2\_BT**: 47944421-47944536 (+), Divergence to consensus: 32.1%  
**26. MER33**: 47945299-47945531 (-), Divergence to consensus: 22.8%  
**27. L1ME4a**: 47945722-47945833 (-), Divergence to consensus: 37.3%  
**28. Bov-tA2**: 47945980-47946171 (-), Divergence to consensus: 23.3%  
**29. LTR16**: 47946475-47946908 (+), Divergence to consensus: 40.9%  
**30. Bov-tA2**: 47947132-47947331 (+), Divergence to consensus: 17.2%

  
Transcription Factor Binding Sites  

**Gata4** (Sequence: AGATAAG (-): 47942851)  
**SOX9** (Sequence: AACAATAG (-): 47926684)  
**SOX9** (Sequence: AACAATAA (-): 47927485)  
**SOX9** (Sequence: AACAATAG (-): 47934668)  
**SOX9** (Sequence: AACAATGG (-): 47935150)  
**SOX9** (Sequence: CCATTGTT (+): 47929953)  
**SOX9** (Sequence: TCATTGTT (+): 47940630)  
**Gata4** (Sequence: CTTATCT (+): 47927470)
