# Supplementary material for: Small RNA sequencing of cryopreserved semen from single bull revealed altered miRNAs and piRNAs expression between High- and Low-motile sperm populations
Source: BMC Genomics. 2017 Jan 4;18:14. doi: 10.1186/s12864-016-3394-7 (PMC5209821; doi:10.1186/s12864-016-3394-7)
Supplement: Additional file 3: — Details for each piRNA clusters found in High Motile (HM) sperm fraction. Genes, repeats, transposable elements and transcription factors binding sites falling within the cluster regions were reported. (ZIP 1896 kb) [file 12864_2016_3394_MOESM3_ESM.zip › 50.html]

piRNA cluster 50


Predicted piRNA cluster no. 50     previous   next
  

Show proTRAC run info
Hide proTRAC run info

================================= proTRAC ====================================  
VERSION: 2.1                                    LAST MODIFIED: 06. October 2015  
  
Please cite:  
Rosenkranz D, Zischler H. proTRAC - a software for probabilistic piRNA cluster  
detection, visualization and analysis. 2012. BMC Bioinformatics 13:5.  
  
and (for proTRAC 2.0 and later):  
Rosenkranz D, Rudloff S, Bastuck K, Ketting RF, Zischler H. Tupaia small RNAs  
provide insights into function and evolution of RNAi-based transposon defense  
in mammals. 2015. RNA 21(5):911-922.  
  
Contact:  
David Rosenkranz  
Institute of Anthropology, small RNA group  
Johannes Gutenberg University Mainz  
email: rosenkranz@uni-mainz.de  
  
You can find the latest proTRAC version at:  
http://sourceforge.net/projects/protrac/files  
http://www.smallRNAgroup-mainz.de/software  
==============================================================================  
  
PARAMETERS:  
Map file: .............../storage/core/barbara/genhome/smallRNA/fertility/Sample\_motile/pirna/Sample\_motile\_26-33\_collapsed.fa.no-dust.map.weighted-10000-1000-b-0  
Genome file: ............/storage/core/barbara/genhome/smallRNA/fertility/Sample\_all/pirna/bt\_311\_chrY.fa  
RepeatMasker annotation: /storage/genomes/bt\_umd31/GCF\_000003055.6\_Bos\_taurus\_UMD\_3.1.1\_repeatMasker\_chr.out  
GeneSet:................./storage/core/barbara/genhome/smallRNA/fertility/Sample\_all/pirna/full.gtf  
  
Significant (p<=0.01) hit density will be calculated based  
on observed hit distribution.  
  
Sliding window size: ........................................ 5000 bp  
Sliding window increament: .................................. 1000 bp  
Normalize each hit by number of genomic hits: ............... 1 [0=no/1=yes]  
Normalize each hit by number of sequence reads: ............. 1 [0=no/1=yes]  
Normalize values (-> per million mapped reads): ............. 1 [0=no/1=yes]  
Min. fraction of hits with 1T(U) or 10A: .................... 0.75  
Alternatively: Min. fraction of hits with 1T(U) and 10A: .... 0.5  
Min. fraction of hits with typical piRNA length: ............ 0.75  
Typical piRNA length: ....................................... 26-33 nt  
Min. size of a piRNA cluster: ............................... 5000 bp.  
Min. number of hits (absolute): ............................. 0  
Min. number of hits (normalized): ........................... 0  
Min. fraction of hits on the mainstrand: .................... 0.75  
Top fraction of mapped sequences (in terms of read counts): . 1%  
Top fraction accounts for max. n% of sequence reads: ........ 90%  
Min. fraction of hits on each arm of a bidirectional cluster: 0.1  
Output image file for each cluster: ......................... 0 [0=no/1=yes]  
Output html file for each cluster: .......................... 1 [0=no/1=yes]  
Output a summary table: ..................................... 1 [0=no/1=yes]  
Output a FASTA file for each cluster (piRNA sequences): ..... 1 [0=no/1=yes]  
Output a FASTA file comprising cluster sequences: ........... 1 [0=no/1=yes]  
Search DNA motifs in clusters: .............................. 1 [0=no/1=yes]  
Output flanking sequences: +/- .............................. 0 bp  
Output ~.pTi file: .......................................... 1 [0=no/1=yes]  
==============================================================================  
  
  
Genome size (without gaps): ............ 2678902517 bp  
Gaps (N/X/-): .......................... 53837044 bp  
Mapped reads: .......................... 658825247023  
Non-identical sequences: ............... 514171  
Genomic hits: .......................... 764233  
Significant densitiy of mapped reads: .. 12867599.5173724 reads/kb

Show proTRAC cluster info
Hide proTRAC cluster info

|  |  |
| --- | --- |
| Location | chr22 |
| Coordinates | 21430284-21436382 |
| Size [bp] | 6099 |
| Sequence hit loci | 97 |
| Mapped reads (normalized) | 119799609 |
| Mapped reads (normalized) per kb | 19642500.2 |
| Normalized reads with 1T (1U) | 82.3% |
| Normalized reads with 10A | 25.4% |
| Normalized reads with length 26-33 nt | 100% |
| Normalized reads on the main strand(s) | 100% |
| Predicted directionality | mono:plus |

100%

0%

1T (1U)  
reads

10A reads

26-33 nt  
reads

reads on mainstrand

**Either the amount of reads with 1T (1U) OR 10A has to exceed 75% (set with option: -1Tor10A)  
Alternatively the amount of reads with 1T (1U) AND 10A has to exceed 50% (set with option: -1Tand10A)  
Minimum amount of reads with preferred size is 75% (set with option: -pisize)  
Minimum amount of reads on the main strand(s) is 75% (set with option: -clstrand)**

Show read coverage
Hide read coverage

WHAT DO I SEE HERE?  
This chart shows the location of mapped sequence reads within a predicted piRNA cluster. The color refers to the number of genomic hits produced by the sequence read in question. A dark red bar indicates that this sequence read produces many other hits elsewhere in the genome. Many adjacent red or yellow bars can indicate the presence of a multi-copy element such as transposons or rRNA genes. A dark green bar indicates that this sequence read maps uniquely to this locus.

1 hit

2-5 hits

6-10 hits

11-20 hits

21-50 hits

51-100 hits

> 100 hits

chr22

21430284

21436382

Gene Set

RepeatMasker

Mapped  
Reads

13.95

plus strand

minus strand

13.95

Region: chr22 12753732-21430290. Max. coverage (+): 4.66. Max coverage (-): 0

Region: chr22 21430291-21430302. Max. coverage (+): 4.66. Max coverage (-): 0

Region: chr22 21430303-21430314. Max. coverage (+): 0. Max coverage (-): 0

Region: chr22 21430315-21430326. Max. coverage (+): 0. Max coverage (-): 0

Region: chr22 21430327-21430338. Max. coverage (+): 0. Max coverage (-): 0

Region: chr22 21430339-21430351. Max. coverage (+): 0. Max coverage (-): 0

Region: chr22 21430352-21430363. Max. coverage (+): 0. Max coverage (-): 0

Region: chr22 21430364-21430375. Max. coverage (+): 0. Max coverage (-): 0

Region: chr22 21430376-21430387. Max. coverage (+): 0. Max coverage (-): 0

Region: chr22 21430388-21430399. Max. coverage (+): 0. Max coverage (-): 0

Region: chr22 21430400-21430412. Max. coverage (+): 2.02. Max coverage (-): 0

Region: chr22 21430413-21430424. Max. coverage (+): 0. Max coverage (-): 0

Region: chr22 21430425-21430436. Max. coverage (+): 0. Max coverage (-): 0

Region: chr22 21430437-21430448. Max. coverage (+): 0. Max coverage (-): 0

Region: chr22 21430449-21430460. Max. coverage (+): 0. Max coverage (-): 0

Region: chr22 21430461-21430473. Max. coverage (+): 0. Max coverage (-): 0

Region: chr22 21430474-21430485. Max. coverage (+): 0. Max coverage (-): 0

Region: chr22 21430486-21430497. Max. coverage (+): 0. Max coverage (-): 0

Region: chr22 21430498-21430509. Max. coverage (+): 0. Max coverage (-): 0

Region: chr22 21430510-21430521. Max. coverage (+): 0. Max coverage (-): 0

Region: chr22 21430522-21430534. Max. coverage (+): 0. Max coverage (-): 0

Region: chr22 21430535-21430546. Max. coverage (+): 0. Max coverage (-): 0

Region: chr22 21430547-21430558. Max. coverage (+): 0. Max coverage (-): 0

Region: chr22 21430559-21430570. Max. coverage (+): 0. Max coverage (-): 0

Region: chr22 21430571-21430582. Max. coverage (+): 0. Max coverage (-): 0

Region: chr22 21430583-21430595. Max. coverage (+): 0. Max coverage (-): 0

Region: chr22 21430596-21430607. Max. coverage (+): 0. Max coverage (-): 0

Region: chr22 21430608-21430619. Max. coverage (+): 0. Max coverage (-): 0

Region: chr22 21430620-21430631. Max. coverage (+): 0. Max coverage (-): 0

Region: chr22 21430632-21430643. Max. coverage (+): 0. Max coverage (-): 0

Region: chr22 21430644-21430656. Max. coverage (+): 0. Max coverage (-): 0

Region: chr22 21430657-21430668. Max. coverage (+): 0. Max coverage (-): 0

Region: chr22 21430669-21430680. Max. coverage (+): 0. Max coverage (-): 0

Region: chr22 21430681-21430692. Max. coverage (+): 0. Max coverage (-): 0

Region: chr22 21430693-21430704. Max. coverage (+): 0. Max coverage (-): 0

Region: chr22 21430705-21430717. Max. coverage (+): 7.63. Max coverage (-): 0

Region: chr22 21430718-21430729. Max. coverage (+): 0. Max coverage (-): 0

Region: chr22 21430730-21430741. Max. coverage (+): 0. Max coverage (-): 0

Region: chr22 21430742-21430753. Max. coverage (+): 0. Max coverage (-): 0

Region: chr22 21430754-21430765. Max. coverage (+): 0. Max coverage (-): 0

Region: chr22 21430766-21430778. Max. coverage (+): 0. Max coverage (-): 0

Region: chr22 21430779-21430790. Max. coverage (+): 0. Max coverage (-): 0

Region: chr22 21430791-21430802. Max. coverage (+): 0. Max coverage (-): 0

Region: chr22 21430803-21430814. Max. coverage (+): 0. Max coverage (-): 0

Region: chr22 21430815-21430826. Max. coverage (+): 0. Max coverage (-): 0

Region: chr22 21430827-21430839. Max. coverage (+): 0. Max coverage (-): 0

Region: chr22 21430840-21430851. Max. coverage (+): 0. Max coverage (-): 0

Region: chr22 21430852-21430863. Max. coverage (+): 0. Max coverage (-): 0

Region: chr22 21430864-21430875. Max. coverage (+): 0. Max coverage (-): 0

Region: chr22 21430876-21430887. Max. coverage (+): 0. Max coverage (-): 0

Region: chr22 21430888-21430899. Max. coverage (+): 0. Max coverage (-): 0

Region: chr22 21430900-21430912. Max. coverage (+): 0. Max coverage (-): 0

Region: chr22 21430913-21430924. Max. coverage (+): 0. Max coverage (-): 0

Region: chr22 21430925-21430936. Max. coverage (+): 0. Max coverage (-): 0

Region: chr22 21430937-21430948. Max. coverage (+): 0. Max coverage (-): 0

Region: chr22 21430949-21430960. Max. coverage (+): 0. Max coverage (-): 0

Region: chr22 21430961-21430973. Max. coverage (+): 0. Max coverage (-): 0

Region: chr22 21430974-21430985. Max. coverage (+): 0. Max coverage (-): 0

Region: chr22 21430986-21430997. Max. coverage (+): 0. Max coverage (-): 0

Region: chr22 21430998-21431009. Max. coverage (+): 0. Max coverage (-): 0

Region: chr22 21431010-21431021. Max. coverage (+): 0. Max coverage (-): 0

Region: chr22 21431022-21431034. Max. coverage (+): 0. Max coverage (-): 0

Region: chr22 21431035-21431046. Max. coverage (+): 0. Max coverage (-): 0

Region: chr22 21431047-21431058. Max. coverage (+): 0. Max coverage (-): 0

Region: chr22 21431059-21431070. Max. coverage (+): 0. Max coverage (-): 0

Region: chr22 21431071-21431082. Max. coverage (+): 0. Max coverage (-): 0

Region: chr22 21431083-21431095. Max. coverage (+): 0. Max coverage (-): 0

Region: chr22 21431096-21431107. Max. coverage (+): 0. Max coverage (-): 0

Region: chr22 21431108-21431119. Max. coverage (+): 0. Max coverage (-): 0

Region: chr22 21431120-21431131. Max. coverage (+): 0. Max coverage (-): 0

Region: chr22 21431132-21431143. Max. coverage (+): 0. Max coverage (-): 0

Region: chr22 21431144-21431156. Max. coverage (+): 0. Max coverage (-): 0

Region: chr22 21431157-21431168. Max. coverage (+): 0. Max coverage (-): 0

Region: chr22 21431169-21431180. Max. coverage (+): 0. Max coverage (-): 0

Region: chr22 21431181-21431192. Max. coverage (+): 0. Max coverage (-): 0

Region: chr22 21431193-21431204. Max. coverage (+): 0. Max coverage (-): 0

Region: chr22 21431205-21431217. Max. coverage (+): 0. Max coverage (-): 0

Region: chr22 21431218-21431229. Max. coverage (+): 0. Max coverage (-): 0

Region: chr22 21431230-21431241. Max. coverage (+): 0. Max coverage (-): 0

Region: chr22 21431242-21431253. Max. coverage (+): 0. Max coverage (-): 0

Region: chr22 21431254-21431265. Max. coverage (+): 0. Max coverage (-): 0

Region: chr22 21431266-21431278. Max. coverage (+): 0. Max coverage (-): 0

Region: chr22 21431279-21431290. Max. coverage (+): 0. Max coverage (-): 0

Region: chr22 21431291-21431302. Max. coverage (+): 0. Max coverage (-): 0

Region: chr22 21431303-21431314. Max. coverage (+): 0. Max coverage (-): 0

Region: chr22 21431315-21431326. Max. coverage (+): 0. Max coverage (-): 0

Region: chr22 21431327-21431339. Max. coverage (+): 0. Max coverage (-): 0

Region: chr22 21431340-21431351. Max. coverage (+): 0. Max coverage (-): 0

Region: chr22 21431352-21431363. Max. coverage (+): 0. Max coverage (-): 0

Region: chr22 21431364-21431375. Max. coverage (+): 0. Max coverage (-): 0

Region: chr22 21431376-21431387. Max. coverage (+): 0. Max coverage (-): 0

Region: chr22 21431388-21431400. Max. coverage (+): 0. Max coverage (-): 0

Region: chr22 21431401-21431412. Max. coverage (+): 0. Max coverage (-): 0

Region: chr22 21431413-21431424. Max. coverage (+): 0. Max coverage (-): 0

Region: chr22 21431425-21431436. Max. coverage (+): 0. Max coverage (-): 0

Region: chr22 21431437-21431448. Max. coverage (+): 0. Max coverage (-): 0

Region: chr22 21431449-21431461. Max. coverage (+): 3.18. Max coverage (-): 0

Region: chr22 21431462-21431473. Max. coverage (+): 1.03. Max coverage (-): 0

Region: chr22 21431474-21431485. Max. coverage (+): 0. Max coverage (-): 0

Region: chr22 21431486-21431497. Max. coverage (+): 0. Max coverage (-): 0

Region: chr22 21431498-21431509. Max. coverage (+): 0. Max coverage (-): 0

Region: chr22 21431510-21431522. Max. coverage (+): 0. Max coverage (-): 0

Region: chr22 21431523-21431534. Max. coverage (+): 0. Max coverage (-): 0

Region: chr22 21431535-21431546. Max. coverage (+): 0. Max coverage (-): 0

Region: chr22 21431547-21431558. Max. coverage (+): 0. Max coverage (-): 0

Region: chr22 21431559-21431570. Max. coverage (+): 0. Max coverage (-): 0

Region: chr22 21431571-21431583. Max. coverage (+): 0. Max coverage (-): 0

Region: chr22 21431584-21431595. Max. coverage (+): 0. Max coverage (-): 0

Region: chr22 21431596-21431607. Max. coverage (+): 0. Max coverage (-): 0

Region: chr22 21431608-21431619. Max. coverage (+): 0. Max coverage (-): 0

Region: chr22 21431620-21431631. Max. coverage (+): 0. Max coverage (-): 0

Region: chr22 21431632-21431644. Max. coverage (+): 0. Max coverage (-): 0

Region: chr22 21431645-21431656. Max. coverage (+): 0. Max coverage (-): 0

Region: chr22 21431657-21431668. Max. coverage (+): 0. Max coverage (-): 0

Region: chr22 21431669-21431680. Max. coverage (+): 0. Max coverage (-): 0

Region: chr22 21431681-21431692. Max. coverage (+): 0. Max coverage (-): 0

Region: chr22 21431693-21431705. Max. coverage (+): 0. Max coverage (-): 0

Region: chr22 21431706-21431717. Max. coverage (+): 0. Max coverage (-): 0

Region: chr22 21431718-21431729. Max. coverage (+): 0. Max coverage (-): 0

Region: chr22 21431730-21431741. Max. coverage (+): 0. Max coverage (-): 0

Region: chr22 21431742-21431753. Max. coverage (+): 0. Max coverage (-): 0

Region: chr22 21431754-21431766. Max. coverage (+): 0. Max coverage (-): 0

Region: chr22 21431767-21431778. Max. coverage (+): 0. Max coverage (-): 0

Region: chr22 21431779-21431790. Max. coverage (+): 0. Max coverage (-): 0

Region: chr22 21431791-21431802. Max. coverage (+): 0. Max coverage (-): 0

Region: chr22 21431803-21431814. Max. coverage (+): 0. Max coverage (-): 0

Region: chr22 21431815-21431827. Max. coverage (+): 0. Max coverage (-): 0

Region: chr22 21431828-21431839. Max. coverage (+): 0. Max coverage (-): 0

Region: chr22 21431840-21431851. Max. coverage (+): 0. Max coverage (-): 0

Region: chr22 21431852-21431863. Max. coverage (+): 0. Max coverage (-): 0

Region: chr22 21431864-21431875. Max. coverage (+): 0. Max coverage (-): 0

Region: chr22 21431876-21431888. Max. coverage (+): 0. Max coverage (-): 0

Region: chr22 21431889-21431900. Max. coverage (+): 0. Max coverage (-): 0

Region: chr22 21431901-21431912. Max. coverage (+): 0. Max coverage (-): 0

Region: chr22 21431913-21431924. Max. coverage (+): 0. Max coverage (-): 0

Region: chr22 21431925-21431936. Max. coverage (+): 0. Max coverage (-): 0

Region: chr22 21431937-21431949. Max. coverage (+): 0. Max coverage (-): 0

Region: chr22 21431950-21431961. Max. coverage (+): 0. Max coverage (-): 0

Region: chr22 21431962-21431973. Max. coverage (+): 0. Max coverage (-): 0

Region: chr22 21431974-21431985. Max. coverage (+): 0. Max coverage (-): 0

Region: chr22 21431986-21431997. Max. coverage (+): 0. Max coverage (-): 0

Region: chr22 21431998-21432010. Max. coverage (+): 0. Max coverage (-): 0

Region: chr22 21432011-21432022. Max. coverage (+): 13.9. Max coverage (-): 0

Region: chr22 21432023-21432034. Max. coverage (+): 0.97. Max coverage (-): 0

Region: chr22 21432035-21432046. Max. coverage (+): 0. Max coverage (-): 0

Region: chr22 21432047-21432058. Max. coverage (+): 0. Max coverage (-): 0

Region: chr22 21432059-21432071. Max. coverage (+): 0. Max coverage (-): 0

Region: chr22 21432072-21432083. Max. coverage (+): 0. Max coverage (-): 0

Region: chr22 21432084-21432095. Max. coverage (+): 0. Max coverage (-): 0

Region: chr22 21432096-21432107. Max. coverage (+): 0. Max coverage (-): 0

Region: chr22 21432108-21432119. Max. coverage (+): 0. Max coverage (-): 0

Region: chr22 21432120-21432131. Max. coverage (+): 0.21. Max coverage (-): 0

Region: chr22 21432132-21432144. Max. coverage (+): 0.21. Max coverage (-): 0

Region: chr22 21432145-21432156. Max. coverage (+): 0. Max coverage (-): 0

Region: chr22 21432157-21432168. Max. coverage (+): 0. Max coverage (-): 0

Region: chr22 21432169-21432180. Max. coverage (+): 0. Max coverage (-): 0

Region: chr22 21432181-21432192. Max. coverage (+): 0. Max coverage (-): 0

Region: chr22 21432193-21432205. Max. coverage (+): 0. Max coverage (-): 0

Region: chr22 21432206-21432217. Max. coverage (+): 0. Max coverage (-): 0

Region: chr22 21432218-21432229. Max. coverage (+): 0. Max coverage (-): 0

Region: chr22 21432230-21432241. Max. coverage (+): 0. Max coverage (-): 0

Region: chr22 21432242-21432253. Max. coverage (+): 0. Max coverage (-): 0

Region: chr22 21432254-21432266. Max. coverage (+): 0. Max coverage (-): 0

Region: chr22 21432267-21432278. Max. coverage (+): 0. Max coverage (-): 0

Region: chr22 21432279-21432290. Max. coverage (+): 0. Max coverage (-): 0

Region: chr22 21432291-21432302. Max. coverage (+): 0. Max coverage (-): 0

Region: chr22 21432303-21432314. Max. coverage (+): 0. Max coverage (-): 0

Region: chr22 21432315-21432327. Max. coverage (+): 0. Max coverage (-): 0

Region: chr22 21432328-21432339. Max. coverage (+): 0. Max coverage (-): 0

Region: chr22 21432340-21432351. Max. coverage (+): 0. Max coverage (-): 0

Region: chr22 21432352-21432363. Max. coverage (+): 0. Max coverage (-): 0

Region: chr22 21432364-21432375. Max. coverage (+): 0. Max coverage (-): 0

Region: chr22 21432376-21432388. Max. coverage (+): 0. Max coverage (-): 0

Region: chr22 21432389-21432400. Max. coverage (+): 0. Max coverage (-): 0

Region: chr22 21432401-21432412. Max. coverage (+): 0. Max coverage (-): 0

Region: chr22 21432413-21432424. Max. coverage (+): 0. Max coverage (-): 0

Region: chr22 21432425-21432436. Max. coverage (+): 0. Max coverage (-): 0

Region: chr22 21432437-21432449. Max. coverage (+): 0. Max coverage (-): 0

Region: chr22 21432450-21432461. Max. coverage (+): 0. Max coverage (-): 0

Region: chr22 21432462-21432473. Max. coverage (+): 0. Max coverage (-): 0

Region: chr22 21432474-21432485. Max. coverage (+): 0. Max coverage (-): 0

Region: chr22 21432486-21432497. Max. coverage (+): 0. Max coverage (-): 0

Region: chr22 21432498-21432510. Max. coverage (+): 0. Max coverage (-): 0

Region: chr22 21432511-21432522. Max. coverage (+): 0. Max coverage (-): 0

Region: chr22 21432523-21432534. Max. coverage (+): 0. Max coverage (-): 0

Region: chr22 21432535-21432546. Max. coverage (+): 0. Max coverage (-): 0

Region: chr22 21432547-21432558. Max. coverage (+): 0. Max coverage (-): 0

Region: chr22 21432559-21432571. Max. coverage (+): 0. Max coverage (-): 0

Region: chr22 21432572-21432583. Max. coverage (+): 0. Max coverage (-): 0

Region: chr22 21432584-21432595. Max. coverage (+): 0. Max coverage (-): 0

Region: chr22 21432596-21432607. Max. coverage (+): 0. Max coverage (-): 0

Region: chr22 21432608-21432619. Max. coverage (+): 0. Max coverage (-): 0

Region: chr22 21432620-21432632. Max. coverage (+): 0. Max coverage (-): 0

Region: chr22 21432633-21432644. Max. coverage (+): 0. Max coverage (-): 0

Region: chr22 21432645-21432656. Max. coverage (+): 0. Max coverage (-): 0

Region: chr22 21432657-21432668. Max. coverage (+): 0. Max coverage (-): 0

Region: chr22 21432669-21432680. Max. coverage (+): 0. Max coverage (-): 0

Region: chr22 21432681-21432693. Max. coverage (+): 0. Max coverage (-): 0

Region: chr22 21432694-21432705. Max. coverage (+): 0. Max coverage (-): 0

Region: chr22 21432706-21432717. Max. coverage (+): 0. Max coverage (-): 0

Region: chr22 21432718-21432729. Max. coverage (+): 0. Max coverage (-): 0

Region: chr22 21432730-21432741. Max. coverage (+): 0. Max coverage (-): 0

Region: chr22 21432742-21432754. Max. coverage (+): 0. Max coverage (-): 0

Region: chr22 21432755-21432766. Max. coverage (+): 0. Max coverage (-): 0

Region: chr22 21432767-21432778. Max. coverage (+): 0. Max coverage (-): 0

Region: chr22 21432779-21432790. Max. coverage (+): 0. Max coverage (-): 0

Region: chr22 21432791-21432802. Max. coverage (+): 0. Max coverage (-): 0

Region: chr22 21432803-21432815. Max. coverage (+): 0. Max coverage (-): 0

Region: chr22 21432816-21432827. Max. coverage (+): 0. Max coverage (-): 0

Region: chr22 21432828-21432839. Max. coverage (+): 0. Max coverage (-): 0

Region: chr22 21432840-21432851. Max. coverage (+): 0. Max coverage (-): 0

Region: chr22 21432852-21432863. Max. coverage (+): 0. Max coverage (-): 0

Region: chr22 21432864-21432876. Max. coverage (+): 0. Max coverage (-): 0

Region: chr22 21432877-21432888. Max. coverage (+): 0. Max coverage (-): 0

Region: chr22 21432889-21432900. Max. coverage (+): 0. Max coverage (-): 0

Region: chr22 21432901-21432912. Max. coverage (+): 0. Max coverage (-): 0

Region: chr22 21432913-21432924. Max. coverage (+): 0. Max coverage (-): 0

Region: chr22 21432925-21432937. Max. coverage (+): 0. Max coverage (-): 0

Region: chr22 21432938-21432949. Max. coverage (+): 0. Max coverage (-): 0

Region: chr22 21432950-21432961. Max. coverage (+): 0. Max coverage (-): 0

Region: chr22 21432962-21432973. Max. coverage (+): 0. Max coverage (-): 0

Region: chr22 21432974-21432985. Max. coverage (+): 0. Max coverage (-): 0

Region: chr22 21432986-21432998. Max. coverage (+): 0.3. Max coverage (-): 0

Region: chr22 21432999-21433010. Max. coverage (+): 0. Max coverage (-): 0

Region: chr22 21433011-21433022. Max. coverage (+): 0. Max coverage (-): 0

Region: chr22 21433023-21433034. Max. coverage (+): 0. Max coverage (-): 0

Region: chr22 21433035-21433046. Max. coverage (+): 0. Max coverage (-): 0

Region: chr22 21433047-21433059. Max. coverage (+): 0. Max coverage (-): 0

Region: chr22 21433060-21433071. Max. coverage (+): 0. Max coverage (-): 0

Region: chr22 21433072-21433083. Max. coverage (+): 0. Max coverage (-): 0

Region: chr22 21433084-21433095. Max. coverage (+): 0. Max coverage (-): 0

Region: chr22 21433096-21433107. Max. coverage (+): 1.64. Max coverage (-): 0

Region: chr22 21433108-21433120. Max. coverage (+): 1.64. Max coverage (-): 0

Region: chr22 21433121-21433132. Max. coverage (+): 0. Max coverage (-): 0

Region: chr22 21433133-21433144. Max. coverage (+): 0. Max coverage (-): 0

Region: chr22 21433145-21433156. Max. coverage (+): 0. Max coverage (-): 0

Region: chr22 21433157-21433168. Max. coverage (+): 0. Max coverage (-): 0

Region: chr22 21433169-21433181. Max. coverage (+): 0. Max coverage (-): 0

Region: chr22 21433182-21433193. Max. coverage (+): 0. Max coverage (-): 0

Region: chr22 21433194-21433205. Max. coverage (+): 0. Max coverage (-): 0

Region: chr22 21433206-21433217. Max. coverage (+): 0. Max coverage (-): 0

Region: chr22 21433218-21433229. Max. coverage (+): 0.4. Max coverage (-): 0

Region: chr22 21433230-21433242. Max. coverage (+): 0.4. Max coverage (-): 0

Region: chr22 21433243-21433254. Max. coverage (+): 0. Max coverage (-): 0

Region: chr22 21433255-21433266. Max. coverage (+): 0. Max coverage (-): 0

Region: chr22 21433267-21433278. Max. coverage (+): 0. Max coverage (-): 0

Region: chr22 21433279-21433290. Max. coverage (+): 0. Max coverage (-): 0

Region: chr22 21433291-21433303. Max. coverage (+): 6.59. Max coverage (-): 0

Region: chr22 21433304-21433315. Max. coverage (+): 6.59. Max coverage (-): 0

Region: chr22 21433316-21433327. Max. coverage (+): 0. Max coverage (-): 0

Region: chr22 21433328-21433339. Max. coverage (+): 0. Max coverage (-): 0

Region: chr22 21433340-21433351. Max. coverage (+): 0. Max coverage (-): 0

Region: chr22 21433352-21433363. Max. coverage (+): 0. Max coverage (-): 0

Region: chr22 21433364-21433376. Max. coverage (+): 0. Max coverage (-): 0

Region: chr22 21433377-21433388. Max. coverage (+): 0. Max coverage (-): 0

Region: chr22 21433389-21433400. Max. coverage (+): 0. Max coverage (-): 0

Region: chr22 21433401-21433412. Max. coverage (+): 0. Max coverage (-): 0

Region: chr22 21433413-21433424. Max. coverage (+): 0. Max coverage (-): 0

Region: chr22 21433425-21433437. Max. coverage (+): 0. Max coverage (-): 0

Region: chr22 21433438-21433449. Max. coverage (+): 1.51. Max coverage (-): 0

Region: chr22 21433450-21433461. Max. coverage (+): 6.43. Max coverage (-): 0

Region: chr22 21433462-21433473. Max. coverage (+): 0.88. Max coverage (-): 0

Region: chr22 21433474-21433485. Max. coverage (+): 0. Max coverage (-): 0

Region: chr22 21433486-21433498. Max. coverage (+): 0. Max coverage (-): 0

Region: chr22 21433499-21433510. Max. coverage (+): 0. Max coverage (-): 0

Region: chr22 21433511-21433522. Max. coverage (+): 0. Max coverage (-): 0

Region: chr22 21433523-21433534. Max. coverage (+): 0. Max coverage (-): 0

Region: chr22 21433535-21433546. Max. coverage (+): 0. Max coverage (-): 0

Region: chr22 21433547-21433559. Max. coverage (+): 0. Max coverage (-): 0

Region: chr22 21433560-21433571. Max. coverage (+): 0. Max coverage (-): 0

Region: chr22 21433572-21433583. Max. coverage (+): 0. Max coverage (-): 0

Region: chr22 21433584-21433595. Max. coverage (+): 0. Max coverage (-): 0

Region: chr22 21433596-21433607. Max. coverage (+): 0. Max coverage (-): 0

Region: chr22 21433608-21433620. Max. coverage (+): 0. Max coverage (-): 0

Region: chr22 21433621-21433632. Max. coverage (+): 0. Max coverage (-): 0

Region: chr22 21433633-21433644. Max. coverage (+): 0. Max coverage (-): 0

Region: chr22 21433645-21433656. Max. coverage (+): 0. Max coverage (-): 0

Region: chr22 21433657-21433668. Max. coverage (+): 0. Max coverage (-): 0

Region: chr22 21433669-21433681. Max. coverage (+): 0. Max coverage (-): 0

Region: chr22 21433682-21433693. Max. coverage (+): 0. Max coverage (-): 0

Region: chr22 21433694-21433705. Max. coverage (+): 0. Max coverage (-): 0

Region: chr22 21433706-21433717. Max. coverage (+): 0. Max coverage (-): 0

Region: chr22 21433718-21433729. Max. coverage (+): 0. Max coverage (-): 0

Region: chr22 21433730-21433742. Max. coverage (+): 0. Max coverage (-): 0

Region: chr22 21433743-21433754. Max. coverage (+): 0. Max coverage (-): 0

Region: chr22 21433755-21433766. Max. coverage (+): 0. Max coverage (-): 0

Region: chr22 21433767-21433778. Max. coverage (+): 0. Max coverage (-): 0

Region: chr22 21433779-21433790. Max. coverage (+): 0. Max coverage (-): 0

Region: chr22 21433791-21433803. Max. coverage (+): 0. Max coverage (-): 0

Region: chr22 21433804-21433815. Max. coverage (+): 0. Max coverage (-): 0

Region: chr22 21433816-21433827. Max. coverage (+): 0. Max coverage (-): 0

Region: chr22 21433828-21433839. Max. coverage (+): 0. Max coverage (-): 0

Region: chr22 21433840-21433851. Max. coverage (+): 0. Max coverage (-): 0

Region: chr22 21433852-21433864. Max. coverage (+): 0. Max coverage (-): 0

Region: chr22 21433865-21433876. Max. coverage (+): 0. Max coverage (-): 0

Region: chr22 21433877-21433888. Max. coverage (+): 0. Max coverage (-): 0

Region: chr22 21433889-21433900. Max. coverage (+): 0. Max coverage (-): 0

Region: chr22 21433901-21433912. Max. coverage (+): 0. Max coverage (-): 0

Region: chr22 21433913-21433925. Max. coverage (+): 0. Max coverage (-): 0

Region: chr22 21433926-21433937. Max. coverage (+): 0. Max coverage (-): 0

Region: chr22 21433938-21433949. Max. coverage (+): 0. Max coverage (-): 0

Region: chr22 21433950-21433961. Max. coverage (+): 0. Max coverage (-): 0

Region: chr22 21433962-21433973. Max. coverage (+): 0. Max coverage (-): 0

Region: chr22 21433974-21433986. Max. coverage (+): 0. Max coverage (-): 0

Region: chr22 21433987-21433998. Max. coverage (+): 0. Max coverage (-): 0

Region: chr22 21433999-21434010. Max. coverage (+): 0. Max coverage (-): 0

Region: chr22 21434011-21434022. Max. coverage (+): 0. Max coverage (-): 0

Region: chr22 21434023-21434034. Max. coverage (+): 0. Max coverage (-): 0

Region: chr22 21434035-21434047. Max. coverage (+): 0. Max coverage (-): 0

Region: chr22 21434048-21434059. Max. coverage (+): 0. Max coverage (-): 0

Region: chr22 21434060-21434071. Max. coverage (+): 0. Max coverage (-): 0

Region: chr22 21434072-21434083. Max. coverage (+): 0. Max coverage (-): 0

Region: chr22 21434084-21434095. Max. coverage (+): 0. Max coverage (-): 0

Region: chr22 21434096-21434108. Max. coverage (+): 0. Max coverage (-): 0

Region: chr22 21434109-21434120. Max. coverage (+): 0. Max coverage (-): 0

Region: chr22 21434121-21434132. Max. coverage (+): 0. Max coverage (-): 0

Region: chr22 21434133-21434144. Max. coverage (+): 0. Max coverage (-): 0

Region: chr22 21434145-21434156. Max. coverage (+): 1.05. Max coverage (-): 0

Region: chr22 21434157-21434169. Max. coverage (+): 0. Max coverage (-): 0

Region: chr22 21434170-21434181. Max. coverage (+): 1.91. Max coverage (-): 0

Region: chr22 21434182-21434193. Max. coverage (+): 0. Max coverage (-): 0

Region: chr22 21434194-21434205. Max. coverage (+): 0. Max coverage (-): 0

Region: chr22 21434206-21434217. Max. coverage (+): 0. Max coverage (-): 0

Region: chr22 21434218-21434230. Max. coverage (+): 4.35. Max coverage (-): 0

Region: chr22 21434231-21434242. Max. coverage (+): 5.72. Max coverage (-): 0

Region: chr22 21434243-21434254. Max. coverage (+): 6.74. Max coverage (-): 0

Region: chr22 21434255-21434266. Max. coverage (+): 6.74. Max coverage (-): 0

Region: chr22 21434267-21434278. Max. coverage (+): 0.67. Max coverage (-): 0

Region: chr22 21434279-21434291. Max. coverage (+): 0. Max coverage (-): 0

Region: chr22 21434292-21434303. Max. coverage (+): 0.16. Max coverage (-): 0

Region: chr22 21434304-21434315. Max. coverage (+): 6.99. Max coverage (-): 0

Region: chr22 21434316-21434327. Max. coverage (+): 1.06. Max coverage (-): 0

Region: chr22 21434328-21434339. Max. coverage (+): 0. Max coverage (-): 0

Region: chr22 21434340-21434352. Max. coverage (+): 0. Max coverage (-): 0

Region: chr22 21434353-21434364. Max. coverage (+): 2.02. Max coverage (-): 0

Region: chr22 21434365-21434376. Max. coverage (+): 6.87. Max coverage (-): 0

Region: chr22 21434377-21434388. Max. coverage (+): 4.85. Max coverage (-): 0

Region: chr22 21434389-21434400. Max. coverage (+): 0. Max coverage (-): 0

Region: chr22 21434401-21434413. Max. coverage (+): 0. Max coverage (-): 0

Region: chr22 21434414-21434425. Max. coverage (+): 0. Max coverage (-): 0

Region: chr22 21434426-21434437. Max. coverage (+): 0. Max coverage (-): 0

Region: chr22 21434438-21434449. Max. coverage (+): 0. Max coverage (-): 0

Region: chr22 21434450-21434461. Max. coverage (+): 0. Max coverage (-): 0

Region: chr22 21434462-21434474. Max. coverage (+): 1.74. Max coverage (-): 0

Region: chr22 21434475-21434486. Max. coverage (+): 0. Max coverage (-): 0

Region: chr22 21434487-21434498. Max. coverage (+): 0. Max coverage (-): 0

Region: chr22 21434499-21434510. Max. coverage (+): 0. Max coverage (-): 0

Region: chr22 21434511-21434522. Max. coverage (+): 3.87. Max coverage (-): 0

Region: chr22 21434523-21434535. Max. coverage (+): 0. Max coverage (-): 0

Region: chr22 21434536-21434547. Max. coverage (+): 0. Max coverage (-): 0

Region: chr22 21434548-21434559. Max. coverage (+): 0. Max coverage (-): 0

Region: chr22 21434560-21434571. Max. coverage (+): 0. Max coverage (-): 0

Region: chr22 21434572-21434583. Max. coverage (+): 0. Max coverage (-): 0

Region: chr22 21434584-21434595. Max. coverage (+): 0. Max coverage (-): 0

Region: chr22 21434596-21434608. Max. coverage (+): 2.07. Max coverage (-): 0

Region: chr22 21434609-21434620. Max. coverage (+): 3.67. Max coverage (-): 0

Region: chr22 21434621-21434632. Max. coverage (+): 3.03. Max coverage (-): 0

Region: chr22 21434633-21434644. Max. coverage (+): 3.03. Max coverage (-): 0

Region: chr22 21434645-21434656. Max. coverage (+): 0. Max coverage (-): 0

Region: chr22 21434657-21434669. Max. coverage (+): 9.47. Max coverage (-): 0

Region: chr22 21434670-21434681. Max. coverage (+): 4.88. Max coverage (-): 0

Region: chr22 21434682-21434693. Max. coverage (+): 4.88. Max coverage (-): 0

Region: chr22 21434694-21434705. Max. coverage (+): 0. Max coverage (-): 0

Region: chr22 21434706-21434717. Max. coverage (+): 0. Max coverage (-): 0

Region: chr22 21434718-21434730. Max. coverage (+): 0. Max coverage (-): 0

Region: chr22 21434731-21434742. Max. coverage (+): 0. Max coverage (-): 0

Region: chr22 21434743-21434754. Max. coverage (+): 0. Max coverage (-): 0

Region: chr22 21434755-21434766. Max. coverage (+): 0. Max coverage (-): 0

Region: chr22 21434767-21434778. Max. coverage (+): 0. Max coverage (-): 0

Region: chr22 21434779-21434791. Max. coverage (+): 0. Max coverage (-): 0

Region: chr22 21434792-21434803. Max. coverage (+): 0.71. Max coverage (-): 0

Region: chr22 21434804-21434815. Max. coverage (+): 0.71. Max coverage (-): 0

Region: chr22 21434816-21434827. Max. coverage (+): 0. Max coverage (-): 0

Region: chr22 21434828-21434839. Max. coverage (+): 0. Max coverage (-): 0

Region: chr22 21434840-21434852. Max. coverage (+): 0. Max coverage (-): 0

Region: chr22 21434853-21434864. Max. coverage (+): 0. Max coverage (-): 0

Region: chr22 21434865-21434876. Max. coverage (+): 0. Max coverage (-): 0

Region: chr22 21434877-21434888. Max. coverage (+): 0. Max coverage (-): 0

Region: chr22 21434889-21434900. Max. coverage (+): 0. Max coverage (-): 0

Region: chr22 21434901-21434913. Max. coverage (+): 0. Max coverage (-): 0

Region: chr22 21434914-21434925. Max. coverage (+): 0. Max coverage (-): 0

Region: chr22 21434926-21434937. Max. coverage (+): 0. Max coverage (-): 0

Region: chr22 21434938-21434949. Max. coverage (+): 0. Max coverage (-): 0

Region: chr22 21434950-21434961. Max. coverage (+): 0. Max coverage (-): 0

Region: chr22 21434962-21434974. Max. coverage (+): 0. Max coverage (-): 0

Region: chr22 21434975-21434986. Max. coverage (+): 0. Max coverage (-): 0

Region: chr22 21434987-21434998. Max. coverage (+): 1.24. Max coverage (-): 0

Region: chr22 21434999-21435010. Max. coverage (+): 1.24. Max coverage (-): 0

Region: chr22 21435011-21435022. Max. coverage (+): 0. Max coverage (-): 0

Region: chr22 21435023-21435035. Max. coverage (+): 0. Max coverage (-): 0

Region: chr22 21435036-21435047. Max. coverage (+): 0. Max coverage (-): 0

Region: chr22 21435048-21435059. Max. coverage (+): 0. Max coverage (-): 0

Region: chr22 21435060-21435071. Max. coverage (+): 0. Max coverage (-): 0

Region: chr22 21435072-21435083. Max. coverage (+): 0. Max coverage (-): 0

Region: chr22 21435084-21435096. Max. coverage (+): 0. Max coverage (-): 0

Region: chr22 21435097-21435108. Max. coverage (+): 0. Max coverage (-): 0

Region: chr22 21435109-21435120. Max. coverage (+): 0. Max coverage (-): 0

Region: chr22 21435121-21435132. Max. coverage (+): 0. Max coverage (-): 0

Region: chr22 21435133-21435144. Max. coverage (+): 0. Max coverage (-): 0

Region: chr22 21435145-21435157. Max. coverage (+): 0. Max coverage (-): 0

Region: chr22 21435158-21435169. Max. coverage (+): 0. Max coverage (-): 0

Region: chr22 21435170-21435181. Max. coverage (+): 0. Max coverage (-): 0

Region: chr22 21435182-21435193. Max. coverage (+): 0. Max coverage (-): 0

Region: chr22 21435194-21435205. Max. coverage (+): 0. Max coverage (-): 0

Region: chr22 21435206-21435218. Max. coverage (+): 0. Max coverage (-): 0

Region: chr22 21435219-21435230. Max. coverage (+): 0. Max coverage (-): 0

Region: chr22 21435231-21435242. Max. coverage (+): 0. Max coverage (-): 0

Region: chr22 21435243-21435254. Max. coverage (+): 0. Max coverage (-): 0

Region: chr22 21435255-21435266. Max. coverage (+): 0. Max coverage (-): 0

Region: chr22 21435267-21435279. Max. coverage (+): 0. Max coverage (-): 0

Region: chr22 21435280-21435291. Max. coverage (+): 0. Max coverage (-): 0

Region: chr22 21435292-21435303. Max. coverage (+): 0. Max coverage (-): 0

Region: chr22 21435304-21435315. Max. coverage (+): 0. Max coverage (-): 0

Region: chr22 21435316-21435327. Max. coverage (+): 0. Max coverage (-): 0

Region: chr22 21435328-21435340. Max. coverage (+): 0. Max coverage (-): 0

Region: chr22 21435341-21435352. Max. coverage (+): 0. Max coverage (-): 0

Region: chr22 21435353-21435364. Max. coverage (+): 0. Max coverage (-): 0

Region: chr22 21435365-21435376. Max. coverage (+): 0. Max coverage (-): 0

Region: chr22 21435377-21435388. Max. coverage (+): 0. Max coverage (-): 0

Region: chr22 21435389-21435401. Max. coverage (+): 0. Max coverage (-): 0

Region: chr22 21435402-21435413. Max. coverage (+): 0. Max coverage (-): 0

Region: chr22 21435414-21435425. Max. coverage (+): 0. Max coverage (-): 0

Region: chr22 21435426-21435437. Max. coverage (+): 0. Max coverage (-): 0

Region: chr22 21435438-21435449. Max. coverage (+): 0. Max coverage (-): 0

Region: chr22 21435450-21435462. Max. coverage (+): 0. Max coverage (-): 0

Region: chr22 21435463-21435474. Max. coverage (+): 0. Max coverage (-): 0

Region: chr22 21435475-21435486. Max. coverage (+): 0. Max coverage (-): 0

Region: chr22 21435487-21435498. Max. coverage (+): 0. Max coverage (-): 0

Region: chr22 21435499-21435510. Max. coverage (+): 0. Max coverage (-): 0

Region: chr22 21435511-21435523. Max. coverage (+): 0. Max coverage (-): 0

Region: chr22 21435524-21435535. Max. coverage (+): 0. Max coverage (-): 0

Region: chr22 21435536-21435547. Max. coverage (+): 6.17. Max coverage (-): 0

Region: chr22 21435548-21435559. Max. coverage (+): 0. Max coverage (-): 0

Region: chr22 21435560-21435571. Max. coverage (+): 0. Max coverage (-): 0

Region: chr22 21435572-21435584. Max. coverage (+): 3.24. Max coverage (-): 0

Region: chr22 21435585-21435596. Max. coverage (+): 0. Max coverage (-): 0

Region: chr22 21435597-21435608. Max. coverage (+): 0. Max coverage (-): 0

Region: chr22 21435609-21435620. Max. coverage (+): 0. Max coverage (-): 0

Region: chr22 21435621-21435632. Max. coverage (+): 0. Max coverage (-): 0

Region: chr22 21435633-21435645. Max. coverage (+): 0. Max coverage (-): 0

Region: chr22 21435646-21435657. Max. coverage (+): 0. Max coverage (-): 0

Region: chr22 21435658-21435669. Max. coverage (+): 4.18. Max coverage (-): 0

Region: chr22 21435670-21435681. Max. coverage (+): 6.07. Max coverage (-): 0

Region: chr22 21435682-21435693. Max. coverage (+): 0. Max coverage (-): 0

Region: chr22 21435694-21435706. Max. coverage (+): 0. Max coverage (-): 0

Region: chr22 21435707-21435718. Max. coverage (+): 0. Max coverage (-): 0

Region: chr22 21435719-21435730. Max. coverage (+): 4.65. Max coverage (-): 0

Region: chr22 21435731-21435742. Max. coverage (+): 3.34. Max coverage (-): 0

Region: chr22 21435743-21435754. Max. coverage (+): 0.69. Max coverage (-): 0

Region: chr22 21435755-21435767. Max. coverage (+): 0.69. Max coverage (-): 0

Region: chr22 21435768-21435779. Max. coverage (+): 0. Max coverage (-): 0

Region: chr22 21435780-21435791. Max. coverage (+): 0. Max coverage (-): 0

Region: chr22 21435792-21435803. Max. coverage (+): 0. Max coverage (-): 0

Region: chr22 21435804-21435815. Max. coverage (+): 0. Max coverage (-): 0

Region: chr22 21435816-21435827. Max. coverage (+): 0. Max coverage (-): 0

Region: chr22 21435828-21435840. Max. coverage (+): 0.93. Max coverage (-): 0

Region: chr22 21435841-21435852. Max. coverage (+): 0.93. Max coverage (-): 0

Region: chr22 21435853-21435864. Max. coverage (+): 0. Max coverage (-): 0

Region: chr22 21435865-21435876. Max. coverage (+): 0. Max coverage (-): 0

Region: chr22 21435877-21435888. Max. coverage (+): 0.67. Max coverage (-): 0

Region: chr22 21435889-21435901. Max. coverage (+): 3.02. Max coverage (-): 0

Region: chr22 21435902-21435913. Max. coverage (+): 4.49. Max coverage (-): 0

Region: chr22 21435914-21435925. Max. coverage (+): 0. Max coverage (-): 0

Region: chr22 21435926-21435937. Max. coverage (+): 0. Max coverage (-): 0

Region: chr22 21435938-21435949. Max. coverage (+): 0. Max coverage (-): 0

Region: chr22 21435950-21435962. Max. coverage (+): 0. Max coverage (-): 0

Region: chr22 21435963-21435974. Max. coverage (+): 0. Max coverage (-): 0

Region: chr22 21435975-21435986. Max. coverage (+): 0. Max coverage (-): 0

Region: chr22 21435987-21435998. Max. coverage (+): 0. Max coverage (-): 0

Region: chr22 21435999-21436010. Max. coverage (+): 0. Max coverage (-): 0

Region: chr22 21436011-21436023. Max. coverage (+): 0. Max coverage (-): 0

Region: chr22 21436024-21436035. Max. coverage (+): 0. Max coverage (-): 0

Region: chr22 21436036-21436047. Max. coverage (+): 0. Max coverage (-): 0

Region: chr22 21436048-21436059. Max. coverage (+): 2.15. Max coverage (-): 0

Region: chr22 21436060-21436071. Max. coverage (+): 2.15. Max coverage (-): 0

Region: chr22 21436072-21436084. Max. coverage (+): 4.81. Max coverage (-): 0

Region: chr22 21436085-21436096. Max. coverage (+): 1.63. Max coverage (-): 0

Region: chr22 21436097-21436108. Max. coverage (+): 0. Max coverage (-): 0

Region: chr22 21436109-21436120. Max. coverage (+): 0. Max coverage (-): 0

Region: chr22 21436121-21436132. Max. coverage (+): 0. Max coverage (-): 0

Region: chr22 21436133-21436145. Max. coverage (+): 3.95. Max coverage (-): 0

Region: chr22 21436146-21436157. Max. coverage (+): 3.95. Max coverage (-): 0

Region: chr22 21436158-21436169. Max. coverage (+): 5.36. Max coverage (-): 0

Region: chr22 21436170-21436181. Max. coverage (+): 0.9. Max coverage (-): 0

Region: chr22 21436182-21436193. Max. coverage (+): 0.51. Max coverage (-): 0

Region: chr22 21436194-21436206. Max. coverage (+): 0. Max coverage (-): 0

Region: chr22 21436207-21436218. Max. coverage (+): 13.95. Max coverage (-): 0

Region: chr22 21436219-21436230. Max. coverage (+): 6.55. Max coverage (-): 0

Region: chr22 21436231-21436242. Max. coverage (+): 0. Max coverage (-): 0

Region: chr22 21436243-21436254. Max. coverage (+): 0. Max coverage (-): 0

Region: chr22 21436255-21436267. Max. coverage (+): 2. Max coverage (-): 0

Region: chr22 21436268-21436279. Max. coverage (+): 0. Max coverage (-): 0

Region: chr22 21436280-21436291. Max. coverage (+): 0. Max coverage (-): 0

Region: chr22 21436292-21436303. Max. coverage (+): 0. Max coverage (-): 0

Region: chr22 21436304-21436315. Max. coverage (+): 0. Max coverage (-): 0

Region: chr22 21436316-21436328. Max. coverage (+): 0. Max coverage (-): 0

Region: chr22 21436329-21436340. Max. coverage (+): 0. Max coverage (-): 0

Region: chr22 21436341-21436352. Max. coverage (+): 0. Max coverage (-): 0

Region: chr22 21436353-21436364. Max. coverage (+): 1.8. Max coverage (-): 0

Region: chr22 21436365-21436376. Max. coverage (+): 0. Max coverage (-): 0

Region: chr22 21436377-. Max. coverage (+): 0. Max coverage (-): 0

RepeatMasker Color Code

**+**

100-98% Identity

<98-95% Identity

<95-90% Identity

<90-85% Identity

<85-80% Identity

<80-75% Identity

<75-70% Identity

<70% Identity

**-**

Gene Set Color Code

**+**

Gene

Pseudogene

**-**

Topology/Coverage Color Code

Coverage Plus Strand

Coverage Minus Strand

Mainstrand: Plus

Mainstrand: Minus

Complementary Strand

Flanking Region  
(if option -flank >0)

Gene Set Annotation  
  
RepeatMasker Annotation  

**1. MER20B**: 21430485-21430704 (-), Divergence to consensus: 36.5%  
**2. MER20B**: 21430811-21430988 (-), Divergence to consensus: 37.1%  
**3. BOV-A2**: 21430990-21431119 (+), Divergence to consensus: 11.5%  
**4. MER20B**: 21431114-21431445 (-), Divergence to consensus: 50.3%  
**5. MIR**: 21431757-21431979 (+), Divergence to consensus: 35.3%  
**6. L2b**: 21432528-21432932 (+), Divergence to consensus: 53.3%  
**7. Bov-tA3**: 21433695-21433881 (-), Divergence to consensus: 10.7%  
**8. MIRb**: 21433913-21434132 (-), Divergence to consensus: 38.3%  
**9. MIRb**: 21434864-21434988 (+), Divergence to consensus: 39.2%  
**10. MIRb**: 21435068-21435309 (+), Divergence to consensus: 41.7%  
**11. Bov-tA2**: 21435327-21435521 (-), Divergence to consensus: 15.4%

  
Transcription Factor Binding Sites  

**Gata4** (Sequence: AGATAAC (-): 21435900)  
**SOX9** (Sequence: CTATTGTT (+): 21434233)  
**Gata4** (Sequence: CTTATCT (+): 21436270)
