# Supplementary material for: Small RNA sequencing of cryopreserved semen from single bull revealed altered miRNAs and piRNAs expression between High- and Low-motile sperm populations
Source: BMC Genomics. 2017 Jan 4;18:14. doi: 10.1186/s12864-016-3394-7 (PMC5209821; doi:10.1186/s12864-016-3394-7)
Supplement: Additional file 3: — Details for each piRNA clusters found in High Motile (HM) sperm fraction. Genes, repeats, transposable elements and transcription factors binding sites falling within the cluster regions were reported. (ZIP 1896 kb) [file 12864_2016_3394_MOESM3_ESM.zip › 51.html]

piRNA cluster 51


Predicted piRNA cluster no. 51     previous   next
  

Show proTRAC run info
Hide proTRAC run info

================================= proTRAC ====================================  
VERSION: 2.1                                    LAST MODIFIED: 06. October 2015  
  
Please cite:  
Rosenkranz D, Zischler H. proTRAC - a software for probabilistic piRNA cluster  
detection, visualization and analysis. 2012. BMC Bioinformatics 13:5.  
  
and (for proTRAC 2.0 and later):  
Rosenkranz D, Rudloff S, Bastuck K, Ketting RF, Zischler H. Tupaia small RNAs  
provide insights into function and evolution of RNAi-based transposon defense  
in mammals. 2015. RNA 21(5):911-922.  
  
Contact:  
David Rosenkranz  
Institute of Anthropology, small RNA group  
Johannes Gutenberg University Mainz  
email: rosenkranz@uni-mainz.de  
  
You can find the latest proTRAC version at:  
http://sourceforge.net/projects/protrac/files  
http://www.smallRNAgroup-mainz.de/software  
==============================================================================  
  
PARAMETERS:  
Map file: .............../storage/core/barbara/genhome/smallRNA/fertility/Sample\_motile/pirna/Sample\_motile\_26-33\_collapsed.fa.no-dust.map.weighted-10000-1000-b-0  
Genome file: ............/storage/core/barbara/genhome/smallRNA/fertility/Sample\_all/pirna/bt\_311\_chrY.fa  
RepeatMasker annotation: /storage/genomes/bt\_umd31/GCF\_000003055.6\_Bos\_taurus\_UMD\_3.1.1\_repeatMasker\_chr.out  
GeneSet:................./storage/core/barbara/genhome/smallRNA/fertility/Sample\_all/pirna/full.gtf  
  
Significant (p<=0.01) hit density will be calculated based  
on observed hit distribution.  
  
Sliding window size: ........................................ 5000 bp  
Sliding window increament: .................................. 1000 bp  
Normalize each hit by number of genomic hits: ............... 1 [0=no/1=yes]  
Normalize each hit by number of sequence reads: ............. 1 [0=no/1=yes]  
Normalize values (-> per million mapped reads): ............. 1 [0=no/1=yes]  
Min. fraction of hits with 1T(U) or 10A: .................... 0.75  
Alternatively: Min. fraction of hits with 1T(U) and 10A: .... 0.5  
Min. fraction of hits with typical piRNA length: ............ 0.75  
Typical piRNA length: ....................................... 26-33 nt  
Min. size of a piRNA cluster: ............................... 5000 bp.  
Min. number of hits (absolute): ............................. 0  
Min. number of hits (normalized): ........................... 0  
Min. fraction of hits on the mainstrand: .................... 0.75  
Top fraction of mapped sequences (in terms of read counts): . 1%  
Top fraction accounts for max. n% of sequence reads: ........ 90%  
Min. fraction of hits on each arm of a bidirectional cluster: 0.1  
Output image file for each cluster: ......................... 0 [0=no/1=yes]  
Output html file for each cluster: .......................... 1 [0=no/1=yes]  
Output a summary table: ..................................... 1 [0=no/1=yes]  
Output a FASTA file for each cluster (piRNA sequences): ..... 1 [0=no/1=yes]  
Output a FASTA file comprising cluster sequences: ........... 1 [0=no/1=yes]  
Search DNA motifs in clusters: .............................. 1 [0=no/1=yes]  
Output flanking sequences: +/- .............................. 0 bp  
Output ~.pTi file: .......................................... 1 [0=no/1=yes]  
==============================================================================  
  
  
Genome size (without gaps): ............ 2678902517 bp  
Gaps (N/X/-): .......................... 53837044 bp  
Mapped reads: .......................... 658825247023  
Non-identical sequences: ............... 514171  
Genomic hits: .......................... 764233  
Significant densitiy of mapped reads: .. 12867599.5173724 reads/kb

Show proTRAC cluster info
Hide proTRAC cluster info

|  |  |
| --- | --- |
| Location | chr22 |
| Coordinates | 44545191-44552499 |
| Size [bp] | 7309 |
| Sequence hit loci | 73 |
| Mapped reads (normalized) | 103400332 |
| Mapped reads (normalized) per kb | 14146987.5 |
| Normalized reads with 1T (1U) | 88.4% |
| Normalized reads with 10A | 36.3% |
| Normalized reads with length 26-33 nt | 100% |
| Normalized reads on the main strand(s) | 99.4% |
| Predicted directionality | mono:plus |

100%

0%

1T (1U)  
reads

10A reads

26-33 nt  
reads

reads on mainstrand

**Either the amount of reads with 1T (1U) OR 10A has to exceed 75% (set with option: -1Tor10A)  
Alternatively the amount of reads with 1T (1U) AND 10A has to exceed 50% (set with option: -1Tand10A)  
Minimum amount of reads with preferred size is 75% (set with option: -pisize)  
Minimum amount of reads on the main strand(s) is 75% (set with option: -clstrand)**

Show read coverage
Hide read coverage

WHAT DO I SEE HERE?  
This chart shows the location of mapped sequence reads within a predicted piRNA cluster. The color refers to the number of genomic hits produced by the sequence read in question. A dark red bar indicates that this sequence read produces many other hits elsewhere in the genome. Many adjacent red or yellow bars can indicate the presence of a multi-copy element such as transposons or rRNA genes. A dark green bar indicates that this sequence read maps uniquely to this locus.

1 hit

2-5 hits

6-10 hits

11-20 hits

21-50 hits

51-100 hits

> 100 hits

chr22

44545191

44552499

Gene Set

RepeatMasker

Mapped  
Reads

17.66

plus strand

minus strand

17.66

Region: chr22 21436378-44545198. Max. coverage (+): 0. Max coverage (-): 0.94

Region: chr22 44545199-44545212. Max. coverage (+): 0. Max coverage (-): 0

Region: chr22 44545213-44545227. Max. coverage (+): 0. Max coverage (-): 0

Region: chr22 44545228-44545242. Max. coverage (+): 0. Max coverage (-): 0

Region: chr22 44545243-44545256. Max. coverage (+): 0. Max coverage (-): 0

Region: chr22 44545257-44545271. Max. coverage (+): 0. Max coverage (-): 0

Region: chr22 44545272-44545286. Max. coverage (+): 0. Max coverage (-): 0

Region: chr22 44545287-44545300. Max. coverage (+): 0. Max coverage (-): 0

Region: chr22 44545301-44545315. Max. coverage (+): 0. Max coverage (-): 0

Region: chr22 44545316-44545329. Max. coverage (+): 0. Max coverage (-): 0

Region: chr22 44545330-44545344. Max. coverage (+): 0. Max coverage (-): 0

Region: chr22 44545345-44545359. Max. coverage (+): 0. Max coverage (-): 0

Region: chr22 44545360-44545373. Max. coverage (+): 0. Max coverage (-): 0

Region: chr22 44545374-44545388. Max. coverage (+): 0. Max coverage (-): 0

Region: chr22 44545389-44545402. Max. coverage (+): 0. Max coverage (-): 0

Region: chr22 44545403-44545417. Max. coverage (+): 0. Max coverage (-): 0

Region: chr22 44545418-44545432. Max. coverage (+): 0. Max coverage (-): 0

Region: chr22 44545433-44545446. Max. coverage (+): 0. Max coverage (-): 0

Region: chr22 44545447-44545461. Max. coverage (+): 0. Max coverage (-): 0

Region: chr22 44545462-44545476. Max. coverage (+): 0. Max coverage (-): 0

Region: chr22 44545477-44545490. Max. coverage (+): 0. Max coverage (-): 0

Region: chr22 44545491-44545505. Max. coverage (+): 0. Max coverage (-): 0

Region: chr22 44545506-44545519. Max. coverage (+): 0. Max coverage (-): 0

Region: chr22 44545520-44545534. Max. coverage (+): 0. Max coverage (-): 0

Region: chr22 44545535-44545549. Max. coverage (+): 0. Max coverage (-): 0

Region: chr22 44545550-44545563. Max. coverage (+): 0. Max coverage (-): 0

Region: chr22 44545564-44545578. Max. coverage (+): 0. Max coverage (-): 0

Region: chr22 44545579-44545592. Max. coverage (+): 0. Max coverage (-): 0

Region: chr22 44545593-44545607. Max. coverage (+): 0. Max coverage (-): 0

Region: chr22 44545608-44545622. Max. coverage (+): 0. Max coverage (-): 0

Region: chr22 44545623-44545636. Max. coverage (+): 0. Max coverage (-): 0

Region: chr22 44545637-44545651. Max. coverage (+): 0. Max coverage (-): 0

Region: chr22 44545652-44545666. Max. coverage (+): 0. Max coverage (-): 0

Region: chr22 44545667-44545680. Max. coverage (+): 0. Max coverage (-): 0

Region: chr22 44545681-44545695. Max. coverage (+): 0. Max coverage (-): 0

Region: chr22 44545696-44545709. Max. coverage (+): 0. Max coverage (-): 0

Region: chr22 44545710-44545724. Max. coverage (+): 0. Max coverage (-): 0

Region: chr22 44545725-44545739. Max. coverage (+): 0. Max coverage (-): 0

Region: chr22 44545740-44545753. Max. coverage (+): 0. Max coverage (-): 0

Region: chr22 44545754-44545768. Max. coverage (+): 0. Max coverage (-): 0

Region: chr22 44545769-44545783. Max. coverage (+): 0. Max coverage (-): 0

Region: chr22 44545784-44545797. Max. coverage (+): 0. Max coverage (-): 0

Region: chr22 44545798-44545812. Max. coverage (+): 0. Max coverage (-): 0

Region: chr22 44545813-44545826. Max. coverage (+): 0. Max coverage (-): 0

Region: chr22 44545827-44545841. Max. coverage (+): 0. Max coverage (-): 0

Region: chr22 44545842-44545856. Max. coverage (+): 0. Max coverage (-): 0

Region: chr22 44545857-44545870. Max. coverage (+): 0. Max coverage (-): 0

Region: chr22 44545871-44545885. Max. coverage (+): 0. Max coverage (-): 0

Region: chr22 44545886-44545899. Max. coverage (+): 0. Max coverage (-): 0

Region: chr22 44545900-44545914. Max. coverage (+): 0. Max coverage (-): 0

Region: chr22 44545915-44545929. Max. coverage (+): 0. Max coverage (-): 0

Region: chr22 44545930-44545943. Max. coverage (+): 0. Max coverage (-): 0

Region: chr22 44545944-44545958. Max. coverage (+): 0. Max coverage (-): 0

Region: chr22 44545959-44545973. Max. coverage (+): 0. Max coverage (-): 0

Region: chr22 44545974-44545987. Max. coverage (+): 0. Max coverage (-): 0

Region: chr22 44545988-44546002. Max. coverage (+): 0. Max coverage (-): 0

Region: chr22 44546003-44546016. Max. coverage (+): 0. Max coverage (-): 0

Region: chr22 44546017-44546031. Max. coverage (+): 0. Max coverage (-): 0

Region: chr22 44546032-44546046. Max. coverage (+): 0. Max coverage (-): 0

Region: chr22 44546047-44546060. Max. coverage (+): 0. Max coverage (-): 0

Region: chr22 44546061-44546075. Max. coverage (+): 0. Max coverage (-): 0

Region: chr22 44546076-44546090. Max. coverage (+): 0. Max coverage (-): 0

Region: chr22 44546091-44546104. Max. coverage (+): 0. Max coverage (-): 0

Region: chr22 44546105-44546119. Max. coverage (+): 0. Max coverage (-): 0

Region: chr22 44546120-44546133. Max. coverage (+): 0. Max coverage (-): 0

Region: chr22 44546134-44546148. Max. coverage (+): 0. Max coverage (-): 0

Region: chr22 44546149-44546163. Max. coverage (+): 0. Max coverage (-): 0

Region: chr22 44546164-44546177. Max. coverage (+): 1.37. Max coverage (-): 0

Region: chr22 44546178-44546192. Max. coverage (+): 0. Max coverage (-): 0

Region: chr22 44546193-44546206. Max. coverage (+): 0. Max coverage (-): 0

Region: chr22 44546207-44546221. Max. coverage (+): 0. Max coverage (-): 0

Region: chr22 44546222-44546236. Max. coverage (+): 0.62. Max coverage (-): 0

Region: chr22 44546237-44546250. Max. coverage (+): 0.62. Max coverage (-): 0

Region: chr22 44546251-44546265. Max. coverage (+): 0. Max coverage (-): 0

Region: chr22 44546266-44546280. Max. coverage (+): 0. Max coverage (-): 0

Region: chr22 44546281-44546294. Max. coverage (+): 0. Max coverage (-): 0

Region: chr22 44546295-44546309. Max. coverage (+): 0. Max coverage (-): 0

Region: chr22 44546310-44546323. Max. coverage (+): 0. Max coverage (-): 0

Region: chr22 44546324-44546338. Max. coverage (+): 0. Max coverage (-): 0

Region: chr22 44546339-44546353. Max. coverage (+): 0. Max coverage (-): 0

Region: chr22 44546354-44546367. Max. coverage (+): 0. Max coverage (-): 0

Region: chr22 44546368-44546382. Max. coverage (+): 0. Max coverage (-): 0

Region: chr22 44546383-44546396. Max. coverage (+): 0. Max coverage (-): 0

Region: chr22 44546397-44546411. Max. coverage (+): 0. Max coverage (-): 0

Region: chr22 44546412-44546426. Max. coverage (+): 0. Max coverage (-): 0

Region: chr22 44546427-44546440. Max. coverage (+): 0. Max coverage (-): 0

Region: chr22 44546441-44546455. Max. coverage (+): 0. Max coverage (-): 0

Region: chr22 44546456-44546470. Max. coverage (+): 0. Max coverage (-): 0

Region: chr22 44546471-44546484. Max. coverage (+): 0. Max coverage (-): 0

Region: chr22 44546485-44546499. Max. coverage (+): 0. Max coverage (-): 0

Region: chr22 44546500-44546513. Max. coverage (+): 0. Max coverage (-): 0

Region: chr22 44546514-44546528. Max. coverage (+): 0. Max coverage (-): 0

Region: chr22 44546529-44546543. Max. coverage (+): 0. Max coverage (-): 0

Region: chr22 44546544-44546557. Max. coverage (+): 0. Max coverage (-): 0

Region: chr22 44546558-44546572. Max. coverage (+): 0. Max coverage (-): 0

Region: chr22 44546573-44546587. Max. coverage (+): 0. Max coverage (-): 0

Region: chr22 44546588-44546601. Max. coverage (+): 0. Max coverage (-): 0

Region: chr22 44546602-44546616. Max. coverage (+): 0. Max coverage (-): 0

Region: chr22 44546617-44546630. Max. coverage (+): 0. Max coverage (-): 0

Region: chr22 44546631-44546645. Max. coverage (+): 0. Max coverage (-): 0

Region: chr22 44546646-44546660. Max. coverage (+): 0. Max coverage (-): 0

Region: chr22 44546661-44546674. Max. coverage (+): 0. Max coverage (-): 0

Region: chr22 44546675-44546689. Max. coverage (+): 0. Max coverage (-): 0

Region: chr22 44546690-44546703. Max. coverage (+): 0. Max coverage (-): 0

Region: chr22 44546704-44546718. Max. coverage (+): 0. Max coverage (-): 0

Region: chr22 44546719-44546733. Max. coverage (+): 0. Max coverage (-): 0

Region: chr22 44546734-44546747. Max. coverage (+): 0. Max coverage (-): 0

Region: chr22 44546748-44546762. Max. coverage (+): 0. Max coverage (-): 0

Region: chr22 44546763-44546777. Max. coverage (+): 0. Max coverage (-): 0

Region: chr22 44546778-44546791. Max. coverage (+): 0. Max coverage (-): 0

Region: chr22 44546792-44546806. Max. coverage (+): 0. Max coverage (-): 0

Region: chr22 44546807-44546820. Max. coverage (+): 0. Max coverage (-): 0

Region: chr22 44546821-44546835. Max. coverage (+): 0. Max coverage (-): 0

Region: chr22 44546836-44546850. Max. coverage (+): 0. Max coverage (-): 0

Region: chr22 44546851-44546864. Max. coverage (+): 0. Max coverage (-): 0

Region: chr22 44546865-44546879. Max. coverage (+): 0. Max coverage (-): 0

Region: chr22 44546880-44546893. Max. coverage (+): 0. Max coverage (-): 0

Region: chr22 44546894-44546908. Max. coverage (+): 0. Max coverage (-): 0

Region: chr22 44546909-44546923. Max. coverage (+): 0. Max coverage (-): 0

Region: chr22 44546924-44546937. Max. coverage (+): 0. Max coverage (-): 0

Region: chr22 44546938-44546952. Max. coverage (+): 0. Max coverage (-): 0

Region: chr22 44546953-44546967. Max. coverage (+): 0. Max coverage (-): 0

Region: chr22 44546968-44546981. Max. coverage (+): 0. Max coverage (-): 0

Region: chr22 44546982-44546996. Max. coverage (+): 0. Max coverage (-): 0

Region: chr22 44546997-44547010. Max. coverage (+): 0. Max coverage (-): 0

Region: chr22 44547011-44547025. Max. coverage (+): 0. Max coverage (-): 0

Region: chr22 44547026-44547040. Max. coverage (+): 0. Max coverage (-): 0

Region: chr22 44547041-44547054. Max. coverage (+): 0. Max coverage (-): 0

Region: chr22 44547055-44547069. Max. coverage (+): 0. Max coverage (-): 0

Region: chr22 44547070-44547084. Max. coverage (+): 0. Max coverage (-): 0

Region: chr22 44547085-44547098. Max. coverage (+): 0. Max coverage (-): 0

Region: chr22 44547099-44547113. Max. coverage (+): 0. Max coverage (-): 0

Region: chr22 44547114-44547127. Max. coverage (+): 0. Max coverage (-): 0

Region: chr22 44547128-44547142. Max. coverage (+): 0. Max coverage (-): 0

Region: chr22 44547143-44547157. Max. coverage (+): 0. Max coverage (-): 0

Region: chr22 44547158-44547171. Max. coverage (+): 0. Max coverage (-): 0

Region: chr22 44547172-44547186. Max. coverage (+): 0. Max coverage (-): 0

Region: chr22 44547187-44547200. Max. coverage (+): 0. Max coverage (-): 0

Region: chr22 44547201-44547215. Max. coverage (+): 0. Max coverage (-): 0

Region: chr22 44547216-44547230. Max. coverage (+): 0. Max coverage (-): 0

Region: chr22 44547231-44547244. Max. coverage (+): 0. Max coverage (-): 0

Region: chr22 44547245-44547259. Max. coverage (+): 0. Max coverage (-): 0

Region: chr22 44547260-44547274. Max. coverage (+): 0. Max coverage (-): 0

Region: chr22 44547275-44547288. Max. coverage (+): 0. Max coverage (-): 0

Region: chr22 44547289-44547303. Max. coverage (+): 0. Max coverage (-): 0

Region: chr22 44547304-44547317. Max. coverage (+): 0. Max coverage (-): 0

Region: chr22 44547318-44547332. Max. coverage (+): 0. Max coverage (-): 0

Region: chr22 44547333-44547347. Max. coverage (+): 0. Max coverage (-): 0

Region: chr22 44547348-44547361. Max. coverage (+): 0. Max coverage (-): 0

Region: chr22 44547362-44547376. Max. coverage (+): 0. Max coverage (-): 0

Region: chr22 44547377-44547391. Max. coverage (+): 4.29. Max coverage (-): 0

Region: chr22 44547392-44547405. Max. coverage (+): 4.29. Max coverage (-): 0

Region: chr22 44547406-44547420. Max. coverage (+): 0. Max coverage (-): 0

Region: chr22 44547421-44547434. Max. coverage (+): 4.06. Max coverage (-): 0

Region: chr22 44547435-44547449. Max. coverage (+): 4.06. Max coverage (-): 0

Region: chr22 44547450-44547464. Max. coverage (+): 0. Max coverage (-): 0

Region: chr22 44547465-44547478. Max. coverage (+): 0. Max coverage (-): 0

Region: chr22 44547479-44547493. Max. coverage (+): 0. Max coverage (-): 0

Region: chr22 44547494-44547507. Max. coverage (+): 0. Max coverage (-): 0

Region: chr22 44547508-44547522. Max. coverage (+): 0. Max coverage (-): 0

Region: chr22 44547523-44547537. Max. coverage (+): 0. Max coverage (-): 0

Region: chr22 44547538-44547551. Max. coverage (+): 0. Max coverage (-): 0

Region: chr22 44547552-44547566. Max. coverage (+): 0. Max coverage (-): 0

Region: chr22 44547567-44547581. Max. coverage (+): 0. Max coverage (-): 0

Region: chr22 44547582-44547595. Max. coverage (+): 0. Max coverage (-): 0

Region: chr22 44547596-44547610. Max. coverage (+): 3.42. Max coverage (-): 0

Region: chr22 44547611-44547624. Max. coverage (+): 0. Max coverage (-): 0

Region: chr22 44547625-44547639. Max. coverage (+): 4.78. Max coverage (-): 0

Region: chr22 44547640-44547654. Max. coverage (+): 9.57. Max coverage (-): 0

Region: chr22 44547655-44547668. Max. coverage (+): 3.03. Max coverage (-): 0

Region: chr22 44547669-44547683. Max. coverage (+): 0. Max coverage (-): 0

Region: chr22 44547684-44547697. Max. coverage (+): 0. Max coverage (-): 0

Region: chr22 44547698-44547712. Max. coverage (+): 3.12. Max coverage (-): 0

Region: chr22 44547713-44547727. Max. coverage (+): 0. Max coverage (-): 0

Region: chr22 44547728-44547741. Max. coverage (+): 0. Max coverage (-): 0

Region: chr22 44547742-44547756. Max. coverage (+): 2.54. Max coverage (-): 0

Region: chr22 44547757-44547771. Max. coverage (+): 2.54. Max coverage (-): 0

Region: chr22 44547772-44547785. Max. coverage (+): 0. Max coverage (-): 0

Region: chr22 44547786-44547800. Max. coverage (+): 0. Max coverage (-): 0

Region: chr22 44547801-44547814. Max. coverage (+): 0. Max coverage (-): 0

Region: chr22 44547815-44547829. Max. coverage (+): 0. Max coverage (-): 0

Region: chr22 44547830-44547844. Max. coverage (+): 4.77. Max coverage (-): 0

Region: chr22 44547845-44547858. Max. coverage (+): 3.78. Max coverage (-): 0

Region: chr22 44547859-44547873. Max. coverage (+): 3.78. Max coverage (-): 0

Region: chr22 44547874-44547888. Max. coverage (+): 0. Max coverage (-): 0

Region: chr22 44547889-44547902. Max. coverage (+): 0. Max coverage (-): 0

Region: chr22 44547903-44547917. Max. coverage (+): 0. Max coverage (-): 0

Region: chr22 44547918-44547931. Max. coverage (+): 0. Max coverage (-): 0

Region: chr22 44547932-44547946. Max. coverage (+): 1.41. Max coverage (-): 0

Region: chr22 44547947-44547961. Max. coverage (+): 1.41. Max coverage (-): 0

Region: chr22 44547962-44547975. Max. coverage (+): 0. Max coverage (-): 0

Region: chr22 44547976-44547990. Max. coverage (+): 0. Max coverage (-): 0

Region: chr22 44547991-44548004. Max. coverage (+): 0. Max coverage (-): 0

Region: chr22 44548005-44548019. Max. coverage (+): 0. Max coverage (-): 0

Region: chr22 44548020-44548034. Max. coverage (+): 0. Max coverage (-): 0

Region: chr22 44548035-44548048. Max. coverage (+): 0. Max coverage (-): 0

Region: chr22 44548049-44548063. Max. coverage (+): 0. Max coverage (-): 0

Region: chr22 44548064-44548078. Max. coverage (+): 0. Max coverage (-): 0

Region: chr22 44548079-44548092. Max. coverage (+): 0. Max coverage (-): 0

Region: chr22 44548093-44548107. Max. coverage (+): 0. Max coverage (-): 0

Region: chr22 44548108-44548121. Max. coverage (+): 0. Max coverage (-): 0

Region: chr22 44548122-44548136. Max. coverage (+): 0. Max coverage (-): 0

Region: chr22 44548137-44548151. Max. coverage (+): 0. Max coverage (-): 0

Region: chr22 44548152-44548165. Max. coverage (+): 0. Max coverage (-): 0

Region: chr22 44548166-44548180. Max. coverage (+): 1.83. Max coverage (-): 0

Region: chr22 44548181-44548194. Max. coverage (+): 0. Max coverage (-): 0

Region: chr22 44548195-44548209. Max. coverage (+): 0. Max coverage (-): 0

Region: chr22 44548210-44548224. Max. coverage (+): 7.23. Max coverage (-): 0

Region: chr22 44548225-44548238. Max. coverage (+): 1.01. Max coverage (-): 0

Region: chr22 44548239-44548253. Max. coverage (+): 0. Max coverage (-): 0

Region: chr22 44548254-44548268. Max. coverage (+): 2.02. Max coverage (-): 0

Region: chr22 44548269-44548282. Max. coverage (+): 0. Max coverage (-): 0

Region: chr22 44548283-44548297. Max. coverage (+): 0. Max coverage (-): 0

Region: chr22 44548298-44548311. Max. coverage (+): 0. Max coverage (-): 0

Region: chr22 44548312-44548326. Max. coverage (+): 0. Max coverage (-): 0

Region: chr22 44548327-44548341. Max. coverage (+): 0. Max coverage (-): 0

Region: chr22 44548342-44548355. Max. coverage (+): 5.14. Max coverage (-): 0

Region: chr22 44548356-44548370. Max. coverage (+): 5.14. Max coverage (-): 0

Region: chr22 44548371-44548385. Max. coverage (+): 0. Max coverage (-): 0

Region: chr22 44548386-44548399. Max. coverage (+): 0. Max coverage (-): 0

Region: chr22 44548400-44548414. Max. coverage (+): 0. Max coverage (-): 0

Region: chr22 44548415-44548428. Max. coverage (+): 0. Max coverage (-): 0

Region: chr22 44548429-44548443. Max. coverage (+): 0. Max coverage (-): 0

Region: chr22 44548444-44548458. Max. coverage (+): 3.12. Max coverage (-): 0

Region: chr22 44548459-44548472. Max. coverage (+): 1.87. Max coverage (-): 0

Region: chr22 44548473-44548487. Max. coverage (+): 0. Max coverage (-): 0

Region: chr22 44548488-44548501. Max. coverage (+): 0. Max coverage (-): 0

Region: chr22 44548502-44548516. Max. coverage (+): 0. Max coverage (-): 0

Region: chr22 44548517-44548531. Max. coverage (+): 0. Max coverage (-): 0

Region: chr22 44548532-44548545. Max. coverage (+): 0. Max coverage (-): 0

Region: chr22 44548546-44548560. Max. coverage (+): 0. Max coverage (-): 0

Region: chr22 44548561-44548575. Max. coverage (+): 0. Max coverage (-): 0

Region: chr22 44548576-44548589. Max. coverage (+): 0. Max coverage (-): 0

Region: chr22 44548590-44548604. Max. coverage (+): 2.22. Max coverage (-): 0

Region: chr22 44548605-44548618. Max. coverage (+): 2.22. Max coverage (-): 0

Region: chr22 44548619-44548633. Max. coverage (+): 3.34. Max coverage (-): 0

Region: chr22 44548634-44548648. Max. coverage (+): 0. Max coverage (-): 0

Region: chr22 44548649-44548662. Max. coverage (+): 0. Max coverage (-): 0

Region: chr22 44548663-44548677. Max. coverage (+): 0. Max coverage (-): 0

Region: chr22 44548678-44548692. Max. coverage (+): 0. Max coverage (-): 0

Region: chr22 44548693-44548706. Max. coverage (+): 0. Max coverage (-): 0

Region: chr22 44548707-44548721. Max. coverage (+): 0. Max coverage (-): 0

Region: chr22 44548722-44548735. Max. coverage (+): 0. Max coverage (-): 0

Region: chr22 44548736-44548750. Max. coverage (+): 0. Max coverage (-): 0

Region: chr22 44548751-44548765. Max. coverage (+): 2.25. Max coverage (-): 0

Region: chr22 44548766-44548779. Max. coverage (+): 5.11. Max coverage (-): 0

Region: chr22 44548780-44548794. Max. coverage (+): 7.48. Max coverage (-): 0

Region: chr22 44548795-44548808. Max. coverage (+): 0. Max coverage (-): 0

Region: chr22 44548809-44548823. Max. coverage (+): 0. Max coverage (-): 0

Region: chr22 44548824-44548838. Max. coverage (+): 0. Max coverage (-): 0

Region: chr22 44548839-44548852. Max. coverage (+): 0. Max coverage (-): 0

Region: chr22 44548853-44548867. Max. coverage (+): 0. Max coverage (-): 0

Region: chr22 44548868-44548882. Max. coverage (+): 0. Max coverage (-): 0

Region: chr22 44548883-44548896. Max. coverage (+): 0. Max coverage (-): 0

Region: chr22 44548897-44548911. Max. coverage (+): 0. Max coverage (-): 0

Region: chr22 44548912-44548925. Max. coverage (+): 0. Max coverage (-): 0

Region: chr22 44548926-44548940. Max. coverage (+): 0. Max coverage (-): 0

Region: chr22 44548941-44548955. Max. coverage (+): 0. Max coverage (-): 0

Region: chr22 44548956-44548969. Max. coverage (+): 0. Max coverage (-): 0

Region: chr22 44548970-44548984. Max. coverage (+): 0. Max coverage (-): 0

Region: chr22 44548985-44548998. Max. coverage (+): 0. Max coverage (-): 0

Region: chr22 44548999-44549013. Max. coverage (+): 0. Max coverage (-): 0

Region: chr22 44549014-44549028. Max. coverage (+): 0. Max coverage (-): 0

Region: chr22 44549029-44549042. Max. coverage (+): 1.04. Max coverage (-): 0

Region: chr22 44549043-44549057. Max. coverage (+): 1.04. Max coverage (-): 0

Region: chr22 44549058-44549072. Max. coverage (+): 1.46. Max coverage (-): 0

Region: chr22 44549073-44549086. Max. coverage (+): 0. Max coverage (-): 0

Region: chr22 44549087-44549101. Max. coverage (+): 0. Max coverage (-): 0

Region: chr22 44549102-44549115. Max. coverage (+): 0. Max coverage (-): 0

Region: chr22 44549116-44549130. Max. coverage (+): 1.92. Max coverage (-): 0

Region: chr22 44549131-44549145. Max. coverage (+): 0. Max coverage (-): 0

Region: chr22 44549146-44549159. Max. coverage (+): 0. Max coverage (-): 0

Region: chr22 44549160-44549174. Max. coverage (+): 6.75. Max coverage (-): 0

Region: chr22 44549175-44549189. Max. coverage (+): 8.01. Max coverage (-): 0

Region: chr22 44549190-44549203. Max. coverage (+): 3.22. Max coverage (-): 0

Region: chr22 44549204-44549218. Max. coverage (+): 0. Max coverage (-): 0

Region: chr22 44549219-44549232. Max. coverage (+): 13.77. Max coverage (-): 0

Region: chr22 44549233-44549247. Max. coverage (+): 17.66. Max coverage (-): 0

Region: chr22 44549248-44549262. Max. coverage (+): 0. Max coverage (-): 0

Region: chr22 44549263-44549276. Max. coverage (+): 0. Max coverage (-): 0

Region: chr22 44549277-44549291. Max. coverage (+): 0. Max coverage (-): 0

Region: chr22 44549292-44549305. Max. coverage (+): 0. Max coverage (-): 0

Region: chr22 44549306-44549320. Max. coverage (+): 0. Max coverage (-): 0

Region: chr22 44549321-44549335. Max. coverage (+): 0. Max coverage (-): 0

Region: chr22 44549336-44549349. Max. coverage (+): 0. Max coverage (-): 0

Region: chr22 44549350-44549364. Max. coverage (+): 0. Max coverage (-): 0

Region: chr22 44549365-44549379. Max. coverage (+): 4.21. Max coverage (-): 0

Region: chr22 44549380-44549393. Max. coverage (+): 4.21. Max coverage (-): 0

Region: chr22 44549394-44549408. Max. coverage (+): 2.57. Max coverage (-): 0

Region: chr22 44549409-44549422. Max. coverage (+): 0. Max coverage (-): 0

Region: chr22 44549423-44549437. Max. coverage (+): 2.72. Max coverage (-): 0

Region: chr22 44549438-44549452. Max. coverage (+): 2.72. Max coverage (-): 0

Region: chr22 44549453-44549466. Max. coverage (+): 0. Max coverage (-): 0

Region: chr22 44549467-44549481. Max. coverage (+): 1.17. Max coverage (-): 0

Region: chr22 44549482-44549496. Max. coverage (+): 1.17. Max coverage (-): 0

Region: chr22 44549497-44549510. Max. coverage (+): 3.85. Max coverage (-): 0

Region: chr22 44549511-44549525. Max. coverage (+): 7.75. Max coverage (-): 0

Region: chr22 44549526-44549539. Max. coverage (+): 0. Max coverage (-): 0

Region: chr22 44549540-44549554. Max. coverage (+): 0. Max coverage (-): 0

Region: chr22 44549555-44549569. Max. coverage (+): 0. Max coverage (-): 0

Region: chr22 44549570-44549583. Max. coverage (+): 0. Max coverage (-): 0

Region: chr22 44549584-44549598. Max. coverage (+): 0. Max coverage (-): 0

Region: chr22 44549599-44549612. Max. coverage (+): 0. Max coverage (-): 0

Region: chr22 44549613-44549627. Max. coverage (+): 0. Max coverage (-): 0

Region: chr22 44549628-44549642. Max. coverage (+): 0. Max coverage (-): 0

Region: chr22 44549643-44549656. Max. coverage (+): 0. Max coverage (-): 0

Region: chr22 44549657-44549671. Max. coverage (+): 0. Max coverage (-): 0

Region: chr22 44549672-44549686. Max. coverage (+): 0. Max coverage (-): 0

Region: chr22 44549687-44549700. Max. coverage (+): 0. Max coverage (-): 0

Region: chr22 44549701-44549715. Max. coverage (+): 0. Max coverage (-): 0

Region: chr22 44549716-44549729. Max. coverage (+): 0. Max coverage (-): 0

Region: chr22 44549730-44549744. Max. coverage (+): 0. Max coverage (-): 0

Region: chr22 44549745-44549759. Max. coverage (+): 0. Max coverage (-): 0

Region: chr22 44549760-44549773. Max. coverage (+): 0. Max coverage (-): 0

Region: chr22 44549774-44549788. Max. coverage (+): 0. Max coverage (-): 0

Region: chr22 44549789-44549802. Max. coverage (+): 0. Max coverage (-): 0

Region: chr22 44549803-44549817. Max. coverage (+): 0. Max coverage (-): 0

Region: chr22 44549818-44549832. Max. coverage (+): 0. Max coverage (-): 0

Region: chr22 44549833-44549846. Max. coverage (+): 0. Max coverage (-): 0

Region: chr22 44549847-44549861. Max. coverage (+): 0. Max coverage (-): 0

Region: chr22 44549862-44549876. Max. coverage (+): 0. Max coverage (-): 0

Region: chr22 44549877-44549890. Max. coverage (+): 0. Max coverage (-): 0

Region: chr22 44549891-44549905. Max. coverage (+): 0. Max coverage (-): 0

Region: chr22 44549906-44549919. Max. coverage (+): 0. Max coverage (-): 0

Region: chr22 44549920-44549934. Max. coverage (+): 0. Max coverage (-): 0

Region: chr22 44549935-44549949. Max. coverage (+): 0. Max coverage (-): 0

Region: chr22 44549950-44549963. Max. coverage (+): 0. Max coverage (-): 0

Region: chr22 44549964-44549978. Max. coverage (+): 0. Max coverage (-): 0

Region: chr22 44549979-44549993. Max. coverage (+): 0. Max coverage (-): 0

Region: chr22 44549994-44550007. Max. coverage (+): 0. Max coverage (-): 0

Region: chr22 44550008-44550022. Max. coverage (+): 0. Max coverage (-): 0

Region: chr22 44550023-44550036. Max. coverage (+): 0. Max coverage (-): 0

Region: chr22 44550037-44550051. Max. coverage (+): 0. Max coverage (-): 0

Region: chr22 44550052-44550066. Max. coverage (+): 0. Max coverage (-): 0

Region: chr22 44550067-44550080. Max. coverage (+): 0. Max coverage (-): 0

Region: chr22 44550081-44550095. Max. coverage (+): 1.25. Max coverage (-): 0

Region: chr22 44550096-44550109. Max. coverage (+): 5.14. Max coverage (-): 0

Region: chr22 44550110-44550124. Max. coverage (+): 1.26. Max coverage (-): 0

Region: chr22 44550125-44550139. Max. coverage (+): 0. Max coverage (-): 0

Region: chr22 44550140-44550153. Max. coverage (+): 0. Max coverage (-): 0

Region: chr22 44550154-44550168. Max. coverage (+): 0. Max coverage (-): 0

Region: chr22 44550169-44550183. Max. coverage (+): 0. Max coverage (-): 0

Region: chr22 44550184-44550197. Max. coverage (+): 0. Max coverage (-): 0

Region: chr22 44550198-44550212. Max. coverage (+): 0. Max coverage (-): 0

Region: chr22 44550213-44550226. Max. coverage (+): 0. Max coverage (-): 0

Region: chr22 44550227-44550241. Max. coverage (+): 0. Max coverage (-): 0

Region: chr22 44550242-44550256. Max. coverage (+): 0. Max coverage (-): 0

Region: chr22 44550257-44550270. Max. coverage (+): 0. Max coverage (-): 0

Region: chr22 44550271-44550285. Max. coverage (+): 0. Max coverage (-): 0

Region: chr22 44550286-44550299. Max. coverage (+): 0. Max coverage (-): 0

Region: chr22 44550300-44550314. Max. coverage (+): 0. Max coverage (-): 0

Region: chr22 44550315-44550329. Max. coverage (+): 0. Max coverage (-): 0

Region: chr22 44550330-44550343. Max. coverage (+): 0. Max coverage (-): 0

Region: chr22 44550344-44550358. Max. coverage (+): 0. Max coverage (-): 0

Region: chr22 44550359-44550373. Max. coverage (+): 0. Max coverage (-): 0

Region: chr22 44550374-44550387. Max. coverage (+): 0. Max coverage (-): 0

Region: chr22 44550388-44550402. Max. coverage (+): 0. Max coverage (-): 0

Region: chr22 44550403-44550416. Max. coverage (+): 0. Max coverage (-): 0

Region: chr22 44550417-44550431. Max. coverage (+): 0. Max coverage (-): 0

Region: chr22 44550432-44550446. Max. coverage (+): 1.39. Max coverage (-): 0

Region: chr22 44550447-44550460. Max. coverage (+): 0. Max coverage (-): 0

Region: chr22 44550461-44550475. Max. coverage (+): 0. Max coverage (-): 0

Region: chr22 44550476-44550490. Max. coverage (+): 0. Max coverage (-): 0

Region: chr22 44550491-44550504. Max. coverage (+): 0. Max coverage (-): 0

Region: chr22 44550505-44550519. Max. coverage (+): 0. Max coverage (-): 0

Region: chr22 44550520-44550533. Max. coverage (+): 0. Max coverage (-): 0

Region: chr22 44550534-44550548. Max. coverage (+): 0. Max coverage (-): 0

Region: chr22 44550549-44550563. Max. coverage (+): 0. Max coverage (-): 0

Region: chr22 44550564-44550577. Max. coverage (+): 0. Max coverage (-): 0

Region: chr22 44550578-44550592. Max. coverage (+): 0. Max coverage (-): 0

Region: chr22 44550593-44550606. Max. coverage (+): 0. Max coverage (-): 0

Region: chr22 44550607-44550621. Max. coverage (+): 0. Max coverage (-): 0

Region: chr22 44550622-44550636. Max. coverage (+): 0. Max coverage (-): 0

Region: chr22 44550637-44550650. Max. coverage (+): 2.73. Max coverage (-): 0

Region: chr22 44550651-44550665. Max. coverage (+): 0. Max coverage (-): 0

Region: chr22 44550666-44550680. Max. coverage (+): 0. Max coverage (-): 0

Region: chr22 44550681-44550694. Max. coverage (+): 0. Max coverage (-): 0

Region: chr22 44550695-44550709. Max. coverage (+): 0. Max coverage (-): 0

Region: chr22 44550710-44550723. Max. coverage (+): 0. Max coverage (-): 0

Region: chr22 44550724-44550738. Max. coverage (+): 0. Max coverage (-): 0

Region: chr22 44550739-44550753. Max. coverage (+): 0. Max coverage (-): 0

Region: chr22 44550754-44550767. Max. coverage (+): 0. Max coverage (-): 0

Region: chr22 44550768-44550782. Max. coverage (+): 0. Max coverage (-): 0

Region: chr22 44550783-44550797. Max. coverage (+): 0. Max coverage (-): 0

Region: chr22 44550798-44550811. Max. coverage (+): 0. Max coverage (-): 0

Region: chr22 44550812-44550826. Max. coverage (+): 0. Max coverage (-): 0

Region: chr22 44550827-44550840. Max. coverage (+): 0. Max coverage (-): 0

Region: chr22 44550841-44550855. Max. coverage (+): 0. Max coverage (-): 0

Region: chr22 44550856-44550870. Max. coverage (+): 0. Max coverage (-): 0

Region: chr22 44550871-44550884. Max. coverage (+): 0. Max coverage (-): 0

Region: chr22 44550885-44550899. Max. coverage (+): 0. Max coverage (-): 0

Region: chr22 44550900-44550913. Max. coverage (+): 0. Max coverage (-): 0

Region: chr22 44550914-44550928. Max. coverage (+): 0. Max coverage (-): 0

Region: chr22 44550929-44550943. Max. coverage (+): 0. Max coverage (-): 0

Region: chr22 44550944-44550957. Max. coverage (+): 0. Max coverage (-): 0

Region: chr22 44550958-44550972. Max. coverage (+): 0. Max coverage (-): 0

Region: chr22 44550973-44550987. Max. coverage (+): 0. Max coverage (-): 0

Region: chr22 44550988-44551001. Max. coverage (+): 0. Max coverage (-): 0

Region: chr22 44551002-44551016. Max. coverage (+): 0. Max coverage (-): 0

Region: chr22 44551017-44551030. Max. coverage (+): 0. Max coverage (-): 0

Region: chr22 44551031-44551045. Max. coverage (+): 0. Max coverage (-): 0

Region: chr22 44551046-44551060. Max. coverage (+): 0. Max coverage (-): 0

Region: chr22 44551061-44551074. Max. coverage (+): 0. Max coverage (-): 0

Region: chr22 44551075-44551089. Max. coverage (+): 0. Max coverage (-): 0

Region: chr22 44551090-44551103. Max. coverage (+): 0. Max coverage (-): 0

Region: chr22 44551104-44551118. Max. coverage (+): 0. Max coverage (-): 0

Region: chr22 44551119-44551133. Max. coverage (+): 0. Max coverage (-): 0

Region: chr22 44551134-44551147. Max. coverage (+): 0. Max coverage (-): 0

Region: chr22 44551148-44551162. Max. coverage (+): 0. Max coverage (-): 0

Region: chr22 44551163-44551177. Max. coverage (+): 0. Max coverage (-): 0

Region: chr22 44551178-44551191. Max. coverage (+): 0. Max coverage (-): 0

Region: chr22 44551192-44551206. Max. coverage (+): 0. Max coverage (-): 0

Region: chr22 44551207-44551220. Max. coverage (+): 0. Max coverage (-): 0

Region: chr22 44551221-44551235. Max. coverage (+): 0. Max coverage (-): 0

Region: chr22 44551236-44551250. Max. coverage (+): 0. Max coverage (-): 0

Region: chr22 44551251-44551264. Max. coverage (+): 0. Max coverage (-): 0

Region: chr22 44551265-44551279. Max. coverage (+): 0. Max coverage (-): 0

Region: chr22 44551280-44551294. Max. coverage (+): 0. Max coverage (-): 0

Region: chr22 44551295-44551308. Max. coverage (+): 0. Max coverage (-): 0

Region: chr22 44551309-44551323. Max. coverage (+): 0. Max coverage (-): 0

Region: chr22 44551324-44551337. Max. coverage (+): 0. Max coverage (-): 0

Region: chr22 44551338-44551352. Max. coverage (+): 0. Max coverage (-): 0

Region: chr22 44551353-44551367. Max. coverage (+): 0. Max coverage (-): 0

Region: chr22 44551368-44551381. Max. coverage (+): 0. Max coverage (-): 0

Region: chr22 44551382-44551396. Max. coverage (+): 0. Max coverage (-): 0

Region: chr22 44551397-44551410. Max. coverage (+): 0. Max coverage (-): 0

Region: chr22 44551411-44551425. Max. coverage (+): 0. Max coverage (-): 0

Region: chr22 44551426-44551440. Max. coverage (+): 0. Max coverage (-): 0

Region: chr22 44551441-44551454. Max. coverage (+): 0. Max coverage (-): 0

Region: chr22 44551455-44551469. Max. coverage (+): 0. Max coverage (-): 0

Region: chr22 44551470-44551484. Max. coverage (+): 0. Max coverage (-): 0

Region: chr22 44551485-44551498. Max. coverage (+): 0. Max coverage (-): 0

Region: chr22 44551499-44551513. Max. coverage (+): 0. Max coverage (-): 0

Region: chr22 44551514-44551527. Max. coverage (+): 0. Max coverage (-): 0

Region: chr22 44551528-44551542. Max. coverage (+): 1.19. Max coverage (-): 0

Region: chr22 44551543-44551557. Max. coverage (+): 1.19. Max coverage (-): 0

Region: chr22 44551558-44551571. Max. coverage (+): 0. Max coverage (-): 0

Region: chr22 44551572-44551586. Max. coverage (+): 0. Max coverage (-): 0

Region: chr22 44551587-44551600. Max. coverage (+): 0. Max coverage (-): 0

Region: chr22 44551601-44551615. Max. coverage (+): 0. Max coverage (-): 0

Region: chr22 44551616-44551630. Max. coverage (+): 0. Max coverage (-): 0

Region: chr22 44551631-44551644. Max. coverage (+): 0. Max coverage (-): 0

Region: chr22 44551645-44551659. Max. coverage (+): 0. Max coverage (-): 0

Region: chr22 44551660-44551674. Max. coverage (+): 0. Max coverage (-): 0

Region: chr22 44551675-44551688. Max. coverage (+): 0. Max coverage (-): 0

Region: chr22 44551689-44551703. Max. coverage (+): 0. Max coverage (-): 0

Region: chr22 44551704-44551717. Max. coverage (+): 0. Max coverage (-): 0

Region: chr22 44551718-44551732. Max. coverage (+): 1.07. Max coverage (-): 0

Region: chr22 44551733-44551747. Max. coverage (+): 0. Max coverage (-): 0

Region: chr22 44551748-44551761. Max. coverage (+): 0. Max coverage (-): 0

Region: chr22 44551762-44551776. Max. coverage (+): 0. Max coverage (-): 0

Region: chr22 44551777-44551791. Max. coverage (+): 0. Max coverage (-): 0

Region: chr22 44551792-44551805. Max. coverage (+): 0. Max coverage (-): 0

Region: chr22 44551806-44551820. Max. coverage (+): 0. Max coverage (-): 0

Region: chr22 44551821-44551834. Max. coverage (+): 0. Max coverage (-): 0

Region: chr22 44551835-44551849. Max. coverage (+): 0. Max coverage (-): 0

Region: chr22 44551850-44551864. Max. coverage (+): 0. Max coverage (-): 0

Region: chr22 44551865-44551878. Max. coverage (+): 0. Max coverage (-): 0

Region: chr22 44551879-44551893. Max. coverage (+): 0. Max coverage (-): 0

Region: chr22 44551894-44551907. Max. coverage (+): 0. Max coverage (-): 0

Region: chr22 44551908-44551922. Max. coverage (+): 0. Max coverage (-): 0

Region: chr22 44551923-44551937. Max. coverage (+): 0. Max coverage (-): 0

Region: chr22 44551938-44551951. Max. coverage (+): 0. Max coverage (-): 0

Region: chr22 44551952-44551966. Max. coverage (+): 0. Max coverage (-): 0

Region: chr22 44551967-44551981. Max. coverage (+): 0. Max coverage (-): 0

Region: chr22 44551982-44551995. Max. coverage (+): 0. Max coverage (-): 0

Region: chr22 44551996-44552010. Max. coverage (+): 0. Max coverage (-): 0

Region: chr22 44552011-44552024. Max. coverage (+): 0. Max coverage (-): 0

Region: chr22 44552025-44552039. Max. coverage (+): 0. Max coverage (-): 0

Region: chr22 44552040-44552054. Max. coverage (+): 0. Max coverage (-): 0

Region: chr22 44552055-44552068. Max. coverage (+): 0. Max coverage (-): 0

Region: chr22 44552069-44552083. Max. coverage (+): 0. Max coverage (-): 0

Region: chr22 44552084-44552098. Max. coverage (+): 0. Max coverage (-): 0

Region: chr22 44552099-44552112. Max. coverage (+): 0. Max coverage (-): 0

Region: chr22 44552113-44552127. Max. coverage (+): 0. Max coverage (-): 0

Region: chr22 44552128-44552141. Max. coverage (+): 0. Max coverage (-): 0

Region: chr22 44552142-44552156. Max. coverage (+): 0. Max coverage (-): 0

Region: chr22 44552157-44552171. Max. coverage (+): 0. Max coverage (-): 0

Region: chr22 44552172-44552185. Max. coverage (+): 0. Max coverage (-): 0

Region: chr22 44552186-44552200. Max. coverage (+): 0. Max coverage (-): 0

Region: chr22 44552201-44552214. Max. coverage (+): 0. Max coverage (-): 0

Region: chr22 44552215-44552229. Max. coverage (+): 0. Max coverage (-): 0

Region: chr22 44552230-44552244. Max. coverage (+): 0. Max coverage (-): 0

Region: chr22 44552245-44552258. Max. coverage (+): 0. Max coverage (-): 0

Region: chr22 44552259-44552273. Max. coverage (+): 0. Max coverage (-): 0

Region: chr22 44552274-44552288. Max. coverage (+): 0. Max coverage (-): 0

Region: chr22 44552289-44552302. Max. coverage (+): 0. Max coverage (-): 0

Region: chr22 44552303-44552317. Max. coverage (+): 0. Max coverage (-): 0

Region: chr22 44552318-44552331. Max. coverage (+): 0. Max coverage (-): 0

Region: chr22 44552332-44552346. Max. coverage (+): 0. Max coverage (-): 0

Region: chr22 44552347-44552361. Max. coverage (+): 0. Max coverage (-): 0

Region: chr22 44552362-44552375. Max. coverage (+): 0. Max coverage (-): 0

Region: chr22 44552376-44552390. Max. coverage (+): 0. Max coverage (-): 0

Region: chr22 44552391-44552404. Max. coverage (+): 0. Max coverage (-): 0

Region: chr22 44552405-44552419. Max. coverage (+): 0. Max coverage (-): 0

Region: chr22 44552420-44552434. Max. coverage (+): 0. Max coverage (-): 0

Region: chr22 44552435-44552448. Max. coverage (+): 0. Max coverage (-): 0

Region: chr22 44552449-44552463. Max. coverage (+): 0. Max coverage (-): 0

Region: chr22 44552464-44552478. Max. coverage (+): 2.12. Max coverage (-): 0

Region: chr22 44552479-44552492. Max. coverage (+): 2.12. Max coverage (-): 0

Region: chr22 44552493-. Max. coverage (+): 0. Max coverage (-): 0

RepeatMasker Color Code

**+**

100-98% Identity

<98-95% Identity

<95-90% Identity

<90-85% Identity

<85-80% Identity

<80-75% Identity

<75-70% Identity

<70% Identity

**-**

Gene Set Color Code

**+**

Gene

Pseudogene

**-**

Topology/Coverage Color Code

Coverage Plus Strand

Coverage Minus Strand

Mainstrand: Plus

Mainstrand: Minus

Complementary Strand

Flanking Region  
(if option -flank >0)

Gene Set Annotation  

**1. IL17RD (protein coding, ENSBTAG00000010999) Tr:00000060984 Ex:12**: 44545601-44546540 (+)  
**2. IL17RD (protein coding, ENSBTAG00000010999) Tr:00000060984 Ex:13**: 44547279-44547391 (+)

  
RepeatMasker Annotation  

**1. MIRb**: 44545313-44545380 (+), Divergence to consensus: 36.8%  
**2. MER46C**: 44546705-44546890 (+), Divergence to consensus: 35.6%  
**3. AT\_rich**: 44550511-44550531 (+), Divergence to consensus: 66.7%  
**4. MER102c**: 44550811-44551067 (-), Divergence to consensus: 42.9%  
**5. Plat\_L3**: 44551896-44552082 (-), Divergence to consensus: 47.8%

  
Transcription Factor Binding Sites  

**SOX9** (Sequence: CCATTGTT (+): 44547870)
