# Supplementary material for: Small RNA sequencing of cryopreserved semen from single bull revealed altered miRNAs and piRNAs expression between High- and Low-motile sperm populations
Source: BMC Genomics. 2017 Jan 4;18:14. doi: 10.1186/s12864-016-3394-7 (PMC5209821; doi:10.1186/s12864-016-3394-7)
Supplement: Additional file 3: — Details for each piRNA clusters found in High Motile (HM) sperm fraction. Genes, repeats, transposable elements and transcription factors binding sites falling within the cluster regions were reported. (ZIP 1896 kb) [file 12864_2016_3394_MOESM3_ESM.zip › 52.html]

piRNA cluster 52


Predicted piRNA cluster no. 52     previous   next
  

Show proTRAC run info
Hide proTRAC run info

================================= proTRAC ====================================  
VERSION: 2.1                                    LAST MODIFIED: 06. October 2015  
  
Please cite:  
Rosenkranz D, Zischler H. proTRAC - a software for probabilistic piRNA cluster  
detection, visualization and analysis. 2012. BMC Bioinformatics 13:5.  
  
and (for proTRAC 2.0 and later):  
Rosenkranz D, Rudloff S, Bastuck K, Ketting RF, Zischler H. Tupaia small RNAs  
provide insights into function and evolution of RNAi-based transposon defense  
in mammals. 2015. RNA 21(5):911-922.  
  
Contact:  
David Rosenkranz  
Institute of Anthropology, small RNA group  
Johannes Gutenberg University Mainz  
email: rosenkranz@uni-mainz.de  
  
You can find the latest proTRAC version at:  
http://sourceforge.net/projects/protrac/files  
http://www.smallRNAgroup-mainz.de/software  
==============================================================================  
  
PARAMETERS:  
Map file: .............../storage/core/barbara/genhome/smallRNA/fertility/Sample\_motile/pirna/Sample\_motile\_26-33\_collapsed.fa.no-dust.map.weighted-10000-1000-b-0  
Genome file: ............/storage/core/barbara/genhome/smallRNA/fertility/Sample\_all/pirna/bt\_311\_chrY.fa  
RepeatMasker annotation: /storage/genomes/bt\_umd31/GCF\_000003055.6\_Bos\_taurus\_UMD\_3.1.1\_repeatMasker\_chr.out  
GeneSet:................./storage/core/barbara/genhome/smallRNA/fertility/Sample\_all/pirna/full.gtf  
  
Significant (p<=0.01) hit density will be calculated based  
on observed hit distribution.  
  
Sliding window size: ........................................ 5000 bp  
Sliding window increament: .................................. 1000 bp  
Normalize each hit by number of genomic hits: ............... 1 [0=no/1=yes]  
Normalize each hit by number of sequence reads: ............. 1 [0=no/1=yes]  
Normalize values (-> per million mapped reads): ............. 1 [0=no/1=yes]  
Min. fraction of hits with 1T(U) or 10A: .................... 0.75  
Alternatively: Min. fraction of hits with 1T(U) and 10A: .... 0.5  
Min. fraction of hits with typical piRNA length: ............ 0.75  
Typical piRNA length: ....................................... 26-33 nt  
Min. size of a piRNA cluster: ............................... 5000 bp.  
Min. number of hits (absolute): ............................. 0  
Min. number of hits (normalized): ........................... 0  
Min. fraction of hits on the mainstrand: .................... 0.75  
Top fraction of mapped sequences (in terms of read counts): . 1%  
Top fraction accounts for max. n% of sequence reads: ........ 90%  
Min. fraction of hits on each arm of a bidirectional cluster: 0.1  
Output image file for each cluster: ......................... 0 [0=no/1=yes]  
Output html file for each cluster: .......................... 1 [0=no/1=yes]  
Output a summary table: ..................................... 1 [0=no/1=yes]  
Output a FASTA file for each cluster (piRNA sequences): ..... 1 [0=no/1=yes]  
Output a FASTA file comprising cluster sequences: ........... 1 [0=no/1=yes]  
Search DNA motifs in clusters: .............................. 1 [0=no/1=yes]  
Output flanking sequences: +/- .............................. 0 bp  
Output ~.pTi file: .......................................... 1 [0=no/1=yes]  
==============================================================================  
  
  
Genome size (without gaps): ............ 2678902517 bp  
Gaps (N/X/-): .......................... 53837044 bp  
Mapped reads: .......................... 658825247023  
Non-identical sequences: ............... 514171  
Genomic hits: .......................... 764233  
Significant densitiy of mapped reads: .. 12867599.5173724 reads/kb

Show proTRAC cluster info
Hide proTRAC cluster info

|  |  |
| --- | --- |
| Location | chr22 |
| Coordinates | 51416535-51443316 |
| Size [bp] | 26782 |
| Sequence hit loci | 914 |
| Mapped reads (normalized) | 1161596730 |
| Mapped reads (normalized) per kb | 43372292.2 |
| Normalized reads with 1T (1U) | 80.1% |
| Normalized reads with 10A | 29.7% |
| Normalized reads with length 26-33 nt | 100% |
| Normalized reads on the main strand(s) | 95.2% |
| Predicted directionality | mono:plus |

100%

0%

1T (1U)  
reads

10A reads

26-33 nt  
reads

reads on mainstrand

**Either the amount of reads with 1T (1U) OR 10A has to exceed 75% (set with option: -1Tor10A)  
Alternatively the amount of reads with 1T (1U) AND 10A has to exceed 50% (set with option: -1Tand10A)  
Minimum amount of reads with preferred size is 75% (set with option: -pisize)  
Minimum amount of reads on the main strand(s) is 75% (set with option: -clstrand)**

Show read coverage
Hide read coverage

WHAT DO I SEE HERE?  
This chart shows the location of mapped sequence reads within a predicted piRNA cluster. The color refers to the number of genomic hits produced by the sequence read in question. A dark red bar indicates that this sequence read produces many other hits elsewhere in the genome. Many adjacent red or yellow bars can indicate the presence of a multi-copy element such as transposons or rRNA genes. A dark green bar indicates that this sequence read maps uniquely to this locus.

1 hit

2-5 hits

6-10 hits

11-20 hits

21-50 hits

51-100 hits

> 100 hits

chr22

51416535

51443316

Gene Set

RepeatMasker

Mapped  
Reads

28.52

plus strand

minus strand

28.52

Region: chr22 44552494-51416561. Max. coverage (+): 1.58. Max coverage (-): 0

Region: chr22 51416562-51416615. Max. coverage (+): 7.81. Max coverage (-): 0

Region: chr22 51416616-51416668. Max. coverage (+): 1.56. Max coverage (-): 0

Region: chr22 51416669-51416722. Max. coverage (+): 4.34. Max coverage (-): 0

Region: chr22 51416723-51416776. Max. coverage (+): 2.84. Max coverage (-): 0

Region: chr22 51416777-51416829. Max. coverage (+): 0. Max coverage (-): 0

Region: chr22 51416830-51416883. Max. coverage (+): 0. Max coverage (-): 0

Region: chr22 51416884-51416936. Max. coverage (+): 0. Max coverage (-): 0

Region: chr22 51416937-51416990. Max. coverage (+): 1.08. Max coverage (-): 0

Region: chr22 51416991-51417043. Max. coverage (+): 0. Max coverage (-): 0

Region: chr22 51417044-51417097. Max. coverage (+): 0. Max coverage (-): 0

Region: chr22 51417098-51417150. Max. coverage (+): 0. Max coverage (-): 0

Region: chr22 51417151-51417204. Max. coverage (+): 0. Max coverage (-): 0

Region: chr22 51417205-51417258. Max. coverage (+): 0. Max coverage (-): 0

Region: chr22 51417259-51417311. Max. coverage (+): 0. Max coverage (-): 0

Region: chr22 51417312-51417365. Max. coverage (+): 0. Max coverage (-): 0

Region: chr22 51417366-51417418. Max. coverage (+): 0. Max coverage (-): 0

Region: chr22 51417419-51417472. Max. coverage (+): 0. Max coverage (-): 0

Region: chr22 51417473-51417525. Max. coverage (+): 0. Max coverage (-): 0

Region: chr22 51417526-51417579. Max. coverage (+): 0. Max coverage (-): 0

Region: chr22 51417580-51417633. Max. coverage (+): 0. Max coverage (-): 0

Region: chr22 51417634-51417686. Max. coverage (+): 0. Max coverage (-): 0

Region: chr22 51417687-51417740. Max. coverage (+): 0. Max coverage (-): 0

Region: chr22 51417741-51417793. Max. coverage (+): 0. Max coverage (-): 0

Region: chr22 51417794-51417847. Max. coverage (+): 0. Max coverage (-): 0

Region: chr22 51417848-51417900. Max. coverage (+): 0. Max coverage (-): 0

Region: chr22 51417901-51417954. Max. coverage (+): 4.56. Max coverage (-): 0

Region: chr22 51417955-51418008. Max. coverage (+): 11.67. Max coverage (-): 7.79

Region: chr22 51418009-51418061. Max. coverage (+): 4.78. Max coverage (-): 0

Region: chr22 51418062-51418115. Max. coverage (+): 12.59. Max coverage (-): 0

Region: chr22 51418116-51418168. Max. coverage (+): 11.31. Max coverage (-): 0

Region: chr22 51418169-51418222. Max. coverage (+): 9.76. Max coverage (-): 0

Region: chr22 51418223-51418275. Max. coverage (+): 10.34. Max coverage (-): 0

Region: chr22 51418276-51418329. Max. coverage (+): 2.06. Max coverage (-): 0

Region: chr22 51418330-51418382. Max. coverage (+): 13.88. Max coverage (-): 0

Region: chr22 51418383-51418436. Max. coverage (+): 9.86. Max coverage (-): 0

Region: chr22 51418437-51418490. Max. coverage (+): 28.52. Max coverage (-): 0

Region: chr22 51418491-51418543. Max. coverage (+): 3.45. Max coverage (-): 0

Region: chr22 51418544-51418597. Max. coverage (+): 1.42. Max coverage (-): 0

Region: chr22 51418598-51418650. Max. coverage (+): 1.43. Max coverage (-): 0

Region: chr22 51418651-51418704. Max. coverage (+): 0. Max coverage (-): 0

Region: chr22 51418705-51418757. Max. coverage (+): 0. Max coverage (-): 0

Region: chr22 51418758-51418811. Max. coverage (+): 0. Max coverage (-): 0

Region: chr22 51418812-51418865. Max. coverage (+): 0.66. Max coverage (-): 0

Region: chr22 51418866-51418918. Max. coverage (+): 4.7. Max coverage (-): 0

Region: chr22 51418919-51418972. Max. coverage (+): 5.49. Max coverage (-): 0

Region: chr22 51418973-51419025. Max. coverage (+): 5.57. Max coverage (-): 0

Region: chr22 51419026-51419079. Max. coverage (+): 0. Max coverage (-): 0

Region: chr22 51419080-51419132. Max. coverage (+): 10.29. Max coverage (-): 0

Region: chr22 51419133-51419186. Max. coverage (+): 0. Max coverage (-): 0

Region: chr22 51419187-51419239. Max. coverage (+): 0. Max coverage (-): 0

Region: chr22 51419240-51419293. Max. coverage (+): 0. Max coverage (-): 0

Region: chr22 51419294-51419347. Max. coverage (+): 0. Max coverage (-): 0

Region: chr22 51419348-51419400. Max. coverage (+): 16.2. Max coverage (-): 0

Region: chr22 51419401-51419454. Max. coverage (+): 4.41. Max coverage (-): 0

Region: chr22 51419455-51419507. Max. coverage (+): 5.1. Max coverage (-): 0

Region: chr22 51419508-51419561. Max. coverage (+): 2.08. Max coverage (-): 0

Region: chr22 51419562-51419614. Max. coverage (+): 1.66. Max coverage (-): 0

Region: chr22 51419615-51419668. Max. coverage (+): 13.4. Max coverage (-): 0

Region: chr22 51419669-51419722. Max. coverage (+): 16.83. Max coverage (-): 0

Region: chr22 51419723-51419775. Max. coverage (+): 0. Max coverage (-): 0

Region: chr22 51419776-51419829. Max. coverage (+): 0. Max coverage (-): 0

Region: chr22 51419830-51419882. Max. coverage (+): 3.16. Max coverage (-): 0

Region: chr22 51419883-51419936. Max. coverage (+): 2.06. Max coverage (-): 0

Region: chr22 51419937-51419989. Max. coverage (+): 0.84. Max coverage (-): 0

Region: chr22 51419990-51420043. Max. coverage (+): 0. Max coverage (-): 0

Region: chr22 51420044-51420097. Max. coverage (+): 6.6. Max coverage (-): 0

Region: chr22 51420098-51420150. Max. coverage (+): 3.9. Max coverage (-): 0

Region: chr22 51420151-51420204. Max. coverage (+): 0. Max coverage (-): 0

Region: chr22 51420205-51420257. Max. coverage (+): 0. Max coverage (-): 0

Region: chr22 51420258-51420311. Max. coverage (+): 0. Max coverage (-): 0

Region: chr22 51420312-51420364. Max. coverage (+): 0. Max coverage (-): 0

Region: chr22 51420365-51420418. Max. coverage (+): 0. Max coverage (-): 0

Region: chr22 51420419-51420471. Max. coverage (+): 5.42. Max coverage (-): 0

Region: chr22 51420472-51420525. Max. coverage (+): 0. Max coverage (-): 0

Region: chr22 51420526-51420579. Max. coverage (+): 6.06. Max coverage (-): 0

Region: chr22 51420580-51420632. Max. coverage (+): 2.91. Max coverage (-): 0

Region: chr22 51420633-51420686. Max. coverage (+): 5.78. Max coverage (-): 0

Region: chr22 51420687-51420739. Max. coverage (+): 5.32. Max coverage (-): 0

Region: chr22 51420740-51420793. Max. coverage (+): 0.83. Max coverage (-): 0

Region: chr22 51420794-51420846. Max. coverage (+): 4.3. Max coverage (-): 0

Region: chr22 51420847-51420900. Max. coverage (+): 0. Max coverage (-): 0

Region: chr22 51420901-51420954. Max. coverage (+): 0. Max coverage (-): 0

Region: chr22 51420955-51421007. Max. coverage (+): 0. Max coverage (-): 0

Region: chr22 51421008-51421061. Max. coverage (+): 0. Max coverage (-): 0

Region: chr22 51421062-51421114. Max. coverage (+): 0. Max coverage (-): 0

Region: chr22 51421115-51421168. Max. coverage (+): 0.15. Max coverage (-): 0

Region: chr22 51421169-51421221. Max. coverage (+): 1.51. Max coverage (-): 0

Region: chr22 51421222-51421275. Max. coverage (+): 5.46. Max coverage (-): 0

Region: chr22 51421276-51421328. Max. coverage (+): 0. Max coverage (-): 0

Region: chr22 51421329-51421382. Max. coverage (+): 0. Max coverage (-): 0

Region: chr22 51421383-51421436. Max. coverage (+): 0. Max coverage (-): 0

Region: chr22 51421437-51421489. Max. coverage (+): 0. Max coverage (-): 0

Region: chr22 51421490-51421543. Max. coverage (+): 0. Max coverage (-): 0

Region: chr22 51421544-51421596. Max. coverage (+): 5.19. Max coverage (-): 0

Region: chr22 51421597-51421650. Max. coverage (+): 14.38. Max coverage (-): 0

Region: chr22 51421651-51421703. Max. coverage (+): 0. Max coverage (-): 0

Region: chr22 51421704-51421757. Max. coverage (+): 5.14. Max coverage (-): 0

Region: chr22 51421758-51421811. Max. coverage (+): 0.79. Max coverage (-): 0

Region: chr22 51421812-51421864. Max. coverage (+): 1.52. Max coverage (-): 0

Region: chr22 51421865-51421918. Max. coverage (+): 0. Max coverage (-): 0

Region: chr22 51421919-51421971. Max. coverage (+): 0. Max coverage (-): 0

Region: chr22 51421972-51422025. Max. coverage (+): 0. Max coverage (-): 0

Region: chr22 51422026-51422078. Max. coverage (+): 0. Max coverage (-): 0

Region: chr22 51422079-51422132. Max. coverage (+): 0. Max coverage (-): 0

Region: chr22 51422133-51422186. Max. coverage (+): 0. Max coverage (-): 0

Region: chr22 51422187-51422239. Max. coverage (+): 2.52. Max coverage (-): 0

Region: chr22 51422240-51422293. Max. coverage (+): 7.73. Max coverage (-): 0

Region: chr22 51422294-51422346. Max. coverage (+): 7.73. Max coverage (-): 0

Region: chr22 51422347-51422400. Max. coverage (+): 8.33. Max coverage (-): 0

Region: chr22 51422401-51422453. Max. coverage (+): 2.99. Max coverage (-): 0

Region: chr22 51422454-51422507. Max. coverage (+): 21.32. Max coverage (-): 0

Region: chr22 51422508-51422560. Max. coverage (+): 4.8. Max coverage (-): 0

Region: chr22 51422561-51422614. Max. coverage (+): 9.29. Max coverage (-): 0

Region: chr22 51422615-51422668. Max. coverage (+): 11.78. Max coverage (-): 0

Region: chr22 51422669-51422721. Max. coverage (+): 9.52. Max coverage (-): 0

Region: chr22 51422722-51422775. Max. coverage (+): 6.36. Max coverage (-): 0

Region: chr22 51422776-51422828. Max. coverage (+): 0.97. Max coverage (-): 0

Region: chr22 51422829-51422882. Max. coverage (+): 5.38. Max coverage (-): 0

Region: chr22 51422883-51422935. Max. coverage (+): 5.38. Max coverage (-): 0

Region: chr22 51422936-51422989. Max. coverage (+): 3.6. Max coverage (-): 0

Region: chr22 51422990-51423043. Max. coverage (+): 3.6. Max coverage (-): 0

Region: chr22 51423044-51423096. Max. coverage (+): 3. Max coverage (-): 0

Region: chr22 51423097-51423150. Max. coverage (+): 22.69. Max coverage (-): 0

Region: chr22 51423151-51423203. Max. coverage (+): 4.51. Max coverage (-): 0

Region: chr22 51423204-51423257. Max. coverage (+): 1.03. Max coverage (-): 0

Region: chr22 51423258-51423310. Max. coverage (+): 10.14. Max coverage (-): 0

Region: chr22 51423311-51423364. Max. coverage (+): 3.19. Max coverage (-): 0

Region: chr22 51423365-51423417. Max. coverage (+): 6.6. Max coverage (-): 0

Region: chr22 51423418-51423471. Max. coverage (+): 3.3. Max coverage (-): 0

Region: chr22 51423472-51423525. Max. coverage (+): 4.15. Max coverage (-): 0

Region: chr22 51423526-51423578. Max. coverage (+): 0. Max coverage (-): 0

Region: chr22 51423579-51423632. Max. coverage (+): 0. Max coverage (-): 0

Region: chr22 51423633-51423685. Max. coverage (+): 0. Max coverage (-): 0

Region: chr22 51423686-51423739. Max. coverage (+): 0. Max coverage (-): 0

Region: chr22 51423740-51423792. Max. coverage (+): 0. Max coverage (-): 0

Region: chr22 51423793-51423846. Max. coverage (+): 0. Max coverage (-): 0

Region: chr22 51423847-51423900. Max. coverage (+): 0. Max coverage (-): 0

Region: chr22 51423901-51423953. Max. coverage (+): 0. Max coverage (-): 0

Region: chr22 51423954-51424007. Max. coverage (+): 0. Max coverage (-): 0

Region: chr22 51424008-51424060. Max. coverage (+): 0. Max coverage (-): 0

Region: chr22 51424061-51424114. Max. coverage (+): 0.66. Max coverage (-): 0

Region: chr22 51424115-51424167. Max. coverage (+): 0. Max coverage (-): 0

Region: chr22 51424168-51424221. Max. coverage (+): 0. Max coverage (-): 0

Region: chr22 51424222-51424274. Max. coverage (+): 0. Max coverage (-): 0

Region: chr22 51424275-51424328. Max. coverage (+): 0. Max coverage (-): 0

Region: chr22 51424329-51424382. Max. coverage (+): 0. Max coverage (-): 0

Region: chr22 51424383-51424435. Max. coverage (+): 0. Max coverage (-): 0

Region: chr22 51424436-51424489. Max. coverage (+): 0. Max coverage (-): 0

Region: chr22 51424490-51424542. Max. coverage (+): 0. Max coverage (-): 0

Region: chr22 51424543-51424596. Max. coverage (+): 0. Max coverage (-): 0

Region: chr22 51424597-51424649. Max. coverage (+): 0. Max coverage (-): 0

Region: chr22 51424650-51424703. Max. coverage (+): 0. Max coverage (-): 0

Region: chr22 51424704-51424757. Max. coverage (+): 0. Max coverage (-): 0

Region: chr22 51424758-51424810. Max. coverage (+): 0. Max coverage (-): 0

Region: chr22 51424811-51424864. Max. coverage (+): 0. Max coverage (-): 0

Region: chr22 51424865-51424917. Max. coverage (+): 0. Max coverage (-): 0

Region: chr22 51424918-51424971. Max. coverage (+): 0. Max coverage (-): 0

Region: chr22 51424972-51425024. Max. coverage (+): 0. Max coverage (-): 0

Region: chr22 51425025-51425078. Max. coverage (+): 0. Max coverage (-): 0

Region: chr22 51425079-51425132. Max. coverage (+): 0. Max coverage (-): 0

Region: chr22 51425133-51425185. Max. coverage (+): 0. Max coverage (-): 0

Region: chr22 51425186-51425239. Max. coverage (+): 0. Max coverage (-): 0

Region: chr22 51425240-51425292. Max. coverage (+): 0. Max coverage (-): 0

Region: chr22 51425293-51425346. Max. coverage (+): 0. Max coverage (-): 0

Region: chr22 51425347-51425399. Max. coverage (+): 0. Max coverage (-): 0

Region: chr22 51425400-51425453. Max. coverage (+): 0. Max coverage (-): 0

Region: chr22 51425454-51425506. Max. coverage (+): 0. Max coverage (-): 0

Region: chr22 51425507-51425560. Max. coverage (+): 0. Max coverage (-): 0

Region: chr22 51425561-51425614. Max. coverage (+): 8.14. Max coverage (-): 0

Region: chr22 51425615-51425667. Max. coverage (+): 8.14. Max coverage (-): 0

Region: chr22 51425668-51425721. Max. coverage (+): 2.74. Max coverage (-): 0

Region: chr22 51425722-51425774. Max. coverage (+): 3.68. Max coverage (-): 0

Region: chr22 51425775-51425828. Max. coverage (+): 1.9. Max coverage (-): 0

Region: chr22 51425829-51425881. Max. coverage (+): 3. Max coverage (-): 0

Region: chr22 51425882-51425935. Max. coverage (+): 5.67. Max coverage (-): 0

Region: chr22 51425936-51425989. Max. coverage (+): 3.83. Max coverage (-): 0

Region: chr22 51425990-51426042. Max. coverage (+): 3.05. Max coverage (-): 0

Region: chr22 51426043-51426096. Max. coverage (+): 11.14. Max coverage (-): 0

Region: chr22 51426097-51426149. Max. coverage (+): 0. Max coverage (-): 0

Region: chr22 51426150-51426203. Max. coverage (+): 9.22. Max coverage (-): 0

Region: chr22 51426204-51426256. Max. coverage (+): 0. Max coverage (-): 0

Region: chr22 51426257-51426310. Max. coverage (+): 0. Max coverage (-): 1.5

Region: chr22 51426311-51426363. Max. coverage (+): 8.5. Max coverage (-): 0

Region: chr22 51426364-51426417. Max. coverage (+): 0. Max coverage (-): 0

Region: chr22 51426418-51426471. Max. coverage (+): 5.23. Max coverage (-): 0

Region: chr22 51426472-51426524. Max. coverage (+): 0. Max coverage (-): 0

Region: chr22 51426525-51426578. Max. coverage (+): 0. Max coverage (-): 0

Region: chr22 51426579-51426631. Max. coverage (+): 3.7. Max coverage (-): 0

Region: chr22 51426632-51426685. Max. coverage (+): 0. Max coverage (-): 0

Region: chr22 51426686-51426738. Max. coverage (+): 0. Max coverage (-): 0

Region: chr22 51426739-51426792. Max. coverage (+): 12.44. Max coverage (-): 0

Region: chr22 51426793-51426846. Max. coverage (+): 4.96. Max coverage (-): 0

Region: chr22 51426847-51426899. Max. coverage (+): 4.96. Max coverage (-): 5.24

Region: chr22 51426900-51426953. Max. coverage (+): 2.18. Max coverage (-): 5.24

Region: chr22 51426954-51427006. Max. coverage (+): 3.29. Max coverage (-): 0

Region: chr22 51427007-51427060. Max. coverage (+): 1.21. Max coverage (-): 0

Region: chr22 51427061-51427113. Max. coverage (+): 0. Max coverage (-): 0

Region: chr22 51427114-51427167. Max. coverage (+): 2.78. Max coverage (-): 0

Region: chr22 51427168-51427221. Max. coverage (+): 2.78. Max coverage (-): 0

Region: chr22 51427222-51427274. Max. coverage (+): 5.51. Max coverage (-): 0

Region: chr22 51427275-51427328. Max. coverage (+): 8.78. Max coverage (-): 0

Region: chr22 51427329-51427381. Max. coverage (+): 3.73. Max coverage (-): 0

Region: chr22 51427382-51427435. Max. coverage (+): 4.61. Max coverage (-): 0

Region: chr22 51427436-51427488. Max. coverage (+): 6.67. Max coverage (-): 0

Region: chr22 51427489-51427542. Max. coverage (+): 0. Max coverage (-): 0

Region: chr22 51427543-51427595. Max. coverage (+): 0. Max coverage (-): 0

Region: chr22 51427596-51427649. Max. coverage (+): 0. Max coverage (-): 0

Region: chr22 51427650-51427703. Max. coverage (+): 0. Max coverage (-): 0

Region: chr22 51427704-51427756. Max. coverage (+): 1.52. Max coverage (-): 0

Region: chr22 51427757-51427810. Max. coverage (+): 1.73. Max coverage (-): 3.97

Region: chr22 51427811-51427863. Max. coverage (+): 1.78. Max coverage (-): 0

Region: chr22 51427864-51427917. Max. coverage (+): 2.18. Max coverage (-): 2.97

Region: chr22 51427918-51427970. Max. coverage (+): 0. Max coverage (-): 11.97

Region: chr22 51427971-51428024. Max. coverage (+): 0. Max coverage (-): 10.68

Region: chr22 51428025-51428078. Max. coverage (+): 2.11. Max coverage (-): 4.56

Region: chr22 51428079-51428131. Max. coverage (+): 4.37. Max coverage (-): 0

Region: chr22 51428132-51428185. Max. coverage (+): 2.19. Max coverage (-): 4.56

Region: chr22 51428186-51428238. Max. coverage (+): 0. Max coverage (-): 0

Region: chr22 51428239-51428292. Max. coverage (+): 1.37. Max coverage (-): 0

Region: chr22 51428293-51428345. Max. coverage (+): 0. Max coverage (-): 0

Region: chr22 51428346-51428399. Max. coverage (+): 0. Max coverage (-): 0

Region: chr22 51428400-51428452. Max. coverage (+): 0. Max coverage (-): 0

Region: chr22 51428453-51428506. Max. coverage (+): 0. Max coverage (-): 2.77

Region: chr22 51428507-51428560. Max. coverage (+): 0. Max coverage (-): 0

Region: chr22 51428561-51428613. Max. coverage (+): 8.99. Max coverage (-): 0

Region: chr22 51428614-51428667. Max. coverage (+): 6.93. Max coverage (-): 0

Region: chr22 51428668-51428720. Max. coverage (+): 6.93. Max coverage (-): 0

Region: chr22 51428721-51428774. Max. coverage (+): 0. Max coverage (-): 0

Region: chr22 51428775-51428827. Max. coverage (+): 0. Max coverage (-): 0.78

Region: chr22 51428828-51428881. Max. coverage (+): 0. Max coverage (-): 6.73

Region: chr22 51428882-51428935. Max. coverage (+): 0. Max coverage (-): 0

Region: chr22 51428936-51428988. Max. coverage (+): 0. Max coverage (-): 0

Region: chr22 51428989-51429042. Max. coverage (+): 1.99. Max coverage (-): 3.75

Region: chr22 51429043-51429095. Max. coverage (+): 3.96. Max coverage (-): 0

Region: chr22 51429096-51429149. Max. coverage (+): 0. Max coverage (-): 0

Region: chr22 51429150-51429202. Max. coverage (+): 6.47. Max coverage (-): 2.29

Region: chr22 51429203-51429256. Max. coverage (+): 0. Max coverage (-): 0

Region: chr22 51429257-51429310. Max. coverage (+): 2.57. Max coverage (-): 0

Region: chr22 51429311-51429363. Max. coverage (+): 2.55. Max coverage (-): 0

Region: chr22 51429364-51429417. Max. coverage (+): 0. Max coverage (-): 0

Region: chr22 51429418-51429470. Max. coverage (+): 9.64. Max coverage (-): 0

Region: chr22 51429471-51429524. Max. coverage (+): 14.17. Max coverage (-): 2.03

Region: chr22 51429525-51429577. Max. coverage (+): 2.35. Max coverage (-): 2.87

Region: chr22 51429578-51429631. Max. coverage (+): 3.93. Max coverage (-): 0.77

Region: chr22 51429632-51429684. Max. coverage (+): 4.97. Max coverage (-): 0

Region: chr22 51429685-51429738. Max. coverage (+): 11.86. Max coverage (-): 0

Region: chr22 51429739-51429792. Max. coverage (+): 0. Max coverage (-): 0

Region: chr22 51429793-51429845. Max. coverage (+): 1.46. Max coverage (-): 0

Region: chr22 51429846-51429899. Max. coverage (+): 0. Max coverage (-): 0

Region: chr22 51429900-51429952. Max. coverage (+): 16.6. Max coverage (-): 0

Region: chr22 51429953-51430006. Max. coverage (+): 1.1. Max coverage (-): 0

Region: chr22 51430007-51430059. Max. coverage (+): 0. Max coverage (-): 0

Region: chr22 51430060-51430113. Max. coverage (+): 4.05. Max coverage (-): 0

Region: chr22 51430114-51430167. Max. coverage (+): 0. Max coverage (-): 0

Region: chr22 51430168-51430220. Max. coverage (+): 8.47. Max coverage (-): 0

Region: chr22 51430221-51430274. Max. coverage (+): 0. Max coverage (-): 0

Region: chr22 51430275-51430327. Max. coverage (+): 0. Max coverage (-): 0

Region: chr22 51430328-51430381. Max. coverage (+): 5.53. Max coverage (-): 0

Region: chr22 51430382-51430434. Max. coverage (+): 0. Max coverage (-): 0

Region: chr22 51430435-51430488. Max. coverage (+): 0. Max coverage (-): 0

Region: chr22 51430489-51430541. Max. coverage (+): 0. Max coverage (-): 0

Region: chr22 51430542-51430595. Max. coverage (+): 2.66. Max coverage (-): 0

Region: chr22 51430596-51430649. Max. coverage (+): 10.92. Max coverage (-): 0

Region: chr22 51430650-51430702. Max. coverage (+): 5.04. Max coverage (-): 0

Region: chr22 51430703-51430756. Max. coverage (+): 0. Max coverage (-): 0

Region: chr22 51430757-51430809. Max. coverage (+): 0. Max coverage (-): 0

Region: chr22 51430810-51430863. Max. coverage (+): 2.64. Max coverage (-): 0

Region: chr22 51430864-51430916. Max. coverage (+): 0. Max coverage (-): 0

Region: chr22 51430917-51430970. Max. coverage (+): 3.63. Max coverage (-): 0

Region: chr22 51430971-51431024. Max. coverage (+): 3.63. Max coverage (-): 0

Region: chr22 51431025-51431077. Max. coverage (+): 0. Max coverage (-): 0

Region: chr22 51431078-51431131. Max. coverage (+): 10.74. Max coverage (-): 0

Region: chr22 51431132-51431184. Max. coverage (+): 0. Max coverage (-): 0

Region: chr22 51431185-51431238. Max. coverage (+): 0. Max coverage (-): 0

Region: chr22 51431239-51431291. Max. coverage (+): 14.87. Max coverage (-): 0

Region: chr22 51431292-51431345. Max. coverage (+): 7.69. Max coverage (-): 0

Region: chr22 51431346-51431399. Max. coverage (+): 1.97. Max coverage (-): 0

Region: chr22 51431400-51431452. Max. coverage (+): 0. Max coverage (-): 0

Region: chr22 51431453-51431506. Max. coverage (+): 1.27. Max coverage (-): 0

Region: chr22 51431507-51431559. Max. coverage (+): 6.66. Max coverage (-): 0

Region: chr22 51431560-51431613. Max. coverage (+): 10.94. Max coverage (-): 0

Region: chr22 51431614-51431666. Max. coverage (+): 6.8. Max coverage (-): 0

Region: chr22 51431667-51431720. Max. coverage (+): 6.35. Max coverage (-): 0

Region: chr22 51431721-51431773. Max. coverage (+): 4.61. Max coverage (-): 0

Region: chr22 51431774-51431827. Max. coverage (+): 0. Max coverage (-): 0

Region: chr22 51431828-51431881. Max. coverage (+): 0. Max coverage (-): 0

Region: chr22 51431882-51431934. Max. coverage (+): 0.2. Max coverage (-): 0

Region: chr22 51431935-51431988. Max. coverage (+): 0. Max coverage (-): 0

Region: chr22 51431989-51432041. Max. coverage (+): 0. Max coverage (-): 0

Region: chr22 51432042-51432095. Max. coverage (+): 0. Max coverage (-): 0

Region: chr22 51432096-51432148. Max. coverage (+): 0. Max coverage (-): 0

Region: chr22 51432149-51432202. Max. coverage (+): 0. Max coverage (-): 0

Region: chr22 51432203-51432256. Max. coverage (+): 0. Max coverage (-): 0

Region: chr22 51432257-51432309. Max. coverage (+): 0. Max coverage (-): 0

Region: chr22 51432310-51432363. Max. coverage (+): 0. Max coverage (-): 0

Region: chr22 51432364-51432416. Max. coverage (+): 0. Max coverage (-): 0

Region: chr22 51432417-51432470. Max. coverage (+): 6.64. Max coverage (-): 0

Region: chr22 51432471-51432523. Max. coverage (+): 0. Max coverage (-): 0

Region: chr22 51432524-51432577. Max. coverage (+): 7.3. Max coverage (-): 0

Region: chr22 51432578-51432630. Max. coverage (+): 5.56. Max coverage (-): 0

Region: chr22 51432631-51432684. Max. coverage (+): 6.23. Max coverage (-): 0

Region: chr22 51432685-51432738. Max. coverage (+): 0. Max coverage (-): 0

Region: chr22 51432739-51432791. Max. coverage (+): 3.65. Max coverage (-): 0

Region: chr22 51432792-51432845. Max. coverage (+): 0. Max coverage (-): 0

Region: chr22 51432846-51432898. Max. coverage (+): 3.4. Max coverage (-): 0

Region: chr22 51432899-51432952. Max. coverage (+): 0.86. Max coverage (-): 0

Region: chr22 51432953-51433005. Max. coverage (+): 5.87. Max coverage (-): 0

Region: chr22 51433006-51433059. Max. coverage (+): 2.46. Max coverage (-): 0

Region: chr22 51433060-51433113. Max. coverage (+): 1.18. Max coverage (-): 0.41

Region: chr22 51433114-51433166. Max. coverage (+): 0. Max coverage (-): 0

Region: chr22 51433167-51433220. Max. coverage (+): 0. Max coverage (-): 3.38

Region: chr22 51433221-51433273. Max. coverage (+): 0. Max coverage (-): 0

Region: chr22 51433274-51433327. Max. coverage (+): 0. Max coverage (-): 0

Region: chr22 51433328-51433380. Max. coverage (+): 0. Max coverage (-): 0

Region: chr22 51433381-51433434. Max. coverage (+): 0. Max coverage (-): 0

Region: chr22 51433435-51433488. Max. coverage (+): 0. Max coverage (-): 0

Region: chr22 51433489-51433541. Max. coverage (+): 0. Max coverage (-): 0

Region: chr22 51433542-51433595. Max. coverage (+): 0. Max coverage (-): 0

Region: chr22 51433596-51433648. Max. coverage (+): 0. Max coverage (-): 0

Region: chr22 51433649-51433702. Max. coverage (+): 0. Max coverage (-): 0

Region: chr22 51433703-51433755. Max. coverage (+): 0. Max coverage (-): 0

Region: chr22 51433756-51433809. Max. coverage (+): 0. Max coverage (-): 0

Region: chr22 51433810-51433862. Max. coverage (+): 0. Max coverage (-): 0

Region: chr22 51433863-51433916. Max. coverage (+): 1.78. Max coverage (-): 0

Region: chr22 51433917-51433970. Max. coverage (+): 0. Max coverage (-): 0

Region: chr22 51433971-51434023. Max. coverage (+): 0. Max coverage (-): 0

Region: chr22 51434024-51434077. Max. coverage (+): 9.22. Max coverage (-): 0

Region: chr22 51434078-51434130. Max. coverage (+): 5.1. Max coverage (-): 0

Region: chr22 51434131-51434184. Max. coverage (+): 0. Max coverage (-): 0

Region: chr22 51434185-51434237. Max. coverage (+): 0. Max coverage (-): 0

Region: chr22 51434238-51434291. Max. coverage (+): 7.19. Max coverage (-): 0

Region: chr22 51434292-51434345. Max. coverage (+): 0. Max coverage (-): 0

Region: chr22 51434346-51434398. Max. coverage (+): 0. Max coverage (-): 0

Region: chr22 51434399-51434452. Max. coverage (+): 0. Max coverage (-): 0

Region: chr22 51434453-51434505. Max. coverage (+): 0. Max coverage (-): 0

Region: chr22 51434506-51434559. Max. coverage (+): 7.52. Max coverage (-): 0

Region: chr22 51434560-51434612. Max. coverage (+): 0. Max coverage (-): 0

Region: chr22 51434613-51434666. Max. coverage (+): 3.59. Max coverage (-): 0

Region: chr22 51434667-51434719. Max. coverage (+): 13.95. Max coverage (-): 0

Region: chr22 51434720-51434773. Max. coverage (+): 5.02. Max coverage (-): 0

Region: chr22 51434774-51434827. Max. coverage (+): 3.74. Max coverage (-): 0

Region: chr22 51434828-51434880. Max. coverage (+): 16.67. Max coverage (-): 0

Region: chr22 51434881-51434934. Max. coverage (+): 6.35. Max coverage (-): 0

Region: chr22 51434935-51434987. Max. coverage (+): 4.84. Max coverage (-): 0

Region: chr22 51434988-51435041. Max. coverage (+): 9.55. Max coverage (-): 0

Region: chr22 51435042-51435094. Max. coverage (+): 9.55. Max coverage (-): 0

Region: chr22 51435095-51435148. Max. coverage (+): 5.71. Max coverage (-): 0

Region: chr22 51435149-51435202. Max. coverage (+): 18.37. Max coverage (-): 0

Region: chr22 51435203-51435255. Max. coverage (+): 1.42. Max coverage (-): 0

Region: chr22 51435256-51435309. Max. coverage (+): 5.73. Max coverage (-): 0

Region: chr22 51435310-51435362. Max. coverage (+): 0. Max coverage (-): 0

Region: chr22 51435363-51435416. Max. coverage (+): 11.22. Max coverage (-): 0

Region: chr22 51435417-51435469. Max. coverage (+): 14.05. Max coverage (-): 0

Region: chr22 51435470-51435523. Max. coverage (+): 1.02. Max coverage (-): 0

Region: chr22 51435524-51435577. Max. coverage (+): 7.79. Max coverage (-): 0

Region: chr22 51435578-51435630. Max. coverage (+): 7.79. Max coverage (-): 0

Region: chr22 51435631-51435684. Max. coverage (+): 12.05. Max coverage (-): 0

Region: chr22 51435685-51435737. Max. coverage (+): 0. Max coverage (-): 0

Region: chr22 51435738-51435791. Max. coverage (+): 0. Max coverage (-): 0

Region: chr22 51435792-51435844. Max. coverage (+): 0. Max coverage (-): 0

Region: chr22 51435845-51435898. Max. coverage (+): 0. Max coverage (-): 0

Region: chr22 51435899-51435951. Max. coverage (+): 0. Max coverage (-): 0

Region: chr22 51435952-51436005. Max. coverage (+): 16.89. Max coverage (-): 0

Region: chr22 51436006-51436059. Max. coverage (+): 5.49. Max coverage (-): 0

Region: chr22 51436060-51436112. Max. coverage (+): 0. Max coverage (-): 0

Region: chr22 51436113-51436166. Max. coverage (+): 1.73. Max coverage (-): 0

Region: chr22 51436167-51436219. Max. coverage (+): 6.27. Max coverage (-): 0

Region: chr22 51436220-51436273. Max. coverage (+): 0. Max coverage (-): 0

Region: chr22 51436274-51436326. Max. coverage (+): 0. Max coverage (-): 0

Region: chr22 51436327-51436380. Max. coverage (+): 5.72. Max coverage (-): 0

Region: chr22 51436381-51436434. Max. coverage (+): 8.5. Max coverage (-): 0

Region: chr22 51436435-51436487. Max. coverage (+): 3.97. Max coverage (-): 0

Region: chr22 51436488-51436541. Max. coverage (+): 0. Max coverage (-): 0

Region: chr22 51436542-51436594. Max. coverage (+): 0. Max coverage (-): 0

Region: chr22 51436595-51436648. Max. coverage (+): 7.62. Max coverage (-): 0

Region: chr22 51436649-51436701. Max. coverage (+): 0. Max coverage (-): 0

Region: chr22 51436702-51436755. Max. coverage (+): 2.18. Max coverage (-): 0

Region: chr22 51436756-51436808. Max. coverage (+): 0. Max coverage (-): 0

Region: chr22 51436809-51436862. Max. coverage (+): 0. Max coverage (-): 0

Region: chr22 51436863-51436916. Max. coverage (+): 0. Max coverage (-): 0

Region: chr22 51436917-51436969. Max. coverage (+): 12.15. Max coverage (-): 0

Region: chr22 51436970-51437023. Max. coverage (+): 12.15. Max coverage (-): 0

Region: chr22 51437024-51437076. Max. coverage (+): 18.07. Max coverage (-): 0

Region: chr22 51437077-51437130. Max. coverage (+): 8.35. Max coverage (-): 0

Region: chr22 51437131-51437183. Max. coverage (+): 1.47. Max coverage (-): 0

Region: chr22 51437184-51437237. Max. coverage (+): 0. Max coverage (-): 0

Region: chr22 51437238-51437291. Max. coverage (+): 0. Max coverage (-): 0

Region: chr22 51437292-51437344. Max. coverage (+): 0. Max coverage (-): 0

Region: chr22 51437345-51437398. Max. coverage (+): 12.64. Max coverage (-): 0

Region: chr22 51437399-51437451. Max. coverage (+): 3.93. Max coverage (-): 0

Region: chr22 51437452-51437505. Max. coverage (+): 26.37. Max coverage (-): 0

Region: chr22 51437506-51437558. Max. coverage (+): 15.16. Max coverage (-): 0

Region: chr22 51437559-51437612. Max. coverage (+): 15.32. Max coverage (-): 0

Region: chr22 51437613-51437665. Max. coverage (+): 1.63. Max coverage (-): 0

Region: chr22 51437666-51437719. Max. coverage (+): 13.67. Max coverage (-): 0

Region: chr22 51437720-51437773. Max. coverage (+): 12.37. Max coverage (-): 0

Region: chr22 51437774-51437826. Max. coverage (+): 3.42. Max coverage (-): 0

Region: chr22 51437827-51437880. Max. coverage (+): 0. Max coverage (-): 0

Region: chr22 51437881-51437933. Max. coverage (+): 0. Max coverage (-): 0

Region: chr22 51437934-51437987. Max. coverage (+): 0. Max coverage (-): 0

Region: chr22 51437988-51438040. Max. coverage (+): 6.47. Max coverage (-): 0

Region: chr22 51438041-51438094. Max. coverage (+): 13.21. Max coverage (-): 0

Region: chr22 51438095-51438148. Max. coverage (+): 3.82. Max coverage (-): 0

Region: chr22 51438149-51438201. Max. coverage (+): 0. Max coverage (-): 0

Region: chr22 51438202-51438255. Max. coverage (+): 0. Max coverage (-): 0

Region: chr22 51438256-51438308. Max. coverage (+): 0. Max coverage (-): 0

Region: chr22 51438309-51438362. Max. coverage (+): 3.05. Max coverage (-): 0

Region: chr22 51438363-51438415. Max. coverage (+): 5.06. Max coverage (-): 0

Region: chr22 51438416-51438469. Max. coverage (+): 0. Max coverage (-): 0

Region: chr22 51438470-51438523. Max. coverage (+): 0. Max coverage (-): 0

Region: chr22 51438524-51438576. Max. coverage (+): 3.52. Max coverage (-): 0

Region: chr22 51438577-51438630. Max. coverage (+): 0. Max coverage (-): 0

Region: chr22 51438631-51438683. Max. coverage (+): 1.86. Max coverage (-): 0

Region: chr22 51438684-51438737. Max. coverage (+): 0. Max coverage (-): 0

Region: chr22 51438738-51438790. Max. coverage (+): 0. Max coverage (-): 0

Region: chr22 51438791-51438844. Max. coverage (+): 0. Max coverage (-): 0

Region: chr22 51438845-51438897. Max. coverage (+): 0. Max coverage (-): 0

Region: chr22 51438898-51438951. Max. coverage (+): 0.83. Max coverage (-): 0

Region: chr22 51438952-51439005. Max. coverage (+): 7.28. Max coverage (-): 0

Region: chr22 51439006-51439058. Max. coverage (+): 0. Max coverage (-): 0

Region: chr22 51439059-51439112. Max. coverage (+): 4.33. Max coverage (-): 0

Region: chr22 51439113-51439165. Max. coverage (+): 5.54. Max coverage (-): 0

Region: chr22 51439166-51439219. Max. coverage (+): 0. Max coverage (-): 0

Region: chr22 51439220-51439272. Max. coverage (+): 0. Max coverage (-): 0

Region: chr22 51439273-51439326. Max. coverage (+): 0. Max coverage (-): 0

Region: chr22 51439327-51439380. Max. coverage (+): 0. Max coverage (-): 0

Region: chr22 51439381-51439433. Max. coverage (+): 0.87. Max coverage (-): 0

Region: chr22 51439434-51439487. Max. coverage (+): 10.73. Max coverage (-): 0

Region: chr22 51439488-51439540. Max. coverage (+): 6.86. Max coverage (-): 0

Region: chr22 51439541-51439594. Max. coverage (+): 0. Max coverage (-): 0

Region: chr22 51439595-51439647. Max. coverage (+): 0. Max coverage (-): 0

Region: chr22 51439648-51439701. Max. coverage (+): 0. Max coverage (-): 0

Region: chr22 51439702-51439754. Max. coverage (+): 0. Max coverage (-): 0

Region: chr22 51439755-51439808. Max. coverage (+): 0. Max coverage (-): 0

Region: chr22 51439809-51439862. Max. coverage (+): 1.57. Max coverage (-): 0

Region: chr22 51439863-51439915. Max. coverage (+): 0. Max coverage (-): 0

Region: chr22 51439916-51439969. Max. coverage (+): 0. Max coverage (-): 0

Region: chr22 51439970-51440022. Max. coverage (+): 0. Max coverage (-): 0

Region: chr22 51440023-51440076. Max. coverage (+): 0. Max coverage (-): 0

Region: chr22 51440077-51440129. Max. coverage (+): 3.38. Max coverage (-): 0

Region: chr22 51440130-51440183. Max. coverage (+): 3.38. Max coverage (-): 0

Region: chr22 51440184-51440237. Max. coverage (+): 0. Max coverage (-): 0

Region: chr22 51440238-51440290. Max. coverage (+): 0. Max coverage (-): 0

Region: chr22 51440291-51440344. Max. coverage (+): 6.74. Max coverage (-): 0

Region: chr22 51440345-51440397. Max. coverage (+): 1.54. Max coverage (-): 0

Region: chr22 51440398-51440451. Max. coverage (+): 0. Max coverage (-): 0

Region: chr22 51440452-51440504. Max. coverage (+): 0. Max coverage (-): 0

Region: chr22 51440505-51440558. Max. coverage (+): 0. Max coverage (-): 0

Region: chr22 51440559-51440612. Max. coverage (+): 3.59. Max coverage (-): 0

Region: chr22 51440613-51440665. Max. coverage (+): 0. Max coverage (-): 0

Region: chr22 51440666-51440719. Max. coverage (+): 0. Max coverage (-): 0

Region: chr22 51440720-51440772. Max. coverage (+): 0. Max coverage (-): 0

Region: chr22 51440773-51440826. Max. coverage (+): 0. Max coverage (-): 0

Region: chr22 51440827-51440879. Max. coverage (+): 0. Max coverage (-): 0

Region: chr22 51440880-51440933. Max. coverage (+): 9.18. Max coverage (-): 0

Region: chr22 51440934-51440986. Max. coverage (+): 1.64. Max coverage (-): 0

Region: chr22 51440987-51441040. Max. coverage (+): 0. Max coverage (-): 0

Region: chr22 51441041-51441094. Max. coverage (+): 2.06. Max coverage (-): 0

Region: chr22 51441095-51441147. Max. coverage (+): 1.61. Max coverage (-): 0

Region: chr22 51441148-51441201. Max. coverage (+): 1.72. Max coverage (-): 0

Region: chr22 51441202-51441254. Max. coverage (+): 4.08. Max coverage (-): 0

Region: chr22 51441255-51441308. Max. coverage (+): 0. Max coverage (-): 0

Region: chr22 51441309-51441361. Max. coverage (+): 0. Max coverage (-): 0

Region: chr22 51441362-51441415. Max. coverage (+): 0. Max coverage (-): 0

Region: chr22 51441416-51441469. Max. coverage (+): 0. Max coverage (-): 0

Region: chr22 51441470-51441522. Max. coverage (+): 0. Max coverage (-): 0

Region: chr22 51441523-51441576. Max. coverage (+): 1.62. Max coverage (-): 0

Region: chr22 51441577-51441629. Max. coverage (+): 0. Max coverage (-): 0

Region: chr22 51441630-51441683. Max. coverage (+): 0. Max coverage (-): 0

Region: chr22 51441684-51441736. Max. coverage (+): 0. Max coverage (-): 0

Region: chr22 51441737-51441790. Max. coverage (+): 0. Max coverage (-): 0

Region: chr22 51441791-51441843. Max. coverage (+): 0. Max coverage (-): 0

Region: chr22 51441844-51441897. Max. coverage (+): 0. Max coverage (-): 0

Region: chr22 51441898-51441951. Max. coverage (+): 0. Max coverage (-): 0

Region: chr22 51441952-51442004. Max. coverage (+): 0. Max coverage (-): 0

Region: chr22 51442005-51442058. Max. coverage (+): 0. Max coverage (-): 0

Region: chr22 51442059-51442111. Max. coverage (+): 0. Max coverage (-): 0

Region: chr22 51442112-51442165. Max. coverage (+): 0. Max coverage (-): 0

Region: chr22 51442166-51442218. Max. coverage (+): 0. Max coverage (-): 0

Region: chr22 51442219-51442272. Max. coverage (+): 0. Max coverage (-): 0

Region: chr22 51442273-51442326. Max. coverage (+): 0. Max coverage (-): 0

Region: chr22 51442327-51442379. Max. coverage (+): 0. Max coverage (-): 0

Region: chr22 51442380-51442433. Max. coverage (+): 3.37. Max coverage (-): 0

Region: chr22 51442434-51442486. Max. coverage (+): 0. Max coverage (-): 0

Region: chr22 51442487-51442540. Max. coverage (+): 0. Max coverage (-): 0

Region: chr22 51442541-51442593. Max. coverage (+): 4.22. Max coverage (-): 0

Region: chr22 51442594-51442647. Max. coverage (+): 0. Max coverage (-): 0

Region: chr22 51442648-51442701. Max. coverage (+): 0. Max coverage (-): 0

Region: chr22 51442702-51442754. Max. coverage (+): 15.53. Max coverage (-): 0

Region: chr22 51442755-51442808. Max. coverage (+): 0. Max coverage (-): 0

Region: chr22 51442809-51442861. Max. coverage (+): 0. Max coverage (-): 0

Region: chr22 51442862-51442915. Max. coverage (+): 0. Max coverage (-): 0

Region: chr22 51442916-51442968. Max. coverage (+): 0. Max coverage (-): 0

Region: chr22 51442969-51443022. Max. coverage (+): 0. Max coverage (-): 0

Region: chr22 51443023-51443075. Max. coverage (+): 0. Max coverage (-): 0

Region: chr22 51443076-51443129. Max. coverage (+): 0. Max coverage (-): 0

Region: chr22 51443130-51443183. Max. coverage (+): 0. Max coverage (-): 0

Region: chr22 51443184-51443236. Max. coverage (+): 0. Max coverage (-): 0

Region: chr22 51443237-51443290. Max. coverage (+): 2.12. Max coverage (-): 0

Region: chr22 51443291-. Max. coverage (+): 2.12. Max coverage (-): 0

RepeatMasker Color Code

**+**

100-98% Identity

<98-95% Identity

<95-90% Identity

<90-85% Identity

<85-80% Identity

<80-75% Identity

<75-70% Identity

<70% Identity

**-**

Gene Set Color Code

**+**

Gene

Pseudogene

**-**

Topology/Coverage Color Code

Coverage Plus Strand

Coverage Minus Strand

Mainstrand: Plus

Mainstrand: Minus

Complementary Strand

Flanking Region  
(if option -flank >0)

Gene Set Annotation  

**1. C3orf84 (protein coding, ENSBTAG00000000982) Tr:00000001300 Ex:1**: 51418820-51418935 (+)  
**2. C3orf84 (protein coding, ENSBTAG00000000982) Tr:00000001300 Ex:2**: 51420789-51420902 (+)  
**3. C3orf84 (protein coding, ENSBTAG00000000982) Tr:00000001300 Ex:3**: 51425932-51425988 (+)  
**4. C3orf84 (protein coding, ENSBTAG00000000982) Tr:00000001300 Ex:4**: 51426395-51426814 (+)  
**5. KLHDC8B (protein coding, ENSBTAG00000000981) Tr:00000001299 Ex:1**: 51432193-51432321 (-)  
**6. KLHDC8B (protein coding, ENSBTAG00000000981) Tr:00000001299 Ex:2**: 51430752-51431261 (-)  
**7. KLHDC8B (protein coding, ENSBTAG00000000981) Tr:00000001299 Ex:3**: 51429530-51429694 (-)  
**8. KLHDC8B (protein coding, ENSBTAG00000000981) Tr:00000001299 Ex:4**: 51428801-51428902 (-)  
**9. KLHDC8B (protein coding, ENSBTAG00000000981) Tr:00000001299 Ex:5**: 51427513-51428371 (-)  
**10. KLHDC8B (protein coding, ENSBTAG00000000981) Tr:00000001298 Ex:1**: 51432193-51432248 (-)  
**11. KLHDC8B (protein coding, ENSBTAG00000000981) Tr:00000001298 Ex:2**: 51430752-51431261 (-)  
**12. KLHDC8B (protein coding, ENSBTAG00000000981) Tr:00000001298 Ex:3**: 51429530-51429694 (-)  
**13. KLHDC8B (protein coding, ENSBTAG00000000981) Tr:00000001298 Ex:4**: 51429004-51429228 (-)  
**14. KLHDC8B (protein coding, ENSBTAG00000000981) Tr:00000001298 Ex:5**: 51428801-51428902 (-)  
**15. KLHDC8B (protein coding, ENSBTAG00000000981) Tr:00000001298 Ex:6**: 51427513-51428371 (-)  
**16. CCDC71 (protein coding, ENSBTAG00000013932) Tr:00000018512 Ex:1**: 51435769-51435809 (+)  
**17. CCDC71 (protein coding, ENSBTAG00000013932) Tr:00000018512 Ex:2**: 51437781-51439453 (+)

  
RepeatMasker Annotation  

**1. AT\_rich**: 51416976-51416996 (+), Divergence to consensus: 47.6%  
**2. L1MC1**: 51417140-51417383 (+), Divergence to consensus: 39%  
**3. L1ME1**: 51417414-51417763 (+), Divergence to consensus: 31.7%  
**4. L1ME3C**: 51417741-51417768 (-), Divergence to consensus: 44.1%  
**5. SINE2-1\_BT**: 51417769-51417887 (-), Divergence to consensus: 19.3%  
**6. L1ME3C**: 51417888-51418147 (-), Divergence to consensus: 44.1%  
**7. L2c**: 51419234-51419351 (+), Divergence to consensus: 40.3%  
**8. L2b**: 51420168-51420296 (+), Divergence to consensus: 36.4%  
**9. GA-rich**: 51420477-51420561 (+), Divergence to consensus: 22.2%  
**10. Bov-tA2**: 51421364-51421577 (+), Divergence to consensus: 18.3%  
**11. LTR33**: 51421877-51421957 (-), Divergence to consensus: 30.1%  
**12. L1MD2**: 51423564-51423761 (-), Divergence to consensus: 24.7%  
**13. SINE2-1\_BT**: 51423779-51423895 (+), Divergence to consensus: 18.8%  
**14. L1-2\_BT**: 51423900-51424081 (-), Divergence to consensus: 42.3%  
**15. AT\_rich**: 51424082-51424117 (+), Divergence to consensus: 61.1%  
**16. Bov-tA2**: 51424200-51424375 (-), Divergence to consensus: 27.8%  
**17. Bov-tA2**: 51424378-51424494 (-), Divergence to consensus: 16.2%  
**18. CHRL**: 51424500-51424636 (-), Divergence to consensus: 17.5%  
**19. L2a**: 51424649-51425611 (-), Divergence to consensus: 45.9%  
**20. MIRc**: 51427523-51427685 (-), Divergence to consensus: 25.2%  
**21. GC\_rich**: 51432192-51432220 (+), Divergence to consensus: 72.4%  
**22. LTR16C**: 51433252-51433552 (-), Divergence to consensus: 33.8%  
**23. CHR-2\_BT**: 51434326-51434533 (+), Divergence to consensus: 22.6%  
**24. L2c**: 51435562-51435634 (+), Divergence to consensus: 40.4%  
**25. ART2A**: 51436833-51436939 (-), Divergence to consensus: 27.1%  
**26. AmnSINE2**: 51437202-51437268 (+), Divergence to consensus: 35.8%

  
Transcription Factor Binding Sites  

**RFX4\_1** (Sequence: CCTGGCAAC (+): 51425833)  
**RFX4\_1** (Sequence: CATGGCAAC (+): 51430297)  
**SPZ1** (Sequence: CTGAAACCCT (-): 51435188)  
**Gata4** (Sequence: AGATAAG (-): 51419425)  
**SOX9** (Sequence: AACAATGA (-): 51419537)  
**SOX9** (Sequence: AACAATGG (-): 51426010)  
**Gata4** (Sequence: CTTATCT (+): 51416951)
