# Supplementary material for: Small RNA sequencing of cryopreserved semen from single bull revealed altered miRNAs and piRNAs expression between High- and Low-motile sperm populations
Source: BMC Genomics. 2017 Jan 4;18:14. doi: 10.1186/s12864-016-3394-7 (PMC5209821; doi:10.1186/s12864-016-3394-7)
Supplement: Additional file 3: — Details for each piRNA clusters found in High Motile (HM) sperm fraction. Genes, repeats, transposable elements and transcription factors binding sites falling within the cluster regions were reported. (ZIP 1896 kb) [file 12864_2016_3394_MOESM3_ESM.zip › 53.html]

piRNA cluster 53


Predicted piRNA cluster no. 53     previous   next
  

Show proTRAC run info
Hide proTRAC run info

================================= proTRAC ====================================  
VERSION: 2.1                                    LAST MODIFIED: 06. October 2015  
  
Please cite:  
Rosenkranz D, Zischler H. proTRAC - a software for probabilistic piRNA cluster  
detection, visualization and analysis. 2012. BMC Bioinformatics 13:5.  
  
and (for proTRAC 2.0 and later):  
Rosenkranz D, Rudloff S, Bastuck K, Ketting RF, Zischler H. Tupaia small RNAs  
provide insights into function and evolution of RNAi-based transposon defense  
in mammals. 2015. RNA 21(5):911-922.  
  
Contact:  
David Rosenkranz  
Institute of Anthropology, small RNA group  
Johannes Gutenberg University Mainz  
email: rosenkranz@uni-mainz.de  
  
You can find the latest proTRAC version at:  
http://sourceforge.net/projects/protrac/files  
http://www.smallRNAgroup-mainz.de/software  
==============================================================================  
  
PARAMETERS:  
Map file: .............../storage/core/barbara/genhome/smallRNA/fertility/Sample\_motile/pirna/Sample\_motile\_26-33\_collapsed.fa.no-dust.map.weighted-10000-1000-b-0  
Genome file: ............/storage/core/barbara/genhome/smallRNA/fertility/Sample\_all/pirna/bt\_311\_chrY.fa  
RepeatMasker annotation: /storage/genomes/bt\_umd31/GCF\_000003055.6\_Bos\_taurus\_UMD\_3.1.1\_repeatMasker\_chr.out  
GeneSet:................./storage/core/barbara/genhome/smallRNA/fertility/Sample\_all/pirna/full.gtf  
  
Significant (p<=0.01) hit density will be calculated based  
on observed hit distribution.  
  
Sliding window size: ........................................ 5000 bp  
Sliding window increament: .................................. 1000 bp  
Normalize each hit by number of genomic hits: ............... 1 [0=no/1=yes]  
Normalize each hit by number of sequence reads: ............. 1 [0=no/1=yes]  
Normalize values (-> per million mapped reads): ............. 1 [0=no/1=yes]  
Min. fraction of hits with 1T(U) or 10A: .................... 0.75  
Alternatively: Min. fraction of hits with 1T(U) and 10A: .... 0.5  
Min. fraction of hits with typical piRNA length: ............ 0.75  
Typical piRNA length: ....................................... 26-33 nt  
Min. size of a piRNA cluster: ............................... 5000 bp.  
Min. number of hits (absolute): ............................. 0  
Min. number of hits (normalized): ........................... 0  
Min. fraction of hits on the mainstrand: .................... 0.75  
Top fraction of mapped sequences (in terms of read counts): . 1%  
Top fraction accounts for max. n% of sequence reads: ........ 90%  
Min. fraction of hits on each arm of a bidirectional cluster: 0.1  
Output image file for each cluster: ......................... 0 [0=no/1=yes]  
Output html file for each cluster: .......................... 1 [0=no/1=yes]  
Output a summary table: ..................................... 1 [0=no/1=yes]  
Output a FASTA file for each cluster (piRNA sequences): ..... 1 [0=no/1=yes]  
Output a FASTA file comprising cluster sequences: ........... 1 [0=no/1=yes]  
Search DNA motifs in clusters: .............................. 1 [0=no/1=yes]  
Output flanking sequences: +/- .............................. 0 bp  
Output ~.pTi file: .......................................... 1 [0=no/1=yes]  
==============================================================================  
  
  
Genome size (without gaps): ............ 2678902517 bp  
Gaps (N/X/-): .......................... 53837044 bp  
Mapped reads: .......................... 658825247023  
Non-identical sequences: ............... 514171  
Genomic hits: .......................... 764233  
Significant densitiy of mapped reads: .. 12867599.5173724 reads/kb

Show proTRAC cluster info
Hide proTRAC cluster info

|  |  |
| --- | --- |
| Location | chr23 |
| Coordinates | 8247311-8258266 |
| Size [bp] | 10956 |
| Sequence hit loci | 176 |
| Mapped reads (normalized) | 238064896 |
| Mapped reads (normalized) per kb | 21729180 |
| Normalized reads with 1T (1U) | 85.4% |
| Normalized reads with 10A | 24.5% |
| Normalized reads with length 26-33 nt | 100% |
| Normalized reads on the main strand(s) | 99.1% |
| Predicted directionality | mono:minus |

100%

0%

1T (1U)  
reads

10A reads

26-33 nt  
reads

reads on mainstrand

**Either the amount of reads with 1T (1U) OR 10A has to exceed 75% (set with option: -1Tor10A)  
Alternatively the amount of reads with 1T (1U) AND 10A has to exceed 50% (set with option: -1Tand10A)  
Minimum amount of reads with preferred size is 75% (set with option: -pisize)  
Minimum amount of reads on the main strand(s) is 75% (set with option: -clstrand)**

Show read coverage
Hide read coverage

WHAT DO I SEE HERE?  
This chart shows the location of mapped sequence reads within a predicted piRNA cluster. The color refers to the number of genomic hits produced by the sequence read in question. A dark red bar indicates that this sequence read produces many other hits elsewhere in the genome. Many adjacent red or yellow bars can indicate the presence of a multi-copy element such as transposons or rRNA genes. A dark green bar indicates that this sequence read maps uniquely to this locus.

1 hit

2-5 hits

6-10 hits

11-20 hits

21-50 hits

51-100 hits

> 100 hits

chr23

8247311

8258266

Gene Set

RepeatMasker

Mapped  
Reads

15.98

plus strand

minus strand

15.98

Region: chr23 51443292-8247321. Max. coverage (+): 0. Max coverage (-): 5.68

Region: chr23 8247322-8247343. Max. coverage (+): 0. Max coverage (-): 4.52

Region: chr23 8247344-8247365. Max. coverage (+): 0. Max coverage (-): 0

Region: chr23 8247366-8247387. Max. coverage (+): 0. Max coverage (-): 0

Region: chr23 8247388-8247409. Max. coverage (+): 0. Max coverage (-): 0

Region: chr23 8247410-8247431. Max. coverage (+): 0. Max coverage (-): 0

Region: chr23 8247432-8247453. Max. coverage (+): 0. Max coverage (-): 0

Region: chr23 8247454-8247475. Max. coverage (+): 0. Max coverage (-): 0

Region: chr23 8247476-8247497. Max. coverage (+): 0. Max coverage (-): 0

Region: chr23 8247498-8247519. Max. coverage (+): 0. Max coverage (-): 0

Region: chr23 8247520-8247541. Max. coverage (+): 0. Max coverage (-): 0

Region: chr23 8247542-8247562. Max. coverage (+): 0. Max coverage (-): 0

Region: chr23 8247563-8247584. Max. coverage (+): 0. Max coverage (-): 0

Region: chr23 8247585-8247606. Max. coverage (+): 0. Max coverage (-): 0

Region: chr23 8247607-8247628. Max. coverage (+): 0. Max coverage (-): 0

Region: chr23 8247629-8247650. Max. coverage (+): 0. Max coverage (-): 0

Region: chr23 8247651-8247672. Max. coverage (+): 0. Max coverage (-): 0

Region: chr23 8247673-8247694. Max. coverage (+): 0. Max coverage (-): 1.39

Region: chr23 8247695-8247716. Max. coverage (+): 0. Max coverage (-): 0

Region: chr23 8247717-8247738. Max. coverage (+): 0. Max coverage (-): 0

Region: chr23 8247739-8247760. Max. coverage (+): 0. Max coverage (-): 0

Region: chr23 8247761-8247782. Max. coverage (+): 0. Max coverage (-): 0

Region: chr23 8247783-8247804. Max. coverage (+): 0. Max coverage (-): 1.17

Region: chr23 8247805-8247825. Max. coverage (+): 0. Max coverage (-): 0

Region: chr23 8247826-8247847. Max. coverage (+): 0. Max coverage (-): 0

Region: chr23 8247848-8247869. Max. coverage (+): 0. Max coverage (-): 0

Region: chr23 8247870-8247891. Max. coverage (+): 0. Max coverage (-): 0

Region: chr23 8247892-8247913. Max. coverage (+): 0. Max coverage (-): 0

Region: chr23 8247914-8247935. Max. coverage (+): 0. Max coverage (-): 0

Region: chr23 8247936-8247957. Max. coverage (+): 0. Max coverage (-): 3.53

Region: chr23 8247958-8247979. Max. coverage (+): 0. Max coverage (-): 3.53

Region: chr23 8247980-8248001. Max. coverage (+): 0. Max coverage (-): 0

Region: chr23 8248002-8248023. Max. coverage (+): 0. Max coverage (-): 0

Region: chr23 8248024-8248045. Max. coverage (+): 0. Max coverage (-): 0

Region: chr23 8248046-8248066. Max. coverage (+): 0. Max coverage (-): 0

Region: chr23 8248067-8248088. Max. coverage (+): 0. Max coverage (-): 0

Region: chr23 8248089-8248110. Max. coverage (+): 0. Max coverage (-): 0

Region: chr23 8248111-8248132. Max. coverage (+): 0. Max coverage (-): 0

Region: chr23 8248133-8248154. Max. coverage (+): 0. Max coverage (-): 0

Region: chr23 8248155-8248176. Max. coverage (+): 0. Max coverage (-): 0

Region: chr23 8248177-8248198. Max. coverage (+): 0. Max coverage (-): 0

Region: chr23 8248199-8248220. Max. coverage (+): 0. Max coverage (-): 0

Region: chr23 8248221-8248242. Max. coverage (+): 0. Max coverage (-): 0

Region: chr23 8248243-8248264. Max. coverage (+): 0. Max coverage (-): 0

Region: chr23 8248265-8248286. Max. coverage (+): 0. Max coverage (-): 0

Region: chr23 8248287-8248307. Max. coverage (+): 0. Max coverage (-): 0

Region: chr23 8248308-8248329. Max. coverage (+): 0. Max coverage (-): 0

Region: chr23 8248330-8248351. Max. coverage (+): 0. Max coverage (-): 0

Region: chr23 8248352-8248373. Max. coverage (+): 0. Max coverage (-): 0

Region: chr23 8248374-8248395. Max. coverage (+): 0. Max coverage (-): 0

Region: chr23 8248396-8248417. Max. coverage (+): 0. Max coverage (-): 0

Region: chr23 8248418-8248439. Max. coverage (+): 0. Max coverage (-): 0

Region: chr23 8248440-8248461. Max. coverage (+): 0. Max coverage (-): 2.72

Region: chr23 8248462-8248483. Max. coverage (+): 0. Max coverage (-): 0

Region: chr23 8248484-8248505. Max. coverage (+): 0. Max coverage (-): 0

Region: chr23 8248506-8248527. Max. coverage (+): 0. Max coverage (-): 0

Region: chr23 8248528-8248549. Max. coverage (+): 0. Max coverage (-): 0

Region: chr23 8248550-8248570. Max. coverage (+): 0. Max coverage (-): 0

Region: chr23 8248571-8248592. Max. coverage (+): 0. Max coverage (-): 0

Region: chr23 8248593-8248614. Max. coverage (+): 0. Max coverage (-): 0

Region: chr23 8248615-8248636. Max. coverage (+): 0. Max coverage (-): 0

Region: chr23 8248637-8248658. Max. coverage (+): 0. Max coverage (-): 0

Region: chr23 8248659-8248680. Max. coverage (+): 0. Max coverage (-): 0

Region: chr23 8248681-8248702. Max. coverage (+): 0. Max coverage (-): 0

Region: chr23 8248703-8248724. Max. coverage (+): 0. Max coverage (-): 0

Region: chr23 8248725-8248746. Max. coverage (+): 0. Max coverage (-): 0

Region: chr23 8248747-8248768. Max. coverage (+): 0. Max coverage (-): 0

Region: chr23 8248769-8248790. Max. coverage (+): 0. Max coverage (-): 0

Region: chr23 8248791-8248811. Max. coverage (+): 0. Max coverage (-): 0

Region: chr23 8248812-8248833. Max. coverage (+): 0. Max coverage (-): 0

Region: chr23 8248834-8248855. Max. coverage (+): 0. Max coverage (-): 0

Region: chr23 8248856-8248877. Max. coverage (+): 0. Max coverage (-): 1.5

Region: chr23 8248878-8248899. Max. coverage (+): 0. Max coverage (-): 0

Region: chr23 8248900-8248921. Max. coverage (+): 0. Max coverage (-): 0

Region: chr23 8248922-8248943. Max. coverage (+): 0. Max coverage (-): 0

Region: chr23 8248944-8248965. Max. coverage (+): 0. Max coverage (-): 0

Region: chr23 8248966-8248987. Max. coverage (+): 0. Max coverage (-): 0

Region: chr23 8248988-8249009. Max. coverage (+): 0. Max coverage (-): 0

Region: chr23 8249010-8249031. Max. coverage (+): 0. Max coverage (-): 0

Region: chr23 8249032-8249053. Max. coverage (+): 0. Max coverage (-): 0

Region: chr23 8249054-8249074. Max. coverage (+): 0. Max coverage (-): 0

Region: chr23 8249075-8249096. Max. coverage (+): 0. Max coverage (-): 1.57

Region: chr23 8249097-8249118. Max. coverage (+): 0. Max coverage (-): 0

Region: chr23 8249119-8249140. Max. coverage (+): 0. Max coverage (-): 0

Region: chr23 8249141-8249162. Max. coverage (+): 0. Max coverage (-): 0

Region: chr23 8249163-8249184. Max. coverage (+): 0. Max coverage (-): 0

Region: chr23 8249185-8249206. Max. coverage (+): 0. Max coverage (-): 0

Region: chr23 8249207-8249228. Max. coverage (+): 0. Max coverage (-): 0

Region: chr23 8249229-8249250. Max. coverage (+): 0. Max coverage (-): 0

Region: chr23 8249251-8249272. Max. coverage (+): 0. Max coverage (-): 0

Region: chr23 8249273-8249294. Max. coverage (+): 0. Max coverage (-): 0

Region: chr23 8249295-8249315. Max. coverage (+): 0. Max coverage (-): 0

Region: chr23 8249316-8249337. Max. coverage (+): 0. Max coverage (-): 0

Region: chr23 8249338-8249359. Max. coverage (+): 0. Max coverage (-): 0

Region: chr23 8249360-8249381. Max. coverage (+): 0. Max coverage (-): 0

Region: chr23 8249382-8249403. Max. coverage (+): 0. Max coverage (-): 0

Region: chr23 8249404-8249425. Max. coverage (+): 0. Max coverage (-): 0

Region: chr23 8249426-8249447. Max. coverage (+): 0. Max coverage (-): 0

Region: chr23 8249448-8249469. Max. coverage (+): 0. Max coverage (-): 0

Region: chr23 8249470-8249491. Max. coverage (+): 0. Max coverage (-): 0

Region: chr23 8249492-8249513. Max. coverage (+): 0. Max coverage (-): 3.53

Region: chr23 8249514-8249535. Max. coverage (+): 0. Max coverage (-): 3.53

Region: chr23 8249536-8249556. Max. coverage (+): 0. Max coverage (-): 8.8

Region: chr23 8249557-8249578. Max. coverage (+): 0. Max coverage (-): 0

Region: chr23 8249579-8249600. Max. coverage (+): 0. Max coverage (-): 7.66

Region: chr23 8249601-8249622. Max. coverage (+): 0. Max coverage (-): 0

Region: chr23 8249623-8249644. Max. coverage (+): 0. Max coverage (-): 0

Region: chr23 8249645-8249666. Max. coverage (+): 0. Max coverage (-): 0

Region: chr23 8249667-8249688. Max. coverage (+): 0. Max coverage (-): 0

Region: chr23 8249689-8249710. Max. coverage (+): 0. Max coverage (-): 0

Region: chr23 8249711-8249732. Max. coverage (+): 0. Max coverage (-): 0

Region: chr23 8249733-8249754. Max. coverage (+): 0. Max coverage (-): 0

Region: chr23 8249755-8249776. Max. coverage (+): 0. Max coverage (-): 0

Region: chr23 8249777-8249798. Max. coverage (+): 0. Max coverage (-): 0

Region: chr23 8249799-8249819. Max. coverage (+): 0. Max coverage (-): 0

Region: chr23 8249820-8249841. Max. coverage (+): 0. Max coverage (-): 0

Region: chr23 8249842-8249863. Max. coverage (+): 0. Max coverage (-): 0

Region: chr23 8249864-8249885. Max. coverage (+): 0. Max coverage (-): 0

Region: chr23 8249886-8249907. Max. coverage (+): 0. Max coverage (-): 0

Region: chr23 8249908-8249929. Max. coverage (+): 0. Max coverage (-): 0

Region: chr23 8249930-8249951. Max. coverage (+): 0. Max coverage (-): 0

Region: chr23 8249952-8249973. Max. coverage (+): 0. Max coverage (-): 2.88

Region: chr23 8249974-8249995. Max. coverage (+): 0. Max coverage (-): 1.75

Region: chr23 8249996-8250017. Max. coverage (+): 0. Max coverage (-): 0

Region: chr23 8250018-8250039. Max. coverage (+): 0. Max coverage (-): 7.59

Region: chr23 8250040-8250060. Max. coverage (+): 0. Max coverage (-): 0

Region: chr23 8250061-8250082. Max. coverage (+): 0. Max coverage (-): 0

Region: chr23 8250083-8250104. Max. coverage (+): 0. Max coverage (-): 0

Region: chr23 8250105-8250126. Max. coverage (+): 0. Max coverage (-): 0

Region: chr23 8250127-8250148. Max. coverage (+): 0. Max coverage (-): 0

Region: chr23 8250149-8250170. Max. coverage (+): 0. Max coverage (-): 0

Region: chr23 8250171-8250192. Max. coverage (+): 0. Max coverage (-): 0

Region: chr23 8250193-8250214. Max. coverage (+): 0. Max coverage (-): 4.29

Region: chr23 8250215-8250236. Max. coverage (+): 0. Max coverage (-): 0

Region: chr23 8250237-8250258. Max. coverage (+): 0. Max coverage (-): 0

Region: chr23 8250259-8250280. Max. coverage (+): 0. Max coverage (-): 1

Region: chr23 8250281-8250301. Max. coverage (+): 0. Max coverage (-): 0

Region: chr23 8250302-8250323. Max. coverage (+): 0. Max coverage (-): 0

Region: chr23 8250324-8250345. Max. coverage (+): 0. Max coverage (-): 0

Region: chr23 8250346-8250367. Max. coverage (+): 0. Max coverage (-): 0

Region: chr23 8250368-8250389. Max. coverage (+): 0. Max coverage (-): 0

Region: chr23 8250390-8250411. Max. coverage (+): 0. Max coverage (-): 0

Region: chr23 8250412-8250433. Max. coverage (+): 0. Max coverage (-): 0

Region: chr23 8250434-8250455. Max. coverage (+): 0. Max coverage (-): 0

Region: chr23 8250456-8250477. Max. coverage (+): 0. Max coverage (-): 0

Region: chr23 8250478-8250499. Max. coverage (+): 0. Max coverage (-): 0

Region: chr23 8250500-8250521. Max. coverage (+): 0. Max coverage (-): 0

Region: chr23 8250522-8250543. Max. coverage (+): 0. Max coverage (-): 0

Region: chr23 8250544-8250564. Max. coverage (+): 0. Max coverage (-): 0

Region: chr23 8250565-8250586. Max. coverage (+): 0. Max coverage (-): 0

Region: chr23 8250587-8250608. Max. coverage (+): 0. Max coverage (-): 0

Region: chr23 8250609-8250630. Max. coverage (+): 0. Max coverage (-): 0

Region: chr23 8250631-8250652. Max. coverage (+): 0. Max coverage (-): 0

Region: chr23 8250653-8250674. Max. coverage (+): 0. Max coverage (-): 9.49

Region: chr23 8250675-8250696. Max. coverage (+): 0. Max coverage (-): 0

Region: chr23 8250697-8250718. Max. coverage (+): 0. Max coverage (-): 0

Region: chr23 8250719-8250740. Max. coverage (+): 0. Max coverage (-): 0

Region: chr23 8250741-8250762. Max. coverage (+): 0. Max coverage (-): 0

Region: chr23 8250763-8250784. Max. coverage (+): 0. Max coverage (-): 2.83

Region: chr23 8250785-8250805. Max. coverage (+): 0. Max coverage (-): 3.75

Region: chr23 8250806-8250827. Max. coverage (+): 0. Max coverage (-): 15.37

Region: chr23 8250828-8250849. Max. coverage (+): 0. Max coverage (-): 3.25

Region: chr23 8250850-8250871. Max. coverage (+): 0. Max coverage (-): 0

Region: chr23 8250872-8250893. Max. coverage (+): 0. Max coverage (-): 0

Region: chr23 8250894-8250915. Max. coverage (+): 0. Max coverage (-): 0

Region: chr23 8250916-8250937. Max. coverage (+): 0. Max coverage (-): 1.67

Region: chr23 8250938-8250959. Max. coverage (+): 0. Max coverage (-): 0

Region: chr23 8250960-8250981. Max. coverage (+): 0. Max coverage (-): 0

Region: chr23 8250982-8251003. Max. coverage (+): 0. Max coverage (-): 8.81

Region: chr23 8251004-8251025. Max. coverage (+): 0. Max coverage (-): 0

Region: chr23 8251026-8251046. Max. coverage (+): 0. Max coverage (-): 0

Region: chr23 8251047-8251068. Max. coverage (+): 0. Max coverage (-): 0

Region: chr23 8251069-8251090. Max. coverage (+): 0. Max coverage (-): 0

Region: chr23 8251091-8251112. Max. coverage (+): 0. Max coverage (-): 0

Region: chr23 8251113-8251134. Max. coverage (+): 0. Max coverage (-): 0

Region: chr23 8251135-8251156. Max. coverage (+): 0. Max coverage (-): 0

Region: chr23 8251157-8251178. Max. coverage (+): 0. Max coverage (-): 0

Region: chr23 8251179-8251200. Max. coverage (+): 0. Max coverage (-): 0

Region: chr23 8251201-8251222. Max. coverage (+): 0. Max coverage (-): 2.49

Region: chr23 8251223-8251244. Max. coverage (+): 0. Max coverage (-): 0

Region: chr23 8251245-8251266. Max. coverage (+): 0. Max coverage (-): 0

Region: chr23 8251267-8251288. Max. coverage (+): 0. Max coverage (-): 0

Region: chr23 8251289-8251309. Max. coverage (+): 0. Max coverage (-): 0

Region: chr23 8251310-8251331. Max. coverage (+): 0. Max coverage (-): 0

Region: chr23 8251332-8251353. Max. coverage (+): 0. Max coverage (-): 0

Region: chr23 8251354-8251375. Max. coverage (+): 0. Max coverage (-): 0

Region: chr23 8251376-8251397. Max. coverage (+): 0. Max coverage (-): 0

Region: chr23 8251398-8251419. Max. coverage (+): 0. Max coverage (-): 0

Region: chr23 8251420-8251441. Max. coverage (+): 0. Max coverage (-): 0

Region: chr23 8251442-8251463. Max. coverage (+): 0. Max coverage (-): 1.44

Region: chr23 8251464-8251485. Max. coverage (+): 0. Max coverage (-): 0

Region: chr23 8251486-8251507. Max. coverage (+): 0. Max coverage (-): 0

Region: chr23 8251508-8251529. Max. coverage (+): 0. Max coverage (-): 0

Region: chr23 8251530-8251550. Max. coverage (+): 0. Max coverage (-): 0

Region: chr23 8251551-8251572. Max. coverage (+): 0. Max coverage (-): 0

Region: chr23 8251573-8251594. Max. coverage (+): 0. Max coverage (-): 0

Region: chr23 8251595-8251616. Max. coverage (+): 0. Max coverage (-): 0

Region: chr23 8251617-8251638. Max. coverage (+): 0. Max coverage (-): 0

Region: chr23 8251639-8251660. Max. coverage (+): 0. Max coverage (-): 0

Region: chr23 8251661-8251682. Max. coverage (+): 0. Max coverage (-): 0

Region: chr23 8251683-8251704. Max. coverage (+): 0. Max coverage (-): 2.3

Region: chr23 8251705-8251726. Max. coverage (+): 0. Max coverage (-): 2.3

Region: chr23 8251727-8251748. Max. coverage (+): 0. Max coverage (-): 0

Region: chr23 8251749-8251770. Max. coverage (+): 0. Max coverage (-): 1.08

Region: chr23 8251771-8251792. Max. coverage (+): 0. Max coverage (-): 2.92

Region: chr23 8251793-8251813. Max. coverage (+): 0. Max coverage (-): 0

Region: chr23 8251814-8251835. Max. coverage (+): 0. Max coverage (-): 0

Region: chr23 8251836-8251857. Max. coverage (+): 0. Max coverage (-): 3.59

Region: chr23 8251858-8251879. Max. coverage (+): 0. Max coverage (-): 0

Region: chr23 8251880-8251901. Max. coverage (+): 0. Max coverage (-): 4.69

Region: chr23 8251902-8251923. Max. coverage (+): 0. Max coverage (-): 2.66

Region: chr23 8251924-8251945. Max. coverage (+): 0. Max coverage (-): 0

Region: chr23 8251946-8251967. Max. coverage (+): 0. Max coverage (-): 0

Region: chr23 8251968-8251989. Max. coverage (+): 0. Max coverage (-): 0

Region: chr23 8251990-8252011. Max. coverage (+): 0. Max coverage (-): 0

Region: chr23 8252012-8252033. Max. coverage (+): 0. Max coverage (-): 0

Region: chr23 8252034-8252054. Max. coverage (+): 0. Max coverage (-): 0

Region: chr23 8252055-8252076. Max. coverage (+): 0. Max coverage (-): 0

Region: chr23 8252077-8252098. Max. coverage (+): 0. Max coverage (-): 0

Region: chr23 8252099-8252120. Max. coverage (+): 0. Max coverage (-): 0

Region: chr23 8252121-8252142. Max. coverage (+): 0. Max coverage (-): 0

Region: chr23 8252143-8252164. Max. coverage (+): 0. Max coverage (-): 0

Region: chr23 8252165-8252186. Max. coverage (+): 0. Max coverage (-): 0

Region: chr23 8252187-8252208. Max. coverage (+): 0. Max coverage (-): 0

Region: chr23 8252209-8252230. Max. coverage (+): 0. Max coverage (-): 0

Region: chr23 8252231-8252252. Max. coverage (+): 0. Max coverage (-): 0

Region: chr23 8252253-8252274. Max. coverage (+): 0. Max coverage (-): 0

Region: chr23 8252275-8252295. Max. coverage (+): 0. Max coverage (-): 0

Region: chr23 8252296-8252317. Max. coverage (+): 0. Max coverage (-): 3.97

Region: chr23 8252318-8252339. Max. coverage (+): 0. Max coverage (-): 3.43

Region: chr23 8252340-8252361. Max. coverage (+): 0. Max coverage (-): 5.17

Region: chr23 8252362-8252383. Max. coverage (+): 0. Max coverage (-): 0.98

Region: chr23 8252384-8252405. Max. coverage (+): 0. Max coverage (-): 9.7

Region: chr23 8252406-8252427. Max. coverage (+): 0. Max coverage (-): 10.09

Region: chr23 8252428-8252449. Max. coverage (+): 0. Max coverage (-): 2.19

Region: chr23 8252450-8252471. Max. coverage (+): 0. Max coverage (-): 0

Region: chr23 8252472-8252493. Max. coverage (+): 0. Max coverage (-): 0

Region: chr23 8252494-8252515. Max. coverage (+): 0. Max coverage (-): 0

Region: chr23 8252516-8252537. Max. coverage (+): 0. Max coverage (-): 1.22

Region: chr23 8252538-8252558. Max. coverage (+): 0. Max coverage (-): 1.22

Region: chr23 8252559-8252580. Max. coverage (+): 0. Max coverage (-): 2

Region: chr23 8252581-8252602. Max. coverage (+): 0. Max coverage (-): 9.55

Region: chr23 8252603-8252624. Max. coverage (+): 0. Max coverage (-): 6.58

Region: chr23 8252625-8252646. Max. coverage (+): 0. Max coverage (-): 0

Region: chr23 8252647-8252668. Max. coverage (+): 0. Max coverage (-): 0

Region: chr23 8252669-8252690. Max. coverage (+): 0. Max coverage (-): 0

Region: chr23 8252691-8252712. Max. coverage (+): 0. Max coverage (-): 0

Region: chr23 8252713-8252734. Max. coverage (+): 0. Max coverage (-): 0

Region: chr23 8252735-8252756. Max. coverage (+): 0. Max coverage (-): 0

Region: chr23 8252757-8252778. Max. coverage (+): 0. Max coverage (-): 0

Region: chr23 8252779-8252799. Max. coverage (+): 0. Max coverage (-): 0

Region: chr23 8252800-8252821. Max. coverage (+): 0. Max coverage (-): 0

Region: chr23 8252822-8252843. Max. coverage (+): 0. Max coverage (-): 0

Region: chr23 8252844-8252865. Max. coverage (+): 0. Max coverage (-): 0

Region: chr23 8252866-8252887. Max. coverage (+): 0. Max coverage (-): 0

Region: chr23 8252888-8252909. Max. coverage (+): 0. Max coverage (-): 0

Region: chr23 8252910-8252931. Max. coverage (+): 0. Max coverage (-): 0

Region: chr23 8252932-8252953. Max. coverage (+): 0. Max coverage (-): 0

Region: chr23 8252954-8252975. Max. coverage (+): 0. Max coverage (-): 0

Region: chr23 8252976-8252997. Max. coverage (+): 0. Max coverage (-): 0

Region: chr23 8252998-8253019. Max. coverage (+): 0. Max coverage (-): 0

Region: chr23 8253020-8253040. Max. coverage (+): 0. Max coverage (-): 0

Region: chr23 8253041-8253062. Max. coverage (+): 0. Max coverage (-): 0

Region: chr23 8253063-8253084. Max. coverage (+): 0. Max coverage (-): 0

Region: chr23 8253085-8253106. Max. coverage (+): 0. Max coverage (-): 0

Region: chr23 8253107-8253128. Max. coverage (+): 0. Max coverage (-): 0

Region: chr23 8253129-8253150. Max. coverage (+): 0. Max coverage (-): 0

Region: chr23 8253151-8253172. Max. coverage (+): 0. Max coverage (-): 0

Region: chr23 8253173-8253194. Max. coverage (+): 0. Max coverage (-): 0

Region: chr23 8253195-8253216. Max. coverage (+): 0. Max coverage (-): 0

Region: chr23 8253217-8253238. Max. coverage (+): 0. Max coverage (-): 0

Region: chr23 8253239-8253260. Max. coverage (+): 0. Max coverage (-): 0

Region: chr23 8253261-8253282. Max. coverage (+): 0. Max coverage (-): 0

Region: chr23 8253283-8253303. Max. coverage (+): 0. Max coverage (-): 0

Region: chr23 8253304-8253325. Max. coverage (+): 0. Max coverage (-): 0

Region: chr23 8253326-8253347. Max. coverage (+): 0. Max coverage (-): 0

Region: chr23 8253348-8253369. Max. coverage (+): 0. Max coverage (-): 0

Region: chr23 8253370-8253391. Max. coverage (+): 0. Max coverage (-): 0

Region: chr23 8253392-8253413. Max. coverage (+): 0. Max coverage (-): 0

Region: chr23 8253414-8253435. Max. coverage (+): 0. Max coverage (-): 0

Region: chr23 8253436-8253457. Max. coverage (+): 0. Max coverage (-): 0

Region: chr23 8253458-8253479. Max. coverage (+): 0. Max coverage (-): 0

Region: chr23 8253480-8253501. Max. coverage (+): 0. Max coverage (-): 0

Region: chr23 8253502-8253523. Max. coverage (+): 0. Max coverage (-): 0

Region: chr23 8253524-8253544. Max. coverage (+): 0. Max coverage (-): 0

Region: chr23 8253545-8253566. Max. coverage (+): 0. Max coverage (-): 0

Region: chr23 8253567-8253588. Max. coverage (+): 0. Max coverage (-): 0

Region: chr23 8253589-8253610. Max. coverage (+): 0. Max coverage (-): 0

Region: chr23 8253611-8253632. Max. coverage (+): 0. Max coverage (-): 0

Region: chr23 8253633-8253654. Max. coverage (+): 0. Max coverage (-): 0

Region: chr23 8253655-8253676. Max. coverage (+): 0. Max coverage (-): 0

Region: chr23 8253677-8253698. Max. coverage (+): 0. Max coverage (-): 3.81

Region: chr23 8253699-8253720. Max. coverage (+): 0. Max coverage (-): 0

Region: chr23 8253721-8253742. Max. coverage (+): 0. Max coverage (-): 0

Region: chr23 8253743-8253764. Max. coverage (+): 0. Max coverage (-): 0

Region: chr23 8253765-8253785. Max. coverage (+): 0. Max coverage (-): 0

Region: chr23 8253786-8253807. Max. coverage (+): 0. Max coverage (-): 0

Region: chr23 8253808-8253829. Max. coverage (+): 0. Max coverage (-): 0

Region: chr23 8253830-8253851. Max. coverage (+): 0. Max coverage (-): 0

Region: chr23 8253852-8253873. Max. coverage (+): 0. Max coverage (-): 0

Region: chr23 8253874-8253895. Max. coverage (+): 0. Max coverage (-): 0

Region: chr23 8253896-8253917. Max. coverage (+): 0. Max coverage (-): 2.05

Region: chr23 8253918-8253939. Max. coverage (+): 0. Max coverage (-): 0

Region: chr23 8253940-8253961. Max. coverage (+): 0. Max coverage (-): 1.18

Region: chr23 8253962-8253983. Max. coverage (+): 0. Max coverage (-): 0

Region: chr23 8253984-8254005. Max. coverage (+): 0. Max coverage (-): 0

Region: chr23 8254006-8254027. Max. coverage (+): 0. Max coverage (-): 0

Region: chr23 8254028-8254048. Max. coverage (+): 0. Max coverage (-): 0

Region: chr23 8254049-8254070. Max. coverage (+): 0. Max coverage (-): 0

Region: chr23 8254071-8254092. Max. coverage (+): 0. Max coverage (-): 0

Region: chr23 8254093-8254114. Max. coverage (+): 0. Max coverage (-): 0

Region: chr23 8254115-8254136. Max. coverage (+): 0. Max coverage (-): 0

Region: chr23 8254137-8254158. Max. coverage (+): 0. Max coverage (-): 0

Region: chr23 8254159-8254180. Max. coverage (+): 0. Max coverage (-): 10.19

Region: chr23 8254181-8254202. Max. coverage (+): 0. Max coverage (-): 10.19

Region: chr23 8254203-8254224. Max. coverage (+): 0. Max coverage (-): 0

Region: chr23 8254225-8254246. Max. coverage (+): 0. Max coverage (-): 0

Region: chr23 8254247-8254268. Max. coverage (+): 0. Max coverage (-): 0

Region: chr23 8254269-8254289. Max. coverage (+): 0. Max coverage (-): 5.95

Region: chr23 8254290-8254311. Max. coverage (+): 0. Max coverage (-): 0

Region: chr23 8254312-8254333. Max. coverage (+): 0. Max coverage (-): 0

Region: chr23 8254334-8254355. Max. coverage (+): 0. Max coverage (-): 0

Region: chr23 8254356-8254377. Max. coverage (+): 0. Max coverage (-): 0

Region: chr23 8254378-8254399. Max. coverage (+): 0. Max coverage (-): 15.98

Region: chr23 8254400-8254421. Max. coverage (+): 0. Max coverage (-): 2.5

Region: chr23 8254422-8254443. Max. coverage (+): 0. Max coverage (-): 0

Region: chr23 8254444-8254465. Max. coverage (+): 0. Max coverage (-): 0

Region: chr23 8254466-8254487. Max. coverage (+): 0. Max coverage (-): 12.37

Region: chr23 8254488-8254509. Max. coverage (+): 0. Max coverage (-): 5.58

Region: chr23 8254510-8254531. Max. coverage (+): 0. Max coverage (-): 4.11

Region: chr23 8254532-8254552. Max. coverage (+): 0. Max coverage (-): 11.54

Region: chr23 8254553-8254574. Max. coverage (+): 0. Max coverage (-): 0

Region: chr23 8254575-8254596. Max. coverage (+): 0. Max coverage (-): 0

Region: chr23 8254597-8254618. Max. coverage (+): 0. Max coverage (-): 0

Region: chr23 8254619-8254640. Max. coverage (+): 0. Max coverage (-): 0

Region: chr23 8254641-8254662. Max. coverage (+): 0. Max coverage (-): 0

Region: chr23 8254663-8254684. Max. coverage (+): 0. Max coverage (-): 0

Region: chr23 8254685-8254706. Max. coverage (+): 0. Max coverage (-): 0

Region: chr23 8254707-8254728. Max. coverage (+): 0. Max coverage (-): 0

Region: chr23 8254729-8254750. Max. coverage (+): 0. Max coverage (-): 0

Region: chr23 8254751-8254772. Max. coverage (+): 0. Max coverage (-): 0

Region: chr23 8254773-8254793. Max. coverage (+): 0. Max coverage (-): 0

Region: chr23 8254794-8254815. Max. coverage (+): 0. Max coverage (-): 0

Region: chr23 8254816-8254837. Max. coverage (+): 0. Max coverage (-): 0

Region: chr23 8254838-8254859. Max. coverage (+): 0. Max coverage (-): 0

Region: chr23 8254860-8254881. Max. coverage (+): 0. Max coverage (-): 0

Region: chr23 8254882-8254903. Max. coverage (+): 0. Max coverage (-): 0

Region: chr23 8254904-8254925. Max. coverage (+): 0. Max coverage (-): 0

Region: chr23 8254926-8254947. Max. coverage (+): 0. Max coverage (-): 0

Region: chr23 8254948-8254969. Max. coverage (+): 0. Max coverage (-): 0

Region: chr23 8254970-8254991. Max. coverage (+): 0. Max coverage (-): 0

Region: chr23 8254992-8255013. Max. coverage (+): 0. Max coverage (-): 0

Region: chr23 8255014-8255034. Max. coverage (+): 0. Max coverage (-): 0

Region: chr23 8255035-8255056. Max. coverage (+): 0. Max coverage (-): 0

Region: chr23 8255057-8255078. Max. coverage (+): 0. Max coverage (-): 0

Region: chr23 8255079-8255100. Max. coverage (+): 0. Max coverage (-): 0

Region: chr23 8255101-8255122. Max. coverage (+): 0. Max coverage (-): 1.1

Region: chr23 8255123-8255144. Max. coverage (+): 0. Max coverage (-): 0

Region: chr23 8255145-8255166. Max. coverage (+): 0. Max coverage (-): 0

Region: chr23 8255167-8255188. Max. coverage (+): 0. Max coverage (-): 1.21

Region: chr23 8255189-8255210. Max. coverage (+): 0. Max coverage (-): 1.79

Region: chr23 8255211-8255232. Max. coverage (+): 0. Max coverage (-): 0

Region: chr23 8255233-8255254. Max. coverage (+): 0. Max coverage (-): 0

Region: chr23 8255255-8255276. Max. coverage (+): 0. Max coverage (-): 0

Region: chr23 8255277-8255297. Max. coverage (+): 0. Max coverage (-): 6.6

Region: chr23 8255298-8255319. Max. coverage (+): 0. Max coverage (-): 1.43

Region: chr23 8255320-8255341. Max. coverage (+): 0. Max coverage (-): 0

Region: chr23 8255342-8255363. Max. coverage (+): 0. Max coverage (-): 0

Region: chr23 8255364-8255385. Max. coverage (+): 0. Max coverage (-): 0

Region: chr23 8255386-8255407. Max. coverage (+): 0. Max coverage (-): 0

Region: chr23 8255408-8255429. Max. coverage (+): 0. Max coverage (-): 0

Region: chr23 8255430-8255451. Max. coverage (+): 0. Max coverage (-): 1.43

Region: chr23 8255452-8255473. Max. coverage (+): 0. Max coverage (-): 1.43

Region: chr23 8255474-8255495. Max. coverage (+): 0. Max coverage (-): 0

Region: chr23 8255496-8255517. Max. coverage (+): 0. Max coverage (-): 0

Region: chr23 8255518-8255538. Max. coverage (+): 0. Max coverage (-): 0

Region: chr23 8255539-8255560. Max. coverage (+): 0. Max coverage (-): 11.89

Region: chr23 8255561-8255582. Max. coverage (+): 0. Max coverage (-): 0

Region: chr23 8255583-8255604. Max. coverage (+): 0. Max coverage (-): 0

Region: chr23 8255605-8255626. Max. coverage (+): 0. Max coverage (-): 0

Region: chr23 8255627-8255648. Max. coverage (+): 0. Max coverage (-): 0

Region: chr23 8255649-8255670. Max. coverage (+): 0. Max coverage (-): 2.43

Region: chr23 8255671-8255692. Max. coverage (+): 0. Max coverage (-): 2.43

Region: chr23 8255693-8255714. Max. coverage (+): 0. Max coverage (-): 1.61

Region: chr23 8255715-8255736. Max. coverage (+): 0. Max coverage (-): 0

Region: chr23 8255737-8255758. Max. coverage (+): 0. Max coverage (-): 1.56

Region: chr23 8255759-8255779. Max. coverage (+): 0. Max coverage (-): 0

Region: chr23 8255780-8255801. Max. coverage (+): 0. Max coverage (-): 4.36

Region: chr23 8255802-8255823. Max. coverage (+): 0. Max coverage (-): 0

Region: chr23 8255824-8255845. Max. coverage (+): 0. Max coverage (-): 0

Region: chr23 8255846-8255867. Max. coverage (+): 0. Max coverage (-): 0

Region: chr23 8255868-8255889. Max. coverage (+): 0. Max coverage (-): 0

Region: chr23 8255890-8255911. Max. coverage (+): 0. Max coverage (-): 0

Region: chr23 8255912-8255933. Max. coverage (+): 0. Max coverage (-): 0

Region: chr23 8255934-8255955. Max. coverage (+): 0. Max coverage (-): 0

Region: chr23 8255956-8255977. Max. coverage (+): 0. Max coverage (-): 0

Region: chr23 8255978-8255999. Max. coverage (+): 0. Max coverage (-): 0

Region: chr23 8256000-8256021. Max. coverage (+): 0. Max coverage (-): 0

Region: chr23 8256022-8256042. Max. coverage (+): 0. Max coverage (-): 0

Region: chr23 8256043-8256064. Max. coverage (+): 0. Max coverage (-): 0

Region: chr23 8256065-8256086. Max. coverage (+): 0. Max coverage (-): 0

Region: chr23 8256087-8256108. Max. coverage (+): 0. Max coverage (-): 0

Region: chr23 8256109-8256130. Max. coverage (+): 0. Max coverage (-): 0

Region: chr23 8256131-8256152. Max. coverage (+): 0. Max coverage (-): 2.16

Region: chr23 8256153-8256174. Max. coverage (+): 0. Max coverage (-): 0

Region: chr23 8256175-8256196. Max. coverage (+): 0. Max coverage (-): 2.43

Region: chr23 8256197-8256218. Max. coverage (+): 0. Max coverage (-): 2.43

Region: chr23 8256219-8256240. Max. coverage (+): 0. Max coverage (-): 0

Region: chr23 8256241-8256262. Max. coverage (+): 0. Max coverage (-): 0

Region: chr23 8256263-8256283. Max. coverage (+): 0. Max coverage (-): 0

Region: chr23 8256284-8256305. Max. coverage (+): 0. Max coverage (-): 0

Region: chr23 8256306-8256327. Max. coverage (+): 0. Max coverage (-): 4.46

Region: chr23 8256328-8256349. Max. coverage (+): 0. Max coverage (-): 0

Region: chr23 8256350-8256371. Max. coverage (+): 0. Max coverage (-): 1.62

Region: chr23 8256372-8256393. Max. coverage (+): 0. Max coverage (-): 0

Region: chr23 8256394-8256415. Max. coverage (+): 0. Max coverage (-): 0

Region: chr23 8256416-8256437. Max. coverage (+): 0. Max coverage (-): 0

Region: chr23 8256438-8256459. Max. coverage (+): 0. Max coverage (-): 0

Region: chr23 8256460-8256481. Max. coverage (+): 0. Max coverage (-): 0

Region: chr23 8256482-8256503. Max. coverage (+): 0. Max coverage (-): 0

Region: chr23 8256504-8256524. Max. coverage (+): 0. Max coverage (-): 0

Region: chr23 8256525-8256546. Max. coverage (+): 0. Max coverage (-): 0

Region: chr23 8256547-8256568. Max. coverage (+): 0. Max coverage (-): 0

Region: chr23 8256569-8256590. Max. coverage (+): 0. Max coverage (-): 0

Region: chr23 8256591-8256612. Max. coverage (+): 0. Max coverage (-): 0

Region: chr23 8256613-8256634. Max. coverage (+): 0. Max coverage (-): 0

Region: chr23 8256635-8256656. Max. coverage (+): 0. Max coverage (-): 0

Region: chr23 8256657-8256678. Max. coverage (+): 0. Max coverage (-): 0

Region: chr23 8256679-8256700. Max. coverage (+): 0. Max coverage (-): 0

Region: chr23 8256701-8256722. Max. coverage (+): 0. Max coverage (-): 0

Region: chr23 8256723-8256744. Max. coverage (+): 0. Max coverage (-): 0

Region: chr23 8256745-8256766. Max. coverage (+): 0. Max coverage (-): 0

Region: chr23 8256767-8256787. Max. coverage (+): 0. Max coverage (-): 0

Region: chr23 8256788-8256809. Max. coverage (+): 0. Max coverage (-): 0

Region: chr23 8256810-8256831. Max. coverage (+): 0. Max coverage (-): 0

Region: chr23 8256832-8256853. Max. coverage (+): 0. Max coverage (-): 0

Region: chr23 8256854-8256875. Max. coverage (+): 0. Max coverage (-): 0

Region: chr23 8256876-8256897. Max. coverage (+): 0. Max coverage (-): 0

Region: chr23 8256898-8256919. Max. coverage (+): 0. Max coverage (-): 0

Region: chr23 8256920-8256941. Max. coverage (+): 0. Max coverage (-): 0

Region: chr23 8256942-8256963. Max. coverage (+): 0. Max coverage (-): 0

Region: chr23 8256964-8256985. Max. coverage (+): 0. Max coverage (-): 0

Region: chr23 8256986-8257007. Max. coverage (+): 0. Max coverage (-): 0

Region: chr23 8257008-8257028. Max. coverage (+): 0. Max coverage (-): 0

Region: chr23 8257029-8257050. Max. coverage (+): 0. Max coverage (-): 0

Region: chr23 8257051-8257072. Max. coverage (+): 0. Max coverage (-): 0

Region: chr23 8257073-8257094. Max. coverage (+): 0. Max coverage (-): 0

Region: chr23 8257095-8257116. Max. coverage (+): 0. Max coverage (-): 0

Region: chr23 8257117-8257138. Max. coverage (+): 0. Max coverage (-): 0

Region: chr23 8257139-8257160. Max. coverage (+): 0. Max coverage (-): 0

Region: chr23 8257161-8257182. Max. coverage (+): 0. Max coverage (-): 0

Region: chr23 8257183-8257204. Max. coverage (+): 0. Max coverage (-): 0

Region: chr23 8257205-8257226. Max. coverage (+): 0. Max coverage (-): 0

Region: chr23 8257227-8257248. Max. coverage (+): 0. Max coverage (-): 0

Region: chr23 8257249-8257270. Max. coverage (+): 0. Max coverage (-): 0

Region: chr23 8257271-8257291. Max. coverage (+): 0. Max coverage (-): 0

Region: chr23 8257292-8257313. Max. coverage (+): 0. Max coverage (-): 0

Region: chr23 8257314-8257335. Max. coverage (+): 0. Max coverage (-): 0

Region: chr23 8257336-8257357. Max. coverage (+): 0. Max coverage (-): 0

Region: chr23 8257358-8257379. Max. coverage (+): 0. Max coverage (-): 0

Region: chr23 8257380-8257401. Max. coverage (+): 0. Max coverage (-): 0

Region: chr23 8257402-8257423. Max. coverage (+): 0. Max coverage (-): 0

Region: chr23 8257424-8257445. Max. coverage (+): 0. Max coverage (-): 0

Region: chr23 8257446-8257467. Max. coverage (+): 0. Max coverage (-): 0

Region: chr23 8257468-8257489. Max. coverage (+): 0. Max coverage (-): 0

Region: chr23 8257490-8257511. Max. coverage (+): 0. Max coverage (-): 0

Region: chr23 8257512-8257532. Max. coverage (+): 0. Max coverage (-): 0

Region: chr23 8257533-8257554. Max. coverage (+): 0. Max coverage (-): 0

Region: chr23 8257555-8257576. Max. coverage (+): 0. Max coverage (-): 0

Region: chr23 8257577-8257598. Max. coverage (+): 0. Max coverage (-): 0

Region: chr23 8257599-8257620. Max. coverage (+): 0. Max coverage (-): 0

Region: chr23 8257621-8257642. Max. coverage (+): 0. Max coverage (-): 0

Region: chr23 8257643-8257664. Max. coverage (+): 0. Max coverage (-): 0

Region: chr23 8257665-8257686. Max. coverage (+): 0. Max coverage (-): 0

Region: chr23 8257687-8257708. Max. coverage (+): 0. Max coverage (-): 0

Region: chr23 8257709-8257730. Max. coverage (+): 0. Max coverage (-): 0

Region: chr23 8257731-8257752. Max. coverage (+): 0. Max coverage (-): 0

Region: chr23 8257753-8257773. Max. coverage (+): 0. Max coverage (-): 0

Region: chr23 8257774-8257795. Max. coverage (+): 0. Max coverage (-): 0

Region: chr23 8257796-8257817. Max. coverage (+): 0. Max coverage (-): 0

Region: chr23 8257818-8257839. Max. coverage (+): 0. Max coverage (-): 0

Region: chr23 8257840-8257861. Max. coverage (+): 0. Max coverage (-): 1.71

Region: chr23 8257862-8257883. Max. coverage (+): 0. Max coverage (-): 1.71

Region: chr23 8257884-8257905. Max. coverage (+): 0. Max coverage (-): 6.91

Region: chr23 8257906-8257927. Max. coverage (+): 0. Max coverage (-): 0

Region: chr23 8257928-8257949. Max. coverage (+): 0. Max coverage (-): 1.74

Region: chr23 8257950-8257971. Max. coverage (+): 0. Max coverage (-): 8.94

Region: chr23 8257972-8257993. Max. coverage (+): 0. Max coverage (-): 4.05

Region: chr23 8257994-8258015. Max. coverage (+): 0. Max coverage (-): 0

Region: chr23 8258016-8258036. Max. coverage (+): 0. Max coverage (-): 0

Region: chr23 8258037-8258058. Max. coverage (+): 0. Max coverage (-): 0

Region: chr23 8258059-8258080. Max. coverage (+): 0. Max coverage (-): 0

Region: chr23 8258081-8258102. Max. coverage (+): 0. Max coverage (-): 8.22

Region: chr23 8258103-8258124. Max. coverage (+): 0. Max coverage (-): 8.71

Region: chr23 8258125-8258146. Max. coverage (+): 1.61. Max coverage (-): 8.71

Region: chr23 8258147-8258168. Max. coverage (+): 0.79. Max coverage (-): 6.86

Region: chr23 8258169-8258190. Max. coverage (+): 0. Max coverage (-): 0

Region: chr23 8258191-8258212. Max. coverage (+): 0. Max coverage (-): 0

Region: chr23 8258213-8258234. Max. coverage (+): 0. Max coverage (-): 3.09

Region: chr23 8258235-8258256. Max. coverage (+): 0.68. Max coverage (-): 3.09

Region: chr23 8258257-. Max. coverage (+): 0. Max coverage (-): 0

RepeatMasker Color Code

**+**

100-98% Identity

<98-95% Identity

<95-90% Identity

<90-85% Identity

<85-80% Identity

<80-75% Identity

<75-70% Identity

<70% Identity

**-**

Gene Set Color Code

**+**

Gene

Pseudogene

**-**

Topology/Coverage Color Code

Coverage Plus Strand

Coverage Minus Strand

Mainstrand: Plus

Mainstrand: Minus

Complementary Strand

Flanking Region  
(if option -flank >0)

Gene Set Annotation  
  
RepeatMasker Annotation  

**1. Bov-tA2**: 8247358-8247553 (+), Divergence to consensus: 14.8%  
**2. LTR33**: 8247601-8247771 (-), Divergence to consensus: 40.9%  
**3. MIR3**: 8248028-8248158 (-), Divergence to consensus: 36.7%  
**4. GC\_rich**: 8248487-8248611 (+), Divergence to consensus: 91.2%  
**5. L2a**: 8249139-8249312 (-), Divergence to consensus: 44.9%  
**6. MER33**: 8249313-8249592 (-), Divergence to consensus: 47.3%  
**7. L2a**: 8249593-8249645 (-), Divergence to consensus: 44.9%  
**8. MIRb**: 8249635-8249782 (+), Divergence to consensus: 40.4%  
**9. L2a**: 8252660-8253247 (-), Divergence to consensus: 44.5%  
**10. LTR67B**: 8253254-8253652 (-), Divergence to consensus: 48.5%  
**11. L2a**: 8253715-8253912 (+), Divergence to consensus: 41.6%  
**12. MIR**: 8253983-8254085 (+), Divergence to consensus: 41.8%  
**13. L1ME3A**: 8254601-8254922 (+), Divergence to consensus: 31.8%  
**14. L1ME3A**: 8254979-8255110 (+), Divergence to consensus: 25%  
**15. Bov-tA2**: 8255376-8255420 (-), Divergence to consensus: 15.6%  
**16. L2a**: 8255837-8256119 (+), Divergence to consensus: 53.7%  
**17. L4\_A\_Mam**: 8256396-8256525 (-), Divergence to consensus: 29.6%  
**18. C-rich**: 8257332-8257479 (+), Divergence to consensus: 33.4%  
**19. AT\_rich**: 8257965-8257986 (+), Divergence to consensus: 72.7%

  
Transcription Factor Binding Sites  

**Gata4** (Sequence: AGATAAG (-): 8249607)  
**SOX9** (Sequence: AACAATAG (-): 8255133)
