# Supplementary material for: Small RNA sequencing of cryopreserved semen from single bull revealed altered miRNAs and piRNAs expression between High- and Low-motile sperm populations
Source: BMC Genomics. 2017 Jan 4;18:14. doi: 10.1186/s12864-016-3394-7 (PMC5209821; doi:10.1186/s12864-016-3394-7)
Supplement: Additional file 3: — Details for each piRNA clusters found in High Motile (HM) sperm fraction. Genes, repeats, transposable elements and transcription factors binding sites falling within the cluster regions were reported. (ZIP 1896 kb) [file 12864_2016_3394_MOESM3_ESM.zip › 54.html]

piRNA cluster 54


Predicted piRNA cluster no. 54     previous   next
  

Show proTRAC run info
Hide proTRAC run info

================================= proTRAC ====================================  
VERSION: 2.1                                    LAST MODIFIED: 06. October 2015  
  
Please cite:  
Rosenkranz D, Zischler H. proTRAC - a software for probabilistic piRNA cluster  
detection, visualization and analysis. 2012. BMC Bioinformatics 13:5.  
  
and (for proTRAC 2.0 and later):  
Rosenkranz D, Rudloff S, Bastuck K, Ketting RF, Zischler H. Tupaia small RNAs  
provide insights into function and evolution of RNAi-based transposon defense  
in mammals. 2015. RNA 21(5):911-922.  
  
Contact:  
David Rosenkranz  
Institute of Anthropology, small RNA group  
Johannes Gutenberg University Mainz  
email: rosenkranz@uni-mainz.de  
  
You can find the latest proTRAC version at:  
http://sourceforge.net/projects/protrac/files  
http://www.smallRNAgroup-mainz.de/software  
==============================================================================  
  
PARAMETERS:  
Map file: .............../storage/core/barbara/genhome/smallRNA/fertility/Sample\_motile/pirna/Sample\_motile\_26-33\_collapsed.fa.no-dust.map.weighted-10000-1000-b-0  
Genome file: ............/storage/core/barbara/genhome/smallRNA/fertility/Sample\_all/pirna/bt\_311\_chrY.fa  
RepeatMasker annotation: /storage/genomes/bt\_umd31/GCF\_000003055.6\_Bos\_taurus\_UMD\_3.1.1\_repeatMasker\_chr.out  
GeneSet:................./storage/core/barbara/genhome/smallRNA/fertility/Sample\_all/pirna/full.gtf  
  
Significant (p<=0.01) hit density will be calculated based  
on observed hit distribution.  
  
Sliding window size: ........................................ 5000 bp  
Sliding window increament: .................................. 1000 bp  
Normalize each hit by number of genomic hits: ............... 1 [0=no/1=yes]  
Normalize each hit by number of sequence reads: ............. 1 [0=no/1=yes]  
Normalize values (-> per million mapped reads): ............. 1 [0=no/1=yes]  
Min. fraction of hits with 1T(U) or 10A: .................... 0.75  
Alternatively: Min. fraction of hits with 1T(U) and 10A: .... 0.5  
Min. fraction of hits with typical piRNA length: ............ 0.75  
Typical piRNA length: ....................................... 26-33 nt  
Min. size of a piRNA cluster: ............................... 5000 bp.  
Min. number of hits (absolute): ............................. 0  
Min. number of hits (normalized): ........................... 0  
Min. fraction of hits on the mainstrand: .................... 0.75  
Top fraction of mapped sequences (in terms of read counts): . 1%  
Top fraction accounts for max. n% of sequence reads: ........ 90%  
Min. fraction of hits on each arm of a bidirectional cluster: 0.1  
Output image file for each cluster: ......................... 0 [0=no/1=yes]  
Output html file for each cluster: .......................... 1 [0=no/1=yes]  
Output a summary table: ..................................... 1 [0=no/1=yes]  
Output a FASTA file for each cluster (piRNA sequences): ..... 1 [0=no/1=yes]  
Output a FASTA file comprising cluster sequences: ........... 1 [0=no/1=yes]  
Search DNA motifs in clusters: .............................. 1 [0=no/1=yes]  
Output flanking sequences: +/- .............................. 0 bp  
Output ~.pTi file: .......................................... 1 [0=no/1=yes]  
==============================================================================  
  
  
Genome size (without gaps): ............ 2678902517 bp  
Gaps (N/X/-): .......................... 53837044 bp  
Mapped reads: .......................... 658825247023  
Non-identical sequences: ............... 514171  
Genomic hits: .......................... 764233  
Significant densitiy of mapped reads: .. 12867599.5173724 reads/kb

Show proTRAC cluster info
Hide proTRAC cluster info

|  |  |
| --- | --- |
| Location | chr23 |
| Coordinates | 17853193-17859260 |
| Size [bp] | 6068 |
| Sequence hit loci | 65 |
| Mapped reads (normalized) | 83903740 |
| Mapped reads (normalized) per kb | 13827247.9 |
| Normalized reads with 1T (1U) | 80.7% |
| Normalized reads with 10A | 32.4% |
| Normalized reads with length 26-33 nt | 100% |
| Normalized reads on the main strand(s) | 88.7% |
| Predicted directionality | mono:minus |

100%

0%

1T (1U)  
reads

10A reads

26-33 nt  
reads

reads on mainstrand

**Either the amount of reads with 1T (1U) OR 10A has to exceed 75% (set with option: -1Tor10A)  
Alternatively the amount of reads with 1T (1U) AND 10A has to exceed 50% (set with option: -1Tand10A)  
Minimum amount of reads with preferred size is 75% (set with option: -pisize)  
Minimum amount of reads on the main strand(s) is 75% (set with option: -clstrand)**

Show read coverage
Hide read coverage

WHAT DO I SEE HERE?  
This chart shows the location of mapped sequence reads within a predicted piRNA cluster. The color refers to the number of genomic hits produced by the sequence read in question. A dark red bar indicates that this sequence read produces many other hits elsewhere in the genome. Many adjacent red or yellow bars can indicate the presence of a multi-copy element such as transposons or rRNA genes. A dark green bar indicates that this sequence read maps uniquely to this locus.

1 hit

2-5 hits

6-10 hits

11-20 hits

21-50 hits

51-100 hits

> 100 hits

chr23

17853193

17859260

Gene Set

RepeatMasker

Mapped  
Reads

14.03

plus strand

minus strand

14.03

Region: chr23 8258258-17853199. Max. coverage (+): 0. Max coverage (-): 1.77

Region: chr23 17853200-17853211. Max. coverage (+): 2.14. Max coverage (-): 1.77

Region: chr23 17853212-17853223. Max. coverage (+): 0. Max coverage (-): 0

Region: chr23 17853224-17853235. Max. coverage (+): 0. Max coverage (-): 0

Region: chr23 17853236-17853247. Max. coverage (+): 0. Max coverage (-): 0

Region: chr23 17853248-17853259. Max. coverage (+): 0. Max coverage (-): 0

Region: chr23 17853260-17853271. Max. coverage (+): 0. Max coverage (-): 0

Region: chr23 17853272-17853284. Max. coverage (+): 0. Max coverage (-): 0

Region: chr23 17853285-17853296. Max. coverage (+): 0. Max coverage (-): 0

Region: chr23 17853297-17853308. Max. coverage (+): 0. Max coverage (-): 0

Region: chr23 17853309-17853320. Max. coverage (+): 0. Max coverage (-): 0

Region: chr23 17853321-17853332. Max. coverage (+): 0. Max coverage (-): 0

Region: chr23 17853333-17853344. Max. coverage (+): 0. Max coverage (-): 0

Region: chr23 17853345-17853356. Max. coverage (+): 0. Max coverage (-): 0

Region: chr23 17853357-17853368. Max. coverage (+): 0. Max coverage (-): 0

Region: chr23 17853369-17853381. Max. coverage (+): 0. Max coverage (-): 0

Region: chr23 17853382-17853393. Max. coverage (+): 0. Max coverage (-): 0

Region: chr23 17853394-17853405. Max. coverage (+): 0. Max coverage (-): 0

Region: chr23 17853406-17853417. Max. coverage (+): 0. Max coverage (-): 0

Region: chr23 17853418-17853429. Max. coverage (+): 0. Max coverage (-): 0

Region: chr23 17853430-17853441. Max. coverage (+): 0. Max coverage (-): 0

Region: chr23 17853442-17853453. Max. coverage (+): 0. Max coverage (-): 0

Region: chr23 17853454-17853466. Max. coverage (+): 0. Max coverage (-): 0

Region: chr23 17853467-17853478. Max. coverage (+): 0. Max coverage (-): 0

Region: chr23 17853479-17853490. Max. coverage (+): 0. Max coverage (-): 0

Region: chr23 17853491-17853502. Max. coverage (+): 0. Max coverage (-): 0

Region: chr23 17853503-17853514. Max. coverage (+): 0. Max coverage (-): 0

Region: chr23 17853515-17853526. Max. coverage (+): 0. Max coverage (-): 0

Region: chr23 17853527-17853538. Max. coverage (+): 0. Max coverage (-): 0

Region: chr23 17853539-17853551. Max. coverage (+): 0. Max coverage (-): 0

Region: chr23 17853552-17853563. Max. coverage (+): 0. Max coverage (-): 0

Region: chr23 17853564-17853575. Max. coverage (+): 0. Max coverage (-): 0

Region: chr23 17853576-17853587. Max. coverage (+): 0. Max coverage (-): 0

Region: chr23 17853588-17853599. Max. coverage (+): 0. Max coverage (-): 0

Region: chr23 17853600-17853611. Max. coverage (+): 0. Max coverage (-): 0

Region: chr23 17853612-17853623. Max. coverage (+): 0. Max coverage (-): 0

Region: chr23 17853624-17853635. Max. coverage (+): 0. Max coverage (-): 0

Region: chr23 17853636-17853648. Max. coverage (+): 0. Max coverage (-): 0

Region: chr23 17853649-17853660. Max. coverage (+): 0. Max coverage (-): 0

Region: chr23 17853661-17853672. Max. coverage (+): 0. Max coverage (-): 0

Region: chr23 17853673-17853684. Max. coverage (+): 0. Max coverage (-): 0

Region: chr23 17853685-17853696. Max. coverage (+): 0. Max coverage (-): 0

Region: chr23 17853697-17853708. Max. coverage (+): 0. Max coverage (-): 0

Region: chr23 17853709-17853720. Max. coverage (+): 0. Max coverage (-): 0

Region: chr23 17853721-17853733. Max. coverage (+): 0. Max coverage (-): 0

Region: chr23 17853734-17853745. Max. coverage (+): 0. Max coverage (-): 0

Region: chr23 17853746-17853757. Max. coverage (+): 0. Max coverage (-): 0

Region: chr23 17853758-17853769. Max. coverage (+): 0. Max coverage (-): 0

Region: chr23 17853770-17853781. Max. coverage (+): 0. Max coverage (-): 0

Region: chr23 17853782-17853793. Max. coverage (+): 0. Max coverage (-): 0

Region: chr23 17853794-17853805. Max. coverage (+): 0. Max coverage (-): 0

Region: chr23 17853806-17853818. Max. coverage (+): 0. Max coverage (-): 0.86

Region: chr23 17853819-17853830. Max. coverage (+): 0. Max coverage (-): 0.86

Region: chr23 17853831-17853842. Max. coverage (+): 0. Max coverage (-): 0

Region: chr23 17853843-17853854. Max. coverage (+): 0. Max coverage (-): 0

Region: chr23 17853855-17853866. Max. coverage (+): 0. Max coverage (-): 0

Region: chr23 17853867-17853878. Max. coverage (+): 0. Max coverage (-): 1.72

Region: chr23 17853879-17853890. Max. coverage (+): 0. Max coverage (-): 1.72

Region: chr23 17853891-17853902. Max. coverage (+): 0. Max coverage (-): 0

Region: chr23 17853903-17853915. Max. coverage (+): 0. Max coverage (-): 0

Region: chr23 17853916-17853927. Max. coverage (+): 0. Max coverage (-): 0

Region: chr23 17853928-17853939. Max. coverage (+): 0. Max coverage (-): 0

Region: chr23 17853940-17853951. Max. coverage (+): 0. Max coverage (-): 0

Region: chr23 17853952-17853963. Max. coverage (+): 0. Max coverage (-): 0

Region: chr23 17853964-17853975. Max. coverage (+): 0. Max coverage (-): 0

Region: chr23 17853976-17853987. Max. coverage (+): 0. Max coverage (-): 0

Region: chr23 17853988-17854000. Max. coverage (+): 0. Max coverage (-): 0

Region: chr23 17854001-17854012. Max. coverage (+): 0. Max coverage (-): 0

Region: chr23 17854013-17854024. Max. coverage (+): 0. Max coverage (-): 0

Region: chr23 17854025-17854036. Max. coverage (+): 0. Max coverage (-): 0

Region: chr23 17854037-17854048. Max. coverage (+): 0. Max coverage (-): 0

Region: chr23 17854049-17854060. Max. coverage (+): 0. Max coverage (-): 0

Region: chr23 17854061-17854072. Max. coverage (+): 0. Max coverage (-): 0

Region: chr23 17854073-17854084. Max. coverage (+): 0. Max coverage (-): 0.63

Region: chr23 17854085-17854097. Max. coverage (+): 0. Max coverage (-): 4.3

Region: chr23 17854098-17854109. Max. coverage (+): 0. Max coverage (-): 0

Region: chr23 17854110-17854121. Max. coverage (+): 0. Max coverage (-): 0

Region: chr23 17854122-17854133. Max. coverage (+): 0. Max coverage (-): 0

Region: chr23 17854134-17854145. Max. coverage (+): 0. Max coverage (-): 0

Region: chr23 17854146-17854157. Max. coverage (+): 0. Max coverage (-): 0

Region: chr23 17854158-17854169. Max. coverage (+): 0. Max coverage (-): 0

Region: chr23 17854170-17854182. Max. coverage (+): 0. Max coverage (-): 0.98

Region: chr23 17854183-17854194. Max. coverage (+): 0. Max coverage (-): 0

Region: chr23 17854195-17854206. Max. coverage (+): 0. Max coverage (-): 0

Region: chr23 17854207-17854218. Max. coverage (+): 0. Max coverage (-): 0

Region: chr23 17854219-17854230. Max. coverage (+): 0. Max coverage (-): 0

Region: chr23 17854231-17854242. Max. coverage (+): 0. Max coverage (-): 0

Region: chr23 17854243-17854254. Max. coverage (+): 0. Max coverage (-): 0

Region: chr23 17854255-17854267. Max. coverage (+): 0. Max coverage (-): 0

Region: chr23 17854268-17854279. Max. coverage (+): 0. Max coverage (-): 0

Region: chr23 17854280-17854291. Max. coverage (+): 0. Max coverage (-): 0

Region: chr23 17854292-17854303. Max. coverage (+): 0. Max coverage (-): 0

Region: chr23 17854304-17854315. Max. coverage (+): 0. Max coverage (-): 0

Region: chr23 17854316-17854327. Max. coverage (+): 0. Max coverage (-): 0

Region: chr23 17854328-17854339. Max. coverage (+): 0. Max coverage (-): 0

Region: chr23 17854340-17854351. Max. coverage (+): 0. Max coverage (-): 0

Region: chr23 17854352-17854364. Max. coverage (+): 0. Max coverage (-): 0

Region: chr23 17854365-17854376. Max. coverage (+): 0. Max coverage (-): 0

Region: chr23 17854377-17854388. Max. coverage (+): 0. Max coverage (-): 0

Region: chr23 17854389-17854400. Max. coverage (+): 0. Max coverage (-): 0

Region: chr23 17854401-17854412. Max. coverage (+): 0. Max coverage (-): 0

Region: chr23 17854413-17854424. Max. coverage (+): 0. Max coverage (-): 0

Region: chr23 17854425-17854436. Max. coverage (+): 0. Max coverage (-): 8

Region: chr23 17854437-17854449. Max. coverage (+): 0. Max coverage (-): 8

Region: chr23 17854450-17854461. Max. coverage (+): 0. Max coverage (-): 0

Region: chr23 17854462-17854473. Max. coverage (+): 0. Max coverage (-): 0

Region: chr23 17854474-17854485. Max. coverage (+): 0. Max coverage (-): 0

Region: chr23 17854486-17854497. Max. coverage (+): 0. Max coverage (-): 0

Region: chr23 17854498-17854509. Max. coverage (+): 0. Max coverage (-): 0

Region: chr23 17854510-17854521. Max. coverage (+): 0. Max coverage (-): 0

Region: chr23 17854522-17854534. Max. coverage (+): 0. Max coverage (-): 0

Region: chr23 17854535-17854546. Max. coverage (+): 0. Max coverage (-): 0

Region: chr23 17854547-17854558. Max. coverage (+): 0. Max coverage (-): 0

Region: chr23 17854559-17854570. Max. coverage (+): 0. Max coverage (-): 0

Region: chr23 17854571-17854582. Max. coverage (+): 0. Max coverage (-): 1.89

Region: chr23 17854583-17854594. Max. coverage (+): 0. Max coverage (-): 0

Region: chr23 17854595-17854606. Max. coverage (+): 0. Max coverage (-): 0

Region: chr23 17854607-17854618. Max. coverage (+): 0. Max coverage (-): 0

Region: chr23 17854619-17854631. Max. coverage (+): 0. Max coverage (-): 0

Region: chr23 17854632-17854643. Max. coverage (+): 0. Max coverage (-): 0

Region: chr23 17854644-17854655. Max. coverage (+): 0. Max coverage (-): 0

Region: chr23 17854656-17854667. Max. coverage (+): 0. Max coverage (-): 0

Region: chr23 17854668-17854679. Max. coverage (+): 0. Max coverage (-): 0

Region: chr23 17854680-17854691. Max. coverage (+): 0. Max coverage (-): 0

Region: chr23 17854692-17854703. Max. coverage (+): 0. Max coverage (-): 0

Region: chr23 17854704-17854716. Max. coverage (+): 0. Max coverage (-): 0

Region: chr23 17854717-17854728. Max. coverage (+): 0. Max coverage (-): 0

Region: chr23 17854729-17854740. Max. coverage (+): 0. Max coverage (-): 0

Region: chr23 17854741-17854752. Max. coverage (+): 0. Max coverage (-): 0

Region: chr23 17854753-17854764. Max. coverage (+): 0. Max coverage (-): 0

Region: chr23 17854765-17854776. Max. coverage (+): 0. Max coverage (-): 0

Region: chr23 17854777-17854788. Max. coverage (+): 0. Max coverage (-): 0

Region: chr23 17854789-17854801. Max. coverage (+): 0. Max coverage (-): 0

Region: chr23 17854802-17854813. Max. coverage (+): 0. Max coverage (-): 0

Region: chr23 17854814-17854825. Max. coverage (+): 0. Max coverage (-): 0

Region: chr23 17854826-17854837. Max. coverage (+): 0. Max coverage (-): 0

Region: chr23 17854838-17854849. Max. coverage (+): 0. Max coverage (-): 0

Region: chr23 17854850-17854861. Max. coverage (+): 0. Max coverage (-): 0

Region: chr23 17854862-17854873. Max. coverage (+): 0. Max coverage (-): 0

Region: chr23 17854874-17854885. Max. coverage (+): 0. Max coverage (-): 0

Region: chr23 17854886-17854898. Max. coverage (+): 0. Max coverage (-): 0

Region: chr23 17854899-17854910. Max. coverage (+): 0. Max coverage (-): 0

Region: chr23 17854911-17854922. Max. coverage (+): 0. Max coverage (-): 0

Region: chr23 17854923-17854934. Max. coverage (+): 0. Max coverage (-): 3.53

Region: chr23 17854935-17854946. Max. coverage (+): 0. Max coverage (-): 3.53

Region: chr23 17854947-17854958. Max. coverage (+): 0. Max coverage (-): 0

Region: chr23 17854959-17854970. Max. coverage (+): 0. Max coverage (-): 0

Region: chr23 17854971-17854983. Max. coverage (+): 0. Max coverage (-): 0

Region: chr23 17854984-17854995. Max. coverage (+): 0. Max coverage (-): 0

Region: chr23 17854996-17855007. Max. coverage (+): 0. Max coverage (-): 0

Region: chr23 17855008-17855019. Max. coverage (+): 0. Max coverage (-): 0

Region: chr23 17855020-17855031. Max. coverage (+): 0. Max coverage (-): 0

Region: chr23 17855032-17855043. Max. coverage (+): 0. Max coverage (-): 0

Region: chr23 17855044-17855055. Max. coverage (+): 0. Max coverage (-): 0

Region: chr23 17855056-17855068. Max. coverage (+): 0. Max coverage (-): 0

Region: chr23 17855069-17855080. Max. coverage (+): 0. Max coverage (-): 0

Region: chr23 17855081-17855092. Max. coverage (+): 0. Max coverage (-): 0

Region: chr23 17855093-17855104. Max. coverage (+): 0. Max coverage (-): 0

Region: chr23 17855105-17855116. Max. coverage (+): 0. Max coverage (-): 0

Region: chr23 17855117-17855128. Max. coverage (+): 0. Max coverage (-): 0

Region: chr23 17855129-17855140. Max. coverage (+): 0. Max coverage (-): 0

Region: chr23 17855141-17855152. Max. coverage (+): 0. Max coverage (-): 0

Region: chr23 17855153-17855165. Max. coverage (+): 0. Max coverage (-): 0

Region: chr23 17855166-17855177. Max. coverage (+): 0. Max coverage (-): 0

Region: chr23 17855178-17855189. Max. coverage (+): 0. Max coverage (-): 0

Region: chr23 17855190-17855201. Max. coverage (+): 0. Max coverage (-): 0

Region: chr23 17855202-17855213. Max. coverage (+): 0. Max coverage (-): 0

Region: chr23 17855214-17855225. Max. coverage (+): 0. Max coverage (-): 0

Region: chr23 17855226-17855237. Max. coverage (+): 0. Max coverage (-): 0

Region: chr23 17855238-17855250. Max. coverage (+): 0. Max coverage (-): 0

Region: chr23 17855251-17855262. Max. coverage (+): 0. Max coverage (-): 0

Region: chr23 17855263-17855274. Max. coverage (+): 0. Max coverage (-): 0

Region: chr23 17855275-17855286. Max. coverage (+): 0. Max coverage (-): 0

Region: chr23 17855287-17855298. Max. coverage (+): 0. Max coverage (-): 0

Region: chr23 17855299-17855310. Max. coverage (+): 0. Max coverage (-): 0

Region: chr23 17855311-17855322. Max. coverage (+): 0. Max coverage (-): 0

Region: chr23 17855323-17855335. Max. coverage (+): 0. Max coverage (-): 0

Region: chr23 17855336-17855347. Max. coverage (+): 0. Max coverage (-): 0

Region: chr23 17855348-17855359. Max. coverage (+): 0. Max coverage (-): 0

Region: chr23 17855360-17855371. Max. coverage (+): 0. Max coverage (-): 0

Region: chr23 17855372-17855383. Max. coverage (+): 0. Max coverage (-): 0

Region: chr23 17855384-17855395. Max. coverage (+): 0. Max coverage (-): 0

Region: chr23 17855396-17855407. Max. coverage (+): 0. Max coverage (-): 0

Region: chr23 17855408-17855419. Max. coverage (+): 0. Max coverage (-): 0

Region: chr23 17855420-17855432. Max. coverage (+): 0. Max coverage (-): 0

Region: chr23 17855433-17855444. Max. coverage (+): 0. Max coverage (-): 0

Region: chr23 17855445-17855456. Max. coverage (+): 0. Max coverage (-): 0

Region: chr23 17855457-17855468. Max. coverage (+): 0. Max coverage (-): 0

Region: chr23 17855469-17855480. Max. coverage (+): 0. Max coverage (-): 0

Region: chr23 17855481-17855492. Max. coverage (+): 0. Max coverage (-): 2.56

Region: chr23 17855493-17855504. Max. coverage (+): 0. Max coverage (-): 2.56

Region: chr23 17855505-17855517. Max. coverage (+): 0. Max coverage (-): 0

Region: chr23 17855518-17855529. Max. coverage (+): 0. Max coverage (-): 0

Region: chr23 17855530-17855541. Max. coverage (+): 0. Max coverage (-): 0

Region: chr23 17855542-17855553. Max. coverage (+): 0. Max coverage (-): 0

Region: chr23 17855554-17855565. Max. coverage (+): 0. Max coverage (-): 0

Region: chr23 17855566-17855577. Max. coverage (+): 0. Max coverage (-): 0

Region: chr23 17855578-17855589. Max. coverage (+): 0. Max coverage (-): 0

Region: chr23 17855590-17855601. Max. coverage (+): 0. Max coverage (-): 0

Region: chr23 17855602-17855614. Max. coverage (+): 0. Max coverage (-): 0

Region: chr23 17855615-17855626. Max. coverage (+): 0. Max coverage (-): 1.28

Region: chr23 17855627-17855638. Max. coverage (+): 0. Max coverage (-): 1.28

Region: chr23 17855639-17855650. Max. coverage (+): 0. Max coverage (-): 0

Region: chr23 17855651-17855662. Max. coverage (+): 0. Max coverage (-): 0

Region: chr23 17855663-17855674. Max. coverage (+): 0. Max coverage (-): 0

Region: chr23 17855675-17855686. Max. coverage (+): 0. Max coverage (-): 0

Region: chr23 17855687-17855699. Max. coverage (+): 0. Max coverage (-): 0

Region: chr23 17855700-17855711. Max. coverage (+): 0. Max coverage (-): 0

Region: chr23 17855712-17855723. Max. coverage (+): 0. Max coverage (-): 1.36

Region: chr23 17855724-17855735. Max. coverage (+): 0. Max coverage (-): 0.37

Region: chr23 17855736-17855747. Max. coverage (+): 0. Max coverage (-): 0

Region: chr23 17855748-17855759. Max. coverage (+): 0. Max coverage (-): 0

Region: chr23 17855760-17855771. Max. coverage (+): 0. Max coverage (-): 0

Region: chr23 17855772-17855784. Max. coverage (+): 0. Max coverage (-): 0

Region: chr23 17855785-17855796. Max. coverage (+): 0. Max coverage (-): 0

Region: chr23 17855797-17855808. Max. coverage (+): 0. Max coverage (-): 0

Region: chr23 17855809-17855820. Max. coverage (+): 0. Max coverage (-): 0

Region: chr23 17855821-17855832. Max. coverage (+): 0. Max coverage (-): 0

Region: chr23 17855833-17855844. Max. coverage (+): 0. Max coverage (-): 0

Region: chr23 17855845-17855856. Max. coverage (+): 0. Max coverage (-): 0

Region: chr23 17855857-17855868. Max. coverage (+): 0. Max coverage (-): 0

Region: chr23 17855869-17855881. Max. coverage (+): 0. Max coverage (-): 0

Region: chr23 17855882-17855893. Max. coverage (+): 0. Max coverage (-): 0

Region: chr23 17855894-17855905. Max. coverage (+): 0. Max coverage (-): 0

Region: chr23 17855906-17855917. Max. coverage (+): 0. Max coverage (-): 0

Region: chr23 17855918-17855929. Max. coverage (+): 0. Max coverage (-): 7.47

Region: chr23 17855930-17855941. Max. coverage (+): 0. Max coverage (-): 7.47

Region: chr23 17855942-17855953. Max. coverage (+): 0. Max coverage (-): 0

Region: chr23 17855954-17855966. Max. coverage (+): 0. Max coverage (-): 0

Region: chr23 17855967-17855978. Max. coverage (+): 0. Max coverage (-): 0

Region: chr23 17855979-17855990. Max. coverage (+): 0. Max coverage (-): 0

Region: chr23 17855991-17856002. Max. coverage (+): 0. Max coverage (-): 0

Region: chr23 17856003-17856014. Max. coverage (+): 0. Max coverage (-): 0

Region: chr23 17856015-17856026. Max. coverage (+): 0. Max coverage (-): 0

Region: chr23 17856027-17856038. Max. coverage (+): 0. Max coverage (-): 0

Region: chr23 17856039-17856051. Max. coverage (+): 0. Max coverage (-): 0

Region: chr23 17856052-17856063. Max. coverage (+): 3.39. Max coverage (-): 0

Region: chr23 17856064-17856075. Max. coverage (+): 3.39. Max coverage (-): 0

Region: chr23 17856076-17856087. Max. coverage (+): 0. Max coverage (-): 0

Region: chr23 17856088-17856099. Max. coverage (+): 0. Max coverage (-): 0

Region: chr23 17856100-17856111. Max. coverage (+): 0. Max coverage (-): 0

Region: chr23 17856112-17856123. Max. coverage (+): 0. Max coverage (-): 0

Region: chr23 17856124-17856135. Max. coverage (+): 0. Max coverage (-): 0

Region: chr23 17856136-17856148. Max. coverage (+): 0. Max coverage (-): 0

Region: chr23 17856149-17856160. Max. coverage (+): 0. Max coverage (-): 0

Region: chr23 17856161-17856172. Max. coverage (+): 0. Max coverage (-): 0

Region: chr23 17856173-17856184. Max. coverage (+): 0. Max coverage (-): 0

Region: chr23 17856185-17856196. Max. coverage (+): 0. Max coverage (-): 0

Region: chr23 17856197-17856208. Max. coverage (+): 0. Max coverage (-): 0

Region: chr23 17856209-17856220. Max. coverage (+): 0. Max coverage (-): 0

Region: chr23 17856221-17856233. Max. coverage (+): 0. Max coverage (-): 0

Region: chr23 17856234-17856245. Max. coverage (+): 0. Max coverage (-): 0

Region: chr23 17856246-17856257. Max. coverage (+): 0. Max coverage (-): 0

Region: chr23 17856258-17856269. Max. coverage (+): 0. Max coverage (-): 0

Region: chr23 17856270-17856281. Max. coverage (+): 0. Max coverage (-): 0

Region: chr23 17856282-17856293. Max. coverage (+): 0. Max coverage (-): 0

Region: chr23 17856294-17856305. Max. coverage (+): 0. Max coverage (-): 0

Region: chr23 17856306-17856318. Max. coverage (+): 0. Max coverage (-): 0

Region: chr23 17856319-17856330. Max. coverage (+): 0. Max coverage (-): 0

Region: chr23 17856331-17856342. Max. coverage (+): 5.13. Max coverage (-): 0

Region: chr23 17856343-17856354. Max. coverage (+): 5.13. Max coverage (-): 0

Region: chr23 17856355-17856366. Max. coverage (+): 0. Max coverage (-): 0

Region: chr23 17856367-17856378. Max. coverage (+): 0. Max coverage (-): 0

Region: chr23 17856379-17856390. Max. coverage (+): 0. Max coverage (-): 0

Region: chr23 17856391-17856402. Max. coverage (+): 0. Max coverage (-): 0

Region: chr23 17856403-17856415. Max. coverage (+): 0. Max coverage (-): 0

Region: chr23 17856416-17856427. Max. coverage (+): 0. Max coverage (-): 0

Region: chr23 17856428-17856439. Max. coverage (+): 0. Max coverage (-): 0

Region: chr23 17856440-17856451. Max. coverage (+): 0. Max coverage (-): 1.08

Region: chr23 17856452-17856463. Max. coverage (+): 0. Max coverage (-): 3.6

Region: chr23 17856464-17856475. Max. coverage (+): 0. Max coverage (-): 0

Region: chr23 17856476-17856487. Max. coverage (+): 0. Max coverage (-): 0

Region: chr23 17856488-17856500. Max. coverage (+): 0. Max coverage (-): 0

Region: chr23 17856501-17856512. Max. coverage (+): 0. Max coverage (-): 0

Region: chr23 17856513-17856524. Max. coverage (+): 0. Max coverage (-): 0

Region: chr23 17856525-17856536. Max. coverage (+): 0. Max coverage (-): 0

Region: chr23 17856537-17856548. Max. coverage (+): 0. Max coverage (-): 0

Region: chr23 17856549-17856560. Max. coverage (+): 0. Max coverage (-): 0

Region: chr23 17856561-17856572. Max. coverage (+): 0. Max coverage (-): 0

Region: chr23 17856573-17856585. Max. coverage (+): 0. Max coverage (-): 0

Region: chr23 17856586-17856597. Max. coverage (+): 0. Max coverage (-): 0

Region: chr23 17856598-17856609. Max. coverage (+): 0. Max coverage (-): 0

Region: chr23 17856610-17856621. Max. coverage (+): 0. Max coverage (-): 0

Region: chr23 17856622-17856633. Max. coverage (+): 0. Max coverage (-): 2.73

Region: chr23 17856634-17856645. Max. coverage (+): 0. Max coverage (-): 2.73

Region: chr23 17856646-17856657. Max. coverage (+): 0. Max coverage (-): 0

Region: chr23 17856658-17856669. Max. coverage (+): 0. Max coverage (-): 0

Region: chr23 17856670-17856682. Max. coverage (+): 0. Max coverage (-): 0

Region: chr23 17856683-17856694. Max. coverage (+): 0. Max coverage (-): 0

Region: chr23 17856695-17856706. Max. coverage (+): 0. Max coverage (-): 0

Region: chr23 17856707-17856718. Max. coverage (+): 0. Max coverage (-): 2

Region: chr23 17856719-17856730. Max. coverage (+): 0. Max coverage (-): 2

Region: chr23 17856731-17856742. Max. coverage (+): 0. Max coverage (-): 0

Region: chr23 17856743-17856754. Max. coverage (+): 0. Max coverage (-): 0

Region: chr23 17856755-17856767. Max. coverage (+): 0. Max coverage (-): 0

Region: chr23 17856768-17856779. Max. coverage (+): 0. Max coverage (-): 0

Region: chr23 17856780-17856791. Max. coverage (+): 0. Max coverage (-): 0

Region: chr23 17856792-17856803. Max. coverage (+): 0. Max coverage (-): 0

Region: chr23 17856804-17856815. Max. coverage (+): 0. Max coverage (-): 0

Region: chr23 17856816-17856827. Max. coverage (+): 0. Max coverage (-): 0

Region: chr23 17856828-17856839. Max. coverage (+): 0. Max coverage (-): 0

Region: chr23 17856840-17856852. Max. coverage (+): 0. Max coverage (-): 0

Region: chr23 17856853-17856864. Max. coverage (+): 0. Max coverage (-): 0

Region: chr23 17856865-17856876. Max. coverage (+): 0. Max coverage (-): 0

Region: chr23 17856877-17856888. Max. coverage (+): 0. Max coverage (-): 0

Region: chr23 17856889-17856900. Max. coverage (+): 0. Max coverage (-): 6.46

Region: chr23 17856901-17856912. Max. coverage (+): 0. Max coverage (-): 0

Region: chr23 17856913-17856924. Max. coverage (+): 0. Max coverage (-): 0

Region: chr23 17856925-17856936. Max. coverage (+): 0.55. Max coverage (-): 4.86

Region: chr23 17856937-17856949. Max. coverage (+): 0.55. Max coverage (-): 4.86

Region: chr23 17856950-17856961. Max. coverage (+): 0. Max coverage (-): 0

Region: chr23 17856962-17856973. Max. coverage (+): 0. Max coverage (-): 2.07

Region: chr23 17856974-17856985. Max. coverage (+): 0. Max coverage (-): 0

Region: chr23 17856986-17856997. Max. coverage (+): 0. Max coverage (-): 1.09

Region: chr23 17856998-17857009. Max. coverage (+): 0. Max coverage (-): 1.09

Region: chr23 17857010-17857021. Max. coverage (+): 0. Max coverage (-): 0

Region: chr23 17857022-17857034. Max. coverage (+): 0. Max coverage (-): 0

Region: chr23 17857035-17857046. Max. coverage (+): 0. Max coverage (-): 0

Region: chr23 17857047-17857058. Max. coverage (+): 0. Max coverage (-): 1.2

Region: chr23 17857059-17857070. Max. coverage (+): 0. Max coverage (-): 1.2

Region: chr23 17857071-17857082. Max. coverage (+): 0. Max coverage (-): 0

Region: chr23 17857083-17857094. Max. coverage (+): 0. Max coverage (-): 0

Region: chr23 17857095-17857106. Max. coverage (+): 0. Max coverage (-): 0

Region: chr23 17857107-17857118. Max. coverage (+): 0. Max coverage (-): 0

Region: chr23 17857119-17857131. Max. coverage (+): 0. Max coverage (-): 0

Region: chr23 17857132-17857143. Max. coverage (+): 0. Max coverage (-): 0

Region: chr23 17857144-17857155. Max. coverage (+): 0. Max coverage (-): 0

Region: chr23 17857156-17857167. Max. coverage (+): 0. Max coverage (-): 0

Region: chr23 17857168-17857179. Max. coverage (+): 0. Max coverage (-): 0

Region: chr23 17857180-17857191. Max. coverage (+): 0. Max coverage (-): 0

Region: chr23 17857192-17857203. Max. coverage (+): 0. Max coverage (-): 0

Region: chr23 17857204-17857216. Max. coverage (+): 0. Max coverage (-): 0

Region: chr23 17857217-17857228. Max. coverage (+): 0. Max coverage (-): 0

Region: chr23 17857229-17857240. Max. coverage (+): 0. Max coverage (-): 0

Region: chr23 17857241-17857252. Max. coverage (+): 0. Max coverage (-): 0

Region: chr23 17857253-17857264. Max. coverage (+): 0. Max coverage (-): 0

Region: chr23 17857265-17857276. Max. coverage (+): 0. Max coverage (-): 0

Region: chr23 17857277-17857288. Max. coverage (+): 0. Max coverage (-): 0

Region: chr23 17857289-17857301. Max. coverage (+): 0. Max coverage (-): 0

Region: chr23 17857302-17857313. Max. coverage (+): 0. Max coverage (-): 0

Region: chr23 17857314-17857325. Max. coverage (+): 0. Max coverage (-): 0

Region: chr23 17857326-17857337. Max. coverage (+): 0. Max coverage (-): 0

Region: chr23 17857338-17857349. Max. coverage (+): 0. Max coverage (-): 0

Region: chr23 17857350-17857361. Max. coverage (+): 0. Max coverage (-): 0

Region: chr23 17857362-17857373. Max. coverage (+): 0. Max coverage (-): 0.61

Region: chr23 17857374-17857385. Max. coverage (+): 3.13. Max coverage (-): 0.61

Region: chr23 17857386-17857398. Max. coverage (+): 3.13. Max coverage (-): 0

Region: chr23 17857399-17857410. Max. coverage (+): 0. Max coverage (-): 0

Region: chr23 17857411-17857422. Max. coverage (+): 0. Max coverage (-): 0

Region: chr23 17857423-17857434. Max. coverage (+): 0. Max coverage (-): 0

Region: chr23 17857435-17857446. Max. coverage (+): 0. Max coverage (-): 0

Region: chr23 17857447-17857458. Max. coverage (+): 0. Max coverage (-): 0

Region: chr23 17857459-17857470. Max. coverage (+): 0. Max coverage (-): 0

Region: chr23 17857471-17857483. Max. coverage (+): 0. Max coverage (-): 0

Region: chr23 17857484-17857495. Max. coverage (+): 0. Max coverage (-): 0

Region: chr23 17857496-17857507. Max. coverage (+): 0. Max coverage (-): 0

Region: chr23 17857508-17857519. Max. coverage (+): 0. Max coverage (-): 3.16

Region: chr23 17857520-17857531. Max. coverage (+): 0. Max coverage (-): 12.9

Region: chr23 17857532-17857543. Max. coverage (+): 0. Max coverage (-): 2.28

Region: chr23 17857544-17857555. Max. coverage (+): 0. Max coverage (-): 3.72

Region: chr23 17857556-17857568. Max. coverage (+): 0. Max coverage (-): 3.94

Region: chr23 17857569-17857580. Max. coverage (+): 0. Max coverage (-): 0

Region: chr23 17857581-17857592. Max. coverage (+): 0. Max coverage (-): 0

Region: chr23 17857593-17857604. Max. coverage (+): 0. Max coverage (-): 0

Region: chr23 17857605-17857616. Max. coverage (+): 0. Max coverage (-): 0

Region: chr23 17857617-17857628. Max. coverage (+): 0. Max coverage (-): 0

Region: chr23 17857629-17857640. Max. coverage (+): 0. Max coverage (-): 0

Region: chr23 17857641-17857652. Max. coverage (+): 0. Max coverage (-): 0

Region: chr23 17857653-17857665. Max. coverage (+): 0. Max coverage (-): 0

Region: chr23 17857666-17857677. Max. coverage (+): 0. Max coverage (-): 0

Region: chr23 17857678-17857689. Max. coverage (+): 0. Max coverage (-): 0.65

Region: chr23 17857690-17857701. Max. coverage (+): 0. Max coverage (-): 0.65

Region: chr23 17857702-17857713. Max. coverage (+): 0. Max coverage (-): 0

Region: chr23 17857714-17857725. Max. coverage (+): 0. Max coverage (-): 0

Region: chr23 17857726-17857737. Max. coverage (+): 0. Max coverage (-): 0

Region: chr23 17857738-17857750. Max. coverage (+): 0. Max coverage (-): 0

Region: chr23 17857751-17857762. Max. coverage (+): 0. Max coverage (-): 0

Region: chr23 17857763-17857774. Max. coverage (+): 0. Max coverage (-): 0

Region: chr23 17857775-17857786. Max. coverage (+): 0. Max coverage (-): 0

Region: chr23 17857787-17857798. Max. coverage (+): 0. Max coverage (-): 0

Region: chr23 17857799-17857810. Max. coverage (+): 0. Max coverage (-): 0

Region: chr23 17857811-17857822. Max. coverage (+): 0. Max coverage (-): 0

Region: chr23 17857823-17857835. Max. coverage (+): 0. Max coverage (-): 0

Region: chr23 17857836-17857847. Max. coverage (+): 0. Max coverage (-): 0

Region: chr23 17857848-17857859. Max. coverage (+): 0. Max coverage (-): 0

Region: chr23 17857860-17857871. Max. coverage (+): 0. Max coverage (-): 0

Region: chr23 17857872-17857883. Max. coverage (+): 0. Max coverage (-): 0

Region: chr23 17857884-17857895. Max. coverage (+): 0. Max coverage (-): 0

Region: chr23 17857896-17857907. Max. coverage (+): 0. Max coverage (-): 0

Region: chr23 17857908-17857919. Max. coverage (+): 0. Max coverage (-): 0

Region: chr23 17857920-17857932. Max. coverage (+): 0. Max coverage (-): 0

Region: chr23 17857933-17857944. Max. coverage (+): 0. Max coverage (-): 0

Region: chr23 17857945-17857956. Max. coverage (+): 0. Max coverage (-): 0

Region: chr23 17857957-17857968. Max. coverage (+): 0. Max coverage (-): 0

Region: chr23 17857969-17857980. Max. coverage (+): 0. Max coverage (-): 0

Region: chr23 17857981-17857992. Max. coverage (+): 0. Max coverage (-): 0

Region: chr23 17857993-17858004. Max. coverage (+): 0. Max coverage (-): 0

Region: chr23 17858005-17858017. Max. coverage (+): 0. Max coverage (-): 0

Region: chr23 17858018-17858029. Max. coverage (+): 0. Max coverage (-): 0

Region: chr23 17858030-17858041. Max. coverage (+): 0. Max coverage (-): 0

Region: chr23 17858042-17858053. Max. coverage (+): 0. Max coverage (-): 0

Region: chr23 17858054-17858065. Max. coverage (+): 0. Max coverage (-): 0

Region: chr23 17858066-17858077. Max. coverage (+): 0. Max coverage (-): 0

Region: chr23 17858078-17858089. Max. coverage (+): 0. Max coverage (-): 0

Region: chr23 17858090-17858102. Max. coverage (+): 0. Max coverage (-): 0

Region: chr23 17858103-17858114. Max. coverage (+): 0. Max coverage (-): 0

Region: chr23 17858115-17858126. Max. coverage (+): 0. Max coverage (-): 0

Region: chr23 17858127-17858138. Max. coverage (+): 0. Max coverage (-): 0

Region: chr23 17858139-17858150. Max. coverage (+): 0. Max coverage (-): 0

Region: chr23 17858151-17858162. Max. coverage (+): 0. Max coverage (-): 0

Region: chr23 17858163-17858174. Max. coverage (+): 0. Max coverage (-): 0

Region: chr23 17858175-17858186. Max. coverage (+): 0. Max coverage (-): 0

Region: chr23 17858187-17858199. Max. coverage (+): 0. Max coverage (-): 0

Region: chr23 17858200-17858211. Max. coverage (+): 0. Max coverage (-): 0

Region: chr23 17858212-17858223. Max. coverage (+): 0. Max coverage (-): 0

Region: chr23 17858224-17858235. Max. coverage (+): 0. Max coverage (-): 0

Region: chr23 17858236-17858247. Max. coverage (+): 0. Max coverage (-): 0

Region: chr23 17858248-17858259. Max. coverage (+): 0. Max coverage (-): 0

Region: chr23 17858260-17858271. Max. coverage (+): 0. Max coverage (-): 0

Region: chr23 17858272-17858284. Max. coverage (+): 0. Max coverage (-): 0

Region: chr23 17858285-17858296. Max. coverage (+): 0. Max coverage (-): 0

Region: chr23 17858297-17858308. Max. coverage (+): 0. Max coverage (-): 0

Region: chr23 17858309-17858320. Max. coverage (+): 0. Max coverage (-): 0.46

Region: chr23 17858321-17858332. Max. coverage (+): 0. Max coverage (-): 0

Region: chr23 17858333-17858344. Max. coverage (+): 0. Max coverage (-): 0

Region: chr23 17858345-17858356. Max. coverage (+): 0. Max coverage (-): 0

Region: chr23 17858357-17858369. Max. coverage (+): 0. Max coverage (-): 0

Region: chr23 17858370-17858381. Max. coverage (+): 0. Max coverage (-): 0

Region: chr23 17858382-17858393. Max. coverage (+): 0. Max coverage (-): 0

Region: chr23 17858394-17858405. Max. coverage (+): 0. Max coverage (-): 0

Region: chr23 17858406-17858417. Max. coverage (+): 0. Max coverage (-): 0

Region: chr23 17858418-17858429. Max. coverage (+): 0. Max coverage (-): 0

Region: chr23 17858430-17858441. Max. coverage (+): 0. Max coverage (-): 0

Region: chr23 17858442-17858453. Max. coverage (+): 0. Max coverage (-): 0

Region: chr23 17858454-17858466. Max. coverage (+): 0. Max coverage (-): 0

Region: chr23 17858467-17858478. Max. coverage (+): 0. Max coverage (-): 0

Region: chr23 17858479-17858490. Max. coverage (+): 0. Max coverage (-): 0

Region: chr23 17858491-17858502. Max. coverage (+): 0. Max coverage (-): 0

Region: chr23 17858503-17858514. Max. coverage (+): 0. Max coverage (-): 0

Region: chr23 17858515-17858526. Max. coverage (+): 0. Max coverage (-): 0

Region: chr23 17858527-17858538. Max. coverage (+): 0. Max coverage (-): 0

Region: chr23 17858539-17858551. Max. coverage (+): 0. Max coverage (-): 0

Region: chr23 17858552-17858563. Max. coverage (+): 0. Max coverage (-): 0

Region: chr23 17858564-17858575. Max. coverage (+): 0. Max coverage (-): 0

Region: chr23 17858576-17858587. Max. coverage (+): 0. Max coverage (-): 0

Region: chr23 17858588-17858599. Max. coverage (+): 0. Max coverage (-): 0

Region: chr23 17858600-17858611. Max. coverage (+): 0. Max coverage (-): 0

Region: chr23 17858612-17858623. Max. coverage (+): 0. Max coverage (-): 0

Region: chr23 17858624-17858635. Max. coverage (+): 0. Max coverage (-): 0

Region: chr23 17858636-17858648. Max. coverage (+): 0. Max coverage (-): 0

Region: chr23 17858649-17858660. Max. coverage (+): 0. Max coverage (-): 0

Region: chr23 17858661-17858672. Max. coverage (+): 0. Max coverage (-): 0

Region: chr23 17858673-17858684. Max. coverage (+): 0. Max coverage (-): 0

Region: chr23 17858685-17858696. Max. coverage (+): 0. Max coverage (-): 0

Region: chr23 17858697-17858708. Max. coverage (+): 0. Max coverage (-): 0

Region: chr23 17858709-17858720. Max. coverage (+): 0. Max coverage (-): 0

Region: chr23 17858721-17858733. Max. coverage (+): 0. Max coverage (-): 0

Region: chr23 17858734-17858745. Max. coverage (+): 0. Max coverage (-): 0

Region: chr23 17858746-17858757. Max. coverage (+): 0. Max coverage (-): 0

Region: chr23 17858758-17858769. Max. coverage (+): 0. Max coverage (-): 3.54

Region: chr23 17858770-17858781. Max. coverage (+): 0. Max coverage (-): 0

Region: chr23 17858782-17858793. Max. coverage (+): 0. Max coverage (-): 0

Region: chr23 17858794-17858805. Max. coverage (+): 0. Max coverage (-): 14.03

Region: chr23 17858806-17858818. Max. coverage (+): 0. Max coverage (-): 14.03

Region: chr23 17858819-17858830. Max. coverage (+): 0. Max coverage (-): 0

Region: chr23 17858831-17858842. Max. coverage (+): 0. Max coverage (-): 0

Region: chr23 17858843-17858854. Max. coverage (+): 0. Max coverage (-): 3.09

Region: chr23 17858855-17858866. Max. coverage (+): 0. Max coverage (-): 0

Region: chr23 17858867-17858878. Max. coverage (+): 0. Max coverage (-): 0

Region: chr23 17858879-17858890. Max. coverage (+): 0. Max coverage (-): 0

Region: chr23 17858891-17858902. Max. coverage (+): 0. Max coverage (-): 0

Region: chr23 17858903-17858915. Max. coverage (+): 0. Max coverage (-): 0

Region: chr23 17858916-17858927. Max. coverage (+): 0. Max coverage (-): 0

Region: chr23 17858928-17858939. Max. coverage (+): 0. Max coverage (-): 0

Region: chr23 17858940-17858951. Max. coverage (+): 0. Max coverage (-): 0

Region: chr23 17858952-17858963. Max. coverage (+): 0. Max coverage (-): 0

Region: chr23 17858964-17858975. Max. coverage (+): 0. Max coverage (-): 0.44

Region: chr23 17858976-17858987. Max. coverage (+): 0. Max coverage (-): 3.39

Region: chr23 17858988-17859000. Max. coverage (+): 0. Max coverage (-): 1.6

Region: chr23 17859001-17859012. Max. coverage (+): 0. Max coverage (-): 0

Region: chr23 17859013-17859024. Max. coverage (+): 0. Max coverage (-): 0

Region: chr23 17859025-17859036. Max. coverage (+): 0. Max coverage (-): 0

Region: chr23 17859037-17859048. Max. coverage (+): 0. Max coverage (-): 0

Region: chr23 17859049-17859060. Max. coverage (+): 0. Max coverage (-): 0

Region: chr23 17859061-17859072. Max. coverage (+): 0. Max coverage (-): 0

Region: chr23 17859073-17859085. Max. coverage (+): 0. Max coverage (-): 0

Region: chr23 17859086-17859097. Max. coverage (+): 0. Max coverage (-): 0

Region: chr23 17859098-17859109. Max. coverage (+): 0. Max coverage (-): 0

Region: chr23 17859110-17859121. Max. coverage (+): 0. Max coverage (-): 0

Region: chr23 17859122-17859133. Max. coverage (+): 0. Max coverage (-): 1.12

Region: chr23 17859134-17859145. Max. coverage (+): 0. Max coverage (-): 1.12

Region: chr23 17859146-17859157. Max. coverage (+): 0. Max coverage (-): 0

Region: chr23 17859158-17859169. Max. coverage (+): 0. Max coverage (-): 0

Region: chr23 17859170-17859182. Max. coverage (+): 0. Max coverage (-): 0

Region: chr23 17859183-17859194. Max. coverage (+): 0. Max coverage (-): 0

Region: chr23 17859195-17859206. Max. coverage (+): 0. Max coverage (-): 0

Region: chr23 17859207-17859218. Max. coverage (+): 0. Max coverage (-): 0

Region: chr23 17859219-17859230. Max. coverage (+): 0. Max coverage (-): 0

Region: chr23 17859231-17859242. Max. coverage (+): 0. Max coverage (-): 3.22

Region: chr23 17859243-17859254. Max. coverage (+): 0. Max coverage (-): 0

Region: chr23 17859255-. Max. coverage (+): 0. Max coverage (-): 0

RepeatMasker Color Code

**+**

100-98% Identity

<98-95% Identity

<95-90% Identity

<90-85% Identity

<85-80% Identity

<80-75% Identity

<75-70% Identity

<70% Identity

**-**

Gene Set Color Code

**+**

Gene

Pseudogene

**-**

Topology/Coverage Color Code

Coverage Plus Strand

Coverage Minus Strand

Mainstrand: Plus

Mainstrand: Minus

Complementary Strand

Flanking Region  
(if option -flank >0)

Gene Set Annotation  

**1. SLC29A1 (protein coding, ENSBTAG00000015131) Tr:00000035955 Ex:8**: 17853142-17853239 (+)  
**2. SLC29A1 (protein coding, ENSBTAG00000015131) Tr:00000035955 Ex:9**: 17853378-17853456 (+)  
**3. SLC29A1 (protein coding, ENSBTAG00000015131) Tr:00000035955 Ex:10**: 17853881-17853978 (+)  
**4. SLC29A1 (protein coding, ENSBTAG00000015131) Tr:00000035955 Ex:11**: 17854198-17854306 (+)  
**5. SLC29A1 (protein coding, ENSBTAG00000015131) Tr:00000035955 Ex:12**: 17854558-17854643 (+)  
**6. SLC29A1 (protein coding, ENSBTAG00000015131) Tr:00000035955 Ex:13**: 17855023-17855222 (+)  
**7. SLC29A1 (protein coding, ENSBTAG00000015131) Tr:00000035955 Ex:14**: 17855542-17856150 (+)

  
RepeatMasker Annotation  

**1. L2**: 17853620-17853677 (-), Divergence to consensus: 33.2%  
**2. C-rich**: 17854006-17854070 (+), Divergence to consensus: 28%  
**3. L1ME3**: 17854743-17854930 (-), Divergence to consensus: 42.7%  
**4. CHRL1\_BT**: 17857753-17857849 (-), Divergence to consensus: 26.8%  
**5. MamRep605**: 17858483-17858605 (+), Divergence to consensus: 32.1%

  
Transcription Factor Binding Sites  

**Gata4** (Sequence: GTTATCT (+): 17853217)  
**Gata4** (Sequence: CTTATCT (+): 17857514)
