# Supplementary material for: Small RNA sequencing of cryopreserved semen from single bull revealed altered miRNAs and piRNAs expression between High- and Low-motile sperm populations
Source: BMC Genomics. 2017 Jan 4;18:14. doi: 10.1186/s12864-016-3394-7 (PMC5209821; doi:10.1186/s12864-016-3394-7)
Supplement: Additional file 3: — Details for each piRNA clusters found in High Motile (HM) sperm fraction. Genes, repeats, transposable elements and transcription factors binding sites falling within the cluster regions were reported. (ZIP 1896 kb) [file 12864_2016_3394_MOESM3_ESM.zip › 55.html]

piRNA cluster 55


Predicted piRNA cluster no. 55     previous   next
  

Show proTRAC run info
Hide proTRAC run info

================================= proTRAC ====================================  
VERSION: 2.1                                    LAST MODIFIED: 06. October 2015  
  
Please cite:  
Rosenkranz D, Zischler H. proTRAC - a software for probabilistic piRNA cluster  
detection, visualization and analysis. 2012. BMC Bioinformatics 13:5.  
  
and (for proTRAC 2.0 and later):  
Rosenkranz D, Rudloff S, Bastuck K, Ketting RF, Zischler H. Tupaia small RNAs  
provide insights into function and evolution of RNAi-based transposon defense  
in mammals. 2015. RNA 21(5):911-922.  
  
Contact:  
David Rosenkranz  
Institute of Anthropology, small RNA group  
Johannes Gutenberg University Mainz  
email: rosenkranz@uni-mainz.de  
  
You can find the latest proTRAC version at:  
http://sourceforge.net/projects/protrac/files  
http://www.smallRNAgroup-mainz.de/software  
==============================================================================  
  
PARAMETERS:  
Map file: .............../storage/core/barbara/genhome/smallRNA/fertility/Sample\_motile/pirna/Sample\_motile\_26-33\_collapsed.fa.no-dust.map.weighted-10000-1000-b-0  
Genome file: ............/storage/core/barbara/genhome/smallRNA/fertility/Sample\_all/pirna/bt\_311\_chrY.fa  
RepeatMasker annotation: /storage/genomes/bt\_umd31/GCF\_000003055.6\_Bos\_taurus\_UMD\_3.1.1\_repeatMasker\_chr.out  
GeneSet:................./storage/core/barbara/genhome/smallRNA/fertility/Sample\_all/pirna/full.gtf  
  
Significant (p<=0.01) hit density will be calculated based  
on observed hit distribution.  
  
Sliding window size: ........................................ 5000 bp  
Sliding window increament: .................................. 1000 bp  
Normalize each hit by number of genomic hits: ............... 1 [0=no/1=yes]  
Normalize each hit by number of sequence reads: ............. 1 [0=no/1=yes]  
Normalize values (-> per million mapped reads): ............. 1 [0=no/1=yes]  
Min. fraction of hits with 1T(U) or 10A: .................... 0.75  
Alternatively: Min. fraction of hits with 1T(U) and 10A: .... 0.5  
Min. fraction of hits with typical piRNA length: ............ 0.75  
Typical piRNA length: ....................................... 26-33 nt  
Min. size of a piRNA cluster: ............................... 5000 bp.  
Min. number of hits (absolute): ............................. 0  
Min. number of hits (normalized): ........................... 0  
Min. fraction of hits on the mainstrand: .................... 0.75  
Top fraction of mapped sequences (in terms of read counts): . 1%  
Top fraction accounts for max. n% of sequence reads: ........ 90%  
Min. fraction of hits on each arm of a bidirectional cluster: 0.1  
Output image file for each cluster: ......................... 0 [0=no/1=yes]  
Output html file for each cluster: .......................... 1 [0=no/1=yes]  
Output a summary table: ..................................... 1 [0=no/1=yes]  
Output a FASTA file for each cluster (piRNA sequences): ..... 1 [0=no/1=yes]  
Output a FASTA file comprising cluster sequences: ........... 1 [0=no/1=yes]  
Search DNA motifs in clusters: .............................. 1 [0=no/1=yes]  
Output flanking sequences: +/- .............................. 0 bp  
Output ~.pTi file: .......................................... 1 [0=no/1=yes]  
==============================================================================  
  
  
Genome size (without gaps): ............ 2678902517 bp  
Gaps (N/X/-): .......................... 53837044 bp  
Mapped reads: .......................... 658825247023  
Non-identical sequences: ............... 514171  
Genomic hits: .......................... 764233  
Significant densitiy of mapped reads: .. 12867599.5173724 reads/kb

Show proTRAC cluster info
Hide proTRAC cluster info

|  |  |
| --- | --- |
| Location | chr23 |
| Coordinates | 47783385-47789344 |
| Size [bp] | 5960 |
| Sequence hit loci | 1666 |
| Mapped reads (normalized) | 1783128115 |
| Mapped reads (normalized) per kb | 299182569.6 |
| Normalized reads with 1T (1U) | 81% |
| Normalized reads with 10A | 27.8% |
| Normalized reads with length 26-33 nt | 100% |
| Normalized reads on the main strand(s) | 100% |
| Predicted directionality | mono:minus |

100%

0%

1T (1U)  
reads

10A reads

26-33 nt  
reads

reads on mainstrand

**Either the amount of reads with 1T (1U) OR 10A has to exceed 75% (set with option: -1Tor10A)  
Alternatively the amount of reads with 1T (1U) AND 10A has to exceed 50% (set with option: -1Tand10A)  
Minimum amount of reads with preferred size is 75% (set with option: -pisize)  
Minimum amount of reads on the main strand(s) is 75% (set with option: -clstrand)**

Show read coverage
Hide read coverage

WHAT DO I SEE HERE?  
This chart shows the location of mapped sequence reads within a predicted piRNA cluster. The color refers to the number of genomic hits produced by the sequence read in question. A dark red bar indicates that this sequence read produces many other hits elsewhere in the genome. Many adjacent red or yellow bars can indicate the presence of a multi-copy element such as transposons or rRNA genes. A dark green bar indicates that this sequence read maps uniquely to this locus.

1 hit

2-5 hits

6-10 hits

11-20 hits

21-50 hits

51-100 hits

> 100 hits

chr23

47783385

47789344

Gene Set

RepeatMasker

Mapped  
Reads

108.41

plus strand

minus strand

108.41

Region: chr23 17859256-47783390. Max. coverage (+): 0. Max coverage (-): 1.42

Region: chr23 47783391-47783402. Max. coverage (+): 0. Max coverage (-): 1.42

Region: chr23 47783403-47783414. Max. coverage (+): 0. Max coverage (-): 1.14

Region: chr23 47783415-47783426. Max. coverage (+): 0. Max coverage (-): 0

Region: chr23 47783427-47783438. Max. coverage (+): 0. Max coverage (-): 0

Region: chr23 47783439-47783450. Max. coverage (+): 0. Max coverage (-): 0

Region: chr23 47783451-47783462. Max. coverage (+): 0. Max coverage (-): 0

Region: chr23 47783463-47783474. Max. coverage (+): 0. Max coverage (-): 0

Region: chr23 47783475-47783486. Max. coverage (+): 0. Max coverage (-): 0

Region: chr23 47783487-47783498. Max. coverage (+): 0. Max coverage (-): 0

Region: chr23 47783499-47783510. Max. coverage (+): 0. Max coverage (-): 0

Region: chr23 47783511-47783522. Max. coverage (+): 0. Max coverage (-): 0

Region: chr23 47783523-47783533. Max. coverage (+): 0. Max coverage (-): 0

Region: chr23 47783534-47783545. Max. coverage (+): 0. Max coverage (-): 0

Region: chr23 47783546-47783557. Max. coverage (+): 0. Max coverage (-): 0

Region: chr23 47783558-47783569. Max. coverage (+): 0. Max coverage (-): 0

Region: chr23 47783570-47783581. Max. coverage (+): 0. Max coverage (-): 0

Region: chr23 47783582-47783593. Max. coverage (+): 0. Max coverage (-): 0

Region: chr23 47783594-47783605. Max. coverage (+): 0. Max coverage (-): 0

Region: chr23 47783606-47783617. Max. coverage (+): 0. Max coverage (-): 0

Region: chr23 47783618-47783629. Max. coverage (+): 0. Max coverage (-): 0

Region: chr23 47783630-47783641. Max. coverage (+): 0. Max coverage (-): 0

Region: chr23 47783642-47783653. Max. coverage (+): 0. Max coverage (-): 0

Region: chr23 47783654-47783665. Max. coverage (+): 0. Max coverage (-): 0

Region: chr23 47783666-47783677. Max. coverage (+): 0. Max coverage (-): 0

Region: chr23 47783678-47783688. Max. coverage (+): 0. Max coverage (-): 0

Region: chr23 47783689-47783700. Max. coverage (+): 0. Max coverage (-): 0

Region: chr23 47783701-47783712. Max. coverage (+): 0. Max coverage (-): 7.92

Region: chr23 47783713-47783724. Max. coverage (+): 0. Max coverage (-): 17.04

Region: chr23 47783725-47783736. Max. coverage (+): 0. Max coverage (-): 3.77

Region: chr23 47783737-47783748. Max. coverage (+): 0. Max coverage (-): 4.07

Region: chr23 47783749-47783760. Max. coverage (+): 0. Max coverage (-): 4.07

Region: chr23 47783761-47783772. Max. coverage (+): 0. Max coverage (-): 4.54

Region: chr23 47783773-47783784. Max. coverage (+): 0. Max coverage (-): 5.03

Region: chr23 47783785-47783796. Max. coverage (+): 0. Max coverage (-): 17.98

Region: chr23 47783797-47783808. Max. coverage (+): 0. Max coverage (-): 17.98

Region: chr23 47783809-47783820. Max. coverage (+): 0. Max coverage (-): 0

Region: chr23 47783821-47783831. Max. coverage (+): 0. Max coverage (-): 0

Region: chr23 47783832-47783843. Max. coverage (+): 0. Max coverage (-): 0

Region: chr23 47783844-47783855. Max. coverage (+): 0. Max coverage (-): 0

Region: chr23 47783856-47783867. Max. coverage (+): 0. Max coverage (-): 7.07

Region: chr23 47783868-47783879. Max. coverage (+): 0. Max coverage (-): 10.63

Region: chr23 47783880-47783891. Max. coverage (+): 0. Max coverage (-): 0

Region: chr23 47783892-47783903. Max. coverage (+): 0. Max coverage (-): 0

Region: chr23 47783904-47783915. Max. coverage (+): 0. Max coverage (-): 1.62

Region: chr23 47783916-47783927. Max. coverage (+): 0. Max coverage (-): 31.09

Region: chr23 47783928-47783939. Max. coverage (+): 0. Max coverage (-): 16.89

Region: chr23 47783940-47783951. Max. coverage (+): 0. Max coverage (-): 13.15

Region: chr23 47783952-47783963. Max. coverage (+): 0. Max coverage (-): 72.46

Region: chr23 47783964-47783975. Max. coverage (+): 0. Max coverage (-): 59.97

Region: chr23 47783976-47783986. Max. coverage (+): 0. Max coverage (-): 2.57

Region: chr23 47783987-47783998. Max. coverage (+): 0. Max coverage (-): 0.18

Region: chr23 47783999-47784010. Max. coverage (+): 0. Max coverage (-): 0

Region: chr23 47784011-47784022. Max. coverage (+): 0. Max coverage (-): 7.15

Region: chr23 47784023-47784034. Max. coverage (+): 0. Max coverage (-): 0

Region: chr23 47784035-47784046. Max. coverage (+): 0. Max coverage (-): 0

Region: chr23 47784047-47784058. Max. coverage (+): 0. Max coverage (-): 92.99

Region: chr23 47784059-47784070. Max. coverage (+): 0. Max coverage (-): 69.68

Region: chr23 47784071-47784082. Max. coverage (+): 0. Max coverage (-): 42.04

Region: chr23 47784083-47784094. Max. coverage (+): 0. Max coverage (-): 21.72

Region: chr23 47784095-47784106. Max. coverage (+): 0. Max coverage (-): 9.91

Region: chr23 47784107-47784118. Max. coverage (+): 0. Max coverage (-): 7.37

Region: chr23 47784119-47784129. Max. coverage (+): 0. Max coverage (-): 4.02

Region: chr23 47784130-47784141. Max. coverage (+): 0. Max coverage (-): 4.01

Region: chr23 47784142-47784153. Max. coverage (+): 0. Max coverage (-): 18.38

Region: chr23 47784154-47784165. Max. coverage (+): 0. Max coverage (-): 9.71

Region: chr23 47784166-47784177. Max. coverage (+): 0. Max coverage (-): 12.44

Region: chr23 47784178-47784189. Max. coverage (+): 0. Max coverage (-): 25.36

Region: chr23 47784190-47784201. Max. coverage (+): 0. Max coverage (-): 30.07

Region: chr23 47784202-47784213. Max. coverage (+): 0. Max coverage (-): 27.14

Region: chr23 47784214-47784225. Max. coverage (+): 0. Max coverage (-): 7.02

Region: chr23 47784226-47784237. Max. coverage (+): 0. Max coverage (-): 6.75

Region: chr23 47784238-47784249. Max. coverage (+): 0. Max coverage (-): 0

Region: chr23 47784250-47784261. Max. coverage (+): 0. Max coverage (-): 0

Region: chr23 47784262-47784273. Max. coverage (+): 0. Max coverage (-): 0

Region: chr23 47784274-47784284. Max. coverage (+): 0. Max coverage (-): 0

Region: chr23 47784285-47784296. Max. coverage (+): 0. Max coverage (-): 0

Region: chr23 47784297-47784308. Max. coverage (+): 0. Max coverage (-): 0

Region: chr23 47784309-47784320. Max. coverage (+): 0. Max coverage (-): 0

Region: chr23 47784321-47784332. Max. coverage (+): 0. Max coverage (-): 0

Region: chr23 47784333-47784344. Max. coverage (+): 0. Max coverage (-): 0

Region: chr23 47784345-47784356. Max. coverage (+): 0. Max coverage (-): 0

Region: chr23 47784357-47784368. Max. coverage (+): 0. Max coverage (-): 0

Region: chr23 47784369-47784380. Max. coverage (+): 0. Max coverage (-): 0

Region: chr23 47784381-47784392. Max. coverage (+): 0. Max coverage (-): 0

Region: chr23 47784393-47784404. Max. coverage (+): 0. Max coverage (-): 0

Region: chr23 47784405-47784416. Max. coverage (+): 0. Max coverage (-): 0

Region: chr23 47784417-47784427. Max. coverage (+): 0. Max coverage (-): 0

Region: chr23 47784428-47784439. Max. coverage (+): 0. Max coverage (-): 0

Region: chr23 47784440-47784451. Max. coverage (+): 0. Max coverage (-): 0

Region: chr23 47784452-47784463. Max. coverage (+): 0. Max coverage (-): 0

Region: chr23 47784464-47784475. Max. coverage (+): 0. Max coverage (-): 0

Region: chr23 47784476-47784487. Max. coverage (+): 0. Max coverage (-): 0

Region: chr23 47784488-47784499. Max. coverage (+): 0. Max coverage (-): 0

Region: chr23 47784500-47784511. Max. coverage (+): 0. Max coverage (-): 0

Region: chr23 47784512-47784523. Max. coverage (+): 0. Max coverage (-): 0

Region: chr23 47784524-47784535. Max. coverage (+): 0. Max coverage (-): 0

Region: chr23 47784536-47784547. Max. coverage (+): 0. Max coverage (-): 0

Region: chr23 47784548-47784559. Max. coverage (+): 0. Max coverage (-): 4.06

Region: chr23 47784560-47784571. Max. coverage (+): 0. Max coverage (-): 8.05

Region: chr23 47784572-47784582. Max. coverage (+): 0. Max coverage (-): 8.49

Region: chr23 47784583-47784594. Max. coverage (+): 0. Max coverage (-): 1.44

Region: chr23 47784595-47784606. Max. coverage (+): 0. Max coverage (-): 2.6

Region: chr23 47784607-47784618. Max. coverage (+): 0. Max coverage (-): 14.79

Region: chr23 47784619-47784630. Max. coverage (+): 0. Max coverage (-): 14.79

Region: chr23 47784631-47784642. Max. coverage (+): 0. Max coverage (-): 26.29

Region: chr23 47784643-47784654. Max. coverage (+): 0. Max coverage (-): 21.05

Region: chr23 47784655-47784666. Max. coverage (+): 0. Max coverage (-): 12.58

Region: chr23 47784667-47784678. Max. coverage (+): 0. Max coverage (-): 0

Region: chr23 47784679-47784690. Max. coverage (+): 0. Max coverage (-): 0

Region: chr23 47784691-47784702. Max. coverage (+): 0. Max coverage (-): 0

Region: chr23 47784703-47784714. Max. coverage (+): 0. Max coverage (-): 0

Region: chr23 47784715-47784725. Max. coverage (+): 0. Max coverage (-): 0

Region: chr23 47784726-47784737. Max. coverage (+): 0. Max coverage (-): 0

Region: chr23 47784738-47784749. Max. coverage (+): 0. Max coverage (-): 0

Region: chr23 47784750-47784761. Max. coverage (+): 0. Max coverage (-): 0

Region: chr23 47784762-47784773. Max. coverage (+): 0. Max coverage (-): 0

Region: chr23 47784774-47784785. Max. coverage (+): 0. Max coverage (-): 0

Region: chr23 47784786-47784797. Max. coverage (+): 0. Max coverage (-): 0

Region: chr23 47784798-47784809. Max. coverage (+): 0. Max coverage (-): 0

Region: chr23 47784810-47784821. Max. coverage (+): 0. Max coverage (-): 0

Region: chr23 47784822-47784833. Max. coverage (+): 0. Max coverage (-): 0

Region: chr23 47784834-47784845. Max. coverage (+): 0. Max coverage (-): 0

Region: chr23 47784846-47784857. Max. coverage (+): 0. Max coverage (-): 0

Region: chr23 47784858-47784869. Max. coverage (+): 0. Max coverage (-): 0

Region: chr23 47784870-47784880. Max. coverage (+): 0. Max coverage (-): 4.92

Region: chr23 47784881-47784892. Max. coverage (+): 0. Max coverage (-): 4.92

Region: chr23 47784893-47784904. Max. coverage (+): 0. Max coverage (-): 0

Region: chr23 47784905-47784916. Max. coverage (+): 0. Max coverage (-): 0

Region: chr23 47784917-47784928. Max. coverage (+): 0. Max coverage (-): 0

Region: chr23 47784929-47784940. Max. coverage (+): 0. Max coverage (-): 0

Region: chr23 47784941-47784952. Max. coverage (+): 0. Max coverage (-): 0

Region: chr23 47784953-47784964. Max. coverage (+): 0. Max coverage (-): 0

Region: chr23 47784965-47784976. Max. coverage (+): 0. Max coverage (-): 0

Region: chr23 47784977-47784988. Max. coverage (+): 0. Max coverage (-): 0

Region: chr23 47784989-47785000. Max. coverage (+): 0. Max coverage (-): 0

Region: chr23 47785001-47785012. Max. coverage (+): 0. Max coverage (-): 0

Region: chr23 47785013-47785023. Max. coverage (+): 0. Max coverage (-): 0

Region: chr23 47785024-47785035. Max. coverage (+): 0. Max coverage (-): 0

Region: chr23 47785036-47785047. Max. coverage (+): 0. Max coverage (-): 0

Region: chr23 47785048-47785059. Max. coverage (+): 0. Max coverage (-): 0

Region: chr23 47785060-47785071. Max. coverage (+): 0. Max coverage (-): 0

Region: chr23 47785072-47785083. Max. coverage (+): 0. Max coverage (-): 0

Region: chr23 47785084-47785095. Max. coverage (+): 0. Max coverage (-): 0

Region: chr23 47785096-47785107. Max. coverage (+): 0. Max coverage (-): 0

Region: chr23 47785108-47785119. Max. coverage (+): 0. Max coverage (-): 0

Region: chr23 47785120-47785131. Max. coverage (+): 0. Max coverage (-): 0

Region: chr23 47785132-47785143. Max. coverage (+): 0. Max coverage (-): 0

Region: chr23 47785144-47785155. Max. coverage (+): 0. Max coverage (-): 0

Region: chr23 47785156-47785167. Max. coverage (+): 0. Max coverage (-): 0

Region: chr23 47785168-47785178. Max. coverage (+): 0. Max coverage (-): 18.67

Region: chr23 47785179-47785190. Max. coverage (+): 0. Max coverage (-): 8.45

Region: chr23 47785191-47785202. Max. coverage (+): 0. Max coverage (-): 0

Region: chr23 47785203-47785214. Max. coverage (+): 0. Max coverage (-): 0

Region: chr23 47785215-47785226. Max. coverage (+): 0. Max coverage (-): 0

Region: chr23 47785227-47785238. Max. coverage (+): 0. Max coverage (-): 1.48

Region: chr23 47785239-47785250. Max. coverage (+): 0. Max coverage (-): 3.33

Region: chr23 47785251-47785262. Max. coverage (+): 0. Max coverage (-): 8.66

Region: chr23 47785263-47785274. Max. coverage (+): 0. Max coverage (-): 12.69

Region: chr23 47785275-47785286. Max. coverage (+): 0. Max coverage (-): 18.56

Region: chr23 47785287-47785298. Max. coverage (+): 0. Max coverage (-): 15.3

Region: chr23 47785299-47785310. Max. coverage (+): 0. Max coverage (-): 2.01

Region: chr23 47785311-47785321. Max. coverage (+): 0. Max coverage (-): 14.43

Region: chr23 47785322-47785333. Max. coverage (+): 0. Max coverage (-): 26.75

Region: chr23 47785334-47785345. Max. coverage (+): 0. Max coverage (-): 0.96

Region: chr23 47785346-47785357. Max. coverage (+): 0. Max coverage (-): 0

Region: chr23 47785358-47785369. Max. coverage (+): 0. Max coverage (-): 0

Region: chr23 47785370-47785381. Max. coverage (+): 0. Max coverage (-): 0

Region: chr23 47785382-47785393. Max. coverage (+): 0. Max coverage (-): 0

Region: chr23 47785394-47785405. Max. coverage (+): 0. Max coverage (-): 0

Region: chr23 47785406-47785417. Max. coverage (+): 0. Max coverage (-): 0

Region: chr23 47785418-47785429. Max. coverage (+): 0. Max coverage (-): 0

Region: chr23 47785430-47785441. Max. coverage (+): 0. Max coverage (-): 0

Region: chr23 47785442-47785453. Max. coverage (+): 0. Max coverage (-): 0

Region: chr23 47785454-47785465. Max. coverage (+): 0. Max coverage (-): 0

Region: chr23 47785466-47785476. Max. coverage (+): 0. Max coverage (-): 4.87

Region: chr23 47785477-47785488. Max. coverage (+): 0. Max coverage (-): 0

Region: chr23 47785489-47785500. Max. coverage (+): 0. Max coverage (-): 0

Region: chr23 47785501-47785512. Max. coverage (+): 0. Max coverage (-): 0

Region: chr23 47785513-47785524. Max. coverage (+): 0. Max coverage (-): 44.16

Region: chr23 47785525-47785536. Max. coverage (+): 0. Max coverage (-): 44.73

Region: chr23 47785537-47785548. Max. coverage (+): 0. Max coverage (-): 19.71

Region: chr23 47785549-47785560. Max. coverage (+): 0. Max coverage (-): 24.8

Region: chr23 47785561-47785572. Max. coverage (+): 0. Max coverage (-): 29.15

Region: chr23 47785573-47785584. Max. coverage (+): 0. Max coverage (-): 0

Region: chr23 47785585-47785596. Max. coverage (+): 0. Max coverage (-): 9.73

Region: chr23 47785597-47785608. Max. coverage (+): 0. Max coverage (-): 17.02

Region: chr23 47785609-47785619. Max. coverage (+): 0. Max coverage (-): 17.02

Region: chr23 47785620-47785631. Max. coverage (+): 0. Max coverage (-): 2.3

Region: chr23 47785632-47785643. Max. coverage (+): 0. Max coverage (-): 5.59

Region: chr23 47785644-47785655. Max. coverage (+): 0. Max coverage (-): 3.31

Region: chr23 47785656-47785667. Max. coverage (+): 0. Max coverage (-): 11.8

Region: chr23 47785668-47785679. Max. coverage (+): 0. Max coverage (-): 4.58

Region: chr23 47785680-47785691. Max. coverage (+): 0. Max coverage (-): 15.27

Region: chr23 47785692-47785703. Max. coverage (+): 0. Max coverage (-): 15.87

Region: chr23 47785704-47785715. Max. coverage (+): 0. Max coverage (-): 0

Region: chr23 47785716-47785727. Max. coverage (+): 0. Max coverage (-): 0

Region: chr23 47785728-47785739. Max. coverage (+): 0. Max coverage (-): 19.76

Region: chr23 47785740-47785751. Max. coverage (+): 0. Max coverage (-): 45.25

Region: chr23 47785752-47785763. Max. coverage (+): 0. Max coverage (-): 48.08

Region: chr23 47785764-47785774. Max. coverage (+): 0. Max coverage (-): 49.06

Region: chr23 47785775-47785786. Max. coverage (+): 0. Max coverage (-): 20.78

Region: chr23 47785787-47785798. Max. coverage (+): 0. Max coverage (-): 2.29

Region: chr23 47785799-47785810. Max. coverage (+): 0. Max coverage (-): 20.07

Region: chr23 47785811-47785822. Max. coverage (+): 0. Max coverage (-): 20.25

Region: chr23 47785823-47785834. Max. coverage (+): 0. Max coverage (-): 9.34

Region: chr23 47785835-47785846. Max. coverage (+): 0. Max coverage (-): 7.84

Region: chr23 47785847-47785858. Max. coverage (+): 0. Max coverage (-): 0

Region: chr23 47785859-47785870. Max. coverage (+): 0. Max coverage (-): 0.73

Region: chr23 47785871-47785882. Max. coverage (+): 0. Max coverage (-): 0

Region: chr23 47785883-47785894. Max. coverage (+): 0. Max coverage (-): 0

Region: chr23 47785895-47785906. Max. coverage (+): 0. Max coverage (-): 0.96

Region: chr23 47785907-47785917. Max. coverage (+): 0. Max coverage (-): 0.96

Region: chr23 47785918-47785929. Max. coverage (+): 0. Max coverage (-): 0

Region: chr23 47785930-47785941. Max. coverage (+): 0. Max coverage (-): 9.71

Region: chr23 47785942-47785953. Max. coverage (+): 0. Max coverage (-): 5.08

Region: chr23 47785954-47785965. Max. coverage (+): 0. Max coverage (-): 0

Region: chr23 47785966-47785977. Max. coverage (+): 0. Max coverage (-): 12.23

Region: chr23 47785978-47785989. Max. coverage (+): 0. Max coverage (-): 5.57

Region: chr23 47785990-47786001. Max. coverage (+): 0. Max coverage (-): 21.23

Region: chr23 47786002-47786013. Max. coverage (+): 0. Max coverage (-): 11.14

Region: chr23 47786014-47786025. Max. coverage (+): 0. Max coverage (-): 7.29

Region: chr23 47786026-47786037. Max. coverage (+): 0. Max coverage (-): 5.14

Region: chr23 47786038-47786049. Max. coverage (+): 0. Max coverage (-): 32.1

Region: chr23 47786050-47786061. Max. coverage (+): 0. Max coverage (-): 50.39

Region: chr23 47786062-47786072. Max. coverage (+): 0. Max coverage (-): 22.9

Region: chr23 47786073-47786084. Max. coverage (+): 0. Max coverage (-): 21.4

Region: chr23 47786085-47786096. Max. coverage (+): 0. Max coverage (-): 6.13

Region: chr23 47786097-47786108. Max. coverage (+): 0. Max coverage (-): 5.08

Region: chr23 47786109-47786120. Max. coverage (+): 0. Max coverage (-): 11.81

Region: chr23 47786121-47786132. Max. coverage (+): 0. Max coverage (-): 9.36

Region: chr23 47786133-47786144. Max. coverage (+): 0. Max coverage (-): 11.14

Region: chr23 47786145-47786156. Max. coverage (+): 0. Max coverage (-): 22.84

Region: chr23 47786157-47786168. Max. coverage (+): 0. Max coverage (-): 18.99

Region: chr23 47786169-47786180. Max. coverage (+): 0. Max coverage (-): 18.99

Region: chr23 47786181-47786192. Max. coverage (+): 0. Max coverage (-): 13.51

Region: chr23 47786193-47786204. Max. coverage (+): 0. Max coverage (-): 8.04

Region: chr23 47786205-47786215. Max. coverage (+): 0. Max coverage (-): 8.04

Region: chr23 47786216-47786227. Max. coverage (+): 0. Max coverage (-): 11.65

Region: chr23 47786228-47786239. Max. coverage (+): 0. Max coverage (-): 2.86

Region: chr23 47786240-47786251. Max. coverage (+): 0. Max coverage (-): 32.34

Region: chr23 47786252-47786263. Max. coverage (+): 0. Max coverage (-): 30.13

Region: chr23 47786264-47786275. Max. coverage (+): 0. Max coverage (-): 17.9

Region: chr23 47786276-47786287. Max. coverage (+): 0. Max coverage (-): 8.97

Region: chr23 47786288-47786299. Max. coverage (+): 0. Max coverage (-): 12.87

Region: chr23 47786300-47786311. Max. coverage (+): 0. Max coverage (-): 2.22

Region: chr23 47786312-47786323. Max. coverage (+): 0. Max coverage (-): 6.76

Region: chr23 47786324-47786335. Max. coverage (+): 0. Max coverage (-): 16.62

Region: chr23 47786336-47786347. Max. coverage (+): 0. Max coverage (-): 1.47

Region: chr23 47786348-47786359. Max. coverage (+): 0. Max coverage (-): 11.19

Region: chr23 47786360-47786370. Max. coverage (+): 0. Max coverage (-): 30.67

Region: chr23 47786371-47786382. Max. coverage (+): 0. Max coverage (-): 29.95

Region: chr23 47786383-47786394. Max. coverage (+): 0. Max coverage (-): 9.65

Region: chr23 47786395-47786406. Max. coverage (+): 0. Max coverage (-): 15.49

Region: chr23 47786407-47786418. Max. coverage (+): 0. Max coverage (-): 18.01

Region: chr23 47786419-47786430. Max. coverage (+): 0. Max coverage (-): 11.8

Region: chr23 47786431-47786442. Max. coverage (+): 0. Max coverage (-): 16.04

Region: chr23 47786443-47786454. Max. coverage (+): 0. Max coverage (-): 7.76

Region: chr23 47786455-47786466. Max. coverage (+): 0. Max coverage (-): 11.01

Region: chr23 47786467-47786478. Max. coverage (+): 0. Max coverage (-): 7.91

Region: chr23 47786479-47786490. Max. coverage (+): 0. Max coverage (-): 11.8

Region: chr23 47786491-47786502. Max. coverage (+): 0. Max coverage (-): 14.44

Region: chr23 47786503-47786513. Max. coverage (+): 0. Max coverage (-): 92.84

Region: chr23 47786514-47786525. Max. coverage (+): 0. Max coverage (-): 108.41

Region: chr23 47786526-47786537. Max. coverage (+): 0. Max coverage (-): 20.53

Region: chr23 47786538-47786549. Max. coverage (+): 0. Max coverage (-): 19.28

Region: chr23 47786550-47786561. Max. coverage (+): 0. Max coverage (-): 21.37

Region: chr23 47786562-47786573. Max. coverage (+): 0. Max coverage (-): 11.32

Region: chr23 47786574-47786585. Max. coverage (+): 0. Max coverage (-): 5.46

Region: chr23 47786586-47786597. Max. coverage (+): 0. Max coverage (-): 4.43

Region: chr23 47786598-47786609. Max. coverage (+): 0. Max coverage (-): 0

Region: chr23 47786610-47786621. Max. coverage (+): 0. Max coverage (-): 3.5

Region: chr23 47786622-47786633. Max. coverage (+): 0. Max coverage (-): 3.5

Region: chr23 47786634-47786645. Max. coverage (+): 0. Max coverage (-): 0

Region: chr23 47786646-47786657. Max. coverage (+): 0. Max coverage (-): 0

Region: chr23 47786658-47786668. Max. coverage (+): 0. Max coverage (-): 0

Region: chr23 47786669-47786680. Max. coverage (+): 0. Max coverage (-): 2.72

Region: chr23 47786681-47786692. Max. coverage (+): 0. Max coverage (-): 2.72

Region: chr23 47786693-47786704. Max. coverage (+): 0. Max coverage (-): 6.37

Region: chr23 47786705-47786716. Max. coverage (+): 0. Max coverage (-): 6.37

Region: chr23 47786717-47786728. Max. coverage (+): 0. Max coverage (-): 0

Region: chr23 47786729-47786740. Max. coverage (+): 0. Max coverage (-): 0.7

Region: chr23 47786741-47786752. Max. coverage (+): 0. Max coverage (-): 0

Region: chr23 47786753-47786764. Max. coverage (+): 0. Max coverage (-): 10.3

Region: chr23 47786765-47786776. Max. coverage (+): 0. Max coverage (-): 11.16

Region: chr23 47786777-47786788. Max. coverage (+): 0. Max coverage (-): 8.58

Region: chr23 47786789-47786800. Max. coverage (+): 0. Max coverage (-): 9.92

Region: chr23 47786801-47786811. Max. coverage (+): 0. Max coverage (-): 2.1

Region: chr23 47786812-47786823. Max. coverage (+): 0. Max coverage (-): 21.85

Region: chr23 47786824-47786835. Max. coverage (+): 0. Max coverage (-): 14.72

Region: chr23 47786836-47786847. Max. coverage (+): 0. Max coverage (-): 0.98

Region: chr23 47786848-47786859. Max. coverage (+): 0. Max coverage (-): 35.44

Region: chr23 47786860-47786871. Max. coverage (+): 0. Max coverage (-): 31.47

Region: chr23 47786872-47786883. Max. coverage (+): 0. Max coverage (-): 4.69

Region: chr23 47786884-47786895. Max. coverage (+): 0. Max coverage (-): 0

Region: chr23 47786896-47786907. Max. coverage (+): 0. Max coverage (-): 1.5

Region: chr23 47786908-47786919. Max. coverage (+): 0. Max coverage (-): 3.23

Region: chr23 47786920-47786931. Max. coverage (+): 0. Max coverage (-): 24.5

Region: chr23 47786932-47786943. Max. coverage (+): 0. Max coverage (-): 14.3

Region: chr23 47786944-47786955. Max. coverage (+): 0. Max coverage (-): 5.42

Region: chr23 47786956-47786966. Max. coverage (+): 0. Max coverage (-): 12.48

Region: chr23 47786967-47786978. Max. coverage (+): 0. Max coverage (-): 53.64

Region: chr23 47786979-47786990. Max. coverage (+): 0. Max coverage (-): 47.97

Region: chr23 47786991-47787002. Max. coverage (+): 0. Max coverage (-): 2.59

Region: chr23 47787003-47787014. Max. coverage (+): 0. Max coverage (-): 20.49

Region: chr23 47787015-47787026. Max. coverage (+): 0. Max coverage (-): 1.53

Region: chr23 47787027-47787038. Max. coverage (+): 0. Max coverage (-): 2.76

Region: chr23 47787039-47787050. Max. coverage (+): 0. Max coverage (-): 48.29

Region: chr23 47787051-47787062. Max. coverage (+): 0. Max coverage (-): 64.21

Region: chr23 47787063-47787074. Max. coverage (+): 0. Max coverage (-): 4.04

Region: chr23 47787075-47787086. Max. coverage (+): 0. Max coverage (-): 5.43

Region: chr23 47787087-47787098. Max. coverage (+): 0. Max coverage (-): 7.84

Region: chr23 47787099-47787109. Max. coverage (+): 0. Max coverage (-): 4.24

Region: chr23 47787110-47787121. Max. coverage (+): 0. Max coverage (-): 6.8

Region: chr23 47787122-47787133. Max. coverage (+): 0. Max coverage (-): 22.72

Region: chr23 47787134-47787145. Max. coverage (+): 0. Max coverage (-): 22.72

Region: chr23 47787146-47787157. Max. coverage (+): 0. Max coverage (-): 19.84

Region: chr23 47787158-47787169. Max. coverage (+): 0. Max coverage (-): 9.91

Region: chr23 47787170-47787181. Max. coverage (+): 0. Max coverage (-): 7.86

Region: chr23 47787182-47787193. Max. coverage (+): 0. Max coverage (-): 12.43

Region: chr23 47787194-47787205. Max. coverage (+): 0. Max coverage (-): 3.94

Region: chr23 47787206-47787217. Max. coverage (+): 0. Max coverage (-): 0

Region: chr23 47787218-47787229. Max. coverage (+): 0. Max coverage (-): 2.48

Region: chr23 47787230-47787241. Max. coverage (+): 0. Max coverage (-): 12.59

Region: chr23 47787242-47787253. Max. coverage (+): 0. Max coverage (-): 12.59

Region: chr23 47787254-47787264. Max. coverage (+): 0. Max coverage (-): 0

Region: chr23 47787265-47787276. Max. coverage (+): 0. Max coverage (-): 0

Region: chr23 47787277-47787288. Max. coverage (+): 0. Max coverage (-): 0

Region: chr23 47787289-47787300. Max. coverage (+): 0. Max coverage (-): 11.35

Region: chr23 47787301-47787312. Max. coverage (+): 0. Max coverage (-): 13.7

Region: chr23 47787313-47787324. Max. coverage (+): 0. Max coverage (-): 42.01

Region: chr23 47787325-47787336. Max. coverage (+): 0. Max coverage (-): 14.83

Region: chr23 47787337-47787348. Max. coverage (+): 0. Max coverage (-): 0

Region: chr23 47787349-47787360. Max. coverage (+): 0. Max coverage (-): 4.42

Region: chr23 47787361-47787372. Max. coverage (+): 0. Max coverage (-): 3.44

Region: chr23 47787373-47787384. Max. coverage (+): 0. Max coverage (-): 0

Region: chr23 47787385-47787396. Max. coverage (+): 0. Max coverage (-): 0

Region: chr23 47787397-47787407. Max. coverage (+): 0. Max coverage (-): 0

Region: chr23 47787408-47787419. Max. coverage (+): 0. Max coverage (-): 0

Region: chr23 47787420-47787431. Max. coverage (+): 0. Max coverage (-): 0

Region: chr23 47787432-47787443. Max. coverage (+): 0. Max coverage (-): 0.25

Region: chr23 47787444-47787455. Max. coverage (+): 0. Max coverage (-): 0

Region: chr23 47787456-47787467. Max. coverage (+): 0. Max coverage (-): 3.79

Region: chr23 47787468-47787479. Max. coverage (+): 0. Max coverage (-): 9.16

Region: chr23 47787480-47787491. Max. coverage (+): 0. Max coverage (-): 14.48

Region: chr23 47787492-47787503. Max. coverage (+): 0. Max coverage (-): 1.02

Region: chr23 47787504-47787515. Max. coverage (+): 0. Max coverage (-): 11.22

Region: chr23 47787516-47787527. Max. coverage (+): 0. Max coverage (-): 18.02

Region: chr23 47787528-47787539. Max. coverage (+): 0. Max coverage (-): 26.92

Region: chr23 47787540-47787551. Max. coverage (+): 0. Max coverage (-): 26.13

Region: chr23 47787552-47787562. Max. coverage (+): 0. Max coverage (-): 19.97

Region: chr23 47787563-47787574. Max. coverage (+): 0. Max coverage (-): 12.28

Region: chr23 47787575-47787586. Max. coverage (+): 0. Max coverage (-): 3.95

Region: chr23 47787587-47787598. Max. coverage (+): 0. Max coverage (-): 3.85

Region: chr23 47787599-47787610. Max. coverage (+): 0. Max coverage (-): 0

Region: chr23 47787611-47787622. Max. coverage (+): 0. Max coverage (-): 0

Region: chr23 47787623-47787634. Max. coverage (+): 0. Max coverage (-): 5.5

Region: chr23 47787635-47787646. Max. coverage (+): 0. Max coverage (-): 39.9

Region: chr23 47787647-47787658. Max. coverage (+): 0. Max coverage (-): 8.89

Region: chr23 47787659-47787670. Max. coverage (+): 0. Max coverage (-): 8.89

Region: chr23 47787671-47787682. Max. coverage (+): 0. Max coverage (-): 30.31

Region: chr23 47787683-47787694. Max. coverage (+): 0. Max coverage (-): 27.79

Region: chr23 47787695-47787705. Max. coverage (+): 0. Max coverage (-): 27.99

Region: chr23 47787706-47787717. Max. coverage (+): 0. Max coverage (-): 2.48

Region: chr23 47787718-47787729. Max. coverage (+): 0. Max coverage (-): 0

Region: chr23 47787730-47787741. Max. coverage (+): 0. Max coverage (-): 0

Region: chr23 47787742-47787753. Max. coverage (+): 0. Max coverage (-): 0

Region: chr23 47787754-47787765. Max. coverage (+): 0. Max coverage (-): 0

Region: chr23 47787766-47787777. Max. coverage (+): 0. Max coverage (-): 0

Region: chr23 47787778-47787789. Max. coverage (+): 0. Max coverage (-): 0

Region: chr23 47787790-47787801. Max. coverage (+): 0. Max coverage (-): 0

Region: chr23 47787802-47787813. Max. coverage (+): 0. Max coverage (-): 0

Region: chr23 47787814-47787825. Max. coverage (+): 0. Max coverage (-): 0

Region: chr23 47787826-47787837. Max. coverage (+): 0. Max coverage (-): 0

Region: chr23 47787838-47787849. Max. coverage (+): 0. Max coverage (-): 0

Region: chr23 47787850-47787860. Max. coverage (+): 0. Max coverage (-): 0

Region: chr23 47787861-47787872. Max. coverage (+): 0. Max coverage (-): 0

Region: chr23 47787873-47787884. Max. coverage (+): 0. Max coverage (-): 0

Region: chr23 47787885-47787896. Max. coverage (+): 0. Max coverage (-): 0

Region: chr23 47787897-47787908. Max. coverage (+): 0. Max coverage (-): 0

Region: chr23 47787909-47787920. Max. coverage (+): 0. Max coverage (-): 0

Region: chr23 47787921-47787932. Max. coverage (+): 0. Max coverage (-): 0

Region: chr23 47787933-47787944. Max. coverage (+): 0. Max coverage (-): 0

Region: chr23 47787945-47787956. Max. coverage (+): 0. Max coverage (-): 0

Region: chr23 47787957-47787968. Max. coverage (+): 0. Max coverage (-): 0

Region: chr23 47787969-47787980. Max. coverage (+): 0. Max coverage (-): 0

Region: chr23 47787981-47787992. Max. coverage (+): 0. Max coverage (-): 0

Region: chr23 47787993-47788003. Max. coverage (+): 0. Max coverage (-): 0

Region: chr23 47788004-47788015. Max. coverage (+): 0. Max coverage (-): 0

Region: chr23 47788016-47788027. Max. coverage (+): 0. Max coverage (-): 0

Region: chr23 47788028-47788039. Max. coverage (+): 0. Max coverage (-): 0

Region: chr23 47788040-47788051. Max. coverage (+): 0. Max coverage (-): 0

Region: chr23 47788052-47788063. Max. coverage (+): 0. Max coverage (-): 0

Region: chr23 47788064-47788075. Max. coverage (+): 0. Max coverage (-): 0

Region: chr23 47788076-47788087. Max. coverage (+): 0. Max coverage (-): 0

Region: chr23 47788088-47788099. Max. coverage (+): 0. Max coverage (-): 0

Region: chr23 47788100-47788111. Max. coverage (+): 0. Max coverage (-): 0

Region: chr23 47788112-47788123. Max. coverage (+): 0. Max coverage (-): 0

Region: chr23 47788124-47788135. Max. coverage (+): 0. Max coverage (-): 0

Region: chr23 47788136-47788147. Max. coverage (+): 0. Max coverage (-): 0

Region: chr23 47788148-47788158. Max. coverage (+): 0. Max coverage (-): 0

Region: chr23 47788159-47788170. Max. coverage (+): 0. Max coverage (-): 0

Region: chr23 47788171-47788182. Max. coverage (+): 0. Max coverage (-): 0

Region: chr23 47788183-47788194. Max. coverage (+): 0. Max coverage (-): 0

Region: chr23 47788195-47788206. Max. coverage (+): 0. Max coverage (-): 0

Region: chr23 47788207-47788218. Max. coverage (+): 0. Max coverage (-): 0

Region: chr23 47788219-47788230. Max. coverage (+): 0. Max coverage (-): 2.67

Region: chr23 47788231-47788242. Max. coverage (+): 0. Max coverage (-): 2.67

Region: chr23 47788243-47788254. Max. coverage (+): 0. Max coverage (-): 0

Region: chr23 47788255-47788266. Max. coverage (+): 0. Max coverage (-): 0

Region: chr23 47788267-47788278. Max. coverage (+): 0. Max coverage (-): 0

Region: chr23 47788279-47788290. Max. coverage (+): 0. Max coverage (-): 0

Region: chr23 47788291-47788301. Max. coverage (+): 0. Max coverage (-): 0

Region: chr23 47788302-47788313. Max. coverage (+): 0. Max coverage (-): 0

Region: chr23 47788314-47788325. Max. coverage (+): 0. Max coverage (-): 0

Region: chr23 47788326-47788337. Max. coverage (+): 0. Max coverage (-): 1.56

Region: chr23 47788338-47788349. Max. coverage (+): 0. Max coverage (-): 14.48

Region: chr23 47788350-47788361. Max. coverage (+): 0. Max coverage (-): 15.7

Region: chr23 47788362-47788373. Max. coverage (+): 0. Max coverage (-): 21.17

Region: chr23 47788374-47788385. Max. coverage (+): 0. Max coverage (-): 1.24

Region: chr23 47788386-47788397. Max. coverage (+): 0. Max coverage (-): 0

Region: chr23 47788398-47788409. Max. coverage (+): 0. Max coverage (-): 0

Region: chr23 47788410-47788421. Max. coverage (+): 0. Max coverage (-): 0

Region: chr23 47788422-47788433. Max. coverage (+): 0. Max coverage (-): 0

Region: chr23 47788434-47788445. Max. coverage (+): 0. Max coverage (-): 0

Region: chr23 47788446-47788456. Max. coverage (+): 0. Max coverage (-): 0

Region: chr23 47788457-47788468. Max. coverage (+): 0. Max coverage (-): 0

Region: chr23 47788469-47788480. Max. coverage (+): 0. Max coverage (-): 0

Region: chr23 47788481-47788492. Max. coverage (+): 0. Max coverage (-): 0

Region: chr23 47788493-47788504. Max. coverage (+): 0. Max coverage (-): 0

Region: chr23 47788505-47788516. Max. coverage (+): 0. Max coverage (-): 0

Region: chr23 47788517-47788528. Max. coverage (+): 0. Max coverage (-): 0

Region: chr23 47788529-47788540. Max. coverage (+): 0. Max coverage (-): 0

Region: chr23 47788541-47788552. Max. coverage (+): 0. Max coverage (-): 0

Region: chr23 47788553-47788564. Max. coverage (+): 0. Max coverage (-): 0

Region: chr23 47788565-47788576. Max. coverage (+): 0. Max coverage (-): 0

Region: chr23 47788577-47788588. Max. coverage (+): 0. Max coverage (-): 0

Region: chr23 47788589-47788599. Max. coverage (+): 0. Max coverage (-): 0

Region: chr23 47788600-47788611. Max. coverage (+): 0. Max coverage (-): 0

Region: chr23 47788612-47788623. Max. coverage (+): 0. Max coverage (-): 0

Region: chr23 47788624-47788635. Max. coverage (+): 0. Max coverage (-): 0

Region: chr23 47788636-47788647. Max. coverage (+): 0. Max coverage (-): 0

Region: chr23 47788648-47788659. Max. coverage (+): 0. Max coverage (-): 0

Region: chr23 47788660-47788671. Max. coverage (+): 0. Max coverage (-): 0

Region: chr23 47788672-47788683. Max. coverage (+): 0. Max coverage (-): 2.12

Region: chr23 47788684-47788695. Max. coverage (+): 0. Max coverage (-): 3.22

Region: chr23 47788696-47788707. Max. coverage (+): 0. Max coverage (-): 3.22

Region: chr23 47788708-47788719. Max. coverage (+): 0. Max coverage (-): 7.16

Region: chr23 47788720-47788731. Max. coverage (+): 0. Max coverage (-): 7.16

Region: chr23 47788732-47788743. Max. coverage (+): 0. Max coverage (-): 0

Region: chr23 47788744-47788754. Max. coverage (+): 0. Max coverage (-): 0

Region: chr23 47788755-47788766. Max. coverage (+): 0. Max coverage (-): 0

Region: chr23 47788767-47788778. Max. coverage (+): 0. Max coverage (-): 0

Region: chr23 47788779-47788790. Max. coverage (+): 0. Max coverage (-): 0

Region: chr23 47788791-47788802. Max. coverage (+): 0. Max coverage (-): 1.62

Region: chr23 47788803-47788814. Max. coverage (+): 0. Max coverage (-): 0

Region: chr23 47788815-47788826. Max. coverage (+): 0. Max coverage (-): 0

Region: chr23 47788827-47788838. Max. coverage (+): 0. Max coverage (-): 0

Region: chr23 47788839-47788850. Max. coverage (+): 0. Max coverage (-): 0

Region: chr23 47788851-47788862. Max. coverage (+): 0. Max coverage (-): 8.19

Region: chr23 47788863-47788874. Max. coverage (+): 0. Max coverage (-): 0

Region: chr23 47788875-47788886. Max. coverage (+): 0. Max coverage (-): 3.02

Region: chr23 47788887-47788897. Max. coverage (+): 0. Max coverage (-): 2.11

Region: chr23 47788898-47788909. Max. coverage (+): 0. Max coverage (-): 0

Region: chr23 47788910-47788921. Max. coverage (+): 0. Max coverage (-): 0

Region: chr23 47788922-47788933. Max. coverage (+): 0. Max coverage (-): 0

Region: chr23 47788934-47788945. Max. coverage (+): 0. Max coverage (-): 0.41

Region: chr23 47788946-47788957. Max. coverage (+): 0. Max coverage (-): 0.78

Region: chr23 47788958-47788969. Max. coverage (+): 0. Max coverage (-): 0.78

Region: chr23 47788970-47788981. Max. coverage (+): 0. Max coverage (-): 0

Region: chr23 47788982-47788993. Max. coverage (+): 0. Max coverage (-): 5.16

Region: chr23 47788994-47789005. Max. coverage (+): 0. Max coverage (-): 5.16

Region: chr23 47789006-47789017. Max. coverage (+): 0. Max coverage (-): 0

Region: chr23 47789018-47789029. Max. coverage (+): 0. Max coverage (-): 0

Region: chr23 47789030-47789041. Max. coverage (+): 0. Max coverage (-): 0

Region: chr23 47789042-47789052. Max. coverage (+): 0. Max coverage (-): 0

Region: chr23 47789053-47789064. Max. coverage (+): 0. Max coverage (-): 0

Region: chr23 47789065-47789076. Max. coverage (+): 0. Max coverage (-): 0

Region: chr23 47789077-47789088. Max. coverage (+): 0. Max coverage (-): 0

Region: chr23 47789089-47789100. Max. coverage (+): 0. Max coverage (-): 0

Region: chr23 47789101-47789112. Max. coverage (+): 0. Max coverage (-): 0

Region: chr23 47789113-47789124. Max. coverage (+): 0. Max coverage (-): 0

Region: chr23 47789125-47789136. Max. coverage (+): 0. Max coverage (-): 0

Region: chr23 47789137-47789148. Max. coverage (+): 0. Max coverage (-): 0

Region: chr23 47789149-47789160. Max. coverage (+): 0. Max coverage (-): 0

Region: chr23 47789161-47789172. Max. coverage (+): 0. Max coverage (-): 0

Region: chr23 47789173-47789184. Max. coverage (+): 0. Max coverage (-): 0.54

Region: chr23 47789185-47789195. Max. coverage (+): 0. Max coverage (-): 0.54

Region: chr23 47789196-47789207. Max. coverage (+): 0. Max coverage (-): 0.63

Region: chr23 47789208-47789219. Max. coverage (+): 0. Max coverage (-): 0

Region: chr23 47789220-47789231. Max. coverage (+): 0. Max coverage (-): 0

Region: chr23 47789232-47789243. Max. coverage (+): 0. Max coverage (-): 0

Region: chr23 47789244-47789255. Max. coverage (+): 0. Max coverage (-): 0

Region: chr23 47789256-47789267. Max. coverage (+): 0. Max coverage (-): 0

Region: chr23 47789268-47789279. Max. coverage (+): 0. Max coverage (-): 0

Region: chr23 47789280-47789291. Max. coverage (+): 0. Max coverage (-): 0

Region: chr23 47789292-47789303. Max. coverage (+): 0. Max coverage (-): 0

Region: chr23 47789304-47789315. Max. coverage (+): 0. Max coverage (-): 0.71

Region: chr23 47789316-47789327. Max. coverage (+): 0. Max coverage (-): 1.87

Region: chr23 47789328-47789339. Max. coverage (+): 0. Max coverage (-): 0

Region: chr23 47789340-. Max. coverage (+): 0. Max coverage (-): 0

RepeatMasker Color Code

**+**

100-98% Identity

<98-95% Identity

<95-90% Identity

<90-85% Identity

<85-80% Identity

<80-75% Identity

<75-70% Identity

<70% Identity

**-**

Gene Set Color Code

**+**

Gene

Pseudogene

**-**

Topology/Coverage Color Code

Coverage Plus Strand

Coverage Minus Strand

Mainstrand: Plus

Mainstrand: Minus

Complementary Strand

Flanking Region  
(if option -flank >0)

Gene Set Annotation  
  
RepeatMasker Annotation  

**1. L2a**: 47783251-47783387 (-), Divergence to consensus: 38.2%  
**2. Tigger1**: 47783438-47783636 (-), Divergence to consensus: 61.5%  
**3. L1MC5a**: 47784258-47784654 (-), Divergence to consensus: 50.8%  
**4. L1MC5a**: 47784698-47784875 (-), Divergence to consensus: 41.8%  
**5. L2a**: 47785036-47785173 (+), Divergence to consensus: 38.6%  
**6. L2a**: 47787738-47787988 (+), Divergence to consensus: 42.7%  
**7. L2a**: 47788001-47788221 (+), Divergence to consensus: 38.2%  
**8. L2a**: 47788268-47788327 (+), Divergence to consensus: 33.3%  
**9. BOV-A2**: 47788404-47788672 (+), Divergence to consensus: 4.8%

  
Transcription Factor Binding Sites
