# Supplementary material for: Small RNA sequencing of cryopreserved semen from single bull revealed altered miRNAs and piRNAs expression between High- and Low-motile sperm populations
Source: BMC Genomics. 2017 Jan 4;18:14. doi: 10.1186/s12864-016-3394-7 (PMC5209821; doi:10.1186/s12864-016-3394-7)
Supplement: Additional file 3: — Details for each piRNA clusters found in High Motile (HM) sperm fraction. Genes, repeats, transposable elements and transcription factors binding sites falling within the cluster regions were reported. (ZIP 1896 kb) [file 12864_2016_3394_MOESM3_ESM.zip › 56.html]

piRNA cluster 56


Predicted piRNA cluster no. 56     previous   next
  

Show proTRAC run info
Hide proTRAC run info

================================= proTRAC ====================================  
VERSION: 2.1                                    LAST MODIFIED: 06. October 2015  
  
Please cite:  
Rosenkranz D, Zischler H. proTRAC - a software for probabilistic piRNA cluster  
detection, visualization and analysis. 2012. BMC Bioinformatics 13:5.  
  
and (for proTRAC 2.0 and later):  
Rosenkranz D, Rudloff S, Bastuck K, Ketting RF, Zischler H. Tupaia small RNAs  
provide insights into function and evolution of RNAi-based transposon defense  
in mammals. 2015. RNA 21(5):911-922.  
  
Contact:  
David Rosenkranz  
Institute of Anthropology, small RNA group  
Johannes Gutenberg University Mainz  
email: rosenkranz@uni-mainz.de  
  
You can find the latest proTRAC version at:  
http://sourceforge.net/projects/protrac/files  
http://www.smallRNAgroup-mainz.de/software  
==============================================================================  
  
PARAMETERS:  
Map file: .............../storage/core/barbara/genhome/smallRNA/fertility/Sample\_motile/pirna/Sample\_motile\_26-33\_collapsed.fa.no-dust.map.weighted-10000-1000-b-0  
Genome file: ............/storage/core/barbara/genhome/smallRNA/fertility/Sample\_all/pirna/bt\_311\_chrY.fa  
RepeatMasker annotation: /storage/genomes/bt\_umd31/GCF\_000003055.6\_Bos\_taurus\_UMD\_3.1.1\_repeatMasker\_chr.out  
GeneSet:................./storage/core/barbara/genhome/smallRNA/fertility/Sample\_all/pirna/full.gtf  
  
Significant (p<=0.01) hit density will be calculated based  
on observed hit distribution.  
  
Sliding window size: ........................................ 5000 bp  
Sliding window increament: .................................. 1000 bp  
Normalize each hit by number of genomic hits: ............... 1 [0=no/1=yes]  
Normalize each hit by number of sequence reads: ............. 1 [0=no/1=yes]  
Normalize values (-> per million mapped reads): ............. 1 [0=no/1=yes]  
Min. fraction of hits with 1T(U) or 10A: .................... 0.75  
Alternatively: Min. fraction of hits with 1T(U) and 10A: .... 0.5  
Min. fraction of hits with typical piRNA length: ............ 0.75  
Typical piRNA length: ....................................... 26-33 nt  
Min. size of a piRNA cluster: ............................... 5000 bp.  
Min. number of hits (absolute): ............................. 0  
Min. number of hits (normalized): ........................... 0  
Min. fraction of hits on the mainstrand: .................... 0.75  
Top fraction of mapped sequences (in terms of read counts): . 1%  
Top fraction accounts for max. n% of sequence reads: ........ 90%  
Min. fraction of hits on each arm of a bidirectional cluster: 0.1  
Output image file for each cluster: ......................... 0 [0=no/1=yes]  
Output html file for each cluster: .......................... 1 [0=no/1=yes]  
Output a summary table: ..................................... 1 [0=no/1=yes]  
Output a FASTA file for each cluster (piRNA sequences): ..... 1 [0=no/1=yes]  
Output a FASTA file comprising cluster sequences: ........... 1 [0=no/1=yes]  
Search DNA motifs in clusters: .............................. 1 [0=no/1=yes]  
Output flanking sequences: +/- .............................. 0 bp  
Output ~.pTi file: .......................................... 1 [0=no/1=yes]  
==============================================================================  
  
  
Genome size (without gaps): ............ 2678902517 bp  
Gaps (N/X/-): .......................... 53837044 bp  
Mapped reads: .......................... 658825247023  
Non-identical sequences: ............... 514171  
Genomic hits: .......................... 764233  
Significant densitiy of mapped reads: .. 12867599.5173724 reads/kb

Show proTRAC cluster info
Hide proTRAC cluster info

|  |  |
| --- | --- |
| Location | chr24 |
| Coordinates | 43003012-43049352 |
| Size [bp] | 46341 |
| Sequence hit loci | 2323 |
| Mapped reads (normalized) | 2697191239 |
| Mapped reads (normalized) per kb | 58203129.8 |
| Normalized reads with 1T (1U) | 81.9% |
| Normalized reads with 10A | 28.6% |
| Normalized reads with length 26-33 nt | 100% |
| Normalized reads on the main strand(s) | 100% |
| Predicted directionality | mono:minus |

100%

0%

1T (1U)  
reads

10A reads

26-33 nt  
reads

reads on mainstrand

**Either the amount of reads with 1T (1U) OR 10A has to exceed 75% (set with option: -1Tor10A)  
Alternatively the amount of reads with 1T (1U) AND 10A has to exceed 50% (set with option: -1Tand10A)  
Minimum amount of reads with preferred size is 75% (set with option: -pisize)  
Minimum amount of reads on the main strand(s) is 75% (set with option: -clstrand)**

Show read coverage
Hide read coverage

WHAT DO I SEE HERE?  
This chart shows the location of mapped sequence reads within a predicted piRNA cluster. The color refers to the number of genomic hits produced by the sequence read in question. A dark red bar indicates that this sequence read produces many other hits elsewhere in the genome. Many adjacent red or yellow bars can indicate the presence of a multi-copy element such as transposons or rRNA genes. A dark green bar indicates that this sequence read maps uniquely to this locus.

1 hit

2-5 hits

6-10 hits

11-20 hits

21-50 hits

51-100 hits

> 100 hits

chr24

43003012

43049352

Gene Set

RepeatMasker

Mapped  
Reads

83.22

plus strand

minus strand

83.22

Region: chr24 47789341-43003058. Max. coverage (+): 0. Max coverage (-): 3.89

Region: chr24 43003059-43003151. Max. coverage (+): 0. Max coverage (-): 0

Region: chr24 43003152-43003243. Max. coverage (+): 0. Max coverage (-): 0

Region: chr24 43003244-43003336. Max. coverage (+): 0. Max coverage (-): 0

Region: chr24 43003337-43003429. Max. coverage (+): 0. Max coverage (-): 0

Region: chr24 43003430-43003521. Max. coverage (+): 0. Max coverage (-): 0

Region: chr24 43003522-43003614. Max. coverage (+): 0. Max coverage (-): 0

Region: chr24 43003615-43003707. Max. coverage (+): 0. Max coverage (-): 0

Region: chr24 43003708-43003799. Max. coverage (+): 0. Max coverage (-): 0

Region: chr24 43003800-43003892. Max. coverage (+): 0. Max coverage (-): 0

Region: chr24 43003893-43003985. Max. coverage (+): 0. Max coverage (-): 0

Region: chr24 43003986-43004077. Max. coverage (+): 0. Max coverage (-): 0

Region: chr24 43004078-43004170. Max. coverage (+): 0. Max coverage (-): 0

Region: chr24 43004171-43004263. Max. coverage (+): 0. Max coverage (-): 0

Region: chr24 43004264-43004355. Max. coverage (+): 0. Max coverage (-): 5.22

Region: chr24 43004356-43004448. Max. coverage (+): 0. Max coverage (-): 3.38

Region: chr24 43004449-43004541. Max. coverage (+): 0. Max coverage (-): 0

Region: chr24 43004542-43004633. Max. coverage (+): 0. Max coverage (-): 0

Region: chr24 43004634-43004726. Max. coverage (+): 0. Max coverage (-): 4.19

Region: chr24 43004727-43004819. Max. coverage (+): 0. Max coverage (-): 2

Region: chr24 43004820-43004911. Max. coverage (+): 0. Max coverage (-): 3.49

Region: chr24 43004912-43005004. Max. coverage (+): 0. Max coverage (-): 10.3

Region: chr24 43005005-43005097. Max. coverage (+): 0. Max coverage (-): 0

Region: chr24 43005098-43005190. Max. coverage (+): 0. Max coverage (-): 6.39

Region: chr24 43005191-43005282. Max. coverage (+): 0. Max coverage (-): 5.54

Region: chr24 43005283-43005375. Max. coverage (+): 0. Max coverage (-): 3.74

Region: chr24 43005376-43005468. Max. coverage (+): 0. Max coverage (-): 2.01

Region: chr24 43005469-43005560. Max. coverage (+): 0. Max coverage (-): 0

Region: chr24 43005561-43005653. Max. coverage (+): 0. Max coverage (-): 0

Region: chr24 43005654-43005746. Max. coverage (+): 0. Max coverage (-): 2.3

Region: chr24 43005747-43005838. Max. coverage (+): 0. Max coverage (-): 2.3

Region: chr24 43005839-43005931. Max. coverage (+): 0. Max coverage (-): 0

Region: chr24 43005932-43006024. Max. coverage (+): 0. Max coverage (-): 0

Region: chr24 43006025-43006116. Max. coverage (+): 0. Max coverage (-): 0

Region: chr24 43006117-43006209. Max. coverage (+): 0. Max coverage (-): 8.08

Region: chr24 43006210-43006302. Max. coverage (+): 0. Max coverage (-): 5.03

Region: chr24 43006303-43006394. Max. coverage (+): 0. Max coverage (-): 0

Region: chr24 43006395-43006487. Max. coverage (+): 0. Max coverage (-): 0

Region: chr24 43006488-43006580. Max. coverage (+): 0. Max coverage (-): 0

Region: chr24 43006581-43006672. Max. coverage (+): 0. Max coverage (-): 0

Region: chr24 43006673-43006765. Max. coverage (+): 0. Max coverage (-): 0

Region: chr24 43006766-43006858. Max. coverage (+): 0. Max coverage (-): 0

Region: chr24 43006859-43006950. Max. coverage (+): 0. Max coverage (-): 0

Region: chr24 43006951-43007043. Max. coverage (+): 0. Max coverage (-): 1.8

Region: chr24 43007044-43007136. Max. coverage (+): 0. Max coverage (-): 3.46

Region: chr24 43007137-43007229. Max. coverage (+): 0. Max coverage (-): 0

Region: chr24 43007230-43007321. Max. coverage (+): 0. Max coverage (-): 5.81

Region: chr24 43007322-43007414. Max. coverage (+): 0. Max coverage (-): 0

Region: chr24 43007415-43007507. Max. coverage (+): 0. Max coverage (-): 3.5

Region: chr24 43007508-43007599. Max. coverage (+): 0. Max coverage (-): 0

Region: chr24 43007600-43007692. Max. coverage (+): 0. Max coverage (-): 0

Region: chr24 43007693-43007785. Max. coverage (+): 0. Max coverage (-): 0

Region: chr24 43007786-43007877. Max. coverage (+): 0. Max coverage (-): 0

Region: chr24 43007878-43007970. Max. coverage (+): 0. Max coverage (-): 0

Region: chr24 43007971-43008063. Max. coverage (+): 0. Max coverage (-): 0

Region: chr24 43008064-43008155. Max. coverage (+): 0. Max coverage (-): 4.97

Region: chr24 43008156-43008248. Max. coverage (+): 0. Max coverage (-): 1.09

Region: chr24 43008249-43008341. Max. coverage (+): 0. Max coverage (-): 0.94

Region: chr24 43008342-43008433. Max. coverage (+): 0. Max coverage (-): 8.79

Region: chr24 43008434-43008526. Max. coverage (+): 0. Max coverage (-): 0

Region: chr24 43008527-43008619. Max. coverage (+): 0. Max coverage (-): 6.24

Region: chr24 43008620-43008711. Max. coverage (+): 0. Max coverage (-): 0

Region: chr24 43008712-43008804. Max. coverage (+): 0. Max coverage (-): 0

Region: chr24 43008805-43008897. Max. coverage (+): 0. Max coverage (-): 2.48

Region: chr24 43008898-43008989. Max. coverage (+): 0. Max coverage (-): 0

Region: chr24 43008990-43009082. Max. coverage (+): 0. Max coverage (-): 9.42

Region: chr24 43009083-43009175. Max. coverage (+): 0. Max coverage (-): 0

Region: chr24 43009176-43009268. Max. coverage (+): 0. Max coverage (-): 1.93

Region: chr24 43009269-43009360. Max. coverage (+): 0. Max coverage (-): 1.83

Region: chr24 43009361-43009453. Max. coverage (+): 0. Max coverage (-): 7.71

Region: chr24 43009454-43009546. Max. coverage (+): 0. Max coverage (-): 6.5

Region: chr24 43009547-43009638. Max. coverage (+): 0. Max coverage (-): 6.91

Region: chr24 43009639-43009731. Max. coverage (+): 0. Max coverage (-): 0

Region: chr24 43009732-43009824. Max. coverage (+): 0. Max coverage (-): 0

Region: chr24 43009825-43009916. Max. coverage (+): 0. Max coverage (-): 0

Region: chr24 43009917-43010009. Max. coverage (+): 0. Max coverage (-): 0

Region: chr24 43010010-43010102. Max. coverage (+): 0. Max coverage (-): 0

Region: chr24 43010103-43010194. Max. coverage (+): 0. Max coverage (-): 2.44

Region: chr24 43010195-43010287. Max. coverage (+): 0. Max coverage (-): 0

Region: chr24 43010288-43010380. Max. coverage (+): 0. Max coverage (-): 0

Region: chr24 43010381-43010472. Max. coverage (+): 0. Max coverage (-): 15.46

Region: chr24 43010473-43010565. Max. coverage (+): 0. Max coverage (-): 0

Region: chr24 43010566-43010658. Max. coverage (+): 0. Max coverage (-): 0

Region: chr24 43010659-43010750. Max. coverage (+): 0. Max coverage (-): 2.87

Region: chr24 43010751-43010843. Max. coverage (+): 0. Max coverage (-): 0.74

Region: chr24 43010844-43010936. Max. coverage (+): 0. Max coverage (-): 7.29

Region: chr24 43010937-43011028. Max. coverage (+): 0. Max coverage (-): 2.22

Region: chr24 43011029-43011121. Max. coverage (+): 0. Max coverage (-): 3.31

Region: chr24 43011122-43011214. Max. coverage (+): 0. Max coverage (-): 3.78

Region: chr24 43011215-43011307. Max. coverage (+): 0. Max coverage (-): 0

Region: chr24 43011308-43011399. Max. coverage (+): 0. Max coverage (-): 1.28

Region: chr24 43011400-43011492. Max. coverage (+): 0. Max coverage (-): 1.51

Region: chr24 43011493-43011585. Max. coverage (+): 0. Max coverage (-): 0

Region: chr24 43011586-43011677. Max. coverage (+): 0. Max coverage (-): 2.6

Region: chr24 43011678-43011770. Max. coverage (+): 0. Max coverage (-): 0

Region: chr24 43011771-43011863. Max. coverage (+): 0. Max coverage (-): 2.18

Region: chr24 43011864-43011955. Max. coverage (+): 0. Max coverage (-): 0

Region: chr24 43011956-43012048. Max. coverage (+): 0. Max coverage (-): 0

Region: chr24 43012049-43012141. Max. coverage (+): 0. Max coverage (-): 0

Region: chr24 43012142-43012233. Max. coverage (+): 0. Max coverage (-): 0.96

Region: chr24 43012234-43012326. Max. coverage (+): 0. Max coverage (-): 13.63

Region: chr24 43012327-43012419. Max. coverage (+): 0. Max coverage (-): 1.94

Region: chr24 43012420-43012511. Max. coverage (+): 0. Max coverage (-): 21.07

Region: chr24 43012512-43012604. Max. coverage (+): 0. Max coverage (-): 0

Region: chr24 43012605-43012697. Max. coverage (+): 0. Max coverage (-): 0

Region: chr24 43012698-43012789. Max. coverage (+): 0. Max coverage (-): 0

Region: chr24 43012790-43012882. Max. coverage (+): 0. Max coverage (-): 0

Region: chr24 43012883-43012975. Max. coverage (+): 0. Max coverage (-): 0

Region: chr24 43012976-43013067. Max. coverage (+): 0. Max coverage (-): 0

Region: chr24 43013068-43013160. Max. coverage (+): 0. Max coverage (-): 0

Region: chr24 43013161-43013253. Max. coverage (+): 0. Max coverage (-): 0

Region: chr24 43013254-43013346. Max. coverage (+): 0. Max coverage (-): 0

Region: chr24 43013347-43013438. Max. coverage (+): 0. Max coverage (-): 0

Region: chr24 43013439-43013531. Max. coverage (+): 0. Max coverage (-): 0

Region: chr24 43013532-43013624. Max. coverage (+): 0. Max coverage (-): 0

Region: chr24 43013625-43013716. Max. coverage (+): 0. Max coverage (-): 0

Region: chr24 43013717-43013809. Max. coverage (+): 0. Max coverage (-): 0

Region: chr24 43013810-43013902. Max. coverage (+): 0. Max coverage (-): 0

Region: chr24 43013903-43013994. Max. coverage (+): 0. Max coverage (-): 0

Region: chr24 43013995-43014087. Max. coverage (+): 0. Max coverage (-): 6.67

Region: chr24 43014088-43014180. Max. coverage (+): 0. Max coverage (-): 0

Region: chr24 43014181-43014272. Max. coverage (+): 0. Max coverage (-): 1.99

Region: chr24 43014273-43014365. Max. coverage (+): 0. Max coverage (-): 8.25

Region: chr24 43014366-43014458. Max. coverage (+): 0. Max coverage (-): 11.38

Region: chr24 43014459-43014550. Max. coverage (+): 0. Max coverage (-): 16.55

Region: chr24 43014551-43014643. Max. coverage (+): 0. Max coverage (-): 6.4

Region: chr24 43014644-43014736. Max. coverage (+): 0. Max coverage (-): 0

Region: chr24 43014737-43014828. Max. coverage (+): 0. Max coverage (-): 0

Region: chr24 43014829-43014921. Max. coverage (+): 0. Max coverage (-): 0

Region: chr24 43014922-43015014. Max. coverage (+): 0. Max coverage (-): 0

Region: chr24 43015015-43015107. Max. coverage (+): 0. Max coverage (-): 0

Region: chr24 43015108-43015199. Max. coverage (+): 0. Max coverage (-): 0

Region: chr24 43015200-43015292. Max. coverage (+): 0. Max coverage (-): 3.55

Region: chr24 43015293-43015385. Max. coverage (+): 0. Max coverage (-): 0

Region: chr24 43015386-43015477. Max. coverage (+): 0. Max coverage (-): 2.01

Region: chr24 43015478-43015570. Max. coverage (+): 0. Max coverage (-): 36.54

Region: chr24 43015571-43015663. Max. coverage (+): 0. Max coverage (-): 31.99

Region: chr24 43015664-43015755. Max. coverage (+): 0. Max coverage (-): 0

Region: chr24 43015756-43015848. Max. coverage (+): 0. Max coverage (-): 0

Region: chr24 43015849-43015941. Max. coverage (+): 0. Max coverage (-): 0

Region: chr24 43015942-43016033. Max. coverage (+): 0. Max coverage (-): 16.82

Region: chr24 43016034-43016126. Max. coverage (+): 0. Max coverage (-): 25.35

Region: chr24 43016127-43016219. Max. coverage (+): 0. Max coverage (-): 11.83

Region: chr24 43016220-43016311. Max. coverage (+): 0. Max coverage (-): 7.74

Region: chr24 43016312-43016404. Max. coverage (+): 0. Max coverage (-): 4.61

Region: chr24 43016405-43016497. Max. coverage (+): 0. Max coverage (-): 33.81

Region: chr24 43016498-43016589. Max. coverage (+): 0. Max coverage (-): 50.61

Region: chr24 43016590-43016682. Max. coverage (+): 0. Max coverage (-): 0

Region: chr24 43016683-43016775. Max. coverage (+): 0. Max coverage (-): 0

Region: chr24 43016776-43016867. Max. coverage (+): 0. Max coverage (-): 0

Region: chr24 43016868-43016960. Max. coverage (+): 0. Max coverage (-): 0

Region: chr24 43016961-43017053. Max. coverage (+): 0. Max coverage (-): 4.35

Region: chr24 43017054-43017146. Max. coverage (+): 0. Max coverage (-): 13.29

Region: chr24 43017147-43017238. Max. coverage (+): 0. Max coverage (-): 2.69

Region: chr24 43017239-43017331. Max. coverage (+): 0. Max coverage (-): 2.05

Region: chr24 43017332-43017424. Max. coverage (+): 0. Max coverage (-): 20.93

Region: chr24 43017425-43017516. Max. coverage (+): 0. Max coverage (-): 12.22

Region: chr24 43017517-43017609. Max. coverage (+): 0. Max coverage (-): 2.78

Region: chr24 43017610-43017702. Max. coverage (+): 0. Max coverage (-): 10.52

Region: chr24 43017703-43017794. Max. coverage (+): 0. Max coverage (-): 7.2

Region: chr24 43017795-43017887. Max. coverage (+): 0. Max coverage (-): 2.3

Region: chr24 43017888-43017980. Max. coverage (+): 0. Max coverage (-): 20.73

Region: chr24 43017981-43018072. Max. coverage (+): 0. Max coverage (-): 0

Region: chr24 43018073-43018165. Max. coverage (+): 0. Max coverage (-): 0

Region: chr24 43018166-43018258. Max. coverage (+): 0. Max coverage (-): 0

Region: chr24 43018259-43018350. Max. coverage (+): 0. Max coverage (-): 0

Region: chr24 43018351-43018443. Max. coverage (+): 0. Max coverage (-): 0

Region: chr24 43018444-43018536. Max. coverage (+): 0. Max coverage (-): 4.21

Region: chr24 43018537-43018628. Max. coverage (+): 0. Max coverage (-): 0

Region: chr24 43018629-43018721. Max. coverage (+): 0. Max coverage (-): 5.97

Region: chr24 43018722-43018814. Max. coverage (+): 0. Max coverage (-): 10.27

Region: chr24 43018815-43018906. Max. coverage (+): 0. Max coverage (-): 9.38

Region: chr24 43018907-43018999. Max. coverage (+): 0. Max coverage (-): 12.07

Region: chr24 43019000-43019092. Max. coverage (+): 0. Max coverage (-): 20.72

Region: chr24 43019093-43019185. Max. coverage (+): 0. Max coverage (-): 8.82

Region: chr24 43019186-43019277. Max. coverage (+): 0. Max coverage (-): 16.37

Region: chr24 43019278-43019370. Max. coverage (+): 0. Max coverage (-): 0

Region: chr24 43019371-43019463. Max. coverage (+): 0. Max coverage (-): 5.47

Region: chr24 43019464-43019555. Max. coverage (+): 0. Max coverage (-): 6.2

Region: chr24 43019556-43019648. Max. coverage (+): 0. Max coverage (-): 0

Region: chr24 43019649-43019741. Max. coverage (+): 0. Max coverage (-): 14.4

Region: chr24 43019742-43019833. Max. coverage (+): 0. Max coverage (-): 25.73

Region: chr24 43019834-43019926. Max. coverage (+): 0. Max coverage (-): 37.62

Region: chr24 43019927-43020019. Max. coverage (+): 0. Max coverage (-): 10.13

Region: chr24 43020020-43020111. Max. coverage (+): 0. Max coverage (-): 19.48

Region: chr24 43020112-43020204. Max. coverage (+): 0. Max coverage (-): 35.29

Region: chr24 43020205-43020297. Max. coverage (+): 0. Max coverage (-): 55.07

Region: chr24 43020298-43020389. Max. coverage (+): 0. Max coverage (-): 28.45

Region: chr24 43020390-43020482. Max. coverage (+): 0. Max coverage (-): 53.26

Region: chr24 43020483-43020575. Max. coverage (+): 0. Max coverage (-): 83.22

Region: chr24 43020576-43020667. Max. coverage (+): 0. Max coverage (-): 44.19

Region: chr24 43020668-43020760. Max. coverage (+): 0. Max coverage (-): 13.38

Region: chr24 43020761-43020853. Max. coverage (+): 0. Max coverage (-): 11.98

Region: chr24 43020854-43020945. Max. coverage (+): 0. Max coverage (-): 20.02

Region: chr24 43020946-43021038. Max. coverage (+): 0. Max coverage (-): 20.54

Region: chr24 43021039-43021131. Max. coverage (+): 0. Max coverage (-): 14.59

Region: chr24 43021132-43021224. Max. coverage (+): 0. Max coverage (-): 73.09

Region: chr24 43021225-43021316. Max. coverage (+): 0. Max coverage (-): 51.86

Region: chr24 43021317-43021409. Max. coverage (+): 0. Max coverage (-): 35.99

Region: chr24 43021410-43021502. Max. coverage (+): 0. Max coverage (-): 72.88

Region: chr24 43021503-43021594. Max. coverage (+): 0. Max coverage (-): 0

Region: chr24 43021595-43021687. Max. coverage (+): 0. Max coverage (-): 13.11

Region: chr24 43021688-43021780. Max. coverage (+): 0. Max coverage (-): 6.24

Region: chr24 43021781-43021872. Max. coverage (+): 0. Max coverage (-): 1.97

Region: chr24 43021873-43021965. Max. coverage (+): 0. Max coverage (-): 1.97

Region: chr24 43021966-43022058. Max. coverage (+): 0. Max coverage (-): 0

Region: chr24 43022059-43022150. Max. coverage (+): 0. Max coverage (-): 0

Region: chr24 43022151-43022243. Max. coverage (+): 0. Max coverage (-): 2.65

Region: chr24 43022244-43022336. Max. coverage (+): 0. Max coverage (-): 0

Region: chr24 43022337-43022428. Max. coverage (+): 0. Max coverage (-): 0

Region: chr24 43022429-43022521. Max. coverage (+): 0. Max coverage (-): 12.55

Region: chr24 43022522-43022614. Max. coverage (+): 0. Max coverage (-): 6.58

Region: chr24 43022615-43022706. Max. coverage (+): 0. Max coverage (-): 20.9

Region: chr24 43022707-43022799. Max. coverage (+): 0. Max coverage (-): 7.95

Region: chr24 43022800-43022892. Max. coverage (+): 0. Max coverage (-): 17.26

Region: chr24 43022893-43022984. Max. coverage (+): 0. Max coverage (-): 3.91

Region: chr24 43022985-43023077. Max. coverage (+): 0. Max coverage (-): 16.21

Region: chr24 43023078-43023170. Max. coverage (+): 0. Max coverage (-): 19.12

Region: chr24 43023171-43023263. Max. coverage (+): 0. Max coverage (-): 17.79

Region: chr24 43023264-43023355. Max. coverage (+): 0. Max coverage (-): 15.47

Region: chr24 43023356-43023448. Max. coverage (+): 0. Max coverage (-): 17.93

Region: chr24 43023449-43023541. Max. coverage (+): 0. Max coverage (-): 0

Region: chr24 43023542-43023633. Max. coverage (+): 0. Max coverage (-): 0

Region: chr24 43023634-43023726. Max. coverage (+): 0. Max coverage (-): 8.17

Region: chr24 43023727-43023819. Max. coverage (+): 0. Max coverage (-): 13.93

Region: chr24 43023820-43023911. Max. coverage (+): 0. Max coverage (-): 8.79

Region: chr24 43023912-43024004. Max. coverage (+): 0. Max coverage (-): 0

Region: chr24 43024005-43024097. Max. coverage (+): 0. Max coverage (-): 0

Region: chr24 43024098-43024189. Max. coverage (+): 0. Max coverage (-): 0

Region: chr24 43024190-43024282. Max. coverage (+): 0. Max coverage (-): 0

Region: chr24 43024283-43024375. Max. coverage (+): 0. Max coverage (-): 0

Region: chr24 43024376-43024467. Max. coverage (+): 0. Max coverage (-): 0

Region: chr24 43024468-43024560. Max. coverage (+): 0. Max coverage (-): 2.44

Region: chr24 43024561-43024653. Max. coverage (+): 0. Max coverage (-): 0

Region: chr24 43024654-43024745. Max. coverage (+): 0. Max coverage (-): 0

Region: chr24 43024746-43024838. Max. coverage (+): 0. Max coverage (-): 0

Region: chr24 43024839-43024931. Max. coverage (+): 0. Max coverage (-): 0

Region: chr24 43024932-43025023. Max. coverage (+): 0. Max coverage (-): 2

Region: chr24 43025024-43025116. Max. coverage (+): 0. Max coverage (-): 0

Region: chr24 43025117-43025209. Max. coverage (+): 0. Max coverage (-): 4.21

Region: chr24 43025210-43025302. Max. coverage (+): 0. Max coverage (-): 6.92

Region: chr24 43025303-43025394. Max. coverage (+): 0. Max coverage (-): 0

Region: chr24 43025395-43025487. Max. coverage (+): 0. Max coverage (-): 0

Region: chr24 43025488-43025580. Max. coverage (+): 0. Max coverage (-): 0

Region: chr24 43025581-43025672. Max. coverage (+): 0. Max coverage (-): 0

Region: chr24 43025673-43025765. Max. coverage (+): 0. Max coverage (-): 0

Region: chr24 43025766-43025858. Max. coverage (+): 0. Max coverage (-): 0

Region: chr24 43025859-43025950. Max. coverage (+): 0. Max coverage (-): 0

Region: chr24 43025951-43026043. Max. coverage (+): 0. Max coverage (-): 0

Region: chr24 43026044-43026136. Max. coverage (+): 0. Max coverage (-): 0

Region: chr24 43026137-43026228. Max. coverage (+): 0. Max coverage (-): 0.51

Region: chr24 43026229-43026321. Max. coverage (+): 0. Max coverage (-): 0

Region: chr24 43026322-43026414. Max. coverage (+): 0. Max coverage (-): 6.39

Region: chr24 43026415-43026506. Max. coverage (+): 0. Max coverage (-): 7.96

Region: chr24 43026507-43026599. Max. coverage (+): 0. Max coverage (-): 0

Region: chr24 43026600-43026692. Max. coverage (+): 0. Max coverage (-): 3.71

Region: chr24 43026693-43026784. Max. coverage (+): 0. Max coverage (-): 1.78

Region: chr24 43026785-43026877. Max. coverage (+): 0. Max coverage (-): 0

Region: chr24 43026878-43026970. Max. coverage (+): 0. Max coverage (-): 1.77

Region: chr24 43026971-43027062. Max. coverage (+): 0. Max coverage (-): 12.82

Region: chr24 43027063-43027155. Max. coverage (+): 0. Max coverage (-): 4.4

Region: chr24 43027156-43027248. Max. coverage (+): 0. Max coverage (-): 13.66

Region: chr24 43027249-43027341. Max. coverage (+): 0. Max coverage (-): 0

Region: chr24 43027342-43027433. Max. coverage (+): 0. Max coverage (-): 11.32

Region: chr24 43027434-43027526. Max. coverage (+): 0. Max coverage (-): 8.4

Region: chr24 43027527-43027619. Max. coverage (+): 0. Max coverage (-): 12.39

Region: chr24 43027620-43027711. Max. coverage (+): 0. Max coverage (-): 12.26

Region: chr24 43027712-43027804. Max. coverage (+): 0. Max coverage (-): 8.84

Region: chr24 43027805-43027897. Max. coverage (+): 0. Max coverage (-): 15.64

Region: chr24 43027898-43027989. Max. coverage (+): 0. Max coverage (-): 6.63

Region: chr24 43027990-43028082. Max. coverage (+): 0. Max coverage (-): 2.55

Region: chr24 43028083-43028175. Max. coverage (+): 0. Max coverage (-): 0

Region: chr24 43028176-43028267. Max. coverage (+): 0. Max coverage (-): 0

Region: chr24 43028268-43028360. Max. coverage (+): 0. Max coverage (-): 0

Region: chr24 43028361-43028453. Max. coverage (+): 0. Max coverage (-): 0

Region: chr24 43028454-43028545. Max. coverage (+): 0. Max coverage (-): 2.17

Region: chr24 43028546-43028638. Max. coverage (+): 0. Max coverage (-): 0

Region: chr24 43028639-43028731. Max. coverage (+): 0. Max coverage (-): 0

Region: chr24 43028732-43028823. Max. coverage (+): 0. Max coverage (-): 0

Region: chr24 43028824-43028916. Max. coverage (+): 0. Max coverage (-): 0

Region: chr24 43028917-43029009. Max. coverage (+): 0. Max coverage (-): 8.11

Region: chr24 43029010-43029101. Max. coverage (+): 0. Max coverage (-): 8.54

Region: chr24 43029102-43029194. Max. coverage (+): 0. Max coverage (-): 0

Region: chr24 43029195-43029287. Max. coverage (+): 0. Max coverage (-): 0.58

Region: chr24 43029288-43029380. Max. coverage (+): 0. Max coverage (-): 6.37

Region: chr24 43029381-43029472. Max. coverage (+): 0. Max coverage (-): 0

Region: chr24 43029473-43029565. Max. coverage (+): 0. Max coverage (-): 0

Region: chr24 43029566-43029658. Max. coverage (+): 0. Max coverage (-): 0

Region: chr24 43029659-43029750. Max. coverage (+): 0. Max coverage (-): 0.79

Region: chr24 43029751-43029843. Max. coverage (+): 0. Max coverage (-): 0

Region: chr24 43029844-43029936. Max. coverage (+): 0. Max coverage (-): 0

Region: chr24 43029937-43030028. Max. coverage (+): 0. Max coverage (-): 0

Region: chr24 43030029-43030121. Max. coverage (+): 0. Max coverage (-): 0

Region: chr24 43030122-43030214. Max. coverage (+): 0. Max coverage (-): 0

Region: chr24 43030215-43030306. Max. coverage (+): 0. Max coverage (-): 0

Region: chr24 43030307-43030399. Max. coverage (+): 0. Max coverage (-): 0

Region: chr24 43030400-43030492. Max. coverage (+): 0. Max coverage (-): 0

Region: chr24 43030493-43030584. Max. coverage (+): 0. Max coverage (-): 0

Region: chr24 43030585-43030677. Max. coverage (+): 0. Max coverage (-): 0

Region: chr24 43030678-43030770. Max. coverage (+): 0. Max coverage (-): 0

Region: chr24 43030771-43030862. Max. coverage (+): 0. Max coverage (-): 0

Region: chr24 43030863-43030955. Max. coverage (+): 0. Max coverage (-): 0

Region: chr24 43030956-43031048. Max. coverage (+): 0. Max coverage (-): 4.86

Region: chr24 43031049-43031140. Max. coverage (+): 0. Max coverage (-): 1.96

Region: chr24 43031141-43031233. Max. coverage (+): 0. Max coverage (-): 0

Region: chr24 43031234-43031326. Max. coverage (+): 0. Max coverage (-): 0

Region: chr24 43031327-43031419. Max. coverage (+): 0. Max coverage (-): 0

Region: chr24 43031420-43031511. Max. coverage (+): 0. Max coverage (-): 0

Region: chr24 43031512-43031604. Max. coverage (+): 0. Max coverage (-): 0

Region: chr24 43031605-43031697. Max. coverage (+): 0. Max coverage (-): 2.84

Region: chr24 43031698-43031789. Max. coverage (+): 0. Max coverage (-): 0

Region: chr24 43031790-43031882. Max. coverage (+): 0. Max coverage (-): 0.55

Region: chr24 43031883-43031975. Max. coverage (+): 0. Max coverage (-): 0

Region: chr24 43031976-43032067. Max. coverage (+): 0. Max coverage (-): 2.98

Region: chr24 43032068-43032160. Max. coverage (+): 0. Max coverage (-): 4.7

Region: chr24 43032161-43032253. Max. coverage (+): 0. Max coverage (-): 0

Region: chr24 43032254-43032345. Max. coverage (+): 0. Max coverage (-): 1.64

Region: chr24 43032346-43032438. Max. coverage (+): 0. Max coverage (-): 2.98

Region: chr24 43032439-43032531. Max. coverage (+): 0. Max coverage (-): 11.77

Region: chr24 43032532-43032623. Max. coverage (+): 0. Max coverage (-): 4.19

Region: chr24 43032624-43032716. Max. coverage (+): 0. Max coverage (-): 2.07

Region: chr24 43032717-43032809. Max. coverage (+): 0. Max coverage (-): 1.23

Region: chr24 43032810-43032901. Max. coverage (+): 0. Max coverage (-): 7.57

Region: chr24 43032902-43032994. Max. coverage (+): 0. Max coverage (-): 5.28

Region: chr24 43032995-43033087. Max. coverage (+): 0. Max coverage (-): 0.8

Region: chr24 43033088-43033179. Max. coverage (+): 0. Max coverage (-): 0

Region: chr24 43033180-43033272. Max. coverage (+): 0. Max coverage (-): 4.56

Region: chr24 43033273-43033365. Max. coverage (+): 0. Max coverage (-): 3.32

Region: chr24 43033366-43033458. Max. coverage (+): 0. Max coverage (-): 1.22

Region: chr24 43033459-43033550. Max. coverage (+): 0. Max coverage (-): 0

Region: chr24 43033551-43033643. Max. coverage (+): 0. Max coverage (-): 0

Region: chr24 43033644-43033736. Max. coverage (+): 0. Max coverage (-): 0

Region: chr24 43033737-43033828. Max. coverage (+): 0. Max coverage (-): 0

Region: chr24 43033829-43033921. Max. coverage (+): 0. Max coverage (-): 0

Region: chr24 43033922-43034014. Max. coverage (+): 0. Max coverage (-): 0

Region: chr24 43034015-43034106. Max. coverage (+): 0. Max coverage (-): 0

Region: chr24 43034107-43034199. Max. coverage (+): 0. Max coverage (-): 0

Region: chr24 43034200-43034292. Max. coverage (+): 0. Max coverage (-): 0

Region: chr24 43034293-43034384. Max. coverage (+): 0. Max coverage (-): 0

Region: chr24 43034385-43034477. Max. coverage (+): 0. Max coverage (-): 0

Region: chr24 43034478-43034570. Max. coverage (+): 0. Max coverage (-): 0

Region: chr24 43034571-43034662. Max. coverage (+): 0. Max coverage (-): 0

Region: chr24 43034663-43034755. Max. coverage (+): 0. Max coverage (-): 0

Region: chr24 43034756-43034848. Max. coverage (+): 0. Max coverage (-): 0

Region: chr24 43034849-43034940. Max. coverage (+): 0. Max coverage (-): 0

Region: chr24 43034941-43035033. Max. coverage (+): 0. Max coverage (-): 0

Region: chr24 43035034-43035126. Max. coverage (+): 0. Max coverage (-): 0

Region: chr24 43035127-43035218. Max. coverage (+): 0. Max coverage (-): 0

Region: chr24 43035219-43035311. Max. coverage (+): 0. Max coverage (-): 0

Region: chr24 43035312-43035404. Max. coverage (+): 0. Max coverage (-): 0

Region: chr24 43035405-43035497. Max. coverage (+): 0. Max coverage (-): 0

Region: chr24 43035498-43035589. Max. coverage (+): 0. Max coverage (-): 0

Region: chr24 43035590-43035682. Max. coverage (+): 0. Max coverage (-): 0

Region: chr24 43035683-43035775. Max. coverage (+): 0. Max coverage (-): 0

Region: chr24 43035776-43035867. Max. coverage (+): 0. Max coverage (-): 0

Region: chr24 43035868-43035960. Max. coverage (+): 0. Max coverage (-): 0

Region: chr24 43035961-43036053. Max. coverage (+): 0. Max coverage (-): 0

Region: chr24 43036054-43036145. Max. coverage (+): 0. Max coverage (-): 0

Region: chr24 43036146-43036238. Max. coverage (+): 0. Max coverage (-): 0

Region: chr24 43036239-43036331. Max. coverage (+): 0. Max coverage (-): 0

Region: chr24 43036332-43036423. Max. coverage (+): 0. Max coverage (-): 0

Region: chr24 43036424-43036516. Max. coverage (+): 0. Max coverage (-): 0

Region: chr24 43036517-43036609. Max. coverage (+): 0. Max coverage (-): 0

Region: chr24 43036610-43036701. Max. coverage (+): 0. Max coverage (-): 0

Region: chr24 43036702-43036794. Max. coverage (+): 0. Max coverage (-): 0

Region: chr24 43036795-43036887. Max. coverage (+): 0. Max coverage (-): 0

Region: chr24 43036888-43036979. Max. coverage (+): 0. Max coverage (-): 0

Region: chr24 43036980-43037072. Max. coverage (+): 0. Max coverage (-): 0

Region: chr24 43037073-43037165. Max. coverage (+): 0. Max coverage (-): 0

Region: chr24 43037166-43037257. Max. coverage (+): 0. Max coverage (-): 0

Region: chr24 43037258-43037350. Max. coverage (+): 0. Max coverage (-): 0

Region: chr24 43037351-43037443. Max. coverage (+): 0. Max coverage (-): 1.81

Region: chr24 43037444-43037536. Max. coverage (+): 0. Max coverage (-): 0

Region: chr24 43037537-43037628. Max. coverage (+): 0. Max coverage (-): 0

Region: chr24 43037629-43037721. Max. coverage (+): 0. Max coverage (-): 0

Region: chr24 43037722-43037814. Max. coverage (+): 0. Max coverage (-): 0

Region: chr24 43037815-43037906. Max. coverage (+): 0. Max coverage (-): 0

Region: chr24 43037907-43037999. Max. coverage (+): 0. Max coverage (-): 0

Region: chr24 43038000-43038092. Max. coverage (+): 0. Max coverage (-): 0

Region: chr24 43038093-43038184. Max. coverage (+): 0. Max coverage (-): 0

Region: chr24 43038185-43038277. Max. coverage (+): 0. Max coverage (-): 0

Region: chr24 43038278-43038370. Max. coverage (+): 0. Max coverage (-): 0

Region: chr24 43038371-43038462. Max. coverage (+): 0. Max coverage (-): 0

Region: chr24 43038463-43038555. Max. coverage (+): 0. Max coverage (-): 0

Region: chr24 43038556-43038648. Max. coverage (+): 0. Max coverage (-): 0

Region: chr24 43038649-43038740. Max. coverage (+): 0. Max coverage (-): 0

Region: chr24 43038741-43038833. Max. coverage (+): 0. Max coverage (-): 2.68

Region: chr24 43038834-43038926. Max. coverage (+): 0. Max coverage (-): 5.55

Region: chr24 43038927-43039018. Max. coverage (+): 0. Max coverage (-): 0

Region: chr24 43039019-43039111. Max. coverage (+): 0. Max coverage (-): 0.5

Region: chr24 43039112-43039204. Max. coverage (+): 0. Max coverage (-): 0

Region: chr24 43039205-43039297. Max. coverage (+): 0. Max coverage (-): 0

Region: chr24 43039298-43039389. Max. coverage (+): 0. Max coverage (-): 0

Region: chr24 43039390-43039482. Max. coverage (+): 0. Max coverage (-): 0

Region: chr24 43039483-43039575. Max. coverage (+): 0. Max coverage (-): 0

Region: chr24 43039576-43039667. Max. coverage (+): 0. Max coverage (-): 0

Region: chr24 43039668-43039760. Max. coverage (+): 0. Max coverage (-): 0

Region: chr24 43039761-43039853. Max. coverage (+): 0. Max coverage (-): 5.71

Region: chr24 43039854-43039945. Max. coverage (+): 0. Max coverage (-): 2.3

Region: chr24 43039946-43040038. Max. coverage (+): 0. Max coverage (-): 0

Region: chr24 43040039-43040131. Max. coverage (+): 0. Max coverage (-): 0

Region: chr24 43040132-43040223. Max. coverage (+): 0. Max coverage (-): 0

Region: chr24 43040224-43040316. Max. coverage (+): 0. Max coverage (-): 0.59

Region: chr24 43040317-43040409. Max. coverage (+): 0. Max coverage (-): 0

Region: chr24 43040410-43040501. Max. coverage (+): 0. Max coverage (-): 0

Region: chr24 43040502-43040594. Max. coverage (+): 0. Max coverage (-): 0

Region: chr24 43040595-43040687. Max. coverage (+): 0. Max coverage (-): 0

Region: chr24 43040688-43040779. Max. coverage (+): 0. Max coverage (-): 0

Region: chr24 43040780-43040872. Max. coverage (+): 0. Max coverage (-): 14.45

Region: chr24 43040873-43040965. Max. coverage (+): 0. Max coverage (-): 4.92

Region: chr24 43040966-43041057. Max. coverage (+): 0. Max coverage (-): 2.8

Region: chr24 43041058-43041150. Max. coverage (+): 0. Max coverage (-): 11.96

Region: chr24 43041151-43041243. Max. coverage (+): 0. Max coverage (-): 0

Region: chr24 43041244-43041336. Max. coverage (+): 0. Max coverage (-): 0

Region: chr24 43041337-43041428. Max. coverage (+): 0. Max coverage (-): 0

Region: chr24 43041429-43041521. Max. coverage (+): 0. Max coverage (-): 1.25

Region: chr24 43041522-43041614. Max. coverage (+): 0. Max coverage (-): 47.89

Region: chr24 43041615-43041706. Max. coverage (+): 0. Max coverage (-): 19.52

Region: chr24 43041707-43041799. Max. coverage (+): 0. Max coverage (-): 8.58

Region: chr24 43041800-43041892. Max. coverage (+): 0. Max coverage (-): 9.89

Region: chr24 43041893-43041984. Max. coverage (+): 0. Max coverage (-): 17.35

Region: chr24 43041985-43042077. Max. coverage (+): 0. Max coverage (-): 7.55

Region: chr24 43042078-43042170. Max. coverage (+): 0. Max coverage (-): 0

Region: chr24 43042171-43042262. Max. coverage (+): 0. Max coverage (-): 0

Region: chr24 43042263-43042355. Max. coverage (+): 0. Max coverage (-): 0

Region: chr24 43042356-43042448. Max. coverage (+): 0. Max coverage (-): 0

Region: chr24 43042449-43042540. Max. coverage (+): 0. Max coverage (-): 0

Region: chr24 43042541-43042633. Max. coverage (+): 0. Max coverage (-): 0

Region: chr24 43042634-43042726. Max. coverage (+): 0. Max coverage (-): 0

Region: chr24 43042727-43042818. Max. coverage (+): 0. Max coverage (-): 0

Region: chr24 43042819-43042911. Max. coverage (+): 0. Max coverage (-): 0

Region: chr24 43042912-43043004. Max. coverage (+): 0. Max coverage (-): 0

Region: chr24 43043005-43043096. Max. coverage (+): 0. Max coverage (-): 0

Region: chr24 43043097-43043189. Max. coverage (+): 0. Max coverage (-): 0

Region: chr24 43043190-43043282. Max. coverage (+): 0. Max coverage (-): 0

Region: chr24 43043283-43043375. Max. coverage (+): 0. Max coverage (-): 0

Region: chr24 43043376-43043467. Max. coverage (+): 0. Max coverage (-): 0

Region: chr24 43043468-43043560. Max. coverage (+): 0. Max coverage (-): 0

Region: chr24 43043561-43043653. Max. coverage (+): 0. Max coverage (-): 0

Region: chr24 43043654-43043745. Max. coverage (+): 0. Max coverage (-): 0

Region: chr24 43043746-43043838. Max. coverage (+): 0. Max coverage (-): 0

Region: chr24 43043839-43043931. Max. coverage (+): 0. Max coverage (-): 0

Region: chr24 43043932-43044023. Max. coverage (+): 0. Max coverage (-): 0

Region: chr24 43044024-43044116. Max. coverage (+): 0. Max coverage (-): 0

Region: chr24 43044117-43044209. Max. coverage (+): 0. Max coverage (-): 0

Region: chr24 43044210-43044301. Max. coverage (+): 0. Max coverage (-): 0

Region: chr24 43044302-43044394. Max. coverage (+): 0. Max coverage (-): 0

Region: chr24 43044395-43044487. Max. coverage (+): 0. Max coverage (-): 0

Region: chr24 43044488-43044579. Max. coverage (+): 0. Max coverage (-): 0

Region: chr24 43044580-43044672. Max. coverage (+): 0. Max coverage (-): 0

Region: chr24 43044673-43044765. Max. coverage (+): 0. Max coverage (-): 0

Region: chr24 43044766-43044857. Max. coverage (+): 0. Max coverage (-): 0

Region: chr24 43044858-43044950. Max. coverage (+): 0. Max coverage (-): 0

Region: chr24 43044951-43045043. Max. coverage (+): 0. Max coverage (-): 0

Region: chr24 43045044-43045135. Max. coverage (+): 0. Max coverage (-): 0

Region: chr24 43045136-43045228. Max. coverage (+): 0. Max coverage (-): 0

Region: chr24 43045229-43045321. Max. coverage (+): 0. Max coverage (-): 0

Region: chr24 43045322-43045414. Max. coverage (+): 0. Max coverage (-): 0

Region: chr24 43045415-43045506. Max. coverage (+): 0. Max coverage (-): 0

Region: chr24 43045507-43045599. Max. coverage (+): 0. Max coverage (-): 4.57

Region: chr24 43045600-43045692. Max. coverage (+): 0. Max coverage (-): 0

Region: chr24 43045693-43045784. Max. coverage (+): 0. Max coverage (-): 0

Region: chr24 43045785-43045877. Max. coverage (+): 0. Max coverage (-): 6.08

Region: chr24 43045878-43045970. Max. coverage (+): 0. Max coverage (-): 19.16

Region: chr24 43045971-43046062. Max. coverage (+): 0. Max coverage (-): 0

Region: chr24 43046063-43046155. Max. coverage (+): 0. Max coverage (-): 5.62

Region: chr24 43046156-43046248. Max. coverage (+): 0. Max coverage (-): 0.77

Region: chr24 43046249-43046340. Max. coverage (+): 0. Max coverage (-): 4.65

Region: chr24 43046341-43046433. Max. coverage (+): 0. Max coverage (-): 1.56

Region: chr24 43046434-43046526. Max. coverage (+): 0. Max coverage (-): 0

Region: chr24 43046527-43046618. Max. coverage (+): 0. Max coverage (-): 1.02

Region: chr24 43046619-43046711. Max. coverage (+): 0. Max coverage (-): 11.91

Region: chr24 43046712-43046804. Max. coverage (+): 0. Max coverage (-): 1.1

Region: chr24 43046805-43046896. Max. coverage (+): 0. Max coverage (-): 0

Region: chr24 43046897-43046989. Max. coverage (+): 0. Max coverage (-): 0.88

Region: chr24 43046990-43047082. Max. coverage (+): 0. Max coverage (-): 2.46

Region: chr24 43047083-43047174. Max. coverage (+): 0. Max coverage (-): 10.95

Region: chr24 43047175-43047267. Max. coverage (+): 0. Max coverage (-): 1.32

Region: chr24 43047268-43047360. Max. coverage (+): 0. Max coverage (-): 5.9

Region: chr24 43047361-43047453. Max. coverage (+): 0. Max coverage (-): 1.94

Region: chr24 43047454-43047545. Max. coverage (+): 0. Max coverage (-): 12.46

Region: chr24 43047546-43047638. Max. coverage (+): 0. Max coverage (-): 6.46

Region: chr24 43047639-43047731. Max. coverage (+): 0. Max coverage (-): 15.04

Region: chr24 43047732-43047823. Max. coverage (+): 0. Max coverage (-): 4.38

Region: chr24 43047824-43047916. Max. coverage (+): 0. Max coverage (-): 1.36

Region: chr24 43047917-43048009. Max. coverage (+): 0. Max coverage (-): 5.62

Region: chr24 43048010-43048101. Max. coverage (+): 0. Max coverage (-): 12.08

Region: chr24 43048102-43048194. Max. coverage (+): 0. Max coverage (-): 0

Region: chr24 43048195-43048287. Max. coverage (+): 0. Max coverage (-): 25.59

Region: chr24 43048288-43048379. Max. coverage (+): 0. Max coverage (-): 0

Region: chr24 43048380-43048472. Max. coverage (+): 0. Max coverage (-): 0

Region: chr24 43048473-43048565. Max. coverage (+): 0. Max coverage (-): 1.71

Region: chr24 43048566-43048657. Max. coverage (+): 0. Max coverage (-): 14.95

Region: chr24 43048658-43048750. Max. coverage (+): 0. Max coverage (-): 19.62

Region: chr24 43048751-43048843. Max. coverage (+): 0. Max coverage (-): 0

Region: chr24 43048844-43048935. Max. coverage (+): 0. Max coverage (-): 9.76

Region: chr24 43048936-43049028. Max. coverage (+): 0. Max coverage (-): 14.5

Region: chr24 43049029-43049121. Max. coverage (+): 0. Max coverage (-): 4.8

Region: chr24 43049122-43049213. Max. coverage (+): 0. Max coverage (-): 20.21

Region: chr24 43049214-43049306. Max. coverage (+): 0. Max coverage (-): 21.62

Region: chr24 43049307-. Max. coverage (+): 0. Max coverage (-): 15.33

RepeatMasker Color Code

**+**

100-98% Identity

<98-95% Identity

<95-90% Identity

<90-85% Identity

<85-80% Identity

<80-75% Identity

<75-70% Identity

<70% Identity

**-**

Gene Set Color Code

**+**

Gene

Pseudogene

**-**

Topology/Coverage Color Code

Coverage Plus Strand

Coverage Minus Strand

Mainstrand: Plus

Mainstrand: Minus

Complementary Strand

Flanking Region  
(if option -flank >0)

Gene Set Annotation  
  
RepeatMasker Annotation  

**1. BOV-A2**: 43003059-43003329 (+), Divergence to consensus: 5.2%  
**2. AT\_rich**: 43003394-43003421 (+), Divergence to consensus: 60.7%  
**3. L1\_BT**: 43003930-43004028 (-), Divergence to consensus: 21.7%  
**4. LTR88a**: 43005558-43005709 (-), Divergence to consensus: 42.2%  
**5. (TG)n**: 43006404-43006503 (+), Divergence to consensus: 15.2%  
**6. Bov-tA1**: 43006551-43006775 (+), Divergence to consensus: 13.3%  
**7. MLT1G3**: 43007535-43008080 (+), Divergence to consensus: 46%  
**8. LTR16A**: 43009117-43009193 (+), Divergence to consensus: 29.3%  
**9. L1-2\_BT**: 43009627-43009717 (+), Divergence to consensus: 18.7%  
**10. (A)n**: 43009722-43009754 (+), Divergence to consensus: 12.1%  
**11. MER20B**: 43009776-43009869 (-), Divergence to consensus: 29%  
**12. MIR3**: 43010259-43010430 (+), Divergence to consensus: 47%  
**13. L2a**: 43010603-43010679 (+), Divergence to consensus: 36.6%  
**14. L3**: 43010790-43010843 (+), Divergence to consensus: 36.8%  
**15. MIRb**: 43011273-43011336 (+), Divergence to consensus: 34.3%  
**16. L1-2\_BT**: 43012524-43012619 (+), Divergence to consensus: 26%  
**17. ART2A**: 43012625-43013157 (-), Divergence to consensus: 15.4%  
**18. BovB**: 43013159-43013741 (-), Divergence to consensus: 15.8%  
**19. A-rich**: 43013758-43013817 (+), Divergence to consensus: 15.3%  
**20. LTR39B\_BT**: 43014626-43015234 (-), Divergence to consensus: 26.7%  
**21. L2b**: 43015665-43015952 (+), Divergence to consensus: 42.9%  
**22. L2b**: 43016357-43016471 (+), Divergence to consensus: 40.7%  
**23. L2b**: 43016587-43016958 (+), Divergence to consensus: 47.7%  
**24. AT\_rich**: 43017564-43017600 (+), Divergence to consensus: 64.9%  
**25. L1MB1**: 43017974-43018176 (-), Divergence to consensus: 28.6%  
**26. L1MA9**: 43018176-43018274 (+), Divergence to consensus: 22.4%  
**27. Bov-tA1**: 43018277-43018491 (+), Divergence to consensus: 16.8%  
**28. Bov-tA2**: 43019298-43019394 (-), Divergence to consensus: 21.6%  
**29. MIRb**: 43020797-43020856 (-), Divergence to consensus: 36.6%  
**30. Bov-tA2**: 43021510-43021622 (+), Divergence to consensus: 18.6%  
**31. Tigger19a**: 43022236-43022340 (-), Divergence to consensus: 44.4%  
**32. L1-3\_BT**: 43023428-43023684 (+), Divergence to consensus: 21.1%  
**33. Bov-tA2**: 43023886-43024086 (+), Divergence to consensus: 14.5%  
**34. AT\_rich**: 43024634-43024654 (+), Divergence to consensus: 57.1%  
**35. L1M4**: 43024659-43024737 (+), Divergence to consensus: 21.7%  
**36. BovB**: 43025342-43025623 (+), Divergence to consensus: 13.1%  
**37. ART2A**: 43025624-43026164 (+), Divergence to consensus: 12.5%  
**38. AT\_rich**: 43026878-43026905 (+), Divergence to consensus: 46.4%  
**39. CHR-2B**: 43028014-43028292 (-), Divergence to consensus: 32.2%  
**40. MER58A**: 43028294-43028467 (+), Divergence to consensus: 39.3%  
**41. BovB**: 43029765-43030372 (+), Divergence to consensus: 3.7%  
**42. ART2A**: 43030373-43030890 (+), Divergence to consensus: 9.9%  
**43. (ATCTG)n**: 43030891-43030914 (+), Divergence to consensus: 4.2%  
**44. Bov-tA2**: 43031287-43031468 (+), Divergence to consensus: 24.2%  
**45. AT\_rich**: 43032223-43032248 (+), Divergence to consensus: 80.8%  
**46. L1\_BT**: 43033501-43033954 (-), Divergence to consensus: 9.5%  
**47. BTLTR1J**: 43033955-43034006 (+), Divergence to consensus: 23.1%  
**48. L1\_BT**: 43034007-43037001 (-), Divergence to consensus: 2.6%  
**49. MIR**: 43037016-43037179 (+), Divergence to consensus: 43.5%  
**50. Bov-tA2**: 43037203-43037325 (+), Divergence to consensus: 16.2%  
**51. SINE2-1\_BT**: 43037541-43037660 (-), Divergence to consensus: 23.3%  
**52. Bov-tA1**: 43037689-43037889 (+), Divergence to consensus: 25.9%  
**53. (TTTG)n**: 43037912-43037939 (+), Divergence to consensus: 3.6%  
**54. MER57LA**: 43037955-43038249 (-), Divergence to consensus: 40.2%  
**55. Bov-tA2**: 43038250-43038439 (-), Divergence to consensus: 21.6%  
**56. MER57LA**: 43038440-43038491 (-), Divergence to consensus: 52.4%  
**57. MIRb**: 43039086-43039167 (-), Divergence to consensus: 29.3%  
**58. MER21B**: 43039184-43039651 (+), Divergence to consensus: 49.2%  
**59. L1MA9**: 43039652-43039801 (-), Divergence to consensus: 23.6%  
**60. Bov-tA2**: 43039940-43040142 (-), Divergence to consensus: 10.5%  
**61. Bov-tA2**: 43040143-43040263 (-), Divergence to consensus: 20.5%  
**62. Bov-tA3**: 43040353-43040583 (+), Divergence to consensus: 18.4%  
**63. MER21B**: 43040589-43040781 (+), Divergence to consensus: 30.5%  
**64. MLT1D**: 43041117-43041506 (+), Divergence to consensus: 47.9%  
**65. 5S**: 43041852-43041893 (-), Divergence to consensus: 19.1%  
**66. BovB**: 43042105-43042826 (+), Divergence to consensus: 9.3%  
**67. BTLTR1**: 43042827-43042897 (-), Divergence to consensus: 12.7%  
**68. BovB**: 43042898-43043750 (+), Divergence to consensus: 5.5%  
**69. ART2A**: 43043751-43044252 (+), Divergence to consensus: 15.2%  
**70. (AACTG)n**: 43044253-43044282 (+), Divergence to consensus: 6.7%  
**71. LTR10A\_BT**: 43044283-43044348 (-), Divergence to consensus: 23.4%  
**72. Bov-tA1**: 43044367-43044563 (-), Divergence to consensus: 14.2%  
**73. LTR10A\_BT**: 43044569-43044770 (-), Divergence to consensus: 26.1%  
**74. L1\_Art**: 43044775-43044869 (+), Divergence to consensus: 23.1%  
**75. LTR10A\_BT**: 43044897-43045016 (-), Divergence to consensus: 16.7%  
**76. Bov-tA1**: 43045030-43045224 (-), Divergence to consensus: 28.2%  
**77. LTR10A\_BT**: 43045225-43045516 (-), Divergence to consensus: 18.4%  
**78. AT\_rich**: 43046818-43046838 (+), Divergence to consensus: 52.4%  
**79. Charlie13a**: 43048057-43048239 (+), Divergence to consensus: 40.7%  
**80. Bov-tA2**: 43048275-43048480 (+), Divergence to consensus: 12.6%  
**81. Charlie13a**: 43048575-43048859 (+), Divergence to consensus: 48.2%

  
Transcription Factor Binding Sites  

**RFX4\_1** (Sequence: GTTGCCAGG (-): 43048591)  
**RFX4\_1** (Sequence: CTTGGCAAC (+): 43003715)  
**Gata4** (Sequence: AGATAAG (-): 43005388)  
**Gata4** (Sequence: AGATAAC (-): 43012391)  
**Gata4** (Sequence: AGATAAC (-): 43031536)  
**Gata4** (Sequence: AGATAAC (-): 43038831)  
**SOX9** (Sequence: AACAATGA (-): 43003377)  
**SOX9** (Sequence: AACAATGG (-): 43014021)  
**SOX9** (Sequence: AACAATAA (-): 43024185)  
**SOX9** (Sequence: AACAATGA (-): 43048549)  
**SOX9** (Sequence: CCATTGTT (+): 43041524)  
**Mybl1\_1** (Sequence: AACCGTTA (+): 43031862)  
**Gata4** (Sequence: GTTATCT (+): 43017650)  
**Gata4** (Sequence: GTTATCT (+): 43018561)  
**Gata4** (Sequence: GTTATCT (+): 43020519)  
**Gata4** (Sequence: CTTATCT (+): 43023194)  
**Gata4** (Sequence: CTTATCT (+): 43047438)  
**Gata4** (Sequence: GTTATCT (+): 43048703)  
**Gata4** (Sequence: CTTATCT (+): 43049288)
