# Supplementary material for: Small RNA sequencing of cryopreserved semen from single bull revealed altered miRNAs and piRNAs expression between High- and Low-motile sperm populations
Source: BMC Genomics. 2017 Jan 4;18:14. doi: 10.1186/s12864-016-3394-7 (PMC5209821; doi:10.1186/s12864-016-3394-7)
Supplement: Additional file 3: — Details for each piRNA clusters found in High Motile (HM) sperm fraction. Genes, repeats, transposable elements and transcription factors binding sites falling within the cluster regions were reported. (ZIP 1896 kb) [file 12864_2016_3394_MOESM3_ESM.zip › 57.html]

piRNA cluster 57


Predicted piRNA cluster no. 57     previous   next
  

Show proTRAC run info
Hide proTRAC run info

================================= proTRAC ====================================  
VERSION: 2.1                                    LAST MODIFIED: 06. October 2015  
  
Please cite:  
Rosenkranz D, Zischler H. proTRAC - a software for probabilistic piRNA cluster  
detection, visualization and analysis. 2012. BMC Bioinformatics 13:5.  
  
and (for proTRAC 2.0 and later):  
Rosenkranz D, Rudloff S, Bastuck K, Ketting RF, Zischler H. Tupaia small RNAs  
provide insights into function and evolution of RNAi-based transposon defense  
in mammals. 2015. RNA 21(5):911-922.  
  
Contact:  
David Rosenkranz  
Institute of Anthropology, small RNA group  
Johannes Gutenberg University Mainz  
email: rosenkranz@uni-mainz.de  
  
You can find the latest proTRAC version at:  
http://sourceforge.net/projects/protrac/files  
http://www.smallRNAgroup-mainz.de/software  
==============================================================================  
  
PARAMETERS:  
Map file: .............../storage/core/barbara/genhome/smallRNA/fertility/Sample\_motile/pirna/Sample\_motile\_26-33\_collapsed.fa.no-dust.map.weighted-10000-1000-b-0  
Genome file: ............/storage/core/barbara/genhome/smallRNA/fertility/Sample\_all/pirna/bt\_311\_chrY.fa  
RepeatMasker annotation: /storage/genomes/bt\_umd31/GCF\_000003055.6\_Bos\_taurus\_UMD\_3.1.1\_repeatMasker\_chr.out  
GeneSet:................./storage/core/barbara/genhome/smallRNA/fertility/Sample\_all/pirna/full.gtf  
  
Significant (p<=0.01) hit density will be calculated based  
on observed hit distribution.  
  
Sliding window size: ........................................ 5000 bp  
Sliding window increament: .................................. 1000 bp  
Normalize each hit by number of genomic hits: ............... 1 [0=no/1=yes]  
Normalize each hit by number of sequence reads: ............. 1 [0=no/1=yes]  
Normalize values (-> per million mapped reads): ............. 1 [0=no/1=yes]  
Min. fraction of hits with 1T(U) or 10A: .................... 0.75  
Alternatively: Min. fraction of hits with 1T(U) and 10A: .... 0.5  
Min. fraction of hits with typical piRNA length: ............ 0.75  
Typical piRNA length: ....................................... 26-33 nt  
Min. size of a piRNA cluster: ............................... 5000 bp.  
Min. number of hits (absolute): ............................. 0  
Min. number of hits (normalized): ........................... 0  
Min. fraction of hits on the mainstrand: .................... 0.75  
Top fraction of mapped sequences (in terms of read counts): . 1%  
Top fraction accounts for max. n% of sequence reads: ........ 90%  
Min. fraction of hits on each arm of a bidirectional cluster: 0.1  
Output image file for each cluster: ......................... 0 [0=no/1=yes]  
Output html file for each cluster: .......................... 1 [0=no/1=yes]  
Output a summary table: ..................................... 1 [0=no/1=yes]  
Output a FASTA file for each cluster (piRNA sequences): ..... 1 [0=no/1=yes]  
Output a FASTA file comprising cluster sequences: ........... 1 [0=no/1=yes]  
Search DNA motifs in clusters: .............................. 1 [0=no/1=yes]  
Output flanking sequences: +/- .............................. 0 bp  
Output ~.pTi file: .......................................... 1 [0=no/1=yes]  
==============================================================================  
  
  
Genome size (without gaps): ............ 2678902517 bp  
Gaps (N/X/-): .......................... 53837044 bp  
Mapped reads: .......................... 658825247023  
Non-identical sequences: ............... 514171  
Genomic hits: .......................... 764233  
Significant densitiy of mapped reads: .. 12867599.5173724 reads/kb

Show proTRAC cluster info
Hide proTRAC cluster info

|  |  |
| --- | --- |
| Location | chr24 |
| Coordinates | 43053379-43076003 |
| Size [bp] | 22625 |
| Sequence hit loci | 5842 |
| Mapped reads (normalized) | 6629135816 |
| Mapped reads (normalized) per kb | 293000478.1 |
| Normalized reads with 1T (1U) | 75.1% |
| Normalized reads with 10A | 28.2% |
| Normalized reads with length 26-33 nt | 100% |
| Normalized reads on the main strand(s) | 98.3% |
| Predicted directionality | bi:minus-plus (split between 43070203 and 43070246) |

100%

0%

1T (1U)  
reads

10A reads

26-33 nt  
reads

reads on mainstrand

**Either the amount of reads with 1T (1U) OR 10A has to exceed 75% (set with option: -1Tor10A)  
Alternatively the amount of reads with 1T (1U) AND 10A has to exceed 50% (set with option: -1Tand10A)  
Minimum amount of reads with preferred size is 75% (set with option: -pisize)  
Minimum amount of reads on the main strand(s) is 75% (set with option: -clstrand)**

Show read coverage
Hide read coverage

WHAT DO I SEE HERE?  
This chart shows the location of mapped sequence reads within a predicted piRNA cluster. The color refers to the number of genomic hits produced by the sequence read in question. A dark red bar indicates that this sequence read produces many other hits elsewhere in the genome. Many adjacent red or yellow bars can indicate the presence of a multi-copy element such as transposons or rRNA genes. A dark green bar indicates that this sequence read maps uniquely to this locus.

1 hit

2-5 hits

6-10 hits

11-20 hits

21-50 hits

51-100 hits

> 100 hits

chr24

43053379

43076003

Gene Set

RepeatMasker

Mapped  
Reads

113.92

plus strand

minus strand

113.92

Region: chr24 43049308-43053401. Max. coverage (+): 0. Max coverage (-): 26.21

Region: chr24 43053402-43053446. Max. coverage (+): 0. Max coverage (-): 7.35

Region: chr24 43053447-43053492. Max. coverage (+): 0. Max coverage (-): 4.83

Region: chr24 43053493-43053537. Max. coverage (+): 0. Max coverage (-): 29.05

Region: chr24 43053538-43053582. Max. coverage (+): 0. Max coverage (-): 20.48

Region: chr24 43053583-43053627. Max. coverage (+): 0. Max coverage (-): 52.16

Region: chr24 43053628-43053673. Max. coverage (+): 0. Max coverage (-): 6.6

Region: chr24 43053674-43053718. Max. coverage (+): 0. Max coverage (-): 22.52

Region: chr24 43053719-43053763. Max. coverage (+): 0. Max coverage (-): 73

Region: chr24 43053764-43053808. Max. coverage (+): 0. Max coverage (-): 19.64

Region: chr24 43053809-43053854. Max. coverage (+): 0. Max coverage (-): 7.42

Region: chr24 43053855-43053899. Max. coverage (+): 0. Max coverage (-): 1.63

Region: chr24 43053900-43053944. Max. coverage (+): 0. Max coverage (-): 5.48

Region: chr24 43053945-43053989. Max. coverage (+): 0. Max coverage (-): 14.69

Region: chr24 43053990-43054035. Max. coverage (+): 0. Max coverage (-): 14.93

Region: chr24 43054036-43054080. Max. coverage (+): 0. Max coverage (-): 20.21

Region: chr24 43054081-43054125. Max. coverage (+): 0. Max coverage (-): 32.81

Region: chr24 43054126-43054170. Max. coverage (+): 0. Max coverage (-): 51.06

Region: chr24 43054171-43054216. Max. coverage (+): 0. Max coverage (-): 44.36

Region: chr24 43054217-43054261. Max. coverage (+): 0. Max coverage (-): 19.56

Region: chr24 43054262-43054306. Max. coverage (+): 0. Max coverage (-): 57.64

Region: chr24 43054307-43054351. Max. coverage (+): 0. Max coverage (-): 13.16

Region: chr24 43054352-43054397. Max. coverage (+): 0. Max coverage (-): 8.7

Region: chr24 43054398-43054442. Max. coverage (+): 0. Max coverage (-): 13.91

Region: chr24 43054443-43054487. Max. coverage (+): 0. Max coverage (-): 35.65

Region: chr24 43054488-43054532. Max. coverage (+): 0. Max coverage (-): 54.02

Region: chr24 43054533-43054578. Max. coverage (+): 0. Max coverage (-): 72.27

Region: chr24 43054579-43054623. Max. coverage (+): 0. Max coverage (-): 57.99

Region: chr24 43054624-43054668. Max. coverage (+): 0. Max coverage (-): 22.66

Region: chr24 43054669-43054713. Max. coverage (+): 0. Max coverage (-): 20.95

Region: chr24 43054714-43054759. Max. coverage (+): 0. Max coverage (-): 0

Region: chr24 43054760-43054804. Max. coverage (+): 0. Max coverage (-): 0

Region: chr24 43054805-43054849. Max. coverage (+): 0. Max coverage (-): 0

Region: chr24 43054850-43054894. Max. coverage (+): 0. Max coverage (-): 0

Region: chr24 43054895-43054940. Max. coverage (+): 0. Max coverage (-): 51.84

Region: chr24 43054941-43054985. Max. coverage (+): 0. Max coverage (-): 50.4

Region: chr24 43054986-43055030. Max. coverage (+): 0. Max coverage (-): 30.59

Region: chr24 43055031-43055075. Max. coverage (+): 0. Max coverage (-): 0

Region: chr24 43055076-43055121. Max. coverage (+): 0. Max coverage (-): 10.05

Region: chr24 43055122-43055166. Max. coverage (+): 0. Max coverage (-): 0

Region: chr24 43055167-43055211. Max. coverage (+): 0. Max coverage (-): 0

Region: chr24 43055212-43055256. Max. coverage (+): 0. Max coverage (-): 8.7

Region: chr24 43055257-43055302. Max. coverage (+): 0. Max coverage (-): 5.67

Region: chr24 43055303-43055347. Max. coverage (+): 0. Max coverage (-): 93.8

Region: chr24 43055348-43055392. Max. coverage (+): 0. Max coverage (-): 72.7

Region: chr24 43055393-43055437. Max. coverage (+): 0. Max coverage (-): 90.43

Region: chr24 43055438-43055483. Max. coverage (+): 0. Max coverage (-): 11.8

Region: chr24 43055484-43055528. Max. coverage (+): 2.13. Max coverage (-): 0

Region: chr24 43055529-43055573. Max. coverage (+): 0. Max coverage (-): 0

Region: chr24 43055574-43055618. Max. coverage (+): 0. Max coverage (-): 39.91

Region: chr24 43055619-43055664. Max. coverage (+): 0. Max coverage (-): 77.56

Region: chr24 43055665-43055709. Max. coverage (+): 0. Max coverage (-): 0

Region: chr24 43055710-43055754. Max. coverage (+): 0. Max coverage (-): 0

Region: chr24 43055755-43055799. Max. coverage (+): 0. Max coverage (-): 0

Region: chr24 43055800-43055845. Max. coverage (+): 0. Max coverage (-): 25.4

Region: chr24 43055846-43055890. Max. coverage (+): 0. Max coverage (-): 54.88

Region: chr24 43055891-43055935. Max. coverage (+): 0. Max coverage (-): 19.52

Region: chr24 43055936-43055980. Max. coverage (+): 0. Max coverage (-): 30.87

Region: chr24 43055981-43056026. Max. coverage (+): 0. Max coverage (-): 30.44

Region: chr24 43056027-43056071. Max. coverage (+): 0.66. Max coverage (-): 16.06

Region: chr24 43056072-43056116. Max. coverage (+): 0. Max coverage (-): 13.97

Region: chr24 43056117-43056161. Max. coverage (+): 0. Max coverage (-): 4.78

Region: chr24 43056162-43056207. Max. coverage (+): 0. Max coverage (-): 0

Region: chr24 43056208-43056252. Max. coverage (+): 0. Max coverage (-): 0

Region: chr24 43056253-43056297. Max. coverage (+): 0. Max coverage (-): 0

Region: chr24 43056298-43056342. Max. coverage (+): 0. Max coverage (-): 0

Region: chr24 43056343-43056388. Max. coverage (+): 0. Max coverage (-): 0

Region: chr24 43056389-43056433. Max. coverage (+): 0. Max coverage (-): 6.97

Region: chr24 43056434-43056478. Max. coverage (+): 0. Max coverage (-): 54.09

Region: chr24 43056479-43056523. Max. coverage (+): 0. Max coverage (-): 17.05

Region: chr24 43056524-43056569. Max. coverage (+): 0. Max coverage (-): 87.17

Region: chr24 43056570-43056614. Max. coverage (+): 0. Max coverage (-): 0

Region: chr24 43056615-43056659. Max. coverage (+): 0. Max coverage (-): 0

Region: chr24 43056660-43056704. Max. coverage (+): 0. Max coverage (-): 0

Region: chr24 43056705-43056750. Max. coverage (+): 0. Max coverage (-): 0

Region: chr24 43056751-43056795. Max. coverage (+): 0. Max coverage (-): 0

Region: chr24 43056796-43056840. Max. coverage (+): 0. Max coverage (-): 0

Region: chr24 43056841-43056885. Max. coverage (+): 0. Max coverage (-): 0

Region: chr24 43056886-43056931. Max. coverage (+): 0. Max coverage (-): 1.1

Region: chr24 43056932-43056976. Max. coverage (+): 0. Max coverage (-): 0

Region: chr24 43056977-43057021. Max. coverage (+): 0. Max coverage (-): 7.44

Region: chr24 43057022-43057066. Max. coverage (+): 0. Max coverage (-): 0.46

Region: chr24 43057067-43057112. Max. coverage (+): 0. Max coverage (-): 2.05

Region: chr24 43057113-43057157. Max. coverage (+): 0. Max coverage (-): 32.18

Region: chr24 43057158-43057202. Max. coverage (+): 0. Max coverage (-): 2.19

Region: chr24 43057203-43057247. Max. coverage (+): 0. Max coverage (-): 10.01

Region: chr24 43057248-43057293. Max. coverage (+): 0. Max coverage (-): 28.74

Region: chr24 43057294-43057338. Max. coverage (+): 0. Max coverage (-): 27.45

Region: chr24 43057339-43057383. Max. coverage (+): 0. Max coverage (-): 17.15

Region: chr24 43057384-43057428. Max. coverage (+): 0. Max coverage (-): 22.07

Region: chr24 43057429-43057474. Max. coverage (+): 0. Max coverage (-): 18.04

Region: chr24 43057475-43057519. Max. coverage (+): 0. Max coverage (-): 31.17

Region: chr24 43057520-43057564. Max. coverage (+): 0. Max coverage (-): 1.02

Region: chr24 43057565-43057609. Max. coverage (+): 0. Max coverage (-): 107.39

Region: chr24 43057610-43057655. Max. coverage (+): 0. Max coverage (-): 99.17

Region: chr24 43057656-43057700. Max. coverage (+): 0. Max coverage (-): 23.29

Region: chr24 43057701-43057745. Max. coverage (+): 0. Max coverage (-): 28.64

Region: chr24 43057746-43057790. Max. coverage (+): 0. Max coverage (-): 18.95

Region: chr24 43057791-43057836. Max. coverage (+): 0. Max coverage (-): 37.72

Region: chr24 43057837-43057881. Max. coverage (+): 0. Max coverage (-): 109.8

Region: chr24 43057882-43057926. Max. coverage (+): 0. Max coverage (-): 51.35

Region: chr24 43057927-43057971. Max. coverage (+): 0. Max coverage (-): 3.59

Region: chr24 43057972-43058017. Max. coverage (+): 0. Max coverage (-): 4.1

Region: chr24 43058018-43058062. Max. coverage (+): 0. Max coverage (-): 16

Region: chr24 43058063-43058107. Max. coverage (+): 0. Max coverage (-): 2.37

Region: chr24 43058108-43058152. Max. coverage (+): 0. Max coverage (-): 6.1

Region: chr24 43058153-43058198. Max. coverage (+): 0. Max coverage (-): 0

Region: chr24 43058199-43058243. Max. coverage (+): 0. Max coverage (-): 14.51

Region: chr24 43058244-43058288. Max. coverage (+): 0. Max coverage (-): 16.13

Region: chr24 43058289-43058333. Max. coverage (+): 0. Max coverage (-): 0

Region: chr24 43058334-43058379. Max. coverage (+): 0. Max coverage (-): 11.03

Region: chr24 43058380-43058424. Max. coverage (+): 0. Max coverage (-): 0

Region: chr24 43058425-43058469. Max. coverage (+): 0. Max coverage (-): 10.37

Region: chr24 43058470-43058514. Max. coverage (+): 0. Max coverage (-): 17.71

Region: chr24 43058515-43058560. Max. coverage (+): 0. Max coverage (-): 28.1

Region: chr24 43058561-43058605. Max. coverage (+): 0. Max coverage (-): 8.88

Region: chr24 43058606-43058650. Max. coverage (+): 0. Max coverage (-): 10.79

Region: chr24 43058651-43058695. Max. coverage (+): 0. Max coverage (-): 2.08

Region: chr24 43058696-43058741. Max. coverage (+): 0. Max coverage (-): 0

Region: chr24 43058742-43058786. Max. coverage (+): 0. Max coverage (-): 2.22

Region: chr24 43058787-43058831. Max. coverage (+): 0. Max coverage (-): 0

Region: chr24 43058832-43058876. Max. coverage (+): 0. Max coverage (-): 1.59

Region: chr24 43058877-43058922. Max. coverage (+): 0. Max coverage (-): 1.59

Region: chr24 43058923-43058967. Max. coverage (+): 0. Max coverage (-): 1.01

Region: chr24 43058968-43059012. Max. coverage (+): 0. Max coverage (-): 0.91

Region: chr24 43059013-43059057. Max. coverage (+): 0. Max coverage (-): 9.11

Region: chr24 43059058-43059103. Max. coverage (+): 0. Max coverage (-): 24.96

Region: chr24 43059104-43059148. Max. coverage (+): 0. Max coverage (-): 10.38

Region: chr24 43059149-43059193. Max. coverage (+): 1.85. Max coverage (-): 0

Region: chr24 43059194-43059238. Max. coverage (+): 0. Max coverage (-): 36.97

Region: chr24 43059239-43059284. Max. coverage (+): 0. Max coverage (-): 0

Region: chr24 43059285-43059329. Max. coverage (+): 0. Max coverage (-): 0

Region: chr24 43059330-43059374. Max. coverage (+): 0. Max coverage (-): 0

Region: chr24 43059375-43059419. Max. coverage (+): 0. Max coverage (-): 5.85

Region: chr24 43059420-43059465. Max. coverage (+): 0. Max coverage (-): 15.19

Region: chr24 43059466-43059510. Max. coverage (+): 0. Max coverage (-): 35.6

Region: chr24 43059511-43059555. Max. coverage (+): 0. Max coverage (-): 4.17

Region: chr24 43059556-43059600. Max. coverage (+): 0. Max coverage (-): 0

Region: chr24 43059601-43059646. Max. coverage (+): 0. Max coverage (-): 1.22

Region: chr24 43059647-43059691. Max. coverage (+): 0. Max coverage (-): 18.69

Region: chr24 43059692-43059736. Max. coverage (+): 0. Max coverage (-): 11.27

Region: chr24 43059737-43059781. Max. coverage (+): 0. Max coverage (-): 15.67

Region: chr24 43059782-43059827. Max. coverage (+): 0. Max coverage (-): 1.91

Region: chr24 43059828-43059872. Max. coverage (+): 0. Max coverage (-): 14.38

Region: chr24 43059873-43059917. Max. coverage (+): 0. Max coverage (-): 14.47

Region: chr24 43059918-43059962. Max. coverage (+): 0. Max coverage (-): 22.66

Region: chr24 43059963-43060008. Max. coverage (+): 0. Max coverage (-): 55.26

Region: chr24 43060009-43060053. Max. coverage (+): 0. Max coverage (-): 37.58

Region: chr24 43060054-43060098. Max. coverage (+): 0. Max coverage (-): 46.41

Region: chr24 43060099-43060143. Max. coverage (+): 0. Max coverage (-): 2.41

Region: chr24 43060144-43060189. Max. coverage (+): 1.09. Max coverage (-): 0

Region: chr24 43060190-43060234. Max. coverage (+): 0. Max coverage (-): 8.68

Region: chr24 43060235-43060279. Max. coverage (+): 5.25. Max coverage (-): 27.81

Region: chr24 43060280-43060324. Max. coverage (+): 5.82. Max coverage (-): 24.51

Region: chr24 43060325-43060370. Max. coverage (+): 0. Max coverage (-): 4.34

Region: chr24 43060371-43060415. Max. coverage (+): 0. Max coverage (-): 3.57

Region: chr24 43060416-43060460. Max. coverage (+): 0. Max coverage (-): 3.57

Region: chr24 43060461-43060505. Max. coverage (+): 0. Max coverage (-): 8.56

Region: chr24 43060506-43060551. Max. coverage (+): 0. Max coverage (-): 8.93

Region: chr24 43060552-43060596. Max. coverage (+): 0. Max coverage (-): 66.51

Region: chr24 43060597-43060641. Max. coverage (+): 0. Max coverage (-): 14.37

Region: chr24 43060642-43060686. Max. coverage (+): 0. Max coverage (-): 8.66

Region: chr24 43060687-43060732. Max. coverage (+): 0. Max coverage (-): 8.15

Region: chr24 43060733-43060777. Max. coverage (+): 0. Max coverage (-): 43.63

Region: chr24 43060778-43060822. Max. coverage (+): 0. Max coverage (-): 48.36

Region: chr24 43060823-43060867. Max. coverage (+): 0. Max coverage (-): 26.44

Region: chr24 43060868-43060913. Max. coverage (+): 1.23. Max coverage (-): 32

Region: chr24 43060914-43060958. Max. coverage (+): 1.39. Max coverage (-): 38.58

Region: chr24 43060959-43061003. Max. coverage (+): 0. Max coverage (-): 17.3

Region: chr24 43061004-43061048. Max. coverage (+): 0. Max coverage (-): 0

Region: chr24 43061049-43061094. Max. coverage (+): 0. Max coverage (-): 3.66

Region: chr24 43061095-43061139. Max. coverage (+): 2.7. Max coverage (-): 28.37

Region: chr24 43061140-43061184. Max. coverage (+): 0. Max coverage (-): 70.51

Region: chr24 43061185-43061229. Max. coverage (+): 0. Max coverage (-): 19.94

Region: chr24 43061230-43061275. Max. coverage (+): 3.06. Max coverage (-): 45.51

Region: chr24 43061276-43061320. Max. coverage (+): 0. Max coverage (-): 50.51

Region: chr24 43061321-43061365. Max. coverage (+): 2.21. Max coverage (-): 21.42

Region: chr24 43061366-43061410. Max. coverage (+): 2.21. Max coverage (-): 5.88

Region: chr24 43061411-43061456. Max. coverage (+): 0. Max coverage (-): 41.55

Region: chr24 43061457-43061501. Max. coverage (+): 2.24. Max coverage (-): 49.32

Region: chr24 43061502-43061546. Max. coverage (+): 0. Max coverage (-): 16.25

Region: chr24 43061547-43061591. Max. coverage (+): 0. Max coverage (-): 12.25

Region: chr24 43061592-43061637. Max. coverage (+): 0. Max coverage (-): 9.34

Region: chr24 43061638-43061682. Max. coverage (+): 0. Max coverage (-): 34.36

Region: chr24 43061683-43061727. Max. coverage (+): 2.59. Max coverage (-): 21.97

Region: chr24 43061728-43061772. Max. coverage (+): 0. Max coverage (-): 16

Region: chr24 43061773-43061818. Max. coverage (+): 0. Max coverage (-): 7.34

Region: chr24 43061819-43061863. Max. coverage (+): 0. Max coverage (-): 9.68

Region: chr24 43061864-43061908. Max. coverage (+): 0. Max coverage (-): 12.31

Region: chr24 43061909-43061953. Max. coverage (+): 0. Max coverage (-): 1.09

Region: chr24 43061954-43061999. Max. coverage (+): 0. Max coverage (-): 2.17

Region: chr24 43062000-43062044. Max. coverage (+): 0.84. Max coverage (-): 0.65

Region: chr24 43062045-43062089. Max. coverage (+): 0. Max coverage (-): 17.58

Region: chr24 43062090-43062134. Max. coverage (+): 0. Max coverage (-): 11.91

Region: chr24 43062135-43062180. Max. coverage (+): 3.05. Max coverage (-): 9.34

Region: chr24 43062181-43062225. Max. coverage (+): 0. Max coverage (-): 14.97

Region: chr24 43062226-43062270. Max. coverage (+): 2.37. Max coverage (-): 33.08

Region: chr24 43062271-43062315. Max. coverage (+): 2.37. Max coverage (-): 39.02

Region: chr24 43062316-43062361. Max. coverage (+): 0. Max coverage (-): 6.06

Region: chr24 43062362-43062406. Max. coverage (+): 0. Max coverage (-): 0

Region: chr24 43062407-43062451. Max. coverage (+): 0. Max coverage (-): 14.99

Region: chr24 43062452-43062496. Max. coverage (+): 0. Max coverage (-): 0.68

Region: chr24 43062497-43062542. Max. coverage (+): 0. Max coverage (-): 16.43

Region: chr24 43062543-43062587. Max. coverage (+): 0. Max coverage (-): 12.75

Region: chr24 43062588-43062632. Max. coverage (+): 0. Max coverage (-): 19.99

Region: chr24 43062633-43062677. Max. coverage (+): 0.44. Max coverage (-): 29.73

Region: chr24 43062678-43062723. Max. coverage (+): 0. Max coverage (-): 0

Region: chr24 43062724-43062768. Max. coverage (+): 0. Max coverage (-): 11.84

Region: chr24 43062769-43062813. Max. coverage (+): 0. Max coverage (-): 38.19

Region: chr24 43062814-43062858. Max. coverage (+): 0. Max coverage (-): 16.71

Region: chr24 43062859-43062904. Max. coverage (+): 0. Max coverage (-): 0

Region: chr24 43062905-43062949. Max. coverage (+): 0. Max coverage (-): 23.42

Region: chr24 43062950-43062994. Max. coverage (+): 2.69. Max coverage (-): 50.21

Region: chr24 43062995-43063039. Max. coverage (+): 0. Max coverage (-): 0

Region: chr24 43063040-43063085. Max. coverage (+): 0. Max coverage (-): 0

Region: chr24 43063086-43063130. Max. coverage (+): 0. Max coverage (-): 0

Region: chr24 43063131-43063175. Max. coverage (+): 0. Max coverage (-): 0

Region: chr24 43063176-43063220. Max. coverage (+): 0. Max coverage (-): 1.34

Region: chr24 43063221-43063266. Max. coverage (+): 0. Max coverage (-): 4.79

Region: chr24 43063267-43063311. Max. coverage (+): 0. Max coverage (-): 0

Region: chr24 43063312-43063356. Max. coverage (+): 0. Max coverage (-): 0

Region: chr24 43063357-43063401. Max. coverage (+): 0. Max coverage (-): 0

Region: chr24 43063402-43063447. Max. coverage (+): 0. Max coverage (-): 3.94

Region: chr24 43063448-43063492. Max. coverage (+): 1.59. Max coverage (-): 2.62

Region: chr24 43063493-43063537. Max. coverage (+): 1.59. Max coverage (-): 8.81

Region: chr24 43063538-43063582. Max. coverage (+): 2.13. Max coverage (-): 6.99

Region: chr24 43063583-43063628. Max. coverage (+): 11.31. Max coverage (-): 3.1

Region: chr24 43063629-43063673. Max. coverage (+): 5.33. Max coverage (-): 0

Region: chr24 43063674-43063718. Max. coverage (+): 0. Max coverage (-): 10.17

Region: chr24 43063719-43063763. Max. coverage (+): 0. Max coverage (-): 7.42

Region: chr24 43063764-43063809. Max. coverage (+): 0. Max coverage (-): 7.86

Region: chr24 43063810-43063854. Max. coverage (+): 0. Max coverage (-): 14.19

Region: chr24 43063855-43063899. Max. coverage (+): 0. Max coverage (-): 9.04

Region: chr24 43063900-43063944. Max. coverage (+): 0. Max coverage (-): 8.26

Region: chr24 43063945-43063990. Max. coverage (+): 0. Max coverage (-): 38.41

Region: chr24 43063991-43064035. Max. coverage (+): 0. Max coverage (-): 2.23

Region: chr24 43064036-43064080. Max. coverage (+): 0. Max coverage (-): 0

Region: chr24 43064081-43064125. Max. coverage (+): 0. Max coverage (-): 0

Region: chr24 43064126-43064171. Max. coverage (+): 1.03. Max coverage (-): 11.22

Region: chr24 43064172-43064216. Max. coverage (+): 0. Max coverage (-): 38.51

Region: chr24 43064217-43064261. Max. coverage (+): 4.56. Max coverage (-): 107.38

Region: chr24 43064262-43064306. Max. coverage (+): 0. Max coverage (-): 1.98

Region: chr24 43064307-43064352. Max. coverage (+): 0. Max coverage (-): 1.49

Region: chr24 43064353-43064397. Max. coverage (+): 0. Max coverage (-): 7.38

Region: chr24 43064398-43064442. Max. coverage (+): 0. Max coverage (-): 12.42

Region: chr24 43064443-43064487. Max. coverage (+): 0. Max coverage (-): 8.68

Region: chr24 43064488-43064533. Max. coverage (+): 0. Max coverage (-): 1.34

Region: chr24 43064534-43064578. Max. coverage (+): 0. Max coverage (-): 0

Region: chr24 43064579-43064623. Max. coverage (+): 0. Max coverage (-): 0

Region: chr24 43064624-43064668. Max. coverage (+): 0. Max coverage (-): 2.23

Region: chr24 43064669-43064714. Max. coverage (+): 0. Max coverage (-): 2.23

Region: chr24 43064715-43064759. Max. coverage (+): 0. Max coverage (-): 9.2

Region: chr24 43064760-43064804. Max. coverage (+): 0. Max coverage (-): 22.31

Region: chr24 43064805-43064849. Max. coverage (+): 0. Max coverage (-): 15.03

Region: chr24 43064850-43064895. Max. coverage (+): 0. Max coverage (-): 7.03

Region: chr24 43064896-43064940. Max. coverage (+): 0. Max coverage (-): 13.17

Region: chr24 43064941-43064985. Max. coverage (+): 0. Max coverage (-): 8.14

Region: chr24 43064986-43065030. Max. coverage (+): 3.28. Max coverage (-): 10.41

Region: chr24 43065031-43065076. Max. coverage (+): 3.28. Max coverage (-): 31.94

Region: chr24 43065077-43065121. Max. coverage (+): 0. Max coverage (-): 8.11

Region: chr24 43065122-43065166. Max. coverage (+): 0. Max coverage (-): 14.12

Region: chr24 43065167-43065211. Max. coverage (+): 0. Max coverage (-): 14.72

Region: chr24 43065212-43065257. Max. coverage (+): 0. Max coverage (-): 9.94

Region: chr24 43065258-43065302. Max. coverage (+): 0. Max coverage (-): 12.3

Region: chr24 43065303-43065347. Max. coverage (+): 0. Max coverage (-): 13.83

Region: chr24 43065348-43065392. Max. coverage (+): 0. Max coverage (-): 20.84

Region: chr24 43065393-43065438. Max. coverage (+): 0. Max coverage (-): 17.44

Region: chr24 43065439-43065483. Max. coverage (+): 0. Max coverage (-): 6.36

Region: chr24 43065484-43065528. Max. coverage (+): 0. Max coverage (-): 13.08

Region: chr24 43065529-43065573. Max. coverage (+): 0. Max coverage (-): 21.32

Region: chr24 43065574-43065619. Max. coverage (+): 0. Max coverage (-): 30.6

Region: chr24 43065620-43065664. Max. coverage (+): 0. Max coverage (-): 29.56

Region: chr24 43065665-43065709. Max. coverage (+): 0. Max coverage (-): 16.64

Region: chr24 43065710-43065754. Max. coverage (+): 0. Max coverage (-): 13.83

Region: chr24 43065755-43065800. Max. coverage (+): 0. Max coverage (-): 22.88

Region: chr24 43065801-43065845. Max. coverage (+): 0. Max coverage (-): 6.96

Region: chr24 43065846-43065890. Max. coverage (+): 0. Max coverage (-): 1.17

Region: chr24 43065891-43065935. Max. coverage (+): 0. Max coverage (-): 0

Region: chr24 43065936-43065981. Max. coverage (+): 0. Max coverage (-): 0

Region: chr24 43065982-43066026. Max. coverage (+): 0. Max coverage (-): 0

Region: chr24 43066027-43066071. Max. coverage (+): 0. Max coverage (-): 0

Region: chr24 43066072-43066116. Max. coverage (+): 0. Max coverage (-): 0

Region: chr24 43066117-43066162. Max. coverage (+): 0. Max coverage (-): 0

Region: chr24 43066163-43066207. Max. coverage (+): 0. Max coverage (-): 2.17

Region: chr24 43066208-43066252. Max. coverage (+): 0. Max coverage (-): 11.38

Region: chr24 43066253-43066297. Max. coverage (+): 0. Max coverage (-): 7.92

Region: chr24 43066298-43066343. Max. coverage (+): 0. Max coverage (-): 9.5

Region: chr24 43066344-43066388. Max. coverage (+): 0. Max coverage (-): 10.38

Region: chr24 43066389-43066433. Max. coverage (+): 0. Max coverage (-): 2.44

Region: chr24 43066434-43066478. Max. coverage (+): 0. Max coverage (-): 0

Region: chr24 43066479-43066524. Max. coverage (+): 0. Max coverage (-): 0

Region: chr24 43066525-43066569. Max. coverage (+): 0. Max coverage (-): 0

Region: chr24 43066570-43066614. Max. coverage (+): 0. Max coverage (-): 0

Region: chr24 43066615-43066659. Max. coverage (+): 0. Max coverage (-): 1.79

Region: chr24 43066660-43066705. Max. coverage (+): 0. Max coverage (-): 0

Region: chr24 43066706-43066750. Max. coverage (+): 0. Max coverage (-): 0

Region: chr24 43066751-43066795. Max. coverage (+): 0. Max coverage (-): 4.72

Region: chr24 43066796-43066840. Max. coverage (+): 0. Max coverage (-): 3.44

Region: chr24 43066841-43066886. Max. coverage (+): 0. Max coverage (-): 10.82

Region: chr24 43066887-43066931. Max. coverage (+): 1.47. Max coverage (-): 11.76

Region: chr24 43066932-43066976. Max. coverage (+): 0.77. Max coverage (-): 17.72

Region: chr24 43066977-43067021. Max. coverage (+): 0. Max coverage (-): 6.68

Region: chr24 43067022-43067067. Max. coverage (+): 0. Max coverage (-): 0

Region: chr24 43067068-43067112. Max. coverage (+): 0. Max coverage (-): 0

Region: chr24 43067113-43067157. Max. coverage (+): 0. Max coverage (-): 0

Region: chr24 43067158-43067202. Max. coverage (+): 0. Max coverage (-): 0

Region: chr24 43067203-43067248. Max. coverage (+): 0. Max coverage (-): 0

Region: chr24 43067249-43067293. Max. coverage (+): 0. Max coverage (-): 12.99

Region: chr24 43067294-43067338. Max. coverage (+): 0. Max coverage (-): 13.99

Region: chr24 43067339-43067383. Max. coverage (+): 0. Max coverage (-): 15.93

Region: chr24 43067384-43067429. Max. coverage (+): 0. Max coverage (-): 13.14

Region: chr24 43067430-43067474. Max. coverage (+): 0. Max coverage (-): 17.17

Region: chr24 43067475-43067519. Max. coverage (+): 0.89. Max coverage (-): 54.08

Region: chr24 43067520-43067564. Max. coverage (+): 0. Max coverage (-): 56.97

Region: chr24 43067565-43067610. Max. coverage (+): 0. Max coverage (-): 6.43

Region: chr24 43067611-43067655. Max. coverage (+): 0. Max coverage (-): 4.76

Region: chr24 43067656-43067700. Max. coverage (+): 0. Max coverage (-): 0

Region: chr24 43067701-43067745. Max. coverage (+): 0. Max coverage (-): 0

Region: chr24 43067746-43067791. Max. coverage (+): 0. Max coverage (-): 4.96

Region: chr24 43067792-43067836. Max. coverage (+): 4.39. Max coverage (-): 34.48

Region: chr24 43067837-43067881. Max. coverage (+): 0. Max coverage (-): 38.23

Region: chr24 43067882-43067926. Max. coverage (+): 0. Max coverage (-): 113.92

Region: chr24 43067927-43067972. Max. coverage (+): 0. Max coverage (-): 46.4

Region: chr24 43067973-43068017. Max. coverage (+): 0. Max coverage (-): 6.26

Region: chr24 43068018-43068062. Max. coverage (+): 0. Max coverage (-): 2.4

Region: chr24 43068063-43068107. Max. coverage (+): 0. Max coverage (-): 14.8

Region: chr24 43068108-43068153. Max. coverage (+): 0. Max coverage (-): 18.41

Region: chr24 43068154-43068198. Max. coverage (+): 0. Max coverage (-): 6.47

Region: chr24 43068199-43068243. Max. coverage (+): 0. Max coverage (-): 35.89

Region: chr24 43068244-43068288. Max. coverage (+): 0. Max coverage (-): 21.12

Region: chr24 43068289-43068334. Max. coverage (+): 0. Max coverage (-): 23.69

Region: chr24 43068335-43068379. Max. coverage (+): 0. Max coverage (-): 10.6

Region: chr24 43068380-43068424. Max. coverage (+): 0. Max coverage (-): 10.72

Region: chr24 43068425-43068469. Max. coverage (+): 0. Max coverage (-): 6.6

Region: chr24 43068470-43068515. Max. coverage (+): 0. Max coverage (-): 11.56

Region: chr24 43068516-43068560. Max. coverage (+): 0. Max coverage (-): 17.83

Region: chr24 43068561-43068605. Max. coverage (+): 0. Max coverage (-): 11.19

Region: chr24 43068606-43068650. Max. coverage (+): 0. Max coverage (-): 7.56

Region: chr24 43068651-43068696. Max. coverage (+): 1.08. Max coverage (-): 9.97

Region: chr24 43068697-43068741. Max. coverage (+): 0. Max coverage (-): 6.12

Region: chr24 43068742-43068786. Max. coverage (+): 0. Max coverage (-): 71.36

Region: chr24 43068787-43068831. Max. coverage (+): 0. Max coverage (-): 22.48

Region: chr24 43068832-43068877. Max. coverage (+): 0. Max coverage (-): 17.2

Region: chr24 43068878-43068922. Max. coverage (+): 0. Max coverage (-): 23.73

Region: chr24 43068923-43068967. Max. coverage (+): 0. Max coverage (-): 3.42

Region: chr24 43068968-43069012. Max. coverage (+): 0. Max coverage (-): 53.76

Region: chr24 43069013-43069058. Max. coverage (+): 0. Max coverage (-): 63.55

Region: chr24 43069059-43069103. Max. coverage (+): 0. Max coverage (-): 84.79

Region: chr24 43069104-43069148. Max. coverage (+): 0. Max coverage (-): 5.98

Region: chr24 43069149-43069193. Max. coverage (+): 4.33. Max coverage (-): 37.73

Region: chr24 43069194-43069239. Max. coverage (+): 0. Max coverage (-): 64.41

Region: chr24 43069240-43069284. Max. coverage (+): 0. Max coverage (-): 29.11

Region: chr24 43069285-43069329. Max. coverage (+): 0. Max coverage (-): 41.45

Region: chr24 43069330-43069374. Max. coverage (+): 0. Max coverage (-): 50.56

Region: chr24 43069375-43069420. Max. coverage (+): 0. Max coverage (-): 17.08

Region: chr24 43069421-43069465. Max. coverage (+): 0. Max coverage (-): 1.27

Region: chr24 43069466-43069510. Max. coverage (+): 1.11. Max coverage (-): 34.81

Region: chr24 43069511-43069555. Max. coverage (+): 0. Max coverage (-): 14.22

Region: chr24 43069556-43069601. Max. coverage (+): 0. Max coverage (-): 25.14

Region: chr24 43069602-43069646. Max. coverage (+): 0. Max coverage (-): 7.65

Region: chr24 43069647-43069691. Max. coverage (+): 2.06. Max coverage (-): 29.2

Region: chr24 43069692-43069736. Max. coverage (+): 0. Max coverage (-): 23.63

Region: chr24 43069737-43069782. Max. coverage (+): 0. Max coverage (-): 1.71

Region: chr24 43069783-43069827. Max. coverage (+): 0. Max coverage (-): 1.57

Region: chr24 43069828-43069872. Max. coverage (+): 0. Max coverage (-): 9.98

Region: chr24 43069873-43069917. Max. coverage (+): 0. Max coverage (-): 0

Region: chr24 43069918-43069963. Max. coverage (+): 0. Max coverage (-): 5.69

Region: chr24 43069964-43070008. Max. coverage (+): 0. Max coverage (-): 0

Region: chr24 43070009-43070053. Max. coverage (+): 0. Max coverage (-): 16.18

Region: chr24 43070054-43070098. Max. coverage (+): 0. Max coverage (-): 14.24

Region: chr24 43070099-43070144. Max. coverage (+): 0. Max coverage (-): 1.94

Region: chr24 43070145-43070189. Max. coverage (+): 0. Max coverage (-): 1.37

Region: chr24 43070190-43070234. Max. coverage (+): 0. Max coverage (-): 1.37

Region: chr24 43070235-43070279. Max. coverage (+): 0. Max coverage (-): 9.24

Region: chr24 43070280-43070325. Max. coverage (+): 0. Max coverage (-): 2.88

Region: chr24 43070326-43070370. Max. coverage (+): 0. Max coverage (-): 2.22

Region: chr24 43070371-43070415. Max. coverage (+): 5.56. Max coverage (-): 2.22

Region: chr24 43070416-43070460. Max. coverage (+): 0. Max coverage (-): 1.55

Region: chr24 43070461-43070506. Max. coverage (+): 4.01. Max coverage (-): 0

Region: chr24 43070507-43070551. Max. coverage (+): 0. Max coverage (-): 0

Region: chr24 43070552-43070596. Max. coverage (+): 5.07. Max coverage (-): 0

Region: chr24 43070597-43070641. Max. coverage (+): 16.16. Max coverage (-): 3.61

Region: chr24 43070642-43070687. Max. coverage (+): 18.81. Max coverage (-): 3.59

Region: chr24 43070688-43070732. Max. coverage (+): 18.07. Max coverage (-): 0

Region: chr24 43070733-43070777. Max. coverage (+): 0. Max coverage (-): 0

Region: chr24 43070778-43070822. Max. coverage (+): 5.35. Max coverage (-): 0

Region: chr24 43070823-43070868. Max. coverage (+): 20.29. Max coverage (-): 3.25

Region: chr24 43070869-43070913. Max. coverage (+): 17.38. Max coverage (-): 0

Region: chr24 43070914-43070958. Max. coverage (+): 0. Max coverage (-): 0

Region: chr24 43070959-43071003. Max. coverage (+): 34.01. Max coverage (-): 0

Region: chr24 43071004-43071049. Max. coverage (+): 20.06. Max coverage (-): 1.91

Region: chr24 43071050-43071094. Max. coverage (+): 14.31. Max coverage (-): 0

Region: chr24 43071095-43071139. Max. coverage (+): 0. Max coverage (-): 1.57

Region: chr24 43071140-43071184. Max. coverage (+): 24.07. Max coverage (-): 3.4

Region: chr24 43071185-43071230. Max. coverage (+): 10.17. Max coverage (-): 3.82

Region: chr24 43071231-43071275. Max. coverage (+): 1.39. Max coverage (-): 2.05

Region: chr24 43071276-43071320. Max. coverage (+): 2.89. Max coverage (-): 3.9

Region: chr24 43071321-43071365. Max. coverage (+): 1.58. Max coverage (-): 0

Region: chr24 43071366-43071411. Max. coverage (+): 17.67. Max coverage (-): 1.39

Region: chr24 43071412-43071456. Max. coverage (+): 15.76. Max coverage (-): 4.52

Region: chr24 43071457-43071501. Max. coverage (+): 27.33. Max coverage (-): 4.52

Region: chr24 43071502-43071546. Max. coverage (+): 25.42. Max coverage (-): 0

Region: chr24 43071547-43071592. Max. coverage (+): 0. Max coverage (-): 0

Region: chr24 43071593-43071637. Max. coverage (+): 0. Max coverage (-): 0

Region: chr24 43071638-43071682. Max. coverage (+): 0. Max coverage (-): 0

Region: chr24 43071683-43071727. Max. coverage (+): 45.78. Max coverage (-): 7.34

Region: chr24 43071728-43071773. Max. coverage (+): 43.84. Max coverage (-): 2.05

Region: chr24 43071774-43071818. Max. coverage (+): 40.67. Max coverage (-): 0

Region: chr24 43071819-43071863. Max. coverage (+): 4.96. Max coverage (-): 0

Region: chr24 43071864-43071908. Max. coverage (+): 0. Max coverage (-): 0

Region: chr24 43071909-43071954. Max. coverage (+): 0. Max coverage (-): 0

Region: chr24 43071955-43071999. Max. coverage (+): 6.53. Max coverage (-): 0

Region: chr24 43072000-43072044. Max. coverage (+): 0. Max coverage (-): 0

Region: chr24 43072045-43072089. Max. coverage (+): 2.48. Max coverage (-): 0

Region: chr24 43072090-43072135. Max. coverage (+): 0. Max coverage (-): 0

Region: chr24 43072136-43072180. Max. coverage (+): 0. Max coverage (-): 1.36

Region: chr24 43072181-43072225. Max. coverage (+): 0. Max coverage (-): 0

Region: chr24 43072226-43072270. Max. coverage (+): 5.64. Max coverage (-): 0

Region: chr24 43072271-43072316. Max. coverage (+): 12.57. Max coverage (-): 1.47

Region: chr24 43072317-43072361. Max. coverage (+): 0. Max coverage (-): 0

Region: chr24 43072362-43072406. Max. coverage (+): 2.59. Max coverage (-): 0

Region: chr24 43072407-43072451. Max. coverage (+): 6.76. Max coverage (-): 0

Region: chr24 43072452-43072497. Max. coverage (+): 15.6. Max coverage (-): 0

Region: chr24 43072498-43072542. Max. coverage (+): 1.75. Max coverage (-): 0

Region: chr24 43072543-43072587. Max. coverage (+): 11.54. Max coverage (-): 0

Region: chr24 43072588-43072632. Max. coverage (+): 3.64. Max coverage (-): 0

Region: chr24 43072633-43072678. Max. coverage (+): 0. Max coverage (-): 0

Region: chr24 43072679-43072723. Max. coverage (+): 0. Max coverage (-): 0

Region: chr24 43072724-43072768. Max. coverage (+): 0. Max coverage (-): 0

Region: chr24 43072769-43072813. Max. coverage (+): 0. Max coverage (-): 0

Region: chr24 43072814-43072859. Max. coverage (+): 0. Max coverage (-): 0

Region: chr24 43072860-43072904. Max. coverage (+): 0. Max coverage (-): 0

Region: chr24 43072905-43072949. Max. coverage (+): 8.52. Max coverage (-): 0

Region: chr24 43072950-43072994. Max. coverage (+): 25.96. Max coverage (-): 0

Region: chr24 43072995-43073040. Max. coverage (+): 10.73. Max coverage (-): 0

Region: chr24 43073041-43073085. Max. coverage (+): 14.35. Max coverage (-): 0

Region: chr24 43073086-43073130. Max. coverage (+): 9.14. Max coverage (-): 0

Region: chr24 43073131-43073175. Max. coverage (+): 8.2. Max coverage (-): 0

Region: chr24 43073176-43073221. Max. coverage (+): 25.18. Max coverage (-): 0

Region: chr24 43073222-43073266. Max. coverage (+): 5. Max coverage (-): 0

Region: chr24 43073267-43073311. Max. coverage (+): 47.46. Max coverage (-): 0

Region: chr24 43073312-43073356. Max. coverage (+): 47.46. Max coverage (-): 0

Region: chr24 43073357-43073402. Max. coverage (+): 10.61. Max coverage (-): 0

Region: chr24 43073403-43073447. Max. coverage (+): 6.42. Max coverage (-): 0

Region: chr24 43073448-43073492. Max. coverage (+): 2.26. Max coverage (-): 0

Region: chr24 43073493-43073537. Max. coverage (+): 0. Max coverage (-): 0

Region: chr24 43073538-43073583. Max. coverage (+): 0. Max coverage (-): 0

Region: chr24 43073584-43073628. Max. coverage (+): 0. Max coverage (-): 0

Region: chr24 43073629-43073673. Max. coverage (+): 0. Max coverage (-): 0

Region: chr24 43073674-43073718. Max. coverage (+): 0. Max coverage (-): 0

Region: chr24 43073719-43073764. Max. coverage (+): 0. Max coverage (-): 0

Region: chr24 43073765-43073809. Max. coverage (+): 0. Max coverage (-): 0

Region: chr24 43073810-43073854. Max. coverage (+): 0. Max coverage (-): 0

Region: chr24 43073855-43073899. Max. coverage (+): 0. Max coverage (-): 0

Region: chr24 43073900-43073945. Max. coverage (+): 0. Max coverage (-): 0

Region: chr24 43073946-43073990. Max. coverage (+): 0. Max coverage (-): 0

Region: chr24 43073991-43074035. Max. coverage (+): 0. Max coverage (-): 0

Region: chr24 43074036-43074080. Max. coverage (+): 0. Max coverage (-): 0

Region: chr24 43074081-43074126. Max. coverage (+): 0. Max coverage (-): 0

Region: chr24 43074127-43074171. Max. coverage (+): 0. Max coverage (-): 0

Region: chr24 43074172-43074216. Max. coverage (+): 7.56. Max coverage (-): 0

Region: chr24 43074217-43074261. Max. coverage (+): 7.34. Max coverage (-): 0

Region: chr24 43074262-43074307. Max. coverage (+): 4.05. Max coverage (-): 8.56

Region: chr24 43074308-43074352. Max. coverage (+): 4.13. Max coverage (-): 0

Region: chr24 43074353-43074397. Max. coverage (+): 0. Max coverage (-): 0

Region: chr24 43074398-43074442. Max. coverage (+): 0. Max coverage (-): 0

Region: chr24 43074443-43074488. Max. coverage (+): 3.37. Max coverage (-): 0

Region: chr24 43074489-43074533. Max. coverage (+): 10.4. Max coverage (-): 0

Region: chr24 43074534-43074578. Max. coverage (+): 4.82. Max coverage (-): 0

Region: chr24 43074579-43074623. Max. coverage (+): 0. Max coverage (-): 0

Region: chr24 43074624-43074669. Max. coverage (+): 0.33. Max coverage (-): 0

Region: chr24 43074670-43074714. Max. coverage (+): 0.33. Max coverage (-): 2.25

Region: chr24 43074715-43074759. Max. coverage (+): 1.45. Max coverage (-): 0

Region: chr24 43074760-43074804. Max. coverage (+): 0. Max coverage (-): 0

Region: chr24 43074805-43074850. Max. coverage (+): 0. Max coverage (-): 0

Region: chr24 43074851-43074895. Max. coverage (+): 0. Max coverage (-): 0

Region: chr24 43074896-43074940. Max. coverage (+): 0. Max coverage (-): 0

Region: chr24 43074941-43074985. Max. coverage (+): 0. Max coverage (-): 0

Region: chr24 43074986-43075031. Max. coverage (+): 0. Max coverage (-): 0

Region: chr24 43075032-43075076. Max. coverage (+): 0. Max coverage (-): 0

Region: chr24 43075077-43075121. Max. coverage (+): 2.54. Max coverage (-): 0

Region: chr24 43075122-43075166. Max. coverage (+): 5.02. Max coverage (-): 0

Region: chr24 43075167-43075212. Max. coverage (+): 5.02. Max coverage (-): 0

Region: chr24 43075213-43075257. Max. coverage (+): 5.25. Max coverage (-): 0

Region: chr24 43075258-43075302. Max. coverage (+): 0. Max coverage (-): 0

Region: chr24 43075303-43075347. Max. coverage (+): 0. Max coverage (-): 0

Region: chr24 43075348-43075393. Max. coverage (+): 5.98. Max coverage (-): 0

Region: chr24 43075394-43075438. Max. coverage (+): 0. Max coverage (-): 0

Region: chr24 43075439-43075483. Max. coverage (+): 0. Max coverage (-): 0

Region: chr24 43075484-43075528. Max. coverage (+): 0. Max coverage (-): 0

Region: chr24 43075529-43075574. Max. coverage (+): 0. Max coverage (-): 0

Region: chr24 43075575-43075619. Max. coverage (+): 0. Max coverage (-): 0

Region: chr24 43075620-43075664. Max. coverage (+): 0. Max coverage (-): 0

Region: chr24 43075665-43075709. Max. coverage (+): 0. Max coverage (-): 0

Region: chr24 43075710-43075755. Max. coverage (+): 0. Max coverage (-): 0

Region: chr24 43075756-43075800. Max. coverage (+): 0. Max coverage (-): 0

Region: chr24 43075801-43075845. Max. coverage (+): 0. Max coverage (-): 0

Region: chr24 43075846-43075890. Max. coverage (+): 0. Max coverage (-): 0

Region: chr24 43075891-43075936. Max. coverage (+): 0. Max coverage (-): 1.67

Region: chr24 43075937-43075981. Max. coverage (+): 8.26. Max coverage (-): 5

Region: chr24 43075982-. Max. coverage (+): 0. Max coverage (-): 5

RepeatMasker Color Code

**+**

100-98% Identity

<98-95% Identity

<95-90% Identity

<90-85% Identity

<85-80% Identity

<80-75% Identity

<75-70% Identity

<70% Identity

**-**

Gene Set Color Code

**+**

Gene

Pseudogene

**-**

Topology/Coverage Color Code

Coverage Plus Strand

Coverage Minus Strand

Mainstrand: Plus

Mainstrand: Minus

Complementary Strand

Flanking Region  
(if option -flank >0)

Gene Set Annotation  
  
RepeatMasker Annotation  

**1. L1M5**: 43054729-43055089 (+), Divergence to consensus: 44.2%  
**2. MamRep38**: 43055155-43055239 (-), Divergence to consensus: 24.1%  
**3. MLT1F**: 43055659-43055889 (+), Divergence to consensus: 36.9%  
**4. MLT1F-int**: 43056202-43056402 (+), Divergence to consensus: 40.2%  
**5. L1\_BT**: 43056583-43056744 (+), Divergence to consensus: 17.3%  
**6. MLT1F-int**: 43056760-43056913 (+), Divergence to consensus: 42%  
**7. MIR3**: 43058808-43058865 (-), Divergence to consensus: 24.1%  
**8. L1M3**: 43059250-43059397 (-), Divergence to consensus: 20.4%  
**9. (TCCCC)n**: 43059791-43059819 (+), Divergence to consensus: 6.9%  
**10. MIRc**: 43063027-43063082 (-), Divergence to consensus: 32.1%  
**11. ART2A**: 43065904-43066205 (+), Divergence to consensus: 18.7%  
**12. Bov-tA2**: 43066418-43066599 (+), Divergence to consensus: 24.7%  
**13. (CACTT)n**: 43066601-43066628 (+), Divergence to consensus: 0%  
**14. MLT1K**: 43067026-43067250 (+), Divergence to consensus: 43%  
**15. LTR16A2**: 43072672-43072774 (+), Divergence to consensus: 28.1%  
**16. SINE2-3\_BT**: 43072815-43072874 (+), Divergence to consensus: 26.7%  
**17. MER63B**: 43073025-43073135 (-), Divergence to consensus: 32.7%  
**18. L2b**: 43073489-43073771 (-), Divergence to consensus: 41.2%  
**19. L2b**: 43073813-43074136 (-), Divergence to consensus: 48.9%  
**20. Bov-tA2**: 43074867-43075054 (-), Divergence to consensus: 14.4%  
**21. HAL1**: 43075402-43075799 (+), Divergence to consensus: 49.6%

  
Transcription Factor Binding Sites  

**RFX4\_2** (Sequence: GTAACCATG (-): 43057874)  
**RFX4\_2** (Sequence: CTTGGTTAC (+): 43065675)  
**Gata4** (Sequence: AGATAAG (-): 43064756)  
**SOX9** (Sequence: AACAATGG (-): 43057333)  
**SOX9** (Sequence: AACAATAA (-): 43075989)  
**SOX9** (Sequence: CCATTGTT (+): 43063980)  
**A-MYB** (Sequence: CCAACTGCCT (-): 43070409)
