# Supplementary material for: Small RNA sequencing of cryopreserved semen from single bull revealed altered miRNAs and piRNAs expression between High- and Low-motile sperm populations
Source: BMC Genomics. 2017 Jan 4;18:14. doi: 10.1186/s12864-016-3394-7 (PMC5209821; doi:10.1186/s12864-016-3394-7)
Supplement: Additional file 3: — Details for each piRNA clusters found in High Motile (HM) sperm fraction. Genes, repeats, transposable elements and transcription factors binding sites falling within the cluster regions were reported. (ZIP 1896 kb) [file 12864_2016_3394_MOESM3_ESM.zip › 58.html]

piRNA cluster 58


Predicted piRNA cluster no. 58     previous   next
  

Show proTRAC run info
Hide proTRAC run info

================================= proTRAC ====================================  
VERSION: 2.1                                    LAST MODIFIED: 06. October 2015  
  
Please cite:  
Rosenkranz D, Zischler H. proTRAC - a software for probabilistic piRNA cluster  
detection, visualization and analysis. 2012. BMC Bioinformatics 13:5.  
  
and (for proTRAC 2.0 and later):  
Rosenkranz D, Rudloff S, Bastuck K, Ketting RF, Zischler H. Tupaia small RNAs  
provide insights into function and evolution of RNAi-based transposon defense  
in mammals. 2015. RNA 21(5):911-922.  
  
Contact:  
David Rosenkranz  
Institute of Anthropology, small RNA group  
Johannes Gutenberg University Mainz  
email: rosenkranz@uni-mainz.de  
  
You can find the latest proTRAC version at:  
http://sourceforge.net/projects/protrac/files  
http://www.smallRNAgroup-mainz.de/software  
==============================================================================  
  
PARAMETERS:  
Map file: .............../storage/core/barbara/genhome/smallRNA/fertility/Sample\_motile/pirna/Sample\_motile\_26-33\_collapsed.fa.no-dust.map.weighted-10000-1000-b-0  
Genome file: ............/storage/core/barbara/genhome/smallRNA/fertility/Sample\_all/pirna/bt\_311\_chrY.fa  
RepeatMasker annotation: /storage/genomes/bt\_umd31/GCF\_000003055.6\_Bos\_taurus\_UMD\_3.1.1\_repeatMasker\_chr.out  
GeneSet:................./storage/core/barbara/genhome/smallRNA/fertility/Sample\_all/pirna/full.gtf  
  
Significant (p<=0.01) hit density will be calculated based  
on observed hit distribution.  
  
Sliding window size: ........................................ 5000 bp  
Sliding window increament: .................................. 1000 bp  
Normalize each hit by number of genomic hits: ............... 1 [0=no/1=yes]  
Normalize each hit by number of sequence reads: ............. 1 [0=no/1=yes]  
Normalize values (-> per million mapped reads): ............. 1 [0=no/1=yes]  
Min. fraction of hits with 1T(U) or 10A: .................... 0.75  
Alternatively: Min. fraction of hits with 1T(U) and 10A: .... 0.5  
Min. fraction of hits with typical piRNA length: ............ 0.75  
Typical piRNA length: ....................................... 26-33 nt  
Min. size of a piRNA cluster: ............................... 5000 bp.  
Min. number of hits (absolute): ............................. 0  
Min. number of hits (normalized): ........................... 0  
Min. fraction of hits on the mainstrand: .................... 0.75  
Top fraction of mapped sequences (in terms of read counts): . 1%  
Top fraction accounts for max. n% of sequence reads: ........ 90%  
Min. fraction of hits on each arm of a bidirectional cluster: 0.1  
Output image file for each cluster: ......................... 0 [0=no/1=yes]  
Output html file for each cluster: .......................... 1 [0=no/1=yes]  
Output a summary table: ..................................... 1 [0=no/1=yes]  
Output a FASTA file for each cluster (piRNA sequences): ..... 1 [0=no/1=yes]  
Output a FASTA file comprising cluster sequences: ........... 1 [0=no/1=yes]  
Search DNA motifs in clusters: .............................. 1 [0=no/1=yes]  
Output flanking sequences: +/- .............................. 0 bp  
Output ~.pTi file: .......................................... 1 [0=no/1=yes]  
==============================================================================  
  
  
Genome size (without gaps): ............ 2678902517 bp  
Gaps (N/X/-): .......................... 53837044 bp  
Mapped reads: .......................... 658825247023  
Non-identical sequences: ............... 514171  
Genomic hits: .......................... 764233  
Significant densitiy of mapped reads: .. 12867599.5173724 reads/kb

Show proTRAC cluster info
Hide proTRAC cluster info

|  |  |
| --- | --- |
| Location | chr25 |
| Coordinates | 28792429-28801027 |
| Size [bp] | 8599 |
| Sequence hit loci | 66 |
| Mapped reads (normalized) | 91529317 |
| Mapped reads (normalized) per kb | 10644181.5 |
| Normalized reads with 1T (1U) | 87.6% |
| Normalized reads with 10A | 40% |
| Normalized reads with length 26-33 nt | 100% |
| Normalized reads on the main strand(s) | 94.8% |
| Predicted directionality | mono:minus |

100%

0%

1T (1U)  
reads

10A reads

26-33 nt  
reads

reads on mainstrand

**Either the amount of reads with 1T (1U) OR 10A has to exceed 75% (set with option: -1Tor10A)  
Alternatively the amount of reads with 1T (1U) AND 10A has to exceed 50% (set with option: -1Tand10A)  
Minimum amount of reads with preferred size is 75% (set with option: -pisize)  
Minimum amount of reads on the main strand(s) is 75% (set with option: -clstrand)**

Show read coverage
Hide read coverage

WHAT DO I SEE HERE?  
This chart shows the location of mapped sequence reads within a predicted piRNA cluster. The color refers to the number of genomic hits produced by the sequence read in question. A dark red bar indicates that this sequence read produces many other hits elsewhere in the genome. Many adjacent red or yellow bars can indicate the presence of a multi-copy element such as transposons or rRNA genes. A dark green bar indicates that this sequence read maps uniquely to this locus.

1 hit

2-5 hits

6-10 hits

11-20 hits

21-50 hits

51-100 hits

> 100 hits

chr25

28792429

28801027

Gene Set

RepeatMasker

Mapped  
Reads

11

plus strand

minus strand

11

Region: chr25 43075983-28792437. Max. coverage (+): 0. Max coverage (-): 7.81

Region: chr25 28792438-28792454. Max. coverage (+): 0. Max coverage (-): 0.95

Region: chr25 28792455-28792471. Max. coverage (+): 0. Max coverage (-): 0

Region: chr25 28792472-28792489. Max. coverage (+): 0. Max coverage (-): 0

Region: chr25 28792490-28792506. Max. coverage (+): 0. Max coverage (-): 0

Region: chr25 28792507-28792523. Max. coverage (+): 0. Max coverage (-): 0

Region: chr25 28792524-28792540. Max. coverage (+): 0. Max coverage (-): 0

Region: chr25 28792541-28792557. Max. coverage (+): 0. Max coverage (-): 0

Region: chr25 28792558-28792575. Max. coverage (+): 0. Max coverage (-): 0

Region: chr25 28792576-28792592. Max. coverage (+): 0. Max coverage (-): 0

Region: chr25 28792593-28792609. Max. coverage (+): 0. Max coverage (-): 0

Region: chr25 28792610-28792626. Max. coverage (+): 0. Max coverage (-): 0

Region: chr25 28792627-28792643. Max. coverage (+): 0. Max coverage (-): 0

Region: chr25 28792644-28792661. Max. coverage (+): 0. Max coverage (-): 0

Region: chr25 28792662-28792678. Max. coverage (+): 0. Max coverage (-): 0

Region: chr25 28792679-28792695. Max. coverage (+): 0. Max coverage (-): 0

Region: chr25 28792696-28792712. Max. coverage (+): 0. Max coverage (-): 0

Region: chr25 28792713-28792729. Max. coverage (+): 0. Max coverage (-): 0

Region: chr25 28792730-28792747. Max. coverage (+): 0. Max coverage (-): 0

Region: chr25 28792748-28792764. Max. coverage (+): 0. Max coverage (-): 0

Region: chr25 28792765-28792781. Max. coverage (+): 0. Max coverage (-): 0

Region: chr25 28792782-28792798. Max. coverage (+): 0. Max coverage (-): 0

Region: chr25 28792799-28792815. Max. coverage (+): 0. Max coverage (-): 0

Region: chr25 28792816-28792833. Max. coverage (+): 0. Max coverage (-): 0

Region: chr25 28792834-28792850. Max. coverage (+): 0. Max coverage (-): 0

Region: chr25 28792851-28792867. Max. coverage (+): 0. Max coverage (-): 0

Region: chr25 28792868-28792884. Max. coverage (+): 0. Max coverage (-): 0

Region: chr25 28792885-28792901. Max. coverage (+): 0. Max coverage (-): 0

Region: chr25 28792902-28792919. Max. coverage (+): 0. Max coverage (-): 0

Region: chr25 28792920-28792936. Max. coverage (+): 0. Max coverage (-): 0

Region: chr25 28792937-28792953. Max. coverage (+): 0. Max coverage (-): 0

Region: chr25 28792954-28792970. Max. coverage (+): 0. Max coverage (-): 0

Region: chr25 28792971-28792987. Max. coverage (+): 0. Max coverage (-): 0

Region: chr25 28792988-28793005. Max. coverage (+): 0. Max coverage (-): 0

Region: chr25 28793006-28793022. Max. coverage (+): 0. Max coverage (-): 0

Region: chr25 28793023-28793039. Max. coverage (+): 0. Max coverage (-): 0

Region: chr25 28793040-28793056. Max. coverage (+): 0. Max coverage (-): 0

Region: chr25 28793057-28793073. Max. coverage (+): 0. Max coverage (-): 0

Region: chr25 28793074-28793091. Max. coverage (+): 0. Max coverage (-): 0

Region: chr25 28793092-28793108. Max. coverage (+): 0. Max coverage (-): 0

Region: chr25 28793109-28793125. Max. coverage (+): 0. Max coverage (-): 0

Region: chr25 28793126-28793142. Max. coverage (+): 0. Max coverage (-): 0

Region: chr25 28793143-28793159. Max. coverage (+): 0. Max coverage (-): 0

Region: chr25 28793160-28793177. Max. coverage (+): 0. Max coverage (-): 0.32

Region: chr25 28793178-28793194. Max. coverage (+): 0. Max coverage (-): 0.32

Region: chr25 28793195-28793211. Max. coverage (+): 0. Max coverage (-): 0

Region: chr25 28793212-28793228. Max. coverage (+): 0. Max coverage (-): 0

Region: chr25 28793229-28793245. Max. coverage (+): 0. Max coverage (-): 0

Region: chr25 28793246-28793263. Max. coverage (+): 0. Max coverage (-): 0

Region: chr25 28793264-28793280. Max. coverage (+): 0. Max coverage (-): 0

Region: chr25 28793281-28793297. Max. coverage (+): 0. Max coverage (-): 0

Region: chr25 28793298-28793314. Max. coverage (+): 0. Max coverage (-): 0

Region: chr25 28793315-28793331. Max. coverage (+): 0. Max coverage (-): 0

Region: chr25 28793332-28793349. Max. coverage (+): 0. Max coverage (-): 0

Region: chr25 28793350-28793366. Max. coverage (+): 0. Max coverage (-): 0

Region: chr25 28793367-28793383. Max. coverage (+): 0. Max coverage (-): 0

Region: chr25 28793384-28793400. Max. coverage (+): 0. Max coverage (-): 0

Region: chr25 28793401-28793417. Max. coverage (+): 0. Max coverage (-): 0

Region: chr25 28793418-28793435. Max. coverage (+): 0. Max coverage (-): 0

Region: chr25 28793436-28793452. Max. coverage (+): 0. Max coverage (-): 0

Region: chr25 28793453-28793469. Max. coverage (+): 0. Max coverage (-): 0

Region: chr25 28793470-28793486. Max. coverage (+): 0. Max coverage (-): 0

Region: chr25 28793487-28793503. Max. coverage (+): 0. Max coverage (-): 0

Region: chr25 28793504-28793521. Max. coverage (+): 0. Max coverage (-): 0

Region: chr25 28793522-28793538. Max. coverage (+): 0. Max coverage (-): 0

Region: chr25 28793539-28793555. Max. coverage (+): 0. Max coverage (-): 0

Region: chr25 28793556-28793572. Max. coverage (+): 0. Max coverage (-): 0

Region: chr25 28793573-28793589. Max. coverage (+): 0. Max coverage (-): 0

Region: chr25 28793590-28793607. Max. coverage (+): 0. Max coverage (-): 0

Region: chr25 28793608-28793624. Max. coverage (+): 0. Max coverage (-): 0

Region: chr25 28793625-28793641. Max. coverage (+): 0. Max coverage (-): 0

Region: chr25 28793642-28793658. Max. coverage (+): 0. Max coverage (-): 0.29

Region: chr25 28793659-28793675. Max. coverage (+): 0. Max coverage (-): 0.29

Region: chr25 28793676-28793693. Max. coverage (+): 0. Max coverage (-): 0

Region: chr25 28793694-28793710. Max. coverage (+): 0. Max coverage (-): 0

Region: chr25 28793711-28793727. Max. coverage (+): 0. Max coverage (-): 0

Region: chr25 28793728-28793744. Max. coverage (+): 0. Max coverage (-): 0

Region: chr25 28793745-28793761. Max. coverage (+): 0. Max coverage (-): 0

Region: chr25 28793762-28793779. Max. coverage (+): 0. Max coverage (-): 0

Region: chr25 28793780-28793796. Max. coverage (+): 0. Max coverage (-): 0

Region: chr25 28793797-28793813. Max. coverage (+): 0. Max coverage (-): 0

Region: chr25 28793814-28793830. Max. coverage (+): 0. Max coverage (-): 0

Region: chr25 28793831-28793847. Max. coverage (+): 0. Max coverage (-): 0

Region: chr25 28793848-28793865. Max. coverage (+): 0. Max coverage (-): 0

Region: chr25 28793866-28793882. Max. coverage (+): 0. Max coverage (-): 0

Region: chr25 28793883-28793899. Max. coverage (+): 0. Max coverage (-): 0

Region: chr25 28793900-28793916. Max. coverage (+): 0. Max coverage (-): 0

Region: chr25 28793917-28793933. Max. coverage (+): 0. Max coverage (-): 0

Region: chr25 28793934-28793951. Max. coverage (+): 0. Max coverage (-): 0

Region: chr25 28793952-28793968. Max. coverage (+): 0. Max coverage (-): 0

Region: chr25 28793969-28793985. Max. coverage (+): 0. Max coverage (-): 0

Region: chr25 28793986-28794002. Max. coverage (+): 0. Max coverage (-): 0

Region: chr25 28794003-28794019. Max. coverage (+): 0. Max coverage (-): 0.6

Region: chr25 28794020-28794037. Max. coverage (+): 0. Max coverage (-): 0.6

Region: chr25 28794038-28794054. Max. coverage (+): 0. Max coverage (-): 0

Region: chr25 28794055-28794071. Max. coverage (+): 0. Max coverage (-): 0

Region: chr25 28794072-28794088. Max. coverage (+): 0. Max coverage (-): 0

Region: chr25 28794089-28794105. Max. coverage (+): 0. Max coverage (-): 0

Region: chr25 28794106-28794123. Max. coverage (+): 0. Max coverage (-): 0

Region: chr25 28794124-28794140. Max. coverage (+): 0. Max coverage (-): 0

Region: chr25 28794141-28794157. Max. coverage (+): 0. Max coverage (-): 0

Region: chr25 28794158-28794174. Max. coverage (+): 0. Max coverage (-): 0

Region: chr25 28794175-28794191. Max. coverage (+): 0. Max coverage (-): 0

Region: chr25 28794192-28794208. Max. coverage (+): 0. Max coverage (-): 0

Region: chr25 28794209-28794226. Max. coverage (+): 0. Max coverage (-): 0

Region: chr25 28794227-28794243. Max. coverage (+): 0. Max coverage (-): 0

Region: chr25 28794244-28794260. Max. coverage (+): 0. Max coverage (-): 0

Region: chr25 28794261-28794277. Max. coverage (+): 0. Max coverage (-): 0

Region: chr25 28794278-28794294. Max. coverage (+): 0. Max coverage (-): 0

Region: chr25 28794295-28794312. Max. coverage (+): 0. Max coverage (-): 0

Region: chr25 28794313-28794329. Max. coverage (+): 0. Max coverage (-): 0

Region: chr25 28794330-28794346. Max. coverage (+): 0. Max coverage (-): 0

Region: chr25 28794347-28794363. Max. coverage (+): 0. Max coverage (-): 0

Region: chr25 28794364-28794380. Max. coverage (+): 0. Max coverage (-): 0

Region: chr25 28794381-28794398. Max. coverage (+): 0. Max coverage (-): 0

Region: chr25 28794399-28794415. Max. coverage (+): 0. Max coverage (-): 0

Region: chr25 28794416-28794432. Max. coverage (+): 0. Max coverage (-): 0

Region: chr25 28794433-28794449. Max. coverage (+): 0. Max coverage (-): 0

Region: chr25 28794450-28794466. Max. coverage (+): 0. Max coverage (-): 0

Region: chr25 28794467-28794484. Max. coverage (+): 0. Max coverage (-): 0

Region: chr25 28794485-28794501. Max. coverage (+): 0. Max coverage (-): 0

Region: chr25 28794502-28794518. Max. coverage (+): 0. Max coverage (-): 0

Region: chr25 28794519-28794535. Max. coverage (+): 0. Max coverage (-): 0

Region: chr25 28794536-28794552. Max. coverage (+): 0. Max coverage (-): 0

Region: chr25 28794553-28794570. Max. coverage (+): 0. Max coverage (-): 0

Region: chr25 28794571-28794587. Max. coverage (+): 0. Max coverage (-): 0

Region: chr25 28794588-28794604. Max. coverage (+): 0. Max coverage (-): 0

Region: chr25 28794605-28794621. Max. coverage (+): 0. Max coverage (-): 0

Region: chr25 28794622-28794638. Max. coverage (+): 0. Max coverage (-): 0

Region: chr25 28794639-28794656. Max. coverage (+): 0. Max coverage (-): 0

Region: chr25 28794657-28794673. Max. coverage (+): 0. Max coverage (-): 0

Region: chr25 28794674-28794690. Max. coverage (+): 0. Max coverage (-): 0

Region: chr25 28794691-28794707. Max. coverage (+): 0. Max coverage (-): 0

Region: chr25 28794708-28794724. Max. coverage (+): 0. Max coverage (-): 0

Region: chr25 28794725-28794742. Max. coverage (+): 0. Max coverage (-): 0

Region: chr25 28794743-28794759. Max. coverage (+): 0. Max coverage (-): 2.45

Region: chr25 28794760-28794776. Max. coverage (+): 7.25. Max coverage (-): 2.45

Region: chr25 28794777-28794793. Max. coverage (+): 7.25. Max coverage (-): 5.5

Region: chr25 28794794-28794810. Max. coverage (+): 0. Max coverage (-): 0

Region: chr25 28794811-28794828. Max. coverage (+): 0. Max coverage (-): 4.56

Region: chr25 28794829-28794845. Max. coverage (+): 0. Max coverage (-): 0

Region: chr25 28794846-28794862. Max. coverage (+): 0. Max coverage (-): 0

Region: chr25 28794863-28794879. Max. coverage (+): 0. Max coverage (-): 0

Region: chr25 28794880-28794896. Max. coverage (+): 0. Max coverage (-): 0

Region: chr25 28794897-28794914. Max. coverage (+): 0. Max coverage (-): 0

Region: chr25 28794915-28794931. Max. coverage (+): 0. Max coverage (-): 0

Region: chr25 28794932-28794948. Max. coverage (+): 0. Max coverage (-): 0

Region: chr25 28794949-28794965. Max. coverage (+): 0. Max coverage (-): 0

Region: chr25 28794966-28794982. Max. coverage (+): 0. Max coverage (-): 0

Region: chr25 28794983-28795000. Max. coverage (+): 0. Max coverage (-): 0

Region: chr25 28795001-28795017. Max. coverage (+): 0. Max coverage (-): 0

Region: chr25 28795018-28795034. Max. coverage (+): 0. Max coverage (-): 0

Region: chr25 28795035-28795051. Max. coverage (+): 0. Max coverage (-): 0

Region: chr25 28795052-28795068. Max. coverage (+): 0. Max coverage (-): 0

Region: chr25 28795069-28795086. Max. coverage (+): 0. Max coverage (-): 0

Region: chr25 28795087-28795103. Max. coverage (+): 0. Max coverage (-): 0

Region: chr25 28795104-28795120. Max. coverage (+): 0. Max coverage (-): 0

Region: chr25 28795121-28795137. Max. coverage (+): 0. Max coverage (-): 0

Region: chr25 28795138-28795154. Max. coverage (+): 0. Max coverage (-): 0

Region: chr25 28795155-28795172. Max. coverage (+): 0. Max coverage (-): 0

Region: chr25 28795173-28795189. Max. coverage (+): 0. Max coverage (-): 0

Region: chr25 28795190-28795206. Max. coverage (+): 0. Max coverage (-): 0

Region: chr25 28795207-28795223. Max. coverage (+): 0. Max coverage (-): 0

Region: chr25 28795224-28795240. Max. coverage (+): 0. Max coverage (-): 0

Region: chr25 28795241-28795258. Max. coverage (+): 0. Max coverage (-): 0

Region: chr25 28795259-28795275. Max. coverage (+): 0. Max coverage (-): 0

Region: chr25 28795276-28795292. Max. coverage (+): 0. Max coverage (-): 0

Region: chr25 28795293-28795309. Max. coverage (+): 0. Max coverage (-): 0

Region: chr25 28795310-28795326. Max. coverage (+): 0. Max coverage (-): 0

Region: chr25 28795327-28795344. Max. coverage (+): 0. Max coverage (-): 0

Region: chr25 28795345-28795361. Max. coverage (+): 0. Max coverage (-): 0

Region: chr25 28795362-28795378. Max. coverage (+): 0. Max coverage (-): 0

Region: chr25 28795379-28795395. Max. coverage (+): 0. Max coverage (-): 0

Region: chr25 28795396-28795412. Max. coverage (+): 0. Max coverage (-): 0

Region: chr25 28795413-28795430. Max. coverage (+): 0. Max coverage (-): 0

Region: chr25 28795431-28795447. Max. coverage (+): 0. Max coverage (-): 0

Region: chr25 28795448-28795464. Max. coverage (+): 0. Max coverage (-): 0

Region: chr25 28795465-28795481. Max. coverage (+): 0. Max coverage (-): 0

Region: chr25 28795482-28795498. Max. coverage (+): 0. Max coverage (-): 0

Region: chr25 28795499-28795516. Max. coverage (+): 0. Max coverage (-): 0

Region: chr25 28795517-28795533. Max. coverage (+): 0. Max coverage (-): 0

Region: chr25 28795534-28795550. Max. coverage (+): 0. Max coverage (-): 0

Region: chr25 28795551-28795567. Max. coverage (+): 0. Max coverage (-): 0

Region: chr25 28795568-28795584. Max. coverage (+): 0. Max coverage (-): 0

Region: chr25 28795585-28795602. Max. coverage (+): 0. Max coverage (-): 0

Region: chr25 28795603-28795619. Max. coverage (+): 0. Max coverage (-): 0

Region: chr25 28795620-28795636. Max. coverage (+): 0. Max coverage (-): 0

Region: chr25 28795637-28795653. Max. coverage (+): 0. Max coverage (-): 0

Region: chr25 28795654-28795670. Max. coverage (+): 0. Max coverage (-): 0

Region: chr25 28795671-28795688. Max. coverage (+): 0. Max coverage (-): 0

Region: chr25 28795689-28795705. Max. coverage (+): 0. Max coverage (-): 0

Region: chr25 28795706-28795722. Max. coverage (+): 0. Max coverage (-): 0

Region: chr25 28795723-28795739. Max. coverage (+): 0. Max coverage (-): 0

Region: chr25 28795740-28795756. Max. coverage (+): 0. Max coverage (-): 0

Region: chr25 28795757-28795774. Max. coverage (+): 0. Max coverage (-): 0

Region: chr25 28795775-28795791. Max. coverage (+): 0. Max coverage (-): 0

Region: chr25 28795792-28795808. Max. coverage (+): 0. Max coverage (-): 0

Region: chr25 28795809-28795825. Max. coverage (+): 0. Max coverage (-): 0

Region: chr25 28795826-28795842. Max. coverage (+): 0. Max coverage (-): 0

Region: chr25 28795843-28795860. Max. coverage (+): 0. Max coverage (-): 0

Region: chr25 28795861-28795877. Max. coverage (+): 0. Max coverage (-): 0

Region: chr25 28795878-28795894. Max. coverage (+): 0. Max coverage (-): 1.87

Region: chr25 28795895-28795911. Max. coverage (+): 0. Max coverage (-): 2.24

Region: chr25 28795912-28795928. Max. coverage (+): 0. Max coverage (-): 2.24

Region: chr25 28795929-28795945. Max. coverage (+): 0. Max coverage (-): 1.78

Region: chr25 28795946-28795963. Max. coverage (+): 0. Max coverage (-): 2.31

Region: chr25 28795964-28795980. Max. coverage (+): 0. Max coverage (-): 0

Region: chr25 28795981-28795997. Max. coverage (+): 0. Max coverage (-): 0

Region: chr25 28795998-28796014. Max. coverage (+): 0. Max coverage (-): 0

Region: chr25 28796015-28796031. Max. coverage (+): 0. Max coverage (-): 0

Region: chr25 28796032-28796049. Max. coverage (+): 0. Max coverage (-): 0

Region: chr25 28796050-28796066. Max. coverage (+): 0. Max coverage (-): 0

Region: chr25 28796067-28796083. Max. coverage (+): 0. Max coverage (-): 4.59

Region: chr25 28796084-28796100. Max. coverage (+): 0. Max coverage (-): 0

Region: chr25 28796101-28796117. Max. coverage (+): 0. Max coverage (-): 0

Region: chr25 28796118-28796135. Max. coverage (+): 0. Max coverage (-): 0

Region: chr25 28796136-28796152. Max. coverage (+): 0. Max coverage (-): 2.61

Region: chr25 28796153-28796169. Max. coverage (+): 0. Max coverage (-): 2.61

Region: chr25 28796170-28796186. Max. coverage (+): 0. Max coverage (-): 0

Region: chr25 28796187-28796203. Max. coverage (+): 0. Max coverage (-): 0

Region: chr25 28796204-28796221. Max. coverage (+): 0. Max coverage (-): 0.43

Region: chr25 28796222-28796238. Max. coverage (+): 0. Max coverage (-): 0

Region: chr25 28796239-28796255. Max. coverage (+): 0. Max coverage (-): 0

Region: chr25 28796256-28796272. Max. coverage (+): 0. Max coverage (-): 0

Region: chr25 28796273-28796289. Max. coverage (+): 0. Max coverage (-): 0

Region: chr25 28796290-28796307. Max. coverage (+): 0. Max coverage (-): 0

Region: chr25 28796308-28796324. Max. coverage (+): 0. Max coverage (-): 3.15

Region: chr25 28796325-28796341. Max. coverage (+): 0. Max coverage (-): 1.48

Region: chr25 28796342-28796358. Max. coverage (+): 0. Max coverage (-): 0

Region: chr25 28796359-28796375. Max. coverage (+): 0. Max coverage (-): 0

Region: chr25 28796376-28796393. Max. coverage (+): 0. Max coverage (-): 0

Region: chr25 28796394-28796410. Max. coverage (+): 0. Max coverage (-): 4.67

Region: chr25 28796411-28796427. Max. coverage (+): 0. Max coverage (-): 6.08

Region: chr25 28796428-28796444. Max. coverage (+): 0. Max coverage (-): 0.42

Region: chr25 28796445-28796461. Max. coverage (+): 0. Max coverage (-): 3.95

Region: chr25 28796462-28796479. Max. coverage (+): 0. Max coverage (-): 0

Region: chr25 28796480-28796496. Max. coverage (+): 0. Max coverage (-): 1.55

Region: chr25 28796497-28796513. Max. coverage (+): 0. Max coverage (-): 0

Region: chr25 28796514-28796530. Max. coverage (+): 0. Max coverage (-): 4.96

Region: chr25 28796531-28796547. Max. coverage (+): 0. Max coverage (-): 4.96

Region: chr25 28796548-28796565. Max. coverage (+): 0. Max coverage (-): 0

Region: chr25 28796566-28796582. Max. coverage (+): 0. Max coverage (-): 0

Region: chr25 28796583-28796599. Max. coverage (+): 0. Max coverage (-): 4.71

Region: chr25 28796600-28796616. Max. coverage (+): 0. Max coverage (-): 0

Region: chr25 28796617-28796633. Max. coverage (+): 0. Max coverage (-): 5.01

Region: chr25 28796634-28796651. Max. coverage (+): 0. Max coverage (-): 0

Region: chr25 28796652-28796668. Max. coverage (+): 0. Max coverage (-): 2.5

Region: chr25 28796669-28796685. Max. coverage (+): 0. Max coverage (-): 0

Region: chr25 28796686-28796702. Max. coverage (+): 0. Max coverage (-): 0

Region: chr25 28796703-28796719. Max. coverage (+): 0. Max coverage (-): 4.02

Region: chr25 28796720-28796737. Max. coverage (+): 0. Max coverage (-): 4.02

Region: chr25 28796738-28796754. Max. coverage (+): 0. Max coverage (-): 0

Region: chr25 28796755-28796771. Max. coverage (+): 0. Max coverage (-): 0

Region: chr25 28796772-28796788. Max. coverage (+): 0. Max coverage (-): 0

Region: chr25 28796789-28796805. Max. coverage (+): 0. Max coverage (-): 0

Region: chr25 28796806-28796823. Max. coverage (+): 0. Max coverage (-): 0

Region: chr25 28796824-28796840. Max. coverage (+): 0. Max coverage (-): 0

Region: chr25 28796841-28796857. Max. coverage (+): 0. Max coverage (-): 1.41

Region: chr25 28796858-28796874. Max. coverage (+): 0. Max coverage (-): 4.54

Region: chr25 28796875-28796891. Max. coverage (+): 0. Max coverage (-): 0

Region: chr25 28796892-28796909. Max. coverage (+): 0. Max coverage (-): 0

Region: chr25 28796910-28796926. Max. coverage (+): 0. Max coverage (-): 0

Region: chr25 28796927-28796943. Max. coverage (+): 0. Max coverage (-): 0

Region: chr25 28796944-28796960. Max. coverage (+): 0. Max coverage (-): 0

Region: chr25 28796961-28796977. Max. coverage (+): 0. Max coverage (-): 0

Region: chr25 28796978-28796995. Max. coverage (+): 0. Max coverage (-): 4.31

Region: chr25 28796996-28797012. Max. coverage (+): 0. Max coverage (-): 2.15

Region: chr25 28797013-28797029. Max. coverage (+): 0. Max coverage (-): 0

Region: chr25 28797030-28797046. Max. coverage (+): 0. Max coverage (-): 0

Region: chr25 28797047-28797063. Max. coverage (+): 0. Max coverage (-): 0

Region: chr25 28797064-28797081. Max. coverage (+): 0. Max coverage (-): 0

Region: chr25 28797082-28797098. Max. coverage (+): 0. Max coverage (-): 0

Region: chr25 28797099-28797115. Max. coverage (+): 0. Max coverage (-): 0

Region: chr25 28797116-28797132. Max. coverage (+): 0. Max coverage (-): 0

Region: chr25 28797133-28797149. Max. coverage (+): 0. Max coverage (-): 0

Region: chr25 28797150-28797167. Max. coverage (+): 0. Max coverage (-): 0

Region: chr25 28797168-28797184. Max. coverage (+): 0. Max coverage (-): 0

Region: chr25 28797185-28797201. Max. coverage (+): 0. Max coverage (-): 0

Region: chr25 28797202-28797218. Max. coverage (+): 0. Max coverage (-): 0

Region: chr25 28797219-28797235. Max. coverage (+): 0. Max coverage (-): 0

Region: chr25 28797236-28797253. Max. coverage (+): 0. Max coverage (-): 0

Region: chr25 28797254-28797270. Max. coverage (+): 0. Max coverage (-): 0

Region: chr25 28797271-28797287. Max. coverage (+): 0. Max coverage (-): 0

Region: chr25 28797288-28797304. Max. coverage (+): 0. Max coverage (-): 0

Region: chr25 28797305-28797321. Max. coverage (+): 0. Max coverage (-): 0

Region: chr25 28797322-28797339. Max. coverage (+): 0. Max coverage (-): 0

Region: chr25 28797340-28797356. Max. coverage (+): 0. Max coverage (-): 0

Region: chr25 28797357-28797373. Max. coverage (+): 0. Max coverage (-): 0

Region: chr25 28797374-28797390. Max. coverage (+): 0. Max coverage (-): 0

Region: chr25 28797391-28797407. Max. coverage (+): 0. Max coverage (-): 0

Region: chr25 28797408-28797425. Max. coverage (+): 0. Max coverage (-): 0

Region: chr25 28797426-28797442. Max. coverage (+): 0. Max coverage (-): 0

Region: chr25 28797443-28797459. Max. coverage (+): 0. Max coverage (-): 2.51

Region: chr25 28797460-28797476. Max. coverage (+): 0. Max coverage (-): 0

Region: chr25 28797477-28797493. Max. coverage (+): 0. Max coverage (-): 0

Region: chr25 28797494-28797511. Max. coverage (+): 0. Max coverage (-): 0

Region: chr25 28797512-28797528. Max. coverage (+): 0. Max coverage (-): 4.32

Region: chr25 28797529-28797545. Max. coverage (+): 0. Max coverage (-): 0

Region: chr25 28797546-28797562. Max. coverage (+): 0. Max coverage (-): 0

Region: chr25 28797563-28797579. Max. coverage (+): 0. Max coverage (-): 0

Region: chr25 28797580-28797596. Max. coverage (+): 0. Max coverage (-): 0

Region: chr25 28797597-28797614. Max. coverage (+): 0. Max coverage (-): 0

Region: chr25 28797615-28797631. Max. coverage (+): 0. Max coverage (-): 0

Region: chr25 28797632-28797648. Max. coverage (+): 0. Max coverage (-): 0

Region: chr25 28797649-28797665. Max. coverage (+): 0. Max coverage (-): 0

Region: chr25 28797666-28797682. Max. coverage (+): 0. Max coverage (-): 0

Region: chr25 28797683-28797700. Max. coverage (+): 0. Max coverage (-): 0

Region: chr25 28797701-28797717. Max. coverage (+): 0. Max coverage (-): 0

Region: chr25 28797718-28797734. Max. coverage (+): 0. Max coverage (-): 0

Region: chr25 28797735-28797751. Max. coverage (+): 0. Max coverage (-): 0

Region: chr25 28797752-28797768. Max. coverage (+): 0. Max coverage (-): 0

Region: chr25 28797769-28797786. Max. coverage (+): 0. Max coverage (-): 0

Region: chr25 28797787-28797803. Max. coverage (+): 0. Max coverage (-): 0

Region: chr25 28797804-28797820. Max. coverage (+): 0. Max coverage (-): 0

Region: chr25 28797821-28797837. Max. coverage (+): 0. Max coverage (-): 0

Region: chr25 28797838-28797854. Max. coverage (+): 0. Max coverage (-): 0

Region: chr25 28797855-28797872. Max. coverage (+): 0. Max coverage (-): 0

Region: chr25 28797873-28797889. Max. coverage (+): 0. Max coverage (-): 0

Region: chr25 28797890-28797906. Max. coverage (+): 0. Max coverage (-): 0

Region: chr25 28797907-28797923. Max. coverage (+): 0. Max coverage (-): 0

Region: chr25 28797924-28797940. Max. coverage (+): 0. Max coverage (-): 0

Region: chr25 28797941-28797958. Max. coverage (+): 0. Max coverage (-): 0

Region: chr25 28797959-28797975. Max. coverage (+): 0. Max coverage (-): 0

Region: chr25 28797976-28797992. Max. coverage (+): 0. Max coverage (-): 0

Region: chr25 28797993-28798009. Max. coverage (+): 0. Max coverage (-): 0

Region: chr25 28798010-28798026. Max. coverage (+): 0. Max coverage (-): 0

Region: chr25 28798027-28798044. Max. coverage (+): 0. Max coverage (-): 0

Region: chr25 28798045-28798061. Max. coverage (+): 0. Max coverage (-): 0

Region: chr25 28798062-28798078. Max. coverage (+): 0. Max coverage (-): 0

Region: chr25 28798079-28798095. Max. coverage (+): 0. Max coverage (-): 0

Region: chr25 28798096-28798112. Max. coverage (+): 0. Max coverage (-): 0

Region: chr25 28798113-28798130. Max. coverage (+): 0. Max coverage (-): 0

Region: chr25 28798131-28798147. Max. coverage (+): 0. Max coverage (-): 0

Region: chr25 28798148-28798164. Max. coverage (+): 0. Max coverage (-): 0

Region: chr25 28798165-28798181. Max. coverage (+): 0. Max coverage (-): 0

Region: chr25 28798182-28798198. Max. coverage (+): 0. Max coverage (-): 0

Region: chr25 28798199-28798216. Max. coverage (+): 0. Max coverage (-): 0

Region: chr25 28798217-28798233. Max. coverage (+): 0. Max coverage (-): 0

Region: chr25 28798234-28798250. Max. coverage (+): 0. Max coverage (-): 0

Region: chr25 28798251-28798267. Max. coverage (+): 0. Max coverage (-): 0

Region: chr25 28798268-28798284. Max. coverage (+): 0. Max coverage (-): 0

Region: chr25 28798285-28798302. Max. coverage (+): 0. Max coverage (-): 0

Region: chr25 28798303-28798319. Max. coverage (+): 0. Max coverage (-): 0

Region: chr25 28798320-28798336. Max. coverage (+): 0. Max coverage (-): 0

Region: chr25 28798337-28798353. Max. coverage (+): 0. Max coverage (-): 0

Region: chr25 28798354-28798370. Max. coverage (+): 0. Max coverage (-): 0

Region: chr25 28798371-28798388. Max. coverage (+): 0. Max coverage (-): 0

Region: chr25 28798389-28798405. Max. coverage (+): 0. Max coverage (-): 0

Region: chr25 28798406-28798422. Max. coverage (+): 0. Max coverage (-): 0

Region: chr25 28798423-28798439. Max. coverage (+): 0. Max coverage (-): 0

Region: chr25 28798440-28798456. Max. coverage (+): 0. Max coverage (-): 0

Region: chr25 28798457-28798474. Max. coverage (+): 0. Max coverage (-): 0

Region: chr25 28798475-28798491. Max. coverage (+): 0. Max coverage (-): 0

Region: chr25 28798492-28798508. Max. coverage (+): 0. Max coverage (-): 2.96

Region: chr25 28798509-28798525. Max. coverage (+): 0. Max coverage (-): 0

Region: chr25 28798526-28798542. Max. coverage (+): 0. Max coverage (-): 0

Region: chr25 28798543-28798560. Max. coverage (+): 0. Max coverage (-): 0

Region: chr25 28798561-28798577. Max. coverage (+): 0. Max coverage (-): 0

Region: chr25 28798578-28798594. Max. coverage (+): 0. Max coverage (-): 0

Region: chr25 28798595-28798611. Max. coverage (+): 0. Max coverage (-): 0

Region: chr25 28798612-28798628. Max. coverage (+): 0. Max coverage (-): 0

Region: chr25 28798629-28798646. Max. coverage (+): 0. Max coverage (-): 0

Region: chr25 28798647-28798663. Max. coverage (+): 0. Max coverage (-): 0

Region: chr25 28798664-28798680. Max. coverage (+): 0. Max coverage (-): 0

Region: chr25 28798681-28798697. Max. coverage (+): 0. Max coverage (-): 0

Region: chr25 28798698-28798714. Max. coverage (+): 0. Max coverage (-): 0

Region: chr25 28798715-28798732. Max. coverage (+): 0. Max coverage (-): 0

Region: chr25 28798733-28798749. Max. coverage (+): 0. Max coverage (-): 0

Region: chr25 28798750-28798766. Max. coverage (+): 0. Max coverage (-): 0

Region: chr25 28798767-28798783. Max. coverage (+): 0. Max coverage (-): 0

Region: chr25 28798784-28798800. Max. coverage (+): 0. Max coverage (-): 0

Region: chr25 28798801-28798818. Max. coverage (+): 0. Max coverage (-): 0

Region: chr25 28798819-28798835. Max. coverage (+): 0. Max coverage (-): 0

Region: chr25 28798836-28798852. Max. coverage (+): 0. Max coverage (-): 0

Region: chr25 28798853-28798869. Max. coverage (+): 0. Max coverage (-): 0

Region: chr25 28798870-28798886. Max. coverage (+): 0. Max coverage (-): 0

Region: chr25 28798887-28798904. Max. coverage (+): 0. Max coverage (-): 0

Region: chr25 28798905-28798921. Max. coverage (+): 0. Max coverage (-): 0

Region: chr25 28798922-28798938. Max. coverage (+): 0. Max coverage (-): 0

Region: chr25 28798939-28798955. Max. coverage (+): 0. Max coverage (-): 0

Region: chr25 28798956-28798972. Max. coverage (+): 0. Max coverage (-): 0

Region: chr25 28798973-28798990. Max. coverage (+): 0. Max coverage (-): 0

Region: chr25 28798991-28799007. Max. coverage (+): 0. Max coverage (-): 0

Region: chr25 28799008-28799024. Max. coverage (+): 0. Max coverage (-): 0

Region: chr25 28799025-28799041. Max. coverage (+): 0. Max coverage (-): 0

Region: chr25 28799042-28799058. Max. coverage (+): 0. Max coverage (-): 0

Region: chr25 28799059-28799076. Max. coverage (+): 0. Max coverage (-): 0

Region: chr25 28799077-28799093. Max. coverage (+): 0. Max coverage (-): 0

Region: chr25 28799094-28799110. Max. coverage (+): 0. Max coverage (-): 0

Region: chr25 28799111-28799127. Max. coverage (+): 0. Max coverage (-): 0

Region: chr25 28799128-28799144. Max. coverage (+): 0. Max coverage (-): 0

Region: chr25 28799145-28799162. Max. coverage (+): 0. Max coverage (-): 0

Region: chr25 28799163-28799179. Max. coverage (+): 0. Max coverage (-): 11

Region: chr25 28799180-28799196. Max. coverage (+): 0. Max coverage (-): 0

Region: chr25 28799197-28799213. Max. coverage (+): 0. Max coverage (-): 0

Region: chr25 28799214-28799230. Max. coverage (+): 0. Max coverage (-): 0

Region: chr25 28799231-28799248. Max. coverage (+): 0. Max coverage (-): 0

Region: chr25 28799249-28799265. Max. coverage (+): 0. Max coverage (-): 1.28

Region: chr25 28799266-28799282. Max. coverage (+): 0. Max coverage (-): 1.28

Region: chr25 28799283-28799299. Max. coverage (+): 0. Max coverage (-): 0

Region: chr25 28799300-28799316. Max. coverage (+): 0. Max coverage (-): 0

Region: chr25 28799317-28799333. Max. coverage (+): 0. Max coverage (-): 0

Region: chr25 28799334-28799351. Max. coverage (+): 0. Max coverage (-): 0

Region: chr25 28799352-28799368. Max. coverage (+): 0. Max coverage (-): 0

Region: chr25 28799369-28799385. Max. coverage (+): 0. Max coverage (-): 0

Region: chr25 28799386-28799402. Max. coverage (+): 0. Max coverage (-): 0

Region: chr25 28799403-28799419. Max. coverage (+): 0. Max coverage (-): 0

Region: chr25 28799420-28799437. Max. coverage (+): 0. Max coverage (-): 0

Region: chr25 28799438-28799454. Max. coverage (+): 0. Max coverage (-): 0

Region: chr25 28799455-28799471. Max. coverage (+): 0. Max coverage (-): 0

Region: chr25 28799472-28799488. Max. coverage (+): 0. Max coverage (-): 0

Region: chr25 28799489-28799505. Max. coverage (+): 0. Max coverage (-): 0

Region: chr25 28799506-28799523. Max. coverage (+): 0. Max coverage (-): 0

Region: chr25 28799524-28799540. Max. coverage (+): 0. Max coverage (-): 0

Region: chr25 28799541-28799557. Max. coverage (+): 0. Max coverage (-): 0

Region: chr25 28799558-28799574. Max. coverage (+): 0. Max coverage (-): 0

Region: chr25 28799575-28799591. Max. coverage (+): 0. Max coverage (-): 0

Region: chr25 28799592-28799609. Max. coverage (+): 0. Max coverage (-): 0

Region: chr25 28799610-28799626. Max. coverage (+): 0. Max coverage (-): 0

Region: chr25 28799627-28799643. Max. coverage (+): 0. Max coverage (-): 0

Region: chr25 28799644-28799660. Max. coverage (+): 0. Max coverage (-): 0

Region: chr25 28799661-28799677. Max. coverage (+): 0. Max coverage (-): 1.03

Region: chr25 28799678-28799695. Max. coverage (+): 0. Max coverage (-): 0

Region: chr25 28799696-28799712. Max. coverage (+): 0. Max coverage (-): 0

Region: chr25 28799713-28799729. Max. coverage (+): 0. Max coverage (-): 0

Region: chr25 28799730-28799746. Max. coverage (+): 0. Max coverage (-): 4.97

Region: chr25 28799747-28799763. Max. coverage (+): 0. Max coverage (-): 4.97

Region: chr25 28799764-28799781. Max. coverage (+): 0. Max coverage (-): 0

Region: chr25 28799782-28799798. Max. coverage (+): 0. Max coverage (-): 0

Region: chr25 28799799-28799815. Max. coverage (+): 0. Max coverage (-): 0

Region: chr25 28799816-28799832. Max. coverage (+): 0. Max coverage (-): 0

Region: chr25 28799833-28799849. Max. coverage (+): 0. Max coverage (-): 0

Region: chr25 28799850-28799867. Max. coverage (+): 0. Max coverage (-): 0

Region: chr25 28799868-28799884. Max. coverage (+): 0. Max coverage (-): 0

Region: chr25 28799885-28799901. Max. coverage (+): 0. Max coverage (-): 0

Region: chr25 28799902-28799918. Max. coverage (+): 0. Max coverage (-): 0

Region: chr25 28799919-28799935. Max. coverage (+): 0. Max coverage (-): 0

Region: chr25 28799936-28799953. Max. coverage (+): 0. Max coverage (-): 0

Region: chr25 28799954-28799970. Max. coverage (+): 0. Max coverage (-): 0

Region: chr25 28799971-28799987. Max. coverage (+): 0. Max coverage (-): 0

Region: chr25 28799988-28800004. Max. coverage (+): 0. Max coverage (-): 0

Region: chr25 28800005-28800021. Max. coverage (+): 0. Max coverage (-): 0

Region: chr25 28800022-28800039. Max. coverage (+): 0. Max coverage (-): 0

Region: chr25 28800040-28800056. Max. coverage (+): 0. Max coverage (-): 0

Region: chr25 28800057-28800073. Max. coverage (+): 0. Max coverage (-): 0

Region: chr25 28800074-28800090. Max. coverage (+): 0. Max coverage (-): 0

Region: chr25 28800091-28800107. Max. coverage (+): 0. Max coverage (-): 0

Region: chr25 28800108-28800125. Max. coverage (+): 0. Max coverage (-): 0

Region: chr25 28800126-28800142. Max. coverage (+): 0. Max coverage (-): 0

Region: chr25 28800143-28800159. Max. coverage (+): 0. Max coverage (-): 0

Region: chr25 28800160-28800176. Max. coverage (+): 0. Max coverage (-): 0

Region: chr25 28800177-28800193. Max. coverage (+): 0. Max coverage (-): 0

Region: chr25 28800194-28800211. Max. coverage (+): 0. Max coverage (-): 0

Region: chr25 28800212-28800228. Max. coverage (+): 0. Max coverage (-): 0

Region: chr25 28800229-28800245. Max. coverage (+): 0. Max coverage (-): 0

Region: chr25 28800246-28800262. Max. coverage (+): 0. Max coverage (-): 0

Region: chr25 28800263-28800279. Max. coverage (+): 0. Max coverage (-): 0

Region: chr25 28800280-28800297. Max. coverage (+): 0. Max coverage (-): 0

Region: chr25 28800298-28800314. Max. coverage (+): 0. Max coverage (-): 0

Region: chr25 28800315-28800331. Max. coverage (+): 0. Max coverage (-): 0

Region: chr25 28800332-28800348. Max. coverage (+): 0. Max coverage (-): 0

Region: chr25 28800349-28800365. Max. coverage (+): 0. Max coverage (-): 0

Region: chr25 28800366-28800383. Max. coverage (+): 0. Max coverage (-): 0

Region: chr25 28800384-28800400. Max. coverage (+): 0. Max coverage (-): 0

Region: chr25 28800401-28800417. Max. coverage (+): 0. Max coverage (-): 0

Region: chr25 28800418-28800434. Max. coverage (+): 0. Max coverage (-): 2.15

Region: chr25 28800435-28800451. Max. coverage (+): 0. Max coverage (-): 0

Region: chr25 28800452-28800469. Max. coverage (+): 0. Max coverage (-): 0

Region: chr25 28800470-28800486. Max. coverage (+): 0. Max coverage (-): 0

Region: chr25 28800487-28800503. Max. coverage (+): 0. Max coverage (-): 0

Region: chr25 28800504-28800520. Max. coverage (+): 0. Max coverage (-): 0

Region: chr25 28800521-28800537. Max. coverage (+): 0. Max coverage (-): 0

Region: chr25 28800538-28800555. Max. coverage (+): 0. Max coverage (-): 0

Region: chr25 28800556-28800572. Max. coverage (+): 0. Max coverage (-): 2.56

Region: chr25 28800573-28800589. Max. coverage (+): 0. Max coverage (-): 2.56

Region: chr25 28800590-28800606. Max. coverage (+): 0. Max coverage (-): 0

Region: chr25 28800607-28800623. Max. coverage (+): 0. Max coverage (-): 0

Region: chr25 28800624-28800641. Max. coverage (+): 0. Max coverage (-): 0

Region: chr25 28800642-28800658. Max. coverage (+): 0. Max coverage (-): 5.96

Region: chr25 28800659-28800675. Max. coverage (+): 0. Max coverage (-): 0

Region: chr25 28800676-28800692. Max. coverage (+): 0. Max coverage (-): 0

Region: chr25 28800693-28800709. Max. coverage (+): 0. Max coverage (-): 0

Region: chr25 28800710-28800727. Max. coverage (+): 0. Max coverage (-): 0

Region: chr25 28800728-28800744. Max. coverage (+): 0. Max coverage (-): 0

Region: chr25 28800745-28800761. Max. coverage (+): 0. Max coverage (-): 0

Region: chr25 28800762-28800778. Max. coverage (+): 0. Max coverage (-): 1.09

Region: chr25 28800779-28800795. Max. coverage (+): 0. Max coverage (-): 0

Region: chr25 28800796-28800813. Max. coverage (+): 0. Max coverage (-): 0

Region: chr25 28800814-28800830. Max. coverage (+): 0. Max coverage (-): 0

Region: chr25 28800831-28800847. Max. coverage (+): 0. Max coverage (-): 0

Region: chr25 28800848-28800864. Max. coverage (+): 0. Max coverage (-): 0

Region: chr25 28800865-28800881. Max. coverage (+): 0. Max coverage (-): 0

Region: chr25 28800882-28800899. Max. coverage (+): 0. Max coverage (-): 0

Region: chr25 28800900-28800916. Max. coverage (+): 0. Max coverage (-): 0

Region: chr25 28800917-28800933. Max. coverage (+): 0. Max coverage (-): 0

Region: chr25 28800934-28800950. Max. coverage (+): 0. Max coverage (-): 0

Region: chr25 28800951-28800967. Max. coverage (+): 0. Max coverage (-): 0

Region: chr25 28800968-28800985. Max. coverage (+): 0. Max coverage (-): 0

Region: chr25 28800986-28801002. Max. coverage (+): 0. Max coverage (-): 2.01

Region: chr25 28801003-28801019. Max. coverage (+): 0. Max coverage (-): 2.01

Region: chr25 28801020-. Max. coverage (+): 0. Max coverage (-): 2.06

RepeatMasker Color Code

**+**

100-98% Identity

<98-95% Identity

<95-90% Identity

<90-85% Identity

<85-80% Identity

<80-75% Identity

<75-70% Identity

<70% Identity

**-**

Gene Set Color Code

**+**

Gene

Pseudogene

**-**

Topology/Coverage Color Code

Coverage Plus Strand

Coverage Minus Strand

Mainstrand: Plus

Mainstrand: Minus

Complementary Strand

Flanking Region  
(if option -flank >0)

Gene Set Annotation  
  
RepeatMasker Annotation  

**1. MLT1L**: 28792760-28793152 (-), Divergence to consensus: 41.8%  
**2. L2b**: 28793288-28793617 (-), Divergence to consensus: 42.9%  
**3. CHR-2\_BT**: 28794853-28795080 (+), Divergence to consensus: 25%  
**4. MER58B**: 28795135-28795240 (+), Divergence to consensus: 26.6%  
**5. Bov-tA1**: 28795254-28795460 (+), Divergence to consensus: 12.1%  
**6. MER58B**: 28795464-28795676 (+), Divergence to consensus: 31.2%  
**7. CHRL**: 28797057-28797226 (+), Divergence to consensus: 15%  
**8. (T)n**: 28797846-28797867 (+), Divergence to consensus: 0%  
**9. L2**: 28798810-28798885 (+), Divergence to consensus: 34.2%  
**10. SINE2-2\_BT**: 28798900-28799010 (-), Divergence to consensus: 23.4%  
**11. MLT1H2**: 28799855-28799922 (+), Divergence to consensus: 32.3%  
**12. Bov-tA1**: 28799933-28800143 (+), Divergence to consensus: 16.6%  
**13. LTR89**: 28800207-28800418 (-), Divergence to consensus: 45.9%

  
Transcription Factor Binding Sites  

**Gata4** (Sequence: CTTATCT (+): 28800497)
