# Supplementary material for: Small RNA sequencing of cryopreserved semen from single bull revealed altered miRNAs and piRNAs expression between High- and Low-motile sperm populations
Source: BMC Genomics. 2017 Jan 4;18:14. doi: 10.1186/s12864-016-3394-7 (PMC5209821; doi:10.1186/s12864-016-3394-7)
Supplement: Additional file 3: — Details for each piRNA clusters found in High Motile (HM) sperm fraction. Genes, repeats, transposable elements and transcription factors binding sites falling within the cluster regions were reported. (ZIP 1896 kb) [file 12864_2016_3394_MOESM3_ESM.zip › 59.html]

piRNA cluster 59


Predicted piRNA cluster no. 59     previous   next
  

Show proTRAC run info
Hide proTRAC run info

================================= proTRAC ====================================  
VERSION: 2.1                                    LAST MODIFIED: 06. October 2015  
  
Please cite:  
Rosenkranz D, Zischler H. proTRAC - a software for probabilistic piRNA cluster  
detection, visualization and analysis. 2012. BMC Bioinformatics 13:5.  
  
and (for proTRAC 2.0 and later):  
Rosenkranz D, Rudloff S, Bastuck K, Ketting RF, Zischler H. Tupaia small RNAs  
provide insights into function and evolution of RNAi-based transposon defense  
in mammals. 2015. RNA 21(5):911-922.  
  
Contact:  
David Rosenkranz  
Institute of Anthropology, small RNA group  
Johannes Gutenberg University Mainz  
email: rosenkranz@uni-mainz.de  
  
You can find the latest proTRAC version at:  
http://sourceforge.net/projects/protrac/files  
http://www.smallRNAgroup-mainz.de/software  
==============================================================================  
  
PARAMETERS:  
Map file: .............../storage/core/barbara/genhome/smallRNA/fertility/Sample\_motile/pirna/Sample\_motile\_26-33\_collapsed.fa.no-dust.map.weighted-10000-1000-b-0  
Genome file: ............/storage/core/barbara/genhome/smallRNA/fertility/Sample\_all/pirna/bt\_311\_chrY.fa  
RepeatMasker annotation: /storage/genomes/bt\_umd31/GCF\_000003055.6\_Bos\_taurus\_UMD\_3.1.1\_repeatMasker\_chr.out  
GeneSet:................./storage/core/barbara/genhome/smallRNA/fertility/Sample\_all/pirna/full.gtf  
  
Significant (p<=0.01) hit density will be calculated based  
on observed hit distribution.  
  
Sliding window size: ........................................ 5000 bp  
Sliding window increament: .................................. 1000 bp  
Normalize each hit by number of genomic hits: ............... 1 [0=no/1=yes]  
Normalize each hit by number of sequence reads: ............. 1 [0=no/1=yes]  
Normalize values (-> per million mapped reads): ............. 1 [0=no/1=yes]  
Min. fraction of hits with 1T(U) or 10A: .................... 0.75  
Alternatively: Min. fraction of hits with 1T(U) and 10A: .... 0.5  
Min. fraction of hits with typical piRNA length: ............ 0.75  
Typical piRNA length: ....................................... 26-33 nt  
Min. size of a piRNA cluster: ............................... 5000 bp.  
Min. number of hits (absolute): ............................. 0  
Min. number of hits (normalized): ........................... 0  
Min. fraction of hits on the mainstrand: .................... 0.75  
Top fraction of mapped sequences (in terms of read counts): . 1%  
Top fraction accounts for max. n% of sequence reads: ........ 90%  
Min. fraction of hits on each arm of a bidirectional cluster: 0.1  
Output image file for each cluster: ......................... 0 [0=no/1=yes]  
Output html file for each cluster: .......................... 1 [0=no/1=yes]  
Output a summary table: ..................................... 1 [0=no/1=yes]  
Output a FASTA file for each cluster (piRNA sequences): ..... 1 [0=no/1=yes]  
Output a FASTA file comprising cluster sequences: ........... 1 [0=no/1=yes]  
Search DNA motifs in clusters: .............................. 1 [0=no/1=yes]  
Output flanking sequences: +/- .............................. 0 bp  
Output ~.pTi file: .......................................... 1 [0=no/1=yes]  
==============================================================================  
  
  
Genome size (without gaps): ............ 2678902517 bp  
Gaps (N/X/-): .......................... 53837044 bp  
Mapped reads: .......................... 658825247023  
Non-identical sequences: ............... 514171  
Genomic hits: .......................... 764233  
Significant densitiy of mapped reads: .. 12867599.5173724 reads/kb

Show proTRAC cluster info
Hide proTRAC cluster info

|  |  |
| --- | --- |
| Location | chr25 |
| Coordinates | 28847615-28861761 |
| Size [bp] | 14147 |
| Sequence hit loci | 170 |
| Mapped reads (normalized) | 229993960 |
| Mapped reads (normalized) per kb | 16257436.9 |
| Normalized reads with 1T (1U) | 89.3% |
| Normalized reads with 10A | 24.7% |
| Normalized reads with length 26-33 nt | 100% |
| Normalized reads on the main strand(s) | 100% |
| Predicted directionality | mono:plus |

100%

0%

1T (1U)  
reads

10A reads

26-33 nt  
reads

reads on mainstrand

**Either the amount of reads with 1T (1U) OR 10A has to exceed 75% (set with option: -1Tor10A)  
Alternatively the amount of reads with 1T (1U) AND 10A has to exceed 50% (set with option: -1Tand10A)  
Minimum amount of reads with preferred size is 75% (set with option: -pisize)  
Minimum amount of reads on the main strand(s) is 75% (set with option: -clstrand)**

Show read coverage
Hide read coverage

WHAT DO I SEE HERE?  
This chart shows the location of mapped sequence reads within a predicted piRNA cluster. The color refers to the number of genomic hits produced by the sequence read in question. A dark red bar indicates that this sequence read produces many other hits elsewhere in the genome. Many adjacent red or yellow bars can indicate the presence of a multi-copy element such as transposons or rRNA genes. A dark green bar indicates that this sequence read maps uniquely to this locus.

1 hit

2-5 hits

6-10 hits

11-20 hits

21-50 hits

51-100 hits

> 100 hits

chr25

28847615

28861761

Gene Set

RepeatMasker

Mapped  
Reads

15.5

plus strand

minus strand

15.5

Region: chr25 28801021-28847629. Max. coverage (+): 2.04. Max coverage (-): 0

Region: chr25 28847630-28847657. Max. coverage (+): 0. Max coverage (-): 0

Region: chr25 28847658-28847685. Max. coverage (+): 0. Max coverage (-): 0

Region: chr25 28847686-28847714. Max. coverage (+): 0. Max coverage (-): 0

Region: chr25 28847715-28847742. Max. coverage (+): 0. Max coverage (-): 0

Region: chr25 28847743-28847770. Max. coverage (+): 0. Max coverage (-): 0

Region: chr25 28847771-28847798. Max. coverage (+): 0. Max coverage (-): 0

Region: chr25 28847799-28847827. Max. coverage (+): 0. Max coverage (-): 0

Region: chr25 28847828-28847855. Max. coverage (+): 1.93. Max coverage (-): 0

Region: chr25 28847856-28847883. Max. coverage (+): 0. Max coverage (-): 0

Region: chr25 28847884-28847912. Max. coverage (+): 0. Max coverage (-): 0

Region: chr25 28847913-28847940. Max. coverage (+): 0. Max coverage (-): 0

Region: chr25 28847941-28847968. Max. coverage (+): 1.59. Max coverage (-): 0

Region: chr25 28847969-28847996. Max. coverage (+): 0. Max coverage (-): 0

Region: chr25 28847997-28848025. Max. coverage (+): 0. Max coverage (-): 0

Region: chr25 28848026-28848053. Max. coverage (+): 0. Max coverage (-): 0

Region: chr25 28848054-28848081. Max. coverage (+): 0. Max coverage (-): 0

Region: chr25 28848082-28848110. Max. coverage (+): 0. Max coverage (-): 0

Region: chr25 28848111-28848138. Max. coverage (+): 0. Max coverage (-): 0

Region: chr25 28848139-28848166. Max. coverage (+): 0. Max coverage (-): 0

Region: chr25 28848167-28848195. Max. coverage (+): 0. Max coverage (-): 0

Region: chr25 28848196-28848223. Max. coverage (+): 0. Max coverage (-): 0

Region: chr25 28848224-28848251. Max. coverage (+): 0. Max coverage (-): 0

Region: chr25 28848252-28848279. Max. coverage (+): 0. Max coverage (-): 0

Region: chr25 28848280-28848308. Max. coverage (+): 0. Max coverage (-): 0

Region: chr25 28848309-28848336. Max. coverage (+): 0. Max coverage (-): 0

Region: chr25 28848337-28848364. Max. coverage (+): 0. Max coverage (-): 0

Region: chr25 28848365-28848393. Max. coverage (+): 0. Max coverage (-): 0

Region: chr25 28848394-28848421. Max. coverage (+): 0. Max coverage (-): 0

Region: chr25 28848422-28848449. Max. coverage (+): 0. Max coverage (-): 0

Region: chr25 28848450-28848477. Max. coverage (+): 9.86. Max coverage (-): 0

Region: chr25 28848478-28848506. Max. coverage (+): 0. Max coverage (-): 0

Region: chr25 28848507-28848534. Max. coverage (+): 12.77. Max coverage (-): 0

Region: chr25 28848535-28848562. Max. coverage (+): 0. Max coverage (-): 0

Region: chr25 28848563-28848591. Max. coverage (+): 0. Max coverage (-): 0

Region: chr25 28848592-28848619. Max. coverage (+): 0. Max coverage (-): 0

Region: chr25 28848620-28848647. Max. coverage (+): 1.64. Max coverage (-): 0

Region: chr25 28848648-28848676. Max. coverage (+): 8.99. Max coverage (-): 0

Region: chr25 28848677-28848704. Max. coverage (+): 0. Max coverage (-): 0

Region: chr25 28848705-28848732. Max. coverage (+): 0. Max coverage (-): 0

Region: chr25 28848733-28848760. Max. coverage (+): 0. Max coverage (-): 0

Region: chr25 28848761-28848789. Max. coverage (+): 0. Max coverage (-): 0

Region: chr25 28848790-28848817. Max. coverage (+): 0. Max coverage (-): 0

Region: chr25 28848818-28848845. Max. coverage (+): 0.42. Max coverage (-): 0

Region: chr25 28848846-28848874. Max. coverage (+): 1.49. Max coverage (-): 0

Region: chr25 28848875-28848902. Max. coverage (+): 0.96. Max coverage (-): 0

Region: chr25 28848903-28848930. Max. coverage (+): 0. Max coverage (-): 0

Region: chr25 28848931-28848958. Max. coverage (+): 2.29. Max coverage (-): 0

Region: chr25 28848959-28848987. Max. coverage (+): 0. Max coverage (-): 0

Region: chr25 28848988-28849015. Max. coverage (+): 0. Max coverage (-): 0

Region: chr25 28849016-28849043. Max. coverage (+): 0. Max coverage (-): 0

Region: chr25 28849044-28849072. Max. coverage (+): 0. Max coverage (-): 0

Region: chr25 28849073-28849100. Max. coverage (+): 0. Max coverage (-): 0

Region: chr25 28849101-28849128. Max. coverage (+): 0. Max coverage (-): 0

Region: chr25 28849129-28849157. Max. coverage (+): 0. Max coverage (-): 0

Region: chr25 28849158-28849185. Max. coverage (+): 3.49. Max coverage (-): 0

Region: chr25 28849186-28849213. Max. coverage (+): 0. Max coverage (-): 0

Region: chr25 28849214-28849241. Max. coverage (+): 0. Max coverage (-): 0

Region: chr25 28849242-28849270. Max. coverage (+): 0. Max coverage (-): 0

Region: chr25 28849271-28849298. Max. coverage (+): 0. Max coverage (-): 0

Region: chr25 28849299-28849326. Max. coverage (+): 0. Max coverage (-): 0

Region: chr25 28849327-28849355. Max. coverage (+): 0. Max coverage (-): 0

Region: chr25 28849356-28849383. Max. coverage (+): 0. Max coverage (-): 0

Region: chr25 28849384-28849411. Max. coverage (+): 0. Max coverage (-): 0

Region: chr25 28849412-28849439. Max. coverage (+): 0. Max coverage (-): 0

Region: chr25 28849440-28849468. Max. coverage (+): 0. Max coverage (-): 0

Region: chr25 28849469-28849496. Max. coverage (+): 4.3. Max coverage (-): 0

Region: chr25 28849497-28849524. Max. coverage (+): 4.3. Max coverage (-): 0

Region: chr25 28849525-28849553. Max. coverage (+): 0. Max coverage (-): 0

Region: chr25 28849554-28849581. Max. coverage (+): 5.52. Max coverage (-): 0

Region: chr25 28849582-28849609. Max. coverage (+): 0.45. Max coverage (-): 0

Region: chr25 28849610-28849638. Max. coverage (+): 1.99. Max coverage (-): 0

Region: chr25 28849639-28849666. Max. coverage (+): 0. Max coverage (-): 0

Region: chr25 28849667-28849694. Max. coverage (+): 2.38. Max coverage (-): 0

Region: chr25 28849695-28849722. Max. coverage (+): 3.04. Max coverage (-): 0

Region: chr25 28849723-28849751. Max. coverage (+): 0.94. Max coverage (-): 0

Region: chr25 28849752-28849779. Max. coverage (+): 0. Max coverage (-): 0

Region: chr25 28849780-28849807. Max. coverage (+): 0. Max coverage (-): 0

Region: chr25 28849808-28849836. Max. coverage (+): 0. Max coverage (-): 0

Region: chr25 28849837-28849864. Max. coverage (+): 0. Max coverage (-): 0

Region: chr25 28849865-28849892. Max. coverage (+): 0. Max coverage (-): 0

Region: chr25 28849893-28849920. Max. coverage (+): 0. Max coverage (-): 0

Region: chr25 28849921-28849949. Max. coverage (+): 0. Max coverage (-): 0

Region: chr25 28849950-28849977. Max. coverage (+): 0. Max coverage (-): 0

Region: chr25 28849978-28850005. Max. coverage (+): 3.01. Max coverage (-): 0

Region: chr25 28850006-28850034. Max. coverage (+): 3.26. Max coverage (-): 0

Region: chr25 28850035-28850062. Max. coverage (+): 0. Max coverage (-): 0

Region: chr25 28850063-28850090. Max. coverage (+): 0. Max coverage (-): 0

Region: chr25 28850091-28850119. Max. coverage (+): 0. Max coverage (-): 0

Region: chr25 28850120-28850147. Max. coverage (+): 0. Max coverage (-): 0

Region: chr25 28850148-28850175. Max. coverage (+): 6.36. Max coverage (-): 0

Region: chr25 28850176-28850203. Max. coverage (+): 6.36. Max coverage (-): 0

Region: chr25 28850204-28850232. Max. coverage (+): 4.96. Max coverage (-): 0

Region: chr25 28850233-28850260. Max. coverage (+): 0. Max coverage (-): 0

Region: chr25 28850261-28850288. Max. coverage (+): 0. Max coverage (-): 0

Region: chr25 28850289-28850317. Max. coverage (+): 0. Max coverage (-): 0

Region: chr25 28850318-28850345. Max. coverage (+): 0. Max coverage (-): 0

Region: chr25 28850346-28850373. Max. coverage (+): 2.6. Max coverage (-): 0

Region: chr25 28850374-28850401. Max. coverage (+): 0. Max coverage (-): 0

Region: chr25 28850402-28850430. Max. coverage (+): 0. Max coverage (-): 0

Region: chr25 28850431-28850458. Max. coverage (+): 0. Max coverage (-): 0

Region: chr25 28850459-28850486. Max. coverage (+): 0. Max coverage (-): 0

Region: chr25 28850487-28850515. Max. coverage (+): 0. Max coverage (-): 0

Region: chr25 28850516-28850543. Max. coverage (+): 0. Max coverage (-): 0

Region: chr25 28850544-28850571. Max. coverage (+): 1.51. Max coverage (-): 0

Region: chr25 28850572-28850600. Max. coverage (+): 0. Max coverage (-): 0

Region: chr25 28850601-28850628. Max. coverage (+): 4.27. Max coverage (-): 0

Region: chr25 28850629-28850656. Max. coverage (+): 0. Max coverage (-): 0

Region: chr25 28850657-28850684. Max. coverage (+): 1.58. Max coverage (-): 0

Region: chr25 28850685-28850713. Max. coverage (+): 0. Max coverage (-): 0

Region: chr25 28850714-28850741. Max. coverage (+): 1.83. Max coverage (-): 0

Region: chr25 28850742-28850769. Max. coverage (+): 13.81. Max coverage (-): 0

Region: chr25 28850770-28850798. Max. coverage (+): 15.5. Max coverage (-): 0

Region: chr25 28850799-28850826. Max. coverage (+): 0. Max coverage (-): 0

Region: chr25 28850827-28850854. Max. coverage (+): 0. Max coverage (-): 0

Region: chr25 28850855-28850882. Max. coverage (+): 0. Max coverage (-): 0

Region: chr25 28850883-28850911. Max. coverage (+): 0. Max coverage (-): 0

Region: chr25 28850912-28850939. Max. coverage (+): 12.66. Max coverage (-): 0

Region: chr25 28850940-28850967. Max. coverage (+): 0. Max coverage (-): 0

Region: chr25 28850968-28850996. Max. coverage (+): 0. Max coverage (-): 0

Region: chr25 28850997-28851024. Max. coverage (+): 0. Max coverage (-): 0

Region: chr25 28851025-28851052. Max. coverage (+): 0.41. Max coverage (-): 0

Region: chr25 28851053-28851081. Max. coverage (+): 2.07. Max coverage (-): 0

Region: chr25 28851082-28851109. Max. coverage (+): 2.07. Max coverage (-): 0

Region: chr25 28851110-28851137. Max. coverage (+): 2. Max coverage (-): 0

Region: chr25 28851138-28851165. Max. coverage (+): 0. Max coverage (-): 0

Region: chr25 28851166-28851194. Max. coverage (+): 2.14. Max coverage (-): 0

Region: chr25 28851195-28851222. Max. coverage (+): 0. Max coverage (-): 0

Region: chr25 28851223-28851250. Max. coverage (+): 2.95. Max coverage (-): 0

Region: chr25 28851251-28851279. Max. coverage (+): 2.95. Max coverage (-): 0

Region: chr25 28851280-28851307. Max. coverage (+): 0. Max coverage (-): 0

Region: chr25 28851308-28851335. Max. coverage (+): 2.26. Max coverage (-): 0

Region: chr25 28851336-28851363. Max. coverage (+): 0. Max coverage (-): 0

Region: chr25 28851364-28851392. Max. coverage (+): 0. Max coverage (-): 0

Region: chr25 28851393-28851420. Max. coverage (+): 2.51. Max coverage (-): 0

Region: chr25 28851421-28851448. Max. coverage (+): 2.51. Max coverage (-): 0

Region: chr25 28851449-28851477. Max. coverage (+): 0. Max coverage (-): 0

Region: chr25 28851478-28851505. Max. coverage (+): 2.58. Max coverage (-): 0

Region: chr25 28851506-28851533. Max. coverage (+): 0. Max coverage (-): 0

Region: chr25 28851534-28851562. Max. coverage (+): 0. Max coverage (-): 0

Region: chr25 28851563-28851590. Max. coverage (+): 3.83. Max coverage (-): 0

Region: chr25 28851591-28851618. Max. coverage (+): 1.48. Max coverage (-): 0

Region: chr25 28851619-28851646. Max. coverage (+): 0. Max coverage (-): 0

Region: chr25 28851647-28851675. Max. coverage (+): 0. Max coverage (-): 0

Region: chr25 28851676-28851703. Max. coverage (+): 0. Max coverage (-): 0

Region: chr25 28851704-28851731. Max. coverage (+): 0. Max coverage (-): 0

Region: chr25 28851732-28851760. Max. coverage (+): 0. Max coverage (-): 0

Region: chr25 28851761-28851788. Max. coverage (+): 4.49. Max coverage (-): 0

Region: chr25 28851789-28851816. Max. coverage (+): 0. Max coverage (-): 0

Region: chr25 28851817-28851844. Max. coverage (+): 0. Max coverage (-): 0

Region: chr25 28851845-28851873. Max. coverage (+): 1.67. Max coverage (-): 0

Region: chr25 28851874-28851901. Max. coverage (+): 1.67. Max coverage (-): 0

Region: chr25 28851902-28851929. Max. coverage (+): 0. Max coverage (-): 0

Region: chr25 28851930-28851958. Max. coverage (+): 0. Max coverage (-): 0

Region: chr25 28851959-28851986. Max. coverage (+): 0. Max coverage (-): 0

Region: chr25 28851987-28852014. Max. coverage (+): 0. Max coverage (-): 0

Region: chr25 28852015-28852043. Max. coverage (+): 0. Max coverage (-): 0

Region: chr25 28852044-28852071. Max. coverage (+): 0. Max coverage (-): 0

Region: chr25 28852072-28852099. Max. coverage (+): 0. Max coverage (-): 0

Region: chr25 28852100-28852127. Max. coverage (+): 0. Max coverage (-): 0

Region: chr25 28852128-28852156. Max. coverage (+): 0. Max coverage (-): 0

Region: chr25 28852157-28852184. Max. coverage (+): 0. Max coverage (-): 0

Region: chr25 28852185-28852212. Max. coverage (+): 0.53. Max coverage (-): 0

Region: chr25 28852213-28852241. Max. coverage (+): 0. Max coverage (-): 0

Region: chr25 28852242-28852269. Max. coverage (+): 0. Max coverage (-): 0

Region: chr25 28852270-28852297. Max. coverage (+): 0.96. Max coverage (-): 0

Region: chr25 28852298-28852325. Max. coverage (+): 0. Max coverage (-): 0

Region: chr25 28852326-28852354. Max. coverage (+): 0. Max coverage (-): 0

Region: chr25 28852355-28852382. Max. coverage (+): 0. Max coverage (-): 0

Region: chr25 28852383-28852410. Max. coverage (+): 5.97. Max coverage (-): 0

Region: chr25 28852411-28852439. Max. coverage (+): 0. Max coverage (-): 0

Region: chr25 28852440-28852467. Max. coverage (+): 0. Max coverage (-): 0

Region: chr25 28852468-28852495. Max. coverage (+): 0. Max coverage (-): 0

Region: chr25 28852496-28852524. Max. coverage (+): 0. Max coverage (-): 0

Region: chr25 28852525-28852552. Max. coverage (+): 0. Max coverage (-): 0

Region: chr25 28852553-28852580. Max. coverage (+): 0. Max coverage (-): 0

Region: chr25 28852581-28852608. Max. coverage (+): 0. Max coverage (-): 0

Region: chr25 28852609-28852637. Max. coverage (+): 0. Max coverage (-): 0

Region: chr25 28852638-28852665. Max. coverage (+): 0. Max coverage (-): 0

Region: chr25 28852666-28852693. Max. coverage (+): 0. Max coverage (-): 0

Region: chr25 28852694-28852722. Max. coverage (+): 0. Max coverage (-): 0

Region: chr25 28852723-28852750. Max. coverage (+): 0. Max coverage (-): 0

Region: chr25 28852751-28852778. Max. coverage (+): 6.46. Max coverage (-): 0

Region: chr25 28852779-28852806. Max. coverage (+): 0. Max coverage (-): 0

Region: chr25 28852807-28852835. Max. coverage (+): 0. Max coverage (-): 0

Region: chr25 28852836-28852863. Max. coverage (+): 0. Max coverage (-): 0

Region: chr25 28852864-28852891. Max. coverage (+): 0. Max coverage (-): 0

Region: chr25 28852892-28852920. Max. coverage (+): 0. Max coverage (-): 0

Region: chr25 28852921-28852948. Max. coverage (+): 0. Max coverage (-): 0

Region: chr25 28852949-28852976. Max. coverage (+): 0. Max coverage (-): 0

Region: chr25 28852977-28853005. Max. coverage (+): 0. Max coverage (-): 0

Region: chr25 28853006-28853033. Max. coverage (+): 0. Max coverage (-): 0

Region: chr25 28853034-28853061. Max. coverage (+): 0. Max coverage (-): 0

Region: chr25 28853062-28853089. Max. coverage (+): 0. Max coverage (-): 0

Region: chr25 28853090-28853118. Max. coverage (+): 0. Max coverage (-): 0

Region: chr25 28853119-28853146. Max. coverage (+): 0. Max coverage (-): 0

Region: chr25 28853147-28853174. Max. coverage (+): 0. Max coverage (-): 0

Region: chr25 28853175-28853203. Max. coverage (+): 0.7. Max coverage (-): 0

Region: chr25 28853204-28853231. Max. coverage (+): 0. Max coverage (-): 0

Region: chr25 28853232-28853259. Max. coverage (+): 0. Max coverage (-): 0

Region: chr25 28853260-28853287. Max. coverage (+): 0. Max coverage (-): 0

Region: chr25 28853288-28853316. Max. coverage (+): 3.6. Max coverage (-): 0

Region: chr25 28853317-28853344. Max. coverage (+): 0. Max coverage (-): 0

Region: chr25 28853345-28853372. Max. coverage (+): 0. Max coverage (-): 0

Region: chr25 28853373-28853401. Max. coverage (+): 0. Max coverage (-): 0

Region: chr25 28853402-28853429. Max. coverage (+): 0. Max coverage (-): 0

Region: chr25 28853430-28853457. Max. coverage (+): 0. Max coverage (-): 0

Region: chr25 28853458-28853486. Max. coverage (+): 0. Max coverage (-): 0

Region: chr25 28853487-28853514. Max. coverage (+): 0. Max coverage (-): 0

Region: chr25 28853515-28853542. Max. coverage (+): 0. Max coverage (-): 0

Region: chr25 28853543-28853570. Max. coverage (+): 0. Max coverage (-): 0

Region: chr25 28853571-28853599. Max. coverage (+): 0. Max coverage (-): 0

Region: chr25 28853600-28853627. Max. coverage (+): 0. Max coverage (-): 0

Region: chr25 28853628-28853655. Max. coverage (+): 0. Max coverage (-): 0

Region: chr25 28853656-28853684. Max. coverage (+): 0. Max coverage (-): 0

Region: chr25 28853685-28853712. Max. coverage (+): 0. Max coverage (-): 0

Region: chr25 28853713-28853740. Max. coverage (+): 0. Max coverage (-): 0

Region: chr25 28853741-28853768. Max. coverage (+): 0. Max coverage (-): 0

Region: chr25 28853769-28853797. Max. coverage (+): 0. Max coverage (-): 0

Region: chr25 28853798-28853825. Max. coverage (+): 0. Max coverage (-): 0

Region: chr25 28853826-28853853. Max. coverage (+): 0. Max coverage (-): 0

Region: chr25 28853854-28853882. Max. coverage (+): 0. Max coverage (-): 0

Region: chr25 28853883-28853910. Max. coverage (+): 0. Max coverage (-): 0

Region: chr25 28853911-28853938. Max. coverage (+): 0. Max coverage (-): 0

Region: chr25 28853939-28853967. Max. coverage (+): 0. Max coverage (-): 0

Region: chr25 28853968-28853995. Max. coverage (+): 0. Max coverage (-): 0

Region: chr25 28853996-28854023. Max. coverage (+): 0. Max coverage (-): 0

Region: chr25 28854024-28854051. Max. coverage (+): 6.44. Max coverage (-): 0

Region: chr25 28854052-28854080. Max. coverage (+): 6.9. Max coverage (-): 0

Region: chr25 28854081-28854108. Max. coverage (+): 3.01. Max coverage (-): 0

Region: chr25 28854109-28854136. Max. coverage (+): 3.01. Max coverage (-): 0

Region: chr25 28854137-28854165. Max. coverage (+): 2.74. Max coverage (-): 0

Region: chr25 28854166-28854193. Max. coverage (+): 0. Max coverage (-): 0

Region: chr25 28854194-28854221. Max. coverage (+): 0.97. Max coverage (-): 0

Region: chr25 28854222-28854249. Max. coverage (+): 0. Max coverage (-): 0

Region: chr25 28854250-28854278. Max. coverage (+): 0. Max coverage (-): 0

Region: chr25 28854279-28854306. Max. coverage (+): 0. Max coverage (-): 0

Region: chr25 28854307-28854334. Max. coverage (+): 0. Max coverage (-): 0

Region: chr25 28854335-28854363. Max. coverage (+): 0. Max coverage (-): 0

Region: chr25 28854364-28854391. Max. coverage (+): 0. Max coverage (-): 0

Region: chr25 28854392-28854419. Max. coverage (+): 0. Max coverage (-): 0

Region: chr25 28854420-28854448. Max. coverage (+): 0. Max coverage (-): 0

Region: chr25 28854449-28854476. Max. coverage (+): 0. Max coverage (-): 0

Region: chr25 28854477-28854504. Max. coverage (+): 0. Max coverage (-): 0

Region: chr25 28854505-28854532. Max. coverage (+): 0. Max coverage (-): 0

Region: chr25 28854533-28854561. Max. coverage (+): 0. Max coverage (-): 0

Region: chr25 28854562-28854589. Max. coverage (+): 0.69. Max coverage (-): 0

Region: chr25 28854590-28854617. Max. coverage (+): 2.2. Max coverage (-): 0

Region: chr25 28854618-28854646. Max. coverage (+): 0. Max coverage (-): 0

Region: chr25 28854647-28854674. Max. coverage (+): 0. Max coverage (-): 0

Region: chr25 28854675-28854702. Max. coverage (+): 0. Max coverage (-): 0

Region: chr25 28854703-28854730. Max. coverage (+): 0. Max coverage (-): 0

Region: chr25 28854731-28854759. Max. coverage (+): 0. Max coverage (-): 0

Region: chr25 28854760-28854787. Max. coverage (+): 0. Max coverage (-): 0

Region: chr25 28854788-28854815. Max. coverage (+): 5.09. Max coverage (-): 0

Region: chr25 28854816-28854844. Max. coverage (+): 5.09. Max coverage (-): 0

Region: chr25 28854845-28854872. Max. coverage (+): 0. Max coverage (-): 0

Region: chr25 28854873-28854900. Max. coverage (+): 0. Max coverage (-): 0

Region: chr25 28854901-28854928. Max. coverage (+): 0. Max coverage (-): 0

Region: chr25 28854929-28854957. Max. coverage (+): 0. Max coverage (-): 0

Region: chr25 28854958-28854985. Max. coverage (+): 0. Max coverage (-): 0

Region: chr25 28854986-28855013. Max. coverage (+): 0. Max coverage (-): 0

Region: chr25 28855014-28855042. Max. coverage (+): 0. Max coverage (-): 0

Region: chr25 28855043-28855070. Max. coverage (+): 0.43. Max coverage (-): 0

Region: chr25 28855071-28855098. Max. coverage (+): 0.43. Max coverage (-): 0

Region: chr25 28855099-28855127. Max. coverage (+): 0. Max coverage (-): 0

Region: chr25 28855128-28855155. Max. coverage (+): 0. Max coverage (-): 0

Region: chr25 28855156-28855183. Max. coverage (+): 0. Max coverage (-): 0

Region: chr25 28855184-28855211. Max. coverage (+): 3.06. Max coverage (-): 0

Region: chr25 28855212-28855240. Max. coverage (+): 3.06. Max coverage (-): 0

Region: chr25 28855241-28855268. Max. coverage (+): 2.48. Max coverage (-): 0

Region: chr25 28855269-28855296. Max. coverage (+): 1.05. Max coverage (-): 0

Region: chr25 28855297-28855325. Max. coverage (+): 1.05. Max coverage (-): 0

Region: chr25 28855326-28855353. Max. coverage (+): 0. Max coverage (-): 0

Region: chr25 28855354-28855381. Max. coverage (+): 0. Max coverage (-): 0

Region: chr25 28855382-28855409. Max. coverage (+): 0. Max coverage (-): 0

Region: chr25 28855410-28855438. Max. coverage (+): 0. Max coverage (-): 0

Region: chr25 28855439-28855466. Max. coverage (+): 0. Max coverage (-): 0

Region: chr25 28855467-28855494. Max. coverage (+): 0. Max coverage (-): 0

Region: chr25 28855495-28855523. Max. coverage (+): 0. Max coverage (-): 0

Region: chr25 28855524-28855551. Max. coverage (+): 0. Max coverage (-): 0

Region: chr25 28855552-28855579. Max. coverage (+): 0. Max coverage (-): 0

Region: chr25 28855580-28855608. Max. coverage (+): 0. Max coverage (-): 0

Region: chr25 28855609-28855636. Max. coverage (+): 0. Max coverage (-): 0

Region: chr25 28855637-28855664. Max. coverage (+): 0. Max coverage (-): 0

Region: chr25 28855665-28855692. Max. coverage (+): 0. Max coverage (-): 0

Region: chr25 28855693-28855721. Max. coverage (+): 0.55. Max coverage (-): 0

Region: chr25 28855722-28855749. Max. coverage (+): 0. Max coverage (-): 0

Region: chr25 28855750-28855777. Max. coverage (+): 0. Max coverage (-): 0

Region: chr25 28855778-28855806. Max. coverage (+): 0. Max coverage (-): 0

Region: chr25 28855807-28855834. Max. coverage (+): 0. Max coverage (-): 0

Region: chr25 28855835-28855862. Max. coverage (+): 0. Max coverage (-): 0

Region: chr25 28855863-28855890. Max. coverage (+): 0. Max coverage (-): 0

Region: chr25 28855891-28855919. Max. coverage (+): 0. Max coverage (-): 0

Region: chr25 28855920-28855947. Max. coverage (+): 0. Max coverage (-): 0

Region: chr25 28855948-28855975. Max. coverage (+): 4.34. Max coverage (-): 0

Region: chr25 28855976-28856004. Max. coverage (+): 4.34. Max coverage (-): 0

Region: chr25 28856005-28856032. Max. coverage (+): 4.34. Max coverage (-): 0

Region: chr25 28856033-28856060. Max. coverage (+): 0. Max coverage (-): 0

Region: chr25 28856061-28856089. Max. coverage (+): 0. Max coverage (-): 0

Region: chr25 28856090-28856117. Max. coverage (+): 0. Max coverage (-): 0

Region: chr25 28856118-28856145. Max. coverage (+): 0. Max coverage (-): 0

Region: chr25 28856146-28856173. Max. coverage (+): 0. Max coverage (-): 0

Region: chr25 28856174-28856202. Max. coverage (+): 0. Max coverage (-): 0

Region: chr25 28856203-28856230. Max. coverage (+): 0. Max coverage (-): 0

Region: chr25 28856231-28856258. Max. coverage (+): 0. Max coverage (-): 0

Region: chr25 28856259-28856287. Max. coverage (+): 0. Max coverage (-): 0

Region: chr25 28856288-28856315. Max. coverage (+): 0. Max coverage (-): 0

Region: chr25 28856316-28856343. Max. coverage (+): 0. Max coverage (-): 0

Region: chr25 28856344-28856371. Max. coverage (+): 0. Max coverage (-): 0

Region: chr25 28856372-28856400. Max. coverage (+): 1.68. Max coverage (-): 0

Region: chr25 28856401-28856428. Max. coverage (+): 0. Max coverage (-): 0

Region: chr25 28856429-28856456. Max. coverage (+): 0. Max coverage (-): 0

Region: chr25 28856457-28856485. Max. coverage (+): 0. Max coverage (-): 0

Region: chr25 28856486-28856513. Max. coverage (+): 0. Max coverage (-): 0

Region: chr25 28856514-28856541. Max. coverage (+): 0. Max coverage (-): 0

Region: chr25 28856542-28856570. Max. coverage (+): 0. Max coverage (-): 0

Region: chr25 28856571-28856598. Max. coverage (+): 0. Max coverage (-): 0

Region: chr25 28856599-28856626. Max. coverage (+): 0. Max coverage (-): 0

Region: chr25 28856627-28856654. Max. coverage (+): 0. Max coverage (-): 0

Region: chr25 28856655-28856683. Max. coverage (+): 0. Max coverage (-): 0

Region: chr25 28856684-28856711. Max. coverage (+): 0. Max coverage (-): 0

Region: chr25 28856712-28856739. Max. coverage (+): 0. Max coverage (-): 0

Region: chr25 28856740-28856768. Max. coverage (+): 0. Max coverage (-): 0

Region: chr25 28856769-28856796. Max. coverage (+): 0. Max coverage (-): 0

Region: chr25 28856797-28856824. Max. coverage (+): 1.84. Max coverage (-): 0

Region: chr25 28856825-28856852. Max. coverage (+): 5.99. Max coverage (-): 0

Region: chr25 28856853-28856881. Max. coverage (+): 0. Max coverage (-): 0

Region: chr25 28856882-28856909. Max. coverage (+): 0. Max coverage (-): 0

Region: chr25 28856910-28856937. Max. coverage (+): 0. Max coverage (-): 0

Region: chr25 28856938-28856966. Max. coverage (+): 0. Max coverage (-): 0

Region: chr25 28856967-28856994. Max. coverage (+): 0. Max coverage (-): 0

Region: chr25 28856995-28857022. Max. coverage (+): 0. Max coverage (-): 0

Region: chr25 28857023-28857051. Max. coverage (+): 0. Max coverage (-): 0

Region: chr25 28857052-28857079. Max. coverage (+): 0. Max coverage (-): 0

Region: chr25 28857080-28857107. Max. coverage (+): 0. Max coverage (-): 0

Region: chr25 28857108-28857135. Max. coverage (+): 0. Max coverage (-): 0

Region: chr25 28857136-28857164. Max. coverage (+): 2.2. Max coverage (-): 0

Region: chr25 28857165-28857192. Max. coverage (+): 0. Max coverage (-): 0

Region: chr25 28857193-28857220. Max. coverage (+): 5.47. Max coverage (-): 0

Region: chr25 28857221-28857249. Max. coverage (+): 1.81. Max coverage (-): 0

Region: chr25 28857250-28857277. Max. coverage (+): 0. Max coverage (-): 0

Region: chr25 28857278-28857305. Max. coverage (+): 0. Max coverage (-): 0

Region: chr25 28857306-28857333. Max. coverage (+): 0. Max coverage (-): 0

Region: chr25 28857334-28857362. Max. coverage (+): 0. Max coverage (-): 0

Region: chr25 28857363-28857390. Max. coverage (+): 0. Max coverage (-): 0

Region: chr25 28857391-28857418. Max. coverage (+): 0. Max coverage (-): 0

Region: chr25 28857419-28857447. Max. coverage (+): 0. Max coverage (-): 0

Region: chr25 28857448-28857475. Max. coverage (+): 0. Max coverage (-): 0

Region: chr25 28857476-28857503. Max. coverage (+): 0. Max coverage (-): 0

Region: chr25 28857504-28857532. Max. coverage (+): 0. Max coverage (-): 0

Region: chr25 28857533-28857560. Max. coverage (+): 0. Max coverage (-): 0

Region: chr25 28857561-28857588. Max. coverage (+): 0. Max coverage (-): 0

Region: chr25 28857589-28857616. Max. coverage (+): 2.1. Max coverage (-): 0

Region: chr25 28857617-28857645. Max. coverage (+): 0. Max coverage (-): 0

Region: chr25 28857646-28857673. Max. coverage (+): 0. Max coverage (-): 0

Region: chr25 28857674-28857701. Max. coverage (+): 0. Max coverage (-): 0

Region: chr25 28857702-28857730. Max. coverage (+): 0. Max coverage (-): 0

Region: chr25 28857731-28857758. Max. coverage (+): 0. Max coverage (-): 0

Region: chr25 28857759-28857786. Max. coverage (+): 0. Max coverage (-): 0

Region: chr25 28857787-28857814. Max. coverage (+): 0. Max coverage (-): 0

Region: chr25 28857815-28857843. Max. coverage (+): 0. Max coverage (-): 0

Region: chr25 28857844-28857871. Max. coverage (+): 0. Max coverage (-): 0

Region: chr25 28857872-28857899. Max. coverage (+): 1.9. Max coverage (-): 0

Region: chr25 28857900-28857928. Max. coverage (+): 0. Max coverage (-): 0

Region: chr25 28857929-28857956. Max. coverage (+): 0. Max coverage (-): 0

Region: chr25 28857957-28857984. Max. coverage (+): 0. Max coverage (-): 0

Region: chr25 28857985-28858013. Max. coverage (+): 0. Max coverage (-): 0

Region: chr25 28858014-28858041. Max. coverage (+): 0. Max coverage (-): 0

Region: chr25 28858042-28858069. Max. coverage (+): 0. Max coverage (-): 0

Region: chr25 28858070-28858097. Max. coverage (+): 0. Max coverage (-): 0

Region: chr25 28858098-28858126. Max. coverage (+): 0. Max coverage (-): 0

Region: chr25 28858127-28858154. Max. coverage (+): 0.69. Max coverage (-): 0

Region: chr25 28858155-28858182. Max. coverage (+): 4.57. Max coverage (-): 0

Region: chr25 28858183-28858211. Max. coverage (+): 0. Max coverage (-): 0

Region: chr25 28858212-28858239. Max. coverage (+): 0. Max coverage (-): 0

Region: chr25 28858240-28858267. Max. coverage (+): 0. Max coverage (-): 0

Region: chr25 28858268-28858295. Max. coverage (+): 0. Max coverage (-): 0

Region: chr25 28858296-28858324. Max. coverage (+): 0. Max coverage (-): 0

Region: chr25 28858325-28858352. Max. coverage (+): 0. Max coverage (-): 0

Region: chr25 28858353-28858380. Max. coverage (+): 2.21. Max coverage (-): 0

Region: chr25 28858381-28858409. Max. coverage (+): 0. Max coverage (-): 0

Region: chr25 28858410-28858437. Max. coverage (+): 1.55. Max coverage (-): 0

Region: chr25 28858438-28858465. Max. coverage (+): 0. Max coverage (-): 0

Region: chr25 28858466-28858494. Max. coverage (+): 0. Max coverage (-): 0

Region: chr25 28858495-28858522. Max. coverage (+): 0. Max coverage (-): 0

Region: chr25 28858523-28858550. Max. coverage (+): 0. Max coverage (-): 0

Region: chr25 28858551-28858578. Max. coverage (+): 0. Max coverage (-): 0

Region: chr25 28858579-28858607. Max. coverage (+): 0. Max coverage (-): 0

Region: chr25 28858608-28858635. Max. coverage (+): 0. Max coverage (-): 0

Region: chr25 28858636-28858663. Max. coverage (+): 4.38. Max coverage (-): 0

Region: chr25 28858664-28858692. Max. coverage (+): 6.51. Max coverage (-): 0

Region: chr25 28858693-28858720. Max. coverage (+): 1.21. Max coverage (-): 0

Region: chr25 28858721-28858748. Max. coverage (+): 1.21. Max coverage (-): 0

Region: chr25 28858749-28858776. Max. coverage (+): 0. Max coverage (-): 0

Region: chr25 28858777-28858805. Max. coverage (+): 0. Max coverage (-): 0

Region: chr25 28858806-28858833. Max. coverage (+): 0. Max coverage (-): 0

Region: chr25 28858834-28858861. Max. coverage (+): 4.3. Max coverage (-): 0

Region: chr25 28858862-28858890. Max. coverage (+): 0. Max coverage (-): 0

Region: chr25 28858891-28858918. Max. coverage (+): 0. Max coverage (-): 0

Region: chr25 28858919-28858946. Max. coverage (+): 0. Max coverage (-): 0

Region: chr25 28858947-28858975. Max. coverage (+): 0. Max coverage (-): 0

Region: chr25 28858976-28859003. Max. coverage (+): 4.55. Max coverage (-): 0

Region: chr25 28859004-28859031. Max. coverage (+): 0. Max coverage (-): 0

Region: chr25 28859032-28859059. Max. coverage (+): 0. Max coverage (-): 0

Region: chr25 28859060-28859088. Max. coverage (+): 0. Max coverage (-): 0

Region: chr25 28859089-28859116. Max. coverage (+): 0. Max coverage (-): 0

Region: chr25 28859117-28859144. Max. coverage (+): 0. Max coverage (-): 0

Region: chr25 28859145-28859173. Max. coverage (+): 0.49. Max coverage (-): 0

Region: chr25 28859174-28859201. Max. coverage (+): 0. Max coverage (-): 0

Region: chr25 28859202-28859229. Max. coverage (+): 2.17. Max coverage (-): 0

Region: chr25 28859230-28859257. Max. coverage (+): 2.14. Max coverage (-): 0

Region: chr25 28859258-28859286. Max. coverage (+): 1.46. Max coverage (-): 0

Region: chr25 28859287-28859314. Max. coverage (+): 5.14. Max coverage (-): 0

Region: chr25 28859315-28859342. Max. coverage (+): 6.54. Max coverage (-): 0

Region: chr25 28859343-28859371. Max. coverage (+): 0. Max coverage (-): 0

Region: chr25 28859372-28859399. Max. coverage (+): 0. Max coverage (-): 0

Region: chr25 28859400-28859427. Max. coverage (+): 0. Max coverage (-): 0

Region: chr25 28859428-28859456. Max. coverage (+): 0. Max coverage (-): 0

Region: chr25 28859457-28859484. Max. coverage (+): 10.45. Max coverage (-): 0

Region: chr25 28859485-28859512. Max. coverage (+): 10.73. Max coverage (-): 0

Region: chr25 28859513-28859540. Max. coverage (+): 2.28. Max coverage (-): 0

Region: chr25 28859541-28859569. Max. coverage (+): 2.28. Max coverage (-): 0

Region: chr25 28859570-28859597. Max. coverage (+): 0. Max coverage (-): 0

Region: chr25 28859598-28859625. Max. coverage (+): 0.24. Max coverage (-): 0

Region: chr25 28859626-28859654. Max. coverage (+): 0. Max coverage (-): 0

Region: chr25 28859655-28859682. Max. coverage (+): 0. Max coverage (-): 0

Region: chr25 28859683-28859710. Max. coverage (+): 0. Max coverage (-): 0

Region: chr25 28859711-28859738. Max. coverage (+): 0. Max coverage (-): 0

Region: chr25 28859739-28859767. Max. coverage (+): 0. Max coverage (-): 0

Region: chr25 28859768-28859795. Max. coverage (+): 0. Max coverage (-): 0

Region: chr25 28859796-28859823. Max. coverage (+): 0. Max coverage (-): 0

Region: chr25 28859824-28859852. Max. coverage (+): 0. Max coverage (-): 0

Region: chr25 28859853-28859880. Max. coverage (+): 0. Max coverage (-): 0

Region: chr25 28859881-28859908. Max. coverage (+): 0. Max coverage (-): 0

Region: chr25 28859909-28859937. Max. coverage (+): 0.93. Max coverage (-): 0

Region: chr25 28859938-28859965. Max. coverage (+): 3.45. Max coverage (-): 0

Region: chr25 28859966-28859993. Max. coverage (+): 0. Max coverage (-): 0

Region: chr25 28859994-28860021. Max. coverage (+): 0. Max coverage (-): 0

Region: chr25 28860022-28860050. Max. coverage (+): 0. Max coverage (-): 0

Region: chr25 28860051-28860078. Max. coverage (+): 0.86. Max coverage (-): 0

Region: chr25 28860079-28860106. Max. coverage (+): 0.86. Max coverage (-): 0

Region: chr25 28860107-28860135. Max. coverage (+): 0. Max coverage (-): 0

Region: chr25 28860136-28860163. Max. coverage (+): 5.38. Max coverage (-): 0

Region: chr25 28860164-28860191. Max. coverage (+): 1.71. Max coverage (-): 0

Region: chr25 28860192-28860219. Max. coverage (+): 1.15. Max coverage (-): 0

Region: chr25 28860220-28860248. Max. coverage (+): 3.7. Max coverage (-): 0

Region: chr25 28860249-28860276. Max. coverage (+): 0. Max coverage (-): 0

Region: chr25 28860277-28860304. Max. coverage (+): 0. Max coverage (-): 0

Region: chr25 28860305-28860333. Max. coverage (+): 0. Max coverage (-): 0

Region: chr25 28860334-28860361. Max. coverage (+): 5.02. Max coverage (-): 0

Region: chr25 28860362-28860389. Max. coverage (+): 0. Max coverage (-): 0

Region: chr25 28860390-28860418. Max. coverage (+): 0. Max coverage (-): 0

Region: chr25 28860419-28860446. Max. coverage (+): 0. Max coverage (-): 0

Region: chr25 28860447-28860474. Max. coverage (+): 0. Max coverage (-): 0

Region: chr25 28860475-28860502. Max. coverage (+): 0. Max coverage (-): 0

Region: chr25 28860503-28860531. Max. coverage (+): 0. Max coverage (-): 0

Region: chr25 28860532-28860559. Max. coverage (+): 0. Max coverage (-): 0

Region: chr25 28860560-28860587. Max. coverage (+): 0. Max coverage (-): 0

Region: chr25 28860588-28860616. Max. coverage (+): 0. Max coverage (-): 0

Region: chr25 28860617-28860644. Max. coverage (+): 0. Max coverage (-): 0

Region: chr25 28860645-28860672. Max. coverage (+): 0. Max coverage (-): 0

Region: chr25 28860673-28860700. Max. coverage (+): 0. Max coverage (-): 0

Region: chr25 28860701-28860729. Max. coverage (+): 0.93. Max coverage (-): 0

Region: chr25 28860730-28860757. Max. coverage (+): 0. Max coverage (-): 0

Region: chr25 28860758-28860785. Max. coverage (+): 0. Max coverage (-): 0

Region: chr25 28860786-28860814. Max. coverage (+): 0. Max coverage (-): 0

Region: chr25 28860815-28860842. Max. coverage (+): 0. Max coverage (-): 0

Region: chr25 28860843-28860870. Max. coverage (+): 0.97. Max coverage (-): 0

Region: chr25 28860871-28860899. Max. coverage (+): 0.97. Max coverage (-): 0

Region: chr25 28860900-28860927. Max. coverage (+): 0. Max coverage (-): 0

Region: chr25 28860928-28860955. Max. coverage (+): 0. Max coverage (-): 0

Region: chr25 28860956-28860983. Max. coverage (+): 0. Max coverage (-): 0

Region: chr25 28860984-28861012. Max. coverage (+): 0. Max coverage (-): 0

Region: chr25 28861013-28861040. Max. coverage (+): 0. Max coverage (-): 0

Region: chr25 28861041-28861068. Max. coverage (+): 0. Max coverage (-): 0

Region: chr25 28861069-28861097. Max. coverage (+): 0. Max coverage (-): 0

Region: chr25 28861098-28861125. Max. coverage (+): 0. Max coverage (-): 0

Region: chr25 28861126-28861153. Max. coverage (+): 0. Max coverage (-): 0

Region: chr25 28861154-28861181. Max. coverage (+): 0. Max coverage (-): 0

Region: chr25 28861182-28861210. Max. coverage (+): 0. Max coverage (-): 0

Region: chr25 28861211-28861238. Max. coverage (+): 0. Max coverage (-): 0

Region: chr25 28861239-28861266. Max. coverage (+): 0. Max coverage (-): 0

Region: chr25 28861267-28861295. Max. coverage (+): 0. Max coverage (-): 0

Region: chr25 28861296-28861323. Max. coverage (+): 0. Max coverage (-): 0

Region: chr25 28861324-28861351. Max. coverage (+): 0. Max coverage (-): 0

Region: chr25 28861352-28861380. Max. coverage (+): 0. Max coverage (-): 0

Region: chr25 28861381-28861408. Max. coverage (+): 6.73. Max coverage (-): 0

Region: chr25 28861409-28861436. Max. coverage (+): 6.73. Max coverage (-): 0

Region: chr25 28861437-28861464. Max. coverage (+): 2.21. Max coverage (-): 0

Region: chr25 28861465-28861493. Max. coverage (+): 0. Max coverage (-): 0

Region: chr25 28861494-28861521. Max. coverage (+): 0. Max coverage (-): 0

Region: chr25 28861522-28861549. Max. coverage (+): 0. Max coverage (-): 0

Region: chr25 28861550-28861578. Max. coverage (+): 0. Max coverage (-): 0

Region: chr25 28861579-28861606. Max. coverage (+): 0. Max coverage (-): 0

Region: chr25 28861607-28861634. Max. coverage (+): 4.67. Max coverage (-): 0

Region: chr25 28861635-28861662. Max. coverage (+): 4.67. Max coverage (-): 0

Region: chr25 28861663-28861691. Max. coverage (+): 0. Max coverage (-): 0

Region: chr25 28861692-28861719. Max. coverage (+): 0. Max coverage (-): 0

Region: chr25 28861720-28861747. Max. coverage (+): 3.54. Max coverage (-): 0

Region: chr25 28861748-. Max. coverage (+): 0. Max coverage (-): 0

RepeatMasker Color Code

**+**

100-98% Identity

<98-95% Identity

<95-90% Identity

<90-85% Identity

<85-80% Identity

<80-75% Identity

<75-70% Identity

<70% Identity

**-**

Gene Set Color Code

**+**

Gene

Pseudogene

**-**

Topology/Coverage Color Code

Coverage Plus Strand

Coverage Minus Strand

Mainstrand: Plus

Mainstrand: Minus

Complementary Strand

Flanking Region  
(if option -flank >0)

Gene Set Annotation  
  
RepeatMasker Annotation  

**1. L2c**: 28848083-28848208 (+), Divergence to consensus: 43.1%  
**2. L2c**: 28849316-28849452 (-), Divergence to consensus: 35.3%  
**3. SINE2-2\_BT**: 28852553-28852674 (-), Divergence to consensus: 20.7%  
**4. CHRL**: 28852972-28853128 (-), Divergence to consensus: 26.9%  
**5. L2c**: 28853455-28853648 (-), Divergence to consensus: 42.8%  
**6. BOV-A2**: 28853649-28853906 (+), Divergence to consensus: 11.2%  
**7. L2c**: 28853907-28854007 (-), Divergence to consensus: 47.7%  
**8. LTR33A**: 28855348-28855542 (-), Divergence to consensus: 36%  
**9. MIR3**: 28857363-28857445 (-), Divergence to consensus: 39.8%  
**10. (TATG)n**: 28858004-28858182 (+), Divergence to consensus: 38.9%  
**11. (CAG)n**: 28858549-28858595 (+), Divergence to consensus: 21.3%  
**12. MIR**: 28860951-28861087 (+), Divergence to consensus: 36.2%  
**13. Bov-tA1**: 28861188-28861403 (-), Divergence to consensus: 12.5%

  
Transcription Factor Binding Sites  

**RFX4\_2** (Sequence: GTATCCATG (-): 28849587)  
**Gata4** (Sequence: AGATAAG (-): 28851095)  
**Gata4** (Sequence: AGATAAG (-): 28854370)  
**Gata4** (Sequence: AGATAAC (-): 28857186)  
**SOX9** (Sequence: AACAATGA (-): 28850516)  
**SOX9** (Sequence: TCATTGTT (+): 28851540)  
**Gata4** (Sequence: CTTATCT (+): 28848008)  
**Gata4** (Sequence: CTTATCT (+): 28856038)  
**Gata4** (Sequence: CTTATCT (+): 28861444)
