# Supplementary material for: Small RNA sequencing of cryopreserved semen from single bull revealed altered miRNAs and piRNAs expression between High- and Low-motile sperm populations
Source: BMC Genomics. 2017 Jan 4;18:14. doi: 10.1186/s12864-016-3394-7 (PMC5209821; doi:10.1186/s12864-016-3394-7)
Supplement: Additional file 3: — Details for each piRNA clusters found in High Motile (HM) sperm fraction. Genes, repeats, transposable elements and transcription factors binding sites falling within the cluster regions were reported. (ZIP 1896 kb) [file 12864_2016_3394_MOESM3_ESM.zip › 6.html]

piRNA cluster 6


Predicted piRNA cluster no. 6     previous   next
  

Show proTRAC run info
Hide proTRAC run info

================================= proTRAC ====================================  
VERSION: 2.1                                    LAST MODIFIED: 06. October 2015  
  
Please cite:  
Rosenkranz D, Zischler H. proTRAC - a software for probabilistic piRNA cluster  
detection, visualization and analysis. 2012. BMC Bioinformatics 13:5.  
  
and (for proTRAC 2.0 and later):  
Rosenkranz D, Rudloff S, Bastuck K, Ketting RF, Zischler H. Tupaia small RNAs  
provide insights into function and evolution of RNAi-based transposon defense  
in mammals. 2015. RNA 21(5):911-922.  
  
Contact:  
David Rosenkranz  
Institute of Anthropology, small RNA group  
Johannes Gutenberg University Mainz  
email: rosenkranz@uni-mainz.de  
  
You can find the latest proTRAC version at:  
http://sourceforge.net/projects/protrac/files  
http://www.smallRNAgroup-mainz.de/software  
==============================================================================  
  
PARAMETERS:  
Map file: .............../storage/core/barbara/genhome/smallRNA/fertility/Sample\_motile/pirna/Sample\_motile\_26-33\_collapsed.fa.no-dust.map.weighted-10000-1000-b-0  
Genome file: ............/storage/core/barbara/genhome/smallRNA/fertility/Sample\_all/pirna/bt\_311\_chrY.fa  
RepeatMasker annotation: /storage/genomes/bt\_umd31/GCF\_000003055.6\_Bos\_taurus\_UMD\_3.1.1\_repeatMasker\_chr.out  
GeneSet:................./storage/core/barbara/genhome/smallRNA/fertility/Sample\_all/pirna/full.gtf  
  
Significant (p<=0.01) hit density will be calculated based  
on observed hit distribution.  
  
Sliding window size: ........................................ 5000 bp  
Sliding window increament: .................................. 1000 bp  
Normalize each hit by number of genomic hits: ............... 1 [0=no/1=yes]  
Normalize each hit by number of sequence reads: ............. 1 [0=no/1=yes]  
Normalize values (-> per million mapped reads): ............. 1 [0=no/1=yes]  
Min. fraction of hits with 1T(U) or 10A: .................... 0.75  
Alternatively: Min. fraction of hits with 1T(U) and 10A: .... 0.5  
Min. fraction of hits with typical piRNA length: ............ 0.75  
Typical piRNA length: ....................................... 26-33 nt  
Min. size of a piRNA cluster: ............................... 5000 bp.  
Min. number of hits (absolute): ............................. 0  
Min. number of hits (normalized): ........................... 0  
Min. fraction of hits on the mainstrand: .................... 0.75  
Top fraction of mapped sequences (in terms of read counts): . 1%  
Top fraction accounts for max. n% of sequence reads: ........ 90%  
Min. fraction of hits on each arm of a bidirectional cluster: 0.1  
Output image file for each cluster: ......................... 0 [0=no/1=yes]  
Output html file for each cluster: .......................... 1 [0=no/1=yes]  
Output a summary table: ..................................... 1 [0=no/1=yes]  
Output a FASTA file for each cluster (piRNA sequences): ..... 1 [0=no/1=yes]  
Output a FASTA file comprising cluster sequences: ........... 1 [0=no/1=yes]  
Search DNA motifs in clusters: .............................. 1 [0=no/1=yes]  
Output flanking sequences: +/- .............................. 0 bp  
Output ~.pTi file: .......................................... 1 [0=no/1=yes]  
==============================================================================  
  
  
Genome size (without gaps): ............ 2678902517 bp  
Gaps (N/X/-): .......................... 53837044 bp  
Mapped reads: .......................... 658825247023  
Non-identical sequences: ............... 514171  
Genomic hits: .......................... 764233  
Significant densitiy of mapped reads: .. 12867599.5173724 reads/kb

Show proTRAC cluster info
Hide proTRAC cluster info

|  |  |
| --- | --- |
| Location | chr10 |
| Coordinates | 59178497-59234823 |
| Size [bp] | 56327 |
| Sequence hit loci | 6953 |
| Mapped reads (normalized) | 8004127836 |
| Mapped reads (normalized) per kb | 142101085.4 |
| Normalized reads with 1T (1U) | 79.3% |
| Normalized reads with 10A | 30.8% |
| Normalized reads with length 26-33 nt | 100% |
| Normalized reads on the main strand(s) | 98.7% |
| Predicted directionality | mono:plus |

100%

0%

1T (1U)  
reads

10A reads

26-33 nt  
reads

reads on mainstrand

**Either the amount of reads with 1T (1U) OR 10A has to exceed 75% (set with option: -1Tor10A)  
Alternatively the amount of reads with 1T (1U) AND 10A has to exceed 50% (set with option: -1Tand10A)  
Minimum amount of reads with preferred size is 75% (set with option: -pisize)  
Minimum amount of reads on the main strand(s) is 75% (set with option: -clstrand)**

Show read coverage
Hide read coverage

WHAT DO I SEE HERE?  
This chart shows the location of mapped sequence reads within a predicted piRNA cluster. The color refers to the number of genomic hits produced by the sequence read in question. A dark red bar indicates that this sequence read produces many other hits elsewhere in the genome. Many adjacent red or yellow bars can indicate the presence of a multi-copy element such as transposons or rRNA genes. A dark green bar indicates that this sequence read maps uniquely to this locus.

1 hit

2-5 hits

6-10 hits

11-20 hits

21-50 hits

51-100 hits

> 100 hits

chr10

59178497

59234823

Gene Set

RepeatMasker

Mapped  
Reads

182.39

plus strand

minus strand

182.39

Region: chr10 47947853-59178553. Max. coverage (+): 0. Max coverage (-): 1

Region: chr10 59178554-59178665. Max. coverage (+): 0. Max coverage (-): 1.56

Region: chr10 59178666-59178778. Max. coverage (+): 0. Max coverage (-): 0

Region: chr10 59178779-59178891. Max. coverage (+): 0. Max coverage (-): 0

Region: chr10 59178892-59179003. Max. coverage (+): 0. Max coverage (-): 0

Region: chr10 59179004-59179116. Max. coverage (+): 0. Max coverage (-): 3.06

Region: chr10 59179117-59179229. Max. coverage (+): 0. Max coverage (-): 5.14

Region: chr10 59179230-59179341. Max. coverage (+): 0. Max coverage (-): 0

Region: chr10 59179342-59179454. Max. coverage (+): 0. Max coverage (-): 2.21

Region: chr10 59179455-59179567. Max. coverage (+): 0. Max coverage (-): 0

Region: chr10 59179568-59179679. Max. coverage (+): 0. Max coverage (-): 0

Region: chr10 59179680-59179792. Max. coverage (+): 0. Max coverage (-): 0

Region: chr10 59179793-59179905. Max. coverage (+): 0. Max coverage (-): 0

Region: chr10 59179906-59180017. Max. coverage (+): 0. Max coverage (-): 0

Region: chr10 59180018-59180130. Max. coverage (+): 0. Max coverage (-): 0

Region: chr10 59180131-59180243. Max. coverage (+): 0. Max coverage (-): 1.3

Region: chr10 59180244-59180355. Max. coverage (+): 0. Max coverage (-): 1.62

Region: chr10 59180356-59180468. Max. coverage (+): 0. Max coverage (-): 1.56

Region: chr10 59180469-59180581. Max. coverage (+): 0. Max coverage (-): 0.8

Region: chr10 59180582-59180693. Max. coverage (+): 0. Max coverage (-): 9.01

Region: chr10 59180694-59180806. Max. coverage (+): 0. Max coverage (-): 4.74

Region: chr10 59180807-59180919. Max. coverage (+): 0. Max coverage (-): 2.56

Region: chr10 59180920-59181031. Max. coverage (+): 0. Max coverage (-): 0

Region: chr10 59181032-59181144. Max. coverage (+): 0. Max coverage (-): 5.94

Region: chr10 59181145-59181257. Max. coverage (+): 0. Max coverage (-): 2.72

Region: chr10 59181258-59181369. Max. coverage (+): 0. Max coverage (-): 0

Region: chr10 59181370-59181482. Max. coverage (+): 0. Max coverage (-): 1.27

Region: chr10 59181483-59181594. Max. coverage (+): 0. Max coverage (-): 1.17

Region: chr10 59181595-59181707. Max. coverage (+): 0. Max coverage (-): 0

Region: chr10 59181708-59181820. Max. coverage (+): 0. Max coverage (-): 7.78

Region: chr10 59181821-59181932. Max. coverage (+): 3.52. Max coverage (-): 1.07

Region: chr10 59181933-59182045. Max. coverage (+): 0. Max coverage (-): 1.47

Region: chr10 59182046-59182158. Max. coverage (+): 0. Max coverage (-): 0

Region: chr10 59182159-59182270. Max. coverage (+): 0. Max coverage (-): 1.65

Region: chr10 59182271-59182383. Max. coverage (+): 4.33. Max coverage (-): 0

Region: chr10 59182384-59182496. Max. coverage (+): 4.33. Max coverage (-): 0

Region: chr10 59182497-59182608. Max. coverage (+): 0.67. Max coverage (-): 0

Region: chr10 59182609-59182721. Max. coverage (+): 0. Max coverage (-): 0

Region: chr10 59182722-59182834. Max. coverage (+): 0. Max coverage (-): 0

Region: chr10 59182835-59182946. Max. coverage (+): 39.62. Max coverage (-): 0

Region: chr10 59182947-59183059. Max. coverage (+): 24.51. Max coverage (-): 0

Region: chr10 59183060-59183172. Max. coverage (+): 16.58. Max coverage (-): 0

Region: chr10 59183173-59183284. Max. coverage (+): 8. Max coverage (-): 0

Region: chr10 59183285-59183397. Max. coverage (+): 20.03. Max coverage (-): 0

Region: chr10 59183398-59183510. Max. coverage (+): 10.57. Max coverage (-): 1.71

Region: chr10 59183511-59183622. Max. coverage (+): 23.23. Max coverage (-): 0

Region: chr10 59183623-59183735. Max. coverage (+): 20.25. Max coverage (-): 0

Region: chr10 59183736-59183848. Max. coverage (+): 10.2. Max coverage (-): 0

Region: chr10 59183849-59183960. Max. coverage (+): 4.86. Max coverage (-): 0

Region: chr10 59183961-59184073. Max. coverage (+): 15.44. Max coverage (-): 0

Region: chr10 59184074-59184186. Max. coverage (+): 12.29. Max coverage (-): 0

Region: chr10 59184187-59184298. Max. coverage (+): 13.1. Max coverage (-): 0

Region: chr10 59184299-59184411. Max. coverage (+): 8.84. Max coverage (-): 0

Region: chr10 59184412-59184523. Max. coverage (+): 0.4. Max coverage (-): 0

Region: chr10 59184524-59184636. Max. coverage (+): 16.53. Max coverage (-): 0

Region: chr10 59184637-59184749. Max. coverage (+): 4.41. Max coverage (-): 0

Region: chr10 59184750-59184861. Max. coverage (+): 9.57. Max coverage (-): 0

Region: chr10 59184862-59184974. Max. coverage (+): 16.42. Max coverage (-): 0

Region: chr10 59184975-59185087. Max. coverage (+): 182.39. Max coverage (-): 0

Region: chr10 59185088-59185199. Max. coverage (+): 60.42. Max coverage (-): 0

Region: chr10 59185200-59185312. Max. coverage (+): 68.58. Max coverage (-): 0

Region: chr10 59185313-59185425. Max. coverage (+): 12.45. Max coverage (-): 0

Region: chr10 59185426-59185537. Max. coverage (+): 19.92. Max coverage (-): 0

Region: chr10 59185538-59185650. Max. coverage (+): 7.61. Max coverage (-): 0

Region: chr10 59185651-59185763. Max. coverage (+): 41.76. Max coverage (-): 0

Region: chr10 59185764-59185875. Max. coverage (+): 9.44. Max coverage (-): 1.05

Region: chr10 59185876-59185988. Max. coverage (+): 43.4. Max coverage (-): 0.7

Region: chr10 59185989-59186101. Max. coverage (+): 33.89. Max coverage (-): 0

Region: chr10 59186102-59186213. Max. coverage (+): 23.25. Max coverage (-): 0

Region: chr10 59186214-59186326. Max. coverage (+): 9.46. Max coverage (-): 0

Region: chr10 59186327-59186439. Max. coverage (+): 10.68. Max coverage (-): 0

Region: chr10 59186440-59186551. Max. coverage (+): 10.53. Max coverage (-): 8.68

Region: chr10 59186552-59186664. Max. coverage (+): 7.49. Max coverage (-): 1.3

Region: chr10 59186665-59186777. Max. coverage (+): 20.01. Max coverage (-): 0

Region: chr10 59186778-59186889. Max. coverage (+): 19.77. Max coverage (-): 0

Region: chr10 59186890-59187002. Max. coverage (+): 0. Max coverage (-): 0

Region: chr10 59187003-59187115. Max. coverage (+): 18.35. Max coverage (-): 1.36

Region: chr10 59187116-59187227. Max. coverage (+): 37.56. Max coverage (-): 0

Region: chr10 59187228-59187340. Max. coverage (+): 14.36. Max coverage (-): 0

Region: chr10 59187341-59187452. Max. coverage (+): 13.04. Max coverage (-): 0

Region: chr10 59187453-59187565. Max. coverage (+): 19.38. Max coverage (-): 0

Region: chr10 59187566-59187678. Max. coverage (+): 29.41. Max coverage (-): 0

Region: chr10 59187679-59187790. Max. coverage (+): 44.5. Max coverage (-): 0

Region: chr10 59187791-59187903. Max. coverage (+): 24.84. Max coverage (-): 0

Region: chr10 59187904-59188016. Max. coverage (+): 34.66. Max coverage (-): 0

Region: chr10 59188017-59188128. Max. coverage (+): 17.11. Max coverage (-): 0

Region: chr10 59188129-59188241. Max. coverage (+): 11.46. Max coverage (-): 0

Region: chr10 59188242-59188354. Max. coverage (+): 44.31. Max coverage (-): 0

Region: chr10 59188355-59188466. Max. coverage (+): 90.21. Max coverage (-): 0

Region: chr10 59188467-59188579. Max. coverage (+): 52.63. Max coverage (-): 6.24

Region: chr10 59188580-59188692. Max. coverage (+): 106.01. Max coverage (-): 0.61

Region: chr10 59188693-59188804. Max. coverage (+): 45.42. Max coverage (-): 0

Region: chr10 59188805-59188917. Max. coverage (+): 0. Max coverage (-): 0

Region: chr10 59188918-59189030. Max. coverage (+): 0. Max coverage (-): 0

Region: chr10 59189031-59189142. Max. coverage (+): 34.01. Max coverage (-): 0

Region: chr10 59189143-59189255. Max. coverage (+): 37.44. Max coverage (-): 0

Region: chr10 59189256-59189368. Max. coverage (+): 8.9. Max coverage (-): 0

Region: chr10 59189369-59189480. Max. coverage (+): 17.36. Max coverage (-): 0

Region: chr10 59189481-59189593. Max. coverage (+): 51.98. Max coverage (-): 0

Region: chr10 59189594-59189706. Max. coverage (+): 14.02. Max coverage (-): 0

Region: chr10 59189707-59189818. Max. coverage (+): 47.23. Max coverage (-): 0

Region: chr10 59189819-59189931. Max. coverage (+): 44.87. Max coverage (-): 0

Region: chr10 59189932-59190044. Max. coverage (+): 41.84. Max coverage (-): 0

Region: chr10 59190045-59190156. Max. coverage (+): 21.53. Max coverage (-): 0

Region: chr10 59190157-59190269. Max. coverage (+): 47.24. Max coverage (-): 0

Region: chr10 59190270-59190381. Max. coverage (+): 50.9. Max coverage (-): 1.87

Region: chr10 59190382-59190494. Max. coverage (+): 11.6. Max coverage (-): 0

Region: chr10 59190495-59190607. Max. coverage (+): 30.24. Max coverage (-): 0

Region: chr10 59190608-59190719. Max. coverage (+): 28.97. Max coverage (-): 0

Region: chr10 59190720-59190832. Max. coverage (+): 8.71. Max coverage (-): 0

Region: chr10 59190833-59190945. Max. coverage (+): 36.19. Max coverage (-): 0

Region: chr10 59190946-59191057. Max. coverage (+): 7.86. Max coverage (-): 0

Region: chr10 59191058-59191170. Max. coverage (+): 9.41. Max coverage (-): 0

Region: chr10 59191171-59191283. Max. coverage (+): 90.9. Max coverage (-): 0

Region: chr10 59191284-59191395. Max. coverage (+): 19.45. Max coverage (-): 0

Region: chr10 59191396-59191508. Max. coverage (+): 12.58. Max coverage (-): 0

Region: chr10 59191509-59191621. Max. coverage (+): 22.3. Max coverage (-): 0

Region: chr10 59191622-59191733. Max. coverage (+): 27.57. Max coverage (-): 0

Region: chr10 59191734-59191846. Max. coverage (+): 4.62. Max coverage (-): 4.35

Region: chr10 59191847-59191959. Max. coverage (+): 11.39. Max coverage (-): 0

Region: chr10 59191960-59192071. Max. coverage (+): 37.59. Max coverage (-): 1.6

Region: chr10 59192072-59192184. Max. coverage (+): 15.22. Max coverage (-): 0

Region: chr10 59192185-59192297. Max. coverage (+): 9.3. Max coverage (-): 0

Region: chr10 59192298-59192409. Max. coverage (+): 9.57. Max coverage (-): 0

Region: chr10 59192410-59192522. Max. coverage (+): 4.28. Max coverage (-): 0

Region: chr10 59192523-59192635. Max. coverage (+): 11.15. Max coverage (-): 0

Region: chr10 59192636-59192747. Max. coverage (+): 1.2. Max coverage (-): 0

Region: chr10 59192748-59192860. Max. coverage (+): 7.28. Max coverage (-): 0

Region: chr10 59192861-59192973. Max. coverage (+): 2.14. Max coverage (-): 0

Region: chr10 59192974-59193085. Max. coverage (+): 5.31. Max coverage (-): 0

Region: chr10 59193086-59193198. Max. coverage (+): 1.99. Max coverage (-): 0

Region: chr10 59193199-59193311. Max. coverage (+): 12.01. Max coverage (-): 0

Region: chr10 59193312-59193423. Max. coverage (+): 5.03. Max coverage (-): 0

Region: chr10 59193424-59193536. Max. coverage (+): 3.95. Max coverage (-): 0

Region: chr10 59193537-59193648. Max. coverage (+): 10.18. Max coverage (-): 0

Region: chr10 59193649-59193761. Max. coverage (+): 4.41. Max coverage (-): 0

Region: chr10 59193762-59193874. Max. coverage (+): 69.23. Max coverage (-): 0

Region: chr10 59193875-59193986. Max. coverage (+): 38.39. Max coverage (-): 0

Region: chr10 59193987-59194099. Max. coverage (+): 51.4. Max coverage (-): 0

Region: chr10 59194100-59194212. Max. coverage (+): 27.8. Max coverage (-): 0

Region: chr10 59194213-59194324. Max. coverage (+): 16.25. Max coverage (-): 0

Region: chr10 59194325-59194437. Max. coverage (+): 13.14. Max coverage (-): 0

Region: chr10 59194438-59194550. Max. coverage (+): 37.96. Max coverage (-): 0

Region: chr10 59194551-59194662. Max. coverage (+): 16.73. Max coverage (-): 0

Region: chr10 59194663-59194775. Max. coverage (+): 62.76. Max coverage (-): 0

Region: chr10 59194776-59194888. Max. coverage (+): 77.4. Max coverage (-): 0

Region: chr10 59194889-59195000. Max. coverage (+): 37.97. Max coverage (-): 0

Region: chr10 59195001-59195113. Max. coverage (+): 18.48. Max coverage (-): 0

Region: chr10 59195114-59195226. Max. coverage (+): 13.22. Max coverage (-): 0

Region: chr10 59195227-59195338. Max. coverage (+): 32.7. Max coverage (-): 0

Region: chr10 59195339-59195451. Max. coverage (+): 6.08. Max coverage (-): 0

Region: chr10 59195452-59195564. Max. coverage (+): 59.49. Max coverage (-): 0

Region: chr10 59195565-59195676. Max. coverage (+): 12.12. Max coverage (-): 0

Region: chr10 59195677-59195789. Max. coverage (+): 6.07. Max coverage (-): 0

Region: chr10 59195790-59195902. Max. coverage (+): 4.55. Max coverage (-): 0

Region: chr10 59195903-59196014. Max. coverage (+): 86.65. Max coverage (-): 0

Region: chr10 59196015-59196127. Max. coverage (+): 38.62. Max coverage (-): 0

Region: chr10 59196128-59196240. Max. coverage (+): 43.43. Max coverage (-): 0

Region: chr10 59196241-59196352. Max. coverage (+): 20. Max coverage (-): 0

Region: chr10 59196353-59196465. Max. coverage (+): 12.5. Max coverage (-): 0

Region: chr10 59196466-59196577. Max. coverage (+): 80.72. Max coverage (-): 0

Region: chr10 59196578-59196690. Max. coverage (+): 11.62. Max coverage (-): 0

Region: chr10 59196691-59196803. Max. coverage (+): 31.87. Max coverage (-): 0

Region: chr10 59196804-59196915. Max. coverage (+): 22.27. Max coverage (-): 0

Region: chr10 59196916-59197028. Max. coverage (+): 10.43. Max coverage (-): 0

Region: chr10 59197029-59197141. Max. coverage (+): 21.85. Max coverage (-): 2.08

Region: chr10 59197142-59197253. Max. coverage (+): 6.17. Max coverage (-): 0

Region: chr10 59197254-59197366. Max. coverage (+): 37.68. Max coverage (-): 0

Region: chr10 59197367-59197479. Max. coverage (+): 17.67. Max coverage (-): 0

Region: chr10 59197480-59197591. Max. coverage (+): 10.2. Max coverage (-): 0

Region: chr10 59197592-59197704. Max. coverage (+): 24.41. Max coverage (-): 0

Region: chr10 59197705-59197817. Max. coverage (+): 1.67. Max coverage (-): 0

Region: chr10 59197818-59197929. Max. coverage (+): 3.8. Max coverage (-): 0

Region: chr10 59197930-59198042. Max. coverage (+): 25.06. Max coverage (-): 0

Region: chr10 59198043-59198155. Max. coverage (+): 53.88. Max coverage (-): 0

Region: chr10 59198156-59198267. Max. coverage (+): 59.2. Max coverage (-): 0

Region: chr10 59198268-59198380. Max. coverage (+): 57.67. Max coverage (-): 0

Region: chr10 59198381-59198493. Max. coverage (+): 22.14. Max coverage (-): 0

Region: chr10 59198494-59198605. Max. coverage (+): 75.07. Max coverage (-): 0

Region: chr10 59198606-59198718. Max. coverage (+): 24.95. Max coverage (-): 0

Region: chr10 59198719-59198831. Max. coverage (+): 51.07. Max coverage (-): 0

Region: chr10 59198832-59198943. Max. coverage (+): 26.69. Max coverage (-): 0

Region: chr10 59198944-59199056. Max. coverage (+): 16.45. Max coverage (-): 0

Region: chr10 59199057-59199169. Max. coverage (+): 50.4. Max coverage (-): 0

Region: chr10 59199170-59199281. Max. coverage (+): 34.72. Max coverage (-): 0

Region: chr10 59199282-59199394. Max. coverage (+): 39.09. Max coverage (-): 0

Region: chr10 59199395-59199506. Max. coverage (+): 12.86. Max coverage (-): 0

Region: chr10 59199507-59199619. Max. coverage (+): 22.9. Max coverage (-): 0

Region: chr10 59199620-59199732. Max. coverage (+): 13.4. Max coverage (-): 0

Region: chr10 59199733-59199844. Max. coverage (+): 27.99. Max coverage (-): 0

Region: chr10 59199845-59199957. Max. coverage (+): 2.36. Max coverage (-): 0

Region: chr10 59199958-59200070. Max. coverage (+): 13.72. Max coverage (-): 0

Region: chr10 59200071-59200182. Max. coverage (+): 9.2. Max coverage (-): 0

Region: chr10 59200183-59200295. Max. coverage (+): 23.38. Max coverage (-): 0

Region: chr10 59200296-59200408. Max. coverage (+): 54.95. Max coverage (-): 0

Region: chr10 59200409-59200520. Max. coverage (+): 4.02. Max coverage (-): 0

Region: chr10 59200521-59200633. Max. coverage (+): 8.42. Max coverage (-): 0

Region: chr10 59200634-59200746. Max. coverage (+): 27.44. Max coverage (-): 0

Region: chr10 59200747-59200858. Max. coverage (+): 4.2. Max coverage (-): 0

Region: chr10 59200859-59200971. Max. coverage (+): 16.72. Max coverage (-): 0

Region: chr10 59200972-59201084. Max. coverage (+): 48.69. Max coverage (-): 0

Region: chr10 59201085-59201196. Max. coverage (+): 4.7. Max coverage (-): 0

Region: chr10 59201197-59201309. Max. coverage (+): 15.08. Max coverage (-): 0

Region: chr10 59201310-59201422. Max. coverage (+): 17.58. Max coverage (-): 0

Region: chr10 59201423-59201534. Max. coverage (+): 8.13. Max coverage (-): 0

Region: chr10 59201535-59201647. Max. coverage (+): 0. Max coverage (-): 3.54

Region: chr10 59201648-59201760. Max. coverage (+): 0. Max coverage (-): 0

Region: chr10 59201761-59201872. Max. coverage (+): 9.74. Max coverage (-): 0

Region: chr10 59201873-59201985. Max. coverage (+): 18.87. Max coverage (-): 0

Region: chr10 59201986-59202098. Max. coverage (+): 1.18. Max coverage (-): 0

Region: chr10 59202099-59202210. Max. coverage (+): 7.59. Max coverage (-): 0

Region: chr10 59202211-59202323. Max. coverage (+): 7.7. Max coverage (-): 0

Region: chr10 59202324-59202435. Max. coverage (+): 5.47. Max coverage (-): 0

Region: chr10 59202436-59202548. Max. coverage (+): 2.56. Max coverage (-): 0

Region: chr10 59202549-59202661. Max. coverage (+): 5.58. Max coverage (-): 0

Region: chr10 59202662-59202773. Max. coverage (+): 13.15. Max coverage (-): 0

Region: chr10 59202774-59202886. Max. coverage (+): 9.11. Max coverage (-): 0

Region: chr10 59202887-59202999. Max. coverage (+): 1.34. Max coverage (-): 0

Region: chr10 59203000-59203111. Max. coverage (+): 3.18. Max coverage (-): 0

Region: chr10 59203112-59203224. Max. coverage (+): 12.98. Max coverage (-): 0

Region: chr10 59203225-59203337. Max. coverage (+): 4.57. Max coverage (-): 0

Region: chr10 59203338-59203449. Max. coverage (+): 7.99. Max coverage (-): 0

Region: chr10 59203450-59203562. Max. coverage (+): 5.2. Max coverage (-): 0

Region: chr10 59203563-59203675. Max. coverage (+): 3.41. Max coverage (-): 0

Region: chr10 59203676-59203787. Max. coverage (+): 6.98. Max coverage (-): 0

Region: chr10 59203788-59203900. Max. coverage (+): 11.53. Max coverage (-): 0

Region: chr10 59203901-59204013. Max. coverage (+): 0. Max coverage (-): 0

Region: chr10 59204014-59204125. Max. coverage (+): 2.79. Max coverage (-): 0

Region: chr10 59204126-59204238. Max. coverage (+): 12.64. Max coverage (-): 0

Region: chr10 59204239-59204351. Max. coverage (+): 8.08. Max coverage (-): 0

Region: chr10 59204352-59204463. Max. coverage (+): 8.6. Max coverage (-): 0

Region: chr10 59204464-59204576. Max. coverage (+): 3.72. Max coverage (-): 0

Region: chr10 59204577-59204689. Max. coverage (+): 5.03. Max coverage (-): 0

Region: chr10 59204690-59204801. Max. coverage (+): 0. Max coverage (-): 0

Region: chr10 59204802-59204914. Max. coverage (+): 9.78. Max coverage (-): 0

Region: chr10 59204915-59205027. Max. coverage (+): 52.57. Max coverage (-): 0

Region: chr10 59205028-59205139. Max. coverage (+): 15.47. Max coverage (-): 0

Region: chr10 59205140-59205252. Max. coverage (+): 34.03. Max coverage (-): 0

Region: chr10 59205253-59205364. Max. coverage (+): 22.19. Max coverage (-): 0

Region: chr10 59205365-59205477. Max. coverage (+): 34.44. Max coverage (-): 0

Region: chr10 59205478-59205590. Max. coverage (+): 15.08. Max coverage (-): 0

Region: chr10 59205591-59205702. Max. coverage (+): 17.25. Max coverage (-): 2.71

Region: chr10 59205703-59205815. Max. coverage (+): 18.99. Max coverage (-): 0

Region: chr10 59205816-59205928. Max. coverage (+): 33.98. Max coverage (-): 0

Region: chr10 59205929-59206040. Max. coverage (+): 6.03. Max coverage (-): 0

Region: chr10 59206041-59206153. Max. coverage (+): 20.24. Max coverage (-): 0

Region: chr10 59206154-59206266. Max. coverage (+): 17.37. Max coverage (-): 0

Region: chr10 59206267-59206378. Max. coverage (+): 18.51. Max coverage (-): 0

Region: chr10 59206379-59206491. Max. coverage (+): 0. Max coverage (-): 0

Region: chr10 59206492-59206604. Max. coverage (+): 5.89. Max coverage (-): 0

Region: chr10 59206605-59206716. Max. coverage (+): 10.47. Max coverage (-): 0

Region: chr10 59206717-59206829. Max. coverage (+): 6.39. Max coverage (-): 0

Region: chr10 59206830-59206942. Max. coverage (+): 22.69. Max coverage (-): 0

Region: chr10 59206943-59207054. Max. coverage (+): 5.49. Max coverage (-): 0

Region: chr10 59207055-59207167. Max. coverage (+): 2.99. Max coverage (-): 0

Region: chr10 59207168-59207280. Max. coverage (+): 0. Max coverage (-): 0

Region: chr10 59207281-59207392. Max. coverage (+): 6.33. Max coverage (-): 0

Region: chr10 59207393-59207505. Max. coverage (+): 5.1. Max coverage (-): 0

Region: chr10 59207506-59207618. Max. coverage (+): 6.2. Max coverage (-): 0

Region: chr10 59207619-59207730. Max. coverage (+): 0. Max coverage (-): 0

Region: chr10 59207731-59207843. Max. coverage (+): 5.1. Max coverage (-): 0

Region: chr10 59207844-59207956. Max. coverage (+): 0. Max coverage (-): 0

Region: chr10 59207957-59208068. Max. coverage (+): 1.31. Max coverage (-): 0

Region: chr10 59208069-59208181. Max. coverage (+): 0. Max coverage (-): 0

Region: chr10 59208182-59208293. Max. coverage (+): 0. Max coverage (-): 0

Region: chr10 59208294-59208406. Max. coverage (+): 3.94. Max coverage (-): 0

Region: chr10 59208407-59208519. Max. coverage (+): 0. Max coverage (-): 0

Region: chr10 59208520-59208631. Max. coverage (+): 5.17. Max coverage (-): 0

Region: chr10 59208632-59208744. Max. coverage (+): 0. Max coverage (-): 0

Region: chr10 59208745-59208857. Max. coverage (+): 0.51. Max coverage (-): 0

Region: chr10 59208858-59208969. Max. coverage (+): 0. Max coverage (-): 0

Region: chr10 59208970-59209082. Max. coverage (+): 2.22. Max coverage (-): 0

Region: chr10 59209083-59209195. Max. coverage (+): 5.81. Max coverage (-): 0

Region: chr10 59209196-59209307. Max. coverage (+): 0. Max coverage (-): 0

Region: chr10 59209308-59209420. Max. coverage (+): 0. Max coverage (-): 0

Region: chr10 59209421-59209533. Max. coverage (+): 0. Max coverage (-): 0

Region: chr10 59209534-59209645. Max. coverage (+): 0. Max coverage (-): 0

Region: chr10 59209646-59209758. Max. coverage (+): 0. Max coverage (-): 0

Region: chr10 59209759-59209871. Max. coverage (+): 0. Max coverage (-): 0

Region: chr10 59209872-59209983. Max. coverage (+): 0. Max coverage (-): 0

Region: chr10 59209984-59210096. Max. coverage (+): 0. Max coverage (-): 0

Region: chr10 59210097-59210209. Max. coverage (+): 0. Max coverage (-): 0

Region: chr10 59210210-59210321. Max. coverage (+): 0. Max coverage (-): 0

Region: chr10 59210322-59210434. Max. coverage (+): 0. Max coverage (-): 0

Region: chr10 59210435-59210547. Max. coverage (+): 0. Max coverage (-): 0

Region: chr10 59210548-59210659. Max. coverage (+): 0. Max coverage (-): 0

Region: chr10 59210660-59210772. Max. coverage (+): 0. Max coverage (-): 0

Region: chr10 59210773-59210885. Max. coverage (+): 0. Max coverage (-): 0

Region: chr10 59210886-59210997. Max. coverage (+): 0. Max coverage (-): 0

Region: chr10 59210998-59211110. Max. coverage (+): 0. Max coverage (-): 0

Region: chr10 59211111-59211222. Max. coverage (+): 0. Max coverage (-): 0

Region: chr10 59211223-59211335. Max. coverage (+): 0. Max coverage (-): 0

Region: chr10 59211336-59211448. Max. coverage (+): 0. Max coverage (-): 0

Region: chr10 59211449-59211560. Max. coverage (+): 0. Max coverage (-): 0

Region: chr10 59211561-59211673. Max. coverage (+): 0. Max coverage (-): 0

Region: chr10 59211674-59211786. Max. coverage (+): 0. Max coverage (-): 0

Region: chr10 59211787-59211898. Max. coverage (+): 0. Max coverage (-): 0

Region: chr10 59211899-59212011. Max. coverage (+): 0. Max coverage (-): 0

Region: chr10 59212012-59212124. Max. coverage (+): 0. Max coverage (-): 0

Region: chr10 59212125-59212236. Max. coverage (+): 0. Max coverage (-): 0

Region: chr10 59212237-59212349. Max. coverage (+): 0. Max coverage (-): 0

Region: chr10 59212350-59212462. Max. coverage (+): 0. Max coverage (-): 0

Region: chr10 59212463-59212574. Max. coverage (+): 63.26. Max coverage (-): 0

Region: chr10 59212575-59212687. Max. coverage (+): 32.51. Max coverage (-): 0

Region: chr10 59212688-59212800. Max. coverage (+): 0. Max coverage (-): 0

Region: chr10 59212801-59212912. Max. coverage (+): 0. Max coverage (-): 0

Region: chr10 59212913-59213025. Max. coverage (+): 14. Max coverage (-): 0

Region: chr10 59213026-59213138. Max. coverage (+): 3.93. Max coverage (-): 0

Region: chr10 59213139-59213250. Max. coverage (+): 5.23. Max coverage (-): 0

Region: chr10 59213251-59213363. Max. coverage (+): 15.48. Max coverage (-): 0

Region: chr10 59213364-59213476. Max. coverage (+): 13.87. Max coverage (-): 0.84

Region: chr10 59213477-59213588. Max. coverage (+): 8.21. Max coverage (-): 0

Region: chr10 59213589-59213701. Max. coverage (+): 13.77. Max coverage (-): 1.32

Region: chr10 59213702-59213814. Max. coverage (+): 6.5. Max coverage (-): 12.91

Region: chr10 59213815-59213926. Max. coverage (+): 8.65. Max coverage (-): 1.77

Region: chr10 59213927-59214039. Max. coverage (+): 14.22. Max coverage (-): 0

Region: chr10 59214040-59214151. Max. coverage (+): 0. Max coverage (-): 0

Region: chr10 59214152-59214264. Max. coverage (+): 28.07. Max coverage (-): 0

Region: chr10 59214265-59214377. Max. coverage (+): 6.97. Max coverage (-): 0

Region: chr10 59214378-59214489. Max. coverage (+): 20.56. Max coverage (-): 0

Region: chr10 59214490-59214602. Max. coverage (+): 23.55. Max coverage (-): 1.04

Region: chr10 59214603-59214715. Max. coverage (+): 0. Max coverage (-): 0

Region: chr10 59214716-59214827. Max. coverage (+): 8.05. Max coverage (-): 0

Region: chr10 59214828-59214940. Max. coverage (+): 0. Max coverage (-): 0

Region: chr10 59214941-59215053. Max. coverage (+): 0. Max coverage (-): 0

Region: chr10 59215054-59215165. Max. coverage (+): 11.79. Max coverage (-): 0

Region: chr10 59215166-59215278. Max. coverage (+): 0.82. Max coverage (-): 0

Region: chr10 59215279-59215391. Max. coverage (+): 0. Max coverage (-): 0

Region: chr10 59215392-59215503. Max. coverage (+): 0. Max coverage (-): 0

Region: chr10 59215504-59215616. Max. coverage (+): 6.81. Max coverage (-): 0

Region: chr10 59215617-59215729. Max. coverage (+): 5.7. Max coverage (-): 0

Region: chr10 59215730-59215841. Max. coverage (+): 10.9. Max coverage (-): 0

Region: chr10 59215842-59215954. Max. coverage (+): 10.78. Max coverage (-): 0

Region: chr10 59215955-59216067. Max. coverage (+): 14.66. Max coverage (-): 0

Region: chr10 59216068-59216179. Max. coverage (+): 0. Max coverage (-): 0

Region: chr10 59216180-59216292. Max. coverage (+): 0. Max coverage (-): 0

Region: chr10 59216293-59216405. Max. coverage (+): 0. Max coverage (-): 0

Region: chr10 59216406-59216517. Max. coverage (+): 0. Max coverage (-): 0

Region: chr10 59216518-59216630. Max. coverage (+): 0. Max coverage (-): 0

Region: chr10 59216631-59216743. Max. coverage (+): 0. Max coverage (-): 0

Region: chr10 59216744-59216855. Max. coverage (+): 0. Max coverage (-): 0

Region: chr10 59216856-59216968. Max. coverage (+): 3.25. Max coverage (-): 0

Region: chr10 59216969-59217080. Max. coverage (+): 3.23. Max coverage (-): 0

Region: chr10 59217081-59217193. Max. coverage (+): 1.07. Max coverage (-): 0

Region: chr10 59217194-59217306. Max. coverage (+): 3.49. Max coverage (-): 0

Region: chr10 59217307-59217418. Max. coverage (+): 2.28. Max coverage (-): 0

Region: chr10 59217419-59217531. Max. coverage (+): 2.62. Max coverage (-): 0

Region: chr10 59217532-59217644. Max. coverage (+): 3.68. Max coverage (-): 0

Region: chr10 59217645-59217756. Max. coverage (+): 2.15. Max coverage (-): 0

Region: chr10 59217757-59217869. Max. coverage (+): 2.3. Max coverage (-): 0

Region: chr10 59217870-59217982. Max. coverage (+): 0. Max coverage (-): 0

Region: chr10 59217983-59218094. Max. coverage (+): 0. Max coverage (-): 0

Region: chr10 59218095-59218207. Max. coverage (+): 6.93. Max coverage (-): 0

Region: chr10 59218208-59218320. Max. coverage (+): 3.14. Max coverage (-): 0

Region: chr10 59218321-59218432. Max. coverage (+): 3.14. Max coverage (-): 0

Region: chr10 59218433-59218545. Max. coverage (+): 0. Max coverage (-): 0

Region: chr10 59218546-59218658. Max. coverage (+): 0. Max coverage (-): 0

Region: chr10 59218659-59218770. Max. coverage (+): 0. Max coverage (-): 0

Region: chr10 59218771-59218883. Max. coverage (+): 5.57. Max coverage (-): 0

Region: chr10 59218884-59218996. Max. coverage (+): 14.08. Max coverage (-): 0

Region: chr10 59218997-59219108. Max. coverage (+): 14.12. Max coverage (-): 0

Region: chr10 59219109-59219221. Max. coverage (+): 3.54. Max coverage (-): 0

Region: chr10 59219222-59219334. Max. coverage (+): 8.58. Max coverage (-): 0

Region: chr10 59219335-59219446. Max. coverage (+): 0. Max coverage (-): 0

Region: chr10 59219447-59219559. Max. coverage (+): 15.66. Max coverage (-): 0

Region: chr10 59219560-59219672. Max. coverage (+): 0. Max coverage (-): 0

Region: chr10 59219673-59219784. Max. coverage (+): 0. Max coverage (-): 0

Region: chr10 59219785-59219897. Max. coverage (+): 0. Max coverage (-): 0

Region: chr10 59219898-59220009. Max. coverage (+): 48.61. Max coverage (-): 0

Region: chr10 59220010-59220122. Max. coverage (+): 15.73. Max coverage (-): 0

Region: chr10 59220123-59220235. Max. coverage (+): 7.43. Max coverage (-): 0

Region: chr10 59220236-59220347. Max. coverage (+): 15.72. Max coverage (-): 0

Region: chr10 59220348-59220460. Max. coverage (+): 15.72. Max coverage (-): 0

Region: chr10 59220461-59220573. Max. coverage (+): 0. Max coverage (-): 0

Region: chr10 59220574-59220685. Max. coverage (+): 11.99. Max coverage (-): 0

Region: chr10 59220686-59220798. Max. coverage (+): 0. Max coverage (-): 0

Region: chr10 59220799-59220911. Max. coverage (+): 0. Max coverage (-): 0

Region: chr10 59220912-59221023. Max. coverage (+): 0. Max coverage (-): 0

Region: chr10 59221024-59221136. Max. coverage (+): 9.4. Max coverage (-): 0

Region: chr10 59221137-59221249. Max. coverage (+): 7.86. Max coverage (-): 0

Region: chr10 59221250-59221361. Max. coverage (+): 9.65. Max coverage (-): 0

Region: chr10 59221362-59221474. Max. coverage (+): 11.23. Max coverage (-): 0

Region: chr10 59221475-59221587. Max. coverage (+): 18.69. Max coverage (-): 0

Region: chr10 59221588-59221699. Max. coverage (+): 5.11. Max coverage (-): 0

Region: chr10 59221700-59221812. Max. coverage (+): 10.77. Max coverage (-): 0

Region: chr10 59221813-59221925. Max. coverage (+): 13.72. Max coverage (-): 0

Region: chr10 59221926-59222037. Max. coverage (+): 2.81. Max coverage (-): 0

Region: chr10 59222038-59222150. Max. coverage (+): 6.56. Max coverage (-): 0

Region: chr10 59222151-59222263. Max. coverage (+): 0. Max coverage (-): 0

Region: chr10 59222264-59222375. Max. coverage (+): 0.21. Max coverage (-): 0

Region: chr10 59222376-59222488. Max. coverage (+): 1.58. Max coverage (-): 0

Region: chr10 59222489-59222601. Max. coverage (+): 8.34. Max coverage (-): 0

Region: chr10 59222602-59222713. Max. coverage (+): 1.48. Max coverage (-): 0

Region: chr10 59222714-59222826. Max. coverage (+): 7.9. Max coverage (-): 0

Region: chr10 59222827-59222939. Max. coverage (+): 0.72. Max coverage (-): 0

Region: chr10 59222940-59223051. Max. coverage (+): 0. Max coverage (-): 0

Region: chr10 59223052-59223164. Max. coverage (+): 2.46. Max coverage (-): 0

Region: chr10 59223165-59223276. Max. coverage (+): 4.68. Max coverage (-): 0

Region: chr10 59223277-59223389. Max. coverage (+): 4.31. Max coverage (-): 0

Region: chr10 59223390-59223502. Max. coverage (+): 4.31. Max coverage (-): 0

Region: chr10 59223503-59223614. Max. coverage (+): 4.95. Max coverage (-): 0

Region: chr10 59223615-59223727. Max. coverage (+): 1.33. Max coverage (-): 0

Region: chr10 59223728-59223840. Max. coverage (+): 0. Max coverage (-): 0

Region: chr10 59223841-59223952. Max. coverage (+): 0. Max coverage (-): 0

Region: chr10 59223953-59224065. Max. coverage (+): 0. Max coverage (-): 0

Region: chr10 59224066-59224178. Max. coverage (+): 0. Max coverage (-): 0

Region: chr10 59224179-59224290. Max. coverage (+): 0. Max coverage (-): 0

Region: chr10 59224291-59224403. Max. coverage (+): 1.14. Max coverage (-): 0

Region: chr10 59224404-59224516. Max. coverage (+): 6.28. Max coverage (-): 0

Region: chr10 59224517-59224628. Max. coverage (+): 5.56. Max coverage (-): 0

Region: chr10 59224629-59224741. Max. coverage (+): 0. Max coverage (-): 0

Region: chr10 59224742-59224854. Max. coverage (+): 0. Max coverage (-): 0

Region: chr10 59224855-59224966. Max. coverage (+): 7.74. Max coverage (-): 0

Region: chr10 59224967-59225079. Max. coverage (+): 5.71. Max coverage (-): 0

Region: chr10 59225080-59225192. Max. coverage (+): 4.51. Max coverage (-): 0

Region: chr10 59225193-59225304. Max. coverage (+): 4.68. Max coverage (-): 0

Region: chr10 59225305-59225417. Max. coverage (+): 1.38. Max coverage (-): 0

Region: chr10 59225418-59225530. Max. coverage (+): 2.85. Max coverage (-): 0

Region: chr10 59225531-59225642. Max. coverage (+): 1.16. Max coverage (-): 0

Region: chr10 59225643-59225755. Max. coverage (+): 0. Max coverage (-): 0

Region: chr10 59225756-59225868. Max. coverage (+): 0. Max coverage (-): 0

Region: chr10 59225869-59225980. Max. coverage (+): 1.58. Max coverage (-): 0

Region: chr10 59225981-59226093. Max. coverage (+): 0. Max coverage (-): 0

Region: chr10 59226094-59226205. Max. coverage (+): 0.91. Max coverage (-): 0

Region: chr10 59226206-59226318. Max. coverage (+): 4.4. Max coverage (-): 0

Region: chr10 59226319-59226431. Max. coverage (+): 1.3. Max coverage (-): 0

Region: chr10 59226432-59226543. Max. coverage (+): 0. Max coverage (-): 0

Region: chr10 59226544-59226656. Max. coverage (+): 10.12. Max coverage (-): 0

Region: chr10 59226657-59226769. Max. coverage (+): 0. Max coverage (-): 0

Region: chr10 59226770-59226881. Max. coverage (+): 0. Max coverage (-): 0

Region: chr10 59226882-59226994. Max. coverage (+): 10.52. Max coverage (-): 0

Region: chr10 59226995-59227107. Max. coverage (+): 1.47. Max coverage (-): 0

Region: chr10 59227108-59227219. Max. coverage (+): 8.41. Max coverage (-): 1.43

Region: chr10 59227220-59227332. Max. coverage (+): 10.58. Max coverage (-): 0

Region: chr10 59227333-59227445. Max. coverage (+): 1.94. Max coverage (-): 0

Region: chr10 59227446-59227557. Max. coverage (+): 8.61. Max coverage (-): 0

Region: chr10 59227558-59227670. Max. coverage (+): 2.39. Max coverage (-): 0

Region: chr10 59227671-59227783. Max. coverage (+): 5.15. Max coverage (-): 0

Region: chr10 59227784-59227895. Max. coverage (+): 2.5. Max coverage (-): 0

Region: chr10 59227896-59228008. Max. coverage (+): 11.38. Max coverage (-): 0

Region: chr10 59228009-59228121. Max. coverage (+): 17.95. Max coverage (-): 0

Region: chr10 59228122-59228233. Max. coverage (+): 3.56. Max coverage (-): 0

Region: chr10 59228234-59228346. Max. coverage (+): 0. Max coverage (-): 0

Region: chr10 59228347-59228459. Max. coverage (+): 8.98. Max coverage (-): 0

Region: chr10 59228460-59228571. Max. coverage (+): 4.75. Max coverage (-): 0

Region: chr10 59228572-59228684. Max. coverage (+): 8.4. Max coverage (-): 0

Region: chr10 59228685-59228797. Max. coverage (+): 33.33. Max coverage (-): 0

Region: chr10 59228798-59228909. Max. coverage (+): 15.68. Max coverage (-): 0

Region: chr10 59228910-59229022. Max. coverage (+): 35.73. Max coverage (-): 0

Region: chr10 59229023-59229134. Max. coverage (+): 5.3. Max coverage (-): 7.94

Region: chr10 59229135-59229247. Max. coverage (+): 6.06. Max coverage (-): 0

Region: chr10 59229248-59229360. Max. coverage (+): 3.86. Max coverage (-): 0

Region: chr10 59229361-59229472. Max. coverage (+): 21.21. Max coverage (-): 0

Region: chr10 59229473-59229585. Max. coverage (+): 6.4. Max coverage (-): 0

Region: chr10 59229586-59229698. Max. coverage (+): 8.61. Max coverage (-): 0

Region: chr10 59229699-59229810. Max. coverage (+): 10.79. Max coverage (-): 0

Region: chr10 59229811-59229923. Max. coverage (+): 8.78. Max coverage (-): 0

Region: chr10 59229924-59230036. Max. coverage (+): 4.8. Max coverage (-): 0

Region: chr10 59230037-59230148. Max. coverage (+): 1.76. Max coverage (-): 0

Region: chr10 59230149-59230261. Max. coverage (+): 3.55. Max coverage (-): 0

Region: chr10 59230262-59230374. Max. coverage (+): 1.15. Max coverage (-): 0

Region: chr10 59230375-59230486. Max. coverage (+): 1.15. Max coverage (-): 0

Region: chr10 59230487-59230599. Max. coverage (+): 4.21. Max coverage (-): 0

Region: chr10 59230600-59230712. Max. coverage (+): 0. Max coverage (-): 0

Region: chr10 59230713-59230824. Max. coverage (+): 0. Max coverage (-): 0

Region: chr10 59230825-59230937. Max. coverage (+): 2.74. Max coverage (-): 0

Region: chr10 59230938-59231050. Max. coverage (+): 2.43. Max coverage (-): 0

Region: chr10 59231051-59231162. Max. coverage (+): 11.55. Max coverage (-): 0

Region: chr10 59231163-59231275. Max. coverage (+): 0. Max coverage (-): 0

Region: chr10 59231276-59231388. Max. coverage (+): 2.17. Max coverage (-): 0

Region: chr10 59231389-59231500. Max. coverage (+): 0. Max coverage (-): 0

Region: chr10 59231501-59231613. Max. coverage (+): 0. Max coverage (-): 0

Region: chr10 59231614-59231726. Max. coverage (+): 0. Max coverage (-): 0

Region: chr10 59231727-59231838. Max. coverage (+): 0. Max coverage (-): 0

Region: chr10 59231839-59231951. Max. coverage (+): 4.46. Max coverage (-): 0.53

Region: chr10 59231952-59232063. Max. coverage (+): 0.93. Max coverage (-): 0

Region: chr10 59232064-59232176. Max. coverage (+): 0. Max coverage (-): 0

Region: chr10 59232177-59232289. Max. coverage (+): 1.75. Max coverage (-): 0

Region: chr10 59232290-59232401. Max. coverage (+): 0. Max coverage (-): 0

Region: chr10 59232402-59232514. Max. coverage (+): 15.26. Max coverage (-): 0.58

Region: chr10 59232515-59232627. Max. coverage (+): 5.71. Max coverage (-): 0

Region: chr10 59232628-59232739. Max. coverage (+): 4.75. Max coverage (-): 0

Region: chr10 59232740-59232852. Max. coverage (+): 2.07. Max coverage (-): 0

Region: chr10 59232853-59232965. Max. coverage (+): 0. Max coverage (-): 0

Region: chr10 59232966-59233077. Max. coverage (+): 0. Max coverage (-): 0

Region: chr10 59233078-59233190. Max. coverage (+): 0. Max coverage (-): 0

Region: chr10 59233191-59233303. Max. coverage (+): 0. Max coverage (-): 0

Region: chr10 59233304-59233415. Max. coverage (+): 9.06. Max coverage (-): 0

Region: chr10 59233416-59233528. Max. coverage (+): 7.1. Max coverage (-): 0

Region: chr10 59233529-59233641. Max. coverage (+): 12.77. Max coverage (-): 0

Region: chr10 59233642-59233753. Max. coverage (+): 7.56. Max coverage (-): 0

Region: chr10 59233754-59233866. Max. coverage (+): 2.5. Max coverage (-): 0

Region: chr10 59233867-59233979. Max. coverage (+): 11.49. Max coverage (-): 0

Region: chr10 59233980-59234091. Max. coverage (+): 5.96. Max coverage (-): 0

Region: chr10 59234092-59234204. Max. coverage (+): 4.98. Max coverage (-): 1.96

Region: chr10 59234205-59234317. Max. coverage (+): 4.9. Max coverage (-): 0

Region: chr10 59234318-59234429. Max. coverage (+): 2.01. Max coverage (-): 0.34

Region: chr10 59234430-59234542. Max. coverage (+): 4.77. Max coverage (-): 0

Region: chr10 59234543-59234655. Max. coverage (+): 0. Max coverage (-): 0

Region: chr10 59234656-59234767. Max. coverage (+): 0. Max coverage (-): 0

Region: chr10 59234768-. Max. coverage (+): 0. Max coverage (-): 3.94

RepeatMasker Color Code

**+**

100-98% Identity

<98-95% Identity

<95-90% Identity

<90-85% Identity

<85-80% Identity

<80-75% Identity

<75-70% Identity

<70% Identity

**-**

Gene Set Color Code

**+**

Gene

Pseudogene

**-**

Topology/Coverage Color Code

Coverage Plus Strand

Coverage Minus Strand

Mainstrand: Plus

Mainstrand: Minus

Complementary Strand

Flanking Region  
(if option -flank >0)

Gene Set Annotation  

**1. CYP19A1 (protein coding, ENSBTAG00000014890) Tr:00000019823 Ex:1**: 59227895-59228038 (+)

  
RepeatMasker Annotation  

**1. Bov-tA1**: 59178813-59179035 (+), Divergence to consensus: 15.7%  
**2. MLT1E1**: 59179463-59179971 (-), Divergence to consensus: 31.4%  
**3. Bov-tA2**: 59179972-59180083 (+), Divergence to consensus: 10%  
**4. L1ME4a**: 59180901-59180980 (-), Divergence to consensus: 35%  
**5. MamTip2**: 59182273-59182377 (+), Divergence to consensus: 40.6%  
**6. L2c**: 59182564-59182882 (-), Divergence to consensus: 49.6%  
**7. L2b**: 59185481-59185601 (+), Divergence to consensus: 39.5%  
**8. MIR3**: 59186817-59187011 (-), Divergence to consensus: 41.1%  
**9. L2c**: 59187538-59187610 (+), Divergence to consensus: 33.2%  
**10. Charlie18a**: 59188809-59189029 (-), Divergence to consensus: 41.7%  
**11. AT\_rich**: 59190374-59190396 (+), Divergence to consensus: 21.7%  
**12. MER91A**: 59191083-59191193 (-), Divergence to consensus: 46.8%  
**13. MIRb**: 59192234-59192342 (+), Divergence to consensus: 42.2%  
**14. L3**: 59192730-59192763 (-), Divergence to consensus: 15.6%  
**15. MIR**: 59193728-59193778 (-), Divergence to consensus: 11.8%  
**16. L2c**: 59193736-59193809 (-), Divergence to consensus: 32.4%  
**17. (TG)n**: 59194985-59195030 (+), Divergence to consensus: 0%  
**18. L2c**: 59195893-59195962 (-), Divergence to consensus: 33.2%  
**19. (A)n**: 59200739-59200772 (+), Divergence to consensus: 14.7%  
**20. (TA)n**: 59201248-59201290 (+), Divergence to consensus: 2.3%  
**21. MamTip2**: 59201558-59201595 (+), Divergence to consensus: 16.2%  
**22. MER94B**: 59203458-59203516 (-), Divergence to consensus: 30.5%  
**23. AT\_rich**: 59203690-59203712 (+), Divergence to consensus: 39.1%  
**24. MIRb**: 59204676-59204875 (-), Divergence to consensus: 42.3%  
**25. MIRc**: 59206311-59206459 (-), Divergence to consensus: 35.3%  
**26. MIRc**: 59206490-59206524 (-), Divergence to consensus: 20%  
**27. MIRb**: 59207024-59207151 (-), Divergence to consensus: 41.7%  
**28. L2c**: 59207192-59207306 (-), Divergence to consensus: 34.6%  
**29. MIRb**: 59208817-59208983 (+), Divergence to consensus: 42%  
**30. AT\_rich**: 59209221-59209243 (+), Divergence to consensus: 60.9%  
**31. L1ME1**: 59209255-59209662 (+), Divergence to consensus: 31.8%  
**32. LTR16B1**: 59209669-59210112 (-), Divergence to consensus: 36%  
**33. CHRL**: 59210118-59210301 (-), Divergence to consensus: 29.7%  
**34. L1ME1**: 59210319-59210971 (+), Divergence to consensus: 35.2%  
**35. SINE2-1\_BT**: 59210972-59211089 (+), Divergence to consensus: 28%  
**36. L1ME1**: 59211090-59211146 (+), Divergence to consensus: 35.2%  
**37. ART2A**: 59211304-59211808 (-), Divergence to consensus: 9.6%  
**38. L1ME1**: 59211809-59212461 (+), Divergence to consensus: 43.1%  
**39. MER45C**: 59212782-59212962 (+), Divergence to consensus: 31%  
**40. MER45C**: 59213073-59213231 (+), Divergence to consensus: 43.4%  
**41. MIR3**: 59214085-59214171 (+), Divergence to consensus: 38%  
**42. MER102b**: 59214590-59214765 (-), Divergence to consensus: 38.8%  
**43. BOV-A2**: 59215279-59215550 (-), Divergence to consensus: 4.8%  
**44. L2**: 59216187-59216647 (+), Divergence to consensus: 46.2%  
**45. (TAGA)n**: 59216648-59216668 (+), Divergence to consensus: 0%  
**46. L2**: 59216669-59216858 (+), Divergence to consensus: 51.7%  
**47. CR1\_Mam**: 59217920-59218151 (-), Divergence to consensus: 39.6%  
**48. AT\_rich**: 59218483-59218517 (+), Divergence to consensus: 65.7%  
**49. MIRc**: 59218551-59218755 (-), Divergence to consensus: 42.8%  
**50. MER91B**: 59219191-59219246 (+), Divergence to consensus: 33.9%  
**51. MIR**: 59219651-59219878 (+), Divergence to consensus: 37.9%  
**52. MIRb**: 59220474-59220585 (+), Divergence to consensus: 43.3%  
**53. MER94**: 59220686-59220797 (-), Divergence to consensus: 32.2%  
**54. MIRb**: 59220893-59221089 (-), Divergence to consensus: 42.1%  
**55. L2c**: 59222447-59222567 (+), Divergence to consensus: 44.4%  
**56. MIRc**: 59222932-59223107 (-), Divergence to consensus: 46.3%  
**57. L1MEd**: 59223763-59224114 (+), Divergence to consensus: 49.9%  
**58. L1MEd**: 59224565-59224805 (+), Divergence to consensus: 45.9%  
**59. MARNA**: 59224848-59225029 (-), Divergence to consensus: 43.3%  
**60. MamTip2**: 59225557-59225601 (+), Divergence to consensus: 33.4%  
**61. MIR**: 59225991-59226162 (-), Divergence to consensus: 35.7%  
**62. Bov-tA2**: 59226427-59226628 (+), Divergence to consensus: 12.9%  
**63. L2a**: 59226655-59226769 (+), Divergence to consensus: 40.9%  
**64. MIR3**: 59227505-59227588 (-), Divergence to consensus: 31.2%  
**65. L2b**: 59227847-59227893 (-), Divergence to consensus: 21.2%  
**66. MIRc**: 59228350-59228444 (-), Divergence to consensus: 29.7%  
**67. Bov-tA3**: 59228552-59228672 (-), Divergence to consensus: 14.9%  
**68. MIR3**: 59229554-59229629 (+), Divergence to consensus: 40.4%  
**69. BOV-A2**: 59230600-59230871 (+), Divergence to consensus: 4.4%  
**70. L2**: 59231551-59231892 (-), Divergence to consensus: 50.4%  
**71. MIR**: 59232306-59232403 (+), Divergence to consensus: 29.6%  
**72. L2**: 59232897-59233371 (+), Divergence to consensus: 48.1%  
**73. MIR**: 59234571-59234795 (+), Divergence to consensus: 35.2%

  
Transcription Factor Binding Sites  

**Mybl1\_1** (Sequence: TAACGGTT (-): 59181159)  
**Mybl1\_1** (Sequence: TAACGGTT (-): 59196938)  
**RFX4\_2** (Sequence: GTAACCATG (-): 59213579)  
**RFX4\_1** (Sequence: GTTGCCAAG (-): 59195076)  
**RFX4\_1** (Sequence: CTTAGCAAC (+): 59202299)  
**RFX4\_2** (Sequence: CGTGGTTAC (+): 59197438)  
**Gata4** (Sequence: AGATAAG (-): 59183862)  
**Gata4** (Sequence: AGATAAG (-): 59212475)  
**Gata4** (Sequence: AGATAAG (-): 59214285)  
**Gata4** (Sequence: AGATAAC (-): 59220076)  
**Gata4** (Sequence: AGATAAG (-): 59227665)  
**Gata4** (Sequence: AGATAAC (-): 59228540)  
**SOX9** (Sequence: AACAATAA (-): 59181291)  
**SOX9** (Sequence: AACAATGA (-): 59183154)  
**SOX9** (Sequence: AACAATGA (-): 59204111)  
**SOX9** (Sequence: AACAATAA (-): 59207638)  
**SOX9** (Sequence: AACAATGG (-): 59207959)  
**SOX9** (Sequence: AACAATGA (-): 59230567)  
**SOX9** (Sequence: TCATTGTT (+): 59182493)  
**SOX9** (Sequence: CTATTGTT (+): 59184581)  
**SOX9** (Sequence: TTATTGTT (+): 59220875)  
**A-MYB** (Sequence: CCAACTGCCA (-): 59200978)  
**A-MYB** (Sequence: TGGCAGTTGG (+): 59199634)  
**SPZ1** (Sequence: AGGGTTTGAG (+): 59185855)  
**SPZ1** (Sequence: AGGGTTAGAG (+): 59207427)  
**Mybl1\_1** (Sequence: AACCGTTA (+): 59193599)  
**Gata4** (Sequence: CTTATCT (+): 59185624)  
**Gata4** (Sequence: GTTATCT (+): 59189535)  
**Gata4** (Sequence: GTTATCT (+): 59192668)  
**Gata4** (Sequence: CTTATCT (+): 59199404)  
**Gata4** (Sequence: CTTATCT (+): 59203382)  
**Gata4** (Sequence: GTTATCT (+): 59230058)
