# Supplementary material for: Small RNA sequencing of cryopreserved semen from single bull revealed altered miRNAs and piRNAs expression between High- and Low-motile sperm populations
Source: BMC Genomics. 2017 Jan 4;18:14. doi: 10.1186/s12864-016-3394-7 (PMC5209821; doi:10.1186/s12864-016-3394-7)
Supplement: Additional file 3: — Details for each piRNA clusters found in High Motile (HM) sperm fraction. Genes, repeats, transposable elements and transcription factors binding sites falling within the cluster regions were reported. (ZIP 1896 kb) [file 12864_2016_3394_MOESM3_ESM.zip › 60.html]

piRNA cluster 60


Predicted piRNA cluster no. 60     previous   next
  

Show proTRAC run info
Hide proTRAC run info

================================= proTRAC ====================================  
VERSION: 2.1                                    LAST MODIFIED: 06. October 2015  
  
Please cite:  
Rosenkranz D, Zischler H. proTRAC - a software for probabilistic piRNA cluster  
detection, visualization and analysis. 2012. BMC Bioinformatics 13:5.  
  
and (for proTRAC 2.0 and later):  
Rosenkranz D, Rudloff S, Bastuck K, Ketting RF, Zischler H. Tupaia small RNAs  
provide insights into function and evolution of RNAi-based transposon defense  
in mammals. 2015. RNA 21(5):911-922.  
  
Contact:  
David Rosenkranz  
Institute of Anthropology, small RNA group  
Johannes Gutenberg University Mainz  
email: rosenkranz@uni-mainz.de  
  
You can find the latest proTRAC version at:  
http://sourceforge.net/projects/protrac/files  
http://www.smallRNAgroup-mainz.de/software  
==============================================================================  
  
PARAMETERS:  
Map file: .............../storage/core/barbara/genhome/smallRNA/fertility/Sample\_motile/pirna/Sample\_motile\_26-33\_collapsed.fa.no-dust.map.weighted-10000-1000-b-0  
Genome file: ............/storage/core/barbara/genhome/smallRNA/fertility/Sample\_all/pirna/bt\_311\_chrY.fa  
RepeatMasker annotation: /storage/genomes/bt\_umd31/GCF\_000003055.6\_Bos\_taurus\_UMD\_3.1.1\_repeatMasker\_chr.out  
GeneSet:................./storage/core/barbara/genhome/smallRNA/fertility/Sample\_all/pirna/full.gtf  
  
Significant (p<=0.01) hit density will be calculated based  
on observed hit distribution.  
  
Sliding window size: ........................................ 5000 bp  
Sliding window increament: .................................. 1000 bp  
Normalize each hit by number of genomic hits: ............... 1 [0=no/1=yes]  
Normalize each hit by number of sequence reads: ............. 1 [0=no/1=yes]  
Normalize values (-> per million mapped reads): ............. 1 [0=no/1=yes]  
Min. fraction of hits with 1T(U) or 10A: .................... 0.75  
Alternatively: Min. fraction of hits with 1T(U) and 10A: .... 0.5  
Min. fraction of hits with typical piRNA length: ............ 0.75  
Typical piRNA length: ....................................... 26-33 nt  
Min. size of a piRNA cluster: ............................... 5000 bp.  
Min. number of hits (absolute): ............................. 0  
Min. number of hits (normalized): ........................... 0  
Min. fraction of hits on the mainstrand: .................... 0.75  
Top fraction of mapped sequences (in terms of read counts): . 1%  
Top fraction accounts for max. n% of sequence reads: ........ 90%  
Min. fraction of hits on each arm of a bidirectional cluster: 0.1  
Output image file for each cluster: ......................... 0 [0=no/1=yes]  
Output html file for each cluster: .......................... 1 [0=no/1=yes]  
Output a summary table: ..................................... 1 [0=no/1=yes]  
Output a FASTA file for each cluster (piRNA sequences): ..... 1 [0=no/1=yes]  
Output a FASTA file comprising cluster sequences: ........... 1 [0=no/1=yes]  
Search DNA motifs in clusters: .............................. 1 [0=no/1=yes]  
Output flanking sequences: +/- .............................. 0 bp  
Output ~.pTi file: .......................................... 1 [0=no/1=yes]  
==============================================================================  
  
  
Genome size (without gaps): ............ 2678902517 bp  
Gaps (N/X/-): .......................... 53837044 bp  
Mapped reads: .......................... 658825247023  
Non-identical sequences: ............... 514171  
Genomic hits: .......................... 764233  
Significant densitiy of mapped reads: .. 12867599.5173724 reads/kb

Show proTRAC cluster info
Hide proTRAC cluster info

|  |  |
| --- | --- |
| Location | chr25 |
| Coordinates | 29348932-29355695 |
| Size [bp] | 6764 |
| Sequence hit loci | 291 |
| Mapped reads (normalized) | 374244481 |
| Mapped reads (normalized) per kb | 55328870.6 |
| Normalized reads with 1T (1U) | 81.7% |
| Normalized reads with 10A | 21.9% |
| Normalized reads with length 26-33 nt | 100% |
| Normalized reads on the main strand(s) | 100% |
| Predicted directionality | mono:plus |

100%

0%

1T (1U)  
reads

10A reads

26-33 nt  
reads

reads on mainstrand

**Either the amount of reads with 1T (1U) OR 10A has to exceed 75% (set with option: -1Tor10A)  
Alternatively the amount of reads with 1T (1U) AND 10A has to exceed 50% (set with option: -1Tand10A)  
Minimum amount of reads with preferred size is 75% (set with option: -pisize)  
Minimum amount of reads on the main strand(s) is 75% (set with option: -clstrand)**

Show read coverage
Hide read coverage

WHAT DO I SEE HERE?  
This chart shows the location of mapped sequence reads within a predicted piRNA cluster. The color refers to the number of genomic hits produced by the sequence read in question. A dark red bar indicates that this sequence read produces many other hits elsewhere in the genome. Many adjacent red or yellow bars can indicate the presence of a multi-copy element such as transposons or rRNA genes. A dark green bar indicates that this sequence read maps uniquely to this locus.

1 hit

2-5 hits

6-10 hits

11-20 hits

21-50 hits

51-100 hits

> 100 hits

chr25

29348932

29355695

Gene Set

RepeatMasker

Mapped  
Reads

30.47

plus strand

minus strand

30.47

Region: chr25 28861749-29348938. Max. coverage (+): 1.58. Max coverage (-): 0

Region: chr25 29348939-29348952. Max. coverage (+): 1.58. Max coverage (-): 0

Region: chr25 29348953-29348965. Max. coverage (+): 0. Max coverage (-): 0

Region: chr25 29348966-29348979. Max. coverage (+): 0.2. Max coverage (-): 0

Region: chr25 29348980-29348992. Max. coverage (+): 0.2. Max coverage (-): 0

Region: chr25 29348993-29349006. Max. coverage (+): 0.8. Max coverage (-): 0

Region: chr25 29349007-29349019. Max. coverage (+): 0. Max coverage (-): 0

Region: chr25 29349020-29349033. Max. coverage (+): 0. Max coverage (-): 0

Region: chr25 29349034-29349046. Max. coverage (+): 0. Max coverage (-): 0

Region: chr25 29349047-29349060. Max. coverage (+): 0. Max coverage (-): 0

Region: chr25 29349061-29349074. Max. coverage (+): 0. Max coverage (-): 0

Region: chr25 29349075-29349087. Max. coverage (+): 0. Max coverage (-): 0

Region: chr25 29349088-29349101. Max. coverage (+): 6.95. Max coverage (-): 0

Region: chr25 29349102-29349114. Max. coverage (+): 6.95. Max coverage (-): 0

Region: chr25 29349115-29349128. Max. coverage (+): 0. Max coverage (-): 0

Region: chr25 29349129-29349141. Max. coverage (+): 0. Max coverage (-): 0

Region: chr25 29349142-29349155. Max. coverage (+): 0. Max coverage (-): 0

Region: chr25 29349156-29349168. Max. coverage (+): 0. Max coverage (-): 0

Region: chr25 29349169-29349182. Max. coverage (+): 0. Max coverage (-): 0

Region: chr25 29349183-29349195. Max. coverage (+): 0. Max coverage (-): 0

Region: chr25 29349196-29349209. Max. coverage (+): 0. Max coverage (-): 0

Region: chr25 29349210-29349222. Max. coverage (+): 1.18. Max coverage (-): 0

Region: chr25 29349223-29349236. Max. coverage (+): 0. Max coverage (-): 0

Region: chr25 29349237-29349249. Max. coverage (+): 0. Max coverage (-): 0

Region: chr25 29349250-29349263. Max. coverage (+): 4.79. Max coverage (-): 0

Region: chr25 29349264-29349276. Max. coverage (+): 0. Max coverage (-): 0

Region: chr25 29349277-29349290. Max. coverage (+): 5.19. Max coverage (-): 0

Region: chr25 29349291-29349304. Max. coverage (+): 5.19. Max coverage (-): 0

Region: chr25 29349305-29349317. Max. coverage (+): 0. Max coverage (-): 0

Region: chr25 29349318-29349331. Max. coverage (+): 0. Max coverage (-): 0

Region: chr25 29349332-29349344. Max. coverage (+): 0. Max coverage (-): 0

Region: chr25 29349345-29349358. Max. coverage (+): 0. Max coverage (-): 0

Region: chr25 29349359-29349371. Max. coverage (+): 0. Max coverage (-): 0

Region: chr25 29349372-29349385. Max. coverage (+): 0. Max coverage (-): 0

Region: chr25 29349386-29349398. Max. coverage (+): 0. Max coverage (-): 0

Region: chr25 29349399-29349412. Max. coverage (+): 0. Max coverage (-): 0

Region: chr25 29349413-29349425. Max. coverage (+): 0. Max coverage (-): 0

Region: chr25 29349426-29349439. Max. coverage (+): 0. Max coverage (-): 0

Region: chr25 29349440-29349452. Max. coverage (+): 0. Max coverage (-): 0

Region: chr25 29349453-29349466. Max. coverage (+): 0. Max coverage (-): 0

Region: chr25 29349467-29349479. Max. coverage (+): 0. Max coverage (-): 0

Region: chr25 29349480-29349493. Max. coverage (+): 4.35. Max coverage (-): 0

Region: chr25 29349494-29349506. Max. coverage (+): 2.23. Max coverage (-): 0

Region: chr25 29349507-29349520. Max. coverage (+): 0. Max coverage (-): 0

Region: chr25 29349521-29349533. Max. coverage (+): 0. Max coverage (-): 0

Region: chr25 29349534-29349547. Max. coverage (+): 0. Max coverage (-): 0

Region: chr25 29349548-29349561. Max. coverage (+): 0. Max coverage (-): 0

Region: chr25 29349562-29349574. Max. coverage (+): 0. Max coverage (-): 0

Region: chr25 29349575-29349588. Max. coverage (+): 0. Max coverage (-): 0

Region: chr25 29349589-29349601. Max. coverage (+): 0. Max coverage (-): 0

Region: chr25 29349602-29349615. Max. coverage (+): 0. Max coverage (-): 0

Region: chr25 29349616-29349628. Max. coverage (+): 0. Max coverage (-): 0

Region: chr25 29349629-29349642. Max. coverage (+): 0. Max coverage (-): 0

Region: chr25 29349643-29349655. Max. coverage (+): 0. Max coverage (-): 0

Region: chr25 29349656-29349669. Max. coverage (+): 0. Max coverage (-): 0

Region: chr25 29349670-29349682. Max. coverage (+): 0. Max coverage (-): 0

Region: chr25 29349683-29349696. Max. coverage (+): 1.8. Max coverage (-): 0

Region: chr25 29349697-29349709. Max. coverage (+): 4.04. Max coverage (-): 0

Region: chr25 29349710-29349723. Max. coverage (+): 2.96. Max coverage (-): 0

Region: chr25 29349724-29349736. Max. coverage (+): 0. Max coverage (-): 0

Region: chr25 29349737-29349750. Max. coverage (+): 0. Max coverage (-): 0

Region: chr25 29349751-29349763. Max. coverage (+): 0. Max coverage (-): 0

Region: chr25 29349764-29349777. Max. coverage (+): 0. Max coverage (-): 0

Region: chr25 29349778-29349791. Max. coverage (+): 0. Max coverage (-): 0

Region: chr25 29349792-29349804. Max. coverage (+): 2.87. Max coverage (-): 0

Region: chr25 29349805-29349818. Max. coverage (+): 11.86. Max coverage (-): 0

Region: chr25 29349819-29349831. Max. coverage (+): 11.86. Max coverage (-): 0

Region: chr25 29349832-29349845. Max. coverage (+): 3.13. Max coverage (-): 0

Region: chr25 29349846-29349858. Max. coverage (+): 0. Max coverage (-): 0

Region: chr25 29349859-29349872. Max. coverage (+): 0. Max coverage (-): 0

Region: chr25 29349873-29349885. Max. coverage (+): 2.95. Max coverage (-): 0

Region: chr25 29349886-29349899. Max. coverage (+): 2.95. Max coverage (-): 0

Region: chr25 29349900-29349912. Max. coverage (+): 10.38. Max coverage (-): 0

Region: chr25 29349913-29349926. Max. coverage (+): 0. Max coverage (-): 0

Region: chr25 29349927-29349939. Max. coverage (+): 0. Max coverage (-): 0

Region: chr25 29349940-29349953. Max. coverage (+): 0. Max coverage (-): 0

Region: chr25 29349954-29349966. Max. coverage (+): 0. Max coverage (-): 0

Region: chr25 29349967-29349980. Max. coverage (+): 0. Max coverage (-): 0

Region: chr25 29349981-29349993. Max. coverage (+): 0. Max coverage (-): 0

Region: chr25 29349994-29350007. Max. coverage (+): 0. Max coverage (-): 0

Region: chr25 29350008-29350021. Max. coverage (+): 0. Max coverage (-): 0

Region: chr25 29350022-29350034. Max. coverage (+): 0. Max coverage (-): 0

Region: chr25 29350035-29350048. Max. coverage (+): 0. Max coverage (-): 0

Region: chr25 29350049-29350061. Max. coverage (+): 0.71. Max coverage (-): 0

Region: chr25 29350062-29350075. Max. coverage (+): 0. Max coverage (-): 0

Region: chr25 29350076-29350088. Max. coverage (+): 0. Max coverage (-): 0

Region: chr25 29350089-29350102. Max. coverage (+): 0. Max coverage (-): 0

Region: chr25 29350103-29350115. Max. coverage (+): 3.82. Max coverage (-): 0

Region: chr25 29350116-29350129. Max. coverage (+): 0. Max coverage (-): 0

Region: chr25 29350130-29350142. Max. coverage (+): 0. Max coverage (-): 0

Region: chr25 29350143-29350156. Max. coverage (+): 0. Max coverage (-): 0

Region: chr25 29350157-29350169. Max. coverage (+): 0. Max coverage (-): 0

Region: chr25 29350170-29350183. Max. coverage (+): 1.54. Max coverage (-): 0

Region: chr25 29350184-29350196. Max. coverage (+): 6.74. Max coverage (-): 0

Region: chr25 29350197-29350210. Max. coverage (+): 12.22. Max coverage (-): 0

Region: chr25 29350211-29350223. Max. coverage (+): 5.23. Max coverage (-): 0

Region: chr25 29350224-29350237. Max. coverage (+): 5.23. Max coverage (-): 0

Region: chr25 29350238-29350250. Max. coverage (+): 1.04. Max coverage (-): 0

Region: chr25 29350251-29350264. Max. coverage (+): 3.72. Max coverage (-): 0

Region: chr25 29350265-29350278. Max. coverage (+): 0. Max coverage (-): 0

Region: chr25 29350279-29350291. Max. coverage (+): 0. Max coverage (-): 0

Region: chr25 29350292-29350305. Max. coverage (+): 0. Max coverage (-): 0

Region: chr25 29350306-29350318. Max. coverage (+): 0. Max coverage (-): 0

Region: chr25 29350319-29350332. Max. coverage (+): 1.88. Max coverage (-): 0

Region: chr25 29350333-29350345. Max. coverage (+): 6.1. Max coverage (-): 0

Region: chr25 29350346-29350359. Max. coverage (+): 2.88. Max coverage (-): 0

Region: chr25 29350360-29350372. Max. coverage (+): 11.52. Max coverage (-): 0

Region: chr25 29350373-29350386. Max. coverage (+): 11.52. Max coverage (-): 0

Region: chr25 29350387-29350399. Max. coverage (+): 8.68. Max coverage (-): 0

Region: chr25 29350400-29350413. Max. coverage (+): 6.52. Max coverage (-): 0

Region: chr25 29350414-29350426. Max. coverage (+): 4.96. Max coverage (-): 0

Region: chr25 29350427-29350440. Max. coverage (+): 4.96. Max coverage (-): 0

Region: chr25 29350441-29350453. Max. coverage (+): 0. Max coverage (-): 0

Region: chr25 29350454-29350467. Max. coverage (+): 0. Max coverage (-): 0

Region: chr25 29350468-29350480. Max. coverage (+): 0. Max coverage (-): 0

Region: chr25 29350481-29350494. Max. coverage (+): 0. Max coverage (-): 0

Region: chr25 29350495-29350508. Max. coverage (+): 3.6. Max coverage (-): 0

Region: chr25 29350509-29350521. Max. coverage (+): 0. Max coverage (-): 0

Region: chr25 29350522-29350535. Max. coverage (+): 0. Max coverage (-): 0

Region: chr25 29350536-29350548. Max. coverage (+): 4.46. Max coverage (-): 0

Region: chr25 29350549-29350562. Max. coverage (+): 4.46. Max coverage (-): 0

Region: chr25 29350563-29350575. Max. coverage (+): 0. Max coverage (-): 0

Region: chr25 29350576-29350589. Max. coverage (+): 0. Max coverage (-): 0

Region: chr25 29350590-29350602. Max. coverage (+): 0. Max coverage (-): 0

Region: chr25 29350603-29350616. Max. coverage (+): 0. Max coverage (-): 0

Region: chr25 29350617-29350629. Max. coverage (+): 3.52. Max coverage (-): 0

Region: chr25 29350630-29350643. Max. coverage (+): 0. Max coverage (-): 0

Region: chr25 29350644-29350656. Max. coverage (+): 0. Max coverage (-): 0

Region: chr25 29350657-29350670. Max. coverage (+): 0. Max coverage (-): 0

Region: chr25 29350671-29350683. Max. coverage (+): 0. Max coverage (-): 0

Region: chr25 29350684-29350697. Max. coverage (+): 0. Max coverage (-): 0

Region: chr25 29350698-29350710. Max. coverage (+): 0. Max coverage (-): 0

Region: chr25 29350711-29350724. Max. coverage (+): 0. Max coverage (-): 0

Region: chr25 29350725-29350737. Max. coverage (+): 3.63. Max coverage (-): 0

Region: chr25 29350738-29350751. Max. coverage (+): 0. Max coverage (-): 0

Region: chr25 29350752-29350765. Max. coverage (+): 0. Max coverage (-): 0

Region: chr25 29350766-29350778. Max. coverage (+): 0. Max coverage (-): 0

Region: chr25 29350779-29350792. Max. coverage (+): 3.53. Max coverage (-): 0

Region: chr25 29350793-29350805. Max. coverage (+): 10.01. Max coverage (-): 0

Region: chr25 29350806-29350819. Max. coverage (+): 10.01. Max coverage (-): 0

Region: chr25 29350820-29350832. Max. coverage (+): 0. Max coverage (-): 0

Region: chr25 29350833-29350846. Max. coverage (+): 0. Max coverage (-): 0

Region: chr25 29350847-29350859. Max. coverage (+): 0. Max coverage (-): 0

Region: chr25 29350860-29350873. Max. coverage (+): 0.55. Max coverage (-): 0

Region: chr25 29350874-29350886. Max. coverage (+): 0.55. Max coverage (-): 0

Region: chr25 29350887-29350900. Max. coverage (+): 0. Max coverage (-): 0

Region: chr25 29350901-29350913. Max. coverage (+): 0. Max coverage (-): 0

Region: chr25 29350914-29350927. Max. coverage (+): 0. Max coverage (-): 0

Region: chr25 29350928-29350940. Max. coverage (+): 0. Max coverage (-): 0

Region: chr25 29350941-29350954. Max. coverage (+): 0. Max coverage (-): 0

Region: chr25 29350955-29350967. Max. coverage (+): 0. Max coverage (-): 0

Region: chr25 29350968-29350981. Max. coverage (+): 0. Max coverage (-): 0

Region: chr25 29350982-29350995. Max. coverage (+): 0. Max coverage (-): 0

Region: chr25 29350996-29351008. Max. coverage (+): 0. Max coverage (-): 0

Region: chr25 29351009-29351022. Max. coverage (+): 11.56. Max coverage (-): 0

Region: chr25 29351023-29351035. Max. coverage (+): 13.19. Max coverage (-): 0

Region: chr25 29351036-29351049. Max. coverage (+): 0. Max coverage (-): 0

Region: chr25 29351050-29351062. Max. coverage (+): 1.15. Max coverage (-): 0

Region: chr25 29351063-29351076. Max. coverage (+): 0.37. Max coverage (-): 0

Region: chr25 29351077-29351089. Max. coverage (+): 0. Max coverage (-): 0

Region: chr25 29351090-29351103. Max. coverage (+): 0. Max coverage (-): 0

Region: chr25 29351104-29351116. Max. coverage (+): 0. Max coverage (-): 0

Region: chr25 29351117-29351130. Max. coverage (+): 1.74. Max coverage (-): 0

Region: chr25 29351131-29351143. Max. coverage (+): 1.74. Max coverage (-): 0

Region: chr25 29351144-29351157. Max. coverage (+): 6.97. Max coverage (-): 0

Region: chr25 29351158-29351170. Max. coverage (+): 0. Max coverage (-): 0

Region: chr25 29351171-29351184. Max. coverage (+): 0. Max coverage (-): 0

Region: chr25 29351185-29351197. Max. coverage (+): 0. Max coverage (-): 0

Region: chr25 29351198-29351211. Max. coverage (+): 0. Max coverage (-): 0

Region: chr25 29351212-29351224. Max. coverage (+): 5.85. Max coverage (-): 0

Region: chr25 29351225-29351238. Max. coverage (+): 0. Max coverage (-): 0

Region: chr25 29351239-29351252. Max. coverage (+): 0.79. Max coverage (-): 0

Region: chr25 29351253-29351265. Max. coverage (+): 0. Max coverage (-): 0

Region: chr25 29351266-29351279. Max. coverage (+): 0. Max coverage (-): 0

Region: chr25 29351280-29351292. Max. coverage (+): 0. Max coverage (-): 0

Region: chr25 29351293-29351306. Max. coverage (+): 0. Max coverage (-): 0

Region: chr25 29351307-29351319. Max. coverage (+): 1.23. Max coverage (-): 0

Region: chr25 29351320-29351333. Max. coverage (+): 4.25. Max coverage (-): 0

Region: chr25 29351334-29351346. Max. coverage (+): 3.06. Max coverage (-): 0

Region: chr25 29351347-29351360. Max. coverage (+): 0. Max coverage (-): 0

Region: chr25 29351361-29351373. Max. coverage (+): 2.3. Max coverage (-): 0

Region: chr25 29351374-29351387. Max. coverage (+): 0. Max coverage (-): 0

Region: chr25 29351388-29351400. Max. coverage (+): 0.6. Max coverage (-): 0

Region: chr25 29351401-29351414. Max. coverage (+): 3.92. Max coverage (-): 0

Region: chr25 29351415-29351427. Max. coverage (+): 4.02. Max coverage (-): 0

Region: chr25 29351428-29351441. Max. coverage (+): 4.02. Max coverage (-): 0

Region: chr25 29351442-29351454. Max. coverage (+): 0. Max coverage (-): 0

Region: chr25 29351455-29351468. Max. coverage (+): 0. Max coverage (-): 0

Region: chr25 29351469-29351482. Max. coverage (+): 0. Max coverage (-): 0

Region: chr25 29351483-29351495. Max. coverage (+): 6.01. Max coverage (-): 0

Region: chr25 29351496-29351509. Max. coverage (+): 2.96. Max coverage (-): 0

Region: chr25 29351510-29351522. Max. coverage (+): 0. Max coverage (-): 0

Region: chr25 29351523-29351536. Max. coverage (+): 0. Max coverage (-): 0

Region: chr25 29351537-29351549. Max. coverage (+): 4.1. Max coverage (-): 0

Region: chr25 29351550-29351563. Max. coverage (+): 5.52. Max coverage (-): 0

Region: chr25 29351564-29351576. Max. coverage (+): 1.4. Max coverage (-): 0

Region: chr25 29351577-29351590. Max. coverage (+): 0. Max coverage (-): 0

Region: chr25 29351591-29351603. Max. coverage (+): 0. Max coverage (-): 0

Region: chr25 29351604-29351617. Max. coverage (+): 2.2. Max coverage (-): 0

Region: chr25 29351618-29351630. Max. coverage (+): 0.5. Max coverage (-): 0

Region: chr25 29351631-29351644. Max. coverage (+): 0. Max coverage (-): 0

Region: chr25 29351645-29351657. Max. coverage (+): 0. Max coverage (-): 0

Region: chr25 29351658-29351671. Max. coverage (+): 0. Max coverage (-): 0

Region: chr25 29351672-29351684. Max. coverage (+): 0. Max coverage (-): 0

Region: chr25 29351685-29351698. Max. coverage (+): 0. Max coverage (-): 0

Region: chr25 29351699-29351712. Max. coverage (+): 0. Max coverage (-): 0

Region: chr25 29351713-29351725. Max. coverage (+): 0. Max coverage (-): 0

Region: chr25 29351726-29351739. Max. coverage (+): 4.02. Max coverage (-): 0

Region: chr25 29351740-29351752. Max. coverage (+): 4.02. Max coverage (-): 0

Region: chr25 29351753-29351766. Max. coverage (+): 1.13. Max coverage (-): 0

Region: chr25 29351767-29351779. Max. coverage (+): 1.13. Max coverage (-): 0

Region: chr25 29351780-29351793. Max. coverage (+): 0. Max coverage (-): 0

Region: chr25 29351794-29351806. Max. coverage (+): 0. Max coverage (-): 0

Region: chr25 29351807-29351820. Max. coverage (+): 0. Max coverage (-): 0

Region: chr25 29351821-29351833. Max. coverage (+): 0. Max coverage (-): 0

Region: chr25 29351834-29351847. Max. coverage (+): 1.15. Max coverage (-): 0

Region: chr25 29351848-29351860. Max. coverage (+): 3.57. Max coverage (-): 0

Region: chr25 29351861-29351874. Max. coverage (+): 2.24. Max coverage (-): 0

Region: chr25 29351875-29351887. Max. coverage (+): 2.24. Max coverage (-): 0

Region: chr25 29351888-29351901. Max. coverage (+): 0. Max coverage (-): 0

Region: chr25 29351902-29351914. Max. coverage (+): 0. Max coverage (-): 0

Region: chr25 29351915-29351928. Max. coverage (+): 0. Max coverage (-): 0

Region: chr25 29351929-29351941. Max. coverage (+): 0. Max coverage (-): 0

Region: chr25 29351942-29351955. Max. coverage (+): 2.67. Max coverage (-): 0

Region: chr25 29351956-29351969. Max. coverage (+): 7.96. Max coverage (-): 0

Region: chr25 29351970-29351982. Max. coverage (+): 7.96. Max coverage (-): 0

Region: chr25 29351983-29351996. Max. coverage (+): 6.1. Max coverage (-): 0

Region: chr25 29351997-29352009. Max. coverage (+): 0. Max coverage (-): 0

Region: chr25 29352010-29352023. Max. coverage (+): 0. Max coverage (-): 0

Region: chr25 29352024-29352036. Max. coverage (+): 6.11. Max coverage (-): 0

Region: chr25 29352037-29352050. Max. coverage (+): 6.11. Max coverage (-): 0

Region: chr25 29352051-29352063. Max. coverage (+): 8.68. Max coverage (-): 0

Region: chr25 29352064-29352077. Max. coverage (+): 0. Max coverage (-): 0

Region: chr25 29352078-29352090. Max. coverage (+): 0. Max coverage (-): 0

Region: chr25 29352091-29352104. Max. coverage (+): 2.26. Max coverage (-): 0

Region: chr25 29352105-29352117. Max. coverage (+): 0. Max coverage (-): 0

Region: chr25 29352118-29352131. Max. coverage (+): 0. Max coverage (-): 0

Region: chr25 29352132-29352144. Max. coverage (+): 0. Max coverage (-): 0

Region: chr25 29352145-29352158. Max. coverage (+): 0. Max coverage (-): 0

Region: chr25 29352159-29352171. Max. coverage (+): 0. Max coverage (-): 0

Region: chr25 29352172-29352185. Max. coverage (+): 0. Max coverage (-): 0

Region: chr25 29352186-29352199. Max. coverage (+): 0. Max coverage (-): 0

Region: chr25 29352200-29352212. Max. coverage (+): 2.27. Max coverage (-): 0

Region: chr25 29352213-29352226. Max. coverage (+): 0. Max coverage (-): 0

Region: chr25 29352227-29352239. Max. coverage (+): 0. Max coverage (-): 0

Region: chr25 29352240-29352253. Max. coverage (+): 0. Max coverage (-): 0

Region: chr25 29352254-29352266. Max. coverage (+): 0. Max coverage (-): 0

Region: chr25 29352267-29352280. Max. coverage (+): 0. Max coverage (-): 0

Region: chr25 29352281-29352293. Max. coverage (+): 0. Max coverage (-): 0

Region: chr25 29352294-29352307. Max. coverage (+): 0. Max coverage (-): 0

Region: chr25 29352308-29352320. Max. coverage (+): 0. Max coverage (-): 0

Region: chr25 29352321-29352334. Max. coverage (+): 0. Max coverage (-): 0

Region: chr25 29352335-29352347. Max. coverage (+): 5.89. Max coverage (-): 0

Region: chr25 29352348-29352361. Max. coverage (+): 5.89. Max coverage (-): 0

Region: chr25 29352362-29352374. Max. coverage (+): 7.35. Max coverage (-): 0

Region: chr25 29352375-29352388. Max. coverage (+): 9.48. Max coverage (-): 0

Region: chr25 29352389-29352401. Max. coverage (+): 5.57. Max coverage (-): 0

Region: chr25 29352402-29352415. Max. coverage (+): 0. Max coverage (-): 0

Region: chr25 29352416-29352428. Max. coverage (+): 0.6. Max coverage (-): 0

Region: chr25 29352429-29352442. Max. coverage (+): 6.04. Max coverage (-): 0

Region: chr25 29352443-29352456. Max. coverage (+): 6.04. Max coverage (-): 0

Region: chr25 29352457-29352469. Max. coverage (+): 0. Max coverage (-): 0

Region: chr25 29352470-29352483. Max. coverage (+): 0. Max coverage (-): 0

Region: chr25 29352484-29352496. Max. coverage (+): 0. Max coverage (-): 0

Region: chr25 29352497-29352510. Max. coverage (+): 0. Max coverage (-): 0

Region: chr25 29352511-29352523. Max. coverage (+): 0. Max coverage (-): 0

Region: chr25 29352524-29352537. Max. coverage (+): 0. Max coverage (-): 0

Region: chr25 29352538-29352550. Max. coverage (+): 5.65. Max coverage (-): 0

Region: chr25 29352551-29352564. Max. coverage (+): 2.35. Max coverage (-): 0

Region: chr25 29352565-29352577. Max. coverage (+): 2.35. Max coverage (-): 0

Region: chr25 29352578-29352591. Max. coverage (+): 1.58. Max coverage (-): 0

Region: chr25 29352592-29352604. Max. coverage (+): 7.42. Max coverage (-): 0

Region: chr25 29352605-29352618. Max. coverage (+): 7.42. Max coverage (-): 0

Region: chr25 29352619-29352631. Max. coverage (+): 4.94. Max coverage (-): 0

Region: chr25 29352632-29352645. Max. coverage (+): 0.95. Max coverage (-): 0

Region: chr25 29352646-29352658. Max. coverage (+): 0.95. Max coverage (-): 0

Region: chr25 29352659-29352672. Max. coverage (+): 0. Max coverage (-): 0

Region: chr25 29352673-29352686. Max. coverage (+): 0. Max coverage (-): 0

Region: chr25 29352687-29352699. Max. coverage (+): 0. Max coverage (-): 0

Region: chr25 29352700-29352713. Max. coverage (+): 0. Max coverage (-): 0

Region: chr25 29352714-29352726. Max. coverage (+): 0. Max coverage (-): 0

Region: chr25 29352727-29352740. Max. coverage (+): 0. Max coverage (-): 0

Region: chr25 29352741-29352753. Max. coverage (+): 6.84. Max coverage (-): 0

Region: chr25 29352754-29352767. Max. coverage (+): 5.01. Max coverage (-): 0

Region: chr25 29352768-29352780. Max. coverage (+): 0. Max coverage (-): 0

Region: chr25 29352781-29352794. Max. coverage (+): 0. Max coverage (-): 0

Region: chr25 29352795-29352807. Max. coverage (+): 2.29. Max coverage (-): 0

Region: chr25 29352808-29352821. Max. coverage (+): 8.8. Max coverage (-): 0

Region: chr25 29352822-29352834. Max. coverage (+): 8.8. Max coverage (-): 0

Region: chr25 29352835-29352848. Max. coverage (+): 1.55. Max coverage (-): 0

Region: chr25 29352849-29352861. Max. coverage (+): 2.22. Max coverage (-): 0

Region: chr25 29352862-29352875. Max. coverage (+): 2.22. Max coverage (-): 0

Region: chr25 29352876-29352888. Max. coverage (+): 0. Max coverage (-): 0

Region: chr25 29352889-29352902. Max. coverage (+): 2.11. Max coverage (-): 0

Region: chr25 29352903-29352915. Max. coverage (+): 2.11. Max coverage (-): 0

Region: chr25 29352916-29352929. Max. coverage (+): 0. Max coverage (-): 0

Region: chr25 29352930-29352943. Max. coverage (+): 12.72. Max coverage (-): 0

Region: chr25 29352944-29352956. Max. coverage (+): 5.86. Max coverage (-): 0

Region: chr25 29352957-29352970. Max. coverage (+): 0. Max coverage (-): 0

Region: chr25 29352971-29352983. Max. coverage (+): 0. Max coverage (-): 0

Region: chr25 29352984-29352997. Max. coverage (+): 0. Max coverage (-): 0

Region: chr25 29352998-29353010. Max. coverage (+): 0. Max coverage (-): 0

Region: chr25 29353011-29353024. Max. coverage (+): 0. Max coverage (-): 0

Region: chr25 29353025-29353037. Max. coverage (+): 10.61. Max coverage (-): 0

Region: chr25 29353038-29353051. Max. coverage (+): 16.16. Max coverage (-): 0

Region: chr25 29353052-29353064. Max. coverage (+): 0. Max coverage (-): 0

Region: chr25 29353065-29353078. Max. coverage (+): 0. Max coverage (-): 0

Region: chr25 29353079-29353091. Max. coverage (+): 0. Max coverage (-): 0

Region: chr25 29353092-29353105. Max. coverage (+): 0. Max coverage (-): 0

Region: chr25 29353106-29353118. Max. coverage (+): 0. Max coverage (-): 0

Region: chr25 29353119-29353132. Max. coverage (+): 0. Max coverage (-): 0

Region: chr25 29353133-29353145. Max. coverage (+): 0. Max coverage (-): 0

Region: chr25 29353146-29353159. Max. coverage (+): 0. Max coverage (-): 0

Region: chr25 29353160-29353173. Max. coverage (+): 0. Max coverage (-): 0

Region: chr25 29353174-29353186. Max. coverage (+): 5.13. Max coverage (-): 0

Region: chr25 29353187-29353200. Max. coverage (+): 0.73. Max coverage (-): 0

Region: chr25 29353201-29353213. Max. coverage (+): 3.97. Max coverage (-): 0

Region: chr25 29353214-29353227. Max. coverage (+): 6.65. Max coverage (-): 0

Region: chr25 29353228-29353240. Max. coverage (+): 0.35. Max coverage (-): 0

Region: chr25 29353241-29353254. Max. coverage (+): 11.9. Max coverage (-): 0

Region: chr25 29353255-29353267. Max. coverage (+): 30.47. Max coverage (-): 0

Region: chr25 29353268-29353281. Max. coverage (+): 14.37. Max coverage (-): 0

Region: chr25 29353282-29353294. Max. coverage (+): 0. Max coverage (-): 0

Region: chr25 29353295-29353308. Max. coverage (+): 0. Max coverage (-): 0

Region: chr25 29353309-29353321. Max. coverage (+): 4.34. Max coverage (-): 0

Region: chr25 29353322-29353335. Max. coverage (+): 5.31. Max coverage (-): 0

Region: chr25 29353336-29353348. Max. coverage (+): 2.51. Max coverage (-): 0

Region: chr25 29353349-29353362. Max. coverage (+): 0. Max coverage (-): 0

Region: chr25 29353363-29353375. Max. coverage (+): 0. Max coverage (-): 0

Region: chr25 29353376-29353389. Max. coverage (+): 0. Max coverage (-): 0

Region: chr25 29353390-29353403. Max. coverage (+): 0. Max coverage (-): 0

Region: chr25 29353404-29353416. Max. coverage (+): 0. Max coverage (-): 0

Region: chr25 29353417-29353430. Max. coverage (+): 0. Max coverage (-): 0

Region: chr25 29353431-29353443. Max. coverage (+): 0. Max coverage (-): 0

Region: chr25 29353444-29353457. Max. coverage (+): 0. Max coverage (-): 0

Region: chr25 29353458-29353470. Max. coverage (+): 0. Max coverage (-): 0

Region: chr25 29353471-29353484. Max. coverage (+): 8.55. Max coverage (-): 0

Region: chr25 29353485-29353497. Max. coverage (+): 8.55. Max coverage (-): 0

Region: chr25 29353498-29353511. Max. coverage (+): 0. Max coverage (-): 0

Region: chr25 29353512-29353524. Max. coverage (+): 0. Max coverage (-): 0

Region: chr25 29353525-29353538. Max. coverage (+): 0. Max coverage (-): 0

Region: chr25 29353539-29353551. Max. coverage (+): 0. Max coverage (-): 0

Region: chr25 29353552-29353565. Max. coverage (+): 0. Max coverage (-): 0

Region: chr25 29353566-29353578. Max. coverage (+): 0. Max coverage (-): 0

Region: chr25 29353579-29353592. Max. coverage (+): 0. Max coverage (-): 0

Region: chr25 29353593-29353605. Max. coverage (+): 1.2. Max coverage (-): 0

Region: chr25 29353606-29353619. Max. coverage (+): 2.64. Max coverage (-): 0

Region: chr25 29353620-29353632. Max. coverage (+): 0. Max coverage (-): 0

Region: chr25 29353633-29353646. Max. coverage (+): 2.16. Max coverage (-): 0

Region: chr25 29353647-29353660. Max. coverage (+): 2.98. Max coverage (-): 0

Region: chr25 29353661-29353673. Max. coverage (+): 5.6. Max coverage (-): 0

Region: chr25 29353674-29353687. Max. coverage (+): 4.13. Max coverage (-): 0

Region: chr25 29353688-29353700. Max. coverage (+): 4.37. Max coverage (-): 0

Region: chr25 29353701-29353714. Max. coverage (+): 6.52. Max coverage (-): 0

Region: chr25 29353715-29353727. Max. coverage (+): 2.26. Max coverage (-): 0

Region: chr25 29353728-29353741. Max. coverage (+): 0. Max coverage (-): 0

Region: chr25 29353742-29353754. Max. coverage (+): 0. Max coverage (-): 0

Region: chr25 29353755-29353768. Max. coverage (+): 0.69. Max coverage (-): 0

Region: chr25 29353769-29353781. Max. coverage (+): 0. Max coverage (-): 0

Region: chr25 29353782-29353795. Max. coverage (+): 0. Max coverage (-): 0

Region: chr25 29353796-29353808. Max. coverage (+): 5.23. Max coverage (-): 0

Region: chr25 29353809-29353822. Max. coverage (+): 6.04. Max coverage (-): 0

Region: chr25 29353823-29353835. Max. coverage (+): 0. Max coverage (-): 0

Region: chr25 29353836-29353849. Max. coverage (+): 0. Max coverage (-): 0

Region: chr25 29353850-29353862. Max. coverage (+): 0. Max coverage (-): 0

Region: chr25 29353863-29353876. Max. coverage (+): 3.87. Max coverage (-): 0

Region: chr25 29353877-29353890. Max. coverage (+): 3.87. Max coverage (-): 0

Region: chr25 29353891-29353903. Max. coverage (+): 0. Max coverage (-): 0

Region: chr25 29353904-29353917. Max. coverage (+): 1.19. Max coverage (-): 0

Region: chr25 29353918-29353930. Max. coverage (+): 0. Max coverage (-): 0

Region: chr25 29353931-29353944. Max. coverage (+): 0. Max coverage (-): 0

Region: chr25 29353945-29353957. Max. coverage (+): 0.48. Max coverage (-): 0

Region: chr25 29353958-29353971. Max. coverage (+): 7.53. Max coverage (-): 0

Region: chr25 29353972-29353984. Max. coverage (+): 7.53. Max coverage (-): 0

Region: chr25 29353985-29353998. Max. coverage (+): 0. Max coverage (-): 0

Region: chr25 29353999-29354011. Max. coverage (+): 0. Max coverage (-): 0

Region: chr25 29354012-29354025. Max. coverage (+): 1.24. Max coverage (-): 0

Region: chr25 29354026-29354038. Max. coverage (+): 0. Max coverage (-): 0

Region: chr25 29354039-29354052. Max. coverage (+): 0. Max coverage (-): 0

Region: chr25 29354053-29354065. Max. coverage (+): 0. Max coverage (-): 0

Region: chr25 29354066-29354079. Max. coverage (+): 0. Max coverage (-): 0

Region: chr25 29354080-29354092. Max. coverage (+): 0. Max coverage (-): 0

Region: chr25 29354093-29354106. Max. coverage (+): 0. Max coverage (-): 0

Region: chr25 29354107-29354119. Max. coverage (+): 0. Max coverage (-): 0

Region: chr25 29354120-29354133. Max. coverage (+): 0. Max coverage (-): 0

Region: chr25 29354134-29354147. Max. coverage (+): 0. Max coverage (-): 0

Region: chr25 29354148-29354160. Max. coverage (+): 0. Max coverage (-): 0

Region: chr25 29354161-29354174. Max. coverage (+): 0. Max coverage (-): 0

Region: chr25 29354175-29354187. Max. coverage (+): 0. Max coverage (-): 0

Region: chr25 29354188-29354201. Max. coverage (+): 0. Max coverage (-): 0

Region: chr25 29354202-29354214. Max. coverage (+): 0. Max coverage (-): 0

Region: chr25 29354215-29354228. Max. coverage (+): 0. Max coverage (-): 0

Region: chr25 29354229-29354241. Max. coverage (+): 0. Max coverage (-): 0

Region: chr25 29354242-29354255. Max. coverage (+): 0. Max coverage (-): 0

Region: chr25 29354256-29354268. Max. coverage (+): 0. Max coverage (-): 0

Region: chr25 29354269-29354282. Max. coverage (+): 0. Max coverage (-): 0

Region: chr25 29354283-29354295. Max. coverage (+): 0. Max coverage (-): 0

Region: chr25 29354296-29354309. Max. coverage (+): 0. Max coverage (-): 0

Region: chr25 29354310-29354322. Max. coverage (+): 0. Max coverage (-): 0

Region: chr25 29354323-29354336. Max. coverage (+): 0. Max coverage (-): 0

Region: chr25 29354337-29354349. Max. coverage (+): 0. Max coverage (-): 0

Region: chr25 29354350-29354363. Max. coverage (+): 0. Max coverage (-): 0

Region: chr25 29354364-29354377. Max. coverage (+): 0. Max coverage (-): 0

Region: chr25 29354378-29354390. Max. coverage (+): 0. Max coverage (-): 0

Region: chr25 29354391-29354404. Max. coverage (+): 0. Max coverage (-): 0

Region: chr25 29354405-29354417. Max. coverage (+): 0. Max coverage (-): 0

Region: chr25 29354418-29354431. Max. coverage (+): 0. Max coverage (-): 0

Region: chr25 29354432-29354444. Max. coverage (+): 11.98. Max coverage (-): 0

Region: chr25 29354445-29354458. Max. coverage (+): 2.54. Max coverage (-): 0

Region: chr25 29354459-29354471. Max. coverage (+): 0. Max coverage (-): 0

Region: chr25 29354472-29354485. Max. coverage (+): 0. Max coverage (-): 0

Region: chr25 29354486-29354498. Max. coverage (+): 0. Max coverage (-): 0

Region: chr25 29354499-29354512. Max. coverage (+): 0. Max coverage (-): 0

Region: chr25 29354513-29354525. Max. coverage (+): 0. Max coverage (-): 0

Region: chr25 29354526-29354539. Max. coverage (+): 0. Max coverage (-): 0

Region: chr25 29354540-29354552. Max. coverage (+): 0. Max coverage (-): 0

Region: chr25 29354553-29354566. Max. coverage (+): 0. Max coverage (-): 0

Region: chr25 29354567-29354579. Max. coverage (+): 0.1. Max coverage (-): 0

Region: chr25 29354580-29354593. Max. coverage (+): 0.1. Max coverage (-): 0

Region: chr25 29354594-29354606. Max. coverage (+): 3.22. Max coverage (-): 0

Region: chr25 29354607-29354620. Max. coverage (+): 0. Max coverage (-): 0

Region: chr25 29354621-29354634. Max. coverage (+): 0. Max coverage (-): 0

Region: chr25 29354635-29354647. Max. coverage (+): 0. Max coverage (-): 0

Region: chr25 29354648-29354661. Max. coverage (+): 0. Max coverage (-): 0

Region: chr25 29354662-29354674. Max. coverage (+): 0. Max coverage (-): 0

Region: chr25 29354675-29354688. Max. coverage (+): 0. Max coverage (-): 0

Region: chr25 29354689-29354701. Max. coverage (+): 0. Max coverage (-): 0

Region: chr25 29354702-29354715. Max. coverage (+): 0. Max coverage (-): 0

Region: chr25 29354716-29354728. Max. coverage (+): 0. Max coverage (-): 0

Region: chr25 29354729-29354742. Max. coverage (+): 0. Max coverage (-): 0

Region: chr25 29354743-29354755. Max. coverage (+): 0. Max coverage (-): 0

Region: chr25 29354756-29354769. Max. coverage (+): 0. Max coverage (-): 0

Region: chr25 29354770-29354782. Max. coverage (+): 0. Max coverage (-): 0

Region: chr25 29354783-29354796. Max. coverage (+): 0. Max coverage (-): 0

Region: chr25 29354797-29354809. Max. coverage (+): 0. Max coverage (-): 0

Region: chr25 29354810-29354823. Max. coverage (+): 4.91. Max coverage (-): 0

Region: chr25 29354824-29354836. Max. coverage (+): 4.91. Max coverage (-): 0

Region: chr25 29354837-29354850. Max. coverage (+): 0. Max coverage (-): 0

Region: chr25 29354851-29354864. Max. coverage (+): 0. Max coverage (-): 0

Region: chr25 29354865-29354877. Max. coverage (+): 0. Max coverage (-): 0

Region: chr25 29354878-29354891. Max. coverage (+): 0. Max coverage (-): 0

Region: chr25 29354892-29354904. Max. coverage (+): 3.93. Max coverage (-): 0

Region: chr25 29354905-29354918. Max. coverage (+): 1.97. Max coverage (-): 0

Region: chr25 29354919-29354931. Max. coverage (+): 0. Max coverage (-): 0

Region: chr25 29354932-29354945. Max. coverage (+): 0. Max coverage (-): 0

Region: chr25 29354946-29354958. Max. coverage (+): 0. Max coverage (-): 0

Region: chr25 29354959-29354972. Max. coverage (+): 0. Max coverage (-): 0

Region: chr25 29354973-29354985. Max. coverage (+): 0. Max coverage (-): 0

Region: chr25 29354986-29354999. Max. coverage (+): 0. Max coverage (-): 0

Region: chr25 29355000-29355012. Max. coverage (+): 0. Max coverage (-): 0

Region: chr25 29355013-29355026. Max. coverage (+): 0. Max coverage (-): 0

Region: chr25 29355027-29355039. Max. coverage (+): 0. Max coverage (-): 0

Region: chr25 29355040-29355053. Max. coverage (+): 0. Max coverage (-): 0

Region: chr25 29355054-29355066. Max. coverage (+): 0. Max coverage (-): 0

Region: chr25 29355067-29355080. Max. coverage (+): 0. Max coverage (-): 0

Region: chr25 29355081-29355094. Max. coverage (+): 0. Max coverage (-): 0

Region: chr25 29355095-29355107. Max. coverage (+): 0. Max coverage (-): 0

Region: chr25 29355108-29355121. Max. coverage (+): 0. Max coverage (-): 0

Region: chr25 29355122-29355134. Max. coverage (+): 0. Max coverage (-): 0

Region: chr25 29355135-29355148. Max. coverage (+): 0. Max coverage (-): 0

Region: chr25 29355149-29355161. Max. coverage (+): 0. Max coverage (-): 0

Region: chr25 29355162-29355175. Max. coverage (+): 0. Max coverage (-): 0

Region: chr25 29355176-29355188. Max. coverage (+): 0. Max coverage (-): 0

Region: chr25 29355189-29355202. Max. coverage (+): 12.63. Max coverage (-): 0

Region: chr25 29355203-29355215. Max. coverage (+): 12.63. Max coverage (-): 0

Region: chr25 29355216-29355229. Max. coverage (+): 0. Max coverage (-): 0

Region: chr25 29355230-29355242. Max. coverage (+): 0. Max coverage (-): 0

Region: chr25 29355243-29355256. Max. coverage (+): 0. Max coverage (-): 0

Region: chr25 29355257-29355269. Max. coverage (+): 1.18. Max coverage (-): 0

Region: chr25 29355270-29355283. Max. coverage (+): 0. Max coverage (-): 0

Region: chr25 29355284-29355296. Max. coverage (+): 0. Max coverage (-): 0

Region: chr25 29355297-29355310. Max. coverage (+): 0. Max coverage (-): 0

Region: chr25 29355311-29355323. Max. coverage (+): 0. Max coverage (-): 0

Region: chr25 29355324-29355337. Max. coverage (+): 0. Max coverage (-): 0

Region: chr25 29355338-29355351. Max. coverage (+): 0. Max coverage (-): 0

Region: chr25 29355352-29355364. Max. coverage (+): 0. Max coverage (-): 0

Region: chr25 29355365-29355378. Max. coverage (+): 0. Max coverage (-): 0

Region: chr25 29355379-29355391. Max. coverage (+): 0. Max coverage (-): 0

Region: chr25 29355392-29355405. Max. coverage (+): 0. Max coverage (-): 0

Region: chr25 29355406-29355418. Max. coverage (+): 0. Max coverage (-): 0

Region: chr25 29355419-29355432. Max. coverage (+): 0. Max coverage (-): 0

Region: chr25 29355433-29355445. Max. coverage (+): 0. Max coverage (-): 0

Region: chr25 29355446-29355459. Max. coverage (+): 0. Max coverage (-): 0

Region: chr25 29355460-29355472. Max. coverage (+): 0. Max coverage (-): 0

Region: chr25 29355473-29355486. Max. coverage (+): 0. Max coverage (-): 0

Region: chr25 29355487-29355499. Max. coverage (+): 0. Max coverage (-): 0

Region: chr25 29355500-29355513. Max. coverage (+): 0. Max coverage (-): 0

Region: chr25 29355514-29355526. Max. coverage (+): 0. Max coverage (-): 0

Region: chr25 29355527-29355540. Max. coverage (+): 0. Max coverage (-): 0

Region: chr25 29355541-29355553. Max. coverage (+): 0. Max coverage (-): 0

Region: chr25 29355554-29355567. Max. coverage (+): 0. Max coverage (-): 0

Region: chr25 29355568-29355581. Max. coverage (+): 0. Max coverage (-): 0

Region: chr25 29355582-29355594. Max. coverage (+): 0. Max coverage (-): 0

Region: chr25 29355595-29355608. Max. coverage (+): 0. Max coverage (-): 0

Region: chr25 29355609-29355621. Max. coverage (+): 0. Max coverage (-): 0

Region: chr25 29355622-29355635. Max. coverage (+): 0. Max coverage (-): 0

Region: chr25 29355636-29355648. Max. coverage (+): 1.3. Max coverage (-): 0

Region: chr25 29355649-29355662. Max. coverage (+): 1.3. Max coverage (-): 0

Region: chr25 29355663-29355675. Max. coverage (+): 0.89. Max coverage (-): 0

Region: chr25 29355676-29355689. Max. coverage (+): 0. Max coverage (-): 0

Region: chr25 29355690-. Max. coverage (+): 0. Max coverage (-): 0

RepeatMasker Color Code

**+**

100-98% Identity

<98-95% Identity

<95-90% Identity

<90-85% Identity

<85-80% Identity

<80-75% Identity

<75-70% Identity

<70% Identity

**-**

Gene Set Color Code

**+**

Gene

Pseudogene

**-**

Topology/Coverage Color Code

Coverage Plus Strand

Coverage Minus Strand

Mainstrand: Plus

Mainstrand: Minus

Complementary Strand

Flanking Region  
(if option -flank >0)

Gene Set Annotation  

**1. CALN1 (protein coding, ENSBTAG00000043969) Tr:00000061255 Ex:6**: 29348906-29349030 (+)

  
RepeatMasker Annotation  

**1. (CCCCCA)n**: 29349432-29349459 (+), Divergence to consensus: 7.1%  
**2. AT\_rich**: 29352254-29352274 (+), Divergence to consensus: 28.6%  
**3. L3b**: 29353365-29353450 (+), Divergence to consensus: 38%  
**4. MIR3**: 29354644-29354764 (+), Divergence to consensus: 45.9%

  
Transcription Factor Binding Sites  

**RFX4\_1** (Sequence: GTTGCTAAG (-): 29351578)  
**RFX4\_1** (Sequence: GTTGCCATG (-): 29352279)  
**Gata4** (Sequence: AGATAAC (-): 29353913)  
**SOX9** (Sequence: TTATTGTT (+): 29354199)  
**SOX9** (Sequence: TCATTGTT (+): 29355558)  
**SPZ1** (Sequence: AGGGTTTGAG (+): 29350808)  
**SPZ1** (Sequence: GGGGTATGAG (+): 29351195)
