# Supplementary material for: Small RNA sequencing of cryopreserved semen from single bull revealed altered miRNAs and piRNAs expression between High- and Low-motile sperm populations
Source: BMC Genomics. 2017 Jan 4;18:14. doi: 10.1186/s12864-016-3394-7 (PMC5209821; doi:10.1186/s12864-016-3394-7)
Supplement: Additional file 3: — Details for each piRNA clusters found in High Motile (HM) sperm fraction. Genes, repeats, transposable elements and transcription factors binding sites falling within the cluster regions were reported. (ZIP 1896 kb) [file 12864_2016_3394_MOESM3_ESM.zip › 61.html]

piRNA cluster 61


Predicted piRNA cluster no. 61     previous   next
  

Show proTRAC run info
Hide proTRAC run info

================================= proTRAC ====================================  
VERSION: 2.1                                    LAST MODIFIED: 06. October 2015  
  
Please cite:  
Rosenkranz D, Zischler H. proTRAC - a software for probabilistic piRNA cluster  
detection, visualization and analysis. 2012. BMC Bioinformatics 13:5.  
  
and (for proTRAC 2.0 and later):  
Rosenkranz D, Rudloff S, Bastuck K, Ketting RF, Zischler H. Tupaia small RNAs  
provide insights into function and evolution of RNAi-based transposon defense  
in mammals. 2015. RNA 21(5):911-922.  
  
Contact:  
David Rosenkranz  
Institute of Anthropology, small RNA group  
Johannes Gutenberg University Mainz  
email: rosenkranz@uni-mainz.de  
  
You can find the latest proTRAC version at:  
http://sourceforge.net/projects/protrac/files  
http://www.smallRNAgroup-mainz.de/software  
==============================================================================  
  
PARAMETERS:  
Map file: .............../storage/core/barbara/genhome/smallRNA/fertility/Sample\_motile/pirna/Sample\_motile\_26-33\_collapsed.fa.no-dust.map.weighted-10000-1000-b-0  
Genome file: ............/storage/core/barbara/genhome/smallRNA/fertility/Sample\_all/pirna/bt\_311\_chrY.fa  
RepeatMasker annotation: /storage/genomes/bt\_umd31/GCF\_000003055.6\_Bos\_taurus\_UMD\_3.1.1\_repeatMasker\_chr.out  
GeneSet:................./storage/core/barbara/genhome/smallRNA/fertility/Sample\_all/pirna/full.gtf  
  
Significant (p<=0.01) hit density will be calculated based  
on observed hit distribution.  
  
Sliding window size: ........................................ 5000 bp  
Sliding window increament: .................................. 1000 bp  
Normalize each hit by number of genomic hits: ............... 1 [0=no/1=yes]  
Normalize each hit by number of sequence reads: ............. 1 [0=no/1=yes]  
Normalize values (-> per million mapped reads): ............. 1 [0=no/1=yes]  
Min. fraction of hits with 1T(U) or 10A: .................... 0.75  
Alternatively: Min. fraction of hits with 1T(U) and 10A: .... 0.5  
Min. fraction of hits with typical piRNA length: ............ 0.75  
Typical piRNA length: ....................................... 26-33 nt  
Min. size of a piRNA cluster: ............................... 5000 bp.  
Min. number of hits (absolute): ............................. 0  
Min. number of hits (normalized): ........................... 0  
Min. fraction of hits on the mainstrand: .................... 0.75  
Top fraction of mapped sequences (in terms of read counts): . 1%  
Top fraction accounts for max. n% of sequence reads: ........ 90%  
Min. fraction of hits on each arm of a bidirectional cluster: 0.1  
Output image file for each cluster: ......................... 0 [0=no/1=yes]  
Output html file for each cluster: .......................... 1 [0=no/1=yes]  
Output a summary table: ..................................... 1 [0=no/1=yes]  
Output a FASTA file for each cluster (piRNA sequences): ..... 1 [0=no/1=yes]  
Output a FASTA file comprising cluster sequences: ........... 1 [0=no/1=yes]  
Search DNA motifs in clusters: .............................. 1 [0=no/1=yes]  
Output flanking sequences: +/- .............................. 0 bp  
Output ~.pTi file: .......................................... 1 [0=no/1=yes]  
==============================================================================  
  
  
Genome size (without gaps): ............ 2678902517 bp  
Gaps (N/X/-): .......................... 53837044 bp  
Mapped reads: .......................... 658825247023  
Non-identical sequences: ............... 514171  
Genomic hits: .......................... 764233  
Significant densitiy of mapped reads: .. 12867599.5173724 reads/kb

Show proTRAC cluster info
Hide proTRAC cluster info

|  |  |
| --- | --- |
| Location | chr25 |
| Coordinates | 34151448-34158599 |
| Size [bp] | 7152 |
| Sequence hit loci | 217 |
| Mapped reads (normalized) | 280384447 |
| Mapped reads (normalized) per kb | 39203641.9 |
| Normalized reads with 1T (1U) | 76.7% |
| Normalized reads with 10A | 37.4% |
| Normalized reads with length 26-33 nt | 100% |
| Normalized reads on the main strand(s) | 100% |
| Predicted directionality | mono:plus |

100%

0%

1T (1U)  
reads

10A reads

26-33 nt  
reads

reads on mainstrand

**Either the amount of reads with 1T (1U) OR 10A has to exceed 75% (set with option: -1Tor10A)  
Alternatively the amount of reads with 1T (1U) AND 10A has to exceed 50% (set with option: -1Tand10A)  
Minimum amount of reads with preferred size is 75% (set with option: -pisize)  
Minimum amount of reads on the main strand(s) is 75% (set with option: -clstrand)**

Show read coverage
Hide read coverage

WHAT DO I SEE HERE?  
This chart shows the location of mapped sequence reads within a predicted piRNA cluster. The color refers to the number of genomic hits produced by the sequence read in question. A dark red bar indicates that this sequence read produces many other hits elsewhere in the genome. Many adjacent red or yellow bars can indicate the presence of a multi-copy element such as transposons or rRNA genes. A dark green bar indicates that this sequence read maps uniquely to this locus.

1 hit

2-5 hits

6-10 hits

11-20 hits

21-50 hits

51-100 hits

> 100 hits

chr25

34151448

34158599

Gene Set

RepeatMasker

Mapped  
Reads

43.82

plus strand

minus strand

43.82

Region: chr25 29355691-34151455. Max. coverage (+): 1.53. Max coverage (-): 0

Region: chr25 34151456-34151469. Max. coverage (+): 0. Max coverage (-): 0

Region: chr25 34151470-34151483. Max. coverage (+): 0. Max coverage (-): 0

Region: chr25 34151484-34151498. Max. coverage (+): 0. Max coverage (-): 0

Region: chr25 34151499-34151512. Max. coverage (+): 0. Max coverage (-): 0

Region: chr25 34151513-34151526. Max. coverage (+): 0. Max coverage (-): 0

Region: chr25 34151527-34151540. Max. coverage (+): 0. Max coverage (-): 0

Region: chr25 34151541-34151555. Max. coverage (+): 0. Max coverage (-): 0

Region: chr25 34151556-34151569. Max. coverage (+): 0. Max coverage (-): 0

Region: chr25 34151570-34151583. Max. coverage (+): 0. Max coverage (-): 0

Region: chr25 34151584-34151598. Max. coverage (+): 0. Max coverage (-): 0

Region: chr25 34151599-34151612. Max. coverage (+): 0. Max coverage (-): 0

Region: chr25 34151613-34151626. Max. coverage (+): 0. Max coverage (-): 0

Region: chr25 34151627-34151641. Max. coverage (+): 0. Max coverage (-): 0

Region: chr25 34151642-34151655. Max. coverage (+): 0. Max coverage (-): 0

Region: chr25 34151656-34151669. Max. coverage (+): 0. Max coverage (-): 0

Region: chr25 34151670-34151684. Max. coverage (+): 0. Max coverage (-): 0

Region: chr25 34151685-34151698. Max. coverage (+): 27.33. Max coverage (-): 0

Region: chr25 34151699-34151712. Max. coverage (+): 27.33. Max coverage (-): 0

Region: chr25 34151713-34151726. Max. coverage (+): 0. Max coverage (-): 0

Region: chr25 34151727-34151741. Max. coverage (+): 0. Max coverage (-): 0

Region: chr25 34151742-34151755. Max. coverage (+): 0. Max coverage (-): 0

Region: chr25 34151756-34151769. Max. coverage (+): 3.71. Max coverage (-): 0

Region: chr25 34151770-34151784. Max. coverage (+): 6.9. Max coverage (-): 0

Region: chr25 34151785-34151798. Max. coverage (+): 0. Max coverage (-): 0

Region: chr25 34151799-34151812. Max. coverage (+): 0. Max coverage (-): 0

Region: chr25 34151813-34151827. Max. coverage (+): 0. Max coverage (-): 0

Region: chr25 34151828-34151841. Max. coverage (+): 0. Max coverage (-): 0

Region: chr25 34151842-34151855. Max. coverage (+): 0. Max coverage (-): 0

Region: chr25 34151856-34151869. Max. coverage (+): 0. Max coverage (-): 0

Region: chr25 34151870-34151884. Max. coverage (+): 0.86. Max coverage (-): 0

Region: chr25 34151885-34151898. Max. coverage (+): 0. Max coverage (-): 0

Region: chr25 34151899-34151912. Max. coverage (+): 0. Max coverage (-): 0

Region: chr25 34151913-34151927. Max. coverage (+): 0. Max coverage (-): 0

Region: chr25 34151928-34151941. Max. coverage (+): 4.75. Max coverage (-): 0

Region: chr25 34151942-34151955. Max. coverage (+): 7.58. Max coverage (-): 0

Region: chr25 34151956-34151970. Max. coverage (+): 0. Max coverage (-): 0

Region: chr25 34151971-34151984. Max. coverage (+): 0. Max coverage (-): 0

Region: chr25 34151985-34151998. Max. coverage (+): 0. Max coverage (-): 0

Region: chr25 34151999-34152013. Max. coverage (+): 12.01. Max coverage (-): 0

Region: chr25 34152014-34152027. Max. coverage (+): 12.01. Max coverage (-): 0

Region: chr25 34152028-34152041. Max. coverage (+): 0. Max coverage (-): 0

Region: chr25 34152042-34152055. Max. coverage (+): 0. Max coverage (-): 0

Region: chr25 34152056-34152070. Max. coverage (+): 0. Max coverage (-): 0

Region: chr25 34152071-34152084. Max. coverage (+): 0. Max coverage (-): 0

Region: chr25 34152085-34152098. Max. coverage (+): 0. Max coverage (-): 0

Region: chr25 34152099-34152113. Max. coverage (+): 0. Max coverage (-): 0

Region: chr25 34152114-34152127. Max. coverage (+): 0. Max coverage (-): 0

Region: chr25 34152128-34152141. Max. coverage (+): 0. Max coverage (-): 0

Region: chr25 34152142-34152156. Max. coverage (+): 0. Max coverage (-): 0

Region: chr25 34152157-34152170. Max. coverage (+): 0. Max coverage (-): 0

Region: chr25 34152171-34152184. Max. coverage (+): 0. Max coverage (-): 0

Region: chr25 34152185-34152198. Max. coverage (+): 0. Max coverage (-): 0

Region: chr25 34152199-34152213. Max. coverage (+): 0. Max coverage (-): 0

Region: chr25 34152214-34152227. Max. coverage (+): 0. Max coverage (-): 0

Region: chr25 34152228-34152241. Max. coverage (+): 0. Max coverage (-): 0

Region: chr25 34152242-34152256. Max. coverage (+): 0. Max coverage (-): 0

Region: chr25 34152257-34152270. Max. coverage (+): 0. Max coverage (-): 0

Region: chr25 34152271-34152284. Max. coverage (+): 0. Max coverage (-): 0

Region: chr25 34152285-34152299. Max. coverage (+): 0. Max coverage (-): 0

Region: chr25 34152300-34152313. Max. coverage (+): 0. Max coverage (-): 0

Region: chr25 34152314-34152327. Max. coverage (+): 0. Max coverage (-): 0

Region: chr25 34152328-34152341. Max. coverage (+): 0. Max coverage (-): 0

Region: chr25 34152342-34152356. Max. coverage (+): 0. Max coverage (-): 0

Region: chr25 34152357-34152370. Max. coverage (+): 0. Max coverage (-): 0

Region: chr25 34152371-34152384. Max. coverage (+): 0. Max coverage (-): 0

Region: chr25 34152385-34152399. Max. coverage (+): 0. Max coverage (-): 0

Region: chr25 34152400-34152413. Max. coverage (+): 0. Max coverage (-): 0

Region: chr25 34152414-34152427. Max. coverage (+): 0. Max coverage (-): 0

Region: chr25 34152428-34152442. Max. coverage (+): 0. Max coverage (-): 0

Region: chr25 34152443-34152456. Max. coverage (+): 0. Max coverage (-): 0

Region: chr25 34152457-34152470. Max. coverage (+): 0. Max coverage (-): 0

Region: chr25 34152471-34152485. Max. coverage (+): 0. Max coverage (-): 0

Region: chr25 34152486-34152499. Max. coverage (+): 0. Max coverage (-): 0

Region: chr25 34152500-34152513. Max. coverage (+): 0. Max coverage (-): 0

Region: chr25 34152514-34152527. Max. coverage (+): 0. Max coverage (-): 0

Region: chr25 34152528-34152542. Max. coverage (+): 0. Max coverage (-): 0

Region: chr25 34152543-34152556. Max. coverage (+): 0. Max coverage (-): 0

Region: chr25 34152557-34152570. Max. coverage (+): 0. Max coverage (-): 0

Region: chr25 34152571-34152585. Max. coverage (+): 0. Max coverage (-): 0

Region: chr25 34152586-34152599. Max. coverage (+): 0. Max coverage (-): 0

Region: chr25 34152600-34152613. Max. coverage (+): 0. Max coverage (-): 0

Region: chr25 34152614-34152628. Max. coverage (+): 0. Max coverage (-): 0

Region: chr25 34152629-34152642. Max. coverage (+): 0. Max coverage (-): 0

Region: chr25 34152643-34152656. Max. coverage (+): 0. Max coverage (-): 0

Region: chr25 34152657-34152670. Max. coverage (+): 0. Max coverage (-): 0

Region: chr25 34152671-34152685. Max. coverage (+): 0. Max coverage (-): 0

Region: chr25 34152686-34152699. Max. coverage (+): 0. Max coverage (-): 0

Region: chr25 34152700-34152713. Max. coverage (+): 0. Max coverage (-): 0

Region: chr25 34152714-34152728. Max. coverage (+): 0. Max coverage (-): 0

Region: chr25 34152729-34152742. Max. coverage (+): 0. Max coverage (-): 0

Region: chr25 34152743-34152756. Max. coverage (+): 0. Max coverage (-): 0

Region: chr25 34152757-34152771. Max. coverage (+): 0. Max coverage (-): 0

Region: chr25 34152772-34152785. Max. coverage (+): 0. Max coverage (-): 0

Region: chr25 34152786-34152799. Max. coverage (+): 0. Max coverage (-): 0

Region: chr25 34152800-34152814. Max. coverage (+): 0. Max coverage (-): 0

Region: chr25 34152815-34152828. Max. coverage (+): 0. Max coverage (-): 0

Region: chr25 34152829-34152842. Max. coverage (+): 0. Max coverage (-): 0

Region: chr25 34152843-34152856. Max. coverage (+): 0. Max coverage (-): 0

Region: chr25 34152857-34152871. Max. coverage (+): 0. Max coverage (-): 0

Region: chr25 34152872-34152885. Max. coverage (+): 0. Max coverage (-): 0

Region: chr25 34152886-34152899. Max. coverage (+): 0. Max coverage (-): 0

Region: chr25 34152900-34152914. Max. coverage (+): 0. Max coverage (-): 0

Region: chr25 34152915-34152928. Max. coverage (+): 0. Max coverage (-): 0

Region: chr25 34152929-34152942. Max. coverage (+): 0. Max coverage (-): 0

Region: chr25 34152943-34152957. Max. coverage (+): 0. Max coverage (-): 0

Region: chr25 34152958-34152971. Max. coverage (+): 0. Max coverage (-): 0

Region: chr25 34152972-34152985. Max. coverage (+): 0. Max coverage (-): 0

Region: chr25 34152986-34152999. Max. coverage (+): 0. Max coverage (-): 0

Region: chr25 34153000-34153014. Max. coverage (+): 0. Max coverage (-): 0

Region: chr25 34153015-34153028. Max. coverage (+): 0. Max coverage (-): 0

Region: chr25 34153029-34153042. Max. coverage (+): 0. Max coverage (-): 0

Region: chr25 34153043-34153057. Max. coverage (+): 0. Max coverage (-): 0

Region: chr25 34153058-34153071. Max. coverage (+): 0. Max coverage (-): 0

Region: chr25 34153072-34153085. Max. coverage (+): 0. Max coverage (-): 0

Region: chr25 34153086-34153100. Max. coverage (+): 2.18. Max coverage (-): 0

Region: chr25 34153101-34153114. Max. coverage (+): 2.18. Max coverage (-): 0

Region: chr25 34153115-34153128. Max. coverage (+): 11.2. Max coverage (-): 0

Region: chr25 34153129-34153143. Max. coverage (+): 0.68. Max coverage (-): 0

Region: chr25 34153144-34153157. Max. coverage (+): 0.68. Max coverage (-): 0

Region: chr25 34153158-34153171. Max. coverage (+): 4.02. Max coverage (-): 0

Region: chr25 34153172-34153185. Max. coverage (+): 4.02. Max coverage (-): 0

Region: chr25 34153186-34153200. Max. coverage (+): 0. Max coverage (-): 0

Region: chr25 34153201-34153214. Max. coverage (+): 0. Max coverage (-): 0

Region: chr25 34153215-34153228. Max. coverage (+): 0. Max coverage (-): 0

Region: chr25 34153229-34153243. Max. coverage (+): 1.79. Max coverage (-): 0

Region: chr25 34153244-34153257. Max. coverage (+): 1.79. Max coverage (-): 0

Region: chr25 34153258-34153271. Max. coverage (+): 0. Max coverage (-): 0

Region: chr25 34153272-34153286. Max. coverage (+): 0. Max coverage (-): 0

Region: chr25 34153287-34153300. Max. coverage (+): 0. Max coverage (-): 0

Region: chr25 34153301-34153314. Max. coverage (+): 0. Max coverage (-): 0

Region: chr25 34153315-34153328. Max. coverage (+): 0. Max coverage (-): 0

Region: chr25 34153329-34153343. Max. coverage (+): 0. Max coverage (-): 0

Region: chr25 34153344-34153357. Max. coverage (+): 0. Max coverage (-): 0

Region: chr25 34153358-34153371. Max. coverage (+): 0. Max coverage (-): 0

Region: chr25 34153372-34153386. Max. coverage (+): 0. Max coverage (-): 0

Region: chr25 34153387-34153400. Max. coverage (+): 0. Max coverage (-): 0

Region: chr25 34153401-34153414. Max. coverage (+): 0. Max coverage (-): 0

Region: chr25 34153415-34153429. Max. coverage (+): 0. Max coverage (-): 0

Region: chr25 34153430-34153443. Max. coverage (+): 1. Max coverage (-): 0

Region: chr25 34153444-34153457. Max. coverage (+): 5.62. Max coverage (-): 0

Region: chr25 34153458-34153472. Max. coverage (+): 0. Max coverage (-): 0

Region: chr25 34153473-34153486. Max. coverage (+): 0. Max coverage (-): 0

Region: chr25 34153487-34153500. Max. coverage (+): 4.71. Max coverage (-): 0

Region: chr25 34153501-34153514. Max. coverage (+): 1.44. Max coverage (-): 0

Region: chr25 34153515-34153529. Max. coverage (+): 1.44. Max coverage (-): 0

Region: chr25 34153530-34153543. Max. coverage (+): 0. Max coverage (-): 0

Region: chr25 34153544-34153557. Max. coverage (+): 1.23. Max coverage (-): 0

Region: chr25 34153558-34153572. Max. coverage (+): 0. Max coverage (-): 0

Region: chr25 34153573-34153586. Max. coverage (+): 0. Max coverage (-): 0

Region: chr25 34153587-34153600. Max. coverage (+): 0. Max coverage (-): 0

Region: chr25 34153601-34153615. Max. coverage (+): 8.44. Max coverage (-): 0

Region: chr25 34153616-34153629. Max. coverage (+): 0. Max coverage (-): 0

Region: chr25 34153630-34153643. Max. coverage (+): 0. Max coverage (-): 0

Region: chr25 34153644-34153657. Max. coverage (+): 0. Max coverage (-): 0

Region: chr25 34153658-34153672. Max. coverage (+): 0. Max coverage (-): 0

Region: chr25 34153673-34153686. Max. coverage (+): 0. Max coverage (-): 0

Region: chr25 34153687-34153700. Max. coverage (+): 0. Max coverage (-): 0

Region: chr25 34153701-34153715. Max. coverage (+): 0. Max coverage (-): 0

Region: chr25 34153716-34153729. Max. coverage (+): 0. Max coverage (-): 0

Region: chr25 34153730-34153743. Max. coverage (+): 0. Max coverage (-): 0

Region: chr25 34153744-34153758. Max. coverage (+): 0. Max coverage (-): 0

Region: chr25 34153759-34153772. Max. coverage (+): 0. Max coverage (-): 0

Region: chr25 34153773-34153786. Max. coverage (+): 0. Max coverage (-): 0

Region: chr25 34153787-34153801. Max. coverage (+): 0. Max coverage (-): 0

Region: chr25 34153802-34153815. Max. coverage (+): 0. Max coverage (-): 0

Region: chr25 34153816-34153829. Max. coverage (+): 0. Max coverage (-): 0

Region: chr25 34153830-34153843. Max. coverage (+): 0. Max coverage (-): 0

Region: chr25 34153844-34153858. Max. coverage (+): 0. Max coverage (-): 0

Region: chr25 34153859-34153872. Max. coverage (+): 0. Max coverage (-): 0

Region: chr25 34153873-34153886. Max. coverage (+): 0. Max coverage (-): 0

Region: chr25 34153887-34153901. Max. coverage (+): 0. Max coverage (-): 0

Region: chr25 34153902-34153915. Max. coverage (+): 0. Max coverage (-): 0

Region: chr25 34153916-34153929. Max. coverage (+): 0. Max coverage (-): 0

Region: chr25 34153930-34153944. Max. coverage (+): 0. Max coverage (-): 0

Region: chr25 34153945-34153958. Max. coverage (+): 0. Max coverage (-): 0

Region: chr25 34153959-34153972. Max. coverage (+): 0. Max coverage (-): 0

Region: chr25 34153973-34153986. Max. coverage (+): 0. Max coverage (-): 0

Region: chr25 34153987-34154001. Max. coverage (+): 0. Max coverage (-): 0

Region: chr25 34154002-34154015. Max. coverage (+): 0. Max coverage (-): 0

Region: chr25 34154016-34154029. Max. coverage (+): 0. Max coverage (-): 0

Region: chr25 34154030-34154044. Max. coverage (+): 0. Max coverage (-): 0

Region: chr25 34154045-34154058. Max. coverage (+): 0. Max coverage (-): 0

Region: chr25 34154059-34154072. Max. coverage (+): 0. Max coverage (-): 0

Region: chr25 34154073-34154087. Max. coverage (+): 0. Max coverage (-): 0

Region: chr25 34154088-34154101. Max. coverage (+): 0. Max coverage (-): 0

Region: chr25 34154102-34154115. Max. coverage (+): 0. Max coverage (-): 0

Region: chr25 34154116-34154129. Max. coverage (+): 0. Max coverage (-): 0

Region: chr25 34154130-34154144. Max. coverage (+): 0. Max coverage (-): 0

Region: chr25 34154145-34154158. Max. coverage (+): 0. Max coverage (-): 0

Region: chr25 34154159-34154172. Max. coverage (+): 0. Max coverage (-): 0

Region: chr25 34154173-34154187. Max. coverage (+): 0. Max coverage (-): 0

Region: chr25 34154188-34154201. Max. coverage (+): 0. Max coverage (-): 0

Region: chr25 34154202-34154215. Max. coverage (+): 0. Max coverage (-): 0

Region: chr25 34154216-34154230. Max. coverage (+): 0. Max coverage (-): 0

Region: chr25 34154231-34154244. Max. coverage (+): 0. Max coverage (-): 0

Region: chr25 34154245-34154258. Max. coverage (+): 0. Max coverage (-): 0

Region: chr25 34154259-34154273. Max. coverage (+): 0. Max coverage (-): 0

Region: chr25 34154274-34154287. Max. coverage (+): 0. Max coverage (-): 0

Region: chr25 34154288-34154301. Max. coverage (+): 0. Max coverage (-): 0

Region: chr25 34154302-34154315. Max. coverage (+): 0. Max coverage (-): 0

Region: chr25 34154316-34154330. Max. coverage (+): 4.72. Max coverage (-): 0

Region: chr25 34154331-34154344. Max. coverage (+): 7.48. Max coverage (-): 0

Region: chr25 34154345-34154358. Max. coverage (+): 0. Max coverage (-): 0

Region: chr25 34154359-34154373. Max. coverage (+): 0. Max coverage (-): 0

Region: chr25 34154374-34154387. Max. coverage (+): 0. Max coverage (-): 0

Region: chr25 34154388-34154401. Max. coverage (+): 0. Max coverage (-): 0

Region: chr25 34154402-34154416. Max. coverage (+): 0. Max coverage (-): 0

Region: chr25 34154417-34154430. Max. coverage (+): 0. Max coverage (-): 0

Region: chr25 34154431-34154444. Max. coverage (+): 0. Max coverage (-): 0

Region: chr25 34154445-34154458. Max. coverage (+): 0. Max coverage (-): 0

Region: chr25 34154459-34154473. Max. coverage (+): 0. Max coverage (-): 0

Region: chr25 34154474-34154487. Max. coverage (+): 0. Max coverage (-): 0

Region: chr25 34154488-34154501. Max. coverage (+): 0. Max coverage (-): 0

Region: chr25 34154502-34154516. Max. coverage (+): 0. Max coverage (-): 0

Region: chr25 34154517-34154530. Max. coverage (+): 0. Max coverage (-): 0

Region: chr25 34154531-34154544. Max. coverage (+): 0. Max coverage (-): 0

Region: chr25 34154545-34154559. Max. coverage (+): 0. Max coverage (-): 0

Region: chr25 34154560-34154573. Max. coverage (+): 0. Max coverage (-): 0

Region: chr25 34154574-34154587. Max. coverage (+): 0. Max coverage (-): 0

Region: chr25 34154588-34154602. Max. coverage (+): 0. Max coverage (-): 0

Region: chr25 34154603-34154616. Max. coverage (+): 0. Max coverage (-): 0

Region: chr25 34154617-34154630. Max. coverage (+): 0. Max coverage (-): 0

Region: chr25 34154631-34154644. Max. coverage (+): 0. Max coverage (-): 0

Region: chr25 34154645-34154659. Max. coverage (+): 0. Max coverage (-): 0

Region: chr25 34154660-34154673. Max. coverage (+): 0. Max coverage (-): 0

Region: chr25 34154674-34154687. Max. coverage (+): 0. Max coverage (-): 0

Region: chr25 34154688-34154702. Max. coverage (+): 0. Max coverage (-): 0

Region: chr25 34154703-34154716. Max. coverage (+): 0. Max coverage (-): 0

Region: chr25 34154717-34154730. Max. coverage (+): 0. Max coverage (-): 0

Region: chr25 34154731-34154745. Max. coverage (+): 0. Max coverage (-): 0

Region: chr25 34154746-34154759. Max. coverage (+): 0. Max coverage (-): 0

Region: chr25 34154760-34154773. Max. coverage (+): 7.93. Max coverage (-): 0

Region: chr25 34154774-34154787. Max. coverage (+): 3.01. Max coverage (-): 0

Region: chr25 34154788-34154802. Max. coverage (+): 0. Max coverage (-): 0

Region: chr25 34154803-34154816. Max. coverage (+): 0. Max coverage (-): 0

Region: chr25 34154817-34154830. Max. coverage (+): 0. Max coverage (-): 0

Region: chr25 34154831-34154845. Max. coverage (+): 0. Max coverage (-): 0

Region: chr25 34154846-34154859. Max. coverage (+): 0. Max coverage (-): 0

Region: chr25 34154860-34154873. Max. coverage (+): 0. Max coverage (-): 0

Region: chr25 34154874-34154888. Max. coverage (+): 0. Max coverage (-): 0

Region: chr25 34154889-34154902. Max. coverage (+): 0. Max coverage (-): 0

Region: chr25 34154903-34154916. Max. coverage (+): 0. Max coverage (-): 0

Region: chr25 34154917-34154931. Max. coverage (+): 0. Max coverage (-): 0

Region: chr25 34154932-34154945. Max. coverage (+): 0. Max coverage (-): 0

Region: chr25 34154946-34154959. Max. coverage (+): 0. Max coverage (-): 0

Region: chr25 34154960-34154973. Max. coverage (+): 0. Max coverage (-): 0

Region: chr25 34154974-34154988. Max. coverage (+): 0. Max coverage (-): 0

Region: chr25 34154989-34155002. Max. coverage (+): 0. Max coverage (-): 0

Region: chr25 34155003-34155016. Max. coverage (+): 0. Max coverage (-): 0

Region: chr25 34155017-34155031. Max. coverage (+): 0. Max coverage (-): 0

Region: chr25 34155032-34155045. Max. coverage (+): 0. Max coverage (-): 0

Region: chr25 34155046-34155059. Max. coverage (+): 0. Max coverage (-): 0

Region: chr25 34155060-34155074. Max. coverage (+): 0. Max coverage (-): 0

Region: chr25 34155075-34155088. Max. coverage (+): 0. Max coverage (-): 0

Region: chr25 34155089-34155102. Max. coverage (+): 0. Max coverage (-): 0

Region: chr25 34155103-34155116. Max. coverage (+): 0. Max coverage (-): 0

Region: chr25 34155117-34155131. Max. coverage (+): 0. Max coverage (-): 0

Region: chr25 34155132-34155145. Max. coverage (+): 0. Max coverage (-): 0

Region: chr25 34155146-34155159. Max. coverage (+): 0. Max coverage (-): 0

Region: chr25 34155160-34155174. Max. coverage (+): 0. Max coverage (-): 0

Region: chr25 34155175-34155188. Max. coverage (+): 0. Max coverage (-): 0

Region: chr25 34155189-34155202. Max. coverage (+): 0. Max coverage (-): 0

Region: chr25 34155203-34155217. Max. coverage (+): 0. Max coverage (-): 0

Region: chr25 34155218-34155231. Max. coverage (+): 0. Max coverage (-): 0

Region: chr25 34155232-34155245. Max. coverage (+): 0. Max coverage (-): 0

Region: chr25 34155246-34155260. Max. coverage (+): 4.9. Max coverage (-): 0

Region: chr25 34155261-34155274. Max. coverage (+): 4.9. Max coverage (-): 0

Region: chr25 34155275-34155288. Max. coverage (+): 0. Max coverage (-): 0

Region: chr25 34155289-34155302. Max. coverage (+): 0. Max coverage (-): 0

Region: chr25 34155303-34155317. Max. coverage (+): 6.75. Max coverage (-): 0

Region: chr25 34155318-34155331. Max. coverage (+): 11.04. Max coverage (-): 0

Region: chr25 34155332-34155345. Max. coverage (+): 5.26. Max coverage (-): 0

Region: chr25 34155346-34155360. Max. coverage (+): 5.26. Max coverage (-): 0

Region: chr25 34155361-34155374. Max. coverage (+): 0. Max coverage (-): 0

Region: chr25 34155375-34155388. Max. coverage (+): 0. Max coverage (-): 0

Region: chr25 34155389-34155403. Max. coverage (+): 0. Max coverage (-): 0

Region: chr25 34155404-34155417. Max. coverage (+): 0. Max coverage (-): 0

Region: chr25 34155418-34155431. Max. coverage (+): 0. Max coverage (-): 0

Region: chr25 34155432-34155445. Max. coverage (+): 0. Max coverage (-): 0

Region: chr25 34155446-34155460. Max. coverage (+): 0. Max coverage (-): 0

Region: chr25 34155461-34155474. Max. coverage (+): 0. Max coverage (-): 0

Region: chr25 34155475-34155488. Max. coverage (+): 0. Max coverage (-): 0

Region: chr25 34155489-34155503. Max. coverage (+): 0. Max coverage (-): 0

Region: chr25 34155504-34155517. Max. coverage (+): 0. Max coverage (-): 0

Region: chr25 34155518-34155531. Max. coverage (+): 0. Max coverage (-): 0

Region: chr25 34155532-34155546. Max. coverage (+): 0. Max coverage (-): 0

Region: chr25 34155547-34155560. Max. coverage (+): 0. Max coverage (-): 0

Region: chr25 34155561-34155574. Max. coverage (+): 0. Max coverage (-): 0

Region: chr25 34155575-34155589. Max. coverage (+): 0. Max coverage (-): 0

Region: chr25 34155590-34155603. Max. coverage (+): 0. Max coverage (-): 0

Region: chr25 34155604-34155617. Max. coverage (+): 0. Max coverage (-): 0

Region: chr25 34155618-34155631. Max. coverage (+): 3.45. Max coverage (-): 0

Region: chr25 34155632-34155646. Max. coverage (+): 4.84. Max coverage (-): 0

Region: chr25 34155647-34155660. Max. coverage (+): 4. Max coverage (-): 0

Region: chr25 34155661-34155674. Max. coverage (+): 14.73. Max coverage (-): 0

Region: chr25 34155675-34155689. Max. coverage (+): 0. Max coverage (-): 0

Region: chr25 34155690-34155703. Max. coverage (+): 13.04. Max coverage (-): 0

Region: chr25 34155704-34155717. Max. coverage (+): 10.94. Max coverage (-): 0

Region: chr25 34155718-34155732. Max. coverage (+): 0. Max coverage (-): 0

Region: chr25 34155733-34155746. Max. coverage (+): 0. Max coverage (-): 0

Region: chr25 34155747-34155760. Max. coverage (+): 5.14. Max coverage (-): 0

Region: chr25 34155761-34155774. Max. coverage (+): 0. Max coverage (-): 0

Region: chr25 34155775-34155789. Max. coverage (+): 5.99. Max coverage (-): 0

Region: chr25 34155790-34155803. Max. coverage (+): 9.26. Max coverage (-): 0

Region: chr25 34155804-34155817. Max. coverage (+): 0.8. Max coverage (-): 0

Region: chr25 34155818-34155832. Max. coverage (+): 3.92. Max coverage (-): 0

Region: chr25 34155833-34155846. Max. coverage (+): 0. Max coverage (-): 0

Region: chr25 34155847-34155860. Max. coverage (+): 0. Max coverage (-): 0

Region: chr25 34155861-34155875. Max. coverage (+): 0. Max coverage (-): 0

Region: chr25 34155876-34155889. Max. coverage (+): 4.88. Max coverage (-): 0

Region: chr25 34155890-34155903. Max. coverage (+): 0. Max coverage (-): 0

Region: chr25 34155904-34155917. Max. coverage (+): 0. Max coverage (-): 0

Region: chr25 34155918-34155932. Max. coverage (+): 0. Max coverage (-): 0

Region: chr25 34155933-34155946. Max. coverage (+): 0. Max coverage (-): 0

Region: chr25 34155947-34155960. Max. coverage (+): 0. Max coverage (-): 0

Region: chr25 34155961-34155975. Max. coverage (+): 0. Max coverage (-): 0

Region: chr25 34155976-34155989. Max. coverage (+): 0.45. Max coverage (-): 0

Region: chr25 34155990-34156003. Max. coverage (+): 0. Max coverage (-): 0

Region: chr25 34156004-34156018. Max. coverage (+): 0. Max coverage (-): 0

Region: chr25 34156019-34156032. Max. coverage (+): 0. Max coverage (-): 0

Region: chr25 34156033-34156046. Max. coverage (+): 0. Max coverage (-): 0

Region: chr25 34156047-34156061. Max. coverage (+): 0. Max coverage (-): 0

Region: chr25 34156062-34156075. Max. coverage (+): 0. Max coverage (-): 0

Region: chr25 34156076-34156089. Max. coverage (+): 0. Max coverage (-): 0

Region: chr25 34156090-34156103. Max. coverage (+): 4.17. Max coverage (-): 0

Region: chr25 34156104-34156118. Max. coverage (+): 4.17. Max coverage (-): 0

Region: chr25 34156119-34156132. Max. coverage (+): 0. Max coverage (-): 0

Region: chr25 34156133-34156146. Max. coverage (+): 0. Max coverage (-): 0

Region: chr25 34156147-34156161. Max. coverage (+): 0. Max coverage (-): 0

Region: chr25 34156162-34156175. Max. coverage (+): 0. Max coverage (-): 0

Region: chr25 34156176-34156189. Max. coverage (+): 0. Max coverage (-): 0

Region: chr25 34156190-34156204. Max. coverage (+): 2.67. Max coverage (-): 0

Region: chr25 34156205-34156218. Max. coverage (+): 4.29. Max coverage (-): 0

Region: chr25 34156219-34156232. Max. coverage (+): 8.91. Max coverage (-): 0

Region: chr25 34156233-34156246. Max. coverage (+): 8.91. Max coverage (-): 0

Region: chr25 34156247-34156261. Max. coverage (+): 4.51. Max coverage (-): 0

Region: chr25 34156262-34156275. Max. coverage (+): 0. Max coverage (-): 0

Region: chr25 34156276-34156289. Max. coverage (+): 0. Max coverage (-): 0

Region: chr25 34156290-34156304. Max. coverage (+): 0. Max coverage (-): 0

Region: chr25 34156305-34156318. Max. coverage (+): 0. Max coverage (-): 0

Region: chr25 34156319-34156332. Max. coverage (+): 0. Max coverage (-): 0

Region: chr25 34156333-34156347. Max. coverage (+): 0. Max coverage (-): 0

Region: chr25 34156348-34156361. Max. coverage (+): 0. Max coverage (-): 0

Region: chr25 34156362-34156375. Max. coverage (+): 0. Max coverage (-): 0

Region: chr25 34156376-34156390. Max. coverage (+): 0. Max coverage (-): 0

Region: chr25 34156391-34156404. Max. coverage (+): 0. Max coverage (-): 0

Region: chr25 34156405-34156418. Max. coverage (+): 0. Max coverage (-): 0

Region: chr25 34156419-34156432. Max. coverage (+): 0. Max coverage (-): 0

Region: chr25 34156433-34156447. Max. coverage (+): 0. Max coverage (-): 0

Region: chr25 34156448-34156461. Max. coverage (+): 0. Max coverage (-): 0

Region: chr25 34156462-34156475. Max. coverage (+): 0. Max coverage (-): 0

Region: chr25 34156476-34156490. Max. coverage (+): 1.54. Max coverage (-): 0

Region: chr25 34156491-34156504. Max. coverage (+): 1.54. Max coverage (-): 0

Region: chr25 34156505-34156518. Max. coverage (+): 0. Max coverage (-): 0

Region: chr25 34156519-34156533. Max. coverage (+): 0. Max coverage (-): 0

Region: chr25 34156534-34156547. Max. coverage (+): 0. Max coverage (-): 0

Region: chr25 34156548-34156561. Max. coverage (+): 0. Max coverage (-): 0

Region: chr25 34156562-34156575. Max. coverage (+): 0. Max coverage (-): 0

Region: chr25 34156576-34156590. Max. coverage (+): 0. Max coverage (-): 0

Region: chr25 34156591-34156604. Max. coverage (+): 0. Max coverage (-): 0

Region: chr25 34156605-34156618. Max. coverage (+): 0. Max coverage (-): 0

Region: chr25 34156619-34156633. Max. coverage (+): 0. Max coverage (-): 0

Region: chr25 34156634-34156647. Max. coverage (+): 0. Max coverage (-): 0

Region: chr25 34156648-34156661. Max. coverage (+): 4.78. Max coverage (-): 0

Region: chr25 34156662-34156676. Max. coverage (+): 0. Max coverage (-): 0

Region: chr25 34156677-34156690. Max. coverage (+): 4.66. Max coverage (-): 0

Region: chr25 34156691-34156704. Max. coverage (+): 4.66. Max coverage (-): 0

Region: chr25 34156705-34156719. Max. coverage (+): 0.96. Max coverage (-): 0

Region: chr25 34156720-34156733. Max. coverage (+): 0.96. Max coverage (-): 0

Region: chr25 34156734-34156747. Max. coverage (+): 0. Max coverage (-): 0

Region: chr25 34156748-34156761. Max. coverage (+): 2.21. Max coverage (-): 0

Region: chr25 34156762-34156776. Max. coverage (+): 2.21. Max coverage (-): 0

Region: chr25 34156777-34156790. Max. coverage (+): 0. Max coverage (-): 0

Region: chr25 34156791-34156804. Max. coverage (+): 1.51. Max coverage (-): 0

Region: chr25 34156805-34156819. Max. coverage (+): 1.51. Max coverage (-): 0

Region: chr25 34156820-34156833. Max. coverage (+): 0. Max coverage (-): 0

Region: chr25 34156834-34156847. Max. coverage (+): 0. Max coverage (-): 0

Region: chr25 34156848-34156862. Max. coverage (+): 0. Max coverage (-): 0

Region: chr25 34156863-34156876. Max. coverage (+): 0. Max coverage (-): 0

Region: chr25 34156877-34156890. Max. coverage (+): 0. Max coverage (-): 0

Region: chr25 34156891-34156904. Max. coverage (+): 0. Max coverage (-): 0

Region: chr25 34156905-34156919. Max. coverage (+): 0. Max coverage (-): 0

Region: chr25 34156920-34156933. Max. coverage (+): 0. Max coverage (-): 0

Region: chr25 34156934-34156947. Max. coverage (+): 0. Max coverage (-): 0

Region: chr25 34156948-34156962. Max. coverage (+): 0. Max coverage (-): 0

Region: chr25 34156963-34156976. Max. coverage (+): 0. Max coverage (-): 0

Region: chr25 34156977-34156990. Max. coverage (+): 0. Max coverage (-): 0

Region: chr25 34156991-34157005. Max. coverage (+): 0. Max coverage (-): 0

Region: chr25 34157006-34157019. Max. coverage (+): 0. Max coverage (-): 0

Region: chr25 34157020-34157033. Max. coverage (+): 2.17. Max coverage (-): 0

Region: chr25 34157034-34157048. Max. coverage (+): 3.07. Max coverage (-): 0

Region: chr25 34157049-34157062. Max. coverage (+): 0. Max coverage (-): 0

Region: chr25 34157063-34157076. Max. coverage (+): 7.08. Max coverage (-): 0

Region: chr25 34157077-34157090. Max. coverage (+): 5.43. Max coverage (-): 0

Region: chr25 34157091-34157105. Max. coverage (+): 2.41. Max coverage (-): 0

Region: chr25 34157106-34157119. Max. coverage (+): 0.29. Max coverage (-): 0

Region: chr25 34157120-34157133. Max. coverage (+): 0.74. Max coverage (-): 0

Region: chr25 34157134-34157148. Max. coverage (+): 3.08. Max coverage (-): 0

Region: chr25 34157149-34157162. Max. coverage (+): 4.54. Max coverage (-): 0

Region: chr25 34157163-34157176. Max. coverage (+): 13.04. Max coverage (-): 0

Region: chr25 34157177-34157191. Max. coverage (+): 3.68. Max coverage (-): 0

Region: chr25 34157192-34157205. Max. coverage (+): 0. Max coverage (-): 0

Region: chr25 34157206-34157219. Max. coverage (+): 0. Max coverage (-): 0

Region: chr25 34157220-34157233. Max. coverage (+): 0. Max coverage (-): 0

Region: chr25 34157234-34157248. Max. coverage (+): 0. Max coverage (-): 0

Region: chr25 34157249-34157262. Max. coverage (+): 0. Max coverage (-): 0

Region: chr25 34157263-34157276. Max. coverage (+): 0. Max coverage (-): 0

Region: chr25 34157277-34157291. Max. coverage (+): 0. Max coverage (-): 0

Region: chr25 34157292-34157305. Max. coverage (+): 0. Max coverage (-): 0

Region: chr25 34157306-34157319. Max. coverage (+): 0. Max coverage (-): 0

Region: chr25 34157320-34157334. Max. coverage (+): 0. Max coverage (-): 0

Region: chr25 34157335-34157348. Max. coverage (+): 0. Max coverage (-): 0

Region: chr25 34157349-34157362. Max. coverage (+): 0. Max coverage (-): 0

Region: chr25 34157363-34157377. Max. coverage (+): 0. Max coverage (-): 0

Region: chr25 34157378-34157391. Max. coverage (+): 0. Max coverage (-): 0

Region: chr25 34157392-34157405. Max. coverage (+): 0. Max coverage (-): 0

Region: chr25 34157406-34157419. Max. coverage (+): 0. Max coverage (-): 0

Region: chr25 34157420-34157434. Max. coverage (+): 0. Max coverage (-): 0

Region: chr25 34157435-34157448. Max. coverage (+): 0. Max coverage (-): 0

Region: chr25 34157449-34157462. Max. coverage (+): 0. Max coverage (-): 0

Region: chr25 34157463-34157477. Max. coverage (+): 0. Max coverage (-): 0

Region: chr25 34157478-34157491. Max. coverage (+): 0. Max coverage (-): 0

Region: chr25 34157492-34157505. Max. coverage (+): 0. Max coverage (-): 0

Region: chr25 34157506-34157520. Max. coverage (+): 0. Max coverage (-): 0

Region: chr25 34157521-34157534. Max. coverage (+): 0. Max coverage (-): 0

Region: chr25 34157535-34157548. Max. coverage (+): 0. Max coverage (-): 0

Region: chr25 34157549-34157562. Max. coverage (+): 0. Max coverage (-): 0

Region: chr25 34157563-34157577. Max. coverage (+): 0. Max coverage (-): 0

Region: chr25 34157578-34157591. Max. coverage (+): 0. Max coverage (-): 0

Region: chr25 34157592-34157605. Max. coverage (+): 0. Max coverage (-): 0

Region: chr25 34157606-34157620. Max. coverage (+): 0. Max coverage (-): 0

Region: chr25 34157621-34157634. Max. coverage (+): 0. Max coverage (-): 0

Region: chr25 34157635-34157648. Max. coverage (+): 0. Max coverage (-): 0

Region: chr25 34157649-34157663. Max. coverage (+): 0. Max coverage (-): 0

Region: chr25 34157664-34157677. Max. coverage (+): 0. Max coverage (-): 0

Region: chr25 34157678-34157691. Max. coverage (+): 0. Max coverage (-): 0

Region: chr25 34157692-34157705. Max. coverage (+): 0. Max coverage (-): 0

Region: chr25 34157706-34157720. Max. coverage (+): 0. Max coverage (-): 0

Region: chr25 34157721-34157734. Max. coverage (+): 0. Max coverage (-): 0

Region: chr25 34157735-34157748. Max. coverage (+): 0. Max coverage (-): 0

Region: chr25 34157749-34157763. Max. coverage (+): 0. Max coverage (-): 0

Region: chr25 34157764-34157777. Max. coverage (+): 0. Max coverage (-): 0

Region: chr25 34157778-34157791. Max. coverage (+): 0. Max coverage (-): 0

Region: chr25 34157792-34157806. Max. coverage (+): 0. Max coverage (-): 0

Region: chr25 34157807-34157820. Max. coverage (+): 0. Max coverage (-): 0

Region: chr25 34157821-34157834. Max. coverage (+): 0. Max coverage (-): 0

Region: chr25 34157835-34157849. Max. coverage (+): 0. Max coverage (-): 0

Region: chr25 34157850-34157863. Max. coverage (+): 0. Max coverage (-): 0

Region: chr25 34157864-34157877. Max. coverage (+): 0. Max coverage (-): 0

Region: chr25 34157878-34157891. Max. coverage (+): 0. Max coverage (-): 0

Region: chr25 34157892-34157906. Max. coverage (+): 0. Max coverage (-): 0

Region: chr25 34157907-34157920. Max. coverage (+): 0. Max coverage (-): 0

Region: chr25 34157921-34157934. Max. coverage (+): 0. Max coverage (-): 0

Region: chr25 34157935-34157949. Max. coverage (+): 0. Max coverage (-): 0

Region: chr25 34157950-34157963. Max. coverage (+): 0. Max coverage (-): 0

Region: chr25 34157964-34157977. Max. coverage (+): 0. Max coverage (-): 0

Region: chr25 34157978-34157992. Max. coverage (+): 0. Max coverage (-): 0

Region: chr25 34157993-34158006. Max. coverage (+): 0. Max coverage (-): 0

Region: chr25 34158007-34158020. Max. coverage (+): 0. Max coverage (-): 0

Region: chr25 34158021-34158034. Max. coverage (+): 0. Max coverage (-): 0

Region: chr25 34158035-34158049. Max. coverage (+): 1.84. Max coverage (-): 0

Region: chr25 34158050-34158063. Max. coverage (+): 0. Max coverage (-): 0

Region: chr25 34158064-34158077. Max. coverage (+): 2.3. Max coverage (-): 0

Region: chr25 34158078-34158092. Max. coverage (+): 2.3. Max coverage (-): 0

Region: chr25 34158093-34158106. Max. coverage (+): 3.74. Max coverage (-): 0

Region: chr25 34158107-34158120. Max. coverage (+): 0. Max coverage (-): 0

Region: chr25 34158121-34158135. Max. coverage (+): 0. Max coverage (-): 0

Region: chr25 34158136-34158149. Max. coverage (+): 0. Max coverage (-): 0

Region: chr25 34158150-34158163. Max. coverage (+): 12.85. Max coverage (-): 0

Region: chr25 34158164-34158178. Max. coverage (+): 9.02. Max coverage (-): 0

Region: chr25 34158179-34158192. Max. coverage (+): 0. Max coverage (-): 0

Region: chr25 34158193-34158206. Max. coverage (+): 0. Max coverage (-): 0

Region: chr25 34158207-34158220. Max. coverage (+): 4.2. Max coverage (-): 0

Region: chr25 34158221-34158235. Max. coverage (+): 9.97. Max coverage (-): 0

Region: chr25 34158236-34158249. Max. coverage (+): 13.57. Max coverage (-): 0

Region: chr25 34158250-34158263. Max. coverage (+): 0. Max coverage (-): 0

Region: chr25 34158264-34158278. Max. coverage (+): 0. Max coverage (-): 0

Region: chr25 34158279-34158292. Max. coverage (+): 0. Max coverage (-): 0

Region: chr25 34158293-34158306. Max. coverage (+): 0. Max coverage (-): 0

Region: chr25 34158307-34158321. Max. coverage (+): 0. Max coverage (-): 0

Region: chr25 34158322-34158335. Max. coverage (+): 0. Max coverage (-): 0

Region: chr25 34158336-34158349. Max. coverage (+): 0. Max coverage (-): 0

Region: chr25 34158350-34158363. Max. coverage (+): 0. Max coverage (-): 0

Region: chr25 34158364-34158378. Max. coverage (+): 0. Max coverage (-): 0

Region: chr25 34158379-34158392. Max. coverage (+): 0. Max coverage (-): 0

Region: chr25 34158393-34158406. Max. coverage (+): 0. Max coverage (-): 0

Region: chr25 34158407-34158421. Max. coverage (+): 0. Max coverage (-): 0

Region: chr25 34158422-34158435. Max. coverage (+): 0.91. Max coverage (-): 0

Region: chr25 34158436-34158449. Max. coverage (+): 26.35. Max coverage (-): 0

Region: chr25 34158450-34158464. Max. coverage (+): 43.82. Max coverage (-): 0

Region: chr25 34158465-34158478. Max. coverage (+): 2.25. Max coverage (-): 0

Region: chr25 34158479-34158492. Max. coverage (+): 25.83. Max coverage (-): 0

Region: chr25 34158493-34158507. Max. coverage (+): 12. Max coverage (-): 0

Region: chr25 34158508-34158521. Max. coverage (+): 0. Max coverage (-): 0

Region: chr25 34158522-34158535. Max. coverage (+): 1.09. Max coverage (-): 0

Region: chr25 34158536-34158549. Max. coverage (+): 5.29. Max coverage (-): 0

Region: chr25 34158550-34158564. Max. coverage (+): 10.48. Max coverage (-): 0

Region: chr25 34158565-34158578. Max. coverage (+): 1.39. Max coverage (-): 0

Region: chr25 34158579-34158592. Max. coverage (+): 1.39. Max coverage (-): 0

Region: chr25 34158593-. Max. coverage (+): 0. Max coverage (-): 0

RepeatMasker Color Code

**+**

100-98% Identity

<98-95% Identity

<95-90% Identity

<90-85% Identity

<85-80% Identity

<80-75% Identity

<75-70% Identity

<70% Identity

**-**

Gene Set Color Code

**+**

Gene

Pseudogene

**-**

Topology/Coverage Color Code

Coverage Plus Strand

Coverage Minus Strand

Mainstrand: Plus

Mainstrand: Minus

Complementary Strand

Flanking Region  
(if option -flank >0)

Gene Set Annotation  

**1. TBL2 (protein coding, ENSBTAG00000000321) Tr:00000000422 Ex:1**: 34151410-34151569 (+)  
**2. TBL2 (protein coding, ENSBTAG00000000321) Tr:00000000422 Ex:2**: 34153085-34153215 (+)  
**3. TBL2 (protein coding, ENSBTAG00000000321) Tr:00000000422 Ex:3**: 34153474-34153658 (+)  
**4. TBL2 (protein coding, ENSBTAG00000000321) Tr:00000000422 Ex:4**: 34154284-34154435 (+)  
**5. TBL2 (protein coding, ENSBTAG00000000321) Tr:00000000422 Ex:5**: 34154755-34154881 (+)  
**6. TBL2 (protein coding, ENSBTAG00000000321) Tr:00000000422 Ex:6**: 34155228-34155380 (+)  
**7. TBL2 (protein coding, ENSBTAG00000000321) Tr:00000000422 Ex:7**: 34155617-34156679 (+)

  
RepeatMasker Annotation  

**1. L2c**: 34152167-34152375 (-), Divergence to consensus: 52.9%  
**2. MER91A**: 34152770-34152860 (+), Divergence to consensus: 41.8%  
**3. MIRb**: 34152884-34152982 (-), Divergence to consensus: 46.6%  
**4. BOV-A2**: 34153850-34153973 (+), Divergence to consensus: 6.7%  
**5. MIRb**: 34154951-34155031 (+), Divergence to consensus: 37%  
**6. MIRc**: 34155062-34155130 (+), Divergence to consensus: 37.7%  
**7. MIR**: 34156579-34156672 (+), Divergence to consensus: 29.4%  
**8. L2c**: 34156907-34157023 (-), Divergence to consensus: 42.8%  
**9. L2a**: 34157240-34158016 (+), Divergence to consensus: 45.3%  
**10. L2a**: 34158335-34158413 (+), Divergence to consensus: 44.3%

  
Transcription Factor Binding Sites  

**RFX4\_1** (Sequence: CTTGGCAAC (+): 34155436)  
**Gata4** (Sequence: CTTATCT (+): 34154636)  
**Gata4** (Sequence: GTTATCT (+): 34156687)
