# Supplementary material for: Small RNA sequencing of cryopreserved semen from single bull revealed altered miRNAs and piRNAs expression between High- and Low-motile sperm populations
Source: BMC Genomics. 2017 Jan 4;18:14. doi: 10.1186/s12864-016-3394-7 (PMC5209821; doi:10.1186/s12864-016-3394-7)
Supplement: Additional file 3: — Details for each piRNA clusters found in High Motile (HM) sperm fraction. Genes, repeats, transposable elements and transcription factors binding sites falling within the cluster regions were reported. (ZIP 1896 kb) [file 12864_2016_3394_MOESM3_ESM.zip › 62.html]

piRNA cluster 62


Predicted piRNA cluster no. 62     previous   next
  

Show proTRAC run info
Hide proTRAC run info

================================= proTRAC ====================================  
VERSION: 2.1                                    LAST MODIFIED: 06. October 2015  
  
Please cite:  
Rosenkranz D, Zischler H. proTRAC - a software for probabilistic piRNA cluster  
detection, visualization and analysis. 2012. BMC Bioinformatics 13:5.  
  
and (for proTRAC 2.0 and later):  
Rosenkranz D, Rudloff S, Bastuck K, Ketting RF, Zischler H. Tupaia small RNAs  
provide insights into function and evolution of RNAi-based transposon defense  
in mammals. 2015. RNA 21(5):911-922.  
  
Contact:  
David Rosenkranz  
Institute of Anthropology, small RNA group  
Johannes Gutenberg University Mainz  
email: rosenkranz@uni-mainz.de  
  
You can find the latest proTRAC version at:  
http://sourceforge.net/projects/protrac/files  
http://www.smallRNAgroup-mainz.de/software  
==============================================================================  
  
PARAMETERS:  
Map file: .............../storage/core/barbara/genhome/smallRNA/fertility/Sample\_motile/pirna/Sample\_motile\_26-33\_collapsed.fa.no-dust.map.weighted-10000-1000-b-0  
Genome file: ............/storage/core/barbara/genhome/smallRNA/fertility/Sample\_all/pirna/bt\_311\_chrY.fa  
RepeatMasker annotation: /storage/genomes/bt\_umd31/GCF\_000003055.6\_Bos\_taurus\_UMD\_3.1.1\_repeatMasker\_chr.out  
GeneSet:................./storage/core/barbara/genhome/smallRNA/fertility/Sample\_all/pirna/full.gtf  
  
Significant (p<=0.01) hit density will be calculated based  
on observed hit distribution.  
  
Sliding window size: ........................................ 5000 bp  
Sliding window increament: .................................. 1000 bp  
Normalize each hit by number of genomic hits: ............... 1 [0=no/1=yes]  
Normalize each hit by number of sequence reads: ............. 1 [0=no/1=yes]  
Normalize values (-> per million mapped reads): ............. 1 [0=no/1=yes]  
Min. fraction of hits with 1T(U) or 10A: .................... 0.75  
Alternatively: Min. fraction of hits with 1T(U) and 10A: .... 0.5  
Min. fraction of hits with typical piRNA length: ............ 0.75  
Typical piRNA length: ....................................... 26-33 nt  
Min. size of a piRNA cluster: ............................... 5000 bp.  
Min. number of hits (absolute): ............................. 0  
Min. number of hits (normalized): ........................... 0  
Min. fraction of hits on the mainstrand: .................... 0.75  
Top fraction of mapped sequences (in terms of read counts): . 1%  
Top fraction accounts for max. n% of sequence reads: ........ 90%  
Min. fraction of hits on each arm of a bidirectional cluster: 0.1  
Output image file for each cluster: ......................... 0 [0=no/1=yes]  
Output html file for each cluster: .......................... 1 [0=no/1=yes]  
Output a summary table: ..................................... 1 [0=no/1=yes]  
Output a FASTA file for each cluster (piRNA sequences): ..... 1 [0=no/1=yes]  
Output a FASTA file comprising cluster sequences: ........... 1 [0=no/1=yes]  
Search DNA motifs in clusters: .............................. 1 [0=no/1=yes]  
Output flanking sequences: +/- .............................. 0 bp  
Output ~.pTi file: .......................................... 1 [0=no/1=yes]  
==============================================================================  
  
  
Genome size (without gaps): ............ 2678902517 bp  
Gaps (N/X/-): .......................... 53837044 bp  
Mapped reads: .......................... 658825247023  
Non-identical sequences: ............... 514171  
Genomic hits: .......................... 764233  
Significant densitiy of mapped reads: .. 12867599.5173724 reads/kb

Show proTRAC cluster info
Hide proTRAC cluster info

|  |  |
| --- | --- |
| Location | chr25 |
| Coordinates | 35548826-35556002 |
| Size [bp] | 7177 |
| Sequence hit loci | 61 |
| Mapped reads (normalized) | 86923259 |
| Mapped reads (normalized) per kb | 12111363.9 |
| Normalized reads with 1T (1U) | 84.2% |
| Normalized reads with 10A | 20.2% |
| Normalized reads with length 26-33 nt | 100% |
| Normalized reads on the main strand(s) | 100% |
| Predicted directionality | mono:minus |

100%

0%

1T (1U)  
reads

10A reads

26-33 nt  
reads

reads on mainstrand

**Either the amount of reads with 1T (1U) OR 10A has to exceed 75% (set with option: -1Tor10A)  
Alternatively the amount of reads with 1T (1U) AND 10A has to exceed 50% (set with option: -1Tand10A)  
Minimum amount of reads with preferred size is 75% (set with option: -pisize)  
Minimum amount of reads on the main strand(s) is 75% (set with option: -clstrand)**

Show read coverage
Hide read coverage

WHAT DO I SEE HERE?  
This chart shows the location of mapped sequence reads within a predicted piRNA cluster. The color refers to the number of genomic hits produced by the sequence read in question. A dark red bar indicates that this sequence read produces many other hits elsewhere in the genome. Many adjacent red or yellow bars can indicate the presence of a multi-copy element such as transposons or rRNA genes. A dark green bar indicates that this sequence read maps uniquely to this locus.

1 hit

2-5 hits

6-10 hits

11-20 hits

21-50 hits

51-100 hits

> 100 hits

chr25

35548826

35556002

Gene Set

RepeatMasker

Mapped  
Reads

10.93

plus strand

minus strand

10.93

Region: chr25 34158594-35548833. Max. coverage (+): 0. Max coverage (-): 4.53

Region: chr25 35548834-35548847. Max. coverage (+): 0. Max coverage (-): 0

Region: chr25 35548848-35548861. Max. coverage (+): 0. Max coverage (-): 0

Region: chr25 35548862-35548876. Max. coverage (+): 0. Max coverage (-): 0

Region: chr25 35548877-35548890. Max. coverage (+): 0. Max coverage (-): 0

Region: chr25 35548891-35548904. Max. coverage (+): 0. Max coverage (-): 0

Region: chr25 35548905-35548919. Max. coverage (+): 0. Max coverage (-): 0

Region: chr25 35548920-35548933. Max. coverage (+): 0. Max coverage (-): 0

Region: chr25 35548934-35548948. Max. coverage (+): 0. Max coverage (-): 0

Region: chr25 35548949-35548962. Max. coverage (+): 0. Max coverage (-): 0

Region: chr25 35548963-35548976. Max. coverage (+): 0. Max coverage (-): 0

Region: chr25 35548977-35548991. Max. coverage (+): 0. Max coverage (-): 0

Region: chr25 35548992-35549005. Max. coverage (+): 0. Max coverage (-): 0

Region: chr25 35549006-35549019. Max. coverage (+): 0. Max coverage (-): 0

Region: chr25 35549020-35549034. Max. coverage (+): 0. Max coverage (-): 0

Region: chr25 35549035-35549048. Max. coverage (+): 0. Max coverage (-): 0

Region: chr25 35549049-35549062. Max. coverage (+): 0. Max coverage (-): 0

Region: chr25 35549063-35549077. Max. coverage (+): 0. Max coverage (-): 0

Region: chr25 35549078-35549091. Max. coverage (+): 0. Max coverage (-): 0

Region: chr25 35549092-35549105. Max. coverage (+): 0. Max coverage (-): 0

Region: chr25 35549106-35549120. Max. coverage (+): 0. Max coverage (-): 0

Region: chr25 35549121-35549134. Max. coverage (+): 0. Max coverage (-): 0

Region: chr25 35549135-35549148. Max. coverage (+): 0. Max coverage (-): 2.03

Region: chr25 35549149-35549163. Max. coverage (+): 0. Max coverage (-): 2.03

Region: chr25 35549164-35549177. Max. coverage (+): 0. Max coverage (-): 0

Region: chr25 35549178-35549192. Max. coverage (+): 0. Max coverage (-): 0

Region: chr25 35549193-35549206. Max. coverage (+): 0. Max coverage (-): 0

Region: chr25 35549207-35549220. Max. coverage (+): 0. Max coverage (-): 0

Region: chr25 35549221-35549235. Max. coverage (+): 0. Max coverage (-): 0

Region: chr25 35549236-35549249. Max. coverage (+): 0. Max coverage (-): 0

Region: chr25 35549250-35549263. Max. coverage (+): 0. Max coverage (-): 0

Region: chr25 35549264-35549278. Max. coverage (+): 0. Max coverage (-): 0

Region: chr25 35549279-35549292. Max. coverage (+): 0. Max coverage (-): 0

Region: chr25 35549293-35549306. Max. coverage (+): 0. Max coverage (-): 0

Region: chr25 35549307-35549321. Max. coverage (+): 0. Max coverage (-): 0

Region: chr25 35549322-35549335. Max. coverage (+): 0. Max coverage (-): 5.02

Region: chr25 35549336-35549349. Max. coverage (+): 0. Max coverage (-): 10.12

Region: chr25 35549350-35549364. Max. coverage (+): 0. Max coverage (-): 2.21

Region: chr25 35549365-35549378. Max. coverage (+): 0. Max coverage (-): 0

Region: chr25 35549379-35549392. Max. coverage (+): 0. Max coverage (-): 0

Region: chr25 35549393-35549407. Max. coverage (+): 0. Max coverage (-): 0

Region: chr25 35549408-35549421. Max. coverage (+): 0. Max coverage (-): 0

Region: chr25 35549422-35549436. Max. coverage (+): 0. Max coverage (-): 0

Region: chr25 35549437-35549450. Max. coverage (+): 0. Max coverage (-): 0

Region: chr25 35549451-35549464. Max. coverage (+): 0. Max coverage (-): 0

Region: chr25 35549465-35549479. Max. coverage (+): 0. Max coverage (-): 0

Region: chr25 35549480-35549493. Max. coverage (+): 0. Max coverage (-): 0

Region: chr25 35549494-35549507. Max. coverage (+): 0. Max coverage (-): 0

Region: chr25 35549508-35549522. Max. coverage (+): 0. Max coverage (-): 0

Region: chr25 35549523-35549536. Max. coverage (+): 0. Max coverage (-): 0

Region: chr25 35549537-35549550. Max. coverage (+): 0. Max coverage (-): 0

Region: chr25 35549551-35549565. Max. coverage (+): 0. Max coverage (-): 0

Region: chr25 35549566-35549579. Max. coverage (+): 0. Max coverage (-): 0

Region: chr25 35549580-35549593. Max. coverage (+): 0. Max coverage (-): 0

Region: chr25 35549594-35549608. Max. coverage (+): 0. Max coverage (-): 0

Region: chr25 35549609-35549622. Max. coverage (+): 0. Max coverage (-): 0

Region: chr25 35549623-35549637. Max. coverage (+): 0. Max coverage (-): 0

Region: chr25 35549638-35549651. Max. coverage (+): 0. Max coverage (-): 0

Region: chr25 35549652-35549665. Max. coverage (+): 0. Max coverage (-): 0

Region: chr25 35549666-35549680. Max. coverage (+): 0. Max coverage (-): 0

Region: chr25 35549681-35549694. Max. coverage (+): 0. Max coverage (-): 0

Region: chr25 35549695-35549708. Max. coverage (+): 0. Max coverage (-): 0

Region: chr25 35549709-35549723. Max. coverage (+): 0. Max coverage (-): 0

Region: chr25 35549724-35549737. Max. coverage (+): 0. Max coverage (-): 0

Region: chr25 35549738-35549751. Max. coverage (+): 0. Max coverage (-): 0

Region: chr25 35549752-35549766. Max. coverage (+): 0. Max coverage (-): 0

Region: chr25 35549767-35549780. Max. coverage (+): 0. Max coverage (-): 0

Region: chr25 35549781-35549794. Max. coverage (+): 0. Max coverage (-): 0

Region: chr25 35549795-35549809. Max. coverage (+): 0. Max coverage (-): 0

Region: chr25 35549810-35549823. Max. coverage (+): 0. Max coverage (-): 0

Region: chr25 35549824-35549837. Max. coverage (+): 0. Max coverage (-): 5

Region: chr25 35549838-35549852. Max. coverage (+): 0. Max coverage (-): 0

Region: chr25 35549853-35549866. Max. coverage (+): 0. Max coverage (-): 0

Region: chr25 35549867-35549881. Max. coverage (+): 0. Max coverage (-): 0

Region: chr25 35549882-35549895. Max. coverage (+): 0. Max coverage (-): 0

Region: chr25 35549896-35549909. Max. coverage (+): 0. Max coverage (-): 0

Region: chr25 35549910-35549924. Max. coverage (+): 0. Max coverage (-): 0

Region: chr25 35549925-35549938. Max. coverage (+): 0. Max coverage (-): 0

Region: chr25 35549939-35549952. Max. coverage (+): 0. Max coverage (-): 0

Region: chr25 35549953-35549967. Max. coverage (+): 0. Max coverage (-): 0

Region: chr25 35549968-35549981. Max. coverage (+): 0. Max coverage (-): 0

Region: chr25 35549982-35549995. Max. coverage (+): 0. Max coverage (-): 0

Region: chr25 35549996-35550010. Max. coverage (+): 0. Max coverage (-): 0

Region: chr25 35550011-35550024. Max. coverage (+): 0. Max coverage (-): 0

Region: chr25 35550025-35550038. Max. coverage (+): 0. Max coverage (-): 0

Region: chr25 35550039-35550053. Max. coverage (+): 0. Max coverage (-): 0

Region: chr25 35550054-35550067. Max. coverage (+): 0. Max coverage (-): 0

Region: chr25 35550068-35550081. Max. coverage (+): 0. Max coverage (-): 0

Region: chr25 35550082-35550096. Max. coverage (+): 0. Max coverage (-): 0

Region: chr25 35550097-35550110. Max. coverage (+): 0. Max coverage (-): 0

Region: chr25 35550111-35550125. Max. coverage (+): 0. Max coverage (-): 0

Region: chr25 35550126-35550139. Max. coverage (+): 0. Max coverage (-): 0

Region: chr25 35550140-35550153. Max. coverage (+): 0. Max coverage (-): 0

Region: chr25 35550154-35550168. Max. coverage (+): 0. Max coverage (-): 0

Region: chr25 35550169-35550182. Max. coverage (+): 0. Max coverage (-): 0

Region: chr25 35550183-35550196. Max. coverage (+): 0. Max coverage (-): 0

Region: chr25 35550197-35550211. Max. coverage (+): 0. Max coverage (-): 0

Region: chr25 35550212-35550225. Max. coverage (+): 0. Max coverage (-): 0

Region: chr25 35550226-35550239. Max. coverage (+): 0. Max coverage (-): 0

Region: chr25 35550240-35550254. Max. coverage (+): 0. Max coverage (-): 0

Region: chr25 35550255-35550268. Max. coverage (+): 0. Max coverage (-): 0

Region: chr25 35550269-35550282. Max. coverage (+): 0. Max coverage (-): 0

Region: chr25 35550283-35550297. Max. coverage (+): 0. Max coverage (-): 0

Region: chr25 35550298-35550311. Max. coverage (+): 0. Max coverage (-): 0

Region: chr25 35550312-35550325. Max. coverage (+): 0. Max coverage (-): 0

Region: chr25 35550326-35550340. Max. coverage (+): 0. Max coverage (-): 0

Region: chr25 35550341-35550354. Max. coverage (+): 0. Max coverage (-): 0

Region: chr25 35550355-35550369. Max. coverage (+): 0. Max coverage (-): 0

Region: chr25 35550370-35550383. Max. coverage (+): 0. Max coverage (-): 0

Region: chr25 35550384-35550397. Max. coverage (+): 0. Max coverage (-): 0

Region: chr25 35550398-35550412. Max. coverage (+): 0. Max coverage (-): 0

Region: chr25 35550413-35550426. Max. coverage (+): 0. Max coverage (-): 0

Region: chr25 35550427-35550440. Max. coverage (+): 0. Max coverage (-): 0

Region: chr25 35550441-35550455. Max. coverage (+): 0. Max coverage (-): 0

Region: chr25 35550456-35550469. Max. coverage (+): 0. Max coverage (-): 0

Region: chr25 35550470-35550483. Max. coverage (+): 0. Max coverage (-): 0

Region: chr25 35550484-35550498. Max. coverage (+): 0. Max coverage (-): 0

Region: chr25 35550499-35550512. Max. coverage (+): 0. Max coverage (-): 0

Region: chr25 35550513-35550526. Max. coverage (+): 0. Max coverage (-): 0

Region: chr25 35550527-35550541. Max. coverage (+): 0. Max coverage (-): 0

Region: chr25 35550542-35550555. Max. coverage (+): 0. Max coverage (-): 0

Region: chr25 35550556-35550570. Max. coverage (+): 0. Max coverage (-): 0

Region: chr25 35550571-35550584. Max. coverage (+): 0. Max coverage (-): 0

Region: chr25 35550585-35550598. Max. coverage (+): 0. Max coverage (-): 2.79

Region: chr25 35550599-35550613. Max. coverage (+): 0. Max coverage (-): 4.78

Region: chr25 35550614-35550627. Max. coverage (+): 0. Max coverage (-): 0

Region: chr25 35550628-35550641. Max. coverage (+): 0. Max coverage (-): 0

Region: chr25 35550642-35550656. Max. coverage (+): 0. Max coverage (-): 0

Region: chr25 35550657-35550670. Max. coverage (+): 0. Max coverage (-): 0

Region: chr25 35550671-35550684. Max. coverage (+): 0. Max coverage (-): 0

Region: chr25 35550685-35550699. Max. coverage (+): 0. Max coverage (-): 0

Region: chr25 35550700-35550713. Max. coverage (+): 0. Max coverage (-): 0

Region: chr25 35550714-35550727. Max. coverage (+): 0. Max coverage (-): 0

Region: chr25 35550728-35550742. Max. coverage (+): 0. Max coverage (-): 0

Region: chr25 35550743-35550756. Max. coverage (+): 0. Max coverage (-): 0

Region: chr25 35550757-35550770. Max. coverage (+): 0. Max coverage (-): 0

Region: chr25 35550771-35550785. Max. coverage (+): 0. Max coverage (-): 0

Region: chr25 35550786-35550799. Max. coverage (+): 0. Max coverage (-): 0

Region: chr25 35550800-35550814. Max. coverage (+): 0. Max coverage (-): 0

Region: chr25 35550815-35550828. Max. coverage (+): 0. Max coverage (-): 0

Region: chr25 35550829-35550842. Max. coverage (+): 0. Max coverage (-): 0

Region: chr25 35550843-35550857. Max. coverage (+): 0. Max coverage (-): 0

Region: chr25 35550858-35550871. Max. coverage (+): 0. Max coverage (-): 0

Region: chr25 35550872-35550885. Max. coverage (+): 0. Max coverage (-): 0

Region: chr25 35550886-35550900. Max. coverage (+): 0. Max coverage (-): 0

Region: chr25 35550901-35550914. Max. coverage (+): 0. Max coverage (-): 0

Region: chr25 35550915-35550928. Max. coverage (+): 0. Max coverage (-): 0

Region: chr25 35550929-35550943. Max. coverage (+): 0. Max coverage (-): 0

Region: chr25 35550944-35550957. Max. coverage (+): 0. Max coverage (-): 0

Region: chr25 35550958-35550971. Max. coverage (+): 0. Max coverage (-): 0

Region: chr25 35550972-35550986. Max. coverage (+): 0. Max coverage (-): 0

Region: chr25 35550987-35551000. Max. coverage (+): 0. Max coverage (-): 0

Region: chr25 35551001-35551014. Max. coverage (+): 0. Max coverage (-): 0

Region: chr25 35551015-35551029. Max. coverage (+): 0. Max coverage (-): 0

Region: chr25 35551030-35551043. Max. coverage (+): 0. Max coverage (-): 0

Region: chr25 35551044-35551058. Max. coverage (+): 0. Max coverage (-): 0

Region: chr25 35551059-35551072. Max. coverage (+): 0. Max coverage (-): 0

Region: chr25 35551073-35551086. Max. coverage (+): 0. Max coverage (-): 0

Region: chr25 35551087-35551101. Max. coverage (+): 0. Max coverage (-): 0

Region: chr25 35551102-35551115. Max. coverage (+): 0. Max coverage (-): 3.71

Region: chr25 35551116-35551129. Max. coverage (+): 0. Max coverage (-): 3.71

Region: chr25 35551130-35551144. Max. coverage (+): 0. Max coverage (-): 0

Region: chr25 35551145-35551158. Max. coverage (+): 0. Max coverage (-): 0

Region: chr25 35551159-35551172. Max. coverage (+): 0. Max coverage (-): 0

Region: chr25 35551173-35551187. Max. coverage (+): 0. Max coverage (-): 0

Region: chr25 35551188-35551201. Max. coverage (+): 0. Max coverage (-): 0

Region: chr25 35551202-35551215. Max. coverage (+): 0. Max coverage (-): 0

Region: chr25 35551216-35551230. Max. coverage (+): 0. Max coverage (-): 1.52

Region: chr25 35551231-35551244. Max. coverage (+): 0. Max coverage (-): 1.52

Region: chr25 35551245-35551259. Max. coverage (+): 0. Max coverage (-): 0

Region: chr25 35551260-35551273. Max. coverage (+): 0. Max coverage (-): 0

Region: chr25 35551274-35551287. Max. coverage (+): 0. Max coverage (-): 0

Region: chr25 35551288-35551302. Max. coverage (+): 0. Max coverage (-): 0

Region: chr25 35551303-35551316. Max. coverage (+): 0. Max coverage (-): 0

Region: chr25 35551317-35551330. Max. coverage (+): 0. Max coverage (-): 0

Region: chr25 35551331-35551345. Max. coverage (+): 0. Max coverage (-): 3.62

Region: chr25 35551346-35551359. Max. coverage (+): 0. Max coverage (-): 3.62

Region: chr25 35551360-35551373. Max. coverage (+): 0. Max coverage (-): 0

Region: chr25 35551374-35551388. Max. coverage (+): 0. Max coverage (-): 0

Region: chr25 35551389-35551402. Max. coverage (+): 0. Max coverage (-): 0

Region: chr25 35551403-35551416. Max. coverage (+): 0. Max coverage (-): 0

Region: chr25 35551417-35551431. Max. coverage (+): 0. Max coverage (-): 0

Region: chr25 35551432-35551445. Max. coverage (+): 0. Max coverage (-): 0

Region: chr25 35551446-35551459. Max. coverage (+): 0. Max coverage (-): 0

Region: chr25 35551460-35551474. Max. coverage (+): 0. Max coverage (-): 0

Region: chr25 35551475-35551488. Max. coverage (+): 0. Max coverage (-): 0

Region: chr25 35551489-35551503. Max. coverage (+): 0. Max coverage (-): 0

Region: chr25 35551504-35551517. Max. coverage (+): 0. Max coverage (-): 0

Region: chr25 35551518-35551531. Max. coverage (+): 0. Max coverage (-): 1.19

Region: chr25 35551532-35551546. Max. coverage (+): 0. Max coverage (-): 2.22

Region: chr25 35551547-35551560. Max. coverage (+): 0. Max coverage (-): 1.96

Region: chr25 35551561-35551574. Max. coverage (+): 0. Max coverage (-): 1.51

Region: chr25 35551575-35551589. Max. coverage (+): 0. Max coverage (-): 0

Region: chr25 35551590-35551603. Max. coverage (+): 0. Max coverage (-): 0

Region: chr25 35551604-35551617. Max. coverage (+): 0. Max coverage (-): 0

Region: chr25 35551618-35551632. Max. coverage (+): 0. Max coverage (-): 0

Region: chr25 35551633-35551646. Max. coverage (+): 0. Max coverage (-): 0

Region: chr25 35551647-35551660. Max. coverage (+): 0. Max coverage (-): 3.63

Region: chr25 35551661-35551675. Max. coverage (+): 0. Max coverage (-): 8.01

Region: chr25 35551676-35551689. Max. coverage (+): 0. Max coverage (-): 1.7

Region: chr25 35551690-35551703. Max. coverage (+): 0. Max coverage (-): 1.3

Region: chr25 35551704-35551718. Max. coverage (+): 0. Max coverage (-): 2.95

Region: chr25 35551719-35551732. Max. coverage (+): 0. Max coverage (-): 6.69

Region: chr25 35551733-35551747. Max. coverage (+): 0. Max coverage (-): 0

Region: chr25 35551748-35551761. Max. coverage (+): 0. Max coverage (-): 0

Region: chr25 35551762-35551775. Max. coverage (+): 0. Max coverage (-): 0

Region: chr25 35551776-35551790. Max. coverage (+): 0. Max coverage (-): 0

Region: chr25 35551791-35551804. Max. coverage (+): 0. Max coverage (-): 0

Region: chr25 35551805-35551818. Max. coverage (+): 0. Max coverage (-): 0

Region: chr25 35551819-35551833. Max. coverage (+): 0. Max coverage (-): 0

Region: chr25 35551834-35551847. Max. coverage (+): 0. Max coverage (-): 0

Region: chr25 35551848-35551861. Max. coverage (+): 0. Max coverage (-): 0

Region: chr25 35551862-35551876. Max. coverage (+): 0. Max coverage (-): 4

Region: chr25 35551877-35551890. Max. coverage (+): 0. Max coverage (-): 4.76

Region: chr25 35551891-35551904. Max. coverage (+): 0. Max coverage (-): 4.76

Region: chr25 35551905-35551919. Max. coverage (+): 0. Max coverage (-): 0

Region: chr25 35551920-35551933. Max. coverage (+): 0. Max coverage (-): 0

Region: chr25 35551934-35551947. Max. coverage (+): 0. Max coverage (-): 0

Region: chr25 35551948-35551962. Max. coverage (+): 0. Max coverage (-): 0

Region: chr25 35551963-35551976. Max. coverage (+): 0. Max coverage (-): 0

Region: chr25 35551977-35551991. Max. coverage (+): 0. Max coverage (-): 4.78

Region: chr25 35551992-35552005. Max. coverage (+): 0. Max coverage (-): 0

Region: chr25 35552006-35552019. Max. coverage (+): 0. Max coverage (-): 0

Region: chr25 35552020-35552034. Max. coverage (+): 0. Max coverage (-): 0

Region: chr25 35552035-35552048. Max. coverage (+): 0. Max coverage (-): 0

Region: chr25 35552049-35552062. Max. coverage (+): 0. Max coverage (-): 0

Region: chr25 35552063-35552077. Max. coverage (+): 0. Max coverage (-): 0

Region: chr25 35552078-35552091. Max. coverage (+): 0. Max coverage (-): 0

Region: chr25 35552092-35552105. Max. coverage (+): 0. Max coverage (-): 0

Region: chr25 35552106-35552120. Max. coverage (+): 0. Max coverage (-): 0

Region: chr25 35552121-35552134. Max. coverage (+): 0. Max coverage (-): 0

Region: chr25 35552135-35552148. Max. coverage (+): 0. Max coverage (-): 0

Region: chr25 35552149-35552163. Max. coverage (+): 0. Max coverage (-): 0

Region: chr25 35552164-35552177. Max. coverage (+): 0. Max coverage (-): 0

Region: chr25 35552178-35552192. Max. coverage (+): 0. Max coverage (-): 10.93

Region: chr25 35552193-35552206. Max. coverage (+): 0. Max coverage (-): 10.93

Region: chr25 35552207-35552220. Max. coverage (+): 0. Max coverage (-): 0

Region: chr25 35552221-35552235. Max. coverage (+): 0. Max coverage (-): 1.73

Region: chr25 35552236-35552249. Max. coverage (+): 0. Max coverage (-): 1.19

Region: chr25 35552250-35552263. Max. coverage (+): 0. Max coverage (-): 0

Region: chr25 35552264-35552278. Max. coverage (+): 0. Max coverage (-): 0

Region: chr25 35552279-35552292. Max. coverage (+): 0. Max coverage (-): 0

Region: chr25 35552293-35552306. Max. coverage (+): 0. Max coverage (-): 0

Region: chr25 35552307-35552321. Max. coverage (+): 0. Max coverage (-): 1.43

Region: chr25 35552322-35552335. Max. coverage (+): 0. Max coverage (-): 1.43

Region: chr25 35552336-35552349. Max. coverage (+): 0. Max coverage (-): 0

Region: chr25 35552350-35552364. Max. coverage (+): 0. Max coverage (-): 0

Region: chr25 35552365-35552378. Max. coverage (+): 0. Max coverage (-): 0

Region: chr25 35552379-35552392. Max. coverage (+): 0. Max coverage (-): 0

Region: chr25 35552393-35552407. Max. coverage (+): 0. Max coverage (-): 0

Region: chr25 35552408-35552421. Max. coverage (+): 0. Max coverage (-): 0

Region: chr25 35552422-35552436. Max. coverage (+): 0. Max coverage (-): 0

Region: chr25 35552437-35552450. Max. coverage (+): 0. Max coverage (-): 0

Region: chr25 35552451-35552464. Max. coverage (+): 0. Max coverage (-): 0

Region: chr25 35552465-35552479. Max. coverage (+): 0. Max coverage (-): 0

Region: chr25 35552480-35552493. Max. coverage (+): 0. Max coverage (-): 0

Region: chr25 35552494-35552507. Max. coverage (+): 0. Max coverage (-): 0

Region: chr25 35552508-35552522. Max. coverage (+): 0. Max coverage (-): 1.22

Region: chr25 35552523-35552536. Max. coverage (+): 0. Max coverage (-): 0

Region: chr25 35552537-35552550. Max. coverage (+): 0. Max coverage (-): 0

Region: chr25 35552551-35552565. Max. coverage (+): 0. Max coverage (-): 0

Region: chr25 35552566-35552579. Max. coverage (+): 0. Max coverage (-): 0

Region: chr25 35552580-35552593. Max. coverage (+): 0. Max coverage (-): 0

Region: chr25 35552594-35552608. Max. coverage (+): 0. Max coverage (-): 0

Region: chr25 35552609-35552622. Max. coverage (+): 0. Max coverage (-): 6.68

Region: chr25 35552623-35552636. Max. coverage (+): 0. Max coverage (-): 0

Region: chr25 35552637-35552651. Max. coverage (+): 0. Max coverage (-): 0

Region: chr25 35552652-35552665. Max. coverage (+): 0. Max coverage (-): 0

Region: chr25 35552666-35552680. Max. coverage (+): 0. Max coverage (-): 0

Region: chr25 35552681-35552694. Max. coverage (+): 0. Max coverage (-): 0

Region: chr25 35552695-35552708. Max. coverage (+): 0. Max coverage (-): 0

Region: chr25 35552709-35552723. Max. coverage (+): 0. Max coverage (-): 0

Region: chr25 35552724-35552737. Max. coverage (+): 0. Max coverage (-): 0

Region: chr25 35552738-35552751. Max. coverage (+): 0. Max coverage (-): 0

Region: chr25 35552752-35552766. Max. coverage (+): 0. Max coverage (-): 0

Region: chr25 35552767-35552780. Max. coverage (+): 0. Max coverage (-): 0

Region: chr25 35552781-35552794. Max. coverage (+): 0. Max coverage (-): 0

Region: chr25 35552795-35552809. Max. coverage (+): 0. Max coverage (-): 0

Region: chr25 35552810-35552823. Max. coverage (+): 0. Max coverage (-): 0

Region: chr25 35552824-35552837. Max. coverage (+): 0. Max coverage (-): 0

Region: chr25 35552838-35552852. Max. coverage (+): 0. Max coverage (-): 0

Region: chr25 35552853-35552866. Max. coverage (+): 0. Max coverage (-): 0

Region: chr25 35552867-35552881. Max. coverage (+): 0. Max coverage (-): 0

Region: chr25 35552882-35552895. Max. coverage (+): 0. Max coverage (-): 0

Region: chr25 35552896-35552909. Max. coverage (+): 0. Max coverage (-): 0

Region: chr25 35552910-35552924. Max. coverage (+): 0. Max coverage (-): 0

Region: chr25 35552925-35552938. Max. coverage (+): 0. Max coverage (-): 0

Region: chr25 35552939-35552952. Max. coverage (+): 0. Max coverage (-): 0

Region: chr25 35552953-35552967. Max. coverage (+): 0. Max coverage (-): 0

Region: chr25 35552968-35552981. Max. coverage (+): 0. Max coverage (-): 0

Region: chr25 35552982-35552995. Max. coverage (+): 0. Max coverage (-): 0

Region: chr25 35552996-35553010. Max. coverage (+): 0. Max coverage (-): 0

Region: chr25 35553011-35553024. Max. coverage (+): 0. Max coverage (-): 0

Region: chr25 35553025-35553038. Max. coverage (+): 0. Max coverage (-): 0

Region: chr25 35553039-35553053. Max. coverage (+): 0. Max coverage (-): 5.34

Region: chr25 35553054-35553067. Max. coverage (+): 0. Max coverage (-): 5.34

Region: chr25 35553068-35553081. Max. coverage (+): 0. Max coverage (-): 0

Region: chr25 35553082-35553096. Max. coverage (+): 0. Max coverage (-): 0

Region: chr25 35553097-35553110. Max. coverage (+): 0. Max coverage (-): 1.81

Region: chr25 35553111-35553125. Max. coverage (+): 0. Max coverage (-): 1.81

Region: chr25 35553126-35553139. Max. coverage (+): 0. Max coverage (-): 0

Region: chr25 35553140-35553153. Max. coverage (+): 0. Max coverage (-): 0

Region: chr25 35553154-35553168. Max. coverage (+): 0. Max coverage (-): 0

Region: chr25 35553169-35553182. Max. coverage (+): 0. Max coverage (-): 0

Region: chr25 35553183-35553196. Max. coverage (+): 0. Max coverage (-): 0

Region: chr25 35553197-35553211. Max. coverage (+): 0. Max coverage (-): 0

Region: chr25 35553212-35553225. Max. coverage (+): 0. Max coverage (-): 0

Region: chr25 35553226-35553239. Max. coverage (+): 0. Max coverage (-): 0

Region: chr25 35553240-35553254. Max. coverage (+): 0. Max coverage (-): 0

Region: chr25 35553255-35553268. Max. coverage (+): 0. Max coverage (-): 0

Region: chr25 35553269-35553282. Max. coverage (+): 0. Max coverage (-): 0

Region: chr25 35553283-35553297. Max. coverage (+): 0. Max coverage (-): 0

Region: chr25 35553298-35553311. Max. coverage (+): 0. Max coverage (-): 0

Region: chr25 35553312-35553325. Max. coverage (+): 0. Max coverage (-): 0

Region: chr25 35553326-35553340. Max. coverage (+): 0. Max coverage (-): 0

Region: chr25 35553341-35553354. Max. coverage (+): 0. Max coverage (-): 0

Region: chr25 35553355-35553369. Max. coverage (+): 0. Max coverage (-): 0

Region: chr25 35553370-35553383. Max. coverage (+): 0. Max coverage (-): 0

Region: chr25 35553384-35553397. Max. coverage (+): 0. Max coverage (-): 0

Region: chr25 35553398-35553412. Max. coverage (+): 0. Max coverage (-): 0

Region: chr25 35553413-35553426. Max. coverage (+): 0. Max coverage (-): 0

Region: chr25 35553427-35553440. Max. coverage (+): 0. Max coverage (-): 0

Region: chr25 35553441-35553455. Max. coverage (+): 0. Max coverage (-): 0

Region: chr25 35553456-35553469. Max. coverage (+): 0. Max coverage (-): 0

Region: chr25 35553470-35553483. Max. coverage (+): 0. Max coverage (-): 0

Region: chr25 35553484-35553498. Max. coverage (+): 0. Max coverage (-): 0

Region: chr25 35553499-35553512. Max. coverage (+): 0. Max coverage (-): 0

Region: chr25 35553513-35553526. Max. coverage (+): 0. Max coverage (-): 0

Region: chr25 35553527-35553541. Max. coverage (+): 0. Max coverage (-): 0

Region: chr25 35553542-35553555. Max. coverage (+): 0. Max coverage (-): 0

Region: chr25 35553556-35553569. Max. coverage (+): 0. Max coverage (-): 0

Region: chr25 35553570-35553584. Max. coverage (+): 0. Max coverage (-): 0

Region: chr25 35553585-35553598. Max. coverage (+): 0. Max coverage (-): 0

Region: chr25 35553599-35553613. Max. coverage (+): 0. Max coverage (-): 0

Region: chr25 35553614-35553627. Max. coverage (+): 0. Max coverage (-): 0

Region: chr25 35553628-35553641. Max. coverage (+): 0. Max coverage (-): 0

Region: chr25 35553642-35553656. Max. coverage (+): 0. Max coverage (-): 0

Region: chr25 35553657-35553670. Max. coverage (+): 0. Max coverage (-): 0

Region: chr25 35553671-35553684. Max. coverage (+): 0. Max coverage (-): 0

Region: chr25 35553685-35553699. Max. coverage (+): 0. Max coverage (-): 0

Region: chr25 35553700-35553713. Max. coverage (+): 0. Max coverage (-): 0

Region: chr25 35553714-35553727. Max. coverage (+): 0. Max coverage (-): 0

Region: chr25 35553728-35553742. Max. coverage (+): 0. Max coverage (-): 0

Region: chr25 35553743-35553756. Max. coverage (+): 0. Max coverage (-): 0

Region: chr25 35553757-35553770. Max. coverage (+): 0. Max coverage (-): 0

Region: chr25 35553771-35553785. Max. coverage (+): 0. Max coverage (-): 0

Region: chr25 35553786-35553799. Max. coverage (+): 0. Max coverage (-): 0

Region: chr25 35553800-35553814. Max. coverage (+): 0. Max coverage (-): 0

Region: chr25 35553815-35553828. Max. coverage (+): 0. Max coverage (-): 1.48

Region: chr25 35553829-35553842. Max. coverage (+): 0. Max coverage (-): 0

Region: chr25 35553843-35553857. Max. coverage (+): 0. Max coverage (-): 0

Region: chr25 35553858-35553871. Max. coverage (+): 0. Max coverage (-): 0

Region: chr25 35553872-35553885. Max. coverage (+): 0. Max coverage (-): 0

Region: chr25 35553886-35553900. Max. coverage (+): 0. Max coverage (-): 0

Region: chr25 35553901-35553914. Max. coverage (+): 0. Max coverage (-): 0

Region: chr25 35553915-35553928. Max. coverage (+): 0. Max coverage (-): 0

Region: chr25 35553929-35553943. Max. coverage (+): 0. Max coverage (-): 0

Region: chr25 35553944-35553957. Max. coverage (+): 0. Max coverage (-): 0

Region: chr25 35553958-35553971. Max. coverage (+): 0. Max coverage (-): 0

Region: chr25 35553972-35553986. Max. coverage (+): 0. Max coverage (-): 0

Region: chr25 35553987-35554000. Max. coverage (+): 0. Max coverage (-): 0

Region: chr25 35554001-35554014. Max. coverage (+): 0. Max coverage (-): 0

Region: chr25 35554015-35554029. Max. coverage (+): 0. Max coverage (-): 0

Region: chr25 35554030-35554043. Max. coverage (+): 0. Max coverage (-): 0

Region: chr25 35554044-35554058. Max. coverage (+): 0. Max coverage (-): 0

Region: chr25 35554059-35554072. Max. coverage (+): 0. Max coverage (-): 0

Region: chr25 35554073-35554086. Max. coverage (+): 0. Max coverage (-): 0

Region: chr25 35554087-35554101. Max. coverage (+): 0. Max coverage (-): 0

Region: chr25 35554102-35554115. Max. coverage (+): 0. Max coverage (-): 0

Region: chr25 35554116-35554129. Max. coverage (+): 0. Max coverage (-): 0

Region: chr25 35554130-35554144. Max. coverage (+): 0. Max coverage (-): 0

Region: chr25 35554145-35554158. Max. coverage (+): 0. Max coverage (-): 0

Region: chr25 35554159-35554172. Max. coverage (+): 0. Max coverage (-): 1.31

Region: chr25 35554173-35554187. Max. coverage (+): 0. Max coverage (-): 1.31

Region: chr25 35554188-35554201. Max. coverage (+): 0. Max coverage (-): 0

Region: chr25 35554202-35554215. Max. coverage (+): 0. Max coverage (-): 0

Region: chr25 35554216-35554230. Max. coverage (+): 0. Max coverage (-): 0

Region: chr25 35554231-35554244. Max. coverage (+): 0. Max coverage (-): 0.29

Region: chr25 35554245-35554258. Max. coverage (+): 0. Max coverage (-): 0.29

Region: chr25 35554259-35554273. Max. coverage (+): 0. Max coverage (-): 5.02

Region: chr25 35554274-35554287. Max. coverage (+): 0. Max coverage (-): 1.16

Region: chr25 35554288-35554302. Max. coverage (+): 0. Max coverage (-): 0

Region: chr25 35554303-35554316. Max. coverage (+): 0. Max coverage (-): 0

Region: chr25 35554317-35554330. Max. coverage (+): 0. Max coverage (-): 0

Region: chr25 35554331-35554345. Max. coverage (+): 0. Max coverage (-): 0

Region: chr25 35554346-35554359. Max. coverage (+): 0. Max coverage (-): 0

Region: chr25 35554360-35554373. Max. coverage (+): 0. Max coverage (-): 0

Region: chr25 35554374-35554388. Max. coverage (+): 0. Max coverage (-): 0

Region: chr25 35554389-35554402. Max. coverage (+): 0. Max coverage (-): 0

Region: chr25 35554403-35554416. Max. coverage (+): 0. Max coverage (-): 0

Region: chr25 35554417-35554431. Max. coverage (+): 0. Max coverage (-): 0

Region: chr25 35554432-35554445. Max. coverage (+): 0. Max coverage (-): 0

Region: chr25 35554446-35554459. Max. coverage (+): 0. Max coverage (-): 1.7

Region: chr25 35554460-35554474. Max. coverage (+): 0. Max coverage (-): 5.69

Region: chr25 35554475-35554488. Max. coverage (+): 0. Max coverage (-): 5.69

Region: chr25 35554489-35554503. Max. coverage (+): 0. Max coverage (-): 0

Region: chr25 35554504-35554517. Max. coverage (+): 0. Max coverage (-): 0

Region: chr25 35554518-35554531. Max. coverage (+): 0. Max coverage (-): 0

Region: chr25 35554532-35554546. Max. coverage (+): 0. Max coverage (-): 0

Region: chr25 35554547-35554560. Max. coverage (+): 0. Max coverage (-): 0

Region: chr25 35554561-35554574. Max. coverage (+): 0. Max coverage (-): 0

Region: chr25 35554575-35554589. Max. coverage (+): 0. Max coverage (-): 0

Region: chr25 35554590-35554603. Max. coverage (+): 0. Max coverage (-): 0

Region: chr25 35554604-35554617. Max. coverage (+): 0. Max coverage (-): 0

Region: chr25 35554618-35554632. Max. coverage (+): 0. Max coverage (-): 0

Region: chr25 35554633-35554646. Max. coverage (+): 0. Max coverage (-): 0

Region: chr25 35554647-35554660. Max. coverage (+): 0. Max coverage (-): 0

Region: chr25 35554661-35554675. Max. coverage (+): 0. Max coverage (-): 0

Region: chr25 35554676-35554689. Max. coverage (+): 0. Max coverage (-): 0

Region: chr25 35554690-35554703. Max. coverage (+): 0. Max coverage (-): 0

Region: chr25 35554704-35554718. Max. coverage (+): 0. Max coverage (-): 0

Region: chr25 35554719-35554732. Max. coverage (+): 0. Max coverage (-): 0

Region: chr25 35554733-35554747. Max. coverage (+): 0. Max coverage (-): 0

Region: chr25 35554748-35554761. Max. coverage (+): 0. Max coverage (-): 0

Region: chr25 35554762-35554775. Max. coverage (+): 0. Max coverage (-): 0

Region: chr25 35554776-35554790. Max. coverage (+): 0. Max coverage (-): 0

Region: chr25 35554791-35554804. Max. coverage (+): 0. Max coverage (-): 0

Region: chr25 35554805-35554818. Max. coverage (+): 0. Max coverage (-): 0

Region: chr25 35554819-35554833. Max. coverage (+): 0. Max coverage (-): 0

Region: chr25 35554834-35554847. Max. coverage (+): 0. Max coverage (-): 0

Region: chr25 35554848-35554861. Max. coverage (+): 0. Max coverage (-): 0

Region: chr25 35554862-35554876. Max. coverage (+): 0. Max coverage (-): 0

Region: chr25 35554877-35554890. Max. coverage (+): 0. Max coverage (-): 0

Region: chr25 35554891-35554904. Max. coverage (+): 0. Max coverage (-): 0

Region: chr25 35554905-35554919. Max. coverage (+): 0. Max coverage (-): 0

Region: chr25 35554920-35554933. Max. coverage (+): 0. Max coverage (-): 0

Region: chr25 35554934-35554947. Max. coverage (+): 0. Max coverage (-): 0

Region: chr25 35554948-35554962. Max. coverage (+): 0. Max coverage (-): 0

Region: chr25 35554963-35554976. Max. coverage (+): 0. Max coverage (-): 0

Region: chr25 35554977-35554991. Max. coverage (+): 0. Max coverage (-): 0

Region: chr25 35554992-35555005. Max. coverage (+): 0. Max coverage (-): 0

Region: chr25 35555006-35555019. Max. coverage (+): 0. Max coverage (-): 0

Region: chr25 35555020-35555034. Max. coverage (+): 0. Max coverage (-): 0

Region: chr25 35555035-35555048. Max. coverage (+): 0. Max coverage (-): 0

Region: chr25 35555049-35555062. Max. coverage (+): 0. Max coverage (-): 0

Region: chr25 35555063-35555077. Max. coverage (+): 0. Max coverage (-): 0

Region: chr25 35555078-35555091. Max. coverage (+): 0. Max coverage (-): 1.03

Region: chr25 35555092-35555105. Max. coverage (+): 0. Max coverage (-): 1.03

Region: chr25 35555106-35555120. Max. coverage (+): 0. Max coverage (-): 0

Region: chr25 35555121-35555134. Max. coverage (+): 0. Max coverage (-): 0

Region: chr25 35555135-35555148. Max. coverage (+): 0. Max coverage (-): 0

Region: chr25 35555149-35555163. Max. coverage (+): 0. Max coverage (-): 0

Region: chr25 35555164-35555177. Max. coverage (+): 0. Max coverage (-): 0

Region: chr25 35555178-35555191. Max. coverage (+): 0. Max coverage (-): 0

Region: chr25 35555192-35555206. Max. coverage (+): 0. Max coverage (-): 0

Region: chr25 35555207-35555220. Max. coverage (+): 0. Max coverage (-): 0

Region: chr25 35555221-35555235. Max. coverage (+): 0. Max coverage (-): 0

Region: chr25 35555236-35555249. Max. coverage (+): 0. Max coverage (-): 1.4

Region: chr25 35555250-35555263. Max. coverage (+): 0. Max coverage (-): 0

Region: chr25 35555264-35555278. Max. coverage (+): 0. Max coverage (-): 0

Region: chr25 35555279-35555292. Max. coverage (+): 0. Max coverage (-): 0

Region: chr25 35555293-35555306. Max. coverage (+): 0. Max coverage (-): 0

Region: chr25 35555307-35555321. Max. coverage (+): 0. Max coverage (-): 0

Region: chr25 35555322-35555335. Max. coverage (+): 0. Max coverage (-): 0

Region: chr25 35555336-35555349. Max. coverage (+): 0. Max coverage (-): 0

Region: chr25 35555350-35555364. Max. coverage (+): 0. Max coverage (-): 4.27

Region: chr25 35555365-35555378. Max. coverage (+): 0. Max coverage (-): 0

Region: chr25 35555379-35555392. Max. coverage (+): 0. Max coverage (-): 0

Region: chr25 35555393-35555407. Max. coverage (+): 0. Max coverage (-): 0

Region: chr25 35555408-35555421. Max. coverage (+): 0. Max coverage (-): 1.89

Region: chr25 35555422-35555436. Max. coverage (+): 0. Max coverage (-): 1.89

Region: chr25 35555437-35555450. Max. coverage (+): 0. Max coverage (-): 0

Region: chr25 35555451-35555464. Max. coverage (+): 0. Max coverage (-): 0

Region: chr25 35555465-35555479. Max. coverage (+): 0. Max coverage (-): 0

Region: chr25 35555480-35555493. Max. coverage (+): 0. Max coverage (-): 0

Region: chr25 35555494-35555507. Max. coverage (+): 0. Max coverage (-): 0

Region: chr25 35555508-35555522. Max. coverage (+): 0. Max coverage (-): 0

Region: chr25 35555523-35555536. Max. coverage (+): 0. Max coverage (-): 0

Region: chr25 35555537-35555550. Max. coverage (+): 0. Max coverage (-): 0

Region: chr25 35555551-35555565. Max. coverage (+): 0. Max coverage (-): 0

Region: chr25 35555566-35555579. Max. coverage (+): 0. Max coverage (-): 0

Region: chr25 35555580-35555593. Max. coverage (+): 0. Max coverage (-): 0

Region: chr25 35555594-35555608. Max. coverage (+): 0. Max coverage (-): 0

Region: chr25 35555609-35555622. Max. coverage (+): 0. Max coverage (-): 0

Region: chr25 35555623-35555636. Max. coverage (+): 0. Max coverage (-): 0

Region: chr25 35555637-35555651. Max. coverage (+): 0. Max coverage (-): 0

Region: chr25 35555652-35555665. Max. coverage (+): 0. Max coverage (-): 0

Region: chr25 35555666-35555680. Max. coverage (+): 0. Max coverage (-): 0

Region: chr25 35555681-35555694. Max. coverage (+): 0. Max coverage (-): 0

Region: chr25 35555695-35555708. Max. coverage (+): 0. Max coverage (-): 0

Region: chr25 35555709-35555723. Max. coverage (+): 0. Max coverage (-): 0

Region: chr25 35555724-35555737. Max. coverage (+): 0. Max coverage (-): 0

Region: chr25 35555738-35555751. Max. coverage (+): 0. Max coverage (-): 0

Region: chr25 35555752-35555766. Max. coverage (+): 0. Max coverage (-): 0

Region: chr25 35555767-35555780. Max. coverage (+): 0. Max coverage (-): 0

Region: chr25 35555781-35555794. Max. coverage (+): 0. Max coverage (-): 0

Region: chr25 35555795-35555809. Max. coverage (+): 0. Max coverage (-): 0

Region: chr25 35555810-35555823. Max. coverage (+): 0. Max coverage (-): 0

Region: chr25 35555824-35555837. Max. coverage (+): 0. Max coverage (-): 0

Region: chr25 35555838-35555852. Max. coverage (+): 0. Max coverage (-): 0

Region: chr25 35555853-35555866. Max. coverage (+): 0. Max coverage (-): 0

Region: chr25 35555867-35555880. Max. coverage (+): 0. Max coverage (-): 0

Region: chr25 35555881-35555895. Max. coverage (+): 0. Max coverage (-): 0

Region: chr25 35555896-35555909. Max. coverage (+): 0. Max coverage (-): 0

Region: chr25 35555910-35555924. Max. coverage (+): 0. Max coverage (-): 0

Region: chr25 35555925-35555938. Max. coverage (+): 0. Max coverage (-): 0

Region: chr25 35555939-35555952. Max. coverage (+): 0. Max coverage (-): 0

Region: chr25 35555953-35555967. Max. coverage (+): 0. Max coverage (-): 0

Region: chr25 35555968-35555981. Max. coverage (+): 0. Max coverage (-): 2.25

Region: chr25 35555982-35555995. Max. coverage (+): 0. Max coverage (-): 0

Region: chr25 35555996-. Max. coverage (+): 0. Max coverage (-): 0

RepeatMasker Color Code

**+**

100-98% Identity

<98-95% Identity

<95-90% Identity

<90-85% Identity

<85-80% Identity

<80-75% Identity

<75-70% Identity

<70% Identity

**-**

Gene Set Color Code

**+**

Gene

Pseudogene

**-**

Topology/Coverage Color Code

Coverage Plus Strand

Coverage Minus Strand

Mainstrand: Plus

Mainstrand: Minus

Complementary Strand

Flanking Region  
(if option -flank >0)

Gene Set Annotation  
  
RepeatMasker Annotation  

**1. ART2A**: 35548981-35549058 (-), Divergence to consensus: 25.6%  
**2. Bov-tA2**: 35548989-35549116 (-), Divergence to consensus: 15.6%  
**3. MIR**: 35550242-35550335 (+), Divergence to consensus: 32.8%  
**4. CHRL**: 35550373-35550548 (+), Divergence to consensus: 24.1%  
**5. Bov-tA2**: 35553169-35553374 (+), Divergence to consensus: 20.1%

  
Transcription Factor Binding Sites  

**RFX4\_1** (Sequence: CGTGGCAAC (+): 35549911)  
**RFX4\_2** (Sequence: CATGGATAC (+): 35549362)  
**Gata4** (Sequence: AGATAAG (-): 35551726)  
**Gata4** (Sequence: AGATAAG (-): 35554174)
