# Supplementary material for: Small RNA sequencing of cryopreserved semen from single bull revealed altered miRNAs and piRNAs expression between High- and Low-motile sperm populations
Source: BMC Genomics. 2017 Jan 4;18:14. doi: 10.1186/s12864-016-3394-7 (PMC5209821; doi:10.1186/s12864-016-3394-7)
Supplement: Additional file 3: — Details for each piRNA clusters found in High Motile (HM) sperm fraction. Genes, repeats, transposable elements and transcription factors binding sites falling within the cluster regions were reported. (ZIP 1896 kb) [file 12864_2016_3394_MOESM3_ESM.zip › 63.html]

piRNA cluster 63


Predicted piRNA cluster no. 63     previous   next
  

Show proTRAC run info
Hide proTRAC run info

================================= proTRAC ====================================  
VERSION: 2.1                                    LAST MODIFIED: 06. October 2015  
  
Please cite:  
Rosenkranz D, Zischler H. proTRAC - a software for probabilistic piRNA cluster  
detection, visualization and analysis. 2012. BMC Bioinformatics 13:5.  
  
and (for proTRAC 2.0 and later):  
Rosenkranz D, Rudloff S, Bastuck K, Ketting RF, Zischler H. Tupaia small RNAs  
provide insights into function and evolution of RNAi-based transposon defense  
in mammals. 2015. RNA 21(5):911-922.  
  
Contact:  
David Rosenkranz  
Institute of Anthropology, small RNA group  
Johannes Gutenberg University Mainz  
email: rosenkranz@uni-mainz.de  
  
You can find the latest proTRAC version at:  
http://sourceforge.net/projects/protrac/files  
http://www.smallRNAgroup-mainz.de/software  
==============================================================================  
  
PARAMETERS:  
Map file: .............../storage/core/barbara/genhome/smallRNA/fertility/Sample\_motile/pirna/Sample\_motile\_26-33\_collapsed.fa.no-dust.map.weighted-10000-1000-b-0  
Genome file: ............/storage/core/barbara/genhome/smallRNA/fertility/Sample\_all/pirna/bt\_311\_chrY.fa  
RepeatMasker annotation: /storage/genomes/bt\_umd31/GCF\_000003055.6\_Bos\_taurus\_UMD\_3.1.1\_repeatMasker\_chr.out  
GeneSet:................./storage/core/barbara/genhome/smallRNA/fertility/Sample\_all/pirna/full.gtf  
  
Significant (p<=0.01) hit density will be calculated based  
on observed hit distribution.  
  
Sliding window size: ........................................ 5000 bp  
Sliding window increament: .................................. 1000 bp  
Normalize each hit by number of genomic hits: ............... 1 [0=no/1=yes]  
Normalize each hit by number of sequence reads: ............. 1 [0=no/1=yes]  
Normalize values (-> per million mapped reads): ............. 1 [0=no/1=yes]  
Min. fraction of hits with 1T(U) or 10A: .................... 0.75  
Alternatively: Min. fraction of hits with 1T(U) and 10A: .... 0.5  
Min. fraction of hits with typical piRNA length: ............ 0.75  
Typical piRNA length: ....................................... 26-33 nt  
Min. size of a piRNA cluster: ............................... 5000 bp.  
Min. number of hits (absolute): ............................. 0  
Min. number of hits (normalized): ........................... 0  
Min. fraction of hits on the mainstrand: .................... 0.75  
Top fraction of mapped sequences (in terms of read counts): . 1%  
Top fraction accounts for max. n% of sequence reads: ........ 90%  
Min. fraction of hits on each arm of a bidirectional cluster: 0.1  
Output image file for each cluster: ......................... 0 [0=no/1=yes]  
Output html file for each cluster: .......................... 1 [0=no/1=yes]  
Output a summary table: ..................................... 1 [0=no/1=yes]  
Output a FASTA file for each cluster (piRNA sequences): ..... 1 [0=no/1=yes]  
Output a FASTA file comprising cluster sequences: ........... 1 [0=no/1=yes]  
Search DNA motifs in clusters: .............................. 1 [0=no/1=yes]  
Output flanking sequences: +/- .............................. 0 bp  
Output ~.pTi file: .......................................... 1 [0=no/1=yes]  
==============================================================================  
  
  
Genome size (without gaps): ............ 2678902517 bp  
Gaps (N/X/-): .......................... 53837044 bp  
Mapped reads: .......................... 658825247023  
Non-identical sequences: ............... 514171  
Genomic hits: .......................... 764233  
Significant densitiy of mapped reads: .. 12867599.5173724 reads/kb

Show proTRAC cluster info
Hide proTRAC cluster info

|  |  |
| --- | --- |
| Location | chr25 |
| Coordinates | 36001474-36008355 |
| Size [bp] | 6882 |
| Sequence hit loci | 357 |
| Mapped reads (normalized) | 424947904 |
| Mapped reads (normalized) per kb | 61747733.8 |
| Normalized reads with 1T (1U) | 81.1% |
| Normalized reads with 10A | 30.7% |
| Normalized reads with length 26-33 nt | 100% |
| Normalized reads on the main strand(s) | 100% |
| Predicted directionality | mono:plus |

100%

0%

1T (1U)  
reads

10A reads

26-33 nt  
reads

reads on mainstrand

**Either the amount of reads with 1T (1U) OR 10A has to exceed 75% (set with option: -1Tor10A)  
Alternatively the amount of reads with 1T (1U) AND 10A has to exceed 50% (set with option: -1Tand10A)  
Minimum amount of reads with preferred size is 75% (set with option: -pisize)  
Minimum amount of reads on the main strand(s) is 75% (set with option: -clstrand)**

Show read coverage
Hide read coverage

WHAT DO I SEE HERE?  
This chart shows the location of mapped sequence reads within a predicted piRNA cluster. The color refers to the number of genomic hits produced by the sequence read in question. A dark red bar indicates that this sequence read produces many other hits elsewhere in the genome. Many adjacent red or yellow bars can indicate the presence of a multi-copy element such as transposons or rRNA genes. A dark green bar indicates that this sequence read maps uniquely to this locus.

1 hit

2-5 hits

6-10 hits

11-20 hits

21-50 hits

51-100 hits

> 100 hits

chr25

36001474

36008355

Gene Set

RepeatMasker

Mapped  
Reads

32.3

plus strand

minus strand

32.3

Region: chr25 35555997-36001480. Max. coverage (+): 5.21. Max coverage (-): 0

Region: chr25 36001481-36001494. Max. coverage (+): 5.21. Max coverage (-): 0

Region: chr25 36001495-36001508. Max. coverage (+): 0. Max coverage (-): 0

Region: chr25 36001509-36001522. Max. coverage (+): 0. Max coverage (-): 0

Region: chr25 36001523-36001535. Max. coverage (+): 0. Max coverage (-): 0

Region: chr25 36001536-36001549. Max. coverage (+): 0. Max coverage (-): 0

Region: chr25 36001550-36001563. Max. coverage (+): 0. Max coverage (-): 0

Region: chr25 36001564-36001577. Max. coverage (+): 0. Max coverage (-): 0

Region: chr25 36001578-36001590. Max. coverage (+): 0. Max coverage (-): 0

Region: chr25 36001591-36001604. Max. coverage (+): 0. Max coverage (-): 0

Region: chr25 36001605-36001618. Max. coverage (+): 0. Max coverage (-): 0

Region: chr25 36001619-36001632. Max. coverage (+): 0. Max coverage (-): 0

Region: chr25 36001633-36001646. Max. coverage (+): 0. Max coverage (-): 0

Region: chr25 36001647-36001659. Max. coverage (+): 0. Max coverage (-): 0

Region: chr25 36001660-36001673. Max. coverage (+): 0. Max coverage (-): 0

Region: chr25 36001674-36001687. Max. coverage (+): 0. Max coverage (-): 0

Region: chr25 36001688-36001701. Max. coverage (+): 0. Max coverage (-): 0

Region: chr25 36001702-36001714. Max. coverage (+): 0. Max coverage (-): 0

Region: chr25 36001715-36001728. Max. coverage (+): 0. Max coverage (-): 0

Region: chr25 36001729-36001742. Max. coverage (+): 0. Max coverage (-): 0

Region: chr25 36001743-36001756. Max. coverage (+): 0. Max coverage (-): 0

Region: chr25 36001757-36001769. Max. coverage (+): 0. Max coverage (-): 0

Region: chr25 36001770-36001783. Max. coverage (+): 0. Max coverage (-): 0

Region: chr25 36001784-36001797. Max. coverage (+): 0. Max coverage (-): 0

Region: chr25 36001798-36001811. Max. coverage (+): 0. Max coverage (-): 0

Region: chr25 36001812-36001824. Max. coverage (+): 0. Max coverage (-): 0

Region: chr25 36001825-36001838. Max. coverage (+): 0. Max coverage (-): 0

Region: chr25 36001839-36001852. Max. coverage (+): 0. Max coverage (-): 0

Region: chr25 36001853-36001866. Max. coverage (+): 0. Max coverage (-): 0

Region: chr25 36001867-36001880. Max. coverage (+): 0. Max coverage (-): 0

Region: chr25 36001881-36001893. Max. coverage (+): 0. Max coverage (-): 0

Region: chr25 36001894-36001907. Max. coverage (+): 0. Max coverage (-): 0

Region: chr25 36001908-36001921. Max. coverage (+): 0. Max coverage (-): 0

Region: chr25 36001922-36001935. Max. coverage (+): 0. Max coverage (-): 0

Region: chr25 36001936-36001948. Max. coverage (+): 0. Max coverage (-): 0

Region: chr25 36001949-36001962. Max. coverage (+): 0. Max coverage (-): 0

Region: chr25 36001963-36001976. Max. coverage (+): 0. Max coverage (-): 0

Region: chr25 36001977-36001990. Max. coverage (+): 0. Max coverage (-): 0

Region: chr25 36001991-36002003. Max. coverage (+): 0. Max coverage (-): 0

Region: chr25 36002004-36002017. Max. coverage (+): 0. Max coverage (-): 0

Region: chr25 36002018-36002031. Max. coverage (+): 0. Max coverage (-): 0

Region: chr25 36002032-36002045. Max. coverage (+): 0. Max coverage (-): 0

Region: chr25 36002046-36002058. Max. coverage (+): 0. Max coverage (-): 0

Region: chr25 36002059-36002072. Max. coverage (+): 0. Max coverage (-): 0

Region: chr25 36002073-36002086. Max. coverage (+): 0. Max coverage (-): 0

Region: chr25 36002087-36002100. Max. coverage (+): 0. Max coverage (-): 0

Region: chr25 36002101-36002114. Max. coverage (+): 0. Max coverage (-): 0

Region: chr25 36002115-36002127. Max. coverage (+): 0. Max coverage (-): 0

Region: chr25 36002128-36002141. Max. coverage (+): 0. Max coverage (-): 0

Region: chr25 36002142-36002155. Max. coverage (+): 0. Max coverage (-): 0

Region: chr25 36002156-36002169. Max. coverage (+): 0. Max coverage (-): 0

Region: chr25 36002170-36002182. Max. coverage (+): 0. Max coverage (-): 0

Region: chr25 36002183-36002196. Max. coverage (+): 0. Max coverage (-): 0

Region: chr25 36002197-36002210. Max. coverage (+): 0. Max coverage (-): 0

Region: chr25 36002211-36002224. Max. coverage (+): 0. Max coverage (-): 0

Region: chr25 36002225-36002237. Max. coverage (+): 0. Max coverage (-): 0

Region: chr25 36002238-36002251. Max. coverage (+): 0. Max coverage (-): 0

Region: chr25 36002252-36002265. Max. coverage (+): 0. Max coverage (-): 0

Region: chr25 36002266-36002279. Max. coverage (+): 0. Max coverage (-): 0

Region: chr25 36002280-36002292. Max. coverage (+): 3.27. Max coverage (-): 0

Region: chr25 36002293-36002306. Max. coverage (+): 3.27. Max coverage (-): 0

Region: chr25 36002307-36002320. Max. coverage (+): 0. Max coverage (-): 0

Region: chr25 36002321-36002334. Max. coverage (+): 3.07. Max coverage (-): 0

Region: chr25 36002335-36002348. Max. coverage (+): 0. Max coverage (-): 0

Region: chr25 36002349-36002361. Max. coverage (+): 0. Max coverage (-): 0

Region: chr25 36002362-36002375. Max. coverage (+): 0. Max coverage (-): 0

Region: chr25 36002376-36002389. Max. coverage (+): 0. Max coverage (-): 0

Region: chr25 36002390-36002403. Max. coverage (+): 0. Max coverage (-): 0

Region: chr25 36002404-36002416. Max. coverage (+): 0. Max coverage (-): 0

Region: chr25 36002417-36002430. Max. coverage (+): 0. Max coverage (-): 0

Region: chr25 36002431-36002444. Max. coverage (+): 0. Max coverage (-): 0

Region: chr25 36002445-36002458. Max. coverage (+): 0. Max coverage (-): 0

Region: chr25 36002459-36002471. Max. coverage (+): 0. Max coverage (-): 0

Region: chr25 36002472-36002485. Max. coverage (+): 0. Max coverage (-): 0

Region: chr25 36002486-36002499. Max. coverage (+): 0. Max coverage (-): 0

Region: chr25 36002500-36002513. Max. coverage (+): 0. Max coverage (-): 0

Region: chr25 36002514-36002526. Max. coverage (+): 0. Max coverage (-): 0

Region: chr25 36002527-36002540. Max. coverage (+): 0. Max coverage (-): 0

Region: chr25 36002541-36002554. Max. coverage (+): 0. Max coverage (-): 0

Region: chr25 36002555-36002568. Max. coverage (+): 0. Max coverage (-): 0

Region: chr25 36002569-36002582. Max. coverage (+): 0. Max coverage (-): 0

Region: chr25 36002583-36002595. Max. coverage (+): 0. Max coverage (-): 0

Region: chr25 36002596-36002609. Max. coverage (+): 0. Max coverage (-): 0

Region: chr25 36002610-36002623. Max. coverage (+): 0. Max coverage (-): 0

Region: chr25 36002624-36002637. Max. coverage (+): 3.12. Max coverage (-): 0

Region: chr25 36002638-36002650. Max. coverage (+): 3.12. Max coverage (-): 0

Region: chr25 36002651-36002664. Max. coverage (+): 0. Max coverage (-): 0

Region: chr25 36002665-36002678. Max. coverage (+): 0. Max coverage (-): 0

Region: chr25 36002679-36002692. Max. coverage (+): 0. Max coverage (-): 0

Region: chr25 36002693-36002705. Max. coverage (+): 0. Max coverage (-): 0

Region: chr25 36002706-36002719. Max. coverage (+): 0. Max coverage (-): 0

Region: chr25 36002720-36002733. Max. coverage (+): 0. Max coverage (-): 0

Region: chr25 36002734-36002747. Max. coverage (+): 0. Max coverage (-): 0

Region: chr25 36002748-36002760. Max. coverage (+): 0. Max coverage (-): 0

Region: chr25 36002761-36002774. Max. coverage (+): 0. Max coverage (-): 0

Region: chr25 36002775-36002788. Max. coverage (+): 0. Max coverage (-): 0

Region: chr25 36002789-36002802. Max. coverage (+): 0. Max coverage (-): 0

Region: chr25 36002803-36002815. Max. coverage (+): 0. Max coverage (-): 0

Region: chr25 36002816-36002829. Max. coverage (+): 0. Max coverage (-): 0

Region: chr25 36002830-36002843. Max. coverage (+): 0. Max coverage (-): 0

Region: chr25 36002844-36002857. Max. coverage (+): 0. Max coverage (-): 0

Region: chr25 36002858-36002871. Max. coverage (+): 0. Max coverage (-): 0

Region: chr25 36002872-36002884. Max. coverage (+): 0. Max coverage (-): 0

Region: chr25 36002885-36002898. Max. coverage (+): 0. Max coverage (-): 0

Region: chr25 36002899-36002912. Max. coverage (+): 0. Max coverage (-): 0

Region: chr25 36002913-36002926. Max. coverage (+): 1.94. Max coverage (-): 0

Region: chr25 36002927-36002939. Max. coverage (+): 3.2. Max coverage (-): 0

Region: chr25 36002940-36002953. Max. coverage (+): 3.19. Max coverage (-): 0

Region: chr25 36002954-36002967. Max. coverage (+): 0. Max coverage (-): 0

Region: chr25 36002968-36002981. Max. coverage (+): 0. Max coverage (-): 0

Region: chr25 36002982-36002994. Max. coverage (+): 0. Max coverage (-): 0

Region: chr25 36002995-36003008. Max. coverage (+): 0. Max coverage (-): 0

Region: chr25 36003009-36003022. Max. coverage (+): 0.28. Max coverage (-): 0

Region: chr25 36003023-36003036. Max. coverage (+): 0.28. Max coverage (-): 0

Region: chr25 36003037-36003049. Max. coverage (+): 0. Max coverage (-): 0

Region: chr25 36003050-36003063. Max. coverage (+): 8.69. Max coverage (-): 0

Region: chr25 36003064-36003077. Max. coverage (+): 0. Max coverage (-): 0

Region: chr25 36003078-36003091. Max. coverage (+): 0. Max coverage (-): 0

Region: chr25 36003092-36003105. Max. coverage (+): 0. Max coverage (-): 0

Region: chr25 36003106-36003118. Max. coverage (+): 0. Max coverage (-): 0

Region: chr25 36003119-36003132. Max. coverage (+): 0. Max coverage (-): 0

Region: chr25 36003133-36003146. Max. coverage (+): 0. Max coverage (-): 0

Region: chr25 36003147-36003160. Max. coverage (+): 0. Max coverage (-): 0

Region: chr25 36003161-36003173. Max. coverage (+): 3.76. Max coverage (-): 0

Region: chr25 36003174-36003187. Max. coverage (+): 2.01. Max coverage (-): 0

Region: chr25 36003188-36003201. Max. coverage (+): 0. Max coverage (-): 0

Region: chr25 36003202-36003215. Max. coverage (+): 0. Max coverage (-): 0

Region: chr25 36003216-36003228. Max. coverage (+): 0. Max coverage (-): 0

Region: chr25 36003229-36003242. Max. coverage (+): 0.63. Max coverage (-): 0

Region: chr25 36003243-36003256. Max. coverage (+): 6.34. Max coverage (-): 0

Region: chr25 36003257-36003270. Max. coverage (+): 14.73. Max coverage (-): 0

Region: chr25 36003271-36003283. Max. coverage (+): 23.24. Max coverage (-): 0

Region: chr25 36003284-36003297. Max. coverage (+): 21.88. Max coverage (-): 0

Region: chr25 36003298-36003311. Max. coverage (+): 8.55. Max coverage (-): 0

Region: chr25 36003312-36003325. Max. coverage (+): 6.01. Max coverage (-): 0

Region: chr25 36003326-36003339. Max. coverage (+): 3.27. Max coverage (-): 0

Region: chr25 36003340-36003352. Max. coverage (+): 0. Max coverage (-): 0

Region: chr25 36003353-36003366. Max. coverage (+): 0. Max coverage (-): 0

Region: chr25 36003367-36003380. Max. coverage (+): 0. Max coverage (-): 0

Region: chr25 36003381-36003394. Max. coverage (+): 0. Max coverage (-): 0

Region: chr25 36003395-36003407. Max. coverage (+): 0. Max coverage (-): 0

Region: chr25 36003408-36003421. Max. coverage (+): 0. Max coverage (-): 0

Region: chr25 36003422-36003435. Max. coverage (+): 0. Max coverage (-): 0

Region: chr25 36003436-36003449. Max. coverage (+): 0. Max coverage (-): 0

Region: chr25 36003450-36003462. Max. coverage (+): 0. Max coverage (-): 0

Region: chr25 36003463-36003476. Max. coverage (+): 0.96. Max coverage (-): 0

Region: chr25 36003477-36003490. Max. coverage (+): 0.96. Max coverage (-): 0

Region: chr25 36003491-36003504. Max. coverage (+): 5.25. Max coverage (-): 0

Region: chr25 36003505-36003517. Max. coverage (+): 5.25. Max coverage (-): 0

Region: chr25 36003518-36003531. Max. coverage (+): 0. Max coverage (-): 0

Region: chr25 36003532-36003545. Max. coverage (+): 3.61. Max coverage (-): 0

Region: chr25 36003546-36003559. Max. coverage (+): 3.61. Max coverage (-): 0

Region: chr25 36003560-36003573. Max. coverage (+): 0.24. Max coverage (-): 0

Region: chr25 36003574-36003586. Max. coverage (+): 0. Max coverage (-): 0

Region: chr25 36003587-36003600. Max. coverage (+): 0. Max coverage (-): 0

Region: chr25 36003601-36003614. Max. coverage (+): 0. Max coverage (-): 0

Region: chr25 36003615-36003628. Max. coverage (+): 0. Max coverage (-): 0

Region: chr25 36003629-36003641. Max. coverage (+): 0. Max coverage (-): 0

Region: chr25 36003642-36003655. Max. coverage (+): 0. Max coverage (-): 0

Region: chr25 36003656-36003669. Max. coverage (+): 0. Max coverage (-): 0

Region: chr25 36003670-36003683. Max. coverage (+): 0. Max coverage (-): 0

Region: chr25 36003684-36003696. Max. coverage (+): 0. Max coverage (-): 0

Region: chr25 36003697-36003710. Max. coverage (+): 1.84. Max coverage (-): 0

Region: chr25 36003711-36003724. Max. coverage (+): 0. Max coverage (-): 0

Region: chr25 36003725-36003738. Max. coverage (+): 0. Max coverage (-): 0

Region: chr25 36003739-36003751. Max. coverage (+): 0. Max coverage (-): 0

Region: chr25 36003752-36003765. Max. coverage (+): 0. Max coverage (-): 0

Region: chr25 36003766-36003779. Max. coverage (+): 0. Max coverage (-): 0

Region: chr25 36003780-36003793. Max. coverage (+): 0. Max coverage (-): 0

Region: chr25 36003794-36003806. Max. coverage (+): 0. Max coverage (-): 0

Region: chr25 36003807-36003820. Max. coverage (+): 0. Max coverage (-): 0

Region: chr25 36003821-36003834. Max. coverage (+): 0. Max coverage (-): 0

Region: chr25 36003835-36003848. Max. coverage (+): 0. Max coverage (-): 0

Region: chr25 36003849-36003862. Max. coverage (+): 0. Max coverage (-): 0

Region: chr25 36003863-36003875. Max. coverage (+): 0. Max coverage (-): 0

Region: chr25 36003876-36003889. Max. coverage (+): 0. Max coverage (-): 0

Region: chr25 36003890-36003903. Max. coverage (+): 0. Max coverage (-): 0

Region: chr25 36003904-36003917. Max. coverage (+): 0. Max coverage (-): 0

Region: chr25 36003918-36003930. Max. coverage (+): 0. Max coverage (-): 0

Region: chr25 36003931-36003944. Max. coverage (+): 0. Max coverage (-): 0

Region: chr25 36003945-36003958. Max. coverage (+): 0. Max coverage (-): 0

Region: chr25 36003959-36003972. Max. coverage (+): 0. Max coverage (-): 0

Region: chr25 36003973-36003985. Max. coverage (+): 0. Max coverage (-): 0

Region: chr25 36003986-36003999. Max. coverage (+): 0. Max coverage (-): 0

Region: chr25 36004000-36004013. Max. coverage (+): 0. Max coverage (-): 0

Region: chr25 36004014-36004027. Max. coverage (+): 0. Max coverage (-): 0

Region: chr25 36004028-36004040. Max. coverage (+): 0. Max coverage (-): 0

Region: chr25 36004041-36004054. Max. coverage (+): 0. Max coverage (-): 0

Region: chr25 36004055-36004068. Max. coverage (+): 0. Max coverage (-): 0

Region: chr25 36004069-36004082. Max. coverage (+): 0. Max coverage (-): 0

Region: chr25 36004083-36004096. Max. coverage (+): 0. Max coverage (-): 0

Region: chr25 36004097-36004109. Max. coverage (+): 0. Max coverage (-): 0

Region: chr25 36004110-36004123. Max. coverage (+): 6.9. Max coverage (-): 0

Region: chr25 36004124-36004137. Max. coverage (+): 6.9. Max coverage (-): 0

Region: chr25 36004138-36004151. Max. coverage (+): 1.08. Max coverage (-): 0

Region: chr25 36004152-36004164. Max. coverage (+): 0. Max coverage (-): 0

Region: chr25 36004165-36004178. Max. coverage (+): 0. Max coverage (-): 0

Region: chr25 36004179-36004192. Max. coverage (+): 0. Max coverage (-): 0

Region: chr25 36004193-36004206. Max. coverage (+): 3.44. Max coverage (-): 0

Region: chr25 36004207-36004219. Max. coverage (+): 3.44. Max coverage (-): 0

Region: chr25 36004220-36004233. Max. coverage (+): 0. Max coverage (-): 0

Region: chr25 36004234-36004247. Max. coverage (+): 0. Max coverage (-): 0

Region: chr25 36004248-36004261. Max. coverage (+): 0. Max coverage (-): 0

Region: chr25 36004262-36004274. Max. coverage (+): 0. Max coverage (-): 0

Region: chr25 36004275-36004288. Max. coverage (+): 0.26. Max coverage (-): 0

Region: chr25 36004289-36004302. Max. coverage (+): 0.26. Max coverage (-): 0

Region: chr25 36004303-36004316. Max. coverage (+): 0. Max coverage (-): 0

Region: chr25 36004317-36004330. Max. coverage (+): 13.92. Max coverage (-): 0

Region: chr25 36004331-36004343. Max. coverage (+): 0. Max coverage (-): 0

Region: chr25 36004344-36004357. Max. coverage (+): 0. Max coverage (-): 0

Region: chr25 36004358-36004371. Max. coverage (+): 0. Max coverage (-): 0

Region: chr25 36004372-36004385. Max. coverage (+): 4.01. Max coverage (-): 0

Region: chr25 36004386-36004398. Max. coverage (+): 4.01. Max coverage (-): 0

Region: chr25 36004399-36004412. Max. coverage (+): 1.89. Max coverage (-): 0

Region: chr25 36004413-36004426. Max. coverage (+): 1.89. Max coverage (-): 0

Region: chr25 36004427-36004440. Max. coverage (+): 5.64. Max coverage (-): 0

Region: chr25 36004441-36004453. Max. coverage (+): 1.35. Max coverage (-): 0

Region: chr25 36004454-36004467. Max. coverage (+): 0. Max coverage (-): 0

Region: chr25 36004468-36004481. Max. coverage (+): 0. Max coverage (-): 0

Region: chr25 36004482-36004495. Max. coverage (+): 0. Max coverage (-): 0

Region: chr25 36004496-36004508. Max. coverage (+): 4.23. Max coverage (-): 0

Region: chr25 36004509-36004522. Max. coverage (+): 6.01. Max coverage (-): 0

Region: chr25 36004523-36004536. Max. coverage (+): 18.96. Max coverage (-): 0

Region: chr25 36004537-36004550. Max. coverage (+): 7.19. Max coverage (-): 0

Region: chr25 36004551-36004564. Max. coverage (+): 6.13. Max coverage (-): 0

Region: chr25 36004565-36004577. Max. coverage (+): 0. Max coverage (-): 0

Region: chr25 36004578-36004591. Max. coverage (+): 0. Max coverage (-): 0

Region: chr25 36004592-36004605. Max. coverage (+): 8.94. Max coverage (-): 0

Region: chr25 36004606-36004619. Max. coverage (+): 25.98. Max coverage (-): 0

Region: chr25 36004620-36004632. Max. coverage (+): 2.97. Max coverage (-): 0

Region: chr25 36004633-36004646. Max. coverage (+): 0. Max coverage (-): 0

Region: chr25 36004647-36004660. Max. coverage (+): 0. Max coverage (-): 0

Region: chr25 36004661-36004674. Max. coverage (+): 0. Max coverage (-): 0

Region: chr25 36004675-36004687. Max. coverage (+): 0.8. Max coverage (-): 0

Region: chr25 36004688-36004701. Max. coverage (+): 0.8. Max coverage (-): 0

Region: chr25 36004702-36004715. Max. coverage (+): 2.17. Max coverage (-): 0

Region: chr25 36004716-36004729. Max. coverage (+): 5.08. Max coverage (-): 0

Region: chr25 36004730-36004742. Max. coverage (+): 4.44. Max coverage (-): 0

Region: chr25 36004743-36004756. Max. coverage (+): 0. Max coverage (-): 0

Region: chr25 36004757-36004770. Max. coverage (+): 0. Max coverage (-): 0

Region: chr25 36004771-36004784. Max. coverage (+): 0. Max coverage (-): 0

Region: chr25 36004785-36004798. Max. coverage (+): 0. Max coverage (-): 0

Region: chr25 36004799-36004811. Max. coverage (+): 0. Max coverage (-): 0

Region: chr25 36004812-36004825. Max. coverage (+): 0. Max coverage (-): 0

Region: chr25 36004826-36004839. Max. coverage (+): 4.6. Max coverage (-): 0

Region: chr25 36004840-36004853. Max. coverage (+): 4.26. Max coverage (-): 0

Region: chr25 36004854-36004866. Max. coverage (+): 0. Max coverage (-): 0

Region: chr25 36004867-36004880. Max. coverage (+): 0. Max coverage (-): 0

Region: chr25 36004881-36004894. Max. coverage (+): 0. Max coverage (-): 0

Region: chr25 36004895-36004908. Max. coverage (+): 16.51. Max coverage (-): 0

Region: chr25 36004909-36004921. Max. coverage (+): 19.36. Max coverage (-): 0

Region: chr25 36004922-36004935. Max. coverage (+): 0. Max coverage (-): 0

Region: chr25 36004936-36004949. Max. coverage (+): 0.68. Max coverage (-): 0

Region: chr25 36004950-36004963. Max. coverage (+): 0. Max coverage (-): 0

Region: chr25 36004964-36004976. Max. coverage (+): 0. Max coverage (-): 0

Region: chr25 36004977-36004990. Max. coverage (+): 4.59. Max coverage (-): 0

Region: chr25 36004991-36005004. Max. coverage (+): 10.45. Max coverage (-): 0

Region: chr25 36005005-36005018. Max. coverage (+): 9.72. Max coverage (-): 0

Region: chr25 36005019-36005031. Max. coverage (+): 0. Max coverage (-): 0

Region: chr25 36005032-36005045. Max. coverage (+): 7.75. Max coverage (-): 0

Region: chr25 36005046-36005059. Max. coverage (+): 7.75. Max coverage (-): 0

Region: chr25 36005060-36005073. Max. coverage (+): 13.21. Max coverage (-): 0

Region: chr25 36005074-36005087. Max. coverage (+): 13.6. Max coverage (-): 0

Region: chr25 36005088-36005100. Max. coverage (+): 0. Max coverage (-): 0

Region: chr25 36005101-36005114. Max. coverage (+): 0. Max coverage (-): 0

Region: chr25 36005115-36005128. Max. coverage (+): 0. Max coverage (-): 0

Region: chr25 36005129-36005142. Max. coverage (+): 0. Max coverage (-): 0

Region: chr25 36005143-36005155. Max. coverage (+): 0. Max coverage (-): 0

Region: chr25 36005156-36005169. Max. coverage (+): 0. Max coverage (-): 0

Region: chr25 36005170-36005183. Max. coverage (+): 0. Max coverage (-): 0

Region: chr25 36005184-36005197. Max. coverage (+): 0. Max coverage (-): 0

Region: chr25 36005198-36005210. Max. coverage (+): 0. Max coverage (-): 0

Region: chr25 36005211-36005224. Max. coverage (+): 0. Max coverage (-): 0

Region: chr25 36005225-36005238. Max. coverage (+): 0. Max coverage (-): 0

Region: chr25 36005239-36005252. Max. coverage (+): 0. Max coverage (-): 0

Region: chr25 36005253-36005265. Max. coverage (+): 0. Max coverage (-): 0

Region: chr25 36005266-36005279. Max. coverage (+): 0. Max coverage (-): 0

Region: chr25 36005280-36005293. Max. coverage (+): 0. Max coverage (-): 0

Region: chr25 36005294-36005307. Max. coverage (+): 0. Max coverage (-): 0

Region: chr25 36005308-36005321. Max. coverage (+): 0. Max coverage (-): 0

Region: chr25 36005322-36005334. Max. coverage (+): 0. Max coverage (-): 0

Region: chr25 36005335-36005348. Max. coverage (+): 0. Max coverage (-): 0

Region: chr25 36005349-36005362. Max. coverage (+): 0. Max coverage (-): 0

Region: chr25 36005363-36005376. Max. coverage (+): 0. Max coverage (-): 0

Region: chr25 36005377-36005389. Max. coverage (+): 0. Max coverage (-): 0

Region: chr25 36005390-36005403. Max. coverage (+): 0. Max coverage (-): 0

Region: chr25 36005404-36005417. Max. coverage (+): 0. Max coverage (-): 0

Region: chr25 36005418-36005431. Max. coverage (+): 0. Max coverage (-): 0

Region: chr25 36005432-36005444. Max. coverage (+): 0. Max coverage (-): 0

Region: chr25 36005445-36005458. Max. coverage (+): 0. Max coverage (-): 0

Region: chr25 36005459-36005472. Max. coverage (+): 0. Max coverage (-): 0

Region: chr25 36005473-36005486. Max. coverage (+): 0. Max coverage (-): 0

Region: chr25 36005487-36005499. Max. coverage (+): 0. Max coverage (-): 0

Region: chr25 36005500-36005513. Max. coverage (+): 0. Max coverage (-): 0

Region: chr25 36005514-36005527. Max. coverage (+): 0. Max coverage (-): 0

Region: chr25 36005528-36005541. Max. coverage (+): 0. Max coverage (-): 0

Region: chr25 36005542-36005555. Max. coverage (+): 0. Max coverage (-): 0

Region: chr25 36005556-36005568. Max. coverage (+): 0. Max coverage (-): 0

Region: chr25 36005569-36005582. Max. coverage (+): 0. Max coverage (-): 0

Region: chr25 36005583-36005596. Max. coverage (+): 0. Max coverage (-): 0

Region: chr25 36005597-36005610. Max. coverage (+): 8.87. Max coverage (-): 0

Region: chr25 36005611-36005623. Max. coverage (+): 5.19. Max coverage (-): 0

Region: chr25 36005624-36005637. Max. coverage (+): 3.99. Max coverage (-): 0

Region: chr25 36005638-36005651. Max. coverage (+): 0. Max coverage (-): 0

Region: chr25 36005652-36005665. Max. coverage (+): 0. Max coverage (-): 0

Region: chr25 36005666-36005678. Max. coverage (+): 0. Max coverage (-): 0

Region: chr25 36005679-36005692. Max. coverage (+): 0. Max coverage (-): 0

Region: chr25 36005693-36005706. Max. coverage (+): 1.43. Max coverage (-): 0

Region: chr25 36005707-36005720. Max. coverage (+): 0. Max coverage (-): 0

Region: chr25 36005721-36005733. Max. coverage (+): 2.43. Max coverage (-): 0

Region: chr25 36005734-36005747. Max. coverage (+): 0. Max coverage (-): 0

Region: chr25 36005748-36005761. Max. coverage (+): 0. Max coverage (-): 0

Region: chr25 36005762-36005775. Max. coverage (+): 0. Max coverage (-): 0

Region: chr25 36005776-36005789. Max. coverage (+): 0. Max coverage (-): 0

Region: chr25 36005790-36005802. Max. coverage (+): 0. Max coverage (-): 0

Region: chr25 36005803-36005816. Max. coverage (+): 1.97. Max coverage (-): 0

Region: chr25 36005817-36005830. Max. coverage (+): 0. Max coverage (-): 0

Region: chr25 36005831-36005844. Max. coverage (+): 2.31. Max coverage (-): 0

Region: chr25 36005845-36005857. Max. coverage (+): 2.31. Max coverage (-): 0

Region: chr25 36005858-36005871. Max. coverage (+): 0. Max coverage (-): 0

Region: chr25 36005872-36005885. Max. coverage (+): 12.39. Max coverage (-): 0

Region: chr25 36005886-36005899. Max. coverage (+): 12.39. Max coverage (-): 0

Region: chr25 36005900-36005912. Max. coverage (+): 0. Max coverage (-): 0

Region: chr25 36005913-36005926. Max. coverage (+): 0. Max coverage (-): 0

Region: chr25 36005927-36005940. Max. coverage (+): 0. Max coverage (-): 0

Region: chr25 36005941-36005954. Max. coverage (+): 0. Max coverage (-): 0

Region: chr25 36005955-36005967. Max. coverage (+): 0. Max coverage (-): 0

Region: chr25 36005968-36005981. Max. coverage (+): 0. Max coverage (-): 0

Region: chr25 36005982-36005995. Max. coverage (+): 0. Max coverage (-): 0

Region: chr25 36005996-36006009. Max. coverage (+): 10.47. Max coverage (-): 0

Region: chr25 36006010-36006023. Max. coverage (+): 10.71. Max coverage (-): 0

Region: chr25 36006024-36006036. Max. coverage (+): 1.02. Max coverage (-): 0

Region: chr25 36006037-36006050. Max. coverage (+): 0. Max coverage (-): 0

Region: chr25 36006051-36006064. Max. coverage (+): 0. Max coverage (-): 0

Region: chr25 36006065-36006078. Max. coverage (+): 0. Max coverage (-): 0

Region: chr25 36006079-36006091. Max. coverage (+): 6.11. Max coverage (-): 0

Region: chr25 36006092-36006105. Max. coverage (+): 6.11. Max coverage (-): 0

Region: chr25 36006106-36006119. Max. coverage (+): 12.15. Max coverage (-): 0

Region: chr25 36006120-36006133. Max. coverage (+): 9.27. Max coverage (-): 0

Region: chr25 36006134-36006146. Max. coverage (+): 7.68. Max coverage (-): 0

Region: chr25 36006147-36006160. Max. coverage (+): 7.68. Max coverage (-): 0

Region: chr25 36006161-36006174. Max. coverage (+): 9.4. Max coverage (-): 0

Region: chr25 36006175-36006188. Max. coverage (+): 0. Max coverage (-): 0

Region: chr25 36006189-36006201. Max. coverage (+): 0. Max coverage (-): 0

Region: chr25 36006202-36006215. Max. coverage (+): 0. Max coverage (-): 0

Region: chr25 36006216-36006229. Max. coverage (+): 0. Max coverage (-): 0

Region: chr25 36006230-36006243. Max. coverage (+): 0. Max coverage (-): 0

Region: chr25 36006244-36006256. Max. coverage (+): 0. Max coverage (-): 0

Region: chr25 36006257-36006270. Max. coverage (+): 10.75. Max coverage (-): 0

Region: chr25 36006271-36006284. Max. coverage (+): 15.24. Max coverage (-): 0

Region: chr25 36006285-36006298. Max. coverage (+): 9.27. Max coverage (-): 0

Region: chr25 36006299-36006312. Max. coverage (+): 3.57. Max coverage (-): 0

Region: chr25 36006313-36006325. Max. coverage (+): 3.57. Max coverage (-): 0

Region: chr25 36006326-36006339. Max. coverage (+): 0.79. Max coverage (-): 0

Region: chr25 36006340-36006353. Max. coverage (+): 0. Max coverage (-): 0

Region: chr25 36006354-36006367. Max. coverage (+): 0. Max coverage (-): 0

Region: chr25 36006368-36006380. Max. coverage (+): 0. Max coverage (-): 0

Region: chr25 36006381-36006394. Max. coverage (+): 32.3. Max coverage (-): 0

Region: chr25 36006395-36006408. Max. coverage (+): 0. Max coverage (-): 0

Region: chr25 36006409-36006422. Max. coverage (+): 0. Max coverage (-): 0

Region: chr25 36006423-36006435. Max. coverage (+): 8.87. Max coverage (-): 0

Region: chr25 36006436-36006449. Max. coverage (+): 10.21. Max coverage (-): 0

Region: chr25 36006450-36006463. Max. coverage (+): 10.21. Max coverage (-): 0

Region: chr25 36006464-36006477. Max. coverage (+): 3.29. Max coverage (-): 0

Region: chr25 36006478-36006490. Max. coverage (+): 0. Max coverage (-): 0

Region: chr25 36006491-36006504. Max. coverage (+): 0. Max coverage (-): 0

Region: chr25 36006505-36006518. Max. coverage (+): 1.73. Max coverage (-): 0

Region: chr25 36006519-36006532. Max. coverage (+): 2.36. Max coverage (-): 0

Region: chr25 36006533-36006546. Max. coverage (+): 5.73. Max coverage (-): 0

Region: chr25 36006547-36006559. Max. coverage (+): 5.73. Max coverage (-): 0

Region: chr25 36006560-36006573. Max. coverage (+): 0. Max coverage (-): 0

Region: chr25 36006574-36006587. Max. coverage (+): 0. Max coverage (-): 0

Region: chr25 36006588-36006601. Max. coverage (+): 0. Max coverage (-): 0

Region: chr25 36006602-36006614. Max. coverage (+): 7.94. Max coverage (-): 0

Region: chr25 36006615-36006628. Max. coverage (+): 7.38. Max coverage (-): 0

Region: chr25 36006629-36006642. Max. coverage (+): 0. Max coverage (-): 0

Region: chr25 36006643-36006656. Max. coverage (+): 7.42. Max coverage (-): 0

Region: chr25 36006657-36006669. Max. coverage (+): 4.16. Max coverage (-): 0

Region: chr25 36006670-36006683. Max. coverage (+): 0.58. Max coverage (-): 0

Region: chr25 36006684-36006697. Max. coverage (+): 0.97. Max coverage (-): 0

Region: chr25 36006698-36006711. Max. coverage (+): 0.97. Max coverage (-): 0

Region: chr25 36006712-36006724. Max. coverage (+): 0. Max coverage (-): 0

Region: chr25 36006725-36006738. Max. coverage (+): 0. Max coverage (-): 0

Region: chr25 36006739-36006752. Max. coverage (+): 0. Max coverage (-): 0

Region: chr25 36006753-36006766. Max. coverage (+): 0. Max coverage (-): 0

Region: chr25 36006767-36006780. Max. coverage (+): 0. Max coverage (-): 0

Region: chr25 36006781-36006793. Max. coverage (+): 0. Max coverage (-): 0

Region: chr25 36006794-36006807. Max. coverage (+): 0. Max coverage (-): 0

Region: chr25 36006808-36006821. Max. coverage (+): 0. Max coverage (-): 0

Region: chr25 36006822-36006835. Max. coverage (+): 0. Max coverage (-): 0

Region: chr25 36006836-36006848. Max. coverage (+): 0. Max coverage (-): 0

Region: chr25 36006849-36006862. Max. coverage (+): 0. Max coverage (-): 0

Region: chr25 36006863-36006876. Max. coverage (+): 0.37. Max coverage (-): 0

Region: chr25 36006877-36006890. Max. coverage (+): 0.37. Max coverage (-): 0

Region: chr25 36006891-36006903. Max. coverage (+): 0. Max coverage (-): 0

Region: chr25 36006904-36006917. Max. coverage (+): 3.9. Max coverage (-): 0

Region: chr25 36006918-36006931. Max. coverage (+): 3.9. Max coverage (-): 0

Region: chr25 36006932-36006945. Max. coverage (+): 1.95. Max coverage (-): 0

Region: chr25 36006946-36006958. Max. coverage (+): 0. Max coverage (-): 0

Region: chr25 36006959-36006972. Max. coverage (+): 0.8. Max coverage (-): 0

Region: chr25 36006973-36006986. Max. coverage (+): 0. Max coverage (-): 0

Region: chr25 36006987-36007000. Max. coverage (+): 0. Max coverage (-): 0

Region: chr25 36007001-36007014. Max. coverage (+): 0. Max coverage (-): 0

Region: chr25 36007015-36007027. Max. coverage (+): 0. Max coverage (-): 0

Region: chr25 36007028-36007041. Max. coverage (+): 0. Max coverage (-): 0

Region: chr25 36007042-36007055. Max. coverage (+): 0. Max coverage (-): 0

Region: chr25 36007056-36007069. Max. coverage (+): 0. Max coverage (-): 0

Region: chr25 36007070-36007082. Max. coverage (+): 2.11. Max coverage (-): 0

Region: chr25 36007083-36007096. Max. coverage (+): 1.68. Max coverage (-): 0

Region: chr25 36007097-36007110. Max. coverage (+): 0. Max coverage (-): 0

Region: chr25 36007111-36007124. Max. coverage (+): 0. Max coverage (-): 0

Region: chr25 36007125-36007137. Max. coverage (+): 0. Max coverage (-): 0

Region: chr25 36007138-36007151. Max. coverage (+): 0. Max coverage (-): 0

Region: chr25 36007152-36007165. Max. coverage (+): 0. Max coverage (-): 0

Region: chr25 36007166-36007179. Max. coverage (+): 0. Max coverage (-): 0

Region: chr25 36007180-36007192. Max. coverage (+): 0. Max coverage (-): 0

Region: chr25 36007193-36007206. Max. coverage (+): 10.13. Max coverage (-): 0

Region: chr25 36007207-36007220. Max. coverage (+): 15.77. Max coverage (-): 0

Region: chr25 36007221-36007234. Max. coverage (+): 3.45. Max coverage (-): 0

Region: chr25 36007235-36007247. Max. coverage (+): 6.12. Max coverage (-): 0

Region: chr25 36007248-36007261. Max. coverage (+): 0. Max coverage (-): 0

Region: chr25 36007262-36007275. Max. coverage (+): 1.38. Max coverage (-): 0

Region: chr25 36007276-36007289. Max. coverage (+): 3.21. Max coverage (-): 0

Region: chr25 36007290-36007303. Max. coverage (+): 4.75. Max coverage (-): 0

Region: chr25 36007304-36007316. Max. coverage (+): 0. Max coverage (-): 0

Region: chr25 36007317-36007330. Max. coverage (+): 3.34. Max coverage (-): 0

Region: chr25 36007331-36007344. Max. coverage (+): 1.99. Max coverage (-): 0

Region: chr25 36007345-36007358. Max. coverage (+): 6.64. Max coverage (-): 0

Region: chr25 36007359-36007371. Max. coverage (+): 0. Max coverage (-): 0

Region: chr25 36007372-36007385. Max. coverage (+): 0. Max coverage (-): 0

Region: chr25 36007386-36007399. Max. coverage (+): 4.5. Max coverage (-): 0

Region: chr25 36007400-36007413. Max. coverage (+): 4.5. Max coverage (-): 0

Region: chr25 36007414-36007426. Max. coverage (+): 3.45. Max coverage (-): 0

Region: chr25 36007427-36007440. Max. coverage (+): 3.45. Max coverage (-): 0

Region: chr25 36007441-36007454. Max. coverage (+): 0. Max coverage (-): 0

Region: chr25 36007455-36007468. Max. coverage (+): 0. Max coverage (-): 0

Region: chr25 36007469-36007481. Max. coverage (+): 0. Max coverage (-): 0

Region: chr25 36007482-36007495. Max. coverage (+): 0. Max coverage (-): 0

Region: chr25 36007496-36007509. Max. coverage (+): 0. Max coverage (-): 0

Region: chr25 36007510-36007523. Max. coverage (+): 0. Max coverage (-): 0

Region: chr25 36007524-36007537. Max. coverage (+): 0. Max coverage (-): 0

Region: chr25 36007538-36007550. Max. coverage (+): 0. Max coverage (-): 0

Region: chr25 36007551-36007564. Max. coverage (+): 0. Max coverage (-): 0

Region: chr25 36007565-36007578. Max. coverage (+): 0. Max coverage (-): 0

Region: chr25 36007579-36007592. Max. coverage (+): 5.81. Max coverage (-): 0

Region: chr25 36007593-36007605. Max. coverage (+): 3.02. Max coverage (-): 0

Region: chr25 36007606-36007619. Max. coverage (+): 0. Max coverage (-): 0

Region: chr25 36007620-36007633. Max. coverage (+): 1.09. Max coverage (-): 0

Region: chr25 36007634-36007647. Max. coverage (+): 0. Max coverage (-): 0

Region: chr25 36007648-36007660. Max. coverage (+): 0. Max coverage (-): 0

Region: chr25 36007661-36007674. Max. coverage (+): 17.44. Max coverage (-): 0

Region: chr25 36007675-36007688. Max. coverage (+): 9.84. Max coverage (-): 0

Region: chr25 36007689-36007702. Max. coverage (+): 5.87. Max coverage (-): 0

Region: chr25 36007703-36007715. Max. coverage (+): 0. Max coverage (-): 0

Region: chr25 36007716-36007729. Max. coverage (+): 0.7. Max coverage (-): 0

Region: chr25 36007730-36007743. Max. coverage (+): 0. Max coverage (-): 0

Region: chr25 36007744-36007757. Max. coverage (+): 1.98. Max coverage (-): 0

Region: chr25 36007758-36007771. Max. coverage (+): 1.98. Max coverage (-): 0

Region: chr25 36007772-36007784. Max. coverage (+): 0. Max coverage (-): 0

Region: chr25 36007785-36007798. Max. coverage (+): 0. Max coverage (-): 0

Region: chr25 36007799-36007812. Max. coverage (+): 0. Max coverage (-): 0

Region: chr25 36007813-36007826. Max. coverage (+): 0. Max coverage (-): 0

Region: chr25 36007827-36007839. Max. coverage (+): 0. Max coverage (-): 0

Region: chr25 36007840-36007853. Max. coverage (+): 0. Max coverage (-): 0

Region: chr25 36007854-36007867. Max. coverage (+): 0. Max coverage (-): 0

Region: chr25 36007868-36007881. Max. coverage (+): 0. Max coverage (-): 0

Region: chr25 36007882-36007894. Max. coverage (+): 0. Max coverage (-): 0

Region: chr25 36007895-36007908. Max. coverage (+): 0.86. Max coverage (-): 0

Region: chr25 36007909-36007922. Max. coverage (+): 0.86. Max coverage (-): 0

Region: chr25 36007923-36007936. Max. coverage (+): 0. Max coverage (-): 0

Region: chr25 36007937-36007949. Max. coverage (+): 0. Max coverage (-): 0

Region: chr25 36007950-36007963. Max. coverage (+): 0. Max coverage (-): 0

Region: chr25 36007964-36007977. Max. coverage (+): 0. Max coverage (-): 0

Region: chr25 36007978-36007991. Max. coverage (+): 0. Max coverage (-): 0

Region: chr25 36007992-36008005. Max. coverage (+): 0. Max coverage (-): 0

Region: chr25 36008006-36008018. Max. coverage (+): 0. Max coverage (-): 0

Region: chr25 36008019-36008032. Max. coverage (+): 0. Max coverage (-): 0

Region: chr25 36008033-36008046. Max. coverage (+): 1.56. Max coverage (-): 0

Region: chr25 36008047-36008060. Max. coverage (+): 1.56. Max coverage (-): 0

Region: chr25 36008061-36008073. Max. coverage (+): 0. Max coverage (-): 0

Region: chr25 36008074-36008087. Max. coverage (+): 0. Max coverage (-): 0

Region: chr25 36008088-36008101. Max. coverage (+): 0. Max coverage (-): 0

Region: chr25 36008102-36008115. Max. coverage (+): 4.74. Max coverage (-): 0

Region: chr25 36008116-36008128. Max. coverage (+): 0. Max coverage (-): 0

Region: chr25 36008129-36008142. Max. coverage (+): 0. Max coverage (-): 0

Region: chr25 36008143-36008156. Max. coverage (+): 0. Max coverage (-): 0

Region: chr25 36008157-36008170. Max. coverage (+): 0. Max coverage (-): 0

Region: chr25 36008171-36008183. Max. coverage (+): 0.73. Max coverage (-): 0

Region: chr25 36008184-36008197. Max. coverage (+): 0.73. Max coverage (-): 0

Region: chr25 36008198-36008211. Max. coverage (+): 1.62. Max coverage (-): 0

Region: chr25 36008212-36008225. Max. coverage (+): 0. Max coverage (-): 0

Region: chr25 36008226-36008239. Max. coverage (+): 2.25. Max coverage (-): 0

Region: chr25 36008240-36008252. Max. coverage (+): 0. Max coverage (-): 0

Region: chr25 36008253-36008266. Max. coverage (+): 0. Max coverage (-): 0

Region: chr25 36008267-36008280. Max. coverage (+): 2.55. Max coverage (-): 0

Region: chr25 36008281-36008294. Max. coverage (+): 2.55. Max coverage (-): 0

Region: chr25 36008295-36008307. Max. coverage (+): 0. Max coverage (-): 0

Region: chr25 36008308-36008321. Max. coverage (+): 0. Max coverage (-): 0

Region: chr25 36008322-36008335. Max. coverage (+): 3.26. Max coverage (-): 0

Region: chr25 36008336-36008349. Max. coverage (+): 0. Max coverage (-): 0

Region: chr25 36008350-. Max. coverage (+): 0. Max coverage (-): 0

RepeatMasker Color Code

**+**

100-98% Identity

<98-95% Identity

<95-90% Identity

<90-85% Identity

<85-80% Identity

<80-75% Identity

<75-70% Identity

<70% Identity

**-**

Gene Set Color Code

**+**

Gene

Pseudogene

**-**

Topology/Coverage Color Code

Coverage Plus Strand

Coverage Minus Strand

Mainstrand: Plus

Mainstrand: Minus

Complementary Strand

Flanking Region  
(if option -flank >0)

Gene Set Annotation  
  
RepeatMasker Annotation  

**1. L2c**: 36002761-36002867 (+), Divergence to consensus: 41%  
**2. MIRb**: 36003936-36004109 (-), Divergence to consensus: 30.6%  
**3. (CA)n**: 36004758-36004800 (+), Divergence to consensus: 2.3%  
**4. MLT1L**: 36005219-36005599 (+), Divergence to consensus: 37.6%  
**5. L2b**: 36006909-36006943 (-), Divergence to consensus: 20%  
**6. (TA)n**: 36007001-36007067 (+), Divergence to consensus: 6%

  
Transcription Factor Binding Sites  

**Gata4** (Sequence: CTTATCT (+): 36006983)
