# Supplementary material for: Small RNA sequencing of cryopreserved semen from single bull revealed altered miRNAs and piRNAs expression between High- and Low-motile sperm populations
Source: BMC Genomics. 2017 Jan 4;18:14. doi: 10.1186/s12864-016-3394-7 (PMC5209821; doi:10.1186/s12864-016-3394-7)
Supplement: Additional file 3: — Details for each piRNA clusters found in High Motile (HM) sperm fraction. Genes, repeats, transposable elements and transcription factors binding sites falling within the cluster regions were reported. (ZIP 1896 kb) [file 12864_2016_3394_MOESM3_ESM.zip › 64.html]

piRNA cluster 64


Predicted piRNA cluster no. 64     previous   next
  

Show proTRAC run info
Hide proTRAC run info

================================= proTRAC ====================================  
VERSION: 2.1                                    LAST MODIFIED: 06. October 2015  
  
Please cite:  
Rosenkranz D, Zischler H. proTRAC - a software for probabilistic piRNA cluster  
detection, visualization and analysis. 2012. BMC Bioinformatics 13:5.  
  
and (for proTRAC 2.0 and later):  
Rosenkranz D, Rudloff S, Bastuck K, Ketting RF, Zischler H. Tupaia small RNAs  
provide insights into function and evolution of RNAi-based transposon defense  
in mammals. 2015. RNA 21(5):911-922.  
  
Contact:  
David Rosenkranz  
Institute of Anthropology, small RNA group  
Johannes Gutenberg University Mainz  
email: rosenkranz@uni-mainz.de  
  
You can find the latest proTRAC version at:  
http://sourceforge.net/projects/protrac/files  
http://www.smallRNAgroup-mainz.de/software  
==============================================================================  
  
PARAMETERS:  
Map file: .............../storage/core/barbara/genhome/smallRNA/fertility/Sample\_motile/pirna/Sample\_motile\_26-33\_collapsed.fa.no-dust.map.weighted-10000-1000-b-0  
Genome file: ............/storage/core/barbara/genhome/smallRNA/fertility/Sample\_all/pirna/bt\_311\_chrY.fa  
RepeatMasker annotation: /storage/genomes/bt\_umd31/GCF\_000003055.6\_Bos\_taurus\_UMD\_3.1.1\_repeatMasker\_chr.out  
GeneSet:................./storage/core/barbara/genhome/smallRNA/fertility/Sample\_all/pirna/full.gtf  
  
Significant (p<=0.01) hit density will be calculated based  
on observed hit distribution.  
  
Sliding window size: ........................................ 5000 bp  
Sliding window increament: .................................. 1000 bp  
Normalize each hit by number of genomic hits: ............... 1 [0=no/1=yes]  
Normalize each hit by number of sequence reads: ............. 1 [0=no/1=yes]  
Normalize values (-> per million mapped reads): ............. 1 [0=no/1=yes]  
Min. fraction of hits with 1T(U) or 10A: .................... 0.75  
Alternatively: Min. fraction of hits with 1T(U) and 10A: .... 0.5  
Min. fraction of hits with typical piRNA length: ............ 0.75  
Typical piRNA length: ....................................... 26-33 nt  
Min. size of a piRNA cluster: ............................... 5000 bp.  
Min. number of hits (absolute): ............................. 0  
Min. number of hits (normalized): ........................... 0  
Min. fraction of hits on the mainstrand: .................... 0.75  
Top fraction of mapped sequences (in terms of read counts): . 1%  
Top fraction accounts for max. n% of sequence reads: ........ 90%  
Min. fraction of hits on each arm of a bidirectional cluster: 0.1  
Output image file for each cluster: ......................... 0 [0=no/1=yes]  
Output html file for each cluster: .......................... 1 [0=no/1=yes]  
Output a summary table: ..................................... 1 [0=no/1=yes]  
Output a FASTA file for each cluster (piRNA sequences): ..... 1 [0=no/1=yes]  
Output a FASTA file comprising cluster sequences: ........... 1 [0=no/1=yes]  
Search DNA motifs in clusters: .............................. 1 [0=no/1=yes]  
Output flanking sequences: +/- .............................. 0 bp  
Output ~.pTi file: .......................................... 1 [0=no/1=yes]  
==============================================================================  
  
  
Genome size (without gaps): ............ 2678902517 bp  
Gaps (N/X/-): .......................... 53837044 bp  
Mapped reads: .......................... 658825247023  
Non-identical sequences: ............... 514171  
Genomic hits: .......................... 764233  
Significant densitiy of mapped reads: .. 12867599.5173724 reads/kb

Show proTRAC cluster info
Hide proTRAC cluster info

|  |  |
| --- | --- |
| Location | chr26 |
| Coordinates | 46666292-46673788 |
| Size [bp] | 7497 |
| Sequence hit loci | 220 |
| Mapped reads (normalized) | 299903515 |
| Mapped reads (normalized) per kb | 40003136.6 |
| Normalized reads with 1T (1U) | 84.2% |
| Normalized reads with 10A | 25.3% |
| Normalized reads with length 26-33 nt | 100% |
| Normalized reads on the main strand(s) | 100% |
| Predicted directionality | mono:minus |

100%

0%

1T (1U)  
reads

10A reads

26-33 nt  
reads

reads on mainstrand

**Either the amount of reads with 1T (1U) OR 10A has to exceed 75% (set with option: -1Tor10A)  
Alternatively the amount of reads with 1T (1U) AND 10A has to exceed 50% (set with option: -1Tand10A)  
Minimum amount of reads with preferred size is 75% (set with option: -pisize)  
Minimum amount of reads on the main strand(s) is 75% (set with option: -clstrand)**

Show read coverage
Hide read coverage

WHAT DO I SEE HERE?  
This chart shows the location of mapped sequence reads within a predicted piRNA cluster. The color refers to the number of genomic hits produced by the sequence read in question. A dark red bar indicates that this sequence read produces many other hits elsewhere in the genome. Many adjacent red or yellow bars can indicate the presence of a multi-copy element such as transposons or rRNA genes. A dark green bar indicates that this sequence read maps uniquely to this locus.

1 hit

2-5 hits

6-10 hits

11-20 hits

21-50 hits

51-100 hits

> 100 hits

chr26

46666292

46673788

Gene Set

RepeatMasker

Mapped  
Reads

26.34

plus strand

minus strand

26.34

Region: chr26 36008351-46666299. Max. coverage (+): 0. Max coverage (-): 2.22

Region: chr26 46666300-46666314. Max. coverage (+): 0. Max coverage (-): 0

Region: chr26 46666315-46666329. Max. coverage (+): 0. Max coverage (-): 0

Region: chr26 46666330-46666344. Max. coverage (+): 0. Max coverage (-): 0

Region: chr26 46666345-46666359. Max. coverage (+): 0. Max coverage (-): 0

Region: chr26 46666360-46666374. Max. coverage (+): 0. Max coverage (-): 0

Region: chr26 46666375-46666389. Max. coverage (+): 0. Max coverage (-): 0

Region: chr26 46666390-46666404. Max. coverage (+): 0. Max coverage (-): 0

Region: chr26 46666405-46666419. Max. coverage (+): 0. Max coverage (-): 0

Region: chr26 46666420-46666434. Max. coverage (+): 0. Max coverage (-): 0

Region: chr26 46666435-46666449. Max. coverage (+): 0. Max coverage (-): 2.91

Region: chr26 46666450-46666464. Max. coverage (+): 0. Max coverage (-): 0

Region: chr26 46666465-46666479. Max. coverage (+): 0. Max coverage (-): 0

Region: chr26 46666480-46666494. Max. coverage (+): 0. Max coverage (-): 0

Region: chr26 46666495-46666509. Max. coverage (+): 0. Max coverage (-): 0

Region: chr26 46666510-46666524. Max. coverage (+): 0. Max coverage (-): 0

Region: chr26 46666525-46666539. Max. coverage (+): 0. Max coverage (-): 0

Region: chr26 46666540-46666554. Max. coverage (+): 0. Max coverage (-): 0

Region: chr26 46666555-46666569. Max. coverage (+): 0. Max coverage (-): 0

Region: chr26 46666570-46666584. Max. coverage (+): 0. Max coverage (-): 0

Region: chr26 46666585-46666599. Max. coverage (+): 0. Max coverage (-): 0

Region: chr26 46666600-46666614. Max. coverage (+): 0. Max coverage (-): 0

Region: chr26 46666615-46666629. Max. coverage (+): 0. Max coverage (-): 0

Region: chr26 46666630-46666644. Max. coverage (+): 0. Max coverage (-): 0

Region: chr26 46666645-46666659. Max. coverage (+): 0. Max coverage (-): 0

Region: chr26 46666660-46666674. Max. coverage (+): 0. Max coverage (-): 0

Region: chr26 46666675-46666689. Max. coverage (+): 0. Max coverage (-): 0

Region: chr26 46666690-46666704. Max. coverage (+): 0. Max coverage (-): 0

Region: chr26 46666705-46666719. Max. coverage (+): 0. Max coverage (-): 0

Region: chr26 46666720-46666734. Max. coverage (+): 0. Max coverage (-): 0

Region: chr26 46666735-46666749. Max. coverage (+): 0. Max coverage (-): 0

Region: chr26 46666750-46666764. Max. coverage (+): 0. Max coverage (-): 0

Region: chr26 46666765-46666779. Max. coverage (+): 0. Max coverage (-): 0

Region: chr26 46666780-46666794. Max. coverage (+): 0. Max coverage (-): 0

Region: chr26 46666795-46666809. Max. coverage (+): 0. Max coverage (-): 0

Region: chr26 46666810-46666824. Max. coverage (+): 0. Max coverage (-): 0

Region: chr26 46666825-46666839. Max. coverage (+): 0. Max coverage (-): 0

Region: chr26 46666840-46666854. Max. coverage (+): 0. Max coverage (-): 0

Region: chr26 46666855-46666869. Max. coverage (+): 0. Max coverage (-): 0

Region: chr26 46666870-46666884. Max. coverage (+): 0. Max coverage (-): 0

Region: chr26 46666885-46666899. Max. coverage (+): 0. Max coverage (-): 0

Region: chr26 46666900-46666914. Max. coverage (+): 0. Max coverage (-): 0

Region: chr26 46666915-46666929. Max. coverage (+): 0. Max coverage (-): 3.66

Region: chr26 46666930-46666944. Max. coverage (+): 0. Max coverage (-): 3.66

Region: chr26 46666945-46666959. Max. coverage (+): 0. Max coverage (-): 5.27

Region: chr26 46666960-46666974. Max. coverage (+): 0. Max coverage (-): 5.27

Region: chr26 46666975-46666989. Max. coverage (+): 0. Max coverage (-): 4.39

Region: chr26 46666990-46667004. Max. coverage (+): 0. Max coverage (-): 0

Region: chr26 46667005-46667019. Max. coverage (+): 0. Max coverage (-): 0

Region: chr26 46667020-46667034. Max. coverage (+): 0. Max coverage (-): 0

Region: chr26 46667035-46667049. Max. coverage (+): 0. Max coverage (-): 0

Region: chr26 46667050-46667064. Max. coverage (+): 0. Max coverage (-): 0

Region: chr26 46667065-46667079. Max. coverage (+): 0. Max coverage (-): 0

Region: chr26 46667080-46667094. Max. coverage (+): 0. Max coverage (-): 0

Region: chr26 46667095-46667109. Max. coverage (+): 0. Max coverage (-): 0

Region: chr26 46667110-46667124. Max. coverage (+): 0. Max coverage (-): 0

Region: chr26 46667125-46667139. Max. coverage (+): 0. Max coverage (-): 0

Region: chr26 46667140-46667154. Max. coverage (+): 0. Max coverage (-): 0

Region: chr26 46667155-46667169. Max. coverage (+): 0. Max coverage (-): 0

Region: chr26 46667170-46667184. Max. coverage (+): 0. Max coverage (-): 0

Region: chr26 46667185-46667199. Max. coverage (+): 0. Max coverage (-): 0

Region: chr26 46667200-46667214. Max. coverage (+): 0. Max coverage (-): 0

Region: chr26 46667215-46667229. Max. coverage (+): 0. Max coverage (-): 0

Region: chr26 46667230-46667244. Max. coverage (+): 0. Max coverage (-): 0

Region: chr26 46667245-46667259. Max. coverage (+): 0. Max coverage (-): 0

Region: chr26 46667260-46667274. Max. coverage (+): 0. Max coverage (-): 0

Region: chr26 46667275-46667289. Max. coverage (+): 0. Max coverage (-): 0

Region: chr26 46667290-46667304. Max. coverage (+): 0. Max coverage (-): 0

Region: chr26 46667305-46667319. Max. coverage (+): 0. Max coverage (-): 3.37

Region: chr26 46667320-46667334. Max. coverage (+): 0. Max coverage (-): 3.37

Region: chr26 46667335-46667349. Max. coverage (+): 0. Max coverage (-): 0

Region: chr26 46667350-46667364. Max. coverage (+): 0. Max coverage (-): 0

Region: chr26 46667365-46667379. Max. coverage (+): 0. Max coverage (-): 0

Region: chr26 46667380-46667394. Max. coverage (+): 0. Max coverage (-): 0.83

Region: chr26 46667395-46667409. Max. coverage (+): 0. Max coverage (-): 9.34

Region: chr26 46667410-46667424. Max. coverage (+): 0. Max coverage (-): 0

Region: chr26 46667425-46667439. Max. coverage (+): 0. Max coverage (-): 0

Region: chr26 46667440-46667454. Max. coverage (+): 0. Max coverage (-): 0

Region: chr26 46667455-46667469. Max. coverage (+): 0. Max coverage (-): 0

Region: chr26 46667470-46667484. Max. coverage (+): 0. Max coverage (-): 0

Region: chr26 46667485-46667499. Max. coverage (+): 0. Max coverage (-): 0

Region: chr26 46667500-46667514. Max. coverage (+): 0. Max coverage (-): 0

Region: chr26 46667515-46667529. Max. coverage (+): 0. Max coverage (-): 0

Region: chr26 46667530-46667543. Max. coverage (+): 0. Max coverage (-): 0

Region: chr26 46667544-46667558. Max. coverage (+): 0. Max coverage (-): 0

Region: chr26 46667559-46667573. Max. coverage (+): 0. Max coverage (-): 0

Region: chr26 46667574-46667588. Max. coverage (+): 0. Max coverage (-): 0

Region: chr26 46667589-46667603. Max. coverage (+): 0. Max coverage (-): 0

Region: chr26 46667604-46667618. Max. coverage (+): 0. Max coverage (-): 0

Region: chr26 46667619-46667633. Max. coverage (+): 0. Max coverage (-): 0

Region: chr26 46667634-46667648. Max. coverage (+): 0. Max coverage (-): 0

Region: chr26 46667649-46667663. Max. coverage (+): 0. Max coverage (-): 0

Region: chr26 46667664-46667678. Max. coverage (+): 0. Max coverage (-): 0

Region: chr26 46667679-46667693. Max. coverage (+): 0. Max coverage (-): 0

Region: chr26 46667694-46667708. Max. coverage (+): 0. Max coverage (-): 0

Region: chr26 46667709-46667723. Max. coverage (+): 0. Max coverage (-): 0

Region: chr26 46667724-46667738. Max. coverage (+): 0. Max coverage (-): 0

Region: chr26 46667739-46667753. Max. coverage (+): 0. Max coverage (-): 2.19

Region: chr26 46667754-46667768. Max. coverage (+): 0. Max coverage (-): 2.19

Region: chr26 46667769-46667783. Max. coverage (+): 0. Max coverage (-): 0

Region: chr26 46667784-46667798. Max. coverage (+): 0. Max coverage (-): 0

Region: chr26 46667799-46667813. Max. coverage (+): 0. Max coverage (-): 0

Region: chr26 46667814-46667828. Max. coverage (+): 0. Max coverage (-): 0

Region: chr26 46667829-46667843. Max. coverage (+): 0. Max coverage (-): 0

Region: chr26 46667844-46667858. Max. coverage (+): 0. Max coverage (-): 0

Region: chr26 46667859-46667873. Max. coverage (+): 0. Max coverage (-): 0

Region: chr26 46667874-46667888. Max. coverage (+): 0. Max coverage (-): 0

Region: chr26 46667889-46667903. Max. coverage (+): 0. Max coverage (-): 0

Region: chr26 46667904-46667918. Max. coverage (+): 0. Max coverage (-): 0

Region: chr26 46667919-46667933. Max. coverage (+): 0. Max coverage (-): 0

Region: chr26 46667934-46667948. Max. coverage (+): 0. Max coverage (-): 0

Region: chr26 46667949-46667963. Max. coverage (+): 0. Max coverage (-): 0

Region: chr26 46667964-46667978. Max. coverage (+): 0. Max coverage (-): 0

Region: chr26 46667979-46667993. Max. coverage (+): 0. Max coverage (-): 0

Region: chr26 46667994-46668008. Max. coverage (+): 0. Max coverage (-): 0

Region: chr26 46668009-46668023. Max. coverage (+): 0. Max coverage (-): 0

Region: chr26 46668024-46668038. Max. coverage (+): 0. Max coverage (-): 0

Region: chr26 46668039-46668053. Max. coverage (+): 0. Max coverage (-): 0

Region: chr26 46668054-46668068. Max. coverage (+): 0. Max coverage (-): 0

Region: chr26 46668069-46668083. Max. coverage (+): 0. Max coverage (-): 0

Region: chr26 46668084-46668098. Max. coverage (+): 0. Max coverage (-): 0

Region: chr26 46668099-46668113. Max. coverage (+): 0. Max coverage (-): 0

Region: chr26 46668114-46668128. Max. coverage (+): 0. Max coverage (-): 0

Region: chr26 46668129-46668143. Max. coverage (+): 0. Max coverage (-): 0

Region: chr26 46668144-46668158. Max. coverage (+): 0. Max coverage (-): 0

Region: chr26 46668159-46668173. Max. coverage (+): 0. Max coverage (-): 0

Region: chr26 46668174-46668188. Max. coverage (+): 0. Max coverage (-): 0

Region: chr26 46668189-46668203. Max. coverage (+): 0. Max coverage (-): 0

Region: chr26 46668204-46668218. Max. coverage (+): 0. Max coverage (-): 0

Region: chr26 46668219-46668233. Max. coverage (+): 0. Max coverage (-): 9.51

Region: chr26 46668234-46668248. Max. coverage (+): 0. Max coverage (-): 0

Region: chr26 46668249-46668263. Max. coverage (+): 0. Max coverage (-): 3.67

Region: chr26 46668264-46668278. Max. coverage (+): 0. Max coverage (-): 0

Region: chr26 46668279-46668293. Max. coverage (+): 0. Max coverage (-): 0

Region: chr26 46668294-46668308. Max. coverage (+): 0. Max coverage (-): 0

Region: chr26 46668309-46668323. Max. coverage (+): 0. Max coverage (-): 0

Region: chr26 46668324-46668338. Max. coverage (+): 0. Max coverage (-): 0

Region: chr26 46668339-46668353. Max. coverage (+): 0. Max coverage (-): 0

Region: chr26 46668354-46668368. Max. coverage (+): 0. Max coverage (-): 0

Region: chr26 46668369-46668383. Max. coverage (+): 0. Max coverage (-): 4.19

Region: chr26 46668384-46668398. Max. coverage (+): 0. Max coverage (-): 1.34

Region: chr26 46668399-46668413. Max. coverage (+): 0. Max coverage (-): 0

Region: chr26 46668414-46668428. Max. coverage (+): 0. Max coverage (-): 0

Region: chr26 46668429-46668443. Max. coverage (+): 0. Max coverage (-): 0

Region: chr26 46668444-46668458. Max. coverage (+): 0. Max coverage (-): 1.69

Region: chr26 46668459-46668473. Max. coverage (+): 0. Max coverage (-): 0

Region: chr26 46668474-46668488. Max. coverage (+): 0. Max coverage (-): 0

Region: chr26 46668489-46668503. Max. coverage (+): 0. Max coverage (-): 0

Region: chr26 46668504-46668518. Max. coverage (+): 0. Max coverage (-): 0

Region: chr26 46668519-46668533. Max. coverage (+): 0. Max coverage (-): 0

Region: chr26 46668534-46668548. Max. coverage (+): 0. Max coverage (-): 6.76

Region: chr26 46668549-46668563. Max. coverage (+): 0. Max coverage (-): 10.7

Region: chr26 46668564-46668578. Max. coverage (+): 0. Max coverage (-): 0

Region: chr26 46668579-46668593. Max. coverage (+): 0. Max coverage (-): 0

Region: chr26 46668594-46668608. Max. coverage (+): 0. Max coverage (-): 0

Region: chr26 46668609-46668623. Max. coverage (+): 0. Max coverage (-): 0

Region: chr26 46668624-46668638. Max. coverage (+): 0. Max coverage (-): 3.7

Region: chr26 46668639-46668653. Max. coverage (+): 0. Max coverage (-): 0

Region: chr26 46668654-46668668. Max. coverage (+): 0. Max coverage (-): 0

Region: chr26 46668669-46668683. Max. coverage (+): 0. Max coverage (-): 0

Region: chr26 46668684-46668698. Max. coverage (+): 0. Max coverage (-): 0

Region: chr26 46668699-46668713. Max. coverage (+): 0. Max coverage (-): 1.96

Region: chr26 46668714-46668728. Max. coverage (+): 0. Max coverage (-): 0.97

Region: chr26 46668729-46668743. Max. coverage (+): 0. Max coverage (-): 0

Region: chr26 46668744-46668758. Max. coverage (+): 0. Max coverage (-): 0

Region: chr26 46668759-46668773. Max. coverage (+): 0. Max coverage (-): 0

Region: chr26 46668774-46668788. Max. coverage (+): 0. Max coverage (-): 0

Region: chr26 46668789-46668803. Max. coverage (+): 0. Max coverage (-): 0

Region: chr26 46668804-46668818. Max. coverage (+): 0. Max coverage (-): 0

Region: chr26 46668819-46668833. Max. coverage (+): 0. Max coverage (-): 0

Region: chr26 46668834-46668848. Max. coverage (+): 0. Max coverage (-): 0

Region: chr26 46668849-46668863. Max. coverage (+): 0. Max coverage (-): 0

Region: chr26 46668864-46668878. Max. coverage (+): 0. Max coverage (-): 0

Region: chr26 46668879-46668893. Max. coverage (+): 0. Max coverage (-): 0

Region: chr26 46668894-46668908. Max. coverage (+): 0. Max coverage (-): 0.88

Region: chr26 46668909-46668923. Max. coverage (+): 0. Max coverage (-): 0.88

Region: chr26 46668924-46668938. Max. coverage (+): 0. Max coverage (-): 0

Region: chr26 46668939-46668953. Max. coverage (+): 0. Max coverage (-): 0

Region: chr26 46668954-46668968. Max. coverage (+): 0. Max coverage (-): 0

Region: chr26 46668969-46668983. Max. coverage (+): 0. Max coverage (-): 7.32

Region: chr26 46668984-46668998. Max. coverage (+): 0. Max coverage (-): 7.32

Region: chr26 46668999-46669013. Max. coverage (+): 0. Max coverage (-): 0.31

Region: chr26 46669014-46669028. Max. coverage (+): 0. Max coverage (-): 0

Region: chr26 46669029-46669043. Max. coverage (+): 0. Max coverage (-): 2.18

Region: chr26 46669044-46669058. Max. coverage (+): 0. Max coverage (-): 2.18

Region: chr26 46669059-46669073. Max. coverage (+): 0. Max coverage (-): 0

Region: chr26 46669074-46669088. Max. coverage (+): 0. Max coverage (-): 0

Region: chr26 46669089-46669103. Max. coverage (+): 0. Max coverage (-): 0

Region: chr26 46669104-46669118. Max. coverage (+): 0. Max coverage (-): 0

Region: chr26 46669119-46669133. Max. coverage (+): 0. Max coverage (-): 0

Region: chr26 46669134-46669148. Max. coverage (+): 0. Max coverage (-): 0

Region: chr26 46669149-46669163. Max. coverage (+): 0. Max coverage (-): 0

Region: chr26 46669164-46669178. Max. coverage (+): 0. Max coverage (-): 4.29

Region: chr26 46669179-46669193. Max. coverage (+): 0. Max coverage (-): 3.8

Region: chr26 46669194-46669208. Max. coverage (+): 0. Max coverage (-): 0.92

Region: chr26 46669209-46669223. Max. coverage (+): 0. Max coverage (-): 0

Region: chr26 46669224-46669238. Max. coverage (+): 0. Max coverage (-): 0

Region: chr26 46669239-46669253. Max. coverage (+): 0. Max coverage (-): 0

Region: chr26 46669254-46669268. Max. coverage (+): 0. Max coverage (-): 0

Region: chr26 46669269-46669283. Max. coverage (+): 0. Max coverage (-): 0

Region: chr26 46669284-46669298. Max. coverage (+): 0. Max coverage (-): 0

Region: chr26 46669299-46669313. Max. coverage (+): 0. Max coverage (-): 0

Region: chr26 46669314-46669328. Max. coverage (+): 0. Max coverage (-): 0

Region: chr26 46669329-46669343. Max. coverage (+): 0. Max coverage (-): 0

Region: chr26 46669344-46669358. Max. coverage (+): 0. Max coverage (-): 0

Region: chr26 46669359-46669373. Max. coverage (+): 0. Max coverage (-): 0

Region: chr26 46669374-46669388. Max. coverage (+): 0. Max coverage (-): 0

Region: chr26 46669389-46669403. Max. coverage (+): 0. Max coverage (-): 0

Region: chr26 46669404-46669418. Max. coverage (+): 0. Max coverage (-): 0

Region: chr26 46669419-46669433. Max. coverage (+): 0. Max coverage (-): 0

Region: chr26 46669434-46669448. Max. coverage (+): 0. Max coverage (-): 0

Region: chr26 46669449-46669463. Max. coverage (+): 0. Max coverage (-): 0

Region: chr26 46669464-46669478. Max. coverage (+): 0. Max coverage (-): 0

Region: chr26 46669479-46669493. Max. coverage (+): 0. Max coverage (-): 0

Region: chr26 46669494-46669508. Max. coverage (+): 0. Max coverage (-): 0

Region: chr26 46669509-46669523. Max. coverage (+): 0. Max coverage (-): 0

Region: chr26 46669524-46669538. Max. coverage (+): 0. Max coverage (-): 0

Region: chr26 46669539-46669553. Max. coverage (+): 0. Max coverage (-): 0

Region: chr26 46669554-46669568. Max. coverage (+): 0. Max coverage (-): 0

Region: chr26 46669569-46669583. Max. coverage (+): 0. Max coverage (-): 0

Region: chr26 46669584-46669598. Max. coverage (+): 0. Max coverage (-): 0

Region: chr26 46669599-46669613. Max. coverage (+): 0. Max coverage (-): 0

Region: chr26 46669614-46669628. Max. coverage (+): 0. Max coverage (-): 0

Region: chr26 46669629-46669643. Max. coverage (+): 0. Max coverage (-): 0

Region: chr26 46669644-46669658. Max. coverage (+): 0. Max coverage (-): 0

Region: chr26 46669659-46669673. Max. coverage (+): 0. Max coverage (-): 0

Region: chr26 46669674-46669688. Max. coverage (+): 0. Max coverage (-): 0

Region: chr26 46669689-46669703. Max. coverage (+): 0. Max coverage (-): 0

Region: chr26 46669704-46669718. Max. coverage (+): 0. Max coverage (-): 0

Region: chr26 46669719-46669733. Max. coverage (+): 0. Max coverage (-): 0

Region: chr26 46669734-46669748. Max. coverage (+): 0. Max coverage (-): 0

Region: chr26 46669749-46669763. Max. coverage (+): 0. Max coverage (-): 0

Region: chr26 46669764-46669778. Max. coverage (+): 0. Max coverage (-): 0

Region: chr26 46669779-46669793. Max. coverage (+): 0. Max coverage (-): 0

Region: chr26 46669794-46669808. Max. coverage (+): 0. Max coverage (-): 0

Region: chr26 46669809-46669823. Max. coverage (+): 0. Max coverage (-): 0

Region: chr26 46669824-46669838. Max. coverage (+): 0. Max coverage (-): 0

Region: chr26 46669839-46669853. Max. coverage (+): 0. Max coverage (-): 3.06

Region: chr26 46669854-46669868. Max. coverage (+): 0. Max coverage (-): 3.06

Region: chr26 46669869-46669883. Max. coverage (+): 0. Max coverage (-): 0

Region: chr26 46669884-46669898. Max. coverage (+): 0. Max coverage (-): 2.31

Region: chr26 46669899-46669913. Max. coverage (+): 0. Max coverage (-): 0

Region: chr26 46669914-46669928. Max. coverage (+): 0. Max coverage (-): 0

Region: chr26 46669929-46669943. Max. coverage (+): 0. Max coverage (-): 1.22

Region: chr26 46669944-46669958. Max. coverage (+): 0. Max coverage (-): 0

Region: chr26 46669959-46669973. Max. coverage (+): 0. Max coverage (-): 0

Region: chr26 46669974-46669988. Max. coverage (+): 0. Max coverage (-): 0

Region: chr26 46669989-46670003. Max. coverage (+): 0. Max coverage (-): 0

Region: chr26 46670004-46670018. Max. coverage (+): 0. Max coverage (-): 0

Region: chr26 46670019-46670033. Max. coverage (+): 0. Max coverage (-): 0

Region: chr26 46670034-46670047. Max. coverage (+): 0. Max coverage (-): 0

Region: chr26 46670048-46670062. Max. coverage (+): 0. Max coverage (-): 0

Region: chr26 46670063-46670077. Max. coverage (+): 0. Max coverage (-): 0

Region: chr26 46670078-46670092. Max. coverage (+): 0. Max coverage (-): 0

Region: chr26 46670093-46670107. Max. coverage (+): 0. Max coverage (-): 0

Region: chr26 46670108-46670122. Max. coverage (+): 0. Max coverage (-): 0

Region: chr26 46670123-46670137. Max. coverage (+): 0. Max coverage (-): 0

Region: chr26 46670138-46670152. Max. coverage (+): 0. Max coverage (-): 0.89

Region: chr26 46670153-46670167. Max. coverage (+): 0. Max coverage (-): 1.59

Region: chr26 46670168-46670182. Max. coverage (+): 0. Max coverage (-): 9.75

Region: chr26 46670183-46670197. Max. coverage (+): 0. Max coverage (-): 1

Region: chr26 46670198-46670212. Max. coverage (+): 0. Max coverage (-): 1

Region: chr26 46670213-46670227. Max. coverage (+): 0. Max coverage (-): 0

Region: chr26 46670228-46670242. Max. coverage (+): 0. Max coverage (-): 4.92

Region: chr26 46670243-46670257. Max. coverage (+): 0. Max coverage (-): 1.57

Region: chr26 46670258-46670272. Max. coverage (+): 0. Max coverage (-): 17.08

Region: chr26 46670273-46670287. Max. coverage (+): 0. Max coverage (-): 17.08

Region: chr26 46670288-46670302. Max. coverage (+): 0. Max coverage (-): 0

Region: chr26 46670303-46670317. Max. coverage (+): 0. Max coverage (-): 0

Region: chr26 46670318-46670332. Max. coverage (+): 0. Max coverage (-): 0

Region: chr26 46670333-46670347. Max. coverage (+): 0. Max coverage (-): 0

Region: chr26 46670348-46670362. Max. coverage (+): 0. Max coverage (-): 0

Region: chr26 46670363-46670377. Max. coverage (+): 0. Max coverage (-): 3.2

Region: chr26 46670378-46670392. Max. coverage (+): 0. Max coverage (-): 0

Region: chr26 46670393-46670407. Max. coverage (+): 0. Max coverage (-): 0

Region: chr26 46670408-46670422. Max. coverage (+): 0. Max coverage (-): 0

Region: chr26 46670423-46670437. Max. coverage (+): 0. Max coverage (-): 0

Region: chr26 46670438-46670452. Max. coverage (+): 0. Max coverage (-): 0

Region: chr26 46670453-46670467. Max. coverage (+): 0. Max coverage (-): 0

Region: chr26 46670468-46670482. Max. coverage (+): 0. Max coverage (-): 0

Region: chr26 46670483-46670497. Max. coverage (+): 0. Max coverage (-): 0

Region: chr26 46670498-46670512. Max. coverage (+): 0. Max coverage (-): 0

Region: chr26 46670513-46670527. Max. coverage (+): 0. Max coverage (-): 0

Region: chr26 46670528-46670542. Max. coverage (+): 0. Max coverage (-): 0

Region: chr26 46670543-46670557. Max. coverage (+): 0. Max coverage (-): 0

Region: chr26 46670558-46670572. Max. coverage (+): 0. Max coverage (-): 0

Region: chr26 46670573-46670587. Max. coverage (+): 0. Max coverage (-): 0

Region: chr26 46670588-46670602. Max. coverage (+): 0. Max coverage (-): 0

Region: chr26 46670603-46670617. Max. coverage (+): 0. Max coverage (-): 0

Region: chr26 46670618-46670632. Max. coverage (+): 0. Max coverage (-): 0

Region: chr26 46670633-46670647. Max. coverage (+): 0. Max coverage (-): 0

Region: chr26 46670648-46670662. Max. coverage (+): 0. Max coverage (-): 0

Region: chr26 46670663-46670677. Max. coverage (+): 0. Max coverage (-): 0

Region: chr26 46670678-46670692. Max. coverage (+): 0. Max coverage (-): 0

Region: chr26 46670693-46670707. Max. coverage (+): 0. Max coverage (-): 3.11

Region: chr26 46670708-46670722. Max. coverage (+): 0. Max coverage (-): 3.11

Region: chr26 46670723-46670737. Max. coverage (+): 0. Max coverage (-): 0

Region: chr26 46670738-46670752. Max. coverage (+): 0. Max coverage (-): 0

Region: chr26 46670753-46670767. Max. coverage (+): 0. Max coverage (-): 0

Region: chr26 46670768-46670782. Max. coverage (+): 0. Max coverage (-): 0

Region: chr26 46670783-46670797. Max. coverage (+): 0. Max coverage (-): 0

Region: chr26 46670798-46670812. Max. coverage (+): 0. Max coverage (-): 0

Region: chr26 46670813-46670827. Max. coverage (+): 0. Max coverage (-): 0

Region: chr26 46670828-46670842. Max. coverage (+): 0. Max coverage (-): 0

Region: chr26 46670843-46670857. Max. coverage (+): 0. Max coverage (-): 0

Region: chr26 46670858-46670872. Max. coverage (+): 0. Max coverage (-): 0

Region: chr26 46670873-46670887. Max. coverage (+): 0. Max coverage (-): 0

Region: chr26 46670888-46670902. Max. coverage (+): 0. Max coverage (-): 0

Region: chr26 46670903-46670917. Max. coverage (+): 0. Max coverage (-): 0

Region: chr26 46670918-46670932. Max. coverage (+): 0. Max coverage (-): 0

Region: chr26 46670933-46670947. Max. coverage (+): 0. Max coverage (-): 0

Region: chr26 46670948-46670962. Max. coverage (+): 0. Max coverage (-): 0

Region: chr26 46670963-46670977. Max. coverage (+): 0. Max coverage (-): 0

Region: chr26 46670978-46670992. Max. coverage (+): 0. Max coverage (-): 0

Region: chr26 46670993-46671007. Max. coverage (+): 0. Max coverage (-): 0

Region: chr26 46671008-46671022. Max. coverage (+): 0. Max coverage (-): 0

Region: chr26 46671023-46671037. Max. coverage (+): 0. Max coverage (-): 0

Region: chr26 46671038-46671052. Max. coverage (+): 0. Max coverage (-): 0

Region: chr26 46671053-46671067. Max. coverage (+): 0. Max coverage (-): 0

Region: chr26 46671068-46671082. Max. coverage (+): 0. Max coverage (-): 0

Region: chr26 46671083-46671097. Max. coverage (+): 0. Max coverage (-): 0

Region: chr26 46671098-46671112. Max. coverage (+): 0. Max coverage (-): 0

Region: chr26 46671113-46671127. Max. coverage (+): 0. Max coverage (-): 0

Region: chr26 46671128-46671142. Max. coverage (+): 0. Max coverage (-): 0

Region: chr26 46671143-46671157. Max. coverage (+): 0. Max coverage (-): 0

Region: chr26 46671158-46671172. Max. coverage (+): 0. Max coverage (-): 1.69

Region: chr26 46671173-46671187. Max. coverage (+): 0. Max coverage (-): 1.69

Region: chr26 46671188-46671202. Max. coverage (+): 0. Max coverage (-): 0

Region: chr26 46671203-46671217. Max. coverage (+): 0. Max coverage (-): 0

Region: chr26 46671218-46671232. Max. coverage (+): 0. Max coverage (-): 0

Region: chr26 46671233-46671247. Max. coverage (+): 0. Max coverage (-): 1.11

Region: chr26 46671248-46671262. Max. coverage (+): 0. Max coverage (-): 1.67

Region: chr26 46671263-46671277. Max. coverage (+): 0. Max coverage (-): 3.06

Region: chr26 46671278-46671292. Max. coverage (+): 0. Max coverage (-): 3.06

Region: chr26 46671293-46671307. Max. coverage (+): 0. Max coverage (-): 7.95

Region: chr26 46671308-46671322. Max. coverage (+): 0. Max coverage (-): 10.32

Region: chr26 46671323-46671337. Max. coverage (+): 0. Max coverage (-): 2.36

Region: chr26 46671338-46671352. Max. coverage (+): 0. Max coverage (-): 6.06

Region: chr26 46671353-46671367. Max. coverage (+): 0. Max coverage (-): 0

Region: chr26 46671368-46671382. Max. coverage (+): 0. Max coverage (-): 6.3

Region: chr26 46671383-46671397. Max. coverage (+): 0. Max coverage (-): 6.3

Region: chr26 46671398-46671412. Max. coverage (+): 0. Max coverage (-): 0

Region: chr26 46671413-46671427. Max. coverage (+): 0. Max coverage (-): 12.77

Region: chr26 46671428-46671442. Max. coverage (+): 0. Max coverage (-): 12.77

Region: chr26 46671443-46671457. Max. coverage (+): 0. Max coverage (-): 4.91

Region: chr26 46671458-46671472. Max. coverage (+): 0. Max coverage (-): 18.04

Region: chr26 46671473-46671487. Max. coverage (+): 0. Max coverage (-): 0

Region: chr26 46671488-46671502. Max. coverage (+): 0. Max coverage (-): 0

Region: chr26 46671503-46671517. Max. coverage (+): 0. Max coverage (-): 0

Region: chr26 46671518-46671532. Max. coverage (+): 0. Max coverage (-): 0

Region: chr26 46671533-46671547. Max. coverage (+): 0. Max coverage (-): 0

Region: chr26 46671548-46671562. Max. coverage (+): 0. Max coverage (-): 0

Region: chr26 46671563-46671577. Max. coverage (+): 0. Max coverage (-): 0

Region: chr26 46671578-46671592. Max. coverage (+): 0. Max coverage (-): 0

Region: chr26 46671593-46671607. Max. coverage (+): 0. Max coverage (-): 1.81

Region: chr26 46671608-46671622. Max. coverage (+): 0. Max coverage (-): 2.57

Region: chr26 46671623-46671637. Max. coverage (+): 0. Max coverage (-): 10.89

Region: chr26 46671638-46671652. Max. coverage (+): 0. Max coverage (-): 26.34

Region: chr26 46671653-46671667. Max. coverage (+): 0. Max coverage (-): 0

Region: chr26 46671668-46671682. Max. coverage (+): 0. Max coverage (-): 0

Region: chr26 46671683-46671697. Max. coverage (+): 0. Max coverage (-): 0

Region: chr26 46671698-46671712. Max. coverage (+): 0. Max coverage (-): 0

Region: chr26 46671713-46671727. Max. coverage (+): 0. Max coverage (-): 0

Region: chr26 46671728-46671742. Max. coverage (+): 0. Max coverage (-): 0

Region: chr26 46671743-46671757. Max. coverage (+): 0. Max coverage (-): 0

Region: chr26 46671758-46671772. Max. coverage (+): 0. Max coverage (-): 0

Region: chr26 46671773-46671787. Max. coverage (+): 0. Max coverage (-): 0

Region: chr26 46671788-46671802. Max. coverage (+): 0. Max coverage (-): 0

Region: chr26 46671803-46671817. Max. coverage (+): 0. Max coverage (-): 0

Region: chr26 46671818-46671832. Max. coverage (+): 0. Max coverage (-): 0

Region: chr26 46671833-46671847. Max. coverage (+): 0. Max coverage (-): 0

Region: chr26 46671848-46671862. Max. coverage (+): 0. Max coverage (-): 0

Region: chr26 46671863-46671877. Max. coverage (+): 0. Max coverage (-): 0

Region: chr26 46671878-46671892. Max. coverage (+): 0. Max coverage (-): 2

Region: chr26 46671893-46671907. Max. coverage (+): 0. Max coverage (-): 2

Region: chr26 46671908-46671922. Max. coverage (+): 0. Max coverage (-): 0

Region: chr26 46671923-46671937. Max. coverage (+): 0. Max coverage (-): 0

Region: chr26 46671938-46671952. Max. coverage (+): 0. Max coverage (-): 0

Region: chr26 46671953-46671967. Max. coverage (+): 0. Max coverage (-): 2.63

Region: chr26 46671968-46671982. Max. coverage (+): 0. Max coverage (-): 0

Region: chr26 46671983-46671997. Max. coverage (+): 0. Max coverage (-): 0

Region: chr26 46671998-46672012. Max. coverage (+): 0. Max coverage (-): 0

Region: chr26 46672013-46672027. Max. coverage (+): 0. Max coverage (-): 0

Region: chr26 46672028-46672042. Max. coverage (+): 0. Max coverage (-): 0

Region: chr26 46672043-46672057. Max. coverage (+): 0. Max coverage (-): 0

Region: chr26 46672058-46672072. Max. coverage (+): 0. Max coverage (-): 0

Region: chr26 46672073-46672087. Max. coverage (+): 0. Max coverage (-): 0

Region: chr26 46672088-46672102. Max. coverage (+): 0. Max coverage (-): 0

Region: chr26 46672103-46672117. Max. coverage (+): 0. Max coverage (-): 0

Region: chr26 46672118-46672132. Max. coverage (+): 0. Max coverage (-): 0

Region: chr26 46672133-46672147. Max. coverage (+): 0. Max coverage (-): 0

Region: chr26 46672148-46672162. Max. coverage (+): 0. Max coverage (-): 0

Region: chr26 46672163-46672177. Max. coverage (+): 0. Max coverage (-): 0

Region: chr26 46672178-46672192. Max. coverage (+): 0. Max coverage (-): 0

Region: chr26 46672193-46672207. Max. coverage (+): 0. Max coverage (-): 0

Region: chr26 46672208-46672222. Max. coverage (+): 0. Max coverage (-): 0

Region: chr26 46672223-46672237. Max. coverage (+): 0. Max coverage (-): 1.95

Region: chr26 46672238-46672252. Max. coverage (+): 0. Max coverage (-): 0

Region: chr26 46672253-46672267. Max. coverage (+): 0. Max coverage (-): 0

Region: chr26 46672268-46672282. Max. coverage (+): 0. Max coverage (-): 0

Region: chr26 46672283-46672297. Max. coverage (+): 0. Max coverage (-): 0

Region: chr26 46672298-46672312. Max. coverage (+): 0. Max coverage (-): 0

Region: chr26 46672313-46672327. Max. coverage (+): 0. Max coverage (-): 10.37

Region: chr26 46672328-46672342. Max. coverage (+): 0. Max coverage (-): 10.37

Region: chr26 46672343-46672357. Max. coverage (+): 0. Max coverage (-): 4.33

Region: chr26 46672358-46672372. Max. coverage (+): 0. Max coverage (-): 0

Region: chr26 46672373-46672387. Max. coverage (+): 0. Max coverage (-): 0

Region: chr26 46672388-46672402. Max. coverage (+): 0. Max coverage (-): 0

Region: chr26 46672403-46672417. Max. coverage (+): 0. Max coverage (-): 3.61

Region: chr26 46672418-46672432. Max. coverage (+): 0. Max coverage (-): 2.95

Region: chr26 46672433-46672447. Max. coverage (+): 0. Max coverage (-): 0

Region: chr26 46672448-46672462. Max. coverage (+): 0. Max coverage (-): 0

Region: chr26 46672463-46672477. Max. coverage (+): 0. Max coverage (-): 0

Region: chr26 46672478-46672492. Max. coverage (+): 0. Max coverage (-): 0

Region: chr26 46672493-46672507. Max. coverage (+): 0. Max coverage (-): 0

Region: chr26 46672508-46672522. Max. coverage (+): 0. Max coverage (-): 0

Region: chr26 46672523-46672537. Max. coverage (+): 0. Max coverage (-): 0.69

Region: chr26 46672538-46672551. Max. coverage (+): 0. Max coverage (-): 0.69

Region: chr26 46672552-46672566. Max. coverage (+): 0. Max coverage (-): 1.43

Region: chr26 46672567-46672581. Max. coverage (+): 0. Max coverage (-): 0

Region: chr26 46672582-46672596. Max. coverage (+): 0. Max coverage (-): 0

Region: chr26 46672597-46672611. Max. coverage (+): 0. Max coverage (-): 6.89

Region: chr26 46672612-46672626. Max. coverage (+): 0. Max coverage (-): 2.1

Region: chr26 46672627-46672641. Max. coverage (+): 0. Max coverage (-): 0

Region: chr26 46672642-46672656. Max. coverage (+): 0. Max coverage (-): 0

Region: chr26 46672657-46672671. Max. coverage (+): 0. Max coverage (-): 0

Region: chr26 46672672-46672686. Max. coverage (+): 0. Max coverage (-): 0

Region: chr26 46672687-46672701. Max. coverage (+): 0. Max coverage (-): 12.89

Region: chr26 46672702-46672716. Max. coverage (+): 0. Max coverage (-): 0

Region: chr26 46672717-46672731. Max. coverage (+): 0. Max coverage (-): 0

Region: chr26 46672732-46672746. Max. coverage (+): 0. Max coverage (-): 0

Region: chr26 46672747-46672761. Max. coverage (+): 0. Max coverage (-): 3.12

Region: chr26 46672762-46672776. Max. coverage (+): 0. Max coverage (-): 5.33

Region: chr26 46672777-46672791. Max. coverage (+): 0. Max coverage (-): 4.11

Region: chr26 46672792-46672806. Max. coverage (+): 0. Max coverage (-): 4.11

Region: chr26 46672807-46672821. Max. coverage (+): 0. Max coverage (-): 3.69

Region: chr26 46672822-46672836. Max. coverage (+): 0. Max coverage (-): 5.71

Region: chr26 46672837-46672851. Max. coverage (+): 0. Max coverage (-): 1.15

Region: chr26 46672852-46672866. Max. coverage (+): 0. Max coverage (-): 2.18

Region: chr26 46672867-46672881. Max. coverage (+): 0. Max coverage (-): 5.57

Region: chr26 46672882-46672896. Max. coverage (+): 0. Max coverage (-): 1.97

Region: chr26 46672897-46672911. Max. coverage (+): 0. Max coverage (-): 0

Region: chr26 46672912-46672926. Max. coverage (+): 0. Max coverage (-): 2.69

Region: chr26 46672927-46672941. Max. coverage (+): 0. Max coverage (-): 8.26

Region: chr26 46672942-46672956. Max. coverage (+): 0. Max coverage (-): 8.26

Region: chr26 46672957-46672971. Max. coverage (+): 0. Max coverage (-): 0

Region: chr26 46672972-46672986. Max. coverage (+): 0. Max coverage (-): 0

Region: chr26 46672987-46673001. Max. coverage (+): 0. Max coverage (-): 0

Region: chr26 46673002-46673016. Max. coverage (+): 0. Max coverage (-): 0

Region: chr26 46673017-46673031. Max. coverage (+): 0. Max coverage (-): 0

Region: chr26 46673032-46673046. Max. coverage (+): 0. Max coverage (-): 0

Region: chr26 46673047-46673061. Max. coverage (+): 0. Max coverage (-): 0

Region: chr26 46673062-46673076. Max. coverage (+): 0. Max coverage (-): 0

Region: chr26 46673077-46673091. Max. coverage (+): 0. Max coverage (-): 0

Region: chr26 46673092-46673106. Max. coverage (+): 0. Max coverage (-): 0

Region: chr26 46673107-46673121. Max. coverage (+): 0. Max coverage (-): 0

Region: chr26 46673122-46673136. Max. coverage (+): 0. Max coverage (-): 0

Region: chr26 46673137-46673151. Max. coverage (+): 0. Max coverage (-): 0

Region: chr26 46673152-46673166. Max. coverage (+): 0. Max coverage (-): 0

Region: chr26 46673167-46673181. Max. coverage (+): 0. Max coverage (-): 0.64

Region: chr26 46673182-46673196. Max. coverage (+): 0. Max coverage (-): 3.86

Region: chr26 46673197-46673211. Max. coverage (+): 0. Max coverage (-): 0

Region: chr26 46673212-46673226. Max. coverage (+): 0. Max coverage (-): 0

Region: chr26 46673227-46673241. Max. coverage (+): 0. Max coverage (-): 0

Region: chr26 46673242-46673256. Max. coverage (+): 0. Max coverage (-): 0

Region: chr26 46673257-46673271. Max. coverage (+): 0. Max coverage (-): 0

Region: chr26 46673272-46673286. Max. coverage (+): 0. Max coverage (-): 0

Region: chr26 46673287-46673301. Max. coverage (+): 0. Max coverage (-): 0

Region: chr26 46673302-46673316. Max. coverage (+): 0. Max coverage (-): 0

Region: chr26 46673317-46673331. Max. coverage (+): 0. Max coverage (-): 0

Region: chr26 46673332-46673346. Max. coverage (+): 0. Max coverage (-): 0

Region: chr26 46673347-46673361. Max. coverage (+): 0. Max coverage (-): 0

Region: chr26 46673362-46673376. Max. coverage (+): 0. Max coverage (-): 0

Region: chr26 46673377-46673391. Max. coverage (+): 0. Max coverage (-): 0

Region: chr26 46673392-46673406. Max. coverage (+): 0. Max coverage (-): 0

Region: chr26 46673407-46673421. Max. coverage (+): 0. Max coverage (-): 18.28

Region: chr26 46673422-46673436. Max. coverage (+): 0. Max coverage (-): 23.24

Region: chr26 46673437-46673451. Max. coverage (+): 0. Max coverage (-): 3.84

Region: chr26 46673452-46673466. Max. coverage (+): 0. Max coverage (-): 0

Region: chr26 46673467-46673481. Max. coverage (+): 0. Max coverage (-): 6.54

Region: chr26 46673482-46673496. Max. coverage (+): 0. Max coverage (-): 4.3

Region: chr26 46673497-46673511. Max. coverage (+): 0. Max coverage (-): 4.17

Region: chr26 46673512-46673526. Max. coverage (+): 0. Max coverage (-): 0

Region: chr26 46673527-46673541. Max. coverage (+): 0. Max coverage (-): 0

Region: chr26 46673542-46673556. Max. coverage (+): 0. Max coverage (-): 0

Region: chr26 46673557-46673571. Max. coverage (+): 0. Max coverage (-): 6.31

Region: chr26 46673572-46673586. Max. coverage (+): 0. Max coverage (-): 4.21

Region: chr26 46673587-46673601. Max. coverage (+): 0. Max coverage (-): 11.58

Region: chr26 46673602-46673616. Max. coverage (+): 0. Max coverage (-): 0

Region: chr26 46673617-46673631. Max. coverage (+): 0. Max coverage (-): 0

Region: chr26 46673632-46673646. Max. coverage (+): 0. Max coverage (-): 0

Region: chr26 46673647-46673661. Max. coverage (+): 0. Max coverage (-): 19.05

Region: chr26 46673662-46673676. Max. coverage (+): 0. Max coverage (-): 21.31

Region: chr26 46673677-46673691. Max. coverage (+): 0. Max coverage (-): 0.39

Region: chr26 46673692-46673706. Max. coverage (+): 0. Max coverage (-): 0

Region: chr26 46673707-46673721. Max. coverage (+): 0. Max coverage (-): 0

Region: chr26 46673722-46673736. Max. coverage (+): 0. Max coverage (-): 6.16

Region: chr26 46673737-46673751. Max. coverage (+): 0. Max coverage (-): 0

Region: chr26 46673752-46673766. Max. coverage (+): 0. Max coverage (-): 4.25

Region: chr26 46673767-46673781. Max. coverage (+): 0. Max coverage (-): 0

Region: chr26 46673782-. Max. coverage (+): 0. Max coverage (-): 0

RepeatMasker Color Code

**+**

100-98% Identity

<98-95% Identity

<95-90% Identity

<90-85% Identity

<85-80% Identity

<80-75% Identity

<75-70% Identity

<70% Identity

**-**

Gene Set Color Code

**+**

Gene

Pseudogene

**-**

Topology/Coverage Color Code

Coverage Plus Strand

Coverage Minus Strand

Mainstrand: Plus

Mainstrand: Minus

Complementary Strand

Flanking Region  
(if option -flank >0)

Gene Set Annotation  
  
RepeatMasker Annotation  

**1. MIRc**: 46666475-46666586 (+), Divergence to consensus: 36.1%  
**2. MIR**: 46667356-46667396 (-), Divergence to consensus: 24.4%  
**3. Helitron3Na\_Mam**: 46669320-46669784 (+), Divergence to consensus: 37.5%  
**4. MER5A1**: 46669993-46670136 (+), Divergence to consensus: 44.3%  
**5. MER58B**: 46670764-46671083 (-), Divergence to consensus: 34.2%  
**6. MIRc**: 46671115-46671156 (+), Divergence to consensus: 26.2%  
**7. L2b**: 46671675-46671745 (-), Divergence to consensus: 22.5%  
**8. (TAGA)n**: 46671796-46671846 (+), Divergence to consensus: 25.5%  
**9. MER102b**: 46672100-46672203 (-), Divergence to consensus: 36.6%  
**10. MIR**: 46672986-46673103 (+), Divergence to consensus: 43.4%

  
Transcription Factor Binding Sites  

**RFX4\_1** (Sequence: GTTGCCATG (-): 46673213)  
**RFX4\_2** (Sequence: CTTAGTTAC (+): 46666351)  
**Gata4** (Sequence: AGATAAG (-): 46668204)  
**Gata4** (Sequence: AGATAAC (-): 46668273)  
**SOX9** (Sequence: AACAATAA (-): 46668777)
