# Supplementary material for: Small RNA sequencing of cryopreserved semen from single bull revealed altered miRNAs and piRNAs expression between High- and Low-motile sperm populations
Source: BMC Genomics. 2017 Jan 4;18:14. doi: 10.1186/s12864-016-3394-7 (PMC5209821; doi:10.1186/s12864-016-3394-7)
Supplement: Additional file 3: — Details for each piRNA clusters found in High Motile (HM) sperm fraction. Genes, repeats, transposable elements and transcription factors binding sites falling within the cluster regions were reported. (ZIP 1896 kb) [file 12864_2016_3394_MOESM3_ESM.zip › 65.html]

piRNA cluster 65


Predicted piRNA cluster no. 65     previous   next
  

Show proTRAC run info
Hide proTRAC run info

================================= proTRAC ====================================  
VERSION: 2.1                                    LAST MODIFIED: 06. October 2015  
  
Please cite:  
Rosenkranz D, Zischler H. proTRAC - a software for probabilistic piRNA cluster  
detection, visualization and analysis. 2012. BMC Bioinformatics 13:5.  
  
and (for proTRAC 2.0 and later):  
Rosenkranz D, Rudloff S, Bastuck K, Ketting RF, Zischler H. Tupaia small RNAs  
provide insights into function and evolution of RNAi-based transposon defense  
in mammals. 2015. RNA 21(5):911-922.  
  
Contact:  
David Rosenkranz  
Institute of Anthropology, small RNA group  
Johannes Gutenberg University Mainz  
email: rosenkranz@uni-mainz.de  
  
You can find the latest proTRAC version at:  
http://sourceforge.net/projects/protrac/files  
http://www.smallRNAgroup-mainz.de/software  
==============================================================================  
  
PARAMETERS:  
Map file: .............../storage/core/barbara/genhome/smallRNA/fertility/Sample\_motile/pirna/Sample\_motile\_26-33\_collapsed.fa.no-dust.map.weighted-10000-1000-b-0  
Genome file: ............/storage/core/barbara/genhome/smallRNA/fertility/Sample\_all/pirna/bt\_311\_chrY.fa  
RepeatMasker annotation: /storage/genomes/bt\_umd31/GCF\_000003055.6\_Bos\_taurus\_UMD\_3.1.1\_repeatMasker\_chr.out  
GeneSet:................./storage/core/barbara/genhome/smallRNA/fertility/Sample\_all/pirna/full.gtf  
  
Significant (p<=0.01) hit density will be calculated based  
on observed hit distribution.  
  
Sliding window size: ........................................ 5000 bp  
Sliding window increament: .................................. 1000 bp  
Normalize each hit by number of genomic hits: ............... 1 [0=no/1=yes]  
Normalize each hit by number of sequence reads: ............. 1 [0=no/1=yes]  
Normalize values (-> per million mapped reads): ............. 1 [0=no/1=yes]  
Min. fraction of hits with 1T(U) or 10A: .................... 0.75  
Alternatively: Min. fraction of hits with 1T(U) and 10A: .... 0.5  
Min. fraction of hits with typical piRNA length: ............ 0.75  
Typical piRNA length: ....................................... 26-33 nt  
Min. size of a piRNA cluster: ............................... 5000 bp.  
Min. number of hits (absolute): ............................. 0  
Min. number of hits (normalized): ........................... 0  
Min. fraction of hits on the mainstrand: .................... 0.75  
Top fraction of mapped sequences (in terms of read counts): . 1%  
Top fraction accounts for max. n% of sequence reads: ........ 90%  
Min. fraction of hits on each arm of a bidirectional cluster: 0.1  
Output image file for each cluster: ......................... 0 [0=no/1=yes]  
Output html file for each cluster: .......................... 1 [0=no/1=yes]  
Output a summary table: ..................................... 1 [0=no/1=yes]  
Output a FASTA file for each cluster (piRNA sequences): ..... 1 [0=no/1=yes]  
Output a FASTA file comprising cluster sequences: ........... 1 [0=no/1=yes]  
Search DNA motifs in clusters: .............................. 1 [0=no/1=yes]  
Output flanking sequences: +/- .............................. 0 bp  
Output ~.pTi file: .......................................... 1 [0=no/1=yes]  
==============================================================================  
  
  
Genome size (without gaps): ............ 2678902517 bp  
Gaps (N/X/-): .......................... 53837044 bp  
Mapped reads: .......................... 658825247023  
Non-identical sequences: ............... 514171  
Genomic hits: .......................... 764233  
Significant densitiy of mapped reads: .. 12867599.5173724 reads/kb

Show proTRAC cluster info
Hide proTRAC cluster info

|  |  |
| --- | --- |
| Location | chr27 |
| Coordinates | 23504625-23512485 |
| Size [bp] | 7861 |
| Sequence hit loci | 249 |
| Mapped reads (normalized) | 289851163 |
| Mapped reads (normalized) per kb | 36872047.2 |
| Normalized reads with 1T (1U) | 81.9% |
| Normalized reads with 10A | 28.5% |
| Normalized reads with length 26-33 nt | 100% |
| Normalized reads on the main strand(s) | 100% |
| Predicted directionality | mono:minus |

100%

0%

1T (1U)  
reads

10A reads

26-33 nt  
reads

reads on mainstrand

**Either the amount of reads with 1T (1U) OR 10A has to exceed 75% (set with option: -1Tor10A)  
Alternatively the amount of reads with 1T (1U) AND 10A has to exceed 50% (set with option: -1Tand10A)  
Minimum amount of reads with preferred size is 75% (set with option: -pisize)  
Minimum amount of reads on the main strand(s) is 75% (set with option: -clstrand)**

Show read coverage
Hide read coverage

WHAT DO I SEE HERE?  
This chart shows the location of mapped sequence reads within a predicted piRNA cluster. The color refers to the number of genomic hits produced by the sequence read in question. A dark red bar indicates that this sequence read produces many other hits elsewhere in the genome. Many adjacent red or yellow bars can indicate the presence of a multi-copy element such as transposons or rRNA genes. A dark green bar indicates that this sequence read maps uniquely to this locus.

1 hit

2-5 hits

6-10 hits

11-20 hits

21-50 hits

51-100 hits

> 100 hits

chr27

23504625

23512485

Gene Set

RepeatMasker

Mapped  
Reads

26.16

plus strand

minus strand

26.16

Region: chr27 46673783-23504632. Max. coverage (+): 0. Max coverage (-): 2.37

Region: chr27 23504633-23504648. Max. coverage (+): 0. Max coverage (-): 0

Region: chr27 23504649-23504664. Max. coverage (+): 0. Max coverage (-): 0

Region: chr27 23504665-23504680. Max. coverage (+): 0. Max coverage (-): 0

Region: chr27 23504681-23504695. Max. coverage (+): 0. Max coverage (-): 0

Region: chr27 23504696-23504711. Max. coverage (+): 0. Max coverage (-): 0

Region: chr27 23504712-23504727. Max. coverage (+): 0. Max coverage (-): 0

Region: chr27 23504728-23504742. Max. coverage (+): 0. Max coverage (-): 0

Region: chr27 23504743-23504758. Max. coverage (+): 0. Max coverage (-): 0

Region: chr27 23504759-23504774. Max. coverage (+): 0. Max coverage (-): 0

Region: chr27 23504775-23504790. Max. coverage (+): 0. Max coverage (-): 0

Region: chr27 23504791-23504805. Max. coverage (+): 0. Max coverage (-): 0

Region: chr27 23504806-23504821. Max. coverage (+): 0. Max coverage (-): 0

Region: chr27 23504822-23504837. Max. coverage (+): 0. Max coverage (-): 0

Region: chr27 23504838-23504852. Max. coverage (+): 0. Max coverage (-): 0

Region: chr27 23504853-23504868. Max. coverage (+): 0. Max coverage (-): 0

Region: chr27 23504869-23504884. Max. coverage (+): 0. Max coverage (-): 0

Region: chr27 23504885-23504900. Max. coverage (+): 0. Max coverage (-): 0

Region: chr27 23504901-23504915. Max. coverage (+): 0. Max coverage (-): 0

Region: chr27 23504916-23504931. Max. coverage (+): 0. Max coverage (-): 0

Region: chr27 23504932-23504947. Max. coverage (+): 0. Max coverage (-): 0

Region: chr27 23504948-23504963. Max. coverage (+): 0. Max coverage (-): 4.8

Region: chr27 23504964-23504978. Max. coverage (+): 0. Max coverage (-): 2.83

Region: chr27 23504979-23504994. Max. coverage (+): 0. Max coverage (-): 0

Region: chr27 23504995-23505010. Max. coverage (+): 0. Max coverage (-): 0

Region: chr27 23505011-23505025. Max. coverage (+): 0. Max coverage (-): 0

Region: chr27 23505026-23505041. Max. coverage (+): 0. Max coverage (-): 0

Region: chr27 23505042-23505057. Max. coverage (+): 0. Max coverage (-): 0

Region: chr27 23505058-23505073. Max. coverage (+): 0. Max coverage (-): 0

Region: chr27 23505074-23505088. Max. coverage (+): 0. Max coverage (-): 4

Region: chr27 23505089-23505104. Max. coverage (+): 0. Max coverage (-): 0

Region: chr27 23505105-23505120. Max. coverage (+): 0. Max coverage (-): 1.45

Region: chr27 23505121-23505135. Max. coverage (+): 0. Max coverage (-): 0

Region: chr27 23505136-23505151. Max. coverage (+): 0. Max coverage (-): 3.72

Region: chr27 23505152-23505167. Max. coverage (+): 0. Max coverage (-): 1.38

Region: chr27 23505168-23505183. Max. coverage (+): 0. Max coverage (-): 0

Region: chr27 23505184-23505198. Max. coverage (+): 0. Max coverage (-): 4.21

Region: chr27 23505199-23505214. Max. coverage (+): 0. Max coverage (-): 0

Region: chr27 23505215-23505230. Max. coverage (+): 0. Max coverage (-): 0

Region: chr27 23505231-23505246. Max. coverage (+): 0. Max coverage (-): 1.26

Region: chr27 23505247-23505261. Max. coverage (+): 0. Max coverage (-): 0

Region: chr27 23505262-23505277. Max. coverage (+): 0. Max coverage (-): 0

Region: chr27 23505278-23505293. Max. coverage (+): 0. Max coverage (-): 0

Region: chr27 23505294-23505308. Max. coverage (+): 0. Max coverage (-): 0

Region: chr27 23505309-23505324. Max. coverage (+): 0. Max coverage (-): 0

Region: chr27 23505325-23505340. Max. coverage (+): 0. Max coverage (-): 0

Region: chr27 23505341-23505356. Max. coverage (+): 0. Max coverage (-): 0

Region: chr27 23505357-23505371. Max. coverage (+): 0. Max coverage (-): 0

Region: chr27 23505372-23505387. Max. coverage (+): 0. Max coverage (-): 0

Region: chr27 23505388-23505403. Max. coverage (+): 0. Max coverage (-): 0

Region: chr27 23505404-23505418. Max. coverage (+): 0. Max coverage (-): 0

Region: chr27 23505419-23505434. Max. coverage (+): 0. Max coverage (-): 0

Region: chr27 23505435-23505450. Max. coverage (+): 0. Max coverage (-): 0

Region: chr27 23505451-23505466. Max. coverage (+): 0. Max coverage (-): 0

Region: chr27 23505467-23505481. Max. coverage (+): 0. Max coverage (-): 0

Region: chr27 23505482-23505497. Max. coverage (+): 0. Max coverage (-): 0

Region: chr27 23505498-23505513. Max. coverage (+): 0. Max coverage (-): 0

Region: chr27 23505514-23505529. Max. coverage (+): 0. Max coverage (-): 0

Region: chr27 23505530-23505544. Max. coverage (+): 0. Max coverage (-): 0

Region: chr27 23505545-23505560. Max. coverage (+): 0. Max coverage (-): 0

Region: chr27 23505561-23505576. Max. coverage (+): 0. Max coverage (-): 0

Region: chr27 23505577-23505591. Max. coverage (+): 0. Max coverage (-): 0

Region: chr27 23505592-23505607. Max. coverage (+): 0. Max coverage (-): 0

Region: chr27 23505608-23505623. Max. coverage (+): 0. Max coverage (-): 0

Region: chr27 23505624-23505639. Max. coverage (+): 0. Max coverage (-): 0

Region: chr27 23505640-23505654. Max. coverage (+): 0. Max coverage (-): 0

Region: chr27 23505655-23505670. Max. coverage (+): 0. Max coverage (-): 0

Region: chr27 23505671-23505686. Max. coverage (+): 0. Max coverage (-): 0

Region: chr27 23505687-23505701. Max. coverage (+): 0. Max coverage (-): 0

Region: chr27 23505702-23505717. Max. coverage (+): 0. Max coverage (-): 0

Region: chr27 23505718-23505733. Max. coverage (+): 0. Max coverage (-): 0

Region: chr27 23505734-23505749. Max. coverage (+): 0. Max coverage (-): 0

Region: chr27 23505750-23505764. Max. coverage (+): 0. Max coverage (-): 0

Region: chr27 23505765-23505780. Max. coverage (+): 0. Max coverage (-): 0

Region: chr27 23505781-23505796. Max. coverage (+): 0. Max coverage (-): 0

Region: chr27 23505797-23505812. Max. coverage (+): 0. Max coverage (-): 0

Region: chr27 23505813-23505827. Max. coverage (+): 0. Max coverage (-): 0

Region: chr27 23505828-23505843. Max. coverage (+): 0. Max coverage (-): 0

Region: chr27 23505844-23505859. Max. coverage (+): 0. Max coverage (-): 0

Region: chr27 23505860-23505874. Max. coverage (+): 0. Max coverage (-): 0

Region: chr27 23505875-23505890. Max. coverage (+): 0. Max coverage (-): 0

Region: chr27 23505891-23505906. Max. coverage (+): 0. Max coverage (-): 0

Region: chr27 23505907-23505922. Max. coverage (+): 0. Max coverage (-): 2.24

Region: chr27 23505923-23505937. Max. coverage (+): 0. Max coverage (-): 0

Region: chr27 23505938-23505953. Max. coverage (+): 0. Max coverage (-): 0

Region: chr27 23505954-23505969. Max. coverage (+): 0. Max coverage (-): 0

Region: chr27 23505970-23505984. Max. coverage (+): 0. Max coverage (-): 2.27

Region: chr27 23505985-23506000. Max. coverage (+): 0. Max coverage (-): 0

Region: chr27 23506001-23506016. Max. coverage (+): 0. Max coverage (-): 0

Region: chr27 23506017-23506032. Max. coverage (+): 0. Max coverage (-): 0

Region: chr27 23506033-23506047. Max. coverage (+): 0. Max coverage (-): 0

Region: chr27 23506048-23506063. Max. coverage (+): 0. Max coverage (-): 0

Region: chr27 23506064-23506079. Max. coverage (+): 0. Max coverage (-): 0

Region: chr27 23506080-23506095. Max. coverage (+): 0. Max coverage (-): 0

Region: chr27 23506096-23506110. Max. coverage (+): 0. Max coverage (-): 0

Region: chr27 23506111-23506126. Max. coverage (+): 0. Max coverage (-): 0

Region: chr27 23506127-23506142. Max. coverage (+): 0. Max coverage (-): 0

Region: chr27 23506143-23506157. Max. coverage (+): 0. Max coverage (-): 0

Region: chr27 23506158-23506173. Max. coverage (+): 0. Max coverage (-): 0

Region: chr27 23506174-23506189. Max. coverage (+): 0. Max coverage (-): 0

Region: chr27 23506190-23506205. Max. coverage (+): 0. Max coverage (-): 0

Region: chr27 23506206-23506220. Max. coverage (+): 0. Max coverage (-): 0

Region: chr27 23506221-23506236. Max. coverage (+): 0. Max coverage (-): 0

Region: chr27 23506237-23506252. Max. coverage (+): 0. Max coverage (-): 0

Region: chr27 23506253-23506267. Max. coverage (+): 0. Max coverage (-): 0

Region: chr27 23506268-23506283. Max. coverage (+): 0. Max coverage (-): 0

Region: chr27 23506284-23506299. Max. coverage (+): 0. Max coverage (-): 0

Region: chr27 23506300-23506315. Max. coverage (+): 0. Max coverage (-): 0

Region: chr27 23506316-23506330. Max. coverage (+): 0. Max coverage (-): 0

Region: chr27 23506331-23506346. Max. coverage (+): 0. Max coverage (-): 0

Region: chr27 23506347-23506362. Max. coverage (+): 0. Max coverage (-): 2.02

Region: chr27 23506363-23506378. Max. coverage (+): 0. Max coverage (-): 0

Region: chr27 23506379-23506393. Max. coverage (+): 0. Max coverage (-): 0.54

Region: chr27 23506394-23506409. Max. coverage (+): 0. Max coverage (-): 1.56

Region: chr27 23506410-23506425. Max. coverage (+): 0. Max coverage (-): 1.08

Region: chr27 23506426-23506440. Max. coverage (+): 0. Max coverage (-): 1.08

Region: chr27 23506441-23506456. Max. coverage (+): 0. Max coverage (-): 1.6

Region: chr27 23506457-23506472. Max. coverage (+): 0. Max coverage (-): 6.04

Region: chr27 23506473-23506488. Max. coverage (+): 0. Max coverage (-): 6.04

Region: chr27 23506489-23506503. Max. coverage (+): 0. Max coverage (-): 0

Region: chr27 23506504-23506519. Max. coverage (+): 0. Max coverage (-): 0

Region: chr27 23506520-23506535. Max. coverage (+): 0. Max coverage (-): 0

Region: chr27 23506536-23506550. Max. coverage (+): 0. Max coverage (-): 0

Region: chr27 23506551-23506566. Max. coverage (+): 0. Max coverage (-): 0

Region: chr27 23506567-23506582. Max. coverage (+): 0. Max coverage (-): 0

Region: chr27 23506583-23506598. Max. coverage (+): 0. Max coverage (-): 0

Region: chr27 23506599-23506613. Max. coverage (+): 0. Max coverage (-): 0

Region: chr27 23506614-23506629. Max. coverage (+): 0. Max coverage (-): 0

Region: chr27 23506630-23506645. Max. coverage (+): 0. Max coverage (-): 0

Region: chr27 23506646-23506660. Max. coverage (+): 0. Max coverage (-): 0

Region: chr27 23506661-23506676. Max. coverage (+): 0. Max coverage (-): 0

Region: chr27 23506677-23506692. Max. coverage (+): 0. Max coverage (-): 0

Region: chr27 23506693-23506708. Max. coverage (+): 0. Max coverage (-): 0

Region: chr27 23506709-23506723. Max. coverage (+): 0. Max coverage (-): 0

Region: chr27 23506724-23506739. Max. coverage (+): 0. Max coverage (-): 0

Region: chr27 23506740-23506755. Max. coverage (+): 0. Max coverage (-): 0

Region: chr27 23506756-23506771. Max. coverage (+): 0. Max coverage (-): 0

Region: chr27 23506772-23506786. Max. coverage (+): 0. Max coverage (-): 0

Region: chr27 23506787-23506802. Max. coverage (+): 0. Max coverage (-): 0

Region: chr27 23506803-23506818. Max. coverage (+): 0. Max coverage (-): 0

Region: chr27 23506819-23506833. Max. coverage (+): 0. Max coverage (-): 0

Region: chr27 23506834-23506849. Max. coverage (+): 0. Max coverage (-): 0

Region: chr27 23506850-23506865. Max. coverage (+): 0. Max coverage (-): 0

Region: chr27 23506866-23506881. Max. coverage (+): 0. Max coverage (-): 0

Region: chr27 23506882-23506896. Max. coverage (+): 0. Max coverage (-): 0

Region: chr27 23506897-23506912. Max. coverage (+): 0. Max coverage (-): 1.01

Region: chr27 23506913-23506928. Max. coverage (+): 0. Max coverage (-): 4.47

Region: chr27 23506929-23506943. Max. coverage (+): 0. Max coverage (-): 0

Region: chr27 23506944-23506959. Max. coverage (+): 0. Max coverage (-): 0

Region: chr27 23506960-23506975. Max. coverage (+): 0. Max coverage (-): 0

Region: chr27 23506976-23506991. Max. coverage (+): 0. Max coverage (-): 0

Region: chr27 23506992-23507006. Max. coverage (+): 0. Max coverage (-): 0

Region: chr27 23507007-23507022. Max. coverage (+): 0. Max coverage (-): 0

Region: chr27 23507023-23507038. Max. coverage (+): 0. Max coverage (-): 0

Region: chr27 23507039-23507054. Max. coverage (+): 0. Max coverage (-): 0

Region: chr27 23507055-23507069. Max. coverage (+): 0. Max coverage (-): 0

Region: chr27 23507070-23507085. Max. coverage (+): 0. Max coverage (-): 0

Region: chr27 23507086-23507101. Max. coverage (+): 0. Max coverage (-): 0

Region: chr27 23507102-23507116. Max. coverage (+): 0. Max coverage (-): 0

Region: chr27 23507117-23507132. Max. coverage (+): 0. Max coverage (-): 0

Region: chr27 23507133-23507148. Max. coverage (+): 0. Max coverage (-): 3.07

Region: chr27 23507149-23507164. Max. coverage (+): 0. Max coverage (-): 3.07

Region: chr27 23507165-23507179. Max. coverage (+): 0. Max coverage (-): 0

Region: chr27 23507180-23507195. Max. coverage (+): 0. Max coverage (-): 0

Region: chr27 23507196-23507211. Max. coverage (+): 0. Max coverage (-): 0

Region: chr27 23507212-23507226. Max. coverage (+): 0. Max coverage (-): 0

Region: chr27 23507227-23507242. Max. coverage (+): 0. Max coverage (-): 0

Region: chr27 23507243-23507258. Max. coverage (+): 0. Max coverage (-): 0

Region: chr27 23507259-23507274. Max. coverage (+): 0. Max coverage (-): 0.51

Region: chr27 23507275-23507289. Max. coverage (+): 0. Max coverage (-): 0

Region: chr27 23507290-23507305. Max. coverage (+): 0. Max coverage (-): 0

Region: chr27 23507306-23507321. Max. coverage (+): 0. Max coverage (-): 0

Region: chr27 23507322-23507337. Max. coverage (+): 0. Max coverage (-): 0

Region: chr27 23507338-23507352. Max. coverage (+): 0. Max coverage (-): 0

Region: chr27 23507353-23507368. Max. coverage (+): 0. Max coverage (-): 0

Region: chr27 23507369-23507384. Max. coverage (+): 0. Max coverage (-): 0

Region: chr27 23507385-23507399. Max. coverage (+): 0. Max coverage (-): 0

Region: chr27 23507400-23507415. Max. coverage (+): 0. Max coverage (-): 0

Region: chr27 23507416-23507431. Max. coverage (+): 0. Max coverage (-): 8.35

Region: chr27 23507432-23507447. Max. coverage (+): 0. Max coverage (-): 0

Region: chr27 23507448-23507462. Max. coverage (+): 0. Max coverage (-): 4.61

Region: chr27 23507463-23507478. Max. coverage (+): 0. Max coverage (-): 4.61

Region: chr27 23507479-23507494. Max. coverage (+): 0. Max coverage (-): 0

Region: chr27 23507495-23507509. Max. coverage (+): 0. Max coverage (-): 0

Region: chr27 23507510-23507525. Max. coverage (+): 0. Max coverage (-): 0

Region: chr27 23507526-23507541. Max. coverage (+): 0. Max coverage (-): 0

Region: chr27 23507542-23507557. Max. coverage (+): 0. Max coverage (-): 0

Region: chr27 23507558-23507572. Max. coverage (+): 0. Max coverage (-): 0

Region: chr27 23507573-23507588. Max. coverage (+): 0. Max coverage (-): 0

Region: chr27 23507589-23507604. Max. coverage (+): 0. Max coverage (-): 0

Region: chr27 23507605-23507620. Max. coverage (+): 0. Max coverage (-): 3.89

Region: chr27 23507621-23507635. Max. coverage (+): 0. Max coverage (-): 5.27

Region: chr27 23507636-23507651. Max. coverage (+): 0. Max coverage (-): 0

Region: chr27 23507652-23507667. Max. coverage (+): 0. Max coverage (-): 0

Region: chr27 23507668-23507682. Max. coverage (+): 0. Max coverage (-): 0

Region: chr27 23507683-23507698. Max. coverage (+): 0. Max coverage (-): 3.06

Region: chr27 23507699-23507714. Max. coverage (+): 0. Max coverage (-): 3.06

Region: chr27 23507715-23507730. Max. coverage (+): 0. Max coverage (-): 0

Region: chr27 23507731-23507745. Max. coverage (+): 0. Max coverage (-): 0

Region: chr27 23507746-23507761. Max. coverage (+): 0. Max coverage (-): 9.56

Region: chr27 23507762-23507777. Max. coverage (+): 0. Max coverage (-): 0

Region: chr27 23507778-23507792. Max. coverage (+): 0. Max coverage (-): 4.42

Region: chr27 23507793-23507808. Max. coverage (+): 0. Max coverage (-): 4.42

Region: chr27 23507809-23507824. Max. coverage (+): 0. Max coverage (-): 0

Region: chr27 23507825-23507840. Max. coverage (+): 0. Max coverage (-): 0

Region: chr27 23507841-23507855. Max. coverage (+): 0. Max coverage (-): 0

Region: chr27 23507856-23507871. Max. coverage (+): 0. Max coverage (-): 0

Region: chr27 23507872-23507887. Max. coverage (+): 0. Max coverage (-): 0

Region: chr27 23507888-23507903. Max. coverage (+): 0. Max coverage (-): 0

Region: chr27 23507904-23507918. Max. coverage (+): 0. Max coverage (-): 0

Region: chr27 23507919-23507934. Max. coverage (+): 0. Max coverage (-): 0

Region: chr27 23507935-23507950. Max. coverage (+): 0. Max coverage (-): 0

Region: chr27 23507951-23507965. Max. coverage (+): 0. Max coverage (-): 0

Region: chr27 23507966-23507981. Max. coverage (+): 0. Max coverage (-): 0

Region: chr27 23507982-23507997. Max. coverage (+): 0. Max coverage (-): 0

Region: chr27 23507998-23508013. Max. coverage (+): 0. Max coverage (-): 0

Region: chr27 23508014-23508028. Max. coverage (+): 0. Max coverage (-): 0

Region: chr27 23508029-23508044. Max. coverage (+): 0. Max coverage (-): 17.18

Region: chr27 23508045-23508060. Max. coverage (+): 0. Max coverage (-): 17.18

Region: chr27 23508061-23508075. Max. coverage (+): 0. Max coverage (-): 0

Region: chr27 23508076-23508091. Max. coverage (+): 0. Max coverage (-): 0

Region: chr27 23508092-23508107. Max. coverage (+): 0. Max coverage (-): 0

Region: chr27 23508108-23508123. Max. coverage (+): 0. Max coverage (-): 0

Region: chr27 23508124-23508138. Max. coverage (+): 0. Max coverage (-): 0

Region: chr27 23508139-23508154. Max. coverage (+): 0. Max coverage (-): 0

Region: chr27 23508155-23508170. Max. coverage (+): 0. Max coverage (-): 0

Region: chr27 23508171-23508186. Max. coverage (+): 0. Max coverage (-): 0

Region: chr27 23508187-23508201. Max. coverage (+): 0. Max coverage (-): 0

Region: chr27 23508202-23508217. Max. coverage (+): 0. Max coverage (-): 4.88

Region: chr27 23508218-23508233. Max. coverage (+): 0. Max coverage (-): 11.75

Region: chr27 23508234-23508248. Max. coverage (+): 0. Max coverage (-): 10.48

Region: chr27 23508249-23508264. Max. coverage (+): 0. Max coverage (-): 3.01

Region: chr27 23508265-23508280. Max. coverage (+): 0. Max coverage (-): 7.49

Region: chr27 23508281-23508296. Max. coverage (+): 0. Max coverage (-): 6.99

Region: chr27 23508297-23508311. Max. coverage (+): 0. Max coverage (-): 12.62

Region: chr27 23508312-23508327. Max. coverage (+): 0. Max coverage (-): 7.28

Region: chr27 23508328-23508343. Max. coverage (+): 0. Max coverage (-): 0

Region: chr27 23508344-23508358. Max. coverage (+): 0. Max coverage (-): 7.09

Region: chr27 23508359-23508374. Max. coverage (+): 0. Max coverage (-): 7.09

Region: chr27 23508375-23508390. Max. coverage (+): 0. Max coverage (-): 2.43

Region: chr27 23508391-23508406. Max. coverage (+): 0. Max coverage (-): 6.06

Region: chr27 23508407-23508421. Max. coverage (+): 0. Max coverage (-): 9.27

Region: chr27 23508422-23508437. Max. coverage (+): 0. Max coverage (-): 0

Region: chr27 23508438-23508453. Max. coverage (+): 0. Max coverage (-): 0

Region: chr27 23508454-23508469. Max. coverage (+): 0. Max coverage (-): 2.57

Region: chr27 23508470-23508484. Max. coverage (+): 0. Max coverage (-): 1.5

Region: chr27 23508485-23508500. Max. coverage (+): 0. Max coverage (-): 7.39

Region: chr27 23508501-23508516. Max. coverage (+): 0. Max coverage (-): 0

Region: chr27 23508517-23508531. Max. coverage (+): 0. Max coverage (-): 0

Region: chr27 23508532-23508547. Max. coverage (+): 0. Max coverage (-): 0

Region: chr27 23508548-23508563. Max. coverage (+): 0. Max coverage (-): 0

Region: chr27 23508564-23508579. Max. coverage (+): 0. Max coverage (-): 0

Region: chr27 23508580-23508594. Max. coverage (+): 0. Max coverage (-): 0

Region: chr27 23508595-23508610. Max. coverage (+): 0. Max coverage (-): 0.72

Region: chr27 23508611-23508626. Max. coverage (+): 0. Max coverage (-): 1.56

Region: chr27 23508627-23508641. Max. coverage (+): 0. Max coverage (-): 0.74

Region: chr27 23508642-23508657. Max. coverage (+): 0. Max coverage (-): 12.3

Region: chr27 23508658-23508673. Max. coverage (+): 0. Max coverage (-): 5.56

Region: chr27 23508674-23508689. Max. coverage (+): 0. Max coverage (-): 6.74

Region: chr27 23508690-23508704. Max. coverage (+): 0. Max coverage (-): 4.25

Region: chr27 23508705-23508720. Max. coverage (+): 0. Max coverage (-): 1.01

Region: chr27 23508721-23508736. Max. coverage (+): 0. Max coverage (-): 0

Region: chr27 23508737-23508752. Max. coverage (+): 0. Max coverage (-): 1.59

Region: chr27 23508753-23508767. Max. coverage (+): 0. Max coverage (-): 6.59

Region: chr27 23508768-23508783. Max. coverage (+): 0. Max coverage (-): 0

Region: chr27 23508784-23508799. Max. coverage (+): 0. Max coverage (-): 9.56

Region: chr27 23508800-23508814. Max. coverage (+): 0. Max coverage (-): 5.72

Region: chr27 23508815-23508830. Max. coverage (+): 0. Max coverage (-): 3.02

Region: chr27 23508831-23508846. Max. coverage (+): 0. Max coverage (-): 1.53

Region: chr27 23508847-23508862. Max. coverage (+): 0. Max coverage (-): 1.45

Region: chr27 23508863-23508877. Max. coverage (+): 0. Max coverage (-): 3.63

Region: chr27 23508878-23508893. Max. coverage (+): 0. Max coverage (-): 3.63

Region: chr27 23508894-23508909. Max. coverage (+): 0. Max coverage (-): 0.66

Region: chr27 23508910-23508924. Max. coverage (+): 0. Max coverage (-): 0.66

Region: chr27 23508925-23508940. Max. coverage (+): 0. Max coverage (-): 0.57

Region: chr27 23508941-23508956. Max. coverage (+): 0. Max coverage (-): 0

Region: chr27 23508957-23508972. Max. coverage (+): 0. Max coverage (-): 0

Region: chr27 23508973-23508987. Max. coverage (+): 0. Max coverage (-): 0

Region: chr27 23508988-23509003. Max. coverage (+): 0. Max coverage (-): 0

Region: chr27 23509004-23509019. Max. coverage (+): 0. Max coverage (-): 0

Region: chr27 23509020-23509035. Max. coverage (+): 0. Max coverage (-): 1.71

Region: chr27 23509036-23509050. Max. coverage (+): 0. Max coverage (-): 0

Region: chr27 23509051-23509066. Max. coverage (+): 0. Max coverage (-): 0

Region: chr27 23509067-23509082. Max. coverage (+): 0. Max coverage (-): 0

Region: chr27 23509083-23509097. Max. coverage (+): 0. Max coverage (-): 0

Region: chr27 23509098-23509113. Max. coverage (+): 0. Max coverage (-): 0.91

Region: chr27 23509114-23509129. Max. coverage (+): 0. Max coverage (-): 0.91

Region: chr27 23509130-23509145. Max. coverage (+): 0. Max coverage (-): 1.03

Region: chr27 23509146-23509160. Max. coverage (+): 0. Max coverage (-): 2.68

Region: chr27 23509161-23509176. Max. coverage (+): 0. Max coverage (-): 0

Region: chr27 23509177-23509192. Max. coverage (+): 0. Max coverage (-): 0

Region: chr27 23509193-23509207. Max. coverage (+): 0. Max coverage (-): 0

Region: chr27 23509208-23509223. Max. coverage (+): 0. Max coverage (-): 0

Region: chr27 23509224-23509239. Max. coverage (+): 0. Max coverage (-): 0

Region: chr27 23509240-23509255. Max. coverage (+): 0. Max coverage (-): 6.71

Region: chr27 23509256-23509270. Max. coverage (+): 0. Max coverage (-): 6.71

Region: chr27 23509271-23509286. Max. coverage (+): 0. Max coverage (-): 1.45

Region: chr27 23509287-23509302. Max. coverage (+): 0. Max coverage (-): 1.45

Region: chr27 23509303-23509318. Max. coverage (+): 0. Max coverage (-): 0

Region: chr27 23509319-23509333. Max. coverage (+): 0. Max coverage (-): 0

Region: chr27 23509334-23509349. Max. coverage (+): 0. Max coverage (-): 0

Region: chr27 23509350-23509365. Max. coverage (+): 0. Max coverage (-): 0

Region: chr27 23509366-23509380. Max. coverage (+): 0. Max coverage (-): 0

Region: chr27 23509381-23509396. Max. coverage (+): 0. Max coverage (-): 0

Region: chr27 23509397-23509412. Max. coverage (+): 0. Max coverage (-): 0

Region: chr27 23509413-23509428. Max. coverage (+): 0. Max coverage (-): 0

Region: chr27 23509429-23509443. Max. coverage (+): 0. Max coverage (-): 0

Region: chr27 23509444-23509459. Max. coverage (+): 0. Max coverage (-): 0

Region: chr27 23509460-23509475. Max. coverage (+): 0. Max coverage (-): 0

Region: chr27 23509476-23509490. Max. coverage (+): 0. Max coverage (-): 0

Region: chr27 23509491-23509506. Max. coverage (+): 0. Max coverage (-): 0

Region: chr27 23509507-23509522. Max. coverage (+): 0. Max coverage (-): 0

Region: chr27 23509523-23509538. Max. coverage (+): 0. Max coverage (-): 0

Region: chr27 23509539-23509553. Max. coverage (+): 0. Max coverage (-): 0

Region: chr27 23509554-23509569. Max. coverage (+): 0. Max coverage (-): 0

Region: chr27 23509570-23509585. Max. coverage (+): 0. Max coverage (-): 0

Region: chr27 23509586-23509601. Max. coverage (+): 0. Max coverage (-): 0

Region: chr27 23509602-23509616. Max. coverage (+): 0. Max coverage (-): 0

Region: chr27 23509617-23509632. Max. coverage (+): 0. Max coverage (-): 0

Region: chr27 23509633-23509648. Max. coverage (+): 0. Max coverage (-): 0

Region: chr27 23509649-23509663. Max. coverage (+): 0. Max coverage (-): 0

Region: chr27 23509664-23509679. Max. coverage (+): 0. Max coverage (-): 0

Region: chr27 23509680-23509695. Max. coverage (+): 0. Max coverage (-): 0

Region: chr27 23509696-23509711. Max. coverage (+): 0. Max coverage (-): 0

Region: chr27 23509712-23509726. Max. coverage (+): 0. Max coverage (-): 0

Region: chr27 23509727-23509742. Max. coverage (+): 0. Max coverage (-): 0

Region: chr27 23509743-23509758. Max. coverage (+): 0. Max coverage (-): 0

Region: chr27 23509759-23509773. Max. coverage (+): 0. Max coverage (-): 0

Region: chr27 23509774-23509789. Max. coverage (+): 0. Max coverage (-): 0

Region: chr27 23509790-23509805. Max. coverage (+): 0. Max coverage (-): 0

Region: chr27 23509806-23509821. Max. coverage (+): 0. Max coverage (-): 0

Region: chr27 23509822-23509836. Max. coverage (+): 0. Max coverage (-): 3.41

Region: chr27 23509837-23509852. Max. coverage (+): 0. Max coverage (-): 3.41

Region: chr27 23509853-23509868. Max. coverage (+): 0. Max coverage (-): 0

Region: chr27 23509869-23509884. Max. coverage (+): 0. Max coverage (-): 0

Region: chr27 23509885-23509899. Max. coverage (+): 0. Max coverage (-): 0

Region: chr27 23509900-23509915. Max. coverage (+): 0. Max coverage (-): 0

Region: chr27 23509916-23509931. Max. coverage (+): 0. Max coverage (-): 0

Region: chr27 23509932-23509946. Max. coverage (+): 0. Max coverage (-): 0

Region: chr27 23509947-23509962. Max. coverage (+): 0. Max coverage (-): 5.65

Region: chr27 23509963-23509978. Max. coverage (+): 0. Max coverage (-): 5.65

Region: chr27 23509979-23509994. Max. coverage (+): 0. Max coverage (-): 0

Region: chr27 23509995-23510009. Max. coverage (+): 0. Max coverage (-): 0

Region: chr27 23510010-23510025. Max. coverage (+): 0. Max coverage (-): 0

Region: chr27 23510026-23510041. Max. coverage (+): 0. Max coverage (-): 0

Region: chr27 23510042-23510056. Max. coverage (+): 0. Max coverage (-): 0

Region: chr27 23510057-23510072. Max. coverage (+): 0. Max coverage (-): 0

Region: chr27 23510073-23510088. Max. coverage (+): 0. Max coverage (-): 0

Region: chr27 23510089-23510104. Max. coverage (+): 0. Max coverage (-): 0

Region: chr27 23510105-23510119. Max. coverage (+): 0. Max coverage (-): 0

Region: chr27 23510120-23510135. Max. coverage (+): 0. Max coverage (-): 0

Region: chr27 23510136-23510151. Max. coverage (+): 0. Max coverage (-): 0

Region: chr27 23510152-23510167. Max. coverage (+): 0. Max coverage (-): 0

Region: chr27 23510168-23510182. Max. coverage (+): 0. Max coverage (-): 0

Region: chr27 23510183-23510198. Max. coverage (+): 0. Max coverage (-): 0

Region: chr27 23510199-23510214. Max. coverage (+): 0. Max coverage (-): 0

Region: chr27 23510215-23510229. Max. coverage (+): 0. Max coverage (-): 0

Region: chr27 23510230-23510245. Max. coverage (+): 0. Max coverage (-): 0

Region: chr27 23510246-23510261. Max. coverage (+): 0. Max coverage (-): 0

Region: chr27 23510262-23510277. Max. coverage (+): 0. Max coverage (-): 0

Region: chr27 23510278-23510292. Max. coverage (+): 0. Max coverage (-): 0

Region: chr27 23510293-23510308. Max. coverage (+): 0. Max coverage (-): 0

Region: chr27 23510309-23510324. Max. coverage (+): 0. Max coverage (-): 0

Region: chr27 23510325-23510339. Max. coverage (+): 0. Max coverage (-): 0

Region: chr27 23510340-23510355. Max. coverage (+): 0. Max coverage (-): 0

Region: chr27 23510356-23510371. Max. coverage (+): 0. Max coverage (-): 0

Region: chr27 23510372-23510387. Max. coverage (+): 0. Max coverage (-): 0

Region: chr27 23510388-23510402. Max. coverage (+): 0. Max coverage (-): 0

Region: chr27 23510403-23510418. Max. coverage (+): 0. Max coverage (-): 0

Region: chr27 23510419-23510434. Max. coverage (+): 0. Max coverage (-): 0

Region: chr27 23510435-23510450. Max. coverage (+): 0. Max coverage (-): 0

Region: chr27 23510451-23510465. Max. coverage (+): 0. Max coverage (-): 0

Region: chr27 23510466-23510481. Max. coverage (+): 0. Max coverage (-): 0

Region: chr27 23510482-23510497. Max. coverage (+): 0. Max coverage (-): 3.54

Region: chr27 23510498-23510512. Max. coverage (+): 0. Max coverage (-): 3.54

Region: chr27 23510513-23510528. Max. coverage (+): 0. Max coverage (-): 1.38

Region: chr27 23510529-23510544. Max. coverage (+): 0. Max coverage (-): 0

Region: chr27 23510545-23510560. Max. coverage (+): 0. Max coverage (-): 0

Region: chr27 23510561-23510575. Max. coverage (+): 0. Max coverage (-): 0

Region: chr27 23510576-23510591. Max. coverage (+): 0. Max coverage (-): 0

Region: chr27 23510592-23510607. Max. coverage (+): 0. Max coverage (-): 0

Region: chr27 23510608-23510622. Max. coverage (+): 0. Max coverage (-): 0

Region: chr27 23510623-23510638. Max. coverage (+): 0. Max coverage (-): 0

Region: chr27 23510639-23510654. Max. coverage (+): 0. Max coverage (-): 0

Region: chr27 23510655-23510670. Max. coverage (+): 0. Max coverage (-): 0

Region: chr27 23510671-23510685. Max. coverage (+): 0. Max coverage (-): 0

Region: chr27 23510686-23510701. Max. coverage (+): 0. Max coverage (-): 0

Region: chr27 23510702-23510717. Max. coverage (+): 0. Max coverage (-): 0

Region: chr27 23510718-23510732. Max. coverage (+): 0. Max coverage (-): 0

Region: chr27 23510733-23510748. Max. coverage (+): 0. Max coverage (-): 0

Region: chr27 23510749-23510764. Max. coverage (+): 0. Max coverage (-): 0

Region: chr27 23510765-23510780. Max. coverage (+): 0. Max coverage (-): 0

Region: chr27 23510781-23510795. Max. coverage (+): 0. Max coverage (-): 0

Region: chr27 23510796-23510811. Max. coverage (+): 0. Max coverage (-): 0

Region: chr27 23510812-23510827. Max. coverage (+): 0. Max coverage (-): 0

Region: chr27 23510828-23510843. Max. coverage (+): 0. Max coverage (-): 0

Region: chr27 23510844-23510858. Max. coverage (+): 0. Max coverage (-): 0

Region: chr27 23510859-23510874. Max. coverage (+): 0. Max coverage (-): 0

Region: chr27 23510875-23510890. Max. coverage (+): 0. Max coverage (-): 1.37

Region: chr27 23510891-23510905. Max. coverage (+): 0. Max coverage (-): 0

Region: chr27 23510906-23510921. Max. coverage (+): 0. Max coverage (-): 0

Region: chr27 23510922-23510937. Max. coverage (+): 0. Max coverage (-): 0

Region: chr27 23510938-23510953. Max. coverage (+): 0. Max coverage (-): 0

Region: chr27 23510954-23510968. Max. coverage (+): 0. Max coverage (-): 0

Region: chr27 23510969-23510984. Max. coverage (+): 0. Max coverage (-): 4.54

Region: chr27 23510985-23511000. Max. coverage (+): 0. Max coverage (-): 4.54

Region: chr27 23511001-23511015. Max. coverage (+): 0. Max coverage (-): 6.1

Region: chr27 23511016-23511031. Max. coverage (+): 0. Max coverage (-): 0

Region: chr27 23511032-23511047. Max. coverage (+): 0. Max coverage (-): 0

Region: chr27 23511048-23511063. Max. coverage (+): 0. Max coverage (-): 3.41

Region: chr27 23511064-23511078. Max. coverage (+): 0. Max coverage (-): 3.41

Region: chr27 23511079-23511094. Max. coverage (+): 0. Max coverage (-): 0

Region: chr27 23511095-23511110. Max. coverage (+): 0. Max coverage (-): 3.94

Region: chr27 23511111-23511126. Max. coverage (+): 0. Max coverage (-): 9.4

Region: chr27 23511127-23511141. Max. coverage (+): 0. Max coverage (-): 10.13

Region: chr27 23511142-23511157. Max. coverage (+): 0. Max coverage (-): 0

Region: chr27 23511158-23511173. Max. coverage (+): 0. Max coverage (-): 7.83

Region: chr27 23511174-23511188. Max. coverage (+): 0. Max coverage (-): 3.26

Region: chr27 23511189-23511204. Max. coverage (+): 0. Max coverage (-): 0

Region: chr27 23511205-23511220. Max. coverage (+): 0. Max coverage (-): 0

Region: chr27 23511221-23511236. Max. coverage (+): 0. Max coverage (-): 0

Region: chr27 23511237-23511251. Max. coverage (+): 0. Max coverage (-): 0

Region: chr27 23511252-23511267. Max. coverage (+): 0. Max coverage (-): 0

Region: chr27 23511268-23511283. Max. coverage (+): 0. Max coverage (-): 0

Region: chr27 23511284-23511298. Max. coverage (+): 0. Max coverage (-): 0

Region: chr27 23511299-23511314. Max. coverage (+): 0. Max coverage (-): 2.91

Region: chr27 23511315-23511330. Max. coverage (+): 0. Max coverage (-): 0.99

Region: chr27 23511331-23511346. Max. coverage (+): 0. Max coverage (-): 0

Region: chr27 23511347-23511361. Max. coverage (+): 0. Max coverage (-): 0

Region: chr27 23511362-23511377. Max. coverage (+): 0. Max coverage (-): 0

Region: chr27 23511378-23511393. Max. coverage (+): 0. Max coverage (-): 0

Region: chr27 23511394-23511409. Max. coverage (+): 0. Max coverage (-): 0

Region: chr27 23511410-23511424. Max. coverage (+): 0. Max coverage (-): 1.32

Region: chr27 23511425-23511440. Max. coverage (+): 0. Max coverage (-): 1.32

Region: chr27 23511441-23511456. Max. coverage (+): 0. Max coverage (-): 0

Region: chr27 23511457-23511471. Max. coverage (+): 0. Max coverage (-): 0

Region: chr27 23511472-23511487. Max. coverage (+): 0. Max coverage (-): 0

Region: chr27 23511488-23511503. Max. coverage (+): 0. Max coverage (-): 0.91

Region: chr27 23511504-23511519. Max. coverage (+): 0. Max coverage (-): 7.43

Region: chr27 23511520-23511534. Max. coverage (+): 0. Max coverage (-): 5.51

Region: chr27 23511535-23511550. Max. coverage (+): 0. Max coverage (-): 0

Region: chr27 23511551-23511566. Max. coverage (+): 0. Max coverage (-): 0

Region: chr27 23511567-23511581. Max. coverage (+): 0. Max coverage (-): 6.6

Region: chr27 23511582-23511597. Max. coverage (+): 0. Max coverage (-): 7.8

Region: chr27 23511598-23511613. Max. coverage (+): 0. Max coverage (-): 26.16

Region: chr27 23511614-23511629. Max. coverage (+): 0. Max coverage (-): 3.9

Region: chr27 23511630-23511644. Max. coverage (+): 0. Max coverage (-): 1.02

Region: chr27 23511645-23511660. Max. coverage (+): 0. Max coverage (-): 5.06

Region: chr27 23511661-23511676. Max. coverage (+): 0. Max coverage (-): 4.66

Region: chr27 23511677-23511692. Max. coverage (+): 0. Max coverage (-): 5.79

Region: chr27 23511693-23511707. Max. coverage (+): 0. Max coverage (-): 1.24

Region: chr27 23511708-23511723. Max. coverage (+): 0. Max coverage (-): 1.24

Region: chr27 23511724-23511739. Max. coverage (+): 0. Max coverage (-): 0

Region: chr27 23511740-23511754. Max. coverage (+): 0. Max coverage (-): 0

Region: chr27 23511755-23511770. Max. coverage (+): 0. Max coverage (-): 0

Region: chr27 23511771-23511786. Max. coverage (+): 0. Max coverage (-): 0

Region: chr27 23511787-23511802. Max. coverage (+): 0. Max coverage (-): 0

Region: chr27 23511803-23511817. Max. coverage (+): 0. Max coverage (-): 0

Region: chr27 23511818-23511833. Max. coverage (+): 0. Max coverage (-): 0

Region: chr27 23511834-23511849. Max. coverage (+): 0. Max coverage (-): 0

Region: chr27 23511850-23511864. Max. coverage (+): 0. Max coverage (-): 0

Region: chr27 23511865-23511880. Max. coverage (+): 0. Max coverage (-): 0

Region: chr27 23511881-23511896. Max. coverage (+): 0. Max coverage (-): 0

Region: chr27 23511897-23511912. Max. coverage (+): 0. Max coverage (-): 0

Region: chr27 23511913-23511927. Max. coverage (+): 0. Max coverage (-): 4.24

Region: chr27 23511928-23511943. Max. coverage (+): 0. Max coverage (-): 8.99

Region: chr27 23511944-23511959. Max. coverage (+): 0. Max coverage (-): 0

Region: chr27 23511960-23511975. Max. coverage (+): 0. Max coverage (-): 0

Region: chr27 23511976-23511990. Max. coverage (+): 0. Max coverage (-): 0

Region: chr27 23511991-23512006. Max. coverage (+): 0. Max coverage (-): 3.98

Region: chr27 23512007-23512022. Max. coverage (+): 0. Max coverage (-): 2.15

Region: chr27 23512023-23512037. Max. coverage (+): 0. Max coverage (-): 6.74

Region: chr27 23512038-23512053. Max. coverage (+): 0. Max coverage (-): 4.58

Region: chr27 23512054-23512069. Max. coverage (+): 0. Max coverage (-): 0.98

Region: chr27 23512070-23512085. Max. coverage (+): 0. Max coverage (-): 3.72

Region: chr27 23512086-23512100. Max. coverage (+): 0. Max coverage (-): 10.3

Region: chr27 23512101-23512116. Max. coverage (+): 0. Max coverage (-): 7.04

Region: chr27 23512117-23512132. Max. coverage (+): 0. Max coverage (-): 7.5

Region: chr27 23512133-23512147. Max. coverage (+): 0. Max coverage (-): 5.67

Region: chr27 23512148-23512163. Max. coverage (+): 0. Max coverage (-): 0

Region: chr27 23512164-23512179. Max. coverage (+): 0. Max coverage (-): 0

Region: chr27 23512180-23512195. Max. coverage (+): 0. Max coverage (-): 0

Region: chr27 23512196-23512210. Max. coverage (+): 0. Max coverage (-): 0

Region: chr27 23512211-23512226. Max. coverage (+): 0. Max coverage (-): 0

Region: chr27 23512227-23512242. Max. coverage (+): 0. Max coverage (-): 0

Region: chr27 23512243-23512258. Max. coverage (+): 0. Max coverage (-): 0

Region: chr27 23512259-23512273. Max. coverage (+): 0. Max coverage (-): 0

Region: chr27 23512274-23512289. Max. coverage (+): 0. Max coverage (-): 0

Region: chr27 23512290-23512305. Max. coverage (+): 0. Max coverage (-): 0

Region: chr27 23512306-23512320. Max. coverage (+): 0. Max coverage (-): 0

Region: chr27 23512321-23512336. Max. coverage (+): 0. Max coverage (-): 0

Region: chr27 23512337-23512352. Max. coverage (+): 0. Max coverage (-): 0

Region: chr27 23512353-23512368. Max. coverage (+): 0. Max coverage (-): 0

Region: chr27 23512369-23512383. Max. coverage (+): 0. Max coverage (-): 0

Region: chr27 23512384-23512399. Max. coverage (+): 0. Max coverage (-): 0

Region: chr27 23512400-23512415. Max. coverage (+): 0. Max coverage (-): 0

Region: chr27 23512416-23512430. Max. coverage (+): 0. Max coverage (-): 0.64

Region: chr27 23512431-23512446. Max. coverage (+): 0. Max coverage (-): 0.64

Region: chr27 23512447-23512462. Max. coverage (+): 0. Max coverage (-): 1.24

Region: chr27 23512463-23512478. Max. coverage (+): 0. Max coverage (-): 1.24

Region: chr27 23512479-. Max. coverage (+): 0. Max coverage (-): 0

RepeatMasker Color Code

**+**

100-98% Identity

<98-95% Identity

<95-90% Identity

<90-85% Identity

<85-80% Identity

<80-75% Identity

<75-70% Identity

<70% Identity

**-**

Gene Set Color Code

**+**

Gene

Pseudogene

**-**

Topology/Coverage Color Code

Coverage Plus Strand

Coverage Minus Strand

Mainstrand: Plus

Mainstrand: Minus

Complementary Strand

Flanking Region  
(if option -flank >0)

Gene Set Annotation  

**1. (protein coding, ENSBTAG00000000357) Tr:00000000463 Ex:1**: 23504854-23505183 (-)

  
RepeatMasker Annotation  

**1. Bov-tA2**: 23505336-23505521 (-), Divergence to consensus: 27.5%  
**2. Bov-tA2**: 23505707-23505913 (-), Divergence to consensus: 13%  
**3. ART2A**: 23506536-23506837 (+), Divergence to consensus: 19.9%  
**4. MIRb**: 23506976-23507083 (-), Divergence to consensus: 43.3%  
**5. (TG)n**: 23509545-23509572 (+), Divergence to consensus: 3.6%  
**6. GC\_rich**: 23510381-23510528 (+), Divergence to consensus: 90.5%  
**7. MIR**: 23511748-23511908 (-), Divergence to consensus: 38.3%  
**8. Bov-tA1**: 23512171-23512369 (-), Divergence to consensus: 18.1%

  
Transcription Factor Binding Sites  

**Gata4** (Sequence: AGATAAC (-): 23508065)  
**SOX9** (Sequence: AACAATGG (-): 23508818)  
**SOX9** (Sequence: CCATTGTT (+): 23507596)
