# Supplementary material for: Small RNA sequencing of cryopreserved semen from single bull revealed altered miRNAs and piRNAs expression between High- and Low-motile sperm populations
Source: BMC Genomics. 2017 Jan 4;18:14. doi: 10.1186/s12864-016-3394-7 (PMC5209821; doi:10.1186/s12864-016-3394-7)
Supplement: Additional file 3: — Details for each piRNA clusters found in High Motile (HM) sperm fraction. Genes, repeats, transposable elements and transcription factors binding sites falling within the cluster regions were reported. (ZIP 1896 kb) [file 12864_2016_3394_MOESM3_ESM.zip › 66.html]

piRNA cluster 66


Predicted piRNA cluster no. 66     previous   next
  

Show proTRAC run info
Hide proTRAC run info

================================= proTRAC ====================================  
VERSION: 2.1                                    LAST MODIFIED: 06. October 2015  
  
Please cite:  
Rosenkranz D, Zischler H. proTRAC - a software for probabilistic piRNA cluster  
detection, visualization and analysis. 2012. BMC Bioinformatics 13:5.  
  
and (for proTRAC 2.0 and later):  
Rosenkranz D, Rudloff S, Bastuck K, Ketting RF, Zischler H. Tupaia small RNAs  
provide insights into function and evolution of RNAi-based transposon defense  
in mammals. 2015. RNA 21(5):911-922.  
  
Contact:  
David Rosenkranz  
Institute of Anthropology, small RNA group  
Johannes Gutenberg University Mainz  
email: rosenkranz@uni-mainz.de  
  
You can find the latest proTRAC version at:  
http://sourceforge.net/projects/protrac/files  
http://www.smallRNAgroup-mainz.de/software  
==============================================================================  
  
PARAMETERS:  
Map file: .............../storage/core/barbara/genhome/smallRNA/fertility/Sample\_motile/pirna/Sample\_motile\_26-33\_collapsed.fa.no-dust.map.weighted-10000-1000-b-0  
Genome file: ............/storage/core/barbara/genhome/smallRNA/fertility/Sample\_all/pirna/bt\_311\_chrY.fa  
RepeatMasker annotation: /storage/genomes/bt\_umd31/GCF\_000003055.6\_Bos\_taurus\_UMD\_3.1.1\_repeatMasker\_chr.out  
GeneSet:................./storage/core/barbara/genhome/smallRNA/fertility/Sample\_all/pirna/full.gtf  
  
Significant (p<=0.01) hit density will be calculated based  
on observed hit distribution.  
  
Sliding window size: ........................................ 5000 bp  
Sliding window increament: .................................. 1000 bp  
Normalize each hit by number of genomic hits: ............... 1 [0=no/1=yes]  
Normalize each hit by number of sequence reads: ............. 1 [0=no/1=yes]  
Normalize values (-> per million mapped reads): ............. 1 [0=no/1=yes]  
Min. fraction of hits with 1T(U) or 10A: .................... 0.75  
Alternatively: Min. fraction of hits with 1T(U) and 10A: .... 0.5  
Min. fraction of hits with typical piRNA length: ............ 0.75  
Typical piRNA length: ....................................... 26-33 nt  
Min. size of a piRNA cluster: ............................... 5000 bp.  
Min. number of hits (absolute): ............................. 0  
Min. number of hits (normalized): ........................... 0  
Min. fraction of hits on the mainstrand: .................... 0.75  
Top fraction of mapped sequences (in terms of read counts): . 1%  
Top fraction accounts for max. n% of sequence reads: ........ 90%  
Min. fraction of hits on each arm of a bidirectional cluster: 0.1  
Output image file for each cluster: ......................... 0 [0=no/1=yes]  
Output html file for each cluster: .......................... 1 [0=no/1=yes]  
Output a summary table: ..................................... 1 [0=no/1=yes]  
Output a FASTA file for each cluster (piRNA sequences): ..... 1 [0=no/1=yes]  
Output a FASTA file comprising cluster sequences: ........... 1 [0=no/1=yes]  
Search DNA motifs in clusters: .............................. 1 [0=no/1=yes]  
Output flanking sequences: +/- .............................. 0 bp  
Output ~.pTi file: .......................................... 1 [0=no/1=yes]  
==============================================================================  
  
  
Genome size (without gaps): ............ 2678902517 bp  
Gaps (N/X/-): .......................... 53837044 bp  
Mapped reads: .......................... 658825247023  
Non-identical sequences: ............... 514171  
Genomic hits: .......................... 764233  
Significant densitiy of mapped reads: .. 12867599.5173724 reads/kb

Show proTRAC cluster info
Hide proTRAC cluster info

|  |  |
| --- | --- |
| Location | chr27 |
| Coordinates | 23580900-23587140 |
| Size [bp] | 6241 |
| Sequence hit loci | 79 |
| Mapped reads (normalized) | 109496718 |
| Mapped reads (normalized) per kb | 17544739.3 |
| Normalized reads with 1T (1U) | 93.5% |
| Normalized reads with 10A | 35% |
| Normalized reads with length 26-33 nt | 100% |
| Normalized reads on the main strand(s) | 100% |
| Predicted directionality | mono:minus |

100%

0%

1T (1U)  
reads

10A reads

26-33 nt  
reads

reads on mainstrand

**Either the amount of reads with 1T (1U) OR 10A has to exceed 75% (set with option: -1Tor10A)  
Alternatively the amount of reads with 1T (1U) AND 10A has to exceed 50% (set with option: -1Tand10A)  
Minimum amount of reads with preferred size is 75% (set with option: -pisize)  
Minimum amount of reads on the main strand(s) is 75% (set with option: -clstrand)**

Show read coverage
Hide read coverage

WHAT DO I SEE HERE?  
This chart shows the location of mapped sequence reads within a predicted piRNA cluster. The color refers to the number of genomic hits produced by the sequence read in question. A dark red bar indicates that this sequence read produces many other hits elsewhere in the genome. Many adjacent red or yellow bars can indicate the presence of a multi-copy element such as transposons or rRNA genes. A dark green bar indicates that this sequence read maps uniquely to this locus.

1 hit

2-5 hits

6-10 hits

11-20 hits

21-50 hits

51-100 hits

> 100 hits

chr27

23580900

23587140

Gene Set

RepeatMasker

Mapped  
Reads

16.47

plus strand

minus strand

16.47

Region: chr27 23512480-23580906. Max. coverage (+): 0. Max coverage (-): 4.61

Region: chr27 23580907-23580918. Max. coverage (+): 0. Max coverage (-): 4.61

Region: chr27 23580919-23580931. Max. coverage (+): 0. Max coverage (-): 0

Region: chr27 23580932-23580943. Max. coverage (+): 0. Max coverage (-): 0

Region: chr27 23580944-23580956. Max. coverage (+): 0. Max coverage (-): 0

Region: chr27 23580957-23580968. Max. coverage (+): 0. Max coverage (-): 0

Region: chr27 23580969-23580981. Max. coverage (+): 0. Max coverage (-): 0

Region: chr27 23580982-23580993. Max. coverage (+): 0. Max coverage (-): 0

Region: chr27 23580994-23581006. Max. coverage (+): 0. Max coverage (-): 0

Region: chr27 23581007-23581018. Max. coverage (+): 0. Max coverage (-): 0

Region: chr27 23581019-23581031. Max. coverage (+): 0. Max coverage (-): 0

Region: chr27 23581032-23581043. Max. coverage (+): 0. Max coverage (-): 0

Region: chr27 23581044-23581056. Max. coverage (+): 0. Max coverage (-): 0

Region: chr27 23581057-23581068. Max. coverage (+): 0. Max coverage (-): 0

Region: chr27 23581069-23581080. Max. coverage (+): 0. Max coverage (-): 0

Region: chr27 23581081-23581093. Max. coverage (+): 0. Max coverage (-): 0

Region: chr27 23581094-23581105. Max. coverage (+): 0. Max coverage (-): 0

Region: chr27 23581106-23581118. Max. coverage (+): 0. Max coverage (-): 0

Region: chr27 23581119-23581130. Max. coverage (+): 0. Max coverage (-): 0

Region: chr27 23581131-23581143. Max. coverage (+): 0. Max coverage (-): 0

Region: chr27 23581144-23581155. Max. coverage (+): 0. Max coverage (-): 0

Region: chr27 23581156-23581168. Max. coverage (+): 0. Max coverage (-): 0

Region: chr27 23581169-23581180. Max. coverage (+): 0. Max coverage (-): 0

Region: chr27 23581181-23581193. Max. coverage (+): 0. Max coverage (-): 0

Region: chr27 23581194-23581205. Max. coverage (+): 0. Max coverage (-): 0

Region: chr27 23581206-23581218. Max. coverage (+): 0. Max coverage (-): 0

Region: chr27 23581219-23581230. Max. coverage (+): 0. Max coverage (-): 0

Region: chr27 23581231-23581243. Max. coverage (+): 0. Max coverage (-): 2.43

Region: chr27 23581244-23581255. Max. coverage (+): 0. Max coverage (-): 6.33

Region: chr27 23581256-23581268. Max. coverage (+): 0. Max coverage (-): 6.33

Region: chr27 23581269-23581280. Max. coverage (+): 0. Max coverage (-): 0

Region: chr27 23581281-23581293. Max. coverage (+): 0. Max coverage (-): 0

Region: chr27 23581294-23581305. Max. coverage (+): 0. Max coverage (-): 0

Region: chr27 23581306-23581318. Max. coverage (+): 0. Max coverage (-): 2.74

Region: chr27 23581319-23581330. Max. coverage (+): 0. Max coverage (-): 0

Region: chr27 23581331-23581343. Max. coverage (+): 0. Max coverage (-): 0

Region: chr27 23581344-23581355. Max. coverage (+): 0. Max coverage (-): 0

Region: chr27 23581356-23581368. Max. coverage (+): 0. Max coverage (-): 0.4

Region: chr27 23581369-23581380. Max. coverage (+): 0. Max coverage (-): 0

Region: chr27 23581381-23581393. Max. coverage (+): 0. Max coverage (-): 0

Region: chr27 23581394-23581405. Max. coverage (+): 0. Max coverage (-): 10.14

Region: chr27 23581406-23581418. Max. coverage (+): 0. Max coverage (-): 10.14

Region: chr27 23581419-23581430. Max. coverage (+): 0. Max coverage (-): 0

Region: chr27 23581431-23581442. Max. coverage (+): 0. Max coverage (-): 0

Region: chr27 23581443-23581455. Max. coverage (+): 0. Max coverage (-): 0

Region: chr27 23581456-23581467. Max. coverage (+): 0. Max coverage (-): 0

Region: chr27 23581468-23581480. Max. coverage (+): 0. Max coverage (-): 0

Region: chr27 23581481-23581492. Max. coverage (+): 0. Max coverage (-): 0

Region: chr27 23581493-23581505. Max. coverage (+): 0. Max coverage (-): 0

Region: chr27 23581506-23581517. Max. coverage (+): 0. Max coverage (-): 0

Region: chr27 23581518-23581530. Max. coverage (+): 0. Max coverage (-): 0

Region: chr27 23581531-23581542. Max. coverage (+): 0. Max coverage (-): 0

Region: chr27 23581543-23581555. Max. coverage (+): 0. Max coverage (-): 0

Region: chr27 23581556-23581567. Max. coverage (+): 0. Max coverage (-): 0

Region: chr27 23581568-23581580. Max. coverage (+): 0. Max coverage (-): 0

Region: chr27 23581581-23581592. Max. coverage (+): 0. Max coverage (-): 0

Region: chr27 23581593-23581605. Max. coverage (+): 0. Max coverage (-): 0

Region: chr27 23581606-23581617. Max. coverage (+): 0. Max coverage (-): 0

Region: chr27 23581618-23581630. Max. coverage (+): 0. Max coverage (-): 0

Region: chr27 23581631-23581642. Max. coverage (+): 0. Max coverage (-): 0

Region: chr27 23581643-23581655. Max. coverage (+): 0. Max coverage (-): 0

Region: chr27 23581656-23581667. Max. coverage (+): 0. Max coverage (-): 0

Region: chr27 23581668-23581680. Max. coverage (+): 0. Max coverage (-): 0

Region: chr27 23581681-23581692. Max. coverage (+): 0. Max coverage (-): 0

Region: chr27 23581693-23581705. Max. coverage (+): 0. Max coverage (-): 5.4

Region: chr27 23581706-23581717. Max. coverage (+): 0. Max coverage (-): 5.4

Region: chr27 23581718-23581730. Max. coverage (+): 0. Max coverage (-): 0

Region: chr27 23581731-23581742. Max. coverage (+): 0. Max coverage (-): 0

Region: chr27 23581743-23581755. Max. coverage (+): 0. Max coverage (-): 0

Region: chr27 23581756-23581767. Max. coverage (+): 0. Max coverage (-): 0

Region: chr27 23581768-23581779. Max. coverage (+): 0. Max coverage (-): 0

Region: chr27 23581780-23581792. Max. coverage (+): 0. Max coverage (-): 0

Region: chr27 23581793-23581804. Max. coverage (+): 0. Max coverage (-): 0

Region: chr27 23581805-23581817. Max. coverage (+): 0. Max coverage (-): 0

Region: chr27 23581818-23581829. Max. coverage (+): 0. Max coverage (-): 0

Region: chr27 23581830-23581842. Max. coverage (+): 0. Max coverage (-): 0

Region: chr27 23581843-23581854. Max. coverage (+): 0. Max coverage (-): 0

Region: chr27 23581855-23581867. Max. coverage (+): 0. Max coverage (-): 0

Region: chr27 23581868-23581879. Max. coverage (+): 0. Max coverage (-): 0

Region: chr27 23581880-23581892. Max. coverage (+): 0. Max coverage (-): 0

Region: chr27 23581893-23581904. Max. coverage (+): 0. Max coverage (-): 0

Region: chr27 23581905-23581917. Max. coverage (+): 0. Max coverage (-): 0

Region: chr27 23581918-23581929. Max. coverage (+): 0. Max coverage (-): 0

Region: chr27 23581930-23581942. Max. coverage (+): 0. Max coverage (-): 0

Region: chr27 23581943-23581954. Max. coverage (+): 0. Max coverage (-): 0

Region: chr27 23581955-23581967. Max. coverage (+): 0. Max coverage (-): 0

Region: chr27 23581968-23581979. Max. coverage (+): 0. Max coverage (-): 0

Region: chr27 23581980-23581992. Max. coverage (+): 0. Max coverage (-): 0

Region: chr27 23581993-23582004. Max. coverage (+): 0. Max coverage (-): 2.4

Region: chr27 23582005-23582017. Max. coverage (+): 0. Max coverage (-): 0

Region: chr27 23582018-23582029. Max. coverage (+): 0. Max coverage (-): 0

Region: chr27 23582030-23582042. Max. coverage (+): 0. Max coverage (-): 0

Region: chr27 23582043-23582054. Max. coverage (+): 0. Max coverage (-): 0

Region: chr27 23582055-23582067. Max. coverage (+): 0. Max coverage (-): 0

Region: chr27 23582068-23582079. Max. coverage (+): 0. Max coverage (-): 0

Region: chr27 23582080-23582092. Max. coverage (+): 0. Max coverage (-): 0

Region: chr27 23582093-23582104. Max. coverage (+): 0. Max coverage (-): 0

Region: chr27 23582105-23582116. Max. coverage (+): 0. Max coverage (-): 0.95

Region: chr27 23582117-23582129. Max. coverage (+): 0. Max coverage (-): 0.95

Region: chr27 23582130-23582141. Max. coverage (+): 0. Max coverage (-): 0

Region: chr27 23582142-23582154. Max. coverage (+): 0. Max coverage (-): 0

Region: chr27 23582155-23582166. Max. coverage (+): 0. Max coverage (-): 0

Region: chr27 23582167-23582179. Max. coverage (+): 0. Max coverage (-): 0

Region: chr27 23582180-23582191. Max. coverage (+): 0. Max coverage (-): 4.7

Region: chr27 23582192-23582204. Max. coverage (+): 0. Max coverage (-): 8.23

Region: chr27 23582205-23582216. Max. coverage (+): 0. Max coverage (-): 0

Region: chr27 23582217-23582229. Max. coverage (+): 0. Max coverage (-): 0

Region: chr27 23582230-23582241. Max. coverage (+): 0. Max coverage (-): 0

Region: chr27 23582242-23582254. Max. coverage (+): 0. Max coverage (-): 0

Region: chr27 23582255-23582266. Max. coverage (+): 0. Max coverage (-): 0

Region: chr27 23582267-23582279. Max. coverage (+): 0. Max coverage (-): 0

Region: chr27 23582280-23582291. Max. coverage (+): 0. Max coverage (-): 0

Region: chr27 23582292-23582304. Max. coverage (+): 0. Max coverage (-): 0

Region: chr27 23582305-23582316. Max. coverage (+): 0. Max coverage (-): 0

Region: chr27 23582317-23582329. Max. coverage (+): 0. Max coverage (-): 0

Region: chr27 23582330-23582341. Max. coverage (+): 0. Max coverage (-): 0

Region: chr27 23582342-23582354. Max. coverage (+): 0. Max coverage (-): 0

Region: chr27 23582355-23582366. Max. coverage (+): 0. Max coverage (-): 0

Region: chr27 23582367-23582379. Max. coverage (+): 0. Max coverage (-): 0

Region: chr27 23582380-23582391. Max. coverage (+): 0. Max coverage (-): 0

Region: chr27 23582392-23582404. Max. coverage (+): 0. Max coverage (-): 0

Region: chr27 23582405-23582416. Max. coverage (+): 0. Max coverage (-): 0

Region: chr27 23582417-23582429. Max. coverage (+): 0. Max coverage (-): 0

Region: chr27 23582430-23582441. Max. coverage (+): 0. Max coverage (-): 0

Region: chr27 23582442-23582454. Max. coverage (+): 0. Max coverage (-): 0

Region: chr27 23582455-23582466. Max. coverage (+): 0. Max coverage (-): 0

Region: chr27 23582467-23582478. Max. coverage (+): 0. Max coverage (-): 0

Region: chr27 23582479-23582491. Max. coverage (+): 0. Max coverage (-): 0

Region: chr27 23582492-23582503. Max. coverage (+): 0. Max coverage (-): 0

Region: chr27 23582504-23582516. Max. coverage (+): 0. Max coverage (-): 0

Region: chr27 23582517-23582528. Max. coverage (+): 0. Max coverage (-): 0

Region: chr27 23582529-23582541. Max. coverage (+): 0. Max coverage (-): 0

Region: chr27 23582542-23582553. Max. coverage (+): 0. Max coverage (-): 0

Region: chr27 23582554-23582566. Max. coverage (+): 0. Max coverage (-): 0

Region: chr27 23582567-23582578. Max. coverage (+): 0. Max coverage (-): 0

Region: chr27 23582579-23582591. Max. coverage (+): 0. Max coverage (-): 0

Region: chr27 23582592-23582603. Max. coverage (+): 0. Max coverage (-): 0

Region: chr27 23582604-23582616. Max. coverage (+): 0. Max coverage (-): 0

Region: chr27 23582617-23582628. Max. coverage (+): 0. Max coverage (-): 0

Region: chr27 23582629-23582641. Max. coverage (+): 0. Max coverage (-): 0

Region: chr27 23582642-23582653. Max. coverage (+): 0. Max coverage (-): 0

Region: chr27 23582654-23582666. Max. coverage (+): 0. Max coverage (-): 0

Region: chr27 23582667-23582678. Max. coverage (+): 0. Max coverage (-): 0

Region: chr27 23582679-23582691. Max. coverage (+): 0. Max coverage (-): 0

Region: chr27 23582692-23582703. Max. coverage (+): 0. Max coverage (-): 0

Region: chr27 23582704-23582716. Max. coverage (+): 0. Max coverage (-): 0

Region: chr27 23582717-23582728. Max. coverage (+): 0. Max coverage (-): 0

Region: chr27 23582729-23582741. Max. coverage (+): 0. Max coverage (-): 0

Region: chr27 23582742-23582753. Max. coverage (+): 0. Max coverage (-): 0

Region: chr27 23582754-23582766. Max. coverage (+): 0. Max coverage (-): 0

Region: chr27 23582767-23582778. Max. coverage (+): 0. Max coverage (-): 0

Region: chr27 23582779-23582791. Max. coverage (+): 0. Max coverage (-): 0

Region: chr27 23582792-23582803. Max. coverage (+): 0. Max coverage (-): 0

Region: chr27 23582804-23582815. Max. coverage (+): 0. Max coverage (-): 0

Region: chr27 23582816-23582828. Max. coverage (+): 0. Max coverage (-): 0

Region: chr27 23582829-23582840. Max. coverage (+): 0. Max coverage (-): 0

Region: chr27 23582841-23582853. Max. coverage (+): 0. Max coverage (-): 0

Region: chr27 23582854-23582865. Max. coverage (+): 0. Max coverage (-): 0

Region: chr27 23582866-23582878. Max. coverage (+): 0. Max coverage (-): 0

Region: chr27 23582879-23582890. Max. coverage (+): 0. Max coverage (-): 0

Region: chr27 23582891-23582903. Max. coverage (+): 0. Max coverage (-): 0

Region: chr27 23582904-23582915. Max. coverage (+): 0. Max coverage (-): 0

Region: chr27 23582916-23582928. Max. coverage (+): 0. Max coverage (-): 0

Region: chr27 23582929-23582940. Max. coverage (+): 0. Max coverage (-): 0

Region: chr27 23582941-23582953. Max. coverage (+): 0. Max coverage (-): 0

Region: chr27 23582954-23582965. Max. coverage (+): 0. Max coverage (-): 0

Region: chr27 23582966-23582978. Max. coverage (+): 0. Max coverage (-): 0

Region: chr27 23582979-23582990. Max. coverage (+): 0. Max coverage (-): 0

Region: chr27 23582991-23583003. Max. coverage (+): 0. Max coverage (-): 0

Region: chr27 23583004-23583015. Max. coverage (+): 0. Max coverage (-): 0

Region: chr27 23583016-23583028. Max. coverage (+): 0. Max coverage (-): 0

Region: chr27 23583029-23583040. Max. coverage (+): 0. Max coverage (-): 0

Region: chr27 23583041-23583053. Max. coverage (+): 0. Max coverage (-): 0

Region: chr27 23583054-23583065. Max. coverage (+): 0. Max coverage (-): 0

Region: chr27 23583066-23583078. Max. coverage (+): 0. Max coverage (-): 0

Region: chr27 23583079-23583090. Max. coverage (+): 0. Max coverage (-): 0

Region: chr27 23583091-23583103. Max. coverage (+): 0. Max coverage (-): 0

Region: chr27 23583104-23583115. Max. coverage (+): 0. Max coverage (-): 0

Region: chr27 23583116-23583128. Max. coverage (+): 0. Max coverage (-): 0

Region: chr27 23583129-23583140. Max. coverage (+): 0. Max coverage (-): 0

Region: chr27 23583141-23583153. Max. coverage (+): 0. Max coverage (-): 0

Region: chr27 23583154-23583165. Max. coverage (+): 0. Max coverage (-): 0

Region: chr27 23583166-23583177. Max. coverage (+): 0. Max coverage (-): 0

Region: chr27 23583178-23583190. Max. coverage (+): 0. Max coverage (-): 3.93

Region: chr27 23583191-23583202. Max. coverage (+): 0. Max coverage (-): 3.41

Region: chr27 23583203-23583215. Max. coverage (+): 0. Max coverage (-): 0

Region: chr27 23583216-23583227. Max. coverage (+): 0. Max coverage (-): 0

Region: chr27 23583228-23583240. Max. coverage (+): 0. Max coverage (-): 0

Region: chr27 23583241-23583252. Max. coverage (+): 0. Max coverage (-): 0

Region: chr27 23583253-23583265. Max. coverage (+): 0. Max coverage (-): 0

Region: chr27 23583266-23583277. Max. coverage (+): 0. Max coverage (-): 4.61

Region: chr27 23583278-23583290. Max. coverage (+): 0. Max coverage (-): 9.72

Region: chr27 23583291-23583302. Max. coverage (+): 0. Max coverage (-): 9.72

Region: chr27 23583303-23583315. Max. coverage (+): 0. Max coverage (-): 0.96

Region: chr27 23583316-23583327. Max. coverage (+): 0. Max coverage (-): 1.68

Region: chr27 23583328-23583340. Max. coverage (+): 0. Max coverage (-): 14.18

Region: chr27 23583341-23583352. Max. coverage (+): 0. Max coverage (-): 7.02

Region: chr27 23583353-23583365. Max. coverage (+): 0. Max coverage (-): 0

Region: chr27 23583366-23583377. Max. coverage (+): 0. Max coverage (-): 0

Region: chr27 23583378-23583390. Max. coverage (+): 0. Max coverage (-): 0

Region: chr27 23583391-23583402. Max. coverage (+): 0. Max coverage (-): 10.32

Region: chr27 23583403-23583415. Max. coverage (+): 0. Max coverage (-): 0.96

Region: chr27 23583416-23583427. Max. coverage (+): 0. Max coverage (-): 1.78

Region: chr27 23583428-23583440. Max. coverage (+): 0. Max coverage (-): 5.92

Region: chr27 23583441-23583452. Max. coverage (+): 0. Max coverage (-): 5.08

Region: chr27 23583453-23583465. Max. coverage (+): 0. Max coverage (-): 14.66

Region: chr27 23583466-23583477. Max. coverage (+): 0. Max coverage (-): 16.47

Region: chr27 23583478-23583490. Max. coverage (+): 0. Max coverage (-): 0

Region: chr27 23583491-23583502. Max. coverage (+): 0. Max coverage (-): 11.74

Region: chr27 23583503-23583514. Max. coverage (+): 0. Max coverage (-): 6.56

Region: chr27 23583515-23583527. Max. coverage (+): 0. Max coverage (-): 0

Region: chr27 23583528-23583539. Max. coverage (+): 0. Max coverage (-): 0

Region: chr27 23583540-23583552. Max. coverage (+): 0. Max coverage (-): 0

Region: chr27 23583553-23583564. Max. coverage (+): 0. Max coverage (-): 0

Region: chr27 23583565-23583577. Max. coverage (+): 0. Max coverage (-): 0

Region: chr27 23583578-23583589. Max. coverage (+): 0. Max coverage (-): 0

Region: chr27 23583590-23583602. Max. coverage (+): 0. Max coverage (-): 0

Region: chr27 23583603-23583614. Max. coverage (+): 0. Max coverage (-): 0

Region: chr27 23583615-23583627. Max. coverage (+): 0. Max coverage (-): 0

Region: chr27 23583628-23583639. Max. coverage (+): 0. Max coverage (-): 0

Region: chr27 23583640-23583652. Max. coverage (+): 0. Max coverage (-): 0

Region: chr27 23583653-23583664. Max. coverage (+): 0. Max coverage (-): 0

Region: chr27 23583665-23583677. Max. coverage (+): 0. Max coverage (-): 0

Region: chr27 23583678-23583689. Max. coverage (+): 0. Max coverage (-): 0

Region: chr27 23583690-23583702. Max. coverage (+): 0. Max coverage (-): 0

Region: chr27 23583703-23583714. Max. coverage (+): 0. Max coverage (-): 0

Region: chr27 23583715-23583727. Max. coverage (+): 0. Max coverage (-): 0

Region: chr27 23583728-23583739. Max. coverage (+): 0. Max coverage (-): 1.42

Region: chr27 23583740-23583752. Max. coverage (+): 0. Max coverage (-): 1.42

Region: chr27 23583753-23583764. Max. coverage (+): 0. Max coverage (-): 0

Region: chr27 23583765-23583777. Max. coverage (+): 0. Max coverage (-): 3.95

Region: chr27 23583778-23583789. Max. coverage (+): 0. Max coverage (-): 3.95

Region: chr27 23583790-23583802. Max. coverage (+): 0. Max coverage (-): 0.35

Region: chr27 23583803-23583814. Max. coverage (+): 0. Max coverage (-): 0

Region: chr27 23583815-23583827. Max. coverage (+): 0. Max coverage (-): 0

Region: chr27 23583828-23583839. Max. coverage (+): 0. Max coverage (-): 2.77

Region: chr27 23583840-23583851. Max. coverage (+): 0. Max coverage (-): 2.77

Region: chr27 23583852-23583864. Max. coverage (+): 0. Max coverage (-): 0

Region: chr27 23583865-23583876. Max. coverage (+): 0. Max coverage (-): 0

Region: chr27 23583877-23583889. Max. coverage (+): 0. Max coverage (-): 0

Region: chr27 23583890-23583901. Max. coverage (+): 0. Max coverage (-): 0

Region: chr27 23583902-23583914. Max. coverage (+): 0. Max coverage (-): 0

Region: chr27 23583915-23583926. Max. coverage (+): 0. Max coverage (-): 0

Region: chr27 23583927-23583939. Max. coverage (+): 0. Max coverage (-): 0

Region: chr27 23583940-23583951. Max. coverage (+): 0. Max coverage (-): 0.6

Region: chr27 23583952-23583964. Max. coverage (+): 0. Max coverage (-): 0

Region: chr27 23583965-23583976. Max. coverage (+): 0. Max coverage (-): 0

Region: chr27 23583977-23583989. Max. coverage (+): 0. Max coverage (-): 0

Region: chr27 23583990-23584001. Max. coverage (+): 0. Max coverage (-): 0

Region: chr27 23584002-23584014. Max. coverage (+): 0. Max coverage (-): 0

Region: chr27 23584015-23584026. Max. coverage (+): 0. Max coverage (-): 0

Region: chr27 23584027-23584039. Max. coverage (+): 0. Max coverage (-): 0

Region: chr27 23584040-23584051. Max. coverage (+): 0. Max coverage (-): 0

Region: chr27 23584052-23584064. Max. coverage (+): 0. Max coverage (-): 0

Region: chr27 23584065-23584076. Max. coverage (+): 0. Max coverage (-): 0

Region: chr27 23584077-23584089. Max. coverage (+): 0. Max coverage (-): 0

Region: chr27 23584090-23584101. Max. coverage (+): 0. Max coverage (-): 0

Region: chr27 23584102-23584114. Max. coverage (+): 0. Max coverage (-): 1.38

Region: chr27 23584115-23584126. Max. coverage (+): 0. Max coverage (-): 1.38

Region: chr27 23584127-23584139. Max. coverage (+): 0. Max coverage (-): 0

Region: chr27 23584140-23584151. Max. coverage (+): 0. Max coverage (-): 0

Region: chr27 23584152-23584164. Max. coverage (+): 0. Max coverage (-): 0

Region: chr27 23584165-23584176. Max. coverage (+): 0. Max coverage (-): 0

Region: chr27 23584177-23584189. Max. coverage (+): 0. Max coverage (-): 0

Region: chr27 23584190-23584201. Max. coverage (+): 0. Max coverage (-): 0

Region: chr27 23584202-23584213. Max. coverage (+): 0. Max coverage (-): 0

Region: chr27 23584214-23584226. Max. coverage (+): 0. Max coverage (-): 0

Region: chr27 23584227-23584238. Max. coverage (+): 0. Max coverage (-): 0

Region: chr27 23584239-23584251. Max. coverage (+): 0. Max coverage (-): 0

Region: chr27 23584252-23584263. Max. coverage (+): 0. Max coverage (-): 0

Region: chr27 23584264-23584276. Max. coverage (+): 0. Max coverage (-): 0

Region: chr27 23584277-23584288. Max. coverage (+): 0. Max coverage (-): 0

Region: chr27 23584289-23584301. Max. coverage (+): 0. Max coverage (-): 0

Region: chr27 23584302-23584313. Max. coverage (+): 0. Max coverage (-): 0

Region: chr27 23584314-23584326. Max. coverage (+): 0. Max coverage (-): 0

Region: chr27 23584327-23584338. Max. coverage (+): 0. Max coverage (-): 0

Region: chr27 23584339-23584351. Max. coverage (+): 0. Max coverage (-): 0

Region: chr27 23584352-23584363. Max. coverage (+): 0. Max coverage (-): 0

Region: chr27 23584364-23584376. Max. coverage (+): 0. Max coverage (-): 7.92

Region: chr27 23584377-23584388. Max. coverage (+): 0. Max coverage (-): 7.92

Region: chr27 23584389-23584401. Max. coverage (+): 0. Max coverage (-): 0.39

Region: chr27 23584402-23584413. Max. coverage (+): 0. Max coverage (-): 4.46

Region: chr27 23584414-23584426. Max. coverage (+): 0. Max coverage (-): 5.6

Region: chr27 23584427-23584438. Max. coverage (+): 0. Max coverage (-): 0

Region: chr27 23584439-23584451. Max. coverage (+): 0. Max coverage (-): 0

Region: chr27 23584452-23584463. Max. coverage (+): 0. Max coverage (-): 0

Region: chr27 23584464-23584476. Max. coverage (+): 0. Max coverage (-): 0

Region: chr27 23584477-23584488. Max. coverage (+): 0. Max coverage (-): 0

Region: chr27 23584489-23584501. Max. coverage (+): 0. Max coverage (-): 0

Region: chr27 23584502-23584513. Max. coverage (+): 0. Max coverage (-): 0

Region: chr27 23584514-23584526. Max. coverage (+): 0. Max coverage (-): 0

Region: chr27 23584527-23584538. Max. coverage (+): 0. Max coverage (-): 0

Region: chr27 23584539-23584550. Max. coverage (+): 0. Max coverage (-): 0

Region: chr27 23584551-23584563. Max. coverage (+): 0. Max coverage (-): 0

Region: chr27 23584564-23584575. Max. coverage (+): 0. Max coverage (-): 0

Region: chr27 23584576-23584588. Max. coverage (+): 0. Max coverage (-): 0

Region: chr27 23584589-23584600. Max. coverage (+): 0. Max coverage (-): 0

Region: chr27 23584601-23584613. Max. coverage (+): 0. Max coverage (-): 0

Region: chr27 23584614-23584625. Max. coverage (+): 0. Max coverage (-): 0

Region: chr27 23584626-23584638. Max. coverage (+): 0. Max coverage (-): 0

Region: chr27 23584639-23584650. Max. coverage (+): 0. Max coverage (-): 0

Region: chr27 23584651-23584663. Max. coverage (+): 0. Max coverage (-): 0

Region: chr27 23584664-23584675. Max. coverage (+): 0. Max coverage (-): 0

Region: chr27 23584676-23584688. Max. coverage (+): 0. Max coverage (-): 0

Region: chr27 23584689-23584700. Max. coverage (+): 0. Max coverage (-): 0

Region: chr27 23584701-23584713. Max. coverage (+): 0. Max coverage (-): 0

Region: chr27 23584714-23584725. Max. coverage (+): 0. Max coverage (-): 0

Region: chr27 23584726-23584738. Max. coverage (+): 0. Max coverage (-): 0

Region: chr27 23584739-23584750. Max. coverage (+): 0. Max coverage (-): 0

Region: chr27 23584751-23584763. Max. coverage (+): 0. Max coverage (-): 0

Region: chr27 23584764-23584775. Max. coverage (+): 0. Max coverage (-): 0

Region: chr27 23584776-23584788. Max. coverage (+): 0. Max coverage (-): 0

Region: chr27 23584789-23584800. Max. coverage (+): 0. Max coverage (-): 0

Region: chr27 23584801-23584813. Max. coverage (+): 0. Max coverage (-): 0

Region: chr27 23584814-23584825. Max. coverage (+): 0. Max coverage (-): 0

Region: chr27 23584826-23584838. Max. coverage (+): 0. Max coverage (-): 0

Region: chr27 23584839-23584850. Max. coverage (+): 0. Max coverage (-): 0

Region: chr27 23584851-23584863. Max. coverage (+): 0. Max coverage (-): 0

Region: chr27 23584864-23584875. Max. coverage (+): 0. Max coverage (-): 0

Region: chr27 23584876-23584887. Max. coverage (+): 0. Max coverage (-): 0

Region: chr27 23584888-23584900. Max. coverage (+): 0. Max coverage (-): 0

Region: chr27 23584901-23584912. Max. coverage (+): 0. Max coverage (-): 0

Region: chr27 23584913-23584925. Max. coverage (+): 0. Max coverage (-): 0

Region: chr27 23584926-23584937. Max. coverage (+): 0. Max coverage (-): 0

Region: chr27 23584938-23584950. Max. coverage (+): 0. Max coverage (-): 0

Region: chr27 23584951-23584962. Max. coverage (+): 0. Max coverage (-): 0

Region: chr27 23584963-23584975. Max. coverage (+): 0. Max coverage (-): 0

Region: chr27 23584976-23584987. Max. coverage (+): 0. Max coverage (-): 0

Region: chr27 23584988-23585000. Max. coverage (+): 0. Max coverage (-): 0

Region: chr27 23585001-23585012. Max. coverage (+): 0. Max coverage (-): 0

Region: chr27 23585013-23585025. Max. coverage (+): 0. Max coverage (-): 0

Region: chr27 23585026-23585037. Max. coverage (+): 0. Max coverage (-): 0

Region: chr27 23585038-23585050. Max. coverage (+): 0. Max coverage (-): 0

Region: chr27 23585051-23585062. Max. coverage (+): 0. Max coverage (-): 0

Region: chr27 23585063-23585075. Max. coverage (+): 0. Max coverage (-): 0

Region: chr27 23585076-23585087. Max. coverage (+): 0. Max coverage (-): 0

Region: chr27 23585088-23585100. Max. coverage (+): 0. Max coverage (-): 0

Region: chr27 23585101-23585112. Max. coverage (+): 0. Max coverage (-): 0

Region: chr27 23585113-23585125. Max. coverage (+): 0. Max coverage (-): 0

Region: chr27 23585126-23585137. Max. coverage (+): 0. Max coverage (-): 0

Region: chr27 23585138-23585150. Max. coverage (+): 0. Max coverage (-): 0

Region: chr27 23585151-23585162. Max. coverage (+): 0. Max coverage (-): 0

Region: chr27 23585163-23585175. Max. coverage (+): 0. Max coverage (-): 0

Region: chr27 23585176-23585187. Max. coverage (+): 0. Max coverage (-): 0

Region: chr27 23585188-23585200. Max. coverage (+): 0. Max coverage (-): 0

Region: chr27 23585201-23585212. Max. coverage (+): 0. Max coverage (-): 0

Region: chr27 23585213-23585225. Max. coverage (+): 0. Max coverage (-): 0

Region: chr27 23585226-23585237. Max. coverage (+): 0. Max coverage (-): 0

Region: chr27 23585238-23585249. Max. coverage (+): 0. Max coverage (-): 0

Region: chr27 23585250-23585262. Max. coverage (+): 0. Max coverage (-): 0

Region: chr27 23585263-23585274. Max. coverage (+): 0. Max coverage (-): 0

Region: chr27 23585275-23585287. Max. coverage (+): 0. Max coverage (-): 0

Region: chr27 23585288-23585299. Max. coverage (+): 0. Max coverage (-): 0

Region: chr27 23585300-23585312. Max. coverage (+): 0. Max coverage (-): 0

Region: chr27 23585313-23585324. Max. coverage (+): 0. Max coverage (-): 0

Region: chr27 23585325-23585337. Max. coverage (+): 0. Max coverage (-): 0

Region: chr27 23585338-23585349. Max. coverage (+): 0. Max coverage (-): 0

Region: chr27 23585350-23585362. Max. coverage (+): 0. Max coverage (-): 0

Region: chr27 23585363-23585374. Max. coverage (+): 0. Max coverage (-): 0

Region: chr27 23585375-23585387. Max. coverage (+): 0. Max coverage (-): 0

Region: chr27 23585388-23585399. Max. coverage (+): 0. Max coverage (-): 0

Region: chr27 23585400-23585412. Max. coverage (+): 0. Max coverage (-): 0

Region: chr27 23585413-23585424. Max. coverage (+): 0. Max coverage (-): 0

Region: chr27 23585425-23585437. Max. coverage (+): 0. Max coverage (-): 0

Region: chr27 23585438-23585449. Max. coverage (+): 0. Max coverage (-): 0

Region: chr27 23585450-23585462. Max. coverage (+): 0. Max coverage (-): 0

Region: chr27 23585463-23585474. Max. coverage (+): 0. Max coverage (-): 0

Region: chr27 23585475-23585487. Max. coverage (+): 0. Max coverage (-): 0

Region: chr27 23585488-23585499. Max. coverage (+): 0. Max coverage (-): 0

Region: chr27 23585500-23585512. Max. coverage (+): 0. Max coverage (-): 0

Region: chr27 23585513-23585524. Max. coverage (+): 0. Max coverage (-): 0

Region: chr27 23585525-23585537. Max. coverage (+): 0. Max coverage (-): 0

Region: chr27 23585538-23585549. Max. coverage (+): 0. Max coverage (-): 0

Region: chr27 23585550-23585562. Max. coverage (+): 0. Max coverage (-): 0

Region: chr27 23585563-23585574. Max. coverage (+): 0. Max coverage (-): 0

Region: chr27 23585575-23585586. Max. coverage (+): 0. Max coverage (-): 0

Region: chr27 23585587-23585599. Max. coverage (+): 0. Max coverage (-): 0

Region: chr27 23585600-23585611. Max. coverage (+): 0. Max coverage (-): 0

Region: chr27 23585612-23585624. Max. coverage (+): 0. Max coverage (-): 0

Region: chr27 23585625-23585636. Max. coverage (+): 0. Max coverage (-): 0

Region: chr27 23585637-23585649. Max. coverage (+): 0. Max coverage (-): 0

Region: chr27 23585650-23585661. Max. coverage (+): 0. Max coverage (-): 0

Region: chr27 23585662-23585674. Max. coverage (+): 0. Max coverage (-): 0

Region: chr27 23585675-23585686. Max. coverage (+): 0. Max coverage (-): 0

Region: chr27 23585687-23585699. Max. coverage (+): 0. Max coverage (-): 0

Region: chr27 23585700-23585711. Max. coverage (+): 0. Max coverage (-): 0

Region: chr27 23585712-23585724. Max. coverage (+): 0. Max coverage (-): 0

Region: chr27 23585725-23585736. Max. coverage (+): 0. Max coverage (-): 0

Region: chr27 23585737-23585749. Max. coverage (+): 0. Max coverage (-): 1.14

Region: chr27 23585750-23585761. Max. coverage (+): 0. Max coverage (-): 1.14

Region: chr27 23585762-23585774. Max. coverage (+): 0. Max coverage (-): 0

Region: chr27 23585775-23585786. Max. coverage (+): 0. Max coverage (-): 0

Region: chr27 23585787-23585799. Max. coverage (+): 0. Max coverage (-): 0

Region: chr27 23585800-23585811. Max. coverage (+): 0. Max coverage (-): 0.6

Region: chr27 23585812-23585824. Max. coverage (+): 0. Max coverage (-): 0.6

Region: chr27 23585825-23585836. Max. coverage (+): 0. Max coverage (-): 0

Region: chr27 23585837-23585849. Max. coverage (+): 0. Max coverage (-): 0

Region: chr27 23585850-23585861. Max. coverage (+): 0. Max coverage (-): 0

Region: chr27 23585862-23585874. Max. coverage (+): 0. Max coverage (-): 0

Region: chr27 23585875-23585886. Max. coverage (+): 0. Max coverage (-): 0

Region: chr27 23585887-23585899. Max. coverage (+): 0. Max coverage (-): 0

Region: chr27 23585900-23585911. Max. coverage (+): 0. Max coverage (-): 0

Region: chr27 23585912-23585924. Max. coverage (+): 0. Max coverage (-): 0

Region: chr27 23585925-23585936. Max. coverage (+): 0. Max coverage (-): 0

Region: chr27 23585937-23585948. Max. coverage (+): 0. Max coverage (-): 0

Region: chr27 23585949-23585961. Max. coverage (+): 0. Max coverage (-): 0

Region: chr27 23585962-23585973. Max. coverage (+): 0. Max coverage (-): 0

Region: chr27 23585974-23585986. Max. coverage (+): 0. Max coverage (-): 0

Region: chr27 23585987-23585998. Max. coverage (+): 0. Max coverage (-): 0

Region: chr27 23585999-23586011. Max. coverage (+): 0. Max coverage (-): 0.58

Region: chr27 23586012-23586023. Max. coverage (+): 0. Max coverage (-): 0.58

Region: chr27 23586024-23586036. Max. coverage (+): 0. Max coverage (-): 0

Region: chr27 23586037-23586048. Max. coverage (+): 0. Max coverage (-): 0

Region: chr27 23586049-23586061. Max. coverage (+): 0. Max coverage (-): 0

Region: chr27 23586062-23586073. Max. coverage (+): 0. Max coverage (-): 0

Region: chr27 23586074-23586086. Max. coverage (+): 0. Max coverage (-): 0

Region: chr27 23586087-23586098. Max. coverage (+): 0. Max coverage (-): 0

Region: chr27 23586099-23586111. Max. coverage (+): 0. Max coverage (-): 0

Region: chr27 23586112-23586123. Max. coverage (+): 0. Max coverage (-): 0

Region: chr27 23586124-23586136. Max. coverage (+): 0. Max coverage (-): 0

Region: chr27 23586137-23586148. Max. coverage (+): 0. Max coverage (-): 0

Region: chr27 23586149-23586161. Max. coverage (+): 0. Max coverage (-): 0

Region: chr27 23586162-23586173. Max. coverage (+): 0. Max coverage (-): 0

Region: chr27 23586174-23586186. Max. coverage (+): 0. Max coverage (-): 0

Region: chr27 23586187-23586198. Max. coverage (+): 0. Max coverage (-): 0

Region: chr27 23586199-23586211. Max. coverage (+): 0. Max coverage (-): 0

Region: chr27 23586212-23586223. Max. coverage (+): 0. Max coverage (-): 0

Region: chr27 23586224-23586236. Max. coverage (+): 0. Max coverage (-): 0

Region: chr27 23586237-23586248. Max. coverage (+): 0. Max coverage (-): 0

Region: chr27 23586249-23586261. Max. coverage (+): 0. Max coverage (-): 0

Region: chr27 23586262-23586273. Max. coverage (+): 0. Max coverage (-): 0

Region: chr27 23586274-23586285. Max. coverage (+): 0. Max coverage (-): 0

Region: chr27 23586286-23586298. Max. coverage (+): 0. Max coverage (-): 0

Region: chr27 23586299-23586310. Max. coverage (+): 0. Max coverage (-): 0

Region: chr27 23586311-23586323. Max. coverage (+): 0. Max coverage (-): 0

Region: chr27 23586324-23586335. Max. coverage (+): 0. Max coverage (-): 0

Region: chr27 23586336-23586348. Max. coverage (+): 0. Max coverage (-): 0

Region: chr27 23586349-23586360. Max. coverage (+): 0. Max coverage (-): 0

Region: chr27 23586361-23586373. Max. coverage (+): 0. Max coverage (-): 0

Region: chr27 23586374-23586385. Max. coverage (+): 0. Max coverage (-): 0

Region: chr27 23586386-23586398. Max. coverage (+): 0. Max coverage (-): 0

Region: chr27 23586399-23586410. Max. coverage (+): 0. Max coverage (-): 0

Region: chr27 23586411-23586423. Max. coverage (+): 0. Max coverage (-): 0

Region: chr27 23586424-23586435. Max. coverage (+): 0. Max coverage (-): 0.52

Region: chr27 23586436-23586448. Max. coverage (+): 0. Max coverage (-): 0.52

Region: chr27 23586449-23586460. Max. coverage (+): 0. Max coverage (-): 0

Region: chr27 23586461-23586473. Max. coverage (+): 0. Max coverage (-): 1.59

Region: chr27 23586474-23586485. Max. coverage (+): 0. Max coverage (-): 0

Region: chr27 23586486-23586498. Max. coverage (+): 0. Max coverage (-): 0

Region: chr27 23586499-23586510. Max. coverage (+): 0. Max coverage (-): 0

Region: chr27 23586511-23586523. Max. coverage (+): 0. Max coverage (-): 0

Region: chr27 23586524-23586535. Max. coverage (+): 0. Max coverage (-): 0

Region: chr27 23586536-23586548. Max. coverage (+): 0. Max coverage (-): 0

Region: chr27 23586549-23586560. Max. coverage (+): 0. Max coverage (-): 0

Region: chr27 23586561-23586573. Max. coverage (+): 0. Max coverage (-): 0

Region: chr27 23586574-23586585. Max. coverage (+): 0. Max coverage (-): 0

Region: chr27 23586586-23586598. Max. coverage (+): 0. Max coverage (-): 0

Region: chr27 23586599-23586610. Max. coverage (+): 0. Max coverage (-): 0

Region: chr27 23586611-23586622. Max. coverage (+): 0. Max coverage (-): 0

Region: chr27 23586623-23586635. Max. coverage (+): 0. Max coverage (-): 0

Region: chr27 23586636-23586647. Max. coverage (+): 0. Max coverage (-): 0

Region: chr27 23586648-23586660. Max. coverage (+): 0. Max coverage (-): 0

Region: chr27 23586661-23586672. Max. coverage (+): 0. Max coverage (-): 0

Region: chr27 23586673-23586685. Max. coverage (+): 0. Max coverage (-): 0

Region: chr27 23586686-23586697. Max. coverage (+): 0. Max coverage (-): 0

Region: chr27 23586698-23586710. Max. coverage (+): 0. Max coverage (-): 0

Region: chr27 23586711-23586722. Max. coverage (+): 0. Max coverage (-): 0

Region: chr27 23586723-23586735. Max. coverage (+): 0. Max coverage (-): 0

Region: chr27 23586736-23586747. Max. coverage (+): 0. Max coverage (-): 0

Region: chr27 23586748-23586760. Max. coverage (+): 0. Max coverage (-): 0

Region: chr27 23586761-23586772. Max. coverage (+): 0. Max coverage (-): 0

Region: chr27 23586773-23586785. Max. coverage (+): 0. Max coverage (-): 0

Region: chr27 23586786-23586797. Max. coverage (+): 0. Max coverage (-): 0

Region: chr27 23586798-23586810. Max. coverage (+): 0. Max coverage (-): 0

Region: chr27 23586811-23586822. Max. coverage (+): 0. Max coverage (-): 0

Region: chr27 23586823-23586835. Max. coverage (+): 0. Max coverage (-): 0

Region: chr27 23586836-23586847. Max. coverage (+): 0. Max coverage (-): 0

Region: chr27 23586848-23586860. Max. coverage (+): 0. Max coverage (-): 0

Region: chr27 23586861-23586872. Max. coverage (+): 0. Max coverage (-): 0

Region: chr27 23586873-23586885. Max. coverage (+): 0. Max coverage (-): 0

Region: chr27 23586886-23586897. Max. coverage (+): 0. Max coverage (-): 0

Region: chr27 23586898-23586910. Max. coverage (+): 0. Max coverage (-): 0

Region: chr27 23586911-23586922. Max. coverage (+): 0. Max coverage (-): 0

Region: chr27 23586923-23586935. Max. coverage (+): 0. Max coverage (-): 0

Region: chr27 23586936-23586947. Max. coverage (+): 0. Max coverage (-): 0

Region: chr27 23586948-23586960. Max. coverage (+): 0. Max coverage (-): 0

Region: chr27 23586961-23586972. Max. coverage (+): 0. Max coverage (-): 0

Region: chr27 23586973-23586984. Max. coverage (+): 0. Max coverage (-): 0

Region: chr27 23586985-23586997. Max. coverage (+): 0. Max coverage (-): 0

Region: chr27 23586998-23587009. Max. coverage (+): 0. Max coverage (-): 1.61

Region: chr27 23587010-23587022. Max. coverage (+): 0. Max coverage (-): 0

Region: chr27 23587023-23587034. Max. coverage (+): 0. Max coverage (-): 0

Region: chr27 23587035-23587047. Max. coverage (+): 0. Max coverage (-): 2.1

Region: chr27 23587048-23587059. Max. coverage (+): 0. Max coverage (-): 0

Region: chr27 23587060-23587072. Max. coverage (+): 0. Max coverage (-): 0

Region: chr27 23587073-23587084. Max. coverage (+): 0. Max coverage (-): 0

Region: chr27 23587085-23587097. Max. coverage (+): 0. Max coverage (-): 0

Region: chr27 23587098-23587109. Max. coverage (+): 0. Max coverage (-): 0

Region: chr27 23587110-23587122. Max. coverage (+): 0. Max coverage (-): 2.44

Region: chr27 23587123-23587134. Max. coverage (+): 0. Max coverage (-): 0

Region: chr27 23587135-. Max. coverage (+): 0. Max coverage (-): 0

RepeatMasker Color Code

**+**

100-98% Identity

<98-95% Identity

<95-90% Identity

<90-85% Identity

<85-80% Identity

<80-75% Identity

<75-70% Identity

<70% Identity

**-**

Gene Set Color Code

**+**

Gene

Pseudogene

**-**

Topology/Coverage Color Code

Coverage Plus Strand

Coverage Minus Strand

Mainstrand: Plus

Mainstrand: Minus

Complementary Strand

Flanking Region  
(if option -flank >0)

Gene Set Annotation  
  
RepeatMasker Annotation  

**1. L1MC3**: 23580931-23581151 (-), Divergence to consensus: 29.7%  
**2. LTR104\_Mam**: 23582494-23582741 (+), Divergence to consensus: 44.4%  
**3. Bov-tA2**: 23582742-23582950 (+), Divergence to consensus: 16.1%  
**4. LTR104\_Mam**: 23582951-23583055 (+), Divergence to consensus: 44.4%  
**5. LTR104\_Mam**: 23583080-23583201 (+), Divergence to consensus: 48.4%  
**6. MIRb**: 23583528-23583622 (-), Divergence to consensus: 39.8%  
**7. AT\_rich**: 23584024-23584044 (+), Divergence to consensus: 38.1%  
**8. MIRb**: 23584504-23584574 (-), Divergence to consensus: 30.4%  
**9. MER21C**: 23584575-23585159 (+), Divergence to consensus: 36%  
**10. BOV-A2**: 23585160-23585430 (+), Divergence to consensus: 5.2%  
**11. MER21C**: 23585431-23585702 (+), Divergence to consensus: 36%  
**12. MIR3**: 23585745-23585818 (-), Divergence to consensus: 35.1%  
**13. (CACCAT)n**: 23586243-23586335 (+), Divergence to consensus: 28.3%  
**14. (CA)n**: 23586665-23586678 (+), Divergence to consensus: 10.5%  
**15. BOV-A2**: 23586679-23586946 (-), Divergence to consensus: 9.4%  
**16. (CA)n**: 23586947-23586969 (+), Divergence to consensus: 10.5%

  
Transcription Factor Binding Sites  

**SOX9** (Sequence: TTATTGTT (+): 23581810)  
**SOX9** (Sequence: CTATTGTT (+): 23585832)  
**Gata4** (Sequence: GTTATCT (+): 23586122)
