# Supplementary material for: Small RNA sequencing of cryopreserved semen from single bull revealed altered miRNAs and piRNAs expression between High- and Low-motile sperm populations
Source: BMC Genomics. 2017 Jan 4;18:14. doi: 10.1186/s12864-016-3394-7 (PMC5209821; doi:10.1186/s12864-016-3394-7)
Supplement: Additional file 3: — Details for each piRNA clusters found in High Motile (HM) sperm fraction. Genes, repeats, transposable elements and transcription factors binding sites falling within the cluster regions were reported. (ZIP 1896 kb) [file 12864_2016_3394_MOESM3_ESM.zip › 67.html]

piRNA cluster 67


Predicted piRNA cluster no. 67     previous   next
  

Show proTRAC run info
Hide proTRAC run info

================================= proTRAC ====================================  
VERSION: 2.1                                    LAST MODIFIED: 06. October 2015  
  
Please cite:  
Rosenkranz D, Zischler H. proTRAC - a software for probabilistic piRNA cluster  
detection, visualization and analysis. 2012. BMC Bioinformatics 13:5.  
  
and (for proTRAC 2.0 and later):  
Rosenkranz D, Rudloff S, Bastuck K, Ketting RF, Zischler H. Tupaia small RNAs  
provide insights into function and evolution of RNAi-based transposon defense  
in mammals. 2015. RNA 21(5):911-922.  
  
Contact:  
David Rosenkranz  
Institute of Anthropology, small RNA group  
Johannes Gutenberg University Mainz  
email: rosenkranz@uni-mainz.de  
  
You can find the latest proTRAC version at:  
http://sourceforge.net/projects/protrac/files  
http://www.smallRNAgroup-mainz.de/software  
==============================================================================  
  
PARAMETERS:  
Map file: .............../storage/core/barbara/genhome/smallRNA/fertility/Sample\_motile/pirna/Sample\_motile\_26-33\_collapsed.fa.no-dust.map.weighted-10000-1000-b-0  
Genome file: ............/storage/core/barbara/genhome/smallRNA/fertility/Sample\_all/pirna/bt\_311\_chrY.fa  
RepeatMasker annotation: /storage/genomes/bt\_umd31/GCF\_000003055.6\_Bos\_taurus\_UMD\_3.1.1\_repeatMasker\_chr.out  
GeneSet:................./storage/core/barbara/genhome/smallRNA/fertility/Sample\_all/pirna/full.gtf  
  
Significant (p<=0.01) hit density will be calculated based  
on observed hit distribution.  
  
Sliding window size: ........................................ 5000 bp  
Sliding window increament: .................................. 1000 bp  
Normalize each hit by number of genomic hits: ............... 1 [0=no/1=yes]  
Normalize each hit by number of sequence reads: ............. 1 [0=no/1=yes]  
Normalize values (-> per million mapped reads): ............. 1 [0=no/1=yes]  
Min. fraction of hits with 1T(U) or 10A: .................... 0.75  
Alternatively: Min. fraction of hits with 1T(U) and 10A: .... 0.5  
Min. fraction of hits with typical piRNA length: ............ 0.75  
Typical piRNA length: ....................................... 26-33 nt  
Min. size of a piRNA cluster: ............................... 5000 bp.  
Min. number of hits (absolute): ............................. 0  
Min. number of hits (normalized): ........................... 0  
Min. fraction of hits on the mainstrand: .................... 0.75  
Top fraction of mapped sequences (in terms of read counts): . 1%  
Top fraction accounts for max. n% of sequence reads: ........ 90%  
Min. fraction of hits on each arm of a bidirectional cluster: 0.1  
Output image file for each cluster: ......................... 0 [0=no/1=yes]  
Output html file for each cluster: .......................... 1 [0=no/1=yes]  
Output a summary table: ..................................... 1 [0=no/1=yes]  
Output a FASTA file for each cluster (piRNA sequences): ..... 1 [0=no/1=yes]  
Output a FASTA file comprising cluster sequences: ........... 1 [0=no/1=yes]  
Search DNA motifs in clusters: .............................. 1 [0=no/1=yes]  
Output flanking sequences: +/- .............................. 0 bp  
Output ~.pTi file: .......................................... 1 [0=no/1=yes]  
==============================================================================  
  
  
Genome size (without gaps): ............ 2678902517 bp  
Gaps (N/X/-): .......................... 53837044 bp  
Mapped reads: .......................... 658825247023  
Non-identical sequences: ............... 514171  
Genomic hits: .......................... 764233  
Significant densitiy of mapped reads: .. 12867599.5173724 reads/kb

Show proTRAC cluster info
Hide proTRAC cluster info

|  |  |
| --- | --- |
| Location | chr27 |
| Coordinates | 38797682-38804809 |
| Size [bp] | 7128 |
| Sequence hit loci | 104 |
| Mapped reads (normalized) | 140851804 |
| Mapped reads (normalized) per kb | 19760354.1 |
| Normalized reads with 1T (1U) | 82.5% |
| Normalized reads with 10A | 24.9% |
| Normalized reads with length 26-33 nt | 100% |
| Normalized reads on the main strand(s) | 100% |
| Predicted directionality | mono:plus |

100%

0%

1T (1U)  
reads

10A reads

26-33 nt  
reads

reads on mainstrand

**Either the amount of reads with 1T (1U) OR 10A has to exceed 75% (set with option: -1Tor10A)  
Alternatively the amount of reads with 1T (1U) AND 10A has to exceed 50% (set with option: -1Tand10A)  
Minimum amount of reads with preferred size is 75% (set with option: -pisize)  
Minimum amount of reads on the main strand(s) is 75% (set with option: -clstrand)**

Show read coverage
Hide read coverage

WHAT DO I SEE HERE?  
This chart shows the location of mapped sequence reads within a predicted piRNA cluster. The color refers to the number of genomic hits produced by the sequence read in question. A dark red bar indicates that this sequence read produces many other hits elsewhere in the genome. Many adjacent red or yellow bars can indicate the presence of a multi-copy element such as transposons or rRNA genes. A dark green bar indicates that this sequence read maps uniquely to this locus.

1 hit

2-5 hits

6-10 hits

11-20 hits

21-50 hits

51-100 hits

> 100 hits

chr27

38797682

38804809

Gene Set

RepeatMasker

Mapped  
Reads

12.11

plus strand

minus strand

12.11

Region: chr27 23587136-38797689. Max. coverage (+): 6.21. Max coverage (-): 0

Region: chr27 38797690-38797703. Max. coverage (+): 2.56. Max coverage (-): 0

Region: chr27 38797704-38797717. Max. coverage (+): 2.56. Max coverage (-): 0

Region: chr27 38797718-38797731. Max. coverage (+): 0. Max coverage (-): 0

Region: chr27 38797732-38797746. Max. coverage (+): 0. Max coverage (-): 0

Region: chr27 38797747-38797760. Max. coverage (+): 0. Max coverage (-): 0

Region: chr27 38797761-38797774. Max. coverage (+): 1.85. Max coverage (-): 0

Region: chr27 38797775-38797788. Max. coverage (+): 0. Max coverage (-): 0

Region: chr27 38797789-38797803. Max. coverage (+): 0. Max coverage (-): 0

Region: chr27 38797804-38797817. Max. coverage (+): 0. Max coverage (-): 0

Region: chr27 38797818-38797831. Max. coverage (+): 4.58. Max coverage (-): 0

Region: chr27 38797832-38797845. Max. coverage (+): 0. Max coverage (-): 0

Region: chr27 38797846-38797860. Max. coverage (+): 0. Max coverage (-): 0

Region: chr27 38797861-38797874. Max. coverage (+): 0. Max coverage (-): 0

Region: chr27 38797875-38797888. Max. coverage (+): 0. Max coverage (-): 0

Region: chr27 38797889-38797902. Max. coverage (+): 0. Max coverage (-): 0

Region: chr27 38797903-38797917. Max. coverage (+): 0. Max coverage (-): 0

Region: chr27 38797918-38797931. Max. coverage (+): 0. Max coverage (-): 0

Region: chr27 38797932-38797945. Max. coverage (+): 0. Max coverage (-): 0

Region: chr27 38797946-38797959. Max. coverage (+): 0. Max coverage (-): 0

Region: chr27 38797960-38797974. Max. coverage (+): 0. Max coverage (-): 0

Region: chr27 38797975-38797988. Max. coverage (+): 0. Max coverage (-): 0

Region: chr27 38797989-38798002. Max. coverage (+): 0. Max coverage (-): 0

Region: chr27 38798003-38798017. Max. coverage (+): 4.91. Max coverage (-): 0

Region: chr27 38798018-38798031. Max. coverage (+): 4.91. Max coverage (-): 0

Region: chr27 38798032-38798045. Max. coverage (+): 0. Max coverage (-): 0

Region: chr27 38798046-38798059. Max. coverage (+): 0. Max coverage (-): 0

Region: chr27 38798060-38798074. Max. coverage (+): 0.5. Max coverage (-): 0

Region: chr27 38798075-38798088. Max. coverage (+): 3.57. Max coverage (-): 0

Region: chr27 38798089-38798102. Max. coverage (+): 0. Max coverage (-): 0

Region: chr27 38798103-38798116. Max. coverage (+): 0. Max coverage (-): 0

Region: chr27 38798117-38798131. Max. coverage (+): 0. Max coverage (-): 0

Region: chr27 38798132-38798145. Max. coverage (+): 0. Max coverage (-): 0

Region: chr27 38798146-38798159. Max. coverage (+): 1.32. Max coverage (-): 0

Region: chr27 38798160-38798173. Max. coverage (+): 1.32. Max coverage (-): 0

Region: chr27 38798174-38798188. Max. coverage (+): 0. Max coverage (-): 0

Region: chr27 38798189-38798202. Max. coverage (+): 0. Max coverage (-): 0

Region: chr27 38798203-38798216. Max. coverage (+): 0. Max coverage (-): 0

Region: chr27 38798217-38798230. Max. coverage (+): 0. Max coverage (-): 0

Region: chr27 38798231-38798245. Max. coverage (+): 0. Max coverage (-): 0

Region: chr27 38798246-38798259. Max. coverage (+): 0. Max coverage (-): 0

Region: chr27 38798260-38798273. Max. coverage (+): 0. Max coverage (-): 0

Region: chr27 38798274-38798287. Max. coverage (+): 0. Max coverage (-): 0

Region: chr27 38798288-38798302. Max. coverage (+): 0. Max coverage (-): 0

Region: chr27 38798303-38798316. Max. coverage (+): 0. Max coverage (-): 0

Region: chr27 38798317-38798330. Max. coverage (+): 0. Max coverage (-): 0

Region: chr27 38798331-38798344. Max. coverage (+): 0. Max coverage (-): 0

Region: chr27 38798345-38798359. Max. coverage (+): 0. Max coverage (-): 0

Region: chr27 38798360-38798373. Max. coverage (+): 0. Max coverage (-): 0

Region: chr27 38798374-38798387. Max. coverage (+): 0. Max coverage (-): 0

Region: chr27 38798388-38798401. Max. coverage (+): 0. Max coverage (-): 0

Region: chr27 38798402-38798416. Max. coverage (+): 0. Max coverage (-): 0

Region: chr27 38798417-38798430. Max. coverage (+): 0. Max coverage (-): 0

Region: chr27 38798431-38798444. Max. coverage (+): 0. Max coverage (-): 0

Region: chr27 38798445-38798458. Max. coverage (+): 0. Max coverage (-): 0

Region: chr27 38798459-38798473. Max. coverage (+): 3.1. Max coverage (-): 0

Region: chr27 38798474-38798487. Max. coverage (+): 0. Max coverage (-): 0

Region: chr27 38798488-38798501. Max. coverage (+): 0. Max coverage (-): 0

Region: chr27 38798502-38798515. Max. coverage (+): 0. Max coverage (-): 0

Region: chr27 38798516-38798530. Max. coverage (+): 0. Max coverage (-): 0

Region: chr27 38798531-38798544. Max. coverage (+): 0. Max coverage (-): 0

Region: chr27 38798545-38798558. Max. coverage (+): 0. Max coverage (-): 0

Region: chr27 38798559-38798572. Max. coverage (+): 0. Max coverage (-): 0

Region: chr27 38798573-38798587. Max. coverage (+): 0. Max coverage (-): 0

Region: chr27 38798588-38798601. Max. coverage (+): 0. Max coverage (-): 0

Region: chr27 38798602-38798615. Max. coverage (+): 0. Max coverage (-): 0

Region: chr27 38798616-38798630. Max. coverage (+): 0. Max coverage (-): 0

Region: chr27 38798631-38798644. Max. coverage (+): 0. Max coverage (-): 0

Region: chr27 38798645-38798658. Max. coverage (+): 0. Max coverage (-): 0

Region: chr27 38798659-38798672. Max. coverage (+): 0. Max coverage (-): 0

Region: chr27 38798673-38798687. Max. coverage (+): 0. Max coverage (-): 0

Region: chr27 38798688-38798701. Max. coverage (+): 0. Max coverage (-): 0

Region: chr27 38798702-38798715. Max. coverage (+): 0. Max coverage (-): 0

Region: chr27 38798716-38798729. Max. coverage (+): 0. Max coverage (-): 0

Region: chr27 38798730-38798744. Max. coverage (+): 1.07. Max coverage (-): 0

Region: chr27 38798745-38798758. Max. coverage (+): 1.07. Max coverage (-): 0

Region: chr27 38798759-38798772. Max. coverage (+): 0. Max coverage (-): 0

Region: chr27 38798773-38798786. Max. coverage (+): 0. Max coverage (-): 0

Region: chr27 38798787-38798801. Max. coverage (+): 0. Max coverage (-): 0

Region: chr27 38798802-38798815. Max. coverage (+): 0. Max coverage (-): 0

Region: chr27 38798816-38798829. Max. coverage (+): 0. Max coverage (-): 0

Region: chr27 38798830-38798843. Max. coverage (+): 0. Max coverage (-): 0

Region: chr27 38798844-38798858. Max. coverage (+): 0. Max coverage (-): 0

Region: chr27 38798859-38798872. Max. coverage (+): 0. Max coverage (-): 0

Region: chr27 38798873-38798886. Max. coverage (+): 0. Max coverage (-): 0

Region: chr27 38798887-38798900. Max. coverage (+): 5.09. Max coverage (-): 0

Region: chr27 38798901-38798915. Max. coverage (+): 5.09. Max coverage (-): 0

Region: chr27 38798916-38798929. Max. coverage (+): 0.47. Max coverage (-): 0

Region: chr27 38798930-38798943. Max. coverage (+): 0. Max coverage (-): 0

Region: chr27 38798944-38798957. Max. coverage (+): 0. Max coverage (-): 0

Region: chr27 38798958-38798972. Max. coverage (+): 0. Max coverage (-): 0

Region: chr27 38798973-38798986. Max. coverage (+): 0. Max coverage (-): 0

Region: chr27 38798987-38799000. Max. coverage (+): 0. Max coverage (-): 0

Region: chr27 38799001-38799014. Max. coverage (+): 0. Max coverage (-): 0

Region: chr27 38799015-38799029. Max. coverage (+): 0. Max coverage (-): 0

Region: chr27 38799030-38799043. Max. coverage (+): 8.04. Max coverage (-): 0

Region: chr27 38799044-38799057. Max. coverage (+): 0. Max coverage (-): 0

Region: chr27 38799058-38799071. Max. coverage (+): 3.43. Max coverage (-): 0

Region: chr27 38799072-38799086. Max. coverage (+): 1.05. Max coverage (-): 0

Region: chr27 38799087-38799100. Max. coverage (+): 0. Max coverage (-): 0

Region: chr27 38799101-38799114. Max. coverage (+): 1.05. Max coverage (-): 0

Region: chr27 38799115-38799128. Max. coverage (+): 0. Max coverage (-): 0

Region: chr27 38799129-38799143. Max. coverage (+): 0. Max coverage (-): 0

Region: chr27 38799144-38799157. Max. coverage (+): 0. Max coverage (-): 0

Region: chr27 38799158-38799171. Max. coverage (+): 0. Max coverage (-): 0

Region: chr27 38799172-38799186. Max. coverage (+): 0. Max coverage (-): 0

Region: chr27 38799187-38799200. Max. coverage (+): 0. Max coverage (-): 0

Region: chr27 38799201-38799214. Max. coverage (+): 12.11. Max coverage (-): 0

Region: chr27 38799215-38799228. Max. coverage (+): 12.11. Max coverage (-): 0

Region: chr27 38799229-38799243. Max. coverage (+): 0. Max coverage (-): 0

Region: chr27 38799244-38799257. Max. coverage (+): 0. Max coverage (-): 0

Region: chr27 38799258-38799271. Max. coverage (+): 0. Max coverage (-): 0

Region: chr27 38799272-38799285. Max. coverage (+): 0. Max coverage (-): 0

Region: chr27 38799286-38799300. Max. coverage (+): 0. Max coverage (-): 0

Region: chr27 38799301-38799314. Max. coverage (+): 0. Max coverage (-): 0

Region: chr27 38799315-38799328. Max. coverage (+): 0. Max coverage (-): 0

Region: chr27 38799329-38799342. Max. coverage (+): 0. Max coverage (-): 0

Region: chr27 38799343-38799357. Max. coverage (+): 0. Max coverage (-): 0

Region: chr27 38799358-38799371. Max. coverage (+): 0. Max coverage (-): 0

Region: chr27 38799372-38799385. Max. coverage (+): 0. Max coverage (-): 0

Region: chr27 38799386-38799399. Max. coverage (+): 0. Max coverage (-): 0

Region: chr27 38799400-38799414. Max. coverage (+): 0. Max coverage (-): 0

Region: chr27 38799415-38799428. Max. coverage (+): 0. Max coverage (-): 0

Region: chr27 38799429-38799442. Max. coverage (+): 0. Max coverage (-): 0

Region: chr27 38799443-38799456. Max. coverage (+): 0. Max coverage (-): 0

Region: chr27 38799457-38799471. Max. coverage (+): 0.67. Max coverage (-): 0

Region: chr27 38799472-38799485. Max. coverage (+): 0.67. Max coverage (-): 0

Region: chr27 38799486-38799499. Max. coverage (+): 0. Max coverage (-): 0

Region: chr27 38799500-38799513. Max. coverage (+): 0. Max coverage (-): 0

Region: chr27 38799514-38799528. Max. coverage (+): 0. Max coverage (-): 0

Region: chr27 38799529-38799542. Max. coverage (+): 0. Max coverage (-): 0

Region: chr27 38799543-38799556. Max. coverage (+): 0. Max coverage (-): 0

Region: chr27 38799557-38799570. Max. coverage (+): 0. Max coverage (-): 0

Region: chr27 38799571-38799585. Max. coverage (+): 0. Max coverage (-): 0

Region: chr27 38799586-38799599. Max. coverage (+): 0. Max coverage (-): 0

Region: chr27 38799600-38799613. Max. coverage (+): 3.19. Max coverage (-): 0

Region: chr27 38799614-38799627. Max. coverage (+): 0. Max coverage (-): 0

Region: chr27 38799628-38799642. Max. coverage (+): 0. Max coverage (-): 0

Region: chr27 38799643-38799656. Max. coverage (+): 2.11. Max coverage (-): 0

Region: chr27 38799657-38799670. Max. coverage (+): 0. Max coverage (-): 0

Region: chr27 38799671-38799684. Max. coverage (+): 0. Max coverage (-): 0

Region: chr27 38799685-38799699. Max. coverage (+): 0. Max coverage (-): 0

Region: chr27 38799700-38799713. Max. coverage (+): 2.49. Max coverage (-): 0

Region: chr27 38799714-38799727. Max. coverage (+): 0. Max coverage (-): 0

Region: chr27 38799728-38799741. Max. coverage (+): 0. Max coverage (-): 0

Region: chr27 38799742-38799756. Max. coverage (+): 5.23. Max coverage (-): 0

Region: chr27 38799757-38799770. Max. coverage (+): 5.23. Max coverage (-): 0

Region: chr27 38799771-38799784. Max. coverage (+): 0.26. Max coverage (-): 0

Region: chr27 38799785-38799799. Max. coverage (+): 0. Max coverage (-): 0

Region: chr27 38799800-38799813. Max. coverage (+): 0. Max coverage (-): 0

Region: chr27 38799814-38799827. Max. coverage (+): 0. Max coverage (-): 0

Region: chr27 38799828-38799841. Max. coverage (+): 0. Max coverage (-): 0

Region: chr27 38799842-38799856. Max. coverage (+): 0.63. Max coverage (-): 0

Region: chr27 38799857-38799870. Max. coverage (+): 0. Max coverage (-): 0

Region: chr27 38799871-38799884. Max. coverage (+): 0. Max coverage (-): 0

Region: chr27 38799885-38799898. Max. coverage (+): 0. Max coverage (-): 0

Region: chr27 38799899-38799913. Max. coverage (+): 0. Max coverage (-): 0

Region: chr27 38799914-38799927. Max. coverage (+): 0. Max coverage (-): 0

Region: chr27 38799928-38799941. Max. coverage (+): 0. Max coverage (-): 0

Region: chr27 38799942-38799955. Max. coverage (+): 0. Max coverage (-): 0

Region: chr27 38799956-38799970. Max. coverage (+): 0. Max coverage (-): 0

Region: chr27 38799971-38799984. Max. coverage (+): 1.7. Max coverage (-): 0

Region: chr27 38799985-38799998. Max. coverage (+): 0. Max coverage (-): 0

Region: chr27 38799999-38800012. Max. coverage (+): 0. Max coverage (-): 0

Region: chr27 38800013-38800027. Max. coverage (+): 0. Max coverage (-): 0

Region: chr27 38800028-38800041. Max. coverage (+): 0. Max coverage (-): 0

Region: chr27 38800042-38800055. Max. coverage (+): 4.97. Max coverage (-): 0

Region: chr27 38800056-38800069. Max. coverage (+): 4.97. Max coverage (-): 0

Region: chr27 38800070-38800084. Max. coverage (+): 7.33. Max coverage (-): 0

Region: chr27 38800085-38800098. Max. coverage (+): 0. Max coverage (-): 0

Region: chr27 38800099-38800112. Max. coverage (+): 0. Max coverage (-): 0

Region: chr27 38800113-38800126. Max. coverage (+): 0. Max coverage (-): 0

Region: chr27 38800127-38800141. Max. coverage (+): 0. Max coverage (-): 0

Region: chr27 38800142-38800155. Max. coverage (+): 0. Max coverage (-): 0

Region: chr27 38800156-38800169. Max. coverage (+): 0. Max coverage (-): 0

Region: chr27 38800170-38800183. Max. coverage (+): 0. Max coverage (-): 0

Region: chr27 38800184-38800198. Max. coverage (+): 0. Max coverage (-): 0

Region: chr27 38800199-38800212. Max. coverage (+): 0. Max coverage (-): 0

Region: chr27 38800213-38800226. Max. coverage (+): 0. Max coverage (-): 0

Region: chr27 38800227-38800240. Max. coverage (+): 0. Max coverage (-): 0

Region: chr27 38800241-38800255. Max. coverage (+): 0. Max coverage (-): 0

Region: chr27 38800256-38800269. Max. coverage (+): 1.78. Max coverage (-): 0

Region: chr27 38800270-38800283. Max. coverage (+): 0. Max coverage (-): 0

Region: chr27 38800284-38800297. Max. coverage (+): 0. Max coverage (-): 0

Region: chr27 38800298-38800312. Max. coverage (+): 0. Max coverage (-): 0

Region: chr27 38800313-38800326. Max. coverage (+): 0. Max coverage (-): 0

Region: chr27 38800327-38800340. Max. coverage (+): 0. Max coverage (-): 0

Region: chr27 38800341-38800354. Max. coverage (+): 0. Max coverage (-): 0

Region: chr27 38800355-38800369. Max. coverage (+): 0. Max coverage (-): 0

Region: chr27 38800370-38800383. Max. coverage (+): 0. Max coverage (-): 0

Region: chr27 38800384-38800397. Max. coverage (+): 0. Max coverage (-): 0

Region: chr27 38800398-38800412. Max. coverage (+): 0. Max coverage (-): 0

Region: chr27 38800413-38800426. Max. coverage (+): 0. Max coverage (-): 0

Region: chr27 38800427-38800440. Max. coverage (+): 0. Max coverage (-): 0

Region: chr27 38800441-38800454. Max. coverage (+): 0. Max coverage (-): 0

Region: chr27 38800455-38800469. Max. coverage (+): 0. Max coverage (-): 0

Region: chr27 38800470-38800483. Max. coverage (+): 0. Max coverage (-): 0

Region: chr27 38800484-38800497. Max. coverage (+): 0. Max coverage (-): 0

Region: chr27 38800498-38800511. Max. coverage (+): 0. Max coverage (-): 0

Region: chr27 38800512-38800526. Max. coverage (+): 0. Max coverage (-): 0

Region: chr27 38800527-38800540. Max. coverage (+): 0. Max coverage (-): 0

Region: chr27 38800541-38800554. Max. coverage (+): 0. Max coverage (-): 0

Region: chr27 38800555-38800568. Max. coverage (+): 0. Max coverage (-): 0

Region: chr27 38800569-38800583. Max. coverage (+): 10.17. Max coverage (-): 0

Region: chr27 38800584-38800597. Max. coverage (+): 0. Max coverage (-): 0

Region: chr27 38800598-38800611. Max. coverage (+): 0. Max coverage (-): 0

Region: chr27 38800612-38800625. Max. coverage (+): 0. Max coverage (-): 0

Region: chr27 38800626-38800640. Max. coverage (+): 0. Max coverage (-): 0

Region: chr27 38800641-38800654. Max. coverage (+): 0. Max coverage (-): 0

Region: chr27 38800655-38800668. Max. coverage (+): 0. Max coverage (-): 0

Region: chr27 38800669-38800682. Max. coverage (+): 0. Max coverage (-): 0

Region: chr27 38800683-38800697. Max. coverage (+): 0. Max coverage (-): 0

Region: chr27 38800698-38800711. Max. coverage (+): 0. Max coverage (-): 0

Region: chr27 38800712-38800725. Max. coverage (+): 0. Max coverage (-): 0

Region: chr27 38800726-38800739. Max. coverage (+): 0. Max coverage (-): 0

Region: chr27 38800740-38800754. Max. coverage (+): 0. Max coverage (-): 0

Region: chr27 38800755-38800768. Max. coverage (+): 4.06. Max coverage (-): 0

Region: chr27 38800769-38800782. Max. coverage (+): 0. Max coverage (-): 0

Region: chr27 38800783-38800796. Max. coverage (+): 0. Max coverage (-): 0

Region: chr27 38800797-38800811. Max. coverage (+): 3.75. Max coverage (-): 0

Region: chr27 38800812-38800825. Max. coverage (+): 0. Max coverage (-): 0

Region: chr27 38800826-38800839. Max. coverage (+): 0. Max coverage (-): 0

Region: chr27 38800840-38800853. Max. coverage (+): 0. Max coverage (-): 0

Region: chr27 38800854-38800868. Max. coverage (+): 0. Max coverage (-): 0

Region: chr27 38800869-38800882. Max. coverage (+): 0. Max coverage (-): 0

Region: chr27 38800883-38800896. Max. coverage (+): 0. Max coverage (-): 0

Region: chr27 38800897-38800910. Max. coverage (+): 0. Max coverage (-): 0

Region: chr27 38800911-38800925. Max. coverage (+): 4.02. Max coverage (-): 0

Region: chr27 38800926-38800939. Max. coverage (+): 5.02. Max coverage (-): 0

Region: chr27 38800940-38800953. Max. coverage (+): 0. Max coverage (-): 0

Region: chr27 38800954-38800968. Max. coverage (+): 0. Max coverage (-): 0

Region: chr27 38800969-38800982. Max. coverage (+): 0. Max coverage (-): 0

Region: chr27 38800983-38800996. Max. coverage (+): 0. Max coverage (-): 0

Region: chr27 38800997-38801010. Max. coverage (+): 0. Max coverage (-): 0

Region: chr27 38801011-38801025. Max. coverage (+): 0. Max coverage (-): 0

Region: chr27 38801026-38801039. Max. coverage (+): 0. Max coverage (-): 0

Region: chr27 38801040-38801053. Max. coverage (+): 0. Max coverage (-): 0

Region: chr27 38801054-38801067. Max. coverage (+): 0. Max coverage (-): 0

Region: chr27 38801068-38801082. Max. coverage (+): 0. Max coverage (-): 0

Region: chr27 38801083-38801096. Max. coverage (+): 0. Max coverage (-): 0

Region: chr27 38801097-38801110. Max. coverage (+): 0. Max coverage (-): 0

Region: chr27 38801111-38801124. Max. coverage (+): 0. Max coverage (-): 0

Region: chr27 38801125-38801139. Max. coverage (+): 0. Max coverage (-): 0

Region: chr27 38801140-38801153. Max. coverage (+): 0. Max coverage (-): 0

Region: chr27 38801154-38801167. Max. coverage (+): 0. Max coverage (-): 0

Region: chr27 38801168-38801181. Max. coverage (+): 0. Max coverage (-): 0

Region: chr27 38801182-38801196. Max. coverage (+): 0. Max coverage (-): 0

Region: chr27 38801197-38801210. Max. coverage (+): 0. Max coverage (-): 0

Region: chr27 38801211-38801224. Max. coverage (+): 0. Max coverage (-): 0

Region: chr27 38801225-38801238. Max. coverage (+): 0. Max coverage (-): 0

Region: chr27 38801239-38801253. Max. coverage (+): 0. Max coverage (-): 0

Region: chr27 38801254-38801267. Max. coverage (+): 0. Max coverage (-): 0

Region: chr27 38801268-38801281. Max. coverage (+): 0. Max coverage (-): 0

Region: chr27 38801282-38801295. Max. coverage (+): 0. Max coverage (-): 0

Region: chr27 38801296-38801310. Max. coverage (+): 0. Max coverage (-): 0

Region: chr27 38801311-38801324. Max. coverage (+): 0. Max coverage (-): 0

Region: chr27 38801325-38801338. Max. coverage (+): 1.73. Max coverage (-): 0

Region: chr27 38801339-38801352. Max. coverage (+): 1.73. Max coverage (-): 0

Region: chr27 38801353-38801367. Max. coverage (+): 0. Max coverage (-): 0

Region: chr27 38801368-38801381. Max. coverage (+): 7.78. Max coverage (-): 0

Region: chr27 38801382-38801395. Max. coverage (+): 4.79. Max coverage (-): 0

Region: chr27 38801396-38801409. Max. coverage (+): 4.79. Max coverage (-): 0

Region: chr27 38801410-38801424. Max. coverage (+): 0. Max coverage (-): 0

Region: chr27 38801425-38801438. Max. coverage (+): 0. Max coverage (-): 0

Region: chr27 38801439-38801452. Max. coverage (+): 0. Max coverage (-): 0

Region: chr27 38801453-38801466. Max. coverage (+): 0. Max coverage (-): 0

Region: chr27 38801467-38801481. Max. coverage (+): 0. Max coverage (-): 0

Region: chr27 38801482-38801495. Max. coverage (+): 0. Max coverage (-): 0

Region: chr27 38801496-38801509. Max. coverage (+): 0. Max coverage (-): 0

Region: chr27 38801510-38801523. Max. coverage (+): 0. Max coverage (-): 0

Region: chr27 38801524-38801538. Max. coverage (+): 0. Max coverage (-): 0

Region: chr27 38801539-38801552. Max. coverage (+): 0. Max coverage (-): 0

Region: chr27 38801553-38801566. Max. coverage (+): 0. Max coverage (-): 0

Region: chr27 38801567-38801581. Max. coverage (+): 0. Max coverage (-): 0

Region: chr27 38801582-38801595. Max. coverage (+): 0. Max coverage (-): 0

Region: chr27 38801596-38801609. Max. coverage (+): 0. Max coverage (-): 0

Region: chr27 38801610-38801623. Max. coverage (+): 0. Max coverage (-): 0

Region: chr27 38801624-38801638. Max. coverage (+): 0. Max coverage (-): 0

Region: chr27 38801639-38801652. Max. coverage (+): 0. Max coverage (-): 0

Region: chr27 38801653-38801666. Max. coverage (+): 0. Max coverage (-): 0

Region: chr27 38801667-38801680. Max. coverage (+): 0. Max coverage (-): 0

Region: chr27 38801681-38801695. Max. coverage (+): 0. Max coverage (-): 0

Region: chr27 38801696-38801709. Max. coverage (+): 0. Max coverage (-): 0

Region: chr27 38801710-38801723. Max. coverage (+): 0. Max coverage (-): 0

Region: chr27 38801724-38801737. Max. coverage (+): 0. Max coverage (-): 0

Region: chr27 38801738-38801752. Max. coverage (+): 0. Max coverage (-): 0

Region: chr27 38801753-38801766. Max. coverage (+): 0. Max coverage (-): 0

Region: chr27 38801767-38801780. Max. coverage (+): 0. Max coverage (-): 0

Region: chr27 38801781-38801794. Max. coverage (+): 0. Max coverage (-): 0

Region: chr27 38801795-38801809. Max. coverage (+): 0. Max coverage (-): 0

Region: chr27 38801810-38801823. Max. coverage (+): 0. Max coverage (-): 0

Region: chr27 38801824-38801837. Max. coverage (+): 0. Max coverage (-): 0

Region: chr27 38801838-38801851. Max. coverage (+): 0. Max coverage (-): 0

Region: chr27 38801852-38801866. Max. coverage (+): 0. Max coverage (-): 0

Region: chr27 38801867-38801880. Max. coverage (+): 0. Max coverage (-): 0

Region: chr27 38801881-38801894. Max. coverage (+): 0. Max coverage (-): 0

Region: chr27 38801895-38801908. Max. coverage (+): 0. Max coverage (-): 0

Region: chr27 38801909-38801923. Max. coverage (+): 4.83. Max coverage (-): 0

Region: chr27 38801924-38801937. Max. coverage (+): 4.83. Max coverage (-): 0

Region: chr27 38801938-38801951. Max. coverage (+): 1. Max coverage (-): 0

Region: chr27 38801952-38801965. Max. coverage (+): 0. Max coverage (-): 0

Region: chr27 38801966-38801980. Max. coverage (+): 0. Max coverage (-): 0

Region: chr27 38801981-38801994. Max. coverage (+): 0. Max coverage (-): 0

Region: chr27 38801995-38802008. Max. coverage (+): 0. Max coverage (-): 0

Region: chr27 38802009-38802022. Max. coverage (+): 0. Max coverage (-): 0

Region: chr27 38802023-38802037. Max. coverage (+): 0. Max coverage (-): 0

Region: chr27 38802038-38802051. Max. coverage (+): 0. Max coverage (-): 0

Region: chr27 38802052-38802065. Max. coverage (+): 0. Max coverage (-): 0

Region: chr27 38802066-38802079. Max. coverage (+): 0. Max coverage (-): 0

Region: chr27 38802080-38802094. Max. coverage (+): 0. Max coverage (-): 0

Region: chr27 38802095-38802108. Max. coverage (+): 0. Max coverage (-): 0

Region: chr27 38802109-38802122. Max. coverage (+): 0. Max coverage (-): 0

Region: chr27 38802123-38802136. Max. coverage (+): 0. Max coverage (-): 0

Region: chr27 38802137-38802151. Max. coverage (+): 0. Max coverage (-): 0

Region: chr27 38802152-38802165. Max. coverage (+): 0. Max coverage (-): 0

Region: chr27 38802166-38802179. Max. coverage (+): 1.94. Max coverage (-): 0

Region: chr27 38802180-38802194. Max. coverage (+): 6.01. Max coverage (-): 0

Region: chr27 38802195-38802208. Max. coverage (+): 0. Max coverage (-): 0

Region: chr27 38802209-38802222. Max. coverage (+): 0. Max coverage (-): 0

Region: chr27 38802223-38802236. Max. coverage (+): 0. Max coverage (-): 0

Region: chr27 38802237-38802251. Max. coverage (+): 0. Max coverage (-): 0

Region: chr27 38802252-38802265. Max. coverage (+): 0. Max coverage (-): 0

Region: chr27 38802266-38802279. Max. coverage (+): 0. Max coverage (-): 0

Region: chr27 38802280-38802293. Max. coverage (+): 0. Max coverage (-): 0

Region: chr27 38802294-38802308. Max. coverage (+): 0. Max coverage (-): 0

Region: chr27 38802309-38802322. Max. coverage (+): 0. Max coverage (-): 0

Region: chr27 38802323-38802336. Max. coverage (+): 0. Max coverage (-): 0

Region: chr27 38802337-38802350. Max. coverage (+): 0. Max coverage (-): 0

Region: chr27 38802351-38802365. Max. coverage (+): 0. Max coverage (-): 0

Region: chr27 38802366-38802379. Max. coverage (+): 0. Max coverage (-): 0

Region: chr27 38802380-38802393. Max. coverage (+): 0. Max coverage (-): 0

Region: chr27 38802394-38802407. Max. coverage (+): 0. Max coverage (-): 0

Region: chr27 38802408-38802422. Max. coverage (+): 0. Max coverage (-): 0

Region: chr27 38802423-38802436. Max. coverage (+): 0. Max coverage (-): 0

Region: chr27 38802437-38802450. Max. coverage (+): 1.67. Max coverage (-): 0

Region: chr27 38802451-38802464. Max. coverage (+): 0. Max coverage (-): 0

Region: chr27 38802465-38802479. Max. coverage (+): 0. Max coverage (-): 0

Region: chr27 38802480-38802493. Max. coverage (+): 0. Max coverage (-): 0

Region: chr27 38802494-38802507. Max. coverage (+): 0. Max coverage (-): 0

Region: chr27 38802508-38802521. Max. coverage (+): 0. Max coverage (-): 0

Region: chr27 38802522-38802536. Max. coverage (+): 0. Max coverage (-): 0

Region: chr27 38802537-38802550. Max. coverage (+): 0. Max coverage (-): 0

Region: chr27 38802551-38802564. Max. coverage (+): 0. Max coverage (-): 0

Region: chr27 38802565-38802578. Max. coverage (+): 0. Max coverage (-): 0

Region: chr27 38802579-38802593. Max. coverage (+): 0. Max coverage (-): 0

Region: chr27 38802594-38802607. Max. coverage (+): 0. Max coverage (-): 0

Region: chr27 38802608-38802621. Max. coverage (+): 0. Max coverage (-): 0

Region: chr27 38802622-38802635. Max. coverage (+): 0. Max coverage (-): 0

Region: chr27 38802636-38802650. Max. coverage (+): 0. Max coverage (-): 0

Region: chr27 38802651-38802664. Max. coverage (+): 0. Max coverage (-): 0

Region: chr27 38802665-38802678. Max. coverage (+): 0. Max coverage (-): 0

Region: chr27 38802679-38802692. Max. coverage (+): 0. Max coverage (-): 0

Region: chr27 38802693-38802707. Max. coverage (+): 0. Max coverage (-): 0

Region: chr27 38802708-38802721. Max. coverage (+): 0. Max coverage (-): 0

Region: chr27 38802722-38802735. Max. coverage (+): 0. Max coverage (-): 0

Region: chr27 38802736-38802750. Max. coverage (+): 0. Max coverage (-): 0

Region: chr27 38802751-38802764. Max. coverage (+): 0. Max coverage (-): 0

Region: chr27 38802765-38802778. Max. coverage (+): 0. Max coverage (-): 0

Region: chr27 38802779-38802792. Max. coverage (+): 0. Max coverage (-): 0

Region: chr27 38802793-38802807. Max. coverage (+): 0. Max coverage (-): 0

Region: chr27 38802808-38802821. Max. coverage (+): 0. Max coverage (-): 0

Region: chr27 38802822-38802835. Max. coverage (+): 0. Max coverage (-): 0

Region: chr27 38802836-38802849. Max. coverage (+): 0. Max coverage (-): 0

Region: chr27 38802850-38802864. Max. coverage (+): 3.72. Max coverage (-): 0

Region: chr27 38802865-38802878. Max. coverage (+): 3.72. Max coverage (-): 0

Region: chr27 38802879-38802892. Max. coverage (+): 0. Max coverage (-): 0

Region: chr27 38802893-38802906. Max. coverage (+): 0. Max coverage (-): 0

Region: chr27 38802907-38802921. Max. coverage (+): 0. Max coverage (-): 0

Region: chr27 38802922-38802935. Max. coverage (+): 1.63. Max coverage (-): 0

Region: chr27 38802936-38802949. Max. coverage (+): 1.63. Max coverage (-): 0

Region: chr27 38802950-38802963. Max. coverage (+): 0. Max coverage (-): 0

Region: chr27 38802964-38802978. Max. coverage (+): 0. Max coverage (-): 0

Region: chr27 38802979-38802992. Max. coverage (+): 0. Max coverage (-): 0

Region: chr27 38802993-38803006. Max. coverage (+): 0. Max coverage (-): 0

Region: chr27 38803007-38803020. Max. coverage (+): 0. Max coverage (-): 0

Region: chr27 38803021-38803035. Max. coverage (+): 0. Max coverage (-): 0

Region: chr27 38803036-38803049. Max. coverage (+): 3.52. Max coverage (-): 0

Region: chr27 38803050-38803063. Max. coverage (+): 0. Max coverage (-): 0

Region: chr27 38803064-38803077. Max. coverage (+): 0. Max coverage (-): 0

Region: chr27 38803078-38803092. Max. coverage (+): 0.52. Max coverage (-): 0

Region: chr27 38803093-38803106. Max. coverage (+): 0. Max coverage (-): 0

Region: chr27 38803107-38803120. Max. coverage (+): 0. Max coverage (-): 0

Region: chr27 38803121-38803134. Max. coverage (+): 0. Max coverage (-): 0

Region: chr27 38803135-38803149. Max. coverage (+): 0. Max coverage (-): 0

Region: chr27 38803150-38803163. Max. coverage (+): 2.25. Max coverage (-): 0

Region: chr27 38803164-38803177. Max. coverage (+): 0. Max coverage (-): 0

Region: chr27 38803178-38803191. Max. coverage (+): 0. Max coverage (-): 0

Region: chr27 38803192-38803206. Max. coverage (+): 0. Max coverage (-): 0

Region: chr27 38803207-38803220. Max. coverage (+): 0. Max coverage (-): 0

Region: chr27 38803221-38803234. Max. coverage (+): 0. Max coverage (-): 0

Region: chr27 38803235-38803248. Max. coverage (+): 0. Max coverage (-): 0

Region: chr27 38803249-38803263. Max. coverage (+): 0. Max coverage (-): 0

Region: chr27 38803264-38803277. Max. coverage (+): 0. Max coverage (-): 0

Region: chr27 38803278-38803291. Max. coverage (+): 0. Max coverage (-): 0

Region: chr27 38803292-38803305. Max. coverage (+): 0. Max coverage (-): 0

Region: chr27 38803306-38803320. Max. coverage (+): 0. Max coverage (-): 0

Region: chr27 38803321-38803334. Max. coverage (+): 0. Max coverage (-): 0

Region: chr27 38803335-38803348. Max. coverage (+): 0. Max coverage (-): 0

Region: chr27 38803349-38803363. Max. coverage (+): 0. Max coverage (-): 0

Region: chr27 38803364-38803377. Max. coverage (+): 0. Max coverage (-): 0

Region: chr27 38803378-38803391. Max. coverage (+): 7.11. Max coverage (-): 0

Region: chr27 38803392-38803405. Max. coverage (+): 6.41. Max coverage (-): 0

Region: chr27 38803406-38803420. Max. coverage (+): 1.92. Max coverage (-): 0

Region: chr27 38803421-38803434. Max. coverage (+): 1.92. Max coverage (-): 0

Region: chr27 38803435-38803448. Max. coverage (+): 0. Max coverage (-): 0

Region: chr27 38803449-38803462. Max. coverage (+): 0. Max coverage (-): 0

Region: chr27 38803463-38803477. Max. coverage (+): 0. Max coverage (-): 0

Region: chr27 38803478-38803491. Max. coverage (+): 0. Max coverage (-): 0

Region: chr27 38803492-38803505. Max. coverage (+): 0. Max coverage (-): 0

Region: chr27 38803506-38803519. Max. coverage (+): 2.34. Max coverage (-): 0

Region: chr27 38803520-38803534. Max. coverage (+): 4.51. Max coverage (-): 0

Region: chr27 38803535-38803548. Max. coverage (+): 0. Max coverage (-): 0

Region: chr27 38803549-38803562. Max. coverage (+): 0. Max coverage (-): 0

Region: chr27 38803563-38803576. Max. coverage (+): 0. Max coverage (-): 0

Region: chr27 38803577-38803591. Max. coverage (+): 0. Max coverage (-): 0

Region: chr27 38803592-38803605. Max. coverage (+): 0. Max coverage (-): 0

Region: chr27 38803606-38803619. Max. coverage (+): 0. Max coverage (-): 0

Region: chr27 38803620-38803633. Max. coverage (+): 0. Max coverage (-): 0

Region: chr27 38803634-38803648. Max. coverage (+): 0. Max coverage (-): 0

Region: chr27 38803649-38803662. Max. coverage (+): 0. Max coverage (-): 0

Region: chr27 38803663-38803676. Max. coverage (+): 0. Max coverage (-): 0

Region: chr27 38803677-38803690. Max. coverage (+): 0. Max coverage (-): 0

Region: chr27 38803691-38803705. Max. coverage (+): 7.28. Max coverage (-): 0

Region: chr27 38803706-38803719. Max. coverage (+): 3.68. Max coverage (-): 0

Region: chr27 38803720-38803733. Max. coverage (+): 8.6. Max coverage (-): 0

Region: chr27 38803734-38803747. Max. coverage (+): 0. Max coverage (-): 0

Region: chr27 38803748-38803762. Max. coverage (+): 0. Max coverage (-): 0

Region: chr27 38803763-38803776. Max. coverage (+): 0. Max coverage (-): 0

Region: chr27 38803777-38803790. Max. coverage (+): 0. Max coverage (-): 0

Region: chr27 38803791-38803804. Max. coverage (+): 0. Max coverage (-): 0

Region: chr27 38803805-38803819. Max. coverage (+): 0. Max coverage (-): 0

Region: chr27 38803820-38803833. Max. coverage (+): 0. Max coverage (-): 0

Region: chr27 38803834-38803847. Max. coverage (+): 0. Max coverage (-): 0

Region: chr27 38803848-38803861. Max. coverage (+): 0. Max coverage (-): 0

Region: chr27 38803862-38803876. Max. coverage (+): 0. Max coverage (-): 0

Region: chr27 38803877-38803890. Max. coverage (+): 0. Max coverage (-): 0

Region: chr27 38803891-38803904. Max. coverage (+): 1.22. Max coverage (-): 0

Region: chr27 38803905-38803918. Max. coverage (+): 0. Max coverage (-): 0

Region: chr27 38803919-38803933. Max. coverage (+): 0. Max coverage (-): 0

Region: chr27 38803934-38803947. Max. coverage (+): 0. Max coverage (-): 0

Region: chr27 38803948-38803961. Max. coverage (+): 0. Max coverage (-): 0

Region: chr27 38803962-38803976. Max. coverage (+): 0. Max coverage (-): 0

Region: chr27 38803977-38803990. Max. coverage (+): 0. Max coverage (-): 0

Region: chr27 38803991-38804004. Max. coverage (+): 0. Max coverage (-): 0

Region: chr27 38804005-38804018. Max. coverage (+): 0. Max coverage (-): 0

Region: chr27 38804019-38804033. Max. coverage (+): 0. Max coverage (-): 0

Region: chr27 38804034-38804047. Max. coverage (+): 0. Max coverage (-): 0

Region: chr27 38804048-38804061. Max. coverage (+): 0. Max coverage (-): 0

Region: chr27 38804062-38804075. Max. coverage (+): 0. Max coverage (-): 0

Region: chr27 38804076-38804090. Max. coverage (+): 0. Max coverage (-): 0

Region: chr27 38804091-38804104. Max. coverage (+): 0. Max coverage (-): 0

Region: chr27 38804105-38804118. Max. coverage (+): 0. Max coverage (-): 0

Region: chr27 38804119-38804132. Max. coverage (+): 0. Max coverage (-): 0

Region: chr27 38804133-38804147. Max. coverage (+): 0. Max coverage (-): 0

Region: chr27 38804148-38804161. Max. coverage (+): 0. Max coverage (-): 0

Region: chr27 38804162-38804175. Max. coverage (+): 0. Max coverage (-): 0

Region: chr27 38804176-38804189. Max. coverage (+): 0. Max coverage (-): 0

Region: chr27 38804190-38804204. Max. coverage (+): 0. Max coverage (-): 0

Region: chr27 38804205-38804218. Max. coverage (+): 0. Max coverage (-): 0

Region: chr27 38804219-38804232. Max. coverage (+): 0. Max coverage (-): 0

Region: chr27 38804233-38804246. Max. coverage (+): 3.06. Max coverage (-): 0

Region: chr27 38804247-38804261. Max. coverage (+): 3.06. Max coverage (-): 0

Region: chr27 38804262-38804275. Max. coverage (+): 0. Max coverage (-): 0

Region: chr27 38804276-38804289. Max. coverage (+): 0. Max coverage (-): 0

Region: chr27 38804290-38804303. Max. coverage (+): 0. Max coverage (-): 0

Region: chr27 38804304-38804318. Max. coverage (+): 0. Max coverage (-): 0

Region: chr27 38804319-38804332. Max. coverage (+): 0. Max coverage (-): 0

Region: chr27 38804333-38804346. Max. coverage (+): 0. Max coverage (-): 0

Region: chr27 38804347-38804360. Max. coverage (+): 0. Max coverage (-): 0

Region: chr27 38804361-38804375. Max. coverage (+): 0. Max coverage (-): 0

Region: chr27 38804376-38804389. Max. coverage (+): 0. Max coverage (-): 0

Region: chr27 38804390-38804403. Max. coverage (+): 0. Max coverage (-): 0

Region: chr27 38804404-38804417. Max. coverage (+): 0. Max coverage (-): 0

Region: chr27 38804418-38804432. Max. coverage (+): 0. Max coverage (-): 0

Region: chr27 38804433-38804446. Max. coverage (+): 0. Max coverage (-): 0

Region: chr27 38804447-38804460. Max. coverage (+): 0. Max coverage (-): 0

Region: chr27 38804461-38804474. Max. coverage (+): 0. Max coverage (-): 0

Region: chr27 38804475-38804489. Max. coverage (+): 0. Max coverage (-): 0

Region: chr27 38804490-38804503. Max. coverage (+): 0. Max coverage (-): 0

Region: chr27 38804504-38804517. Max. coverage (+): 0. Max coverage (-): 0

Region: chr27 38804518-38804532. Max. coverage (+): 0. Max coverage (-): 0

Region: chr27 38804533-38804546. Max. coverage (+): 0. Max coverage (-): 0

Region: chr27 38804547-38804560. Max. coverage (+): 0. Max coverage (-): 0

Region: chr27 38804561-38804574. Max. coverage (+): 0. Max coverage (-): 0

Region: chr27 38804575-38804589. Max. coverage (+): 0. Max coverage (-): 0

Region: chr27 38804590-38804603. Max. coverage (+): 0. Max coverage (-): 0

Region: chr27 38804604-38804617. Max. coverage (+): 0. Max coverage (-): 0

Region: chr27 38804618-38804631. Max. coverage (+): 0. Max coverage (-): 0

Region: chr27 38804632-38804646. Max. coverage (+): 0. Max coverage (-): 0

Region: chr27 38804647-38804660. Max. coverage (+): 0. Max coverage (-): 0

Region: chr27 38804661-38804674. Max. coverage (+): 0. Max coverage (-): 0

Region: chr27 38804675-38804688. Max. coverage (+): 0. Max coverage (-): 0

Region: chr27 38804689-38804703. Max. coverage (+): 1.89. Max coverage (-): 0

Region: chr27 38804704-38804717. Max. coverage (+): 0. Max coverage (-): 0

Region: chr27 38804718-38804731. Max. coverage (+): 0. Max coverage (-): 0

Region: chr27 38804732-38804745. Max. coverage (+): 0. Max coverage (-): 0

Region: chr27 38804746-38804760. Max. coverage (+): 0. Max coverage (-): 0

Region: chr27 38804761-38804774. Max. coverage (+): 0. Max coverage (-): 0

Region: chr27 38804775-38804788. Max. coverage (+): 5.98. Max coverage (-): 0

Region: chr27 38804789-38804802. Max. coverage (+): 0. Max coverage (-): 0

Region: chr27 38804803-. Max. coverage (+): 0. Max coverage (-): 0

RepeatMasker Color Code

**+**

100-98% Identity

<98-95% Identity

<95-90% Identity

<90-85% Identity

<85-80% Identity

<80-75% Identity

<75-70% Identity

<70% Identity

**-**

Gene Set Color Code

**+**

Gene

Pseudogene

**-**

Topology/Coverage Color Code

Coverage Plus Strand

Coverage Minus Strand

Mainstrand: Plus

Mainstrand: Minus

Complementary Strand

Flanking Region  
(if option -flank >0)

Gene Set Annotation  

**1. PSD3 (protein coding, ENSBTAG00000033137) Tr:00000039724 Ex:14**: 38797673-38797972 (+)

  
RepeatMasker Annotation  

**1. (TG)n**: 38799906-38799953 (+), Divergence to consensus: 12.8%  
**2. AT\_rich**: 38802474-38802534 (+), Divergence to consensus: 78.7%  
**3. (A)n**: 38802667-38802693 (+), Divergence to consensus: 7.4%

  
Transcription Factor Binding Sites  

**RFX4\_2** (Sequence: GTAACCAGG (-): 38804498)  
**RFX4\_1** (Sequence: CTTAGCAAC (+): 38802714)  
**SPZ1** (Sequence: CTCTAACCCT (-): 38799599)  
**SOX9** (Sequence: TTATTGTT (+): 38802802)  
**SOX9** (Sequence: CTATTGTT (+): 38803418)  
**Gata4** (Sequence: CTTATCT (+): 38803157)
