# Supplementary material for: Small RNA sequencing of cryopreserved semen from single bull revealed altered miRNAs and piRNAs expression between High- and Low-motile sperm populations
Source: BMC Genomics. 2017 Jan 4;18:14. doi: 10.1186/s12864-016-3394-7 (PMC5209821; doi:10.1186/s12864-016-3394-7)
Supplement: Additional file 3: — Details for each piRNA clusters found in High Motile (HM) sperm fraction. Genes, repeats, transposable elements and transcription factors binding sites falling within the cluster regions were reported. (ZIP 1896 kb) [file 12864_2016_3394_MOESM3_ESM.zip › 68.html]

piRNA cluster 68


Predicted piRNA cluster no. 68     previous   next
  

Show proTRAC run info
Hide proTRAC run info

================================= proTRAC ====================================  
VERSION: 2.1                                    LAST MODIFIED: 06. October 2015  
  
Please cite:  
Rosenkranz D, Zischler H. proTRAC - a software for probabilistic piRNA cluster  
detection, visualization and analysis. 2012. BMC Bioinformatics 13:5.  
  
and (for proTRAC 2.0 and later):  
Rosenkranz D, Rudloff S, Bastuck K, Ketting RF, Zischler H. Tupaia small RNAs  
provide insights into function and evolution of RNAi-based transposon defense  
in mammals. 2015. RNA 21(5):911-922.  
  
Contact:  
David Rosenkranz  
Institute of Anthropology, small RNA group  
Johannes Gutenberg University Mainz  
email: rosenkranz@uni-mainz.de  
  
You can find the latest proTRAC version at:  
http://sourceforge.net/projects/protrac/files  
http://www.smallRNAgroup-mainz.de/software  
==============================================================================  
  
PARAMETERS:  
Map file: .............../storage/core/barbara/genhome/smallRNA/fertility/Sample\_motile/pirna/Sample\_motile\_26-33\_collapsed.fa.no-dust.map.weighted-10000-1000-b-0  
Genome file: ............/storage/core/barbara/genhome/smallRNA/fertility/Sample\_all/pirna/bt\_311\_chrY.fa  
RepeatMasker annotation: /storage/genomes/bt\_umd31/GCF\_000003055.6\_Bos\_taurus\_UMD\_3.1.1\_repeatMasker\_chr.out  
GeneSet:................./storage/core/barbara/genhome/smallRNA/fertility/Sample\_all/pirna/full.gtf  
  
Significant (p<=0.01) hit density will be calculated based  
on observed hit distribution.  
  
Sliding window size: ........................................ 5000 bp  
Sliding window increament: .................................. 1000 bp  
Normalize each hit by number of genomic hits: ............... 1 [0=no/1=yes]  
Normalize each hit by number of sequence reads: ............. 1 [0=no/1=yes]  
Normalize values (-> per million mapped reads): ............. 1 [0=no/1=yes]  
Min. fraction of hits with 1T(U) or 10A: .................... 0.75  
Alternatively: Min. fraction of hits with 1T(U) and 10A: .... 0.5  
Min. fraction of hits with typical piRNA length: ............ 0.75  
Typical piRNA length: ....................................... 26-33 nt  
Min. size of a piRNA cluster: ............................... 5000 bp.  
Min. number of hits (absolute): ............................. 0  
Min. number of hits (normalized): ........................... 0  
Min. fraction of hits on the mainstrand: .................... 0.75  
Top fraction of mapped sequences (in terms of read counts): . 1%  
Top fraction accounts for max. n% of sequence reads: ........ 90%  
Min. fraction of hits on each arm of a bidirectional cluster: 0.1  
Output image file for each cluster: ......................... 0 [0=no/1=yes]  
Output html file for each cluster: .......................... 1 [0=no/1=yes]  
Output a summary table: ..................................... 1 [0=no/1=yes]  
Output a FASTA file for each cluster (piRNA sequences): ..... 1 [0=no/1=yes]  
Output a FASTA file comprising cluster sequences: ........... 1 [0=no/1=yes]  
Search DNA motifs in clusters: .............................. 1 [0=no/1=yes]  
Output flanking sequences: +/- .............................. 0 bp  
Output ~.pTi file: .......................................... 1 [0=no/1=yes]  
==============================================================================  
  
  
Genome size (without gaps): ............ 2678902517 bp  
Gaps (N/X/-): .......................... 53837044 bp  
Mapped reads: .......................... 658825247023  
Non-identical sequences: ............... 514171  
Genomic hits: .......................... 764233  
Significant densitiy of mapped reads: .. 12867599.5173724 reads/kb

Show proTRAC cluster info
Hide proTRAC cluster info

|  |  |
| --- | --- |
| Location | chr28 |
| Coordinates | 1795135-1801561 |
| Size [bp] | 6427 |
| Sequence hit loci | 949 |
| Mapped reads (normalized) | 596597444.5 |
| Mapped reads (normalized) per kb | 92826737.9 |
| Normalized reads with 1T (1U) | 78.2% |
| Normalized reads with 10A | 29.7% |
| Normalized reads with length 26-33 nt | 100% |
| Normalized reads on the main strand(s) | 99.1% |
| Predicted directionality | mono:plus |

100%

0%

1T (1U)  
reads

10A reads

26-33 nt  
reads

reads on mainstrand

**Either the amount of reads with 1T (1U) OR 10A has to exceed 75% (set with option: -1Tor10A)  
Alternatively the amount of reads with 1T (1U) AND 10A has to exceed 50% (set with option: -1Tand10A)  
Minimum amount of reads with preferred size is 75% (set with option: -pisize)  
Minimum amount of reads on the main strand(s) is 75% (set with option: -clstrand)**

Show read coverage
Hide read coverage

WHAT DO I SEE HERE?  
This chart shows the location of mapped sequence reads within a predicted piRNA cluster. The color refers to the number of genomic hits produced by the sequence read in question. A dark red bar indicates that this sequence read produces many other hits elsewhere in the genome. Many adjacent red or yellow bars can indicate the presence of a multi-copy element such as transposons or rRNA genes. A dark green bar indicates that this sequence read maps uniquely to this locus.

1 hit

2-5 hits

6-10 hits

11-20 hits

21-50 hits

51-100 hits

> 100 hits

chr28

1795135

1801561

Gene Set

RepeatMasker

Mapped  
Reads

53.71

plus strand

minus strand

53.71

Region: chr28 38804804-1795141. Max. coverage (+): 2.01. Max coverage (-): 0

Region: chr28 1795142-1795154. Max. coverage (+): 0. Max coverage (-): 0

Region: chr28 1795155-1795167. Max. coverage (+): 4.39. Max coverage (-): 0

Region: chr28 1795168-1795179. Max. coverage (+): 5.33. Max coverage (-): 0

Region: chr28 1795180-1795192. Max. coverage (+): 0. Max coverage (-): 0

Region: chr28 1795193-1795205. Max. coverage (+): 0. Max coverage (-): 0

Region: chr28 1795206-1795218. Max. coverage (+): 0. Max coverage (-): 0

Region: chr28 1795219-1795231. Max. coverage (+): 0. Max coverage (-): 0

Region: chr28 1795232-1795244. Max. coverage (+): 0. Max coverage (-): 0

Region: chr28 1795245-1795257. Max. coverage (+): 0. Max coverage (-): 0

Region: chr28 1795258-1795269. Max. coverage (+): 0. Max coverage (-): 0

Region: chr28 1795270-1795282. Max. coverage (+): 0. Max coverage (-): 0

Region: chr28 1795283-1795295. Max. coverage (+): 0. Max coverage (-): 0

Region: chr28 1795296-1795308. Max. coverage (+): 0. Max coverage (-): 0

Region: chr28 1795309-1795321. Max. coverage (+): 0. Max coverage (-): 0

Region: chr28 1795322-1795334. Max. coverage (+): 0. Max coverage (-): 0

Region: chr28 1795335-1795347. Max. coverage (+): 0. Max coverage (-): 0

Region: chr28 1795348-1795359. Max. coverage (+): 0. Max coverage (-): 0

Region: chr28 1795360-1795372. Max. coverage (+): 0. Max coverage (-): 0

Region: chr28 1795373-1795385. Max. coverage (+): 0. Max coverage (-): 0

Region: chr28 1795386-1795398. Max. coverage (+): 0. Max coverage (-): 0

Region: chr28 1795399-1795411. Max. coverage (+): 0. Max coverage (-): 0

Region: chr28 1795412-1795424. Max. coverage (+): 0. Max coverage (-): 0

Region: chr28 1795425-1795437. Max. coverage (+): 0. Max coverage (-): 0

Region: chr28 1795438-1795449. Max. coverage (+): 0. Max coverage (-): 0

Region: chr28 1795450-1795462. Max. coverage (+): 0. Max coverage (-): 0

Region: chr28 1795463-1795475. Max. coverage (+): 1.33. Max coverage (-): 0

Region: chr28 1795476-1795488. Max. coverage (+): 1.33. Max coverage (-): 0

Region: chr28 1795489-1795501. Max. coverage (+): 0. Max coverage (-): 0

Region: chr28 1795502-1795514. Max. coverage (+): 0. Max coverage (-): 0

Region: chr28 1795515-1795527. Max. coverage (+): 0. Max coverage (-): 0

Region: chr28 1795528-1795539. Max. coverage (+): 6.06. Max coverage (-): 0

Region: chr28 1795540-1795552. Max. coverage (+): 6.06. Max coverage (-): 0

Region: chr28 1795553-1795565. Max. coverage (+): 0. Max coverage (-): 0

Region: chr28 1795566-1795578. Max. coverage (+): 0. Max coverage (-): 0

Region: chr28 1795579-1795591. Max. coverage (+): 0. Max coverage (-): 0

Region: chr28 1795592-1795604. Max. coverage (+): 0. Max coverage (-): 0

Region: chr28 1795605-1795617. Max. coverage (+): 0. Max coverage (-): 0

Region: chr28 1795618-1795629. Max. coverage (+): 0. Max coverage (-): 0

Region: chr28 1795630-1795642. Max. coverage (+): 0. Max coverage (-): 0

Region: chr28 1795643-1795655. Max. coverage (+): 8.59. Max coverage (-): 0

Region: chr28 1795656-1795668. Max. coverage (+): 0. Max coverage (-): 0

Region: chr28 1795669-1795681. Max. coverage (+): 0. Max coverage (-): 0

Region: chr28 1795682-1795694. Max. coverage (+): 0. Max coverage (-): 0

Region: chr28 1795695-1795707. Max. coverage (+): 0. Max coverage (-): 0

Region: chr28 1795708-1795719. Max. coverage (+): 0. Max coverage (-): 0

Region: chr28 1795720-1795732. Max. coverage (+): 0. Max coverage (-): 0

Region: chr28 1795733-1795745. Max. coverage (+): 0. Max coverage (-): 0

Region: chr28 1795746-1795758. Max. coverage (+): 0. Max coverage (-): 0

Region: chr28 1795759-1795771. Max. coverage (+): 0. Max coverage (-): 0

Region: chr28 1795772-1795784. Max. coverage (+): 0. Max coverage (-): 0

Region: chr28 1795785-1795796. Max. coverage (+): 0. Max coverage (-): 0

Region: chr28 1795797-1795809. Max. coverage (+): 0. Max coverage (-): 0

Region: chr28 1795810-1795822. Max. coverage (+): 0. Max coverage (-): 0

Region: chr28 1795823-1795835. Max. coverage (+): 2.04. Max coverage (-): 0

Region: chr28 1795836-1795848. Max. coverage (+): 0. Max coverage (-): 0

Region: chr28 1795849-1795861. Max. coverage (+): 0. Max coverage (-): 0

Region: chr28 1795862-1795874. Max. coverage (+): 0. Max coverage (-): 0

Region: chr28 1795875-1795886. Max. coverage (+): 0. Max coverage (-): 0

Region: chr28 1795887-1795899. Max. coverage (+): 3.86. Max coverage (-): 0

Region: chr28 1795900-1795912. Max. coverage (+): 3.86. Max coverage (-): 0

Region: chr28 1795913-1795925. Max. coverage (+): 0. Max coverage (-): 0

Region: chr28 1795926-1795938. Max. coverage (+): 0. Max coverage (-): 0

Region: chr28 1795939-1795951. Max. coverage (+): 1.93. Max coverage (-): 0

Region: chr28 1795952-1795964. Max. coverage (+): 0.96. Max coverage (-): 0

Region: chr28 1795965-1795976. Max. coverage (+): 0.96. Max coverage (-): 0

Region: chr28 1795977-1795989. Max. coverage (+): 0. Max coverage (-): 0

Region: chr28 1795990-1796002. Max. coverage (+): 0. Max coverage (-): 0

Region: chr28 1796003-1796015. Max. coverage (+): 0. Max coverage (-): 0

Region: chr28 1796016-1796028. Max. coverage (+): 0. Max coverage (-): 0

Region: chr28 1796029-1796041. Max. coverage (+): 0. Max coverage (-): 0

Region: chr28 1796042-1796054. Max. coverage (+): 0. Max coverage (-): 0

Region: chr28 1796055-1796066. Max. coverage (+): 0.51. Max coverage (-): 0

Region: chr28 1796067-1796079. Max. coverage (+): 0.51. Max coverage (-): 0

Region: chr28 1796080-1796092. Max. coverage (+): 0. Max coverage (-): 0

Region: chr28 1796093-1796105. Max. coverage (+): 0. Max coverage (-): 0

Region: chr28 1796106-1796118. Max. coverage (+): 0. Max coverage (-): 0

Region: chr28 1796119-1796131. Max. coverage (+): 0. Max coverage (-): 0

Region: chr28 1796132-1796144. Max. coverage (+): 0. Max coverage (-): 0

Region: chr28 1796145-1796156. Max. coverage (+): 0. Max coverage (-): 0

Region: chr28 1796157-1796169. Max. coverage (+): 0. Max coverage (-): 0

Region: chr28 1796170-1796182. Max. coverage (+): 0. Max coverage (-): 0

Region: chr28 1796183-1796195. Max. coverage (+): 0. Max coverage (-): 0

Region: chr28 1796196-1796208. Max. coverage (+): 0. Max coverage (-): 0

Region: chr28 1796209-1796221. Max. coverage (+): 0. Max coverage (-): 0

Region: chr28 1796222-1796234. Max. coverage (+): 0. Max coverage (-): 0

Region: chr28 1796235-1796246. Max. coverage (+): 0. Max coverage (-): 0

Region: chr28 1796247-1796259. Max. coverage (+): 0. Max coverage (-): 0

Region: chr28 1796260-1796272. Max. coverage (+): 0.57. Max coverage (-): 0

Region: chr28 1796273-1796285. Max. coverage (+): 0. Max coverage (-): 0

Region: chr28 1796286-1796298. Max. coverage (+): 0. Max coverage (-): 0

Region: chr28 1796299-1796311. Max. coverage (+): 0. Max coverage (-): 0

Region: chr28 1796312-1796323. Max. coverage (+): 0. Max coverage (-): 0

Region: chr28 1796324-1796336. Max. coverage (+): 0. Max coverage (-): 0

Region: chr28 1796337-1796349. Max. coverage (+): 0. Max coverage (-): 0

Region: chr28 1796350-1796362. Max. coverage (+): 0. Max coverage (-): 0

Region: chr28 1796363-1796375. Max. coverage (+): 7.43. Max coverage (-): 0

Region: chr28 1796376-1796388. Max. coverage (+): 0. Max coverage (-): 0

Region: chr28 1796389-1796401. Max. coverage (+): 0. Max coverage (-): 0

Region: chr28 1796402-1796413. Max. coverage (+): 0. Max coverage (-): 0

Region: chr28 1796414-1796426. Max. coverage (+): 0. Max coverage (-): 0

Region: chr28 1796427-1796439. Max. coverage (+): 0. Max coverage (-): 0

Region: chr28 1796440-1796452. Max. coverage (+): 0. Max coverage (-): 0

Region: chr28 1796453-1796465. Max. coverage (+): 0. Max coverage (-): 0

Region: chr28 1796466-1796478. Max. coverage (+): 0. Max coverage (-): 0

Region: chr28 1796479-1796491. Max. coverage (+): 0. Max coverage (-): 0

Region: chr28 1796492-1796503. Max. coverage (+): 0. Max coverage (-): 0

Region: chr28 1796504-1796516. Max. coverage (+): 0. Max coverage (-): 0

Region: chr28 1796517-1796529. Max. coverage (+): 0. Max coverage (-): 0

Region: chr28 1796530-1796542. Max. coverage (+): 0. Max coverage (-): 0

Region: chr28 1796543-1796555. Max. coverage (+): 0. Max coverage (-): 0

Region: chr28 1796556-1796568. Max. coverage (+): 0. Max coverage (-): 0

Region: chr28 1796569-1796581. Max. coverage (+): 0. Max coverage (-): 0

Region: chr28 1796582-1796593. Max. coverage (+): 0. Max coverage (-): 0

Region: chr28 1796594-1796606. Max. coverage (+): 0. Max coverage (-): 0

Region: chr28 1796607-1796619. Max. coverage (+): 0. Max coverage (-): 0

Region: chr28 1796620-1796632. Max. coverage (+): 0. Max coverage (-): 0

Region: chr28 1796633-1796645. Max. coverage (+): 0. Max coverage (-): 0

Region: chr28 1796646-1796658. Max. coverage (+): 0. Max coverage (-): 0

Region: chr28 1796659-1796671. Max. coverage (+): 2.19. Max coverage (-): 0

Region: chr28 1796672-1796683. Max. coverage (+): 2.19. Max coverage (-): 0

Region: chr28 1796684-1796696. Max. coverage (+): 0. Max coverage (-): 0

Region: chr28 1796697-1796709. Max. coverage (+): 0. Max coverage (-): 0

Region: chr28 1796710-1796722. Max. coverage (+): 0. Max coverage (-): 0

Region: chr28 1796723-1796735. Max. coverage (+): 0. Max coverage (-): 0

Region: chr28 1796736-1796748. Max. coverage (+): 0. Max coverage (-): 0

Region: chr28 1796749-1796761. Max. coverage (+): 0. Max coverage (-): 0

Region: chr28 1796762-1796773. Max. coverage (+): 0. Max coverage (-): 0

Region: chr28 1796774-1796786. Max. coverage (+): 0. Max coverage (-): 0

Region: chr28 1796787-1796799. Max. coverage (+): 0. Max coverage (-): 0

Region: chr28 1796800-1796812. Max. coverage (+): 0. Max coverage (-): 0

Region: chr28 1796813-1796825. Max. coverage (+): 0. Max coverage (-): 0

Region: chr28 1796826-1796838. Max. coverage (+): 0. Max coverage (-): 0

Region: chr28 1796839-1796851. Max. coverage (+): 0. Max coverage (-): 0

Region: chr28 1796852-1796863. Max. coverage (+): 0. Max coverage (-): 0

Region: chr28 1796864-1796876. Max. coverage (+): 0. Max coverage (-): 0

Region: chr28 1796877-1796889. Max. coverage (+): 0. Max coverage (-): 0

Region: chr28 1796890-1796902. Max. coverage (+): 0. Max coverage (-): 0

Region: chr28 1796903-1796915. Max. coverage (+): 0. Max coverage (-): 0

Region: chr28 1796916-1796928. Max. coverage (+): 0. Max coverage (-): 0

Region: chr28 1796929-1796940. Max. coverage (+): 0. Max coverage (-): 0

Region: chr28 1796941-1796953. Max. coverage (+): 0. Max coverage (-): 0

Region: chr28 1796954-1796966. Max. coverage (+): 0. Max coverage (-): 0

Region: chr28 1796967-1796979. Max. coverage (+): 0. Max coverage (-): 0

Region: chr28 1796980-1796992. Max. coverage (+): 0. Max coverage (-): 0

Region: chr28 1796993-1797005. Max. coverage (+): 0. Max coverage (-): 0

Region: chr28 1797006-1797018. Max. coverage (+): 0. Max coverage (-): 0

Region: chr28 1797019-1797030. Max. coverage (+): 0. Max coverage (-): 0

Region: chr28 1797031-1797043. Max. coverage (+): 0. Max coverage (-): 0

Region: chr28 1797044-1797056. Max. coverage (+): 0. Max coverage (-): 0

Region: chr28 1797057-1797069. Max. coverage (+): 0. Max coverage (-): 0

Region: chr28 1797070-1797082. Max. coverage (+): 0. Max coverage (-): 0

Region: chr28 1797083-1797095. Max. coverage (+): 0. Max coverage (-): 0

Region: chr28 1797096-1797108. Max. coverage (+): 0. Max coverage (-): 0

Region: chr28 1797109-1797120. Max. coverage (+): 0. Max coverage (-): 0

Region: chr28 1797121-1797133. Max. coverage (+): 0. Max coverage (-): 0

Region: chr28 1797134-1797146. Max. coverage (+): 0. Max coverage (-): 0

Region: chr28 1797147-1797159. Max. coverage (+): 0. Max coverage (-): 0

Region: chr28 1797160-1797172. Max. coverage (+): 0. Max coverage (-): 0

Region: chr28 1797173-1797185. Max. coverage (+): 0. Max coverage (-): 0

Region: chr28 1797186-1797198. Max. coverage (+): 0. Max coverage (-): 0

Region: chr28 1797199-1797210. Max. coverage (+): 0. Max coverage (-): 0

Region: chr28 1797211-1797223. Max. coverage (+): 0. Max coverage (-): 0

Region: chr28 1797224-1797236. Max. coverage (+): 0. Max coverage (-): 0

Region: chr28 1797237-1797249. Max. coverage (+): 0. Max coverage (-): 0

Region: chr28 1797250-1797262. Max. coverage (+): 0. Max coverage (-): 0

Region: chr28 1797263-1797275. Max. coverage (+): 0. Max coverage (-): 0

Region: chr28 1797276-1797288. Max. coverage (+): 0. Max coverage (-): 0

Region: chr28 1797289-1797300. Max. coverage (+): 0. Max coverage (-): 0

Region: chr28 1797301-1797313. Max. coverage (+): 0. Max coverage (-): 0

Region: chr28 1797314-1797326. Max. coverage (+): 0. Max coverage (-): 0

Region: chr28 1797327-1797339. Max. coverage (+): 0. Max coverage (-): 0

Region: chr28 1797340-1797352. Max. coverage (+): 0. Max coverage (-): 0

Region: chr28 1797353-1797365. Max. coverage (+): 0. Max coverage (-): 0

Region: chr28 1797366-1797378. Max. coverage (+): 0. Max coverage (-): 0

Region: chr28 1797379-1797390. Max. coverage (+): 0. Max coverage (-): 0

Region: chr28 1797391-1797403. Max. coverage (+): 0. Max coverage (-): 0

Region: chr28 1797404-1797416. Max. coverage (+): 0. Max coverage (-): 0

Region: chr28 1797417-1797429. Max. coverage (+): 0. Max coverage (-): 0

Region: chr28 1797430-1797442. Max. coverage (+): 0. Max coverage (-): 0

Region: chr28 1797443-1797455. Max. coverage (+): 0. Max coverage (-): 0

Region: chr28 1797456-1797468. Max. coverage (+): 0. Max coverage (-): 0

Region: chr28 1797469-1797480. Max. coverage (+): 0. Max coverage (-): 0

Region: chr28 1797481-1797493. Max. coverage (+): 0. Max coverage (-): 0

Region: chr28 1797494-1797506. Max. coverage (+): 0. Max coverage (-): 0

Region: chr28 1797507-1797519. Max. coverage (+): 0. Max coverage (-): 0

Region: chr28 1797520-1797532. Max. coverage (+): 0. Max coverage (-): 0

Region: chr28 1797533-1797545. Max. coverage (+): 0. Max coverage (-): 0

Region: chr28 1797546-1797557. Max. coverage (+): 1.72. Max coverage (-): 0

Region: chr28 1797558-1797570. Max. coverage (+): 1.72. Max coverage (-): 0

Region: chr28 1797571-1797583. Max. coverage (+): 0.78. Max coverage (-): 0

Region: chr28 1797584-1797596. Max. coverage (+): 0. Max coverage (-): 0

Region: chr28 1797597-1797609. Max. coverage (+): 0. Max coverage (-): 0

Region: chr28 1797610-1797622. Max. coverage (+): 0. Max coverage (-): 0

Region: chr28 1797623-1797635. Max. coverage (+): 0. Max coverage (-): 0

Region: chr28 1797636-1797647. Max. coverage (+): 0. Max coverage (-): 0

Region: chr28 1797648-1797660. Max. coverage (+): 0. Max coverage (-): 0

Region: chr28 1797661-1797673. Max. coverage (+): 0. Max coverage (-): 0

Region: chr28 1797674-1797686. Max. coverage (+): 0. Max coverage (-): 0

Region: chr28 1797687-1797699. Max. coverage (+): 0. Max coverage (-): 0

Region: chr28 1797700-1797712. Max. coverage (+): 0. Max coverage (-): 0

Region: chr28 1797713-1797725. Max. coverage (+): 0. Max coverage (-): 0

Region: chr28 1797726-1797737. Max. coverage (+): 0. Max coverage (-): 0

Region: chr28 1797738-1797750. Max. coverage (+): 0. Max coverage (-): 0

Region: chr28 1797751-1797763. Max. coverage (+): 0. Max coverage (-): 0

Region: chr28 1797764-1797776. Max. coverage (+): 0. Max coverage (-): 0

Region: chr28 1797777-1797789. Max. coverage (+): 0. Max coverage (-): 0

Region: chr28 1797790-1797802. Max. coverage (+): 0. Max coverage (-): 0

Region: chr28 1797803-1797815. Max. coverage (+): 0. Max coverage (-): 0

Region: chr28 1797816-1797827. Max. coverage (+): 0. Max coverage (-): 0

Region: chr28 1797828-1797840. Max. coverage (+): 0. Max coverage (-): 0

Region: chr28 1797841-1797853. Max. coverage (+): 0. Max coverage (-): 0

Region: chr28 1797854-1797866. Max. coverage (+): 0. Max coverage (-): 0

Region: chr28 1797867-1797879. Max. coverage (+): 0. Max coverage (-): 0

Region: chr28 1797880-1797892. Max. coverage (+): 0. Max coverage (-): 0

Region: chr28 1797893-1797905. Max. coverage (+): 0. Max coverage (-): 0

Region: chr28 1797906-1797917. Max. coverage (+): 0. Max coverage (-): 0

Region: chr28 1797918-1797930. Max. coverage (+): 0. Max coverage (-): 0

Region: chr28 1797931-1797943. Max. coverage (+): 0. Max coverage (-): 0

Region: chr28 1797944-1797956. Max. coverage (+): 0. Max coverage (-): 0

Region: chr28 1797957-1797969. Max. coverage (+): 0. Max coverage (-): 0

Region: chr28 1797970-1797982. Max. coverage (+): 0. Max coverage (-): 0

Region: chr28 1797983-1797995. Max. coverage (+): 0. Max coverage (-): 0

Region: chr28 1797996-1798007. Max. coverage (+): 0. Max coverage (-): 0

Region: chr28 1798008-1798020. Max. coverage (+): 0. Max coverage (-): 0

Region: chr28 1798021-1798033. Max. coverage (+): 0. Max coverage (-): 0

Region: chr28 1798034-1798046. Max. coverage (+): 0. Max coverage (-): 0

Region: chr28 1798047-1798059. Max. coverage (+): 0. Max coverage (-): 0

Region: chr28 1798060-1798072. Max. coverage (+): 0. Max coverage (-): 0

Region: chr28 1798073-1798084. Max. coverage (+): 0. Max coverage (-): 0

Region: chr28 1798085-1798097. Max. coverage (+): 0. Max coverage (-): 0

Region: chr28 1798098-1798110. Max. coverage (+): 0. Max coverage (-): 0

Region: chr28 1798111-1798123. Max. coverage (+): 0. Max coverage (-): 0

Region: chr28 1798124-1798136. Max. coverage (+): 0. Max coverage (-): 1.44

Region: chr28 1798137-1798149. Max. coverage (+): 0. Max coverage (-): 1.44

Region: chr28 1798150-1798162. Max. coverage (+): 0. Max coverage (-): 0

Region: chr28 1798163-1798174. Max. coverage (+): 0. Max coverage (-): 0

Region: chr28 1798175-1798187. Max. coverage (+): 0. Max coverage (-): 0

Region: chr28 1798188-1798200. Max. coverage (+): 0. Max coverage (-): 0

Region: chr28 1798201-1798213. Max. coverage (+): 0. Max coverage (-): 0

Region: chr28 1798214-1798226. Max. coverage (+): 0. Max coverage (-): 0

Region: chr28 1798227-1798239. Max. coverage (+): 0. Max coverage (-): 0

Region: chr28 1798240-1798252. Max. coverage (+): 0. Max coverage (-): 0

Region: chr28 1798253-1798264. Max. coverage (+): 0. Max coverage (-): 0

Region: chr28 1798265-1798277. Max. coverage (+): 0. Max coverage (-): 0

Region: chr28 1798278-1798290. Max. coverage (+): 0. Max coverage (-): 0

Region: chr28 1798291-1798303. Max. coverage (+): 0. Max coverage (-): 0

Region: chr28 1798304-1798316. Max. coverage (+): 0. Max coverage (-): 0

Region: chr28 1798317-1798329. Max. coverage (+): 0. Max coverage (-): 0

Region: chr28 1798330-1798342. Max. coverage (+): 0. Max coverage (-): 0

Region: chr28 1798343-1798354. Max. coverage (+): 0. Max coverage (-): 0

Region: chr28 1798355-1798367. Max. coverage (+): 0. Max coverage (-): 0

Region: chr28 1798368-1798380. Max. coverage (+): 0. Max coverage (-): 0

Region: chr28 1798381-1798393. Max. coverage (+): 0. Max coverage (-): 0

Region: chr28 1798394-1798406. Max. coverage (+): 0. Max coverage (-): 0

Region: chr28 1798407-1798419. Max. coverage (+): 0. Max coverage (-): 0

Region: chr28 1798420-1798432. Max. coverage (+): 0. Max coverage (-): 0

Region: chr28 1798433-1798444. Max. coverage (+): 0. Max coverage (-): 0

Region: chr28 1798445-1798457. Max. coverage (+): 0. Max coverage (-): 0

Region: chr28 1798458-1798470. Max. coverage (+): 0. Max coverage (-): 0

Region: chr28 1798471-1798483. Max. coverage (+): 0. Max coverage (-): 0

Region: chr28 1798484-1798496. Max. coverage (+): 0. Max coverage (-): 0

Region: chr28 1798497-1798509. Max. coverage (+): 0. Max coverage (-): 0

Region: chr28 1798510-1798522. Max. coverage (+): 0. Max coverage (-): 0

Region: chr28 1798523-1798534. Max. coverage (+): 0. Max coverage (-): 0

Region: chr28 1798535-1798547. Max. coverage (+): 0. Max coverage (-): 0

Region: chr28 1798548-1798560. Max. coverage (+): 0. Max coverage (-): 0

Region: chr28 1798561-1798573. Max. coverage (+): 0. Max coverage (-): 0

Region: chr28 1798574-1798586. Max. coverage (+): 0. Max coverage (-): 0

Region: chr28 1798587-1798599. Max. coverage (+): 0. Max coverage (-): 0

Region: chr28 1798600-1798612. Max. coverage (+): 0. Max coverage (-): 0

Region: chr28 1798613-1798624. Max. coverage (+): 0. Max coverage (-): 0

Region: chr28 1798625-1798637. Max. coverage (+): 0. Max coverage (-): 0

Region: chr28 1798638-1798650. Max. coverage (+): 0. Max coverage (-): 0

Region: chr28 1798651-1798663. Max. coverage (+): 0. Max coverage (-): 0

Region: chr28 1798664-1798676. Max. coverage (+): 3.55. Max coverage (-): 0

Region: chr28 1798677-1798689. Max. coverage (+): 3.55. Max coverage (-): 0

Region: chr28 1798690-1798701. Max. coverage (+): 0. Max coverage (-): 0

Region: chr28 1798702-1798714. Max. coverage (+): 0. Max coverage (-): 0

Region: chr28 1798715-1798727. Max. coverage (+): 0. Max coverage (-): 0

Region: chr28 1798728-1798740. Max. coverage (+): 0. Max coverage (-): 0

Region: chr28 1798741-1798753. Max. coverage (+): 1.07. Max coverage (-): 0

Region: chr28 1798754-1798766. Max. coverage (+): 10.07. Max coverage (-): 0

Region: chr28 1798767-1798779. Max. coverage (+): 0. Max coverage (-): 0

Region: chr28 1798780-1798791. Max. coverage (+): 0. Max coverage (-): 0

Region: chr28 1798792-1798804. Max. coverage (+): 0. Max coverage (-): 0

Region: chr28 1798805-1798817. Max. coverage (+): 0. Max coverage (-): 0

Region: chr28 1798818-1798830. Max. coverage (+): 3.66. Max coverage (-): 0

Region: chr28 1798831-1798843. Max. coverage (+): 3.66. Max coverage (-): 0

Region: chr28 1798844-1798856. Max. coverage (+): 5.64. Max coverage (-): 0

Region: chr28 1798857-1798869. Max. coverage (+): 0. Max coverage (-): 0

Region: chr28 1798870-1798881. Max. coverage (+): 0. Max coverage (-): 0

Region: chr28 1798882-1798894. Max. coverage (+): 10.82. Max coverage (-): 0

Region: chr28 1798895-1798907. Max. coverage (+): 3.54. Max coverage (-): 0

Region: chr28 1798908-1798920. Max. coverage (+): 0. Max coverage (-): 0

Region: chr28 1798921-1798933. Max. coverage (+): 0. Max coverage (-): 0

Region: chr28 1798934-1798946. Max. coverage (+): 0. Max coverage (-): 0

Region: chr28 1798947-1798959. Max. coverage (+): 0. Max coverage (-): 0

Region: chr28 1798960-1798971. Max. coverage (+): 0.55. Max coverage (-): 0

Region: chr28 1798972-1798984. Max. coverage (+): 5.2. Max coverage (-): 0

Region: chr28 1798985-1798997. Max. coverage (+): 5.2. Max coverage (-): 0

Region: chr28 1798998-1799010. Max. coverage (+): 0. Max coverage (-): 0

Region: chr28 1799011-1799023. Max. coverage (+): 0. Max coverage (-): 0

Region: chr28 1799024-1799036. Max. coverage (+): 0. Max coverage (-): 0

Region: chr28 1799037-1799049. Max. coverage (+): 0. Max coverage (-): 0

Region: chr28 1799050-1799061. Max. coverage (+): 0.01. Max coverage (-): 0

Region: chr28 1799062-1799074. Max. coverage (+): 3.51. Max coverage (-): 0

Region: chr28 1799075-1799087. Max. coverage (+): 0. Max coverage (-): 0

Region: chr28 1799088-1799100. Max. coverage (+): 0. Max coverage (-): 0

Region: chr28 1799101-1799113. Max. coverage (+): 0. Max coverage (-): 0

Region: chr28 1799114-1799126. Max. coverage (+): 0. Max coverage (-): 0

Region: chr28 1799127-1799139. Max. coverage (+): 0. Max coverage (-): 0

Region: chr28 1799140-1799151. Max. coverage (+): 0. Max coverage (-): 0

Region: chr28 1799152-1799164. Max. coverage (+): 0. Max coverage (-): 0

Region: chr28 1799165-1799177. Max. coverage (+): 0. Max coverage (-): 0

Region: chr28 1799178-1799190. Max. coverage (+): 0. Max coverage (-): 0

Region: chr28 1799191-1799203. Max. coverage (+): 0. Max coverage (-): 0

Region: chr28 1799204-1799216. Max. coverage (+): 0. Max coverage (-): 0

Region: chr28 1799217-1799228. Max. coverage (+): 0. Max coverage (-): 0

Region: chr28 1799229-1799241. Max. coverage (+): 0. Max coverage (-): 0

Region: chr28 1799242-1799254. Max. coverage (+): 0. Max coverage (-): 0

Region: chr28 1799255-1799267. Max. coverage (+): 0. Max coverage (-): 0

Region: chr28 1799268-1799280. Max. coverage (+): 0. Max coverage (-): 0

Region: chr28 1799281-1799293. Max. coverage (+): 0. Max coverage (-): 0

Region: chr28 1799294-1799306. Max. coverage (+): 0. Max coverage (-): 0

Region: chr28 1799307-1799318. Max. coverage (+): 0. Max coverage (-): 0

Region: chr28 1799319-1799331. Max. coverage (+): 0. Max coverage (-): 0

Region: chr28 1799332-1799344. Max. coverage (+): 0. Max coverage (-): 0

Region: chr28 1799345-1799357. Max. coverage (+): 0. Max coverage (-): 0

Region: chr28 1799358-1799370. Max. coverage (+): 0. Max coverage (-): 0

Region: chr28 1799371-1799383. Max. coverage (+): 0. Max coverage (-): 0

Region: chr28 1799384-1799396. Max. coverage (+): 0. Max coverage (-): 0

Region: chr28 1799397-1799408. Max. coverage (+): 9.18. Max coverage (-): 0

Region: chr28 1799409-1799421. Max. coverage (+): 6.15. Max coverage (-): 0.74

Region: chr28 1799422-1799434. Max. coverage (+): 10.46. Max coverage (-): 0.74

Region: chr28 1799435-1799447. Max. coverage (+): 2.73. Max coverage (-): 0

Region: chr28 1799448-1799460. Max. coverage (+): 0. Max coverage (-): 0

Region: chr28 1799461-1799473. Max. coverage (+): 0. Max coverage (-): 0

Region: chr28 1799474-1799486. Max. coverage (+): 9.95. Max coverage (-): 0

Region: chr28 1799487-1799498. Max. coverage (+): 2.45. Max coverage (-): 0

Region: chr28 1799499-1799511. Max. coverage (+): 6.72. Max coverage (-): 0

Region: chr28 1799512-1799524. Max. coverage (+): 19.12. Max coverage (-): 0

Region: chr28 1799525-1799537. Max. coverage (+): 4.36. Max coverage (-): 0

Region: chr28 1799538-1799550. Max. coverage (+): 0. Max coverage (-): 0

Region: chr28 1799551-1799563. Max. coverage (+): 0. Max coverage (-): 0

Region: chr28 1799564-1799576. Max. coverage (+): 6.37. Max coverage (-): 0

Region: chr28 1799577-1799588. Max. coverage (+): 7.39. Max coverage (-): 0

Region: chr28 1799589-1799601. Max. coverage (+): 11.87. Max coverage (-): 0

Region: chr28 1799602-1799614. Max. coverage (+): 8.57. Max coverage (-): 0

Region: chr28 1799615-1799627. Max. coverage (+): 17.72. Max coverage (-): 0

Region: chr28 1799628-1799640. Max. coverage (+): 12.87. Max coverage (-): 0

Region: chr28 1799641-1799653. Max. coverage (+): 12.54. Max coverage (-): 0

Region: chr28 1799654-1799666. Max. coverage (+): 4.3. Max coverage (-): 2.13

Region: chr28 1799667-1799678. Max. coverage (+): 0. Max coverage (-): 0

Region: chr28 1799679-1799691. Max. coverage (+): 0. Max coverage (-): 0

Region: chr28 1799692-1799704. Max. coverage (+): 1.98. Max coverage (-): 0

Region: chr28 1799705-1799717. Max. coverage (+): 3.3. Max coverage (-): 0

Region: chr28 1799718-1799730. Max. coverage (+): 11.24. Max coverage (-): 0

Region: chr28 1799731-1799743. Max. coverage (+): 9.01. Max coverage (-): 0

Region: chr28 1799744-1799756. Max. coverage (+): 9.87. Max coverage (-): 0

Region: chr28 1799757-1799768. Max. coverage (+): 7.58. Max coverage (-): 0

Region: chr28 1799769-1799781. Max. coverage (+): 0.71. Max coverage (-): 0

Region: chr28 1799782-1799794. Max. coverage (+): 0.71. Max coverage (-): 0

Region: chr28 1799795-1799807. Max. coverage (+): 0. Max coverage (-): 0

Region: chr28 1799808-1799820. Max. coverage (+): 0. Max coverage (-): 0

Region: chr28 1799821-1799833. Max. coverage (+): 0. Max coverage (-): 0

Region: chr28 1799834-1799845. Max. coverage (+): 3.79. Max coverage (-): 0

Region: chr28 1799846-1799858. Max. coverage (+): 0. Max coverage (-): 0

Region: chr28 1799859-1799871. Max. coverage (+): 0. Max coverage (-): 0

Region: chr28 1799872-1799884. Max. coverage (+): 0. Max coverage (-): 0

Region: chr28 1799885-1799897. Max. coverage (+): 8.45. Max coverage (-): 0

Region: chr28 1799898-1799910. Max. coverage (+): 23.98. Max coverage (-): 0

Region: chr28 1799911-1799923. Max. coverage (+): 7.93. Max coverage (-): 0

Region: chr28 1799924-1799935. Max. coverage (+): 4.45. Max coverage (-): 0

Region: chr28 1799936-1799948. Max. coverage (+): 53.71. Max coverage (-): 0

Region: chr28 1799949-1799961. Max. coverage (+): 46.27. Max coverage (-): 0

Region: chr28 1799962-1799974. Max. coverage (+): 0. Max coverage (-): 0

Region: chr28 1799975-1799987. Max. coverage (+): 4.62. Max coverage (-): 0

Region: chr28 1799988-1800000. Max. coverage (+): 6.44. Max coverage (-): 0

Region: chr28 1800001-1800013. Max. coverage (+): 6.44. Max coverage (-): 0

Region: chr28 1800014-1800025. Max. coverage (+): 0. Max coverage (-): 0

Region: chr28 1800026-1800038. Max. coverage (+): 3.91. Max coverage (-): 0

Region: chr28 1800039-1800051. Max. coverage (+): 31.37. Max coverage (-): 0

Region: chr28 1800052-1800064. Max. coverage (+): 31.37. Max coverage (-): 0

Region: chr28 1800065-1800077. Max. coverage (+): 34.53. Max coverage (-): 0

Region: chr28 1800078-1800090. Max. coverage (+): 5.54. Max coverage (-): 0

Region: chr28 1800091-1800103. Max. coverage (+): 0. Max coverage (-): 0

Region: chr28 1800104-1800115. Max. coverage (+): 4.94. Max coverage (-): 0

Region: chr28 1800116-1800128. Max. coverage (+): 35.52. Max coverage (-): 0

Region: chr28 1800129-1800141. Max. coverage (+): 13.14. Max coverage (-): 0

Region: chr28 1800142-1800154. Max. coverage (+): 6.3. Max coverage (-): 0

Region: chr28 1800155-1800167. Max. coverage (+): 0. Max coverage (-): 0

Region: chr28 1800168-1800180. Max. coverage (+): 0. Max coverage (-): 0

Region: chr28 1800181-1800193. Max. coverage (+): 0. Max coverage (-): 0

Region: chr28 1800194-1800205. Max. coverage (+): 0. Max coverage (-): 0

Region: chr28 1800206-1800218. Max. coverage (+): 0. Max coverage (-): 0

Region: chr28 1800219-1800231. Max. coverage (+): 0. Max coverage (-): 0

Region: chr28 1800232-1800244. Max. coverage (+): 0. Max coverage (-): 0

Region: chr28 1800245-1800257. Max. coverage (+): 5.36. Max coverage (-): 0

Region: chr28 1800258-1800270. Max. coverage (+): 5.36. Max coverage (-): 0

Region: chr28 1800271-1800283. Max. coverage (+): 0. Max coverage (-): 0

Region: chr28 1800284-1800295. Max. coverage (+): 0. Max coverage (-): 0

Region: chr28 1800296-1800308. Max. coverage (+): 0. Max coverage (-): 0

Region: chr28 1800309-1800321. Max. coverage (+): 0. Max coverage (-): 0

Region: chr28 1800322-1800334. Max. coverage (+): 0. Max coverage (-): 0

Region: chr28 1800335-1800347. Max. coverage (+): 0. Max coverage (-): 0

Region: chr28 1800348-1800360. Max. coverage (+): 0. Max coverage (-): 0

Region: chr28 1800361-1800373. Max. coverage (+): 1.44. Max coverage (-): 0

Region: chr28 1800374-1800385. Max. coverage (+): 5.57. Max coverage (-): 2.37

Region: chr28 1800386-1800398. Max. coverage (+): 20.89. Max coverage (-): 1.9

Region: chr28 1800399-1800411. Max. coverage (+): 23.9. Max coverage (-): 0

Region: chr28 1800412-1800424. Max. coverage (+): 1.32. Max coverage (-): 0

Region: chr28 1800425-1800437. Max. coverage (+): 1.32. Max coverage (-): 0

Region: chr28 1800438-1800450. Max. coverage (+): 21.39. Max coverage (-): 0

Region: chr28 1800451-1800462. Max. coverage (+): 2.04. Max coverage (-): 0

Region: chr28 1800463-1800475. Max. coverage (+): 5.22. Max coverage (-): 0

Region: chr28 1800476-1800488. Max. coverage (+): 1.71. Max coverage (-): 0

Region: chr28 1800489-1800501. Max. coverage (+): 0. Max coverage (-): 0

Region: chr28 1800502-1800514. Max. coverage (+): 0.46. Max coverage (-): 0

Region: chr28 1800515-1800527. Max. coverage (+): 0.46. Max coverage (-): 0

Region: chr28 1800528-1800540. Max. coverage (+): 0.12. Max coverage (-): 0

Region: chr28 1800541-1800552. Max. coverage (+): 7.19. Max coverage (-): 0

Region: chr28 1800553-1800565. Max. coverage (+): 4.76. Max coverage (-): 0

Region: chr28 1800566-1800578. Max. coverage (+): 10.83. Max coverage (-): 0

Region: chr28 1800579-1800591. Max. coverage (+): 0. Max coverage (-): 0

Region: chr28 1800592-1800604. Max. coverage (+): 0. Max coverage (-): 0

Region: chr28 1800605-1800617. Max. coverage (+): 8.76. Max coverage (-): 0

Region: chr28 1800618-1800630. Max. coverage (+): 2.62. Max coverage (-): 0

Region: chr28 1800631-1800642. Max. coverage (+): 0. Max coverage (-): 0

Region: chr28 1800643-1800655. Max. coverage (+): 0. Max coverage (-): 0

Region: chr28 1800656-1800668. Max. coverage (+): 1.02. Max coverage (-): 0

Region: chr28 1800669-1800681. Max. coverage (+): 1.02. Max coverage (-): 0

Region: chr28 1800682-1800694. Max. coverage (+): 0. Max coverage (-): 0

Region: chr28 1800695-1800707. Max. coverage (+): 0.76. Max coverage (-): 0

Region: chr28 1800708-1800720. Max. coverage (+): 2.02. Max coverage (-): 0

Region: chr28 1800721-1800732. Max. coverage (+): 9.91. Max coverage (-): 0

Region: chr28 1800733-1800745. Max. coverage (+): 0. Max coverage (-): 0

Region: chr28 1800746-1800758. Max. coverage (+): 6.81. Max coverage (-): 0

Region: chr28 1800759-1800771. Max. coverage (+): 26.75. Max coverage (-): 0

Region: chr28 1800772-1800784. Max. coverage (+): 0. Max coverage (-): 0

Region: chr28 1800785-1800797. Max. coverage (+): 0. Max coverage (-): 0

Region: chr28 1800798-1800810. Max. coverage (+): 2.9. Max coverage (-): 0

Region: chr28 1800811-1800822. Max. coverage (+): 14.29. Max coverage (-): 0

Region: chr28 1800823-1800835. Max. coverage (+): 12.67. Max coverage (-): 0

Region: chr28 1800836-1800848. Max. coverage (+): 10.1. Max coverage (-): 0

Region: chr28 1800849-1800861. Max. coverage (+): 0. Max coverage (-): 0

Region: chr28 1800862-1800874. Max. coverage (+): 2.06. Max coverage (-): 0

Region: chr28 1800875-1800887. Max. coverage (+): 5.58. Max coverage (-): 0

Region: chr28 1800888-1800900. Max. coverage (+): 17.42. Max coverage (-): 0

Region: chr28 1800901-1800912. Max. coverage (+): 2.59. Max coverage (-): 0

Region: chr28 1800913-1800925. Max. coverage (+): 1.46. Max coverage (-): 0

Region: chr28 1800926-1800938. Max. coverage (+): 2.77. Max coverage (-): 0

Region: chr28 1800939-1800951. Max. coverage (+): 2.77. Max coverage (-): 0

Region: chr28 1800952-1800964. Max. coverage (+): 0.36. Max coverage (-): 0

Region: chr28 1800965-1800977. Max. coverage (+): 1.67. Max coverage (-): 0

Region: chr28 1800978-1800989. Max. coverage (+): 1.98. Max coverage (-): 0

Region: chr28 1800990-1801002. Max. coverage (+): 9.88. Max coverage (-): 0

Region: chr28 1801003-1801015. Max. coverage (+): 5.6. Max coverage (-): 0

Region: chr28 1801016-1801028. Max. coverage (+): 4.67. Max coverage (-): 0

Region: chr28 1801029-1801041. Max. coverage (+): 5.86. Max coverage (-): 0

Region: chr28 1801042-1801054. Max. coverage (+): 4.87. Max coverage (-): 0

Region: chr28 1801055-1801067. Max. coverage (+): 0. Max coverage (-): 0

Region: chr28 1801068-1801079. Max. coverage (+): 0. Max coverage (-): 0

Region: chr28 1801080-1801092. Max. coverage (+): 0. Max coverage (-): 0

Region: chr28 1801093-1801105. Max. coverage (+): 0. Max coverage (-): 0

Region: chr28 1801106-1801118. Max. coverage (+): 0. Max coverage (-): 0

Region: chr28 1801119-1801131. Max. coverage (+): 0. Max coverage (-): 0

Region: chr28 1801132-1801144. Max. coverage (+): 0.5. Max coverage (-): 0

Region: chr28 1801145-1801157. Max. coverage (+): 0. Max coverage (-): 0

Region: chr28 1801158-1801169. Max. coverage (+): 0. Max coverage (-): 0

Region: chr28 1801170-1801182. Max. coverage (+): 2.88. Max coverage (-): 0

Region: chr28 1801183-1801195. Max. coverage (+): 2.88. Max coverage (-): 0

Region: chr28 1801196-1801208. Max. coverage (+): 3.8. Max coverage (-): 0

Region: chr28 1801209-1801221. Max. coverage (+): 7.87. Max coverage (-): 0

Region: chr28 1801222-1801234. Max. coverage (+): 0.58. Max coverage (-): 0

Region: chr28 1801235-1801247. Max. coverage (+): 7.37. Max coverage (-): 0

Region: chr28 1801248-1801259. Max. coverage (+): 13.49. Max coverage (-): 0

Region: chr28 1801260-1801272. Max. coverage (+): 6. Max coverage (-): 0

Region: chr28 1801273-1801285. Max. coverage (+): 3.46. Max coverage (-): 0

Region: chr28 1801286-1801298. Max. coverage (+): 6.1. Max coverage (-): 0

Region: chr28 1801299-1801311. Max. coverage (+): 3.59. Max coverage (-): 0

Region: chr28 1801312-1801324. Max. coverage (+): 0. Max coverage (-): 0

Region: chr28 1801325-1801337. Max. coverage (+): 20.41. Max coverage (-): 0

Region: chr28 1801338-1801349. Max. coverage (+): 8.85. Max coverage (-): 0

Region: chr28 1801350-1801362. Max. coverage (+): 4.87. Max coverage (-): 0

Region: chr28 1801363-1801375. Max. coverage (+): 0. Max coverage (-): 0

Region: chr28 1801376-1801388. Max. coverage (+): 1.89. Max coverage (-): 0

Region: chr28 1801389-1801401. Max. coverage (+): 0. Max coverage (-): 0

Region: chr28 1801402-1801414. Max. coverage (+): 2.97. Max coverage (-): 0

Region: chr28 1801415-1801427. Max. coverage (+): 2.97. Max coverage (-): 0

Region: chr28 1801428-1801439. Max. coverage (+): 3.92. Max coverage (-): 0

Region: chr28 1801440-1801452. Max. coverage (+): 3.1. Max coverage (-): 0

Region: chr28 1801453-1801465. Max. coverage (+): 0. Max coverage (-): 0

Region: chr28 1801466-1801478. Max. coverage (+): 0. Max coverage (-): 0

Region: chr28 1801479-1801491. Max. coverage (+): 2.66. Max coverage (-): 0

Region: chr28 1801492-1801504. Max. coverage (+): 5.06. Max coverage (-): 0

Region: chr28 1801505-1801517. Max. coverage (+): 2.76. Max coverage (-): 0

Region: chr28 1801518-1801529. Max. coverage (+): 0. Max coverage (-): 0

Region: chr28 1801530-1801542. Max. coverage (+): 1.91. Max coverage (-): 0

Region: chr28 1801543-1801555. Max. coverage (+): 0. Max coverage (-): 0

Region: chr28 1801556-. Max. coverage (+): 0. Max coverage (-): 0

RepeatMasker Color Code

**+**

100-98% Identity

<98-95% Identity

<95-90% Identity

<90-85% Identity

<85-80% Identity

<80-75% Identity

<75-70% Identity

<70% Identity

**-**

Gene Set Color Code

**+**

Gene

Pseudogene

**-**

Topology/Coverage Color Code

Coverage Plus Strand

Coverage Minus Strand

Mainstrand: Plus

Mainstrand: Minus

Complementary Strand

Flanking Region  
(if option -flank >0)

Gene Set Annotation  
  
RepeatMasker Annotation  

**1. L2c**: 1796613-1796653 (-), Divergence to consensus: 21.9%  
**2. AT\_rich**: 1796802-1796825 (+), Divergence to consensus: 50%  
**3. SINE2-1\_BT**: 1796841-1796947 (-), Divergence to consensus: 27.1%  
**4. MER74A**: 1796973-1797287 (+), Divergence to consensus: 38.6%  
**5. Bov-tA3**: 1799194-1799396 (+), Divergence to consensus: 11.3%

  
Transcription Factor Binding Sites  

**Gata4** (Sequence: AGATAAC (-): 1795231)
