# Supplementary material for: Small RNA sequencing of cryopreserved semen from single bull revealed altered miRNAs and piRNAs expression between High- and Low-motile sperm populations
Source: BMC Genomics. 2017 Jan 4;18:14. doi: 10.1186/s12864-016-3394-7 (PMC5209821; doi:10.1186/s12864-016-3394-7)
Supplement: Additional file 3: — Details for each piRNA clusters found in High Motile (HM) sperm fraction. Genes, repeats, transposable elements and transcription factors binding sites falling within the cluster regions were reported. (ZIP 1896 kb) [file 12864_2016_3394_MOESM3_ESM.zip › 69.html]

piRNA cluster 69


Predicted piRNA cluster no. 69     previous   next
  

Show proTRAC run info
Hide proTRAC run info

================================= proTRAC ====================================  
VERSION: 2.1                                    LAST MODIFIED: 06. October 2015  
  
Please cite:  
Rosenkranz D, Zischler H. proTRAC - a software for probabilistic piRNA cluster  
detection, visualization and analysis. 2012. BMC Bioinformatics 13:5.  
  
and (for proTRAC 2.0 and later):  
Rosenkranz D, Rudloff S, Bastuck K, Ketting RF, Zischler H. Tupaia small RNAs  
provide insights into function and evolution of RNAi-based transposon defense  
in mammals. 2015. RNA 21(5):911-922.  
  
Contact:  
David Rosenkranz  
Institute of Anthropology, small RNA group  
Johannes Gutenberg University Mainz  
email: rosenkranz@uni-mainz.de  
  
You can find the latest proTRAC version at:  
http://sourceforge.net/projects/protrac/files  
http://www.smallRNAgroup-mainz.de/software  
==============================================================================  
  
PARAMETERS:  
Map file: .............../storage/core/barbara/genhome/smallRNA/fertility/Sample\_motile/pirna/Sample\_motile\_26-33\_collapsed.fa.no-dust.map.weighted-10000-1000-b-0  
Genome file: ............/storage/core/barbara/genhome/smallRNA/fertility/Sample\_all/pirna/bt\_311\_chrY.fa  
RepeatMasker annotation: /storage/genomes/bt\_umd31/GCF\_000003055.6\_Bos\_taurus\_UMD\_3.1.1\_repeatMasker\_chr.out  
GeneSet:................./storage/core/barbara/genhome/smallRNA/fertility/Sample\_all/pirna/full.gtf  
  
Significant (p<=0.01) hit density will be calculated based  
on observed hit distribution.  
  
Sliding window size: ........................................ 5000 bp  
Sliding window increament: .................................. 1000 bp  
Normalize each hit by number of genomic hits: ............... 1 [0=no/1=yes]  
Normalize each hit by number of sequence reads: ............. 1 [0=no/1=yes]  
Normalize values (-> per million mapped reads): ............. 1 [0=no/1=yes]  
Min. fraction of hits with 1T(U) or 10A: .................... 0.75  
Alternatively: Min. fraction of hits with 1T(U) and 10A: .... 0.5  
Min. fraction of hits with typical piRNA length: ............ 0.75  
Typical piRNA length: ....................................... 26-33 nt  
Min. size of a piRNA cluster: ............................... 5000 bp.  
Min. number of hits (absolute): ............................. 0  
Min. number of hits (normalized): ........................... 0  
Min. fraction of hits on the mainstrand: .................... 0.75  
Top fraction of mapped sequences (in terms of read counts): . 1%  
Top fraction accounts for max. n% of sequence reads: ........ 90%  
Min. fraction of hits on each arm of a bidirectional cluster: 0.1  
Output image file for each cluster: ......................... 0 [0=no/1=yes]  
Output html file for each cluster: .......................... 1 [0=no/1=yes]  
Output a summary table: ..................................... 1 [0=no/1=yes]  
Output a FASTA file for each cluster (piRNA sequences): ..... 1 [0=no/1=yes]  
Output a FASTA file comprising cluster sequences: ........... 1 [0=no/1=yes]  
Search DNA motifs in clusters: .............................. 1 [0=no/1=yes]  
Output flanking sequences: +/- .............................. 0 bp  
Output ~.pTi file: .......................................... 1 [0=no/1=yes]  
==============================================================================  
  
  
Genome size (without gaps): ............ 2678902517 bp  
Gaps (N/X/-): .......................... 53837044 bp  
Mapped reads: .......................... 658825247023  
Non-identical sequences: ............... 514171  
Genomic hits: .......................... 764233  
Significant densitiy of mapped reads: .. 12867599.5173724 reads/kb

Show proTRAC cluster info
Hide proTRAC cluster info

|  |  |
| --- | --- |
| Location | chr28 |
| Coordinates | 4511466-4517429 |
| Size [bp] | 5964 |
| Sequence hit loci | 1076 |
| Mapped reads (normalized) | 694520087.8 |
| Mapped reads (normalized) per kb | 116452060.3 |
| Normalized reads with 1T (1U) | 75.7% |
| Normalized reads with 10A | 22.8% |
| Normalized reads with length 26-33 nt | 100% |
| Normalized reads on the main strand(s) | 99.9% |
| Predicted directionality | mono:plus |

100%

0%

1T (1U)  
reads

10A reads

26-33 nt  
reads

reads on mainstrand

**Either the amount of reads with 1T (1U) OR 10A has to exceed 75% (set with option: -1Tor10A)  
Alternatively the amount of reads with 1T (1U) AND 10A has to exceed 50% (set with option: -1Tand10A)  
Minimum amount of reads with preferred size is 75% (set with option: -pisize)  
Minimum amount of reads on the main strand(s) is 75% (set with option: -clstrand)**

Show read coverage
Hide read coverage

WHAT DO I SEE HERE?  
This chart shows the location of mapped sequence reads within a predicted piRNA cluster. The color refers to the number of genomic hits produced by the sequence read in question. A dark red bar indicates that this sequence read produces many other hits elsewhere in the genome. Many adjacent red or yellow bars can indicate the presence of a multi-copy element such as transposons or rRNA genes. A dark green bar indicates that this sequence read maps uniquely to this locus.

1 hit

2-5 hits

6-10 hits

11-20 hits

21-50 hits

51-100 hits

> 100 hits

chr28

4511466

4517429

Gene Set

RepeatMasker

Mapped  
Reads

57.4

plus strand

minus strand

57.4

Region: chr28 1801557-4511471. Max. coverage (+): 1.64. Max coverage (-): 0

Region: chr28 4511472-4511483. Max. coverage (+): 1.64. Max coverage (-): 0

Region: chr28 4511484-4511495. Max. coverage (+): 0. Max coverage (-): 0

Region: chr28 4511496-4511507. Max. coverage (+): 0. Max coverage (-): 0

Region: chr28 4511508-4511519. Max. coverage (+): 0. Max coverage (-): 0

Region: chr28 4511520-4511531. Max. coverage (+): 0. Max coverage (-): 0

Region: chr28 4511532-4511543. Max. coverage (+): 0. Max coverage (-): 0

Region: chr28 4511544-4511555. Max. coverage (+): 0. Max coverage (-): 0

Region: chr28 4511556-4511567. Max. coverage (+): 0. Max coverage (-): 0

Region: chr28 4511568-4511579. Max. coverage (+): 0. Max coverage (-): 0

Region: chr28 4511580-4511591. Max. coverage (+): 0. Max coverage (-): 0

Region: chr28 4511592-4511603. Max. coverage (+): 0. Max coverage (-): 0

Region: chr28 4511604-4511615. Max. coverage (+): 0. Max coverage (-): 0

Region: chr28 4511616-4511627. Max. coverage (+): 0. Max coverage (-): 0

Region: chr28 4511628-4511638. Max. coverage (+): 0. Max coverage (-): 0

Region: chr28 4511639-4511650. Max. coverage (+): 0. Max coverage (-): 0

Region: chr28 4511651-4511662. Max. coverage (+): 0. Max coverage (-): 0

Region: chr28 4511663-4511674. Max. coverage (+): 0. Max coverage (-): 0

Region: chr28 4511675-4511686. Max. coverage (+): 0. Max coverage (-): 0

Region: chr28 4511687-4511698. Max. coverage (+): 0. Max coverage (-): 0

Region: chr28 4511699-4511710. Max. coverage (+): 0. Max coverage (-): 0

Region: chr28 4511711-4511722. Max. coverage (+): 0. Max coverage (-): 0

Region: chr28 4511723-4511734. Max. coverage (+): 0. Max coverage (-): 0

Region: chr28 4511735-4511746. Max. coverage (+): 0. Max coverage (-): 0

Region: chr28 4511747-4511758. Max. coverage (+): 2.07. Max coverage (-): 0

Region: chr28 4511759-4511770. Max. coverage (+): 2.07. Max coverage (-): 0

Region: chr28 4511771-4511782. Max. coverage (+): 0. Max coverage (-): 0

Region: chr28 4511783-4511794. Max. coverage (+): 0. Max coverage (-): 0

Region: chr28 4511795-4511805. Max. coverage (+): 0. Max coverage (-): 0

Region: chr28 4511806-4511817. Max. coverage (+): 0. Max coverage (-): 0

Region: chr28 4511818-4511829. Max. coverage (+): 0.55. Max coverage (-): 0

Region: chr28 4511830-4511841. Max. coverage (+): 0.55. Max coverage (-): 0

Region: chr28 4511842-4511853. Max. coverage (+): 0. Max coverage (-): 0

Region: chr28 4511854-4511865. Max. coverage (+): 0. Max coverage (-): 0

Region: chr28 4511866-4511877. Max. coverage (+): 0. Max coverage (-): 0

Region: chr28 4511878-4511889. Max. coverage (+): 0. Max coverage (-): 0

Region: chr28 4511890-4511901. Max. coverage (+): 0. Max coverage (-): 0

Region: chr28 4511902-4511913. Max. coverage (+): 0. Max coverage (-): 0

Region: chr28 4511914-4511925. Max. coverage (+): 0. Max coverage (-): 0

Region: chr28 4511926-4511937. Max. coverage (+): 0. Max coverage (-): 0

Region: chr28 4511938-4511949. Max. coverage (+): 0. Max coverage (-): 0

Region: chr28 4511950-4511961. Max. coverage (+): 0. Max coverage (-): 0

Region: chr28 4511962-4511972. Max. coverage (+): 0. Max coverage (-): 0

Region: chr28 4511973-4511984. Max. coverage (+): 0. Max coverage (-): 0

Region: chr28 4511985-4511996. Max. coverage (+): 0.52. Max coverage (-): 0

Region: chr28 4511997-4512008. Max. coverage (+): 0. Max coverage (-): 0

Region: chr28 4512009-4512020. Max. coverage (+): 0. Max coverage (-): 0

Region: chr28 4512021-4512032. Max. coverage (+): 0. Max coverage (-): 0

Region: chr28 4512033-4512044. Max. coverage (+): 0. Max coverage (-): 0

Region: chr28 4512045-4512056. Max. coverage (+): 0. Max coverage (-): 0

Region: chr28 4512057-4512068. Max. coverage (+): 0. Max coverage (-): 0

Region: chr28 4512069-4512080. Max. coverage (+): 0. Max coverage (-): 0

Region: chr28 4512081-4512092. Max. coverage (+): 0. Max coverage (-): 0

Region: chr28 4512093-4512104. Max. coverage (+): 0. Max coverage (-): 0

Region: chr28 4512105-4512116. Max. coverage (+): 0. Max coverage (-): 0

Region: chr28 4512117-4512128. Max. coverage (+): 0. Max coverage (-): 0

Region: chr28 4512129-4512139. Max. coverage (+): 0. Max coverage (-): 0

Region: chr28 4512140-4512151. Max. coverage (+): 0. Max coverage (-): 0

Region: chr28 4512152-4512163. Max. coverage (+): 0. Max coverage (-): 0

Region: chr28 4512164-4512175. Max. coverage (+): 0. Max coverage (-): 0

Region: chr28 4512176-4512187. Max. coverage (+): 0.58. Max coverage (-): 0

Region: chr28 4512188-4512199. Max. coverage (+): 0.58. Max coverage (-): 0

Region: chr28 4512200-4512211. Max. coverage (+): 0. Max coverage (-): 0

Region: chr28 4512212-4512223. Max. coverage (+): 0. Max coverage (-): 0

Region: chr28 4512224-4512235. Max. coverage (+): 0. Max coverage (-): 0

Region: chr28 4512236-4512247. Max. coverage (+): 0. Max coverage (-): 0

Region: chr28 4512248-4512259. Max. coverage (+): 0. Max coverage (-): 0

Region: chr28 4512260-4512271. Max. coverage (+): 0. Max coverage (-): 0

Region: chr28 4512272-4512283. Max. coverage (+): 0. Max coverage (-): 0

Region: chr28 4512284-4512294. Max. coverage (+): 0. Max coverage (-): 0

Region: chr28 4512295-4512306. Max. coverage (+): 0. Max coverage (-): 0

Region: chr28 4512307-4512318. Max. coverage (+): 0. Max coverage (-): 0

Region: chr28 4512319-4512330. Max. coverage (+): 0. Max coverage (-): 0

Region: chr28 4512331-4512342. Max. coverage (+): 0. Max coverage (-): 0

Region: chr28 4512343-4512354. Max. coverage (+): 0. Max coverage (-): 0

Region: chr28 4512355-4512366. Max. coverage (+): 0. Max coverage (-): 0

Region: chr28 4512367-4512378. Max. coverage (+): 0. Max coverage (-): 0

Region: chr28 4512379-4512390. Max. coverage (+): 0. Max coverage (-): 0

Region: chr28 4512391-4512402. Max. coverage (+): 0. Max coverage (-): 0

Region: chr28 4512403-4512414. Max. coverage (+): 0. Max coverage (-): 0

Region: chr28 4512415-4512426. Max. coverage (+): 0. Max coverage (-): 0

Region: chr28 4512427-4512438. Max. coverage (+): 0. Max coverage (-): 0

Region: chr28 4512439-4512450. Max. coverage (+): 0. Max coverage (-): 0

Region: chr28 4512451-4512461. Max. coverage (+): 0. Max coverage (-): 0

Region: chr28 4512462-4512473. Max. coverage (+): 0. Max coverage (-): 0

Region: chr28 4512474-4512485. Max. coverage (+): 0. Max coverage (-): 0

Region: chr28 4512486-4512497. Max. coverage (+): 0. Max coverage (-): 0

Region: chr28 4512498-4512509. Max. coverage (+): 0. Max coverage (-): 0

Region: chr28 4512510-4512521. Max. coverage (+): 0. Max coverage (-): 0

Region: chr28 4512522-4512533. Max. coverage (+): 0. Max coverage (-): 0

Region: chr28 4512534-4512545. Max. coverage (+): 0. Max coverage (-): 0

Region: chr28 4512546-4512557. Max. coverage (+): 0. Max coverage (-): 0

Region: chr28 4512558-4512569. Max. coverage (+): 0. Max coverage (-): 0

Region: chr28 4512570-4512581. Max. coverage (+): 0. Max coverage (-): 0

Region: chr28 4512582-4512593. Max. coverage (+): 0. Max coverage (-): 0

Region: chr28 4512594-4512605. Max. coverage (+): 2.26. Max coverage (-): 0

Region: chr28 4512606-4512617. Max. coverage (+): 0. Max coverage (-): 0

Region: chr28 4512618-4512628. Max. coverage (+): 0. Max coverage (-): 0

Region: chr28 4512629-4512640. Max. coverage (+): 0. Max coverage (-): 0

Region: chr28 4512641-4512652. Max. coverage (+): 0. Max coverage (-): 0

Region: chr28 4512653-4512664. Max. coverage (+): 0. Max coverage (-): 0

Region: chr28 4512665-4512676. Max. coverage (+): 0. Max coverage (-): 0

Region: chr28 4512677-4512688. Max. coverage (+): 0. Max coverage (-): 0

Region: chr28 4512689-4512700. Max. coverage (+): 0. Max coverage (-): 0

Region: chr28 4512701-4512712. Max. coverage (+): 0. Max coverage (-): 0

Region: chr28 4512713-4512724. Max. coverage (+): 0. Max coverage (-): 0

Region: chr28 4512725-4512736. Max. coverage (+): 0. Max coverage (-): 0

Region: chr28 4512737-4512748. Max. coverage (+): 0. Max coverage (-): 0

Region: chr28 4512749-4512760. Max. coverage (+): 0. Max coverage (-): 0

Region: chr28 4512761-4512772. Max. coverage (+): 0. Max coverage (-): 0

Region: chr28 4512773-4512784. Max. coverage (+): 0. Max coverage (-): 0

Region: chr28 4512785-4512795. Max. coverage (+): 0. Max coverage (-): 0

Region: chr28 4512796-4512807. Max. coverage (+): 0. Max coverage (-): 0

Region: chr28 4512808-4512819. Max. coverage (+): 0. Max coverage (-): 0

Region: chr28 4512820-4512831. Max. coverage (+): 0. Max coverage (-): 0

Region: chr28 4512832-4512843. Max. coverage (+): 0. Max coverage (-): 0

Region: chr28 4512844-4512855. Max. coverage (+): 0. Max coverage (-): 0

Region: chr28 4512856-4512867. Max. coverage (+): 0. Max coverage (-): 0

Region: chr28 4512868-4512879. Max. coverage (+): 0. Max coverage (-): 0

Region: chr28 4512880-4512891. Max. coverage (+): 0. Max coverage (-): 0

Region: chr28 4512892-4512903. Max. coverage (+): 0. Max coverage (-): 0

Region: chr28 4512904-4512915. Max. coverage (+): 0. Max coverage (-): 0

Region: chr28 4512916-4512927. Max. coverage (+): 0. Max coverage (-): 0

Region: chr28 4512928-4512939. Max. coverage (+): 0. Max coverage (-): 0

Region: chr28 4512940-4512951. Max. coverage (+): 0. Max coverage (-): 0

Region: chr28 4512952-4512962. Max. coverage (+): 0. Max coverage (-): 0

Region: chr28 4512963-4512974. Max. coverage (+): 0. Max coverage (-): 0

Region: chr28 4512975-4512986. Max. coverage (+): 0. Max coverage (-): 0

Region: chr28 4512987-4512998. Max. coverage (+): 0. Max coverage (-): 0

Region: chr28 4512999-4513010. Max. coverage (+): 0. Max coverage (-): 0

Region: chr28 4513011-4513022. Max. coverage (+): 0. Max coverage (-): 0

Region: chr28 4513023-4513034. Max. coverage (+): 0. Max coverage (-): 0

Region: chr28 4513035-4513046. Max. coverage (+): 0. Max coverage (-): 0

Region: chr28 4513047-4513058. Max. coverage (+): 0. Max coverage (-): 0

Region: chr28 4513059-4513070. Max. coverage (+): 0. Max coverage (-): 0

Region: chr28 4513071-4513082. Max. coverage (+): 0. Max coverage (-): 0

Region: chr28 4513083-4513094. Max. coverage (+): 0. Max coverage (-): 0

Region: chr28 4513095-4513106. Max. coverage (+): 0. Max coverage (-): 0

Region: chr28 4513107-4513118. Max. coverage (+): 0. Max coverage (-): 0

Region: chr28 4513119-4513129. Max. coverage (+): 0. Max coverage (-): 0

Region: chr28 4513130-4513141. Max. coverage (+): 0. Max coverage (-): 0

Region: chr28 4513142-4513153. Max. coverage (+): 0. Max coverage (-): 0

Region: chr28 4513154-4513165. Max. coverage (+): 0. Max coverage (-): 0

Region: chr28 4513166-4513177. Max. coverage (+): 0. Max coverage (-): 0

Region: chr28 4513178-4513189. Max. coverage (+): 0. Max coverage (-): 0

Region: chr28 4513190-4513201. Max. coverage (+): 0. Max coverage (-): 0

Region: chr28 4513202-4513213. Max. coverage (+): 0. Max coverage (-): 0

Region: chr28 4513214-4513225. Max. coverage (+): 0. Max coverage (-): 0

Region: chr28 4513226-4513237. Max. coverage (+): 0. Max coverage (-): 0

Region: chr28 4513238-4513249. Max. coverage (+): 0. Max coverage (-): 0

Region: chr28 4513250-4513261. Max. coverage (+): 0. Max coverage (-): 0

Region: chr28 4513262-4513273. Max. coverage (+): 0. Max coverage (-): 0

Region: chr28 4513274-4513285. Max. coverage (+): 0. Max coverage (-): 0

Region: chr28 4513286-4513296. Max. coverage (+): 0. Max coverage (-): 0

Region: chr28 4513297-4513308. Max. coverage (+): 0. Max coverage (-): 0

Region: chr28 4513309-4513320. Max. coverage (+): 0. Max coverage (-): 0

Region: chr28 4513321-4513332. Max. coverage (+): 0. Max coverage (-): 0

Region: chr28 4513333-4513344. Max. coverage (+): 0. Max coverage (-): 0

Region: chr28 4513345-4513356. Max. coverage (+): 0. Max coverage (-): 0

Region: chr28 4513357-4513368. Max. coverage (+): 0. Max coverage (-): 0

Region: chr28 4513369-4513380. Max. coverage (+): 0. Max coverage (-): 0

Region: chr28 4513381-4513392. Max. coverage (+): 0. Max coverage (-): 0

Region: chr28 4513393-4513404. Max. coverage (+): 0. Max coverage (-): 0

Region: chr28 4513405-4513416. Max. coverage (+): 0. Max coverage (-): 0

Region: chr28 4513417-4513428. Max. coverage (+): 0. Max coverage (-): 0

Region: chr28 4513429-4513440. Max. coverage (+): 0. Max coverage (-): 0

Region: chr28 4513441-4513452. Max. coverage (+): 0. Max coverage (-): 0

Region: chr28 4513453-4513463. Max. coverage (+): 0.81. Max coverage (-): 0

Region: chr28 4513464-4513475. Max. coverage (+): 0. Max coverage (-): 0

Region: chr28 4513476-4513487. Max. coverage (+): 0. Max coverage (-): 0

Region: chr28 4513488-4513499. Max. coverage (+): 0. Max coverage (-): 0

Region: chr28 4513500-4513511. Max. coverage (+): 0. Max coverage (-): 0

Region: chr28 4513512-4513523. Max. coverage (+): 0. Max coverage (-): 0

Region: chr28 4513524-4513535. Max. coverage (+): 0. Max coverage (-): 0

Region: chr28 4513536-4513547. Max. coverage (+): 0. Max coverage (-): 0

Region: chr28 4513548-4513559. Max. coverage (+): 0. Max coverage (-): 0

Region: chr28 4513560-4513571. Max. coverage (+): 0. Max coverage (-): 0

Region: chr28 4513572-4513583. Max. coverage (+): 0. Max coverage (-): 0

Region: chr28 4513584-4513595. Max. coverage (+): 0. Max coverage (-): 0

Region: chr28 4513596-4513607. Max. coverage (+): 0. Max coverage (-): 0

Region: chr28 4513608-4513619. Max. coverage (+): 0. Max coverage (-): 0

Region: chr28 4513620-4513630. Max. coverage (+): 0. Max coverage (-): 0

Region: chr28 4513631-4513642. Max. coverage (+): 0. Max coverage (-): 0

Region: chr28 4513643-4513654. Max. coverage (+): 0. Max coverage (-): 0

Region: chr28 4513655-4513666. Max. coverage (+): 0. Max coverage (-): 0

Region: chr28 4513667-4513678. Max. coverage (+): 0. Max coverage (-): 0

Region: chr28 4513679-4513690. Max. coverage (+): 0. Max coverage (-): 0

Region: chr28 4513691-4513702. Max. coverage (+): 0. Max coverage (-): 0

Region: chr28 4513703-4513714. Max. coverage (+): 0. Max coverage (-): 0

Region: chr28 4513715-4513726. Max. coverage (+): 0. Max coverage (-): 0

Region: chr28 4513727-4513738. Max. coverage (+): 0. Max coverage (-): 0

Region: chr28 4513739-4513750. Max. coverage (+): 0. Max coverage (-): 0

Region: chr28 4513751-4513762. Max. coverage (+): 0. Max coverage (-): 0

Region: chr28 4513763-4513774. Max. coverage (+): 0. Max coverage (-): 0

Region: chr28 4513775-4513785. Max. coverage (+): 0. Max coverage (-): 0

Region: chr28 4513786-4513797. Max. coverage (+): 0. Max coverage (-): 0

Region: chr28 4513798-4513809. Max. coverage (+): 0. Max coverage (-): 0

Region: chr28 4513810-4513821. Max. coverage (+): 0. Max coverage (-): 0

Region: chr28 4513822-4513833. Max. coverage (+): 0. Max coverage (-): 0

Region: chr28 4513834-4513845. Max. coverage (+): 0. Max coverage (-): 0

Region: chr28 4513846-4513857. Max. coverage (+): 0. Max coverage (-): 0

Region: chr28 4513858-4513869. Max. coverage (+): 0. Max coverage (-): 0

Region: chr28 4513870-4513881. Max. coverage (+): 0. Max coverage (-): 0

Region: chr28 4513882-4513893. Max. coverage (+): 0. Max coverage (-): 0

Region: chr28 4513894-4513905. Max. coverage (+): 0. Max coverage (-): 0

Region: chr28 4513906-4513917. Max. coverage (+): 0. Max coverage (-): 0

Region: chr28 4513918-4513929. Max. coverage (+): 0. Max coverage (-): 0

Region: chr28 4513930-4513941. Max. coverage (+): 0. Max coverage (-): 0

Region: chr28 4513942-4513952. Max. coverage (+): 0. Max coverage (-): 0

Region: chr28 4513953-4513964. Max. coverage (+): 0. Max coverage (-): 0

Region: chr28 4513965-4513976. Max. coverage (+): 0. Max coverage (-): 0

Region: chr28 4513977-4513988. Max. coverage (+): 0. Max coverage (-): 0

Region: chr28 4513989-4514000. Max. coverage (+): 0. Max coverage (-): 0

Region: chr28 4514001-4514012. Max. coverage (+): 0. Max coverage (-): 0

Region: chr28 4514013-4514024. Max. coverage (+): 0. Max coverage (-): 0

Region: chr28 4514025-4514036. Max. coverage (+): 0. Max coverage (-): 0

Region: chr28 4514037-4514048. Max. coverage (+): 0. Max coverage (-): 0

Region: chr28 4514049-4514060. Max. coverage (+): 0. Max coverage (-): 0

Region: chr28 4514061-4514072. Max. coverage (+): 0. Max coverage (-): 0

Region: chr28 4514073-4514084. Max. coverage (+): 0. Max coverage (-): 0

Region: chr28 4514085-4514096. Max. coverage (+): 0. Max coverage (-): 0

Region: chr28 4514097-4514108. Max. coverage (+): 0. Max coverage (-): 0

Region: chr28 4514109-4514119. Max. coverage (+): 0. Max coverage (-): 0

Region: chr28 4514120-4514131. Max. coverage (+): 0. Max coverage (-): 0

Region: chr28 4514132-4514143. Max. coverage (+): 0. Max coverage (-): 0

Region: chr28 4514144-4514155. Max. coverage (+): 0. Max coverage (-): 0

Region: chr28 4514156-4514167. Max. coverage (+): 0. Max coverage (-): 0

Region: chr28 4514168-4514179. Max. coverage (+): 0. Max coverage (-): 0

Region: chr28 4514180-4514191. Max. coverage (+): 0. Max coverage (-): 0

Region: chr28 4514192-4514203. Max. coverage (+): 0. Max coverage (-): 0

Region: chr28 4514204-4514215. Max. coverage (+): 0. Max coverage (-): 0

Region: chr28 4514216-4514227. Max. coverage (+): 0. Max coverage (-): 0

Region: chr28 4514228-4514239. Max. coverage (+): 0. Max coverage (-): 0

Region: chr28 4514240-4514251. Max. coverage (+): 0. Max coverage (-): 0

Region: chr28 4514252-4514263. Max. coverage (+): 0. Max coverage (-): 0

Region: chr28 4514264-4514275. Max. coverage (+): 0. Max coverage (-): 0

Region: chr28 4514276-4514286. Max. coverage (+): 0. Max coverage (-): 0

Region: chr28 4514287-4514298. Max. coverage (+): 0. Max coverage (-): 0

Region: chr28 4514299-4514310. Max. coverage (+): 0. Max coverage (-): 0

Region: chr28 4514311-4514322. Max. coverage (+): 0. Max coverage (-): 0

Region: chr28 4514323-4514334. Max. coverage (+): 0. Max coverage (-): 0

Region: chr28 4514335-4514346. Max. coverage (+): 0. Max coverage (-): 0

Region: chr28 4514347-4514358. Max. coverage (+): 0. Max coverage (-): 0

Region: chr28 4514359-4514370. Max. coverage (+): 0. Max coverage (-): 0

Region: chr28 4514371-4514382. Max. coverage (+): 0. Max coverage (-): 0

Region: chr28 4514383-4514394. Max. coverage (+): 0. Max coverage (-): 0

Region: chr28 4514395-4514406. Max. coverage (+): 0. Max coverage (-): 0

Region: chr28 4514407-4514418. Max. coverage (+): 0. Max coverage (-): 0

Region: chr28 4514419-4514430. Max. coverage (+): 0. Max coverage (-): 0

Region: chr28 4514431-4514442. Max. coverage (+): 0. Max coverage (-): 0

Region: chr28 4514443-4514453. Max. coverage (+): 0. Max coverage (-): 0

Region: chr28 4514454-4514465. Max. coverage (+): 0. Max coverage (-): 0

Region: chr28 4514466-4514477. Max. coverage (+): 0. Max coverage (-): 0

Region: chr28 4514478-4514489. Max. coverage (+): 0. Max coverage (-): 0

Region: chr28 4514490-4514501. Max. coverage (+): 0. Max coverage (-): 0

Region: chr28 4514502-4514513. Max. coverage (+): 0. Max coverage (-): 0

Region: chr28 4514514-4514525. Max. coverage (+): 0. Max coverage (-): 0

Region: chr28 4514526-4514537. Max. coverage (+): 0. Max coverage (-): 0

Region: chr28 4514538-4514549. Max. coverage (+): 0. Max coverage (-): 0

Region: chr28 4514550-4514561. Max. coverage (+): 10.55. Max coverage (-): 0

Region: chr28 4514562-4514573. Max. coverage (+): 2.12. Max coverage (-): 0

Region: chr28 4514574-4514585. Max. coverage (+): 0. Max coverage (-): 0

Region: chr28 4514586-4514597. Max. coverage (+): 4.15. Max coverage (-): 0

Region: chr28 4514598-4514609. Max. coverage (+): 4.15. Max coverage (-): 0

Region: chr28 4514610-4514620. Max. coverage (+): 1.47. Max coverage (-): 0

Region: chr28 4514621-4514632. Max. coverage (+): 0. Max coverage (-): 0

Region: chr28 4514633-4514644. Max. coverage (+): 1.4. Max coverage (-): 0

Region: chr28 4514645-4514656. Max. coverage (+): 0. Max coverage (-): 0

Region: chr28 4514657-4514668. Max. coverage (+): 0. Max coverage (-): 0

Region: chr28 4514669-4514680. Max. coverage (+): 0. Max coverage (-): 0

Region: chr28 4514681-4514692. Max. coverage (+): 0. Max coverage (-): 0

Region: chr28 4514693-4514704. Max. coverage (+): 0. Max coverage (-): 0

Region: chr28 4514705-4514716. Max. coverage (+): 3.72. Max coverage (-): 0

Region: chr28 4514717-4514728. Max. coverage (+): 5.27. Max coverage (-): 0

Region: chr28 4514729-4514740. Max. coverage (+): 5.27. Max coverage (-): 0

Region: chr28 4514741-4514752. Max. coverage (+): 0. Max coverage (-): 0

Region: chr28 4514753-4514764. Max. coverage (+): 0. Max coverage (-): 0

Region: chr28 4514765-4514776. Max. coverage (+): 10.76. Max coverage (-): 0

Region: chr28 4514777-4514787. Max. coverage (+): 10.76. Max coverage (-): 0

Region: chr28 4514788-4514799. Max. coverage (+): 2.58. Max coverage (-): 0

Region: chr28 4514800-4514811. Max. coverage (+): 2.58. Max coverage (-): 0

Region: chr28 4514812-4514823. Max. coverage (+): 0. Max coverage (-): 0

Region: chr28 4514824-4514835. Max. coverage (+): 0. Max coverage (-): 0

Region: chr28 4514836-4514847. Max. coverage (+): 0. Max coverage (-): 0

Region: chr28 4514848-4514859. Max. coverage (+): 0. Max coverage (-): 0

Region: chr28 4514860-4514871. Max. coverage (+): 2.04. Max coverage (-): 0

Region: chr28 4514872-4514883. Max. coverage (+): 0. Max coverage (-): 0

Region: chr28 4514884-4514895. Max. coverage (+): 0. Max coverage (-): 0

Region: chr28 4514896-4514907. Max. coverage (+): 0. Max coverage (-): 0

Region: chr28 4514908-4514919. Max. coverage (+): 11.65. Max coverage (-): 0

Region: chr28 4514920-4514931. Max. coverage (+): 11.65. Max coverage (-): 0

Region: chr28 4514932-4514943. Max. coverage (+): 0. Max coverage (-): 0

Region: chr28 4514944-4514954. Max. coverage (+): 0. Max coverage (-): 0

Region: chr28 4514955-4514966. Max. coverage (+): 0. Max coverage (-): 0

Region: chr28 4514967-4514978. Max. coverage (+): 0. Max coverage (-): 0

Region: chr28 4514979-4514990. Max. coverage (+): 0. Max coverage (-): 0

Region: chr28 4514991-4515002. Max. coverage (+): 0.19. Max coverage (-): 0

Region: chr28 4515003-4515014. Max. coverage (+): 0. Max coverage (-): 0

Region: chr28 4515015-4515026. Max. coverage (+): 0. Max coverage (-): 0

Region: chr28 4515027-4515038. Max. coverage (+): 0. Max coverage (-): 0

Region: chr28 4515039-4515050. Max. coverage (+): 0. Max coverage (-): 0

Region: chr28 4515051-4515062. Max. coverage (+): 0. Max coverage (-): 0

Region: chr28 4515063-4515074. Max. coverage (+): 0. Max coverage (-): 0

Region: chr28 4515075-4515086. Max. coverage (+): 0. Max coverage (-): 0

Region: chr28 4515087-4515098. Max. coverage (+): 0. Max coverage (-): 0

Region: chr28 4515099-4515110. Max. coverage (+): 0. Max coverage (-): 0

Region: chr28 4515111-4515121. Max. coverage (+): 0. Max coverage (-): 0

Region: chr28 4515122-4515133. Max. coverage (+): 0. Max coverage (-): 0

Region: chr28 4515134-4515145. Max. coverage (+): 0. Max coverage (-): 0

Region: chr28 4515146-4515157. Max. coverage (+): 0. Max coverage (-): 0

Region: chr28 4515158-4515169. Max. coverage (+): 0. Max coverage (-): 0

Region: chr28 4515170-4515181. Max. coverage (+): 0. Max coverage (-): 0

Region: chr28 4515182-4515193. Max. coverage (+): 0. Max coverage (-): 0

Region: chr28 4515194-4515205. Max. coverage (+): 0. Max coverage (-): 0

Region: chr28 4515206-4515217. Max. coverage (+): 0. Max coverage (-): 0

Region: chr28 4515218-4515229. Max. coverage (+): 0. Max coverage (-): 0

Region: chr28 4515230-4515241. Max. coverage (+): 0. Max coverage (-): 0

Region: chr28 4515242-4515253. Max. coverage (+): 0. Max coverage (-): 0

Region: chr28 4515254-4515265. Max. coverage (+): 0. Max coverage (-): 0

Region: chr28 4515266-4515276. Max. coverage (+): 6.25. Max coverage (-): 0

Region: chr28 4515277-4515288. Max. coverage (+): 9.81. Max coverage (-): 0

Region: chr28 4515289-4515300. Max. coverage (+): 6.57. Max coverage (-): 0.79

Region: chr28 4515301-4515312. Max. coverage (+): 11.18. Max coverage (-): 0.79

Region: chr28 4515313-4515324. Max. coverage (+): 2.92. Max coverage (-): 0

Region: chr28 4515325-4515336. Max. coverage (+): 0. Max coverage (-): 0

Region: chr28 4515337-4515348. Max. coverage (+): 4.06. Max coverage (-): 0

Region: chr28 4515349-4515360. Max. coverage (+): 10.64. Max coverage (-): 0

Region: chr28 4515361-4515372. Max. coverage (+): 7.63. Max coverage (-): 0

Region: chr28 4515373-4515384. Max. coverage (+): 0. Max coverage (-): 0

Region: chr28 4515385-4515396. Max. coverage (+): 20.43. Max coverage (-): 0

Region: chr28 4515397-4515408. Max. coverage (+): 9.84. Max coverage (-): 0

Region: chr28 4515409-4515420. Max. coverage (+): 2.46. Max coverage (-): 0

Region: chr28 4515421-4515432. Max. coverage (+): 0. Max coverage (-): 0

Region: chr28 4515433-4515443. Max. coverage (+): 0. Max coverage (-): 0

Region: chr28 4515444-4515455. Max. coverage (+): 6.8. Max coverage (-): 0

Region: chr28 4515456-4515467. Max. coverage (+): 7.9. Max coverage (-): 0

Region: chr28 4515468-4515479. Max. coverage (+): 12.68. Max coverage (-): 0

Region: chr28 4515480-4515491. Max. coverage (+): 9.16. Max coverage (-): 0

Region: chr28 4515492-4515503. Max. coverage (+): 18.94. Max coverage (-): 0

Region: chr28 4515504-4515515. Max. coverage (+): 8.18. Max coverage (-): 0

Region: chr28 4515516-4515527. Max. coverage (+): 16.09. Max coverage (-): 0

Region: chr28 4515528-4515539. Max. coverage (+): 6.05. Max coverage (-): 0

Region: chr28 4515540-4515551. Max. coverage (+): 2.8. Max coverage (-): 0

Region: chr28 4515552-4515563. Max. coverage (+): 2.8. Max coverage (-): 0

Region: chr28 4515564-4515575. Max. coverage (+): 0. Max coverage (-): 0

Region: chr28 4515576-4515587. Max. coverage (+): 1.81. Max coverage (-): 0

Region: chr28 4515588-4515599. Max. coverage (+): 3.53. Max coverage (-): 0

Region: chr28 4515600-4515610. Max. coverage (+): 12.01. Max coverage (-): 0

Region: chr28 4515611-4515622. Max. coverage (+): 9.62. Max coverage (-): 0

Region: chr28 4515623-4515634. Max. coverage (+): 14.66. Max coverage (-): 0

Region: chr28 4515635-4515646. Max. coverage (+): 14.66. Max coverage (-): 0

Region: chr28 4515647-4515658. Max. coverage (+): 0. Max coverage (-): 0

Region: chr28 4515659-4515670. Max. coverage (+): 0.76. Max coverage (-): 0

Region: chr28 4515671-4515682. Max. coverage (+): 0. Max coverage (-): 0

Region: chr28 4515683-4515694. Max. coverage (+): 0. Max coverage (-): 0

Region: chr28 4515695-4515706. Max. coverage (+): 0. Max coverage (-): 0

Region: chr28 4515707-4515718. Max. coverage (+): 4.05. Max coverage (-): 0

Region: chr28 4515719-4515730. Max. coverage (+): 4.05. Max coverage (-): 0

Region: chr28 4515731-4515742. Max. coverage (+): 0. Max coverage (-): 0

Region: chr28 4515743-4515754. Max. coverage (+): 0. Max coverage (-): 0

Region: chr28 4515755-4515766. Max. coverage (+): 0. Max coverage (-): 0

Region: chr28 4515767-4515777. Max. coverage (+): 8.22. Max coverage (-): 0

Region: chr28 4515778-4515789. Max. coverage (+): 11.77. Max coverage (-): 0

Region: chr28 4515790-4515801. Max. coverage (+): 0. Max coverage (-): 0

Region: chr28 4515802-4515813. Max. coverage (+): 4.76. Max coverage (-): 0

Region: chr28 4515814-4515825. Max. coverage (+): 57.4. Max coverage (-): 0

Region: chr28 4515826-4515837. Max. coverage (+): 49.45. Max coverage (-): 0

Region: chr28 4515838-4515849. Max. coverage (+): 0. Max coverage (-): 0

Region: chr28 4515850-4515861. Max. coverage (+): 0.63. Max coverage (-): 0

Region: chr28 4515862-4515873. Max. coverage (+): 30.17. Max coverage (-): 0

Region: chr28 4515874-4515885. Max. coverage (+): 21.28. Max coverage (-): 0

Region: chr28 4515886-4515897. Max. coverage (+): 14.51. Max coverage (-): 0

Region: chr28 4515898-4515909. Max. coverage (+): 17. Max coverage (-): 0

Region: chr28 4515910-4515921. Max. coverage (+): 41.34. Max coverage (-): 0

Region: chr28 4515922-4515933. Max. coverage (+): 56.99. Max coverage (-): 0

Region: chr28 4515934-4515944. Max. coverage (+): 42.3. Max coverage (-): 0

Region: chr28 4515945-4515956. Max. coverage (+): 11.87. Max coverage (-): 0

Region: chr28 4515957-4515968. Max. coverage (+): 5.01. Max coverage (-): 0

Region: chr28 4515969-4515980. Max. coverage (+): 2.49. Max coverage (-): 0

Region: chr28 4515981-4515992. Max. coverage (+): 0. Max coverage (-): 0

Region: chr28 4515993-4516004. Max. coverage (+): 20.49. Max coverage (-): 0

Region: chr28 4516005-4516016. Max. coverage (+): 11.65. Max coverage (-): 0

Region: chr28 4516017-4516028. Max. coverage (+): 3.02. Max coverage (-): 0

Region: chr28 4516029-4516040. Max. coverage (+): 0. Max coverage (-): 0

Region: chr28 4516041-4516052. Max. coverage (+): 2.54. Max coverage (-): 0

Region: chr28 4516053-4516064. Max. coverage (+): 0. Max coverage (-): 0

Region: chr28 4516065-4516076. Max. coverage (+): 0. Max coverage (-): 0

Region: chr28 4516077-4516088. Max. coverage (+): 0. Max coverage (-): 0

Region: chr28 4516089-4516100. Max. coverage (+): 0. Max coverage (-): 0

Region: chr28 4516101-4516111. Max. coverage (+): 0. Max coverage (-): 0

Region: chr28 4516112-4516123. Max. coverage (+): 0. Max coverage (-): 0

Region: chr28 4516124-4516135. Max. coverage (+): 0. Max coverage (-): 0

Region: chr28 4516136-4516147. Max. coverage (+): 7.91. Max coverage (-): 0

Region: chr28 4516148-4516159. Max. coverage (+): 2.18. Max coverage (-): 0

Region: chr28 4516160-4516171. Max. coverage (+): 4.24. Max coverage (-): 0

Region: chr28 4516172-4516183. Max. coverage (+): 0. Max coverage (-): 0

Region: chr28 4516184-4516195. Max. coverage (+): 21.42. Max coverage (-): 0

Region: chr28 4516196-4516207. Max. coverage (+): 21.42. Max coverage (-): 0

Region: chr28 4516208-4516219. Max. coverage (+): 0. Max coverage (-): 0

Region: chr28 4516220-4516231. Max. coverage (+): 0. Max coverage (-): 0

Region: chr28 4516232-4516243. Max. coverage (+): 0. Max coverage (-): 0

Region: chr28 4516244-4516255. Max. coverage (+): 7.95. Max coverage (-): 0

Region: chr28 4516256-4516267. Max. coverage (+): 14.48. Max coverage (-): 0

Region: chr28 4516268-4516278. Max. coverage (+): 21.57. Max coverage (-): 0

Region: chr28 4516279-4516290. Max. coverage (+): 17.25. Max coverage (-): 0

Region: chr28 4516291-4516302. Max. coverage (+): 2.93. Max coverage (-): 0

Region: chr28 4516303-4516314. Max. coverage (+): 1.42. Max coverage (-): 0

Region: chr28 4516315-4516326. Max. coverage (+): 23.07. Max coverage (-): 0

Region: chr28 4516327-4516338. Max. coverage (+): 22.96. Max coverage (-): 0

Region: chr28 4516339-4516350. Max. coverage (+): 5.63. Max coverage (-): 0

Region: chr28 4516351-4516362. Max. coverage (+): 2.33. Max coverage (-): 0

Region: chr28 4516363-4516374. Max. coverage (+): 0. Max coverage (-): 0

Region: chr28 4516375-4516386. Max. coverage (+): 0. Max coverage (-): 0

Region: chr28 4516387-4516398. Max. coverage (+): 0.5. Max coverage (-): 0

Region: chr28 4516399-4516410. Max. coverage (+): 0.5. Max coverage (-): 0

Region: chr28 4516411-4516422. Max. coverage (+): 0.57. Max coverage (-): 0

Region: chr28 4516423-4516434. Max. coverage (+): 16.48. Max coverage (-): 0

Region: chr28 4516435-4516445. Max. coverage (+): 0.37. Max coverage (-): 0

Region: chr28 4516446-4516457. Max. coverage (+): 0.37. Max coverage (-): 0

Region: chr28 4516458-4516469. Max. coverage (+): 0. Max coverage (-): 0

Region: chr28 4516470-4516481. Max. coverage (+): 0. Max coverage (-): 0

Region: chr28 4516482-4516493. Max. coverage (+): 9.45. Max coverage (-): 0

Region: chr28 4516494-4516505. Max. coverage (+): 9.45. Max coverage (-): 0

Region: chr28 4516506-4516517. Max. coverage (+): 2.83. Max coverage (-): 0

Region: chr28 4516518-4516529. Max. coverage (+): 0. Max coverage (-): 0

Region: chr28 4516530-4516541. Max. coverage (+): 0. Max coverage (-): 0

Region: chr28 4516542-4516553. Max. coverage (+): 1.1. Max coverage (-): 0

Region: chr28 4516554-4516565. Max. coverage (+): 0. Max coverage (-): 0

Region: chr28 4516566-4516577. Max. coverage (+): 0. Max coverage (-): 0

Region: chr28 4516578-4516589. Max. coverage (+): 1.68. Max coverage (-): 0

Region: chr28 4516590-4516601. Max. coverage (+): 10.76. Max coverage (-): 0

Region: chr28 4516602-4516612. Max. coverage (+): 12.14. Max coverage (-): 0

Region: chr28 4516613-4516624. Max. coverage (+): 3.45. Max coverage (-): 0

Region: chr28 4516625-4516636. Max. coverage (+): 5.2. Max coverage (-): 0

Region: chr28 4516637-4516648. Max. coverage (+): 18.78. Max coverage (-): 0

Region: chr28 4516649-4516660. Max. coverage (+): 12.28. Max coverage (-): 0

Region: chr28 4516661-4516672. Max. coverage (+): 8.98. Max coverage (-): 0

Region: chr28 4516673-4516684. Max. coverage (+): 0. Max coverage (-): 0

Region: chr28 4516685-4516696. Max. coverage (+): 15.84. Max coverage (-): 0

Region: chr28 4516697-4516708. Max. coverage (+): 13.26. Max coverage (-): 0

Region: chr28 4516709-4516720. Max. coverage (+): 29.56. Max coverage (-): 0

Region: chr28 4516721-4516732. Max. coverage (+): 19.45. Max coverage (-): 0

Region: chr28 4516733-4516744. Max. coverage (+): 0. Max coverage (-): 0

Region: chr28 4516745-4516756. Max. coverage (+): 2.22. Max coverage (-): 0

Region: chr28 4516757-4516767. Max. coverage (+): 5.43. Max coverage (-): 0

Region: chr28 4516768-4516779. Max. coverage (+): 18.8. Max coverage (-): 0

Region: chr28 4516780-4516791. Max. coverage (+): 2.79. Max coverage (-): 0

Region: chr28 4516792-4516803. Max. coverage (+): 0. Max coverage (-): 0

Region: chr28 4516804-4516815. Max. coverage (+): 1.57. Max coverage (-): 0

Region: chr28 4516816-4516827. Max. coverage (+): 2.99. Max coverage (-): 0

Region: chr28 4516828-4516839. Max. coverage (+): 0. Max coverage (-): 0

Region: chr28 4516840-4516851. Max. coverage (+): 1.8. Max coverage (-): 0

Region: chr28 4516852-4516863. Max. coverage (+): 3.96. Max coverage (-): 0

Region: chr28 4516864-4516875. Max. coverage (+): 1.86. Max coverage (-): 0

Region: chr28 4516876-4516887. Max. coverage (+): 3.47. Max coverage (-): 0

Region: chr28 4516888-4516899. Max. coverage (+): 1.72. Max coverage (-): 0

Region: chr28 4516900-4516911. Max. coverage (+): 6.33. Max coverage (-): 0

Region: chr28 4516912-4516923. Max. coverage (+): 4.63. Max coverage (-): 0

Region: chr28 4516924-4516934. Max. coverage (+): 5.25. Max coverage (-): 0

Region: chr28 4516935-4516946. Max. coverage (+): 0. Max coverage (-): 0

Region: chr28 4516947-4516958. Max. coverage (+): 0. Max coverage (-): 0

Region: chr28 4516959-4516970. Max. coverage (+): 0. Max coverage (-): 0

Region: chr28 4516971-4516982. Max. coverage (+): 0. Max coverage (-): 0

Region: chr28 4516983-4516994. Max. coverage (+): 0. Max coverage (-): 0

Region: chr28 4516995-4517006. Max. coverage (+): 0. Max coverage (-): 0

Region: chr28 4517007-4517018. Max. coverage (+): 0.54. Max coverage (-): 0

Region: chr28 4517019-4517030. Max. coverage (+): 0.54. Max coverage (-): 0

Region: chr28 4517031-4517042. Max. coverage (+): 0. Max coverage (-): 0

Region: chr28 4517043-4517054. Max. coverage (+): 0. Max coverage (-): 0

Region: chr28 4517055-4517066. Max. coverage (+): 3.11. Max coverage (-): 0

Region: chr28 4517067-4517078. Max. coverage (+): 0. Max coverage (-): 0

Region: chr28 4517079-4517090. Max. coverage (+): 4.1. Max coverage (-): 0

Region: chr28 4517091-4517101. Max. coverage (+): 8.48. Max coverage (-): 0

Region: chr28 4517102-4517113. Max. coverage (+): 2.75. Max coverage (-): 0

Region: chr28 4517114-4517125. Max. coverage (+): 6.71. Max coverage (-): 0

Region: chr28 4517126-4517137. Max. coverage (+): 14.55. Max coverage (-): 0

Region: chr28 4517138-4517149. Max. coverage (+): 9.06. Max coverage (-): 0

Region: chr28 4517150-4517161. Max. coverage (+): 3.73. Max coverage (-): 0

Region: chr28 4517162-4517173. Max. coverage (+): 6.58. Max coverage (-): 0

Region: chr28 4517174-4517185. Max. coverage (+): 6.36. Max coverage (-): 0

Region: chr28 4517186-4517197. Max. coverage (+): 0. Max coverage (-): 0

Region: chr28 4517198-4517209. Max. coverage (+): 22.01. Max coverage (-): 0

Region: chr28 4517210-4517221. Max. coverage (+): 16. Max coverage (-): 0

Region: chr28 4517222-4517233. Max. coverage (+): 5.25. Max coverage (-): 0

Region: chr28 4517234-4517245. Max. coverage (+): 0. Max coverage (-): 0

Region: chr28 4517246-4517257. Max. coverage (+): 2.04. Max coverage (-): 0

Region: chr28 4517258-4517268. Max. coverage (+): 1.62. Max coverage (-): 0

Region: chr28 4517269-4517280. Max. coverage (+): 0. Max coverage (-): 0

Region: chr28 4517281-4517292. Max. coverage (+): 10.1. Max coverage (-): 0

Region: chr28 4517293-4517304. Max. coverage (+): 9.7. Max coverage (-): 0

Region: chr28 4517305-4517316. Max. coverage (+): 5.39. Max coverage (-): 0

Region: chr28 4517317-4517328. Max. coverage (+): 3.37. Max coverage (-): 0

Region: chr28 4517329-4517340. Max. coverage (+): 0. Max coverage (-): 0

Region: chr28 4517341-4517352. Max. coverage (+): 0. Max coverage (-): 0

Region: chr28 4517353-4517364. Max. coverage (+): 2.89. Max coverage (-): 0

Region: chr28 4517365-4517376. Max. coverage (+): 5.51. Max coverage (-): 0

Region: chr28 4517377-4517388. Max. coverage (+): 3.95. Max coverage (-): 0

Region: chr28 4517389-4517400. Max. coverage (+): 0. Max coverage (-): 0

Region: chr28 4517401-4517412. Max. coverage (+): 0.84. Max coverage (-): 0

Region: chr28 4517413-4517424. Max. coverage (+): 0. Max coverage (-): 0

Region: chr28 4517425-. Max. coverage (+): 0. Max coverage (-): 0

RepeatMasker Color Code

**+**

100-98% Identity

<98-95% Identity

<95-90% Identity

<90-85% Identity

<85-80% Identity

<80-75% Identity

<75-70% Identity

<70% Identity

**-**

Gene Set Color Code

**+**

Gene

Pseudogene

**-**

Topology/Coverage Color Code

Coverage Plus Strand

Coverage Minus Strand

Mainstrand: Plus

Mainstrand: Minus

Complementary Strand

Flanking Region  
(if option -flank >0)

Gene Set Annotation  
  
RepeatMasker Annotation  

**1. L2c**: 4512505-4512582 (-), Divergence to consensus: 36.4%  
**2. SINE2-1\_BT**: 4512721-4512830 (-), Divergence to consensus: 23.4%  
**3. MER74A**: 4512856-4513168 (+), Divergence to consensus: 37.6%  
**4. Bov-tA3**: 4515071-4515274 (+), Divergence to consensus: 10.8%

  
Transcription Factor Binding Sites
