# Supplementary material for: Small RNA sequencing of cryopreserved semen from single bull revealed altered miRNAs and piRNAs expression between High- and Low-motile sperm populations
Source: BMC Genomics. 2017 Jan 4;18:14. doi: 10.1186/s12864-016-3394-7 (PMC5209821; doi:10.1186/s12864-016-3394-7)
Supplement: Additional file 3: — Details for each piRNA clusters found in High Motile (HM) sperm fraction. Genes, repeats, transposable elements and transcription factors binding sites falling within the cluster regions were reported. (ZIP 1896 kb) [file 12864_2016_3394_MOESM3_ESM.zip › 7.html]

piRNA cluster 7


Predicted piRNA cluster no. 7     previous   next
  

Show proTRAC run info
Hide proTRAC run info

================================= proTRAC ====================================  
VERSION: 2.1                                    LAST MODIFIED: 06. October 2015  
  
Please cite:  
Rosenkranz D, Zischler H. proTRAC - a software for probabilistic piRNA cluster  
detection, visualization and analysis. 2012. BMC Bioinformatics 13:5.  
  
and (for proTRAC 2.0 and later):  
Rosenkranz D, Rudloff S, Bastuck K, Ketting RF, Zischler H. Tupaia small RNAs  
provide insights into function and evolution of RNAi-based transposon defense  
in mammals. 2015. RNA 21(5):911-922.  
  
Contact:  
David Rosenkranz  
Institute of Anthropology, small RNA group  
Johannes Gutenberg University Mainz  
email: rosenkranz@uni-mainz.de  
  
You can find the latest proTRAC version at:  
http://sourceforge.net/projects/protrac/files  
http://www.smallRNAgroup-mainz.de/software  
==============================================================================  
  
PARAMETERS:  
Map file: .............../storage/core/barbara/genhome/smallRNA/fertility/Sample\_motile/pirna/Sample\_motile\_26-33\_collapsed.fa.no-dust.map.weighted-10000-1000-b-0  
Genome file: ............/storage/core/barbara/genhome/smallRNA/fertility/Sample\_all/pirna/bt\_311\_chrY.fa  
RepeatMasker annotation: /storage/genomes/bt\_umd31/GCF\_000003055.6\_Bos\_taurus\_UMD\_3.1.1\_repeatMasker\_chr.out  
GeneSet:................./storage/core/barbara/genhome/smallRNA/fertility/Sample\_all/pirna/full.gtf  
  
Significant (p<=0.01) hit density will be calculated based  
on observed hit distribution.  
  
Sliding window size: ........................................ 5000 bp  
Sliding window increament: .................................. 1000 bp  
Normalize each hit by number of genomic hits: ............... 1 [0=no/1=yes]  
Normalize each hit by number of sequence reads: ............. 1 [0=no/1=yes]  
Normalize values (-> per million mapped reads): ............. 1 [0=no/1=yes]  
Min. fraction of hits with 1T(U) or 10A: .................... 0.75  
Alternatively: Min. fraction of hits with 1T(U) and 10A: .... 0.5  
Min. fraction of hits with typical piRNA length: ............ 0.75  
Typical piRNA length: ....................................... 26-33 nt  
Min. size of a piRNA cluster: ............................... 5000 bp.  
Min. number of hits (absolute): ............................. 0  
Min. number of hits (normalized): ........................... 0  
Min. fraction of hits on the mainstrand: .................... 0.75  
Top fraction of mapped sequences (in terms of read counts): . 1%  
Top fraction accounts for max. n% of sequence reads: ........ 90%  
Min. fraction of hits on each arm of a bidirectional cluster: 0.1  
Output image file for each cluster: ......................... 0 [0=no/1=yes]  
Output html file for each cluster: .......................... 1 [0=no/1=yes]  
Output a summary table: ..................................... 1 [0=no/1=yes]  
Output a FASTA file for each cluster (piRNA sequences): ..... 1 [0=no/1=yes]  
Output a FASTA file comprising cluster sequences: ........... 1 [0=no/1=yes]  
Search DNA motifs in clusters: .............................. 1 [0=no/1=yes]  
Output flanking sequences: +/- .............................. 0 bp  
Output ~.pTi file: .......................................... 1 [0=no/1=yes]  
==============================================================================  
  
  
Genome size (without gaps): ............ 2678902517 bp  
Gaps (N/X/-): .......................... 53837044 bp  
Mapped reads: .......................... 658825247023  
Non-identical sequences: ............... 514171  
Genomic hits: .......................... 764233  
Significant densitiy of mapped reads: .. 12867599.5173724 reads/kb

Show proTRAC cluster info
Hide proTRAC cluster info

|  |  |
| --- | --- |
| Location | chr10 |
| Coordinates | 100785383-100790862 |
| Size [bp] | 5480 |
| Sequence hit loci | 72 |
| Mapped reads (normalized) | 91956908 |
| Mapped reads (normalized) per kb | 16780457.7 |
| Normalized reads with 1T (1U) | 79.4% |
| Normalized reads with 10A | 33.6% |
| Normalized reads with length 26-33 nt | 100% |
| Normalized reads on the main strand(s) | 98.9% |
| Predicted directionality | mono:minus |

100%

0%

1T (1U)  
reads

10A reads

26-33 nt  
reads

reads on mainstrand

**Either the amount of reads with 1T (1U) OR 10A has to exceed 75% (set with option: -1Tor10A)  
Alternatively the amount of reads with 1T (1U) AND 10A has to exceed 50% (set with option: -1Tand10A)  
Minimum amount of reads with preferred size is 75% (set with option: -pisize)  
Minimum amount of reads on the main strand(s) is 75% (set with option: -clstrand)**

Show read coverage
Hide read coverage

WHAT DO I SEE HERE?  
This chart shows the location of mapped sequence reads within a predicted piRNA cluster. The color refers to the number of genomic hits produced by the sequence read in question. A dark red bar indicates that this sequence read produces many other hits elsewhere in the genome. Many adjacent red or yellow bars can indicate the presence of a multi-copy element such as transposons or rRNA genes. A dark green bar indicates that this sequence read maps uniquely to this locus.

1 hit

2-5 hits

6-10 hits

11-20 hits

21-50 hits

51-100 hits

> 100 hits

chr10

100785383

100790862

Gene Set

RepeatMasker

Mapped  
Reads

19.82

plus strand

minus strand

19.82

Region: chr10 59234769-100785388. Max. coverage (+): 0. Max coverage (-): 1.99

Region: chr10 100785389-100785399. Max. coverage (+): 0. Max coverage (-): 1.99

Region: chr10 100785400-100785410. Max. coverage (+): 0. Max coverage (-): 0

Region: chr10 100785411-100785421. Max. coverage (+): 0. Max coverage (-): 0

Region: chr10 100785422-100785432. Max. coverage (+): 0. Max coverage (-): 0

Region: chr10 100785433-100785443. Max. coverage (+): 0. Max coverage (-): 0

Region: chr10 100785444-100785454. Max. coverage (+): 0. Max coverage (-): 0

Region: chr10 100785455-100785465. Max. coverage (+): 0. Max coverage (-): 0

Region: chr10 100785466-100785476. Max. coverage (+): 0. Max coverage (-): 0

Region: chr10 100785477-100785487. Max. coverage (+): 0. Max coverage (-): 0

Region: chr10 100785488-100785498. Max. coverage (+): 0. Max coverage (-): 0

Region: chr10 100785499-100785509. Max. coverage (+): 0. Max coverage (-): 0

Region: chr10 100785510-100785519. Max. coverage (+): 0. Max coverage (-): 0

Region: chr10 100785520-100785530. Max. coverage (+): 0. Max coverage (-): 0

Region: chr10 100785531-100785541. Max. coverage (+): 0. Max coverage (-): 0

Region: chr10 100785542-100785552. Max. coverage (+): 0. Max coverage (-): 0

Region: chr10 100785553-100785563. Max. coverage (+): 0. Max coverage (-): 0

Region: chr10 100785564-100785574. Max. coverage (+): 0. Max coverage (-): 0

Region: chr10 100785575-100785585. Max. coverage (+): 0. Max coverage (-): 0

Region: chr10 100785586-100785596. Max. coverage (+): 0. Max coverage (-): 0

Region: chr10 100785597-100785607. Max. coverage (+): 0. Max coverage (-): 0

Region: chr10 100785608-100785618. Max. coverage (+): 0. Max coverage (-): 0

Region: chr10 100785619-100785629. Max. coverage (+): 0. Max coverage (-): 0

Region: chr10 100785630-100785640. Max. coverage (+): 0. Max coverage (-): 0

Region: chr10 100785641-100785651. Max. coverage (+): 0. Max coverage (-): 0

Region: chr10 100785652-100785662. Max. coverage (+): 0. Max coverage (-): 0

Region: chr10 100785663-100785673. Max. coverage (+): 0. Max coverage (-): 0

Region: chr10 100785674-100785684. Max. coverage (+): 0. Max coverage (-): 0

Region: chr10 100785685-100785695. Max. coverage (+): 0. Max coverage (-): 0

Region: chr10 100785696-100785706. Max. coverage (+): 0. Max coverage (-): 0

Region: chr10 100785707-100785717. Max. coverage (+): 0. Max coverage (-): 0

Region: chr10 100785718-100785728. Max. coverage (+): 0. Max coverage (-): 0

Region: chr10 100785729-100785739. Max. coverage (+): 0. Max coverage (-): 0

Region: chr10 100785740-100785750. Max. coverage (+): 0. Max coverage (-): 0

Region: chr10 100785751-100785761. Max. coverage (+): 0. Max coverage (-): 0

Region: chr10 100785762-100785772. Max. coverage (+): 0. Max coverage (-): 0

Region: chr10 100785773-100785783. Max. coverage (+): 0. Max coverage (-): 0

Region: chr10 100785784-100785793. Max. coverage (+): 0. Max coverage (-): 0

Region: chr10 100785794-100785804. Max. coverage (+): 0. Max coverage (-): 0

Region: chr10 100785805-100785815. Max. coverage (+): 0. Max coverage (-): 0

Region: chr10 100785816-100785826. Max. coverage (+): 0. Max coverage (-): 0

Region: chr10 100785827-100785837. Max. coverage (+): 0. Max coverage (-): 0

Region: chr10 100785838-100785848. Max. coverage (+): 0. Max coverage (-): 0

Region: chr10 100785849-100785859. Max. coverage (+): 0. Max coverage (-): 0

Region: chr10 100785860-100785870. Max. coverage (+): 0. Max coverage (-): 0

Region: chr10 100785871-100785881. Max. coverage (+): 0. Max coverage (-): 0

Region: chr10 100785882-100785892. Max. coverage (+): 0. Max coverage (-): 0

Region: chr10 100785893-100785903. Max. coverage (+): 0. Max coverage (-): 0

Region: chr10 100785904-100785914. Max. coverage (+): 0. Max coverage (-): 0

Region: chr10 100785915-100785925. Max. coverage (+): 0. Max coverage (-): 0

Region: chr10 100785926-100785936. Max. coverage (+): 0. Max coverage (-): 0

Region: chr10 100785937-100785947. Max. coverage (+): 0. Max coverage (-): 0

Region: chr10 100785948-100785958. Max. coverage (+): 0. Max coverage (-): 0

Region: chr10 100785959-100785969. Max. coverage (+): 0. Max coverage (-): 0

Region: chr10 100785970-100785980. Max. coverage (+): 0. Max coverage (-): 0

Region: chr10 100785981-100785991. Max. coverage (+): 0. Max coverage (-): 0

Region: chr10 100785992-100786002. Max. coverage (+): 0. Max coverage (-): 0

Region: chr10 100786003-100786013. Max. coverage (+): 0. Max coverage (-): 0

Region: chr10 100786014-100786024. Max. coverage (+): 0. Max coverage (-): 0

Region: chr10 100786025-100786035. Max. coverage (+): 0. Max coverage (-): 0

Region: chr10 100786036-100786046. Max. coverage (+): 0. Max coverage (-): 0

Region: chr10 100786047-100786057. Max. coverage (+): 0. Max coverage (-): 0

Region: chr10 100786058-100786067. Max. coverage (+): 0. Max coverage (-): 0

Region: chr10 100786068-100786078. Max. coverage (+): 0. Max coverage (-): 0

Region: chr10 100786079-100786089. Max. coverage (+): 0. Max coverage (-): 0

Region: chr10 100786090-100786100. Max. coverage (+): 0. Max coverage (-): 0

Region: chr10 100786101-100786111. Max. coverage (+): 0. Max coverage (-): 0

Region: chr10 100786112-100786122. Max. coverage (+): 0. Max coverage (-): 0

Region: chr10 100786123-100786133. Max. coverage (+): 0. Max coverage (-): 0

Region: chr10 100786134-100786144. Max. coverage (+): 0. Max coverage (-): 0

Region: chr10 100786145-100786155. Max. coverage (+): 0. Max coverage (-): 0

Region: chr10 100786156-100786166. Max. coverage (+): 0. Max coverage (-): 0

Region: chr10 100786167-100786177. Max. coverage (+): 0. Max coverage (-): 0

Region: chr10 100786178-100786188. Max. coverage (+): 0. Max coverage (-): 0

Region: chr10 100786189-100786199. Max. coverage (+): 0. Max coverage (-): 0

Region: chr10 100786200-100786210. Max. coverage (+): 0. Max coverage (-): 0

Region: chr10 100786211-100786221. Max. coverage (+): 0. Max coverage (-): 0

Region: chr10 100786222-100786232. Max. coverage (+): 0. Max coverage (-): 0

Region: chr10 100786233-100786243. Max. coverage (+): 0. Max coverage (-): 0

Region: chr10 100786244-100786254. Max. coverage (+): 0. Max coverage (-): 0

Region: chr10 100786255-100786265. Max. coverage (+): 0. Max coverage (-): 0

Region: chr10 100786266-100786276. Max. coverage (+): 0. Max coverage (-): 0

Region: chr10 100786277-100786287. Max. coverage (+): 0. Max coverage (-): 0

Region: chr10 100786288-100786298. Max. coverage (+): 0. Max coverage (-): 0

Region: chr10 100786299-100786309. Max. coverage (+): 0. Max coverage (-): 0

Region: chr10 100786310-100786320. Max. coverage (+): 0. Max coverage (-): 0

Region: chr10 100786321-100786331. Max. coverage (+): 0. Max coverage (-): 0

Region: chr10 100786332-100786341. Max. coverage (+): 0. Max coverage (-): 0

Region: chr10 100786342-100786352. Max. coverage (+): 0. Max coverage (-): 0

Region: chr10 100786353-100786363. Max. coverage (+): 0. Max coverage (-): 0

Region: chr10 100786364-100786374. Max. coverage (+): 0. Max coverage (-): 0

Region: chr10 100786375-100786385. Max. coverage (+): 0. Max coverage (-): 0

Region: chr10 100786386-100786396. Max. coverage (+): 0. Max coverage (-): 0

Region: chr10 100786397-100786407. Max. coverage (+): 0. Max coverage (-): 0

Region: chr10 100786408-100786418. Max. coverage (+): 0. Max coverage (-): 0

Region: chr10 100786419-100786429. Max. coverage (+): 0. Max coverage (-): 0

Region: chr10 100786430-100786440. Max. coverage (+): 0. Max coverage (-): 0

Region: chr10 100786441-100786451. Max. coverage (+): 0. Max coverage (-): 0

Region: chr10 100786452-100786462. Max. coverage (+): 0. Max coverage (-): 0

Region: chr10 100786463-100786473. Max. coverage (+): 0. Max coverage (-): 0

Region: chr10 100786474-100786484. Max. coverage (+): 0. Max coverage (-): 0

Region: chr10 100786485-100786495. Max. coverage (+): 0. Max coverage (-): 0

Region: chr10 100786496-100786506. Max. coverage (+): 0. Max coverage (-): 0

Region: chr10 100786507-100786517. Max. coverage (+): 0. Max coverage (-): 0

Region: chr10 100786518-100786528. Max. coverage (+): 0. Max coverage (-): 0

Region: chr10 100786529-100786539. Max. coverage (+): 0. Max coverage (-): 0

Region: chr10 100786540-100786550. Max. coverage (+): 0. Max coverage (-): 0

Region: chr10 100786551-100786561. Max. coverage (+): 0. Max coverage (-): 0

Region: chr10 100786562-100786572. Max. coverage (+): 0. Max coverage (-): 0

Region: chr10 100786573-100786583. Max. coverage (+): 0. Max coverage (-): 0

Region: chr10 100786584-100786594. Max. coverage (+): 0. Max coverage (-): 0

Region: chr10 100786595-100786605. Max. coverage (+): 0. Max coverage (-): 0

Region: chr10 100786606-100786615. Max. coverage (+): 0. Max coverage (-): 0

Region: chr10 100786616-100786626. Max. coverage (+): 0. Max coverage (-): 0

Region: chr10 100786627-100786637. Max. coverage (+): 0. Max coverage (-): 0

Region: chr10 100786638-100786648. Max. coverage (+): 0. Max coverage (-): 0

Region: chr10 100786649-100786659. Max. coverage (+): 0. Max coverage (-): 0

Region: chr10 100786660-100786670. Max. coverage (+): 0. Max coverage (-): 0

Region: chr10 100786671-100786681. Max. coverage (+): 0. Max coverage (-): 0

Region: chr10 100786682-100786692. Max. coverage (+): 0. Max coverage (-): 0

Region: chr10 100786693-100786703. Max. coverage (+): 0. Max coverage (-): 0

Region: chr10 100786704-100786714. Max. coverage (+): 0. Max coverage (-): 0

Region: chr10 100786715-100786725. Max. coverage (+): 0. Max coverage (-): 0

Region: chr10 100786726-100786736. Max. coverage (+): 0. Max coverage (-): 0

Region: chr10 100786737-100786747. Max. coverage (+): 0. Max coverage (-): 0

Region: chr10 100786748-100786758. Max. coverage (+): 0. Max coverage (-): 0

Region: chr10 100786759-100786769. Max. coverage (+): 0. Max coverage (-): 0

Region: chr10 100786770-100786780. Max. coverage (+): 0. Max coverage (-): 0

Region: chr10 100786781-100786791. Max. coverage (+): 0. Max coverage (-): 0

Region: chr10 100786792-100786802. Max. coverage (+): 0. Max coverage (-): 0

Region: chr10 100786803-100786813. Max. coverage (+): 0. Max coverage (-): 0

Region: chr10 100786814-100786824. Max. coverage (+): 0. Max coverage (-): 0

Region: chr10 100786825-100786835. Max. coverage (+): 0. Max coverage (-): 0

Region: chr10 100786836-100786846. Max. coverage (+): 0. Max coverage (-): 0

Region: chr10 100786847-100786857. Max. coverage (+): 0. Max coverage (-): 0

Region: chr10 100786858-100786868. Max. coverage (+): 0. Max coverage (-): 0

Region: chr10 100786869-100786879. Max. coverage (+): 0. Max coverage (-): 0

Region: chr10 100786880-100786889. Max. coverage (+): 0. Max coverage (-): 0

Region: chr10 100786890-100786900. Max. coverage (+): 0. Max coverage (-): 0

Region: chr10 100786901-100786911. Max. coverage (+): 0. Max coverage (-): 0

Region: chr10 100786912-100786922. Max. coverage (+): 0. Max coverage (-): 6.06

Region: chr10 100786923-100786933. Max. coverage (+): 0. Max coverage (-): 7.79

Region: chr10 100786934-100786944. Max. coverage (+): 0. Max coverage (-): 0

Region: chr10 100786945-100786955. Max. coverage (+): 0. Max coverage (-): 0

Region: chr10 100786956-100786966. Max. coverage (+): 0. Max coverage (-): 0

Region: chr10 100786967-100786977. Max. coverage (+): 0. Max coverage (-): 0

Region: chr10 100786978-100786988. Max. coverage (+): 0. Max coverage (-): 0

Region: chr10 100786989-100786999. Max. coverage (+): 0. Max coverage (-): 0

Region: chr10 100787000-100787010. Max. coverage (+): 0. Max coverage (-): 0

Region: chr10 100787011-100787021. Max. coverage (+): 0. Max coverage (-): 0

Region: chr10 100787022-100787032. Max. coverage (+): 0. Max coverage (-): 0

Region: chr10 100787033-100787043. Max. coverage (+): 0. Max coverage (-): 0

Region: chr10 100787044-100787054. Max. coverage (+): 0. Max coverage (-): 3.2

Region: chr10 100787055-100787065. Max. coverage (+): 0. Max coverage (-): 3.2

Region: chr10 100787066-100787076. Max. coverage (+): 0. Max coverage (-): 14.71

Region: chr10 100787077-100787087. Max. coverage (+): 0. Max coverage (-): 3.84

Region: chr10 100787088-100787098. Max. coverage (+): 0. Max coverage (-): 1.23

Region: chr10 100787099-100787109. Max. coverage (+): 0. Max coverage (-): 0

Region: chr10 100787110-100787120. Max. coverage (+): 0. Max coverage (-): 0

Region: chr10 100787121-100787131. Max. coverage (+): 0. Max coverage (-): 0.68

Region: chr10 100787132-100787142. Max. coverage (+): 0. Max coverage (-): 4.66

Region: chr10 100787143-100787153. Max. coverage (+): 0. Max coverage (-): 0

Region: chr10 100787154-100787163. Max. coverage (+): 0. Max coverage (-): 0

Region: chr10 100787164-100787174. Max. coverage (+): 0. Max coverage (-): 0

Region: chr10 100787175-100787185. Max. coverage (+): 0. Max coverage (-): 0

Region: chr10 100787186-100787196. Max. coverage (+): 0. Max coverage (-): 0

Region: chr10 100787197-100787207. Max. coverage (+): 0. Max coverage (-): 0

Region: chr10 100787208-100787218. Max. coverage (+): 0. Max coverage (-): 0

Region: chr10 100787219-100787229. Max. coverage (+): 0. Max coverage (-): 0

Region: chr10 100787230-100787240. Max. coverage (+): 0. Max coverage (-): 0

Region: chr10 100787241-100787251. Max. coverage (+): 0. Max coverage (-): 0

Region: chr10 100787252-100787262. Max. coverage (+): 0. Max coverage (-): 0

Region: chr10 100787263-100787273. Max. coverage (+): 0. Max coverage (-): 0

Region: chr10 100787274-100787284. Max. coverage (+): 0. Max coverage (-): 0

Region: chr10 100787285-100787295. Max. coverage (+): 0. Max coverage (-): 0

Region: chr10 100787296-100787306. Max. coverage (+): 0. Max coverage (-): 0

Region: chr10 100787307-100787317. Max. coverage (+): 0. Max coverage (-): 0

Region: chr10 100787318-100787328. Max. coverage (+): 0. Max coverage (-): 0

Region: chr10 100787329-100787339. Max. coverage (+): 0. Max coverage (-): 0

Region: chr10 100787340-100787350. Max. coverage (+): 0. Max coverage (-): 0

Region: chr10 100787351-100787361. Max. coverage (+): 0. Max coverage (-): 0

Region: chr10 100787362-100787372. Max. coverage (+): 0. Max coverage (-): 0

Region: chr10 100787373-100787383. Max. coverage (+): 0. Max coverage (-): 0

Region: chr10 100787384-100787394. Max. coverage (+): 0. Max coverage (-): 0

Region: chr10 100787395-100787405. Max. coverage (+): 0. Max coverage (-): 0

Region: chr10 100787406-100787416. Max. coverage (+): 0. Max coverage (-): 0

Region: chr10 100787417-100787427. Max. coverage (+): 0. Max coverage (-): 0

Region: chr10 100787428-100787437. Max. coverage (+): 0. Max coverage (-): 0

Region: chr10 100787438-100787448. Max. coverage (+): 0. Max coverage (-): 0

Region: chr10 100787449-100787459. Max. coverage (+): 0. Max coverage (-): 0

Region: chr10 100787460-100787470. Max. coverage (+): 0. Max coverage (-): 0

Region: chr10 100787471-100787481. Max. coverage (+): 0. Max coverage (-): 0

Region: chr10 100787482-100787492. Max. coverage (+): 0. Max coverage (-): 0

Region: chr10 100787493-100787503. Max. coverage (+): 0. Max coverage (-): 0

Region: chr10 100787504-100787514. Max. coverage (+): 0. Max coverage (-): 0

Region: chr10 100787515-100787525. Max. coverage (+): 0. Max coverage (-): 0

Region: chr10 100787526-100787536. Max. coverage (+): 0. Max coverage (-): 0

Region: chr10 100787537-100787547. Max. coverage (+): 0. Max coverage (-): 0

Region: chr10 100787548-100787558. Max. coverage (+): 0. Max coverage (-): 0

Region: chr10 100787559-100787569. Max. coverage (+): 0. Max coverage (-): 0

Region: chr10 100787570-100787580. Max. coverage (+): 0. Max coverage (-): 0

Region: chr10 100787581-100787591. Max. coverage (+): 0. Max coverage (-): 0

Region: chr10 100787592-100787602. Max. coverage (+): 0. Max coverage (-): 0

Region: chr10 100787603-100787613. Max. coverage (+): 0. Max coverage (-): 0

Region: chr10 100787614-100787624. Max. coverage (+): 0. Max coverage (-): 0

Region: chr10 100787625-100787635. Max. coverage (+): 0. Max coverage (-): 0

Region: chr10 100787636-100787646. Max. coverage (+): 0. Max coverage (-): 0

Region: chr10 100787647-100787657. Max. coverage (+): 0. Max coverage (-): 0

Region: chr10 100787658-100787668. Max. coverage (+): 0. Max coverage (-): 0

Region: chr10 100787669-100787679. Max. coverage (+): 0. Max coverage (-): 0

Region: chr10 100787680-100787690. Max. coverage (+): 0. Max coverage (-): 0

Region: chr10 100787691-100787701. Max. coverage (+): 0. Max coverage (-): 0

Region: chr10 100787702-100787711. Max. coverage (+): 0. Max coverage (-): 0

Region: chr10 100787712-100787722. Max. coverage (+): 0. Max coverage (-): 0

Region: chr10 100787723-100787733. Max. coverage (+): 0. Max coverage (-): 0

Region: chr10 100787734-100787744. Max. coverage (+): 0. Max coverage (-): 4.67

Region: chr10 100787745-100787755. Max. coverage (+): 0. Max coverage (-): 2.29

Region: chr10 100787756-100787766. Max. coverage (+): 0. Max coverage (-): 2.2

Region: chr10 100787767-100787777. Max. coverage (+): 0. Max coverage (-): 1.29

Region: chr10 100787778-100787788. Max. coverage (+): 0. Max coverage (-): 5.16

Region: chr10 100787789-100787799. Max. coverage (+): 0. Max coverage (-): 0

Region: chr10 100787800-100787810. Max. coverage (+): 0. Max coverage (-): 5.69

Region: chr10 100787811-100787821. Max. coverage (+): 0. Max coverage (-): 5.69

Region: chr10 100787822-100787832. Max. coverage (+): 0. Max coverage (-): 2.22

Region: chr10 100787833-100787843. Max. coverage (+): 0. Max coverage (-): 8.71

Region: chr10 100787844-100787854. Max. coverage (+): 0. Max coverage (-): 4.06

Region: chr10 100787855-100787865. Max. coverage (+): 0. Max coverage (-): 0

Region: chr10 100787866-100787876. Max. coverage (+): 0. Max coverage (-): 0

Region: chr10 100787877-100787887. Max. coverage (+): 0. Max coverage (-): 0

Region: chr10 100787888-100787898. Max. coverage (+): 0. Max coverage (-): 0

Region: chr10 100787899-100787909. Max. coverage (+): 0. Max coverage (-): 3.34

Region: chr10 100787910-100787920. Max. coverage (+): 0. Max coverage (-): 0.84

Region: chr10 100787921-100787931. Max. coverage (+): 0. Max coverage (-): 0.84

Region: chr10 100787932-100787942. Max. coverage (+): 0. Max coverage (-): 0

Region: chr10 100787943-100787953. Max. coverage (+): 0. Max coverage (-): 2.2

Region: chr10 100787954-100787964. Max. coverage (+): 0. Max coverage (-): 2.2

Region: chr10 100787965-100787975. Max. coverage (+): 0. Max coverage (-): 1.34

Region: chr10 100787976-100787985. Max. coverage (+): 0. Max coverage (-): 1.34

Region: chr10 100787986-100787996. Max. coverage (+): 0. Max coverage (-): 0.48

Region: chr10 100787997-100788007. Max. coverage (+): 0. Max coverage (-): 6.34

Region: chr10 100788008-100788018. Max. coverage (+): 0. Max coverage (-): 0.92

Region: chr10 100788019-100788029. Max. coverage (+): 0. Max coverage (-): 0

Region: chr10 100788030-100788040. Max. coverage (+): 0. Max coverage (-): 0

Region: chr10 100788041-100788051. Max. coverage (+): 0. Max coverage (-): 0

Region: chr10 100788052-100788062. Max. coverage (+): 0. Max coverage (-): 2.23

Region: chr10 100788063-100788073. Max. coverage (+): 0. Max coverage (-): 2.23

Region: chr10 100788074-100788084. Max. coverage (+): 0. Max coverage (-): 0

Region: chr10 100788085-100788095. Max. coverage (+): 0. Max coverage (-): 4.37

Region: chr10 100788096-100788106. Max. coverage (+): 0. Max coverage (-): 4.37

Region: chr10 100788107-100788117. Max. coverage (+): 0. Max coverage (-): 0

Region: chr10 100788118-100788128. Max. coverage (+): 0. Max coverage (-): 0

Region: chr10 100788129-100788139. Max. coverage (+): 0. Max coverage (-): 0

Region: chr10 100788140-100788150. Max. coverage (+): 0. Max coverage (-): 0

Region: chr10 100788151-100788161. Max. coverage (+): 0. Max coverage (-): 0

Region: chr10 100788162-100788172. Max. coverage (+): 0. Max coverage (-): 0

Region: chr10 100788173-100788183. Max. coverage (+): 0. Max coverage (-): 0

Region: chr10 100788184-100788194. Max. coverage (+): 0. Max coverage (-): 0

Region: chr10 100788195-100788205. Max. coverage (+): 0. Max coverage (-): 0

Region: chr10 100788206-100788216. Max. coverage (+): 0. Max coverage (-): 0

Region: chr10 100788217-100788227. Max. coverage (+): 0. Max coverage (-): 0

Region: chr10 100788228-100788238. Max. coverage (+): 0. Max coverage (-): 0

Region: chr10 100788239-100788249. Max. coverage (+): 0. Max coverage (-): 0

Region: chr10 100788250-100788259. Max. coverage (+): 0. Max coverage (-): 0

Region: chr10 100788260-100788270. Max. coverage (+): 0. Max coverage (-): 0

Region: chr10 100788271-100788281. Max. coverage (+): 0. Max coverage (-): 0

Region: chr10 100788282-100788292. Max. coverage (+): 0. Max coverage (-): 0

Region: chr10 100788293-100788303. Max. coverage (+): 0. Max coverage (-): 0

Region: chr10 100788304-100788314. Max. coverage (+): 0. Max coverage (-): 0

Region: chr10 100788315-100788325. Max. coverage (+): 0. Max coverage (-): 0

Region: chr10 100788326-100788336. Max. coverage (+): 0. Max coverage (-): 0

Region: chr10 100788337-100788347. Max. coverage (+): 0. Max coverage (-): 0

Region: chr10 100788348-100788358. Max. coverage (+): 0. Max coverage (-): 0

Region: chr10 100788359-100788369. Max. coverage (+): 0. Max coverage (-): 0

Region: chr10 100788370-100788380. Max. coverage (+): 0. Max coverage (-): 0

Region: chr10 100788381-100788391. Max. coverage (+): 0. Max coverage (-): 0

Region: chr10 100788392-100788402. Max. coverage (+): 0. Max coverage (-): 0

Region: chr10 100788403-100788413. Max. coverage (+): 0. Max coverage (-): 0

Region: chr10 100788414-100788424. Max. coverage (+): 0. Max coverage (-): 0

Region: chr10 100788425-100788435. Max. coverage (+): 0. Max coverage (-): 0

Region: chr10 100788436-100788446. Max. coverage (+): 0. Max coverage (-): 0

Region: chr10 100788447-100788457. Max. coverage (+): 0. Max coverage (-): 0

Region: chr10 100788458-100788468. Max. coverage (+): 0. Max coverage (-): 0

Region: chr10 100788469-100788479. Max. coverage (+): 0. Max coverage (-): 0

Region: chr10 100788480-100788490. Max. coverage (+): 0. Max coverage (-): 0

Region: chr10 100788491-100788501. Max. coverage (+): 0. Max coverage (-): 0

Region: chr10 100788502-100788512. Max. coverage (+): 0. Max coverage (-): 0

Region: chr10 100788513-100788523. Max. coverage (+): 0. Max coverage (-): 0

Region: chr10 100788524-100788533. Max. coverage (+): 0. Max coverage (-): 0

Region: chr10 100788534-100788544. Max. coverage (+): 0. Max coverage (-): 0

Region: chr10 100788545-100788555. Max. coverage (+): 0. Max coverage (-): 0

Region: chr10 100788556-100788566. Max. coverage (+): 0. Max coverage (-): 0

Region: chr10 100788567-100788577. Max. coverage (+): 0. Max coverage (-): 0

Region: chr10 100788578-100788588. Max. coverage (+): 0. Max coverage (-): 0

Region: chr10 100788589-100788599. Max. coverage (+): 0. Max coverage (-): 0

Region: chr10 100788600-100788610. Max. coverage (+): 0. Max coverage (-): 0

Region: chr10 100788611-100788621. Max. coverage (+): 0. Max coverage (-): 0

Region: chr10 100788622-100788632. Max. coverage (+): 0. Max coverage (-): 0

Region: chr10 100788633-100788643. Max. coverage (+): 0. Max coverage (-): 0

Region: chr10 100788644-100788654. Max. coverage (+): 0. Max coverage (-): 0

Region: chr10 100788655-100788665. Max. coverage (+): 0. Max coverage (-): 0

Region: chr10 100788666-100788676. Max. coverage (+): 0. Max coverage (-): 1.84

Region: chr10 100788677-100788687. Max. coverage (+): 0. Max coverage (-): 1.84

Region: chr10 100788688-100788698. Max. coverage (+): 0. Max coverage (-): 0

Region: chr10 100788699-100788709. Max. coverage (+): 0. Max coverage (-): 0

Region: chr10 100788710-100788720. Max. coverage (+): 0. Max coverage (-): 0

Region: chr10 100788721-100788731. Max. coverage (+): 0. Max coverage (-): 0

Region: chr10 100788732-100788742. Max. coverage (+): 0. Max coverage (-): 0

Region: chr10 100788743-100788753. Max. coverage (+): 0. Max coverage (-): 0

Region: chr10 100788754-100788764. Max. coverage (+): 0. Max coverage (-): 0

Region: chr10 100788765-100788775. Max. coverage (+): 0. Max coverage (-): 0

Region: chr10 100788776-100788786. Max. coverage (+): 0. Max coverage (-): 0

Region: chr10 100788787-100788797. Max. coverage (+): 0. Max coverage (-): 0

Region: chr10 100788798-100788807. Max. coverage (+): 0. Max coverage (-): 0

Region: chr10 100788808-100788818. Max. coverage (+): 0. Max coverage (-): 0

Region: chr10 100788819-100788829. Max. coverage (+): 0. Max coverage (-): 0

Region: chr10 100788830-100788840. Max. coverage (+): 0. Max coverage (-): 0

Region: chr10 100788841-100788851. Max. coverage (+): 0. Max coverage (-): 0

Region: chr10 100788852-100788862. Max. coverage (+): 0. Max coverage (-): 0.92

Region: chr10 100788863-100788873. Max. coverage (+): 0. Max coverage (-): 0

Region: chr10 100788874-100788884. Max. coverage (+): 0. Max coverage (-): 0

Region: chr10 100788885-100788895. Max. coverage (+): 0. Max coverage (-): 0

Region: chr10 100788896-100788906. Max. coverage (+): 0. Max coverage (-): 0

Region: chr10 100788907-100788917. Max. coverage (+): 0. Max coverage (-): 0

Region: chr10 100788918-100788928. Max. coverage (+): 0. Max coverage (-): 1.88

Region: chr10 100788929-100788939. Max. coverage (+): 0. Max coverage (-): 19.82

Region: chr10 100788940-100788950. Max. coverage (+): 0. Max coverage (-): 18.84

Region: chr10 100788951-100788961. Max. coverage (+): 0. Max coverage (-): 0

Region: chr10 100788962-100788972. Max. coverage (+): 0. Max coverage (-): 0

Region: chr10 100788973-100788983. Max. coverage (+): 0. Max coverage (-): 0

Region: chr10 100788984-100788994. Max. coverage (+): 0. Max coverage (-): 0

Region: chr10 100788995-100789005. Max. coverage (+): 0. Max coverage (-): 0

Region: chr10 100789006-100789016. Max. coverage (+): 0. Max coverage (-): 0

Region: chr10 100789017-100789027. Max. coverage (+): 0. Max coverage (-): 0

Region: chr10 100789028-100789038. Max. coverage (+): 0. Max coverage (-): 0

Region: chr10 100789039-100789049. Max. coverage (+): 0. Max coverage (-): 0

Region: chr10 100789050-100789060. Max. coverage (+): 0. Max coverage (-): 0.34

Region: chr10 100789061-100789071. Max. coverage (+): 0. Max coverage (-): 0

Region: chr10 100789072-100789081. Max. coverage (+): 0. Max coverage (-): 0

Region: chr10 100789082-100789092. Max. coverage (+): 0. Max coverage (-): 2.1

Region: chr10 100789093-100789103. Max. coverage (+): 0. Max coverage (-): 6.98

Region: chr10 100789104-100789114. Max. coverage (+): 0. Max coverage (-): 6.98

Region: chr10 100789115-100789125. Max. coverage (+): 0. Max coverage (-): 0

Region: chr10 100789126-100789136. Max. coverage (+): 0. Max coverage (-): 0

Region: chr10 100789137-100789147. Max. coverage (+): 0. Max coverage (-): 0

Region: chr10 100789148-100789158. Max. coverage (+): 0. Max coverage (-): 0

Region: chr10 100789159-100789169. Max. coverage (+): 0. Max coverage (-): 0

Region: chr10 100789170-100789180. Max. coverage (+): 0. Max coverage (-): 0

Region: chr10 100789181-100789191. Max. coverage (+): 0. Max coverage (-): 0

Region: chr10 100789192-100789202. Max. coverage (+): 0. Max coverage (-): 0

Region: chr10 100789203-100789213. Max. coverage (+): 0. Max coverage (-): 0

Region: chr10 100789214-100789224. Max. coverage (+): 0. Max coverage (-): 3.73

Region: chr10 100789225-100789235. Max. coverage (+): 0. Max coverage (-): 3.73

Region: chr10 100789236-100789246. Max. coverage (+): 0. Max coverage (-): 0

Region: chr10 100789247-100789257. Max. coverage (+): 0. Max coverage (-): 5.25

Region: chr10 100789258-100789268. Max. coverage (+): 0. Max coverage (-): 5.25

Region: chr10 100789269-100789279. Max. coverage (+): 0. Max coverage (-): 0

Region: chr10 100789280-100789290. Max. coverage (+): 0. Max coverage (-): 0

Region: chr10 100789291-100789301. Max. coverage (+): 0. Max coverage (-): 0

Region: chr10 100789302-100789312. Max. coverage (+): 0. Max coverage (-): 0

Region: chr10 100789313-100789323. Max. coverage (+): 0. Max coverage (-): 0.74

Region: chr10 100789324-100789334. Max. coverage (+): 0. Max coverage (-): 1.98

Region: chr10 100789335-100789345. Max. coverage (+): 0. Max coverage (-): 0

Region: chr10 100789346-100789355. Max. coverage (+): 0. Max coverage (-): 4

Region: chr10 100789356-100789366. Max. coverage (+): 0. Max coverage (-): 4

Region: chr10 100789367-100789377. Max. coverage (+): 0. Max coverage (-): 0.49

Region: chr10 100789378-100789388. Max. coverage (+): 0. Max coverage (-): 0

Region: chr10 100789389-100789399. Max. coverage (+): 0. Max coverage (-): 0

Region: chr10 100789400-100789410. Max. coverage (+): 0. Max coverage (-): 0

Region: chr10 100789411-100789421. Max. coverage (+): 0. Max coverage (-): 0

Region: chr10 100789422-100789432. Max. coverage (+): 0. Max coverage (-): 0

Region: chr10 100789433-100789443. Max. coverage (+): 0. Max coverage (-): 0

Region: chr10 100789444-100789454. Max. coverage (+): 0. Max coverage (-): 0

Region: chr10 100789455-100789465. Max. coverage (+): 0. Max coverage (-): 0

Region: chr10 100789466-100789476. Max. coverage (+): 0. Max coverage (-): 0

Region: chr10 100789477-100789487. Max. coverage (+): 0. Max coverage (-): 0

Region: chr10 100789488-100789498. Max. coverage (+): 0. Max coverage (-): 0

Region: chr10 100789499-100789509. Max. coverage (+): 0. Max coverage (-): 0

Region: chr10 100789510-100789520. Max. coverage (+): 0. Max coverage (-): 0

Region: chr10 100789521-100789531. Max. coverage (+): 0. Max coverage (-): 0

Region: chr10 100789532-100789542. Max. coverage (+): 0. Max coverage (-): 0

Region: chr10 100789543-100789553. Max. coverage (+): 0. Max coverage (-): 0

Region: chr10 100789554-100789564. Max. coverage (+): 0. Max coverage (-): 0

Region: chr10 100789565-100789575. Max. coverage (+): 0. Max coverage (-): 0

Region: chr10 100789576-100789586. Max. coverage (+): 0. Max coverage (-): 0

Region: chr10 100789587-100789597. Max. coverage (+): 0. Max coverage (-): 0

Region: chr10 100789598-100789608. Max. coverage (+): 0. Max coverage (-): 0

Region: chr10 100789609-100789619. Max. coverage (+): 0. Max coverage (-): 0

Region: chr10 100789620-100789629. Max. coverage (+): 0. Max coverage (-): 0

Region: chr10 100789630-100789640. Max. coverage (+): 0. Max coverage (-): 0

Region: chr10 100789641-100789651. Max. coverage (+): 0. Max coverage (-): 0

Region: chr10 100789652-100789662. Max. coverage (+): 0. Max coverage (-): 0

Region: chr10 100789663-100789673. Max. coverage (+): 0. Max coverage (-): 0

Region: chr10 100789674-100789684. Max. coverage (+): 0. Max coverage (-): 0

Region: chr10 100789685-100789695. Max. coverage (+): 0. Max coverage (-): 0

Region: chr10 100789696-100789706. Max. coverage (+): 0. Max coverage (-): 0

Region: chr10 100789707-100789717. Max. coverage (+): 0. Max coverage (-): 0

Region: chr10 100789718-100789728. Max. coverage (+): 0. Max coverage (-): 0

Region: chr10 100789729-100789739. Max. coverage (+): 0. Max coverage (-): 0

Region: chr10 100789740-100789750. Max. coverage (+): 0. Max coverage (-): 0

Region: chr10 100789751-100789761. Max. coverage (+): 0. Max coverage (-): 0

Region: chr10 100789762-100789772. Max. coverage (+): 0. Max coverage (-): 0

Region: chr10 100789773-100789783. Max. coverage (+): 0. Max coverage (-): 0

Region: chr10 100789784-100789794. Max. coverage (+): 0. Max coverage (-): 0

Region: chr10 100789795-100789805. Max. coverage (+): 0. Max coverage (-): 0

Region: chr10 100789806-100789816. Max. coverage (+): 0. Max coverage (-): 0

Region: chr10 100789817-100789827. Max. coverage (+): 0. Max coverage (-): 0

Region: chr10 100789828-100789838. Max. coverage (+): 0. Max coverage (-): 0

Region: chr10 100789839-100789849. Max. coverage (+): 0. Max coverage (-): 0

Region: chr10 100789850-100789860. Max. coverage (+): 0. Max coverage (-): 0

Region: chr10 100789861-100789871. Max. coverage (+): 0. Max coverage (-): 0

Region: chr10 100789872-100789882. Max. coverage (+): 0. Max coverage (-): 0

Region: chr10 100789883-100789893. Max. coverage (+): 0. Max coverage (-): 0

Region: chr10 100789894-100789903. Max. coverage (+): 0. Max coverage (-): 0

Region: chr10 100789904-100789914. Max. coverage (+): 0. Max coverage (-): 0

Region: chr10 100789915-100789925. Max. coverage (+): 0. Max coverage (-): 0

Region: chr10 100789926-100789936. Max. coverage (+): 0. Max coverage (-): 0

Region: chr10 100789937-100789947. Max. coverage (+): 0. Max coverage (-): 0

Region: chr10 100789948-100789958. Max. coverage (+): 0. Max coverage (-): 0

Region: chr10 100789959-100789969. Max. coverage (+): 0. Max coverage (-): 0

Region: chr10 100789970-100789980. Max. coverage (+): 0. Max coverage (-): 0

Region: chr10 100789981-100789991. Max. coverage (+): 0. Max coverage (-): 0

Region: chr10 100789992-100790002. Max. coverage (+): 0. Max coverage (-): 0

Region: chr10 100790003-100790013. Max. coverage (+): 0. Max coverage (-): 0

Region: chr10 100790014-100790024. Max. coverage (+): 0. Max coverage (-): 0

Region: chr10 100790025-100790035. Max. coverage (+): 0. Max coverage (-): 0

Region: chr10 100790036-100790046. Max. coverage (+): 0. Max coverage (-): 0

Region: chr10 100790047-100790057. Max. coverage (+): 0. Max coverage (-): 0

Region: chr10 100790058-100790068. Max. coverage (+): 0. Max coverage (-): 0

Region: chr10 100790069-100790079. Max. coverage (+): 0. Max coverage (-): 0

Region: chr10 100790080-100790090. Max. coverage (+): 0. Max coverage (-): 0

Region: chr10 100790091-100790101. Max. coverage (+): 0. Max coverage (-): 0

Region: chr10 100790102-100790112. Max. coverage (+): 0. Max coverage (-): 0

Region: chr10 100790113-100790123. Max. coverage (+): 0. Max coverage (-): 0

Region: chr10 100790124-100790134. Max. coverage (+): 0. Max coverage (-): 0

Region: chr10 100790135-100790145. Max. coverage (+): 0. Max coverage (-): 0

Region: chr10 100790146-100790156. Max. coverage (+): 0. Max coverage (-): 0

Region: chr10 100790157-100790167. Max. coverage (+): 0. Max coverage (-): 0

Region: chr10 100790168-100790177. Max. coverage (+): 0. Max coverage (-): 0

Region: chr10 100790178-100790188. Max. coverage (+): 0. Max coverage (-): 0

Region: chr10 100790189-100790199. Max. coverage (+): 0. Max coverage (-): 0

Region: chr10 100790200-100790210. Max. coverage (+): 0. Max coverage (-): 0

Region: chr10 100790211-100790221. Max. coverage (+): 0. Max coverage (-): 0

Region: chr10 100790222-100790232. Max. coverage (+): 0. Max coverage (-): 0

Region: chr10 100790233-100790243. Max. coverage (+): 0. Max coverage (-): 0

Region: chr10 100790244-100790254. Max. coverage (+): 0. Max coverage (-): 0

Region: chr10 100790255-100790265. Max. coverage (+): 0. Max coverage (-): 0

Region: chr10 100790266-100790276. Max. coverage (+): 0. Max coverage (-): 0

Region: chr10 100790277-100790287. Max. coverage (+): 0. Max coverage (-): 0

Region: chr10 100790288-100790298. Max. coverage (+): 0. Max coverage (-): 0

Region: chr10 100790299-100790309. Max. coverage (+): 0. Max coverage (-): 0

Region: chr10 100790310-100790320. Max. coverage (+): 0. Max coverage (-): 0

Region: chr10 100790321-100790331. Max. coverage (+): 0. Max coverage (-): 0

Region: chr10 100790332-100790342. Max. coverage (+): 0. Max coverage (-): 0

Region: chr10 100790343-100790353. Max. coverage (+): 0. Max coverage (-): 0

Region: chr10 100790354-100790364. Max. coverage (+): 0. Max coverage (-): 0

Region: chr10 100790365-100790375. Max. coverage (+): 0. Max coverage (-): 0

Region: chr10 100790376-100790386. Max. coverage (+): 0. Max coverage (-): 0

Region: chr10 100790387-100790397. Max. coverage (+): 0. Max coverage (-): 0

Region: chr10 100790398-100790408. Max. coverage (+): 0. Max coverage (-): 0

Region: chr10 100790409-100790419. Max. coverage (+): 0. Max coverage (-): 0

Region: chr10 100790420-100790430. Max. coverage (+): 0. Max coverage (-): 0

Region: chr10 100790431-100790441. Max. coverage (+): 0. Max coverage (-): 0

Region: chr10 100790442-100790451. Max. coverage (+): 0. Max coverage (-): 0

Region: chr10 100790452-100790462. Max. coverage (+): 0. Max coverage (-): 0

Region: chr10 100790463-100790473. Max. coverage (+): 0. Max coverage (-): 0

Region: chr10 100790474-100790484. Max. coverage (+): 0. Max coverage (-): 0

Region: chr10 100790485-100790495. Max. coverage (+): 0. Max coverage (-): 0

Region: chr10 100790496-100790506. Max. coverage (+): 0. Max coverage (-): 0

Region: chr10 100790507-100790517. Max. coverage (+): 0. Max coverage (-): 0

Region: chr10 100790518-100790528. Max. coverage (+): 0. Max coverage (-): 0

Region: chr10 100790529-100790539. Max. coverage (+): 0. Max coverage (-): 0

Region: chr10 100790540-100790550. Max. coverage (+): 0. Max coverage (-): 0

Region: chr10 100790551-100790561. Max. coverage (+): 0. Max coverage (-): 0

Region: chr10 100790562-100790572. Max. coverage (+): 0. Max coverage (-): 0

Region: chr10 100790573-100790583. Max. coverage (+): 0. Max coverage (-): 0

Region: chr10 100790584-100790594. Max. coverage (+): 0. Max coverage (-): 0

Region: chr10 100790595-100790605. Max. coverage (+): 0. Max coverage (-): 0

Region: chr10 100790606-100790616. Max. coverage (+): 0. Max coverage (-): 0

Region: chr10 100790617-100790627. Max. coverage (+): 0. Max coverage (-): 0

Region: chr10 100790628-100790638. Max. coverage (+): 0. Max coverage (-): 0

Region: chr10 100790639-100790649. Max. coverage (+): 0. Max coverage (-): 0

Region: chr10 100790650-100790660. Max. coverage (+): 0. Max coverage (-): 0

Region: chr10 100790661-100790671. Max. coverage (+): 0. Max coverage (-): 0

Region: chr10 100790672-100790682. Max. coverage (+): 0. Max coverage (-): 0

Region: chr10 100790683-100790693. Max. coverage (+): 0. Max coverage (-): 0

Region: chr10 100790694-100790704. Max. coverage (+): 0. Max coverage (-): 0

Region: chr10 100790705-100790715. Max. coverage (+): 0. Max coverage (-): 0

Region: chr10 100790716-100790725. Max. coverage (+): 0. Max coverage (-): 0

Region: chr10 100790726-100790736. Max. coverage (+): 0. Max coverage (-): 0

Region: chr10 100790737-100790747. Max. coverage (+): 0. Max coverage (-): 0

Region: chr10 100790748-100790758. Max. coverage (+): 0. Max coverage (-): 0

Region: chr10 100790759-100790769. Max. coverage (+): 0. Max coverage (-): 0

Region: chr10 100790770-100790780. Max. coverage (+): 0. Max coverage (-): 0

Region: chr10 100790781-100790791. Max. coverage (+): 0. Max coverage (-): 0

Region: chr10 100790792-100790802. Max. coverage (+): 0. Max coverage (-): 0

Region: chr10 100790803-100790813. Max. coverage (+): 0. Max coverage (-): 0

Region: chr10 100790814-100790824. Max. coverage (+): 0. Max coverage (-): 0

Region: chr10 100790825-100790835. Max. coverage (+): 1.49. Max coverage (-): 0

Region: chr10 100790836-100790846. Max. coverage (+): 1.49. Max coverage (-): 0

Region: chr10 100790847-100790857. Max. coverage (+): 0. Max coverage (-): 0

Region: chr10 100790858-. Max. coverage (+): 0. Max coverage (-): 0

RepeatMasker Color Code

**+**

100-98% Identity

<98-95% Identity

<95-90% Identity

<90-85% Identity

<85-80% Identity

<80-75% Identity

<75-70% Identity

<70% Identity

**-**

Gene Set Color Code

**+**

Gene

Pseudogene

**-**

Topology/Coverage Color Code

Coverage Plus Strand

Coverage Minus Strand

Mainstrand: Plus

Mainstrand: Minus

Complementary Strand

Flanking Region  
(if option -flank >0)

Gene Set Annotation  

**1. GALC (protein coding, ENSBTAG00000001124) Tr:00000001495 Ex:1**: 100789571-100789765 (-)

  
RepeatMasker Annotation  

**1. ART2A**: 100784984-100785384 (+), Divergence to consensus: 17%  
**2. Bov-tA2**: 100785425-100785522 (-), Divergence to consensus: 12.2%  
**3. CHR-2A**: 100785472-100785532 (-), Divergence to consensus: 27.8%  
**4. Bov-tA2**: 100785557-100785666 (+), Divergence to consensus: 15.8%  
**5. MER34B-int**: 100785671-100786125 (+), Divergence to consensus: 25%  
**6. Bov-tA3**: 100786138-100786297 (-), Divergence to consensus: 10.6%  
**7. MER34B-int**: 100786300-100786860 (+), Divergence to consensus: 34.5%  
**8. LTR9A\_EC**: 100787218-100787733 (+), Divergence to consensus: 32.3%  
**9. Bov-tA3**: 100788146-100788300 (+), Divergence to consensus: 10.4%  
**10. L1MC3**: 100788344-100788634 (+), Divergence to consensus: 27.7%  
**11. MER5A**: 100788728-100788845 (+), Divergence to consensus: 33%  
**12. BovB**: 100790089-100790273 (+), Divergence to consensus: 26.8%  
**13. ART2A**: 100790275-100790805 (+), Divergence to consensus: 15.2%

  
Transcription Factor Binding Sites  

**RFX4\_1** (Sequence: GTTGCCAGG (-): 100789732)  
**Gata4** (Sequence: AGATAAG (-): 100788324)
