# Supplementary material for: Small RNA sequencing of cryopreserved semen from single bull revealed altered miRNAs and piRNAs expression between High- and Low-motile sperm populations
Source: BMC Genomics. 2017 Jan 4;18:14. doi: 10.1186/s12864-016-3394-7 (PMC5209821; doi:10.1186/s12864-016-3394-7)
Supplement: Additional file 3: — Details for each piRNA clusters found in High Motile (HM) sperm fraction. Genes, repeats, transposable elements and transcription factors binding sites falling within the cluster regions were reported. (ZIP 1896 kb) [file 12864_2016_3394_MOESM3_ESM.zip › 70.html]

piRNA cluster 70


Predicted piRNA cluster no. 70     previous   next
  

Show proTRAC run info
Hide proTRAC run info

================================= proTRAC ====================================  
VERSION: 2.1                                    LAST MODIFIED: 06. October 2015  
  
Please cite:  
Rosenkranz D, Zischler H. proTRAC - a software for probabilistic piRNA cluster  
detection, visualization and analysis. 2012. BMC Bioinformatics 13:5.  
  
and (for proTRAC 2.0 and later):  
Rosenkranz D, Rudloff S, Bastuck K, Ketting RF, Zischler H. Tupaia small RNAs  
provide insights into function and evolution of RNAi-based transposon defense  
in mammals. 2015. RNA 21(5):911-922.  
  
Contact:  
David Rosenkranz  
Institute of Anthropology, small RNA group  
Johannes Gutenberg University Mainz  
email: rosenkranz@uni-mainz.de  
  
You can find the latest proTRAC version at:  
http://sourceforge.net/projects/protrac/files  
http://www.smallRNAgroup-mainz.de/software  
==============================================================================  
  
PARAMETERS:  
Map file: .............../storage/core/barbara/genhome/smallRNA/fertility/Sample\_motile/pirna/Sample\_motile\_26-33\_collapsed.fa.no-dust.map.weighted-10000-1000-b-0  
Genome file: ............/storage/core/barbara/genhome/smallRNA/fertility/Sample\_all/pirna/bt\_311\_chrY.fa  
RepeatMasker annotation: /storage/genomes/bt\_umd31/GCF\_000003055.6\_Bos\_taurus\_UMD\_3.1.1\_repeatMasker\_chr.out  
GeneSet:................./storage/core/barbara/genhome/smallRNA/fertility/Sample\_all/pirna/full.gtf  
  
Significant (p<=0.01) hit density will be calculated based  
on observed hit distribution.  
  
Sliding window size: ........................................ 5000 bp  
Sliding window increament: .................................. 1000 bp  
Normalize each hit by number of genomic hits: ............... 1 [0=no/1=yes]  
Normalize each hit by number of sequence reads: ............. 1 [0=no/1=yes]  
Normalize values (-> per million mapped reads): ............. 1 [0=no/1=yes]  
Min. fraction of hits with 1T(U) or 10A: .................... 0.75  
Alternatively: Min. fraction of hits with 1T(U) and 10A: .... 0.5  
Min. fraction of hits with typical piRNA length: ............ 0.75  
Typical piRNA length: ....................................... 26-33 nt  
Min. size of a piRNA cluster: ............................... 5000 bp.  
Min. number of hits (absolute): ............................. 0  
Min. number of hits (normalized): ........................... 0  
Min. fraction of hits on the mainstrand: .................... 0.75  
Top fraction of mapped sequences (in terms of read counts): . 1%  
Top fraction accounts for max. n% of sequence reads: ........ 90%  
Min. fraction of hits on each arm of a bidirectional cluster: 0.1  
Output image file for each cluster: ......................... 0 [0=no/1=yes]  
Output html file for each cluster: .......................... 1 [0=no/1=yes]  
Output a summary table: ..................................... 1 [0=no/1=yes]  
Output a FASTA file for each cluster (piRNA sequences): ..... 1 [0=no/1=yes]  
Output a FASTA file comprising cluster sequences: ........... 1 [0=no/1=yes]  
Search DNA motifs in clusters: .............................. 1 [0=no/1=yes]  
Output flanking sequences: +/- .............................. 0 bp  
Output ~.pTi file: .......................................... 1 [0=no/1=yes]  
==============================================================================  
  
  
Genome size (without gaps): ............ 2678902517 bp  
Gaps (N/X/-): .......................... 53837044 bp  
Mapped reads: .......................... 658825247023  
Non-identical sequences: ............... 514171  
Genomic hits: .......................... 764233  
Significant densitiy of mapped reads: .. 12867599.5173724 reads/kb

Show proTRAC cluster info
Hide proTRAC cluster info

|  |  |
| --- | --- |
| Location | chr28 |
| Coordinates | 25294706-25335576 |
| Size [bp] | 40871 |
| Sequence hit loci | 4386 |
| Mapped reads (normalized) | 5098040524 |
| Mapped reads (normalized) per kb | 124734910.4 |
| Normalized reads with 1T (1U) | 78.7% |
| Normalized reads with 10A | 32.7% |
| Normalized reads with length 26-33 nt | 100% |
| Normalized reads on the main strand(s) | 99.9% |
| Predicted directionality | mono:minus |

100%

0%

1T (1U)  
reads

10A reads

26-33 nt  
reads

reads on mainstrand

**Either the amount of reads with 1T (1U) OR 10A has to exceed 75% (set with option: -1Tor10A)  
Alternatively the amount of reads with 1T (1U) AND 10A has to exceed 50% (set with option: -1Tand10A)  
Minimum amount of reads with preferred size is 75% (set with option: -pisize)  
Minimum amount of reads on the main strand(s) is 75% (set with option: -clstrand)**

Show read coverage
Hide read coverage

WHAT DO I SEE HERE?  
This chart shows the location of mapped sequence reads within a predicted piRNA cluster. The color refers to the number of genomic hits produced by the sequence read in question. A dark red bar indicates that this sequence read produces many other hits elsewhere in the genome. Many adjacent red or yellow bars can indicate the presence of a multi-copy element such as transposons or rRNA genes. A dark green bar indicates that this sequence read maps uniquely to this locus.

1 hit

2-5 hits

6-10 hits

11-20 hits

21-50 hits

51-100 hits

> 100 hits

chr28

25294706

25335576

Gene Set

RepeatMasker

Mapped  
Reads

241.24

plus strand

minus strand

241.24

Region: chr28 4517426-25294746. Max. coverage (+): 0. Max coverage (-): 7.68

Region: chr28 25294747-25294828. Max. coverage (+): 0. Max coverage (-): 13.74

Region: chr28 25294829-25294910. Max. coverage (+): 0. Max coverage (-): 4.81

Region: chr28 25294911-25294992. Max. coverage (+): 0. Max coverage (-): 3.09

Region: chr28 25294993-25295073. Max. coverage (+): 0. Max coverage (-): 6.66

Region: chr28 25295074-25295155. Max. coverage (+): 0. Max coverage (-): 11.21

Region: chr28 25295156-25295237. Max. coverage (+): 0. Max coverage (-): 15.53

Region: chr28 25295238-25295319. Max. coverage (+): 0. Max coverage (-): 0

Region: chr28 25295320-25295400. Max. coverage (+): 0. Max coverage (-): 0

Region: chr28 25295401-25295482. Max. coverage (+): 0. Max coverage (-): 0

Region: chr28 25295483-25295564. Max. coverage (+): 0. Max coverage (-): 2.15

Region: chr28 25295565-25295646. Max. coverage (+): 0. Max coverage (-): 2.15

Region: chr28 25295647-25295727. Max. coverage (+): 0. Max coverage (-): 1.05

Region: chr28 25295728-25295809. Max. coverage (+): 0. Max coverage (-): 0

Region: chr28 25295810-25295891. Max. coverage (+): 0. Max coverage (-): 0

Region: chr28 25295892-25295973. Max. coverage (+): 0. Max coverage (-): 0.77

Region: chr28 25295974-25296054. Max. coverage (+): 0. Max coverage (-): 3.15

Region: chr28 25296055-25296136. Max. coverage (+): 0. Max coverage (-): 0

Region: chr28 25296137-25296218. Max. coverage (+): 0. Max coverage (-): 0

Region: chr28 25296219-25296299. Max. coverage (+): 0. Max coverage (-): 1.01

Region: chr28 25296300-25296381. Max. coverage (+): 0. Max coverage (-): 0

Region: chr28 25296382-25296463. Max. coverage (+): 0. Max coverage (-): 0

Region: chr28 25296464-25296545. Max. coverage (+): 0. Max coverage (-): 4.66

Region: chr28 25296546-25296626. Max. coverage (+): 0. Max coverage (-): 1.46

Region: chr28 25296627-25296708. Max. coverage (+): 0. Max coverage (-): 2.77

Region: chr28 25296709-25296790. Max. coverage (+): 0. Max coverage (-): 0

Region: chr28 25296791-25296872. Max. coverage (+): 0. Max coverage (-): 0

Region: chr28 25296873-25296953. Max. coverage (+): 0. Max coverage (-): 0

Region: chr28 25296954-25297035. Max. coverage (+): 0. Max coverage (-): 0

Region: chr28 25297036-25297117. Max. coverage (+): 0. Max coverage (-): 0

Region: chr28 25297118-25297199. Max. coverage (+): 0. Max coverage (-): 0

Region: chr28 25297200-25297280. Max. coverage (+): 0. Max coverage (-): 0

Region: chr28 25297281-25297362. Max. coverage (+): 0. Max coverage (-): 0

Region: chr28 25297363-25297444. Max. coverage (+): 0. Max coverage (-): 0

Region: chr28 25297445-25297526. Max. coverage (+): 0. Max coverage (-): 0

Region: chr28 25297527-25297607. Max. coverage (+): 0. Max coverage (-): 0

Region: chr28 25297608-25297689. Max. coverage (+): 0. Max coverage (-): 0

Region: chr28 25297690-25297771. Max. coverage (+): 0. Max coverage (-): 0

Region: chr28 25297772-25297853. Max. coverage (+): 0. Max coverage (-): 0

Region: chr28 25297854-25297934. Max. coverage (+): 0. Max coverage (-): 0

Region: chr28 25297935-25298016. Max. coverage (+): 0. Max coverage (-): 0

Region: chr28 25298017-25298098. Max. coverage (+): 0. Max coverage (-): 0

Region: chr28 25298099-25298180. Max. coverage (+): 0. Max coverage (-): 0

Region: chr28 25298181-25298261. Max. coverage (+): 0. Max coverage (-): 0

Region: chr28 25298262-25298343. Max. coverage (+): 0. Max coverage (-): 0

Region: chr28 25298344-25298425. Max. coverage (+): 0. Max coverage (-): 0

Region: chr28 25298426-25298507. Max. coverage (+): 0. Max coverage (-): 0

Region: chr28 25298508-25298588. Max. coverage (+): 0. Max coverage (-): 0

Region: chr28 25298589-25298670. Max. coverage (+): 0. Max coverage (-): 0

Region: chr28 25298671-25298752. Max. coverage (+): 0. Max coverage (-): 0

Region: chr28 25298753-25298833. Max. coverage (+): 0. Max coverage (-): 0

Region: chr28 25298834-25298915. Max. coverage (+): 0. Max coverage (-): 0

Region: chr28 25298916-25298997. Max. coverage (+): 0. Max coverage (-): 0

Region: chr28 25298998-25299079. Max. coverage (+): 0. Max coverage (-): 0

Region: chr28 25299080-25299160. Max. coverage (+): 0. Max coverage (-): 0

Region: chr28 25299161-25299242. Max. coverage (+): 0. Max coverage (-): 0

Region: chr28 25299243-25299324. Max. coverage (+): 0. Max coverage (-): 0

Region: chr28 25299325-25299406. Max. coverage (+): 0. Max coverage (-): 0

Region: chr28 25299407-25299487. Max. coverage (+): 0. Max coverage (-): 0

Region: chr28 25299488-25299569. Max. coverage (+): 0. Max coverage (-): 0

Region: chr28 25299570-25299651. Max. coverage (+): 0. Max coverage (-): 0

Region: chr28 25299652-25299733. Max. coverage (+): 0. Max coverage (-): 0

Region: chr28 25299734-25299814. Max. coverage (+): 0. Max coverage (-): 0

Region: chr28 25299815-25299896. Max. coverage (+): 0. Max coverage (-): 0.65

Region: chr28 25299897-25299978. Max. coverage (+): 0. Max coverage (-): 3.19

Region: chr28 25299979-25300060. Max. coverage (+): 0. Max coverage (-): 0

Region: chr28 25300061-25300141. Max. coverage (+): 0. Max coverage (-): 0

Region: chr28 25300142-25300223. Max. coverage (+): 0. Max coverage (-): 0

Region: chr28 25300224-25300305. Max. coverage (+): 0. Max coverage (-): 0

Region: chr28 25300306-25300387. Max. coverage (+): 0. Max coverage (-): 0

Region: chr28 25300388-25300468. Max. coverage (+): 0. Max coverage (-): 0

Region: chr28 25300469-25300550. Max. coverage (+): 0. Max coverage (-): 0

Region: chr28 25300551-25300632. Max. coverage (+): 0. Max coverage (-): 0

Region: chr28 25300633-25300714. Max. coverage (+): 0. Max coverage (-): 0

Region: chr28 25300715-25300795. Max. coverage (+): 0. Max coverage (-): 0

Region: chr28 25300796-25300877. Max. coverage (+): 0. Max coverage (-): 2.1

Region: chr28 25300878-25300959. Max. coverage (+): 0. Max coverage (-): 13.48

Region: chr28 25300960-25301041. Max. coverage (+): 0. Max coverage (-): 5.87

Region: chr28 25301042-25301122. Max. coverage (+): 0. Max coverage (-): 0

Region: chr28 25301123-25301204. Max. coverage (+): 0. Max coverage (-): 0

Region: chr28 25301205-25301286. Max. coverage (+): 0. Max coverage (-): 0

Region: chr28 25301287-25301367. Max. coverage (+): 0. Max coverage (-): 0

Region: chr28 25301368-25301449. Max. coverage (+): 0. Max coverage (-): 0

Region: chr28 25301450-25301531. Max. coverage (+): 0. Max coverage (-): 0

Region: chr28 25301532-25301613. Max. coverage (+): 0. Max coverage (-): 0

Region: chr28 25301614-25301694. Max. coverage (+): 0. Max coverage (-): 0

Region: chr28 25301695-25301776. Max. coverage (+): 0. Max coverage (-): 0

Region: chr28 25301777-25301858. Max. coverage (+): 0. Max coverage (-): 0

Region: chr28 25301859-25301940. Max. coverage (+): 0. Max coverage (-): 0

Region: chr28 25301941-25302021. Max. coverage (+): 0. Max coverage (-): 0

Region: chr28 25302022-25302103. Max. coverage (+): 0. Max coverage (-): 0

Region: chr28 25302104-25302185. Max. coverage (+): 0. Max coverage (-): 0

Region: chr28 25302186-25302267. Max. coverage (+): 0. Max coverage (-): 0

Region: chr28 25302268-25302348. Max. coverage (+): 0. Max coverage (-): 0

Region: chr28 25302349-25302430. Max. coverage (+): 0. Max coverage (-): 1.54

Region: chr28 25302431-25302512. Max. coverage (+): 0. Max coverage (-): 0

Region: chr28 25302513-25302594. Max. coverage (+): 0. Max coverage (-): 0

Region: chr28 25302595-25302675. Max. coverage (+): 0. Max coverage (-): 0

Region: chr28 25302676-25302757. Max. coverage (+): 0. Max coverage (-): 3.22

Region: chr28 25302758-25302839. Max. coverage (+): 0. Max coverage (-): 27.92

Region: chr28 25302840-25302921. Max. coverage (+): 0. Max coverage (-): 5.2

Region: chr28 25302922-25303002. Max. coverage (+): 0. Max coverage (-): 0

Region: chr28 25303003-25303084. Max. coverage (+): 0. Max coverage (-): 0

Region: chr28 25303085-25303166. Max. coverage (+): 0. Max coverage (-): 0

Region: chr28 25303167-25303248. Max. coverage (+): 0. Max coverage (-): 0

Region: chr28 25303249-25303329. Max. coverage (+): 0. Max coverage (-): 0

Region: chr28 25303330-25303411. Max. coverage (+): 0. Max coverage (-): 0

Region: chr28 25303412-25303493. Max. coverage (+): 0. Max coverage (-): 0

Region: chr28 25303494-25303575. Max. coverage (+): 0. Max coverage (-): 4.11

Region: chr28 25303576-25303656. Max. coverage (+): 0. Max coverage (-): 3.85

Region: chr28 25303657-25303738. Max. coverage (+): 0. Max coverage (-): 17.69

Region: chr28 25303739-25303820. Max. coverage (+): 0. Max coverage (-): 18.87

Region: chr28 25303821-25303901. Max. coverage (+): 0. Max coverage (-): 6.69

Region: chr28 25303902-25303983. Max. coverage (+): 0. Max coverage (-): 9.07

Region: chr28 25303984-25304065. Max. coverage (+): 0. Max coverage (-): 0

Region: chr28 25304066-25304147. Max. coverage (+): 0. Max coverage (-): 0

Region: chr28 25304148-25304228. Max. coverage (+): 0. Max coverage (-): 0

Region: chr28 25304229-25304310. Max. coverage (+): 0. Max coverage (-): 0

Region: chr28 25304311-25304392. Max. coverage (+): 0. Max coverage (-): 0

Region: chr28 25304393-25304474. Max. coverage (+): 0. Max coverage (-): 0

Region: chr28 25304475-25304555. Max. coverage (+): 0. Max coverage (-): 0

Region: chr28 25304556-25304637. Max. coverage (+): 0. Max coverage (-): 0

Region: chr28 25304638-25304719. Max. coverage (+): 0. Max coverage (-): 17.77

Region: chr28 25304720-25304801. Max. coverage (+): 0. Max coverage (-): 3.39

Region: chr28 25304802-25304882. Max. coverage (+): 0. Max coverage (-): 0

Region: chr28 25304883-25304964. Max. coverage (+): 0. Max coverage (-): 0

Region: chr28 25304965-25305046. Max. coverage (+): 0. Max coverage (-): 0

Region: chr28 25305047-25305128. Max. coverage (+): 0. Max coverage (-): 0

Region: chr28 25305129-25305209. Max. coverage (+): 0. Max coverage (-): 0

Region: chr28 25305210-25305291. Max. coverage (+): 0. Max coverage (-): 0

Region: chr28 25305292-25305373. Max. coverage (+): 0. Max coverage (-): 0

Region: chr28 25305374-25305455. Max. coverage (+): 0. Max coverage (-): 0

Region: chr28 25305456-25305536. Max. coverage (+): 0. Max coverage (-): 21.08

Region: chr28 25305537-25305618. Max. coverage (+): 0. Max coverage (-): 30.6

Region: chr28 25305619-25305700. Max. coverage (+): 0. Max coverage (-): 47.16

Region: chr28 25305701-25305782. Max. coverage (+): 0. Max coverage (-): 27.11

Region: chr28 25305783-25305863. Max. coverage (+): 0. Max coverage (-): 0

Region: chr28 25305864-25305945. Max. coverage (+): 0. Max coverage (-): 0

Region: chr28 25305946-25306027. Max. coverage (+): 0. Max coverage (-): 0

Region: chr28 25306028-25306109. Max. coverage (+): 0. Max coverage (-): 0

Region: chr28 25306110-25306190. Max. coverage (+): 0. Max coverage (-): 0

Region: chr28 25306191-25306272. Max. coverage (+): 0. Max coverage (-): 0

Region: chr28 25306273-25306354. Max. coverage (+): 0. Max coverage (-): 0

Region: chr28 25306355-25306435. Max. coverage (+): 0. Max coverage (-): 41.17

Region: chr28 25306436-25306517. Max. coverage (+): 0. Max coverage (-): 1.87

Region: chr28 25306518-25306599. Max. coverage (+): 0. Max coverage (-): 0

Region: chr28 25306600-25306681. Max. coverage (+): 0. Max coverage (-): 0

Region: chr28 25306682-25306762. Max. coverage (+): 0. Max coverage (-): 0

Region: chr28 25306763-25306844. Max. coverage (+): 0. Max coverage (-): 8.66

Region: chr28 25306845-25306926. Max. coverage (+): 0. Max coverage (-): 20.89

Region: chr28 25306927-25307008. Max. coverage (+): 0. Max coverage (-): 241.24

Region: chr28 25307009-25307089. Max. coverage (+): 0. Max coverage (-): 32.03

Region: chr28 25307090-25307171. Max. coverage (+): 0. Max coverage (-): 35.2

Region: chr28 25307172-25307253. Max. coverage (+): 0. Max coverage (-): 63.32

Region: chr28 25307254-25307335. Max. coverage (+): 0. Max coverage (-): 56.75

Region: chr28 25307336-25307416. Max. coverage (+): 0. Max coverage (-): 8.29

Region: chr28 25307417-25307498. Max. coverage (+): 0. Max coverage (-): 0

Region: chr28 25307499-25307580. Max. coverage (+): 0. Max coverage (-): 0

Region: chr28 25307581-25307662. Max. coverage (+): 0. Max coverage (-): 0

Region: chr28 25307663-25307743. Max. coverage (+): 0. Max coverage (-): 0

Region: chr28 25307744-25307825. Max. coverage (+): 0. Max coverage (-): 0

Region: chr28 25307826-25307907. Max. coverage (+): 0. Max coverage (-): 4.18

Region: chr28 25307908-25307989. Max. coverage (+): 0. Max coverage (-): 8.99

Region: chr28 25307990-25308070. Max. coverage (+): 0. Max coverage (-): 6.74

Region: chr28 25308071-25308152. Max. coverage (+): 0. Max coverage (-): 6.74

Region: chr28 25308153-25308234. Max. coverage (+): 0. Max coverage (-): 3.45

Region: chr28 25308235-25308316. Max. coverage (+): 0. Max coverage (-): 8.03

Region: chr28 25308317-25308397. Max. coverage (+): 0. Max coverage (-): 1.12

Region: chr28 25308398-25308479. Max. coverage (+): 0. Max coverage (-): 0

Region: chr28 25308480-25308561. Max. coverage (+): 0. Max coverage (-): 14.81

Region: chr28 25308562-25308643. Max. coverage (+): 0. Max coverage (-): 48.79

Region: chr28 25308644-25308724. Max. coverage (+): 0. Max coverage (-): 14.17

Region: chr28 25308725-25308806. Max. coverage (+): 0. Max coverage (-): 14.66

Region: chr28 25308807-25308888. Max. coverage (+): 0. Max coverage (-): 2

Region: chr28 25308889-25308969. Max. coverage (+): 0. Max coverage (-): 4.2

Region: chr28 25308970-25309051. Max. coverage (+): 0. Max coverage (-): 13.37

Region: chr28 25309052-25309133. Max. coverage (+): 0. Max coverage (-): 11.76

Region: chr28 25309134-25309215. Max. coverage (+): 0. Max coverage (-): 17.9

Region: chr28 25309216-25309296. Max. coverage (+): 0. Max coverage (-): 5.2

Region: chr28 25309297-25309378. Max. coverage (+): 0. Max coverage (-): 7.42

Region: chr28 25309379-25309460. Max. coverage (+): 0. Max coverage (-): 8.52

Region: chr28 25309461-25309542. Max. coverage (+): 0. Max coverage (-): 1.69

Region: chr28 25309543-25309623. Max. coverage (+): 0. Max coverage (-): 2.19

Region: chr28 25309624-25309705. Max. coverage (+): 0. Max coverage (-): 9.61

Region: chr28 25309706-25309787. Max. coverage (+): 0. Max coverage (-): 20.46

Region: chr28 25309788-25309869. Max. coverage (+): 0. Max coverage (-): 1.84

Region: chr28 25309870-25309950. Max. coverage (+): 0. Max coverage (-): 14.34

Region: chr28 25309951-25310032. Max. coverage (+): 0. Max coverage (-): 2.8

Region: chr28 25310033-25310114. Max. coverage (+): 0. Max coverage (-): 2.46

Region: chr28 25310115-25310196. Max. coverage (+): 0. Max coverage (-): 0

Region: chr28 25310197-25310277. Max. coverage (+): 0. Max coverage (-): 0

Region: chr28 25310278-25310359. Max. coverage (+): 0. Max coverage (-): 0

Region: chr28 25310360-25310441. Max. coverage (+): 0. Max coverage (-): 0

Region: chr28 25310442-25310523. Max. coverage (+): 0. Max coverage (-): 6.27

Region: chr28 25310524-25310604. Max. coverage (+): 0. Max coverage (-): 26.59

Region: chr28 25310605-25310686. Max. coverage (+): 0. Max coverage (-): 21.12

Region: chr28 25310687-25310768. Max. coverage (+): 0. Max coverage (-): 0

Region: chr28 25310769-25310850. Max. coverage (+): 0. Max coverage (-): 0

Region: chr28 25310851-25310931. Max. coverage (+): 0. Max coverage (-): 22.72

Region: chr28 25310932-25311013. Max. coverage (+): 0. Max coverage (-): 9.81

Region: chr28 25311014-25311095. Max. coverage (+): 0. Max coverage (-): 15.83

Region: chr28 25311096-25311177. Max. coverage (+): 0. Max coverage (-): 6.73

Region: chr28 25311178-25311258. Max. coverage (+): 0. Max coverage (-): 3.34

Region: chr28 25311259-25311340. Max. coverage (+): 0. Max coverage (-): 13.24

Region: chr28 25311341-25311422. Max. coverage (+): 0. Max coverage (-): 0

Region: chr28 25311423-25311503. Max. coverage (+): 0. Max coverage (-): 0

Region: chr28 25311504-25311585. Max. coverage (+): 0. Max coverage (-): 21.19

Region: chr28 25311586-25311667. Max. coverage (+): 0. Max coverage (-): 30.54

Region: chr28 25311668-25311749. Max. coverage (+): 0. Max coverage (-): 9.35

Region: chr28 25311750-25311830. Max. coverage (+): 0. Max coverage (-): 46.53

Region: chr28 25311831-25311912. Max. coverage (+): 0. Max coverage (-): 5.25

Region: chr28 25311913-25311994. Max. coverage (+): 0. Max coverage (-): 20.39

Region: chr28 25311995-25312076. Max. coverage (+): 0. Max coverage (-): 1.99

Region: chr28 25312077-25312157. Max. coverage (+): 0. Max coverage (-): 0

Region: chr28 25312158-25312239. Max. coverage (+): 0. Max coverage (-): 14.2

Region: chr28 25312240-25312321. Max. coverage (+): 0. Max coverage (-): 27.97

Region: chr28 25312322-25312403. Max. coverage (+): 0. Max coverage (-): 28.82

Region: chr28 25312404-25312484. Max. coverage (+): 0. Max coverage (-): 12.06

Region: chr28 25312485-25312566. Max. coverage (+): 0. Max coverage (-): 6.46

Region: chr28 25312567-25312648. Max. coverage (+): 0. Max coverage (-): 0

Region: chr28 25312649-25312730. Max. coverage (+): 0. Max coverage (-): 0

Region: chr28 25312731-25312811. Max. coverage (+): 0. Max coverage (-): 0

Region: chr28 25312812-25312893. Max. coverage (+): 0. Max coverage (-): 0

Region: chr28 25312894-25312975. Max. coverage (+): 0. Max coverage (-): 0

Region: chr28 25312976-25313057. Max. coverage (+): 0. Max coverage (-): 15.54

Region: chr28 25313058-25313138. Max. coverage (+): 0. Max coverage (-): 12.94

Region: chr28 25313139-25313220. Max. coverage (+): 0. Max coverage (-): 25.32

Region: chr28 25313221-25313302. Max. coverage (+): 0. Max coverage (-): 24.01

Region: chr28 25313303-25313384. Max. coverage (+): 0. Max coverage (-): 34.75

Region: chr28 25313385-25313465. Max. coverage (+): 0. Max coverage (-): 20.4

Region: chr28 25313466-25313547. Max. coverage (+): 0. Max coverage (-): 11.5

Region: chr28 25313548-25313629. Max. coverage (+): 0. Max coverage (-): 17.54

Region: chr28 25313630-25313711. Max. coverage (+): 0. Max coverage (-): 49.87

Region: chr28 25313712-25313792. Max. coverage (+): 0. Max coverage (-): 8.81

Region: chr28 25313793-25313874. Max. coverage (+): 0. Max coverage (-): 47

Region: chr28 25313875-25313956. Max. coverage (+): 0. Max coverage (-): 25.72

Region: chr28 25313957-25314037. Max. coverage (+): 0. Max coverage (-): 15.07

Region: chr28 25314038-25314119. Max. coverage (+): 0. Max coverage (-): 17.54

Region: chr28 25314120-25314201. Max. coverage (+): 0. Max coverage (-): 26.39

Region: chr28 25314202-25314283. Max. coverage (+): 0. Max coverage (-): 8.54

Region: chr28 25314284-25314364. Max. coverage (+): 0. Max coverage (-): 12.57

Region: chr28 25314365-25314446. Max. coverage (+): 0. Max coverage (-): 16.05

Region: chr28 25314447-25314528. Max. coverage (+): 0. Max coverage (-): 24.05

Region: chr28 25314529-25314610. Max. coverage (+): 0. Max coverage (-): 7.47

Region: chr28 25314611-25314691. Max. coverage (+): 0. Max coverage (-): 5.44

Region: chr28 25314692-25314773. Max. coverage (+): 0. Max coverage (-): 17.42

Region: chr28 25314774-25314855. Max. coverage (+): 0. Max coverage (-): 19.88

Region: chr28 25314856-25314937. Max. coverage (+): 0. Max coverage (-): 16.32

Region: chr28 25314938-25315018. Max. coverage (+): 0. Max coverage (-): 23.71

Region: chr28 25315019-25315100. Max. coverage (+): 0. Max coverage (-): 11.82

Region: chr28 25315101-25315182. Max. coverage (+): 0. Max coverage (-): 11.92

Region: chr28 25315183-25315264. Max. coverage (+): 0. Max coverage (-): 19.13

Region: chr28 25315265-25315345. Max. coverage (+): 0. Max coverage (-): 73.41

Region: chr28 25315346-25315427. Max. coverage (+): 0. Max coverage (-): 22.31

Region: chr28 25315428-25315509. Max. coverage (+): 0. Max coverage (-): 16.9

Region: chr28 25315510-25315591. Max. coverage (+): 0. Max coverage (-): 10.21

Region: chr28 25315592-25315672. Max. coverage (+): 0. Max coverage (-): 43.95

Region: chr28 25315673-25315754. Max. coverage (+): 0. Max coverage (-): 19.12

Region: chr28 25315755-25315836. Max. coverage (+): 0. Max coverage (-): 16.3

Region: chr28 25315837-25315918. Max. coverage (+): 0. Max coverage (-): 38.33

Region: chr28 25315919-25315999. Max. coverage (+): 0. Max coverage (-): 27.93

Region: chr28 25316000-25316081. Max. coverage (+): 0. Max coverage (-): 126.89

Region: chr28 25316082-25316163. Max. coverage (+): 0. Max coverage (-): 27.01

Region: chr28 25316164-25316245. Max. coverage (+): 0. Max coverage (-): 30.36

Region: chr28 25316246-25316326. Max. coverage (+): 0. Max coverage (-): 6.73

Region: chr28 25316327-25316408. Max. coverage (+): 0. Max coverage (-): 5.16

Region: chr28 25316409-25316490. Max. coverage (+): 0. Max coverage (-): 11.5

Region: chr28 25316491-25316571. Max. coverage (+): 0. Max coverage (-): 34.66

Region: chr28 25316572-25316653. Max. coverage (+): 0. Max coverage (-): 27.87

Region: chr28 25316654-25316735. Max. coverage (+): 0. Max coverage (-): 20.52

Region: chr28 25316736-25316817. Max. coverage (+): 0. Max coverage (-): 46.61

Region: chr28 25316818-25316898. Max. coverage (+): 0. Max coverage (-): 40.19

Region: chr28 25316899-25316980. Max. coverage (+): 0. Max coverage (-): 61.32

Region: chr28 25316981-25317062. Max. coverage (+): 0. Max coverage (-): 49.26

Region: chr28 25317063-25317144. Max. coverage (+): 0. Max coverage (-): 20.46

Region: chr28 25317145-25317225. Max. coverage (+): 0. Max coverage (-): 70.3

Region: chr28 25317226-25317307. Max. coverage (+): 0. Max coverage (-): 27.38

Region: chr28 25317308-25317389. Max. coverage (+): 0. Max coverage (-): 17.44

Region: chr28 25317390-25317471. Max. coverage (+): 0. Max coverage (-): 9.45

Region: chr28 25317472-25317552. Max. coverage (+): 0. Max coverage (-): 14.89

Region: chr28 25317553-25317634. Max. coverage (+): 0. Max coverage (-): 12.81

Region: chr28 25317635-25317716. Max. coverage (+): 0. Max coverage (-): 25.17

Region: chr28 25317717-25317798. Max. coverage (+): 0. Max coverage (-): 67.15

Region: chr28 25317799-25317879. Max. coverage (+): 0. Max coverage (-): 7.64

Region: chr28 25317880-25317961. Max. coverage (+): 0. Max coverage (-): 0

Region: chr28 25317962-25318043. Max. coverage (+): 0. Max coverage (-): 1.22

Region: chr28 25318044-25318125. Max. coverage (+): 0. Max coverage (-): 9.21

Region: chr28 25318126-25318206. Max. coverage (+): 0. Max coverage (-): 18.88

Region: chr28 25318207-25318288. Max. coverage (+): 0. Max coverage (-): 9.15

Region: chr28 25318289-25318370. Max. coverage (+): 0. Max coverage (-): 12.88

Region: chr28 25318371-25318452. Max. coverage (+): 0. Max coverage (-): 14.35

Region: chr28 25318453-25318533. Max. coverage (+): 0. Max coverage (-): 19.1

Region: chr28 25318534-25318615. Max. coverage (+): 0. Max coverage (-): 10.16

Region: chr28 25318616-25318697. Max. coverage (+): 0. Max coverage (-): 0

Region: chr28 25318698-25318779. Max. coverage (+): 0. Max coverage (-): 0

Region: chr28 25318780-25318860. Max. coverage (+): 0. Max coverage (-): 0

Region: chr28 25318861-25318942. Max. coverage (+): 0. Max coverage (-): 14.41

Region: chr28 25318943-25319024. Max. coverage (+): 0. Max coverage (-): 11.97

Region: chr28 25319025-25319105. Max. coverage (+): 0. Max coverage (-): 0

Region: chr28 25319106-25319187. Max. coverage (+): 0. Max coverage (-): 0

Region: chr28 25319188-25319269. Max. coverage (+): 0. Max coverage (-): 4.33

Region: chr28 25319270-25319351. Max. coverage (+): 0. Max coverage (-): 5.86

Region: chr28 25319352-25319432. Max. coverage (+): 0. Max coverage (-): 0

Region: chr28 25319433-25319514. Max. coverage (+): 0. Max coverage (-): 0

Region: chr28 25319515-25319596. Max. coverage (+): 0. Max coverage (-): 0

Region: chr28 25319597-25319678. Max. coverage (+): 0. Max coverage (-): 0

Region: chr28 25319679-25319759. Max. coverage (+): 0. Max coverage (-): 0

Region: chr28 25319760-25319841. Max. coverage (+): 0. Max coverage (-): 0

Region: chr28 25319842-25319923. Max. coverage (+): 0. Max coverage (-): 0

Region: chr28 25319924-25320005. Max. coverage (+): 0. Max coverage (-): 0

Region: chr28 25320006-25320086. Max. coverage (+): 0. Max coverage (-): 0

Region: chr28 25320087-25320168. Max. coverage (+): 0. Max coverage (-): 0

Region: chr28 25320169-25320250. Max. coverage (+): 0. Max coverage (-): 0

Region: chr28 25320251-25320332. Max. coverage (+): 0. Max coverage (-): 0

Region: chr28 25320333-25320413. Max. coverage (+): 0. Max coverage (-): 0

Region: chr28 25320414-25320495. Max. coverage (+): 0. Max coverage (-): 0

Region: chr28 25320496-25320577. Max. coverage (+): 0. Max coverage (-): 4.81

Region: chr28 25320578-25320659. Max. coverage (+): 0. Max coverage (-): 4.81

Region: chr28 25320660-25320740. Max. coverage (+): 0. Max coverage (-): 0

Region: chr28 25320741-25320822. Max. coverage (+): 0. Max coverage (-): 0

Region: chr28 25320823-25320904. Max. coverage (+): 0. Max coverage (-): 0

Region: chr28 25320905-25320986. Max. coverage (+): 0. Max coverage (-): 5.86

Region: chr28 25320987-25321067. Max. coverage (+): 0. Max coverage (-): 4.3

Region: chr28 25321068-25321149. Max. coverage (+): 0. Max coverage (-): 4.8

Region: chr28 25321150-25321231. Max. coverage (+): 0. Max coverage (-): 0

Region: chr28 25321232-25321313. Max. coverage (+): 0. Max coverage (-): 0

Region: chr28 25321314-25321394. Max. coverage (+): 0. Max coverage (-): 0

Region: chr28 25321395-25321476. Max. coverage (+): 0. Max coverage (-): 0

Region: chr28 25321477-25321558. Max. coverage (+): 0. Max coverage (-): 3.03

Region: chr28 25321559-25321639. Max. coverage (+): 0. Max coverage (-): 2.04

Region: chr28 25321640-25321721. Max. coverage (+): 0. Max coverage (-): 2.04

Region: chr28 25321722-25321803. Max. coverage (+): 0. Max coverage (-): 0

Region: chr28 25321804-25321885. Max. coverage (+): 0. Max coverage (-): 3.36

Region: chr28 25321886-25321966. Max. coverage (+): 0. Max coverage (-): 3.12

Region: chr28 25321967-25322048. Max. coverage (+): 0. Max coverage (-): 0

Region: chr28 25322049-25322130. Max. coverage (+): 0. Max coverage (-): 0

Region: chr28 25322131-25322212. Max. coverage (+): 0. Max coverage (-): 0

Region: chr28 25322213-25322293. Max. coverage (+): 0. Max coverage (-): 4.58

Region: chr28 25322294-25322375. Max. coverage (+): 0. Max coverage (-): 5.67

Region: chr28 25322376-25322457. Max. coverage (+): 0. Max coverage (-): 0

Region: chr28 25322458-25322539. Max. coverage (+): 0. Max coverage (-): 0

Region: chr28 25322540-25322620. Max. coverage (+): 0. Max coverage (-): 0

Region: chr28 25322621-25322702. Max. coverage (+): 0. Max coverage (-): 0

Region: chr28 25322703-25322784. Max. coverage (+): 0. Max coverage (-): 0

Region: chr28 25322785-25322866. Max. coverage (+): 0. Max coverage (-): 10.65

Region: chr28 25322867-25322947. Max. coverage (+): 0. Max coverage (-): 0

Region: chr28 25322948-25323029. Max. coverage (+): 0. Max coverage (-): 0

Region: chr28 25323030-25323111. Max. coverage (+): 0. Max coverage (-): 0.76

Region: chr28 25323112-25323193. Max. coverage (+): 0. Max coverage (-): 26.11

Region: chr28 25323194-25323274. Max. coverage (+): 0. Max coverage (-): 5.11

Region: chr28 25323275-25323356. Max. coverage (+): 0. Max coverage (-): 21.25

Region: chr28 25323357-25323438. Max. coverage (+): 0. Max coverage (-): 22.78

Region: chr28 25323439-25323520. Max. coverage (+): 0. Max coverage (-): 6.5

Region: chr28 25323521-25323601. Max. coverage (+): 0. Max coverage (-): 14.14

Region: chr28 25323602-25323683. Max. coverage (+): 0. Max coverage (-): 6.32

Region: chr28 25323684-25323765. Max. coverage (+): 0. Max coverage (-): 1.08

Region: chr28 25323766-25323847. Max. coverage (+): 0. Max coverage (-): 6.42

Region: chr28 25323848-25323928. Max. coverage (+): 0. Max coverage (-): 4.61

Region: chr28 25323929-25324010. Max. coverage (+): 0. Max coverage (-): 2.8

Region: chr28 25324011-25324092. Max. coverage (+): 0. Max coverage (-): 8.64

Region: chr28 25324093-25324173. Max. coverage (+): 0. Max coverage (-): 3

Region: chr28 25324174-25324255. Max. coverage (+): 0. Max coverage (-): 4.32

Region: chr28 25324256-25324337. Max. coverage (+): 0. Max coverage (-): 4.42

Region: chr28 25324338-25324419. Max. coverage (+): 0. Max coverage (-): 9

Region: chr28 25324420-25324500. Max. coverage (+): 0. Max coverage (-): 6.34

Region: chr28 25324501-25324582. Max. coverage (+): 0. Max coverage (-): 5.72

Region: chr28 25324583-25324664. Max. coverage (+): 0. Max coverage (-): 3.66

Region: chr28 25324665-25324746. Max. coverage (+): 0. Max coverage (-): 9.76

Region: chr28 25324747-25324827. Max. coverage (+): 0. Max coverage (-): 17.76

Region: chr28 25324828-25324909. Max. coverage (+): 0. Max coverage (-): 16.61

Region: chr28 25324910-25324991. Max. coverage (+): 0. Max coverage (-): 15

Region: chr28 25324992-25325073. Max. coverage (+): 0. Max coverage (-): 20.64

Region: chr28 25325074-25325154. Max. coverage (+): 0. Max coverage (-): 4.53

Region: chr28 25325155-25325236. Max. coverage (+): 0. Max coverage (-): 9.98

Region: chr28 25325237-25325318. Max. coverage (+): 0. Max coverage (-): 19.23

Region: chr28 25325319-25325400. Max. coverage (+): 0. Max coverage (-): 11.51

Region: chr28 25325401-25325481. Max. coverage (+): 0. Max coverage (-): 5.2

Region: chr28 25325482-25325563. Max. coverage (+): 0. Max coverage (-): 6.75

Region: chr28 25325564-25325645. Max. coverage (+): 0. Max coverage (-): 3.26

Region: chr28 25325646-25325727. Max. coverage (+): 0. Max coverage (-): 2.08

Region: chr28 25325728-25325808. Max. coverage (+): 0. Max coverage (-): 4.06

Region: chr28 25325809-25325890. Max. coverage (+): 0. Max coverage (-): 14.77

Region: chr28 25325891-25325972. Max. coverage (+): 0. Max coverage (-): 1.23

Region: chr28 25325973-25326054. Max. coverage (+): 0. Max coverage (-): 0

Region: chr28 25326055-25326135. Max. coverage (+): 0. Max coverage (-): 0

Region: chr28 25326136-25326217. Max. coverage (+): 0. Max coverage (-): 0

Region: chr28 25326218-25326299. Max. coverage (+): 0. Max coverage (-): 2.25

Region: chr28 25326300-25326381. Max. coverage (+): 0. Max coverage (-): 0

Region: chr28 25326382-25326462. Max. coverage (+): 0. Max coverage (-): 0.42

Region: chr28 25326463-25326544. Max. coverage (+): 0. Max coverage (-): 0.42

Region: chr28 25326545-25326626. Max. coverage (+): 0. Max coverage (-): 0

Region: chr28 25326627-25326707. Max. coverage (+): 0. Max coverage (-): 0

Region: chr28 25326708-25326789. Max. coverage (+): 0. Max coverage (-): 0

Region: chr28 25326790-25326871. Max. coverage (+): 0. Max coverage (-): 0

Region: chr28 25326872-25326953. Max. coverage (+): 0. Max coverage (-): 0

Region: chr28 25326954-25327034. Max. coverage (+): 0. Max coverage (-): 3.17

Region: chr28 25327035-25327116. Max. coverage (+): 0. Max coverage (-): 0

Region: chr28 25327117-25327198. Max. coverage (+): 0. Max coverage (-): 4.53

Region: chr28 25327199-25327280. Max. coverage (+): 0. Max coverage (-): 0

Region: chr28 25327281-25327361. Max. coverage (+): 0. Max coverage (-): 0

Region: chr28 25327362-25327443. Max. coverage (+): 0. Max coverage (-): 0

Region: chr28 25327444-25327525. Max. coverage (+): 0. Max coverage (-): 3.86

Region: chr28 25327526-25327607. Max. coverage (+): 0. Max coverage (-): 7.19

Region: chr28 25327608-25327688. Max. coverage (+): 0. Max coverage (-): 3.06

Region: chr28 25327689-25327770. Max. coverage (+): 0. Max coverage (-): 4.19

Region: chr28 25327771-25327852. Max. coverage (+): 0. Max coverage (-): 4.5

Region: chr28 25327853-25327934. Max. coverage (+): 0. Max coverage (-): 0

Region: chr28 25327935-25328015. Max. coverage (+): 0. Max coverage (-): 4.78

Region: chr28 25328016-25328097. Max. coverage (+): 0. Max coverage (-): 0

Region: chr28 25328098-25328179. Max. coverage (+): 0. Max coverage (-): 4.83

Region: chr28 25328180-25328261. Max. coverage (+): 0. Max coverage (-): 0.77

Region: chr28 25328262-25328342. Max. coverage (+): 0. Max coverage (-): 0

Region: chr28 25328343-25328424. Max. coverage (+): 0. Max coverage (-): 0

Region: chr28 25328425-25328506. Max. coverage (+): 0. Max coverage (-): 0

Region: chr28 25328507-25328588. Max. coverage (+): 0. Max coverage (-): 0

Region: chr28 25328589-25328669. Max. coverage (+): 0. Max coverage (-): 0

Region: chr28 25328670-25328751. Max. coverage (+): 0. Max coverage (-): 0

Region: chr28 25328752-25328833. Max. coverage (+): 0. Max coverage (-): 0

Region: chr28 25328834-25328915. Max. coverage (+): 0. Max coverage (-): 0

Region: chr28 25328916-25328996. Max. coverage (+): 0. Max coverage (-): 0

Region: chr28 25328997-25329078. Max. coverage (+): 0. Max coverage (-): 0

Region: chr28 25329079-25329160. Max. coverage (+): 0. Max coverage (-): 0

Region: chr28 25329161-25329241. Max. coverage (+): 0. Max coverage (-): 0

Region: chr28 25329242-25329323. Max. coverage (+): 0. Max coverage (-): 0.68

Region: chr28 25329324-25329405. Max. coverage (+): 0. Max coverage (-): 0

Region: chr28 25329406-25329487. Max. coverage (+): 0. Max coverage (-): 0

Region: chr28 25329488-25329568. Max. coverage (+): 0. Max coverage (-): 5.05

Region: chr28 25329569-25329650. Max. coverage (+): 0. Max coverage (-): 0

Region: chr28 25329651-25329732. Max. coverage (+): 0. Max coverage (-): 0

Region: chr28 25329733-25329814. Max. coverage (+): 0. Max coverage (-): 0

Region: chr28 25329815-25329895. Max. coverage (+): 0. Max coverage (-): 0.77

Region: chr28 25329896-25329977. Max. coverage (+): 0. Max coverage (-): 0

Region: chr28 25329978-25330059. Max. coverage (+): 0. Max coverage (-): 1.7

Region: chr28 25330060-25330141. Max. coverage (+): 0. Max coverage (-): 4.57

Region: chr28 25330142-25330222. Max. coverage (+): 0. Max coverage (-): 4.21

Region: chr28 25330223-25330304. Max. coverage (+): 0. Max coverage (-): 8.58

Region: chr28 25330305-25330386. Max. coverage (+): 0. Max coverage (-): 0

Region: chr28 25330387-25330468. Max. coverage (+): 0. Max coverage (-): 0

Region: chr28 25330469-25330549. Max. coverage (+): 0. Max coverage (-): 0

Region: chr28 25330550-25330631. Max. coverage (+): 0. Max coverage (-): 0

Region: chr28 25330632-25330713. Max. coverage (+): 0. Max coverage (-): 0

Region: chr28 25330714-25330795. Max. coverage (+): 0. Max coverage (-): 0

Region: chr28 25330796-25330876. Max. coverage (+): 3.99. Max coverage (-): 2.07

Region: chr28 25330877-25330958. Max. coverage (+): 0. Max coverage (-): 0

Region: chr28 25330959-25331040. Max. coverage (+): 0. Max coverage (-): 0

Region: chr28 25331041-25331122. Max. coverage (+): 0. Max coverage (-): 0

Region: chr28 25331123-25331203. Max. coverage (+): 0. Max coverage (-): 0

Region: chr28 25331204-25331285. Max. coverage (+): 0. Max coverage (-): 5.82

Region: chr28 25331286-25331367. Max. coverage (+): 0. Max coverage (-): 10.4

Region: chr28 25331368-25331449. Max. coverage (+): 0. Max coverage (-): 8.02

Region: chr28 25331450-25331530. Max. coverage (+): 0. Max coverage (-): 0

Region: chr28 25331531-25331612. Max. coverage (+): 0. Max coverage (-): 0

Region: chr28 25331613-25331694. Max. coverage (+): 0. Max coverage (-): 0

Region: chr28 25331695-25331775. Max. coverage (+): 0. Max coverage (-): 0

Region: chr28 25331776-25331857. Max. coverage (+): 0. Max coverage (-): 4.11

Region: chr28 25331858-25331939. Max. coverage (+): 0. Max coverage (-): 10.28

Region: chr28 25331940-25332021. Max. coverage (+): 0. Max coverage (-): 5.2

Region: chr28 25332022-25332102. Max. coverage (+): 0. Max coverage (-): 7.19

Region: chr28 25332103-25332184. Max. coverage (+): 0. Max coverage (-): 13.24

Region: chr28 25332185-25332266. Max. coverage (+): 0. Max coverage (-): 9.69

Region: chr28 25332267-25332348. Max. coverage (+): 0. Max coverage (-): 5.48

Region: chr28 25332349-25332429. Max. coverage (+): 0. Max coverage (-): 21.25

Region: chr28 25332430-25332511. Max. coverage (+): 0. Max coverage (-): 19.65

Region: chr28 25332512-25332593. Max. coverage (+): 0. Max coverage (-): 9.19

Region: chr28 25332594-25332675. Max. coverage (+): 0. Max coverage (-): 39.75

Region: chr28 25332676-25332756. Max. coverage (+): 0. Max coverage (-): 14.76

Region: chr28 25332757-25332838. Max. coverage (+): 0. Max coverage (-): 72.32

Region: chr28 25332839-25332920. Max. coverage (+): 0. Max coverage (-): 63.93

Region: chr28 25332921-25333002. Max. coverage (+): 0. Max coverage (-): 10.61

Region: chr28 25333003-25333083. Max. coverage (+): 0. Max coverage (-): 0

Region: chr28 25333084-25333165. Max. coverage (+): 0. Max coverage (-): 4.98

Region: chr28 25333166-25333247. Max. coverage (+): 0. Max coverage (-): 14.44

Region: chr28 25333248-25333329. Max. coverage (+): 0. Max coverage (-): 9.54

Region: chr28 25333330-25333410. Max. coverage (+): 0. Max coverage (-): 36.27

Region: chr28 25333411-25333492. Max. coverage (+): 0. Max coverage (-): 35.03

Region: chr28 25333493-25333574. Max. coverage (+): 0. Max coverage (-): 1.68

Region: chr28 25333575-25333656. Max. coverage (+): 0. Max coverage (-): 12.4

Region: chr28 25333657-25333737. Max. coverage (+): 0. Max coverage (-): 8.3

Region: chr28 25333738-25333819. Max. coverage (+): 0. Max coverage (-): 49.88

Region: chr28 25333820-25333901. Max. coverage (+): 0. Max coverage (-): 15.42

Region: chr28 25333902-25333983. Max. coverage (+): 0. Max coverage (-): 0

Region: chr28 25333984-25334064. Max. coverage (+): 0. Max coverage (-): 0

Region: chr28 25334065-25334146. Max. coverage (+): 0. Max coverage (-): 0

Region: chr28 25334147-25334228. Max. coverage (+): 0. Max coverage (-): 0

Region: chr28 25334229-25334309. Max. coverage (+): 0. Max coverage (-): 0

Region: chr28 25334310-25334391. Max. coverage (+): 0. Max coverage (-): 2.72

Region: chr28 25334392-25334473. Max. coverage (+): 0. Max coverage (-): 1.11

Region: chr28 25334474-25334555. Max. coverage (+): 0. Max coverage (-): 0

Region: chr28 25334556-25334636. Max. coverage (+): 0. Max coverage (-): 0

Region: chr28 25334637-25334718. Max. coverage (+): 0. Max coverage (-): 0

Region: chr28 25334719-25334800. Max. coverage (+): 0. Max coverage (-): 2.51

Region: chr28 25334801-25334882. Max. coverage (+): 0. Max coverage (-): 2.51

Region: chr28 25334883-25334963. Max. coverage (+): 0. Max coverage (-): 0

Region: chr28 25334964-25335045. Max. coverage (+): 0. Max coverage (-): 0

Region: chr28 25335046-25335127. Max. coverage (+): 0. Max coverage (-): 0

Region: chr28 25335128-25335209. Max. coverage (+): 0. Max coverage (-): 0

Region: chr28 25335210-25335290. Max. coverage (+): 0. Max coverage (-): 0

Region: chr28 25335291-25335372. Max. coverage (+): 0. Max coverage (-): 0

Region: chr28 25335373-25335454. Max. coverage (+): 0. Max coverage (-): 0

Region: chr28 25335455-25335536. Max. coverage (+): 0. Max coverage (-): 0

Region: chr28 25335537-. Max. coverage (+): 1.41. Max coverage (-): 0

RepeatMasker Color Code

**+**

100-98% Identity

<98-95% Identity

<95-90% Identity

<90-85% Identity

<85-80% Identity

<80-75% Identity

<75-70% Identity

<70% Identity

**-**

Gene Set Color Code

**+**

Gene

Pseudogene

**-**

Topology/Coverage Color Code

Coverage Plus Strand

Coverage Minus Strand

Mainstrand: Plus

Mainstrand: Minus

Complementary Strand

Flanking Region  
(if option -flank >0)

Gene Set Annotation  

**1. STOX1 (protein coding, ENSBTAG00000019028) Tr:00000025324 Ex:2**: 25320940-25321092 (+)  
**2. STOX1 (protein coding, ENSBTAG00000019028) Tr:00000025324 Ex:3**: 25323161-25325504 (+)  
**3. STOX1 (protein coding, ENSBTAG00000019028) Tr:00000025324 Ex:4**: 25328127-25328274 (+)  
**4. DDX50 (protein coding, ENSBTAG00000033413) Tr:00000022002 Ex:1**: 25335074-25335160 (+)

  
RepeatMasker Annotation  

**1. BOV-A2**: 25295224-25295494 (+), Divergence to consensus: 7%  
**2. MER113**: 25296413-25296575 (+), Divergence to consensus: 44.2%  
**3. ART2A**: 25296671-25297210 (-), Divergence to consensus: 13.6%  
**4. BovB**: 25297211-25298051 (-), Divergence to consensus: 5.2%  
**5. BTLTR1**: 25298052-25298121 (+), Divergence to consensus: 10%  
**6. BovB**: 25298123-25299669 (-), Divergence to consensus: 6.7%  
**7. Bov-tA1**: 25299670-25299713 (-), Divergence to consensus: 16.3%  
**8. MER21C**: 25299997-25300216 (-), Divergence to consensus: 36.8%  
**9. Bov-tA1**: 25300217-25300431 (+), Divergence to consensus: 19.9%  
**10. MER21C**: 25300432-25300837 (-), Divergence to consensus: 36.8%  
**11. MER21C\_BT**: 25300770-25300846 (-), Divergence to consensus: 17.1%  
**12. CHRL1\_BT**: 25301061-25301192 (-), Divergence to consensus: 23%  
**13. CHRL1\_BT**: 25301215-25301348 (-), Divergence to consensus: 27.6%  
**14. LTR68**: 25301350-25301431 (+), Divergence to consensus: 24.9%  
**15. L1M4b**: 25301432-25301529 (-), Divergence to consensus: 23.7%  
**16. L1M4b**: 25301536-25301676 (-), Divergence to consensus: 26.5%  
**17. L1M4b**: 25301681-25301821 (-), Divergence to consensus: 30.3%  
**18. L1M4b**: 25301837-25302392 (-), Divergence to consensus: 35.6%  
**19. LTR68**: 25302426-25302720 (+), Divergence to consensus: 42.9%  
**20. HERVL40-int**: 25302913-25303126 (-), Divergence to consensus: 40%  
**21. Bov-tA2**: 25303144-25303345 (+), Divergence to consensus: 15.4%  
**22. Bov-tA1**: 25303367-25303552 (+), Divergence to consensus: 12.4%  
**23. MER68-int**: 25303997-25304875 (-), Divergence to consensus: 46.7%  
**24. MER41\_BT**: 25304876-25305442 (-), Divergence to consensus: 29.4%  
**25. MER68-int**: 25305443-25306389 (-), Divergence to consensus: 53.8%  
**26. MER68-int**: 25306482-25306757 (-), Divergence to consensus: 36.6%  
**27. LTR68**: 25307367-25307842 (+), Divergence to consensus: 39.9%  
**28. MLT1L**: 25310066-25310316 (-), Divergence to consensus: 39.1%  
**29. Bov-tA3**: 25310650-25310890 (+), Divergence to consensus: 12.9%  
**30. MIRc**: 25311147-25311247 (-), Divergence to consensus: 38.7%  
**31. L2c**: 25311365-25311535 (+), Divergence to consensus: 31.6%  
**32. MER103C**: 25311854-25312050 (+), Divergence to consensus: 50.4%  
**33. MIR3**: 25312051-25312170 (-), Divergence to consensus: 39.6%  
**34. MLT1E2**: 25312532-25313033 (+), Divergence to consensus: 27%  
**35. AT\_rich**: 25315574-25315597 (+), Divergence to consensus: 54.2%  
**36. MER5A1**: 25317868-25318034 (+), Divergence to consensus: 32.1%  
**37. MER20**: 25318641-25318825 (+), Divergence to consensus: 34.5%  
**38. MER81**: 25318828-25318907 (+), Divergence to consensus: 23.7%  
**39. SINE2-1\_BT**: 25319049-25319151 (-), Divergence to consensus: 50.2%  
**40. AT\_rich**: 25319169-25319189 (+), Divergence to consensus: 23.8%  
**41. SINE2-3\_BT**: 25319199-25319253 (-), Divergence to consensus: 26.7%  
**42. BovB**: 25319336-25319976 (+), Divergence to consensus: 12.4%  
**43. ART2A**: 25319978-25320509 (+), Divergence to consensus: 20.8%  
**44. CHR-2B**: 25321209-25321508 (-), Divergence to consensus: 25.2%  
**45. SINE2-2\_BT**: 25321706-25321824 (-), Divergence to consensus: 29.6%  
**46. Bov-tA1**: 25321962-25322185 (+), Divergence to consensus: 20.5%  
**47. Bov-tA1**: 25322398-25322620 (+), Divergence to consensus: 20.5%  
**48. SINE2-2\_BT**: 25325962-25326065 (-), Divergence to consensus: 40.7%  
**49. L1ME4a**: 25326079-25326284 (-), Divergence to consensus: 45.9%  
**50. (TA)n**: 25326295-25326319 (+), Divergence to consensus: 8%  
**51. L1ME4a**: 25326575-25326959 (-), Divergence to consensus: 40.4%  
**52. SINE2-2\_BT**: 25327187-25327276 (-), Divergence to consensus: 24.4%  
**53. L2c**: 25327379-25327569 (-), Divergence to consensus: 42.8%  
**54. L4\_C\_Mam**: 25329104-25329264 (-), Divergence to consensus: 41.3%  
**55. L3**: 25329607-25329825 (+), Divergence to consensus: 47.5%  
**56. MIR**: 25330358-25330557 (+), Divergence to consensus: 43.8%  
**57. MamTip2**: 25330654-25330788 (+), Divergence to consensus: 46.1%  
**58. Charlie19a**: 25330874-25331227 (+), Divergence to consensus: 44.1%  
**59. Bov-tA2**: 25331449-25331623 (-), Divergence to consensus: 11.1%  
**60. Bov-tA2**: 25331623-25331706 (-), Divergence to consensus: 15.5%  
**61. BovB**: 25333466-25333505 (-), Divergence to consensus: 15%  
**62. MIR3**: 25333963-25334074 (+), Divergence to consensus: 41.8%  
**63. Bov-tA2**: 25334075-25334283 (-), Divergence to consensus: 12.6%  
**64. MIR3**: 25334284-25334327 (+), Divergence to consensus: 41.8%

  
Transcription Factor Binding Sites  

**RFX4\_1** (Sequence: CTTAGCAAC (+): 25316186)  
**RFX4\_2** (Sequence: CATAGATAC (+): 25328683)  
**Gata4** (Sequence: AGATAAC (-): 25313619)  
**Gata4** (Sequence: AGATAAC (-): 25323735)  
**SOX9** (Sequence: AACAATGA (-): 25332997)  
**SOX9** (Sequence: TTATTGTT (+): 25315196)  
**SOX9** (Sequence: TCATTGTT (+): 25315557)  
**A-MYB** (Sequence: TGACAGTTGG (+): 25329449)  
**SPZ1** (Sequence: GGGGTTTCAG (+): 25333422)  
**Gata4** (Sequence: CTTATCT (+): 25309701)  
**Gata4** (Sequence: CTTATCT (+): 25320583)  
**Gata4** (Sequence: GTTATCT (+): 25325570)  
**Gata4** (Sequence: GTTATCT (+): 25329548)  
**Gata4** (Sequence: GTTATCT (+): 25332689)
