# Supplementary material for: Small RNA sequencing of cryopreserved semen from single bull revealed altered miRNAs and piRNAs expression between High- and Low-motile sperm populations
Source: BMC Genomics. 2017 Jan 4;18:14. doi: 10.1186/s12864-016-3394-7 (PMC5209821; doi:10.1186/s12864-016-3394-7)
Supplement: Additional file 3: — Details for each piRNA clusters found in High Motile (HM) sperm fraction. Genes, repeats, transposable elements and transcription factors binding sites falling within the cluster regions were reported. (ZIP 1896 kb) [file 12864_2016_3394_MOESM3_ESM.zip › 71.html]

piRNA cluster 71


Predicted piRNA cluster no. 71     previous   next
  

Show proTRAC run info
Hide proTRAC run info

================================= proTRAC ====================================  
VERSION: 2.1                                    LAST MODIFIED: 06. October 2015  
  
Please cite:  
Rosenkranz D, Zischler H. proTRAC - a software for probabilistic piRNA cluster  
detection, visualization and analysis. 2012. BMC Bioinformatics 13:5.  
  
and (for proTRAC 2.0 and later):  
Rosenkranz D, Rudloff S, Bastuck K, Ketting RF, Zischler H. Tupaia small RNAs  
provide insights into function and evolution of RNAi-based transposon defense  
in mammals. 2015. RNA 21(5):911-922.  
  
Contact:  
David Rosenkranz  
Institute of Anthropology, small RNA group  
Johannes Gutenberg University Mainz  
email: rosenkranz@uni-mainz.de  
  
You can find the latest proTRAC version at:  
http://sourceforge.net/projects/protrac/files  
http://www.smallRNAgroup-mainz.de/software  
==============================================================================  
  
PARAMETERS:  
Map file: .............../storage/core/barbara/genhome/smallRNA/fertility/Sample\_motile/pirna/Sample\_motile\_26-33\_collapsed.fa.no-dust.map.weighted-10000-1000-b-0  
Genome file: ............/storage/core/barbara/genhome/smallRNA/fertility/Sample\_all/pirna/bt\_311\_chrY.fa  
RepeatMasker annotation: /storage/genomes/bt\_umd31/GCF\_000003055.6\_Bos\_taurus\_UMD\_3.1.1\_repeatMasker\_chr.out  
GeneSet:................./storage/core/barbara/genhome/smallRNA/fertility/Sample\_all/pirna/full.gtf  
  
Significant (p<=0.01) hit density will be calculated based  
on observed hit distribution.  
  
Sliding window size: ........................................ 5000 bp  
Sliding window increament: .................................. 1000 bp  
Normalize each hit by number of genomic hits: ............... 1 [0=no/1=yes]  
Normalize each hit by number of sequence reads: ............. 1 [0=no/1=yes]  
Normalize values (-> per million mapped reads): ............. 1 [0=no/1=yes]  
Min. fraction of hits with 1T(U) or 10A: .................... 0.75  
Alternatively: Min. fraction of hits with 1T(U) and 10A: .... 0.5  
Min. fraction of hits with typical piRNA length: ............ 0.75  
Typical piRNA length: ....................................... 26-33 nt  
Min. size of a piRNA cluster: ............................... 5000 bp.  
Min. number of hits (absolute): ............................. 0  
Min. number of hits (normalized): ........................... 0  
Min. fraction of hits on the mainstrand: .................... 0.75  
Top fraction of mapped sequences (in terms of read counts): . 1%  
Top fraction accounts for max. n% of sequence reads: ........ 90%  
Min. fraction of hits on each arm of a bidirectional cluster: 0.1  
Output image file for each cluster: ......................... 0 [0=no/1=yes]  
Output html file for each cluster: .......................... 1 [0=no/1=yes]  
Output a summary table: ..................................... 1 [0=no/1=yes]  
Output a FASTA file for each cluster (piRNA sequences): ..... 1 [0=no/1=yes]  
Output a FASTA file comprising cluster sequences: ........... 1 [0=no/1=yes]  
Search DNA motifs in clusters: .............................. 1 [0=no/1=yes]  
Output flanking sequences: +/- .............................. 0 bp  
Output ~.pTi file: .......................................... 1 [0=no/1=yes]  
==============================================================================  
  
  
Genome size (without gaps): ............ 2678902517 bp  
Gaps (N/X/-): .......................... 53837044 bp  
Mapped reads: .......................... 658825247023  
Non-identical sequences: ............... 514171  
Genomic hits: .......................... 764233  
Significant densitiy of mapped reads: .. 12867599.5173724 reads/kb

Show proTRAC cluster info
Hide proTRAC cluster info

|  |  |
| --- | --- |
| Location | chr28 |
| Coordinates | 26785217-26791016 |
| Size [bp] | 5800 |
| Sequence hit loci | 80 |
| Mapped reads (normalized) | 106179924 |
| Mapped reads (normalized) per kb | 18306883.4 |
| Normalized reads with 1T (1U) | 88.6% |
| Normalized reads with 10A | 34.4% |
| Normalized reads with length 26-33 nt | 100% |
| Normalized reads on the main strand(s) | 100% |
| Predicted directionality | mono:plus |

100%

0%

1T (1U)  
reads

10A reads

26-33 nt  
reads

reads on mainstrand

**Either the amount of reads with 1T (1U) OR 10A has to exceed 75% (set with option: -1Tor10A)  
Alternatively the amount of reads with 1T (1U) AND 10A has to exceed 50% (set with option: -1Tand10A)  
Minimum amount of reads with preferred size is 75% (set with option: -pisize)  
Minimum amount of reads on the main strand(s) is 75% (set with option: -clstrand)**

Show read coverage
Hide read coverage

WHAT DO I SEE HERE?  
This chart shows the location of mapped sequence reads within a predicted piRNA cluster. The color refers to the number of genomic hits produced by the sequence read in question. A dark red bar indicates that this sequence read produces many other hits elsewhere in the genome. Many adjacent red or yellow bars can indicate the presence of a multi-copy element such as transposons or rRNA genes. A dark green bar indicates that this sequence read maps uniquely to this locus.

1 hit

2-5 hits

6-10 hits

11-20 hits

21-50 hits

51-100 hits

> 100 hits

chr28

26785217

26791016

Gene Set

RepeatMasker

Mapped  
Reads

11.13

plus strand

minus strand

11.13

Region: chr28 25335538-26785222. Max. coverage (+): 1.43. Max coverage (-): 0

Region: chr28 26785223-26785234. Max. coverage (+): 1.43. Max coverage (-): 0

Region: chr28 26785235-26785245. Max. coverage (+): 0. Max coverage (-): 0

Region: chr28 26785246-26785257. Max. coverage (+): 0. Max coverage (-): 0

Region: chr28 26785258-26785269. Max. coverage (+): 0. Max coverage (-): 0

Region: chr28 26785270-26785280. Max. coverage (+): 0. Max coverage (-): 0

Region: chr28 26785281-26785292. Max. coverage (+): 0. Max coverage (-): 0

Region: chr28 26785293-26785303. Max. coverage (+): 0. Max coverage (-): 0

Region: chr28 26785304-26785315. Max. coverage (+): 0. Max coverage (-): 0

Region: chr28 26785316-26785327. Max. coverage (+): 0. Max coverage (-): 0

Region: chr28 26785328-26785338. Max. coverage (+): 0. Max coverage (-): 0

Region: chr28 26785339-26785350. Max. coverage (+): 0. Max coverage (-): 0

Region: chr28 26785351-26785361. Max. coverage (+): 0. Max coverage (-): 0

Region: chr28 26785362-26785373. Max. coverage (+): 0. Max coverage (-): 0

Region: chr28 26785374-26785385. Max. coverage (+): 0. Max coverage (-): 0

Region: chr28 26785386-26785396. Max. coverage (+): 0. Max coverage (-): 0

Region: chr28 26785397-26785408. Max. coverage (+): 0. Max coverage (-): 0

Region: chr28 26785409-26785419. Max. coverage (+): 0. Max coverage (-): 0

Region: chr28 26785420-26785431. Max. coverage (+): 0. Max coverage (-): 0

Region: chr28 26785432-26785443. Max. coverage (+): 0. Max coverage (-): 0

Region: chr28 26785444-26785454. Max. coverage (+): 0. Max coverage (-): 0

Region: chr28 26785455-26785466. Max. coverage (+): 0. Max coverage (-): 0

Region: chr28 26785467-26785477. Max. coverage (+): 0. Max coverage (-): 0

Region: chr28 26785478-26785489. Max. coverage (+): 0. Max coverage (-): 0

Region: chr28 26785490-26785501. Max. coverage (+): 0. Max coverage (-): 0

Region: chr28 26785502-26785512. Max. coverage (+): 0. Max coverage (-): 0

Region: chr28 26785513-26785524. Max. coverage (+): 0. Max coverage (-): 0

Region: chr28 26785525-26785535. Max. coverage (+): 0. Max coverage (-): 0

Region: chr28 26785536-26785547. Max. coverage (+): 0. Max coverage (-): 0

Region: chr28 26785548-26785559. Max. coverage (+): 0. Max coverage (-): 0

Region: chr28 26785560-26785570. Max. coverage (+): 0. Max coverage (-): 0

Region: chr28 26785571-26785582. Max. coverage (+): 0. Max coverage (-): 0

Region: chr28 26785583-26785593. Max. coverage (+): 0. Max coverage (-): 0

Region: chr28 26785594-26785605. Max. coverage (+): 0. Max coverage (-): 0

Region: chr28 26785606-26785617. Max. coverage (+): 0. Max coverage (-): 0

Region: chr28 26785618-26785628. Max. coverage (+): 0. Max coverage (-): 0

Region: chr28 26785629-26785640. Max. coverage (+): 0. Max coverage (-): 0

Region: chr28 26785641-26785651. Max. coverage (+): 0. Max coverage (-): 0

Region: chr28 26785652-26785663. Max. coverage (+): 0. Max coverage (-): 0

Region: chr28 26785664-26785675. Max. coverage (+): 0. Max coverage (-): 0

Region: chr28 26785676-26785686. Max. coverage (+): 0. Max coverage (-): 0

Region: chr28 26785687-26785698. Max. coverage (+): 0. Max coverage (-): 0

Region: chr28 26785699-26785709. Max. coverage (+): 0. Max coverage (-): 0

Region: chr28 26785710-26785721. Max. coverage (+): 0. Max coverage (-): 0

Region: chr28 26785722-26785733. Max. coverage (+): 0. Max coverage (-): 0

Region: chr28 26785734-26785744. Max. coverage (+): 0. Max coverage (-): 0

Region: chr28 26785745-26785756. Max. coverage (+): 0. Max coverage (-): 0

Region: chr28 26785757-26785767. Max. coverage (+): 0. Max coverage (-): 0

Region: chr28 26785768-26785779. Max. coverage (+): 0.92. Max coverage (-): 0

Region: chr28 26785780-26785791. Max. coverage (+): 0. Max coverage (-): 0

Region: chr28 26785792-26785802. Max. coverage (+): 0. Max coverage (-): 0

Region: chr28 26785803-26785814. Max. coverage (+): 0. Max coverage (-): 0

Region: chr28 26785815-26785825. Max. coverage (+): 0. Max coverage (-): 0

Region: chr28 26785826-26785837. Max. coverage (+): 0. Max coverage (-): 0

Region: chr28 26785838-26785849. Max. coverage (+): 4.87. Max coverage (-): 0

Region: chr28 26785850-26785860. Max. coverage (+): 4.87. Max coverage (-): 0

Region: chr28 26785861-26785872. Max. coverage (+): 0. Max coverage (-): 0

Region: chr28 26785873-26785883. Max. coverage (+): 0. Max coverage (-): 0

Region: chr28 26785884-26785895. Max. coverage (+): 0. Max coverage (-): 0

Region: chr28 26785896-26785907. Max. coverage (+): 0. Max coverage (-): 0

Region: chr28 26785908-26785918. Max. coverage (+): 0. Max coverage (-): 0

Region: chr28 26785919-26785930. Max. coverage (+): 0. Max coverage (-): 0

Region: chr28 26785931-26785941. Max. coverage (+): 0. Max coverage (-): 0

Region: chr28 26785942-26785953. Max. coverage (+): 0. Max coverage (-): 0

Region: chr28 26785954-26785965. Max. coverage (+): 0. Max coverage (-): 0

Region: chr28 26785966-26785976. Max. coverage (+): 0. Max coverage (-): 0

Region: chr28 26785977-26785988. Max. coverage (+): 0. Max coverage (-): 0

Region: chr28 26785989-26785999. Max. coverage (+): 0. Max coverage (-): 0

Region: chr28 26786000-26786011. Max. coverage (+): 0. Max coverage (-): 0

Region: chr28 26786012-26786023. Max. coverage (+): 0. Max coverage (-): 0

Region: chr28 26786024-26786034. Max. coverage (+): 0. Max coverage (-): 0

Region: chr28 26786035-26786046. Max. coverage (+): 0. Max coverage (-): 0

Region: chr28 26786047-26786057. Max. coverage (+): 0. Max coverage (-): 0

Region: chr28 26786058-26786069. Max. coverage (+): 0. Max coverage (-): 0

Region: chr28 26786070-26786081. Max. coverage (+): 0. Max coverage (-): 0

Region: chr28 26786082-26786092. Max. coverage (+): 0. Max coverage (-): 0

Region: chr28 26786093-26786104. Max. coverage (+): 0.38. Max coverage (-): 0

Region: chr28 26786105-26786115. Max. coverage (+): 0. Max coverage (-): 0

Region: chr28 26786116-26786127. Max. coverage (+): 1.99. Max coverage (-): 0

Region: chr28 26786128-26786139. Max. coverage (+): 3.81. Max coverage (-): 0

Region: chr28 26786140-26786150. Max. coverage (+): 3.81. Max coverage (-): 0

Region: chr28 26786151-26786162. Max. coverage (+): 0. Max coverage (-): 0

Region: chr28 26786163-26786173. Max. coverage (+): 0. Max coverage (-): 0

Region: chr28 26786174-26786185. Max. coverage (+): 0. Max coverage (-): 0

Region: chr28 26786186-26786197. Max. coverage (+): 0. Max coverage (-): 0

Region: chr28 26786198-26786208. Max. coverage (+): 0. Max coverage (-): 0

Region: chr28 26786209-26786220. Max. coverage (+): 0. Max coverage (-): 0

Region: chr28 26786221-26786231. Max. coverage (+): 0. Max coverage (-): 0

Region: chr28 26786232-26786243. Max. coverage (+): 0. Max coverage (-): 0

Region: chr28 26786244-26786255. Max. coverage (+): 0. Max coverage (-): 0

Region: chr28 26786256-26786266. Max. coverage (+): 0. Max coverage (-): 0

Region: chr28 26786267-26786278. Max. coverage (+): 0. Max coverage (-): 0

Region: chr28 26786279-26786289. Max. coverage (+): 0. Max coverage (-): 0

Region: chr28 26786290-26786301. Max. coverage (+): 1.9. Max coverage (-): 0

Region: chr28 26786302-26786313. Max. coverage (+): 0. Max coverage (-): 0

Region: chr28 26786314-26786324. Max. coverage (+): 0. Max coverage (-): 0

Region: chr28 26786325-26786336. Max. coverage (+): 0. Max coverage (-): 0

Region: chr28 26786337-26786347. Max. coverage (+): 0. Max coverage (-): 0

Region: chr28 26786348-26786359. Max. coverage (+): 6.06. Max coverage (-): 0

Region: chr28 26786360-26786371. Max. coverage (+): 1.84. Max coverage (-): 0

Region: chr28 26786372-26786382. Max. coverage (+): 0. Max coverage (-): 0

Region: chr28 26786383-26786394. Max. coverage (+): 0. Max coverage (-): 0

Region: chr28 26786395-26786405. Max. coverage (+): 0. Max coverage (-): 0

Region: chr28 26786406-26786417. Max. coverage (+): 0. Max coverage (-): 0

Region: chr28 26786418-26786429. Max. coverage (+): 0. Max coverage (-): 0

Region: chr28 26786430-26786440. Max. coverage (+): 0. Max coverage (-): 0

Region: chr28 26786441-26786452. Max. coverage (+): 0. Max coverage (-): 0

Region: chr28 26786453-26786463. Max. coverage (+): 0. Max coverage (-): 0

Region: chr28 26786464-26786475. Max. coverage (+): 0. Max coverage (-): 0

Region: chr28 26786476-26786487. Max. coverage (+): 0. Max coverage (-): 0

Region: chr28 26786488-26786498. Max. coverage (+): 0. Max coverage (-): 0

Region: chr28 26786499-26786510. Max. coverage (+): 0. Max coverage (-): 0

Region: chr28 26786511-26786521. Max. coverage (+): 0. Max coverage (-): 0

Region: chr28 26786522-26786533. Max. coverage (+): 0. Max coverage (-): 0

Region: chr28 26786534-26786545. Max. coverage (+): 0. Max coverage (-): 0

Region: chr28 26786546-26786556. Max. coverage (+): 0. Max coverage (-): 0

Region: chr28 26786557-26786568. Max. coverage (+): 0. Max coverage (-): 0

Region: chr28 26786569-26786579. Max. coverage (+): 0. Max coverage (-): 0

Region: chr28 26786580-26786591. Max. coverage (+): 0. Max coverage (-): 0

Region: chr28 26786592-26786603. Max. coverage (+): 0. Max coverage (-): 0

Region: chr28 26786604-26786614. Max. coverage (+): 0. Max coverage (-): 0

Region: chr28 26786615-26786626. Max. coverage (+): 0. Max coverage (-): 0

Region: chr28 26786627-26786637. Max. coverage (+): 0. Max coverage (-): 0

Region: chr28 26786638-26786649. Max. coverage (+): 0. Max coverage (-): 0

Region: chr28 26786650-26786661. Max. coverage (+): 0. Max coverage (-): 0

Region: chr28 26786662-26786672. Max. coverage (+): 0. Max coverage (-): 0

Region: chr28 26786673-26786684. Max. coverage (+): 0. Max coverage (-): 0

Region: chr28 26786685-26786695. Max. coverage (+): 0. Max coverage (-): 0

Region: chr28 26786696-26786707. Max. coverage (+): 0. Max coverage (-): 0

Region: chr28 26786708-26786719. Max. coverage (+): 0. Max coverage (-): 0

Region: chr28 26786720-26786730. Max. coverage (+): 8.9. Max coverage (-): 0

Region: chr28 26786731-26786742. Max. coverage (+): 0. Max coverage (-): 0

Region: chr28 26786743-26786753. Max. coverage (+): 0. Max coverage (-): 0

Region: chr28 26786754-26786765. Max. coverage (+): 0. Max coverage (-): 0

Region: chr28 26786766-26786777. Max. coverage (+): 2.27. Max coverage (-): 0

Region: chr28 26786778-26786788. Max. coverage (+): 2.27. Max coverage (-): 0

Region: chr28 26786789-26786800. Max. coverage (+): 0. Max coverage (-): 0

Region: chr28 26786801-26786811. Max. coverage (+): 0. Max coverage (-): 0

Region: chr28 26786812-26786823. Max. coverage (+): 4.84. Max coverage (-): 0

Region: chr28 26786824-26786835. Max. coverage (+): 1.19. Max coverage (-): 0

Region: chr28 26786836-26786846. Max. coverage (+): 0. Max coverage (-): 0

Region: chr28 26786847-26786858. Max. coverage (+): 0. Max coverage (-): 0

Region: chr28 26786859-26786869. Max. coverage (+): 0. Max coverage (-): 0

Region: chr28 26786870-26786881. Max. coverage (+): 0. Max coverage (-): 0

Region: chr28 26786882-26786893. Max. coverage (+): 1.55. Max coverage (-): 0

Region: chr28 26786894-26786904. Max. coverage (+): 0. Max coverage (-): 0

Region: chr28 26786905-26786916. Max. coverage (+): 0. Max coverage (-): 0

Region: chr28 26786917-26786927. Max. coverage (+): 4.55. Max coverage (-): 0

Region: chr28 26786928-26786939. Max. coverage (+): 5.87. Max coverage (-): 0

Region: chr28 26786940-26786951. Max. coverage (+): 0. Max coverage (-): 0

Region: chr28 26786952-26786962. Max. coverage (+): 0. Max coverage (-): 0

Region: chr28 26786963-26786974. Max. coverage (+): 4.86. Max coverage (-): 0

Region: chr28 26786975-26786985. Max. coverage (+): 4.86. Max coverage (-): 0

Region: chr28 26786986-26786997. Max. coverage (+): 6.65. Max coverage (-): 0

Region: chr28 26786998-26787009. Max. coverage (+): 6.65. Max coverage (-): 0

Region: chr28 26787010-26787020. Max. coverage (+): 0. Max coverage (-): 0

Region: chr28 26787021-26787032. Max. coverage (+): 0. Max coverage (-): 0

Region: chr28 26787033-26787043. Max. coverage (+): 0. Max coverage (-): 0

Region: chr28 26787044-26787055. Max. coverage (+): 0. Max coverage (-): 0

Region: chr28 26787056-26787067. Max. coverage (+): 0. Max coverage (-): 0

Region: chr28 26787068-26787078. Max. coverage (+): 0. Max coverage (-): 0

Region: chr28 26787079-26787090. Max. coverage (+): 0.68. Max coverage (-): 0

Region: chr28 26787091-26787101. Max. coverage (+): 0. Max coverage (-): 0

Region: chr28 26787102-26787113. Max. coverage (+): 0. Max coverage (-): 0

Region: chr28 26787114-26787125. Max. coverage (+): 0. Max coverage (-): 0

Region: chr28 26787126-26787136. Max. coverage (+): 0. Max coverage (-): 0

Region: chr28 26787137-26787148. Max. coverage (+): 0. Max coverage (-): 0

Region: chr28 26787149-26787159. Max. coverage (+): 0. Max coverage (-): 0

Region: chr28 26787160-26787171. Max. coverage (+): 0. Max coverage (-): 0

Region: chr28 26787172-26787183. Max. coverage (+): 0. Max coverage (-): 0

Region: chr28 26787184-26787194. Max. coverage (+): 0. Max coverage (-): 0

Region: chr28 26787195-26787206. Max. coverage (+): 0. Max coverage (-): 0

Region: chr28 26787207-26787217. Max. coverage (+): 0. Max coverage (-): 0

Region: chr28 26787218-26787229. Max. coverage (+): 0. Max coverage (-): 0

Region: chr28 26787230-26787241. Max. coverage (+): 2.27. Max coverage (-): 0

Region: chr28 26787242-26787252. Max. coverage (+): 0. Max coverage (-): 0

Region: chr28 26787253-26787264. Max. coverage (+): 0. Max coverage (-): 0

Region: chr28 26787265-26787275. Max. coverage (+): 0. Max coverage (-): 0

Region: chr28 26787276-26787287. Max. coverage (+): 0. Max coverage (-): 0

Region: chr28 26787288-26787299. Max. coverage (+): 0. Max coverage (-): 0

Region: chr28 26787300-26787310. Max. coverage (+): 0. Max coverage (-): 0

Region: chr28 26787311-26787322. Max. coverage (+): 0. Max coverage (-): 0

Region: chr28 26787323-26787333. Max. coverage (+): 0. Max coverage (-): 0

Region: chr28 26787334-26787345. Max. coverage (+): 0. Max coverage (-): 0

Region: chr28 26787346-26787357. Max. coverage (+): 0. Max coverage (-): 0

Region: chr28 26787358-26787368. Max. coverage (+): 0. Max coverage (-): 0

Region: chr28 26787369-26787380. Max. coverage (+): 0. Max coverage (-): 0

Region: chr28 26787381-26787391. Max. coverage (+): 0. Max coverage (-): 0

Region: chr28 26787392-26787403. Max. coverage (+): 0. Max coverage (-): 0

Region: chr28 26787404-26787415. Max. coverage (+): 0. Max coverage (-): 0

Region: chr28 26787416-26787426. Max. coverage (+): 0. Max coverage (-): 0

Region: chr28 26787427-26787438. Max. coverage (+): 0. Max coverage (-): 0

Region: chr28 26787439-26787449. Max. coverage (+): 0. Max coverage (-): 0

Region: chr28 26787450-26787461. Max. coverage (+): 0. Max coverage (-): 0

Region: chr28 26787462-26787473. Max. coverage (+): 1.04. Max coverage (-): 0

Region: chr28 26787474-26787484. Max. coverage (+): 0. Max coverage (-): 0

Region: chr28 26787485-26787496. Max. coverage (+): 0. Max coverage (-): 0

Region: chr28 26787497-26787507. Max. coverage (+): 0. Max coverage (-): 0

Region: chr28 26787508-26787519. Max. coverage (+): 0. Max coverage (-): 0

Region: chr28 26787520-26787531. Max. coverage (+): 0. Max coverage (-): 0

Region: chr28 26787532-26787542. Max. coverage (+): 0. Max coverage (-): 0

Region: chr28 26787543-26787554. Max. coverage (+): 0. Max coverage (-): 0

Region: chr28 26787555-26787565. Max. coverage (+): 0. Max coverage (-): 0

Region: chr28 26787566-26787577. Max. coverage (+): 0. Max coverage (-): 0

Region: chr28 26787578-26787589. Max. coverage (+): 0. Max coverage (-): 0

Region: chr28 26787590-26787600. Max. coverage (+): 0. Max coverage (-): 0

Region: chr28 26787601-26787612. Max. coverage (+): 0. Max coverage (-): 0

Region: chr28 26787613-26787623. Max. coverage (+): 0. Max coverage (-): 0

Region: chr28 26787624-26787635. Max. coverage (+): 0. Max coverage (-): 0

Region: chr28 26787636-26787647. Max. coverage (+): 0. Max coverage (-): 0

Region: chr28 26787648-26787658. Max. coverage (+): 0. Max coverage (-): 0

Region: chr28 26787659-26787670. Max. coverage (+): 0. Max coverage (-): 0

Region: chr28 26787671-26787681. Max. coverage (+): 0. Max coverage (-): 0

Region: chr28 26787682-26787693. Max. coverage (+): 0. Max coverage (-): 0

Region: chr28 26787694-26787705. Max. coverage (+): 0. Max coverage (-): 0

Region: chr28 26787706-26787716. Max. coverage (+): 0. Max coverage (-): 0

Region: chr28 26787717-26787728. Max. coverage (+): 0. Max coverage (-): 0

Region: chr28 26787729-26787739. Max. coverage (+): 0. Max coverage (-): 0

Region: chr28 26787740-26787751. Max. coverage (+): 0. Max coverage (-): 0

Region: chr28 26787752-26787763. Max. coverage (+): 0. Max coverage (-): 0

Region: chr28 26787764-26787774. Max. coverage (+): 0. Max coverage (-): 0

Region: chr28 26787775-26787786. Max. coverage (+): 0. Max coverage (-): 0

Region: chr28 26787787-26787797. Max. coverage (+): 0. Max coverage (-): 0

Region: chr28 26787798-26787809. Max. coverage (+): 0. Max coverage (-): 0

Region: chr28 26787810-26787821. Max. coverage (+): 0. Max coverage (-): 0

Region: chr28 26787822-26787832. Max. coverage (+): 0. Max coverage (-): 0

Region: chr28 26787833-26787844. Max. coverage (+): 0. Max coverage (-): 0

Region: chr28 26787845-26787855. Max. coverage (+): 0. Max coverage (-): 0

Region: chr28 26787856-26787867. Max. coverage (+): 0. Max coverage (-): 0

Region: chr28 26787868-26787879. Max. coverage (+): 0. Max coverage (-): 0

Region: chr28 26787880-26787890. Max. coverage (+): 0. Max coverage (-): 0

Region: chr28 26787891-26787902. Max. coverage (+): 0. Max coverage (-): 0

Region: chr28 26787903-26787913. Max. coverage (+): 0. Max coverage (-): 0

Region: chr28 26787914-26787925. Max. coverage (+): 0. Max coverage (-): 0

Region: chr28 26787926-26787937. Max. coverage (+): 0. Max coverage (-): 0

Region: chr28 26787938-26787948. Max. coverage (+): 0. Max coverage (-): 0

Region: chr28 26787949-26787960. Max. coverage (+): 0. Max coverage (-): 0

Region: chr28 26787961-26787971. Max. coverage (+): 0. Max coverage (-): 0

Region: chr28 26787972-26787983. Max. coverage (+): 0. Max coverage (-): 0

Region: chr28 26787984-26787995. Max. coverage (+): 0. Max coverage (-): 0

Region: chr28 26787996-26788006. Max. coverage (+): 0. Max coverage (-): 0

Region: chr28 26788007-26788018. Max. coverage (+): 0. Max coverage (-): 0

Region: chr28 26788019-26788029. Max. coverage (+): 0. Max coverage (-): 0

Region: chr28 26788030-26788041. Max. coverage (+): 0. Max coverage (-): 0

Region: chr28 26788042-26788053. Max. coverage (+): 0. Max coverage (-): 0

Region: chr28 26788054-26788064. Max. coverage (+): 0. Max coverage (-): 0

Region: chr28 26788065-26788076. Max. coverage (+): 5.07. Max coverage (-): 0

Region: chr28 26788077-26788087. Max. coverage (+): 5.07. Max coverage (-): 0

Region: chr28 26788088-26788099. Max. coverage (+): 0. Max coverage (-): 0

Region: chr28 26788100-26788111. Max. coverage (+): 2.54. Max coverage (-): 0

Region: chr28 26788112-26788122. Max. coverage (+): 7.46. Max coverage (-): 0

Region: chr28 26788123-26788134. Max. coverage (+): 0. Max coverage (-): 0

Region: chr28 26788135-26788145. Max. coverage (+): 0. Max coverage (-): 0

Region: chr28 26788146-26788157. Max. coverage (+): 0. Max coverage (-): 0

Region: chr28 26788158-26788169. Max. coverage (+): 0. Max coverage (-): 0

Region: chr28 26788170-26788180. Max. coverage (+): 0. Max coverage (-): 0

Region: chr28 26788181-26788192. Max. coverage (+): 0. Max coverage (-): 0

Region: chr28 26788193-26788203. Max. coverage (+): 0. Max coverage (-): 0

Region: chr28 26788204-26788215. Max. coverage (+): 0. Max coverage (-): 0

Region: chr28 26788216-26788227. Max. coverage (+): 0. Max coverage (-): 0

Region: chr28 26788228-26788238. Max. coverage (+): 0. Max coverage (-): 0

Region: chr28 26788239-26788250. Max. coverage (+): 6.32. Max coverage (-): 0

Region: chr28 26788251-26788261. Max. coverage (+): 6.32. Max coverage (-): 0

Region: chr28 26788262-26788273. Max. coverage (+): 0. Max coverage (-): 0

Region: chr28 26788274-26788285. Max. coverage (+): 0. Max coverage (-): 0

Region: chr28 26788286-26788296. Max. coverage (+): 0. Max coverage (-): 0

Region: chr28 26788297-26788308. Max. coverage (+): 0. Max coverage (-): 0

Region: chr28 26788309-26788319. Max. coverage (+): 0. Max coverage (-): 0

Region: chr28 26788320-26788331. Max. coverage (+): 0. Max coverage (-): 0

Region: chr28 26788332-26788343. Max. coverage (+): 0. Max coverage (-): 0

Region: chr28 26788344-26788354. Max. coverage (+): 0. Max coverage (-): 0

Region: chr28 26788355-26788366. Max. coverage (+): 0. Max coverage (-): 0

Region: chr28 26788367-26788377. Max. coverage (+): 3.65. Max coverage (-): 0

Region: chr28 26788378-26788389. Max. coverage (+): 3.65. Max coverage (-): 0

Region: chr28 26788390-26788401. Max. coverage (+): 0. Max coverage (-): 0

Region: chr28 26788402-26788412. Max. coverage (+): 0. Max coverage (-): 0

Region: chr28 26788413-26788424. Max. coverage (+): 0. Max coverage (-): 0

Region: chr28 26788425-26788435. Max. coverage (+): 0. Max coverage (-): 0

Region: chr28 26788436-26788447. Max. coverage (+): 0. Max coverage (-): 0

Region: chr28 26788448-26788459. Max. coverage (+): 0. Max coverage (-): 0

Region: chr28 26788460-26788470. Max. coverage (+): 0. Max coverage (-): 0

Region: chr28 26788471-26788482. Max. coverage (+): 0. Max coverage (-): 0

Region: chr28 26788483-26788493. Max. coverage (+): 0. Max coverage (-): 0

Region: chr28 26788494-26788505. Max. coverage (+): 0. Max coverage (-): 0

Region: chr28 26788506-26788517. Max. coverage (+): 0. Max coverage (-): 0

Region: chr28 26788518-26788528. Max. coverage (+): 0. Max coverage (-): 0

Region: chr28 26788529-26788540. Max. coverage (+): 0. Max coverage (-): 0

Region: chr28 26788541-26788551. Max. coverage (+): 0. Max coverage (-): 0

Region: chr28 26788552-26788563. Max. coverage (+): 0. Max coverage (-): 0

Region: chr28 26788564-26788575. Max. coverage (+): 0. Max coverage (-): 0

Region: chr28 26788576-26788586. Max. coverage (+): 0. Max coverage (-): 0

Region: chr28 26788587-26788598. Max. coverage (+): 0. Max coverage (-): 0

Region: chr28 26788599-26788609. Max. coverage (+): 0. Max coverage (-): 0

Region: chr28 26788610-26788621. Max. coverage (+): 0. Max coverage (-): 0

Region: chr28 26788622-26788633. Max. coverage (+): 0. Max coverage (-): 0

Region: chr28 26788634-26788644. Max. coverage (+): 0. Max coverage (-): 0

Region: chr28 26788645-26788656. Max. coverage (+): 0. Max coverage (-): 0

Region: chr28 26788657-26788667. Max. coverage (+): 0. Max coverage (-): 0

Region: chr28 26788668-26788679. Max. coverage (+): 0. Max coverage (-): 0

Region: chr28 26788680-26788691. Max. coverage (+): 0. Max coverage (-): 0

Region: chr28 26788692-26788702. Max. coverage (+): 0. Max coverage (-): 0

Region: chr28 26788703-26788714. Max. coverage (+): 0. Max coverage (-): 0

Region: chr28 26788715-26788725. Max. coverage (+): 0. Max coverage (-): 0

Region: chr28 26788726-26788737. Max. coverage (+): 0. Max coverage (-): 0

Region: chr28 26788738-26788749. Max. coverage (+): 0. Max coverage (-): 0

Region: chr28 26788750-26788760. Max. coverage (+): 0. Max coverage (-): 0

Region: chr28 26788761-26788772. Max. coverage (+): 0. Max coverage (-): 0

Region: chr28 26788773-26788783. Max. coverage (+): 0. Max coverage (-): 0

Region: chr28 26788784-26788795. Max. coverage (+): 0. Max coverage (-): 0

Region: chr28 26788796-26788807. Max. coverage (+): 0. Max coverage (-): 0

Region: chr28 26788808-26788818. Max. coverage (+): 0. Max coverage (-): 0

Region: chr28 26788819-26788830. Max. coverage (+): 0. Max coverage (-): 0

Region: chr28 26788831-26788841. Max. coverage (+): 0. Max coverage (-): 0

Region: chr28 26788842-26788853. Max. coverage (+): 0. Max coverage (-): 0

Region: chr28 26788854-26788865. Max. coverage (+): 0. Max coverage (-): 0

Region: chr28 26788866-26788876. Max. coverage (+): 0. Max coverage (-): 0

Region: chr28 26788877-26788888. Max. coverage (+): 0. Max coverage (-): 0

Region: chr28 26788889-26788899. Max. coverage (+): 0. Max coverage (-): 0

Region: chr28 26788900-26788911. Max. coverage (+): 0. Max coverage (-): 0

Region: chr28 26788912-26788923. Max. coverage (+): 0. Max coverage (-): 0

Region: chr28 26788924-26788934. Max. coverage (+): 0. Max coverage (-): 0

Region: chr28 26788935-26788946. Max. coverage (+): 0. Max coverage (-): 0

Region: chr28 26788947-26788957. Max. coverage (+): 0. Max coverage (-): 0

Region: chr28 26788958-26788969. Max. coverage (+): 0. Max coverage (-): 0

Region: chr28 26788970-26788981. Max. coverage (+): 0. Max coverage (-): 0

Region: chr28 26788982-26788992. Max. coverage (+): 0. Max coverage (-): 0

Region: chr28 26788993-26789004. Max. coverage (+): 0. Max coverage (-): 0

Region: chr28 26789005-26789015. Max. coverage (+): 0. Max coverage (-): 0

Region: chr28 26789016-26789027. Max. coverage (+): 2.37. Max coverage (-): 0

Region: chr28 26789028-26789039. Max. coverage (+): 0. Max coverage (-): 0

Region: chr28 26789040-26789050. Max. coverage (+): 2.88. Max coverage (-): 0

Region: chr28 26789051-26789062. Max. coverage (+): 2.88. Max coverage (-): 0

Region: chr28 26789063-26789073. Max. coverage (+): 0. Max coverage (-): 0

Region: chr28 26789074-26789085. Max. coverage (+): 0. Max coverage (-): 0

Region: chr28 26789086-26789097. Max. coverage (+): 0. Max coverage (-): 0

Region: chr28 26789098-26789108. Max. coverage (+): 0. Max coverage (-): 0

Region: chr28 26789109-26789120. Max. coverage (+): 0. Max coverage (-): 0

Region: chr28 26789121-26789131. Max. coverage (+): 0. Max coverage (-): 0

Region: chr28 26789132-26789143. Max. coverage (+): 0. Max coverage (-): 0

Region: chr28 26789144-26789155. Max. coverage (+): 0. Max coverage (-): 0

Region: chr28 26789156-26789166. Max. coverage (+): 0. Max coverage (-): 0

Region: chr28 26789167-26789178. Max. coverage (+): 0. Max coverage (-): 0

Region: chr28 26789179-26789189. Max. coverage (+): 1.47. Max coverage (-): 0

Region: chr28 26789190-26789201. Max. coverage (+): 0. Max coverage (-): 0

Region: chr28 26789202-26789213. Max. coverage (+): 0. Max coverage (-): 0

Region: chr28 26789214-26789224. Max. coverage (+): 0. Max coverage (-): 0

Region: chr28 26789225-26789236. Max. coverage (+): 0. Max coverage (-): 0

Region: chr28 26789237-26789247. Max. coverage (+): 0. Max coverage (-): 0

Region: chr28 26789248-26789259. Max. coverage (+): 0. Max coverage (-): 0

Region: chr28 26789260-26789271. Max. coverage (+): 0. Max coverage (-): 0

Region: chr28 26789272-26789282. Max. coverage (+): 0. Max coverage (-): 0

Region: chr28 26789283-26789294. Max. coverage (+): 0. Max coverage (-): 0

Region: chr28 26789295-26789305. Max. coverage (+): 0. Max coverage (-): 0

Region: chr28 26789306-26789317. Max. coverage (+): 0. Max coverage (-): 0

Region: chr28 26789318-26789329. Max. coverage (+): 1.76. Max coverage (-): 0

Region: chr28 26789330-26789340. Max. coverage (+): 7.5. Max coverage (-): 0

Region: chr28 26789341-26789352. Max. coverage (+): 9.54. Max coverage (-): 0

Region: chr28 26789353-26789363. Max. coverage (+): 0. Max coverage (-): 0

Region: chr28 26789364-26789375. Max. coverage (+): 0. Max coverage (-): 0

Region: chr28 26789376-26789387. Max. coverage (+): 0. Max coverage (-): 0

Region: chr28 26789388-26789398. Max. coverage (+): 0. Max coverage (-): 0

Region: chr28 26789399-26789410. Max. coverage (+): 0. Max coverage (-): 0

Region: chr28 26789411-26789421. Max. coverage (+): 0. Max coverage (-): 0

Region: chr28 26789422-26789433. Max. coverage (+): 0. Max coverage (-): 0

Region: chr28 26789434-26789445. Max. coverage (+): 0. Max coverage (-): 0

Region: chr28 26789446-26789456. Max. coverage (+): 0. Max coverage (-): 0

Region: chr28 26789457-26789468. Max. coverage (+): 0. Max coverage (-): 0

Region: chr28 26789469-26789479. Max. coverage (+): 0. Max coverage (-): 0

Region: chr28 26789480-26789491. Max. coverage (+): 0. Max coverage (-): 0

Region: chr28 26789492-26789503. Max. coverage (+): 1.33. Max coverage (-): 0

Region: chr28 26789504-26789514. Max. coverage (+): 1.33. Max coverage (-): 0

Region: chr28 26789515-26789526. Max. coverage (+): 0. Max coverage (-): 0

Region: chr28 26789527-26789537. Max. coverage (+): 4.6. Max coverage (-): 0

Region: chr28 26789538-26789549. Max. coverage (+): 4.88. Max coverage (-): 0

Region: chr28 26789550-26789561. Max. coverage (+): 4.88. Max coverage (-): 0

Region: chr28 26789562-26789572. Max. coverage (+): 0. Max coverage (-): 0

Region: chr28 26789573-26789584. Max. coverage (+): 0. Max coverage (-): 0

Region: chr28 26789585-26789595. Max. coverage (+): 0. Max coverage (-): 0

Region: chr28 26789596-26789607. Max. coverage (+): 3.26. Max coverage (-): 0

Region: chr28 26789608-26789619. Max. coverage (+): 0. Max coverage (-): 0

Region: chr28 26789620-26789630. Max. coverage (+): 0. Max coverage (-): 0

Region: chr28 26789631-26789642. Max. coverage (+): 0. Max coverage (-): 0

Region: chr28 26789643-26789653. Max. coverage (+): 0. Max coverage (-): 0

Region: chr28 26789654-26789665. Max. coverage (+): 2.54. Max coverage (-): 0

Region: chr28 26789666-26789677. Max. coverage (+): 8.78. Max coverage (-): 0

Region: chr28 26789678-26789688. Max. coverage (+): 0. Max coverage (-): 0

Region: chr28 26789689-26789700. Max. coverage (+): 0. Max coverage (-): 0

Region: chr28 26789701-26789711. Max. coverage (+): 0. Max coverage (-): 0

Region: chr28 26789712-26789723. Max. coverage (+): 0. Max coverage (-): 0

Region: chr28 26789724-26789735. Max. coverage (+): 0. Max coverage (-): 0

Region: chr28 26789736-26789746. Max. coverage (+): 4.42. Max coverage (-): 0

Region: chr28 26789747-26789758. Max. coverage (+): 4.42. Max coverage (-): 0

Region: chr28 26789759-26789769. Max. coverage (+): 0. Max coverage (-): 0

Region: chr28 26789770-26789781. Max. coverage (+): 0. Max coverage (-): 0

Region: chr28 26789782-26789793. Max. coverage (+): 0. Max coverage (-): 0

Region: chr28 26789794-26789804. Max. coverage (+): 0. Max coverage (-): 0

Region: chr28 26789805-26789816. Max. coverage (+): 0. Max coverage (-): 0

Region: chr28 26789817-26789827. Max. coverage (+): 0. Max coverage (-): 0

Region: chr28 26789828-26789839. Max. coverage (+): 0. Max coverage (-): 0

Region: chr28 26789840-26789851. Max. coverage (+): 0. Max coverage (-): 0

Region: chr28 26789852-26789862. Max. coverage (+): 0. Max coverage (-): 0

Region: chr28 26789863-26789874. Max. coverage (+): 1.64. Max coverage (-): 0

Region: chr28 26789875-26789885. Max. coverage (+): 1.64. Max coverage (-): 0

Region: chr28 26789886-26789897. Max. coverage (+): 0. Max coverage (-): 0

Region: chr28 26789898-26789909. Max. coverage (+): 0. Max coverage (-): 0

Region: chr28 26789910-26789920. Max. coverage (+): 0. Max coverage (-): 0

Region: chr28 26789921-26789932. Max. coverage (+): 0. Max coverage (-): 0

Region: chr28 26789933-26789943. Max. coverage (+): 0. Max coverage (-): 0

Region: chr28 26789944-26789955. Max. coverage (+): 0. Max coverage (-): 0

Region: chr28 26789956-26789967. Max. coverage (+): 0. Max coverage (-): 0

Region: chr28 26789968-26789978. Max. coverage (+): 0. Max coverage (-): 0

Region: chr28 26789979-26789990. Max. coverage (+): 11.13. Max coverage (-): 0

Region: chr28 26789991-26790001. Max. coverage (+): 0. Max coverage (-): 0

Region: chr28 26790002-26790013. Max. coverage (+): 1.58. Max coverage (-): 0

Region: chr28 26790014-26790025. Max. coverage (+): 2.21. Max coverage (-): 0

Region: chr28 26790026-26790036. Max. coverage (+): 2.21. Max coverage (-): 0

Region: chr28 26790037-26790048. Max. coverage (+): 0. Max coverage (-): 0

Region: chr28 26790049-26790059. Max. coverage (+): 0. Max coverage (-): 0

Region: chr28 26790060-26790071. Max. coverage (+): 0. Max coverage (-): 0

Region: chr28 26790072-26790083. Max. coverage (+): 0. Max coverage (-): 0

Region: chr28 26790084-26790094. Max. coverage (+): 0. Max coverage (-): 0

Region: chr28 26790095-26790106. Max. coverage (+): 0. Max coverage (-): 0

Region: chr28 26790107-26790117. Max. coverage (+): 0. Max coverage (-): 0

Region: chr28 26790118-26790129. Max. coverage (+): 0. Max coverage (-): 0

Region: chr28 26790130-26790141. Max. coverage (+): 0. Max coverage (-): 0

Region: chr28 26790142-26790152. Max. coverage (+): 0. Max coverage (-): 0

Region: chr28 26790153-26790164. Max. coverage (+): 0. Max coverage (-): 0

Region: chr28 26790165-26790175. Max. coverage (+): 1.8. Max coverage (-): 0

Region: chr28 26790176-26790187. Max. coverage (+): 1.8. Max coverage (-): 0

Region: chr28 26790188-26790199. Max. coverage (+): 0. Max coverage (-): 0

Region: chr28 26790200-26790210. Max. coverage (+): 0. Max coverage (-): 0

Region: chr28 26790211-26790222. Max. coverage (+): 0. Max coverage (-): 0

Region: chr28 26790223-26790233. Max. coverage (+): 0. Max coverage (-): 0

Region: chr28 26790234-26790245. Max. coverage (+): 0. Max coverage (-): 0

Region: chr28 26790246-26790257. Max. coverage (+): 0. Max coverage (-): 0

Region: chr28 26790258-26790268. Max. coverage (+): 3.17. Max coverage (-): 0

Region: chr28 26790269-26790280. Max. coverage (+): 3.17. Max coverage (-): 0

Region: chr28 26790281-26790291. Max. coverage (+): 0. Max coverage (-): 0

Region: chr28 26790292-26790303. Max. coverage (+): 0. Max coverage (-): 0

Region: chr28 26790304-26790315. Max. coverage (+): 2.1. Max coverage (-): 0

Region: chr28 26790316-26790326. Max. coverage (+): 2.1. Max coverage (-): 0

Region: chr28 26790327-26790338. Max. coverage (+): 0. Max coverage (-): 0

Region: chr28 26790339-26790349. Max. coverage (+): 0. Max coverage (-): 0

Region: chr28 26790350-26790361. Max. coverage (+): 0. Max coverage (-): 0

Region: chr28 26790362-26790373. Max. coverage (+): 0. Max coverage (-): 0

Region: chr28 26790374-26790384. Max. coverage (+): 0. Max coverage (-): 0

Region: chr28 26790385-26790396. Max. coverage (+): 0. Max coverage (-): 0

Region: chr28 26790397-26790407. Max. coverage (+): 0. Max coverage (-): 0

Region: chr28 26790408-26790419. Max. coverage (+): 0. Max coverage (-): 0

Region: chr28 26790420-26790431. Max. coverage (+): 0. Max coverage (-): 0

Region: chr28 26790432-26790442. Max. coverage (+): 0. Max coverage (-): 0

Region: chr28 26790443-26790454. Max. coverage (+): 0. Max coverage (-): 0

Region: chr28 26790455-26790465. Max. coverage (+): 0. Max coverage (-): 0

Region: chr28 26790466-26790477. Max. coverage (+): 0. Max coverage (-): 0

Region: chr28 26790478-26790489. Max. coverage (+): 0. Max coverage (-): 0

Region: chr28 26790490-26790500. Max. coverage (+): 0. Max coverage (-): 0

Region: chr28 26790501-26790512. Max. coverage (+): 0. Max coverage (-): 0

Region: chr28 26790513-26790523. Max. coverage (+): 0. Max coverage (-): 0

Region: chr28 26790524-26790535. Max. coverage (+): 5.18. Max coverage (-): 0

Region: chr28 26790536-26790547. Max. coverage (+): 5.18. Max coverage (-): 0

Region: chr28 26790548-26790558. Max. coverage (+): 0. Max coverage (-): 0

Region: chr28 26790559-26790570. Max. coverage (+): 0. Max coverage (-): 0

Region: chr28 26790571-26790581. Max. coverage (+): 0. Max coverage (-): 0

Region: chr28 26790582-26790593. Max. coverage (+): 0. Max coverage (-): 0

Region: chr28 26790594-26790605. Max. coverage (+): 0. Max coverage (-): 0

Region: chr28 26790606-26790616. Max. coverage (+): 0. Max coverage (-): 0

Region: chr28 26790617-26790628. Max. coverage (+): 0. Max coverage (-): 0

Region: chr28 26790629-26790639. Max. coverage (+): 0. Max coverage (-): 0

Region: chr28 26790640-26790651. Max. coverage (+): 0.99. Max coverage (-): 0

Region: chr28 26790652-26790663. Max. coverage (+): 0.99. Max coverage (-): 0

Region: chr28 26790664-26790674. Max. coverage (+): 0. Max coverage (-): 0

Region: chr28 26790675-26790686. Max. coverage (+): 0. Max coverage (-): 0

Region: chr28 26790687-26790697. Max. coverage (+): 0. Max coverage (-): 0

Region: chr28 26790698-26790709. Max. coverage (+): 0. Max coverage (-): 0

Region: chr28 26790710-26790721. Max. coverage (+): 0. Max coverage (-): 0

Region: chr28 26790722-26790732. Max. coverage (+): 0. Max coverage (-): 0

Region: chr28 26790733-26790744. Max. coverage (+): 0. Max coverage (-): 0

Region: chr28 26790745-26790755. Max. coverage (+): 0. Max coverage (-): 0

Region: chr28 26790756-26790767. Max. coverage (+): 0.87. Max coverage (-): 0

Region: chr28 26790768-26790779. Max. coverage (+): 0.87. Max coverage (-): 0

Region: chr28 26790780-26790790. Max. coverage (+): 0. Max coverage (-): 0

Region: chr28 26790791-26790802. Max. coverage (+): 0. Max coverage (-): 0

Region: chr28 26790803-26790813. Max. coverage (+): 0. Max coverage (-): 0

Region: chr28 26790814-26790825. Max. coverage (+): 0. Max coverage (-): 0

Region: chr28 26790826-26790837. Max. coverage (+): 0. Max coverage (-): 0

Region: chr28 26790838-26790848. Max. coverage (+): 0. Max coverage (-): 0

Region: chr28 26790849-26790860. Max. coverage (+): 0. Max coverage (-): 0

Region: chr28 26790861-26790871. Max. coverage (+): 0. Max coverage (-): 0

Region: chr28 26790872-26790883. Max. coverage (+): 0. Max coverage (-): 0

Region: chr28 26790884-26790895. Max. coverage (+): 0. Max coverage (-): 0

Region: chr28 26790896-26790906. Max. coverage (+): 0. Max coverage (-): 0

Region: chr28 26790907-26790918. Max. coverage (+): 0. Max coverage (-): 0

Region: chr28 26790919-26790929. Max. coverage (+): 0. Max coverage (-): 0

Region: chr28 26790930-26790941. Max. coverage (+): 0. Max coverage (-): 0

Region: chr28 26790942-26790953. Max. coverage (+): 0. Max coverage (-): 0

Region: chr28 26790954-26790964. Max. coverage (+): 0. Max coverage (-): 0

Region: chr28 26790965-26790976. Max. coverage (+): 0. Max coverage (-): 0

Region: chr28 26790977-26790987. Max. coverage (+): 0.42. Max coverage (-): 0

Region: chr28 26790988-26790999. Max. coverage (+): 0.42. Max coverage (-): 0

Region: chr28 26791000-26791011. Max. coverage (+): 0. Max coverage (-): 0

Region: chr28 26791012-. Max. coverage (+): 0. Max coverage (-): 0

RepeatMasker Color Code

**+**

100-98% Identity

<98-95% Identity

<95-90% Identity

<90-85% Identity

<85-80% Identity

<80-75% Identity

<75-70% Identity

<70% Identity

**-**

Gene Set Color Code

**+**

Gene

Pseudogene

**-**

Topology/Coverage Color Code

Coverage Plus Strand

Coverage Minus Strand

Mainstrand: Plus

Mainstrand: Minus

Complementary Strand

Flanking Region  
(if option -flank >0)

Gene Set Annotation  

**1. EIF4EBP2 (protein coding, ENSBTAG00000019124) Tr:00000025454 Ex:3**: 26785176-26786028 (+)

  
RepeatMasker Annotation  

**1. Bov-tA1**: 26787867-26788065 (-), Divergence to consensus: 23.1%  
**2. MLT1F1**: 26788447-26788927 (-), Divergence to consensus: 34.3%

  
Transcription Factor Binding Sites  

**Gata4** (Sequence: AGATAAG (-): 26786309)  
**SOX9** (Sequence: TCATTGTT (+): 26786172)  
**SPZ1** (Sequence: GGGGTTTCAG (+): 26789714)  
**SPZ1** (Sequence: GGGGTATCAG (+): 26790168)  
**Gata4** (Sequence: CTTATCT (+): 26786475)  
**Gata4** (Sequence: CTTATCT (+): 26789779)
