# Supplementary material for: Small RNA sequencing of cryopreserved semen from single bull revealed altered miRNAs and piRNAs expression between High- and Low-motile sperm populations
Source: BMC Genomics. 2017 Jan 4;18:14. doi: 10.1186/s12864-016-3394-7 (PMC5209821; doi:10.1186/s12864-016-3394-7)
Supplement: Additional file 3: — Details for each piRNA clusters found in High Motile (HM) sperm fraction. Genes, repeats, transposable elements and transcription factors binding sites falling within the cluster regions were reported. (ZIP 1896 kb) [file 12864_2016_3394_MOESM3_ESM.zip › 72.html]

piRNA cluster 72


Predicted piRNA cluster no. 72     previous   next
  

Show proTRAC run info
Hide proTRAC run info

================================= proTRAC ====================================  
VERSION: 2.1                                    LAST MODIFIED: 06. October 2015  
  
Please cite:  
Rosenkranz D, Zischler H. proTRAC - a software for probabilistic piRNA cluster  
detection, visualization and analysis. 2012. BMC Bioinformatics 13:5.  
  
and (for proTRAC 2.0 and later):  
Rosenkranz D, Rudloff S, Bastuck K, Ketting RF, Zischler H. Tupaia small RNAs  
provide insights into function and evolution of RNAi-based transposon defense  
in mammals. 2015. RNA 21(5):911-922.  
  
Contact:  
David Rosenkranz  
Institute of Anthropology, small RNA group  
Johannes Gutenberg University Mainz  
email: rosenkranz@uni-mainz.de  
  
You can find the latest proTRAC version at:  
http://sourceforge.net/projects/protrac/files  
http://www.smallRNAgroup-mainz.de/software  
==============================================================================  
  
PARAMETERS:  
Map file: .............../storage/core/barbara/genhome/smallRNA/fertility/Sample\_motile/pirna/Sample\_motile\_26-33\_collapsed.fa.no-dust.map.weighted-10000-1000-b-0  
Genome file: ............/storage/core/barbara/genhome/smallRNA/fertility/Sample\_all/pirna/bt\_311\_chrY.fa  
RepeatMasker annotation: /storage/genomes/bt\_umd31/GCF\_000003055.6\_Bos\_taurus\_UMD\_3.1.1\_repeatMasker\_chr.out  
GeneSet:................./storage/core/barbara/genhome/smallRNA/fertility/Sample\_all/pirna/full.gtf  
  
Significant (p<=0.01) hit density will be calculated based  
on observed hit distribution.  
  
Sliding window size: ........................................ 5000 bp  
Sliding window increament: .................................. 1000 bp  
Normalize each hit by number of genomic hits: ............... 1 [0=no/1=yes]  
Normalize each hit by number of sequence reads: ............. 1 [0=no/1=yes]  
Normalize values (-> per million mapped reads): ............. 1 [0=no/1=yes]  
Min. fraction of hits with 1T(U) or 10A: .................... 0.75  
Alternatively: Min. fraction of hits with 1T(U) and 10A: .... 0.5  
Min. fraction of hits with typical piRNA length: ............ 0.75  
Typical piRNA length: ....................................... 26-33 nt  
Min. size of a piRNA cluster: ............................... 5000 bp.  
Min. number of hits (absolute): ............................. 0  
Min. number of hits (normalized): ........................... 0  
Min. fraction of hits on the mainstrand: .................... 0.75  
Top fraction of mapped sequences (in terms of read counts): . 1%  
Top fraction accounts for max. n% of sequence reads: ........ 90%  
Min. fraction of hits on each arm of a bidirectional cluster: 0.1  
Output image file for each cluster: ......................... 0 [0=no/1=yes]  
Output html file for each cluster: .......................... 1 [0=no/1=yes]  
Output a summary table: ..................................... 1 [0=no/1=yes]  
Output a FASTA file for each cluster (piRNA sequences): ..... 1 [0=no/1=yes]  
Output a FASTA file comprising cluster sequences: ........... 1 [0=no/1=yes]  
Search DNA motifs in clusters: .............................. 1 [0=no/1=yes]  
Output flanking sequences: +/- .............................. 0 bp  
Output ~.pTi file: .......................................... 1 [0=no/1=yes]  
==============================================================================  
  
  
Genome size (without gaps): ............ 2678902517 bp  
Gaps (N/X/-): .......................... 53837044 bp  
Mapped reads: .......................... 658825247023  
Non-identical sequences: ............... 514171  
Genomic hits: .......................... 764233  
Significant densitiy of mapped reads: .. 12867599.5173724 reads/kb

Show proTRAC cluster info
Hide proTRAC cluster info

|  |  |
| --- | --- |
| Location | chr28 |
| Coordinates | 28538628-28543987 |
| Size [bp] | 5360 |
| Sequence hit loci | 98 |
| Mapped reads (normalized) | 130178232 |
| Mapped reads (normalized) per kb | 24286983.6 |
| Normalized reads with 1T (1U) | 91.7% |
| Normalized reads with 10A | 34.1% |
| Normalized reads with length 26-33 nt | 100% |
| Normalized reads on the main strand(s) | 100% |
| Predicted directionality | mono:minus |

100%

0%

1T (1U)  
reads

10A reads

26-33 nt  
reads

reads on mainstrand

**Either the amount of reads with 1T (1U) OR 10A has to exceed 75% (set with option: -1Tor10A)  
Alternatively the amount of reads with 1T (1U) AND 10A has to exceed 50% (set with option: -1Tand10A)  
Minimum amount of reads with preferred size is 75% (set with option: -pisize)  
Minimum amount of reads on the main strand(s) is 75% (set with option: -clstrand)**

Show read coverage
Hide read coverage

WHAT DO I SEE HERE?  
This chart shows the location of mapped sequence reads within a predicted piRNA cluster. The color refers to the number of genomic hits produced by the sequence read in question. A dark red bar indicates that this sequence read produces many other hits elsewhere in the genome. Many adjacent red or yellow bars can indicate the presence of a multi-copy element such as transposons or rRNA genes. A dark green bar indicates that this sequence read maps uniquely to this locus.

1 hit

2-5 hits

6-10 hits

11-20 hits

21-50 hits

51-100 hits

> 100 hits

chr28

28538628

28543987

Gene Set

RepeatMasker

Mapped  
Reads

16.74

plus strand

minus strand

16.74

Region: chr28 26791013-28538633. Max. coverage (+): 0. Max coverage (-): 2.77

Region: chr28 28538634-28538644. Max. coverage (+): 0. Max coverage (-): 2.77

Region: chr28 28538645-28538654. Max. coverage (+): 0. Max coverage (-): 0

Region: chr28 28538655-28538665. Max. coverage (+): 0. Max coverage (-): 0

Region: chr28 28538666-28538676. Max. coverage (+): 0. Max coverage (-): 0

Region: chr28 28538677-28538686. Max. coverage (+): 0. Max coverage (-): 0

Region: chr28 28538687-28538697. Max. coverage (+): 0. Max coverage (-): 0

Region: chr28 28538698-28538708. Max. coverage (+): 0. Max coverage (-): 0

Region: chr28 28538709-28538719. Max. coverage (+): 0. Max coverage (-): 0

Region: chr28 28538720-28538729. Max. coverage (+): 0. Max coverage (-): 0

Region: chr28 28538730-28538740. Max. coverage (+): 0. Max coverage (-): 0

Region: chr28 28538741-28538751. Max. coverage (+): 0. Max coverage (-): 0

Region: chr28 28538752-28538761. Max. coverage (+): 0. Max coverage (-): 0

Region: chr28 28538762-28538772. Max. coverage (+): 0. Max coverage (-): 0

Region: chr28 28538773-28538783. Max. coverage (+): 0. Max coverage (-): 0

Region: chr28 28538784-28538794. Max. coverage (+): 0. Max coverage (-): 0

Region: chr28 28538795-28538804. Max. coverage (+): 0. Max coverage (-): 0

Region: chr28 28538805-28538815. Max. coverage (+): 0. Max coverage (-): 0

Region: chr28 28538816-28538826. Max. coverage (+): 0. Max coverage (-): 0

Region: chr28 28538827-28538837. Max. coverage (+): 0. Max coverage (-): 0

Region: chr28 28538838-28538847. Max. coverage (+): 0. Max coverage (-): 0

Region: chr28 28538848-28538858. Max. coverage (+): 0. Max coverage (-): 0

Region: chr28 28538859-28538869. Max. coverage (+): 0. Max coverage (-): 0

Region: chr28 28538870-28538879. Max. coverage (+): 0. Max coverage (-): 0

Region: chr28 28538880-28538890. Max. coverage (+): 0. Max coverage (-): 0

Region: chr28 28538891-28538901. Max. coverage (+): 0. Max coverage (-): 0

Region: chr28 28538902-28538912. Max. coverage (+): 0. Max coverage (-): 0

Region: chr28 28538913-28538922. Max. coverage (+): 0. Max coverage (-): 0

Region: chr28 28538923-28538933. Max. coverage (+): 0. Max coverage (-): 0

Region: chr28 28538934-28538944. Max. coverage (+): 0. Max coverage (-): 0

Region: chr28 28538945-28538954. Max. coverage (+): 0. Max coverage (-): 0

Region: chr28 28538955-28538965. Max. coverage (+): 0. Max coverage (-): 0

Region: chr28 28538966-28538976. Max. coverage (+): 0. Max coverage (-): 0

Region: chr28 28538977-28538987. Max. coverage (+): 0. Max coverage (-): 0

Region: chr28 28538988-28538997. Max. coverage (+): 0. Max coverage (-): 0

Region: chr28 28538998-28539008. Max. coverage (+): 0. Max coverage (-): 0

Region: chr28 28539009-28539019. Max. coverage (+): 0. Max coverage (-): 0

Region: chr28 28539020-28539029. Max. coverage (+): 0. Max coverage (-): 0

Region: chr28 28539030-28539040. Max. coverage (+): 0. Max coverage (-): 0

Region: chr28 28539041-28539051. Max. coverage (+): 0. Max coverage (-): 0

Region: chr28 28539052-28539062. Max. coverage (+): 0. Max coverage (-): 0

Region: chr28 28539063-28539072. Max. coverage (+): 0. Max coverage (-): 0

Region: chr28 28539073-28539083. Max. coverage (+): 0. Max coverage (-): 0

Region: chr28 28539084-28539094. Max. coverage (+): 0. Max coverage (-): 0

Region: chr28 28539095-28539105. Max. coverage (+): 0. Max coverage (-): 0

Region: chr28 28539106-28539115. Max. coverage (+): 0. Max coverage (-): 0

Region: chr28 28539116-28539126. Max. coverage (+): 0. Max coverage (-): 0

Region: chr28 28539127-28539137. Max. coverage (+): 0. Max coverage (-): 0

Region: chr28 28539138-28539147. Max. coverage (+): 0. Max coverage (-): 0

Region: chr28 28539148-28539158. Max. coverage (+): 0. Max coverage (-): 0

Region: chr28 28539159-28539169. Max. coverage (+): 0. Max coverage (-): 0

Region: chr28 28539170-28539180. Max. coverage (+): 0. Max coverage (-): 0

Region: chr28 28539181-28539190. Max. coverage (+): 0. Max coverage (-): 0

Region: chr28 28539191-28539201. Max. coverage (+): 0. Max coverage (-): 0

Region: chr28 28539202-28539212. Max. coverage (+): 0. Max coverage (-): 0

Region: chr28 28539213-28539222. Max. coverage (+): 0. Max coverage (-): 0

Region: chr28 28539223-28539233. Max. coverage (+): 0. Max coverage (-): 0

Region: chr28 28539234-28539244. Max. coverage (+): 0. Max coverage (-): 0

Region: chr28 28539245-28539255. Max. coverage (+): 0. Max coverage (-): 0

Region: chr28 28539256-28539265. Max. coverage (+): 0. Max coverage (-): 0

Region: chr28 28539266-28539276. Max. coverage (+): 0. Max coverage (-): 0

Region: chr28 28539277-28539287. Max. coverage (+): 0. Max coverage (-): 0

Region: chr28 28539288-28539297. Max. coverage (+): 0. Max coverage (-): 0

Region: chr28 28539298-28539308. Max. coverage (+): 0. Max coverage (-): 0

Region: chr28 28539309-28539319. Max. coverage (+): 0. Max coverage (-): 0

Region: chr28 28539320-28539330. Max. coverage (+): 0. Max coverage (-): 0

Region: chr28 28539331-28539340. Max. coverage (+): 0. Max coverage (-): 0

Region: chr28 28539341-28539351. Max. coverage (+): 0. Max coverage (-): 0

Region: chr28 28539352-28539362. Max. coverage (+): 0. Max coverage (-): 0

Region: chr28 28539363-28539373. Max. coverage (+): 0. Max coverage (-): 0

Region: chr28 28539374-28539383. Max. coverage (+): 0. Max coverage (-): 0

Region: chr28 28539384-28539394. Max. coverage (+): 0. Max coverage (-): 0

Region: chr28 28539395-28539405. Max. coverage (+): 0. Max coverage (-): 0

Region: chr28 28539406-28539415. Max. coverage (+): 0. Max coverage (-): 0

Region: chr28 28539416-28539426. Max. coverage (+): 0. Max coverage (-): 0

Region: chr28 28539427-28539437. Max. coverage (+): 0. Max coverage (-): 0

Region: chr28 28539438-28539448. Max. coverage (+): 0. Max coverage (-): 9.23

Region: chr28 28539449-28539458. Max. coverage (+): 0. Max coverage (-): 0.52

Region: chr28 28539459-28539469. Max. coverage (+): 0. Max coverage (-): 0

Region: chr28 28539470-28539480. Max. coverage (+): 0. Max coverage (-): 0

Region: chr28 28539481-28539490. Max. coverage (+): 0. Max coverage (-): 0

Region: chr28 28539491-28539501. Max. coverage (+): 0. Max coverage (-): 0

Region: chr28 28539502-28539512. Max. coverage (+): 0. Max coverage (-): 0

Region: chr28 28539513-28539523. Max. coverage (+): 0. Max coverage (-): 0

Region: chr28 28539524-28539533. Max. coverage (+): 0. Max coverage (-): 0

Region: chr28 28539534-28539544. Max. coverage (+): 0. Max coverage (-): 0

Region: chr28 28539545-28539555. Max. coverage (+): 0. Max coverage (-): 0

Region: chr28 28539556-28539565. Max. coverage (+): 0. Max coverage (-): 0

Region: chr28 28539566-28539576. Max. coverage (+): 0. Max coverage (-): 0

Region: chr28 28539577-28539587. Max. coverage (+): 0. Max coverage (-): 0

Region: chr28 28539588-28539598. Max. coverage (+): 0. Max coverage (-): 0

Region: chr28 28539599-28539608. Max. coverage (+): 0. Max coverage (-): 2.25

Region: chr28 28539609-28539619. Max. coverage (+): 0. Max coverage (-): 3.67

Region: chr28 28539620-28539630. Max. coverage (+): 0. Max coverage (-): 2.17

Region: chr28 28539631-28539641. Max. coverage (+): 0. Max coverage (-): 0

Region: chr28 28539642-28539651. Max. coverage (+): 0. Max coverage (-): 0

Region: chr28 28539652-28539662. Max. coverage (+): 0. Max coverage (-): 3.61

Region: chr28 28539663-28539673. Max. coverage (+): 0. Max coverage (-): 3.61

Region: chr28 28539674-28539683. Max. coverage (+): 0. Max coverage (-): 0

Region: chr28 28539684-28539694. Max. coverage (+): 0. Max coverage (-): 0

Region: chr28 28539695-28539705. Max. coverage (+): 0. Max coverage (-): 0

Region: chr28 28539706-28539716. Max. coverage (+): 0. Max coverage (-): 0

Region: chr28 28539717-28539726. Max. coverage (+): 0. Max coverage (-): 0

Region: chr28 28539727-28539737. Max. coverage (+): 0. Max coverage (-): 0

Region: chr28 28539738-28539748. Max. coverage (+): 0. Max coverage (-): 0

Region: chr28 28539749-28539758. Max. coverage (+): 0. Max coverage (-): 0

Region: chr28 28539759-28539769. Max. coverage (+): 0. Max coverage (-): 0

Region: chr28 28539770-28539780. Max. coverage (+): 0. Max coverage (-): 0

Region: chr28 28539781-28539791. Max. coverage (+): 0. Max coverage (-): 0

Region: chr28 28539792-28539801. Max. coverage (+): 0. Max coverage (-): 0

Region: chr28 28539802-28539812. Max. coverage (+): 0. Max coverage (-): 0

Region: chr28 28539813-28539823. Max. coverage (+): 0. Max coverage (-): 0

Region: chr28 28539824-28539833. Max. coverage (+): 0. Max coverage (-): 0

Region: chr28 28539834-28539844. Max. coverage (+): 0. Max coverage (-): 0

Region: chr28 28539845-28539855. Max. coverage (+): 0. Max coverage (-): 0

Region: chr28 28539856-28539866. Max. coverage (+): 0. Max coverage (-): 0

Region: chr28 28539867-28539876. Max. coverage (+): 0. Max coverage (-): 0

Region: chr28 28539877-28539887. Max. coverage (+): 0. Max coverage (-): 0

Region: chr28 28539888-28539898. Max. coverage (+): 0. Max coverage (-): 0

Region: chr28 28539899-28539909. Max. coverage (+): 0. Max coverage (-): 0

Region: chr28 28539910-28539919. Max. coverage (+): 0. Max coverage (-): 2.07

Region: chr28 28539920-28539930. Max. coverage (+): 0. Max coverage (-): 0

Region: chr28 28539931-28539941. Max. coverage (+): 0. Max coverage (-): 0

Region: chr28 28539942-28539951. Max. coverage (+): 0. Max coverage (-): 1.05

Region: chr28 28539952-28539962. Max. coverage (+): 0. Max coverage (-): 1.05

Region: chr28 28539963-28539973. Max. coverage (+): 0. Max coverage (-): 0

Region: chr28 28539974-28539984. Max. coverage (+): 0. Max coverage (-): 0

Region: chr28 28539985-28539994. Max. coverage (+): 0. Max coverage (-): 0

Region: chr28 28539995-28540005. Max. coverage (+): 0. Max coverage (-): 3.21

Region: chr28 28540006-28540016. Max. coverage (+): 0. Max coverage (-): 3.21

Region: chr28 28540017-28540026. Max. coverage (+): 0. Max coverage (-): 0

Region: chr28 28540027-28540037. Max. coverage (+): 0. Max coverage (-): 0

Region: chr28 28540038-28540048. Max. coverage (+): 0. Max coverage (-): 0

Region: chr28 28540049-28540059. Max. coverage (+): 0. Max coverage (-): 0

Region: chr28 28540060-28540069. Max. coverage (+): 0. Max coverage (-): 0

Region: chr28 28540070-28540080. Max. coverage (+): 0. Max coverage (-): 0

Region: chr28 28540081-28540091. Max. coverage (+): 0. Max coverage (-): 0

Region: chr28 28540092-28540101. Max. coverage (+): 0. Max coverage (-): 0

Region: chr28 28540102-28540112. Max. coverage (+): 0. Max coverage (-): 0

Region: chr28 28540113-28540123. Max. coverage (+): 0. Max coverage (-): 0

Region: chr28 28540124-28540134. Max. coverage (+): 0. Max coverage (-): 0

Region: chr28 28540135-28540144. Max. coverage (+): 0. Max coverage (-): 0

Region: chr28 28540145-28540155. Max. coverage (+): 0. Max coverage (-): 0

Region: chr28 28540156-28540166. Max. coverage (+): 0. Max coverage (-): 0

Region: chr28 28540167-28540177. Max. coverage (+): 0. Max coverage (-): 0

Region: chr28 28540178-28540187. Max. coverage (+): 0. Max coverage (-): 0

Region: chr28 28540188-28540198. Max. coverage (+): 0. Max coverage (-): 0

Region: chr28 28540199-28540209. Max. coverage (+): 0. Max coverage (-): 0

Region: chr28 28540210-28540219. Max. coverage (+): 0. Max coverage (-): 0

Region: chr28 28540220-28540230. Max. coverage (+): 0. Max coverage (-): 0

Region: chr28 28540231-28540241. Max. coverage (+): 0. Max coverage (-): 0

Region: chr28 28540242-28540252. Max. coverage (+): 0. Max coverage (-): 0

Region: chr28 28540253-28540262. Max. coverage (+): 0. Max coverage (-): 9.34

Region: chr28 28540263-28540273. Max. coverage (+): 0. Max coverage (-): 9.34

Region: chr28 28540274-28540284. Max. coverage (+): 0. Max coverage (-): 2.24

Region: chr28 28540285-28540294. Max. coverage (+): 0. Max coverage (-): 2.24

Region: chr28 28540295-28540305. Max. coverage (+): 0. Max coverage (-): 0

Region: chr28 28540306-28540316. Max. coverage (+): 0. Max coverage (-): 0

Region: chr28 28540317-28540327. Max. coverage (+): 0. Max coverage (-): 0

Region: chr28 28540328-28540337. Max. coverage (+): 0. Max coverage (-): 0

Region: chr28 28540338-28540348. Max. coverage (+): 0. Max coverage (-): 0

Region: chr28 28540349-28540359. Max. coverage (+): 0. Max coverage (-): 0

Region: chr28 28540360-28540369. Max. coverage (+): 0. Max coverage (-): 0

Region: chr28 28540370-28540380. Max. coverage (+): 0. Max coverage (-): 0

Region: chr28 28540381-28540391. Max. coverage (+): 0. Max coverage (-): 0

Region: chr28 28540392-28540402. Max. coverage (+): 0. Max coverage (-): 0

Region: chr28 28540403-28540412. Max. coverage (+): 0. Max coverage (-): 0

Region: chr28 28540413-28540423. Max. coverage (+): 0. Max coverage (-): 0

Region: chr28 28540424-28540434. Max. coverage (+): 0. Max coverage (-): 0

Region: chr28 28540435-28540445. Max. coverage (+): 0. Max coverage (-): 6.95

Region: chr28 28540446-28540455. Max. coverage (+): 0. Max coverage (-): 5.7

Region: chr28 28540456-28540466. Max. coverage (+): 0. Max coverage (-): 0

Region: chr28 28540467-28540477. Max. coverage (+): 0. Max coverage (-): 0

Region: chr28 28540478-28540487. Max. coverage (+): 0. Max coverage (-): 0

Region: chr28 28540488-28540498. Max. coverage (+): 0. Max coverage (-): 0

Region: chr28 28540499-28540509. Max. coverage (+): 0. Max coverage (-): 0

Region: chr28 28540510-28540520. Max. coverage (+): 0. Max coverage (-): 0

Region: chr28 28540521-28540530. Max. coverage (+): 0. Max coverage (-): 0

Region: chr28 28540531-28540541. Max. coverage (+): 0. Max coverage (-): 0

Region: chr28 28540542-28540552. Max. coverage (+): 0. Max coverage (-): 0

Region: chr28 28540553-28540562. Max. coverage (+): 0. Max coverage (-): 0

Region: chr28 28540563-28540573. Max. coverage (+): 0. Max coverage (-): 1.5

Region: chr28 28540574-28540584. Max. coverage (+): 0. Max coverage (-): 3.04

Region: chr28 28540585-28540595. Max. coverage (+): 0. Max coverage (-): 0

Region: chr28 28540596-28540605. Max. coverage (+): 0. Max coverage (-): 0

Region: chr28 28540606-28540616. Max. coverage (+): 0. Max coverage (-): 0.72

Region: chr28 28540617-28540627. Max. coverage (+): 0. Max coverage (-): 0.72

Region: chr28 28540628-28540637. Max. coverage (+): 0. Max coverage (-): 0

Region: chr28 28540638-28540648. Max. coverage (+): 0. Max coverage (-): 0

Region: chr28 28540649-28540659. Max. coverage (+): 0. Max coverage (-): 0

Region: chr28 28540660-28540670. Max. coverage (+): 0. Max coverage (-): 0

Region: chr28 28540671-28540680. Max. coverage (+): 0. Max coverage (-): 0

Region: chr28 28540681-28540691. Max. coverage (+): 0. Max coverage (-): 0

Region: chr28 28540692-28540702. Max. coverage (+): 0. Max coverage (-): 0

Region: chr28 28540703-28540713. Max. coverage (+): 0. Max coverage (-): 0

Region: chr28 28540714-28540723. Max. coverage (+): 0. Max coverage (-): 0

Region: chr28 28540724-28540734. Max. coverage (+): 0. Max coverage (-): 0

Region: chr28 28540735-28540745. Max. coverage (+): 0. Max coverage (-): 0

Region: chr28 28540746-28540755. Max. coverage (+): 0. Max coverage (-): 0

Region: chr28 28540756-28540766. Max. coverage (+): 0. Max coverage (-): 0

Region: chr28 28540767-28540777. Max. coverage (+): 0. Max coverage (-): 0

Region: chr28 28540778-28540788. Max. coverage (+): 0. Max coverage (-): 0

Region: chr28 28540789-28540798. Max. coverage (+): 0. Max coverage (-): 0

Region: chr28 28540799-28540809. Max. coverage (+): 0. Max coverage (-): 0

Region: chr28 28540810-28540820. Max. coverage (+): 0. Max coverage (-): 0

Region: chr28 28540821-28540830. Max. coverage (+): 0. Max coverage (-): 0

Region: chr28 28540831-28540841. Max. coverage (+): 0. Max coverage (-): 0

Region: chr28 28540842-28540852. Max. coverage (+): 0. Max coverage (-): 0

Region: chr28 28540853-28540863. Max. coverage (+): 0. Max coverage (-): 0

Region: chr28 28540864-28540873. Max. coverage (+): 0. Max coverage (-): 0

Region: chr28 28540874-28540884. Max. coverage (+): 0. Max coverage (-): 0

Region: chr28 28540885-28540895. Max. coverage (+): 0. Max coverage (-): 0

Region: chr28 28540896-28540905. Max. coverage (+): 0. Max coverage (-): 0

Region: chr28 28540906-28540916. Max. coverage (+): 0. Max coverage (-): 0

Region: chr28 28540917-28540927. Max. coverage (+): 0. Max coverage (-): 0

Region: chr28 28540928-28540938. Max. coverage (+): 0. Max coverage (-): 0

Region: chr28 28540939-28540948. Max. coverage (+): 0. Max coverage (-): 0

Region: chr28 28540949-28540959. Max. coverage (+): 0. Max coverage (-): 0

Region: chr28 28540960-28540970. Max. coverage (+): 0. Max coverage (-): 0

Region: chr28 28540971-28540981. Max. coverage (+): 0. Max coverage (-): 2.03

Region: chr28 28540982-28540991. Max. coverage (+): 0. Max coverage (-): 0

Region: chr28 28540992-28541002. Max. coverage (+): 0. Max coverage (-): 0

Region: chr28 28541003-28541013. Max. coverage (+): 0. Max coverage (-): 0

Region: chr28 28541014-28541023. Max. coverage (+): 0. Max coverage (-): 0

Region: chr28 28541024-28541034. Max. coverage (+): 0. Max coverage (-): 0

Region: chr28 28541035-28541045. Max. coverage (+): 0. Max coverage (-): 1.2

Region: chr28 28541046-28541056. Max. coverage (+): 0. Max coverage (-): 0

Region: chr28 28541057-28541066. Max. coverage (+): 0. Max coverage (-): 0

Region: chr28 28541067-28541077. Max. coverage (+): 0. Max coverage (-): 0

Region: chr28 28541078-28541088. Max. coverage (+): 0. Max coverage (-): 0

Region: chr28 28541089-28541098. Max. coverage (+): 0. Max coverage (-): 0

Region: chr28 28541099-28541109. Max. coverage (+): 0. Max coverage (-): 0

Region: chr28 28541110-28541120. Max. coverage (+): 0. Max coverage (-): 0

Region: chr28 28541121-28541131. Max. coverage (+): 0. Max coverage (-): 0

Region: chr28 28541132-28541141. Max. coverage (+): 0. Max coverage (-): 0

Region: chr28 28541142-28541152. Max. coverage (+): 0. Max coverage (-): 0

Region: chr28 28541153-28541163. Max. coverage (+): 0. Max coverage (-): 0

Region: chr28 28541164-28541173. Max. coverage (+): 0. Max coverage (-): 0

Region: chr28 28541174-28541184. Max. coverage (+): 0. Max coverage (-): 0

Region: chr28 28541185-28541195. Max. coverage (+): 0. Max coverage (-): 0

Region: chr28 28541196-28541206. Max. coverage (+): 0. Max coverage (-): 0

Region: chr28 28541207-28541216. Max. coverage (+): 0. Max coverage (-): 0

Region: chr28 28541217-28541227. Max. coverage (+): 0. Max coverage (-): 0

Region: chr28 28541228-28541238. Max. coverage (+): 0. Max coverage (-): 0

Region: chr28 28541239-28541249. Max. coverage (+): 0. Max coverage (-): 0

Region: chr28 28541250-28541259. Max. coverage (+): 0. Max coverage (-): 0

Region: chr28 28541260-28541270. Max. coverage (+): 0. Max coverage (-): 0

Region: chr28 28541271-28541281. Max. coverage (+): 0. Max coverage (-): 0

Region: chr28 28541282-28541291. Max. coverage (+): 0. Max coverage (-): 0

Region: chr28 28541292-28541302. Max. coverage (+): 0. Max coverage (-): 0

Region: chr28 28541303-28541313. Max. coverage (+): 0. Max coverage (-): 0

Region: chr28 28541314-28541324. Max. coverage (+): 0. Max coverage (-): 0

Region: chr28 28541325-28541334. Max. coverage (+): 0. Max coverage (-): 0

Region: chr28 28541335-28541345. Max. coverage (+): 0. Max coverage (-): 0.95

Region: chr28 28541346-28541356. Max. coverage (+): 0. Max coverage (-): 0.95

Region: chr28 28541357-28541366. Max. coverage (+): 0. Max coverage (-): 0

Region: chr28 28541367-28541377. Max. coverage (+): 0. Max coverage (-): 0

Region: chr28 28541378-28541388. Max. coverage (+): 0. Max coverage (-): 0

Region: chr28 28541389-28541399. Max. coverage (+): 0. Max coverage (-): 0

Region: chr28 28541400-28541409. Max. coverage (+): 0. Max coverage (-): 0

Region: chr28 28541410-28541420. Max. coverage (+): 0. Max coverage (-): 0

Region: chr28 28541421-28541431. Max. coverage (+): 0. Max coverage (-): 0

Region: chr28 28541432-28541441. Max. coverage (+): 0. Max coverage (-): 0

Region: chr28 28541442-28541452. Max. coverage (+): 0. Max coverage (-): 0

Region: chr28 28541453-28541463. Max. coverage (+): 0. Max coverage (-): 0

Region: chr28 28541464-28541474. Max. coverage (+): 0. Max coverage (-): 0

Region: chr28 28541475-28541484. Max. coverage (+): 0. Max coverage (-): 0

Region: chr28 28541485-28541495. Max. coverage (+): 0. Max coverage (-): 0

Region: chr28 28541496-28541506. Max. coverage (+): 0. Max coverage (-): 0

Region: chr28 28541507-28541517. Max. coverage (+): 0. Max coverage (-): 0

Region: chr28 28541518-28541527. Max. coverage (+): 0. Max coverage (-): 0

Region: chr28 28541528-28541538. Max. coverage (+): 0. Max coverage (-): 0

Region: chr28 28541539-28541549. Max. coverage (+): 0. Max coverage (-): 0

Region: chr28 28541550-28541559. Max. coverage (+): 0. Max coverage (-): 0

Region: chr28 28541560-28541570. Max. coverage (+): 0. Max coverage (-): 0

Region: chr28 28541571-28541581. Max. coverage (+): 0. Max coverage (-): 0

Region: chr28 28541582-28541592. Max. coverage (+): 0. Max coverage (-): 0

Region: chr28 28541593-28541602. Max. coverage (+): 0. Max coverage (-): 0

Region: chr28 28541603-28541613. Max. coverage (+): 0. Max coverage (-): 0

Region: chr28 28541614-28541624. Max. coverage (+): 0. Max coverage (-): 0

Region: chr28 28541625-28541634. Max. coverage (+): 0. Max coverage (-): 0

Region: chr28 28541635-28541645. Max. coverage (+): 0. Max coverage (-): 0

Region: chr28 28541646-28541656. Max. coverage (+): 0. Max coverage (-): 5.16

Region: chr28 28541657-28541667. Max. coverage (+): 0. Max coverage (-): 5.16

Region: chr28 28541668-28541677. Max. coverage (+): 0. Max coverage (-): 0

Region: chr28 28541678-28541688. Max. coverage (+): 0. Max coverage (-): 0

Region: chr28 28541689-28541699. Max. coverage (+): 0. Max coverage (-): 7.68

Region: chr28 28541700-28541709. Max. coverage (+): 0. Max coverage (-): 3.95

Region: chr28 28541710-28541720. Max. coverage (+): 0. Max coverage (-): 0

Region: chr28 28541721-28541731. Max. coverage (+): 0. Max coverage (-): 0

Region: chr28 28541732-28541742. Max. coverage (+): 0. Max coverage (-): 0

Region: chr28 28541743-28541752. Max. coverage (+): 0. Max coverage (-): 0

Region: chr28 28541753-28541763. Max. coverage (+): 0. Max coverage (-): 0

Region: chr28 28541764-28541774. Max. coverage (+): 0. Max coverage (-): 2.05

Region: chr28 28541775-28541785. Max. coverage (+): 0. Max coverage (-): 0

Region: chr28 28541786-28541795. Max. coverage (+): 0. Max coverage (-): 0

Region: chr28 28541796-28541806. Max. coverage (+): 0. Max coverage (-): 0

Region: chr28 28541807-28541817. Max. coverage (+): 0. Max coverage (-): 0

Region: chr28 28541818-28541827. Max. coverage (+): 0. Max coverage (-): 0

Region: chr28 28541828-28541838. Max. coverage (+): 0. Max coverage (-): 0

Region: chr28 28541839-28541849. Max. coverage (+): 0. Max coverage (-): 0

Region: chr28 28541850-28541860. Max. coverage (+): 0. Max coverage (-): 0

Region: chr28 28541861-28541870. Max. coverage (+): 0. Max coverage (-): 0

Region: chr28 28541871-28541881. Max. coverage (+): 0. Max coverage (-): 0

Region: chr28 28541882-28541892. Max. coverage (+): 0. Max coverage (-): 0

Region: chr28 28541893-28541902. Max. coverage (+): 0. Max coverage (-): 0

Region: chr28 28541903-28541913. Max. coverage (+): 0. Max coverage (-): 0

Region: chr28 28541914-28541924. Max. coverage (+): 0. Max coverage (-): 0

Region: chr28 28541925-28541935. Max. coverage (+): 0. Max coverage (-): 0

Region: chr28 28541936-28541945. Max. coverage (+): 0. Max coverage (-): 0

Region: chr28 28541946-28541956. Max. coverage (+): 0. Max coverage (-): 0

Region: chr28 28541957-28541967. Max. coverage (+): 0. Max coverage (-): 0

Region: chr28 28541968-28541977. Max. coverage (+): 0. Max coverage (-): 0

Region: chr28 28541978-28541988. Max. coverage (+): 0. Max coverage (-): 1.21

Region: chr28 28541989-28541999. Max. coverage (+): 0. Max coverage (-): 1.21

Region: chr28 28542000-28542010. Max. coverage (+): 0. Max coverage (-): 0

Region: chr28 28542011-28542020. Max. coverage (+): 0. Max coverage (-): 0

Region: chr28 28542021-28542031. Max. coverage (+): 0. Max coverage (-): 0

Region: chr28 28542032-28542042. Max. coverage (+): 0. Max coverage (-): 0

Region: chr28 28542043-28542053. Max. coverage (+): 0. Max coverage (-): 0

Region: chr28 28542054-28542063. Max. coverage (+): 0. Max coverage (-): 0

Region: chr28 28542064-28542074. Max. coverage (+): 0. Max coverage (-): 0

Region: chr28 28542075-28542085. Max. coverage (+): 0. Max coverage (-): 0

Region: chr28 28542086-28542095. Max. coverage (+): 0. Max coverage (-): 0

Region: chr28 28542096-28542106. Max. coverage (+): 0. Max coverage (-): 0

Region: chr28 28542107-28542117. Max. coverage (+): 0. Max coverage (-): 0

Region: chr28 28542118-28542128. Max. coverage (+): 0. Max coverage (-): 0

Region: chr28 28542129-28542138. Max. coverage (+): 0. Max coverage (-): 0

Region: chr28 28542139-28542149. Max. coverage (+): 0. Max coverage (-): 0

Region: chr28 28542150-28542160. Max. coverage (+): 0. Max coverage (-): 0

Region: chr28 28542161-28542170. Max. coverage (+): 0. Max coverage (-): 0

Region: chr28 28542171-28542181. Max. coverage (+): 0. Max coverage (-): 0

Region: chr28 28542182-28542192. Max. coverage (+): 0. Max coverage (-): 0

Region: chr28 28542193-28542203. Max. coverage (+): 0. Max coverage (-): 0

Region: chr28 28542204-28542213. Max. coverage (+): 0. Max coverage (-): 0

Region: chr28 28542214-28542224. Max. coverage (+): 0. Max coverage (-): 0

Region: chr28 28542225-28542235. Max. coverage (+): 0. Max coverage (-): 0

Region: chr28 28542236-28542245. Max. coverage (+): 0. Max coverage (-): 0

Region: chr28 28542246-28542256. Max. coverage (+): 0. Max coverage (-): 0

Region: chr28 28542257-28542267. Max. coverage (+): 0. Max coverage (-): 0

Region: chr28 28542268-28542278. Max. coverage (+): 0. Max coverage (-): 0

Region: chr28 28542279-28542288. Max. coverage (+): 0. Max coverage (-): 0

Region: chr28 28542289-28542299. Max. coverage (+): 0. Max coverage (-): 0

Region: chr28 28542300-28542310. Max. coverage (+): 0. Max coverage (-): 0

Region: chr28 28542311-28542321. Max. coverage (+): 0. Max coverage (-): 0

Region: chr28 28542322-28542331. Max. coverage (+): 0. Max coverage (-): 0

Region: chr28 28542332-28542342. Max. coverage (+): 0. Max coverage (-): 0

Region: chr28 28542343-28542353. Max. coverage (+): 0. Max coverage (-): 0

Region: chr28 28542354-28542363. Max. coverage (+): 0. Max coverage (-): 0

Region: chr28 28542364-28542374. Max. coverage (+): 0. Max coverage (-): 0

Region: chr28 28542375-28542385. Max. coverage (+): 0. Max coverage (-): 0

Region: chr28 28542386-28542396. Max. coverage (+): 0. Max coverage (-): 0

Region: chr28 28542397-28542406. Max. coverage (+): 0. Max coverage (-): 1.85

Region: chr28 28542407-28542417. Max. coverage (+): 0. Max coverage (-): 3.2

Region: chr28 28542418-28542428. Max. coverage (+): 0. Max coverage (-): 5.62

Region: chr28 28542429-28542438. Max. coverage (+): 0. Max coverage (-): 4.98

Region: chr28 28542439-28542449. Max. coverage (+): 0. Max coverage (-): 0

Region: chr28 28542450-28542460. Max. coverage (+): 0. Max coverage (-): 0

Region: chr28 28542461-28542471. Max. coverage (+): 0. Max coverage (-): 1.78

Region: chr28 28542472-28542481. Max. coverage (+): 0. Max coverage (-): 0

Region: chr28 28542482-28542492. Max. coverage (+): 0. Max coverage (-): 11.85

Region: chr28 28542493-28542503. Max. coverage (+): 0. Max coverage (-): 16.74

Region: chr28 28542504-28542513. Max. coverage (+): 0. Max coverage (-): 1.3

Region: chr28 28542514-28542524. Max. coverage (+): 0. Max coverage (-): 0

Region: chr28 28542525-28542535. Max. coverage (+): 0. Max coverage (-): 0

Region: chr28 28542536-28542546. Max. coverage (+): 0. Max coverage (-): 1.41

Region: chr28 28542547-28542556. Max. coverage (+): 0. Max coverage (-): 0

Region: chr28 28542557-28542567. Max. coverage (+): 0. Max coverage (-): 0

Region: chr28 28542568-28542578. Max. coverage (+): 0. Max coverage (-): 0

Region: chr28 28542579-28542589. Max. coverage (+): 0. Max coverage (-): 0

Region: chr28 28542590-28542599. Max. coverage (+): 0. Max coverage (-): 4.04

Region: chr28 28542600-28542610. Max. coverage (+): 0. Max coverage (-): 0

Region: chr28 28542611-28542621. Max. coverage (+): 0. Max coverage (-): 0

Region: chr28 28542622-28542631. Max. coverage (+): 0. Max coverage (-): 0

Region: chr28 28542632-28542642. Max. coverage (+): 0. Max coverage (-): 0

Region: chr28 28542643-28542653. Max. coverage (+): 0. Max coverage (-): 0

Region: chr28 28542654-28542664. Max. coverage (+): 0. Max coverage (-): 0

Region: chr28 28542665-28542674. Max. coverage (+): 0. Max coverage (-): 0

Region: chr28 28542675-28542685. Max. coverage (+): 0. Max coverage (-): 0

Region: chr28 28542686-28542696. Max. coverage (+): 0. Max coverage (-): 0

Region: chr28 28542697-28542706. Max. coverage (+): 0. Max coverage (-): 0

Region: chr28 28542707-28542717. Max. coverage (+): 0. Max coverage (-): 0

Region: chr28 28542718-28542728. Max. coverage (+): 0. Max coverage (-): 1.99

Region: chr28 28542729-28542739. Max. coverage (+): 0. Max coverage (-): 0

Region: chr28 28542740-28542749. Max. coverage (+): 0. Max coverage (-): 0

Region: chr28 28542750-28542760. Max. coverage (+): 0. Max coverage (-): 0

Region: chr28 28542761-28542771. Max. coverage (+): 0. Max coverage (-): 0

Region: chr28 28542772-28542781. Max. coverage (+): 0. Max coverage (-): 13.22

Region: chr28 28542782-28542792. Max. coverage (+): 0. Max coverage (-): 13.22

Region: chr28 28542793-28542803. Max. coverage (+): 0. Max coverage (-): 1.92

Region: chr28 28542804-28542814. Max. coverage (+): 0. Max coverage (-): 0.49

Region: chr28 28542815-28542824. Max. coverage (+): 0. Max coverage (-): 6.04

Region: chr28 28542825-28542835. Max. coverage (+): 0. Max coverage (-): 5.55

Region: chr28 28542836-28542846. Max. coverage (+): 0. Max coverage (-): 0

Region: chr28 28542847-28542857. Max. coverage (+): 0. Max coverage (-): 5.14

Region: chr28 28542858-28542867. Max. coverage (+): 0. Max coverage (-): 5.14

Region: chr28 28542868-28542878. Max. coverage (+): 0. Max coverage (-): 0

Region: chr28 28542879-28542889. Max. coverage (+): 0. Max coverage (-): 1.4

Region: chr28 28542890-28542899. Max. coverage (+): 0. Max coverage (-): 4.69

Region: chr28 28542900-28542910. Max. coverage (+): 0. Max coverage (-): 0

Region: chr28 28542911-28542921. Max. coverage (+): 0. Max coverage (-): 0

Region: chr28 28542922-28542932. Max. coverage (+): 0. Max coverage (-): 4.31

Region: chr28 28542933-28542942. Max. coverage (+): 0. Max coverage (-): 4.31

Region: chr28 28542943-28542953. Max. coverage (+): 0. Max coverage (-): 0

Region: chr28 28542954-28542964. Max. coverage (+): 0. Max coverage (-): 2.08

Region: chr28 28542965-28542974. Max. coverage (+): 0. Max coverage (-): 2.08

Region: chr28 28542975-28542985. Max. coverage (+): 0. Max coverage (-): 4.18

Region: chr28 28542986-28542996. Max. coverage (+): 0. Max coverage (-): 15.41

Region: chr28 28542997-28543007. Max. coverage (+): 0. Max coverage (-): 15.41

Region: chr28 28543008-28543017. Max. coverage (+): 0. Max coverage (-): 0

Region: chr28 28543018-28543028. Max. coverage (+): 0. Max coverage (-): 0

Region: chr28 28543029-28543039. Max. coverage (+): 0. Max coverage (-): 0

Region: chr28 28543040-28543049. Max. coverage (+): 0. Max coverage (-): 0

Region: chr28 28543050-28543060. Max. coverage (+): 0. Max coverage (-): 0

Region: chr28 28543061-28543071. Max. coverage (+): 0. Max coverage (-): 0

Region: chr28 28543072-28543082. Max. coverage (+): 0. Max coverage (-): 0

Region: chr28 28543083-28543092. Max. coverage (+): 0. Max coverage (-): 0

Region: chr28 28543093-28543103. Max. coverage (+): 0. Max coverage (-): 0

Region: chr28 28543104-28543114. Max. coverage (+): 0. Max coverage (-): 0

Region: chr28 28543115-28543125. Max. coverage (+): 0. Max coverage (-): 0

Region: chr28 28543126-28543135. Max. coverage (+): 0. Max coverage (-): 0

Region: chr28 28543136-28543146. Max. coverage (+): 0. Max coverage (-): 0

Region: chr28 28543147-28543157. Max. coverage (+): 0. Max coverage (-): 0

Region: chr28 28543158-28543167. Max. coverage (+): 0. Max coverage (-): 0

Region: chr28 28543168-28543178. Max. coverage (+): 0. Max coverage (-): 0

Region: chr28 28543179-28543189. Max. coverage (+): 0. Max coverage (-): 0

Region: chr28 28543190-28543200. Max. coverage (+): 0. Max coverage (-): 0

Region: chr28 28543201-28543210. Max. coverage (+): 0. Max coverage (-): 0

Region: chr28 28543211-28543221. Max. coverage (+): 0. Max coverage (-): 0

Region: chr28 28543222-28543232. Max. coverage (+): 0. Max coverage (-): 0

Region: chr28 28543233-28543242. Max. coverage (+): 0. Max coverage (-): 0

Region: chr28 28543243-28543253. Max. coverage (+): 0. Max coverage (-): 0

Region: chr28 28543254-28543264. Max. coverage (+): 0. Max coverage (-): 0

Region: chr28 28543265-28543275. Max. coverage (+): 0. Max coverage (-): 0

Region: chr28 28543276-28543285. Max. coverage (+): 0. Max coverage (-): 0

Region: chr28 28543286-28543296. Max. coverage (+): 0. Max coverage (-): 0

Region: chr28 28543297-28543307. Max. coverage (+): 0. Max coverage (-): 0

Region: chr28 28543308-28543317. Max. coverage (+): 0. Max coverage (-): 0

Region: chr28 28543318-28543328. Max. coverage (+): 0. Max coverage (-): 0

Region: chr28 28543329-28543339. Max. coverage (+): 0. Max coverage (-): 0

Region: chr28 28543340-28543350. Max. coverage (+): 0. Max coverage (-): 0

Region: chr28 28543351-28543360. Max. coverage (+): 0. Max coverage (-): 0

Region: chr28 28543361-28543371. Max. coverage (+): 0. Max coverage (-): 0

Region: chr28 28543372-28543382. Max. coverage (+): 0. Max coverage (-): 0

Region: chr28 28543383-28543393. Max. coverage (+): 0. Max coverage (-): 0

Region: chr28 28543394-28543403. Max. coverage (+): 0. Max coverage (-): 0

Region: chr28 28543404-28543414. Max. coverage (+): 0. Max coverage (-): 0

Region: chr28 28543415-28543425. Max. coverage (+): 0. Max coverage (-): 0

Region: chr28 28543426-28543435. Max. coverage (+): 0. Max coverage (-): 0

Region: chr28 28543436-28543446. Max. coverage (+): 0. Max coverage (-): 0

Region: chr28 28543447-28543457. Max. coverage (+): 0. Max coverage (-): 0

Region: chr28 28543458-28543468. Max. coverage (+): 0. Max coverage (-): 0

Region: chr28 28543469-28543478. Max. coverage (+): 0. Max coverage (-): 0

Region: chr28 28543479-28543489. Max. coverage (+): 0. Max coverage (-): 0

Region: chr28 28543490-28543500. Max. coverage (+): 0. Max coverage (-): 0

Region: chr28 28543501-28543510. Max. coverage (+): 0. Max coverage (-): 0

Region: chr28 28543511-28543521. Max. coverage (+): 0. Max coverage (-): 0

Region: chr28 28543522-28543532. Max. coverage (+): 0. Max coverage (-): 0

Region: chr28 28543533-28543543. Max. coverage (+): 0. Max coverage (-): 0

Region: chr28 28543544-28543553. Max. coverage (+): 0. Max coverage (-): 0

Region: chr28 28543554-28543564. Max. coverage (+): 0. Max coverage (-): 0

Region: chr28 28543565-28543575. Max. coverage (+): 0. Max coverage (-): 0

Region: chr28 28543576-28543585. Max. coverage (+): 0. Max coverage (-): 0

Region: chr28 28543586-28543596. Max. coverage (+): 0. Max coverage (-): 0

Region: chr28 28543597-28543607. Max. coverage (+): 0. Max coverage (-): 7.6

Region: chr28 28543608-28543618. Max. coverage (+): 0. Max coverage (-): 7.6

Region: chr28 28543619-28543628. Max. coverage (+): 0. Max coverage (-): 0

Region: chr28 28543629-28543639. Max. coverage (+): 0. Max coverage (-): 0

Region: chr28 28543640-28543650. Max. coverage (+): 0. Max coverage (-): 0

Region: chr28 28543651-28543661. Max. coverage (+): 0. Max coverage (-): 0

Region: chr28 28543662-28543671. Max. coverage (+): 0. Max coverage (-): 0

Region: chr28 28543672-28543682. Max. coverage (+): 0. Max coverage (-): 0

Region: chr28 28543683-28543693. Max. coverage (+): 0. Max coverage (-): 0

Region: chr28 28543694-28543703. Max. coverage (+): 0. Max coverage (-): 0

Region: chr28 28543704-28543714. Max. coverage (+): 0. Max coverage (-): 0

Region: chr28 28543715-28543725. Max. coverage (+): 0. Max coverage (-): 0

Region: chr28 28543726-28543736. Max. coverage (+): 0. Max coverage (-): 0

Region: chr28 28543737-28543746. Max. coverage (+): 0. Max coverage (-): 0

Region: chr28 28543747-28543757. Max. coverage (+): 0. Max coverage (-): 0

Region: chr28 28543758-28543768. Max. coverage (+): 0. Max coverage (-): 3.67

Region: chr28 28543769-28543778. Max. coverage (+): 0. Max coverage (-): 10.58

Region: chr28 28543779-28543789. Max. coverage (+): 0. Max coverage (-): 10.58

Region: chr28 28543790-28543800. Max. coverage (+): 0. Max coverage (-): 0

Region: chr28 28543801-28543811. Max. coverage (+): 0. Max coverage (-): 0

Region: chr28 28543812-28543821. Max. coverage (+): 0. Max coverage (-): 0

Region: chr28 28543822-28543832. Max. coverage (+): 0. Max coverage (-): 0

Region: chr28 28543833-28543843. Max. coverage (+): 0. Max coverage (-): 0

Region: chr28 28543844-28543853. Max. coverage (+): 0. Max coverage (-): 5.04

Region: chr28 28543854-28543864. Max. coverage (+): 0. Max coverage (-): 0

Region: chr28 28543865-28543875. Max. coverage (+): 0. Max coverage (-): 0

Region: chr28 28543876-28543886. Max. coverage (+): 0. Max coverage (-): 0

Region: chr28 28543887-28543896. Max. coverage (+): 0. Max coverage (-): 0

Region: chr28 28543897-28543907. Max. coverage (+): 0. Max coverage (-): 0

Region: chr28 28543908-28543918. Max. coverage (+): 0. Max coverage (-): 0

Region: chr28 28543919-28543929. Max. coverage (+): 0. Max coverage (-): 0

Region: chr28 28543930-28543939. Max. coverage (+): 0. Max coverage (-): 0

Region: chr28 28543940-28543950. Max. coverage (+): 0. Max coverage (-): 2.6

Region: chr28 28543951-28543961. Max. coverage (+): 0. Max coverage (-): 2.76

Region: chr28 28543962-28543971. Max. coverage (+): 0. Max coverage (-): 2.76

Region: chr28 28543972-28543982. Max. coverage (+): 0. Max coverage (-): 0

Region: chr28 28543983-. Max. coverage (+): 0. Max coverage (-): 0

RepeatMasker Color Code

**+**

100-98% Identity

<98-95% Identity

<95-90% Identity

<90-85% Identity

<85-80% Identity

<80-75% Identity

<75-70% Identity

<70% Identity

**-**

Gene Set Color Code

**+**

Gene

Pseudogene

**-**

Topology/Coverage Color Code

Coverage Plus Strand

Coverage Minus Strand

Mainstrand: Plus

Mainstrand: Minus

Complementary Strand

Flanking Region  
(if option -flank >0)

Gene Set Annotation  
  
RepeatMasker Annotation  

**1. (TG)n**: 28538656-28538683 (+), Divergence to consensus: 0%  
**2. Bov-tA3**: 28538690-28538810 (-), Divergence to consensus: 19.8%  
**3. MIRb**: 28538821-28538901 (+), Divergence to consensus: 34.9%  
**4. MIRb**: 28539608-28539664 (+), Divergence to consensus: 34.1%  
**5. MIR**: 28539795-28539903 (+), Divergence to consensus: 37.3%  
**6. MIR**: 28540052-28540236 (+), Divergence to consensus: 55.9%  
**7. L2b**: 28542057-28542186 (+), Divergence to consensus: 44.9%  
**8. L2c**: 28542199-28542338 (+), Divergence to consensus: 42.4%  
**9. MIRc**: 28543049-28543200 (+), Divergence to consensus: 36.2%  
**10. MIR3**: 28543223-28543278 (-), Divergence to consensus: 28.6%

  
Transcription Factor Binding Sites  

**RFX4\_2** (Sequence: CTTGGTTAC (+): 28540456)
