# Supplementary material for: Small RNA sequencing of cryopreserved semen from single bull revealed altered miRNAs and piRNAs expression between High- and Low-motile sperm populations
Source: BMC Genomics. 2017 Jan 4;18:14. doi: 10.1186/s12864-016-3394-7 (PMC5209821; doi:10.1186/s12864-016-3394-7)
Supplement: Additional file 3: — Details for each piRNA clusters found in High Motile (HM) sperm fraction. Genes, repeats, transposable elements and transcription factors binding sites falling within the cluster regions were reported. (ZIP 1896 kb) [file 12864_2016_3394_MOESM3_ESM.zip › 73.html]

piRNA cluster 73


Predicted piRNA cluster no. 73     previous   next
  

Show proTRAC run info
Hide proTRAC run info

================================= proTRAC ====================================  
VERSION: 2.1                                    LAST MODIFIED: 06. October 2015  
  
Please cite:  
Rosenkranz D, Zischler H. proTRAC - a software for probabilistic piRNA cluster  
detection, visualization and analysis. 2012. BMC Bioinformatics 13:5.  
  
and (for proTRAC 2.0 and later):  
Rosenkranz D, Rudloff S, Bastuck K, Ketting RF, Zischler H. Tupaia small RNAs  
provide insights into function and evolution of RNAi-based transposon defense  
in mammals. 2015. RNA 21(5):911-922.  
  
Contact:  
David Rosenkranz  
Institute of Anthropology, small RNA group  
Johannes Gutenberg University Mainz  
email: rosenkranz@uni-mainz.de  
  
You can find the latest proTRAC version at:  
http://sourceforge.net/projects/protrac/files  
http://www.smallRNAgroup-mainz.de/software  
==============================================================================  
  
PARAMETERS:  
Map file: .............../storage/core/barbara/genhome/smallRNA/fertility/Sample\_motile/pirna/Sample\_motile\_26-33\_collapsed.fa.no-dust.map.weighted-10000-1000-b-0  
Genome file: ............/storage/core/barbara/genhome/smallRNA/fertility/Sample\_all/pirna/bt\_311\_chrY.fa  
RepeatMasker annotation: /storage/genomes/bt\_umd31/GCF\_000003055.6\_Bos\_taurus\_UMD\_3.1.1\_repeatMasker\_chr.out  
GeneSet:................./storage/core/barbara/genhome/smallRNA/fertility/Sample\_all/pirna/full.gtf  
  
Significant (p<=0.01) hit density will be calculated based  
on observed hit distribution.  
  
Sliding window size: ........................................ 5000 bp  
Sliding window increament: .................................. 1000 bp  
Normalize each hit by number of genomic hits: ............... 1 [0=no/1=yes]  
Normalize each hit by number of sequence reads: ............. 1 [0=no/1=yes]  
Normalize values (-> per million mapped reads): ............. 1 [0=no/1=yes]  
Min. fraction of hits with 1T(U) or 10A: .................... 0.75  
Alternatively: Min. fraction of hits with 1T(U) and 10A: .... 0.5  
Min. fraction of hits with typical piRNA length: ............ 0.75  
Typical piRNA length: ....................................... 26-33 nt  
Min. size of a piRNA cluster: ............................... 5000 bp.  
Min. number of hits (absolute): ............................. 0  
Min. number of hits (normalized): ........................... 0  
Min. fraction of hits on the mainstrand: .................... 0.75  
Top fraction of mapped sequences (in terms of read counts): . 1%  
Top fraction accounts for max. n% of sequence reads: ........ 90%  
Min. fraction of hits on each arm of a bidirectional cluster: 0.1  
Output image file for each cluster: ......................... 0 [0=no/1=yes]  
Output html file for each cluster: .......................... 1 [0=no/1=yes]  
Output a summary table: ..................................... 1 [0=no/1=yes]  
Output a FASTA file for each cluster (piRNA sequences): ..... 1 [0=no/1=yes]  
Output a FASTA file comprising cluster sequences: ........... 1 [0=no/1=yes]  
Search DNA motifs in clusters: .............................. 1 [0=no/1=yes]  
Output flanking sequences: +/- .............................. 0 bp  
Output ~.pTi file: .......................................... 1 [0=no/1=yes]  
==============================================================================  
  
  
Genome size (without gaps): ............ 2678902517 bp  
Gaps (N/X/-): .......................... 53837044 bp  
Mapped reads: .......................... 658825247023  
Non-identical sequences: ............... 514171  
Genomic hits: .......................... 764233  
Significant densitiy of mapped reads: .. 12867599.5173724 reads/kb

Show proTRAC cluster info
Hide proTRAC cluster info

|  |  |
| --- | --- |
| Location | chr28 |
| Coordinates | 33682298-33738603 |
| Size [bp] | 56306 |
| Sequence hit loci | 8831 |
| Mapped reads (normalized) | 10133352992 |
| Mapped reads (normalized) per kb | 179969328.2 |
| Normalized reads with 1T (1U) | 75.9% |
| Normalized reads with 10A | 31.6% |
| Normalized reads with length 26-33 nt | 100% |
| Normalized reads on the main strand(s) | 98.7% |
| Predicted directionality | mono:plus |

100%

0%

1T (1U)  
reads

10A reads

26-33 nt  
reads

reads on mainstrand

**Either the amount of reads with 1T (1U) OR 10A has to exceed 75% (set with option: -1Tor10A)  
Alternatively the amount of reads with 1T (1U) AND 10A has to exceed 50% (set with option: -1Tand10A)  
Minimum amount of reads with preferred size is 75% (set with option: -pisize)  
Minimum amount of reads on the main strand(s) is 75% (set with option: -clstrand)**

Show read coverage
Hide read coverage

WHAT DO I SEE HERE?  
This chart shows the location of mapped sequence reads within a predicted piRNA cluster. The color refers to the number of genomic hits produced by the sequence read in question. A dark red bar indicates that this sequence read produces many other hits elsewhere in the genome. Many adjacent red or yellow bars can indicate the presence of a multi-copy element such as transposons or rRNA genes. A dark green bar indicates that this sequence read maps uniquely to this locus.

1 hit

2-5 hits

6-10 hits

11-20 hits

21-50 hits

51-100 hits

> 100 hits

chr28

33682298

33738603

Gene Set

RepeatMasker

Mapped  
Reads

225.1

plus strand

minus strand

225.1

Region: chr28 28543984-33682354. Max. coverage (+): 0. Max coverage (-): 2.32

Region: chr28 33682355-33682466. Max. coverage (+): 0. Max coverage (-): 0

Region: chr28 33682467-33682579. Max. coverage (+): 0. Max coverage (-): 0

Region: chr28 33682580-33682692. Max. coverage (+): 0. Max coverage (-): 1.08

Region: chr28 33682693-33682804. Max. coverage (+): 0. Max coverage (-): 1.08

Region: chr28 33682805-33682917. Max. coverage (+): 0. Max coverage (-): 0

Region: chr28 33682918-33683029. Max. coverage (+): 0. Max coverage (-): 0

Region: chr28 33683030-33683142. Max. coverage (+): 0. Max coverage (-): 0

Region: chr28 33683143-33683255. Max. coverage (+): 0. Max coverage (-): 0

Region: chr28 33683256-33683367. Max. coverage (+): 0. Max coverage (-): 0

Region: chr28 33683368-33683480. Max. coverage (+): 0. Max coverage (-): 0

Region: chr28 33683481-33683593. Max. coverage (+): 0. Max coverage (-): 0

Region: chr28 33683594-33683705. Max. coverage (+): 0. Max coverage (-): 4.22

Region: chr28 33683706-33683818. Max. coverage (+): 0. Max coverage (-): 0.77

Region: chr28 33683819-33683930. Max. coverage (+): 0. Max coverage (-): 1.35

Region: chr28 33683931-33684043. Max. coverage (+): 0. Max coverage (-): 0

Region: chr28 33684044-33684156. Max. coverage (+): 0. Max coverage (-): 0

Region: chr28 33684157-33684268. Max. coverage (+): 0. Max coverage (-): 0

Region: chr28 33684269-33684381. Max. coverage (+): 0. Max coverage (-): 0

Region: chr28 33684382-33684493. Max. coverage (+): 0. Max coverage (-): 0

Region: chr28 33684494-33684606. Max. coverage (+): 0. Max coverage (-): 1.64

Region: chr28 33684607-33684719. Max. coverage (+): 2.66. Max coverage (-): 5.17

Region: chr28 33684720-33684831. Max. coverage (+): 0. Max coverage (-): 16.26

Region: chr28 33684832-33684944. Max. coverage (+): 0. Max coverage (-): 1.25

Region: chr28 33684945-33685056. Max. coverage (+): 0. Max coverage (-): 0

Region: chr28 33685057-33685169. Max. coverage (+): 0. Max coverage (-): 0

Region: chr28 33685170-33685282. Max. coverage (+): 0. Max coverage (-): 2.05

Region: chr28 33685283-33685394. Max. coverage (+): 0. Max coverage (-): 1.07

Region: chr28 33685395-33685507. Max. coverage (+): 0. Max coverage (-): 0

Region: chr28 33685508-33685620. Max. coverage (+): 0. Max coverage (-): 0

Region: chr28 33685621-33685732. Max. coverage (+): 0. Max coverage (-): 0

Region: chr28 33685733-33685845. Max. coverage (+): 4.71. Max coverage (-): 0.76

Region: chr28 33685846-33685957. Max. coverage (+): 0. Max coverage (-): 5.74

Region: chr28 33685958-33686070. Max. coverage (+): 0. Max coverage (-): 5.35

Region: chr28 33686071-33686183. Max. coverage (+): 0. Max coverage (-): 10.84

Region: chr28 33686184-33686295. Max. coverage (+): 0. Max coverage (-): 8.16

Region: chr28 33686296-33686408. Max. coverage (+): 0. Max coverage (-): 7.82

Region: chr28 33686409-33686520. Max. coverage (+): 0. Max coverage (-): 0

Region: chr28 33686521-33686633. Max. coverage (+): 0. Max coverage (-): 0

Region: chr28 33686634-33686746. Max. coverage (+): 0. Max coverage (-): 0

Region: chr28 33686747-33686858. Max. coverage (+): 0. Max coverage (-): 0

Region: chr28 33686859-33686971. Max. coverage (+): 0. Max coverage (-): 0

Region: chr28 33686972-33687084. Max. coverage (+): 0. Max coverage (-): 0

Region: chr28 33687085-33687196. Max. coverage (+): 0. Max coverage (-): 0

Region: chr28 33687197-33687309. Max. coverage (+): 0. Max coverage (-): 0

Region: chr28 33687310-33687421. Max. coverage (+): 0. Max coverage (-): 0

Region: chr28 33687422-33687534. Max. coverage (+): 0. Max coverage (-): 0

Region: chr28 33687535-33687647. Max. coverage (+): 0. Max coverage (-): 0

Region: chr28 33687648-33687759. Max. coverage (+): 0. Max coverage (-): 3.63

Region: chr28 33687760-33687872. Max. coverage (+): 0. Max coverage (-): 5.08

Region: chr28 33687873-33687984. Max. coverage (+): 0. Max coverage (-): 0

Region: chr28 33687985-33688097. Max. coverage (+): 0. Max coverage (-): 0

Region: chr28 33688098-33688210. Max. coverage (+): 0. Max coverage (-): 0

Region: chr28 33688211-33688322. Max. coverage (+): 0. Max coverage (-): 0

Region: chr28 33688323-33688435. Max. coverage (+): 0. Max coverage (-): 0

Region: chr28 33688436-33688547. Max. coverage (+): 0. Max coverage (-): 0

Region: chr28 33688548-33688660. Max. coverage (+): 0. Max coverage (-): 0

Region: chr28 33688661-33688773. Max. coverage (+): 0. Max coverage (-): 0

Region: chr28 33688774-33688885. Max. coverage (+): 0. Max coverage (-): 0

Region: chr28 33688886-33688998. Max. coverage (+): 0. Max coverage (-): 0

Region: chr28 33688999-33689111. Max. coverage (+): 0. Max coverage (-): 0

Region: chr28 33689112-33689223. Max. coverage (+): 0. Max coverage (-): 0

Region: chr28 33689224-33689336. Max. coverage (+): 0. Max coverage (-): 0

Region: chr28 33689337-33689448. Max. coverage (+): 0. Max coverage (-): 0

Region: chr28 33689449-33689561. Max. coverage (+): 0. Max coverage (-): 0

Region: chr28 33689562-33689674. Max. coverage (+): 0. Max coverage (-): 0

Region: chr28 33689675-33689786. Max. coverage (+): 0. Max coverage (-): 0

Region: chr28 33689787-33689899. Max. coverage (+): 0. Max coverage (-): 0

Region: chr28 33689900-33690011. Max. coverage (+): 0. Max coverage (-): 0

Region: chr28 33690012-33690124. Max. coverage (+): 0. Max coverage (-): 0

Region: chr28 33690125-33690237. Max. coverage (+): 0. Max coverage (-): 0

Region: chr28 33690238-33690349. Max. coverage (+): 0. Max coverage (-): 0

Region: chr28 33690350-33690462. Max. coverage (+): 0. Max coverage (-): 0

Region: chr28 33690463-33690574. Max. coverage (+): 0. Max coverage (-): 0

Region: chr28 33690575-33690687. Max. coverage (+): 0. Max coverage (-): 0

Region: chr28 33690688-33690800. Max. coverage (+): 0. Max coverage (-): 0

Region: chr28 33690801-33690912. Max. coverage (+): 0. Max coverage (-): 0

Region: chr28 33690913-33691025. Max. coverage (+): 0.59. Max coverage (-): 10.79

Region: chr28 33691026-33691138. Max. coverage (+): 0. Max coverage (-): 0

Region: chr28 33691139-33691250. Max. coverage (+): 0. Max coverage (-): 4.53

Region: chr28 33691251-33691363. Max. coverage (+): 0. Max coverage (-): 0

Region: chr28 33691364-33691475. Max. coverage (+): 13.72. Max coverage (-): 0

Region: chr28 33691476-33691588. Max. coverage (+): 11.45. Max coverage (-): 0

Region: chr28 33691589-33691701. Max. coverage (+): 32.51. Max coverage (-): 0

Region: chr28 33691702-33691813. Max. coverage (+): 36.48. Max coverage (-): 0

Region: chr28 33691814-33691926. Max. coverage (+): 18.81. Max coverage (-): 0

Region: chr28 33691927-33692038. Max. coverage (+): 27.41. Max coverage (-): 0

Region: chr28 33692039-33692151. Max. coverage (+): 3.15. Max coverage (-): 0

Region: chr28 33692152-33692264. Max. coverage (+): 32.38. Max coverage (-): 0

Region: chr28 33692265-33692376. Max. coverage (+): 26.16. Max coverage (-): 1.24

Region: chr28 33692377-33692489. Max. coverage (+): 26.16. Max coverage (-): 0

Region: chr28 33692490-33692601. Max. coverage (+): 16.24. Max coverage (-): 0

Region: chr28 33692602-33692714. Max. coverage (+): 12.95. Max coverage (-): 0

Region: chr28 33692715-33692827. Max. coverage (+): 26.06. Max coverage (-): 1.89

Region: chr28 33692828-33692939. Max. coverage (+): 12.61. Max coverage (-): 0

Region: chr28 33692940-33693052. Max. coverage (+): 13.2. Max coverage (-): 0

Region: chr28 33693053-33693165. Max. coverage (+): 16.34. Max coverage (-): 4.95

Region: chr28 33693166-33693277. Max. coverage (+): 74.87. Max coverage (-): 0

Region: chr28 33693278-33693390. Max. coverage (+): 47.22. Max coverage (-): 6.45

Region: chr28 33693391-33693502. Max. coverage (+): 11.73. Max coverage (-): 2.54

Region: chr28 33693503-33693615. Max. coverage (+): 25.53. Max coverage (-): 4.86

Region: chr28 33693616-33693728. Max. coverage (+): 9.3. Max coverage (-): 0

Region: chr28 33693729-33693840. Max. coverage (+): 23.39. Max coverage (-): 0

Region: chr28 33693841-33693953. Max. coverage (+): 49.96. Max coverage (-): 0.6

Region: chr28 33693954-33694065. Max. coverage (+): 0. Max coverage (-): 0

Region: chr28 33694066-33694178. Max. coverage (+): 37.81. Max coverage (-): 4.02

Region: chr28 33694179-33694291. Max. coverage (+): 70.51. Max coverage (-): 4.02

Region: chr28 33694292-33694403. Max. coverage (+): 10.36. Max coverage (-): 0

Region: chr28 33694404-33694516. Max. coverage (+): 0. Max coverage (-): 0

Region: chr28 33694517-33694629. Max. coverage (+): 44.3. Max coverage (-): 0

Region: chr28 33694630-33694741. Max. coverage (+): 11.07. Max coverage (-): 0

Region: chr28 33694742-33694854. Max. coverage (+): 85.47. Max coverage (-): 0

Region: chr28 33694855-33694966. Max. coverage (+): 67.97. Max coverage (-): 0

Region: chr28 33694967-33695079. Max. coverage (+): 85.53. Max coverage (-): 0

Region: chr28 33695080-33695192. Max. coverage (+): 40.14. Max coverage (-): 0

Region: chr28 33695193-33695304. Max. coverage (+): 17.27. Max coverage (-): 0

Region: chr28 33695305-33695417. Max. coverage (+): 72.48. Max coverage (-): 0.91

Region: chr28 33695418-33695529. Max. coverage (+): 56.32. Max coverage (-): 0

Region: chr28 33695530-33695642. Max. coverage (+): 57.62. Max coverage (-): 0

Region: chr28 33695643-33695755. Max. coverage (+): 19.68. Max coverage (-): 0

Region: chr28 33695756-33695867. Max. coverage (+): 27.86. Max coverage (-): 0

Region: chr28 33695868-33695980. Max. coverage (+): 98.55. Max coverage (-): 0

Region: chr28 33695981-33696092. Max. coverage (+): 0. Max coverage (-): 0

Region: chr28 33696093-33696205. Max. coverage (+): 0. Max coverage (-): 0

Region: chr28 33696206-33696318. Max. coverage (+): 0. Max coverage (-): 0

Region: chr28 33696319-33696430. Max. coverage (+): 31.31. Max coverage (-): 0

Region: chr28 33696431-33696543. Max. coverage (+): 0. Max coverage (-): 0

Region: chr28 33696544-33696656. Max. coverage (+): 18.28. Max coverage (-): 0

Region: chr28 33696657-33696768. Max. coverage (+): 46.08. Max coverage (-): 0

Region: chr28 33696769-33696881. Max. coverage (+): 13.77. Max coverage (-): 0

Region: chr28 33696882-33696993. Max. coverage (+): 14.03. Max coverage (-): 0

Region: chr28 33696994-33697106. Max. coverage (+): 9.96. Max coverage (-): 0

Region: chr28 33697107-33697219. Max. coverage (+): 6.28. Max coverage (-): 1.93

Region: chr28 33697220-33697331. Max. coverage (+): 137.25. Max coverage (-): 1.13

Region: chr28 33697332-33697444. Max. coverage (+): 3.17. Max coverage (-): 0

Region: chr28 33697445-33697556. Max. coverage (+): 20.89. Max coverage (-): 0

Region: chr28 33697557-33697669. Max. coverage (+): 20.89. Max coverage (-): 0

Region: chr28 33697670-33697782. Max. coverage (+): 29.23. Max coverage (-): 0

Region: chr28 33697783-33697894. Max. coverage (+): 0.78. Max coverage (-): 0

Region: chr28 33697895-33698007. Max. coverage (+): 65.15. Max coverage (-): 0

Region: chr28 33698008-33698119. Max. coverage (+): 124.32. Max coverage (-): 0

Region: chr28 33698120-33698232. Max. coverage (+): 38.71. Max coverage (-): 0

Region: chr28 33698233-33698345. Max. coverage (+): 61.91. Max coverage (-): 0

Region: chr28 33698346-33698457. Max. coverage (+): 74.89. Max coverage (-): 0

Region: chr28 33698458-33698570. Max. coverage (+): 37.26. Max coverage (-): 0

Region: chr28 33698571-33698683. Max. coverage (+): 41.69. Max coverage (-): 0

Region: chr28 33698684-33698795. Max. coverage (+): 38.02. Max coverage (-): 0

Region: chr28 33698796-33698908. Max. coverage (+): 10.33. Max coverage (-): 0

Region: chr28 33698909-33699020. Max. coverage (+): 5.57. Max coverage (-): 0

Region: chr28 33699021-33699133. Max. coverage (+): 0. Max coverage (-): 0

Region: chr28 33699134-33699246. Max. coverage (+): 54.78. Max coverage (-): 1.56

Region: chr28 33699247-33699358. Max. coverage (+): 37.03. Max coverage (-): 0

Region: chr28 33699359-33699471. Max. coverage (+): 68.8. Max coverage (-): 0

Region: chr28 33699472-33699583. Max. coverage (+): 0. Max coverage (-): 0

Region: chr28 33699584-33699696. Max. coverage (+): 0. Max coverage (-): 0

Region: chr28 33699697-33699809. Max. coverage (+): 0. Max coverage (-): 0

Region: chr28 33699810-33699921. Max. coverage (+): 0. Max coverage (-): 0

Region: chr28 33699922-33700034. Max. coverage (+): 0. Max coverage (-): 0

Region: chr28 33700035-33700147. Max. coverage (+): 0. Max coverage (-): 0

Region: chr28 33700148-33700259. Max. coverage (+): 0. Max coverage (-): 0

Region: chr28 33700260-33700372. Max. coverage (+): 0. Max coverage (-): 0

Region: chr28 33700373-33700484. Max. coverage (+): 0. Max coverage (-): 0

Region: chr28 33700485-33700597. Max. coverage (+): 0. Max coverage (-): 0

Region: chr28 33700598-33700710. Max. coverage (+): 0. Max coverage (-): 0

Region: chr28 33700711-33700822. Max. coverage (+): 5.25. Max coverage (-): 0

Region: chr28 33700823-33700935. Max. coverage (+): 4.58. Max coverage (-): 0

Region: chr28 33700936-33701047. Max. coverage (+): 3.76. Max coverage (-): 0

Region: chr28 33701048-33701160. Max. coverage (+): 6.18. Max coverage (-): 0

Region: chr28 33701161-33701273. Max. coverage (+): 16.87. Max coverage (-): 0

Region: chr28 33701274-33701385. Max. coverage (+): 11.48. Max coverage (-): 0

Region: chr28 33701386-33701498. Max. coverage (+): 3.53. Max coverage (-): 0

Region: chr28 33701499-33701610. Max. coverage (+): 3.4. Max coverage (-): 0

Region: chr28 33701611-33701723. Max. coverage (+): 0. Max coverage (-): 0

Region: chr28 33701724-33701836. Max. coverage (+): 0. Max coverage (-): 0

Region: chr28 33701837-33701948. Max. coverage (+): 0. Max coverage (-): 0

Region: chr28 33701949-33702061. Max. coverage (+): 0. Max coverage (-): 0

Region: chr28 33702062-33702174. Max. coverage (+): 0. Max coverage (-): 0

Region: chr28 33702175-33702286. Max. coverage (+): 0. Max coverage (-): 0

Region: chr28 33702287-33702399. Max. coverage (+): 0. Max coverage (-): 0

Region: chr28 33702400-33702511. Max. coverage (+): 0. Max coverage (-): 0

Region: chr28 33702512-33702624. Max. coverage (+): 0. Max coverage (-): 0

Region: chr28 33702625-33702737. Max. coverage (+): 0. Max coverage (-): 0

Region: chr28 33702738-33702849. Max. coverage (+): 0. Max coverage (-): 0

Region: chr28 33702850-33702962. Max. coverage (+): 0. Max coverage (-): 0

Region: chr28 33702963-33703074. Max. coverage (+): 0. Max coverage (-): 0

Region: chr28 33703075-33703187. Max. coverage (+): 0. Max coverage (-): 0

Region: chr28 33703188-33703300. Max. coverage (+): 0. Max coverage (-): 0

Region: chr28 33703301-33703412. Max. coverage (+): 0. Max coverage (-): 0

Region: chr28 33703413-33703525. Max. coverage (+): 0. Max coverage (-): 0

Region: chr28 33703526-33703637. Max. coverage (+): 0. Max coverage (-): 0

Region: chr28 33703638-33703750. Max. coverage (+): 0. Max coverage (-): 0

Region: chr28 33703751-33703863. Max. coverage (+): 20.07. Max coverage (-): 0

Region: chr28 33703864-33703975. Max. coverage (+): 22.17. Max coverage (-): 0

Region: chr28 33703976-33704088. Max. coverage (+): 0. Max coverage (-): 0

Region: chr28 33704089-33704201. Max. coverage (+): 0.64. Max coverage (-): 0

Region: chr28 33704202-33704313. Max. coverage (+): 0. Max coverage (-): 0

Region: chr28 33704314-33704426. Max. coverage (+): 6.84. Max coverage (-): 0

Region: chr28 33704427-33704538. Max. coverage (+): 16.56. Max coverage (-): 0

Region: chr28 33704539-33704651. Max. coverage (+): 24.34. Max coverage (-): 0

Region: chr28 33704652-33704764. Max. coverage (+): 19.67. Max coverage (-): 0

Region: chr28 33704765-33704876. Max. coverage (+): 23.86. Max coverage (-): 0.96

Region: chr28 33704877-33704989. Max. coverage (+): 61.17. Max coverage (-): 0

Region: chr28 33704990-33705101. Max. coverage (+): 14.22. Max coverage (-): 5.09

Region: chr28 33705102-33705214. Max. coverage (+): 64.15. Max coverage (-): 0

Region: chr28 33705215-33705327. Max. coverage (+): 86.29. Max coverage (-): 0

Region: chr28 33705328-33705439. Max. coverage (+): 52.51. Max coverage (-): 0

Region: chr28 33705440-33705552. Max. coverage (+): 22.23. Max coverage (-): 1.6

Region: chr28 33705553-33705664. Max. coverage (+): 55.01. Max coverage (-): 0

Region: chr28 33705665-33705777. Max. coverage (+): 16.6. Max coverage (-): 0

Region: chr28 33705778-33705890. Max. coverage (+): 41.59. Max coverage (-): 0

Region: chr28 33705891-33706002. Max. coverage (+): 67.1. Max coverage (-): 0

Region: chr28 33706003-33706115. Max. coverage (+): 5.19. Max coverage (-): 0

Region: chr28 33706116-33706228. Max. coverage (+): 30.71. Max coverage (-): 0

Region: chr28 33706229-33706340. Max. coverage (+): 38.61. Max coverage (-): 0

Region: chr28 33706341-33706453. Max. coverage (+): 225.1. Max coverage (-): 0

Region: chr28 33706454-33706565. Max. coverage (+): 14.01. Max coverage (-): 0

Region: chr28 33706566-33706678. Max. coverage (+): 22.22. Max coverage (-): 0

Region: chr28 33706679-33706791. Max. coverage (+): 89.46. Max coverage (-): 0

Region: chr28 33706792-33706903. Max. coverage (+): 39. Max coverage (-): 0

Region: chr28 33706904-33707016. Max. coverage (+): 47.63. Max coverage (-): 0

Region: chr28 33707017-33707128. Max. coverage (+): 63.44. Max coverage (-): 0

Region: chr28 33707129-33707241. Max. coverage (+): 75.47. Max coverage (-): 5.41

Region: chr28 33707242-33707354. Max. coverage (+): 69.05. Max coverage (-): 0

Region: chr28 33707355-33707466. Max. coverage (+): 69.27. Max coverage (-): 0

Region: chr28 33707467-33707579. Max. coverage (+): 10.22. Max coverage (-): 0

Region: chr28 33707580-33707692. Max. coverage (+): 12.78. Max coverage (-): 0

Region: chr28 33707693-33707804. Max. coverage (+): 4.17. Max coverage (-): 0

Region: chr28 33707805-33707917. Max. coverage (+): 0. Max coverage (-): 0

Region: chr28 33707918-33708029. Max. coverage (+): 46.99. Max coverage (-): 0

Region: chr28 33708030-33708142. Max. coverage (+): 86.31. Max coverage (-): 0

Region: chr28 33708143-33708255. Max. coverage (+): 15.66. Max coverage (-): 0

Region: chr28 33708256-33708367. Max. coverage (+): 29.46. Max coverage (-): 0

Region: chr28 33708368-33708480. Max. coverage (+): 33.9. Max coverage (-): 0

Region: chr28 33708481-33708592. Max. coverage (+): 80.53. Max coverage (-): 0

Region: chr28 33708593-33708705. Max. coverage (+): 70.59. Max coverage (-): 0

Region: chr28 33708706-33708818. Max. coverage (+): 12.37. Max coverage (-): 0

Region: chr28 33708819-33708930. Max. coverage (+): 44.81. Max coverage (-): 0

Region: chr28 33708931-33709043. Max. coverage (+): 12.25. Max coverage (-): 2.09

Region: chr28 33709044-33709155. Max. coverage (+): 0. Max coverage (-): 0

Region: chr28 33709156-33709268. Max. coverage (+): 0. Max coverage (-): 0

Region: chr28 33709269-33709381. Max. coverage (+): 0. Max coverage (-): 0

Region: chr28 33709382-33709493. Max. coverage (+): 10.56. Max coverage (-): 0

Region: chr28 33709494-33709606. Max. coverage (+): 21.21. Max coverage (-): 0

Region: chr28 33709607-33709719. Max. coverage (+): 5.91. Max coverage (-): 0

Region: chr28 33709720-33709831. Max. coverage (+): 0. Max coverage (-): 0

Region: chr28 33709832-33709944. Max. coverage (+): 20.21. Max coverage (-): 0

Region: chr28 33709945-33710056. Max. coverage (+): 32.3. Max coverage (-): 0

Region: chr28 33710057-33710169. Max. coverage (+): 19.2. Max coverage (-): 0

Region: chr28 33710170-33710282. Max. coverage (+): 29.44. Max coverage (-): 0

Region: chr28 33710283-33710394. Max. coverage (+): 27.8. Max coverage (-): 0

Region: chr28 33710395-33710507. Max. coverage (+): 25.95. Max coverage (-): 0

Region: chr28 33710508-33710619. Max. coverage (+): 8.47. Max coverage (-): 0

Region: chr28 33710620-33710732. Max. coverage (+): 15.29. Max coverage (-): 0

Region: chr28 33710733-33710845. Max. coverage (+): 18.51. Max coverage (-): 0

Region: chr28 33710846-33710957. Max. coverage (+): 13.5. Max coverage (-): 0

Region: chr28 33710958-33711070. Max. coverage (+): 13.22. Max coverage (-): 0

Region: chr28 33711071-33711182. Max. coverage (+): 9.7. Max coverage (-): 0

Region: chr28 33711183-33711295. Max. coverage (+): 0. Max coverage (-): 0

Region: chr28 33711296-33711408. Max. coverage (+): 0. Max coverage (-): 0

Region: chr28 33711409-33711520. Max. coverage (+): 18.42. Max coverage (-): 0

Region: chr28 33711521-33711633. Max. coverage (+): 45.65. Max coverage (-): 0

Region: chr28 33711634-33711746. Max. coverage (+): 49.29. Max coverage (-): 0

Region: chr28 33711747-33711858. Max. coverage (+): 151.45. Max coverage (-): 0

Region: chr28 33711859-33711971. Max. coverage (+): 14.46. Max coverage (-): 0

Region: chr28 33711972-33712083. Max. coverage (+): 87.37. Max coverage (-): 0

Region: chr28 33712084-33712196. Max. coverage (+): 41.09. Max coverage (-): 0

Region: chr28 33712197-33712309. Max. coverage (+): 37.91. Max coverage (-): 0

Region: chr28 33712310-33712421. Max. coverage (+): 161.05. Max coverage (-): 0

Region: chr28 33712422-33712534. Max. coverage (+): 101.27. Max coverage (-): 0

Region: chr28 33712535-33712646. Max. coverage (+): 40.42. Max coverage (-): 0

Region: chr28 33712647-33712759. Max. coverage (+): 37.94. Max coverage (-): 0

Region: chr28 33712760-33712872. Max. coverage (+): 33.42. Max coverage (-): 0

Region: chr28 33712873-33712984. Max. coverage (+): 28.86. Max coverage (-): 0

Region: chr28 33712985-33713097. Max. coverage (+): 40.17. Max coverage (-): 0

Region: chr28 33713098-33713209. Max. coverage (+): 28.45. Max coverage (-): 0

Region: chr28 33713210-33713322. Max. coverage (+): 6.88. Max coverage (-): 0

Region: chr28 33713323-33713435. Max. coverage (+): 51.77. Max coverage (-): 0

Region: chr28 33713436-33713547. Max. coverage (+): 27.16. Max coverage (-): 0

Region: chr28 33713548-33713660. Max. coverage (+): 9.07. Max coverage (-): 0

Region: chr28 33713661-33713773. Max. coverage (+): 19.04. Max coverage (-): 0

Region: chr28 33713774-33713885. Max. coverage (+): 14.7. Max coverage (-): 0

Region: chr28 33713886-33713998. Max. coverage (+): 25.84. Max coverage (-): 0

Region: chr28 33713999-33714110. Max. coverage (+): 17.92. Max coverage (-): 0

Region: chr28 33714111-33714223. Max. coverage (+): 0. Max coverage (-): 0

Region: chr28 33714224-33714336. Max. coverage (+): 0. Max coverage (-): 0

Region: chr28 33714337-33714448. Max. coverage (+): 6.16. Max coverage (-): 0

Region: chr28 33714449-33714561. Max. coverage (+): 173.21. Max coverage (-): 0

Region: chr28 33714562-33714673. Max. coverage (+): 38.82. Max coverage (-): 0

Region: chr28 33714674-33714786. Max. coverage (+): 36.38. Max coverage (-): 0

Region: chr28 33714787-33714899. Max. coverage (+): 137.66. Max coverage (-): 0

Region: chr28 33714900-33715011. Max. coverage (+): 19.39. Max coverage (-): 0

Region: chr28 33715012-33715124. Max. coverage (+): 14.92. Max coverage (-): 0

Region: chr28 33715125-33715237. Max. coverage (+): 14.04. Max coverage (-): 0

Region: chr28 33715238-33715349. Max. coverage (+): 15.42. Max coverage (-): 0

Region: chr28 33715350-33715462. Max. coverage (+): 15.76. Max coverage (-): 0

Region: chr28 33715463-33715574. Max. coverage (+): 7.84. Max coverage (-): 0

Region: chr28 33715575-33715687. Max. coverage (+): 4.14. Max coverage (-): 0

Region: chr28 33715688-33715800. Max. coverage (+): 4. Max coverage (-): 0

Region: chr28 33715801-33715912. Max. coverage (+): 0. Max coverage (-): 0

Region: chr28 33715913-33716025. Max. coverage (+): 0. Max coverage (-): 0

Region: chr28 33716026-33716137. Max. coverage (+): 0. Max coverage (-): 0

Region: chr28 33716138-33716250. Max. coverage (+): 6.86. Max coverage (-): 0

Region: chr28 33716251-33716363. Max. coverage (+): 0. Max coverage (-): 0

Region: chr28 33716364-33716475. Max. coverage (+): 2.93. Max coverage (-): 0

Region: chr28 33716476-33716588. Max. coverage (+): 0. Max coverage (-): 0

Region: chr28 33716589-33716700. Max. coverage (+): 0. Max coverage (-): 0

Region: chr28 33716701-33716813. Max. coverage (+): 3.11. Max coverage (-): 0

Region: chr28 33716814-33716926. Max. coverage (+): 0. Max coverage (-): 0

Region: chr28 33716927-33717038. Max. coverage (+): 8.32. Max coverage (-): 0

Region: chr28 33717039-33717151. Max. coverage (+): 0.83. Max coverage (-): 0

Region: chr28 33717152-33717264. Max. coverage (+): 0. Max coverage (-): 0

Region: chr28 33717265-33717376. Max. coverage (+): 8.08. Max coverage (-): 0

Region: chr28 33717377-33717489. Max. coverage (+): 0. Max coverage (-): 0

Region: chr28 33717490-33717601. Max. coverage (+): 0. Max coverage (-): 0

Region: chr28 33717602-33717714. Max. coverage (+): 18.51. Max coverage (-): 0

Region: chr28 33717715-33717827. Max. coverage (+): 32.34. Max coverage (-): 0

Region: chr28 33717828-33717939. Max. coverage (+): 13.11. Max coverage (-): 0

Region: chr28 33717940-33718052. Max. coverage (+): 9.62. Max coverage (-): 0

Region: chr28 33718053-33718164. Max. coverage (+): 4.47. Max coverage (-): 0

Region: chr28 33718165-33718277. Max. coverage (+): 5.08. Max coverage (-): 0

Region: chr28 33718278-33718390. Max. coverage (+): 1.57. Max coverage (-): 0

Region: chr28 33718391-33718502. Max. coverage (+): 3.09. Max coverage (-): 0

Region: chr28 33718503-33718615. Max. coverage (+): 29.49. Max coverage (-): 0

Region: chr28 33718616-33718727. Max. coverage (+): 17.46. Max coverage (-): 0

Region: chr28 33718728-33718840. Max. coverage (+): 11.46. Max coverage (-): 0

Region: chr28 33718841-33718953. Max. coverage (+): 5.85. Max coverage (-): 0

Region: chr28 33718954-33719065. Max. coverage (+): 7.11. Max coverage (-): 0

Region: chr28 33719066-33719178. Max. coverage (+): 7.11. Max coverage (-): 0

Region: chr28 33719179-33719291. Max. coverage (+): 0. Max coverage (-): 0

Region: chr28 33719292-33719403. Max. coverage (+): 8.55. Max coverage (-): 0

Region: chr28 33719404-33719516. Max. coverage (+): 23.1. Max coverage (-): 0

Region: chr28 33719517-33719628. Max. coverage (+): 8.04. Max coverage (-): 0

Region: chr28 33719629-33719741. Max. coverage (+): 2.21. Max coverage (-): 0

Region: chr28 33719742-33719854. Max. coverage (+): 2.15. Max coverage (-): 0

Region: chr28 33719855-33719966. Max. coverage (+): 7.83. Max coverage (-): 0

Region: chr28 33719967-33720079. Max. coverage (+): 5.81. Max coverage (-): 0

Region: chr28 33720080-33720191. Max. coverage (+): 1.52. Max coverage (-): 0

Region: chr28 33720192-33720304. Max. coverage (+): 8.69. Max coverage (-): 0

Region: chr28 33720305-33720417. Max. coverage (+): 11.99. Max coverage (-): 0

Region: chr28 33720418-33720529. Max. coverage (+): 4.57. Max coverage (-): 0

Region: chr28 33720530-33720642. Max. coverage (+): 5.48. Max coverage (-): 0

Region: chr28 33720643-33720754. Max. coverage (+): 2.28. Max coverage (-): 0

Region: chr28 33720755-33720867. Max. coverage (+): 5.54. Max coverage (-): 0

Region: chr28 33720868-33720980. Max. coverage (+): 8.21. Max coverage (-): 0

Region: chr28 33720981-33721092. Max. coverage (+): 6.76. Max coverage (-): 0

Region: chr28 33721093-33721205. Max. coverage (+): 3.99. Max coverage (-): 0

Region: chr28 33721206-33721318. Max. coverage (+): 0. Max coverage (-): 0

Region: chr28 33721319-33721430. Max. coverage (+): 8.23. Max coverage (-): 0

Region: chr28 33721431-33721543. Max. coverage (+): 2.44. Max coverage (-): 0

Region: chr28 33721544-33721655. Max. coverage (+): 15.78. Max coverage (-): 0

Region: chr28 33721656-33721768. Max. coverage (+): 0.75. Max coverage (-): 0

Region: chr28 33721769-33721881. Max. coverage (+): 4.67. Max coverage (-): 0

Region: chr28 33721882-33721993. Max. coverage (+): 1.29. Max coverage (-): 0

Region: chr28 33721994-33722106. Max. coverage (+): 0. Max coverage (-): 0

Region: chr28 33722107-33722218. Max. coverage (+): 0. Max coverage (-): 0

Region: chr28 33722219-33722331. Max. coverage (+): 11.48. Max coverage (-): 0

Region: chr28 33722332-33722444. Max. coverage (+): 5.11. Max coverage (-): 0

Region: chr28 33722445-33722556. Max. coverage (+): 4.25. Max coverage (-): 0

Region: chr28 33722557-33722669. Max. coverage (+): 17.33. Max coverage (-): 0

Region: chr28 33722670-33722782. Max. coverage (+): 3.94. Max coverage (-): 0

Region: chr28 33722783-33722894. Max. coverage (+): 4.28. Max coverage (-): 0

Region: chr28 33722895-33723007. Max. coverage (+): 2.24. Max coverage (-): 0

Region: chr28 33723008-33723119. Max. coverage (+): 4.89. Max coverage (-): 0

Region: chr28 33723120-33723232. Max. coverage (+): 0. Max coverage (-): 0

Region: chr28 33723233-33723345. Max. coverage (+): 0. Max coverage (-): 0

Region: chr28 33723346-33723457. Max. coverage (+): 8.74. Max coverage (-): 0

Region: chr28 33723458-33723570. Max. coverage (+): 5.27. Max coverage (-): 0

Region: chr28 33723571-33723682. Max. coverage (+): 0.26. Max coverage (-): 0

Region: chr28 33723683-33723795. Max. coverage (+): 0. Max coverage (-): 0

Region: chr28 33723796-33723908. Max. coverage (+): 6.26. Max coverage (-): 0

Region: chr28 33723909-33724020. Max. coverage (+): 1.5. Max coverage (-): 0

Region: chr28 33724021-33724133. Max. coverage (+): 8.81. Max coverage (-): 0

Region: chr28 33724134-33724245. Max. coverage (+): 10.39. Max coverage (-): 0

Region: chr28 33724246-33724358. Max. coverage (+): 13.34. Max coverage (-): 0

Region: chr28 33724359-33724471. Max. coverage (+): 16.2. Max coverage (-): 0

Region: chr28 33724472-33724583. Max. coverage (+): 3.96. Max coverage (-): 0

Region: chr28 33724584-33724696. Max. coverage (+): 1.21. Max coverage (-): 0

Region: chr28 33724697-33724809. Max. coverage (+): 4.1. Max coverage (-): 0

Region: chr28 33724810-33724921. Max. coverage (+): 5.39. Max coverage (-): 0

Region: chr28 33724922-33725034. Max. coverage (+): 7.25. Max coverage (-): 0

Region: chr28 33725035-33725146. Max. coverage (+): 0. Max coverage (-): 0

Region: chr28 33725147-33725259. Max. coverage (+): 0. Max coverage (-): 0

Region: chr28 33725260-33725372. Max. coverage (+): 2.41. Max coverage (-): 0

Region: chr28 33725373-33725484. Max. coverage (+): 8.94. Max coverage (-): 0

Region: chr28 33725485-33725597. Max. coverage (+): 0. Max coverage (-): 0

Region: chr28 33725598-33725709. Max. coverage (+): 0. Max coverage (-): 0

Region: chr28 33725710-33725822. Max. coverage (+): 0. Max coverage (-): 0

Region: chr28 33725823-33725935. Max. coverage (+): 0. Max coverage (-): 0

Region: chr28 33725936-33726047. Max. coverage (+): 4.53. Max coverage (-): 0

Region: chr28 33726048-33726160. Max. coverage (+): 4.53. Max coverage (-): 0

Region: chr28 33726161-33726272. Max. coverage (+): 0. Max coverage (-): 0

Region: chr28 33726273-33726385. Max. coverage (+): 0. Max coverage (-): 0

Region: chr28 33726386-33726498. Max. coverage (+): 0. Max coverage (-): 0

Region: chr28 33726499-33726610. Max. coverage (+): 0. Max coverage (-): 0

Region: chr28 33726611-33726723. Max. coverage (+): 0. Max coverage (-): 0

Region: chr28 33726724-33726836. Max. coverage (+): 0. Max coverage (-): 0

Region: chr28 33726837-33726948. Max. coverage (+): 0. Max coverage (-): 0

Region: chr28 33726949-33727061. Max. coverage (+): 0. Max coverage (-): 0

Region: chr28 33727062-33727173. Max. coverage (+): 0. Max coverage (-): 0

Region: chr28 33727174-33727286. Max. coverage (+): 0. Max coverage (-): 0

Region: chr28 33727287-33727399. Max. coverage (+): 0. Max coverage (-): 0

Region: chr28 33727400-33727511. Max. coverage (+): 0. Max coverage (-): 0

Region: chr28 33727512-33727624. Max. coverage (+): 0. Max coverage (-): 0

Region: chr28 33727625-33727736. Max. coverage (+): 0. Max coverage (-): 0

Region: chr28 33727737-33727849. Max. coverage (+): 0. Max coverage (-): 0

Region: chr28 33727850-33727962. Max. coverage (+): 0. Max coverage (-): 0

Region: chr28 33727963-33728074. Max. coverage (+): 0. Max coverage (-): 0

Region: chr28 33728075-33728187. Max. coverage (+): 0. Max coverage (-): 0

Region: chr28 33728188-33728300. Max. coverage (+): 0. Max coverage (-): 0

Region: chr28 33728301-33728412. Max. coverage (+): 0. Max coverage (-): 0

Region: chr28 33728413-33728525. Max. coverage (+): 0. Max coverage (-): 0

Region: chr28 33728526-33728637. Max. coverage (+): 0. Max coverage (-): 0

Region: chr28 33728638-33728750. Max. coverage (+): 0. Max coverage (-): 0

Region: chr28 33728751-33728863. Max. coverage (+): 0. Max coverage (-): 0

Region: chr28 33728864-33728975. Max. coverage (+): 0. Max coverage (-): 0

Region: chr28 33728976-33729088. Max. coverage (+): 2.45. Max coverage (-): 0

Region: chr28 33729089-33729200. Max. coverage (+): 1.88. Max coverage (-): 0

Region: chr28 33729201-33729313. Max. coverage (+): 0. Max coverage (-): 0

Region: chr28 33729314-33729426. Max. coverage (+): 0. Max coverage (-): 0

Region: chr28 33729427-33729538. Max. coverage (+): 0. Max coverage (-): 0

Region: chr28 33729539-33729651. Max. coverage (+): 1.99. Max coverage (-): 0

Region: chr28 33729652-33729763. Max. coverage (+): 1.99. Max coverage (-): 0

Region: chr28 33729764-33729876. Max. coverage (+): 0. Max coverage (-): 0

Region: chr28 33729877-33729989. Max. coverage (+): 0. Max coverage (-): 0

Region: chr28 33729990-33730101. Max. coverage (+): 8.93. Max coverage (-): 0

Region: chr28 33730102-33730214. Max. coverage (+): 0. Max coverage (-): 0

Region: chr28 33730215-33730327. Max. coverage (+): 1.68. Max coverage (-): 0

Region: chr28 33730328-33730439. Max. coverage (+): 4.58. Max coverage (-): 0

Region: chr28 33730440-33730552. Max. coverage (+): 0. Max coverage (-): 0

Region: chr28 33730553-33730664. Max. coverage (+): 0. Max coverage (-): 0

Region: chr28 33730665-33730777. Max. coverage (+): 0. Max coverage (-): 0

Region: chr28 33730778-33730890. Max. coverage (+): 0. Max coverage (-): 0

Region: chr28 33730891-33731002. Max. coverage (+): 0. Max coverage (-): 0

Region: chr28 33731003-33731115. Max. coverage (+): 0. Max coverage (-): 0

Region: chr28 33731116-33731227. Max. coverage (+): 1.27. Max coverage (-): 0

Region: chr28 33731228-33731340. Max. coverage (+): 3.37. Max coverage (-): 0

Region: chr28 33731341-33731453. Max. coverage (+): 6.15. Max coverage (-): 0

Region: chr28 33731454-33731565. Max. coverage (+): 0. Max coverage (-): 0

Region: chr28 33731566-33731678. Max. coverage (+): 0. Max coverage (-): 0

Region: chr28 33731679-33731790. Max. coverage (+): 0.24. Max coverage (-): 0

Region: chr28 33731791-33731903. Max. coverage (+): 13.17. Max coverage (-): 0

Region: chr28 33731904-33732016. Max. coverage (+): 3.84. Max coverage (-): 0

Region: chr28 33732017-33732128. Max. coverage (+): 16.45. Max coverage (-): 0

Region: chr28 33732129-33732241. Max. coverage (+): 5.17. Max coverage (-): 0

Region: chr28 33732242-33732354. Max. coverage (+): 1.5. Max coverage (-): 0

Region: chr28 33732355-33732466. Max. coverage (+): 0. Max coverage (-): 0

Region: chr28 33732467-33732579. Max. coverage (+): 0. Max coverage (-): 0

Region: chr28 33732580-33732691. Max. coverage (+): 3.01. Max coverage (-): 0

Region: chr28 33732692-33732804. Max. coverage (+): 3.18. Max coverage (-): 0

Region: chr28 33732805-33732917. Max. coverage (+): 1.32. Max coverage (-): 0

Region: chr28 33732918-33733029. Max. coverage (+): 17.15. Max coverage (-): 0

Region: chr28 33733030-33733142. Max. coverage (+): 0. Max coverage (-): 0

Region: chr28 33733143-33733254. Max. coverage (+): 0. Max coverage (-): 0

Region: chr28 33733255-33733367. Max. coverage (+): 0. Max coverage (-): 0

Region: chr28 33733368-33733480. Max. coverage (+): 0. Max coverage (-): 0

Region: chr28 33733481-33733592. Max. coverage (+): 0. Max coverage (-): 0

Region: chr28 33733593-33733705. Max. coverage (+): 0. Max coverage (-): 0

Region: chr28 33733706-33733817. Max. coverage (+): 0. Max coverage (-): 0

Region: chr28 33733818-33733930. Max. coverage (+): 0. Max coverage (-): 0

Region: chr28 33733931-33734043. Max. coverage (+): 0. Max coverage (-): 0

Region: chr28 33734044-33734155. Max. coverage (+): 0. Max coverage (-): 0

Region: chr28 33734156-33734268. Max. coverage (+): 0. Max coverage (-): 0

Region: chr28 33734269-33734381. Max. coverage (+): 0. Max coverage (-): 0

Region: chr28 33734382-33734493. Max. coverage (+): 0. Max coverage (-): 0

Region: chr28 33734494-33734606. Max. coverage (+): 0. Max coverage (-): 0

Region: chr28 33734607-33734718. Max. coverage (+): 5.49. Max coverage (-): 0

Region: chr28 33734719-33734831. Max. coverage (+): 0. Max coverage (-): 0

Region: chr28 33734832-33734944. Max. coverage (+): 8.52. Max coverage (-): 0

Region: chr28 33734945-33735056. Max. coverage (+): 7.43. Max coverage (-): 0

Region: chr28 33735057-33735169. Max. coverage (+): 0.73. Max coverage (-): 0

Region: chr28 33735170-33735281. Max. coverage (+): 2.5. Max coverage (-): 0

Region: chr28 33735282-33735394. Max. coverage (+): 1.37. Max coverage (-): 0

Region: chr28 33735395-33735507. Max. coverage (+): 1.18. Max coverage (-): 0

Region: chr28 33735508-33735619. Max. coverage (+): 2.39. Max coverage (-): 0

Region: chr28 33735620-33735732. Max. coverage (+): 0. Max coverage (-): 0

Region: chr28 33735733-33735845. Max. coverage (+): 0. Max coverage (-): 0

Region: chr28 33735846-33735957. Max. coverage (+): 1.87. Max coverage (-): 0

Region: chr28 33735958-33736070. Max. coverage (+): 1.87. Max coverage (-): 0

Region: chr28 33736071-33736182. Max. coverage (+): 0. Max coverage (-): 0

Region: chr28 33736183-33736295. Max. coverage (+): 12.17. Max coverage (-): 0

Region: chr28 33736296-33736408. Max. coverage (+): 0. Max coverage (-): 0

Region: chr28 33736409-33736520. Max. coverage (+): 3.37. Max coverage (-): 0

Region: chr28 33736521-33736633. Max. coverage (+): 4.59. Max coverage (-): 0

Region: chr28 33736634-33736745. Max. coverage (+): 10.57. Max coverage (-): 0

Region: chr28 33736746-33736858. Max. coverage (+): 0. Max coverage (-): 0

Region: chr28 33736859-33736971. Max. coverage (+): 0. Max coverage (-): 0

Region: chr28 33736972-33737083. Max. coverage (+): 3.94. Max coverage (-): 0

Region: chr28 33737084-33737196. Max. coverage (+): 1.25. Max coverage (-): 0

Region: chr28 33737197-33737308. Max. coverage (+): 0. Max coverage (-): 0

Region: chr28 33737309-33737421. Max. coverage (+): 10.23. Max coverage (-): 0

Region: chr28 33737422-33737534. Max. coverage (+): 15.04. Max coverage (-): 0

Region: chr28 33737535-33737646. Max. coverage (+): 0.98. Max coverage (-): 0

Region: chr28 33737647-33737759. Max. coverage (+): 0. Max coverage (-): 0

Region: chr28 33737760-33737872. Max. coverage (+): 0. Max coverage (-): 0

Region: chr28 33737873-33737984. Max. coverage (+): 0. Max coverage (-): 0

Region: chr28 33737985-33738097. Max. coverage (+): 3.4. Max coverage (-): 0

Region: chr28 33738098-33738209. Max. coverage (+): 5.87. Max coverage (-): 0

Region: chr28 33738210-33738322. Max. coverage (+): 5.04. Max coverage (-): 0

Region: chr28 33738323-33738435. Max. coverage (+): 5.82. Max coverage (-): 0

Region: chr28 33738436-33738547. Max. coverage (+): 2.43. Max coverage (-): 0

Region: chr28 33738548-. Max. coverage (+): 1.54. Max coverage (-): 0

RepeatMasker Color Code

**+**

100-98% Identity

<98-95% Identity

<95-90% Identity

<90-85% Identity

<85-80% Identity

<80-75% Identity

<75-70% Identity

<70% Identity

**-**

Gene Set Color Code

**+**

Gene

Pseudogene

**-**

Topology/Coverage Color Code

Coverage Plus Strand

Coverage Minus Strand

Mainstrand: Plus

Mainstrand: Minus

Complementary Strand

Flanking Region  
(if option -flank >0)

Gene Set Annotation  

**1. DLG5 (protein coding, ENSBTAG00000013187) Tr:00000017554 Ex:21**: 33736370-33736532 (-)  
**2. DLG5 (protein coding, ENSBTAG00000013187) Tr:00000017554 Ex:22**: 33736128-33736261 (-)  
**3. DLG5 (protein coding, ENSBTAG00000013187) Tr:00000017554 Ex:23**: 33735320-33735466 (-)  
**4. DLG5 (protein coding, ENSBTAG00000013187) Tr:00000017554 Ex:24**: 33732997-33733180 (-)  
**5. DLG5 (protein coding, ENSBTAG00000013187) Tr:00000017554 Ex:25**: 33732269-33732417 (-)  
**6. DLG5 (protein coding, ENSBTAG00000013187) Tr:00000017554 Ex:26**: 33731287-33731457 (-)  
**7. DLG5 (protein coding, ENSBTAG00000013187) Tr:00000017554 Ex:27**: 33730201-33730397 (-)  
**8. DLG5 (protein coding, ENSBTAG00000013187) Tr:00000017554 Ex:28**: 33724499-33724642 (-)  
**9. DLG5 (protein coding, ENSBTAG00000013187) Tr:00000017554 Ex:29**: 33724131-33724258 (-)  
**10. DLG5 (protein coding, ENSBTAG00000013187) Tr:00000017554 Ex:30**: 33723633-33723742 (-)  
**11. DLG5 (protein coding, ENSBTAG00000013187) Tr:00000017554 Ex:31**: 33722861-33722970 (-)  
**12. DLG5 (protein coding, ENSBTAG00000013187) Tr:00000017554 Ex:32**: 33719674-33721398 (-)

  
RepeatMasker Annotation  

**1. CHR-2B**: 33682409-33682687 (-), Divergence to consensus: 31.2%  
**2. BovB**: 33682739-33683008 (+), Divergence to consensus: 3%  
**3. ART2A**: 33683009-33683539 (+), Divergence to consensus: 9.1%  
**4. L2c**: 33684063-33684138 (+), Divergence to consensus: 29.7%  
**5. MLT1N2**: 33684243-33684362 (+), Divergence to consensus: 36.8%  
**6. MLT1J2**: 33684363-33684543 (-), Divergence to consensus: 40%  
**7. MLT1J2**: 33684561-33684728 (-), Divergence to consensus: 45.3%  
**8. MIR**: 33684872-33684965 (+), Divergence to consensus: 31.2%  
**9. Bov-tA2**: 33684970-33685175 (-), Divergence to consensus: 14.7%  
**10. SINE2-1\_BT**: 33685460-33685578 (-), Divergence to consensus: 21.9%  
**11. Bov-tA3**: 33686483-33686636 (-), Divergence to consensus: 22.1%  
**12. CHR-2A**: 33686589-33686644 (-), Divergence to consensus: 21.4%  
**13. L1\_BT**: 33686646-33686939 (-), Divergence to consensus: 14.4%  
**14. L1MC1**: 33686940-33687737 (+), Divergence to consensus: 30.5%  
**15. MER58A**: 33687740-33687853 (+), Divergence to consensus: 35%  
**16. L1MD**: 33687903-33688281 (+), Divergence to consensus: 24.8%  
**17. Bov-tA2**: 33688282-33688400 (+), Divergence to consensus: 19%  
**18. Bov-tA2**: 33688401-33688571 (+), Divergence to consensus: 29.3%  
**19. L1MD**: 33688592-33688832 (+), Divergence to consensus: 31.5%  
**20. L1MA9**: 33688834-33688950 (-), Divergence to consensus: 18.9%  
**21. Bov-tA1**: 33688951-33689174 (+), Divergence to consensus: 16.5%  
**22. L1MA9**: 33689175-33689362 (-), Divergence to consensus: 18.9%  
**23. Bov-tA2**: 33689368-33689435 (+), Divergence to consensus: 16.2%  
**24. L1MA9**: 33689442-33689735 (-), Divergence to consensus: 25.9%  
**25. L1ME1**: 33689735-33689800 (+), Divergence to consensus: 20%  
**26. L1ME1**: 33689797-33690219 (+), Divergence to consensus: 43.6%  
**27. L1ME1**: 33690218-33690682 (+), Divergence to consensus: 35.6%  
**28. BOV-A2**: 33691282-33691410 (+), Divergence to consensus: 8.5%  
**29. L4\_C\_Mam**: 33691990-33692131 (-), Divergence to consensus: 35.6%  
**30. Arthur1A**: 33693070-33693221 (-), Divergence to consensus: 37.6%  
**31. (TTCA)n**: 33693935-33693965 (+), Divergence to consensus: 6.5%  
**32. G-rich**: 33695677-33695769 (+), Divergence to consensus: 20.1%  
**33. AT\_rich**: 33695959-33695987 (+), Divergence to consensus: 79.3%  
**34. L1MEd**: 33696006-33696398 (+), Divergence to consensus: 43.4%  
**35. L1MEd**: 33696418-33696647 (+), Divergence to consensus: 40.3%  
**36. L2c**: 33697080-33697133 (+), Divergence to consensus: 33.5%  
**37. L2c**: 33699039-33699136 (+), Divergence to consensus: 28.5%  
**38. BOV-A2**: 33699494-33699572 (+), Divergence to consensus: 11.4%  
**39. BTLTR1C**: 33699573-33700780 (-), Divergence to consensus: 30.7%  
**40. Tigger15a**: 33700795-33700898 (-), Divergence to consensus: 43%  
**41. L1ME3G**: 33701353-33701566 (+), Divergence to consensus: 47.4%  
**42. L1MCc**: 33701552-33702657 (+), Divergence to consensus: 38.9%  
**43. LTR13B\_BT**: 33702752-33703028 (+), Divergence to consensus: 19.3%  
**44. L1MB5**: 33703050-33703365 (+), Divergence to consensus: 27.5%  
**45. CHRL**: 33703366-33703516 (+), Divergence to consensus: 37.7%  
**46. L1MB5**: 33703517-33703760 (+), Divergence to consensus: 27.5%  
**47. L1MCc**: 33703948-33704098 (+), Divergence to consensus: 28.4%  
**48. MER110**: 33704144-33704372 (+), Divergence to consensus: 40.3%  
**49. MIR3**: 33705231-33705287 (-), Divergence to consensus: 31.6%  
**50. L3**: 33706073-33706159 (-), Divergence to consensus: 39.1%  
**51. MamGypLTR3a**: 33706538-33706652 (-), Divergence to consensus: 39.9%  
**52. MamGypLTR3a**: 33706895-33706960 (-), Divergence to consensus: 31.8%  
**53. MIR**: 33707773-33707952 (+), Divergence to consensus: 35%  
**54. LTR16B1**: 33709018-33709465 (+), Divergence to consensus: 34.5%  
**55. L1ME4a**: 33709500-33709647 (-), Divergence to consensus: 37.2%  
**56. MLT1N2**: 33709701-33709852 (+), Divergence to consensus: 36.4%  
**57. MIR**: 33709933-33710021 (-), Divergence to consensus: 40.2%  
**58. MIRb**: 33710179-33710249 (+), Divergence to consensus: 32.7%  
**59. MER89**: 33711101-33711470 (+), Divergence to consensus: 21.7%  
**60. BOV-A2**: 33714161-33714432 (-), Divergence to consensus: 4.1%  
**61. MLT2C1**: 33715759-33715967 (+), Divergence to consensus: 45.2%  
**62. MLT2C1**: 33716076-33716179 (+), Divergence to consensus: 46.8%  
**63. Bov-tA2**: 33716180-33716369 (+), Divergence to consensus: 20%  
**64. MLT2C1**: 33716370-33716402 (+), Divergence to consensus: 46.8%  
**65. HAL1**: 33716582-33716755 (+), Divergence to consensus: 36.7%  
**66. HAL1**: 33716885-33716998 (+), Divergence to consensus: 30.9%  
**67. HAL1**: 33717086-33717238 (+), Divergence to consensus: 47%  
**68. ART2A**: 33717322-33717630 (+), Divergence to consensus: 21%  
**69. L1ME3D**: 33718129-33718340 (+), Divergence to consensus: 48.7%  
**70. MER5C**: 33722042-33722235 (+), Divergence to consensus: 30.1%  
**71. MER81**: 33724786-33724848 (+), Divergence to consensus: 20.6%  
**72. MLT1J**: 33725051-33725314 (+), Divergence to consensus: 43.1%  
**73. Bov-tA2**: 33725642-33725705 (+), Divergence to consensus: 6.2%  
**74. L1M4c**: 33726250-33727012 (+), Divergence to consensus: 30.6%  
**75. Bov-tA1**: 33727013-33727224 (-), Divergence to consensus: 14.6%  
**76. L1M4c**: 33727225-33727297 (+), Divergence to consensus: 28.3%  
**77. MLT1D**: 33727302-33727503 (+), Divergence to consensus: 31.9%  
**78. L1MEg**: 33727505-33727585 (+), Divergence to consensus: 28.6%  
**79. Bov-tA2**: 33727672-33727727 (+), Divergence to consensus: 18.2%  
**80. L1MA9**: 33727742-33727869 (+), Divergence to consensus: 31.7%  
**81. L1MEg**: 33727867-33728370 (+), Divergence to consensus: 44.8%  
**82. L1MEg**: 33728462-33728726 (+), Divergence to consensus: 38.2%  
**83. Bov-tA1**: 33728744-33729000 (-), Divergence to consensus: 31.8%  
**84. MER58C**: 33729172-33729299 (-), Divergence to consensus: 29%  
**85. MLT1J**: 33729742-33729988 (+), Divergence to consensus: 44.5%  
**86. L1MB8**: 33730684-33731073 (+), Divergence to consensus: 33.6%  
**87. L2b**: 33733565-33733615 (-), Divergence to consensus: 31.3%  
**88. BovB**: 33733770-33734048 (+), Divergence to consensus: 9.3%  
**89. ART2A**: 33734050-33734564 (+), Divergence to consensus: 18.7%  
**90. GC\_rich**: 33737733-33737759 (+), Divergence to consensus: 48.1%  
**91. Charlie19a**: 33737796-33737836 (-), Divergence to consensus: 17.1%

  
Transcription Factor Binding Sites  

**RFX4\_1** (Sequence: GTTGCCAGG (-): 33691230)  
**RFX4\_1** (Sequence: GTTGCTAGG (-): 33692553)  
**RFX4\_1** (Sequence: CCTGGCAAC (+): 33718603)  
**RFX4\_2** (Sequence: CCTGGTTAC (+): 33719431)  
**RFX4\_2** (Sequence: CCTGGTTAC (+): 33729684)  
**Gata4** (Sequence: AGATAAG (-): 33682333)  
**Gata4** (Sequence: AGATAAG (-): 33685449)  
**Gata4** (Sequence: AGATAAC (-): 33694539)  
**Gata4** (Sequence: AGATAAC (-): 33696922)  
**Gata4** (Sequence: AGATAAG (-): 33714447)  
**SOX9** (Sequence: AACAATAG (-): 33693135)  
**SOX9** (Sequence: AACAATGA (-): 33701499)  
**SOX9** (Sequence: AACAATAA (-): 33715680)  
**SOX9** (Sequence: AACAATAA (-): 33717290)  
**SOX9** (Sequence: AACAATAA (-): 33737546)  
**A-MYB** (Sequence: CCAACTGTCA (-): 33694548)  
**Gata4** (Sequence: GTTATCT (+): 33685348)  
**Gata4** (Sequence: GTTATCT (+): 33685637)  
**Gata4** (Sequence: CTTATCT (+): 33696965)
