# Supplementary material for: Small RNA sequencing of cryopreserved semen from single bull revealed altered miRNAs and piRNAs expression between High- and Low-motile sperm populations
Source: BMC Genomics. 2017 Jan 4;18:14. doi: 10.1186/s12864-016-3394-7 (PMC5209821; doi:10.1186/s12864-016-3394-7)
Supplement: Additional file 3: — Details for each piRNA clusters found in High Motile (HM) sperm fraction. Genes, repeats, transposable elements and transcription factors binding sites falling within the cluster regions were reported. (ZIP 1896 kb) [file 12864_2016_3394_MOESM3_ESM.zip › 74.html]

piRNA cluster 74


Predicted piRNA cluster no. 74     previous   next
  

Show proTRAC run info
Hide proTRAC run info

================================= proTRAC ====================================  
VERSION: 2.1                                    LAST MODIFIED: 06. October 2015  
  
Please cite:  
Rosenkranz D, Zischler H. proTRAC - a software for probabilistic piRNA cluster  
detection, visualization and analysis. 2012. BMC Bioinformatics 13:5.  
  
and (for proTRAC 2.0 and later):  
Rosenkranz D, Rudloff S, Bastuck K, Ketting RF, Zischler H. Tupaia small RNAs  
provide insights into function and evolution of RNAi-based transposon defense  
in mammals. 2015. RNA 21(5):911-922.  
  
Contact:  
David Rosenkranz  
Institute of Anthropology, small RNA group  
Johannes Gutenberg University Mainz  
email: rosenkranz@uni-mainz.de  
  
You can find the latest proTRAC version at:  
http://sourceforge.net/projects/protrac/files  
http://www.smallRNAgroup-mainz.de/software  
==============================================================================  
  
PARAMETERS:  
Map file: .............../storage/core/barbara/genhome/smallRNA/fertility/Sample\_motile/pirna/Sample\_motile\_26-33\_collapsed.fa.no-dust.map.weighted-10000-1000-b-0  
Genome file: ............/storage/core/barbara/genhome/smallRNA/fertility/Sample\_all/pirna/bt\_311\_chrY.fa  
RepeatMasker annotation: /storage/genomes/bt\_umd31/GCF\_000003055.6\_Bos\_taurus\_UMD\_3.1.1\_repeatMasker\_chr.out  
GeneSet:................./storage/core/barbara/genhome/smallRNA/fertility/Sample\_all/pirna/full.gtf  
  
Significant (p<=0.01) hit density will be calculated based  
on observed hit distribution.  
  
Sliding window size: ........................................ 5000 bp  
Sliding window increament: .................................. 1000 bp  
Normalize each hit by number of genomic hits: ............... 1 [0=no/1=yes]  
Normalize each hit by number of sequence reads: ............. 1 [0=no/1=yes]  
Normalize values (-> per million mapped reads): ............. 1 [0=no/1=yes]  
Min. fraction of hits with 1T(U) or 10A: .................... 0.75  
Alternatively: Min. fraction of hits with 1T(U) and 10A: .... 0.5  
Min. fraction of hits with typical piRNA length: ............ 0.75  
Typical piRNA length: ....................................... 26-33 nt  
Min. size of a piRNA cluster: ............................... 5000 bp.  
Min. number of hits (absolute): ............................. 0  
Min. number of hits (normalized): ........................... 0  
Min. fraction of hits on the mainstrand: .................... 0.75  
Top fraction of mapped sequences (in terms of read counts): . 1%  
Top fraction accounts for max. n% of sequence reads: ........ 90%  
Min. fraction of hits on each arm of a bidirectional cluster: 0.1  
Output image file for each cluster: ......................... 0 [0=no/1=yes]  
Output html file for each cluster: .......................... 1 [0=no/1=yes]  
Output a summary table: ..................................... 1 [0=no/1=yes]  
Output a FASTA file for each cluster (piRNA sequences): ..... 1 [0=no/1=yes]  
Output a FASTA file comprising cluster sequences: ........... 1 [0=no/1=yes]  
Search DNA motifs in clusters: .............................. 1 [0=no/1=yes]  
Output flanking sequences: +/- .............................. 0 bp  
Output ~.pTi file: .......................................... 1 [0=no/1=yes]  
==============================================================================  
  
  
Genome size (without gaps): ............ 2678902517 bp  
Gaps (N/X/-): .......................... 53837044 bp  
Mapped reads: .......................... 658825247023  
Non-identical sequences: ............... 514171  
Genomic hits: .......................... 764233  
Significant densitiy of mapped reads: .. 12867599.5173724 reads/kb

Show proTRAC cluster info
Hide proTRAC cluster info

|  |  |
| --- | --- |
| Location | chr29 |
| Coordinates | 45305693-45312322 |
| Size [bp] | 6630 |
| Sequence hit loci | 86 |
| Mapped reads (normalized) | 114952101 |
| Mapped reads (normalized) per kb | 17338175.1 |
| Normalized reads with 1T (1U) | 80% |
| Normalized reads with 10A | 21.8% |
| Normalized reads with length 26-33 nt | 100% |
| Normalized reads on the main strand(s) | 100% |
| Predicted directionality | mono:plus |

100%

0%

1T (1U)  
reads

10A reads

26-33 nt  
reads

reads on mainstrand

**Either the amount of reads with 1T (1U) OR 10A has to exceed 75% (set with option: -1Tor10A)  
Alternatively the amount of reads with 1T (1U) AND 10A has to exceed 50% (set with option: -1Tand10A)  
Minimum amount of reads with preferred size is 75% (set with option: -pisize)  
Minimum amount of reads on the main strand(s) is 75% (set with option: -clstrand)**

Show read coverage
Hide read coverage

WHAT DO I SEE HERE?  
This chart shows the location of mapped sequence reads within a predicted piRNA cluster. The color refers to the number of genomic hits produced by the sequence read in question. A dark red bar indicates that this sequence read produces many other hits elsewhere in the genome. Many adjacent red or yellow bars can indicate the presence of a multi-copy element such as transposons or rRNA genes. A dark green bar indicates that this sequence read maps uniquely to this locus.

1 hit

2-5 hits

6-10 hits

11-20 hits

21-50 hits

51-100 hits

> 100 hits

chr29

45305693

45312322

Gene Set

RepeatMasker

Mapped  
Reads

11.34

plus strand

minus strand

11.34

Region: chr29 33738549-45305699. Max. coverage (+): 1.66. Max coverage (-): 0

Region: chr29 45305700-45305712. Max. coverage (+): 1.66. Max coverage (-): 0

Region: chr29 45305713-45305726. Max. coverage (+): 0. Max coverage (-): 0

Region: chr29 45305727-45305739. Max. coverage (+): 0. Max coverage (-): 0

Region: chr29 45305740-45305752. Max. coverage (+): 0. Max coverage (-): 0

Region: chr29 45305753-45305765. Max. coverage (+): 0. Max coverage (-): 0

Region: chr29 45305766-45305779. Max. coverage (+): 0. Max coverage (-): 0

Region: chr29 45305780-45305792. Max. coverage (+): 0. Max coverage (-): 0

Region: chr29 45305793-45305805. Max. coverage (+): 0. Max coverage (-): 0

Region: chr29 45305806-45305818. Max. coverage (+): 0. Max coverage (-): 0

Region: chr29 45305819-45305832. Max. coverage (+): 7.59. Max coverage (-): 0

Region: chr29 45305833-45305845. Max. coverage (+): 0. Max coverage (-): 0

Region: chr29 45305846-45305858. Max. coverage (+): 0. Max coverage (-): 0

Region: chr29 45305859-45305872. Max. coverage (+): 0. Max coverage (-): 0

Region: chr29 45305873-45305885. Max. coverage (+): 0.28. Max coverage (-): 0

Region: chr29 45305886-45305898. Max. coverage (+): 11.34. Max coverage (-): 0

Region: chr29 45305899-45305911. Max. coverage (+): 9.15. Max coverage (-): 0

Region: chr29 45305912-45305925. Max. coverage (+): 0. Max coverage (-): 0

Region: chr29 45305926-45305938. Max. coverage (+): 0. Max coverage (-): 0

Region: chr29 45305939-45305951. Max. coverage (+): 0. Max coverage (-): 0

Region: chr29 45305952-45305964. Max. coverage (+): 0. Max coverage (-): 0

Region: chr29 45305965-45305978. Max. coverage (+): 0. Max coverage (-): 0

Region: chr29 45305979-45305991. Max. coverage (+): 0. Max coverage (-): 0

Region: chr29 45305992-45306004. Max. coverage (+): 0. Max coverage (-): 0

Region: chr29 45306005-45306017. Max. coverage (+): 0. Max coverage (-): 0

Region: chr29 45306018-45306031. Max. coverage (+): 0. Max coverage (-): 0

Region: chr29 45306032-45306044. Max. coverage (+): 0. Max coverage (-): 0

Region: chr29 45306045-45306057. Max. coverage (+): 1. Max coverage (-): 0

Region: chr29 45306058-45306070. Max. coverage (+): 1. Max coverage (-): 0

Region: chr29 45306071-45306084. Max. coverage (+): 0. Max coverage (-): 0

Region: chr29 45306085-45306097. Max. coverage (+): 0. Max coverage (-): 0

Region: chr29 45306098-45306110. Max. coverage (+): 0. Max coverage (-): 0

Region: chr29 45306111-45306123. Max. coverage (+): 0. Max coverage (-): 0

Region: chr29 45306124-45306137. Max. coverage (+): 0. Max coverage (-): 0

Region: chr29 45306138-45306150. Max. coverage (+): 0. Max coverage (-): 0

Region: chr29 45306151-45306163. Max. coverage (+): 0. Max coverage (-): 0

Region: chr29 45306164-45306176. Max. coverage (+): 0. Max coverage (-): 0

Region: chr29 45306177-45306190. Max. coverage (+): 0. Max coverage (-): 0

Region: chr29 45306191-45306203. Max. coverage (+): 0. Max coverage (-): 0

Region: chr29 45306204-45306216. Max. coverage (+): 0. Max coverage (-): 0

Region: chr29 45306217-45306230. Max. coverage (+): 0. Max coverage (-): 0

Region: chr29 45306231-45306243. Max. coverage (+): 0. Max coverage (-): 0

Region: chr29 45306244-45306256. Max. coverage (+): 0. Max coverage (-): 0

Region: chr29 45306257-45306269. Max. coverage (+): 0. Max coverage (-): 0

Region: chr29 45306270-45306283. Max. coverage (+): 0. Max coverage (-): 0

Region: chr29 45306284-45306296. Max. coverage (+): 0. Max coverage (-): 0

Region: chr29 45306297-45306309. Max. coverage (+): 0. Max coverage (-): 0

Region: chr29 45306310-45306322. Max. coverage (+): 0. Max coverage (-): 0

Region: chr29 45306323-45306336. Max. coverage (+): 0. Max coverage (-): 0

Region: chr29 45306337-45306349. Max. coverage (+): 2.46. Max coverage (-): 0

Region: chr29 45306350-45306362. Max. coverage (+): 2.46. Max coverage (-): 0

Region: chr29 45306363-45306375. Max. coverage (+): 0. Max coverage (-): 0

Region: chr29 45306376-45306389. Max. coverage (+): 0. Max coverage (-): 0

Region: chr29 45306390-45306402. Max. coverage (+): 0. Max coverage (-): 0

Region: chr29 45306403-45306415. Max. coverage (+): 0. Max coverage (-): 0

Region: chr29 45306416-45306428. Max. coverage (+): 0. Max coverage (-): 0

Region: chr29 45306429-45306442. Max. coverage (+): 0. Max coverage (-): 0

Region: chr29 45306443-45306455. Max. coverage (+): 0. Max coverage (-): 0

Region: chr29 45306456-45306468. Max. coverage (+): 0. Max coverage (-): 0

Region: chr29 45306469-45306481. Max. coverage (+): 5.56. Max coverage (-): 0

Region: chr29 45306482-45306495. Max. coverage (+): 0. Max coverage (-): 0

Region: chr29 45306496-45306508. Max. coverage (+): 0. Max coverage (-): 0

Region: chr29 45306509-45306521. Max. coverage (+): 0. Max coverage (-): 0

Region: chr29 45306522-45306535. Max. coverage (+): 0. Max coverage (-): 0

Region: chr29 45306536-45306548. Max. coverage (+): 2.15. Max coverage (-): 0

Region: chr29 45306549-45306561. Max. coverage (+): 0. Max coverage (-): 0

Region: chr29 45306562-45306574. Max. coverage (+): 0. Max coverage (-): 0

Region: chr29 45306575-45306588. Max. coverage (+): 0. Max coverage (-): 0

Region: chr29 45306589-45306601. Max. coverage (+): 0. Max coverage (-): 0

Region: chr29 45306602-45306614. Max. coverage (+): 0. Max coverage (-): 0

Region: chr29 45306615-45306627. Max. coverage (+): 0. Max coverage (-): 0

Region: chr29 45306628-45306641. Max. coverage (+): 0. Max coverage (-): 0

Region: chr29 45306642-45306654. Max. coverage (+): 0. Max coverage (-): 0

Region: chr29 45306655-45306667. Max. coverage (+): 0. Max coverage (-): 0

Region: chr29 45306668-45306680. Max. coverage (+): 0. Max coverage (-): 0

Region: chr29 45306681-45306694. Max. coverage (+): 0. Max coverage (-): 0

Region: chr29 45306695-45306707. Max. coverage (+): 0. Max coverage (-): 0

Region: chr29 45306708-45306720. Max. coverage (+): 0. Max coverage (-): 0

Region: chr29 45306721-45306733. Max. coverage (+): 0. Max coverage (-): 0

Region: chr29 45306734-45306747. Max. coverage (+): 0. Max coverage (-): 0

Region: chr29 45306748-45306760. Max. coverage (+): 0. Max coverage (-): 0

Region: chr29 45306761-45306773. Max. coverage (+): 0.37. Max coverage (-): 0

Region: chr29 45306774-45306786. Max. coverage (+): 0. Max coverage (-): 0

Region: chr29 45306787-45306800. Max. coverage (+): 0. Max coverage (-): 0

Region: chr29 45306801-45306813. Max. coverage (+): 3.8. Max coverage (-): 0

Region: chr29 45306814-45306826. Max. coverage (+): 3.8. Max coverage (-): 0

Region: chr29 45306827-45306839. Max. coverage (+): 0. Max coverage (-): 0

Region: chr29 45306840-45306853. Max. coverage (+): 0. Max coverage (-): 0

Region: chr29 45306854-45306866. Max. coverage (+): 0. Max coverage (-): 0

Region: chr29 45306867-45306879. Max. coverage (+): 0. Max coverage (-): 0

Region: chr29 45306880-45306893. Max. coverage (+): 0. Max coverage (-): 0

Region: chr29 45306894-45306906. Max. coverage (+): 0. Max coverage (-): 0

Region: chr29 45306907-45306919. Max. coverage (+): 0. Max coverage (-): 0

Region: chr29 45306920-45306932. Max. coverage (+): 0. Max coverage (-): 0

Region: chr29 45306933-45306946. Max. coverage (+): 0. Max coverage (-): 0

Region: chr29 45306947-45306959. Max. coverage (+): 0. Max coverage (-): 0

Region: chr29 45306960-45306972. Max. coverage (+): 0. Max coverage (-): 0

Region: chr29 45306973-45306985. Max. coverage (+): 0. Max coverage (-): 0

Region: chr29 45306986-45306999. Max. coverage (+): 0. Max coverage (-): 0

Region: chr29 45307000-45307012. Max. coverage (+): 0. Max coverage (-): 0

Region: chr29 45307013-45307025. Max. coverage (+): 0. Max coverage (-): 0

Region: chr29 45307026-45307038. Max. coverage (+): 1.99. Max coverage (-): 0

Region: chr29 45307039-45307052. Max. coverage (+): 1.99. Max coverage (-): 0

Region: chr29 45307053-45307065. Max. coverage (+): 0. Max coverage (-): 0

Region: chr29 45307066-45307078. Max. coverage (+): 0. Max coverage (-): 0

Region: chr29 45307079-45307091. Max. coverage (+): 0. Max coverage (-): 0

Region: chr29 45307092-45307105. Max. coverage (+): 0. Max coverage (-): 0

Region: chr29 45307106-45307118. Max. coverage (+): 0. Max coverage (-): 0

Region: chr29 45307119-45307131. Max. coverage (+): 0. Max coverage (-): 0

Region: chr29 45307132-45307144. Max. coverage (+): 0. Max coverage (-): 0

Region: chr29 45307145-45307158. Max. coverage (+): 0. Max coverage (-): 0

Region: chr29 45307159-45307171. Max. coverage (+): 0. Max coverage (-): 0

Region: chr29 45307172-45307184. Max. coverage (+): 0. Max coverage (-): 0

Region: chr29 45307185-45307198. Max. coverage (+): 0. Max coverage (-): 0

Region: chr29 45307199-45307211. Max. coverage (+): 0. Max coverage (-): 0

Region: chr29 45307212-45307224. Max. coverage (+): 0. Max coverage (-): 0

Region: chr29 45307225-45307237. Max. coverage (+): 0. Max coverage (-): 0

Region: chr29 45307238-45307251. Max. coverage (+): 0. Max coverage (-): 0

Region: chr29 45307252-45307264. Max. coverage (+): 0. Max coverage (-): 0

Region: chr29 45307265-45307277. Max. coverage (+): 0. Max coverage (-): 0

Region: chr29 45307278-45307290. Max. coverage (+): 0. Max coverage (-): 0

Region: chr29 45307291-45307304. Max. coverage (+): 0. Max coverage (-): 0

Region: chr29 45307305-45307317. Max. coverage (+): 0. Max coverage (-): 0

Region: chr29 45307318-45307330. Max. coverage (+): 2.9. Max coverage (-): 0

Region: chr29 45307331-45307343. Max. coverage (+): 2.9. Max coverage (-): 0

Region: chr29 45307344-45307357. Max. coverage (+): 0. Max coverage (-): 0

Region: chr29 45307358-45307370. Max. coverage (+): 0. Max coverage (-): 0

Region: chr29 45307371-45307383. Max. coverage (+): 2.7. Max coverage (-): 0

Region: chr29 45307384-45307396. Max. coverage (+): 2.7. Max coverage (-): 0

Region: chr29 45307397-45307410. Max. coverage (+): 0. Max coverage (-): 0

Region: chr29 45307411-45307423. Max. coverage (+): 0. Max coverage (-): 0

Region: chr29 45307424-45307436. Max. coverage (+): 0. Max coverage (-): 0

Region: chr29 45307437-45307449. Max. coverage (+): 0. Max coverage (-): 0

Region: chr29 45307450-45307463. Max. coverage (+): 0. Max coverage (-): 0

Region: chr29 45307464-45307476. Max. coverage (+): 0. Max coverage (-): 0

Region: chr29 45307477-45307489. Max. coverage (+): 0. Max coverage (-): 0

Region: chr29 45307490-45307502. Max. coverage (+): 5.24. Max coverage (-): 0

Region: chr29 45307503-45307516. Max. coverage (+): 3.98. Max coverage (-): 0

Region: chr29 45307517-45307529. Max. coverage (+): 0. Max coverage (-): 0

Region: chr29 45307530-45307542. Max. coverage (+): 0. Max coverage (-): 0

Region: chr29 45307543-45307556. Max. coverage (+): 0. Max coverage (-): 0

Region: chr29 45307557-45307569. Max. coverage (+): 0. Max coverage (-): 0

Region: chr29 45307570-45307582. Max. coverage (+): 0. Max coverage (-): 0

Region: chr29 45307583-45307595. Max. coverage (+): 1.64. Max coverage (-): 0

Region: chr29 45307596-45307609. Max. coverage (+): 0. Max coverage (-): 0

Region: chr29 45307610-45307622. Max. coverage (+): 0. Max coverage (-): 0

Region: chr29 45307623-45307635. Max. coverage (+): 0. Max coverage (-): 0

Region: chr29 45307636-45307648. Max. coverage (+): 0. Max coverage (-): 0

Region: chr29 45307649-45307662. Max. coverage (+): 3.14. Max coverage (-): 0

Region: chr29 45307663-45307675. Max. coverage (+): 3.14. Max coverage (-): 0

Region: chr29 45307676-45307688. Max. coverage (+): 1.34. Max coverage (-): 0

Region: chr29 45307689-45307701. Max. coverage (+): 1.34. Max coverage (-): 0

Region: chr29 45307702-45307715. Max. coverage (+): 0. Max coverage (-): 0

Region: chr29 45307716-45307728. Max. coverage (+): 2.96. Max coverage (-): 0

Region: chr29 45307729-45307741. Max. coverage (+): 2.96. Max coverage (-): 0

Region: chr29 45307742-45307754. Max. coverage (+): 0. Max coverage (-): 0

Region: chr29 45307755-45307768. Max. coverage (+): 0. Max coverage (-): 0

Region: chr29 45307769-45307781. Max. coverage (+): 0. Max coverage (-): 0

Region: chr29 45307782-45307794. Max. coverage (+): 0. Max coverage (-): 0

Region: chr29 45307795-45307807. Max. coverage (+): 0. Max coverage (-): 0

Region: chr29 45307808-45307821. Max. coverage (+): 6.66. Max coverage (-): 0

Region: chr29 45307822-45307834. Max. coverage (+): 4.74. Max coverage (-): 0

Region: chr29 45307835-45307847. Max. coverage (+): 4.74. Max coverage (-): 0

Region: chr29 45307848-45307861. Max. coverage (+): 0. Max coverage (-): 0

Region: chr29 45307862-45307874. Max. coverage (+): 1.16. Max coverage (-): 0

Region: chr29 45307875-45307887. Max. coverage (+): 0.95. Max coverage (-): 0

Region: chr29 45307888-45307900. Max. coverage (+): 0.34. Max coverage (-): 0

Region: chr29 45307901-45307914. Max. coverage (+): 0. Max coverage (-): 0

Region: chr29 45307915-45307927. Max. coverage (+): 0.67. Max coverage (-): 0

Region: chr29 45307928-45307940. Max. coverage (+): 0.67. Max coverage (-): 0

Region: chr29 45307941-45307953. Max. coverage (+): 0. Max coverage (-): 0

Region: chr29 45307954-45307967. Max. coverage (+): 0. Max coverage (-): 0

Region: chr29 45307968-45307980. Max. coverage (+): 0. Max coverage (-): 0

Region: chr29 45307981-45307993. Max. coverage (+): 0. Max coverage (-): 0

Region: chr29 45307994-45308006. Max. coverage (+): 0. Max coverage (-): 0

Region: chr29 45308007-45308020. Max. coverage (+): 0. Max coverage (-): 0

Region: chr29 45308021-45308033. Max. coverage (+): 0. Max coverage (-): 0

Region: chr29 45308034-45308046. Max. coverage (+): 0. Max coverage (-): 0

Region: chr29 45308047-45308059. Max. coverage (+): 0. Max coverage (-): 0

Region: chr29 45308060-45308073. Max. coverage (+): 0. Max coverage (-): 0

Region: chr29 45308074-45308086. Max. coverage (+): 0. Max coverage (-): 0

Region: chr29 45308087-45308099. Max. coverage (+): 0. Max coverage (-): 0

Region: chr29 45308100-45308112. Max. coverage (+): 0. Max coverage (-): 0

Region: chr29 45308113-45308126. Max. coverage (+): 0. Max coverage (-): 0

Region: chr29 45308127-45308139. Max. coverage (+): 0. Max coverage (-): 0

Region: chr29 45308140-45308152. Max. coverage (+): 0. Max coverage (-): 0

Region: chr29 45308153-45308165. Max. coverage (+): 0. Max coverage (-): 0

Region: chr29 45308166-45308179. Max. coverage (+): 0. Max coverage (-): 0

Region: chr29 45308180-45308192. Max. coverage (+): 0. Max coverage (-): 0

Region: chr29 45308193-45308205. Max. coverage (+): 0. Max coverage (-): 0

Region: chr29 45308206-45308219. Max. coverage (+): 0.98. Max coverage (-): 0

Region: chr29 45308220-45308232. Max. coverage (+): 0.98. Max coverage (-): 0

Region: chr29 45308233-45308245. Max. coverage (+): 0. Max coverage (-): 0

Region: chr29 45308246-45308258. Max. coverage (+): 0. Max coverage (-): 0

Region: chr29 45308259-45308272. Max. coverage (+): 1.61. Max coverage (-): 0

Region: chr29 45308273-45308285. Max. coverage (+): 1.61. Max coverage (-): 0

Region: chr29 45308286-45308298. Max. coverage (+): 0. Max coverage (-): 0

Region: chr29 45308299-45308311. Max. coverage (+): 0. Max coverage (-): 0

Region: chr29 45308312-45308325. Max. coverage (+): 7.69. Max coverage (-): 0

Region: chr29 45308326-45308338. Max. coverage (+): 0. Max coverage (-): 0

Region: chr29 45308339-45308351. Max. coverage (+): 0. Max coverage (-): 0

Region: chr29 45308352-45308364. Max. coverage (+): 0. Max coverage (-): 0

Region: chr29 45308365-45308378. Max. coverage (+): 0. Max coverage (-): 0

Region: chr29 45308379-45308391. Max. coverage (+): 0. Max coverage (-): 0

Region: chr29 45308392-45308404. Max. coverage (+): 0. Max coverage (-): 0

Region: chr29 45308405-45308417. Max. coverage (+): 0. Max coverage (-): 0

Region: chr29 45308418-45308431. Max. coverage (+): 0. Max coverage (-): 0

Region: chr29 45308432-45308444. Max. coverage (+): 0. Max coverage (-): 0

Region: chr29 45308445-45308457. Max. coverage (+): 0. Max coverage (-): 0

Region: chr29 45308458-45308470. Max. coverage (+): 0. Max coverage (-): 0

Region: chr29 45308471-45308484. Max. coverage (+): 0. Max coverage (-): 0

Region: chr29 45308485-45308497. Max. coverage (+): 0. Max coverage (-): 0

Region: chr29 45308498-45308510. Max. coverage (+): 0. Max coverage (-): 0

Region: chr29 45308511-45308524. Max. coverage (+): 4.08. Max coverage (-): 0

Region: chr29 45308525-45308537. Max. coverage (+): 4.08. Max coverage (-): 0

Region: chr29 45308538-45308550. Max. coverage (+): 0. Max coverage (-): 0

Region: chr29 45308551-45308563. Max. coverage (+): 0. Max coverage (-): 0

Region: chr29 45308564-45308577. Max. coverage (+): 0. Max coverage (-): 0

Region: chr29 45308578-45308590. Max. coverage (+): 0. Max coverage (-): 0

Region: chr29 45308591-45308603. Max. coverage (+): 0. Max coverage (-): 0

Region: chr29 45308604-45308616. Max. coverage (+): 0. Max coverage (-): 0

Region: chr29 45308617-45308630. Max. coverage (+): 0. Max coverage (-): 0

Region: chr29 45308631-45308643. Max. coverage (+): 0. Max coverage (-): 0

Region: chr29 45308644-45308656. Max. coverage (+): 0. Max coverage (-): 0

Region: chr29 45308657-45308669. Max. coverage (+): 0. Max coverage (-): 0

Region: chr29 45308670-45308683. Max. coverage (+): 3.15. Max coverage (-): 0

Region: chr29 45308684-45308696. Max. coverage (+): 3.15. Max coverage (-): 0

Region: chr29 45308697-45308709. Max. coverage (+): 0. Max coverage (-): 0

Region: chr29 45308710-45308722. Max. coverage (+): 4.08. Max coverage (-): 0

Region: chr29 45308723-45308736. Max. coverage (+): 4.08. Max coverage (-): 0

Region: chr29 45308737-45308749. Max. coverage (+): 3.3. Max coverage (-): 0

Region: chr29 45308750-45308762. Max. coverage (+): 1.27. Max coverage (-): 0

Region: chr29 45308763-45308775. Max. coverage (+): 2.38. Max coverage (-): 0

Region: chr29 45308776-45308789. Max. coverage (+): 4.65. Max coverage (-): 0

Region: chr29 45308790-45308802. Max. coverage (+): 0. Max coverage (-): 0

Region: chr29 45308803-45308815. Max. coverage (+): 0. Max coverage (-): 0

Region: chr29 45308816-45308828. Max. coverage (+): 4.66. Max coverage (-): 0

Region: chr29 45308829-45308842. Max. coverage (+): 4.66. Max coverage (-): 0

Region: chr29 45308843-45308855. Max. coverage (+): 0. Max coverage (-): 0

Region: chr29 45308856-45308868. Max. coverage (+): 0. Max coverage (-): 0

Region: chr29 45308869-45308882. Max. coverage (+): 0. Max coverage (-): 0

Region: chr29 45308883-45308895. Max. coverage (+): 0. Max coverage (-): 0

Region: chr29 45308896-45308908. Max. coverage (+): 0. Max coverage (-): 0

Region: chr29 45308909-45308921. Max. coverage (+): 4.24. Max coverage (-): 0

Region: chr29 45308922-45308935. Max. coverage (+): 0. Max coverage (-): 0

Region: chr29 45308936-45308948. Max. coverage (+): 0. Max coverage (-): 0

Region: chr29 45308949-45308961. Max. coverage (+): 0. Max coverage (-): 0

Region: chr29 45308962-45308974. Max. coverage (+): 0. Max coverage (-): 0

Region: chr29 45308975-45308988. Max. coverage (+): 0. Max coverage (-): 0

Region: chr29 45308989-45309001. Max. coverage (+): 0. Max coverage (-): 0

Region: chr29 45309002-45309014. Max. coverage (+): 0. Max coverage (-): 0

Region: chr29 45309015-45309027. Max. coverage (+): 0. Max coverage (-): 0

Region: chr29 45309028-45309041. Max. coverage (+): 0. Max coverage (-): 0

Region: chr29 45309042-45309054. Max. coverage (+): 0. Max coverage (-): 0

Region: chr29 45309055-45309067. Max. coverage (+): 0. Max coverage (-): 0

Region: chr29 45309068-45309080. Max. coverage (+): 0. Max coverage (-): 0

Region: chr29 45309081-45309094. Max. coverage (+): 0. Max coverage (-): 0

Region: chr29 45309095-45309107. Max. coverage (+): 0. Max coverage (-): 0

Region: chr29 45309108-45309120. Max. coverage (+): 4.4. Max coverage (-): 0

Region: chr29 45309121-45309133. Max. coverage (+): 4.4. Max coverage (-): 0

Region: chr29 45309134-45309147. Max. coverage (+): 0. Max coverage (-): 0

Region: chr29 45309148-45309160. Max. coverage (+): 0. Max coverage (-): 0

Region: chr29 45309161-45309173. Max. coverage (+): 0. Max coverage (-): 0

Region: chr29 45309174-45309187. Max. coverage (+): 0. Max coverage (-): 0

Region: chr29 45309188-45309200. Max. coverage (+): 0. Max coverage (-): 0

Region: chr29 45309201-45309213. Max. coverage (+): 0. Max coverage (-): 0

Region: chr29 45309214-45309226. Max. coverage (+): 6.19. Max coverage (-): 0

Region: chr29 45309227-45309240. Max. coverage (+): 6.19. Max coverage (-): 0

Region: chr29 45309241-45309253. Max. coverage (+): 0. Max coverage (-): 0

Region: chr29 45309254-45309266. Max. coverage (+): 0. Max coverage (-): 0

Region: chr29 45309267-45309279. Max. coverage (+): 0. Max coverage (-): 0

Region: chr29 45309280-45309293. Max. coverage (+): 0. Max coverage (-): 0

Region: chr29 45309294-45309306. Max. coverage (+): 0. Max coverage (-): 0

Region: chr29 45309307-45309319. Max. coverage (+): 0. Max coverage (-): 0

Region: chr29 45309320-45309332. Max. coverage (+): 0. Max coverage (-): 0

Region: chr29 45309333-45309346. Max. coverage (+): 2.92. Max coverage (-): 0

Region: chr29 45309347-45309359. Max. coverage (+): 2.92. Max coverage (-): 0

Region: chr29 45309360-45309372. Max. coverage (+): 0. Max coverage (-): 0

Region: chr29 45309373-45309385. Max. coverage (+): 0. Max coverage (-): 0

Region: chr29 45309386-45309399. Max. coverage (+): 0. Max coverage (-): 0

Region: chr29 45309400-45309412. Max. coverage (+): 0. Max coverage (-): 0

Region: chr29 45309413-45309425. Max. coverage (+): 0. Max coverage (-): 0

Region: chr29 45309426-45309438. Max. coverage (+): 0. Max coverage (-): 0

Region: chr29 45309439-45309452. Max. coverage (+): 0. Max coverage (-): 0

Region: chr29 45309453-45309465. Max. coverage (+): 0. Max coverage (-): 0

Region: chr29 45309466-45309478. Max. coverage (+): 0. Max coverage (-): 0

Region: chr29 45309479-45309491. Max. coverage (+): 0. Max coverage (-): 0

Region: chr29 45309492-45309505. Max. coverage (+): 0. Max coverage (-): 0

Region: chr29 45309506-45309518. Max. coverage (+): 0. Max coverage (-): 0

Region: chr29 45309519-45309531. Max. coverage (+): 0. Max coverage (-): 0

Region: chr29 45309532-45309545. Max. coverage (+): 0. Max coverage (-): 0

Region: chr29 45309546-45309558. Max. coverage (+): 0. Max coverage (-): 0

Region: chr29 45309559-45309571. Max. coverage (+): 0. Max coverage (-): 0

Region: chr29 45309572-45309584. Max. coverage (+): 0. Max coverage (-): 0

Region: chr29 45309585-45309598. Max. coverage (+): 1.62. Max coverage (-): 0

Region: chr29 45309599-45309611. Max. coverage (+): 0. Max coverage (-): 0

Region: chr29 45309612-45309624. Max. coverage (+): 0. Max coverage (-): 0

Region: chr29 45309625-45309637. Max. coverage (+): 0. Max coverage (-): 0

Region: chr29 45309638-45309651. Max. coverage (+): 0. Max coverage (-): 0

Region: chr29 45309652-45309664. Max. coverage (+): 0. Max coverage (-): 0

Region: chr29 45309665-45309677. Max. coverage (+): 0. Max coverage (-): 0

Region: chr29 45309678-45309690. Max. coverage (+): 0. Max coverage (-): 0

Region: chr29 45309691-45309704. Max. coverage (+): 0. Max coverage (-): 0

Region: chr29 45309705-45309717. Max. coverage (+): 0. Max coverage (-): 0

Region: chr29 45309718-45309730. Max. coverage (+): 7.41. Max coverage (-): 0

Region: chr29 45309731-45309743. Max. coverage (+): 7.41. Max coverage (-): 0

Region: chr29 45309744-45309757. Max. coverage (+): 0. Max coverage (-): 0

Region: chr29 45309758-45309770. Max. coverage (+): 0. Max coverage (-): 0

Region: chr29 45309771-45309783. Max. coverage (+): 0. Max coverage (-): 0

Region: chr29 45309784-45309796. Max. coverage (+): 0. Max coverage (-): 0

Region: chr29 45309797-45309810. Max. coverage (+): 0. Max coverage (-): 0

Region: chr29 45309811-45309823. Max. coverage (+): 0. Max coverage (-): 0

Region: chr29 45309824-45309836. Max. coverage (+): 0. Max coverage (-): 0

Region: chr29 45309837-45309850. Max. coverage (+): 0. Max coverage (-): 0

Region: chr29 45309851-45309863. Max. coverage (+): 0. Max coverage (-): 0

Region: chr29 45309864-45309876. Max. coverage (+): 0. Max coverage (-): 0

Region: chr29 45309877-45309889. Max. coverage (+): 0. Max coverage (-): 0

Region: chr29 45309890-45309903. Max. coverage (+): 0. Max coverage (-): 0

Region: chr29 45309904-45309916. Max. coverage (+): 1.95. Max coverage (-): 0

Region: chr29 45309917-45309929. Max. coverage (+): 1.95. Max coverage (-): 0

Region: chr29 45309930-45309942. Max. coverage (+): 0. Max coverage (-): 0

Region: chr29 45309943-45309956. Max. coverage (+): 0. Max coverage (-): 0

Region: chr29 45309957-45309969. Max. coverage (+): 0. Max coverage (-): 0

Region: chr29 45309970-45309982. Max. coverage (+): 0. Max coverage (-): 0

Region: chr29 45309983-45309995. Max. coverage (+): 0. Max coverage (-): 0

Region: chr29 45309996-45310009. Max. coverage (+): 0. Max coverage (-): 0

Region: chr29 45310010-45310022. Max. coverage (+): 0. Max coverage (-): 0

Region: chr29 45310023-45310035. Max. coverage (+): 0. Max coverage (-): 0

Region: chr29 45310036-45310048. Max. coverage (+): 0. Max coverage (-): 0

Region: chr29 45310049-45310062. Max. coverage (+): 0. Max coverage (-): 0

Region: chr29 45310063-45310075. Max. coverage (+): 0. Max coverage (-): 0

Region: chr29 45310076-45310088. Max. coverage (+): 0. Max coverage (-): 0

Region: chr29 45310089-45310101. Max. coverage (+): 0. Max coverage (-): 0

Region: chr29 45310102-45310115. Max. coverage (+): 0. Max coverage (-): 0

Region: chr29 45310116-45310128. Max. coverage (+): 1.94. Max coverage (-): 0

Region: chr29 45310129-45310141. Max. coverage (+): 0. Max coverage (-): 0

Region: chr29 45310142-45310154. Max. coverage (+): 0. Max coverage (-): 0

Region: chr29 45310155-45310168. Max. coverage (+): 0. Max coverage (-): 0

Region: chr29 45310169-45310181. Max. coverage (+): 0. Max coverage (-): 0

Region: chr29 45310182-45310194. Max. coverage (+): 0. Max coverage (-): 0

Region: chr29 45310195-45310208. Max. coverage (+): 0. Max coverage (-): 0

Region: chr29 45310209-45310221. Max. coverage (+): 0. Max coverage (-): 0

Region: chr29 45310222-45310234. Max. coverage (+): 0. Max coverage (-): 0

Region: chr29 45310235-45310247. Max. coverage (+): 0. Max coverage (-): 0

Region: chr29 45310248-45310261. Max. coverage (+): 0.93. Max coverage (-): 0

Region: chr29 45310262-45310274. Max. coverage (+): 0. Max coverage (-): 0

Region: chr29 45310275-45310287. Max. coverage (+): 0. Max coverage (-): 0

Region: chr29 45310288-45310300. Max. coverage (+): 0. Max coverage (-): 0

Region: chr29 45310301-45310314. Max. coverage (+): 0.77. Max coverage (-): 0

Region: chr29 45310315-45310327. Max. coverage (+): 0. Max coverage (-): 0

Region: chr29 45310328-45310340. Max. coverage (+): 0. Max coverage (-): 0

Region: chr29 45310341-45310353. Max. coverage (+): 0.49. Max coverage (-): 0

Region: chr29 45310354-45310367. Max. coverage (+): 0.49. Max coverage (-): 0

Region: chr29 45310368-45310380. Max. coverage (+): 0. Max coverage (-): 0

Region: chr29 45310381-45310393. Max. coverage (+): 0. Max coverage (-): 0

Region: chr29 45310394-45310406. Max. coverage (+): 8.88. Max coverage (-): 0

Region: chr29 45310407-45310420. Max. coverage (+): 7.38. Max coverage (-): 0

Region: chr29 45310421-45310433. Max. coverage (+): 0. Max coverage (-): 0

Region: chr29 45310434-45310446. Max. coverage (+): 0. Max coverage (-): 0

Region: chr29 45310447-45310459. Max. coverage (+): 0. Max coverage (-): 0

Region: chr29 45310460-45310473. Max. coverage (+): 0. Max coverage (-): 0

Region: chr29 45310474-45310486. Max. coverage (+): 0. Max coverage (-): 0

Region: chr29 45310487-45310499. Max. coverage (+): 0. Max coverage (-): 0

Region: chr29 45310500-45310513. Max. coverage (+): 0.81. Max coverage (-): 0

Region: chr29 45310514-45310526. Max. coverage (+): 0.81. Max coverage (-): 0

Region: chr29 45310527-45310539. Max. coverage (+): 0.67. Max coverage (-): 0

Region: chr29 45310540-45310552. Max. coverage (+): 0. Max coverage (-): 0

Region: chr29 45310553-45310566. Max. coverage (+): 2.9. Max coverage (-): 0

Region: chr29 45310567-45310579. Max. coverage (+): 0. Max coverage (-): 0

Region: chr29 45310580-45310592. Max. coverage (+): 0. Max coverage (-): 0

Region: chr29 45310593-45310605. Max. coverage (+): 0. Max coverage (-): 0

Region: chr29 45310606-45310619. Max. coverage (+): 0. Max coverage (-): 0

Region: chr29 45310620-45310632. Max. coverage (+): 0. Max coverage (-): 0

Region: chr29 45310633-45310645. Max. coverage (+): 0. Max coverage (-): 0

Region: chr29 45310646-45310658. Max. coverage (+): 0. Max coverage (-): 0

Region: chr29 45310659-45310672. Max. coverage (+): 0. Max coverage (-): 0

Region: chr29 45310673-45310685. Max. coverage (+): 0. Max coverage (-): 0

Region: chr29 45310686-45310698. Max. coverage (+): 0. Max coverage (-): 0

Region: chr29 45310699-45310711. Max. coverage (+): 4.72. Max coverage (-): 0

Region: chr29 45310712-45310725. Max. coverage (+): 4.72. Max coverage (-): 0

Region: chr29 45310726-45310738. Max. coverage (+): 0. Max coverage (-): 0

Region: chr29 45310739-45310751. Max. coverage (+): 0. Max coverage (-): 0

Region: chr29 45310752-45310764. Max. coverage (+): 0. Max coverage (-): 0

Region: chr29 45310765-45310778. Max. coverage (+): 0. Max coverage (-): 0

Region: chr29 45310779-45310791. Max. coverage (+): 0. Max coverage (-): 0

Region: chr29 45310792-45310804. Max. coverage (+): 0. Max coverage (-): 0

Region: chr29 45310805-45310817. Max. coverage (+): 0. Max coverage (-): 0

Region: chr29 45310818-45310831. Max. coverage (+): 0. Max coverage (-): 0

Region: chr29 45310832-45310844. Max. coverage (+): 5.14. Max coverage (-): 0

Region: chr29 45310845-45310857. Max. coverage (+): 5.14. Max coverage (-): 0

Region: chr29 45310858-45310871. Max. coverage (+): 0. Max coverage (-): 0

Region: chr29 45310872-45310884. Max. coverage (+): 0. Max coverage (-): 0

Region: chr29 45310885-45310897. Max. coverage (+): 0. Max coverage (-): 0

Region: chr29 45310898-45310910. Max. coverage (+): 0. Max coverage (-): 0

Region: chr29 45310911-45310924. Max. coverage (+): 0. Max coverage (-): 0

Region: chr29 45310925-45310937. Max. coverage (+): 0. Max coverage (-): 0

Region: chr29 45310938-45310950. Max. coverage (+): 0. Max coverage (-): 0

Region: chr29 45310951-45310963. Max. coverage (+): 0. Max coverage (-): 0

Region: chr29 45310964-45310977. Max. coverage (+): 0. Max coverage (-): 0

Region: chr29 45310978-45310990. Max. coverage (+): 0. Max coverage (-): 0

Region: chr29 45310991-45311003. Max. coverage (+): 0. Max coverage (-): 0

Region: chr29 45311004-45311016. Max. coverage (+): 0. Max coverage (-): 0

Region: chr29 45311017-45311030. Max. coverage (+): 0. Max coverage (-): 0

Region: chr29 45311031-45311043. Max. coverage (+): 0. Max coverage (-): 0

Region: chr29 45311044-45311056. Max. coverage (+): 0. Max coverage (-): 0

Region: chr29 45311057-45311069. Max. coverage (+): 0. Max coverage (-): 0

Region: chr29 45311070-45311083. Max. coverage (+): 0. Max coverage (-): 0

Region: chr29 45311084-45311096. Max. coverage (+): 0. Max coverage (-): 0

Region: chr29 45311097-45311109. Max. coverage (+): 0. Max coverage (-): 0

Region: chr29 45311110-45311122. Max. coverage (+): 0. Max coverage (-): 0

Region: chr29 45311123-45311136. Max. coverage (+): 0. Max coverage (-): 0

Region: chr29 45311137-45311149. Max. coverage (+): 0. Max coverage (-): 0

Region: chr29 45311150-45311162. Max. coverage (+): 0. Max coverage (-): 0

Region: chr29 45311163-45311176. Max. coverage (+): 0. Max coverage (-): 0

Region: chr29 45311177-45311189. Max. coverage (+): 0. Max coverage (-): 0

Region: chr29 45311190-45311202. Max. coverage (+): 0.44. Max coverage (-): 0

Region: chr29 45311203-45311215. Max. coverage (+): 0. Max coverage (-): 0

Region: chr29 45311216-45311229. Max. coverage (+): 0. Max coverage (-): 0

Region: chr29 45311230-45311242. Max. coverage (+): 0. Max coverage (-): 0

Region: chr29 45311243-45311255. Max. coverage (+): 0. Max coverage (-): 0

Region: chr29 45311256-45311268. Max. coverage (+): 0. Max coverage (-): 0

Region: chr29 45311269-45311282. Max. coverage (+): 0. Max coverage (-): 0

Region: chr29 45311283-45311295. Max. coverage (+): 0. Max coverage (-): 0

Region: chr29 45311296-45311308. Max. coverage (+): 0. Max coverage (-): 0

Region: chr29 45311309-45311321. Max. coverage (+): 0. Max coverage (-): 0

Region: chr29 45311322-45311335. Max. coverage (+): 0. Max coverage (-): 0

Region: chr29 45311336-45311348. Max. coverage (+): 0. Max coverage (-): 0

Region: chr29 45311349-45311361. Max. coverage (+): 0. Max coverage (-): 0

Region: chr29 45311362-45311374. Max. coverage (+): 0. Max coverage (-): 0

Region: chr29 45311375-45311388. Max. coverage (+): 0. Max coverage (-): 0

Region: chr29 45311389-45311401. Max. coverage (+): 0. Max coverage (-): 0

Region: chr29 45311402-45311414. Max. coverage (+): 0. Max coverage (-): 0

Region: chr29 45311415-45311427. Max. coverage (+): 0. Max coverage (-): 0

Region: chr29 45311428-45311441. Max. coverage (+): 0. Max coverage (-): 0

Region: chr29 45311442-45311454. Max. coverage (+): 0. Max coverage (-): 0

Region: chr29 45311455-45311467. Max. coverage (+): 3.05. Max coverage (-): 0

Region: chr29 45311468-45311480. Max. coverage (+): 3.05. Max coverage (-): 0

Region: chr29 45311481-45311494. Max. coverage (+): 0. Max coverage (-): 0

Region: chr29 45311495-45311507. Max. coverage (+): 2.18. Max coverage (-): 0

Region: chr29 45311508-45311520. Max. coverage (+): 2.18. Max coverage (-): 0

Region: chr29 45311521-45311534. Max. coverage (+): 0. Max coverage (-): 0

Region: chr29 45311535-45311547. Max. coverage (+): 0. Max coverage (-): 0

Region: chr29 45311548-45311560. Max. coverage (+): 0. Max coverage (-): 0

Region: chr29 45311561-45311573. Max. coverage (+): 0. Max coverage (-): 0

Region: chr29 45311574-45311587. Max. coverage (+): 0. Max coverage (-): 0

Region: chr29 45311588-45311600. Max. coverage (+): 0. Max coverage (-): 0

Region: chr29 45311601-45311613. Max. coverage (+): 0. Max coverage (-): 0

Region: chr29 45311614-45311626. Max. coverage (+): 0. Max coverage (-): 0

Region: chr29 45311627-45311640. Max. coverage (+): 0. Max coverage (-): 0

Region: chr29 45311641-45311653. Max. coverage (+): 0. Max coverage (-): 0

Region: chr29 45311654-45311666. Max. coverage (+): 0. Max coverage (-): 0

Region: chr29 45311667-45311679. Max. coverage (+): 0. Max coverage (-): 0

Region: chr29 45311680-45311693. Max. coverage (+): 0. Max coverage (-): 0

Region: chr29 45311694-45311706. Max. coverage (+): 0. Max coverage (-): 0

Region: chr29 45311707-45311719. Max. coverage (+): 0. Max coverage (-): 0

Region: chr29 45311720-45311732. Max. coverage (+): 0. Max coverage (-): 0

Region: chr29 45311733-45311746. Max. coverage (+): 0. Max coverage (-): 0

Region: chr29 45311747-45311759. Max. coverage (+): 0. Max coverage (-): 0

Region: chr29 45311760-45311772. Max. coverage (+): 0. Max coverage (-): 0

Region: chr29 45311773-45311785. Max. coverage (+): 0. Max coverage (-): 0

Region: chr29 45311786-45311799. Max. coverage (+): 0. Max coverage (-): 0

Region: chr29 45311800-45311812. Max. coverage (+): 0. Max coverage (-): 0

Region: chr29 45311813-45311825. Max. coverage (+): 0. Max coverage (-): 0

Region: chr29 45311826-45311839. Max. coverage (+): 0. Max coverage (-): 0

Region: chr29 45311840-45311852. Max. coverage (+): 0. Max coverage (-): 0

Region: chr29 45311853-45311865. Max. coverage (+): 0. Max coverage (-): 0

Region: chr29 45311866-45311878. Max. coverage (+): 0. Max coverage (-): 0

Region: chr29 45311879-45311892. Max. coverage (+): 0. Max coverage (-): 0

Region: chr29 45311893-45311905. Max. coverage (+): 0. Max coverage (-): 0

Region: chr29 45311906-45311918. Max. coverage (+): 0. Max coverage (-): 0

Region: chr29 45311919-45311931. Max. coverage (+): 0. Max coverage (-): 0

Region: chr29 45311932-45311945. Max. coverage (+): 0. Max coverage (-): 0

Region: chr29 45311946-45311958. Max. coverage (+): 0. Max coverage (-): 0

Region: chr29 45311959-45311971. Max. coverage (+): 0. Max coverage (-): 0

Region: chr29 45311972-45311984. Max. coverage (+): 0. Max coverage (-): 0

Region: chr29 45311985-45311998. Max. coverage (+): 0. Max coverage (-): 0

Region: chr29 45311999-45312011. Max. coverage (+): 0. Max coverage (-): 0

Region: chr29 45312012-45312024. Max. coverage (+): 0. Max coverage (-): 0

Region: chr29 45312025-45312037. Max. coverage (+): 0. Max coverage (-): 0

Region: chr29 45312038-45312051. Max. coverage (+): 0. Max coverage (-): 0

Region: chr29 45312052-45312064. Max. coverage (+): 0. Max coverage (-): 0

Region: chr29 45312065-45312077. Max. coverage (+): 0. Max coverage (-): 0

Region: chr29 45312078-45312090. Max. coverage (+): 0. Max coverage (-): 0

Region: chr29 45312091-45312104. Max. coverage (+): 0. Max coverage (-): 0

Region: chr29 45312105-45312117. Max. coverage (+): 0. Max coverage (-): 0

Region: chr29 45312118-45312130. Max. coverage (+): 0. Max coverage (-): 0

Region: chr29 45312131-45312143. Max. coverage (+): 0. Max coverage (-): 0

Region: chr29 45312144-45312157. Max. coverage (+): 0. Max coverage (-): 0

Region: chr29 45312158-45312170. Max. coverage (+): 0. Max coverage (-): 0

Region: chr29 45312171-45312183. Max. coverage (+): 0. Max coverage (-): 0

Region: chr29 45312184-45312197. Max. coverage (+): 0. Max coverage (-): 0

Region: chr29 45312198-45312210. Max. coverage (+): 0. Max coverage (-): 0

Region: chr29 45312211-45312223. Max. coverage (+): 0. Max coverage (-): 0

Region: chr29 45312224-45312236. Max. coverage (+): 0. Max coverage (-): 0

Region: chr29 45312237-45312250. Max. coverage (+): 0. Max coverage (-): 0

Region: chr29 45312251-45312263. Max. coverage (+): 0. Max coverage (-): 0

Region: chr29 45312264-45312276. Max. coverage (+): 0. Max coverage (-): 0

Region: chr29 45312277-45312289. Max. coverage (+): 0. Max coverage (-): 0

Region: chr29 45312290-45312303. Max. coverage (+): 2.4. Max coverage (-): 0

Region: chr29 45312304-45312316. Max. coverage (+): 0. Max coverage (-): 0

Region: chr29 45312317-. Max. coverage (+): 0. Max coverage (-): 0

RepeatMasker Color Code

**+**

100-98% Identity

<98-95% Identity

<95-90% Identity

<90-85% Identity

<85-80% Identity

<80-75% Identity

<75-70% Identity

<70% Identity

**-**

Gene Set Color Code

**+**

Gene

Pseudogene

**-**

Topology/Coverage Color Code

Coverage Plus Strand

Coverage Minus Strand

Mainstrand: Plus

Mainstrand: Minus

Complementary Strand

Flanking Region  
(if option -flank >0)

Gene Set Annotation  

**1. RBM14 (protein coding, ENSBTAG00000001225) Tr:00000001619 Ex:2**: 45305606-45307070 (+)  
**2. RBM14 (protein coding, ENSBTAG00000001225) Tr:00000001619 Ex:3**: 45307845-45308722 (+)

  
RepeatMasker Annotation  

**1. SINE2-3\_BT**: 45311299-45311416 (+), Divergence to consensus: 28.9%  
**2. CHR-2\_BT**: 45311553-45311713 (+), Divergence to consensus: 22.2%  
**3. MIRb**: 45311731-45311887 (+), Divergence to consensus: 37%

  
Transcription Factor Binding Sites  

**RFX4\_2** (Sequence: CCTAGTTAC (+): 45311269)  
**A-MYB** (Sequence: CCAACTGTCT (-): 45307820)
