# Supplementary material for: Small RNA sequencing of cryopreserved semen from single bull revealed altered miRNAs and piRNAs expression between High- and Low-motile sperm populations
Source: BMC Genomics. 2017 Jan 4;18:14. doi: 10.1186/s12864-016-3394-7 (PMC5209821; doi:10.1186/s12864-016-3394-7)
Supplement: Additional file 3: — Details for each piRNA clusters found in High Motile (HM) sperm fraction. Genes, repeats, transposable elements and transcription factors binding sites falling within the cluster regions were reported. (ZIP 1896 kb) [file 12864_2016_3394_MOESM3_ESM.zip › 75.html]

piRNA cluster 75


Predicted piRNA cluster no. 75     previous   next
  

Show proTRAC run info
Hide proTRAC run info

================================= proTRAC ====================================  
VERSION: 2.1                                    LAST MODIFIED: 06. October 2015  
  
Please cite:  
Rosenkranz D, Zischler H. proTRAC - a software for probabilistic piRNA cluster  
detection, visualization and analysis. 2012. BMC Bioinformatics 13:5.  
  
and (for proTRAC 2.0 and later):  
Rosenkranz D, Rudloff S, Bastuck K, Ketting RF, Zischler H. Tupaia small RNAs  
provide insights into function and evolution of RNAi-based transposon defense  
in mammals. 2015. RNA 21(5):911-922.  
  
Contact:  
David Rosenkranz  
Institute of Anthropology, small RNA group  
Johannes Gutenberg University Mainz  
email: rosenkranz@uni-mainz.de  
  
You can find the latest proTRAC version at:  
http://sourceforge.net/projects/protrac/files  
http://www.smallRNAgroup-mainz.de/software  
==============================================================================  
  
PARAMETERS:  
Map file: .............../storage/core/barbara/genhome/smallRNA/fertility/Sample\_motile/pirna/Sample\_motile\_26-33\_collapsed.fa.no-dust.map.weighted-10000-1000-b-0  
Genome file: ............/storage/core/barbara/genhome/smallRNA/fertility/Sample\_all/pirna/bt\_311\_chrY.fa  
RepeatMasker annotation: /storage/genomes/bt\_umd31/GCF\_000003055.6\_Bos\_taurus\_UMD\_3.1.1\_repeatMasker\_chr.out  
GeneSet:................./storage/core/barbara/genhome/smallRNA/fertility/Sample\_all/pirna/full.gtf  
  
Significant (p<=0.01) hit density will be calculated based  
on observed hit distribution.  
  
Sliding window size: ........................................ 5000 bp  
Sliding window increament: .................................. 1000 bp  
Normalize each hit by number of genomic hits: ............... 1 [0=no/1=yes]  
Normalize each hit by number of sequence reads: ............. 1 [0=no/1=yes]  
Normalize values (-> per million mapped reads): ............. 1 [0=no/1=yes]  
Min. fraction of hits with 1T(U) or 10A: .................... 0.75  
Alternatively: Min. fraction of hits with 1T(U) and 10A: .... 0.5  
Min. fraction of hits with typical piRNA length: ............ 0.75  
Typical piRNA length: ....................................... 26-33 nt  
Min. size of a piRNA cluster: ............................... 5000 bp.  
Min. number of hits (absolute): ............................. 0  
Min. number of hits (normalized): ........................... 0  
Min. fraction of hits on the mainstrand: .................... 0.75  
Top fraction of mapped sequences (in terms of read counts): . 1%  
Top fraction accounts for max. n% of sequence reads: ........ 90%  
Min. fraction of hits on each arm of a bidirectional cluster: 0.1  
Output image file for each cluster: ......................... 0 [0=no/1=yes]  
Output html file for each cluster: .......................... 1 [0=no/1=yes]  
Output a summary table: ..................................... 1 [0=no/1=yes]  
Output a FASTA file for each cluster (piRNA sequences): ..... 1 [0=no/1=yes]  
Output a FASTA file comprising cluster sequences: ........... 1 [0=no/1=yes]  
Search DNA motifs in clusters: .............................. 1 [0=no/1=yes]  
Output flanking sequences: +/- .............................. 0 bp  
Output ~.pTi file: .......................................... 1 [0=no/1=yes]  
==============================================================================  
  
  
Genome size (without gaps): ............ 2678902517 bp  
Gaps (N/X/-): .......................... 53837044 bp  
Mapped reads: .......................... 658825247023  
Non-identical sequences: ............... 514171  
Genomic hits: .......................... 764233  
Significant densitiy of mapped reads: .. 12867599.5173724 reads/kb

Show proTRAC cluster info
Hide proTRAC cluster info

|  |  |
| --- | --- |
| Location | chr29 |
| Coordinates | 47923886-47937860 |
| Size [bp] | 13975 |
| Sequence hit loci | 274 |
| Mapped reads (normalized) | 336970148.1 |
| Mapped reads (normalized) per kb | 24112354.1 |
| Normalized reads with 1T (1U) | 76.8% |
| Normalized reads with 10A | 35.5% |
| Normalized reads with length 26-33 nt | 100% |
| Normalized reads on the main strand(s) | 100% |
| Predicted directionality | mono:minus |

100%

0%

1T (1U)  
reads

10A reads

26-33 nt  
reads

reads on mainstrand

**Either the amount of reads with 1T (1U) OR 10A has to exceed 75% (set with option: -1Tor10A)  
Alternatively the amount of reads with 1T (1U) AND 10A has to exceed 50% (set with option: -1Tand10A)  
Minimum amount of reads with preferred size is 75% (set with option: -pisize)  
Minimum amount of reads on the main strand(s) is 75% (set with option: -clstrand)**

Show read coverage
Hide read coverage

WHAT DO I SEE HERE?  
This chart shows the location of mapped sequence reads within a predicted piRNA cluster. The color refers to the number of genomic hits produced by the sequence read in question. A dark red bar indicates that this sequence read produces many other hits elsewhere in the genome. Many adjacent red or yellow bars can indicate the presence of a multi-copy element such as transposons or rRNA genes. A dark green bar indicates that this sequence read maps uniquely to this locus.

1 hit

2-5 hits

6-10 hits

11-20 hits

21-50 hits

51-100 hits

> 100 hits

chr29

47923886

47937860

Gene Set

RepeatMasker

Mapped  
Reads

26.7

plus strand

minus strand

26.7

Region: chr29 45312318-47923899. Max. coverage (+): 0. Max coverage (-): 4.4

Region: chr29 47923900-47923927. Max. coverage (+): 0. Max coverage (-): 0

Region: chr29 47923928-47923955. Max. coverage (+): 0. Max coverage (-): 0

Region: chr29 47923956-47923983. Max. coverage (+): 0. Max coverage (-): 0

Region: chr29 47923984-47924011. Max. coverage (+): 0. Max coverage (-): 0

Region: chr29 47924012-47924039. Max. coverage (+): 0. Max coverage (-): 0

Region: chr29 47924040-47924067. Max. coverage (+): 0. Max coverage (-): 0

Region: chr29 47924068-47924095. Max. coverage (+): 0. Max coverage (-): 0

Region: chr29 47924096-47924123. Max. coverage (+): 0. Max coverage (-): 2.89

Region: chr29 47924124-47924151. Max. coverage (+): 0. Max coverage (-): 1.45

Region: chr29 47924152-47924179. Max. coverage (+): 0. Max coverage (-): 0

Region: chr29 47924180-47924207. Max. coverage (+): 0. Max coverage (-): 5.18

Region: chr29 47924208-47924235. Max. coverage (+): 0. Max coverage (-): 2.57

Region: chr29 47924236-47924263. Max. coverage (+): 0. Max coverage (-): 0

Region: chr29 47924264-47924291. Max. coverage (+): 0. Max coverage (-): 0

Region: chr29 47924292-47924319. Max. coverage (+): 0. Max coverage (-): 0

Region: chr29 47924320-47924347. Max. coverage (+): 0. Max coverage (-): 0

Region: chr29 47924348-47924375. Max. coverage (+): 0. Max coverage (-): 0

Region: chr29 47924376-47924403. Max. coverage (+): 0. Max coverage (-): 0

Region: chr29 47924404-47924431. Max. coverage (+): 0. Max coverage (-): 0

Region: chr29 47924432-47924458. Max. coverage (+): 0. Max coverage (-): 1.77

Region: chr29 47924459-47924486. Max. coverage (+): 0. Max coverage (-): 4.34

Region: chr29 47924487-47924514. Max. coverage (+): 0. Max coverage (-): 0

Region: chr29 47924515-47924542. Max. coverage (+): 0. Max coverage (-): 0

Region: chr29 47924543-47924570. Max. coverage (+): 0. Max coverage (-): 0

Region: chr29 47924571-47924598. Max. coverage (+): 0. Max coverage (-): 0

Region: chr29 47924599-47924626. Max. coverage (+): 0. Max coverage (-): 0

Region: chr29 47924627-47924654. Max. coverage (+): 0. Max coverage (-): 0

Region: chr29 47924655-47924682. Max. coverage (+): 0. Max coverage (-): 6.06

Region: chr29 47924683-47924710. Max. coverage (+): 0. Max coverage (-): 0

Region: chr29 47924711-47924738. Max. coverage (+): 0. Max coverage (-): 0

Region: chr29 47924739-47924766. Max. coverage (+): 0. Max coverage (-): 0

Region: chr29 47924767-47924794. Max. coverage (+): 0. Max coverage (-): 0.74

Region: chr29 47924795-47924822. Max. coverage (+): 0. Max coverage (-): 0

Region: chr29 47924823-47924850. Max. coverage (+): 0. Max coverage (-): 1.77

Region: chr29 47924851-47924878. Max. coverage (+): 0. Max coverage (-): 0

Region: chr29 47924879-47924906. Max. coverage (+): 0. Max coverage (-): 1.38

Region: chr29 47924907-47924934. Max. coverage (+): 0. Max coverage (-): 0

Region: chr29 47924935-47924962. Max. coverage (+): 0. Max coverage (-): 4.25

Region: chr29 47924963-47924990. Max. coverage (+): 0. Max coverage (-): 3.69

Region: chr29 47924991-47925017. Max. coverage (+): 0. Max coverage (-): 0

Region: chr29 47925018-47925045. Max. coverage (+): 0. Max coverage (-): 0

Region: chr29 47925046-47925073. Max. coverage (+): 0. Max coverage (-): 0

Region: chr29 47925074-47925101. Max. coverage (+): 0. Max coverage (-): 0

Region: chr29 47925102-47925129. Max. coverage (+): 0. Max coverage (-): 1.13

Region: chr29 47925130-47925157. Max. coverage (+): 0. Max coverage (-): 5.14

Region: chr29 47925158-47925185. Max. coverage (+): 0. Max coverage (-): 5.14

Region: chr29 47925186-47925213. Max. coverage (+): 0. Max coverage (-): 0

Region: chr29 47925214-47925241. Max. coverage (+): 0. Max coverage (-): 0

Region: chr29 47925242-47925269. Max. coverage (+): 0. Max coverage (-): 0

Region: chr29 47925270-47925297. Max. coverage (+): 0. Max coverage (-): 0

Region: chr29 47925298-47925325. Max. coverage (+): 0. Max coverage (-): 0

Region: chr29 47925326-47925353. Max. coverage (+): 0. Max coverage (-): 0

Region: chr29 47925354-47925381. Max. coverage (+): 0. Max coverage (-): 0

Region: chr29 47925382-47925409. Max. coverage (+): 0. Max coverage (-): 0

Region: chr29 47925410-47925437. Max. coverage (+): 0. Max coverage (-): 0

Region: chr29 47925438-47925465. Max. coverage (+): 0. Max coverage (-): 0

Region: chr29 47925466-47925493. Max. coverage (+): 0. Max coverage (-): 0

Region: chr29 47925494-47925521. Max. coverage (+): 0. Max coverage (-): 0

Region: chr29 47925522-47925549. Max. coverage (+): 0. Max coverage (-): 0

Region: chr29 47925550-47925576. Max. coverage (+): 0. Max coverage (-): 0

Region: chr29 47925577-47925604. Max. coverage (+): 0. Max coverage (-): 0

Region: chr29 47925605-47925632. Max. coverage (+): 0. Max coverage (-): 0

Region: chr29 47925633-47925660. Max. coverage (+): 0. Max coverage (-): 0

Region: chr29 47925661-47925688. Max. coverage (+): 0. Max coverage (-): 0

Region: chr29 47925689-47925716. Max. coverage (+): 0. Max coverage (-): 0

Region: chr29 47925717-47925744. Max. coverage (+): 0. Max coverage (-): 0

Region: chr29 47925745-47925772. Max. coverage (+): 0. Max coverage (-): 0

Region: chr29 47925773-47925800. Max. coverage (+): 0. Max coverage (-): 0

Region: chr29 47925801-47925828. Max. coverage (+): 0. Max coverage (-): 0

Region: chr29 47925829-47925856. Max. coverage (+): 0. Max coverage (-): 0

Region: chr29 47925857-47925884. Max. coverage (+): 0. Max coverage (-): 0

Region: chr29 47925885-47925912. Max. coverage (+): 0. Max coverage (-): 0

Region: chr29 47925913-47925940. Max. coverage (+): 0. Max coverage (-): 0

Region: chr29 47925941-47925968. Max. coverage (+): 0. Max coverage (-): 0

Region: chr29 47925969-47925996. Max. coverage (+): 0. Max coverage (-): 0

Region: chr29 47925997-47926024. Max. coverage (+): 0. Max coverage (-): 0

Region: chr29 47926025-47926052. Max. coverage (+): 0. Max coverage (-): 0

Region: chr29 47926053-47926080. Max. coverage (+): 0. Max coverage (-): 0

Region: chr29 47926081-47926108. Max. coverage (+): 0. Max coverage (-): 0

Region: chr29 47926109-47926135. Max. coverage (+): 0. Max coverage (-): 0

Region: chr29 47926136-47926163. Max. coverage (+): 0. Max coverage (-): 0

Region: chr29 47926164-47926191. Max. coverage (+): 0. Max coverage (-): 0

Region: chr29 47926192-47926219. Max. coverage (+): 0. Max coverage (-): 0

Region: chr29 47926220-47926247. Max. coverage (+): 0. Max coverage (-): 0

Region: chr29 47926248-47926275. Max. coverage (+): 0. Max coverage (-): 0

Region: chr29 47926276-47926303. Max. coverage (+): 0. Max coverage (-): 0

Region: chr29 47926304-47926331. Max. coverage (+): 0. Max coverage (-): 0

Region: chr29 47926332-47926359. Max. coverage (+): 0. Max coverage (-): 0

Region: chr29 47926360-47926387. Max. coverage (+): 0. Max coverage (-): 0

Region: chr29 47926388-47926415. Max. coverage (+): 0. Max coverage (-): 0

Region: chr29 47926416-47926443. Max. coverage (+): 0. Max coverage (-): 0

Region: chr29 47926444-47926471. Max. coverage (+): 0. Max coverage (-): 0

Region: chr29 47926472-47926499. Max. coverage (+): 0. Max coverage (-): 0

Region: chr29 47926500-47926527. Max. coverage (+): 0. Max coverage (-): 0

Region: chr29 47926528-47926555. Max. coverage (+): 0. Max coverage (-): 0

Region: chr29 47926556-47926583. Max. coverage (+): 0. Max coverage (-): 0

Region: chr29 47926584-47926611. Max. coverage (+): 0. Max coverage (-): 0

Region: chr29 47926612-47926639. Max. coverage (+): 0. Max coverage (-): 0

Region: chr29 47926640-47926667. Max. coverage (+): 0. Max coverage (-): 0

Region: chr29 47926668-47926694. Max. coverage (+): 0. Max coverage (-): 0

Region: chr29 47926695-47926722. Max. coverage (+): 0. Max coverage (-): 0

Region: chr29 47926723-47926750. Max. coverage (+): 0. Max coverage (-): 0

Region: chr29 47926751-47926778. Max. coverage (+): 0. Max coverage (-): 0

Region: chr29 47926779-47926806. Max. coverage (+): 0. Max coverage (-): 0

Region: chr29 47926807-47926834. Max. coverage (+): 0. Max coverage (-): 0

Region: chr29 47926835-47926862. Max. coverage (+): 0. Max coverage (-): 1.49

Region: chr29 47926863-47926890. Max. coverage (+): 0. Max coverage (-): 1.49

Region: chr29 47926891-47926918. Max. coverage (+): 0. Max coverage (-): 0

Region: chr29 47926919-47926946. Max. coverage (+): 0. Max coverage (-): 0

Region: chr29 47926947-47926974. Max. coverage (+): 0. Max coverage (-): 11.82

Region: chr29 47926975-47927002. Max. coverage (+): 0. Max coverage (-): 3.56

Region: chr29 47927003-47927030. Max. coverage (+): 0. Max coverage (-): 0.31

Region: chr29 47927031-47927058. Max. coverage (+): 0. Max coverage (-): 24.51

Region: chr29 47927059-47927086. Max. coverage (+): 0. Max coverage (-): 26.7

Region: chr29 47927087-47927114. Max. coverage (+): 0. Max coverage (-): 0

Region: chr29 47927115-47927142. Max. coverage (+): 0. Max coverage (-): 0

Region: chr29 47927143-47927170. Max. coverage (+): 0. Max coverage (-): 0

Region: chr29 47927171-47927198. Max. coverage (+): 0. Max coverage (-): 0

Region: chr29 47927199-47927226. Max. coverage (+): 0. Max coverage (-): 0

Region: chr29 47927227-47927253. Max. coverage (+): 0. Max coverage (-): 0

Region: chr29 47927254-47927281. Max. coverage (+): 0. Max coverage (-): 0

Region: chr29 47927282-47927309. Max. coverage (+): 0. Max coverage (-): 0

Region: chr29 47927310-47927337. Max. coverage (+): 0. Max coverage (-): 0

Region: chr29 47927338-47927365. Max. coverage (+): 0. Max coverage (-): 0

Region: chr29 47927366-47927393. Max. coverage (+): 0. Max coverage (-): 0

Region: chr29 47927394-47927421. Max. coverage (+): 0. Max coverage (-): 0

Region: chr29 47927422-47927449. Max. coverage (+): 0. Max coverage (-): 0

Region: chr29 47927450-47927477. Max. coverage (+): 0. Max coverage (-): 0

Region: chr29 47927478-47927505. Max. coverage (+): 0. Max coverage (-): 0

Region: chr29 47927506-47927533. Max. coverage (+): 0. Max coverage (-): 0

Region: chr29 47927534-47927561. Max. coverage (+): 0. Max coverage (-): 0

Region: chr29 47927562-47927589. Max. coverage (+): 0. Max coverage (-): 0

Region: chr29 47927590-47927617. Max. coverage (+): 0. Max coverage (-): 0

Region: chr29 47927618-47927645. Max. coverage (+): 0. Max coverage (-): 0

Region: chr29 47927646-47927673. Max. coverage (+): 0. Max coverage (-): 0

Region: chr29 47927674-47927701. Max. coverage (+): 0. Max coverage (-): 0

Region: chr29 47927702-47927729. Max. coverage (+): 0. Max coverage (-): 0

Region: chr29 47927730-47927757. Max. coverage (+): 0. Max coverage (-): 0

Region: chr29 47927758-47927785. Max. coverage (+): 0. Max coverage (-): 0

Region: chr29 47927786-47927812. Max. coverage (+): 0. Max coverage (-): 0

Region: chr29 47927813-47927840. Max. coverage (+): 0. Max coverage (-): 0

Region: chr29 47927841-47927868. Max. coverage (+): 0. Max coverage (-): 0

Region: chr29 47927869-47927896. Max. coverage (+): 0. Max coverage (-): 0.83

Region: chr29 47927897-47927924. Max. coverage (+): 0. Max coverage (-): 10.36

Region: chr29 47927925-47927952. Max. coverage (+): 0. Max coverage (-): 5.2

Region: chr29 47927953-47927980. Max. coverage (+): 0. Max coverage (-): 0

Region: chr29 47927981-47928008. Max. coverage (+): 0. Max coverage (-): 0

Region: chr29 47928009-47928036. Max. coverage (+): 0. Max coverage (-): 0

Region: chr29 47928037-47928064. Max. coverage (+): 0. Max coverage (-): 0

Region: chr29 47928065-47928092. Max. coverage (+): 0. Max coverage (-): 0

Region: chr29 47928093-47928120. Max. coverage (+): 0. Max coverage (-): 0

Region: chr29 47928121-47928148. Max. coverage (+): 0. Max coverage (-): 0

Region: chr29 47928149-47928176. Max. coverage (+): 0. Max coverage (-): 0

Region: chr29 47928177-47928204. Max. coverage (+): 0. Max coverage (-): 0

Region: chr29 47928205-47928232. Max. coverage (+): 0. Max coverage (-): 0

Region: chr29 47928233-47928260. Max. coverage (+): 0. Max coverage (-): 0

Region: chr29 47928261-47928288. Max. coverage (+): 0. Max coverage (-): 0

Region: chr29 47928289-47928316. Max. coverage (+): 0. Max coverage (-): 0

Region: chr29 47928317-47928344. Max. coverage (+): 0. Max coverage (-): 0

Region: chr29 47928345-47928371. Max. coverage (+): 0. Max coverage (-): 0

Region: chr29 47928372-47928399. Max. coverage (+): 0. Max coverage (-): 7.97

Region: chr29 47928400-47928427. Max. coverage (+): 0. Max coverage (-): 5.3

Region: chr29 47928428-47928455. Max. coverage (+): 0. Max coverage (-): 0

Region: chr29 47928456-47928483. Max. coverage (+): 0. Max coverage (-): 0

Region: chr29 47928484-47928511. Max. coverage (+): 0. Max coverage (-): 0

Region: chr29 47928512-47928539. Max. coverage (+): 0. Max coverage (-): 0

Region: chr29 47928540-47928567. Max. coverage (+): 0. Max coverage (-): 0

Region: chr29 47928568-47928595. Max. coverage (+): 0. Max coverage (-): 3

Region: chr29 47928596-47928623. Max. coverage (+): 0. Max coverage (-): 4.48

Region: chr29 47928624-47928651. Max. coverage (+): 0. Max coverage (-): 2.08

Region: chr29 47928652-47928679. Max. coverage (+): 0. Max coverage (-): 0

Region: chr29 47928680-47928707. Max. coverage (+): 0. Max coverage (-): 0

Region: chr29 47928708-47928735. Max. coverage (+): 0. Max coverage (-): 0

Region: chr29 47928736-47928763. Max. coverage (+): 0. Max coverage (-): 0

Region: chr29 47928764-47928791. Max. coverage (+): 0. Max coverage (-): 0

Region: chr29 47928792-47928819. Max. coverage (+): 0. Max coverage (-): 0

Region: chr29 47928820-47928847. Max. coverage (+): 0. Max coverage (-): 0

Region: chr29 47928848-47928875. Max. coverage (+): 0. Max coverage (-): 0

Region: chr29 47928876-47928903. Max. coverage (+): 0. Max coverage (-): 0

Region: chr29 47928904-47928930. Max. coverage (+): 0. Max coverage (-): 0

Region: chr29 47928931-47928958. Max. coverage (+): 0. Max coverage (-): 0

Region: chr29 47928959-47928986. Max. coverage (+): 0. Max coverage (-): 0

Region: chr29 47928987-47929014. Max. coverage (+): 0. Max coverage (-): 0

Region: chr29 47929015-47929042. Max. coverage (+): 0. Max coverage (-): 0

Region: chr29 47929043-47929070. Max. coverage (+): 0. Max coverage (-): 0

Region: chr29 47929071-47929098. Max. coverage (+): 0. Max coverage (-): 0

Region: chr29 47929099-47929126. Max. coverage (+): 0. Max coverage (-): 0

Region: chr29 47929127-47929154. Max. coverage (+): 0. Max coverage (-): 0

Region: chr29 47929155-47929182. Max. coverage (+): 0. Max coverage (-): 0

Region: chr29 47929183-47929210. Max. coverage (+): 0. Max coverage (-): 0

Region: chr29 47929211-47929238. Max. coverage (+): 0. Max coverage (-): 0

Region: chr29 47929239-47929266. Max. coverage (+): 0. Max coverage (-): 0

Region: chr29 47929267-47929294. Max. coverage (+): 0. Max coverage (-): 0

Region: chr29 47929295-47929322. Max. coverage (+): 0. Max coverage (-): 0

Region: chr29 47929323-47929350. Max. coverage (+): 0. Max coverage (-): 0

Region: chr29 47929351-47929378. Max. coverage (+): 0. Max coverage (-): 0

Region: chr29 47929379-47929406. Max. coverage (+): 0. Max coverage (-): 0

Region: chr29 47929407-47929434. Max. coverage (+): 0. Max coverage (-): 0

Region: chr29 47929435-47929462. Max. coverage (+): 0. Max coverage (-): 0

Region: chr29 47929463-47929489. Max. coverage (+): 0. Max coverage (-): 0

Region: chr29 47929490-47929517. Max. coverage (+): 0. Max coverage (-): 0

Region: chr29 47929518-47929545. Max. coverage (+): 0. Max coverage (-): 0

Region: chr29 47929546-47929573. Max. coverage (+): 0. Max coverage (-): 0

Region: chr29 47929574-47929601. Max. coverage (+): 0. Max coverage (-): 0

Region: chr29 47929602-47929629. Max. coverage (+): 0. Max coverage (-): 0

Region: chr29 47929630-47929657. Max. coverage (+): 0. Max coverage (-): 0

Region: chr29 47929658-47929685. Max. coverage (+): 0. Max coverage (-): 0

Region: chr29 47929686-47929713. Max. coverage (+): 0. Max coverage (-): 0

Region: chr29 47929714-47929741. Max. coverage (+): 0. Max coverage (-): 1.36

Region: chr29 47929742-47929769. Max. coverage (+): 0. Max coverage (-): 1.36

Region: chr29 47929770-47929797. Max. coverage (+): 0. Max coverage (-): 0

Region: chr29 47929798-47929825. Max. coverage (+): 0. Max coverage (-): 2.85

Region: chr29 47929826-47929853. Max. coverage (+): 0. Max coverage (-): 0

Region: chr29 47929854-47929881. Max. coverage (+): 0. Max coverage (-): 0

Region: chr29 47929882-47929909. Max. coverage (+): 0. Max coverage (-): 0

Region: chr29 47929910-47929937. Max. coverage (+): 0. Max coverage (-): 2.6

Region: chr29 47929938-47929965. Max. coverage (+): 0. Max coverage (-): 3.06

Region: chr29 47929966-47929993. Max. coverage (+): 0. Max coverage (-): 0

Region: chr29 47929994-47930021. Max. coverage (+): 0. Max coverage (-): 0

Region: chr29 47930022-47930048. Max. coverage (+): 0. Max coverage (-): 0

Region: chr29 47930049-47930076. Max. coverage (+): 0. Max coverage (-): 0

Region: chr29 47930077-47930104. Max. coverage (+): 0. Max coverage (-): 0

Region: chr29 47930105-47930132. Max. coverage (+): 0. Max coverage (-): 0

Region: chr29 47930133-47930160. Max. coverage (+): 0. Max coverage (-): 0

Region: chr29 47930161-47930188. Max. coverage (+): 0. Max coverage (-): 0.9

Region: chr29 47930189-47930216. Max. coverage (+): 0. Max coverage (-): 1.57

Region: chr29 47930217-47930244. Max. coverage (+): 0. Max coverage (-): 1.57

Region: chr29 47930245-47930272. Max. coverage (+): 0. Max coverage (-): 0

Region: chr29 47930273-47930300. Max. coverage (+): 0. Max coverage (-): 0

Region: chr29 47930301-47930328. Max. coverage (+): 0. Max coverage (-): 0.5

Region: chr29 47930329-47930356. Max. coverage (+): 0. Max coverage (-): 0.59

Region: chr29 47930357-47930384. Max. coverage (+): 0. Max coverage (-): 1.06

Region: chr29 47930385-47930412. Max. coverage (+): 0. Max coverage (-): 0

Region: chr29 47930413-47930440. Max. coverage (+): 0. Max coverage (-): 1.52

Region: chr29 47930441-47930468. Max. coverage (+): 0. Max coverage (-): 1.52

Region: chr29 47930469-47930496. Max. coverage (+): 0. Max coverage (-): 0

Region: chr29 47930497-47930524. Max. coverage (+): 0. Max coverage (-): 0

Region: chr29 47930525-47930552. Max. coverage (+): 0. Max coverage (-): 0

Region: chr29 47930553-47930580. Max. coverage (+): 0. Max coverage (-): 0

Region: chr29 47930581-47930607. Max. coverage (+): 0. Max coverage (-): 4.75

Region: chr29 47930608-47930635. Max. coverage (+): 0. Max coverage (-): 4.75

Region: chr29 47930636-47930663. Max. coverage (+): 0. Max coverage (-): 2.75

Region: chr29 47930664-47930691. Max. coverage (+): 0. Max coverage (-): 1.32

Region: chr29 47930692-47930719. Max. coverage (+): 0. Max coverage (-): 0

Region: chr29 47930720-47930747. Max. coverage (+): 0. Max coverage (-): 4.23

Region: chr29 47930748-47930775. Max. coverage (+): 0. Max coverage (-): 0

Region: chr29 47930776-47930803. Max. coverage (+): 0. Max coverage (-): 6.66

Region: chr29 47930804-47930831. Max. coverage (+): 0. Max coverage (-): 0

Region: chr29 47930832-47930859. Max. coverage (+): 0. Max coverage (-): 0

Region: chr29 47930860-47930887. Max. coverage (+): 0. Max coverage (-): 0

Region: chr29 47930888-47930915. Max. coverage (+): 0. Max coverage (-): 0

Region: chr29 47930916-47930943. Max. coverage (+): 0. Max coverage (-): 0

Region: chr29 47930944-47930971. Max. coverage (+): 0. Max coverage (-): 0

Region: chr29 47930972-47930999. Max. coverage (+): 0. Max coverage (-): 0

Region: chr29 47931000-47931027. Max. coverage (+): 0. Max coverage (-): 0

Region: chr29 47931028-47931055. Max. coverage (+): 0. Max coverage (-): 2.22

Region: chr29 47931056-47931083. Max. coverage (+): 0. Max coverage (-): 0

Region: chr29 47931084-47931111. Max. coverage (+): 0. Max coverage (-): 0

Region: chr29 47931112-47931139. Max. coverage (+): 0. Max coverage (-): 0

Region: chr29 47931140-47931166. Max. coverage (+): 0. Max coverage (-): 0

Region: chr29 47931167-47931194. Max. coverage (+): 0. Max coverage (-): 0

Region: chr29 47931195-47931222. Max. coverage (+): 0. Max coverage (-): 0

Region: chr29 47931223-47931250. Max. coverage (+): 0. Max coverage (-): 0

Region: chr29 47931251-47931278. Max. coverage (+): 0. Max coverage (-): 0

Region: chr29 47931279-47931306. Max. coverage (+): 0. Max coverage (-): 8.01

Region: chr29 47931307-47931334. Max. coverage (+): 0. Max coverage (-): 0

Region: chr29 47931335-47931362. Max. coverage (+): 0. Max coverage (-): 11.48

Region: chr29 47931363-47931390. Max. coverage (+): 0. Max coverage (-): 0

Region: chr29 47931391-47931418. Max. coverage (+): 0. Max coverage (-): 0

Region: chr29 47931419-47931446. Max. coverage (+): 0. Max coverage (-): 0

Region: chr29 47931447-47931474. Max. coverage (+): 0. Max coverage (-): 2.58

Region: chr29 47931475-47931502. Max. coverage (+): 0. Max coverage (-): 3.95

Region: chr29 47931503-47931530. Max. coverage (+): 0. Max coverage (-): 13.18

Region: chr29 47931531-47931558. Max. coverage (+): 0. Max coverage (-): 0

Region: chr29 47931559-47931586. Max. coverage (+): 0. Max coverage (-): 0

Region: chr29 47931587-47931614. Max. coverage (+): 0. Max coverage (-): 0

Region: chr29 47931615-47931642. Max. coverage (+): 0. Max coverage (-): 0

Region: chr29 47931643-47931670. Max. coverage (+): 0. Max coverage (-): 0

Region: chr29 47931671-47931698. Max. coverage (+): 0. Max coverage (-): 0

Region: chr29 47931699-47931725. Max. coverage (+): 0. Max coverage (-): 0

Region: chr29 47931726-47931753. Max. coverage (+): 0. Max coverage (-): 0

Region: chr29 47931754-47931781. Max. coverage (+): 0. Max coverage (-): 0

Region: chr29 47931782-47931809. Max. coverage (+): 0. Max coverage (-): 0

Region: chr29 47931810-47931837. Max. coverage (+): 0. Max coverage (-): 0

Region: chr29 47931838-47931865. Max. coverage (+): 0. Max coverage (-): 0

Region: chr29 47931866-47931893. Max. coverage (+): 0. Max coverage (-): 0

Region: chr29 47931894-47931921. Max. coverage (+): 0. Max coverage (-): 0

Region: chr29 47931922-47931949. Max. coverage (+): 0. Max coverage (-): 0

Region: chr29 47931950-47931977. Max. coverage (+): 0. Max coverage (-): 0

Region: chr29 47931978-47932005. Max. coverage (+): 0. Max coverage (-): 0

Region: chr29 47932006-47932033. Max. coverage (+): 0. Max coverage (-): 0

Region: chr29 47932034-47932061. Max. coverage (+): 0. Max coverage (-): 0

Region: chr29 47932062-47932089. Max. coverage (+): 0. Max coverage (-): 0

Region: chr29 47932090-47932117. Max. coverage (+): 0. Max coverage (-): 0

Region: chr29 47932118-47932145. Max. coverage (+): 0. Max coverage (-): 1.91

Region: chr29 47932146-47932173. Max. coverage (+): 0. Max coverage (-): 0

Region: chr29 47932174-47932201. Max. coverage (+): 0. Max coverage (-): 0

Region: chr29 47932202-47932229. Max. coverage (+): 0. Max coverage (-): 5.32

Region: chr29 47932230-47932257. Max. coverage (+): 0. Max coverage (-): 0

Region: chr29 47932258-47932284. Max. coverage (+): 0. Max coverage (-): 0

Region: chr29 47932285-47932312. Max. coverage (+): 0. Max coverage (-): 0

Region: chr29 47932313-47932340. Max. coverage (+): 0. Max coverage (-): 0

Region: chr29 47932341-47932368. Max. coverage (+): 0. Max coverage (-): 0

Region: chr29 47932369-47932396. Max. coverage (+): 0. Max coverage (-): 1.12

Region: chr29 47932397-47932424. Max. coverage (+): 0. Max coverage (-): 0

Region: chr29 47932425-47932452. Max. coverage (+): 0. Max coverage (-): 0

Region: chr29 47932453-47932480. Max. coverage (+): 0. Max coverage (-): 0.55

Region: chr29 47932481-47932508. Max. coverage (+): 0. Max coverage (-): 0

Region: chr29 47932509-47932536. Max. coverage (+): 0. Max coverage (-): 0

Region: chr29 47932537-47932564. Max. coverage (+): 0. Max coverage (-): 5.51

Region: chr29 47932565-47932592. Max. coverage (+): 0. Max coverage (-): 0

Region: chr29 47932593-47932620. Max. coverage (+): 0. Max coverage (-): 0

Region: chr29 47932621-47932648. Max. coverage (+): 0. Max coverage (-): 0

Region: chr29 47932649-47932676. Max. coverage (+): 0. Max coverage (-): 0

Region: chr29 47932677-47932704. Max. coverage (+): 0. Max coverage (-): 0

Region: chr29 47932705-47932732. Max. coverage (+): 0. Max coverage (-): 0

Region: chr29 47932733-47932760. Max. coverage (+): 0. Max coverage (-): 0

Region: chr29 47932761-47932788. Max. coverage (+): 0. Max coverage (-): 0

Region: chr29 47932789-47932816. Max. coverage (+): 0. Max coverage (-): 0

Region: chr29 47932817-47932843. Max. coverage (+): 0. Max coverage (-): 0

Region: chr29 47932844-47932871. Max. coverage (+): 0. Max coverage (-): 0

Region: chr29 47932872-47932899. Max. coverage (+): 0. Max coverage (-): 0

Region: chr29 47932900-47932927. Max. coverage (+): 0. Max coverage (-): 0

Region: chr29 47932928-47932955. Max. coverage (+): 0. Max coverage (-): 0

Region: chr29 47932956-47932983. Max. coverage (+): 0. Max coverage (-): 0

Region: chr29 47932984-47933011. Max. coverage (+): 0. Max coverage (-): 0

Region: chr29 47933012-47933039. Max. coverage (+): 0. Max coverage (-): 0

Region: chr29 47933040-47933067. Max. coverage (+): 0. Max coverage (-): 0

Region: chr29 47933068-47933095. Max. coverage (+): 0. Max coverage (-): 0

Region: chr29 47933096-47933123. Max. coverage (+): 0. Max coverage (-): 0

Region: chr29 47933124-47933151. Max. coverage (+): 0. Max coverage (-): 0

Region: chr29 47933152-47933179. Max. coverage (+): 0. Max coverage (-): 0

Region: chr29 47933180-47933207. Max. coverage (+): 0. Max coverage (-): 0

Region: chr29 47933208-47933235. Max. coverage (+): 0. Max coverage (-): 0

Region: chr29 47933236-47933263. Max. coverage (+): 0. Max coverage (-): 0

Region: chr29 47933264-47933291. Max. coverage (+): 0. Max coverage (-): 0

Region: chr29 47933292-47933319. Max. coverage (+): 0. Max coverage (-): 0

Region: chr29 47933320-47933347. Max. coverage (+): 0. Max coverage (-): 0

Region: chr29 47933348-47933375. Max. coverage (+): 0. Max coverage (-): 2.46

Region: chr29 47933376-47933402. Max. coverage (+): 0. Max coverage (-): 0

Region: chr29 47933403-47933430. Max. coverage (+): 0. Max coverage (-): 0

Region: chr29 47933431-47933458. Max. coverage (+): 0. Max coverage (-): 0

Region: chr29 47933459-47933486. Max. coverage (+): 0. Max coverage (-): 0

Region: chr29 47933487-47933514. Max. coverage (+): 0. Max coverage (-): 0

Region: chr29 47933515-47933542. Max. coverage (+): 0. Max coverage (-): 0

Region: chr29 47933543-47933570. Max. coverage (+): 0. Max coverage (-): 0

Region: chr29 47933571-47933598. Max. coverage (+): 0. Max coverage (-): 0

Region: chr29 47933599-47933626. Max. coverage (+): 0. Max coverage (-): 7.66

Region: chr29 47933627-47933654. Max. coverage (+): 0. Max coverage (-): 2.29

Region: chr29 47933655-47933682. Max. coverage (+): 0. Max coverage (-): 0

Region: chr29 47933683-47933710. Max. coverage (+): 0. Max coverage (-): 1.53

Region: chr29 47933711-47933738. Max. coverage (+): 0. Max coverage (-): 1.57

Region: chr29 47933739-47933766. Max. coverage (+): 0. Max coverage (-): 0

Region: chr29 47933767-47933794. Max. coverage (+): 0. Max coverage (-): 0

Region: chr29 47933795-47933822. Max. coverage (+): 0. Max coverage (-): 0

Region: chr29 47933823-47933850. Max. coverage (+): 0. Max coverage (-): 0

Region: chr29 47933851-47933878. Max. coverage (+): 0. Max coverage (-): 5.62

Region: chr29 47933879-47933906. Max. coverage (+): 0. Max coverage (-): 4.52

Region: chr29 47933907-47933934. Max. coverage (+): 0. Max coverage (-): 0

Region: chr29 47933935-47933961. Max. coverage (+): 0. Max coverage (-): 0

Region: chr29 47933962-47933989. Max. coverage (+): 0. Max coverage (-): 5.62

Region: chr29 47933990-47934017. Max. coverage (+): 0. Max coverage (-): 2.83

Region: chr29 47934018-47934045. Max. coverage (+): 0. Max coverage (-): 16.15

Region: chr29 47934046-47934073. Max. coverage (+): 0. Max coverage (-): 0

Region: chr29 47934074-47934101. Max. coverage (+): 0. Max coverage (-): 4.48

Region: chr29 47934102-47934129. Max. coverage (+): 0. Max coverage (-): 0

Region: chr29 47934130-47934157. Max. coverage (+): 0. Max coverage (-): 0.63

Region: chr29 47934158-47934185. Max. coverage (+): 0. Max coverage (-): 1.13

Region: chr29 47934186-47934213. Max. coverage (+): 0. Max coverage (-): 0

Region: chr29 47934214-47934241. Max. coverage (+): 0. Max coverage (-): 0

Region: chr29 47934242-47934269. Max. coverage (+): 0. Max coverage (-): 1.09

Region: chr29 47934270-47934297. Max. coverage (+): 0. Max coverage (-): 0.51

Region: chr29 47934298-47934325. Max. coverage (+): 0. Max coverage (-): 0.51

Region: chr29 47934326-47934353. Max. coverage (+): 0. Max coverage (-): 0

Region: chr29 47934354-47934381. Max. coverage (+): 0. Max coverage (-): 1.11

Region: chr29 47934382-47934409. Max. coverage (+): 0. Max coverage (-): 3.61

Region: chr29 47934410-47934437. Max. coverage (+): 0. Max coverage (-): 3.61

Region: chr29 47934438-47934465. Max. coverage (+): 0. Max coverage (-): 0

Region: chr29 47934466-47934493. Max. coverage (+): 0. Max coverage (-): 0

Region: chr29 47934494-47934520. Max. coverage (+): 0. Max coverage (-): 0

Region: chr29 47934521-47934548. Max. coverage (+): 0. Max coverage (-): 0

Region: chr29 47934549-47934576. Max. coverage (+): 0. Max coverage (-): 0

Region: chr29 47934577-47934604. Max. coverage (+): 0. Max coverage (-): 0

Region: chr29 47934605-47934632. Max. coverage (+): 0. Max coverage (-): 0

Region: chr29 47934633-47934660. Max. coverage (+): 0. Max coverage (-): 0

Region: chr29 47934661-47934688. Max. coverage (+): 0. Max coverage (-): 0

Region: chr29 47934689-47934716. Max. coverage (+): 0. Max coverage (-): 0

Region: chr29 47934717-47934744. Max. coverage (+): 0. Max coverage (-): 0

Region: chr29 47934745-47934772. Max. coverage (+): 0. Max coverage (-): 0

Region: chr29 47934773-47934800. Max. coverage (+): 0. Max coverage (-): 0

Region: chr29 47934801-47934828. Max. coverage (+): 0. Max coverage (-): 0

Region: chr29 47934829-47934856. Max. coverage (+): 0. Max coverage (-): 0

Region: chr29 47934857-47934884. Max. coverage (+): 0. Max coverage (-): 0

Region: chr29 47934885-47934912. Max. coverage (+): 0. Max coverage (-): 0

Region: chr29 47934913-47934940. Max. coverage (+): 0. Max coverage (-): 7.44

Region: chr29 47934941-47934968. Max. coverage (+): 0. Max coverage (-): 1.6

Region: chr29 47934969-47934996. Max. coverage (+): 0. Max coverage (-): 2.59

Region: chr29 47934997-47935024. Max. coverage (+): 0. Max coverage (-): 22.03

Region: chr29 47935025-47935052. Max. coverage (+): 0. Max coverage (-): 0

Region: chr29 47935053-47935079. Max. coverage (+): 0. Max coverage (-): 0.84

Region: chr29 47935080-47935107. Max. coverage (+): 0. Max coverage (-): 6.61

Region: chr29 47935108-47935135. Max. coverage (+): 0. Max coverage (-): 16.34

Region: chr29 47935136-47935163. Max. coverage (+): 0. Max coverage (-): 18.17

Region: chr29 47935164-47935191. Max. coverage (+): 0. Max coverage (-): 0

Region: chr29 47935192-47935219. Max. coverage (+): 0. Max coverage (-): 0.49

Region: chr29 47935220-47935247. Max. coverage (+): 0. Max coverage (-): 0

Region: chr29 47935248-47935275. Max. coverage (+): 0. Max coverage (-): 0

Region: chr29 47935276-47935303. Max. coverage (+): 0. Max coverage (-): 0

Region: chr29 47935304-47935331. Max. coverage (+): 0. Max coverage (-): 0

Region: chr29 47935332-47935359. Max. coverage (+): 0. Max coverage (-): 0

Region: chr29 47935360-47935387. Max. coverage (+): 0. Max coverage (-): 4.12

Region: chr29 47935388-47935415. Max. coverage (+): 0. Max coverage (-): 0.57

Region: chr29 47935416-47935443. Max. coverage (+): 0. Max coverage (-): 1.74

Region: chr29 47935444-47935471. Max. coverage (+): 0. Max coverage (-): 0

Region: chr29 47935472-47935499. Max. coverage (+): 0. Max coverage (-): 8.68

Region: chr29 47935500-47935527. Max. coverage (+): 0. Max coverage (-): 10.59

Region: chr29 47935528-47935555. Max. coverage (+): 0. Max coverage (-): 10.59

Region: chr29 47935556-47935583. Max. coverage (+): 0. Max coverage (-): 2.01

Region: chr29 47935584-47935611. Max. coverage (+): 0. Max coverage (-): 8.23

Region: chr29 47935612-47935638. Max. coverage (+): 0. Max coverage (-): 0

Region: chr29 47935639-47935666. Max. coverage (+): 0. Max coverage (-): 0

Region: chr29 47935667-47935694. Max. coverage (+): 0. Max coverage (-): 0

Region: chr29 47935695-47935722. Max. coverage (+): 0. Max coverage (-): 0

Region: chr29 47935723-47935750. Max. coverage (+): 0. Max coverage (-): 0

Region: chr29 47935751-47935778. Max. coverage (+): 0. Max coverage (-): 0

Region: chr29 47935779-47935806. Max. coverage (+): 0. Max coverage (-): 8.65

Region: chr29 47935807-47935834. Max. coverage (+): 0. Max coverage (-): 12.52

Region: chr29 47935835-47935862. Max. coverage (+): 0. Max coverage (-): 0

Region: chr29 47935863-47935890. Max. coverage (+): 0. Max coverage (-): 0

Region: chr29 47935891-47935918. Max. coverage (+): 0. Max coverage (-): 0

Region: chr29 47935919-47935946. Max. coverage (+): 0. Max coverage (-): 0

Region: chr29 47935947-47935974. Max. coverage (+): 0. Max coverage (-): 0

Region: chr29 47935975-47936002. Max. coverage (+): 0. Max coverage (-): 0

Region: chr29 47936003-47936030. Max. coverage (+): 0. Max coverage (-): 0

Region: chr29 47936031-47936058. Max. coverage (+): 0. Max coverage (-): 0

Region: chr29 47936059-47936086. Max. coverage (+): 0. Max coverage (-): 0

Region: chr29 47936087-47936114. Max. coverage (+): 0. Max coverage (-): 0

Region: chr29 47936115-47936142. Max. coverage (+): 0. Max coverage (-): 0

Region: chr29 47936143-47936170. Max. coverage (+): 0. Max coverage (-): 0

Region: chr29 47936171-47936197. Max. coverage (+): 0. Max coverage (-): 0

Region: chr29 47936198-47936225. Max. coverage (+): 0. Max coverage (-): 0

Region: chr29 47936226-47936253. Max. coverage (+): 0. Max coverage (-): 0

Region: chr29 47936254-47936281. Max. coverage (+): 0. Max coverage (-): 16.8

Region: chr29 47936282-47936309. Max. coverage (+): 0. Max coverage (-): 0

Region: chr29 47936310-47936337. Max. coverage (+): 0. Max coverage (-): 0

Region: chr29 47936338-47936365. Max. coverage (+): 0. Max coverage (-): 0

Region: chr29 47936366-47936393. Max. coverage (+): 0. Max coverage (-): 0

Region: chr29 47936394-47936421. Max. coverage (+): 0. Max coverage (-): 0

Region: chr29 47936422-47936449. Max. coverage (+): 0. Max coverage (-): 0

Region: chr29 47936450-47936477. Max. coverage (+): 0. Max coverage (-): 0

Region: chr29 47936478-47936505. Max. coverage (+): 0. Max coverage (-): 0

Region: chr29 47936506-47936533. Max. coverage (+): 0. Max coverage (-): 0

Region: chr29 47936534-47936561. Max. coverage (+): 0. Max coverage (-): 0

Region: chr29 47936562-47936589. Max. coverage (+): 0. Max coverage (-): 0

Region: chr29 47936590-47936617. Max. coverage (+): 0. Max coverage (-): 0

Region: chr29 47936618-47936645. Max. coverage (+): 0. Max coverage (-): 0

Region: chr29 47936646-47936673. Max. coverage (+): 0. Max coverage (-): 0

Region: chr29 47936674-47936701. Max. coverage (+): 0. Max coverage (-): 0

Region: chr29 47936702-47936729. Max. coverage (+): 0. Max coverage (-): 0

Region: chr29 47936730-47936756. Max. coverage (+): 0. Max coverage (-): 0

Region: chr29 47936757-47936784. Max. coverage (+): 0. Max coverage (-): 0

Region: chr29 47936785-47936812. Max. coverage (+): 0. Max coverage (-): 0

Region: chr29 47936813-47936840. Max. coverage (+): 0. Max coverage (-): 0

Region: chr29 47936841-47936868. Max. coverage (+): 0. Max coverage (-): 0

Region: chr29 47936869-47936896. Max. coverage (+): 0. Max coverage (-): 0

Region: chr29 47936897-47936924. Max. coverage (+): 0. Max coverage (-): 0

Region: chr29 47936925-47936952. Max. coverage (+): 0. Max coverage (-): 0

Region: chr29 47936953-47936980. Max. coverage (+): 0. Max coverage (-): 0

Region: chr29 47936981-47937008. Max. coverage (+): 0. Max coverage (-): 6.52

Region: chr29 47937009-47937036. Max. coverage (+): 0. Max coverage (-): 0

Region: chr29 47937037-47937064. Max. coverage (+): 0. Max coverage (-): 0

Region: chr29 47937065-47937092. Max. coverage (+): 0. Max coverage (-): 0

Region: chr29 47937093-47937120. Max. coverage (+): 0. Max coverage (-): 0

Region: chr29 47937121-47937148. Max. coverage (+): 0. Max coverage (-): 0

Region: chr29 47937149-47937176. Max. coverage (+): 0. Max coverage (-): 0

Region: chr29 47937177-47937204. Max. coverage (+): 0. Max coverage (-): 0

Region: chr29 47937205-47937232. Max. coverage (+): 0. Max coverage (-): 0.36

Region: chr29 47937233-47937260. Max. coverage (+): 0. Max coverage (-): 3.83

Region: chr29 47937261-47937288. Max. coverage (+): 0. Max coverage (-): 0

Region: chr29 47937289-47937315. Max. coverage (+): 0. Max coverage (-): 0

Region: chr29 47937316-47937343. Max. coverage (+): 0. Max coverage (-): 0

Region: chr29 47937344-47937371. Max. coverage (+): 0. Max coverage (-): 0

Region: chr29 47937372-47937399. Max. coverage (+): 0. Max coverage (-): 0

Region: chr29 47937400-47937427. Max. coverage (+): 0. Max coverage (-): 8.4

Region: chr29 47937428-47937455. Max. coverage (+): 0. Max coverage (-): 10.48

Region: chr29 47937456-47937483. Max. coverage (+): 0. Max coverage (-): 10.75

Region: chr29 47937484-47937511. Max. coverage (+): 0. Max coverage (-): 0

Region: chr29 47937512-47937539. Max. coverage (+): 0. Max coverage (-): 0

Region: chr29 47937540-47937567. Max. coverage (+): 0. Max coverage (-): 0

Region: chr29 47937568-47937595. Max. coverage (+): 0. Max coverage (-): 0

Region: chr29 47937596-47937623. Max. coverage (+): 0. Max coverage (-): 0

Region: chr29 47937624-47937651. Max. coverage (+): 0. Max coverage (-): 0

Region: chr29 47937652-47937679. Max. coverage (+): 0. Max coverage (-): 0

Region: chr29 47937680-47937707. Max. coverage (+): 0. Max coverage (-): 0

Region: chr29 47937708-47937735. Max. coverage (+): 0. Max coverage (-): 12.73

Region: chr29 47937736-47937763. Max. coverage (+): 0. Max coverage (-): 15.09

Region: chr29 47937764-47937791. Max. coverage (+): 0. Max coverage (-): 0

Region: chr29 47937792-47937819. Max. coverage (+): 0. Max coverage (-): 0

Region: chr29 47937820-47937847. Max. coverage (+): 0. Max coverage (-): 0.6

Region: chr29 47937848-. Max. coverage (+): 0. Max coverage (-): 0

RepeatMasker Color Code

**+**

100-98% Identity

<98-95% Identity

<95-90% Identity

<90-85% Identity

<85-80% Identity

<80-75% Identity

<75-70% Identity

<70% Identity

**-**

Gene Set Color Code

**+**

Gene

Pseudogene

**-**

Topology/Coverage Color Code

Coverage Plus Strand

Coverage Minus Strand

Mainstrand: Plus

Mainstrand: Minus

Complementary Strand

Flanking Region  
(if option -flank >0)

Gene Set Annotation  
  
RepeatMasker Annotation  

**1. Bov-tA3**: 47924243-47924414 (-), Divergence to consensus: 15.2%  
**2. BovB**: 47925267-47926014 (+), Divergence to consensus: 17.9%  
**3. ART2A**: 47926016-47926539 (+), Divergence to consensus: 18.2%  
**4. Bov-tA1**: 47926597-47926813 (-), Divergence to consensus: 23%  
**5. MER21C**: 47927111-47927887 (-), Divergence to consensus: 31.3%  
**6. SINE2-2\_BT**: 47927986-47928097 (-), Divergence to consensus: 17.9%  
**7. Bov-tA2**: 47928734-47928917 (-), Divergence to consensus: 21.7%  
**8. MLT1C**: 47929331-47929726 (-), Divergence to consensus: 39.2%  
**9. MIR3**: 47931117-47931254 (+), Divergence to consensus: 32.8%  
**10. MIRc**: 47931584-47931731 (-), Divergence to consensus: 38.7%  
**11. ART2A**: 47931732-47932114 (+), Divergence to consensus: 20.9%  
**12. MamTip2**: 47932243-47932299 (+), Divergence to consensus: 24.6%  
**13. MER63A**: 47932576-47932672 (+), Divergence to consensus: 35%  
**14. L1MEd**: 47932764-47932879 (-), Divergence to consensus: 31.9%  
**15. MIRb**: 47935949-47936067 (-), Divergence to consensus: 46.2%  
**16. MLT1C**: 47936391-47936836 (+), Divergence to consensus: 48.8%  
**17. MIR**: 47936907-47936977 (+), Divergence to consensus: 33.4%

  
Transcription Factor Binding Sites  

**SOX9** (Sequence: AACAATGA (-): 47928555)  
**SOX9** (Sequence: TTATTGTT (+): 47929208)  
**SOX9** (Sequence: TCATTGTT (+): 47937008)  
**A-MYB** (Sequence: AGACAGTTGG (+): 47931353)  
**SPZ1** (Sequence: AGGGTTTCAG (+): 47927939)  
**Gata4** (Sequence: GTTATCT (+): 47937789)
