# Supplementary material for: Small RNA sequencing of cryopreserved semen from single bull revealed altered miRNAs and piRNAs expression between High- and Low-motile sperm populations
Source: BMC Genomics. 2017 Jan 4;18:14. doi: 10.1186/s12864-016-3394-7 (PMC5209821; doi:10.1186/s12864-016-3394-7)
Supplement: Additional file 3: — Details for each piRNA clusters found in High Motile (HM) sperm fraction. Genes, repeats, transposable elements and transcription factors binding sites falling within the cluster regions were reported. (ZIP 1896 kb) [file 12864_2016_3394_MOESM3_ESM.zip › 76.html]

piRNA cluster 76


Predicted piRNA cluster no. 76     previous   next
  

Show proTRAC run info
Hide proTRAC run info

================================= proTRAC ====================================  
VERSION: 2.1                                    LAST MODIFIED: 06. October 2015  
  
Please cite:  
Rosenkranz D, Zischler H. proTRAC - a software for probabilistic piRNA cluster  
detection, visualization and analysis. 2012. BMC Bioinformatics 13:5.  
  
and (for proTRAC 2.0 and later):  
Rosenkranz D, Rudloff S, Bastuck K, Ketting RF, Zischler H. Tupaia small RNAs  
provide insights into function and evolution of RNAi-based transposon defense  
in mammals. 2015. RNA 21(5):911-922.  
  
Contact:  
David Rosenkranz  
Institute of Anthropology, small RNA group  
Johannes Gutenberg University Mainz  
email: rosenkranz@uni-mainz.de  
  
You can find the latest proTRAC version at:  
http://sourceforge.net/projects/protrac/files  
http://www.smallRNAgroup-mainz.de/software  
==============================================================================  
  
PARAMETERS:  
Map file: .............../storage/core/barbara/genhome/smallRNA/fertility/Sample\_motile/pirna/Sample\_motile\_26-33\_collapsed.fa.no-dust.map.weighted-10000-1000-b-0  
Genome file: ............/storage/core/barbara/genhome/smallRNA/fertility/Sample\_all/pirna/bt\_311\_chrY.fa  
RepeatMasker annotation: /storage/genomes/bt\_umd31/GCF\_000003055.6\_Bos\_taurus\_UMD\_3.1.1\_repeatMasker\_chr.out  
GeneSet:................./storage/core/barbara/genhome/smallRNA/fertility/Sample\_all/pirna/full.gtf  
  
Significant (p<=0.01) hit density will be calculated based  
on observed hit distribution.  
  
Sliding window size: ........................................ 5000 bp  
Sliding window increament: .................................. 1000 bp  
Normalize each hit by number of genomic hits: ............... 1 [0=no/1=yes]  
Normalize each hit by number of sequence reads: ............. 1 [0=no/1=yes]  
Normalize values (-> per million mapped reads): ............. 1 [0=no/1=yes]  
Min. fraction of hits with 1T(U) or 10A: .................... 0.75  
Alternatively: Min. fraction of hits with 1T(U) and 10A: .... 0.5  
Min. fraction of hits with typical piRNA length: ............ 0.75  
Typical piRNA length: ....................................... 26-33 nt  
Min. size of a piRNA cluster: ............................... 5000 bp.  
Min. number of hits (absolute): ............................. 0  
Min. number of hits (normalized): ........................... 0  
Min. fraction of hits on the mainstrand: .................... 0.75  
Top fraction of mapped sequences (in terms of read counts): . 1%  
Top fraction accounts for max. n% of sequence reads: ........ 90%  
Min. fraction of hits on each arm of a bidirectional cluster: 0.1  
Output image file for each cluster: ......................... 0 [0=no/1=yes]  
Output html file for each cluster: .......................... 1 [0=no/1=yes]  
Output a summary table: ..................................... 1 [0=no/1=yes]  
Output a FASTA file for each cluster (piRNA sequences): ..... 1 [0=no/1=yes]  
Output a FASTA file comprising cluster sequences: ........... 1 [0=no/1=yes]  
Search DNA motifs in clusters: .............................. 1 [0=no/1=yes]  
Output flanking sequences: +/- .............................. 0 bp  
Output ~.pTi file: .......................................... 1 [0=no/1=yes]  
==============================================================================  
  
  
Genome size (without gaps): ............ 2678902517 bp  
Gaps (N/X/-): .......................... 53837044 bp  
Mapped reads: .......................... 658825247023  
Non-identical sequences: ............... 514171  
Genomic hits: .......................... 764233  
Significant densitiy of mapped reads: .. 12867599.5173724 reads/kb

Show proTRAC cluster info
Hide proTRAC cluster info

|  |  |
| --- | --- |
| Location | chr3 |
| Coordinates | 49516713-49522035 |
| Size [bp] | 5323 |
| Sequence hit loci | 100 |
| Mapped reads (normalized) | 103667994 |
| Mapped reads (normalized) per kb | 19475482.6 |
| Normalized reads with 1T (1U) | 85.7% |
| Normalized reads with 10A | 27.8% |
| Normalized reads with length 26-33 nt | 100% |
| Normalized reads on the main strand(s) | 100% |
| Predicted directionality | mono:plus |

100%

0%

1T (1U)  
reads

10A reads

26-33 nt  
reads

reads on mainstrand

**Either the amount of reads with 1T (1U) OR 10A has to exceed 75% (set with option: -1Tor10A)  
Alternatively the amount of reads with 1T (1U) AND 10A has to exceed 50% (set with option: -1Tand10A)  
Minimum amount of reads with preferred size is 75% (set with option: -pisize)  
Minimum amount of reads on the main strand(s) is 75% (set with option: -clstrand)**

Show read coverage
Hide read coverage

WHAT DO I SEE HERE?  
This chart shows the location of mapped sequence reads within a predicted piRNA cluster. The color refers to the number of genomic hits produced by the sequence read in question. A dark red bar indicates that this sequence read produces many other hits elsewhere in the genome. Many adjacent red or yellow bars can indicate the presence of a multi-copy element such as transposons or rRNA genes. A dark green bar indicates that this sequence read maps uniquely to this locus.

1 hit

2-5 hits

6-10 hits

11-20 hits

21-50 hits

51-100 hits

> 100 hits

chr3

49516713

49522035

Gene Set

RepeatMasker

Mapped  
Reads

15.41

plus strand

minus strand

15.41

Region: chr3 47937849-49516718. Max. coverage (+): 4.26. Max coverage (-): 0

Region: chr3 49516719-49516728. Max. coverage (+): 4.26. Max coverage (-): 0

Region: chr3 49516729-49516739. Max. coverage (+): 1.87. Max coverage (-): 0

Region: chr3 49516740-49516750. Max. coverage (+): 0. Max coverage (-): 0

Region: chr3 49516751-49516760. Max. coverage (+): 1.2. Max coverage (-): 0

Region: chr3 49516761-49516771. Max. coverage (+): 5.38. Max coverage (-): 0

Region: chr3 49516772-49516782. Max. coverage (+): 5.38. Max coverage (-): 0

Region: chr3 49516783-49516792. Max. coverage (+): 0. Max coverage (-): 0

Region: chr3 49516793-49516803. Max. coverage (+): 0. Max coverage (-): 0

Region: chr3 49516804-49516814. Max. coverage (+): 0. Max coverage (-): 0

Region: chr3 49516815-49516824. Max. coverage (+): 0. Max coverage (-): 0

Region: chr3 49516825-49516835. Max. coverage (+): 1.51. Max coverage (-): 0

Region: chr3 49516836-49516846. Max. coverage (+): 1.51. Max coverage (-): 0

Region: chr3 49516847-49516856. Max. coverage (+): 0. Max coverage (-): 0

Region: chr3 49516857-49516867. Max. coverage (+): 3.44. Max coverage (-): 0

Region: chr3 49516868-49516878. Max. coverage (+): 0. Max coverage (-): 0

Region: chr3 49516879-49516888. Max. coverage (+): 1.08. Max coverage (-): 0

Region: chr3 49516889-49516899. Max. coverage (+): 0. Max coverage (-): 0

Region: chr3 49516900-49516909. Max. coverage (+): 0. Max coverage (-): 0

Region: chr3 49516910-49516920. Max. coverage (+): 0. Max coverage (-): 0

Region: chr3 49516921-49516931. Max. coverage (+): 0. Max coverage (-): 0

Region: chr3 49516932-49516941. Max. coverage (+): 0. Max coverage (-): 0

Region: chr3 49516942-49516952. Max. coverage (+): 0. Max coverage (-): 0

Region: chr3 49516953-49516963. Max. coverage (+): 0. Max coverage (-): 0

Region: chr3 49516964-49516973. Max. coverage (+): 0. Max coverage (-): 0

Region: chr3 49516974-49516984. Max. coverage (+): 0. Max coverage (-): 0

Region: chr3 49516985-49516995. Max. coverage (+): 0. Max coverage (-): 0

Region: chr3 49516996-49517005. Max. coverage (+): 2.91. Max coverage (-): 0

Region: chr3 49517006-49517016. Max. coverage (+): 3.48. Max coverage (-): 0

Region: chr3 49517017-49517027. Max. coverage (+): 2.25. Max coverage (-): 0

Region: chr3 49517028-49517037. Max. coverage (+): 2.97. Max coverage (-): 0

Region: chr3 49517038-49517048. Max. coverage (+): 0.71. Max coverage (-): 0

Region: chr3 49517049-49517058. Max. coverage (+): 0. Max coverage (-): 0

Region: chr3 49517059-49517069. Max. coverage (+): 4.34. Max coverage (-): 0

Region: chr3 49517070-49517080. Max. coverage (+): 0. Max coverage (-): 0

Region: chr3 49517081-49517090. Max. coverage (+): 0. Max coverage (-): 0

Region: chr3 49517091-49517101. Max. coverage (+): 0. Max coverage (-): 0

Region: chr3 49517102-49517112. Max. coverage (+): 0.36. Max coverage (-): 0

Region: chr3 49517113-49517122. Max. coverage (+): 0.36. Max coverage (-): 0

Region: chr3 49517123-49517133. Max. coverage (+): 0. Max coverage (-): 0

Region: chr3 49517134-49517144. Max. coverage (+): 0. Max coverage (-): 0

Region: chr3 49517145-49517154. Max. coverage (+): 0. Max coverage (-): 0

Region: chr3 49517155-49517165. Max. coverage (+): 0. Max coverage (-): 0

Region: chr3 49517166-49517176. Max. coverage (+): 0. Max coverage (-): 0

Region: chr3 49517177-49517186. Max. coverage (+): 0. Max coverage (-): 0

Region: chr3 49517187-49517197. Max. coverage (+): 0. Max coverage (-): 0

Region: chr3 49517198-49517208. Max. coverage (+): 0. Max coverage (-): 0

Region: chr3 49517209-49517218. Max. coverage (+): 0. Max coverage (-): 0

Region: chr3 49517219-49517229. Max. coverage (+): 0. Max coverage (-): 0

Region: chr3 49517230-49517239. Max. coverage (+): 0. Max coverage (-): 0

Region: chr3 49517240-49517250. Max. coverage (+): 0. Max coverage (-): 0

Region: chr3 49517251-49517261. Max. coverage (+): 0. Max coverage (-): 0

Region: chr3 49517262-49517271. Max. coverage (+): 0. Max coverage (-): 0

Region: chr3 49517272-49517282. Max. coverage (+): 1.77. Max coverage (-): 0

Region: chr3 49517283-49517293. Max. coverage (+): 1.77. Max coverage (-): 0

Region: chr3 49517294-49517303. Max. coverage (+): 0. Max coverage (-): 0

Region: chr3 49517304-49517314. Max. coverage (+): 0. Max coverage (-): 0

Region: chr3 49517315-49517325. Max. coverage (+): 0. Max coverage (-): 0

Region: chr3 49517326-49517335. Max. coverage (+): 0. Max coverage (-): 0

Region: chr3 49517336-49517346. Max. coverage (+): 0. Max coverage (-): 0

Region: chr3 49517347-49517357. Max. coverage (+): 0. Max coverage (-): 0

Region: chr3 49517358-49517367. Max. coverage (+): 0. Max coverage (-): 0

Region: chr3 49517368-49517378. Max. coverage (+): 0. Max coverage (-): 0

Region: chr3 49517379-49517389. Max. coverage (+): 0. Max coverage (-): 0

Region: chr3 49517390-49517399. Max. coverage (+): 0. Max coverage (-): 0

Region: chr3 49517400-49517410. Max. coverage (+): 0. Max coverage (-): 0

Region: chr3 49517411-49517420. Max. coverage (+): 0. Max coverage (-): 0

Region: chr3 49517421-49517431. Max. coverage (+): 0. Max coverage (-): 0

Region: chr3 49517432-49517442. Max. coverage (+): 0. Max coverage (-): 0

Region: chr3 49517443-49517452. Max. coverage (+): 0. Max coverage (-): 0

Region: chr3 49517453-49517463. Max. coverage (+): 0. Max coverage (-): 0

Region: chr3 49517464-49517474. Max. coverage (+): 0. Max coverage (-): 0

Region: chr3 49517475-49517484. Max. coverage (+): 0. Max coverage (-): 0

Region: chr3 49517485-49517495. Max. coverage (+): 0. Max coverage (-): 0

Region: chr3 49517496-49517506. Max. coverage (+): 0. Max coverage (-): 0

Region: chr3 49517507-49517516. Max. coverage (+): 0. Max coverage (-): 0

Region: chr3 49517517-49517527. Max. coverage (+): 0. Max coverage (-): 0

Region: chr3 49517528-49517538. Max. coverage (+): 0. Max coverage (-): 0

Region: chr3 49517539-49517548. Max. coverage (+): 0. Max coverage (-): 0

Region: chr3 49517549-49517559. Max. coverage (+): 0. Max coverage (-): 0

Region: chr3 49517560-49517570. Max. coverage (+): 0. Max coverage (-): 0

Region: chr3 49517571-49517580. Max. coverage (+): 0. Max coverage (-): 0

Region: chr3 49517581-49517591. Max. coverage (+): 0. Max coverage (-): 0

Region: chr3 49517592-49517601. Max. coverage (+): 0. Max coverage (-): 0

Region: chr3 49517602-49517612. Max. coverage (+): 0. Max coverage (-): 0

Region: chr3 49517613-49517623. Max. coverage (+): 0. Max coverage (-): 0

Region: chr3 49517624-49517633. Max. coverage (+): 0. Max coverage (-): 0

Region: chr3 49517634-49517644. Max. coverage (+): 0. Max coverage (-): 0

Region: chr3 49517645-49517655. Max. coverage (+): 0. Max coverage (-): 0

Region: chr3 49517656-49517665. Max. coverage (+): 0. Max coverage (-): 0

Region: chr3 49517666-49517676. Max. coverage (+): 0. Max coverage (-): 0

Region: chr3 49517677-49517687. Max. coverage (+): 0. Max coverage (-): 0

Region: chr3 49517688-49517697. Max. coverage (+): 0. Max coverage (-): 0

Region: chr3 49517698-49517708. Max. coverage (+): 0. Max coverage (-): 0

Region: chr3 49517709-49517719. Max. coverage (+): 0. Max coverage (-): 0

Region: chr3 49517720-49517729. Max. coverage (+): 0. Max coverage (-): 0

Region: chr3 49517730-49517740. Max. coverage (+): 0. Max coverage (-): 0

Region: chr3 49517741-49517750. Max. coverage (+): 0. Max coverage (-): 0

Region: chr3 49517751-49517761. Max. coverage (+): 0. Max coverage (-): 0

Region: chr3 49517762-49517772. Max. coverage (+): 0. Max coverage (-): 0

Region: chr3 49517773-49517782. Max. coverage (+): 0. Max coverage (-): 0

Region: chr3 49517783-49517793. Max. coverage (+): 0. Max coverage (-): 0

Region: chr3 49517794-49517804. Max. coverage (+): 0. Max coverage (-): 0

Region: chr3 49517805-49517814. Max. coverage (+): 0. Max coverage (-): 0

Region: chr3 49517815-49517825. Max. coverage (+): 0. Max coverage (-): 0

Region: chr3 49517826-49517836. Max. coverage (+): 0. Max coverage (-): 0

Region: chr3 49517837-49517846. Max. coverage (+): 0. Max coverage (-): 0

Region: chr3 49517847-49517857. Max. coverage (+): 0. Max coverage (-): 0

Region: chr3 49517858-49517868. Max. coverage (+): 0. Max coverage (-): 0

Region: chr3 49517869-49517878. Max. coverage (+): 4.22. Max coverage (-): 0

Region: chr3 49517879-49517889. Max. coverage (+): 3.18. Max coverage (-): 0

Region: chr3 49517890-49517900. Max. coverage (+): 0. Max coverage (-): 0

Region: chr3 49517901-49517910. Max. coverage (+): 0. Max coverage (-): 0

Region: chr3 49517911-49517921. Max. coverage (+): 0. Max coverage (-): 0

Region: chr3 49517922-49517931. Max. coverage (+): 0. Max coverage (-): 0

Region: chr3 49517932-49517942. Max. coverage (+): 0. Max coverage (-): 0

Region: chr3 49517943-49517953. Max. coverage (+): 0. Max coverage (-): 0

Region: chr3 49517954-49517963. Max. coverage (+): 0. Max coverage (-): 0

Region: chr3 49517964-49517974. Max. coverage (+): 0. Max coverage (-): 0

Region: chr3 49517975-49517985. Max. coverage (+): 0. Max coverage (-): 0

Region: chr3 49517986-49517995. Max. coverage (+): 0. Max coverage (-): 0

Region: chr3 49517996-49518006. Max. coverage (+): 0. Max coverage (-): 0

Region: chr3 49518007-49518017. Max. coverage (+): 0. Max coverage (-): 0

Region: chr3 49518018-49518027. Max. coverage (+): 0. Max coverage (-): 0

Region: chr3 49518028-49518038. Max. coverage (+): 0. Max coverage (-): 0

Region: chr3 49518039-49518049. Max. coverage (+): 0. Max coverage (-): 0

Region: chr3 49518050-49518059. Max. coverage (+): 0. Max coverage (-): 0

Region: chr3 49518060-49518070. Max. coverage (+): 0. Max coverage (-): 0

Region: chr3 49518071-49518081. Max. coverage (+): 0. Max coverage (-): 0

Region: chr3 49518082-49518091. Max. coverage (+): 0. Max coverage (-): 0

Region: chr3 49518092-49518102. Max. coverage (+): 0. Max coverage (-): 0

Region: chr3 49518103-49518112. Max. coverage (+): 0. Max coverage (-): 0

Region: chr3 49518113-49518123. Max. coverage (+): 0. Max coverage (-): 0

Region: chr3 49518124-49518134. Max. coverage (+): 0. Max coverage (-): 0

Region: chr3 49518135-49518144. Max. coverage (+): 0. Max coverage (-): 0

Region: chr3 49518145-49518155. Max. coverage (+): 0. Max coverage (-): 0

Region: chr3 49518156-49518166. Max. coverage (+): 0. Max coverage (-): 0

Region: chr3 49518167-49518176. Max. coverage (+): 0. Max coverage (-): 0

Region: chr3 49518177-49518187. Max. coverage (+): 0. Max coverage (-): 0

Region: chr3 49518188-49518198. Max. coverage (+): 0. Max coverage (-): 0

Region: chr3 49518199-49518208. Max. coverage (+): 0. Max coverage (-): 0

Region: chr3 49518209-49518219. Max. coverage (+): 0. Max coverage (-): 0

Region: chr3 49518220-49518230. Max. coverage (+): 0. Max coverage (-): 0

Region: chr3 49518231-49518240. Max. coverage (+): 0. Max coverage (-): 0

Region: chr3 49518241-49518251. Max. coverage (+): 0. Max coverage (-): 0

Region: chr3 49518252-49518261. Max. coverage (+): 0. Max coverage (-): 0

Region: chr3 49518262-49518272. Max. coverage (+): 0. Max coverage (-): 0

Region: chr3 49518273-49518283. Max. coverage (+): 0. Max coverage (-): 0

Region: chr3 49518284-49518293. Max. coverage (+): 0. Max coverage (-): 0

Region: chr3 49518294-49518304. Max. coverage (+): 0. Max coverage (-): 0

Region: chr3 49518305-49518315. Max. coverage (+): 0. Max coverage (-): 0

Region: chr3 49518316-49518325. Max. coverage (+): 0. Max coverage (-): 0

Region: chr3 49518326-49518336. Max. coverage (+): 0. Max coverage (-): 0

Region: chr3 49518337-49518347. Max. coverage (+): 0. Max coverage (-): 0

Region: chr3 49518348-49518357. Max. coverage (+): 0. Max coverage (-): 0

Region: chr3 49518358-49518368. Max. coverage (+): 0. Max coverage (-): 0

Region: chr3 49518369-49518379. Max. coverage (+): 0. Max coverage (-): 0

Region: chr3 49518380-49518389. Max. coverage (+): 0. Max coverage (-): 0

Region: chr3 49518390-49518400. Max. coverage (+): 0. Max coverage (-): 0

Region: chr3 49518401-49518411. Max. coverage (+): 0. Max coverage (-): 0

Region: chr3 49518412-49518421. Max. coverage (+): 0. Max coverage (-): 0

Region: chr3 49518422-49518432. Max. coverage (+): 0. Max coverage (-): 0

Region: chr3 49518433-49518442. Max. coverage (+): 0. Max coverage (-): 0

Region: chr3 49518443-49518453. Max. coverage (+): 0. Max coverage (-): 0

Region: chr3 49518454-49518464. Max. coverage (+): 0. Max coverage (-): 0

Region: chr3 49518465-49518474. Max. coverage (+): 0. Max coverage (-): 0

Region: chr3 49518475-49518485. Max. coverage (+): 0. Max coverage (-): 0

Region: chr3 49518486-49518496. Max. coverage (+): 0. Max coverage (-): 0

Region: chr3 49518497-49518506. Max. coverage (+): 0. Max coverage (-): 0

Region: chr3 49518507-49518517. Max. coverage (+): 0. Max coverage (-): 0

Region: chr3 49518518-49518528. Max. coverage (+): 0. Max coverage (-): 0

Region: chr3 49518529-49518538. Max. coverage (+): 0. Max coverage (-): 0

Region: chr3 49518539-49518549. Max. coverage (+): 0. Max coverage (-): 0

Region: chr3 49518550-49518560. Max. coverage (+): 0. Max coverage (-): 0

Region: chr3 49518561-49518570. Max. coverage (+): 0. Max coverage (-): 0

Region: chr3 49518571-49518581. Max. coverage (+): 0. Max coverage (-): 0

Region: chr3 49518582-49518592. Max. coverage (+): 0. Max coverage (-): 0

Region: chr3 49518593-49518602. Max. coverage (+): 0. Max coverage (-): 0

Region: chr3 49518603-49518613. Max. coverage (+): 0. Max coverage (-): 0

Region: chr3 49518614-49518623. Max. coverage (+): 0.75. Max coverage (-): 0

Region: chr3 49518624-49518634. Max. coverage (+): 0.75. Max coverage (-): 0

Region: chr3 49518635-49518645. Max. coverage (+): 0. Max coverage (-): 0

Region: chr3 49518646-49518655. Max. coverage (+): 0. Max coverage (-): 0

Region: chr3 49518656-49518666. Max. coverage (+): 0. Max coverage (-): 0

Region: chr3 49518667-49518677. Max. coverage (+): 0. Max coverage (-): 0

Region: chr3 49518678-49518687. Max. coverage (+): 0. Max coverage (-): 0

Region: chr3 49518688-49518698. Max. coverage (+): 0. Max coverage (-): 0

Region: chr3 49518699-49518709. Max. coverage (+): 0. Max coverage (-): 0

Region: chr3 49518710-49518719. Max. coverage (+): 0. Max coverage (-): 0

Region: chr3 49518720-49518730. Max. coverage (+): 0. Max coverage (-): 0

Region: chr3 49518731-49518741. Max. coverage (+): 0. Max coverage (-): 0

Region: chr3 49518742-49518751. Max. coverage (+): 0. Max coverage (-): 0

Region: chr3 49518752-49518762. Max. coverage (+): 0. Max coverage (-): 0

Region: chr3 49518763-49518773. Max. coverage (+): 0. Max coverage (-): 0

Region: chr3 49518774-49518783. Max. coverage (+): 0. Max coverage (-): 0

Region: chr3 49518784-49518794. Max. coverage (+): 0. Max coverage (-): 0

Region: chr3 49518795-49518804. Max. coverage (+): 0. Max coverage (-): 0

Region: chr3 49518805-49518815. Max. coverage (+): 0. Max coverage (-): 0

Region: chr3 49518816-49518826. Max. coverage (+): 0. Max coverage (-): 0

Region: chr3 49518827-49518836. Max. coverage (+): 0. Max coverage (-): 0

Region: chr3 49518837-49518847. Max. coverage (+): 0. Max coverage (-): 0

Region: chr3 49518848-49518858. Max. coverage (+): 0. Max coverage (-): 0

Region: chr3 49518859-49518868. Max. coverage (+): 0. Max coverage (-): 0

Region: chr3 49518869-49518879. Max. coverage (+): 0. Max coverage (-): 0

Region: chr3 49518880-49518890. Max. coverage (+): 0. Max coverage (-): 0

Region: chr3 49518891-49518900. Max. coverage (+): 0. Max coverage (-): 0

Region: chr3 49518901-49518911. Max. coverage (+): 0. Max coverage (-): 0

Region: chr3 49518912-49518922. Max. coverage (+): 0. Max coverage (-): 0

Region: chr3 49518923-49518932. Max. coverage (+): 0. Max coverage (-): 0

Region: chr3 49518933-49518943. Max. coverage (+): 0. Max coverage (-): 0

Region: chr3 49518944-49518953. Max. coverage (+): 3.4. Max coverage (-): 0

Region: chr3 49518954-49518964. Max. coverage (+): 1.41. Max coverage (-): 0

Region: chr3 49518965-49518975. Max. coverage (+): 1.19. Max coverage (-): 0

Region: chr3 49518976-49518985. Max. coverage (+): 0. Max coverage (-): 0

Region: chr3 49518986-49518996. Max. coverage (+): 0. Max coverage (-): 0

Region: chr3 49518997-49519007. Max. coverage (+): 1.46. Max coverage (-): 0

Region: chr3 49519008-49519017. Max. coverage (+): 2.59. Max coverage (-): 0

Region: chr3 49519018-49519028. Max. coverage (+): 0. Max coverage (-): 0

Region: chr3 49519029-49519039. Max. coverage (+): 0. Max coverage (-): 0

Region: chr3 49519040-49519049. Max. coverage (+): 0. Max coverage (-): 0

Region: chr3 49519050-49519060. Max. coverage (+): 0. Max coverage (-): 0

Region: chr3 49519061-49519071. Max. coverage (+): 0. Max coverage (-): 0

Region: chr3 49519072-49519081. Max. coverage (+): 0. Max coverage (-): 0

Region: chr3 49519082-49519092. Max. coverage (+): 3.52. Max coverage (-): 0

Region: chr3 49519093-49519103. Max. coverage (+): 3.52. Max coverage (-): 0

Region: chr3 49519104-49519113. Max. coverage (+): 1.4. Max coverage (-): 0

Region: chr3 49519114-49519124. Max. coverage (+): 1.4. Max coverage (-): 0

Region: chr3 49519125-49519134. Max. coverage (+): 0. Max coverage (-): 0

Region: chr3 49519135-49519145. Max. coverage (+): 0. Max coverage (-): 0

Region: chr3 49519146-49519156. Max. coverage (+): 0. Max coverage (-): 0

Region: chr3 49519157-49519166. Max. coverage (+): 0. Max coverage (-): 0

Region: chr3 49519167-49519177. Max. coverage (+): 0.96. Max coverage (-): 0

Region: chr3 49519178-49519188. Max. coverage (+): 0. Max coverage (-): 0

Region: chr3 49519189-49519198. Max. coverage (+): 0. Max coverage (-): 0

Region: chr3 49519199-49519209. Max. coverage (+): 1.09. Max coverage (-): 0

Region: chr3 49519210-49519220. Max. coverage (+): 1.09. Max coverage (-): 0

Region: chr3 49519221-49519230. Max. coverage (+): 0. Max coverage (-): 0

Region: chr3 49519231-49519241. Max. coverage (+): 0. Max coverage (-): 0

Region: chr3 49519242-49519252. Max. coverage (+): 0. Max coverage (-): 0

Region: chr3 49519253-49519262. Max. coverage (+): 0. Max coverage (-): 0

Region: chr3 49519263-49519273. Max. coverage (+): 0. Max coverage (-): 0

Region: chr3 49519274-49519284. Max. coverage (+): 0. Max coverage (-): 0

Region: chr3 49519285-49519294. Max. coverage (+): 0. Max coverage (-): 0

Region: chr3 49519295-49519305. Max. coverage (+): 2.98. Max coverage (-): 0

Region: chr3 49519306-49519315. Max. coverage (+): 2.98. Max coverage (-): 0

Region: chr3 49519316-49519326. Max. coverage (+): 0. Max coverage (-): 0

Region: chr3 49519327-49519337. Max. coverage (+): 0. Max coverage (-): 0

Region: chr3 49519338-49519347. Max. coverage (+): 0. Max coverage (-): 0

Region: chr3 49519348-49519358. Max. coverage (+): 0. Max coverage (-): 0

Region: chr3 49519359-49519369. Max. coverage (+): 0. Max coverage (-): 0

Region: chr3 49519370-49519379. Max. coverage (+): 0. Max coverage (-): 0

Region: chr3 49519380-49519390. Max. coverage (+): 0. Max coverage (-): 0

Region: chr3 49519391-49519401. Max. coverage (+): 0. Max coverage (-): 0

Region: chr3 49519402-49519411. Max. coverage (+): 0. Max coverage (-): 0

Region: chr3 49519412-49519422. Max. coverage (+): 0. Max coverage (-): 0

Region: chr3 49519423-49519433. Max. coverage (+): 0. Max coverage (-): 0

Region: chr3 49519434-49519443. Max. coverage (+): 0. Max coverage (-): 0

Region: chr3 49519444-49519454. Max. coverage (+): 0. Max coverage (-): 0

Region: chr3 49519455-49519464. Max. coverage (+): 0. Max coverage (-): 0

Region: chr3 49519465-49519475. Max. coverage (+): 0. Max coverage (-): 0

Region: chr3 49519476-49519486. Max. coverage (+): 0. Max coverage (-): 0

Region: chr3 49519487-49519496. Max. coverage (+): 0. Max coverage (-): 0

Region: chr3 49519497-49519507. Max. coverage (+): 0. Max coverage (-): 0

Region: chr3 49519508-49519518. Max. coverage (+): 0. Max coverage (-): 0

Region: chr3 49519519-49519528. Max. coverage (+): 0. Max coverage (-): 0

Region: chr3 49519529-49519539. Max. coverage (+): 0. Max coverage (-): 0

Region: chr3 49519540-49519550. Max. coverage (+): 1.98. Max coverage (-): 0

Region: chr3 49519551-49519560. Max. coverage (+): 3.94. Max coverage (-): 0

Region: chr3 49519561-49519571. Max. coverage (+): 3.94. Max coverage (-): 0

Region: chr3 49519572-49519582. Max. coverage (+): 0. Max coverage (-): 0

Region: chr3 49519583-49519592. Max. coverage (+): 0. Max coverage (-): 0

Region: chr3 49519593-49519603. Max. coverage (+): 0. Max coverage (-): 0

Region: chr3 49519604-49519614. Max. coverage (+): 0.95. Max coverage (-): 0

Region: chr3 49519615-49519624. Max. coverage (+): 0.95. Max coverage (-): 0

Region: chr3 49519625-49519635. Max. coverage (+): 0. Max coverage (-): 0

Region: chr3 49519636-49519645. Max. coverage (+): 0. Max coverage (-): 0

Region: chr3 49519646-49519656. Max. coverage (+): 0. Max coverage (-): 0

Region: chr3 49519657-49519667. Max. coverage (+): 0. Max coverage (-): 0

Region: chr3 49519668-49519677. Max. coverage (+): 0. Max coverage (-): 0

Region: chr3 49519678-49519688. Max. coverage (+): 0. Max coverage (-): 0

Region: chr3 49519689-49519699. Max. coverage (+): 0. Max coverage (-): 0

Region: chr3 49519700-49519709. Max. coverage (+): 0. Max coverage (-): 0

Region: chr3 49519710-49519720. Max. coverage (+): 0. Max coverage (-): 0

Region: chr3 49519721-49519731. Max. coverage (+): 1.2. Max coverage (-): 0

Region: chr3 49519732-49519741. Max. coverage (+): 1.2. Max coverage (-): 0

Region: chr3 49519742-49519752. Max. coverage (+): 0. Max coverage (-): 0

Region: chr3 49519753-49519763. Max. coverage (+): 3.76. Max coverage (-): 0

Region: chr3 49519764-49519773. Max. coverage (+): 0. Max coverage (-): 0

Region: chr3 49519774-49519784. Max. coverage (+): 1.49. Max coverage (-): 0

Region: chr3 49519785-49519795. Max. coverage (+): 1.49. Max coverage (-): 0

Region: chr3 49519796-49519805. Max. coverage (+): 0. Max coverage (-): 0

Region: chr3 49519806-49519816. Max. coverage (+): 0. Max coverage (-): 0

Region: chr3 49519817-49519826. Max. coverage (+): 0. Max coverage (-): 0

Region: chr3 49519827-49519837. Max. coverage (+): 2.04. Max coverage (-): 0

Region: chr3 49519838-49519848. Max. coverage (+): 3.93. Max coverage (-): 0

Region: chr3 49519849-49519858. Max. coverage (+): 0. Max coverage (-): 0

Region: chr3 49519859-49519869. Max. coverage (+): 0. Max coverage (-): 0

Region: chr3 49519870-49519880. Max. coverage (+): 0. Max coverage (-): 0

Region: chr3 49519881-49519890. Max. coverage (+): 0. Max coverage (-): 0

Region: chr3 49519891-49519901. Max. coverage (+): 1.83. Max coverage (-): 0

Region: chr3 49519902-49519912. Max. coverage (+): 4.33. Max coverage (-): 0

Region: chr3 49519913-49519922. Max. coverage (+): 0. Max coverage (-): 0

Region: chr3 49519923-49519933. Max. coverage (+): 0. Max coverage (-): 0

Region: chr3 49519934-49519944. Max. coverage (+): 0. Max coverage (-): 0

Region: chr3 49519945-49519954. Max. coverage (+): 0. Max coverage (-): 0

Region: chr3 49519955-49519965. Max. coverage (+): 0. Max coverage (-): 0

Region: chr3 49519966-49519975. Max. coverage (+): 0. Max coverage (-): 0

Region: chr3 49519976-49519986. Max. coverage (+): 0. Max coverage (-): 0

Region: chr3 49519987-49519997. Max. coverage (+): 0. Max coverage (-): 0

Region: chr3 49519998-49520007. Max. coverage (+): 0. Max coverage (-): 0

Region: chr3 49520008-49520018. Max. coverage (+): 0. Max coverage (-): 0

Region: chr3 49520019-49520029. Max. coverage (+): 0. Max coverage (-): 0

Region: chr3 49520030-49520039. Max. coverage (+): 0. Max coverage (-): 0

Region: chr3 49520040-49520050. Max. coverage (+): 0. Max coverage (-): 0

Region: chr3 49520051-49520061. Max. coverage (+): 0. Max coverage (-): 0

Region: chr3 49520062-49520071. Max. coverage (+): 0. Max coverage (-): 0

Region: chr3 49520072-49520082. Max. coverage (+): 0. Max coverage (-): 0

Region: chr3 49520083-49520093. Max. coverage (+): 0. Max coverage (-): 0

Region: chr3 49520094-49520103. Max. coverage (+): 3.15. Max coverage (-): 0

Region: chr3 49520104-49520114. Max. coverage (+): 3.15. Max coverage (-): 0

Region: chr3 49520115-49520125. Max. coverage (+): 0. Max coverage (-): 0

Region: chr3 49520126-49520135. Max. coverage (+): 0.95. Max coverage (-): 0

Region: chr3 49520136-49520146. Max. coverage (+): 0.95. Max coverage (-): 0

Region: chr3 49520147-49520156. Max. coverage (+): 0. Max coverage (-): 0

Region: chr3 49520157-49520167. Max. coverage (+): 0. Max coverage (-): 0

Region: chr3 49520168-49520178. Max. coverage (+): 0. Max coverage (-): 0

Region: chr3 49520179-49520188. Max. coverage (+): 0. Max coverage (-): 0

Region: chr3 49520189-49520199. Max. coverage (+): 0. Max coverage (-): 0

Region: chr3 49520200-49520210. Max. coverage (+): 0. Max coverage (-): 0

Region: chr3 49520211-49520220. Max. coverage (+): 0. Max coverage (-): 0

Region: chr3 49520221-49520231. Max. coverage (+): 0. Max coverage (-): 0

Region: chr3 49520232-49520242. Max. coverage (+): 0. Max coverage (-): 0

Region: chr3 49520243-49520252. Max. coverage (+): 0. Max coverage (-): 0

Region: chr3 49520253-49520263. Max. coverage (+): 0. Max coverage (-): 0

Region: chr3 49520264-49520274. Max. coverage (+): 0. Max coverage (-): 0

Region: chr3 49520275-49520284. Max. coverage (+): 0. Max coverage (-): 0

Region: chr3 49520285-49520295. Max. coverage (+): 0. Max coverage (-): 0

Region: chr3 49520296-49520306. Max. coverage (+): 0. Max coverage (-): 0

Region: chr3 49520307-49520316. Max. coverage (+): 0. Max coverage (-): 0

Region: chr3 49520317-49520327. Max. coverage (+): 0. Max coverage (-): 0

Region: chr3 49520328-49520337. Max. coverage (+): 0. Max coverage (-): 0

Region: chr3 49520338-49520348. Max. coverage (+): 0. Max coverage (-): 0

Region: chr3 49520349-49520359. Max. coverage (+): 0. Max coverage (-): 0

Region: chr3 49520360-49520369. Max. coverage (+): 0. Max coverage (-): 0

Region: chr3 49520370-49520380. Max. coverage (+): 0. Max coverage (-): 0

Region: chr3 49520381-49520391. Max. coverage (+): 0. Max coverage (-): 0

Region: chr3 49520392-49520401. Max. coverage (+): 0. Max coverage (-): 0

Region: chr3 49520402-49520412. Max. coverage (+): 0. Max coverage (-): 0

Region: chr3 49520413-49520423. Max. coverage (+): 0. Max coverage (-): 0

Region: chr3 49520424-49520433. Max. coverage (+): 0. Max coverage (-): 0

Region: chr3 49520434-49520444. Max. coverage (+): 0. Max coverage (-): 0

Region: chr3 49520445-49520455. Max. coverage (+): 0. Max coverage (-): 0

Region: chr3 49520456-49520465. Max. coverage (+): 0. Max coverage (-): 0

Region: chr3 49520466-49520476. Max. coverage (+): 0. Max coverage (-): 0

Region: chr3 49520477-49520487. Max. coverage (+): 0. Max coverage (-): 0

Region: chr3 49520488-49520497. Max. coverage (+): 0. Max coverage (-): 0

Region: chr3 49520498-49520508. Max. coverage (+): 15.41. Max coverage (-): 0

Region: chr3 49520509-49520518. Max. coverage (+): 3.5. Max coverage (-): 0

Region: chr3 49520519-49520529. Max. coverage (+): 0.92. Max coverage (-): 0

Region: chr3 49520530-49520540. Max. coverage (+): 0.92. Max coverage (-): 0

Region: chr3 49520541-49520550. Max. coverage (+): 0.24. Max coverage (-): 0

Region: chr3 49520551-49520561. Max. coverage (+): 0. Max coverage (-): 0

Region: chr3 49520562-49520572. Max. coverage (+): 0. Max coverage (-): 0

Region: chr3 49520573-49520582. Max. coverage (+): 0. Max coverage (-): 0

Region: chr3 49520583-49520593. Max. coverage (+): 0. Max coverage (-): 0

Region: chr3 49520594-49520604. Max. coverage (+): 2.32. Max coverage (-): 0

Region: chr3 49520605-49520614. Max. coverage (+): 0. Max coverage (-): 0

Region: chr3 49520615-49520625. Max. coverage (+): 3.43. Max coverage (-): 0

Region: chr3 49520626-49520636. Max. coverage (+): 0. Max coverage (-): 0

Region: chr3 49520637-49520646. Max. coverage (+): 0. Max coverage (-): 0

Region: chr3 49520647-49520657. Max. coverage (+): 1.85. Max coverage (-): 0

Region: chr3 49520658-49520667. Max. coverage (+): 1.85. Max coverage (-): 0

Region: chr3 49520668-49520678. Max. coverage (+): 0. Max coverage (-): 0

Region: chr3 49520679-49520689. Max. coverage (+): 4.48. Max coverage (-): 0

Region: chr3 49520690-49520699. Max. coverage (+): 4.48. Max coverage (-): 0

Region: chr3 49520700-49520710. Max. coverage (+): 0. Max coverage (-): 0

Region: chr3 49520711-49520721. Max. coverage (+): 0. Max coverage (-): 0

Region: chr3 49520722-49520731. Max. coverage (+): 0. Max coverage (-): 0

Region: chr3 49520732-49520742. Max. coverage (+): 8.22. Max coverage (-): 0

Region: chr3 49520743-49520753. Max. coverage (+): 8.22. Max coverage (-): 0

Region: chr3 49520754-49520763. Max. coverage (+): 0. Max coverage (-): 0

Region: chr3 49520764-49520774. Max. coverage (+): 0.92. Max coverage (-): 0

Region: chr3 49520775-49520785. Max. coverage (+): 2.63. Max coverage (-): 0

Region: chr3 49520786-49520795. Max. coverage (+): 0. Max coverage (-): 0

Region: chr3 49520796-49520806. Max. coverage (+): 0. Max coverage (-): 0

Region: chr3 49520807-49520817. Max. coverage (+): 0. Max coverage (-): 0

Region: chr3 49520818-49520827. Max. coverage (+): 0. Max coverage (-): 0

Region: chr3 49520828-49520838. Max. coverage (+): 2. Max coverage (-): 0

Region: chr3 49520839-49520848. Max. coverage (+): 2. Max coverage (-): 0

Region: chr3 49520849-49520859. Max. coverage (+): 0. Max coverage (-): 0

Region: chr3 49520860-49520870. Max. coverage (+): 0. Max coverage (-): 0

Region: chr3 49520871-49520880. Max. coverage (+): 0. Max coverage (-): 0

Region: chr3 49520881-49520891. Max. coverage (+): 0. Max coverage (-): 0

Region: chr3 49520892-49520902. Max. coverage (+): 0. Max coverage (-): 0

Region: chr3 49520903-49520912. Max. coverage (+): 0. Max coverage (-): 0

Region: chr3 49520913-49520923. Max. coverage (+): 0. Max coverage (-): 0

Region: chr3 49520924-49520934. Max. coverage (+): 0. Max coverage (-): 0

Region: chr3 49520935-49520944. Max. coverage (+): 0. Max coverage (-): 0

Region: chr3 49520945-49520955. Max. coverage (+): 0. Max coverage (-): 0

Region: chr3 49520956-49520966. Max. coverage (+): 4.34. Max coverage (-): 0

Region: chr3 49520967-49520976. Max. coverage (+): 0. Max coverage (-): 0

Region: chr3 49520977-49520987. Max. coverage (+): 0. Max coverage (-): 0

Region: chr3 49520988-49520998. Max. coverage (+): 0. Max coverage (-): 0

Region: chr3 49520999-49521008. Max. coverage (+): 0. Max coverage (-): 0

Region: chr3 49521009-49521019. Max. coverage (+): 0. Max coverage (-): 0

Region: chr3 49521020-49521029. Max. coverage (+): 0. Max coverage (-): 0

Region: chr3 49521030-49521040. Max. coverage (+): 0. Max coverage (-): 0

Region: chr3 49521041-49521051. Max. coverage (+): 0. Max coverage (-): 0

Region: chr3 49521052-49521061. Max. coverage (+): 0. Max coverage (-): 0

Region: chr3 49521062-49521072. Max. coverage (+): 0. Max coverage (-): 0

Region: chr3 49521073-49521083. Max. coverage (+): 0. Max coverage (-): 0

Region: chr3 49521084-49521093. Max. coverage (+): 0. Max coverage (-): 0

Region: chr3 49521094-49521104. Max. coverage (+): 0. Max coverage (-): 0

Region: chr3 49521105-49521115. Max. coverage (+): 2.74. Max coverage (-): 0

Region: chr3 49521116-49521125. Max. coverage (+): 2.74. Max coverage (-): 0

Region: chr3 49521126-49521136. Max. coverage (+): 0. Max coverage (-): 0

Region: chr3 49521137-49521147. Max. coverage (+): 0. Max coverage (-): 0

Region: chr3 49521148-49521157. Max. coverage (+): 0. Max coverage (-): 0

Region: chr3 49521158-49521168. Max. coverage (+): 2.58. Max coverage (-): 0

Region: chr3 49521169-49521178. Max. coverage (+): 2.58. Max coverage (-): 0

Region: chr3 49521179-49521189. Max. coverage (+): 0. Max coverage (-): 0

Region: chr3 49521190-49521200. Max. coverage (+): 0. Max coverage (-): 0

Region: chr3 49521201-49521210. Max. coverage (+): 0. Max coverage (-): 0

Region: chr3 49521211-49521221. Max. coverage (+): 0. Max coverage (-): 0

Region: chr3 49521222-49521232. Max. coverage (+): 0. Max coverage (-): 0

Region: chr3 49521233-49521242. Max. coverage (+): 0. Max coverage (-): 0

Region: chr3 49521243-49521253. Max. coverage (+): 0. Max coverage (-): 0

Region: chr3 49521254-49521264. Max. coverage (+): 0. Max coverage (-): 0

Region: chr3 49521265-49521274. Max. coverage (+): 0. Max coverage (-): 0

Region: chr3 49521275-49521285. Max. coverage (+): 0. Max coverage (-): 0

Region: chr3 49521286-49521296. Max. coverage (+): 0. Max coverage (-): 0

Region: chr3 49521297-49521306. Max. coverage (+): 0. Max coverage (-): 0

Region: chr3 49521307-49521317. Max. coverage (+): 0. Max coverage (-): 0

Region: chr3 49521318-49521328. Max. coverage (+): 0. Max coverage (-): 0

Region: chr3 49521329-49521338. Max. coverage (+): 0. Max coverage (-): 0

Region: chr3 49521339-49521349. Max. coverage (+): 0. Max coverage (-): 0

Region: chr3 49521350-49521359. Max. coverage (+): 0. Max coverage (-): 0

Region: chr3 49521360-49521370. Max. coverage (+): 0. Max coverage (-): 0

Region: chr3 49521371-49521381. Max. coverage (+): 0. Max coverage (-): 0

Region: chr3 49521382-49521391. Max. coverage (+): 0. Max coverage (-): 0

Region: chr3 49521392-49521402. Max. coverage (+): 0. Max coverage (-): 0

Region: chr3 49521403-49521413. Max. coverage (+): 0. Max coverage (-): 0

Region: chr3 49521414-49521423. Max. coverage (+): 0. Max coverage (-): 0

Region: chr3 49521424-49521434. Max. coverage (+): 4.74. Max coverage (-): 0

Region: chr3 49521435-49521445. Max. coverage (+): 0. Max coverage (-): 0

Region: chr3 49521446-49521455. Max. coverage (+): 0. Max coverage (-): 0

Region: chr3 49521456-49521466. Max. coverage (+): 0. Max coverage (-): 0

Region: chr3 49521467-49521477. Max. coverage (+): 0. Max coverage (-): 0

Region: chr3 49521478-49521487. Max. coverage (+): 0. Max coverage (-): 0

Region: chr3 49521488-49521498. Max. coverage (+): 0. Max coverage (-): 0

Region: chr3 49521499-49521509. Max. coverage (+): 7.28. Max coverage (-): 0

Region: chr3 49521510-49521519. Max. coverage (+): 7.28. Max coverage (-): 0

Region: chr3 49521520-49521530. Max. coverage (+): 0. Max coverage (-): 0

Region: chr3 49521531-49521540. Max. coverage (+): 0. Max coverage (-): 0

Region: chr3 49521541-49521551. Max. coverage (+): 0. Max coverage (-): 0

Region: chr3 49521552-49521562. Max. coverage (+): 0. Max coverage (-): 0

Region: chr3 49521563-49521572. Max. coverage (+): 0. Max coverage (-): 0

Region: chr3 49521573-49521583. Max. coverage (+): 0. Max coverage (-): 0

Region: chr3 49521584-49521594. Max. coverage (+): 0. Max coverage (-): 0

Region: chr3 49521595-49521604. Max. coverage (+): 0. Max coverage (-): 0

Region: chr3 49521605-49521615. Max. coverage (+): 0. Max coverage (-): 0

Region: chr3 49521616-49521626. Max. coverage (+): 0. Max coverage (-): 0

Region: chr3 49521627-49521636. Max. coverage (+): 0. Max coverage (-): 0

Region: chr3 49521637-49521647. Max. coverage (+): 0. Max coverage (-): 0

Region: chr3 49521648-49521658. Max. coverage (+): 0. Max coverage (-): 0

Region: chr3 49521659-49521668. Max. coverage (+): 0. Max coverage (-): 0

Region: chr3 49521669-49521679. Max. coverage (+): 0. Max coverage (-): 0

Region: chr3 49521680-49521690. Max. coverage (+): 1.99. Max coverage (-): 0

Region: chr3 49521691-49521700. Max. coverage (+): 1.99. Max coverage (-): 0

Region: chr3 49521701-49521711. Max. coverage (+): 0. Max coverage (-): 0

Region: chr3 49521712-49521721. Max. coverage (+): 0. Max coverage (-): 0

Region: chr3 49521722-49521732. Max. coverage (+): 0. Max coverage (-): 0

Region: chr3 49521733-49521743. Max. coverage (+): 0. Max coverage (-): 0

Region: chr3 49521744-49521753. Max. coverage (+): 0. Max coverage (-): 0

Region: chr3 49521754-49521764. Max. coverage (+): 0. Max coverage (-): 0

Region: chr3 49521765-49521775. Max. coverage (+): 0. Max coverage (-): 0

Region: chr3 49521776-49521785. Max. coverage (+): 0. Max coverage (-): 0

Region: chr3 49521786-49521796. Max. coverage (+): 0. Max coverage (-): 0

Region: chr3 49521797-49521807. Max. coverage (+): 0.76. Max coverage (-): 0

Region: chr3 49521808-49521817. Max. coverage (+): 0.76. Max coverage (-): 0

Region: chr3 49521818-49521828. Max. coverage (+): 0. Max coverage (-): 0

Region: chr3 49521829-49521839. Max. coverage (+): 0. Max coverage (-): 0

Region: chr3 49521840-49521849. Max. coverage (+): 0. Max coverage (-): 0

Region: chr3 49521850-49521860. Max. coverage (+): 0. Max coverage (-): 0

Region: chr3 49521861-49521870. Max. coverage (+): 0. Max coverage (-): 0

Region: chr3 49521871-49521881. Max. coverage (+): 0. Max coverage (-): 0

Region: chr3 49521882-49521892. Max. coverage (+): 0. Max coverage (-): 0

Region: chr3 49521893-49521902. Max. coverage (+): 0. Max coverage (-): 0

Region: chr3 49521903-49521913. Max. coverage (+): 0. Max coverage (-): 0

Region: chr3 49521914-49521924. Max. coverage (+): 0. Max coverage (-): 0

Region: chr3 49521925-49521934. Max. coverage (+): 0. Max coverage (-): 0

Region: chr3 49521935-49521945. Max. coverage (+): 0. Max coverage (-): 0

Region: chr3 49521946-49521956. Max. coverage (+): 0. Max coverage (-): 0

Region: chr3 49521957-49521966. Max. coverage (+): 4.84. Max coverage (-): 0

Region: chr3 49521967-49521977. Max. coverage (+): 4.84. Max coverage (-): 0

Region: chr3 49521978-49521988. Max. coverage (+): 0. Max coverage (-): 0

Region: chr3 49521989-49521998. Max. coverage (+): 0. Max coverage (-): 0

Region: chr3 49521999-49522009. Max. coverage (+): 0.72. Max coverage (-): 0

Region: chr3 49522010-49522020. Max. coverage (+): 0.72. Max coverage (-): 0

Region: chr3 49522021-49522030. Max. coverage (+): 0. Max coverage (-): 0

Region: chr3 49522031-. Max. coverage (+): 0. Max coverage (-): 0

RepeatMasker Color Code

**+**

100-98% Identity

<98-95% Identity

<95-90% Identity

<90-85% Identity

<85-80% Identity

<80-75% Identity

<75-70% Identity

<70% Identity

**-**

Gene Set Color Code

**+**

Gene

Pseudogene

**-**

Topology/Coverage Color Code

Coverage Plus Strand

Coverage Minus Strand

Mainstrand: Plus

Mainstrand: Minus

Complementary Strand

Flanking Region  
(if option -flank >0)

Gene Set Annotation  
  
RepeatMasker Annotation  

**1. ERV1-3-I\_BT-int**: 49517289-49517641 (-), Divergence to consensus: 39.8%  
**2. Bov-tA1**: 49517642-49517857 (+), Divergence to consensus: 14.3%  
**3. Bov-tA2**: 49517959-49518158 (+), Divergence to consensus: 13.1%  
**4. ERV1-2-I\_BT-int**: 49518240-49518546 (-), Divergence to consensus: 38.8%  
**5. Bov-tA3**: 49518762-49518925 (+), Divergence to consensus: 18.3%  
**6. ERV1-3-I\_BT-int**: 49518932-49519199 (-), Divergence to consensus: 45.7%  
**7. ERV1-3-I\_BT-int**: 49519208-49519322 (-), Divergence to consensus: 36.6%  
**8. Bov-tA2**: 49520296-49520484 (-), Divergence to consensus: 20.7%  
**9. MER66-int**: 49521429-49521538 (-), Divergence to consensus: 35.1%

  
Transcription Factor Binding Sites  

**Gata4** (Sequence: AGATAAG (-): 49521164)  
**SOX9** (Sequence: CTATTGTT (+): 49519833)
