# Supplementary material for: Small RNA sequencing of cryopreserved semen from single bull revealed altered miRNAs and piRNAs expression between High- and Low-motile sperm populations
Source: BMC Genomics. 2017 Jan 4;18:14. doi: 10.1186/s12864-016-3394-7 (PMC5209821; doi:10.1186/s12864-016-3394-7)
Supplement: Additional file 3: — Details for each piRNA clusters found in High Motile (HM) sperm fraction. Genes, repeats, transposable elements and transcription factors binding sites falling within the cluster regions were reported. (ZIP 1896 kb) [file 12864_2016_3394_MOESM3_ESM.zip › 77.html]

piRNA cluster 77


Predicted piRNA cluster no. 77     previous   next
  

Show proTRAC run info
Hide proTRAC run info

================================= proTRAC ====================================  
VERSION: 2.1                                    LAST MODIFIED: 06. October 2015  
  
Please cite:  
Rosenkranz D, Zischler H. proTRAC - a software for probabilistic piRNA cluster  
detection, visualization and analysis. 2012. BMC Bioinformatics 13:5.  
  
and (for proTRAC 2.0 and later):  
Rosenkranz D, Rudloff S, Bastuck K, Ketting RF, Zischler H. Tupaia small RNAs  
provide insights into function and evolution of RNAi-based transposon defense  
in mammals. 2015. RNA 21(5):911-922.  
  
Contact:  
David Rosenkranz  
Institute of Anthropology, small RNA group  
Johannes Gutenberg University Mainz  
email: rosenkranz@uni-mainz.de  
  
You can find the latest proTRAC version at:  
http://sourceforge.net/projects/protrac/files  
http://www.smallRNAgroup-mainz.de/software  
==============================================================================  
  
PARAMETERS:  
Map file: .............../storage/core/barbara/genhome/smallRNA/fertility/Sample\_motile/pirna/Sample\_motile\_26-33\_collapsed.fa.no-dust.map.weighted-10000-1000-b-0  
Genome file: ............/storage/core/barbara/genhome/smallRNA/fertility/Sample\_all/pirna/bt\_311\_chrY.fa  
RepeatMasker annotation: /storage/genomes/bt\_umd31/GCF\_000003055.6\_Bos\_taurus\_UMD\_3.1.1\_repeatMasker\_chr.out  
GeneSet:................./storage/core/barbara/genhome/smallRNA/fertility/Sample\_all/pirna/full.gtf  
  
Significant (p<=0.01) hit density will be calculated based  
on observed hit distribution.  
  
Sliding window size: ........................................ 5000 bp  
Sliding window increament: .................................. 1000 bp  
Normalize each hit by number of genomic hits: ............... 1 [0=no/1=yes]  
Normalize each hit by number of sequence reads: ............. 1 [0=no/1=yes]  
Normalize values (-> per million mapped reads): ............. 1 [0=no/1=yes]  
Min. fraction of hits with 1T(U) or 10A: .................... 0.75  
Alternatively: Min. fraction of hits with 1T(U) and 10A: .... 0.5  
Min. fraction of hits with typical piRNA length: ............ 0.75  
Typical piRNA length: ....................................... 26-33 nt  
Min. size of a piRNA cluster: ............................... 5000 bp.  
Min. number of hits (absolute): ............................. 0  
Min. number of hits (normalized): ........................... 0  
Min. fraction of hits on the mainstrand: .................... 0.75  
Top fraction of mapped sequences (in terms of read counts): . 1%  
Top fraction accounts for max. n% of sequence reads: ........ 90%  
Min. fraction of hits on each arm of a bidirectional cluster: 0.1  
Output image file for each cluster: ......................... 0 [0=no/1=yes]  
Output html file for each cluster: .......................... 1 [0=no/1=yes]  
Output a summary table: ..................................... 1 [0=no/1=yes]  
Output a FASTA file for each cluster (piRNA sequences): ..... 1 [0=no/1=yes]  
Output a FASTA file comprising cluster sequences: ........... 1 [0=no/1=yes]  
Search DNA motifs in clusters: .............................. 1 [0=no/1=yes]  
Output flanking sequences: +/- .............................. 0 bp  
Output ~.pTi file: .......................................... 1 [0=no/1=yes]  
==============================================================================  
  
  
Genome size (without gaps): ............ 2678902517 bp  
Gaps (N/X/-): .......................... 53837044 bp  
Mapped reads: .......................... 658825247023  
Non-identical sequences: ............... 514171  
Genomic hits: .......................... 764233  
Significant densitiy of mapped reads: .. 12867599.5173724 reads/kb

Show proTRAC cluster info
Hide proTRAC cluster info

|  |  |
| --- | --- |
| Location | chr3 |
| Coordinates | 50438059-50444617 |
| Size [bp] | 6559 |
| Sequence hit loci | 111 |
| Mapped reads (normalized) | 143349034.7 |
| Mapped reads (normalized) per kb | 21855318.6 |
| Normalized reads with 1T (1U) | 83.3% |
| Normalized reads with 10A | 26.4% |
| Normalized reads with length 26-33 nt | 100% |
| Normalized reads on the main strand(s) | 94.6% |
| Predicted directionality | mono:minus |

100%

0%

1T (1U)  
reads

10A reads

26-33 nt  
reads

reads on mainstrand

**Either the amount of reads with 1T (1U) OR 10A has to exceed 75% (set with option: -1Tor10A)  
Alternatively the amount of reads with 1T (1U) AND 10A has to exceed 50% (set with option: -1Tand10A)  
Minimum amount of reads with preferred size is 75% (set with option: -pisize)  
Minimum amount of reads on the main strand(s) is 75% (set with option: -clstrand)**

Show read coverage
Hide read coverage

WHAT DO I SEE HERE?  
This chart shows the location of mapped sequence reads within a predicted piRNA cluster. The color refers to the number of genomic hits produced by the sequence read in question. A dark red bar indicates that this sequence read produces many other hits elsewhere in the genome. Many adjacent red or yellow bars can indicate the presence of a multi-copy element such as transposons or rRNA genes. A dark green bar indicates that this sequence read maps uniquely to this locus.

1 hit

2-5 hits

6-10 hits

11-20 hits

21-50 hits

51-100 hits

> 100 hits

chr3

50438059

50444617

Gene Set

RepeatMasker

Mapped  
Reads

35.16

plus strand

minus strand

35.16

Region: chr3 49522032-50438065. Max. coverage (+): 0.02. Max coverage (-): 0

Region: chr3 50438066-50438078. Max. coverage (+): 0.02. Max coverage (-): 0

Region: chr3 50438079-50438091. Max. coverage (+): 0. Max coverage (-): 0

Region: chr3 50438092-50438104. Max. coverage (+): 0. Max coverage (-): 0

Region: chr3 50438105-50438118. Max. coverage (+): 0. Max coverage (-): 0

Region: chr3 50438119-50438131. Max. coverage (+): 0. Max coverage (-): 0

Region: chr3 50438132-50438144. Max. coverage (+): 0. Max coverage (-): 0

Region: chr3 50438145-50438157. Max. coverage (+): 0. Max coverage (-): 0

Region: chr3 50438158-50438170. Max. coverage (+): 0. Max coverage (-): 0

Region: chr3 50438171-50438183. Max. coverage (+): 0.1. Max coverage (-): 0

Region: chr3 50438184-50438196. Max. coverage (+): 0.1. Max coverage (-): 0

Region: chr3 50438197-50438209. Max. coverage (+): 0. Max coverage (-): 0

Region: chr3 50438210-50438222. Max. coverage (+): 0. Max coverage (-): 0

Region: chr3 50438223-50438236. Max. coverage (+): 0. Max coverage (-): 0

Region: chr3 50438237-50438249. Max. coverage (+): 0. Max coverage (-): 0

Region: chr3 50438250-50438262. Max. coverage (+): 0. Max coverage (-): 0

Region: chr3 50438263-50438275. Max. coverage (+): 0. Max coverage (-): 0

Region: chr3 50438276-50438288. Max. coverage (+): 0. Max coverage (-): 0

Region: chr3 50438289-50438301. Max. coverage (+): 0. Max coverage (-): 0

Region: chr3 50438302-50438314. Max. coverage (+): 0. Max coverage (-): 0

Region: chr3 50438315-50438327. Max. coverage (+): 0. Max coverage (-): 0

Region: chr3 50438328-50438341. Max. coverage (+): 0. Max coverage (-): 0

Region: chr3 50438342-50438354. Max. coverage (+): 0. Max coverage (-): 0

Region: chr3 50438355-50438367. Max. coverage (+): 0. Max coverage (-): 0

Region: chr3 50438368-50438380. Max. coverage (+): 0. Max coverage (-): 0

Region: chr3 50438381-50438393. Max. coverage (+): 0. Max coverage (-): 0

Region: chr3 50438394-50438406. Max. coverage (+): 0. Max coverage (-): 0

Region: chr3 50438407-50438419. Max. coverage (+): 0. Max coverage (-): 4.81

Region: chr3 50438420-50438432. Max. coverage (+): 0. Max coverage (-): 0

Region: chr3 50438433-50438445. Max. coverage (+): 0. Max coverage (-): 0

Region: chr3 50438446-50438459. Max. coverage (+): 0. Max coverage (-): 0

Region: chr3 50438460-50438472. Max. coverage (+): 0. Max coverage (-): 0

Region: chr3 50438473-50438485. Max. coverage (+): 0.01. Max coverage (-): 0

Region: chr3 50438486-50438498. Max. coverage (+): 0. Max coverage (-): 0

Region: chr3 50438499-50438511. Max. coverage (+): 0. Max coverage (-): 0

Region: chr3 50438512-50438524. Max. coverage (+): 0. Max coverage (-): 0

Region: chr3 50438525-50438537. Max. coverage (+): 0.01. Max coverage (-): 0.01

Region: chr3 50438538-50438550. Max. coverage (+): 0.02. Max coverage (-): 0.01

Region: chr3 50438551-50438564. Max. coverage (+): 0. Max coverage (-): 0

Region: chr3 50438565-50438577. Max. coverage (+): 0. Max coverage (-): 0

Region: chr3 50438578-50438590. Max. coverage (+): 0. Max coverage (-): 0

Region: chr3 50438591-50438603. Max. coverage (+): 0. Max coverage (-): 0

Region: chr3 50438604-50438616. Max. coverage (+): 0. Max coverage (-): 0

Region: chr3 50438617-50438629. Max. coverage (+): 0. Max coverage (-): 0

Region: chr3 50438630-50438642. Max. coverage (+): 0. Max coverage (-): 0

Region: chr3 50438643-50438655. Max. coverage (+): 0. Max coverage (-): 1.12

Region: chr3 50438656-50438668. Max. coverage (+): 0. Max coverage (-): 0

Region: chr3 50438669-50438682. Max. coverage (+): 0. Max coverage (-): 0

Region: chr3 50438683-50438695. Max. coverage (+): 0. Max coverage (-): 0

Region: chr3 50438696-50438708. Max. coverage (+): 0. Max coverage (-): 0

Region: chr3 50438709-50438721. Max. coverage (+): 0. Max coverage (-): 0

Region: chr3 50438722-50438734. Max. coverage (+): 0. Max coverage (-): 0

Region: chr3 50438735-50438747. Max. coverage (+): 0. Max coverage (-): 0

Region: chr3 50438748-50438760. Max. coverage (+): 0. Max coverage (-): 4.3

Region: chr3 50438761-50438773. Max. coverage (+): 0. Max coverage (-): 0

Region: chr3 50438774-50438787. Max. coverage (+): 0. Max coverage (-): 0

Region: chr3 50438788-50438800. Max. coverage (+): 0. Max coverage (-): 0

Region: chr3 50438801-50438813. Max. coverage (+): 0. Max coverage (-): 0

Region: chr3 50438814-50438826. Max. coverage (+): 0. Max coverage (-): 0

Region: chr3 50438827-50438839. Max. coverage (+): 0. Max coverage (-): 0

Region: chr3 50438840-50438852. Max. coverage (+): 0. Max coverage (-): 0

Region: chr3 50438853-50438865. Max. coverage (+): 0. Max coverage (-): 0

Region: chr3 50438866-50438878. Max. coverage (+): 0. Max coverage (-): 1.41

Region: chr3 50438879-50438891. Max. coverage (+): 0. Max coverage (-): 6.25

Region: chr3 50438892-50438905. Max. coverage (+): 0. Max coverage (-): 6.04

Region: chr3 50438906-50438918. Max. coverage (+): 0. Max coverage (-): 6.04

Region: chr3 50438919-50438931. Max. coverage (+): 0. Max coverage (-): 0

Region: chr3 50438932-50438944. Max. coverage (+): 0. Max coverage (-): 0

Region: chr3 50438945-50438957. Max. coverage (+): 0. Max coverage (-): 0

Region: chr3 50438958-50438970. Max. coverage (+): 0. Max coverage (-): 0.36

Region: chr3 50438971-50438983. Max. coverage (+): 0. Max coverage (-): 0

Region: chr3 50438984-50438996. Max. coverage (+): 0. Max coverage (-): 0

Region: chr3 50438997-50439010. Max. coverage (+): 0. Max coverage (-): 3.72

Region: chr3 50439011-50439023. Max. coverage (+): 0. Max coverage (-): 3.72

Region: chr3 50439024-50439036. Max. coverage (+): 0. Max coverage (-): 0

Region: chr3 50439037-50439049. Max. coverage (+): 0. Max coverage (-): 0

Region: chr3 50439050-50439062. Max. coverage (+): 0. Max coverage (-): 0

Region: chr3 50439063-50439075. Max. coverage (+): 0. Max coverage (-): 0

Region: chr3 50439076-50439088. Max. coverage (+): 0. Max coverage (-): 4.99

Region: chr3 50439089-50439101. Max. coverage (+): 0. Max coverage (-): 4.99

Region: chr3 50439102-50439114. Max. coverage (+): 0. Max coverage (-): 0

Region: chr3 50439115-50439128. Max. coverage (+): 0. Max coverage (-): 0

Region: chr3 50439129-50439141. Max. coverage (+): 0. Max coverage (-): 0

Region: chr3 50439142-50439154. Max. coverage (+): 0. Max coverage (-): 0

Region: chr3 50439155-50439167. Max. coverage (+): 0. Max coverage (-): 0

Region: chr3 50439168-50439180. Max. coverage (+): 0. Max coverage (-): 0

Region: chr3 50439181-50439193. Max. coverage (+): 0. Max coverage (-): 0

Region: chr3 50439194-50439206. Max. coverage (+): 0. Max coverage (-): 1.12

Region: chr3 50439207-50439219. Max. coverage (+): 0. Max coverage (-): 1.12

Region: chr3 50439220-50439233. Max. coverage (+): 0. Max coverage (-): 0

Region: chr3 50439234-50439246. Max. coverage (+): 0. Max coverage (-): 0

Region: chr3 50439247-50439259. Max. coverage (+): 0. Max coverage (-): 0

Region: chr3 50439260-50439272. Max. coverage (+): 0. Max coverage (-): 0

Region: chr3 50439273-50439285. Max. coverage (+): 0. Max coverage (-): 0

Region: chr3 50439286-50439298. Max. coverage (+): 0. Max coverage (-): 0

Region: chr3 50439299-50439311. Max. coverage (+): 0. Max coverage (-): 0

Region: chr3 50439312-50439324. Max. coverage (+): 0. Max coverage (-): 0

Region: chr3 50439325-50439338. Max. coverage (+): 0. Max coverage (-): 0

Region: chr3 50439339-50439351. Max. coverage (+): 0. Max coverage (-): 0.63

Region: chr3 50439352-50439364. Max. coverage (+): 0. Max coverage (-): 0.63

Region: chr3 50439365-50439377. Max. coverage (+): 0. Max coverage (-): 0

Region: chr3 50439378-50439390. Max. coverage (+): 0. Max coverage (-): 0

Region: chr3 50439391-50439403. Max. coverage (+): 0. Max coverage (-): 0

Region: chr3 50439404-50439416. Max. coverage (+): 0. Max coverage (-): 0

Region: chr3 50439417-50439429. Max. coverage (+): 0. Max coverage (-): 2.3

Region: chr3 50439430-50439442. Max. coverage (+): 0. Max coverage (-): 2.3

Region: chr3 50439443-50439456. Max. coverage (+): 0. Max coverage (-): 0

Region: chr3 50439457-50439469. Max. coverage (+): 0. Max coverage (-): 0

Region: chr3 50439470-50439482. Max. coverage (+): 0. Max coverage (-): 3.29

Region: chr3 50439483-50439495. Max. coverage (+): 0. Max coverage (-): 0

Region: chr3 50439496-50439508. Max. coverage (+): 0. Max coverage (-): 0

Region: chr3 50439509-50439521. Max. coverage (+): 0. Max coverage (-): 0

Region: chr3 50439522-50439534. Max. coverage (+): 0. Max coverage (-): 0

Region: chr3 50439535-50439547. Max. coverage (+): 0. Max coverage (-): 0

Region: chr3 50439548-50439561. Max. coverage (+): 0. Max coverage (-): 0

Region: chr3 50439562-50439574. Max. coverage (+): 0. Max coverage (-): 0

Region: chr3 50439575-50439587. Max. coverage (+): 0. Max coverage (-): 0

Region: chr3 50439588-50439600. Max. coverage (+): 0. Max coverage (-): 0

Region: chr3 50439601-50439613. Max. coverage (+): 0. Max coverage (-): 0

Region: chr3 50439614-50439626. Max. coverage (+): 0. Max coverage (-): 3.25

Region: chr3 50439627-50439639. Max. coverage (+): 0. Max coverage (-): 3.37

Region: chr3 50439640-50439652. Max. coverage (+): 0. Max coverage (-): 4.11

Region: chr3 50439653-50439665. Max. coverage (+): 0. Max coverage (-): 0

Region: chr3 50439666-50439679. Max. coverage (+): 0. Max coverage (-): 0

Region: chr3 50439680-50439692. Max. coverage (+): 0. Max coverage (-): 5.04

Region: chr3 50439693-50439705. Max. coverage (+): 0. Max coverage (-): 0

Region: chr3 50439706-50439718. Max. coverage (+): 0. Max coverage (-): 0

Region: chr3 50439719-50439731. Max. coverage (+): 0. Max coverage (-): 0

Region: chr3 50439732-50439744. Max. coverage (+): 0. Max coverage (-): 0

Region: chr3 50439745-50439757. Max. coverage (+): 0. Max coverage (-): 1.15

Region: chr3 50439758-50439770. Max. coverage (+): 0. Max coverage (-): 4.53

Region: chr3 50439771-50439784. Max. coverage (+): 0. Max coverage (-): 4.53

Region: chr3 50439785-50439797. Max. coverage (+): 0. Max coverage (-): 0

Region: chr3 50439798-50439810. Max. coverage (+): 0. Max coverage (-): 0

Region: chr3 50439811-50439823. Max. coverage (+): 0. Max coverage (-): 0

Region: chr3 50439824-50439836. Max. coverage (+): 0. Max coverage (-): 0

Region: chr3 50439837-50439849. Max. coverage (+): 0. Max coverage (-): 0

Region: chr3 50439850-50439862. Max. coverage (+): 0. Max coverage (-): 0

Region: chr3 50439863-50439875. Max. coverage (+): 0. Max coverage (-): 0

Region: chr3 50439876-50439888. Max. coverage (+): 0. Max coverage (-): 0

Region: chr3 50439889-50439902. Max. coverage (+): 0. Max coverage (-): 0

Region: chr3 50439903-50439915. Max. coverage (+): 0. Max coverage (-): 0

Region: chr3 50439916-50439928. Max. coverage (+): 0. Max coverage (-): 0

Region: chr3 50439929-50439941. Max. coverage (+): 0. Max coverage (-): 0

Region: chr3 50439942-50439954. Max. coverage (+): 0. Max coverage (-): 0

Region: chr3 50439955-50439967. Max. coverage (+): 0. Max coverage (-): 0

Region: chr3 50439968-50439980. Max. coverage (+): 0. Max coverage (-): 0

Region: chr3 50439981-50439993. Max. coverage (+): 0. Max coverage (-): 0

Region: chr3 50439994-50440007. Max. coverage (+): 0. Max coverage (-): 0

Region: chr3 50440008-50440020. Max. coverage (+): 0. Max coverage (-): 0

Region: chr3 50440021-50440033. Max. coverage (+): 2.3. Max coverage (-): 0

Region: chr3 50440034-50440046. Max. coverage (+): 0. Max coverage (-): 0

Region: chr3 50440047-50440059. Max. coverage (+): 0. Max coverage (-): 0

Region: chr3 50440060-50440072. Max. coverage (+): 0. Max coverage (-): 0

Region: chr3 50440073-50440085. Max. coverage (+): 0. Max coverage (-): 0

Region: chr3 50440086-50440098. Max. coverage (+): 0. Max coverage (-): 0

Region: chr3 50440099-50440111. Max. coverage (+): 0. Max coverage (-): 2.07

Region: chr3 50440112-50440125. Max. coverage (+): 0. Max coverage (-): 2.07

Region: chr3 50440126-50440138. Max. coverage (+): 0. Max coverage (-): 1.03

Region: chr3 50440139-50440151. Max. coverage (+): 0. Max coverage (-): 0

Region: chr3 50440152-50440164. Max. coverage (+): 0. Max coverage (-): 2.25

Region: chr3 50440165-50440177. Max. coverage (+): 0. Max coverage (-): 0

Region: chr3 50440178-50440190. Max. coverage (+): 0. Max coverage (-): 0

Region: chr3 50440191-50440203. Max. coverage (+): 0. Max coverage (-): 0

Region: chr3 50440204-50440216. Max. coverage (+): 0. Max coverage (-): 0

Region: chr3 50440217-50440230. Max. coverage (+): 0. Max coverage (-): 0

Region: chr3 50440231-50440243. Max. coverage (+): 0. Max coverage (-): 0

Region: chr3 50440244-50440256. Max. coverage (+): 0. Max coverage (-): 0

Region: chr3 50440257-50440269. Max. coverage (+): 0. Max coverage (-): 0

Region: chr3 50440270-50440282. Max. coverage (+): 0. Max coverage (-): 0

Region: chr3 50440283-50440295. Max. coverage (+): 0. Max coverage (-): 0

Region: chr3 50440296-50440308. Max. coverage (+): 0. Max coverage (-): 2.63

Region: chr3 50440309-50440321. Max. coverage (+): 0. Max coverage (-): 2.63

Region: chr3 50440322-50440334. Max. coverage (+): 0. Max coverage (-): 0

Region: chr3 50440335-50440348. Max. coverage (+): 0. Max coverage (-): 1.71

Region: chr3 50440349-50440361. Max. coverage (+): 0. Max coverage (-): 1.71

Region: chr3 50440362-50440374. Max. coverage (+): 0. Max coverage (-): 0

Region: chr3 50440375-50440387. Max. coverage (+): 0. Max coverage (-): 0

Region: chr3 50440388-50440400. Max. coverage (+): 0. Max coverage (-): 0

Region: chr3 50440401-50440413. Max. coverage (+): 0. Max coverage (-): 0

Region: chr3 50440414-50440426. Max. coverage (+): 0. Max coverage (-): 0

Region: chr3 50440427-50440439. Max. coverage (+): 0. Max coverage (-): 0

Region: chr3 50440440-50440453. Max. coverage (+): 0. Max coverage (-): 0

Region: chr3 50440454-50440466. Max. coverage (+): 0. Max coverage (-): 0

Region: chr3 50440467-50440479. Max. coverage (+): 0. Max coverage (-): 0

Region: chr3 50440480-50440492. Max. coverage (+): 0. Max coverage (-): 0

Region: chr3 50440493-50440505. Max. coverage (+): 0. Max coverage (-): 2.28

Region: chr3 50440506-50440518. Max. coverage (+): 0. Max coverage (-): 7.96

Region: chr3 50440519-50440531. Max. coverage (+): 2.53. Max coverage (-): 0.37

Region: chr3 50440532-50440544. Max. coverage (+): 0.79. Max coverage (-): 0.37

Region: chr3 50440545-50440557. Max. coverage (+): 0. Max coverage (-): 0

Region: chr3 50440558-50440571. Max. coverage (+): 0. Max coverage (-): 1.85

Region: chr3 50440572-50440584. Max. coverage (+): 4.51. Max coverage (-): 1.85

Region: chr3 50440585-50440597. Max. coverage (+): 4.51. Max coverage (-): 0

Region: chr3 50440598-50440610. Max. coverage (+): 1.23. Max coverage (-): 0

Region: chr3 50440611-50440623. Max. coverage (+): 0. Max coverage (-): 0

Region: chr3 50440624-50440636. Max. coverage (+): 0. Max coverage (-): 0

Region: chr3 50440637-50440649. Max. coverage (+): 0. Max coverage (-): 2.98

Region: chr3 50440650-50440662. Max. coverage (+): 0. Max coverage (-): 0

Region: chr3 50440663-50440676. Max. coverage (+): 0. Max coverage (-): 0

Region: chr3 50440677-50440689. Max. coverage (+): 0. Max coverage (-): 0

Region: chr3 50440690-50440702. Max. coverage (+): 0. Max coverage (-): 0

Region: chr3 50440703-50440715. Max. coverage (+): 0. Max coverage (-): 0

Region: chr3 50440716-50440728. Max. coverage (+): 0. Max coverage (-): 0

Region: chr3 50440729-50440741. Max. coverage (+): 0. Max coverage (-): 0

Region: chr3 50440742-50440754. Max. coverage (+): 0. Max coverage (-): 0

Region: chr3 50440755-50440767. Max. coverage (+): 0. Max coverage (-): 0

Region: chr3 50440768-50440780. Max. coverage (+): 0. Max coverage (-): 0

Region: chr3 50440781-50440794. Max. coverage (+): 0. Max coverage (-): 0

Region: chr3 50440795-50440807. Max. coverage (+): 0. Max coverage (-): 0

Region: chr3 50440808-50440820. Max. coverage (+): 0. Max coverage (-): 0

Region: chr3 50440821-50440833. Max. coverage (+): 0. Max coverage (-): 0

Region: chr3 50440834-50440846. Max. coverage (+): 0. Max coverage (-): 0

Region: chr3 50440847-50440859. Max. coverage (+): 0. Max coverage (-): 2.19

Region: chr3 50440860-50440872. Max. coverage (+): 0. Max coverage (-): 4.6

Region: chr3 50440873-50440885. Max. coverage (+): 0. Max coverage (-): 0

Region: chr3 50440886-50440899. Max. coverage (+): 0. Max coverage (-): 0

Region: chr3 50440900-50440912. Max. coverage (+): 0. Max coverage (-): 0

Region: chr3 50440913-50440925. Max. coverage (+): 0. Max coverage (-): 0

Region: chr3 50440926-50440938. Max. coverage (+): 0. Max coverage (-): 0

Region: chr3 50440939-50440951. Max. coverage (+): 0. Max coverage (-): 0

Region: chr3 50440952-50440964. Max. coverage (+): 0. Max coverage (-): 3.23

Region: chr3 50440965-50440977. Max. coverage (+): 0. Max coverage (-): 3.23

Region: chr3 50440978-50440990. Max. coverage (+): 0. Max coverage (-): 0

Region: chr3 50440991-50441003. Max. coverage (+): 0. Max coverage (-): 2.97

Region: chr3 50441004-50441017. Max. coverage (+): 0. Max coverage (-): 2.97

Region: chr3 50441018-50441030. Max. coverage (+): 0. Max coverage (-): 0

Region: chr3 50441031-50441043. Max. coverage (+): 0. Max coverage (-): 0

Region: chr3 50441044-50441056. Max. coverage (+): 0. Max coverage (-): 3.39

Region: chr3 50441057-50441069. Max. coverage (+): 0. Max coverage (-): 3.39

Region: chr3 50441070-50441082. Max. coverage (+): 0. Max coverage (-): 7.29

Region: chr3 50441083-50441095. Max. coverage (+): 0. Max coverage (-): 7.29

Region: chr3 50441096-50441108. Max. coverage (+): 0. Max coverage (-): 0

Region: chr3 50441109-50441122. Max. coverage (+): 0. Max coverage (-): 0

Region: chr3 50441123-50441135. Max. coverage (+): 0. Max coverage (-): 0

Region: chr3 50441136-50441148. Max. coverage (+): 0. Max coverage (-): 0

Region: chr3 50441149-50441161. Max. coverage (+): 0. Max coverage (-): 0

Region: chr3 50441162-50441174. Max. coverage (+): 0. Max coverage (-): 0

Region: chr3 50441175-50441187. Max. coverage (+): 0. Max coverage (-): 0

Region: chr3 50441188-50441200. Max. coverage (+): 0. Max coverage (-): 0

Region: chr3 50441201-50441213. Max. coverage (+): 0. Max coverage (-): 0

Region: chr3 50441214-50441226. Max. coverage (+): 0. Max coverage (-): 0

Region: chr3 50441227-50441240. Max. coverage (+): 0. Max coverage (-): 0

Region: chr3 50441241-50441253. Max. coverage (+): 0. Max coverage (-): 0

Region: chr3 50441254-50441266. Max. coverage (+): 0. Max coverage (-): 0

Region: chr3 50441267-50441279. Max. coverage (+): 0. Max coverage (-): 0

Region: chr3 50441280-50441292. Max. coverage (+): 0. Max coverage (-): 0

Region: chr3 50441293-50441305. Max. coverage (+): 0. Max coverage (-): 0

Region: chr3 50441306-50441318. Max. coverage (+): 0. Max coverage (-): 0

Region: chr3 50441319-50441331. Max. coverage (+): 0. Max coverage (-): 0

Region: chr3 50441332-50441345. Max. coverage (+): 0. Max coverage (-): 0

Region: chr3 50441346-50441358. Max. coverage (+): 0. Max coverage (-): 0

Region: chr3 50441359-50441371. Max. coverage (+): 0. Max coverage (-): 0

Region: chr3 50441372-50441384. Max. coverage (+): 0. Max coverage (-): 0

Region: chr3 50441385-50441397. Max. coverage (+): 0. Max coverage (-): 0

Region: chr3 50441398-50441410. Max. coverage (+): 0. Max coverage (-): 0

Region: chr3 50441411-50441423. Max. coverage (+): 0. Max coverage (-): 0

Region: chr3 50441424-50441436. Max. coverage (+): 0. Max coverage (-): 0

Region: chr3 50441437-50441450. Max. coverage (+): 0. Max coverage (-): 1.66

Region: chr3 50441451-50441463. Max. coverage (+): 0. Max coverage (-): 1.66

Region: chr3 50441464-50441476. Max. coverage (+): 0. Max coverage (-): 0

Region: chr3 50441477-50441489. Max. coverage (+): 0. Max coverage (-): 0

Region: chr3 50441490-50441502. Max. coverage (+): 0. Max coverage (-): 0

Region: chr3 50441503-50441515. Max. coverage (+): 0. Max coverage (-): 0

Region: chr3 50441516-50441528. Max. coverage (+): 0. Max coverage (-): 0

Region: chr3 50441529-50441541. Max. coverage (+): 0. Max coverage (-): 0

Region: chr3 50441542-50441554. Max. coverage (+): 0. Max coverage (-): 0

Region: chr3 50441555-50441568. Max. coverage (+): 0. Max coverage (-): 0

Region: chr3 50441569-50441581. Max. coverage (+): 0. Max coverage (-): 9.03

Region: chr3 50441582-50441594. Max. coverage (+): 0. Max coverage (-): 9.03

Region: chr3 50441595-50441607. Max. coverage (+): 0. Max coverage (-): 0

Region: chr3 50441608-50441620. Max. coverage (+): 0. Max coverage (-): 4.73

Region: chr3 50441621-50441633. Max. coverage (+): 0. Max coverage (-): 0

Region: chr3 50441634-50441646. Max. coverage (+): 0. Max coverage (-): 0

Region: chr3 50441647-50441659. Max. coverage (+): 0. Max coverage (-): 0

Region: chr3 50441660-50441673. Max. coverage (+): 0. Max coverage (-): 0.86

Region: chr3 50441674-50441686. Max. coverage (+): 0. Max coverage (-): 0.86

Region: chr3 50441687-50441699. Max. coverage (+): 0. Max coverage (-): 0

Region: chr3 50441700-50441712. Max. coverage (+): 0. Max coverage (-): 0

Region: chr3 50441713-50441725. Max. coverage (+): 0. Max coverage (-): 0

Region: chr3 50441726-50441738. Max. coverage (+): 0. Max coverage (-): 0

Region: chr3 50441739-50441751. Max. coverage (+): 0. Max coverage (-): 0

Region: chr3 50441752-50441764. Max. coverage (+): 0. Max coverage (-): 0

Region: chr3 50441765-50441777. Max. coverage (+): 0. Max coverage (-): 0

Region: chr3 50441778-50441791. Max. coverage (+): 0. Max coverage (-): 0

Region: chr3 50441792-50441804. Max. coverage (+): 0. Max coverage (-): 0

Region: chr3 50441805-50441817. Max. coverage (+): 0. Max coverage (-): 0

Region: chr3 50441818-50441830. Max. coverage (+): 0. Max coverage (-): 4.44

Region: chr3 50441831-50441843. Max. coverage (+): 0. Max coverage (-): 0

Region: chr3 50441844-50441856. Max. coverage (+): 0. Max coverage (-): 0

Region: chr3 50441857-50441869. Max. coverage (+): 0. Max coverage (-): 0

Region: chr3 50441870-50441882. Max. coverage (+): 0. Max coverage (-): 5.22

Region: chr3 50441883-50441896. Max. coverage (+): 0. Max coverage (-): 35.16

Region: chr3 50441897-50441909. Max. coverage (+): 0. Max coverage (-): 2.04

Region: chr3 50441910-50441922. Max. coverage (+): 0. Max coverage (-): 0

Region: chr3 50441923-50441935. Max. coverage (+): 0. Max coverage (-): 0

Region: chr3 50441936-50441948. Max. coverage (+): 0. Max coverage (-): 0

Region: chr3 50441949-50441961. Max. coverage (+): 0. Max coverage (-): 0

Region: chr3 50441962-50441974. Max. coverage (+): 0. Max coverage (-): 0

Region: chr3 50441975-50441987. Max. coverage (+): 0. Max coverage (-): 0

Region: chr3 50441988-50442000. Max. coverage (+): 0. Max coverage (-): 0

Region: chr3 50442001-50442014. Max. coverage (+): 0. Max coverage (-): 0

Region: chr3 50442015-50442027. Max. coverage (+): 0. Max coverage (-): 0

Region: chr3 50442028-50442040. Max. coverage (+): 0. Max coverage (-): 0

Region: chr3 50442041-50442053. Max. coverage (+): 0. Max coverage (-): 0

Region: chr3 50442054-50442066. Max. coverage (+): 0. Max coverage (-): 0

Region: chr3 50442067-50442079. Max. coverage (+): 0. Max coverage (-): 0

Region: chr3 50442080-50442092. Max. coverage (+): 0. Max coverage (-): 0

Region: chr3 50442093-50442105. Max. coverage (+): 0. Max coverage (-): 0

Region: chr3 50442106-50442119. Max. coverage (+): 0. Max coverage (-): 0

Region: chr3 50442120-50442132. Max. coverage (+): 0. Max coverage (-): 1.83

Region: chr3 50442133-50442145. Max. coverage (+): 0. Max coverage (-): 0

Region: chr3 50442146-50442158. Max. coverage (+): 0. Max coverage (-): 1.94

Region: chr3 50442159-50442171. Max. coverage (+): 0. Max coverage (-): 1.94

Region: chr3 50442172-50442184. Max. coverage (+): 0. Max coverage (-): 0

Region: chr3 50442185-50442197. Max. coverage (+): 0. Max coverage (-): 0

Region: chr3 50442198-50442210. Max. coverage (+): 0. Max coverage (-): 0

Region: chr3 50442211-50442223. Max. coverage (+): 0. Max coverage (-): 0

Region: chr3 50442224-50442237. Max. coverage (+): 0. Max coverage (-): 0

Region: chr3 50442238-50442250. Max. coverage (+): 0. Max coverage (-): 0

Region: chr3 50442251-50442263. Max. coverage (+): 0. Max coverage (-): 0

Region: chr3 50442264-50442276. Max. coverage (+): 0. Max coverage (-): 0

Region: chr3 50442277-50442289. Max. coverage (+): 0. Max coverage (-): 0

Region: chr3 50442290-50442302. Max. coverage (+): 0. Max coverage (-): 0

Region: chr3 50442303-50442315. Max. coverage (+): 0. Max coverage (-): 0

Region: chr3 50442316-50442328. Max. coverage (+): 0. Max coverage (-): 0

Region: chr3 50442329-50442342. Max. coverage (+): 0. Max coverage (-): 0

Region: chr3 50442343-50442355. Max. coverage (+): 0. Max coverage (-): 13.03

Region: chr3 50442356-50442368. Max. coverage (+): 0. Max coverage (-): 5.12

Region: chr3 50442369-50442381. Max. coverage (+): 0. Max coverage (-): 0

Region: chr3 50442382-50442394. Max. coverage (+): 0. Max coverage (-): 0

Region: chr3 50442395-50442407. Max. coverage (+): 0. Max coverage (-): 0.29

Region: chr3 50442408-50442420. Max. coverage (+): 0. Max coverage (-): 0.29

Region: chr3 50442421-50442433. Max. coverage (+): 0. Max coverage (-): 4.73

Region: chr3 50442434-50442446. Max. coverage (+): 0. Max coverage (-): 4.73

Region: chr3 50442447-50442460. Max. coverage (+): 0. Max coverage (-): 0

Region: chr3 50442461-50442473. Max. coverage (+): 0. Max coverage (-): 0

Region: chr3 50442474-50442486. Max. coverage (+): 0. Max coverage (-): 6.42

Region: chr3 50442487-50442499. Max. coverage (+): 0. Max coverage (-): 6.42

Region: chr3 50442500-50442512. Max. coverage (+): 0. Max coverage (-): 0

Region: chr3 50442513-50442525. Max. coverage (+): 0. Max coverage (-): 0

Region: chr3 50442526-50442538. Max. coverage (+): 0. Max coverage (-): 4.09

Region: chr3 50442539-50442551. Max. coverage (+): 0. Max coverage (-): 4.09

Region: chr3 50442552-50442565. Max. coverage (+): 0. Max coverage (-): 0.69

Region: chr3 50442566-50442578. Max. coverage (+): 0. Max coverage (-): 0.69

Region: chr3 50442579-50442591. Max. coverage (+): 0. Max coverage (-): 0

Region: chr3 50442592-50442604. Max. coverage (+): 0. Max coverage (-): 0

Region: chr3 50442605-50442617. Max. coverage (+): 0. Max coverage (-): 0

Region: chr3 50442618-50442630. Max. coverage (+): 0. Max coverage (-): 0

Region: chr3 50442631-50442643. Max. coverage (+): 0. Max coverage (-): 0

Region: chr3 50442644-50442656. Max. coverage (+): 0. Max coverage (-): 0

Region: chr3 50442657-50442669. Max. coverage (+): 0. Max coverage (-): 0

Region: chr3 50442670-50442683. Max. coverage (+): 0. Max coverage (-): 0

Region: chr3 50442684-50442696. Max. coverage (+): 0. Max coverage (-): 0

Region: chr3 50442697-50442709. Max. coverage (+): 0. Max coverage (-): 0

Region: chr3 50442710-50442722. Max. coverage (+): 0. Max coverage (-): 0

Region: chr3 50442723-50442735. Max. coverage (+): 0. Max coverage (-): 0

Region: chr3 50442736-50442748. Max. coverage (+): 0. Max coverage (-): 0

Region: chr3 50442749-50442761. Max. coverage (+): 0. Max coverage (-): 0

Region: chr3 50442762-50442774. Max. coverage (+): 0. Max coverage (-): 0

Region: chr3 50442775-50442788. Max. coverage (+): 0. Max coverage (-): 0

Region: chr3 50442789-50442801. Max. coverage (+): 0. Max coverage (-): 0

Region: chr3 50442802-50442814. Max. coverage (+): 0. Max coverage (-): 0

Region: chr3 50442815-50442827. Max. coverage (+): 0. Max coverage (-): 0

Region: chr3 50442828-50442840. Max. coverage (+): 0. Max coverage (-): 0

Region: chr3 50442841-50442853. Max. coverage (+): 0. Max coverage (-): 0

Region: chr3 50442854-50442866. Max. coverage (+): 0. Max coverage (-): 0

Region: chr3 50442867-50442879. Max. coverage (+): 0. Max coverage (-): 0

Region: chr3 50442880-50442892. Max. coverage (+): 0. Max coverage (-): 0

Region: chr3 50442893-50442906. Max. coverage (+): 0. Max coverage (-): 0

Region: chr3 50442907-50442919. Max. coverage (+): 0. Max coverage (-): 0

Region: chr3 50442920-50442932. Max. coverage (+): 0. Max coverage (-): 0

Region: chr3 50442933-50442945. Max. coverage (+): 0. Max coverage (-): 3.64

Region: chr3 50442946-50442958. Max. coverage (+): 0. Max coverage (-): 0

Region: chr3 50442959-50442971. Max. coverage (+): 0. Max coverage (-): 0

Region: chr3 50442972-50442984. Max. coverage (+): 0. Max coverage (-): 0

Region: chr3 50442985-50442997. Max. coverage (+): 0. Max coverage (-): 0

Region: chr3 50442998-50443011. Max. coverage (+): 0. Max coverage (-): 0

Region: chr3 50443012-50443024. Max. coverage (+): 0. Max coverage (-): 0

Region: chr3 50443025-50443037. Max. coverage (+): 0. Max coverage (-): 0

Region: chr3 50443038-50443050. Max. coverage (+): 0. Max coverage (-): 0

Region: chr3 50443051-50443063. Max. coverage (+): 0. Max coverage (-): 0

Region: chr3 50443064-50443076. Max. coverage (+): 0. Max coverage (-): 0

Region: chr3 50443077-50443089. Max. coverage (+): 0. Max coverage (-): 0

Region: chr3 50443090-50443102. Max. coverage (+): 0. Max coverage (-): 0

Region: chr3 50443103-50443115. Max. coverage (+): 0. Max coverage (-): 0

Region: chr3 50443116-50443129. Max. coverage (+): 0. Max coverage (-): 0

Region: chr3 50443130-50443142. Max. coverage (+): 0. Max coverage (-): 0

Region: chr3 50443143-50443155. Max. coverage (+): 0. Max coverage (-): 0

Region: chr3 50443156-50443168. Max. coverage (+): 0. Max coverage (-): 0

Region: chr3 50443169-50443181. Max. coverage (+): 0. Max coverage (-): 0

Region: chr3 50443182-50443194. Max. coverage (+): 0. Max coverage (-): 0

Region: chr3 50443195-50443207. Max. coverage (+): 0. Max coverage (-): 0

Region: chr3 50443208-50443220. Max. coverage (+): 0. Max coverage (-): 0

Region: chr3 50443221-50443234. Max. coverage (+): 0. Max coverage (-): 0

Region: chr3 50443235-50443247. Max. coverage (+): 0. Max coverage (-): 0

Region: chr3 50443248-50443260. Max. coverage (+): 0. Max coverage (-): 0

Region: chr3 50443261-50443273. Max. coverage (+): 0. Max coverage (-): 0

Region: chr3 50443274-50443286. Max. coverage (+): 0. Max coverage (-): 0

Region: chr3 50443287-50443299. Max. coverage (+): 0. Max coverage (-): 0

Region: chr3 50443300-50443312. Max. coverage (+): 0. Max coverage (-): 0

Region: chr3 50443313-50443325. Max. coverage (+): 0. Max coverage (-): 0

Region: chr3 50443326-50443338. Max. coverage (+): 0. Max coverage (-): 0

Region: chr3 50443339-50443352. Max. coverage (+): 0. Max coverage (-): 0

Region: chr3 50443353-50443365. Max. coverage (+): 0. Max coverage (-): 0

Region: chr3 50443366-50443378. Max. coverage (+): 0. Max coverage (-): 0

Region: chr3 50443379-50443391. Max. coverage (+): 0. Max coverage (-): 0

Region: chr3 50443392-50443404. Max. coverage (+): 0. Max coverage (-): 0

Region: chr3 50443405-50443417. Max. coverage (+): 0. Max coverage (-): 0

Region: chr3 50443418-50443430. Max. coverage (+): 0. Max coverage (-): 0

Region: chr3 50443431-50443443. Max. coverage (+): 0. Max coverage (-): 0

Region: chr3 50443444-50443457. Max. coverage (+): 0. Max coverage (-): 0

Region: chr3 50443458-50443470. Max. coverage (+): 0. Max coverage (-): 0

Region: chr3 50443471-50443483. Max. coverage (+): 0. Max coverage (-): 0

Region: chr3 50443484-50443496. Max. coverage (+): 0. Max coverage (-): 0

Region: chr3 50443497-50443509. Max. coverage (+): 0. Max coverage (-): 0

Region: chr3 50443510-50443522. Max. coverage (+): 0. Max coverage (-): 0

Region: chr3 50443523-50443535. Max. coverage (+): 0. Max coverage (-): 0

Region: chr3 50443536-50443548. Max. coverage (+): 0. Max coverage (-): 0

Region: chr3 50443549-50443562. Max. coverage (+): 0. Max coverage (-): 6.24

Region: chr3 50443563-50443575. Max. coverage (+): 0. Max coverage (-): 0

Region: chr3 50443576-50443588. Max. coverage (+): 0. Max coverage (-): 0

Region: chr3 50443589-50443601. Max. coverage (+): 0. Max coverage (-): 0

Region: chr3 50443602-50443614. Max. coverage (+): 0. Max coverage (-): 0

Region: chr3 50443615-50443627. Max. coverage (+): 0. Max coverage (-): 0

Region: chr3 50443628-50443640. Max. coverage (+): 0. Max coverage (-): 0

Region: chr3 50443641-50443653. Max. coverage (+): 0. Max coverage (-): 0

Region: chr3 50443654-50443666. Max. coverage (+): 0. Max coverage (-): 0.49

Region: chr3 50443667-50443680. Max. coverage (+): 0. Max coverage (-): 0.49

Region: chr3 50443681-50443693. Max. coverage (+): 0. Max coverage (-): 0

Region: chr3 50443694-50443706. Max. coverage (+): 0. Max coverage (-): 0

Region: chr3 50443707-50443719. Max. coverage (+): 0. Max coverage (-): 0

Region: chr3 50443720-50443732. Max. coverage (+): 0. Max coverage (-): 0

Region: chr3 50443733-50443745. Max. coverage (+): 0. Max coverage (-): 0

Region: chr3 50443746-50443758. Max. coverage (+): 0. Max coverage (-): 0

Region: chr3 50443759-50443771. Max. coverage (+): 0. Max coverage (-): 0

Region: chr3 50443772-50443785. Max. coverage (+): 0. Max coverage (-): 0

Region: chr3 50443786-50443798. Max. coverage (+): 0. Max coverage (-): 0

Region: chr3 50443799-50443811. Max. coverage (+): 0. Max coverage (-): 0

Region: chr3 50443812-50443824. Max. coverage (+): 0. Max coverage (-): 0

Region: chr3 50443825-50443837. Max. coverage (+): 0. Max coverage (-): 0

Region: chr3 50443838-50443850. Max. coverage (+): 0. Max coverage (-): 0

Region: chr3 50443851-50443863. Max. coverage (+): 0. Max coverage (-): 0

Region: chr3 50443864-50443876. Max. coverage (+): 0. Max coverage (-): 0

Region: chr3 50443877-50443889. Max. coverage (+): 0. Max coverage (-): 0

Region: chr3 50443890-50443903. Max. coverage (+): 0. Max coverage (-): 0

Region: chr3 50443904-50443916. Max. coverage (+): 0. Max coverage (-): 0

Region: chr3 50443917-50443929. Max. coverage (+): 0. Max coverage (-): 0

Region: chr3 50443930-50443942. Max. coverage (+): 0. Max coverage (-): 0

Region: chr3 50443943-50443955. Max. coverage (+): 0. Max coverage (-): 0

Region: chr3 50443956-50443968. Max. coverage (+): 0. Max coverage (-): 0

Region: chr3 50443969-50443981. Max. coverage (+): 0. Max coverage (-): 0

Region: chr3 50443982-50443994. Max. coverage (+): 0. Max coverage (-): 0

Region: chr3 50443995-50444008. Max. coverage (+): 0. Max coverage (-): 0

Region: chr3 50444009-50444021. Max. coverage (+): 0. Max coverage (-): 0

Region: chr3 50444022-50444034. Max. coverage (+): 0. Max coverage (-): 0

Region: chr3 50444035-50444047. Max. coverage (+): 0. Max coverage (-): 0

Region: chr3 50444048-50444060. Max. coverage (+): 0. Max coverage (-): 0

Region: chr3 50444061-50444073. Max. coverage (+): 0. Max coverage (-): 0

Region: chr3 50444074-50444086. Max. coverage (+): 0. Max coverage (-): 0

Region: chr3 50444087-50444099. Max. coverage (+): 0. Max coverage (-): 0

Region: chr3 50444100-50444112. Max. coverage (+): 0. Max coverage (-): 0

Region: chr3 50444113-50444126. Max. coverage (+): 0.41. Max coverage (-): 0

Region: chr3 50444127-50444139. Max. coverage (+): 0.41. Max coverage (-): 0

Region: chr3 50444140-50444152. Max. coverage (+): 0. Max coverage (-): 0

Region: chr3 50444153-50444165. Max. coverage (+): 0. Max coverage (-): 0

Region: chr3 50444166-50444178. Max. coverage (+): 0. Max coverage (-): 0

Region: chr3 50444179-50444191. Max. coverage (+): 0. Max coverage (-): 0

Region: chr3 50444192-50444204. Max. coverage (+): 0. Max coverage (-): 0

Region: chr3 50444205-50444217. Max. coverage (+): 0. Max coverage (-): 0

Region: chr3 50444218-50444231. Max. coverage (+): 0. Max coverage (-): 0

Region: chr3 50444232-50444244. Max. coverage (+): 0. Max coverage (-): 0

Region: chr3 50444245-50444257. Max. coverage (+): 0. Max coverage (-): 0

Region: chr3 50444258-50444270. Max. coverage (+): 0. Max coverage (-): 0

Region: chr3 50444271-50444283. Max. coverage (+): 0. Max coverage (-): 0

Region: chr3 50444284-50444296. Max. coverage (+): 0. Max coverage (-): 0

Region: chr3 50444297-50444309. Max. coverage (+): 0. Max coverage (-): 0

Region: chr3 50444310-50444322. Max. coverage (+): 0. Max coverage (-): 0

Region: chr3 50444323-50444335. Max. coverage (+): 0. Max coverage (-): 0

Region: chr3 50444336-50444349. Max. coverage (+): 0. Max coverage (-): 0

Region: chr3 50444350-50444362. Max. coverage (+): 0. Max coverage (-): 0

Region: chr3 50444363-50444375. Max. coverage (+): 0. Max coverage (-): 0

Region: chr3 50444376-50444388. Max. coverage (+): 0. Max coverage (-): 0

Region: chr3 50444389-50444401. Max. coverage (+): 0. Max coverage (-): 0

Region: chr3 50444402-50444414. Max. coverage (+): 0. Max coverage (-): 0

Region: chr3 50444415-50444427. Max. coverage (+): 0. Max coverage (-): 0

Region: chr3 50444428-50444440. Max. coverage (+): 0. Max coverage (-): 0

Region: chr3 50444441-50444454. Max. coverage (+): 0. Max coverage (-): 0

Region: chr3 50444455-50444467. Max. coverage (+): 0. Max coverage (-): 0

Region: chr3 50444468-50444480. Max. coverage (+): 0. Max coverage (-): 0

Region: chr3 50444481-50444493. Max. coverage (+): 0. Max coverage (-): 0

Region: chr3 50444494-50444506. Max. coverage (+): 0. Max coverage (-): 0

Region: chr3 50444507-50444519. Max. coverage (+): 0. Max coverage (-): 0

Region: chr3 50444520-50444532. Max. coverage (+): 0.21. Max coverage (-): 0

Region: chr3 50444533-50444545. Max. coverage (+): 0.21. Max coverage (-): 0

Region: chr3 50444546-50444558. Max. coverage (+): 0. Max coverage (-): 0

Region: chr3 50444559-50444572. Max. coverage (+): 0. Max coverage (-): 0

Region: chr3 50444573-50444585. Max. coverage (+): 0.15. Max coverage (-): 0.34

Region: chr3 50444586-50444598. Max. coverage (+): 0.48. Max coverage (-): 0.34

Region: chr3 50444599-50444611. Max. coverage (+): 0. Max coverage (-): 0

Region: chr3 50444612-. Max. coverage (+): 0. Max coverage (-): 0

RepeatMasker Color Code

**+**

100-98% Identity

<98-95% Identity

<95-90% Identity

<90-85% Identity

<85-80% Identity

<80-75% Identity

<75-70% Identity

<70% Identity

**-**

Gene Set Color Code

**+**

Gene

Pseudogene

**-**

Topology/Coverage Color Code

Coverage Plus Strand

Coverage Minus Strand

Mainstrand: Plus

Mainstrand: Minus

Complementary Strand

Flanking Region  
(if option -flank >0)

Gene Set Annotation  
  
RepeatMasker Annotation  

**1. ERV1-2-I\_BT-int**: 50441944-50442107 (+), Divergence to consensus: 38.1%  
**2. BOV-A2**: 50442637-50442908 (-), Divergence to consensus: 4%  
**3. ERV1-1-I\_BT-int**: 50443166-50443292 (+), Divergence to consensus: 32.4%

  
Transcription Factor Binding Sites  

**RFX4\_2** (Sequence: GTATCTAGG (-): 50438191)  
**RFX4\_2** (Sequence: GTAACTAAG (-): 50442484)  
**Gata4** (Sequence: AGATAAG (-): 50441611)  
**SPZ1** (Sequence: GGGGTATCAG (+): 50440577)  
**Gata4** (Sequence: CTTATCT (+): 50443788)
