# Supplementary material for: Small RNA sequencing of cryopreserved semen from single bull revealed altered miRNAs and piRNAs expression between High- and Low-motile sperm populations
Source: BMC Genomics. 2017 Jan 4;18:14. doi: 10.1186/s12864-016-3394-7 (PMC5209821; doi:10.1186/s12864-016-3394-7)
Supplement: Additional file 3: — Details for each piRNA clusters found in High Motile (HM) sperm fraction. Genes, repeats, transposable elements and transcription factors binding sites falling within the cluster regions were reported. (ZIP 1896 kb) [file 12864_2016_3394_MOESM3_ESM.zip › 78.html]

piRNA cluster 78


Predicted piRNA cluster no. 78     previous   next
  

Show proTRAC run info
Hide proTRAC run info

================================= proTRAC ====================================  
VERSION: 2.1                                    LAST MODIFIED: 06. October 2015  
  
Please cite:  
Rosenkranz D, Zischler H. proTRAC - a software for probabilistic piRNA cluster  
detection, visualization and analysis. 2012. BMC Bioinformatics 13:5.  
  
and (for proTRAC 2.0 and later):  
Rosenkranz D, Rudloff S, Bastuck K, Ketting RF, Zischler H. Tupaia small RNAs  
provide insights into function and evolution of RNAi-based transposon defense  
in mammals. 2015. RNA 21(5):911-922.  
  
Contact:  
David Rosenkranz  
Institute of Anthropology, small RNA group  
Johannes Gutenberg University Mainz  
email: rosenkranz@uni-mainz.de  
  
You can find the latest proTRAC version at:  
http://sourceforge.net/projects/protrac/files  
http://www.smallRNAgroup-mainz.de/software  
==============================================================================  
  
PARAMETERS:  
Map file: .............../storage/core/barbara/genhome/smallRNA/fertility/Sample\_motile/pirna/Sample\_motile\_26-33\_collapsed.fa.no-dust.map.weighted-10000-1000-b-0  
Genome file: ............/storage/core/barbara/genhome/smallRNA/fertility/Sample\_all/pirna/bt\_311\_chrY.fa  
RepeatMasker annotation: /storage/genomes/bt\_umd31/GCF\_000003055.6\_Bos\_taurus\_UMD\_3.1.1\_repeatMasker\_chr.out  
GeneSet:................./storage/core/barbara/genhome/smallRNA/fertility/Sample\_all/pirna/full.gtf  
  
Significant (p<=0.01) hit density will be calculated based  
on observed hit distribution.  
  
Sliding window size: ........................................ 5000 bp  
Sliding window increament: .................................. 1000 bp  
Normalize each hit by number of genomic hits: ............... 1 [0=no/1=yes]  
Normalize each hit by number of sequence reads: ............. 1 [0=no/1=yes]  
Normalize values (-> per million mapped reads): ............. 1 [0=no/1=yes]  
Min. fraction of hits with 1T(U) or 10A: .................... 0.75  
Alternatively: Min. fraction of hits with 1T(U) and 10A: .... 0.5  
Min. fraction of hits with typical piRNA length: ............ 0.75  
Typical piRNA length: ....................................... 26-33 nt  
Min. size of a piRNA cluster: ............................... 5000 bp.  
Min. number of hits (absolute): ............................. 0  
Min. number of hits (normalized): ........................... 0  
Min. fraction of hits on the mainstrand: .................... 0.75  
Top fraction of mapped sequences (in terms of read counts): . 1%  
Top fraction accounts for max. n% of sequence reads: ........ 90%  
Min. fraction of hits on each arm of a bidirectional cluster: 0.1  
Output image file for each cluster: ......................... 0 [0=no/1=yes]  
Output html file for each cluster: .......................... 1 [0=no/1=yes]  
Output a summary table: ..................................... 1 [0=no/1=yes]  
Output a FASTA file for each cluster (piRNA sequences): ..... 1 [0=no/1=yes]  
Output a FASTA file comprising cluster sequences: ........... 1 [0=no/1=yes]  
Search DNA motifs in clusters: .............................. 1 [0=no/1=yes]  
Output flanking sequences: +/- .............................. 0 bp  
Output ~.pTi file: .......................................... 1 [0=no/1=yes]  
==============================================================================  
  
  
Genome size (without gaps): ............ 2678902517 bp  
Gaps (N/X/-): .......................... 53837044 bp  
Mapped reads: .......................... 658825247023  
Non-identical sequences: ............... 514171  
Genomic hits: .......................... 764233  
Significant densitiy of mapped reads: .. 12867599.5173724 reads/kb

Show proTRAC cluster info
Hide proTRAC cluster info

|  |  |
| --- | --- |
| Location | chr3 |
| Coordinates | 57098542-57105141 |
| Size [bp] | 6600 |
| Sequence hit loci | 508 |
| Mapped reads (normalized) | 573781789.4 |
| Mapped reads (normalized) per kb | 86936634.8 |
| Normalized reads with 1T (1U) | 84.3% |
| Normalized reads with 10A | 29.9% |
| Normalized reads with length 26-33 nt | 100% |
| Normalized reads on the main strand(s) | 99.5% |
| Predicted directionality | mono:minus |

100%

0%

1T (1U)  
reads

10A reads

26-33 nt  
reads

reads on mainstrand

**Either the amount of reads with 1T (1U) OR 10A has to exceed 75% (set with option: -1Tor10A)  
Alternatively the amount of reads with 1T (1U) AND 10A has to exceed 50% (set with option: -1Tand10A)  
Minimum amount of reads with preferred size is 75% (set with option: -pisize)  
Minimum amount of reads on the main strand(s) is 75% (set with option: -clstrand)**

Show read coverage
Hide read coverage

WHAT DO I SEE HERE?  
This chart shows the location of mapped sequence reads within a predicted piRNA cluster. The color refers to the number of genomic hits produced by the sequence read in question. A dark red bar indicates that this sequence read produces many other hits elsewhere in the genome. Many adjacent red or yellow bars can indicate the presence of a multi-copy element such as transposons or rRNA genes. A dark green bar indicates that this sequence read maps uniquely to this locus.

1 hit

2-5 hits

6-10 hits

11-20 hits

21-50 hits

51-100 hits

> 100 hits

chr3

57098542

57105141

Gene Set

RepeatMasker

Mapped  
Reads

53.08

plus strand

minus strand

53.08

Region: chr3 50444613-57098548. Max. coverage (+): 0. Max coverage (-): 1.38

Region: chr3 57098549-57098561. Max. coverage (+): 0. Max coverage (-): 1.38

Region: chr3 57098562-57098574. Max. coverage (+): 0. Max coverage (-): 0

Region: chr3 57098575-57098588. Max. coverage (+): 0. Max coverage (-): 0

Region: chr3 57098589-57098601. Max. coverage (+): 0. Max coverage (-): 0

Region: chr3 57098602-57098614. Max. coverage (+): 0. Max coverage (-): 0

Region: chr3 57098615-57098627. Max. coverage (+): 0. Max coverage (-): 0

Region: chr3 57098628-57098640. Max. coverage (+): 0. Max coverage (-): 0

Region: chr3 57098641-57098654. Max. coverage (+): 0. Max coverage (-): 0

Region: chr3 57098655-57098667. Max. coverage (+): 0. Max coverage (-): 0

Region: chr3 57098668-57098680. Max. coverage (+): 0. Max coverage (-): 0

Region: chr3 57098681-57098693. Max. coverage (+): 0. Max coverage (-): 0

Region: chr3 57098694-57098706. Max. coverage (+): 0. Max coverage (-): 0

Region: chr3 57098707-57098720. Max. coverage (+): 0. Max coverage (-): 0

Region: chr3 57098721-57098733. Max. coverage (+): 0. Max coverage (-): 0

Region: chr3 57098734-57098746. Max. coverage (+): 0. Max coverage (-): 0

Region: chr3 57098747-57098759. Max. coverage (+): 0. Max coverage (-): 0

Region: chr3 57098760-57098772. Max. coverage (+): 0. Max coverage (-): 0

Region: chr3 57098773-57098786. Max. coverage (+): 0. Max coverage (-): 0

Region: chr3 57098787-57098799. Max. coverage (+): 0. Max coverage (-): 0

Region: chr3 57098800-57098812. Max. coverage (+): 0. Max coverage (-): 0

Region: chr3 57098813-57098825. Max. coverage (+): 0. Max coverage (-): 0

Region: chr3 57098826-57098838. Max. coverage (+): 0. Max coverage (-): 0

Region: chr3 57098839-57098852. Max. coverage (+): 0. Max coverage (-): 0

Region: chr3 57098853-57098865. Max. coverage (+): 0. Max coverage (-): 0

Region: chr3 57098866-57098878. Max. coverage (+): 0. Max coverage (-): 0

Region: chr3 57098879-57098891. Max. coverage (+): 0. Max coverage (-): 0

Region: chr3 57098892-57098904. Max. coverage (+): 0. Max coverage (-): 0

Region: chr3 57098905-57098918. Max. coverage (+): 0. Max coverage (-): 0

Region: chr3 57098919-57098931. Max. coverage (+): 0. Max coverage (-): 0

Region: chr3 57098932-57098944. Max. coverage (+): 0. Max coverage (-): 0

Region: chr3 57098945-57098957. Max. coverage (+): 0. Max coverage (-): 0

Region: chr3 57098958-57098970. Max. coverage (+): 0. Max coverage (-): 0

Region: chr3 57098971-57098984. Max. coverage (+): 0. Max coverage (-): 0

Region: chr3 57098985-57098997. Max. coverage (+): 0. Max coverage (-): 0

Region: chr3 57098998-57099010. Max. coverage (+): 0. Max coverage (-): 0

Region: chr3 57099011-57099023. Max. coverage (+): 0. Max coverage (-): 0

Region: chr3 57099024-57099036. Max. coverage (+): 0. Max coverage (-): 0

Region: chr3 57099037-57099050. Max. coverage (+): 0. Max coverage (-): 0

Region: chr3 57099051-57099063. Max. coverage (+): 0. Max coverage (-): 0

Region: chr3 57099064-57099076. Max. coverage (+): 0. Max coverage (-): 0

Region: chr3 57099077-57099089. Max. coverage (+): 0. Max coverage (-): 0

Region: chr3 57099090-57099102. Max. coverage (+): 0. Max coverage (-): 0

Region: chr3 57099103-57099116. Max. coverage (+): 0. Max coverage (-): 0

Region: chr3 57099117-57099129. Max. coverage (+): 0. Max coverage (-): 0

Region: chr3 57099130-57099142. Max. coverage (+): 0. Max coverage (-): 0

Region: chr3 57099143-57099155. Max. coverage (+): 0. Max coverage (-): 0

Region: chr3 57099156-57099168. Max. coverage (+): 0. Max coverage (-): 0

Region: chr3 57099169-57099182. Max. coverage (+): 0. Max coverage (-): 0

Region: chr3 57099183-57099195. Max. coverage (+): 0. Max coverage (-): 0

Region: chr3 57099196-57099208. Max. coverage (+): 0. Max coverage (-): 0

Region: chr3 57099209-57099221. Max. coverage (+): 0. Max coverage (-): 0

Region: chr3 57099222-57099234. Max. coverage (+): 0. Max coverage (-): 0

Region: chr3 57099235-57099248. Max. coverage (+): 0. Max coverage (-): 0

Region: chr3 57099249-57099261. Max. coverage (+): 0. Max coverage (-): 0

Region: chr3 57099262-57099274. Max. coverage (+): 0. Max coverage (-): 5.69

Region: chr3 57099275-57099287. Max. coverage (+): 0. Max coverage (-): 0.67

Region: chr3 57099288-57099300. Max. coverage (+): 0. Max coverage (-): 7.84

Region: chr3 57099301-57099314. Max. coverage (+): 0. Max coverage (-): 0

Region: chr3 57099315-57099327. Max. coverage (+): 0. Max coverage (-): 0

Region: chr3 57099328-57099340. Max. coverage (+): 0. Max coverage (-): 0

Region: chr3 57099341-57099353. Max. coverage (+): 0. Max coverage (-): 0

Region: chr3 57099354-57099366. Max. coverage (+): 0. Max coverage (-): 0

Region: chr3 57099367-57099380. Max. coverage (+): 0. Max coverage (-): 0

Region: chr3 57099381-57099393. Max. coverage (+): 0. Max coverage (-): 0

Region: chr3 57099394-57099406. Max. coverage (+): 0. Max coverage (-): 0

Region: chr3 57099407-57099419. Max. coverage (+): 0. Max coverage (-): 0

Region: chr3 57099420-57099432. Max. coverage (+): 0. Max coverage (-): 0

Region: chr3 57099433-57099446. Max. coverage (+): 0. Max coverage (-): 0

Region: chr3 57099447-57099459. Max. coverage (+): 0. Max coverage (-): 0

Region: chr3 57099460-57099472. Max. coverage (+): 0. Max coverage (-): 0

Region: chr3 57099473-57099485. Max. coverage (+): 0. Max coverage (-): 0

Region: chr3 57099486-57099498. Max. coverage (+): 0. Max coverage (-): 0

Region: chr3 57099499-57099512. Max. coverage (+): 0. Max coverage (-): 0

Region: chr3 57099513-57099525. Max. coverage (+): 0. Max coverage (-): 0

Region: chr3 57099526-57099538. Max. coverage (+): 0. Max coverage (-): 0

Region: chr3 57099539-57099551. Max. coverage (+): 0. Max coverage (-): 0

Region: chr3 57099552-57099564. Max. coverage (+): 0. Max coverage (-): 0

Region: chr3 57099565-57099578. Max. coverage (+): 0. Max coverage (-): 0

Region: chr3 57099579-57099591. Max. coverage (+): 0. Max coverage (-): 0

Region: chr3 57099592-57099604. Max. coverage (+): 0. Max coverage (-): 0

Region: chr3 57099605-57099617. Max. coverage (+): 0. Max coverage (-): 0

Region: chr3 57099618-57099630. Max. coverage (+): 0. Max coverage (-): 0

Region: chr3 57099631-57099644. Max. coverage (+): 0. Max coverage (-): 0

Region: chr3 57099645-57099657. Max. coverage (+): 0. Max coverage (-): 0

Region: chr3 57099658-57099670. Max. coverage (+): 0. Max coverage (-): 0

Region: chr3 57099671-57099683. Max. coverage (+): 0. Max coverage (-): 0

Region: chr3 57099684-57099696. Max. coverage (+): 0. Max coverage (-): 0

Region: chr3 57099697-57099710. Max. coverage (+): 0. Max coverage (-): 0

Region: chr3 57099711-57099723. Max. coverage (+): 0. Max coverage (-): 0

Region: chr3 57099724-57099736. Max. coverage (+): 0. Max coverage (-): 0.77

Region: chr3 57099737-57099749. Max. coverage (+): 0. Max coverage (-): 0

Region: chr3 57099750-57099762. Max. coverage (+): 0. Max coverage (-): 0

Region: chr3 57099763-57099776. Max. coverage (+): 0. Max coverage (-): 0

Region: chr3 57099777-57099789. Max. coverage (+): 0. Max coverage (-): 6.47

Region: chr3 57099790-57099802. Max. coverage (+): 0. Max coverage (-): 0.57

Region: chr3 57099803-57099815. Max. coverage (+): 0. Max coverage (-): 0

Region: chr3 57099816-57099828. Max. coverage (+): 0. Max coverage (-): 2.27

Region: chr3 57099829-57099842. Max. coverage (+): 0. Max coverage (-): 2.27

Region: chr3 57099843-57099855. Max. coverage (+): 0. Max coverage (-): 1.17

Region: chr3 57099856-57099868. Max. coverage (+): 0. Max coverage (-): 0

Region: chr3 57099869-57099881. Max. coverage (+): 0. Max coverage (-): 0

Region: chr3 57099882-57099894. Max. coverage (+): 0. Max coverage (-): 0

Region: chr3 57099895-57099908. Max. coverage (+): 0. Max coverage (-): 0

Region: chr3 57099909-57099921. Max. coverage (+): 0. Max coverage (-): 0

Region: chr3 57099922-57099934. Max. coverage (+): 0. Max coverage (-): 0

Region: chr3 57099935-57099947. Max. coverage (+): 0. Max coverage (-): 0

Region: chr3 57099948-57099960. Max. coverage (+): 0. Max coverage (-): 0

Region: chr3 57099961-57099974. Max. coverage (+): 0. Max coverage (-): 0

Region: chr3 57099975-57099987. Max. coverage (+): 0. Max coverage (-): 0

Region: chr3 57099988-57100000. Max. coverage (+): 0. Max coverage (-): 3.96

Region: chr3 57100001-57100013. Max. coverage (+): 0. Max coverage (-): 1.23

Region: chr3 57100014-57100026. Max. coverage (+): 0. Max coverage (-): 0

Region: chr3 57100027-57100040. Max. coverage (+): 0. Max coverage (-): 0

Region: chr3 57100041-57100053. Max. coverage (+): 0. Max coverage (-): 0

Region: chr3 57100054-57100066. Max. coverage (+): 0. Max coverage (-): 0

Region: chr3 57100067-57100079. Max. coverage (+): 0. Max coverage (-): 2.05

Region: chr3 57100080-57100092. Max. coverage (+): 0. Max coverage (-): 8.8

Region: chr3 57100093-57100106. Max. coverage (+): 0. Max coverage (-): 2.14

Region: chr3 57100107-57100119. Max. coverage (+): 0. Max coverage (-): 0

Region: chr3 57100120-57100132. Max. coverage (+): 0. Max coverage (-): 0

Region: chr3 57100133-57100145. Max. coverage (+): 0. Max coverage (-): 0

Region: chr3 57100146-57100158. Max. coverage (+): 0. Max coverage (-): 0

Region: chr3 57100159-57100172. Max. coverage (+): 0. Max coverage (-): 0

Region: chr3 57100173-57100185. Max. coverage (+): 0. Max coverage (-): 0

Region: chr3 57100186-57100198. Max. coverage (+): 0. Max coverage (-): 0

Region: chr3 57100199-57100211. Max. coverage (+): 0. Max coverage (-): 0

Region: chr3 57100212-57100224. Max. coverage (+): 0. Max coverage (-): 0

Region: chr3 57100225-57100238. Max. coverage (+): 0. Max coverage (-): 0

Region: chr3 57100239-57100251. Max. coverage (+): 0. Max coverage (-): 0

Region: chr3 57100252-57100264. Max. coverage (+): 0. Max coverage (-): 0

Region: chr3 57100265-57100277. Max. coverage (+): 0. Max coverage (-): 0

Region: chr3 57100278-57100290. Max. coverage (+): 0. Max coverage (-): 0

Region: chr3 57100291-57100304. Max. coverage (+): 0. Max coverage (-): 0

Region: chr3 57100305-57100317. Max. coverage (+): 0. Max coverage (-): 0

Region: chr3 57100318-57100330. Max. coverage (+): 0. Max coverage (-): 0

Region: chr3 57100331-57100343. Max. coverage (+): 0. Max coverage (-): 0

Region: chr3 57100344-57100356. Max. coverage (+): 0. Max coverage (-): 0

Region: chr3 57100357-57100370. Max. coverage (+): 0. Max coverage (-): 0

Region: chr3 57100371-57100383. Max. coverage (+): 0. Max coverage (-): 0

Region: chr3 57100384-57100396. Max. coverage (+): 0. Max coverage (-): 0

Region: chr3 57100397-57100409. Max. coverage (+): 0. Max coverage (-): 0

Region: chr3 57100410-57100422. Max. coverage (+): 0. Max coverage (-): 0

Region: chr3 57100423-57100436. Max. coverage (+): 0. Max coverage (-): 0

Region: chr3 57100437-57100449. Max. coverage (+): 0. Max coverage (-): 3.95

Region: chr3 57100450-57100462. Max. coverage (+): 0. Max coverage (-): 8.52

Region: chr3 57100463-57100475. Max. coverage (+): 0. Max coverage (-): 4.13

Region: chr3 57100476-57100488. Max. coverage (+): 0. Max coverage (-): 0

Region: chr3 57100489-57100502. Max. coverage (+): 0. Max coverage (-): 0

Region: chr3 57100503-57100515. Max. coverage (+): 0. Max coverage (-): 0

Region: chr3 57100516-57100528. Max. coverage (+): 0. Max coverage (-): 0

Region: chr3 57100529-57100541. Max. coverage (+): 0. Max coverage (-): 0

Region: chr3 57100542-57100554. Max. coverage (+): 0. Max coverage (-): 0

Region: chr3 57100555-57100568. Max. coverage (+): 0. Max coverage (-): 0

Region: chr3 57100569-57100581. Max. coverage (+): 0. Max coverage (-): 0

Region: chr3 57100582-57100594. Max. coverage (+): 0. Max coverage (-): 0

Region: chr3 57100595-57100607. Max. coverage (+): 0. Max coverage (-): 0

Region: chr3 57100608-57100620. Max. coverage (+): 0. Max coverage (-): 0

Region: chr3 57100621-57100634. Max. coverage (+): 0. Max coverage (-): 0

Region: chr3 57100635-57100647. Max. coverage (+): 0. Max coverage (-): 0

Region: chr3 57100648-57100660. Max. coverage (+): 0. Max coverage (-): 0

Region: chr3 57100661-57100673. Max. coverage (+): 0. Max coverage (-): 3.78

Region: chr3 57100674-57100686. Max. coverage (+): 0. Max coverage (-): 2.98

Region: chr3 57100687-57100700. Max. coverage (+): 0. Max coverage (-): 0.4

Region: chr3 57100701-57100713. Max. coverage (+): 0. Max coverage (-): 0.4

Region: chr3 57100714-57100726. Max. coverage (+): 0. Max coverage (-): 15.18

Region: chr3 57100727-57100739. Max. coverage (+): 0. Max coverage (-): 2.33

Region: chr3 57100740-57100752. Max. coverage (+): 0. Max coverage (-): 3.69

Region: chr3 57100753-57100766. Max. coverage (+): 0. Max coverage (-): 4.58

Region: chr3 57100767-57100779. Max. coverage (+): 0. Max coverage (-): 4.58

Region: chr3 57100780-57100792. Max. coverage (+): 0. Max coverage (-): 2.62

Region: chr3 57100793-57100805. Max. coverage (+): 0. Max coverage (-): 0

Region: chr3 57100806-57100818. Max. coverage (+): 0. Max coverage (-): 0

Region: chr3 57100819-57100832. Max. coverage (+): 0. Max coverage (-): 4.78

Region: chr3 57100833-57100845. Max. coverage (+): 0. Max coverage (-): 0

Region: chr3 57100846-57100858. Max. coverage (+): 0. Max coverage (-): 9.36

Region: chr3 57100859-57100871. Max. coverage (+): 0. Max coverage (-): 9.36

Region: chr3 57100872-57100884. Max. coverage (+): 0. Max coverage (-): 0.54

Region: chr3 57100885-57100898. Max. coverage (+): 0. Max coverage (-): 0

Region: chr3 57100899-57100911. Max. coverage (+): 0. Max coverage (-): 1.68

Region: chr3 57100912-57100924. Max. coverage (+): 0. Max coverage (-): 5.51

Region: chr3 57100925-57100937. Max. coverage (+): 0. Max coverage (-): 26.49

Region: chr3 57100938-57100950. Max. coverage (+): 0. Max coverage (-): 24.06

Region: chr3 57100951-57100964. Max. coverage (+): 0. Max coverage (-): 1.6

Region: chr3 57100965-57100977. Max. coverage (+): 0. Max coverage (-): 2.06

Region: chr3 57100978-57100990. Max. coverage (+): 0. Max coverage (-): 0

Region: chr3 57100991-57101003. Max. coverage (+): 0. Max coverage (-): 0

Region: chr3 57101004-57101016. Max. coverage (+): 0. Max coverage (-): 7.49

Region: chr3 57101017-57101030. Max. coverage (+): 0. Max coverage (-): 3.97

Region: chr3 57101031-57101043. Max. coverage (+): 0. Max coverage (-): 0

Region: chr3 57101044-57101056. Max. coverage (+): 0. Max coverage (-): 13.2

Region: chr3 57101057-57101069. Max. coverage (+): 0. Max coverage (-): 24.56

Region: chr3 57101070-57101082. Max. coverage (+): 0. Max coverage (-): 9.78

Region: chr3 57101083-57101096. Max. coverage (+): 0. Max coverage (-): 7.81

Region: chr3 57101097-57101109. Max. coverage (+): 0. Max coverage (-): 4.73

Region: chr3 57101110-57101122. Max. coverage (+): 0. Max coverage (-): 6.09

Region: chr3 57101123-57101135. Max. coverage (+): 0. Max coverage (-): 0

Region: chr3 57101136-57101148. Max. coverage (+): 0. Max coverage (-): 2.23

Region: chr3 57101149-57101162. Max. coverage (+): 0. Max coverage (-): 2.23

Region: chr3 57101163-57101175. Max. coverage (+): 0. Max coverage (-): 0

Region: chr3 57101176-57101188. Max. coverage (+): 0. Max coverage (-): 0

Region: chr3 57101189-57101201. Max. coverage (+): 0. Max coverage (-): 13.49

Region: chr3 57101202-57101214. Max. coverage (+): 0. Max coverage (-): 0

Region: chr3 57101215-57101228. Max. coverage (+): 0. Max coverage (-): 12.61

Region: chr3 57101229-57101241. Max. coverage (+): 0. Max coverage (-): 53.08

Region: chr3 57101242-57101254. Max. coverage (+): 0. Max coverage (-): 30.44

Region: chr3 57101255-57101267. Max. coverage (+): 0. Max coverage (-): 21.82

Region: chr3 57101268-57101280. Max. coverage (+): 0. Max coverage (-): 4.84

Region: chr3 57101281-57101294. Max. coverage (+): 0. Max coverage (-): 29.26

Region: chr3 57101295-57101307. Max. coverage (+): 0. Max coverage (-): 14.46

Region: chr3 57101308-57101320. Max. coverage (+): 0. Max coverage (-): 6.07

Region: chr3 57101321-57101333. Max. coverage (+): 0. Max coverage (-): 17.65

Region: chr3 57101334-57101346. Max. coverage (+): 0. Max coverage (-): 3.16

Region: chr3 57101347-57101360. Max. coverage (+): 0. Max coverage (-): 0

Region: chr3 57101361-57101373. Max. coverage (+): 4.01. Max coverage (-): 0

Region: chr3 57101374-57101386. Max. coverage (+): 0. Max coverage (-): 4.9

Region: chr3 57101387-57101399. Max. coverage (+): 0. Max coverage (-): 10.01

Region: chr3 57101400-57101412. Max. coverage (+): 0. Max coverage (-): 10.01

Region: chr3 57101413-57101426. Max. coverage (+): 0. Max coverage (-): 13.59

Region: chr3 57101427-57101439. Max. coverage (+): 0. Max coverage (-): 0

Region: chr3 57101440-57101452. Max. coverage (+): 0. Max coverage (-): 0

Region: chr3 57101453-57101465. Max. coverage (+): 0. Max coverage (-): 0

Region: chr3 57101466-57101478. Max. coverage (+): 0. Max coverage (-): 0.82

Region: chr3 57101479-57101492. Max. coverage (+): 0. Max coverage (-): 0

Region: chr3 57101493-57101505. Max. coverage (+): 0. Max coverage (-): 2.2

Region: chr3 57101506-57101518. Max. coverage (+): 0. Max coverage (-): 3.48

Region: chr3 57101519-57101531. Max. coverage (+): 0. Max coverage (-): 0

Region: chr3 57101532-57101544. Max. coverage (+): 0. Max coverage (-): 0

Region: chr3 57101545-57101558. Max. coverage (+): 0. Max coverage (-): 10.71

Region: chr3 57101559-57101571. Max. coverage (+): 0. Max coverage (-): 4.78

Region: chr3 57101572-57101584. Max. coverage (+): 0. Max coverage (-): 4.78

Region: chr3 57101585-57101597. Max. coverage (+): 0. Max coverage (-): 1.3

Region: chr3 57101598-57101610. Max. coverage (+): 0. Max coverage (-): 0

Region: chr3 57101611-57101624. Max. coverage (+): 0. Max coverage (-): 0

Region: chr3 57101625-57101637. Max. coverage (+): 0. Max coverage (-): 3.59

Region: chr3 57101638-57101650. Max. coverage (+): 0. Max coverage (-): 3.59

Region: chr3 57101651-57101663. Max. coverage (+): 0. Max coverage (-): 5.21

Region: chr3 57101664-57101676. Max. coverage (+): 0. Max coverage (-): 1

Region: chr3 57101677-57101690. Max. coverage (+): 0. Max coverage (-): 0

Region: chr3 57101691-57101703. Max. coverage (+): 0. Max coverage (-): 2.07

Region: chr3 57101704-57101716. Max. coverage (+): 0. Max coverage (-): 0.32

Region: chr3 57101717-57101729. Max. coverage (+): 0. Max coverage (-): 0.32

Region: chr3 57101730-57101742. Max. coverage (+): 0. Max coverage (-): 0

Region: chr3 57101743-57101756. Max. coverage (+): 0. Max coverage (-): 15.3

Region: chr3 57101757-57101769. Max. coverage (+): 0. Max coverage (-): 19.16

Region: chr3 57101770-57101782. Max. coverage (+): 0. Max coverage (-): 1.88

Region: chr3 57101783-57101795. Max. coverage (+): 0. Max coverage (-): 0

Region: chr3 57101796-57101808. Max. coverage (+): 0. Max coverage (-): 0

Region: chr3 57101809-57101822. Max. coverage (+): 0. Max coverage (-): 0

Region: chr3 57101823-57101835. Max. coverage (+): 0. Max coverage (-): 0

Region: chr3 57101836-57101848. Max. coverage (+): 0. Max coverage (-): 5.48

Region: chr3 57101849-57101861. Max. coverage (+): 0. Max coverage (-): 6.87

Region: chr3 57101862-57101874. Max. coverage (+): 0. Max coverage (-): 5.46

Region: chr3 57101875-57101888. Max. coverage (+): 0. Max coverage (-): 5.46

Region: chr3 57101889-57101901. Max. coverage (+): 0. Max coverage (-): 1.15

Region: chr3 57101902-57101914. Max. coverage (+): 0. Max coverage (-): 0

Region: chr3 57101915-57101927. Max. coverage (+): 0. Max coverage (-): 0

Region: chr3 57101928-57101940. Max. coverage (+): 0. Max coverage (-): 0.43

Region: chr3 57101941-57101954. Max. coverage (+): 0. Max coverage (-): 1.39

Region: chr3 57101955-57101967. Max. coverage (+): 0. Max coverage (-): 11.9

Region: chr3 57101968-57101980. Max. coverage (+): 0. Max coverage (-): 11.9

Region: chr3 57101981-57101993. Max. coverage (+): 0. Max coverage (-): 1.37

Region: chr3 57101994-57102006. Max. coverage (+): 0. Max coverage (-): 4.26

Region: chr3 57102007-57102020. Max. coverage (+): 0. Max coverage (-): 4.26

Region: chr3 57102021-57102033. Max. coverage (+): 0. Max coverage (-): 4.29

Region: chr3 57102034-57102046. Max. coverage (+): 0. Max coverage (-): 4.29

Region: chr3 57102047-57102059. Max. coverage (+): 0. Max coverage (-): 0

Region: chr3 57102060-57102072. Max. coverage (+): 0. Max coverage (-): 0

Region: chr3 57102073-57102086. Max. coverage (+): 0. Max coverage (-): 2.25

Region: chr3 57102087-57102099. Max. coverage (+): 0. Max coverage (-): 2.25

Region: chr3 57102100-57102112. Max. coverage (+): 0. Max coverage (-): 4.23

Region: chr3 57102113-57102125. Max. coverage (+): 0. Max coverage (-): 6.17

Region: chr3 57102126-57102138. Max. coverage (+): 0. Max coverage (-): 8.18

Region: chr3 57102139-57102152. Max. coverage (+): 0. Max coverage (-): 18.12

Region: chr3 57102153-57102165. Max. coverage (+): 0. Max coverage (-): 1.03

Region: chr3 57102166-57102178. Max. coverage (+): 0. Max coverage (-): 0.39

Region: chr3 57102179-57102191. Max. coverage (+): 0. Max coverage (-): 0.39

Region: chr3 57102192-57102204. Max. coverage (+): 0. Max coverage (-): 0

Region: chr3 57102205-57102218. Max. coverage (+): 0. Max coverage (-): 1.05

Region: chr3 57102219-57102231. Max. coverage (+): 0. Max coverage (-): 1.13

Region: chr3 57102232-57102244. Max. coverage (+): 0. Max coverage (-): 1.13

Region: chr3 57102245-57102257. Max. coverage (+): 0. Max coverage (-): 3.28

Region: chr3 57102258-57102270. Max. coverage (+): 0. Max coverage (-): 2.44

Region: chr3 57102271-57102284. Max. coverage (+): 0. Max coverage (-): 3.68

Region: chr3 57102285-57102297. Max. coverage (+): 0. Max coverage (-): 1.56

Region: chr3 57102298-57102310. Max. coverage (+): 0. Max coverage (-): 9.06

Region: chr3 57102311-57102323. Max. coverage (+): 0. Max coverage (-): 9.06

Region: chr3 57102324-57102336. Max. coverage (+): 0. Max coverage (-): 0

Region: chr3 57102337-57102350. Max. coverage (+): 0. Max coverage (-): 1.17

Region: chr3 57102351-57102363. Max. coverage (+): 0. Max coverage (-): 0

Region: chr3 57102364-57102376. Max. coverage (+): 0. Max coverage (-): 0

Region: chr3 57102377-57102389. Max. coverage (+): 0. Max coverage (-): 0

Region: chr3 57102390-57102402. Max. coverage (+): 0. Max coverage (-): 0

Region: chr3 57102403-57102416. Max. coverage (+): 0. Max coverage (-): 0

Region: chr3 57102417-57102429. Max. coverage (+): 0. Max coverage (-): 0

Region: chr3 57102430-57102442. Max. coverage (+): 0. Max coverage (-): 0

Region: chr3 57102443-57102455. Max. coverage (+): 0. Max coverage (-): 0

Region: chr3 57102456-57102468. Max. coverage (+): 0. Max coverage (-): 0

Region: chr3 57102469-57102482. Max. coverage (+): 0. Max coverage (-): 0

Region: chr3 57102483-57102495. Max. coverage (+): 0. Max coverage (-): 0

Region: chr3 57102496-57102508. Max. coverage (+): 0. Max coverage (-): 0

Region: chr3 57102509-57102521. Max. coverage (+): 0. Max coverage (-): 0

Region: chr3 57102522-57102534. Max. coverage (+): 0. Max coverage (-): 0

Region: chr3 57102535-57102548. Max. coverage (+): 0. Max coverage (-): 0

Region: chr3 57102549-57102561. Max. coverage (+): 0. Max coverage (-): 0

Region: chr3 57102562-57102574. Max. coverage (+): 0. Max coverage (-): 0

Region: chr3 57102575-57102587. Max. coverage (+): 0. Max coverage (-): 0

Region: chr3 57102588-57102600. Max. coverage (+): 0. Max coverage (-): 0

Region: chr3 57102601-57102614. Max. coverage (+): 0. Max coverage (-): 0

Region: chr3 57102615-57102627. Max. coverage (+): 0. Max coverage (-): 0

Region: chr3 57102628-57102640. Max. coverage (+): 0. Max coverage (-): 0

Region: chr3 57102641-57102653. Max. coverage (+): 0. Max coverage (-): 0

Region: chr3 57102654-57102666. Max. coverage (+): 0. Max coverage (-): 0

Region: chr3 57102667-57102680. Max. coverage (+): 0. Max coverage (-): 1.69

Region: chr3 57102681-57102693. Max. coverage (+): 0. Max coverage (-): 1.69

Region: chr3 57102694-57102706. Max. coverage (+): 0. Max coverage (-): 3.68

Region: chr3 57102707-57102719. Max. coverage (+): 0. Max coverage (-): 3.68

Region: chr3 57102720-57102732. Max. coverage (+): 0. Max coverage (-): 0

Region: chr3 57102733-57102746. Max. coverage (+): 0. Max coverage (-): 0

Region: chr3 57102747-57102759. Max. coverage (+): 0. Max coverage (-): 1

Region: chr3 57102760-57102772. Max. coverage (+): 0. Max coverage (-): 1.84

Region: chr3 57102773-57102785. Max. coverage (+): 0. Max coverage (-): 1.65

Region: chr3 57102786-57102798. Max. coverage (+): 0. Max coverage (-): 0

Region: chr3 57102799-57102812. Max. coverage (+): 0. Max coverage (-): 0

Region: chr3 57102813-57102825. Max. coverage (+): 0. Max coverage (-): 14.59

Region: chr3 57102826-57102838. Max. coverage (+): 0. Max coverage (-): 0

Region: chr3 57102839-57102851. Max. coverage (+): 0. Max coverage (-): 13.21

Region: chr3 57102852-57102864. Max. coverage (+): 0. Max coverage (-): 18.06

Region: chr3 57102865-57102878. Max. coverage (+): 0. Max coverage (-): 8.03

Region: chr3 57102879-57102891. Max. coverage (+): 0. Max coverage (-): 6.88

Region: chr3 57102892-57102904. Max. coverage (+): 0. Max coverage (-): 6.88

Region: chr3 57102905-57102917. Max. coverage (+): 0. Max coverage (-): 0

Region: chr3 57102918-57102930. Max. coverage (+): 0. Max coverage (-): 11.81

Region: chr3 57102931-57102944. Max. coverage (+): 0. Max coverage (-): 8.44

Region: chr3 57102945-57102957. Max. coverage (+): 0. Max coverage (-): 2.36

Region: chr3 57102958-57102970. Max. coverage (+): 0. Max coverage (-): 0.45

Region: chr3 57102971-57102983. Max. coverage (+): 0. Max coverage (-): 0.45

Region: chr3 57102984-57102996. Max. coverage (+): 0. Max coverage (-): 19.75

Region: chr3 57102997-57103010. Max. coverage (+): 0. Max coverage (-): 19.75

Region: chr3 57103011-57103023. Max. coverage (+): 0. Max coverage (-): 0

Region: chr3 57103024-57103036. Max. coverage (+): 0. Max coverage (-): 0

Region: chr3 57103037-57103049. Max. coverage (+): 0. Max coverage (-): 12

Region: chr3 57103050-57103062. Max. coverage (+): 0. Max coverage (-): 12

Region: chr3 57103063-57103076. Max. coverage (+): 0. Max coverage (-): 0

Region: chr3 57103077-57103089. Max. coverage (+): 0. Max coverage (-): 6.36

Region: chr3 57103090-57103102. Max. coverage (+): 0. Max coverage (-): 6.36

Region: chr3 57103103-57103115. Max. coverage (+): 0. Max coverage (-): 4.35

Region: chr3 57103116-57103128. Max. coverage (+): 0. Max coverage (-): 0.89

Region: chr3 57103129-57103142. Max. coverage (+): 0. Max coverage (-): 2.26

Region: chr3 57103143-57103155. Max. coverage (+): 0. Max coverage (-): 2.26

Region: chr3 57103156-57103168. Max. coverage (+): 0. Max coverage (-): 16.52

Region: chr3 57103169-57103181. Max. coverage (+): 0. Max coverage (-): 11.25

Region: chr3 57103182-57103194. Max. coverage (+): 0. Max coverage (-): 3.58

Region: chr3 57103195-57103208. Max. coverage (+): 0. Max coverage (-): 4.15

Region: chr3 57103209-57103221. Max. coverage (+): 0. Max coverage (-): 0.89

Region: chr3 57103222-57103234. Max. coverage (+): 0. Max coverage (-): 0

Region: chr3 57103235-57103247. Max. coverage (+): 0. Max coverage (-): 3.32

Region: chr3 57103248-57103260. Max. coverage (+): 0. Max coverage (-): 6.21

Region: chr3 57103261-57103274. Max. coverage (+): 0. Max coverage (-): 8.97

Region: chr3 57103275-57103287. Max. coverage (+): 0. Max coverage (-): 5.76

Region: chr3 57103288-57103300. Max. coverage (+): 0. Max coverage (-): 0

Region: chr3 57103301-57103313. Max. coverage (+): 0. Max coverage (-): 0

Region: chr3 57103314-57103326. Max. coverage (+): 0. Max coverage (-): 0

Region: chr3 57103327-57103340. Max. coverage (+): 0. Max coverage (-): 0

Region: chr3 57103341-57103353. Max. coverage (+): 0. Max coverage (-): 0

Region: chr3 57103354-57103366. Max. coverage (+): 0. Max coverage (-): 0

Region: chr3 57103367-57103379. Max. coverage (+): 0. Max coverage (-): 0.51

Region: chr3 57103380-57103392. Max. coverage (+): 0. Max coverage (-): 0

Region: chr3 57103393-57103406. Max. coverage (+): 0. Max coverage (-): 0

Region: chr3 57103407-57103419. Max. coverage (+): 0. Max coverage (-): 6.45

Region: chr3 57103420-57103432. Max. coverage (+): 0. Max coverage (-): 3.14

Region: chr3 57103433-57103445. Max. coverage (+): 0. Max coverage (-): 5.23

Region: chr3 57103446-57103458. Max. coverage (+): 0. Max coverage (-): 5.23

Region: chr3 57103459-57103472. Max. coverage (+): 0. Max coverage (-): 0

Region: chr3 57103473-57103485. Max. coverage (+): 0. Max coverage (-): 0

Region: chr3 57103486-57103498. Max. coverage (+): 0. Max coverage (-): 0

Region: chr3 57103499-57103511. Max. coverage (+): 0. Max coverage (-): 0

Region: chr3 57103512-57103524. Max. coverage (+): 0. Max coverage (-): 0.37

Region: chr3 57103525-57103538. Max. coverage (+): 0. Max coverage (-): 3.6

Region: chr3 57103539-57103551. Max. coverage (+): 0. Max coverage (-): 13.07

Region: chr3 57103552-57103564. Max. coverage (+): 0. Max coverage (-): 12.91

Region: chr3 57103565-57103577. Max. coverage (+): 0. Max coverage (-): 0

Region: chr3 57103578-57103590. Max. coverage (+): 0. Max coverage (-): 0

Region: chr3 57103591-57103604. Max. coverage (+): 0. Max coverage (-): 0

Region: chr3 57103605-57103617. Max. coverage (+): 0. Max coverage (-): 0

Region: chr3 57103618-57103630. Max. coverage (+): 0. Max coverage (-): 0

Region: chr3 57103631-57103643. Max. coverage (+): 0. Max coverage (-): 0

Region: chr3 57103644-57103656. Max. coverage (+): 0. Max coverage (-): 0

Region: chr3 57103657-57103670. Max. coverage (+): 0. Max coverage (-): 0

Region: chr3 57103671-57103683. Max. coverage (+): 0. Max coverage (-): 0

Region: chr3 57103684-57103696. Max. coverage (+): 0. Max coverage (-): 0

Region: chr3 57103697-57103709. Max. coverage (+): 0. Max coverage (-): 0

Region: chr3 57103710-57103722. Max. coverage (+): 0. Max coverage (-): 0

Region: chr3 57103723-57103736. Max. coverage (+): 0. Max coverage (-): 0

Region: chr3 57103737-57103749. Max. coverage (+): 0. Max coverage (-): 0

Region: chr3 57103750-57103762. Max. coverage (+): 0. Max coverage (-): 0

Region: chr3 57103763-57103775. Max. coverage (+): 0. Max coverage (-): 0

Region: chr3 57103776-57103788. Max. coverage (+): 0. Max coverage (-): 0

Region: chr3 57103789-57103802. Max. coverage (+): 0. Max coverage (-): 0

Region: chr3 57103803-57103815. Max. coverage (+): 0. Max coverage (-): 0

Region: chr3 57103816-57103828. Max. coverage (+): 0. Max coverage (-): 0

Region: chr3 57103829-57103841. Max. coverage (+): 0. Max coverage (-): 0

Region: chr3 57103842-57103854. Max. coverage (+): 0. Max coverage (-): 0

Region: chr3 57103855-57103868. Max. coverage (+): 0. Max coverage (-): 0

Region: chr3 57103869-57103881. Max. coverage (+): 0. Max coverage (-): 0

Region: chr3 57103882-57103894. Max. coverage (+): 0. Max coverage (-): 0

Region: chr3 57103895-57103907. Max. coverage (+): 0. Max coverage (-): 0

Region: chr3 57103908-57103920. Max. coverage (+): 0. Max coverage (-): 0

Region: chr3 57103921-57103934. Max. coverage (+): 0. Max coverage (-): 11.81

Region: chr3 57103935-57103947. Max. coverage (+): 0. Max coverage (-): 2.23

Region: chr3 57103948-57103960. Max. coverage (+): 0. Max coverage (-): 0

Region: chr3 57103961-57103973. Max. coverage (+): 0. Max coverage (-): 4.01

Region: chr3 57103974-57103986. Max. coverage (+): 0. Max coverage (-): 0

Region: chr3 57103987-57104000. Max. coverage (+): 0. Max coverage (-): 0

Region: chr3 57104001-57104013. Max. coverage (+): 0. Max coverage (-): 0

Region: chr3 57104014-57104026. Max. coverage (+): 0. Max coverage (-): 0

Region: chr3 57104027-57104039. Max. coverage (+): 0. Max coverage (-): 0

Region: chr3 57104040-57104052. Max. coverage (+): 0. Max coverage (-): 0

Region: chr3 57104053-57104066. Max. coverage (+): 0. Max coverage (-): 0

Region: chr3 57104067-57104079. Max. coverage (+): 0. Max coverage (-): 0

Region: chr3 57104080-57104092. Max. coverage (+): 0. Max coverage (-): 6.73

Region: chr3 57104093-57104105. Max. coverage (+): 0. Max coverage (-): 19.45

Region: chr3 57104106-57104118. Max. coverage (+): 0. Max coverage (-): 0

Region: chr3 57104119-57104132. Max. coverage (+): 0. Max coverage (-): 0

Region: chr3 57104133-57104145. Max. coverage (+): 0. Max coverage (-): 0

Region: chr3 57104146-57104158. Max. coverage (+): 0. Max coverage (-): 0

Region: chr3 57104159-57104171. Max. coverage (+): 0. Max coverage (-): 17.19

Region: chr3 57104172-57104184. Max. coverage (+): 0. Max coverage (-): 20.73

Region: chr3 57104185-57104198. Max. coverage (+): 0. Max coverage (-): 2.06

Region: chr3 57104199-57104211. Max. coverage (+): 0. Max coverage (-): 2.06

Region: chr3 57104212-57104224. Max. coverage (+): 0. Max coverage (-): 1.96

Region: chr3 57104225-57104237. Max. coverage (+): 0. Max coverage (-): 0

Region: chr3 57104238-57104250. Max. coverage (+): 0. Max coverage (-): 0

Region: chr3 57104251-57104264. Max. coverage (+): 0. Max coverage (-): 0

Region: chr3 57104265-57104277. Max. coverage (+): 0. Max coverage (-): 0

Region: chr3 57104278-57104290. Max. coverage (+): 0. Max coverage (-): 0

Region: chr3 57104291-57104303. Max. coverage (+): 0. Max coverage (-): 0

Region: chr3 57104304-57104316. Max. coverage (+): 0. Max coverage (-): 0

Region: chr3 57104317-57104330. Max. coverage (+): 0. Max coverage (-): 0

Region: chr3 57104331-57104343. Max. coverage (+): 0. Max coverage (-): 0

Region: chr3 57104344-57104356. Max. coverage (+): 0. Max coverage (-): 0

Region: chr3 57104357-57104369. Max. coverage (+): 0. Max coverage (-): 0

Region: chr3 57104370-57104382. Max. coverage (+): 0. Max coverage (-): 0

Region: chr3 57104383-57104396. Max. coverage (+): 0. Max coverage (-): 0

Region: chr3 57104397-57104409. Max. coverage (+): 0. Max coverage (-): 0

Region: chr3 57104410-57104422. Max. coverage (+): 0. Max coverage (-): 0

Region: chr3 57104423-57104435. Max. coverage (+): 0. Max coverage (-): 0

Region: chr3 57104436-57104448. Max. coverage (+): 0. Max coverage (-): 0

Region: chr3 57104449-57104462. Max. coverage (+): 0. Max coverage (-): 0

Region: chr3 57104463-57104475. Max. coverage (+): 0. Max coverage (-): 0

Region: chr3 57104476-57104488. Max. coverage (+): 0. Max coverage (-): 0

Region: chr3 57104489-57104501. Max. coverage (+): 0. Max coverage (-): 0

Region: chr3 57104502-57104514. Max. coverage (+): 0. Max coverage (-): 0

Region: chr3 57104515-57104528. Max. coverage (+): 0. Max coverage (-): 1.31

Region: chr3 57104529-57104541. Max. coverage (+): 0. Max coverage (-): 1.31

Region: chr3 57104542-57104554. Max. coverage (+): 0. Max coverage (-): 0

Region: chr3 57104555-57104567. Max. coverage (+): 0. Max coverage (-): 0

Region: chr3 57104568-57104580. Max. coverage (+): 0. Max coverage (-): 0

Region: chr3 57104581-57104594. Max. coverage (+): 0. Max coverage (-): 0

Region: chr3 57104595-57104607. Max. coverage (+): 0. Max coverage (-): 0

Region: chr3 57104608-57104620. Max. coverage (+): 0. Max coverage (-): 0

Region: chr3 57104621-57104633. Max. coverage (+): 0. Max coverage (-): 0

Region: chr3 57104634-57104646. Max. coverage (+): 0. Max coverage (-): 0

Region: chr3 57104647-57104660. Max. coverage (+): 0. Max coverage (-): 0

Region: chr3 57104661-57104673. Max. coverage (+): 0. Max coverage (-): 0

Region: chr3 57104674-57104686. Max. coverage (+): 0. Max coverage (-): 0

Region: chr3 57104687-57104699. Max. coverage (+): 0. Max coverage (-): 0

Region: chr3 57104700-57104712. Max. coverage (+): 0. Max coverage (-): 0

Region: chr3 57104713-57104726. Max. coverage (+): 0. Max coverage (-): 0

Region: chr3 57104727-57104739. Max. coverage (+): 0. Max coverage (-): 0

Region: chr3 57104740-57104752. Max. coverage (+): 0. Max coverage (-): 0

Region: chr3 57104753-57104765. Max. coverage (+): 0. Max coverage (-): 0

Region: chr3 57104766-57104778. Max. coverage (+): 0. Max coverage (-): 0

Region: chr3 57104779-57104792. Max. coverage (+): 0. Max coverage (-): 0

Region: chr3 57104793-57104805. Max. coverage (+): 0. Max coverage (-): 0

Region: chr3 57104806-57104818. Max. coverage (+): 0. Max coverage (-): 0

Region: chr3 57104819-57104831. Max. coverage (+): 0. Max coverage (-): 0

Region: chr3 57104832-57104844. Max. coverage (+): 0. Max coverage (-): 0

Region: chr3 57104845-57104858. Max. coverage (+): 0. Max coverage (-): 0

Region: chr3 57104859-57104871. Max. coverage (+): 0. Max coverage (-): 0

Region: chr3 57104872-57104884. Max. coverage (+): 0. Max coverage (-): 0

Region: chr3 57104885-57104897. Max. coverage (+): 0. Max coverage (-): 0

Region: chr3 57104898-57104910. Max. coverage (+): 0. Max coverage (-): 0

Region: chr3 57104911-57104924. Max. coverage (+): 0. Max coverage (-): 0

Region: chr3 57104925-57104937. Max. coverage (+): 0. Max coverage (-): 0

Region: chr3 57104938-57104950. Max. coverage (+): 0. Max coverage (-): 2.25

Region: chr3 57104951-57104963. Max. coverage (+): 0. Max coverage (-): 0

Region: chr3 57104964-57104976. Max. coverage (+): 0. Max coverage (-): 0

Region: chr3 57104977-57104990. Max. coverage (+): 0. Max coverage (-): 0

Region: chr3 57104991-57105003. Max. coverage (+): 0. Max coverage (-): 0

Region: chr3 57105004-57105016. Max. coverage (+): 0. Max coverage (-): 0

Region: chr3 57105017-57105029. Max. coverage (+): 0. Max coverage (-): 0

Region: chr3 57105030-57105042. Max. coverage (+): 0. Max coverage (-): 0

Region: chr3 57105043-57105056. Max. coverage (+): 0. Max coverage (-): 0

Region: chr3 57105057-57105069. Max. coverage (+): 0. Max coverage (-): 0

Region: chr3 57105070-57105082. Max. coverage (+): 0. Max coverage (-): 0

Region: chr3 57105083-57105095. Max. coverage (+): 0. Max coverage (-): 0

Region: chr3 57105096-57105108. Max. coverage (+): 0. Max coverage (-): 1.2

Region: chr3 57105109-57105122. Max. coverage (+): 0. Max coverage (-): 3.93

Region: chr3 57105123-57105135. Max. coverage (+): 0. Max coverage (-): 0

Region: chr3 57105136-. Max. coverage (+): 0. Max coverage (-): 0

RepeatMasker Color Code

**+**

100-98% Identity

<98-95% Identity

<95-90% Identity

<90-85% Identity

<85-80% Identity

<80-75% Identity

<75-70% Identity

<70% Identity

**-**

Gene Set Color Code

**+**

Gene

Pseudogene

**-**

Topology/Coverage Color Code

Coverage Plus Strand

Coverage Minus Strand

Mainstrand: Plus

Mainstrand: Minus

Complementary Strand

Flanking Region  
(if option -flank >0)

Gene Set Annotation  

**1. (protein coding, ENSBTAG00000017595) Tr:00000023395 Ex:8**: 57105060-57105286 (-)

  
RepeatMasker Annotation  

**1. MIRc**: 57099478-57099731 (-), Divergence to consensus: 18.2%  
**2. L1\_BT**: 57102370-57102390 (+), Divergence to consensus: 24.8%  
**3. (GGGAGA)n**: 57102391-57102411 (+), Divergence to consensus: 0%  
**4. L1\_BT**: 57102412-57102679 (+), Divergence to consensus: 24.8%  
**5. ART2A**: 57103620-57103918 (+), Divergence to consensus: 21.2%

  
Transcription Factor Binding Sites  

**RFX4\_2** (Sequence: CTTAGATAC (+): 57104431)
