# Supplementary material for: Small RNA sequencing of cryopreserved semen from single bull revealed altered miRNAs and piRNAs expression between High- and Low-motile sperm populations
Source: BMC Genomics. 2017 Jan 4;18:14. doi: 10.1186/s12864-016-3394-7 (PMC5209821; doi:10.1186/s12864-016-3394-7)
Supplement: Additional file 3: — Details for each piRNA clusters found in High Motile (HM) sperm fraction. Genes, repeats, transposable elements and transcription factors binding sites falling within the cluster regions were reported. (ZIP 1896 kb) [file 12864_2016_3394_MOESM3_ESM.zip › 79.html]

piRNA cluster 79


Predicted piRNA cluster no. 79     previous   next
  

Show proTRAC run info
Hide proTRAC run info

================================= proTRAC ====================================  
VERSION: 2.1                                    LAST MODIFIED: 06. October 2015  
  
Please cite:  
Rosenkranz D, Zischler H. proTRAC - a software for probabilistic piRNA cluster  
detection, visualization and analysis. 2012. BMC Bioinformatics 13:5.  
  
and (for proTRAC 2.0 and later):  
Rosenkranz D, Rudloff S, Bastuck K, Ketting RF, Zischler H. Tupaia small RNAs  
provide insights into function and evolution of RNAi-based transposon defense  
in mammals. 2015. RNA 21(5):911-922.  
  
Contact:  
David Rosenkranz  
Institute of Anthropology, small RNA group  
Johannes Gutenberg University Mainz  
email: rosenkranz@uni-mainz.de  
  
You can find the latest proTRAC version at:  
http://sourceforge.net/projects/protrac/files  
http://www.smallRNAgroup-mainz.de/software  
==============================================================================  
  
PARAMETERS:  
Map file: .............../storage/core/barbara/genhome/smallRNA/fertility/Sample\_motile/pirna/Sample\_motile\_26-33\_collapsed.fa.no-dust.map.weighted-10000-1000-b-0  
Genome file: ............/storage/core/barbara/genhome/smallRNA/fertility/Sample\_all/pirna/bt\_311\_chrY.fa  
RepeatMasker annotation: /storage/genomes/bt\_umd31/GCF\_000003055.6\_Bos\_taurus\_UMD\_3.1.1\_repeatMasker\_chr.out  
GeneSet:................./storage/core/barbara/genhome/smallRNA/fertility/Sample\_all/pirna/full.gtf  
  
Significant (p<=0.01) hit density will be calculated based  
on observed hit distribution.  
  
Sliding window size: ........................................ 5000 bp  
Sliding window increament: .................................. 1000 bp  
Normalize each hit by number of genomic hits: ............... 1 [0=no/1=yes]  
Normalize each hit by number of sequence reads: ............. 1 [0=no/1=yes]  
Normalize values (-> per million mapped reads): ............. 1 [0=no/1=yes]  
Min. fraction of hits with 1T(U) or 10A: .................... 0.75  
Alternatively: Min. fraction of hits with 1T(U) and 10A: .... 0.5  
Min. fraction of hits with typical piRNA length: ............ 0.75  
Typical piRNA length: ....................................... 26-33 nt  
Min. size of a piRNA cluster: ............................... 5000 bp.  
Min. number of hits (absolute): ............................. 0  
Min. number of hits (normalized): ........................... 0  
Min. fraction of hits on the mainstrand: .................... 0.75  
Top fraction of mapped sequences (in terms of read counts): . 1%  
Top fraction accounts for max. n% of sequence reads: ........ 90%  
Min. fraction of hits on each arm of a bidirectional cluster: 0.1  
Output image file for each cluster: ......................... 0 [0=no/1=yes]  
Output html file for each cluster: .......................... 1 [0=no/1=yes]  
Output a summary table: ..................................... 1 [0=no/1=yes]  
Output a FASTA file for each cluster (piRNA sequences): ..... 1 [0=no/1=yes]  
Output a FASTA file comprising cluster sequences: ........... 1 [0=no/1=yes]  
Search DNA motifs in clusters: .............................. 1 [0=no/1=yes]  
Output flanking sequences: +/- .............................. 0 bp  
Output ~.pTi file: .......................................... 1 [0=no/1=yes]  
==============================================================================  
  
  
Genome size (without gaps): ............ 2678902517 bp  
Gaps (N/X/-): .......................... 53837044 bp  
Mapped reads: .......................... 658825247023  
Non-identical sequences: ............... 514171  
Genomic hits: .......................... 764233  
Significant densitiy of mapped reads: .. 12867599.5173724 reads/kb

Show proTRAC cluster info
Hide proTRAC cluster info

|  |  |
| --- | --- |
| Location | chr3 |
| Coordinates | 94040986-94049385 |
| Size [bp] | 8400 |
| Sequence hit loci | 973 |
| Mapped reads (normalized) | 1155305149 |
| Mapped reads (normalized) per kb | 137536327.3 |
| Normalized reads with 1T (1U) | 82.7% |
| Normalized reads with 10A | 22.3% |
| Normalized reads with length 26-33 nt | 100% |
| Normalized reads on the main strand(s) | 100% |
| Predicted directionality | mono:minus |

100%

0%

1T (1U)  
reads

10A reads

26-33 nt  
reads

reads on mainstrand

**Either the amount of reads with 1T (1U) OR 10A has to exceed 75% (set with option: -1Tor10A)  
Alternatively the amount of reads with 1T (1U) AND 10A has to exceed 50% (set with option: -1Tand10A)  
Minimum amount of reads with preferred size is 75% (set with option: -pisize)  
Minimum amount of reads on the main strand(s) is 75% (set with option: -clstrand)**

Show read coverage
Hide read coverage

WHAT DO I SEE HERE?  
This chart shows the location of mapped sequence reads within a predicted piRNA cluster. The color refers to the number of genomic hits produced by the sequence read in question. A dark red bar indicates that this sequence read produces many other hits elsewhere in the genome. Many adjacent red or yellow bars can indicate the presence of a multi-copy element such as transposons or rRNA genes. A dark green bar indicates that this sequence read maps uniquely to this locus.

1 hit

2-5 hits

6-10 hits

11-20 hits

21-50 hits

51-100 hits

> 100 hits

chr3

94040986

94049385

Gene Set

RepeatMasker

Mapped  
Reads

51.54

plus strand

minus strand

51.54

Region: chr3 57105137-94040994. Max. coverage (+): 0. Max coverage (-): 1.58

Region: chr3 94040995-94041011. Max. coverage (+): 0. Max coverage (-): 0

Region: chr3 94041012-94041027. Max. coverage (+): 0. Max coverage (-): 0

Region: chr3 94041028-94041044. Max. coverage (+): 0. Max coverage (-): 4.22

Region: chr3 94041045-94041061. Max. coverage (+): 0. Max coverage (-): 0

Region: chr3 94041062-94041078. Max. coverage (+): 0. Max coverage (-): 0

Region: chr3 94041079-94041095. Max. coverage (+): 0. Max coverage (-): 4.28

Region: chr3 94041096-94041111. Max. coverage (+): 0. Max coverage (-): 0

Region: chr3 94041112-94041128. Max. coverage (+): 0. Max coverage (-): 0

Region: chr3 94041129-94041145. Max. coverage (+): 0. Max coverage (-): 0

Region: chr3 94041146-94041162. Max. coverage (+): 0. Max coverage (-): 0

Region: chr3 94041163-94041179. Max. coverage (+): 0. Max coverage (-): 0

Region: chr3 94041180-94041195. Max. coverage (+): 0. Max coverage (-): 0

Region: chr3 94041196-94041212. Max. coverage (+): 0. Max coverage (-): 0

Region: chr3 94041213-94041229. Max. coverage (+): 0. Max coverage (-): 0

Region: chr3 94041230-94041246. Max. coverage (+): 0. Max coverage (-): 0

Region: chr3 94041247-94041263. Max. coverage (+): 0. Max coverage (-): 0

Region: chr3 94041264-94041279. Max. coverage (+): 0. Max coverage (-): 0

Region: chr3 94041280-94041296. Max. coverage (+): 0. Max coverage (-): 0

Region: chr3 94041297-94041313. Max. coverage (+): 0. Max coverage (-): 0

Region: chr3 94041314-94041330. Max. coverage (+): 0. Max coverage (-): 9.1

Region: chr3 94041331-94041347. Max. coverage (+): 0. Max coverage (-): 5.87

Region: chr3 94041348-94041363. Max. coverage (+): 0. Max coverage (-): 0

Region: chr3 94041364-94041380. Max. coverage (+): 0. Max coverage (-): 0

Region: chr3 94041381-94041397. Max. coverage (+): 0. Max coverage (-): 0

Region: chr3 94041398-94041414. Max. coverage (+): 0. Max coverage (-): 0

Region: chr3 94041415-94041431. Max. coverage (+): 0. Max coverage (-): 0

Region: chr3 94041432-94041447. Max. coverage (+): 0. Max coverage (-): 3.58

Region: chr3 94041448-94041464. Max. coverage (+): 0. Max coverage (-): 4.1

Region: chr3 94041465-94041481. Max. coverage (+): 0. Max coverage (-): 1.26

Region: chr3 94041482-94041498. Max. coverage (+): 0. Max coverage (-): 0

Region: chr3 94041499-94041515. Max. coverage (+): 0. Max coverage (-): 0

Region: chr3 94041516-94041531. Max. coverage (+): 0. Max coverage (-): 0

Region: chr3 94041532-94041548. Max. coverage (+): 0. Max coverage (-): 0

Region: chr3 94041549-94041565. Max. coverage (+): 0. Max coverage (-): 4.33

Region: chr3 94041566-94041582. Max. coverage (+): 0. Max coverage (-): 4.33

Region: chr3 94041583-94041599. Max. coverage (+): 0. Max coverage (-): 11.49

Region: chr3 94041600-94041615. Max. coverage (+): 0. Max coverage (-): 10.31

Region: chr3 94041616-94041632. Max. coverage (+): 0. Max coverage (-): 4.52

Region: chr3 94041633-94041649. Max. coverage (+): 0. Max coverage (-): 0

Region: chr3 94041650-94041666. Max. coverage (+): 0. Max coverage (-): 0

Region: chr3 94041667-94041683. Max. coverage (+): 0. Max coverage (-): 0

Region: chr3 94041684-94041699. Max. coverage (+): 0. Max coverage (-): 0

Region: chr3 94041700-94041716. Max. coverage (+): 0. Max coverage (-): 0

Region: chr3 94041717-94041733. Max. coverage (+): 0. Max coverage (-): 0

Region: chr3 94041734-94041750. Max. coverage (+): 0. Max coverage (-): 0

Region: chr3 94041751-94041767. Max. coverage (+): 0. Max coverage (-): 0

Region: chr3 94041768-94041783. Max. coverage (+): 0. Max coverage (-): 0

Region: chr3 94041784-94041800. Max. coverage (+): 0. Max coverage (-): 0

Region: chr3 94041801-94041817. Max. coverage (+): 0. Max coverage (-): 0

Region: chr3 94041818-94041834. Max. coverage (+): 0. Max coverage (-): 0

Region: chr3 94041835-94041851. Max. coverage (+): 0. Max coverage (-): 0

Region: chr3 94041852-94041867. Max. coverage (+): 0. Max coverage (-): 0

Region: chr3 94041868-94041884. Max. coverage (+): 0. Max coverage (-): 0

Region: chr3 94041885-94041901. Max. coverage (+): 0. Max coverage (-): 0

Region: chr3 94041902-94041918. Max. coverage (+): 0. Max coverage (-): 0

Region: chr3 94041919-94041935. Max. coverage (+): 0. Max coverage (-): 0

Region: chr3 94041936-94041951. Max. coverage (+): 0. Max coverage (-): 8.01

Region: chr3 94041952-94041968. Max. coverage (+): 0. Max coverage (-): 5.29

Region: chr3 94041969-94041985. Max. coverage (+): 0. Max coverage (-): 2.81

Region: chr3 94041986-94042002. Max. coverage (+): 0. Max coverage (-): 1.28

Region: chr3 94042003-94042019. Max. coverage (+): 0. Max coverage (-): 0

Region: chr3 94042020-94042035. Max. coverage (+): 0. Max coverage (-): 0

Region: chr3 94042036-94042052. Max. coverage (+): 0. Max coverage (-): 0

Region: chr3 94042053-94042069. Max. coverage (+): 0. Max coverage (-): 0

Region: chr3 94042070-94042086. Max. coverage (+): 0. Max coverage (-): 0

Region: chr3 94042087-94042103. Max. coverage (+): 0. Max coverage (-): 0

Region: chr3 94042104-94042119. Max. coverage (+): 0. Max coverage (-): 0

Region: chr3 94042120-94042136. Max. coverage (+): 0. Max coverage (-): 0

Region: chr3 94042137-94042153. Max. coverage (+): 0. Max coverage (-): 0

Region: chr3 94042154-94042170. Max. coverage (+): 0. Max coverage (-): 0

Region: chr3 94042171-94042187. Max. coverage (+): 0. Max coverage (-): 0

Region: chr3 94042188-94042203. Max. coverage (+): 0. Max coverage (-): 0

Region: chr3 94042204-94042220. Max. coverage (+): 0. Max coverage (-): 0

Region: chr3 94042221-94042237. Max. coverage (+): 0. Max coverage (-): 0

Region: chr3 94042238-94042254. Max. coverage (+): 0. Max coverage (-): 0

Region: chr3 94042255-94042271. Max. coverage (+): 0. Max coverage (-): 0

Region: chr3 94042272-94042287. Max. coverage (+): 0. Max coverage (-): 0.72

Region: chr3 94042288-94042304. Max. coverage (+): 0. Max coverage (-): 6.25

Region: chr3 94042305-94042321. Max. coverage (+): 0. Max coverage (-): 19.83

Region: chr3 94042322-94042338. Max. coverage (+): 0. Max coverage (-): 1.44

Region: chr3 94042339-94042355. Max. coverage (+): 0. Max coverage (-): 18.17

Region: chr3 94042356-94042371. Max. coverage (+): 0. Max coverage (-): 6.09

Region: chr3 94042372-94042388. Max. coverage (+): 0. Max coverage (-): 9.61

Region: chr3 94042389-94042405. Max. coverage (+): 0. Max coverage (-): 10.5

Region: chr3 94042406-94042422. Max. coverage (+): 0. Max coverage (-): 0

Region: chr3 94042423-94042439. Max. coverage (+): 0. Max coverage (-): 0

Region: chr3 94042440-94042455. Max. coverage (+): 0. Max coverage (-): 0

Region: chr3 94042456-94042472. Max. coverage (+): 0. Max coverage (-): 0

Region: chr3 94042473-94042489. Max. coverage (+): 0. Max coverage (-): 0

Region: chr3 94042490-94042506. Max. coverage (+): 0. Max coverage (-): 0

Region: chr3 94042507-94042523. Max. coverage (+): 0. Max coverage (-): 0

Region: chr3 94042524-94042539. Max. coverage (+): 0. Max coverage (-): 0

Region: chr3 94042540-94042556. Max. coverage (+): 0. Max coverage (-): 0

Region: chr3 94042557-94042573. Max. coverage (+): 0. Max coverage (-): 0

Region: chr3 94042574-94042590. Max. coverage (+): 0. Max coverage (-): 0

Region: chr3 94042591-94042607. Max. coverage (+): 0. Max coverage (-): 0

Region: chr3 94042608-94042623. Max. coverage (+): 0. Max coverage (-): 0

Region: chr3 94042624-94042640. Max. coverage (+): 0. Max coverage (-): 0

Region: chr3 94042641-94042657. Max. coverage (+): 0. Max coverage (-): 0

Region: chr3 94042658-94042674. Max. coverage (+): 0. Max coverage (-): 0

Region: chr3 94042675-94042691. Max. coverage (+): 0. Max coverage (-): 0

Region: chr3 94042692-94042707. Max. coverage (+): 0. Max coverage (-): 0

Region: chr3 94042708-94042724. Max. coverage (+): 0. Max coverage (-): 0

Region: chr3 94042725-94042741. Max. coverage (+): 0. Max coverage (-): 0

Region: chr3 94042742-94042758. Max. coverage (+): 0. Max coverage (-): 0

Region: chr3 94042759-94042775. Max. coverage (+): 0. Max coverage (-): 0

Region: chr3 94042776-94042791. Max. coverage (+): 0. Max coverage (-): 0

Region: chr3 94042792-94042808. Max. coverage (+): 0. Max coverage (-): 0

Region: chr3 94042809-94042825. Max. coverage (+): 0. Max coverage (-): 0

Region: chr3 94042826-94042842. Max. coverage (+): 0. Max coverage (-): 0

Region: chr3 94042843-94042859. Max. coverage (+): 0. Max coverage (-): 0

Region: chr3 94042860-94042875. Max. coverage (+): 0. Max coverage (-): 0

Region: chr3 94042876-94042892. Max. coverage (+): 0. Max coverage (-): 0

Region: chr3 94042893-94042909. Max. coverage (+): 0. Max coverage (-): 0

Region: chr3 94042910-94042926. Max. coverage (+): 0. Max coverage (-): 0

Region: chr3 94042927-94042943. Max. coverage (+): 0. Max coverage (-): 0

Region: chr3 94042944-94042959. Max. coverage (+): 0. Max coverage (-): 0

Region: chr3 94042960-94042976. Max. coverage (+): 0. Max coverage (-): 0

Region: chr3 94042977-94042993. Max. coverage (+): 0. Max coverage (-): 0

Region: chr3 94042994-94043010. Max. coverage (+): 0. Max coverage (-): 0

Region: chr3 94043011-94043027. Max. coverage (+): 0. Max coverage (-): 0

Region: chr3 94043028-94043043. Max. coverage (+): 0. Max coverage (-): 0

Region: chr3 94043044-94043060. Max. coverage (+): 0. Max coverage (-): 0

Region: chr3 94043061-94043077. Max. coverage (+): 0. Max coverage (-): 2.06

Region: chr3 94043078-94043094. Max. coverage (+): 0. Max coverage (-): 2.06

Region: chr3 94043095-94043111. Max. coverage (+): 0. Max coverage (-): 0

Region: chr3 94043112-94043127. Max. coverage (+): 0. Max coverage (-): 0

Region: chr3 94043128-94043144. Max. coverage (+): 0. Max coverage (-): 0

Region: chr3 94043145-94043161. Max. coverage (+): 0. Max coverage (-): 8.16

Region: chr3 94043162-94043178. Max. coverage (+): 0. Max coverage (-): 4.35

Region: chr3 94043179-94043195. Max. coverage (+): 0. Max coverage (-): 0

Region: chr3 94043196-94043211. Max. coverage (+): 0. Max coverage (-): 5.39

Region: chr3 94043212-94043228. Max. coverage (+): 0. Max coverage (-): 5.39

Region: chr3 94043229-94043245. Max. coverage (+): 0. Max coverage (-): 0

Region: chr3 94043246-94043262. Max. coverage (+): 0. Max coverage (-): 0

Region: chr3 94043263-94043279. Max. coverage (+): 0. Max coverage (-): 3.75

Region: chr3 94043280-94043295. Max. coverage (+): 0. Max coverage (-): 3.75

Region: chr3 94043296-94043312. Max. coverage (+): 0. Max coverage (-): 0

Region: chr3 94043313-94043329. Max. coverage (+): 0. Max coverage (-): 4.52

Region: chr3 94043330-94043346. Max. coverage (+): 0. Max coverage (-): 4.52

Region: chr3 94043347-94043363. Max. coverage (+): 0. Max coverage (-): 0.45

Region: chr3 94043364-94043379. Max. coverage (+): 0. Max coverage (-): 1.09

Region: chr3 94043380-94043396. Max. coverage (+): 0. Max coverage (-): 1.09

Region: chr3 94043397-94043413. Max. coverage (+): 0. Max coverage (-): 1.02

Region: chr3 94043414-94043430. Max. coverage (+): 0. Max coverage (-): 0

Region: chr3 94043431-94043447. Max. coverage (+): 0. Max coverage (-): 0

Region: chr3 94043448-94043463. Max. coverage (+): 0. Max coverage (-): 0

Region: chr3 94043464-94043480. Max. coverage (+): 0. Max coverage (-): 0

Region: chr3 94043481-94043497. Max. coverage (+): 0. Max coverage (-): 10

Region: chr3 94043498-94043514. Max. coverage (+): 0. Max coverage (-): 0

Region: chr3 94043515-94043531. Max. coverage (+): 0. Max coverage (-): 0

Region: chr3 94043532-94043547. Max. coverage (+): 0. Max coverage (-): 0

Region: chr3 94043548-94043564. Max. coverage (+): 0. Max coverage (-): 0

Region: chr3 94043565-94043581. Max. coverage (+): 0. Max coverage (-): 0

Region: chr3 94043582-94043598. Max. coverage (+): 0. Max coverage (-): 0

Region: chr3 94043599-94043615. Max. coverage (+): 0. Max coverage (-): 0

Region: chr3 94043616-94043631. Max. coverage (+): 0. Max coverage (-): 0

Region: chr3 94043632-94043648. Max. coverage (+): 0. Max coverage (-): 0

Region: chr3 94043649-94043665. Max. coverage (+): 0. Max coverage (-): 0

Region: chr3 94043666-94043682. Max. coverage (+): 0. Max coverage (-): 4.45

Region: chr3 94043683-94043699. Max. coverage (+): 0. Max coverage (-): 8.03

Region: chr3 94043700-94043715. Max. coverage (+): 0. Max coverage (-): 13.12

Region: chr3 94043716-94043732. Max. coverage (+): 0. Max coverage (-): 3.79

Region: chr3 94043733-94043749. Max. coverage (+): 0. Max coverage (-): 8.26

Region: chr3 94043750-94043766. Max. coverage (+): 0. Max coverage (-): 8.26

Region: chr3 94043767-94043783. Max. coverage (+): 0. Max coverage (-): 7.68

Region: chr3 94043784-94043799. Max. coverage (+): 0. Max coverage (-): 4.08

Region: chr3 94043800-94043816. Max. coverage (+): 0. Max coverage (-): 4.71

Region: chr3 94043817-94043833. Max. coverage (+): 0. Max coverage (-): 2.61

Region: chr3 94043834-94043850. Max. coverage (+): 0. Max coverage (-): 3.08

Region: chr3 94043851-94043867. Max. coverage (+): 0. Max coverage (-): 3.08

Region: chr3 94043868-94043883. Max. coverage (+): 0. Max coverage (-): 0

Region: chr3 94043884-94043900. Max. coverage (+): 0. Max coverage (-): 0.69

Region: chr3 94043901-94043917. Max. coverage (+): 0. Max coverage (-): 0.69

Region: chr3 94043918-94043934. Max. coverage (+): 0. Max coverage (-): 0

Region: chr3 94043935-94043951. Max. coverage (+): 0. Max coverage (-): 3.77

Region: chr3 94043952-94043967. Max. coverage (+): 0. Max coverage (-): 0

Region: chr3 94043968-94043984. Max. coverage (+): 0. Max coverage (-): 2.33

Region: chr3 94043985-94044001. Max. coverage (+): 0. Max coverage (-): 18.54

Region: chr3 94044002-94044018. Max. coverage (+): 0. Max coverage (-): 29.65

Region: chr3 94044019-94044035. Max. coverage (+): 0. Max coverage (-): 9.85

Region: chr3 94044036-94044051. Max. coverage (+): 0. Max coverage (-): 10.77

Region: chr3 94044052-94044068. Max. coverage (+): 0. Max coverage (-): 0

Region: chr3 94044069-94044085. Max. coverage (+): 0. Max coverage (-): 0

Region: chr3 94044086-94044102. Max. coverage (+): 0. Max coverage (-): 26.27

Region: chr3 94044103-94044119. Max. coverage (+): 0. Max coverage (-): 17.81

Region: chr3 94044120-94044135. Max. coverage (+): 0. Max coverage (-): 9.73

Region: chr3 94044136-94044152. Max. coverage (+): 0. Max coverage (-): 9.73

Region: chr3 94044153-94044169. Max. coverage (+): 0. Max coverage (-): 3.54

Region: chr3 94044170-94044186. Max. coverage (+): 0. Max coverage (-): 4.77

Region: chr3 94044187-94044203. Max. coverage (+): 0. Max coverage (-): 25.45

Region: chr3 94044204-94044219. Max. coverage (+): 0. Max coverage (-): 39.32

Region: chr3 94044220-94044236. Max. coverage (+): 0. Max coverage (-): 0

Region: chr3 94044237-94044253. Max. coverage (+): 0. Max coverage (-): 0

Region: chr3 94044254-94044270. Max. coverage (+): 0. Max coverage (-): 0

Region: chr3 94044271-94044287. Max. coverage (+): 0. Max coverage (-): 0

Region: chr3 94044288-94044303. Max. coverage (+): 0. Max coverage (-): 0

Region: chr3 94044304-94044320. Max. coverage (+): 0. Max coverage (-): 0

Region: chr3 94044321-94044337. Max. coverage (+): 0. Max coverage (-): 0

Region: chr3 94044338-94044354. Max. coverage (+): 0. Max coverage (-): 0

Region: chr3 94044355-94044371. Max. coverage (+): 0. Max coverage (-): 0

Region: chr3 94044372-94044387. Max. coverage (+): 0. Max coverage (-): 0

Region: chr3 94044388-94044404. Max. coverage (+): 0. Max coverage (-): 0

Region: chr3 94044405-94044421. Max. coverage (+): 0. Max coverage (-): 0

Region: chr3 94044422-94044438. Max. coverage (+): 0. Max coverage (-): 4.49

Region: chr3 94044439-94044455. Max. coverage (+): 0. Max coverage (-): 8.32

Region: chr3 94044456-94044471. Max. coverage (+): 0. Max coverage (-): 4.94

Region: chr3 94044472-94044488. Max. coverage (+): 0. Max coverage (-): 1.61

Region: chr3 94044489-94044505. Max. coverage (+): 0. Max coverage (-): 0

Region: chr3 94044506-94044522. Max. coverage (+): 0. Max coverage (-): 0

Region: chr3 94044523-94044539. Max. coverage (+): 0. Max coverage (-): 0

Region: chr3 94044540-94044555. Max. coverage (+): 0. Max coverage (-): 4.9

Region: chr3 94044556-94044572. Max. coverage (+): 0. Max coverage (-): 15.36

Region: chr3 94044573-94044589. Max. coverage (+): 0. Max coverage (-): 31.64

Region: chr3 94044590-94044606. Max. coverage (+): 0. Max coverage (-): 17.75

Region: chr3 94044607-94044623. Max. coverage (+): 0. Max coverage (-): 4.7

Region: chr3 94044624-94044639. Max. coverage (+): 0. Max coverage (-): 0.23

Region: chr3 94044640-94044656. Max. coverage (+): 0. Max coverage (-): 6.47

Region: chr3 94044657-94044673. Max. coverage (+): 0. Max coverage (-): 4.45

Region: chr3 94044674-94044690. Max. coverage (+): 0. Max coverage (-): 7.93

Region: chr3 94044691-94044707. Max. coverage (+): 0. Max coverage (-): 16.7

Region: chr3 94044708-94044723. Max. coverage (+): 0. Max coverage (-): 27.1

Region: chr3 94044724-94044740. Max. coverage (+): 0. Max coverage (-): 50.52

Region: chr3 94044741-94044757. Max. coverage (+): 0. Max coverage (-): 1.66

Region: chr3 94044758-94044774. Max. coverage (+): 0. Max coverage (-): 3.08

Region: chr3 94044775-94044791. Max. coverage (+): 0. Max coverage (-): 0

Region: chr3 94044792-94044807. Max. coverage (+): 0. Max coverage (-): 0

Region: chr3 94044808-94044824. Max. coverage (+): 0. Max coverage (-): 0

Region: chr3 94044825-94044841. Max. coverage (+): 0. Max coverage (-): 0

Region: chr3 94044842-94044858. Max. coverage (+): 0. Max coverage (-): 0

Region: chr3 94044859-94044875. Max. coverage (+): 0. Max coverage (-): 0

Region: chr3 94044876-94044891. Max. coverage (+): 0. Max coverage (-): 0

Region: chr3 94044892-94044908. Max. coverage (+): 0. Max coverage (-): 0

Region: chr3 94044909-94044925. Max. coverage (+): 0. Max coverage (-): 0

Region: chr3 94044926-94044942. Max. coverage (+): 0. Max coverage (-): 0

Region: chr3 94044943-94044959. Max. coverage (+): 0. Max coverage (-): 0

Region: chr3 94044960-94044975. Max. coverage (+): 0. Max coverage (-): 0

Region: chr3 94044976-94044992. Max. coverage (+): 0. Max coverage (-): 12.29

Region: chr3 94044993-94045009. Max. coverage (+): 0. Max coverage (-): 1.4

Region: chr3 94045010-94045026. Max. coverage (+): 0. Max coverage (-): 5.38

Region: chr3 94045027-94045043. Max. coverage (+): 0. Max coverage (-): 15.36

Region: chr3 94045044-94045059. Max. coverage (+): 0. Max coverage (-): 6.47

Region: chr3 94045060-94045076. Max. coverage (+): 0. Max coverage (-): 16.35

Region: chr3 94045077-94045093. Max. coverage (+): 0. Max coverage (-): 14.98

Region: chr3 94045094-94045110. Max. coverage (+): 0. Max coverage (-): 0

Region: chr3 94045111-94045127. Max. coverage (+): 0. Max coverage (-): 0.9

Region: chr3 94045128-94045143. Max. coverage (+): 0. Max coverage (-): 0.9

Region: chr3 94045144-94045160. Max. coverage (+): 0. Max coverage (-): 1.89

Region: chr3 94045161-94045177. Max. coverage (+): 0. Max coverage (-): 0

Region: chr3 94045178-94045194. Max. coverage (+): 0. Max coverage (-): 0

Region: chr3 94045195-94045211. Max. coverage (+): 0. Max coverage (-): 3.49

Region: chr3 94045212-94045227. Max. coverage (+): 0. Max coverage (-): 3.19

Region: chr3 94045228-94045244. Max. coverage (+): 0. Max coverage (-): 1.47

Region: chr3 94045245-94045261. Max. coverage (+): 0. Max coverage (-): 1.37

Region: chr3 94045262-94045278. Max. coverage (+): 0. Max coverage (-): 13.44

Region: chr3 94045279-94045295. Max. coverage (+): 0. Max coverage (-): 13.44

Region: chr3 94045296-94045311. Max. coverage (+): 0. Max coverage (-): 13.3

Region: chr3 94045312-94045328. Max. coverage (+): 0. Max coverage (-): 20.54

Region: chr3 94045329-94045345. Max. coverage (+): 0. Max coverage (-): 6.89

Region: chr3 94045346-94045362. Max. coverage (+): 0. Max coverage (-): 24.82

Region: chr3 94045363-94045379. Max. coverage (+): 0. Max coverage (-): 0

Region: chr3 94045380-94045395. Max. coverage (+): 0. Max coverage (-): 0

Region: chr3 94045396-94045412. Max. coverage (+): 0. Max coverage (-): 0

Region: chr3 94045413-94045429. Max. coverage (+): 0. Max coverage (-): 4.25

Region: chr3 94045430-94045446. Max. coverage (+): 0. Max coverage (-): 4.31

Region: chr3 94045447-94045463. Max. coverage (+): 0. Max coverage (-): 6.62

Region: chr3 94045464-94045479. Max. coverage (+): 0. Max coverage (-): 7.59

Region: chr3 94045480-94045496. Max. coverage (+): 0. Max coverage (-): 0

Region: chr3 94045497-94045513. Max. coverage (+): 0. Max coverage (-): 8.94

Region: chr3 94045514-94045530. Max. coverage (+): 0. Max coverage (-): 2.42

Region: chr3 94045531-94045547. Max. coverage (+): 0. Max coverage (-): 25.94

Region: chr3 94045548-94045563. Max. coverage (+): 0. Max coverage (-): 17.92

Region: chr3 94045564-94045580. Max. coverage (+): 0. Max coverage (-): 10.03

Region: chr3 94045581-94045597. Max. coverage (+): 0. Max coverage (-): 5.76

Region: chr3 94045598-94045614. Max. coverage (+): 0. Max coverage (-): 6.54

Region: chr3 94045615-94045631. Max. coverage (+): 0. Max coverage (-): 1.72

Region: chr3 94045632-94045647. Max. coverage (+): 0. Max coverage (-): 0.46

Region: chr3 94045648-94045664. Max. coverage (+): 0. Max coverage (-): 17.35

Region: chr3 94045665-94045681. Max. coverage (+): 0. Max coverage (-): 0

Region: chr3 94045682-94045698. Max. coverage (+): 0. Max coverage (-): 0.97

Region: chr3 94045699-94045715. Max. coverage (+): 0. Max coverage (-): 12.2

Region: chr3 94045716-94045731. Max. coverage (+): 0. Max coverage (-): 5.67

Region: chr3 94045732-94045748. Max. coverage (+): 0. Max coverage (-): 20.08

Region: chr3 94045749-94045765. Max. coverage (+): 0. Max coverage (-): 17.4

Region: chr3 94045766-94045782. Max. coverage (+): 0. Max coverage (-): 0

Region: chr3 94045783-94045799. Max. coverage (+): 0. Max coverage (-): 0

Region: chr3 94045800-94045815. Max. coverage (+): 0. Max coverage (-): 5.9

Region: chr3 94045816-94045832. Max. coverage (+): 0. Max coverage (-): 8.92

Region: chr3 94045833-94045849. Max. coverage (+): 0. Max coverage (-): 10.41

Region: chr3 94045850-94045866. Max. coverage (+): 0. Max coverage (-): 5.18

Region: chr3 94045867-94045883. Max. coverage (+): 0. Max coverage (-): 4.04

Region: chr3 94045884-94045899. Max. coverage (+): 0. Max coverage (-): 17.8

Region: chr3 94045900-94045916. Max. coverage (+): 0. Max coverage (-): 24.18

Region: chr3 94045917-94045933. Max. coverage (+): 0. Max coverage (-): 51.54

Region: chr3 94045934-94045950. Max. coverage (+): 0. Max coverage (-): 40.32

Region: chr3 94045951-94045967. Max. coverage (+): 0. Max coverage (-): 10.74

Region: chr3 94045968-94045983. Max. coverage (+): 0. Max coverage (-): 8.44

Region: chr3 94045984-94046000. Max. coverage (+): 0. Max coverage (-): 6.82

Region: chr3 94046001-94046017. Max. coverage (+): 0. Max coverage (-): 20.03

Region: chr3 94046018-94046034. Max. coverage (+): 0. Max coverage (-): 1.88

Region: chr3 94046035-94046051. Max. coverage (+): 0. Max coverage (-): 11.95

Region: chr3 94046052-94046067. Max. coverage (+): 0. Max coverage (-): 0

Region: chr3 94046068-94046084. Max. coverage (+): 0. Max coverage (-): 3.69

Region: chr3 94046085-94046101. Max. coverage (+): 0. Max coverage (-): 4.9

Region: chr3 94046102-94046118. Max. coverage (+): 0. Max coverage (-): 2.59

Region: chr3 94046119-94046135. Max. coverage (+): 0. Max coverage (-): 0

Region: chr3 94046136-94046151. Max. coverage (+): 0. Max coverage (-): 0

Region: chr3 94046152-94046168. Max. coverage (+): 0. Max coverage (-): 0

Region: chr3 94046169-94046185. Max. coverage (+): 0. Max coverage (-): 9.46

Region: chr3 94046186-94046202. Max. coverage (+): 0. Max coverage (-): 6.63

Region: chr3 94046203-94046219. Max. coverage (+): 0. Max coverage (-): 0

Region: chr3 94046220-94046235. Max. coverage (+): 0. Max coverage (-): 5.34

Region: chr3 94046236-94046252. Max. coverage (+): 0. Max coverage (-): 0.75

Region: chr3 94046253-94046269. Max. coverage (+): 0. Max coverage (-): 1.23

Region: chr3 94046270-94046286. Max. coverage (+): 0. Max coverage (-): 2.83

Region: chr3 94046287-94046303. Max. coverage (+): 0. Max coverage (-): 0

Region: chr3 94046304-94046319. Max. coverage (+): 0. Max coverage (-): 0

Region: chr3 94046320-94046336. Max. coverage (+): 0. Max coverage (-): 4.35

Region: chr3 94046337-94046353. Max. coverage (+): 0. Max coverage (-): 0

Region: chr3 94046354-94046370. Max. coverage (+): 0. Max coverage (-): 2.9

Region: chr3 94046371-94046387. Max. coverage (+): 0. Max coverage (-): 2.9

Region: chr3 94046388-94046403. Max. coverage (+): 0. Max coverage (-): 2.07

Region: chr3 94046404-94046420. Max. coverage (+): 0. Max coverage (-): 8.93

Region: chr3 94046421-94046437. Max. coverage (+): 0. Max coverage (-): 10.76

Region: chr3 94046438-94046454. Max. coverage (+): 0. Max coverage (-): 2.82

Region: chr3 94046455-94046471. Max. coverage (+): 0. Max coverage (-): 0

Region: chr3 94046472-94046487. Max. coverage (+): 0. Max coverage (-): 0

Region: chr3 94046488-94046504. Max. coverage (+): 0. Max coverage (-): 0

Region: chr3 94046505-94046521. Max. coverage (+): 0. Max coverage (-): 0

Region: chr3 94046522-94046538. Max. coverage (+): 0. Max coverage (-): 0

Region: chr3 94046539-94046555. Max. coverage (+): 0. Max coverage (-): 0

Region: chr3 94046556-94046571. Max. coverage (+): 0. Max coverage (-): 0

Region: chr3 94046572-94046588. Max. coverage (+): 0. Max coverage (-): 0

Region: chr3 94046589-94046605. Max. coverage (+): 0. Max coverage (-): 0

Region: chr3 94046606-94046622. Max. coverage (+): 0. Max coverage (-): 0

Region: chr3 94046623-94046639. Max. coverage (+): 0. Max coverage (-): 0

Region: chr3 94046640-94046655. Max. coverage (+): 0. Max coverage (-): 0.51

Region: chr3 94046656-94046672. Max. coverage (+): 0. Max coverage (-): 3.38

Region: chr3 94046673-94046689. Max. coverage (+): 0. Max coverage (-): 0

Region: chr3 94046690-94046706. Max. coverage (+): 0. Max coverage (-): 2.74

Region: chr3 94046707-94046723. Max. coverage (+): 0. Max coverage (-): 10.34

Region: chr3 94046724-94046739. Max. coverage (+): 0. Max coverage (-): 10.34

Region: chr3 94046740-94046756. Max. coverage (+): 0. Max coverage (-): 27.87

Region: chr3 94046757-94046773. Max. coverage (+): 0. Max coverage (-): 25.95

Region: chr3 94046774-94046790. Max. coverage (+): 0. Max coverage (-): 0

Region: chr3 94046791-94046807. Max. coverage (+): 0. Max coverage (-): 11.54

Region: chr3 94046808-94046823. Max. coverage (+): 0. Max coverage (-): 0

Region: chr3 94046824-94046840. Max. coverage (+): 0. Max coverage (-): 7.22

Region: chr3 94046841-94046857. Max. coverage (+): 0. Max coverage (-): 6.37

Region: chr3 94046858-94046874. Max. coverage (+): 0. Max coverage (-): 3.89

Region: chr3 94046875-94046891. Max. coverage (+): 0. Max coverage (-): 0.49

Region: chr3 94046892-94046907. Max. coverage (+): 0. Max coverage (-): 0

Region: chr3 94046908-94046924. Max. coverage (+): 0. Max coverage (-): 0

Region: chr3 94046925-94046941. Max. coverage (+): 0. Max coverage (-): 0

Region: chr3 94046942-94046958. Max. coverage (+): 0. Max coverage (-): 0

Region: chr3 94046959-94046975. Max. coverage (+): 0. Max coverage (-): 0

Region: chr3 94046976-94046991. Max. coverage (+): 0. Max coverage (-): 0

Region: chr3 94046992-94047008. Max. coverage (+): 0. Max coverage (-): 0

Region: chr3 94047009-94047025. Max. coverage (+): 0. Max coverage (-): 0

Region: chr3 94047026-94047042. Max. coverage (+): 0. Max coverage (-): 0

Region: chr3 94047043-94047059. Max. coverage (+): 0. Max coverage (-): 0

Region: chr3 94047060-94047075. Max. coverage (+): 0. Max coverage (-): 1.02

Region: chr3 94047076-94047092. Max. coverage (+): 0. Max coverage (-): 9.38

Region: chr3 94047093-94047109. Max. coverage (+): 0. Max coverage (-): 25.54

Region: chr3 94047110-94047126. Max. coverage (+): 0. Max coverage (-): 25.85

Region: chr3 94047127-94047143. Max. coverage (+): 0. Max coverage (-): 3.75

Region: chr3 94047144-94047159. Max. coverage (+): 0. Max coverage (-): 0.81

Region: chr3 94047160-94047176. Max. coverage (+): 0. Max coverage (-): 19.65

Region: chr3 94047177-94047193. Max. coverage (+): 0. Max coverage (-): 6.25

Region: chr3 94047194-94047210. Max. coverage (+): 0. Max coverage (-): 14.13

Region: chr3 94047211-94047227. Max. coverage (+): 0. Max coverage (-): 13.54

Region: chr3 94047228-94047243. Max. coverage (+): 0. Max coverage (-): 2.76

Region: chr3 94047244-94047260. Max. coverage (+): 0. Max coverage (-): 2.76

Region: chr3 94047261-94047277. Max. coverage (+): 0. Max coverage (-): 0

Region: chr3 94047278-94047294. Max. coverage (+): 0. Max coverage (-): 0

Region: chr3 94047295-94047311. Max. coverage (+): 0. Max coverage (-): 16.1

Region: chr3 94047312-94047327. Max. coverage (+): 0. Max coverage (-): 28.62

Region: chr3 94047328-94047344. Max. coverage (+): 0. Max coverage (-): 25.39

Region: chr3 94047345-94047361. Max. coverage (+): 0. Max coverage (-): 12.74

Region: chr3 94047362-94047378. Max. coverage (+): 0. Max coverage (-): 5.21

Region: chr3 94047379-94047395. Max. coverage (+): 0. Max coverage (-): 2.27

Region: chr3 94047396-94047411. Max. coverage (+): 0. Max coverage (-): 19.15

Region: chr3 94047412-94047428. Max. coverage (+): 0. Max coverage (-): 14.4

Region: chr3 94047429-94047445. Max. coverage (+): 0. Max coverage (-): 13.56

Region: chr3 94047446-94047462. Max. coverage (+): 0. Max coverage (-): 21.19

Region: chr3 94047463-94047479. Max. coverage (+): 0. Max coverage (-): 31.84

Region: chr3 94047480-94047495. Max. coverage (+): 0. Max coverage (-): 11.64

Region: chr3 94047496-94047512. Max. coverage (+): 0. Max coverage (-): 9.98

Region: chr3 94047513-94047529. Max. coverage (+): 0. Max coverage (-): 19.81

Region: chr3 94047530-94047546. Max. coverage (+): 0. Max coverage (-): 21.4

Region: chr3 94047547-94047563. Max. coverage (+): 0. Max coverage (-): 2.41

Region: chr3 94047564-94047579. Max. coverage (+): 0. Max coverage (-): 13.79

Region: chr3 94047580-94047596. Max. coverage (+): 0. Max coverage (-): 1.73

Region: chr3 94047597-94047613. Max. coverage (+): 0. Max coverage (-): 0

Region: chr3 94047614-94047630. Max. coverage (+): 0. Max coverage (-): 0

Region: chr3 94047631-94047647. Max. coverage (+): 0. Max coverage (-): 0

Region: chr3 94047648-94047663. Max. coverage (+): 0. Max coverage (-): 0

Region: chr3 94047664-94047680. Max. coverage (+): 0. Max coverage (-): 0

Region: chr3 94047681-94047697. Max. coverage (+): 0. Max coverage (-): 0

Region: chr3 94047698-94047714. Max. coverage (+): 0. Max coverage (-): 0

Region: chr3 94047715-94047731. Max. coverage (+): 0. Max coverage (-): 0

Region: chr3 94047732-94047747. Max. coverage (+): 0. Max coverage (-): 0

Region: chr3 94047748-94047764. Max. coverage (+): 0. Max coverage (-): 0

Region: chr3 94047765-94047781. Max. coverage (+): 0. Max coverage (-): 0

Region: chr3 94047782-94047798. Max. coverage (+): 0. Max coverage (-): 0

Region: chr3 94047799-94047815. Max. coverage (+): 0. Max coverage (-): 0

Region: chr3 94047816-94047831. Max. coverage (+): 0. Max coverage (-): 0

Region: chr3 94047832-94047848. Max. coverage (+): 0. Max coverage (-): 11.21

Region: chr3 94047849-94047865. Max. coverage (+): 0. Max coverage (-): 0

Region: chr3 94047866-94047882. Max. coverage (+): 0. Max coverage (-): 0.94

Region: chr3 94047883-94047899. Max. coverage (+): 0. Max coverage (-): 4.81

Region: chr3 94047900-94047915. Max. coverage (+): 0. Max coverage (-): 1.43

Region: chr3 94047916-94047932. Max. coverage (+): 0. Max coverage (-): 2.75

Region: chr3 94047933-94047949. Max. coverage (+): 0. Max coverage (-): 7.19

Region: chr3 94047950-94047966. Max. coverage (+): 0. Max coverage (-): 1.06

Region: chr3 94047967-94047983. Max. coverage (+): 0. Max coverage (-): 3.09

Region: chr3 94047984-94047999. Max. coverage (+): 0. Max coverage (-): 0

Region: chr3 94048000-94048016. Max. coverage (+): 0. Max coverage (-): 0

Region: chr3 94048017-94048033. Max. coverage (+): 0. Max coverage (-): 0

Region: chr3 94048034-94048050. Max. coverage (+): 0. Max coverage (-): 0

Region: chr3 94048051-94048067. Max. coverage (+): 0. Max coverage (-): 0

Region: chr3 94048068-94048083. Max. coverage (+): 0. Max coverage (-): 0

Region: chr3 94048084-94048100. Max. coverage (+): 0. Max coverage (-): 0

Region: chr3 94048101-94048117. Max. coverage (+): 0. Max coverage (-): 0

Region: chr3 94048118-94048134. Max. coverage (+): 0. Max coverage (-): 0

Region: chr3 94048135-94048151. Max. coverage (+): 0. Max coverage (-): 0

Region: chr3 94048152-94048167. Max. coverage (+): 0. Max coverage (-): 5.61

Region: chr3 94048168-94048184. Max. coverage (+): 0. Max coverage (-): 5.61

Region: chr3 94048185-94048201. Max. coverage (+): 0. Max coverage (-): 0

Region: chr3 94048202-94048218. Max. coverage (+): 0. Max coverage (-): 0

Region: chr3 94048219-94048235. Max. coverage (+): 0. Max coverage (-): 0

Region: chr3 94048236-94048251. Max. coverage (+): 0. Max coverage (-): 0

Region: chr3 94048252-94048268. Max. coverage (+): 0. Max coverage (-): 0

Region: chr3 94048269-94048285. Max. coverage (+): 0. Max coverage (-): 0

Region: chr3 94048286-94048302. Max. coverage (+): 0. Max coverage (-): 0

Region: chr3 94048303-94048319. Max. coverage (+): 0. Max coverage (-): 0

Region: chr3 94048320-94048335. Max. coverage (+): 0. Max coverage (-): 0

Region: chr3 94048336-94048352. Max. coverage (+): 0. Max coverage (-): 0

Region: chr3 94048353-94048369. Max. coverage (+): 0. Max coverage (-): 6.36

Region: chr3 94048370-94048386. Max. coverage (+): 0. Max coverage (-): 15.35

Region: chr3 94048387-94048403. Max. coverage (+): 0. Max coverage (-): 15.35

Region: chr3 94048404-94048419. Max. coverage (+): 0. Max coverage (-): 0

Region: chr3 94048420-94048436. Max. coverage (+): 0. Max coverage (-): 0

Region: chr3 94048437-94048453. Max. coverage (+): 0. Max coverage (-): 5.95

Region: chr3 94048454-94048470. Max. coverage (+): 0. Max coverage (-): 5.03

Region: chr3 94048471-94048487. Max. coverage (+): 0. Max coverage (-): 0

Region: chr3 94048488-94048503. Max. coverage (+): 0. Max coverage (-): 0

Region: chr3 94048504-94048520. Max. coverage (+): 0. Max coverage (-): 11.02

Region: chr3 94048521-94048537. Max. coverage (+): 0. Max coverage (-): 0

Region: chr3 94048538-94048554. Max. coverage (+): 0. Max coverage (-): 0

Region: chr3 94048555-94048571. Max. coverage (+): 0. Max coverage (-): 1.02

Region: chr3 94048572-94048587. Max. coverage (+): 0. Max coverage (-): 6.25

Region: chr3 94048588-94048604. Max. coverage (+): 0. Max coverage (-): 0

Region: chr3 94048605-94048621. Max. coverage (+): 0. Max coverage (-): 0

Region: chr3 94048622-94048638. Max. coverage (+): 0. Max coverage (-): 8.92

Region: chr3 94048639-94048655. Max. coverage (+): 0. Max coverage (-): 0

Region: chr3 94048656-94048671. Max. coverage (+): 0. Max coverage (-): 0

Region: chr3 94048672-94048688. Max. coverage (+): 0. Max coverage (-): 1.37

Region: chr3 94048689-94048705. Max. coverage (+): 0. Max coverage (-): 0

Region: chr3 94048706-94048722. Max. coverage (+): 0. Max coverage (-): 0

Region: chr3 94048723-94048739. Max. coverage (+): 0. Max coverage (-): 0

Region: chr3 94048740-94048755. Max. coverage (+): 0. Max coverage (-): 0

Region: chr3 94048756-94048772. Max. coverage (+): 0. Max coverage (-): 0

Region: chr3 94048773-94048789. Max. coverage (+): 0. Max coverage (-): 0

Region: chr3 94048790-94048806. Max. coverage (+): 0. Max coverage (-): 0

Region: chr3 94048807-94048823. Max. coverage (+): 0. Max coverage (-): 1.37

Region: chr3 94048824-94048839. Max. coverage (+): 0. Max coverage (-): 0

Region: chr3 94048840-94048856. Max. coverage (+): 0. Max coverage (-): 0.7

Region: chr3 94048857-94048873. Max. coverage (+): 0. Max coverage (-): 0

Region: chr3 94048874-94048890. Max. coverage (+): 0. Max coverage (-): 0

Region: chr3 94048891-94048907. Max. coverage (+): 0. Max coverage (-): 0

Region: chr3 94048908-94048923. Max. coverage (+): 0. Max coverage (-): 2.72

Region: chr3 94048924-94048940. Max. coverage (+): 0. Max coverage (-): 2.72

Region: chr3 94048941-94048957. Max. coverage (+): 0. Max coverage (-): 0

Region: chr3 94048958-94048974. Max. coverage (+): 0. Max coverage (-): 0

Region: chr3 94048975-94048991. Max. coverage (+): 0. Max coverage (-): 0

Region: chr3 94048992-94049007. Max. coverage (+): 0. Max coverage (-): 1.53

Region: chr3 94049008-94049024. Max. coverage (+): 0. Max coverage (-): 1.74

Region: chr3 94049025-94049041. Max. coverage (+): 0. Max coverage (-): 1.74

Region: chr3 94049042-94049058. Max. coverage (+): 0. Max coverage (-): 0

Region: chr3 94049059-94049075. Max. coverage (+): 0. Max coverage (-): 0

Region: chr3 94049076-94049091. Max. coverage (+): 0. Max coverage (-): 0

Region: chr3 94049092-94049108. Max. coverage (+): 0. Max coverage (-): 0

Region: chr3 94049109-94049125. Max. coverage (+): 0. Max coverage (-): 3.92

Region: chr3 94049126-94049142. Max. coverage (+): 0. Max coverage (-): 6.93

Region: chr3 94049143-94049159. Max. coverage (+): 0. Max coverage (-): 4.46

Region: chr3 94049160-94049175. Max. coverage (+): 0. Max coverage (-): 0

Region: chr3 94049176-94049192. Max. coverage (+): 0. Max coverage (-): 0

Region: chr3 94049193-94049209. Max. coverage (+): 0. Max coverage (-): 0

Region: chr3 94049210-94049226. Max. coverage (+): 0. Max coverage (-): 0

Region: chr3 94049227-94049243. Max. coverage (+): 0. Max coverage (-): 0

Region: chr3 94049244-94049259. Max. coverage (+): 0. Max coverage (-): 0

Region: chr3 94049260-94049276. Max. coverage (+): 0. Max coverage (-): 0

Region: chr3 94049277-94049293. Max. coverage (+): 0. Max coverage (-): 0

Region: chr3 94049294-94049310. Max. coverage (+): 0. Max coverage (-): 0

Region: chr3 94049311-94049327. Max. coverage (+): 0. Max coverage (-): 0

Region: chr3 94049328-94049343. Max. coverage (+): 0. Max coverage (-): 0

Region: chr3 94049344-94049360. Max. coverage (+): 0. Max coverage (-): 0.65

Region: chr3 94049361-94049377. Max. coverage (+): 0. Max coverage (-): 0.65

Region: chr3 94049378-. Max. coverage (+): 0. Max coverage (-): 0

RepeatMasker Color Code

**+**

100-98% Identity

<98-95% Identity

<95-90% Identity

<90-85% Identity

<85-80% Identity

<80-75% Identity

<75-70% Identity

<70% Identity

**-**

Gene Set Color Code

**+**

Gene

Pseudogene

**-**

Topology/Coverage Color Code

Coverage Plus Strand

Coverage Minus Strand

Mainstrand: Plus

Mainstrand: Minus

Complementary Strand

Flanking Region  
(if option -flank >0)

Gene Set Annotation  

**1. (protein coding, ENSBTAG00000002910) Tr:00000003779 Ex:8**: 94043983-94044033 (+)  
**2. (protein coding, ENSBTAG00000002910) Tr:00000003779 Ex:9**: 94045451-94045498 (+)  
**3. (protein coding, ENSBTAG00000002910) Tr:00000003779 Ex:10**: 94046306-94046956 (+)  
**4. ZYG11A (protein coding, ENSBTAG00000026032) Tr:00000052828 Ex:14**: 94049248-94049414 (-)

  
RepeatMasker Annotation  

**1. Tigger2a\_Art**: 94041673-94041864 (+), Divergence to consensus: 39.1%  
**2. MER81**: 94042034-94042115 (+), Divergence to consensus: 37.8%  
**3. L1ME4b**: 94042146-94042245 (-), Divergence to consensus: 35%  
**4. L1MC4a**: 94042448-94042987 (-), Divergence to consensus: 43.5%  
**5. (TA)n**: 94043007-94043062 (+), Divergence to consensus: 3.6%  
**6. MIRb**: 94043426-94043477 (-), Divergence to consensus: 26.9%  
**7. MIR3**: 94043543-94043649 (+), Divergence to consensus: 40%  
**8. MIR3**: 94044247-94044379 (-), Divergence to consensus: 42.6%  
**9. L2c**: 94044506-94044546 (-), Divergence to consensus: 24.4%  
**10. MIR**: 94044793-94044940 (+), Divergence to consensus: 33.1%  
**11. MIR3**: 94046486-94046532 (+), Divergence to consensus: 23.4%  
**12. MIRc**: 94046987-94047054 (+), Divergence to consensus: 28.4%  
**13. Bov-tA3**: 94047607-94047810 (-), Divergence to consensus: 11.3%  
**14. AT\_rich**: 94048699-94048722 (+), Divergence to consensus: 54.2%

  
Transcription Factor Binding Sites  

**RFX4\_1** (Sequence: GTTGCCAGG (-): 94045021)  
**SOX9** (Sequence: AACAATAA (-): 94046670)  
**Gata4** (Sequence: CTTATCT (+): 94047591)
