# Supplementary material for: Small RNA sequencing of cryopreserved semen from single bull revealed altered miRNAs and piRNAs expression between High- and Low-motile sperm populations
Source: BMC Genomics. 2017 Jan 4;18:14. doi: 10.1186/s12864-016-3394-7 (PMC5209821; doi:10.1186/s12864-016-3394-7)
Supplement: Additional file 3: — Details for each piRNA clusters found in High Motile (HM) sperm fraction. Genes, repeats, transposable elements and transcription factors binding sites falling within the cluster regions were reported. (ZIP 1896 kb) [file 12864_2016_3394_MOESM3_ESM.zip › 8.html]

piRNA cluster 8


Predicted piRNA cluster no. 8     previous   next
  

Show proTRAC run info
Hide proTRAC run info

================================= proTRAC ====================================  
VERSION: 2.1                                    LAST MODIFIED: 06. October 2015  
  
Please cite:  
Rosenkranz D, Zischler H. proTRAC - a software for probabilistic piRNA cluster  
detection, visualization and analysis. 2012. BMC Bioinformatics 13:5.  
  
and (for proTRAC 2.0 and later):  
Rosenkranz D, Rudloff S, Bastuck K, Ketting RF, Zischler H. Tupaia small RNAs  
provide insights into function and evolution of RNAi-based transposon defense  
in mammals. 2015. RNA 21(5):911-922.  
  
Contact:  
David Rosenkranz  
Institute of Anthropology, small RNA group  
Johannes Gutenberg University Mainz  
email: rosenkranz@uni-mainz.de  
  
You can find the latest proTRAC version at:  
http://sourceforge.net/projects/protrac/files  
http://www.smallRNAgroup-mainz.de/software  
==============================================================================  
  
PARAMETERS:  
Map file: .............../storage/core/barbara/genhome/smallRNA/fertility/Sample\_motile/pirna/Sample\_motile\_26-33\_collapsed.fa.no-dust.map.weighted-10000-1000-b-0  
Genome file: ............/storage/core/barbara/genhome/smallRNA/fertility/Sample\_all/pirna/bt\_311\_chrY.fa  
RepeatMasker annotation: /storage/genomes/bt\_umd31/GCF\_000003055.6\_Bos\_taurus\_UMD\_3.1.1\_repeatMasker\_chr.out  
GeneSet:................./storage/core/barbara/genhome/smallRNA/fertility/Sample\_all/pirna/full.gtf  
  
Significant (p<=0.01) hit density will be calculated based  
on observed hit distribution.  
  
Sliding window size: ........................................ 5000 bp  
Sliding window increament: .................................. 1000 bp  
Normalize each hit by number of genomic hits: ............... 1 [0=no/1=yes]  
Normalize each hit by number of sequence reads: ............. 1 [0=no/1=yes]  
Normalize values (-> per million mapped reads): ............. 1 [0=no/1=yes]  
Min. fraction of hits with 1T(U) or 10A: .................... 0.75  
Alternatively: Min. fraction of hits with 1T(U) and 10A: .... 0.5  
Min. fraction of hits with typical piRNA length: ............ 0.75  
Typical piRNA length: ....................................... 26-33 nt  
Min. size of a piRNA cluster: ............................... 5000 bp.  
Min. number of hits (absolute): ............................. 0  
Min. number of hits (normalized): ........................... 0  
Min. fraction of hits on the mainstrand: .................... 0.75  
Top fraction of mapped sequences (in terms of read counts): . 1%  
Top fraction accounts for max. n% of sequence reads: ........ 90%  
Min. fraction of hits on each arm of a bidirectional cluster: 0.1  
Output image file for each cluster: ......................... 0 [0=no/1=yes]  
Output html file for each cluster: .......................... 1 [0=no/1=yes]  
Output a summary table: ..................................... 1 [0=no/1=yes]  
Output a FASTA file for each cluster (piRNA sequences): ..... 1 [0=no/1=yes]  
Output a FASTA file comprising cluster sequences: ........... 1 [0=no/1=yes]  
Search DNA motifs in clusters: .............................. 1 [0=no/1=yes]  
Output flanking sequences: +/- .............................. 0 bp  
Output ~.pTi file: .......................................... 1 [0=no/1=yes]  
==============================================================================  
  
  
Genome size (without gaps): ............ 2678902517 bp  
Gaps (N/X/-): .......................... 53837044 bp  
Mapped reads: .......................... 658825247023  
Non-identical sequences: ............... 514171  
Genomic hits: .......................... 764233  
Significant densitiy of mapped reads: .. 12867599.5173724 reads/kb

Show proTRAC cluster info
Hide proTRAC cluster info

|  |  |
| --- | --- |
| Location | chr10 |
| Coordinates | 100888851-100939949 |
| Size [bp] | 51099 |
| Sequence hit loci | 14211 |
| Mapped reads (normalized) | 15940590148 |
| Mapped reads (normalized) per kb | 311955031.4 |
| Normalized reads with 1T (1U) | 76.4% |
| Normalized reads with 10A | 29.6% |
| Normalized reads with length 26-33 nt | 100% |
| Normalized reads on the main strand(s) | 99.6% |
| Predicted directionality | bi:minus-plus (split between 100933553 and 100933615) |

100%

0%

1T (1U)  
reads

10A reads

26-33 nt  
reads

reads on mainstrand

**Either the amount of reads with 1T (1U) OR 10A has to exceed 75% (set with option: -1Tor10A)  
Alternatively the amount of reads with 1T (1U) AND 10A has to exceed 50% (set with option: -1Tand10A)  
Minimum amount of reads with preferred size is 75% (set with option: -pisize)  
Minimum amount of reads on the main strand(s) is 75% (set with option: -clstrand)**

Show read coverage
Hide read coverage

WHAT DO I SEE HERE?  
This chart shows the location of mapped sequence reads within a predicted piRNA cluster. The color refers to the number of genomic hits produced by the sequence read in question. A dark red bar indicates that this sequence read produces many other hits elsewhere in the genome. Many adjacent red or yellow bars can indicate the presence of a multi-copy element such as transposons or rRNA genes. A dark green bar indicates that this sequence read maps uniquely to this locus.

1 hit

2-5 hits

6-10 hits

11-20 hits

21-50 hits

51-100 hits

> 100 hits

chr10

100888851

100939949

Gene Set

RepeatMasker

Mapped  
Reads

335.78

plus strand

minus strand

335.78

Region: chr10 100790859-100888902. Max. coverage (+): 0. Max coverage (-): 6.78

Region: chr10 100888903-100889004. Max. coverage (+): 0. Max coverage (-): 0

Region: chr10 100889005-100889106. Max. coverage (+): 0. Max coverage (-): 0

Region: chr10 100889107-100889208. Max. coverage (+): 0. Max coverage (-): 1.19

Region: chr10 100889209-100889310. Max. coverage (+): 0. Max coverage (-): 8.71

Region: chr10 100889311-100889413. Max. coverage (+): 0. Max coverage (-): 8.7

Region: chr10 100889414-100889515. Max. coverage (+): 0. Max coverage (-): 2.63

Region: chr10 100889516-100889617. Max. coverage (+): 0. Max coverage (-): 17.56

Region: chr10 100889618-100889719. Max. coverage (+): 0. Max coverage (-): 8.3

Region: chr10 100889720-100889821. Max. coverage (+): 0. Max coverage (-): 10.63

Region: chr10 100889822-100889924. Max. coverage (+): 0. Max coverage (-): 4.65

Region: chr10 100889925-100890026. Max. coverage (+): 0. Max coverage (-): 0

Region: chr10 100890027-100890128. Max. coverage (+): 0. Max coverage (-): 0

Region: chr10 100890129-100890230. Max. coverage (+): 0. Max coverage (-): 0

Region: chr10 100890231-100890332. Max. coverage (+): 0. Max coverage (-): 0

Region: chr10 100890333-100890435. Max. coverage (+): 0. Max coverage (-): 0

Region: chr10 100890436-100890537. Max. coverage (+): 0. Max coverage (-): 0

Region: chr10 100890538-100890639. Max. coverage (+): 0. Max coverage (-): 0

Region: chr10 100890640-100890741. Max. coverage (+): 0. Max coverage (-): 0

Region: chr10 100890742-100890843. Max. coverage (+): 0. Max coverage (-): 0

Region: chr10 100890844-100890946. Max. coverage (+): 0. Max coverage (-): 0

Region: chr10 100890947-100891048. Max. coverage (+): 0. Max coverage (-): 0

Region: chr10 100891049-100891150. Max. coverage (+): 0. Max coverage (-): 0

Region: chr10 100891151-100891252. Max. coverage (+): 0. Max coverage (-): 0

Region: chr10 100891253-100891354. Max. coverage (+): 0. Max coverage (-): 0

Region: chr10 100891355-100891457. Max. coverage (+): 0. Max coverage (-): 0

Region: chr10 100891458-100891559. Max. coverage (+): 0. Max coverage (-): 0

Region: chr10 100891560-100891661. Max. coverage (+): 0. Max coverage (-): 0

Region: chr10 100891662-100891763. Max. coverage (+): 0. Max coverage (-): 0

Region: chr10 100891764-100891865. Max. coverage (+): 0. Max coverage (-): 0

Region: chr10 100891866-100891968. Max. coverage (+): 0. Max coverage (-): 0

Region: chr10 100891969-100892070. Max. coverage (+): 0. Max coverage (-): 0

Region: chr10 100892071-100892172. Max. coverage (+): 0. Max coverage (-): 0

Region: chr10 100892173-100892274. Max. coverage (+): 0. Max coverage (-): 0

Region: chr10 100892275-100892376. Max. coverage (+): 0. Max coverage (-): 0

Region: chr10 100892377-100892479. Max. coverage (+): 0. Max coverage (-): 0

Region: chr10 100892480-100892581. Max. coverage (+): 0. Max coverage (-): 0

Region: chr10 100892582-100892683. Max. coverage (+): 0. Max coverage (-): 0

Region: chr10 100892684-100892785. Max. coverage (+): 0. Max coverage (-): 0

Region: chr10 100892786-100892887. Max. coverage (+): 0. Max coverage (-): 0

Region: chr10 100892888-100892990. Max. coverage (+): 0. Max coverage (-): 0

Region: chr10 100892991-100893092. Max. coverage (+): 0. Max coverage (-): 0

Region: chr10 100893093-100893194. Max. coverage (+): 0. Max coverage (-): 0

Region: chr10 100893195-100893296. Max. coverage (+): 0. Max coverage (-): 0

Region: chr10 100893297-100893398. Max. coverage (+): 0. Max coverage (-): 0

Region: chr10 100893399-100893501. Max. coverage (+): 0. Max coverage (-): 0

Region: chr10 100893502-100893603. Max. coverage (+): 0. Max coverage (-): 0

Region: chr10 100893604-100893705. Max. coverage (+): 0. Max coverage (-): 0

Region: chr10 100893706-100893807. Max. coverage (+): 0. Max coverage (-): 10.36

Region: chr10 100893808-100893909. Max. coverage (+): 0. Max coverage (-): 0

Region: chr10 100893910-100894011. Max. coverage (+): 0. Max coverage (-): 0

Region: chr10 100894012-100894114. Max. coverage (+): 0. Max coverage (-): 0

Region: chr10 100894115-100894216. Max. coverage (+): 0. Max coverage (-): 0

Region: chr10 100894217-100894318. Max. coverage (+): 0. Max coverage (-): 0

Region: chr10 100894319-100894420. Max. coverage (+): 0. Max coverage (-): 0

Region: chr10 100894421-100894522. Max. coverage (+): 0. Max coverage (-): 4.22

Region: chr10 100894523-100894625. Max. coverage (+): 0. Max coverage (-): 0

Region: chr10 100894626-100894727. Max. coverage (+): 0. Max coverage (-): 0

Region: chr10 100894728-100894829. Max. coverage (+): 0. Max coverage (-): 0

Region: chr10 100894830-100894931. Max. coverage (+): 0. Max coverage (-): 0

Region: chr10 100894932-100895033. Max. coverage (+): 0. Max coverage (-): 18.52

Region: chr10 100895034-100895136. Max. coverage (+): 0. Max coverage (-): 1.43

Region: chr10 100895137-100895238. Max. coverage (+): 0. Max coverage (-): 8.57

Region: chr10 100895239-100895340. Max. coverage (+): 0. Max coverage (-): 4.12

Region: chr10 100895341-100895442. Max. coverage (+): 0. Max coverage (-): 0

Region: chr10 100895443-100895544. Max. coverage (+): 0. Max coverage (-): 26.26

Region: chr10 100895545-100895647. Max. coverage (+): 0. Max coverage (-): 37.95

Region: chr10 100895648-100895749. Max. coverage (+): 0. Max coverage (-): 29.66

Region: chr10 100895750-100895851. Max. coverage (+): 0. Max coverage (-): 0.95

Region: chr10 100895852-100895953. Max. coverage (+): 0. Max coverage (-): 0

Region: chr10 100895954-100896055. Max. coverage (+): 0. Max coverage (-): 0

Region: chr10 100896056-100896158. Max. coverage (+): 0. Max coverage (-): 0

Region: chr10 100896159-100896260. Max. coverage (+): 0. Max coverage (-): 0

Region: chr10 100896261-100896362. Max. coverage (+): 0. Max coverage (-): 0

Region: chr10 100896363-100896464. Max. coverage (+): 0. Max coverage (-): 18.95

Region: chr10 100896465-100896566. Max. coverage (+): 0. Max coverage (-): 0

Region: chr10 100896567-100896669. Max. coverage (+): 0. Max coverage (-): 0

Region: chr10 100896670-100896771. Max. coverage (+): 0. Max coverage (-): 0

Region: chr10 100896772-100896873. Max. coverage (+): 0. Max coverage (-): 0

Region: chr10 100896874-100896975. Max. coverage (+): 0. Max coverage (-): 0

Region: chr10 100896976-100897077. Max. coverage (+): 0. Max coverage (-): 0

Region: chr10 100897078-100897180. Max. coverage (+): 0. Max coverage (-): 0

Region: chr10 100897181-100897282. Max. coverage (+): 0. Max coverage (-): 0

Region: chr10 100897283-100897384. Max. coverage (+): 0. Max coverage (-): 0

Region: chr10 100897385-100897486. Max. coverage (+): 0. Max coverage (-): 2.02

Region: chr10 100897487-100897588. Max. coverage (+): 0. Max coverage (-): 0

Region: chr10 100897589-100897691. Max. coverage (+): 0. Max coverage (-): 0

Region: chr10 100897692-100897793. Max. coverage (+): 0. Max coverage (-): 0

Region: chr10 100897794-100897895. Max. coverage (+): 0. Max coverage (-): 0

Region: chr10 100897896-100897997. Max. coverage (+): 0. Max coverage (-): 0

Region: chr10 100897998-100898099. Max. coverage (+): 0. Max coverage (-): 0

Region: chr10 100898100-100898202. Max. coverage (+): 0. Max coverage (-): 0

Region: chr10 100898203-100898304. Max. coverage (+): 0. Max coverage (-): 0

Region: chr10 100898305-100898406. Max. coverage (+): 0. Max coverage (-): 0

Region: chr10 100898407-100898508. Max. coverage (+): 0. Max coverage (-): 0

Region: chr10 100898509-100898610. Max. coverage (+): 0. Max coverage (-): 0

Region: chr10 100898611-100898713. Max. coverage (+): 0. Max coverage (-): 0

Region: chr10 100898714-100898815. Max. coverage (+): 0. Max coverage (-): 0

Region: chr10 100898816-100898917. Max. coverage (+): 0. Max coverage (-): 0

Region: chr10 100898918-100899019. Max. coverage (+): 0. Max coverage (-): 4.1

Region: chr10 100899020-100899121. Max. coverage (+): 0. Max coverage (-): 15.91

Region: chr10 100899122-100899224. Max. coverage (+): 0. Max coverage (-): 3.57

Region: chr10 100899225-100899326. Max. coverage (+): 0. Max coverage (-): 0

Region: chr10 100899327-100899428. Max. coverage (+): 0. Max coverage (-): 0

Region: chr10 100899429-100899530. Max. coverage (+): 0. Max coverage (-): 0

Region: chr10 100899531-100899632. Max. coverage (+): 0. Max coverage (-): 0

Region: chr10 100899633-100899735. Max. coverage (+): 0. Max coverage (-): 0

Region: chr10 100899736-100899837. Max. coverage (+): 0. Max coverage (-): 0

Region: chr10 100899838-100899939. Max. coverage (+): 0. Max coverage (-): 0

Region: chr10 100899940-100900041. Max. coverage (+): 0. Max coverage (-): 0

Region: chr10 100900042-100900143. Max. coverage (+): 0. Max coverage (-): 0

Region: chr10 100900144-100900246. Max. coverage (+): 0. Max coverage (-): 0

Region: chr10 100900247-100900348. Max. coverage (+): 0. Max coverage (-): 0

Region: chr10 100900349-100900450. Max. coverage (+): 0. Max coverage (-): 0

Region: chr10 100900451-100900552. Max. coverage (+): 0. Max coverage (-): 0

Region: chr10 100900553-100900654. Max. coverage (+): 0. Max coverage (-): 0

Region: chr10 100900655-100900757. Max. coverage (+): 0. Max coverage (-): 0

Region: chr10 100900758-100900859. Max. coverage (+): 0. Max coverage (-): 0

Region: chr10 100900860-100900961. Max. coverage (+): 0. Max coverage (-): 0

Region: chr10 100900962-100901063. Max. coverage (+): 0. Max coverage (-): 0

Region: chr10 100901064-100901165. Max. coverage (+): 0. Max coverage (-): 6.43

Region: chr10 100901166-100901268. Max. coverage (+): 0. Max coverage (-): 0

Region: chr10 100901269-100901370. Max. coverage (+): 0. Max coverage (-): 0

Region: chr10 100901371-100901472. Max. coverage (+): 0. Max coverage (-): 0

Region: chr10 100901473-100901574. Max. coverage (+): 0. Max coverage (-): 0.86

Region: chr10 100901575-100901676. Max. coverage (+): 0. Max coverage (-): 3.5

Region: chr10 100901677-100901779. Max. coverage (+): 0. Max coverage (-): 0

Region: chr10 100901780-100901881. Max. coverage (+): 0. Max coverage (-): 0

Region: chr10 100901882-100901983. Max. coverage (+): 0. Max coverage (-): 0

Region: chr10 100901984-100902085. Max. coverage (+): 0. Max coverage (-): 0

Region: chr10 100902086-100902187. Max. coverage (+): 0. Max coverage (-): 0

Region: chr10 100902188-100902290. Max. coverage (+): 0. Max coverage (-): 3.6

Region: chr10 100902291-100902392. Max. coverage (+): 0. Max coverage (-): 1.43

Region: chr10 100902393-100902494. Max. coverage (+): 0. Max coverage (-): 0

Region: chr10 100902495-100902596. Max. coverage (+): 0. Max coverage (-): 4.94

Region: chr10 100902597-100902698. Max. coverage (+): 0. Max coverage (-): 10.45

Region: chr10 100902699-100902801. Max. coverage (+): 0. Max coverage (-): 1.06

Region: chr10 100902802-100902903. Max. coverage (+): 0. Max coverage (-): 0

Region: chr10 100902904-100903005. Max. coverage (+): 0. Max coverage (-): 0

Region: chr10 100903006-100903107. Max. coverage (+): 0. Max coverage (-): 0

Region: chr10 100903108-100903209. Max. coverage (+): 0. Max coverage (-): 0

Region: chr10 100903210-100903312. Max. coverage (+): 0. Max coverage (-): 0

Region: chr10 100903313-100903414. Max. coverage (+): 0. Max coverage (-): 0

Region: chr10 100903415-100903516. Max. coverage (+): 0. Max coverage (-): 0

Region: chr10 100903517-100903618. Max. coverage (+): 0. Max coverage (-): 3.96

Region: chr10 100903619-100903720. Max. coverage (+): 0. Max coverage (-): 0

Region: chr10 100903721-100903823. Max. coverage (+): 0. Max coverage (-): 0

Region: chr10 100903824-100903925. Max. coverage (+): 0. Max coverage (-): 0

Region: chr10 100903926-100904027. Max. coverage (+): 0. Max coverage (-): 0

Region: chr10 100904028-100904129. Max. coverage (+): 0. Max coverage (-): 4.15

Region: chr10 100904130-100904231. Max. coverage (+): 0. Max coverage (-): 0

Region: chr10 100904232-100904333. Max. coverage (+): 0. Max coverage (-): 0

Region: chr10 100904334-100904436. Max. coverage (+): 0. Max coverage (-): 0

Region: chr10 100904437-100904538. Max. coverage (+): 0. Max coverage (-): 0

Region: chr10 100904539-100904640. Max. coverage (+): 0. Max coverage (-): 0

Region: chr10 100904641-100904742. Max. coverage (+): 0. Max coverage (-): 9.14

Region: chr10 100904743-100904844. Max. coverage (+): 0. Max coverage (-): 4.41

Region: chr10 100904845-100904947. Max. coverage (+): 0. Max coverage (-): 4.52

Region: chr10 100904948-100905049. Max. coverage (+): 0. Max coverage (-): 1.65

Region: chr10 100905050-100905151. Max. coverage (+): 0. Max coverage (-): 0

Region: chr10 100905152-100905253. Max. coverage (+): 0. Max coverage (-): 0

Region: chr10 100905254-100905355. Max. coverage (+): 0. Max coverage (-): 0

Region: chr10 100905356-100905458. Max. coverage (+): 0. Max coverage (-): 0

Region: chr10 100905459-100905560. Max. coverage (+): 0. Max coverage (-): 6.16

Region: chr10 100905561-100905662. Max. coverage (+): 0. Max coverage (-): 0

Region: chr10 100905663-100905764. Max. coverage (+): 0. Max coverage (-): 0

Region: chr10 100905765-100905866. Max. coverage (+): 0. Max coverage (-): 28.85

Region: chr10 100905867-100905969. Max. coverage (+): 0. Max coverage (-): 0

Region: chr10 100905970-100906071. Max. coverage (+): 0. Max coverage (-): 0

Region: chr10 100906072-100906173. Max. coverage (+): 0. Max coverage (-): 5.69

Region: chr10 100906174-100906275. Max. coverage (+): 0. Max coverage (-): 5.69

Region: chr10 100906276-100906377. Max. coverage (+): 0. Max coverage (-): 1.29

Region: chr10 100906378-100906480. Max. coverage (+): 0. Max coverage (-): 0

Region: chr10 100906481-100906582. Max. coverage (+): 0. Max coverage (-): 3.08

Region: chr10 100906583-100906684. Max. coverage (+): 0. Max coverage (-): 11.52

Region: chr10 100906685-100906786. Max. coverage (+): 0. Max coverage (-): 13.41

Region: chr10 100906787-100906888. Max. coverage (+): 0. Max coverage (-): 6.4

Region: chr10 100906889-100906991. Max. coverage (+): 0. Max coverage (-): 8.71

Region: chr10 100906992-100907093. Max. coverage (+): 0. Max coverage (-): 16.56

Region: chr10 100907094-100907195. Max. coverage (+): 0. Max coverage (-): 17.2

Region: chr10 100907196-100907297. Max. coverage (+): 0. Max coverage (-): 8.52

Region: chr10 100907298-100907399. Max. coverage (+): 0. Max coverage (-): 13.6

Region: chr10 100907400-100907502. Max. coverage (+): 0. Max coverage (-): 0

Region: chr10 100907503-100907604. Max. coverage (+): 0. Max coverage (-): 0

Region: chr10 100907605-100907706. Max. coverage (+): 0. Max coverage (-): 0

Region: chr10 100907707-100907808. Max. coverage (+): 0. Max coverage (-): 0

Region: chr10 100907809-100907910. Max. coverage (+): 0. Max coverage (-): 0

Region: chr10 100907911-100908013. Max. coverage (+): 0. Max coverage (-): 1.4

Region: chr10 100908014-100908115. Max. coverage (+): 0. Max coverage (-): 8.57

Region: chr10 100908116-100908217. Max. coverage (+): 0. Max coverage (-): 9.82

Region: chr10 100908218-100908319. Max. coverage (+): 0. Max coverage (-): 8.53

Region: chr10 100908320-100908421. Max. coverage (+): 0. Max coverage (-): 24.99

Region: chr10 100908422-100908524. Max. coverage (+): 0. Max coverage (-): 15.67

Region: chr10 100908525-100908626. Max. coverage (+): 0. Max coverage (-): 18.57

Region: chr10 100908627-100908728. Max. coverage (+): 0. Max coverage (-): 10.52

Region: chr10 100908729-100908830. Max. coverage (+): 0. Max coverage (-): 42.12

Region: chr10 100908831-100908932. Max. coverage (+): 0. Max coverage (-): 16.63

Region: chr10 100908933-100909035. Max. coverage (+): 0. Max coverage (-): 24.46

Region: chr10 100909036-100909137. Max. coverage (+): 0. Max coverage (-): 9.41

Region: chr10 100909138-100909239. Max. coverage (+): 0. Max coverage (-): 0

Region: chr10 100909240-100909341. Max. coverage (+): 0. Max coverage (-): 0

Region: chr10 100909342-100909443. Max. coverage (+): 0. Max coverage (-): 0

Region: chr10 100909444-100909546. Max. coverage (+): 0. Max coverage (-): 0

Region: chr10 100909547-100909648. Max. coverage (+): 0. Max coverage (-): 0

Region: chr10 100909649-100909750. Max. coverage (+): 0. Max coverage (-): 10.49

Region: chr10 100909751-100909852. Max. coverage (+): 0. Max coverage (-): 29.81

Region: chr10 100909853-100909954. Max. coverage (+): 0. Max coverage (-): 21.19

Region: chr10 100909955-100910057. Max. coverage (+): 0. Max coverage (-): 25.48

Region: chr10 100910058-100910159. Max. coverage (+): 0. Max coverage (-): 34.55

Region: chr10 100910160-100910261. Max. coverage (+): 0. Max coverage (-): 17.78

Region: chr10 100910262-100910363. Max. coverage (+): 0. Max coverage (-): 0

Region: chr10 100910364-100910465. Max. coverage (+): 0. Max coverage (-): 0

Region: chr10 100910466-100910568. Max. coverage (+): 0. Max coverage (-): 37.47

Region: chr10 100910569-100910670. Max. coverage (+): 0. Max coverage (-): 31.3

Region: chr10 100910671-100910772. Max. coverage (+): 0. Max coverage (-): 37.07

Region: chr10 100910773-100910874. Max. coverage (+): 0. Max coverage (-): 57.24

Region: chr10 100910875-100910976. Max. coverage (+): 0. Max coverage (-): 14.72

Region: chr10 100910977-100911079. Max. coverage (+): 0. Max coverage (-): 66.67

Region: chr10 100911080-100911181. Max. coverage (+): 0. Max coverage (-): 19.71

Region: chr10 100911182-100911283. Max. coverage (+): 0. Max coverage (-): 85.26

Region: chr10 100911284-100911385. Max. coverage (+): 0. Max coverage (-): 60.33

Region: chr10 100911386-100911487. Max. coverage (+): 0. Max coverage (-): 38.05

Region: chr10 100911488-100911590. Max. coverage (+): 0. Max coverage (-): 30.29

Region: chr10 100911591-100911692. Max. coverage (+): 0. Max coverage (-): 26.24

Region: chr10 100911693-100911794. Max. coverage (+): 0. Max coverage (-): 62.77

Region: chr10 100911795-100911896. Max. coverage (+): 0. Max coverage (-): 32.67

Region: chr10 100911897-100911998. Max. coverage (+): 0. Max coverage (-): 49.36

Region: chr10 100911999-100912101. Max. coverage (+): 0. Max coverage (-): 6.26

Region: chr10 100912102-100912203. Max. coverage (+): 0. Max coverage (-): 1.79

Region: chr10 100912204-100912305. Max. coverage (+): 0. Max coverage (-): 40.04

Region: chr10 100912306-100912407. Max. coverage (+): 0.63. Max coverage (-): 24.86

Region: chr10 100912408-100912509. Max. coverage (+): 3.92. Max coverage (-): 117.38

Region: chr10 100912510-100912612. Max. coverage (+): 0. Max coverage (-): 0

Region: chr10 100912613-100912714. Max. coverage (+): 4.47. Max coverage (-): 8.46

Region: chr10 100912715-100912816. Max. coverage (+): 3.33. Max coverage (-): 0.53

Region: chr10 100912817-100912918. Max. coverage (+): 0. Max coverage (-): 72.33

Region: chr10 100912919-100913020. Max. coverage (+): 2.37. Max coverage (-): 21.82

Region: chr10 100913021-100913123. Max. coverage (+): 0. Max coverage (-): 8.16

Region: chr10 100913124-100913225. Max. coverage (+): 2.37. Max coverage (-): 56.95

Region: chr10 100913226-100913327. Max. coverage (+): 0. Max coverage (-): 99.72

Region: chr10 100913328-100913429. Max. coverage (+): 0. Max coverage (-): 22.34

Region: chr10 100913430-100913531. Max. coverage (+): 0. Max coverage (-): 16.08

Region: chr10 100913532-100913634. Max. coverage (+): 0. Max coverage (-): 27.53

Region: chr10 100913635-100913736. Max. coverage (+): 0. Max coverage (-): 6.94

Region: chr10 100913737-100913838. Max. coverage (+): 0. Max coverage (-): 41.1

Region: chr10 100913839-100913940. Max. coverage (+): 0. Max coverage (-): 23.69

Region: chr10 100913941-100914042. Max. coverage (+): 0. Max coverage (-): 42.39

Region: chr10 100914043-100914145. Max. coverage (+): 1.18. Max coverage (-): 14.29

Region: chr10 100914146-100914247. Max. coverage (+): 1.42. Max coverage (-): 66.94

Region: chr10 100914248-100914349. Max. coverage (+): 0.92. Max coverage (-): 50.27

Region: chr10 100914350-100914451. Max. coverage (+): 0. Max coverage (-): 105.13

Region: chr10 100914452-100914553. Max. coverage (+): 0. Max coverage (-): 19.54

Region: chr10 100914554-100914655. Max. coverage (+): 0. Max coverage (-): 33.54

Region: chr10 100914656-100914758. Max. coverage (+): 0. Max coverage (-): 20.08

Region: chr10 100914759-100914860. Max. coverage (+): 0. Max coverage (-): 77.79

Region: chr10 100914861-100914962. Max. coverage (+): 8.2. Max coverage (-): 10.44

Region: chr10 100914963-100915064. Max. coverage (+): 0. Max coverage (-): 6.71

Region: chr10 100915065-100915166. Max. coverage (+): 0. Max coverage (-): 10.24

Region: chr10 100915167-100915269. Max. coverage (+): 0. Max coverage (-): 8.51

Region: chr10 100915270-100915371. Max. coverage (+): 0. Max coverage (-): 78.7

Region: chr10 100915372-100915473. Max. coverage (+): 0. Max coverage (-): 35.29

Region: chr10 100915474-100915575. Max. coverage (+): 0. Max coverage (-): 22.99

Region: chr10 100915576-100915677. Max. coverage (+): 0. Max coverage (-): 67.47

Region: chr10 100915678-100915780. Max. coverage (+): 0. Max coverage (-): 62.55

Region: chr10 100915781-100915882. Max. coverage (+): 0.33. Max coverage (-): 107.93

Region: chr10 100915883-100915984. Max. coverage (+): 0. Max coverage (-): 20.37

Region: chr10 100915985-100916086. Max. coverage (+): 0. Max coverage (-): 4.64

Region: chr10 100916087-100916188. Max. coverage (+): 0. Max coverage (-): 34.59

Region: chr10 100916189-100916291. Max. coverage (+): 0. Max coverage (-): 45.47

Region: chr10 100916292-100916393. Max. coverage (+): 0. Max coverage (-): 24.81

Region: chr10 100916394-100916495. Max. coverage (+): 2.33. Max coverage (-): 41.53

Region: chr10 100916496-100916597. Max. coverage (+): 0. Max coverage (-): 19.08

Region: chr10 100916598-100916699. Max. coverage (+): 0. Max coverage (-): 16.72

Region: chr10 100916700-100916802. Max. coverage (+): 0. Max coverage (-): 17.14

Region: chr10 100916803-100916904. Max. coverage (+): 0. Max coverage (-): 16.26

Region: chr10 100916905-100917006. Max. coverage (+): 0. Max coverage (-): 15.19

Region: chr10 100917007-100917108. Max. coverage (+): 0. Max coverage (-): 111.72

Region: chr10 100917109-100917210. Max. coverage (+): 0. Max coverage (-): 28.07

Region: chr10 100917211-100917313. Max. coverage (+): 2.29. Max coverage (-): 28.15

Region: chr10 100917314-100917415. Max. coverage (+): 0. Max coverage (-): 44.98

Region: chr10 100917416-100917517. Max. coverage (+): 0. Max coverage (-): 44.26

Region: chr10 100917518-100917619. Max. coverage (+): 0. Max coverage (-): 14.32

Region: chr10 100917620-100917721. Max. coverage (+): 0. Max coverage (-): 148.35

Region: chr10 100917722-100917824. Max. coverage (+): 0. Max coverage (-): 80.6

Region: chr10 100917825-100917926. Max. coverage (+): 0. Max coverage (-): 18.26

Region: chr10 100917927-100918028. Max. coverage (+): 0. Max coverage (-): 7.25

Region: chr10 100918029-100918130. Max. coverage (+): 0. Max coverage (-): 1.26

Region: chr10 100918131-100918232. Max. coverage (+): 0. Max coverage (-): 17.25

Region: chr10 100918233-100918335. Max. coverage (+): 0. Max coverage (-): 41.25

Region: chr10 100918336-100918437. Max. coverage (+): 1.09. Max coverage (-): 28.13

Region: chr10 100918438-100918539. Max. coverage (+): 0. Max coverage (-): 38.15

Region: chr10 100918540-100918641. Max. coverage (+): 0. Max coverage (-): 34.52

Region: chr10 100918642-100918743. Max. coverage (+): 0. Max coverage (-): 45.38

Region: chr10 100918744-100918846. Max. coverage (+): 0. Max coverage (-): 48.62

Region: chr10 100918847-100918948. Max. coverage (+): 0. Max coverage (-): 18.12

Region: chr10 100918949-100919050. Max. coverage (+): 0. Max coverage (-): 11.44

Region: chr10 100919051-100919152. Max. coverage (+): 0. Max coverage (-): 7.03

Region: chr10 100919153-100919254. Max. coverage (+): 3.33. Max coverage (-): 35.33

Region: chr10 100919255-100919357. Max. coverage (+): 0. Max coverage (-): 103.57

Region: chr10 100919358-100919459. Max. coverage (+): 0. Max coverage (-): 3.08

Region: chr10 100919460-100919561. Max. coverage (+): 0. Max coverage (-): 53.53

Region: chr10 100919562-100919663. Max. coverage (+): 0. Max coverage (-): 31.29

Region: chr10 100919664-100919765. Max. coverage (+): 1.84. Max coverage (-): 15.17

Region: chr10 100919766-100919868. Max. coverage (+): 1.84. Max coverage (-): 6.05

Region: chr10 100919869-100919970. Max. coverage (+): 0. Max coverage (-): 15.82

Region: chr10 100919971-100920072. Max. coverage (+): 0. Max coverage (-): 28.17

Region: chr10 100920073-100920174. Max. coverage (+): 0. Max coverage (-): 12.74

Region: chr10 100920175-100920276. Max. coverage (+): 0. Max coverage (-): 64.24

Region: chr10 100920277-100920379. Max. coverage (+): 0. Max coverage (-): 16.23

Region: chr10 100920380-100920481. Max. coverage (+): 0. Max coverage (-): 37.79

Region: chr10 100920482-100920583. Max. coverage (+): 0. Max coverage (-): 21.03

Region: chr10 100920584-100920685. Max. coverage (+): 0. Max coverage (-): 21.5

Region: chr10 100920686-100920787. Max. coverage (+): 0. Max coverage (-): 45.12

Region: chr10 100920788-100920890. Max. coverage (+): 0. Max coverage (-): 70.95

Region: chr10 100920891-100920992. Max. coverage (+): 1.11. Max coverage (-): 35.76

Region: chr10 100920993-100921094. Max. coverage (+): 0. Max coverage (-): 77.27

Region: chr10 100921095-100921196. Max. coverage (+): 0. Max coverage (-): 12.9

Region: chr10 100921197-100921298. Max. coverage (+): 0. Max coverage (-): 47.26

Region: chr10 100921299-100921401. Max. coverage (+): 0. Max coverage (-): 35.63

Region: chr10 100921402-100921503. Max. coverage (+): 0. Max coverage (-): 22.63

Region: chr10 100921504-100921605. Max. coverage (+): 0. Max coverage (-): 55.27

Region: chr10 100921606-100921707. Max. coverage (+): 0. Max coverage (-): 68.57

Region: chr10 100921708-100921809. Max. coverage (+): 0. Max coverage (-): 1.85

Region: chr10 100921810-100921912. Max. coverage (+): 0. Max coverage (-): 0

Region: chr10 100921913-100922014. Max. coverage (+): 0. Max coverage (-): 2.6

Region: chr10 100922015-100922116. Max. coverage (+): 0. Max coverage (-): 2.26

Region: chr10 100922117-100922218. Max. coverage (+): 0. Max coverage (-): 27.28

Region: chr10 100922219-100922320. Max. coverage (+): 0. Max coverage (-): 24.9

Region: chr10 100922321-100922423. Max. coverage (+): 0. Max coverage (-): 4.21

Region: chr10 100922424-100922525. Max. coverage (+): 0.91. Max coverage (-): 17.14

Region: chr10 100922526-100922627. Max. coverage (+): 0. Max coverage (-): 11.86

Region: chr10 100922628-100922729. Max. coverage (+): 0. Max coverage (-): 8.24

Region: chr10 100922730-100922831. Max. coverage (+): 0. Max coverage (-): 42.29

Region: chr10 100922832-100922934. Max. coverage (+): 0. Max coverage (-): 38.63

Region: chr10 100922935-100923036. Max. coverage (+): 0. Max coverage (-): 43.12

Region: chr10 100923037-100923138. Max. coverage (+): 0. Max coverage (-): 0

Region: chr10 100923139-100923240. Max. coverage (+): 0. Max coverage (-): 0

Region: chr10 100923241-100923342. Max. coverage (+): 0. Max coverage (-): 14.81

Region: chr10 100923343-100923445. Max. coverage (+): 0. Max coverage (-): 20.17

Region: chr10 100923446-100923547. Max. coverage (+): 0. Max coverage (-): 21.26

Region: chr10 100923548-100923649. Max. coverage (+): 0. Max coverage (-): 18.28

Region: chr10 100923650-100923751. Max. coverage (+): 0. Max coverage (-): 46.08

Region: chr10 100923752-100923853. Max. coverage (+): 0. Max coverage (-): 52.21

Region: chr10 100923854-100923956. Max. coverage (+): 0. Max coverage (-): 0

Region: chr10 100923957-100924058. Max. coverage (+): 0. Max coverage (-): 0

Region: chr10 100924059-100924160. Max. coverage (+): 0. Max coverage (-): 0

Region: chr10 100924161-100924262. Max. coverage (+): 0. Max coverage (-): 9.84

Region: chr10 100924263-100924364. Max. coverage (+): 0. Max coverage (-): 14.26

Region: chr10 100924365-100924467. Max. coverage (+): 0. Max coverage (-): 41.39

Region: chr10 100924468-100924569. Max. coverage (+): 0. Max coverage (-): 11.08

Region: chr10 100924570-100924671. Max. coverage (+): 0. Max coverage (-): 40.1

Region: chr10 100924672-100924773. Max. coverage (+): 0. Max coverage (-): 63.81

Region: chr10 100924774-100924875. Max. coverage (+): 0. Max coverage (-): 48.42

Region: chr10 100924876-100924977. Max. coverage (+): 0. Max coverage (-): 82.44

Region: chr10 100924978-100925080. Max. coverage (+): 0. Max coverage (-): 100.11

Region: chr10 100925081-100925182. Max. coverage (+): 0. Max coverage (-): 115.45

Region: chr10 100925183-100925284. Max. coverage (+): 0. Max coverage (-): 140.1

Region: chr10 100925285-100925386. Max. coverage (+): 0. Max coverage (-): 36.37

Region: chr10 100925387-100925488. Max. coverage (+): 0. Max coverage (-): 0

Region: chr10 100925489-100925591. Max. coverage (+): 0. Max coverage (-): 10.45

Region: chr10 100925592-100925693. Max. coverage (+): 0. Max coverage (-): 26.07

Region: chr10 100925694-100925795. Max. coverage (+): 0. Max coverage (-): 21.3

Region: chr10 100925796-100925897. Max. coverage (+): 0. Max coverage (-): 34.89

Region: chr10 100925898-100925999. Max. coverage (+): 0. Max coverage (-): 22.56

Region: chr10 100926000-100926102. Max. coverage (+): 0. Max coverage (-): 44.17

Region: chr10 100926103-100926204. Max. coverage (+): 0. Max coverage (-): 32.13

Region: chr10 100926205-100926306. Max. coverage (+): 0. Max coverage (-): 21.97

Region: chr10 100926307-100926408. Max. coverage (+): 0. Max coverage (-): 31.61

Region: chr10 100926409-100926510. Max. coverage (+): 0. Max coverage (-): 22.8

Region: chr10 100926511-100926613. Max. coverage (+): 0. Max coverage (-): 33.13

Region: chr10 100926614-100926715. Max. coverage (+): 0. Max coverage (-): 84.88

Region: chr10 100926716-100926817. Max. coverage (+): 0. Max coverage (-): 56.6

Region: chr10 100926818-100926919. Max. coverage (+): 0. Max coverage (-): 64.41

Region: chr10 100926920-100927021. Max. coverage (+): 0. Max coverage (-): 40.62

Region: chr10 100927022-100927124. Max. coverage (+): 0. Max coverage (-): 31.6

Region: chr10 100927125-100927226. Max. coverage (+): 0. Max coverage (-): 82.15

Region: chr10 100927227-100927328. Max. coverage (+): 0. Max coverage (-): 94.39

Region: chr10 100927329-100927430. Max. coverage (+): 0. Max coverage (-): 0

Region: chr10 100927431-100927532. Max. coverage (+): 0. Max coverage (-): 16.97

Region: chr10 100927533-100927635. Max. coverage (+): 0. Max coverage (-): 13.16

Region: chr10 100927636-100927737. Max. coverage (+): 0. Max coverage (-): 9.85

Region: chr10 100927738-100927839. Max. coverage (+): 0. Max coverage (-): 16.78

Region: chr10 100927840-100927941. Max. coverage (+): 0. Max coverage (-): 32.9

Region: chr10 100927942-100928043. Max. coverage (+): 0. Max coverage (-): 2

Region: chr10 100928044-100928146. Max. coverage (+): 0. Max coverage (-): 12.56

Region: chr10 100928147-100928248. Max. coverage (+): 0. Max coverage (-): 36.28

Region: chr10 100928249-100928350. Max. coverage (+): 0. Max coverage (-): 53.07

Region: chr10 100928351-100928452. Max. coverage (+): 0. Max coverage (-): 165.19

Region: chr10 100928453-100928554. Max. coverage (+): 0. Max coverage (-): 0

Region: chr10 100928555-100928657. Max. coverage (+): 0. Max coverage (-): 0

Region: chr10 100928658-100928759. Max. coverage (+): 0. Max coverage (-): 15.67

Region: chr10 100928760-100928861. Max. coverage (+): 0. Max coverage (-): 6.43

Region: chr10 100928862-100928963. Max. coverage (+): 0. Max coverage (-): 0

Region: chr10 100928964-100929065. Max. coverage (+): 3.27. Max coverage (-): 9.17

Region: chr10 100929066-100929168. Max. coverage (+): 0. Max coverage (-): 43.94

Region: chr10 100929169-100929270. Max. coverage (+): 0. Max coverage (-): 9.94

Region: chr10 100929271-100929372. Max. coverage (+): 0. Max coverage (-): 12.9

Region: chr10 100929373-100929474. Max. coverage (+): 1.52. Max coverage (-): 21.64

Region: chr10 100929475-100929576. Max. coverage (+): 0. Max coverage (-): 30.95

Region: chr10 100929577-100929679. Max. coverage (+): 0. Max coverage (-): 69.39

Region: chr10 100929680-100929781. Max. coverage (+): 0. Max coverage (-): 51.05

Region: chr10 100929782-100929883. Max. coverage (+): 0. Max coverage (-): 96.41

Region: chr10 100929884-100929985. Max. coverage (+): 0. Max coverage (-): 150.07

Region: chr10 100929986-100930087. Max. coverage (+): 0. Max coverage (-): 55.18

Region: chr10 100930088-100930190. Max. coverage (+): 0. Max coverage (-): 19.9

Region: chr10 100930191-100930292. Max. coverage (+): 0. Max coverage (-): 76.72

Region: chr10 100930293-100930394. Max. coverage (+): 0. Max coverage (-): 17.7

Region: chr10 100930395-100930496. Max. coverage (+): 0. Max coverage (-): 48.68

Region: chr10 100930497-100930598. Max. coverage (+): 4.84. Max coverage (-): 54.66

Region: chr10 100930599-100930701. Max. coverage (+): 0. Max coverage (-): 84.93

Region: chr10 100930702-100930803. Max. coverage (+): 0. Max coverage (-): 5.91

Region: chr10 100930804-100930905. Max. coverage (+): 0. Max coverage (-): 1.29

Region: chr10 100930906-100931007. Max. coverage (+): 0. Max coverage (-): 39.15

Region: chr10 100931008-100931109. Max. coverage (+): 0. Max coverage (-): 9.86

Region: chr10 100931110-100931212. Max. coverage (+): 0. Max coverage (-): 14.37

Region: chr10 100931213-100931314. Max. coverage (+): 0. Max coverage (-): 57

Region: chr10 100931315-100931416. Max. coverage (+): 0. Max coverage (-): 18.18

Region: chr10 100931417-100931518. Max. coverage (+): 0. Max coverage (-): 66.71

Region: chr10 100931519-100931620. Max. coverage (+): 2.23. Max coverage (-): 13.02

Region: chr10 100931621-100931723. Max. coverage (+): 0. Max coverage (-): 45.19

Region: chr10 100931724-100931825. Max. coverage (+): 0. Max coverage (-): 276.37

Region: chr10 100931826-100931927. Max. coverage (+): 0. Max coverage (-): 335.78

Region: chr10 100931928-100932029. Max. coverage (+): 0. Max coverage (-): 0

Region: chr10 100932030-100932131. Max. coverage (+): 0. Max coverage (-): 12.64

Region: chr10 100932132-100932234. Max. coverage (+): 0. Max coverage (-): 4.86

Region: chr10 100932235-100932336. Max. coverage (+): 0. Max coverage (-): 20.2

Region: chr10 100932337-100932438. Max. coverage (+): 0. Max coverage (-): 7.29

Region: chr10 100932439-100932540. Max. coverage (+): 0. Max coverage (-): 15.68

Region: chr10 100932541-100932642. Max. coverage (+): 0. Max coverage (-): 5.44

Region: chr10 100932643-100932745. Max. coverage (+): 0. Max coverage (-): 53.97

Region: chr10 100932746-100932847. Max. coverage (+): 0. Max coverage (-): 11.94

Region: chr10 100932848-100932949. Max. coverage (+): 0. Max coverage (-): 11.24

Region: chr10 100932950-100933051. Max. coverage (+): 0. Max coverage (-): 27.94

Region: chr10 100933052-100933153. Max. coverage (+): 0.28. Max coverage (-): 65.39

Region: chr10 100933154-100933256. Max. coverage (+): 0.79. Max coverage (-): 34.65

Region: chr10 100933257-100933358. Max. coverage (+): 0. Max coverage (-): 0

Region: chr10 100933359-100933460. Max. coverage (+): 0. Max coverage (-): 28.24

Region: chr10 100933461-100933562. Max. coverage (+): 0.44. Max coverage (-): 2.03

Region: chr10 100933563-100933664. Max. coverage (+): 20.8. Max coverage (-): 0

Region: chr10 100933665-100933767. Max. coverage (+): 28.54. Max coverage (-): 0

Region: chr10 100933768-100933869. Max. coverage (+): 34.31. Max coverage (-): 0

Region: chr10 100933870-100933971. Max. coverage (+): 16.89. Max coverage (-): 3.04

Region: chr10 100933972-100934073. Max. coverage (+): 0. Max coverage (-): 0

Region: chr10 100934074-100934175. Max. coverage (+): 2.69. Max coverage (-): 0

Region: chr10 100934176-100934278. Max. coverage (+): 38.1. Max coverage (-): 0

Region: chr10 100934279-100934380. Max. coverage (+): 20.97. Max coverage (-): 1.14

Region: chr10 100934381-100934482. Max. coverage (+): 30.18. Max coverage (-): 1.8

Region: chr10 100934483-100934584. Max. coverage (+): 39.81. Max coverage (-): 0

Region: chr10 100934585-100934686. Max. coverage (+): 26.68. Max coverage (-): 0

Region: chr10 100934687-100934789. Max. coverage (+): 1.78. Max coverage (-): 0

Region: chr10 100934790-100934891. Max. coverage (+): 9.56. Max coverage (-): 0

Region: chr10 100934892-100934993. Max. coverage (+): 20.48. Max coverage (-): 5.18

Region: chr10 100934994-100935095. Max. coverage (+): 60.35. Max coverage (-): 3.45

Region: chr10 100935096-100935197. Max. coverage (+): 24.35. Max coverage (-): 2.18

Region: chr10 100935198-100935299. Max. coverage (+): 46.52. Max coverage (-): 0

Region: chr10 100935300-100935402. Max. coverage (+): 0. Max coverage (-): 0

Region: chr10 100935403-100935504. Max. coverage (+): 0. Max coverage (-): 0

Region: chr10 100935505-100935606. Max. coverage (+): 0. Max coverage (-): 0

Region: chr10 100935607-100935708. Max. coverage (+): 60.99. Max coverage (-): 0.9

Region: chr10 100935709-100935810. Max. coverage (+): 127.35. Max coverage (-): 0

Region: chr10 100935811-100935913. Max. coverage (+): 12.57. Max coverage (-): 0

Region: chr10 100935914-100936015. Max. coverage (+): 48.43. Max coverage (-): 0

Region: chr10 100936016-100936117. Max. coverage (+): 16.21. Max coverage (-): 0

Region: chr10 100936118-100936219. Max. coverage (+): 11.38. Max coverage (-): 0

Region: chr10 100936220-100936321. Max. coverage (+): 5.71. Max coverage (-): 0

Region: chr10 100936322-100936424. Max. coverage (+): 25.1. Max coverage (-): 0

Region: chr10 100936425-100936526. Max. coverage (+): 36.08. Max coverage (-): 0

Region: chr10 100936527-100936628. Max. coverage (+): 107.29. Max coverage (-): 0

Region: chr10 100936629-100936730. Max. coverage (+): 92.82. Max coverage (-): 0

Region: chr10 100936731-100936832. Max. coverage (+): 49.79. Max coverage (-): 0

Region: chr10 100936833-100936935. Max. coverage (+): 95.01. Max coverage (-): 0

Region: chr10 100936936-100937037. Max. coverage (+): 59.65. Max coverage (-): 0

Region: chr10 100937038-100937139. Max. coverage (+): 32.4. Max coverage (-): 0

Region: chr10 100937140-100937241. Max. coverage (+): 52.76. Max coverage (-): 0

Region: chr10 100937242-100937343. Max. coverage (+): 51.23. Max coverage (-): 0

Region: chr10 100937344-100937446. Max. coverage (+): 72.27. Max coverage (-): 0

Region: chr10 100937447-100937548. Max. coverage (+): 34.94. Max coverage (-): 0

Region: chr10 100937549-100937650. Max. coverage (+): 7.32. Max coverage (-): 0

Region: chr10 100937651-100937752. Max. coverage (+): 89.01. Max coverage (-): 0

Region: chr10 100937753-100937854. Max. coverage (+): 33.32. Max coverage (-): 1.62

Region: chr10 100937855-100937957. Max. coverage (+): 6.55. Max coverage (-): 0

Region: chr10 100937958-100938059. Max. coverage (+): 53.19. Max coverage (-): 0

Region: chr10 100938060-100938161. Max. coverage (+): 38.77. Max coverage (-): 0

Region: chr10 100938162-100938263. Max. coverage (+): 9.87. Max coverage (-): 0

Region: chr10 100938264-100938365. Max. coverage (+): 29.05. Max coverage (-): 0

Region: chr10 100938366-100938468. Max. coverage (+): 71.34. Max coverage (-): 5.27

Region: chr10 100938469-100938570. Max. coverage (+): 34.75. Max coverage (-): 0

Region: chr10 100938571-100938672. Max. coverage (+): 97.7. Max coverage (-): 0

Region: chr10 100938673-100938774. Max. coverage (+): 69.85. Max coverage (-): 0

Region: chr10 100938775-100938876. Max. coverage (+): 17.12. Max coverage (-): 0

Region: chr10 100938877-100938979. Max. coverage (+): 18.99. Max coverage (-): 0.69

Region: chr10 100938980-100939081. Max. coverage (+): 44.88. Max coverage (-): 0

Region: chr10 100939082-100939183. Max. coverage (+): 6.84. Max coverage (-): 0

Region: chr10 100939184-100939285. Max. coverage (+): 35.45. Max coverage (-): 0

Region: chr10 100939286-100939387. Max. coverage (+): 37.92. Max coverage (-): 0

Region: chr10 100939388-100939490. Max. coverage (+): 27.28. Max coverage (-): 0

Region: chr10 100939491-100939592. Max. coverage (+): 34.87. Max coverage (-): 0

Region: chr10 100939593-100939694. Max. coverage (+): 45.49. Max coverage (-): 2.05

Region: chr10 100939695-100939796. Max. coverage (+): 15.76. Max coverage (-): 1.34

Region: chr10 100939797-100939898. Max. coverage (+): 62.82. Max coverage (-): 1.52

Region: chr10 100939899-. Max. coverage (+): 3.81. Max coverage (-): 0

RepeatMasker Color Code

**+**

100-98% Identity

<98-95% Identity

<95-90% Identity

<90-85% Identity

<85-80% Identity

<80-75% Identity

<75-70% Identity

<70% Identity

**-**

Gene Set Color Code

**+**

Gene

Pseudogene

**-**

Topology/Coverage Color Code

Coverage Plus Strand

Coverage Minus Strand

Mainstrand: Plus

Mainstrand: Minus

Complementary Strand

Flanking Region  
(if option -flank >0)

Gene Set Annotation  
  
RepeatMasker Annotation  

**1. Bov-tA1**: 100888938-100889118 (-), Divergence to consensus: 19.1%  
**2. Tigger1**: 100889953-100890343 (+), Divergence to consensus: 19%  
**3. BovB**: 100890358-100890445 (+), Divergence to consensus: 20.6%  
**4. BTLTR1**: 100890446-100890515 (-), Divergence to consensus: 8.6%  
**5. BovB**: 100890516-100891363 (+), Divergence to consensus: 12.5%  
**6. ART2A**: 100891365-100891875 (+), Divergence to consensus: 16.5%  
**7. Tigger1**: 100891874-100892075 (+), Divergence to consensus: 27%  
**8. Bov-tA2**: 100892075-100892274 (-), Divergence to consensus: 15.5%  
**9. Tigger1**: 100892277-100893689 (+), Divergence to consensus: 24.5%  
**10. MLT1E2**: 100893829-100894423 (-), Divergence to consensus: 30.3%  
**11. Charlie2a**: 100894503-100894637 (-), Divergence to consensus: 45.8%  
**12. Bov-tA1**: 100894638-100894841 (+), Divergence to consensus: 26%  
**13. Charlie2a**: 100894842-100895302 (-), Divergence to consensus: 45.8%  
**14. LTR102\_Mam**: 100895316-100895522 (-), Divergence to consensus: 23.8%  
**15. ORSL**: 100895524-100895732 (+), Divergence to consensus: 44.8%  
**16. LTR102\_Mam**: 100895813-100896371 (-), Divergence to consensus: 43.3%  
**17. L1-2\_BT**: 100896483-100897000 (+), Divergence to consensus: 30%  
**18. Bov-tA2**: 100897017-100897205 (-), Divergence to consensus: 16.4%  
**19. Bov-tA2**: 100897206-100897326 (-), Divergence to consensus: 21.6%  
**20. AT\_rich**: 100897343-100897377 (+), Divergence to consensus: 65.7%  
**21. Charlie2a**: 100897673-100897766 (-), Divergence to consensus: 46.5%  
**22. BOV-A2**: 100897767-100898037 (-), Divergence to consensus: 4.4%  
**23. Charlie2a**: 100898038-100898185 (-), Divergence to consensus: 46.7%  
**24. BTLTR1J3**: 100898186-100898595 (+), Divergence to consensus: 10.6%  
**25. Charlie2a**: 100898596-100898960 (-), Divergence to consensus: 46.7%  
**26. ART2A**: 100899155-100899579 (+), Divergence to consensus: 19.4%  
**27. BTLTR1**: 100899580-100900824 (+), Divergence to consensus: 3.8%  
**28. ART2A**: 100900825-100900919 (+), Divergence to consensus: 16.7%  
**29. L2b**: 100900998-100901077 (-), Divergence to consensus: 31.3%  
**30. MER58D**: 100901226-100901307 (-), Divergence to consensus: 35%  
**31. HAL1**: 100901740-100902188 (-), Divergence to consensus: 47.4%  
**32. MLT1J2**: 100902398-100902564 (-), Divergence to consensus: 35.6%  
**33. MLT1J2-int**: 100902780-100902822 (-), Divergence to consensus: 41.9%  
**34. BOV-A2**: 100902823-100903081 (-), Divergence to consensus: 11.6%  
**35. MLT1J2-int**: 100903082-100903328 (-), Divergence to consensus: 37.1%  
**36. Bov-tA2**: 100903349-100903533 (-), Divergence to consensus: 35.9%  
**37. MLT1J2-int**: 100903796-100903963 (-), Divergence to consensus: 38.9%  
**38. MLT1J2**: 100903979-100904072 (-), Divergence to consensus: 36.4%  
**39. MLT1J2**: 100904164-100904308 (-), Divergence to consensus: 34.7%  
**40. HAL1**: 100904343-100904592 (-), Divergence to consensus: 37.8%  
**41. HAL1**: 100904597-100904932 (-), Divergence to consensus: 49.7%  
**42. HAL1**: 100904983-100905459 (-), Divergence to consensus: 44.5%  
**43. MER115**: 100905546-100905753 (+), Divergence to consensus: 40.7%  
**44. Zaphod**: 100905863-100906007 (+), Divergence to consensus: 31.9%  
**45. HAL1**: 100906015-100906058 (-), Divergence to consensus: 22.7%  
**46. HAL1**: 100906058-100906109 (-), Divergence to consensus: 23.1%  
**47. (TTTTG)n**: 100906334-100906357 (+), Divergence to consensus: 4.2%  
**48. L1\_BT**: 100907359-100907485 (+), Divergence to consensus: 4.7%  
**49. BTLTR1J**: 100907486-100907556 (-), Divergence to consensus: 28.2%  
**50. L1\_BT**: 100907557-100907971 (+), Divergence to consensus: 18.7%  
**51. AT\_rich**: 100907972-100907993 (+), Divergence to consensus: 50%  
**52. LSU-rRNA\_Hsa**: 100909090-100909358 (+), Divergence to consensus: 38.3%  
**53. LSU-rRNA\_Hsa**: 100909469-100909665 (+), Divergence to consensus: 42%  
**54. LTR78B**: 100909844-100910137 (-), Divergence to consensus: 46.9%  
**55. LTR78B**: 100910311-100910463 (-), Divergence to consensus: 40.5%  
**56. LTR78B**: 100910885-100910964 (-), Divergence to consensus: 35%  
**57. MamRep1894**: 100912514-100912592 (+), Divergence to consensus: 20.2%  
**58. MER94B**: 100916564-100916610 (+), Divergence to consensus: 21.3%  
**59. Bov-tA2**: 100921784-100921973 (-), Divergence to consensus: 27.5%  
**60. MIRb**: 100923010-100923220 (+), Divergence to consensus: 31.7%  
**61. L2b**: 100923866-100924023 (-), Divergence to consensus: 46.1%  
**62. (A)n**: 100924161-100924187 (+), Divergence to consensus: 11.1%  
**63. Bov-tA2**: 100925399-100925536 (+), Divergence to consensus: 13%  
**64. BOV-A2**: 100927300-100927462 (+), Divergence to consensus: 5.5%  
**65. Bov-tA2**: 100928490-100928669 (+), Divergence to consensus: 16.1%  
**66. MER5A1**: 100928791-100928939 (-), Divergence to consensus: 36.4%  
**67. AT\_rich**: 100930324-100930348 (+), Divergence to consensus: 32%  
**68. MIR**: 100931886-100932107 (-), Divergence to consensus: 39%  
**69. L2b**: 100933982-100934084 (+), Divergence to consensus: 40%  
**70. MIR3**: 100934088-100934132 (+), Divergence to consensus: 24.4%  
**71. GA-rich**: 100934728-100934801 (+), Divergence to consensus: 22%  
**72. L2c**: 100935305-100935640 (+), Divergence to consensus: 46.6%  
**73. L3**: 100936053-100936159 (+), Divergence to consensus: 42.8%  
**74. G-rich**: 100937115-100937166 (+), Divergence to consensus: 23.1%  
**75. GC\_rich**: 100939546-100939567 (+), Divergence to consensus: 50%  
**76. GC\_rich**: 100939548-100939585 (+), Divergence to consensus: 65.8%

  
Transcription Factor Binding Sites  

**RFX4\_2** (Sequence: GTATCTAAG (-): 100907130)  
**RFX4\_2** (Sequence: GTATCTAAG (-): 100930933)  
**RFX4\_2** (Sequence: GTAACCATG (-): 100938759)  
**RFX4\_1** (Sequence: GTTGCTATG (-): 100908054)  
**RFX4\_1** (Sequence: GTTGCCACG (-): 100912973)  
**RFX4\_1** (Sequence: GTTGCCAAG (-): 100917775)  
**SPZ1** (Sequence: CTGAAACCCT (-): 100908736)  
**SPZ1** (Sequence: CTGATACCCT (-): 100914108)  
**RFX4\_2** (Sequence: CTTAGTTAC (+): 100895110)  
**RFX4\_2** (Sequence: CTTGGATAC (+): 100923398)  
**Gata4** (Sequence: AGATAAC (-): 100910564)  
**Gata4** (Sequence: AGATAAG (-): 100911433)  
**Gata4** (Sequence: AGATAAG (-): 100929022)  
**Gata4** (Sequence: AGATAAG (-): 100938434)  
**SOX9** (Sequence: TTATTGTT (+): 100927146)  
**Gata4** (Sequence: GTTATCT (+): 100906553)  
**Gata4** (Sequence: CTTATCT (+): 100923460)  
**Gata4** (Sequence: CTTATCT (+): 100924957)  
**Gata4** (Sequence: CTTATCT (+): 100932396)
