# Supplementary material for: Small RNA sequencing of cryopreserved semen from single bull revealed altered miRNAs and piRNAs expression between High- and Low-motile sperm populations
Source: BMC Genomics. 2017 Jan 4;18:14. doi: 10.1186/s12864-016-3394-7 (PMC5209821; doi:10.1186/s12864-016-3394-7)
Supplement: Additional file 3: — Details for each piRNA clusters found in High Motile (HM) sperm fraction. Genes, repeats, transposable elements and transcription factors binding sites falling within the cluster regions were reported. (ZIP 1896 kb) [file 12864_2016_3394_MOESM3_ESM.zip › 80.html]

piRNA cluster 80


Predicted piRNA cluster no. 80     previous   next
  

Show proTRAC run info
Hide proTRAC run info

================================= proTRAC ====================================  
VERSION: 2.1                                    LAST MODIFIED: 06. October 2015  
  
Please cite:  
Rosenkranz D, Zischler H. proTRAC - a software for probabilistic piRNA cluster  
detection, visualization and analysis. 2012. BMC Bioinformatics 13:5.  
  
and (for proTRAC 2.0 and later):  
Rosenkranz D, Rudloff S, Bastuck K, Ketting RF, Zischler H. Tupaia small RNAs  
provide insights into function and evolution of RNAi-based transposon defense  
in mammals. 2015. RNA 21(5):911-922.  
  
Contact:  
David Rosenkranz  
Institute of Anthropology, small RNA group  
Johannes Gutenberg University Mainz  
email: rosenkranz@uni-mainz.de  
  
You can find the latest proTRAC version at:  
http://sourceforge.net/projects/protrac/files  
http://www.smallRNAgroup-mainz.de/software  
==============================================================================  
  
PARAMETERS:  
Map file: .............../storage/core/barbara/genhome/smallRNA/fertility/Sample\_motile/pirna/Sample\_motile\_26-33\_collapsed.fa.no-dust.map.weighted-10000-1000-b-0  
Genome file: ............/storage/core/barbara/genhome/smallRNA/fertility/Sample\_all/pirna/bt\_311\_chrY.fa  
RepeatMasker annotation: /storage/genomes/bt\_umd31/GCF\_000003055.6\_Bos\_taurus\_UMD\_3.1.1\_repeatMasker\_chr.out  
GeneSet:................./storage/core/barbara/genhome/smallRNA/fertility/Sample\_all/pirna/full.gtf  
  
Significant (p<=0.01) hit density will be calculated based  
on observed hit distribution.  
  
Sliding window size: ........................................ 5000 bp  
Sliding window increament: .................................. 1000 bp  
Normalize each hit by number of genomic hits: ............... 1 [0=no/1=yes]  
Normalize each hit by number of sequence reads: ............. 1 [0=no/1=yes]  
Normalize values (-> per million mapped reads): ............. 1 [0=no/1=yes]  
Min. fraction of hits with 1T(U) or 10A: .................... 0.75  
Alternatively: Min. fraction of hits with 1T(U) and 10A: .... 0.5  
Min. fraction of hits with typical piRNA length: ............ 0.75  
Typical piRNA length: ....................................... 26-33 nt  
Min. size of a piRNA cluster: ............................... 5000 bp.  
Min. number of hits (absolute): ............................. 0  
Min. number of hits (normalized): ........................... 0  
Min. fraction of hits on the mainstrand: .................... 0.75  
Top fraction of mapped sequences (in terms of read counts): . 1%  
Top fraction accounts for max. n% of sequence reads: ........ 90%  
Min. fraction of hits on each arm of a bidirectional cluster: 0.1  
Output image file for each cluster: ......................... 0 [0=no/1=yes]  
Output html file for each cluster: .......................... 1 [0=no/1=yes]  
Output a summary table: ..................................... 1 [0=no/1=yes]  
Output a FASTA file for each cluster (piRNA sequences): ..... 1 [0=no/1=yes]  
Output a FASTA file comprising cluster sequences: ........... 1 [0=no/1=yes]  
Search DNA motifs in clusters: .............................. 1 [0=no/1=yes]  
Output flanking sequences: +/- .............................. 0 bp  
Output ~.pTi file: .......................................... 1 [0=no/1=yes]  
==============================================================================  
  
  
Genome size (without gaps): ............ 2678902517 bp  
Gaps (N/X/-): .......................... 53837044 bp  
Mapped reads: .......................... 658825247023  
Non-identical sequences: ............... 514171  
Genomic hits: .......................... 764233  
Significant densitiy of mapped reads: .. 12867599.5173724 reads/kb

Show proTRAC cluster info
Hide proTRAC cluster info

|  |  |
| --- | --- |
| Location | chr3 |
| Coordinates | 118174589-118179806 |
| Size [bp] | 5218 |
| Sequence hit loci | 560 |
| Mapped reads (normalized) | 665969010 |
| Mapped reads (normalized) per kb | 127629170.2 |
| Normalized reads with 1T (1U) | 81.9% |
| Normalized reads with 10A | 32.8% |
| Normalized reads with length 26-33 nt | 100% |
| Normalized reads on the main strand(s) | 99.8% |
| Predicted directionality | mono:minus |

100%

0%

1T (1U)  
reads

10A reads

26-33 nt  
reads

reads on mainstrand

**Either the amount of reads with 1T (1U) OR 10A has to exceed 75% (set with option: -1Tor10A)  
Alternatively the amount of reads with 1T (1U) AND 10A has to exceed 50% (set with option: -1Tand10A)  
Minimum amount of reads with preferred size is 75% (set with option: -pisize)  
Minimum amount of reads on the main strand(s) is 75% (set with option: -clstrand)**

Show read coverage
Hide read coverage

WHAT DO I SEE HERE?  
This chart shows the location of mapped sequence reads within a predicted piRNA cluster. The color refers to the number of genomic hits produced by the sequence read in question. A dark red bar indicates that this sequence read produces many other hits elsewhere in the genome. Many adjacent red or yellow bars can indicate the presence of a multi-copy element such as transposons or rRNA genes. A dark green bar indicates that this sequence read maps uniquely to this locus.

1 hit

2-5 hits

6-10 hits

11-20 hits

21-50 hits

51-100 hits

> 100 hits

chr3

118174589

118179806

Gene Set

RepeatMasker

Mapped  
Reads

159.05

plus strand

minus strand

159.05

Region: chr3 94049379-118174594. Max. coverage (+): 0. Max coverage (-): 1.03

Region: chr3 118174595-118174604. Max. coverage (+): 0. Max coverage (-): 1.03

Region: chr3 118174605-118174615. Max. coverage (+): 0. Max coverage (-): 2.09

Region: chr3 118174616-118174625. Max. coverage (+): 0. Max coverage (-): 2.09

Region: chr3 118174626-118174635. Max. coverage (+): 0. Max coverage (-): 0

Region: chr3 118174636-118174646. Max. coverage (+): 0. Max coverage (-): 0

Region: chr3 118174647-118174656. Max. coverage (+): 0. Max coverage (-): 0

Region: chr3 118174657-118174667. Max. coverage (+): 0. Max coverage (-): 0

Region: chr3 118174668-118174677. Max. coverage (+): 0. Max coverage (-): 0

Region: chr3 118174678-118174688. Max. coverage (+): 0. Max coverage (-): 0

Region: chr3 118174689-118174698. Max. coverage (+): 0. Max coverage (-): 0

Region: chr3 118174699-118174709. Max. coverage (+): 0. Max coverage (-): 1.07

Region: chr3 118174710-118174719. Max. coverage (+): 0. Max coverage (-): 1.07

Region: chr3 118174720-118174729. Max. coverage (+): 0. Max coverage (-): 0

Region: chr3 118174730-118174740. Max. coverage (+): 0. Max coverage (-): 0

Region: chr3 118174741-118174750. Max. coverage (+): 0. Max coverage (-): 0

Region: chr3 118174751-118174761. Max. coverage (+): 0. Max coverage (-): 0

Region: chr3 118174762-118174771. Max. coverage (+): 0. Max coverage (-): 0

Region: chr3 118174772-118174782. Max. coverage (+): 0. Max coverage (-): 0

Region: chr3 118174783-118174792. Max. coverage (+): 0. Max coverage (-): 0

Region: chr3 118174793-118174802. Max. coverage (+): 0. Max coverage (-): 0

Region: chr3 118174803-118174813. Max. coverage (+): 0. Max coverage (-): 0

Region: chr3 118174814-118174823. Max. coverage (+): 0. Max coverage (-): 0

Region: chr3 118174824-118174834. Max. coverage (+): 0. Max coverage (-): 0

Region: chr3 118174835-118174844. Max. coverage (+): 0. Max coverage (-): 0

Region: chr3 118174845-118174855. Max. coverage (+): 0. Max coverage (-): 0

Region: chr3 118174856-118174865. Max. coverage (+): 0. Max coverage (-): 0

Region: chr3 118174866-118174875. Max. coverage (+): 0. Max coverage (-): 0

Region: chr3 118174876-118174886. Max. coverage (+): 0. Max coverage (-): 0

Region: chr3 118174887-118174896. Max. coverage (+): 0. Max coverage (-): 0

Region: chr3 118174897-118174907. Max. coverage (+): 0. Max coverage (-): 0

Region: chr3 118174908-118174917. Max. coverage (+): 0. Max coverage (-): 0

Region: chr3 118174918-118174928. Max. coverage (+): 0. Max coverage (-): 0

Region: chr3 118174929-118174938. Max. coverage (+): 0. Max coverage (-): 0

Region: chr3 118174939-118174949. Max. coverage (+): 0. Max coverage (-): 0

Region: chr3 118174950-118174959. Max. coverage (+): 0. Max coverage (-): 0

Region: chr3 118174960-118174969. Max. coverage (+): 0. Max coverage (-): 0

Region: chr3 118174970-118174980. Max. coverage (+): 0. Max coverage (-): 0

Region: chr3 118174981-118174990. Max. coverage (+): 0. Max coverage (-): 0

Region: chr3 118174991-118175001. Max. coverage (+): 0. Max coverage (-): 0

Region: chr3 118175002-118175011. Max. coverage (+): 0. Max coverage (-): 0

Region: chr3 118175012-118175022. Max. coverage (+): 0. Max coverage (-): 0

Region: chr3 118175023-118175032. Max. coverage (+): 0. Max coverage (-): 0

Region: chr3 118175033-118175042. Max. coverage (+): 0. Max coverage (-): 0

Region: chr3 118175043-118175053. Max. coverage (+): 0. Max coverage (-): 0

Region: chr3 118175054-118175063. Max. coverage (+): 0. Max coverage (-): 0

Region: chr3 118175064-118175074. Max. coverage (+): 0. Max coverage (-): 0

Region: chr3 118175075-118175084. Max. coverage (+): 0. Max coverage (-): 0

Region: chr3 118175085-118175095. Max. coverage (+): 0. Max coverage (-): 0

Region: chr3 118175096-118175105. Max. coverage (+): 0. Max coverage (-): 0

Region: chr3 118175106-118175116. Max. coverage (+): 0. Max coverage (-): 0

Region: chr3 118175117-118175126. Max. coverage (+): 0. Max coverage (-): 0

Region: chr3 118175127-118175136. Max. coverage (+): 0. Max coverage (-): 0

Region: chr3 118175137-118175147. Max. coverage (+): 0. Max coverage (-): 0

Region: chr3 118175148-118175157. Max. coverage (+): 0. Max coverage (-): 0

Region: chr3 118175158-118175168. Max. coverage (+): 0. Max coverage (-): 0

Region: chr3 118175169-118175178. Max. coverage (+): 0. Max coverage (-): 0

Region: chr3 118175179-118175189. Max. coverage (+): 0. Max coverage (-): 0

Region: chr3 118175190-118175199. Max. coverage (+): 0. Max coverage (-): 0

Region: chr3 118175200-118175209. Max. coverage (+): 0. Max coverage (-): 0

Region: chr3 118175210-118175220. Max. coverage (+): 0. Max coverage (-): 0

Region: chr3 118175221-118175230. Max. coverage (+): 0. Max coverage (-): 0

Region: chr3 118175231-118175241. Max. coverage (+): 0. Max coverage (-): 0

Region: chr3 118175242-118175251. Max. coverage (+): 0. Max coverage (-): 0

Region: chr3 118175252-118175262. Max. coverage (+): 0. Max coverage (-): 0

Region: chr3 118175263-118175272. Max. coverage (+): 0. Max coverage (-): 0

Region: chr3 118175273-118175282. Max. coverage (+): 0. Max coverage (-): 0

Region: chr3 118175283-118175293. Max. coverage (+): 0. Max coverage (-): 0

Region: chr3 118175294-118175303. Max. coverage (+): 0. Max coverage (-): 0.47

Region: chr3 118175304-118175314. Max. coverage (+): 0. Max coverage (-): 5.78

Region: chr3 118175315-118175324. Max. coverage (+): 0. Max coverage (-): 15.04

Region: chr3 118175325-118175335. Max. coverage (+): 0. Max coverage (-): 0

Region: chr3 118175336-118175345. Max. coverage (+): 0. Max coverage (-): 4.35

Region: chr3 118175346-118175356. Max. coverage (+): 0. Max coverage (-): 12.13

Region: chr3 118175357-118175366. Max. coverage (+): 0. Max coverage (-): 5.58

Region: chr3 118175367-118175376. Max. coverage (+): 0. Max coverage (-): 0

Region: chr3 118175377-118175387. Max. coverage (+): 0. Max coverage (-): 1.06

Region: chr3 118175388-118175397. Max. coverage (+): 0. Max coverage (-): 2.76

Region: chr3 118175398-118175408. Max. coverage (+): 0. Max coverage (-): 2.12

Region: chr3 118175409-118175418. Max. coverage (+): 0. Max coverage (-): 0

Region: chr3 118175419-118175429. Max. coverage (+): 0. Max coverage (-): 11.28

Region: chr3 118175430-118175439. Max. coverage (+): 0. Max coverage (-): 11.28

Region: chr3 118175440-118175449. Max. coverage (+): 0. Max coverage (-): 0

Region: chr3 118175450-118175460. Max. coverage (+): 0. Max coverage (-): 1.81

Region: chr3 118175461-118175470. Max. coverage (+): 0. Max coverage (-): 1.81

Region: chr3 118175471-118175481. Max. coverage (+): 0. Max coverage (-): 0

Region: chr3 118175482-118175491. Max. coverage (+): 0. Max coverage (-): 0

Region: chr3 118175492-118175502. Max. coverage (+): 0. Max coverage (-): 1.99

Region: chr3 118175503-118175512. Max. coverage (+): 0. Max coverage (-): 1.99

Region: chr3 118175513-118175523. Max. coverage (+): 0. Max coverage (-): 0

Region: chr3 118175524-118175533. Max. coverage (+): 0. Max coverage (-): 0

Region: chr3 118175534-118175543. Max. coverage (+): 0. Max coverage (-): 0

Region: chr3 118175544-118175554. Max. coverage (+): 0. Max coverage (-): 2.05

Region: chr3 118175555-118175564. Max. coverage (+): 0. Max coverage (-): 1.68

Region: chr3 118175565-118175575. Max. coverage (+): 0. Max coverage (-): 0

Region: chr3 118175576-118175585. Max. coverage (+): 0. Max coverage (-): 1.04

Region: chr3 118175586-118175596. Max. coverage (+): 0. Max coverage (-): 0

Region: chr3 118175597-118175606. Max. coverage (+): 0. Max coverage (-): 0

Region: chr3 118175607-118175616. Max. coverage (+): 0. Max coverage (-): 0

Region: chr3 118175617-118175627. Max. coverage (+): 0. Max coverage (-): 0

Region: chr3 118175628-118175637. Max. coverage (+): 0. Max coverage (-): 0

Region: chr3 118175638-118175648. Max. coverage (+): 0. Max coverage (-): 0

Region: chr3 118175649-118175658. Max. coverage (+): 0. Max coverage (-): 0

Region: chr3 118175659-118175669. Max. coverage (+): 0. Max coverage (-): 0

Region: chr3 118175670-118175679. Max. coverage (+): 0. Max coverage (-): 0

Region: chr3 118175680-118175689. Max. coverage (+): 0. Max coverage (-): 0

Region: chr3 118175690-118175700. Max. coverage (+): 0. Max coverage (-): 0

Region: chr3 118175701-118175710. Max. coverage (+): 0. Max coverage (-): 98.2

Region: chr3 118175711-118175721. Max. coverage (+): 0. Max coverage (-): 88.91

Region: chr3 118175722-118175731. Max. coverage (+): 0. Max coverage (-): 18.76

Region: chr3 118175732-118175742. Max. coverage (+): 0. Max coverage (-): 15.22

Region: chr3 118175743-118175752. Max. coverage (+): 0. Max coverage (-): 21.91

Region: chr3 118175753-118175763. Max. coverage (+): 0. Max coverage (-): 25.1

Region: chr3 118175764-118175773. Max. coverage (+): 0. Max coverage (-): 10.86

Region: chr3 118175774-118175783. Max. coverage (+): 0. Max coverage (-): 0

Region: chr3 118175784-118175794. Max. coverage (+): 0. Max coverage (-): 0

Region: chr3 118175795-118175804. Max. coverage (+): 0. Max coverage (-): 0.73

Region: chr3 118175805-118175815. Max. coverage (+): 0. Max coverage (-): 0.73

Region: chr3 118175816-118175825. Max. coverage (+): 0. Max coverage (-): 0

Region: chr3 118175826-118175836. Max. coverage (+): 0. Max coverage (-): 0

Region: chr3 118175837-118175846. Max. coverage (+): 0. Max coverage (-): 0

Region: chr3 118175847-118175856. Max. coverage (+): 0. Max coverage (-): 0

Region: chr3 118175857-118175867. Max. coverage (+): 0. Max coverage (-): 0

Region: chr3 118175868-118175877. Max. coverage (+): 0. Max coverage (-): 0

Region: chr3 118175878-118175888. Max. coverage (+): 0. Max coverage (-): 0

Region: chr3 118175889-118175898. Max. coverage (+): 0. Max coverage (-): 0

Region: chr3 118175899-118175909. Max. coverage (+): 0. Max coverage (-): 0

Region: chr3 118175910-118175919. Max. coverage (+): 0. Max coverage (-): 0

Region: chr3 118175920-118175930. Max. coverage (+): 0. Max coverage (-): 0

Region: chr3 118175931-118175940. Max. coverage (+): 0. Max coverage (-): 0

Region: chr3 118175941-118175950. Max. coverage (+): 0. Max coverage (-): 5.17

Region: chr3 118175951-118175961. Max. coverage (+): 0. Max coverage (-): 15.58

Region: chr3 118175962-118175971. Max. coverage (+): 0. Max coverage (-): 14.18

Region: chr3 118175972-118175982. Max. coverage (+): 0. Max coverage (-): 0

Region: chr3 118175983-118175992. Max. coverage (+): 0. Max coverage (-): 0

Region: chr3 118175993-118176003. Max. coverage (+): 0. Max coverage (-): 0

Region: chr3 118176004-118176013. Max. coverage (+): 0. Max coverage (-): 4.01

Region: chr3 118176014-118176023. Max. coverage (+): 0. Max coverage (-): 0

Region: chr3 118176024-118176034. Max. coverage (+): 0. Max coverage (-): 2.21

Region: chr3 118176035-118176044. Max. coverage (+): 0. Max coverage (-): 2.21

Region: chr3 118176045-118176055. Max. coverage (+): 0. Max coverage (-): 0

Region: chr3 118176056-118176065. Max. coverage (+): 0. Max coverage (-): 25.46

Region: chr3 118176066-118176076. Max. coverage (+): 0. Max coverage (-): 27.67

Region: chr3 118176077-118176086. Max. coverage (+): 0. Max coverage (-): 25.16

Region: chr3 118176087-118176097. Max. coverage (+): 0. Max coverage (-): 9.13

Region: chr3 118176098-118176107. Max. coverage (+): 0. Max coverage (-): 0

Region: chr3 118176108-118176117. Max. coverage (+): 0. Max coverage (-): 0

Region: chr3 118176118-118176128. Max. coverage (+): 0. Max coverage (-): 0

Region: chr3 118176129-118176138. Max. coverage (+): 0. Max coverage (-): 0

Region: chr3 118176139-118176149. Max. coverage (+): 0. Max coverage (-): 0

Region: chr3 118176150-118176159. Max. coverage (+): 0. Max coverage (-): 0

Region: chr3 118176160-118176170. Max. coverage (+): 0. Max coverage (-): 0

Region: chr3 118176171-118176180. Max. coverage (+): 0. Max coverage (-): 0

Region: chr3 118176181-118176190. Max. coverage (+): 0. Max coverage (-): 1.4

Region: chr3 118176191-118176201. Max. coverage (+): 0. Max coverage (-): 3.94

Region: chr3 118176202-118176211. Max. coverage (+): 0. Max coverage (-): 3.94

Region: chr3 118176212-118176222. Max. coverage (+): 0. Max coverage (-): 16.24

Region: chr3 118176223-118176232. Max. coverage (+): 0. Max coverage (-): 15.63

Region: chr3 118176233-118176243. Max. coverage (+): 0. Max coverage (-): 5.99

Region: chr3 118176244-118176253. Max. coverage (+): 0. Max coverage (-): 45.21

Region: chr3 118176254-118176263. Max. coverage (+): 0. Max coverage (-): 87.36

Region: chr3 118176264-118176274. Max. coverage (+): 0. Max coverage (-): 57.49

Region: chr3 118176275-118176284. Max. coverage (+): 0. Max coverage (-): 10.33

Region: chr3 118176285-118176295. Max. coverage (+): 0. Max coverage (-): 0

Region: chr3 118176296-118176305. Max. coverage (+): 0. Max coverage (-): 17.12

Region: chr3 118176306-118176316. Max. coverage (+): 0. Max coverage (-): 17.12

Region: chr3 118176317-118176326. Max. coverage (+): 0. Max coverage (-): 0

Region: chr3 118176327-118176337. Max. coverage (+): 0. Max coverage (-): 0

Region: chr3 118176338-118176347. Max. coverage (+): 0. Max coverage (-): 0.57

Region: chr3 118176348-118176357. Max. coverage (+): 0. Max coverage (-): 0

Region: chr3 118176358-118176368. Max. coverage (+): 0. Max coverage (-): 0

Region: chr3 118176369-118176378. Max. coverage (+): 0. Max coverage (-): 0

Region: chr3 118176379-118176389. Max. coverage (+): 0. Max coverage (-): 0

Region: chr3 118176390-118176399. Max. coverage (+): 0. Max coverage (-): 0

Region: chr3 118176400-118176410. Max. coverage (+): 0. Max coverage (-): 0

Region: chr3 118176411-118176420. Max. coverage (+): 0. Max coverage (-): 19.07

Region: chr3 118176421-118176430. Max. coverage (+): 0. Max coverage (-): 29.52

Region: chr3 118176431-118176441. Max. coverage (+): 0. Max coverage (-): 0.8

Region: chr3 118176442-118176451. Max. coverage (+): 0. Max coverage (-): 10.55

Region: chr3 118176452-118176462. Max. coverage (+): 0. Max coverage (-): 6.44

Region: chr3 118176463-118176472. Max. coverage (+): 0. Max coverage (-): 0

Region: chr3 118176473-118176483. Max. coverage (+): 0. Max coverage (-): 0

Region: chr3 118176484-118176493. Max. coverage (+): 0. Max coverage (-): 0

Region: chr3 118176494-118176504. Max. coverage (+): 0. Max coverage (-): 0

Region: chr3 118176505-118176514. Max. coverage (+): 0. Max coverage (-): 0

Region: chr3 118176515-118176524. Max. coverage (+): 0. Max coverage (-): 0

Region: chr3 118176525-118176535. Max. coverage (+): 0. Max coverage (-): 0

Region: chr3 118176536-118176545. Max. coverage (+): 0. Max coverage (-): 0

Region: chr3 118176546-118176556. Max. coverage (+): 0. Max coverage (-): 0

Region: chr3 118176557-118176566. Max. coverage (+): 0. Max coverage (-): 0

Region: chr3 118176567-118176577. Max. coverage (+): 0. Max coverage (-): 0

Region: chr3 118176578-118176587. Max. coverage (+): 0. Max coverage (-): 0

Region: chr3 118176588-118176597. Max. coverage (+): 0. Max coverage (-): 0

Region: chr3 118176598-118176608. Max. coverage (+): 0. Max coverage (-): 0

Region: chr3 118176609-118176618. Max. coverage (+): 0. Max coverage (-): 0

Region: chr3 118176619-118176629. Max. coverage (+): 0. Max coverage (-): 0

Region: chr3 118176630-118176639. Max. coverage (+): 0. Max coverage (-): 0

Region: chr3 118176640-118176650. Max. coverage (+): 0. Max coverage (-): 0

Region: chr3 118176651-118176660. Max. coverage (+): 0. Max coverage (-): 0

Region: chr3 118176661-118176670. Max. coverage (+): 0. Max coverage (-): 0

Region: chr3 118176671-118176681. Max. coverage (+): 0. Max coverage (-): 0

Region: chr3 118176682-118176691. Max. coverage (+): 0. Max coverage (-): 0

Region: chr3 118176692-118176702. Max. coverage (+): 0. Max coverage (-): 0

Region: chr3 118176703-118176712. Max. coverage (+): 0. Max coverage (-): 0

Region: chr3 118176713-118176723. Max. coverage (+): 0. Max coverage (-): 0

Region: chr3 118176724-118176733. Max. coverage (+): 0. Max coverage (-): 0

Region: chr3 118176734-118176744. Max. coverage (+): 0. Max coverage (-): 0

Region: chr3 118176745-118176754. Max. coverage (+): 0. Max coverage (-): 0

Region: chr3 118176755-118176764. Max. coverage (+): 0. Max coverage (-): 0

Region: chr3 118176765-118176775. Max. coverage (+): 0. Max coverage (-): 4.85

Region: chr3 118176776-118176785. Max. coverage (+): 0. Max coverage (-): 7.49

Region: chr3 118176786-118176796. Max. coverage (+): 0. Max coverage (-): 8.06

Region: chr3 118176797-118176806. Max. coverage (+): 0. Max coverage (-): 0

Region: chr3 118176807-118176817. Max. coverage (+): 0. Max coverage (-): 0

Region: chr3 118176818-118176827. Max. coverage (+): 0. Max coverage (-): 0

Region: chr3 118176828-118176837. Max. coverage (+): 0. Max coverage (-): 0

Region: chr3 118176838-118176848. Max. coverage (+): 0. Max coverage (-): 0

Region: chr3 118176849-118176858. Max. coverage (+): 0. Max coverage (-): 0

Region: chr3 118176859-118176869. Max. coverage (+): 0. Max coverage (-): 0

Region: chr3 118176870-118176879. Max. coverage (+): 0. Max coverage (-): 0

Region: chr3 118176880-118176890. Max. coverage (+): 0. Max coverage (-): 0

Region: chr3 118176891-118176900. Max. coverage (+): 0. Max coverage (-): 0

Region: chr3 118176901-118176911. Max. coverage (+): 0. Max coverage (-): 0

Region: chr3 118176912-118176921. Max. coverage (+): 0. Max coverage (-): 0

Region: chr3 118176922-118176931. Max. coverage (+): 0. Max coverage (-): 0

Region: chr3 118176932-118176942. Max. coverage (+): 0. Max coverage (-): 0

Region: chr3 118176943-118176952. Max. coverage (+): 0. Max coverage (-): 0

Region: chr3 118176953-118176963. Max. coverage (+): 0. Max coverage (-): 0

Region: chr3 118176964-118176973. Max. coverage (+): 0. Max coverage (-): 0

Region: chr3 118176974-118176984. Max. coverage (+): 0. Max coverage (-): 0

Region: chr3 118176985-118176994. Max. coverage (+): 0. Max coverage (-): 0

Region: chr3 118176995-118177004. Max. coverage (+): 0. Max coverage (-): 0

Region: chr3 118177005-118177015. Max. coverage (+): 0. Max coverage (-): 0

Region: chr3 118177016-118177025. Max. coverage (+): 0. Max coverage (-): 0

Region: chr3 118177026-118177036. Max. coverage (+): 0. Max coverage (-): 0

Region: chr3 118177037-118177046. Max. coverage (+): 0. Max coverage (-): 0

Region: chr3 118177047-118177057. Max. coverage (+): 0. Max coverage (-): 0

Region: chr3 118177058-118177067. Max. coverage (+): 0. Max coverage (-): 0

Region: chr3 118177068-118177077. Max. coverage (+): 0. Max coverage (-): 0

Region: chr3 118177078-118177088. Max. coverage (+): 0. Max coverage (-): 0

Region: chr3 118177089-118177098. Max. coverage (+): 0. Max coverage (-): 0

Region: chr3 118177099-118177109. Max. coverage (+): 0. Max coverage (-): 0

Region: chr3 118177110-118177119. Max. coverage (+): 0. Max coverage (-): 0

Region: chr3 118177120-118177130. Max. coverage (+): 0. Max coverage (-): 0

Region: chr3 118177131-118177140. Max. coverage (+): 0. Max coverage (-): 0

Region: chr3 118177141-118177151. Max. coverage (+): 0. Max coverage (-): 0

Region: chr3 118177152-118177161. Max. coverage (+): 0. Max coverage (-): 0

Region: chr3 118177162-118177171. Max. coverage (+): 0. Max coverage (-): 0

Region: chr3 118177172-118177182. Max. coverage (+): 0. Max coverage (-): 0

Region: chr3 118177183-118177192. Max. coverage (+): 0. Max coverage (-): 0

Region: chr3 118177193-118177203. Max. coverage (+): 0. Max coverage (-): 0

Region: chr3 118177204-118177213. Max. coverage (+): 0. Max coverage (-): 0

Region: chr3 118177214-118177224. Max. coverage (+): 0. Max coverage (-): 4.67

Region: chr3 118177225-118177234. Max. coverage (+): 0. Max coverage (-): 4.67

Region: chr3 118177235-118177244. Max. coverage (+): 0. Max coverage (-): 0

Region: chr3 118177245-118177255. Max. coverage (+): 0. Max coverage (-): 0

Region: chr3 118177256-118177265. Max. coverage (+): 0. Max coverage (-): 0

Region: chr3 118177266-118177276. Max. coverage (+): 0. Max coverage (-): 0

Region: chr3 118177277-118177286. Max. coverage (+): 0. Max coverage (-): 0

Region: chr3 118177287-118177297. Max. coverage (+): 0. Max coverage (-): 0

Region: chr3 118177298-118177307. Max. coverage (+): 0. Max coverage (-): 0

Region: chr3 118177308-118177318. Max. coverage (+): 0. Max coverage (-): 0

Region: chr3 118177319-118177328. Max. coverage (+): 0. Max coverage (-): 0

Region: chr3 118177329-118177338. Max. coverage (+): 0. Max coverage (-): 0

Region: chr3 118177339-118177349. Max. coverage (+): 0. Max coverage (-): 0

Region: chr3 118177350-118177359. Max. coverage (+): 0. Max coverage (-): 0

Region: chr3 118177360-118177370. Max. coverage (+): 0. Max coverage (-): 0

Region: chr3 118177371-118177380. Max. coverage (+): 0. Max coverage (-): 0

Region: chr3 118177381-118177391. Max. coverage (+): 0. Max coverage (-): 0

Region: chr3 118177392-118177401. Max. coverage (+): 0. Max coverage (-): 0

Region: chr3 118177402-118177411. Max. coverage (+): 0. Max coverage (-): 0

Region: chr3 118177412-118177422. Max. coverage (+): 0. Max coverage (-): 0

Region: chr3 118177423-118177432. Max. coverage (+): 0. Max coverage (-): 0

Region: chr3 118177433-118177443. Max. coverage (+): 0. Max coverage (-): 0

Region: chr3 118177444-118177453. Max. coverage (+): 0. Max coverage (-): 0

Region: chr3 118177454-118177464. Max. coverage (+): 0. Max coverage (-): 0

Region: chr3 118177465-118177474. Max. coverage (+): 0. Max coverage (-): 0

Region: chr3 118177475-118177484. Max. coverage (+): 0. Max coverage (-): 0

Region: chr3 118177485-118177495. Max. coverage (+): 0. Max coverage (-): 0

Region: chr3 118177496-118177505. Max. coverage (+): 0. Max coverage (-): 0

Region: chr3 118177506-118177516. Max. coverage (+): 0. Max coverage (-): 0

Region: chr3 118177517-118177526. Max. coverage (+): 0. Max coverage (-): 0

Region: chr3 118177527-118177537. Max. coverage (+): 0. Max coverage (-): 0

Region: chr3 118177538-118177547. Max. coverage (+): 0. Max coverage (-): 0

Region: chr3 118177548-118177558. Max. coverage (+): 0. Max coverage (-): 0

Region: chr3 118177559-118177568. Max. coverage (+): 0. Max coverage (-): 0

Region: chr3 118177569-118177578. Max. coverage (+): 0. Max coverage (-): 0

Region: chr3 118177579-118177589. Max. coverage (+): 0. Max coverage (-): 0

Region: chr3 118177590-118177599. Max. coverage (+): 0. Max coverage (-): 0

Region: chr3 118177600-118177610. Max. coverage (+): 0. Max coverage (-): 0

Region: chr3 118177611-118177620. Max. coverage (+): 0. Max coverage (-): 0

Region: chr3 118177621-118177631. Max. coverage (+): 0. Max coverage (-): 0

Region: chr3 118177632-118177641. Max. coverage (+): 0. Max coverage (-): 0

Region: chr3 118177642-118177651. Max. coverage (+): 0. Max coverage (-): 0

Region: chr3 118177652-118177662. Max. coverage (+): 0. Max coverage (-): 0

Region: chr3 118177663-118177672. Max. coverage (+): 0. Max coverage (-): 0

Region: chr3 118177673-118177683. Max. coverage (+): 0. Max coverage (-): 0

Region: chr3 118177684-118177693. Max. coverage (+): 0. Max coverage (-): 0

Region: chr3 118177694-118177704. Max. coverage (+): 0. Max coverage (-): 3.82

Region: chr3 118177705-118177714. Max. coverage (+): 0. Max coverage (-): 0

Region: chr3 118177715-118177725. Max. coverage (+): 0. Max coverage (-): 0

Region: chr3 118177726-118177735. Max. coverage (+): 0. Max coverage (-): 0

Region: chr3 118177736-118177745. Max. coverage (+): 0. Max coverage (-): 0

Region: chr3 118177746-118177756. Max. coverage (+): 0. Max coverage (-): 0

Region: chr3 118177757-118177766. Max. coverage (+): 0. Max coverage (-): 0

Region: chr3 118177767-118177777. Max. coverage (+): 0. Max coverage (-): 0

Region: chr3 118177778-118177787. Max. coverage (+): 0. Max coverage (-): 7.48

Region: chr3 118177788-118177798. Max. coverage (+): 0. Max coverage (-): 12.03

Region: chr3 118177799-118177808. Max. coverage (+): 0. Max coverage (-): 6.13

Region: chr3 118177809-118177818. Max. coverage (+): 0. Max coverage (-): 0

Region: chr3 118177819-118177829. Max. coverage (+): 0. Max coverage (-): 0

Region: chr3 118177830-118177839. Max. coverage (+): 0. Max coverage (-): 0

Region: chr3 118177840-118177850. Max. coverage (+): 0. Max coverage (-): 0

Region: chr3 118177851-118177860. Max. coverage (+): 0. Max coverage (-): 0

Region: chr3 118177861-118177871. Max. coverage (+): 0. Max coverage (-): 5.09

Region: chr3 118177872-118177881. Max. coverage (+): 0. Max coverage (-): 5.09

Region: chr3 118177882-118177891. Max. coverage (+): 0. Max coverage (-): 0

Region: chr3 118177892-118177902. Max. coverage (+): 0. Max coverage (-): 0

Region: chr3 118177903-118177912. Max. coverage (+): 0. Max coverage (-): 0

Region: chr3 118177913-118177923. Max. coverage (+): 0. Max coverage (-): 0

Region: chr3 118177924-118177933. Max. coverage (+): 0. Max coverage (-): 0

Region: chr3 118177934-118177944. Max. coverage (+): 0. Max coverage (-): 0

Region: chr3 118177945-118177954. Max. coverage (+): 0. Max coverage (-): 0

Region: chr3 118177955-118177965. Max. coverage (+): 0. Max coverage (-): 0

Region: chr3 118177966-118177975. Max. coverage (+): 0. Max coverage (-): 0

Region: chr3 118177976-118177985. Max. coverage (+): 0. Max coverage (-): 0

Region: chr3 118177986-118177996. Max. coverage (+): 0. Max coverage (-): 0

Region: chr3 118177997-118178006. Max. coverage (+): 0. Max coverage (-): 0

Region: chr3 118178007-118178017. Max. coverage (+): 0. Max coverage (-): 0

Region: chr3 118178018-118178027. Max. coverage (+): 0. Max coverage (-): 0

Region: chr3 118178028-118178038. Max. coverage (+): 0. Max coverage (-): 0

Region: chr3 118178039-118178048. Max. coverage (+): 0. Max coverage (-): 0

Region: chr3 118178049-118178058. Max. coverage (+): 0. Max coverage (-): 0

Region: chr3 118178059-118178069. Max. coverage (+): 0. Max coverage (-): 0

Region: chr3 118178070-118178079. Max. coverage (+): 0. Max coverage (-): 0

Region: chr3 118178080-118178090. Max. coverage (+): 0. Max coverage (-): 0

Region: chr3 118178091-118178100. Max. coverage (+): 0. Max coverage (-): 0

Region: chr3 118178101-118178111. Max. coverage (+): 0. Max coverage (-): 0

Region: chr3 118178112-118178121. Max. coverage (+): 0. Max coverage (-): 0

Region: chr3 118178122-118178132. Max. coverage (+): 0. Max coverage (-): 0

Region: chr3 118178133-118178142. Max. coverage (+): 0. Max coverage (-): 0

Region: chr3 118178143-118178152. Max. coverage (+): 0. Max coverage (-): 0

Region: chr3 118178153-118178163. Max. coverage (+): 0. Max coverage (-): 0

Region: chr3 118178164-118178173. Max. coverage (+): 0. Max coverage (-): 0

Region: chr3 118178174-118178184. Max. coverage (+): 0. Max coverage (-): 0

Region: chr3 118178185-118178194. Max. coverage (+): 0. Max coverage (-): 0

Region: chr3 118178195-118178205. Max. coverage (+): 0. Max coverage (-): 0

Region: chr3 118178206-118178215. Max. coverage (+): 0. Max coverage (-): 0

Region: chr3 118178216-118178225. Max. coverage (+): 0. Max coverage (-): 0

Region: chr3 118178226-118178236. Max. coverage (+): 0. Max coverage (-): 0

Region: chr3 118178237-118178246. Max. coverage (+): 0. Max coverage (-): 0

Region: chr3 118178247-118178257. Max. coverage (+): 0. Max coverage (-): 0

Region: chr3 118178258-118178267. Max. coverage (+): 0. Max coverage (-): 0

Region: chr3 118178268-118178278. Max. coverage (+): 0. Max coverage (-): 0

Region: chr3 118178279-118178288. Max. coverage (+): 0. Max coverage (-): 0

Region: chr3 118178289-118178298. Max. coverage (+): 0. Max coverage (-): 0

Region: chr3 118178299-118178309. Max. coverage (+): 0. Max coverage (-): 0

Region: chr3 118178310-118178319. Max. coverage (+): 0. Max coverage (-): 0

Region: chr3 118178320-118178330. Max. coverage (+): 0. Max coverage (-): 0

Region: chr3 118178331-118178340. Max. coverage (+): 0. Max coverage (-): 0

Region: chr3 118178341-118178351. Max. coverage (+): 0. Max coverage (-): 0

Region: chr3 118178352-118178361. Max. coverage (+): 0. Max coverage (-): 0

Region: chr3 118178362-118178372. Max. coverage (+): 0. Max coverage (-): 0

Region: chr3 118178373-118178382. Max. coverage (+): 0. Max coverage (-): 0

Region: chr3 118178383-118178392. Max. coverage (+): 0. Max coverage (-): 0

Region: chr3 118178393-118178403. Max. coverage (+): 0. Max coverage (-): 0

Region: chr3 118178404-118178413. Max. coverage (+): 0. Max coverage (-): 0

Region: chr3 118178414-118178424. Max. coverage (+): 0. Max coverage (-): 0

Region: chr3 118178425-118178434. Max. coverage (+): 0. Max coverage (-): 0

Region: chr3 118178435-118178445. Max. coverage (+): 0. Max coverage (-): 0

Region: chr3 118178446-118178455. Max. coverage (+): 0. Max coverage (-): 0

Region: chr3 118178456-118178465. Max. coverage (+): 0. Max coverage (-): 0

Region: chr3 118178466-118178476. Max. coverage (+): 0. Max coverage (-): 0

Region: chr3 118178477-118178486. Max. coverage (+): 0. Max coverage (-): 0

Region: chr3 118178487-118178497. Max. coverage (+): 0. Max coverage (-): 0

Region: chr3 118178498-118178507. Max. coverage (+): 0. Max coverage (-): 0

Region: chr3 118178508-118178518. Max. coverage (+): 0. Max coverage (-): 0

Region: chr3 118178519-118178528. Max. coverage (+): 0. Max coverage (-): 0

Region: chr3 118178529-118178539. Max. coverage (+): 0. Max coverage (-): 0

Region: chr3 118178540-118178549. Max. coverage (+): 0. Max coverage (-): 0

Region: chr3 118178550-118178559. Max. coverage (+): 0. Max coverage (-): 0

Region: chr3 118178560-118178570. Max. coverage (+): 0. Max coverage (-): 0

Region: chr3 118178571-118178580. Max. coverage (+): 0. Max coverage (-): 0

Region: chr3 118178581-118178591. Max. coverage (+): 0. Max coverage (-): 0

Region: chr3 118178592-118178601. Max. coverage (+): 0. Max coverage (-): 0

Region: chr3 118178602-118178612. Max. coverage (+): 0. Max coverage (-): 0

Region: chr3 118178613-118178622. Max. coverage (+): 0. Max coverage (-): 0

Region: chr3 118178623-118178632. Max. coverage (+): 0. Max coverage (-): 0

Region: chr3 118178633-118178643. Max. coverage (+): 0. Max coverage (-): 0

Region: chr3 118178644-118178653. Max. coverage (+): 0. Max coverage (-): 0

Region: chr3 118178654-118178664. Max. coverage (+): 0. Max coverage (-): 0

Region: chr3 118178665-118178674. Max. coverage (+): 0. Max coverage (-): 0

Region: chr3 118178675-118178685. Max. coverage (+): 0. Max coverage (-): 0

Region: chr3 118178686-118178695. Max. coverage (+): 0. Max coverage (-): 0

Region: chr3 118178696-118178706. Max. coverage (+): 0. Max coverage (-): 0

Region: chr3 118178707-118178716. Max. coverage (+): 0. Max coverage (-): 0

Region: chr3 118178717-118178726. Max. coverage (+): 0. Max coverage (-): 0

Region: chr3 118178727-118178737. Max. coverage (+): 0. Max coverage (-): 0

Region: chr3 118178738-118178747. Max. coverage (+): 0. Max coverage (-): 0

Region: chr3 118178748-118178758. Max. coverage (+): 0. Max coverage (-): 0

Region: chr3 118178759-118178768. Max. coverage (+): 0. Max coverage (-): 0

Region: chr3 118178769-118178779. Max. coverage (+): 0. Max coverage (-): 0

Region: chr3 118178780-118178789. Max. coverage (+): 0. Max coverage (-): 0

Region: chr3 118178790-118178799. Max. coverage (+): 0. Max coverage (-): 0

Region: chr3 118178800-118178810. Max. coverage (+): 0. Max coverage (-): 0

Region: chr3 118178811-118178820. Max. coverage (+): 0. Max coverage (-): 0

Region: chr3 118178821-118178831. Max. coverage (+): 0. Max coverage (-): 0

Region: chr3 118178832-118178841. Max. coverage (+): 0. Max coverage (-): 0

Region: chr3 118178842-118178852. Max. coverage (+): 0. Max coverage (-): 0

Region: chr3 118178853-118178862. Max. coverage (+): 0. Max coverage (-): 0

Region: chr3 118178863-118178872. Max. coverage (+): 0. Max coverage (-): 0

Region: chr3 118178873-118178883. Max. coverage (+): 0. Max coverage (-): 0

Region: chr3 118178884-118178893. Max. coverage (+): 0. Max coverage (-): 0

Region: chr3 118178894-118178904. Max. coverage (+): 0. Max coverage (-): 0

Region: chr3 118178905-118178914. Max. coverage (+): 0. Max coverage (-): 0

Region: chr3 118178915-118178925. Max. coverage (+): 0. Max coverage (-): 0

Region: chr3 118178926-118178935. Max. coverage (+): 0. Max coverage (-): 0

Region: chr3 118178936-118178946. Max. coverage (+): 0. Max coverage (-): 0

Region: chr3 118178947-118178956. Max. coverage (+): 0. Max coverage (-): 0

Region: chr3 118178957-118178966. Max. coverage (+): 0. Max coverage (-): 0

Region: chr3 118178967-118178977. Max. coverage (+): 0. Max coverage (-): 0

Region: chr3 118178978-118178987. Max. coverage (+): 0. Max coverage (-): 0

Region: chr3 118178988-118178998. Max. coverage (+): 0. Max coverage (-): 0

Region: chr3 118178999-118179008. Max. coverage (+): 0. Max coverage (-): 0

Region: chr3 118179009-118179019. Max. coverage (+): 0. Max coverage (-): 0

Region: chr3 118179020-118179029. Max. coverage (+): 0. Max coverage (-): 0

Region: chr3 118179030-118179039. Max. coverage (+): 0. Max coverage (-): 0

Region: chr3 118179040-118179050. Max. coverage (+): 0. Max coverage (-): 0

Region: chr3 118179051-118179060. Max. coverage (+): 0. Max coverage (-): 0

Region: chr3 118179061-118179071. Max. coverage (+): 0. Max coverage (-): 0

Region: chr3 118179072-118179081. Max. coverage (+): 0. Max coverage (-): 0

Region: chr3 118179082-118179092. Max. coverage (+): 0. Max coverage (-): 0

Region: chr3 118179093-118179102. Max. coverage (+): 0. Max coverage (-): 0

Region: chr3 118179103-118179113. Max. coverage (+): 0. Max coverage (-): 0

Region: chr3 118179114-118179123. Max. coverage (+): 0. Max coverage (-): 0

Region: chr3 118179124-118179133. Max. coverage (+): 0. Max coverage (-): 0

Region: chr3 118179134-118179144. Max. coverage (+): 0. Max coverage (-): 0

Region: chr3 118179145-118179154. Max. coverage (+): 0. Max coverage (-): 0

Region: chr3 118179155-118179165. Max. coverage (+): 0. Max coverage (-): 0

Region: chr3 118179166-118179175. Max. coverage (+): 0. Max coverage (-): 0

Region: chr3 118179176-118179186. Max. coverage (+): 0. Max coverage (-): 0

Region: chr3 118179187-118179196. Max. coverage (+): 0. Max coverage (-): 0

Region: chr3 118179197-118179206. Max. coverage (+): 0. Max coverage (-): 0

Region: chr3 118179207-118179217. Max. coverage (+): 0. Max coverage (-): 0

Region: chr3 118179218-118179227. Max. coverage (+): 0. Max coverage (-): 0

Region: chr3 118179228-118179238. Max. coverage (+): 0. Max coverage (-): 0

Region: chr3 118179239-118179248. Max. coverage (+): 0. Max coverage (-): 0

Region: chr3 118179249-118179259. Max. coverage (+): 0. Max coverage (-): 0

Region: chr3 118179260-118179269. Max. coverage (+): 0. Max coverage (-): 0

Region: chr3 118179270-118179279. Max. coverage (+): 0. Max coverage (-): 0

Region: chr3 118179280-118179290. Max. coverage (+): 0. Max coverage (-): 0

Region: chr3 118179291-118179300. Max. coverage (+): 0. Max coverage (-): 2.12

Region: chr3 118179301-118179311. Max. coverage (+): 0. Max coverage (-): 0

Region: chr3 118179312-118179321. Max. coverage (+): 0. Max coverage (-): 0

Region: chr3 118179322-118179332. Max. coverage (+): 0. Max coverage (-): 0

Region: chr3 118179333-118179342. Max. coverage (+): 0. Max coverage (-): 0

Region: chr3 118179343-118179353. Max. coverage (+): 0. Max coverage (-): 0

Region: chr3 118179354-118179363. Max. coverage (+): 0. Max coverage (-): 0

Region: chr3 118179364-118179373. Max. coverage (+): 0. Max coverage (-): 0

Region: chr3 118179374-118179384. Max. coverage (+): 0. Max coverage (-): 0

Region: chr3 118179385-118179394. Max. coverage (+): 0. Max coverage (-): 0

Region: chr3 118179395-118179405. Max. coverage (+): 0. Max coverage (-): 0

Region: chr3 118179406-118179415. Max. coverage (+): 0. Max coverage (-): 0

Region: chr3 118179416-118179426. Max. coverage (+): 0. Max coverage (-): 0

Region: chr3 118179427-118179436. Max. coverage (+): 0. Max coverage (-): 0

Region: chr3 118179437-118179446. Max. coverage (+): 0. Max coverage (-): 0

Region: chr3 118179447-118179457. Max. coverage (+): 0. Max coverage (-): 0

Region: chr3 118179458-118179467. Max. coverage (+): 0. Max coverage (-): 0

Region: chr3 118179468-118179478. Max. coverage (+): 0. Max coverage (-): 0

Region: chr3 118179479-118179488. Max. coverage (+): 0. Max coverage (-): 0

Region: chr3 118179489-118179499. Max. coverage (+): 0. Max coverage (-): 0

Region: chr3 118179500-118179509. Max. coverage (+): 0. Max coverage (-): 0

Region: chr3 118179510-118179520. Max. coverage (+): 0. Max coverage (-): 0

Region: chr3 118179521-118179530. Max. coverage (+): 0. Max coverage (-): 0

Region: chr3 118179531-118179540. Max. coverage (+): 0. Max coverage (-): 47.56

Region: chr3 118179541-118179551. Max. coverage (+): 0. Max coverage (-): 68.8

Region: chr3 118179552-118179561. Max. coverage (+): 2.01. Max coverage (-): 16.52

Region: chr3 118179562-118179572. Max. coverage (+): 2.01. Max coverage (-): 8.38

Region: chr3 118179573-118179582. Max. coverage (+): 0. Max coverage (-): 65.26

Region: chr3 118179583-118179593. Max. coverage (+): 0. Max coverage (-): 71.3

Region: chr3 118179594-118179603. Max. coverage (+): 0. Max coverage (-): 0

Region: chr3 118179604-118179613. Max. coverage (+): 0. Max coverage (-): 15.22

Region: chr3 118179614-118179624. Max. coverage (+): 0. Max coverage (-): 15.22

Region: chr3 118179625-118179634. Max. coverage (+): 0. Max coverage (-): 0

Region: chr3 118179635-118179645. Max. coverage (+): 0. Max coverage (-): 0

Region: chr3 118179646-118179655. Max. coverage (+): 0. Max coverage (-): 0

Region: chr3 118179656-118179666. Max. coverage (+): 0. Max coverage (-): 0

Region: chr3 118179667-118179676. Max. coverage (+): 0. Max coverage (-): 0

Region: chr3 118179677-118179686. Max. coverage (+): 0. Max coverage (-): 0

Region: chr3 118179687-118179697. Max. coverage (+): 0. Max coverage (-): 0

Region: chr3 118179698-118179707. Max. coverage (+): 0. Max coverage (-): 0

Region: chr3 118179708-118179718. Max. coverage (+): 0. Max coverage (-): 0

Region: chr3 118179719-118179728. Max. coverage (+): 0. Max coverage (-): 2.86

Region: chr3 118179729-118179739. Max. coverage (+): 0. Max coverage (-): 149.4

Region: chr3 118179740-118179749. Max. coverage (+): 0. Max coverage (-): 159.05

Region: chr3 118179750-118179760. Max. coverage (+): 0. Max coverage (-): 13.47

Region: chr3 118179761-118179770. Max. coverage (+): 0. Max coverage (-): 13.47

Region: chr3 118179771-118179780. Max. coverage (+): 0. Max coverage (-): 68.79

Region: chr3 118179781-118179791. Max. coverage (+): 0. Max coverage (-): 68.79

Region: chr3 118179792-118179801. Max. coverage (+): 0. Max coverage (-): 0

Region: chr3 118179802-. Max. coverage (+): 0. Max coverage (-): 0

RepeatMasker Color Code

**+**

100-98% Identity

<98-95% Identity

<95-90% Identity

<90-85% Identity

<85-80% Identity

<80-75% Identity

<75-70% Identity

<70% Identity

**-**

Gene Set Color Code

**+**

Gene

Pseudogene

**-**

Topology/Coverage Color Code

Coverage Plus Strand

Coverage Minus Strand

Mainstrand: Plus

Mainstrand: Minus

Complementary Strand

Flanking Region  
(if option -flank >0)

Gene Set Annotation  
  
RepeatMasker Annotation  

**1. Bov-tA3**: 118174802-118175001 (+), Divergence to consensus: 14%  
**2. L1ME2z**: 118175037-118175423 (+), Divergence to consensus: 38%  
**3. LTR75**: 118175946-118176006 (-), Divergence to consensus: 24.9%  
**4. MLT1F2**: 118176502-118176754 (+), Divergence to consensus: 45.4%  
**5. Bov-tA3**: 118178165-118178376 (+), Divergence to consensus: 11.3%  
**6. L2a**: 118178455-118178707 (+), Divergence to consensus: 48.5%

  
Transcription Factor Binding Sites  

**SPZ1** (Sequence: CTCAAACCCC (-): 118177188)  
**A-MYB** (Sequence: CCAACTGCCA (-): 118176888)
