# Supplementary material for: Small RNA sequencing of cryopreserved semen from single bull revealed altered miRNAs and piRNAs expression between High- and Low-motile sperm populations
Source: BMC Genomics. 2017 Jan 4;18:14. doi: 10.1186/s12864-016-3394-7 (PMC5209821; doi:10.1186/s12864-016-3394-7)
Supplement: Additional file 3: — Details for each piRNA clusters found in High Motile (HM) sperm fraction. Genes, repeats, transposable elements and transcription factors binding sites falling within the cluster regions were reported. (ZIP 1896 kb) [file 12864_2016_3394_MOESM3_ESM.zip › 81.html]

piRNA cluster 81


Predicted piRNA cluster no. 81     previous   next
  

Show proTRAC run info
Hide proTRAC run info

================================= proTRAC ====================================  
VERSION: 2.1                                    LAST MODIFIED: 06. October 2015  
  
Please cite:  
Rosenkranz D, Zischler H. proTRAC - a software for probabilistic piRNA cluster  
detection, visualization and analysis. 2012. BMC Bioinformatics 13:5.  
  
and (for proTRAC 2.0 and later):  
Rosenkranz D, Rudloff S, Bastuck K, Ketting RF, Zischler H. Tupaia small RNAs  
provide insights into function and evolution of RNAi-based transposon defense  
in mammals. 2015. RNA 21(5):911-922.  
  
Contact:  
David Rosenkranz  
Institute of Anthropology, small RNA group  
Johannes Gutenberg University Mainz  
email: rosenkranz@uni-mainz.de  
  
You can find the latest proTRAC version at:  
http://sourceforge.net/projects/protrac/files  
http://www.smallRNAgroup-mainz.de/software  
==============================================================================  
  
PARAMETERS:  
Map file: .............../storage/core/barbara/genhome/smallRNA/fertility/Sample\_motile/pirna/Sample\_motile\_26-33\_collapsed.fa.no-dust.map.weighted-10000-1000-b-0  
Genome file: ............/storage/core/barbara/genhome/smallRNA/fertility/Sample\_all/pirna/bt\_311\_chrY.fa  
RepeatMasker annotation: /storage/genomes/bt\_umd31/GCF\_000003055.6\_Bos\_taurus\_UMD\_3.1.1\_repeatMasker\_chr.out  
GeneSet:................./storage/core/barbara/genhome/smallRNA/fertility/Sample\_all/pirna/full.gtf  
  
Significant (p<=0.01) hit density will be calculated based  
on observed hit distribution.  
  
Sliding window size: ........................................ 5000 bp  
Sliding window increament: .................................. 1000 bp  
Normalize each hit by number of genomic hits: ............... 1 [0=no/1=yes]  
Normalize each hit by number of sequence reads: ............. 1 [0=no/1=yes]  
Normalize values (-> per million mapped reads): ............. 1 [0=no/1=yes]  
Min. fraction of hits with 1T(U) or 10A: .................... 0.75  
Alternatively: Min. fraction of hits with 1T(U) and 10A: .... 0.5  
Min. fraction of hits with typical piRNA length: ............ 0.75  
Typical piRNA length: ....................................... 26-33 nt  
Min. size of a piRNA cluster: ............................... 5000 bp.  
Min. number of hits (absolute): ............................. 0  
Min. number of hits (normalized): ........................... 0  
Min. fraction of hits on the mainstrand: .................... 0.75  
Top fraction of mapped sequences (in terms of read counts): . 1%  
Top fraction accounts for max. n% of sequence reads: ........ 90%  
Min. fraction of hits on each arm of a bidirectional cluster: 0.1  
Output image file for each cluster: ......................... 0 [0=no/1=yes]  
Output html file for each cluster: .......................... 1 [0=no/1=yes]  
Output a summary table: ..................................... 1 [0=no/1=yes]  
Output a FASTA file for each cluster (piRNA sequences): ..... 1 [0=no/1=yes]  
Output a FASTA file comprising cluster sequences: ........... 1 [0=no/1=yes]  
Search DNA motifs in clusters: .............................. 1 [0=no/1=yes]  
Output flanking sequences: +/- .............................. 0 bp  
Output ~.pTi file: .......................................... 1 [0=no/1=yes]  
==============================================================================  
  
  
Genome size (without gaps): ............ 2678902517 bp  
Gaps (N/X/-): .......................... 53837044 bp  
Mapped reads: .......................... 658825247023  
Non-identical sequences: ............... 514171  
Genomic hits: .......................... 764233  
Significant densitiy of mapped reads: .. 12867599.5173724 reads/kb

Show proTRAC cluster info
Hide proTRAC cluster info

|  |  |
| --- | --- |
| Location | chr3 |
| Coordinates | 118372297-118377741 |
| Size [bp] | 5445 |
| Sequence hit loci | 378 |
| Mapped reads (normalized) | 478044707 |
| Mapped reads (normalized) per kb | 87795171.2 |
| Normalized reads with 1T (1U) | 86.8% |
| Normalized reads with 10A | 26.8% |
| Normalized reads with length 26-33 nt | 100% |
| Normalized reads on the main strand(s) | 99.9% |
| Predicted directionality | mono:plus |

100%

0%

1T (1U)  
reads

10A reads

26-33 nt  
reads

reads on mainstrand

**Either the amount of reads with 1T (1U) OR 10A has to exceed 75% (set with option: -1Tor10A)  
Alternatively the amount of reads with 1T (1U) AND 10A has to exceed 50% (set with option: -1Tand10A)  
Minimum amount of reads with preferred size is 75% (set with option: -pisize)  
Minimum amount of reads on the main strand(s) is 75% (set with option: -clstrand)**

Show read coverage
Hide read coverage

WHAT DO I SEE HERE?  
This chart shows the location of mapped sequence reads within a predicted piRNA cluster. The color refers to the number of genomic hits produced by the sequence read in question. A dark red bar indicates that this sequence read produces many other hits elsewhere in the genome. Many adjacent red or yellow bars can indicate the presence of a multi-copy element such as transposons or rRNA genes. A dark green bar indicates that this sequence read maps uniquely to this locus.

1 hit

2-5 hits

6-10 hits

11-20 hits

21-50 hits

51-100 hits

> 100 hits

chr3

118372297

118377741

Gene Set

RepeatMasker

Mapped  
Reads

32.24

plus strand

minus strand

32.24

Region: chr3 118179803-118372302. Max. coverage (+): 22.95. Max coverage (-): 0

Region: chr3 118372303-118372313. Max. coverage (+): 32.24. Max coverage (-): 0

Region: chr3 118372314-118372324. Max. coverage (+): 13.82. Max coverage (-): 0

Region: chr3 118372325-118372335. Max. coverage (+): 0. Max coverage (-): 0

Region: chr3 118372336-118372346. Max. coverage (+): 0. Max coverage (-): 0

Region: chr3 118372347-118372356. Max. coverage (+): 0. Max coverage (-): 0

Region: chr3 118372357-118372367. Max. coverage (+): 20.16. Max coverage (-): 0

Region: chr3 118372368-118372378. Max. coverage (+): 29.79. Max coverage (-): 0

Region: chr3 118372379-118372389. Max. coverage (+): 0.61. Max coverage (-): 0

Region: chr3 118372390-118372400. Max. coverage (+): 0. Max coverage (-): 0

Region: chr3 118372401-118372411. Max. coverage (+): 0. Max coverage (-): 0

Region: chr3 118372412-118372422. Max. coverage (+): 0. Max coverage (-): 0

Region: chr3 118372423-118372433. Max. coverage (+): 0. Max coverage (-): 0

Region: chr3 118372434-118372444. Max. coverage (+): 0. Max coverage (-): 0

Region: chr3 118372445-118372454. Max. coverage (+): 6.8. Max coverage (-): 0

Region: chr3 118372455-118372465. Max. coverage (+): 6.8. Max coverage (-): 0

Region: chr3 118372466-118372476. Max. coverage (+): 1.54. Max coverage (-): 0

Region: chr3 118372477-118372487. Max. coverage (+): 3.16. Max coverage (-): 0

Region: chr3 118372488-118372498. Max. coverage (+): 3.16. Max coverage (-): 0

Region: chr3 118372499-118372509. Max. coverage (+): 0. Max coverage (-): 0

Region: chr3 118372510-118372520. Max. coverage (+): 0. Max coverage (-): 0

Region: chr3 118372521-118372531. Max. coverage (+): 0. Max coverage (-): 0

Region: chr3 118372532-118372542. Max. coverage (+): 3.57. Max coverage (-): 0

Region: chr3 118372543-118372552. Max. coverage (+): 6.28. Max coverage (-): 0

Region: chr3 118372553-118372563. Max. coverage (+): 9.99. Max coverage (-): 0

Region: chr3 118372564-118372574. Max. coverage (+): 7.32. Max coverage (-): 0

Region: chr3 118372575-118372585. Max. coverage (+): 0. Max coverage (-): 0

Region: chr3 118372586-118372596. Max. coverage (+): 5.2. Max coverage (-): 0

Region: chr3 118372597-118372607. Max. coverage (+): 0. Max coverage (-): 0

Region: chr3 118372608-118372618. Max. coverage (+): 0. Max coverage (-): 0

Region: chr3 118372619-118372629. Max. coverage (+): 10.24. Max coverage (-): 0

Region: chr3 118372630-118372640. Max. coverage (+): 18.2. Max coverage (-): 0

Region: chr3 118372641-118372650. Max. coverage (+): 4.01. Max coverage (-): 0

Region: chr3 118372651-118372661. Max. coverage (+): 0. Max coverage (-): 0

Region: chr3 118372662-118372672. Max. coverage (+): 0. Max coverage (-): 0

Region: chr3 118372673-118372683. Max. coverage (+): 8.01. Max coverage (-): 0

Region: chr3 118372684-118372694. Max. coverage (+): 4.95. Max coverage (-): 0

Region: chr3 118372695-118372705. Max. coverage (+): 1.44. Max coverage (-): 0

Region: chr3 118372706-118372716. Max. coverage (+): 4.59. Max coverage (-): 0

Region: chr3 118372717-118372727. Max. coverage (+): 16.15. Max coverage (-): 0

Region: chr3 118372728-118372738. Max. coverage (+): 25.52. Max coverage (-): 0

Region: chr3 118372739-118372748. Max. coverage (+): 5.26. Max coverage (-): 0

Region: chr3 118372749-118372759. Max. coverage (+): 5.58. Max coverage (-): 0

Region: chr3 118372760-118372770. Max. coverage (+): 4.63. Max coverage (-): 0

Region: chr3 118372771-118372781. Max. coverage (+): 0. Max coverage (-): 0

Region: chr3 118372782-118372792. Max. coverage (+): 0. Max coverage (-): 0

Region: chr3 118372793-118372803. Max. coverage (+): 0. Max coverage (-): 0

Region: chr3 118372804-118372814. Max. coverage (+): 0. Max coverage (-): 0

Region: chr3 118372815-118372825. Max. coverage (+): 0. Max coverage (-): 0

Region: chr3 118372826-118372836. Max. coverage (+): 0. Max coverage (-): 0

Region: chr3 118372837-118372846. Max. coverage (+): 0. Max coverage (-): 0

Region: chr3 118372847-118372857. Max. coverage (+): 0. Max coverage (-): 0

Region: chr3 118372858-118372868. Max. coverage (+): 0. Max coverage (-): 0

Region: chr3 118372869-118372879. Max. coverage (+): 0. Max coverage (-): 0

Region: chr3 118372880-118372890. Max. coverage (+): 4.61. Max coverage (-): 0

Region: chr3 118372891-118372901. Max. coverage (+): 0. Max coverage (-): 0

Region: chr3 118372902-118372912. Max. coverage (+): 0. Max coverage (-): 0

Region: chr3 118372913-118372923. Max. coverage (+): 0. Max coverage (-): 0

Region: chr3 118372924-118372934. Max. coverage (+): 0. Max coverage (-): 0

Region: chr3 118372935-118372944. Max. coverage (+): 0. Max coverage (-): 0

Region: chr3 118372945-118372955. Max. coverage (+): 0. Max coverage (-): 0

Region: chr3 118372956-118372966. Max. coverage (+): 0.71. Max coverage (-): 0

Region: chr3 118372967-118372977. Max. coverage (+): 9.48. Max coverage (-): 0

Region: chr3 118372978-118372988. Max. coverage (+): 2.96. Max coverage (-): 0

Region: chr3 118372989-118372999. Max. coverage (+): 2.96. Max coverage (-): 0

Region: chr3 118373000-118373010. Max. coverage (+): 0. Max coverage (-): 0

Region: chr3 118373011-118373021. Max. coverage (+): 1.95. Max coverage (-): 0

Region: chr3 118373022-118373032. Max. coverage (+): 8.61. Max coverage (-): 0

Region: chr3 118373033-118373042. Max. coverage (+): 12.94. Max coverage (-): 0.75

Region: chr3 118373043-118373053. Max. coverage (+): 20.57. Max coverage (-): 0.75

Region: chr3 118373054-118373064. Max. coverage (+): 21.74. Max coverage (-): 0

Region: chr3 118373065-118373075. Max. coverage (+): 4.11. Max coverage (-): 0

Region: chr3 118373076-118373086. Max. coverage (+): 0. Max coverage (-): 0

Region: chr3 118373087-118373097. Max. coverage (+): 0. Max coverage (-): 0

Region: chr3 118373098-118373108. Max. coverage (+): 2.37. Max coverage (-): 0

Region: chr3 118373109-118373119. Max. coverage (+): 6.45. Max coverage (-): 0

Region: chr3 118373120-118373130. Max. coverage (+): 0. Max coverage (-): 0

Region: chr3 118373131-118373140. Max. coverage (+): 2.21. Max coverage (-): 0

Region: chr3 118373141-118373151. Max. coverage (+): 2.21. Max coverage (-): 0

Region: chr3 118373152-118373162. Max. coverage (+): 0. Max coverage (-): 0

Region: chr3 118373163-118373173. Max. coverage (+): 7.54. Max coverage (-): 0

Region: chr3 118373174-118373184. Max. coverage (+): 7.54. Max coverage (-): 0

Region: chr3 118373185-118373195. Max. coverage (+): 0. Max coverage (-): 0

Region: chr3 118373196-118373206. Max. coverage (+): 2.06. Max coverage (-): 0

Region: chr3 118373207-118373217. Max. coverage (+): 2.06. Max coverage (-): 0

Region: chr3 118373218-118373228. Max. coverage (+): 3.33. Max coverage (-): 0

Region: chr3 118373229-118373238. Max. coverage (+): 2.87. Max coverage (-): 0

Region: chr3 118373239-118373249. Max. coverage (+): 6.01. Max coverage (-): 0

Region: chr3 118373250-118373260. Max. coverage (+): 10.68. Max coverage (-): 0

Region: chr3 118373261-118373271. Max. coverage (+): 9.34. Max coverage (-): 0

Region: chr3 118373272-118373282. Max. coverage (+): 17.66. Max coverage (-): 0

Region: chr3 118373283-118373293. Max. coverage (+): 5.9. Max coverage (-): 0

Region: chr3 118373294-118373304. Max. coverage (+): 5.72. Max coverage (-): 0

Region: chr3 118373305-118373315. Max. coverage (+): 5.72. Max coverage (-): 0

Region: chr3 118373316-118373326. Max. coverage (+): 0. Max coverage (-): 0

Region: chr3 118373327-118373336. Max. coverage (+): 0. Max coverage (-): 0

Region: chr3 118373337-118373347. Max. coverage (+): 0. Max coverage (-): 0

Region: chr3 118373348-118373358. Max. coverage (+): 0. Max coverage (-): 0

Region: chr3 118373359-118373369. Max. coverage (+): 0. Max coverage (-): 0

Region: chr3 118373370-118373380. Max. coverage (+): 0. Max coverage (-): 0

Region: chr3 118373381-118373391. Max. coverage (+): 0.82. Max coverage (-): 0

Region: chr3 118373392-118373402. Max. coverage (+): 0. Max coverage (-): 0

Region: chr3 118373403-118373413. Max. coverage (+): 0. Max coverage (-): 0

Region: chr3 118373414-118373424. Max. coverage (+): 0. Max coverage (-): 0

Region: chr3 118373425-118373435. Max. coverage (+): 4.57. Max coverage (-): 0

Region: chr3 118373436-118373445. Max. coverage (+): 0. Max coverage (-): 0

Region: chr3 118373446-118373456. Max. coverage (+): 0. Max coverage (-): 0

Region: chr3 118373457-118373467. Max. coverage (+): 0. Max coverage (-): 0

Region: chr3 118373468-118373478. Max. coverage (+): 0.61. Max coverage (-): 0

Region: chr3 118373479-118373489. Max. coverage (+): 0.61. Max coverage (-): 0

Region: chr3 118373490-118373500. Max. coverage (+): 0.49. Max coverage (-): 0

Region: chr3 118373501-118373511. Max. coverage (+): 0. Max coverage (-): 0

Region: chr3 118373512-118373522. Max. coverage (+): 16.1. Max coverage (-): 0

Region: chr3 118373523-118373533. Max. coverage (+): 16.1. Max coverage (-): 0

Region: chr3 118373534-118373543. Max. coverage (+): 10.99. Max coverage (-): 0

Region: chr3 118373544-118373554. Max. coverage (+): 2.73. Max coverage (-): 0

Region: chr3 118373555-118373565. Max. coverage (+): 0. Max coverage (-): 0

Region: chr3 118373566-118373576. Max. coverage (+): 2.93. Max coverage (-): 0

Region: chr3 118373577-118373587. Max. coverage (+): 7.14. Max coverage (-): 0

Region: chr3 118373588-118373598. Max. coverage (+): 4.53. Max coverage (-): 0

Region: chr3 118373599-118373609. Max. coverage (+): 5.04. Max coverage (-): 0

Region: chr3 118373610-118373620. Max. coverage (+): 4.7. Max coverage (-): 0

Region: chr3 118373621-118373631. Max. coverage (+): 3.92. Max coverage (-): 0

Region: chr3 118373632-118373641. Max. coverage (+): 2.71. Max coverage (-): 0

Region: chr3 118373642-118373652. Max. coverage (+): 2.79. Max coverage (-): 0

Region: chr3 118373653-118373663. Max. coverage (+): 0. Max coverage (-): 0

Region: chr3 118373664-118373674. Max. coverage (+): 5.91. Max coverage (-): 0

Region: chr3 118373675-118373685. Max. coverage (+): 8.34. Max coverage (-): 0

Region: chr3 118373686-118373696. Max. coverage (+): 1.63. Max coverage (-): 0

Region: chr3 118373697-118373707. Max. coverage (+): 1.63. Max coverage (-): 0

Region: chr3 118373708-118373718. Max. coverage (+): 0.51. Max coverage (-): 0

Region: chr3 118373719-118373729. Max. coverage (+): 0. Max coverage (-): 0

Region: chr3 118373730-118373739. Max. coverage (+): 0. Max coverage (-): 0

Region: chr3 118373740-118373750. Max. coverage (+): 0. Max coverage (-): 0

Region: chr3 118373751-118373761. Max. coverage (+): 0. Max coverage (-): 0

Region: chr3 118373762-118373772. Max. coverage (+): 9.31. Max coverage (-): 0

Region: chr3 118373773-118373783. Max. coverage (+): 1.36. Max coverage (-): 0

Region: chr3 118373784-118373794. Max. coverage (+): 0. Max coverage (-): 0

Region: chr3 118373795-118373805. Max. coverage (+): 0. Max coverage (-): 0

Region: chr3 118373806-118373816. Max. coverage (+): 0. Max coverage (-): 0

Region: chr3 118373817-118373827. Max. coverage (+): 0. Max coverage (-): 0

Region: chr3 118373828-118373837. Max. coverage (+): 0. Max coverage (-): 0

Region: chr3 118373838-118373848. Max. coverage (+): 0. Max coverage (-): 0

Region: chr3 118373849-118373859. Max. coverage (+): 0. Max coverage (-): 0

Region: chr3 118373860-118373870. Max. coverage (+): 0. Max coverage (-): 0

Region: chr3 118373871-118373881. Max. coverage (+): 0. Max coverage (-): 0

Region: chr3 118373882-118373892. Max. coverage (+): 0. Max coverage (-): 0

Region: chr3 118373893-118373903. Max. coverage (+): 0. Max coverage (-): 0

Region: chr3 118373904-118373914. Max. coverage (+): 0. Max coverage (-): 0

Region: chr3 118373915-118373925. Max. coverage (+): 3.25. Max coverage (-): 0

Region: chr3 118373926-118373935. Max. coverage (+): 3.25. Max coverage (-): 0

Region: chr3 118373936-118373946. Max. coverage (+): 0. Max coverage (-): 0

Region: chr3 118373947-118373957. Max. coverage (+): 0. Max coverage (-): 0

Region: chr3 118373958-118373968. Max. coverage (+): 0. Max coverage (-): 0

Region: chr3 118373969-118373979. Max. coverage (+): 0. Max coverage (-): 0

Region: chr3 118373980-118373990. Max. coverage (+): 0. Max coverage (-): 0

Region: chr3 118373991-118374001. Max. coverage (+): 16.58. Max coverage (-): 0

Region: chr3 118374002-118374012. Max. coverage (+): 8.53. Max coverage (-): 0

Region: chr3 118374013-118374023. Max. coverage (+): 6.25. Max coverage (-): 0

Region: chr3 118374024-118374033. Max. coverage (+): 0. Max coverage (-): 0

Region: chr3 118374034-118374044. Max. coverage (+): 0. Max coverage (-): 0

Region: chr3 118374045-118374055. Max. coverage (+): 0. Max coverage (-): 0

Region: chr3 118374056-118374066. Max. coverage (+): 0. Max coverage (-): 0

Region: chr3 118374067-118374077. Max. coverage (+): 0. Max coverage (-): 0

Region: chr3 118374078-118374088. Max. coverage (+): 0. Max coverage (-): 0

Region: chr3 118374089-118374099. Max. coverage (+): 0. Max coverage (-): 0

Region: chr3 118374100-118374110. Max. coverage (+): 0. Max coverage (-): 0

Region: chr3 118374111-118374121. Max. coverage (+): 7.14. Max coverage (-): 0

Region: chr3 118374122-118374131. Max. coverage (+): 7.14. Max coverage (-): 0

Region: chr3 118374132-118374142. Max. coverage (+): 3.25. Max coverage (-): 0

Region: chr3 118374143-118374153. Max. coverage (+): 0. Max coverage (-): 0

Region: chr3 118374154-118374164. Max. coverage (+): 0. Max coverage (-): 0

Region: chr3 118374165-118374175. Max. coverage (+): 0. Max coverage (-): 0

Region: chr3 118374176-118374186. Max. coverage (+): 0. Max coverage (-): 0

Region: chr3 118374187-118374197. Max. coverage (+): 0. Max coverage (-): 0

Region: chr3 118374198-118374208. Max. coverage (+): 0. Max coverage (-): 0

Region: chr3 118374209-118374219. Max. coverage (+): 0. Max coverage (-): 0

Region: chr3 118374220-118374229. Max. coverage (+): 0. Max coverage (-): 0

Region: chr3 118374230-118374240. Max. coverage (+): 5.61. Max coverage (-): 0

Region: chr3 118374241-118374251. Max. coverage (+): 10.31. Max coverage (-): 0

Region: chr3 118374252-118374262. Max. coverage (+): 0. Max coverage (-): 0

Region: chr3 118374263-118374273. Max. coverage (+): 0. Max coverage (-): 0

Region: chr3 118374274-118374284. Max. coverage (+): 1.5. Max coverage (-): 0

Region: chr3 118374285-118374295. Max. coverage (+): 1.5. Max coverage (-): 0

Region: chr3 118374296-118374306. Max. coverage (+): 0. Max coverage (-): 0

Region: chr3 118374307-118374317. Max. coverage (+): 0. Max coverage (-): 0

Region: chr3 118374318-118374327. Max. coverage (+): 0. Max coverage (-): 0

Region: chr3 118374328-118374338. Max. coverage (+): 0. Max coverage (-): 0

Region: chr3 118374339-118374349. Max. coverage (+): 0. Max coverage (-): 0

Region: chr3 118374350-118374360. Max. coverage (+): 0. Max coverage (-): 0

Region: chr3 118374361-118374371. Max. coverage (+): 0. Max coverage (-): 0

Region: chr3 118374372-118374382. Max. coverage (+): 0. Max coverage (-): 0

Region: chr3 118374383-118374393. Max. coverage (+): 0. Max coverage (-): 0

Region: chr3 118374394-118374404. Max. coverage (+): 0. Max coverage (-): 0

Region: chr3 118374405-118374415. Max. coverage (+): 0. Max coverage (-): 0

Region: chr3 118374416-118374425. Max. coverage (+): 0. Max coverage (-): 0

Region: chr3 118374426-118374436. Max. coverage (+): 3.42. Max coverage (-): 0

Region: chr3 118374437-118374447. Max. coverage (+): 3.42. Max coverage (-): 0

Region: chr3 118374448-118374458. Max. coverage (+): 0. Max coverage (-): 0

Region: chr3 118374459-118374469. Max. coverage (+): 0. Max coverage (-): 0

Region: chr3 118374470-118374480. Max. coverage (+): 0. Max coverage (-): 0

Region: chr3 118374481-118374491. Max. coverage (+): 0. Max coverage (-): 0

Region: chr3 118374492-118374502. Max. coverage (+): 0. Max coverage (-): 0

Region: chr3 118374503-118374513. Max. coverage (+): 0. Max coverage (-): 0

Region: chr3 118374514-118374524. Max. coverage (+): 0. Max coverage (-): 0

Region: chr3 118374525-118374534. Max. coverage (+): 0. Max coverage (-): 0

Region: chr3 118374535-118374545. Max. coverage (+): 0. Max coverage (-): 0

Region: chr3 118374546-118374556. Max. coverage (+): 0. Max coverage (-): 0

Region: chr3 118374557-118374567. Max. coverage (+): 0. Max coverage (-): 0

Region: chr3 118374568-118374578. Max. coverage (+): 2.41. Max coverage (-): 0

Region: chr3 118374579-118374589. Max. coverage (+): 9.54. Max coverage (-): 0

Region: chr3 118374590-118374600. Max. coverage (+): 9.54. Max coverage (-): 0

Region: chr3 118374601-118374611. Max. coverage (+): 0.8. Max coverage (-): 0

Region: chr3 118374612-118374622. Max. coverage (+): 0.8. Max coverage (-): 0

Region: chr3 118374623-118374632. Max. coverage (+): 0. Max coverage (-): 0

Region: chr3 118374633-118374643. Max. coverage (+): 0. Max coverage (-): 0

Region: chr3 118374644-118374654. Max. coverage (+): 0. Max coverage (-): 0

Region: chr3 118374655-118374665. Max. coverage (+): 0. Max coverage (-): 0

Region: chr3 118374666-118374676. Max. coverage (+): 0. Max coverage (-): 0

Region: chr3 118374677-118374687. Max. coverage (+): 0. Max coverage (-): 0

Region: chr3 118374688-118374698. Max. coverage (+): 0. Max coverage (-): 0

Region: chr3 118374699-118374709. Max. coverage (+): 0. Max coverage (-): 0

Region: chr3 118374710-118374720. Max. coverage (+): 0. Max coverage (-): 0

Region: chr3 118374721-118374730. Max. coverage (+): 0. Max coverage (-): 0

Region: chr3 118374731-118374741. Max. coverage (+): 0. Max coverage (-): 0

Region: chr3 118374742-118374752. Max. coverage (+): 0. Max coverage (-): 0

Region: chr3 118374753-118374763. Max. coverage (+): 0. Max coverage (-): 0

Region: chr3 118374764-118374774. Max. coverage (+): 0. Max coverage (-): 0

Region: chr3 118374775-118374785. Max. coverage (+): 0. Max coverage (-): 0

Region: chr3 118374786-118374796. Max. coverage (+): 0. Max coverage (-): 0

Region: chr3 118374797-118374807. Max. coverage (+): 10.44. Max coverage (-): 0

Region: chr3 118374808-118374818. Max. coverage (+): 6.49. Max coverage (-): 0

Region: chr3 118374819-118374828. Max. coverage (+): 4.29. Max coverage (-): 0

Region: chr3 118374829-118374839. Max. coverage (+): 16.63. Max coverage (-): 0

Region: chr3 118374840-118374850. Max. coverage (+): 16.63. Max coverage (-): 0

Region: chr3 118374851-118374861. Max. coverage (+): 5.01. Max coverage (-): 0

Region: chr3 118374862-118374872. Max. coverage (+): 8.45. Max coverage (-): 0

Region: chr3 118374873-118374883. Max. coverage (+): 8.45. Max coverage (-): 0

Region: chr3 118374884-118374894. Max. coverage (+): 0.19. Max coverage (-): 0

Region: chr3 118374895-118374905. Max. coverage (+): 0.19. Max coverage (-): 0

Region: chr3 118374906-118374916. Max. coverage (+): 1.96. Max coverage (-): 0

Region: chr3 118374917-118374926. Max. coverage (+): 0. Max coverage (-): 0

Region: chr3 118374927-118374937. Max. coverage (+): 2.77. Max coverage (-): 0

Region: chr3 118374938-118374948. Max. coverage (+): 6.12. Max coverage (-): 0

Region: chr3 118374949-118374959. Max. coverage (+): 3.35. Max coverage (-): 0

Region: chr3 118374960-118374970. Max. coverage (+): 0. Max coverage (-): 0

Region: chr3 118374971-118374981. Max. coverage (+): 1.84. Max coverage (-): 0

Region: chr3 118374982-118374992. Max. coverage (+): 0. Max coverage (-): 0

Region: chr3 118374993-118375003. Max. coverage (+): 0. Max coverage (-): 0

Region: chr3 118375004-118375014. Max. coverage (+): 0. Max coverage (-): 0

Region: chr3 118375015-118375024. Max. coverage (+): 0. Max coverage (-): 0

Region: chr3 118375025-118375035. Max. coverage (+): 0. Max coverage (-): 0

Region: chr3 118375036-118375046. Max. coverage (+): 0. Max coverage (-): 0

Region: chr3 118375047-118375057. Max. coverage (+): 0. Max coverage (-): 0

Region: chr3 118375058-118375068. Max. coverage (+): 0. Max coverage (-): 0

Region: chr3 118375069-118375079. Max. coverage (+): 0. Max coverage (-): 0

Region: chr3 118375080-118375090. Max. coverage (+): 0. Max coverage (-): 0

Region: chr3 118375091-118375101. Max. coverage (+): 0. Max coverage (-): 0

Region: chr3 118375102-118375112. Max. coverage (+): 0. Max coverage (-): 0

Region: chr3 118375113-118375122. Max. coverage (+): 0. Max coverage (-): 0

Region: chr3 118375123-118375133. Max. coverage (+): 0. Max coverage (-): 0

Region: chr3 118375134-118375144. Max. coverage (+): 0. Max coverage (-): 0

Region: chr3 118375145-118375155. Max. coverage (+): 0. Max coverage (-): 0

Region: chr3 118375156-118375166. Max. coverage (+): 0. Max coverage (-): 0

Region: chr3 118375167-118375177. Max. coverage (+): 0. Max coverage (-): 0

Region: chr3 118375178-118375188. Max. coverage (+): 0. Max coverage (-): 0

Region: chr3 118375189-118375199. Max. coverage (+): 0. Max coverage (-): 0

Region: chr3 118375200-118375210. Max. coverage (+): 0. Max coverage (-): 0

Region: chr3 118375211-118375220. Max. coverage (+): 0. Max coverage (-): 0

Region: chr3 118375221-118375231. Max. coverage (+): 0. Max coverage (-): 0

Region: chr3 118375232-118375242. Max. coverage (+): 7.71. Max coverage (-): 0

Region: chr3 118375243-118375253. Max. coverage (+): 7.71. Max coverage (-): 0

Region: chr3 118375254-118375264. Max. coverage (+): 0. Max coverage (-): 0

Region: chr3 118375265-118375275. Max. coverage (+): 0. Max coverage (-): 0

Region: chr3 118375276-118375286. Max. coverage (+): 3.54. Max coverage (-): 0

Region: chr3 118375287-118375297. Max. coverage (+): 19.79. Max coverage (-): 0

Region: chr3 118375298-118375308. Max. coverage (+): 18.86. Max coverage (-): 0

Region: chr3 118375309-118375318. Max. coverage (+): 1.91. Max coverage (-): 0

Region: chr3 118375319-118375329. Max. coverage (+): 0. Max coverage (-): 0

Region: chr3 118375330-118375340. Max. coverage (+): 0. Max coverage (-): 0

Region: chr3 118375341-118375351. Max. coverage (+): 0. Max coverage (-): 0

Region: chr3 118375352-118375362. Max. coverage (+): 0. Max coverage (-): 0

Region: chr3 118375363-118375373. Max. coverage (+): 0. Max coverage (-): 0

Region: chr3 118375374-118375384. Max. coverage (+): 0. Max coverage (-): 0

Region: chr3 118375385-118375395. Max. coverage (+): 0. Max coverage (-): 0

Region: chr3 118375396-118375406. Max. coverage (+): 0. Max coverage (-): 0

Region: chr3 118375407-118375416. Max. coverage (+): 18.4. Max coverage (-): 0

Region: chr3 118375417-118375427. Max. coverage (+): 5.54. Max coverage (-): 0

Region: chr3 118375428-118375438. Max. coverage (+): 6.3. Max coverage (-): 0

Region: chr3 118375439-118375449. Max. coverage (+): 5.82. Max coverage (-): 0

Region: chr3 118375450-118375460. Max. coverage (+): 5.82. Max coverage (-): 0

Region: chr3 118375461-118375471. Max. coverage (+): 7.99. Max coverage (-): 0

Region: chr3 118375472-118375482. Max. coverage (+): 7.99. Max coverage (-): 0

Region: chr3 118375483-118375493. Max. coverage (+): 0. Max coverage (-): 0

Region: chr3 118375494-118375504. Max. coverage (+): 0. Max coverage (-): 0

Region: chr3 118375505-118375514. Max. coverage (+): 3.97. Max coverage (-): 0

Region: chr3 118375515-118375525. Max. coverage (+): 15.73. Max coverage (-): 0

Region: chr3 118375526-118375536. Max. coverage (+): 4.15. Max coverage (-): 0

Region: chr3 118375537-118375547. Max. coverage (+): 0. Max coverage (-): 0

Region: chr3 118375548-118375558. Max. coverage (+): 0. Max coverage (-): 0

Region: chr3 118375559-118375569. Max. coverage (+): 1.73. Max coverage (-): 0

Region: chr3 118375570-118375580. Max. coverage (+): 1.73. Max coverage (-): 0

Region: chr3 118375581-118375591. Max. coverage (+): 0. Max coverage (-): 0

Region: chr3 118375592-118375602. Max. coverage (+): 0. Max coverage (-): 0

Region: chr3 118375603-118375613. Max. coverage (+): 0. Max coverage (-): 0

Region: chr3 118375614-118375623. Max. coverage (+): 0. Max coverage (-): 0

Region: chr3 118375624-118375634. Max. coverage (+): 0. Max coverage (-): 0

Region: chr3 118375635-118375645. Max. coverage (+): 0. Max coverage (-): 0

Region: chr3 118375646-118375656. Max. coverage (+): 0. Max coverage (-): 0

Region: chr3 118375657-118375667. Max. coverage (+): 0. Max coverage (-): 0

Region: chr3 118375668-118375678. Max. coverage (+): 0. Max coverage (-): 0

Region: chr3 118375679-118375689. Max. coverage (+): 0. Max coverage (-): 0

Region: chr3 118375690-118375700. Max. coverage (+): 0. Max coverage (-): 0

Region: chr3 118375701-118375711. Max. coverage (+): 0. Max coverage (-): 0

Region: chr3 118375712-118375721. Max. coverage (+): 0. Max coverage (-): 0

Region: chr3 118375722-118375732. Max. coverage (+): 0. Max coverage (-): 0

Region: chr3 118375733-118375743. Max. coverage (+): 0. Max coverage (-): 0

Region: chr3 118375744-118375754. Max. coverage (+): 0. Max coverage (-): 0

Region: chr3 118375755-118375765. Max. coverage (+): 0. Max coverage (-): 0

Region: chr3 118375766-118375776. Max. coverage (+): 0. Max coverage (-): 0

Region: chr3 118375777-118375787. Max. coverage (+): 0. Max coverage (-): 0

Region: chr3 118375788-118375798. Max. coverage (+): 0. Max coverage (-): 0

Region: chr3 118375799-118375809. Max. coverage (+): 0.94. Max coverage (-): 0

Region: chr3 118375810-118375819. Max. coverage (+): 2.03. Max coverage (-): 0

Region: chr3 118375820-118375830. Max. coverage (+): 2.03. Max coverage (-): 0

Region: chr3 118375831-118375841. Max. coverage (+): 3.9. Max coverage (-): 0

Region: chr3 118375842-118375852. Max. coverage (+): 3.9. Max coverage (-): 0

Region: chr3 118375853-118375863. Max. coverage (+): 0. Max coverage (-): 0

Region: chr3 118375864-118375874. Max. coverage (+): 0. Max coverage (-): 0

Region: chr3 118375875-118375885. Max. coverage (+): 0. Max coverage (-): 0

Region: chr3 118375886-118375896. Max. coverage (+): 0. Max coverage (-): 0

Region: chr3 118375897-118375907. Max. coverage (+): 0. Max coverage (-): 0

Region: chr3 118375908-118375917. Max. coverage (+): 0. Max coverage (-): 0

Region: chr3 118375918-118375928. Max. coverage (+): 0. Max coverage (-): 0

Region: chr3 118375929-118375939. Max. coverage (+): 0. Max coverage (-): 0

Region: chr3 118375940-118375950. Max. coverage (+): 0. Max coverage (-): 0

Region: chr3 118375951-118375961. Max. coverage (+): 0. Max coverage (-): 0

Region: chr3 118375962-118375972. Max. coverage (+): 0. Max coverage (-): 0

Region: chr3 118375973-118375983. Max. coverage (+): 0. Max coverage (-): 0

Region: chr3 118375984-118375994. Max. coverage (+): 0. Max coverage (-): 0

Region: chr3 118375995-118376005. Max. coverage (+): 0. Max coverage (-): 0

Region: chr3 118376006-118376015. Max. coverage (+): 0. Max coverage (-): 0

Region: chr3 118376016-118376026. Max. coverage (+): 0. Max coverage (-): 0

Region: chr3 118376027-118376037. Max. coverage (+): 0. Max coverage (-): 0

Region: chr3 118376038-118376048. Max. coverage (+): 0. Max coverage (-): 0

Region: chr3 118376049-118376059. Max. coverage (+): 0. Max coverage (-): 0

Region: chr3 118376060-118376070. Max. coverage (+): 0. Max coverage (-): 0

Region: chr3 118376071-118376081. Max. coverage (+): 0. Max coverage (-): 0

Region: chr3 118376082-118376092. Max. coverage (+): 0. Max coverage (-): 0

Region: chr3 118376093-118376103. Max. coverage (+): 0. Max coverage (-): 0

Region: chr3 118376104-118376113. Max. coverage (+): 0. Max coverage (-): 0

Region: chr3 118376114-118376124. Max. coverage (+): 0. Max coverage (-): 0

Region: chr3 118376125-118376135. Max. coverage (+): 0. Max coverage (-): 0

Region: chr3 118376136-118376146. Max. coverage (+): 0. Max coverage (-): 0

Region: chr3 118376147-118376157. Max. coverage (+): 0. Max coverage (-): 0

Region: chr3 118376158-118376168. Max. coverage (+): 0. Max coverage (-): 0

Region: chr3 118376169-118376179. Max. coverage (+): 12.8. Max coverage (-): 0

Region: chr3 118376180-118376190. Max. coverage (+): 12.8. Max coverage (-): 0

Region: chr3 118376191-118376201. Max. coverage (+): 2.86. Max coverage (-): 0

Region: chr3 118376202-118376211. Max. coverage (+): 21.58. Max coverage (-): 0

Region: chr3 118376212-118376222. Max. coverage (+): 21.58. Max coverage (-): 0

Region: chr3 118376223-118376233. Max. coverage (+): 6.89. Max coverage (-): 0

Region: chr3 118376234-118376244. Max. coverage (+): 0. Max coverage (-): 0

Region: chr3 118376245-118376255. Max. coverage (+): 0. Max coverage (-): 0

Region: chr3 118376256-118376266. Max. coverage (+): 0. Max coverage (-): 0

Region: chr3 118376267-118376277. Max. coverage (+): 0. Max coverage (-): 0

Region: chr3 118376278-118376288. Max. coverage (+): 0. Max coverage (-): 0

Region: chr3 118376289-118376299. Max. coverage (+): 0. Max coverage (-): 0

Region: chr3 118376300-118376309. Max. coverage (+): 0. Max coverage (-): 0

Region: chr3 118376310-118376320. Max. coverage (+): 0. Max coverage (-): 0

Region: chr3 118376321-118376331. Max. coverage (+): 0. Max coverage (-): 0

Region: chr3 118376332-118376342. Max. coverage (+): 0. Max coverage (-): 0

Region: chr3 118376343-118376353. Max. coverage (+): 0. Max coverage (-): 0

Region: chr3 118376354-118376364. Max. coverage (+): 0. Max coverage (-): 0

Region: chr3 118376365-118376375. Max. coverage (+): 0. Max coverage (-): 0

Region: chr3 118376376-118376386. Max. coverage (+): 0. Max coverage (-): 0

Region: chr3 118376387-118376397. Max. coverage (+): 0. Max coverage (-): 0

Region: chr3 118376398-118376407. Max. coverage (+): 0. Max coverage (-): 0

Region: chr3 118376408-118376418. Max. coverage (+): 0. Max coverage (-): 0

Region: chr3 118376419-118376429. Max. coverage (+): 0. Max coverage (-): 0

Region: chr3 118376430-118376440. Max. coverage (+): 0. Max coverage (-): 0

Region: chr3 118376441-118376451. Max. coverage (+): 0. Max coverage (-): 0

Region: chr3 118376452-118376462. Max. coverage (+): 0. Max coverage (-): 0

Region: chr3 118376463-118376473. Max. coverage (+): 0. Max coverage (-): 0

Region: chr3 118376474-118376484. Max. coverage (+): 0. Max coverage (-): 0

Region: chr3 118376485-118376495. Max. coverage (+): 0. Max coverage (-): 0

Region: chr3 118376496-118376505. Max. coverage (+): 0. Max coverage (-): 0

Region: chr3 118376506-118376516. Max. coverage (+): 0. Max coverage (-): 0

Region: chr3 118376517-118376527. Max. coverage (+): 0. Max coverage (-): 0

Region: chr3 118376528-118376538. Max. coverage (+): 0. Max coverage (-): 0

Region: chr3 118376539-118376549. Max. coverage (+): 0. Max coverage (-): 0

Region: chr3 118376550-118376560. Max. coverage (+): 0. Max coverage (-): 0

Region: chr3 118376561-118376571. Max. coverage (+): 0. Max coverage (-): 0

Region: chr3 118376572-118376582. Max. coverage (+): 0. Max coverage (-): 0

Region: chr3 118376583-118376593. Max. coverage (+): 0. Max coverage (-): 0

Region: chr3 118376594-118376603. Max. coverage (+): 0. Max coverage (-): 0

Region: chr3 118376604-118376614. Max. coverage (+): 6.64. Max coverage (-): 0

Region: chr3 118376615-118376625. Max. coverage (+): 0. Max coverage (-): 0

Region: chr3 118376626-118376636. Max. coverage (+): 0. Max coverage (-): 0

Region: chr3 118376637-118376647. Max. coverage (+): 0. Max coverage (-): 0

Region: chr3 118376648-118376658. Max. coverage (+): 0. Max coverage (-): 0

Region: chr3 118376659-118376669. Max. coverage (+): 0. Max coverage (-): 0

Region: chr3 118376670-118376680. Max. coverage (+): 0. Max coverage (-): 0

Region: chr3 118376681-118376691. Max. coverage (+): 0. Max coverage (-): 0

Region: chr3 118376692-118376702. Max. coverage (+): 0. Max coverage (-): 0

Region: chr3 118376703-118376712. Max. coverage (+): 1.63. Max coverage (-): 0

Region: chr3 118376713-118376723. Max. coverage (+): 9.67. Max coverage (-): 0

Region: chr3 118376724-118376734. Max. coverage (+): 0. Max coverage (-): 0

Region: chr3 118376735-118376745. Max. coverage (+): 0. Max coverage (-): 0

Region: chr3 118376746-118376756. Max. coverage (+): 0. Max coverage (-): 0

Region: chr3 118376757-118376767. Max. coverage (+): 0. Max coverage (-): 0

Region: chr3 118376768-118376778. Max. coverage (+): 0. Max coverage (-): 0

Region: chr3 118376779-118376789. Max. coverage (+): 0. Max coverage (-): 0

Region: chr3 118376790-118376800. Max. coverage (+): 0. Max coverage (-): 0

Region: chr3 118376801-118376810. Max. coverage (+): 2.58. Max coverage (-): 0

Region: chr3 118376811-118376821. Max. coverage (+): 0. Max coverage (-): 0

Region: chr3 118376822-118376832. Max. coverage (+): 0. Max coverage (-): 0

Region: chr3 118376833-118376843. Max. coverage (+): 0. Max coverage (-): 0

Region: chr3 118376844-118376854. Max. coverage (+): 0. Max coverage (-): 0

Region: chr3 118376855-118376865. Max. coverage (+): 0. Max coverage (-): 0

Region: chr3 118376866-118376876. Max. coverage (+): 0. Max coverage (-): 0

Region: chr3 118376877-118376887. Max. coverage (+): 0. Max coverage (-): 0

Region: chr3 118376888-118376898. Max. coverage (+): 0. Max coverage (-): 0

Region: chr3 118376899-118376908. Max. coverage (+): 0. Max coverage (-): 0

Region: chr3 118376909-118376919. Max. coverage (+): 0. Max coverage (-): 0

Region: chr3 118376920-118376930. Max. coverage (+): 2.95. Max coverage (-): 0

Region: chr3 118376931-118376941. Max. coverage (+): 17.03. Max coverage (-): 0

Region: chr3 118376942-118376952. Max. coverage (+): 15.17. Max coverage (-): 0

Region: chr3 118376953-118376963. Max. coverage (+): 0. Max coverage (-): 0

Region: chr3 118376964-118376974. Max. coverage (+): 0. Max coverage (-): 0

Region: chr3 118376975-118376985. Max. coverage (+): 0. Max coverage (-): 0

Region: chr3 118376986-118376996. Max. coverage (+): 0. Max coverage (-): 0

Region: chr3 118376997-118377006. Max. coverage (+): 0. Max coverage (-): 0

Region: chr3 118377007-118377017. Max. coverage (+): 0. Max coverage (-): 0

Region: chr3 118377018-118377028. Max. coverage (+): 0. Max coverage (-): 0

Region: chr3 118377029-118377039. Max. coverage (+): 0. Max coverage (-): 0

Region: chr3 118377040-118377050. Max. coverage (+): 0. Max coverage (-): 0

Region: chr3 118377051-118377061. Max. coverage (+): 0. Max coverage (-): 0

Region: chr3 118377062-118377072. Max. coverage (+): 0. Max coverage (-): 0

Region: chr3 118377073-118377083. Max. coverage (+): 0. Max coverage (-): 0

Region: chr3 118377084-118377094. Max. coverage (+): 0. Max coverage (-): 0

Region: chr3 118377095-118377104. Max. coverage (+): 7.97. Max coverage (-): 0

Region: chr3 118377105-118377115. Max. coverage (+): 0. Max coverage (-): 0

Region: chr3 118377116-118377126. Max. coverage (+): 0. Max coverage (-): 0

Region: chr3 118377127-118377137. Max. coverage (+): 0. Max coverage (-): 0

Region: chr3 118377138-118377148. Max. coverage (+): 0. Max coverage (-): 0

Region: chr3 118377149-118377159. Max. coverage (+): 0. Max coverage (-): 0

Region: chr3 118377160-118377170. Max. coverage (+): 0. Max coverage (-): 0

Region: chr3 118377171-118377181. Max. coverage (+): 0. Max coverage (-): 0

Region: chr3 118377182-118377192. Max. coverage (+): 7.27. Max coverage (-): 0

Region: chr3 118377193-118377202. Max. coverage (+): 9.42. Max coverage (-): 0

Region: chr3 118377203-118377213. Max. coverage (+): 3.55. Max coverage (-): 0

Region: chr3 118377214-118377224. Max. coverage (+): 0. Max coverage (-): 0

Region: chr3 118377225-118377235. Max. coverage (+): 0. Max coverage (-): 0

Region: chr3 118377236-118377246. Max. coverage (+): 0. Max coverage (-): 0

Region: chr3 118377247-118377257. Max. coverage (+): 0. Max coverage (-): 0

Region: chr3 118377258-118377268. Max. coverage (+): 0.51. Max coverage (-): 0

Region: chr3 118377269-118377279. Max. coverage (+): 0.51. Max coverage (-): 0

Region: chr3 118377280-118377290. Max. coverage (+): 0.81. Max coverage (-): 0

Region: chr3 118377291-118377300. Max. coverage (+): 0.9. Max coverage (-): 0

Region: chr3 118377301-118377311. Max. coverage (+): 0.9. Max coverage (-): 0

Region: chr3 118377312-118377322. Max. coverage (+): 0.24. Max coverage (-): 0

Region: chr3 118377323-118377333. Max. coverage (+): 0. Max coverage (-): 0

Region: chr3 118377334-118377344. Max. coverage (+): 0. Max coverage (-): 0

Region: chr3 118377345-118377355. Max. coverage (+): 7.09. Max coverage (-): 0

Region: chr3 118377356-118377366. Max. coverage (+): 7.09. Max coverage (-): 0

Region: chr3 118377367-118377377. Max. coverage (+): 1.56. Max coverage (-): 0

Region: chr3 118377378-118377388. Max. coverage (+): 1.56. Max coverage (-): 0

Region: chr3 118377389-118377398. Max. coverage (+): 2.25. Max coverage (-): 0

Region: chr3 118377399-118377409. Max. coverage (+): 0. Max coverage (-): 0

Region: chr3 118377410-118377420. Max. coverage (+): 0. Max coverage (-): 0

Region: chr3 118377421-118377431. Max. coverage (+): 0. Max coverage (-): 0

Region: chr3 118377432-118377442. Max. coverage (+): 0. Max coverage (-): 0

Region: chr3 118377443-118377453. Max. coverage (+): 0. Max coverage (-): 0

Region: chr3 118377454-118377464. Max. coverage (+): 0. Max coverage (-): 0

Region: chr3 118377465-118377475. Max. coverage (+): 0. Max coverage (-): 0

Region: chr3 118377476-118377486. Max. coverage (+): 0. Max coverage (-): 0

Region: chr3 118377487-118377496. Max. coverage (+): 0. Max coverage (-): 0

Region: chr3 118377497-118377507. Max. coverage (+): 0. Max coverage (-): 0

Region: chr3 118377508-118377518. Max. coverage (+): 0. Max coverage (-): 0

Region: chr3 118377519-118377529. Max. coverage (+): 0. Max coverage (-): 0

Region: chr3 118377530-118377540. Max. coverage (+): 0. Max coverage (-): 0

Region: chr3 118377541-118377551. Max. coverage (+): 0. Max coverage (-): 0

Region: chr3 118377552-118377562. Max. coverage (+): 0. Max coverage (-): 0

Region: chr3 118377563-118377573. Max. coverage (+): 0. Max coverage (-): 0

Region: chr3 118377574-118377584. Max. coverage (+): 0. Max coverage (-): 0

Region: chr3 118377585-118377594. Max. coverage (+): 0. Max coverage (-): 0

Region: chr3 118377595-118377605. Max. coverage (+): 0. Max coverage (-): 0

Region: chr3 118377606-118377616. Max. coverage (+): 0. Max coverage (-): 0

Region: chr3 118377617-118377627. Max. coverage (+): 0. Max coverage (-): 0

Region: chr3 118377628-118377638. Max. coverage (+): 0. Max coverage (-): 0

Region: chr3 118377639-118377649. Max. coverage (+): 0. Max coverage (-): 0

Region: chr3 118377650-118377660. Max. coverage (+): 0. Max coverage (-): 0

Region: chr3 118377661-118377671. Max. coverage (+): 0. Max coverage (-): 0

Region: chr3 118377672-118377682. Max. coverage (+): 0. Max coverage (-): 0

Region: chr3 118377683-118377692. Max. coverage (+): 0. Max coverage (-): 0

Region: chr3 118377693-118377703. Max. coverage (+): 0. Max coverage (-): 0

Region: chr3 118377704-118377714. Max. coverage (+): 1.51. Max coverage (-): 0

Region: chr3 118377715-118377725. Max. coverage (+): 1.51. Max coverage (-): 0

Region: chr3 118377726-118377736. Max. coverage (+): 0. Max coverage (-): 0

Region: chr3 118377737-. Max. coverage (+): 0. Max coverage (-): 0

RepeatMasker Color Code

**+**

100-98% Identity

<98-95% Identity

<95-90% Identity

<90-85% Identity

<85-80% Identity

<80-75% Identity

<75-70% Identity

<70% Identity

**-**

Gene Set Color Code

**+**

Gene

Pseudogene

**-**

Topology/Coverage Color Code

Coverage Plus Strand

Coverage Minus Strand

Mainstrand: Plus

Mainstrand: Minus

Complementary Strand

Flanking Region  
(if option -flank >0)

Gene Set Annotation  

**1. ASB1 (protein coding, ENSBTAG00000003376) Tr:00000004378 Ex:5**: 118372187-118372314 (+)

  
RepeatMasker Annotation  

**1. (CA)n**: 118372806-118372833 (+), Divergence to consensus: 10.7%  
**2. L4\_A\_Mam**: 118375357-118375400 (-), Divergence to consensus: 13.9%  
**3. MIRc**: 118375701-118375763 (+), Divergence to consensus: 30.2%  
**4. Charlie18a**: 118375973-118376166 (+), Divergence to consensus: 26.2%

  
Transcription Factor Binding Sites  

**RFX4\_2** (Sequence: GTATCCAGG (-): 118373476)
