# Supplementary material for: Small RNA sequencing of cryopreserved semen from single bull revealed altered miRNAs and piRNAs expression between High- and Low-motile sperm populations
Source: BMC Genomics. 2017 Jan 4;18:14. doi: 10.1186/s12864-016-3394-7 (PMC5209821; doi:10.1186/s12864-016-3394-7)
Supplement: Additional file 3: — Details for each piRNA clusters found in High Motile (HM) sperm fraction. Genes, repeats, transposable elements and transcription factors binding sites falling within the cluster regions were reported. (ZIP 1896 kb) [file 12864_2016_3394_MOESM3_ESM.zip › 82.html]

piRNA cluster 82


Predicted piRNA cluster no. 82     previous   next
  

Show proTRAC run info
Hide proTRAC run info

================================= proTRAC ====================================  
VERSION: 2.1                                    LAST MODIFIED: 06. October 2015  
  
Please cite:  
Rosenkranz D, Zischler H. proTRAC - a software for probabilistic piRNA cluster  
detection, visualization and analysis. 2012. BMC Bioinformatics 13:5.  
  
and (for proTRAC 2.0 and later):  
Rosenkranz D, Rudloff S, Bastuck K, Ketting RF, Zischler H. Tupaia small RNAs  
provide insights into function and evolution of RNAi-based transposon defense  
in mammals. 2015. RNA 21(5):911-922.  
  
Contact:  
David Rosenkranz  
Institute of Anthropology, small RNA group  
Johannes Gutenberg University Mainz  
email: rosenkranz@uni-mainz.de  
  
You can find the latest proTRAC version at:  
http://sourceforge.net/projects/protrac/files  
http://www.smallRNAgroup-mainz.de/software  
==============================================================================  
  
PARAMETERS:  
Map file: .............../storage/core/barbara/genhome/smallRNA/fertility/Sample\_motile/pirna/Sample\_motile\_26-33\_collapsed.fa.no-dust.map.weighted-10000-1000-b-0  
Genome file: ............/storage/core/barbara/genhome/smallRNA/fertility/Sample\_all/pirna/bt\_311\_chrY.fa  
RepeatMasker annotation: /storage/genomes/bt\_umd31/GCF\_000003055.6\_Bos\_taurus\_UMD\_3.1.1\_repeatMasker\_chr.out  
GeneSet:................./storage/core/barbara/genhome/smallRNA/fertility/Sample\_all/pirna/full.gtf  
  
Significant (p<=0.01) hit density will be calculated based  
on observed hit distribution.  
  
Sliding window size: ........................................ 5000 bp  
Sliding window increament: .................................. 1000 bp  
Normalize each hit by number of genomic hits: ............... 1 [0=no/1=yes]  
Normalize each hit by number of sequence reads: ............. 1 [0=no/1=yes]  
Normalize values (-> per million mapped reads): ............. 1 [0=no/1=yes]  
Min. fraction of hits with 1T(U) or 10A: .................... 0.75  
Alternatively: Min. fraction of hits with 1T(U) and 10A: .... 0.5  
Min. fraction of hits with typical piRNA length: ............ 0.75  
Typical piRNA length: ....................................... 26-33 nt  
Min. size of a piRNA cluster: ............................... 5000 bp.  
Min. number of hits (absolute): ............................. 0  
Min. number of hits (normalized): ........................... 0  
Min. fraction of hits on the mainstrand: .................... 0.75  
Top fraction of mapped sequences (in terms of read counts): . 1%  
Top fraction accounts for max. n% of sequence reads: ........ 90%  
Min. fraction of hits on each arm of a bidirectional cluster: 0.1  
Output image file for each cluster: ......................... 0 [0=no/1=yes]  
Output html file for each cluster: .......................... 1 [0=no/1=yes]  
Output a summary table: ..................................... 1 [0=no/1=yes]  
Output a FASTA file for each cluster (piRNA sequences): ..... 1 [0=no/1=yes]  
Output a FASTA file comprising cluster sequences: ........... 1 [0=no/1=yes]  
Search DNA motifs in clusters: .............................. 1 [0=no/1=yes]  
Output flanking sequences: +/- .............................. 0 bp  
Output ~.pTi file: .......................................... 1 [0=no/1=yes]  
==============================================================================  
  
  
Genome size (without gaps): ............ 2678902517 bp  
Gaps (N/X/-): .......................... 53837044 bp  
Mapped reads: .......................... 658825247023  
Non-identical sequences: ............... 514171  
Genomic hits: .......................... 764233  
Significant densitiy of mapped reads: .. 12867599.5173724 reads/kb

Show proTRAC cluster info
Hide proTRAC cluster info

|  |  |
| --- | --- |
| Location | chr4 |
| Coordinates | 69091262-69097100 |
| Size [bp] | 5839 |
| Sequence hit loci | 59 |
| Mapped reads (normalized) | 98834497 |
| Mapped reads (normalized) per kb | 16926613.6 |
| Normalized reads with 1T (1U) | 83.8% |
| Normalized reads with 10A | 45% |
| Normalized reads with length 26-33 nt | 100% |
| Normalized reads on the main strand(s) | 100% |
| Predicted directionality | mono:minus |

100%

0%

1T (1U)  
reads

10A reads

26-33 nt  
reads

reads on mainstrand

**Either the amount of reads with 1T (1U) OR 10A has to exceed 75% (set with option: -1Tor10A)  
Alternatively the amount of reads with 1T (1U) AND 10A has to exceed 50% (set with option: -1Tand10A)  
Minimum amount of reads with preferred size is 75% (set with option: -pisize)  
Minimum amount of reads on the main strand(s) is 75% (set with option: -clstrand)**

Show read coverage
Hide read coverage

WHAT DO I SEE HERE?  
This chart shows the location of mapped sequence reads within a predicted piRNA cluster. The color refers to the number of genomic hits produced by the sequence read in question. A dark red bar indicates that this sequence read produces many other hits elsewhere in the genome. Many adjacent red or yellow bars can indicate the presence of a multi-copy element such as transposons or rRNA genes. A dark green bar indicates that this sequence read maps uniquely to this locus.

1 hit

2-5 hits

6-10 hits

11-20 hits

21-50 hits

51-100 hits

> 100 hits

chr4

69091262

69097100

Gene Set

RepeatMasker

Mapped  
Reads

14.26

plus strand

minus strand

14.26

Region: chr4 118377738-69091267. Max. coverage (+): 0. Max coverage (-): 1.39

Region: chr4 69091268-69091279. Max. coverage (+): 0. Max coverage (-): 1.39

Region: chr4 69091280-69091291. Max. coverage (+): 0. Max coverage (-): 0

Region: chr4 69091292-69091302. Max. coverage (+): 0. Max coverage (-): 0

Region: chr4 69091303-69091314. Max. coverage (+): 0. Max coverage (-): 0

Region: chr4 69091315-69091326. Max. coverage (+): 0. Max coverage (-): 0

Region: chr4 69091327-69091337. Max. coverage (+): 0. Max coverage (-): 0

Region: chr4 69091338-69091349. Max. coverage (+): 0. Max coverage (-): 0

Region: chr4 69091350-69091361. Max. coverage (+): 0. Max coverage (-): 0

Region: chr4 69091362-69091372. Max. coverage (+): 0. Max coverage (-): 0

Region: chr4 69091373-69091384. Max. coverage (+): 0. Max coverage (-): 0

Region: chr4 69091385-69091396. Max. coverage (+): 0. Max coverage (-): 0

Region: chr4 69091397-69091407. Max. coverage (+): 0. Max coverage (-): 0

Region: chr4 69091408-69091419. Max. coverage (+): 0. Max coverage (-): 0

Region: chr4 69091420-69091431. Max. coverage (+): 0. Max coverage (-): 0

Region: chr4 69091432-69091443. Max. coverage (+): 0. Max coverage (-): 0

Region: chr4 69091444-69091454. Max. coverage (+): 0. Max coverage (-): 0

Region: chr4 69091455-69091466. Max. coverage (+): 0. Max coverage (-): 0

Region: chr4 69091467-69091478. Max. coverage (+): 0. Max coverage (-): 0

Region: chr4 69091479-69091489. Max. coverage (+): 0. Max coverage (-): 0

Region: chr4 69091490-69091501. Max. coverage (+): 0. Max coverage (-): 0

Region: chr4 69091502-69091513. Max. coverage (+): 0. Max coverage (-): 0

Region: chr4 69091514-69091524. Max. coverage (+): 0. Max coverage (-): 0

Region: chr4 69091525-69091536. Max. coverage (+): 0. Max coverage (-): 0

Region: chr4 69091537-69091548. Max. coverage (+): 0. Max coverage (-): 0

Region: chr4 69091549-69091559. Max. coverage (+): 0. Max coverage (-): 0

Region: chr4 69091560-69091571. Max. coverage (+): 0. Max coverage (-): 0

Region: chr4 69091572-69091583. Max. coverage (+): 0. Max coverage (-): 0

Region: chr4 69091584-69091594. Max. coverage (+): 0. Max coverage (-): 0

Region: chr4 69091595-69091606. Max. coverage (+): 0. Max coverage (-): 0

Region: chr4 69091607-69091618. Max. coverage (+): 0. Max coverage (-): 0

Region: chr4 69091619-69091629. Max. coverage (+): 0. Max coverage (-): 0

Region: chr4 69091630-69091641. Max. coverage (+): 0. Max coverage (-): 0

Region: chr4 69091642-69091653. Max. coverage (+): 0. Max coverage (-): 0

Region: chr4 69091654-69091664. Max. coverage (+): 0. Max coverage (-): 0

Region: chr4 69091665-69091676. Max. coverage (+): 0. Max coverage (-): 0

Region: chr4 69091677-69091688. Max. coverage (+): 0. Max coverage (-): 0

Region: chr4 69091689-69091699. Max. coverage (+): 0. Max coverage (-): 0

Region: chr4 69091700-69091711. Max. coverage (+): 0. Max coverage (-): 0

Region: chr4 69091712-69091723. Max. coverage (+): 0. Max coverage (-): 0

Region: chr4 69091724-69091734. Max. coverage (+): 0. Max coverage (-): 0

Region: chr4 69091735-69091746. Max. coverage (+): 0. Max coverage (-): 0

Region: chr4 69091747-69091758. Max. coverage (+): 0. Max coverage (-): 0

Region: chr4 69091759-69091769. Max. coverage (+): 0. Max coverage (-): 0

Region: chr4 69091770-69091781. Max. coverage (+): 0. Max coverage (-): 2.54

Region: chr4 69091782-69091793. Max. coverage (+): 0. Max coverage (-): 2.54

Region: chr4 69091794-69091805. Max. coverage (+): 0. Max coverage (-): 0

Region: chr4 69091806-69091816. Max. coverage (+): 0. Max coverage (-): 0

Region: chr4 69091817-69091828. Max. coverage (+): 0. Max coverage (-): 0

Region: chr4 69091829-69091840. Max. coverage (+): 0. Max coverage (-): 0

Region: chr4 69091841-69091851. Max. coverage (+): 0. Max coverage (-): 0

Region: chr4 69091852-69091863. Max. coverage (+): 0. Max coverage (-): 0

Region: chr4 69091864-69091875. Max. coverage (+): 0. Max coverage (-): 0

Region: chr4 69091876-69091886. Max. coverage (+): 0. Max coverage (-): 0

Region: chr4 69091887-69091898. Max. coverage (+): 0. Max coverage (-): 0

Region: chr4 69091899-69091910. Max. coverage (+): 0. Max coverage (-): 0

Region: chr4 69091911-69091921. Max. coverage (+): 0. Max coverage (-): 0

Region: chr4 69091922-69091933. Max. coverage (+): 0. Max coverage (-): 0

Region: chr4 69091934-69091945. Max. coverage (+): 0. Max coverage (-): 0

Region: chr4 69091946-69091956. Max. coverage (+): 0. Max coverage (-): 0

Region: chr4 69091957-69091968. Max. coverage (+): 0. Max coverage (-): 4.82

Region: chr4 69091969-69091980. Max. coverage (+): 0. Max coverage (-): 4.82

Region: chr4 69091981-69091991. Max. coverage (+): 0. Max coverage (-): 0

Region: chr4 69091992-69092003. Max. coverage (+): 0. Max coverage (-): 0

Region: chr4 69092004-69092015. Max. coverage (+): 0. Max coverage (-): 0

Region: chr4 69092016-69092026. Max. coverage (+): 0. Max coverage (-): 0

Region: chr4 69092027-69092038. Max. coverage (+): 0. Max coverage (-): 0

Region: chr4 69092039-69092050. Max. coverage (+): 0. Max coverage (-): 0

Region: chr4 69092051-69092061. Max. coverage (+): 0. Max coverage (-): 0

Region: chr4 69092062-69092073. Max. coverage (+): 0. Max coverage (-): 0

Region: chr4 69092074-69092085. Max. coverage (+): 0. Max coverage (-): 0

Region: chr4 69092086-69092096. Max. coverage (+): 0. Max coverage (-): 0

Region: chr4 69092097-69092108. Max. coverage (+): 0. Max coverage (-): 0

Region: chr4 69092109-69092120. Max. coverage (+): 0. Max coverage (-): 0

Region: chr4 69092121-69092132. Max. coverage (+): 0. Max coverage (-): 0

Region: chr4 69092133-69092143. Max. coverage (+): 0. Max coverage (-): 0

Region: chr4 69092144-69092155. Max. coverage (+): 0. Max coverage (-): 0

Region: chr4 69092156-69092167. Max. coverage (+): 0. Max coverage (-): 0

Region: chr4 69092168-69092178. Max. coverage (+): 0. Max coverage (-): 0

Region: chr4 69092179-69092190. Max. coverage (+): 0. Max coverage (-): 0

Region: chr4 69092191-69092202. Max. coverage (+): 0. Max coverage (-): 0

Region: chr4 69092203-69092213. Max. coverage (+): 0. Max coverage (-): 0

Region: chr4 69092214-69092225. Max. coverage (+): 0. Max coverage (-): 0

Region: chr4 69092226-69092237. Max. coverage (+): 0. Max coverage (-): 0

Region: chr4 69092238-69092248. Max. coverage (+): 0. Max coverage (-): 0

Region: chr4 69092249-69092260. Max. coverage (+): 0. Max coverage (-): 0

Region: chr4 69092261-69092272. Max. coverage (+): 0. Max coverage (-): 0

Region: chr4 69092273-69092283. Max. coverage (+): 0. Max coverage (-): 0

Region: chr4 69092284-69092295. Max. coverage (+): 0. Max coverage (-): 0

Region: chr4 69092296-69092307. Max. coverage (+): 0. Max coverage (-): 0

Region: chr4 69092308-69092318. Max. coverage (+): 0. Max coverage (-): 0

Region: chr4 69092319-69092330. Max. coverage (+): 0. Max coverage (-): 0

Region: chr4 69092331-69092342. Max. coverage (+): 0. Max coverage (-): 0

Region: chr4 69092343-69092353. Max. coverage (+): 0. Max coverage (-): 0

Region: chr4 69092354-69092365. Max. coverage (+): 0. Max coverage (-): 0

Region: chr4 69092366-69092377. Max. coverage (+): 0. Max coverage (-): 0

Region: chr4 69092378-69092388. Max. coverage (+): 0. Max coverage (-): 0

Region: chr4 69092389-69092400. Max. coverage (+): 0. Max coverage (-): 0

Region: chr4 69092401-69092412. Max. coverage (+): 0. Max coverage (-): 0

Region: chr4 69092413-69092423. Max. coverage (+): 0. Max coverage (-): 0

Region: chr4 69092424-69092435. Max. coverage (+): 0. Max coverage (-): 0

Region: chr4 69092436-69092447. Max. coverage (+): 0. Max coverage (-): 0

Region: chr4 69092448-69092458. Max. coverage (+): 0. Max coverage (-): 0

Region: chr4 69092459-69092470. Max. coverage (+): 0. Max coverage (-): 0

Region: chr4 69092471-69092482. Max. coverage (+): 0. Max coverage (-): 0

Region: chr4 69092483-69092494. Max. coverage (+): 0. Max coverage (-): 0

Region: chr4 69092495-69092505. Max. coverage (+): 0. Max coverage (-): 0

Region: chr4 69092506-69092517. Max. coverage (+): 0. Max coverage (-): 0

Region: chr4 69092518-69092529. Max. coverage (+): 0. Max coverage (-): 0

Region: chr4 69092530-69092540. Max. coverage (+): 0. Max coverage (-): 0

Region: chr4 69092541-69092552. Max. coverage (+): 0. Max coverage (-): 0

Region: chr4 69092553-69092564. Max. coverage (+): 0. Max coverage (-): 0

Region: chr4 69092565-69092575. Max. coverage (+): 0. Max coverage (-): 0

Region: chr4 69092576-69092587. Max. coverage (+): 0. Max coverage (-): 0

Region: chr4 69092588-69092599. Max. coverage (+): 0. Max coverage (-): 0

Region: chr4 69092600-69092610. Max. coverage (+): 0. Max coverage (-): 0

Region: chr4 69092611-69092622. Max. coverage (+): 0. Max coverage (-): 0

Region: chr4 69092623-69092634. Max. coverage (+): 0. Max coverage (-): 0

Region: chr4 69092635-69092645. Max. coverage (+): 0. Max coverage (-): 0

Region: chr4 69092646-69092657. Max. coverage (+): 0. Max coverage (-): 0

Region: chr4 69092658-69092669. Max. coverage (+): 0. Max coverage (-): 0

Region: chr4 69092670-69092680. Max. coverage (+): 0. Max coverage (-): 0

Region: chr4 69092681-69092692. Max. coverage (+): 0. Max coverage (-): 0

Region: chr4 69092693-69092704. Max. coverage (+): 0. Max coverage (-): 0

Region: chr4 69092705-69092715. Max. coverage (+): 0. Max coverage (-): 0

Region: chr4 69092716-69092727. Max. coverage (+): 0. Max coverage (-): 0

Region: chr4 69092728-69092739. Max. coverage (+): 0. Max coverage (-): 0

Region: chr4 69092740-69092750. Max. coverage (+): 0. Max coverage (-): 8.51

Region: chr4 69092751-69092762. Max. coverage (+): 0. Max coverage (-): 0

Region: chr4 69092763-69092774. Max. coverage (+): 0. Max coverage (-): 0.79

Region: chr4 69092775-69092785. Max. coverage (+): 0. Max coverage (-): 0.79

Region: chr4 69092786-69092797. Max. coverage (+): 0. Max coverage (-): 0

Region: chr4 69092798-69092809. Max. coverage (+): 0. Max coverage (-): 0

Region: chr4 69092810-69092821. Max. coverage (+): 0. Max coverage (-): 0

Region: chr4 69092822-69092832. Max. coverage (+): 0. Max coverage (-): 0

Region: chr4 69092833-69092844. Max. coverage (+): 0. Max coverage (-): 0

Region: chr4 69092845-69092856. Max. coverage (+): 0. Max coverage (-): 0

Region: chr4 69092857-69092867. Max. coverage (+): 0. Max coverage (-): 0

Region: chr4 69092868-69092879. Max. coverage (+): 0. Max coverage (-): 0

Region: chr4 69092880-69092891. Max. coverage (+): 0. Max coverage (-): 0

Region: chr4 69092892-69092902. Max. coverage (+): 0. Max coverage (-): 0

Region: chr4 69092903-69092914. Max. coverage (+): 0. Max coverage (-): 0

Region: chr4 69092915-69092926. Max. coverage (+): 0. Max coverage (-): 0

Region: chr4 69092927-69092937. Max. coverage (+): 0. Max coverage (-): 0

Region: chr4 69092938-69092949. Max. coverage (+): 0. Max coverage (-): 0

Region: chr4 69092950-69092961. Max. coverage (+): 0. Max coverage (-): 0

Region: chr4 69092962-69092972. Max. coverage (+): 0. Max coverage (-): 0

Region: chr4 69092973-69092984. Max. coverage (+): 0. Max coverage (-): 0

Region: chr4 69092985-69092996. Max. coverage (+): 0. Max coverage (-): 0

Region: chr4 69092997-69093007. Max. coverage (+): 0. Max coverage (-): 0

Region: chr4 69093008-69093019. Max. coverage (+): 0. Max coverage (-): 0

Region: chr4 69093020-69093031. Max. coverage (+): 0. Max coverage (-): 0

Region: chr4 69093032-69093042. Max. coverage (+): 0. Max coverage (-): 0

Region: chr4 69093043-69093054. Max. coverage (+): 0. Max coverage (-): 0

Region: chr4 69093055-69093066. Max. coverage (+): 0. Max coverage (-): 0

Region: chr4 69093067-69093077. Max. coverage (+): 0. Max coverage (-): 0

Region: chr4 69093078-69093089. Max. coverage (+): 0. Max coverage (-): 0

Region: chr4 69093090-69093101. Max. coverage (+): 0. Max coverage (-): 0

Region: chr4 69093102-69093112. Max. coverage (+): 0. Max coverage (-): 0

Region: chr4 69093113-69093124. Max. coverage (+): 0. Max coverage (-): 0

Region: chr4 69093125-69093136. Max. coverage (+): 0. Max coverage (-): 0

Region: chr4 69093137-69093147. Max. coverage (+): 0. Max coverage (-): 0

Region: chr4 69093148-69093159. Max. coverage (+): 0. Max coverage (-): 3.16

Region: chr4 69093160-69093171. Max. coverage (+): 0. Max coverage (-): 0

Region: chr4 69093172-69093183. Max. coverage (+): 0. Max coverage (-): 0

Region: chr4 69093184-69093194. Max. coverage (+): 0. Max coverage (-): 0

Region: chr4 69093195-69093206. Max. coverage (+): 0. Max coverage (-): 0

Region: chr4 69093207-69093218. Max. coverage (+): 0. Max coverage (-): 2.04

Region: chr4 69093219-69093229. Max. coverage (+): 0. Max coverage (-): 0

Region: chr4 69093230-69093241. Max. coverage (+): 0. Max coverage (-): 0

Region: chr4 69093242-69093253. Max. coverage (+): 0. Max coverage (-): 0

Region: chr4 69093254-69093264. Max. coverage (+): 0. Max coverage (-): 0

Region: chr4 69093265-69093276. Max. coverage (+): 0. Max coverage (-): 0

Region: chr4 69093277-69093288. Max. coverage (+): 0. Max coverage (-): 0

Region: chr4 69093289-69093299. Max. coverage (+): 0. Max coverage (-): 0

Region: chr4 69093300-69093311. Max. coverage (+): 0. Max coverage (-): 0

Region: chr4 69093312-69093323. Max. coverage (+): 0. Max coverage (-): 0

Region: chr4 69093324-69093334. Max. coverage (+): 0. Max coverage (-): 0

Region: chr4 69093335-69093346. Max. coverage (+): 0. Max coverage (-): 0

Region: chr4 69093347-69093358. Max. coverage (+): 0. Max coverage (-): 0

Region: chr4 69093359-69093369. Max. coverage (+): 0. Max coverage (-): 0

Region: chr4 69093370-69093381. Max. coverage (+): 0. Max coverage (-): 0

Region: chr4 69093382-69093393. Max. coverage (+): 0. Max coverage (-): 0

Region: chr4 69093394-69093404. Max. coverage (+): 0. Max coverage (-): 0

Region: chr4 69093405-69093416. Max. coverage (+): 0. Max coverage (-): 0

Region: chr4 69093417-69093428. Max. coverage (+): 0. Max coverage (-): 0

Region: chr4 69093429-69093439. Max. coverage (+): 0. Max coverage (-): 0

Region: chr4 69093440-69093451. Max. coverage (+): 0. Max coverage (-): 0

Region: chr4 69093452-69093463. Max. coverage (+): 0. Max coverage (-): 0

Region: chr4 69093464-69093474. Max. coverage (+): 0. Max coverage (-): 0

Region: chr4 69093475-69093486. Max. coverage (+): 0. Max coverage (-): 0

Region: chr4 69093487-69093498. Max. coverage (+): 0. Max coverage (-): 0

Region: chr4 69093499-69093510. Max. coverage (+): 0. Max coverage (-): 0

Region: chr4 69093511-69093521. Max. coverage (+): 0. Max coverage (-): 0

Region: chr4 69093522-69093533. Max. coverage (+): 0. Max coverage (-): 0

Region: chr4 69093534-69093545. Max. coverage (+): 0. Max coverage (-): 0

Region: chr4 69093546-69093556. Max. coverage (+): 0. Max coverage (-): 0

Region: chr4 69093557-69093568. Max. coverage (+): 0. Max coverage (-): 0

Region: chr4 69093569-69093580. Max. coverage (+): 0. Max coverage (-): 0

Region: chr4 69093581-69093591. Max. coverage (+): 0. Max coverage (-): 0

Region: chr4 69093592-69093603. Max. coverage (+): 0. Max coverage (-): 0

Region: chr4 69093604-69093615. Max. coverage (+): 0. Max coverage (-): 0

Region: chr4 69093616-69093626. Max. coverage (+): 0. Max coverage (-): 0

Region: chr4 69093627-69093638. Max. coverage (+): 0. Max coverage (-): 0

Region: chr4 69093639-69093650. Max. coverage (+): 0. Max coverage (-): 0

Region: chr4 69093651-69093661. Max. coverage (+): 0. Max coverage (-): 0

Region: chr4 69093662-69093673. Max. coverage (+): 0. Max coverage (-): 0

Region: chr4 69093674-69093685. Max. coverage (+): 0. Max coverage (-): 0.84

Region: chr4 69093686-69093696. Max. coverage (+): 0. Max coverage (-): 0.84

Region: chr4 69093697-69093708. Max. coverage (+): 0. Max coverage (-): 0

Region: chr4 69093709-69093720. Max. coverage (+): 0. Max coverage (-): 0

Region: chr4 69093721-69093731. Max. coverage (+): 0. Max coverage (-): 0

Region: chr4 69093732-69093743. Max. coverage (+): 0. Max coverage (-): 0.73

Region: chr4 69093744-69093755. Max. coverage (+): 0. Max coverage (-): 0

Region: chr4 69093756-69093766. Max. coverage (+): 0. Max coverage (-): 0

Region: chr4 69093767-69093778. Max. coverage (+): 0. Max coverage (-): 0

Region: chr4 69093779-69093790. Max. coverage (+): 0. Max coverage (-): 0

Region: chr4 69093791-69093801. Max. coverage (+): 0. Max coverage (-): 0

Region: chr4 69093802-69093813. Max. coverage (+): 0. Max coverage (-): 0

Region: chr4 69093814-69093825. Max. coverage (+): 0. Max coverage (-): 0

Region: chr4 69093826-69093836. Max. coverage (+): 0. Max coverage (-): 0

Region: chr4 69093837-69093848. Max. coverage (+): 0. Max coverage (-): 0

Region: chr4 69093849-69093860. Max. coverage (+): 0. Max coverage (-): 0

Region: chr4 69093861-69093872. Max. coverage (+): 0. Max coverage (-): 6.27

Region: chr4 69093873-69093883. Max. coverage (+): 0. Max coverage (-): 6.27

Region: chr4 69093884-69093895. Max. coverage (+): 0. Max coverage (-): 0

Region: chr4 69093896-69093907. Max. coverage (+): 0. Max coverage (-): 0

Region: chr4 69093908-69093918. Max. coverage (+): 0. Max coverage (-): 0

Region: chr4 69093919-69093930. Max. coverage (+): 0. Max coverage (-): 0

Region: chr4 69093931-69093942. Max. coverage (+): 0. Max coverage (-): 0

Region: chr4 69093943-69093953. Max. coverage (+): 0. Max coverage (-): 0

Region: chr4 69093954-69093965. Max. coverage (+): 0. Max coverage (-): 0

Region: chr4 69093966-69093977. Max. coverage (+): 0. Max coverage (-): 0

Region: chr4 69093978-69093988. Max. coverage (+): 0. Max coverage (-): 9.73

Region: chr4 69093989-69094000. Max. coverage (+): 0. Max coverage (-): 9.73

Region: chr4 69094001-69094012. Max. coverage (+): 0. Max coverage (-): 0

Region: chr4 69094013-69094023. Max. coverage (+): 0. Max coverage (-): 0

Region: chr4 69094024-69094035. Max. coverage (+): 0. Max coverage (-): 0

Region: chr4 69094036-69094047. Max. coverage (+): 0. Max coverage (-): 0

Region: chr4 69094048-69094058. Max. coverage (+): 0. Max coverage (-): 0

Region: chr4 69094059-69094070. Max. coverage (+): 0. Max coverage (-): 13.18

Region: chr4 69094071-69094082. Max. coverage (+): 0. Max coverage (-): 14.26

Region: chr4 69094083-69094093. Max. coverage (+): 0. Max coverage (-): 5.18

Region: chr4 69094094-69094105. Max. coverage (+): 0. Max coverage (-): 4.95

Region: chr4 69094106-69094117. Max. coverage (+): 0. Max coverage (-): 8.95

Region: chr4 69094118-69094128. Max. coverage (+): 0. Max coverage (-): 0

Region: chr4 69094129-69094140. Max. coverage (+): 0. Max coverage (-): 0

Region: chr4 69094141-69094152. Max. coverage (+): 0. Max coverage (-): 5.5

Region: chr4 69094153-69094163. Max. coverage (+): 0. Max coverage (-): 5.08

Region: chr4 69094164-69094175. Max. coverage (+): 0. Max coverage (-): 0

Region: chr4 69094176-69094187. Max. coverage (+): 0. Max coverage (-): 0

Region: chr4 69094188-69094199. Max. coverage (+): 0. Max coverage (-): 0

Region: chr4 69094200-69094210. Max. coverage (+): 0. Max coverage (-): 0

Region: chr4 69094211-69094222. Max. coverage (+): 0. Max coverage (-): 0

Region: chr4 69094223-69094234. Max. coverage (+): 0. Max coverage (-): 0

Region: chr4 69094235-69094245. Max. coverage (+): 0. Max coverage (-): 0

Region: chr4 69094246-69094257. Max. coverage (+): 0. Max coverage (-): 0

Region: chr4 69094258-69094269. Max. coverage (+): 0. Max coverage (-): 0

Region: chr4 69094270-69094280. Max. coverage (+): 0. Max coverage (-): 0

Region: chr4 69094281-69094292. Max. coverage (+): 0. Max coverage (-): 0

Region: chr4 69094293-69094304. Max. coverage (+): 0. Max coverage (-): 0

Region: chr4 69094305-69094315. Max. coverage (+): 0. Max coverage (-): 0

Region: chr4 69094316-69094327. Max. coverage (+): 0. Max coverage (-): 0

Region: chr4 69094328-69094339. Max. coverage (+): 0. Max coverage (-): 0

Region: chr4 69094340-69094350. Max. coverage (+): 0. Max coverage (-): 0

Region: chr4 69094351-69094362. Max. coverage (+): 0. Max coverage (-): 0

Region: chr4 69094363-69094374. Max. coverage (+): 0. Max coverage (-): 0

Region: chr4 69094375-69094385. Max. coverage (+): 0. Max coverage (-): 0

Region: chr4 69094386-69094397. Max. coverage (+): 0. Max coverage (-): 1.52

Region: chr4 69094398-69094409. Max. coverage (+): 0. Max coverage (-): 1.52

Region: chr4 69094410-69094420. Max. coverage (+): 0. Max coverage (-): 0

Region: chr4 69094421-69094432. Max. coverage (+): 0. Max coverage (-): 0

Region: chr4 69094433-69094444. Max. coverage (+): 0. Max coverage (-): 0

Region: chr4 69094445-69094455. Max. coverage (+): 0. Max coverage (-): 0

Region: chr4 69094456-69094467. Max. coverage (+): 0. Max coverage (-): 0

Region: chr4 69094468-69094479. Max. coverage (+): 0. Max coverage (-): 0

Region: chr4 69094480-69094490. Max. coverage (+): 0. Max coverage (-): 0

Region: chr4 69094491-69094502. Max. coverage (+): 0. Max coverage (-): 0

Region: chr4 69094503-69094514. Max. coverage (+): 0. Max coverage (-): 0

Region: chr4 69094515-69094526. Max. coverage (+): 0. Max coverage (-): 0

Region: chr4 69094527-69094537. Max. coverage (+): 0. Max coverage (-): 0

Region: chr4 69094538-69094549. Max. coverage (+): 0. Max coverage (-): 0

Region: chr4 69094550-69094561. Max. coverage (+): 0. Max coverage (-): 0

Region: chr4 69094562-69094572. Max. coverage (+): 0. Max coverage (-): 0

Region: chr4 69094573-69094584. Max. coverage (+): 0. Max coverage (-): 0

Region: chr4 69094585-69094596. Max. coverage (+): 0. Max coverage (-): 0

Region: chr4 69094597-69094607. Max. coverage (+): 0. Max coverage (-): 0

Region: chr4 69094608-69094619. Max. coverage (+): 0. Max coverage (-): 0

Region: chr4 69094620-69094631. Max. coverage (+): 0. Max coverage (-): 0

Region: chr4 69094632-69094642. Max. coverage (+): 0. Max coverage (-): 0

Region: chr4 69094643-69094654. Max. coverage (+): 0. Max coverage (-): 0

Region: chr4 69094655-69094666. Max. coverage (+): 0. Max coverage (-): 0

Region: chr4 69094667-69094677. Max. coverage (+): 0. Max coverage (-): 0

Region: chr4 69094678-69094689. Max. coverage (+): 0. Max coverage (-): 0

Region: chr4 69094690-69094701. Max. coverage (+): 0. Max coverage (-): 2.1

Region: chr4 69094702-69094712. Max. coverage (+): 0. Max coverage (-): 0

Region: chr4 69094713-69094724. Max. coverage (+): 0. Max coverage (-): 0

Region: chr4 69094725-69094736. Max. coverage (+): 0. Max coverage (-): 0

Region: chr4 69094737-69094747. Max. coverage (+): 0. Max coverage (-): 0

Region: chr4 69094748-69094759. Max. coverage (+): 0. Max coverage (-): 0

Region: chr4 69094760-69094771. Max. coverage (+): 0. Max coverage (-): 0

Region: chr4 69094772-69094782. Max. coverage (+): 0. Max coverage (-): 0

Region: chr4 69094783-69094794. Max. coverage (+): 0. Max coverage (-): 0

Region: chr4 69094795-69094806. Max. coverage (+): 0. Max coverage (-): 0

Region: chr4 69094807-69094817. Max. coverage (+): 0. Max coverage (-): 0

Region: chr4 69094818-69094829. Max. coverage (+): 0. Max coverage (-): 2.21

Region: chr4 69094830-69094841. Max. coverage (+): 0. Max coverage (-): 10.93

Region: chr4 69094842-69094852. Max. coverage (+): 0. Max coverage (-): 9.33

Region: chr4 69094853-69094864. Max. coverage (+): 0. Max coverage (-): 0

Region: chr4 69094865-69094876. Max. coverage (+): 0. Max coverage (-): 0

Region: chr4 69094877-69094888. Max. coverage (+): 0. Max coverage (-): 0

Region: chr4 69094889-69094899. Max. coverage (+): 0. Max coverage (-): 0

Region: chr4 69094900-69094911. Max. coverage (+): 0. Max coverage (-): 0

Region: chr4 69094912-69094923. Max. coverage (+): 0. Max coverage (-): 0

Region: chr4 69094924-69094934. Max. coverage (+): 0. Max coverage (-): 0

Region: chr4 69094935-69094946. Max. coverage (+): 0. Max coverage (-): 0

Region: chr4 69094947-69094958. Max. coverage (+): 0. Max coverage (-): 0

Region: chr4 69094959-69094969. Max. coverage (+): 0. Max coverage (-): 6.75

Region: chr4 69094970-69094981. Max. coverage (+): 0. Max coverage (-): 5.99

Region: chr4 69094982-69094993. Max. coverage (+): 0. Max coverage (-): 5.99

Region: chr4 69094994-69095004. Max. coverage (+): 0. Max coverage (-): 1.17

Region: chr4 69095005-69095016. Max. coverage (+): 0. Max coverage (-): 4.83

Region: chr4 69095017-69095028. Max. coverage (+): 0. Max coverage (-): 0

Region: chr4 69095029-69095039. Max. coverage (+): 0. Max coverage (-): 0

Region: chr4 69095040-69095051. Max. coverage (+): 0. Max coverage (-): 0

Region: chr4 69095052-69095063. Max. coverage (+): 0. Max coverage (-): 0

Region: chr4 69095064-69095074. Max. coverage (+): 0. Max coverage (-): 0

Region: chr4 69095075-69095086. Max. coverage (+): 0. Max coverage (-): 0

Region: chr4 69095087-69095098. Max. coverage (+): 0. Max coverage (-): 8.47

Region: chr4 69095099-69095109. Max. coverage (+): 0. Max coverage (-): 0

Region: chr4 69095110-69095121. Max. coverage (+): 0. Max coverage (-): 0

Region: chr4 69095122-69095133. Max. coverage (+): 0. Max coverage (-): 0

Region: chr4 69095134-69095144. Max. coverage (+): 0. Max coverage (-): 0

Region: chr4 69095145-69095156. Max. coverage (+): 0. Max coverage (-): 0

Region: chr4 69095157-69095168. Max. coverage (+): 0. Max coverage (-): 0

Region: chr4 69095169-69095179. Max. coverage (+): 0. Max coverage (-): 0

Region: chr4 69095180-69095191. Max. coverage (+): 0. Max coverage (-): 0

Region: chr4 69095192-69095203. Max. coverage (+): 0. Max coverage (-): 0

Region: chr4 69095204-69095215. Max. coverage (+): 0. Max coverage (-): 0

Region: chr4 69095216-69095226. Max. coverage (+): 0. Max coverage (-): 0

Region: chr4 69095227-69095238. Max. coverage (+): 0. Max coverage (-): 0

Region: chr4 69095239-69095250. Max. coverage (+): 0. Max coverage (-): 0

Region: chr4 69095251-69095261. Max. coverage (+): 0. Max coverage (-): 0

Region: chr4 69095262-69095273. Max. coverage (+): 0. Max coverage (-): 0

Region: chr4 69095274-69095285. Max. coverage (+): 0. Max coverage (-): 0

Region: chr4 69095286-69095296. Max. coverage (+): 0. Max coverage (-): 0

Region: chr4 69095297-69095308. Max. coverage (+): 0. Max coverage (-): 0

Region: chr4 69095309-69095320. Max. coverage (+): 0. Max coverage (-): 0

Region: chr4 69095321-69095331. Max. coverage (+): 0. Max coverage (-): 0

Region: chr4 69095332-69095343. Max. coverage (+): 0. Max coverage (-): 0

Region: chr4 69095344-69095355. Max. coverage (+): 0. Max coverage (-): 3.88

Region: chr4 69095356-69095366. Max. coverage (+): 0. Max coverage (-): 0

Region: chr4 69095367-69095378. Max. coverage (+): 0. Max coverage (-): 0

Region: chr4 69095379-69095390. Max. coverage (+): 0. Max coverage (-): 0

Region: chr4 69095391-69095401. Max. coverage (+): 0. Max coverage (-): 0

Region: chr4 69095402-69095413. Max. coverage (+): 0. Max coverage (-): 0

Region: chr4 69095414-69095425. Max. coverage (+): 0. Max coverage (-): 0

Region: chr4 69095426-69095436. Max. coverage (+): 0. Max coverage (-): 0

Region: chr4 69095437-69095448. Max. coverage (+): 0. Max coverage (-): 0

Region: chr4 69095449-69095460. Max. coverage (+): 0. Max coverage (-): 0

Region: chr4 69095461-69095471. Max. coverage (+): 0. Max coverage (-): 0

Region: chr4 69095472-69095483. Max. coverage (+): 0. Max coverage (-): 0

Region: chr4 69095484-69095495. Max. coverage (+): 0. Max coverage (-): 0

Region: chr4 69095496-69095506. Max. coverage (+): 0. Max coverage (-): 1.83

Region: chr4 69095507-69095518. Max. coverage (+): 0. Max coverage (-): 1.83

Region: chr4 69095519-69095530. Max. coverage (+): 0. Max coverage (-): 0

Region: chr4 69095531-69095541. Max. coverage (+): 0. Max coverage (-): 0

Region: chr4 69095542-69095553. Max. coverage (+): 0. Max coverage (-): 0

Region: chr4 69095554-69095565. Max. coverage (+): 0. Max coverage (-): 0

Region: chr4 69095566-69095577. Max. coverage (+): 0. Max coverage (-): 0

Region: chr4 69095578-69095588. Max. coverage (+): 0. Max coverage (-): 0

Region: chr4 69095589-69095600. Max. coverage (+): 0. Max coverage (-): 0

Region: chr4 69095601-69095612. Max. coverage (+): 0. Max coverage (-): 0

Region: chr4 69095613-69095623. Max. coverage (+): 0. Max coverage (-): 0

Region: chr4 69095624-69095635. Max. coverage (+): 0. Max coverage (-): 0

Region: chr4 69095636-69095647. Max. coverage (+): 0. Max coverage (-): 0

Region: chr4 69095648-69095658. Max. coverage (+): 0. Max coverage (-): 0

Region: chr4 69095659-69095670. Max. coverage (+): 0. Max coverage (-): 0

Region: chr4 69095671-69095682. Max. coverage (+): 0. Max coverage (-): 0

Region: chr4 69095683-69095693. Max. coverage (+): 0. Max coverage (-): 0

Region: chr4 69095694-69095705. Max. coverage (+): 0. Max coverage (-): 0

Region: chr4 69095706-69095717. Max. coverage (+): 0. Max coverage (-): 0

Region: chr4 69095718-69095728. Max. coverage (+): 0. Max coverage (-): 0

Region: chr4 69095729-69095740. Max. coverage (+): 0. Max coverage (-): 0

Region: chr4 69095741-69095752. Max. coverage (+): 0. Max coverage (-): 0

Region: chr4 69095753-69095763. Max. coverage (+): 0. Max coverage (-): 0

Region: chr4 69095764-69095775. Max. coverage (+): 0. Max coverage (-): 0

Region: chr4 69095776-69095787. Max. coverage (+): 0. Max coverage (-): 0

Region: chr4 69095788-69095798. Max. coverage (+): 0. Max coverage (-): 4.38

Region: chr4 69095799-69095810. Max. coverage (+): 0. Max coverage (-): 4.38

Region: chr4 69095811-69095822. Max. coverage (+): 0. Max coverage (-): 0

Region: chr4 69095823-69095833. Max. coverage (+): 0. Max coverage (-): 0

Region: chr4 69095834-69095845. Max. coverage (+): 0. Max coverage (-): 0

Region: chr4 69095846-69095857. Max. coverage (+): 0. Max coverage (-): 0

Region: chr4 69095858-69095868. Max. coverage (+): 0. Max coverage (-): 0

Region: chr4 69095869-69095880. Max. coverage (+): 0. Max coverage (-): 0

Region: chr4 69095881-69095892. Max. coverage (+): 0. Max coverage (-): 0

Region: chr4 69095893-69095904. Max. coverage (+): 0. Max coverage (-): 0

Region: chr4 69095905-69095915. Max. coverage (+): 0. Max coverage (-): 0

Region: chr4 69095916-69095927. Max. coverage (+): 0. Max coverage (-): 0

Region: chr4 69095928-69095939. Max. coverage (+): 0. Max coverage (-): 0

Region: chr4 69095940-69095950. Max. coverage (+): 0. Max coverage (-): 0

Region: chr4 69095951-69095962. Max. coverage (+): 0. Max coverage (-): 0

Region: chr4 69095963-69095974. Max. coverage (+): 0. Max coverage (-): 0

Region: chr4 69095975-69095985. Max. coverage (+): 0. Max coverage (-): 0

Region: chr4 69095986-69095997. Max. coverage (+): 0. Max coverage (-): 0

Region: chr4 69095998-69096009. Max. coverage (+): 0. Max coverage (-): 0

Region: chr4 69096010-69096020. Max. coverage (+): 0. Max coverage (-): 0

Region: chr4 69096021-69096032. Max. coverage (+): 0. Max coverage (-): 0

Region: chr4 69096033-69096044. Max. coverage (+): 0. Max coverage (-): 0

Region: chr4 69096045-69096055. Max. coverage (+): 0. Max coverage (-): 0

Region: chr4 69096056-69096067. Max. coverage (+): 0. Max coverage (-): 0

Region: chr4 69096068-69096079. Max. coverage (+): 0. Max coverage (-): 0

Region: chr4 69096080-69096090. Max. coverage (+): 0. Max coverage (-): 0

Region: chr4 69096091-69096102. Max. coverage (+): 0. Max coverage (-): 0

Region: chr4 69096103-69096114. Max. coverage (+): 0. Max coverage (-): 0

Region: chr4 69096115-69096125. Max. coverage (+): 0. Max coverage (-): 0

Region: chr4 69096126-69096137. Max. coverage (+): 0. Max coverage (-): 0

Region: chr4 69096138-69096149. Max. coverage (+): 0. Max coverage (-): 0

Region: chr4 69096150-69096160. Max. coverage (+): 0. Max coverage (-): 0

Region: chr4 69096161-69096172. Max. coverage (+): 0. Max coverage (-): 0

Region: chr4 69096173-69096184. Max. coverage (+): 0. Max coverage (-): 0

Region: chr4 69096185-69096195. Max. coverage (+): 0. Max coverage (-): 0

Region: chr4 69096196-69096207. Max. coverage (+): 0. Max coverage (-): 0

Region: chr4 69096208-69096219. Max. coverage (+): 0. Max coverage (-): 0

Region: chr4 69096220-69096230. Max. coverage (+): 0. Max coverage (-): 0

Region: chr4 69096231-69096242. Max. coverage (+): 0. Max coverage (-): 0

Region: chr4 69096243-69096254. Max. coverage (+): 0. Max coverage (-): 0

Region: chr4 69096255-69096266. Max. coverage (+): 0. Max coverage (-): 0

Region: chr4 69096267-69096277. Max. coverage (+): 0. Max coverage (-): 0

Region: chr4 69096278-69096289. Max. coverage (+): 0. Max coverage (-): 0

Region: chr4 69096290-69096301. Max. coverage (+): 0. Max coverage (-): 1.29

Region: chr4 69096302-69096312. Max. coverage (+): 0. Max coverage (-): 1.4

Region: chr4 69096313-69096324. Max. coverage (+): 0. Max coverage (-): 1.4

Region: chr4 69096325-69096336. Max. coverage (+): 0. Max coverage (-): 0

Region: chr4 69096337-69096347. Max. coverage (+): 0. Max coverage (-): 0

Region: chr4 69096348-69096359. Max. coverage (+): 0. Max coverage (-): 0

Region: chr4 69096360-69096371. Max. coverage (+): 0. Max coverage (-): 0

Region: chr4 69096372-69096382. Max. coverage (+): 0. Max coverage (-): 0

Region: chr4 69096383-69096394. Max. coverage (+): 0. Max coverage (-): 0

Region: chr4 69096395-69096406. Max. coverage (+): 0. Max coverage (-): 0

Region: chr4 69096407-69096417. Max. coverage (+): 0. Max coverage (-): 0

Region: chr4 69096418-69096429. Max. coverage (+): 0. Max coverage (-): 0

Region: chr4 69096430-69096441. Max. coverage (+): 0. Max coverage (-): 0

Region: chr4 69096442-69096452. Max. coverage (+): 0. Max coverage (-): 0

Region: chr4 69096453-69096464. Max. coverage (+): 0. Max coverage (-): 0

Region: chr4 69096465-69096476. Max. coverage (+): 0. Max coverage (-): 0

Region: chr4 69096477-69096487. Max. coverage (+): 0. Max coverage (-): 0

Region: chr4 69096488-69096499. Max. coverage (+): 0. Max coverage (-): 0

Region: chr4 69096500-69096511. Max. coverage (+): 0. Max coverage (-): 0

Region: chr4 69096512-69096522. Max. coverage (+): 0. Max coverage (-): 0

Region: chr4 69096523-69096534. Max. coverage (+): 0. Max coverage (-): 0

Region: chr4 69096535-69096546. Max. coverage (+): 0. Max coverage (-): 0

Region: chr4 69096547-69096557. Max. coverage (+): 0. Max coverage (-): 0

Region: chr4 69096558-69096569. Max. coverage (+): 0. Max coverage (-): 0

Region: chr4 69096570-69096581. Max. coverage (+): 0. Max coverage (-): 0

Region: chr4 69096582-69096593. Max. coverage (+): 0. Max coverage (-): 0

Region: chr4 69096594-69096604. Max. coverage (+): 0. Max coverage (-): 0

Region: chr4 69096605-69096616. Max. coverage (+): 0. Max coverage (-): 0

Region: chr4 69096617-69096628. Max. coverage (+): 0. Max coverage (-): 0

Region: chr4 69096629-69096639. Max. coverage (+): 0. Max coverage (-): 0

Region: chr4 69096640-69096651. Max. coverage (+): 0. Max coverage (-): 0

Region: chr4 69096652-69096663. Max. coverage (+): 0. Max coverage (-): 0

Region: chr4 69096664-69096674. Max. coverage (+): 0. Max coverage (-): 0

Region: chr4 69096675-69096686. Max. coverage (+): 0. Max coverage (-): 0

Region: chr4 69096687-69096698. Max. coverage (+): 0. Max coverage (-): 0

Region: chr4 69096699-69096709. Max. coverage (+): 0. Max coverage (-): 0

Region: chr4 69096710-69096721. Max. coverage (+): 0. Max coverage (-): 0

Region: chr4 69096722-69096733. Max. coverage (+): 0. Max coverage (-): 0

Region: chr4 69096734-69096744. Max. coverage (+): 0. Max coverage (-): 0

Region: chr4 69096745-69096756. Max. coverage (+): 0. Max coverage (-): 0

Region: chr4 69096757-69096768. Max. coverage (+): 0. Max coverage (-): 0

Region: chr4 69096769-69096779. Max. coverage (+): 0. Max coverage (-): 0

Region: chr4 69096780-69096791. Max. coverage (+): 0. Max coverage (-): 1.76

Region: chr4 69096792-69096803. Max. coverage (+): 0. Max coverage (-): 2.29

Region: chr4 69096804-69096814. Max. coverage (+): 0. Max coverage (-): 2.29

Region: chr4 69096815-69096826. Max. coverage (+): 0. Max coverage (-): 0

Region: chr4 69096827-69096838. Max. coverage (+): 0. Max coverage (-): 0

Region: chr4 69096839-69096849. Max. coverage (+): 0. Max coverage (-): 0

Region: chr4 69096850-69096861. Max. coverage (+): 0. Max coverage (-): 0

Region: chr4 69096862-69096873. Max. coverage (+): 0. Max coverage (-): 0

Region: chr4 69096874-69096884. Max. coverage (+): 0. Max coverage (-): 0

Region: chr4 69096885-69096896. Max. coverage (+): 0. Max coverage (-): 0

Region: chr4 69096897-69096908. Max. coverage (+): 0. Max coverage (-): 0

Region: chr4 69096909-69096919. Max. coverage (+): 0. Max coverage (-): 0

Region: chr4 69096920-69096931. Max. coverage (+): 0. Max coverage (-): 0

Region: chr4 69096932-69096943. Max. coverage (+): 0. Max coverage (-): 0

Region: chr4 69096944-69096955. Max. coverage (+): 0. Max coverage (-): 1.11

Region: chr4 69096956-69096966. Max. coverage (+): 0. Max coverage (-): 1.11

Region: chr4 69096967-69096978. Max. coverage (+): 0. Max coverage (-): 0

Region: chr4 69096979-69096990. Max. coverage (+): 0. Max coverage (-): 4.52

Region: chr4 69096991-69097001. Max. coverage (+): 0. Max coverage (-): 4.52

Region: chr4 69097002-69097013. Max. coverage (+): 0. Max coverage (-): 0

Region: chr4 69097014-69097025. Max. coverage (+): 0. Max coverage (-): 0

Region: chr4 69097026-69097036. Max. coverage (+): 0. Max coverage (-): 0

Region: chr4 69097037-69097048. Max. coverage (+): 0. Max coverage (-): 0

Region: chr4 69097049-69097060. Max. coverage (+): 0. Max coverage (-): 0

Region: chr4 69097061-69097071. Max. coverage (+): 0. Max coverage (-): 0.99

Region: chr4 69097072-69097083. Max. coverage (+): 0. Max coverage (-): 0.99

Region: chr4 69097084-69097095. Max. coverage (+): 0. Max coverage (-): 0

Region: chr4 69097096-. Max. coverage (+): 0. Max coverage (-): 0

RepeatMasker Color Code

**+**

100-98% Identity

<98-95% Identity

<95-90% Identity

<90-85% Identity

<85-80% Identity

<80-75% Identity

<75-70% Identity

<70% Identity

**-**

Gene Set Color Code

**+**

Gene

Pseudogene

**-**

Topology/Coverage Color Code

Coverage Plus Strand

Coverage Minus Strand

Mainstrand: Plus

Mainstrand: Minus

Complementary Strand

Flanking Region  
(if option -flank >0)

Gene Set Annotation  
  
RepeatMasker Annotation  

**1. C-rich**: 69091587-69091659 (+), Divergence to consensus: 26%  
**2. MamTip2**: 69092180-69092396 (+), Divergence to consensus: 46.7%  
**3. Bov-tA1**: 69092421-69092643 (+), Divergence to consensus: 20.6%  
**4. MLT1L**: 69093362-69093412 (+), Divergence to consensus: 21.6%  
**5. MER5A1**: 69093494-69093645 (+), Divergence to consensus: 20.6%  
**6. L2c**: 69094257-69094291 (+), Divergence to consensus: 22.9%  
**7. MIR3**: 69094506-69094651 (+), Divergence to consensus: 42.3%  
**8. MIRb**: 69096004-69096199 (+), Divergence to consensus: 39.8%  
**9. MER5B**: 69096603-69096713 (+), Divergence to consensus: 26.1%

  
Transcription Factor Binding Sites  

**Gata4** (Sequence: AGATAAG (-): 69095946)  
**SOX9** (Sequence: AACAATGG (-): 69095967)
