# Supplementary material for: Small RNA sequencing of cryopreserved semen from single bull revealed altered miRNAs and piRNAs expression between High- and Low-motile sperm populations
Source: BMC Genomics. 2017 Jan 4;18:14. doi: 10.1186/s12864-016-3394-7 (PMC5209821; doi:10.1186/s12864-016-3394-7)
Supplement: Additional file 3: — Details for each piRNA clusters found in High Motile (HM) sperm fraction. Genes, repeats, transposable elements and transcription factors binding sites falling within the cluster regions were reported. (ZIP 1896 kb) [file 12864_2016_3394_MOESM3_ESM.zip › 83.html]

piRNA cluster 83


Predicted piRNA cluster no. 83     previous   next
  

Show proTRAC run info
Hide proTRAC run info

================================= proTRAC ====================================  
VERSION: 2.1                                    LAST MODIFIED: 06. October 2015  
  
Please cite:  
Rosenkranz D, Zischler H. proTRAC - a software for probabilistic piRNA cluster  
detection, visualization and analysis. 2012. BMC Bioinformatics 13:5.  
  
and (for proTRAC 2.0 and later):  
Rosenkranz D, Rudloff S, Bastuck K, Ketting RF, Zischler H. Tupaia small RNAs  
provide insights into function and evolution of RNAi-based transposon defense  
in mammals. 2015. RNA 21(5):911-922.  
  
Contact:  
David Rosenkranz  
Institute of Anthropology, small RNA group  
Johannes Gutenberg University Mainz  
email: rosenkranz@uni-mainz.de  
  
You can find the latest proTRAC version at:  
http://sourceforge.net/projects/protrac/files  
http://www.smallRNAgroup-mainz.de/software  
==============================================================================  
  
PARAMETERS:  
Map file: .............../storage/core/barbara/genhome/smallRNA/fertility/Sample\_motile/pirna/Sample\_motile\_26-33\_collapsed.fa.no-dust.map.weighted-10000-1000-b-0  
Genome file: ............/storage/core/barbara/genhome/smallRNA/fertility/Sample\_all/pirna/bt\_311\_chrY.fa  
RepeatMasker annotation: /storage/genomes/bt\_umd31/GCF\_000003055.6\_Bos\_taurus\_UMD\_3.1.1\_repeatMasker\_chr.out  
GeneSet:................./storage/core/barbara/genhome/smallRNA/fertility/Sample\_all/pirna/full.gtf  
  
Significant (p<=0.01) hit density will be calculated based  
on observed hit distribution.  
  
Sliding window size: ........................................ 5000 bp  
Sliding window increament: .................................. 1000 bp  
Normalize each hit by number of genomic hits: ............... 1 [0=no/1=yes]  
Normalize each hit by number of sequence reads: ............. 1 [0=no/1=yes]  
Normalize values (-> per million mapped reads): ............. 1 [0=no/1=yes]  
Min. fraction of hits with 1T(U) or 10A: .................... 0.75  
Alternatively: Min. fraction of hits with 1T(U) and 10A: .... 0.5  
Min. fraction of hits with typical piRNA length: ............ 0.75  
Typical piRNA length: ....................................... 26-33 nt  
Min. size of a piRNA cluster: ............................... 5000 bp.  
Min. number of hits (absolute): ............................. 0  
Min. number of hits (normalized): ........................... 0  
Min. fraction of hits on the mainstrand: .................... 0.75  
Top fraction of mapped sequences (in terms of read counts): . 1%  
Top fraction accounts for max. n% of sequence reads: ........ 90%  
Min. fraction of hits on each arm of a bidirectional cluster: 0.1  
Output image file for each cluster: ......................... 0 [0=no/1=yes]  
Output html file for each cluster: .......................... 1 [0=no/1=yes]  
Output a summary table: ..................................... 1 [0=no/1=yes]  
Output a FASTA file for each cluster (piRNA sequences): ..... 1 [0=no/1=yes]  
Output a FASTA file comprising cluster sequences: ........... 1 [0=no/1=yes]  
Search DNA motifs in clusters: .............................. 1 [0=no/1=yes]  
Output flanking sequences: +/- .............................. 0 bp  
Output ~.pTi file: .......................................... 1 [0=no/1=yes]  
==============================================================================  
  
  
Genome size (without gaps): ............ 2678902517 bp  
Gaps (N/X/-): .......................... 53837044 bp  
Mapped reads: .......................... 658825247023  
Non-identical sequences: ............... 514171  
Genomic hits: .......................... 764233  
Significant densitiy of mapped reads: .. 12867599.5173724 reads/kb

Show proTRAC cluster info
Hide proTRAC cluster info

|  |  |
| --- | --- |
| Location | chr5 |
| Coordinates | 25971171-25978903 |
| Size [bp] | 7733 |
| Sequence hit loci | 168 |
| Mapped reads (normalized) | 206110259 |
| Mapped reads (normalized) per kb | 26653337.5 |
| Normalized reads with 1T (1U) | 94.2% |
| Normalized reads with 10A | 28.7% |
| Normalized reads with length 26-33 nt | 100% |
| Normalized reads on the main strand(s) | 100% |
| Predicted directionality | mono:plus |

100%

0%

1T (1U)  
reads

10A reads

26-33 nt  
reads

reads on mainstrand

**Either the amount of reads with 1T (1U) OR 10A has to exceed 75% (set with option: -1Tor10A)  
Alternatively the amount of reads with 1T (1U) AND 10A has to exceed 50% (set with option: -1Tand10A)  
Minimum amount of reads with preferred size is 75% (set with option: -pisize)  
Minimum amount of reads on the main strand(s) is 75% (set with option: -clstrand)**

Show read coverage
Hide read coverage

WHAT DO I SEE HERE?  
This chart shows the location of mapped sequence reads within a predicted piRNA cluster. The color refers to the number of genomic hits produced by the sequence read in question. A dark red bar indicates that this sequence read produces many other hits elsewhere in the genome. Many adjacent red or yellow bars can indicate the presence of a multi-copy element such as transposons or rRNA genes. A dark green bar indicates that this sequence read maps uniquely to this locus.

1 hit

2-5 hits

6-10 hits

11-20 hits

21-50 hits

51-100 hits

> 100 hits

chr5

25971171

25978903

Gene Set

RepeatMasker

Mapped  
Reads

16.77

plus strand

minus strand

16.77

Region: chr5 69097097-25971178. Max. coverage (+): 10.15. Max coverage (-): 0

Region: chr5 25971179-25971194. Max. coverage (+): 8.84. Max coverage (-): 0

Region: chr5 25971195-25971209. Max. coverage (+): 6.27. Max coverage (-): 0

Region: chr5 25971210-25971225. Max. coverage (+): 6.27. Max coverage (-): 0

Region: chr5 25971226-25971240. Max. coverage (+): 0. Max coverage (-): 0

Region: chr5 25971241-25971256. Max. coverage (+): 0. Max coverage (-): 0

Region: chr5 25971257-25971271. Max. coverage (+): 0. Max coverage (-): 0

Region: chr5 25971272-25971286. Max. coverage (+): 0. Max coverage (-): 0

Region: chr5 25971287-25971302. Max. coverage (+): 0. Max coverage (-): 0

Region: chr5 25971303-25971317. Max. coverage (+): 0. Max coverage (-): 0

Region: chr5 25971318-25971333. Max. coverage (+): 0. Max coverage (-): 0

Region: chr5 25971334-25971348. Max. coverage (+): 0. Max coverage (-): 0

Region: chr5 25971349-25971364. Max. coverage (+): 3.91. Max coverage (-): 0

Region: chr5 25971365-25971379. Max. coverage (+): 0. Max coverage (-): 0

Region: chr5 25971380-25971395. Max. coverage (+): 0. Max coverage (-): 0

Region: chr5 25971396-25971410. Max. coverage (+): 0.58. Max coverage (-): 0

Region: chr5 25971411-25971426. Max. coverage (+): 0.58. Max coverage (-): 0

Region: chr5 25971427-25971441. Max. coverage (+): 0. Max coverage (-): 0

Region: chr5 25971442-25971457. Max. coverage (+): 0. Max coverage (-): 0

Region: chr5 25971458-25971472. Max. coverage (+): 2.62. Max coverage (-): 0

Region: chr5 25971473-25971488. Max. coverage (+): 2.62. Max coverage (-): 0

Region: chr5 25971489-25971503. Max. coverage (+): 0. Max coverage (-): 0

Region: chr5 25971504-25971518. Max. coverage (+): 0. Max coverage (-): 0

Region: chr5 25971519-25971534. Max. coverage (+): 0. Max coverage (-): 0

Region: chr5 25971535-25971549. Max. coverage (+): 4.91. Max coverage (-): 0

Region: chr5 25971550-25971565. Max. coverage (+): 4.91. Max coverage (-): 0

Region: chr5 25971566-25971580. Max. coverage (+): 0. Max coverage (-): 0

Region: chr5 25971581-25971596. Max. coverage (+): 0. Max coverage (-): 0

Region: chr5 25971597-25971611. Max. coverage (+): 0. Max coverage (-): 0

Region: chr5 25971612-25971627. Max. coverage (+): 0. Max coverage (-): 0

Region: chr5 25971628-25971642. Max. coverage (+): 0. Max coverage (-): 0

Region: chr5 25971643-25971658. Max. coverage (+): 8.35. Max coverage (-): 0

Region: chr5 25971659-25971673. Max. coverage (+): 2.56. Max coverage (-): 0

Region: chr5 25971674-25971689. Max. coverage (+): 2.06. Max coverage (-): 0

Region: chr5 25971690-25971704. Max. coverage (+): 2.06. Max coverage (-): 0

Region: chr5 25971705-25971720. Max. coverage (+): 0. Max coverage (-): 0

Region: chr5 25971721-25971735. Max. coverage (+): 0. Max coverage (-): 0

Region: chr5 25971736-25971750. Max. coverage (+): 0. Max coverage (-): 0

Region: chr5 25971751-25971766. Max. coverage (+): 0. Max coverage (-): 0

Region: chr5 25971767-25971781. Max. coverage (+): 0. Max coverage (-): 0

Region: chr5 25971782-25971797. Max. coverage (+): 0. Max coverage (-): 0

Region: chr5 25971798-25971812. Max. coverage (+): 0. Max coverage (-): 0

Region: chr5 25971813-25971828. Max. coverage (+): 0. Max coverage (-): 0

Region: chr5 25971829-25971843. Max. coverage (+): 0. Max coverage (-): 0

Region: chr5 25971844-25971859. Max. coverage (+): 0. Max coverage (-): 0

Region: chr5 25971860-25971874. Max. coverage (+): 0. Max coverage (-): 0

Region: chr5 25971875-25971890. Max. coverage (+): 0. Max coverage (-): 0

Region: chr5 25971891-25971905. Max. coverage (+): 0. Max coverage (-): 0

Region: chr5 25971906-25971921. Max. coverage (+): 0. Max coverage (-): 0

Region: chr5 25971922-25971936. Max. coverage (+): 0. Max coverage (-): 0

Region: chr5 25971937-25971952. Max. coverage (+): 0. Max coverage (-): 0

Region: chr5 25971953-25971967. Max. coverage (+): 0. Max coverage (-): 0

Region: chr5 25971968-25971982. Max. coverage (+): 0. Max coverage (-): 0

Region: chr5 25971983-25971998. Max. coverage (+): 0.67. Max coverage (-): 0

Region: chr5 25971999-25972013. Max. coverage (+): 0.67. Max coverage (-): 0

Region: chr5 25972014-25972029. Max. coverage (+): 0. Max coverage (-): 0

Region: chr5 25972030-25972044. Max. coverage (+): 0. Max coverage (-): 0

Region: chr5 25972045-25972060. Max. coverage (+): 0. Max coverage (-): 0

Region: chr5 25972061-25972075. Max. coverage (+): 0. Max coverage (-): 0

Region: chr5 25972076-25972091. Max. coverage (+): 0. Max coverage (-): 0

Region: chr5 25972092-25972106. Max. coverage (+): 0. Max coverage (-): 0

Region: chr5 25972107-25972122. Max. coverage (+): 0. Max coverage (-): 0

Region: chr5 25972123-25972137. Max. coverage (+): 0. Max coverage (-): 0

Region: chr5 25972138-25972153. Max. coverage (+): 0. Max coverage (-): 0

Region: chr5 25972154-25972168. Max. coverage (+): 0. Max coverage (-): 0

Region: chr5 25972169-25972184. Max. coverage (+): 0. Max coverage (-): 0

Region: chr5 25972185-25972199. Max. coverage (+): 0. Max coverage (-): 0

Region: chr5 25972200-25972214. Max. coverage (+): 0. Max coverage (-): 0

Region: chr5 25972215-25972230. Max. coverage (+): 0. Max coverage (-): 0

Region: chr5 25972231-25972245. Max. coverage (+): 0. Max coverage (-): 0

Region: chr5 25972246-25972261. Max. coverage (+): 0. Max coverage (-): 0

Region: chr5 25972262-25972276. Max. coverage (+): 0. Max coverage (-): 0

Region: chr5 25972277-25972292. Max. coverage (+): 0. Max coverage (-): 0

Region: chr5 25972293-25972307. Max. coverage (+): 0. Max coverage (-): 0

Region: chr5 25972308-25972323. Max. coverage (+): 0. Max coverage (-): 0

Region: chr5 25972324-25972338. Max. coverage (+): 0. Max coverage (-): 0

Region: chr5 25972339-25972354. Max. coverage (+): 1.52. Max coverage (-): 0

Region: chr5 25972355-25972369. Max. coverage (+): 1.52. Max coverage (-): 0

Region: chr5 25972370-25972385. Max. coverage (+): 4.82. Max coverage (-): 0

Region: chr5 25972386-25972400. Max. coverage (+): 0. Max coverage (-): 0

Region: chr5 25972401-25972416. Max. coverage (+): 0. Max coverage (-): 0

Region: chr5 25972417-25972431. Max. coverage (+): 0. Max coverage (-): 0

Region: chr5 25972432-25972446. Max. coverage (+): 0. Max coverage (-): 0

Region: chr5 25972447-25972462. Max. coverage (+): 0. Max coverage (-): 0

Region: chr5 25972463-25972477. Max. coverage (+): 6.52. Max coverage (-): 0

Region: chr5 25972478-25972493. Max. coverage (+): 0.7. Max coverage (-): 0

Region: chr5 25972494-25972508. Max. coverage (+): 1.67. Max coverage (-): 0

Region: chr5 25972509-25972524. Max. coverage (+): 2.11. Max coverage (-): 0

Region: chr5 25972525-25972539. Max. coverage (+): 0. Max coverage (-): 0

Region: chr5 25972540-25972555. Max. coverage (+): 0. Max coverage (-): 0

Region: chr5 25972556-25972570. Max. coverage (+): 2.13. Max coverage (-): 0

Region: chr5 25972571-25972586. Max. coverage (+): 2.13. Max coverage (-): 0

Region: chr5 25972587-25972601. Max. coverage (+): 6.21. Max coverage (-): 0

Region: chr5 25972602-25972617. Max. coverage (+): 4.03. Max coverage (-): 0

Region: chr5 25972618-25972632. Max. coverage (+): 0. Max coverage (-): 0

Region: chr5 25972633-25972648. Max. coverage (+): 0. Max coverage (-): 0

Region: chr5 25972649-25972663. Max. coverage (+): 0. Max coverage (-): 0

Region: chr5 25972664-25972678. Max. coverage (+): 0. Max coverage (-): 0

Region: chr5 25972679-25972694. Max. coverage (+): 0. Max coverage (-): 0

Region: chr5 25972695-25972709. Max. coverage (+): 0. Max coverage (-): 0

Region: chr5 25972710-25972725. Max. coverage (+): 0. Max coverage (-): 0

Region: chr5 25972726-25972740. Max. coverage (+): 0. Max coverage (-): 0

Region: chr5 25972741-25972756. Max. coverage (+): 0. Max coverage (-): 0

Region: chr5 25972757-25972771. Max. coverage (+): 0. Max coverage (-): 0

Region: chr5 25972772-25972787. Max. coverage (+): 0. Max coverage (-): 0

Region: chr5 25972788-25972802. Max. coverage (+): 0. Max coverage (-): 0

Region: chr5 25972803-25972818. Max. coverage (+): 0.73. Max coverage (-): 0

Region: chr5 25972819-25972833. Max. coverage (+): 0.73. Max coverage (-): 0

Region: chr5 25972834-25972849. Max. coverage (+): 0. Max coverage (-): 0

Region: chr5 25972850-25972864. Max. coverage (+): 0. Max coverage (-): 0

Region: chr5 25972865-25972879. Max. coverage (+): 0. Max coverage (-): 0

Region: chr5 25972880-25972895. Max. coverage (+): 0. Max coverage (-): 0

Region: chr5 25972896-25972910. Max. coverage (+): 0. Max coverage (-): 0

Region: chr5 25972911-25972926. Max. coverage (+): 0. Max coverage (-): 0

Region: chr5 25972927-25972941. Max. coverage (+): 0. Max coverage (-): 0

Region: chr5 25972942-25972957. Max. coverage (+): 0. Max coverage (-): 0

Region: chr5 25972958-25972972. Max. coverage (+): 0. Max coverage (-): 0

Region: chr5 25972973-25972988. Max. coverage (+): 0. Max coverage (-): 0

Region: chr5 25972989-25973003. Max. coverage (+): 0. Max coverage (-): 0

Region: chr5 25973004-25973019. Max. coverage (+): 0. Max coverage (-): 0

Region: chr5 25973020-25973034. Max. coverage (+): 0. Max coverage (-): 0

Region: chr5 25973035-25973050. Max. coverage (+): 0. Max coverage (-): 0

Region: chr5 25973051-25973065. Max. coverage (+): 0. Max coverage (-): 0

Region: chr5 25973066-25973081. Max. coverage (+): 0. Max coverage (-): 0

Region: chr5 25973082-25973096. Max. coverage (+): 0. Max coverage (-): 0

Region: chr5 25973097-25973111. Max. coverage (+): 0. Max coverage (-): 0

Region: chr5 25973112-25973127. Max. coverage (+): 0. Max coverage (-): 0

Region: chr5 25973128-25973142. Max. coverage (+): 0. Max coverage (-): 0

Region: chr5 25973143-25973158. Max. coverage (+): 0. Max coverage (-): 0

Region: chr5 25973159-25973173. Max. coverage (+): 0. Max coverage (-): 0

Region: chr5 25973174-25973189. Max. coverage (+): 0. Max coverage (-): 0

Region: chr5 25973190-25973204. Max. coverage (+): 0. Max coverage (-): 0

Region: chr5 25973205-25973220. Max. coverage (+): 1.88. Max coverage (-): 0

Region: chr5 25973221-25973235. Max. coverage (+): 1.42. Max coverage (-): 0

Region: chr5 25973236-25973251. Max. coverage (+): 1.91. Max coverage (-): 0

Region: chr5 25973252-25973266. Max. coverage (+): 4.87. Max coverage (-): 0

Region: chr5 25973267-25973282. Max. coverage (+): 0. Max coverage (-): 0

Region: chr5 25973283-25973297. Max. coverage (+): 2.51. Max coverage (-): 0

Region: chr5 25973298-25973313. Max. coverage (+): 2.51. Max coverage (-): 0

Region: chr5 25973314-25973328. Max. coverage (+): 1.63. Max coverage (-): 0

Region: chr5 25973329-25973343. Max. coverage (+): 5.58. Max coverage (-): 0

Region: chr5 25973344-25973359. Max. coverage (+): 0. Max coverage (-): 0

Region: chr5 25973360-25973374. Max. coverage (+): 0. Max coverage (-): 0

Region: chr5 25973375-25973390. Max. coverage (+): 0. Max coverage (-): 0

Region: chr5 25973391-25973405. Max. coverage (+): 0. Max coverage (-): 0

Region: chr5 25973406-25973421. Max. coverage (+): 0. Max coverage (-): 0

Region: chr5 25973422-25973436. Max. coverage (+): 0. Max coverage (-): 0

Region: chr5 25973437-25973452. Max. coverage (+): 0. Max coverage (-): 0

Region: chr5 25973453-25973467. Max. coverage (+): 4.71. Max coverage (-): 0

Region: chr5 25973468-25973483. Max. coverage (+): 4.9. Max coverage (-): 0

Region: chr5 25973484-25973498. Max. coverage (+): 3.59. Max coverage (-): 0

Region: chr5 25973499-25973514. Max. coverage (+): 0. Max coverage (-): 0

Region: chr5 25973515-25973529. Max. coverage (+): 16.77. Max coverage (-): 0

Region: chr5 25973530-25973545. Max. coverage (+): 10.19. Max coverage (-): 0

Region: chr5 25973546-25973560. Max. coverage (+): 0. Max coverage (-): 0

Region: chr5 25973561-25973575. Max. coverage (+): 0. Max coverage (-): 0

Region: chr5 25973576-25973591. Max. coverage (+): 0. Max coverage (-): 0

Region: chr5 25973592-25973606. Max. coverage (+): 0. Max coverage (-): 0

Region: chr5 25973607-25973622. Max. coverage (+): 0. Max coverage (-): 0

Region: chr5 25973623-25973637. Max. coverage (+): 0. Max coverage (-): 0

Region: chr5 25973638-25973653. Max. coverage (+): 0. Max coverage (-): 0

Region: chr5 25973654-25973668. Max. coverage (+): 0. Max coverage (-): 0

Region: chr5 25973669-25973684. Max. coverage (+): 0. Max coverage (-): 0

Region: chr5 25973685-25973699. Max. coverage (+): 4.27. Max coverage (-): 0

Region: chr5 25973700-25973715. Max. coverage (+): 3.37. Max coverage (-): 0

Region: chr5 25973716-25973730. Max. coverage (+): 0.57. Max coverage (-): 0

Region: chr5 25973731-25973746. Max. coverage (+): 0. Max coverage (-): 0

Region: chr5 25973747-25973761. Max. coverage (+): 0. Max coverage (-): 0

Region: chr5 25973762-25973777. Max. coverage (+): 0. Max coverage (-): 0

Region: chr5 25973778-25973792. Max. coverage (+): 0. Max coverage (-): 0

Region: chr5 25973793-25973807. Max. coverage (+): 0. Max coverage (-): 0

Region: chr5 25973808-25973823. Max. coverage (+): 0. Max coverage (-): 0

Region: chr5 25973824-25973838. Max. coverage (+): 0. Max coverage (-): 0

Region: chr5 25973839-25973854. Max. coverage (+): 0. Max coverage (-): 0

Region: chr5 25973855-25973869. Max. coverage (+): 0.49. Max coverage (-): 0

Region: chr5 25973870-25973885. Max. coverage (+): 0. Max coverage (-): 0

Region: chr5 25973886-25973900. Max. coverage (+): 3.81. Max coverage (-): 0

Region: chr5 25973901-25973916. Max. coverage (+): 0. Max coverage (-): 0

Region: chr5 25973917-25973931. Max. coverage (+): 0. Max coverage (-): 0

Region: chr5 25973932-25973947. Max. coverage (+): 0. Max coverage (-): 0

Region: chr5 25973948-25973962. Max. coverage (+): 0. Max coverage (-): 0

Region: chr5 25973963-25973978. Max. coverage (+): 1.51. Max coverage (-): 0

Region: chr5 25973979-25973993. Max. coverage (+): 1.51. Max coverage (-): 0

Region: chr5 25973994-25974009. Max. coverage (+): 4.41. Max coverage (-): 0

Region: chr5 25974010-25974024. Max. coverage (+): 2.98. Max coverage (-): 0

Region: chr5 25974025-25974039. Max. coverage (+): 0. Max coverage (-): 0

Region: chr5 25974040-25974055. Max. coverage (+): 0. Max coverage (-): 0

Region: chr5 25974056-25974070. Max. coverage (+): 0. Max coverage (-): 0

Region: chr5 25974071-25974086. Max. coverage (+): 0.41. Max coverage (-): 0

Region: chr5 25974087-25974101. Max. coverage (+): 1.69. Max coverage (-): 0

Region: chr5 25974102-25974117. Max. coverage (+): 0. Max coverage (-): 0

Region: chr5 25974118-25974132. Max. coverage (+): 0. Max coverage (-): 0

Region: chr5 25974133-25974148. Max. coverage (+): 0. Max coverage (-): 0

Region: chr5 25974149-25974163. Max. coverage (+): 0. Max coverage (-): 0

Region: chr5 25974164-25974179. Max. coverage (+): 0. Max coverage (-): 0

Region: chr5 25974180-25974194. Max. coverage (+): 9.33. Max coverage (-): 0

Region: chr5 25974195-25974210. Max. coverage (+): 1.68. Max coverage (-): 0

Region: chr5 25974211-25974225. Max. coverage (+): 0. Max coverage (-): 0

Region: chr5 25974226-25974241. Max. coverage (+): 4.33. Max coverage (-): 0

Region: chr5 25974242-25974256. Max. coverage (+): 4.33. Max coverage (-): 0

Region: chr5 25974257-25974271. Max. coverage (+): 4.72. Max coverage (-): 0

Region: chr5 25974272-25974287. Max. coverage (+): 6.88. Max coverage (-): 0

Region: chr5 25974288-25974302. Max. coverage (+): 0.77. Max coverage (-): 0

Region: chr5 25974303-25974318. Max. coverage (+): 0. Max coverage (-): 0

Region: chr5 25974319-25974333. Max. coverage (+): 1.29. Max coverage (-): 0

Region: chr5 25974334-25974349. Max. coverage (+): 0. Max coverage (-): 0

Region: chr5 25974350-25974364. Max. coverage (+): 2.65. Max coverage (-): 0

Region: chr5 25974365-25974380. Max. coverage (+): 2.65. Max coverage (-): 0

Region: chr5 25974381-25974395. Max. coverage (+): 3.51. Max coverage (-): 0

Region: chr5 25974396-25974411. Max. coverage (+): 0. Max coverage (-): 0

Region: chr5 25974412-25974426. Max. coverage (+): 0. Max coverage (-): 0

Region: chr5 25974427-25974442. Max. coverage (+): 0. Max coverage (-): 0

Region: chr5 25974443-25974457. Max. coverage (+): 0. Max coverage (-): 0

Region: chr5 25974458-25974472. Max. coverage (+): 0. Max coverage (-): 0

Region: chr5 25974473-25974488. Max. coverage (+): 0. Max coverage (-): 0

Region: chr5 25974489-25974503. Max. coverage (+): 0. Max coverage (-): 0

Region: chr5 25974504-25974519. Max. coverage (+): 0. Max coverage (-): 0

Region: chr5 25974520-25974534. Max. coverage (+): 0. Max coverage (-): 0

Region: chr5 25974535-25974550. Max. coverage (+): 0. Max coverage (-): 0

Region: chr5 25974551-25974565. Max. coverage (+): 0. Max coverage (-): 0

Region: chr5 25974566-25974581. Max. coverage (+): 0. Max coverage (-): 0

Region: chr5 25974582-25974596. Max. coverage (+): 0. Max coverage (-): 0

Region: chr5 25974597-25974612. Max. coverage (+): 0. Max coverage (-): 0

Region: chr5 25974613-25974627. Max. coverage (+): 0. Max coverage (-): 0

Region: chr5 25974628-25974643. Max. coverage (+): 0. Max coverage (-): 0

Region: chr5 25974644-25974658. Max. coverage (+): 0. Max coverage (-): 0

Region: chr5 25974659-25974674. Max. coverage (+): 0. Max coverage (-): 0

Region: chr5 25974675-25974689. Max. coverage (+): 0. Max coverage (-): 0

Region: chr5 25974690-25974704. Max. coverage (+): 0. Max coverage (-): 0

Region: chr5 25974705-25974720. Max. coverage (+): 0. Max coverage (-): 0

Region: chr5 25974721-25974735. Max. coverage (+): 1.17. Max coverage (-): 0

Region: chr5 25974736-25974751. Max. coverage (+): 2.15. Max coverage (-): 0

Region: chr5 25974752-25974766. Max. coverage (+): 0. Max coverage (-): 0

Region: chr5 25974767-25974782. Max. coverage (+): 0. Max coverage (-): 0

Region: chr5 25974783-25974797. Max. coverage (+): 0. Max coverage (-): 0

Region: chr5 25974798-25974813. Max. coverage (+): 0. Max coverage (-): 0

Region: chr5 25974814-25974828. Max. coverage (+): 0. Max coverage (-): 0

Region: chr5 25974829-25974844. Max. coverage (+): 0. Max coverage (-): 0

Region: chr5 25974845-25974859. Max. coverage (+): 0. Max coverage (-): 0

Region: chr5 25974860-25974875. Max. coverage (+): 0. Max coverage (-): 0

Region: chr5 25974876-25974890. Max. coverage (+): 0. Max coverage (-): 0

Region: chr5 25974891-25974906. Max. coverage (+): 0. Max coverage (-): 0

Region: chr5 25974907-25974921. Max. coverage (+): 0. Max coverage (-): 0

Region: chr5 25974922-25974936. Max. coverage (+): 0. Max coverage (-): 0

Region: chr5 25974937-25974952. Max. coverage (+): 0. Max coverage (-): 0

Region: chr5 25974953-25974967. Max. coverage (+): 0. Max coverage (-): 0

Region: chr5 25974968-25974983. Max. coverage (+): 0. Max coverage (-): 0

Region: chr5 25974984-25974998. Max. coverage (+): 0. Max coverage (-): 0

Region: chr5 25974999-25975014. Max. coverage (+): 0. Max coverage (-): 0

Region: chr5 25975015-25975029. Max. coverage (+): 0. Max coverage (-): 0

Region: chr5 25975030-25975045. Max. coverage (+): 0. Max coverage (-): 0

Region: chr5 25975046-25975060. Max. coverage (+): 0. Max coverage (-): 0

Region: chr5 25975061-25975076. Max. coverage (+): 0. Max coverage (-): 0

Region: chr5 25975077-25975091. Max. coverage (+): 0. Max coverage (-): 0

Region: chr5 25975092-25975107. Max. coverage (+): 0. Max coverage (-): 0

Region: chr5 25975108-25975122. Max. coverage (+): 0. Max coverage (-): 0

Region: chr5 25975123-25975138. Max. coverage (+): 0. Max coverage (-): 0

Region: chr5 25975139-25975153. Max. coverage (+): 0. Max coverage (-): 0

Region: chr5 25975154-25975168. Max. coverage (+): 0. Max coverage (-): 0

Region: chr5 25975169-25975184. Max. coverage (+): 0. Max coverage (-): 0

Region: chr5 25975185-25975199. Max. coverage (+): 0. Max coverage (-): 0

Region: chr5 25975200-25975215. Max. coverage (+): 0. Max coverage (-): 0

Region: chr5 25975216-25975230. Max. coverage (+): 4.25. Max coverage (-): 0

Region: chr5 25975231-25975246. Max. coverage (+): 0. Max coverage (-): 0

Region: chr5 25975247-25975261. Max. coverage (+): 0. Max coverage (-): 0

Region: chr5 25975262-25975277. Max. coverage (+): 0. Max coverage (-): 0

Region: chr5 25975278-25975292. Max. coverage (+): 0. Max coverage (-): 0

Region: chr5 25975293-25975308. Max. coverage (+): 1.8. Max coverage (-): 0

Region: chr5 25975309-25975323. Max. coverage (+): 0. Max coverage (-): 0

Region: chr5 25975324-25975339. Max. coverage (+): 0. Max coverage (-): 0

Region: chr5 25975340-25975354. Max. coverage (+): 0. Max coverage (-): 0

Region: chr5 25975355-25975370. Max. coverage (+): 0. Max coverage (-): 0

Region: chr5 25975371-25975385. Max. coverage (+): 0. Max coverage (-): 0

Region: chr5 25975386-25975400. Max. coverage (+): 0. Max coverage (-): 0

Region: chr5 25975401-25975416. Max. coverage (+): 0. Max coverage (-): 0

Region: chr5 25975417-25975431. Max. coverage (+): 0. Max coverage (-): 0

Region: chr5 25975432-25975447. Max. coverage (+): 0. Max coverage (-): 0

Region: chr5 25975448-25975462. Max. coverage (+): 0. Max coverage (-): 0

Region: chr5 25975463-25975478. Max. coverage (+): 0. Max coverage (-): 0

Region: chr5 25975479-25975493. Max. coverage (+): 0. Max coverage (-): 0

Region: chr5 25975494-25975509. Max. coverage (+): 0. Max coverage (-): 0

Region: chr5 25975510-25975524. Max. coverage (+): 0. Max coverage (-): 0

Region: chr5 25975525-25975540. Max. coverage (+): 0. Max coverage (-): 0

Region: chr5 25975541-25975555. Max. coverage (+): 0. Max coverage (-): 0

Region: chr5 25975556-25975571. Max. coverage (+): 0. Max coverage (-): 0

Region: chr5 25975572-25975586. Max. coverage (+): 0. Max coverage (-): 0

Region: chr5 25975587-25975602. Max. coverage (+): 0. Max coverage (-): 0

Region: chr5 25975603-25975617. Max. coverage (+): 0. Max coverage (-): 0

Region: chr5 25975618-25975632. Max. coverage (+): 0. Max coverage (-): 0

Region: chr5 25975633-25975648. Max. coverage (+): 0. Max coverage (-): 0

Region: chr5 25975649-25975663. Max. coverage (+): 0. Max coverage (-): 0

Region: chr5 25975664-25975679. Max. coverage (+): 0. Max coverage (-): 0

Region: chr5 25975680-25975694. Max. coverage (+): 0. Max coverage (-): 0

Region: chr5 25975695-25975710. Max. coverage (+): 0. Max coverage (-): 0

Region: chr5 25975711-25975725. Max. coverage (+): 0. Max coverage (-): 0

Region: chr5 25975726-25975741. Max. coverage (+): 0. Max coverage (-): 0

Region: chr5 25975742-25975756. Max. coverage (+): 0. Max coverage (-): 0

Region: chr5 25975757-25975772. Max. coverage (+): 0. Max coverage (-): 0

Region: chr5 25975773-25975787. Max. coverage (+): 0. Max coverage (-): 0

Region: chr5 25975788-25975803. Max. coverage (+): 3.05. Max coverage (-): 0

Region: chr5 25975804-25975818. Max. coverage (+): 3.05. Max coverage (-): 0

Region: chr5 25975819-25975833. Max. coverage (+): 0. Max coverage (-): 0

Region: chr5 25975834-25975849. Max. coverage (+): 0. Max coverage (-): 0

Region: chr5 25975850-25975864. Max. coverage (+): 0. Max coverage (-): 0

Region: chr5 25975865-25975880. Max. coverage (+): 0. Max coverage (-): 0

Region: chr5 25975881-25975895. Max. coverage (+): 3.46. Max coverage (-): 0

Region: chr5 25975896-25975911. Max. coverage (+): 3.46. Max coverage (-): 0

Region: chr5 25975912-25975926. Max. coverage (+): 0. Max coverage (-): 0

Region: chr5 25975927-25975942. Max. coverage (+): 0. Max coverage (-): 0

Region: chr5 25975943-25975957. Max. coverage (+): 0. Max coverage (-): 0

Region: chr5 25975958-25975973. Max. coverage (+): 0. Max coverage (-): 0

Region: chr5 25975974-25975988. Max. coverage (+): 0.92. Max coverage (-): 0

Region: chr5 25975989-25976004. Max. coverage (+): 0.92. Max coverage (-): 0

Region: chr5 25976005-25976019. Max. coverage (+): 0. Max coverage (-): 0

Region: chr5 25976020-25976035. Max. coverage (+): 4.52. Max coverage (-): 0

Region: chr5 25976036-25976050. Max. coverage (+): 0. Max coverage (-): 0

Region: chr5 25976051-25976065. Max. coverage (+): 0. Max coverage (-): 0

Region: chr5 25976066-25976081. Max. coverage (+): 0. Max coverage (-): 0

Region: chr5 25976082-25976096. Max. coverage (+): 0. Max coverage (-): 0

Region: chr5 25976097-25976112. Max. coverage (+): 0. Max coverage (-): 0

Region: chr5 25976113-25976127. Max. coverage (+): 0. Max coverage (-): 0

Region: chr5 25976128-25976143. Max. coverage (+): 2.04. Max coverage (-): 0

Region: chr5 25976144-25976158. Max. coverage (+): 2.04. Max coverage (-): 0

Region: chr5 25976159-25976174. Max. coverage (+): 0. Max coverage (-): 0

Region: chr5 25976175-25976189. Max. coverage (+): 0. Max coverage (-): 0

Region: chr5 25976190-25976205. Max. coverage (+): 1.64. Max coverage (-): 0

Region: chr5 25976206-25976220. Max. coverage (+): 0. Max coverage (-): 0

Region: chr5 25976221-25976236. Max. coverage (+): 0. Max coverage (-): 0

Region: chr5 25976237-25976251. Max. coverage (+): 2.43. Max coverage (-): 0

Region: chr5 25976252-25976267. Max. coverage (+): 0.71. Max coverage (-): 0

Region: chr5 25976268-25976282. Max. coverage (+): 0. Max coverage (-): 0

Region: chr5 25976283-25976297. Max. coverage (+): 0. Max coverage (-): 0

Region: chr5 25976298-25976313. Max. coverage (+): 0. Max coverage (-): 0

Region: chr5 25976314-25976328. Max. coverage (+): 0. Max coverage (-): 0

Region: chr5 25976329-25976344. Max. coverage (+): 0. Max coverage (-): 0

Region: chr5 25976345-25976359. Max. coverage (+): 0. Max coverage (-): 0

Region: chr5 25976360-25976375. Max. coverage (+): 0. Max coverage (-): 0

Region: chr5 25976376-25976390. Max. coverage (+): 2.21. Max coverage (-): 0

Region: chr5 25976391-25976406. Max. coverage (+): 0. Max coverage (-): 0

Region: chr5 25976407-25976421. Max. coverage (+): 0.92. Max coverage (-): 0

Region: chr5 25976422-25976437. Max. coverage (+): 5.09. Max coverage (-): 0

Region: chr5 25976438-25976452. Max. coverage (+): 0. Max coverage (-): 0

Region: chr5 25976453-25976468. Max. coverage (+): 0. Max coverage (-): 0

Region: chr5 25976469-25976483. Max. coverage (+): 0. Max coverage (-): 0

Region: chr5 25976484-25976499. Max. coverage (+): 7.12. Max coverage (-): 0

Region: chr5 25976500-25976514. Max. coverage (+): 2.2. Max coverage (-): 0

Region: chr5 25976515-25976529. Max. coverage (+): 4.86. Max coverage (-): 0

Region: chr5 25976530-25976545. Max. coverage (+): 4.86. Max coverage (-): 0

Region: chr5 25976546-25976560. Max. coverage (+): 0. Max coverage (-): 0

Region: chr5 25976561-25976576. Max. coverage (+): 0. Max coverage (-): 0

Region: chr5 25976577-25976591. Max. coverage (+): 0. Max coverage (-): 0

Region: chr5 25976592-25976607. Max. coverage (+): 5.14. Max coverage (-): 0

Region: chr5 25976608-25976622. Max. coverage (+): 2.93. Max coverage (-): 0

Region: chr5 25976623-25976638. Max. coverage (+): 0.96. Max coverage (-): 0

Region: chr5 25976639-25976653. Max. coverage (+): 4.4. Max coverage (-): 0

Region: chr5 25976654-25976669. Max. coverage (+): 0.97. Max coverage (-): 0

Region: chr5 25976670-25976684. Max. coverage (+): 0.97. Max coverage (-): 0

Region: chr5 25976685-25976700. Max. coverage (+): 0. Max coverage (-): 0

Region: chr5 25976701-25976715. Max. coverage (+): 0. Max coverage (-): 0

Region: chr5 25976716-25976731. Max. coverage (+): 0. Max coverage (-): 0

Region: chr5 25976732-25976746. Max. coverage (+): 0. Max coverage (-): 0

Region: chr5 25976747-25976761. Max. coverage (+): 0. Max coverage (-): 0

Region: chr5 25976762-25976777. Max. coverage (+): 0. Max coverage (-): 0

Region: chr5 25976778-25976792. Max. coverage (+): 0. Max coverage (-): 0

Region: chr5 25976793-25976808. Max. coverage (+): 0.9. Max coverage (-): 0

Region: chr5 25976809-25976823. Max. coverage (+): 1.61. Max coverage (-): 0

Region: chr5 25976824-25976839. Max. coverage (+): 0. Max coverage (-): 0

Region: chr5 25976840-25976854. Max. coverage (+): 0. Max coverage (-): 0

Region: chr5 25976855-25976870. Max. coverage (+): 0. Max coverage (-): 0

Region: chr5 25976871-25976885. Max. coverage (+): 0. Max coverage (-): 0

Region: chr5 25976886-25976901. Max. coverage (+): 3.44. Max coverage (-): 0

Region: chr5 25976902-25976916. Max. coverage (+): 0. Max coverage (-): 0

Region: chr5 25976917-25976932. Max. coverage (+): 0. Max coverage (-): 0

Region: chr5 25976933-25976947. Max. coverage (+): 0.74. Max coverage (-): 0

Region: chr5 25976948-25976963. Max. coverage (+): 3.14. Max coverage (-): 0

Region: chr5 25976964-25976978. Max. coverage (+): 0. Max coverage (-): 0

Region: chr5 25976979-25976993. Max. coverage (+): 1.29. Max coverage (-): 0

Region: chr5 25976994-25977009. Max. coverage (+): 9.37. Max coverage (-): 0

Region: chr5 25977010-25977024. Max. coverage (+): 5.27. Max coverage (-): 0

Region: chr5 25977025-25977040. Max. coverage (+): 0. Max coverage (-): 0

Region: chr5 25977041-25977055. Max. coverage (+): 2.05. Max coverage (-): 0

Region: chr5 25977056-25977071. Max. coverage (+): 0. Max coverage (-): 0

Region: chr5 25977072-25977086. Max. coverage (+): 0. Max coverage (-): 0

Region: chr5 25977087-25977102. Max. coverage (+): 0. Max coverage (-): 0

Region: chr5 25977103-25977117. Max. coverage (+): 4.2. Max coverage (-): 0

Region: chr5 25977118-25977133. Max. coverage (+): 6.05. Max coverage (-): 0

Region: chr5 25977134-25977148. Max. coverage (+): 2.39. Max coverage (-): 0

Region: chr5 25977149-25977164. Max. coverage (+): 0.39. Max coverage (-): 0

Region: chr5 25977165-25977179. Max. coverage (+): 4.36. Max coverage (-): 0

Region: chr5 25977180-25977195. Max. coverage (+): 5.59. Max coverage (-): 0

Region: chr5 25977196-25977210. Max. coverage (+): 1.55. Max coverage (-): 0

Region: chr5 25977211-25977225. Max. coverage (+): 0. Max coverage (-): 0

Region: chr5 25977226-25977241. Max. coverage (+): 0. Max coverage (-): 0

Region: chr5 25977242-25977256. Max. coverage (+): 0. Max coverage (-): 0

Region: chr5 25977257-25977272. Max. coverage (+): 0. Max coverage (-): 0

Region: chr5 25977273-25977287. Max. coverage (+): 0. Max coverage (-): 0

Region: chr5 25977288-25977303. Max. coverage (+): 0. Max coverage (-): 0

Region: chr5 25977304-25977318. Max. coverage (+): 0. Max coverage (-): 0

Region: chr5 25977319-25977334. Max. coverage (+): 0. Max coverage (-): 0

Region: chr5 25977335-25977349. Max. coverage (+): 0. Max coverage (-): 0

Region: chr5 25977350-25977365. Max. coverage (+): 0. Max coverage (-): 0

Region: chr5 25977366-25977380. Max. coverage (+): 0. Max coverage (-): 0

Region: chr5 25977381-25977396. Max. coverage (+): 0. Max coverage (-): 0

Region: chr5 25977397-25977411. Max. coverage (+): 0. Max coverage (-): 0

Region: chr5 25977412-25977426. Max. coverage (+): 0. Max coverage (-): 0

Region: chr5 25977427-25977442. Max. coverage (+): 0. Max coverage (-): 0

Region: chr5 25977443-25977457. Max. coverage (+): 0. Max coverage (-): 0

Region: chr5 25977458-25977473. Max. coverage (+): 0. Max coverage (-): 0

Region: chr5 25977474-25977488. Max. coverage (+): 0. Max coverage (-): 0

Region: chr5 25977489-25977504. Max. coverage (+): 0. Max coverage (-): 0

Region: chr5 25977505-25977519. Max. coverage (+): 3.3. Max coverage (-): 0

Region: chr5 25977520-25977535. Max. coverage (+): 3.3. Max coverage (-): 0

Region: chr5 25977536-25977550. Max. coverage (+): 3.55. Max coverage (-): 0

Region: chr5 25977551-25977566. Max. coverage (+): 0. Max coverage (-): 0

Region: chr5 25977567-25977581. Max. coverage (+): 0. Max coverage (-): 0

Region: chr5 25977582-25977597. Max. coverage (+): 0. Max coverage (-): 0

Region: chr5 25977598-25977612. Max. coverage (+): 0. Max coverage (-): 0

Region: chr5 25977613-25977628. Max. coverage (+): 0. Max coverage (-): 0

Region: chr5 25977629-25977643. Max. coverage (+): 0. Max coverage (-): 0

Region: chr5 25977644-25977658. Max. coverage (+): 0. Max coverage (-): 0

Region: chr5 25977659-25977674. Max. coverage (+): 0. Max coverage (-): 0

Region: chr5 25977675-25977689. Max. coverage (+): 0. Max coverage (-): 0

Region: chr5 25977690-25977705. Max. coverage (+): 0. Max coverage (-): 0

Region: chr5 25977706-25977720. Max. coverage (+): 0. Max coverage (-): 0

Region: chr5 25977721-25977736. Max. coverage (+): 0. Max coverage (-): 0

Region: chr5 25977737-25977751. Max. coverage (+): 0. Max coverage (-): 0

Region: chr5 25977752-25977767. Max. coverage (+): 0. Max coverage (-): 0

Region: chr5 25977768-25977782. Max. coverage (+): 0.56. Max coverage (-): 0

Region: chr5 25977783-25977798. Max. coverage (+): 0.56. Max coverage (-): 0

Region: chr5 25977799-25977813. Max. coverage (+): 0. Max coverage (-): 0

Region: chr5 25977814-25977829. Max. coverage (+): 0. Max coverage (-): 0

Region: chr5 25977830-25977844. Max. coverage (+): 0. Max coverage (-): 0

Region: chr5 25977845-25977860. Max. coverage (+): 0. Max coverage (-): 0

Region: chr5 25977861-25977875. Max. coverage (+): 0. Max coverage (-): 0

Region: chr5 25977876-25977890. Max. coverage (+): 0. Max coverage (-): 0

Region: chr5 25977891-25977906. Max. coverage (+): 0. Max coverage (-): 0

Region: chr5 25977907-25977921. Max. coverage (+): 0. Max coverage (-): 0

Region: chr5 25977922-25977937. Max. coverage (+): 0. Max coverage (-): 0

Region: chr5 25977938-25977952. Max. coverage (+): 0. Max coverage (-): 0

Region: chr5 25977953-25977968. Max. coverage (+): 0. Max coverage (-): 0

Region: chr5 25977969-25977983. Max. coverage (+): 0. Max coverage (-): 0

Region: chr5 25977984-25977999. Max. coverage (+): 0. Max coverage (-): 0

Region: chr5 25978000-25978014. Max. coverage (+): 0. Max coverage (-): 0

Region: chr5 25978015-25978030. Max. coverage (+): 0. Max coverage (-): 0

Region: chr5 25978031-25978045. Max. coverage (+): 0. Max coverage (-): 0

Region: chr5 25978046-25978061. Max. coverage (+): 5.74. Max coverage (-): 0

Region: chr5 25978062-25978076. Max. coverage (+): 0. Max coverage (-): 0

Region: chr5 25978077-25978092. Max. coverage (+): 0.88. Max coverage (-): 0

Region: chr5 25978093-25978107. Max. coverage (+): 8.27. Max coverage (-): 0

Region: chr5 25978108-25978122. Max. coverage (+): 0. Max coverage (-): 0

Region: chr5 25978123-25978138. Max. coverage (+): 0. Max coverage (-): 0

Region: chr5 25978139-25978153. Max. coverage (+): 0. Max coverage (-): 0

Region: chr5 25978154-25978169. Max. coverage (+): 2.86. Max coverage (-): 0

Region: chr5 25978170-25978184. Max. coverage (+): 2.86. Max coverage (-): 0

Region: chr5 25978185-25978200. Max. coverage (+): 0. Max coverage (-): 0

Region: chr5 25978201-25978215. Max. coverage (+): 0. Max coverage (-): 0

Region: chr5 25978216-25978231. Max. coverage (+): 6.46. Max coverage (-): 0

Region: chr5 25978232-25978246. Max. coverage (+): 6.46. Max coverage (-): 0

Region: chr5 25978247-25978262. Max. coverage (+): 0. Max coverage (-): 0

Region: chr5 25978263-25978277. Max. coverage (+): 0.68. Max coverage (-): 0

Region: chr5 25978278-25978293. Max. coverage (+): 0. Max coverage (-): 0

Region: chr5 25978294-25978308. Max. coverage (+): 0. Max coverage (-): 0

Region: chr5 25978309-25978324. Max. coverage (+): 0. Max coverage (-): 0

Region: chr5 25978325-25978339. Max. coverage (+): 0. Max coverage (-): 0

Region: chr5 25978340-25978354. Max. coverage (+): 0. Max coverage (-): 0

Region: chr5 25978355-25978370. Max. coverage (+): 0. Max coverage (-): 0

Region: chr5 25978371-25978385. Max. coverage (+): 0.73. Max coverage (-): 0

Region: chr5 25978386-25978401. Max. coverage (+): 0.73. Max coverage (-): 0

Region: chr5 25978402-25978416. Max. coverage (+): 0. Max coverage (-): 0

Region: chr5 25978417-25978432. Max. coverage (+): 0. Max coverage (-): 0

Region: chr5 25978433-25978447. Max. coverage (+): 0.84. Max coverage (-): 0

Region: chr5 25978448-25978463. Max. coverage (+): 0. Max coverage (-): 0

Region: chr5 25978464-25978478. Max. coverage (+): 0. Max coverage (-): 0

Region: chr5 25978479-25978494. Max. coverage (+): 0. Max coverage (-): 0

Region: chr5 25978495-25978509. Max. coverage (+): 0. Max coverage (-): 0

Region: chr5 25978510-25978525. Max. coverage (+): 0. Max coverage (-): 0

Region: chr5 25978526-25978540. Max. coverage (+): 0. Max coverage (-): 0

Region: chr5 25978541-25978556. Max. coverage (+): 0. Max coverage (-): 0

Region: chr5 25978557-25978571. Max. coverage (+): 0. Max coverage (-): 0

Region: chr5 25978572-25978586. Max. coverage (+): 0. Max coverage (-): 0

Region: chr5 25978587-25978602. Max. coverage (+): 0. Max coverage (-): 0

Region: chr5 25978603-25978617. Max. coverage (+): 0. Max coverage (-): 0

Region: chr5 25978618-25978633. Max. coverage (+): 0. Max coverage (-): 0

Region: chr5 25978634-25978648. Max. coverage (+): 0. Max coverage (-): 0

Region: chr5 25978649-25978664. Max. coverage (+): 0. Max coverage (-): 0

Region: chr5 25978665-25978679. Max. coverage (+): 0. Max coverage (-): 0

Region: chr5 25978680-25978695. Max. coverage (+): 0. Max coverage (-): 0

Region: chr5 25978696-25978710. Max. coverage (+): 0. Max coverage (-): 0

Region: chr5 25978711-25978726. Max. coverage (+): 0. Max coverage (-): 0

Region: chr5 25978727-25978741. Max. coverage (+): 0. Max coverage (-): 0

Region: chr5 25978742-25978757. Max. coverage (+): 0. Max coverage (-): 0

Region: chr5 25978758-25978772. Max. coverage (+): 0. Max coverage (-): 0

Region: chr5 25978773-25978788. Max. coverage (+): 0. Max coverage (-): 0

Region: chr5 25978789-25978803. Max. coverage (+): 0. Max coverage (-): 0

Region: chr5 25978804-25978818. Max. coverage (+): 0. Max coverage (-): 0

Region: chr5 25978819-25978834. Max. coverage (+): 0. Max coverage (-): 0

Region: chr5 25978835-25978849. Max. coverage (+): 0. Max coverage (-): 0

Region: chr5 25978850-25978865. Max. coverage (+): 4.7. Max coverage (-): 0

Region: chr5 25978866-25978880. Max. coverage (+): 4.7. Max coverage (-): 0

Region: chr5 25978881-25978896. Max. coverage (+): 0. Max coverage (-): 0

Region: chr5 25978897-. Max. coverage (+): 0. Max coverage (-): 0

RepeatMasker Color Code

**+**

100-98% Identity

<98-95% Identity

<95-90% Identity

<90-85% Identity

<85-80% Identity

<80-75% Identity

<75-70% Identity

<70% Identity

**-**

Gene Set Color Code

**+**

Gene

Pseudogene

**-**

Topology/Coverage Color Code

Coverage Plus Strand

Coverage Minus Strand

Mainstrand: Plus

Mainstrand: Minus

Complementary Strand

Flanking Region  
(if option -flank >0)

Gene Set Annotation  

**1. CBX5 (protein coding, ENSBTAG00000006246) Tr:00000008198 Ex:5**: 25971103-25971390 (+)

  
RepeatMasker Annotation  

**1. L2c**: 25975047-25975098 (-), Divergence to consensus: 26.9%  
**2. MIR**: 25977834-25977998 (+), Divergence to consensus: 29.8%

  
Transcription Factor Binding Sites  

**RFX4\_2** (Sequence: GTAACTAAG (-): 25973305)  
**RFX4\_1** (Sequence: GTTGCCAGG (-): 25973562)  
**Gata4** (Sequence: AGATAAC (-): 25975301)  
**SOX9** (Sequence: CTATTGTT (+): 25974337)
