# Supplementary material for: Small RNA sequencing of cryopreserved semen from single bull revealed altered miRNAs and piRNAs expression between High- and Low-motile sperm populations
Source: BMC Genomics. 2017 Jan 4;18:14. doi: 10.1186/s12864-016-3394-7 (PMC5209821; doi:10.1186/s12864-016-3394-7)
Supplement: Additional file 3: — Details for each piRNA clusters found in High Motile (HM) sperm fraction. Genes, repeats, transposable elements and transcription factors binding sites falling within the cluster regions were reported. (ZIP 1896 kb) [file 12864_2016_3394_MOESM3_ESM.zip › 84.html]

piRNA cluster 84


Predicted piRNA cluster no. 84     previous   next
  

Show proTRAC run info
Hide proTRAC run info

================================= proTRAC ====================================  
VERSION: 2.1                                    LAST MODIFIED: 06. October 2015  
  
Please cite:  
Rosenkranz D, Zischler H. proTRAC - a software for probabilistic piRNA cluster  
detection, visualization and analysis. 2012. BMC Bioinformatics 13:5.  
  
and (for proTRAC 2.0 and later):  
Rosenkranz D, Rudloff S, Bastuck K, Ketting RF, Zischler H. Tupaia small RNAs  
provide insights into function and evolution of RNAi-based transposon defense  
in mammals. 2015. RNA 21(5):911-922.  
  
Contact:  
David Rosenkranz  
Institute of Anthropology, small RNA group  
Johannes Gutenberg University Mainz  
email: rosenkranz@uni-mainz.de  
  
You can find the latest proTRAC version at:  
http://sourceforge.net/projects/protrac/files  
http://www.smallRNAgroup-mainz.de/software  
==============================================================================  
  
PARAMETERS:  
Map file: .............../storage/core/barbara/genhome/smallRNA/fertility/Sample\_motile/pirna/Sample\_motile\_26-33\_collapsed.fa.no-dust.map.weighted-10000-1000-b-0  
Genome file: ............/storage/core/barbara/genhome/smallRNA/fertility/Sample\_all/pirna/bt\_311\_chrY.fa  
RepeatMasker annotation: /storage/genomes/bt\_umd31/GCF\_000003055.6\_Bos\_taurus\_UMD\_3.1.1\_repeatMasker\_chr.out  
GeneSet:................./storage/core/barbara/genhome/smallRNA/fertility/Sample\_all/pirna/full.gtf  
  
Significant (p<=0.01) hit density will be calculated based  
on observed hit distribution.  
  
Sliding window size: ........................................ 5000 bp  
Sliding window increament: .................................. 1000 bp  
Normalize each hit by number of genomic hits: ............... 1 [0=no/1=yes]  
Normalize each hit by number of sequence reads: ............. 1 [0=no/1=yes]  
Normalize values (-> per million mapped reads): ............. 1 [0=no/1=yes]  
Min. fraction of hits with 1T(U) or 10A: .................... 0.75  
Alternatively: Min. fraction of hits with 1T(U) and 10A: .... 0.5  
Min. fraction of hits with typical piRNA length: ............ 0.75  
Typical piRNA length: ....................................... 26-33 nt  
Min. size of a piRNA cluster: ............................... 5000 bp.  
Min. number of hits (absolute): ............................. 0  
Min. number of hits (normalized): ........................... 0  
Min. fraction of hits on the mainstrand: .................... 0.75  
Top fraction of mapped sequences (in terms of read counts): . 1%  
Top fraction accounts for max. n% of sequence reads: ........ 90%  
Min. fraction of hits on each arm of a bidirectional cluster: 0.1  
Output image file for each cluster: ......................... 0 [0=no/1=yes]  
Output html file for each cluster: .......................... 1 [0=no/1=yes]  
Output a summary table: ..................................... 1 [0=no/1=yes]  
Output a FASTA file for each cluster (piRNA sequences): ..... 1 [0=no/1=yes]  
Output a FASTA file comprising cluster sequences: ........... 1 [0=no/1=yes]  
Search DNA motifs in clusters: .............................. 1 [0=no/1=yes]  
Output flanking sequences: +/- .............................. 0 bp  
Output ~.pTi file: .......................................... 1 [0=no/1=yes]  
==============================================================================  
  
  
Genome size (without gaps): ............ 2678902517 bp  
Gaps (N/X/-): .......................... 53837044 bp  
Mapped reads: .......................... 658825247023  
Non-identical sequences: ............... 514171  
Genomic hits: .......................... 764233  
Significant densitiy of mapped reads: .. 12867599.5173724 reads/kb

Show proTRAC cluster info
Hide proTRAC cluster info

|  |  |
| --- | --- |
| Location | chr5 |
| Coordinates | 28463403-28468924 |
| Size [bp] | 5522 |
| Sequence hit loci | 112 |
| Mapped reads (normalized) | 160686214 |
| Mapped reads (normalized) per kb | 29099278.2 |
| Normalized reads with 1T (1U) | 82.7% |
| Normalized reads with 10A | 25% |
| Normalized reads with length 26-33 nt | 100% |
| Normalized reads on the main strand(s) | 99.2% |
| Predicted directionality | mono:minus |

100%

0%

1T (1U)  
reads

10A reads

26-33 nt  
reads

reads on mainstrand

**Either the amount of reads with 1T (1U) OR 10A has to exceed 75% (set with option: -1Tor10A)  
Alternatively the amount of reads with 1T (1U) AND 10A has to exceed 50% (set with option: -1Tand10A)  
Minimum amount of reads with preferred size is 75% (set with option: -pisize)  
Minimum amount of reads on the main strand(s) is 75% (set with option: -clstrand)**

Show read coverage
Hide read coverage

WHAT DO I SEE HERE?  
This chart shows the location of mapped sequence reads within a predicted piRNA cluster. The color refers to the number of genomic hits produced by the sequence read in question. A dark red bar indicates that this sequence read produces many other hits elsewhere in the genome. Many adjacent red or yellow bars can indicate the presence of a multi-copy element such as transposons or rRNA genes. A dark green bar indicates that this sequence read maps uniquely to this locus.

1 hit

2-5 hits

6-10 hits

11-20 hits

21-50 hits

51-100 hits

> 100 hits

chr5

28463403

28468924

Gene Set

RepeatMasker

Mapped  
Reads

18.51

plus strand

minus strand

18.51

Region: chr5 25978898-28463408. Max. coverage (+): 1.86. Max coverage (-): 0

Region: chr5 28463409-28463419. Max. coverage (+): 1.86. Max coverage (-): 0

Region: chr5 28463420-28463430. Max. coverage (+): 0. Max coverage (-): 0

Region: chr5 28463431-28463441. Max. coverage (+): 0. Max coverage (-): 0

Region: chr5 28463442-28463452. Max. coverage (+): 0. Max coverage (-): 0

Region: chr5 28463453-28463463. Max. coverage (+): 0. Max coverage (-): 0

Region: chr5 28463464-28463474. Max. coverage (+): 0. Max coverage (-): 0

Region: chr5 28463475-28463485. Max. coverage (+): 0. Max coverage (-): 0

Region: chr5 28463486-28463496. Max. coverage (+): 0. Max coverage (-): 0

Region: chr5 28463497-28463507. Max. coverage (+): 0. Max coverage (-): 0

Region: chr5 28463508-28463518. Max. coverage (+): 0. Max coverage (-): 0

Region: chr5 28463519-28463530. Max. coverage (+): 0. Max coverage (-): 0

Region: chr5 28463531-28463541. Max. coverage (+): 0. Max coverage (-): 0

Region: chr5 28463542-28463552. Max. coverage (+): 0. Max coverage (-): 0

Region: chr5 28463553-28463563. Max. coverage (+): 0. Max coverage (-): 0

Region: chr5 28463564-28463574. Max. coverage (+): 0. Max coverage (-): 0

Region: chr5 28463575-28463585. Max. coverage (+): 0. Max coverage (-): 0

Region: chr5 28463586-28463596. Max. coverage (+): 0. Max coverage (-): 0

Region: chr5 28463597-28463607. Max. coverage (+): 0. Max coverage (-): 0

Region: chr5 28463608-28463618. Max. coverage (+): 0. Max coverage (-): 0

Region: chr5 28463619-28463629. Max. coverage (+): 0. Max coverage (-): 0

Region: chr5 28463630-28463640. Max. coverage (+): 0. Max coverage (-): 0

Region: chr5 28463641-28463651. Max. coverage (+): 0. Max coverage (-): 0

Region: chr5 28463652-28463662. Max. coverage (+): 0. Max coverage (-): 0

Region: chr5 28463663-28463673. Max. coverage (+): 0. Max coverage (-): 0

Region: chr5 28463674-28463684. Max. coverage (+): 0. Max coverage (-): 0

Region: chr5 28463685-28463695. Max. coverage (+): 0. Max coverage (-): 0

Region: chr5 28463696-28463706. Max. coverage (+): 0. Max coverage (-): 0

Region: chr5 28463707-28463717. Max. coverage (+): 0. Max coverage (-): 0

Region: chr5 28463718-28463728. Max. coverage (+): 0. Max coverage (-): 0

Region: chr5 28463729-28463739. Max. coverage (+): 0. Max coverage (-): 0

Region: chr5 28463740-28463750. Max. coverage (+): 0. Max coverage (-): 0

Region: chr5 28463751-28463761. Max. coverage (+): 0. Max coverage (-): 0

Region: chr5 28463762-28463772. Max. coverage (+): 0. Max coverage (-): 0

Region: chr5 28463773-28463784. Max. coverage (+): 0. Max coverage (-): 0

Region: chr5 28463785-28463795. Max. coverage (+): 0. Max coverage (-): 0

Region: chr5 28463796-28463806. Max. coverage (+): 0. Max coverage (-): 0

Region: chr5 28463807-28463817. Max. coverage (+): 0. Max coverage (-): 0

Region: chr5 28463818-28463828. Max. coverage (+): 0. Max coverage (-): 0

Region: chr5 28463829-28463839. Max. coverage (+): 0. Max coverage (-): 0

Region: chr5 28463840-28463850. Max. coverage (+): 0. Max coverage (-): 0

Region: chr5 28463851-28463861. Max. coverage (+): 0. Max coverage (-): 0

Region: chr5 28463862-28463872. Max. coverage (+): 0. Max coverage (-): 0

Region: chr5 28463873-28463883. Max. coverage (+): 0. Max coverage (-): 0

Region: chr5 28463884-28463894. Max. coverage (+): 0. Max coverage (-): 0

Region: chr5 28463895-28463905. Max. coverage (+): 0. Max coverage (-): 0

Region: chr5 28463906-28463916. Max. coverage (+): 0. Max coverage (-): 0

Region: chr5 28463917-28463927. Max. coverage (+): 0. Max coverage (-): 0

Region: chr5 28463928-28463938. Max. coverage (+): 0. Max coverage (-): 0

Region: chr5 28463939-28463949. Max. coverage (+): 0. Max coverage (-): 0

Region: chr5 28463950-28463960. Max. coverage (+): 0. Max coverage (-): 0

Region: chr5 28463961-28463971. Max. coverage (+): 0. Max coverage (-): 0

Region: chr5 28463972-28463982. Max. coverage (+): 0. Max coverage (-): 0

Region: chr5 28463983-28463993. Max. coverage (+): 0. Max coverage (-): 0

Region: chr5 28463994-28464004. Max. coverage (+): 0. Max coverage (-): 0

Region: chr5 28464005-28464015. Max. coverage (+): 0. Max coverage (-): 0

Region: chr5 28464016-28464026. Max. coverage (+): 0. Max coverage (-): 0

Region: chr5 28464027-28464038. Max. coverage (+): 0. Max coverage (-): 2.41

Region: chr5 28464039-28464049. Max. coverage (+): 0. Max coverage (-): 0

Region: chr5 28464050-28464060. Max. coverage (+): 0. Max coverage (-): 0

Region: chr5 28464061-28464071. Max. coverage (+): 0. Max coverage (-): 0

Region: chr5 28464072-28464082. Max. coverage (+): 0. Max coverage (-): 0

Region: chr5 28464083-28464093. Max. coverage (+): 0. Max coverage (-): 0

Region: chr5 28464094-28464104. Max. coverage (+): 0. Max coverage (-): 0

Region: chr5 28464105-28464115. Max. coverage (+): 0. Max coverage (-): 0

Region: chr5 28464116-28464126. Max. coverage (+): 0. Max coverage (-): 0

Region: chr5 28464127-28464137. Max. coverage (+): 0. Max coverage (-): 0

Region: chr5 28464138-28464148. Max. coverage (+): 0. Max coverage (-): 0

Region: chr5 28464149-28464159. Max. coverage (+): 0. Max coverage (-): 0

Region: chr5 28464160-28464170. Max. coverage (+): 0. Max coverage (-): 0

Region: chr5 28464171-28464181. Max. coverage (+): 0. Max coverage (-): 0

Region: chr5 28464182-28464192. Max. coverage (+): 0. Max coverage (-): 0

Region: chr5 28464193-28464203. Max. coverage (+): 0. Max coverage (-): 0

Region: chr5 28464204-28464214. Max. coverage (+): 0. Max coverage (-): 0

Region: chr5 28464215-28464225. Max. coverage (+): 0. Max coverage (-): 0

Region: chr5 28464226-28464236. Max. coverage (+): 0. Max coverage (-): 0

Region: chr5 28464237-28464247. Max. coverage (+): 0. Max coverage (-): 0

Region: chr5 28464248-28464258. Max. coverage (+): 0. Max coverage (-): 0

Region: chr5 28464259-28464269. Max. coverage (+): 0. Max coverage (-): 0

Region: chr5 28464270-28464280. Max. coverage (+): 0. Max coverage (-): 0

Region: chr5 28464281-28464292. Max. coverage (+): 0. Max coverage (-): 0

Region: chr5 28464293-28464303. Max. coverage (+): 0. Max coverage (-): 0

Region: chr5 28464304-28464314. Max. coverage (+): 0. Max coverage (-): 0

Region: chr5 28464315-28464325. Max. coverage (+): 0. Max coverage (-): 0

Region: chr5 28464326-28464336. Max. coverage (+): 0. Max coverage (-): 0

Region: chr5 28464337-28464347. Max. coverage (+): 0. Max coverage (-): 0

Region: chr5 28464348-28464358. Max. coverage (+): 0. Max coverage (-): 0

Region: chr5 28464359-28464369. Max. coverage (+): 0. Max coverage (-): 0

Region: chr5 28464370-28464380. Max. coverage (+): 0. Max coverage (-): 0

Region: chr5 28464381-28464391. Max. coverage (+): 0. Max coverage (-): 0

Region: chr5 28464392-28464402. Max. coverage (+): 0. Max coverage (-): 0

Region: chr5 28464403-28464413. Max. coverage (+): 0. Max coverage (-): 0

Region: chr5 28464414-28464424. Max. coverage (+): 0. Max coverage (-): 0

Region: chr5 28464425-28464435. Max. coverage (+): 0. Max coverage (-): 0

Region: chr5 28464436-28464446. Max. coverage (+): 0. Max coverage (-): 0

Region: chr5 28464447-28464457. Max. coverage (+): 0. Max coverage (-): 0

Region: chr5 28464458-28464468. Max. coverage (+): 0. Max coverage (-): 4.69

Region: chr5 28464469-28464479. Max. coverage (+): 0. Max coverage (-): 4.69

Region: chr5 28464480-28464490. Max. coverage (+): 0. Max coverage (-): 0

Region: chr5 28464491-28464501. Max. coverage (+): 0. Max coverage (-): 0

Region: chr5 28464502-28464512. Max. coverage (+): 0. Max coverage (-): 0

Region: chr5 28464513-28464523. Max. coverage (+): 0. Max coverage (-): 0

Region: chr5 28464524-28464535. Max. coverage (+): 0. Max coverage (-): 0

Region: chr5 28464536-28464546. Max. coverage (+): 0. Max coverage (-): 0

Region: chr5 28464547-28464557. Max. coverage (+): 0. Max coverage (-): 0

Region: chr5 28464558-28464568. Max. coverage (+): 0. Max coverage (-): 0

Region: chr5 28464569-28464579. Max. coverage (+): 0. Max coverage (-): 0

Region: chr5 28464580-28464590. Max. coverage (+): 0. Max coverage (-): 0

Region: chr5 28464591-28464601. Max. coverage (+): 0. Max coverage (-): 0

Region: chr5 28464602-28464612. Max. coverage (+): 0. Max coverage (-): 0

Region: chr5 28464613-28464623. Max. coverage (+): 0. Max coverage (-): 0

Region: chr5 28464624-28464634. Max. coverage (+): 0. Max coverage (-): 0

Region: chr5 28464635-28464645. Max. coverage (+): 0. Max coverage (-): 0

Region: chr5 28464646-28464656. Max. coverage (+): 0. Max coverage (-): 0

Region: chr5 28464657-28464667. Max. coverage (+): 0. Max coverage (-): 0

Region: chr5 28464668-28464678. Max. coverage (+): 0. Max coverage (-): 0

Region: chr5 28464679-28464689. Max. coverage (+): 0. Max coverage (-): 0

Region: chr5 28464690-28464700. Max. coverage (+): 0. Max coverage (-): 0

Region: chr5 28464701-28464711. Max. coverage (+): 0. Max coverage (-): 0

Region: chr5 28464712-28464722. Max. coverage (+): 0. Max coverage (-): 0

Region: chr5 28464723-28464733. Max. coverage (+): 0. Max coverage (-): 0

Region: chr5 28464734-28464744. Max. coverage (+): 0. Max coverage (-): 0

Region: chr5 28464745-28464755. Max. coverage (+): 0. Max coverage (-): 0

Region: chr5 28464756-28464766. Max. coverage (+): 0. Max coverage (-): 0

Region: chr5 28464767-28464777. Max. coverage (+): 0. Max coverage (-): 0

Region: chr5 28464778-28464789. Max. coverage (+): 0. Max coverage (-): 0

Region: chr5 28464790-28464800. Max. coverage (+): 0. Max coverage (-): 0

Region: chr5 28464801-28464811. Max. coverage (+): 0. Max coverage (-): 0

Region: chr5 28464812-28464822. Max. coverage (+): 0. Max coverage (-): 0

Region: chr5 28464823-28464833. Max. coverage (+): 0. Max coverage (-): 0

Region: chr5 28464834-28464844. Max. coverage (+): 0. Max coverage (-): 0

Region: chr5 28464845-28464855. Max. coverage (+): 0. Max coverage (-): 0

Region: chr5 28464856-28464866. Max. coverage (+): 0. Max coverage (-): 0

Region: chr5 28464867-28464877. Max. coverage (+): 0. Max coverage (-): 0

Region: chr5 28464878-28464888. Max. coverage (+): 0. Max coverage (-): 0

Region: chr5 28464889-28464899. Max. coverage (+): 0. Max coverage (-): 0

Region: chr5 28464900-28464910. Max. coverage (+): 0. Max coverage (-): 0

Region: chr5 28464911-28464921. Max. coverage (+): 0. Max coverage (-): 0

Region: chr5 28464922-28464932. Max. coverage (+): 0. Max coverage (-): 0

Region: chr5 28464933-28464943. Max. coverage (+): 0. Max coverage (-): 0

Region: chr5 28464944-28464954. Max. coverage (+): 0. Max coverage (-): 0

Region: chr5 28464955-28464965. Max. coverage (+): 0. Max coverage (-): 0

Region: chr5 28464966-28464976. Max. coverage (+): 0. Max coverage (-): 0

Region: chr5 28464977-28464987. Max. coverage (+): 0. Max coverage (-): 0

Region: chr5 28464988-28464998. Max. coverage (+): 0. Max coverage (-): 2.56

Region: chr5 28464999-28465009. Max. coverage (+): 0. Max coverage (-): 2.56

Region: chr5 28465010-28465020. Max. coverage (+): 0. Max coverage (-): 0

Region: chr5 28465021-28465031. Max. coverage (+): 0. Max coverage (-): 2.98

Region: chr5 28465032-28465043. Max. coverage (+): 0. Max coverage (-): 2.98

Region: chr5 28465044-28465054. Max. coverage (+): 0. Max coverage (-): 4.75

Region: chr5 28465055-28465065. Max. coverage (+): 0. Max coverage (-): 0

Region: chr5 28465066-28465076. Max. coverage (+): 0. Max coverage (-): 0.59

Region: chr5 28465077-28465087. Max. coverage (+): 0. Max coverage (-): 0.59

Region: chr5 28465088-28465098. Max. coverage (+): 0. Max coverage (-): 0

Region: chr5 28465099-28465109. Max. coverage (+): 0. Max coverage (-): 0

Region: chr5 28465110-28465120. Max. coverage (+): 0. Max coverage (-): 0

Region: chr5 28465121-28465131. Max. coverage (+): 0. Max coverage (-): 0

Region: chr5 28465132-28465142. Max. coverage (+): 0. Max coverage (-): 0

Region: chr5 28465143-28465153. Max. coverage (+): 0. Max coverage (-): 0

Region: chr5 28465154-28465164. Max. coverage (+): 0. Max coverage (-): 0

Region: chr5 28465165-28465175. Max. coverage (+): 0. Max coverage (-): 0

Region: chr5 28465176-28465186. Max. coverage (+): 0. Max coverage (-): 0

Region: chr5 28465187-28465197. Max. coverage (+): 0. Max coverage (-): 0

Region: chr5 28465198-28465208. Max. coverage (+): 0. Max coverage (-): 0

Region: chr5 28465209-28465219. Max. coverage (+): 0. Max coverage (-): 0.77

Region: chr5 28465220-28465230. Max. coverage (+): 0. Max coverage (-): 0

Region: chr5 28465231-28465241. Max. coverage (+): 0. Max coverage (-): 0

Region: chr5 28465242-28465252. Max. coverage (+): 0. Max coverage (-): 0

Region: chr5 28465253-28465263. Max. coverage (+): 0. Max coverage (-): 4.38

Region: chr5 28465264-28465274. Max. coverage (+): 0. Max coverage (-): 0.35

Region: chr5 28465275-28465286. Max. coverage (+): 0. Max coverage (-): 0

Region: chr5 28465287-28465297. Max. coverage (+): 0. Max coverage (-): 0

Region: chr5 28465298-28465308. Max. coverage (+): 0. Max coverage (-): 0

Region: chr5 28465309-28465319. Max. coverage (+): 0. Max coverage (-): 0

Region: chr5 28465320-28465330. Max. coverage (+): 0. Max coverage (-): 0.77

Region: chr5 28465331-28465341. Max. coverage (+): 0. Max coverage (-): 0

Region: chr5 28465342-28465352. Max. coverage (+): 0. Max coverage (-): 0

Region: chr5 28465353-28465363. Max. coverage (+): 0. Max coverage (-): 10.63

Region: chr5 28465364-28465374. Max. coverage (+): 0. Max coverage (-): 12.28

Region: chr5 28465375-28465385. Max. coverage (+): 0. Max coverage (-): 0

Region: chr5 28465386-28465396. Max. coverage (+): 0. Max coverage (-): 8.84

Region: chr5 28465397-28465407. Max. coverage (+): 0. Max coverage (-): 8.84

Region: chr5 28465408-28465418. Max. coverage (+): 0. Max coverage (-): 0

Region: chr5 28465419-28465429. Max. coverage (+): 0. Max coverage (-): 0

Region: chr5 28465430-28465440. Max. coverage (+): 0. Max coverage (-): 0

Region: chr5 28465441-28465451. Max. coverage (+): 0. Max coverage (-): 0

Region: chr5 28465452-28465462. Max. coverage (+): 0. Max coverage (-): 0

Region: chr5 28465463-28465473. Max. coverage (+): 0. Max coverage (-): 0.75

Region: chr5 28465474-28465484. Max. coverage (+): 0. Max coverage (-): 0.75

Region: chr5 28465485-28465495. Max. coverage (+): 0. Max coverage (-): 3.16

Region: chr5 28465496-28465506. Max. coverage (+): 0. Max coverage (-): 8.21

Region: chr5 28465507-28465517. Max. coverage (+): 0. Max coverage (-): 5.05

Region: chr5 28465518-28465528. Max. coverage (+): 0. Max coverage (-): 0

Region: chr5 28465529-28465540. Max. coverage (+): 0. Max coverage (-): 0

Region: chr5 28465541-28465551. Max. coverage (+): 0. Max coverage (-): 0

Region: chr5 28465552-28465562. Max. coverage (+): 0. Max coverage (-): 0

Region: chr5 28465563-28465573. Max. coverage (+): 0. Max coverage (-): 0

Region: chr5 28465574-28465584. Max. coverage (+): 0. Max coverage (-): 0

Region: chr5 28465585-28465595. Max. coverage (+): 0. Max coverage (-): 0

Region: chr5 28465596-28465606. Max. coverage (+): 0. Max coverage (-): 0

Region: chr5 28465607-28465617. Max. coverage (+): 0. Max coverage (-): 0

Region: chr5 28465618-28465628. Max. coverage (+): 0. Max coverage (-): 0

Region: chr5 28465629-28465639. Max. coverage (+): 0. Max coverage (-): 1.54

Region: chr5 28465640-28465650. Max. coverage (+): 0. Max coverage (-): 0

Region: chr5 28465651-28465661. Max. coverage (+): 0. Max coverage (-): 0

Region: chr5 28465662-28465672. Max. coverage (+): 0. Max coverage (-): 0

Region: chr5 28465673-28465683. Max. coverage (+): 0. Max coverage (-): 0

Region: chr5 28465684-28465694. Max. coverage (+): 0. Max coverage (-): 2.88

Region: chr5 28465695-28465705. Max. coverage (+): 0. Max coverage (-): 2.88

Region: chr5 28465706-28465716. Max. coverage (+): 0. Max coverage (-): 0

Region: chr5 28465717-28465727. Max. coverage (+): 0. Max coverage (-): 0

Region: chr5 28465728-28465738. Max. coverage (+): 0. Max coverage (-): 0

Region: chr5 28465739-28465749. Max. coverage (+): 0. Max coverage (-): 0

Region: chr5 28465750-28465760. Max. coverage (+): 0. Max coverage (-): 0

Region: chr5 28465761-28465771. Max. coverage (+): 0. Max coverage (-): 0

Region: chr5 28465772-28465782. Max. coverage (+): 0. Max coverage (-): 0

Region: chr5 28465783-28465794. Max. coverage (+): 0. Max coverage (-): 0

Region: chr5 28465795-28465805. Max. coverage (+): 0. Max coverage (-): 2.24

Region: chr5 28465806-28465816. Max. coverage (+): 0. Max coverage (-): 2.24

Region: chr5 28465817-28465827. Max. coverage (+): 0. Max coverage (-): 0

Region: chr5 28465828-28465838. Max. coverage (+): 0. Max coverage (-): 0

Region: chr5 28465839-28465849. Max. coverage (+): 0. Max coverage (-): 1.01

Region: chr5 28465850-28465860. Max. coverage (+): 0. Max coverage (-): 1.01

Region: chr5 28465861-28465871. Max. coverage (+): 0. Max coverage (-): 0

Region: chr5 28465872-28465882. Max. coverage (+): 0. Max coverage (-): 0

Region: chr5 28465883-28465893. Max. coverage (+): 0. Max coverage (-): 0

Region: chr5 28465894-28465904. Max. coverage (+): 0. Max coverage (-): 0

Region: chr5 28465905-28465915. Max. coverage (+): 0. Max coverage (-): 0

Region: chr5 28465916-28465926. Max. coverage (+): 0. Max coverage (-): 0

Region: chr5 28465927-28465937. Max. coverage (+): 0. Max coverage (-): 0

Region: chr5 28465938-28465948. Max. coverage (+): 0. Max coverage (-): 0

Region: chr5 28465949-28465959. Max. coverage (+): 0. Max coverage (-): 0

Region: chr5 28465960-28465970. Max. coverage (+): 0. Max coverage (-): 1.58

Region: chr5 28465971-28465981. Max. coverage (+): 0. Max coverage (-): 1.58

Region: chr5 28465982-28465992. Max. coverage (+): 0. Max coverage (-): 0

Region: chr5 28465993-28466003. Max. coverage (+): 0. Max coverage (-): 0

Region: chr5 28466004-28466014. Max. coverage (+): 0. Max coverage (-): 0

Region: chr5 28466015-28466025. Max. coverage (+): 0. Max coverage (-): 5.85

Region: chr5 28466026-28466036. Max. coverage (+): 0. Max coverage (-): 0

Region: chr5 28466037-28466048. Max. coverage (+): 0. Max coverage (-): 0

Region: chr5 28466049-28466059. Max. coverage (+): 0. Max coverage (-): 0

Region: chr5 28466060-28466070. Max. coverage (+): 0. Max coverage (-): 0

Region: chr5 28466071-28466081. Max. coverage (+): 0. Max coverage (-): 0

Region: chr5 28466082-28466092. Max. coverage (+): 0. Max coverage (-): 0

Region: chr5 28466093-28466103. Max. coverage (+): 0. Max coverage (-): 0

Region: chr5 28466104-28466114. Max. coverage (+): 0. Max coverage (-): 0.28

Region: chr5 28466115-28466125. Max. coverage (+): 0. Max coverage (-): 0.28

Region: chr5 28466126-28466136. Max. coverage (+): 0. Max coverage (-): 0

Region: chr5 28466137-28466147. Max. coverage (+): 0. Max coverage (-): 0

Region: chr5 28466148-28466158. Max. coverage (+): 0. Max coverage (-): 0

Region: chr5 28466159-28466169. Max. coverage (+): 0. Max coverage (-): 0

Region: chr5 28466170-28466180. Max. coverage (+): 0. Max coverage (-): 0

Region: chr5 28466181-28466191. Max. coverage (+): 0. Max coverage (-): 0

Region: chr5 28466192-28466202. Max. coverage (+): 0. Max coverage (-): 0

Region: chr5 28466203-28466213. Max. coverage (+): 0. Max coverage (-): 0

Region: chr5 28466214-28466224. Max. coverage (+): 0. Max coverage (-): 0

Region: chr5 28466225-28466235. Max. coverage (+): 0. Max coverage (-): 0

Region: chr5 28466236-28466246. Max. coverage (+): 0. Max coverage (-): 14.17

Region: chr5 28466247-28466257. Max. coverage (+): 0. Max coverage (-): 7.54

Region: chr5 28466258-28466268. Max. coverage (+): 0. Max coverage (-): 0

Region: chr5 28466269-28466279. Max. coverage (+): 0. Max coverage (-): 0

Region: chr5 28466280-28466291. Max. coverage (+): 0. Max coverage (-): 0

Region: chr5 28466292-28466302. Max. coverage (+): 0. Max coverage (-): 0

Region: chr5 28466303-28466313. Max. coverage (+): 0. Max coverage (-): 10.65

Region: chr5 28466314-28466324. Max. coverage (+): 0. Max coverage (-): 18.51

Region: chr5 28466325-28466335. Max. coverage (+): 0. Max coverage (-): 5.28

Region: chr5 28466336-28466346. Max. coverage (+): 0. Max coverage (-): 7.35

Region: chr5 28466347-28466357. Max. coverage (+): 0. Max coverage (-): 0

Region: chr5 28466358-28466368. Max. coverage (+): 0. Max coverage (-): 0

Region: chr5 28466369-28466379. Max. coverage (+): 0. Max coverage (-): 2.47

Region: chr5 28466380-28466390. Max. coverage (+): 0. Max coverage (-): 4.56

Region: chr5 28466391-28466401. Max. coverage (+): 0. Max coverage (-): 4.56

Region: chr5 28466402-28466412. Max. coverage (+): 0. Max coverage (-): 5.36

Region: chr5 28466413-28466423. Max. coverage (+): 0. Max coverage (-): 5.36

Region: chr5 28466424-28466434. Max. coverage (+): 0. Max coverage (-): 2.05

Region: chr5 28466435-28466445. Max. coverage (+): 0. Max coverage (-): 0

Region: chr5 28466446-28466456. Max. coverage (+): 0. Max coverage (-): 0

Region: chr5 28466457-28466467. Max. coverage (+): 0. Max coverage (-): 0

Region: chr5 28466468-28466478. Max. coverage (+): 0. Max coverage (-): 0

Region: chr5 28466479-28466489. Max. coverage (+): 0. Max coverage (-): 0

Region: chr5 28466490-28466500. Max. coverage (+): 0. Max coverage (-): 0

Region: chr5 28466501-28466511. Max. coverage (+): 0. Max coverage (-): 7.13

Region: chr5 28466512-28466522. Max. coverage (+): 0. Max coverage (-): 3.92

Region: chr5 28466523-28466533. Max. coverage (+): 0. Max coverage (-): 0

Region: chr5 28466534-28466545. Max. coverage (+): 0. Max coverage (-): 0

Region: chr5 28466546-28466556. Max. coverage (+): 0. Max coverage (-): 0

Region: chr5 28466557-28466567. Max. coverage (+): 0. Max coverage (-): 0

Region: chr5 28466568-28466578. Max. coverage (+): 0. Max coverage (-): 0

Region: chr5 28466579-28466589. Max. coverage (+): 0. Max coverage (-): 0

Region: chr5 28466590-28466600. Max. coverage (+): 0. Max coverage (-): 0

Region: chr5 28466601-28466611. Max. coverage (+): 0. Max coverage (-): 0

Region: chr5 28466612-28466622. Max. coverage (+): 0. Max coverage (-): 0

Region: chr5 28466623-28466633. Max. coverage (+): 0. Max coverage (-): 0

Region: chr5 28466634-28466644. Max. coverage (+): 0. Max coverage (-): 0

Region: chr5 28466645-28466655. Max. coverage (+): 0. Max coverage (-): 0

Region: chr5 28466656-28466666. Max. coverage (+): 0. Max coverage (-): 0

Region: chr5 28466667-28466677. Max. coverage (+): 0. Max coverage (-): 0

Region: chr5 28466678-28466688. Max. coverage (+): 0. Max coverage (-): 0

Region: chr5 28466689-28466699. Max. coverage (+): 0. Max coverage (-): 0

Region: chr5 28466700-28466710. Max. coverage (+): 0. Max coverage (-): 0

Region: chr5 28466711-28466721. Max. coverage (+): 0. Max coverage (-): 0

Region: chr5 28466722-28466732. Max. coverage (+): 0. Max coverage (-): 0

Region: chr5 28466733-28466743. Max. coverage (+): 0. Max coverage (-): 0

Region: chr5 28466744-28466754. Max. coverage (+): 0. Max coverage (-): 1.75

Region: chr5 28466755-28466765. Max. coverage (+): 0. Max coverage (-): 3.48

Region: chr5 28466766-28466776. Max. coverage (+): 0. Max coverage (-): 6.97

Region: chr5 28466777-28466787. Max. coverage (+): 0. Max coverage (-): 0

Region: chr5 28466788-28466799. Max. coverage (+): 0. Max coverage (-): 0

Region: chr5 28466800-28466810. Max. coverage (+): 0. Max coverage (-): 0

Region: chr5 28466811-28466821. Max. coverage (+): 0. Max coverage (-): 1.48

Region: chr5 28466822-28466832. Max. coverage (+): 0. Max coverage (-): 1.48

Region: chr5 28466833-28466843. Max. coverage (+): 0. Max coverage (-): 0

Region: chr5 28466844-28466854. Max. coverage (+): 0. Max coverage (-): 0

Region: chr5 28466855-28466865. Max. coverage (+): 0. Max coverage (-): 0

Region: chr5 28466866-28466876. Max. coverage (+): 0. Max coverage (-): 0

Region: chr5 28466877-28466887. Max. coverage (+): 0. Max coverage (-): 0

Region: chr5 28466888-28466898. Max. coverage (+): 0. Max coverage (-): 0

Region: chr5 28466899-28466909. Max. coverage (+): 0. Max coverage (-): 0

Region: chr5 28466910-28466920. Max. coverage (+): 0. Max coverage (-): 0

Region: chr5 28466921-28466931. Max. coverage (+): 0. Max coverage (-): 0

Region: chr5 28466932-28466942. Max. coverage (+): 0. Max coverage (-): 0

Region: chr5 28466943-28466953. Max. coverage (+): 0. Max coverage (-): 0

Region: chr5 28466954-28466964. Max. coverage (+): 0. Max coverage (-): 0

Region: chr5 28466965-28466975. Max. coverage (+): 0. Max coverage (-): 0

Region: chr5 28466976-28466986. Max. coverage (+): 0. Max coverage (-): 0

Region: chr5 28466987-28466997. Max. coverage (+): 0. Max coverage (-): 4.88

Region: chr5 28466998-28467008. Max. coverage (+): 0. Max coverage (-): 12.46

Region: chr5 28467009-28467019. Max. coverage (+): 0. Max coverage (-): 12.46

Region: chr5 28467020-28467030. Max. coverage (+): 0. Max coverage (-): 0

Region: chr5 28467031-28467041. Max. coverage (+): 0. Max coverage (-): 0

Region: chr5 28467042-28467053. Max. coverage (+): 0. Max coverage (-): 0.37

Region: chr5 28467054-28467064. Max. coverage (+): 0. Max coverage (-): 0.37

Region: chr5 28467065-28467075. Max. coverage (+): 0. Max coverage (-): 0

Region: chr5 28467076-28467086. Max. coverage (+): 0. Max coverage (-): 0

Region: chr5 28467087-28467097. Max. coverage (+): 0. Max coverage (-): 10.56

Region: chr5 28467098-28467108. Max. coverage (+): 0. Max coverage (-): 8.95

Region: chr5 28467109-28467119. Max. coverage (+): 0. Max coverage (-): 0

Region: chr5 28467120-28467130. Max. coverage (+): 0. Max coverage (-): 2.38

Region: chr5 28467131-28467141. Max. coverage (+): 0. Max coverage (-): 4.32

Region: chr5 28467142-28467152. Max. coverage (+): 0. Max coverage (-): 4.32

Region: chr5 28467153-28467163. Max. coverage (+): 0. Max coverage (-): 0

Region: chr5 28467164-28467174. Max. coverage (+): 0. Max coverage (-): 0

Region: chr5 28467175-28467185. Max. coverage (+): 0. Max coverage (-): 3.23

Region: chr5 28467186-28467196. Max. coverage (+): 0. Max coverage (-): 5.05

Region: chr5 28467197-28467207. Max. coverage (+): 0. Max coverage (-): 3.36

Region: chr5 28467208-28467218. Max. coverage (+): 0. Max coverage (-): 3.36

Region: chr5 28467219-28467229. Max. coverage (+): 0. Max coverage (-): 6.18

Region: chr5 28467230-28467240. Max. coverage (+): 0. Max coverage (-): 4.6

Region: chr5 28467241-28467251. Max. coverage (+): 0. Max coverage (-): 0

Region: chr5 28467252-28467262. Max. coverage (+): 0. Max coverage (-): 0

Region: chr5 28467263-28467273. Max. coverage (+): 0. Max coverage (-): 1.02

Region: chr5 28467274-28467284. Max. coverage (+): 0. Max coverage (-): 8.71

Region: chr5 28467285-28467296. Max. coverage (+): 0. Max coverage (-): 8.71

Region: chr5 28467297-28467307. Max. coverage (+): 0. Max coverage (-): 0

Region: chr5 28467308-28467318. Max. coverage (+): 0. Max coverage (-): 0

Region: chr5 28467319-28467329. Max. coverage (+): 0. Max coverage (-): 0

Region: chr5 28467330-28467340. Max. coverage (+): 0. Max coverage (-): 0

Region: chr5 28467341-28467351. Max. coverage (+): 0. Max coverage (-): 0

Region: chr5 28467352-28467362. Max. coverage (+): 0. Max coverage (-): 0

Region: chr5 28467363-28467373. Max. coverage (+): 0. Max coverage (-): 0

Region: chr5 28467374-28467384. Max. coverage (+): 0. Max coverage (-): 0

Region: chr5 28467385-28467395. Max. coverage (+): 0. Max coverage (-): 0

Region: chr5 28467396-28467406. Max. coverage (+): 0. Max coverage (-): 0

Region: chr5 28467407-28467417. Max. coverage (+): 0. Max coverage (-): 0

Region: chr5 28467418-28467428. Max. coverage (+): 0. Max coverage (-): 0

Region: chr5 28467429-28467439. Max. coverage (+): 0. Max coverage (-): 0

Region: chr5 28467440-28467450. Max. coverage (+): 0. Max coverage (-): 0

Region: chr5 28467451-28467461. Max. coverage (+): 0. Max coverage (-): 0

Region: chr5 28467462-28467472. Max. coverage (+): 0. Max coverage (-): 0

Region: chr5 28467473-28467483. Max. coverage (+): 0. Max coverage (-): 0

Region: chr5 28467484-28467494. Max. coverage (+): 0. Max coverage (-): 0

Region: chr5 28467495-28467505. Max. coverage (+): 0. Max coverage (-): 0

Region: chr5 28467506-28467516. Max. coverage (+): 0. Max coverage (-): 0

Region: chr5 28467517-28467527. Max. coverage (+): 0. Max coverage (-): 0.99

Region: chr5 28467528-28467538. Max. coverage (+): 0. Max coverage (-): 0.99

Region: chr5 28467539-28467550. Max. coverage (+): 0. Max coverage (-): 0

Region: chr5 28467551-28467561. Max. coverage (+): 0. Max coverage (-): 0

Region: chr5 28467562-28467572. Max. coverage (+): 0. Max coverage (-): 0

Region: chr5 28467573-28467583. Max. coverage (+): 0. Max coverage (-): 1.19

Region: chr5 28467584-28467594. Max. coverage (+): 0. Max coverage (-): 1.19

Region: chr5 28467595-28467605. Max. coverage (+): 0. Max coverage (-): 0

Region: chr5 28467606-28467616. Max. coverage (+): 0. Max coverage (-): 0

Region: chr5 28467617-28467627. Max. coverage (+): 0. Max coverage (-): 0

Region: chr5 28467628-28467638. Max. coverage (+): 0. Max coverage (-): 0

Region: chr5 28467639-28467649. Max. coverage (+): 0. Max coverage (-): 0

Region: chr5 28467650-28467660. Max. coverage (+): 0. Max coverage (-): 0

Region: chr5 28467661-28467671. Max. coverage (+): 0. Max coverage (-): 0

Region: chr5 28467672-28467682. Max. coverage (+): 0. Max coverage (-): 0

Region: chr5 28467683-28467693. Max. coverage (+): 0. Max coverage (-): 0

Region: chr5 28467694-28467704. Max. coverage (+): 0. Max coverage (-): 0

Region: chr5 28467705-28467715. Max. coverage (+): 0. Max coverage (-): 0

Region: chr5 28467716-28467726. Max. coverage (+): 0. Max coverage (-): 0

Region: chr5 28467727-28467737. Max. coverage (+): 0. Max coverage (-): 0

Region: chr5 28467738-28467748. Max. coverage (+): 0. Max coverage (-): 0

Region: chr5 28467749-28467759. Max. coverage (+): 0. Max coverage (-): 0

Region: chr5 28467760-28467770. Max. coverage (+): 0. Max coverage (-): 0

Region: chr5 28467771-28467781. Max. coverage (+): 0. Max coverage (-): 0

Region: chr5 28467782-28467792. Max. coverage (+): 0. Max coverage (-): 0

Region: chr5 28467793-28467804. Max. coverage (+): 0. Max coverage (-): 0

Region: chr5 28467805-28467815. Max. coverage (+): 0. Max coverage (-): 0

Region: chr5 28467816-28467826. Max. coverage (+): 0. Max coverage (-): 0

Region: chr5 28467827-28467837. Max. coverage (+): 0. Max coverage (-): 0

Region: chr5 28467838-28467848. Max. coverage (+): 0. Max coverage (-): 0

Region: chr5 28467849-28467859. Max. coverage (+): 0. Max coverage (-): 0

Region: chr5 28467860-28467870. Max. coverage (+): 0. Max coverage (-): 0

Region: chr5 28467871-28467881. Max. coverage (+): 0. Max coverage (-): 0

Region: chr5 28467882-28467892. Max. coverage (+): 0. Max coverage (-): 1.9

Region: chr5 28467893-28467903. Max. coverage (+): 0. Max coverage (-): 1.9

Region: chr5 28467904-28467914. Max. coverage (+): 0. Max coverage (-): 4.8

Region: chr5 28467915-28467925. Max. coverage (+): 0. Max coverage (-): 5.65

Region: chr5 28467926-28467936. Max. coverage (+): 0. Max coverage (-): 5.65

Region: chr5 28467937-28467947. Max. coverage (+): 0. Max coverage (-): 0

Region: chr5 28467948-28467958. Max. coverage (+): 0. Max coverage (-): 0

Region: chr5 28467959-28467969. Max. coverage (+): 0. Max coverage (-): 0

Region: chr5 28467970-28467980. Max. coverage (+): 0. Max coverage (-): 0

Region: chr5 28467981-28467991. Max. coverage (+): 0. Max coverage (-): 0

Region: chr5 28467992-28468002. Max. coverage (+): 0. Max coverage (-): 0

Region: chr5 28468003-28468013. Max. coverage (+): 0. Max coverage (-): 0

Region: chr5 28468014-28468024. Max. coverage (+): 0. Max coverage (-): 0

Region: chr5 28468025-28468035. Max. coverage (+): 0. Max coverage (-): 0

Region: chr5 28468036-28468047. Max. coverage (+): 0. Max coverage (-): 0

Region: chr5 28468048-28468058. Max. coverage (+): 0. Max coverage (-): 0

Region: chr5 28468059-28468069. Max. coverage (+): 0. Max coverage (-): 0

Region: chr5 28468070-28468080. Max. coverage (+): 0. Max coverage (-): 0

Region: chr5 28468081-28468091. Max. coverage (+): 0. Max coverage (-): 0

Region: chr5 28468092-28468102. Max. coverage (+): 0. Max coverage (-): 0

Region: chr5 28468103-28468113. Max. coverage (+): 0. Max coverage (-): 0

Region: chr5 28468114-28468124. Max. coverage (+): 0. Max coverage (-): 0

Region: chr5 28468125-28468135. Max. coverage (+): 0. Max coverage (-): 0

Region: chr5 28468136-28468146. Max. coverage (+): 0. Max coverage (-): 0

Region: chr5 28468147-28468157. Max. coverage (+): 0. Max coverage (-): 0

Region: chr5 28468158-28468168. Max. coverage (+): 0. Max coverage (-): 0

Region: chr5 28468169-28468179. Max. coverage (+): 0. Max coverage (-): 0

Region: chr5 28468180-28468190. Max. coverage (+): 0. Max coverage (-): 0

Region: chr5 28468191-28468201. Max. coverage (+): 0. Max coverage (-): 0

Region: chr5 28468202-28468212. Max. coverage (+): 0. Max coverage (-): 0

Region: chr5 28468213-28468223. Max. coverage (+): 0. Max coverage (-): 0

Region: chr5 28468224-28468234. Max. coverage (+): 0. Max coverage (-): 0

Region: chr5 28468235-28468245. Max. coverage (+): 0. Max coverage (-): 0

Region: chr5 28468246-28468256. Max. coverage (+): 0. Max coverage (-): 0

Region: chr5 28468257-28468267. Max. coverage (+): 0. Max coverage (-): 0

Region: chr5 28468268-28468278. Max. coverage (+): 0. Max coverage (-): 0

Region: chr5 28468279-28468289. Max. coverage (+): 0. Max coverage (-): 0

Region: chr5 28468290-28468301. Max. coverage (+): 0. Max coverage (-): 0

Region: chr5 28468302-28468312. Max. coverage (+): 0. Max coverage (-): 0

Region: chr5 28468313-28468323. Max. coverage (+): 0. Max coverage (-): 0

Region: chr5 28468324-28468334. Max. coverage (+): 0. Max coverage (-): 0

Region: chr5 28468335-28468345. Max. coverage (+): 0. Max coverage (-): 2.75

Region: chr5 28468346-28468356. Max. coverage (+): 0. Max coverage (-): 2.75

Region: chr5 28468357-28468367. Max. coverage (+): 0. Max coverage (-): 0

Region: chr5 28468368-28468378. Max. coverage (+): 0. Max coverage (-): 0

Region: chr5 28468379-28468389. Max. coverage (+): 0. Max coverage (-): 0

Region: chr5 28468390-28468400. Max. coverage (+): 0. Max coverage (-): 0

Region: chr5 28468401-28468411. Max. coverage (+): 0. Max coverage (-): 0

Region: chr5 28468412-28468422. Max. coverage (+): 0. Max coverage (-): 0

Region: chr5 28468423-28468433. Max. coverage (+): 0. Max coverage (-): 0

Region: chr5 28468434-28468444. Max. coverage (+): 0. Max coverage (-): 0

Region: chr5 28468445-28468455. Max. coverage (+): 0. Max coverage (-): 0

Region: chr5 28468456-28468466. Max. coverage (+): 0. Max coverage (-): 0

Region: chr5 28468467-28468477. Max. coverage (+): 0. Max coverage (-): 0

Region: chr5 28468478-28468488. Max. coverage (+): 0. Max coverage (-): 0

Region: chr5 28468489-28468499. Max. coverage (+): 0. Max coverage (-): 0

Region: chr5 28468500-28468510. Max. coverage (+): 0. Max coverage (-): 0

Region: chr5 28468511-28468521. Max. coverage (+): 0. Max coverage (-): 0

Region: chr5 28468522-28468532. Max. coverage (+): 0. Max coverage (-): 0

Region: chr5 28468533-28468543. Max. coverage (+): 0. Max coverage (-): 0

Region: chr5 28468544-28468555. Max. coverage (+): 0. Max coverage (-): 0

Region: chr5 28468556-28468566. Max. coverage (+): 0. Max coverage (-): 0

Region: chr5 28468567-28468577. Max. coverage (+): 0. Max coverage (-): 0

Region: chr5 28468578-28468588. Max. coverage (+): 0. Max coverage (-): 0

Region: chr5 28468589-28468599. Max. coverage (+): 0. Max coverage (-): 0

Region: chr5 28468600-28468610. Max. coverage (+): 0. Max coverage (-): 0

Region: chr5 28468611-28468621. Max. coverage (+): 0. Max coverage (-): 0

Region: chr5 28468622-28468632. Max. coverage (+): 0. Max coverage (-): 0

Region: chr5 28468633-28468643. Max. coverage (+): 0. Max coverage (-): 0

Region: chr5 28468644-28468654. Max. coverage (+): 0. Max coverage (-): 0

Region: chr5 28468655-28468665. Max. coverage (+): 0. Max coverage (-): 0

Region: chr5 28468666-28468676. Max. coverage (+): 0. Max coverage (-): 0

Region: chr5 28468677-28468687. Max. coverage (+): 0. Max coverage (-): 0

Region: chr5 28468688-28468698. Max. coverage (+): 0. Max coverage (-): 0

Region: chr5 28468699-28468709. Max. coverage (+): 0. Max coverage (-): 0

Region: chr5 28468710-28468720. Max. coverage (+): 0. Max coverage (-): 0

Region: chr5 28468721-28468731. Max. coverage (+): 0. Max coverage (-): 0

Region: chr5 28468732-28468742. Max. coverage (+): 0. Max coverage (-): 0.56

Region: chr5 28468743-28468753. Max. coverage (+): 0. Max coverage (-): 0.56

Region: chr5 28468754-28468764. Max. coverage (+): 0. Max coverage (-): 0

Region: chr5 28468765-28468775. Max. coverage (+): 0. Max coverage (-): 0

Region: chr5 28468776-28468786. Max. coverage (+): 0. Max coverage (-): 0

Region: chr5 28468787-28468797. Max. coverage (+): 0. Max coverage (-): 0

Region: chr5 28468798-28468809. Max. coverage (+): 0. Max coverage (-): 0

Region: chr5 28468810-28468820. Max. coverage (+): 0. Max coverage (-): 0

Region: chr5 28468821-28468831. Max. coverage (+): 0. Max coverage (-): 0

Region: chr5 28468832-28468842. Max. coverage (+): 0. Max coverage (-): 0

Region: chr5 28468843-28468853. Max. coverage (+): 0. Max coverage (-): 0

Region: chr5 28468854-28468864. Max. coverage (+): 0. Max coverage (-): 0

Region: chr5 28468865-28468875. Max. coverage (+): 0. Max coverage (-): 0

Region: chr5 28468876-28468886. Max. coverage (+): 0. Max coverage (-): 0

Region: chr5 28468887-28468897. Max. coverage (+): 0. Max coverage (-): 6.82

Region: chr5 28468898-28468908. Max. coverage (+): 0. Max coverage (-): 6.82

Region: chr5 28468909-28468919. Max. coverage (+): 0. Max coverage (-): 0

Region: chr5 28468920-. Max. coverage (+): 0. Max coverage (-): 0

RepeatMasker Color Code

**+**

100-98% Identity

<98-95% Identity

<95-90% Identity

<90-85% Identity

<85-80% Identity

<80-75% Identity

<75-70% Identity

<70% Identity

**-**

Gene Set Color Code

**+**

Gene

Pseudogene

**-**

Topology/Coverage Color Code

Coverage Plus Strand

Coverage Minus Strand

Mainstrand: Plus

Mainstrand: Minus

Complementary Strand

Flanking Region  
(if option -flank >0)

Gene Set Annotation  
  
RepeatMasker Annotation  

**1. Tigger19a**: 28464701-28464808 (-), Divergence to consensus: 39.7%  
**2. (CA)n**: 28466460-28466494 (+), Divergence to consensus: 20%  
**3. MamRep1894**: 28467351-28467472 (-), Divergence to consensus: 24.6%  
**4. L3b**: 28468164-28468282 (-), Divergence to consensus: 36.3%

  
Transcription Factor Binding Sites  

**RFX4\_1** (Sequence: CGTAGCAAC (+): 28467617)  
**Gata4** (Sequence: CTTATCT (+): 28468095)
