# Supplementary material for: Small RNA sequencing of cryopreserved semen from single bull revealed altered miRNAs and piRNAs expression between High- and Low-motile sperm populations
Source: BMC Genomics. 2017 Jan 4;18:14. doi: 10.1186/s12864-016-3394-7 (PMC5209821; doi:10.1186/s12864-016-3394-7)
Supplement: Additional file 3: — Details for each piRNA clusters found in High Motile (HM) sperm fraction. Genes, repeats, transposable elements and transcription factors binding sites falling within the cluster regions were reported. (ZIP 1896 kb) [file 12864_2016_3394_MOESM3_ESM.zip › 85.html]

piRNA cluster 85


Predicted piRNA cluster no. 85     previous   next
  

Show proTRAC run info
Hide proTRAC run info

================================= proTRAC ====================================  
VERSION: 2.1                                    LAST MODIFIED: 06. October 2015  
  
Please cite:  
Rosenkranz D, Zischler H. proTRAC - a software for probabilistic piRNA cluster  
detection, visualization and analysis. 2012. BMC Bioinformatics 13:5.  
  
and (for proTRAC 2.0 and later):  
Rosenkranz D, Rudloff S, Bastuck K, Ketting RF, Zischler H. Tupaia small RNAs  
provide insights into function and evolution of RNAi-based transposon defense  
in mammals. 2015. RNA 21(5):911-922.  
  
Contact:  
David Rosenkranz  
Institute of Anthropology, small RNA group  
Johannes Gutenberg University Mainz  
email: rosenkranz@uni-mainz.de  
  
You can find the latest proTRAC version at:  
http://sourceforge.net/projects/protrac/files  
http://www.smallRNAgroup-mainz.de/software  
==============================================================================  
  
PARAMETERS:  
Map file: .............../storage/core/barbara/genhome/smallRNA/fertility/Sample\_motile/pirna/Sample\_motile\_26-33\_collapsed.fa.no-dust.map.weighted-10000-1000-b-0  
Genome file: ............/storage/core/barbara/genhome/smallRNA/fertility/Sample\_all/pirna/bt\_311\_chrY.fa  
RepeatMasker annotation: /storage/genomes/bt\_umd31/GCF\_000003055.6\_Bos\_taurus\_UMD\_3.1.1\_repeatMasker\_chr.out  
GeneSet:................./storage/core/barbara/genhome/smallRNA/fertility/Sample\_all/pirna/full.gtf  
  
Significant (p<=0.01) hit density will be calculated based  
on observed hit distribution.  
  
Sliding window size: ........................................ 5000 bp  
Sliding window increament: .................................. 1000 bp  
Normalize each hit by number of genomic hits: ............... 1 [0=no/1=yes]  
Normalize each hit by number of sequence reads: ............. 1 [0=no/1=yes]  
Normalize values (-> per million mapped reads): ............. 1 [0=no/1=yes]  
Min. fraction of hits with 1T(U) or 10A: .................... 0.75  
Alternatively: Min. fraction of hits with 1T(U) and 10A: .... 0.5  
Min. fraction of hits with typical piRNA length: ............ 0.75  
Typical piRNA length: ....................................... 26-33 nt  
Min. size of a piRNA cluster: ............................... 5000 bp.  
Min. number of hits (absolute): ............................. 0  
Min. number of hits (normalized): ........................... 0  
Min. fraction of hits on the mainstrand: .................... 0.75  
Top fraction of mapped sequences (in terms of read counts): . 1%  
Top fraction accounts for max. n% of sequence reads: ........ 90%  
Min. fraction of hits on each arm of a bidirectional cluster: 0.1  
Output image file for each cluster: ......................... 0 [0=no/1=yes]  
Output html file for each cluster: .......................... 1 [0=no/1=yes]  
Output a summary table: ..................................... 1 [0=no/1=yes]  
Output a FASTA file for each cluster (piRNA sequences): ..... 1 [0=no/1=yes]  
Output a FASTA file comprising cluster sequences: ........... 1 [0=no/1=yes]  
Search DNA motifs in clusters: .............................. 1 [0=no/1=yes]  
Output flanking sequences: +/- .............................. 0 bp  
Output ~.pTi file: .......................................... 1 [0=no/1=yes]  
==============================================================================  
  
  
Genome size (without gaps): ............ 2678902517 bp  
Gaps (N/X/-): .......................... 53837044 bp  
Mapped reads: .......................... 658825247023  
Non-identical sequences: ............... 514171  
Genomic hits: .......................... 764233  
Significant densitiy of mapped reads: .. 12867599.5173724 reads/kb

Show proTRAC cluster info
Hide proTRAC cluster info

|  |  |
| --- | --- |
| Location | chr5 |
| Coordinates | 56702801-56708565 |
| Size [bp] | 5765 |
| Sequence hit loci | 63 |
| Mapped reads (normalized) | 85787903 |
| Mapped reads (normalized) per kb | 14880815.8 |
| Normalized reads with 1T (1U) | 80.2% |
| Normalized reads with 10A | 45.9% |
| Normalized reads with length 26-33 nt | 100% |
| Normalized reads on the main strand(s) | 100% |
| Predicted directionality | mono:plus |

100%

0%

1T (1U)  
reads

10A reads

26-33 nt  
reads

reads on mainstrand

**Either the amount of reads with 1T (1U) OR 10A has to exceed 75% (set with option: -1Tor10A)  
Alternatively the amount of reads with 1T (1U) AND 10A has to exceed 50% (set with option: -1Tand10A)  
Minimum amount of reads with preferred size is 75% (set with option: -pisize)  
Minimum amount of reads on the main strand(s) is 75% (set with option: -clstrand)**

Show read coverage
Hide read coverage

WHAT DO I SEE HERE?  
This chart shows the location of mapped sequence reads within a predicted piRNA cluster. The color refers to the number of genomic hits produced by the sequence read in question. A dark red bar indicates that this sequence read produces many other hits elsewhere in the genome. Many adjacent red or yellow bars can indicate the presence of a multi-copy element such as transposons or rRNA genes. A dark green bar indicates that this sequence read maps uniquely to this locus.

1 hit

2-5 hits

6-10 hits

11-20 hits

21-50 hits

51-100 hits

> 100 hits

chr5

56702801

56708565

Gene Set

RepeatMasker

Mapped  
Reads

16.91

plus strand

minus strand

16.91

Region: chr5 28468921-56702806. Max. coverage (+): 1.73. Max coverage (-): 0

Region: chr5 56702807-56702818. Max. coverage (+): 1.73. Max coverage (-): 0

Region: chr5 56702819-56702829. Max. coverage (+): 0. Max coverage (-): 0

Region: chr5 56702830-56702841. Max. coverage (+): 1.16. Max coverage (-): 0

Region: chr5 56702842-56702852. Max. coverage (+): 1.16. Max coverage (-): 0

Region: chr5 56702853-56702864. Max. coverage (+): 0. Max coverage (-): 0

Region: chr5 56702865-56702875. Max. coverage (+): 0. Max coverage (-): 0

Region: chr5 56702876-56702887. Max. coverage (+): 0. Max coverage (-): 0

Region: chr5 56702888-56702899. Max. coverage (+): 0. Max coverage (-): 0

Region: chr5 56702900-56702910. Max. coverage (+): 0. Max coverage (-): 0

Region: chr5 56702911-56702922. Max. coverage (+): 0. Max coverage (-): 0

Region: chr5 56702923-56702933. Max. coverage (+): 0. Max coverage (-): 0

Region: chr5 56702934-56702945. Max. coverage (+): 0. Max coverage (-): 0

Region: chr5 56702946-56702956. Max. coverage (+): 0. Max coverage (-): 0

Region: chr5 56702957-56702968. Max. coverage (+): 0. Max coverage (-): 0

Region: chr5 56702969-56702979. Max. coverage (+): 0. Max coverage (-): 0

Region: chr5 56702980-56702991. Max. coverage (+): 0. Max coverage (-): 0

Region: chr5 56702992-56703002. Max. coverage (+): 0. Max coverage (-): 0

Region: chr5 56703003-56703014. Max. coverage (+): 0. Max coverage (-): 0

Region: chr5 56703015-56703025. Max. coverage (+): 0. Max coverage (-): 0

Region: chr5 56703026-56703037. Max. coverage (+): 0. Max coverage (-): 0

Region: chr5 56703038-56703048. Max. coverage (+): 0. Max coverage (-): 0

Region: chr5 56703049-56703060. Max. coverage (+): 0. Max coverage (-): 0

Region: chr5 56703061-56703071. Max. coverage (+): 0. Max coverage (-): 0

Region: chr5 56703072-56703083. Max. coverage (+): 0. Max coverage (-): 0

Region: chr5 56703084-56703095. Max. coverage (+): 0. Max coverage (-): 0

Region: chr5 56703096-56703106. Max. coverage (+): 0. Max coverage (-): 0

Region: chr5 56703107-56703118. Max. coverage (+): 0. Max coverage (-): 0

Region: chr5 56703119-56703129. Max. coverage (+): 0. Max coverage (-): 0

Region: chr5 56703130-56703141. Max. coverage (+): 0. Max coverage (-): 0

Region: chr5 56703142-56703152. Max. coverage (+): 0. Max coverage (-): 0

Region: chr5 56703153-56703164. Max. coverage (+): 0. Max coverage (-): 0

Region: chr5 56703165-56703175. Max. coverage (+): 0. Max coverage (-): 0

Region: chr5 56703176-56703187. Max. coverage (+): 0. Max coverage (-): 0

Region: chr5 56703188-56703198. Max. coverage (+): 0. Max coverage (-): 0

Region: chr5 56703199-56703210. Max. coverage (+): 0. Max coverage (-): 0

Region: chr5 56703211-56703221. Max. coverage (+): 0. Max coverage (-): 0

Region: chr5 56703222-56703233. Max. coverage (+): 0. Max coverage (-): 0

Region: chr5 56703234-56703244. Max. coverage (+): 0. Max coverage (-): 0

Region: chr5 56703245-56703256. Max. coverage (+): 0. Max coverage (-): 0

Region: chr5 56703257-56703267. Max. coverage (+): 0. Max coverage (-): 0

Region: chr5 56703268-56703279. Max. coverage (+): 0. Max coverage (-): 0

Region: chr5 56703280-56703291. Max. coverage (+): 0. Max coverage (-): 0

Region: chr5 56703292-56703302. Max. coverage (+): 0. Max coverage (-): 0

Region: chr5 56703303-56703314. Max. coverage (+): 0. Max coverage (-): 0

Region: chr5 56703315-56703325. Max. coverage (+): 0. Max coverage (-): 0

Region: chr5 56703326-56703337. Max. coverage (+): 0. Max coverage (-): 0

Region: chr5 56703338-56703348. Max. coverage (+): 0. Max coverage (-): 0

Region: chr5 56703349-56703360. Max. coverage (+): 0. Max coverage (-): 0

Region: chr5 56703361-56703371. Max. coverage (+): 0. Max coverage (-): 0

Region: chr5 56703372-56703383. Max. coverage (+): 0. Max coverage (-): 0

Region: chr5 56703384-56703394. Max. coverage (+): 0. Max coverage (-): 0

Region: chr5 56703395-56703406. Max. coverage (+): 0. Max coverage (-): 0

Region: chr5 56703407-56703417. Max. coverage (+): 0. Max coverage (-): 0

Region: chr5 56703418-56703429. Max. coverage (+): 0. Max coverage (-): 0

Region: chr5 56703430-56703440. Max. coverage (+): 0. Max coverage (-): 0

Region: chr5 56703441-56703452. Max. coverage (+): 0. Max coverage (-): 0

Region: chr5 56703453-56703463. Max. coverage (+): 0. Max coverage (-): 0

Region: chr5 56703464-56703475. Max. coverage (+): 0. Max coverage (-): 0

Region: chr5 56703476-56703487. Max. coverage (+): 0. Max coverage (-): 0

Region: chr5 56703488-56703498. Max. coverage (+): 0. Max coverage (-): 0

Region: chr5 56703499-56703510. Max. coverage (+): 0. Max coverage (-): 0

Region: chr5 56703511-56703521. Max. coverage (+): 0. Max coverage (-): 0

Region: chr5 56703522-56703533. Max. coverage (+): 0. Max coverage (-): 0

Region: chr5 56703534-56703544. Max. coverage (+): 0. Max coverage (-): 0

Region: chr5 56703545-56703556. Max. coverage (+): 0. Max coverage (-): 0

Region: chr5 56703557-56703567. Max. coverage (+): 0. Max coverage (-): 0

Region: chr5 56703568-56703579. Max. coverage (+): 0. Max coverage (-): 0

Region: chr5 56703580-56703590. Max. coverage (+): 0. Max coverage (-): 0

Region: chr5 56703591-56703602. Max. coverage (+): 0. Max coverage (-): 0

Region: chr5 56703603-56703613. Max. coverage (+): 0. Max coverage (-): 0

Region: chr5 56703614-56703625. Max. coverage (+): 0. Max coverage (-): 0

Region: chr5 56703626-56703636. Max. coverage (+): 0. Max coverage (-): 0

Region: chr5 56703637-56703648. Max. coverage (+): 0. Max coverage (-): 0

Region: chr5 56703649-56703659. Max. coverage (+): 0. Max coverage (-): 0

Region: chr5 56703660-56703671. Max. coverage (+): 0. Max coverage (-): 0

Region: chr5 56703672-56703683. Max. coverage (+): 0. Max coverage (-): 0

Region: chr5 56703684-56703694. Max. coverage (+): 0. Max coverage (-): 0

Region: chr5 56703695-56703706. Max. coverage (+): 0. Max coverage (-): 0

Region: chr5 56703707-56703717. Max. coverage (+): 0. Max coverage (-): 0

Region: chr5 56703718-56703729. Max. coverage (+): 0. Max coverage (-): 0

Region: chr5 56703730-56703740. Max. coverage (+): 0. Max coverage (-): 0

Region: chr5 56703741-56703752. Max. coverage (+): 0. Max coverage (-): 0

Region: chr5 56703753-56703763. Max. coverage (+): 0. Max coverage (-): 0

Region: chr5 56703764-56703775. Max. coverage (+): 0. Max coverage (-): 0

Region: chr5 56703776-56703786. Max. coverage (+): 3.88. Max coverage (-): 0

Region: chr5 56703787-56703798. Max. coverage (+): 0. Max coverage (-): 0

Region: chr5 56703799-56703809. Max. coverage (+): 0. Max coverage (-): 0

Region: chr5 56703810-56703821. Max. coverage (+): 7.17. Max coverage (-): 0

Region: chr5 56703822-56703832. Max. coverage (+): 7.17. Max coverage (-): 0

Region: chr5 56703833-56703844. Max. coverage (+): 0. Max coverage (-): 0

Region: chr5 56703845-56703855. Max. coverage (+): 0. Max coverage (-): 0

Region: chr5 56703856-56703867. Max. coverage (+): 0. Max coverage (-): 0

Region: chr5 56703868-56703879. Max. coverage (+): 0. Max coverage (-): 0

Region: chr5 56703880-56703890. Max. coverage (+): 6.39. Max coverage (-): 0

Region: chr5 56703891-56703902. Max. coverage (+): 0. Max coverage (-): 0

Region: chr5 56703903-56703913. Max. coverage (+): 0. Max coverage (-): 0

Region: chr5 56703914-56703925. Max. coverage (+): 0. Max coverage (-): 0

Region: chr5 56703926-56703936. Max. coverage (+): 0. Max coverage (-): 0

Region: chr5 56703937-56703948. Max. coverage (+): 0. Max coverage (-): 0

Region: chr5 56703949-56703959. Max. coverage (+): 0. Max coverage (-): 0

Region: chr5 56703960-56703971. Max. coverage (+): 0. Max coverage (-): 0

Region: chr5 56703972-56703982. Max. coverage (+): 0. Max coverage (-): 0

Region: chr5 56703983-56703994. Max. coverage (+): 0. Max coverage (-): 0

Region: chr5 56703995-56704005. Max. coverage (+): 0. Max coverage (-): 0

Region: chr5 56704006-56704017. Max. coverage (+): 0. Max coverage (-): 0

Region: chr5 56704018-56704028. Max. coverage (+): 0. Max coverage (-): 0

Region: chr5 56704029-56704040. Max. coverage (+): 0. Max coverage (-): 0

Region: chr5 56704041-56704052. Max. coverage (+): 0. Max coverage (-): 0

Region: chr5 56704053-56704063. Max. coverage (+): 0. Max coverage (-): 0

Region: chr5 56704064-56704075. Max. coverage (+): 0. Max coverage (-): 0

Region: chr5 56704076-56704086. Max. coverage (+): 0. Max coverage (-): 0

Region: chr5 56704087-56704098. Max. coverage (+): 0. Max coverage (-): 0

Region: chr5 56704099-56704109. Max. coverage (+): 0. Max coverage (-): 0

Region: chr5 56704110-56704121. Max. coverage (+): 0. Max coverage (-): 0

Region: chr5 56704122-56704132. Max. coverage (+): 0. Max coverage (-): 0

Region: chr5 56704133-56704144. Max. coverage (+): 0. Max coverage (-): 0

Region: chr5 56704145-56704155. Max. coverage (+): 0. Max coverage (-): 0

Region: chr5 56704156-56704167. Max. coverage (+): 0. Max coverage (-): 0

Region: chr5 56704168-56704178. Max. coverage (+): 0. Max coverage (-): 0

Region: chr5 56704179-56704190. Max. coverage (+): 0. Max coverage (-): 0

Region: chr5 56704191-56704201. Max. coverage (+): 0. Max coverage (-): 0

Region: chr5 56704202-56704213. Max. coverage (+): 0. Max coverage (-): 0

Region: chr5 56704214-56704224. Max. coverage (+): 0. Max coverage (-): 0

Region: chr5 56704225-56704236. Max. coverage (+): 0. Max coverage (-): 0

Region: chr5 56704237-56704248. Max. coverage (+): 0. Max coverage (-): 0

Region: chr5 56704249-56704259. Max. coverage (+): 0. Max coverage (-): 0

Region: chr5 56704260-56704271. Max. coverage (+): 0. Max coverage (-): 0

Region: chr5 56704272-56704282. Max. coverage (+): 0. Max coverage (-): 0

Region: chr5 56704283-56704294. Max. coverage (+): 0. Max coverage (-): 0

Region: chr5 56704295-56704305. Max. coverage (+): 0. Max coverage (-): 0

Region: chr5 56704306-56704317. Max. coverage (+): 0. Max coverage (-): 0

Region: chr5 56704318-56704328. Max. coverage (+): 0. Max coverage (-): 0

Region: chr5 56704329-56704340. Max. coverage (+): 0. Max coverage (-): 0

Region: chr5 56704341-56704351. Max. coverage (+): 0. Max coverage (-): 0

Region: chr5 56704352-56704363. Max. coverage (+): 0. Max coverage (-): 0

Region: chr5 56704364-56704374. Max. coverage (+): 0. Max coverage (-): 0

Region: chr5 56704375-56704386. Max. coverage (+): 0. Max coverage (-): 0

Region: chr5 56704387-56704397. Max. coverage (+): 0. Max coverage (-): 0

Region: chr5 56704398-56704409. Max. coverage (+): 0. Max coverage (-): 0

Region: chr5 56704410-56704420. Max. coverage (+): 0. Max coverage (-): 0

Region: chr5 56704421-56704432. Max. coverage (+): 0. Max coverage (-): 0

Region: chr5 56704433-56704444. Max. coverage (+): 0. Max coverage (-): 0

Region: chr5 56704445-56704455. Max. coverage (+): 0. Max coverage (-): 0

Region: chr5 56704456-56704467. Max. coverage (+): 0. Max coverage (-): 0

Region: chr5 56704468-56704478. Max. coverage (+): 0. Max coverage (-): 0

Region: chr5 56704479-56704490. Max. coverage (+): 0. Max coverage (-): 0

Region: chr5 56704491-56704501. Max. coverage (+): 0. Max coverage (-): 0

Region: chr5 56704502-56704513. Max. coverage (+): 0. Max coverage (-): 0

Region: chr5 56704514-56704524. Max. coverage (+): 0. Max coverage (-): 0

Region: chr5 56704525-56704536. Max. coverage (+): 0. Max coverage (-): 0

Region: chr5 56704537-56704547. Max. coverage (+): 0. Max coverage (-): 0

Region: chr5 56704548-56704559. Max. coverage (+): 0. Max coverage (-): 0

Region: chr5 56704560-56704570. Max. coverage (+): 0. Max coverage (-): 0

Region: chr5 56704571-56704582. Max. coverage (+): 0. Max coverage (-): 0

Region: chr5 56704583-56704593. Max. coverage (+): 0. Max coverage (-): 0

Region: chr5 56704594-56704605. Max. coverage (+): 0. Max coverage (-): 0

Region: chr5 56704606-56704616. Max. coverage (+): 0. Max coverage (-): 0

Region: chr5 56704617-56704628. Max. coverage (+): 0. Max coverage (-): 0

Region: chr5 56704629-56704640. Max. coverage (+): 0. Max coverage (-): 0

Region: chr5 56704641-56704651. Max. coverage (+): 0. Max coverage (-): 0

Region: chr5 56704652-56704663. Max. coverage (+): 0. Max coverage (-): 0

Region: chr5 56704664-56704674. Max. coverage (+): 0. Max coverage (-): 0

Region: chr5 56704675-56704686. Max. coverage (+): 0. Max coverage (-): 0

Region: chr5 56704687-56704697. Max. coverage (+): 0. Max coverage (-): 0

Region: chr5 56704698-56704709. Max. coverage (+): 0. Max coverage (-): 0

Region: chr5 56704710-56704720. Max. coverage (+): 0. Max coverage (-): 0

Region: chr5 56704721-56704732. Max. coverage (+): 0. Max coverage (-): 0

Region: chr5 56704733-56704743. Max. coverage (+): 0. Max coverage (-): 0

Region: chr5 56704744-56704755. Max. coverage (+): 0. Max coverage (-): 0

Region: chr5 56704756-56704766. Max. coverage (+): 0. Max coverage (-): 0

Region: chr5 56704767-56704778. Max. coverage (+): 0. Max coverage (-): 0

Region: chr5 56704779-56704789. Max. coverage (+): 0. Max coverage (-): 0

Region: chr5 56704790-56704801. Max. coverage (+): 0. Max coverage (-): 0

Region: chr5 56704802-56704812. Max. coverage (+): 0. Max coverage (-): 0

Region: chr5 56704813-56704824. Max. coverage (+): 0. Max coverage (-): 0

Region: chr5 56704825-56704836. Max. coverage (+): 1.46. Max coverage (-): 0

Region: chr5 56704837-56704847. Max. coverage (+): 0.55. Max coverage (-): 0

Region: chr5 56704848-56704859. Max. coverage (+): 1.27. Max coverage (-): 0

Region: chr5 56704860-56704870. Max. coverage (+): 0. Max coverage (-): 0

Region: chr5 56704871-56704882. Max. coverage (+): 1.13. Max coverage (-): 0

Region: chr5 56704883-56704893. Max. coverage (+): 1.13. Max coverage (-): 0

Region: chr5 56704894-56704905. Max. coverage (+): 0. Max coverage (-): 0

Region: chr5 56704906-56704916. Max. coverage (+): 0. Max coverage (-): 0

Region: chr5 56704917-56704928. Max. coverage (+): 0. Max coverage (-): 0

Region: chr5 56704929-56704939. Max. coverage (+): 0. Max coverage (-): 0

Region: chr5 56704940-56704951. Max. coverage (+): 0. Max coverage (-): 0

Region: chr5 56704952-56704962. Max. coverage (+): 0. Max coverage (-): 0

Region: chr5 56704963-56704974. Max. coverage (+): 7.64. Max coverage (-): 0

Region: chr5 56704975-56704985. Max. coverage (+): 0. Max coverage (-): 0

Region: chr5 56704986-56704997. Max. coverage (+): 0. Max coverage (-): 0

Region: chr5 56704998-56705008. Max. coverage (+): 0. Max coverage (-): 0

Region: chr5 56705009-56705020. Max. coverage (+): 0. Max coverage (-): 0

Region: chr5 56705021-56705032. Max. coverage (+): 0. Max coverage (-): 0

Region: chr5 56705033-56705043. Max. coverage (+): 0. Max coverage (-): 0

Region: chr5 56705044-56705055. Max. coverage (+): 0. Max coverage (-): 0

Region: chr5 56705056-56705066. Max. coverage (+): 0. Max coverage (-): 0

Region: chr5 56705067-56705078. Max. coverage (+): 4.46. Max coverage (-): 0

Region: chr5 56705079-56705089. Max. coverage (+): 0. Max coverage (-): 0

Region: chr5 56705090-56705101. Max. coverage (+): 0. Max coverage (-): 0

Region: chr5 56705102-56705112. Max. coverage (+): 11.16. Max coverage (-): 0

Region: chr5 56705113-56705124. Max. coverage (+): 11.16. Max coverage (-): 0

Region: chr5 56705125-56705135. Max. coverage (+): 0. Max coverage (-): 0

Region: chr5 56705136-56705147. Max. coverage (+): 0. Max coverage (-): 0

Region: chr5 56705148-56705158. Max. coverage (+): 0. Max coverage (-): 0

Region: chr5 56705159-56705170. Max. coverage (+): 0. Max coverage (-): 0

Region: chr5 56705171-56705181. Max. coverage (+): 0. Max coverage (-): 0

Region: chr5 56705182-56705193. Max. coverage (+): 0. Max coverage (-): 0

Region: chr5 56705194-56705205. Max. coverage (+): 4.41. Max coverage (-): 0

Region: chr5 56705206-56705216. Max. coverage (+): 0. Max coverage (-): 0

Region: chr5 56705217-56705228. Max. coverage (+): 0. Max coverage (-): 0

Region: chr5 56705229-56705239. Max. coverage (+): 0. Max coverage (-): 0

Region: chr5 56705240-56705251. Max. coverage (+): 0. Max coverage (-): 0

Region: chr5 56705252-56705262. Max. coverage (+): 0. Max coverage (-): 0

Region: chr5 56705263-56705274. Max. coverage (+): 0. Max coverage (-): 0

Region: chr5 56705275-56705285. Max. coverage (+): 0. Max coverage (-): 0

Region: chr5 56705286-56705297. Max. coverage (+): 1.69. Max coverage (-): 0

Region: chr5 56705298-56705308. Max. coverage (+): 1.69. Max coverage (-): 0

Region: chr5 56705309-56705320. Max. coverage (+): 0. Max coverage (-): 0

Region: chr5 56705321-56705331. Max. coverage (+): 0. Max coverage (-): 0

Region: chr5 56705332-56705343. Max. coverage (+): 0. Max coverage (-): 0

Region: chr5 56705344-56705354. Max. coverage (+): 0. Max coverage (-): 0

Region: chr5 56705355-56705366. Max. coverage (+): 0. Max coverage (-): 0

Region: chr5 56705367-56705377. Max. coverage (+): 0. Max coverage (-): 0

Region: chr5 56705378-56705389. Max. coverage (+): 0. Max coverage (-): 0

Region: chr5 56705390-56705401. Max. coverage (+): 0. Max coverage (-): 0

Region: chr5 56705402-56705412. Max. coverage (+): 0. Max coverage (-): 0

Region: chr5 56705413-56705424. Max. coverage (+): 0. Max coverage (-): 0

Region: chr5 56705425-56705435. Max. coverage (+): 0. Max coverage (-): 0

Region: chr5 56705436-56705447. Max. coverage (+): 0. Max coverage (-): 0

Region: chr5 56705448-56705458. Max. coverage (+): 0. Max coverage (-): 0

Region: chr5 56705459-56705470. Max. coverage (+): 0. Max coverage (-): 0

Region: chr5 56705471-56705481. Max. coverage (+): 0. Max coverage (-): 0

Region: chr5 56705482-56705493. Max. coverage (+): 0. Max coverage (-): 0

Region: chr5 56705494-56705504. Max. coverage (+): 0. Max coverage (-): 0

Region: chr5 56705505-56705516. Max. coverage (+): 0. Max coverage (-): 0

Region: chr5 56705517-56705527. Max. coverage (+): 0. Max coverage (-): 0

Region: chr5 56705528-56705539. Max. coverage (+): 0. Max coverage (-): 0

Region: chr5 56705540-56705550. Max. coverage (+): 0. Max coverage (-): 0

Region: chr5 56705551-56705562. Max. coverage (+): 0. Max coverage (-): 0

Region: chr5 56705563-56705573. Max. coverage (+): 2.13. Max coverage (-): 0

Region: chr5 56705574-56705585. Max. coverage (+): 2.13. Max coverage (-): 0

Region: chr5 56705586-56705597. Max. coverage (+): 2.78. Max coverage (-): 0

Region: chr5 56705598-56705608. Max. coverage (+): 2.78. Max coverage (-): 0

Region: chr5 56705609-56705620. Max. coverage (+): 1.62. Max coverage (-): 0

Region: chr5 56705621-56705631. Max. coverage (+): 1.62. Max coverage (-): 0

Region: chr5 56705632-56705643. Max. coverage (+): 0. Max coverage (-): 0

Region: chr5 56705644-56705654. Max. coverage (+): 0. Max coverage (-): 0

Region: chr5 56705655-56705666. Max. coverage (+): 0. Max coverage (-): 0

Region: chr5 56705667-56705677. Max. coverage (+): 0. Max coverage (-): 0

Region: chr5 56705678-56705689. Max. coverage (+): 0. Max coverage (-): 0

Region: chr5 56705690-56705700. Max. coverage (+): 0. Max coverage (-): 0

Region: chr5 56705701-56705712. Max. coverage (+): 0. Max coverage (-): 0

Region: chr5 56705713-56705723. Max. coverage (+): 0. Max coverage (-): 0

Region: chr5 56705724-56705735. Max. coverage (+): 0. Max coverage (-): 0

Region: chr5 56705736-56705746. Max. coverage (+): 0. Max coverage (-): 0

Region: chr5 56705747-56705758. Max. coverage (+): 0. Max coverage (-): 0

Region: chr5 56705759-56705769. Max. coverage (+): 0. Max coverage (-): 0

Region: chr5 56705770-56705781. Max. coverage (+): 0. Max coverage (-): 0

Region: chr5 56705782-56705793. Max. coverage (+): 0.78. Max coverage (-): 0

Region: chr5 56705794-56705804. Max. coverage (+): 0.78. Max coverage (-): 0

Region: chr5 56705805-56705816. Max. coverage (+): 0. Max coverage (-): 0

Region: chr5 56705817-56705827. Max. coverage (+): 0. Max coverage (-): 0

Region: chr5 56705828-56705839. Max. coverage (+): 0. Max coverage (-): 0

Region: chr5 56705840-56705850. Max. coverage (+): 0. Max coverage (-): 0

Region: chr5 56705851-56705862. Max. coverage (+): 0. Max coverage (-): 0

Region: chr5 56705863-56705873. Max. coverage (+): 0. Max coverage (-): 0

Region: chr5 56705874-56705885. Max. coverage (+): 0. Max coverage (-): 0

Region: chr5 56705886-56705896. Max. coverage (+): 3.49. Max coverage (-): 0

Region: chr5 56705897-56705908. Max. coverage (+): 3.49. Max coverage (-): 0

Region: chr5 56705909-56705919. Max. coverage (+): 0. Max coverage (-): 0

Region: chr5 56705920-56705931. Max. coverage (+): 0. Max coverage (-): 0

Region: chr5 56705932-56705942. Max. coverage (+): 0. Max coverage (-): 0

Region: chr5 56705943-56705954. Max. coverage (+): 0. Max coverage (-): 0

Region: chr5 56705955-56705965. Max. coverage (+): 0. Max coverage (-): 0

Region: chr5 56705966-56705977. Max. coverage (+): 0. Max coverage (-): 0

Region: chr5 56705978-56705989. Max. coverage (+): 0. Max coverage (-): 0

Region: chr5 56705990-56706000. Max. coverage (+): 0. Max coverage (-): 0

Region: chr5 56706001-56706012. Max. coverage (+): 0. Max coverage (-): 0

Region: chr5 56706013-56706023. Max. coverage (+): 0. Max coverage (-): 0

Region: chr5 56706024-56706035. Max. coverage (+): 0. Max coverage (-): 0

Region: chr5 56706036-56706046. Max. coverage (+): 0. Max coverage (-): 0

Region: chr5 56706047-56706058. Max. coverage (+): 0. Max coverage (-): 0

Region: chr5 56706059-56706069. Max. coverage (+): 0. Max coverage (-): 0

Region: chr5 56706070-56706081. Max. coverage (+): 0.54. Max coverage (-): 0

Region: chr5 56706082-56706092. Max. coverage (+): 0. Max coverage (-): 0

Region: chr5 56706093-56706104. Max. coverage (+): 0. Max coverage (-): 0

Region: chr5 56706105-56706115. Max. coverage (+): 0. Max coverage (-): 0

Region: chr5 56706116-56706127. Max. coverage (+): 0. Max coverage (-): 0

Region: chr5 56706128-56706138. Max. coverage (+): 0. Max coverage (-): 0

Region: chr5 56706139-56706150. Max. coverage (+): 0. Max coverage (-): 0

Region: chr5 56706151-56706161. Max. coverage (+): 2.98. Max coverage (-): 0

Region: chr5 56706162-56706173. Max. coverage (+): 0. Max coverage (-): 0

Region: chr5 56706174-56706185. Max. coverage (+): 0. Max coverage (-): 0

Region: chr5 56706186-56706196. Max. coverage (+): 0. Max coverage (-): 0

Region: chr5 56706197-56706208. Max. coverage (+): 7.33. Max coverage (-): 0

Region: chr5 56706209-56706219. Max. coverage (+): 1.26. Max coverage (-): 0

Region: chr5 56706220-56706231. Max. coverage (+): 1.67. Max coverage (-): 0

Region: chr5 56706232-56706242. Max. coverage (+): 0. Max coverage (-): 0

Region: chr5 56706243-56706254. Max. coverage (+): 0. Max coverage (-): 0

Region: chr5 56706255-56706265. Max. coverage (+): 0. Max coverage (-): 0

Region: chr5 56706266-56706277. Max. coverage (+): 0. Max coverage (-): 0

Region: chr5 56706278-56706288. Max. coverage (+): 0. Max coverage (-): 0

Region: chr5 56706289-56706300. Max. coverage (+): 0. Max coverage (-): 0

Region: chr5 56706301-56706311. Max. coverage (+): 0. Max coverage (-): 0

Region: chr5 56706312-56706323. Max. coverage (+): 0. Max coverage (-): 0

Region: chr5 56706324-56706334. Max. coverage (+): 0. Max coverage (-): 0

Region: chr5 56706335-56706346. Max. coverage (+): 0. Max coverage (-): 0

Region: chr5 56706347-56706358. Max. coverage (+): 0. Max coverage (-): 0

Region: chr5 56706359-56706369. Max. coverage (+): 0. Max coverage (-): 0

Region: chr5 56706370-56706381. Max. coverage (+): 16.91. Max coverage (-): 0

Region: chr5 56706382-56706392. Max. coverage (+): 7.52. Max coverage (-): 0

Region: chr5 56706393-56706404. Max. coverage (+): 0. Max coverage (-): 0

Region: chr5 56706405-56706415. Max. coverage (+): 0. Max coverage (-): 0

Region: chr5 56706416-56706427. Max. coverage (+): 0. Max coverage (-): 0

Region: chr5 56706428-56706438. Max. coverage (+): 0. Max coverage (-): 0

Region: chr5 56706439-56706450. Max. coverage (+): 0. Max coverage (-): 0

Region: chr5 56706451-56706461. Max. coverage (+): 0. Max coverage (-): 0

Region: chr5 56706462-56706473. Max. coverage (+): 0. Max coverage (-): 0

Region: chr5 56706474-56706484. Max. coverage (+): 0. Max coverage (-): 0

Region: chr5 56706485-56706496. Max. coverage (+): 0. Max coverage (-): 0

Region: chr5 56706497-56706507. Max. coverage (+): 0. Max coverage (-): 0

Region: chr5 56706508-56706519. Max. coverage (+): 0. Max coverage (-): 0

Region: chr5 56706520-56706530. Max. coverage (+): 0.83. Max coverage (-): 0

Region: chr5 56706531-56706542. Max. coverage (+): 0.83. Max coverage (-): 0

Region: chr5 56706543-56706554. Max. coverage (+): 0. Max coverage (-): 0

Region: chr5 56706555-56706565. Max. coverage (+): 0. Max coverage (-): 0

Region: chr5 56706566-56706577. Max. coverage (+): 0. Max coverage (-): 0

Region: chr5 56706578-56706588. Max. coverage (+): 0. Max coverage (-): 0

Region: chr5 56706589-56706600. Max. coverage (+): 0. Max coverage (-): 0

Region: chr5 56706601-56706611. Max. coverage (+): 0. Max coverage (-): 0

Region: chr5 56706612-56706623. Max. coverage (+): 0. Max coverage (-): 0

Region: chr5 56706624-56706634. Max. coverage (+): 0. Max coverage (-): 0

Region: chr5 56706635-56706646. Max. coverage (+): 2.57. Max coverage (-): 0

Region: chr5 56706647-56706657. Max. coverage (+): 0. Max coverage (-): 0

Region: chr5 56706658-56706669. Max. coverage (+): 0. Max coverage (-): 0

Region: chr5 56706670-56706680. Max. coverage (+): 0. Max coverage (-): 0

Region: chr5 56706681-56706692. Max. coverage (+): 0. Max coverage (-): 0

Region: chr5 56706693-56706703. Max. coverage (+): 0. Max coverage (-): 0

Region: chr5 56706704-56706715. Max. coverage (+): 0. Max coverage (-): 0

Region: chr5 56706716-56706726. Max. coverage (+): 0. Max coverage (-): 0

Region: chr5 56706727-56706738. Max. coverage (+): 0. Max coverage (-): 0

Region: chr5 56706739-56706750. Max. coverage (+): 0. Max coverage (-): 0

Region: chr5 56706751-56706761. Max. coverage (+): 0. Max coverage (-): 0

Region: chr5 56706762-56706773. Max. coverage (+): 0. Max coverage (-): 0

Region: chr5 56706774-56706784. Max. coverage (+): 0. Max coverage (-): 0

Region: chr5 56706785-56706796. Max. coverage (+): 0. Max coverage (-): 0

Region: chr5 56706797-56706807. Max. coverage (+): 0. Max coverage (-): 0

Region: chr5 56706808-56706819. Max. coverage (+): 0. Max coverage (-): 0

Region: chr5 56706820-56706830. Max. coverage (+): 0. Max coverage (-): 0

Region: chr5 56706831-56706842. Max. coverage (+): 0. Max coverage (-): 0

Region: chr5 56706843-56706853. Max. coverage (+): 0. Max coverage (-): 0

Region: chr5 56706854-56706865. Max. coverage (+): 0. Max coverage (-): 0

Region: chr5 56706866-56706876. Max. coverage (+): 0. Max coverage (-): 0

Region: chr5 56706877-56706888. Max. coverage (+): 0. Max coverage (-): 0

Region: chr5 56706889-56706899. Max. coverage (+): 0. Max coverage (-): 0

Region: chr5 56706900-56706911. Max. coverage (+): 0. Max coverage (-): 0

Region: chr5 56706912-56706922. Max. coverage (+): 0. Max coverage (-): 0

Region: chr5 56706923-56706934. Max. coverage (+): 0. Max coverage (-): 0

Region: chr5 56706935-56706946. Max. coverage (+): 0. Max coverage (-): 0

Region: chr5 56706947-56706957. Max. coverage (+): 0. Max coverage (-): 0

Region: chr5 56706958-56706969. Max. coverage (+): 0. Max coverage (-): 0

Region: chr5 56706970-56706980. Max. coverage (+): 0. Max coverage (-): 0

Region: chr5 56706981-56706992. Max. coverage (+): 0. Max coverage (-): 0

Region: chr5 56706993-56707003. Max. coverage (+): 0. Max coverage (-): 0

Region: chr5 56707004-56707015. Max. coverage (+): 0. Max coverage (-): 0

Region: chr5 56707016-56707026. Max. coverage (+): 0. Max coverage (-): 0

Region: chr5 56707027-56707038. Max. coverage (+): 0. Max coverage (-): 0

Region: chr5 56707039-56707049. Max. coverage (+): 0. Max coverage (-): 0

Region: chr5 56707050-56707061. Max. coverage (+): 0. Max coverage (-): 0

Region: chr5 56707062-56707072. Max. coverage (+): 0. Max coverage (-): 0

Region: chr5 56707073-56707084. Max. coverage (+): 0. Max coverage (-): 0

Region: chr5 56707085-56707095. Max. coverage (+): 0. Max coverage (-): 0

Region: chr5 56707096-56707107. Max. coverage (+): 0. Max coverage (-): 0

Region: chr5 56707108-56707118. Max. coverage (+): 13.12. Max coverage (-): 0

Region: chr5 56707119-56707130. Max. coverage (+): 13.12. Max coverage (-): 0

Region: chr5 56707131-56707142. Max. coverage (+): 0. Max coverage (-): 0

Region: chr5 56707143-56707153. Max. coverage (+): 0. Max coverage (-): 0

Region: chr5 56707154-56707165. Max. coverage (+): 2.25. Max coverage (-): 0

Region: chr5 56707166-56707176. Max. coverage (+): 2.25. Max coverage (-): 0

Region: chr5 56707177-56707188. Max. coverage (+): 0. Max coverage (-): 0

Region: chr5 56707189-56707199. Max. coverage (+): 0. Max coverage (-): 0

Region: chr5 56707200-56707211. Max. coverage (+): 0. Max coverage (-): 0

Region: chr5 56707212-56707222. Max. coverage (+): 0. Max coverage (-): 0

Region: chr5 56707223-56707234. Max. coverage (+): 0. Max coverage (-): 0

Region: chr5 56707235-56707245. Max. coverage (+): 1.66. Max coverage (-): 0

Region: chr5 56707246-56707257. Max. coverage (+): 0. Max coverage (-): 0

Region: chr5 56707258-56707268. Max. coverage (+): 0. Max coverage (-): 0

Region: chr5 56707269-56707280. Max. coverage (+): 0. Max coverage (-): 0

Region: chr5 56707281-56707291. Max. coverage (+): 0. Max coverage (-): 0

Region: chr5 56707292-56707303. Max. coverage (+): 0. Max coverage (-): 0

Region: chr5 56707304-56707314. Max. coverage (+): 0. Max coverage (-): 0

Region: chr5 56707315-56707326. Max. coverage (+): 0. Max coverage (-): 0

Region: chr5 56707327-56707338. Max. coverage (+): 0. Max coverage (-): 0

Region: chr5 56707339-56707349. Max. coverage (+): 1.78. Max coverage (-): 0

Region: chr5 56707350-56707361. Max. coverage (+): 0. Max coverage (-): 0

Region: chr5 56707362-56707372. Max. coverage (+): 0. Max coverage (-): 0

Region: chr5 56707373-56707384. Max. coverage (+): 0. Max coverage (-): 0

Region: chr5 56707385-56707395. Max. coverage (+): 0. Max coverage (-): 0

Region: chr5 56707396-56707407. Max. coverage (+): 0. Max coverage (-): 0

Region: chr5 56707408-56707418. Max. coverage (+): 0. Max coverage (-): 0

Region: chr5 56707419-56707430. Max. coverage (+): 0. Max coverage (-): 0

Region: chr5 56707431-56707441. Max. coverage (+): 0. Max coverage (-): 0

Region: chr5 56707442-56707453. Max. coverage (+): 0. Max coverage (-): 0

Region: chr5 56707454-56707464. Max. coverage (+): 0. Max coverage (-): 0

Region: chr5 56707465-56707476. Max. coverage (+): 0. Max coverage (-): 0

Region: chr5 56707477-56707487. Max. coverage (+): 0. Max coverage (-): 0

Region: chr5 56707488-56707499. Max. coverage (+): 0. Max coverage (-): 0

Region: chr5 56707500-56707511. Max. coverage (+): 0. Max coverage (-): 0

Region: chr5 56707512-56707522. Max. coverage (+): 0. Max coverage (-): 0

Region: chr5 56707523-56707534. Max. coverage (+): 0. Max coverage (-): 0

Region: chr5 56707535-56707545. Max. coverage (+): 0. Max coverage (-): 0

Region: chr5 56707546-56707557. Max. coverage (+): 0. Max coverage (-): 0

Region: chr5 56707558-56707568. Max. coverage (+): 0. Max coverage (-): 0

Region: chr5 56707569-56707580. Max. coverage (+): 0. Max coverage (-): 0

Region: chr5 56707581-56707591. Max. coverage (+): 0. Max coverage (-): 0

Region: chr5 56707592-56707603. Max. coverage (+): 0. Max coverage (-): 0

Region: chr5 56707604-56707614. Max. coverage (+): 0. Max coverage (-): 0

Region: chr5 56707615-56707626. Max. coverage (+): 0. Max coverage (-): 0

Region: chr5 56707627-56707637. Max. coverage (+): 0. Max coverage (-): 0

Region: chr5 56707638-56707649. Max. coverage (+): 0. Max coverage (-): 0

Region: chr5 56707650-56707660. Max. coverage (+): 0. Max coverage (-): 0

Region: chr5 56707661-56707672. Max. coverage (+): 0. Max coverage (-): 0

Region: chr5 56707673-56707683. Max. coverage (+): 0. Max coverage (-): 0

Region: chr5 56707684-56707695. Max. coverage (+): 0. Max coverage (-): 0

Region: chr5 56707696-56707707. Max. coverage (+): 0. Max coverage (-): 0

Region: chr5 56707708-56707718. Max. coverage (+): 0. Max coverage (-): 0

Region: chr5 56707719-56707730. Max. coverage (+): 0. Max coverage (-): 0

Region: chr5 56707731-56707741. Max. coverage (+): 0. Max coverage (-): 0

Region: chr5 56707742-56707753. Max. coverage (+): 0. Max coverage (-): 0

Region: chr5 56707754-56707764. Max. coverage (+): 0. Max coverage (-): 0

Region: chr5 56707765-56707776. Max. coverage (+): 0. Max coverage (-): 0

Region: chr5 56707777-56707787. Max. coverage (+): 0. Max coverage (-): 0

Region: chr5 56707788-56707799. Max. coverage (+): 0. Max coverage (-): 0

Region: chr5 56707800-56707810. Max. coverage (+): 0. Max coverage (-): 0

Region: chr5 56707811-56707822. Max. coverage (+): 3.77. Max coverage (-): 0

Region: chr5 56707823-56707833. Max. coverage (+): 3.77. Max coverage (-): 0

Region: chr5 56707834-56707845. Max. coverage (+): 0. Max coverage (-): 0

Region: chr5 56707846-56707856. Max. coverage (+): 0. Max coverage (-): 0

Region: chr5 56707857-56707868. Max. coverage (+): 0. Max coverage (-): 0

Region: chr5 56707869-56707879. Max. coverage (+): 0. Max coverage (-): 0

Region: chr5 56707880-56707891. Max. coverage (+): 0. Max coverage (-): 0

Region: chr5 56707892-56707903. Max. coverage (+): 0. Max coverage (-): 0

Region: chr5 56707904-56707914. Max. coverage (+): 1.61. Max coverage (-): 0

Region: chr5 56707915-56707926. Max. coverage (+): 1.61. Max coverage (-): 0

Region: chr5 56707927-56707937. Max. coverage (+): 0. Max coverage (-): 0

Region: chr5 56707938-56707949. Max. coverage (+): 0. Max coverage (-): 0

Region: chr5 56707950-56707960. Max. coverage (+): 0. Max coverage (-): 0

Region: chr5 56707961-56707972. Max. coverage (+): 0. Max coverage (-): 0

Region: chr5 56707973-56707983. Max. coverage (+): 0. Max coverage (-): 0

Region: chr5 56707984-56707995. Max. coverage (+): 0. Max coverage (-): 0

Region: chr5 56707996-56708006. Max. coverage (+): 0. Max coverage (-): 0

Region: chr5 56708007-56708018. Max. coverage (+): 1.57. Max coverage (-): 0

Region: chr5 56708019-56708029. Max. coverage (+): 3.61. Max coverage (-): 0

Region: chr5 56708030-56708041. Max. coverage (+): 0. Max coverage (-): 0

Region: chr5 56708042-56708052. Max. coverage (+): 0. Max coverage (-): 0

Region: chr5 56708053-56708064. Max. coverage (+): 0. Max coverage (-): 0

Region: chr5 56708065-56708075. Max. coverage (+): 0. Max coverage (-): 0

Region: chr5 56708076-56708087. Max. coverage (+): 0.62. Max coverage (-): 0

Region: chr5 56708088-56708099. Max. coverage (+): 0.62. Max coverage (-): 0

Region: chr5 56708100-56708110. Max. coverage (+): 0. Max coverage (-): 0

Region: chr5 56708111-56708122. Max. coverage (+): 0. Max coverage (-): 0

Region: chr5 56708123-56708133. Max. coverage (+): 0. Max coverage (-): 0

Region: chr5 56708134-56708145. Max. coverage (+): 0. Max coverage (-): 0

Region: chr5 56708146-56708156. Max. coverage (+): 0. Max coverage (-): 0

Region: chr5 56708157-56708168. Max. coverage (+): 0. Max coverage (-): 0

Region: chr5 56708169-56708179. Max. coverage (+): 0. Max coverage (-): 0

Region: chr5 56708180-56708191. Max. coverage (+): 0. Max coverage (-): 0

Region: chr5 56708192-56708202. Max. coverage (+): 0. Max coverage (-): 0

Region: chr5 56708203-56708214. Max. coverage (+): 0. Max coverage (-): 0

Region: chr5 56708215-56708225. Max. coverage (+): 0. Max coverage (-): 0

Region: chr5 56708226-56708237. Max. coverage (+): 0. Max coverage (-): 0

Region: chr5 56708238-56708248. Max. coverage (+): 0. Max coverage (-): 0

Region: chr5 56708249-56708260. Max. coverage (+): 0. Max coverage (-): 0

Region: chr5 56708261-56708271. Max. coverage (+): 0. Max coverage (-): 0

Region: chr5 56708272-56708283. Max. coverage (+): 0. Max coverage (-): 0

Region: chr5 56708284-56708295. Max. coverage (+): 0. Max coverage (-): 0

Region: chr5 56708296-56708306. Max. coverage (+): 0. Max coverage (-): 0

Region: chr5 56708307-56708318. Max. coverage (+): 0. Max coverage (-): 0

Region: chr5 56708319-56708329. Max. coverage (+): 0. Max coverage (-): 0

Region: chr5 56708330-56708341. Max. coverage (+): 0. Max coverage (-): 0

Region: chr5 56708342-56708352. Max. coverage (+): 0. Max coverage (-): 0

Region: chr5 56708353-56708364. Max. coverage (+): 0. Max coverage (-): 0

Region: chr5 56708365-56708375. Max. coverage (+): 0. Max coverage (-): 0

Region: chr5 56708376-56708387. Max. coverage (+): 0. Max coverage (-): 0

Region: chr5 56708388-56708398. Max. coverage (+): 0. Max coverage (-): 0

Region: chr5 56708399-56708410. Max. coverage (+): 0. Max coverage (-): 0

Region: chr5 56708411-56708421. Max. coverage (+): 0. Max coverage (-): 0

Region: chr5 56708422-56708433. Max. coverage (+): 0. Max coverage (-): 0

Region: chr5 56708434-56708444. Max. coverage (+): 0. Max coverage (-): 0

Region: chr5 56708445-56708456. Max. coverage (+): 0. Max coverage (-): 0

Region: chr5 56708457-56708467. Max. coverage (+): 0. Max coverage (-): 0

Region: chr5 56708468-56708479. Max. coverage (+): 0. Max coverage (-): 0

Region: chr5 56708480-56708491. Max. coverage (+): 0. Max coverage (-): 0

Region: chr5 56708492-56708502. Max. coverage (+): 0. Max coverage (-): 0

Region: chr5 56708503-56708514. Max. coverage (+): 0. Max coverage (-): 0

Region: chr5 56708515-56708525. Max. coverage (+): 0. Max coverage (-): 0

Region: chr5 56708526-56708537. Max. coverage (+): 4.08. Max coverage (-): 0

Region: chr5 56708538-56708548. Max. coverage (+): 4.08. Max coverage (-): 0

Region: chr5 56708549-56708560. Max. coverage (+): 0. Max coverage (-): 0

Region: chr5 56708561-. Max. coverage (+): 0. Max coverage (-): 0

RepeatMasker Color Code

**+**

100-98% Identity

<98-95% Identity

<95-90% Identity

<90-85% Identity

<85-80% Identity

<80-75% Identity

<75-70% Identity

<70% Identity

**-**

Gene Set Color Code

**+**

Gene

Pseudogene

**-**

Topology/Coverage Color Code

Coverage Plus Strand

Coverage Minus Strand

Mainstrand: Plus

Mainstrand: Minus

Complementary Strand

Flanking Region  
(if option -flank >0)

Gene Set Annotation  

**1. NEMP1 (protein coding, ENSBTAG00000014659) Tr:00000019519 Ex:6**: 56702764-56702989 (+)  
**2. NEMP1 (protein coding, ENSBTAG00000014659) Tr:00000019519 Ex:7**: 56703776-56703949 (+)  
**3. NEMP1 (protein coding, ENSBTAG00000014659) Tr:00000019519 Ex:8**: 56704697-56705119 (+)

  
RepeatMasker Annotation  
  
Transcription Factor Binding Sites  

**Gata4** (Sequence: AGATAAC (-): 56703716)  
**Gata4** (Sequence: AGATAAC (-): 56704393)
