# Supplementary material for: Small RNA sequencing of cryopreserved semen from single bull revealed altered miRNAs and piRNAs expression between High- and Low-motile sperm populations
Source: BMC Genomics. 2017 Jan 4;18:14. doi: 10.1186/s12864-016-3394-7 (PMC5209821; doi:10.1186/s12864-016-3394-7)
Supplement: Additional file 3: — Details for each piRNA clusters found in High Motile (HM) sperm fraction. Genes, repeats, transposable elements and transcription factors binding sites falling within the cluster regions were reported. (ZIP 1896 kb) [file 12864_2016_3394_MOESM3_ESM.zip › 86.html]

piRNA cluster 86


Predicted piRNA cluster no. 86     previous   next
  

Show proTRAC run info
Hide proTRAC run info

================================= proTRAC ====================================  
VERSION: 2.1                                    LAST MODIFIED: 06. October 2015  
  
Please cite:  
Rosenkranz D, Zischler H. proTRAC - a software for probabilistic piRNA cluster  
detection, visualization and analysis. 2012. BMC Bioinformatics 13:5.  
  
and (for proTRAC 2.0 and later):  
Rosenkranz D, Rudloff S, Bastuck K, Ketting RF, Zischler H. Tupaia small RNAs  
provide insights into function and evolution of RNAi-based transposon defense  
in mammals. 2015. RNA 21(5):911-922.  
  
Contact:  
David Rosenkranz  
Institute of Anthropology, small RNA group  
Johannes Gutenberg University Mainz  
email: rosenkranz@uni-mainz.de  
  
You can find the latest proTRAC version at:  
http://sourceforge.net/projects/protrac/files  
http://www.smallRNAgroup-mainz.de/software  
==============================================================================  
  
PARAMETERS:  
Map file: .............../storage/core/barbara/genhome/smallRNA/fertility/Sample\_motile/pirna/Sample\_motile\_26-33\_collapsed.fa.no-dust.map.weighted-10000-1000-b-0  
Genome file: ............/storage/core/barbara/genhome/smallRNA/fertility/Sample\_all/pirna/bt\_311\_chrY.fa  
RepeatMasker annotation: /storage/genomes/bt\_umd31/GCF\_000003055.6\_Bos\_taurus\_UMD\_3.1.1\_repeatMasker\_chr.out  
GeneSet:................./storage/core/barbara/genhome/smallRNA/fertility/Sample\_all/pirna/full.gtf  
  
Significant (p<=0.01) hit density will be calculated based  
on observed hit distribution.  
  
Sliding window size: ........................................ 5000 bp  
Sliding window increament: .................................. 1000 bp  
Normalize each hit by number of genomic hits: ............... 1 [0=no/1=yes]  
Normalize each hit by number of sequence reads: ............. 1 [0=no/1=yes]  
Normalize values (-> per million mapped reads): ............. 1 [0=no/1=yes]  
Min. fraction of hits with 1T(U) or 10A: .................... 0.75  
Alternatively: Min. fraction of hits with 1T(U) and 10A: .... 0.5  
Min. fraction of hits with typical piRNA length: ............ 0.75  
Typical piRNA length: ....................................... 26-33 nt  
Min. size of a piRNA cluster: ............................... 5000 bp.  
Min. number of hits (absolute): ............................. 0  
Min. number of hits (normalized): ........................... 0  
Min. fraction of hits on the mainstrand: .................... 0.75  
Top fraction of mapped sequences (in terms of read counts): . 1%  
Top fraction accounts for max. n% of sequence reads: ........ 90%  
Min. fraction of hits on each arm of a bidirectional cluster: 0.1  
Output image file for each cluster: ......................... 0 [0=no/1=yes]  
Output html file for each cluster: .......................... 1 [0=no/1=yes]  
Output a summary table: ..................................... 1 [0=no/1=yes]  
Output a FASTA file for each cluster (piRNA sequences): ..... 1 [0=no/1=yes]  
Output a FASTA file comprising cluster sequences: ........... 1 [0=no/1=yes]  
Search DNA motifs in clusters: .............................. 1 [0=no/1=yes]  
Output flanking sequences: +/- .............................. 0 bp  
Output ~.pTi file: .......................................... 1 [0=no/1=yes]  
==============================================================================  
  
  
Genome size (without gaps): ............ 2678902517 bp  
Gaps (N/X/-): .......................... 53837044 bp  
Mapped reads: .......................... 658825247023  
Non-identical sequences: ............... 514171  
Genomic hits: .......................... 764233  
Significant densitiy of mapped reads: .. 12867599.5173724 reads/kb

Show proTRAC cluster info
Hide proTRAC cluster info

|  |  |
| --- | --- |
| Location | chr5 |
| Coordinates | 56763771-56770303 |
| Size [bp] | 6533 |
| Sequence hit loci | 465 |
| Mapped reads (normalized) | 549455706 |
| Mapped reads (normalized) per kb | 84104654.2 |
| Normalized reads with 1T (1U) | 84.2% |
| Normalized reads with 10A | 30.7% |
| Normalized reads with length 26-33 nt | 100% |
| Normalized reads on the main strand(s) | 100% |
| Predicted directionality | mono:plus |

100%

0%

1T (1U)  
reads

10A reads

26-33 nt  
reads

reads on mainstrand

**Either the amount of reads with 1T (1U) OR 10A has to exceed 75% (set with option: -1Tor10A)  
Alternatively the amount of reads with 1T (1U) AND 10A has to exceed 50% (set with option: -1Tand10A)  
Minimum amount of reads with preferred size is 75% (set with option: -pisize)  
Minimum amount of reads on the main strand(s) is 75% (set with option: -clstrand)**

Show read coverage
Hide read coverage

WHAT DO I SEE HERE?  
This chart shows the location of mapped sequence reads within a predicted piRNA cluster. The color refers to the number of genomic hits produced by the sequence read in question. A dark red bar indicates that this sequence read produces many other hits elsewhere in the genome. Many adjacent red or yellow bars can indicate the presence of a multi-copy element such as transposons or rRNA genes. A dark green bar indicates that this sequence read maps uniquely to this locus.

1 hit

2-5 hits

6-10 hits

11-20 hits

21-50 hits

51-100 hits

> 100 hits

chr5

56763771

56770303

Gene Set

RepeatMasker

Mapped  
Reads

38.71

plus strand

minus strand

38.71

Region: chr5 56708562-56763777. Max. coverage (+): 0.43. Max coverage (-): 0

Region: chr5 56763778-56763790. Max. coverage (+): 0.43. Max coverage (-): 0

Region: chr5 56763791-56763803. Max. coverage (+): 0. Max coverage (-): 0

Region: chr5 56763804-56763816. Max. coverage (+): 0. Max coverage (-): 0

Region: chr5 56763817-56763829. Max. coverage (+): 0. Max coverage (-): 0

Region: chr5 56763830-56763842. Max. coverage (+): 0. Max coverage (-): 0

Region: chr5 56763843-56763855. Max. coverage (+): 0. Max coverage (-): 0

Region: chr5 56763856-56763868. Max. coverage (+): 0. Max coverage (-): 0

Region: chr5 56763869-56763882. Max. coverage (+): 0. Max coverage (-): 0

Region: chr5 56763883-56763895. Max. coverage (+): 4.46. Max coverage (-): 0

Region: chr5 56763896-56763908. Max. coverage (+): 0. Max coverage (-): 0

Region: chr5 56763909-56763921. Max. coverage (+): 0. Max coverage (-): 0

Region: chr5 56763922-56763934. Max. coverage (+): 0. Max coverage (-): 0

Region: chr5 56763935-56763947. Max. coverage (+): 1.63. Max coverage (-): 0

Region: chr5 56763948-56763960. Max. coverage (+): 0. Max coverage (-): 0

Region: chr5 56763961-56763973. Max. coverage (+): 0. Max coverage (-): 0

Region: chr5 56763974-56763986. Max. coverage (+): 0. Max coverage (-): 0

Region: chr5 56763987-56763999. Max. coverage (+): 0. Max coverage (-): 0

Region: chr5 56764000-56764012. Max. coverage (+): 0. Max coverage (-): 0

Region: chr5 56764013-56764025. Max. coverage (+): 0. Max coverage (-): 0

Region: chr5 56764026-56764038. Max. coverage (+): 0. Max coverage (-): 0

Region: chr5 56764039-56764051. Max. coverage (+): 0. Max coverage (-): 0

Region: chr5 56764052-56764064. Max. coverage (+): 0. Max coverage (-): 0

Region: chr5 56764065-56764078. Max. coverage (+): 0. Max coverage (-): 0

Region: chr5 56764079-56764091. Max. coverage (+): 0. Max coverage (-): 0

Region: chr5 56764092-56764104. Max. coverage (+): 0. Max coverage (-): 0

Region: chr5 56764105-56764117. Max. coverage (+): 0. Max coverage (-): 0

Region: chr5 56764118-56764130. Max. coverage (+): 0. Max coverage (-): 0

Region: chr5 56764131-56764143. Max. coverage (+): 0. Max coverage (-): 0

Region: chr5 56764144-56764156. Max. coverage (+): 0. Max coverage (-): 0

Region: chr5 56764157-56764169. Max. coverage (+): 0. Max coverage (-): 0

Region: chr5 56764170-56764182. Max. coverage (+): 0. Max coverage (-): 0

Region: chr5 56764183-56764195. Max. coverage (+): 0. Max coverage (-): 0

Region: chr5 56764196-56764208. Max. coverage (+): 0. Max coverage (-): 0

Region: chr5 56764209-56764221. Max. coverage (+): 0. Max coverage (-): 0

Region: chr5 56764222-56764234. Max. coverage (+): 0. Max coverage (-): 0

Region: chr5 56764235-56764247. Max. coverage (+): 0. Max coverage (-): 0

Region: chr5 56764248-56764260. Max. coverage (+): 0. Max coverage (-): 0

Region: chr5 56764261-56764274. Max. coverage (+): 0. Max coverage (-): 0

Region: chr5 56764275-56764287. Max. coverage (+): 0. Max coverage (-): 0

Region: chr5 56764288-56764300. Max. coverage (+): 0. Max coverage (-): 0

Region: chr5 56764301-56764313. Max. coverage (+): 0. Max coverage (-): 0

Region: chr5 56764314-56764326. Max. coverage (+): 0. Max coverage (-): 0

Region: chr5 56764327-56764339. Max. coverage (+): 0. Max coverage (-): 0

Region: chr5 56764340-56764352. Max. coverage (+): 0. Max coverage (-): 0

Region: chr5 56764353-56764365. Max. coverage (+): 0. Max coverage (-): 0

Region: chr5 56764366-56764378. Max. coverage (+): 0. Max coverage (-): 0

Region: chr5 56764379-56764391. Max. coverage (+): 0. Max coverage (-): 0

Region: chr5 56764392-56764404. Max. coverage (+): 0. Max coverage (-): 0

Region: chr5 56764405-56764417. Max. coverage (+): 0. Max coverage (-): 0

Region: chr5 56764418-56764430. Max. coverage (+): 0. Max coverage (-): 0

Region: chr5 56764431-56764443. Max. coverage (+): 0. Max coverage (-): 0

Region: chr5 56764444-56764456. Max. coverage (+): 0. Max coverage (-): 0

Region: chr5 56764457-56764470. Max. coverage (+): 0. Max coverage (-): 0

Region: chr5 56764471-56764483. Max. coverage (+): 0. Max coverage (-): 0

Region: chr5 56764484-56764496. Max. coverage (+): 0. Max coverage (-): 0

Region: chr5 56764497-56764509. Max. coverage (+): 0.67. Max coverage (-): 0

Region: chr5 56764510-56764522. Max. coverage (+): 0.67. Max coverage (-): 0

Region: chr5 56764523-56764535. Max. coverage (+): 0. Max coverage (-): 0

Region: chr5 56764536-56764548. Max. coverage (+): 0. Max coverage (-): 0

Region: chr5 56764549-56764561. Max. coverage (+): 0. Max coverage (-): 0

Region: chr5 56764562-56764574. Max. coverage (+): 0. Max coverage (-): 0

Region: chr5 56764575-56764587. Max. coverage (+): 0. Max coverage (-): 0

Region: chr5 56764588-56764600. Max. coverage (+): 0. Max coverage (-): 0

Region: chr5 56764601-56764613. Max. coverage (+): 0. Max coverage (-): 0

Region: chr5 56764614-56764626. Max. coverage (+): 0. Max coverage (-): 0

Region: chr5 56764627-56764639. Max. coverage (+): 0. Max coverage (-): 0

Region: chr5 56764640-56764652. Max. coverage (+): 0. Max coverage (-): 0

Region: chr5 56764653-56764666. Max. coverage (+): 0. Max coverage (-): 0

Region: chr5 56764667-56764679. Max. coverage (+): 0. Max coverage (-): 0

Region: chr5 56764680-56764692. Max. coverage (+): 0. Max coverage (-): 0

Region: chr5 56764693-56764705. Max. coverage (+): 0. Max coverage (-): 0

Region: chr5 56764706-56764718. Max. coverage (+): 2.02. Max coverage (-): 0

Region: chr5 56764719-56764731. Max. coverage (+): 2.16. Max coverage (-): 0

Region: chr5 56764732-56764744. Max. coverage (+): 0. Max coverage (-): 0

Region: chr5 56764745-56764757. Max. coverage (+): 0. Max coverage (-): 0

Region: chr5 56764758-56764770. Max. coverage (+): 0. Max coverage (-): 0

Region: chr5 56764771-56764783. Max. coverage (+): 0. Max coverage (-): 0

Region: chr5 56764784-56764796. Max. coverage (+): 0. Max coverage (-): 0

Region: chr5 56764797-56764809. Max. coverage (+): 0. Max coverage (-): 0

Region: chr5 56764810-56764822. Max. coverage (+): 0. Max coverage (-): 0

Region: chr5 56764823-56764835. Max. coverage (+): 1.27. Max coverage (-): 0

Region: chr5 56764836-56764848. Max. coverage (+): 0. Max coverage (-): 0

Region: chr5 56764849-56764862. Max. coverage (+): 0. Max coverage (-): 0

Region: chr5 56764863-56764875. Max. coverage (+): 0. Max coverage (-): 0

Region: chr5 56764876-56764888. Max. coverage (+): 4.1. Max coverage (-): 0

Region: chr5 56764889-56764901. Max. coverage (+): 4.1. Max coverage (-): 0

Region: chr5 56764902-56764914. Max. coverage (+): 0. Max coverage (-): 0

Region: chr5 56764915-56764927. Max. coverage (+): 1.88. Max coverage (-): 0

Region: chr5 56764928-56764940. Max. coverage (+): 1.88. Max coverage (-): 0

Region: chr5 56764941-56764953. Max. coverage (+): 0. Max coverage (-): 0

Region: chr5 56764954-56764966. Max. coverage (+): 1.31. Max coverage (-): 0

Region: chr5 56764967-56764979. Max. coverage (+): 1.31. Max coverage (-): 0

Region: chr5 56764980-56764992. Max. coverage (+): 0. Max coverage (-): 0

Region: chr5 56764993-56765005. Max. coverage (+): 0. Max coverage (-): 0

Region: chr5 56765006-56765018. Max. coverage (+): 0. Max coverage (-): 0

Region: chr5 56765019-56765031. Max. coverage (+): 1.47. Max coverage (-): 0

Region: chr5 56765032-56765044. Max. coverage (+): 1.47. Max coverage (-): 0

Region: chr5 56765045-56765058. Max. coverage (+): 0. Max coverage (-): 0

Region: chr5 56765059-56765071. Max. coverage (+): 0. Max coverage (-): 0

Region: chr5 56765072-56765084. Max. coverage (+): 0. Max coverage (-): 0

Region: chr5 56765085-56765097. Max. coverage (+): 0. Max coverage (-): 0

Region: chr5 56765098-56765110. Max. coverage (+): 0. Max coverage (-): 0

Region: chr5 56765111-56765123. Max. coverage (+): 0. Max coverage (-): 0

Region: chr5 56765124-56765136. Max. coverage (+): 0. Max coverage (-): 0

Region: chr5 56765137-56765149. Max. coverage (+): 0. Max coverage (-): 0

Region: chr5 56765150-56765162. Max. coverage (+): 0. Max coverage (-): 0

Region: chr5 56765163-56765175. Max. coverage (+): 0. Max coverage (-): 0

Region: chr5 56765176-56765188. Max. coverage (+): 0. Max coverage (-): 0

Region: chr5 56765189-56765201. Max. coverage (+): 0. Max coverage (-): 0

Region: chr5 56765202-56765214. Max. coverage (+): 0.9. Max coverage (-): 0

Region: chr5 56765215-56765227. Max. coverage (+): 0. Max coverage (-): 0

Region: chr5 56765228-56765240. Max. coverage (+): 0. Max coverage (-): 0

Region: chr5 56765241-56765253. Max. coverage (+): 0. Max coverage (-): 0

Region: chr5 56765254-56765267. Max. coverage (+): 0. Max coverage (-): 0

Region: chr5 56765268-56765280. Max. coverage (+): 1.49. Max coverage (-): 0

Region: chr5 56765281-56765293. Max. coverage (+): 0. Max coverage (-): 0

Region: chr5 56765294-56765306. Max. coverage (+): 0. Max coverage (-): 0

Region: chr5 56765307-56765319. Max. coverage (+): 0. Max coverage (-): 0

Region: chr5 56765320-56765332. Max. coverage (+): 0. Max coverage (-): 0

Region: chr5 56765333-56765345. Max. coverage (+): 0. Max coverage (-): 0

Region: chr5 56765346-56765358. Max. coverage (+): 3.34. Max coverage (-): 0

Region: chr5 56765359-56765371. Max. coverage (+): 0. Max coverage (-): 0

Region: chr5 56765372-56765384. Max. coverage (+): 0. Max coverage (-): 0

Region: chr5 56765385-56765397. Max. coverage (+): 0. Max coverage (-): 0

Region: chr5 56765398-56765410. Max. coverage (+): 5.26. Max coverage (-): 0

Region: chr5 56765411-56765423. Max. coverage (+): 7.59. Max coverage (-): 0

Region: chr5 56765424-56765436. Max. coverage (+): 6.25. Max coverage (-): 0

Region: chr5 56765437-56765449. Max. coverage (+): 3.26. Max coverage (-): 0

Region: chr5 56765450-56765463. Max. coverage (+): 0.68. Max coverage (-): 0

Region: chr5 56765464-56765476. Max. coverage (+): 0. Max coverage (-): 0

Region: chr5 56765477-56765489. Max. coverage (+): 0. Max coverage (-): 0

Region: chr5 56765490-56765502. Max. coverage (+): 0. Max coverage (-): 0

Region: chr5 56765503-56765515. Max. coverage (+): 0.96. Max coverage (-): 0

Region: chr5 56765516-56765528. Max. coverage (+): 0.96. Max coverage (-): 0

Region: chr5 56765529-56765541. Max. coverage (+): 0. Max coverage (-): 0

Region: chr5 56765542-56765554. Max. coverage (+): 0. Max coverage (-): 0

Region: chr5 56765555-56765567. Max. coverage (+): 0. Max coverage (-): 0

Region: chr5 56765568-56765580. Max. coverage (+): 0. Max coverage (-): 0

Region: chr5 56765581-56765593. Max. coverage (+): 0. Max coverage (-): 0

Region: chr5 56765594-56765606. Max. coverage (+): 0. Max coverage (-): 0

Region: chr5 56765607-56765619. Max. coverage (+): 5.88. Max coverage (-): 0

Region: chr5 56765620-56765632. Max. coverage (+): 6.93. Max coverage (-): 0

Region: chr5 56765633-56765645. Max. coverage (+): 0. Max coverage (-): 0

Region: chr5 56765646-56765659. Max. coverage (+): 0.63. Max coverage (-): 0

Region: chr5 56765660-56765672. Max. coverage (+): 4.59. Max coverage (-): 0

Region: chr5 56765673-56765685. Max. coverage (+): 0. Max coverage (-): 0

Region: chr5 56765686-56765698. Max. coverage (+): 0. Max coverage (-): 0

Region: chr5 56765699-56765711. Max. coverage (+): 2.24. Max coverage (-): 0

Region: chr5 56765712-56765724. Max. coverage (+): 2.85. Max coverage (-): 0

Region: chr5 56765725-56765737. Max. coverage (+): 0. Max coverage (-): 0

Region: chr5 56765738-56765750. Max. coverage (+): 0. Max coverage (-): 0

Region: chr5 56765751-56765763. Max. coverage (+): 1.32. Max coverage (-): 0

Region: chr5 56765764-56765776. Max. coverage (+): 0. Max coverage (-): 0

Region: chr5 56765777-56765789. Max. coverage (+): 0. Max coverage (-): 0

Region: chr5 56765790-56765802. Max. coverage (+): 0. Max coverage (-): 0

Region: chr5 56765803-56765815. Max. coverage (+): 0. Max coverage (-): 0

Region: chr5 56765816-56765828. Max. coverage (+): 0. Max coverage (-): 0

Region: chr5 56765829-56765841. Max. coverage (+): 0. Max coverage (-): 0

Region: chr5 56765842-56765855. Max. coverage (+): 9.86. Max coverage (-): 0

Region: chr5 56765856-56765868. Max. coverage (+): 10.43. Max coverage (-): 0

Region: chr5 56765869-56765881. Max. coverage (+): 0. Max coverage (-): 0

Region: chr5 56765882-56765894. Max. coverage (+): 0. Max coverage (-): 0

Region: chr5 56765895-56765907. Max. coverage (+): 0. Max coverage (-): 0

Region: chr5 56765908-56765920. Max. coverage (+): 0. Max coverage (-): 0

Region: chr5 56765921-56765933. Max. coverage (+): 0. Max coverage (-): 0

Region: chr5 56765934-56765946. Max. coverage (+): 2.31. Max coverage (-): 0

Region: chr5 56765947-56765959. Max. coverage (+): 2.31. Max coverage (-): 0

Region: chr5 56765960-56765972. Max. coverage (+): 0. Max coverage (-): 0

Region: chr5 56765973-56765985. Max. coverage (+): 7.8. Max coverage (-): 0

Region: chr5 56765986-56765998. Max. coverage (+): 7.8. Max coverage (-): 0

Region: chr5 56765999-56766011. Max. coverage (+): 1.05. Max coverage (-): 0

Region: chr5 56766012-56766024. Max. coverage (+): 1.05. Max coverage (-): 0

Region: chr5 56766025-56766037. Max. coverage (+): 0. Max coverage (-): 0

Region: chr5 56766038-56766051. Max. coverage (+): 0. Max coverage (-): 0

Region: chr5 56766052-56766064. Max. coverage (+): 0. Max coverage (-): 0

Region: chr5 56766065-56766077. Max. coverage (+): 0. Max coverage (-): 0

Region: chr5 56766078-56766090. Max. coverage (+): 0. Max coverage (-): 0

Region: chr5 56766091-56766103. Max. coverage (+): 0. Max coverage (-): 0

Region: chr5 56766104-56766116. Max. coverage (+): 0. Max coverage (-): 0

Region: chr5 56766117-56766129. Max. coverage (+): 0. Max coverage (-): 0

Region: chr5 56766130-56766142. Max. coverage (+): 2.75. Max coverage (-): 0

Region: chr5 56766143-56766155. Max. coverage (+): 2.75. Max coverage (-): 0

Region: chr5 56766156-56766168. Max. coverage (+): 2.02. Max coverage (-): 0

Region: chr5 56766169-56766181. Max. coverage (+): 0. Max coverage (-): 0

Region: chr5 56766182-56766194. Max. coverage (+): 0. Max coverage (-): 0

Region: chr5 56766195-56766207. Max. coverage (+): 0. Max coverage (-): 0

Region: chr5 56766208-56766220. Max. coverage (+): 1.13. Max coverage (-): 0

Region: chr5 56766221-56766233. Max. coverage (+): 2.14. Max coverage (-): 0

Region: chr5 56766234-56766247. Max. coverage (+): 0. Max coverage (-): 0

Region: chr5 56766248-56766260. Max. coverage (+): 0. Max coverage (-): 0

Region: chr5 56766261-56766273. Max. coverage (+): 0. Max coverage (-): 0

Region: chr5 56766274-56766286. Max. coverage (+): 0. Max coverage (-): 0

Region: chr5 56766287-56766299. Max. coverage (+): 0. Max coverage (-): 0

Region: chr5 56766300-56766312. Max. coverage (+): 0. Max coverage (-): 0

Region: chr5 56766313-56766325. Max. coverage (+): 0. Max coverage (-): 0

Region: chr5 56766326-56766338. Max. coverage (+): 0. Max coverage (-): 0

Region: chr5 56766339-56766351. Max. coverage (+): 0. Max coverage (-): 0

Region: chr5 56766352-56766364. Max. coverage (+): 0. Max coverage (-): 0

Region: chr5 56766365-56766377. Max. coverage (+): 0. Max coverage (-): 0

Region: chr5 56766378-56766390. Max. coverage (+): 0. Max coverage (-): 0

Region: chr5 56766391-56766403. Max. coverage (+): 0. Max coverage (-): 0

Region: chr5 56766404-56766416. Max. coverage (+): 0. Max coverage (-): 0

Region: chr5 56766417-56766429. Max. coverage (+): 0. Max coverage (-): 0

Region: chr5 56766430-56766442. Max. coverage (+): 0. Max coverage (-): 0

Region: chr5 56766443-56766456. Max. coverage (+): 0. Max coverage (-): 0

Region: chr5 56766457-56766469. Max. coverage (+): 6. Max coverage (-): 0

Region: chr5 56766470-56766482. Max. coverage (+): 0.82. Max coverage (-): 0

Region: chr5 56766483-56766495. Max. coverage (+): 0. Max coverage (-): 0

Region: chr5 56766496-56766508. Max. coverage (+): 0. Max coverage (-): 0

Region: chr5 56766509-56766521. Max. coverage (+): 0. Max coverage (-): 0

Region: chr5 56766522-56766534. Max. coverage (+): 0. Max coverage (-): 0

Region: chr5 56766535-56766547. Max. coverage (+): 0. Max coverage (-): 0

Region: chr5 56766548-56766560. Max. coverage (+): 2.46. Max coverage (-): 0

Region: chr5 56766561-56766573. Max. coverage (+): 1.11. Max coverage (-): 0

Region: chr5 56766574-56766586. Max. coverage (+): 0. Max coverage (-): 0

Region: chr5 56766587-56766599. Max. coverage (+): 0. Max coverage (-): 0

Region: chr5 56766600-56766612. Max. coverage (+): 0. Max coverage (-): 0

Region: chr5 56766613-56766625. Max. coverage (+): 1.49. Max coverage (-): 0

Region: chr5 56766626-56766638. Max. coverage (+): 6.92. Max coverage (-): 0

Region: chr5 56766639-56766652. Max. coverage (+): 0. Max coverage (-): 0

Region: chr5 56766653-56766665. Max. coverage (+): 7.83. Max coverage (-): 0

Region: chr5 56766666-56766678. Max. coverage (+): 8.49. Max coverage (-): 0

Region: chr5 56766679-56766691. Max. coverage (+): 18.49. Max coverage (-): 0

Region: chr5 56766692-56766704. Max. coverage (+): 0. Max coverage (-): 0

Region: chr5 56766705-56766717. Max. coverage (+): 8.38. Max coverage (-): 0

Region: chr5 56766718-56766730. Max. coverage (+): 8.77. Max coverage (-): 0

Region: chr5 56766731-56766743. Max. coverage (+): 1.2. Max coverage (-): 0

Region: chr5 56766744-56766756. Max. coverage (+): 1.11. Max coverage (-): 0

Region: chr5 56766757-56766769. Max. coverage (+): 0. Max coverage (-): 0

Region: chr5 56766770-56766782. Max. coverage (+): 1.71. Max coverage (-): 0

Region: chr5 56766783-56766795. Max. coverage (+): 1.71. Max coverage (-): 0

Region: chr5 56766796-56766808. Max. coverage (+): 0. Max coverage (-): 0

Region: chr5 56766809-56766821. Max. coverage (+): 0. Max coverage (-): 0

Region: chr5 56766822-56766834. Max. coverage (+): 0. Max coverage (-): 0

Region: chr5 56766835-56766848. Max. coverage (+): 0. Max coverage (-): 0

Region: chr5 56766849-56766861. Max. coverage (+): 0. Max coverage (-): 0

Region: chr5 56766862-56766874. Max. coverage (+): 0. Max coverage (-): 0

Region: chr5 56766875-56766887. Max. coverage (+): 4.56. Max coverage (-): 0

Region: chr5 56766888-56766900. Max. coverage (+): 4.56. Max coverage (-): 0

Region: chr5 56766901-56766913. Max. coverage (+): 0. Max coverage (-): 0

Region: chr5 56766914-56766926. Max. coverage (+): 0.59. Max coverage (-): 0

Region: chr5 56766927-56766939. Max. coverage (+): 10.37. Max coverage (-): 0

Region: chr5 56766940-56766952. Max. coverage (+): 4.63. Max coverage (-): 0

Region: chr5 56766953-56766965. Max. coverage (+): 0. Max coverage (-): 0

Region: chr5 56766966-56766978. Max. coverage (+): 0. Max coverage (-): 0

Region: chr5 56766979-56766991. Max. coverage (+): 1.43. Max coverage (-): 0

Region: chr5 56766992-56767004. Max. coverage (+): 1.43. Max coverage (-): 0

Region: chr5 56767005-56767017. Max. coverage (+): 0. Max coverage (-): 0

Region: chr5 56767018-56767030. Max. coverage (+): 0. Max coverage (-): 0

Region: chr5 56767031-56767044. Max. coverage (+): 2.98. Max coverage (-): 0

Region: chr5 56767045-56767057. Max. coverage (+): 2.98. Max coverage (-): 0

Region: chr5 56767058-56767070. Max. coverage (+): 0. Max coverage (-): 0

Region: chr5 56767071-56767083. Max. coverage (+): 0. Max coverage (-): 0

Region: chr5 56767084-56767096. Max. coverage (+): 0. Max coverage (-): 0

Region: chr5 56767097-56767109. Max. coverage (+): 0. Max coverage (-): 0

Region: chr5 56767110-56767122. Max. coverage (+): 0. Max coverage (-): 0

Region: chr5 56767123-56767135. Max. coverage (+): 0. Max coverage (-): 0

Region: chr5 56767136-56767148. Max. coverage (+): 0. Max coverage (-): 0

Region: chr5 56767149-56767161. Max. coverage (+): 10.82. Max coverage (-): 0

Region: chr5 56767162-56767174. Max. coverage (+): 0. Max coverage (-): 0

Region: chr5 56767175-56767187. Max. coverage (+): 0.43. Max coverage (-): 0

Region: chr5 56767188-56767200. Max. coverage (+): 0.43. Max coverage (-): 0

Region: chr5 56767201-56767213. Max. coverage (+): 11.03. Max coverage (-): 0

Region: chr5 56767214-56767226. Max. coverage (+): 12.91. Max coverage (-): 0

Region: chr5 56767227-56767240. Max. coverage (+): 0. Max coverage (-): 0

Region: chr5 56767241-56767253. Max. coverage (+): 0. Max coverage (-): 0

Region: chr5 56767254-56767266. Max. coverage (+): 0. Max coverage (-): 0

Region: chr5 56767267-56767279. Max. coverage (+): 0. Max coverage (-): 0

Region: chr5 56767280-56767292. Max. coverage (+): 0. Max coverage (-): 0

Region: chr5 56767293-56767305. Max. coverage (+): 0. Max coverage (-): 0

Region: chr5 56767306-56767318. Max. coverage (+): 0. Max coverage (-): 0

Region: chr5 56767319-56767331. Max. coverage (+): 0. Max coverage (-): 0

Region: chr5 56767332-56767344. Max. coverage (+): 0. Max coverage (-): 0

Region: chr5 56767345-56767357. Max. coverage (+): 0. Max coverage (-): 0

Region: chr5 56767358-56767370. Max. coverage (+): 0. Max coverage (-): 0

Region: chr5 56767371-56767383. Max. coverage (+): 0. Max coverage (-): 0

Region: chr5 56767384-56767396. Max. coverage (+): 0. Max coverage (-): 0

Region: chr5 56767397-56767409. Max. coverage (+): 8.25. Max coverage (-): 0

Region: chr5 56767410-56767422. Max. coverage (+): 6.11. Max coverage (-): 0

Region: chr5 56767423-56767436. Max. coverage (+): 0. Max coverage (-): 0

Region: chr5 56767437-56767449. Max. coverage (+): 2.13. Max coverage (-): 0

Region: chr5 56767450-56767462. Max. coverage (+): 0. Max coverage (-): 0

Region: chr5 56767463-56767475. Max. coverage (+): 2.18. Max coverage (-): 0

Region: chr5 56767476-56767488. Max. coverage (+): 12.57. Max coverage (-): 0

Region: chr5 56767489-56767501. Max. coverage (+): 12.57. Max coverage (-): 0

Region: chr5 56767502-56767514. Max. coverage (+): 0.94. Max coverage (-): 0

Region: chr5 56767515-56767527. Max. coverage (+): 10.61. Max coverage (-): 0

Region: chr5 56767528-56767540. Max. coverage (+): 10.7. Max coverage (-): 0

Region: chr5 56767541-56767553. Max. coverage (+): 0. Max coverage (-): 0

Region: chr5 56767554-56767566. Max. coverage (+): 0. Max coverage (-): 0

Region: chr5 56767567-56767579. Max. coverage (+): 4.53. Max coverage (-): 0

Region: chr5 56767580-56767592. Max. coverage (+): 19.88. Max coverage (-): 0

Region: chr5 56767593-56767605. Max. coverage (+): 4.94. Max coverage (-): 0

Region: chr5 56767606-56767618. Max. coverage (+): 0. Max coverage (-): 0

Region: chr5 56767619-56767632. Max. coverage (+): 0. Max coverage (-): 0

Region: chr5 56767633-56767645. Max. coverage (+): 0. Max coverage (-): 0

Region: chr5 56767646-56767658. Max. coverage (+): 0. Max coverage (-): 0

Region: chr5 56767659-56767671. Max. coverage (+): 0. Max coverage (-): 0

Region: chr5 56767672-56767684. Max. coverage (+): 0. Max coverage (-): 0

Region: chr5 56767685-56767697. Max. coverage (+): 0. Max coverage (-): 0

Region: chr5 56767698-56767710. Max. coverage (+): 9.7. Max coverage (-): 0

Region: chr5 56767711-56767723. Max. coverage (+): 1.68. Max coverage (-): 0

Region: chr5 56767724-56767736. Max. coverage (+): 0. Max coverage (-): 0

Region: chr5 56767737-56767749. Max. coverage (+): 0. Max coverage (-): 0

Region: chr5 56767750-56767762. Max. coverage (+): 0. Max coverage (-): 0

Region: chr5 56767763-56767775. Max. coverage (+): 0. Max coverage (-): 0

Region: chr5 56767776-56767788. Max. coverage (+): 0. Max coverage (-): 0

Region: chr5 56767789-56767801. Max. coverage (+): 0. Max coverage (-): 0

Region: chr5 56767802-56767814. Max. coverage (+): 5.86. Max coverage (-): 0

Region: chr5 56767815-56767827. Max. coverage (+): 5.86. Max coverage (-): 0

Region: chr5 56767828-56767841. Max. coverage (+): 0. Max coverage (-): 0

Region: chr5 56767842-56767854. Max. coverage (+): 0. Max coverage (-): 0

Region: chr5 56767855-56767867. Max. coverage (+): 10.75. Max coverage (-): 0

Region: chr5 56767868-56767880. Max. coverage (+): 10.75. Max coverage (-): 0

Region: chr5 56767881-56767893. Max. coverage (+): 7.14. Max coverage (-): 0

Region: chr5 56767894-56767906. Max. coverage (+): 19.97. Max coverage (-): 0

Region: chr5 56767907-56767919. Max. coverage (+): 0. Max coverage (-): 0

Region: chr5 56767920-56767932. Max. coverage (+): 0. Max coverage (-): 0

Region: chr5 56767933-56767945. Max. coverage (+): 0. Max coverage (-): 0

Region: chr5 56767946-56767958. Max. coverage (+): 0. Max coverage (-): 0

Region: chr5 56767959-56767971. Max. coverage (+): 0. Max coverage (-): 0

Region: chr5 56767972-56767984. Max. coverage (+): 1.28. Max coverage (-): 0

Region: chr5 56767985-56767997. Max. coverage (+): 3.39. Max coverage (-): 0

Region: chr5 56767998-56768010. Max. coverage (+): 0. Max coverage (-): 0

Region: chr5 56768011-56768023. Max. coverage (+): 7.58. Max coverage (-): 0

Region: chr5 56768024-56768037. Max. coverage (+): 5.89. Max coverage (-): 0

Region: chr5 56768038-56768050. Max. coverage (+): 5.89. Max coverage (-): 0

Region: chr5 56768051-56768063. Max. coverage (+): 1.06. Max coverage (-): 0

Region: chr5 56768064-56768076. Max. coverage (+): 24.57. Max coverage (-): 0

Region: chr5 56768077-56768089. Max. coverage (+): 36.62. Max coverage (-): 0

Region: chr5 56768090-56768102. Max. coverage (+): 35.98. Max coverage (-): 0

Region: chr5 56768103-56768115. Max. coverage (+): 8.46. Max coverage (-): 0

Region: chr5 56768116-56768128. Max. coverage (+): 0. Max coverage (-): 0

Region: chr5 56768129-56768141. Max. coverage (+): 0. Max coverage (-): 0

Region: chr5 56768142-56768154. Max. coverage (+): 0. Max coverage (-): 0

Region: chr5 56768155-56768167. Max. coverage (+): 0. Max coverage (-): 0

Region: chr5 56768168-56768180. Max. coverage (+): 0. Max coverage (-): 0

Region: chr5 56768181-56768193. Max. coverage (+): 1.74. Max coverage (-): 0

Region: chr5 56768194-56768206. Max. coverage (+): 1.51. Max coverage (-): 0

Region: chr5 56768207-56768219. Max. coverage (+): 0. Max coverage (-): 0

Region: chr5 56768220-56768233. Max. coverage (+): 12.82. Max coverage (-): 0

Region: chr5 56768234-56768246. Max. coverage (+): 19.01. Max coverage (-): 0

Region: chr5 56768247-56768259. Max. coverage (+): 0.8. Max coverage (-): 0

Region: chr5 56768260-56768272. Max. coverage (+): 0. Max coverage (-): 0

Region: chr5 56768273-56768285. Max. coverage (+): 0. Max coverage (-): 0

Region: chr5 56768286-56768298. Max. coverage (+): 18.52. Max coverage (-): 0

Region: chr5 56768299-56768311. Max. coverage (+): 6.13. Max coverage (-): 0

Region: chr5 56768312-56768324. Max. coverage (+): 12.56. Max coverage (-): 0

Region: chr5 56768325-56768337. Max. coverage (+): 12.56. Max coverage (-): 0

Region: chr5 56768338-56768350. Max. coverage (+): 0. Max coverage (-): 0

Region: chr5 56768351-56768363. Max. coverage (+): 0. Max coverage (-): 0

Region: chr5 56768364-56768376. Max. coverage (+): 0. Max coverage (-): 0

Region: chr5 56768377-56768389. Max. coverage (+): 14.74. Max coverage (-): 0

Region: chr5 56768390-56768402. Max. coverage (+): 9.81. Max coverage (-): 0

Region: chr5 56768403-56768415. Max. coverage (+): 2.48. Max coverage (-): 0

Region: chr5 56768416-56768429. Max. coverage (+): 0. Max coverage (-): 0

Region: chr5 56768430-56768442. Max. coverage (+): 3.08. Max coverage (-): 0

Region: chr5 56768443-56768455. Max. coverage (+): 3.4. Max coverage (-): 0

Region: chr5 56768456-56768468. Max. coverage (+): 3.4. Max coverage (-): 0

Region: chr5 56768469-56768481. Max. coverage (+): 2.3. Max coverage (-): 0

Region: chr5 56768482-56768494. Max. coverage (+): 3.99. Max coverage (-): 0

Region: chr5 56768495-56768507. Max. coverage (+): 0. Max coverage (-): 0

Region: chr5 56768508-56768520. Max. coverage (+): 0. Max coverage (-): 0

Region: chr5 56768521-56768533. Max. coverage (+): 0. Max coverage (-): 0

Region: chr5 56768534-56768546. Max. coverage (+): 0. Max coverage (-): 0

Region: chr5 56768547-56768559. Max. coverage (+): 0. Max coverage (-): 0

Region: chr5 56768560-56768572. Max. coverage (+): 10.38. Max coverage (-): 0

Region: chr5 56768573-56768585. Max. coverage (+): 10.38. Max coverage (-): 0

Region: chr5 56768586-56768598. Max. coverage (+): 4.2. Max coverage (-): 0

Region: chr5 56768599-56768611. Max. coverage (+): 8.73. Max coverage (-): 0

Region: chr5 56768612-56768625. Max. coverage (+): 1.66. Max coverage (-): 0

Region: chr5 56768626-56768638. Max. coverage (+): 0. Max coverage (-): 0

Region: chr5 56768639-56768651. Max. coverage (+): 16.17. Max coverage (-): 0

Region: chr5 56768652-56768664. Max. coverage (+): 9.84. Max coverage (-): 0

Region: chr5 56768665-56768677. Max. coverage (+): 17.74. Max coverage (-): 0

Region: chr5 56768678-56768690. Max. coverage (+): 6.61. Max coverage (-): 0

Region: chr5 56768691-56768703. Max. coverage (+): 0. Max coverage (-): 0

Region: chr5 56768704-56768716. Max. coverage (+): 1.34. Max coverage (-): 0

Region: chr5 56768717-56768729. Max. coverage (+): 1.34. Max coverage (-): 0

Region: chr5 56768730-56768742. Max. coverage (+): 0.42. Max coverage (-): 0

Region: chr5 56768743-56768755. Max. coverage (+): 0. Max coverage (-): 0

Region: chr5 56768756-56768768. Max. coverage (+): 5.9. Max coverage (-): 0

Region: chr5 56768769-56768781. Max. coverage (+): 5.9. Max coverage (-): 0

Region: chr5 56768782-56768794. Max. coverage (+): 2.56. Max coverage (-): 0

Region: chr5 56768795-56768807. Max. coverage (+): 1.76. Max coverage (-): 0

Region: chr5 56768808-56768821. Max. coverage (+): 4.5. Max coverage (-): 0

Region: chr5 56768822-56768834. Max. coverage (+): 4.5. Max coverage (-): 0

Region: chr5 56768835-56768847. Max. coverage (+): 0. Max coverage (-): 0

Region: chr5 56768848-56768860. Max. coverage (+): 9.45. Max coverage (-): 0

Region: chr5 56768861-56768873. Max. coverage (+): 15.06. Max coverage (-): 0

Region: chr5 56768874-56768886. Max. coverage (+): 1.07. Max coverage (-): 0

Region: chr5 56768887-56768899. Max. coverage (+): 4.23. Max coverage (-): 0

Region: chr5 56768900-56768912. Max. coverage (+): 3.15. Max coverage (-): 0

Region: chr5 56768913-56768925. Max. coverage (+): 0.94. Max coverage (-): 0

Region: chr5 56768926-56768938. Max. coverage (+): 15.16. Max coverage (-): 0

Region: chr5 56768939-56768951. Max. coverage (+): 9.63. Max coverage (-): 0

Region: chr5 56768952-56768964. Max. coverage (+): 0. Max coverage (-): 0

Region: chr5 56768965-56768977. Max. coverage (+): 1.9. Max coverage (-): 0

Region: chr5 56768978-56768990. Max. coverage (+): 1.87. Max coverage (-): 0

Region: chr5 56768991-56769003. Max. coverage (+): 8.56. Max coverage (-): 0

Region: chr5 56769004-56769016. Max. coverage (+): 38.71. Max coverage (-): 0

Region: chr5 56769017-56769030. Max. coverage (+): 18.98. Max coverage (-): 0

Region: chr5 56769031-56769043. Max. coverage (+): 0. Max coverage (-): 0

Region: chr5 56769044-56769056. Max. coverage (+): 0. Max coverage (-): 0

Region: chr5 56769057-56769069. Max. coverage (+): 0. Max coverage (-): 0

Region: chr5 56769070-56769082. Max. coverage (+): 0. Max coverage (-): 0

Region: chr5 56769083-56769095. Max. coverage (+): 0. Max coverage (-): 0

Region: chr5 56769096-56769108. Max. coverage (+): 1.92. Max coverage (-): 0

Region: chr5 56769109-56769121. Max. coverage (+): 1.92. Max coverage (-): 0

Region: chr5 56769122-56769134. Max. coverage (+): 0. Max coverage (-): 0

Region: chr5 56769135-56769147. Max. coverage (+): 1.03. Max coverage (-): 0

Region: chr5 56769148-56769160. Max. coverage (+): 13.69. Max coverage (-): 0

Region: chr5 56769161-56769173. Max. coverage (+): 4.91. Max coverage (-): 0

Region: chr5 56769174-56769186. Max. coverage (+): 6.51. Max coverage (-): 0

Region: chr5 56769187-56769199. Max. coverage (+): 6.08. Max coverage (-): 0

Region: chr5 56769200-56769212. Max. coverage (+): 6.08. Max coverage (-): 0

Region: chr5 56769213-56769226. Max. coverage (+): 10.69. Max coverage (-): 0

Region: chr5 56769227-56769239. Max. coverage (+): 0.34. Max coverage (-): 0

Region: chr5 56769240-56769252. Max. coverage (+): 0.34. Max coverage (-): 0

Region: chr5 56769253-56769265. Max. coverage (+): 0. Max coverage (-): 0

Region: chr5 56769266-56769278. Max. coverage (+): 0. Max coverage (-): 0

Region: chr5 56769279-56769291. Max. coverage (+): 0. Max coverage (-): 0

Region: chr5 56769292-56769304. Max. coverage (+): 2.26. Max coverage (-): 0

Region: chr5 56769305-56769317. Max. coverage (+): 6.32. Max coverage (-): 0

Region: chr5 56769318-56769330. Max. coverage (+): 0. Max coverage (-): 0

Region: chr5 56769331-56769343. Max. coverage (+): 0. Max coverage (-): 0

Region: chr5 56769344-56769356. Max. coverage (+): 0. Max coverage (-): 0

Region: chr5 56769357-56769369. Max. coverage (+): 5.12. Max coverage (-): 0

Region: chr5 56769370-56769382. Max. coverage (+): 31.15. Max coverage (-): 0

Region: chr5 56769383-56769395. Max. coverage (+): 0. Max coverage (-): 0

Region: chr5 56769396-56769408. Max. coverage (+): 0.58. Max coverage (-): 0

Region: chr5 56769409-56769422. Max. coverage (+): 5.52. Max coverage (-): 0

Region: chr5 56769423-56769435. Max. coverage (+): 0. Max coverage (-): 0

Region: chr5 56769436-56769448. Max. coverage (+): 0. Max coverage (-): 0

Region: chr5 56769449-56769461. Max. coverage (+): 0. Max coverage (-): 0

Region: chr5 56769462-56769474. Max. coverage (+): 0. Max coverage (-): 0

Region: chr5 56769475-56769487. Max. coverage (+): 0. Max coverage (-): 0

Region: chr5 56769488-56769500. Max. coverage (+): 0. Max coverage (-): 0

Region: chr5 56769501-56769513. Max. coverage (+): 0. Max coverage (-): 0

Region: chr5 56769514-56769526. Max. coverage (+): 0. Max coverage (-): 0

Region: chr5 56769527-56769539. Max. coverage (+): 0. Max coverage (-): 0

Region: chr5 56769540-56769552. Max. coverage (+): 0. Max coverage (-): 0

Region: chr5 56769553-56769565. Max. coverage (+): 0. Max coverage (-): 0

Region: chr5 56769566-56769578. Max. coverage (+): 0. Max coverage (-): 0

Region: chr5 56769579-56769591. Max. coverage (+): 0. Max coverage (-): 0

Region: chr5 56769592-56769604. Max. coverage (+): 0. Max coverage (-): 0

Region: chr5 56769605-56769618. Max. coverage (+): 0. Max coverage (-): 0

Region: chr5 56769619-56769631. Max. coverage (+): 0. Max coverage (-): 0

Region: chr5 56769632-56769644. Max. coverage (+): 0. Max coverage (-): 0

Region: chr5 56769645-56769657. Max. coverage (+): 0. Max coverage (-): 0

Region: chr5 56769658-56769670. Max. coverage (+): 0. Max coverage (-): 0

Region: chr5 56769671-56769683. Max. coverage (+): 0. Max coverage (-): 0

Region: chr5 56769684-56769696. Max. coverage (+): 0. Max coverage (-): 0

Region: chr5 56769697-56769709. Max. coverage (+): 0. Max coverage (-): 0

Region: chr5 56769710-56769722. Max. coverage (+): 0. Max coverage (-): 0

Region: chr5 56769723-56769735. Max. coverage (+): 0. Max coverage (-): 0

Region: chr5 56769736-56769748. Max. coverage (+): 0. Max coverage (-): 0

Region: chr5 56769749-56769761. Max. coverage (+): 0. Max coverage (-): 0

Region: chr5 56769762-56769774. Max. coverage (+): 0. Max coverage (-): 0

Region: chr5 56769775-56769787. Max. coverage (+): 0. Max coverage (-): 0

Region: chr5 56769788-56769800. Max. coverage (+): 0. Max coverage (-): 0

Region: chr5 56769801-56769814. Max. coverage (+): 9.39. Max coverage (-): 0

Region: chr5 56769815-56769827. Max. coverage (+): 7.94. Max coverage (-): 0

Region: chr5 56769828-56769840. Max. coverage (+): 0. Max coverage (-): 0

Region: chr5 56769841-56769853. Max. coverage (+): 0. Max coverage (-): 0

Region: chr5 56769854-56769866. Max. coverage (+): 0. Max coverage (-): 0

Region: chr5 56769867-56769879. Max. coverage (+): 0. Max coverage (-): 0

Region: chr5 56769880-56769892. Max. coverage (+): 1.92. Max coverage (-): 0

Region: chr5 56769893-56769905. Max. coverage (+): 0. Max coverage (-): 0

Region: chr5 56769906-56769918. Max. coverage (+): 0. Max coverage (-): 0

Region: chr5 56769919-56769931. Max. coverage (+): 0. Max coverage (-): 0

Region: chr5 56769932-56769944. Max. coverage (+): 0. Max coverage (-): 0

Region: chr5 56769945-56769957. Max. coverage (+): 0. Max coverage (-): 0

Region: chr5 56769958-56769970. Max. coverage (+): 0. Max coverage (-): 0

Region: chr5 56769971-56769983. Max. coverage (+): 0. Max coverage (-): 0

Region: chr5 56769984-56769996. Max. coverage (+): 0. Max coverage (-): 0

Region: chr5 56769997-56770010. Max. coverage (+): 0. Max coverage (-): 0

Region: chr5 56770011-56770023. Max. coverage (+): 0. Max coverage (-): 0

Region: chr5 56770024-56770036. Max. coverage (+): 0. Max coverage (-): 0

Region: chr5 56770037-56770049. Max. coverage (+): 0. Max coverage (-): 0

Region: chr5 56770050-56770062. Max. coverage (+): 0. Max coverage (-): 0

Region: chr5 56770063-56770075. Max. coverage (+): 0. Max coverage (-): 0

Region: chr5 56770076-56770088. Max. coverage (+): 0. Max coverage (-): 0

Region: chr5 56770089-56770101. Max. coverage (+): 0. Max coverage (-): 0

Region: chr5 56770102-56770114. Max. coverage (+): 0. Max coverage (-): 0

Region: chr5 56770115-56770127. Max. coverage (+): 0. Max coverage (-): 0

Region: chr5 56770128-56770140. Max. coverage (+): 0. Max coverage (-): 0

Region: chr5 56770141-56770153. Max. coverage (+): 0. Max coverage (-): 0

Region: chr5 56770154-56770166. Max. coverage (+): 0. Max coverage (-): 0

Region: chr5 56770167-56770179. Max. coverage (+): 0. Max coverage (-): 0

Region: chr5 56770180-56770192. Max. coverage (+): 0. Max coverage (-): 0

Region: chr5 56770193-56770206. Max. coverage (+): 0. Max coverage (-): 0

Region: chr5 56770207-56770219. Max. coverage (+): 0. Max coverage (-): 0

Region: chr5 56770220-56770232. Max. coverage (+): 0. Max coverage (-): 0

Region: chr5 56770233-56770245. Max. coverage (+): 0. Max coverage (-): 0

Region: chr5 56770246-56770258. Max. coverage (+): 0. Max coverage (-): 0

Region: chr5 56770259-56770271. Max. coverage (+): 0. Max coverage (-): 0

Region: chr5 56770272-56770284. Max. coverage (+): 1.22. Max coverage (-): 0

Region: chr5 56770285-56770297. Max. coverage (+): 0. Max coverage (-): 0

Region: chr5 56770298-. Max. coverage (+): 0. Max coverage (-): 0

RepeatMasker Color Code

**+**

100-98% Identity

<98-95% Identity

<95-90% Identity

<90-85% Identity

<85-80% Identity

<80-75% Identity

<75-70% Identity

<70% Identity

**-**

Gene Set Color Code

**+**

Gene

Pseudogene

**-**

Topology/Coverage Color Code

Coverage Plus Strand

Coverage Minus Strand

Mainstrand: Plus

Mainstrand: Minus

Complementary Strand

Flanking Region  
(if option -flank >0)

Gene Set Annotation  

**1. ZBTB39 (protein coding, ENSBTAG00000038498) Tr:00000056411 Ex:1**: 56764459-56766582 (+)

  
RepeatMasker Annotation  

**1. AT\_rich**: 56768041-56768061 (+), Divergence to consensus: 38.1%  
**2. MER45A**: 56769484-56769554 (-), Divergence to consensus: 29.5%  
**3. Bov-tA2**: 56769597-56769805 (-), Divergence to consensus: 10.6%

  
Transcription Factor Binding Sites  

**A-MYB** (Sequence: AGGCAGTTGG (+): 56766855)
