# Supplementary material for: Small RNA sequencing of cryopreserved semen from single bull revealed altered miRNAs and piRNAs expression between High- and Low-motile sperm populations
Source: BMC Genomics. 2017 Jan 4;18:14. doi: 10.1186/s12864-016-3394-7 (PMC5209821; doi:10.1186/s12864-016-3394-7)
Supplement: Additional file 3: — Details for each piRNA clusters found in High Motile (HM) sperm fraction. Genes, repeats, transposable elements and transcription factors binding sites falling within the cluster regions were reported. (ZIP 1896 kb) [file 12864_2016_3394_MOESM3_ESM.zip › 87.html]

piRNA cluster 87


Predicted piRNA cluster no. 87     previous   next
  

Show proTRAC run info
Hide proTRAC run info

================================= proTRAC ====================================  
VERSION: 2.1                                    LAST MODIFIED: 06. October 2015  
  
Please cite:  
Rosenkranz D, Zischler H. proTRAC - a software for probabilistic piRNA cluster  
detection, visualization and analysis. 2012. BMC Bioinformatics 13:5.  
  
and (for proTRAC 2.0 and later):  
Rosenkranz D, Rudloff S, Bastuck K, Ketting RF, Zischler H. Tupaia small RNAs  
provide insights into function and evolution of RNAi-based transposon defense  
in mammals. 2015. RNA 21(5):911-922.  
  
Contact:  
David Rosenkranz  
Institute of Anthropology, small RNA group  
Johannes Gutenberg University Mainz  
email: rosenkranz@uni-mainz.de  
  
You can find the latest proTRAC version at:  
http://sourceforge.net/projects/protrac/files  
http://www.smallRNAgroup-mainz.de/software  
==============================================================================  
  
PARAMETERS:  
Map file: .............../storage/core/barbara/genhome/smallRNA/fertility/Sample\_motile/pirna/Sample\_motile\_26-33\_collapsed.fa.no-dust.map.weighted-10000-1000-b-0  
Genome file: ............/storage/core/barbara/genhome/smallRNA/fertility/Sample\_all/pirna/bt\_311\_chrY.fa  
RepeatMasker annotation: /storage/genomes/bt\_umd31/GCF\_000003055.6\_Bos\_taurus\_UMD\_3.1.1\_repeatMasker\_chr.out  
GeneSet:................./storage/core/barbara/genhome/smallRNA/fertility/Sample\_all/pirna/full.gtf  
  
Significant (p<=0.01) hit density will be calculated based  
on observed hit distribution.  
  
Sliding window size: ........................................ 5000 bp  
Sliding window increament: .................................. 1000 bp  
Normalize each hit by number of genomic hits: ............... 1 [0=no/1=yes]  
Normalize each hit by number of sequence reads: ............. 1 [0=no/1=yes]  
Normalize values (-> per million mapped reads): ............. 1 [0=no/1=yes]  
Min. fraction of hits with 1T(U) or 10A: .................... 0.75  
Alternatively: Min. fraction of hits with 1T(U) and 10A: .... 0.5  
Min. fraction of hits with typical piRNA length: ............ 0.75  
Typical piRNA length: ....................................... 26-33 nt  
Min. size of a piRNA cluster: ............................... 5000 bp.  
Min. number of hits (absolute): ............................. 0  
Min. number of hits (normalized): ........................... 0  
Min. fraction of hits on the mainstrand: .................... 0.75  
Top fraction of mapped sequences (in terms of read counts): . 1%  
Top fraction accounts for max. n% of sequence reads: ........ 90%  
Min. fraction of hits on each arm of a bidirectional cluster: 0.1  
Output image file for each cluster: ......................... 0 [0=no/1=yes]  
Output html file for each cluster: .......................... 1 [0=no/1=yes]  
Output a summary table: ..................................... 1 [0=no/1=yes]  
Output a FASTA file for each cluster (piRNA sequences): ..... 1 [0=no/1=yes]  
Output a FASTA file comprising cluster sequences: ........... 1 [0=no/1=yes]  
Search DNA motifs in clusters: .............................. 1 [0=no/1=yes]  
Output flanking sequences: +/- .............................. 0 bp  
Output ~.pTi file: .......................................... 1 [0=no/1=yes]  
==============================================================================  
  
  
Genome size (without gaps): ............ 2678902517 bp  
Gaps (N/X/-): .......................... 53837044 bp  
Mapped reads: .......................... 658825247023  
Non-identical sequences: ............... 514171  
Genomic hits: .......................... 764233  
Significant densitiy of mapped reads: .. 12867599.5173724 reads/kb

Show proTRAC cluster info
Hide proTRAC cluster info

|  |  |
| --- | --- |
| Location | chr5 |
| Coordinates | 76153176-76177962 |
| Size [bp] | 24787 |
| Sequence hit loci | 4615 |
| Mapped reads (normalized) | 4956484298.1 |
| Mapped reads (normalized) per kb | 199963057.2 |
| Normalized reads with 1T (1U) | 77.7% |
| Normalized reads with 10A | 27.5% |
| Normalized reads with length 26-33 nt | 100% |
| Normalized reads on the main strand(s) | 98.4% |
| Predicted directionality | mono:minus |

100%

0%

1T (1U)  
reads

10A reads

26-33 nt  
reads

reads on mainstrand

**Either the amount of reads with 1T (1U) OR 10A has to exceed 75% (set with option: -1Tor10A)  
Alternatively the amount of reads with 1T (1U) AND 10A has to exceed 50% (set with option: -1Tand10A)  
Minimum amount of reads with preferred size is 75% (set with option: -pisize)  
Minimum amount of reads on the main strand(s) is 75% (set with option: -clstrand)**

Show read coverage
Hide read coverage

WHAT DO I SEE HERE?  
This chart shows the location of mapped sequence reads within a predicted piRNA cluster. The color refers to the number of genomic hits produced by the sequence read in question. A dark red bar indicates that this sequence read produces many other hits elsewhere in the genome. Many adjacent red or yellow bars can indicate the presence of a multi-copy element such as transposons or rRNA genes. A dark green bar indicates that this sequence read maps uniquely to this locus.

1 hit

2-5 hits

6-10 hits

11-20 hits

21-50 hits

51-100 hits

> 100 hits

chr5

76153176

76177962

Gene Set

RepeatMasker

Mapped  
Reads

134.21

plus strand

minus strand

134.21

Region: chr5 56770299-76153200. Max. coverage (+): 0. Max coverage (-): 1.3

Region: chr5 76153201-76153250. Max. coverage (+): 0. Max coverage (-): 0

Region: chr5 76153251-76153299. Max. coverage (+): 0. Max coverage (-): 0

Region: chr5 76153300-76153349. Max. coverage (+): 0. Max coverage (-): 0

Region: chr5 76153350-76153399. Max. coverage (+): 0. Max coverage (-): 0

Region: chr5 76153400-76153448. Max. coverage (+): 0. Max coverage (-): 0

Region: chr5 76153449-76153498. Max. coverage (+): 0. Max coverage (-): 0

Region: chr5 76153499-76153547. Max. coverage (+): 0. Max coverage (-): 0

Region: chr5 76153548-76153597. Max. coverage (+): 0. Max coverage (-): 0

Region: chr5 76153598-76153646. Max. coverage (+): 0. Max coverage (-): 0

Region: chr5 76153647-76153696. Max. coverage (+): 0. Max coverage (-): 0

Region: chr5 76153697-76153746. Max. coverage (+): 0. Max coverage (-): 0

Region: chr5 76153747-76153795. Max. coverage (+): 0. Max coverage (-): 0

Region: chr5 76153796-76153845. Max. coverage (+): 0. Max coverage (-): 0

Region: chr5 76153846-76153894. Max. coverage (+): 0. Max coverage (-): 0

Region: chr5 76153895-76153944. Max. coverage (+): 0. Max coverage (-): 2.79

Region: chr5 76153945-76153993. Max. coverage (+): 0. Max coverage (-): 2.79

Region: chr5 76153994-76154043. Max. coverage (+): 0. Max coverage (-): 2.72

Region: chr5 76154044-76154093. Max. coverage (+): 0. Max coverage (-): 0

Region: chr5 76154094-76154142. Max. coverage (+): 0. Max coverage (-): 1.67

Region: chr5 76154143-76154192. Max. coverage (+): 0. Max coverage (-): 0

Region: chr5 76154193-76154241. Max. coverage (+): 0. Max coverage (-): 0

Region: chr5 76154242-76154291. Max. coverage (+): 0. Max coverage (-): 7.3

Region: chr5 76154292-76154340. Max. coverage (+): 0. Max coverage (-): 7.26

Region: chr5 76154341-76154390. Max. coverage (+): 0. Max coverage (-): 5.18

Region: chr5 76154391-76154440. Max. coverage (+): 0. Max coverage (-): 4.57

Region: chr5 76154441-76154489. Max. coverage (+): 0. Max coverage (-): 0

Region: chr5 76154490-76154539. Max. coverage (+): 0. Max coverage (-): 0

Region: chr5 76154540-76154588. Max. coverage (+): 0. Max coverage (-): 8.24

Region: chr5 76154589-76154638. Max. coverage (+): 0. Max coverage (-): 0

Region: chr5 76154639-76154688. Max. coverage (+): 0. Max coverage (-): 0

Region: chr5 76154689-76154737. Max. coverage (+): 0. Max coverage (-): 0

Region: chr5 76154738-76154787. Max. coverage (+): 0. Max coverage (-): 0

Region: chr5 76154788-76154836. Max. coverage (+): 0. Max coverage (-): 7.72

Region: chr5 76154837-76154886. Max. coverage (+): 0. Max coverage (-): 0

Region: chr5 76154887-76154935. Max. coverage (+): 0. Max coverage (-): 0

Region: chr5 76154936-76154985. Max. coverage (+): 0. Max coverage (-): 0

Region: chr5 76154986-76155035. Max. coverage (+): 0. Max coverage (-): 0

Region: chr5 76155036-76155084. Max. coverage (+): 0. Max coverage (-): 0

Region: chr5 76155085-76155134. Max. coverage (+): 0. Max coverage (-): 0

Region: chr5 76155135-76155183. Max. coverage (+): 0. Max coverage (-): 0

Region: chr5 76155184-76155233. Max. coverage (+): 0. Max coverage (-): 0

Region: chr5 76155234-76155282. Max. coverage (+): 0. Max coverage (-): 0

Region: chr5 76155283-76155332. Max. coverage (+): 0. Max coverage (-): 0

Region: chr5 76155333-76155382. Max. coverage (+): 0. Max coverage (-): 0

Region: chr5 76155383-76155431. Max. coverage (+): 0. Max coverage (-): 0

Region: chr5 76155432-76155481. Max. coverage (+): 0. Max coverage (-): 0

Region: chr5 76155482-76155530. Max. coverage (+): 0. Max coverage (-): 0

Region: chr5 76155531-76155580. Max. coverage (+): 0. Max coverage (-): 0

Region: chr5 76155581-76155629. Max. coverage (+): 0. Max coverage (-): 0

Region: chr5 76155630-76155679. Max. coverage (+): 0. Max coverage (-): 0

Region: chr5 76155680-76155729. Max. coverage (+): 0. Max coverage (-): 0

Region: chr5 76155730-76155778. Max. coverage (+): 0. Max coverage (-): 0

Region: chr5 76155779-76155828. Max. coverage (+): 0. Max coverage (-): 0

Region: chr5 76155829-76155877. Max. coverage (+): 0. Max coverage (-): 10.12

Region: chr5 76155878-76155927. Max. coverage (+): 0. Max coverage (-): 12.88

Region: chr5 76155928-76155976. Max. coverage (+): 0. Max coverage (-): 14.18

Region: chr5 76155977-76156026. Max. coverage (+): 0. Max coverage (-): 9.43

Region: chr5 76156027-76156076. Max. coverage (+): 0. Max coverage (-): 3.63

Region: chr5 76156077-76156125. Max. coverage (+): 0. Max coverage (-): 18.67

Region: chr5 76156126-76156175. Max. coverage (+): 0. Max coverage (-): 0

Region: chr5 76156176-76156224. Max. coverage (+): 0. Max coverage (-): 1.51

Region: chr5 76156225-76156274. Max. coverage (+): 0. Max coverage (-): 3.2

Region: chr5 76156275-76156323. Max. coverage (+): 0. Max coverage (-): 14.59

Region: chr5 76156324-76156373. Max. coverage (+): 0. Max coverage (-): 0

Region: chr5 76156374-76156423. Max. coverage (+): 0. Max coverage (-): 0

Region: chr5 76156424-76156472. Max. coverage (+): 0. Max coverage (-): 0

Region: chr5 76156473-76156522. Max. coverage (+): 0. Max coverage (-): 0

Region: chr5 76156523-76156571. Max. coverage (+): 0. Max coverage (-): 1.33

Region: chr5 76156572-76156621. Max. coverage (+): 0. Max coverage (-): 7.12

Region: chr5 76156622-76156670. Max. coverage (+): 0. Max coverage (-): 1.2

Region: chr5 76156671-76156720. Max. coverage (+): 0. Max coverage (-): 0

Region: chr5 76156721-76156770. Max. coverage (+): 0. Max coverage (-): 0

Region: chr5 76156771-76156819. Max. coverage (+): 0. Max coverage (-): 0

Region: chr5 76156820-76156869. Max. coverage (+): 0. Max coverage (-): 0

Region: chr5 76156870-76156918. Max. coverage (+): 0. Max coverage (-): 0

Region: chr5 76156919-76156968. Max. coverage (+): 0. Max coverage (-): 5.25

Region: chr5 76156969-76157017. Max. coverage (+): 0. Max coverage (-): 10.97

Region: chr5 76157018-76157067. Max. coverage (+): 0. Max coverage (-): 20.12

Region: chr5 76157068-76157117. Max. coverage (+): 0. Max coverage (-): 20.41

Region: chr5 76157118-76157166. Max. coverage (+): 0. Max coverage (-): 7.34

Region: chr5 76157167-76157216. Max. coverage (+): 0. Max coverage (-): 23.16

Region: chr5 76157217-76157265. Max. coverage (+): 0. Max coverage (-): 5.77

Region: chr5 76157266-76157315. Max. coverage (+): 0. Max coverage (-): 16.79

Region: chr5 76157316-76157365. Max. coverage (+): 0. Max coverage (-): 10.4

Region: chr5 76157366-76157414. Max. coverage (+): 0. Max coverage (-): 5.46

Region: chr5 76157415-76157464. Max. coverage (+): 0. Max coverage (-): 0

Region: chr5 76157465-76157513. Max. coverage (+): 0. Max coverage (-): 14.22

Region: chr5 76157514-76157563. Max. coverage (+): 0. Max coverage (-): 17.81

Region: chr5 76157564-76157612. Max. coverage (+): 0. Max coverage (-): 31.87

Region: chr5 76157613-76157662. Max. coverage (+): 0. Max coverage (-): 10.37

Region: chr5 76157663-76157712. Max. coverage (+): 0. Max coverage (-): 31.56

Region: chr5 76157713-76157761. Max. coverage (+): 0. Max coverage (-): 15.45

Region: chr5 76157762-76157811. Max. coverage (+): 0. Max coverage (-): 13.97

Region: chr5 76157812-76157860. Max. coverage (+): 0. Max coverage (-): 14.2

Region: chr5 76157861-76157910. Max. coverage (+): 0. Max coverage (-): 12.48

Region: chr5 76157911-76157959. Max. coverage (+): 0. Max coverage (-): 23.87

Region: chr5 76157960-76158009. Max. coverage (+): 0. Max coverage (-): 5.06

Region: chr5 76158010-76158059. Max. coverage (+): 0. Max coverage (-): 0

Region: chr5 76158060-76158108. Max. coverage (+): 0. Max coverage (-): 0

Region: chr5 76158109-76158158. Max. coverage (+): 0. Max coverage (-): 0

Region: chr5 76158159-76158207. Max. coverage (+): 0. Max coverage (-): 0

Region: chr5 76158208-76158257. Max. coverage (+): 0. Max coverage (-): 0

Region: chr5 76158258-76158306. Max. coverage (+): 0. Max coverage (-): 0

Region: chr5 76158307-76158356. Max. coverage (+): 0. Max coverage (-): 0

Region: chr5 76158357-76158406. Max. coverage (+): 0. Max coverage (-): 0

Region: chr5 76158407-76158455. Max. coverage (+): 0. Max coverage (-): 0

Region: chr5 76158456-76158505. Max. coverage (+): 0. Max coverage (-): 0

Region: chr5 76158506-76158554. Max. coverage (+): 0. Max coverage (-): 0

Region: chr5 76158555-76158604. Max. coverage (+): 0. Max coverage (-): 0

Region: chr5 76158605-76158653. Max. coverage (+): 0. Max coverage (-): 2.85

Region: chr5 76158654-76158703. Max. coverage (+): 0. Max coverage (-): 10.42

Region: chr5 76158704-76158753. Max. coverage (+): 0. Max coverage (-): 9.48

Region: chr5 76158754-76158802. Max. coverage (+): 0. Max coverage (-): 24.01

Region: chr5 76158803-76158852. Max. coverage (+): 0. Max coverage (-): 24.52

Region: chr5 76158853-76158901. Max. coverage (+): 0. Max coverage (-): 6.87

Region: chr5 76158902-76158951. Max. coverage (+): 0. Max coverage (-): 25.78

Region: chr5 76158952-76159000. Max. coverage (+): 0. Max coverage (-): 10.56

Region: chr5 76159001-76159050. Max. coverage (+): 0. Max coverage (-): 24.28

Region: chr5 76159051-76159100. Max. coverage (+): 0. Max coverage (-): 0

Region: chr5 76159101-76159149. Max. coverage (+): 0. Max coverage (-): 0

Region: chr5 76159150-76159199. Max. coverage (+): 0. Max coverage (-): 0

Region: chr5 76159200-76159248. Max. coverage (+): 0. Max coverage (-): 17.71

Region: chr5 76159249-76159298. Max. coverage (+): 0. Max coverage (-): 0

Region: chr5 76159299-76159347. Max. coverage (+): 0. Max coverage (-): 0

Region: chr5 76159348-76159397. Max. coverage (+): 0. Max coverage (-): 0

Region: chr5 76159398-76159447. Max. coverage (+): 0. Max coverage (-): 3.66

Region: chr5 76159448-76159496. Max. coverage (+): 0. Max coverage (-): 8.73

Region: chr5 76159497-76159546. Max. coverage (+): 0. Max coverage (-): 0

Region: chr5 76159547-76159595. Max. coverage (+): 0. Max coverage (-): 0

Region: chr5 76159596-76159645. Max. coverage (+): 0. Max coverage (-): 0

Region: chr5 76159646-76159694. Max. coverage (+): 0. Max coverage (-): 0

Region: chr5 76159695-76159744. Max. coverage (+): 0. Max coverage (-): 50.83

Region: chr5 76159745-76159794. Max. coverage (+): 0. Max coverage (-): 13.98

Region: chr5 76159795-76159843. Max. coverage (+): 0. Max coverage (-): 27.06

Region: chr5 76159844-76159893. Max. coverage (+): 0. Max coverage (-): 18.18

Region: chr5 76159894-76159942. Max. coverage (+): 0. Max coverage (-): 4.67

Region: chr5 76159943-76159992. Max. coverage (+): 0. Max coverage (-): 9.59

Region: chr5 76159993-76160041. Max. coverage (+): 0. Max coverage (-): 62.11

Region: chr5 76160042-76160091. Max. coverage (+): 0. Max coverage (-): 14.52

Region: chr5 76160092-76160141. Max. coverage (+): 0. Max coverage (-): 4.44

Region: chr5 76160142-76160190. Max. coverage (+): 0. Max coverage (-): 4.58

Region: chr5 76160191-76160240. Max. coverage (+): 0. Max coverage (-): 4.97

Region: chr5 76160241-76160289. Max. coverage (+): 0. Max coverage (-): 38.62

Region: chr5 76160290-76160339. Max. coverage (+): 0. Max coverage (-): 8.4

Region: chr5 76160340-76160389. Max. coverage (+): 0. Max coverage (-): 21.62

Region: chr5 76160390-76160438. Max. coverage (+): 0. Max coverage (-): 55.55

Region: chr5 76160439-76160488. Max. coverage (+): 0. Max coverage (-): 54.32

Region: chr5 76160489-76160537. Max. coverage (+): 0. Max coverage (-): 12.42

Region: chr5 76160538-76160587. Max. coverage (+): 0. Max coverage (-): 21.43

Region: chr5 76160588-76160636. Max. coverage (+): 0. Max coverage (-): 28.12

Region: chr5 76160637-76160686. Max. coverage (+): 0. Max coverage (-): 0

Region: chr5 76160687-76160736. Max. coverage (+): 0. Max coverage (-): 0

Region: chr5 76160737-76160785. Max. coverage (+): 0. Max coverage (-): 0

Region: chr5 76160786-76160835. Max. coverage (+): 0. Max coverage (-): 0

Region: chr5 76160836-76160884. Max. coverage (+): 0. Max coverage (-): 0

Region: chr5 76160885-76160934. Max. coverage (+): 0. Max coverage (-): 13.06

Region: chr5 76160935-76160983. Max. coverage (+): 0. Max coverage (-): 14.04

Region: chr5 76160984-76161033. Max. coverage (+): 0. Max coverage (-): 6.08

Region: chr5 76161034-76161083. Max. coverage (+): 0. Max coverage (-): 5.24

Region: chr5 76161084-76161132. Max. coverage (+): 0. Max coverage (-): 4.81

Region: chr5 76161133-76161182. Max. coverage (+): 0. Max coverage (-): 3.63

Region: chr5 76161183-76161231. Max. coverage (+): 0. Max coverage (-): 0

Region: chr5 76161232-76161281. Max. coverage (+): 0. Max coverage (-): 0

Region: chr5 76161282-76161330. Max. coverage (+): 0. Max coverage (-): 0

Region: chr5 76161331-76161380. Max. coverage (+): 0. Max coverage (-): 0

Region: chr5 76161381-76161430. Max. coverage (+): 0. Max coverage (-): 0

Region: chr5 76161431-76161479. Max. coverage (+): 0. Max coverage (-): 0

Region: chr5 76161480-76161529. Max. coverage (+): 0. Max coverage (-): 7.07

Region: chr5 76161530-76161578. Max. coverage (+): 0. Max coverage (-): 16.36

Region: chr5 76161579-76161628. Max. coverage (+): 0. Max coverage (-): 5.9

Region: chr5 76161629-76161677. Max. coverage (+): 0. Max coverage (-): 9.2

Region: chr5 76161678-76161727. Max. coverage (+): 0. Max coverage (-): 21.07

Region: chr5 76161728-76161777. Max. coverage (+): 0. Max coverage (-): 0

Region: chr5 76161778-76161826. Max. coverage (+): 0. Max coverage (-): 2.21

Region: chr5 76161827-76161876. Max. coverage (+): 0. Max coverage (-): 0

Region: chr5 76161877-76161925. Max. coverage (+): 0. Max coverage (-): 17.1

Region: chr5 76161926-76161975. Max. coverage (+): 0. Max coverage (-): 2.57

Region: chr5 76161976-76162024. Max. coverage (+): 0. Max coverage (-): 10.86

Region: chr5 76162025-76162074. Max. coverage (+): 0. Max coverage (-): 3.63

Region: chr5 76162075-76162124. Max. coverage (+): 0. Max coverage (-): 5.23

Region: chr5 76162125-76162173. Max. coverage (+): 0. Max coverage (-): 4.7

Region: chr5 76162174-76162223. Max. coverage (+): 0. Max coverage (-): 5.83

Region: chr5 76162224-76162272. Max. coverage (+): 0. Max coverage (-): 13.67

Region: chr5 76162273-76162322. Max. coverage (+): 0. Max coverage (-): 30.05

Region: chr5 76162323-76162371. Max. coverage (+): 0. Max coverage (-): 0

Region: chr5 76162372-76162421. Max. coverage (+): 0. Max coverage (-): 0

Region: chr5 76162422-76162471. Max. coverage (+): 0. Max coverage (-): 0

Region: chr5 76162472-76162520. Max. coverage (+): 0. Max coverage (-): 0

Region: chr5 76162521-76162570. Max. coverage (+): 0. Max coverage (-): 0

Region: chr5 76162571-76162619. Max. coverage (+): 0. Max coverage (-): 0

Region: chr5 76162620-76162669. Max. coverage (+): 0. Max coverage (-): 0

Region: chr5 76162670-76162718. Max. coverage (+): 0. Max coverage (-): 0

Region: chr5 76162719-76162768. Max. coverage (+): 0. Max coverage (-): 0

Region: chr5 76162769-76162818. Max. coverage (+): 0. Max coverage (-): 0

Region: chr5 76162819-76162867. Max. coverage (+): 0. Max coverage (-): 0

Region: chr5 76162868-76162917. Max. coverage (+): 0. Max coverage (-): 0

Region: chr5 76162918-76162966. Max. coverage (+): 0. Max coverage (-): 0

Region: chr5 76162967-76163016. Max. coverage (+): 0. Max coverage (-): 9.11

Region: chr5 76163017-76163066. Max. coverage (+): 0. Max coverage (-): 10.62

Region: chr5 76163067-76163115. Max. coverage (+): 0. Max coverage (-): 23.01

Region: chr5 76163116-76163165. Max. coverage (+): 0. Max coverage (-): 42.29

Region: chr5 76163166-76163214. Max. coverage (+): 0. Max coverage (-): 15.56

Region: chr5 76163215-76163264. Max. coverage (+): 0. Max coverage (-): 1.7

Region: chr5 76163265-76163313. Max. coverage (+): 0. Max coverage (-): 25.69

Region: chr5 76163314-76163363. Max. coverage (+): 0. Max coverage (-): 18.36

Region: chr5 76163364-76163413. Max. coverage (+): 0. Max coverage (-): 23.19

Region: chr5 76163414-76163462. Max. coverage (+): 0. Max coverage (-): 28.25

Region: chr5 76163463-76163512. Max. coverage (+): 0. Max coverage (-): 7.96

Region: chr5 76163513-76163561. Max. coverage (+): 0. Max coverage (-): 16.35

Region: chr5 76163562-76163611. Max. coverage (+): 0. Max coverage (-): 3.95

Region: chr5 76163612-76163660. Max. coverage (+): 0. Max coverage (-): 9.45

Region: chr5 76163661-76163710. Max. coverage (+): 0. Max coverage (-): 2.45

Region: chr5 76163711-76163760. Max. coverage (+): 0. Max coverage (-): 5.55

Region: chr5 76163761-76163809. Max. coverage (+): 0. Max coverage (-): 23.62

Region: chr5 76163810-76163859. Max. coverage (+): 0. Max coverage (-): 21.61

Region: chr5 76163860-76163908. Max. coverage (+): 0. Max coverage (-): 35.91

Region: chr5 76163909-76163958. Max. coverage (+): 0. Max coverage (-): 27.29

Region: chr5 76163959-76164007. Max. coverage (+): 0. Max coverage (-): 76.75

Region: chr5 76164008-76164057. Max. coverage (+): 0. Max coverage (-): 5.81

Region: chr5 76164058-76164107. Max. coverage (+): 0. Max coverage (-): 3.04

Region: chr5 76164108-76164156. Max. coverage (+): 0. Max coverage (-): 4.11

Region: chr5 76164157-76164206. Max. coverage (+): 0. Max coverage (-): 47.64

Region: chr5 76164207-76164255. Max. coverage (+): 0. Max coverage (-): 6.76

Region: chr5 76164256-76164305. Max. coverage (+): 0. Max coverage (-): 25.54

Region: chr5 76164306-76164354. Max. coverage (+): 0. Max coverage (-): 31.08

Region: chr5 76164355-76164404. Max. coverage (+): 0. Max coverage (-): 21.17

Region: chr5 76164405-76164454. Max. coverage (+): 0. Max coverage (-): 9.71

Region: chr5 76164455-76164503. Max. coverage (+): 0. Max coverage (-): 45.6

Region: chr5 76164504-76164553. Max. coverage (+): 0. Max coverage (-): 23.88

Region: chr5 76164554-76164602. Max. coverage (+): 0. Max coverage (-): 0

Region: chr5 76164603-76164652. Max. coverage (+): 0. Max coverage (-): 0

Region: chr5 76164653-76164701. Max. coverage (+): 0. Max coverage (-): 0

Region: chr5 76164702-76164751. Max. coverage (+): 0. Max coverage (-): 0

Region: chr5 76164752-76164801. Max. coverage (+): 0. Max coverage (-): 0

Region: chr5 76164802-76164850. Max. coverage (+): 0. Max coverage (-): 7.61

Region: chr5 76164851-76164900. Max. coverage (+): 0. Max coverage (-): 14.56

Region: chr5 76164901-76164949. Max. coverage (+): 0. Max coverage (-): 8.9

Region: chr5 76164950-76164999. Max. coverage (+): 0. Max coverage (-): 13.86

Region: chr5 76165000-76165048. Max. coverage (+): 0. Max coverage (-): 3.43

Region: chr5 76165049-76165098. Max. coverage (+): 0. Max coverage (-): 3.58

Region: chr5 76165099-76165148. Max. coverage (+): 0. Max coverage (-): 10.96

Region: chr5 76165149-76165197. Max. coverage (+): 0. Max coverage (-): 5.5

Region: chr5 76165198-76165247. Max. coverage (+): 0. Max coverage (-): 0.35

Region: chr5 76165248-76165296. Max. coverage (+): 0. Max coverage (-): 1.23

Region: chr5 76165297-76165346. Max. coverage (+): 0. Max coverage (-): 15.65

Region: chr5 76165347-76165395. Max. coverage (+): 0. Max coverage (-): 48.44

Region: chr5 76165396-76165445. Max. coverage (+): 0. Max coverage (-): 4.54

Region: chr5 76165446-76165495. Max. coverage (+): 0. Max coverage (-): 4.72

Region: chr5 76165496-76165544. Max. coverage (+): 0. Max coverage (-): 34.38

Region: chr5 76165545-76165594. Max. coverage (+): 0. Max coverage (-): 55.18

Region: chr5 76165595-76165643. Max. coverage (+): 0. Max coverage (-): 49.47

Region: chr5 76165644-76165693. Max. coverage (+): 0. Max coverage (-): 10.61

Region: chr5 76165694-76165743. Max. coverage (+): 0. Max coverage (-): 4.24

Region: chr5 76165744-76165792. Max. coverage (+): 0. Max coverage (-): 4.13

Region: chr5 76165793-76165842. Max. coverage (+): 0. Max coverage (-): 20.89

Region: chr5 76165843-76165891. Max. coverage (+): 0. Max coverage (-): 110.93

Region: chr5 76165892-76165941. Max. coverage (+): 0. Max coverage (-): 87.87

Region: chr5 76165942-76165990. Max. coverage (+): 0. Max coverage (-): 10.25

Region: chr5 76165991-76166040. Max. coverage (+): 0. Max coverage (-): 31.62

Region: chr5 76166041-76166090. Max. coverage (+): 0. Max coverage (-): 23.85

Region: chr5 76166091-76166139. Max. coverage (+): 0. Max coverage (-): 28.44

Region: chr5 76166140-76166189. Max. coverage (+): 0. Max coverage (-): 5.12

Region: chr5 76166190-76166238. Max. coverage (+): 0. Max coverage (-): 7

Region: chr5 76166239-76166288. Max. coverage (+): 5.19. Max coverage (-): 0

Region: chr5 76166289-76166337. Max. coverage (+): 5.19. Max coverage (-): 9.36

Region: chr5 76166338-76166387. Max. coverage (+): 0. Max coverage (-): 26.77

Region: chr5 76166388-76166437. Max. coverage (+): 0. Max coverage (-): 30.4

Region: chr5 76166438-76166486. Max. coverage (+): 0. Max coverage (-): 78.5

Region: chr5 76166487-76166536. Max. coverage (+): 0. Max coverage (-): 42.22

Region: chr5 76166537-76166585. Max. coverage (+): 0. Max coverage (-): 36.8

Region: chr5 76166586-76166635. Max. coverage (+): 0. Max coverage (-): 24.79

Region: chr5 76166636-76166684. Max. coverage (+): 0. Max coverage (-): 22.21

Region: chr5 76166685-76166734. Max. coverage (+): 0. Max coverage (-): 0

Region: chr5 76166735-76166784. Max. coverage (+): 0. Max coverage (-): 0

Region: chr5 76166785-76166833. Max. coverage (+): 0. Max coverage (-): 0

Region: chr5 76166834-76166883. Max. coverage (+): 0. Max coverage (-): 0

Region: chr5 76166884-76166932. Max. coverage (+): 0. Max coverage (-): 23.69

Region: chr5 76166933-76166982. Max. coverage (+): 0. Max coverage (-): 2.99

Region: chr5 76166983-76167031. Max. coverage (+): 0. Max coverage (-): 1.39

Region: chr5 76167032-76167081. Max. coverage (+): 0. Max coverage (-): 20.59

Region: chr5 76167082-76167131. Max. coverage (+): 0. Max coverage (-): 45.44

Region: chr5 76167132-76167180. Max. coverage (+): 0. Max coverage (-): 54.7

Region: chr5 76167181-76167230. Max. coverage (+): 0. Max coverage (-): 14.16

Region: chr5 76167231-76167279. Max. coverage (+): 0. Max coverage (-): 2.19

Region: chr5 76167280-76167329. Max. coverage (+): 0. Max coverage (-): 4.35

Region: chr5 76167330-76167378. Max. coverage (+): 0. Max coverage (-): 19.51

Region: chr5 76167379-76167428. Max. coverage (+): 0. Max coverage (-): 3.32

Region: chr5 76167429-76167478. Max. coverage (+): 0. Max coverage (-): 12.39

Region: chr5 76167479-76167527. Max. coverage (+): 0. Max coverage (-): 5.97

Region: chr5 76167528-76167577. Max. coverage (+): 0. Max coverage (-): 5.97

Region: chr5 76167578-76167626. Max. coverage (+): 0. Max coverage (-): 5.01

Region: chr5 76167627-76167676. Max. coverage (+): 0. Max coverage (-): 19.5

Region: chr5 76167677-76167725. Max. coverage (+): 0. Max coverage (-): 31.99

Region: chr5 76167726-76167775. Max. coverage (+): 0. Max coverage (-): 25.41

Region: chr5 76167776-76167825. Max. coverage (+): 0. Max coverage (-): 13.25

Region: chr5 76167826-76167874. Max. coverage (+): 2.93. Max coverage (-): 133.3

Region: chr5 76167875-76167924. Max. coverage (+): 0. Max coverage (-): 14.84

Region: chr5 76167925-76167973. Max. coverage (+): 0. Max coverage (-): 43.98

Region: chr5 76167974-76168023. Max. coverage (+): 4.51. Max coverage (-): 0

Region: chr5 76168024-76168072. Max. coverage (+): 0. Max coverage (-): 26.89

Region: chr5 76168073-76168122. Max. coverage (+): 0. Max coverage (-): 52.76

Region: chr5 76168123-76168172. Max. coverage (+): 0. Max coverage (-): 26.99

Region: chr5 76168173-76168221. Max. coverage (+): 0. Max coverage (-): 11.86

Region: chr5 76168222-76168271. Max. coverage (+): 0. Max coverage (-): 0

Region: chr5 76168272-76168320. Max. coverage (+): 0. Max coverage (-): 0

Region: chr5 76168321-76168370. Max. coverage (+): 0. Max coverage (-): 7.8

Region: chr5 76168371-76168420. Max. coverage (+): 0. Max coverage (-): 6.45

Region: chr5 76168421-76168469. Max. coverage (+): 0. Max coverage (-): 7.01

Region: chr5 76168470-76168519. Max. coverage (+): 0. Max coverage (-): 26.25

Region: chr5 76168520-76168568. Max. coverage (+): 0. Max coverage (-): 28.5

Region: chr5 76168569-76168618. Max. coverage (+): 0. Max coverage (-): 60.93

Region: chr5 76168619-76168667. Max. coverage (+): 0. Max coverage (-): 0

Region: chr5 76168668-76168717. Max. coverage (+): 0. Max coverage (-): 13.32

Region: chr5 76168718-76168767. Max. coverage (+): 0. Max coverage (-): 29.31

Region: chr5 76168768-76168816. Max. coverage (+): 0. Max coverage (-): 5.73

Region: chr5 76168817-76168866. Max. coverage (+): 0. Max coverage (-): 5.73

Region: chr5 76168867-76168915. Max. coverage (+): 0. Max coverage (-): 18.2

Region: chr5 76168916-76168965. Max. coverage (+): 0. Max coverage (-): 7.02

Region: chr5 76168966-76169014. Max. coverage (+): 0. Max coverage (-): 3.91

Region: chr5 76169015-76169064. Max. coverage (+): 0. Max coverage (-): 50.94

Region: chr5 76169065-76169114. Max. coverage (+): 0. Max coverage (-): 27.1

Region: chr5 76169115-76169163. Max. coverage (+): 0. Max coverage (-): 44.45

Region: chr5 76169164-76169213. Max. coverage (+): 0. Max coverage (-): 1.41

Region: chr5 76169214-76169262. Max. coverage (+): 0. Max coverage (-): 0

Region: chr5 76169263-76169312. Max. coverage (+): 0. Max coverage (-): 7.6

Region: chr5 76169313-76169361. Max. coverage (+): 0. Max coverage (-): 0

Region: chr5 76169362-76169411. Max. coverage (+): 0. Max coverage (-): 10.75

Region: chr5 76169412-76169461. Max. coverage (+): 0. Max coverage (-): 10.99

Region: chr5 76169462-76169510. Max. coverage (+): 0. Max coverage (-): 62.07

Region: chr5 76169511-76169560. Max. coverage (+): 0. Max coverage (-): 30.94

Region: chr5 76169561-76169609. Max. coverage (+): 0. Max coverage (-): 134.21

Region: chr5 76169610-76169659. Max. coverage (+): 0. Max coverage (-): 2.05

Region: chr5 76169660-76169708. Max. coverage (+): 0. Max coverage (-): 4.65

Region: chr5 76169709-76169758. Max. coverage (+): 0. Max coverage (-): 16.73

Region: chr5 76169759-76169808. Max. coverage (+): 0. Max coverage (-): 2.24

Region: chr5 76169809-76169857. Max. coverage (+): 0. Max coverage (-): 6.19

Region: chr5 76169858-76169907. Max. coverage (+): 0. Max coverage (-): 8.82

Region: chr5 76169908-76169956. Max. coverage (+): 0. Max coverage (-): 4.91

Region: chr5 76169957-76170006. Max. coverage (+): 0. Max coverage (-): 1.41

Region: chr5 76170007-76170055. Max. coverage (+): 1.11. Max coverage (-): 0

Region: chr5 76170056-76170105. Max. coverage (+): 0. Max coverage (-): 4.51

Region: chr5 76170106-76170155. Max. coverage (+): 0. Max coverage (-): 37.83

Region: chr5 76170156-76170204. Max. coverage (+): 0. Max coverage (-): 7.31

Region: chr5 76170205-76170254. Max. coverage (+): 0. Max coverage (-): 26.25

Region: chr5 76170255-76170303. Max. coverage (+): 0. Max coverage (-): 11.86

Region: chr5 76170304-76170353. Max. coverage (+): 0. Max coverage (-): 0

Region: chr5 76170354-76170402. Max. coverage (+): 0. Max coverage (-): 44.75

Region: chr5 76170403-76170452. Max. coverage (+): 0. Max coverage (-): 14.31

Region: chr5 76170453-76170502. Max. coverage (+): 2.35. Max coverage (-): 25.65

Region: chr5 76170503-76170551. Max. coverage (+): 0. Max coverage (-): 6.81

Region: chr5 76170552-76170601. Max. coverage (+): 0. Max coverage (-): 36.49

Region: chr5 76170602-76170650. Max. coverage (+): 0. Max coverage (-): 19.02

Region: chr5 76170651-76170700. Max. coverage (+): 0. Max coverage (-): 19.78

Region: chr5 76170701-76170749. Max. coverage (+): 0. Max coverage (-): 50.48

Region: chr5 76170750-76170799. Max. coverage (+): 0. Max coverage (-): 4.94

Region: chr5 76170800-76170849. Max. coverage (+): 0. Max coverage (-): 1.33

Region: chr5 76170850-76170898. Max. coverage (+): 6.82. Max coverage (-): 15.66

Region: chr5 76170899-76170948. Max. coverage (+): 1.58. Max coverage (-): 0

Region: chr5 76170949-76170997. Max. coverage (+): 0. Max coverage (-): 0

Region: chr5 76170998-76171047. Max. coverage (+): 0. Max coverage (-): 0

Region: chr5 76171048-76171097. Max. coverage (+): 0. Max coverage (-): 33.99

Region: chr5 76171098-76171146. Max. coverage (+): 0. Max coverage (-): 32.94

Region: chr5 76171147-76171196. Max. coverage (+): 0. Max coverage (-): 54.56

Region: chr5 76171197-76171245. Max. coverage (+): 0. Max coverage (-): 1.38

Region: chr5 76171246-76171295. Max. coverage (+): 0. Max coverage (-): 46.16

Region: chr5 76171296-76171344. Max. coverage (+): 0. Max coverage (-): 27.71

Region: chr5 76171345-76171394. Max. coverage (+): 0. Max coverage (-): 50.03

Region: chr5 76171395-76171444. Max. coverage (+): 0. Max coverage (-): 3.06

Region: chr5 76171445-76171493. Max. coverage (+): 0. Max coverage (-): 36.24

Region: chr5 76171494-76171543. Max. coverage (+): 0. Max coverage (-): 13.13

Region: chr5 76171544-76171592. Max. coverage (+): 0. Max coverage (-): 7.03

Region: chr5 76171593-76171642. Max. coverage (+): 0. Max coverage (-): 11.44

Region: chr5 76171643-76171691. Max. coverage (+): 0. Max coverage (-): 22.18

Region: chr5 76171692-76171741. Max. coverage (+): 0. Max coverage (-): 17.17

Region: chr5 76171742-76171791. Max. coverage (+): 0. Max coverage (-): 15.31

Region: chr5 76171792-76171840. Max. coverage (+): 0. Max coverage (-): 33.9

Region: chr5 76171841-76171890. Max. coverage (+): 0. Max coverage (-): 4.28

Region: chr5 76171891-76171939. Max. coverage (+): 0. Max coverage (-): 6.21

Region: chr5 76171940-76171989. Max. coverage (+): 0. Max coverage (-): 0

Region: chr5 76171990-76172038. Max. coverage (+): 0. Max coverage (-): 0

Region: chr5 76172039-76172088. Max. coverage (+): 0. Max coverage (-): 0

Region: chr5 76172089-76172138. Max. coverage (+): 0. Max coverage (-): 3.82

Region: chr5 76172139-76172187. Max. coverage (+): 0. Max coverage (-): 0

Region: chr5 76172188-76172237. Max. coverage (+): 0. Max coverage (-): 1.7

Region: chr5 76172238-76172286. Max. coverage (+): 0. Max coverage (-): 1.98

Region: chr5 76172287-76172336. Max. coverage (+): 0. Max coverage (-): 3.15

Region: chr5 76172337-76172385. Max. coverage (+): 0. Max coverage (-): 0

Region: chr5 76172386-76172435. Max. coverage (+): 0. Max coverage (-): 0

Region: chr5 76172436-76172485. Max. coverage (+): 0. Max coverage (-): 8.57

Region: chr5 76172486-76172534. Max. coverage (+): 0. Max coverage (-): 0

Region: chr5 76172535-76172584. Max. coverage (+): 0. Max coverage (-): 0

Region: chr5 76172585-76172633. Max. coverage (+): 2.72. Max coverage (-): 1.18

Region: chr5 76172634-76172683. Max. coverage (+): 0. Max coverage (-): 0

Region: chr5 76172684-76172732. Max. coverage (+): 0. Max coverage (-): 0

Region: chr5 76172733-76172782. Max. coverage (+): 0. Max coverage (-): 3.25

Region: chr5 76172783-76172832. Max. coverage (+): 6.16. Max coverage (-): 2.57

Region: chr5 76172833-76172881. Max. coverage (+): 0. Max coverage (-): 12.39

Region: chr5 76172882-76172931. Max. coverage (+): 0. Max coverage (-): 0

Region: chr5 76172932-76172980. Max. coverage (+): 0. Max coverage (-): 0

Region: chr5 76172981-76173030. Max. coverage (+): 0. Max coverage (-): 5.64

Region: chr5 76173031-76173079. Max. coverage (+): 0. Max coverage (-): 0

Region: chr5 76173080-76173129. Max. coverage (+): 0. Max coverage (-): 0

Region: chr5 76173130-76173179. Max. coverage (+): 0. Max coverage (-): 0

Region: chr5 76173180-76173228. Max. coverage (+): 0. Max coverage (-): 3.92

Region: chr5 76173229-76173278. Max. coverage (+): 0. Max coverage (-): 31.19

Region: chr5 76173279-76173327. Max. coverage (+): 0. Max coverage (-): 7.18

Region: chr5 76173328-76173377. Max. coverage (+): 0. Max coverage (-): 1.78

Region: chr5 76173378-76173426. Max. coverage (+): 0. Max coverage (-): 0

Region: chr5 76173427-76173476. Max. coverage (+): 0. Max coverage (-): 0

Region: chr5 76173477-76173526. Max. coverage (+): 0. Max coverage (-): 0

Region: chr5 76173527-76173575. Max. coverage (+): 8.21. Max coverage (-): 1.92

Region: chr5 76173576-76173625. Max. coverage (+): 0. Max coverage (-): 0

Region: chr5 76173626-76173674. Max. coverage (+): 0. Max coverage (-): 0

Region: chr5 76173675-76173724. Max. coverage (+): 0. Max coverage (-): 0

Region: chr5 76173725-76173773. Max. coverage (+): 0. Max coverage (-): 0

Region: chr5 76173774-76173823. Max. coverage (+): 0. Max coverage (-): 0

Region: chr5 76173824-76173873. Max. coverage (+): 0. Max coverage (-): 0

Region: chr5 76173874-76173922. Max. coverage (+): 0. Max coverage (-): 0

Region: chr5 76173923-76173972. Max. coverage (+): 23.93. Max coverage (-): 0

Region: chr5 76173973-76174021. Max. coverage (+): 13.23. Max coverage (-): 0

Region: chr5 76174022-76174071. Max. coverage (+): 4.03. Max coverage (-): 0

Region: chr5 76174072-76174121. Max. coverage (+): 0. Max coverage (-): 0

Region: chr5 76174122-76174170. Max. coverage (+): 0. Max coverage (-): 0

Region: chr5 76174171-76174220. Max. coverage (+): 0. Max coverage (-): 0

Region: chr5 76174221-76174269. Max. coverage (+): 0. Max coverage (-): 0

Region: chr5 76174270-76174319. Max. coverage (+): 3.13. Max coverage (-): 0

Region: chr5 76174320-76174368. Max. coverage (+): 3.13. Max coverage (-): 0

Region: chr5 76174369-76174418. Max. coverage (+): 0. Max coverage (-): 0

Region: chr5 76174419-76174468. Max. coverage (+): 0. Max coverage (-): 0

Region: chr5 76174469-76174517. Max. coverage (+): 0. Max coverage (-): 0

Region: chr5 76174518-76174567. Max. coverage (+): 0. Max coverage (-): 0

Region: chr5 76174568-76174616. Max. coverage (+): 0. Max coverage (-): 0

Region: chr5 76174617-76174666. Max. coverage (+): 0. Max coverage (-): 0

Region: chr5 76174667-76174715. Max. coverage (+): 0. Max coverage (-): 0

Region: chr5 76174716-76174765. Max. coverage (+): 0. Max coverage (-): 0

Region: chr5 76174766-76174815. Max. coverage (+): 0. Max coverage (-): 0

Region: chr5 76174816-76174864. Max. coverage (+): 2.19. Max coverage (-): 0

Region: chr5 76174865-76174914. Max. coverage (+): 0. Max coverage (-): 0

Region: chr5 76174915-76174963. Max. coverage (+): 0. Max coverage (-): 0

Region: chr5 76174964-76175013. Max. coverage (+): 0. Max coverage (-): 0

Region: chr5 76175014-76175062. Max. coverage (+): 0. Max coverage (-): 0

Region: chr5 76175063-76175112. Max. coverage (+): 0. Max coverage (-): 0

Region: chr5 76175113-76175162. Max. coverage (+): 0. Max coverage (-): 0

Region: chr5 76175163-76175211. Max. coverage (+): 0. Max coverage (-): 0

Region: chr5 76175212-76175261. Max. coverage (+): 0. Max coverage (-): 0

Region: chr5 76175262-76175310. Max. coverage (+): 0. Max coverage (-): 0

Region: chr5 76175311-76175360. Max. coverage (+): 4.78. Max coverage (-): 0

Region: chr5 76175361-76175409. Max. coverage (+): 0. Max coverage (-): 0

Region: chr5 76175410-76175459. Max. coverage (+): 0. Max coverage (-): 0

Region: chr5 76175460-76175509. Max. coverage (+): 0.58. Max coverage (-): 0

Region: chr5 76175510-76175558. Max. coverage (+): 0. Max coverage (-): 0

Region: chr5 76175559-76175608. Max. coverage (+): 0. Max coverage (-): 0

Region: chr5 76175609-76175657. Max. coverage (+): 0. Max coverage (-): 0

Region: chr5 76175658-76175707. Max. coverage (+): 0. Max coverage (-): 0

Region: chr5 76175708-76175756. Max. coverage (+): 0.57. Max coverage (-): 0

Region: chr5 76175757-76175806. Max. coverage (+): 0. Max coverage (-): 0

Region: chr5 76175807-76175856. Max. coverage (+): 0. Max coverage (-): 0

Region: chr5 76175857-76175905. Max. coverage (+): 0. Max coverage (-): 0

Region: chr5 76175906-76175955. Max. coverage (+): 0. Max coverage (-): 0

Region: chr5 76175956-76176004. Max. coverage (+): 0. Max coverage (-): 0

Region: chr5 76176005-76176054. Max. coverage (+): 0. Max coverage (-): 0

Region: chr5 76176055-76176103. Max. coverage (+): 0. Max coverage (-): 0

Region: chr5 76176104-76176153. Max. coverage (+): 0. Max coverage (-): 0

Region: chr5 76176154-76176203. Max. coverage (+): 0. Max coverage (-): 0

Region: chr5 76176204-76176252. Max. coverage (+): 0. Max coverage (-): 0

Region: chr5 76176253-76176302. Max. coverage (+): 0. Max coverage (-): 0

Region: chr5 76176303-76176351. Max. coverage (+): 0. Max coverage (-): 0

Region: chr5 76176352-76176401. Max. coverage (+): 0. Max coverage (-): 0

Region: chr5 76176402-76176450. Max. coverage (+): 0. Max coverage (-): 0

Region: chr5 76176451-76176500. Max. coverage (+): 0. Max coverage (-): 0

Region: chr5 76176501-76176550. Max. coverage (+): 0. Max coverage (-): 0

Region: chr5 76176551-76176599. Max. coverage (+): 0. Max coverage (-): 0

Region: chr5 76176600-76176649. Max. coverage (+): 0. Max coverage (-): 0

Region: chr5 76176650-76176698. Max. coverage (+): 0. Max coverage (-): 0

Region: chr5 76176699-76176748. Max. coverage (+): 3.41. Max coverage (-): 0

Region: chr5 76176749-76176798. Max. coverage (+): 3.41. Max coverage (-): 0

Region: chr5 76176799-76176847. Max. coverage (+): 0. Max coverage (-): 0

Region: chr5 76176848-76176897. Max. coverage (+): 0. Max coverage (-): 0

Region: chr5 76176898-76176946. Max. coverage (+): 0. Max coverage (-): 0

Region: chr5 76176947-76176996. Max. coverage (+): 0. Max coverage (-): 0

Region: chr5 76176997-76177045. Max. coverage (+): 1.39. Max coverage (-): 0

Region: chr5 76177046-76177095. Max. coverage (+): 0. Max coverage (-): 0

Region: chr5 76177096-76177145. Max. coverage (+): 0. Max coverage (-): 0

Region: chr5 76177146-76177194. Max. coverage (+): 1.8. Max coverage (-): 0

Region: chr5 76177195-76177244. Max. coverage (+): 0. Max coverage (-): 0

Region: chr5 76177245-76177293. Max. coverage (+): 0. Max coverage (-): 0

Region: chr5 76177294-76177343. Max. coverage (+): 0. Max coverage (-): 0

Region: chr5 76177344-76177392. Max. coverage (+): 0. Max coverage (-): 0

Region: chr5 76177393-76177442. Max. coverage (+): 0. Max coverage (-): 0

Region: chr5 76177443-76177492. Max. coverage (+): 0. Max coverage (-): 0

Region: chr5 76177493-76177541. Max. coverage (+): 0. Max coverage (-): 0

Region: chr5 76177542-76177591. Max. coverage (+): 0. Max coverage (-): 0

Region: chr5 76177592-76177640. Max. coverage (+): 23.74. Max coverage (-): 0

Region: chr5 76177641-76177690. Max. coverage (+): 0. Max coverage (-): 0

Region: chr5 76177691-76177739. Max. coverage (+): 0. Max coverage (-): 0

Region: chr5 76177740-76177789. Max. coverage (+): 0. Max coverage (-): 0

Region: chr5 76177790-76177839. Max. coverage (+): 2.12. Max coverage (-): 0

Region: chr5 76177840-76177888. Max. coverage (+): 0. Max coverage (-): 0

Region: chr5 76177889-76177938. Max. coverage (+): 0.77. Max coverage (-): 0

Region: chr5 76177939-. Max. coverage (+): 0.77. Max coverage (-): 0

RepeatMasker Color Code

**+**

100-98% Identity

<98-95% Identity

<95-90% Identity

<90-85% Identity

<85-80% Identity

<80-75% Identity

<75-70% Identity

<70% Identity

**-**

Gene Set Color Code

**+**

Gene

Pseudogene

**-**

Topology/Coverage Color Code

Coverage Plus Strand

Coverage Minus Strand

Mainstrand: Plus

Mainstrand: Minus

Complementary Strand

Flanking Region  
(if option -flank >0)

Gene Set Annotation  

**1. ELFN2 (protein coding, ENSBTAG00000007259) Tr:00000009548 Ex:1**: 76173495-76175969 (-)

  
RepeatMasker Annotation  

**1. (TG)n**: 76153209-76153253 (+), Divergence to consensus: 2.2%  
**2. MIR**: 76153267-76153401 (-), Divergence to consensus: 43.9%  
**3. MLT1J**: 76153464-76153921 (-), Divergence to consensus: 42.9%  
**4. MIR**: 76154108-76154215 (+), Divergence to consensus: 44.8%  
**5. Plat\_L3**: 76154480-76154545 (-), Divergence to consensus: 24.5%  
**6. L3**: 76154550-76154782 (-), Divergence to consensus: 46%  
**7. MLT1L**: 76155132-76155284 (-), Divergence to consensus: 38.8%  
**8. X7B\_LINE**: 76155287-76155359 (+), Divergence to consensus: 38.4%  
**9. L2c**: 76155373-76155424 (-), Divergence to consensus: 32.7%  
**10. L2c**: 76155472-76155789 (-), Divergence to consensus: 43.9%  
**11. L2c**: 76155803-76155871 (-), Divergence to consensus: 23.2%  
**12. L3**: 76156307-76156350 (-), Divergence to consensus: 29.6%  
**13. MIR**: 76156379-76156513 (-), Divergence to consensus: 39.5%  
**14. MIRb**: 76156693-76156868 (+), Divergence to consensus: 49.5%  
**15. C-rich**: 76157421-76157464 (+), Divergence to consensus: 22%  
**16. MER115**: 76158059-76158633 (-), Divergence to consensus: 45.2%  
**17. MIRb**: 76159044-76159208 (+), Divergence to consensus: 41.2%  
**18. LTR67B**: 76159257-76159407 (+), Divergence to consensus: 38.4%  
**19. LTR67B**: 76159526-76159710 (+), Divergence to consensus: 39.5%  
**20. Bov-tA2**: 76160658-76160719 (-), Divergence to consensus: 17.7%  
**21. BovB**: 76160720-76160873 (-), Divergence to consensus: 19.9%  
**22. ART2A**: 76161187-76161513 (+), Divergence to consensus: 17.9%  
**23. MER41\_BT**: 76162340-76162380 (-), Divergence to consensus: 14.6%  
**24. MER41\_BT**: 76162367-76162939 (-), Divergence to consensus: 25.7%  
**25. Bov-tA3**: 76164553-76164777 (+), Divergence to consensus: 13.5%  
**26. MamRep1151**: 76166342-76166962 (+), Divergence to consensus: 44.7%  
**27. MIRb**: 76170924-76170987 (+), Divergence to consensus: 41.7%  
**28. MIRb**: 76172881-76173007 (+), Divergence to consensus: 40.2%  
**29. (CGTG)n**: 76173350-76173385 (+), Divergence to consensus: 11.1%  
**30. GC\_rich**: 76173399-76173433 (+), Divergence to consensus: 68.6%  
**31. GC\_rich**: 76176009-76176032 (+), Divergence to consensus: 50%  
**32. MIR3**: 76177322-76177410 (-), Divergence to consensus: 29.7%  
**33. MIRc**: 76177477-76177549 (+), Divergence to consensus: 30.4%

  
Transcription Factor Binding Sites  

**RFX4\_1** (Sequence: GTTGCTAGG (-): 76157073)  
**RFX4\_1** (Sequence: GTTGCCAAG (-): 76164539)  
**RFX4\_1** (Sequence: GTTGCCATG (-): 76172115)  
**RFX4\_1** (Sequence: CCTGGCAAC (+): 76160130)  
**RFX4\_2** (Sequence: CTTGGTTAC (+): 76175346)  
**SOX9** (Sequence: AACAATGA (-): 76160489)  
**SOX9** (Sequence: TTATTGTT (+): 76157251)  
**A-MYB** (Sequence: CCAACTGCCA (-): 76172097)  
**A-MYB** (Sequence: CCAACTGTCA (-): 76176139)
