# Supplementary material for: Small RNA sequencing of cryopreserved semen from single bull revealed altered miRNAs and piRNAs expression between High- and Low-motile sperm populations
Source: BMC Genomics. 2017 Jan 4;18:14. doi: 10.1186/s12864-016-3394-7 (PMC5209821; doi:10.1186/s12864-016-3394-7)
Supplement: Additional file 3: — Details for each piRNA clusters found in High Motile (HM) sperm fraction. Genes, repeats, transposable elements and transcription factors binding sites falling within the cluster regions were reported. (ZIP 1896 kb) [file 12864_2016_3394_MOESM3_ESM.zip › 88.html]

piRNA cluster 88


Predicted piRNA cluster no. 88     previous   next
  

Show proTRAC run info
Hide proTRAC run info

================================= proTRAC ====================================  
VERSION: 2.1                                    LAST MODIFIED: 06. October 2015  
  
Please cite:  
Rosenkranz D, Zischler H. proTRAC - a software for probabilistic piRNA cluster  
detection, visualization and analysis. 2012. BMC Bioinformatics 13:5.  
  
and (for proTRAC 2.0 and later):  
Rosenkranz D, Rudloff S, Bastuck K, Ketting RF, Zischler H. Tupaia small RNAs  
provide insights into function and evolution of RNAi-based transposon defense  
in mammals. 2015. RNA 21(5):911-922.  
  
Contact:  
David Rosenkranz  
Institute of Anthropology, small RNA group  
Johannes Gutenberg University Mainz  
email: rosenkranz@uni-mainz.de  
  
You can find the latest proTRAC version at:  
http://sourceforge.net/projects/protrac/files  
http://www.smallRNAgroup-mainz.de/software  
==============================================================================  
  
PARAMETERS:  
Map file: .............../storage/core/barbara/genhome/smallRNA/fertility/Sample\_motile/pirna/Sample\_motile\_26-33\_collapsed.fa.no-dust.map.weighted-10000-1000-b-0  
Genome file: ............/storage/core/barbara/genhome/smallRNA/fertility/Sample\_all/pirna/bt\_311\_chrY.fa  
RepeatMasker annotation: /storage/genomes/bt\_umd31/GCF\_000003055.6\_Bos\_taurus\_UMD\_3.1.1\_repeatMasker\_chr.out  
GeneSet:................./storage/core/barbara/genhome/smallRNA/fertility/Sample\_all/pirna/full.gtf  
  
Significant (p<=0.01) hit density will be calculated based  
on observed hit distribution.  
  
Sliding window size: ........................................ 5000 bp  
Sliding window increament: .................................. 1000 bp  
Normalize each hit by number of genomic hits: ............... 1 [0=no/1=yes]  
Normalize each hit by number of sequence reads: ............. 1 [0=no/1=yes]  
Normalize values (-> per million mapped reads): ............. 1 [0=no/1=yes]  
Min. fraction of hits with 1T(U) or 10A: .................... 0.75  
Alternatively: Min. fraction of hits with 1T(U) and 10A: .... 0.5  
Min. fraction of hits with typical piRNA length: ............ 0.75  
Typical piRNA length: ....................................... 26-33 nt  
Min. size of a piRNA cluster: ............................... 5000 bp.  
Min. number of hits (absolute): ............................. 0  
Min. number of hits (normalized): ........................... 0  
Min. fraction of hits on the mainstrand: .................... 0.75  
Top fraction of mapped sequences (in terms of read counts): . 1%  
Top fraction accounts for max. n% of sequence reads: ........ 90%  
Min. fraction of hits on each arm of a bidirectional cluster: 0.1  
Output image file for each cluster: ......................... 0 [0=no/1=yes]  
Output html file for each cluster: .......................... 1 [0=no/1=yes]  
Output a summary table: ..................................... 1 [0=no/1=yes]  
Output a FASTA file for each cluster (piRNA sequences): ..... 1 [0=no/1=yes]  
Output a FASTA file comprising cluster sequences: ........... 1 [0=no/1=yes]  
Search DNA motifs in clusters: .............................. 1 [0=no/1=yes]  
Output flanking sequences: +/- .............................. 0 bp  
Output ~.pTi file: .......................................... 1 [0=no/1=yes]  
==============================================================================  
  
  
Genome size (without gaps): ............ 2678902517 bp  
Gaps (N/X/-): .......................... 53837044 bp  
Mapped reads: .......................... 658825247023  
Non-identical sequences: ............... 514171  
Genomic hits: .......................... 764233  
Significant densitiy of mapped reads: .. 12867599.5173724 reads/kb

Show proTRAC cluster info
Hide proTRAC cluster info

|  |  |
| --- | --- |
| Location | chr5 |
| Coordinates | 117255378-117261860 |
| Size [bp] | 6483 |
| Sequence hit loci | 183 |
| Mapped reads (normalized) | 143768859.9 |
| Mapped reads (normalized) per kb | 22176285.7 |
| Normalized reads with 1T (1U) | 83.7% |
| Normalized reads with 10A | 32.4% |
| Normalized reads with length 26-33 nt | 100% |
| Normalized reads on the main strand(s) | 96.5% |
| Predicted directionality | mono:minus |

100%

0%

1T (1U)  
reads

10A reads

26-33 nt  
reads

reads on mainstrand

**Either the amount of reads with 1T (1U) OR 10A has to exceed 75% (set with option: -1Tor10A)  
Alternatively the amount of reads with 1T (1U) AND 10A has to exceed 50% (set with option: -1Tand10A)  
Minimum amount of reads with preferred size is 75% (set with option: -pisize)  
Minimum amount of reads on the main strand(s) is 75% (set with option: -clstrand)**

Show read coverage
Hide read coverage

WHAT DO I SEE HERE?  
This chart shows the location of mapped sequence reads within a predicted piRNA cluster. The color refers to the number of genomic hits produced by the sequence read in question. A dark red bar indicates that this sequence read produces many other hits elsewhere in the genome. Many adjacent red or yellow bars can indicate the presence of a multi-copy element such as transposons or rRNA genes. A dark green bar indicates that this sequence read maps uniquely to this locus.

1 hit

2-5 hits

6-10 hits

11-20 hits

21-50 hits

51-100 hits

> 100 hits

chr5

117255378

117261860

Gene Set

RepeatMasker

Mapped  
Reads

12.24

plus strand

minus strand

12.24

Region: chr5 76177940-117255384. Max. coverage (+): 0. Max coverage (-): 4.59

Region: chr5 117255385-117255397. Max. coverage (+): 0. Max coverage (-): 4.59

Region: chr5 117255398-117255410. Max. coverage (+): 0. Max coverage (-): 0

Region: chr5 117255411-117255423. Max. coverage (+): 0. Max coverage (-): 0

Region: chr5 117255424-117255436. Max. coverage (+): 0. Max coverage (-): 0

Region: chr5 117255437-117255449. Max. coverage (+): 0. Max coverage (-): 0

Region: chr5 117255450-117255462. Max. coverage (+): 0. Max coverage (-): 0

Region: chr5 117255463-117255475. Max. coverage (+): 0. Max coverage (-): 0

Region: chr5 117255476-117255488. Max. coverage (+): 0. Max coverage (-): 0

Region: chr5 117255489-117255501. Max. coverage (+): 0. Max coverage (-): 0

Region: chr5 117255502-117255514. Max. coverage (+): 0. Max coverage (-): 0

Region: chr5 117255515-117255527. Max. coverage (+): 0. Max coverage (-): 0

Region: chr5 117255528-117255540. Max. coverage (+): 0. Max coverage (-): 0

Region: chr5 117255541-117255553. Max. coverage (+): 0. Max coverage (-): 0

Region: chr5 117255554-117255566. Max. coverage (+): 0. Max coverage (-): 0

Region: chr5 117255567-117255578. Max. coverage (+): 0. Max coverage (-): 0

Region: chr5 117255579-117255591. Max. coverage (+): 0. Max coverage (-): 0

Region: chr5 117255592-117255604. Max. coverage (+): 0. Max coverage (-): 0

Region: chr5 117255605-117255617. Max. coverage (+): 0. Max coverage (-): 0

Region: chr5 117255618-117255630. Max. coverage (+): 0. Max coverage (-): 0

Region: chr5 117255631-117255643. Max. coverage (+): 0. Max coverage (-): 0

Region: chr5 117255644-117255656. Max. coverage (+): 0. Max coverage (-): 0

Region: chr5 117255657-117255669. Max. coverage (+): 0. Max coverage (-): 0

Region: chr5 117255670-117255682. Max. coverage (+): 0. Max coverage (-): 0

Region: chr5 117255683-117255695. Max. coverage (+): 0. Max coverage (-): 0

Region: chr5 117255696-117255708. Max. coverage (+): 0. Max coverage (-): 0

Region: chr5 117255709-117255721. Max. coverage (+): 0. Max coverage (-): 0

Region: chr5 117255722-117255734. Max. coverage (+): 0. Max coverage (-): 0

Region: chr5 117255735-117255747. Max. coverage (+): 0. Max coverage (-): 0

Region: chr5 117255748-117255760. Max. coverage (+): 0. Max coverage (-): 0

Region: chr5 117255761-117255773. Max. coverage (+): 0. Max coverage (-): 0

Region: chr5 117255774-117255786. Max. coverage (+): 0. Max coverage (-): 0

Region: chr5 117255787-117255799. Max. coverage (+): 0. Max coverage (-): 0

Region: chr5 117255800-117255812. Max. coverage (+): 0. Max coverage (-): 0

Region: chr5 117255813-117255825. Max. coverage (+): 0. Max coverage (-): 0

Region: chr5 117255826-117255838. Max. coverage (+): 0. Max coverage (-): 0

Region: chr5 117255839-117255851. Max. coverage (+): 0. Max coverage (-): 0

Region: chr5 117255852-117255864. Max. coverage (+): 0. Max coverage (-): 0

Region: chr5 117255865-117255877. Max. coverage (+): 0. Max coverage (-): 0

Region: chr5 117255878-117255890. Max. coverage (+): 0. Max coverage (-): 0

Region: chr5 117255891-117255903. Max. coverage (+): 0. Max coverage (-): 0

Region: chr5 117255904-117255916. Max. coverage (+): 0. Max coverage (-): 0

Region: chr5 117255917-117255929. Max. coverage (+): 0. Max coverage (-): 0

Region: chr5 117255930-117255942. Max. coverage (+): 0. Max coverage (-): 0

Region: chr5 117255943-117255954. Max. coverage (+): 0. Max coverage (-): 0

Region: chr5 117255955-117255967. Max. coverage (+): 0. Max coverage (-): 0

Region: chr5 117255968-117255980. Max. coverage (+): 0. Max coverage (-): 0

Region: chr5 117255981-117255993. Max. coverage (+): 0. Max coverage (-): 0

Region: chr5 117255994-117256006. Max. coverage (+): 0. Max coverage (-): 0

Region: chr5 117256007-117256019. Max. coverage (+): 0. Max coverage (-): 0

Region: chr5 117256020-117256032. Max. coverage (+): 0. Max coverage (-): 0

Region: chr5 117256033-117256045. Max. coverage (+): 0. Max coverage (-): 0

Region: chr5 117256046-117256058. Max. coverage (+): 0. Max coverage (-): 0

Region: chr5 117256059-117256071. Max. coverage (+): 0. Max coverage (-): 0

Region: chr5 117256072-117256084. Max. coverage (+): 0. Max coverage (-): 0

Region: chr5 117256085-117256097. Max. coverage (+): 0. Max coverage (-): 0

Region: chr5 117256098-117256110. Max. coverage (+): 0. Max coverage (-): 0

Region: chr5 117256111-117256123. Max. coverage (+): 0. Max coverage (-): 0

Region: chr5 117256124-117256136. Max. coverage (+): 0. Max coverage (-): 0

Region: chr5 117256137-117256149. Max. coverage (+): 0. Max coverage (-): 0

Region: chr5 117256150-117256162. Max. coverage (+): 0. Max coverage (-): 0

Region: chr5 117256163-117256175. Max. coverage (+): 0. Max coverage (-): 0

Region: chr5 117256176-117256188. Max. coverage (+): 0. Max coverage (-): 0

Region: chr5 117256189-117256201. Max. coverage (+): 0. Max coverage (-): 0

Region: chr5 117256202-117256214. Max. coverage (+): 0. Max coverage (-): 0

Region: chr5 117256215-117256227. Max. coverage (+): 0. Max coverage (-): 0

Region: chr5 117256228-117256240. Max. coverage (+): 0. Max coverage (-): 0

Region: chr5 117256241-117256253. Max. coverage (+): 0. Max coverage (-): 0

Region: chr5 117256254-117256266. Max. coverage (+): 0. Max coverage (-): 0

Region: chr5 117256267-117256279. Max. coverage (+): 0. Max coverage (-): 0

Region: chr5 117256280-117256292. Max. coverage (+): 0. Max coverage (-): 0

Region: chr5 117256293-117256305. Max. coverage (+): 0. Max coverage (-): 0

Region: chr5 117256306-117256318. Max. coverage (+): 0. Max coverage (-): 0

Region: chr5 117256319-117256331. Max. coverage (+): 0. Max coverage (-): 0

Region: chr5 117256332-117256343. Max. coverage (+): 0. Max coverage (-): 0

Region: chr5 117256344-117256356. Max. coverage (+): 0. Max coverage (-): 0

Region: chr5 117256357-117256369. Max. coverage (+): 0. Max coverage (-): 0

Region: chr5 117256370-117256382. Max. coverage (+): 0. Max coverage (-): 0

Region: chr5 117256383-117256395. Max. coverage (+): 0. Max coverage (-): 0

Region: chr5 117256396-117256408. Max. coverage (+): 0. Max coverage (-): 0

Region: chr5 117256409-117256421. Max. coverage (+): 0. Max coverage (-): 0

Region: chr5 117256422-117256434. Max. coverage (+): 0. Max coverage (-): 0

Region: chr5 117256435-117256447. Max. coverage (+): 0. Max coverage (-): 0

Region: chr5 117256448-117256460. Max. coverage (+): 0. Max coverage (-): 0

Region: chr5 117256461-117256473. Max. coverage (+): 0. Max coverage (-): 0

Region: chr5 117256474-117256486. Max. coverage (+): 0. Max coverage (-): 0

Region: chr5 117256487-117256499. Max. coverage (+): 0. Max coverage (-): 0

Region: chr5 117256500-117256512. Max. coverage (+): 0. Max coverage (-): 0

Region: chr5 117256513-117256525. Max. coverage (+): 0. Max coverage (-): 0

Region: chr5 117256526-117256538. Max. coverage (+): 0. Max coverage (-): 0

Region: chr5 117256539-117256551. Max. coverage (+): 0. Max coverage (-): 0

Region: chr5 117256552-117256564. Max. coverage (+): 0. Max coverage (-): 0

Region: chr5 117256565-117256577. Max. coverage (+): 0. Max coverage (-): 0

Region: chr5 117256578-117256590. Max. coverage (+): 0. Max coverage (-): 0

Region: chr5 117256591-117256603. Max. coverage (+): 0. Max coverage (-): 0

Region: chr5 117256604-117256616. Max. coverage (+): 0. Max coverage (-): 0

Region: chr5 117256617-117256629. Max. coverage (+): 0. Max coverage (-): 0

Region: chr5 117256630-117256642. Max. coverage (+): 0. Max coverage (-): 0

Region: chr5 117256643-117256655. Max. coverage (+): 0. Max coverage (-): 0

Region: chr5 117256656-117256668. Max. coverage (+): 0. Max coverage (-): 0

Region: chr5 117256669-117256681. Max. coverage (+): 0. Max coverage (-): 1.24

Region: chr5 117256682-117256694. Max. coverage (+): 0. Max coverage (-): 0

Region: chr5 117256695-117256707. Max. coverage (+): 0. Max coverage (-): 0

Region: chr5 117256708-117256719. Max. coverage (+): 0. Max coverage (-): 0

Region: chr5 117256720-117256732. Max. coverage (+): 0. Max coverage (-): 0

Region: chr5 117256733-117256745. Max. coverage (+): 0. Max coverage (-): 0

Region: chr5 117256746-117256758. Max. coverage (+): 0. Max coverage (-): 0

Region: chr5 117256759-117256771. Max. coverage (+): 0. Max coverage (-): 0

Region: chr5 117256772-117256784. Max. coverage (+): 0. Max coverage (-): 0

Region: chr5 117256785-117256797. Max. coverage (+): 0. Max coverage (-): 0

Region: chr5 117256798-117256810. Max. coverage (+): 0. Max coverage (-): 0

Region: chr5 117256811-117256823. Max. coverage (+): 0. Max coverage (-): 0.79

Region: chr5 117256824-117256836. Max. coverage (+): 0. Max coverage (-): 0

Region: chr5 117256837-117256849. Max. coverage (+): 0. Max coverage (-): 0

Region: chr5 117256850-117256862. Max. coverage (+): 0. Max coverage (-): 0

Region: chr5 117256863-117256875. Max. coverage (+): 0. Max coverage (-): 0

Region: chr5 117256876-117256888. Max. coverage (+): 0. Max coverage (-): 0

Region: chr5 117256889-117256901. Max. coverage (+): 0. Max coverage (-): 0

Region: chr5 117256902-117256914. Max. coverage (+): 0. Max coverage (-): 0

Region: chr5 117256915-117256927. Max. coverage (+): 0. Max coverage (-): 0

Region: chr5 117256928-117256940. Max. coverage (+): 0. Max coverage (-): 0

Region: chr5 117256941-117256953. Max. coverage (+): 0. Max coverage (-): 0

Region: chr5 117256954-117256966. Max. coverage (+): 0. Max coverage (-): 0

Region: chr5 117256967-117256979. Max. coverage (+): 0. Max coverage (-): 0

Region: chr5 117256980-117256992. Max. coverage (+): 0. Max coverage (-): 0

Region: chr5 117256993-117257005. Max. coverage (+): 0. Max coverage (-): 0

Region: chr5 117257006-117257018. Max. coverage (+): 0. Max coverage (-): 0

Region: chr5 117257019-117257031. Max. coverage (+): 0. Max coverage (-): 0

Region: chr5 117257032-117257044. Max. coverage (+): 0. Max coverage (-): 0

Region: chr5 117257045-117257057. Max. coverage (+): 0. Max coverage (-): 0

Region: chr5 117257058-117257070. Max. coverage (+): 0. Max coverage (-): 0

Region: chr5 117257071-117257083. Max. coverage (+): 0. Max coverage (-): 0

Region: chr5 117257084-117257095. Max. coverage (+): 0. Max coverage (-): 0

Region: chr5 117257096-117257108. Max. coverage (+): 0. Max coverage (-): 0

Region: chr5 117257109-117257121. Max. coverage (+): 0. Max coverage (-): 0

Region: chr5 117257122-117257134. Max. coverage (+): 0. Max coverage (-): 0

Region: chr5 117257135-117257147. Max. coverage (+): 0. Max coverage (-): 0

Region: chr5 117257148-117257160. Max. coverage (+): 0. Max coverage (-): 0

Region: chr5 117257161-117257173. Max. coverage (+): 0. Max coverage (-): 0

Region: chr5 117257174-117257186. Max. coverage (+): 0. Max coverage (-): 0

Region: chr5 117257187-117257199. Max. coverage (+): 0. Max coverage (-): 0

Region: chr5 117257200-117257212. Max. coverage (+): 0. Max coverage (-): 0

Region: chr5 117257213-117257225. Max. coverage (+): 0. Max coverage (-): 0

Region: chr5 117257226-117257238. Max. coverage (+): 0. Max coverage (-): 0

Region: chr5 117257239-117257251. Max. coverage (+): 0. Max coverage (-): 0

Region: chr5 117257252-117257264. Max. coverage (+): 0. Max coverage (-): 0

Region: chr5 117257265-117257277. Max. coverage (+): 0. Max coverage (-): 0

Region: chr5 117257278-117257290. Max. coverage (+): 0. Max coverage (-): 0

Region: chr5 117257291-117257303. Max. coverage (+): 0. Max coverage (-): 0

Region: chr5 117257304-117257316. Max. coverage (+): 0. Max coverage (-): 0.25

Region: chr5 117257317-117257329. Max. coverage (+): 0. Max coverage (-): 0

Region: chr5 117257330-117257342. Max. coverage (+): 0. Max coverage (-): 0

Region: chr5 117257343-117257355. Max. coverage (+): 0. Max coverage (-): 0.29

Region: chr5 117257356-117257368. Max. coverage (+): 0. Max coverage (-): 0

Region: chr5 117257369-117257381. Max. coverage (+): 0. Max coverage (-): 0

Region: chr5 117257382-117257394. Max. coverage (+): 0. Max coverage (-): 0

Region: chr5 117257395-117257407. Max. coverage (+): 0. Max coverage (-): 1.02

Region: chr5 117257408-117257420. Max. coverage (+): 0. Max coverage (-): 1.02

Region: chr5 117257421-117257433. Max. coverage (+): 0. Max coverage (-): 0

Region: chr5 117257434-117257446. Max. coverage (+): 0. Max coverage (-): 0

Region: chr5 117257447-117257459. Max. coverage (+): 0. Max coverage (-): 0

Region: chr5 117257460-117257472. Max. coverage (+): 0. Max coverage (-): 0

Region: chr5 117257473-117257484. Max. coverage (+): 0. Max coverage (-): 0

Region: chr5 117257485-117257497. Max. coverage (+): 0. Max coverage (-): 0

Region: chr5 117257498-117257510. Max. coverage (+): 0. Max coverage (-): 0

Region: chr5 117257511-117257523. Max. coverage (+): 0. Max coverage (-): 0

Region: chr5 117257524-117257536. Max. coverage (+): 0. Max coverage (-): 0

Region: chr5 117257537-117257549. Max. coverage (+): 0. Max coverage (-): 0

Region: chr5 117257550-117257562. Max. coverage (+): 0. Max coverage (-): 0.15

Region: chr5 117257563-117257575. Max. coverage (+): 0. Max coverage (-): 0.15

Region: chr5 117257576-117257588. Max. coverage (+): 0. Max coverage (-): 0

Region: chr5 117257589-117257601. Max. coverage (+): 0. Max coverage (-): 0

Region: chr5 117257602-117257614. Max. coverage (+): 0. Max coverage (-): 0

Region: chr5 117257615-117257627. Max. coverage (+): 0. Max coverage (-): 0

Region: chr5 117257628-117257640. Max. coverage (+): 0. Max coverage (-): 0

Region: chr5 117257641-117257653. Max. coverage (+): 0. Max coverage (-): 0

Region: chr5 117257654-117257666. Max. coverage (+): 0. Max coverage (-): 0

Region: chr5 117257667-117257679. Max. coverage (+): 0. Max coverage (-): 0

Region: chr5 117257680-117257692. Max. coverage (+): 0. Max coverage (-): 0

Region: chr5 117257693-117257705. Max. coverage (+): 0. Max coverage (-): 0

Region: chr5 117257706-117257718. Max. coverage (+): 0. Max coverage (-): 0

Region: chr5 117257719-117257731. Max. coverage (+): 0. Max coverage (-): 0

Region: chr5 117257732-117257744. Max. coverage (+): 0. Max coverage (-): 0

Region: chr5 117257745-117257757. Max. coverage (+): 0. Max coverage (-): 0

Region: chr5 117257758-117257770. Max. coverage (+): 0. Max coverage (-): 0

Region: chr5 117257771-117257783. Max. coverage (+): 0. Max coverage (-): 0

Region: chr5 117257784-117257796. Max. coverage (+): 0. Max coverage (-): 0

Region: chr5 117257797-117257809. Max. coverage (+): 0. Max coverage (-): 0

Region: chr5 117257810-117257822. Max. coverage (+): 0. Max coverage (-): 0

Region: chr5 117257823-117257835. Max. coverage (+): 0. Max coverage (-): 0

Region: chr5 117257836-117257848. Max. coverage (+): 0. Max coverage (-): 0

Region: chr5 117257849-117257860. Max. coverage (+): 0. Max coverage (-): 0

Region: chr5 117257861-117257873. Max. coverage (+): 0. Max coverage (-): 0

Region: chr5 117257874-117257886. Max. coverage (+): 0. Max coverage (-): 0

Region: chr5 117257887-117257899. Max. coverage (+): 0. Max coverage (-): 0

Region: chr5 117257900-117257912. Max. coverage (+): 0. Max coverage (-): 0

Region: chr5 117257913-117257925. Max. coverage (+): 0. Max coverage (-): 0

Region: chr5 117257926-117257938. Max. coverage (+): 0. Max coverage (-): 0

Region: chr5 117257939-117257951. Max. coverage (+): 0. Max coverage (-): 0

Region: chr5 117257952-117257964. Max. coverage (+): 0. Max coverage (-): 0

Region: chr5 117257965-117257977. Max. coverage (+): 0. Max coverage (-): 0

Region: chr5 117257978-117257990. Max. coverage (+): 0. Max coverage (-): 0

Region: chr5 117257991-117258003. Max. coverage (+): 0. Max coverage (-): 0

Region: chr5 117258004-117258016. Max. coverage (+): 0. Max coverage (-): 0

Region: chr5 117258017-117258029. Max. coverage (+): 0. Max coverage (-): 0

Region: chr5 117258030-117258042. Max. coverage (+): 0. Max coverage (-): 0

Region: chr5 117258043-117258055. Max. coverage (+): 0. Max coverage (-): 0

Region: chr5 117258056-117258068. Max. coverage (+): 0. Max coverage (-): 0

Region: chr5 117258069-117258081. Max. coverage (+): 0. Max coverage (-): 0

Region: chr5 117258082-117258094. Max. coverage (+): 0. Max coverage (-): 0

Region: chr5 117258095-117258107. Max. coverage (+): 0. Max coverage (-): 0

Region: chr5 117258108-117258120. Max. coverage (+): 0. Max coverage (-): 0

Region: chr5 117258121-117258133. Max. coverage (+): 0. Max coverage (-): 2.94

Region: chr5 117258134-117258146. Max. coverage (+): 0. Max coverage (-): 2.94

Region: chr5 117258147-117258159. Max. coverage (+): 0. Max coverage (-): 0

Region: chr5 117258160-117258172. Max. coverage (+): 0. Max coverage (-): 4.46

Region: chr5 117258173-117258185. Max. coverage (+): 0. Max coverage (-): 4.46

Region: chr5 117258186-117258198. Max. coverage (+): 0. Max coverage (-): 2.06

Region: chr5 117258199-117258211. Max. coverage (+): 0. Max coverage (-): 0

Region: chr5 117258212-117258224. Max. coverage (+): 0. Max coverage (-): 3.54

Region: chr5 117258225-117258237. Max. coverage (+): 0. Max coverage (-): 3.54

Region: chr5 117258238-117258249. Max. coverage (+): 0. Max coverage (-): 0

Region: chr5 117258250-117258262. Max. coverage (+): 0. Max coverage (-): 0

Region: chr5 117258263-117258275. Max. coverage (+): 0. Max coverage (-): 0

Region: chr5 117258276-117258288. Max. coverage (+): 0. Max coverage (-): 0.96

Region: chr5 117258289-117258301. Max. coverage (+): 0. Max coverage (-): 0

Region: chr5 117258302-117258314. Max. coverage (+): 0. Max coverage (-): 0

Region: chr5 117258315-117258327. Max. coverage (+): 0. Max coverage (-): 0

Region: chr5 117258328-117258340. Max. coverage (+): 0. Max coverage (-): 0

Region: chr5 117258341-117258353. Max. coverage (+): 0. Max coverage (-): 0

Region: chr5 117258354-117258366. Max. coverage (+): 0. Max coverage (-): 0

Region: chr5 117258367-117258379. Max. coverage (+): 0. Max coverage (-): 0

Region: chr5 117258380-117258392. Max. coverage (+): 0. Max coverage (-): 0

Region: chr5 117258393-117258405. Max. coverage (+): 0. Max coverage (-): 7.37

Region: chr5 117258406-117258418. Max. coverage (+): 0. Max coverage (-): 0

Region: chr5 117258419-117258431. Max. coverage (+): 0. Max coverage (-): 4.44

Region: chr5 117258432-117258444. Max. coverage (+): 0. Max coverage (-): 2.81

Region: chr5 117258445-117258457. Max. coverage (+): 0. Max coverage (-): 0

Region: chr5 117258458-117258470. Max. coverage (+): 0. Max coverage (-): 0

Region: chr5 117258471-117258483. Max. coverage (+): 0. Max coverage (-): 11.44

Region: chr5 117258484-117258496. Max. coverage (+): 0. Max coverage (-): 12.24

Region: chr5 117258497-117258509. Max. coverage (+): 0. Max coverage (-): 0

Region: chr5 117258510-117258522. Max. coverage (+): 0. Max coverage (-): 0

Region: chr5 117258523-117258535. Max. coverage (+): 0. Max coverage (-): 0

Region: chr5 117258536-117258548. Max. coverage (+): 0. Max coverage (-): 0

Region: chr5 117258549-117258561. Max. coverage (+): 0. Max coverage (-): 0

Region: chr5 117258562-117258574. Max. coverage (+): 0. Max coverage (-): 0

Region: chr5 117258575-117258587. Max. coverage (+): 0. Max coverage (-): 0

Region: chr5 117258588-117258600. Max. coverage (+): 0. Max coverage (-): 0

Region: chr5 117258601-117258613. Max. coverage (+): 0. Max coverage (-): 0

Region: chr5 117258614-117258625. Max. coverage (+): 0. Max coverage (-): 0

Region: chr5 117258626-117258638. Max. coverage (+): 0. Max coverage (-): 0

Region: chr5 117258639-117258651. Max. coverage (+): 0. Max coverage (-): 0

Region: chr5 117258652-117258664. Max. coverage (+): 0. Max coverage (-): 0

Region: chr5 117258665-117258677. Max. coverage (+): 0. Max coverage (-): 0

Region: chr5 117258678-117258690. Max. coverage (+): 0. Max coverage (-): 0

Region: chr5 117258691-117258703. Max. coverage (+): 0. Max coverage (-): 0

Region: chr5 117258704-117258716. Max. coverage (+): 0. Max coverage (-): 0

Region: chr5 117258717-117258729. Max. coverage (+): 0. Max coverage (-): 0

Region: chr5 117258730-117258742. Max. coverage (+): 0. Max coverage (-): 0

Region: chr5 117258743-117258755. Max. coverage (+): 0. Max coverage (-): 0

Region: chr5 117258756-117258768. Max. coverage (+): 0. Max coverage (-): 0

Region: chr5 117258769-117258781. Max. coverage (+): 0. Max coverage (-): 0

Region: chr5 117258782-117258794. Max. coverage (+): 0. Max coverage (-): 0

Region: chr5 117258795-117258807. Max. coverage (+): 0. Max coverage (-): 0

Region: chr5 117258808-117258820. Max. coverage (+): 0. Max coverage (-): 0

Region: chr5 117258821-117258833. Max. coverage (+): 0. Max coverage (-): 0

Region: chr5 117258834-117258846. Max. coverage (+): 0. Max coverage (-): 0

Region: chr5 117258847-117258859. Max. coverage (+): 0. Max coverage (-): 0

Region: chr5 117258860-117258872. Max. coverage (+): 0. Max coverage (-): 0

Region: chr5 117258873-117258885. Max. coverage (+): 0. Max coverage (-): 0

Region: chr5 117258886-117258898. Max. coverage (+): 0. Max coverage (-): 0

Region: chr5 117258899-117258911. Max. coverage (+): 0. Max coverage (-): 0

Region: chr5 117258912-117258924. Max. coverage (+): 0. Max coverage (-): 0

Region: chr5 117258925-117258937. Max. coverage (+): 0. Max coverage (-): 0

Region: chr5 117258938-117258950. Max. coverage (+): 0. Max coverage (-): 0

Region: chr5 117258951-117258963. Max. coverage (+): 0. Max coverage (-): 0

Region: chr5 117258964-117258976. Max. coverage (+): 0. Max coverage (-): 0

Region: chr5 117258977-117258989. Max. coverage (+): 0. Max coverage (-): 0.68

Region: chr5 117258990-117259001. Max. coverage (+): 0. Max coverage (-): 3.66

Region: chr5 117259002-117259014. Max. coverage (+): 0. Max coverage (-): 4.61

Region: chr5 117259015-117259027. Max. coverage (+): 0. Max coverage (-): 5.57

Region: chr5 117259028-117259040. Max. coverage (+): 0. Max coverage (-): 2.05

Region: chr5 117259041-117259053. Max. coverage (+): 0. Max coverage (-): 5.04

Region: chr5 117259054-117259066. Max. coverage (+): 0. Max coverage (-): 0

Region: chr5 117259067-117259079. Max. coverage (+): 0. Max coverage (-): 0

Region: chr5 117259080-117259092. Max. coverage (+): 0. Max coverage (-): 0

Region: chr5 117259093-117259105. Max. coverage (+): 0. Max coverage (-): 0.29

Region: chr5 117259106-117259118. Max. coverage (+): 0. Max coverage (-): 0.29

Region: chr5 117259119-117259131. Max. coverage (+): 0. Max coverage (-): 0

Region: chr5 117259132-117259144. Max. coverage (+): 0. Max coverage (-): 0

Region: chr5 117259145-117259157. Max. coverage (+): 0. Max coverage (-): 0

Region: chr5 117259158-117259170. Max. coverage (+): 0. Max coverage (-): 0

Region: chr5 117259171-117259183. Max. coverage (+): 0. Max coverage (-): 0.64

Region: chr5 117259184-117259196. Max. coverage (+): 0. Max coverage (-): 0

Region: chr5 117259197-117259209. Max. coverage (+): 0. Max coverage (-): 0.36

Region: chr5 117259210-117259222. Max. coverage (+): 0. Max coverage (-): 0.36

Region: chr5 117259223-117259235. Max. coverage (+): 0. Max coverage (-): 2.27

Region: chr5 117259236-117259248. Max. coverage (+): 0. Max coverage (-): 0

Region: chr5 117259249-117259261. Max. coverage (+): 0. Max coverage (-): 0

Region: chr5 117259262-117259274. Max. coverage (+): 0. Max coverage (-): 0

Region: chr5 117259275-117259287. Max. coverage (+): 0. Max coverage (-): 0

Region: chr5 117259288-117259300. Max. coverage (+): 0. Max coverage (-): 0

Region: chr5 117259301-117259313. Max. coverage (+): 0. Max coverage (-): 0

Region: chr5 117259314-117259326. Max. coverage (+): 0. Max coverage (-): 0

Region: chr5 117259327-117259339. Max. coverage (+): 0. Max coverage (-): 0

Region: chr5 117259340-117259352. Max. coverage (+): 0. Max coverage (-): 0

Region: chr5 117259353-117259365. Max. coverage (+): 0. Max coverage (-): 0

Region: chr5 117259366-117259378. Max. coverage (+): 0. Max coverage (-): 0.49

Region: chr5 117259379-117259390. Max. coverage (+): 0. Max coverage (-): 5.33

Region: chr5 117259391-117259403. Max. coverage (+): 0. Max coverage (-): 4.84

Region: chr5 117259404-117259416. Max. coverage (+): 0. Max coverage (-): 0

Region: chr5 117259417-117259429. Max. coverage (+): 0. Max coverage (-): 0

Region: chr5 117259430-117259442. Max. coverage (+): 0. Max coverage (-): 0

Region: chr5 117259443-117259455. Max. coverage (+): 0. Max coverage (-): 0

Region: chr5 117259456-117259468. Max. coverage (+): 0. Max coverage (-): 4.77

Region: chr5 117259469-117259481. Max. coverage (+): 0. Max coverage (-): 4.77

Region: chr5 117259482-117259494. Max. coverage (+): 0. Max coverage (-): 0

Region: chr5 117259495-117259507. Max. coverage (+): 0. Max coverage (-): 4.37

Region: chr5 117259508-117259520. Max. coverage (+): 0. Max coverage (-): 4.37

Region: chr5 117259521-117259533. Max. coverage (+): 0. Max coverage (-): 0

Region: chr5 117259534-117259546. Max. coverage (+): 0. Max coverage (-): 0

Region: chr5 117259547-117259559. Max. coverage (+): 0. Max coverage (-): 0

Region: chr5 117259560-117259572. Max. coverage (+): 0. Max coverage (-): 0.15

Region: chr5 117259573-117259585. Max. coverage (+): 0. Max coverage (-): 0

Region: chr5 117259586-117259598. Max. coverage (+): 0. Max coverage (-): 3.58

Region: chr5 117259599-117259611. Max. coverage (+): 0. Max coverage (-): 0

Region: chr5 117259612-117259624. Max. coverage (+): 0. Max coverage (-): 1.29

Region: chr5 117259625-117259637. Max. coverage (+): 0. Max coverage (-): 4.19

Region: chr5 117259638-117259650. Max. coverage (+): 0. Max coverage (-): 0

Region: chr5 117259651-117259663. Max. coverage (+): 0. Max coverage (-): 0

Region: chr5 117259664-117259676. Max. coverage (+): 0. Max coverage (-): 0

Region: chr5 117259677-117259689. Max. coverage (+): 0. Max coverage (-): 0

Region: chr5 117259690-117259702. Max. coverage (+): 0. Max coverage (-): 1.16

Region: chr5 117259703-117259715. Max. coverage (+): 0. Max coverage (-): 1.16

Region: chr5 117259716-117259728. Max. coverage (+): 0. Max coverage (-): 0

Region: chr5 117259729-117259741. Max. coverage (+): 0. Max coverage (-): 0

Region: chr5 117259742-117259754. Max. coverage (+): 0. Max coverage (-): 0

Region: chr5 117259755-117259766. Max. coverage (+): 0. Max coverage (-): 0

Region: chr5 117259767-117259779. Max. coverage (+): 0. Max coverage (-): 0

Region: chr5 117259780-117259792. Max. coverage (+): 0. Max coverage (-): 0

Region: chr5 117259793-117259805. Max. coverage (+): 0. Max coverage (-): 0

Region: chr5 117259806-117259818. Max. coverage (+): 0. Max coverage (-): 0

Region: chr5 117259819-117259831. Max. coverage (+): 0. Max coverage (-): 0

Region: chr5 117259832-117259844. Max. coverage (+): 0. Max coverage (-): 0

Region: chr5 117259845-117259857. Max. coverage (+): 0. Max coverage (-): 0

Region: chr5 117259858-117259870. Max. coverage (+): 0. Max coverage (-): 0

Region: chr5 117259871-117259883. Max. coverage (+): 0. Max coverage (-): 0

Region: chr5 117259884-117259896. Max. coverage (+): 0. Max coverage (-): 0

Region: chr5 117259897-117259909. Max. coverage (+): 0. Max coverage (-): 0

Region: chr5 117259910-117259922. Max. coverage (+): 0. Max coverage (-): 0

Region: chr5 117259923-117259935. Max. coverage (+): 0. Max coverage (-): 0

Region: chr5 117259936-117259948. Max. coverage (+): 0. Max coverage (-): 0

Region: chr5 117259949-117259961. Max. coverage (+): 0. Max coverage (-): 1.2

Region: chr5 117259962-117259974. Max. coverage (+): 0. Max coverage (-): 1.2

Region: chr5 117259975-117259987. Max. coverage (+): 0. Max coverage (-): 4.34

Region: chr5 117259988-117260000. Max. coverage (+): 0. Max coverage (-): 4.34

Region: chr5 117260001-117260013. Max. coverage (+): 0. Max coverage (-): 0

Region: chr5 117260014-117260026. Max. coverage (+): 0. Max coverage (-): 5.15

Region: chr5 117260027-117260039. Max. coverage (+): 0. Max coverage (-): 10.3

Region: chr5 117260040-117260052. Max. coverage (+): 0. Max coverage (-): 0

Region: chr5 117260053-117260065. Max. coverage (+): 0. Max coverage (-): 0

Region: chr5 117260066-117260078. Max. coverage (+): 0. Max coverage (-): 2.54

Region: chr5 117260079-117260091. Max. coverage (+): 0. Max coverage (-): 0

Region: chr5 117260092-117260104. Max. coverage (+): 0. Max coverage (-): 0

Region: chr5 117260105-117260117. Max. coverage (+): 0. Max coverage (-): 0.94

Region: chr5 117260118-117260130. Max. coverage (+): 0. Max coverage (-): 0.94

Region: chr5 117260131-117260143. Max. coverage (+): 0. Max coverage (-): 0

Region: chr5 117260144-117260155. Max. coverage (+): 0. Max coverage (-): 0

Region: chr5 117260156-117260168. Max. coverage (+): 0. Max coverage (-): 0

Region: chr5 117260169-117260181. Max. coverage (+): 0. Max coverage (-): 4.58

Region: chr5 117260182-117260194. Max. coverage (+): 0. Max coverage (-): 3.36

Region: chr5 117260195-117260207. Max. coverage (+): 0. Max coverage (-): 0

Region: chr5 117260208-117260220. Max. coverage (+): 0. Max coverage (-): 0

Region: chr5 117260221-117260233. Max. coverage (+): 0. Max coverage (-): 0

Region: chr5 117260234-117260246. Max. coverage (+): 0. Max coverage (-): 0

Region: chr5 117260247-117260259. Max. coverage (+): 0. Max coverage (-): 0

Region: chr5 117260260-117260272. Max. coverage (+): 0. Max coverage (-): 1.31

Region: chr5 117260273-117260285. Max. coverage (+): 0. Max coverage (-): 0

Region: chr5 117260286-117260298. Max. coverage (+): 0. Max coverage (-): 0

Region: chr5 117260299-117260311. Max. coverage (+): 0. Max coverage (-): 0

Region: chr5 117260312-117260324. Max. coverage (+): 0. Max coverage (-): 0

Region: chr5 117260325-117260337. Max. coverage (+): 0. Max coverage (-): 2.4

Region: chr5 117260338-117260350. Max. coverage (+): 0. Max coverage (-): 0.47

Region: chr5 117260351-117260363. Max. coverage (+): 0. Max coverage (-): 0

Region: chr5 117260364-117260376. Max. coverage (+): 0. Max coverage (-): 0

Region: chr5 117260377-117260389. Max. coverage (+): 0. Max coverage (-): 0

Region: chr5 117260390-117260402. Max. coverage (+): 0. Max coverage (-): 0

Region: chr5 117260403-117260415. Max. coverage (+): 0. Max coverage (-): 0

Region: chr5 117260416-117260428. Max. coverage (+): 0. Max coverage (-): 0

Region: chr5 117260429-117260441. Max. coverage (+): 0. Max coverage (-): 0

Region: chr5 117260442-117260454. Max. coverage (+): 0. Max coverage (-): 0

Region: chr5 117260455-117260467. Max. coverage (+): 0. Max coverage (-): 0

Region: chr5 117260468-117260480. Max. coverage (+): 0. Max coverage (-): 2.5

Region: chr5 117260481-117260493. Max. coverage (+): 0. Max coverage (-): 1.4

Region: chr5 117260494-117260506. Max. coverage (+): 0. Max coverage (-): 0

Region: chr5 117260507-117260519. Max. coverage (+): 0. Max coverage (-): 0

Region: chr5 117260520-117260531. Max. coverage (+): 0. Max coverage (-): 0

Region: chr5 117260532-117260544. Max. coverage (+): 1.35. Max coverage (-): 0

Region: chr5 117260545-117260557. Max. coverage (+): 0. Max coverage (-): 2.35

Region: chr5 117260558-117260570. Max. coverage (+): 0. Max coverage (-): 1.15

Region: chr5 117260571-117260583. Max. coverage (+): 0.49. Max coverage (-): 0

Region: chr5 117260584-117260596. Max. coverage (+): 0.49. Max coverage (-): 0

Region: chr5 117260597-117260609. Max. coverage (+): 0. Max coverage (-): 0

Region: chr5 117260610-117260622. Max. coverage (+): 0. Max coverage (-): 0

Region: chr5 117260623-117260635. Max. coverage (+): 0. Max coverage (-): 0

Region: chr5 117260636-117260648. Max. coverage (+): 0. Max coverage (-): 0

Region: chr5 117260649-117260661. Max. coverage (+): 0. Max coverage (-): 0

Region: chr5 117260662-117260674. Max. coverage (+): 0. Max coverage (-): 2.85

Region: chr5 117260675-117260687. Max. coverage (+): 0. Max coverage (-): 5.37

Region: chr5 117260688-117260700. Max. coverage (+): 1.06. Max coverage (-): 5.01

Region: chr5 117260701-117260713. Max. coverage (+): 0. Max coverage (-): 0.43

Region: chr5 117260714-117260726. Max. coverage (+): 0. Max coverage (-): 0

Region: chr5 117260727-117260739. Max. coverage (+): 0. Max coverage (-): 2.04

Region: chr5 117260740-117260752. Max. coverage (+): 0. Max coverage (-): 2.04

Region: chr5 117260753-117260765. Max. coverage (+): 0. Max coverage (-): 0

Region: chr5 117260766-117260778. Max. coverage (+): 0. Max coverage (-): 0

Region: chr5 117260779-117260791. Max. coverage (+): 0. Max coverage (-): 0

Region: chr5 117260792-117260804. Max. coverage (+): 0. Max coverage (-): 0.46

Region: chr5 117260805-117260817. Max. coverage (+): 0. Max coverage (-): 0

Region: chr5 117260818-117260830. Max. coverage (+): 0. Max coverage (-): 1.68

Region: chr5 117260831-117260843. Max. coverage (+): 0. Max coverage (-): 1.68

Region: chr5 117260844-117260856. Max. coverage (+): 0. Max coverage (-): 0

Region: chr5 117260857-117260869. Max. coverage (+): 0. Max coverage (-): 0

Region: chr5 117260870-117260882. Max. coverage (+): 0. Max coverage (-): 6.32

Region: chr5 117260883-117260895. Max. coverage (+): 0. Max coverage (-): 0.67

Region: chr5 117260896-117260907. Max. coverage (+): 0.45. Max coverage (-): 1.95

Region: chr5 117260908-117260920. Max. coverage (+): 0.45. Max coverage (-): 2.21

Region: chr5 117260921-117260933. Max. coverage (+): 0. Max coverage (-): 1.29

Region: chr5 117260934-117260946. Max. coverage (+): 0. Max coverage (-): 0.98

Region: chr5 117260947-117260959. Max. coverage (+): 0. Max coverage (-): 0

Region: chr5 117260960-117260972. Max. coverage (+): 0. Max coverage (-): 0

Region: chr5 117260973-117260985. Max. coverage (+): 0. Max coverage (-): 0

Region: chr5 117260986-117260998. Max. coverage (+): 0. Max coverage (-): 0

Region: chr5 117260999-117261011. Max. coverage (+): 0. Max coverage (-): 0

Region: chr5 117261012-117261024. Max. coverage (+): 0. Max coverage (-): 0

Region: chr5 117261025-117261037. Max. coverage (+): 0. Max coverage (-): 0

Region: chr5 117261038-117261050. Max. coverage (+): 0. Max coverage (-): 0

Region: chr5 117261051-117261063. Max. coverage (+): 0. Max coverage (-): 0

Region: chr5 117261064-117261076. Max. coverage (+): 0. Max coverage (-): 0.92

Region: chr5 117261077-117261089. Max. coverage (+): 0. Max coverage (-): 0.34

Region: chr5 117261090-117261102. Max. coverage (+): 0.66. Max coverage (-): 0.81

Region: chr5 117261103-117261115. Max. coverage (+): 0.66. Max coverage (-): 5.69

Region: chr5 117261116-117261128. Max. coverage (+): 0. Max coverage (-): 8.26

Region: chr5 117261129-117261141. Max. coverage (+): 0. Max coverage (-): 8.26

Region: chr5 117261142-117261154. Max. coverage (+): 0. Max coverage (-): 0.46

Region: chr5 117261155-117261167. Max. coverage (+): 0. Max coverage (-): 0.78

Region: chr5 117261168-117261180. Max. coverage (+): 0. Max coverage (-): 11.47

Region: chr5 117261181-117261193. Max. coverage (+): 0. Max coverage (-): 11.47

Region: chr5 117261194-117261206. Max. coverage (+): 0. Max coverage (-): 2.94

Region: chr5 117261207-117261219. Max. coverage (+): 0. Max coverage (-): 2.55

Region: chr5 117261220-117261232. Max. coverage (+): 0. Max coverage (-): 2.14

Region: chr5 117261233-117261245. Max. coverage (+): 0. Max coverage (-): 0

Region: chr5 117261246-117261258. Max. coverage (+): 0. Max coverage (-): 0

Region: chr5 117261259-117261271. Max. coverage (+): 0. Max coverage (-): 1.55

Region: chr5 117261272-117261284. Max. coverage (+): 0. Max coverage (-): 1.13

Region: chr5 117261285-117261296. Max. coverage (+): 0. Max coverage (-): 0.86

Region: chr5 117261297-117261309. Max. coverage (+): 0. Max coverage (-): 0

Region: chr5 117261310-117261322. Max. coverage (+): 0. Max coverage (-): 0

Region: chr5 117261323-117261335. Max. coverage (+): 0. Max coverage (-): 0

Region: chr5 117261336-117261348. Max. coverage (+): 0. Max coverage (-): 0

Region: chr5 117261349-117261361. Max. coverage (+): 0. Max coverage (-): 0

Region: chr5 117261362-117261374. Max. coverage (+): 0. Max coverage (-): 6.25

Region: chr5 117261375-117261387. Max. coverage (+): 0. Max coverage (-): 8.39

Region: chr5 117261388-117261400. Max. coverage (+): 0. Max coverage (-): 0

Region: chr5 117261401-117261413. Max. coverage (+): 0. Max coverage (-): 0

Region: chr5 117261414-117261426. Max. coverage (+): 0. Max coverage (-): 0

Region: chr5 117261427-117261439. Max. coverage (+): 0. Max coverage (-): 0

Region: chr5 117261440-117261452. Max. coverage (+): 0. Max coverage (-): 0

Region: chr5 117261453-117261465. Max. coverage (+): 0. Max coverage (-): 0

Region: chr5 117261466-117261478. Max. coverage (+): 0. Max coverage (-): 0

Region: chr5 117261479-117261491. Max. coverage (+): 0. Max coverage (-): 0

Region: chr5 117261492-117261504. Max. coverage (+): 0. Max coverage (-): 0

Region: chr5 117261505-117261517. Max. coverage (+): 0. Max coverage (-): 0

Region: chr5 117261518-117261530. Max. coverage (+): 0. Max coverage (-): 0

Region: chr5 117261531-117261543. Max. coverage (+): 0. Max coverage (-): 1.81

Region: chr5 117261544-117261556. Max. coverage (+): 0.77. Max coverage (-): 0

Region: chr5 117261557-117261569. Max. coverage (+): 0.77. Max coverage (-): 0

Region: chr5 117261570-117261582. Max. coverage (+): 0. Max coverage (-): 0.61

Region: chr5 117261583-117261595. Max. coverage (+): 0. Max coverage (-): 1.44

Region: chr5 117261596-117261608. Max. coverage (+): 0. Max coverage (-): 1.44

Region: chr5 117261609-117261621. Max. coverage (+): 0. Max coverage (-): 0.75

Region: chr5 117261622-117261634. Max. coverage (+): 0. Max coverage (-): 0.43

Region: chr5 117261635-117261647. Max. coverage (+): 0. Max coverage (-): 0

Region: chr5 117261648-117261660. Max. coverage (+): 0. Max coverage (-): 0

Region: chr5 117261661-117261672. Max. coverage (+): 0. Max coverage (-): 0

Region: chr5 117261673-117261685. Max. coverage (+): 0. Max coverage (-): 3.29

Region: chr5 117261686-117261698. Max. coverage (+): 0. Max coverage (-): 0

Region: chr5 117261699-117261711. Max. coverage (+): 0. Max coverage (-): 0

Region: chr5 117261712-117261724. Max. coverage (+): 0. Max coverage (-): 0

Region: chr5 117261725-117261737. Max. coverage (+): 0. Max coverage (-): 0

Region: chr5 117261738-117261750. Max. coverage (+): 0. Max coverage (-): 0

Region: chr5 117261751-117261763. Max. coverage (+): 0. Max coverage (-): 0

Region: chr5 117261764-117261776. Max. coverage (+): 0. Max coverage (-): 1.88

Region: chr5 117261777-117261789. Max. coverage (+): 1.81. Max coverage (-): 1.4

Region: chr5 117261790-117261802. Max. coverage (+): 1.81. Max coverage (-): 1.09

Region: chr5 117261803-117261815. Max. coverage (+): 0. Max coverage (-): 0

Region: chr5 117261816-117261828. Max. coverage (+): 0. Max coverage (-): 1.31

Region: chr5 117261829-117261841. Max. coverage (+): 1.09. Max coverage (-): 1.45

Region: chr5 117261842-117261854. Max. coverage (+): 0. Max coverage (-): 0

Region: chr5 117261855-. Max. coverage (+): 0. Max coverage (-): 0

RepeatMasker Color Code

**+**

100-98% Identity

<98-95% Identity

<95-90% Identity

<90-85% Identity

<85-80% Identity

<80-75% Identity

<75-70% Identity

<70% Identity

**-**

Gene Set Color Code

**+**

Gene

Pseudogene

**-**

Topology/Coverage Color Code

Coverage Plus Strand

Coverage Minus Strand

Mainstrand: Plus

Mainstrand: Minus

Complementary Strand

Flanking Region  
(if option -flank >0)

Gene Set Annotation  
  
RepeatMasker Annotation  

**1. MIRc**: 117256222-117256302 (+), Divergence to consensus: 40.9%  
**2. Bov-tA2**: 117256423-117256549 (-), Divergence to consensus: 18.4%  
**3. AmnSINE1**: 117258545-117258616 (+), Divergence to consensus: 34.7%

  
Transcription Factor Binding Sites  

**SPZ1** (Sequence: CTCTAACCCT (-): 117257323)  
**Gata4** (Sequence: AGATAAG (-): 117257785)  
**Gata4** (Sequence: AGATAAC (-): 117260815)  
**SOX9** (Sequence: AACAATAA (-): 117256808)  
**Gata4** (Sequence: CTTATCT (+): 117255952)  
**Gata4** (Sequence: CTTATCT (+): 117260463)
