# Supplementary material for: Small RNA sequencing of cryopreserved semen from single bull revealed altered miRNAs and piRNAs expression between High- and Low-motile sperm populations
Source: BMC Genomics. 2017 Jan 4;18:14. doi: 10.1186/s12864-016-3394-7 (PMC5209821; doi:10.1186/s12864-016-3394-7)
Supplement: Additional file 3: — Details for each piRNA clusters found in High Motile (HM) sperm fraction. Genes, repeats, transposable elements and transcription factors binding sites falling within the cluster regions were reported. (ZIP 1896 kb) [file 12864_2016_3394_MOESM3_ESM.zip › 89.html]

piRNA cluster 89


Predicted piRNA cluster no. 89     previous   next
  

Show proTRAC run info
Hide proTRAC run info

================================= proTRAC ====================================  
VERSION: 2.1                                    LAST MODIFIED: 06. October 2015  
  
Please cite:  
Rosenkranz D, Zischler H. proTRAC - a software for probabilistic piRNA cluster  
detection, visualization and analysis. 2012. BMC Bioinformatics 13:5.  
  
and (for proTRAC 2.0 and later):  
Rosenkranz D, Rudloff S, Bastuck K, Ketting RF, Zischler H. Tupaia small RNAs  
provide insights into function and evolution of RNAi-based transposon defense  
in mammals. 2015. RNA 21(5):911-922.  
  
Contact:  
David Rosenkranz  
Institute of Anthropology, small RNA group  
Johannes Gutenberg University Mainz  
email: rosenkranz@uni-mainz.de  
  
You can find the latest proTRAC version at:  
http://sourceforge.net/projects/protrac/files  
http://www.smallRNAgroup-mainz.de/software  
==============================================================================  
  
PARAMETERS:  
Map file: .............../storage/core/barbara/genhome/smallRNA/fertility/Sample\_motile/pirna/Sample\_motile\_26-33\_collapsed.fa.no-dust.map.weighted-10000-1000-b-0  
Genome file: ............/storage/core/barbara/genhome/smallRNA/fertility/Sample\_all/pirna/bt\_311\_chrY.fa  
RepeatMasker annotation: /storage/genomes/bt\_umd31/GCF\_000003055.6\_Bos\_taurus\_UMD\_3.1.1\_repeatMasker\_chr.out  
GeneSet:................./storage/core/barbara/genhome/smallRNA/fertility/Sample\_all/pirna/full.gtf  
  
Significant (p<=0.01) hit density will be calculated based  
on observed hit distribution.  
  
Sliding window size: ........................................ 5000 bp  
Sliding window increament: .................................. 1000 bp  
Normalize each hit by number of genomic hits: ............... 1 [0=no/1=yes]  
Normalize each hit by number of sequence reads: ............. 1 [0=no/1=yes]  
Normalize values (-> per million mapped reads): ............. 1 [0=no/1=yes]  
Min. fraction of hits with 1T(U) or 10A: .................... 0.75  
Alternatively: Min. fraction of hits with 1T(U) and 10A: .... 0.5  
Min. fraction of hits with typical piRNA length: ............ 0.75  
Typical piRNA length: ....................................... 26-33 nt  
Min. size of a piRNA cluster: ............................... 5000 bp.  
Min. number of hits (absolute): ............................. 0  
Min. number of hits (normalized): ........................... 0  
Min. fraction of hits on the mainstrand: .................... 0.75  
Top fraction of mapped sequences (in terms of read counts): . 1%  
Top fraction accounts for max. n% of sequence reads: ........ 90%  
Min. fraction of hits on each arm of a bidirectional cluster: 0.1  
Output image file for each cluster: ......................... 0 [0=no/1=yes]  
Output html file for each cluster: .......................... 1 [0=no/1=yes]  
Output a summary table: ..................................... 1 [0=no/1=yes]  
Output a FASTA file for each cluster (piRNA sequences): ..... 1 [0=no/1=yes]  
Output a FASTA file comprising cluster sequences: ........... 1 [0=no/1=yes]  
Search DNA motifs in clusters: .............................. 1 [0=no/1=yes]  
Output flanking sequences: +/- .............................. 0 bp  
Output ~.pTi file: .......................................... 1 [0=no/1=yes]  
==============================================================================  
  
  
Genome size (without gaps): ............ 2678902517 bp  
Gaps (N/X/-): .......................... 53837044 bp  
Mapped reads: .......................... 658825247023  
Non-identical sequences: ............... 514171  
Genomic hits: .......................... 764233  
Significant densitiy of mapped reads: .. 12867599.5173724 reads/kb

Show proTRAC cluster info
Hide proTRAC cluster info

|  |  |
| --- | --- |
| Location | chr6 |
| Coordinates | 73878366-73884632 |
| Size [bp] | 6267 |
| Sequence hit loci | 64 |
| Mapped reads (normalized) | 92165201.8 |
| Mapped reads (normalized) per kb | 14706430.8 |
| Normalized reads with 1T (1U) | 87.8% |
| Normalized reads with 10A | 22.3% |
| Normalized reads with length 26-33 nt | 100% |
| Normalized reads on the main strand(s) | 100% |
| Predicted directionality | mono:minus |

100%

0%

1T (1U)  
reads

10A reads

26-33 nt  
reads

reads on mainstrand

**Either the amount of reads with 1T (1U) OR 10A has to exceed 75% (set with option: -1Tor10A)  
Alternatively the amount of reads with 1T (1U) AND 10A has to exceed 50% (set with option: -1Tand10A)  
Minimum amount of reads with preferred size is 75% (set with option: -pisize)  
Minimum amount of reads on the main strand(s) is 75% (set with option: -clstrand)**

Show read coverage
Hide read coverage

WHAT DO I SEE HERE?  
This chart shows the location of mapped sequence reads within a predicted piRNA cluster. The color refers to the number of genomic hits produced by the sequence read in question. A dark red bar indicates that this sequence read produces many other hits elsewhere in the genome. Many adjacent red or yellow bars can indicate the presence of a multi-copy element such as transposons or rRNA genes. A dark green bar indicates that this sequence read maps uniquely to this locus.

1 hit

2-5 hits

6-10 hits

11-20 hits

21-50 hits

51-100 hits

> 100 hits

chr6

73878366

73884632

Gene Set

RepeatMasker

Mapped  
Reads

21.9

plus strand

minus strand

21.9

Region: chr6 117261856-73878372. Max. coverage (+): 0. Max coverage (-): 3.64

Region: chr6 73878373-73878384. Max. coverage (+): 0. Max coverage (-): 3.64

Region: chr6 73878385-73878397. Max. coverage (+): 0. Max coverage (-): 4.1

Region: chr6 73878398-73878409. Max. coverage (+): 0. Max coverage (-): 4.1

Region: chr6 73878410-73878422. Max. coverage (+): 0. Max coverage (-): 0

Region: chr6 73878423-73878434. Max. coverage (+): 0. Max coverage (-): 0

Region: chr6 73878435-73878447. Max. coverage (+): 0. Max coverage (-): 0

Region: chr6 73878448-73878460. Max. coverage (+): 0. Max coverage (-): 0

Region: chr6 73878461-73878472. Max. coverage (+): 0. Max coverage (-): 0

Region: chr6 73878473-73878485. Max. coverage (+): 0. Max coverage (-): 0

Region: chr6 73878486-73878497. Max. coverage (+): 0. Max coverage (-): 0

Region: chr6 73878498-73878510. Max. coverage (+): 0. Max coverage (-): 0

Region: chr6 73878511-73878522. Max. coverage (+): 0. Max coverage (-): 0

Region: chr6 73878523-73878535. Max. coverage (+): 0. Max coverage (-): 0

Region: chr6 73878536-73878547. Max. coverage (+): 0. Max coverage (-): 0

Region: chr6 73878548-73878560. Max. coverage (+): 0. Max coverage (-): 0

Region: chr6 73878561-73878572. Max. coverage (+): 0. Max coverage (-): 0

Region: chr6 73878573-73878585. Max. coverage (+): 0. Max coverage (-): 0

Region: chr6 73878586-73878597. Max. coverage (+): 0. Max coverage (-): 0

Region: chr6 73878598-73878610. Max. coverage (+): 0. Max coverage (-): 0

Region: chr6 73878611-73878622. Max. coverage (+): 0. Max coverage (-): 0

Region: chr6 73878623-73878635. Max. coverage (+): 0. Max coverage (-): 0

Region: chr6 73878636-73878648. Max. coverage (+): 0. Max coverage (-): 0

Region: chr6 73878649-73878660. Max. coverage (+): 0. Max coverage (-): 0

Region: chr6 73878661-73878673. Max. coverage (+): 0. Max coverage (-): 0

Region: chr6 73878674-73878685. Max. coverage (+): 0. Max coverage (-): 0

Region: chr6 73878686-73878698. Max. coverage (+): 0. Max coverage (-): 0

Region: chr6 73878699-73878710. Max. coverage (+): 0. Max coverage (-): 0

Region: chr6 73878711-73878723. Max. coverage (+): 0. Max coverage (-): 0

Region: chr6 73878724-73878735. Max. coverage (+): 0. Max coverage (-): 0

Region: chr6 73878736-73878748. Max. coverage (+): 0. Max coverage (-): 0

Region: chr6 73878749-73878760. Max. coverage (+): 0. Max coverage (-): 0

Region: chr6 73878761-73878773. Max. coverage (+): 0. Max coverage (-): 0

Region: chr6 73878774-73878785. Max. coverage (+): 0. Max coverage (-): 0

Region: chr6 73878786-73878798. Max. coverage (+): 0. Max coverage (-): 0

Region: chr6 73878799-73878810. Max. coverage (+): 0. Max coverage (-): 0

Region: chr6 73878811-73878823. Max. coverage (+): 0. Max coverage (-): 0

Region: chr6 73878824-73878836. Max. coverage (+): 0. Max coverage (-): 0

Region: chr6 73878837-73878848. Max. coverage (+): 0. Max coverage (-): 0

Region: chr6 73878849-73878861. Max. coverage (+): 0. Max coverage (-): 0

Region: chr6 73878862-73878873. Max. coverage (+): 0. Max coverage (-): 3.9

Region: chr6 73878874-73878886. Max. coverage (+): 0. Max coverage (-): 3.9

Region: chr6 73878887-73878898. Max. coverage (+): 0. Max coverage (-): 8.06

Region: chr6 73878899-73878911. Max. coverage (+): 0. Max coverage (-): 0

Region: chr6 73878912-73878923. Max. coverage (+): 0. Max coverage (-): 0

Region: chr6 73878924-73878936. Max. coverage (+): 0. Max coverage (-): 0

Region: chr6 73878937-73878948. Max. coverage (+): 0. Max coverage (-): 5.73

Region: chr6 73878949-73878961. Max. coverage (+): 0. Max coverage (-): 5.73

Region: chr6 73878962-73878973. Max. coverage (+): 0. Max coverage (-): 0

Region: chr6 73878974-73878986. Max. coverage (+): 0. Max coverage (-): 0

Region: chr6 73878987-73878998. Max. coverage (+): 0. Max coverage (-): 0

Region: chr6 73878999-73879011. Max. coverage (+): 0. Max coverage (-): 0

Region: chr6 73879012-73879024. Max. coverage (+): 0. Max coverage (-): 0

Region: chr6 73879025-73879036. Max. coverage (+): 0. Max coverage (-): 0

Region: chr6 73879037-73879049. Max. coverage (+): 0. Max coverage (-): 0

Region: chr6 73879050-73879061. Max. coverage (+): 0. Max coverage (-): 0

Region: chr6 73879062-73879074. Max. coverage (+): 0. Max coverage (-): 0

Region: chr6 73879075-73879086. Max. coverage (+): 0. Max coverage (-): 0

Region: chr6 73879087-73879099. Max. coverage (+): 0. Max coverage (-): 0

Region: chr6 73879100-73879111. Max. coverage (+): 0. Max coverage (-): 0

Region: chr6 73879112-73879124. Max. coverage (+): 0. Max coverage (-): 0

Region: chr6 73879125-73879136. Max. coverage (+): 0. Max coverage (-): 0

Region: chr6 73879137-73879149. Max. coverage (+): 0. Max coverage (-): 0

Region: chr6 73879150-73879161. Max. coverage (+): 0. Max coverage (-): 0

Region: chr6 73879162-73879174. Max. coverage (+): 0. Max coverage (-): 0

Region: chr6 73879175-73879186. Max. coverage (+): 0. Max coverage (-): 0

Region: chr6 73879187-73879199. Max. coverage (+): 0. Max coverage (-): 0

Region: chr6 73879200-73879212. Max. coverage (+): 0. Max coverage (-): 0

Region: chr6 73879213-73879224. Max. coverage (+): 0. Max coverage (-): 0

Region: chr6 73879225-73879237. Max. coverage (+): 0. Max coverage (-): 0

Region: chr6 73879238-73879249. Max. coverage (+): 0. Max coverage (-): 0

Region: chr6 73879250-73879262. Max. coverage (+): 0. Max coverage (-): 0

Region: chr6 73879263-73879274. Max. coverage (+): 0. Max coverage (-): 0

Region: chr6 73879275-73879287. Max. coverage (+): 0. Max coverage (-): 0

Region: chr6 73879288-73879299. Max. coverage (+): 0. Max coverage (-): 0

Region: chr6 73879300-73879312. Max. coverage (+): 0. Max coverage (-): 0

Region: chr6 73879313-73879324. Max. coverage (+): 0. Max coverage (-): 0

Region: chr6 73879325-73879337. Max. coverage (+): 0. Max coverage (-): 0

Region: chr6 73879338-73879349. Max. coverage (+): 0. Max coverage (-): 0

Region: chr6 73879350-73879362. Max. coverage (+): 0. Max coverage (-): 0

Region: chr6 73879363-73879374. Max. coverage (+): 0. Max coverage (-): 0

Region: chr6 73879375-73879387. Max. coverage (+): 0. Max coverage (-): 0

Region: chr6 73879388-73879400. Max. coverage (+): 0. Max coverage (-): 0

Region: chr6 73879401-73879412. Max. coverage (+): 0. Max coverage (-): 0

Region: chr6 73879413-73879425. Max. coverage (+): 0. Max coverage (-): 0

Region: chr6 73879426-73879437. Max. coverage (+): 0. Max coverage (-): 0

Region: chr6 73879438-73879450. Max. coverage (+): 0. Max coverage (-): 0

Region: chr6 73879451-73879462. Max. coverage (+): 0. Max coverage (-): 0

Region: chr6 73879463-73879475. Max. coverage (+): 0. Max coverage (-): 0

Region: chr6 73879476-73879487. Max. coverage (+): 0. Max coverage (-): 0

Region: chr6 73879488-73879500. Max. coverage (+): 0. Max coverage (-): 0

Region: chr6 73879501-73879512. Max. coverage (+): 0. Max coverage (-): 0

Region: chr6 73879513-73879525. Max. coverage (+): 0. Max coverage (-): 0

Region: chr6 73879526-73879537. Max. coverage (+): 0. Max coverage (-): 0

Region: chr6 73879538-73879550. Max. coverage (+): 0. Max coverage (-): 0

Region: chr6 73879551-73879562. Max. coverage (+): 0. Max coverage (-): 0

Region: chr6 73879563-73879575. Max. coverage (+): 0. Max coverage (-): 0

Region: chr6 73879576-73879588. Max. coverage (+): 0. Max coverage (-): 0

Region: chr6 73879589-73879600. Max. coverage (+): 0. Max coverage (-): 3.34

Region: chr6 73879601-73879613. Max. coverage (+): 0. Max coverage (-): 3.34

Region: chr6 73879614-73879625. Max. coverage (+): 0. Max coverage (-): 0

Region: chr6 73879626-73879638. Max. coverage (+): 0. Max coverage (-): 0

Region: chr6 73879639-73879650. Max. coverage (+): 0. Max coverage (-): 0

Region: chr6 73879651-73879663. Max. coverage (+): 0. Max coverage (-): 0

Region: chr6 73879664-73879675. Max. coverage (+): 0. Max coverage (-): 0

Region: chr6 73879676-73879688. Max. coverage (+): 0. Max coverage (-): 0

Region: chr6 73879689-73879700. Max. coverage (+): 0. Max coverage (-): 0

Region: chr6 73879701-73879713. Max. coverage (+): 0. Max coverage (-): 0

Region: chr6 73879714-73879725. Max. coverage (+): 0. Max coverage (-): 0

Region: chr6 73879726-73879738. Max. coverage (+): 0. Max coverage (-): 0

Region: chr6 73879739-73879751. Max. coverage (+): 0. Max coverage (-): 0

Region: chr6 73879752-73879763. Max. coverage (+): 0. Max coverage (-): 0

Region: chr6 73879764-73879776. Max. coverage (+): 0. Max coverage (-): 0

Region: chr6 73879777-73879788. Max. coverage (+): 0. Max coverage (-): 0

Region: chr6 73879789-73879801. Max. coverage (+): 0. Max coverage (-): 0

Region: chr6 73879802-73879813. Max. coverage (+): 0. Max coverage (-): 0

Region: chr6 73879814-73879826. Max. coverage (+): 0. Max coverage (-): 0

Region: chr6 73879827-73879838. Max. coverage (+): 0. Max coverage (-): 0

Region: chr6 73879839-73879851. Max. coverage (+): 0. Max coverage (-): 0

Region: chr6 73879852-73879863. Max. coverage (+): 0. Max coverage (-): 0

Region: chr6 73879864-73879876. Max. coverage (+): 0. Max coverage (-): 0

Region: chr6 73879877-73879888. Max. coverage (+): 0. Max coverage (-): 0

Region: chr6 73879889-73879901. Max. coverage (+): 0. Max coverage (-): 0

Region: chr6 73879902-73879913. Max. coverage (+): 0. Max coverage (-): 0

Region: chr6 73879914-73879926. Max. coverage (+): 0. Max coverage (-): 0

Region: chr6 73879927-73879939. Max. coverage (+): 0. Max coverage (-): 0

Region: chr6 73879940-73879951. Max. coverage (+): 0. Max coverage (-): 0

Region: chr6 73879952-73879964. Max. coverage (+): 0. Max coverage (-): 0

Region: chr6 73879965-73879976. Max. coverage (+): 0. Max coverage (-): 0

Region: chr6 73879977-73879989. Max. coverage (+): 0. Max coverage (-): 0

Region: chr6 73879990-73880001. Max. coverage (+): 0. Max coverage (-): 0

Region: chr6 73880002-73880014. Max. coverage (+): 0. Max coverage (-): 0

Region: chr6 73880015-73880026. Max. coverage (+): 0. Max coverage (-): 0

Region: chr6 73880027-73880039. Max. coverage (+): 0. Max coverage (-): 0

Region: chr6 73880040-73880051. Max. coverage (+): 0. Max coverage (-): 0

Region: chr6 73880052-73880064. Max. coverage (+): 0. Max coverage (-): 0

Region: chr6 73880065-73880076. Max. coverage (+): 0. Max coverage (-): 0

Region: chr6 73880077-73880089. Max. coverage (+): 0. Max coverage (-): 0

Region: chr6 73880090-73880101. Max. coverage (+): 0. Max coverage (-): 0

Region: chr6 73880102-73880114. Max. coverage (+): 0. Max coverage (-): 0

Region: chr6 73880115-73880127. Max. coverage (+): 0. Max coverage (-): 0

Region: chr6 73880128-73880139. Max. coverage (+): 0. Max coverage (-): 0

Region: chr6 73880140-73880152. Max. coverage (+): 0. Max coverage (-): 0

Region: chr6 73880153-73880164. Max. coverage (+): 0. Max coverage (-): 0

Region: chr6 73880165-73880177. Max. coverage (+): 0. Max coverage (-): 0

Region: chr6 73880178-73880189. Max. coverage (+): 0. Max coverage (-): 0

Region: chr6 73880190-73880202. Max. coverage (+): 0. Max coverage (-): 0

Region: chr6 73880203-73880214. Max. coverage (+): 0. Max coverage (-): 0

Region: chr6 73880215-73880227. Max. coverage (+): 0. Max coverage (-): 0

Region: chr6 73880228-73880239. Max. coverage (+): 0. Max coverage (-): 0

Region: chr6 73880240-73880252. Max. coverage (+): 0. Max coverage (-): 0

Region: chr6 73880253-73880264. Max. coverage (+): 0. Max coverage (-): 0

Region: chr6 73880265-73880277. Max. coverage (+): 0. Max coverage (-): 0

Region: chr6 73880278-73880289. Max. coverage (+): 0. Max coverage (-): 0

Region: chr6 73880290-73880302. Max. coverage (+): 0. Max coverage (-): 0

Region: chr6 73880303-73880315. Max. coverage (+): 0. Max coverage (-): 0

Region: chr6 73880316-73880327. Max. coverage (+): 0. Max coverage (-): 0

Region: chr6 73880328-73880340. Max. coverage (+): 0. Max coverage (-): 0

Region: chr6 73880341-73880352. Max. coverage (+): 0. Max coverage (-): 0

Region: chr6 73880353-73880365. Max. coverage (+): 0. Max coverage (-): 0

Region: chr6 73880366-73880377. Max. coverage (+): 0. Max coverage (-): 0

Region: chr6 73880378-73880390. Max. coverage (+): 0. Max coverage (-): 0

Region: chr6 73880391-73880402. Max. coverage (+): 0. Max coverage (-): 0

Region: chr6 73880403-73880415. Max. coverage (+): 0. Max coverage (-): 0

Region: chr6 73880416-73880427. Max. coverage (+): 0. Max coverage (-): 0

Region: chr6 73880428-73880440. Max. coverage (+): 0. Max coverage (-): 0

Region: chr6 73880441-73880452. Max. coverage (+): 0. Max coverage (-): 0

Region: chr6 73880453-73880465. Max. coverage (+): 0. Max coverage (-): 0

Region: chr6 73880466-73880477. Max. coverage (+): 0. Max coverage (-): 0

Region: chr6 73880478-73880490. Max. coverage (+): 0. Max coverage (-): 0

Region: chr6 73880491-73880503. Max. coverage (+): 0. Max coverage (-): 4.05

Region: chr6 73880504-73880515. Max. coverage (+): 0. Max coverage (-): 4.05

Region: chr6 73880516-73880528. Max. coverage (+): 0. Max coverage (-): 0

Region: chr6 73880529-73880540. Max. coverage (+): 0. Max coverage (-): 0

Region: chr6 73880541-73880553. Max. coverage (+): 0. Max coverage (-): 0

Region: chr6 73880554-73880565. Max. coverage (+): 0. Max coverage (-): 0

Region: chr6 73880566-73880578. Max. coverage (+): 0. Max coverage (-): 0

Region: chr6 73880579-73880590. Max. coverage (+): 0. Max coverage (-): 0

Region: chr6 73880591-73880603. Max. coverage (+): 0. Max coverage (-): 0

Region: chr6 73880604-73880615. Max. coverage (+): 0. Max coverage (-): 0

Region: chr6 73880616-73880628. Max. coverage (+): 0. Max coverage (-): 0

Region: chr6 73880629-73880640. Max. coverage (+): 0. Max coverage (-): 0

Region: chr6 73880641-73880653. Max. coverage (+): 0. Max coverage (-): 0

Region: chr6 73880654-73880665. Max. coverage (+): 0. Max coverage (-): 0

Region: chr6 73880666-73880678. Max. coverage (+): 0. Max coverage (-): 1.62

Region: chr6 73880679-73880691. Max. coverage (+): 0. Max coverage (-): 0

Region: chr6 73880692-73880703. Max. coverage (+): 0. Max coverage (-): 0

Region: chr6 73880704-73880716. Max. coverage (+): 0. Max coverage (-): 0

Region: chr6 73880717-73880728. Max. coverage (+): 0. Max coverage (-): 0

Region: chr6 73880729-73880741. Max. coverage (+): 0. Max coverage (-): 0

Region: chr6 73880742-73880753. Max. coverage (+): 0. Max coverage (-): 0

Region: chr6 73880754-73880766. Max. coverage (+): 0. Max coverage (-): 0

Region: chr6 73880767-73880778. Max. coverage (+): 0. Max coverage (-): 0

Region: chr6 73880779-73880791. Max. coverage (+): 0. Max coverage (-): 0

Region: chr6 73880792-73880803. Max. coverage (+): 0. Max coverage (-): 0

Region: chr6 73880804-73880816. Max. coverage (+): 0. Max coverage (-): 0

Region: chr6 73880817-73880828. Max. coverage (+): 0. Max coverage (-): 0

Region: chr6 73880829-73880841. Max. coverage (+): 0. Max coverage (-): 0

Region: chr6 73880842-73880853. Max. coverage (+): 0. Max coverage (-): 0

Region: chr6 73880854-73880866. Max. coverage (+): 0. Max coverage (-): 0

Region: chr6 73880867-73880879. Max. coverage (+): 0. Max coverage (-): 0

Region: chr6 73880880-73880891. Max. coverage (+): 0. Max coverage (-): 0

Region: chr6 73880892-73880904. Max. coverage (+): 0. Max coverage (-): 0

Region: chr6 73880905-73880916. Max. coverage (+): 0. Max coverage (-): 0

Region: chr6 73880917-73880929. Max. coverage (+): 0. Max coverage (-): 0

Region: chr6 73880930-73880941. Max. coverage (+): 0. Max coverage (-): 0

Region: chr6 73880942-73880954. Max. coverage (+): 0. Max coverage (-): 0

Region: chr6 73880955-73880966. Max. coverage (+): 0. Max coverage (-): 0

Region: chr6 73880967-73880979. Max. coverage (+): 0. Max coverage (-): 0

Region: chr6 73880980-73880991. Max. coverage (+): 0. Max coverage (-): 0

Region: chr6 73880992-73881004. Max. coverage (+): 0. Max coverage (-): 0

Region: chr6 73881005-73881016. Max. coverage (+): 0. Max coverage (-): 0

Region: chr6 73881017-73881029. Max. coverage (+): 0. Max coverage (-): 0

Region: chr6 73881030-73881042. Max. coverage (+): 0. Max coverage (-): 0

Region: chr6 73881043-73881054. Max. coverage (+): 0. Max coverage (-): 0

Region: chr6 73881055-73881067. Max. coverage (+): 0. Max coverage (-): 0

Region: chr6 73881068-73881079. Max. coverage (+): 0. Max coverage (-): 0

Region: chr6 73881080-73881092. Max. coverage (+): 0. Max coverage (-): 0

Region: chr6 73881093-73881104. Max. coverage (+): 0. Max coverage (-): 0

Region: chr6 73881105-73881117. Max. coverage (+): 0. Max coverage (-): 0

Region: chr6 73881118-73881129. Max. coverage (+): 0. Max coverage (-): 0

Region: chr6 73881130-73881142. Max. coverage (+): 0. Max coverage (-): 0

Region: chr6 73881143-73881154. Max. coverage (+): 0. Max coverage (-): 0

Region: chr6 73881155-73881167. Max. coverage (+): 0. Max coverage (-): 0

Region: chr6 73881168-73881179. Max. coverage (+): 0. Max coverage (-): 0

Region: chr6 73881180-73881192. Max. coverage (+): 0. Max coverage (-): 0

Region: chr6 73881193-73881204. Max. coverage (+): 0. Max coverage (-): 0

Region: chr6 73881205-73881217. Max. coverage (+): 0. Max coverage (-): 0

Region: chr6 73881218-73881230. Max. coverage (+): 0. Max coverage (-): 0

Region: chr6 73881231-73881242. Max. coverage (+): 0. Max coverage (-): 0

Region: chr6 73881243-73881255. Max. coverage (+): 0. Max coverage (-): 0

Region: chr6 73881256-73881267. Max. coverage (+): 0. Max coverage (-): 0

Region: chr6 73881268-73881280. Max. coverage (+): 0. Max coverage (-): 0

Region: chr6 73881281-73881292. Max. coverage (+): 0. Max coverage (-): 0

Region: chr6 73881293-73881305. Max. coverage (+): 0. Max coverage (-): 0

Region: chr6 73881306-73881317. Max. coverage (+): 0. Max coverage (-): 0

Region: chr6 73881318-73881330. Max. coverage (+): 0. Max coverage (-): 0

Region: chr6 73881331-73881342. Max. coverage (+): 0. Max coverage (-): 2.38

Region: chr6 73881343-73881355. Max. coverage (+): 0. Max coverage (-): 5.63

Region: chr6 73881356-73881367. Max. coverage (+): 0. Max coverage (-): 0

Region: chr6 73881368-73881380. Max. coverage (+): 0. Max coverage (-): 0

Region: chr6 73881381-73881392. Max. coverage (+): 0. Max coverage (-): 0

Region: chr6 73881393-73881405. Max. coverage (+): 0. Max coverage (-): 2.2

Region: chr6 73881406-73881418. Max. coverage (+): 0. Max coverage (-): 2.2

Region: chr6 73881419-73881430. Max. coverage (+): 0. Max coverage (-): 0

Region: chr6 73881431-73881443. Max. coverage (+): 0. Max coverage (-): 0

Region: chr6 73881444-73881455. Max. coverage (+): 0. Max coverage (-): 0

Region: chr6 73881456-73881468. Max. coverage (+): 0. Max coverage (-): 0

Region: chr6 73881469-73881480. Max. coverage (+): 0. Max coverage (-): 0

Region: chr6 73881481-73881493. Max. coverage (+): 0. Max coverage (-): 0

Region: chr6 73881494-73881505. Max. coverage (+): 0. Max coverage (-): 0

Region: chr6 73881506-73881518. Max. coverage (+): 0. Max coverage (-): 0

Region: chr6 73881519-73881530. Max. coverage (+): 0. Max coverage (-): 0

Region: chr6 73881531-73881543. Max. coverage (+): 0. Max coverage (-): 0

Region: chr6 73881544-73881555. Max. coverage (+): 0. Max coverage (-): 0

Region: chr6 73881556-73881568. Max. coverage (+): 0. Max coverage (-): 0

Region: chr6 73881569-73881580. Max. coverage (+): 0. Max coverage (-): 0

Region: chr6 73881581-73881593. Max. coverage (+): 0. Max coverage (-): 0

Region: chr6 73881594-73881606. Max. coverage (+): 0. Max coverage (-): 0

Region: chr6 73881607-73881618. Max. coverage (+): 0. Max coverage (-): 0

Region: chr6 73881619-73881631. Max. coverage (+): 0. Max coverage (-): 0

Region: chr6 73881632-73881643. Max. coverage (+): 0. Max coverage (-): 0

Region: chr6 73881644-73881656. Max. coverage (+): 0. Max coverage (-): 0

Region: chr6 73881657-73881668. Max. coverage (+): 0. Max coverage (-): 0

Region: chr6 73881669-73881681. Max. coverage (+): 0. Max coverage (-): 0

Region: chr6 73881682-73881693. Max. coverage (+): 0. Max coverage (-): 0

Region: chr6 73881694-73881706. Max. coverage (+): 0. Max coverage (-): 0

Region: chr6 73881707-73881718. Max. coverage (+): 0. Max coverage (-): 0

Region: chr6 73881719-73881731. Max. coverage (+): 0. Max coverage (-): 0

Region: chr6 73881732-73881743. Max. coverage (+): 0. Max coverage (-): 0

Region: chr6 73881744-73881756. Max. coverage (+): 0. Max coverage (-): 0

Region: chr6 73881757-73881768. Max. coverage (+): 0. Max coverage (-): 0

Region: chr6 73881769-73881781. Max. coverage (+): 0. Max coverage (-): 0

Region: chr6 73881782-73881794. Max. coverage (+): 0. Max coverage (-): 0

Region: chr6 73881795-73881806. Max. coverage (+): 0. Max coverage (-): 0

Region: chr6 73881807-73881819. Max. coverage (+): 0. Max coverage (-): 0

Region: chr6 73881820-73881831. Max. coverage (+): 0. Max coverage (-): 0

Region: chr6 73881832-73881844. Max. coverage (+): 0. Max coverage (-): 4.9

Region: chr6 73881845-73881856. Max. coverage (+): 0. Max coverage (-): 0

Region: chr6 73881857-73881869. Max. coverage (+): 0. Max coverage (-): 0

Region: chr6 73881870-73881881. Max. coverage (+): 0. Max coverage (-): 0

Region: chr6 73881882-73881894. Max. coverage (+): 0. Max coverage (-): 0

Region: chr6 73881895-73881906. Max. coverage (+): 0. Max coverage (-): 0

Region: chr6 73881907-73881919. Max. coverage (+): 0. Max coverage (-): 0

Region: chr6 73881920-73881931. Max. coverage (+): 0. Max coverage (-): 0

Region: chr6 73881932-73881944. Max. coverage (+): 0. Max coverage (-): 0

Region: chr6 73881945-73881956. Max. coverage (+): 0. Max coverage (-): 0

Region: chr6 73881957-73881969. Max. coverage (+): 0. Max coverage (-): 0

Region: chr6 73881970-73881982. Max. coverage (+): 0. Max coverage (-): 0

Region: chr6 73881983-73881994. Max. coverage (+): 0. Max coverage (-): 0

Region: chr6 73881995-73882007. Max. coverage (+): 0. Max coverage (-): 0

Region: chr6 73882008-73882019. Max. coverage (+): 0. Max coverage (-): 0

Region: chr6 73882020-73882032. Max. coverage (+): 0. Max coverage (-): 0

Region: chr6 73882033-73882044. Max. coverage (+): 0. Max coverage (-): 0

Region: chr6 73882045-73882057. Max. coverage (+): 0. Max coverage (-): 0

Region: chr6 73882058-73882069. Max. coverage (+): 0. Max coverage (-): 0

Region: chr6 73882070-73882082. Max. coverage (+): 0. Max coverage (-): 0

Region: chr6 73882083-73882094. Max. coverage (+): 0. Max coverage (-): 0

Region: chr6 73882095-73882107. Max. coverage (+): 0. Max coverage (-): 0

Region: chr6 73882108-73882119. Max. coverage (+): 0. Max coverage (-): 0

Region: chr6 73882120-73882132. Max. coverage (+): 0. Max coverage (-): 0

Region: chr6 73882133-73882145. Max. coverage (+): 0. Max coverage (-): 0

Region: chr6 73882146-73882157. Max. coverage (+): 0. Max coverage (-): 0

Region: chr6 73882158-73882170. Max. coverage (+): 0. Max coverage (-): 0

Region: chr6 73882171-73882182. Max. coverage (+): 0. Max coverage (-): 0

Region: chr6 73882183-73882195. Max. coverage (+): 0. Max coverage (-): 0

Region: chr6 73882196-73882207. Max. coverage (+): 0. Max coverage (-): 0

Region: chr6 73882208-73882220. Max. coverage (+): 0. Max coverage (-): 0

Region: chr6 73882221-73882232. Max. coverage (+): 0. Max coverage (-): 0

Region: chr6 73882233-73882245. Max. coverage (+): 0. Max coverage (-): 0

Region: chr6 73882246-73882257. Max. coverage (+): 0. Max coverage (-): 0

Region: chr6 73882258-73882270. Max. coverage (+): 0. Max coverage (-): 0

Region: chr6 73882271-73882282. Max. coverage (+): 0. Max coverage (-): 0

Region: chr6 73882283-73882295. Max. coverage (+): 0. Max coverage (-): 0

Region: chr6 73882296-73882307. Max. coverage (+): 0. Max coverage (-): 0

Region: chr6 73882308-73882320. Max. coverage (+): 0. Max coverage (-): 0

Region: chr6 73882321-73882333. Max. coverage (+): 0. Max coverage (-): 0

Region: chr6 73882334-73882345. Max. coverage (+): 0. Max coverage (-): 0

Region: chr6 73882346-73882358. Max. coverage (+): 0. Max coverage (-): 0

Region: chr6 73882359-73882370. Max. coverage (+): 0. Max coverage (-): 0

Region: chr6 73882371-73882383. Max. coverage (+): 0. Max coverage (-): 0

Region: chr6 73882384-73882395. Max. coverage (+): 0. Max coverage (-): 0

Region: chr6 73882396-73882408. Max. coverage (+): 0. Max coverage (-): 5.22

Region: chr6 73882409-73882420. Max. coverage (+): 0. Max coverage (-): 5.22

Region: chr6 73882421-73882433. Max. coverage (+): 0. Max coverage (-): 0

Region: chr6 73882434-73882445. Max. coverage (+): 0. Max coverage (-): 0

Region: chr6 73882446-73882458. Max. coverage (+): 0. Max coverage (-): 0

Region: chr6 73882459-73882470. Max. coverage (+): 0. Max coverage (-): 0

Region: chr6 73882471-73882483. Max. coverage (+): 0. Max coverage (-): 0

Region: chr6 73882484-73882495. Max. coverage (+): 0. Max coverage (-): 0

Region: chr6 73882496-73882508. Max. coverage (+): 0. Max coverage (-): 0

Region: chr6 73882509-73882521. Max. coverage (+): 0. Max coverage (-): 0

Region: chr6 73882522-73882533. Max. coverage (+): 0. Max coverage (-): 0

Region: chr6 73882534-73882546. Max. coverage (+): 0. Max coverage (-): 0

Region: chr6 73882547-73882558. Max. coverage (+): 0. Max coverage (-): 0

Region: chr6 73882559-73882571. Max. coverage (+): 0. Max coverage (-): 5.65

Region: chr6 73882572-73882583. Max. coverage (+): 0. Max coverage (-): 0

Region: chr6 73882584-73882596. Max. coverage (+): 0. Max coverage (-): 0

Region: chr6 73882597-73882608. Max. coverage (+): 0. Max coverage (-): 0

Region: chr6 73882609-73882621. Max. coverage (+): 0. Max coverage (-): 9.93

Region: chr6 73882622-73882633. Max. coverage (+): 0. Max coverage (-): 21.9

Region: chr6 73882634-73882646. Max. coverage (+): 0. Max coverage (-): 0

Region: chr6 73882647-73882658. Max. coverage (+): 0. Max coverage (-): 0

Region: chr6 73882659-73882671. Max. coverage (+): 0. Max coverage (-): 4.45

Region: chr6 73882672-73882683. Max. coverage (+): 0. Max coverage (-): 3.69

Region: chr6 73882684-73882696. Max. coverage (+): 0. Max coverage (-): 3.69

Region: chr6 73882697-73882709. Max. coverage (+): 0. Max coverage (-): 1.58

Region: chr6 73882710-73882721. Max. coverage (+): 0. Max coverage (-): 0

Region: chr6 73882722-73882734. Max. coverage (+): 0. Max coverage (-): 1.23

Region: chr6 73882735-73882746. Max. coverage (+): 0. Max coverage (-): 6.52

Region: chr6 73882747-73882759. Max. coverage (+): 0. Max coverage (-): 5.47

Region: chr6 73882760-73882771. Max. coverage (+): 0. Max coverage (-): 0

Region: chr6 73882772-73882784. Max. coverage (+): 0. Max coverage (-): 1.29

Region: chr6 73882785-73882796. Max. coverage (+): 0. Max coverage (-): 7.64

Region: chr6 73882797-73882809. Max. coverage (+): 0. Max coverage (-): 7.07

Region: chr6 73882810-73882821. Max. coverage (+): 0. Max coverage (-): 0

Region: chr6 73882822-73882834. Max. coverage (+): 0. Max coverage (-): 0

Region: chr6 73882835-73882846. Max. coverage (+): 0. Max coverage (-): 4.67

Region: chr6 73882847-73882859. Max. coverage (+): 0. Max coverage (-): 4.67

Region: chr6 73882860-73882871. Max. coverage (+): 0. Max coverage (-): 4.62

Region: chr6 73882872-73882884. Max. coverage (+): 0. Max coverage (-): 7.1

Region: chr6 73882885-73882897. Max. coverage (+): 0. Max coverage (-): 4.93

Region: chr6 73882898-73882909. Max. coverage (+): 0. Max coverage (-): 0

Region: chr6 73882910-73882922. Max. coverage (+): 0. Max coverage (-): 0

Region: chr6 73882923-73882934. Max. coverage (+): 0. Max coverage (-): 0

Region: chr6 73882935-73882947. Max. coverage (+): 0. Max coverage (-): 0

Region: chr6 73882948-73882959. Max. coverage (+): 0. Max coverage (-): 0

Region: chr6 73882960-73882972. Max. coverage (+): 0. Max coverage (-): 0

Region: chr6 73882973-73882984. Max. coverage (+): 0. Max coverage (-): 0

Region: chr6 73882985-73882997. Max. coverage (+): 0. Max coverage (-): 0

Region: chr6 73882998-73883009. Max. coverage (+): 0. Max coverage (-): 0

Region: chr6 73883010-73883022. Max. coverage (+): 0. Max coverage (-): 0

Region: chr6 73883023-73883034. Max. coverage (+): 0. Max coverage (-): 0

Region: chr6 73883035-73883047. Max. coverage (+): 0. Max coverage (-): 0

Region: chr6 73883048-73883059. Max. coverage (+): 0. Max coverage (-): 0

Region: chr6 73883060-73883072. Max. coverage (+): 0. Max coverage (-): 0

Region: chr6 73883073-73883085. Max. coverage (+): 0. Max coverage (-): 0

Region: chr6 73883086-73883097. Max. coverage (+): 0. Max coverage (-): 0

Region: chr6 73883098-73883110. Max. coverage (+): 0. Max coverage (-): 0

Region: chr6 73883111-73883122. Max. coverage (+): 0. Max coverage (-): 0

Region: chr6 73883123-73883135. Max. coverage (+): 0. Max coverage (-): 0

Region: chr6 73883136-73883147. Max. coverage (+): 0. Max coverage (-): 0

Region: chr6 73883148-73883160. Max. coverage (+): 0. Max coverage (-): 0

Region: chr6 73883161-73883172. Max. coverage (+): 0. Max coverage (-): 5.21

Region: chr6 73883173-73883185. Max. coverage (+): 0. Max coverage (-): 5.21

Region: chr6 73883186-73883197. Max. coverage (+): 0. Max coverage (-): 0

Region: chr6 73883198-73883210. Max. coverage (+): 0. Max coverage (-): 0

Region: chr6 73883211-73883222. Max. coverage (+): 0. Max coverage (-): 0

Region: chr6 73883223-73883235. Max. coverage (+): 0. Max coverage (-): 0

Region: chr6 73883236-73883247. Max. coverage (+): 0. Max coverage (-): 0

Region: chr6 73883248-73883260. Max. coverage (+): 0. Max coverage (-): 5.33

Region: chr6 73883261-73883273. Max. coverage (+): 0. Max coverage (-): 5.33

Region: chr6 73883274-73883285. Max. coverage (+): 0. Max coverage (-): 0

Region: chr6 73883286-73883298. Max. coverage (+): 0. Max coverage (-): 0

Region: chr6 73883299-73883310. Max. coverage (+): 0. Max coverage (-): 0

Region: chr6 73883311-73883323. Max. coverage (+): 0. Max coverage (-): 0

Region: chr6 73883324-73883335. Max. coverage (+): 0. Max coverage (-): 0

Region: chr6 73883336-73883348. Max. coverage (+): 0. Max coverage (-): 0

Region: chr6 73883349-73883360. Max. coverage (+): 0. Max coverage (-): 0

Region: chr6 73883361-73883373. Max. coverage (+): 0. Max coverage (-): 0

Region: chr6 73883374-73883385. Max. coverage (+): 0. Max coverage (-): 0

Region: chr6 73883386-73883398. Max. coverage (+): 0. Max coverage (-): 0

Region: chr6 73883399-73883410. Max. coverage (+): 0. Max coverage (-): 0

Region: chr6 73883411-73883423. Max. coverage (+): 0. Max coverage (-): 0

Region: chr6 73883424-73883436. Max. coverage (+): 0. Max coverage (-): 0

Region: chr6 73883437-73883448. Max. coverage (+): 0. Max coverage (-): 0

Region: chr6 73883449-73883461. Max. coverage (+): 0. Max coverage (-): 0

Region: chr6 73883462-73883473. Max. coverage (+): 0. Max coverage (-): 0

Region: chr6 73883474-73883486. Max. coverage (+): 0. Max coverage (-): 0

Region: chr6 73883487-73883498. Max. coverage (+): 0. Max coverage (-): 0

Region: chr6 73883499-73883511. Max. coverage (+): 0. Max coverage (-): 0

Region: chr6 73883512-73883523. Max. coverage (+): 0. Max coverage (-): 0

Region: chr6 73883524-73883536. Max. coverage (+): 0. Max coverage (-): 0

Region: chr6 73883537-73883548. Max. coverage (+): 0. Max coverage (-): 0

Region: chr6 73883549-73883561. Max. coverage (+): 0. Max coverage (-): 0

Region: chr6 73883562-73883573. Max. coverage (+): 0. Max coverage (-): 0

Region: chr6 73883574-73883586. Max. coverage (+): 0. Max coverage (-): 0

Region: chr6 73883587-73883598. Max. coverage (+): 0. Max coverage (-): 0

Region: chr6 73883599-73883611. Max. coverage (+): 0. Max coverage (-): 0

Region: chr6 73883612-73883624. Max. coverage (+): 0. Max coverage (-): 0

Region: chr6 73883625-73883636. Max. coverage (+): 0. Max coverage (-): 0

Region: chr6 73883637-73883649. Max. coverage (+): 0. Max coverage (-): 0

Region: chr6 73883650-73883661. Max. coverage (+): 0. Max coverage (-): 0

Region: chr6 73883662-73883674. Max. coverage (+): 0. Max coverage (-): 0

Region: chr6 73883675-73883686. Max. coverage (+): 0. Max coverage (-): 0

Region: chr6 73883687-73883699. Max. coverage (+): 0. Max coverage (-): 0

Region: chr6 73883700-73883711. Max. coverage (+): 0. Max coverage (-): 0

Region: chr6 73883712-73883724. Max. coverage (+): 0. Max coverage (-): 0

Region: chr6 73883725-73883736. Max. coverage (+): 0. Max coverage (-): 0

Region: chr6 73883737-73883749. Max. coverage (+): 0. Max coverage (-): 0

Region: chr6 73883750-73883761. Max. coverage (+): 0. Max coverage (-): 0

Region: chr6 73883762-73883774. Max. coverage (+): 0. Max coverage (-): 0

Region: chr6 73883775-73883786. Max. coverage (+): 0. Max coverage (-): 0

Region: chr6 73883787-73883799. Max. coverage (+): 0. Max coverage (-): 0

Region: chr6 73883800-73883812. Max. coverage (+): 0. Max coverage (-): 0

Region: chr6 73883813-73883824. Max. coverage (+): 0. Max coverage (-): 0

Region: chr6 73883825-73883837. Max. coverage (+): 0. Max coverage (-): 0

Region: chr6 73883838-73883849. Max. coverage (+): 0. Max coverage (-): 0

Region: chr6 73883850-73883862. Max. coverage (+): 0. Max coverage (-): 0

Region: chr6 73883863-73883874. Max. coverage (+): 0. Max coverage (-): 0

Region: chr6 73883875-73883887. Max. coverage (+): 0. Max coverage (-): 0

Region: chr6 73883888-73883899. Max. coverage (+): 0. Max coverage (-): 0

Region: chr6 73883900-73883912. Max. coverage (+): 0. Max coverage (-): 0

Region: chr6 73883913-73883924. Max. coverage (+): 0. Max coverage (-): 0

Region: chr6 73883925-73883937. Max. coverage (+): 0. Max coverage (-): 0

Region: chr6 73883938-73883949. Max. coverage (+): 0. Max coverage (-): 0

Region: chr6 73883950-73883962. Max. coverage (+): 0. Max coverage (-): 0

Region: chr6 73883963-73883974. Max. coverage (+): 0. Max coverage (-): 0

Region: chr6 73883975-73883987. Max. coverage (+): 0. Max coverage (-): 0

Region: chr6 73883988-73884000. Max. coverage (+): 0. Max coverage (-): 0

Region: chr6 73884001-73884012. Max. coverage (+): 0. Max coverage (-): 0

Region: chr6 73884013-73884025. Max. coverage (+): 0. Max coverage (-): 0

Region: chr6 73884026-73884037. Max. coverage (+): 0. Max coverage (-): 0

Region: chr6 73884038-73884050. Max. coverage (+): 0. Max coverage (-): 0

Region: chr6 73884051-73884062. Max. coverage (+): 0. Max coverage (-): 0

Region: chr6 73884063-73884075. Max. coverage (+): 0. Max coverage (-): 0

Region: chr6 73884076-73884087. Max. coverage (+): 0. Max coverage (-): 0

Region: chr6 73884088-73884100. Max. coverage (+): 0. Max coverage (-): 0

Region: chr6 73884101-73884112. Max. coverage (+): 0. Max coverage (-): 0

Region: chr6 73884113-73884125. Max. coverage (+): 0. Max coverage (-): 0

Region: chr6 73884126-73884137. Max. coverage (+): 0. Max coverage (-): 0

Region: chr6 73884138-73884150. Max. coverage (+): 0. Max coverage (-): 0

Region: chr6 73884151-73884162. Max. coverage (+): 0. Max coverage (-): 0

Region: chr6 73884163-73884175. Max. coverage (+): 0. Max coverage (-): 0

Region: chr6 73884176-73884188. Max. coverage (+): 0. Max coverage (-): 0

Region: chr6 73884189-73884200. Max. coverage (+): 0. Max coverage (-): 0

Region: chr6 73884201-73884213. Max. coverage (+): 0. Max coverage (-): 0

Region: chr6 73884214-73884225. Max. coverage (+): 0. Max coverage (-): 0

Region: chr6 73884226-73884238. Max. coverage (+): 0. Max coverage (-): 0

Region: chr6 73884239-73884250. Max. coverage (+): 0. Max coverage (-): 0

Region: chr6 73884251-73884263. Max. coverage (+): 0. Max coverage (-): 0

Region: chr6 73884264-73884275. Max. coverage (+): 0. Max coverage (-): 0

Region: chr6 73884276-73884288. Max. coverage (+): 0. Max coverage (-): 0

Region: chr6 73884289-73884300. Max. coverage (+): 0. Max coverage (-): 0

Region: chr6 73884301-73884313. Max. coverage (+): 0. Max coverage (-): 0

Region: chr6 73884314-73884325. Max. coverage (+): 0. Max coverage (-): 0

Region: chr6 73884326-73884338. Max. coverage (+): 0. Max coverage (-): 0

Region: chr6 73884339-73884350. Max. coverage (+): 0. Max coverage (-): 0

Region: chr6 73884351-73884363. Max. coverage (+): 0. Max coverage (-): 0

Region: chr6 73884364-73884376. Max. coverage (+): 0. Max coverage (-): 0

Region: chr6 73884377-73884388. Max. coverage (+): 0. Max coverage (-): 0

Region: chr6 73884389-73884401. Max. coverage (+): 0. Max coverage (-): 0

Region: chr6 73884402-73884413. Max. coverage (+): 0. Max coverage (-): 0

Region: chr6 73884414-73884426. Max. coverage (+): 0. Max coverage (-): 0

Region: chr6 73884427-73884438. Max. coverage (+): 0. Max coverage (-): 0

Region: chr6 73884439-73884451. Max. coverage (+): 0. Max coverage (-): 0

Region: chr6 73884452-73884463. Max. coverage (+): 0. Max coverage (-): 0

Region: chr6 73884464-73884476. Max. coverage (+): 0. Max coverage (-): 0

Region: chr6 73884477-73884488. Max. coverage (+): 0. Max coverage (-): 0

Region: chr6 73884489-73884501. Max. coverage (+): 0. Max coverage (-): 0

Region: chr6 73884502-73884513. Max. coverage (+): 0. Max coverage (-): 0

Region: chr6 73884514-73884526. Max. coverage (+): 0. Max coverage (-): 0

Region: chr6 73884527-73884538. Max. coverage (+): 0. Max coverage (-): 0

Region: chr6 73884539-73884551. Max. coverage (+): 0. Max coverage (-): 0

Region: chr6 73884552-73884564. Max. coverage (+): 0. Max coverage (-): 0

Region: chr6 73884565-73884576. Max. coverage (+): 0. Max coverage (-): 0

Region: chr6 73884577-73884589. Max. coverage (+): 0. Max coverage (-): 0

Region: chr6 73884590-73884601. Max. coverage (+): 0. Max coverage (-): 0.66

Region: chr6 73884602-73884614. Max. coverage (+): 0. Max coverage (-): 1.71

Region: chr6 73884615-73884626. Max. coverage (+): 0. Max coverage (-): 0

Region: chr6 73884627-. Max. coverage (+): 0. Max coverage (-): 0

RepeatMasker Color Code

**+**

100-98% Identity

<98-95% Identity

<95-90% Identity

<90-85% Identity

<85-80% Identity

<80-75% Identity

<75-70% Identity

<70% Identity

**-**

Gene Set Color Code

**+**

Gene

Pseudogene

**-**

Topology/Coverage Color Code

Coverage Plus Strand

Coverage Minus Strand

Mainstrand: Plus

Mainstrand: Minus

Complementary Strand

Flanking Region  
(if option -flank >0)

Gene Set Annotation  
  
RepeatMasker Annotation  

**1. MER20**: 73878640-73878828 (-), Divergence to consensus: 34.9%  
**2. MIR**: 73879446-73879552 (-), Divergence to consensus: 33.9%  
**3. MER20**: 73879887-73880026 (-), Divergence to consensus: 24.3%  
**4. MER3**: 73880068-73880204 (+), Divergence to consensus: 25.4%  
**5. CHR-2B**: 73880753-73881058 (+), Divergence to consensus: 23.2%  
**6. L2a**: 73881172-73881258 (+), Divergence to consensus: 39%  
**7. (CTG)n**: 73882134-73882213 (+), Divergence to consensus: 30%  
**8. AT\_rich**: 73882790-73882818 (+), Divergence to consensus: 48.3%  
**9. Bov-tA2**: 73882949-73883144 (-), Divergence to consensus: 20.9%  
**10. (TG)n**: 73883414-73883476 (+), Divergence to consensus: 1.6%  
**11. CHR-2B**: 73883653-73883704 (+), Divergence to consensus: 21.1%  
**12. Bov-tA3**: 73883661-73883829 (+), Divergence to consensus: 10.2%  
**13. Bov-tA2**: 73883857-73884058 (+), Divergence to consensus: 22.3%

  
Transcription Factor Binding Sites  

**RFX4\_2** (Sequence: GTATCTATG (-): 73884622)  
**Mybl1\_1** (Sequence: AACCGTTA (+): 73883517)
