# Supplementary material for: Small RNA sequencing of cryopreserved semen from single bull revealed altered miRNAs and piRNAs expression between High- and Low-motile sperm populations
Source: BMC Genomics. 2017 Jan 4;18:14. doi: 10.1186/s12864-016-3394-7 (PMC5209821; doi:10.1186/s12864-016-3394-7)
Supplement: Additional file 3: — Details for each piRNA clusters found in High Motile (HM) sperm fraction. Genes, repeats, transposable elements and transcription factors binding sites falling within the cluster regions were reported. (ZIP 1896 kb) [file 12864_2016_3394_MOESM3_ESM.zip › 9.html]

piRNA cluster 9


Predicted piRNA cluster no. 9     previous   next
  

Show proTRAC run info
Hide proTRAC run info

================================= proTRAC ====================================  
VERSION: 2.1                                    LAST MODIFIED: 06. October 2015  
  
Please cite:  
Rosenkranz D, Zischler H. proTRAC - a software for probabilistic piRNA cluster  
detection, visualization and analysis. 2012. BMC Bioinformatics 13:5.  
  
and (for proTRAC 2.0 and later):  
Rosenkranz D, Rudloff S, Bastuck K, Ketting RF, Zischler H. Tupaia small RNAs  
provide insights into function and evolution of RNAi-based transposon defense  
in mammals. 2015. RNA 21(5):911-922.  
  
Contact:  
David Rosenkranz  
Institute of Anthropology, small RNA group  
Johannes Gutenberg University Mainz  
email: rosenkranz@uni-mainz.de  
  
You can find the latest proTRAC version at:  
http://sourceforge.net/projects/protrac/files  
http://www.smallRNAgroup-mainz.de/software  
==============================================================================  
  
PARAMETERS:  
Map file: .............../storage/core/barbara/genhome/smallRNA/fertility/Sample\_motile/pirna/Sample\_motile\_26-33\_collapsed.fa.no-dust.map.weighted-10000-1000-b-0  
Genome file: ............/storage/core/barbara/genhome/smallRNA/fertility/Sample\_all/pirna/bt\_311\_chrY.fa  
RepeatMasker annotation: /storage/genomes/bt\_umd31/GCF\_000003055.6\_Bos\_taurus\_UMD\_3.1.1\_repeatMasker\_chr.out  
GeneSet:................./storage/core/barbara/genhome/smallRNA/fertility/Sample\_all/pirna/full.gtf  
  
Significant (p<=0.01) hit density will be calculated based  
on observed hit distribution.  
  
Sliding window size: ........................................ 5000 bp  
Sliding window increament: .................................. 1000 bp  
Normalize each hit by number of genomic hits: ............... 1 [0=no/1=yes]  
Normalize each hit by number of sequence reads: ............. 1 [0=no/1=yes]  
Normalize values (-> per million mapped reads): ............. 1 [0=no/1=yes]  
Min. fraction of hits with 1T(U) or 10A: .................... 0.75  
Alternatively: Min. fraction of hits with 1T(U) and 10A: .... 0.5  
Min. fraction of hits with typical piRNA length: ............ 0.75  
Typical piRNA length: ....................................... 26-33 nt  
Min. size of a piRNA cluster: ............................... 5000 bp.  
Min. number of hits (absolute): ............................. 0  
Min. number of hits (normalized): ........................... 0  
Min. fraction of hits on the mainstrand: .................... 0.75  
Top fraction of mapped sequences (in terms of read counts): . 1%  
Top fraction accounts for max. n% of sequence reads: ........ 90%  
Min. fraction of hits on each arm of a bidirectional cluster: 0.1  
Output image file for each cluster: ......................... 0 [0=no/1=yes]  
Output html file for each cluster: .......................... 1 [0=no/1=yes]  
Output a summary table: ..................................... 1 [0=no/1=yes]  
Output a FASTA file for each cluster (piRNA sequences): ..... 1 [0=no/1=yes]  
Output a FASTA file comprising cluster sequences: ........... 1 [0=no/1=yes]  
Search DNA motifs in clusters: .............................. 1 [0=no/1=yes]  
Output flanking sequences: +/- .............................. 0 bp  
Output ~.pTi file: .......................................... 1 [0=no/1=yes]  
==============================================================================  
  
  
Genome size (without gaps): ............ 2678902517 bp  
Gaps (N/X/-): .......................... 53837044 bp  
Mapped reads: .......................... 658825247023  
Non-identical sequences: ............... 514171  
Genomic hits: .......................... 764233  
Significant densitiy of mapped reads: .. 12867599.5173724 reads/kb

Show proTRAC cluster info
Hide proTRAC cluster info

|  |  |
| --- | --- |
| Location | chr10 |
| Coordinates | 100968663-100991989 |
| Size [bp] | 23327 |
| Sequence hit loci | 1514 |
| Mapped reads (normalized) | 1877073347 |
| Mapped reads (normalized) per kb | 80467841.9 |
| Normalized reads with 1T (1U) | 82% |
| Normalized reads with 10A | 30.8% |
| Normalized reads with length 26-33 nt | 100% |
| Normalized reads on the main strand(s) | 99.7% |
| Predicted directionality | mono:plus |

100%

0%

1T (1U)  
reads

10A reads

26-33 nt  
reads

reads on mainstrand

**Either the amount of reads with 1T (1U) OR 10A has to exceed 75% (set with option: -1Tor10A)  
Alternatively the amount of reads with 1T (1U) AND 10A has to exceed 50% (set with option: -1Tand10A)  
Minimum amount of reads with preferred size is 75% (set with option: -pisize)  
Minimum amount of reads on the main strand(s) is 75% (set with option: -clstrand)**

Show read coverage
Hide read coverage

WHAT DO I SEE HERE?  
This chart shows the location of mapped sequence reads within a predicted piRNA cluster. The color refers to the number of genomic hits produced by the sequence read in question. A dark red bar indicates that this sequence read produces many other hits elsewhere in the genome. Many adjacent red or yellow bars can indicate the presence of a multi-copy element such as transposons or rRNA genes. A dark green bar indicates that this sequence read maps uniquely to this locus.

1 hit

2-5 hits

6-10 hits

11-20 hits

21-50 hits

51-100 hits

> 100 hits

chr10

100968663

100991989

Gene Set

RepeatMasker

Mapped  
Reads

77.35

plus strand

minus strand

77.35

Region: chr10 100939900-100968686. Max. coverage (+): 4.99. Max coverage (-): 0

Region: chr10 100968687-100968732. Max. coverage (+): 0. Max coverage (-): 4.47

Region: chr10 100968733-100968779. Max. coverage (+): 0. Max coverage (-): 0

Region: chr10 100968780-100968826. Max. coverage (+): 14.98. Max coverage (-): 0

Region: chr10 100968827-100968872. Max. coverage (+): 6.66. Max coverage (-): 0

Region: chr10 100968873-100968919. Max. coverage (+): 8.17. Max coverage (-): 0

Region: chr10 100968920-100968966. Max. coverage (+): 13.25. Max coverage (-): 0

Region: chr10 100968967-100969012. Max. coverage (+): 0.75. Max coverage (-): 0

Region: chr10 100969013-100969059. Max. coverage (+): 5.24. Max coverage (-): 0

Region: chr10 100969060-100969106. Max. coverage (+): 0. Max coverage (-): 0

Region: chr10 100969107-100969152. Max. coverage (+): 0. Max coverage (-): 0

Region: chr10 100969153-100969199. Max. coverage (+): 2.53. Max coverage (-): 1.76

Region: chr10 100969200-100969246. Max. coverage (+): 9.73. Max coverage (-): 0

Region: chr10 100969247-100969292. Max. coverage (+): 3.13. Max coverage (-): 0

Region: chr10 100969293-100969339. Max. coverage (+): 68.8. Max coverage (-): 1.51

Region: chr10 100969340-100969386. Max. coverage (+): 68.8. Max coverage (-): 0

Region: chr10 100969387-100969432. Max. coverage (+): 0. Max coverage (-): 0

Region: chr10 100969433-100969479. Max. coverage (+): 0. Max coverage (-): 0

Region: chr10 100969480-100969526. Max. coverage (+): 8.47. Max coverage (-): 0

Region: chr10 100969527-100969572. Max. coverage (+): 40.32. Max coverage (-): 0

Region: chr10 100969573-100969619. Max. coverage (+): 0. Max coverage (-): 0

Region: chr10 100969620-100969666. Max. coverage (+): 5.19. Max coverage (-): 0

Region: chr10 100969667-100969712. Max. coverage (+): 6.05. Max coverage (-): 0

Region: chr10 100969713-100969759. Max. coverage (+): 1.79. Max coverage (-): 0

Region: chr10 100969760-100969806. Max. coverage (+): 10.17. Max coverage (-): 0

Region: chr10 100969807-100969852. Max. coverage (+): 9.95. Max coverage (-): 0

Region: chr10 100969853-100969899. Max. coverage (+): 18.98. Max coverage (-): 0

Region: chr10 100969900-100969945. Max. coverage (+): 27.14. Max coverage (-): 0

Region: chr10 100969946-100969992. Max. coverage (+): 29.06. Max coverage (-): 0

Region: chr10 100969993-100970039. Max. coverage (+): 17.6. Max coverage (-): 0

Region: chr10 100970040-100970085. Max. coverage (+): 9.29. Max coverage (-): 0

Region: chr10 100970086-100970132. Max. coverage (+): 0. Max coverage (-): 0

Region: chr10 100970133-100970179. Max. coverage (+): 26.11. Max coverage (-): 0

Region: chr10 100970180-100970225. Max. coverage (+): 26.11. Max coverage (-): 0

Region: chr10 100970226-100970272. Max. coverage (+): 18.9. Max coverage (-): 0

Region: chr10 100970273-100970319. Max. coverage (+): 26.88. Max coverage (-): 0

Region: chr10 100970320-100970365. Max. coverage (+): 11.4. Max coverage (-): 0

Region: chr10 100970366-100970412. Max. coverage (+): 6.26. Max coverage (-): 0

Region: chr10 100970413-100970459. Max. coverage (+): 13.9. Max coverage (-): 0

Region: chr10 100970460-100970505. Max. coverage (+): 40.56. Max coverage (-): 0

Region: chr10 100970506-100970552. Max. coverage (+): 28.81. Max coverage (-): 0

Region: chr10 100970553-100970599. Max. coverage (+): 8.64. Max coverage (-): 0

Region: chr10 100970600-100970645. Max. coverage (+): 0. Max coverage (-): 0

Region: chr10 100970646-100970692. Max. coverage (+): 0. Max coverage (-): 0

Region: chr10 100970693-100970739. Max. coverage (+): 77.35. Max coverage (-): 0

Region: chr10 100970740-100970785. Max. coverage (+): 20.44. Max coverage (-): 0

Region: chr10 100970786-100970832. Max. coverage (+): 3.72. Max coverage (-): 0

Region: chr10 100970833-100970879. Max. coverage (+): 0. Max coverage (-): 0

Region: chr10 100970880-100970925. Max. coverage (+): 0. Max coverage (-): 0

Region: chr10 100970926-100970972. Max. coverage (+): 0. Max coverage (-): 0

Region: chr10 100970973-100971019. Max. coverage (+): 13.18. Max coverage (-): 0

Region: chr10 100971020-100971065. Max. coverage (+): 0. Max coverage (-): 0

Region: chr10 100971066-100971112. Max. coverage (+): 0. Max coverage (-): 0

Region: chr10 100971113-100971158. Max. coverage (+): 0. Max coverage (-): 0

Region: chr10 100971159-100971205. Max. coverage (+): 0. Max coverage (-): 0

Region: chr10 100971206-100971252. Max. coverage (+): 2.79. Max coverage (-): 0

Region: chr10 100971253-100971298. Max. coverage (+): 2.26. Max coverage (-): 0

Region: chr10 100971299-100971345. Max. coverage (+): 14.25. Max coverage (-): 0

Region: chr10 100971346-100971392. Max. coverage (+): 34.53. Max coverage (-): 0

Region: chr10 100971393-100971438. Max. coverage (+): 45.61. Max coverage (-): 0

Region: chr10 100971439-100971485. Max. coverage (+): 3.32. Max coverage (-): 0

Region: chr10 100971486-100971532. Max. coverage (+): 22.84. Max coverage (-): 0

Region: chr10 100971533-100971578. Max. coverage (+): 4.58. Max coverage (-): 0

Region: chr10 100971579-100971625. Max. coverage (+): 31.03. Max coverage (-): 0

Region: chr10 100971626-100971672. Max. coverage (+): 0.6. Max coverage (-): 0

Region: chr10 100971673-100971718. Max. coverage (+): 26.12. Max coverage (-): 0

Region: chr10 100971719-100971765. Max. coverage (+): 13.6. Max coverage (-): 0

Region: chr10 100971766-100971812. Max. coverage (+): 13.27. Max coverage (-): 0

Region: chr10 100971813-100971858. Max. coverage (+): 21.26. Max coverage (-): 0

Region: chr10 100971859-100971905. Max. coverage (+): 10.71. Max coverage (-): 0

Region: chr10 100971906-100971952. Max. coverage (+): 15.88. Max coverage (-): 0

Region: chr10 100971953-100971998. Max. coverage (+): 3.2. Max coverage (-): 0

Region: chr10 100971999-100972045. Max. coverage (+): 10.94. Max coverage (-): 0

Region: chr10 100972046-100972092. Max. coverage (+): 0. Max coverage (-): 0

Region: chr10 100972093-100972138. Max. coverage (+): 2.17. Max coverage (-): 0

Region: chr10 100972139-100972185. Max. coverage (+): 0. Max coverage (-): 0

Region: chr10 100972186-100972232. Max. coverage (+): 0. Max coverage (-): 0

Region: chr10 100972233-100972278. Max. coverage (+): 0. Max coverage (-): 0

Region: chr10 100972279-100972325. Max. coverage (+): 0. Max coverage (-): 0

Region: chr10 100972326-100972371. Max. coverage (+): 22.41. Max coverage (-): 0

Region: chr10 100972372-100972418. Max. coverage (+): 17.76. Max coverage (-): 0

Region: chr10 100972419-100972465. Max. coverage (+): 4.95. Max coverage (-): 0

Region: chr10 100972466-100972511. Max. coverage (+): 4.95. Max coverage (-): 0

Region: chr10 100972512-100972558. Max. coverage (+): 2.38. Max coverage (-): 0

Region: chr10 100972559-100972605. Max. coverage (+): 1.94. Max coverage (-): 0

Region: chr10 100972606-100972651. Max. coverage (+): 1.02. Max coverage (-): 0

Region: chr10 100972652-100972698. Max. coverage (+): 3.69. Max coverage (-): 0

Region: chr10 100972699-100972745. Max. coverage (+): 3.69. Max coverage (-): 0

Region: chr10 100972746-100972791. Max. coverage (+): 0. Max coverage (-): 0

Region: chr10 100972792-100972838. Max. coverage (+): 0. Max coverage (-): 0

Region: chr10 100972839-100972885. Max. coverage (+): 0. Max coverage (-): 0

Region: chr10 100972886-100972931. Max. coverage (+): 0. Max coverage (-): 0

Region: chr10 100972932-100972978. Max. coverage (+): 23.66. Max coverage (-): 0

Region: chr10 100972979-100973025. Max. coverage (+): 0. Max coverage (-): 0

Region: chr10 100973026-100973071. Max. coverage (+): 0. Max coverage (-): 0

Region: chr10 100973072-100973118. Max. coverage (+): 0. Max coverage (-): 0

Region: chr10 100973119-100973165. Max. coverage (+): 0. Max coverage (-): 0

Region: chr10 100973166-100973211. Max. coverage (+): 0. Max coverage (-): 0

Region: chr10 100973212-100973258. Max. coverage (+): 0. Max coverage (-): 0

Region: chr10 100973259-100973305. Max. coverage (+): 0. Max coverage (-): 0

Region: chr10 100973306-100973351. Max. coverage (+): 0. Max coverage (-): 0

Region: chr10 100973352-100973398. Max. coverage (+): 0. Max coverage (-): 0

Region: chr10 100973399-100973445. Max. coverage (+): 1.69. Max coverage (-): 0

Region: chr10 100973446-100973491. Max. coverage (+): 5.92. Max coverage (-): 0

Region: chr10 100973492-100973538. Max. coverage (+): 13.27. Max coverage (-): 0

Region: chr10 100973539-100973584. Max. coverage (+): 4.63. Max coverage (-): 0

Region: chr10 100973585-100973631. Max. coverage (+): 13.51. Max coverage (-): 0

Region: chr10 100973632-100973678. Max. coverage (+): 20.48. Max coverage (-): 0

Region: chr10 100973679-100973724. Max. coverage (+): 0. Max coverage (-): 0

Region: chr10 100973725-100973771. Max. coverage (+): 0. Max coverage (-): 0

Region: chr10 100973772-100973818. Max. coverage (+): 14.28. Max coverage (-): 0

Region: chr10 100973819-100973864. Max. coverage (+): 0. Max coverage (-): 0

Region: chr10 100973865-100973911. Max. coverage (+): 1.25. Max coverage (-): 0

Region: chr10 100973912-100973958. Max. coverage (+): 12.67. Max coverage (-): 0

Region: chr10 100973959-100974004. Max. coverage (+): 12.09. Max coverage (-): 0

Region: chr10 100974005-100974051. Max. coverage (+): 0. Max coverage (-): 0

Region: chr10 100974052-100974098. Max. coverage (+): 0. Max coverage (-): 0

Region: chr10 100974099-100974144. Max. coverage (+): 13.91. Max coverage (-): 0

Region: chr10 100974145-100974191. Max. coverage (+): 6.2. Max coverage (-): 0

Region: chr10 100974192-100974238. Max. coverage (+): 7.78. Max coverage (-): 0

Region: chr10 100974239-100974284. Max. coverage (+): 0. Max coverage (-): 0

Region: chr10 100974285-100974331. Max. coverage (+): 0. Max coverage (-): 0

Region: chr10 100974332-100974378. Max. coverage (+): 0. Max coverage (-): 0

Region: chr10 100974379-100974424. Max. coverage (+): 0. Max coverage (-): 0

Region: chr10 100974425-100974471. Max. coverage (+): 1.61. Max coverage (-): 0

Region: chr10 100974472-100974518. Max. coverage (+): 0. Max coverage (-): 0

Region: chr10 100974519-100974564. Max. coverage (+): 0. Max coverage (-): 0

Region: chr10 100974565-100974611. Max. coverage (+): 0. Max coverage (-): 0

Region: chr10 100974612-100974658. Max. coverage (+): 0. Max coverage (-): 0

Region: chr10 100974659-100974704. Max. coverage (+): 0. Max coverage (-): 0

Region: chr10 100974705-100974751. Max. coverage (+): 0. Max coverage (-): 0

Region: chr10 100974752-100974798. Max. coverage (+): 0. Max coverage (-): 0

Region: chr10 100974799-100974844. Max. coverage (+): 5.24. Max coverage (-): 0

Region: chr10 100974845-100974891. Max. coverage (+): 7.27. Max coverage (-): 0

Region: chr10 100974892-100974937. Max. coverage (+): 7.27. Max coverage (-): 0

Region: chr10 100974938-100974984. Max. coverage (+): 0. Max coverage (-): 0

Region: chr10 100974985-100975031. Max. coverage (+): 7.01. Max coverage (-): 0

Region: chr10 100975032-100975077. Max. coverage (+): 4.79. Max coverage (-): 0

Region: chr10 100975078-100975124. Max. coverage (+): 4.79. Max coverage (-): 0

Region: chr10 100975125-100975171. Max. coverage (+): 0. Max coverage (-): 0

Region: chr10 100975172-100975217. Max. coverage (+): 2.34. Max coverage (-): 0

Region: chr10 100975218-100975264. Max. coverage (+): 5.12. Max coverage (-): 0

Region: chr10 100975265-100975311. Max. coverage (+): 1.93. Max coverage (-): 0

Region: chr10 100975312-100975357. Max. coverage (+): 0. Max coverage (-): 0

Region: chr10 100975358-100975404. Max. coverage (+): 1.14. Max coverage (-): 0

Region: chr10 100975405-100975451. Max. coverage (+): 4.75. Max coverage (-): 0

Region: chr10 100975452-100975497. Max. coverage (+): 0. Max coverage (-): 0

Region: chr10 100975498-100975544. Max. coverage (+): 0. Max coverage (-): 0

Region: chr10 100975545-100975591. Max. coverage (+): 0. Max coverage (-): 0

Region: chr10 100975592-100975637. Max. coverage (+): 0. Max coverage (-): 0

Region: chr10 100975638-100975684. Max. coverage (+): 0. Max coverage (-): 0

Region: chr10 100975685-100975731. Max. coverage (+): 0. Max coverage (-): 0

Region: chr10 100975732-100975777. Max. coverage (+): 0. Max coverage (-): 0

Region: chr10 100975778-100975824. Max. coverage (+): 9.56. Max coverage (-): 0

Region: chr10 100975825-100975871. Max. coverage (+): 1.29. Max coverage (-): 0

Region: chr10 100975872-100975917. Max. coverage (+): 0. Max coverage (-): 0

Region: chr10 100975918-100975964. Max. coverage (+): 8.32. Max coverage (-): 0

Region: chr10 100975965-100976011. Max. coverage (+): 0. Max coverage (-): 0

Region: chr10 100976012-100976057. Max. coverage (+): 0. Max coverage (-): 0

Region: chr10 100976058-100976104. Max. coverage (+): 0. Max coverage (-): 0

Region: chr10 100976105-100976150. Max. coverage (+): 7.24. Max coverage (-): 0

Region: chr10 100976151-100976197. Max. coverage (+): 7.24. Max coverage (-): 0

Region: chr10 100976198-100976244. Max. coverage (+): 1.52. Max coverage (-): 0

Region: chr10 100976245-100976290. Max. coverage (+): 0. Max coverage (-): 0

Region: chr10 100976291-100976337. Max. coverage (+): 3.16. Max coverage (-): 0

Region: chr10 100976338-100976384. Max. coverage (+): 0.27. Max coverage (-): 0

Region: chr10 100976385-100976430. Max. coverage (+): 3.16. Max coverage (-): 0

Region: chr10 100976431-100976477. Max. coverage (+): 2.16. Max coverage (-): 0

Region: chr10 100976478-100976524. Max. coverage (+): 3.75. Max coverage (-): 0

Region: chr10 100976525-100976570. Max. coverage (+): 0. Max coverage (-): 0

Region: chr10 100976571-100976617. Max. coverage (+): 0. Max coverage (-): 0

Region: chr10 100976618-100976664. Max. coverage (+): 0. Max coverage (-): 0

Region: chr10 100976665-100976710. Max. coverage (+): 3.51. Max coverage (-): 0

Region: chr10 100976711-100976757. Max. coverage (+): 0. Max coverage (-): 0

Region: chr10 100976758-100976804. Max. coverage (+): 0. Max coverage (-): 0

Region: chr10 100976805-100976850. Max. coverage (+): 0.99. Max coverage (-): 0

Region: chr10 100976851-100976897. Max. coverage (+): 0. Max coverage (-): 0

Region: chr10 100976898-100976944. Max. coverage (+): 0. Max coverage (-): 0

Region: chr10 100976945-100976990. Max. coverage (+): 0. Max coverage (-): 0

Region: chr10 100976991-100977037. Max. coverage (+): 0. Max coverage (-): 0

Region: chr10 100977038-100977084. Max. coverage (+): 1.75. Max coverage (-): 0

Region: chr10 100977085-100977130. Max. coverage (+): 0. Max coverage (-): 0

Region: chr10 100977131-100977177. Max. coverage (+): 0. Max coverage (-): 0

Region: chr10 100977178-100977224. Max. coverage (+): 0. Max coverage (-): 0

Region: chr10 100977225-100977270. Max. coverage (+): 1.53. Max coverage (-): 0

Region: chr10 100977271-100977317. Max. coverage (+): 4.34. Max coverage (-): 0

Region: chr10 100977318-100977363. Max. coverage (+): 3.32. Max coverage (-): 0

Region: chr10 100977364-100977410. Max. coverage (+): 23.62. Max coverage (-): 0

Region: chr10 100977411-100977457. Max. coverage (+): 0. Max coverage (-): 0

Region: chr10 100977458-100977503. Max. coverage (+): 0. Max coverage (-): 0

Region: chr10 100977504-100977550. Max. coverage (+): 0. Max coverage (-): 0

Region: chr10 100977551-100977597. Max. coverage (+): 0. Max coverage (-): 0

Region: chr10 100977598-100977643. Max. coverage (+): 11.19. Max coverage (-): 0

Region: chr10 100977644-100977690. Max. coverage (+): 0.77. Max coverage (-): 0

Region: chr10 100977691-100977737. Max. coverage (+): 0.77. Max coverage (-): 0

Region: chr10 100977738-100977783. Max. coverage (+): 0. Max coverage (-): 0

Region: chr10 100977784-100977830. Max. coverage (+): 0. Max coverage (-): 0

Region: chr10 100977831-100977877. Max. coverage (+): 0. Max coverage (-): 0

Region: chr10 100977878-100977923. Max. coverage (+): 0. Max coverage (-): 0

Region: chr10 100977924-100977970. Max. coverage (+): 4.85. Max coverage (-): 0

Region: chr10 100977971-100978017. Max. coverage (+): 19. Max coverage (-): 0

Region: chr10 100978018-100978063. Max. coverage (+): 11.85. Max coverage (-): 0

Region: chr10 100978064-100978110. Max. coverage (+): 0. Max coverage (-): 0

Region: chr10 100978111-100978157. Max. coverage (+): 0. Max coverage (-): 0

Region: chr10 100978158-100978203. Max. coverage (+): 9.35. Max coverage (-): 0

Region: chr10 100978204-100978250. Max. coverage (+): 7.09. Max coverage (-): 0

Region: chr10 100978251-100978297. Max. coverage (+): 2.83. Max coverage (-): 0

Region: chr10 100978298-100978343. Max. coverage (+): 1.96. Max coverage (-): 0

Region: chr10 100978344-100978390. Max. coverage (+): 18.81. Max coverage (-): 0

Region: chr10 100978391-100978437. Max. coverage (+): 4.67. Max coverage (-): 0

Region: chr10 100978438-100978483. Max. coverage (+): 2.72. Max coverage (-): 0

Region: chr10 100978484-100978530. Max. coverage (+): 0. Max coverage (-): 0

Region: chr10 100978531-100978576. Max. coverage (+): 0. Max coverage (-): 0

Region: chr10 100978577-100978623. Max. coverage (+): 0. Max coverage (-): 0

Region: chr10 100978624-100978670. Max. coverage (+): 0. Max coverage (-): 0

Region: chr10 100978671-100978716. Max. coverage (+): 0. Max coverage (-): 0

Region: chr10 100978717-100978763. Max. coverage (+): 0. Max coverage (-): 0

Region: chr10 100978764-100978810. Max. coverage (+): 1.06. Max coverage (-): 0

Region: chr10 100978811-100978856. Max. coverage (+): 1.06. Max coverage (-): 0

Region: chr10 100978857-100978903. Max. coverage (+): 26.97. Max coverage (-): 0

Region: chr10 100978904-100978950. Max. coverage (+): 7.29. Max coverage (-): 0

Region: chr10 100978951-100978996. Max. coverage (+): 0. Max coverage (-): 0

Region: chr10 100978997-100979043. Max. coverage (+): 0. Max coverage (-): 0

Region: chr10 100979044-100979090. Max. coverage (+): 0. Max coverage (-): 0

Region: chr10 100979091-100979136. Max. coverage (+): 0. Max coverage (-): 0

Region: chr10 100979137-100979183. Max. coverage (+): 13.09. Max coverage (-): 0

Region: chr10 100979184-100979230. Max. coverage (+): 24.55. Max coverage (-): 0

Region: chr10 100979231-100979276. Max. coverage (+): 6.88. Max coverage (-): 0

Region: chr10 100979277-100979323. Max. coverage (+): 0. Max coverage (-): 0

Region: chr10 100979324-100979370. Max. coverage (+): 10. Max coverage (-): 0

Region: chr10 100979371-100979416. Max. coverage (+): 0. Max coverage (-): 0

Region: chr10 100979417-100979463. Max. coverage (+): 0. Max coverage (-): 0

Region: chr10 100979464-100979510. Max. coverage (+): 0. Max coverage (-): 0

Region: chr10 100979511-100979556. Max. coverage (+): 0. Max coverage (-): 0

Region: chr10 100979557-100979603. Max. coverage (+): 4.82. Max coverage (-): 0

Region: chr10 100979604-100979650. Max. coverage (+): 4.82. Max coverage (-): 0

Region: chr10 100979651-100979696. Max. coverage (+): 1.42. Max coverage (-): 0

Region: chr10 100979697-100979743. Max. coverage (+): 0. Max coverage (-): 0

Region: chr10 100979744-100979789. Max. coverage (+): 14.31. Max coverage (-): 0

Region: chr10 100979790-100979836. Max. coverage (+): 7.16. Max coverage (-): 0

Region: chr10 100979837-100979883. Max. coverage (+): 13.46. Max coverage (-): 0

Region: chr10 100979884-100979929. Max. coverage (+): 0. Max coverage (-): 0

Region: chr10 100979930-100979976. Max. coverage (+): 5.09. Max coverage (-): 0

Region: chr10 100979977-100980023. Max. coverage (+): 1.52. Max coverage (-): 0

Region: chr10 100980024-100980069. Max. coverage (+): 12.04. Max coverage (-): 0

Region: chr10 100980070-100980116. Max. coverage (+): 0. Max coverage (-): 0

Region: chr10 100980117-100980163. Max. coverage (+): 6.01. Max coverage (-): 0

Region: chr10 100980164-100980209. Max. coverage (+): 2.19. Max coverage (-): 0

Region: chr10 100980210-100980256. Max. coverage (+): 3.44. Max coverage (-): 0

Region: chr10 100980257-100980303. Max. coverage (+): 0. Max coverage (-): 0

Region: chr10 100980304-100980349. Max. coverage (+): 5.22. Max coverage (-): 0

Region: chr10 100980350-100980396. Max. coverage (+): 13.69. Max coverage (-): 0

Region: chr10 100980397-100980443. Max. coverage (+): 1.98. Max coverage (-): 0

Region: chr10 100980444-100980489. Max. coverage (+): 13.34. Max coverage (-): 0

Region: chr10 100980490-100980536. Max. coverage (+): 13.38. Max coverage (-): 0

Region: chr10 100980537-100980583. Max. coverage (+): 2.04. Max coverage (-): 0

Region: chr10 100980584-100980629. Max. coverage (+): 11.87. Max coverage (-): 0

Region: chr10 100980630-100980676. Max. coverage (+): 3.32. Max coverage (-): 0

Region: chr10 100980677-100980723. Max. coverage (+): 13.79. Max coverage (-): 0

Region: chr10 100980724-100980769. Max. coverage (+): 17.15. Max coverage (-): 0

Region: chr10 100980770-100980816. Max. coverage (+): 10.31. Max coverage (-): 0

Region: chr10 100980817-100980863. Max. coverage (+): 8.17. Max coverage (-): 0

Region: chr10 100980864-100980909. Max. coverage (+): 0. Max coverage (-): 0

Region: chr10 100980910-100980956. Max. coverage (+): 0. Max coverage (-): 0

Region: chr10 100980957-100981002. Max. coverage (+): 10.58. Max coverage (-): 0

Region: chr10 100981003-100981049. Max. coverage (+): 1.81. Max coverage (-): 0

Region: chr10 100981050-100981096. Max. coverage (+): 0. Max coverage (-): 0

Region: chr10 100981097-100981142. Max. coverage (+): 0. Max coverage (-): 0

Region: chr10 100981143-100981189. Max. coverage (+): 6.87. Max coverage (-): 0

Region: chr10 100981190-100981236. Max. coverage (+): 6.73. Max coverage (-): 0

Region: chr10 100981237-100981282. Max. coverage (+): 7.48. Max coverage (-): 0

Region: chr10 100981283-100981329. Max. coverage (+): 6.51. Max coverage (-): 0

Region: chr10 100981330-100981376. Max. coverage (+): 6.52. Max coverage (-): 0

Region: chr10 100981377-100981422. Max. coverage (+): 0. Max coverage (-): 0

Region: chr10 100981423-100981469. Max. coverage (+): 0. Max coverage (-): 0

Region: chr10 100981470-100981516. Max. coverage (+): 0. Max coverage (-): 0

Region: chr10 100981517-100981562. Max. coverage (+): 0. Max coverage (-): 0

Region: chr10 100981563-100981609. Max. coverage (+): 0. Max coverage (-): 0

Region: chr10 100981610-100981656. Max. coverage (+): 9.61. Max coverage (-): 0

Region: chr10 100981657-100981702. Max. coverage (+): 0. Max coverage (-): 0

Region: chr10 100981703-100981749. Max. coverage (+): 0. Max coverage (-): 0

Region: chr10 100981750-100981796. Max. coverage (+): 2.29. Max coverage (-): 0

Region: chr10 100981797-100981842. Max. coverage (+): 2.29. Max coverage (-): 0

Region: chr10 100981843-100981889. Max. coverage (+): 0. Max coverage (-): 0

Region: chr10 100981890-100981936. Max. coverage (+): 0.87. Max coverage (-): 0

Region: chr10 100981937-100981982. Max. coverage (+): 0.87. Max coverage (-): 0

Region: chr10 100981983-100982029. Max. coverage (+): 0. Max coverage (-): 0

Region: chr10 100982030-100982076. Max. coverage (+): 0. Max coverage (-): 0

Region: chr10 100982077-100982122. Max. coverage (+): 0. Max coverage (-): 0

Region: chr10 100982123-100982169. Max. coverage (+): 0. Max coverage (-): 0

Region: chr10 100982170-100982215. Max. coverage (+): 0. Max coverage (-): 0

Region: chr10 100982216-100982262. Max. coverage (+): 0. Max coverage (-): 0

Region: chr10 100982263-100982309. Max. coverage (+): 0. Max coverage (-): 0

Region: chr10 100982310-100982355. Max. coverage (+): 0. Max coverage (-): 0

Region: chr10 100982356-100982402. Max. coverage (+): 0. Max coverage (-): 0

Region: chr10 100982403-100982449. Max. coverage (+): 0. Max coverage (-): 0

Region: chr10 100982450-100982495. Max. coverage (+): 0. Max coverage (-): 0

Region: chr10 100982496-100982542. Max. coverage (+): 0.36. Max coverage (-): 0

Region: chr10 100982543-100982589. Max. coverage (+): 0. Max coverage (-): 0

Region: chr10 100982590-100982635. Max. coverage (+): 0. Max coverage (-): 0

Region: chr10 100982636-100982682. Max. coverage (+): 0. Max coverage (-): 0

Region: chr10 100982683-100982729. Max. coverage (+): 0. Max coverage (-): 0

Region: chr10 100982730-100982775. Max. coverage (+): 0. Max coverage (-): 0

Region: chr10 100982776-100982822. Max. coverage (+): 0. Max coverage (-): 0

Region: chr10 100982823-100982869. Max. coverage (+): 0. Max coverage (-): 0

Region: chr10 100982870-100982915. Max. coverage (+): 0.8. Max coverage (-): 0

Region: chr10 100982916-100982962. Max. coverage (+): 14.73. Max coverage (-): 0

Region: chr10 100982963-100983009. Max. coverage (+): 5.18. Max coverage (-): 0

Region: chr10 100983010-100983055. Max. coverage (+): 3.65. Max coverage (-): 0

Region: chr10 100983056-100983102. Max. coverage (+): 0.6. Max coverage (-): 0

Region: chr10 100983103-100983149. Max. coverage (+): 3.82. Max coverage (-): 0

Region: chr10 100983150-100983195. Max. coverage (+): 0. Max coverage (-): 0

Region: chr10 100983196-100983242. Max. coverage (+): 0. Max coverage (-): 0

Region: chr10 100983243-100983289. Max. coverage (+): 0. Max coverage (-): 0

Region: chr10 100983290-100983335. Max. coverage (+): 0. Max coverage (-): 0

Region: chr10 100983336-100983382. Max. coverage (+): 20.55. Max coverage (-): 0

Region: chr10 100983383-100983428. Max. coverage (+): 17.45. Max coverage (-): 0

Region: chr10 100983429-100983475. Max. coverage (+): 0. Max coverage (-): 0

Region: chr10 100983476-100983522. Max. coverage (+): 7.89. Max coverage (-): 0

Region: chr10 100983523-100983568. Max. coverage (+): 0. Max coverage (-): 0

Region: chr10 100983569-100983615. Max. coverage (+): 11.91. Max coverage (-): 0

Region: chr10 100983616-100983662. Max. coverage (+): 0. Max coverage (-): 0

Region: chr10 100983663-100983708. Max. coverage (+): 1.52. Max coverage (-): 0

Region: chr10 100983709-100983755. Max. coverage (+): 1.52. Max coverage (-): 0

Region: chr10 100983756-100983802. Max. coverage (+): 0. Max coverage (-): 0

Region: chr10 100983803-100983848. Max. coverage (+): 0. Max coverage (-): 0

Region: chr10 100983849-100983895. Max. coverage (+): 0. Max coverage (-): 0

Region: chr10 100983896-100983942. Max. coverage (+): 0. Max coverage (-): 0

Region: chr10 100983943-100983988. Max. coverage (+): 0. Max coverage (-): 0

Region: chr10 100983989-100984035. Max. coverage (+): 0. Max coverage (-): 0

Region: chr10 100984036-100984082. Max. coverage (+): 0. Max coverage (-): 0

Region: chr10 100984083-100984128. Max. coverage (+): 0. Max coverage (-): 0

Region: chr10 100984129-100984175. Max. coverage (+): 0. Max coverage (-): 0

Region: chr10 100984176-100984222. Max. coverage (+): 30.13. Max coverage (-): 0

Region: chr10 100984223-100984268. Max. coverage (+): 8.65. Max coverage (-): 0

Region: chr10 100984269-100984315. Max. coverage (+): 13.66. Max coverage (-): 0

Region: chr10 100984316-100984362. Max. coverage (+): 1.63. Max coverage (-): 0

Region: chr10 100984363-100984408. Max. coverage (+): 1.63. Max coverage (-): 0

Region: chr10 100984409-100984455. Max. coverage (+): 3.54. Max coverage (-): 0

Region: chr10 100984456-100984502. Max. coverage (+): 7.58. Max coverage (-): 0

Region: chr10 100984503-100984548. Max. coverage (+): 0. Max coverage (-): 0

Region: chr10 100984549-100984595. Max. coverage (+): 0. Max coverage (-): 0

Region: chr10 100984596-100984641. Max. coverage (+): 0. Max coverage (-): 0

Region: chr10 100984642-100984688. Max. coverage (+): 0. Max coverage (-): 0

Region: chr10 100984689-100984735. Max. coverage (+): 0. Max coverage (-): 0

Region: chr10 100984736-100984781. Max. coverage (+): 0. Max coverage (-): 0

Region: chr10 100984782-100984828. Max. coverage (+): 26.8. Max coverage (-): 0

Region: chr10 100984829-100984875. Max. coverage (+): 11.31. Max coverage (-): 0

Region: chr10 100984876-100984921. Max. coverage (+): 10.91. Max coverage (-): 0

Region: chr10 100984922-100984968. Max. coverage (+): 0. Max coverage (-): 0

Region: chr10 100984969-100985015. Max. coverage (+): 0. Max coverage (-): 0

Region: chr10 100985016-100985061. Max. coverage (+): 14.08. Max coverage (-): 0

Region: chr10 100985062-100985108. Max. coverage (+): 8.93. Max coverage (-): 0

Region: chr10 100985109-100985155. Max. coverage (+): 20.81. Max coverage (-): 0

Region: chr10 100985156-100985201. Max. coverage (+): 6.55. Max coverage (-): 0

Region: chr10 100985202-100985248. Max. coverage (+): 0. Max coverage (-): 0

Region: chr10 100985249-100985295. Max. coverage (+): 0. Max coverage (-): 0

Region: chr10 100985296-100985341. Max. coverage (+): 3.31. Max coverage (-): 0

Region: chr10 100985342-100985388. Max. coverage (+): 32.14. Max coverage (-): 0

Region: chr10 100985389-100985435. Max. coverage (+): 0. Max coverage (-): 0

Region: chr10 100985436-100985481. Max. coverage (+): 0. Max coverage (-): 0

Region: chr10 100985482-100985528. Max. coverage (+): 0.47. Max coverage (-): 0

Region: chr10 100985529-100985575. Max. coverage (+): 3.03. Max coverage (-): 0

Region: chr10 100985576-100985621. Max. coverage (+): 4.96. Max coverage (-): 0

Region: chr10 100985622-100985668. Max. coverage (+): 1.46. Max coverage (-): 0

Region: chr10 100985669-100985715. Max. coverage (+): 0. Max coverage (-): 0

Region: chr10 100985716-100985761. Max. coverage (+): 0. Max coverage (-): 0

Region: chr10 100985762-100985808. Max. coverage (+): 26.22. Max coverage (-): 0

Region: chr10 100985809-100985854. Max. coverage (+): 1.73. Max coverage (-): 0

Region: chr10 100985855-100985901. Max. coverage (+): 0. Max coverage (-): 0

Region: chr10 100985902-100985948. Max. coverage (+): 0. Max coverage (-): 0

Region: chr10 100985949-100985994. Max. coverage (+): 1.02. Max coverage (-): 0

Region: chr10 100985995-100986041. Max. coverage (+): 4.93. Max coverage (-): 0

Region: chr10 100986042-100986088. Max. coverage (+): 0. Max coverage (-): 0

Region: chr10 100986089-100986134. Max. coverage (+): 0. Max coverage (-): 0

Region: chr10 100986135-100986181. Max. coverage (+): 4.09. Max coverage (-): 0

Region: chr10 100986182-100986228. Max. coverage (+): 5.44. Max coverage (-): 0

Region: chr10 100986229-100986274. Max. coverage (+): 0. Max coverage (-): 0

Region: chr10 100986275-100986321. Max. coverage (+): 0. Max coverage (-): 0

Region: chr10 100986322-100986368. Max. coverage (+): 0. Max coverage (-): 0

Region: chr10 100986369-100986414. Max. coverage (+): 3.07. Max coverage (-): 0

Region: chr10 100986415-100986461. Max. coverage (+): 0. Max coverage (-): 0

Region: chr10 100986462-100986508. Max. coverage (+): 0. Max coverage (-): 0

Region: chr10 100986509-100986554. Max. coverage (+): 0. Max coverage (-): 0

Region: chr10 100986555-100986601. Max. coverage (+): 0. Max coverage (-): 0

Region: chr10 100986602-100986648. Max. coverage (+): 0. Max coverage (-): 0

Region: chr10 100986649-100986694. Max. coverage (+): 1.29. Max coverage (-): 0

Region: chr10 100986695-100986741. Max. coverage (+): 6.46. Max coverage (-): 0

Region: chr10 100986742-100986788. Max. coverage (+): 14.11. Max coverage (-): 0

Region: chr10 100986789-100986834. Max. coverage (+): 14.08. Max coverage (-): 0

Region: chr10 100986835-100986881. Max. coverage (+): 0. Max coverage (-): 0

Region: chr10 100986882-100986928. Max. coverage (+): 0. Max coverage (-): 0

Region: chr10 100986929-100986974. Max. coverage (+): 0.39. Max coverage (-): 0

Region: chr10 100986975-100987021. Max. coverage (+): 0.76. Max coverage (-): 0

Region: chr10 100987022-100987068. Max. coverage (+): 7.55. Max coverage (-): 0

Region: chr10 100987069-100987114. Max. coverage (+): 0. Max coverage (-): 0

Region: chr10 100987115-100987161. Max. coverage (+): 0. Max coverage (-): 0

Region: chr10 100987162-100987207. Max. coverage (+): 0. Max coverage (-): 0

Region: chr10 100987208-100987254. Max. coverage (+): 0. Max coverage (-): 0

Region: chr10 100987255-100987301. Max. coverage (+): 0.85. Max coverage (-): 0

Region: chr10 100987302-100987347. Max. coverage (+): 4.01. Max coverage (-): 0

Region: chr10 100987348-100987394. Max. coverage (+): 0. Max coverage (-): 0

Region: chr10 100987395-100987441. Max. coverage (+): 0. Max coverage (-): 0

Region: chr10 100987442-100987487. Max. coverage (+): 0. Max coverage (-): 0

Region: chr10 100987488-100987534. Max. coverage (+): 0. Max coverage (-): 0

Region: chr10 100987535-100987581. Max. coverage (+): 1.13. Max coverage (-): 0

Region: chr10 100987582-100987627. Max. coverage (+): 3.63. Max coverage (-): 0

Region: chr10 100987628-100987674. Max. coverage (+): 0. Max coverage (-): 0

Region: chr10 100987675-100987721. Max. coverage (+): 3.2. Max coverage (-): 0

Region: chr10 100987722-100987767. Max. coverage (+): 8.39. Max coverage (-): 0

Region: chr10 100987768-100987814. Max. coverage (+): 4.37. Max coverage (-): 0

Region: chr10 100987815-100987861. Max. coverage (+): 0. Max coverage (-): 0

Region: chr10 100987862-100987907. Max. coverage (+): 0. Max coverage (-): 0

Region: chr10 100987908-100987954. Max. coverage (+): 0. Max coverage (-): 0

Region: chr10 100987955-100988001. Max. coverage (+): 3.52. Max coverage (-): 0

Region: chr10 100988002-100988047. Max. coverage (+): 0. Max coverage (-): 0

Region: chr10 100988048-100988094. Max. coverage (+): 0. Max coverage (-): 0

Region: chr10 100988095-100988141. Max. coverage (+): 0. Max coverage (-): 0

Region: chr10 100988142-100988187. Max. coverage (+): 0. Max coverage (-): 0

Region: chr10 100988188-100988234. Max. coverage (+): 5.13. Max coverage (-): 0

Region: chr10 100988235-100988281. Max. coverage (+): 8.4. Max coverage (-): 0

Region: chr10 100988282-100988327. Max. coverage (+): 5.64. Max coverage (-): 0

Region: chr10 100988328-100988374. Max. coverage (+): 0. Max coverage (-): 0

Region: chr10 100988375-100988420. Max. coverage (+): 0. Max coverage (-): 0

Region: chr10 100988421-100988467. Max. coverage (+): 1.19. Max coverage (-): 0

Region: chr10 100988468-100988514. Max. coverage (+): 0. Max coverage (-): 0

Region: chr10 100988515-100988560. Max. coverage (+): 0. Max coverage (-): 0

Region: chr10 100988561-100988607. Max. coverage (+): 0. Max coverage (-): 0

Region: chr10 100988608-100988654. Max. coverage (+): 0. Max coverage (-): 0

Region: chr10 100988655-100988700. Max. coverage (+): 0. Max coverage (-): 0

Region: chr10 100988701-100988747. Max. coverage (+): 9.55. Max coverage (-): 0

Region: chr10 100988748-100988794. Max. coverage (+): 3.72. Max coverage (-): 0

Region: chr10 100988795-100988840. Max. coverage (+): 0.99. Max coverage (-): 0

Region: chr10 100988841-100988887. Max. coverage (+): 0. Max coverage (-): 0

Region: chr10 100988888-100988934. Max. coverage (+): 0. Max coverage (-): 0

Region: chr10 100988935-100988980. Max. coverage (+): 4.44. Max coverage (-): 0

Region: chr10 100988981-100989027. Max. coverage (+): 0. Max coverage (-): 0

Region: chr10 100989028-100989074. Max. coverage (+): 0. Max coverage (-): 0

Region: chr10 100989075-100989120. Max. coverage (+): 0. Max coverage (-): 0

Region: chr10 100989121-100989167. Max. coverage (+): 0. Max coverage (-): 0

Region: chr10 100989168-100989214. Max. coverage (+): 0.45. Max coverage (-): 0

Region: chr10 100989215-100989260. Max. coverage (+): 2.48. Max coverage (-): 0

Region: chr10 100989261-100989307. Max. coverage (+): 2.48. Max coverage (-): 0

Region: chr10 100989308-100989354. Max. coverage (+): 0. Max coverage (-): 0

Region: chr10 100989355-100989400. Max. coverage (+): 0. Max coverage (-): 0

Region: chr10 100989401-100989447. Max. coverage (+): 0. Max coverage (-): 0

Region: chr10 100989448-100989494. Max. coverage (+): 0. Max coverage (-): 0

Region: chr10 100989495-100989540. Max. coverage (+): 0. Max coverage (-): 0

Region: chr10 100989541-100989587. Max. coverage (+): 0. Max coverage (-): 0

Region: chr10 100989588-100989633. Max. coverage (+): 3.39. Max coverage (-): 0

Region: chr10 100989634-100989680. Max. coverage (+): 0. Max coverage (-): 0

Region: chr10 100989681-100989727. Max. coverage (+): 3.54. Max coverage (-): 0

Region: chr10 100989728-100989773. Max. coverage (+): 0. Max coverage (-): 0

Region: chr10 100989774-100989820. Max. coverage (+): 0. Max coverage (-): 0

Region: chr10 100989821-100989867. Max. coverage (+): 0. Max coverage (-): 0

Region: chr10 100989868-100989913. Max. coverage (+): 0. Max coverage (-): 0

Region: chr10 100989914-100989960. Max. coverage (+): 0. Max coverage (-): 0

Region: chr10 100989961-100990007. Max. coverage (+): 0. Max coverage (-): 0

Region: chr10 100990008-100990053. Max. coverage (+): 0. Max coverage (-): 0

Region: chr10 100990054-100990100. Max. coverage (+): 0. Max coverage (-): 0

Region: chr10 100990101-100990147. Max. coverage (+): 0. Max coverage (-): 0

Region: chr10 100990148-100990193. Max. coverage (+): 0. Max coverage (-): 0

Region: chr10 100990194-100990240. Max. coverage (+): 0. Max coverage (-): 0

Region: chr10 100990241-100990287. Max. coverage (+): 0. Max coverage (-): 0

Region: chr10 100990288-100990333. Max. coverage (+): 0. Max coverage (-): 0

Region: chr10 100990334-100990380. Max. coverage (+): 0. Max coverage (-): 0

Region: chr10 100990381-100990427. Max. coverage (+): 0. Max coverage (-): 0

Region: chr10 100990428-100990473. Max. coverage (+): 0. Max coverage (-): 0

Region: chr10 100990474-100990520. Max. coverage (+): 0. Max coverage (-): 0

Region: chr10 100990521-100990567. Max. coverage (+): 0. Max coverage (-): 0

Region: chr10 100990568-100990613. Max. coverage (+): 0. Max coverage (-): 0

Region: chr10 100990614-100990660. Max. coverage (+): 0. Max coverage (-): 0

Region: chr10 100990661-100990707. Max. coverage (+): 0. Max coverage (-): 0

Region: chr10 100990708-100990753. Max. coverage (+): 0. Max coverage (-): 0

Region: chr10 100990754-100990800. Max. coverage (+): 0. Max coverage (-): 0

Region: chr10 100990801-100990846. Max. coverage (+): 0. Max coverage (-): 0

Region: chr10 100990847-100990893. Max. coverage (+): 0. Max coverage (-): 0

Region: chr10 100990894-100990940. Max. coverage (+): 0.81. Max coverage (-): 0

Region: chr10 100990941-100990986. Max. coverage (+): 0. Max coverage (-): 0

Region: chr10 100990987-100991033. Max. coverage (+): 0. Max coverage (-): 0

Region: chr10 100991034-100991080. Max. coverage (+): 4.29. Max coverage (-): 0

Region: chr10 100991081-100991126. Max. coverage (+): 4.8. Max coverage (-): 0

Region: chr10 100991127-100991173. Max. coverage (+): 0. Max coverage (-): 0

Region: chr10 100991174-100991220. Max. coverage (+): 0. Max coverage (-): 0

Region: chr10 100991221-100991266. Max. coverage (+): 0. Max coverage (-): 0

Region: chr10 100991267-100991313. Max. coverage (+): 0. Max coverage (-): 0

Region: chr10 100991314-100991360. Max. coverage (+): 0. Max coverage (-): 0

Region: chr10 100991361-100991406. Max. coverage (+): 0. Max coverage (-): 0

Region: chr10 100991407-100991453. Max. coverage (+): 0. Max coverage (-): 0

Region: chr10 100991454-100991500. Max. coverage (+): 0. Max coverage (-): 0

Region: chr10 100991501-100991546. Max. coverage (+): 0. Max coverage (-): 0

Region: chr10 100991547-100991593. Max. coverage (+): 0. Max coverage (-): 0

Region: chr10 100991594-100991640. Max. coverage (+): 0. Max coverage (-): 0

Region: chr10 100991641-100991686. Max. coverage (+): 0. Max coverage (-): 0

Region: chr10 100991687-100991733. Max. coverage (+): 0. Max coverage (-): 0

Region: chr10 100991734-100991780. Max. coverage (+): 0. Max coverage (-): 0

Region: chr10 100991781-100991826. Max. coverage (+): 0. Max coverage (-): 0

Region: chr10 100991827-100991873. Max. coverage (+): 0. Max coverage (-): 0

Region: chr10 100991874-100991920. Max. coverage (+): 0. Max coverage (-): 0

Region: chr10 100991921-100991966. Max. coverage (+): 1.31. Max coverage (-): 0

Region: chr10 100991967-. Max. coverage (+): 1.31. Max coverage (-): 0

RepeatMasker Color Code

**+**

100-98% Identity

<98-95% Identity

<95-90% Identity

<90-85% Identity

<85-80% Identity

<80-75% Identity

<75-70% Identity

<70% Identity

**-**

Gene Set Color Code

**+**

Gene

Pseudogene

**-**

Topology/Coverage Color Code

Coverage Plus Strand

Coverage Minus Strand

Mainstrand: Plus

Mainstrand: Minus

Complementary Strand

Flanking Region  
(if option -flank >0)

Gene Set Annotation  

**1. KCNK10 (protein coding, ENSBTAG00000019355) Tr:00000025779 Ex:4**: 100988552-100988738 (-)  
**2. KCNK10 (protein coding, ENSBTAG00000019355) Tr:00000025779 Ex:5**: 100984235-100984377 (-)  
**3. KCNK10 (protein coding, ENSBTAG00000019355) Tr:00000025779 Ex:6**: 100981353-100981970 (-)

  
RepeatMasker Annotation  

**1. L2c**: 100970875-100970973 (+), Divergence to consensus: 32.6%  
**2. Bov-tA3**: 100971033-100971227 (-), Divergence to consensus: 10.2%  
**3. AT\_rich**: 100971767-100971790 (+), Divergence to consensus: 50%  
**4. AT\_rich**: 100971769-100971792 (+), Divergence to consensus: 50%  
**5. L2c**: 100972188-100972319 (-), Divergence to consensus: 42.5%  
**6. Bov-tA1**: 100972720-100972933 (-), Divergence to consensus: 21.5%  
**7. Charlie1b**: 100972969-100973096 (-), Divergence to consensus: 35.2%  
**8. MER117**: 100973102-100973237 (+), Divergence to consensus: 39.2%  
**9. MIRb**: 100974467-100974599 (+), Divergence to consensus: 46%  
**10. Bov-tA2**: 100974609-100974797 (+), Divergence to consensus: 19.1%  
**11. ART2A**: 100974751-100974804 (+), Divergence to consensus: 27.8%  
**12. AT\_rich**: 100976323-100976343 (+), Divergence to consensus: 42.9%  
**13. MIR3**: 100977044-100977182 (-), Divergence to consensus: 44.8%  
**14. Bov-tA1**: 100977709-100977916 (-), Divergence to consensus: 22.2%  
**15. MIRb**: 100978562-100978787 (+), Divergence to consensus: 39.5%  
**16. Bov-tA2**: 100978970-100979154 (-), Divergence to consensus: 13%  
**17. GC\_rich**: 100981406-100981443 (+), Divergence to consensus: 76.3%  
**18. C-rich**: 100982027-100982191 (+), Divergence to consensus: 33%  
**19. T-rich**: 100983263-100983348 (+), Divergence to consensus: 30.2%  
**20. (CTG)n**: 100983910-100983934 (+), Divergence to consensus: 0%  
**21. BOV-A2**: 100983935-100984195 (-), Divergence to consensus: 5.5%  
**22. Bov-tA2**: 100984623-100984807 (-), Divergence to consensus: 26.5%  
**23. MIR3**: 100985225-100985306 (-), Divergence to consensus: 31%  
**24. L1-2\_BT**: 100986537-100986659 (+), Divergence to consensus: 38.2%  
**25. Charlie24**: 100988830-100988912 (-), Divergence to consensus: 22.2%  
**26. Bov-tA2**: 100990649-100990852 (-), Divergence to consensus: 15.8%  
**27. AT\_rich**: 100991201-100991221 (+), Divergence to consensus: 52.4%  
**28. Tigger19a**: 100991584-100991690 (+), Divergence to consensus: 40.2%

  
Transcription Factor Binding Sites  

**RFX4\_2** (Sequence: GTAACCAAG (-): 100985441)  
**RFX4\_1** (Sequence: GTTGCCATG (-): 100972539)  
**RFX4\_1** (Sequence: GTTGCCAGG (-): 100979705)  
**Gata4** (Sequence: AGATAAC (-): 100971605)  
**SOX9** (Sequence: AACAATGG (-): 100977392)  
**Gata4** (Sequence: CTTATCT (+): 100983326)
