# Supplementary material for: Small RNA sequencing of cryopreserved semen from single bull revealed altered miRNAs and piRNAs expression between High- and Low-motile sperm populations
Source: BMC Genomics. 2017 Jan 4;18:14. doi: 10.1186/s12864-016-3394-7 (PMC5209821; doi:10.1186/s12864-016-3394-7)
Supplement: Additional file 3: — Details for each piRNA clusters found in High Motile (HM) sperm fraction. Genes, repeats, transposable elements and transcription factors binding sites falling within the cluster regions were reported. (ZIP 1896 kb) [file 12864_2016_3394_MOESM3_ESM.zip › 90.html]

piRNA cluster 90


Predicted piRNA cluster no. 90     previous   next
  

Show proTRAC run info
Hide proTRAC run info

================================= proTRAC ====================================  
VERSION: 2.1                                    LAST MODIFIED: 06. October 2015  
  
Please cite:  
Rosenkranz D, Zischler H. proTRAC - a software for probabilistic piRNA cluster  
detection, visualization and analysis. 2012. BMC Bioinformatics 13:5.  
  
and (for proTRAC 2.0 and later):  
Rosenkranz D, Rudloff S, Bastuck K, Ketting RF, Zischler H. Tupaia small RNAs  
provide insights into function and evolution of RNAi-based transposon defense  
in mammals. 2015. RNA 21(5):911-922.  
  
Contact:  
David Rosenkranz  
Institute of Anthropology, small RNA group  
Johannes Gutenberg University Mainz  
email: rosenkranz@uni-mainz.de  
  
You can find the latest proTRAC version at:  
http://sourceforge.net/projects/protrac/files  
http://www.smallRNAgroup-mainz.de/software  
==============================================================================  
  
PARAMETERS:  
Map file: .............../storage/core/barbara/genhome/smallRNA/fertility/Sample\_motile/pirna/Sample\_motile\_26-33\_collapsed.fa.no-dust.map.weighted-10000-1000-b-0  
Genome file: ............/storage/core/barbara/genhome/smallRNA/fertility/Sample\_all/pirna/bt\_311\_chrY.fa  
RepeatMasker annotation: /storage/genomes/bt\_umd31/GCF\_000003055.6\_Bos\_taurus\_UMD\_3.1.1\_repeatMasker\_chr.out  
GeneSet:................./storage/core/barbara/genhome/smallRNA/fertility/Sample\_all/pirna/full.gtf  
  
Significant (p<=0.01) hit density will be calculated based  
on observed hit distribution.  
  
Sliding window size: ........................................ 5000 bp  
Sliding window increament: .................................. 1000 bp  
Normalize each hit by number of genomic hits: ............... 1 [0=no/1=yes]  
Normalize each hit by number of sequence reads: ............. 1 [0=no/1=yes]  
Normalize values (-> per million mapped reads): ............. 1 [0=no/1=yes]  
Min. fraction of hits with 1T(U) or 10A: .................... 0.75  
Alternatively: Min. fraction of hits with 1T(U) and 10A: .... 0.5  
Min. fraction of hits with typical piRNA length: ............ 0.75  
Typical piRNA length: ....................................... 26-33 nt  
Min. size of a piRNA cluster: ............................... 5000 bp.  
Min. number of hits (absolute): ............................. 0  
Min. number of hits (normalized): ........................... 0  
Min. fraction of hits on the mainstrand: .................... 0.75  
Top fraction of mapped sequences (in terms of read counts): . 1%  
Top fraction accounts for max. n% of sequence reads: ........ 90%  
Min. fraction of hits on each arm of a bidirectional cluster: 0.1  
Output image file for each cluster: ......................... 0 [0=no/1=yes]  
Output html file for each cluster: .......................... 1 [0=no/1=yes]  
Output a summary table: ..................................... 1 [0=no/1=yes]  
Output a FASTA file for each cluster (piRNA sequences): ..... 1 [0=no/1=yes]  
Output a FASTA file comprising cluster sequences: ........... 1 [0=no/1=yes]  
Search DNA motifs in clusters: .............................. 1 [0=no/1=yes]  
Output flanking sequences: +/- .............................. 0 bp  
Output ~.pTi file: .......................................... 1 [0=no/1=yes]  
==============================================================================  
  
  
Genome size (without gaps): ............ 2678902517 bp  
Gaps (N/X/-): .......................... 53837044 bp  
Mapped reads: .......................... 658825247023  
Non-identical sequences: ............... 514171  
Genomic hits: .......................... 764233  
Significant densitiy of mapped reads: .. 12867599.5173724 reads/kb

Show proTRAC cluster info
Hide proTRAC cluster info

|  |  |
| --- | --- |
| Location | chr7 |
| Coordinates | 15469507-15482110 |
| Size [bp] | 12604 |
| Sequence hit loci | 701 |
| Mapped reads (normalized) | 823250794.2 |
| Mapped reads (normalized) per kb | 65316629.2 |
| Normalized reads with 1T (1U) | 87.5% |
| Normalized reads with 10A | 25.6% |
| Normalized reads with length 26-33 nt | 100% |
| Normalized reads on the main strand(s) | 99.6% |
| Predicted directionality | mono:minus |

100%

0%

1T (1U)  
reads

10A reads

26-33 nt  
reads

reads on mainstrand

**Either the amount of reads with 1T (1U) OR 10A has to exceed 75% (set with option: -1Tor10A)  
Alternatively the amount of reads with 1T (1U) AND 10A has to exceed 50% (set with option: -1Tand10A)  
Minimum amount of reads with preferred size is 75% (set with option: -pisize)  
Minimum amount of reads on the main strand(s) is 75% (set with option: -clstrand)**

Show read coverage
Hide read coverage

WHAT DO I SEE HERE?  
This chart shows the location of mapped sequence reads within a predicted piRNA cluster. The color refers to the number of genomic hits produced by the sequence read in question. A dark red bar indicates that this sequence read produces many other hits elsewhere in the genome. Many adjacent red or yellow bars can indicate the presence of a multi-copy element such as transposons or rRNA genes. A dark green bar indicates that this sequence read maps uniquely to this locus.

1 hit

2-5 hits

6-10 hits

11-20 hits

21-50 hits

51-100 hits

> 100 hits

chr7

15469507

15482110

Gene Set

RepeatMasker

Mapped  
Reads

50.68

plus strand

minus strand

50.68

Region: chr7 73884628-15469519. Max. coverage (+): 0. Max coverage (-): 3.86

Region: chr7 15469520-15469544. Max. coverage (+): 0. Max coverage (-): 0

Region: chr7 15469545-15469570. Max. coverage (+): 0. Max coverage (-): 0

Region: chr7 15469571-15469595. Max. coverage (+): 0. Max coverage (-): 0

Region: chr7 15469596-15469620. Max. coverage (+): 0. Max coverage (-): 0

Region: chr7 15469621-15469645. Max. coverage (+): 0. Max coverage (-): 0

Region: chr7 15469646-15469670. Max. coverage (+): 0. Max coverage (-): 0

Region: chr7 15469671-15469696. Max. coverage (+): 0. Max coverage (-): 0

Region: chr7 15469697-15469721. Max. coverage (+): 0. Max coverage (-): 0

Region: chr7 15469722-15469746. Max. coverage (+): 0. Max coverage (-): 0

Region: chr7 15469747-15469771. Max. coverage (+): 0. Max coverage (-): 0

Region: chr7 15469772-15469796. Max. coverage (+): 0. Max coverage (-): 0

Region: chr7 15469797-15469822. Max. coverage (+): 0. Max coverage (-): 0

Region: chr7 15469823-15469847. Max. coverage (+): 0. Max coverage (-): 0

Region: chr7 15469848-15469872. Max. coverage (+): 0. Max coverage (-): 0

Region: chr7 15469873-15469897. Max. coverage (+): 0. Max coverage (-): 0

Region: chr7 15469898-15469922. Max. coverage (+): 0. Max coverage (-): 0

Region: chr7 15469923-15469948. Max. coverage (+): 0. Max coverage (-): 0

Region: chr7 15469949-15469973. Max. coverage (+): 0. Max coverage (-): 0

Region: chr7 15469974-15469998. Max. coverage (+): 0. Max coverage (-): 0

Region: chr7 15469999-15470023. Max. coverage (+): 0. Max coverage (-): 0

Region: chr7 15470024-15470048. Max. coverage (+): 0. Max coverage (-): 0

Region: chr7 15470049-15470074. Max. coverage (+): 0. Max coverage (-): 0

Region: chr7 15470075-15470099. Max. coverage (+): 0. Max coverage (-): 0

Region: chr7 15470100-15470124. Max. coverage (+): 0. Max coverage (-): 0

Region: chr7 15470125-15470149. Max. coverage (+): 0. Max coverage (-): 0

Region: chr7 15470150-15470175. Max. coverage (+): 0. Max coverage (-): 0

Region: chr7 15470176-15470200. Max. coverage (+): 0. Max coverage (-): 0

Region: chr7 15470201-15470225. Max. coverage (+): 0. Max coverage (-): 0

Region: chr7 15470226-15470250. Max. coverage (+): 0. Max coverage (-): 0

Region: chr7 15470251-15470275. Max. coverage (+): 0. Max coverage (-): 0

Region: chr7 15470276-15470301. Max. coverage (+): 0. Max coverage (-): 0

Region: chr7 15470302-15470326. Max. coverage (+): 0. Max coverage (-): 0

Region: chr7 15470327-15470351. Max. coverage (+): 0. Max coverage (-): 0

Region: chr7 15470352-15470376. Max. coverage (+): 0. Max coverage (-): 0

Region: chr7 15470377-15470401. Max. coverage (+): 0. Max coverage (-): 0

Region: chr7 15470402-15470427. Max. coverage (+): 0. Max coverage (-): 0

Region: chr7 15470428-15470452. Max. coverage (+): 0. Max coverage (-): 0

Region: chr7 15470453-15470477. Max. coverage (+): 0. Max coverage (-): 0

Region: chr7 15470478-15470502. Max. coverage (+): 0. Max coverage (-): 0

Region: chr7 15470503-15470527. Max. coverage (+): 0. Max coverage (-): 0

Region: chr7 15470528-15470553. Max. coverage (+): 0. Max coverage (-): 0

Region: chr7 15470554-15470578. Max. coverage (+): 0. Max coverage (-): 0

Region: chr7 15470579-15470603. Max. coverage (+): 0. Max coverage (-): 0

Region: chr7 15470604-15470628. Max. coverage (+): 0. Max coverage (-): 0

Region: chr7 15470629-15470653. Max. coverage (+): 0. Max coverage (-): 0

Region: chr7 15470654-15470679. Max. coverage (+): 0. Max coverage (-): 0

Region: chr7 15470680-15470704. Max. coverage (+): 0. Max coverage (-): 0

Region: chr7 15470705-15470729. Max. coverage (+): 0. Max coverage (-): 0

Region: chr7 15470730-15470754. Max. coverage (+): 0. Max coverage (-): 0

Region: chr7 15470755-15470780. Max. coverage (+): 0. Max coverage (-): 0

Region: chr7 15470781-15470805. Max. coverage (+): 0. Max coverage (-): 0

Region: chr7 15470806-15470830. Max. coverage (+): 0. Max coverage (-): 0

Region: chr7 15470831-15470855. Max. coverage (+): 0. Max coverage (-): 0

Region: chr7 15470856-15470880. Max. coverage (+): 0. Max coverage (-): 0

Region: chr7 15470881-15470906. Max. coverage (+): 0. Max coverage (-): 0

Region: chr7 15470907-15470931. Max. coverage (+): 0. Max coverage (-): 0

Region: chr7 15470932-15470956. Max. coverage (+): 0. Max coverage (-): 3.57

Region: chr7 15470957-15470981. Max. coverage (+): 0. Max coverage (-): 3.57

Region: chr7 15470982-15471006. Max. coverage (+): 0. Max coverage (-): 0

Region: chr7 15471007-15471032. Max. coverage (+): 0. Max coverage (-): 0

Region: chr7 15471033-15471057. Max. coverage (+): 0. Max coverage (-): 0

Region: chr7 15471058-15471082. Max. coverage (+): 0. Max coverage (-): 0

Region: chr7 15471083-15471107. Max. coverage (+): 0. Max coverage (-): 0

Region: chr7 15471108-15471132. Max. coverage (+): 0. Max coverage (-): 0

Region: chr7 15471133-15471158. Max. coverage (+): 0. Max coverage (-): 0

Region: chr7 15471159-15471183. Max. coverage (+): 0. Max coverage (-): 0

Region: chr7 15471184-15471208. Max. coverage (+): 0. Max coverage (-): 0

Region: chr7 15471209-15471233. Max. coverage (+): 0. Max coverage (-): 0

Region: chr7 15471234-15471258. Max. coverage (+): 0. Max coverage (-): 0

Region: chr7 15471259-15471284. Max. coverage (+): 0. Max coverage (-): 0

Region: chr7 15471285-15471309. Max. coverage (+): 0. Max coverage (-): 0

Region: chr7 15471310-15471334. Max. coverage (+): 0. Max coverage (-): 0

Region: chr7 15471335-15471359. Max. coverage (+): 0. Max coverage (-): 0

Region: chr7 15471360-15471384. Max. coverage (+): 0. Max coverage (-): 0

Region: chr7 15471385-15471410. Max. coverage (+): 0. Max coverage (-): 2.5

Region: chr7 15471411-15471435. Max. coverage (+): 0. Max coverage (-): 0

Region: chr7 15471436-15471460. Max. coverage (+): 0. Max coverage (-): 0

Region: chr7 15471461-15471485. Max. coverage (+): 0. Max coverage (-): 0

Region: chr7 15471486-15471511. Max. coverage (+): 0. Max coverage (-): 0

Region: chr7 15471512-15471536. Max. coverage (+): 0. Max coverage (-): 0

Region: chr7 15471537-15471561. Max. coverage (+): 0. Max coverage (-): 0

Region: chr7 15471562-15471586. Max. coverage (+): 0. Max coverage (-): 0

Region: chr7 15471587-15471611. Max. coverage (+): 0. Max coverage (-): 0

Region: chr7 15471612-15471637. Max. coverage (+): 0. Max coverage (-): 0

Region: chr7 15471638-15471662. Max. coverage (+): 0. Max coverage (-): 0

Region: chr7 15471663-15471687. Max. coverage (+): 0. Max coverage (-): 0

Region: chr7 15471688-15471712. Max. coverage (+): 0. Max coverage (-): 0

Region: chr7 15471713-15471737. Max. coverage (+): 0. Max coverage (-): 0

Region: chr7 15471738-15471763. Max. coverage (+): 0. Max coverage (-): 0

Region: chr7 15471764-15471788. Max. coverage (+): 0. Max coverage (-): 0

Region: chr7 15471789-15471813. Max. coverage (+): 0. Max coverage (-): 0

Region: chr7 15471814-15471838. Max. coverage (+): 0. Max coverage (-): 0

Region: chr7 15471839-15471863. Max. coverage (+): 0. Max coverage (-): 0

Region: chr7 15471864-15471889. Max. coverage (+): 0. Max coverage (-): 0

Region: chr7 15471890-15471914. Max. coverage (+): 0. Max coverage (-): 0

Region: chr7 15471915-15471939. Max. coverage (+): 0. Max coverage (-): 0

Region: chr7 15471940-15471964. Max. coverage (+): 0. Max coverage (-): 0

Region: chr7 15471965-15471989. Max. coverage (+): 0. Max coverage (-): 0

Region: chr7 15471990-15472015. Max. coverage (+): 0. Max coverage (-): 0

Region: chr7 15472016-15472040. Max. coverage (+): 0. Max coverage (-): 0

Region: chr7 15472041-15472065. Max. coverage (+): 0. Max coverage (-): 0

Region: chr7 15472066-15472090. Max. coverage (+): 0. Max coverage (-): 0

Region: chr7 15472091-15472116. Max. coverage (+): 0. Max coverage (-): 0

Region: chr7 15472117-15472141. Max. coverage (+): 0. Max coverage (-): 0

Region: chr7 15472142-15472166. Max. coverage (+): 0. Max coverage (-): 0

Region: chr7 15472167-15472191. Max. coverage (+): 0. Max coverage (-): 0

Region: chr7 15472192-15472216. Max. coverage (+): 0. Max coverage (-): 0

Region: chr7 15472217-15472242. Max. coverage (+): 0. Max coverage (-): 0

Region: chr7 15472243-15472267. Max. coverage (+): 0. Max coverage (-): 0

Region: chr7 15472268-15472292. Max. coverage (+): 0. Max coverage (-): 0

Region: chr7 15472293-15472317. Max. coverage (+): 0. Max coverage (-): 0

Region: chr7 15472318-15472342. Max. coverage (+): 0. Max coverage (-): 0

Region: chr7 15472343-15472368. Max. coverage (+): 0. Max coverage (-): 0

Region: chr7 15472369-15472393. Max. coverage (+): 0. Max coverage (-): 0

Region: chr7 15472394-15472418. Max. coverage (+): 0. Max coverage (-): 0

Region: chr7 15472419-15472443. Max. coverage (+): 0. Max coverage (-): 3.32

Region: chr7 15472444-15472468. Max. coverage (+): 0. Max coverage (-): 9.25

Region: chr7 15472469-15472494. Max. coverage (+): 0. Max coverage (-): 9.25

Region: chr7 15472495-15472519. Max. coverage (+): 0. Max coverage (-): 0

Region: chr7 15472520-15472544. Max. coverage (+): 1.84. Max coverage (-): 3.36

Region: chr7 15472545-15472569. Max. coverage (+): 0. Max coverage (-): 0

Region: chr7 15472570-15472594. Max. coverage (+): 0. Max coverage (-): 0

Region: chr7 15472595-15472620. Max. coverage (+): 0. Max coverage (-): 0

Region: chr7 15472621-15472645. Max. coverage (+): 0. Max coverage (-): 0

Region: chr7 15472646-15472670. Max. coverage (+): 0. Max coverage (-): 0

Region: chr7 15472671-15472695. Max. coverage (+): 0. Max coverage (-): 0

Region: chr7 15472696-15472721. Max. coverage (+): 0. Max coverage (-): 12.12

Region: chr7 15472722-15472746. Max. coverage (+): 0. Max coverage (-): 2.46

Region: chr7 15472747-15472771. Max. coverage (+): 0. Max coverage (-): 0

Region: chr7 15472772-15472796. Max. coverage (+): 0. Max coverage (-): 0

Region: chr7 15472797-15472821. Max. coverage (+): 0. Max coverage (-): 0

Region: chr7 15472822-15472847. Max. coverage (+): 0. Max coverage (-): 0

Region: chr7 15472848-15472872. Max. coverage (+): 0. Max coverage (-): 0

Region: chr7 15472873-15472897. Max. coverage (+): 0. Max coverage (-): 0

Region: chr7 15472898-15472922. Max. coverage (+): 0. Max coverage (-): 0

Region: chr7 15472923-15472947. Max. coverage (+): 0. Max coverage (-): 0

Region: chr7 15472948-15472973. Max. coverage (+): 0. Max coverage (-): 3.64

Region: chr7 15472974-15472998. Max. coverage (+): 0. Max coverage (-): 11.01

Region: chr7 15472999-15473023. Max. coverage (+): 0.36. Max coverage (-): 17.7

Region: chr7 15473024-15473048. Max. coverage (+): 0.36. Max coverage (-): 24.68

Region: chr7 15473049-15473073. Max. coverage (+): 0. Max coverage (-): 4.45

Region: chr7 15473074-15473099. Max. coverage (+): 0. Max coverage (-): 0

Region: chr7 15473100-15473124. Max. coverage (+): 0. Max coverage (-): 0

Region: chr7 15473125-15473149. Max. coverage (+): 0. Max coverage (-): 0

Region: chr7 15473150-15473174. Max. coverage (+): 0. Max coverage (-): 0

Region: chr7 15473175-15473199. Max. coverage (+): 0. Max coverage (-): 0

Region: chr7 15473200-15473225. Max. coverage (+): 0. Max coverage (-): 0

Region: chr7 15473226-15473250. Max. coverage (+): 0. Max coverage (-): 0

Region: chr7 15473251-15473275. Max. coverage (+): 0. Max coverage (-): 0

Region: chr7 15473276-15473300. Max. coverage (+): 0. Max coverage (-): 0

Region: chr7 15473301-15473326. Max. coverage (+): 0. Max coverage (-): 6.23

Region: chr7 15473327-15473351. Max. coverage (+): 0. Max coverage (-): 0

Region: chr7 15473352-15473376. Max. coverage (+): 0. Max coverage (-): 0

Region: chr7 15473377-15473401. Max. coverage (+): 0. Max coverage (-): 0

Region: chr7 15473402-15473426. Max. coverage (+): 0. Max coverage (-): 0

Region: chr7 15473427-15473452. Max. coverage (+): 0. Max coverage (-): 0

Region: chr7 15473453-15473477. Max. coverage (+): 0. Max coverage (-): 0

Region: chr7 15473478-15473502. Max. coverage (+): 0. Max coverage (-): 0

Region: chr7 15473503-15473527. Max. coverage (+): 0. Max coverage (-): 0

Region: chr7 15473528-15473552. Max. coverage (+): 0. Max coverage (-): 0

Region: chr7 15473553-15473578. Max. coverage (+): 0. Max coverage (-): 0

Region: chr7 15473579-15473603. Max. coverage (+): 0. Max coverage (-): 0

Region: chr7 15473604-15473628. Max. coverage (+): 0. Max coverage (-): 10.03

Region: chr7 15473629-15473653. Max. coverage (+): 0. Max coverage (-): 1.39

Region: chr7 15473654-15473678. Max. coverage (+): 0. Max coverage (-): 11.12

Region: chr7 15473679-15473704. Max. coverage (+): 0. Max coverage (-): 28.16

Region: chr7 15473705-15473729. Max. coverage (+): 0. Max coverage (-): 0

Region: chr7 15473730-15473754. Max. coverage (+): 0. Max coverage (-): 0

Region: chr7 15473755-15473779. Max. coverage (+): 0. Max coverage (-): 0

Region: chr7 15473780-15473804. Max. coverage (+): 0. Max coverage (-): 0

Region: chr7 15473805-15473830. Max. coverage (+): 0. Max coverage (-): 0

Region: chr7 15473831-15473855. Max. coverage (+): 0. Max coverage (-): 0

Region: chr7 15473856-15473880. Max. coverage (+): 0. Max coverage (-): 0

Region: chr7 15473881-15473905. Max. coverage (+): 0. Max coverage (-): 0

Region: chr7 15473906-15473931. Max. coverage (+): 0. Max coverage (-): 0

Region: chr7 15473932-15473956. Max. coverage (+): 0. Max coverage (-): 0

Region: chr7 15473957-15473981. Max. coverage (+): 0. Max coverage (-): 0

Region: chr7 15473982-15474006. Max. coverage (+): 0. Max coverage (-): 0

Region: chr7 15474007-15474031. Max. coverage (+): 0. Max coverage (-): 15.08

Region: chr7 15474032-15474057. Max. coverage (+): 0. Max coverage (-): 6.7

Region: chr7 15474058-15474082. Max. coverage (+): 0. Max coverage (-): 11.09

Region: chr7 15474083-15474107. Max. coverage (+): 0. Max coverage (-): 0.12

Region: chr7 15474108-15474132. Max. coverage (+): 0. Max coverage (-): 0

Region: chr7 15474133-15474157. Max. coverage (+): 0. Max coverage (-): 0

Region: chr7 15474158-15474183. Max. coverage (+): 0. Max coverage (-): 0

Region: chr7 15474184-15474208. Max. coverage (+): 0. Max coverage (-): 0

Region: chr7 15474209-15474233. Max. coverage (+): 0. Max coverage (-): 0

Region: chr7 15474234-15474258. Max. coverage (+): 0. Max coverage (-): 0

Region: chr7 15474259-15474283. Max. coverage (+): 0. Max coverage (-): 0

Region: chr7 15474284-15474309. Max. coverage (+): 0. Max coverage (-): 0

Region: chr7 15474310-15474334. Max. coverage (+): 0. Max coverage (-): 0

Region: chr7 15474335-15474359. Max. coverage (+): 0. Max coverage (-): 0

Region: chr7 15474360-15474384. Max. coverage (+): 0. Max coverage (-): 0

Region: chr7 15474385-15474409. Max. coverage (+): 0. Max coverage (-): 0

Region: chr7 15474410-15474435. Max. coverage (+): 0. Max coverage (-): 0

Region: chr7 15474436-15474460. Max. coverage (+): 0. Max coverage (-): 0

Region: chr7 15474461-15474485. Max. coverage (+): 0. Max coverage (-): 0

Region: chr7 15474486-15474510. Max. coverage (+): 0. Max coverage (-): 0

Region: chr7 15474511-15474535. Max. coverage (+): 0. Max coverage (-): 0

Region: chr7 15474536-15474561. Max. coverage (+): 0. Max coverage (-): 0

Region: chr7 15474562-15474586. Max. coverage (+): 0. Max coverage (-): 0

Region: chr7 15474587-15474611. Max. coverage (+): 0. Max coverage (-): 0

Region: chr7 15474612-15474636. Max. coverage (+): 0. Max coverage (-): 0

Region: chr7 15474637-15474662. Max. coverage (+): 0. Max coverage (-): 11.86

Region: chr7 15474663-15474687. Max. coverage (+): 0. Max coverage (-): 13.81

Region: chr7 15474688-15474712. Max. coverage (+): 0. Max coverage (-): 13.81

Region: chr7 15474713-15474737. Max. coverage (+): 0. Max coverage (-): 6.81

Region: chr7 15474738-15474762. Max. coverage (+): 0. Max coverage (-): 13.82

Region: chr7 15474763-15474788. Max. coverage (+): 0. Max coverage (-): 19.75

Region: chr7 15474789-15474813. Max. coverage (+): 0. Max coverage (-): 12.91

Region: chr7 15474814-15474838. Max. coverage (+): 0. Max coverage (-): 9.02

Region: chr7 15474839-15474863. Max. coverage (+): 0. Max coverage (-): 20.9

Region: chr7 15474864-15474888. Max. coverage (+): 0. Max coverage (-): 21.49

Region: chr7 15474889-15474914. Max. coverage (+): 0. Max coverage (-): 31.61

Region: chr7 15474915-15474939. Max. coverage (+): 0. Max coverage (-): 0

Region: chr7 15474940-15474964. Max. coverage (+): 0. Max coverage (-): 0

Region: chr7 15474965-15474989. Max. coverage (+): 0. Max coverage (-): 0

Region: chr7 15474990-15475014. Max. coverage (+): 0. Max coverage (-): 0

Region: chr7 15475015-15475040. Max. coverage (+): 0. Max coverage (-): 0

Region: chr7 15475041-15475065. Max. coverage (+): 0. Max coverage (-): 0

Region: chr7 15475066-15475090. Max. coverage (+): 0. Max coverage (-): 1.96

Region: chr7 15475091-15475115. Max. coverage (+): 0. Max coverage (-): 5.24

Region: chr7 15475116-15475140. Max. coverage (+): 0. Max coverage (-): 2.24

Region: chr7 15475141-15475166. Max. coverage (+): 0. Max coverage (-): 12.92

Region: chr7 15475167-15475191. Max. coverage (+): 0. Max coverage (-): 12.92

Region: chr7 15475192-15475216. Max. coverage (+): 0. Max coverage (-): 0

Region: chr7 15475217-15475241. Max. coverage (+): 0. Max coverage (-): 0

Region: chr7 15475242-15475267. Max. coverage (+): 0. Max coverage (-): 0

Region: chr7 15475268-15475292. Max. coverage (+): 0. Max coverage (-): 0

Region: chr7 15475293-15475317. Max. coverage (+): 0. Max coverage (-): 0

Region: chr7 15475318-15475342. Max. coverage (+): 0. Max coverage (-): 0

Region: chr7 15475343-15475367. Max. coverage (+): 0. Max coverage (-): 0

Region: chr7 15475368-15475393. Max. coverage (+): 0. Max coverage (-): 0

Region: chr7 15475394-15475418. Max. coverage (+): 0. Max coverage (-): 0

Region: chr7 15475419-15475443. Max. coverage (+): 0. Max coverage (-): 0

Region: chr7 15475444-15475468. Max. coverage (+): 0. Max coverage (-): 0

Region: chr7 15475469-15475493. Max. coverage (+): 0. Max coverage (-): 0

Region: chr7 15475494-15475519. Max. coverage (+): 0. Max coverage (-): 0

Region: chr7 15475520-15475544. Max. coverage (+): 0. Max coverage (-): 0

Region: chr7 15475545-15475569. Max. coverage (+): 0. Max coverage (-): 0

Region: chr7 15475570-15475594. Max. coverage (+): 0. Max coverage (-): 0

Region: chr7 15475595-15475619. Max. coverage (+): 0. Max coverage (-): 0

Region: chr7 15475620-15475645. Max. coverage (+): 0. Max coverage (-): 0

Region: chr7 15475646-15475670. Max. coverage (+): 0. Max coverage (-): 27.89

Region: chr7 15475671-15475695. Max. coverage (+): 0. Max coverage (-): 9.37

Region: chr7 15475696-15475720. Max. coverage (+): 0. Max coverage (-): 0

Region: chr7 15475721-15475745. Max. coverage (+): 0. Max coverage (-): 0

Region: chr7 15475746-15475771. Max. coverage (+): 0. Max coverage (-): 0

Region: chr7 15475772-15475796. Max. coverage (+): 0. Max coverage (-): 0

Region: chr7 15475797-15475821. Max. coverage (+): 0. Max coverage (-): 0

Region: chr7 15475822-15475846. Max. coverage (+): 0. Max coverage (-): 0

Region: chr7 15475847-15475872. Max. coverage (+): 0. Max coverage (-): 0

Region: chr7 15475873-15475897. Max. coverage (+): 0. Max coverage (-): 0

Region: chr7 15475898-15475922. Max. coverage (+): 0. Max coverage (-): 0

Region: chr7 15475923-15475947. Max. coverage (+): 0. Max coverage (-): 0

Region: chr7 15475948-15475972. Max. coverage (+): 0. Max coverage (-): 4.58

Region: chr7 15475973-15475998. Max. coverage (+): 0. Max coverage (-): 0

Region: chr7 15475999-15476023. Max. coverage (+): 0. Max coverage (-): 0

Region: chr7 15476024-15476048. Max. coverage (+): 0. Max coverage (-): 0

Region: chr7 15476049-15476073. Max. coverage (+): 0. Max coverage (-): 0

Region: chr7 15476074-15476098. Max. coverage (+): 0. Max coverage (-): 0

Region: chr7 15476099-15476124. Max. coverage (+): 0. Max coverage (-): 10.18

Region: chr7 15476125-15476149. Max. coverage (+): 0. Max coverage (-): 46.31

Region: chr7 15476150-15476174. Max. coverage (+): 0. Max coverage (-): 50.68

Region: chr7 15476175-15476199. Max. coverage (+): 0. Max coverage (-): 11.96

Region: chr7 15476200-15476224. Max. coverage (+): 0. Max coverage (-): 10.08

Region: chr7 15476225-15476250. Max. coverage (+): 0. Max coverage (-): 0

Region: chr7 15476251-15476275. Max. coverage (+): 0. Max coverage (-): 0

Region: chr7 15476276-15476300. Max. coverage (+): 0. Max coverage (-): 0

Region: chr7 15476301-15476325. Max. coverage (+): 0. Max coverage (-): 0

Region: chr7 15476326-15476350. Max. coverage (+): 0. Max coverage (-): 0

Region: chr7 15476351-15476376. Max. coverage (+): 0. Max coverage (-): 0

Region: chr7 15476377-15476401. Max. coverage (+): 0. Max coverage (-): 0

Region: chr7 15476402-15476426. Max. coverage (+): 0. Max coverage (-): 0

Region: chr7 15476427-15476451. Max. coverage (+): 0. Max coverage (-): 0

Region: chr7 15476452-15476477. Max. coverage (+): 0. Max coverage (-): 0

Region: chr7 15476478-15476502. Max. coverage (+): 0. Max coverage (-): 0

Region: chr7 15476503-15476527. Max. coverage (+): 0. Max coverage (-): 0

Region: chr7 15476528-15476552. Max. coverage (+): 0. Max coverage (-): 0

Region: chr7 15476553-15476577. Max. coverage (+): 0. Max coverage (-): 0

Region: chr7 15476578-15476603. Max. coverage (+): 0. Max coverage (-): 0

Region: chr7 15476604-15476628. Max. coverage (+): 0. Max coverage (-): 0

Region: chr7 15476629-15476653. Max. coverage (+): 0. Max coverage (-): 0

Region: chr7 15476654-15476678. Max. coverage (+): 0. Max coverage (-): 0

Region: chr7 15476679-15476703. Max. coverage (+): 0. Max coverage (-): 0

Region: chr7 15476704-15476729. Max. coverage (+): 0. Max coverage (-): 15.93

Region: chr7 15476730-15476754. Max. coverage (+): 0. Max coverage (-): 15.93

Region: chr7 15476755-15476779. Max. coverage (+): 0. Max coverage (-): 8

Region: chr7 15476780-15476804. Max. coverage (+): 0. Max coverage (-): 4.29

Region: chr7 15476805-15476829. Max. coverage (+): 0. Max coverage (-): 4.11

Region: chr7 15476830-15476855. Max. coverage (+): 0. Max coverage (-): 9.47

Region: chr7 15476856-15476880. Max. coverage (+): 0. Max coverage (-): 8.37

Region: chr7 15476881-15476905. Max. coverage (+): 0. Max coverage (-): 16.33

Region: chr7 15476906-15476930. Max. coverage (+): 0. Max coverage (-): 28.89

Region: chr7 15476931-15476955. Max. coverage (+): 0. Max coverage (-): 14.68

Region: chr7 15476956-15476981. Max. coverage (+): 0. Max coverage (-): 17.85

Region: chr7 15476982-15477006. Max. coverage (+): 0. Max coverage (-): 14.4

Region: chr7 15477007-15477031. Max. coverage (+): 0. Max coverage (-): 37.44

Region: chr7 15477032-15477056. Max. coverage (+): 0. Max coverage (-): 6.52

Region: chr7 15477057-15477082. Max. coverage (+): 0. Max coverage (-): 17.72

Region: chr7 15477083-15477107. Max. coverage (+): 2.56. Max coverage (-): 29.29

Region: chr7 15477108-15477132. Max. coverage (+): 0. Max coverage (-): 9.53

Region: chr7 15477133-15477157. Max. coverage (+): 0. Max coverage (-): 15.12

Region: chr7 15477158-15477182. Max. coverage (+): 0. Max coverage (-): 17.72

Region: chr7 15477183-15477208. Max. coverage (+): 0. Max coverage (-): 0

Region: chr7 15477209-15477233. Max. coverage (+): 0. Max coverage (-): 0

Region: chr7 15477234-15477258. Max. coverage (+): 0. Max coverage (-): 0

Region: chr7 15477259-15477283. Max. coverage (+): 0. Max coverage (-): 0

Region: chr7 15477284-15477308. Max. coverage (+): 0. Max coverage (-): 0

Region: chr7 15477309-15477334. Max. coverage (+): 0. Max coverage (-): 0

Region: chr7 15477335-15477359. Max. coverage (+): 0. Max coverage (-): 0

Region: chr7 15477360-15477384. Max. coverage (+): 0. Max coverage (-): 0

Region: chr7 15477385-15477409. Max. coverage (+): 0. Max coverage (-): 0

Region: chr7 15477410-15477434. Max. coverage (+): 0. Max coverage (-): 0

Region: chr7 15477435-15477460. Max. coverage (+): 0. Max coverage (-): 0

Region: chr7 15477461-15477485. Max. coverage (+): 0. Max coverage (-): 0

Region: chr7 15477486-15477510. Max. coverage (+): 0. Max coverage (-): 0

Region: chr7 15477511-15477535. Max. coverage (+): 0. Max coverage (-): 0

Region: chr7 15477536-15477560. Max. coverage (+): 0. Max coverage (-): 0

Region: chr7 15477561-15477586. Max. coverage (+): 0. Max coverage (-): 0

Region: chr7 15477587-15477611. Max. coverage (+): 0. Max coverage (-): 0

Region: chr7 15477612-15477636. Max. coverage (+): 0. Max coverage (-): 0

Region: chr7 15477637-15477661. Max. coverage (+): 0. Max coverage (-): 0

Region: chr7 15477662-15477686. Max. coverage (+): 0. Max coverage (-): 0

Region: chr7 15477687-15477712. Max. coverage (+): 0. Max coverage (-): 0

Region: chr7 15477713-15477737. Max. coverage (+): 0. Max coverage (-): 0

Region: chr7 15477738-15477762. Max. coverage (+): 0. Max coverage (-): 0

Region: chr7 15477763-15477787. Max. coverage (+): 0. Max coverage (-): 0

Region: chr7 15477788-15477813. Max. coverage (+): 0. Max coverage (-): 0

Region: chr7 15477814-15477838. Max. coverage (+): 0. Max coverage (-): 0

Region: chr7 15477839-15477863. Max. coverage (+): 0. Max coverage (-): 0

Region: chr7 15477864-15477888. Max. coverage (+): 0. Max coverage (-): 0

Region: chr7 15477889-15477913. Max. coverage (+): 0. Max coverage (-): 0

Region: chr7 15477914-15477939. Max. coverage (+): 0. Max coverage (-): 0

Region: chr7 15477940-15477964. Max. coverage (+): 0. Max coverage (-): 0

Region: chr7 15477965-15477989. Max. coverage (+): 0. Max coverage (-): 0

Region: chr7 15477990-15478014. Max. coverage (+): 0. Max coverage (-): 0

Region: chr7 15478015-15478039. Max. coverage (+): 0. Max coverage (-): 0

Region: chr7 15478040-15478065. Max. coverage (+): 0. Max coverage (-): 1.69

Region: chr7 15478066-15478090. Max. coverage (+): 0. Max coverage (-): 1.69

Region: chr7 15478091-15478115. Max. coverage (+): 0. Max coverage (-): 1.71

Region: chr7 15478116-15478140. Max. coverage (+): 0. Max coverage (-): 2.92

Region: chr7 15478141-15478165. Max. coverage (+): 0. Max coverage (-): 0

Region: chr7 15478166-15478191. Max. coverage (+): 0. Max coverage (-): 0

Region: chr7 15478192-15478216. Max. coverage (+): 0. Max coverage (-): 0

Region: chr7 15478217-15478241. Max. coverage (+): 0. Max coverage (-): 0

Region: chr7 15478242-15478266. Max. coverage (+): 0. Max coverage (-): 0

Region: chr7 15478267-15478291. Max. coverage (+): 0. Max coverage (-): 4.97

Region: chr7 15478292-15478317. Max. coverage (+): 0. Max coverage (-): 4.97

Region: chr7 15478318-15478342. Max. coverage (+): 0. Max coverage (-): 0

Region: chr7 15478343-15478367. Max. coverage (+): 0. Max coverage (-): 3.01

Region: chr7 15478368-15478392. Max. coverage (+): 0. Max coverage (-): 6.01

Region: chr7 15478393-15478418. Max. coverage (+): 0. Max coverage (-): 0

Region: chr7 15478419-15478443. Max. coverage (+): 0. Max coverage (-): 0

Region: chr7 15478444-15478468. Max. coverage (+): 0. Max coverage (-): 0

Region: chr7 15478469-15478493. Max. coverage (+): 0. Max coverage (-): 0

Region: chr7 15478494-15478518. Max. coverage (+): 0. Max coverage (-): 0

Region: chr7 15478519-15478544. Max. coverage (+): 0. Max coverage (-): 0

Region: chr7 15478545-15478569. Max. coverage (+): 0. Max coverage (-): 0

Region: chr7 15478570-15478594. Max. coverage (+): 0. Max coverage (-): 0

Region: chr7 15478595-15478619. Max. coverage (+): 0. Max coverage (-): 0

Region: chr7 15478620-15478644. Max. coverage (+): 0. Max coverage (-): 2.3

Region: chr7 15478645-15478670. Max. coverage (+): 0. Max coverage (-): 7.65

Region: chr7 15478671-15478695. Max. coverage (+): 0. Max coverage (-): 0

Region: chr7 15478696-15478720. Max. coverage (+): 0. Max coverage (-): 0

Region: chr7 15478721-15478745. Max. coverage (+): 0. Max coverage (-): 0

Region: chr7 15478746-15478770. Max. coverage (+): 0. Max coverage (-): 0

Region: chr7 15478771-15478796. Max. coverage (+): 0. Max coverage (-): 0

Region: chr7 15478797-15478821. Max. coverage (+): 0. Max coverage (-): 0

Region: chr7 15478822-15478846. Max. coverage (+): 0. Max coverage (-): 0

Region: chr7 15478847-15478871. Max. coverage (+): 0. Max coverage (-): 4.54

Region: chr7 15478872-15478896. Max. coverage (+): 0. Max coverage (-): 4.54

Region: chr7 15478897-15478922. Max. coverage (+): 0. Max coverage (-): 0.45

Region: chr7 15478923-15478947. Max. coverage (+): 0. Max coverage (-): 3.45

Region: chr7 15478948-15478972. Max. coverage (+): 0. Max coverage (-): 6.41

Region: chr7 15478973-15478997. Max. coverage (+): 0. Max coverage (-): 0.94

Region: chr7 15478998-15479023. Max. coverage (+): 0. Max coverage (-): 0.76

Region: chr7 15479024-15479048. Max. coverage (+): 0. Max coverage (-): 4.02

Region: chr7 15479049-15479073. Max. coverage (+): 0. Max coverage (-): 4.02

Region: chr7 15479074-15479098. Max. coverage (+): 0. Max coverage (-): 0

Region: chr7 15479099-15479123. Max. coverage (+): 0. Max coverage (-): 0

Region: chr7 15479124-15479149. Max. coverage (+): 0. Max coverage (-): 0

Region: chr7 15479150-15479174. Max. coverage (+): 0. Max coverage (-): 0

Region: chr7 15479175-15479199. Max. coverage (+): 0. Max coverage (-): 0

Region: chr7 15479200-15479224. Max. coverage (+): 0. Max coverage (-): 0

Region: chr7 15479225-15479249. Max. coverage (+): 0. Max coverage (-): 0

Region: chr7 15479250-15479275. Max. coverage (+): 0. Max coverage (-): 5.96

Region: chr7 15479276-15479300. Max. coverage (+): 0. Max coverage (-): 2.26

Region: chr7 15479301-15479325. Max. coverage (+): 0. Max coverage (-): 0

Region: chr7 15479326-15479350. Max. coverage (+): 0. Max coverage (-): 0

Region: chr7 15479351-15479375. Max. coverage (+): 0. Max coverage (-): 0

Region: chr7 15479376-15479401. Max. coverage (+): 0. Max coverage (-): 0

Region: chr7 15479402-15479426. Max. coverage (+): 0. Max coverage (-): 0

Region: chr7 15479427-15479451. Max. coverage (+): 0. Max coverage (-): 9.86

Region: chr7 15479452-15479476. Max. coverage (+): 0. Max coverage (-): 11.52

Region: chr7 15479477-15479501. Max. coverage (+): 0. Max coverage (-): 0.86

Region: chr7 15479502-15479527. Max. coverage (+): 0. Max coverage (-): 16.25

Region: chr7 15479528-15479552. Max. coverage (+): 0. Max coverage (-): 15.19

Region: chr7 15479553-15479577. Max. coverage (+): 0. Max coverage (-): 11.94

Region: chr7 15479578-15479602. Max. coverage (+): 0. Max coverage (-): 2.3

Region: chr7 15479603-15479628. Max. coverage (+): 0. Max coverage (-): 9.17

Region: chr7 15479629-15479653. Max. coverage (+): 0. Max coverage (-): 9.17

Region: chr7 15479654-15479678. Max. coverage (+): 0. Max coverage (-): 4.53

Region: chr7 15479679-15479703. Max. coverage (+): 0. Max coverage (-): 13.48

Region: chr7 15479704-15479728. Max. coverage (+): 0. Max coverage (-): 8.63

Region: chr7 15479729-15479754. Max. coverage (+): 0. Max coverage (-): 1.77

Region: chr7 15479755-15479779. Max. coverage (+): 0. Max coverage (-): 7.02

Region: chr7 15479780-15479804. Max. coverage (+): 0. Max coverage (-): 0

Region: chr7 15479805-15479829. Max. coverage (+): 0. Max coverage (-): 0

Region: chr7 15479830-15479854. Max. coverage (+): 0. Max coverage (-): 0

Region: chr7 15479855-15479880. Max. coverage (+): 0. Max coverage (-): 0

Region: chr7 15479881-15479905. Max. coverage (+): 0. Max coverage (-): 0

Region: chr7 15479906-15479930. Max. coverage (+): 0. Max coverage (-): 0

Region: chr7 15479931-15479955. Max. coverage (+): 0. Max coverage (-): 0

Region: chr7 15479956-15479980. Max. coverage (+): 0. Max coverage (-): 0

Region: chr7 15479981-15480006. Max. coverage (+): 0. Max coverage (-): 0

Region: chr7 15480007-15480031. Max. coverage (+): 0. Max coverage (-): 0

Region: chr7 15480032-15480056. Max. coverage (+): 0. Max coverage (-): 0

Region: chr7 15480057-15480081. Max. coverage (+): 0. Max coverage (-): 4.23

Region: chr7 15480082-15480106. Max. coverage (+): 0. Max coverage (-): 3.42

Region: chr7 15480107-15480132. Max. coverage (+): 0. Max coverage (-): 10.34

Region: chr7 15480133-15480157. Max. coverage (+): 0. Max coverage (-): 0

Region: chr7 15480158-15480182. Max. coverage (+): 0. Max coverage (-): 0

Region: chr7 15480183-15480207. Max. coverage (+): 0. Max coverage (-): 0

Region: chr7 15480208-15480233. Max. coverage (+): 0. Max coverage (-): 0

Region: chr7 15480234-15480258. Max. coverage (+): 0. Max coverage (-): 0

Region: chr7 15480259-15480283. Max. coverage (+): 0. Max coverage (-): 0

Region: chr7 15480284-15480308. Max. coverage (+): 0. Max coverage (-): 0

Region: chr7 15480309-15480333. Max. coverage (+): 0. Max coverage (-): 0

Region: chr7 15480334-15480359. Max. coverage (+): 0. Max coverage (-): 0

Region: chr7 15480360-15480384. Max. coverage (+): 0. Max coverage (-): 0

Region: chr7 15480385-15480409. Max. coverage (+): 0. Max coverage (-): 0

Region: chr7 15480410-15480434. Max. coverage (+): 0. Max coverage (-): 0

Region: chr7 15480435-15480459. Max. coverage (+): 0. Max coverage (-): 0

Region: chr7 15480460-15480485. Max. coverage (+): 0. Max coverage (-): 2.14

Region: chr7 15480486-15480510. Max. coverage (+): 0. Max coverage (-): 2.04

Region: chr7 15480511-15480535. Max. coverage (+): 0. Max coverage (-): 2.04

Region: chr7 15480536-15480560. Max. coverage (+): 0. Max coverage (-): 0

Region: chr7 15480561-15480585. Max. coverage (+): 0. Max coverage (-): 3.34

Region: chr7 15480586-15480611. Max. coverage (+): 0. Max coverage (-): 0.76

Region: chr7 15480612-15480636. Max. coverage (+): 0. Max coverage (-): 2.56

Region: chr7 15480637-15480661. Max. coverage (+): 0. Max coverage (-): 0

Region: chr7 15480662-15480686. Max. coverage (+): 0. Max coverage (-): 0

Region: chr7 15480687-15480711. Max. coverage (+): 0. Max coverage (-): 7.87

Region: chr7 15480712-15480737. Max. coverage (+): 0. Max coverage (-): 24.88

Region: chr7 15480738-15480762. Max. coverage (+): 0. Max coverage (-): 7.96

Region: chr7 15480763-15480787. Max. coverage (+): 0. Max coverage (-): 5.42

Region: chr7 15480788-15480812. Max. coverage (+): 0. Max coverage (-): 0

Region: chr7 15480813-15480837. Max. coverage (+): 0. Max coverage (-): 0

Region: chr7 15480838-15480863. Max. coverage (+): 0. Max coverage (-): 0

Region: chr7 15480864-15480888. Max. coverage (+): 0. Max coverage (-): 0

Region: chr7 15480889-15480913. Max. coverage (+): 0. Max coverage (-): 4.17

Region: chr7 15480914-15480938. Max. coverage (+): 0. Max coverage (-): 2.78

Region: chr7 15480939-15480964. Max. coverage (+): 0. Max coverage (-): 4.95

Region: chr7 15480965-15480989. Max. coverage (+): 0. Max coverage (-): 4.95

Region: chr7 15480990-15481014. Max. coverage (+): 0. Max coverage (-): 2.68

Region: chr7 15481015-15481039. Max. coverage (+): 0. Max coverage (-): 18.16

Region: chr7 15481040-15481064. Max. coverage (+): 0. Max coverage (-): 18.16

Region: chr7 15481065-15481090. Max. coverage (+): 0. Max coverage (-): 0

Region: chr7 15481091-15481115. Max. coverage (+): 0. Max coverage (-): 0

Region: chr7 15481116-15481140. Max. coverage (+): 0. Max coverage (-): 0.7

Region: chr7 15481141-15481165. Max. coverage (+): 0. Max coverage (-): 0.7

Region: chr7 15481166-15481190. Max. coverage (+): 0. Max coverage (-): 2.24

Region: chr7 15481191-15481216. Max. coverage (+): 0. Max coverage (-): 2.24

Region: chr7 15481217-15481241. Max. coverage (+): 0. Max coverage (-): 0

Region: chr7 15481242-15481266. Max. coverage (+): 0. Max coverage (-): 0

Region: chr7 15481267-15481291. Max. coverage (+): 0. Max coverage (-): 0

Region: chr7 15481292-15481316. Max. coverage (+): 0. Max coverage (-): 0

Region: chr7 15481317-15481342. Max. coverage (+): 0. Max coverage (-): 0

Region: chr7 15481343-15481367. Max. coverage (+): 0. Max coverage (-): 0

Region: chr7 15481368-15481392. Max. coverage (+): 0. Max coverage (-): 0

Region: chr7 15481393-15481417. Max. coverage (+): 0. Max coverage (-): 0

Region: chr7 15481418-15481442. Max. coverage (+): 0. Max coverage (-): 0

Region: chr7 15481443-15481468. Max. coverage (+): 0. Max coverage (-): 0

Region: chr7 15481469-15481493. Max. coverage (+): 0. Max coverage (-): 0

Region: chr7 15481494-15481518. Max. coverage (+): 0. Max coverage (-): 0

Region: chr7 15481519-15481543. Max. coverage (+): 0. Max coverage (-): 0

Region: chr7 15481544-15481569. Max. coverage (+): 0. Max coverage (-): 0

Region: chr7 15481570-15481594. Max. coverage (+): 0. Max coverage (-): 0

Region: chr7 15481595-15481619. Max. coverage (+): 0. Max coverage (-): 0

Region: chr7 15481620-15481644. Max. coverage (+): 0. Max coverage (-): 0

Region: chr7 15481645-15481669. Max. coverage (+): 0. Max coverage (-): 0

Region: chr7 15481670-15481695. Max. coverage (+): 0. Max coverage (-): 0

Region: chr7 15481696-15481720. Max. coverage (+): 0. Max coverage (-): 0

Region: chr7 15481721-15481745. Max. coverage (+): 0. Max coverage (-): 0

Region: chr7 15481746-15481770. Max. coverage (+): 0. Max coverage (-): 0

Region: chr7 15481771-15481795. Max. coverage (+): 0. Max coverage (-): 0

Region: chr7 15481796-15481821. Max. coverage (+): 0. Max coverage (-): 0

Region: chr7 15481822-15481846. Max. coverage (+): 0. Max coverage (-): 0

Region: chr7 15481847-15481871. Max. coverage (+): 0. Max coverage (-): 0

Region: chr7 15481872-15481896. Max. coverage (+): 0. Max coverage (-): 0

Region: chr7 15481897-15481921. Max. coverage (+): 0. Max coverage (-): 0

Region: chr7 15481922-15481947. Max. coverage (+): 0. Max coverage (-): 0

Region: chr7 15481948-15481972. Max. coverage (+): 0. Max coverage (-): 0

Region: chr7 15481973-15481997. Max. coverage (+): 0. Max coverage (-): 0

Region: chr7 15481998-15482022. Max. coverage (+): 0. Max coverage (-): 0

Region: chr7 15482023-15482047. Max. coverage (+): 0. Max coverage (-): 0

Region: chr7 15482048-15482073. Max. coverage (+): 0. Max coverage (-): 1.75

Region: chr7 15482074-15482098. Max. coverage (+): 0. Max coverage (-): 3.42

Region: chr7 15482099-. Max. coverage (+): 0. Max coverage (-): 0

RepeatMasker Color Code

**+**

100-98% Identity

<98-95% Identity

<95-90% Identity

<90-85% Identity

<85-80% Identity

<80-75% Identity

<75-70% Identity

<70% Identity

**-**

Gene Set Color Code

**+**

Gene

Pseudogene

**-**

Topology/Coverage Color Code

Coverage Plus Strand

Coverage Minus Strand

Mainstrand: Plus

Mainstrand: Minus

Complementary Strand

Flanking Region  
(if option -flank >0)

Gene Set Annotation  

**1. (protein coding, ENSBTAG00000039523) Tr:00000054432 Ex:5**: 15471211-15472604 (+)

  
RepeatMasker Annotation  

**1. MLT1J1**: 15469572-15469624 (+), Divergence to consensus: 18.9%  
**2. Charlie1a**: 15469912-15470323 (-), Divergence to consensus: 27.5%  
**3. L1M5**: 15470328-15470463 (+), Divergence to consensus: 35.2%  
**4. L1M5**: 15470523-15470672 (+), Divergence to consensus: 32.2%  
**5. L1ME3Cz**: 15470749-15470934 (+), Divergence to consensus: 30.5%  
**6. L1MC4a**: 15470994-15471199 (-), Divergence to consensus: 29.6%  
**7. Bov-tA2**: 15471224-15471343 (+), Divergence to consensus: 11.6%  
**8. BovB**: 15471490-15471890 (+), Divergence to consensus: 6%  
**9. ART2A**: 15471891-15472417 (+), Divergence to consensus: 7.8%  
**10. L2**: 15472608-15472715 (+), Divergence to consensus: 32.4%  
**11. LTR16B**: 15472754-15473038 (-), Divergence to consensus: 44.5%  
**12. CHR-2\_BT**: 15473088-15473304 (+), Divergence to consensus: 21.4%  
**13. CHR-2A**: 15473717-15473989 (+), Divergence to consensus: 34.8%  
**14. L1M5**: 15474131-15474456 (+), Divergence to consensus: 40.7%  
**15. L1-2\_BT**: 15474475-15474564 (+), Divergence to consensus: 30%  
**16. SINE2-1\_BT**: 15474936-15475051 (-), Divergence to consensus: 25.8%  
**17. AT\_rich**: 15475057-15475095 (+), Divergence to consensus: 79.5%  
**18. LTR40a**: 15475218-15475648 (+), Divergence to consensus: 42.3%  
**19. Bov-tA2**: 15475737-15475924 (+), Divergence to consensus: 12.9%  
**20. L1M5**: 15475999-15476106 (+), Divergence to consensus: 41.7%  
**21. L2a**: 15476251-15476335 (-), Divergence to consensus: 25.3%  
**22. L1M5**: 15476336-15476715 (+), Divergence to consensus: 38.2%  
**23. L1MB7**: 15477212-15477293 (-), Divergence to consensus: 27.1%  
**24. Bov-tA3**: 15477295-15477458 (+), Divergence to consensus: 9.8%  
**25. L1MB7**: 15477482-15477720 (-), Divergence to consensus: 34.8%  
**26. SINE2-2\_BT**: 15477721-15477843 (+), Divergence to consensus: 30.4%  
**27. L1MB7**: 15477844-15478015 (-), Divergence to consensus: 34.8%  
**28. AT\_rich**: 15478138-15478162 (+), Divergence to consensus: 44%  
**29. AT\_rich**: 15478140-15478165 (+), Divergence to consensus: 69.2%  
**30. SINE2-1\_BT**: 15478167-15478279 (-), Divergence to consensus: 17.7%  
**31. MER58A**: 15478437-15478624 (+), Divergence to consensus: 27.2%  
**32. CHRL1\_BT**: 15478688-15478839 (+), Divergence to consensus: 22.3%  
**33. SINE2-1\_BT**: 15479105-15479218 (-), Divergence to consensus: 27.5%  
**34. L1ME2**: 15479317-15479439 (+), Divergence to consensus: 23.6%  
**35. LTR65**: 15479830-15480070 (-), Divergence to consensus: 29.7%  
**36. Bov-tA2**: 15480144-15480337 (+), Divergence to consensus: 20.2%  
**37. L1M5**: 15480346-15480466 (+), Divergence to consensus: 35.5%  
**38. Bov-tA2**: 15481489-15481690 (-), Divergence to consensus: 13.4%

  
Transcription Factor Binding Sites  

**Gata4** (Sequence: AGATAAC (-): 15473601)  
**SOX9** (Sequence: AACAATGG (-): 15479690)  
**SOX9** (Sequence: TTATTGTT (+): 15471356)  
**SOX9** (Sequence: CTATTGTT (+): 15478842)  
**Gata4** (Sequence: GTTATCT (+): 15472725)  
**Gata4** (Sequence: CTTATCT (+): 15481915)
