# Supplementary material for: Small RNA sequencing of cryopreserved semen from single bull revealed altered miRNAs and piRNAs expression between High- and Low-motile sperm populations
Source: BMC Genomics. 2017 Jan 4;18:14. doi: 10.1186/s12864-016-3394-7 (PMC5209821; doi:10.1186/s12864-016-3394-7)
Supplement: Additional file 3: — Details for each piRNA clusters found in High Motile (HM) sperm fraction. Genes, repeats, transposable elements and transcription factors binding sites falling within the cluster regions were reported. (ZIP 1896 kb) [file 12864_2016_3394_MOESM3_ESM.zip › 91.html]

piRNA cluster 91


Predicted piRNA cluster no. 91     previous   next
  

Show proTRAC run info
Hide proTRAC run info

================================= proTRAC ====================================  
VERSION: 2.1                                    LAST MODIFIED: 06. October 2015  
  
Please cite:  
Rosenkranz D, Zischler H. proTRAC - a software for probabilistic piRNA cluster  
detection, visualization and analysis. 2012. BMC Bioinformatics 13:5.  
  
and (for proTRAC 2.0 and later):  
Rosenkranz D, Rudloff S, Bastuck K, Ketting RF, Zischler H. Tupaia small RNAs  
provide insights into function and evolution of RNAi-based transposon defense  
in mammals. 2015. RNA 21(5):911-922.  
  
Contact:  
David Rosenkranz  
Institute of Anthropology, small RNA group  
Johannes Gutenberg University Mainz  
email: rosenkranz@uni-mainz.de  
  
You can find the latest proTRAC version at:  
http://sourceforge.net/projects/protrac/files  
http://www.smallRNAgroup-mainz.de/software  
==============================================================================  
  
PARAMETERS:  
Map file: .............../storage/core/barbara/genhome/smallRNA/fertility/Sample\_motile/pirna/Sample\_motile\_26-33\_collapsed.fa.no-dust.map.weighted-10000-1000-b-0  
Genome file: ............/storage/core/barbara/genhome/smallRNA/fertility/Sample\_all/pirna/bt\_311\_chrY.fa  
RepeatMasker annotation: /storage/genomes/bt\_umd31/GCF\_000003055.6\_Bos\_taurus\_UMD\_3.1.1\_repeatMasker\_chr.out  
GeneSet:................./storage/core/barbara/genhome/smallRNA/fertility/Sample\_all/pirna/full.gtf  
  
Significant (p<=0.01) hit density will be calculated based  
on observed hit distribution.  
  
Sliding window size: ........................................ 5000 bp  
Sliding window increament: .................................. 1000 bp  
Normalize each hit by number of genomic hits: ............... 1 [0=no/1=yes]  
Normalize each hit by number of sequence reads: ............. 1 [0=no/1=yes]  
Normalize values (-> per million mapped reads): ............. 1 [0=no/1=yes]  
Min. fraction of hits with 1T(U) or 10A: .................... 0.75  
Alternatively: Min. fraction of hits with 1T(U) and 10A: .... 0.5  
Min. fraction of hits with typical piRNA length: ............ 0.75  
Typical piRNA length: ....................................... 26-33 nt  
Min. size of a piRNA cluster: ............................... 5000 bp.  
Min. number of hits (absolute): ............................. 0  
Min. number of hits (normalized): ........................... 0  
Min. fraction of hits on the mainstrand: .................... 0.75  
Top fraction of mapped sequences (in terms of read counts): . 1%  
Top fraction accounts for max. n% of sequence reads: ........ 90%  
Min. fraction of hits on each arm of a bidirectional cluster: 0.1  
Output image file for each cluster: ......................... 0 [0=no/1=yes]  
Output html file for each cluster: .......................... 1 [0=no/1=yes]  
Output a summary table: ..................................... 1 [0=no/1=yes]  
Output a FASTA file for each cluster (piRNA sequences): ..... 1 [0=no/1=yes]  
Output a FASTA file comprising cluster sequences: ........... 1 [0=no/1=yes]  
Search DNA motifs in clusters: .............................. 1 [0=no/1=yes]  
Output flanking sequences: +/- .............................. 0 bp  
Output ~.pTi file: .......................................... 1 [0=no/1=yes]  
==============================================================================  
  
  
Genome size (without gaps): ............ 2678902517 bp  
Gaps (N/X/-): .......................... 53837044 bp  
Mapped reads: .......................... 658825247023  
Non-identical sequences: ............... 514171  
Genomic hits: .......................... 764233  
Significant densitiy of mapped reads: .. 12867599.5173724 reads/kb

Show proTRAC cluster info
Hide proTRAC cluster info

|  |  |
| --- | --- |
| Location | chr8 |
| Coordinates | 9491185-9498875 |
| Size [bp] | 7691 |
| Sequence hit loci | 79 |
| Mapped reads (normalized) | 108202969 |
| Mapped reads (normalized) per kb | 14068777.7 |
| Normalized reads with 1T (1U) | 80.9% |
| Normalized reads with 10A | 40.3% |
| Normalized reads with length 26-33 nt | 100% |
| Normalized reads on the main strand(s) | 95.9% |
| Predicted directionality | mono:minus |

100%

0%

1T (1U)  
reads

10A reads

26-33 nt  
reads

reads on mainstrand

**Either the amount of reads with 1T (1U) OR 10A has to exceed 75% (set with option: -1Tor10A)  
Alternatively the amount of reads with 1T (1U) AND 10A has to exceed 50% (set with option: -1Tand10A)  
Minimum amount of reads with preferred size is 75% (set with option: -pisize)  
Minimum amount of reads on the main strand(s) is 75% (set with option: -clstrand)**

Show read coverage
Hide read coverage

WHAT DO I SEE HERE?  
This chart shows the location of mapped sequence reads within a predicted piRNA cluster. The color refers to the number of genomic hits produced by the sequence read in question. A dark red bar indicates that this sequence read produces many other hits elsewhere in the genome. Many adjacent red or yellow bars can indicate the presence of a multi-copy element such as transposons or rRNA genes. A dark green bar indicates that this sequence read maps uniquely to this locus.

1 hit

2-5 hits

6-10 hits

11-20 hits

21-50 hits

51-100 hits

> 100 hits

chr8

9491185

9498875

Gene Set

RepeatMasker

Mapped  
Reads

13.34

plus strand

minus strand

13.34

Region: chr8 15482100-9491192. Max. coverage (+): 0. Max coverage (-): 1.48

Region: chr8 9491193-9491208. Max. coverage (+): 0. Max coverage (-): 0

Region: chr8 9491209-9491223. Max. coverage (+): 0. Max coverage (-): 0

Region: chr8 9491224-9491238. Max. coverage (+): 0. Max coverage (-): 0

Region: chr8 9491239-9491254. Max. coverage (+): 0. Max coverage (-): 0.74

Region: chr8 9491255-9491269. Max. coverage (+): 0. Max coverage (-): 6.15

Region: chr8 9491270-9491284. Max. coverage (+): 0. Max coverage (-): 0

Region: chr8 9491285-9491300. Max. coverage (+): 0. Max coverage (-): 0

Region: chr8 9491301-9491315. Max. coverage (+): 0. Max coverage (-): 0

Region: chr8 9491316-9491331. Max. coverage (+): 0. Max coverage (-): 0

Region: chr8 9491332-9491346. Max. coverage (+): 0. Max coverage (-): 0

Region: chr8 9491347-9491361. Max. coverage (+): 0. Max coverage (-): 0

Region: chr8 9491362-9491377. Max. coverage (+): 0. Max coverage (-): 0

Region: chr8 9491378-9491392. Max. coverage (+): 0. Max coverage (-): 0

Region: chr8 9491393-9491408. Max. coverage (+): 0. Max coverage (-): 0

Region: chr8 9491409-9491423. Max. coverage (+): 0. Max coverage (-): 0

Region: chr8 9491424-9491438. Max. coverage (+): 0. Max coverage (-): 0

Region: chr8 9491439-9491454. Max. coverage (+): 0. Max coverage (-): 0

Region: chr8 9491455-9491469. Max. coverage (+): 0. Max coverage (-): 0

Region: chr8 9491470-9491484. Max. coverage (+): 0. Max coverage (-): 0

Region: chr8 9491485-9491500. Max. coverage (+): 0. Max coverage (-): 0

Region: chr8 9491501-9491515. Max. coverage (+): 0. Max coverage (-): 0

Region: chr8 9491516-9491531. Max. coverage (+): 0. Max coverage (-): 0

Region: chr8 9491532-9491546. Max. coverage (+): 0. Max coverage (-): 0

Region: chr8 9491547-9491561. Max. coverage (+): 0. Max coverage (-): 0

Region: chr8 9491562-9491577. Max. coverage (+): 0. Max coverage (-): 0

Region: chr8 9491578-9491592. Max. coverage (+): 0. Max coverage (-): 0

Region: chr8 9491593-9491608. Max. coverage (+): 0. Max coverage (-): 0

Region: chr8 9491609-9491623. Max. coverage (+): 0.94. Max coverage (-): 0

Region: chr8 9491624-9491638. Max. coverage (+): 0.94. Max coverage (-): 0

Region: chr8 9491639-9491654. Max. coverage (+): 0. Max coverage (-): 0

Region: chr8 9491655-9491669. Max. coverage (+): 0. Max coverage (-): 0

Region: chr8 9491670-9491684. Max. coverage (+): 0. Max coverage (-): 0

Region: chr8 9491685-9491700. Max. coverage (+): 0. Max coverage (-): 0

Region: chr8 9491701-9491715. Max. coverage (+): 0. Max coverage (-): 0

Region: chr8 9491716-9491731. Max. coverage (+): 0. Max coverage (-): 0

Region: chr8 9491732-9491746. Max. coverage (+): 0. Max coverage (-): 0

Region: chr8 9491747-9491761. Max. coverage (+): 0. Max coverage (-): 0

Region: chr8 9491762-9491777. Max. coverage (+): 0. Max coverage (-): 0

Region: chr8 9491778-9491792. Max. coverage (+): 0. Max coverage (-): 0

Region: chr8 9491793-9491807. Max. coverage (+): 0. Max coverage (-): 0

Region: chr8 9491808-9491823. Max. coverage (+): 0. Max coverage (-): 0

Region: chr8 9491824-9491838. Max. coverage (+): 0. Max coverage (-): 0

Region: chr8 9491839-9491854. Max. coverage (+): 0. Max coverage (-): 0

Region: chr8 9491855-9491869. Max. coverage (+): 0. Max coverage (-): 1.7

Region: chr8 9491870-9491884. Max. coverage (+): 0. Max coverage (-): 1.7

Region: chr8 9491885-9491900. Max. coverage (+): 0. Max coverage (-): 0

Region: chr8 9491901-9491915. Max. coverage (+): 0. Max coverage (-): 2.27

Region: chr8 9491916-9491931. Max. coverage (+): 0. Max coverage (-): 0

Region: chr8 9491932-9491946. Max. coverage (+): 0. Max coverage (-): 0

Region: chr8 9491947-9491961. Max. coverage (+): 0. Max coverage (-): 0

Region: chr8 9491962-9491977. Max. coverage (+): 0. Max coverage (-): 0

Region: chr8 9491978-9491992. Max. coverage (+): 0. Max coverage (-): 0

Region: chr8 9491993-9492007. Max. coverage (+): 0. Max coverage (-): 0

Region: chr8 9492008-9492023. Max. coverage (+): 0. Max coverage (-): 0

Region: chr8 9492024-9492038. Max. coverage (+): 0. Max coverage (-): 0

Region: chr8 9492039-9492054. Max. coverage (+): 0. Max coverage (-): 0

Region: chr8 9492055-9492069. Max. coverage (+): 0. Max coverage (-): 0

Region: chr8 9492070-9492084. Max. coverage (+): 0. Max coverage (-): 0

Region: chr8 9492085-9492100. Max. coverage (+): 0. Max coverage (-): 5.19

Region: chr8 9492101-9492115. Max. coverage (+): 0. Max coverage (-): 0

Region: chr8 9492116-9492130. Max. coverage (+): 0. Max coverage (-): 0

Region: chr8 9492131-9492146. Max. coverage (+): 0. Max coverage (-): 0

Region: chr8 9492147-9492161. Max. coverage (+): 0. Max coverage (-): 0

Region: chr8 9492162-9492177. Max. coverage (+): 0. Max coverage (-): 4.51

Region: chr8 9492178-9492192. Max. coverage (+): 0. Max coverage (-): 4.51

Region: chr8 9492193-9492207. Max. coverage (+): 0. Max coverage (-): 0

Region: chr8 9492208-9492223. Max. coverage (+): 0. Max coverage (-): 0

Region: chr8 9492224-9492238. Max. coverage (+): 0. Max coverage (-): 0

Region: chr8 9492239-9492254. Max. coverage (+): 0. Max coverage (-): 0

Region: chr8 9492255-9492269. Max. coverage (+): 0. Max coverage (-): 0

Region: chr8 9492270-9492284. Max. coverage (+): 0. Max coverage (-): 0

Region: chr8 9492285-9492300. Max. coverage (+): 0. Max coverage (-): 1.11

Region: chr8 9492301-9492315. Max. coverage (+): 0. Max coverage (-): 10.09

Region: chr8 9492316-9492330. Max. coverage (+): 0. Max coverage (-): 7.28

Region: chr8 9492331-9492346. Max. coverage (+): 0. Max coverage (-): 0

Region: chr8 9492347-9492361. Max. coverage (+): 0. Max coverage (-): 0

Region: chr8 9492362-9492377. Max. coverage (+): 0. Max coverage (-): 0

Region: chr8 9492378-9492392. Max. coverage (+): 0. Max coverage (-): 0

Region: chr8 9492393-9492407. Max. coverage (+): 0. Max coverage (-): 0

Region: chr8 9492408-9492423. Max. coverage (+): 0. Max coverage (-): 0

Region: chr8 9492424-9492438. Max. coverage (+): 0. Max coverage (-): 0

Region: chr8 9492439-9492454. Max. coverage (+): 0. Max coverage (-): 0

Region: chr8 9492455-9492469. Max. coverage (+): 0. Max coverage (-): 0

Region: chr8 9492470-9492484. Max. coverage (+): 0. Max coverage (-): 0

Region: chr8 9492485-9492500. Max. coverage (+): 0. Max coverage (-): 0

Region: chr8 9492501-9492515. Max. coverage (+): 0. Max coverage (-): 0

Region: chr8 9492516-9492530. Max. coverage (+): 0. Max coverage (-): 0

Region: chr8 9492531-9492546. Max. coverage (+): 0. Max coverage (-): 0

Region: chr8 9492547-9492561. Max. coverage (+): 0. Max coverage (-): 0

Region: chr8 9492562-9492577. Max. coverage (+): 0. Max coverage (-): 0

Region: chr8 9492578-9492592. Max. coverage (+): 0. Max coverage (-): 0

Region: chr8 9492593-9492607. Max. coverage (+): 0. Max coverage (-): 0

Region: chr8 9492608-9492623. Max. coverage (+): 0. Max coverage (-): 0

Region: chr8 9492624-9492638. Max. coverage (+): 0. Max coverage (-): 0

Region: chr8 9492639-9492653. Max. coverage (+): 0. Max coverage (-): 0

Region: chr8 9492654-9492669. Max. coverage (+): 0. Max coverage (-): 0

Region: chr8 9492670-9492684. Max. coverage (+): 0. Max coverage (-): 0

Region: chr8 9492685-9492700. Max. coverage (+): 0. Max coverage (-): 0

Region: chr8 9492701-9492715. Max. coverage (+): 0.62. Max coverage (-): 0

Region: chr8 9492716-9492730. Max. coverage (+): 0.53. Max coverage (-): 0

Region: chr8 9492731-9492746. Max. coverage (+): 0. Max coverage (-): 0

Region: chr8 9492747-9492761. Max. coverage (+): 0. Max coverage (-): 0

Region: chr8 9492762-9492777. Max. coverage (+): 0. Max coverage (-): 0

Region: chr8 9492778-9492792. Max. coverage (+): 0. Max coverage (-): 0

Region: chr8 9492793-9492807. Max. coverage (+): 0. Max coverage (-): 0

Region: chr8 9492808-9492823. Max. coverage (+): 0. Max coverage (-): 1.66

Region: chr8 9492824-9492838. Max. coverage (+): 0. Max coverage (-): 1.66

Region: chr8 9492839-9492853. Max. coverage (+): 0. Max coverage (-): 0

Region: chr8 9492854-9492869. Max. coverage (+): 0. Max coverage (-): 0

Region: chr8 9492870-9492884. Max. coverage (+): 0. Max coverage (-): 0

Region: chr8 9492885-9492900. Max. coverage (+): 0. Max coverage (-): 0

Region: chr8 9492901-9492915. Max. coverage (+): 0. Max coverage (-): 0

Region: chr8 9492916-9492930. Max. coverage (+): 0. Max coverage (-): 0

Region: chr8 9492931-9492946. Max. coverage (+): 0. Max coverage (-): 0

Region: chr8 9492947-9492961. Max. coverage (+): 0. Max coverage (-): 0

Region: chr8 9492962-9492977. Max. coverage (+): 0. Max coverage (-): 0

Region: chr8 9492978-9492992. Max. coverage (+): 0. Max coverage (-): 0

Region: chr8 9492993-9493007. Max. coverage (+): 0. Max coverage (-): 0

Region: chr8 9493008-9493023. Max. coverage (+): 0. Max coverage (-): 8.53

Region: chr8 9493024-9493038. Max. coverage (+): 1.47. Max coverage (-): 0

Region: chr8 9493039-9493053. Max. coverage (+): 1.47. Max coverage (-): 0

Region: chr8 9493054-9493069. Max. coverage (+): 0. Max coverage (-): 0

Region: chr8 9493070-9493084. Max. coverage (+): 0. Max coverage (-): 0

Region: chr8 9493085-9493100. Max. coverage (+): 0. Max coverage (-): 0

Region: chr8 9493101-9493115. Max. coverage (+): 0. Max coverage (-): 0

Region: chr8 9493116-9493130. Max. coverage (+): 0. Max coverage (-): 0

Region: chr8 9493131-9493146. Max. coverage (+): 0. Max coverage (-): 0

Region: chr8 9493147-9493161. Max. coverage (+): 1.34. Max coverage (-): 0

Region: chr8 9493162-9493176. Max. coverage (+): 0. Max coverage (-): 0

Region: chr8 9493177-9493192. Max. coverage (+): 0. Max coverage (-): 0

Region: chr8 9493193-9493207. Max. coverage (+): 0. Max coverage (-): 0

Region: chr8 9493208-9493223. Max. coverage (+): 0. Max coverage (-): 0

Region: chr8 9493224-9493238. Max. coverage (+): 0. Max coverage (-): 0

Region: chr8 9493239-9493253. Max. coverage (+): 0. Max coverage (-): 0

Region: chr8 9493254-9493269. Max. coverage (+): 0. Max coverage (-): 0

Region: chr8 9493270-9493284. Max. coverage (+): 0. Max coverage (-): 0

Region: chr8 9493285-9493300. Max. coverage (+): 0. Max coverage (-): 0

Region: chr8 9493301-9493315. Max. coverage (+): 0. Max coverage (-): 0

Region: chr8 9493316-9493330. Max. coverage (+): 0. Max coverage (-): 0

Region: chr8 9493331-9493346. Max. coverage (+): 0. Max coverage (-): 0

Region: chr8 9493347-9493361. Max. coverage (+): 0. Max coverage (-): 0

Region: chr8 9493362-9493376. Max. coverage (+): 0. Max coverage (-): 0

Region: chr8 9493377-9493392. Max. coverage (+): 0. Max coverage (-): 5.38

Region: chr8 9493393-9493407. Max. coverage (+): 0. Max coverage (-): 1.77

Region: chr8 9493408-9493423. Max. coverage (+): 0. Max coverage (-): 0

Region: chr8 9493424-9493438. Max. coverage (+): 0. Max coverage (-): 0

Region: chr8 9493439-9493453. Max. coverage (+): 0. Max coverage (-): 0

Region: chr8 9493454-9493469. Max. coverage (+): 0. Max coverage (-): 0

Region: chr8 9493470-9493484. Max. coverage (+): 0. Max coverage (-): 0

Region: chr8 9493485-9493499. Max. coverage (+): 0. Max coverage (-): 0

Region: chr8 9493500-9493515. Max. coverage (+): 0. Max coverage (-): 1.16

Region: chr8 9493516-9493530. Max. coverage (+): 0. Max coverage (-): 1.16

Region: chr8 9493531-9493546. Max. coverage (+): 0. Max coverage (-): 0

Region: chr8 9493547-9493561. Max. coverage (+): 0. Max coverage (-): 0

Region: chr8 9493562-9493576. Max. coverage (+): 0. Max coverage (-): 0

Region: chr8 9493577-9493592. Max. coverage (+): 0. Max coverage (-): 0

Region: chr8 9493593-9493607. Max. coverage (+): 0. Max coverage (-): 0

Region: chr8 9493608-9493623. Max. coverage (+): 0. Max coverage (-): 0

Region: chr8 9493624-9493638. Max. coverage (+): 0. Max coverage (-): 0

Region: chr8 9493639-9493653. Max. coverage (+): 0. Max coverage (-): 0

Region: chr8 9493654-9493669. Max. coverage (+): 0. Max coverage (-): 0

Region: chr8 9493670-9493684. Max. coverage (+): 0. Max coverage (-): 0

Region: chr8 9493685-9493699. Max. coverage (+): 0. Max coverage (-): 0

Region: chr8 9493700-9493715. Max. coverage (+): 0. Max coverage (-): 0

Region: chr8 9493716-9493730. Max. coverage (+): 0. Max coverage (-): 0

Region: chr8 9493731-9493746. Max. coverage (+): 0. Max coverage (-): 0

Region: chr8 9493747-9493761. Max. coverage (+): 0. Max coverage (-): 0

Region: chr8 9493762-9493776. Max. coverage (+): 0. Max coverage (-): 0

Region: chr8 9493777-9493792. Max. coverage (+): 0. Max coverage (-): 0

Region: chr8 9493793-9493807. Max. coverage (+): 0. Max coverage (-): 0

Region: chr8 9493808-9493823. Max. coverage (+): 0. Max coverage (-): 0

Region: chr8 9493824-9493838. Max. coverage (+): 0. Max coverage (-): 0

Region: chr8 9493839-9493853. Max. coverage (+): 0. Max coverage (-): 1.6

Region: chr8 9493854-9493869. Max. coverage (+): 0. Max coverage (-): 0

Region: chr8 9493870-9493884. Max. coverage (+): 0. Max coverage (-): 0

Region: chr8 9493885-9493899. Max. coverage (+): 0. Max coverage (-): 0

Region: chr8 9493900-9493915. Max. coverage (+): 0. Max coverage (-): 0

Region: chr8 9493916-9493930. Max. coverage (+): 0. Max coverage (-): 0

Region: chr8 9493931-9493946. Max. coverage (+): 0. Max coverage (-): 0

Region: chr8 9493947-9493961. Max. coverage (+): 0. Max coverage (-): 0

Region: chr8 9493962-9493976. Max. coverage (+): 0. Max coverage (-): 0

Region: chr8 9493977-9493992. Max. coverage (+): 0. Max coverage (-): 0

Region: chr8 9493993-9494007. Max. coverage (+): 0. Max coverage (-): 0

Region: chr8 9494008-9494022. Max. coverage (+): 0. Max coverage (-): 0

Region: chr8 9494023-9494038. Max. coverage (+): 0. Max coverage (-): 0

Region: chr8 9494039-9494053. Max. coverage (+): 0. Max coverage (-): 0

Region: chr8 9494054-9494069. Max. coverage (+): 0. Max coverage (-): 0

Region: chr8 9494070-9494084. Max. coverage (+): 0. Max coverage (-): 0

Region: chr8 9494085-9494099. Max. coverage (+): 0. Max coverage (-): 0

Region: chr8 9494100-9494115. Max. coverage (+): 0. Max coverage (-): 0

Region: chr8 9494116-9494130. Max. coverage (+): 0. Max coverage (-): 0

Region: chr8 9494131-9494146. Max. coverage (+): 0. Max coverage (-): 0

Region: chr8 9494147-9494161. Max. coverage (+): 0. Max coverage (-): 0

Region: chr8 9494162-9494176. Max. coverage (+): 0. Max coverage (-): 0

Region: chr8 9494177-9494192. Max. coverage (+): 0. Max coverage (-): 0

Region: chr8 9494193-9494207. Max. coverage (+): 0. Max coverage (-): 0

Region: chr8 9494208-9494222. Max. coverage (+): 0. Max coverage (-): 4.81

Region: chr8 9494223-9494238. Max. coverage (+): 0. Max coverage (-): 1.12

Region: chr8 9494239-9494253. Max. coverage (+): 0. Max coverage (-): 0

Region: chr8 9494254-9494269. Max. coverage (+): 0. Max coverage (-): 0

Region: chr8 9494270-9494284. Max. coverage (+): 0. Max coverage (-): 0

Region: chr8 9494285-9494299. Max. coverage (+): 0. Max coverage (-): 0

Region: chr8 9494300-9494315. Max. coverage (+): 0. Max coverage (-): 5.01

Region: chr8 9494316-9494330. Max. coverage (+): 0. Max coverage (-): 0

Region: chr8 9494331-9494346. Max. coverage (+): 0. Max coverage (-): 0.32

Region: chr8 9494347-9494361. Max. coverage (+): 0. Max coverage (-): 0

Region: chr8 9494362-9494376. Max. coverage (+): 0. Max coverage (-): 0

Region: chr8 9494377-9494392. Max. coverage (+): 0. Max coverage (-): 0

Region: chr8 9494393-9494407. Max. coverage (+): 0. Max coverage (-): 0

Region: chr8 9494408-9494422. Max. coverage (+): 0. Max coverage (-): 0

Region: chr8 9494423-9494438. Max. coverage (+): 0. Max coverage (-): 0

Region: chr8 9494439-9494453. Max. coverage (+): 0. Max coverage (-): 0

Region: chr8 9494454-9494469. Max. coverage (+): 0. Max coverage (-): 2.14

Region: chr8 9494470-9494484. Max. coverage (+): 0. Max coverage (-): 2.14

Region: chr8 9494485-9494499. Max. coverage (+): 0. Max coverage (-): 0.78

Region: chr8 9494500-9494515. Max. coverage (+): 0. Max coverage (-): 0

Region: chr8 9494516-9494530. Max. coverage (+): 0. Max coverage (-): 0

Region: chr8 9494531-9494545. Max. coverage (+): 0. Max coverage (-): 0

Region: chr8 9494546-9494561. Max. coverage (+): 0. Max coverage (-): 0

Region: chr8 9494562-9494576. Max. coverage (+): 0. Max coverage (-): 0

Region: chr8 9494577-9494592. Max. coverage (+): 0. Max coverage (-): 0

Region: chr8 9494593-9494607. Max. coverage (+): 0. Max coverage (-): 0

Region: chr8 9494608-9494622. Max. coverage (+): 0. Max coverage (-): 0

Region: chr8 9494623-9494638. Max. coverage (+): 0. Max coverage (-): 0

Region: chr8 9494639-9494653. Max. coverage (+): 0. Max coverage (-): 0

Region: chr8 9494654-9494669. Max. coverage (+): 0. Max coverage (-): 0

Region: chr8 9494670-9494684. Max. coverage (+): 0. Max coverage (-): 0

Region: chr8 9494685-9494699. Max. coverage (+): 0. Max coverage (-): 0

Region: chr8 9494700-9494715. Max. coverage (+): 0. Max coverage (-): 0

Region: chr8 9494716-9494730. Max. coverage (+): 0. Max coverage (-): 0

Region: chr8 9494731-9494745. Max. coverage (+): 0. Max coverage (-): 0

Region: chr8 9494746-9494761. Max. coverage (+): 0. Max coverage (-): 0

Region: chr8 9494762-9494776. Max. coverage (+): 0. Max coverage (-): 3.1

Region: chr8 9494777-9494792. Max. coverage (+): 0. Max coverage (-): 4.29

Region: chr8 9494793-9494807. Max. coverage (+): 0. Max coverage (-): 4.29

Region: chr8 9494808-9494822. Max. coverage (+): 0. Max coverage (-): 0

Region: chr8 9494823-9494838. Max. coverage (+): 0. Max coverage (-): 1.66

Region: chr8 9494839-9494853. Max. coverage (+): 0. Max coverage (-): 0

Region: chr8 9494854-9494868. Max. coverage (+): 0. Max coverage (-): 0

Region: chr8 9494869-9494884. Max. coverage (+): 0. Max coverage (-): 0

Region: chr8 9494885-9494899. Max. coverage (+): 0. Max coverage (-): 0

Region: chr8 9494900-9494915. Max. coverage (+): 0. Max coverage (-): 0

Region: chr8 9494916-9494930. Max. coverage (+): 0. Max coverage (-): 0

Region: chr8 9494931-9494945. Max. coverage (+): 0. Max coverage (-): 0

Region: chr8 9494946-9494961. Max. coverage (+): 1.86. Max coverage (-): 0

Region: chr8 9494962-9494976. Max. coverage (+): 1.86. Max coverage (-): 0

Region: chr8 9494977-9494992. Max. coverage (+): 0. Max coverage (-): 0

Region: chr8 9494993-9495007. Max. coverage (+): 0. Max coverage (-): 2.06

Region: chr8 9495008-9495022. Max. coverage (+): 0. Max coverage (-): 2.06

Region: chr8 9495023-9495038. Max. coverage (+): 0. Max coverage (-): 0

Region: chr8 9495039-9495053. Max. coverage (+): 0. Max coverage (-): 0

Region: chr8 9495054-9495068. Max. coverage (+): 0. Max coverage (-): 0

Region: chr8 9495069-9495084. Max. coverage (+): 0. Max coverage (-): 0

Region: chr8 9495085-9495099. Max. coverage (+): 0. Max coverage (-): 0

Region: chr8 9495100-9495115. Max. coverage (+): 0. Max coverage (-): 0

Region: chr8 9495116-9495130. Max. coverage (+): 0. Max coverage (-): 3.96

Region: chr8 9495131-9495145. Max. coverage (+): 0. Max coverage (-): 3.96

Region: chr8 9495146-9495161. Max. coverage (+): 0. Max coverage (-): 0

Region: chr8 9495162-9495176. Max. coverage (+): 0. Max coverage (-): 0

Region: chr8 9495177-9495192. Max. coverage (+): 0. Max coverage (-): 0

Region: chr8 9495193-9495207. Max. coverage (+): 0. Max coverage (-): 0

Region: chr8 9495208-9495222. Max. coverage (+): 0. Max coverage (-): 0

Region: chr8 9495223-9495238. Max. coverage (+): 0. Max coverage (-): 1.37

Region: chr8 9495239-9495253. Max. coverage (+): 0. Max coverage (-): 0

Region: chr8 9495254-9495268. Max. coverage (+): 0. Max coverage (-): 0.62

Region: chr8 9495269-9495284. Max. coverage (+): 0. Max coverage (-): 0

Region: chr8 9495285-9495299. Max. coverage (+): 0. Max coverage (-): 0

Region: chr8 9495300-9495315. Max. coverage (+): 0. Max coverage (-): 0

Region: chr8 9495316-9495330. Max. coverage (+): 0. Max coverage (-): 0

Region: chr8 9495331-9495345. Max. coverage (+): 0. Max coverage (-): 0

Region: chr8 9495346-9495361. Max. coverage (+): 0. Max coverage (-): 0

Region: chr8 9495362-9495376. Max. coverage (+): 0. Max coverage (-): 0

Region: chr8 9495377-9495391. Max. coverage (+): 0. Max coverage (-): 0

Region: chr8 9495392-9495407. Max. coverage (+): 0. Max coverage (-): 0

Region: chr8 9495408-9495422. Max. coverage (+): 0. Max coverage (-): 0

Region: chr8 9495423-9495438. Max. coverage (+): 0. Max coverage (-): 0

Region: chr8 9495439-9495453. Max. coverage (+): 0. Max coverage (-): 4.4

Region: chr8 9495454-9495468. Max. coverage (+): 0. Max coverage (-): 0

Region: chr8 9495469-9495484. Max. coverage (+): 0. Max coverage (-): 0

Region: chr8 9495485-9495499. Max. coverage (+): 0. Max coverage (-): 0

Region: chr8 9495500-9495515. Max. coverage (+): 0. Max coverage (-): 0

Region: chr8 9495516-9495530. Max. coverage (+): 0. Max coverage (-): 0

Region: chr8 9495531-9495545. Max. coverage (+): 0. Max coverage (-): 0

Region: chr8 9495546-9495561. Max. coverage (+): 0. Max coverage (-): 0

Region: chr8 9495562-9495576. Max. coverage (+): 0. Max coverage (-): 0

Region: chr8 9495577-9495591. Max. coverage (+): 0. Max coverage (-): 1.92

Region: chr8 9495592-9495607. Max. coverage (+): 0. Max coverage (-): 0

Region: chr8 9495608-9495622. Max. coverage (+): 0. Max coverage (-): 0

Region: chr8 9495623-9495638. Max. coverage (+): 0. Max coverage (-): 0

Region: chr8 9495639-9495653. Max. coverage (+): 0. Max coverage (-): 0

Region: chr8 9495654-9495668. Max. coverage (+): 0. Max coverage (-): 0

Region: chr8 9495669-9495684. Max. coverage (+): 0. Max coverage (-): 0

Region: chr8 9495685-9495699. Max. coverage (+): 0. Max coverage (-): 0

Region: chr8 9495700-9495714. Max. coverage (+): 0. Max coverage (-): 0

Region: chr8 9495715-9495730. Max. coverage (+): 0. Max coverage (-): 0

Region: chr8 9495731-9495745. Max. coverage (+): 0. Max coverage (-): 0

Region: chr8 9495746-9495761. Max. coverage (+): 0. Max coverage (-): 0

Region: chr8 9495762-9495776. Max. coverage (+): 0. Max coverage (-): 13.34

Region: chr8 9495777-9495791. Max. coverage (+): 0. Max coverage (-): 13.34

Region: chr8 9495792-9495807. Max. coverage (+): 0. Max coverage (-): 0

Region: chr8 9495808-9495822. Max. coverage (+): 0. Max coverage (-): 0

Region: chr8 9495823-9495838. Max. coverage (+): 0. Max coverage (-): 3.02

Region: chr8 9495839-9495853. Max. coverage (+): 0. Max coverage (-): 3.02

Region: chr8 9495854-9495868. Max. coverage (+): 0. Max coverage (-): 0

Region: chr8 9495869-9495884. Max. coverage (+): 0. Max coverage (-): 0

Region: chr8 9495885-9495899. Max. coverage (+): 0. Max coverage (-): 0

Region: chr8 9495900-9495914. Max. coverage (+): 0. Max coverage (-): 0

Region: chr8 9495915-9495930. Max. coverage (+): 0. Max coverage (-): 0

Region: chr8 9495931-9495945. Max. coverage (+): 0. Max coverage (-): 0

Region: chr8 9495946-9495961. Max. coverage (+): 0. Max coverage (-): 0

Region: chr8 9495962-9495976. Max. coverage (+): 0. Max coverage (-): 0

Region: chr8 9495977-9495991. Max. coverage (+): 0. Max coverage (-): 4.11

Region: chr8 9495992-9496007. Max. coverage (+): 0. Max coverage (-): 1.09

Region: chr8 9496008-9496022. Max. coverage (+): 0. Max coverage (-): 1.09

Region: chr8 9496023-9496038. Max. coverage (+): 0. Max coverage (-): 5.95

Region: chr8 9496039-9496053. Max. coverage (+): 0. Max coverage (-): 5.95

Region: chr8 9496054-9496068. Max. coverage (+): 0. Max coverage (-): 0

Region: chr8 9496069-9496084. Max. coverage (+): 0. Max coverage (-): 0

Region: chr8 9496085-9496099. Max. coverage (+): 0. Max coverage (-): 0

Region: chr8 9496100-9496114. Max. coverage (+): 0. Max coverage (-): 0

Region: chr8 9496115-9496130. Max. coverage (+): 0. Max coverage (-): 0

Region: chr8 9496131-9496145. Max. coverage (+): 0. Max coverage (-): 0

Region: chr8 9496146-9496161. Max. coverage (+): 0. Max coverage (-): 0

Region: chr8 9496162-9496176. Max. coverage (+): 0. Max coverage (-): 0

Region: chr8 9496177-9496191. Max. coverage (+): 0. Max coverage (-): 0

Region: chr8 9496192-9496207. Max. coverage (+): 0. Max coverage (-): 0

Region: chr8 9496208-9496222. Max. coverage (+): 0. Max coverage (-): 0

Region: chr8 9496223-9496237. Max. coverage (+): 0. Max coverage (-): 0

Region: chr8 9496238-9496253. Max. coverage (+): 0. Max coverage (-): 0

Region: chr8 9496254-9496268. Max. coverage (+): 0. Max coverage (-): 0

Region: chr8 9496269-9496284. Max. coverage (+): 0. Max coverage (-): 0

Region: chr8 9496285-9496299. Max. coverage (+): 0. Max coverage (-): 0

Region: chr8 9496300-9496314. Max. coverage (+): 0. Max coverage (-): 0

Region: chr8 9496315-9496330. Max. coverage (+): 0. Max coverage (-): 0

Region: chr8 9496331-9496345. Max. coverage (+): 0. Max coverage (-): 0

Region: chr8 9496346-9496361. Max. coverage (+): 0. Max coverage (-): 0

Region: chr8 9496362-9496376. Max. coverage (+): 0. Max coverage (-): 0

Region: chr8 9496377-9496391. Max. coverage (+): 0. Max coverage (-): 0

Region: chr8 9496392-9496407. Max. coverage (+): 0. Max coverage (-): 0

Region: chr8 9496408-9496422. Max. coverage (+): 0. Max coverage (-): 0

Region: chr8 9496423-9496437. Max. coverage (+): 0. Max coverage (-): 0

Region: chr8 9496438-9496453. Max. coverage (+): 0. Max coverage (-): 0

Region: chr8 9496454-9496468. Max. coverage (+): 0. Max coverage (-): 0

Region: chr8 9496469-9496484. Max. coverage (+): 0. Max coverage (-): 0

Region: chr8 9496485-9496499. Max. coverage (+): 0. Max coverage (-): 0

Region: chr8 9496500-9496514. Max. coverage (+): 0. Max coverage (-): 0

Region: chr8 9496515-9496530. Max. coverage (+): 0. Max coverage (-): 0

Region: chr8 9496531-9496545. Max. coverage (+): 0. Max coverage (-): 0

Region: chr8 9496546-9496561. Max. coverage (+): 0. Max coverage (-): 0

Region: chr8 9496562-9496576. Max. coverage (+): 0. Max coverage (-): 0

Region: chr8 9496577-9496591. Max. coverage (+): 0. Max coverage (-): 0

Region: chr8 9496592-9496607. Max. coverage (+): 0. Max coverage (-): 0

Region: chr8 9496608-9496622. Max. coverage (+): 0. Max coverage (-): 0

Region: chr8 9496623-9496637. Max. coverage (+): 0. Max coverage (-): 0

Region: chr8 9496638-9496653. Max. coverage (+): 0. Max coverage (-): 0

Region: chr8 9496654-9496668. Max. coverage (+): 0. Max coverage (-): 0

Region: chr8 9496669-9496684. Max. coverage (+): 0. Max coverage (-): 0

Region: chr8 9496685-9496699. Max. coverage (+): 0. Max coverage (-): 0

Region: chr8 9496700-9496714. Max. coverage (+): 0. Max coverage (-): 0

Region: chr8 9496715-9496730. Max. coverage (+): 0. Max coverage (-): 0

Region: chr8 9496731-9496745. Max. coverage (+): 0. Max coverage (-): 0

Region: chr8 9496746-9496760. Max. coverage (+): 0. Max coverage (-): 0

Region: chr8 9496761-9496776. Max. coverage (+): 0. Max coverage (-): 0

Region: chr8 9496777-9496791. Max. coverage (+): 0. Max coverage (-): 0

Region: chr8 9496792-9496807. Max. coverage (+): 0. Max coverage (-): 0

Region: chr8 9496808-9496822. Max. coverage (+): 0. Max coverage (-): 0

Region: chr8 9496823-9496837. Max. coverage (+): 0. Max coverage (-): 0

Region: chr8 9496838-9496853. Max. coverage (+): 0. Max coverage (-): 0

Region: chr8 9496854-9496868. Max. coverage (+): 0. Max coverage (-): 0

Region: chr8 9496869-9496884. Max. coverage (+): 0. Max coverage (-): 0

Region: chr8 9496885-9496899. Max. coverage (+): 0. Max coverage (-): 0

Region: chr8 9496900-9496914. Max. coverage (+): 0. Max coverage (-): 0

Region: chr8 9496915-9496930. Max. coverage (+): 0. Max coverage (-): 0

Region: chr8 9496931-9496945. Max. coverage (+): 0. Max coverage (-): 0

Region: chr8 9496946-9496960. Max. coverage (+): 0. Max coverage (-): 0

Region: chr8 9496961-9496976. Max. coverage (+): 0. Max coverage (-): 0

Region: chr8 9496977-9496991. Max. coverage (+): 0. Max coverage (-): 0.95

Region: chr8 9496992-9497007. Max. coverage (+): 0. Max coverage (-): 0

Region: chr8 9497008-9497022. Max. coverage (+): 0. Max coverage (-): 0

Region: chr8 9497023-9497037. Max. coverage (+): 0. Max coverage (-): 0

Region: chr8 9497038-9497053. Max. coverage (+): 0. Max coverage (-): 0

Region: chr8 9497054-9497068. Max. coverage (+): 0. Max coverage (-): 0

Region: chr8 9497069-9497083. Max. coverage (+): 0. Max coverage (-): 0

Region: chr8 9497084-9497099. Max. coverage (+): 0. Max coverage (-): 0

Region: chr8 9497100-9497114. Max. coverage (+): 0. Max coverage (-): 0

Region: chr8 9497115-9497130. Max. coverage (+): 0. Max coverage (-): 0

Region: chr8 9497131-9497145. Max. coverage (+): 0. Max coverage (-): 0

Region: chr8 9497146-9497160. Max. coverage (+): 0. Max coverage (-): 0

Region: chr8 9497161-9497176. Max. coverage (+): 0. Max coverage (-): 0

Region: chr8 9497177-9497191. Max. coverage (+): 0. Max coverage (-): 1.74

Region: chr8 9497192-9497207. Max. coverage (+): 0. Max coverage (-): 0

Region: chr8 9497208-9497222. Max. coverage (+): 0. Max coverage (-): 0

Region: chr8 9497223-9497237. Max. coverage (+): 0. Max coverage (-): 0

Region: chr8 9497238-9497253. Max. coverage (+): 0. Max coverage (-): 0

Region: chr8 9497254-9497268. Max. coverage (+): 0. Max coverage (-): 0

Region: chr8 9497269-9497283. Max. coverage (+): 0. Max coverage (-): 6

Region: chr8 9497284-9497299. Max. coverage (+): 0. Max coverage (-): 6

Region: chr8 9497300-9497314. Max. coverage (+): 0. Max coverage (-): 0

Region: chr8 9497315-9497330. Max. coverage (+): 0. Max coverage (-): 0

Region: chr8 9497331-9497345. Max. coverage (+): 0. Max coverage (-): 4.02

Region: chr8 9497346-9497360. Max. coverage (+): 0. Max coverage (-): 4.02

Region: chr8 9497361-9497376. Max. coverage (+): 0. Max coverage (-): 4.61

Region: chr8 9497377-9497391. Max. coverage (+): 0. Max coverage (-): 4.61

Region: chr8 9497392-9497407. Max. coverage (+): 0. Max coverage (-): 0

Region: chr8 9497408-9497422. Max. coverage (+): 0. Max coverage (-): 0

Region: chr8 9497423-9497437. Max. coverage (+): 0. Max coverage (-): 0

Region: chr8 9497438-9497453. Max. coverage (+): 0. Max coverage (-): 0

Region: chr8 9497454-9497468. Max. coverage (+): 0. Max coverage (-): 0

Region: chr8 9497469-9497483. Max. coverage (+): 0. Max coverage (-): 0

Region: chr8 9497484-9497499. Max. coverage (+): 0. Max coverage (-): 0

Region: chr8 9497500-9497514. Max. coverage (+): 0. Max coverage (-): 0

Region: chr8 9497515-9497530. Max. coverage (+): 0. Max coverage (-): 0

Region: chr8 9497531-9497545. Max. coverage (+): 0. Max coverage (-): 0

Region: chr8 9497546-9497560. Max. coverage (+): 0. Max coverage (-): 0

Region: chr8 9497561-9497576. Max. coverage (+): 0. Max coverage (-): 1.53

Region: chr8 9497577-9497591. Max. coverage (+): 0. Max coverage (-): 0

Region: chr8 9497592-9497606. Max. coverage (+): 0. Max coverage (-): 5.13

Region: chr8 9497607-9497622. Max. coverage (+): 0. Max coverage (-): 0

Region: chr8 9497623-9497637. Max. coverage (+): 0. Max coverage (-): 0

Region: chr8 9497638-9497653. Max. coverage (+): 0. Max coverage (-): 0

Region: chr8 9497654-9497668. Max. coverage (+): 0. Max coverage (-): 0

Region: chr8 9497669-9497683. Max. coverage (+): 0. Max coverage (-): 0

Region: chr8 9497684-9497699. Max. coverage (+): 0. Max coverage (-): 0

Region: chr8 9497700-9497714. Max. coverage (+): 0. Max coverage (-): 0

Region: chr8 9497715-9497730. Max. coverage (+): 0. Max coverage (-): 0

Region: chr8 9497731-9497745. Max. coverage (+): 0. Max coverage (-): 0

Region: chr8 9497746-9497760. Max. coverage (+): 0. Max coverage (-): 0

Region: chr8 9497761-9497776. Max. coverage (+): 0. Max coverage (-): 3.75

Region: chr8 9497777-9497791. Max. coverage (+): 0. Max coverage (-): 2.68

Region: chr8 9497792-9497806. Max. coverage (+): 0. Max coverage (-): 0

Region: chr8 9497807-9497822. Max. coverage (+): 0. Max coverage (-): 0

Region: chr8 9497823-9497837. Max. coverage (+): 0. Max coverage (-): 0

Region: chr8 9497838-9497853. Max. coverage (+): 0. Max coverage (-): 0

Region: chr8 9497854-9497868. Max. coverage (+): 0. Max coverage (-): 0

Region: chr8 9497869-9497883. Max. coverage (+): 0. Max coverage (-): 2.63

Region: chr8 9497884-9497899. Max. coverage (+): 0. Max coverage (-): 2.63

Region: chr8 9497900-9497914. Max. coverage (+): 0. Max coverage (-): 0

Region: chr8 9497915-9497930. Max. coverage (+): 0. Max coverage (-): 0

Region: chr8 9497931-9497945. Max. coverage (+): 0. Max coverage (-): 0

Region: chr8 9497946-9497960. Max. coverage (+): 0. Max coverage (-): 0

Region: chr8 9497961-9497976. Max. coverage (+): 0. Max coverage (-): 0

Region: chr8 9497977-9497991. Max. coverage (+): 0. Max coverage (-): 0

Region: chr8 9497992-9498006. Max. coverage (+): 0. Max coverage (-): 0

Region: chr8 9498007-9498022. Max. coverage (+): 0. Max coverage (-): 0

Region: chr8 9498023-9498037. Max. coverage (+): 0. Max coverage (-): 0

Region: chr8 9498038-9498053. Max. coverage (+): 0. Max coverage (-): 0

Region: chr8 9498054-9498068. Max. coverage (+): 0. Max coverage (-): 0

Region: chr8 9498069-9498083. Max. coverage (+): 0. Max coverage (-): 0

Region: chr8 9498084-9498099. Max. coverage (+): 0. Max coverage (-): 0

Region: chr8 9498100-9498114. Max. coverage (+): 0. Max coverage (-): 0

Region: chr8 9498115-9498129. Max. coverage (+): 0. Max coverage (-): 0

Region: chr8 9498130-9498145. Max. coverage (+): 0. Max coverage (-): 0

Region: chr8 9498146-9498160. Max. coverage (+): 0. Max coverage (-): 0

Region: chr8 9498161-9498176. Max. coverage (+): 0. Max coverage (-): 0

Region: chr8 9498177-9498191. Max. coverage (+): 0. Max coverage (-): 0

Region: chr8 9498192-9498206. Max. coverage (+): 0. Max coverage (-): 1.74

Region: chr8 9498207-9498222. Max. coverage (+): 0. Max coverage (-): 0

Region: chr8 9498223-9498237. Max. coverage (+): 0. Max coverage (-): 0

Region: chr8 9498238-9498253. Max. coverage (+): 0. Max coverage (-): 0

Region: chr8 9498254-9498268. Max. coverage (+): 0. Max coverage (-): 0

Region: chr8 9498269-9498283. Max. coverage (+): 0. Max coverage (-): 0

Region: chr8 9498284-9498299. Max. coverage (+): 0. Max coverage (-): 0

Region: chr8 9498300-9498314. Max. coverage (+): 0. Max coverage (-): 0

Region: chr8 9498315-9498329. Max. coverage (+): 0. Max coverage (-): 0

Region: chr8 9498330-9498345. Max. coverage (+): 0. Max coverage (-): 0

Region: chr8 9498346-9498360. Max. coverage (+): 0. Max coverage (-): 0

Region: chr8 9498361-9498376. Max. coverage (+): 0. Max coverage (-): 0

Region: chr8 9498377-9498391. Max. coverage (+): 0. Max coverage (-): 0

Region: chr8 9498392-9498406. Max. coverage (+): 0. Max coverage (-): 0

Region: chr8 9498407-9498422. Max. coverage (+): 0. Max coverage (-): 0

Region: chr8 9498423-9498437. Max. coverage (+): 0. Max coverage (-): 0

Region: chr8 9498438-9498452. Max. coverage (+): 0. Max coverage (-): 0

Region: chr8 9498453-9498468. Max. coverage (+): 0. Max coverage (-): 0

Region: chr8 9498469-9498483. Max. coverage (+): 0. Max coverage (-): 0

Region: chr8 9498484-9498499. Max. coverage (+): 0. Max coverage (-): 1.68

Region: chr8 9498500-9498514. Max. coverage (+): 0. Max coverage (-): 0

Region: chr8 9498515-9498529. Max. coverage (+): 0. Max coverage (-): 0

Region: chr8 9498530-9498545. Max. coverage (+): 0. Max coverage (-): 0

Region: chr8 9498546-9498560. Max. coverage (+): 0. Max coverage (-): 0

Region: chr8 9498561-9498576. Max. coverage (+): 0. Max coverage (-): 0

Region: chr8 9498577-9498591. Max. coverage (+): 0. Max coverage (-): 0

Region: chr8 9498592-9498606. Max. coverage (+): 0. Max coverage (-): 0

Region: chr8 9498607-9498622. Max. coverage (+): 0. Max coverage (-): 0

Region: chr8 9498623-9498637. Max. coverage (+): 0. Max coverage (-): 0

Region: chr8 9498638-9498652. Max. coverage (+): 0. Max coverage (-): 0

Region: chr8 9498653-9498668. Max. coverage (+): 0. Max coverage (-): 0

Region: chr8 9498669-9498683. Max. coverage (+): 0. Max coverage (-): 0

Region: chr8 9498684-9498699. Max. coverage (+): 0. Max coverage (-): 0

Region: chr8 9498700-9498714. Max. coverage (+): 0. Max coverage (-): 0

Region: chr8 9498715-9498729. Max. coverage (+): 0. Max coverage (-): 0

Region: chr8 9498730-9498745. Max. coverage (+): 0. Max coverage (-): 0

Region: chr8 9498746-9498760. Max. coverage (+): 0. Max coverage (-): 0

Region: chr8 9498761-9498776. Max. coverage (+): 0. Max coverage (-): 0

Region: chr8 9498777-9498791. Max. coverage (+): 0. Max coverage (-): 0

Region: chr8 9498792-9498806. Max. coverage (+): 0. Max coverage (-): 0

Region: chr8 9498807-9498822. Max. coverage (+): 0. Max coverage (-): 0

Region: chr8 9498823-9498837. Max. coverage (+): 0. Max coverage (-): 0

Region: chr8 9498838-9498852. Max. coverage (+): 0. Max coverage (-): 0.88

Region: chr8 9498853-9498868. Max. coverage (+): 0. Max coverage (-): 0

Region: chr8 9498869-. Max. coverage (+): 0. Max coverage (-): 0

RepeatMasker Color Code

**+**

100-98% Identity

<98-95% Identity

<95-90% Identity

<90-85% Identity

<85-80% Identity

<80-75% Identity

<75-70% Identity

<70% Identity

**-**

Gene Set Color Code

**+**

Gene

Pseudogene

**-**

Topology/Coverage Color Code

Coverage Plus Strand

Coverage Minus Strand

Mainstrand: Plus

Mainstrand: Minus

Complementary Strand

Flanking Region  
(if option -flank >0)

Gene Set Annotation  
  
RepeatMasker Annotation  

**1. Bov-tA1**: 9491389-9491563 (-), Divergence to consensus: 13.2%  
**2. BOV-A2**: 9492353-9492624 (-), Divergence to consensus: 5.5%  
**3. BovB**: 9496079-9496184 (+), Divergence to consensus: 13.2%  
**4. ART2A**: 9496185-9496716 (+), Divergence to consensus: 9.5%

  
Transcription Factor Binding Sites  

**RFX4\_2** (Sequence: GTATCCAGG (-): 9493736)  
**RFX4\_2** (Sequence: GTAACTAAG (-): 9495340)  
**RFX4\_2** (Sequence: CTTGGATAC (+): 9491965)  
**RFX4\_2** (Sequence: CATGGTTAC (+): 9494894)
