# Supplementary material for: Small RNA sequencing of cryopreserved semen from single bull revealed altered miRNAs and piRNAs expression between High- and Low-motile sperm populations
Source: BMC Genomics. 2017 Jan 4;18:14. doi: 10.1186/s12864-016-3394-7 (PMC5209821; doi:10.1186/s12864-016-3394-7)
Supplement: Additional file 3: — Details for each piRNA clusters found in High Motile (HM) sperm fraction. Genes, repeats, transposable elements and transcription factors binding sites falling within the cluster regions were reported. (ZIP 1896 kb) [file 12864_2016_3394_MOESM3_ESM.zip › 92.html]

piRNA cluster 92


Predicted piRNA cluster no. 92     previous   next
  

Show proTRAC run info
Hide proTRAC run info

================================= proTRAC ====================================  
VERSION: 2.1                                    LAST MODIFIED: 06. October 2015  
  
Please cite:  
Rosenkranz D, Zischler H. proTRAC - a software for probabilistic piRNA cluster  
detection, visualization and analysis. 2012. BMC Bioinformatics 13:5.  
  
and (for proTRAC 2.0 and later):  
Rosenkranz D, Rudloff S, Bastuck K, Ketting RF, Zischler H. Tupaia small RNAs  
provide insights into function and evolution of RNAi-based transposon defense  
in mammals. 2015. RNA 21(5):911-922.  
  
Contact:  
David Rosenkranz  
Institute of Anthropology, small RNA group  
Johannes Gutenberg University Mainz  
email: rosenkranz@uni-mainz.de  
  
You can find the latest proTRAC version at:  
http://sourceforge.net/projects/protrac/files  
http://www.smallRNAgroup-mainz.de/software  
==============================================================================  
  
PARAMETERS:  
Map file: .............../storage/core/barbara/genhome/smallRNA/fertility/Sample\_motile/pirna/Sample\_motile\_26-33\_collapsed.fa.no-dust.map.weighted-10000-1000-b-0  
Genome file: ............/storage/core/barbara/genhome/smallRNA/fertility/Sample\_all/pirna/bt\_311\_chrY.fa  
RepeatMasker annotation: /storage/genomes/bt\_umd31/GCF\_000003055.6\_Bos\_taurus\_UMD\_3.1.1\_repeatMasker\_chr.out  
GeneSet:................./storage/core/barbara/genhome/smallRNA/fertility/Sample\_all/pirna/full.gtf  
  
Significant (p<=0.01) hit density will be calculated based  
on observed hit distribution.  
  
Sliding window size: ........................................ 5000 bp  
Sliding window increament: .................................. 1000 bp  
Normalize each hit by number of genomic hits: ............... 1 [0=no/1=yes]  
Normalize each hit by number of sequence reads: ............. 1 [0=no/1=yes]  
Normalize values (-> per million mapped reads): ............. 1 [0=no/1=yes]  
Min. fraction of hits with 1T(U) or 10A: .................... 0.75  
Alternatively: Min. fraction of hits with 1T(U) and 10A: .... 0.5  
Min. fraction of hits with typical piRNA length: ............ 0.75  
Typical piRNA length: ....................................... 26-33 nt  
Min. size of a piRNA cluster: ............................... 5000 bp.  
Min. number of hits (absolute): ............................. 0  
Min. number of hits (normalized): ........................... 0  
Min. fraction of hits on the mainstrand: .................... 0.75  
Top fraction of mapped sequences (in terms of read counts): . 1%  
Top fraction accounts for max. n% of sequence reads: ........ 90%  
Min. fraction of hits on each arm of a bidirectional cluster: 0.1  
Output image file for each cluster: ......................... 0 [0=no/1=yes]  
Output html file for each cluster: .......................... 1 [0=no/1=yes]  
Output a summary table: ..................................... 1 [0=no/1=yes]  
Output a FASTA file for each cluster (piRNA sequences): ..... 1 [0=no/1=yes]  
Output a FASTA file comprising cluster sequences: ........... 1 [0=no/1=yes]  
Search DNA motifs in clusters: .............................. 1 [0=no/1=yes]  
Output flanking sequences: +/- .............................. 0 bp  
Output ~.pTi file: .......................................... 1 [0=no/1=yes]  
==============================================================================  
  
  
Genome size (without gaps): ............ 2678902517 bp  
Gaps (N/X/-): .......................... 53837044 bp  
Mapped reads: .......................... 658825247023  
Non-identical sequences: ............... 514171  
Genomic hits: .......................... 764233  
Significant densitiy of mapped reads: .. 12867599.5173724 reads/kb

Show proTRAC cluster info
Hide proTRAC cluster info

|  |  |
| --- | --- |
| Location | chr8 |
| Coordinates | 12685158-12692120 |
| Size [bp] | 6963 |
| Sequence hit loci | 281 |
| Mapped reads (normalized) | 341157046 |
| Mapped reads (normalized) per kb | 48995698.1 |
| Normalized reads with 1T (1U) | 86.4% |
| Normalized reads with 10A | 28.8% |
| Normalized reads with length 26-33 nt | 100% |
| Normalized reads on the main strand(s) | 93.9% |
| Predicted directionality | mono:plus |

100%

0%

1T (1U)  
reads

10A reads

26-33 nt  
reads

reads on mainstrand

**Either the amount of reads with 1T (1U) OR 10A has to exceed 75% (set with option: -1Tor10A)  
Alternatively the amount of reads with 1T (1U) AND 10A has to exceed 50% (set with option: -1Tand10A)  
Minimum amount of reads with preferred size is 75% (set with option: -pisize)  
Minimum amount of reads on the main strand(s) is 75% (set with option: -clstrand)**

Show read coverage
Hide read coverage

WHAT DO I SEE HERE?  
This chart shows the location of mapped sequence reads within a predicted piRNA cluster. The color refers to the number of genomic hits produced by the sequence read in question. A dark red bar indicates that this sequence read produces many other hits elsewhere in the genome. Many adjacent red or yellow bars can indicate the presence of a multi-copy element such as transposons or rRNA genes. A dark green bar indicates that this sequence read maps uniquely to this locus.

1 hit

2-5 hits

6-10 hits

11-20 hits

21-50 hits

51-100 hits

> 100 hits

chr8

12685158

12692120

Gene Set

RepeatMasker

Mapped  
Reads

58.09

plus strand

minus strand

58.09

Region: chr8 9498870-12685164. Max. coverage (+): 0. Max coverage (-): 0.91

Region: chr8 12685165-12685178. Max. coverage (+): 0. Max coverage (-): 0.91

Region: chr8 12685179-12685192. Max. coverage (+): 0. Max coverage (-): 0

Region: chr8 12685193-12685206. Max. coverage (+): 0. Max coverage (-): 0

Region: chr8 12685207-12685220. Max. coverage (+): 0. Max coverage (-): 0

Region: chr8 12685221-12685234. Max. coverage (+): 0. Max coverage (-): 0

Region: chr8 12685235-12685248. Max. coverage (+): 0. Max coverage (-): 0

Region: chr8 12685249-12685262. Max. coverage (+): 0. Max coverage (-): 0

Region: chr8 12685263-12685276. Max. coverage (+): 0. Max coverage (-): 0

Region: chr8 12685277-12685290. Max. coverage (+): 0. Max coverage (-): 0

Region: chr8 12685291-12685304. Max. coverage (+): 0. Max coverage (-): 0

Region: chr8 12685305-12685318. Max. coverage (+): 0. Max coverage (-): 0

Region: chr8 12685319-12685332. Max. coverage (+): 0. Max coverage (-): 0

Region: chr8 12685333-12685346. Max. coverage (+): 0. Max coverage (-): 0

Region: chr8 12685347-12685359. Max. coverage (+): 0. Max coverage (-): 0

Region: chr8 12685360-12685373. Max. coverage (+): 0. Max coverage (-): 0

Region: chr8 12685374-12685387. Max. coverage (+): 0. Max coverage (-): 0

Region: chr8 12685388-12685401. Max. coverage (+): 0. Max coverage (-): 0

Region: chr8 12685402-12685415. Max. coverage (+): 0. Max coverage (-): 0

Region: chr8 12685416-12685429. Max. coverage (+): 0. Max coverage (-): 0

Region: chr8 12685430-12685443. Max. coverage (+): 0. Max coverage (-): 0

Region: chr8 12685444-12685457. Max. coverage (+): 0. Max coverage (-): 0

Region: chr8 12685458-12685471. Max. coverage (+): 0. Max coverage (-): 0

Region: chr8 12685472-12685485. Max. coverage (+): 0. Max coverage (-): 0

Region: chr8 12685486-12685499. Max. coverage (+): 0. Max coverage (-): 0

Region: chr8 12685500-12685513. Max. coverage (+): 0. Max coverage (-): 0

Region: chr8 12685514-12685527. Max. coverage (+): 0. Max coverage (-): 0

Region: chr8 12685528-12685540. Max. coverage (+): 0. Max coverage (-): 3.37

Region: chr8 12685541-12685554. Max. coverage (+): 0. Max coverage (-): 0

Region: chr8 12685555-12685568. Max. coverage (+): 0. Max coverage (-): 0

Region: chr8 12685569-12685582. Max. coverage (+): 0. Max coverage (-): 0

Region: chr8 12685583-12685596. Max. coverage (+): 0. Max coverage (-): 0

Region: chr8 12685597-12685610. Max. coverage (+): 0. Max coverage (-): 0

Region: chr8 12685611-12685624. Max. coverage (+): 0. Max coverage (-): 0

Region: chr8 12685625-12685638. Max. coverage (+): 0. Max coverage (-): 0

Region: chr8 12685639-12685652. Max. coverage (+): 0. Max coverage (-): 0

Region: chr8 12685653-12685666. Max. coverage (+): 0. Max coverage (-): 0

Region: chr8 12685667-12685680. Max. coverage (+): 0. Max coverage (-): 0

Region: chr8 12685681-12685694. Max. coverage (+): 0. Max coverage (-): 0

Region: chr8 12685695-12685708. Max. coverage (+): 0. Max coverage (-): 0

Region: chr8 12685709-12685722. Max. coverage (+): 0. Max coverage (-): 0

Region: chr8 12685723-12685735. Max. coverage (+): 0. Max coverage (-): 0

Region: chr8 12685736-12685749. Max. coverage (+): 0. Max coverage (-): 0

Region: chr8 12685750-12685763. Max. coverage (+): 0. Max coverage (-): 0

Region: chr8 12685764-12685777. Max. coverage (+): 0. Max coverage (-): 0

Region: chr8 12685778-12685791. Max. coverage (+): 0. Max coverage (-): 0

Region: chr8 12685792-12685805. Max. coverage (+): 0. Max coverage (-): 0

Region: chr8 12685806-12685819. Max. coverage (+): 0. Max coverage (-): 0

Region: chr8 12685820-12685833. Max. coverage (+): 0. Max coverage (-): 0

Region: chr8 12685834-12685847. Max. coverage (+): 0. Max coverage (-): 0

Region: chr8 12685848-12685861. Max. coverage (+): 0. Max coverage (-): 0

Region: chr8 12685862-12685875. Max. coverage (+): 0. Max coverage (-): 0

Region: chr8 12685876-12685889. Max. coverage (+): 0. Max coverage (-): 0

Region: chr8 12685890-12685903. Max. coverage (+): 0. Max coverage (-): 0

Region: chr8 12685904-12685916. Max. coverage (+): 0. Max coverage (-): 0

Region: chr8 12685917-12685930. Max. coverage (+): 0. Max coverage (-): 0

Region: chr8 12685931-12685944. Max. coverage (+): 0. Max coverage (-): 0

Region: chr8 12685945-12685958. Max. coverage (+): 0. Max coverage (-): 0

Region: chr8 12685959-12685972. Max. coverage (+): 0. Max coverage (-): 0

Region: chr8 12685973-12685986. Max. coverage (+): 0. Max coverage (-): 0

Region: chr8 12685987-12686000. Max. coverage (+): 0. Max coverage (-): 0

Region: chr8 12686001-12686014. Max. coverage (+): 0. Max coverage (-): 0

Region: chr8 12686015-12686028. Max. coverage (+): 0. Max coverage (-): 0

Region: chr8 12686029-12686042. Max. coverage (+): 0. Max coverage (-): 0

Region: chr8 12686043-12686056. Max. coverage (+): 0. Max coverage (-): 0

Region: chr8 12686057-12686070. Max. coverage (+): 0. Max coverage (-): 0

Region: chr8 12686071-12686084. Max. coverage (+): 0. Max coverage (-): 0

Region: chr8 12686085-12686098. Max. coverage (+): 0. Max coverage (-): 0

Region: chr8 12686099-12686111. Max. coverage (+): 0. Max coverage (-): 0

Region: chr8 12686112-12686125. Max. coverage (+): 0. Max coverage (-): 0

Region: chr8 12686126-12686139. Max. coverage (+): 0. Max coverage (-): 0

Region: chr8 12686140-12686153. Max. coverage (+): 0. Max coverage (-): 0

Region: chr8 12686154-12686167. Max. coverage (+): 0. Max coverage (-): 0

Region: chr8 12686168-12686181. Max. coverage (+): 0. Max coverage (-): 0

Region: chr8 12686182-12686195. Max. coverage (+): 0. Max coverage (-): 0

Region: chr8 12686196-12686209. Max. coverage (+): 0. Max coverage (-): 0

Region: chr8 12686210-12686223. Max. coverage (+): 0. Max coverage (-): 0

Region: chr8 12686224-12686237. Max. coverage (+): 0. Max coverage (-): 0

Region: chr8 12686238-12686251. Max. coverage (+): 0. Max coverage (-): 0

Region: chr8 12686252-12686265. Max. coverage (+): 0. Max coverage (-): 0

Region: chr8 12686266-12686279. Max. coverage (+): 0. Max coverage (-): 0

Region: chr8 12686280-12686292. Max. coverage (+): 0. Max coverage (-): 0

Region: chr8 12686293-12686306. Max. coverage (+): 0. Max coverage (-): 0

Region: chr8 12686307-12686320. Max. coverage (+): 0. Max coverage (-): 0

Region: chr8 12686321-12686334. Max. coverage (+): 0. Max coverage (-): 0

Region: chr8 12686335-12686348. Max. coverage (+): 0. Max coverage (-): 0

Region: chr8 12686349-12686362. Max. coverage (+): 0. Max coverage (-): 0

Region: chr8 12686363-12686376. Max. coverage (+): 0. Max coverage (-): 0

Region: chr8 12686377-12686390. Max. coverage (+): 0. Max coverage (-): 0

Region: chr8 12686391-12686404. Max. coverage (+): 0. Max coverage (-): 0

Region: chr8 12686405-12686418. Max. coverage (+): 0. Max coverage (-): 0

Region: chr8 12686419-12686432. Max. coverage (+): 0. Max coverage (-): 0

Region: chr8 12686433-12686446. Max. coverage (+): 0. Max coverage (-): 0

Region: chr8 12686447-12686460. Max. coverage (+): 0. Max coverage (-): 0

Region: chr8 12686461-12686474. Max. coverage (+): 0. Max coverage (-): 0

Region: chr8 12686475-12686487. Max. coverage (+): 0. Max coverage (-): 0

Region: chr8 12686488-12686501. Max. coverage (+): 0. Max coverage (-): 0

Region: chr8 12686502-12686515. Max. coverage (+): 0. Max coverage (-): 0

Region: chr8 12686516-12686529. Max. coverage (+): 0. Max coverage (-): 0

Region: chr8 12686530-12686543. Max. coverage (+): 0. Max coverage (-): 0

Region: chr8 12686544-12686557. Max. coverage (+): 0. Max coverage (-): 0

Region: chr8 12686558-12686571. Max. coverage (+): 0. Max coverage (-): 0

Region: chr8 12686572-12686585. Max. coverage (+): 0. Max coverage (-): 0

Region: chr8 12686586-12686599. Max. coverage (+): 0. Max coverage (-): 0

Region: chr8 12686600-12686613. Max. coverage (+): 0. Max coverage (-): 0

Region: chr8 12686614-12686627. Max. coverage (+): 0. Max coverage (-): 0

Region: chr8 12686628-12686641. Max. coverage (+): 0. Max coverage (-): 0

Region: chr8 12686642-12686655. Max. coverage (+): 0. Max coverage (-): 0

Region: chr8 12686656-12686668. Max. coverage (+): 0. Max coverage (-): 0

Region: chr8 12686669-12686682. Max. coverage (+): 0. Max coverage (-): 0

Region: chr8 12686683-12686696. Max. coverage (+): 0. Max coverage (-): 0

Region: chr8 12686697-12686710. Max. coverage (+): 0. Max coverage (-): 0

Region: chr8 12686711-12686724. Max. coverage (+): 0. Max coverage (-): 0

Region: chr8 12686725-12686738. Max. coverage (+): 0. Max coverage (-): 0

Region: chr8 12686739-12686752. Max. coverage (+): 0. Max coverage (-): 0

Region: chr8 12686753-12686766. Max. coverage (+): 0. Max coverage (-): 0

Region: chr8 12686767-12686780. Max. coverage (+): 0. Max coverage (-): 0

Region: chr8 12686781-12686794. Max. coverage (+): 0. Max coverage (-): 0

Region: chr8 12686795-12686808. Max. coverage (+): 0. Max coverage (-): 0

Region: chr8 12686809-12686822. Max. coverage (+): 0. Max coverage (-): 0

Region: chr8 12686823-12686836. Max. coverage (+): 0. Max coverage (-): 0

Region: chr8 12686837-12686850. Max. coverage (+): 0. Max coverage (-): 0

Region: chr8 12686851-12686863. Max. coverage (+): 0. Max coverage (-): 0

Region: chr8 12686864-12686877. Max. coverage (+): 0. Max coverage (-): 0

Region: chr8 12686878-12686891. Max. coverage (+): 1.59. Max coverage (-): 0

Region: chr8 12686892-12686905. Max. coverage (+): 1.59. Max coverage (-): 0

Region: chr8 12686906-12686919. Max. coverage (+): 6.56. Max coverage (-): 1.24

Region: chr8 12686920-12686933. Max. coverage (+): 6.56. Max coverage (-): 0

Region: chr8 12686934-12686947. Max. coverage (+): 15.28. Max coverage (-): 0

Region: chr8 12686948-12686961. Max. coverage (+): 15.28. Max coverage (-): 0

Region: chr8 12686962-12686975. Max. coverage (+): 0. Max coverage (-): 0

Region: chr8 12686976-12686989. Max. coverage (+): 0. Max coverage (-): 4.56

Region: chr8 12686990-12687003. Max. coverage (+): 1.05. Max coverage (-): 0

Region: chr8 12687004-12687017. Max. coverage (+): 11.46. Max coverage (-): 0

Region: chr8 12687018-12687031. Max. coverage (+): 15.36. Max coverage (-): 0

Region: chr8 12687032-12687044. Max. coverage (+): 23.36. Max coverage (-): 0

Region: chr8 12687045-12687058. Max. coverage (+): 20.84. Max coverage (-): 0

Region: chr8 12687059-12687072. Max. coverage (+): 0. Max coverage (-): 1.49

Region: chr8 12687073-12687086. Max. coverage (+): 0. Max coverage (-): 1.49

Region: chr8 12687087-12687100. Max. coverage (+): 5.3. Max coverage (-): 0

Region: chr8 12687101-12687114. Max. coverage (+): 5.3. Max coverage (-): 0

Region: chr8 12687115-12687128. Max. coverage (+): 0. Max coverage (-): 0

Region: chr8 12687129-12687142. Max. coverage (+): 0. Max coverage (-): 0

Region: chr8 12687143-12687156. Max. coverage (+): 0. Max coverage (-): 7.7

Region: chr8 12687157-12687170. Max. coverage (+): 0. Max coverage (-): 3.92

Region: chr8 12687171-12687184. Max. coverage (+): 0. Max coverage (-): 0

Region: chr8 12687185-12687198. Max. coverage (+): 0. Max coverage (-): 0.71

Region: chr8 12687199-12687212. Max. coverage (+): 0. Max coverage (-): 1.64

Region: chr8 12687213-12687226. Max. coverage (+): 2.4. Max coverage (-): 0

Region: chr8 12687227-12687239. Max. coverage (+): 2.4. Max coverage (-): 0

Region: chr8 12687240-12687253. Max. coverage (+): 40.09. Max coverage (-): 0

Region: chr8 12687254-12687267. Max. coverage (+): 41.58. Max coverage (-): 0

Region: chr8 12687268-12687281. Max. coverage (+): 0. Max coverage (-): 0

Region: chr8 12687282-12687295. Max. coverage (+): 0. Max coverage (-): 0

Region: chr8 12687296-12687309. Max. coverage (+): 1.11. Max coverage (-): 4.48

Region: chr8 12687310-12687323. Max. coverage (+): 1.11. Max coverage (-): 4.48

Region: chr8 12687324-12687337. Max. coverage (+): 0. Max coverage (-): 0

Region: chr8 12687338-12687351. Max. coverage (+): 0. Max coverage (-): 0

Region: chr8 12687352-12687365. Max. coverage (+): 0. Max coverage (-): 0

Region: chr8 12687366-12687379. Max. coverage (+): 0. Max coverage (-): 0

Region: chr8 12687380-12687393. Max. coverage (+): 0. Max coverage (-): 0

Region: chr8 12687394-12687407. Max. coverage (+): 0. Max coverage (-): 0

Region: chr8 12687408-12687420. Max. coverage (+): 0. Max coverage (-): 0

Region: chr8 12687421-12687434. Max. coverage (+): 0. Max coverage (-): 0

Region: chr8 12687435-12687448. Max. coverage (+): 0. Max coverage (-): 0

Region: chr8 12687449-12687462. Max. coverage (+): 0. Max coverage (-): 0

Region: chr8 12687463-12687476. Max. coverage (+): 4.6. Max coverage (-): 0

Region: chr8 12687477-12687490. Max. coverage (+): 0. Max coverage (-): 0

Region: chr8 12687491-12687504. Max. coverage (+): 3.69. Max coverage (-): 0

Region: chr8 12687505-12687518. Max. coverage (+): 0. Max coverage (-): 0.79

Region: chr8 12687519-12687532. Max. coverage (+): 0. Max coverage (-): 0.79

Region: chr8 12687533-12687546. Max. coverage (+): 0. Max coverage (-): 0

Region: chr8 12687547-12687560. Max. coverage (+): 0. Max coverage (-): 0

Region: chr8 12687561-12687574. Max. coverage (+): 0. Max coverage (-): 0

Region: chr8 12687575-12687588. Max. coverage (+): 0. Max coverage (-): 0

Region: chr8 12687589-12687602. Max. coverage (+): 0. Max coverage (-): 0

Region: chr8 12687603-12687615. Max. coverage (+): 0. Max coverage (-): 0

Region: chr8 12687616-12687629. Max. coverage (+): 0. Max coverage (-): 0

Region: chr8 12687630-12687643. Max. coverage (+): 0. Max coverage (-): 0

Region: chr8 12687644-12687657. Max. coverage (+): 0. Max coverage (-): 1.32

Region: chr8 12687658-12687671. Max. coverage (+): 0. Max coverage (-): 1.32

Region: chr8 12687672-12687685. Max. coverage (+): 0. Max coverage (-): 0

Region: chr8 12687686-12687699. Max. coverage (+): 0.93. Max coverage (-): 0

Region: chr8 12687700-12687713. Max. coverage (+): 0.93. Max coverage (-): 0

Region: chr8 12687714-12687727. Max. coverage (+): 0. Max coverage (-): 0

Region: chr8 12687728-12687741. Max. coverage (+): 0. Max coverage (-): 0

Region: chr8 12687742-12687755. Max. coverage (+): 0. Max coverage (-): 0

Region: chr8 12687756-12687769. Max. coverage (+): 0. Max coverage (-): 0

Region: chr8 12687770-12687783. Max. coverage (+): 0. Max coverage (-): 2.08

Region: chr8 12687784-12687796. Max. coverage (+): 0. Max coverage (-): 0

Region: chr8 12687797-12687810. Max. coverage (+): 0. Max coverage (-): 0

Region: chr8 12687811-12687824. Max. coverage (+): 0. Max coverage (-): 0

Region: chr8 12687825-12687838. Max. coverage (+): 0. Max coverage (-): 0

Region: chr8 12687839-12687852. Max. coverage (+): 0. Max coverage (-): 0

Region: chr8 12687853-12687866. Max. coverage (+): 0. Max coverage (-): 0

Region: chr8 12687867-12687880. Max. coverage (+): 0. Max coverage (-): 0

Region: chr8 12687881-12687894. Max. coverage (+): 0. Max coverage (-): 0

Region: chr8 12687895-12687908. Max. coverage (+): 0. Max coverage (-): 0

Region: chr8 12687909-12687922. Max. coverage (+): 0. Max coverage (-): 0

Region: chr8 12687923-12687936. Max. coverage (+): 0. Max coverage (-): 0

Region: chr8 12687937-12687950. Max. coverage (+): 0. Max coverage (-): 0

Region: chr8 12687951-12687964. Max. coverage (+): 0. Max coverage (-): 0

Region: chr8 12687965-12687978. Max. coverage (+): 0. Max coverage (-): 0

Region: chr8 12687979-12687991. Max. coverage (+): 0. Max coverage (-): 0

Region: chr8 12687992-12688005. Max. coverage (+): 0. Max coverage (-): 0

Region: chr8 12688006-12688019. Max. coverage (+): 0. Max coverage (-): 0

Region: chr8 12688020-12688033. Max. coverage (+): 0. Max coverage (-): 0

Region: chr8 12688034-12688047. Max. coverage (+): 0. Max coverage (-): 0

Region: chr8 12688048-12688061. Max. coverage (+): 0. Max coverage (-): 0

Region: chr8 12688062-12688075. Max. coverage (+): 0. Max coverage (-): 0

Region: chr8 12688076-12688089. Max. coverage (+): 0. Max coverage (-): 0

Region: chr8 12688090-12688103. Max. coverage (+): 0. Max coverage (-): 0

Region: chr8 12688104-12688117. Max. coverage (+): 0. Max coverage (-): 0

Region: chr8 12688118-12688131. Max. coverage (+): 0. Max coverage (-): 0

Region: chr8 12688132-12688145. Max. coverage (+): 0. Max coverage (-): 0

Region: chr8 12688146-12688159. Max. coverage (+): 0. Max coverage (-): 0

Region: chr8 12688160-12688172. Max. coverage (+): 0. Max coverage (-): 0

Region: chr8 12688173-12688186. Max. coverage (+): 0. Max coverage (-): 0

Region: chr8 12688187-12688200. Max. coverage (+): 5.95. Max coverage (-): 0

Region: chr8 12688201-12688214. Max. coverage (+): 8.66. Max coverage (-): 0

Region: chr8 12688215-12688228. Max. coverage (+): 4.79. Max coverage (-): 0

Region: chr8 12688229-12688242. Max. coverage (+): 7.05. Max coverage (-): 0

Region: chr8 12688243-12688256. Max. coverage (+): 7.05. Max coverage (-): 0

Region: chr8 12688257-12688270. Max. coverage (+): 0. Max coverage (-): 0

Region: chr8 12688271-12688284. Max. coverage (+): 24.11. Max coverage (-): 0

Region: chr8 12688285-12688298. Max. coverage (+): 58.09. Max coverage (-): 0

Region: chr8 12688299-12688312. Max. coverage (+): 17.51. Max coverage (-): 0

Region: chr8 12688313-12688326. Max. coverage (+): 0. Max coverage (-): 0

Region: chr8 12688327-12688340. Max. coverage (+): 2.22. Max coverage (-): 0

Region: chr8 12688341-12688354. Max. coverage (+): 2.22. Max coverage (-): 0

Region: chr8 12688355-12688367. Max. coverage (+): 21.58. Max coverage (-): 0

Region: chr8 12688368-12688381. Max. coverage (+): 35.57. Max coverage (-): 0

Region: chr8 12688382-12688395. Max. coverage (+): 5.61. Max coverage (-): 0

Region: chr8 12688396-12688409. Max. coverage (+): 6.36. Max coverage (-): 0

Region: chr8 12688410-12688423. Max. coverage (+): 4.71. Max coverage (-): 0

Region: chr8 12688424-12688437. Max. coverage (+): 5.06. Max coverage (-): 0

Region: chr8 12688438-12688451. Max. coverage (+): 9.71. Max coverage (-): 0

Region: chr8 12688452-12688465. Max. coverage (+): 29.64. Max coverage (-): 0

Region: chr8 12688466-12688479. Max. coverage (+): 15.93. Max coverage (-): 0

Region: chr8 12688480-12688493. Max. coverage (+): 0. Max coverage (-): 0

Region: chr8 12688494-12688507. Max. coverage (+): 4.69. Max coverage (-): 0

Region: chr8 12688508-12688521. Max. coverage (+): 10.52. Max coverage (-): 0

Region: chr8 12688522-12688535. Max. coverage (+): 0. Max coverage (-): 0

Region: chr8 12688536-12688548. Max. coverage (+): 0. Max coverage (-): 0

Region: chr8 12688549-12688562. Max. coverage (+): 8.59. Max coverage (-): 0

Region: chr8 12688563-12688576. Max. coverage (+): 11.74. Max coverage (-): 0

Region: chr8 12688577-12688590. Max. coverage (+): 14.29. Max coverage (-): 0

Region: chr8 12688591-12688604. Max. coverage (+): 13.95. Max coverage (-): 0

Region: chr8 12688605-12688618. Max. coverage (+): 3.62. Max coverage (-): 0

Region: chr8 12688619-12688632. Max. coverage (+): 0. Max coverage (-): 0

Region: chr8 12688633-12688646. Max. coverage (+): 0. Max coverage (-): 0

Region: chr8 12688647-12688660. Max. coverage (+): 0. Max coverage (-): 0

Region: chr8 12688661-12688674. Max. coverage (+): 0. Max coverage (-): 0

Region: chr8 12688675-12688688. Max. coverage (+): 0. Max coverage (-): 0

Region: chr8 12688689-12688702. Max. coverage (+): 0. Max coverage (-): 0

Region: chr8 12688703-12688716. Max. coverage (+): 0. Max coverage (-): 0

Region: chr8 12688717-12688730. Max. coverage (+): 0. Max coverage (-): 0

Region: chr8 12688731-12688743. Max. coverage (+): 0. Max coverage (-): 0

Region: chr8 12688744-12688757. Max. coverage (+): 0. Max coverage (-): 0

Region: chr8 12688758-12688771. Max. coverage (+): 0. Max coverage (-): 0

Region: chr8 12688772-12688785. Max. coverage (+): 0. Max coverage (-): 0

Region: chr8 12688786-12688799. Max. coverage (+): 0. Max coverage (-): 0

Region: chr8 12688800-12688813. Max. coverage (+): 0. Max coverage (-): 0

Region: chr8 12688814-12688827. Max. coverage (+): 0. Max coverage (-): 0

Region: chr8 12688828-12688841. Max. coverage (+): 0. Max coverage (-): 0

Region: chr8 12688842-12688855. Max. coverage (+): 0. Max coverage (-): 0

Region: chr8 12688856-12688869. Max. coverage (+): 0. Max coverage (-): 0

Region: chr8 12688870-12688883. Max. coverage (+): 0. Max coverage (-): 0

Region: chr8 12688884-12688897. Max. coverage (+): 0. Max coverage (-): 0

Region: chr8 12688898-12688911. Max. coverage (+): 0. Max coverage (-): 0

Region: chr8 12688912-12688924. Max. coverage (+): 0. Max coverage (-): 0

Region: chr8 12688925-12688938. Max. coverage (+): 0. Max coverage (-): 0

Region: chr8 12688939-12688952. Max. coverage (+): 0. Max coverage (-): 0

Region: chr8 12688953-12688966. Max. coverage (+): 0. Max coverage (-): 0

Region: chr8 12688967-12688980. Max. coverage (+): 0. Max coverage (-): 0

Region: chr8 12688981-12688994. Max. coverage (+): 2.14. Max coverage (-): 0

Region: chr8 12688995-12689008. Max. coverage (+): 0. Max coverage (-): 0

Region: chr8 12689009-12689022. Max. coverage (+): 0. Max coverage (-): 0

Region: chr8 12689023-12689036. Max. coverage (+): 0. Max coverage (-): 0

Region: chr8 12689037-12689050. Max. coverage (+): 0. Max coverage (-): 0

Region: chr8 12689051-12689064. Max. coverage (+): 0. Max coverage (-): 0

Region: chr8 12689065-12689078. Max. coverage (+): 0. Max coverage (-): 0

Region: chr8 12689079-12689092. Max. coverage (+): 0. Max coverage (-): 0

Region: chr8 12689093-12689106. Max. coverage (+): 0. Max coverage (-): 0

Region: chr8 12689107-12689119. Max. coverage (+): 0. Max coverage (-): 0

Region: chr8 12689120-12689133. Max. coverage (+): 0. Max coverage (-): 0

Region: chr8 12689134-12689147. Max. coverage (+): 0. Max coverage (-): 0

Region: chr8 12689148-12689161. Max. coverage (+): 0. Max coverage (-): 0

Region: chr8 12689162-12689175. Max. coverage (+): 0. Max coverage (-): 0

Region: chr8 12689176-12689189. Max. coverage (+): 0. Max coverage (-): 0

Region: chr8 12689190-12689203. Max. coverage (+): 0. Max coverage (-): 0

Region: chr8 12689204-12689217. Max. coverage (+): 0. Max coverage (-): 0

Region: chr8 12689218-12689231. Max. coverage (+): 0. Max coverage (-): 0

Region: chr8 12689232-12689245. Max. coverage (+): 0. Max coverage (-): 0

Region: chr8 12689246-12689259. Max. coverage (+): 0. Max coverage (-): 0

Region: chr8 12689260-12689273. Max. coverage (+): 0. Max coverage (-): 0

Region: chr8 12689274-12689287. Max. coverage (+): 0. Max coverage (-): 0

Region: chr8 12689288-12689300. Max. coverage (+): 0. Max coverage (-): 0

Region: chr8 12689301-12689314. Max. coverage (+): 0. Max coverage (-): 0

Region: chr8 12689315-12689328. Max. coverage (+): 0. Max coverage (-): 0

Region: chr8 12689329-12689342. Max. coverage (+): 0. Max coverage (-): 0

Region: chr8 12689343-12689356. Max. coverage (+): 0. Max coverage (-): 0

Region: chr8 12689357-12689370. Max. coverage (+): 0. Max coverage (-): 0

Region: chr8 12689371-12689384. Max. coverage (+): 0. Max coverage (-): 0

Region: chr8 12689385-12689398. Max. coverage (+): 0. Max coverage (-): 0

Region: chr8 12689399-12689412. Max. coverage (+): 0. Max coverage (-): 0

Region: chr8 12689413-12689426. Max. coverage (+): 0. Max coverage (-): 0

Region: chr8 12689427-12689440. Max. coverage (+): 0. Max coverage (-): 0

Region: chr8 12689441-12689454. Max. coverage (+): 0. Max coverage (-): 0

Region: chr8 12689455-12689468. Max. coverage (+): 0. Max coverage (-): 0

Region: chr8 12689469-12689482. Max. coverage (+): 0. Max coverage (-): 0

Region: chr8 12689483-12689495. Max. coverage (+): 4.56. Max coverage (-): 0

Region: chr8 12689496-12689509. Max. coverage (+): 4.56. Max coverage (-): 0

Region: chr8 12689510-12689523. Max. coverage (+): 0. Max coverage (-): 0

Region: chr8 12689524-12689537. Max. coverage (+): 0. Max coverage (-): 0

Region: chr8 12689538-12689551. Max. coverage (+): 0. Max coverage (-): 0

Region: chr8 12689552-12689565. Max. coverage (+): 0. Max coverage (-): 0

Region: chr8 12689566-12689579. Max. coverage (+): 0. Max coverage (-): 0

Region: chr8 12689580-12689593. Max. coverage (+): 0. Max coverage (-): 0

Region: chr8 12689594-12689607. Max. coverage (+): 0. Max coverage (-): 0

Region: chr8 12689608-12689621. Max. coverage (+): 0. Max coverage (-): 0

Region: chr8 12689622-12689635. Max. coverage (+): 0. Max coverage (-): 0

Region: chr8 12689636-12689649. Max. coverage (+): 0. Max coverage (-): 0

Region: chr8 12689650-12689663. Max. coverage (+): 0. Max coverage (-): 0

Region: chr8 12689664-12689676. Max. coverage (+): 0. Max coverage (-): 0

Region: chr8 12689677-12689690. Max. coverage (+): 0. Max coverage (-): 0

Region: chr8 12689691-12689704. Max. coverage (+): 0. Max coverage (-): 0

Region: chr8 12689705-12689718. Max. coverage (+): 0. Max coverage (-): 0

Region: chr8 12689719-12689732. Max. coverage (+): 0. Max coverage (-): 0

Region: chr8 12689733-12689746. Max. coverage (+): 0. Max coverage (-): 0

Region: chr8 12689747-12689760. Max. coverage (+): 0. Max coverage (-): 0

Region: chr8 12689761-12689774. Max. coverage (+): 0. Max coverage (-): 0

Region: chr8 12689775-12689788. Max. coverage (+): 0. Max coverage (-): 0

Region: chr8 12689789-12689802. Max. coverage (+): 0. Max coverage (-): 0

Region: chr8 12689803-12689816. Max. coverage (+): 0. Max coverage (-): 0

Region: chr8 12689817-12689830. Max. coverage (+): 0. Max coverage (-): 0

Region: chr8 12689831-12689844. Max. coverage (+): 0. Max coverage (-): 0

Region: chr8 12689845-12689858. Max. coverage (+): 2.23. Max coverage (-): 0

Region: chr8 12689859-12689871. Max. coverage (+): 2.23. Max coverage (-): 0

Region: chr8 12689872-12689885. Max. coverage (+): 0. Max coverage (-): 0

Region: chr8 12689886-12689899. Max. coverage (+): 0. Max coverage (-): 0

Region: chr8 12689900-12689913. Max. coverage (+): 0. Max coverage (-): 0

Region: chr8 12689914-12689927. Max. coverage (+): 0. Max coverage (-): 0

Region: chr8 12689928-12689941. Max. coverage (+): 0. Max coverage (-): 0

Region: chr8 12689942-12689955. Max. coverage (+): 0. Max coverage (-): 0

Region: chr8 12689956-12689969. Max. coverage (+): 0. Max coverage (-): 0

Region: chr8 12689970-12689983. Max. coverage (+): 0. Max coverage (-): 0

Region: chr8 12689984-12689997. Max. coverage (+): 0. Max coverage (-): 0

Region: chr8 12689998-12690011. Max. coverage (+): 0. Max coverage (-): 0

Region: chr8 12690012-12690025. Max. coverage (+): 0. Max coverage (-): 0

Region: chr8 12690026-12690039. Max. coverage (+): 0. Max coverage (-): 0

Region: chr8 12690040-12690052. Max. coverage (+): 0. Max coverage (-): 0

Region: chr8 12690053-12690066. Max. coverage (+): 0. Max coverage (-): 0

Region: chr8 12690067-12690080. Max. coverage (+): 0. Max coverage (-): 0

Region: chr8 12690081-12690094. Max. coverage (+): 0. Max coverage (-): 0

Region: chr8 12690095-12690108. Max. coverage (+): 0. Max coverage (-): 0

Region: chr8 12690109-12690122. Max. coverage (+): 0. Max coverage (-): 0

Region: chr8 12690123-12690136. Max. coverage (+): 10.14. Max coverage (-): 0

Region: chr8 12690137-12690150. Max. coverage (+): 12.28. Max coverage (-): 0

Region: chr8 12690151-12690164. Max. coverage (+): 0. Max coverage (-): 0

Region: chr8 12690165-12690178. Max. coverage (+): 3.73. Max coverage (-): 0

Region: chr8 12690179-12690192. Max. coverage (+): 3.73. Max coverage (-): 0

Region: chr8 12690193-12690206. Max. coverage (+): 0. Max coverage (-): 0

Region: chr8 12690207-12690220. Max. coverage (+): 0. Max coverage (-): 0

Region: chr8 12690221-12690234. Max. coverage (+): 0. Max coverage (-): 0

Region: chr8 12690235-12690247. Max. coverage (+): 0. Max coverage (-): 0

Region: chr8 12690248-12690261. Max. coverage (+): 0. Max coverage (-): 0

Region: chr8 12690262-12690275. Max. coverage (+): 1.4. Max coverage (-): 0

Region: chr8 12690276-12690289. Max. coverage (+): 2.11. Max coverage (-): 0

Region: chr8 12690290-12690303. Max. coverage (+): 0. Max coverage (-): 0

Region: chr8 12690304-12690317. Max. coverage (+): 0. Max coverage (-): 0

Region: chr8 12690318-12690331. Max. coverage (+): 0. Max coverage (-): 0

Region: chr8 12690332-12690345. Max. coverage (+): 0. Max coverage (-): 0

Region: chr8 12690346-12690359. Max. coverage (+): 0. Max coverage (-): 0

Region: chr8 12690360-12690373. Max. coverage (+): 0. Max coverage (-): 0

Region: chr8 12690374-12690387. Max. coverage (+): 0. Max coverage (-): 0

Region: chr8 12690388-12690401. Max. coverage (+): 1.63. Max coverage (-): 0

Region: chr8 12690402-12690415. Max. coverage (+): 1.63. Max coverage (-): 0

Region: chr8 12690416-12690428. Max. coverage (+): 0. Max coverage (-): 0

Region: chr8 12690429-12690442. Max. coverage (+): 0. Max coverage (-): 0

Region: chr8 12690443-12690456. Max. coverage (+): 0. Max coverage (-): 0

Region: chr8 12690457-12690470. Max. coverage (+): 0. Max coverage (-): 0

Region: chr8 12690471-12690484. Max. coverage (+): 0. Max coverage (-): 0

Region: chr8 12690485-12690498. Max. coverage (+): 0. Max coverage (-): 0

Region: chr8 12690499-12690512. Max. coverage (+): 0. Max coverage (-): 0

Region: chr8 12690513-12690526. Max. coverage (+): 0. Max coverage (-): 0

Region: chr8 12690527-12690540. Max. coverage (+): 0. Max coverage (-): 0

Region: chr8 12690541-12690554. Max. coverage (+): 0. Max coverage (-): 0

Region: chr8 12690555-12690568. Max. coverage (+): 0. Max coverage (-): 0

Region: chr8 12690569-12690582. Max. coverage (+): 0. Max coverage (-): 0

Region: chr8 12690583-12690596. Max. coverage (+): 0. Max coverage (-): 0

Region: chr8 12690597-12690610. Max. coverage (+): 0. Max coverage (-): 0

Region: chr8 12690611-12690623. Max. coverage (+): 0. Max coverage (-): 0

Region: chr8 12690624-12690637. Max. coverage (+): 0. Max coverage (-): 0

Region: chr8 12690638-12690651. Max. coverage (+): 0. Max coverage (-): 0

Region: chr8 12690652-12690665. Max. coverage (+): 0. Max coverage (-): 0

Region: chr8 12690666-12690679. Max. coverage (+): 0. Max coverage (-): 0

Region: chr8 12690680-12690693. Max. coverage (+): 0. Max coverage (-): 0

Region: chr8 12690694-12690707. Max. coverage (+): 0. Max coverage (-): 0

Region: chr8 12690708-12690721. Max. coverage (+): 0. Max coverage (-): 0

Region: chr8 12690722-12690735. Max. coverage (+): 0. Max coverage (-): 0

Region: chr8 12690736-12690749. Max. coverage (+): 0. Max coverage (-): 0

Region: chr8 12690750-12690763. Max. coverage (+): 0. Max coverage (-): 0

Region: chr8 12690764-12690777. Max. coverage (+): 0. Max coverage (-): 0

Region: chr8 12690778-12690791. Max. coverage (+): 0. Max coverage (-): 0

Region: chr8 12690792-12690804. Max. coverage (+): 0. Max coverage (-): 0

Region: chr8 12690805-12690818. Max. coverage (+): 0. Max coverage (-): 0

Region: chr8 12690819-12690832. Max. coverage (+): 0. Max coverage (-): 0

Region: chr8 12690833-12690846. Max. coverage (+): 0. Max coverage (-): 0

Region: chr8 12690847-12690860. Max. coverage (+): 0. Max coverage (-): 0

Region: chr8 12690861-12690874. Max. coverage (+): 0. Max coverage (-): 0

Region: chr8 12690875-12690888. Max. coverage (+): 0. Max coverage (-): 0

Region: chr8 12690889-12690902. Max. coverage (+): 0. Max coverage (-): 0

Region: chr8 12690903-12690916. Max. coverage (+): 3.83. Max coverage (-): 0

Region: chr8 12690917-12690930. Max. coverage (+): 3.83. Max coverage (-): 0

Region: chr8 12690931-12690944. Max. coverage (+): 0. Max coverage (-): 0

Region: chr8 12690945-12690958. Max. coverage (+): 0. Max coverage (-): 0

Region: chr8 12690959-12690972. Max. coverage (+): 0. Max coverage (-): 0

Region: chr8 12690973-12690986. Max. coverage (+): 0. Max coverage (-): 0

Region: chr8 12690987-12690999. Max. coverage (+): 0. Max coverage (-): 0

Region: chr8 12691000-12691013. Max. coverage (+): 0. Max coverage (-): 0

Region: chr8 12691014-12691027. Max. coverage (+): 0. Max coverage (-): 0

Region: chr8 12691028-12691041. Max. coverage (+): 0. Max coverage (-): 0

Region: chr8 12691042-12691055. Max. coverage (+): 0. Max coverage (-): 0

Region: chr8 12691056-12691069. Max. coverage (+): 0. Max coverage (-): 0

Region: chr8 12691070-12691083. Max. coverage (+): 0. Max coverage (-): 0

Region: chr8 12691084-12691097. Max. coverage (+): 0. Max coverage (-): 0

Region: chr8 12691098-12691111. Max. coverage (+): 0. Max coverage (-): 0

Region: chr8 12691112-12691125. Max. coverage (+): 0. Max coverage (-): 0

Region: chr8 12691126-12691139. Max. coverage (+): 0. Max coverage (-): 0

Region: chr8 12691140-12691153. Max. coverage (+): 0. Max coverage (-): 0

Region: chr8 12691154-12691167. Max. coverage (+): 0. Max coverage (-): 0

Region: chr8 12691168-12691180. Max. coverage (+): 0. Max coverage (-): 0

Region: chr8 12691181-12691194. Max. coverage (+): 0. Max coverage (-): 0

Region: chr8 12691195-12691208. Max. coverage (+): 0. Max coverage (-): 0

Region: chr8 12691209-12691222. Max. coverage (+): 0. Max coverage (-): 0

Region: chr8 12691223-12691236. Max. coverage (+): 0. Max coverage (-): 0

Region: chr8 12691237-12691250. Max. coverage (+): 0. Max coverage (-): 0

Region: chr8 12691251-12691264. Max. coverage (+): 0. Max coverage (-): 0

Region: chr8 12691265-12691278. Max. coverage (+): 0. Max coverage (-): 0

Region: chr8 12691279-12691292. Max. coverage (+): 0. Max coverage (-): 0

Region: chr8 12691293-12691306. Max. coverage (+): 0. Max coverage (-): 0

Region: chr8 12691307-12691320. Max. coverage (+): 0. Max coverage (-): 0

Region: chr8 12691321-12691334. Max. coverage (+): 0. Max coverage (-): 0

Region: chr8 12691335-12691348. Max. coverage (+): 0. Max coverage (-): 0

Region: chr8 12691349-12691362. Max. coverage (+): 0. Max coverage (-): 0

Region: chr8 12691363-12691375. Max. coverage (+): 0. Max coverage (-): 0

Region: chr8 12691376-12691389. Max. coverage (+): 0. Max coverage (-): 0

Region: chr8 12691390-12691403. Max. coverage (+): 0. Max coverage (-): 0

Region: chr8 12691404-12691417. Max. coverage (+): 0. Max coverage (-): 0

Region: chr8 12691418-12691431. Max. coverage (+): 0. Max coverage (-): 0

Region: chr8 12691432-12691445. Max. coverage (+): 0. Max coverage (-): 0

Region: chr8 12691446-12691459. Max. coverage (+): 0. Max coverage (-): 0

Region: chr8 12691460-12691473. Max. coverage (+): 0. Max coverage (-): 0

Region: chr8 12691474-12691487. Max. coverage (+): 0. Max coverage (-): 0

Region: chr8 12691488-12691501. Max. coverage (+): 0. Max coverage (-): 0

Region: chr8 12691502-12691515. Max. coverage (+): 0. Max coverage (-): 0

Region: chr8 12691516-12691529. Max. coverage (+): 0. Max coverage (-): 0

Region: chr8 12691530-12691543. Max. coverage (+): 0. Max coverage (-): 0

Region: chr8 12691544-12691556. Max. coverage (+): 0. Max coverage (-): 0

Region: chr8 12691557-12691570. Max. coverage (+): 0. Max coverage (-): 0

Region: chr8 12691571-12691584. Max. coverage (+): 0. Max coverage (-): 0

Region: chr8 12691585-12691598. Max. coverage (+): 0. Max coverage (-): 0

Region: chr8 12691599-12691612. Max. coverage (+): 0. Max coverage (-): 0

Region: chr8 12691613-12691626. Max. coverage (+): 0. Max coverage (-): 0

Region: chr8 12691627-12691640. Max. coverage (+): 0. Max coverage (-): 0

Region: chr8 12691641-12691654. Max. coverage (+): 0. Max coverage (-): 0

Region: chr8 12691655-12691668. Max. coverage (+): 0. Max coverage (-): 0

Region: chr8 12691669-12691682. Max. coverage (+): 0. Max coverage (-): 0

Region: chr8 12691683-12691696. Max. coverage (+): 0. Max coverage (-): 0

Region: chr8 12691697-12691710. Max. coverage (+): 0. Max coverage (-): 0

Region: chr8 12691711-12691724. Max. coverage (+): 0. Max coverage (-): 0

Region: chr8 12691725-12691738. Max. coverage (+): 0. Max coverage (-): 0

Region: chr8 12691739-12691751. Max. coverage (+): 0. Max coverage (-): 0

Region: chr8 12691752-12691765. Max. coverage (+): 0. Max coverage (-): 0

Region: chr8 12691766-12691779. Max. coverage (+): 0. Max coverage (-): 0

Region: chr8 12691780-12691793. Max. coverage (+): 0. Max coverage (-): 0

Region: chr8 12691794-12691807. Max. coverage (+): 0. Max coverage (-): 0

Region: chr8 12691808-12691821. Max. coverage (+): 0. Max coverage (-): 0

Region: chr8 12691822-12691835. Max. coverage (+): 0. Max coverage (-): 0

Region: chr8 12691836-12691849. Max. coverage (+): 0. Max coverage (-): 0

Region: chr8 12691850-12691863. Max. coverage (+): 0. Max coverage (-): 0

Region: chr8 12691864-12691877. Max. coverage (+): 0. Max coverage (-): 0

Region: chr8 12691878-12691891. Max. coverage (+): 0. Max coverage (-): 0

Region: chr8 12691892-12691905. Max. coverage (+): 0. Max coverage (-): 0

Region: chr8 12691906-12691919. Max. coverage (+): 0. Max coverage (-): 0

Region: chr8 12691920-12691932. Max. coverage (+): 0. Max coverage (-): 0

Region: chr8 12691933-12691946. Max. coverage (+): 0. Max coverage (-): 0

Region: chr8 12691947-12691960. Max. coverage (+): 0. Max coverage (-): 0

Region: chr8 12691961-12691974. Max. coverage (+): 0. Max coverage (-): 0

Region: chr8 12691975-12691988. Max. coverage (+): 12.08. Max coverage (-): 0

Region: chr8 12691989-12692002. Max. coverage (+): 6.79. Max coverage (-): 0

Region: chr8 12692003-12692016. Max. coverage (+): 0. Max coverage (-): 0

Region: chr8 12692017-12692030. Max. coverage (+): 0. Max coverage (-): 0

Region: chr8 12692031-12692044. Max. coverage (+): 0. Max coverage (-): 0

Region: chr8 12692045-12692058. Max. coverage (+): 2.12. Max coverage (-): 0

Region: chr8 12692059-12692072. Max. coverage (+): 12.79. Max coverage (-): 0

Region: chr8 12692073-12692086. Max. coverage (+): 2.86. Max coverage (-): 0

Region: chr8 12692087-12692100. Max. coverage (+): 2.86. Max coverage (-): 0

Region: chr8 12692101-12692114. Max. coverage (+): 0. Max coverage (-): 0

Region: chr8 12692115-. Max. coverage (+): 0. Max coverage (-): 0

RepeatMasker Color Code

**+**

100-98% Identity

<98-95% Identity

<95-90% Identity

<90-85% Identity

<85-80% Identity

<80-75% Identity

<75-70% Identity

<70% Identity

**-**

Gene Set Color Code

**+**

Gene

Pseudogene

**-**

Topology/Coverage Color Code

Coverage Plus Strand

Coverage Minus Strand

Mainstrand: Plus

Mainstrand: Minus

Complementary Strand

Flanking Region  
(if option -flank >0)

Gene Set Annotation  
  
RepeatMasker Annotation  

**1. L1\_Art**: 12685305-12685452 (-), Divergence to consensus: 22.3%  
**2. (CA)n**: 12685766-12685797 (+), Divergence to consensus: 9.4%  
**3. BOV-A2**: 12689524-12689642 (+), Divergence to consensus: 8.4%  
**4. (CAG)n**: 12689643-12689663 (+), Divergence to consensus: 0%  
**5. Bov-tA2**: 12690490-12690690 (-), Divergence to consensus: 17.9%  
**6. L1-2\_BT**: 12691315-12691630 (+), Divergence to consensus: 22.8%

  
Transcription Factor Binding Sites  

**Gata4** (Sequence: AGATAAG (-): 12687124)  
**Gata4** (Sequence: AGATAAG (-): 12691750)  
**SOX9** (Sequence: AACAATAA (-): 12689766)  
**A-MYB** (Sequence: AGACAGTTGG (+): 12686759)  
**Gata4** (Sequence: CTTATCT (+): 12688279)  
**Gata4** (Sequence: GTTATCT (+): 12688903)
