# Supplementary material for: Small RNA sequencing of cryopreserved semen from single bull revealed altered miRNAs and piRNAs expression between High- and Low-motile sperm populations
Source: BMC Genomics. 2017 Jan 4;18:14. doi: 10.1186/s12864-016-3394-7 (PMC5209821; doi:10.1186/s12864-016-3394-7)
Supplement: Additional file 3: — Details for each piRNA clusters found in High Motile (HM) sperm fraction. Genes, repeats, transposable elements and transcription factors binding sites falling within the cluster regions were reported. (ZIP 1896 kb) [file 12864_2016_3394_MOESM3_ESM.zip › 93.html]

piRNA cluster 93


Predicted piRNA cluster no. 93     previous   next
  

Show proTRAC run info
Hide proTRAC run info

================================= proTRAC ====================================  
VERSION: 2.1                                    LAST MODIFIED: 06. October 2015  
  
Please cite:  
Rosenkranz D, Zischler H. proTRAC - a software for probabilistic piRNA cluster  
detection, visualization and analysis. 2012. BMC Bioinformatics 13:5.  
  
and (for proTRAC 2.0 and later):  
Rosenkranz D, Rudloff S, Bastuck K, Ketting RF, Zischler H. Tupaia small RNAs  
provide insights into function and evolution of RNAi-based transposon defense  
in mammals. 2015. RNA 21(5):911-922.  
  
Contact:  
David Rosenkranz  
Institute of Anthropology, small RNA group  
Johannes Gutenberg University Mainz  
email: rosenkranz@uni-mainz.de  
  
You can find the latest proTRAC version at:  
http://sourceforge.net/projects/protrac/files  
http://www.smallRNAgroup-mainz.de/software  
==============================================================================  
  
PARAMETERS:  
Map file: .............../storage/core/barbara/genhome/smallRNA/fertility/Sample\_motile/pirna/Sample\_motile\_26-33\_collapsed.fa.no-dust.map.weighted-10000-1000-b-0  
Genome file: ............/storage/core/barbara/genhome/smallRNA/fertility/Sample\_all/pirna/bt\_311\_chrY.fa  
RepeatMasker annotation: /storage/genomes/bt\_umd31/GCF\_000003055.6\_Bos\_taurus\_UMD\_3.1.1\_repeatMasker\_chr.out  
GeneSet:................./storage/core/barbara/genhome/smallRNA/fertility/Sample\_all/pirna/full.gtf  
  
Significant (p<=0.01) hit density will be calculated based  
on observed hit distribution.  
  
Sliding window size: ........................................ 5000 bp  
Sliding window increament: .................................. 1000 bp  
Normalize each hit by number of genomic hits: ............... 1 [0=no/1=yes]  
Normalize each hit by number of sequence reads: ............. 1 [0=no/1=yes]  
Normalize values (-> per million mapped reads): ............. 1 [0=no/1=yes]  
Min. fraction of hits with 1T(U) or 10A: .................... 0.75  
Alternatively: Min. fraction of hits with 1T(U) and 10A: .... 0.5  
Min. fraction of hits with typical piRNA length: ............ 0.75  
Typical piRNA length: ....................................... 26-33 nt  
Min. size of a piRNA cluster: ............................... 5000 bp.  
Min. number of hits (absolute): ............................. 0  
Min. number of hits (normalized): ........................... 0  
Min. fraction of hits on the mainstrand: .................... 0.75  
Top fraction of mapped sequences (in terms of read counts): . 1%  
Top fraction accounts for max. n% of sequence reads: ........ 90%  
Min. fraction of hits on each arm of a bidirectional cluster: 0.1  
Output image file for each cluster: ......................... 0 [0=no/1=yes]  
Output html file for each cluster: .......................... 1 [0=no/1=yes]  
Output a summary table: ..................................... 1 [0=no/1=yes]  
Output a FASTA file for each cluster (piRNA sequences): ..... 1 [0=no/1=yes]  
Output a FASTA file comprising cluster sequences: ........... 1 [0=no/1=yes]  
Search DNA motifs in clusters: .............................. 1 [0=no/1=yes]  
Output flanking sequences: +/- .............................. 0 bp  
Output ~.pTi file: .......................................... 1 [0=no/1=yes]  
==============================================================================  
  
  
Genome size (without gaps): ............ 2678902517 bp  
Gaps (N/X/-): .......................... 53837044 bp  
Mapped reads: .......................... 658825247023  
Non-identical sequences: ............... 514171  
Genomic hits: .......................... 764233  
Significant densitiy of mapped reads: .. 12867599.5173724 reads/kb

Show proTRAC cluster info
Hide proTRAC cluster info

|  |  |
| --- | --- |
| Location | chr8 |
| Coordinates | 17575005-17626374 |
| Size [bp] | 51370 |
| Sequence hit loci | 14719 |
| Mapped reads (normalized) | 16290429267 |
| Mapped reads (normalized) per kb | 317119510.7 |
| Normalized reads with 1T (1U) | 76% |
| Normalized reads with 10A | 28.8% |
| Normalized reads with length 26-33 nt | 100% |
| Normalized reads on the main strand(s) | 95.9% |
| Predicted directionality | mono:plus |

100%

0%

1T (1U)  
reads

10A reads

26-33 nt  
reads

reads on mainstrand

**Either the amount of reads with 1T (1U) OR 10A has to exceed 75% (set with option: -1Tor10A)  
Alternatively the amount of reads with 1T (1U) AND 10A has to exceed 50% (set with option: -1Tand10A)  
Minimum amount of reads with preferred size is 75% (set with option: -pisize)  
Minimum amount of reads on the main strand(s) is 75% (set with option: -clstrand)**

Show read coverage
Hide read coverage

WHAT DO I SEE HERE?  
This chart shows the location of mapped sequence reads within a predicted piRNA cluster. The color refers to the number of genomic hits produced by the sequence read in question. A dark red bar indicates that this sequence read produces many other hits elsewhere in the genome. Many adjacent red or yellow bars can indicate the presence of a multi-copy element such as transposons or rRNA genes. A dark green bar indicates that this sequence read maps uniquely to this locus.

1 hit

2-5 hits

6-10 hits

11-20 hits

21-50 hits

51-100 hits

> 100 hits

chr8

17575005

17626374

Gene Set

RepeatMasker

Mapped  
Reads

555.76

plus strand

minus strand

555.76

Region: chr8 12692116-17575056. Max. coverage (+): 0. Max coverage (-): 4.61

Region: chr8 17575057-17575159. Max. coverage (+): 0. Max coverage (-): 0

Region: chr8 17575160-17575261. Max. coverage (+): 0. Max coverage (-): 0

Region: chr8 17575262-17575364. Max. coverage (+): 0. Max coverage (-): 0

Region: chr8 17575365-17575467. Max. coverage (+): 0. Max coverage (-): 0

Region: chr8 17575468-17575570. Max. coverage (+): 0. Max coverage (-): 0

Region: chr8 17575571-17575672. Max. coverage (+): 0. Max coverage (-): 0

Region: chr8 17575673-17575775. Max. coverage (+): 0. Max coverage (-): 0

Region: chr8 17575776-17575878. Max. coverage (+): 0. Max coverage (-): 0

Region: chr8 17575879-17575981. Max. coverage (+): 0. Max coverage (-): 3.53

Region: chr8 17575982-17576083. Max. coverage (+): 0. Max coverage (-): 1.53

Region: chr8 17576084-17576186. Max. coverage (+): 4.9. Max coverage (-): 0

Region: chr8 17576187-17576289. Max. coverage (+): 0. Max coverage (-): 1.24

Region: chr8 17576290-17576391. Max. coverage (+): 0. Max coverage (-): 0

Region: chr8 17576392-17576494. Max. coverage (+): 0. Max coverage (-): 0

Region: chr8 17576495-17576597. Max. coverage (+): 0. Max coverage (-): 0

Region: chr8 17576598-17576700. Max. coverage (+): 0. Max coverage (-): 0

Region: chr8 17576701-17576802. Max. coverage (+): 0. Max coverage (-): 0

Region: chr8 17576803-17576905. Max. coverage (+): 0. Max coverage (-): 0

Region: chr8 17576906-17577008. Max. coverage (+): 0. Max coverage (-): 0.84

Region: chr8 17577009-17577111. Max. coverage (+): 1.1. Max coverage (-): 0

Region: chr8 17577112-17577213. Max. coverage (+): 4.77. Max coverage (-): 0

Region: chr8 17577214-17577316. Max. coverage (+): 3.26. Max coverage (-): 2.96

Region: chr8 17577317-17577419. Max. coverage (+): 8.37. Max coverage (-): 0

Region: chr8 17577420-17577522. Max. coverage (+): 1.33. Max coverage (-): 3.21

Region: chr8 17577523-17577624. Max. coverage (+): 2.41. Max coverage (-): 0

Region: chr8 17577625-17577727. Max. coverage (+): 0. Max coverage (-): 0

Region: chr8 17577728-17577830. Max. coverage (+): 0. Max coverage (-): 0

Region: chr8 17577831-17577933. Max. coverage (+): 0. Max coverage (-): 0

Region: chr8 17577934-17578035. Max. coverage (+): 0. Max coverage (-): 0

Region: chr8 17578036-17578138. Max. coverage (+): 0. Max coverage (-): 0

Region: chr8 17578139-17578241. Max. coverage (+): 0. Max coverage (-): 0

Region: chr8 17578242-17578344. Max. coverage (+): 3.32. Max coverage (-): 0

Region: chr8 17578345-17578446. Max. coverage (+): 9.22. Max coverage (-): 3.18

Region: chr8 17578447-17578549. Max. coverage (+): 7.9. Max coverage (-): 0

Region: chr8 17578550-17578652. Max. coverage (+): 19.16. Max coverage (-): 3.05

Region: chr8 17578653-17578755. Max. coverage (+): 32.59. Max coverage (-): 2.24

Region: chr8 17578756-17578857. Max. coverage (+): 13.39. Max coverage (-): 0

Region: chr8 17578858-17578960. Max. coverage (+): 16.27. Max coverage (-): 0

Region: chr8 17578961-17579063. Max. coverage (+): 59.39. Max coverage (-): 0

Region: chr8 17579064-17579165. Max. coverage (+): 13.63. Max coverage (-): 0

Region: chr8 17579166-17579268. Max. coverage (+): 13.95. Max coverage (-): 0

Region: chr8 17579269-17579371. Max. coverage (+): 23.67. Max coverage (-): 5.08

Region: chr8 17579372-17579474. Max. coverage (+): 31.66. Max coverage (-): 7.41

Region: chr8 17579475-17579576. Max. coverage (+): 33.8. Max coverage (-): 2.88

Region: chr8 17579577-17579679. Max. coverage (+): 51.14. Max coverage (-): 1.94

Region: chr8 17579680-17579782. Max. coverage (+): 47.05. Max coverage (-): 0

Region: chr8 17579783-17579885. Max. coverage (+): 39.49. Max coverage (-): 0

Region: chr8 17579886-17579987. Max. coverage (+): 16.54. Max coverage (-): 0

Region: chr8 17579988-17580090. Max. coverage (+): 21.45. Max coverage (-): 1.73

Region: chr8 17580091-17580193. Max. coverage (+): 26.03. Max coverage (-): 0

Region: chr8 17580194-17580296. Max. coverage (+): 26.65. Max coverage (-): 0

Region: chr8 17580297-17580398. Max. coverage (+): 22.97. Max coverage (-): 3.32

Region: chr8 17580399-17580501. Max. coverage (+): 4.18. Max coverage (-): 4.85

Region: chr8 17580502-17580604. Max. coverage (+): 23.27. Max coverage (-): 1.79

Region: chr8 17580605-17580707. Max. coverage (+): 13.37. Max coverage (-): 1.72

Region: chr8 17580708-17580809. Max. coverage (+): 19.88. Max coverage (-): 0

Region: chr8 17580810-17580912. Max. coverage (+): 52.33. Max coverage (-): 0

Region: chr8 17580913-17581015. Max. coverage (+): 63.33. Max coverage (-): 0

Region: chr8 17581016-17581118. Max. coverage (+): 60.28. Max coverage (-): 0

Region: chr8 17581119-17581220. Max. coverage (+): 54.09. Max coverage (-): 0

Region: chr8 17581221-17581323. Max. coverage (+): 17.12. Max coverage (-): 1.82

Region: chr8 17581324-17581426. Max. coverage (+): 0. Max coverage (-): 0

Region: chr8 17581427-17581528. Max. coverage (+): 67.62. Max coverage (-): 4.69

Region: chr8 17581529-17581631. Max. coverage (+): 56.01. Max coverage (-): 0

Region: chr8 17581632-17581734. Max. coverage (+): 38.61. Max coverage (-): 0

Region: chr8 17581735-17581837. Max. coverage (+): 21.14. Max coverage (-): 10.4

Region: chr8 17581838-17581939. Max. coverage (+): 15.83. Max coverage (-): 10.4

Region: chr8 17581940-17582042. Max. coverage (+): 24.78. Max coverage (-): 0

Region: chr8 17582043-17582145. Max. coverage (+): 53.57. Max coverage (-): 0

Region: chr8 17582146-17582248. Max. coverage (+): 55.32. Max coverage (-): 0

Region: chr8 17582249-17582350. Max. coverage (+): 25.07. Max coverage (-): 0

Region: chr8 17582351-17582453. Max. coverage (+): 22.29. Max coverage (-): 0

Region: chr8 17582454-17582556. Max. coverage (+): 21.67. Max coverage (-): 0

Region: chr8 17582557-17582659. Max. coverage (+): 34.13. Max coverage (-): 0

Region: chr8 17582660-17582761. Max. coverage (+): 24.35. Max coverage (-): 0

Region: chr8 17582762-17582864. Max. coverage (+): 37.81. Max coverage (-): 0

Region: chr8 17582865-17582967. Max. coverage (+): 11.57. Max coverage (-): 0

Region: chr8 17582968-17583070. Max. coverage (+): 4.31. Max coverage (-): 0

Region: chr8 17583071-17583172. Max. coverage (+): 7.85. Max coverage (-): 0

Region: chr8 17583173-17583275. Max. coverage (+): 5.42. Max coverage (-): 0

Region: chr8 17583276-17583378. Max. coverage (+): 0. Max coverage (-): 0

Region: chr8 17583379-17583481. Max. coverage (+): 0. Max coverage (-): 0

Region: chr8 17583482-17583583. Max. coverage (+): 68.66. Max coverage (-): 0

Region: chr8 17583584-17583686. Max. coverage (+): 22.39. Max coverage (-): 4.13

Region: chr8 17583687-17583789. Max. coverage (+): 45.49. Max coverage (-): 0

Region: chr8 17583790-17583892. Max. coverage (+): 44.83. Max coverage (-): 0

Region: chr8 17583893-17583994. Max. coverage (+): 55.42. Max coverage (-): 2.23

Region: chr8 17583995-17584097. Max. coverage (+): 31.78. Max coverage (-): 3.18

Region: chr8 17584098-17584200. Max. coverage (+): 61.54. Max coverage (-): 0

Region: chr8 17584201-17584302. Max. coverage (+): 34.33. Max coverage (-): 0

Region: chr8 17584303-17584405. Max. coverage (+): 21.52. Max coverage (-): 1.88

Region: chr8 17584406-17584508. Max. coverage (+): 10.25. Max coverage (-): 0

Region: chr8 17584509-17584611. Max. coverage (+): 16.89. Max coverage (-): 0

Region: chr8 17584612-17584713. Max. coverage (+): 12.07. Max coverage (-): 0

Region: chr8 17584714-17584816. Max. coverage (+): 3.91. Max coverage (-): 15.58

Region: chr8 17584817-17584919. Max. coverage (+): 15.11. Max coverage (-): 0

Region: chr8 17584920-17585022. Max. coverage (+): 55.68. Max coverage (-): 1.59

Region: chr8 17585023-17585124. Max. coverage (+): 102.29. Max coverage (-): 6.04

Region: chr8 17585125-17585227. Max. coverage (+): 26.55. Max coverage (-): 0

Region: chr8 17585228-17585330. Max. coverage (+): 181.46. Max coverage (-): 0

Region: chr8 17585331-17585433. Max. coverage (+): 73.65. Max coverage (-): 0

Region: chr8 17585434-17585535. Max. coverage (+): 72.52. Max coverage (-): 0

Region: chr8 17585536-17585638. Max. coverage (+): 37.8. Max coverage (-): 0

Region: chr8 17585639-17585741. Max. coverage (+): 29.68. Max coverage (-): 0

Region: chr8 17585742-17585844. Max. coverage (+): 249.49. Max coverage (-): 1.98

Region: chr8 17585845-17585946. Max. coverage (+): 46.07. Max coverage (-): 0

Region: chr8 17585947-17586049. Max. coverage (+): 555.76. Max coverage (-): 1.21

Region: chr8 17586050-17586152. Max. coverage (+): 88.95. Max coverage (-): 3.38

Region: chr8 17586153-17586255. Max. coverage (+): 80.49. Max coverage (-): 0

Region: chr8 17586256-17586357. Max. coverage (+): 34.1. Max coverage (-): 0.95

Region: chr8 17586358-17586460. Max. coverage (+): 35.34. Max coverage (-): 0

Region: chr8 17586461-17586563. Max. coverage (+): 138.74. Max coverage (-): 0.74

Region: chr8 17586564-17586665. Max. coverage (+): 34.5. Max coverage (-): 1.72

Region: chr8 17586666-17586768. Max. coverage (+): 34.01. Max coverage (-): 2.48

Region: chr8 17586769-17586871. Max. coverage (+): 114.15. Max coverage (-): 0

Region: chr8 17586872-17586974. Max. coverage (+): 44.29. Max coverage (-): 0

Region: chr8 17586975-17587076. Max. coverage (+): 40.86. Max coverage (-): 6.17

Region: chr8 17587077-17587179. Max. coverage (+): 36.68. Max coverage (-): 12.95

Region: chr8 17587180-17587282. Max. coverage (+): 39.96. Max coverage (-): 15.04

Region: chr8 17587283-17587385. Max. coverage (+): 85.6. Max coverage (-): 7.46

Region: chr8 17587386-17587487. Max. coverage (+): 27.18. Max coverage (-): 5.67

Region: chr8 17587488-17587590. Max. coverage (+): 25.41. Max coverage (-): 22.35

Region: chr8 17587591-17587693. Max. coverage (+): 41.72. Max coverage (-): 15.53

Region: chr8 17587694-17587796. Max. coverage (+): 15.56. Max coverage (-): 23.51

Region: chr8 17587797-17587898. Max. coverage (+): 6.85. Max coverage (-): 0

Region: chr8 17587899-17588001. Max. coverage (+): 11.01. Max coverage (-): 2.01

Region: chr8 17588002-17588104. Max. coverage (+): 137.37. Max coverage (-): 23.43

Region: chr8 17588105-17588207. Max. coverage (+): 18.09. Max coverage (-): 3.71

Region: chr8 17588208-17588309. Max. coverage (+): 95.17. Max coverage (-): 20.49

Region: chr8 17588310-17588412. Max. coverage (+): 43.45. Max coverage (-): 5.4

Region: chr8 17588413-17588515. Max. coverage (+): 7.28. Max coverage (-): 5.4

Region: chr8 17588516-17588618. Max. coverage (+): 2.12. Max coverage (-): 0

Region: chr8 17588619-17588720. Max. coverage (+): 14.32. Max coverage (-): 13.62

Region: chr8 17588721-17588823. Max. coverage (+): 0.54. Max coverage (-): 3.48

Region: chr8 17588824-17588926. Max. coverage (+): 1.49. Max coverage (-): 0

Region: chr8 17588927-17589029. Max. coverage (+): 66.74. Max coverage (-): 4.94

Region: chr8 17589030-17589131. Max. coverage (+): 76.38. Max coverage (-): 12.83

Region: chr8 17589132-17589234. Max. coverage (+): 19.73. Max coverage (-): 5.14

Region: chr8 17589235-17589337. Max. coverage (+): 115.79. Max coverage (-): 8.07

Region: chr8 17589338-17589439. Max. coverage (+): 98.97. Max coverage (-): 3.3

Region: chr8 17589440-17589542. Max. coverage (+): 99.85. Max coverage (-): 0

Region: chr8 17589543-17589645. Max. coverage (+): 91.12. Max coverage (-): 0

Region: chr8 17589646-17589748. Max. coverage (+): 118.99. Max coverage (-): 0

Region: chr8 17589749-17589850. Max. coverage (+): 109.44. Max coverage (-): 0

Region: chr8 17589851-17589953. Max. coverage (+): 50.15. Max coverage (-): 0

Region: chr8 17589954-17590056. Max. coverage (+): 41.49. Max coverage (-): 0

Region: chr8 17590057-17590159. Max. coverage (+): 37.53. Max coverage (-): 0

Region: chr8 17590160-17590261. Max. coverage (+): 85.05. Max coverage (-): 0

Region: chr8 17590262-17590364. Max. coverage (+): 43.64. Max coverage (-): 12.9

Region: chr8 17590365-17590467. Max. coverage (+): 22.62. Max coverage (-): 5.9

Region: chr8 17590468-17590570. Max. coverage (+): 47.06. Max coverage (-): 4.5

Region: chr8 17590571-17590672. Max. coverage (+): 39.01. Max coverage (-): 0

Region: chr8 17590673-17590775. Max. coverage (+): 19.43. Max coverage (-): 2.46

Region: chr8 17590776-17590878. Max. coverage (+): 37.24. Max coverage (-): 4.68

Region: chr8 17590879-17590981. Max. coverage (+): 92.57. Max coverage (-): 0.63

Region: chr8 17590982-17591083. Max. coverage (+): 15.44. Max coverage (-): 5.6

Region: chr8 17591084-17591186. Max. coverage (+): 13.01. Max coverage (-): 1.83

Region: chr8 17591187-17591289. Max. coverage (+): 7.61. Max coverage (-): 2.03

Region: chr8 17591290-17591392. Max. coverage (+): 58. Max coverage (-): 1.6

Region: chr8 17591393-17591494. Max. coverage (+): 62.06. Max coverage (-): 1.87

Region: chr8 17591495-17591597. Max. coverage (+): 22.39. Max coverage (-): 10.48

Region: chr8 17591598-17591700. Max. coverage (+): 23.75. Max coverage (-): 7.76

Region: chr8 17591701-17591802. Max. coverage (+): 59.32. Max coverage (-): 8.28

Region: chr8 17591803-17591905. Max. coverage (+): 40.38. Max coverage (-): 7.87

Region: chr8 17591906-17592008. Max. coverage (+): 14.27. Max coverage (-): 1.39

Region: chr8 17592009-17592111. Max. coverage (+): 19.01. Max coverage (-): 7.34

Region: chr8 17592112-17592213. Max. coverage (+): 20.8. Max coverage (-): 4.13

Region: chr8 17592214-17592316. Max. coverage (+): 146.27. Max coverage (-): 10.19

Region: chr8 17592317-17592419. Max. coverage (+): 169.64. Max coverage (-): 12.97

Region: chr8 17592420-17592522. Max. coverage (+): 79.97. Max coverage (-): 24.68

Region: chr8 17592523-17592624. Max. coverage (+): 20.99. Max coverage (-): 4.94

Region: chr8 17592625-17592727. Max. coverage (+): 51.03. Max coverage (-): 21.03

Region: chr8 17592728-17592830. Max. coverage (+): 140.55. Max coverage (-): 37.11

Region: chr8 17592831-17592933. Max. coverage (+): 30.42. Max coverage (-): 10.12

Region: chr8 17592934-17593035. Max. coverage (+): 77.92. Max coverage (-): 10.12

Region: chr8 17593036-17593138. Max. coverage (+): 49.5. Max coverage (-): 0

Region: chr8 17593139-17593241. Max. coverage (+): 116.89. Max coverage (-): 0

Region: chr8 17593242-17593344. Max. coverage (+): 17.17. Max coverage (-): 0

Region: chr8 17593345-17593446. Max. coverage (+): 16.39. Max coverage (-): 0

Region: chr8 17593447-17593549. Max. coverage (+): 20.44. Max coverage (-): 0

Region: chr8 17593550-17593652. Max. coverage (+): 13.73. Max coverage (-): 0

Region: chr8 17593653-17593755. Max. coverage (+): 14.96. Max coverage (-): 0

Region: chr8 17593756-17593857. Max. coverage (+): 28.46. Max coverage (-): 0

Region: chr8 17593858-17593960. Max. coverage (+): 27.99. Max coverage (-): 0

Region: chr8 17593961-17594063. Max. coverage (+): 13.09. Max coverage (-): 0

Region: chr8 17594064-17594166. Max. coverage (+): 0. Max coverage (-): 0

Region: chr8 17594167-17594268. Max. coverage (+): 16.18. Max coverage (-): 0

Region: chr8 17594269-17594371. Max. coverage (+): 33.22. Max coverage (-): 0

Region: chr8 17594372-17594474. Max. coverage (+): 23.42. Max coverage (-): 0

Region: chr8 17594475-17594576. Max. coverage (+): 26.88. Max coverage (-): 0

Region: chr8 17594577-17594679. Max. coverage (+): 11.21. Max coverage (-): 0

Region: chr8 17594680-17594782. Max. coverage (+): 7.18. Max coverage (-): 0

Region: chr8 17594783-17594885. Max. coverage (+): 50.74. Max coverage (-): 2.19

Region: chr8 17594886-17594987. Max. coverage (+): 40.84. Max coverage (-): 0

Region: chr8 17594988-17595090. Max. coverage (+): 118.53. Max coverage (-): 0

Region: chr8 17595091-17595193. Max. coverage (+): 40.56. Max coverage (-): 0

Region: chr8 17595194-17595296. Max. coverage (+): 23.99. Max coverage (-): 0

Region: chr8 17595297-17595398. Max. coverage (+): 23.99. Max coverage (-): 0

Region: chr8 17595399-17595501. Max. coverage (+): 23.22. Max coverage (-): 0

Region: chr8 17595502-17595604. Max. coverage (+): 14.67. Max coverage (-): 0

Region: chr8 17595605-17595707. Max. coverage (+): 14.71. Max coverage (-): 0

Region: chr8 17595708-17595809. Max. coverage (+): 6. Max coverage (-): 0

Region: chr8 17595810-17595912. Max. coverage (+): 14.35. Max coverage (-): 0

Region: chr8 17595913-17596015. Max. coverage (+): 0. Max coverage (-): 0

Region: chr8 17596016-17596118. Max. coverage (+): 0. Max coverage (-): 0

Region: chr8 17596119-17596220. Max. coverage (+): 1.55. Max coverage (-): 0

Region: chr8 17596221-17596323. Max. coverage (+): 13.96. Max coverage (-): 0

Region: chr8 17596324-17596426. Max. coverage (+): 2.52. Max coverage (-): 0

Region: chr8 17596427-17596529. Max. coverage (+): 0. Max coverage (-): 0

Region: chr8 17596530-17596631. Max. coverage (+): 0. Max coverage (-): 0

Region: chr8 17596632-17596734. Max. coverage (+): 0. Max coverage (-): 0

Region: chr8 17596735-17596837. Max. coverage (+): 0. Max coverage (-): 0

Region: chr8 17596838-17596939. Max. coverage (+): 0. Max coverage (-): 0

Region: chr8 17596940-17597042. Max. coverage (+): 26.66. Max coverage (-): 0

Region: chr8 17597043-17597145. Max. coverage (+): 44.25. Max coverage (-): 0

Region: chr8 17597146-17597248. Max. coverage (+): 31.47. Max coverage (-): 0

Region: chr8 17597249-17597350. Max. coverage (+): 79.47. Max coverage (-): 0

Region: chr8 17597351-17597453. Max. coverage (+): 75.13. Max coverage (-): 0

Region: chr8 17597454-17597556. Max. coverage (+): 66.24. Max coverage (-): 0

Region: chr8 17597557-17597659. Max. coverage (+): 50.21. Max coverage (-): 0

Region: chr8 17597660-17597761. Max. coverage (+): 25.25. Max coverage (-): 0

Region: chr8 17597762-17597864. Max. coverage (+): 76.47. Max coverage (-): 0

Region: chr8 17597865-17597967. Max. coverage (+): 68.3. Max coverage (-): 0

Region: chr8 17597968-17598070. Max. coverage (+): 33.32. Max coverage (-): 0

Region: chr8 17598071-17598172. Max. coverage (+): 55.22. Max coverage (-): 0

Region: chr8 17598173-17598275. Max. coverage (+): 30.72. Max coverage (-): 0

Region: chr8 17598276-17598378. Max. coverage (+): 42.01. Max coverage (-): 0

Region: chr8 17598379-17598481. Max. coverage (+): 73.45. Max coverage (-): 0

Region: chr8 17598482-17598583. Max. coverage (+): 35.17. Max coverage (-): 0

Region: chr8 17598584-17598686. Max. coverage (+): 31.2. Max coverage (-): 0

Region: chr8 17598687-17598789. Max. coverage (+): 40.12. Max coverage (-): 0

Region: chr8 17598790-17598892. Max. coverage (+): 50.78. Max coverage (-): 0

Region: chr8 17598893-17598994. Max. coverage (+): 41.05. Max coverage (-): 0

Region: chr8 17598995-17599097. Max. coverage (+): 25.65. Max coverage (-): 0

Region: chr8 17599098-17599200. Max. coverage (+): 28.84. Max coverage (-): 0

Region: chr8 17599201-17599303. Max. coverage (+): 14.38. Max coverage (-): 0

Region: chr8 17599304-17599405. Max. coverage (+): 46.74. Max coverage (-): 0

Region: chr8 17599406-17599508. Max. coverage (+): 25.13. Max coverage (-): 0

Region: chr8 17599509-17599611. Max. coverage (+): 32.82. Max coverage (-): 0

Region: chr8 17599612-17599713. Max. coverage (+): 38.54. Max coverage (-): 0

Region: chr8 17599714-17599816. Max. coverage (+): 61.83. Max coverage (-): 0

Region: chr8 17599817-17599919. Max. coverage (+): 95.61. Max coverage (-): 0

Region: chr8 17599920-17600022. Max. coverage (+): 40.18. Max coverage (-): 0

Region: chr8 17600023-17600124. Max. coverage (+): 25.01. Max coverage (-): 0

Region: chr8 17600125-17600227. Max. coverage (+): 19.11. Max coverage (-): 0

Region: chr8 17600228-17600330. Max. coverage (+): 61.72. Max coverage (-): 0

Region: chr8 17600331-17600433. Max. coverage (+): 3.4. Max coverage (-): 0

Region: chr8 17600434-17600535. Max. coverage (+): 0. Max coverage (-): 0

Region: chr8 17600536-17600638. Max. coverage (+): 9.72. Max coverage (-): 0

Region: chr8 17600639-17600741. Max. coverage (+): 27.56. Max coverage (-): 0

Region: chr8 17600742-17600844. Max. coverage (+): 67.84. Max coverage (-): 0

Region: chr8 17600845-17600946. Max. coverage (+): 30.02. Max coverage (-): 0

Region: chr8 17600947-17601049. Max. coverage (+): 11.13. Max coverage (-): 0

Region: chr8 17601050-17601152. Max. coverage (+): 29.87. Max coverage (-): 0

Region: chr8 17601153-17601255. Max. coverage (+): 15.6. Max coverage (-): 0

Region: chr8 17601256-17601357. Max. coverage (+): 13.49. Max coverage (-): 0

Region: chr8 17601358-17601460. Max. coverage (+): 66.8. Max coverage (-): 0

Region: chr8 17601461-17601563. Max. coverage (+): 11.91. Max coverage (-): 0

Region: chr8 17601564-17601666. Max. coverage (+): 10.33. Max coverage (-): 0

Region: chr8 17601667-17601768. Max. coverage (+): 11.35. Max coverage (-): 0

Region: chr8 17601769-17601871. Max. coverage (+): 10.75. Max coverage (-): 0

Region: chr8 17601872-17601974. Max. coverage (+): 6.66. Max coverage (-): 0

Region: chr8 17601975-17602076. Max. coverage (+): 18.71. Max coverage (-): 0

Region: chr8 17602077-17602179. Max. coverage (+): 17.69. Max coverage (-): 0

Region: chr8 17602180-17602282. Max. coverage (+): 7.74. Max coverage (-): 0

Region: chr8 17602283-17602385. Max. coverage (+): 4.45. Max coverage (-): 0

Region: chr8 17602386-17602487. Max. coverage (+): 0. Max coverage (-): 0

Region: chr8 17602488-17602590. Max. coverage (+): 0. Max coverage (-): 0

Region: chr8 17602591-17602693. Max. coverage (+): 15.44. Max coverage (-): 0

Region: chr8 17602694-17602796. Max. coverage (+): 18.14. Max coverage (-): 0

Region: chr8 17602797-17602898. Max. coverage (+): 1.25. Max coverage (-): 0

Region: chr8 17602899-17603001. Max. coverage (+): 0. Max coverage (-): 0

Region: chr8 17603002-17603104. Max. coverage (+): 6.49. Max coverage (-): 0

Region: chr8 17603105-17603207. Max. coverage (+): 10.1. Max coverage (-): 0

Region: chr8 17603208-17603309. Max. coverage (+): 6.22. Max coverage (-): 0

Region: chr8 17603310-17603412. Max. coverage (+): 3.58. Max coverage (-): 0

Region: chr8 17603413-17603515. Max. coverage (+): 9.68. Max coverage (-): 0

Region: chr8 17603516-17603618. Max. coverage (+): 4.96. Max coverage (-): 0

Region: chr8 17603619-17603720. Max. coverage (+): 6.99. Max coverage (-): 0

Region: chr8 17603721-17603823. Max. coverage (+): 2.67. Max coverage (-): 0

Region: chr8 17603824-17603926. Max. coverage (+): 7.72. Max coverage (-): 0

Region: chr8 17603927-17604029. Max. coverage (+): 4.81. Max coverage (-): 0

Region: chr8 17604030-17604131. Max. coverage (+): 13.87. Max coverage (-): 0

Region: chr8 17604132-17604234. Max. coverage (+): 0. Max coverage (-): 0

Region: chr8 17604235-17604337. Max. coverage (+): 0. Max coverage (-): 0

Region: chr8 17604338-17604440. Max. coverage (+): 7.02. Max coverage (-): 0

Region: chr8 17604441-17604542. Max. coverage (+): 0. Max coverage (-): 0

Region: chr8 17604543-17604645. Max. coverage (+): 0. Max coverage (-): 0

Region: chr8 17604646-17604748. Max. coverage (+): 0. Max coverage (-): 0

Region: chr8 17604749-17604850. Max. coverage (+): 0. Max coverage (-): 0

Region: chr8 17604851-17604953. Max. coverage (+): 0. Max coverage (-): 0

Region: chr8 17604954-17605056. Max. coverage (+): 0. Max coverage (-): 0

Region: chr8 17605057-17605159. Max. coverage (+): 0. Max coverage (-): 0

Region: chr8 17605160-17605261. Max. coverage (+): 0. Max coverage (-): 0

Region: chr8 17605262-17605364. Max. coverage (+): 0. Max coverage (-): 0

Region: chr8 17605365-17605467. Max. coverage (+): 0. Max coverage (-): 0

Region: chr8 17605468-17605570. Max. coverage (+): 0. Max coverage (-): 0

Region: chr8 17605571-17605672. Max. coverage (+): 0. Max coverage (-): 0

Region: chr8 17605673-17605775. Max. coverage (+): 0. Max coverage (-): 0

Region: chr8 17605776-17605878. Max. coverage (+): 0. Max coverage (-): 0

Region: chr8 17605879-17605981. Max. coverage (+): 0. Max coverage (-): 0

Region: chr8 17605982-17606083. Max. coverage (+): 0. Max coverage (-): 0

Region: chr8 17606084-17606186. Max. coverage (+): 0. Max coverage (-): 0

Region: chr8 17606187-17606289. Max. coverage (+): 0. Max coverage (-): 0

Region: chr8 17606290-17606392. Max. coverage (+): 0. Max coverage (-): 0

Region: chr8 17606393-17606494. Max. coverage (+): 0. Max coverage (-): 0

Region: chr8 17606495-17606597. Max. coverage (+): 0. Max coverage (-): 0

Region: chr8 17606598-17606700. Max. coverage (+): 0. Max coverage (-): 0

Region: chr8 17606701-17606803. Max. coverage (+): 0. Max coverage (-): 0

Region: chr8 17606804-17606905. Max. coverage (+): 0. Max coverage (-): 0

Region: chr8 17606906-17607008. Max. coverage (+): 0. Max coverage (-): 0

Region: chr8 17607009-17607111. Max. coverage (+): 0. Max coverage (-): 0

Region: chr8 17607112-17607213. Max. coverage (+): 0. Max coverage (-): 0

Region: chr8 17607214-17607316. Max. coverage (+): 0. Max coverage (-): 0

Region: chr8 17607317-17607419. Max. coverage (+): 0. Max coverage (-): 0

Region: chr8 17607420-17607522. Max. coverage (+): 0. Max coverage (-): 0

Region: chr8 17607523-17607624. Max. coverage (+): 0. Max coverage (-): 0

Region: chr8 17607625-17607727. Max. coverage (+): 0. Max coverage (-): 0

Region: chr8 17607728-17607830. Max. coverage (+): 4.48. Max coverage (-): 0

Region: chr8 17607831-17607933. Max. coverage (+): 5.84. Max coverage (-): 0

Region: chr8 17607934-17608035. Max. coverage (+): 0. Max coverage (-): 0

Region: chr8 17608036-17608138. Max. coverage (+): 0. Max coverage (-): 0

Region: chr8 17608139-17608241. Max. coverage (+): 0. Max coverage (-): 0

Region: chr8 17608242-17608344. Max. coverage (+): 0. Max coverage (-): 0

Region: chr8 17608345-17608446. Max. coverage (+): 4.32. Max coverage (-): 0

Region: chr8 17608447-17608549. Max. coverage (+): 4.32. Max coverage (-): 0

Region: chr8 17608550-17608652. Max. coverage (+): 0. Max coverage (-): 0

Region: chr8 17608653-17608755. Max. coverage (+): 2.22. Max coverage (-): 0

Region: chr8 17608756-17608857. Max. coverage (+): 0. Max coverage (-): 0

Region: chr8 17608858-17608960. Max. coverage (+): 0. Max coverage (-): 0

Region: chr8 17608961-17609063. Max. coverage (+): 1.47. Max coverage (-): 0

Region: chr8 17609064-17609166. Max. coverage (+): 4.82. Max coverage (-): 0

Region: chr8 17609167-17609268. Max. coverage (+): 4.82. Max coverage (-): 0

Region: chr8 17609269-17609371. Max. coverage (+): 3.95. Max coverage (-): 0

Region: chr8 17609372-17609474. Max. coverage (+): 0. Max coverage (-): 0

Region: chr8 17609475-17609577. Max. coverage (+): 0. Max coverage (-): 0

Region: chr8 17609578-17609679. Max. coverage (+): 0. Max coverage (-): 0

Region: chr8 17609680-17609782. Max. coverage (+): 0. Max coverage (-): 0

Region: chr8 17609783-17609885. Max. coverage (+): 0. Max coverage (-): 0

Region: chr8 17609886-17609987. Max. coverage (+): 0. Max coverage (-): 0

Region: chr8 17609988-17610090. Max. coverage (+): 14.65. Max coverage (-): 0

Region: chr8 17610091-17610193. Max. coverage (+): 8.32. Max coverage (-): 0

Region: chr8 17610194-17610296. Max. coverage (+): 41.74. Max coverage (-): 0

Region: chr8 17610297-17610398. Max. coverage (+): 71.36. Max coverage (-): 0

Region: chr8 17610399-17610501. Max. coverage (+): 49.94. Max coverage (-): 0

Region: chr8 17610502-17610604. Max. coverage (+): 47.75. Max coverage (-): 0

Region: chr8 17610605-17610707. Max. coverage (+): 44.54. Max coverage (-): 0

Region: chr8 17610708-17610809. Max. coverage (+): 42.05. Max coverage (-): 0

Region: chr8 17610810-17610912. Max. coverage (+): 0. Max coverage (-): 0

Region: chr8 17610913-17611015. Max. coverage (+): 0. Max coverage (-): 0

Region: chr8 17611016-17611118. Max. coverage (+): 27.96. Max coverage (-): 0

Region: chr8 17611119-17611220. Max. coverage (+): 13.98. Max coverage (-): 0

Region: chr8 17611221-17611323. Max. coverage (+): 26.33. Max coverage (-): 0

Region: chr8 17611324-17611426. Max. coverage (+): 12.88. Max coverage (-): 0

Region: chr8 17611427-17611529. Max. coverage (+): 17.75. Max coverage (-): 0

Region: chr8 17611530-17611631. Max. coverage (+): 0. Max coverage (-): 0

Region: chr8 17611632-17611734. Max. coverage (+): 0. Max coverage (-): 0

Region: chr8 17611735-17611837. Max. coverage (+): 0. Max coverage (-): 0

Region: chr8 17611838-17611940. Max. coverage (+): 0. Max coverage (-): 0

Region: chr8 17611941-17612042. Max. coverage (+): 0. Max coverage (-): 0

Region: chr8 17612043-17612145. Max. coverage (+): 0. Max coverage (-): 0

Region: chr8 17612146-17612248. Max. coverage (+): 0. Max coverage (-): 0

Region: chr8 17612249-17612350. Max. coverage (+): 0. Max coverage (-): 0

Region: chr8 17612351-17612453. Max. coverage (+): 0. Max coverage (-): 0

Region: chr8 17612454-17612556. Max. coverage (+): 0. Max coverage (-): 0

Region: chr8 17612557-17612659. Max. coverage (+): 0. Max coverage (-): 0

Region: chr8 17612660-17612761. Max. coverage (+): 0. Max coverage (-): 0

Region: chr8 17612762-17612864. Max. coverage (+): 0. Max coverage (-): 0

Region: chr8 17612865-17612967. Max. coverage (+): 0. Max coverage (-): 0

Region: chr8 17612968-17613070. Max. coverage (+): 0. Max coverage (-): 0

Region: chr8 17613071-17613172. Max. coverage (+): 0. Max coverage (-): 0

Region: chr8 17613173-17613275. Max. coverage (+): 0. Max coverage (-): 0

Region: chr8 17613276-17613378. Max. coverage (+): 0. Max coverage (-): 0

Region: chr8 17613379-17613481. Max. coverage (+): 0. Max coverage (-): 0

Region: chr8 17613482-17613583. Max. coverage (+): 0. Max coverage (-): 0

Region: chr8 17613584-17613686. Max. coverage (+): 0. Max coverage (-): 0

Region: chr8 17613687-17613789. Max. coverage (+): 0. Max coverage (-): 0

Region: chr8 17613790-17613892. Max. coverage (+): 0. Max coverage (-): 0

Region: chr8 17613893-17613994. Max. coverage (+): 0. Max coverage (-): 0

Region: chr8 17613995-17614097. Max. coverage (+): 0. Max coverage (-): 0

Region: chr8 17614098-17614200. Max. coverage (+): 0. Max coverage (-): 0

Region: chr8 17614201-17614303. Max. coverage (+): 0. Max coverage (-): 0

Region: chr8 17614304-17614405. Max. coverage (+): 0. Max coverage (-): 0

Region: chr8 17614406-17614508. Max. coverage (+): 0. Max coverage (-): 0

Region: chr8 17614509-17614611. Max. coverage (+): 0. Max coverage (-): 0

Region: chr8 17614612-17614714. Max. coverage (+): 0. Max coverage (-): 0

Region: chr8 17614715-17614816. Max. coverage (+): 0. Max coverage (-): 0

Region: chr8 17614817-17614919. Max. coverage (+): 2.72. Max coverage (-): 0

Region: chr8 17614920-17615022. Max. coverage (+): 0. Max coverage (-): 0

Region: chr8 17615023-17615124. Max. coverage (+): 0. Max coverage (-): 0

Region: chr8 17615125-17615227. Max. coverage (+): 0. Max coverage (-): 0

Region: chr8 17615228-17615330. Max. coverage (+): 0. Max coverage (-): 0

Region: chr8 17615331-17615433. Max. coverage (+): 4.67. Max coverage (-): 0

Region: chr8 17615434-17615535. Max. coverage (+): 10.71. Max coverage (-): 0

Region: chr8 17615536-17615638. Max. coverage (+): 11.21. Max coverage (-): 0

Region: chr8 17615639-17615741. Max. coverage (+): 6.16. Max coverage (-): 0

Region: chr8 17615742-17615844. Max. coverage (+): 7.07. Max coverage (-): 0

Region: chr8 17615845-17615946. Max. coverage (+): 0. Max coverage (-): 0

Region: chr8 17615947-17616049. Max. coverage (+): 0. Max coverage (-): 0

Region: chr8 17616050-17616152. Max. coverage (+): 0. Max coverage (-): 0

Region: chr8 17616153-17616255. Max. coverage (+): 0. Max coverage (-): 0

Region: chr8 17616256-17616357. Max. coverage (+): 0. Max coverage (-): 0

Region: chr8 17616358-17616460. Max. coverage (+): 0. Max coverage (-): 0

Region: chr8 17616461-17616563. Max. coverage (+): 5.96. Max coverage (-): 0

Region: chr8 17616564-17616666. Max. coverage (+): 0. Max coverage (-): 0

Region: chr8 17616667-17616768. Max. coverage (+): 8.47. Max coverage (-): 0

Region: chr8 17616769-17616871. Max. coverage (+): 13.04. Max coverage (-): 0

Region: chr8 17616872-17616974. Max. coverage (+): 10.46. Max coverage (-): 0

Region: chr8 17616975-17617077. Max. coverage (+): 7.16. Max coverage (-): 0

Region: chr8 17617078-17617179. Max. coverage (+): 21.82. Max coverage (-): 0

Region: chr8 17617180-17617282. Max. coverage (+): 0. Max coverage (-): 0

Region: chr8 17617283-17617385. Max. coverage (+): 0. Max coverage (-): 0

Region: chr8 17617386-17617487. Max. coverage (+): 0. Max coverage (-): 0

Region: chr8 17617488-17617590. Max. coverage (+): 0. Max coverage (-): 0

Region: chr8 17617591-17617693. Max. coverage (+): 0. Max coverage (-): 0

Region: chr8 17617694-17617796. Max. coverage (+): 0. Max coverage (-): 0

Region: chr8 17617797-17617898. Max. coverage (+): 0. Max coverage (-): 0

Region: chr8 17617899-17618001. Max. coverage (+): 0. Max coverage (-): 0

Region: chr8 17618002-17618104. Max. coverage (+): 0. Max coverage (-): 0

Region: chr8 17618105-17618207. Max. coverage (+): 0. Max coverage (-): 0

Region: chr8 17618208-17618309. Max. coverage (+): 0. Max coverage (-): 0

Region: chr8 17618310-17618412. Max. coverage (+): 0. Max coverage (-): 0

Region: chr8 17618413-17618515. Max. coverage (+): 0. Max coverage (-): 0

Region: chr8 17618516-17618618. Max. coverage (+): 0. Max coverage (-): 0

Region: chr8 17618619-17618720. Max. coverage (+): 0. Max coverage (-): 0

Region: chr8 17618721-17618823. Max. coverage (+): 0. Max coverage (-): 0

Region: chr8 17618824-17618926. Max. coverage (+): 0. Max coverage (-): 0

Region: chr8 17618927-17619029. Max. coverage (+): 0. Max coverage (-): 0

Region: chr8 17619030-17619131. Max. coverage (+): 0. Max coverage (-): 0

Region: chr8 17619132-17619234. Max. coverage (+): 0. Max coverage (-): 0

Region: chr8 17619235-17619337. Max. coverage (+): 0. Max coverage (-): 0

Region: chr8 17619338-17619440. Max. coverage (+): 0. Max coverage (-): 0

Region: chr8 17619441-17619542. Max. coverage (+): 0. Max coverage (-): 0

Region: chr8 17619543-17619645. Max. coverage (+): 0. Max coverage (-): 0

Region: chr8 17619646-17619748. Max. coverage (+): 11.71. Max coverage (-): 0

Region: chr8 17619749-17619851. Max. coverage (+): 2.18. Max coverage (-): 0

Region: chr8 17619852-17619953. Max. coverage (+): 2.62. Max coverage (-): 0

Region: chr8 17619954-17620056. Max. coverage (+): 1.26. Max coverage (-): 0

Region: chr8 17620057-17620159. Max. coverage (+): 2.31. Max coverage (-): 0

Region: chr8 17620160-17620261. Max. coverage (+): 7.23. Max coverage (-): 0

Region: chr8 17620262-17620364. Max. coverage (+): 5.01. Max coverage (-): 0

Region: chr8 17620365-17620467. Max. coverage (+): 5.01. Max coverage (-): 0

Region: chr8 17620468-17620570. Max. coverage (+): 2.22. Max coverage (-): 0

Region: chr8 17620571-17620672. Max. coverage (+): 8.22. Max coverage (-): 0

Region: chr8 17620673-17620775. Max. coverage (+): 17.94. Max coverage (-): 0

Region: chr8 17620776-17620878. Max. coverage (+): 10.07. Max coverage (-): 0

Region: chr8 17620879-17620981. Max. coverage (+): 11.1. Max coverage (-): 0

Region: chr8 17620982-17621083. Max. coverage (+): 4.36. Max coverage (-): 0

Region: chr8 17621084-17621186. Max. coverage (+): 2.63. Max coverage (-): 0

Region: chr8 17621187-17621289. Max. coverage (+): 0. Max coverage (-): 0

Region: chr8 17621290-17621392. Max. coverage (+): 0. Max coverage (-): 0

Region: chr8 17621393-17621494. Max. coverage (+): 0. Max coverage (-): 0

Region: chr8 17621495-17621597. Max. coverage (+): 0. Max coverage (-): 0

Region: chr8 17621598-17621700. Max. coverage (+): 0. Max coverage (-): 0

Region: chr8 17621701-17621803. Max. coverage (+): 0. Max coverage (-): 0

Region: chr8 17621804-17621905. Max. coverage (+): 0. Max coverage (-): 0

Region: chr8 17621906-17622008. Max. coverage (+): 0. Max coverage (-): 0

Region: chr8 17622009-17622111. Max. coverage (+): 0. Max coverage (-): 0

Region: chr8 17622112-17622214. Max. coverage (+): 0. Max coverage (-): 0

Region: chr8 17622215-17622316. Max. coverage (+): 0. Max coverage (-): 0

Region: chr8 17622317-17622419. Max. coverage (+): 1.12. Max coverage (-): 0

Region: chr8 17622420-17622522. Max. coverage (+): 6.34. Max coverage (-): 0

Region: chr8 17622523-17622624. Max. coverage (+): 2.8. Max coverage (-): 0

Region: chr8 17622625-17622727. Max. coverage (+): 4. Max coverage (-): 0

Region: chr8 17622728-17622830. Max. coverage (+): 11.57. Max coverage (-): 0

Region: chr8 17622831-17622933. Max. coverage (+): 1.35. Max coverage (-): 0

Region: chr8 17622934-17623035. Max. coverage (+): 0. Max coverage (-): 0

Region: chr8 17623036-17623138. Max. coverage (+): 7.76. Max coverage (-): 0

Region: chr8 17623139-17623241. Max. coverage (+): 0. Max coverage (-): 0

Region: chr8 17623242-17623344. Max. coverage (+): 0. Max coverage (-): 0

Region: chr8 17623345-17623446. Max. coverage (+): 5.69. Max coverage (-): 0

Region: chr8 17623447-17623549. Max. coverage (+): 1.26. Max coverage (-): 0

Region: chr8 17623550-17623652. Max. coverage (+): 0. Max coverage (-): 0

Region: chr8 17623653-17623755. Max. coverage (+): 0. Max coverage (-): 0

Region: chr8 17623756-17623857. Max. coverage (+): 0. Max coverage (-): 0

Region: chr8 17623858-17623960. Max. coverage (+): 4.87. Max coverage (-): 0

Region: chr8 17623961-17624063. Max. coverage (+): 4.38. Max coverage (-): 0

Region: chr8 17624064-17624166. Max. coverage (+): 4.38. Max coverage (-): 0

Region: chr8 17624167-17624268. Max. coverage (+): 0. Max coverage (-): 0

Region: chr8 17624269-17624371. Max. coverage (+): 5.35. Max coverage (-): 0

Region: chr8 17624372-17624474. Max. coverage (+): 2.81. Max coverage (-): 0

Region: chr8 17624475-17624577. Max. coverage (+): 16.06. Max coverage (-): 0

Region: chr8 17624578-17624679. Max. coverage (+): 5. Max coverage (-): 0

Region: chr8 17624680-17624782. Max. coverage (+): 7.04. Max coverage (-): 0

Region: chr8 17624783-17624885. Max. coverage (+): 7.04. Max coverage (-): 0

Region: chr8 17624886-17624988. Max. coverage (+): 7.42. Max coverage (-): 0

Region: chr8 17624989-17625090. Max. coverage (+): 0.95. Max coverage (-): 0

Region: chr8 17625091-17625193. Max. coverage (+): 4.49. Max coverage (-): 0

Region: chr8 17625194-17625296. Max. coverage (+): 1.26. Max coverage (-): 0

Region: chr8 17625297-17625398. Max. coverage (+): 1.23. Max coverage (-): 0

Region: chr8 17625399-17625501. Max. coverage (+): 2.06. Max coverage (-): 0

Region: chr8 17625502-17625604. Max. coverage (+): 10. Max coverage (-): 0

Region: chr8 17625605-17625707. Max. coverage (+): 12.17. Max coverage (-): 0

Region: chr8 17625708-17625809. Max. coverage (+): 0. Max coverage (-): 0

Region: chr8 17625810-17625912. Max. coverage (+): 6.46. Max coverage (-): 0

Region: chr8 17625913-17626015. Max. coverage (+): 3.48. Max coverage (-): 0

Region: chr8 17626016-17626118. Max. coverage (+): 2.06. Max coverage (-): 0

Region: chr8 17626119-17626220. Max. coverage (+): 0. Max coverage (-): 0

Region: chr8 17626221-17626323. Max. coverage (+): 0.17. Max coverage (-): 0

Region: chr8 17626324-. Max. coverage (+): 0.17. Max coverage (-): 0

RepeatMasker Color Code

**+**

100-98% Identity

<98-95% Identity

<95-90% Identity

<90-85% Identity

<85-80% Identity

<80-75% Identity

<75-70% Identity

<70% Identity

**-**

Gene Set Color Code

**+**

Gene

Pseudogene

**-**

Topology/Coverage Color Code

Coverage Plus Strand

Coverage Minus Strand

Mainstrand: Plus

Mainstrand: Minus

Complementary Strand

Flanking Region  
(if option -flank >0)

Gene Set Annotation  
  
RepeatMasker Annotation  

**1. AT\_rich**: 17575026-17575062 (+), Divergence to consensus: 73%  
**2. L1-2\_BT**: 17575063-17575241 (-), Divergence to consensus: 27.9%  
**3. MIRc**: 17576459-17576558 (+), Divergence to consensus: 47.6%  
**4. BOV-A2**: 17576559-17576829 (-), Divergence to consensus: 2.2%  
**5. MIRc**: 17576830-17576925 (+), Divergence to consensus: 47.6%  
**6. L1ME4a**: 17577730-17578253 (-), Divergence to consensus: 41.7%  
**7. L2c**: 17579075-17579133 (+), Divergence to consensus: 30.5%  
**8. MIRb**: 17579196-17579331 (-), Divergence to consensus: 33%  
**9. L2a**: 17581334-17581446 (+), Divergence to consensus: 38.5%  
**10. (TG)n**: 17582375-17582406 (+), Divergence to consensus: 6.2%  
**11. ART2A**: 17583268-17583517 (+), Divergence to consensus: 14.5%  
**12. Bov-tA3**: 17594040-17594252 (+), Divergence to consensus: 13.8%  
**13. Bov-tA3**: 17594660-17594772 (+), Divergence to consensus: 7.1%  
**14. L2c**: 17595532-17595612 (+), Divergence to consensus: 37.5%  
**15. BOV-A2**: 17596407-17596647 (-), Divergence to consensus: 15.4%  
**16. L1\_BT**: 17596648-17596965 (+), Divergence to consensus: 20.5%  
**17. Bov-tA2**: 17596996-17597030 (-), Divergence to consensus: 2.9%  
**18. MIR**: 17599298-17599392 (+), Divergence to consensus: 28.1%  
**19. L1ME4c**: 17600319-17600551 (-), Divergence to consensus: 43.4%  
**20. AT\_rich**: 17600966-17601005 (+), Divergence to consensus: 70%  
**21. MIRb**: 17602370-17602610 (-), Divergence to consensus: 40.5%  
**22. MIRb**: 17602980-17603069 (-), Divergence to consensus: 37.8%  
**23. MIRc**: 17604003-17604096 (-), Divergence to consensus: 37.2%  
**24. Bov-tA3**: 17604147-17604342 (-), Divergence to consensus: 18.3%  
**25. L1ME2**: 17604422-17604557 (+), Divergence to consensus: 30.8%  
**26. L1MEc**: 17604590-17605107 (+), Divergence to consensus: 43.6%  
**27. Bov-tA2**: 17605111-17605311 (-), Divergence to consensus: 38.5%  
**28. ART2A**: 17605335-17605855 (-), Divergence to consensus: 20.1%  
**29. BovB**: 17605856-17606691 (-), Divergence to consensus: 10.8%  
**30. BTLTR1**: 17606693-17606763 (+), Divergence to consensus: 17.5%  
**31. BovB**: 17606765-17607525 (-), Divergence to consensus: 11.9%  
**32. L1MEc**: 17607521-17607762 (+), Divergence to consensus: 29.8%  
**33. L1ME3**: 17607762-17607930 (+), Divergence to consensus: 43.4%  
**34. Bov-tA2**: 17607948-17608155 (-), Divergence to consensus: 18.6%  
**35. BOV-A2**: 17608167-17608431 (-), Divergence to consensus: 5.7%  
**36. BovB**: 17609333-17609462 (+), Divergence to consensus: 4.6%  
**37. ART2A**: 17609463-17609979 (+), Divergence to consensus: 9.7%  
**38. (AACTG)n**: 17609980-17609999 (+), Divergence to consensus: 0%  
**39. BOV-A2**: 17610145-17610273 (-), Divergence to consensus: 7.8%  
**40. MLT1H**: 17610820-17610878 (+), Divergence to consensus: 20.3%  
**41. MLT1H**: 17610891-17611015 (+), Divergence to consensus: 35.5%  
**42. MLT1C**: 17611507-17611644 (-), Divergence to consensus: 24.9%  
**43. ART2A**: 17611644-17612060 (+), Divergence to consensus: 9.4%  
**44. (AACTG)n**: 17612061-17612080 (+), Divergence to consensus: 0%  
**45. MLT1C**: 17612083-17612393 (-), Divergence to consensus: 28.7%  
**46. L1M4**: 17612397-17612982 (-), Divergence to consensus: 43.5%  
**47. SINE2-2\_BT**: 17612983-17613083 (+), Divergence to consensus: 32.7%  
**48. (TA)n**: 17613089-17613110 (+), Divergence to consensus: 0%  
**49. L1M4**: 17613111-17613364 (-), Divergence to consensus: 47.2%  
**50. Bov-tA2**: 17613380-17613566 (-), Divergence to consensus: 14.6%  
**51. L1M4**: 17613672-17614088 (-), Divergence to consensus: 45.9%  
**52. (TA)n**: 17614152-17614260 (+), Divergence to consensus: 25.8%  
**53. L1MEi**: 17614322-17614878 (-), Divergence to consensus: 45.1%  
**54. L1MEi**: 17614935-17615379 (-), Divergence to consensus: 40.7%  
**55. L1MEi**: 17615614-17615770 (-), Divergence to consensus: 44.7%  
**56. MER68B**: 17615781-17616066 (-), Divergence to consensus: 35.4%  
**57. MLT1C**: 17616067-17616518 (-), Divergence to consensus: 36.2%  
**58. MER68B**: 17616551-17616716 (-), Divergence to consensus: 25.9%  
**59. MER68-int**: 17617180-17617337 (-), Divergence to consensus: 28.5%  
**60. Bov-tA2**: 17617345-17617531 (+), Divergence to consensus: 18.2%  
**61. MER68-int**: 17617570-17619079 (-), Divergence to consensus: 35.7%  
**62. MER68B**: 17619098-17619646 (-), Divergence to consensus: 35.3%  
**63. L1MEi**: 17619888-17620068 (-), Divergence to consensus: 48.5%  
**64. LTR88c**: 17620145-17620339 (-), Divergence to consensus: 43.9%  
**65. ART2A**: 17621166-17621696 (-), Divergence to consensus: 12.4%  
**66. BovB**: 17621697-17622220 (-), Divergence to consensus: 6.5%  
**67. L3**: 17622255-17622333 (-), Divergence to consensus: 38%  
**68. MamGypLTR1a**: 17623219-17623328 (+), Divergence to consensus: 33.6%  
**69. MLT2B4**: 17623577-17623859 (-), Divergence to consensus: 28.4%

  
Transcription Factor Binding Sites  

**RFX4\_1** (Sequence: CGTGGCAAC (+): 17625652)  
**RFX4\_2** (Sequence: CCTGGATAC (+): 17597290)  
**RFX4\_2** (Sequence: CATGGATAC (+): 17597300)  
**Gata4** (Sequence: AGATAAG (-): 17594841)  
**Gata4** (Sequence: AGATAAG (-): 17600114)  
**Gata4** (Sequence: AGATAAG (-): 17623497)  
**Gata4** (Sequence: AGATAAC (-): 17624370)  
**SOX9** (Sequence: AACAATGA (-): 17580126)  
**SOX9** (Sequence: AACAATAA (-): 17581264)  
**SOX9** (Sequence: AACAATGA (-): 17592501)  
**SOX9** (Sequence: AACAATGA (-): 17593454)  
**SOX9** (Sequence: AACAATAA (-): 17593477)  
**SOX9** (Sequence: AACAATGA (-): 17610561)  
**SOX9** (Sequence: AACAATAA (-): 17625134)  
**SOX9** (Sequence: TTATTGTT (+): 17584659)  
**SOX9** (Sequence: TTATTGTT (+): 17590137)  
**SOX9** (Sequence: CCATTGTT (+): 17600388)  
**SOX9** (Sequence: CTATTGTT (+): 17608985)  
**Gata4** (Sequence: GTTATCT (+): 17577568)  
**Gata4** (Sequence: CTTATCT (+): 17592792)  
**Gata4** (Sequence: GTTATCT (+): 17595751)  
**Gata4** (Sequence: GTTATCT (+): 17598558)  
**Gata4** (Sequence: CTTATCT (+): 17603517)  
**Gata4** (Sequence: GTTATCT (+): 17611382)
