# Supplementary material for: Small RNA sequencing of cryopreserved semen from single bull revealed altered miRNAs and piRNAs expression between High- and Low-motile sperm populations
Source: BMC Genomics. 2017 Jan 4;18:14. doi: 10.1186/s12864-016-3394-7 (PMC5209821; doi:10.1186/s12864-016-3394-7)
Supplement: Additional file 3: — Details for each piRNA clusters found in High Motile (HM) sperm fraction. Genes, repeats, transposable elements and transcription factors binding sites falling within the cluster regions were reported. (ZIP 1896 kb) [file 12864_2016_3394_MOESM3_ESM.zip › 94.html]

piRNA cluster 94


Predicted piRNA cluster no. 94     previous   next
  

Show proTRAC run info
Hide proTRAC run info

================================= proTRAC ====================================  
VERSION: 2.1                                    LAST MODIFIED: 06. October 2015  
  
Please cite:  
Rosenkranz D, Zischler H. proTRAC - a software for probabilistic piRNA cluster  
detection, visualization and analysis. 2012. BMC Bioinformatics 13:5.  
  
and (for proTRAC 2.0 and later):  
Rosenkranz D, Rudloff S, Bastuck K, Ketting RF, Zischler H. Tupaia small RNAs  
provide insights into function and evolution of RNAi-based transposon defense  
in mammals. 2015. RNA 21(5):911-922.  
  
Contact:  
David Rosenkranz  
Institute of Anthropology, small RNA group  
Johannes Gutenberg University Mainz  
email: rosenkranz@uni-mainz.de  
  
You can find the latest proTRAC version at:  
http://sourceforge.net/projects/protrac/files  
http://www.smallRNAgroup-mainz.de/software  
==============================================================================  
  
PARAMETERS:  
Map file: .............../storage/core/barbara/genhome/smallRNA/fertility/Sample\_motile/pirna/Sample\_motile\_26-33\_collapsed.fa.no-dust.map.weighted-10000-1000-b-0  
Genome file: ............/storage/core/barbara/genhome/smallRNA/fertility/Sample\_all/pirna/bt\_311\_chrY.fa  
RepeatMasker annotation: /storage/genomes/bt\_umd31/GCF\_000003055.6\_Bos\_taurus\_UMD\_3.1.1\_repeatMasker\_chr.out  
GeneSet:................./storage/core/barbara/genhome/smallRNA/fertility/Sample\_all/pirna/full.gtf  
  
Significant (p<=0.01) hit density will be calculated based  
on observed hit distribution.  
  
Sliding window size: ........................................ 5000 bp  
Sliding window increament: .................................. 1000 bp  
Normalize each hit by number of genomic hits: ............... 1 [0=no/1=yes]  
Normalize each hit by number of sequence reads: ............. 1 [0=no/1=yes]  
Normalize values (-> per million mapped reads): ............. 1 [0=no/1=yes]  
Min. fraction of hits with 1T(U) or 10A: .................... 0.75  
Alternatively: Min. fraction of hits with 1T(U) and 10A: .... 0.5  
Min. fraction of hits with typical piRNA length: ............ 0.75  
Typical piRNA length: ....................................... 26-33 nt  
Min. size of a piRNA cluster: ............................... 5000 bp.  
Min. number of hits (absolute): ............................. 0  
Min. number of hits (normalized): ........................... 0  
Min. fraction of hits on the mainstrand: .................... 0.75  
Top fraction of mapped sequences (in terms of read counts): . 1%  
Top fraction accounts for max. n% of sequence reads: ........ 90%  
Min. fraction of hits on each arm of a bidirectional cluster: 0.1  
Output image file for each cluster: ......................... 0 [0=no/1=yes]  
Output html file for each cluster: .......................... 1 [0=no/1=yes]  
Output a summary table: ..................................... 1 [0=no/1=yes]  
Output a FASTA file for each cluster (piRNA sequences): ..... 1 [0=no/1=yes]  
Output a FASTA file comprising cluster sequences: ........... 1 [0=no/1=yes]  
Search DNA motifs in clusters: .............................. 1 [0=no/1=yes]  
Output flanking sequences: +/- .............................. 0 bp  
Output ~.pTi file: .......................................... 1 [0=no/1=yes]  
==============================================================================  
  
  
Genome size (without gaps): ............ 2678902517 bp  
Gaps (N/X/-): .......................... 53837044 bp  
Mapped reads: .......................... 658825247023  
Non-identical sequences: ............... 514171  
Genomic hits: .......................... 764233  
Significant densitiy of mapped reads: .. 12867599.5173724 reads/kb

Show proTRAC cluster info
Hide proTRAC cluster info

|  |  |
| --- | --- |
| Location | chr8 |
| Coordinates | 63687889-63698441 |
| Size [bp] | 10553 |
| Sequence hit loci | 223 |
| Mapped reads (normalized) | 279452226 |
| Mapped reads (normalized) per kb | 26480832.6 |
| Normalized reads with 1T (1U) | 78.6% |
| Normalized reads with 10A | 34.5% |
| Normalized reads with length 26-33 nt | 100% |
| Normalized reads on the main strand(s) | 100% |
| Predicted directionality | mono:minus |

100%

0%

1T (1U)  
reads

10A reads

26-33 nt  
reads

reads on mainstrand

**Either the amount of reads with 1T (1U) OR 10A has to exceed 75% (set with option: -1Tor10A)  
Alternatively the amount of reads with 1T (1U) AND 10A has to exceed 50% (set with option: -1Tand10A)  
Minimum amount of reads with preferred size is 75% (set with option: -pisize)  
Minimum amount of reads on the main strand(s) is 75% (set with option: -clstrand)**

Show read coverage
Hide read coverage

WHAT DO I SEE HERE?  
This chart shows the location of mapped sequence reads within a predicted piRNA cluster. The color refers to the number of genomic hits produced by the sequence read in question. A dark red bar indicates that this sequence read produces many other hits elsewhere in the genome. Many adjacent red or yellow bars can indicate the presence of a multi-copy element such as transposons or rRNA genes. A dark green bar indicates that this sequence read maps uniquely to this locus.

1 hit

2-5 hits

6-10 hits

11-20 hits

21-50 hits

51-100 hits

> 100 hits

chr8

63687889

63698441

Gene Set

RepeatMasker

Mapped  
Reads

60.41

plus strand

minus strand

60.41

Region: chr8 17626325-63687899. Max. coverage (+): 0. Max coverage (-): 1.35

Region: chr8 63687900-63687920. Max. coverage (+): 0. Max coverage (-): 4.85

Region: chr8 63687921-63687941. Max. coverage (+): 0. Max coverage (-): 8.94

Region: chr8 63687942-63687962. Max. coverage (+): 0. Max coverage (-): 8.37

Region: chr8 63687963-63687983. Max. coverage (+): 0. Max coverage (-): 0.87

Region: chr8 63687984-63688005. Max. coverage (+): 0. Max coverage (-): 8.88

Region: chr8 63688006-63688026. Max. coverage (+): 0. Max coverage (-): 6.9

Region: chr8 63688027-63688047. Max. coverage (+): 0. Max coverage (-): 57.89

Region: chr8 63688048-63688068. Max. coverage (+): 0. Max coverage (-): 50.42

Region: chr8 63688069-63688089. Max. coverage (+): 0. Max coverage (-): 60.41

Region: chr8 63688090-63688110. Max. coverage (+): 0. Max coverage (-): 2.1

Region: chr8 63688111-63688131. Max. coverage (+): 0. Max coverage (-): 0

Region: chr8 63688132-63688152. Max. coverage (+): 0. Max coverage (-): 0

Region: chr8 63688153-63688173. Max. coverage (+): 0. Max coverage (-): 0

Region: chr8 63688174-63688195. Max. coverage (+): 0. Max coverage (-): 0

Region: chr8 63688196-63688216. Max. coverage (+): 0. Max coverage (-): 0

Region: chr8 63688217-63688237. Max. coverage (+): 0. Max coverage (-): 0

Region: chr8 63688238-63688258. Max. coverage (+): 0. Max coverage (-): 0

Region: chr8 63688259-63688279. Max. coverage (+): 0. Max coverage (-): 0

Region: chr8 63688280-63688300. Max. coverage (+): 0. Max coverage (-): 0

Region: chr8 63688301-63688321. Max. coverage (+): 0. Max coverage (-): 0

Region: chr8 63688322-63688342. Max. coverage (+): 0. Max coverage (-): 0

Region: chr8 63688343-63688363. Max. coverage (+): 0. Max coverage (-): 0

Region: chr8 63688364-63688384. Max. coverage (+): 0. Max coverage (-): 0

Region: chr8 63688385-63688406. Max. coverage (+): 0. Max coverage (-): 0

Region: chr8 63688407-63688427. Max. coverage (+): 0. Max coverage (-): 0

Region: chr8 63688428-63688448. Max. coverage (+): 0. Max coverage (-): 0

Region: chr8 63688449-63688469. Max. coverage (+): 0. Max coverage (-): 0

Region: chr8 63688470-63688490. Max. coverage (+): 0. Max coverage (-): 0

Region: chr8 63688491-63688511. Max. coverage (+): 0. Max coverage (-): 0

Region: chr8 63688512-63688532. Max. coverage (+): 0. Max coverage (-): 0

Region: chr8 63688533-63688553. Max. coverage (+): 0. Max coverage (-): 0

Region: chr8 63688554-63688574. Max. coverage (+): 0. Max coverage (-): 0

Region: chr8 63688575-63688596. Max. coverage (+): 0. Max coverage (-): 0

Region: chr8 63688597-63688617. Max. coverage (+): 0. Max coverage (-): 0

Region: chr8 63688618-63688638. Max. coverage (+): 0. Max coverage (-): 0

Region: chr8 63688639-63688659. Max. coverage (+): 0. Max coverage (-): 0

Region: chr8 63688660-63688680. Max. coverage (+): 0. Max coverage (-): 0

Region: chr8 63688681-63688701. Max. coverage (+): 0. Max coverage (-): 0

Region: chr8 63688702-63688722. Max. coverage (+): 0. Max coverage (-): 0

Region: chr8 63688723-63688743. Max. coverage (+): 0. Max coverage (-): 0

Region: chr8 63688744-63688764. Max. coverage (+): 0. Max coverage (-): 0

Region: chr8 63688765-63688786. Max. coverage (+): 0. Max coverage (-): 0

Region: chr8 63688787-63688807. Max. coverage (+): 0. Max coverage (-): 0

Region: chr8 63688808-63688828. Max. coverage (+): 0. Max coverage (-): 0

Region: chr8 63688829-63688849. Max. coverage (+): 0. Max coverage (-): 0

Region: chr8 63688850-63688870. Max. coverage (+): 0. Max coverage (-): 0

Region: chr8 63688871-63688891. Max. coverage (+): 0. Max coverage (-): 0

Region: chr8 63688892-63688912. Max. coverage (+): 0. Max coverage (-): 0

Region: chr8 63688913-63688933. Max. coverage (+): 0. Max coverage (-): 0

Region: chr8 63688934-63688954. Max. coverage (+): 0. Max coverage (-): 0

Region: chr8 63688955-63688975. Max. coverage (+): 0. Max coverage (-): 0

Region: chr8 63688976-63688997. Max. coverage (+): 0. Max coverage (-): 0

Region: chr8 63688998-63689018. Max. coverage (+): 0. Max coverage (-): 0

Region: chr8 63689019-63689039. Max. coverage (+): 0. Max coverage (-): 0

Region: chr8 63689040-63689060. Max. coverage (+): 0. Max coverage (-): 0

Region: chr8 63689061-63689081. Max. coverage (+): 0. Max coverage (-): 0

Region: chr8 63689082-63689102. Max. coverage (+): 0. Max coverage (-): 0

Region: chr8 63689103-63689123. Max. coverage (+): 0. Max coverage (-): 0

Region: chr8 63689124-63689144. Max. coverage (+): 0. Max coverage (-): 0

Region: chr8 63689145-63689165. Max. coverage (+): 0. Max coverage (-): 0

Region: chr8 63689166-63689187. Max. coverage (+): 0. Max coverage (-): 0

Region: chr8 63689188-63689208. Max. coverage (+): 0. Max coverage (-): 0

Region: chr8 63689209-63689229. Max. coverage (+): 0. Max coverage (-): 0

Region: chr8 63689230-63689250. Max. coverage (+): 0. Max coverage (-): 0

Region: chr8 63689251-63689271. Max. coverage (+): 0. Max coverage (-): 0

Region: chr8 63689272-63689292. Max. coverage (+): 0. Max coverage (-): 0

Region: chr8 63689293-63689313. Max. coverage (+): 0. Max coverage (-): 0

Region: chr8 63689314-63689334. Max. coverage (+): 0. Max coverage (-): 0

Region: chr8 63689335-63689355. Max. coverage (+): 0. Max coverage (-): 0

Region: chr8 63689356-63689376. Max. coverage (+): 0. Max coverage (-): 0

Region: chr8 63689377-63689398. Max. coverage (+): 0. Max coverage (-): 0

Region: chr8 63689399-63689419. Max. coverage (+): 0. Max coverage (-): 0

Region: chr8 63689420-63689440. Max. coverage (+): 0. Max coverage (-): 0

Region: chr8 63689441-63689461. Max. coverage (+): 0. Max coverage (-): 0

Region: chr8 63689462-63689482. Max. coverage (+): 0. Max coverage (-): 0

Region: chr8 63689483-63689503. Max. coverage (+): 0. Max coverage (-): 0

Region: chr8 63689504-63689524. Max. coverage (+): 0. Max coverage (-): 0

Region: chr8 63689525-63689545. Max. coverage (+): 0. Max coverage (-): 0

Region: chr8 63689546-63689566. Max. coverage (+): 0. Max coverage (-): 0

Region: chr8 63689567-63689588. Max. coverage (+): 0. Max coverage (-): 0

Region: chr8 63689589-63689609. Max. coverage (+): 0. Max coverage (-): 0

Region: chr8 63689610-63689630. Max. coverage (+): 0. Max coverage (-): 0

Region: chr8 63689631-63689651. Max. coverage (+): 0. Max coverage (-): 0

Region: chr8 63689652-63689672. Max. coverage (+): 0. Max coverage (-): 0

Region: chr8 63689673-63689693. Max. coverage (+): 0. Max coverage (-): 0

Region: chr8 63689694-63689714. Max. coverage (+): 0. Max coverage (-): 0

Region: chr8 63689715-63689735. Max. coverage (+): 0. Max coverage (-): 0

Region: chr8 63689736-63689756. Max. coverage (+): 0. Max coverage (-): 0

Region: chr8 63689757-63689777. Max. coverage (+): 0. Max coverage (-): 0

Region: chr8 63689778-63689799. Max. coverage (+): 0. Max coverage (-): 0

Region: chr8 63689800-63689820. Max. coverage (+): 0. Max coverage (-): 0

Region: chr8 63689821-63689841. Max. coverage (+): 0. Max coverage (-): 0

Region: chr8 63689842-63689862. Max. coverage (+): 0. Max coverage (-): 0

Region: chr8 63689863-63689883. Max. coverage (+): 0. Max coverage (-): 0

Region: chr8 63689884-63689904. Max. coverage (+): 0. Max coverage (-): 0

Region: chr8 63689905-63689925. Max. coverage (+): 0. Max coverage (-): 0

Region: chr8 63689926-63689946. Max. coverage (+): 0. Max coverage (-): 0

Region: chr8 63689947-63689967. Max. coverage (+): 0. Max coverage (-): 0

Region: chr8 63689968-63689989. Max. coverage (+): 0. Max coverage (-): 0

Region: chr8 63689990-63690010. Max. coverage (+): 0. Max coverage (-): 0

Region: chr8 63690011-63690031. Max. coverage (+): 0. Max coverage (-): 0

Region: chr8 63690032-63690052. Max. coverage (+): 0. Max coverage (-): 0

Region: chr8 63690053-63690073. Max. coverage (+): 0. Max coverage (-): 0

Region: chr8 63690074-63690094. Max. coverage (+): 0. Max coverage (-): 0

Region: chr8 63690095-63690115. Max. coverage (+): 0. Max coverage (-): 0

Region: chr8 63690116-63690136. Max. coverage (+): 0. Max coverage (-): 0

Region: chr8 63690137-63690157. Max. coverage (+): 0. Max coverage (-): 0

Region: chr8 63690158-63690179. Max. coverage (+): 0. Max coverage (-): 0

Region: chr8 63690180-63690200. Max. coverage (+): 0. Max coverage (-): 0

Region: chr8 63690201-63690221. Max. coverage (+): 0. Max coverage (-): 0

Region: chr8 63690222-63690242. Max. coverage (+): 0. Max coverage (-): 0

Region: chr8 63690243-63690263. Max. coverage (+): 0. Max coverage (-): 0

Region: chr8 63690264-63690284. Max. coverage (+): 0. Max coverage (-): 0

Region: chr8 63690285-63690305. Max. coverage (+): 0. Max coverage (-): 0

Region: chr8 63690306-63690326. Max. coverage (+): 0. Max coverage (-): 0

Region: chr8 63690327-63690347. Max. coverage (+): 0. Max coverage (-): 0

Region: chr8 63690348-63690368. Max. coverage (+): 0. Max coverage (-): 0

Region: chr8 63690369-63690390. Max. coverage (+): 0. Max coverage (-): 0

Region: chr8 63690391-63690411. Max. coverage (+): 0. Max coverage (-): 0

Region: chr8 63690412-63690432. Max. coverage (+): 0. Max coverage (-): 0

Region: chr8 63690433-63690453. Max. coverage (+): 0. Max coverage (-): 0

Region: chr8 63690454-63690474. Max. coverage (+): 0. Max coverage (-): 0

Region: chr8 63690475-63690495. Max. coverage (+): 0. Max coverage (-): 0

Region: chr8 63690496-63690516. Max. coverage (+): 0. Max coverage (-): 0

Region: chr8 63690517-63690537. Max. coverage (+): 0. Max coverage (-): 0

Region: chr8 63690538-63690558. Max. coverage (+): 0. Max coverage (-): 0

Region: chr8 63690559-63690580. Max. coverage (+): 0. Max coverage (-): 0

Region: chr8 63690581-63690601. Max. coverage (+): 0. Max coverage (-): 0

Region: chr8 63690602-63690622. Max. coverage (+): 0. Max coverage (-): 0

Region: chr8 63690623-63690643. Max. coverage (+): 0. Max coverage (-): 0

Region: chr8 63690644-63690664. Max. coverage (+): 0. Max coverage (-): 0

Region: chr8 63690665-63690685. Max. coverage (+): 0. Max coverage (-): 0

Region: chr8 63690686-63690706. Max. coverage (+): 0. Max coverage (-): 0

Region: chr8 63690707-63690727. Max. coverage (+): 0. Max coverage (-): 0

Region: chr8 63690728-63690748. Max. coverage (+): 0. Max coverage (-): 0

Region: chr8 63690749-63690769. Max. coverage (+): 0. Max coverage (-): 0

Region: chr8 63690770-63690791. Max. coverage (+): 0. Max coverage (-): 0

Region: chr8 63690792-63690812. Max. coverage (+): 0. Max coverage (-): 0

Region: chr8 63690813-63690833. Max. coverage (+): 0. Max coverage (-): 0

Region: chr8 63690834-63690854. Max. coverage (+): 0. Max coverage (-): 0

Region: chr8 63690855-63690875. Max. coverage (+): 0. Max coverage (-): 0

Region: chr8 63690876-63690896. Max. coverage (+): 0. Max coverage (-): 0

Region: chr8 63690897-63690917. Max. coverage (+): 0. Max coverage (-): 0

Region: chr8 63690918-63690938. Max. coverage (+): 0. Max coverage (-): 0

Region: chr8 63690939-63690959. Max. coverage (+): 0. Max coverage (-): 0

Region: chr8 63690960-63690981. Max. coverage (+): 0. Max coverage (-): 0

Region: chr8 63690982-63691002. Max. coverage (+): 0. Max coverage (-): 0

Region: chr8 63691003-63691023. Max. coverage (+): 0. Max coverage (-): 0

Region: chr8 63691024-63691044. Max. coverage (+): 0. Max coverage (-): 0

Region: chr8 63691045-63691065. Max. coverage (+): 0. Max coverage (-): 0

Region: chr8 63691066-63691086. Max. coverage (+): 0. Max coverage (-): 0

Region: chr8 63691087-63691107. Max. coverage (+): 0. Max coverage (-): 0

Region: chr8 63691108-63691128. Max. coverage (+): 0. Max coverage (-): 0

Region: chr8 63691129-63691149. Max. coverage (+): 0. Max coverage (-): 0

Region: chr8 63691150-63691170. Max. coverage (+): 0. Max coverage (-): 0

Region: chr8 63691171-63691192. Max. coverage (+): 0. Max coverage (-): 0

Region: chr8 63691193-63691213. Max. coverage (+): 0. Max coverage (-): 0

Region: chr8 63691214-63691234. Max. coverage (+): 0. Max coverage (-): 0

Region: chr8 63691235-63691255. Max. coverage (+): 0. Max coverage (-): 0

Region: chr8 63691256-63691276. Max. coverage (+): 0. Max coverage (-): 0

Region: chr8 63691277-63691297. Max. coverage (+): 0. Max coverage (-): 0

Region: chr8 63691298-63691318. Max. coverage (+): 0. Max coverage (-): 0

Region: chr8 63691319-63691339. Max. coverage (+): 0. Max coverage (-): 0

Region: chr8 63691340-63691360. Max. coverage (+): 0. Max coverage (-): 0

Region: chr8 63691361-63691382. Max. coverage (+): 0. Max coverage (-): 0

Region: chr8 63691383-63691403. Max. coverage (+): 0. Max coverage (-): 0

Region: chr8 63691404-63691424. Max. coverage (+): 0. Max coverage (-): 0

Region: chr8 63691425-63691445. Max. coverage (+): 0. Max coverage (-): 0

Region: chr8 63691446-63691466. Max. coverage (+): 0. Max coverage (-): 0

Region: chr8 63691467-63691487. Max. coverage (+): 0. Max coverage (-): 0

Region: chr8 63691488-63691508. Max. coverage (+): 0. Max coverage (-): 0

Region: chr8 63691509-63691529. Max. coverage (+): 0. Max coverage (-): 0

Region: chr8 63691530-63691550. Max. coverage (+): 0. Max coverage (-): 0

Region: chr8 63691551-63691571. Max. coverage (+): 0. Max coverage (-): 0

Region: chr8 63691572-63691593. Max. coverage (+): 0. Max coverage (-): 0

Region: chr8 63691594-63691614. Max. coverage (+): 0. Max coverage (-): 0

Region: chr8 63691615-63691635. Max. coverage (+): 0. Max coverage (-): 0

Region: chr8 63691636-63691656. Max. coverage (+): 0. Max coverage (-): 0

Region: chr8 63691657-63691677. Max. coverage (+): 0. Max coverage (-): 0

Region: chr8 63691678-63691698. Max. coverage (+): 0. Max coverage (-): 0

Region: chr8 63691699-63691719. Max. coverage (+): 0. Max coverage (-): 0

Region: chr8 63691720-63691740. Max. coverage (+): 0. Max coverage (-): 0

Region: chr8 63691741-63691761. Max. coverage (+): 0. Max coverage (-): 0

Region: chr8 63691762-63691783. Max. coverage (+): 0. Max coverage (-): 0

Region: chr8 63691784-63691804. Max. coverage (+): 0. Max coverage (-): 0

Region: chr8 63691805-63691825. Max. coverage (+): 0. Max coverage (-): 0

Region: chr8 63691826-63691846. Max. coverage (+): 0. Max coverage (-): 0

Region: chr8 63691847-63691867. Max. coverage (+): 0. Max coverage (-): 0

Region: chr8 63691868-63691888. Max. coverage (+): 0. Max coverage (-): 0

Region: chr8 63691889-63691909. Max. coverage (+): 0. Max coverage (-): 0

Region: chr8 63691910-63691930. Max. coverage (+): 0. Max coverage (-): 0

Region: chr8 63691931-63691951. Max. coverage (+): 0. Max coverage (-): 0

Region: chr8 63691952-63691973. Max. coverage (+): 0. Max coverage (-): 0

Region: chr8 63691974-63691994. Max. coverage (+): 0. Max coverage (-): 0

Region: chr8 63691995-63692015. Max. coverage (+): 0. Max coverage (-): 0

Region: chr8 63692016-63692036. Max. coverage (+): 0. Max coverage (-): 0

Region: chr8 63692037-63692057. Max. coverage (+): 0. Max coverage (-): 0

Region: chr8 63692058-63692078. Max. coverage (+): 0. Max coverage (-): 0

Region: chr8 63692079-63692099. Max. coverage (+): 0. Max coverage (-): 0

Region: chr8 63692100-63692120. Max. coverage (+): 0. Max coverage (-): 0

Region: chr8 63692121-63692141. Max. coverage (+): 0. Max coverage (-): 0

Region: chr8 63692142-63692162. Max. coverage (+): 0. Max coverage (-): 0

Region: chr8 63692163-63692184. Max. coverage (+): 0. Max coverage (-): 0

Region: chr8 63692185-63692205. Max. coverage (+): 0. Max coverage (-): 0

Region: chr8 63692206-63692226. Max. coverage (+): 0. Max coverage (-): 0

Region: chr8 63692227-63692247. Max. coverage (+): 0. Max coverage (-): 0

Region: chr8 63692248-63692268. Max. coverage (+): 0. Max coverage (-): 0

Region: chr8 63692269-63692289. Max. coverage (+): 0. Max coverage (-): 0

Region: chr8 63692290-63692310. Max. coverage (+): 0. Max coverage (-): 0

Region: chr8 63692311-63692331. Max. coverage (+): 0. Max coverage (-): 0

Region: chr8 63692332-63692352. Max. coverage (+): 0. Max coverage (-): 0

Region: chr8 63692353-63692374. Max. coverage (+): 0. Max coverage (-): 0

Region: chr8 63692375-63692395. Max. coverage (+): 0. Max coverage (-): 0

Region: chr8 63692396-63692416. Max. coverage (+): 0. Max coverage (-): 0

Region: chr8 63692417-63692437. Max. coverage (+): 0. Max coverage (-): 0

Region: chr8 63692438-63692458. Max. coverage (+): 0. Max coverage (-): 0

Region: chr8 63692459-63692479. Max. coverage (+): 0. Max coverage (-): 0

Region: chr8 63692480-63692500. Max. coverage (+): 0. Max coverage (-): 0

Region: chr8 63692501-63692521. Max. coverage (+): 0. Max coverage (-): 0

Region: chr8 63692522-63692542. Max. coverage (+): 0. Max coverage (-): 0

Region: chr8 63692543-63692563. Max. coverage (+): 0. Max coverage (-): 0

Region: chr8 63692564-63692585. Max. coverage (+): 0. Max coverage (-): 0

Region: chr8 63692586-63692606. Max. coverage (+): 0. Max coverage (-): 0

Region: chr8 63692607-63692627. Max. coverage (+): 0. Max coverage (-): 0

Region: chr8 63692628-63692648. Max. coverage (+): 0. Max coverage (-): 0

Region: chr8 63692649-63692669. Max. coverage (+): 0. Max coverage (-): 0

Region: chr8 63692670-63692690. Max. coverage (+): 0. Max coverage (-): 0

Region: chr8 63692691-63692711. Max. coverage (+): 0. Max coverage (-): 0

Region: chr8 63692712-63692732. Max. coverage (+): 0. Max coverage (-): 0

Region: chr8 63692733-63692753. Max. coverage (+): 0. Max coverage (-): 0

Region: chr8 63692754-63692775. Max. coverage (+): 0. Max coverage (-): 0

Region: chr8 63692776-63692796. Max. coverage (+): 0. Max coverage (-): 0

Region: chr8 63692797-63692817. Max. coverage (+): 0. Max coverage (-): 0

Region: chr8 63692818-63692838. Max. coverage (+): 0. Max coverage (-): 0

Region: chr8 63692839-63692859. Max. coverage (+): 0. Max coverage (-): 0

Region: chr8 63692860-63692880. Max. coverage (+): 0. Max coverage (-): 0

Region: chr8 63692881-63692901. Max. coverage (+): 0. Max coverage (-): 0

Region: chr8 63692902-63692922. Max. coverage (+): 0. Max coverage (-): 0

Region: chr8 63692923-63692943. Max. coverage (+): 0. Max coverage (-): 0

Region: chr8 63692944-63692964. Max. coverage (+): 0. Max coverage (-): 0

Region: chr8 63692965-63692986. Max. coverage (+): 0. Max coverage (-): 0

Region: chr8 63692987-63693007. Max. coverage (+): 0. Max coverage (-): 0

Region: chr8 63693008-63693028. Max. coverage (+): 0. Max coverage (-): 0

Region: chr8 63693029-63693049. Max. coverage (+): 0. Max coverage (-): 0

Region: chr8 63693050-63693070. Max. coverage (+): 0. Max coverage (-): 0

Region: chr8 63693071-63693091. Max. coverage (+): 0. Max coverage (-): 0

Region: chr8 63693092-63693112. Max. coverage (+): 0. Max coverage (-): 0

Region: chr8 63693113-63693133. Max. coverage (+): 0. Max coverage (-): 0

Region: chr8 63693134-63693154. Max. coverage (+): 0. Max coverage (-): 0

Region: chr8 63693155-63693176. Max. coverage (+): 0. Max coverage (-): 0

Region: chr8 63693177-63693197. Max. coverage (+): 0. Max coverage (-): 0

Region: chr8 63693198-63693218. Max. coverage (+): 0. Max coverage (-): 0

Region: chr8 63693219-63693239. Max. coverage (+): 0. Max coverage (-): 0

Region: chr8 63693240-63693260. Max. coverage (+): 0. Max coverage (-): 0

Region: chr8 63693261-63693281. Max. coverage (+): 0. Max coverage (-): 0

Region: chr8 63693282-63693302. Max. coverage (+): 0. Max coverage (-): 0

Region: chr8 63693303-63693323. Max. coverage (+): 0. Max coverage (-): 0

Region: chr8 63693324-63693344. Max. coverage (+): 0. Max coverage (-): 0

Region: chr8 63693345-63693366. Max. coverage (+): 0. Max coverage (-): 0

Region: chr8 63693367-63693387. Max. coverage (+): 0. Max coverage (-): 0

Region: chr8 63693388-63693408. Max. coverage (+): 0. Max coverage (-): 0

Region: chr8 63693409-63693429. Max. coverage (+): 0. Max coverage (-): 0

Region: chr8 63693430-63693450. Max. coverage (+): 0. Max coverage (-): 0

Region: chr8 63693451-63693471. Max. coverage (+): 0. Max coverage (-): 0

Region: chr8 63693472-63693492. Max. coverage (+): 0. Max coverage (-): 0

Region: chr8 63693493-63693513. Max. coverage (+): 0. Max coverage (-): 0

Region: chr8 63693514-63693534. Max. coverage (+): 0. Max coverage (-): 0

Region: chr8 63693535-63693555. Max. coverage (+): 0. Max coverage (-): 0

Region: chr8 63693556-63693577. Max. coverage (+): 0. Max coverage (-): 0

Region: chr8 63693578-63693598. Max. coverage (+): 0. Max coverage (-): 0

Region: chr8 63693599-63693619. Max. coverage (+): 0. Max coverage (-): 0

Region: chr8 63693620-63693640. Max. coverage (+): 0. Max coverage (-): 0

Region: chr8 63693641-63693661. Max. coverage (+): 0. Max coverage (-): 0

Region: chr8 63693662-63693682. Max. coverage (+): 0. Max coverage (-): 0

Region: chr8 63693683-63693703. Max. coverage (+): 0. Max coverage (-): 0

Region: chr8 63693704-63693724. Max. coverage (+): 0. Max coverage (-): 0

Region: chr8 63693725-63693745. Max. coverage (+): 0. Max coverage (-): 0

Region: chr8 63693746-63693767. Max. coverage (+): 0. Max coverage (-): 0

Region: chr8 63693768-63693788. Max. coverage (+): 0. Max coverage (-): 0

Region: chr8 63693789-63693809. Max. coverage (+): 0. Max coverage (-): 0

Region: chr8 63693810-63693830. Max. coverage (+): 0. Max coverage (-): 0

Region: chr8 63693831-63693851. Max. coverage (+): 0. Max coverage (-): 0

Region: chr8 63693852-63693872. Max. coverage (+): 0. Max coverage (-): 0

Region: chr8 63693873-63693893. Max. coverage (+): 0. Max coverage (-): 0

Region: chr8 63693894-63693914. Max. coverage (+): 0. Max coverage (-): 0

Region: chr8 63693915-63693935. Max. coverage (+): 0. Max coverage (-): 0

Region: chr8 63693936-63693956. Max. coverage (+): 0. Max coverage (-): 0

Region: chr8 63693957-63693978. Max. coverage (+): 0. Max coverage (-): 0

Region: chr8 63693979-63693999. Max. coverage (+): 0. Max coverage (-): 0

Region: chr8 63694000-63694020. Max. coverage (+): 0. Max coverage (-): 0

Region: chr8 63694021-63694041. Max. coverage (+): 0. Max coverage (-): 0

Region: chr8 63694042-63694062. Max. coverage (+): 0. Max coverage (-): 0

Region: chr8 63694063-63694083. Max. coverage (+): 0. Max coverage (-): 0

Region: chr8 63694084-63694104. Max. coverage (+): 0. Max coverage (-): 0

Region: chr8 63694105-63694125. Max. coverage (+): 0. Max coverage (-): 0.62

Region: chr8 63694126-63694146. Max. coverage (+): 0. Max coverage (-): 0

Region: chr8 63694147-63694168. Max. coverage (+): 0. Max coverage (-): 0

Region: chr8 63694169-63694189. Max. coverage (+): 0. Max coverage (-): 0

Region: chr8 63694190-63694210. Max. coverage (+): 0. Max coverage (-): 0

Region: chr8 63694211-63694231. Max. coverage (+): 0. Max coverage (-): 0

Region: chr8 63694232-63694252. Max. coverage (+): 0. Max coverage (-): 0

Region: chr8 63694253-63694273. Max. coverage (+): 0. Max coverage (-): 0

Region: chr8 63694274-63694294. Max. coverage (+): 0. Max coverage (-): 0

Region: chr8 63694295-63694315. Max. coverage (+): 0. Max coverage (-): 0

Region: chr8 63694316-63694336. Max. coverage (+): 0. Max coverage (-): 0

Region: chr8 63694337-63694357. Max. coverage (+): 0. Max coverage (-): 0

Region: chr8 63694358-63694379. Max. coverage (+): 0. Max coverage (-): 0

Region: chr8 63694380-63694400. Max. coverage (+): 0. Max coverage (-): 0

Region: chr8 63694401-63694421. Max. coverage (+): 0. Max coverage (-): 0

Region: chr8 63694422-63694442. Max. coverage (+): 0. Max coverage (-): 0

Region: chr8 63694443-63694463. Max. coverage (+): 0. Max coverage (-): 0

Region: chr8 63694464-63694484. Max. coverage (+): 0. Max coverage (-): 0

Region: chr8 63694485-63694505. Max. coverage (+): 0. Max coverage (-): 0

Region: chr8 63694506-63694526. Max. coverage (+): 0. Max coverage (-): 0

Region: chr8 63694527-63694547. Max. coverage (+): 0. Max coverage (-): 0

Region: chr8 63694548-63694569. Max. coverage (+): 0. Max coverage (-): 0

Region: chr8 63694570-63694590. Max. coverage (+): 0. Max coverage (-): 0

Region: chr8 63694591-63694611. Max. coverage (+): 0. Max coverage (-): 0

Region: chr8 63694612-63694632. Max. coverage (+): 0. Max coverage (-): 0

Region: chr8 63694633-63694653. Max. coverage (+): 0. Max coverage (-): 0

Region: chr8 63694654-63694674. Max. coverage (+): 0. Max coverage (-): 0

Region: chr8 63694675-63694695. Max. coverage (+): 0. Max coverage (-): 0

Region: chr8 63694696-63694716. Max. coverage (+): 0. Max coverage (-): 0

Region: chr8 63694717-63694737. Max. coverage (+): 0. Max coverage (-): 0

Region: chr8 63694738-63694759. Max. coverage (+): 0. Max coverage (-): 0

Region: chr8 63694760-63694780. Max. coverage (+): 0. Max coverage (-): 0

Region: chr8 63694781-63694801. Max. coverage (+): 0. Max coverage (-): 0

Region: chr8 63694802-63694822. Max. coverage (+): 0. Max coverage (-): 0

Region: chr8 63694823-63694843. Max. coverage (+): 0. Max coverage (-): 0

Region: chr8 63694844-63694864. Max. coverage (+): 0. Max coverage (-): 0

Region: chr8 63694865-63694885. Max. coverage (+): 0. Max coverage (-): 0

Region: chr8 63694886-63694906. Max. coverage (+): 0. Max coverage (-): 0

Region: chr8 63694907-63694927. Max. coverage (+): 0. Max coverage (-): 0

Region: chr8 63694928-63694948. Max. coverage (+): 0. Max coverage (-): 0

Region: chr8 63694949-63694970. Max. coverage (+): 0. Max coverage (-): 0

Region: chr8 63694971-63694991. Max. coverage (+): 0. Max coverage (-): 0

Region: chr8 63694992-63695012. Max. coverage (+): 0. Max coverage (-): 0

Region: chr8 63695013-63695033. Max. coverage (+): 0. Max coverage (-): 0

Region: chr8 63695034-63695054. Max. coverage (+): 0. Max coverage (-): 0

Region: chr8 63695055-63695075. Max. coverage (+): 0. Max coverage (-): 0

Region: chr8 63695076-63695096. Max. coverage (+): 0. Max coverage (-): 0

Region: chr8 63695097-63695117. Max. coverage (+): 0. Max coverage (-): 0

Region: chr8 63695118-63695138. Max. coverage (+): 0. Max coverage (-): 0

Region: chr8 63695139-63695160. Max. coverage (+): 0. Max coverage (-): 0

Region: chr8 63695161-63695181. Max. coverage (+): 0. Max coverage (-): 0

Region: chr8 63695182-63695202. Max. coverage (+): 0. Max coverage (-): 0

Region: chr8 63695203-63695223. Max. coverage (+): 0. Max coverage (-): 0

Region: chr8 63695224-63695244. Max. coverage (+): 0. Max coverage (-): 0

Region: chr8 63695245-63695265. Max. coverage (+): 0. Max coverage (-): 0

Region: chr8 63695266-63695286. Max. coverage (+): 0. Max coverage (-): 0

Region: chr8 63695287-63695307. Max. coverage (+): 0. Max coverage (-): 0

Region: chr8 63695308-63695328. Max. coverage (+): 0. Max coverage (-): 0

Region: chr8 63695329-63695349. Max. coverage (+): 0. Max coverage (-): 0

Region: chr8 63695350-63695371. Max. coverage (+): 0. Max coverage (-): 0

Region: chr8 63695372-63695392. Max. coverage (+): 0. Max coverage (-): 0

Region: chr8 63695393-63695413. Max. coverage (+): 0. Max coverage (-): 0

Region: chr8 63695414-63695434. Max. coverage (+): 0. Max coverage (-): 0

Region: chr8 63695435-63695455. Max. coverage (+): 0. Max coverage (-): 0

Region: chr8 63695456-63695476. Max. coverage (+): 0. Max coverage (-): 0

Region: chr8 63695477-63695497. Max. coverage (+): 0. Max coverage (-): 0

Region: chr8 63695498-63695518. Max. coverage (+): 0. Max coverage (-): 0

Region: chr8 63695519-63695539. Max. coverage (+): 0. Max coverage (-): 0

Region: chr8 63695540-63695561. Max. coverage (+): 0. Max coverage (-): 5.26

Region: chr8 63695562-63695582. Max. coverage (+): 0. Max coverage (-): 10.49

Region: chr8 63695583-63695603. Max. coverage (+): 0. Max coverage (-): 0.33

Region: chr8 63695604-63695624. Max. coverage (+): 0. Max coverage (-): 0

Region: chr8 63695625-63695645. Max. coverage (+): 0. Max coverage (-): 0

Region: chr8 63695646-63695666. Max. coverage (+): 0. Max coverage (-): 0

Region: chr8 63695667-63695687. Max. coverage (+): 0. Max coverage (-): 0

Region: chr8 63695688-63695708. Max. coverage (+): 0. Max coverage (-): 0

Region: chr8 63695709-63695729. Max. coverage (+): 0. Max coverage (-): 0

Region: chr8 63695730-63695750. Max. coverage (+): 0. Max coverage (-): 0

Region: chr8 63695751-63695772. Max. coverage (+): 0. Max coverage (-): 0

Region: chr8 63695773-63695793. Max. coverage (+): 0. Max coverage (-): 0

Region: chr8 63695794-63695814. Max. coverage (+): 0. Max coverage (-): 0

Region: chr8 63695815-63695835. Max. coverage (+): 0. Max coverage (-): 0

Region: chr8 63695836-63695856. Max. coverage (+): 0. Max coverage (-): 0

Region: chr8 63695857-63695877. Max. coverage (+): 0. Max coverage (-): 0

Region: chr8 63695878-63695898. Max. coverage (+): 0. Max coverage (-): 0

Region: chr8 63695899-63695919. Max. coverage (+): 0. Max coverage (-): 0

Region: chr8 63695920-63695940. Max. coverage (+): 0. Max coverage (-): 0

Region: chr8 63695941-63695962. Max. coverage (+): 0. Max coverage (-): 0

Region: chr8 63695963-63695983. Max. coverage (+): 0. Max coverage (-): 0

Region: chr8 63695984-63696004. Max. coverage (+): 0. Max coverage (-): 0

Region: chr8 63696005-63696025. Max. coverage (+): 0. Max coverage (-): 0

Region: chr8 63696026-63696046. Max. coverage (+): 0. Max coverage (-): 0

Region: chr8 63696047-63696067. Max. coverage (+): 0. Max coverage (-): 0

Region: chr8 63696068-63696088. Max. coverage (+): 0. Max coverage (-): 0

Region: chr8 63696089-63696109. Max. coverage (+): 0. Max coverage (-): 0

Region: chr8 63696110-63696130. Max. coverage (+): 0. Max coverage (-): 0

Region: chr8 63696131-63696151. Max. coverage (+): 0. Max coverage (-): 0

Region: chr8 63696152-63696173. Max. coverage (+): 0. Max coverage (-): 0

Region: chr8 63696174-63696194. Max. coverage (+): 0. Max coverage (-): 0

Region: chr8 63696195-63696215. Max. coverage (+): 0. Max coverage (-): 0

Region: chr8 63696216-63696236. Max. coverage (+): 0. Max coverage (-): 0

Region: chr8 63696237-63696257. Max. coverage (+): 0. Max coverage (-): 0

Region: chr8 63696258-63696278. Max. coverage (+): 0. Max coverage (-): 0

Region: chr8 63696279-63696299. Max. coverage (+): 0. Max coverage (-): 0

Region: chr8 63696300-63696320. Max. coverage (+): 0. Max coverage (-): 0

Region: chr8 63696321-63696341. Max. coverage (+): 0. Max coverage (-): 0

Region: chr8 63696342-63696363. Max. coverage (+): 0. Max coverage (-): 2.2

Region: chr8 63696364-63696384. Max. coverage (+): 0. Max coverage (-): 2.2

Region: chr8 63696385-63696405. Max. coverage (+): 0. Max coverage (-): 2.76

Region: chr8 63696406-63696426. Max. coverage (+): 0. Max coverage (-): 0

Region: chr8 63696427-63696447. Max. coverage (+): 0. Max coverage (-): 0

Region: chr8 63696448-63696468. Max. coverage (+): 0. Max coverage (-): 0

Region: chr8 63696469-63696489. Max. coverage (+): 0. Max coverage (-): 0

Region: chr8 63696490-63696510. Max. coverage (+): 0. Max coverage (-): 0

Region: chr8 63696511-63696531. Max. coverage (+): 0. Max coverage (-): 0

Region: chr8 63696532-63696553. Max. coverage (+): 0. Max coverage (-): 0

Region: chr8 63696554-63696574. Max. coverage (+): 0. Max coverage (-): 0

Region: chr8 63696575-63696595. Max. coverage (+): 0. Max coverage (-): 0

Region: chr8 63696596-63696616. Max. coverage (+): 0. Max coverage (-): 0

Region: chr8 63696617-63696637. Max. coverage (+): 0. Max coverage (-): 0

Region: chr8 63696638-63696658. Max. coverage (+): 0. Max coverage (-): 0

Region: chr8 63696659-63696679. Max. coverage (+): 0. Max coverage (-): 0

Region: chr8 63696680-63696700. Max. coverage (+): 0. Max coverage (-): 0

Region: chr8 63696701-63696721. Max. coverage (+): 0. Max coverage (-): 5.83

Region: chr8 63696722-63696742. Max. coverage (+): 0. Max coverage (-): 5.83

Region: chr8 63696743-63696764. Max. coverage (+): 0. Max coverage (-): 0

Region: chr8 63696765-63696785. Max. coverage (+): 0. Max coverage (-): 3.24

Region: chr8 63696786-63696806. Max. coverage (+): 0. Max coverage (-): 20.38

Region: chr8 63696807-63696827. Max. coverage (+): 0. Max coverage (-): 3.99

Region: chr8 63696828-63696848. Max. coverage (+): 0. Max coverage (-): 3.99

Region: chr8 63696849-63696869. Max. coverage (+): 0. Max coverage (-): 0

Region: chr8 63696870-63696890. Max. coverage (+): 0. Max coverage (-): 0

Region: chr8 63696891-63696911. Max. coverage (+): 0. Max coverage (-): 0

Region: chr8 63696912-63696932. Max. coverage (+): 0. Max coverage (-): 0

Region: chr8 63696933-63696954. Max. coverage (+): 0. Max coverage (-): 0

Region: chr8 63696955-63696975. Max. coverage (+): 0. Max coverage (-): 0

Region: chr8 63696976-63696996. Max. coverage (+): 0. Max coverage (-): 0

Region: chr8 63696997-63697017. Max. coverage (+): 0. Max coverage (-): 0

Region: chr8 63697018-63697038. Max. coverage (+): 0. Max coverage (-): 0

Region: chr8 63697039-63697059. Max. coverage (+): 0. Max coverage (-): 0

Region: chr8 63697060-63697080. Max. coverage (+): 0. Max coverage (-): 0

Region: chr8 63697081-63697101. Max. coverage (+): 0. Max coverage (-): 0

Region: chr8 63697102-63697122. Max. coverage (+): 0. Max coverage (-): 0

Region: chr8 63697123-63697143. Max. coverage (+): 0. Max coverage (-): 0

Region: chr8 63697144-63697165. Max. coverage (+): 0. Max coverage (-): 2.31

Region: chr8 63697166-63697186. Max. coverage (+): 0. Max coverage (-): 8.26

Region: chr8 63697187-63697207. Max. coverage (+): 0. Max coverage (-): 2.58

Region: chr8 63697208-63697228. Max. coverage (+): 0. Max coverage (-): 2.84

Region: chr8 63697229-63697249. Max. coverage (+): 0. Max coverage (-): 2.84

Region: chr8 63697250-63697270. Max. coverage (+): 0. Max coverage (-): 5.96

Region: chr8 63697271-63697291. Max. coverage (+): 0. Max coverage (-): 1.77

Region: chr8 63697292-63697312. Max. coverage (+): 0. Max coverage (-): 2.25

Region: chr8 63697313-63697333. Max. coverage (+): 0. Max coverage (-): 7.74

Region: chr8 63697334-63697355. Max. coverage (+): 0. Max coverage (-): 43.32

Region: chr8 63697356-63697376. Max. coverage (+): 0. Max coverage (-): 30.82

Region: chr8 63697377-63697397. Max. coverage (+): 0. Max coverage (-): 14.71

Region: chr8 63697398-63697418. Max. coverage (+): 0. Max coverage (-): 0

Region: chr8 63697419-63697439. Max. coverage (+): 0. Max coverage (-): 0

Region: chr8 63697440-63697460. Max. coverage (+): 0. Max coverage (-): 0

Region: chr8 63697461-63697481. Max. coverage (+): 0. Max coverage (-): 0

Region: chr8 63697482-63697502. Max. coverage (+): 0. Max coverage (-): 0

Region: chr8 63697503-63697523. Max. coverage (+): 0. Max coverage (-): 0

Region: chr8 63697524-63697544. Max. coverage (+): 0. Max coverage (-): 0

Region: chr8 63697545-63697566. Max. coverage (+): 0. Max coverage (-): 0

Region: chr8 63697567-63697587. Max. coverage (+): 0. Max coverage (-): 0

Region: chr8 63697588-63697608. Max. coverage (+): 0. Max coverage (-): 2.96

Region: chr8 63697609-63697629. Max. coverage (+): 0. Max coverage (-): 1.01

Region: chr8 63697630-63697650. Max. coverage (+): 0. Max coverage (-): 2.94

Region: chr8 63697651-63697671. Max. coverage (+): 0. Max coverage (-): 0

Region: chr8 63697672-63697692. Max. coverage (+): 0. Max coverage (-): 0

Region: chr8 63697693-63697713. Max. coverage (+): 0. Max coverage (-): 0

Region: chr8 63697714-63697734. Max. coverage (+): 0. Max coverage (-): 1.91

Region: chr8 63697735-63697756. Max. coverage (+): 0. Max coverage (-): 2.01

Region: chr8 63697757-63697777. Max. coverage (+): 0. Max coverage (-): 0

Region: chr8 63697778-63697798. Max. coverage (+): 0. Max coverage (-): 0

Region: chr8 63697799-63697819. Max. coverage (+): 0. Max coverage (-): 0

Region: chr8 63697820-63697840. Max. coverage (+): 0. Max coverage (-): 0

Region: chr8 63697841-63697861. Max. coverage (+): 0. Max coverage (-): 0

Region: chr8 63697862-63697882. Max. coverage (+): 0. Max coverage (-): 0

Region: chr8 63697883-63697903. Max. coverage (+): 0. Max coverage (-): 0

Region: chr8 63697904-63697924. Max. coverage (+): 0. Max coverage (-): 0

Region: chr8 63697925-63697946. Max. coverage (+): 0. Max coverage (-): 0

Region: chr8 63697947-63697967. Max. coverage (+): 0. Max coverage (-): 0

Region: chr8 63697968-63697988. Max. coverage (+): 0. Max coverage (-): 0

Region: chr8 63697989-63698009. Max. coverage (+): 0. Max coverage (-): 0

Region: chr8 63698010-63698030. Max. coverage (+): 0. Max coverage (-): 0

Region: chr8 63698031-63698051. Max. coverage (+): 0. Max coverage (-): 0

Region: chr8 63698052-63698072. Max. coverage (+): 0. Max coverage (-): 0

Region: chr8 63698073-63698093. Max. coverage (+): 0. Max coverage (-): 0

Region: chr8 63698094-63698114. Max. coverage (+): 0. Max coverage (-): 0

Region: chr8 63698115-63698135. Max. coverage (+): 0. Max coverage (-): 0

Region: chr8 63698136-63698157. Max. coverage (+): 0. Max coverage (-): 0

Region: chr8 63698158-63698178. Max. coverage (+): 0. Max coverage (-): 0

Region: chr8 63698179-63698199. Max. coverage (+): 0. Max coverage (-): 0

Region: chr8 63698200-63698220. Max. coverage (+): 0. Max coverage (-): 0

Region: chr8 63698221-63698241. Max. coverage (+): 0. Max coverage (-): 0

Region: chr8 63698242-63698262. Max. coverage (+): 0. Max coverage (-): 2.59

Region: chr8 63698263-63698283. Max. coverage (+): 0. Max coverage (-): 0

Region: chr8 63698284-63698304. Max. coverage (+): 0. Max coverage (-): 0

Region: chr8 63698305-63698325. Max. coverage (+): 0. Max coverage (-): 0

Region: chr8 63698326-63698347. Max. coverage (+): 0. Max coverage (-): 0.72

Region: chr8 63698348-63698368. Max. coverage (+): 0. Max coverage (-): 0

Region: chr8 63698369-63698389. Max. coverage (+): 0. Max coverage (-): 0

Region: chr8 63698390-63698410. Max. coverage (+): 0. Max coverage (-): 0

Region: chr8 63698411-63698431. Max. coverage (+): 0. Max coverage (-): 4.95

Region: chr8 63698432-. Max. coverage (+): 0. Max coverage (-): 0

RepeatMasker Color Code

**+**

100-98% Identity

<98-95% Identity

<95-90% Identity

<90-85% Identity

<85-80% Identity

<80-75% Identity

<75-70% Identity

<70% Identity

**-**

Gene Set Color Code

**+**

Gene

Pseudogene

**-**

Topology/Coverage Color Code

Coverage Plus Strand

Coverage Minus Strand

Mainstrand: Plus

Mainstrand: Minus

Complementary Strand

Flanking Region  
(if option -flank >0)

Gene Set Annotation  

**1. TRIM14 (protein coding, ENSBTAG00000024851) Tr:00000034608 Ex:2**: 63695562-63695657 (-)  
**2. TRIM14 (protein coding, ENSBTAG00000024851) Tr:00000034608 Ex:3**: 63687888-63688121 (-)

  
RepeatMasker Annotation  

**1. L1ME4a**: 63688152-63688535 (+), Divergence to consensus: 39.2%  
**2. SINE2-1\_BT**: 63688620-63688737 (+), Divergence to consensus: 23.7%  
**3. Bov-tA2**: 63688774-63688977 (-), Divergence to consensus: 13.4%  
**4. (TA)n**: 63689063-63689166 (+), Divergence to consensus: 25%  
**5. L1MC5**: 63689258-63689374 (+), Divergence to consensus: 27%  
**6. L1MC5**: 63689383-63689520 (+), Divergence to consensus: 31.9%  
**7. L1MB4**: 63690015-63690657 (+), Divergence to consensus: 33.8%  
**8. BOV-A2**: 63690658-63690926 (+), Divergence to consensus: 4.5%  
**9. L1MB4**: 63690927-63691099 (+), Divergence to consensus: 25.8%  
**10. L1MB4**: 63691139-63691212 (+), Divergence to consensus: 16.3%  
**11. L1ME3G**: 63691225-63691554 (+), Divergence to consensus: 47.2%  
**12. AT\_rich**: 63692288-63692309 (+), Divergence to consensus: 40.9%  
**13. Bov-tA2**: 63692311-63692490 (-), Divergence to consensus: 15%  
**14. L1ME4a**: 63693002-63693262 (+), Divergence to consensus: 44.4%  
**15. Bov-tA2**: 63693319-63693519 (-), Divergence to consensus: 24.9%  
**16. Bov-tA2**: 63693539-63693662 (-), Divergence to consensus: 7.3%  
**17. Bov-tA2**: 63694186-63694388 (-), Divergence to consensus: 15.3%  
**18. L3**: 63694413-63694511 (-), Divergence to consensus: 32%  
**19. MER94**: 63694647-63694726 (-), Divergence to consensus: 35%  
**20. Arthur1A**: 63695308-63695452 (+), Divergence to consensus: 33.8%  
**21. MIR3**: 63695712-63695852 (-), Divergence to consensus: 35.2%  
**22. MIRb**: 63696425-63696656 (+), Divergence to consensus: 46.4%  
**23. L2c**: 63696858-63697085 (+), Divergence to consensus: 44.5%  
**24. ART2A**: 63697400-63697589 (-), Divergence to consensus: 16.2%

  
Transcription Factor Binding Sites  

**RFX4\_2** (Sequence: GTATCCAGG (-): 63691629)  
**Gata4** (Sequence: AGATAAC (-): 63695705)  
**Gata4** (Sequence: AGATAAG (-): 63697264)  
**SOX9** (Sequence: CTATTGTT (+): 63697240)  
**Gata4** (Sequence: CTTATCT (+): 63689628)  
**Gata4** (Sequence: GTTATCT (+): 63697190)
