# Supplementary material for: Small RNA sequencing of cryopreserved semen from single bull revealed altered miRNAs and piRNAs expression between High- and Low-motile sperm populations
Source: BMC Genomics. 2017 Jan 4;18:14. doi: 10.1186/s12864-016-3394-7 (PMC5209821; doi:10.1186/s12864-016-3394-7)
Supplement: Additional file 3: — Details for each piRNA clusters found in High Motile (HM) sperm fraction. Genes, repeats, transposable elements and transcription factors binding sites falling within the cluster regions were reported. (ZIP 1896 kb) [file 12864_2016_3394_MOESM3_ESM.zip › 95.html]

piRNA cluster 95


Predicted piRNA cluster no. 95     previous   next
  

Show proTRAC run info
Hide proTRAC run info

================================= proTRAC ====================================  
VERSION: 2.1                                    LAST MODIFIED: 06. October 2015  
  
Please cite:  
Rosenkranz D, Zischler H. proTRAC - a software for probabilistic piRNA cluster  
detection, visualization and analysis. 2012. BMC Bioinformatics 13:5.  
  
and (for proTRAC 2.0 and later):  
Rosenkranz D, Rudloff S, Bastuck K, Ketting RF, Zischler H. Tupaia small RNAs  
provide insights into function and evolution of RNAi-based transposon defense  
in mammals. 2015. RNA 21(5):911-922.  
  
Contact:  
David Rosenkranz  
Institute of Anthropology, small RNA group  
Johannes Gutenberg University Mainz  
email: rosenkranz@uni-mainz.de  
  
You can find the latest proTRAC version at:  
http://sourceforge.net/projects/protrac/files  
http://www.smallRNAgroup-mainz.de/software  
==============================================================================  
  
PARAMETERS:  
Map file: .............../storage/core/barbara/genhome/smallRNA/fertility/Sample\_motile/pirna/Sample\_motile\_26-33\_collapsed.fa.no-dust.map.weighted-10000-1000-b-0  
Genome file: ............/storage/core/barbara/genhome/smallRNA/fertility/Sample\_all/pirna/bt\_311\_chrY.fa  
RepeatMasker annotation: /storage/genomes/bt\_umd31/GCF\_000003055.6\_Bos\_taurus\_UMD\_3.1.1\_repeatMasker\_chr.out  
GeneSet:................./storage/core/barbara/genhome/smallRNA/fertility/Sample\_all/pirna/full.gtf  
  
Significant (p<=0.01) hit density will be calculated based  
on observed hit distribution.  
  
Sliding window size: ........................................ 5000 bp  
Sliding window increament: .................................. 1000 bp  
Normalize each hit by number of genomic hits: ............... 1 [0=no/1=yes]  
Normalize each hit by number of sequence reads: ............. 1 [0=no/1=yes]  
Normalize values (-> per million mapped reads): ............. 1 [0=no/1=yes]  
Min. fraction of hits with 1T(U) or 10A: .................... 0.75  
Alternatively: Min. fraction of hits with 1T(U) and 10A: .... 0.5  
Min. fraction of hits with typical piRNA length: ............ 0.75  
Typical piRNA length: ....................................... 26-33 nt  
Min. size of a piRNA cluster: ............................... 5000 bp.  
Min. number of hits (absolute): ............................. 0  
Min. number of hits (normalized): ........................... 0  
Min. fraction of hits on the mainstrand: .................... 0.75  
Top fraction of mapped sequences (in terms of read counts): . 1%  
Top fraction accounts for max. n% of sequence reads: ........ 90%  
Min. fraction of hits on each arm of a bidirectional cluster: 0.1  
Output image file for each cluster: ......................... 0 [0=no/1=yes]  
Output html file for each cluster: .......................... 1 [0=no/1=yes]  
Output a summary table: ..................................... 1 [0=no/1=yes]  
Output a FASTA file for each cluster (piRNA sequences): ..... 1 [0=no/1=yes]  
Output a FASTA file comprising cluster sequences: ........... 1 [0=no/1=yes]  
Search DNA motifs in clusters: .............................. 1 [0=no/1=yes]  
Output flanking sequences: +/- .............................. 0 bp  
Output ~.pTi file: .......................................... 1 [0=no/1=yes]  
==============================================================================  
  
  
Genome size (without gaps): ............ 2678902517 bp  
Gaps (N/X/-): .......................... 53837044 bp  
Mapped reads: .......................... 658825247023  
Non-identical sequences: ............... 514171  
Genomic hits: .......................... 764233  
Significant densitiy of mapped reads: .. 12867599.5173724 reads/kb

Show proTRAC cluster info
Hide proTRAC cluster info

|  |  |
| --- | --- |
| Location | chr8 |
| Coordinates | 85687285-85692721 |
| Size [bp] | 5437 |
| Sequence hit loci | 64 |
| Mapped reads (normalized) | 81287880 |
| Mapped reads (normalized) per kb | 14950870 |
| Normalized reads with 1T (1U) | 87.4% |
| Normalized reads with 10A | 36.4% |
| Normalized reads with length 26-33 nt | 100% |
| Normalized reads on the main strand(s) | 94.8% |
| Predicted directionality | mono:minus |

100%

0%

1T (1U)  
reads

10A reads

26-33 nt  
reads

reads on mainstrand

**Either the amount of reads with 1T (1U) OR 10A has to exceed 75% (set with option: -1Tor10A)  
Alternatively the amount of reads with 1T (1U) AND 10A has to exceed 50% (set with option: -1Tand10A)  
Minimum amount of reads with preferred size is 75% (set with option: -pisize)  
Minimum amount of reads on the main strand(s) is 75% (set with option: -clstrand)**

Show read coverage
Hide read coverage

WHAT DO I SEE HERE?  
This chart shows the location of mapped sequence reads within a predicted piRNA cluster. The color refers to the number of genomic hits produced by the sequence read in question. A dark red bar indicates that this sequence read produces many other hits elsewhere in the genome. Many adjacent red or yellow bars can indicate the presence of a multi-copy element such as transposons or rRNA genes. A dark green bar indicates that this sequence read maps uniquely to this locus.

1 hit

2-5 hits

6-10 hits

11-20 hits

21-50 hits

51-100 hits

> 100 hits

chr8

85687285

85692721

Gene Set

RepeatMasker

Mapped  
Reads

11.8

plus strand

minus strand

11.8

Region: chr8 63698433-85687290. Max. coverage (+): 4.54. Max coverage (-): 0

Region: chr8 85687291-85687301. Max. coverage (+): 4.54. Max coverage (-): 0

Region: chr8 85687302-85687312. Max. coverage (+): 0. Max coverage (-): 0

Region: chr8 85687313-85687323. Max. coverage (+): 0. Max coverage (-): 0

Region: chr8 85687324-85687333. Max. coverage (+): 0. Max coverage (-): 0

Region: chr8 85687334-85687344. Max. coverage (+): 0. Max coverage (-): 0

Region: chr8 85687345-85687355. Max. coverage (+): 0. Max coverage (-): 0

Region: chr8 85687356-85687366. Max. coverage (+): 0. Max coverage (-): 0

Region: chr8 85687367-85687377. Max. coverage (+): 0. Max coverage (-): 0

Region: chr8 85687378-85687388. Max. coverage (+): 0. Max coverage (-): 0

Region: chr8 85687389-85687399. Max. coverage (+): 0. Max coverage (-): 0

Region: chr8 85687400-85687410. Max. coverage (+): 0. Max coverage (-): 0

Region: chr8 85687411-85687420. Max. coverage (+): 0. Max coverage (-): 0

Region: chr8 85687421-85687431. Max. coverage (+): 0. Max coverage (-): 0

Region: chr8 85687432-85687442. Max. coverage (+): 0. Max coverage (-): 0

Region: chr8 85687443-85687453. Max. coverage (+): 0. Max coverage (-): 0

Region: chr8 85687454-85687464. Max. coverage (+): 0. Max coverage (-): 0

Region: chr8 85687465-85687475. Max. coverage (+): 0. Max coverage (-): 0

Region: chr8 85687476-85687486. Max. coverage (+): 0. Max coverage (-): 0

Region: chr8 85687487-85687497. Max. coverage (+): 0. Max coverage (-): 0

Region: chr8 85687498-85687507. Max. coverage (+): 0. Max coverage (-): 0

Region: chr8 85687508-85687518. Max. coverage (+): 0. Max coverage (-): 0

Region: chr8 85687519-85687529. Max. coverage (+): 0. Max coverage (-): 0

Region: chr8 85687530-85687540. Max. coverage (+): 0. Max coverage (-): 0

Region: chr8 85687541-85687551. Max. coverage (+): 0. Max coverage (-): 0

Region: chr8 85687552-85687562. Max. coverage (+): 0. Max coverage (-): 0

Region: chr8 85687563-85687573. Max. coverage (+): 0. Max coverage (-): 0

Region: chr8 85687574-85687584. Max. coverage (+): 0. Max coverage (-): 0

Region: chr8 85687585-85687594. Max. coverage (+): 0. Max coverage (-): 0

Region: chr8 85687595-85687605. Max. coverage (+): 0. Max coverage (-): 0

Region: chr8 85687606-85687616. Max. coverage (+): 0. Max coverage (-): 0

Region: chr8 85687617-85687627. Max. coverage (+): 0. Max coverage (-): 0

Region: chr8 85687628-85687638. Max. coverage (+): 0. Max coverage (-): 0

Region: chr8 85687639-85687649. Max. coverage (+): 0. Max coverage (-): 0

Region: chr8 85687650-85687660. Max. coverage (+): 0. Max coverage (-): 0

Region: chr8 85687661-85687671. Max. coverage (+): 0. Max coverage (-): 0

Region: chr8 85687672-85687681. Max. coverage (+): 0. Max coverage (-): 0

Region: chr8 85687682-85687692. Max. coverage (+): 0. Max coverage (-): 0

Region: chr8 85687693-85687703. Max. coverage (+): 0. Max coverage (-): 0

Region: chr8 85687704-85687714. Max. coverage (+): 0. Max coverage (-): 0

Region: chr8 85687715-85687725. Max. coverage (+): 0. Max coverage (-): 0

Region: chr8 85687726-85687736. Max. coverage (+): 0. Max coverage (-): 0

Region: chr8 85687737-85687747. Max. coverage (+): 0. Max coverage (-): 0

Region: chr8 85687748-85687758. Max. coverage (+): 0. Max coverage (-): 0

Region: chr8 85687759-85687768. Max. coverage (+): 0. Max coverage (-): 0

Region: chr8 85687769-85687779. Max. coverage (+): 0. Max coverage (-): 0

Region: chr8 85687780-85687790. Max. coverage (+): 0. Max coverage (-): 0

Region: chr8 85687791-85687801. Max. coverage (+): 0. Max coverage (-): 0

Region: chr8 85687802-85687812. Max. coverage (+): 0. Max coverage (-): 0

Region: chr8 85687813-85687823. Max. coverage (+): 0. Max coverage (-): 0

Region: chr8 85687824-85687834. Max. coverage (+): 0. Max coverage (-): 0

Region: chr8 85687835-85687845. Max. coverage (+): 0. Max coverage (-): 0

Region: chr8 85687846-85687855. Max. coverage (+): 0. Max coverage (-): 0

Region: chr8 85687856-85687866. Max. coverage (+): 0. Max coverage (-): 0

Region: chr8 85687867-85687877. Max. coverage (+): 0. Max coverage (-): 0

Region: chr8 85687878-85687888. Max. coverage (+): 0. Max coverage (-): 0

Region: chr8 85687889-85687899. Max. coverage (+): 0. Max coverage (-): 0

Region: chr8 85687900-85687910. Max. coverage (+): 0. Max coverage (-): 0

Region: chr8 85687911-85687921. Max. coverage (+): 0. Max coverage (-): 0

Region: chr8 85687922-85687932. Max. coverage (+): 0. Max coverage (-): 0

Region: chr8 85687933-85687942. Max. coverage (+): 0. Max coverage (-): 0

Region: chr8 85687943-85687953. Max. coverage (+): 0. Max coverage (-): 0

Region: chr8 85687954-85687964. Max. coverage (+): 0. Max coverage (-): 0

Region: chr8 85687965-85687975. Max. coverage (+): 0. Max coverage (-): 0

Region: chr8 85687976-85687986. Max. coverage (+): 0. Max coverage (-): 0

Region: chr8 85687987-85687997. Max. coverage (+): 0. Max coverage (-): 0

Region: chr8 85687998-85688008. Max. coverage (+): 0. Max coverage (-): 0

Region: chr8 85688009-85688018. Max. coverage (+): 0. Max coverage (-): 0

Region: chr8 85688019-85688029. Max. coverage (+): 0. Max coverage (-): 0

Region: chr8 85688030-85688040. Max. coverage (+): 0. Max coverage (-): 0

Region: chr8 85688041-85688051. Max. coverage (+): 0. Max coverage (-): 0

Region: chr8 85688052-85688062. Max. coverage (+): 0. Max coverage (-): 0

Region: chr8 85688063-85688073. Max. coverage (+): 0. Max coverage (-): 0

Region: chr8 85688074-85688084. Max. coverage (+): 0. Max coverage (-): 0

Region: chr8 85688085-85688095. Max. coverage (+): 0. Max coverage (-): 0

Region: chr8 85688096-85688105. Max. coverage (+): 0. Max coverage (-): 0

Region: chr8 85688106-85688116. Max. coverage (+): 0. Max coverage (-): 0

Region: chr8 85688117-85688127. Max. coverage (+): 0. Max coverage (-): 0

Region: chr8 85688128-85688138. Max. coverage (+): 0. Max coverage (-): 0

Region: chr8 85688139-85688149. Max. coverage (+): 0. Max coverage (-): 0

Region: chr8 85688150-85688160. Max. coverage (+): 0. Max coverage (-): 0

Region: chr8 85688161-85688171. Max. coverage (+): 0. Max coverage (-): 0

Region: chr8 85688172-85688182. Max. coverage (+): 0. Max coverage (-): 0

Region: chr8 85688183-85688192. Max. coverage (+): 0. Max coverage (-): 0

Region: chr8 85688193-85688203. Max. coverage (+): 0. Max coverage (-): 0

Region: chr8 85688204-85688214. Max. coverage (+): 0. Max coverage (-): 0

Region: chr8 85688215-85688225. Max. coverage (+): 0. Max coverage (-): 0

Region: chr8 85688226-85688236. Max. coverage (+): 0. Max coverage (-): 0

Region: chr8 85688237-85688247. Max. coverage (+): 0. Max coverage (-): 0

Region: chr8 85688248-85688258. Max. coverage (+): 0. Max coverage (-): 0

Region: chr8 85688259-85688269. Max. coverage (+): 0. Max coverage (-): 0

Region: chr8 85688270-85688279. Max. coverage (+): 0. Max coverage (-): 0

Region: chr8 85688280-85688290. Max. coverage (+): 0. Max coverage (-): 0

Region: chr8 85688291-85688301. Max. coverage (+): 0. Max coverage (-): 0

Region: chr8 85688302-85688312. Max. coverage (+): 0. Max coverage (-): 0

Region: chr8 85688313-85688323. Max. coverage (+): 0. Max coverage (-): 0

Region: chr8 85688324-85688334. Max. coverage (+): 0. Max coverage (-): 0

Region: chr8 85688335-85688345. Max. coverage (+): 0. Max coverage (-): 0

Region: chr8 85688346-85688356. Max. coverage (+): 0. Max coverage (-): 0

Region: chr8 85688357-85688366. Max. coverage (+): 0. Max coverage (-): 0

Region: chr8 85688367-85688377. Max. coverage (+): 0. Max coverage (-): 0

Region: chr8 85688378-85688388. Max. coverage (+): 0. Max coverage (-): 0

Region: chr8 85688389-85688399. Max. coverage (+): 0. Max coverage (-): 0

Region: chr8 85688400-85688410. Max. coverage (+): 0. Max coverage (-): 0

Region: chr8 85688411-85688421. Max. coverage (+): 0. Max coverage (-): 0

Region: chr8 85688422-85688432. Max. coverage (+): 0. Max coverage (-): 0

Region: chr8 85688433-85688443. Max. coverage (+): 0. Max coverage (-): 0

Region: chr8 85688444-85688453. Max. coverage (+): 0. Max coverage (-): 0

Region: chr8 85688454-85688464. Max. coverage (+): 0. Max coverage (-): 0

Region: chr8 85688465-85688475. Max. coverage (+): 0. Max coverage (-): 0

Region: chr8 85688476-85688486. Max. coverage (+): 0. Max coverage (-): 0

Region: chr8 85688487-85688497. Max. coverage (+): 0. Max coverage (-): 0

Region: chr8 85688498-85688508. Max. coverage (+): 0. Max coverage (-): 0

Region: chr8 85688509-85688519. Max. coverage (+): 0. Max coverage (-): 0

Region: chr8 85688520-85688530. Max. coverage (+): 0. Max coverage (-): 0

Region: chr8 85688531-85688540. Max. coverage (+): 0. Max coverage (-): 0

Region: chr8 85688541-85688551. Max. coverage (+): 0. Max coverage (-): 0

Region: chr8 85688552-85688562. Max. coverage (+): 0. Max coverage (-): 0

Region: chr8 85688563-85688573. Max. coverage (+): 0. Max coverage (-): 0

Region: chr8 85688574-85688584. Max. coverage (+): 0. Max coverage (-): 0

Region: chr8 85688585-85688595. Max. coverage (+): 0. Max coverage (-): 0

Region: chr8 85688596-85688606. Max. coverage (+): 0. Max coverage (-): 0

Region: chr8 85688607-85688617. Max. coverage (+): 0. Max coverage (-): 0

Region: chr8 85688618-85688627. Max. coverage (+): 0. Max coverage (-): 2.15

Region: chr8 85688628-85688638. Max. coverage (+): 0. Max coverage (-): 2.15

Region: chr8 85688639-85688649. Max. coverage (+): 0. Max coverage (-): 0

Region: chr8 85688650-85688660. Max. coverage (+): 0. Max coverage (-): 0

Region: chr8 85688661-85688671. Max. coverage (+): 0. Max coverage (-): 0

Region: chr8 85688672-85688682. Max. coverage (+): 0. Max coverage (-): 0

Region: chr8 85688683-85688693. Max. coverage (+): 0. Max coverage (-): 0.91

Region: chr8 85688694-85688704. Max. coverage (+): 0. Max coverage (-): 0.91

Region: chr8 85688705-85688714. Max. coverage (+): 0. Max coverage (-): 0

Region: chr8 85688715-85688725. Max. coverage (+): 0. Max coverage (-): 0

Region: chr8 85688726-85688736. Max. coverage (+): 0. Max coverage (-): 0

Region: chr8 85688737-85688747. Max. coverage (+): 0. Max coverage (-): 0

Region: chr8 85688748-85688758. Max. coverage (+): 0. Max coverage (-): 1.7

Region: chr8 85688759-85688769. Max. coverage (+): 0. Max coverage (-): 1.7

Region: chr8 85688770-85688780. Max. coverage (+): 0. Max coverage (-): 0

Region: chr8 85688781-85688791. Max. coverage (+): 0. Max coverage (-): 0

Region: chr8 85688792-85688801. Max. coverage (+): 0. Max coverage (-): 0

Region: chr8 85688802-85688812. Max. coverage (+): 0. Max coverage (-): 0

Region: chr8 85688813-85688823. Max. coverage (+): 0. Max coverage (-): 0

Region: chr8 85688824-85688834. Max. coverage (+): 0. Max coverage (-): 0

Region: chr8 85688835-85688845. Max. coverage (+): 0. Max coverage (-): 0

Region: chr8 85688846-85688856. Max. coverage (+): 0. Max coverage (-): 0

Region: chr8 85688857-85688867. Max. coverage (+): 0. Max coverage (-): 0

Region: chr8 85688868-85688878. Max. coverage (+): 0. Max coverage (-): 0

Region: chr8 85688879-85688888. Max. coverage (+): 0. Max coverage (-): 0

Region: chr8 85688889-85688899. Max. coverage (+): 0. Max coverage (-): 0

Region: chr8 85688900-85688910. Max. coverage (+): 0. Max coverage (-): 0

Region: chr8 85688911-85688921. Max. coverage (+): 0. Max coverage (-): 0

Region: chr8 85688922-85688932. Max. coverage (+): 0. Max coverage (-): 0

Region: chr8 85688933-85688943. Max. coverage (+): 0. Max coverage (-): 0

Region: chr8 85688944-85688954. Max. coverage (+): 0. Max coverage (-): 0

Region: chr8 85688955-85688965. Max. coverage (+): 0. Max coverage (-): 0

Region: chr8 85688966-85688975. Max. coverage (+): 0. Max coverage (-): 0

Region: chr8 85688976-85688986. Max. coverage (+): 0. Max coverage (-): 0

Region: chr8 85688987-85688997. Max. coverage (+): 0. Max coverage (-): 0

Region: chr8 85688998-85689008. Max. coverage (+): 0. Max coverage (-): 0

Region: chr8 85689009-85689019. Max. coverage (+): 0. Max coverage (-): 0

Region: chr8 85689020-85689030. Max. coverage (+): 0. Max coverage (-): 0

Region: chr8 85689031-85689041. Max. coverage (+): 0. Max coverage (-): 0

Region: chr8 85689042-85689052. Max. coverage (+): 0. Max coverage (-): 0

Region: chr8 85689053-85689062. Max. coverage (+): 0. Max coverage (-): 0

Region: chr8 85689063-85689073. Max. coverage (+): 0. Max coverage (-): 0

Region: chr8 85689074-85689084. Max. coverage (+): 0. Max coverage (-): 0

Region: chr8 85689085-85689095. Max. coverage (+): 0. Max coverage (-): 0

Region: chr8 85689096-85689106. Max. coverage (+): 0. Max coverage (-): 3.33

Region: chr8 85689107-85689117. Max. coverage (+): 0. Max coverage (-): 3.33

Region: chr8 85689118-85689128. Max. coverage (+): 0. Max coverage (-): 0

Region: chr8 85689129-85689139. Max. coverage (+): 0. Max coverage (-): 0

Region: chr8 85689140-85689149. Max. coverage (+): 0. Max coverage (-): 0

Region: chr8 85689150-85689160. Max. coverage (+): 0. Max coverage (-): 0

Region: chr8 85689161-85689171. Max. coverage (+): 0. Max coverage (-): 0

Region: chr8 85689172-85689182. Max. coverage (+): 0. Max coverage (-): 0

Region: chr8 85689183-85689193. Max. coverage (+): 0. Max coverage (-): 0

Region: chr8 85689194-85689204. Max. coverage (+): 0. Max coverage (-): 0

Region: chr8 85689205-85689215. Max. coverage (+): 0. Max coverage (-): 0

Region: chr8 85689216-85689226. Max. coverage (+): 0. Max coverage (-): 0

Region: chr8 85689227-85689236. Max. coverage (+): 0. Max coverage (-): 0

Region: chr8 85689237-85689247. Max. coverage (+): 0. Max coverage (-): 0

Region: chr8 85689248-85689258. Max. coverage (+): 0. Max coverage (-): 0

Region: chr8 85689259-85689269. Max. coverage (+): 0. Max coverage (-): 0

Region: chr8 85689270-85689280. Max. coverage (+): 0. Max coverage (-): 0

Region: chr8 85689281-85689291. Max. coverage (+): 0. Max coverage (-): 0

Region: chr8 85689292-85689302. Max. coverage (+): 0. Max coverage (-): 0

Region: chr8 85689303-85689313. Max. coverage (+): 0. Max coverage (-): 0

Region: chr8 85689314-85689323. Max. coverage (+): 0. Max coverage (-): 0

Region: chr8 85689324-85689334. Max. coverage (+): 0. Max coverage (-): 0

Region: chr8 85689335-85689345. Max. coverage (+): 0. Max coverage (-): 0

Region: chr8 85689346-85689356. Max. coverage (+): 0. Max coverage (-): 0

Region: chr8 85689357-85689367. Max. coverage (+): 0. Max coverage (-): 0

Region: chr8 85689368-85689378. Max. coverage (+): 0. Max coverage (-): 0

Region: chr8 85689379-85689389. Max. coverage (+): 0. Max coverage (-): 0

Region: chr8 85689390-85689399. Max. coverage (+): 0. Max coverage (-): 0

Region: chr8 85689400-85689410. Max. coverage (+): 0. Max coverage (-): 0

Region: chr8 85689411-85689421. Max. coverage (+): 0. Max coverage (-): 3.6

Region: chr8 85689422-85689432. Max. coverage (+): 0. Max coverage (-): 3.6

Region: chr8 85689433-85689443. Max. coverage (+): 0. Max coverage (-): 0

Region: chr8 85689444-85689454. Max. coverage (+): 0. Max coverage (-): 0

Region: chr8 85689455-85689465. Max. coverage (+): 0. Max coverage (-): 0

Region: chr8 85689466-85689476. Max. coverage (+): 0. Max coverage (-): 0

Region: chr8 85689477-85689486. Max. coverage (+): 0. Max coverage (-): 0

Region: chr8 85689487-85689497. Max. coverage (+): 0. Max coverage (-): 0

Region: chr8 85689498-85689508. Max. coverage (+): 0. Max coverage (-): 0

Region: chr8 85689509-85689519. Max. coverage (+): 0. Max coverage (-): 0

Region: chr8 85689520-85689530. Max. coverage (+): 0. Max coverage (-): 0

Region: chr8 85689531-85689541. Max. coverage (+): 0. Max coverage (-): 0

Region: chr8 85689542-85689552. Max. coverage (+): 0. Max coverage (-): 0

Region: chr8 85689553-85689563. Max. coverage (+): 0. Max coverage (-): 1.34

Region: chr8 85689564-85689573. Max. coverage (+): 0. Max coverage (-): 0

Region: chr8 85689574-85689584. Max. coverage (+): 0. Max coverage (-): 0

Region: chr8 85689585-85689595. Max. coverage (+): 0. Max coverage (-): 0

Region: chr8 85689596-85689606. Max. coverage (+): 0. Max coverage (-): 0

Region: chr8 85689607-85689617. Max. coverage (+): 0. Max coverage (-): 0

Region: chr8 85689618-85689628. Max. coverage (+): 0. Max coverage (-): 0

Region: chr8 85689629-85689639. Max. coverage (+): 0. Max coverage (-): 0

Region: chr8 85689640-85689650. Max. coverage (+): 0. Max coverage (-): 0

Region: chr8 85689651-85689660. Max. coverage (+): 0. Max coverage (-): 0

Region: chr8 85689661-85689671. Max. coverage (+): 0. Max coverage (-): 0

Region: chr8 85689672-85689682. Max. coverage (+): 0. Max coverage (-): 1.08

Region: chr8 85689683-85689693. Max. coverage (+): 0. Max coverage (-): 1.08

Region: chr8 85689694-85689704. Max. coverage (+): 0. Max coverage (-): 0.27

Region: chr8 85689705-85689715. Max. coverage (+): 0. Max coverage (-): 0.27

Region: chr8 85689716-85689726. Max. coverage (+): 0. Max coverage (-): 0

Region: chr8 85689727-85689737. Max. coverage (+): 0. Max coverage (-): 0

Region: chr8 85689738-85689747. Max. coverage (+): 0. Max coverage (-): 0

Region: chr8 85689748-85689758. Max. coverage (+): 0. Max coverage (-): 0

Region: chr8 85689759-85689769. Max. coverage (+): 0. Max coverage (-): 0

Region: chr8 85689770-85689780. Max. coverage (+): 0. Max coverage (-): 0

Region: chr8 85689781-85689791. Max. coverage (+): 0. Max coverage (-): 2.74

Region: chr8 85689792-85689802. Max. coverage (+): 0. Max coverage (-): 2.74

Region: chr8 85689803-85689813. Max. coverage (+): 0. Max coverage (-): 0

Region: chr8 85689814-85689824. Max. coverage (+): 0. Max coverage (-): 0

Region: chr8 85689825-85689834. Max. coverage (+): 0. Max coverage (-): 6.84

Region: chr8 85689835-85689845. Max. coverage (+): 0. Max coverage (-): 6.84

Region: chr8 85689846-85689856. Max. coverage (+): 0. Max coverage (-): 0

Region: chr8 85689857-85689867. Max. coverage (+): 0. Max coverage (-): 0

Region: chr8 85689868-85689878. Max. coverage (+): 0. Max coverage (-): 0

Region: chr8 85689879-85689889. Max. coverage (+): 0. Max coverage (-): 0

Region: chr8 85689890-85689900. Max. coverage (+): 0. Max coverage (-): 0

Region: chr8 85689901-85689911. Max. coverage (+): 0. Max coverage (-): 0

Region: chr8 85689912-85689921. Max. coverage (+): 0. Max coverage (-): 0

Region: chr8 85689922-85689932. Max. coverage (+): 0. Max coverage (-): 0

Region: chr8 85689933-85689943. Max. coverage (+): 0. Max coverage (-): 1.46

Region: chr8 85689944-85689954. Max. coverage (+): 0. Max coverage (-): 6.27

Region: chr8 85689955-85689965. Max. coverage (+): 0. Max coverage (-): 1.09

Region: chr8 85689966-85689976. Max. coverage (+): 0. Max coverage (-): 2.57

Region: chr8 85689977-85689987. Max. coverage (+): 0. Max coverage (-): 0

Region: chr8 85689988-85689998. Max. coverage (+): 0. Max coverage (-): 0

Region: chr8 85689999-85690008. Max. coverage (+): 0. Max coverage (-): 2.31

Region: chr8 85690009-85690019. Max. coverage (+): 0. Max coverage (-): 2.31

Region: chr8 85690020-85690030. Max. coverage (+): 0. Max coverage (-): 0

Region: chr8 85690031-85690041. Max. coverage (+): 0. Max coverage (-): 6.13

Region: chr8 85690042-85690052. Max. coverage (+): 0. Max coverage (-): 7.54

Region: chr8 85690053-85690063. Max. coverage (+): 0. Max coverage (-): 0

Region: chr8 85690064-85690074. Max. coverage (+): 0. Max coverage (-): 0

Region: chr8 85690075-85690085. Max. coverage (+): 0. Max coverage (-): 0

Region: chr8 85690086-85690095. Max. coverage (+): 0. Max coverage (-): 0

Region: chr8 85690096-85690106. Max. coverage (+): 0. Max coverage (-): 0

Region: chr8 85690107-85690117. Max. coverage (+): 0. Max coverage (-): 0

Region: chr8 85690118-85690128. Max. coverage (+): 0. Max coverage (-): 0

Region: chr8 85690129-85690139. Max. coverage (+): 0. Max coverage (-): 0

Region: chr8 85690140-85690150. Max. coverage (+): 0. Max coverage (-): 0.41

Region: chr8 85690151-85690161. Max. coverage (+): 0. Max coverage (-): 0.41

Region: chr8 85690162-85690172. Max. coverage (+): 0. Max coverage (-): 0

Region: chr8 85690173-85690182. Max. coverage (+): 0. Max coverage (-): 0

Region: chr8 85690183-85690193. Max. coverage (+): 0. Max coverage (-): 0

Region: chr8 85690194-85690204. Max. coverage (+): 0. Max coverage (-): 0

Region: chr8 85690205-85690215. Max. coverage (+): 0. Max coverage (-): 0

Region: chr8 85690216-85690226. Max. coverage (+): 0. Max coverage (-): 0

Region: chr8 85690227-85690237. Max. coverage (+): 0. Max coverage (-): 6.33

Region: chr8 85690238-85690248. Max. coverage (+): 0. Max coverage (-): 4.02

Region: chr8 85690249-85690259. Max. coverage (+): 0. Max coverage (-): 0

Region: chr8 85690260-85690269. Max. coverage (+): 0. Max coverage (-): 0.81

Region: chr8 85690270-85690280. Max. coverage (+): 0. Max coverage (-): 11.8

Region: chr8 85690281-85690291. Max. coverage (+): 0. Max coverage (-): 7

Region: chr8 85690292-85690302. Max. coverage (+): 0. Max coverage (-): 0

Region: chr8 85690303-85690313. Max. coverage (+): 0. Max coverage (-): 0

Region: chr8 85690314-85690324. Max. coverage (+): 0. Max coverage (-): 0

Region: chr8 85690325-85690335. Max. coverage (+): 0. Max coverage (-): 0

Region: chr8 85690336-85690346. Max. coverage (+): 0. Max coverage (-): 0

Region: chr8 85690347-85690356. Max. coverage (+): 0. Max coverage (-): 0

Region: chr8 85690357-85690367. Max. coverage (+): 0. Max coverage (-): 5.05

Region: chr8 85690368-85690378. Max. coverage (+): 0. Max coverage (-): 0

Region: chr8 85690379-85690389. Max. coverage (+): 0. Max coverage (-): 0

Region: chr8 85690390-85690400. Max. coverage (+): 0. Max coverage (-): 0

Region: chr8 85690401-85690411. Max. coverage (+): 0. Max coverage (-): 0

Region: chr8 85690412-85690422. Max. coverage (+): 0. Max coverage (-): 0

Region: chr8 85690423-85690433. Max. coverage (+): 0. Max coverage (-): 0

Region: chr8 85690434-85690443. Max. coverage (+): 0. Max coverage (-): 0

Region: chr8 85690444-85690454. Max. coverage (+): 0. Max coverage (-): 0.61

Region: chr8 85690455-85690465. Max. coverage (+): 0. Max coverage (-): 0.61

Region: chr8 85690466-85690476. Max. coverage (+): 0. Max coverage (-): 0

Region: chr8 85690477-85690487. Max. coverage (+): 0. Max coverage (-): 0

Region: chr8 85690488-85690498. Max. coverage (+): 0. Max coverage (-): 0

Region: chr8 85690499-85690509. Max. coverage (+): 0. Max coverage (-): 0

Region: chr8 85690510-85690520. Max. coverage (+): 0. Max coverage (-): 0

Region: chr8 85690521-85690530. Max. coverage (+): 0. Max coverage (-): 0

Region: chr8 85690531-85690541. Max. coverage (+): 0. Max coverage (-): 0

Region: chr8 85690542-85690552. Max. coverage (+): 0. Max coverage (-): 0

Region: chr8 85690553-85690563. Max. coverage (+): 0. Max coverage (-): 0

Region: chr8 85690564-85690574. Max. coverage (+): 0. Max coverage (-): 2.69

Region: chr8 85690575-85690585. Max. coverage (+): 0. Max coverage (-): 0

Region: chr8 85690586-85690596. Max. coverage (+): 0. Max coverage (-): 0

Region: chr8 85690597-85690607. Max. coverage (+): 0. Max coverage (-): 0

Region: chr8 85690608-85690617. Max. coverage (+): 0. Max coverage (-): 0

Region: chr8 85690618-85690628. Max. coverage (+): 0. Max coverage (-): 4.57

Region: chr8 85690629-85690639. Max. coverage (+): 0. Max coverage (-): 0

Region: chr8 85690640-85690650. Max. coverage (+): 0. Max coverage (-): 0

Region: chr8 85690651-85690661. Max. coverage (+): 0. Max coverage (-): 4.69

Region: chr8 85690662-85690672. Max. coverage (+): 0. Max coverage (-): 2.86

Region: chr8 85690673-85690683. Max. coverage (+): 0. Max coverage (-): 0

Region: chr8 85690684-85690693. Max. coverage (+): 0. Max coverage (-): 0

Region: chr8 85690694-85690704. Max. coverage (+): 0. Max coverage (-): 0

Region: chr8 85690705-85690715. Max. coverage (+): 0. Max coverage (-): 0

Region: chr8 85690716-85690726. Max. coverage (+): 0. Max coverage (-): 0.4

Region: chr8 85690727-85690737. Max. coverage (+): 0. Max coverage (-): 0

Region: chr8 85690738-85690748. Max. coverage (+): 0. Max coverage (-): 0

Region: chr8 85690749-85690759. Max. coverage (+): 0. Max coverage (-): 0

Region: chr8 85690760-85690770. Max. coverage (+): 0. Max coverage (-): 0

Region: chr8 85690771-85690780. Max. coverage (+): 0. Max coverage (-): 0

Region: chr8 85690781-85690791. Max. coverage (+): 0. Max coverage (-): 0

Region: chr8 85690792-85690802. Max. coverage (+): 0. Max coverage (-): 0

Region: chr8 85690803-85690813. Max. coverage (+): 0. Max coverage (-): 0

Region: chr8 85690814-85690824. Max. coverage (+): 0. Max coverage (-): 0

Region: chr8 85690825-85690835. Max. coverage (+): 0. Max coverage (-): 0

Region: chr8 85690836-85690846. Max. coverage (+): 0. Max coverage (-): 0

Region: chr8 85690847-85690857. Max. coverage (+): 0. Max coverage (-): 0

Region: chr8 85690858-85690867. Max. coverage (+): 0. Max coverage (-): 0

Region: chr8 85690868-85690878. Max. coverage (+): 0. Max coverage (-): 0

Region: chr8 85690879-85690889. Max. coverage (+): 0. Max coverage (-): 0

Region: chr8 85690890-85690900. Max. coverage (+): 0. Max coverage (-): 0

Region: chr8 85690901-85690911. Max. coverage (+): 0. Max coverage (-): 0

Region: chr8 85690912-85690922. Max. coverage (+): 0. Max coverage (-): 0

Region: chr8 85690923-85690933. Max. coverage (+): 0. Max coverage (-): 0

Region: chr8 85690934-85690944. Max. coverage (+): 0. Max coverage (-): 0

Region: chr8 85690945-85690954. Max. coverage (+): 0. Max coverage (-): 0

Region: chr8 85690955-85690965. Max. coverage (+): 0. Max coverage (-): 0

Region: chr8 85690966-85690976. Max. coverage (+): 0. Max coverage (-): 0

Region: chr8 85690977-85690987. Max. coverage (+): 0. Max coverage (-): 0

Region: chr8 85690988-85690998. Max. coverage (+): 0. Max coverage (-): 0

Region: chr8 85690999-85691009. Max. coverage (+): 0. Max coverage (-): 0

Region: chr8 85691010-85691020. Max. coverage (+): 0. Max coverage (-): 0

Region: chr8 85691021-85691031. Max. coverage (+): 0. Max coverage (-): 0

Region: chr8 85691032-85691041. Max. coverage (+): 0. Max coverage (-): 0

Region: chr8 85691042-85691052. Max. coverage (+): 0. Max coverage (-): 0

Region: chr8 85691053-85691063. Max. coverage (+): 0. Max coverage (-): 2.84

Region: chr8 85691064-85691074. Max. coverage (+): 0. Max coverage (-): 2.84

Region: chr8 85691075-85691085. Max. coverage (+): 0. Max coverage (-): 0

Region: chr8 85691086-85691096. Max. coverage (+): 0. Max coverage (-): 0

Region: chr8 85691097-85691107. Max. coverage (+): 0. Max coverage (-): 0

Region: chr8 85691108-85691118. Max. coverage (+): 0. Max coverage (-): 0

Region: chr8 85691119-85691128. Max. coverage (+): 0. Max coverage (-): 0

Region: chr8 85691129-85691139. Max. coverage (+): 0. Max coverage (-): 0

Region: chr8 85691140-85691150. Max. coverage (+): 0. Max coverage (-): 0

Region: chr8 85691151-85691161. Max. coverage (+): 0. Max coverage (-): 0

Region: chr8 85691162-85691172. Max. coverage (+): 0. Max coverage (-): 0

Region: chr8 85691173-85691183. Max. coverage (+): 0. Max coverage (-): 0

Region: chr8 85691184-85691194. Max. coverage (+): 0. Max coverage (-): 0

Region: chr8 85691195-85691205. Max. coverage (+): 0. Max coverage (-): 0

Region: chr8 85691206-85691215. Max. coverage (+): 0. Max coverage (-): 0

Region: chr8 85691216-85691226. Max. coverage (+): 0. Max coverage (-): 0

Region: chr8 85691227-85691237. Max. coverage (+): 0. Max coverage (-): 0

Region: chr8 85691238-85691248. Max. coverage (+): 0. Max coverage (-): 0

Region: chr8 85691249-85691259. Max. coverage (+): 0. Max coverage (-): 0.42

Region: chr8 85691260-85691270. Max. coverage (+): 0. Max coverage (-): 0

Region: chr8 85691271-85691281. Max. coverage (+): 0. Max coverage (-): 0

Region: chr8 85691282-85691292. Max. coverage (+): 0. Max coverage (-): 0

Region: chr8 85691293-85691302. Max. coverage (+): 0. Max coverage (-): 0

Region: chr8 85691303-85691313. Max. coverage (+): 0. Max coverage (-): 0

Region: chr8 85691314-85691324. Max. coverage (+): 0. Max coverage (-): 0

Region: chr8 85691325-85691335. Max. coverage (+): 0. Max coverage (-): 1.02

Region: chr8 85691336-85691346. Max. coverage (+): 0. Max coverage (-): 1.02

Region: chr8 85691347-85691357. Max. coverage (+): 0. Max coverage (-): 0

Region: chr8 85691358-85691368. Max. coverage (+): 0. Max coverage (-): 0

Region: chr8 85691369-85691379. Max. coverage (+): 0. Max coverage (-): 0

Region: chr8 85691380-85691389. Max. coverage (+): 0. Max coverage (-): 0

Region: chr8 85691390-85691400. Max. coverage (+): 0. Max coverage (-): 0

Region: chr8 85691401-85691411. Max. coverage (+): 0. Max coverage (-): 1.09

Region: chr8 85691412-85691422. Max. coverage (+): 0. Max coverage (-): 0

Region: chr8 85691423-85691433. Max. coverage (+): 0. Max coverage (-): 0

Region: chr8 85691434-85691444. Max. coverage (+): 0. Max coverage (-): 0

Region: chr8 85691445-85691455. Max. coverage (+): 0. Max coverage (-): 0

Region: chr8 85691456-85691466. Max. coverage (+): 0. Max coverage (-): 0

Region: chr8 85691467-85691476. Max. coverage (+): 0. Max coverage (-): 0

Region: chr8 85691477-85691487. Max. coverage (+): 0. Max coverage (-): 0

Region: chr8 85691488-85691498. Max. coverage (+): 0. Max coverage (-): 0

Region: chr8 85691499-85691509. Max. coverage (+): 0. Max coverage (-): 0

Region: chr8 85691510-85691520. Max. coverage (+): 0. Max coverage (-): 0

Region: chr8 85691521-85691531. Max. coverage (+): 0. Max coverage (-): 0

Region: chr8 85691532-85691542. Max. coverage (+): 0. Max coverage (-): 0

Region: chr8 85691543-85691553. Max. coverage (+): 0. Max coverage (-): 0

Region: chr8 85691554-85691563. Max. coverage (+): 0. Max coverage (-): 0

Region: chr8 85691564-85691574. Max. coverage (+): 0. Max coverage (-): 0

Region: chr8 85691575-85691585. Max. coverage (+): 0. Max coverage (-): 0

Region: chr8 85691586-85691596. Max. coverage (+): 0. Max coverage (-): 0

Region: chr8 85691597-85691607. Max. coverage (+): 0. Max coverage (-): 3.25

Region: chr8 85691608-85691618. Max. coverage (+): 0. Max coverage (-): 0

Region: chr8 85691619-85691629. Max. coverage (+): 0. Max coverage (-): 0.88

Region: chr8 85691630-85691640. Max. coverage (+): 0. Max coverage (-): 0.88

Region: chr8 85691641-85691650. Max. coverage (+): 0. Max coverage (-): 0

Region: chr8 85691651-85691661. Max. coverage (+): 0. Max coverage (-): 1.59

Region: chr8 85691662-85691672. Max. coverage (+): 0. Max coverage (-): 1.59

Region: chr8 85691673-85691683. Max. coverage (+): 0. Max coverage (-): 0.24

Region: chr8 85691684-85691694. Max. coverage (+): 0. Max coverage (-): 0

Region: chr8 85691695-85691705. Max. coverage (+): 0. Max coverage (-): 0

Region: chr8 85691706-85691716. Max. coverage (+): 0. Max coverage (-): 0

Region: chr8 85691717-85691727. Max. coverage (+): 0. Max coverage (-): 0

Region: chr8 85691728-85691737. Max. coverage (+): 0. Max coverage (-): 0

Region: chr8 85691738-85691748. Max. coverage (+): 0. Max coverage (-): 0

Region: chr8 85691749-85691759. Max. coverage (+): 0. Max coverage (-): 7.51

Region: chr8 85691760-85691770. Max. coverage (+): 0. Max coverage (-): 1.29

Region: chr8 85691771-85691781. Max. coverage (+): 0. Max coverage (-): 0

Region: chr8 85691782-85691792. Max. coverage (+): 0. Max coverage (-): 0

Region: chr8 85691793-85691803. Max. coverage (+): 0. Max coverage (-): 0

Region: chr8 85691804-85691814. Max. coverage (+): 0. Max coverage (-): 0

Region: chr8 85691815-85691824. Max. coverage (+): 0. Max coverage (-): 0

Region: chr8 85691825-85691835. Max. coverage (+): 0. Max coverage (-): 0

Region: chr8 85691836-85691846. Max. coverage (+): 0. Max coverage (-): 0

Region: chr8 85691847-85691857. Max. coverage (+): 0. Max coverage (-): 0

Region: chr8 85691858-85691868. Max. coverage (+): 0. Max coverage (-): 0

Region: chr8 85691869-85691879. Max. coverage (+): 0. Max coverage (-): 0

Region: chr8 85691880-85691890. Max. coverage (+): 0. Max coverage (-): 0

Region: chr8 85691891-85691901. Max. coverage (+): 0. Max coverage (-): 2.53

Region: chr8 85691902-85691911. Max. coverage (+): 0. Max coverage (-): 3.94

Region: chr8 85691912-85691922. Max. coverage (+): 0. Max coverage (-): 1.41

Region: chr8 85691923-85691933. Max. coverage (+): 0. Max coverage (-): 0

Region: chr8 85691934-85691944. Max. coverage (+): 0. Max coverage (-): 2.84

Region: chr8 85691945-85691955. Max. coverage (+): 0. Max coverage (-): 0

Region: chr8 85691956-85691966. Max. coverage (+): 0. Max coverage (-): 4.92

Region: chr8 85691967-85691977. Max. coverage (+): 0. Max coverage (-): 4.92

Region: chr8 85691978-85691988. Max. coverage (+): 0. Max coverage (-): 0

Region: chr8 85691989-85691998. Max. coverage (+): 1.93. Max coverage (-): 0

Region: chr8 85691999-85692009. Max. coverage (+): 1.93. Max coverage (-): 0

Region: chr8 85692010-85692020. Max. coverage (+): 0. Max coverage (-): 0

Region: chr8 85692021-85692031. Max. coverage (+): 0. Max coverage (-): 0

Region: chr8 85692032-85692042. Max. coverage (+): 0. Max coverage (-): 0

Region: chr8 85692043-85692053. Max. coverage (+): 0. Max coverage (-): 0

Region: chr8 85692054-85692064. Max. coverage (+): 0. Max coverage (-): 0

Region: chr8 85692065-85692074. Max. coverage (+): 0. Max coverage (-): 0

Region: chr8 85692075-85692085. Max. coverage (+): 0. Max coverage (-): 0

Region: chr8 85692086-85692096. Max. coverage (+): 0. Max coverage (-): 0

Region: chr8 85692097-85692107. Max. coverage (+): 0. Max coverage (-): 0

Region: chr8 85692108-85692118. Max. coverage (+): 0. Max coverage (-): 0

Region: chr8 85692119-85692129. Max. coverage (+): 0. Max coverage (-): 0

Region: chr8 85692130-85692140. Max. coverage (+): 0. Max coverage (-): 0

Region: chr8 85692141-85692151. Max. coverage (+): 0. Max coverage (-): 0

Region: chr8 85692152-85692161. Max. coverage (+): 0. Max coverage (-): 0

Region: chr8 85692162-85692172. Max. coverage (+): 0. Max coverage (-): 0

Region: chr8 85692173-85692183. Max. coverage (+): 0. Max coverage (-): 0

Region: chr8 85692184-85692194. Max. coverage (+): 0. Max coverage (-): 0

Region: chr8 85692195-85692205. Max. coverage (+): 0. Max coverage (-): 0

Region: chr8 85692206-85692216. Max. coverage (+): 0. Max coverage (-): 0

Region: chr8 85692217-85692227. Max. coverage (+): 0. Max coverage (-): 0

Region: chr8 85692228-85692238. Max. coverage (+): 0. Max coverage (-): 0

Region: chr8 85692239-85692248. Max. coverage (+): 0. Max coverage (-): 0

Region: chr8 85692249-85692259. Max. coverage (+): 0. Max coverage (-): 0

Region: chr8 85692260-85692270. Max. coverage (+): 0. Max coverage (-): 0

Region: chr8 85692271-85692281. Max. coverage (+): 0. Max coverage (-): 0

Region: chr8 85692282-85692292. Max. coverage (+): 0. Max coverage (-): 0

Region: chr8 85692293-85692303. Max. coverage (+): 0. Max coverage (-): 0

Region: chr8 85692304-85692314. Max. coverage (+): 0. Max coverage (-): 0

Region: chr8 85692315-85692325. Max. coverage (+): 0. Max coverage (-): 0

Region: chr8 85692326-85692335. Max. coverage (+): 0. Max coverage (-): 0

Region: chr8 85692336-85692346. Max. coverage (+): 0. Max coverage (-): 0

Region: chr8 85692347-85692357. Max. coverage (+): 0. Max coverage (-): 0

Region: chr8 85692358-85692368. Max. coverage (+): 0. Max coverage (-): 0

Region: chr8 85692369-85692379. Max. coverage (+): 0. Max coverage (-): 0

Region: chr8 85692380-85692390. Max. coverage (+): 0. Max coverage (-): 0

Region: chr8 85692391-85692401. Max. coverage (+): 0. Max coverage (-): 0

Region: chr8 85692402-85692412. Max. coverage (+): 0. Max coverage (-): 0

Region: chr8 85692413-85692422. Max. coverage (+): 0. Max coverage (-): 0

Region: chr8 85692423-85692433. Max. coverage (+): 0. Max coverage (-): 0

Region: chr8 85692434-85692444. Max. coverage (+): 0. Max coverage (-): 0

Region: chr8 85692445-85692455. Max. coverage (+): 0. Max coverage (-): 0

Region: chr8 85692456-85692466. Max. coverage (+): 0. Max coverage (-): 0

Region: chr8 85692467-85692477. Max. coverage (+): 0. Max coverage (-): 0

Region: chr8 85692478-85692488. Max. coverage (+): 0. Max coverage (-): 0

Region: chr8 85692489-85692499. Max. coverage (+): 0. Max coverage (-): 0

Region: chr8 85692500-85692509. Max. coverage (+): 0. Max coverage (-): 0

Region: chr8 85692510-85692520. Max. coverage (+): 0. Max coverage (-): 0

Region: chr8 85692521-85692531. Max. coverage (+): 0. Max coverage (-): 0

Region: chr8 85692532-85692542. Max. coverage (+): 0. Max coverage (-): 0

Region: chr8 85692543-85692553. Max. coverage (+): 0. Max coverage (-): 0

Region: chr8 85692554-85692564. Max. coverage (+): 0. Max coverage (-): 0

Region: chr8 85692565-85692575. Max. coverage (+): 0. Max coverage (-): 0

Region: chr8 85692576-85692586. Max. coverage (+): 0. Max coverage (-): 0

Region: chr8 85692587-85692596. Max. coverage (+): 0. Max coverage (-): 0

Region: chr8 85692597-85692607. Max. coverage (+): 0. Max coverage (-): 0

Region: chr8 85692608-85692618. Max. coverage (+): 0. Max coverage (-): 0

Region: chr8 85692619-85692629. Max. coverage (+): 0. Max coverage (-): 0

Region: chr8 85692630-85692640. Max. coverage (+): 0. Max coverage (-): 0

Region: chr8 85692641-85692651. Max. coverage (+): 0. Max coverage (-): 0

Region: chr8 85692652-85692662. Max. coverage (+): 0. Max coverage (-): 0

Region: chr8 85692663-85692673. Max. coverage (+): 0. Max coverage (-): 0

Region: chr8 85692674-85692683. Max. coverage (+): 0. Max coverage (-): 0

Region: chr8 85692684-85692694. Max. coverage (+): 0. Max coverage (-): 0.95

Region: chr8 85692695-85692705. Max. coverage (+): 0. Max coverage (-): 0.95

Region: chr8 85692706-85692716. Max. coverage (+): 0. Max coverage (-): 0

Region: chr8 85692717-. Max. coverage (+): 0. Max coverage (-): 0

RepeatMasker Color Code

**+**

100-98% Identity

<98-95% Identity

<95-90% Identity

<90-85% Identity

<85-80% Identity

<80-75% Identity

<75-70% Identity

<70% Identity

**-**

Gene Set Color Code

**+**

Gene

Pseudogene

**-**

Topology/Coverage Color Code

Coverage Plus Strand

Coverage Minus Strand

Mainstrand: Plus

Mainstrand: Minus

Complementary Strand

Flanking Region  
(if option -flank >0)

Gene Set Annotation  

**1. BICD2 (protein coding, ENSBTAG00000046549) Tr:00000065971 Ex:7**: 85692416-85692725 (-)

  
RepeatMasker Annotation  

**1. AT\_rich**: 85688479-85688499 (+), Divergence to consensus: 23.8%

  
Transcription Factor Binding Sites
