# Supplementary material for: Small RNA sequencing of cryopreserved semen from single bull revealed altered miRNAs and piRNAs expression between High- and Low-motile sperm populations
Source: BMC Genomics. 2017 Jan 4;18:14. doi: 10.1186/s12864-016-3394-7 (PMC5209821; doi:10.1186/s12864-016-3394-7)
Supplement: Additional file 3: — Details for each piRNA clusters found in High Motile (HM) sperm fraction. Genes, repeats, transposable elements and transcription factors binding sites falling within the cluster regions were reported. (ZIP 1896 kb) [file 12864_2016_3394_MOESM3_ESM.zip › 96.html]

piRNA cluster 96


Predicted piRNA cluster no. 96     previous   next
  

Show proTRAC run info
Hide proTRAC run info

================================= proTRAC ====================================  
VERSION: 2.1                                    LAST MODIFIED: 06. October 2015  
  
Please cite:  
Rosenkranz D, Zischler H. proTRAC - a software for probabilistic piRNA cluster  
detection, visualization and analysis. 2012. BMC Bioinformatics 13:5.  
  
and (for proTRAC 2.0 and later):  
Rosenkranz D, Rudloff S, Bastuck K, Ketting RF, Zischler H. Tupaia small RNAs  
provide insights into function and evolution of RNAi-based transposon defense  
in mammals. 2015. RNA 21(5):911-922.  
  
Contact:  
David Rosenkranz  
Institute of Anthropology, small RNA group  
Johannes Gutenberg University Mainz  
email: rosenkranz@uni-mainz.de  
  
You can find the latest proTRAC version at:  
http://sourceforge.net/projects/protrac/files  
http://www.smallRNAgroup-mainz.de/software  
==============================================================================  
  
PARAMETERS:  
Map file: .............../storage/core/barbara/genhome/smallRNA/fertility/Sample\_motile/pirna/Sample\_motile\_26-33\_collapsed.fa.no-dust.map.weighted-10000-1000-b-0  
Genome file: ............/storage/core/barbara/genhome/smallRNA/fertility/Sample\_all/pirna/bt\_311\_chrY.fa  
RepeatMasker annotation: /storage/genomes/bt\_umd31/GCF\_000003055.6\_Bos\_taurus\_UMD\_3.1.1\_repeatMasker\_chr.out  
GeneSet:................./storage/core/barbara/genhome/smallRNA/fertility/Sample\_all/pirna/full.gtf  
  
Significant (p<=0.01) hit density will be calculated based  
on observed hit distribution.  
  
Sliding window size: ........................................ 5000 bp  
Sliding window increament: .................................. 1000 bp  
Normalize each hit by number of genomic hits: ............... 1 [0=no/1=yes]  
Normalize each hit by number of sequence reads: ............. 1 [0=no/1=yes]  
Normalize values (-> per million mapped reads): ............. 1 [0=no/1=yes]  
Min. fraction of hits with 1T(U) or 10A: .................... 0.75  
Alternatively: Min. fraction of hits with 1T(U) and 10A: .... 0.5  
Min. fraction of hits with typical piRNA length: ............ 0.75  
Typical piRNA length: ....................................... 26-33 nt  
Min. size of a piRNA cluster: ............................... 5000 bp.  
Min. number of hits (absolute): ............................. 0  
Min. number of hits (normalized): ........................... 0  
Min. fraction of hits on the mainstrand: .................... 0.75  
Top fraction of mapped sequences (in terms of read counts): . 1%  
Top fraction accounts for max. n% of sequence reads: ........ 90%  
Min. fraction of hits on each arm of a bidirectional cluster: 0.1  
Output image file for each cluster: ......................... 0 [0=no/1=yes]  
Output html file for each cluster: .......................... 1 [0=no/1=yes]  
Output a summary table: ..................................... 1 [0=no/1=yes]  
Output a FASTA file for each cluster (piRNA sequences): ..... 1 [0=no/1=yes]  
Output a FASTA file comprising cluster sequences: ........... 1 [0=no/1=yes]  
Search DNA motifs in clusters: .............................. 1 [0=no/1=yes]  
Output flanking sequences: +/- .............................. 0 bp  
Output ~.pTi file: .......................................... 1 [0=no/1=yes]  
==============================================================================  
  
  
Genome size (without gaps): ............ 2678902517 bp  
Gaps (N/X/-): .......................... 53837044 bp  
Mapped reads: .......................... 658825247023  
Non-identical sequences: ............... 514171  
Genomic hits: .......................... 764233  
Significant densitiy of mapped reads: .. 12867599.5173724 reads/kb

Show proTRAC cluster info
Hide proTRAC cluster info

|  |  |
| --- | --- |
| Location | chr8 |
| Coordinates | 100099738-100113039 |
| Size [bp] | 13302 |
| Sequence hit loci | 1420 |
| Mapped reads (normalized) | 1660820172 |
| Mapped reads (normalized) per kb | 124854922 |
| Normalized reads with 1T (1U) | 75.2% |
| Normalized reads with 10A | 36.6% |
| Normalized reads with length 26-33 nt | 100% |
| Normalized reads on the main strand(s) | 100% |
| Predicted directionality | mono:plus |

100%

0%

1T (1U)  
reads

10A reads

26-33 nt  
reads

reads on mainstrand

**Either the amount of reads with 1T (1U) OR 10A has to exceed 75% (set with option: -1Tor10A)  
Alternatively the amount of reads with 1T (1U) AND 10A has to exceed 50% (set with option: -1Tand10A)  
Minimum amount of reads with preferred size is 75% (set with option: -pisize)  
Minimum amount of reads on the main strand(s) is 75% (set with option: -clstrand)**

Show read coverage
Hide read coverage

WHAT DO I SEE HERE?  
This chart shows the location of mapped sequence reads within a predicted piRNA cluster. The color refers to the number of genomic hits produced by the sequence read in question. A dark red bar indicates that this sequence read produces many other hits elsewhere in the genome. Many adjacent red or yellow bars can indicate the presence of a multi-copy element such as transposons or rRNA genes. A dark green bar indicates that this sequence read maps uniquely to this locus.

1 hit

2-5 hits

6-10 hits

11-20 hits

21-50 hits

51-100 hits

> 100 hits

chr8

100099738

100113039

Gene Set

RepeatMasker

Mapped  
Reads

138.77

plus strand

minus strand

138.77

Region: chr8 85692718-100099751. Max. coverage (+): 5.1. Max coverage (-): 0

Region: chr8 100099752-100099777. Max. coverage (+): 4.5. Max coverage (-): 0

Region: chr8 100099778-100099804. Max. coverage (+): 0. Max coverage (-): 0

Region: chr8 100099805-100099831. Max. coverage (+): 0. Max coverage (-): 0

Region: chr8 100099832-100099857. Max. coverage (+): 13.92. Max coverage (-): 0

Region: chr8 100099858-100099884. Max. coverage (+): 10.29. Max coverage (-): 0

Region: chr8 100099885-100099910. Max. coverage (+): 0. Max coverage (-): 0

Region: chr8 100099911-100099937. Max. coverage (+): 2.79. Max coverage (-): 0

Region: chr8 100099938-100099964. Max. coverage (+): 0. Max coverage (-): 0

Region: chr8 100099965-100099990. Max. coverage (+): 0. Max coverage (-): 0

Region: chr8 100099991-100100017. Max. coverage (+): 4. Max coverage (-): 0

Region: chr8 100100018-100100043. Max. coverage (+): 0. Max coverage (-): 0

Region: chr8 100100044-100100070. Max. coverage (+): 0. Max coverage (-): 0

Region: chr8 100100071-100100097. Max. coverage (+): 0. Max coverage (-): 0

Region: chr8 100100098-100100123. Max. coverage (+): 0. Max coverage (-): 0

Region: chr8 100100124-100100150. Max. coverage (+): 7.42. Max coverage (-): 0

Region: chr8 100100151-100100176. Max. coverage (+): 0. Max coverage (-): 0

Region: chr8 100100177-100100203. Max. coverage (+): 0. Max coverage (-): 0

Region: chr8 100100204-100100230. Max. coverage (+): 0. Max coverage (-): 0

Region: chr8 100100231-100100256. Max. coverage (+): 0. Max coverage (-): 0

Region: chr8 100100257-100100283. Max. coverage (+): 0. Max coverage (-): 0

Region: chr8 100100284-100100309. Max. coverage (+): 4.04. Max coverage (-): 0

Region: chr8 100100310-100100336. Max. coverage (+): 0. Max coverage (-): 0

Region: chr8 100100337-100100363. Max. coverage (+): 0. Max coverage (-): 0

Region: chr8 100100364-100100389. Max. coverage (+): 0. Max coverage (-): 0

Region: chr8 100100390-100100416. Max. coverage (+): 0. Max coverage (-): 0

Region: chr8 100100417-100100443. Max. coverage (+): 0. Max coverage (-): 0

Region: chr8 100100444-100100469. Max. coverage (+): 0. Max coverage (-): 0

Region: chr8 100100470-100100496. Max. coverage (+): 0. Max coverage (-): 0

Region: chr8 100100497-100100522. Max. coverage (+): 0. Max coverage (-): 0

Region: chr8 100100523-100100549. Max. coverage (+): 0. Max coverage (-): 0

Region: chr8 100100550-100100576. Max. coverage (+): 0. Max coverage (-): 0

Region: chr8 100100577-100100602. Max. coverage (+): 0. Max coverage (-): 0

Region: chr8 100100603-100100629. Max. coverage (+): 0. Max coverage (-): 0

Region: chr8 100100630-100100655. Max. coverage (+): 28.6. Max coverage (-): 0

Region: chr8 100100656-100100682. Max. coverage (+): 14.13. Max coverage (-): 0

Region: chr8 100100683-100100709. Max. coverage (+): 12.62. Max coverage (-): 0

Region: chr8 100100710-100100735. Max. coverage (+): 17.01. Max coverage (-): 0

Region: chr8 100100736-100100762. Max. coverage (+): 0. Max coverage (-): 0

Region: chr8 100100763-100100788. Max. coverage (+): 0. Max coverage (-): 0

Region: chr8 100100789-100100815. Max. coverage (+): 0. Max coverage (-): 0

Region: chr8 100100816-100100842. Max. coverage (+): 0. Max coverage (-): 0

Region: chr8 100100843-100100868. Max. coverage (+): 0. Max coverage (-): 0

Region: chr8 100100869-100100895. Max. coverage (+): 0. Max coverage (-): 0

Region: chr8 100100896-100100921. Max. coverage (+): 0. Max coverage (-): 0

Region: chr8 100100922-100100948. Max. coverage (+): 0. Max coverage (-): 0

Region: chr8 100100949-100100975. Max. coverage (+): 0. Max coverage (-): 0

Region: chr8 100100976-100101001. Max. coverage (+): 0. Max coverage (-): 0

Region: chr8 100101002-100101028. Max. coverage (+): 0. Max coverage (-): 0

Region: chr8 100101029-100101054. Max. coverage (+): 0. Max coverage (-): 0

Region: chr8 100101055-100101081. Max. coverage (+): 0. Max coverage (-): 0

Region: chr8 100101082-100101108. Max. coverage (+): 0. Max coverage (-): 0

Region: chr8 100101109-100101134. Max. coverage (+): 0. Max coverage (-): 0

Region: chr8 100101135-100101161. Max. coverage (+): 0. Max coverage (-): 0

Region: chr8 100101162-100101187. Max. coverage (+): 0. Max coverage (-): 0

Region: chr8 100101188-100101214. Max. coverage (+): 0. Max coverage (-): 0

Region: chr8 100101215-100101241. Max. coverage (+): 0. Max coverage (-): 0

Region: chr8 100101242-100101267. Max. coverage (+): 6.93. Max coverage (-): 0

Region: chr8 100101268-100101294. Max. coverage (+): 7.18. Max coverage (-): 0

Region: chr8 100101295-100101320. Max. coverage (+): 9.87. Max coverage (-): 0

Region: chr8 100101321-100101347. Max. coverage (+): 3.04. Max coverage (-): 0

Region: chr8 100101348-100101374. Max. coverage (+): 0. Max coverage (-): 0

Region: chr8 100101375-100101400. Max. coverage (+): 0. Max coverage (-): 0

Region: chr8 100101401-100101427. Max. coverage (+): 1.69. Max coverage (-): 0

Region: chr8 100101428-100101453. Max. coverage (+): 0. Max coverage (-): 0

Region: chr8 100101454-100101480. Max. coverage (+): 0. Max coverage (-): 0

Region: chr8 100101481-100101507. Max. coverage (+): 0. Max coverage (-): 0

Region: chr8 100101508-100101533. Max. coverage (+): 0. Max coverage (-): 0

Region: chr8 100101534-100101560. Max. coverage (+): 0. Max coverage (-): 0

Region: chr8 100101561-100101586. Max. coverage (+): 0. Max coverage (-): 0

Region: chr8 100101587-100101613. Max. coverage (+): 0. Max coverage (-): 0

Region: chr8 100101614-100101640. Max. coverage (+): 0. Max coverage (-): 0

Region: chr8 100101641-100101666. Max. coverage (+): 0. Max coverage (-): 0

Region: chr8 100101667-100101693. Max. coverage (+): 0. Max coverage (-): 0

Region: chr8 100101694-100101719. Max. coverage (+): 0. Max coverage (-): 0

Region: chr8 100101720-100101746. Max. coverage (+): 0. Max coverage (-): 0

Region: chr8 100101747-100101773. Max. coverage (+): 0. Max coverage (-): 0

Region: chr8 100101774-100101799. Max. coverage (+): 0. Max coverage (-): 0

Region: chr8 100101800-100101826. Max. coverage (+): 0. Max coverage (-): 0

Region: chr8 100101827-100101853. Max. coverage (+): 0. Max coverage (-): 0

Region: chr8 100101854-100101879. Max. coverage (+): 0. Max coverage (-): 0

Region: chr8 100101880-100101906. Max. coverage (+): 0. Max coverage (-): 0

Region: chr8 100101907-100101932. Max. coverage (+): 0. Max coverage (-): 0

Region: chr8 100101933-100101959. Max. coverage (+): 0. Max coverage (-): 0

Region: chr8 100101960-100101986. Max. coverage (+): 0. Max coverage (-): 0

Region: chr8 100101987-100102012. Max. coverage (+): 0. Max coverage (-): 0

Region: chr8 100102013-100102039. Max. coverage (+): 0. Max coverage (-): 0

Region: chr8 100102040-100102065. Max. coverage (+): 0. Max coverage (-): 0

Region: chr8 100102066-100102092. Max. coverage (+): 0. Max coverage (-): 0

Region: chr8 100102093-100102119. Max. coverage (+): 1.99. Max coverage (-): 0

Region: chr8 100102120-100102145. Max. coverage (+): 0. Max coverage (-): 0

Region: chr8 100102146-100102172. Max. coverage (+): 0. Max coverage (-): 0

Region: chr8 100102173-100102198. Max. coverage (+): 0. Max coverage (-): 0

Region: chr8 100102199-100102225. Max. coverage (+): 0. Max coverage (-): 0

Region: chr8 100102226-100102252. Max. coverage (+): 0. Max coverage (-): 0

Region: chr8 100102253-100102278. Max. coverage (+): 0. Max coverage (-): 0

Region: chr8 100102279-100102305. Max. coverage (+): 0. Max coverage (-): 0

Region: chr8 100102306-100102331. Max. coverage (+): 0. Max coverage (-): 0

Region: chr8 100102332-100102358. Max. coverage (+): 0. Max coverage (-): 0

Region: chr8 100102359-100102385. Max. coverage (+): 0. Max coverage (-): 0

Region: chr8 100102386-100102411. Max. coverage (+): 0. Max coverage (-): 0

Region: chr8 100102412-100102438. Max. coverage (+): 0. Max coverage (-): 0

Region: chr8 100102439-100102464. Max. coverage (+): 0. Max coverage (-): 0

Region: chr8 100102465-100102491. Max. coverage (+): 0. Max coverage (-): 0

Region: chr8 100102492-100102518. Max. coverage (+): 0. Max coverage (-): 0

Region: chr8 100102519-100102544. Max. coverage (+): 0. Max coverage (-): 0

Region: chr8 100102545-100102571. Max. coverage (+): 0. Max coverage (-): 0

Region: chr8 100102572-100102597. Max. coverage (+): 0. Max coverage (-): 0

Region: chr8 100102598-100102624. Max. coverage (+): 0. Max coverage (-): 0

Region: chr8 100102625-100102651. Max. coverage (+): 0. Max coverage (-): 0

Region: chr8 100102652-100102677. Max. coverage (+): 0. Max coverage (-): 0

Region: chr8 100102678-100102704. Max. coverage (+): 0. Max coverage (-): 0

Region: chr8 100102705-100102730. Max. coverage (+): 0. Max coverage (-): 0

Region: chr8 100102731-100102757. Max. coverage (+): 0. Max coverage (-): 0

Region: chr8 100102758-100102784. Max. coverage (+): 0. Max coverage (-): 0

Region: chr8 100102785-100102810. Max. coverage (+): 0. Max coverage (-): 0

Region: chr8 100102811-100102837. Max. coverage (+): 0. Max coverage (-): 0

Region: chr8 100102838-100102863. Max. coverage (+): 0. Max coverage (-): 0

Region: chr8 100102864-100102890. Max. coverage (+): 0. Max coverage (-): 0

Region: chr8 100102891-100102917. Max. coverage (+): 0. Max coverage (-): 0

Region: chr8 100102918-100102943. Max. coverage (+): 0. Max coverage (-): 0

Region: chr8 100102944-100102970. Max. coverage (+): 0. Max coverage (-): 0

Region: chr8 100102971-100102996. Max. coverage (+): 0. Max coverage (-): 0

Region: chr8 100102997-100103023. Max. coverage (+): 0. Max coverage (-): 0

Region: chr8 100103024-100103050. Max. coverage (+): 0. Max coverage (-): 0

Region: chr8 100103051-100103076. Max. coverage (+): 0. Max coverage (-): 0

Region: chr8 100103077-100103103. Max. coverage (+): 0. Max coverage (-): 0

Region: chr8 100103104-100103130. Max. coverage (+): 0. Max coverage (-): 0

Region: chr8 100103131-100103156. Max. coverage (+): 0. Max coverage (-): 0

Region: chr8 100103157-100103183. Max. coverage (+): 0. Max coverage (-): 0

Region: chr8 100103184-100103209. Max. coverage (+): 0. Max coverage (-): 0

Region: chr8 100103210-100103236. Max. coverage (+): 0. Max coverage (-): 0

Region: chr8 100103237-100103263. Max. coverage (+): 0. Max coverage (-): 0

Region: chr8 100103264-100103289. Max. coverage (+): 0. Max coverage (-): 0

Region: chr8 100103290-100103316. Max. coverage (+): 0. Max coverage (-): 0

Region: chr8 100103317-100103342. Max. coverage (+): 0. Max coverage (-): 0

Region: chr8 100103343-100103369. Max. coverage (+): 0. Max coverage (-): 0

Region: chr8 100103370-100103396. Max. coverage (+): 0. Max coverage (-): 0

Region: chr8 100103397-100103422. Max. coverage (+): 0. Max coverage (-): 0

Region: chr8 100103423-100103449. Max. coverage (+): 0. Max coverage (-): 0

Region: chr8 100103450-100103475. Max. coverage (+): 0. Max coverage (-): 0

Region: chr8 100103476-100103502. Max. coverage (+): 0. Max coverage (-): 0

Region: chr8 100103503-100103529. Max. coverage (+): 0. Max coverage (-): 0

Region: chr8 100103530-100103555. Max. coverage (+): 1.72. Max coverage (-): 0

Region: chr8 100103556-100103582. Max. coverage (+): 0. Max coverage (-): 0

Region: chr8 100103583-100103608. Max. coverage (+): 0. Max coverage (-): 0

Region: chr8 100103609-100103635. Max. coverage (+): 0. Max coverage (-): 0

Region: chr8 100103636-100103662. Max. coverage (+): 0. Max coverage (-): 0

Region: chr8 100103663-100103688. Max. coverage (+): 0. Max coverage (-): 0

Region: chr8 100103689-100103715. Max. coverage (+): 0. Max coverage (-): 0

Region: chr8 100103716-100103741. Max. coverage (+): 0. Max coverage (-): 0

Region: chr8 100103742-100103768. Max. coverage (+): 0. Max coverage (-): 0

Region: chr8 100103769-100103795. Max. coverage (+): 0. Max coverage (-): 0

Region: chr8 100103796-100103821. Max. coverage (+): 0. Max coverage (-): 0

Region: chr8 100103822-100103848. Max. coverage (+): 0. Max coverage (-): 0

Region: chr8 100103849-100103874. Max. coverage (+): 0. Max coverage (-): 0

Region: chr8 100103875-100103901. Max. coverage (+): 0. Max coverage (-): 0

Region: chr8 100103902-100103928. Max. coverage (+): 0. Max coverage (-): 0

Region: chr8 100103929-100103954. Max. coverage (+): 0. Max coverage (-): 0

Region: chr8 100103955-100103981. Max. coverage (+): 0. Max coverage (-): 0

Region: chr8 100103982-100104007. Max. coverage (+): 0. Max coverage (-): 0

Region: chr8 100104008-100104034. Max. coverage (+): 0. Max coverage (-): 0

Region: chr8 100104035-100104061. Max. coverage (+): 0. Max coverage (-): 0

Region: chr8 100104062-100104087. Max. coverage (+): 0. Max coverage (-): 0

Region: chr8 100104088-100104114. Max. coverage (+): 0. Max coverage (-): 0

Region: chr8 100104115-100104140. Max. coverage (+): 0. Max coverage (-): 0

Region: chr8 100104141-100104167. Max. coverage (+): 0. Max coverage (-): 0

Region: chr8 100104168-100104194. Max. coverage (+): 0. Max coverage (-): 0

Region: chr8 100104195-100104220. Max. coverage (+): 0. Max coverage (-): 0

Region: chr8 100104221-100104247. Max. coverage (+): 0. Max coverage (-): 0

Region: chr8 100104248-100104273. Max. coverage (+): 0. Max coverage (-): 0

Region: chr8 100104274-100104300. Max. coverage (+): 0. Max coverage (-): 0

Region: chr8 100104301-100104327. Max. coverage (+): 0. Max coverage (-): 0

Region: chr8 100104328-100104353. Max. coverage (+): 0. Max coverage (-): 0

Region: chr8 100104354-100104380. Max. coverage (+): 0. Max coverage (-): 0

Region: chr8 100104381-100104407. Max. coverage (+): 0. Max coverage (-): 0

Region: chr8 100104408-100104433. Max. coverage (+): 0. Max coverage (-): 0

Region: chr8 100104434-100104460. Max. coverage (+): 0. Max coverage (-): 0

Region: chr8 100104461-100104486. Max. coverage (+): 0. Max coverage (-): 0

Region: chr8 100104487-100104513. Max. coverage (+): 0. Max coverage (-): 0

Region: chr8 100104514-100104540. Max. coverage (+): 0. Max coverage (-): 0

Region: chr8 100104541-100104566. Max. coverage (+): 0. Max coverage (-): 0

Region: chr8 100104567-100104593. Max. coverage (+): 0. Max coverage (-): 0

Region: chr8 100104594-100104619. Max. coverage (+): 0. Max coverage (-): 0

Region: chr8 100104620-100104646. Max. coverage (+): 0. Max coverage (-): 0

Region: chr8 100104647-100104673. Max. coverage (+): 0. Max coverage (-): 0

Region: chr8 100104674-100104699. Max. coverage (+): 0. Max coverage (-): 0

Region: chr8 100104700-100104726. Max. coverage (+): 0. Max coverage (-): 0

Region: chr8 100104727-100104752. Max. coverage (+): 0. Max coverage (-): 0

Region: chr8 100104753-100104779. Max. coverage (+): 0. Max coverage (-): 0

Region: chr8 100104780-100104806. Max. coverage (+): 0. Max coverage (-): 0

Region: chr8 100104807-100104832. Max. coverage (+): 0. Max coverage (-): 0

Region: chr8 100104833-100104859. Max. coverage (+): 0. Max coverage (-): 0

Region: chr8 100104860-100104885. Max. coverage (+): 0. Max coverage (-): 0

Region: chr8 100104886-100104912. Max. coverage (+): 0. Max coverage (-): 0

Region: chr8 100104913-100104939. Max. coverage (+): 0. Max coverage (-): 0

Region: chr8 100104940-100104965. Max. coverage (+): 0. Max coverage (-): 0

Region: chr8 100104966-100104992. Max. coverage (+): 0. Max coverage (-): 0

Region: chr8 100104993-100105018. Max. coverage (+): 0. Max coverage (-): 0

Region: chr8 100105019-100105045. Max. coverage (+): 0. Max coverage (-): 0

Region: chr8 100105046-100105072. Max. coverage (+): 0. Max coverage (-): 0

Region: chr8 100105073-100105098. Max. coverage (+): 0. Max coverage (-): 0

Region: chr8 100105099-100105125. Max. coverage (+): 0. Max coverage (-): 0

Region: chr8 100105126-100105151. Max. coverage (+): 0. Max coverage (-): 0

Region: chr8 100105152-100105178. Max. coverage (+): 0. Max coverage (-): 0

Region: chr8 100105179-100105205. Max. coverage (+): 0. Max coverage (-): 0

Region: chr8 100105206-100105231. Max. coverage (+): 0. Max coverage (-): 0

Region: chr8 100105232-100105258. Max. coverage (+): 0. Max coverage (-): 0

Region: chr8 100105259-100105284. Max. coverage (+): 0. Max coverage (-): 0

Region: chr8 100105285-100105311. Max. coverage (+): 0. Max coverage (-): 0

Region: chr8 100105312-100105338. Max. coverage (+): 0. Max coverage (-): 0

Region: chr8 100105339-100105364. Max. coverage (+): 0. Max coverage (-): 0

Region: chr8 100105365-100105391. Max. coverage (+): 0. Max coverage (-): 0

Region: chr8 100105392-100105417. Max. coverage (+): 0. Max coverage (-): 0

Region: chr8 100105418-100105444. Max. coverage (+): 0. Max coverage (-): 0

Region: chr8 100105445-100105471. Max. coverage (+): 0. Max coverage (-): 0

Region: chr8 100105472-100105497. Max. coverage (+): 0. Max coverage (-): 0

Region: chr8 100105498-100105524. Max. coverage (+): 0. Max coverage (-): 0

Region: chr8 100105525-100105550. Max. coverage (+): 0. Max coverage (-): 0

Region: chr8 100105551-100105577. Max. coverage (+): 0. Max coverage (-): 0

Region: chr8 100105578-100105604. Max. coverage (+): 0. Max coverage (-): 0

Region: chr8 100105605-100105630. Max. coverage (+): 0. Max coverage (-): 0

Region: chr8 100105631-100105657. Max. coverage (+): 0. Max coverage (-): 0

Region: chr8 100105658-100105683. Max. coverage (+): 0. Max coverage (-): 0

Region: chr8 100105684-100105710. Max. coverage (+): 0. Max coverage (-): 0

Region: chr8 100105711-100105737. Max. coverage (+): 0. Max coverage (-): 0

Region: chr8 100105738-100105763. Max. coverage (+): 0. Max coverage (-): 0

Region: chr8 100105764-100105790. Max. coverage (+): 0. Max coverage (-): 0

Region: chr8 100105791-100105817. Max. coverage (+): 0. Max coverage (-): 0

Region: chr8 100105818-100105843. Max. coverage (+): 0. Max coverage (-): 0

Region: chr8 100105844-100105870. Max. coverage (+): 0. Max coverage (-): 0

Region: chr8 100105871-100105896. Max. coverage (+): 0. Max coverage (-): 0

Region: chr8 100105897-100105923. Max. coverage (+): 0. Max coverage (-): 0

Region: chr8 100105924-100105950. Max. coverage (+): 0. Max coverage (-): 0

Region: chr8 100105951-100105976. Max. coverage (+): 0. Max coverage (-): 0

Region: chr8 100105977-100106003. Max. coverage (+): 0. Max coverage (-): 0

Region: chr8 100106004-100106029. Max. coverage (+): 0. Max coverage (-): 0

Region: chr8 100106030-100106056. Max. coverage (+): 0. Max coverage (-): 0

Region: chr8 100106057-100106083. Max. coverage (+): 0. Max coverage (-): 0

Region: chr8 100106084-100106109. Max. coverage (+): 0. Max coverage (-): 0

Region: chr8 100106110-100106136. Max. coverage (+): 0. Max coverage (-): 0

Region: chr8 100106137-100106162. Max. coverage (+): 0. Max coverage (-): 0

Region: chr8 100106163-100106189. Max. coverage (+): 0. Max coverage (-): 0

Region: chr8 100106190-100106216. Max. coverage (+): 0. Max coverage (-): 0

Region: chr8 100106217-100106242. Max. coverage (+): 0. Max coverage (-): 0

Region: chr8 100106243-100106269. Max. coverage (+): 0. Max coverage (-): 0

Region: chr8 100106270-100106295. Max. coverage (+): 0. Max coverage (-): 0

Region: chr8 100106296-100106322. Max. coverage (+): 0. Max coverage (-): 0

Region: chr8 100106323-100106349. Max. coverage (+): 0. Max coverage (-): 0

Region: chr8 100106350-100106375. Max. coverage (+): 0. Max coverage (-): 0

Region: chr8 100106376-100106402. Max. coverage (+): 0. Max coverage (-): 0

Region: chr8 100106403-100106428. Max. coverage (+): 0. Max coverage (-): 0

Region: chr8 100106429-100106455. Max. coverage (+): 0. Max coverage (-): 0

Region: chr8 100106456-100106482. Max. coverage (+): 0. Max coverage (-): 0

Region: chr8 100106483-100106508. Max. coverage (+): 0. Max coverage (-): 0

Region: chr8 100106509-100106535. Max. coverage (+): 0. Max coverage (-): 0

Region: chr8 100106536-100106561. Max. coverage (+): 0. Max coverage (-): 0

Region: chr8 100106562-100106588. Max. coverage (+): 0. Max coverage (-): 0

Region: chr8 100106589-100106615. Max. coverage (+): 0. Max coverage (-): 0

Region: chr8 100106616-100106641. Max. coverage (+): 0. Max coverage (-): 0

Region: chr8 100106642-100106668. Max. coverage (+): 0. Max coverage (-): 0

Region: chr8 100106669-100106694. Max. coverage (+): 0. Max coverage (-): 0

Region: chr8 100106695-100106721. Max. coverage (+): 0. Max coverage (-): 0

Region: chr8 100106722-100106748. Max. coverage (+): 0. Max coverage (-): 0

Region: chr8 100106749-100106774. Max. coverage (+): 0. Max coverage (-): 0

Region: chr8 100106775-100106801. Max. coverage (+): 0. Max coverage (-): 0

Region: chr8 100106802-100106827. Max. coverage (+): 0. Max coverage (-): 0

Region: chr8 100106828-100106854. Max. coverage (+): 0. Max coverage (-): 0

Region: chr8 100106855-100106881. Max. coverage (+): 0. Max coverage (-): 0

Region: chr8 100106882-100106907. Max. coverage (+): 0. Max coverage (-): 0

Region: chr8 100106908-100106934. Max. coverage (+): 0. Max coverage (-): 0

Region: chr8 100106935-100106960. Max. coverage (+): 0. Max coverage (-): 0

Region: chr8 100106961-100106987. Max. coverage (+): 0. Max coverage (-): 0

Region: chr8 100106988-100107014. Max. coverage (+): 0. Max coverage (-): 0

Region: chr8 100107015-100107040. Max. coverage (+): 0. Max coverage (-): 0

Region: chr8 100107041-100107067. Max. coverage (+): 0. Max coverage (-): 0

Region: chr8 100107068-100107094. Max. coverage (+): 0. Max coverage (-): 0

Region: chr8 100107095-100107120. Max. coverage (+): 0. Max coverage (-): 0

Region: chr8 100107121-100107147. Max. coverage (+): 0. Max coverage (-): 0

Region: chr8 100107148-100107173. Max. coverage (+): 0. Max coverage (-): 0

Region: chr8 100107174-100107200. Max. coverage (+): 0. Max coverage (-): 0

Region: chr8 100107201-100107227. Max. coverage (+): 0. Max coverage (-): 0

Region: chr8 100107228-100107253. Max. coverage (+): 0. Max coverage (-): 0

Region: chr8 100107254-100107280. Max. coverage (+): 0. Max coverage (-): 0

Region: chr8 100107281-100107306. Max. coverage (+): 0. Max coverage (-): 0

Region: chr8 100107307-100107333. Max. coverage (+): 0. Max coverage (-): 0

Region: chr8 100107334-100107360. Max. coverage (+): 0. Max coverage (-): 0

Region: chr8 100107361-100107386. Max. coverage (+): 0. Max coverage (-): 0

Region: chr8 100107387-100107413. Max. coverage (+): 0. Max coverage (-): 0

Region: chr8 100107414-100107439. Max. coverage (+): 0. Max coverage (-): 0

Region: chr8 100107440-100107466. Max. coverage (+): 0. Max coverage (-): 0

Region: chr8 100107467-100107493. Max. coverage (+): 0. Max coverage (-): 0

Region: chr8 100107494-100107519. Max. coverage (+): 0. Max coverage (-): 0

Region: chr8 100107520-100107546. Max. coverage (+): 0. Max coverage (-): 0

Region: chr8 100107547-100107572. Max. coverage (+): 0. Max coverage (-): 0

Region: chr8 100107573-100107599. Max. coverage (+): 0. Max coverage (-): 0

Region: chr8 100107600-100107626. Max. coverage (+): 0. Max coverage (-): 0

Region: chr8 100107627-100107652. Max. coverage (+): 0. Max coverage (-): 0

Region: chr8 100107653-100107679. Max. coverage (+): 0. Max coverage (-): 0

Region: chr8 100107680-100107705. Max. coverage (+): 0. Max coverage (-): 0

Region: chr8 100107706-100107732. Max. coverage (+): 0. Max coverage (-): 0

Region: chr8 100107733-100107759. Max. coverage (+): 0. Max coverage (-): 0

Region: chr8 100107760-100107785. Max. coverage (+): 0. Max coverage (-): 0

Region: chr8 100107786-100107812. Max. coverage (+): 0. Max coverage (-): 0

Region: chr8 100107813-100107838. Max. coverage (+): 0. Max coverage (-): 0

Region: chr8 100107839-100107865. Max. coverage (+): 0. Max coverage (-): 0

Region: chr8 100107866-100107892. Max. coverage (+): 0. Max coverage (-): 0

Region: chr8 100107893-100107918. Max. coverage (+): 0. Max coverage (-): 0

Region: chr8 100107919-100107945. Max. coverage (+): 0. Max coverage (-): 0

Region: chr8 100107946-100107971. Max. coverage (+): 0. Max coverage (-): 0

Region: chr8 100107972-100107998. Max. coverage (+): 0. Max coverage (-): 0

Region: chr8 100107999-100108025. Max. coverage (+): 0. Max coverage (-): 0

Region: chr8 100108026-100108051. Max. coverage (+): 0. Max coverage (-): 0

Region: chr8 100108052-100108078. Max. coverage (+): 0. Max coverage (-): 0

Region: chr8 100108079-100108104. Max. coverage (+): 0. Max coverage (-): 0

Region: chr8 100108105-100108131. Max. coverage (+): 0. Max coverage (-): 0

Region: chr8 100108132-100108158. Max. coverage (+): 0. Max coverage (-): 0

Region: chr8 100108159-100108184. Max. coverage (+): 0. Max coverage (-): 0

Region: chr8 100108185-100108211. Max. coverage (+): 0. Max coverage (-): 0

Region: chr8 100108212-100108237. Max. coverage (+): 0. Max coverage (-): 0

Region: chr8 100108238-100108264. Max. coverage (+): 0. Max coverage (-): 0

Region: chr8 100108265-100108291. Max. coverage (+): 0. Max coverage (-): 0

Region: chr8 100108292-100108317. Max. coverage (+): 0. Max coverage (-): 0

Region: chr8 100108318-100108344. Max. coverage (+): 0. Max coverage (-): 0

Region: chr8 100108345-100108370. Max. coverage (+): 0. Max coverage (-): 0

Region: chr8 100108371-100108397. Max. coverage (+): 0. Max coverage (-): 0

Region: chr8 100108398-100108424. Max. coverage (+): 26.36. Max coverage (-): 0

Region: chr8 100108425-100108450. Max. coverage (+): 14.05. Max coverage (-): 0

Region: chr8 100108451-100108477. Max. coverage (+): 12.12. Max coverage (-): 0

Region: chr8 100108478-100108504. Max. coverage (+): 9.73. Max coverage (-): 0

Region: chr8 100108505-100108530. Max. coverage (+): 55.71. Max coverage (-): 0

Region: chr8 100108531-100108557. Max. coverage (+): 6.66. Max coverage (-): 0

Region: chr8 100108558-100108583. Max. coverage (+): 0. Max coverage (-): 0

Region: chr8 100108584-100108610. Max. coverage (+): 0. Max coverage (-): 0

Region: chr8 100108611-100108637. Max. coverage (+): 0. Max coverage (-): 0

Region: chr8 100108638-100108663. Max. coverage (+): 0. Max coverage (-): 0

Region: chr8 100108664-100108690. Max. coverage (+): 0. Max coverage (-): 0

Region: chr8 100108691-100108716. Max. coverage (+): 0. Max coverage (-): 0

Region: chr8 100108717-100108743. Max. coverage (+): 0. Max coverage (-): 0

Region: chr8 100108744-100108770. Max. coverage (+): 0. Max coverage (-): 0

Region: chr8 100108771-100108796. Max. coverage (+): 0. Max coverage (-): 0

Region: chr8 100108797-100108823. Max. coverage (+): 0. Max coverage (-): 0

Region: chr8 100108824-100108849. Max. coverage (+): 0. Max coverage (-): 0

Region: chr8 100108850-100108876. Max. coverage (+): 0. Max coverage (-): 0

Region: chr8 100108877-100108903. Max. coverage (+): 0. Max coverage (-): 0

Region: chr8 100108904-100108929. Max. coverage (+): 0. Max coverage (-): 0

Region: chr8 100108930-100108956. Max. coverage (+): 0. Max coverage (-): 0

Region: chr8 100108957-100108982. Max. coverage (+): 0. Max coverage (-): 0

Region: chr8 100108983-100109009. Max. coverage (+): 0. Max coverage (-): 0

Region: chr8 100109010-100109036. Max. coverage (+): 0. Max coverage (-): 0

Region: chr8 100109037-100109062. Max. coverage (+): 0. Max coverage (-): 0

Region: chr8 100109063-100109089. Max. coverage (+): 0. Max coverage (-): 0

Region: chr8 100109090-100109115. Max. coverage (+): 0. Max coverage (-): 0

Region: chr8 100109116-100109142. Max. coverage (+): 9.51. Max coverage (-): 0

Region: chr8 100109143-100109169. Max. coverage (+): 8.98. Max coverage (-): 0

Region: chr8 100109170-100109195. Max. coverage (+): 0. Max coverage (-): 0

Region: chr8 100109196-100109222. Max. coverage (+): 0. Max coverage (-): 0

Region: chr8 100109223-100109248. Max. coverage (+): 0. Max coverage (-): 0

Region: chr8 100109249-100109275. Max. coverage (+): 0. Max coverage (-): 0

Region: chr8 100109276-100109302. Max. coverage (+): 17.94. Max coverage (-): 0

Region: chr8 100109303-100109328. Max. coverage (+): 53.57. Max coverage (-): 0

Region: chr8 100109329-100109355. Max. coverage (+): 0. Max coverage (-): 0

Region: chr8 100109356-100109381. Max. coverage (+): 15.85. Max coverage (-): 0

Region: chr8 100109382-100109408. Max. coverage (+): 0. Max coverage (-): 0

Region: chr8 100109409-100109435. Max. coverage (+): 9.4. Max coverage (-): 0

Region: chr8 100109436-100109461. Max. coverage (+): 10.84. Max coverage (-): 0

Region: chr8 100109462-100109488. Max. coverage (+): 58.17. Max coverage (-): 0

Region: chr8 100109489-100109514. Max. coverage (+): 3.38. Max coverage (-): 0

Region: chr8 100109515-100109541. Max. coverage (+): 3.48. Max coverage (-): 0

Region: chr8 100109542-100109568. Max. coverage (+): 5.04. Max coverage (-): 0

Region: chr8 100109569-100109594. Max. coverage (+): 8.2. Max coverage (-): 0

Region: chr8 100109595-100109621. Max. coverage (+): 0. Max coverage (-): 0

Region: chr8 100109622-100109647. Max. coverage (+): 31.15. Max coverage (-): 0

Region: chr8 100109648-100109674. Max. coverage (+): 25.42. Max coverage (-): 0

Region: chr8 100109675-100109701. Max. coverage (+): 10.92. Max coverage (-): 0

Region: chr8 100109702-100109727. Max. coverage (+): 10.92. Max coverage (-): 0

Region: chr8 100109728-100109754. Max. coverage (+): 8.75. Max coverage (-): 0

Region: chr8 100109755-100109781. Max. coverage (+): 8.75. Max coverage (-): 0

Region: chr8 100109782-100109807. Max. coverage (+): 12.37. Max coverage (-): 0

Region: chr8 100109808-100109834. Max. coverage (+): 18.93. Max coverage (-): 0

Region: chr8 100109835-100109860. Max. coverage (+): 21.37. Max coverage (-): 0

Region: chr8 100109861-100109887. Max. coverage (+): 23.33. Max coverage (-): 0

Region: chr8 100109888-100109914. Max. coverage (+): 2.33. Max coverage (-): 0

Region: chr8 100109915-100109940. Max. coverage (+): 15.08. Max coverage (-): 0

Region: chr8 100109941-100109967. Max. coverage (+): 0.63. Max coverage (-): 0

Region: chr8 100109968-100109993. Max. coverage (+): 11.7. Max coverage (-): 0

Region: chr8 100109994-100110020. Max. coverage (+): 14.58. Max coverage (-): 0

Region: chr8 100110021-100110047. Max. coverage (+): 9.34. Max coverage (-): 0

Region: chr8 100110048-100110073. Max. coverage (+): 6.54. Max coverage (-): 0

Region: chr8 100110074-100110100. Max. coverage (+): 10.99. Max coverage (-): 0

Region: chr8 100110101-100110126. Max. coverage (+): 41.29. Max coverage (-): 0

Region: chr8 100110127-100110153. Max. coverage (+): 80.02. Max coverage (-): 0

Region: chr8 100110154-100110180. Max. coverage (+): 20.62. Max coverage (-): 0

Region: chr8 100110181-100110206. Max. coverage (+): 1.45. Max coverage (-): 0

Region: chr8 100110207-100110233. Max. coverage (+): 0. Max coverage (-): 0

Region: chr8 100110234-100110259. Max. coverage (+): 0. Max coverage (-): 0

Region: chr8 100110260-100110286. Max. coverage (+): 0. Max coverage (-): 0

Region: chr8 100110287-100110313. Max. coverage (+): 0. Max coverage (-): 0

Region: chr8 100110314-100110339. Max. coverage (+): 0. Max coverage (-): 0

Region: chr8 100110340-100110366. Max. coverage (+): 19.84. Max coverage (-): 0

Region: chr8 100110367-100110392. Max. coverage (+): 0. Max coverage (-): 0

Region: chr8 100110393-100110419. Max. coverage (+): 138.77. Max coverage (-): 0

Region: chr8 100110420-100110446. Max. coverage (+): 132.23. Max coverage (-): 0

Region: chr8 100110447-100110472. Max. coverage (+): 86.01. Max coverage (-): 0

Region: chr8 100110473-100110499. Max. coverage (+): 18.19. Max coverage (-): 0

Region: chr8 100110500-100110525. Max. coverage (+): 42.13. Max coverage (-): 0

Region: chr8 100110526-100110552. Max. coverage (+): 88.32. Max coverage (-): 0

Region: chr8 100110553-100110579. Max. coverage (+): 98.89. Max coverage (-): 0

Region: chr8 100110580-100110605. Max. coverage (+): 20.7. Max coverage (-): 0

Region: chr8 100110606-100110632. Max. coverage (+): 30.88. Max coverage (-): 0

Region: chr8 100110633-100110658. Max. coverage (+): 31.99. Max coverage (-): 0

Region: chr8 100110659-100110685. Max. coverage (+): 16.72. Max coverage (-): 0

Region: chr8 100110686-100110712. Max. coverage (+): 31.59. Max coverage (-): 0

Region: chr8 100110713-100110738. Max. coverage (+): 19.37. Max coverage (-): 0

Region: chr8 100110739-100110765. Max. coverage (+): 4.22. Max coverage (-): 0

Region: chr8 100110766-100110791. Max. coverage (+): 5.17. Max coverage (-): 0

Region: chr8 100110792-100110818. Max. coverage (+): 26.27. Max coverage (-): 0

Region: chr8 100110819-100110845. Max. coverage (+): 33.17. Max coverage (-): 0

Region: chr8 100110846-100110871. Max. coverage (+): 20.92. Max coverage (-): 0

Region: chr8 100110872-100110898. Max. coverage (+): 33.71. Max coverage (-): 0

Region: chr8 100110899-100110924. Max. coverage (+): 33.08. Max coverage (-): 0

Region: chr8 100110925-100110951. Max. coverage (+): 33.08. Max coverage (-): 0

Region: chr8 100110952-100110978. Max. coverage (+): 13.83. Max coverage (-): 0

Region: chr8 100110979-100111004. Max. coverage (+): 3.25. Max coverage (-): 0

Region: chr8 100111005-100111031. Max. coverage (+): 17.35. Max coverage (-): 0

Region: chr8 100111032-100111058. Max. coverage (+): 108.52. Max coverage (-): 0

Region: chr8 100111059-100111084. Max. coverage (+): 4.08. Max coverage (-): 0

Region: chr8 100111085-100111111. Max. coverage (+): 22.12. Max coverage (-): 0

Region: chr8 100111112-100111137. Max. coverage (+): 22.12. Max coverage (-): 0

Region: chr8 100111138-100111164. Max. coverage (+): 10.61. Max coverage (-): 0

Region: chr8 100111165-100111191. Max. coverage (+): 30.92. Max coverage (-): 0

Region: chr8 100111192-100111217. Max. coverage (+): 7.75. Max coverage (-): 0

Region: chr8 100111218-100111244. Max. coverage (+): 5.4. Max coverage (-): 0

Region: chr8 100111245-100111270. Max. coverage (+): 1.71. Max coverage (-): 0

Region: chr8 100111271-100111297. Max. coverage (+): 8.47. Max coverage (-): 0

Region: chr8 100111298-100111324. Max. coverage (+): 7.55. Max coverage (-): 0

Region: chr8 100111325-100111350. Max. coverage (+): 7.4. Max coverage (-): 0

Region: chr8 100111351-100111377. Max. coverage (+): 26.28. Max coverage (-): 0

Region: chr8 100111378-100111403. Max. coverage (+): 47.32. Max coverage (-): 0

Region: chr8 100111404-100111430. Max. coverage (+): 13.38. Max coverage (-): 0

Region: chr8 100111431-100111457. Max. coverage (+): 13.38. Max coverage (-): 0

Region: chr8 100111458-100111483. Max. coverage (+): 0. Max coverage (-): 0

Region: chr8 100111484-100111510. Max. coverage (+): 0. Max coverage (-): 0

Region: chr8 100111511-100111536. Max. coverage (+): 5.1. Max coverage (-): 0

Region: chr8 100111537-100111563. Max. coverage (+): 0. Max coverage (-): 0

Region: chr8 100111564-100111590. Max. coverage (+): 0. Max coverage (-): 0

Region: chr8 100111591-100111616. Max. coverage (+): 32.32. Max coverage (-): 0

Region: chr8 100111617-100111643. Max. coverage (+): 22.03. Max coverage (-): 0

Region: chr8 100111644-100111669. Max. coverage (+): 39.61. Max coverage (-): 0

Region: chr8 100111670-100111696. Max. coverage (+): 44.02. Max coverage (-): 0

Region: chr8 100111697-100111723. Max. coverage (+): 14.46. Max coverage (-): 0

Region: chr8 100111724-100111749. Max. coverage (+): 7.63. Max coverage (-): 0

Region: chr8 100111750-100111776. Max. coverage (+): 10.22. Max coverage (-): 0

Region: chr8 100111777-100111802. Max. coverage (+): 18.5. Max coverage (-): 0

Region: chr8 100111803-100111829. Max. coverage (+): 5.61. Max coverage (-): 0

Region: chr8 100111830-100111856. Max. coverage (+): 10.9. Max coverage (-): 0

Region: chr8 100111857-100111882. Max. coverage (+): 0. Max coverage (-): 0

Region: chr8 100111883-100111909. Max. coverage (+): 6.44. Max coverage (-): 0

Region: chr8 100111910-100111935. Max. coverage (+): 2.45. Max coverage (-): 0

Region: chr8 100111936-100111962. Max. coverage (+): 0. Max coverage (-): 0

Region: chr8 100111963-100111989. Max. coverage (+): 3.82. Max coverage (-): 0

Region: chr8 100111990-100112015. Max. coverage (+): 8.18. Max coverage (-): 0

Region: chr8 100112016-100112042. Max. coverage (+): 0. Max coverage (-): 0

Region: chr8 100112043-100112068. Max. coverage (+): 0. Max coverage (-): 0

Region: chr8 100112069-100112095. Max. coverage (+): 4.77. Max coverage (-): 0

Region: chr8 100112096-100112122. Max. coverage (+): 0. Max coverage (-): 0

Region: chr8 100112123-100112148. Max. coverage (+): 0. Max coverage (-): 0

Region: chr8 100112149-100112175. Max. coverage (+): 0. Max coverage (-): 0

Region: chr8 100112176-100112201. Max. coverage (+): 0. Max coverage (-): 0

Region: chr8 100112202-100112228. Max. coverage (+): 0. Max coverage (-): 0

Region: chr8 100112229-100112255. Max. coverage (+): 0. Max coverage (-): 0

Region: chr8 100112256-100112281. Max. coverage (+): 0. Max coverage (-): 0

Region: chr8 100112282-100112308. Max. coverage (+): 0. Max coverage (-): 0

Region: chr8 100112309-100112334. Max. coverage (+): 0. Max coverage (-): 0

Region: chr8 100112335-100112361. Max. coverage (+): 0. Max coverage (-): 0

Region: chr8 100112362-100112388. Max. coverage (+): 0. Max coverage (-): 0

Region: chr8 100112389-100112414. Max. coverage (+): 0. Max coverage (-): 0

Region: chr8 100112415-100112441. Max. coverage (+): 0. Max coverage (-): 0

Region: chr8 100112442-100112468. Max. coverage (+): 0. Max coverage (-): 0

Region: chr8 100112469-100112494. Max. coverage (+): 0. Max coverage (-): 0

Region: chr8 100112495-100112521. Max. coverage (+): 0. Max coverage (-): 0

Region: chr8 100112522-100112547. Max. coverage (+): 0. Max coverage (-): 0

Region: chr8 100112548-100112574. Max. coverage (+): 0. Max coverage (-): 0

Region: chr8 100112575-100112601. Max. coverage (+): 0. Max coverage (-): 0

Region: chr8 100112602-100112627. Max. coverage (+): 0. Max coverage (-): 0

Region: chr8 100112628-100112654. Max. coverage (+): 0. Max coverage (-): 0

Region: chr8 100112655-100112680. Max. coverage (+): 0. Max coverage (-): 0

Region: chr8 100112681-100112707. Max. coverage (+): 0. Max coverage (-): 0

Region: chr8 100112708-100112734. Max. coverage (+): 0. Max coverage (-): 0

Region: chr8 100112735-100112760. Max. coverage (+): 0. Max coverage (-): 0

Region: chr8 100112761-100112787. Max. coverage (+): 0. Max coverage (-): 0

Region: chr8 100112788-100112813. Max. coverage (+): 0. Max coverage (-): 0

Region: chr8 100112814-100112840. Max. coverage (+): 0. Max coverage (-): 0

Region: chr8 100112841-100112867. Max. coverage (+): 0. Max coverage (-): 0

Region: chr8 100112868-100112893. Max. coverage (+): 0. Max coverage (-): 0

Region: chr8 100112894-100112920. Max. coverage (+): 0. Max coverage (-): 0

Region: chr8 100112921-100112946. Max. coverage (+): 0. Max coverage (-): 0

Region: chr8 100112947-100112973. Max. coverage (+): 0. Max coverage (-): 0

Region: chr8 100112974-100113000. Max. coverage (+): 0. Max coverage (-): 0

Region: chr8 100113001-100113026. Max. coverage (+): 1.28. Max coverage (-): 0

Region: chr8 100113027-. Max. coverage (+): 0. Max coverage (-): 0

RepeatMasker Color Code

**+**

100-98% Identity

<98-95% Identity

<95-90% Identity

<90-85% Identity

<85-80% Identity

<80-75% Identity

<75-70% Identity

<70% Identity

**-**

Gene Set Color Code

**+**

Gene

Pseudogene

**-**

Topology/Coverage Color Code

Coverage Plus Strand

Coverage Minus Strand

Mainstrand: Plus

Mainstrand: Minus

Complementary Strand

Flanking Region  
(if option -flank >0)

Gene Set Annotation  
  
RepeatMasker Annotation  

**1. L2a**: 100100165-100100237 (+), Divergence to consensus: 33.2%  
**2. LTR65**: 100102802-100103416 (+), Divergence to consensus: 30.8%  
**3. ART2A**: 100103696-100104213 (-), Divergence to consensus: 15.9%  
**4. BovB**: 100104215-100105064 (-), Divergence to consensus: 10.2%  
**5. BTLTR1**: 100105065-100105136 (+), Divergence to consensus: 12.5%  
**6. BovB**: 100105137-100105203 (-), Divergence to consensus: 9.1%  
**7. Bov-tA2**: 100106221-100106338 (+), Divergence to consensus: 16.1%  
**8. Bov-tA2**: 100106338-100106440 (+), Divergence to consensus: 14.6%  
**9. ART2A**: 100106439-100106558 (-), Divergence to consensus: 3.3%  
**10. BovB**: 100106559-100106791 (-), Divergence to consensus: 6%  
**11. ART2A**: 100106792-100107208 (+), Divergence to consensus: 9.3%  
**12. Bov-tA3**: 100107208-100107300 (+), Divergence to consensus: 29%  
**13. AT\_rich**: 100107347-100107367 (+), Divergence to consensus: 33.3%  
**14. MER110-int**: 100108567-100109112 (+), Divergence to consensus: 47.7%  
**15. SINE2-1\_BT**: 100109169-100109289 (+), Divergence to consensus: 27.9%  
**16. MIRc**: 100110204-100110325 (-), Divergence to consensus: 29.5%  
**17. L1MC5a**: 100112128-100112591 (-), Divergence to consensus: 43.2%  
**18. MIRb**: 100112663-100112862 (+), Divergence to consensus: 44.5%

  
Transcription Factor Binding Sites  

**RFX4\_2** (Sequence: GTAACTATG (-): 100109388)  
**RFX4\_2** (Sequence: CATAGATAC (+): 100111618)  
**Gata4** (Sequence: AGATAAC (-): 100099856)  
**Gata4** (Sequence: AGATAAC (-): 100105793)  
**SOX9** (Sequence: TCATTGTT (+): 100107462)  
**Mybl1\_1** (Sequence: AACCGTTA (+): 100099984)
