# Supplementary material for: Small RNA sequencing of cryopreserved semen from single bull revealed altered miRNAs and piRNAs expression between High- and Low-motile sperm populations
Source: BMC Genomics. 2017 Jan 4;18:14. doi: 10.1186/s12864-016-3394-7 (PMC5209821; doi:10.1186/s12864-016-3394-7)
Supplement: Additional file 3: — Details for each piRNA clusters found in High Motile (HM) sperm fraction. Genes, repeats, transposable elements and transcription factors binding sites falling within the cluster regions were reported. (ZIP 1896 kb) [file 12864_2016_3394_MOESM3_ESM.zip › 97.html]

piRNA cluster 97


Predicted piRNA cluster no. 97     previous   next
  

Show proTRAC run info
Hide proTRAC run info

================================= proTRAC ====================================  
VERSION: 2.1                                    LAST MODIFIED: 06. October 2015  
  
Please cite:  
Rosenkranz D, Zischler H. proTRAC - a software for probabilistic piRNA cluster  
detection, visualization and analysis. 2012. BMC Bioinformatics 13:5.  
  
and (for proTRAC 2.0 and later):  
Rosenkranz D, Rudloff S, Bastuck K, Ketting RF, Zischler H. Tupaia small RNAs  
provide insights into function and evolution of RNAi-based transposon defense  
in mammals. 2015. RNA 21(5):911-922.  
  
Contact:  
David Rosenkranz  
Institute of Anthropology, small RNA group  
Johannes Gutenberg University Mainz  
email: rosenkranz@uni-mainz.de  
  
You can find the latest proTRAC version at:  
http://sourceforge.net/projects/protrac/files  
http://www.smallRNAgroup-mainz.de/software  
==============================================================================  
  
PARAMETERS:  
Map file: .............../storage/core/barbara/genhome/smallRNA/fertility/Sample\_motile/pirna/Sample\_motile\_26-33\_collapsed.fa.no-dust.map.weighted-10000-1000-b-0  
Genome file: ............/storage/core/barbara/genhome/smallRNA/fertility/Sample\_all/pirna/bt\_311\_chrY.fa  
RepeatMasker annotation: /storage/genomes/bt\_umd31/GCF\_000003055.6\_Bos\_taurus\_UMD\_3.1.1\_repeatMasker\_chr.out  
GeneSet:................./storage/core/barbara/genhome/smallRNA/fertility/Sample\_all/pirna/full.gtf  
  
Significant (p<=0.01) hit density will be calculated based  
on observed hit distribution.  
  
Sliding window size: ........................................ 5000 bp  
Sliding window increament: .................................. 1000 bp  
Normalize each hit by number of genomic hits: ............... 1 [0=no/1=yes]  
Normalize each hit by number of sequence reads: ............. 1 [0=no/1=yes]  
Normalize values (-> per million mapped reads): ............. 1 [0=no/1=yes]  
Min. fraction of hits with 1T(U) or 10A: .................... 0.75  
Alternatively: Min. fraction of hits with 1T(U) and 10A: .... 0.5  
Min. fraction of hits with typical piRNA length: ............ 0.75  
Typical piRNA length: ....................................... 26-33 nt  
Min. size of a piRNA cluster: ............................... 5000 bp.  
Min. number of hits (absolute): ............................. 0  
Min. number of hits (normalized): ........................... 0  
Min. fraction of hits on the mainstrand: .................... 0.75  
Top fraction of mapped sequences (in terms of read counts): . 1%  
Top fraction accounts for max. n% of sequence reads: ........ 90%  
Min. fraction of hits on each arm of a bidirectional cluster: 0.1  
Output image file for each cluster: ......................... 0 [0=no/1=yes]  
Output html file for each cluster: .......................... 1 [0=no/1=yes]  
Output a summary table: ..................................... 1 [0=no/1=yes]  
Output a FASTA file for each cluster (piRNA sequences): ..... 1 [0=no/1=yes]  
Output a FASTA file comprising cluster sequences: ........... 1 [0=no/1=yes]  
Search DNA motifs in clusters: .............................. 1 [0=no/1=yes]  
Output flanking sequences: +/- .............................. 0 bp  
Output ~.pTi file: .......................................... 1 [0=no/1=yes]  
==============================================================================  
  
  
Genome size (without gaps): ............ 2678902517 bp  
Gaps (N/X/-): .......................... 53837044 bp  
Mapped reads: .......................... 658825247023  
Non-identical sequences: ............... 514171  
Genomic hits: .......................... 764233  
Significant densitiy of mapped reads: .. 12867599.5173724 reads/kb

Show proTRAC cluster info
Hide proTRAC cluster info

|  |  |
| --- | --- |
| Location | chr8 |
| Coordinates | 100784742-100793005 |
| Size [bp] | 8264 |
| Sequence hit loci | 659 |
| Mapped reads (normalized) | 746732968.5 |
| Mapped reads (normalized) per kb | 90359749.3 |
| Normalized reads with 1T (1U) | 80.4% |
| Normalized reads with 10A | 37.4% |
| Normalized reads with length 26-33 nt | 100% |
| Normalized reads on the main strand(s) | 100% |
| Predicted directionality | mono:minus |

100%

0%

1T (1U)  
reads

10A reads

26-33 nt  
reads

reads on mainstrand

**Either the amount of reads with 1T (1U) OR 10A has to exceed 75% (set with option: -1Tor10A)  
Alternatively the amount of reads with 1T (1U) AND 10A has to exceed 50% (set with option: -1Tand10A)  
Minimum amount of reads with preferred size is 75% (set with option: -pisize)  
Minimum amount of reads on the main strand(s) is 75% (set with option: -clstrand)**

Show read coverage
Hide read coverage

WHAT DO I SEE HERE?  
This chart shows the location of mapped sequence reads within a predicted piRNA cluster. The color refers to the number of genomic hits produced by the sequence read in question. A dark red bar indicates that this sequence read produces many other hits elsewhere in the genome. Many adjacent red or yellow bars can indicate the presence of a multi-copy element such as transposons or rRNA genes. A dark green bar indicates that this sequence read maps uniquely to this locus.

1 hit

2-5 hits

6-10 hits

11-20 hits

21-50 hits

51-100 hits

> 100 hits

chr8

100784742

100793005

Gene Set

RepeatMasker

Mapped  
Reads

68.22

plus strand

minus strand

68.22

Region: chr8 100113028-100784750. Max. coverage (+): 0. Max coverage (-): 2.35

Region: chr8 100784751-100784766. Max. coverage (+): 0. Max coverage (-): 0.28

Region: chr8 100784767-100784783. Max. coverage (+): 0. Max coverage (-): 0

Region: chr8 100784784-100784799. Max. coverage (+): 0. Max coverage (-): 0

Region: chr8 100784800-100784816. Max. coverage (+): 0. Max coverage (-): 0

Region: chr8 100784817-100784832. Max. coverage (+): 0. Max coverage (-): 0

Region: chr8 100784833-100784849. Max. coverage (+): 0. Max coverage (-): 0

Region: chr8 100784850-100784865. Max. coverage (+): 0. Max coverage (-): 0

Region: chr8 100784866-100784882. Max. coverage (+): 0. Max coverage (-): 0

Region: chr8 100784883-100784899. Max. coverage (+): 0. Max coverage (-): 0

Region: chr8 100784900-100784915. Max. coverage (+): 0. Max coverage (-): 0

Region: chr8 100784916-100784932. Max. coverage (+): 0. Max coverage (-): 0

Region: chr8 100784933-100784948. Max. coverage (+): 0. Max coverage (-): 0

Region: chr8 100784949-100784965. Max. coverage (+): 0. Max coverage (-): 0

Region: chr8 100784966-100784981. Max. coverage (+): 0. Max coverage (-): 0

Region: chr8 100784982-100784998. Max. coverage (+): 0. Max coverage (-): 0

Region: chr8 100784999-100785014. Max. coverage (+): 0. Max coverage (-): 2.13

Region: chr8 100785015-100785031. Max. coverage (+): 0. Max coverage (-): 0

Region: chr8 100785032-100785047. Max. coverage (+): 0. Max coverage (-): 0

Region: chr8 100785048-100785064. Max. coverage (+): 0. Max coverage (-): 0

Region: chr8 100785065-100785080. Max. coverage (+): 0. Max coverage (-): 0

Region: chr8 100785081-100785097. Max. coverage (+): 0. Max coverage (-): 0

Region: chr8 100785098-100785113. Max. coverage (+): 0. Max coverage (-): 0

Region: chr8 100785114-100785130. Max. coverage (+): 0. Max coverage (-): 0

Region: chr8 100785131-100785146. Max. coverage (+): 0. Max coverage (-): 0

Region: chr8 100785147-100785163. Max. coverage (+): 0. Max coverage (-): 1.69

Region: chr8 100785164-100785179. Max. coverage (+): 0. Max coverage (-): 1.69

Region: chr8 100785180-100785196. Max. coverage (+): 0. Max coverage (-): 0

Region: chr8 100785197-100785213. Max. coverage (+): 0. Max coverage (-): 0

Region: chr8 100785214-100785229. Max. coverage (+): 0. Max coverage (-): 0

Region: chr8 100785230-100785246. Max. coverage (+): 0. Max coverage (-): 0

Region: chr8 100785247-100785262. Max. coverage (+): 0. Max coverage (-): 0

Region: chr8 100785263-100785279. Max. coverage (+): 0. Max coverage (-): 0

Region: chr8 100785280-100785295. Max. coverage (+): 0. Max coverage (-): 0

Region: chr8 100785296-100785312. Max. coverage (+): 0. Max coverage (-): 0

Region: chr8 100785313-100785328. Max. coverage (+): 0. Max coverage (-): 0

Region: chr8 100785329-100785345. Max. coverage (+): 0. Max coverage (-): 0

Region: chr8 100785346-100785361. Max. coverage (+): 0. Max coverage (-): 0

Region: chr8 100785362-100785378. Max. coverage (+): 0. Max coverage (-): 0

Region: chr8 100785379-100785394. Max. coverage (+): 0. Max coverage (-): 0

Region: chr8 100785395-100785411. Max. coverage (+): 0. Max coverage (-): 0

Region: chr8 100785412-100785427. Max. coverage (+): 0. Max coverage (-): 0

Region: chr8 100785428-100785444. Max. coverage (+): 0. Max coverage (-): 0

Region: chr8 100785445-100785460. Max. coverage (+): 0. Max coverage (-): 0

Region: chr8 100785461-100785477. Max. coverage (+): 0. Max coverage (-): 0

Region: chr8 100785478-100785494. Max. coverage (+): 0. Max coverage (-): 0

Region: chr8 100785495-100785510. Max. coverage (+): 0. Max coverage (-): 0

Region: chr8 100785511-100785527. Max. coverage (+): 0. Max coverage (-): 0

Region: chr8 100785528-100785543. Max. coverage (+): 0. Max coverage (-): 0

Region: chr8 100785544-100785560. Max. coverage (+): 0. Max coverage (-): 0

Region: chr8 100785561-100785576. Max. coverage (+): 0. Max coverage (-): 0

Region: chr8 100785577-100785593. Max. coverage (+): 0. Max coverage (-): 0

Region: chr8 100785594-100785609. Max. coverage (+): 0. Max coverage (-): 0

Region: chr8 100785610-100785626. Max. coverage (+): 0. Max coverage (-): 0

Region: chr8 100785627-100785642. Max. coverage (+): 0. Max coverage (-): 0

Region: chr8 100785643-100785659. Max. coverage (+): 0. Max coverage (-): 0

Region: chr8 100785660-100785675. Max. coverage (+): 0. Max coverage (-): 0

Region: chr8 100785676-100785692. Max. coverage (+): 0. Max coverage (-): 0

Region: chr8 100785693-100785708. Max. coverage (+): 0. Max coverage (-): 0

Region: chr8 100785709-100785725. Max. coverage (+): 0. Max coverage (-): 0

Region: chr8 100785726-100785741. Max. coverage (+): 0. Max coverage (-): 0

Region: chr8 100785742-100785758. Max. coverage (+): 0. Max coverage (-): 0

Region: chr8 100785759-100785774. Max. coverage (+): 0. Max coverage (-): 0

Region: chr8 100785775-100785791. Max. coverage (+): 0. Max coverage (-): 0

Region: chr8 100785792-100785808. Max. coverage (+): 0. Max coverage (-): 0

Region: chr8 100785809-100785824. Max. coverage (+): 0. Max coverage (-): 0

Region: chr8 100785825-100785841. Max. coverage (+): 0. Max coverage (-): 0

Region: chr8 100785842-100785857. Max. coverage (+): 0. Max coverage (-): 8.25

Region: chr8 100785858-100785874. Max. coverage (+): 0. Max coverage (-): 0

Region: chr8 100785875-100785890. Max. coverage (+): 0. Max coverage (-): 0

Region: chr8 100785891-100785907. Max. coverage (+): 0. Max coverage (-): 0

Region: chr8 100785908-100785923. Max. coverage (+): 0. Max coverage (-): 0

Region: chr8 100785924-100785940. Max. coverage (+): 0. Max coverage (-): 0

Region: chr8 100785941-100785956. Max. coverage (+): 0. Max coverage (-): 0

Region: chr8 100785957-100785973. Max. coverage (+): 0. Max coverage (-): 0

Region: chr8 100785974-100785989. Max. coverage (+): 0. Max coverage (-): 0

Region: chr8 100785990-100786006. Max. coverage (+): 0. Max coverage (-): 0

Region: chr8 100786007-100786022. Max. coverage (+): 0. Max coverage (-): 0

Region: chr8 100786023-100786039. Max. coverage (+): 0. Max coverage (-): 0

Region: chr8 100786040-100786055. Max. coverage (+): 0. Max coverage (-): 0

Region: chr8 100786056-100786072. Max. coverage (+): 0. Max coverage (-): 0

Region: chr8 100786073-100786089. Max. coverage (+): 0. Max coverage (-): 0

Region: chr8 100786090-100786105. Max. coverage (+): 0. Max coverage (-): 0

Region: chr8 100786106-100786122. Max. coverage (+): 0. Max coverage (-): 0

Region: chr8 100786123-100786138. Max. coverage (+): 0. Max coverage (-): 0

Region: chr8 100786139-100786155. Max. coverage (+): 0. Max coverage (-): 4.06

Region: chr8 100786156-100786171. Max. coverage (+): 0. Max coverage (-): 0

Region: chr8 100786172-100786188. Max. coverage (+): 0. Max coverage (-): 0

Region: chr8 100786189-100786204. Max. coverage (+): 0. Max coverage (-): 0

Region: chr8 100786205-100786221. Max. coverage (+): 0. Max coverage (-): 0

Region: chr8 100786222-100786237. Max. coverage (+): 0. Max coverage (-): 0

Region: chr8 100786238-100786254. Max. coverage (+): 0. Max coverage (-): 0

Region: chr8 100786255-100786270. Max. coverage (+): 0. Max coverage (-): 0

Region: chr8 100786271-100786287. Max. coverage (+): 0. Max coverage (-): 0

Region: chr8 100786288-100786303. Max. coverage (+): 0. Max coverage (-): 0

Region: chr8 100786304-100786320. Max. coverage (+): 0. Max coverage (-): 0

Region: chr8 100786321-100786336. Max. coverage (+): 0. Max coverage (-): 0

Region: chr8 100786337-100786353. Max. coverage (+): 0. Max coverage (-): 4.95

Region: chr8 100786354-100786370. Max. coverage (+): 0. Max coverage (-): 4.73

Region: chr8 100786371-100786386. Max. coverage (+): 0. Max coverage (-): 4.73

Region: chr8 100786387-100786403. Max. coverage (+): 0. Max coverage (-): 0

Region: chr8 100786404-100786419. Max. coverage (+): 0. Max coverage (-): 4.49

Region: chr8 100786420-100786436. Max. coverage (+): 0. Max coverage (-): 9.42

Region: chr8 100786437-100786452. Max. coverage (+): 0. Max coverage (-): 4.43

Region: chr8 100786453-100786469. Max. coverage (+): 0. Max coverage (-): 0

Region: chr8 100786470-100786485. Max. coverage (+): 0. Max coverage (-): 0

Region: chr8 100786486-100786502. Max. coverage (+): 0. Max coverage (-): 0

Region: chr8 100786503-100786518. Max. coverage (+): 0. Max coverage (-): 0

Region: chr8 100786519-100786535. Max. coverage (+): 0. Max coverage (-): 0

Region: chr8 100786536-100786551. Max. coverage (+): 0. Max coverage (-): 0

Region: chr8 100786552-100786568. Max. coverage (+): 0. Max coverage (-): 0

Region: chr8 100786569-100786584. Max. coverage (+): 0. Max coverage (-): 0

Region: chr8 100786585-100786601. Max. coverage (+): 0. Max coverage (-): 0

Region: chr8 100786602-100786617. Max. coverage (+): 0. Max coverage (-): 0

Region: chr8 100786618-100786634. Max. coverage (+): 0. Max coverage (-): 0

Region: chr8 100786635-100786650. Max. coverage (+): 0. Max coverage (-): 0

Region: chr8 100786651-100786667. Max. coverage (+): 0. Max coverage (-): 0

Region: chr8 100786668-100786684. Max. coverage (+): 0. Max coverage (-): 0

Region: chr8 100786685-100786700. Max. coverage (+): 0. Max coverage (-): 0

Region: chr8 100786701-100786717. Max. coverage (+): 0. Max coverage (-): 0

Region: chr8 100786718-100786733. Max. coverage (+): 0. Max coverage (-): 0

Region: chr8 100786734-100786750. Max. coverage (+): 0. Max coverage (-): 0

Region: chr8 100786751-100786766. Max. coverage (+): 0. Max coverage (-): 0

Region: chr8 100786767-100786783. Max. coverage (+): 0. Max coverage (-): 0

Region: chr8 100786784-100786799. Max. coverage (+): 0. Max coverage (-): 0

Region: chr8 100786800-100786816. Max. coverage (+): 0. Max coverage (-): 0

Region: chr8 100786817-100786832. Max. coverage (+): 0. Max coverage (-): 0

Region: chr8 100786833-100786849. Max. coverage (+): 0. Max coverage (-): 0

Region: chr8 100786850-100786865. Max. coverage (+): 0. Max coverage (-): 0

Region: chr8 100786866-100786882. Max. coverage (+): 0. Max coverage (-): 0

Region: chr8 100786883-100786898. Max. coverage (+): 0. Max coverage (-): 0

Region: chr8 100786899-100786915. Max. coverage (+): 0. Max coverage (-): 0

Region: chr8 100786916-100786931. Max. coverage (+): 0. Max coverage (-): 0

Region: chr8 100786932-100786948. Max. coverage (+): 0. Max coverage (-): 0

Region: chr8 100786949-100786965. Max. coverage (+): 0. Max coverage (-): 0

Region: chr8 100786966-100786981. Max. coverage (+): 0. Max coverage (-): 0

Region: chr8 100786982-100786998. Max. coverage (+): 0. Max coverage (-): 0

Region: chr8 100786999-100787014. Max. coverage (+): 0. Max coverage (-): 9.71

Region: chr8 100787015-100787031. Max. coverage (+): 0. Max coverage (-): 12.93

Region: chr8 100787032-100787047. Max. coverage (+): 0. Max coverage (-): 0

Region: chr8 100787048-100787064. Max. coverage (+): 0. Max coverage (-): 0

Region: chr8 100787065-100787080. Max. coverage (+): 0. Max coverage (-): 0

Region: chr8 100787081-100787097. Max. coverage (+): 0. Max coverage (-): 0

Region: chr8 100787098-100787113. Max. coverage (+): 0. Max coverage (-): 0

Region: chr8 100787114-100787130. Max. coverage (+): 0. Max coverage (-): 0

Region: chr8 100787131-100787146. Max. coverage (+): 0. Max coverage (-): 0

Region: chr8 100787147-100787163. Max. coverage (+): 0. Max coverage (-): 0

Region: chr8 100787164-100787179. Max. coverage (+): 0. Max coverage (-): 0

Region: chr8 100787180-100787196. Max. coverage (+): 0. Max coverage (-): 0

Region: chr8 100787197-100787212. Max. coverage (+): 0. Max coverage (-): 0

Region: chr8 100787213-100787229. Max. coverage (+): 0. Max coverage (-): 0

Region: chr8 100787230-100787245. Max. coverage (+): 0. Max coverage (-): 0

Region: chr8 100787246-100787262. Max. coverage (+): 0. Max coverage (-): 0

Region: chr8 100787263-100787279. Max. coverage (+): 0. Max coverage (-): 0

Region: chr8 100787280-100787295. Max. coverage (+): 0. Max coverage (-): 0

Region: chr8 100787296-100787312. Max. coverage (+): 0. Max coverage (-): 0

Region: chr8 100787313-100787328. Max. coverage (+): 0. Max coverage (-): 0

Region: chr8 100787329-100787345. Max. coverage (+): 0. Max coverage (-): 0

Region: chr8 100787346-100787361. Max. coverage (+): 0. Max coverage (-): 0

Region: chr8 100787362-100787378. Max. coverage (+): 0. Max coverage (-): 0

Region: chr8 100787379-100787394. Max. coverage (+): 0. Max coverage (-): 0

Region: chr8 100787395-100787411. Max. coverage (+): 0. Max coverage (-): 0

Region: chr8 100787412-100787427. Max. coverage (+): 0. Max coverage (-): 0

Region: chr8 100787428-100787444. Max. coverage (+): 0. Max coverage (-): 4.53

Region: chr8 100787445-100787460. Max. coverage (+): 0. Max coverage (-): 4.53

Region: chr8 100787461-100787477. Max. coverage (+): 0. Max coverage (-): 4.33

Region: chr8 100787478-100787493. Max. coverage (+): 0. Max coverage (-): 0

Region: chr8 100787494-100787510. Max. coverage (+): 0. Max coverage (-): 1.15

Region: chr8 100787511-100787526. Max. coverage (+): 0. Max coverage (-): 0

Region: chr8 100787527-100787543. Max. coverage (+): 0. Max coverage (-): 1.89

Region: chr8 100787544-100787560. Max. coverage (+): 0. Max coverage (-): 0

Region: chr8 100787561-100787576. Max. coverage (+): 0. Max coverage (-): 0

Region: chr8 100787577-100787593. Max. coverage (+): 0. Max coverage (-): 0

Region: chr8 100787594-100787609. Max. coverage (+): 0. Max coverage (-): 5.42

Region: chr8 100787610-100787626. Max. coverage (+): 0. Max coverage (-): 0

Region: chr8 100787627-100787642. Max. coverage (+): 0. Max coverage (-): 0

Region: chr8 100787643-100787659. Max. coverage (+): 0. Max coverage (-): 0

Region: chr8 100787660-100787675. Max. coverage (+): 0. Max coverage (-): 0

Region: chr8 100787676-100787692. Max. coverage (+): 0. Max coverage (-): 3.02

Region: chr8 100787693-100787708. Max. coverage (+): 0. Max coverage (-): 0

Region: chr8 100787709-100787725. Max. coverage (+): 0. Max coverage (-): 0

Region: chr8 100787726-100787741. Max. coverage (+): 0. Max coverage (-): 1.05

Region: chr8 100787742-100787758. Max. coverage (+): 0. Max coverage (-): 0

Region: chr8 100787759-100787774. Max. coverage (+): 0. Max coverage (-): 0

Region: chr8 100787775-100787791. Max. coverage (+): 0. Max coverage (-): 0

Region: chr8 100787792-100787807. Max. coverage (+): 0. Max coverage (-): 3.56

Region: chr8 100787808-100787824. Max. coverage (+): 0. Max coverage (-): 5.33

Region: chr8 100787825-100787840. Max. coverage (+): 0. Max coverage (-): 0

Region: chr8 100787841-100787857. Max. coverage (+): 0. Max coverage (-): 2.04

Region: chr8 100787858-100787874. Max. coverage (+): 0. Max coverage (-): 2.42

Region: chr8 100787875-100787890. Max. coverage (+): 0. Max coverage (-): 0

Region: chr8 100787891-100787907. Max. coverage (+): 0. Max coverage (-): 0

Region: chr8 100787908-100787923. Max. coverage (+): 0. Max coverage (-): 0

Region: chr8 100787924-100787940. Max. coverage (+): 0. Max coverage (-): 0

Region: chr8 100787941-100787956. Max. coverage (+): 0. Max coverage (-): 0

Region: chr8 100787957-100787973. Max. coverage (+): 0. Max coverage (-): 0

Region: chr8 100787974-100787989. Max. coverage (+): 0. Max coverage (-): 0

Region: chr8 100787990-100788006. Max. coverage (+): 0. Max coverage (-): 0

Region: chr8 100788007-100788022. Max. coverage (+): 0. Max coverage (-): 0

Region: chr8 100788023-100788039. Max. coverage (+): 0. Max coverage (-): 0

Region: chr8 100788040-100788055. Max. coverage (+): 0. Max coverage (-): 0

Region: chr8 100788056-100788072. Max. coverage (+): 0. Max coverage (-): 0

Region: chr8 100788073-100788088. Max. coverage (+): 0. Max coverage (-): 0

Region: chr8 100788089-100788105. Max. coverage (+): 0. Max coverage (-): 0

Region: chr8 100788106-100788121. Max. coverage (+): 0. Max coverage (-): 6.92

Region: chr8 100788122-100788138. Max. coverage (+): 0. Max coverage (-): 6.92

Region: chr8 100788139-100788155. Max. coverage (+): 0. Max coverage (-): 0

Region: chr8 100788156-100788171. Max. coverage (+): 0. Max coverage (-): 0

Region: chr8 100788172-100788188. Max. coverage (+): 0. Max coverage (-): 0

Region: chr8 100788189-100788204. Max. coverage (+): 0. Max coverage (-): 0

Region: chr8 100788205-100788221. Max. coverage (+): 0. Max coverage (-): 0

Region: chr8 100788222-100788237. Max. coverage (+): 0. Max coverage (-): 4.37

Region: chr8 100788238-100788254. Max. coverage (+): 0. Max coverage (-): 7.92

Region: chr8 100788255-100788270. Max. coverage (+): 0. Max coverage (-): 0

Region: chr8 100788271-100788287. Max. coverage (+): 0. Max coverage (-): 0

Region: chr8 100788288-100788303. Max. coverage (+): 0. Max coverage (-): 0

Region: chr8 100788304-100788320. Max. coverage (+): 0. Max coverage (-): 0

Region: chr8 100788321-100788336. Max. coverage (+): 0. Max coverage (-): 0

Region: chr8 100788337-100788353. Max. coverage (+): 0. Max coverage (-): 0

Region: chr8 100788354-100788369. Max. coverage (+): 0. Max coverage (-): 0

Region: chr8 100788370-100788386. Max. coverage (+): 0. Max coverage (-): 0

Region: chr8 100788387-100788402. Max. coverage (+): 0. Max coverage (-): 0

Region: chr8 100788403-100788419. Max. coverage (+): 0. Max coverage (-): 0

Region: chr8 100788420-100788436. Max. coverage (+): 0. Max coverage (-): 0

Region: chr8 100788437-100788452. Max. coverage (+): 0. Max coverage (-): 4.04

Region: chr8 100788453-100788469. Max. coverage (+): 0. Max coverage (-): 2.33

Region: chr8 100788470-100788485. Max. coverage (+): 0. Max coverage (-): 2.33

Region: chr8 100788486-100788502. Max. coverage (+): 0. Max coverage (-): 2.29

Region: chr8 100788503-100788518. Max. coverage (+): 0. Max coverage (-): 1.87

Region: chr8 100788519-100788535. Max. coverage (+): 0. Max coverage (-): 1.74

Region: chr8 100788536-100788551. Max. coverage (+): 0. Max coverage (-): 0

Region: chr8 100788552-100788568. Max. coverage (+): 0. Max coverage (-): 0

Region: chr8 100788569-100788584. Max. coverage (+): 0. Max coverage (-): 0

Region: chr8 100788585-100788601. Max. coverage (+): 0. Max coverage (-): 0

Region: chr8 100788602-100788617. Max. coverage (+): 0. Max coverage (-): 0

Region: chr8 100788618-100788634. Max. coverage (+): 0. Max coverage (-): 0

Region: chr8 100788635-100788650. Max. coverage (+): 0. Max coverage (-): 0

Region: chr8 100788651-100788667. Max. coverage (+): 0. Max coverage (-): 0

Region: chr8 100788668-100788683. Max. coverage (+): 0. Max coverage (-): 0

Region: chr8 100788684-100788700. Max. coverage (+): 0. Max coverage (-): 0

Region: chr8 100788701-100788716. Max. coverage (+): 0. Max coverage (-): 0

Region: chr8 100788717-100788733. Max. coverage (+): 0. Max coverage (-): 1.44

Region: chr8 100788734-100788750. Max. coverage (+): 0. Max coverage (-): 2.98

Region: chr8 100788751-100788766. Max. coverage (+): 0. Max coverage (-): 2.98

Region: chr8 100788767-100788783. Max. coverage (+): 0. Max coverage (-): 1.11

Region: chr8 100788784-100788799. Max. coverage (+): 0. Max coverage (-): 1.11

Region: chr8 100788800-100788816. Max. coverage (+): 0. Max coverage (-): 2.06

Region: chr8 100788817-100788832. Max. coverage (+): 0. Max coverage (-): 6.91

Region: chr8 100788833-100788849. Max. coverage (+): 0. Max coverage (-): 0

Region: chr8 100788850-100788865. Max. coverage (+): 0. Max coverage (-): 0

Region: chr8 100788866-100788882. Max. coverage (+): 0. Max coverage (-): 9.41

Region: chr8 100788883-100788898. Max. coverage (+): 0. Max coverage (-): 9.41

Region: chr8 100788899-100788915. Max. coverage (+): 0. Max coverage (-): 0

Region: chr8 100788916-100788931. Max. coverage (+): 0. Max coverage (-): 0

Region: chr8 100788932-100788948. Max. coverage (+): 0. Max coverage (-): 0

Region: chr8 100788949-100788964. Max. coverage (+): 0. Max coverage (-): 0

Region: chr8 100788965-100788981. Max. coverage (+): 0. Max coverage (-): 0

Region: chr8 100788982-100788997. Max. coverage (+): 0. Max coverage (-): 0

Region: chr8 100788998-100789014. Max. coverage (+): 0. Max coverage (-): 0

Region: chr8 100789015-100789031. Max. coverage (+): 0. Max coverage (-): 0

Region: chr8 100789032-100789047. Max. coverage (+): 0. Max coverage (-): 0

Region: chr8 100789048-100789064. Max. coverage (+): 0. Max coverage (-): 1.05

Region: chr8 100789065-100789080. Max. coverage (+): 0. Max coverage (-): 1.05

Region: chr8 100789081-100789097. Max. coverage (+): 0. Max coverage (-): 2.82

Region: chr8 100789098-100789113. Max. coverage (+): 0. Max coverage (-): 2.82

Region: chr8 100789114-100789130. Max. coverage (+): 0. Max coverage (-): 0

Region: chr8 100789131-100789146. Max. coverage (+): 0. Max coverage (-): 13.16

Region: chr8 100789147-100789163. Max. coverage (+): 0. Max coverage (-): 5.98

Region: chr8 100789164-100789179. Max. coverage (+): 0. Max coverage (-): 0

Region: chr8 100789180-100789196. Max. coverage (+): 0. Max coverage (-): 0

Region: chr8 100789197-100789212. Max. coverage (+): 0. Max coverage (-): 4.63

Region: chr8 100789213-100789229. Max. coverage (+): 0. Max coverage (-): 0

Region: chr8 100789230-100789245. Max. coverage (+): 0. Max coverage (-): 0

Region: chr8 100789246-100789262. Max. coverage (+): 0. Max coverage (-): 0

Region: chr8 100789263-100789278. Max. coverage (+): 0. Max coverage (-): 9.2

Region: chr8 100789279-100789295. Max. coverage (+): 0. Max coverage (-): 14.45

Region: chr8 100789296-100789311. Max. coverage (+): 0. Max coverage (-): 0

Region: chr8 100789312-100789328. Max. coverage (+): 0. Max coverage (-): 0

Region: chr8 100789329-100789345. Max. coverage (+): 0. Max coverage (-): 0

Region: chr8 100789346-100789361. Max. coverage (+): 0. Max coverage (-): 0

Region: chr8 100789362-100789378. Max. coverage (+): 0. Max coverage (-): 0

Region: chr8 100789379-100789394. Max. coverage (+): 0. Max coverage (-): 0

Region: chr8 100789395-100789411. Max. coverage (+): 0. Max coverage (-): 0

Region: chr8 100789412-100789427. Max. coverage (+): 0. Max coverage (-): 0

Region: chr8 100789428-100789444. Max. coverage (+): 0. Max coverage (-): 0

Region: chr8 100789445-100789460. Max. coverage (+): 0. Max coverage (-): 0

Region: chr8 100789461-100789477. Max. coverage (+): 0. Max coverage (-): 0

Region: chr8 100789478-100789493. Max. coverage (+): 0. Max coverage (-): 0

Region: chr8 100789494-100789510. Max. coverage (+): 0. Max coverage (-): 0

Region: chr8 100789511-100789526. Max. coverage (+): 0. Max coverage (-): 0

Region: chr8 100789527-100789543. Max. coverage (+): 0. Max coverage (-): 0

Region: chr8 100789544-100789559. Max. coverage (+): 0. Max coverage (-): 0

Region: chr8 100789560-100789576. Max. coverage (+): 0. Max coverage (-): 0

Region: chr8 100789577-100789592. Max. coverage (+): 0. Max coverage (-): 0

Region: chr8 100789593-100789609. Max. coverage (+): 0. Max coverage (-): 0

Region: chr8 100789610-100789626. Max. coverage (+): 0. Max coverage (-): 0

Region: chr8 100789627-100789642. Max. coverage (+): 0. Max coverage (-): 0

Region: chr8 100789643-100789659. Max. coverage (+): 0. Max coverage (-): 0.78

Region: chr8 100789660-100789675. Max. coverage (+): 0. Max coverage (-): 0

Region: chr8 100789676-100789692. Max. coverage (+): 0. Max coverage (-): 0.86

Region: chr8 100789693-100789708. Max. coverage (+): 0. Max coverage (-): 3

Region: chr8 100789709-100789725. Max. coverage (+): 0. Max coverage (-): 5.32

Region: chr8 100789726-100789741. Max. coverage (+): 0. Max coverage (-): 5.32

Region: chr8 100789742-100789758. Max. coverage (+): 0. Max coverage (-): 2.34

Region: chr8 100789759-100789774. Max. coverage (+): 0. Max coverage (-): 6.55

Region: chr8 100789775-100789791. Max. coverage (+): 0. Max coverage (-): 0

Region: chr8 100789792-100789807. Max. coverage (+): 0. Max coverage (-): 0

Region: chr8 100789808-100789824. Max. coverage (+): 0. Max coverage (-): 0

Region: chr8 100789825-100789840. Max. coverage (+): 0. Max coverage (-): 0

Region: chr8 100789841-100789857. Max. coverage (+): 0. Max coverage (-): 1.09

Region: chr8 100789858-100789873. Max. coverage (+): 0. Max coverage (-): 6.1

Region: chr8 100789874-100789890. Max. coverage (+): 0. Max coverage (-): 6.1

Region: chr8 100789891-100789906. Max. coverage (+): 0. Max coverage (-): 0

Region: chr8 100789907-100789923. Max. coverage (+): 0. Max coverage (-): 5

Region: chr8 100789924-100789940. Max. coverage (+): 0. Max coverage (-): 6.11

Region: chr8 100789941-100789956. Max. coverage (+): 0. Max coverage (-): 2.41

Region: chr8 100789957-100789973. Max. coverage (+): 0. Max coverage (-): 0

Region: chr8 100789974-100789989. Max. coverage (+): 0. Max coverage (-): 0

Region: chr8 100789990-100790006. Max. coverage (+): 0. Max coverage (-): 0

Region: chr8 100790007-100790022. Max. coverage (+): 0. Max coverage (-): 0

Region: chr8 100790023-100790039. Max. coverage (+): 0. Max coverage (-): 0

Region: chr8 100790040-100790055. Max. coverage (+): 0. Max coverage (-): 0

Region: chr8 100790056-100790072. Max. coverage (+): 0. Max coverage (-): 0

Region: chr8 100790073-100790088. Max. coverage (+): 0. Max coverage (-): 0

Region: chr8 100790089-100790105. Max. coverage (+): 0. Max coverage (-): 1.07

Region: chr8 100790106-100790121. Max. coverage (+): 0. Max coverage (-): 4.35

Region: chr8 100790122-100790138. Max. coverage (+): 0. Max coverage (-): 0

Region: chr8 100790139-100790154. Max. coverage (+): 0. Max coverage (-): 2.27

Region: chr8 100790155-100790171. Max. coverage (+): 0. Max coverage (-): 0.93

Region: chr8 100790172-100790187. Max. coverage (+): 0. Max coverage (-): 2.58

Region: chr8 100790188-100790204. Max. coverage (+): 0. Max coverage (-): 0

Region: chr8 100790205-100790221. Max. coverage (+): 0. Max coverage (-): 1.29

Region: chr8 100790222-100790237. Max. coverage (+): 0. Max coverage (-): 15.82

Region: chr8 100790238-100790254. Max. coverage (+): 0. Max coverage (-): 7.77

Region: chr8 100790255-100790270. Max. coverage (+): 0. Max coverage (-): 10.98

Region: chr8 100790271-100790287. Max. coverage (+): 0. Max coverage (-): 6.43

Region: chr8 100790288-100790303. Max. coverage (+): 0. Max coverage (-): 2.25

Region: chr8 100790304-100790320. Max. coverage (+): 0. Max coverage (-): 5.5

Region: chr8 100790321-100790336. Max. coverage (+): 0. Max coverage (-): 0.72

Region: chr8 100790337-100790353. Max. coverage (+): 0. Max coverage (-): 17.81

Region: chr8 100790354-100790369. Max. coverage (+): 0. Max coverage (-): 0

Region: chr8 100790370-100790386. Max. coverage (+): 0. Max coverage (-): 0

Region: chr8 100790387-100790402. Max. coverage (+): 0. Max coverage (-): 0

Region: chr8 100790403-100790419. Max. coverage (+): 0. Max coverage (-): 0

Region: chr8 100790420-100790435. Max. coverage (+): 0. Max coverage (-): 0

Region: chr8 100790436-100790452. Max. coverage (+): 0. Max coverage (-): 0

Region: chr8 100790453-100790468. Max. coverage (+): 0. Max coverage (-): 0

Region: chr8 100790469-100790485. Max. coverage (+): 0. Max coverage (-): 0

Region: chr8 100790486-100790502. Max. coverage (+): 0. Max coverage (-): 0

Region: chr8 100790503-100790518. Max. coverage (+): 0. Max coverage (-): 0

Region: chr8 100790519-100790535. Max. coverage (+): 0. Max coverage (-): 0

Region: chr8 100790536-100790551. Max. coverage (+): 0. Max coverage (-): 0

Region: chr8 100790552-100790568. Max. coverage (+): 0. Max coverage (-): 0

Region: chr8 100790569-100790584. Max. coverage (+): 0. Max coverage (-): 0

Region: chr8 100790585-100790601. Max. coverage (+): 0. Max coverage (-): 0

Region: chr8 100790602-100790617. Max. coverage (+): 0. Max coverage (-): 0

Region: chr8 100790618-100790634. Max. coverage (+): 0. Max coverage (-): 0

Region: chr8 100790635-100790650. Max. coverage (+): 0. Max coverage (-): 0

Region: chr8 100790651-100790667. Max. coverage (+): 0. Max coverage (-): 0

Region: chr8 100790668-100790683. Max. coverage (+): 0. Max coverage (-): 0

Region: chr8 100790684-100790700. Max. coverage (+): 0. Max coverage (-): 0

Region: chr8 100790701-100790716. Max. coverage (+): 0. Max coverage (-): 0

Region: chr8 100790717-100790733. Max. coverage (+): 0. Max coverage (-): 0

Region: chr8 100790734-100790749. Max. coverage (+): 0. Max coverage (-): 0

Region: chr8 100790750-100790766. Max. coverage (+): 0. Max coverage (-): 0

Region: chr8 100790767-100790782. Max. coverage (+): 0. Max coverage (-): 0

Region: chr8 100790783-100790799. Max. coverage (+): 0. Max coverage (-): 0

Region: chr8 100790800-100790816. Max. coverage (+): 0. Max coverage (-): 0

Region: chr8 100790817-100790832. Max. coverage (+): 0. Max coverage (-): 0

Region: chr8 100790833-100790849. Max. coverage (+): 0. Max coverage (-): 0

Region: chr8 100790850-100790865. Max. coverage (+): 0. Max coverage (-): 0

Region: chr8 100790866-100790882. Max. coverage (+): 0. Max coverage (-): 0

Region: chr8 100790883-100790898. Max. coverage (+): 0. Max coverage (-): 0

Region: chr8 100790899-100790915. Max. coverage (+): 0. Max coverage (-): 0

Region: chr8 100790916-100790931. Max. coverage (+): 0. Max coverage (-): 0

Region: chr8 100790932-100790948. Max. coverage (+): 0. Max coverage (-): 0

Region: chr8 100790949-100790964. Max. coverage (+): 0. Max coverage (-): 0

Region: chr8 100790965-100790981. Max. coverage (+): 0. Max coverage (-): 0

Region: chr8 100790982-100790997. Max. coverage (+): 0. Max coverage (-): 0

Region: chr8 100790998-100791014. Max. coverage (+): 0. Max coverage (-): 0

Region: chr8 100791015-100791030. Max. coverage (+): 0. Max coverage (-): 0

Region: chr8 100791031-100791047. Max. coverage (+): 0. Max coverage (-): 0

Region: chr8 100791048-100791063. Max. coverage (+): 0. Max coverage (-): 0

Region: chr8 100791064-100791080. Max. coverage (+): 0. Max coverage (-): 0

Region: chr8 100791081-100791097. Max. coverage (+): 0. Max coverage (-): 0

Region: chr8 100791098-100791113. Max. coverage (+): 0. Max coverage (-): 0

Region: chr8 100791114-100791130. Max. coverage (+): 0. Max coverage (-): 1.14

Region: chr8 100791131-100791146. Max. coverage (+): 0. Max coverage (-): 1.43

Region: chr8 100791147-100791163. Max. coverage (+): 0. Max coverage (-): 0

Region: chr8 100791164-100791179. Max. coverage (+): 0. Max coverage (-): 3.01

Region: chr8 100791180-100791196. Max. coverage (+): 0. Max coverage (-): 0

Region: chr8 100791197-100791212. Max. coverage (+): 0. Max coverage (-): 1.45

Region: chr8 100791213-100791229. Max. coverage (+): 0. Max coverage (-): 0

Region: chr8 100791230-100791245. Max. coverage (+): 0. Max coverage (-): 0

Region: chr8 100791246-100791262. Max. coverage (+): 0. Max coverage (-): 0

Region: chr8 100791263-100791278. Max. coverage (+): 0. Max coverage (-): 0

Region: chr8 100791279-100791295. Max. coverage (+): 0. Max coverage (-): 5.46

Region: chr8 100791296-100791311. Max. coverage (+): 0. Max coverage (-): 3.49

Region: chr8 100791312-100791328. Max. coverage (+): 0. Max coverage (-): 1.73

Region: chr8 100791329-100791344. Max. coverage (+): 0. Max coverage (-): 1.73

Region: chr8 100791345-100791361. Max. coverage (+): 0. Max coverage (-): 2.48

Region: chr8 100791362-100791377. Max. coverage (+): 0. Max coverage (-): 2.48

Region: chr8 100791378-100791394. Max. coverage (+): 0. Max coverage (-): 2.64

Region: chr8 100791395-100791411. Max. coverage (+): 0. Max coverage (-): 2.64

Region: chr8 100791412-100791427. Max. coverage (+): 0. Max coverage (-): 2.38

Region: chr8 100791428-100791444. Max. coverage (+): 0. Max coverage (-): 0

Region: chr8 100791445-100791460. Max. coverage (+): 0. Max coverage (-): 0

Region: chr8 100791461-100791477. Max. coverage (+): 0. Max coverage (-): 0

Region: chr8 100791478-100791493. Max. coverage (+): 0. Max coverage (-): 0

Region: chr8 100791494-100791510. Max. coverage (+): 0. Max coverage (-): 3.73

Region: chr8 100791511-100791526. Max. coverage (+): 0. Max coverage (-): 13.02

Region: chr8 100791527-100791543. Max. coverage (+): 0. Max coverage (-): 0

Region: chr8 100791544-100791559. Max. coverage (+): 0. Max coverage (-): 24.69

Region: chr8 100791560-100791576. Max. coverage (+): 0. Max coverage (-): 1.67

Region: chr8 100791577-100791592. Max. coverage (+): 0. Max coverage (-): 7.36

Region: chr8 100791593-100791609. Max. coverage (+): 0. Max coverage (-): 5.51

Region: chr8 100791610-100791625. Max. coverage (+): 0. Max coverage (-): 6.11

Region: chr8 100791626-100791642. Max. coverage (+): 0. Max coverage (-): 1.97

Region: chr8 100791643-100791658. Max. coverage (+): 0. Max coverage (-): 4.44

Region: chr8 100791659-100791675. Max. coverage (+): 0. Max coverage (-): 5.27

Region: chr8 100791676-100791692. Max. coverage (+): 0. Max coverage (-): 14.1

Region: chr8 100791693-100791708. Max. coverage (+): 0. Max coverage (-): 11.39

Region: chr8 100791709-100791725. Max. coverage (+): 0. Max coverage (-): 30.89

Region: chr8 100791726-100791741. Max. coverage (+): 0. Max coverage (-): 18.48

Region: chr8 100791742-100791758. Max. coverage (+): 0. Max coverage (-): 13.31

Region: chr8 100791759-100791774. Max. coverage (+): 0. Max coverage (-): 19.96

Region: chr8 100791775-100791791. Max. coverage (+): 0. Max coverage (-): 0

Region: chr8 100791792-100791807. Max. coverage (+): 0. Max coverage (-): 5.71

Region: chr8 100791808-100791824. Max. coverage (+): 0. Max coverage (-): 18.84

Region: chr8 100791825-100791840. Max. coverage (+): 0. Max coverage (-): 0.62

Region: chr8 100791841-100791857. Max. coverage (+): 0. Max coverage (-): 15.42

Region: chr8 100791858-100791873. Max. coverage (+): 0. Max coverage (-): 15.42

Region: chr8 100791874-100791890. Max. coverage (+): 0. Max coverage (-): 13.12

Region: chr8 100791891-100791906. Max. coverage (+): 0. Max coverage (-): 16.39

Region: chr8 100791907-100791923. Max. coverage (+): 0. Max coverage (-): 17.01

Region: chr8 100791924-100791939. Max. coverage (+): 0. Max coverage (-): 10.32

Region: chr8 100791940-100791956. Max. coverage (+): 0. Max coverage (-): 35.41

Region: chr8 100791957-100791972. Max. coverage (+): 0. Max coverage (-): 20.54

Region: chr8 100791973-100791989. Max. coverage (+): 0. Max coverage (-): 15.43

Region: chr8 100791990-100792006. Max. coverage (+): 0. Max coverage (-): 35.78

Region: chr8 100792007-100792022. Max. coverage (+): 0. Max coverage (-): 3.82

Region: chr8 100792023-100792039. Max. coverage (+): 0. Max coverage (-): 2.4

Region: chr8 100792040-100792055. Max. coverage (+): 0. Max coverage (-): 0

Region: chr8 100792056-100792072. Max. coverage (+): 0. Max coverage (-): 12.77

Region: chr8 100792073-100792088. Max. coverage (+): 0. Max coverage (-): 24.24

Region: chr8 100792089-100792105. Max. coverage (+): 0. Max coverage (-): 24.94

Region: chr8 100792106-100792121. Max. coverage (+): 0. Max coverage (-): 19.22

Region: chr8 100792122-100792138. Max. coverage (+): 0. Max coverage (-): 68.22

Region: chr8 100792139-100792154. Max. coverage (+): 0. Max coverage (-): 3.21

Region: chr8 100792155-100792171. Max. coverage (+): 0. Max coverage (-): 0

Region: chr8 100792172-100792187. Max. coverage (+): 0. Max coverage (-): 0

Region: chr8 100792188-100792204. Max. coverage (+): 0. Max coverage (-): 2.22

Region: chr8 100792205-100792220. Max. coverage (+): 0. Max coverage (-): 3.64

Region: chr8 100792221-100792237. Max. coverage (+): 0. Max coverage (-): 2.07

Region: chr8 100792238-100792253. Max. coverage (+): 0. Max coverage (-): 27.65

Region: chr8 100792254-100792270. Max. coverage (+): 0. Max coverage (-): 15.24

Region: chr8 100792271-100792287. Max. coverage (+): 0. Max coverage (-): 0

Region: chr8 100792288-100792303. Max. coverage (+): 0. Max coverage (-): 0

Region: chr8 100792304-100792320. Max. coverage (+): 0. Max coverage (-): 0

Region: chr8 100792321-100792336. Max. coverage (+): 0. Max coverage (-): 9.22

Region: chr8 100792337-100792353. Max. coverage (+): 0. Max coverage (-): 17.42

Region: chr8 100792354-100792369. Max. coverage (+): 0. Max coverage (-): 16.69

Region: chr8 100792370-100792386. Max. coverage (+): 0. Max coverage (-): 16.69

Region: chr8 100792387-100792402. Max. coverage (+): 0. Max coverage (-): 18.42

Region: chr8 100792403-100792419. Max. coverage (+): 0. Max coverage (-): 22.83

Region: chr8 100792420-100792435. Max. coverage (+): 0. Max coverage (-): 5.23

Region: chr8 100792436-100792452. Max. coverage (+): 0. Max coverage (-): 0

Region: chr8 100792453-100792468. Max. coverage (+): 0. Max coverage (-): 0

Region: chr8 100792469-100792485. Max. coverage (+): 0. Max coverage (-): 0

Region: chr8 100792486-100792501. Max. coverage (+): 0. Max coverage (-): 7.88

Region: chr8 100792502-100792518. Max. coverage (+): 0. Max coverage (-): 0

Region: chr8 100792519-100792534. Max. coverage (+): 0. Max coverage (-): 2.61

Region: chr8 100792535-100792551. Max. coverage (+): 0. Max coverage (-): 3.79

Region: chr8 100792552-100792568. Max. coverage (+): 0. Max coverage (-): 1.69

Region: chr8 100792569-100792584. Max. coverage (+): 0. Max coverage (-): 1.69

Region: chr8 100792585-100792601. Max. coverage (+): 0. Max coverage (-): 6.66

Region: chr8 100792602-100792617. Max. coverage (+): 0. Max coverage (-): 48.77

Region: chr8 100792618-100792634. Max. coverage (+): 0. Max coverage (-): 3.29

Region: chr8 100792635-100792650. Max. coverage (+): 0. Max coverage (-): 0

Region: chr8 100792651-100792667. Max. coverage (+): 0. Max coverage (-): 12.06

Region: chr8 100792668-100792683. Max. coverage (+): 0. Max coverage (-): 12.63

Region: chr8 100792684-100792700. Max. coverage (+): 0. Max coverage (-): 14.68

Region: chr8 100792701-100792716. Max. coverage (+): 0. Max coverage (-): 29.89

Region: chr8 100792717-100792733. Max. coverage (+): 0. Max coverage (-): 0

Region: chr8 100792734-100792749. Max. coverage (+): 0. Max coverage (-): 3.42

Region: chr8 100792750-100792766. Max. coverage (+): 0. Max coverage (-): 3.59

Region: chr8 100792767-100792782. Max. coverage (+): 0. Max coverage (-): 1.53

Region: chr8 100792783-100792799. Max. coverage (+): 0. Max coverage (-): 0

Region: chr8 100792800-100792815. Max. coverage (+): 0. Max coverage (-): 4.8

Region: chr8 100792816-100792832. Max. coverage (+): 0. Max coverage (-): 47.79

Region: chr8 100792833-100792848. Max. coverage (+): 0. Max coverage (-): 41.84

Region: chr8 100792849-100792865. Max. coverage (+): 0. Max coverage (-): 0

Region: chr8 100792866-100792882. Max. coverage (+): 0. Max coverage (-): 0

Region: chr8 100792883-100792898. Max. coverage (+): 0. Max coverage (-): 5.83

Region: chr8 100792899-100792915. Max. coverage (+): 0. Max coverage (-): 0

Region: chr8 100792916-100792931. Max. coverage (+): 0. Max coverage (-): 0

Region: chr8 100792932-100792948. Max. coverage (+): 0. Max coverage (-): 0

Region: chr8 100792949-100792964. Max. coverage (+): 0. Max coverage (-): 1.95

Region: chr8 100792965-100792981. Max. coverage (+): 0. Max coverage (-): 8.1

Region: chr8 100792982-100792997. Max. coverage (+): 0. Max coverage (-): 5.76

Region: chr8 100792998-. Max. coverage (+): 0. Max coverage (-): 0

RepeatMasker Color Code

**+**

100-98% Identity

<98-95% Identity

<95-90% Identity

<90-85% Identity

<85-80% Identity

<80-75% Identity

<75-70% Identity

<70% Identity

**-**

Gene Set Color Code

**+**

Gene

Pseudogene

**-**

Topology/Coverage Color Code

Coverage Plus Strand

Coverage Minus Strand

Mainstrand: Plus

Mainstrand: Minus

Complementary Strand

Flanking Region  
(if option -flank >0)

Gene Set Annotation  
  
RepeatMasker Annotation  

**1. L3**: 100785197-100785307 (-), Divergence to consensus: 32.5%  
**2. A-rich**: 100785360-100785392 (+), Divergence to consensus: 15.2%  
**3. MLT1K**: 100785603-100785783 (-), Divergence to consensus: 43.5%  
**4. L3**: 100785877-100786040 (-), Divergence to consensus: 48.2%  
**5. CHRL1\_BT**: 100786447-100786612 (-), Divergence to consensus: 29%  
**6. MLT1J**: 100786623-100786994 (-), Divergence to consensus: 49.4%  
**7. AT\_rich**: 100787756-100787806 (+), Divergence to consensus: 80.4%  
**8. MIR**: 100788857-100789011 (-), Divergence to consensus: 47.9%  
**9. MIRc**: 100789040-100789137 (-), Divergence to consensus: 45.6%  
**10. L2a**: 100789352-100789637 (+), Divergence to consensus: 45.1%  
**11. L1MC4a**: 100789816-100790370 (-), Divergence to consensus: 53.6%  
**12. LTR68**: 100790374-100790531 (-), Divergence to consensus: 24.7%  
**13. (CAGTT)n**: 100790532-100790574 (+), Divergence to consensus: 9.4%  
**14. ART2A**: 100790575-100790777 (-), Divergence to consensus: 10.9%  
**15. ART2A**: 100790752-100790826 (-), Divergence to consensus: 22.6%  
**16. LTR68**: 100790819-100791226 (-), Divergence to consensus: 35.2%  
**17. L1MC4a**: 100791448-100791545 (-), Divergence to consensus: 25.2%

  
Transcription Factor Binding Sites  

**Gata4** (Sequence: AGATAAC (-): 100786094)  
**SOX9** (Sequence: AACAATAG (-): 100788682)  
**SOX9** (Sequence: AACAATAA (-): 100788839)  
**SOX9** (Sequence: CCATTGTT (+): 100787680)  
**SOX9** (Sequence: TCATTGTT (+): 100789937)
