# Supplementary material for: Small RNA sequencing of cryopreserved semen from single bull revealed altered miRNAs and piRNAs expression between High- and Low-motile sperm populations
Source: BMC Genomics. 2017 Jan 4;18:14. doi: 10.1186/s12864-016-3394-7 (PMC5209821; doi:10.1186/s12864-016-3394-7)
Supplement: Additional file 3: — Details for each piRNA clusters found in High Motile (HM) sperm fraction. Genes, repeats, transposable elements and transcription factors binding sites falling within the cluster regions were reported. (ZIP 1896 kb) [file 12864_2016_3394_MOESM3_ESM.zip › 98.html]

piRNA cluster 98


Predicted piRNA cluster no. 98     previous   next
  

Show proTRAC run info
Hide proTRAC run info

================================= proTRAC ====================================  
VERSION: 2.1                                    LAST MODIFIED: 06. October 2015  
  
Please cite:  
Rosenkranz D, Zischler H. proTRAC - a software for probabilistic piRNA cluster  
detection, visualization and analysis. 2012. BMC Bioinformatics 13:5.  
  
and (for proTRAC 2.0 and later):  
Rosenkranz D, Rudloff S, Bastuck K, Ketting RF, Zischler H. Tupaia small RNAs  
provide insights into function and evolution of RNAi-based transposon defense  
in mammals. 2015. RNA 21(5):911-922.  
  
Contact:  
David Rosenkranz  
Institute of Anthropology, small RNA group  
Johannes Gutenberg University Mainz  
email: rosenkranz@uni-mainz.de  
  
You can find the latest proTRAC version at:  
http://sourceforge.net/projects/protrac/files  
http://www.smallRNAgroup-mainz.de/software  
==============================================================================  
  
PARAMETERS:  
Map file: .............../storage/core/barbara/genhome/smallRNA/fertility/Sample\_motile/pirna/Sample\_motile\_26-33\_collapsed.fa.no-dust.map.weighted-10000-1000-b-0  
Genome file: ............/storage/core/barbara/genhome/smallRNA/fertility/Sample\_all/pirna/bt\_311\_chrY.fa  
RepeatMasker annotation: /storage/genomes/bt\_umd31/GCF\_000003055.6\_Bos\_taurus\_UMD\_3.1.1\_repeatMasker\_chr.out  
GeneSet:................./storage/core/barbara/genhome/smallRNA/fertility/Sample\_all/pirna/full.gtf  
  
Significant (p<=0.01) hit density will be calculated based  
on observed hit distribution.  
  
Sliding window size: ........................................ 5000 bp  
Sliding window increament: .................................. 1000 bp  
Normalize each hit by number of genomic hits: ............... 1 [0=no/1=yes]  
Normalize each hit by number of sequence reads: ............. 1 [0=no/1=yes]  
Normalize values (-> per million mapped reads): ............. 1 [0=no/1=yes]  
Min. fraction of hits with 1T(U) or 10A: .................... 0.75  
Alternatively: Min. fraction of hits with 1T(U) and 10A: .... 0.5  
Min. fraction of hits with typical piRNA length: ............ 0.75  
Typical piRNA length: ....................................... 26-33 nt  
Min. size of a piRNA cluster: ............................... 5000 bp.  
Min. number of hits (absolute): ............................. 0  
Min. number of hits (normalized): ........................... 0  
Min. fraction of hits on the mainstrand: .................... 0.75  
Top fraction of mapped sequences (in terms of read counts): . 1%  
Top fraction accounts for max. n% of sequence reads: ........ 90%  
Min. fraction of hits on each arm of a bidirectional cluster: 0.1  
Output image file for each cluster: ......................... 0 [0=no/1=yes]  
Output html file for each cluster: .......................... 1 [0=no/1=yes]  
Output a summary table: ..................................... 1 [0=no/1=yes]  
Output a FASTA file for each cluster (piRNA sequences): ..... 1 [0=no/1=yes]  
Output a FASTA file comprising cluster sequences: ........... 1 [0=no/1=yes]  
Search DNA motifs in clusters: .............................. 1 [0=no/1=yes]  
Output flanking sequences: +/- .............................. 0 bp  
Output ~.pTi file: .......................................... 1 [0=no/1=yes]  
==============================================================================  
  
  
Genome size (without gaps): ............ 2678902517 bp  
Gaps (N/X/-): .......................... 53837044 bp  
Mapped reads: .......................... 658825247023  
Non-identical sequences: ............... 514171  
Genomic hits: .......................... 764233  
Significant densitiy of mapped reads: .. 12867599.5173724 reads/kb

Show proTRAC cluster info
Hide proTRAC cluster info

|  |  |
| --- | --- |
| Location | chr9 |
| Coordinates | 13062673-13068884 |
| Size [bp] | 6212 |
| Sequence hit loci | 75 |
| Mapped reads (normalized) | 99374329.9 |
| Mapped reads (normalized) per kb | 15997155.5 |
| Normalized reads with 1T (1U) | 85.1% |
| Normalized reads with 10A | 23.6% |
| Normalized reads with length 26-33 nt | 100% |
| Normalized reads on the main strand(s) | 98.4% |
| Predicted directionality | mono:plus |

100%

0%

1T (1U)  
reads

10A reads

26-33 nt  
reads

reads on mainstrand

**Either the amount of reads with 1T (1U) OR 10A has to exceed 75% (set with option: -1Tor10A)  
Alternatively the amount of reads with 1T (1U) AND 10A has to exceed 50% (set with option: -1Tand10A)  
Minimum amount of reads with preferred size is 75% (set with option: -pisize)  
Minimum amount of reads on the main strand(s) is 75% (set with option: -clstrand)**

Show read coverage
Hide read coverage

WHAT DO I SEE HERE?  
This chart shows the location of mapped sequence reads within a predicted piRNA cluster. The color refers to the number of genomic hits produced by the sequence read in question. A dark red bar indicates that this sequence read produces many other hits elsewhere in the genome. Many adjacent red or yellow bars can indicate the presence of a multi-copy element such as transposons or rRNA genes. A dark green bar indicates that this sequence read maps uniquely to this locus.

1 hit

2-5 hits

6-10 hits

11-20 hits

21-50 hits

51-100 hits

> 100 hits

chr9

13062673

13068884

Gene Set

RepeatMasker

Mapped  
Reads

17.69

plus strand

minus strand

17.69

Region: chr9 100792999-13062679. Max. coverage (+): 15.41. Max coverage (-): 0

Region: chr9 13062680-13062691. Max. coverage (+): 17.69. Max coverage (-): 0

Region: chr9 13062692-13062704. Max. coverage (+): 0. Max coverage (-): 0

Region: chr9 13062705-13062716. Max. coverage (+): 0. Max coverage (-): 0

Region: chr9 13062717-13062728. Max. coverage (+): 0. Max coverage (-): 0

Region: chr9 13062729-13062741. Max. coverage (+): 9.32. Max coverage (-): 0

Region: chr9 13062742-13062753. Max. coverage (+): 9.95. Max coverage (-): 0

Region: chr9 13062754-13062766. Max. coverage (+): 0. Max coverage (-): 0

Region: chr9 13062767-13062778. Max. coverage (+): 1.08. Max coverage (-): 0

Region: chr9 13062779-13062791. Max. coverage (+): 1.08. Max coverage (-): 0

Region: chr9 13062792-13062803. Max. coverage (+): 0. Max coverage (-): 0

Region: chr9 13062804-13062815. Max. coverage (+): 0. Max coverage (-): 0

Region: chr9 13062816-13062828. Max. coverage (+): 0. Max coverage (-): 0

Region: chr9 13062829-13062840. Max. coverage (+): 1.09. Max coverage (-): 0

Region: chr9 13062841-13062853. Max. coverage (+): 2.86. Max coverage (-): 0

Region: chr9 13062854-13062865. Max. coverage (+): 0. Max coverage (-): 0

Region: chr9 13062866-13062877. Max. coverage (+): 0. Max coverage (-): 0

Region: chr9 13062878-13062890. Max. coverage (+): 0. Max coverage (-): 0

Region: chr9 13062891-13062902. Max. coverage (+): 0. Max coverage (-): 0

Region: chr9 13062903-13062915. Max. coverage (+): 0. Max coverage (-): 0

Region: chr9 13062916-13062927. Max. coverage (+): 0. Max coverage (-): 0

Region: chr9 13062928-13062940. Max. coverage (+): 0. Max coverage (-): 0

Region: chr9 13062941-13062952. Max. coverage (+): 5.19. Max coverage (-): 0

Region: chr9 13062953-13062964. Max. coverage (+): 5.19. Max coverage (-): 0

Region: chr9 13062965-13062977. Max. coverage (+): 0. Max coverage (-): 0

Region: chr9 13062978-13062989. Max. coverage (+): 0. Max coverage (-): 0

Region: chr9 13062990-13063002. Max. coverage (+): 0. Max coverage (-): 0

Region: chr9 13063003-13063014. Max. coverage (+): 0. Max coverage (-): 0

Region: chr9 13063015-13063027. Max. coverage (+): 0. Max coverage (-): 0

Region: chr9 13063028-13063039. Max. coverage (+): 5.18. Max coverage (-): 0

Region: chr9 13063040-13063051. Max. coverage (+): 0. Max coverage (-): 0

Region: chr9 13063052-13063064. Max. coverage (+): 0. Max coverage (-): 0

Region: chr9 13063065-13063076. Max. coverage (+): 0. Max coverage (-): 0

Region: chr9 13063077-13063089. Max. coverage (+): 1.38. Max coverage (-): 0

Region: chr9 13063090-13063101. Max. coverage (+): 1.38. Max coverage (-): 0

Region: chr9 13063102-13063114. Max. coverage (+): 0. Max coverage (-): 0

Region: chr9 13063115-13063126. Max. coverage (+): 0. Max coverage (-): 0

Region: chr9 13063127-13063138. Max. coverage (+): 0. Max coverage (-): 0

Region: chr9 13063139-13063151. Max. coverage (+): 0. Max coverage (-): 0

Region: chr9 13063152-13063163. Max. coverage (+): 0. Max coverage (-): 0

Region: chr9 13063164-13063176. Max. coverage (+): 0. Max coverage (-): 0

Region: chr9 13063177-13063188. Max. coverage (+): 0. Max coverage (-): 0

Region: chr9 13063189-13063201. Max. coverage (+): 0. Max coverage (-): 0

Region: chr9 13063202-13063213. Max. coverage (+): 0. Max coverage (-): 0

Region: chr9 13063214-13063225. Max. coverage (+): 0. Max coverage (-): 0

Region: chr9 13063226-13063238. Max. coverage (+): 0. Max coverage (-): 0

Region: chr9 13063239-13063250. Max. coverage (+): 0. Max coverage (-): 0

Region: chr9 13063251-13063263. Max. coverage (+): 0. Max coverage (-): 0

Region: chr9 13063264-13063275. Max. coverage (+): 0. Max coverage (-): 0

Region: chr9 13063276-13063287. Max. coverage (+): 0. Max coverage (-): 0

Region: chr9 13063288-13063300. Max. coverage (+): 0. Max coverage (-): 0

Region: chr9 13063301-13063312. Max. coverage (+): 0. Max coverage (-): 0

Region: chr9 13063313-13063325. Max. coverage (+): 0. Max coverage (-): 0

Region: chr9 13063326-13063337. Max. coverage (+): 0. Max coverage (-): 0

Region: chr9 13063338-13063350. Max. coverage (+): 0. Max coverage (-): 0

Region: chr9 13063351-13063362. Max. coverage (+): 0. Max coverage (-): 0

Region: chr9 13063363-13063374. Max. coverage (+): 0. Max coverage (-): 0

Region: chr9 13063375-13063387. Max. coverage (+): 0. Max coverage (-): 0

Region: chr9 13063388-13063399. Max. coverage (+): 0. Max coverage (-): 0

Region: chr9 13063400-13063412. Max. coverage (+): 0. Max coverage (-): 0

Region: chr9 13063413-13063424. Max. coverage (+): 0. Max coverage (-): 0

Region: chr9 13063425-13063437. Max. coverage (+): 0. Max coverage (-): 0

Region: chr9 13063438-13063449. Max. coverage (+): 0. Max coverage (-): 0

Region: chr9 13063450-13063461. Max. coverage (+): 0. Max coverage (-): 0

Region: chr9 13063462-13063474. Max. coverage (+): 0. Max coverage (-): 0

Region: chr9 13063475-13063486. Max. coverage (+): 0. Max coverage (-): 0

Region: chr9 13063487-13063499. Max. coverage (+): 0. Max coverage (-): 0

Region: chr9 13063500-13063511. Max. coverage (+): 0. Max coverage (-): 0

Region: chr9 13063512-13063524. Max. coverage (+): 0. Max coverage (-): 0

Region: chr9 13063525-13063536. Max. coverage (+): 0. Max coverage (-): 0

Region: chr9 13063537-13063548. Max. coverage (+): 0. Max coverage (-): 0

Region: chr9 13063549-13063561. Max. coverage (+): 0. Max coverage (-): 0

Region: chr9 13063562-13063573. Max. coverage (+): 0. Max coverage (-): 0

Region: chr9 13063574-13063586. Max. coverage (+): 0. Max coverage (-): 0

Region: chr9 13063587-13063598. Max. coverage (+): 0. Max coverage (-): 0

Region: chr9 13063599-13063611. Max. coverage (+): 0. Max coverage (-): 0

Region: chr9 13063612-13063623. Max. coverage (+): 0. Max coverage (-): 0

Region: chr9 13063624-13063635. Max. coverage (+): 0. Max coverage (-): 0

Region: chr9 13063636-13063648. Max. coverage (+): 0. Max coverage (-): 0

Region: chr9 13063649-13063660. Max. coverage (+): 0. Max coverage (-): 0

Region: chr9 13063661-13063673. Max. coverage (+): 0. Max coverage (-): 0

Region: chr9 13063674-13063685. Max. coverage (+): 0. Max coverage (-): 0

Region: chr9 13063686-13063697. Max. coverage (+): 0. Max coverage (-): 0

Region: chr9 13063698-13063710. Max. coverage (+): 0. Max coverage (-): 0

Region: chr9 13063711-13063722. Max. coverage (+): 0. Max coverage (-): 0

Region: chr9 13063723-13063735. Max. coverage (+): 0. Max coverage (-): 0

Region: chr9 13063736-13063747. Max. coverage (+): 0. Max coverage (-): 0

Region: chr9 13063748-13063760. Max. coverage (+): 0. Max coverage (-): 0

Region: chr9 13063761-13063772. Max. coverage (+): 0. Max coverage (-): 0

Region: chr9 13063773-13063784. Max. coverage (+): 0. Max coverage (-): 0

Region: chr9 13063785-13063797. Max. coverage (+): 0. Max coverage (-): 0

Region: chr9 13063798-13063809. Max. coverage (+): 0. Max coverage (-): 0

Region: chr9 13063810-13063822. Max. coverage (+): 0. Max coverage (-): 0

Region: chr9 13063823-13063834. Max. coverage (+): 0. Max coverage (-): 0

Region: chr9 13063835-13063847. Max. coverage (+): 0. Max coverage (-): 0

Region: chr9 13063848-13063859. Max. coverage (+): 0. Max coverage (-): 0

Region: chr9 13063860-13063871. Max. coverage (+): 0. Max coverage (-): 0

Region: chr9 13063872-13063884. Max. coverage (+): 0. Max coverage (-): 0

Region: chr9 13063885-13063896. Max. coverage (+): 5.11. Max coverage (-): 0

Region: chr9 13063897-13063909. Max. coverage (+): 5.11. Max coverage (-): 0

Region: chr9 13063910-13063921. Max. coverage (+): 0. Max coverage (-): 0

Region: chr9 13063922-13063934. Max. coverage (+): 0. Max coverage (-): 0

Region: chr9 13063935-13063946. Max. coverage (+): 0. Max coverage (-): 0

Region: chr9 13063947-13063958. Max. coverage (+): 0. Max coverage (-): 0

Region: chr9 13063959-13063971. Max. coverage (+): 0. Max coverage (-): 0

Region: chr9 13063972-13063983. Max. coverage (+): 0. Max coverage (-): 0

Region: chr9 13063984-13063996. Max. coverage (+): 0. Max coverage (-): 0

Region: chr9 13063997-13064008. Max. coverage (+): 0. Max coverage (-): 0

Region: chr9 13064009-13064021. Max. coverage (+): 0. Max coverage (-): 0

Region: chr9 13064022-13064033. Max. coverage (+): 0. Max coverage (-): 0

Region: chr9 13064034-13064045. Max. coverage (+): 0. Max coverage (-): 0

Region: chr9 13064046-13064058. Max. coverage (+): 0. Max coverage (-): 0

Region: chr9 13064059-13064070. Max. coverage (+): 0. Max coverage (-): 0

Region: chr9 13064071-13064083. Max. coverage (+): 0. Max coverage (-): 0

Region: chr9 13064084-13064095. Max. coverage (+): 0. Max coverage (-): 0

Region: chr9 13064096-13064107. Max. coverage (+): 0. Max coverage (-): 0

Region: chr9 13064108-13064120. Max. coverage (+): 0. Max coverage (-): 0

Region: chr9 13064121-13064132. Max. coverage (+): 0. Max coverage (-): 0

Region: chr9 13064133-13064145. Max. coverage (+): 0. Max coverage (-): 0

Region: chr9 13064146-13064157. Max. coverage (+): 0. Max coverage (-): 0

Region: chr9 13064158-13064170. Max. coverage (+): 0. Max coverage (-): 0

Region: chr9 13064171-13064182. Max. coverage (+): 0. Max coverage (-): 0

Region: chr9 13064183-13064194. Max. coverage (+): 0. Max coverage (-): 0

Region: chr9 13064195-13064207. Max. coverage (+): 0. Max coverage (-): 0

Region: chr9 13064208-13064219. Max. coverage (+): 0. Max coverage (-): 0

Region: chr9 13064220-13064232. Max. coverage (+): 0. Max coverage (-): 0

Region: chr9 13064233-13064244. Max. coverage (+): 0. Max coverage (-): 0

Region: chr9 13064245-13064257. Max. coverage (+): 0. Max coverage (-): 0

Region: chr9 13064258-13064269. Max. coverage (+): 0. Max coverage (-): 0

Region: chr9 13064270-13064281. Max. coverage (+): 0. Max coverage (-): 0

Region: chr9 13064282-13064294. Max. coverage (+): 0. Max coverage (-): 0

Region: chr9 13064295-13064306. Max. coverage (+): 0. Max coverage (-): 0

Region: chr9 13064307-13064319. Max. coverage (+): 0. Max coverage (-): 0

Region: chr9 13064320-13064331. Max. coverage (+): 0. Max coverage (-): 0

Region: chr9 13064332-13064344. Max. coverage (+): 0. Max coverage (-): 0

Region: chr9 13064345-13064356. Max. coverage (+): 0. Max coverage (-): 0

Region: chr9 13064357-13064368. Max. coverage (+): 0. Max coverage (-): 0

Region: chr9 13064369-13064381. Max. coverage (+): 0. Max coverage (-): 0

Region: chr9 13064382-13064393. Max. coverage (+): 0. Max coverage (-): 0

Region: chr9 13064394-13064406. Max. coverage (+): 0. Max coverage (-): 0

Region: chr9 13064407-13064418. Max. coverage (+): 0. Max coverage (-): 0

Region: chr9 13064419-13064430. Max. coverage (+): 0. Max coverage (-): 0

Region: chr9 13064431-13064443. Max. coverage (+): 0. Max coverage (-): 0

Region: chr9 13064444-13064455. Max. coverage (+): 0. Max coverage (-): 0

Region: chr9 13064456-13064468. Max. coverage (+): 0. Max coverage (-): 0

Region: chr9 13064469-13064480. Max. coverage (+): 0. Max coverage (-): 0

Region: chr9 13064481-13064493. Max. coverage (+): 0. Max coverage (-): 0

Region: chr9 13064494-13064505. Max. coverage (+): 0. Max coverage (-): 0

Region: chr9 13064506-13064517. Max. coverage (+): 0. Max coverage (-): 0

Region: chr9 13064518-13064530. Max. coverage (+): 0. Max coverage (-): 0

Region: chr9 13064531-13064542. Max. coverage (+): 0. Max coverage (-): 0

Region: chr9 13064543-13064555. Max. coverage (+): 0. Max coverage (-): 0

Region: chr9 13064556-13064567. Max. coverage (+): 0. Max coverage (-): 0

Region: chr9 13064568-13064580. Max. coverage (+): 0. Max coverage (-): 0

Region: chr9 13064581-13064592. Max. coverage (+): 5.16. Max coverage (-): 0

Region: chr9 13064593-13064604. Max. coverage (+): 5.16. Max coverage (-): 0

Region: chr9 13064605-13064617. Max. coverage (+): 0. Max coverage (-): 0

Region: chr9 13064618-13064629. Max. coverage (+): 0. Max coverage (-): 0

Region: chr9 13064630-13064642. Max. coverage (+): 0. Max coverage (-): 0

Region: chr9 13064643-13064654. Max. coverage (+): 0. Max coverage (-): 0

Region: chr9 13064655-13064667. Max. coverage (+): 0. Max coverage (-): 0

Region: chr9 13064668-13064679. Max. coverage (+): 0. Max coverage (-): 0

Region: chr9 13064680-13064691. Max. coverage (+): 0. Max coverage (-): 0

Region: chr9 13064692-13064704. Max. coverage (+): 0. Max coverage (-): 0

Region: chr9 13064705-13064716. Max. coverage (+): 0. Max coverage (-): 0

Region: chr9 13064717-13064729. Max. coverage (+): 0. Max coverage (-): 0

Region: chr9 13064730-13064741. Max. coverage (+): 0. Max coverage (-): 0

Region: chr9 13064742-13064754. Max. coverage (+): 0. Max coverage (-): 0

Region: chr9 13064755-13064766. Max. coverage (+): 0. Max coverage (-): 0

Region: chr9 13064767-13064778. Max. coverage (+): 0. Max coverage (-): 0

Region: chr9 13064779-13064791. Max. coverage (+): 0. Max coverage (-): 0

Region: chr9 13064792-13064803. Max. coverage (+): 0. Max coverage (-): 0

Region: chr9 13064804-13064816. Max. coverage (+): 0. Max coverage (-): 0

Region: chr9 13064817-13064828. Max. coverage (+): 0. Max coverage (-): 0

Region: chr9 13064829-13064840. Max. coverage (+): 0. Max coverage (-): 0

Region: chr9 13064841-13064853. Max. coverage (+): 0. Max coverage (-): 0

Region: chr9 13064854-13064865. Max. coverage (+): 0. Max coverage (-): 0

Region: chr9 13064866-13064878. Max. coverage (+): 0. Max coverage (-): 0

Region: chr9 13064879-13064890. Max. coverage (+): 0. Max coverage (-): 0

Region: chr9 13064891-13064903. Max. coverage (+): 0. Max coverage (-): 0

Region: chr9 13064904-13064915. Max. coverage (+): 0. Max coverage (-): 0

Region: chr9 13064916-13064927. Max. coverage (+): 0. Max coverage (-): 0

Region: chr9 13064928-13064940. Max. coverage (+): 0. Max coverage (-): 0

Region: chr9 13064941-13064952. Max. coverage (+): 0. Max coverage (-): 0

Region: chr9 13064953-13064965. Max. coverage (+): 0. Max coverage (-): 0

Region: chr9 13064966-13064977. Max. coverage (+): 0. Max coverage (-): 0

Region: chr9 13064978-13064990. Max. coverage (+): 0. Max coverage (-): 0

Region: chr9 13064991-13065002. Max. coverage (+): 0. Max coverage (-): 0

Region: chr9 13065003-13065014. Max. coverage (+): 0. Max coverage (-): 0

Region: chr9 13065015-13065027. Max. coverage (+): 0. Max coverage (-): 0

Region: chr9 13065028-13065039. Max. coverage (+): 0. Max coverage (-): 0

Region: chr9 13065040-13065052. Max. coverage (+): 0. Max coverage (-): 0

Region: chr9 13065053-13065064. Max. coverage (+): 0. Max coverage (-): 0

Region: chr9 13065065-13065077. Max. coverage (+): 0. Max coverage (-): 0

Region: chr9 13065078-13065089. Max. coverage (+): 0. Max coverage (-): 0

Region: chr9 13065090-13065101. Max. coverage (+): 0. Max coverage (-): 0

Region: chr9 13065102-13065114. Max. coverage (+): 0. Max coverage (-): 0

Region: chr9 13065115-13065126. Max. coverage (+): 0. Max coverage (-): 0

Region: chr9 13065127-13065139. Max. coverage (+): 0. Max coverage (-): 0

Region: chr9 13065140-13065151. Max. coverage (+): 0. Max coverage (-): 0

Region: chr9 13065152-13065164. Max. coverage (+): 0. Max coverage (-): 0

Region: chr9 13065165-13065176. Max. coverage (+): 2.28. Max coverage (-): 0

Region: chr9 13065177-13065188. Max. coverage (+): 2.28. Max coverage (-): 0

Region: chr9 13065189-13065201. Max. coverage (+): 0. Max coverage (-): 0

Region: chr9 13065202-13065213. Max. coverage (+): 0. Max coverage (-): 0

Region: chr9 13065214-13065226. Max. coverage (+): 0. Max coverage (-): 0

Region: chr9 13065227-13065238. Max. coverage (+): 0. Max coverage (-): 0

Region: chr9 13065239-13065250. Max. coverage (+): 0. Max coverage (-): 0

Region: chr9 13065251-13065263. Max. coverage (+): 0. Max coverage (-): 0

Region: chr9 13065264-13065275. Max. coverage (+): 0. Max coverage (-): 0

Region: chr9 13065276-13065288. Max. coverage (+): 0. Max coverage (-): 0

Region: chr9 13065289-13065300. Max. coverage (+): 0. Max coverage (-): 0

Region: chr9 13065301-13065313. Max. coverage (+): 0. Max coverage (-): 0

Region: chr9 13065314-13065325. Max. coverage (+): 0. Max coverage (-): 0

Region: chr9 13065326-13065337. Max. coverage (+): 0. Max coverage (-): 0

Region: chr9 13065338-13065350. Max. coverage (+): 0. Max coverage (-): 0

Region: chr9 13065351-13065362. Max. coverage (+): 0. Max coverage (-): 0

Region: chr9 13065363-13065375. Max. coverage (+): 0. Max coverage (-): 0

Region: chr9 13065376-13065387. Max. coverage (+): 0. Max coverage (-): 0

Region: chr9 13065388-13065400. Max. coverage (+): 1.58. Max coverage (-): 0

Region: chr9 13065401-13065412. Max. coverage (+): 1.58. Max coverage (-): 0

Region: chr9 13065413-13065424. Max. coverage (+): 4.75. Max coverage (-): 0

Region: chr9 13065425-13065437. Max. coverage (+): 2.25. Max coverage (-): 0

Region: chr9 13065438-13065449. Max. coverage (+): 2.25. Max coverage (-): 0

Region: chr9 13065450-13065462. Max. coverage (+): 0. Max coverage (-): 0

Region: chr9 13065463-13065474. Max. coverage (+): 0. Max coverage (-): 0

Region: chr9 13065475-13065487. Max. coverage (+): 0. Max coverage (-): 0

Region: chr9 13065488-13065499. Max. coverage (+): 0. Max coverage (-): 0

Region: chr9 13065500-13065511. Max. coverage (+): 0.77. Max coverage (-): 0

Region: chr9 13065512-13065524. Max. coverage (+): 0. Max coverage (-): 0

Region: chr9 13065525-13065536. Max. coverage (+): 0. Max coverage (-): 0

Region: chr9 13065537-13065549. Max. coverage (+): 0. Max coverage (-): 0

Region: chr9 13065550-13065561. Max. coverage (+): 0. Max coverage (-): 0

Region: chr9 13065562-13065574. Max. coverage (+): 0. Max coverage (-): 0

Region: chr9 13065575-13065586. Max. coverage (+): 0. Max coverage (-): 0

Region: chr9 13065587-13065598. Max. coverage (+): 0. Max coverage (-): 0

Region: chr9 13065599-13065611. Max. coverage (+): 0. Max coverage (-): 0

Region: chr9 13065612-13065623. Max. coverage (+): 0. Max coverage (-): 0

Region: chr9 13065624-13065636. Max. coverage (+): 0. Max coverage (-): 0

Region: chr9 13065637-13065648. Max. coverage (+): 0. Max coverage (-): 0

Region: chr9 13065649-13065660. Max. coverage (+): 0. Max coverage (-): 0

Region: chr9 13065661-13065673. Max. coverage (+): 0. Max coverage (-): 0

Region: chr9 13065674-13065685. Max. coverage (+): 0. Max coverage (-): 0

Region: chr9 13065686-13065698. Max. coverage (+): 1.96. Max coverage (-): 0

Region: chr9 13065699-13065710. Max. coverage (+): 0. Max coverage (-): 0

Region: chr9 13065711-13065723. Max. coverage (+): 0. Max coverage (-): 0

Region: chr9 13065724-13065735. Max. coverage (+): 0. Max coverage (-): 0

Region: chr9 13065736-13065747. Max. coverage (+): 0. Max coverage (-): 0

Region: chr9 13065748-13065760. Max. coverage (+): 0. Max coverage (-): 0

Region: chr9 13065761-13065772. Max. coverage (+): 0. Max coverage (-): 0

Region: chr9 13065773-13065785. Max. coverage (+): 0. Max coverage (-): 0

Region: chr9 13065786-13065797. Max. coverage (+): 1.1. Max coverage (-): 0

Region: chr9 13065798-13065810. Max. coverage (+): 0. Max coverage (-): 0

Region: chr9 13065811-13065822. Max. coverage (+): 0. Max coverage (-): 0

Region: chr9 13065823-13065834. Max. coverage (+): 0. Max coverage (-): 0

Region: chr9 13065835-13065847. Max. coverage (+): 0. Max coverage (-): 0

Region: chr9 13065848-13065859. Max. coverage (+): 0. Max coverage (-): 0

Region: chr9 13065860-13065872. Max. coverage (+): 0. Max coverage (-): 0

Region: chr9 13065873-13065884. Max. coverage (+): 0. Max coverage (-): 0

Region: chr9 13065885-13065897. Max. coverage (+): 0. Max coverage (-): 0

Region: chr9 13065898-13065909. Max. coverage (+): 2.56. Max coverage (-): 0

Region: chr9 13065910-13065921. Max. coverage (+): 6.17. Max coverage (-): 0

Region: chr9 13065922-13065934. Max. coverage (+): 6.17. Max coverage (-): 0

Region: chr9 13065935-13065946. Max. coverage (+): 0. Max coverage (-): 0

Region: chr9 13065947-13065959. Max. coverage (+): 0. Max coverage (-): 0

Region: chr9 13065960-13065971. Max. coverage (+): 0. Max coverage (-): 0

Region: chr9 13065972-13065983. Max. coverage (+): 0. Max coverage (-): 0

Region: chr9 13065984-13065996. Max. coverage (+): 0. Max coverage (-): 0

Region: chr9 13065997-13066008. Max. coverage (+): 0. Max coverage (-): 0

Region: chr9 13066009-13066021. Max. coverage (+): 0. Max coverage (-): 0

Region: chr9 13066022-13066033. Max. coverage (+): 0. Max coverage (-): 0

Region: chr9 13066034-13066046. Max. coverage (+): 1.14. Max coverage (-): 0

Region: chr9 13066047-13066058. Max. coverage (+): 1.14. Max coverage (-): 0

Region: chr9 13066059-13066070. Max. coverage (+): 0. Max coverage (-): 0

Region: chr9 13066071-13066083. Max. coverage (+): 0. Max coverage (-): 0

Region: chr9 13066084-13066095. Max. coverage (+): 0. Max coverage (-): 0

Region: chr9 13066096-13066108. Max. coverage (+): 0. Max coverage (-): 0

Region: chr9 13066109-13066120. Max. coverage (+): 0. Max coverage (-): 0

Region: chr9 13066121-13066133. Max. coverage (+): 0. Max coverage (-): 0

Region: chr9 13066134-13066145. Max. coverage (+): 0. Max coverage (-): 0

Region: chr9 13066146-13066157. Max. coverage (+): 0. Max coverage (-): 0

Region: chr9 13066158-13066170. Max. coverage (+): 0. Max coverage (-): 0

Region: chr9 13066171-13066182. Max. coverage (+): 0. Max coverage (-): 0

Region: chr9 13066183-13066195. Max. coverage (+): 0. Max coverage (-): 0

Region: chr9 13066196-13066207. Max. coverage (+): 0. Max coverage (-): 0

Region: chr9 13066208-13066220. Max. coverage (+): 0. Max coverage (-): 0

Region: chr9 13066221-13066232. Max. coverage (+): 0. Max coverage (-): 0

Region: chr9 13066233-13066244. Max. coverage (+): 0. Max coverage (-): 0

Region: chr9 13066245-13066257. Max. coverage (+): 0. Max coverage (-): 0

Region: chr9 13066258-13066269. Max. coverage (+): 0. Max coverage (-): 0

Region: chr9 13066270-13066282. Max. coverage (+): 0. Max coverage (-): 0

Region: chr9 13066283-13066294. Max. coverage (+): 0. Max coverage (-): 0

Region: chr9 13066295-13066307. Max. coverage (+): 0. Max coverage (-): 0

Region: chr9 13066308-13066319. Max. coverage (+): 0. Max coverage (-): 0

Region: chr9 13066320-13066331. Max. coverage (+): 0. Max coverage (-): 0

Region: chr9 13066332-13066344. Max. coverage (+): 0. Max coverage (-): 0

Region: chr9 13066345-13066356. Max. coverage (+): 0. Max coverage (-): 0

Region: chr9 13066357-13066369. Max. coverage (+): 0. Max coverage (-): 0

Region: chr9 13066370-13066381. Max. coverage (+): 0. Max coverage (-): 0

Region: chr9 13066382-13066393. Max. coverage (+): 0. Max coverage (-): 0

Region: chr9 13066394-13066406. Max. coverage (+): 0. Max coverage (-): 0

Region: chr9 13066407-13066418. Max. coverage (+): 0. Max coverage (-): 0

Region: chr9 13066419-13066431. Max. coverage (+): 0. Max coverage (-): 0

Region: chr9 13066432-13066443. Max. coverage (+): 0. Max coverage (-): 0

Region: chr9 13066444-13066456. Max. coverage (+): 0. Max coverage (-): 0

Region: chr9 13066457-13066468. Max. coverage (+): 0. Max coverage (-): 0

Region: chr9 13066469-13066480. Max. coverage (+): 0. Max coverage (-): 0

Region: chr9 13066481-13066493. Max. coverage (+): 0. Max coverage (-): 0

Region: chr9 13066494-13066505. Max. coverage (+): 0. Max coverage (-): 0

Region: chr9 13066506-13066518. Max. coverage (+): 0. Max coverage (-): 0

Region: chr9 13066519-13066530. Max. coverage (+): 0. Max coverage (-): 0

Region: chr9 13066531-13066543. Max. coverage (+): 0. Max coverage (-): 0

Region: chr9 13066544-13066555. Max. coverage (+): 0. Max coverage (-): 0

Region: chr9 13066556-13066567. Max. coverage (+): 0. Max coverage (-): 0

Region: chr9 13066568-13066580. Max. coverage (+): 0. Max coverage (-): 0

Region: chr9 13066581-13066592. Max. coverage (+): 0. Max coverage (-): 0

Region: chr9 13066593-13066605. Max. coverage (+): 0. Max coverage (-): 0

Region: chr9 13066606-13066617. Max. coverage (+): 0. Max coverage (-): 0

Region: chr9 13066618-13066630. Max. coverage (+): 0. Max coverage (-): 0

Region: chr9 13066631-13066642. Max. coverage (+): 0. Max coverage (-): 0

Region: chr9 13066643-13066654. Max. coverage (+): 0. Max coverage (-): 0

Region: chr9 13066655-13066667. Max. coverage (+): 0. Max coverage (-): 0

Region: chr9 13066668-13066679. Max. coverage (+): 0. Max coverage (-): 0

Region: chr9 13066680-13066692. Max. coverage (+): 0. Max coverage (-): 0

Region: chr9 13066693-13066704. Max. coverage (+): 0. Max coverage (-): 0

Region: chr9 13066705-13066717. Max. coverage (+): 0. Max coverage (-): 0

Region: chr9 13066718-13066729. Max. coverage (+): 0. Max coverage (-): 0

Region: chr9 13066730-13066741. Max. coverage (+): 0. Max coverage (-): 0

Region: chr9 13066742-13066754. Max. coverage (+): 0. Max coverage (-): 0

Region: chr9 13066755-13066766. Max. coverage (+): 0. Max coverage (-): 0

Region: chr9 13066767-13066779. Max. coverage (+): 0. Max coverage (-): 0

Region: chr9 13066780-13066791. Max. coverage (+): 0. Max coverage (-): 0

Region: chr9 13066792-13066803. Max. coverage (+): 0. Max coverage (-): 0

Region: chr9 13066804-13066816. Max. coverage (+): 0. Max coverage (-): 0

Region: chr9 13066817-13066828. Max. coverage (+): 0. Max coverage (-): 0

Region: chr9 13066829-13066841. Max. coverage (+): 13.62. Max coverage (-): 0

Region: chr9 13066842-13066853. Max. coverage (+): 13.62. Max coverage (-): 2.45

Region: chr9 13066854-13066866. Max. coverage (+): 0. Max coverage (-): 0

Region: chr9 13066867-13066878. Max. coverage (+): 0. Max coverage (-): 0

Region: chr9 13066879-13066890. Max. coverage (+): 0. Max coverage (-): 0

Region: chr9 13066891-13066903. Max. coverage (+): 0. Max coverage (-): 0

Region: chr9 13066904-13066915. Max. coverage (+): 0. Max coverage (-): 0

Region: chr9 13066916-13066928. Max. coverage (+): 0. Max coverage (-): 0

Region: chr9 13066929-13066940. Max. coverage (+): 0. Max coverage (-): 0

Region: chr9 13066941-13066953. Max. coverage (+): 4.7. Max coverage (-): 0

Region: chr9 13066954-13066965. Max. coverage (+): 4.7. Max coverage (-): 0

Region: chr9 13066966-13066977. Max. coverage (+): 0. Max coverage (-): 0

Region: chr9 13066978-13066990. Max. coverage (+): 0. Max coverage (-): 0

Region: chr9 13066991-13067002. Max. coverage (+): 0. Max coverage (-): 0

Region: chr9 13067003-13067015. Max. coverage (+): 0. Max coverage (-): 0

Region: chr9 13067016-13067027. Max. coverage (+): 0. Max coverage (-): 0

Region: chr9 13067028-13067040. Max. coverage (+): 9.02. Max coverage (-): 0

Region: chr9 13067041-13067052. Max. coverage (+): 4.75. Max coverage (-): 0

Region: chr9 13067053-13067064. Max. coverage (+): 0. Max coverage (-): 0

Region: chr9 13067065-13067077. Max. coverage (+): 0. Max coverage (-): 0

Region: chr9 13067078-13067089. Max. coverage (+): 0. Max coverage (-): 0

Region: chr9 13067090-13067102. Max. coverage (+): 5.78. Max coverage (-): 0

Region: chr9 13067103-13067114. Max. coverage (+): 4.84. Max coverage (-): 0

Region: chr9 13067115-13067127. Max. coverage (+): 0. Max coverage (-): 0

Region: chr9 13067128-13067139. Max. coverage (+): 1.76. Max coverage (-): 0

Region: chr9 13067140-13067151. Max. coverage (+): 1.76. Max coverage (-): 0

Region: chr9 13067152-13067164. Max. coverage (+): 0. Max coverage (-): 0

Region: chr9 13067165-13067176. Max. coverage (+): 0. Max coverage (-): 0

Region: chr9 13067177-13067189. Max. coverage (+): 0. Max coverage (-): 0

Region: chr9 13067190-13067201. Max. coverage (+): 0. Max coverage (-): 0

Region: chr9 13067202-13067213. Max. coverage (+): 0. Max coverage (-): 0

Region: chr9 13067214-13067226. Max. coverage (+): 0. Max coverage (-): 0

Region: chr9 13067227-13067238. Max. coverage (+): 0. Max coverage (-): 0

Region: chr9 13067239-13067251. Max. coverage (+): 0. Max coverage (-): 0

Region: chr9 13067252-13067263. Max. coverage (+): 0. Max coverage (-): 0

Region: chr9 13067264-13067276. Max. coverage (+): 0. Max coverage (-): 0

Region: chr9 13067277-13067288. Max. coverage (+): 0. Max coverage (-): 0

Region: chr9 13067289-13067300. Max. coverage (+): 0. Max coverage (-): 0

Region: chr9 13067301-13067313. Max. coverage (+): 0. Max coverage (-): 0

Region: chr9 13067314-13067325. Max. coverage (+): 0. Max coverage (-): 0

Region: chr9 13067326-13067338. Max. coverage (+): 0. Max coverage (-): 0

Region: chr9 13067339-13067350. Max. coverage (+): 0. Max coverage (-): 0

Region: chr9 13067351-13067363. Max. coverage (+): 0. Max coverage (-): 0

Region: chr9 13067364-13067375. Max. coverage (+): 0. Max coverage (-): 0

Region: chr9 13067376-13067387. Max. coverage (+): 0. Max coverage (-): 0

Region: chr9 13067388-13067400. Max. coverage (+): 0. Max coverage (-): 0

Region: chr9 13067401-13067412. Max. coverage (+): 0. Max coverage (-): 0

Region: chr9 13067413-13067425. Max. coverage (+): 0. Max coverage (-): 0

Region: chr9 13067426-13067437. Max. coverage (+): 0. Max coverage (-): 0

Region: chr9 13067438-13067450. Max. coverage (+): 0. Max coverage (-): 0

Region: chr9 13067451-13067462. Max. coverage (+): 0. Max coverage (-): 0

Region: chr9 13067463-13067474. Max. coverage (+): 0. Max coverage (-): 0

Region: chr9 13067475-13067487. Max. coverage (+): 7.41. Max coverage (-): 0

Region: chr9 13067488-13067499. Max. coverage (+): 0. Max coverage (-): 0

Region: chr9 13067500-13067512. Max. coverage (+): 0. Max coverage (-): 0

Region: chr9 13067513-13067524. Max. coverage (+): 0. Max coverage (-): 0

Region: chr9 13067525-13067536. Max. coverage (+): 0. Max coverage (-): 0

Region: chr9 13067537-13067549. Max. coverage (+): 0. Max coverage (-): 0

Region: chr9 13067550-13067561. Max. coverage (+): 0. Max coverage (-): 0

Region: chr9 13067562-13067574. Max. coverage (+): 0. Max coverage (-): 0

Region: chr9 13067575-13067586. Max. coverage (+): 0. Max coverage (-): 0

Region: chr9 13067587-13067599. Max. coverage (+): 0. Max coverage (-): 0

Region: chr9 13067600-13067611. Max. coverage (+): 0. Max coverage (-): 0

Region: chr9 13067612-13067623. Max. coverage (+): 0. Max coverage (-): 0

Region: chr9 13067624-13067636. Max. coverage (+): 0. Max coverage (-): 0

Region: chr9 13067637-13067648. Max. coverage (+): 0. Max coverage (-): 0

Region: chr9 13067649-13067661. Max. coverage (+): 0. Max coverage (-): 0

Region: chr9 13067662-13067673. Max. coverage (+): 0. Max coverage (-): 0

Region: chr9 13067674-13067686. Max. coverage (+): 1.94. Max coverage (-): 0

Region: chr9 13067687-13067698. Max. coverage (+): 1.94. Max coverage (-): 0

Region: chr9 13067699-13067710. Max. coverage (+): 0. Max coverage (-): 0

Region: chr9 13067711-13067723. Max. coverage (+): 0. Max coverage (-): 0

Region: chr9 13067724-13067735. Max. coverage (+): 1.77. Max coverage (-): 0

Region: chr9 13067736-13067748. Max. coverage (+): 1.77. Max coverage (-): 0

Region: chr9 13067749-13067760. Max. coverage (+): 0. Max coverage (-): 0

Region: chr9 13067761-13067773. Max. coverage (+): 0. Max coverage (-): 0

Region: chr9 13067774-13067785. Max. coverage (+): 0. Max coverage (-): 0

Region: chr9 13067786-13067797. Max. coverage (+): 0. Max coverage (-): 0

Region: chr9 13067798-13067810. Max. coverage (+): 0. Max coverage (-): 0

Region: chr9 13067811-13067822. Max. coverage (+): 0. Max coverage (-): 0

Region: chr9 13067823-13067835. Max. coverage (+): 0. Max coverage (-): 0

Region: chr9 13067836-13067847. Max. coverage (+): 0. Max coverage (-): 0

Region: chr9 13067848-13067860. Max. coverage (+): 0. Max coverage (-): 0

Region: chr9 13067861-13067872. Max. coverage (+): 0. Max coverage (-): 0

Region: chr9 13067873-13067884. Max. coverage (+): 0. Max coverage (-): 0

Region: chr9 13067885-13067897. Max. coverage (+): 0. Max coverage (-): 0

Region: chr9 13067898-13067909. Max. coverage (+): 0. Max coverage (-): 0

Region: chr9 13067910-13067922. Max. coverage (+): 0. Max coverage (-): 0

Region: chr9 13067923-13067934. Max. coverage (+): 0. Max coverage (-): 0

Region: chr9 13067935-13067946. Max. coverage (+): 0. Max coverage (-): 0

Region: chr9 13067947-13067959. Max. coverage (+): 0. Max coverage (-): 0

Region: chr9 13067960-13067971. Max. coverage (+): 0. Max coverage (-): 0

Region: chr9 13067972-13067984. Max. coverage (+): 0. Max coverage (-): 0

Region: chr9 13067985-13067996. Max. coverage (+): 0. Max coverage (-): 0

Region: chr9 13067997-13068009. Max. coverage (+): 0. Max coverage (-): 0

Region: chr9 13068010-13068021. Max. coverage (+): 5.54. Max coverage (-): 0

Region: chr9 13068022-13068033. Max. coverage (+): 0. Max coverage (-): 0

Region: chr9 13068034-13068046. Max. coverage (+): 0. Max coverage (-): 0

Region: chr9 13068047-13068058. Max. coverage (+): 0. Max coverage (-): 0

Region: chr9 13068059-13068071. Max. coverage (+): 0. Max coverage (-): 0

Region: chr9 13068072-13068083. Max. coverage (+): 0. Max coverage (-): 0

Region: chr9 13068084-13068096. Max. coverage (+): 0. Max coverage (-): 0

Region: chr9 13068097-13068108. Max. coverage (+): 0. Max coverage (-): 0

Region: chr9 13068109-13068120. Max. coverage (+): 0. Max coverage (-): 0

Region: chr9 13068121-13068133. Max. coverage (+): 0. Max coverage (-): 0

Region: chr9 13068134-13068145. Max. coverage (+): 0. Max coverage (-): 0

Region: chr9 13068146-13068158. Max. coverage (+): 0. Max coverage (-): 0

Region: chr9 13068159-13068170. Max. coverage (+): 0. Max coverage (-): 0

Region: chr9 13068171-13068183. Max. coverage (+): 0. Max coverage (-): 0

Region: chr9 13068184-13068195. Max. coverage (+): 0. Max coverage (-): 0

Region: chr9 13068196-13068207. Max. coverage (+): 0. Max coverage (-): 0

Region: chr9 13068208-13068220. Max. coverage (+): 0. Max coverage (-): 0

Region: chr9 13068221-13068232. Max. coverage (+): 0. Max coverage (-): 0

Region: chr9 13068233-13068245. Max. coverage (+): 0. Max coverage (-): 0

Region: chr9 13068246-13068257. Max. coverage (+): 0. Max coverage (-): 0

Region: chr9 13068258-13068270. Max. coverage (+): 0. Max coverage (-): 0

Region: chr9 13068271-13068282. Max. coverage (+): 0. Max coverage (-): 0

Region: chr9 13068283-13068294. Max. coverage (+): 0. Max coverage (-): 0

Region: chr9 13068295-13068307. Max. coverage (+): 0. Max coverage (-): 0

Region: chr9 13068308-13068319. Max. coverage (+): 0. Max coverage (-): 0

Region: chr9 13068320-13068332. Max. coverage (+): 0. Max coverage (-): 0

Region: chr9 13068333-13068344. Max. coverage (+): 0. Max coverage (-): 0

Region: chr9 13068345-13068356. Max. coverage (+): 0. Max coverage (-): 0

Region: chr9 13068357-13068369. Max. coverage (+): 0. Max coverage (-): 0

Region: chr9 13068370-13068381. Max. coverage (+): 0. Max coverage (-): 0

Region: chr9 13068382-13068394. Max. coverage (+): 0. Max coverage (-): 0

Region: chr9 13068395-13068406. Max. coverage (+): 0. Max coverage (-): 0

Region: chr9 13068407-13068419. Max. coverage (+): 0. Max coverage (-): 0

Region: chr9 13068420-13068431. Max. coverage (+): 0. Max coverage (-): 0

Region: chr9 13068432-13068443. Max. coverage (+): 1.72. Max coverage (-): 0

Region: chr9 13068444-13068456. Max. coverage (+): 0. Max coverage (-): 0

Region: chr9 13068457-13068468. Max. coverage (+): 0. Max coverage (-): 0

Region: chr9 13068469-13068481. Max. coverage (+): 0. Max coverage (-): 0

Region: chr9 13068482-13068493. Max. coverage (+): 0. Max coverage (-): 0

Region: chr9 13068494-13068506. Max. coverage (+): 0.52. Max coverage (-): 0

Region: chr9 13068507-13068518. Max. coverage (+): 0.52. Max coverage (-): 0

Region: chr9 13068519-13068530. Max. coverage (+): 0. Max coverage (-): 0

Region: chr9 13068531-13068543. Max. coverage (+): 0. Max coverage (-): 0

Region: chr9 13068544-13068555. Max. coverage (+): 0. Max coverage (-): 0

Region: chr9 13068556-13068568. Max. coverage (+): 0. Max coverage (-): 0

Region: chr9 13068569-13068580. Max. coverage (+): 0. Max coverage (-): 0

Region: chr9 13068581-13068593. Max. coverage (+): 0. Max coverage (-): 0

Region: chr9 13068594-13068605. Max. coverage (+): 0. Max coverage (-): 0

Region: chr9 13068606-13068617. Max. coverage (+): 0. Max coverage (-): 0

Region: chr9 13068618-13068630. Max. coverage (+): 0. Max coverage (-): 0

Region: chr9 13068631-13068642. Max. coverage (+): 0. Max coverage (-): 0

Region: chr9 13068643-13068655. Max. coverage (+): 0. Max coverage (-): 0

Region: chr9 13068656-13068667. Max. coverage (+): 0. Max coverage (-): 0

Region: chr9 13068668-13068680. Max. coverage (+): 0. Max coverage (-): 0

Region: chr9 13068681-13068692. Max. coverage (+): 0. Max coverage (-): 0

Region: chr9 13068693-13068704. Max. coverage (+): 0. Max coverage (-): 0

Region: chr9 13068705-13068717. Max. coverage (+): 0. Max coverage (-): 0

Region: chr9 13068718-13068729. Max. coverage (+): 0. Max coverage (-): 0

Region: chr9 13068730-13068742. Max. coverage (+): 0. Max coverage (-): 0

Region: chr9 13068743-13068754. Max. coverage (+): 0. Max coverage (-): 0

Region: chr9 13068755-13068766. Max. coverage (+): 0. Max coverage (-): 0

Region: chr9 13068767-13068779. Max. coverage (+): 0. Max coverage (-): 0

Region: chr9 13068780-13068791. Max. coverage (+): 0. Max coverage (-): 0

Region: chr9 13068792-13068804. Max. coverage (+): 0. Max coverage (-): 0

Region: chr9 13068805-13068816. Max. coverage (+): 1.03. Max coverage (-): 0

Region: chr9 13068817-13068829. Max. coverage (+): 1.03. Max coverage (-): 0

Region: chr9 13068830-13068841. Max. coverage (+): 0. Max coverage (-): 0

Region: chr9 13068842-13068853. Max. coverage (+): 6.54. Max coverage (-): 0

Region: chr9 13068854-13068866. Max. coverage (+): 9.37. Max coverage (-): 0

Region: chr9 13068867-13068878. Max. coverage (+): 0. Max coverage (-): 0

Region: chr9 13068879-. Max. coverage (+): 0. Max coverage (-): 0

RepeatMasker Color Code

**+**

100-98% Identity

<98-95% Identity

<95-90% Identity

<90-85% Identity

<85-80% Identity

<80-75% Identity

<75-70% Identity

<70% Identity

**-**

Gene Set Color Code

**+**

Gene

Pseudogene

**-**

Topology/Coverage Color Code

Coverage Plus Strand

Coverage Minus Strand

Mainstrand: Plus

Mainstrand: Minus

Complementary Strand

Flanking Region  
(if option -flank >0)

Gene Set Annotation  
  
RepeatMasker Annotation  

**1. L1M3**: 13063383-13063580 (-), Divergence to consensus: 34.9%  
**2. Bov-tA2**: 13063672-13063882 (+), Divergence to consensus: 23.6%  
**3. L1-2\_BT**: 13063935-13064563 (-), Divergence to consensus: 37.5%  
**4. L1MC**: 13064703-13064988 (-), Divergence to consensus: 42.6%  
**5. L1MB5**: 13066164-13066372 (+), Divergence to consensus: 36.7%

  
Transcription Factor Binding Sites  

**RFX4\_2** (Sequence: CCTGGATAC (+): 13067989)  
**SOX9** (Sequence: CTATTGTT (+): 13063284)
