# Supplementary material for: Small RNA sequencing of cryopreserved semen from single bull revealed altered miRNAs and piRNAs expression between High- and Low-motile sperm populations
Source: BMC Genomics. 2017 Jan 4;18:14. doi: 10.1186/s12864-016-3394-7 (PMC5209821; doi:10.1186/s12864-016-3394-7)
Supplement: Additional file 3: — Details for each piRNA clusters found in High Motile (HM) sperm fraction. Genes, repeats, transposable elements and transcription factors binding sites falling within the cluster regions were reported. (ZIP 1896 kb) [file 12864_2016_3394_MOESM3_ESM.zip › 99.html]

piRNA cluster 99


Predicted piRNA cluster no. 99     previous   next
  

Show proTRAC run info
Hide proTRAC run info

================================= proTRAC ====================================  
VERSION: 2.1                                    LAST MODIFIED: 06. October 2015  
  
Please cite:  
Rosenkranz D, Zischler H. proTRAC - a software for probabilistic piRNA cluster  
detection, visualization and analysis. 2012. BMC Bioinformatics 13:5.  
  
and (for proTRAC 2.0 and later):  
Rosenkranz D, Rudloff S, Bastuck K, Ketting RF, Zischler H. Tupaia small RNAs  
provide insights into function and evolution of RNAi-based transposon defense  
in mammals. 2015. RNA 21(5):911-922.  
  
Contact:  
David Rosenkranz  
Institute of Anthropology, small RNA group  
Johannes Gutenberg University Mainz  
email: rosenkranz@uni-mainz.de  
  
You can find the latest proTRAC version at:  
http://sourceforge.net/projects/protrac/files  
http://www.smallRNAgroup-mainz.de/software  
==============================================================================  
  
PARAMETERS:  
Map file: .............../storage/core/barbara/genhome/smallRNA/fertility/Sample\_motile/pirna/Sample\_motile\_26-33\_collapsed.fa.no-dust.map.weighted-10000-1000-b-0  
Genome file: ............/storage/core/barbara/genhome/smallRNA/fertility/Sample\_all/pirna/bt\_311\_chrY.fa  
RepeatMasker annotation: /storage/genomes/bt\_umd31/GCF\_000003055.6\_Bos\_taurus\_UMD\_3.1.1\_repeatMasker\_chr.out  
GeneSet:................./storage/core/barbara/genhome/smallRNA/fertility/Sample\_all/pirna/full.gtf  
  
Significant (p<=0.01) hit density will be calculated based  
on observed hit distribution.  
  
Sliding window size: ........................................ 5000 bp  
Sliding window increament: .................................. 1000 bp  
Normalize each hit by number of genomic hits: ............... 1 [0=no/1=yes]  
Normalize each hit by number of sequence reads: ............. 1 [0=no/1=yes]  
Normalize values (-> per million mapped reads): ............. 1 [0=no/1=yes]  
Min. fraction of hits with 1T(U) or 10A: .................... 0.75  
Alternatively: Min. fraction of hits with 1T(U) and 10A: .... 0.5  
Min. fraction of hits with typical piRNA length: ............ 0.75  
Typical piRNA length: ....................................... 26-33 nt  
Min. size of a piRNA cluster: ............................... 5000 bp.  
Min. number of hits (absolute): ............................. 0  
Min. number of hits (normalized): ........................... 0  
Min. fraction of hits on the mainstrand: .................... 0.75  
Top fraction of mapped sequences (in terms of read counts): . 1%  
Top fraction accounts for max. n% of sequence reads: ........ 90%  
Min. fraction of hits on each arm of a bidirectional cluster: 0.1  
Output image file for each cluster: ......................... 0 [0=no/1=yes]  
Output html file for each cluster: .......................... 1 [0=no/1=yes]  
Output a summary table: ..................................... 1 [0=no/1=yes]  
Output a FASTA file for each cluster (piRNA sequences): ..... 1 [0=no/1=yes]  
Output a FASTA file comprising cluster sequences: ........... 1 [0=no/1=yes]  
Search DNA motifs in clusters: .............................. 1 [0=no/1=yes]  
Output flanking sequences: +/- .............................. 0 bp  
Output ~.pTi file: .......................................... 1 [0=no/1=yes]  
==============================================================================  
  
  
Genome size (without gaps): ............ 2678902517 bp  
Gaps (N/X/-): .......................... 53837044 bp  
Mapped reads: .......................... 658825247023  
Non-identical sequences: ............... 514171  
Genomic hits: .......................... 764233  
Significant densitiy of mapped reads: .. 12867599.5173724 reads/kb

Show proTRAC cluster info
Hide proTRAC cluster info

|  |  |
| --- | --- |
| Location | chr9 |
| Coordinates | 103967028-103972444 |
| Size [bp] | 5417 |
| Sequence hit loci | 56 |
| Mapped reads (normalized) | 83315920 |
| Mapped reads (normalized) per kb | 15380454.1 |
| Normalized reads with 1T (1U) | 83.6% |
| Normalized reads with 10A | 26.9% |
| Normalized reads with length 26-33 nt | 100% |
| Normalized reads on the main strand(s) | 91.7% |
| Predicted directionality | mono:minus |

100%

0%

1T (1U)  
reads

10A reads

26-33 nt  
reads

reads on mainstrand

**Either the amount of reads with 1T (1U) OR 10A has to exceed 75% (set with option: -1Tor10A)  
Alternatively the amount of reads with 1T (1U) AND 10A has to exceed 50% (set with option: -1Tand10A)  
Minimum amount of reads with preferred size is 75% (set with option: -pisize)  
Minimum amount of reads on the main strand(s) is 75% (set with option: -clstrand)**

Show read coverage
Hide read coverage

WHAT DO I SEE HERE?  
This chart shows the location of mapped sequence reads within a predicted piRNA cluster. The color refers to the number of genomic hits produced by the sequence read in question. A dark red bar indicates that this sequence read produces many other hits elsewhere in the genome. Many adjacent red or yellow bars can indicate the presence of a multi-copy element such as transposons or rRNA genes. A dark green bar indicates that this sequence read maps uniquely to this locus.

1 hit

2-5 hits

6-10 hits

11-20 hits

21-50 hits

51-100 hits

> 100 hits

chr9

103967028

103972444

Gene Set

RepeatMasker

Mapped  
Reads

12.96

plus strand

minus strand

12.96

Region: chr9 13068880-103967033. Max. coverage (+): 0. Max coverage (-): 1.33

Region: chr9 103967034-103967044. Max. coverage (+): 0. Max coverage (-): 1.33

Region: chr9 103967045-103967055. Max. coverage (+): 0. Max coverage (-): 0

Region: chr9 103967056-103967065. Max. coverage (+): 0. Max coverage (-): 0

Region: chr9 103967066-103967076. Max. coverage (+): 0. Max coverage (-): 0

Region: chr9 103967077-103967087. Max. coverage (+): 0. Max coverage (-): 0

Region: chr9 103967088-103967098. Max. coverage (+): 0. Max coverage (-): 0

Region: chr9 103967099-103967109. Max. coverage (+): 0. Max coverage (-): 0

Region: chr9 103967110-103967120. Max. coverage (+): 0. Max coverage (-): 0

Region: chr9 103967121-103967130. Max. coverage (+): 0. Max coverage (-): 0

Region: chr9 103967131-103967141. Max. coverage (+): 0. Max coverage (-): 0

Region: chr9 103967142-103967152. Max. coverage (+): 0. Max coverage (-): 0

Region: chr9 103967153-103967163. Max. coverage (+): 0. Max coverage (-): 0

Region: chr9 103967164-103967174. Max. coverage (+): 0. Max coverage (-): 0

Region: chr9 103967175-103967185. Max. coverage (+): 0. Max coverage (-): 0

Region: chr9 103967186-103967195. Max. coverage (+): 0. Max coverage (-): 0

Region: chr9 103967196-103967206. Max. coverage (+): 0. Max coverage (-): 0

Region: chr9 103967207-103967217. Max. coverage (+): 0. Max coverage (-): 0

Region: chr9 103967218-103967228. Max. coverage (+): 0. Max coverage (-): 0

Region: chr9 103967229-103967239. Max. coverage (+): 0. Max coverage (-): 0

Region: chr9 103967240-103967250. Max. coverage (+): 0. Max coverage (-): 0

Region: chr9 103967251-103967260. Max. coverage (+): 0. Max coverage (-): 0

Region: chr9 103967261-103967271. Max. coverage (+): 0. Max coverage (-): 0

Region: chr9 103967272-103967282. Max. coverage (+): 0. Max coverage (-): 0

Region: chr9 103967283-103967293. Max. coverage (+): 0. Max coverage (-): 0

Region: chr9 103967294-103967304. Max. coverage (+): 0. Max coverage (-): 0

Region: chr9 103967305-103967315. Max. coverage (+): 0. Max coverage (-): 0

Region: chr9 103967316-103967325. Max. coverage (+): 0. Max coverage (-): 0

Region: chr9 103967326-103967336. Max. coverage (+): 0. Max coverage (-): 0

Region: chr9 103967337-103967347. Max. coverage (+): 0. Max coverage (-): 0

Region: chr9 103967348-103967358. Max. coverage (+): 0. Max coverage (-): 0

Region: chr9 103967359-103967369. Max. coverage (+): 0. Max coverage (-): 0

Region: chr9 103967370-103967380. Max. coverage (+): 0. Max coverage (-): 0

Region: chr9 103967381-103967390. Max. coverage (+): 0. Max coverage (-): 0

Region: chr9 103967391-103967401. Max. coverage (+): 0. Max coverage (-): 0

Region: chr9 103967402-103967412. Max. coverage (+): 0. Max coverage (-): 0

Region: chr9 103967413-103967423. Max. coverage (+): 0. Max coverage (-): 0

Region: chr9 103967424-103967434. Max. coverage (+): 0. Max coverage (-): 0

Region: chr9 103967435-103967445. Max. coverage (+): 0. Max coverage (-): 0

Region: chr9 103967446-103967455. Max. coverage (+): 0. Max coverage (-): 0

Region: chr9 103967456-103967466. Max. coverage (+): 0. Max coverage (-): 0

Region: chr9 103967467-103967477. Max. coverage (+): 0. Max coverage (-): 0

Region: chr9 103967478-103967488. Max. coverage (+): 0. Max coverage (-): 0

Region: chr9 103967489-103967499. Max. coverage (+): 0. Max coverage (-): 0

Region: chr9 103967500-103967510. Max. coverage (+): 0. Max coverage (-): 0

Region: chr9 103967511-103967520. Max. coverage (+): 0. Max coverage (-): 4.41

Region: chr9 103967521-103967531. Max. coverage (+): 0. Max coverage (-): 4.41

Region: chr9 103967532-103967542. Max. coverage (+): 0. Max coverage (-): 0

Region: chr9 103967543-103967553. Max. coverage (+): 0. Max coverage (-): 0

Region: chr9 103967554-103967564. Max. coverage (+): 0. Max coverage (-): 0

Region: chr9 103967565-103967575. Max. coverage (+): 0. Max coverage (-): 0

Region: chr9 103967576-103967585. Max. coverage (+): 0. Max coverage (-): 1.12

Region: chr9 103967586-103967596. Max. coverage (+): 0. Max coverage (-): 1.12

Region: chr9 103967597-103967607. Max. coverage (+): 0. Max coverage (-): 0

Region: chr9 103967608-103967618. Max. coverage (+): 0. Max coverage (-): 0

Region: chr9 103967619-103967629. Max. coverage (+): 0. Max coverage (-): 0

Region: chr9 103967630-103967640. Max. coverage (+): 0. Max coverage (-): 0

Region: chr9 103967641-103967650. Max. coverage (+): 0. Max coverage (-): 0

Region: chr9 103967651-103967661. Max. coverage (+): 0. Max coverage (-): 0

Region: chr9 103967662-103967672. Max. coverage (+): 0. Max coverage (-): 0

Region: chr9 103967673-103967683. Max. coverage (+): 0. Max coverage (-): 0

Region: chr9 103967684-103967694. Max. coverage (+): 0. Max coverage (-): 0

Region: chr9 103967695-103967705. Max. coverage (+): 0. Max coverage (-): 0

Region: chr9 103967706-103967715. Max. coverage (+): 0. Max coverage (-): 0.94

Region: chr9 103967716-103967726. Max. coverage (+): 0. Max coverage (-): 0

Region: chr9 103967727-103967737. Max. coverage (+): 0. Max coverage (-): 0

Region: chr9 103967738-103967748. Max. coverage (+): 0. Max coverage (-): 0

Region: chr9 103967749-103967759. Max. coverage (+): 0. Max coverage (-): 0

Region: chr9 103967760-103967770. Max. coverage (+): 0. Max coverage (-): 0

Region: chr9 103967771-103967780. Max. coverage (+): 0. Max coverage (-): 0

Region: chr9 103967781-103967791. Max. coverage (+): 0. Max coverage (-): 0

Region: chr9 103967792-103967802. Max. coverage (+): 0. Max coverage (-): 0

Region: chr9 103967803-103967813. Max. coverage (+): 0. Max coverage (-): 0

Region: chr9 103967814-103967824. Max. coverage (+): 0. Max coverage (-): 0

Region: chr9 103967825-103967835. Max. coverage (+): 0. Max coverage (-): 0

Region: chr9 103967836-103967845. Max. coverage (+): 0. Max coverage (-): 0

Region: chr9 103967846-103967856. Max. coverage (+): 0. Max coverage (-): 0

Region: chr9 103967857-103967867. Max. coverage (+): 0. Max coverage (-): 0

Region: chr9 103967868-103967878. Max. coverage (+): 0. Max coverage (-): 0

Region: chr9 103967879-103967889. Max. coverage (+): 0. Max coverage (-): 0

Region: chr9 103967890-103967900. Max. coverage (+): 0. Max coverage (-): 0

Region: chr9 103967901-103967910. Max. coverage (+): 0. Max coverage (-): 0.54

Region: chr9 103967911-103967921. Max. coverage (+): 0. Max coverage (-): 0.54

Region: chr9 103967922-103967932. Max. coverage (+): 0. Max coverage (-): 0

Region: chr9 103967933-103967943. Max. coverage (+): 0. Max coverage (-): 0

Region: chr9 103967944-103967954. Max. coverage (+): 0. Max coverage (-): 0

Region: chr9 103967955-103967965. Max. coverage (+): 0. Max coverage (-): 0

Region: chr9 103967966-103967975. Max. coverage (+): 0. Max coverage (-): 0

Region: chr9 103967976-103967986. Max. coverage (+): 0. Max coverage (-): 0

Region: chr9 103967987-103967997. Max. coverage (+): 0. Max coverage (-): 0

Region: chr9 103967998-103968008. Max. coverage (+): 0. Max coverage (-): 0

Region: chr9 103968009-103968019. Max. coverage (+): 0. Max coverage (-): 0

Region: chr9 103968020-103968030. Max. coverage (+): 0. Max coverage (-): 0

Region: chr9 103968031-103968040. Max. coverage (+): 0. Max coverage (-): 0

Region: chr9 103968041-103968051. Max. coverage (+): 0. Max coverage (-): 0

Region: chr9 103968052-103968062. Max. coverage (+): 0. Max coverage (-): 0

Region: chr9 103968063-103968073. Max. coverage (+): 0. Max coverage (-): 0

Region: chr9 103968074-103968084. Max. coverage (+): 0. Max coverage (-): 0

Region: chr9 103968085-103968095. Max. coverage (+): 0. Max coverage (-): 0

Region: chr9 103968096-103968105. Max. coverage (+): 0. Max coverage (-): 0

Region: chr9 103968106-103968116. Max. coverage (+): 0. Max coverage (-): 0

Region: chr9 103968117-103968127. Max. coverage (+): 0. Max coverage (-): 0

Region: chr9 103968128-103968138. Max. coverage (+): 0. Max coverage (-): 0

Region: chr9 103968139-103968149. Max. coverage (+): 0. Max coverage (-): 0

Region: chr9 103968150-103968160. Max. coverage (+): 0. Max coverage (-): 0

Region: chr9 103968161-103968170. Max. coverage (+): 0. Max coverage (-): 0

Region: chr9 103968171-103968181. Max. coverage (+): 0. Max coverage (-): 0

Region: chr9 103968182-103968192. Max. coverage (+): 0. Max coverage (-): 0

Region: chr9 103968193-103968203. Max. coverage (+): 0. Max coverage (-): 0

Region: chr9 103968204-103968214. Max. coverage (+): 0. Max coverage (-): 0

Region: chr9 103968215-103968225. Max. coverage (+): 0. Max coverage (-): 0

Region: chr9 103968226-103968235. Max. coverage (+): 0. Max coverage (-): 0

Region: chr9 103968236-103968246. Max. coverage (+): 0. Max coverage (-): 0

Region: chr9 103968247-103968257. Max. coverage (+): 0. Max coverage (-): 0

Region: chr9 103968258-103968268. Max. coverage (+): 0. Max coverage (-): 0

Region: chr9 103968269-103968279. Max. coverage (+): 0. Max coverage (-): 0

Region: chr9 103968280-103968290. Max. coverage (+): 0. Max coverage (-): 0

Region: chr9 103968291-103968300. Max. coverage (+): 0. Max coverage (-): 0

Region: chr9 103968301-103968311. Max. coverage (+): 0. Max coverage (-): 0

Region: chr9 103968312-103968322. Max. coverage (+): 0. Max coverage (-): 0

Region: chr9 103968323-103968333. Max. coverage (+): 0. Max coverage (-): 0

Region: chr9 103968334-103968344. Max. coverage (+): 0. Max coverage (-): 0

Region: chr9 103968345-103968355. Max. coverage (+): 0. Max coverage (-): 0

Region: chr9 103968356-103968365. Max. coverage (+): 0. Max coverage (-): 0

Region: chr9 103968366-103968376. Max. coverage (+): 0. Max coverage (-): 0

Region: chr9 103968377-103968387. Max. coverage (+): 0. Max coverage (-): 0

Region: chr9 103968388-103968398. Max. coverage (+): 0. Max coverage (-): 0

Region: chr9 103968399-103968409. Max. coverage (+): 0. Max coverage (-): 0

Region: chr9 103968410-103968420. Max. coverage (+): 0. Max coverage (-): 0

Region: chr9 103968421-103968431. Max. coverage (+): 0. Max coverage (-): 0

Region: chr9 103968432-103968441. Max. coverage (+): 0. Max coverage (-): 0

Region: chr9 103968442-103968452. Max. coverage (+): 0. Max coverage (-): 0

Region: chr9 103968453-103968463. Max. coverage (+): 0. Max coverage (-): 0

Region: chr9 103968464-103968474. Max. coverage (+): 0. Max coverage (-): 0

Region: chr9 103968475-103968485. Max. coverage (+): 0. Max coverage (-): 0

Region: chr9 103968486-103968496. Max. coverage (+): 0. Max coverage (-): 0

Region: chr9 103968497-103968506. Max. coverage (+): 0. Max coverage (-): 0

Region: chr9 103968507-103968517. Max. coverage (+): 0. Max coverage (-): 0

Region: chr9 103968518-103968528. Max. coverage (+): 0. Max coverage (-): 3.59

Region: chr9 103968529-103968539. Max. coverage (+): 0. Max coverage (-): 3.59

Region: chr9 103968540-103968550. Max. coverage (+): 0. Max coverage (-): 0

Region: chr9 103968551-103968561. Max. coverage (+): 0. Max coverage (-): 0

Region: chr9 103968562-103968571. Max. coverage (+): 0. Max coverage (-): 0

Region: chr9 103968572-103968582. Max. coverage (+): 0. Max coverage (-): 0

Region: chr9 103968583-103968593. Max. coverage (+): 0. Max coverage (-): 0

Region: chr9 103968594-103968604. Max. coverage (+): 0. Max coverage (-): 0

Region: chr9 103968605-103968615. Max. coverage (+): 0. Max coverage (-): 0

Region: chr9 103968616-103968626. Max. coverage (+): 0. Max coverage (-): 0

Region: chr9 103968627-103968636. Max. coverage (+): 0. Max coverage (-): 0

Region: chr9 103968637-103968647. Max. coverage (+): 0. Max coverage (-): 0

Region: chr9 103968648-103968658. Max. coverage (+): 0. Max coverage (-): 0

Region: chr9 103968659-103968669. Max. coverage (+): 0. Max coverage (-): 0

Region: chr9 103968670-103968680. Max. coverage (+): 0. Max coverage (-): 0

Region: chr9 103968681-103968691. Max. coverage (+): 0. Max coverage (-): 0

Region: chr9 103968692-103968701. Max. coverage (+): 0. Max coverage (-): 0

Region: chr9 103968702-103968712. Max. coverage (+): 0. Max coverage (-): 0

Region: chr9 103968713-103968723. Max. coverage (+): 0. Max coverage (-): 0

Region: chr9 103968724-103968734. Max. coverage (+): 0. Max coverage (-): 0

Region: chr9 103968735-103968745. Max. coverage (+): 0. Max coverage (-): 0

Region: chr9 103968746-103968756. Max. coverage (+): 0. Max coverage (-): 0

Region: chr9 103968757-103968766. Max. coverage (+): 0. Max coverage (-): 0

Region: chr9 103968767-103968777. Max. coverage (+): 0. Max coverage (-): 0

Region: chr9 103968778-103968788. Max. coverage (+): 0. Max coverage (-): 0

Region: chr9 103968789-103968799. Max. coverage (+): 0. Max coverage (-): 0

Region: chr9 103968800-103968810. Max. coverage (+): 0. Max coverage (-): 0

Region: chr9 103968811-103968821. Max. coverage (+): 0. Max coverage (-): 0

Region: chr9 103968822-103968831. Max. coverage (+): 0. Max coverage (-): 0

Region: chr9 103968832-103968842. Max. coverage (+): 0. Max coverage (-): 0

Region: chr9 103968843-103968853. Max. coverage (+): 0. Max coverage (-): 0

Region: chr9 103968854-103968864. Max. coverage (+): 0. Max coverage (-): 0

Region: chr9 103968865-103968875. Max. coverage (+): 0. Max coverage (-): 0

Region: chr9 103968876-103968886. Max. coverage (+): 0. Max coverage (-): 0

Region: chr9 103968887-103968896. Max. coverage (+): 0. Max coverage (-): 0

Region: chr9 103968897-103968907. Max. coverage (+): 0. Max coverage (-): 0

Region: chr9 103968908-103968918. Max. coverage (+): 0. Max coverage (-): 1

Region: chr9 103968919-103968929. Max. coverage (+): 0. Max coverage (-): 4.07

Region: chr9 103968930-103968940. Max. coverage (+): 0. Max coverage (-): 3.07

Region: chr9 103968941-103968951. Max. coverage (+): 0. Max coverage (-): 0

Region: chr9 103968952-103968961. Max. coverage (+): 0. Max coverage (-): 0

Region: chr9 103968962-103968972. Max. coverage (+): 0. Max coverage (-): 0

Region: chr9 103968973-103968983. Max. coverage (+): 0. Max coverage (-): 0

Region: chr9 103968984-103968994. Max. coverage (+): 0. Max coverage (-): 0

Region: chr9 103968995-103969005. Max. coverage (+): 0. Max coverage (-): 0

Region: chr9 103969006-103969016. Max. coverage (+): 0. Max coverage (-): 0

Region: chr9 103969017-103969026. Max. coverage (+): 0. Max coverage (-): 0

Region: chr9 103969027-103969037. Max. coverage (+): 0. Max coverage (-): 0

Region: chr9 103969038-103969048. Max. coverage (+): 0. Max coverage (-): 0

Region: chr9 103969049-103969059. Max. coverage (+): 0. Max coverage (-): 0

Region: chr9 103969060-103969070. Max. coverage (+): 0. Max coverage (-): 0

Region: chr9 103969071-103969081. Max. coverage (+): 0. Max coverage (-): 0

Region: chr9 103969082-103969091. Max. coverage (+): 0. Max coverage (-): 4.74

Region: chr9 103969092-103969102. Max. coverage (+): 0. Max coverage (-): 4.74

Region: chr9 103969103-103969113. Max. coverage (+): 0. Max coverage (-): 0

Region: chr9 103969114-103969124. Max. coverage (+): 0. Max coverage (-): 0

Region: chr9 103969125-103969135. Max. coverage (+): 0. Max coverage (-): 0

Region: chr9 103969136-103969146. Max. coverage (+): 0. Max coverage (-): 0

Region: chr9 103969147-103969156. Max. coverage (+): 0. Max coverage (-): 0

Region: chr9 103969157-103969167. Max. coverage (+): 0. Max coverage (-): 0

Region: chr9 103969168-103969178. Max. coverage (+): 0. Max coverage (-): 0

Region: chr9 103969179-103969189. Max. coverage (+): 0. Max coverage (-): 0

Region: chr9 103969190-103969200. Max. coverage (+): 0. Max coverage (-): 3.65

Region: chr9 103969201-103969211. Max. coverage (+): 0. Max coverage (-): 3.65

Region: chr9 103969212-103969221. Max. coverage (+): 0. Max coverage (-): 4.74

Region: chr9 103969222-103969232. Max. coverage (+): 0. Max coverage (-): 4.74

Region: chr9 103969233-103969243. Max. coverage (+): 0. Max coverage (-): 0

Region: chr9 103969244-103969254. Max. coverage (+): 0. Max coverage (-): 0

Region: chr9 103969255-103969265. Max. coverage (+): 0. Max coverage (-): 0

Region: chr9 103969266-103969276. Max. coverage (+): 0. Max coverage (-): 0

Region: chr9 103969277-103969286. Max. coverage (+): 0. Max coverage (-): 0

Region: chr9 103969287-103969297. Max. coverage (+): 0. Max coverage (-): 0

Region: chr9 103969298-103969308. Max. coverage (+): 0. Max coverage (-): 0

Region: chr9 103969309-103969319. Max. coverage (+): 0. Max coverage (-): 0

Region: chr9 103969320-103969330. Max. coverage (+): 0. Max coverage (-): 0

Region: chr9 103969331-103969341. Max. coverage (+): 0. Max coverage (-): 0

Region: chr9 103969342-103969351. Max. coverage (+): 0. Max coverage (-): 0

Region: chr9 103969352-103969362. Max. coverage (+): 0. Max coverage (-): 0

Region: chr9 103969363-103969373. Max. coverage (+): 0. Max coverage (-): 0

Region: chr9 103969374-103969384. Max. coverage (+): 0. Max coverage (-): 0

Region: chr9 103969385-103969395. Max. coverage (+): 0. Max coverage (-): 0

Region: chr9 103969396-103969406. Max. coverage (+): 0. Max coverage (-): 0

Region: chr9 103969407-103969416. Max. coverage (+): 0. Max coverage (-): 0

Region: chr9 103969417-103969427. Max. coverage (+): 0. Max coverage (-): 0

Region: chr9 103969428-103969438. Max. coverage (+): 0. Max coverage (-): 0

Region: chr9 103969439-103969449. Max. coverage (+): 0. Max coverage (-): 0

Region: chr9 103969450-103969460. Max. coverage (+): 0. Max coverage (-): 0

Region: chr9 103969461-103969471. Max. coverage (+): 0. Max coverage (-): 0

Region: chr9 103969472-103969481. Max. coverage (+): 0. Max coverage (-): 0

Region: chr9 103969482-103969492. Max. coverage (+): 0. Max coverage (-): 0

Region: chr9 103969493-103969503. Max. coverage (+): 0. Max coverage (-): 0

Region: chr9 103969504-103969514. Max. coverage (+): 0. Max coverage (-): 0

Region: chr9 103969515-103969525. Max. coverage (+): 0. Max coverage (-): 0

Region: chr9 103969526-103969536. Max. coverage (+): 0. Max coverage (-): 1.69

Region: chr9 103969537-103969546. Max. coverage (+): 0. Max coverage (-): 0

Region: chr9 103969547-103969557. Max. coverage (+): 0. Max coverage (-): 0

Region: chr9 103969558-103969568. Max. coverage (+): 0. Max coverage (-): 0

Region: chr9 103969569-103969579. Max. coverage (+): 0. Max coverage (-): 0

Region: chr9 103969580-103969590. Max. coverage (+): 0. Max coverage (-): 5.14

Region: chr9 103969591-103969601. Max. coverage (+): 0. Max coverage (-): 5.14

Region: chr9 103969602-103969611. Max. coverage (+): 0. Max coverage (-): 0

Region: chr9 103969612-103969622. Max. coverage (+): 0. Max coverage (-): 0

Region: chr9 103969623-103969633. Max. coverage (+): 0. Max coverage (-): 0

Region: chr9 103969634-103969644. Max. coverage (+): 0. Max coverage (-): 0

Region: chr9 103969645-103969655. Max. coverage (+): 0. Max coverage (-): 0

Region: chr9 103969656-103969666. Max. coverage (+): 0. Max coverage (-): 0

Region: chr9 103969667-103969676. Max. coverage (+): 0. Max coverage (-): 0.6

Region: chr9 103969677-103969687. Max. coverage (+): 0. Max coverage (-): 0.6

Region: chr9 103969688-103969698. Max. coverage (+): 0. Max coverage (-): 0

Region: chr9 103969699-103969709. Max. coverage (+): 0. Max coverage (-): 0

Region: chr9 103969710-103969720. Max. coverage (+): 0. Max coverage (-): 0

Region: chr9 103969721-103969731. Max. coverage (+): 0. Max coverage (-): 0

Region: chr9 103969732-103969741. Max. coverage (+): 0. Max coverage (-): 0

Region: chr9 103969742-103969752. Max. coverage (+): 0. Max coverage (-): 0

Region: chr9 103969753-103969763. Max. coverage (+): 0. Max coverage (-): 0

Region: chr9 103969764-103969774. Max. coverage (+): 7.22. Max coverage (-): 0

Region: chr9 103969775-103969785. Max. coverage (+): 7.22. Max coverage (-): 0

Region: chr9 103969786-103969796. Max. coverage (+): 0. Max coverage (-): 0

Region: chr9 103969797-103969806. Max. coverage (+): 0. Max coverage (-): 0.84

Region: chr9 103969807-103969817. Max. coverage (+): 0. Max coverage (-): 0

Region: chr9 103969818-103969828. Max. coverage (+): 0. Max coverage (-): 0

Region: chr9 103969829-103969839. Max. coverage (+): 0. Max coverage (-): 0

Region: chr9 103969840-103969850. Max. coverage (+): 0. Max coverage (-): 0

Region: chr9 103969851-103969861. Max. coverage (+): 0. Max coverage (-): 0

Region: chr9 103969862-103969871. Max. coverage (+): 0. Max coverage (-): 0

Region: chr9 103969872-103969882. Max. coverage (+): 0. Max coverage (-): 0

Region: chr9 103969883-103969893. Max. coverage (+): 0. Max coverage (-): 0

Region: chr9 103969894-103969904. Max. coverage (+): 0. Max coverage (-): 0

Region: chr9 103969905-103969915. Max. coverage (+): 0. Max coverage (-): 0

Region: chr9 103969916-103969926. Max. coverage (+): 0. Max coverage (-): 0

Region: chr9 103969927-103969936. Max. coverage (+): 0. Max coverage (-): 0

Region: chr9 103969937-103969947. Max. coverage (+): 0. Max coverage (-): 0

Region: chr9 103969948-103969958. Max. coverage (+): 0. Max coverage (-): 0

Region: chr9 103969959-103969969. Max. coverage (+): 0. Max coverage (-): 0

Region: chr9 103969970-103969980. Max. coverage (+): 0. Max coverage (-): 0

Region: chr9 103969981-103969991. Max. coverage (+): 0. Max coverage (-): 0

Region: chr9 103969992-103970001. Max. coverage (+): 0. Max coverage (-): 0

Region: chr9 103970002-103970012. Max. coverage (+): 1.46. Max coverage (-): 0

Region: chr9 103970013-103970023. Max. coverage (+): 0. Max coverage (-): 7.34

Region: chr9 103970024-103970034. Max. coverage (+): 0. Max coverage (-): 7.34

Region: chr9 103970035-103970045. Max. coverage (+): 0. Max coverage (-): 0

Region: chr9 103970046-103970056. Max. coverage (+): 0. Max coverage (-): 0

Region: chr9 103970057-103970066. Max. coverage (+): 0. Max coverage (-): 0

Region: chr9 103970067-103970077. Max. coverage (+): 0. Max coverage (-): 0

Region: chr9 103970078-103970088. Max. coverage (+): 0. Max coverage (-): 0

Region: chr9 103970089-103970099. Max. coverage (+): 0. Max coverage (-): 0

Region: chr9 103970100-103970110. Max. coverage (+): 0. Max coverage (-): 0

Region: chr9 103970111-103970121. Max. coverage (+): 0. Max coverage (-): 0

Region: chr9 103970122-103970131. Max. coverage (+): 0. Max coverage (-): 0

Region: chr9 103970132-103970142. Max. coverage (+): 0. Max coverage (-): 0

Region: chr9 103970143-103970153. Max. coverage (+): 0. Max coverage (-): 0

Region: chr9 103970154-103970164. Max. coverage (+): 0. Max coverage (-): 0

Region: chr9 103970165-103970175. Max. coverage (+): 0. Max coverage (-): 0

Region: chr9 103970176-103970186. Max. coverage (+): 0. Max coverage (-): 0

Region: chr9 103970187-103970196. Max. coverage (+): 0. Max coverage (-): 0

Region: chr9 103970197-103970207. Max. coverage (+): 0. Max coverage (-): 0

Region: chr9 103970208-103970218. Max. coverage (+): 0. Max coverage (-): 0

Region: chr9 103970219-103970229. Max. coverage (+): 0. Max coverage (-): 0

Region: chr9 103970230-103970240. Max. coverage (+): 0. Max coverage (-): 0

Region: chr9 103970241-103970251. Max. coverage (+): 0. Max coverage (-): 0

Region: chr9 103970252-103970261. Max. coverage (+): 0. Max coverage (-): 1.41

Region: chr9 103970262-103970272. Max. coverage (+): 1.87. Max coverage (-): 1.41

Region: chr9 103970273-103970283. Max. coverage (+): 1.87. Max coverage (-): 0

Region: chr9 103970284-103970294. Max. coverage (+): 0. Max coverage (-): 0

Region: chr9 103970295-103970305. Max. coverage (+): 0. Max coverage (-): 0

Region: chr9 103970306-103970316. Max. coverage (+): 0. Max coverage (-): 0

Region: chr9 103970317-103970326. Max. coverage (+): 0. Max coverage (-): 0

Region: chr9 103970327-103970337. Max. coverage (+): 0. Max coverage (-): 0

Region: chr9 103970338-103970348. Max. coverage (+): 0. Max coverage (-): 0

Region: chr9 103970349-103970359. Max. coverage (+): 0. Max coverage (-): 0

Region: chr9 103970360-103970370. Max. coverage (+): 0. Max coverage (-): 0

Region: chr9 103970371-103970381. Max. coverage (+): 0. Max coverage (-): 0

Region: chr9 103970382-103970391. Max. coverage (+): 0. Max coverage (-): 0

Region: chr9 103970392-103970402. Max. coverage (+): 0. Max coverage (-): 0

Region: chr9 103970403-103970413. Max. coverage (+): 0. Max coverage (-): 0

Region: chr9 103970414-103970424. Max. coverage (+): 0. Max coverage (-): 0

Region: chr9 103970425-103970435. Max. coverage (+): 0. Max coverage (-): 0

Region: chr9 103970436-103970446. Max. coverage (+): 0. Max coverage (-): 0

Region: chr9 103970447-103970456. Max. coverage (+): 0. Max coverage (-): 0

Region: chr9 103970457-103970467. Max. coverage (+): 0. Max coverage (-): 0

Region: chr9 103970468-103970478. Max. coverage (+): 0. Max coverage (-): 0

Region: chr9 103970479-103970489. Max. coverage (+): 0. Max coverage (-): 0

Region: chr9 103970490-103970500. Max. coverage (+): 0. Max coverage (-): 0

Region: chr9 103970501-103970511. Max. coverage (+): 0. Max coverage (-): 0

Region: chr9 103970512-103970521. Max. coverage (+): 0. Max coverage (-): 0

Region: chr9 103970522-103970532. Max. coverage (+): 0. Max coverage (-): 0

Region: chr9 103970533-103970543. Max. coverage (+): 0. Max coverage (-): 0

Region: chr9 103970544-103970554. Max. coverage (+): 0. Max coverage (-): 0

Region: chr9 103970555-103970565. Max. coverage (+): 0. Max coverage (-): 0

Region: chr9 103970566-103970576. Max. coverage (+): 0. Max coverage (-): 0

Region: chr9 103970577-103970586. Max. coverage (+): 0. Max coverage (-): 0

Region: chr9 103970587-103970597. Max. coverage (+): 0. Max coverage (-): 0

Region: chr9 103970598-103970608. Max. coverage (+): 0. Max coverage (-): 0

Region: chr9 103970609-103970619. Max. coverage (+): 0. Max coverage (-): 0

Region: chr9 103970620-103970630. Max. coverage (+): 0. Max coverage (-): 3.51

Region: chr9 103970631-103970641. Max. coverage (+): 0. Max coverage (-): 11.67

Region: chr9 103970642-103970651. Max. coverage (+): 0. Max coverage (-): 11.67

Region: chr9 103970652-103970662. Max. coverage (+): 0. Max coverage (-): 0

Region: chr9 103970663-103970673. Max. coverage (+): 0. Max coverage (-): 0

Region: chr9 103970674-103970684. Max. coverage (+): 0. Max coverage (-): 0

Region: chr9 103970685-103970695. Max. coverage (+): 0. Max coverage (-): 0

Region: chr9 103970696-103970706. Max. coverage (+): 0. Max coverage (-): 0

Region: chr9 103970707-103970716. Max. coverage (+): 0. Max coverage (-): 0

Region: chr9 103970717-103970727. Max. coverage (+): 0. Max coverage (-): 0

Region: chr9 103970728-103970738. Max. coverage (+): 0. Max coverage (-): 0

Region: chr9 103970739-103970749. Max. coverage (+): 0. Max coverage (-): 0

Region: chr9 103970750-103970760. Max. coverage (+): 0. Max coverage (-): 0

Region: chr9 103970761-103970771. Max. coverage (+): 0. Max coverage (-): 0

Region: chr9 103970772-103970781. Max. coverage (+): 0. Max coverage (-): 0

Region: chr9 103970782-103970792. Max. coverage (+): 0. Max coverage (-): 0

Region: chr9 103970793-103970803. Max. coverage (+): 0. Max coverage (-): 1.03

Region: chr9 103970804-103970814. Max. coverage (+): 0. Max coverage (-): 0

Region: chr9 103970815-103970825. Max. coverage (+): 0. Max coverage (-): 0

Region: chr9 103970826-103970836. Max. coverage (+): 0. Max coverage (-): 0

Region: chr9 103970837-103970846. Max. coverage (+): 0. Max coverage (-): 0

Region: chr9 103970847-103970857. Max. coverage (+): 0. Max coverage (-): 0

Region: chr9 103970858-103970868. Max. coverage (+): 0. Max coverage (-): 0

Region: chr9 103970869-103970879. Max. coverage (+): 0. Max coverage (-): 0

Region: chr9 103970880-103970890. Max. coverage (+): 0. Max coverage (-): 1.57

Region: chr9 103970891-103970901. Max. coverage (+): 0. Max coverage (-): 1.57

Region: chr9 103970902-103970911. Max. coverage (+): 0. Max coverage (-): 0

Region: chr9 103970912-103970922. Max. coverage (+): 0. Max coverage (-): 0

Region: chr9 103970923-103970933. Max. coverage (+): 0. Max coverage (-): 0

Region: chr9 103970934-103970944. Max. coverage (+): 0. Max coverage (-): 0

Region: chr9 103970945-103970955. Max. coverage (+): 0. Max coverage (-): 0

Region: chr9 103970956-103970966. Max. coverage (+): 0. Max coverage (-): 0

Region: chr9 103970967-103970976. Max. coverage (+): 0. Max coverage (-): 0

Region: chr9 103970977-103970987. Max. coverage (+): 0. Max coverage (-): 1.74

Region: chr9 103970988-103970998. Max. coverage (+): 0. Max coverage (-): 0

Region: chr9 103970999-103971009. Max. coverage (+): 0. Max coverage (-): 0

Region: chr9 103971010-103971020. Max. coverage (+): 0. Max coverage (-): 0

Region: chr9 103971021-103971031. Max. coverage (+): 0. Max coverage (-): 0

Region: chr9 103971032-103971041. Max. coverage (+): 0. Max coverage (-): 0

Region: chr9 103971042-103971052. Max. coverage (+): 0. Max coverage (-): 0

Region: chr9 103971053-103971063. Max. coverage (+): 0. Max coverage (-): 0

Region: chr9 103971064-103971074. Max. coverage (+): 0. Max coverage (-): 0

Region: chr9 103971075-103971085. Max. coverage (+): 0. Max coverage (-): 0

Region: chr9 103971086-103971096. Max. coverage (+): 0. Max coverage (-): 0

Region: chr9 103971097-103971107. Max. coverage (+): 0. Max coverage (-): 0

Region: chr9 103971108-103971117. Max. coverage (+): 0. Max coverage (-): 0

Region: chr9 103971118-103971128. Max. coverage (+): 0. Max coverage (-): 0

Region: chr9 103971129-103971139. Max. coverage (+): 0. Max coverage (-): 0

Region: chr9 103971140-103971150. Max. coverage (+): 0. Max coverage (-): 0

Region: chr9 103971151-103971161. Max. coverage (+): 0. Max coverage (-): 0

Region: chr9 103971162-103971172. Max. coverage (+): 0. Max coverage (-): 0

Region: chr9 103971173-103971182. Max. coverage (+): 0. Max coverage (-): 0

Region: chr9 103971183-103971193. Max. coverage (+): 0. Max coverage (-): 0

Region: chr9 103971194-103971204. Max. coverage (+): 0. Max coverage (-): 0

Region: chr9 103971205-103971215. Max. coverage (+): 0. Max coverage (-): 0

Region: chr9 103971216-103971226. Max. coverage (+): 0. Max coverage (-): 0

Region: chr9 103971227-103971237. Max. coverage (+): 0. Max coverage (-): 0

Region: chr9 103971238-103971247. Max. coverage (+): 0. Max coverage (-): 0

Region: chr9 103971248-103971258. Max. coverage (+): 0. Max coverage (-): 0

Region: chr9 103971259-103971269. Max. coverage (+): 0. Max coverage (-): 0

Region: chr9 103971270-103971280. Max. coverage (+): 0. Max coverage (-): 0

Region: chr9 103971281-103971291. Max. coverage (+): 0. Max coverage (-): 0

Region: chr9 103971292-103971302. Max. coverage (+): 0. Max coverage (-): 0

Region: chr9 103971303-103971312. Max. coverage (+): 0. Max coverage (-): 0

Region: chr9 103971313-103971323. Max. coverage (+): 0. Max coverage (-): 0

Region: chr9 103971324-103971334. Max. coverage (+): 0. Max coverage (-): 0

Region: chr9 103971335-103971345. Max. coverage (+): 0. Max coverage (-): 0

Region: chr9 103971346-103971356. Max. coverage (+): 0. Max coverage (-): 0

Region: chr9 103971357-103971367. Max. coverage (+): 0. Max coverage (-): 0

Region: chr9 103971368-103971377. Max. coverage (+): 0. Max coverage (-): 0

Region: chr9 103971378-103971388. Max. coverage (+): 0. Max coverage (-): 0

Region: chr9 103971389-103971399. Max. coverage (+): 0. Max coverage (-): 0

Region: chr9 103971400-103971410. Max. coverage (+): 0. Max coverage (-): 0

Region: chr9 103971411-103971421. Max. coverage (+): 0. Max coverage (-): 0

Region: chr9 103971422-103971432. Max. coverage (+): 0. Max coverage (-): 0

Region: chr9 103971433-103971442. Max. coverage (+): 0. Max coverage (-): 0

Region: chr9 103971443-103971453. Max. coverage (+): 0. Max coverage (-): 0

Region: chr9 103971454-103971464. Max. coverage (+): 0. Max coverage (-): 0

Region: chr9 103971465-103971475. Max. coverage (+): 0. Max coverage (-): 0

Region: chr9 103971476-103971486. Max. coverage (+): 0. Max coverage (-): 0

Region: chr9 103971487-103971497. Max. coverage (+): 0. Max coverage (-): 0

Region: chr9 103971498-103971507. Max. coverage (+): 0. Max coverage (-): 0

Region: chr9 103971508-103971518. Max. coverage (+): 0. Max coverage (-): 4.73

Region: chr9 103971519-103971529. Max. coverage (+): 0. Max coverage (-): 10.82

Region: chr9 103971530-103971540. Max. coverage (+): 0. Max coverage (-): 5.09

Region: chr9 103971541-103971551. Max. coverage (+): 0. Max coverage (-): 5.09

Region: chr9 103971552-103971562. Max. coverage (+): 0. Max coverage (-): 0

Region: chr9 103971563-103971572. Max. coverage (+): 0. Max coverage (-): 2.26

Region: chr9 103971573-103971583. Max. coverage (+): 0. Max coverage (-): 0

Region: chr9 103971584-103971594. Max. coverage (+): 0. Max coverage (-): 0

Region: chr9 103971595-103971605. Max. coverage (+): 0. Max coverage (-): 0

Region: chr9 103971606-103971616. Max. coverage (+): 0. Max coverage (-): 0

Region: chr9 103971617-103971627. Max. coverage (+): 0. Max coverage (-): 0

Region: chr9 103971628-103971637. Max. coverage (+): 0. Max coverage (-): 0

Region: chr9 103971638-103971648. Max. coverage (+): 0. Max coverage (-): 0

Region: chr9 103971649-103971659. Max. coverage (+): 0. Max coverage (-): 0

Region: chr9 103971660-103971670. Max. coverage (+): 0. Max coverage (-): 0

Region: chr9 103971671-103971681. Max. coverage (+): 0. Max coverage (-): 2.08

Region: chr9 103971682-103971692. Max. coverage (+): 0. Max coverage (-): 2.08

Region: chr9 103971693-103971702. Max. coverage (+): 0. Max coverage (-): 0

Region: chr9 103971703-103971713. Max. coverage (+): 0. Max coverage (-): 0

Region: chr9 103971714-103971724. Max. coverage (+): 0. Max coverage (-): 0

Region: chr9 103971725-103971735. Max. coverage (+): 0. Max coverage (-): 0

Region: chr9 103971736-103971746. Max. coverage (+): 0. Max coverage (-): 0

Region: chr9 103971747-103971757. Max. coverage (+): 0. Max coverage (-): 1.05

Region: chr9 103971758-103971767. Max. coverage (+): 0. Max coverage (-): 12.96

Region: chr9 103971768-103971778. Max. coverage (+): 0. Max coverage (-): 8.26

Region: chr9 103971779-103971789. Max. coverage (+): 0. Max coverage (-): 0

Region: chr9 103971790-103971800. Max. coverage (+): 0. Max coverage (-): 0

Region: chr9 103971801-103971811. Max. coverage (+): 0. Max coverage (-): 0

Region: chr9 103971812-103971822. Max. coverage (+): 0. Max coverage (-): 0

Region: chr9 103971823-103971832. Max. coverage (+): 0. Max coverage (-): 0

Region: chr9 103971833-103971843. Max. coverage (+): 0. Max coverage (-): 0

Region: chr9 103971844-103971854. Max. coverage (+): 0. Max coverage (-): 0

Region: chr9 103971855-103971865. Max. coverage (+): 0. Max coverage (-): 0

Region: chr9 103971866-103971876. Max. coverage (+): 0. Max coverage (-): 0

Region: chr9 103971877-103971887. Max. coverage (+): 0. Max coverage (-): 1.16

Region: chr9 103971888-103971897. Max. coverage (+): 0. Max coverage (-): 1.66

Region: chr9 103971898-103971908. Max. coverage (+): 0. Max coverage (-): 3.17

Region: chr9 103971909-103971919. Max. coverage (+): 0. Max coverage (-): 0.98

Region: chr9 103971920-103971930. Max. coverage (+): 0. Max coverage (-): 0

Region: chr9 103971931-103971941. Max. coverage (+): 0. Max coverage (-): 0

Region: chr9 103971942-103971952. Max. coverage (+): 0. Max coverage (-): 0

Region: chr9 103971953-103971962. Max. coverage (+): 0. Max coverage (-): 0

Region: chr9 103971963-103971973. Max. coverage (+): 0. Max coverage (-): 0

Region: chr9 103971974-103971984. Max. coverage (+): 0. Max coverage (-): 0

Region: chr9 103971985-103971995. Max. coverage (+): 0. Max coverage (-): 0

Region: chr9 103971996-103972006. Max. coverage (+): 0. Max coverage (-): 0

Region: chr9 103972007-103972017. Max. coverage (+): 0. Max coverage (-): 0

Region: chr9 103972018-103972027. Max. coverage (+): 0. Max coverage (-): 0

Region: chr9 103972028-103972038. Max. coverage (+): 0. Max coverage (-): 0

Region: chr9 103972039-103972049. Max. coverage (+): 0. Max coverage (-): 0

Region: chr9 103972050-103972060. Max. coverage (+): 0. Max coverage (-): 0

Region: chr9 103972061-103972071. Max. coverage (+): 0. Max coverage (-): 0

Region: chr9 103972072-103972082. Max. coverage (+): 0. Max coverage (-): 0

Region: chr9 103972083-103972092. Max. coverage (+): 0. Max coverage (-): 0

Region: chr9 103972093-103972103. Max. coverage (+): 0. Max coverage (-): 0

Region: chr9 103972104-103972114. Max. coverage (+): 0. Max coverage (-): 0

Region: chr9 103972115-103972125. Max. coverage (+): 0. Max coverage (-): 0

Region: chr9 103972126-103972136. Max. coverage (+): 0. Max coverage (-): 0

Region: chr9 103972137-103972147. Max. coverage (+): 0. Max coverage (-): 0

Region: chr9 103972148-103972157. Max. coverage (+): 0. Max coverage (-): 0

Region: chr9 103972158-103972168. Max. coverage (+): 0. Max coverage (-): 0

Region: chr9 103972169-103972179. Max. coverage (+): 0. Max coverage (-): 0

Region: chr9 103972180-103972190. Max. coverage (+): 0. Max coverage (-): 0

Region: chr9 103972191-103972201. Max. coverage (+): 0. Max coverage (-): 0

Region: chr9 103972202-103972212. Max. coverage (+): 0. Max coverage (-): 5.01

Region: chr9 103972213-103972222. Max. coverage (+): 0. Max coverage (-): 5.01

Region: chr9 103972223-103972233. Max. coverage (+): 0. Max coverage (-): 0

Region: chr9 103972234-103972244. Max. coverage (+): 0. Max coverage (-): 0

Region: chr9 103972245-103972255. Max. coverage (+): 0. Max coverage (-): 0

Region: chr9 103972256-103972266. Max. coverage (+): 0. Max coverage (-): 0

Region: chr9 103972267-103972277. Max. coverage (+): 0. Max coverage (-): 0

Region: chr9 103972278-103972287. Max. coverage (+): 0. Max coverage (-): 0

Region: chr9 103972288-103972298. Max. coverage (+): 0. Max coverage (-): 0

Region: chr9 103972299-103972309. Max. coverage (+): 0. Max coverage (-): 0

Region: chr9 103972310-103972320. Max. coverage (+): 0. Max coverage (-): 0

Region: chr9 103972321-103972331. Max. coverage (+): 0. Max coverage (-): 0

Region: chr9 103972332-103972342. Max. coverage (+): 0. Max coverage (-): 0

Region: chr9 103972343-103972352. Max. coverage (+): 0. Max coverage (-): 0

Region: chr9 103972353-103972363. Max. coverage (+): 0. Max coverage (-): 0

Region: chr9 103972364-103972374. Max. coverage (+): 0. Max coverage (-): 0

Region: chr9 103972375-103972385. Max. coverage (+): 0. Max coverage (-): 0

Region: chr9 103972386-103972396. Max. coverage (+): 0. Max coverage (-): 0

Region: chr9 103972397-103972407. Max. coverage (+): 0. Max coverage (-): 0

Region: chr9 103972408-103972417. Max. coverage (+): 0. Max coverage (-): 5.66

Region: chr9 103972418-103972428. Max. coverage (+): 0. Max coverage (-): 5.66

Region: chr9 103972429-103972439. Max. coverage (+): 0. Max coverage (-): 0

Region: chr9 103972440-. Max. coverage (+): 0. Max coverage (-): 0

RepeatMasker Color Code

**+**

100-98% Identity

<98-95% Identity

<95-90% Identity

<90-85% Identity

<85-80% Identity

<80-75% Identity

<75-70% Identity

<70% Identity

**-**

Gene Set Color Code

**+**

Gene

Pseudogene

**-**

Topology/Coverage Color Code

Coverage Plus Strand

Coverage Minus Strand

Mainstrand: Plus

Mainstrand: Minus

Complementary Strand

Flanking Region  
(if option -flank >0)

Gene Set Annotation  

**1. KIF25 (protein coding, ENSBTAG00000007694) Tr:00000010118 Ex:11**: 103969515-103969589 (+)  
**2. KIF25 (protein coding, ENSBTAG00000007694) Tr:00000010118 Ex:12**: 103971913-103972062 (+)

  
RepeatMasker Annotation  

**1. L1MC5**: 103967147-103967189 (-), Divergence to consensus: 13.9%  
**2. (CTG)n**: 103968010-103968036 (+), Divergence to consensus: 0%  
**3. BOV-A2**: 103968037-103968070 (-), Divergence to consensus: 0%  
**4. L1MC5**: 103968089-103968240 (+), Divergence to consensus: 34.3%  
**5. Bov-tA2**: 103969251-103969456 (+), Divergence to consensus: 8.7%  
**6. Bov-tA2**: 103969841-103969942 (-), Divergence to consensus: 35.8%  
**7. CHR-2A**: 103971218-103971454 (+), Divergence to consensus: 38.5%

  
Transcription Factor Binding Sites
