# Supplementary material for: Small RNA sequencing of cryopreserved semen from single bull revealed altered miRNAs and piRNAs expression between High- and Low-motile sperm populations
Source: BMC Genomics. 2017 Jan 4;18:14. doi: 10.1186/s12864-016-3394-7 (PMC5209821; doi:10.1186/s12864-016-3394-7)
Supplement: Additional file 4: — Details for each piRNA clusters found in Low Motile (LM) sperm fraction. Genes, repeats, transposable elements and transcription factors binding sites falling within the cluster regions were reported. (ZIP 1034 kb) [file 12864_2016_3394_MOESM4_ESM.zip › 1.html]

piRNA cluster 1


Predicted piRNA cluster no. 1     previous   next
  

Show proTRAC run info
Hide proTRAC run info

================================= proTRAC ====================================  
VERSION: 2.1                                    LAST MODIFIED: 06. October 2015  
  
Please cite:  
Rosenkranz D, Zischler H. proTRAC - a software for probabilistic piRNA cluster  
detection, visualization and analysis. 2012. BMC Bioinformatics 13:5.  
  
and (for proTRAC 2.0 and later):  
Rosenkranz D, Rudloff S, Bastuck K, Ketting RF, Zischler H. Tupaia small RNAs  
provide insights into function and evolution of RNAi-based transposon defense  
in mammals. 2015. RNA 21(5):911-922.  
  
Contact:  
David Rosenkranz  
Institute of Anthropology, small RNA group  
Johannes Gutenberg University Mainz  
email: rosenkranz@uni-mainz.de  
  
You can find the latest proTRAC version at:  
http://sourceforge.net/projects/protrac/files  
http://www.smallRNAgroup-mainz.de/software  
==============================================================================  
  
PARAMETERS:  
Map file: .............../storage/core/barbara/genhome/smallRNA/fertility/Sample\_not\_motile/pirna/Sample\_not\_motile\_26-33\_collapsed.fa.no-dust.map.weighted-10000-1000-b-0  
Genome file: ............/storage/core/barbara/genhome/smallRNA/fertility/Sample\_all/pirna/bt\_311\_chrY.fa  
RepeatMasker annotation: /storage/genomes/bt\_umd31/GCF\_000003055.6\_Bos\_taurus\_UMD\_3.1.1\_repeatMasker\_chr.out  
GeneSet:................./storage/core/barbara/genhome/smallRNA/fertility/Sample\_all/pirna/full.gtf  
  
Significant (p<=0.01) hit density will be calculated based  
on observed hit distribution.  
  
Sliding window size: ........................................ 5000 bp  
Sliding window increament: .................................. 1000 bp  
Normalize each hit by number of genomic hits: ............... 1 [0=no/1=yes]  
Normalize each hit by number of sequence reads: ............. 1 [0=no/1=yes]  
Normalize values (-> per million mapped reads): ............. 1 [0=no/1=yes]  
Min. fraction of hits with 1T(U) or 10A: .................... 0.75  
Alternatively: Min. fraction of hits with 1T(U) and 10A: .... 0.5  
Min. fraction of hits with typical piRNA length: ............ 0.75  
Typical piRNA length: ....................................... 26-33 nt  
Min. size of a piRNA cluster: ............................... 5000 bp.  
Min. number of hits (absolute): ............................. 0  
Min. number of hits (normalized): ........................... 0  
Min. fraction of hits on the mainstrand: .................... 0.75  
Top fraction of mapped sequences (in terms of read counts): . 1%  
Top fraction accounts for max. n% of sequence reads: ........ 90%  
Min. fraction of hits on each arm of a bidirectional cluster: 0.1  
Output image file for each cluster: ......................... 0 [0=no/1=yes]  
Output html file for each cluster: .......................... 1 [0=no/1=yes]  
Output a summary table: ..................................... 1 [0=no/1=yes]  
Output a FASTA file for each cluster (piRNA sequences): ..... 1 [0=no/1=yes]  
Output a FASTA file comprising cluster sequences: ........... 1 [0=no/1=yes]  
Search DNA motifs in clusters: .............................. 1 [0=no/1=yes]  
Output flanking sequences: +/- .............................. 0 bp  
Output ~.pTi file: .......................................... 1 [0=no/1=yes]  
==============================================================================  
  
  
Genome size (without gaps): ............ 2678902517 bp  
Gaps (N/X/-): .......................... 53837044 bp  
Mapped reads: .......................... 738059667487  
Non-identical sequences: ............... 277001  
Genomic hits: .......................... 533816  
Significant densitiy of mapped reads: .. 15118061 reads/kb

Show proTRAC cluster info
Hide proTRAC cluster info

|  |  |
| --- | --- |
| Location | chr1 |
| Coordinates | 80730867-80736004 |
| Size [bp] | 5138 |
| Sequence hit loci | 98 |
| Mapped reads (normalized) | 269835101 |
| Mapped reads (normalized) per kb | 52517536.2 |
| Normalized reads with 1T (1U) | 96.2% |
| Normalized reads with 10A | 30.9% |
| Normalized reads with length 26-33 nt | 100% |
| Normalized reads on the main strand(s) | 100% |
| Predicted directionality | mono:plus |

100%

0%

1T (1U)  
reads

10A reads

26-33 nt  
reads

reads on mainstrand

**Either the amount of reads with 1T (1U) OR 10A has to exceed 75% (set with option: -1Tor10A)  
Alternatively the amount of reads with 1T (1U) AND 10A has to exceed 50% (set with option: -1Tand10A)  
Minimum amount of reads with preferred size is 75% (set with option: -pisize)  
Minimum amount of reads on the main strand(s) is 75% (set with option: -clstrand)**

Show read coverage
Hide read coverage

WHAT DO I SEE HERE?  
This chart shows the location of mapped sequence reads within a predicted piRNA cluster. The color refers to the number of genomic hits produced by the sequence read in question. A dark red bar indicates that this sequence read produces many other hits elsewhere in the genome. Many adjacent red or yellow bars can indicate the presence of a multi-copy element such as transposons or rRNA genes. A dark green bar indicates that this sequence read maps uniquely to this locus.

1 hit

2-5 hits

6-10 hits

11-20 hits

21-50 hits

51-100 hits

> 100 hits

chr1

80730867

80736004

Gene Set

RepeatMasker

Mapped  
Reads

38.69

plus strand

minus strand

38.69

Region: chr1 1-80730872. Max. coverage (+): 2.9. Max coverage (-): 0

Region: chr1 80730873-80730882. Max. coverage (+): 2.9. Max coverage (-): 0

Region: chr1 80730883-80730892. Max. coverage (+): 0. Max coverage (-): 0

Region: chr1 80730893-80730902. Max. coverage (+): 0. Max coverage (-): 0

Region: chr1 80730903-80730913. Max. coverage (+): 0. Max coverage (-): 0

Region: chr1 80730914-80730923. Max. coverage (+): 0. Max coverage (-): 0

Region: chr1 80730924-80730933. Max. coverage (+): 0. Max coverage (-): 0

Region: chr1 80730934-80730944. Max. coverage (+): 0. Max coverage (-): 0

Region: chr1 80730945-80730954. Max. coverage (+): 0. Max coverage (-): 0

Region: chr1 80730955-80730964. Max. coverage (+): 0. Max coverage (-): 0

Region: chr1 80730965-80730974. Max. coverage (+): 0. Max coverage (-): 0

Region: chr1 80730975-80730985. Max. coverage (+): 0. Max coverage (-): 0

Region: chr1 80730986-80730995. Max. coverage (+): 0. Max coverage (-): 0

Region: chr1 80730996-80731005. Max. coverage (+): 0. Max coverage (-): 0

Region: chr1 80731006-80731016. Max. coverage (+): 0. Max coverage (-): 0

Region: chr1 80731017-80731026. Max. coverage (+): 0. Max coverage (-): 0

Region: chr1 80731027-80731036. Max. coverage (+): 0. Max coverage (-): 0

Region: chr1 80731037-80731046. Max. coverage (+): 0. Max coverage (-): 0

Region: chr1 80731047-80731057. Max. coverage (+): 0. Max coverage (-): 0

Region: chr1 80731058-80731067. Max. coverage (+): 0. Max coverage (-): 0

Region: chr1 80731068-80731077. Max. coverage (+): 0. Max coverage (-): 0

Region: chr1 80731078-80731087. Max. coverage (+): 0. Max coverage (-): 0

Region: chr1 80731088-80731098. Max. coverage (+): 0. Max coverage (-): 0

Region: chr1 80731099-80731108. Max. coverage (+): 0. Max coverage (-): 0

Region: chr1 80731109-80731118. Max. coverage (+): 0. Max coverage (-): 0

Region: chr1 80731119-80731129. Max. coverage (+): 0. Max coverage (-): 0

Region: chr1 80731130-80731139. Max. coverage (+): 0. Max coverage (-): 0

Region: chr1 80731140-80731149. Max. coverage (+): 0. Max coverage (-): 0

Region: chr1 80731150-80731159. Max. coverage (+): 0. Max coverage (-): 0

Region: chr1 80731160-80731170. Max. coverage (+): 0. Max coverage (-): 0

Region: chr1 80731171-80731180. Max. coverage (+): 0. Max coverage (-): 0

Region: chr1 80731181-80731190. Max. coverage (+): 0. Max coverage (-): 0

Region: chr1 80731191-80731200. Max. coverage (+): 0. Max coverage (-): 0

Region: chr1 80731201-80731211. Max. coverage (+): 0. Max coverage (-): 0

Region: chr1 80731212-80731221. Max. coverage (+): 0. Max coverage (-): 0

Region: chr1 80731222-80731231. Max. coverage (+): 0. Max coverage (-): 0

Region: chr1 80731232-80731242. Max. coverage (+): 0. Max coverage (-): 0

Region: chr1 80731243-80731252. Max. coverage (+): 0. Max coverage (-): 0

Region: chr1 80731253-80731262. Max. coverage (+): 0. Max coverage (-): 0

Region: chr1 80731263-80731272. Max. coverage (+): 0. Max coverage (-): 0

Region: chr1 80731273-80731283. Max. coverage (+): 0. Max coverage (-): 0

Region: chr1 80731284-80731293. Max. coverage (+): 0. Max coverage (-): 0

Region: chr1 80731294-80731303. Max. coverage (+): 0. Max coverage (-): 0

Region: chr1 80731304-80731314. Max. coverage (+): 0. Max coverage (-): 0

Region: chr1 80731315-80731324. Max. coverage (+): 0. Max coverage (-): 0

Region: chr1 80731325-80731334. Max. coverage (+): 0. Max coverage (-): 0

Region: chr1 80731335-80731344. Max. coverage (+): 0. Max coverage (-): 0

Region: chr1 80731345-80731355. Max. coverage (+): 0. Max coverage (-): 0

Region: chr1 80731356-80731365. Max. coverage (+): 0. Max coverage (-): 0

Region: chr1 80731366-80731375. Max. coverage (+): 0. Max coverage (-): 0

Region: chr1 80731376-80731385. Max. coverage (+): 0. Max coverage (-): 0

Region: chr1 80731386-80731396. Max. coverage (+): 0. Max coverage (-): 0

Region: chr1 80731397-80731406. Max. coverage (+): 0. Max coverage (-): 0

Region: chr1 80731407-80731416. Max. coverage (+): 0. Max coverage (-): 0

Region: chr1 80731417-80731427. Max. coverage (+): 0. Max coverage (-): 0

Region: chr1 80731428-80731437. Max. coverage (+): 0. Max coverage (-): 0

Region: chr1 80731438-80731447. Max. coverage (+): 0. Max coverage (-): 0

Region: chr1 80731448-80731457. Max. coverage (+): 0. Max coverage (-): 0

Region: chr1 80731458-80731468. Max. coverage (+): 0. Max coverage (-): 0

Region: chr1 80731469-80731478. Max. coverage (+): 0. Max coverage (-): 0

Region: chr1 80731479-80731488. Max. coverage (+): 0. Max coverage (-): 0

Region: chr1 80731489-80731498. Max. coverage (+): 0. Max coverage (-): 0

Region: chr1 80731499-80731509. Max. coverage (+): 0. Max coverage (-): 0

Region: chr1 80731510-80731519. Max. coverage (+): 0. Max coverage (-): 0

Region: chr1 80731520-80731529. Max. coverage (+): 0. Max coverage (-): 0

Region: chr1 80731530-80731540. Max. coverage (+): 0. Max coverage (-): 0

Region: chr1 80731541-80731550. Max. coverage (+): 0. Max coverage (-): 0

Region: chr1 80731551-80731560. Max. coverage (+): 0. Max coverage (-): 0

Region: chr1 80731561-80731570. Max. coverage (+): 0. Max coverage (-): 0

Region: chr1 80731571-80731581. Max. coverage (+): 0. Max coverage (-): 0

Region: chr1 80731582-80731591. Max. coverage (+): 0. Max coverage (-): 0

Region: chr1 80731592-80731601. Max. coverage (+): 0. Max coverage (-): 0

Region: chr1 80731602-80731612. Max. coverage (+): 0. Max coverage (-): 0

Region: chr1 80731613-80731622. Max. coverage (+): 0. Max coverage (-): 0

Region: chr1 80731623-80731632. Max. coverage (+): 0. Max coverage (-): 0

Region: chr1 80731633-80731642. Max. coverage (+): 0. Max coverage (-): 0

Region: chr1 80731643-80731653. Max. coverage (+): 0. Max coverage (-): 0

Region: chr1 80731654-80731663. Max. coverage (+): 0. Max coverage (-): 0

Region: chr1 80731664-80731673. Max. coverage (+): 0. Max coverage (-): 0

Region: chr1 80731674-80731683. Max. coverage (+): 0. Max coverage (-): 0

Region: chr1 80731684-80731694. Max. coverage (+): 0. Max coverage (-): 0

Region: chr1 80731695-80731704. Max. coverage (+): 0. Max coverage (-): 0

Region: chr1 80731705-80731714. Max. coverage (+): 0. Max coverage (-): 0

Region: chr1 80731715-80731725. Max. coverage (+): 0. Max coverage (-): 0

Region: chr1 80731726-80731735. Max. coverage (+): 0. Max coverage (-): 0

Region: chr1 80731736-80731745. Max. coverage (+): 0. Max coverage (-): 0

Region: chr1 80731746-80731755. Max. coverage (+): 0. Max coverage (-): 0

Region: chr1 80731756-80731766. Max. coverage (+): 7.19. Max coverage (-): 0

Region: chr1 80731767-80731776. Max. coverage (+): 0. Max coverage (-): 0

Region: chr1 80731777-80731786. Max. coverage (+): 0. Max coverage (-): 0

Region: chr1 80731787-80731796. Max. coverage (+): 0. Max coverage (-): 0

Region: chr1 80731797-80731807. Max. coverage (+): 0. Max coverage (-): 0

Region: chr1 80731808-80731817. Max. coverage (+): 0. Max coverage (-): 0

Region: chr1 80731818-80731827. Max. coverage (+): 0. Max coverage (-): 0

Region: chr1 80731828-80731838. Max. coverage (+): 0. Max coverage (-): 0

Region: chr1 80731839-80731848. Max. coverage (+): 0. Max coverage (-): 0

Region: chr1 80731849-80731858. Max. coverage (+): 0. Max coverage (-): 0

Region: chr1 80731859-80731868. Max. coverage (+): 0. Max coverage (-): 0

Region: chr1 80731869-80731879. Max. coverage (+): 0. Max coverage (-): 0

Region: chr1 80731880-80731889. Max. coverage (+): 0. Max coverage (-): 0

Region: chr1 80731890-80731899. Max. coverage (+): 0. Max coverage (-): 0

Region: chr1 80731900-80731910. Max. coverage (+): 0. Max coverage (-): 0

Region: chr1 80731911-80731920. Max. coverage (+): 0. Max coverage (-): 0

Region: chr1 80731921-80731930. Max. coverage (+): 0. Max coverage (-): 0

Region: chr1 80731931-80731940. Max. coverage (+): 0. Max coverage (-): 0

Region: chr1 80731941-80731951. Max. coverage (+): 0. Max coverage (-): 0

Region: chr1 80731952-80731961. Max. coverage (+): 0. Max coverage (-): 0

Region: chr1 80731962-80731971. Max. coverage (+): 0. Max coverage (-): 0

Region: chr1 80731972-80731981. Max. coverage (+): 0. Max coverage (-): 0

Region: chr1 80731982-80731992. Max. coverage (+): 0. Max coverage (-): 0

Region: chr1 80731993-80732002. Max. coverage (+): 0. Max coverage (-): 0

Region: chr1 80732003-80732012. Max. coverage (+): 0. Max coverage (-): 0

Region: chr1 80732013-80732023. Max. coverage (+): 0. Max coverage (-): 0

Region: chr1 80732024-80732033. Max. coverage (+): 0. Max coverage (-): 0

Region: chr1 80732034-80732043. Max. coverage (+): 0. Max coverage (-): 0

Region: chr1 80732044-80732053. Max. coverage (+): 0. Max coverage (-): 0

Region: chr1 80732054-80732064. Max. coverage (+): 0. Max coverage (-): 0

Region: chr1 80732065-80732074. Max. coverage (+): 0. Max coverage (-): 0

Region: chr1 80732075-80732084. Max. coverage (+): 0. Max coverage (-): 0

Region: chr1 80732085-80732094. Max. coverage (+): 0. Max coverage (-): 0

Region: chr1 80732095-80732105. Max. coverage (+): 0. Max coverage (-): 0

Region: chr1 80732106-80732115. Max. coverage (+): 0. Max coverage (-): 0

Region: chr1 80732116-80732125. Max. coverage (+): 0. Max coverage (-): 0

Region: chr1 80732126-80732136. Max. coverage (+): 0. Max coverage (-): 0

Region: chr1 80732137-80732146. Max. coverage (+): 0. Max coverage (-): 0

Region: chr1 80732147-80732156. Max. coverage (+): 0. Max coverage (-): 0

Region: chr1 80732157-80732166. Max. coverage (+): 0. Max coverage (-): 0

Region: chr1 80732167-80732177. Max. coverage (+): 0. Max coverage (-): 0

Region: chr1 80732178-80732187. Max. coverage (+): 0. Max coverage (-): 0

Region: chr1 80732188-80732197. Max. coverage (+): 0. Max coverage (-): 0

Region: chr1 80732198-80732208. Max. coverage (+): 0. Max coverage (-): 0

Region: chr1 80732209-80732218. Max. coverage (+): 0. Max coverage (-): 0

Region: chr1 80732219-80732228. Max. coverage (+): 0. Max coverage (-): 0

Region: chr1 80732229-80732238. Max. coverage (+): 0. Max coverage (-): 0

Region: chr1 80732239-80732249. Max. coverage (+): 0. Max coverage (-): 0

Region: chr1 80732250-80732259. Max. coverage (+): 0. Max coverage (-): 0

Region: chr1 80732260-80732269. Max. coverage (+): 0. Max coverage (-): 0

Region: chr1 80732270-80732279. Max. coverage (+): 0. Max coverage (-): 0

Region: chr1 80732280-80732290. Max. coverage (+): 0. Max coverage (-): 0

Region: chr1 80732291-80732300. Max. coverage (+): 0. Max coverage (-): 0

Region: chr1 80732301-80732310. Max. coverage (+): 0. Max coverage (-): 0

Region: chr1 80732311-80732321. Max. coverage (+): 0. Max coverage (-): 0

Region: chr1 80732322-80732331. Max. coverage (+): 0. Max coverage (-): 0

Region: chr1 80732332-80732341. Max. coverage (+): 0. Max coverage (-): 0

Region: chr1 80732342-80732351. Max. coverage (+): 0. Max coverage (-): 0

Region: chr1 80732352-80732362. Max. coverage (+): 0. Max coverage (-): 0

Region: chr1 80732363-80732372. Max. coverage (+): 0. Max coverage (-): 0

Region: chr1 80732373-80732382. Max. coverage (+): 0. Max coverage (-): 0

Region: chr1 80732383-80732392. Max. coverage (+): 0. Max coverage (-): 0

Region: chr1 80732393-80732403. Max. coverage (+): 0. Max coverage (-): 0

Region: chr1 80732404-80732413. Max. coverage (+): 0. Max coverage (-): 0

Region: chr1 80732414-80732423. Max. coverage (+): 0. Max coverage (-): 0

Region: chr1 80732424-80732434. Max. coverage (+): 0. Max coverage (-): 0

Region: chr1 80732435-80732444. Max. coverage (+): 0. Max coverage (-): 0

Region: chr1 80732445-80732454. Max. coverage (+): 0. Max coverage (-): 0

Region: chr1 80732455-80732464. Max. coverage (+): 0. Max coverage (-): 0

Region: chr1 80732465-80732475. Max. coverage (+): 0. Max coverage (-): 0

Region: chr1 80732476-80732485. Max. coverage (+): 0. Max coverage (-): 0

Region: chr1 80732486-80732495. Max. coverage (+): 0. Max coverage (-): 0

Region: chr1 80732496-80732506. Max. coverage (+): 0. Max coverage (-): 0

Region: chr1 80732507-80732516. Max. coverage (+): 0. Max coverage (-): 0

Region: chr1 80732517-80732526. Max. coverage (+): 0. Max coverage (-): 0

Region: chr1 80732527-80732536. Max. coverage (+): 0. Max coverage (-): 0

Region: chr1 80732537-80732547. Max. coverage (+): 0. Max coverage (-): 0

Region: chr1 80732548-80732557. Max. coverage (+): 12.85. Max coverage (-): 0

Region: chr1 80732558-80732567. Max. coverage (+): 2.57. Max coverage (-): 0

Region: chr1 80732568-80732577. Max. coverage (+): 0. Max coverage (-): 0

Region: chr1 80732578-80732588. Max. coverage (+): 0. Max coverage (-): 0

Region: chr1 80732589-80732598. Max. coverage (+): 0. Max coverage (-): 0

Region: chr1 80732599-80732608. Max. coverage (+): 0. Max coverage (-): 0

Region: chr1 80732609-80732619. Max. coverage (+): 0. Max coverage (-): 0

Region: chr1 80732620-80732629. Max. coverage (+): 0. Max coverage (-): 0

Region: chr1 80732630-80732639. Max. coverage (+): 0. Max coverage (-): 0

Region: chr1 80732640-80732649. Max. coverage (+): 0. Max coverage (-): 0

Region: chr1 80732650-80732660. Max. coverage (+): 0. Max coverage (-): 0

Region: chr1 80732661-80732670. Max. coverage (+): 0. Max coverage (-): 0

Region: chr1 80732671-80732680. Max. coverage (+): 0. Max coverage (-): 0

Region: chr1 80732681-80732690. Max. coverage (+): 0. Max coverage (-): 0

Region: chr1 80732691-80732701. Max. coverage (+): 0. Max coverage (-): 0

Region: chr1 80732702-80732711. Max. coverage (+): 0. Max coverage (-): 0

Region: chr1 80732712-80732721. Max. coverage (+): 0. Max coverage (-): 0

Region: chr1 80732722-80732732. Max. coverage (+): 0. Max coverage (-): 0

Region: chr1 80732733-80732742. Max. coverage (+): 0. Max coverage (-): 0

Region: chr1 80732743-80732752. Max. coverage (+): 0. Max coverage (-): 0

Region: chr1 80732753-80732762. Max. coverage (+): 0. Max coverage (-): 0

Region: chr1 80732763-80732773. Max. coverage (+): 2.79. Max coverage (-): 0

Region: chr1 80732774-80732783. Max. coverage (+): 38.69. Max coverage (-): 0

Region: chr1 80732784-80732793. Max. coverage (+): 0. Max coverage (-): 0

Region: chr1 80732794-80732804. Max. coverage (+): 2.07. Max coverage (-): 0

Region: chr1 80732805-80732814. Max. coverage (+): 2.07. Max coverage (-): 0

Region: chr1 80732815-80732824. Max. coverage (+): 0. Max coverage (-): 0

Region: chr1 80732825-80732834. Max. coverage (+): 10.63. Max coverage (-): 0

Region: chr1 80732835-80732845. Max. coverage (+): 20.36. Max coverage (-): 0

Region: chr1 80732846-80732855. Max. coverage (+): 6.93. Max coverage (-): 0

Region: chr1 80732856-80732865. Max. coverage (+): 0. Max coverage (-): 0

Region: chr1 80732866-80732875. Max. coverage (+): 0. Max coverage (-): 0

Region: chr1 80732876-80732886. Max. coverage (+): 0. Max coverage (-): 0

Region: chr1 80732887-80732896. Max. coverage (+): 0. Max coverage (-): 0

Region: chr1 80732897-80732906. Max. coverage (+): 0. Max coverage (-): 0

Region: chr1 80732907-80732917. Max. coverage (+): 0. Max coverage (-): 0

Region: chr1 80732918-80732927. Max. coverage (+): 0. Max coverage (-): 0

Region: chr1 80732928-80732937. Max. coverage (+): 0. Max coverage (-): 0

Region: chr1 80732938-80732947. Max. coverage (+): 0. Max coverage (-): 0

Region: chr1 80732948-80732958. Max. coverage (+): 0. Max coverage (-): 0

Region: chr1 80732959-80732968. Max. coverage (+): 0. Max coverage (-): 0

Region: chr1 80732969-80732978. Max. coverage (+): 0. Max coverage (-): 0

Region: chr1 80732979-80732988. Max. coverage (+): 0. Max coverage (-): 0

Region: chr1 80732989-80732999. Max. coverage (+): 0. Max coverage (-): 0

Region: chr1 80733000-80733009. Max. coverage (+): 0. Max coverage (-): 0

Region: chr1 80733010-80733019. Max. coverage (+): 0. Max coverage (-): 0

Region: chr1 80733020-80733030. Max. coverage (+): 0. Max coverage (-): 0

Region: chr1 80733031-80733040. Max. coverage (+): 0. Max coverage (-): 0

Region: chr1 80733041-80733050. Max. coverage (+): 0. Max coverage (-): 0

Region: chr1 80733051-80733060. Max. coverage (+): 0. Max coverage (-): 0

Region: chr1 80733061-80733071. Max. coverage (+): 0. Max coverage (-): 0

Region: chr1 80733072-80733081. Max. coverage (+): 0. Max coverage (-): 0

Region: chr1 80733082-80733091. Max. coverage (+): 0. Max coverage (-): 0

Region: chr1 80733092-80733102. Max. coverage (+): 0. Max coverage (-): 0

Region: chr1 80733103-80733112. Max. coverage (+): 0. Max coverage (-): 0

Region: chr1 80733113-80733122. Max. coverage (+): 0. Max coverage (-): 0

Region: chr1 80733123-80733132. Max. coverage (+): 0. Max coverage (-): 0

Region: chr1 80733133-80733143. Max. coverage (+): 0. Max coverage (-): 0

Region: chr1 80733144-80733153. Max. coverage (+): 8.62. Max coverage (-): 0

Region: chr1 80733154-80733163. Max. coverage (+): 8.62. Max coverage (-): 0

Region: chr1 80733164-80733173. Max. coverage (+): 0. Max coverage (-): 0

Region: chr1 80733174-80733184. Max. coverage (+): 0. Max coverage (-): 0

Region: chr1 80733185-80733194. Max. coverage (+): 0. Max coverage (-): 0

Region: chr1 80733195-80733204. Max. coverage (+): 0. Max coverage (-): 0

Region: chr1 80733205-80733215. Max. coverage (+): 0. Max coverage (-): 0

Region: chr1 80733216-80733225. Max. coverage (+): 0. Max coverage (-): 0

Region: chr1 80733226-80733235. Max. coverage (+): 0. Max coverage (-): 0

Region: chr1 80733236-80733245. Max. coverage (+): 6.13. Max coverage (-): 0

Region: chr1 80733246-80733256. Max. coverage (+): 6.13. Max coverage (-): 0

Region: chr1 80733257-80733266. Max. coverage (+): 0. Max coverage (-): 0

Region: chr1 80733267-80733276. Max. coverage (+): 6.01. Max coverage (-): 0

Region: chr1 80733277-80733286. Max. coverage (+): 0. Max coverage (-): 0

Region: chr1 80733287-80733297. Max. coverage (+): 0. Max coverage (-): 0

Region: chr1 80733298-80733307. Max. coverage (+): 0. Max coverage (-): 0

Region: chr1 80733308-80733317. Max. coverage (+): 0. Max coverage (-): 0

Region: chr1 80733318-80733328. Max. coverage (+): 0. Max coverage (-): 0

Region: chr1 80733329-80733338. Max. coverage (+): 0. Max coverage (-): 0

Region: chr1 80733339-80733348. Max. coverage (+): 0. Max coverage (-): 0

Region: chr1 80733349-80733358. Max. coverage (+): 0. Max coverage (-): 0

Region: chr1 80733359-80733369. Max. coverage (+): 0. Max coverage (-): 0

Region: chr1 80733370-80733379. Max. coverage (+): 0. Max coverage (-): 0

Region: chr1 80733380-80733389. Max. coverage (+): 0. Max coverage (-): 0

Region: chr1 80733390-80733400. Max. coverage (+): 0. Max coverage (-): 0

Region: chr1 80733401-80733410. Max. coverage (+): 0. Max coverage (-): 0

Region: chr1 80733411-80733420. Max. coverage (+): 7.17. Max coverage (-): 0

Region: chr1 80733421-80733430. Max. coverage (+): 0. Max coverage (-): 0

Region: chr1 80733431-80733441. Max. coverage (+): 0. Max coverage (-): 0

Region: chr1 80733442-80733451. Max. coverage (+): 0. Max coverage (-): 0

Region: chr1 80733452-80733461. Max. coverage (+): 0. Max coverage (-): 0

Region: chr1 80733462-80733471. Max. coverage (+): 0. Max coverage (-): 0

Region: chr1 80733472-80733482. Max. coverage (+): 0. Max coverage (-): 0

Region: chr1 80733483-80733492. Max. coverage (+): 0. Max coverage (-): 0

Region: chr1 80733493-80733502. Max. coverage (+): 0. Max coverage (-): 0

Region: chr1 80733503-80733513. Max. coverage (+): 5.32. Max coverage (-): 0

Region: chr1 80733514-80733523. Max. coverage (+): 5.32. Max coverage (-): 0

Region: chr1 80733524-80733533. Max. coverage (+): 5.95. Max coverage (-): 0

Region: chr1 80733534-80733543. Max. coverage (+): 5.95. Max coverage (-): 0

Region: chr1 80733544-80733554. Max. coverage (+): 2.68. Max coverage (-): 0

Region: chr1 80733555-80733564. Max. coverage (+): 0. Max coverage (-): 0

Region: chr1 80733565-80733574. Max. coverage (+): 6.12. Max coverage (-): 0

Region: chr1 80733575-80733585. Max. coverage (+): 6.12. Max coverage (-): 0

Region: chr1 80733586-80733595. Max. coverage (+): 0. Max coverage (-): 0

Region: chr1 80733596-80733605. Max. coverage (+): 0. Max coverage (-): 0

Region: chr1 80733606-80733615. Max. coverage (+): 0. Max coverage (-): 0

Region: chr1 80733616-80733626. Max. coverage (+): 0. Max coverage (-): 0

Region: chr1 80733627-80733636. Max. coverage (+): 0. Max coverage (-): 0

Region: chr1 80733637-80733646. Max. coverage (+): 0. Max coverage (-): 0

Region: chr1 80733647-80733656. Max. coverage (+): 0. Max coverage (-): 0

Region: chr1 80733657-80733667. Max. coverage (+): 18.33. Max coverage (-): 0

Region: chr1 80733668-80733677. Max. coverage (+): 11.73. Max coverage (-): 0

Region: chr1 80733678-80733687. Max. coverage (+): 0. Max coverage (-): 0

Region: chr1 80733688-80733698. Max. coverage (+): 0. Max coverage (-): 0

Region: chr1 80733699-80733708. Max. coverage (+): 0. Max coverage (-): 0

Region: chr1 80733709-80733718. Max. coverage (+): 0. Max coverage (-): 0

Region: chr1 80733719-80733728. Max. coverage (+): 0.63. Max coverage (-): 0

Region: chr1 80733729-80733739. Max. coverage (+): 0.87. Max coverage (-): 0

Region: chr1 80733740-80733749. Max. coverage (+): 0. Max coverage (-): 0

Region: chr1 80733750-80733759. Max. coverage (+): 1.69. Max coverage (-): 0

Region: chr1 80733760-80733769. Max. coverage (+): 1.69. Max coverage (-): 0

Region: chr1 80733770-80733780. Max. coverage (+): 0. Max coverage (-): 0

Region: chr1 80733781-80733790. Max. coverage (+): 0. Max coverage (-): 0

Region: chr1 80733791-80733800. Max. coverage (+): 0. Max coverage (-): 0

Region: chr1 80733801-80733811. Max. coverage (+): 0. Max coverage (-): 0

Region: chr1 80733812-80733821. Max. coverage (+): 0. Max coverage (-): 0

Region: chr1 80733822-80733831. Max. coverage (+): 0. Max coverage (-): 0

Region: chr1 80733832-80733841. Max. coverage (+): 0. Max coverage (-): 0

Region: chr1 80733842-80733852. Max. coverage (+): 0. Max coverage (-): 0

Region: chr1 80733853-80733862. Max. coverage (+): 0. Max coverage (-): 0

Region: chr1 80733863-80733872. Max. coverage (+): 10.27. Max coverage (-): 0

Region: chr1 80733873-80733883. Max. coverage (+): 10.27. Max coverage (-): 0

Region: chr1 80733884-80733893. Max. coverage (+): 0. Max coverage (-): 0

Region: chr1 80733894-80733903. Max. coverage (+): 0. Max coverage (-): 0

Region: chr1 80733904-80733913. Max. coverage (+): 0. Max coverage (-): 0

Region: chr1 80733914-80733924. Max. coverage (+): 0. Max coverage (-): 0

Region: chr1 80733925-80733934. Max. coverage (+): 0. Max coverage (-): 0

Region: chr1 80733935-80733944. Max. coverage (+): 0. Max coverage (-): 0

Region: chr1 80733945-80733954. Max. coverage (+): 0. Max coverage (-): 0

Region: chr1 80733955-80733965. Max. coverage (+): 0. Max coverage (-): 0

Region: chr1 80733966-80733975. Max. coverage (+): 0. Max coverage (-): 0

Region: chr1 80733976-80733985. Max. coverage (+): 0. Max coverage (-): 0

Region: chr1 80733986-80733996. Max. coverage (+): 1.18. Max coverage (-): 0

Region: chr1 80733997-80734006. Max. coverage (+): 16.72. Max coverage (-): 0

Region: chr1 80734007-80734016. Max. coverage (+): 0. Max coverage (-): 0

Region: chr1 80734017-80734026. Max. coverage (+): 0. Max coverage (-): 0

Region: chr1 80734027-80734037. Max. coverage (+): 0. Max coverage (-): 0

Region: chr1 80734038-80734047. Max. coverage (+): 0. Max coverage (-): 0

Region: chr1 80734048-80734057. Max. coverage (+): 0. Max coverage (-): 0

Region: chr1 80734058-80734067. Max. coverage (+): 6.6. Max coverage (-): 0

Region: chr1 80734068-80734078. Max. coverage (+): 6.6. Max coverage (-): 0

Region: chr1 80734079-80734088. Max. coverage (+): 0. Max coverage (-): 0

Region: chr1 80734089-80734098. Max. coverage (+): 0. Max coverage (-): 0

Region: chr1 80734099-80734109. Max. coverage (+): 0. Max coverage (-): 0

Region: chr1 80734110-80734119. Max. coverage (+): 1.28. Max coverage (-): 0

Region: chr1 80734120-80734129. Max. coverage (+): 1.28. Max coverage (-): 0

Region: chr1 80734130-80734139. Max. coverage (+): 1.6. Max coverage (-): 0

Region: chr1 80734140-80734150. Max. coverage (+): 1.6. Max coverage (-): 0

Region: chr1 80734151-80734160. Max. coverage (+): 0. Max coverage (-): 0

Region: chr1 80734161-80734170. Max. coverage (+): 0. Max coverage (-): 0

Region: chr1 80734171-80734181. Max. coverage (+): 0. Max coverage (-): 0

Region: chr1 80734182-80734191. Max. coverage (+): 0. Max coverage (-): 0

Region: chr1 80734192-80734201. Max. coverage (+): 0. Max coverage (-): 0

Region: chr1 80734202-80734211. Max. coverage (+): 0. Max coverage (-): 0

Region: chr1 80734212-80734222. Max. coverage (+): 0. Max coverage (-): 0

Region: chr1 80734223-80734232. Max. coverage (+): 6.08. Max coverage (-): 0

Region: chr1 80734233-80734242. Max. coverage (+): 8.87. Max coverage (-): 0

Region: chr1 80734243-80734252. Max. coverage (+): 4.73. Max coverage (-): 0

Region: chr1 80734253-80734263. Max. coverage (+): 4.73. Max coverage (-): 0

Region: chr1 80734264-80734273. Max. coverage (+): 7.99. Max coverage (-): 0

Region: chr1 80734274-80734283. Max. coverage (+): 7.99. Max coverage (-): 0

Region: chr1 80734284-80734294. Max. coverage (+): 4.26. Max coverage (-): 0

Region: chr1 80734295-80734304. Max. coverage (+): 11.23. Max coverage (-): 0

Region: chr1 80734305-80734314. Max. coverage (+): 11.23. Max coverage (-): 0

Region: chr1 80734315-80734324. Max. coverage (+): 6.97. Max coverage (-): 0

Region: chr1 80734325-80734335. Max. coverage (+): 0. Max coverage (-): 0

Region: chr1 80734336-80734345. Max. coverage (+): 0. Max coverage (-): 0

Region: chr1 80734346-80734355. Max. coverage (+): 35.49. Max coverage (-): 0

Region: chr1 80734356-80734365. Max. coverage (+): 36.99. Max coverage (-): 0

Region: chr1 80734366-80734376. Max. coverage (+): 10.43. Max coverage (-): 0

Region: chr1 80734377-80734386. Max. coverage (+): 0. Max coverage (-): 0

Region: chr1 80734387-80734396. Max. coverage (+): 0. Max coverage (-): 0

Region: chr1 80734397-80734407. Max. coverage (+): 0. Max coverage (-): 0

Region: chr1 80734408-80734417. Max. coverage (+): 3.58. Max coverage (-): 0

Region: chr1 80734418-80734427. Max. coverage (+): 3.58. Max coverage (-): 0

Region: chr1 80734428-80734437. Max. coverage (+): 0. Max coverage (-): 0

Region: chr1 80734438-80734448. Max. coverage (+): 0.66. Max coverage (-): 0

Region: chr1 80734449-80734458. Max. coverage (+): 0.66. Max coverage (-): 0

Region: chr1 80734459-80734468. Max. coverage (+): 0. Max coverage (-): 0

Region: chr1 80734469-80734479. Max. coverage (+): 0. Max coverage (-): 0

Region: chr1 80734480-80734489. Max. coverage (+): 0. Max coverage (-): 0

Region: chr1 80734490-80734499. Max. coverage (+): 0. Max coverage (-): 0

Region: chr1 80734500-80734509. Max. coverage (+): 0. Max coverage (-): 0

Region: chr1 80734510-80734520. Max. coverage (+): 0. Max coverage (-): 0

Region: chr1 80734521-80734530. Max. coverage (+): 0. Max coverage (-): 0

Region: chr1 80734531-80734540. Max. coverage (+): 0. Max coverage (-): 0

Region: chr1 80734541-80734550. Max. coverage (+): 0. Max coverage (-): 0

Region: chr1 80734551-80734561. Max. coverage (+): 0. Max coverage (-): 0

Region: chr1 80734562-80734571. Max. coverage (+): 0. Max coverage (-): 0

Region: chr1 80734572-80734581. Max. coverage (+): 0. Max coverage (-): 0

Region: chr1 80734582-80734592. Max. coverage (+): 1.68. Max coverage (-): 0

Region: chr1 80734593-80734602. Max. coverage (+): 0. Max coverage (-): 0

Region: chr1 80734603-80734612. Max. coverage (+): 0. Max coverage (-): 0

Region: chr1 80734613-80734622. Max. coverage (+): 0. Max coverage (-): 0

Region: chr1 80734623-80734633. Max. coverage (+): 0. Max coverage (-): 0

Region: chr1 80734634-80734643. Max. coverage (+): 0. Max coverage (-): 0

Region: chr1 80734644-80734653. Max. coverage (+): 0. Max coverage (-): 0

Region: chr1 80734654-80734663. Max. coverage (+): 0. Max coverage (-): 0

Region: chr1 80734664-80734674. Max. coverage (+): 0. Max coverage (-): 0

Region: chr1 80734675-80734684. Max. coverage (+): 0. Max coverage (-): 0

Region: chr1 80734685-80734694. Max. coverage (+): 3.35. Max coverage (-): 0

Region: chr1 80734695-80734705. Max. coverage (+): 3.35. Max coverage (-): 0

Region: chr1 80734706-80734715. Max. coverage (+): 0. Max coverage (-): 0

Region: chr1 80734716-80734725. Max. coverage (+): 0. Max coverage (-): 0

Region: chr1 80734726-80734735. Max. coverage (+): 0. Max coverage (-): 0

Region: chr1 80734736-80734746. Max. coverage (+): 0. Max coverage (-): 0

Region: chr1 80734747-80734756. Max. coverage (+): 0. Max coverage (-): 0

Region: chr1 80734757-80734766. Max. coverage (+): 0. Max coverage (-): 0

Region: chr1 80734767-80734777. Max. coverage (+): 0. Max coverage (-): 0

Region: chr1 80734778-80734787. Max. coverage (+): 0. Max coverage (-): 0

Region: chr1 80734788-80734797. Max. coverage (+): 0. Max coverage (-): 0

Region: chr1 80734798-80734807. Max. coverage (+): 0. Max coverage (-): 0

Region: chr1 80734808-80734818. Max. coverage (+): 0. Max coverage (-): 0

Region: chr1 80734819-80734828. Max. coverage (+): 0. Max coverage (-): 0

Region: chr1 80734829-80734838. Max. coverage (+): 0. Max coverage (-): 0

Region: chr1 80734839-80734848. Max. coverage (+): 0. Max coverage (-): 0

Region: chr1 80734849-80734859. Max. coverage (+): 0. Max coverage (-): 0

Region: chr1 80734860-80734869. Max. coverage (+): 0. Max coverage (-): 0

Region: chr1 80734870-80734879. Max. coverage (+): 0. Max coverage (-): 0

Region: chr1 80734880-80734890. Max. coverage (+): 0. Max coverage (-): 0

Region: chr1 80734891-80734900. Max. coverage (+): 0. Max coverage (-): 0

Region: chr1 80734901-80734910. Max. coverage (+): 0. Max coverage (-): 0

Region: chr1 80734911-80734920. Max. coverage (+): 0. Max coverage (-): 0

Region: chr1 80734921-80734931. Max. coverage (+): 0. Max coverage (-): 0

Region: chr1 80734932-80734941. Max. coverage (+): 0. Max coverage (-): 0

Region: chr1 80734942-80734951. Max. coverage (+): 0. Max coverage (-): 0

Region: chr1 80734952-80734961. Max. coverage (+): 0. Max coverage (-): 0

Region: chr1 80734962-80734972. Max. coverage (+): 0. Max coverage (-): 0

Region: chr1 80734973-80734982. Max. coverage (+): 0. Max coverage (-): 0

Region: chr1 80734983-80734992. Max. coverage (+): 0. Max coverage (-): 0

Region: chr1 80734993-80735003. Max. coverage (+): 0. Max coverage (-): 0

Region: chr1 80735004-80735013. Max. coverage (+): 0. Max coverage (-): 0

Region: chr1 80735014-80735023. Max. coverage (+): 0. Max coverage (-): 0

Region: chr1 80735024-80735033. Max. coverage (+): 0. Max coverage (-): 0

Region: chr1 80735034-80735044. Max. coverage (+): 0. Max coverage (-): 0

Region: chr1 80735045-80735054. Max. coverage (+): 0. Max coverage (-): 0

Region: chr1 80735055-80735064. Max. coverage (+): 0. Max coverage (-): 0

Region: chr1 80735065-80735075. Max. coverage (+): 0. Max coverage (-): 0

Region: chr1 80735076-80735085. Max. coverage (+): 0. Max coverage (-): 0

Region: chr1 80735086-80735095. Max. coverage (+): 0. Max coverage (-): 0

Region: chr1 80735096-80735105. Max. coverage (+): 0. Max coverage (-): 0

Region: chr1 80735106-80735116. Max. coverage (+): 0. Max coverage (-): 0

Region: chr1 80735117-80735126. Max. coverage (+): 0. Max coverage (-): 0

Region: chr1 80735127-80735136. Max. coverage (+): 0. Max coverage (-): 0

Region: chr1 80735137-80735146. Max. coverage (+): 0. Max coverage (-): 0

Region: chr1 80735147-80735157. Max. coverage (+): 0. Max coverage (-): 0

Region: chr1 80735158-80735167. Max. coverage (+): 0. Max coverage (-): 0

Region: chr1 80735168-80735177. Max. coverage (+): 0. Max coverage (-): 0

Region: chr1 80735178-80735188. Max. coverage (+): 0. Max coverage (-): 0

Region: chr1 80735189-80735198. Max. coverage (+): 0. Max coverage (-): 0

Region: chr1 80735199-80735208. Max. coverage (+): 0. Max coverage (-): 0

Region: chr1 80735209-80735218. Max. coverage (+): 0. Max coverage (-): 0

Region: chr1 80735219-80735229. Max. coverage (+): 0. Max coverage (-): 0

Region: chr1 80735230-80735239. Max. coverage (+): 0. Max coverage (-): 0

Region: chr1 80735240-80735249. Max. coverage (+): 0. Max coverage (-): 0

Region: chr1 80735250-80735259. Max. coverage (+): 0. Max coverage (-): 0

Region: chr1 80735260-80735270. Max. coverage (+): 0. Max coverage (-): 0

Region: chr1 80735271-80735280. Max. coverage (+): 0. Max coverage (-): 0

Region: chr1 80735281-80735290. Max. coverage (+): 0. Max coverage (-): 0

Region: chr1 80735291-80735301. Max. coverage (+): 0. Max coverage (-): 0

Region: chr1 80735302-80735311. Max. coverage (+): 0. Max coverage (-): 0

Region: chr1 80735312-80735321. Max. coverage (+): 0. Max coverage (-): 0

Region: chr1 80735322-80735331. Max. coverage (+): 0. Max coverage (-): 0

Region: chr1 80735332-80735342. Max. coverage (+): 0. Max coverage (-): 0

Region: chr1 80735343-80735352. Max. coverage (+): 0. Max coverage (-): 0

Region: chr1 80735353-80735362. Max. coverage (+): 0. Max coverage (-): 0

Region: chr1 80735363-80735373. Max. coverage (+): 0. Max coverage (-): 0

Region: chr1 80735374-80735383. Max. coverage (+): 0. Max coverage (-): 0

Region: chr1 80735384-80735393. Max. coverage (+): 0. Max coverage (-): 0

Region: chr1 80735394-80735403. Max. coverage (+): 0. Max coverage (-): 0

Region: chr1 80735404-80735414. Max. coverage (+): 0. Max coverage (-): 0

Region: chr1 80735415-80735424. Max. coverage (+): 0. Max coverage (-): 0

Region: chr1 80735425-80735434. Max. coverage (+): 0. Max coverage (-): 0

Region: chr1 80735435-80735444. Max. coverage (+): 0. Max coverage (-): 0

Region: chr1 80735445-80735455. Max. coverage (+): 11.48. Max coverage (-): 0

Region: chr1 80735456-80735465. Max. coverage (+): 5.1. Max coverage (-): 0

Region: chr1 80735466-80735475. Max. coverage (+): 0. Max coverage (-): 0

Region: chr1 80735476-80735486. Max. coverage (+): 0. Max coverage (-): 0

Region: chr1 80735487-80735496. Max. coverage (+): 0. Max coverage (-): 0

Region: chr1 80735497-80735506. Max. coverage (+): 3.8. Max coverage (-): 0

Region: chr1 80735507-80735516. Max. coverage (+): 0. Max coverage (-): 0

Region: chr1 80735517-80735527. Max. coverage (+): 0. Max coverage (-): 0

Region: chr1 80735528-80735537. Max. coverage (+): 0. Max coverage (-): 0

Region: chr1 80735538-80735547. Max. coverage (+): 0. Max coverage (-): 0

Region: chr1 80735548-80735557. Max. coverage (+): 0. Max coverage (-): 0

Region: chr1 80735558-80735568. Max. coverage (+): 0. Max coverage (-): 0

Region: chr1 80735569-80735578. Max. coverage (+): 0. Max coverage (-): 0

Region: chr1 80735579-80735588. Max. coverage (+): 0. Max coverage (-): 0

Region: chr1 80735589-80735599. Max. coverage (+): 0. Max coverage (-): 0

Region: chr1 80735600-80735609. Max. coverage (+): 0. Max coverage (-): 0

Region: chr1 80735610-80735619. Max. coverage (+): 0. Max coverage (-): 0

Region: chr1 80735620-80735629. Max. coverage (+): 0. Max coverage (-): 0

Region: chr1 80735630-80735640. Max. coverage (+): 0. Max coverage (-): 0

Region: chr1 80735641-80735650. Max. coverage (+): 0.35. Max coverage (-): 0

Region: chr1 80735651-80735660. Max. coverage (+): 0. Max coverage (-): 0

Region: chr1 80735661-80735671. Max. coverage (+): 0. Max coverage (-): 0

Region: chr1 80735672-80735681. Max. coverage (+): 0. Max coverage (-): 0

Region: chr1 80735682-80735691. Max. coverage (+): 3.08. Max coverage (-): 0

Region: chr1 80735692-80735701. Max. coverage (+): 0. Max coverage (-): 0

Region: chr1 80735702-80735712. Max. coverage (+): 0. Max coverage (-): 0

Region: chr1 80735713-80735722. Max. coverage (+): 0. Max coverage (-): 0

Region: chr1 80735723-80735732. Max. coverage (+): 0. Max coverage (-): 0

Region: chr1 80735733-80735742. Max. coverage (+): 0. Max coverage (-): 0

Region: chr1 80735743-80735753. Max. coverage (+): 0. Max coverage (-): 0

Region: chr1 80735754-80735763. Max. coverage (+): 6.73. Max coverage (-): 0

Region: chr1 80735764-80735773. Max. coverage (+): 0. Max coverage (-): 0

Region: chr1 80735774-80735784. Max. coverage (+): 0. Max coverage (-): 0

Region: chr1 80735785-80735794. Max. coverage (+): 0. Max coverage (-): 0

Region: chr1 80735795-80735804. Max. coverage (+): 0. Max coverage (-): 0

Region: chr1 80735805-80735814. Max. coverage (+): 4.76. Max coverage (-): 0

Region: chr1 80735815-80735825. Max. coverage (+): 4.76. Max coverage (-): 0

Region: chr1 80735826-80735835. Max. coverage (+): 0. Max coverage (-): 0

Region: chr1 80735836-80735845. Max. coverage (+): 0. Max coverage (-): 0

Region: chr1 80735846-80735855. Max. coverage (+): 0. Max coverage (-): 0

Region: chr1 80735856-80735866. Max. coverage (+): 0. Max coverage (-): 0

Region: chr1 80735867-80735876. Max. coverage (+): 0. Max coverage (-): 0

Region: chr1 80735877-80735886. Max. coverage (+): 0. Max coverage (-): 0

Region: chr1 80735887-80735897. Max. coverage (+): 0. Max coverage (-): 0

Region: chr1 80735898-80735907. Max. coverage (+): 0. Max coverage (-): 0

Region: chr1 80735908-80735917. Max. coverage (+): 0. Max coverage (-): 0

Region: chr1 80735918-80735927. Max. coverage (+): 0. Max coverage (-): 0

Region: chr1 80735928-80735938. Max. coverage (+): 0. Max coverage (-): 0

Region: chr1 80735939-80735948. Max. coverage (+): 0. Max coverage (-): 0

Region: chr1 80735949-80735958. Max. coverage (+): 0. Max coverage (-): 0

Region: chr1 80735959-80735969. Max. coverage (+): 0. Max coverage (-): 0

Region: chr1 80735970-80735979. Max. coverage (+): 6.91. Max coverage (-): 0

Region: chr1 80735980-80735989. Max. coverage (+): 6.91. Max coverage (-): 0

Region: chr1 80735990-80735999. Max. coverage (+): 0. Max coverage (-): 0

Region: chr1 80736000-. Max. coverage (+): 0. Max coverage (-): 0

RepeatMasker Color Code

**+**

100-98% Identity

<98-95% Identity

<95-90% Identity

<90-85% Identity

<85-80% Identity

<80-75% Identity

<75-70% Identity

<70% Identity

**-**

Gene Set Color Code

**+**

Gene

Pseudogene

**-**

Topology/Coverage Color Code

Coverage Plus Strand

Coverage Minus Strand

Mainstrand: Plus

Mainstrand: Minus

Complementary Strand

Flanking Region  
(if option -flank >0)

Gene Set Annotation  
  
RepeatMasker Annotation  

**1. L1ME3B**: 80731990-80732139 (-), Divergence to consensus: 42.1%  
**2. MER5A**: 80732154-80732234 (+), Divergence to consensus: 29.6%  
**3. MER5A**: 80732292-80732330 (-), Divergence to consensus: 17.9%  
**4. BOV-A2**: 80732328-80732448 (-), Divergence to consensus: 8.2%  
**5. MER5A**: 80732449-80732499 (-), Divergence to consensus: 29.4%  
**6. MERX**: 80732645-80732691 (+), Divergence to consensus: 19.1%  
**7. L1MEg**: 80732938-80733114 (-), Divergence to consensus: 39.6%  
**8. ART2A**: 80735087-80735226 (-), Divergence to consensus: 19.4%  
**9. (TATG)n**: 80735253-80735352 (+), Divergence to consensus: 26.5%

  
Transcription Factor Binding Sites  

**Gata4** (Sequence: AGATAAC (-): 80730957)  
**Gata4** (Sequence: CTTATCT (+): 80734328)
