# Supplementary material for: Small RNA sequencing of cryopreserved semen from single bull revealed altered miRNAs and piRNAs expression between High- and Low-motile sperm populations
Source: BMC Genomics. 2017 Jan 4;18:14. doi: 10.1186/s12864-016-3394-7 (PMC5209821; doi:10.1186/s12864-016-3394-7)
Supplement: Additional file 4: — Details for each piRNA clusters found in Low Motile (LM) sperm fraction. Genes, repeats, transposable elements and transcription factors binding sites falling within the cluster regions were reported. (ZIP 1034 kb) [file 12864_2016_3394_MOESM4_ESM.zip › 10.html]

piRNA cluster 10


Predicted piRNA cluster no. 10     previous   next
  

Show proTRAC run info
Hide proTRAC run info

================================= proTRAC ====================================  
VERSION: 2.1                                    LAST MODIFIED: 06. October 2015  
  
Please cite:  
Rosenkranz D, Zischler H. proTRAC - a software for probabilistic piRNA cluster  
detection, visualization and analysis. 2012. BMC Bioinformatics 13:5.  
  
and (for proTRAC 2.0 and later):  
Rosenkranz D, Rudloff S, Bastuck K, Ketting RF, Zischler H. Tupaia small RNAs  
provide insights into function and evolution of RNAi-based transposon defense  
in mammals. 2015. RNA 21(5):911-922.  
  
Contact:  
David Rosenkranz  
Institute of Anthropology, small RNA group  
Johannes Gutenberg University Mainz  
email: rosenkranz@uni-mainz.de  
  
You can find the latest proTRAC version at:  
http://sourceforge.net/projects/protrac/files  
http://www.smallRNAgroup-mainz.de/software  
==============================================================================  
  
PARAMETERS:  
Map file: .............../storage/core/barbara/genhome/smallRNA/fertility/Sample\_not\_motile/pirna/Sample\_not\_motile\_26-33\_collapsed.fa.no-dust.map.weighted-10000-1000-b-0  
Genome file: ............/storage/core/barbara/genhome/smallRNA/fertility/Sample\_all/pirna/bt\_311\_chrY.fa  
RepeatMasker annotation: /storage/genomes/bt\_umd31/GCF\_000003055.6\_Bos\_taurus\_UMD\_3.1.1\_repeatMasker\_chr.out  
GeneSet:................./storage/core/barbara/genhome/smallRNA/fertility/Sample\_all/pirna/full.gtf  
  
Significant (p<=0.01) hit density will be calculated based  
on observed hit distribution.  
  
Sliding window size: ........................................ 5000 bp  
Sliding window increament: .................................. 1000 bp  
Normalize each hit by number of genomic hits: ............... 1 [0=no/1=yes]  
Normalize each hit by number of sequence reads: ............. 1 [0=no/1=yes]  
Normalize values (-> per million mapped reads): ............. 1 [0=no/1=yes]  
Min. fraction of hits with 1T(U) or 10A: .................... 0.75  
Alternatively: Min. fraction of hits with 1T(U) and 10A: .... 0.5  
Min. fraction of hits with typical piRNA length: ............ 0.75  
Typical piRNA length: ....................................... 26-33 nt  
Min. size of a piRNA cluster: ............................... 5000 bp.  
Min. number of hits (absolute): ............................. 0  
Min. number of hits (normalized): ........................... 0  
Min. fraction of hits on the mainstrand: .................... 0.75  
Top fraction of mapped sequences (in terms of read counts): . 1%  
Top fraction accounts for max. n% of sequence reads: ........ 90%  
Min. fraction of hits on each arm of a bidirectional cluster: 0.1  
Output image file for each cluster: ......................... 0 [0=no/1=yes]  
Output html file for each cluster: .......................... 1 [0=no/1=yes]  
Output a summary table: ..................................... 1 [0=no/1=yes]  
Output a FASTA file for each cluster (piRNA sequences): ..... 1 [0=no/1=yes]  
Output a FASTA file comprising cluster sequences: ........... 1 [0=no/1=yes]  
Search DNA motifs in clusters: .............................. 1 [0=no/1=yes]  
Output flanking sequences: +/- .............................. 0 bp  
Output ~.pTi file: .......................................... 1 [0=no/1=yes]  
==============================================================================  
  
  
Genome size (without gaps): ............ 2678902517 bp  
Gaps (N/X/-): .......................... 53837044 bp  
Mapped reads: .......................... 738059667487  
Non-identical sequences: ............... 277001  
Genomic hits: .......................... 533816  
Significant densitiy of mapped reads: .. 15118061 reads/kb

Show proTRAC cluster info
Hide proTRAC cluster info

|  |  |
| --- | --- |
| Location | chr13 |
| Coordinates | 38147008-38153269 |
| Size [bp] | 6262 |
| Sequence hit loci | 183 |
| Mapped reads (normalized) | 447363385.9 |
| Mapped reads (normalized) per kb | 71440975.1 |
| Normalized reads with 1T (1U) | 82.9% |
| Normalized reads with 10A | 36.3% |
| Normalized reads with length 26-33 nt | 100% |
| Normalized reads on the main strand(s) | 100% |
| Predicted directionality | mono:minus |

100%

0%

1T (1U)  
reads

10A reads

26-33 nt  
reads

reads on mainstrand

**Either the amount of reads with 1T (1U) OR 10A has to exceed 75% (set with option: -1Tor10A)  
Alternatively the amount of reads with 1T (1U) AND 10A has to exceed 50% (set with option: -1Tand10A)  
Minimum amount of reads with preferred size is 75% (set with option: -pisize)  
Minimum amount of reads on the main strand(s) is 75% (set with option: -clstrand)**

Show read coverage
Hide read coverage

WHAT DO I SEE HERE?  
This chart shows the location of mapped sequence reads within a predicted piRNA cluster. The color refers to the number of genomic hits produced by the sequence read in question. A dark red bar indicates that this sequence read produces many other hits elsewhere in the genome. Many adjacent red or yellow bars can indicate the presence of a multi-copy element such as transposons or rRNA genes. A dark green bar indicates that this sequence read maps uniquely to this locus.

1 hit

2-5 hits

6-10 hits

11-20 hits

21-50 hits

51-100 hits

> 100 hits

chr13

38147008

38153269

Gene Set

RepeatMasker

Mapped  
Reads

35.48

plus strand

minus strand

35.48

Region: chr13 29637378-38147014. Max. coverage (+): 0. Max coverage (-): 15.02

Region: chr13 38147015-38147026. Max. coverage (+): 0. Max coverage (-): 15.02

Region: chr13 38147027-38147039. Max. coverage (+): 0. Max coverage (-): 8.22

Region: chr13 38147040-38147051. Max. coverage (+): 0. Max coverage (-): 0

Region: chr13 38147052-38147064. Max. coverage (+): 0. Max coverage (-): 0

Region: chr13 38147065-38147076. Max. coverage (+): 0. Max coverage (-): 0

Region: chr13 38147077-38147089. Max. coverage (+): 0. Max coverage (-): 0

Region: chr13 38147090-38147101. Max. coverage (+): 0. Max coverage (-): 33.57

Region: chr13 38147102-38147114. Max. coverage (+): 0. Max coverage (-): 19.16

Region: chr13 38147115-38147126. Max. coverage (+): 0. Max coverage (-): 6.33

Region: chr13 38147127-38147139. Max. coverage (+): 0. Max coverage (-): 0

Region: chr13 38147140-38147152. Max. coverage (+): 0. Max coverage (-): 0

Region: chr13 38147153-38147164. Max. coverage (+): 0. Max coverage (-): 0

Region: chr13 38147165-38147177. Max. coverage (+): 0. Max coverage (-): 0

Region: chr13 38147178-38147189. Max. coverage (+): 0. Max coverage (-): 0

Region: chr13 38147190-38147202. Max. coverage (+): 0. Max coverage (-): 0

Region: chr13 38147203-38147214. Max. coverage (+): 0. Max coverage (-): 0

Region: chr13 38147215-38147227. Max. coverage (+): 0. Max coverage (-): 0

Region: chr13 38147228-38147239. Max. coverage (+): 0. Max coverage (-): 0

Region: chr13 38147240-38147252. Max. coverage (+): 0. Max coverage (-): 0

Region: chr13 38147253-38147264. Max. coverage (+): 0. Max coverage (-): 0

Region: chr13 38147265-38147277. Max. coverage (+): 0. Max coverage (-): 0.77

Region: chr13 38147278-38147289. Max. coverage (+): 0. Max coverage (-): 0.77

Region: chr13 38147290-38147302. Max. coverage (+): 0. Max coverage (-): 0

Region: chr13 38147303-38147314. Max. coverage (+): 0. Max coverage (-): 12.35

Region: chr13 38147315-38147327. Max. coverage (+): 0. Max coverage (-): 19.29

Region: chr13 38147328-38147339. Max. coverage (+): 0. Max coverage (-): 0

Region: chr13 38147340-38147352. Max. coverage (+): 0. Max coverage (-): 0

Region: chr13 38147353-38147364. Max. coverage (+): 0. Max coverage (-): 0

Region: chr13 38147365-38147377. Max. coverage (+): 0. Max coverage (-): 0

Region: chr13 38147378-38147389. Max. coverage (+): 0. Max coverage (-): 0

Region: chr13 38147390-38147402. Max. coverage (+): 0. Max coverage (-): 0

Region: chr13 38147403-38147415. Max. coverage (+): 0. Max coverage (-): 0

Region: chr13 38147416-38147427. Max. coverage (+): 0. Max coverage (-): 0

Region: chr13 38147428-38147440. Max. coverage (+): 0. Max coverage (-): 0

Region: chr13 38147441-38147452. Max. coverage (+): 0. Max coverage (-): 0

Region: chr13 38147453-38147465. Max. coverage (+): 0. Max coverage (-): 2.16

Region: chr13 38147466-38147477. Max. coverage (+): 0. Max coverage (-): 0

Region: chr13 38147478-38147490. Max. coverage (+): 0. Max coverage (-): 0

Region: chr13 38147491-38147502. Max. coverage (+): 0. Max coverage (-): 0

Region: chr13 38147503-38147515. Max. coverage (+): 0. Max coverage (-): 0

Region: chr13 38147516-38147527. Max. coverage (+): 0. Max coverage (-): 0

Region: chr13 38147528-38147540. Max. coverage (+): 0. Max coverage (-): 0

Region: chr13 38147541-38147552. Max. coverage (+): 0. Max coverage (-): 1.53

Region: chr13 38147553-38147565. Max. coverage (+): 0. Max coverage (-): 9.26

Region: chr13 38147566-38147577. Max. coverage (+): 0. Max coverage (-): 9.26

Region: chr13 38147578-38147590. Max. coverage (+): 0. Max coverage (-): 0

Region: chr13 38147591-38147602. Max. coverage (+): 0. Max coverage (-): 0

Region: chr13 38147603-38147615. Max. coverage (+): 0. Max coverage (-): 0

Region: chr13 38147616-38147627. Max. coverage (+): 0. Max coverage (-): 0

Region: chr13 38147628-38147640. Max. coverage (+): 0. Max coverage (-): 0

Region: chr13 38147641-38147652. Max. coverage (+): 0. Max coverage (-): 0

Region: chr13 38147653-38147665. Max. coverage (+): 0. Max coverage (-): 1.56

Region: chr13 38147666-38147678. Max. coverage (+): 0. Max coverage (-): 1.56

Region: chr13 38147679-38147690. Max. coverage (+): 0. Max coverage (-): 5.03

Region: chr13 38147691-38147703. Max. coverage (+): 0. Max coverage (-): 19.87

Region: chr13 38147704-38147715. Max. coverage (+): 0. Max coverage (-): 19.87

Region: chr13 38147716-38147728. Max. coverage (+): 0. Max coverage (-): 6.32

Region: chr13 38147729-38147740. Max. coverage (+): 0. Max coverage (-): 0

Region: chr13 38147741-38147753. Max. coverage (+): 0. Max coverage (-): 0

Region: chr13 38147754-38147765. Max. coverage (+): 0. Max coverage (-): 0

Region: chr13 38147766-38147778. Max. coverage (+): 0. Max coverage (-): 0

Region: chr13 38147779-38147790. Max. coverage (+): 0. Max coverage (-): 6.63

Region: chr13 38147791-38147803. Max. coverage (+): 0. Max coverage (-): 11.65

Region: chr13 38147804-38147815. Max. coverage (+): 0. Max coverage (-): 0

Region: chr13 38147816-38147828. Max. coverage (+): 0. Max coverage (-): 0

Region: chr13 38147829-38147840. Max. coverage (+): 0. Max coverage (-): 10.93

Region: chr13 38147841-38147853. Max. coverage (+): 0. Max coverage (-): 3.3

Region: chr13 38147854-38147865. Max. coverage (+): 0. Max coverage (-): 0

Region: chr13 38147866-38147878. Max. coverage (+): 0. Max coverage (-): 0

Region: chr13 38147879-38147890. Max. coverage (+): 0. Max coverage (-): 0

Region: chr13 38147891-38147903. Max. coverage (+): 0. Max coverage (-): 0

Region: chr13 38147904-38147915. Max. coverage (+): 0. Max coverage (-): 0

Region: chr13 38147916-38147928. Max. coverage (+): 0. Max coverage (-): 0

Region: chr13 38147929-38147941. Max. coverage (+): 0. Max coverage (-): 0

Region: chr13 38147942-38147953. Max. coverage (+): 0. Max coverage (-): 0

Region: chr13 38147954-38147966. Max. coverage (+): 0. Max coverage (-): 22.7

Region: chr13 38147967-38147978. Max. coverage (+): 0. Max coverage (-): 22.7

Region: chr13 38147979-38147991. Max. coverage (+): 0. Max coverage (-): 0.88

Region: chr13 38147992-38148003. Max. coverage (+): 0. Max coverage (-): 16.02

Region: chr13 38148004-38148016. Max. coverage (+): 0. Max coverage (-): 16.99

Region: chr13 38148017-38148028. Max. coverage (+): 0. Max coverage (-): 0

Region: chr13 38148029-38148041. Max. coverage (+): 0. Max coverage (-): 2.3

Region: chr13 38148042-38148053. Max. coverage (+): 0. Max coverage (-): 9.44

Region: chr13 38148054-38148066. Max. coverage (+): 0. Max coverage (-): 1.37

Region: chr13 38148067-38148078. Max. coverage (+): 0. Max coverage (-): 11.45

Region: chr13 38148079-38148091. Max. coverage (+): 0. Max coverage (-): 0

Region: chr13 38148092-38148103. Max. coverage (+): 0. Max coverage (-): 0

Region: chr13 38148104-38148116. Max. coverage (+): 0. Max coverage (-): 0

Region: chr13 38148117-38148128. Max. coverage (+): 0. Max coverage (-): 0

Region: chr13 38148129-38148141. Max. coverage (+): 0. Max coverage (-): 6.21

Region: chr13 38148142-38148153. Max. coverage (+): 0. Max coverage (-): 10.12

Region: chr13 38148154-38148166. Max. coverage (+): 0. Max coverage (-): 2.86

Region: chr13 38148167-38148178. Max. coverage (+): 0. Max coverage (-): 0

Region: chr13 38148179-38148191. Max. coverage (+): 0. Max coverage (-): 0

Region: chr13 38148192-38148204. Max. coverage (+): 0. Max coverage (-): 0

Region: chr13 38148205-38148216. Max. coverage (+): 0. Max coverage (-): 0

Region: chr13 38148217-38148229. Max. coverage (+): 0. Max coverage (-): 0

Region: chr13 38148230-38148241. Max. coverage (+): 0. Max coverage (-): 2.79

Region: chr13 38148242-38148254. Max. coverage (+): 0. Max coverage (-): 5.36

Region: chr13 38148255-38148266. Max. coverage (+): 0. Max coverage (-): 2.94

Region: chr13 38148267-38148279. Max. coverage (+): 0. Max coverage (-): 0

Region: chr13 38148280-38148291. Max. coverage (+): 0. Max coverage (-): 3.94

Region: chr13 38148292-38148304. Max. coverage (+): 0. Max coverage (-): 3.94

Region: chr13 38148305-38148316. Max. coverage (+): 0. Max coverage (-): 0

Region: chr13 38148317-38148329. Max. coverage (+): 0. Max coverage (-): 0.34

Region: chr13 38148330-38148341. Max. coverage (+): 0. Max coverage (-): 0

Region: chr13 38148342-38148354. Max. coverage (+): 0. Max coverage (-): 0

Region: chr13 38148355-38148366. Max. coverage (+): 0. Max coverage (-): 0

Region: chr13 38148367-38148379. Max. coverage (+): 0. Max coverage (-): 0

Region: chr13 38148380-38148391. Max. coverage (+): 0. Max coverage (-): 0

Region: chr13 38148392-38148404. Max. coverage (+): 0. Max coverage (-): 0

Region: chr13 38148405-38148416. Max. coverage (+): 0. Max coverage (-): 0

Region: chr13 38148417-38148429. Max. coverage (+): 0. Max coverage (-): 0

Region: chr13 38148430-38148441. Max. coverage (+): 0. Max coverage (-): 0

Region: chr13 38148442-38148454. Max. coverage (+): 0. Max coverage (-): 0

Region: chr13 38148455-38148467. Max. coverage (+): 0. Max coverage (-): 0

Region: chr13 38148468-38148479. Max. coverage (+): 0. Max coverage (-): 0

Region: chr13 38148480-38148492. Max. coverage (+): 0. Max coverage (-): 0

Region: chr13 38148493-38148504. Max. coverage (+): 0. Max coverage (-): 0

Region: chr13 38148505-38148517. Max. coverage (+): 0. Max coverage (-): 0

Region: chr13 38148518-38148529. Max. coverage (+): 0. Max coverage (-): 0

Region: chr13 38148530-38148542. Max. coverage (+): 0. Max coverage (-): 0

Region: chr13 38148543-38148554. Max. coverage (+): 0. Max coverage (-): 0

Region: chr13 38148555-38148567. Max. coverage (+): 0. Max coverage (-): 0

Region: chr13 38148568-38148579. Max. coverage (+): 0. Max coverage (-): 0

Region: chr13 38148580-38148592. Max. coverage (+): 0. Max coverage (-): 0

Region: chr13 38148593-38148604. Max. coverage (+): 0. Max coverage (-): 5.02

Region: chr13 38148605-38148617. Max. coverage (+): 0. Max coverage (-): 0

Region: chr13 38148618-38148629. Max. coverage (+): 0. Max coverage (-): 0

Region: chr13 38148630-38148642. Max. coverage (+): 0. Max coverage (-): 0

Region: chr13 38148643-38148654. Max. coverage (+): 0. Max coverage (-): 0

Region: chr13 38148655-38148667. Max. coverage (+): 0. Max coverage (-): 0

Region: chr13 38148668-38148679. Max. coverage (+): 0. Max coverage (-): 0

Region: chr13 38148680-38148692. Max. coverage (+): 0. Max coverage (-): 0

Region: chr13 38148693-38148705. Max. coverage (+): 0. Max coverage (-): 0

Region: chr13 38148706-38148717. Max. coverage (+): 0. Max coverage (-): 0

Region: chr13 38148718-38148730. Max. coverage (+): 0. Max coverage (-): 0

Region: chr13 38148731-38148742. Max. coverage (+): 0. Max coverage (-): 0

Region: chr13 38148743-38148755. Max. coverage (+): 0. Max coverage (-): 0

Region: chr13 38148756-38148767. Max. coverage (+): 0. Max coverage (-): 0

Region: chr13 38148768-38148780. Max. coverage (+): 0. Max coverage (-): 0

Region: chr13 38148781-38148792. Max. coverage (+): 0. Max coverage (-): 0

Region: chr13 38148793-38148805. Max. coverage (+): 0. Max coverage (-): 0.26

Region: chr13 38148806-38148817. Max. coverage (+): 0. Max coverage (-): 0.26

Region: chr13 38148818-38148830. Max. coverage (+): 0. Max coverage (-): 0

Region: chr13 38148831-38148842. Max. coverage (+): 0. Max coverage (-): 0

Region: chr13 38148843-38148855. Max. coverage (+): 0. Max coverage (-): 0

Region: chr13 38148856-38148867. Max. coverage (+): 0. Max coverage (-): 6.46

Region: chr13 38148868-38148880. Max. coverage (+): 0. Max coverage (-): 6.53

Region: chr13 38148881-38148892. Max. coverage (+): 0. Max coverage (-): 0.8

Region: chr13 38148893-38148905. Max. coverage (+): 0. Max coverage (-): 10.5

Region: chr13 38148906-38148917. Max. coverage (+): 0. Max coverage (-): 6.21

Region: chr13 38148918-38148930. Max. coverage (+): 0. Max coverage (-): 0

Region: chr13 38148931-38148942. Max. coverage (+): 0. Max coverage (-): 0

Region: chr13 38148943-38148955. Max. coverage (+): 0. Max coverage (-): 9.44

Region: chr13 38148956-38148968. Max. coverage (+): 0. Max coverage (-): 2.04

Region: chr13 38148969-38148980. Max. coverage (+): 0. Max coverage (-): 1.37

Region: chr13 38148981-38148993. Max. coverage (+): 0. Max coverage (-): 0

Region: chr13 38148994-38149005. Max. coverage (+): 0. Max coverage (-): 0

Region: chr13 38149006-38149018. Max. coverage (+): 0. Max coverage (-): 0

Region: chr13 38149019-38149030. Max. coverage (+): 0. Max coverage (-): 0

Region: chr13 38149031-38149043. Max. coverage (+): 0. Max coverage (-): 2.35

Region: chr13 38149044-38149055. Max. coverage (+): 0. Max coverage (-): 3.87

Region: chr13 38149056-38149068. Max. coverage (+): 0. Max coverage (-): 3.87

Region: chr13 38149069-38149080. Max. coverage (+): 0. Max coverage (-): 0

Region: chr13 38149081-38149093. Max. coverage (+): 0. Max coverage (-): 0

Region: chr13 38149094-38149105. Max. coverage (+): 0. Max coverage (-): 0

Region: chr13 38149106-38149118. Max. coverage (+): 0. Max coverage (-): 0

Region: chr13 38149119-38149130. Max. coverage (+): 0. Max coverage (-): 0

Region: chr13 38149131-38149143. Max. coverage (+): 0. Max coverage (-): 0

Region: chr13 38149144-38149155. Max. coverage (+): 0. Max coverage (-): 5.3

Region: chr13 38149156-38149168. Max. coverage (+): 0. Max coverage (-): 2.91

Region: chr13 38149169-38149180. Max. coverage (+): 0. Max coverage (-): 0

Region: chr13 38149181-38149193. Max. coverage (+): 0. Max coverage (-): 0

Region: chr13 38149194-38149205. Max. coverage (+): 0. Max coverage (-): 0

Region: chr13 38149206-38149218. Max. coverage (+): 0. Max coverage (-): 0

Region: chr13 38149219-38149231. Max. coverage (+): 0. Max coverage (-): 0.34

Region: chr13 38149232-38149243. Max. coverage (+): 0. Max coverage (-): 0.34

Region: chr13 38149244-38149256. Max. coverage (+): 0. Max coverage (-): 6.72

Region: chr13 38149257-38149268. Max. coverage (+): 0. Max coverage (-): 0

Region: chr13 38149269-38149281. Max. coverage (+): 0. Max coverage (-): 0

Region: chr13 38149282-38149293. Max. coverage (+): 0. Max coverage (-): 0

Region: chr13 38149294-38149306. Max. coverage (+): 0. Max coverage (-): 0

Region: chr13 38149307-38149318. Max. coverage (+): 0. Max coverage (-): 0

Region: chr13 38149319-38149331. Max. coverage (+): 0. Max coverage (-): 0

Region: chr13 38149332-38149343. Max. coverage (+): 0. Max coverage (-): 0

Region: chr13 38149344-38149356. Max. coverage (+): 0. Max coverage (-): 0

Region: chr13 38149357-38149368. Max. coverage (+): 0. Max coverage (-): 0

Region: chr13 38149369-38149381. Max. coverage (+): 0. Max coverage (-): 0

Region: chr13 38149382-38149393. Max. coverage (+): 0. Max coverage (-): 0

Region: chr13 38149394-38149406. Max. coverage (+): 0. Max coverage (-): 0

Region: chr13 38149407-38149418. Max. coverage (+): 0. Max coverage (-): 0

Region: chr13 38149419-38149431. Max. coverage (+): 0. Max coverage (-): 0

Region: chr13 38149432-38149443. Max. coverage (+): 0. Max coverage (-): 0

Region: chr13 38149444-38149456. Max. coverage (+): 0. Max coverage (-): 0

Region: chr13 38149457-38149468. Max. coverage (+): 0. Max coverage (-): 0

Region: chr13 38149469-38149481. Max. coverage (+): 0. Max coverage (-): 0

Region: chr13 38149482-38149494. Max. coverage (+): 0. Max coverage (-): 5.32

Region: chr13 38149495-38149506. Max. coverage (+): 0. Max coverage (-): 5.32

Region: chr13 38149507-38149519. Max. coverage (+): 0. Max coverage (-): 1

Region: chr13 38149520-38149531. Max. coverage (+): 0. Max coverage (-): 28.84

Region: chr13 38149532-38149544. Max. coverage (+): 0. Max coverage (-): 17.18

Region: chr13 38149545-38149556. Max. coverage (+): 0. Max coverage (-): 0

Region: chr13 38149557-38149569. Max. coverage (+): 0. Max coverage (-): 0

Region: chr13 38149570-38149581. Max. coverage (+): 0. Max coverage (-): 0

Region: chr13 38149582-38149594. Max. coverage (+): 0. Max coverage (-): 0

Region: chr13 38149595-38149606. Max. coverage (+): 0. Max coverage (-): 0

Region: chr13 38149607-38149619. Max. coverage (+): 0. Max coverage (-): 1.8

Region: chr13 38149620-38149631. Max. coverage (+): 0. Max coverage (-): 1.8

Region: chr13 38149632-38149644. Max. coverage (+): 0. Max coverage (-): 0

Region: chr13 38149645-38149656. Max. coverage (+): 0. Max coverage (-): 0

Region: chr13 38149657-38149669. Max. coverage (+): 0. Max coverage (-): 0

Region: chr13 38149670-38149681. Max. coverage (+): 0. Max coverage (-): 0

Region: chr13 38149682-38149694. Max. coverage (+): 0. Max coverage (-): 0

Region: chr13 38149695-38149706. Max. coverage (+): 0. Max coverage (-): 0

Region: chr13 38149707-38149719. Max. coverage (+): 0. Max coverage (-): 0.26

Region: chr13 38149720-38149731. Max. coverage (+): 0. Max coverage (-): 0.26

Region: chr13 38149732-38149744. Max. coverage (+): 0. Max coverage (-): 0

Region: chr13 38149745-38149757. Max. coverage (+): 0. Max coverage (-): 0

Region: chr13 38149758-38149769. Max. coverage (+): 0. Max coverage (-): 0

Region: chr13 38149770-38149782. Max. coverage (+): 0. Max coverage (-): 5.08

Region: chr13 38149783-38149794. Max. coverage (+): 0. Max coverage (-): 5.08

Region: chr13 38149795-38149807. Max. coverage (+): 0. Max coverage (-): 15.76

Region: chr13 38149808-38149819. Max. coverage (+): 0. Max coverage (-): 6.87

Region: chr13 38149820-38149832. Max. coverage (+): 0. Max coverage (-): 0

Region: chr13 38149833-38149844. Max. coverage (+): 0. Max coverage (-): 35.48

Region: chr13 38149845-38149857. Max. coverage (+): 0. Max coverage (-): 30.12

Region: chr13 38149858-38149869. Max. coverage (+): 0. Max coverage (-): 12.97

Region: chr13 38149870-38149882. Max. coverage (+): 0. Max coverage (-): 1.35

Region: chr13 38149883-38149894. Max. coverage (+): 0. Max coverage (-): 0

Region: chr13 38149895-38149907. Max. coverage (+): 0. Max coverage (-): 0

Region: chr13 38149908-38149919. Max. coverage (+): 0. Max coverage (-): 0

Region: chr13 38149920-38149932. Max. coverage (+): 0. Max coverage (-): 0

Region: chr13 38149933-38149944. Max. coverage (+): 0. Max coverage (-): 0

Region: chr13 38149945-38149957. Max. coverage (+): 0. Max coverage (-): 0

Region: chr13 38149958-38149969. Max. coverage (+): 0. Max coverage (-): 0

Region: chr13 38149970-38149982. Max. coverage (+): 0. Max coverage (-): 0

Region: chr13 38149983-38149994. Max. coverage (+): 0. Max coverage (-): 0

Region: chr13 38149995-38150007. Max. coverage (+): 0. Max coverage (-): 0

Region: chr13 38150008-38150020. Max. coverage (+): 0. Max coverage (-): 0

Region: chr13 38150021-38150032. Max. coverage (+): 0. Max coverage (-): 0

Region: chr13 38150033-38150045. Max. coverage (+): 0. Max coverage (-): 0

Region: chr13 38150046-38150057. Max. coverage (+): 0. Max coverage (-): 0

Region: chr13 38150058-38150070. Max. coverage (+): 0. Max coverage (-): 0

Region: chr13 38150071-38150082. Max. coverage (+): 0. Max coverage (-): 0

Region: chr13 38150083-38150095. Max. coverage (+): 0. Max coverage (-): 0

Region: chr13 38150096-38150107. Max. coverage (+): 0. Max coverage (-): 0

Region: chr13 38150108-38150120. Max. coverage (+): 0. Max coverage (-): 0

Region: chr13 38150121-38150132. Max. coverage (+): 0. Max coverage (-): 0

Region: chr13 38150133-38150145. Max. coverage (+): 0. Max coverage (-): 0

Region: chr13 38150146-38150157. Max. coverage (+): 0. Max coverage (-): 0.63

Region: chr13 38150158-38150170. Max. coverage (+): 0. Max coverage (-): 0

Region: chr13 38150171-38150182. Max. coverage (+): 0. Max coverage (-): 0

Region: chr13 38150183-38150195. Max. coverage (+): 0. Max coverage (-): 0

Region: chr13 38150196-38150207. Max. coverage (+): 0. Max coverage (-): 0

Region: chr13 38150208-38150220. Max. coverage (+): 0. Max coverage (-): 0

Region: chr13 38150221-38150232. Max. coverage (+): 0. Max coverage (-): 0

Region: chr13 38150233-38150245. Max. coverage (+): 0. Max coverage (-): 0

Region: chr13 38150246-38150257. Max. coverage (+): 0. Max coverage (-): 0

Region: chr13 38150258-38150270. Max. coverage (+): 0. Max coverage (-): 0

Region: chr13 38150271-38150283. Max. coverage (+): 0. Max coverage (-): 0

Region: chr13 38150284-38150295. Max. coverage (+): 0. Max coverage (-): 0

Region: chr13 38150296-38150308. Max. coverage (+): 0. Max coverage (-): 0

Region: chr13 38150309-38150320. Max. coverage (+): 0. Max coverage (-): 0

Region: chr13 38150321-38150333. Max. coverage (+): 0. Max coverage (-): 0

Region: chr13 38150334-38150345. Max. coverage (+): 0. Max coverage (-): 0

Region: chr13 38150346-38150358. Max. coverage (+): 0. Max coverage (-): 0

Region: chr13 38150359-38150370. Max. coverage (+): 0. Max coverage (-): 0

Region: chr13 38150371-38150383. Max. coverage (+): 0. Max coverage (-): 0

Region: chr13 38150384-38150395. Max. coverage (+): 0. Max coverage (-): 0

Region: chr13 38150396-38150408. Max. coverage (+): 0. Max coverage (-): 0

Region: chr13 38150409-38150420. Max. coverage (+): 0. Max coverage (-): 0

Region: chr13 38150421-38150433. Max. coverage (+): 0. Max coverage (-): 0

Region: chr13 38150434-38150445. Max. coverage (+): 0. Max coverage (-): 0

Region: chr13 38150446-38150458. Max. coverage (+): 0. Max coverage (-): 0

Region: chr13 38150459-38150470. Max. coverage (+): 0. Max coverage (-): 0

Region: chr13 38150471-38150483. Max. coverage (+): 0. Max coverage (-): 0

Region: chr13 38150484-38150495. Max. coverage (+): 0. Max coverage (-): 6.64

Region: chr13 38150496-38150508. Max. coverage (+): 0. Max coverage (-): 0

Region: chr13 38150509-38150520. Max. coverage (+): 0. Max coverage (-): 0

Region: chr13 38150521-38150533. Max. coverage (+): 0. Max coverage (-): 6.83

Region: chr13 38150534-38150546. Max. coverage (+): 0. Max coverage (-): 5.91

Region: chr13 38150547-38150558. Max. coverage (+): 0. Max coverage (-): 18.43

Region: chr13 38150559-38150571. Max. coverage (+): 0. Max coverage (-): 21.96

Region: chr13 38150572-38150583. Max. coverage (+): 0. Max coverage (-): 4.97

Region: chr13 38150584-38150596. Max. coverage (+): 0. Max coverage (-): 4.42

Region: chr13 38150597-38150608. Max. coverage (+): 0. Max coverage (-): 0

Region: chr13 38150609-38150621. Max. coverage (+): 0. Max coverage (-): 0

Region: chr13 38150622-38150633. Max. coverage (+): 0. Max coverage (-): 0

Region: chr13 38150634-38150646. Max. coverage (+): 0. Max coverage (-): 0

Region: chr13 38150647-38150658. Max. coverage (+): 0. Max coverage (-): 0

Region: chr13 38150659-38150671. Max. coverage (+): 0. Max coverage (-): 0

Region: chr13 38150672-38150683. Max. coverage (+): 0. Max coverage (-): 0

Region: chr13 38150684-38150696. Max. coverage (+): 0. Max coverage (-): 0

Region: chr13 38150697-38150708. Max. coverage (+): 0. Max coverage (-): 0

Region: chr13 38150709-38150721. Max. coverage (+): 0. Max coverage (-): 0

Region: chr13 38150722-38150733. Max. coverage (+): 0. Max coverage (-): 0

Region: chr13 38150734-38150746. Max. coverage (+): 0. Max coverage (-): 0

Region: chr13 38150747-38150758. Max. coverage (+): 0. Max coverage (-): 0

Region: chr13 38150759-38150771. Max. coverage (+): 0. Max coverage (-): 0

Region: chr13 38150772-38150783. Max. coverage (+): 0. Max coverage (-): 0

Region: chr13 38150784-38150796. Max. coverage (+): 0. Max coverage (-): 0

Region: chr13 38150797-38150809. Max. coverage (+): 0. Max coverage (-): 0

Region: chr13 38150810-38150821. Max. coverage (+): 0. Max coverage (-): 4.85

Region: chr13 38150822-38150834. Max. coverage (+): 0. Max coverage (-): 11.63

Region: chr13 38150835-38150846. Max. coverage (+): 0. Max coverage (-): 0

Region: chr13 38150847-38150859. Max. coverage (+): 0. Max coverage (-): 0

Region: chr13 38150860-38150871. Max. coverage (+): 0. Max coverage (-): 0

Region: chr13 38150872-38150884. Max. coverage (+): 0. Max coverage (-): 0

Region: chr13 38150885-38150896. Max. coverage (+): 0. Max coverage (-): 0

Region: chr13 38150897-38150909. Max. coverage (+): 0. Max coverage (-): 0

Region: chr13 38150910-38150921. Max. coverage (+): 0. Max coverage (-): 0

Region: chr13 38150922-38150934. Max. coverage (+): 0. Max coverage (-): 0

Region: chr13 38150935-38150946. Max. coverage (+): 0. Max coverage (-): 0

Region: chr13 38150947-38150959. Max. coverage (+): 0. Max coverage (-): 0

Region: chr13 38150960-38150971. Max. coverage (+): 0. Max coverage (-): 0

Region: chr13 38150972-38150984. Max. coverage (+): 0. Max coverage (-): 0

Region: chr13 38150985-38150996. Max. coverage (+): 0. Max coverage (-): 0

Region: chr13 38150997-38151009. Max. coverage (+): 0. Max coverage (-): 0

Region: chr13 38151010-38151021. Max. coverage (+): 0. Max coverage (-): 0

Region: chr13 38151022-38151034. Max. coverage (+): 0. Max coverage (-): 0

Region: chr13 38151035-38151046. Max. coverage (+): 0. Max coverage (-): 0

Region: chr13 38151047-38151059. Max. coverage (+): 0. Max coverage (-): 0

Region: chr13 38151060-38151072. Max. coverage (+): 0. Max coverage (-): 0

Region: chr13 38151073-38151084. Max. coverage (+): 0. Max coverage (-): 0

Region: chr13 38151085-38151097. Max. coverage (+): 0. Max coverage (-): 0

Region: chr13 38151098-38151109. Max. coverage (+): 0. Max coverage (-): 0

Region: chr13 38151110-38151122. Max. coverage (+): 0. Max coverage (-): 0

Region: chr13 38151123-38151134. Max. coverage (+): 0. Max coverage (-): 0

Region: chr13 38151135-38151147. Max. coverage (+): 0. Max coverage (-): 0

Region: chr13 38151148-38151159. Max. coverage (+): 0. Max coverage (-): 0

Region: chr13 38151160-38151172. Max. coverage (+): 0. Max coverage (-): 0

Region: chr13 38151173-38151184. Max. coverage (+): 0. Max coverage (-): 0

Region: chr13 38151185-38151197. Max. coverage (+): 0. Max coverage (-): 0

Region: chr13 38151198-38151209. Max. coverage (+): 0. Max coverage (-): 0

Region: chr13 38151210-38151222. Max. coverage (+): 0. Max coverage (-): 0

Region: chr13 38151223-38151234. Max. coverage (+): 0. Max coverage (-): 0

Region: chr13 38151235-38151247. Max. coverage (+): 0. Max coverage (-): 0

Region: chr13 38151248-38151259. Max. coverage (+): 0. Max coverage (-): 0

Region: chr13 38151260-38151272. Max. coverage (+): 0. Max coverage (-): 0

Region: chr13 38151273-38151284. Max. coverage (+): 0. Max coverage (-): 0

Region: chr13 38151285-38151297. Max. coverage (+): 0. Max coverage (-): 0

Region: chr13 38151298-38151309. Max. coverage (+): 0. Max coverage (-): 0

Region: chr13 38151310-38151322. Max. coverage (+): 0. Max coverage (-): 0

Region: chr13 38151323-38151335. Max. coverage (+): 0. Max coverage (-): 0

Region: chr13 38151336-38151347. Max. coverage (+): 0. Max coverage (-): 0

Region: chr13 38151348-38151360. Max. coverage (+): 0. Max coverage (-): 0

Region: chr13 38151361-38151372. Max. coverage (+): 0. Max coverage (-): 0

Region: chr13 38151373-38151385. Max. coverage (+): 0. Max coverage (-): 0

Region: chr13 38151386-38151397. Max. coverage (+): 0. Max coverage (-): 0

Region: chr13 38151398-38151410. Max. coverage (+): 0. Max coverage (-): 8.89

Region: chr13 38151411-38151422. Max. coverage (+): 0. Max coverage (-): 4.54

Region: chr13 38151423-38151435. Max. coverage (+): 0. Max coverage (-): 5.21

Region: chr13 38151436-38151447. Max. coverage (+): 0. Max coverage (-): 5.21

Region: chr13 38151448-38151460. Max. coverage (+): 0. Max coverage (-): 3.3

Region: chr13 38151461-38151472. Max. coverage (+): 0. Max coverage (-): 3.3

Region: chr13 38151473-38151485. Max. coverage (+): 0. Max coverage (-): 0

Region: chr13 38151486-38151497. Max. coverage (+): 0. Max coverage (-): 9.6

Region: chr13 38151498-38151510. Max. coverage (+): 0. Max coverage (-): 14.61

Region: chr13 38151511-38151522. Max. coverage (+): 0. Max coverage (-): 0

Region: chr13 38151523-38151535. Max. coverage (+): 0. Max coverage (-): 0

Region: chr13 38151536-38151547. Max. coverage (+): 0. Max coverage (-): 0

Region: chr13 38151548-38151560. Max. coverage (+): 0. Max coverage (-): 0

Region: chr13 38151561-38151572. Max. coverage (+): 0. Max coverage (-): 0

Region: chr13 38151573-38151585. Max. coverage (+): 0. Max coverage (-): 0

Region: chr13 38151586-38151598. Max. coverage (+): 0. Max coverage (-): 0

Region: chr13 38151599-38151610. Max. coverage (+): 0. Max coverage (-): 2.22

Region: chr13 38151611-38151623. Max. coverage (+): 0. Max coverage (-): 7.45

Region: chr13 38151624-38151635. Max. coverage (+): 0. Max coverage (-): 0

Region: chr13 38151636-38151648. Max. coverage (+): 0. Max coverage (-): 0

Region: chr13 38151649-38151660. Max. coverage (+): 0. Max coverage (-): 0

Region: chr13 38151661-38151673. Max. coverage (+): 0. Max coverage (-): 0

Region: chr13 38151674-38151685. Max. coverage (+): 0. Max coverage (-): 0

Region: chr13 38151686-38151698. Max. coverage (+): 0. Max coverage (-): 0

Region: chr13 38151699-38151710. Max. coverage (+): 0. Max coverage (-): 8.8

Region: chr13 38151711-38151723. Max. coverage (+): 0. Max coverage (-): 8.8

Region: chr13 38151724-38151735. Max. coverage (+): 0. Max coverage (-): 0

Region: chr13 38151736-38151748. Max. coverage (+): 0. Max coverage (-): 0

Region: chr13 38151749-38151760. Max. coverage (+): 0. Max coverage (-): 0

Region: chr13 38151761-38151773. Max. coverage (+): 0. Max coverage (-): 0

Region: chr13 38151774-38151785. Max. coverage (+): 0. Max coverage (-): 0

Region: chr13 38151786-38151798. Max. coverage (+): 0. Max coverage (-): 0.63

Region: chr13 38151799-38151810. Max. coverage (+): 0. Max coverage (-): 0

Region: chr13 38151811-38151823. Max. coverage (+): 0. Max coverage (-): 0

Region: chr13 38151824-38151836. Max. coverage (+): 0. Max coverage (-): 5.5

Region: chr13 38151837-38151848. Max. coverage (+): 0. Max coverage (-): 6.6

Region: chr13 38151849-38151861. Max. coverage (+): 0. Max coverage (-): 12.87

Region: chr13 38151862-38151873. Max. coverage (+): 0. Max coverage (-): 0

Region: chr13 38151874-38151886. Max. coverage (+): 0. Max coverage (-): 0

Region: chr13 38151887-38151898. Max. coverage (+): 0. Max coverage (-): 0

Region: chr13 38151899-38151911. Max. coverage (+): 0. Max coverage (-): 0

Region: chr13 38151912-38151923. Max. coverage (+): 0. Max coverage (-): 16.39

Region: chr13 38151924-38151936. Max. coverage (+): 0. Max coverage (-): 16.39

Region: chr13 38151937-38151948. Max. coverage (+): 0. Max coverage (-): 0

Region: chr13 38151949-38151961. Max. coverage (+): 0. Max coverage (-): 0

Region: chr13 38151962-38151973. Max. coverage (+): 0. Max coverage (-): 0

Region: chr13 38151974-38151986. Max. coverage (+): 0. Max coverage (-): 0

Region: chr13 38151987-38151998. Max. coverage (+): 0. Max coverage (-): 0

Region: chr13 38151999-38152011. Max. coverage (+): 0. Max coverage (-): 3.22

Region: chr13 38152012-38152023. Max. coverage (+): 0. Max coverage (-): 3.22

Region: chr13 38152024-38152036. Max. coverage (+): 0. Max coverage (-): 0

Region: chr13 38152037-38152048. Max. coverage (+): 0. Max coverage (-): 0

Region: chr13 38152049-38152061. Max. coverage (+): 0. Max coverage (-): 0

Region: chr13 38152062-38152073. Max. coverage (+): 0. Max coverage (-): 0

Region: chr13 38152074-38152086. Max. coverage (+): 0. Max coverage (-): 0

Region: chr13 38152087-38152099. Max. coverage (+): 0. Max coverage (-): 0

Region: chr13 38152100-38152111. Max. coverage (+): 0. Max coverage (-): 4.96

Region: chr13 38152112-38152124. Max. coverage (+): 0. Max coverage (-): 4.96

Region: chr13 38152125-38152136. Max. coverage (+): 0. Max coverage (-): 1.18

Region: chr13 38152137-38152149. Max. coverage (+): 0. Max coverage (-): 0

Region: chr13 38152150-38152161. Max. coverage (+): 0. Max coverage (-): 0

Region: chr13 38152162-38152174. Max. coverage (+): 0. Max coverage (-): 0

Region: chr13 38152175-38152186. Max. coverage (+): 0. Max coverage (-): 0

Region: chr13 38152187-38152199. Max. coverage (+): 0. Max coverage (-): 0

Region: chr13 38152200-38152211. Max. coverage (+): 0. Max coverage (-): 0

Region: chr13 38152212-38152224. Max. coverage (+): 0. Max coverage (-): 0

Region: chr13 38152225-38152236. Max. coverage (+): 0. Max coverage (-): 0

Region: chr13 38152237-38152249. Max. coverage (+): 0. Max coverage (-): 0

Region: chr13 38152250-38152261. Max. coverage (+): 0. Max coverage (-): 0

Region: chr13 38152262-38152274. Max. coverage (+): 0. Max coverage (-): 0

Region: chr13 38152275-38152286. Max. coverage (+): 0. Max coverage (-): 0

Region: chr13 38152287-38152299. Max. coverage (+): 0. Max coverage (-): 0

Region: chr13 38152300-38152311. Max. coverage (+): 0. Max coverage (-): 0

Region: chr13 38152312-38152324. Max. coverage (+): 0. Max coverage (-): 0

Region: chr13 38152325-38152336. Max. coverage (+): 0. Max coverage (-): 0

Region: chr13 38152337-38152349. Max. coverage (+): 0. Max coverage (-): 1.08

Region: chr13 38152350-38152362. Max. coverage (+): 0. Max coverage (-): 1.08

Region: chr13 38152363-38152374. Max. coverage (+): 0. Max coverage (-): 0

Region: chr13 38152375-38152387. Max. coverage (+): 0. Max coverage (-): 0

Region: chr13 38152388-38152399. Max. coverage (+): 0. Max coverage (-): 0

Region: chr13 38152400-38152412. Max. coverage (+): 0. Max coverage (-): 4.27

Region: chr13 38152413-38152424. Max. coverage (+): 0. Max coverage (-): 0

Region: chr13 38152425-38152437. Max. coverage (+): 0. Max coverage (-): 0

Region: chr13 38152438-38152449. Max. coverage (+): 0. Max coverage (-): 0

Region: chr13 38152450-38152462. Max. coverage (+): 0. Max coverage (-): 0

Region: chr13 38152463-38152474. Max. coverage (+): 0. Max coverage (-): 0

Region: chr13 38152475-38152487. Max. coverage (+): 0. Max coverage (-): 0

Region: chr13 38152488-38152499. Max. coverage (+): 0. Max coverage (-): 0

Region: chr13 38152500-38152512. Max. coverage (+): 0. Max coverage (-): 0

Region: chr13 38152513-38152524. Max. coverage (+): 0. Max coverage (-): 0

Region: chr13 38152525-38152537. Max. coverage (+): 0. Max coverage (-): 4.24

Region: chr13 38152538-38152549. Max. coverage (+): 0. Max coverage (-): 0

Region: chr13 38152550-38152562. Max. coverage (+): 0. Max coverage (-): 0

Region: chr13 38152563-38152574. Max. coverage (+): 0. Max coverage (-): 0

Region: chr13 38152575-38152587. Max. coverage (+): 0. Max coverage (-): 0

Region: chr13 38152588-38152599. Max. coverage (+): 0. Max coverage (-): 0

Region: chr13 38152600-38152612. Max. coverage (+): 0. Max coverage (-): 0

Region: chr13 38152613-38152625. Max. coverage (+): 0. Max coverage (-): 0

Region: chr13 38152626-38152637. Max. coverage (+): 0. Max coverage (-): 0

Region: chr13 38152638-38152650. Max. coverage (+): 0. Max coverage (-): 0

Region: chr13 38152651-38152662. Max. coverage (+): 0. Max coverage (-): 0

Region: chr13 38152663-38152675. Max. coverage (+): 0. Max coverage (-): 0

Region: chr13 38152676-38152687. Max. coverage (+): 0. Max coverage (-): 0

Region: chr13 38152688-38152700. Max. coverage (+): 0. Max coverage (-): 0

Region: chr13 38152701-38152712. Max. coverage (+): 0. Max coverage (-): 0

Region: chr13 38152713-38152725. Max. coverage (+): 0. Max coverage (-): 0

Region: chr13 38152726-38152737. Max. coverage (+): 0. Max coverage (-): 0

Region: chr13 38152738-38152750. Max. coverage (+): 0. Max coverage (-): 0

Region: chr13 38152751-38152762. Max. coverage (+): 0. Max coverage (-): 0

Region: chr13 38152763-38152775. Max. coverage (+): 0. Max coverage (-): 0

Region: chr13 38152776-38152787. Max. coverage (+): 0. Max coverage (-): 0

Region: chr13 38152788-38152800. Max. coverage (+): 0. Max coverage (-): 0

Region: chr13 38152801-38152812. Max. coverage (+): 0. Max coverage (-): 0

Region: chr13 38152813-38152825. Max. coverage (+): 0. Max coverage (-): 0

Region: chr13 38152826-38152837. Max. coverage (+): 0. Max coverage (-): 0

Region: chr13 38152838-38152850. Max. coverage (+): 0. Max coverage (-): 0

Region: chr13 38152851-38152862. Max. coverage (+): 0. Max coverage (-): 0

Region: chr13 38152863-38152875. Max. coverage (+): 0. Max coverage (-): 0

Region: chr13 38152876-38152888. Max. coverage (+): 0. Max coverage (-): 0

Region: chr13 38152889-38152900. Max. coverage (+): 0. Max coverage (-): 0

Region: chr13 38152901-38152913. Max. coverage (+): 0. Max coverage (-): 0

Region: chr13 38152914-38152925. Max. coverage (+): 0. Max coverage (-): 0

Region: chr13 38152926-38152938. Max. coverage (+): 0. Max coverage (-): 0

Region: chr13 38152939-38152950. Max. coverage (+): 0. Max coverage (-): 0

Region: chr13 38152951-38152963. Max. coverage (+): 0. Max coverage (-): 0

Region: chr13 38152964-38152975. Max. coverage (+): 0. Max coverage (-): 0

Region: chr13 38152976-38152988. Max. coverage (+): 0. Max coverage (-): 0

Region: chr13 38152989-38153000. Max. coverage (+): 0. Max coverage (-): 0

Region: chr13 38153001-38153013. Max. coverage (+): 0. Max coverage (-): 0

Region: chr13 38153014-38153025. Max. coverage (+): 0. Max coverage (-): 0

Region: chr13 38153026-38153038. Max. coverage (+): 0. Max coverage (-): 0

Region: chr13 38153039-38153050. Max. coverage (+): 0. Max coverage (-): 0

Region: chr13 38153051-38153063. Max. coverage (+): 0. Max coverage (-): 0

Region: chr13 38153064-38153075. Max. coverage (+): 0. Max coverage (-): 0

Region: chr13 38153076-38153088. Max. coverage (+): 0. Max coverage (-): 0

Region: chr13 38153089-38153100. Max. coverage (+): 0. Max coverage (-): 0

Region: chr13 38153101-38153113. Max. coverage (+): 0. Max coverage (-): 0

Region: chr13 38153114-38153125. Max. coverage (+): 0. Max coverage (-): 0

Region: chr13 38153126-38153138. Max. coverage (+): 0. Max coverage (-): 0

Region: chr13 38153139-38153151. Max. coverage (+): 0. Max coverage (-): 0

Region: chr13 38153152-38153163. Max. coverage (+): 0. Max coverage (-): 0

Region: chr13 38153164-38153176. Max. coverage (+): 0. Max coverage (-): 0

Region: chr13 38153177-38153188. Max. coverage (+): 0. Max coverage (-): 0

Region: chr13 38153189-38153201. Max. coverage (+): 0. Max coverage (-): 0

Region: chr13 38153202-38153213. Max. coverage (+): 0. Max coverage (-): 0

Region: chr13 38153214-38153226. Max. coverage (+): 0. Max coverage (-): 0

Region: chr13 38153227-38153238. Max. coverage (+): 0. Max coverage (-): 2.83

Region: chr13 38153239-38153251. Max. coverage (+): 0. Max coverage (-): 2.83

Region: chr13 38153252-38153263. Max. coverage (+): 0. Max coverage (-): 0

Region: chr13 38153264-. Max. coverage (+): 0. Max coverage (-): 0

RepeatMasker Color Code

**+**

100-98% Identity

<98-95% Identity

<95-90% Identity

<90-85% Identity

<85-80% Identity

<80-75% Identity

<75-70% Identity

<70% Identity

**-**

Gene Set Color Code

**+**

Gene

Pseudogene

**-**

Topology/Coverage Color Code

Coverage Plus Strand

Coverage Minus Strand

Mainstrand: Plus

Mainstrand: Minus

Complementary Strand

Flanking Region  
(if option -flank >0)

Gene Set Annotation  

**1. SLC6A9 (protein coding, ENSBTAG00000006043) Tr:00000011456 Ex:2**: 38153204-38153318 (-)

  
RepeatMasker Annotation  

**1. L2b**: 38147496-38147536 (-), Divergence to consensus: 19.5%  
**2. L2**: 38147609-38147675 (-), Divergence to consensus: 40.3%  
**3. MIRb**: 38150210-38150375 (+), Divergence to consensus: 40.5%

  
Transcription Factor Binding Sites  

**RFX4\_2** (Sequence: GTATCCAAG (-): 38148015)
