# Supplementary material for: Small RNA sequencing of cryopreserved semen from single bull revealed altered miRNAs and piRNAs expression between High- and Low-motile sperm populations
Source: BMC Genomics. 2017 Jan 4;18:14. doi: 10.1186/s12864-016-3394-7 (PMC5209821; doi:10.1186/s12864-016-3394-7)
Supplement: Additional file 4: — Details for each piRNA clusters found in Low Motile (LM) sperm fraction. Genes, repeats, transposable elements and transcription factors binding sites falling within the cluster regions were reported. (ZIP 1034 kb) [file 12864_2016_3394_MOESM4_ESM.zip › 11.html]

piRNA cluster 11


Predicted piRNA cluster no. 11     previous   next
  

Show proTRAC run info
Hide proTRAC run info

================================= proTRAC ====================================  
VERSION: 2.1                                    LAST MODIFIED: 06. October 2015  
  
Please cite:  
Rosenkranz D, Zischler H. proTRAC - a software for probabilistic piRNA cluster  
detection, visualization and analysis. 2012. BMC Bioinformatics 13:5.  
  
and (for proTRAC 2.0 and later):  
Rosenkranz D, Rudloff S, Bastuck K, Ketting RF, Zischler H. Tupaia small RNAs  
provide insights into function and evolution of RNAi-based transposon defense  
in mammals. 2015. RNA 21(5):911-922.  
  
Contact:  
David Rosenkranz  
Institute of Anthropology, small RNA group  
Johannes Gutenberg University Mainz  
email: rosenkranz@uni-mainz.de  
  
You can find the latest proTRAC version at:  
http://sourceforge.net/projects/protrac/files  
http://www.smallRNAgroup-mainz.de/software  
==============================================================================  
  
PARAMETERS:  
Map file: .............../storage/core/barbara/genhome/smallRNA/fertility/Sample\_not\_motile/pirna/Sample\_not\_motile\_26-33\_collapsed.fa.no-dust.map.weighted-10000-1000-b-0  
Genome file: ............/storage/core/barbara/genhome/smallRNA/fertility/Sample\_all/pirna/bt\_311\_chrY.fa  
RepeatMasker annotation: /storage/genomes/bt\_umd31/GCF\_000003055.6\_Bos\_taurus\_UMD\_3.1.1\_repeatMasker\_chr.out  
GeneSet:................./storage/core/barbara/genhome/smallRNA/fertility/Sample\_all/pirna/full.gtf  
  
Significant (p<=0.01) hit density will be calculated based  
on observed hit distribution.  
  
Sliding window size: ........................................ 5000 bp  
Sliding window increament: .................................. 1000 bp  
Normalize each hit by number of genomic hits: ............... 1 [0=no/1=yes]  
Normalize each hit by number of sequence reads: ............. 1 [0=no/1=yes]  
Normalize values (-> per million mapped reads): ............. 1 [0=no/1=yes]  
Min. fraction of hits with 1T(U) or 10A: .................... 0.75  
Alternatively: Min. fraction of hits with 1T(U) and 10A: .... 0.5  
Min. fraction of hits with typical piRNA length: ............ 0.75  
Typical piRNA length: ....................................... 26-33 nt  
Min. size of a piRNA cluster: ............................... 5000 bp.  
Min. number of hits (absolute): ............................. 0  
Min. number of hits (normalized): ........................... 0  
Min. fraction of hits on the mainstrand: .................... 0.75  
Top fraction of mapped sequences (in terms of read counts): . 1%  
Top fraction accounts for max. n% of sequence reads: ........ 90%  
Min. fraction of hits on each arm of a bidirectional cluster: 0.1  
Output image file for each cluster: ......................... 0 [0=no/1=yes]  
Output html file for each cluster: .......................... 1 [0=no/1=yes]  
Output a summary table: ..................................... 1 [0=no/1=yes]  
Output a FASTA file for each cluster (piRNA sequences): ..... 1 [0=no/1=yes]  
Output a FASTA file comprising cluster sequences: ........... 1 [0=no/1=yes]  
Search DNA motifs in clusters: .............................. 1 [0=no/1=yes]  
Output flanking sequences: +/- .............................. 0 bp  
Output ~.pTi file: .......................................... 1 [0=no/1=yes]  
==============================================================================  
  
  
Genome size (without gaps): ............ 2678902517 bp  
Gaps (N/X/-): .......................... 53837044 bp  
Mapped reads: .......................... 738059667487  
Non-identical sequences: ............... 277001  
Genomic hits: .......................... 533816  
Significant densitiy of mapped reads: .. 15118061 reads/kb

Show proTRAC cluster info
Hide proTRAC cluster info

|  |  |
| --- | --- |
| Location | chr14 |
| Coordinates | 479293-487355 |
| Size [bp] | 8063 |
| Sequence hit loci | 403 |
| Mapped reads (normalized) | 918825717 |
| Mapped reads (normalized) per kb | 113955812.6 |
| Normalized reads with 1T (1U) | 76.3% |
| Normalized reads with 10A | 27.7% |
| Normalized reads with length 26-33 nt | 100% |
| Normalized reads on the main strand(s) | 92.6% |
| Predicted directionality | mono:plus |

100%

0%

1T (1U)  
reads

10A reads

26-33 nt  
reads

reads on mainstrand

**Either the amount of reads with 1T (1U) OR 10A has to exceed 75% (set with option: -1Tor10A)  
Alternatively the amount of reads with 1T (1U) AND 10A has to exceed 50% (set with option: -1Tand10A)  
Minimum amount of reads with preferred size is 75% (set with option: -pisize)  
Minimum amount of reads on the main strand(s) is 75% (set with option: -clstrand)**

Show read coverage
Hide read coverage

WHAT DO I SEE HERE?  
This chart shows the location of mapped sequence reads within a predicted piRNA cluster. The color refers to the number of genomic hits produced by the sequence read in question. A dark red bar indicates that this sequence read produces many other hits elsewhere in the genome. Many adjacent red or yellow bars can indicate the presence of a multi-copy element such as transposons or rRNA genes. A dark green bar indicates that this sequence read maps uniquely to this locus.

1 hit

2-5 hits

6-10 hits

11-20 hits

21-50 hits

51-100 hits

> 100 hits

chr14

479293

487355

Gene Set

RepeatMasker

Mapped  
Reads

120.2

plus strand

minus strand

120.2

Region: chr14 38153265-479301. Max. coverage (+): 0. Max coverage (-): 1.2

Region: chr14 479302-479317. Max. coverage (+): 0. Max coverage (-): 0

Region: chr14 479318-479333. Max. coverage (+): 0. Max coverage (-): 0

Region: chr14 479334-479349. Max. coverage (+): 0. Max coverage (-): 0

Region: chr14 479350-479365. Max. coverage (+): 0. Max coverage (-): 0

Region: chr14 479366-479381. Max. coverage (+): 0. Max coverage (-): 0

Region: chr14 479382-479397. Max. coverage (+): 0. Max coverage (-): 0

Region: chr14 479398-479413. Max. coverage (+): 0. Max coverage (-): 0

Region: chr14 479414-479430. Max. coverage (+): 0. Max coverage (-): 0

Region: chr14 479431-479446. Max. coverage (+): 0. Max coverage (-): 0

Region: chr14 479447-479462. Max. coverage (+): 0. Max coverage (-): 0

Region: chr14 479463-479478. Max. coverage (+): 0. Max coverage (-): 0

Region: chr14 479479-479494. Max. coverage (+): 0. Max coverage (-): 0

Region: chr14 479495-479510. Max. coverage (+): 0. Max coverage (-): 0

Region: chr14 479511-479526. Max. coverage (+): 0. Max coverage (-): 0

Region: chr14 479527-479542. Max. coverage (+): 0. Max coverage (-): 0

Region: chr14 479543-479559. Max. coverage (+): 0. Max coverage (-): 0

Region: chr14 479560-479575. Max. coverage (+): 0. Max coverage (-): 0

Region: chr14 479576-479591. Max. coverage (+): 0. Max coverage (-): 0

Region: chr14 479592-479607. Max. coverage (+): 0. Max coverage (-): 0

Region: chr14 479608-479623. Max. coverage (+): 0. Max coverage (-): 0

Region: chr14 479624-479639. Max. coverage (+): 0. Max coverage (-): 0

Region: chr14 479640-479655. Max. coverage (+): 0. Max coverage (-): 0

Region: chr14 479656-479671. Max. coverage (+): 0. Max coverage (-): 0

Region: chr14 479672-479688. Max. coverage (+): 0. Max coverage (-): 0

Region: chr14 479689-479704. Max. coverage (+): 0. Max coverage (-): 0

Region: chr14 479705-479720. Max. coverage (+): 0. Max coverage (-): 0

Region: chr14 479721-479736. Max. coverage (+): 0. Max coverage (-): 0

Region: chr14 479737-479752. Max. coverage (+): 0. Max coverage (-): 0

Region: chr14 479753-479768. Max. coverage (+): 0. Max coverage (-): 0

Region: chr14 479769-479784. Max. coverage (+): 0. Max coverage (-): 0

Region: chr14 479785-479800. Max. coverage (+): 0. Max coverage (-): 0

Region: chr14 479801-479817. Max. coverage (+): 0. Max coverage (-): 0.1

Region: chr14 479818-479833. Max. coverage (+): 0. Max coverage (-): 0

Region: chr14 479834-479849. Max. coverage (+): 0. Max coverage (-): 0

Region: chr14 479850-479865. Max. coverage (+): 0. Max coverage (-): 0

Region: chr14 479866-479881. Max. coverage (+): 0. Max coverage (-): 0

Region: chr14 479882-479897. Max. coverage (+): 0. Max coverage (-): 0

Region: chr14 479898-479913. Max. coverage (+): 0. Max coverage (-): 0

Region: chr14 479914-479929. Max. coverage (+): 0. Max coverage (-): 0

Region: chr14 479930-479946. Max. coverage (+): 0. Max coverage (-): 0

Region: chr14 479947-479962. Max. coverage (+): 0. Max coverage (-): 0

Region: chr14 479963-479978. Max. coverage (+): 0. Max coverage (-): 0

Region: chr14 479979-479994. Max. coverage (+): 0. Max coverage (-): 0

Region: chr14 479995-480010. Max. coverage (+): 0. Max coverage (-): 0

Region: chr14 480011-480026. Max. coverage (+): 0. Max coverage (-): 0

Region: chr14 480027-480042. Max. coverage (+): 0. Max coverage (-): 0

Region: chr14 480043-480058. Max. coverage (+): 0. Max coverage (-): 6.53

Region: chr14 480059-480075. Max. coverage (+): 0. Max coverage (-): 5.9

Region: chr14 480076-480091. Max. coverage (+): 0. Max coverage (-): 0.09

Region: chr14 480092-480107. Max. coverage (+): 0. Max coverage (-): 0

Region: chr14 480108-480123. Max. coverage (+): 0. Max coverage (-): 0

Region: chr14 480124-480139. Max. coverage (+): 0. Max coverage (-): 0

Region: chr14 480140-480155. Max. coverage (+): 0. Max coverage (-): 0

Region: chr14 480156-480171. Max. coverage (+): 0. Max coverage (-): 0

Region: chr14 480172-480187. Max. coverage (+): 0. Max coverage (-): 0

Region: chr14 480188-480204. Max. coverage (+): 0. Max coverage (-): 0

Region: chr14 480205-480220. Max. coverage (+): 0. Max coverage (-): 0

Region: chr14 480221-480236. Max. coverage (+): 0. Max coverage (-): 0

Region: chr14 480237-480252. Max. coverage (+): 0. Max coverage (-): 0

Region: chr14 480253-480268. Max. coverage (+): 0. Max coverage (-): 0

Region: chr14 480269-480284. Max. coverage (+): 0. Max coverage (-): 0

Region: chr14 480285-480300. Max. coverage (+): 0. Max coverage (-): 0

Region: chr14 480301-480317. Max. coverage (+): 0. Max coverage (-): 0

Region: chr14 480318-480333. Max. coverage (+): 0. Max coverage (-): 0

Region: chr14 480334-480349. Max. coverage (+): 0. Max coverage (-): 0

Region: chr14 480350-480365. Max. coverage (+): 0. Max coverage (-): 0

Region: chr14 480366-480381. Max. coverage (+): 0. Max coverage (-): 0

Region: chr14 480382-480397. Max. coverage (+): 0. Max coverage (-): 0

Region: chr14 480398-480413. Max. coverage (+): 0. Max coverage (-): 0

Region: chr14 480414-480429. Max. coverage (+): 0. Max coverage (-): 0

Region: chr14 480430-480446. Max. coverage (+): 0. Max coverage (-): 0

Region: chr14 480447-480462. Max. coverage (+): 0. Max coverage (-): 0

Region: chr14 480463-480478. Max. coverage (+): 0. Max coverage (-): 0

Region: chr14 480479-480494. Max. coverage (+): 0. Max coverage (-): 0

Region: chr14 480495-480510. Max. coverage (+): 0. Max coverage (-): 0

Region: chr14 480511-480526. Max. coverage (+): 0. Max coverage (-): 0

Region: chr14 480527-480542. Max. coverage (+): 0. Max coverage (-): 6.49

Region: chr14 480543-480558. Max. coverage (+): 0. Max coverage (-): 0

Region: chr14 480559-480575. Max. coverage (+): 0. Max coverage (-): 0

Region: chr14 480576-480591. Max. coverage (+): 0. Max coverage (-): 0

Region: chr14 480592-480607. Max. coverage (+): 0. Max coverage (-): 0

Region: chr14 480608-480623. Max. coverage (+): 0. Max coverage (-): 0

Region: chr14 480624-480639. Max. coverage (+): 0. Max coverage (-): 0

Region: chr14 480640-480655. Max. coverage (+): 0. Max coverage (-): 0

Region: chr14 480656-480671. Max. coverage (+): 0. Max coverage (-): 0

Region: chr14 480672-480687. Max. coverage (+): 0. Max coverage (-): 0

Region: chr14 480688-480704. Max. coverage (+): 0. Max coverage (-): 0

Region: chr14 480705-480720. Max. coverage (+): 0. Max coverage (-): 0

Region: chr14 480721-480736. Max. coverage (+): 0. Max coverage (-): 0

Region: chr14 480737-480752. Max. coverage (+): 0. Max coverage (-): 0

Region: chr14 480753-480768. Max. coverage (+): 0. Max coverage (-): 0

Region: chr14 480769-480784. Max. coverage (+): 0. Max coverage (-): 0

Region: chr14 480785-480800. Max. coverage (+): 0. Max coverage (-): 0

Region: chr14 480801-480816. Max. coverage (+): 0. Max coverage (-): 0

Region: chr14 480817-480833. Max. coverage (+): 0. Max coverage (-): 0

Region: chr14 480834-480849. Max. coverage (+): 0. Max coverage (-): 0.97

Region: chr14 480850-480865. Max. coverage (+): 0. Max coverage (-): 0

Region: chr14 480866-480881. Max. coverage (+): 0. Max coverage (-): 0

Region: chr14 480882-480897. Max. coverage (+): 0. Max coverage (-): 0

Region: chr14 480898-480913. Max. coverage (+): 0. Max coverage (-): 0

Region: chr14 480914-480929. Max. coverage (+): 0. Max coverage (-): 0

Region: chr14 480930-480945. Max. coverage (+): 0. Max coverage (-): 0

Region: chr14 480946-480962. Max. coverage (+): 0. Max coverage (-): 0

Region: chr14 480963-480978. Max. coverage (+): 0. Max coverage (-): 0

Region: chr14 480979-480994. Max. coverage (+): 0. Max coverage (-): 0

Region: chr14 480995-481010. Max. coverage (+): 0. Max coverage (-): 0

Region: chr14 481011-481026. Max. coverage (+): 0. Max coverage (-): 0

Region: chr14 481027-481042. Max. coverage (+): 0. Max coverage (-): 0

Region: chr14 481043-481058. Max. coverage (+): 0. Max coverage (-): 0

Region: chr14 481059-481074. Max. coverage (+): 0. Max coverage (-): 0

Region: chr14 481075-481091. Max. coverage (+): 0. Max coverage (-): 0

Region: chr14 481092-481107. Max. coverage (+): 0. Max coverage (-): 0

Region: chr14 481108-481123. Max. coverage (+): 0. Max coverage (-): 0

Region: chr14 481124-481139. Max. coverage (+): 0. Max coverage (-): 0

Region: chr14 481140-481155. Max. coverage (+): 0. Max coverage (-): 0

Region: chr14 481156-481171. Max. coverage (+): 0. Max coverage (-): 0

Region: chr14 481172-481187. Max. coverage (+): 0. Max coverage (-): 0

Region: chr14 481188-481203. Max. coverage (+): 0. Max coverage (-): 0

Region: chr14 481204-481220. Max. coverage (+): 0. Max coverage (-): 0

Region: chr14 481221-481236. Max. coverage (+): 0. Max coverage (-): 0

Region: chr14 481237-481252. Max. coverage (+): 0. Max coverage (-): 0

Region: chr14 481253-481268. Max. coverage (+): 0. Max coverage (-): 0

Region: chr14 481269-481284. Max. coverage (+): 0. Max coverage (-): 0

Region: chr14 481285-481300. Max. coverage (+): 0. Max coverage (-): 0

Region: chr14 481301-481316. Max. coverage (+): 0. Max coverage (-): 0

Region: chr14 481317-481332. Max. coverage (+): 0. Max coverage (-): 0

Region: chr14 481333-481349. Max. coverage (+): 0. Max coverage (-): 0

Region: chr14 481350-481365. Max. coverage (+): 0. Max coverage (-): 0

Region: chr14 481366-481381. Max. coverage (+): 0. Max coverage (-): 0

Region: chr14 481382-481397. Max. coverage (+): 0. Max coverage (-): 0

Region: chr14 481398-481413. Max. coverage (+): 0. Max coverage (-): 0

Region: chr14 481414-481429. Max. coverage (+): 0. Max coverage (-): 0

Region: chr14 481430-481445. Max. coverage (+): 0. Max coverage (-): 0

Region: chr14 481446-481461. Max. coverage (+): 0. Max coverage (-): 0

Region: chr14 481462-481478. Max. coverage (+): 0. Max coverage (-): 0

Region: chr14 481479-481494. Max. coverage (+): 0. Max coverage (-): 0

Region: chr14 481495-481510. Max. coverage (+): 0. Max coverage (-): 0

Region: chr14 481511-481526. Max. coverage (+): 0. Max coverage (-): 0

Region: chr14 481527-481542. Max. coverage (+): 0. Max coverage (-): 0

Region: chr14 481543-481558. Max. coverage (+): 0. Max coverage (-): 0

Region: chr14 481559-481574. Max. coverage (+): 0. Max coverage (-): 0

Region: chr14 481575-481590. Max. coverage (+): 0. Max coverage (-): 0

Region: chr14 481591-481607. Max. coverage (+): 0. Max coverage (-): 0

Region: chr14 481608-481623. Max. coverage (+): 0. Max coverage (-): 0

Region: chr14 481624-481639. Max. coverage (+): 0. Max coverage (-): 0

Region: chr14 481640-481655. Max. coverage (+): 0. Max coverage (-): 0

Region: chr14 481656-481671. Max. coverage (+): 0. Max coverage (-): 0

Region: chr14 481672-481687. Max. coverage (+): 0. Max coverage (-): 0.74

Region: chr14 481688-481703. Max. coverage (+): 0. Max coverage (-): 0

Region: chr14 481704-481719. Max. coverage (+): 0. Max coverage (-): 0

Region: chr14 481720-481736. Max. coverage (+): 0. Max coverage (-): 0

Region: chr14 481737-481752. Max. coverage (+): 0. Max coverage (-): 4.79

Region: chr14 481753-481768. Max. coverage (+): 0. Max coverage (-): 0

Region: chr14 481769-481784. Max. coverage (+): 0. Max coverage (-): 0

Region: chr14 481785-481800. Max. coverage (+): 0. Max coverage (-): 0

Region: chr14 481801-481816. Max. coverage (+): 0. Max coverage (-): 0

Region: chr14 481817-481832. Max. coverage (+): 0. Max coverage (-): 0

Region: chr14 481833-481848. Max. coverage (+): 0. Max coverage (-): 0.27

Region: chr14 481849-481865. Max. coverage (+): 0. Max coverage (-): 0.27

Region: chr14 481866-481881. Max. coverage (+): 0. Max coverage (-): 0

Region: chr14 481882-481897. Max. coverage (+): 0. Max coverage (-): 0

Region: chr14 481898-481913. Max. coverage (+): 0. Max coverage (-): 2.57

Region: chr14 481914-481929. Max. coverage (+): 0. Max coverage (-): 0

Region: chr14 481930-481945. Max. coverage (+): 0. Max coverage (-): 0

Region: chr14 481946-481961. Max. coverage (+): 0. Max coverage (-): 0

Region: chr14 481962-481977. Max. coverage (+): 0. Max coverage (-): 0

Region: chr14 481978-481994. Max. coverage (+): 0. Max coverage (-): 0

Region: chr14 481995-482010. Max. coverage (+): 0. Max coverage (-): 0

Region: chr14 482011-482026. Max. coverage (+): 0. Max coverage (-): 0

Region: chr14 482027-482042. Max. coverage (+): 0. Max coverage (-): 0

Region: chr14 482043-482058. Max. coverage (+): 0. Max coverage (-): 0

Region: chr14 482059-482074. Max. coverage (+): 0. Max coverage (-): 0

Region: chr14 482075-482090. Max. coverage (+): 0. Max coverage (-): 0

Region: chr14 482091-482106. Max. coverage (+): 0. Max coverage (-): 0

Region: chr14 482107-482123. Max. coverage (+): 0. Max coverage (-): 0

Region: chr14 482124-482139. Max. coverage (+): 0. Max coverage (-): 0

Region: chr14 482140-482155. Max. coverage (+): 0. Max coverage (-): 0

Region: chr14 482156-482171. Max. coverage (+): 0. Max coverage (-): 0

Region: chr14 482172-482187. Max. coverage (+): 0. Max coverage (-): 0

Region: chr14 482188-482203. Max. coverage (+): 0. Max coverage (-): 0

Region: chr14 482204-482219. Max. coverage (+): 0. Max coverage (-): 0

Region: chr14 482220-482235. Max. coverage (+): 0. Max coverage (-): 0

Region: chr14 482236-482252. Max. coverage (+): 0. Max coverage (-): 0.17

Region: chr14 482253-482268. Max. coverage (+): 0. Max coverage (-): 0

Region: chr14 482269-482284. Max. coverage (+): 0. Max coverage (-): 0

Region: chr14 482285-482300. Max. coverage (+): 0. Max coverage (-): 0

Region: chr14 482301-482316. Max. coverage (+): 0. Max coverage (-): 0

Region: chr14 482317-482332. Max. coverage (+): 0. Max coverage (-): 0

Region: chr14 482333-482348. Max. coverage (+): 0. Max coverage (-): 0

Region: chr14 482349-482365. Max. coverage (+): 0. Max coverage (-): 0

Region: chr14 482366-482381. Max. coverage (+): 0. Max coverage (-): 0

Region: chr14 482382-482397. Max. coverage (+): 0. Max coverage (-): 0

Region: chr14 482398-482413. Max. coverage (+): 0. Max coverage (-): 0

Region: chr14 482414-482429. Max. coverage (+): 0. Max coverage (-): 0

Region: chr14 482430-482445. Max. coverage (+): 0. Max coverage (-): 0

Region: chr14 482446-482461. Max. coverage (+): 0. Max coverage (-): 0

Region: chr14 482462-482477. Max. coverage (+): 0. Max coverage (-): 0

Region: chr14 482478-482494. Max. coverage (+): 0. Max coverage (-): 0

Region: chr14 482495-482510. Max. coverage (+): 0. Max coverage (-): 0

Region: chr14 482511-482526. Max. coverage (+): 0. Max coverage (-): 0

Region: chr14 482527-482542. Max. coverage (+): 0. Max coverage (-): 0

Region: chr14 482543-482558. Max. coverage (+): 0. Max coverage (-): 0

Region: chr14 482559-482574. Max. coverage (+): 0. Max coverage (-): 0

Region: chr14 482575-482590. Max. coverage (+): 0. Max coverage (-): 6.44

Region: chr14 482591-482606. Max. coverage (+): 0. Max coverage (-): 14.92

Region: chr14 482607-482623. Max. coverage (+): 0. Max coverage (-): 0

Region: chr14 482624-482639. Max. coverage (+): 0. Max coverage (-): 0

Region: chr14 482640-482655. Max. coverage (+): 0. Max coverage (-): 0

Region: chr14 482656-482671. Max. coverage (+): 0. Max coverage (-): 0

Region: chr14 482672-482687. Max. coverage (+): 0. Max coverage (-): 0

Region: chr14 482688-482703. Max. coverage (+): 0. Max coverage (-): 0

Region: chr14 482704-482719. Max. coverage (+): 0. Max coverage (-): 0

Region: chr14 482720-482735. Max. coverage (+): 0. Max coverage (-): 0

Region: chr14 482736-482752. Max. coverage (+): 0. Max coverage (-): 0

Region: chr14 482753-482768. Max. coverage (+): 0. Max coverage (-): 0

Region: chr14 482769-482784. Max. coverage (+): 0. Max coverage (-): 0

Region: chr14 482785-482800. Max. coverage (+): 0. Max coverage (-): 0

Region: chr14 482801-482816. Max. coverage (+): 0. Max coverage (-): 0

Region: chr14 482817-482832. Max. coverage (+): 0. Max coverage (-): 0

Region: chr14 482833-482848. Max. coverage (+): 0. Max coverage (-): 0

Region: chr14 482849-482864. Max. coverage (+): 0. Max coverage (-): 0

Region: chr14 482865-482881. Max. coverage (+): 0. Max coverage (-): 0

Region: chr14 482882-482897. Max. coverage (+): 0. Max coverage (-): 0

Region: chr14 482898-482913. Max. coverage (+): 0. Max coverage (-): 0

Region: chr14 482914-482929. Max. coverage (+): 0. Max coverage (-): 0

Region: chr14 482930-482945. Max. coverage (+): 0. Max coverage (-): 0

Region: chr14 482946-482961. Max. coverage (+): 0. Max coverage (-): 0

Region: chr14 482962-482977. Max. coverage (+): 0. Max coverage (-): 0.74

Region: chr14 482978-482993. Max. coverage (+): 0. Max coverage (-): 0

Region: chr14 482994-483010. Max. coverage (+): 0. Max coverage (-): 0

Region: chr14 483011-483026. Max. coverage (+): 0. Max coverage (-): 0

Region: chr14 483027-483042. Max. coverage (+): 3.64. Max coverage (-): 10.2

Region: chr14 483043-483058. Max. coverage (+): 3.19. Max coverage (-): 10.2

Region: chr14 483059-483074. Max. coverage (+): 2.88. Max coverage (-): 6.85

Region: chr14 483075-483090. Max. coverage (+): 0. Max coverage (-): 0

Region: chr14 483091-483106. Max. coverage (+): 0. Max coverage (-): 0

Region: chr14 483107-483122. Max. coverage (+): 0. Max coverage (-): 0

Region: chr14 483123-483139. Max. coverage (+): 0. Max coverage (-): 0

Region: chr14 483140-483155. Max. coverage (+): 0. Max coverage (-): 0

Region: chr14 483156-483171. Max. coverage (+): 14.07. Max coverage (-): 0

Region: chr14 483172-483187. Max. coverage (+): 14.07. Max coverage (-): 0

Region: chr14 483188-483203. Max. coverage (+): 0. Max coverage (-): 0

Region: chr14 483204-483219. Max. coverage (+): 0. Max coverage (-): 0

Region: chr14 483220-483235. Max. coverage (+): 0. Max coverage (-): 0

Region: chr14 483236-483251. Max. coverage (+): 0. Max coverage (-): 0

Region: chr14 483252-483268. Max. coverage (+): 0. Max coverage (-): 0

Region: chr14 483269-483284. Max. coverage (+): 0. Max coverage (-): 0

Region: chr14 483285-483300. Max. coverage (+): 5.25. Max coverage (-): 0

Region: chr14 483301-483316. Max. coverage (+): 0. Max coverage (-): 0

Region: chr14 483317-483332. Max. coverage (+): 0. Max coverage (-): 0

Region: chr14 483333-483348. Max. coverage (+): 0. Max coverage (-): 0

Region: chr14 483349-483364. Max. coverage (+): 0. Max coverage (-): 0

Region: chr14 483365-483380. Max. coverage (+): 0. Max coverage (-): 0

Region: chr14 483381-483397. Max. coverage (+): 0. Max coverage (-): 0

Region: chr14 483398-483413. Max. coverage (+): 0. Max coverage (-): 0

Region: chr14 483414-483429. Max. coverage (+): 0. Max coverage (-): 0

Region: chr14 483430-483445. Max. coverage (+): 0.44. Max coverage (-): 0.29

Region: chr14 483446-483461. Max. coverage (+): 2.36. Max coverage (-): 0.29

Region: chr14 483462-483477. Max. coverage (+): 42.3. Max coverage (-): 0

Region: chr14 483478-483493. Max. coverage (+): 0. Max coverage (-): 8.11

Region: chr14 483494-483509. Max. coverage (+): 0. Max coverage (-): 2.11

Region: chr14 483510-483526. Max. coverage (+): 0. Max coverage (-): 0

Region: chr14 483527-483542. Max. coverage (+): 0. Max coverage (-): 0

Region: chr14 483543-483558. Max. coverage (+): 6.27. Max coverage (-): 0.25

Region: chr14 483559-483574. Max. coverage (+): 5.64. Max coverage (-): 0

Region: chr14 483575-483590. Max. coverage (+): 0.07. Max coverage (-): 0

Region: chr14 483591-483606. Max. coverage (+): 0. Max coverage (-): 0

Region: chr14 483607-483622. Max. coverage (+): 0. Max coverage (-): 0

Region: chr14 483623-483638. Max. coverage (+): 0. Max coverage (-): 0

Region: chr14 483639-483655. Max. coverage (+): 0. Max coverage (-): 0

Region: chr14 483656-483671. Max. coverage (+): 0. Max coverage (-): 0

Region: chr14 483672-483687. Max. coverage (+): 6.96. Max coverage (-): 0

Region: chr14 483688-483703. Max. coverage (+): 0. Max coverage (-): 0

Region: chr14 483704-483719. Max. coverage (+): 0. Max coverage (-): 0

Region: chr14 483720-483735. Max. coverage (+): 0. Max coverage (-): 0

Region: chr14 483736-483751. Max. coverage (+): 0. Max coverage (-): 0

Region: chr14 483752-483767. Max. coverage (+): 0. Max coverage (-): 0

Region: chr14 483768-483784. Max. coverage (+): 0. Max coverage (-): 0

Region: chr14 483785-483800. Max. coverage (+): 0. Max coverage (-): 0

Region: chr14 483801-483816. Max. coverage (+): 0. Max coverage (-): 0

Region: chr14 483817-483832. Max. coverage (+): 0. Max coverage (-): 0

Region: chr14 483833-483848. Max. coverage (+): 0. Max coverage (-): 0

Region: chr14 483849-483864. Max. coverage (+): 0. Max coverage (-): 0

Region: chr14 483865-483880. Max. coverage (+): 0. Max coverage (-): 0

Region: chr14 483881-483896. Max. coverage (+): 0. Max coverage (-): 0

Region: chr14 483897-483913. Max. coverage (+): 0. Max coverage (-): 0

Region: chr14 483914-483929. Max. coverage (+): 0. Max coverage (-): 0

Region: chr14 483930-483945. Max. coverage (+): 0. Max coverage (-): 0

Region: chr14 483946-483961. Max. coverage (+): 0. Max coverage (-): 0

Region: chr14 483962-483977. Max. coverage (+): 0. Max coverage (-): 0

Region: chr14 483978-483993. Max. coverage (+): 0. Max coverage (-): 0

Region: chr14 483994-484009. Max. coverage (+): 4.56. Max coverage (-): 0

Region: chr14 484010-484025. Max. coverage (+): 4.56. Max coverage (-): 0

Region: chr14 484026-484042. Max. coverage (+): 0. Max coverage (-): 0

Region: chr14 484043-484058. Max. coverage (+): 0. Max coverage (-): 0

Region: chr14 484059-484074. Max. coverage (+): 2.47. Max coverage (-): 0

Region: chr14 484075-484090. Max. coverage (+): 7.68. Max coverage (-): 0

Region: chr14 484091-484106. Max. coverage (+): 0. Max coverage (-): 0

Region: chr14 484107-484122. Max. coverage (+): 0. Max coverage (-): 0

Region: chr14 484123-484138. Max. coverage (+): 0. Max coverage (-): 0

Region: chr14 484139-484154. Max. coverage (+): 0. Max coverage (-): 0

Region: chr14 484155-484171. Max. coverage (+): 1.28. Max coverage (-): 0

Region: chr14 484172-484187. Max. coverage (+): 1.28. Max coverage (-): 0

Region: chr14 484188-484203. Max. coverage (+): 7.51. Max coverage (-): 0

Region: chr14 484204-484219. Max. coverage (+): 0. Max coverage (-): 0

Region: chr14 484220-484235. Max. coverage (+): 0. Max coverage (-): 0

Region: chr14 484236-484251. Max. coverage (+): 0. Max coverage (-): 0

Region: chr14 484252-484267. Max. coverage (+): 0. Max coverage (-): 0

Region: chr14 484268-484283. Max. coverage (+): 43.41. Max coverage (-): 0

Region: chr14 484284-484300. Max. coverage (+): 38.71. Max coverage (-): 0

Region: chr14 484301-484316. Max. coverage (+): 1.79. Max coverage (-): 0

Region: chr14 484317-484332. Max. coverage (+): 0. Max coverage (-): 0

Region: chr14 484333-484348. Max. coverage (+): 25.65. Max coverage (-): 0

Region: chr14 484349-484364. Max. coverage (+): 17.9. Max coverage (-): 0

Region: chr14 484365-484380. Max. coverage (+): 0. Max coverage (-): 0

Region: chr14 484381-484396. Max. coverage (+): 7.46. Max coverage (-): 7.86

Region: chr14 484397-484413. Max. coverage (+): 47.95. Max coverage (-): 0

Region: chr14 484414-484429. Max. coverage (+): 49.65. Max coverage (-): 2.51

Region: chr14 484430-484445. Max. coverage (+): 94.59. Max coverage (-): 2.51

Region: chr14 484446-484461. Max. coverage (+): 83.88. Max coverage (-): 0

Region: chr14 484462-484477. Max. coverage (+): 0. Max coverage (-): 0

Region: chr14 484478-484493. Max. coverage (+): 0. Max coverage (-): 0

Region: chr14 484494-484509. Max. coverage (+): 0. Max coverage (-): 0

Region: chr14 484510-484525. Max. coverage (+): 0. Max coverage (-): 4.93

Region: chr14 484526-484542. Max. coverage (+): 11.23. Max coverage (-): 4.93

Region: chr14 484543-484558. Max. coverage (+): 7.8. Max coverage (-): 0

Region: chr14 484559-484574. Max. coverage (+): 1.81. Max coverage (-): 0

Region: chr14 484575-484590. Max. coverage (+): 14.6. Max coverage (-): 0

Region: chr14 484591-484606. Max. coverage (+): 1.58. Max coverage (-): 3.73

Region: chr14 484607-484622. Max. coverage (+): 1.58. Max coverage (-): 3.73

Region: chr14 484623-484638. Max. coverage (+): 32.81. Max coverage (-): 0

Region: chr14 484639-484654. Max. coverage (+): 0. Max coverage (-): 0

Region: chr14 484655-484671. Max. coverage (+): 5.56. Max coverage (-): 0

Region: chr14 484672-484687. Max. coverage (+): 5.56. Max coverage (-): 0

Region: chr14 484688-484703. Max. coverage (+): 0. Max coverage (-): 0

Region: chr14 484704-484719. Max. coverage (+): 44.78. Max coverage (-): 0

Region: chr14 484720-484735. Max. coverage (+): 16.37. Max coverage (-): 0

Region: chr14 484736-484751. Max. coverage (+): 0. Max coverage (-): 0

Region: chr14 484752-484767. Max. coverage (+): 0. Max coverage (-): 0

Region: chr14 484768-484783. Max. coverage (+): 0. Max coverage (-): 0

Region: chr14 484784-484800. Max. coverage (+): 7.73. Max coverage (-): 0

Region: chr14 484801-484816. Max. coverage (+): 14.4. Max coverage (-): 0

Region: chr14 484817-484832. Max. coverage (+): 5.4. Max coverage (-): 0

Region: chr14 484833-484848. Max. coverage (+): 1.86. Max coverage (-): 0

Region: chr14 484849-484864. Max. coverage (+): 2.38. Max coverage (-): 0

Region: chr14 484865-484880. Max. coverage (+): 9.86. Max coverage (-): 0

Region: chr14 484881-484896. Max. coverage (+): 9.86. Max coverage (-): 0

Region: chr14 484897-484912. Max. coverage (+): 0. Max coverage (-): 0

Region: chr14 484913-484929. Max. coverage (+): 0. Max coverage (-): 0

Region: chr14 484930-484945. Max. coverage (+): 0. Max coverage (-): 0

Region: chr14 484946-484961. Max. coverage (+): 0. Max coverage (-): 0

Region: chr14 484962-484977. Max. coverage (+): 0. Max coverage (-): 0

Region: chr14 484978-484993. Max. coverage (+): 0. Max coverage (-): 0

Region: chr14 484994-485009. Max. coverage (+): 0. Max coverage (-): 0

Region: chr14 485010-485025. Max. coverage (+): 5.2. Max coverage (-): 0

Region: chr14 485026-485041. Max. coverage (+): 6.03. Max coverage (-): 0

Region: chr14 485042-485058. Max. coverage (+): 6.03. Max coverage (-): 0

Region: chr14 485059-485074. Max. coverage (+): 0. Max coverage (-): 0

Region: chr14 485075-485090. Max. coverage (+): 72.96. Max coverage (-): 0

Region: chr14 485091-485106. Max. coverage (+): 120.2. Max coverage (-): 0

Region: chr14 485107-485122. Max. coverage (+): 7.72. Max coverage (-): 0

Region: chr14 485123-485138. Max. coverage (+): 7.72. Max coverage (-): 0

Region: chr14 485139-485154. Max. coverage (+): 6.06. Max coverage (-): 0

Region: chr14 485155-485170. Max. coverage (+): 6.96. Max coverage (-): 0

Region: chr14 485171-485187. Max. coverage (+): 0. Max coverage (-): 0

Region: chr14 485188-485203. Max. coverage (+): 0. Max coverage (-): 0

Region: chr14 485204-485219. Max. coverage (+): 33.39. Max coverage (-): 0

Region: chr14 485220-485235. Max. coverage (+): 23.67. Max coverage (-): 0

Region: chr14 485236-485251. Max. coverage (+): 4.76. Max coverage (-): 0

Region: chr14 485252-485267. Max. coverage (+): 3.99. Max coverage (-): 0

Region: chr14 485268-485283. Max. coverage (+): 0. Max coverage (-): 0

Region: chr14 485284-485299. Max. coverage (+): 0. Max coverage (-): 0

Region: chr14 485300-485316. Max. coverage (+): 44.18. Max coverage (-): 0

Region: chr14 485317-485332. Max. coverage (+): 4.94. Max coverage (-): 0

Region: chr14 485333-485348. Max. coverage (+): 0. Max coverage (-): 0

Region: chr14 485349-485364. Max. coverage (+): 0. Max coverage (-): 0

Region: chr14 485365-485380. Max. coverage (+): 0. Max coverage (-): 0

Region: chr14 485381-485396. Max. coverage (+): 8.08. Max coverage (-): 0

Region: chr14 485397-485412. Max. coverage (+): 0. Max coverage (-): 0

Region: chr14 485413-485428. Max. coverage (+): 2.77. Max coverage (-): 0

Region: chr14 485429-485445. Max. coverage (+): 30.9. Max coverage (-): 0

Region: chr14 485446-485461. Max. coverage (+): 8.73. Max coverage (-): 0

Region: chr14 485462-485477. Max. coverage (+): 8.73. Max coverage (-): 0

Region: chr14 485478-485493. Max. coverage (+): 7.17. Max coverage (-): 0

Region: chr14 485494-485509. Max. coverage (+): 16.17. Max coverage (-): 0

Region: chr14 485510-485525. Max. coverage (+): 12.17. Max coverage (-): 0

Region: chr14 485526-485541. Max. coverage (+): 0. Max coverage (-): 0

Region: chr14 485542-485557. Max. coverage (+): 0. Max coverage (-): 0

Region: chr14 485558-485574. Max. coverage (+): 0. Max coverage (-): 0

Region: chr14 485575-485590. Max. coverage (+): 0. Max coverage (-): 0

Region: chr14 485591-485606. Max. coverage (+): 20.27. Max coverage (-): 0

Region: chr14 485607-485622. Max. coverage (+): 20.27. Max coverage (-): 0

Region: chr14 485623-485638. Max. coverage (+): 0. Max coverage (-): 0

Region: chr14 485639-485654. Max. coverage (+): 13.44. Max coverage (-): 0

Region: chr14 485655-485670. Max. coverage (+): 79.35. Max coverage (-): 0

Region: chr14 485671-485686. Max. coverage (+): 4.64. Max coverage (-): 0

Region: chr14 485687-485703. Max. coverage (+): 1.51. Max coverage (-): 0

Region: chr14 485704-485719. Max. coverage (+): 1.51. Max coverage (-): 0

Region: chr14 485720-485735. Max. coverage (+): 0. Max coverage (-): 0

Region: chr14 485736-485751. Max. coverage (+): 0. Max coverage (-): 0

Region: chr14 485752-485767. Max. coverage (+): 0. Max coverage (-): 0

Region: chr14 485768-485783. Max. coverage (+): 6.94. Max coverage (-): 0

Region: chr14 485784-485799. Max. coverage (+): 10.23. Max coverage (-): 0

Region: chr14 485800-485815. Max. coverage (+): 0. Max coverage (-): 0

Region: chr14 485816-485832. Max. coverage (+): 0. Max coverage (-): 0

Region: chr14 485833-485848. Max. coverage (+): 0. Max coverage (-): 0

Region: chr14 485849-485864. Max. coverage (+): 0. Max coverage (-): 0

Region: chr14 485865-485880. Max. coverage (+): 0. Max coverage (-): 0

Region: chr14 485881-485896. Max. coverage (+): 4.11. Max coverage (-): 0

Region: chr14 485897-485912. Max. coverage (+): 35.2. Max coverage (-): 0

Region: chr14 485913-485928. Max. coverage (+): 0. Max coverage (-): 0

Region: chr14 485929-485944. Max. coverage (+): 1.22. Max coverage (-): 0

Region: chr14 485945-485961. Max. coverage (+): 0. Max coverage (-): 0

Region: chr14 485962-485977. Max. coverage (+): 0. Max coverage (-): 0

Region: chr14 485978-485993. Max. coverage (+): 0. Max coverage (-): 0

Region: chr14 485994-486009. Max. coverage (+): 0. Max coverage (-): 0

Region: chr14 486010-486025. Max. coverage (+): 0. Max coverage (-): 0

Region: chr14 486026-486041. Max. coverage (+): 0. Max coverage (-): 0

Region: chr14 486042-486057. Max. coverage (+): 0. Max coverage (-): 0

Region: chr14 486058-486073. Max. coverage (+): 0. Max coverage (-): 0

Region: chr14 486074-486090. Max. coverage (+): 0. Max coverage (-): 0

Region: chr14 486091-486106. Max. coverage (+): 1.36. Max coverage (-): 0

Region: chr14 486107-486122. Max. coverage (+): 1.36. Max coverage (-): 0

Region: chr14 486123-486138. Max. coverage (+): 1.5. Max coverage (-): 0

Region: chr14 486139-486154. Max. coverage (+): 1.5. Max coverage (-): 0

Region: chr14 486155-486170. Max. coverage (+): 9.73. Max coverage (-): 0

Region: chr14 486171-486186. Max. coverage (+): 10.94. Max coverage (-): 1.41

Region: chr14 486187-486202. Max. coverage (+): 0. Max coverage (-): 1.41

Region: chr14 486203-486219. Max. coverage (+): 0. Max coverage (-): 0

Region: chr14 486220-486235. Max. coverage (+): 0. Max coverage (-): 0

Region: chr14 486236-486251. Max. coverage (+): 5.81. Max coverage (-): 0

Region: chr14 486252-486267. Max. coverage (+): 0. Max coverage (-): 0

Region: chr14 486268-486283. Max. coverage (+): 7.45. Max coverage (-): 0

Region: chr14 486284-486299. Max. coverage (+): 20.63. Max coverage (-): 0

Region: chr14 486300-486315. Max. coverage (+): 0. Max coverage (-): 0

Region: chr14 486316-486331. Max. coverage (+): 0. Max coverage (-): 0

Region: chr14 486332-486348. Max. coverage (+): 0. Max coverage (-): 0

Region: chr14 486349-486364. Max. coverage (+): 0. Max coverage (-): 0

Region: chr14 486365-486380. Max. coverage (+): 0. Max coverage (-): 0

Region: chr14 486381-486396. Max. coverage (+): 0. Max coverage (-): 0

Region: chr14 486397-486412. Max. coverage (+): 0. Max coverage (-): 0

Region: chr14 486413-486428. Max. coverage (+): 0. Max coverage (-): 0

Region: chr14 486429-486444. Max. coverage (+): 0. Max coverage (-): 0

Region: chr14 486445-486461. Max. coverage (+): 0. Max coverage (-): 0

Region: chr14 486462-486477. Max. coverage (+): 0. Max coverage (-): 0

Region: chr14 486478-486493. Max. coverage (+): 0. Max coverage (-): 0

Region: chr14 486494-486509. Max. coverage (+): 0.11. Max coverage (-): 0

Region: chr14 486510-486525. Max. coverage (+): 0. Max coverage (-): 0

Region: chr14 486526-486541. Max. coverage (+): 0. Max coverage (-): 0

Region: chr14 486542-486557. Max. coverage (+): 0. Max coverage (-): 0

Region: chr14 486558-486573. Max. coverage (+): 0. Max coverage (-): 0

Region: chr14 486574-486590. Max. coverage (+): 0. Max coverage (-): 0

Region: chr14 486591-486606. Max. coverage (+): 0. Max coverage (-): 0

Region: chr14 486607-486622. Max. coverage (+): 0. Max coverage (-): 0

Region: chr14 486623-486638. Max. coverage (+): 0. Max coverage (-): 0

Region: chr14 486639-486654. Max. coverage (+): 0. Max coverage (-): 0

Region: chr14 486655-486670. Max. coverage (+): 0. Max coverage (-): 0

Region: chr14 486671-486686. Max. coverage (+): 0. Max coverage (-): 0

Region: chr14 486687-486702. Max. coverage (+): 0. Max coverage (-): 0

Region: chr14 486703-486719. Max. coverage (+): 0. Max coverage (-): 0

Region: chr14 486720-486735. Max. coverage (+): 0. Max coverage (-): 0

Region: chr14 486736-486751. Max. coverage (+): 0. Max coverage (-): 0

Region: chr14 486752-486767. Max. coverage (+): 0. Max coverage (-): 0

Region: chr14 486768-486783. Max. coverage (+): 0. Max coverage (-): 0

Region: chr14 486784-486799. Max. coverage (+): 0. Max coverage (-): 0

Region: chr14 486800-486815. Max. coverage (+): 0. Max coverage (-): 0

Region: chr14 486816-486831. Max. coverage (+): 0. Max coverage (-): 0

Region: chr14 486832-486848. Max. coverage (+): 0. Max coverage (-): 0

Region: chr14 486849-486864. Max. coverage (+): 0. Max coverage (-): 0

Region: chr14 486865-486880. Max. coverage (+): 0. Max coverage (-): 0

Region: chr14 486881-486896. Max. coverage (+): 0. Max coverage (-): 0

Region: chr14 486897-486912. Max. coverage (+): 0. Max coverage (-): 0

Region: chr14 486913-486928. Max. coverage (+): 0. Max coverage (-): 0

Region: chr14 486929-486944. Max. coverage (+): 0. Max coverage (-): 0

Region: chr14 486945-486960. Max. coverage (+): 0. Max coverage (-): 0

Region: chr14 486961-486977. Max. coverage (+): 0. Max coverage (-): 0

Region: chr14 486978-486993. Max. coverage (+): 0. Max coverage (-): 0

Region: chr14 486994-487009. Max. coverage (+): 0. Max coverage (-): 0

Region: chr14 487010-487025. Max. coverage (+): 0. Max coverage (-): 0

Region: chr14 487026-487041. Max. coverage (+): 0. Max coverage (-): 0

Region: chr14 487042-487057. Max. coverage (+): 0. Max coverage (-): 0

Region: chr14 487058-487073. Max. coverage (+): 0. Max coverage (-): 0

Region: chr14 487074-487089. Max. coverage (+): 0. Max coverage (-): 0

Region: chr14 487090-487106. Max. coverage (+): 0. Max coverage (-): 0

Region: chr14 487107-487122. Max. coverage (+): 0. Max coverage (-): 0

Region: chr14 487123-487138. Max. coverage (+): 0. Max coverage (-): 0

Region: chr14 487139-487154. Max. coverage (+): 0. Max coverage (-): 0

Region: chr14 487155-487170. Max. coverage (+): 0. Max coverage (-): 0

Region: chr14 487171-487186. Max. coverage (+): 0. Max coverage (-): 0

Region: chr14 487187-487202. Max. coverage (+): 0. Max coverage (-): 0

Region: chr14 487203-487218. Max. coverage (+): 0. Max coverage (-): 0

Region: chr14 487219-487235. Max. coverage (+): 0. Max coverage (-): 0

Region: chr14 487236-487251. Max. coverage (+): 0. Max coverage (-): 0

Region: chr14 487252-487267. Max. coverage (+): 0. Max coverage (-): 0

Region: chr14 487268-487283. Max. coverage (+): 0. Max coverage (-): 0

Region: chr14 487284-487299. Max. coverage (+): 0. Max coverage (-): 0

Region: chr14 487300-487315. Max. coverage (+): 0. Max coverage (-): 0

Region: chr14 487316-487331. Max. coverage (+): 2.83. Max coverage (-): 0

Region: chr14 487332-487347. Max. coverage (+): 0. Max coverage (-): 0

Region: chr14 487348-. Max. coverage (+): 0. Max coverage (-): 0

RepeatMasker Color Code

**+**

100-98% Identity

<98-95% Identity

<95-90% Identity

<90-85% Identity

<85-80% Identity

<80-75% Identity

<75-70% Identity

<70% Identity

**-**

Gene Set Color Code

**+**

Gene

Pseudogene

**-**

Topology/Coverage Color Code

Coverage Plus Strand

Coverage Minus Strand

Mainstrand: Plus

Mainstrand: Minus

Complementary Strand

Flanking Region  
(if option -flank >0)

Gene Set Annotation  
  
RepeatMasker Annotation  

**1. Tigger12c**: 479354-479513 (-), Divergence to consensus: 40.2%  
**2. MER33**: 480592-480728 (+), Divergence to consensus: 40.9%  
**3. ART2A**: 480989-481102 (-), Divergence to consensus: 13.3%  
**4. L1MC5**: 481271-481386 (+), Divergence to consensus: 25%  
**5. ART2A**: 481408-481596 (-), Divergence to consensus: 18%  
**6. BovB**: 481598-481680 (-), Divergence to consensus: 18.1%  
**7. (GCTG)n**: 483360-483383 (+), Divergence to consensus: 4.2%  
**8. L1\_Art**: 483721-483866 (+), Divergence to consensus: 47.7%  
**9. (TAGA)n**: 483867-483890 (+), Divergence to consensus: 0%  
**10. L1\_Art**: 483891-483945 (+), Divergence to consensus: 47.7%  
**11. (TGAA)n**: 483951-483977 (+), Divergence to consensus: 7.4%  
**12. SINE2-2\_BT**: 484909-485019 (+), Divergence to consensus: 28.1%  
**13. L1MB3**: 485823-485869 (+), Divergence to consensus: 10.6%  
**14. L2a**: 485991-486050 (+), Divergence to consensus: 36.3%  
**15. BTLTR1D2**: 486540-487189 (-), Divergence to consensus: 14.4%

  
Transcription Factor Binding Sites  

**SOX9** (Sequence: AACAATGA (-): 479570)  
**SOX9** (Sequence: AACAATGA (-): 479930)  
**Gata4** (Sequence: GTTATCT (+): 480766)
