# Supplementary material for: Small RNA sequencing of cryopreserved semen from single bull revealed altered miRNAs and piRNAs expression between High- and Low-motile sperm populations
Source: BMC Genomics. 2017 Jan 4;18:14. doi: 10.1186/s12864-016-3394-7 (PMC5209821; doi:10.1186/s12864-016-3394-7)
Supplement: Additional file 4: — Details for each piRNA clusters found in Low Motile (LM) sperm fraction. Genes, repeats, transposable elements and transcription factors binding sites falling within the cluster regions were reported. (ZIP 1034 kb) [file 12864_2016_3394_MOESM4_ESM.zip › 12.html]

piRNA cluster 12


Predicted piRNA cluster no. 12     previous   next
  

Show proTRAC run info
Hide proTRAC run info

================================= proTRAC ====================================  
VERSION: 2.1                                    LAST MODIFIED: 06. October 2015  
  
Please cite:  
Rosenkranz D, Zischler H. proTRAC - a software for probabilistic piRNA cluster  
detection, visualization and analysis. 2012. BMC Bioinformatics 13:5.  
  
and (for proTRAC 2.0 and later):  
Rosenkranz D, Rudloff S, Bastuck K, Ketting RF, Zischler H. Tupaia small RNAs  
provide insights into function and evolution of RNAi-based transposon defense  
in mammals. 2015. RNA 21(5):911-922.  
  
Contact:  
David Rosenkranz  
Institute of Anthropology, small RNA group  
Johannes Gutenberg University Mainz  
email: rosenkranz@uni-mainz.de  
  
You can find the latest proTRAC version at:  
http://sourceforge.net/projects/protrac/files  
http://www.smallRNAgroup-mainz.de/software  
==============================================================================  
  
PARAMETERS:  
Map file: .............../storage/core/barbara/genhome/smallRNA/fertility/Sample\_not\_motile/pirna/Sample\_not\_motile\_26-33\_collapsed.fa.no-dust.map.weighted-10000-1000-b-0  
Genome file: ............/storage/core/barbara/genhome/smallRNA/fertility/Sample\_all/pirna/bt\_311\_chrY.fa  
RepeatMasker annotation: /storage/genomes/bt\_umd31/GCF\_000003055.6\_Bos\_taurus\_UMD\_3.1.1\_repeatMasker\_chr.out  
GeneSet:................./storage/core/barbara/genhome/smallRNA/fertility/Sample\_all/pirna/full.gtf  
  
Significant (p<=0.01) hit density will be calculated based  
on observed hit distribution.  
  
Sliding window size: ........................................ 5000 bp  
Sliding window increament: .................................. 1000 bp  
Normalize each hit by number of genomic hits: ............... 1 [0=no/1=yes]  
Normalize each hit by number of sequence reads: ............. 1 [0=no/1=yes]  
Normalize values (-> per million mapped reads): ............. 1 [0=no/1=yes]  
Min. fraction of hits with 1T(U) or 10A: .................... 0.75  
Alternatively: Min. fraction of hits with 1T(U) and 10A: .... 0.5  
Min. fraction of hits with typical piRNA length: ............ 0.75  
Typical piRNA length: ....................................... 26-33 nt  
Min. size of a piRNA cluster: ............................... 5000 bp.  
Min. number of hits (absolute): ............................. 0  
Min. number of hits (normalized): ........................... 0  
Min. fraction of hits on the mainstrand: .................... 0.75  
Top fraction of mapped sequences (in terms of read counts): . 1%  
Top fraction accounts for max. n% of sequence reads: ........ 90%  
Min. fraction of hits on each arm of a bidirectional cluster: 0.1  
Output image file for each cluster: ......................... 0 [0=no/1=yes]  
Output html file for each cluster: .......................... 1 [0=no/1=yes]  
Output a summary table: ..................................... 1 [0=no/1=yes]  
Output a FASTA file for each cluster (piRNA sequences): ..... 1 [0=no/1=yes]  
Output a FASTA file comprising cluster sequences: ........... 1 [0=no/1=yes]  
Search DNA motifs in clusters: .............................. 1 [0=no/1=yes]  
Output flanking sequences: +/- .............................. 0 bp  
Output ~.pTi file: .......................................... 1 [0=no/1=yes]  
==============================================================================  
  
  
Genome size (without gaps): ............ 2678902517 bp  
Gaps (N/X/-): .......................... 53837044 bp  
Mapped reads: .......................... 738059667487  
Non-identical sequences: ............... 277001  
Genomic hits: .......................... 533816  
Significant densitiy of mapped reads: .. 15118061 reads/kb

Show proTRAC cluster info
Hide proTRAC cluster info

|  |  |
| --- | --- |
| Location | chr14 |
| Coordinates | 15173921-15195809 |
| Size [bp] | 21889 |
| Sequence hit loci | 1337 |
| Mapped reads (normalized) | 1482799636.8 |
| Mapped reads (normalized) per kb | 67741771.5 |
| Normalized reads with 1T (1U) | 82.3% |
| Normalized reads with 10A | 36.3% |
| Normalized reads with length 26-33 nt | 100% |
| Normalized reads on the main strand(s) | 93.6% |
| Predicted directionality | mono:minus |

100%

0%

1T (1U)  
reads

10A reads

26-33 nt  
reads

reads on mainstrand

**Either the amount of reads with 1T (1U) OR 10A has to exceed 75% (set with option: -1Tor10A)  
Alternatively the amount of reads with 1T (1U) AND 10A has to exceed 50% (set with option: -1Tand10A)  
Minimum amount of reads with preferred size is 75% (set with option: -pisize)  
Minimum amount of reads on the main strand(s) is 75% (set with option: -clstrand)**

Show read coverage
Hide read coverage

WHAT DO I SEE HERE?  
This chart shows the location of mapped sequence reads within a predicted piRNA cluster. The color refers to the number of genomic hits produced by the sequence read in question. A dark red bar indicates that this sequence read produces many other hits elsewhere in the genome. Many adjacent red or yellow bars can indicate the presence of a multi-copy element such as transposons or rRNA genes. A dark green bar indicates that this sequence read maps uniquely to this locus.

1 hit

2-5 hits

6-10 hits

11-20 hits

21-50 hits

51-100 hits

> 100 hits

chr14

15173921

15195809

Gene Set

RepeatMasker

Mapped  
Reads

71.71

plus strand

minus strand

71.71

Region: chr14 487349-15173942. Max. coverage (+): 0. Max coverage (-): 1.11

Region: chr14 15173943-15173986. Max. coverage (+): 0. Max coverage (-): 0

Region: chr14 15173987-15174030. Max. coverage (+): 0. Max coverage (-): 5.48

Region: chr14 15174031-15174074. Max. coverage (+): 0. Max coverage (-): 0

Region: chr14 15174075-15174118. Max. coverage (+): 0. Max coverage (-): 3.27

Region: chr14 15174119-15174161. Max. coverage (+): 0. Max coverage (-): 0

Region: chr14 15174162-15174205. Max. coverage (+): 0. Max coverage (-): 0

Region: chr14 15174206-15174249. Max. coverage (+): 5.81. Max coverage (-): 0

Region: chr14 15174250-15174293. Max. coverage (+): 0. Max coverage (-): 0

Region: chr14 15174294-15174336. Max. coverage (+): 0. Max coverage (-): 0

Region: chr14 15174337-15174380. Max. coverage (+): 0. Max coverage (-): 0

Region: chr14 15174381-15174424. Max. coverage (+): 0. Max coverage (-): 0

Region: chr14 15174425-15174468. Max. coverage (+): 0. Max coverage (-): 0

Region: chr14 15174469-15174512. Max. coverage (+): 0. Max coverage (-): 3.73

Region: chr14 15174513-15174555. Max. coverage (+): 0. Max coverage (-): 0

Region: chr14 15174556-15174599. Max. coverage (+): 0. Max coverage (-): 0

Region: chr14 15174600-15174643. Max. coverage (+): 0. Max coverage (-): 0

Region: chr14 15174644-15174687. Max. coverage (+): 0. Max coverage (-): 0

Region: chr14 15174688-15174730. Max. coverage (+): 0. Max coverage (-): 0

Region: chr14 15174731-15174774. Max. coverage (+): 0.88. Max coverage (-): 0

Region: chr14 15174775-15174818. Max. coverage (+): 0. Max coverage (-): 0

Region: chr14 15174819-15174862. Max. coverage (+): 0. Max coverage (-): 0

Region: chr14 15174863-15174906. Max. coverage (+): 0. Max coverage (-): 0.53

Region: chr14 15174907-15174949. Max. coverage (+): 0. Max coverage (-): 0

Region: chr14 15174950-15174993. Max. coverage (+): 0. Max coverage (-): 0

Region: chr14 15174994-15175037. Max. coverage (+): 0. Max coverage (-): 0

Region: chr14 15175038-15175081. Max. coverage (+): 0. Max coverage (-): 0

Region: chr14 15175082-15175124. Max. coverage (+): 0. Max coverage (-): 0

Region: chr14 15175125-15175168. Max. coverage (+): 0. Max coverage (-): 0

Region: chr14 15175169-15175212. Max. coverage (+): 0. Max coverage (-): 0

Region: chr14 15175213-15175256. Max. coverage (+): 0. Max coverage (-): 0

Region: chr14 15175257-15175300. Max. coverage (+): 0. Max coverage (-): 0

Region: chr14 15175301-15175343. Max. coverage (+): 0. Max coverage (-): 0

Region: chr14 15175344-15175387. Max. coverage (+): 0. Max coverage (-): 4.47

Region: chr14 15175388-15175431. Max. coverage (+): 0. Max coverage (-): 0

Region: chr14 15175432-15175475. Max. coverage (+): 0. Max coverage (-): 0

Region: chr14 15175476-15175518. Max. coverage (+): 0. Max coverage (-): 0

Region: chr14 15175519-15175562. Max. coverage (+): 0. Max coverage (-): 0

Region: chr14 15175563-15175606. Max. coverage (+): 0. Max coverage (-): 0

Region: chr14 15175607-15175650. Max. coverage (+): 1.05. Max coverage (-): 0

Region: chr14 15175651-15175694. Max. coverage (+): 0. Max coverage (-): 0

Region: chr14 15175695-15175737. Max. coverage (+): 0. Max coverage (-): 0

Region: chr14 15175738-15175781. Max. coverage (+): 0. Max coverage (-): 0

Region: chr14 15175782-15175825. Max. coverage (+): 0. Max coverage (-): 0.64

Region: chr14 15175826-15175869. Max. coverage (+): 0. Max coverage (-): 0

Region: chr14 15175870-15175912. Max. coverage (+): 0. Max coverage (-): 0

Region: chr14 15175913-15175956. Max. coverage (+): 0. Max coverage (-): 0

Region: chr14 15175957-15176000. Max. coverage (+): 0. Max coverage (-): 0

Region: chr14 15176001-15176044. Max. coverage (+): 0. Max coverage (-): 0

Region: chr14 15176045-15176088. Max. coverage (+): 0. Max coverage (-): 0

Region: chr14 15176089-15176131. Max. coverage (+): 0. Max coverage (-): 0

Region: chr14 15176132-15176175. Max. coverage (+): 0. Max coverage (-): 0

Region: chr14 15176176-15176219. Max. coverage (+): 0. Max coverage (-): 0

Region: chr14 15176220-15176263. Max. coverage (+): 0. Max coverage (-): 0

Region: chr14 15176264-15176306. Max. coverage (+): 0. Max coverage (-): 4.21

Region: chr14 15176307-15176350. Max. coverage (+): 0. Max coverage (-): 0

Region: chr14 15176351-15176394. Max. coverage (+): 0. Max coverage (-): 0

Region: chr14 15176395-15176438. Max. coverage (+): 0. Max coverage (-): 0

Region: chr14 15176439-15176482. Max. coverage (+): 0. Max coverage (-): 0

Region: chr14 15176483-15176525. Max. coverage (+): 0. Max coverage (-): 0

Region: chr14 15176526-15176569. Max. coverage (+): 0. Max coverage (-): 0

Region: chr14 15176570-15176613. Max. coverage (+): 0. Max coverage (-): 0

Region: chr14 15176614-15176657. Max. coverage (+): 0. Max coverage (-): 0

Region: chr14 15176658-15176700. Max. coverage (+): 0. Max coverage (-): 0

Region: chr14 15176701-15176744. Max. coverage (+): 0. Max coverage (-): 0

Region: chr14 15176745-15176788. Max. coverage (+): 0. Max coverage (-): 0

Region: chr14 15176789-15176832. Max. coverage (+): 0. Max coverage (-): 0

Region: chr14 15176833-15176876. Max. coverage (+): 0. Max coverage (-): 0

Region: chr14 15176877-15176919. Max. coverage (+): 0. Max coverage (-): 0

Region: chr14 15176920-15176963. Max. coverage (+): 0. Max coverage (-): 0

Region: chr14 15176964-15177007. Max. coverage (+): 0. Max coverage (-): 0

Region: chr14 15177008-15177051. Max. coverage (+): 0. Max coverage (-): 0

Region: chr14 15177052-15177094. Max. coverage (+): 0. Max coverage (-): 0

Region: chr14 15177095-15177138. Max. coverage (+): 0. Max coverage (-): 0

Region: chr14 15177139-15177182. Max. coverage (+): 0. Max coverage (-): 0

Region: chr14 15177183-15177226. Max. coverage (+): 0. Max coverage (-): 0

Region: chr14 15177227-15177270. Max. coverage (+): 0. Max coverage (-): 0

Region: chr14 15177271-15177313. Max. coverage (+): 0. Max coverage (-): 0

Region: chr14 15177314-15177357. Max. coverage (+): 0. Max coverage (-): 0

Region: chr14 15177358-15177401. Max. coverage (+): 0. Max coverage (-): 13.6

Region: chr14 15177402-15177445. Max. coverage (+): 0. Max coverage (-): 0

Region: chr14 15177446-15177488. Max. coverage (+): 0. Max coverage (-): 0

Region: chr14 15177489-15177532. Max. coverage (+): 0. Max coverage (-): 6.23

Region: chr14 15177533-15177576. Max. coverage (+): 0. Max coverage (-): 0

Region: chr14 15177577-15177620. Max. coverage (+): 0. Max coverage (-): 0

Region: chr14 15177621-15177664. Max. coverage (+): 0. Max coverage (-): 0

Region: chr14 15177665-15177707. Max. coverage (+): 0. Max coverage (-): 0

Region: chr14 15177708-15177751. Max. coverage (+): 0. Max coverage (-): 50.59

Region: chr14 15177752-15177795. Max. coverage (+): 0. Max coverage (-): 0

Region: chr14 15177796-15177839. Max. coverage (+): 0. Max coverage (-): 0

Region: chr14 15177840-15177882. Max. coverage (+): 0. Max coverage (-): 0

Region: chr14 15177883-15177926. Max. coverage (+): 0. Max coverage (-): 0

Region: chr14 15177927-15177970. Max. coverage (+): 0. Max coverage (-): 0

Region: chr14 15177971-15178014. Max. coverage (+): 0. Max coverage (-): 0

Region: chr14 15178015-15178058. Max. coverage (+): 0. Max coverage (-): 0

Region: chr14 15178059-15178101. Max. coverage (+): 0. Max coverage (-): 0

Region: chr14 15178102-15178145. Max. coverage (+): 0. Max coverage (-): 0

Region: chr14 15178146-15178189. Max. coverage (+): 0. Max coverage (-): 0

Region: chr14 15178190-15178233. Max. coverage (+): 0. Max coverage (-): 0

Region: chr14 15178234-15178276. Max. coverage (+): 0. Max coverage (-): 0

Region: chr14 15178277-15178320. Max. coverage (+): 0. Max coverage (-): 0

Region: chr14 15178321-15178364. Max. coverage (+): 0. Max coverage (-): 0

Region: chr14 15178365-15178408. Max. coverage (+): 0. Max coverage (-): 0

Region: chr14 15178409-15178452. Max. coverage (+): 0. Max coverage (-): 0

Region: chr14 15178453-15178495. Max. coverage (+): 0. Max coverage (-): 0

Region: chr14 15178496-15178539. Max. coverage (+): 0. Max coverage (-): 0

Region: chr14 15178540-15178583. Max. coverage (+): 0. Max coverage (-): 23.76

Region: chr14 15178584-15178627. Max. coverage (+): 2.86. Max coverage (-): 23.76

Region: chr14 15178628-15178670. Max. coverage (+): 0. Max coverage (-): 34.81

Region: chr14 15178671-15178714. Max. coverage (+): 0. Max coverage (-): 27.85

Region: chr14 15178715-15178758. Max. coverage (+): 0. Max coverage (-): 15.59

Region: chr14 15178759-15178802. Max. coverage (+): 0. Max coverage (-): 9.04

Region: chr14 15178803-15178846. Max. coverage (+): 0. Max coverage (-): 0

Region: chr14 15178847-15178889. Max. coverage (+): 0. Max coverage (-): 0

Region: chr14 15178890-15178933. Max. coverage (+): 0. Max coverage (-): 0

Region: chr14 15178934-15178977. Max. coverage (+): 0. Max coverage (-): 0

Region: chr14 15178978-15179021. Max. coverage (+): 0. Max coverage (-): 0

Region: chr14 15179022-15179064. Max. coverage (+): 0. Max coverage (-): 0

Region: chr14 15179065-15179108. Max. coverage (+): 0. Max coverage (-): 0

Region: chr14 15179109-15179152. Max. coverage (+): 0. Max coverage (-): 0

Region: chr14 15179153-15179196. Max. coverage (+): 0. Max coverage (-): 5.39

Region: chr14 15179197-15179240. Max. coverage (+): 0. Max coverage (-): 0

Region: chr14 15179241-15179283. Max. coverage (+): 0. Max coverage (-): 10.37

Region: chr14 15179284-15179327. Max. coverage (+): 2.8. Max coverage (-): 6.4

Region: chr14 15179328-15179371. Max. coverage (+): 0. Max coverage (-): 2.71

Region: chr14 15179372-15179415. Max. coverage (+): 1.7. Max coverage (-): 20.9

Region: chr14 15179416-15179458. Max. coverage (+): 0. Max coverage (-): 12.88

Region: chr14 15179459-15179502. Max. coverage (+): 0. Max coverage (-): 1.83

Region: chr14 15179503-15179546. Max. coverage (+): 1.39. Max coverage (-): 0

Region: chr14 15179547-15179590. Max. coverage (+): 0. Max coverage (-): 0.81

Region: chr14 15179591-15179634. Max. coverage (+): 0. Max coverage (-): 2.3

Region: chr14 15179635-15179677. Max. coverage (+): 0. Max coverage (-): 6.32

Region: chr14 15179678-15179721. Max. coverage (+): 0. Max coverage (-): 4.03

Region: chr14 15179722-15179765. Max. coverage (+): 0. Max coverage (-): 12.65

Region: chr14 15179766-15179809. Max. coverage (+): 0. Max coverage (-): 57.68

Region: chr14 15179810-15179852. Max. coverage (+): 0. Max coverage (-): 14.93

Region: chr14 15179853-15179896. Max. coverage (+): 2.34. Max coverage (-): 0

Region: chr14 15179897-15179940. Max. coverage (+): 2.34. Max coverage (-): 8.27

Region: chr14 15179941-15179984. Max. coverage (+): 0. Max coverage (-): 1.32

Region: chr14 15179985-15180028. Max. coverage (+): 0. Max coverage (-): 0

Region: chr14 15180029-15180071. Max. coverage (+): 0. Max coverage (-): 0

Region: chr14 15180072-15180115. Max. coverage (+): 0. Max coverage (-): 0

Region: chr14 15180116-15180159. Max. coverage (+): 1.61. Max coverage (-): 19.88

Region: chr14 15180160-15180203. Max. coverage (+): 0. Max coverage (-): 0

Region: chr14 15180204-15180246. Max. coverage (+): 0. Max coverage (-): 1.74

Region: chr14 15180247-15180290. Max. coverage (+): 1.32. Max coverage (-): 0

Region: chr14 15180291-15180334. Max. coverage (+): 0. Max coverage (-): 0.77

Region: chr14 15180335-15180378. Max. coverage (+): 0. Max coverage (-): 2.3

Region: chr14 15180379-15180422. Max. coverage (+): 0. Max coverage (-): 6.32

Region: chr14 15180423-15180465. Max. coverage (+): 0. Max coverage (-): 4.03

Region: chr14 15180466-15180509. Max. coverage (+): 0. Max coverage (-): 12.65

Region: chr14 15180510-15180553. Max. coverage (+): 0. Max coverage (-): 0

Region: chr14 15180554-15180597. Max. coverage (+): 0. Max coverage (-): 14.93

Region: chr14 15180598-15180640. Max. coverage (+): 0. Max coverage (-): 0

Region: chr14 15180641-15180684. Max. coverage (+): 0. Max coverage (-): 0

Region: chr14 15180685-15180728. Max. coverage (+): 0. Max coverage (-): 0

Region: chr14 15180729-15180772. Max. coverage (+): 0. Max coverage (-): 0

Region: chr14 15180773-15180816. Max. coverage (+): 0. Max coverage (-): 0

Region: chr14 15180817-15180859. Max. coverage (+): 0. Max coverage (-): 0

Region: chr14 15180860-15180903. Max. coverage (+): 0. Max coverage (-): 0

Region: chr14 15180904-15180947. Max. coverage (+): 0. Max coverage (-): 0

Region: chr14 15180948-15180991. Max. coverage (+): 0. Max coverage (-): 0

Region: chr14 15180992-15181034. Max. coverage (+): 0. Max coverage (-): 0

Region: chr14 15181035-15181078. Max. coverage (+): 0. Max coverage (-): 0

Region: chr14 15181079-15181122. Max. coverage (+): 0. Max coverage (-): 0

Region: chr14 15181123-15181166. Max. coverage (+): 0. Max coverage (-): 0

Region: chr14 15181167-15181210. Max. coverage (+): 0. Max coverage (-): 0

Region: chr14 15181211-15181253. Max. coverage (+): 0. Max coverage (-): 0

Region: chr14 15181254-15181297. Max. coverage (+): 0. Max coverage (-): 0

Region: chr14 15181298-15181341. Max. coverage (+): 0. Max coverage (-): 0

Region: chr14 15181342-15181385. Max. coverage (+): 0. Max coverage (-): 0

Region: chr14 15181386-15181428. Max. coverage (+): 0. Max coverage (-): 0

Region: chr14 15181429-15181472. Max. coverage (+): 0. Max coverage (-): 0

Region: chr14 15181473-15181516. Max. coverage (+): 0. Max coverage (-): 0

Region: chr14 15181517-15181560. Max. coverage (+): 0. Max coverage (-): 0

Region: chr14 15181561-15181604. Max. coverage (+): 0. Max coverage (-): 0

Region: chr14 15181605-15181647. Max. coverage (+): 0. Max coverage (-): 0

Region: chr14 15181648-15181691. Max. coverage (+): 0. Max coverage (-): 0

Region: chr14 15181692-15181735. Max. coverage (+): 0. Max coverage (-): 0

Region: chr14 15181736-15181779. Max. coverage (+): 0. Max coverage (-): 0

Region: chr14 15181780-15181822. Max. coverage (+): 2.13. Max coverage (-): 7.91

Region: chr14 15181823-15181866. Max. coverage (+): 2.13. Max coverage (-): 4.88

Region: chr14 15181867-15181910. Max. coverage (+): 1.29. Max coverage (-): 2.07

Region: chr14 15181911-15181954. Max. coverage (+): 1.29. Max coverage (-): 15.93

Region: chr14 15181955-15181998. Max. coverage (+): 0. Max coverage (-): 7.84

Region: chr14 15181999-15182041. Max. coverage (+): 1.06. Max coverage (-): 1.4

Region: chr14 15182042-15182085. Max. coverage (+): 1.06. Max coverage (-): 0

Region: chr14 15182086-15182129. Max. coverage (+): 0. Max coverage (-): 1.84

Region: chr14 15182130-15182173. Max. coverage (+): 0. Max coverage (-): 2.71

Region: chr14 15182174-15182216. Max. coverage (+): 0. Max coverage (-): 5.06

Region: chr14 15182217-15182260. Max. coverage (+): 0. Max coverage (-): 6.1

Region: chr14 15182261-15182304. Max. coverage (+): 0. Max coverage (-): 10.13

Region: chr14 15182305-15182348. Max. coverage (+): 0. Max coverage (-): 46.22

Region: chr14 15182349-15182392. Max. coverage (+): 0. Max coverage (-): 11.96

Region: chr14 15182393-15182435. Max. coverage (+): 1.88. Max coverage (-): 6.63

Region: chr14 15182436-15182479. Max. coverage (+): 0. Max coverage (-): 4.7

Region: chr14 15182480-15182523. Max. coverage (+): 0. Max coverage (-): 5.58

Region: chr14 15182524-15182567. Max. coverage (+): 0.15. Max coverage (-): 18.95

Region: chr14 15182568-15182610. Max. coverage (+): 0. Max coverage (-): 17.67

Region: chr14 15182611-15182654. Max. coverage (+): 10.8. Max coverage (-): 38.44

Region: chr14 15182655-15182698. Max. coverage (+): 0. Max coverage (-): 8.33

Region: chr14 15182699-15182742. Max. coverage (+): 10.8. Max coverage (-): 38.44

Region: chr14 15182743-15182786. Max. coverage (+): 0. Max coverage (-): 8.33

Region: chr14 15182787-15182829. Max. coverage (+): 0. Max coverage (-): 8.33

Region: chr14 15182830-15182873. Max. coverage (+): 9.61. Max coverage (-): 34.22

Region: chr14 15182874-15182917. Max. coverage (+): 0. Max coverage (-): 7.41

Region: chr14 15182918-15182961. Max. coverage (+): 9.61. Max coverage (-): 34.22

Region: chr14 15182962-15183004. Max. coverage (+): 4.2. Max coverage (-): 7.49

Region: chr14 15183005-15183048. Max. coverage (+): 0. Max coverage (-): 18.74

Region: chr14 15183049-15183092. Max. coverage (+): 0. Max coverage (-): 6.65

Region: chr14 15183093-15183136. Max. coverage (+): 0. Max coverage (-): 0

Region: chr14 15183137-15183180. Max. coverage (+): 0. Max coverage (-): 0

Region: chr14 15183181-15183223. Max. coverage (+): 0.11. Max coverage (-): 0

Region: chr14 15183224-15183267. Max. coverage (+): 0. Max coverage (-): 18.97

Region: chr14 15183268-15183311. Max. coverage (+): 7.56. Max coverage (-): 20.13

Region: chr14 15183312-15183355. Max. coverage (+): 3.82. Max coverage (-): 4.17

Region: chr14 15183356-15183398. Max. coverage (+): 0. Max coverage (-): 0

Region: chr14 15183399-15183442. Max. coverage (+): 0. Max coverage (-): 7.97

Region: chr14 15183443-15183486. Max. coverage (+): 0. Max coverage (-): 0

Region: chr14 15183487-15183530. Max. coverage (+): 0. Max coverage (-): 16.01

Region: chr14 15183531-15183574. Max. coverage (+): 0. Max coverage (-): 2.76

Region: chr14 15183575-15183617. Max. coverage (+): 0. Max coverage (-): 2.76

Region: chr14 15183618-15183661. Max. coverage (+): 0. Max coverage (-): 0.44

Region: chr14 15183662-15183705. Max. coverage (+): 1.49. Max coverage (-): 6.45

Region: chr14 15183706-15183749. Max. coverage (+): 1.49. Max coverage (-): 11.42

Region: chr14 15183750-15183792. Max. coverage (+): 0. Max coverage (-): 0

Region: chr14 15183793-15183836. Max. coverage (+): 0. Max coverage (-): 1.27

Region: chr14 15183837-15183880. Max. coverage (+): 0. Max coverage (-): 0

Region: chr14 15183881-15183924. Max. coverage (+): 0. Max coverage (-): 0

Region: chr14 15183925-15183968. Max. coverage (+): 0. Max coverage (-): 0

Region: chr14 15183969-15184011. Max. coverage (+): 0. Max coverage (-): 0

Region: chr14 15184012-15184055. Max. coverage (+): 0. Max coverage (-): 0

Region: chr14 15184056-15184099. Max. coverage (+): 0. Max coverage (-): 0

Region: chr14 15184100-15184143. Max. coverage (+): 0. Max coverage (-): 4.54

Region: chr14 15184144-15184186. Max. coverage (+): 0. Max coverage (-): 0

Region: chr14 15184187-15184230. Max. coverage (+): 0. Max coverage (-): 0

Region: chr14 15184231-15184274. Max. coverage (+): 0. Max coverage (-): 0

Region: chr14 15184275-15184318. Max. coverage (+): 0. Max coverage (-): 0

Region: chr14 15184319-15184362. Max. coverage (+): 0. Max coverage (-): 0

Region: chr14 15184363-15184405. Max. coverage (+): 0. Max coverage (-): 0

Region: chr14 15184406-15184449. Max. coverage (+): 0. Max coverage (-): 0

Region: chr14 15184450-15184493. Max. coverage (+): 0. Max coverage (-): 0

Region: chr14 15184494-15184537. Max. coverage (+): 0. Max coverage (-): 0

Region: chr14 15184538-15184580. Max. coverage (+): 0. Max coverage (-): 0

Region: chr14 15184581-15184624. Max. coverage (+): 0. Max coverage (-): 0

Region: chr14 15184625-15184668. Max. coverage (+): 0. Max coverage (-): 11.38

Region: chr14 15184669-15184712. Max. coverage (+): 0. Max coverage (-): 0

Region: chr14 15184713-15184756. Max. coverage (+): 0. Max coverage (-): 0

Region: chr14 15184757-15184799. Max. coverage (+): 0. Max coverage (-): 0

Region: chr14 15184800-15184843. Max. coverage (+): 0. Max coverage (-): 0

Region: chr14 15184844-15184887. Max. coverage (+): 0. Max coverage (-): 0

Region: chr14 15184888-15184931. Max. coverage (+): 0. Max coverage (-): 0

Region: chr14 15184932-15184974. Max. coverage (+): 0. Max coverage (-): 0

Region: chr14 15184975-15185018. Max. coverage (+): 0. Max coverage (-): 0

Region: chr14 15185019-15185062. Max. coverage (+): 0. Max coverage (-): 0

Region: chr14 15185063-15185106. Max. coverage (+): 0. Max coverage (-): 0

Region: chr14 15185107-15185150. Max. coverage (+): 0. Max coverage (-): 0

Region: chr14 15185151-15185193. Max. coverage (+): 0. Max coverage (-): 0

Region: chr14 15185194-15185237. Max. coverage (+): 0. Max coverage (-): 0

Region: chr14 15185238-15185281. Max. coverage (+): 0. Max coverage (-): 0

Region: chr14 15185282-15185325. Max. coverage (+): 0. Max coverage (-): 0

Region: chr14 15185326-15185368. Max. coverage (+): 0. Max coverage (-): 0

Region: chr14 15185369-15185412. Max. coverage (+): 0. Max coverage (-): 0

Region: chr14 15185413-15185456. Max. coverage (+): 0. Max coverage (-): 0

Region: chr14 15185457-15185500. Max. coverage (+): 0. Max coverage (-): 0

Region: chr14 15185501-15185544. Max. coverage (+): 0. Max coverage (-): 0

Region: chr14 15185545-15185587. Max. coverage (+): 0. Max coverage (-): 0

Region: chr14 15185588-15185631. Max. coverage (+): 0. Max coverage (-): 0

Region: chr14 15185632-15185675. Max. coverage (+): 0. Max coverage (-): 0

Region: chr14 15185676-15185719. Max. coverage (+): 0. Max coverage (-): 0

Region: chr14 15185720-15185762. Max. coverage (+): 0. Max coverage (-): 0

Region: chr14 15185763-15185806. Max. coverage (+): 0. Max coverage (-): 0

Region: chr14 15185807-15185850. Max. coverage (+): 0. Max coverage (-): 0

Region: chr14 15185851-15185894. Max. coverage (+): 0. Max coverage (-): 0

Region: chr14 15185895-15185938. Max. coverage (+): 0. Max coverage (-): 0

Region: chr14 15185939-15185981. Max. coverage (+): 0. Max coverage (-): 6.58

Region: chr14 15185982-15186025. Max. coverage (+): 0. Max coverage (-): 1.4

Region: chr14 15186026-15186069. Max. coverage (+): 0. Max coverage (-): 2.46

Region: chr14 15186070-15186113. Max. coverage (+): 0. Max coverage (-): 13.23

Region: chr14 15186114-15186156. Max. coverage (+): 0. Max coverage (-): 2.28

Region: chr14 15186157-15186200. Max. coverage (+): 0. Max coverage (-): 0.37

Region: chr14 15186201-15186244. Max. coverage (+): 0. Max coverage (-): 0.43

Region: chr14 15186245-15186288. Max. coverage (+): 1.23. Max coverage (-): 9.44

Region: chr14 15186289-15186332. Max. coverage (+): 1.43. Max coverage (-): 1.21

Region: chr14 15186333-15186375. Max. coverage (+): 1.82. Max coverage (-): 1.05

Region: chr14 15186376-15186419. Max. coverage (+): 0.31. Max coverage (-): 1.64

Region: chr14 15186420-15186463. Max. coverage (+): 0. Max coverage (-): 3.03

Region: chr14 15186464-15186507. Max. coverage (+): 0. Max coverage (-): 0

Region: chr14 15186508-15186550. Max. coverage (+): 0. Max coverage (-): 0.78

Region: chr14 15186551-15186594. Max. coverage (+): 0. Max coverage (-): 0

Region: chr14 15186595-15186638. Max. coverage (+): 0. Max coverage (-): 0

Region: chr14 15186639-15186682. Max. coverage (+): 0. Max coverage (-): 0.19

Region: chr14 15186683-15186726. Max. coverage (+): 0.54. Max coverage (-): 0

Region: chr14 15186727-15186769. Max. coverage (+): 0. Max coverage (-): 6.35

Region: chr14 15186770-15186813. Max. coverage (+): 0. Max coverage (-): 9.72

Region: chr14 15186814-15186857. Max. coverage (+): 0. Max coverage (-): 0

Region: chr14 15186858-15186901. Max. coverage (+): 0. Max coverage (-): 0

Region: chr14 15186902-15186944. Max. coverage (+): 0. Max coverage (-): 0

Region: chr14 15186945-15186988. Max. coverage (+): 0. Max coverage (-): 0

Region: chr14 15186989-15187032. Max. coverage (+): 0. Max coverage (-): 11.04

Region: chr14 15187033-15187076. Max. coverage (+): 0. Max coverage (-): 2.34

Region: chr14 15187077-15187120. Max. coverage (+): 0. Max coverage (-): 0.37

Region: chr14 15187121-15187163. Max. coverage (+): 0. Max coverage (-): 0.44

Region: chr14 15187164-15187207. Max. coverage (+): 1.26. Max coverage (-): 9.67

Region: chr14 15187208-15187251. Max. coverage (+): 1.46. Max coverage (-): 1.24

Region: chr14 15187252-15187295. Max. coverage (+): 1.86. Max coverage (-): 1.08

Region: chr14 15187296-15187338. Max. coverage (+): 0.32. Max coverage (-): 1.68

Region: chr14 15187339-15187382. Max. coverage (+): 0. Max coverage (-): 3.11

Region: chr14 15187383-15187426. Max. coverage (+): 0. Max coverage (-): 1.34

Region: chr14 15187427-15187470. Max. coverage (+): 0. Max coverage (-): 0.8

Region: chr14 15187471-15187514. Max. coverage (+): 0. Max coverage (-): 0

Region: chr14 15187515-15187557. Max. coverage (+): 0. Max coverage (-): 0

Region: chr14 15187558-15187601. Max. coverage (+): 0. Max coverage (-): 0

Region: chr14 15187602-15187645. Max. coverage (+): 0.55. Max coverage (-): 0

Region: chr14 15187646-15187689. Max. coverage (+): 0.55. Max coverage (-): 6.51

Region: chr14 15187690-15187732. Max. coverage (+): 0. Max coverage (-): 9.96

Region: chr14 15187733-15187776. Max. coverage (+): 0. Max coverage (-): 0

Region: chr14 15187777-15187820. Max. coverage (+): 0. Max coverage (-): 0

Region: chr14 15187821-15187864. Max. coverage (+): 1.3. Max coverage (-): 1.45

Region: chr14 15187865-15187908. Max. coverage (+): 1.8. Max coverage (-): 0

Region: chr14 15187909-15187951. Max. coverage (+): 0.79. Max coverage (-): 3.95

Region: chr14 15187952-15187995. Max. coverage (+): 0. Max coverage (-): 0

Region: chr14 15187996-15188039. Max. coverage (+): 0. Max coverage (-): 0

Region: chr14 15188040-15188083. Max. coverage (+): 0. Max coverage (-): 0

Region: chr14 15188084-15188126. Max. coverage (+): 0. Max coverage (-): 14.16

Region: chr14 15188127-15188170. Max. coverage (+): 0. Max coverage (-): 1.9

Region: chr14 15188171-15188214. Max. coverage (+): 0. Max coverage (-): 0

Region: chr14 15188215-15188258. Max. coverage (+): 0. Max coverage (-): 0.39

Region: chr14 15188259-15188302. Max. coverage (+): 1.31. Max coverage (-): 10.1

Region: chr14 15188303-15188345. Max. coverage (+): 1.53. Max coverage (-): 5.29

Region: chr14 15188346-15188389. Max. coverage (+): 0. Max coverage (-): 1.13

Region: chr14 15188390-15188433. Max. coverage (+): 1.95. Max coverage (-): 1.76

Region: chr14 15188434-15188477. Max. coverage (+): 0.33. Max coverage (-): 0.91

Region: chr14 15188478-15188520. Max. coverage (+): 4.2. Max coverage (-): 3.22

Region: chr14 15188521-15188564. Max. coverage (+): 0. Max coverage (-): 1.17

Region: chr14 15188565-15188608. Max. coverage (+): 0. Max coverage (-): 0

Region: chr14 15188609-15188652. Max. coverage (+): 0. Max coverage (-): 0

Region: chr14 15188653-15188696. Max. coverage (+): 0. Max coverage (-): 0.2

Region: chr14 15188697-15188739. Max. coverage (+): 0. Max coverage (-): 0

Region: chr14 15188740-15188783. Max. coverage (+): 0.58. Max coverage (-): 1.07

Region: chr14 15188784-15188827. Max. coverage (+): 0. Max coverage (-): 8.09

Region: chr14 15188828-15188871. Max. coverage (+): 0. Max coverage (-): 10.4

Region: chr14 15188872-15188914. Max. coverage (+): 0. Max coverage (-): 0

Region: chr14 15188915-15188958. Max. coverage (+): 1.36. Max coverage (-): 1.52

Region: chr14 15188959-15189002. Max. coverage (+): 1.88. Max coverage (-): 0

Region: chr14 15189003-15189046. Max. coverage (+): 1.36. Max coverage (-): 4.13

Region: chr14 15189047-15189090. Max. coverage (+): 0. Max coverage (-): 0

Region: chr14 15189091-15189133. Max. coverage (+): 0. Max coverage (-): 0

Region: chr14 15189134-15189177. Max. coverage (+): 0. Max coverage (-): 0

Region: chr14 15189178-15189221. Max. coverage (+): 0. Max coverage (-): 5.99

Region: chr14 15189222-15189265. Max. coverage (+): 0. Max coverage (-): 35.56

Region: chr14 15189266-15189308. Max. coverage (+): 0. Max coverage (-): 0

Region: chr14 15189309-15189352. Max. coverage (+): 0. Max coverage (-): 1.69

Region: chr14 15189353-15189396. Max. coverage (+): 3.01. Max coverage (-): 1.69

Region: chr14 15189397-15189440. Max. coverage (+): 0. Max coverage (-): 3.09

Region: chr14 15189441-15189484. Max. coverage (+): 0. Max coverage (-): 2.29

Region: chr14 15189485-15189527. Max. coverage (+): 0. Max coverage (-): 0

Region: chr14 15189528-15189571. Max. coverage (+): 0. Max coverage (-): 2.02

Region: chr14 15189572-15189615. Max. coverage (+): 0. Max coverage (-): 0

Region: chr14 15189616-15189659. Max. coverage (+): 0. Max coverage (-): 0

Region: chr14 15189660-15189702. Max. coverage (+): 0. Max coverage (-): 0.47

Region: chr14 15189703-15189746. Max. coverage (+): 1.33. Max coverage (-): 10.25

Region: chr14 15189747-15189790. Max. coverage (+): 0. Max coverage (-): 0

Region: chr14 15189791-15189834. Max. coverage (+): 1.98. Max coverage (-): 1.14

Region: chr14 15189835-15189878. Max. coverage (+): 0.34. Max coverage (-): 1.78

Region: chr14 15189879-15189921. Max. coverage (+): 0. Max coverage (-): 3.29

Region: chr14 15189922-15189965. Max. coverage (+): 0. Max coverage (-): 3.29

Region: chr14 15189966-15190009. Max. coverage (+): 0. Max coverage (-): 0.85

Region: chr14 15190010-15190053. Max. coverage (+): 0. Max coverage (-): 0

Region: chr14 15190054-15190096. Max. coverage (+): 0. Max coverage (-): 0

Region: chr14 15190097-15190140. Max. coverage (+): 0. Max coverage (-): 0.21

Region: chr14 15190141-15190184. Max. coverage (+): 0. Max coverage (-): 0

Region: chr14 15190185-15190228. Max. coverage (+): 0. Max coverage (-): 0

Region: chr14 15190229-15190272. Max. coverage (+): 0. Max coverage (-): 0

Region: chr14 15190273-15190315. Max. coverage (+): 0. Max coverage (-): 0

Region: chr14 15190316-15190359. Max. coverage (+): 0. Max coverage (-): 0

Region: chr14 15190360-15190403. Max. coverage (+): 0. Max coverage (-): 0

Region: chr14 15190404-15190447. Max. coverage (+): 0. Max coverage (-): 0

Region: chr14 15190448-15190490. Max. coverage (+): 0. Max coverage (-): 0

Region: chr14 15190491-15190534. Max. coverage (+): 0. Max coverage (-): 4.83

Region: chr14 15190535-15190578. Max. coverage (+): 0. Max coverage (-): 0

Region: chr14 15190579-15190622. Max. coverage (+): 0. Max coverage (-): 18.93

Region: chr14 15190623-15190666. Max. coverage (+): 0. Max coverage (-): 0

Region: chr14 15190667-15190709. Max. coverage (+): 0. Max coverage (-): 0

Region: chr14 15190710-15190753. Max. coverage (+): 0. Max coverage (-): 0

Region: chr14 15190754-15190797. Max. coverage (+): 0. Max coverage (-): 0

Region: chr14 15190798-15190841. Max. coverage (+): 0. Max coverage (-): 0

Region: chr14 15190842-15190884. Max. coverage (+): 0. Max coverage (-): 0

Region: chr14 15190885-15190928. Max. coverage (+): 0. Max coverage (-): 0

Region: chr14 15190929-15190972. Max. coverage (+): 0. Max coverage (-): 0

Region: chr14 15190973-15191016. Max. coverage (+): 0. Max coverage (-): 0

Region: chr14 15191017-15191060. Max. coverage (+): 0. Max coverage (-): 0

Region: chr14 15191061-15191103. Max. coverage (+): 0. Max coverage (-): 0

Region: chr14 15191104-15191147. Max. coverage (+): 0. Max coverage (-): 0

Region: chr14 15191148-15191191. Max. coverage (+): 0. Max coverage (-): 0

Region: chr14 15191192-15191235. Max. coverage (+): 0. Max coverage (-): 0

Region: chr14 15191236-15191278. Max. coverage (+): 0. Max coverage (-): 5.64

Region: chr14 15191279-15191322. Max. coverage (+): 0. Max coverage (-): 33.46

Region: chr14 15191323-15191366. Max. coverage (+): 0. Max coverage (-): 0

Region: chr14 15191367-15191410. Max. coverage (+): 0. Max coverage (-): 0

Region: chr14 15191411-15191454. Max. coverage (+): 2.83. Max coverage (-): 1.59

Region: chr14 15191455-15191497. Max. coverage (+): 0. Max coverage (-): 0.89

Region: chr14 15191498-15191541. Max. coverage (+): 0. Max coverage (-): 2.91

Region: chr14 15191542-15191585. Max. coverage (+): 0. Max coverage (-): 0

Region: chr14 15191586-15191629. Max. coverage (+): 0.12. Max coverage (-): 2.77

Region: chr14 15191630-15191672. Max. coverage (+): 0.4. Max coverage (-): 11.6

Region: chr14 15191673-15191716. Max. coverage (+): 4.19. Max coverage (-): 71.71

Region: chr14 15191717-15191760. Max. coverage (+): 0. Max coverage (-): 6.44

Region: chr14 15191761-15191804. Max. coverage (+): 0. Max coverage (-): 2.38

Region: chr14 15191805-15191848. Max. coverage (+): 0. Max coverage (-): 3.53

Region: chr14 15191849-15191891. Max. coverage (+): 0. Max coverage (-): 5.8

Region: chr14 15191892-15191935. Max. coverage (+): 0. Max coverage (-): 13.01

Region: chr14 15191936-15191979. Max. coverage (+): 0. Max coverage (-): 10.04

Region: chr14 15191980-15192023. Max. coverage (+): 0. Max coverage (-): 3.73

Region: chr14 15192024-15192066. Max. coverage (+): 0. Max coverage (-): 0

Region: chr14 15192067-15192110. Max. coverage (+): 0. Max coverage (-): 0

Region: chr14 15192111-15192154. Max. coverage (+): 0. Max coverage (-): 0

Region: chr14 15192155-15192198. Max. coverage (+): 0. Max coverage (-): 0

Region: chr14 15192199-15192242. Max. coverage (+): 0. Max coverage (-): 2.55

Region: chr14 15192243-15192285. Max. coverage (+): 0. Max coverage (-): 8.82

Region: chr14 15192286-15192329. Max. coverage (+): 0. Max coverage (-): 0

Region: chr14 15192330-15192373. Max. coverage (+): 0. Max coverage (-): 0

Region: chr14 15192374-15192417. Max. coverage (+): 0. Max coverage (-): 4.03

Region: chr14 15192418-15192460. Max. coverage (+): 0. Max coverage (-): 4.03

Region: chr14 15192461-15192504. Max. coverage (+): 0. Max coverage (-): 0

Region: chr14 15192505-15192548. Max. coverage (+): 0. Max coverage (-): 28.18

Region: chr14 15192549-15192592. Max. coverage (+): 0. Max coverage (-): 7.96

Region: chr14 15192593-15192636. Max. coverage (+): 0. Max coverage (-): 0

Region: chr14 15192637-15192679. Max. coverage (+): 0.41. Max coverage (-): 4.17

Region: chr14 15192680-15192723. Max. coverage (+): 0. Max coverage (-): 20.47

Region: chr14 15192724-15192767. Max. coverage (+): 0. Max coverage (-): 0

Region: chr14 15192768-15192811. Max. coverage (+): 0. Max coverage (-): 0

Region: chr14 15192812-15192854. Max. coverage (+): 0. Max coverage (-): 0

Region: chr14 15192855-15192898. Max. coverage (+): 0. Max coverage (-): 3.43

Region: chr14 15192899-15192942. Max. coverage (+): 0. Max coverage (-): 3.43

Region: chr14 15192943-15192986. Max. coverage (+): 0. Max coverage (-): 0

Region: chr14 15192987-15193030. Max. coverage (+): 0. Max coverage (-): 0

Region: chr14 15193031-15193073. Max. coverage (+): 0. Max coverage (-): 0

Region: chr14 15193074-15193117. Max. coverage (+): 0. Max coverage (-): 0

Region: chr14 15193118-15193161. Max. coverage (+): 0. Max coverage (-): 8.1

Region: chr14 15193162-15193205. Max. coverage (+): 0. Max coverage (-): 0

Region: chr14 15193206-15193248. Max. coverage (+): 0. Max coverage (-): 0

Region: chr14 15193249-15193292. Max. coverage (+): 0. Max coverage (-): 0

Region: chr14 15193293-15193336. Max. coverage (+): 0. Max coverage (-): 4.03

Region: chr14 15193337-15193380. Max. coverage (+): 0. Max coverage (-): 0.7

Region: chr14 15193381-15193424. Max. coverage (+): 0. Max coverage (-): 25.11

Region: chr14 15193425-15193467. Max. coverage (+): 0. Max coverage (-): 14.75

Region: chr14 15193468-15193511. Max. coverage (+): 0. Max coverage (-): 7.09

Region: chr14 15193512-15193555. Max. coverage (+): 0. Max coverage (-): 3.71

Region: chr14 15193556-15193599. Max. coverage (+): 0. Max coverage (-): 3.71

Region: chr14 15193600-15193642. Max. coverage (+): 0. Max coverage (-): 0

Region: chr14 15193643-15193686. Max. coverage (+): 0. Max coverage (-): 0

Region: chr14 15193687-15193730. Max. coverage (+): 0. Max coverage (-): 0

Region: chr14 15193731-15193774. Max. coverage (+): 0. Max coverage (-): 0

Region: chr14 15193775-15193818. Max. coverage (+): 0. Max coverage (-): 0

Region: chr14 15193819-15193861. Max. coverage (+): 0. Max coverage (-): 0

Region: chr14 15193862-15193905. Max. coverage (+): 0. Max coverage (-): 0

Region: chr14 15193906-15193949. Max. coverage (+): 0. Max coverage (-): 0

Region: chr14 15193950-15193993. Max. coverage (+): 0. Max coverage (-): 1.66

Region: chr14 15193994-15194036. Max. coverage (+): 0. Max coverage (-): 0

Region: chr14 15194037-15194080. Max. coverage (+): 0. Max coverage (-): 0

Region: chr14 15194081-15194124. Max. coverage (+): 0. Max coverage (-): 0

Region: chr14 15194125-15194168. Max. coverage (+): 0. Max coverage (-): 0

Region: chr14 15194169-15194212. Max. coverage (+): 0. Max coverage (-): 0

Region: chr14 15194213-15194255. Max. coverage (+): 0. Max coverage (-): 0

Region: chr14 15194256-15194299. Max. coverage (+): 0. Max coverage (-): 4.4

Region: chr14 15194300-15194343. Max. coverage (+): 0. Max coverage (-): 0

Region: chr14 15194344-15194387. Max. coverage (+): 0.36. Max coverage (-): 3.71

Region: chr14 15194388-15194430. Max. coverage (+): 0. Max coverage (-): 3.71

Region: chr14 15194431-15194474. Max. coverage (+): 0. Max coverage (-): 0

Region: chr14 15194475-15194518. Max. coverage (+): 0. Max coverage (-): 0

Region: chr14 15194519-15194562. Max. coverage (+): 0. Max coverage (-): 0

Region: chr14 15194563-15194606. Max. coverage (+): 0. Max coverage (-): 0

Region: chr14 15194607-15194649. Max. coverage (+): 0. Max coverage (-): 0

Region: chr14 15194650-15194693. Max. coverage (+): 0. Max coverage (-): 0

Region: chr14 15194694-15194737. Max. coverage (+): 0. Max coverage (-): 0

Region: chr14 15194738-15194781. Max. coverage (+): 0. Max coverage (-): 0

Region: chr14 15194782-15194824. Max. coverage (+): 0. Max coverage (-): 1.31

Region: chr14 15194825-15194868. Max. coverage (+): 0. Max coverage (-): 0

Region: chr14 15194869-15194912. Max. coverage (+): 0. Max coverage (-): 0

Region: chr14 15194913-15194956. Max. coverage (+): 0. Max coverage (-): 0

Region: chr14 15194957-15195000. Max. coverage (+): 0. Max coverage (-): 0

Region: chr14 15195001-15195043. Max. coverage (+): 0. Max coverage (-): 0

Region: chr14 15195044-15195087. Max. coverage (+): 0. Max coverage (-): 0

Region: chr14 15195088-15195131. Max. coverage (+): 0. Max coverage (-): 0

Region: chr14 15195132-15195175. Max. coverage (+): 0. Max coverage (-): 0

Region: chr14 15195176-15195218. Max. coverage (+): 0. Max coverage (-): 0

Region: chr14 15195219-15195262. Max. coverage (+): 0. Max coverage (-): 0

Region: chr14 15195263-15195306. Max. coverage (+): 0. Max coverage (-): 0

Region: chr14 15195307-15195350. Max. coverage (+): 0. Max coverage (-): 0

Region: chr14 15195351-15195394. Max. coverage (+): 0. Max coverage (-): 0

Region: chr14 15195395-15195437. Max. coverage (+): 0. Max coverage (-): 0

Region: chr14 15195438-15195481. Max. coverage (+): 0. Max coverage (-): 0

Region: chr14 15195482-15195525. Max. coverage (+): 0. Max coverage (-): 0

Region: chr14 15195526-15195569. Max. coverage (+): 0. Max coverage (-): 0

Region: chr14 15195570-15195612. Max. coverage (+): 0. Max coverage (-): 0

Region: chr14 15195613-15195656. Max. coverage (+): 0. Max coverage (-): 0

Region: chr14 15195657-15195700. Max. coverage (+): 0. Max coverage (-): 0

Region: chr14 15195701-15195744. Max. coverage (+): 0. Max coverage (-): 0

Region: chr14 15195745-15195788. Max. coverage (+): 0. Max coverage (-): 1.31

Region: chr14 15195789-. Max. coverage (+): 0. Max coverage (-): 0

RepeatMasker Color Code

**+**

100-98% Identity

<98-95% Identity

<95-90% Identity

<90-85% Identity

<85-80% Identity

<80-75% Identity

<75-70% Identity

<70% Identity

**-**

Gene Set Color Code

**+**

Gene

Pseudogene

**-**

Topology/Coverage Color Code

Coverage Plus Strand

Coverage Minus Strand

Mainstrand: Plus

Mainstrand: Minus

Complementary Strand

Flanking Region  
(if option -flank >0)

Gene Set Annotation  
  
RepeatMasker Annotation  

**1. BOV-A2**: 15174270-15174322 (+), Divergence to consensus: 13.2%  
**2. L2a**: 15174355-15174458 (+), Divergence to consensus: 34%  
**3. L2a**: 15175243-15175346 (+), Divergence to consensus: 34%  
**4. MIR**: 15176364-15176484 (-), Divergence to consensus: 34.1%  
**5. ERV1-2-I\_BT-int**: 15176485-15177020 (+), Divergence to consensus: 24.7%  
**6. ERV1-2-I\_BT-int**: 15177121-15177371 (+), Divergence to consensus: 26.3%  
**7. BOV-A2**: 15177798-15177930 (+), Divergence to consensus: 9%  
**8. ERV1-2-I\_BT-int**: 15178056-15178177 (+), Divergence to consensus: 26.7%  
**9. BOV-A2**: 15178801-15178933 (+), Divergence to consensus: 7.5%  
**10. ERV1-2-I\_BT-int**: 15179059-15179180 (+), Divergence to consensus: 25.9%  
**11. SINE2-3\_BT**: 15192024-15192202 (-), Divergence to consensus: 18%  
**12. (T)n**: 15192616-15192645 (+), Divergence to consensus: 13.3%  
**13. SINE2-3\_BT**: 15192928-15193106 (-), Divergence to consensus: 18%  
**14. T-rich**: 15193519-15193549 (+), Divergence to consensus: 16.1%  
**15. LTR103\_Mam**: 15193611-15193779 (-), Divergence to consensus: 45.4%  
**16. L1MC4**: 15194094-15194171 (+), Divergence to consensus: 24.7%  
**17. LTR103\_Mam**: 15194443-15194611 (-), Divergence to consensus: 45.4%  
**18. L1MC4**: 15194926-15195084 (+), Divergence to consensus: 38.1%  
**19. Bov-tA2**: 15195085-15195295 (+), Divergence to consensus: 16.5%  
**20. L1MC4**: 15195296-15195356 (+), Divergence to consensus: 38.1%

  
Transcription Factor Binding Sites  

**RFX4\_1** (Sequence: CTTAGCAAC (+): 15181078)  
**RFX4\_1** (Sequence: CTTAGCAAC (+): 15183303)  
**RFX4\_1** (Sequence: CTTAGCAAC (+): 15185016)  
**RFX4\_1** (Sequence: CTTAGCAAC (+): 15185854)  
**RFX4\_2** (Sequence: CCTAGATAC (+): 15174780)  
**RFX4\_2** (Sequence: CCTAGATAC (+): 15175668)  
**Gata4** (Sequence: AGATAAC (-): 15176138)  
**Gata4** (Sequence: AGATAAG (-): 15176253)  
**Gata4** (Sequence: AGATAAC (-): 15181319)  
**Gata4** (Sequence: AGATAAC (-): 15183544)  
**Gata4** (Sequence: AGATAAC (-): 15185258)  
**Gata4** (Sequence: AGATAAC (-): 15186095)  
**Gata4** (Sequence: AGATAAC (-): 15187018)  
**Gata4** (Sequence: AGATAAC (-): 15188133)  
**SOX9** (Sequence: AACAATAA (-): 15180807)  
**SOX9** (Sequence: AACAATAA (-): 15182590)  
**SOX9** (Sequence: AACAATAA (-): 15183032)  
**SOX9** (Sequence: AACAATAA (-): 15184745)  
**SOX9** (Sequence: AACAATAA (-): 15185583)
