# Supplementary material for: Small RNA sequencing of cryopreserved semen from single bull revealed altered miRNAs and piRNAs expression between High- and Low-motile sperm populations
Source: BMC Genomics. 2017 Jan 4;18:14. doi: 10.1186/s12864-016-3394-7 (PMC5209821; doi:10.1186/s12864-016-3394-7)
Supplement: Additional file 4: — Details for each piRNA clusters found in Low Motile (LM) sperm fraction. Genes, repeats, transposable elements and transcription factors binding sites falling within the cluster regions were reported. (ZIP 1034 kb) [file 12864_2016_3394_MOESM4_ESM.zip › 13.html]

piRNA cluster 13


Predicted piRNA cluster no. 13     previous   next
  

Show proTRAC run info
Hide proTRAC run info

================================= proTRAC ====================================  
VERSION: 2.1                                    LAST MODIFIED: 06. October 2015  
  
Please cite:  
Rosenkranz D, Zischler H. proTRAC - a software for probabilistic piRNA cluster  
detection, visualization and analysis. 2012. BMC Bioinformatics 13:5.  
  
and (for proTRAC 2.0 and later):  
Rosenkranz D, Rudloff S, Bastuck K, Ketting RF, Zischler H. Tupaia small RNAs  
provide insights into function and evolution of RNAi-based transposon defense  
in mammals. 2015. RNA 21(5):911-922.  
  
Contact:  
David Rosenkranz  
Institute of Anthropology, small RNA group  
Johannes Gutenberg University Mainz  
email: rosenkranz@uni-mainz.de  
  
You can find the latest proTRAC version at:  
http://sourceforge.net/projects/protrac/files  
http://www.smallRNAgroup-mainz.de/software  
==============================================================================  
  
PARAMETERS:  
Map file: .............../storage/core/barbara/genhome/smallRNA/fertility/Sample\_not\_motile/pirna/Sample\_not\_motile\_26-33\_collapsed.fa.no-dust.map.weighted-10000-1000-b-0  
Genome file: ............/storage/core/barbara/genhome/smallRNA/fertility/Sample\_all/pirna/bt\_311\_chrY.fa  
RepeatMasker annotation: /storage/genomes/bt\_umd31/GCF\_000003055.6\_Bos\_taurus\_UMD\_3.1.1\_repeatMasker\_chr.out  
GeneSet:................./storage/core/barbara/genhome/smallRNA/fertility/Sample\_all/pirna/full.gtf  
  
Significant (p<=0.01) hit density will be calculated based  
on observed hit distribution.  
  
Sliding window size: ........................................ 5000 bp  
Sliding window increament: .................................. 1000 bp  
Normalize each hit by number of genomic hits: ............... 1 [0=no/1=yes]  
Normalize each hit by number of sequence reads: ............. 1 [0=no/1=yes]  
Normalize values (-> per million mapped reads): ............. 1 [0=no/1=yes]  
Min. fraction of hits with 1T(U) or 10A: .................... 0.75  
Alternatively: Min. fraction of hits with 1T(U) and 10A: .... 0.5  
Min. fraction of hits with typical piRNA length: ............ 0.75  
Typical piRNA length: ....................................... 26-33 nt  
Min. size of a piRNA cluster: ............................... 5000 bp.  
Min. number of hits (absolute): ............................. 0  
Min. number of hits (normalized): ........................... 0  
Min. fraction of hits on the mainstrand: .................... 0.75  
Top fraction of mapped sequences (in terms of read counts): . 1%  
Top fraction accounts for max. n% of sequence reads: ........ 90%  
Min. fraction of hits on each arm of a bidirectional cluster: 0.1  
Output image file for each cluster: ......................... 0 [0=no/1=yes]  
Output html file for each cluster: .......................... 1 [0=no/1=yes]  
Output a summary table: ..................................... 1 [0=no/1=yes]  
Output a FASTA file for each cluster (piRNA sequences): ..... 1 [0=no/1=yes]  
Output a FASTA file comprising cluster sequences: ........... 1 [0=no/1=yes]  
Search DNA motifs in clusters: .............................. 1 [0=no/1=yes]  
Output flanking sequences: +/- .............................. 0 bp  
Output ~.pTi file: .......................................... 1 [0=no/1=yes]  
==============================================================================  
  
  
Genome size (without gaps): ............ 2678902517 bp  
Gaps (N/X/-): .......................... 53837044 bp  
Mapped reads: .......................... 738059667487  
Non-identical sequences: ............... 277001  
Genomic hits: .......................... 533816  
Significant densitiy of mapped reads: .. 15118061 reads/kb

Show proTRAC cluster info
Hide proTRAC cluster info

|  |  |
| --- | --- |
| Location | chr14 |
| Coordinates | 16791335-16809679 |
| Size [bp] | 18345 |
| Sequence hit loci | 404 |
| Mapped reads (normalized) | 1092657743 |
| Mapped reads (normalized) per kb | 59561610.4 |
| Normalized reads with 1T (1U) | 88.7% |
| Normalized reads with 10A | 32.9% |
| Normalized reads with length 26-33 nt | 100% |
| Normalized reads on the main strand(s) | 100% |
| Predicted directionality | mono:plus |

100%

0%

1T (1U)  
reads

10A reads

26-33 nt  
reads

reads on mainstrand

**Either the amount of reads with 1T (1U) OR 10A has to exceed 75% (set with option: -1Tor10A)  
Alternatively the amount of reads with 1T (1U) AND 10A has to exceed 50% (set with option: -1Tand10A)  
Minimum amount of reads with preferred size is 75% (set with option: -pisize)  
Minimum amount of reads on the main strand(s) is 75% (set with option: -clstrand)**

Show read coverage
Hide read coverage

WHAT DO I SEE HERE?  
This chart shows the location of mapped sequence reads within a predicted piRNA cluster. The color refers to the number of genomic hits produced by the sequence read in question. A dark red bar indicates that this sequence read produces many other hits elsewhere in the genome. Many adjacent red or yellow bars can indicate the presence of a multi-copy element such as transposons or rRNA genes. A dark green bar indicates that this sequence read maps uniquely to this locus.

1 hit

2-5 hits

6-10 hits

11-20 hits

21-50 hits

51-100 hits

> 100 hits

chr14

16791335

16809679

Gene Set

RepeatMasker

Mapped  
Reads

72.63

plus strand

minus strand

72.63

Region: chr14 15195790-16791353. Max. coverage (+): 4.55. Max coverage (-): 0

Region: chr14 16791354-16791390. Max. coverage (+): 0. Max coverage (-): 0

Region: chr14 16791391-16791426. Max. coverage (+): 0. Max coverage (-): 0

Region: chr14 16791427-16791463. Max. coverage (+): 0. Max coverage (-): 0

Region: chr14 16791464-16791500. Max. coverage (+): 0. Max coverage (-): 0

Region: chr14 16791501-16791536. Max. coverage (+): 0. Max coverage (-): 0

Region: chr14 16791537-16791573. Max. coverage (+): 0. Max coverage (-): 0

Region: chr14 16791574-16791610. Max. coverage (+): 0. Max coverage (-): 0

Region: chr14 16791611-16791646. Max. coverage (+): 0. Max coverage (-): 0

Region: chr14 16791647-16791683. Max. coverage (+): 0. Max coverage (-): 0

Region: chr14 16791684-16791720. Max. coverage (+): 0. Max coverage (-): 0

Region: chr14 16791721-16791756. Max. coverage (+): 0. Max coverage (-): 0

Region: chr14 16791757-16791793. Max. coverage (+): 0. Max coverage (-): 0

Region: chr14 16791794-16791830. Max. coverage (+): 0. Max coverage (-): 0

Region: chr14 16791831-16791867. Max. coverage (+): 0. Max coverage (-): 0

Region: chr14 16791868-16791903. Max. coverage (+): 0. Max coverage (-): 0

Region: chr14 16791904-16791940. Max. coverage (+): 0. Max coverage (-): 0

Region: chr14 16791941-16791977. Max. coverage (+): 0. Max coverage (-): 0

Region: chr14 16791978-16792013. Max. coverage (+): 0. Max coverage (-): 0

Region: chr14 16792014-16792050. Max. coverage (+): 0. Max coverage (-): 0

Region: chr14 16792051-16792087. Max. coverage (+): 0. Max coverage (-): 0

Region: chr14 16792088-16792123. Max. coverage (+): 0. Max coverage (-): 0

Region: chr14 16792124-16792160. Max. coverage (+): 0. Max coverage (-): 0

Region: chr14 16792161-16792197. Max. coverage (+): 0. Max coverage (-): 0

Region: chr14 16792198-16792233. Max. coverage (+): 0. Max coverage (-): 0

Region: chr14 16792234-16792270. Max. coverage (+): 0. Max coverage (-): 0

Region: chr14 16792271-16792307. Max. coverage (+): 0. Max coverage (-): 0

Region: chr14 16792308-16792343. Max. coverage (+): 0. Max coverage (-): 0

Region: chr14 16792344-16792380. Max. coverage (+): 0. Max coverage (-): 0

Region: chr14 16792381-16792417. Max. coverage (+): 0. Max coverage (-): 0

Region: chr14 16792418-16792454. Max. coverage (+): 0. Max coverage (-): 0

Region: chr14 16792455-16792490. Max. coverage (+): 0. Max coverage (-): 0

Region: chr14 16792491-16792527. Max. coverage (+): 0. Max coverage (-): 0

Region: chr14 16792528-16792564. Max. coverage (+): 0. Max coverage (-): 0

Region: chr14 16792565-16792600. Max. coverage (+): 0. Max coverage (-): 0

Region: chr14 16792601-16792637. Max. coverage (+): 0. Max coverage (-): 0

Region: chr14 16792638-16792674. Max. coverage (+): 0. Max coverage (-): 0

Region: chr14 16792675-16792710. Max. coverage (+): 0. Max coverage (-): 0

Region: chr14 16792711-16792747. Max. coverage (+): 0. Max coverage (-): 0

Region: chr14 16792748-16792784. Max. coverage (+): 0. Max coverage (-): 0

Region: chr14 16792785-16792820. Max. coverage (+): 0. Max coverage (-): 0

Region: chr14 16792821-16792857. Max. coverage (+): 0. Max coverage (-): 0

Region: chr14 16792858-16792894. Max. coverage (+): 0. Max coverage (-): 0

Region: chr14 16792895-16792931. Max. coverage (+): 0. Max coverage (-): 0

Region: chr14 16792932-16792967. Max. coverage (+): 5.48. Max coverage (-): 0

Region: chr14 16792968-16793004. Max. coverage (+): 2.61. Max coverage (-): 0

Region: chr14 16793005-16793041. Max. coverage (+): 2.46. Max coverage (-): 0

Region: chr14 16793042-16793077. Max. coverage (+): 8.52. Max coverage (-): 0

Region: chr14 16793078-16793114. Max. coverage (+): 3.49. Max coverage (-): 0

Region: chr14 16793115-16793151. Max. coverage (+): 17.93. Max coverage (-): 0

Region: chr14 16793152-16793187. Max. coverage (+): 3.1. Max coverage (-): 0

Region: chr14 16793188-16793224. Max. coverage (+): 11.22. Max coverage (-): 0

Region: chr14 16793225-16793261. Max. coverage (+): 0. Max coverage (-): 0

Region: chr14 16793262-16793297. Max. coverage (+): 0. Max coverage (-): 0

Region: chr14 16793298-16793334. Max. coverage (+): 0. Max coverage (-): 0

Region: chr14 16793335-16793371. Max. coverage (+): 0. Max coverage (-): 0

Region: chr14 16793372-16793407. Max. coverage (+): 0. Max coverage (-): 0

Region: chr14 16793408-16793444. Max. coverage (+): 0. Max coverage (-): 0

Region: chr14 16793445-16793481. Max. coverage (+): 0. Max coverage (-): 0

Region: chr14 16793482-16793518. Max. coverage (+): 0. Max coverage (-): 0

Region: chr14 16793519-16793554. Max. coverage (+): 0. Max coverage (-): 0

Region: chr14 16793555-16793591. Max. coverage (+): 0. Max coverage (-): 0

Region: chr14 16793592-16793628. Max. coverage (+): 0. Max coverage (-): 0

Region: chr14 16793629-16793664. Max. coverage (+): 0. Max coverage (-): 0

Region: chr14 16793665-16793701. Max. coverage (+): 0. Max coverage (-): 0

Region: chr14 16793702-16793738. Max. coverage (+): 0. Max coverage (-): 0

Region: chr14 16793739-16793774. Max. coverage (+): 0. Max coverage (-): 0

Region: chr14 16793775-16793811. Max. coverage (+): 0. Max coverage (-): 0

Region: chr14 16793812-16793848. Max. coverage (+): 0. Max coverage (-): 0

Region: chr14 16793849-16793884. Max. coverage (+): 0. Max coverage (-): 0

Region: chr14 16793885-16793921. Max. coverage (+): 0. Max coverage (-): 0

Region: chr14 16793922-16793958. Max. coverage (+): 0. Max coverage (-): 0

Region: chr14 16793959-16793995. Max. coverage (+): 0. Max coverage (-): 0

Region: chr14 16793996-16794031. Max. coverage (+): 0. Max coverage (-): 0

Region: chr14 16794032-16794068. Max. coverage (+): 0. Max coverage (-): 0

Region: chr14 16794069-16794105. Max. coverage (+): 0. Max coverage (-): 0

Region: chr14 16794106-16794141. Max. coverage (+): 0. Max coverage (-): 0

Region: chr14 16794142-16794178. Max. coverage (+): 0. Max coverage (-): 0

Region: chr14 16794179-16794215. Max. coverage (+): 0. Max coverage (-): 0

Region: chr14 16794216-16794251. Max. coverage (+): 0. Max coverage (-): 0

Region: chr14 16794252-16794288. Max. coverage (+): 0. Max coverage (-): 0

Region: chr14 16794289-16794325. Max. coverage (+): 0. Max coverage (-): 0

Region: chr14 16794326-16794361. Max. coverage (+): 0.1. Max coverage (-): 0

Region: chr14 16794362-16794398. Max. coverage (+): 0. Max coverage (-): 0

Region: chr14 16794399-16794435. Max. coverage (+): 0. Max coverage (-): 0

Region: chr14 16794436-16794471. Max. coverage (+): 0. Max coverage (-): 0

Region: chr14 16794472-16794508. Max. coverage (+): 0. Max coverage (-): 0

Region: chr14 16794509-16794545. Max. coverage (+): 0. Max coverage (-): 0

Region: chr14 16794546-16794582. Max. coverage (+): 0. Max coverage (-): 0

Region: chr14 16794583-16794618. Max. coverage (+): 0. Max coverage (-): 0

Region: chr14 16794619-16794655. Max. coverage (+): 17.92. Max coverage (-): 0

Region: chr14 16794656-16794692. Max. coverage (+): 45.66. Max coverage (-): 0

Region: chr14 16794693-16794728. Max. coverage (+): 6.5. Max coverage (-): 0

Region: chr14 16794729-16794765. Max. coverage (+): 4.21. Max coverage (-): 0

Region: chr14 16794766-16794802. Max. coverage (+): 11.35. Max coverage (-): 0

Region: chr14 16794803-16794838. Max. coverage (+): 4.08. Max coverage (-): 0

Region: chr14 16794839-16794875. Max. coverage (+): 0. Max coverage (-): 0

Region: chr14 16794876-16794912. Max. coverage (+): 0. Max coverage (-): 0

Region: chr14 16794913-16794948. Max. coverage (+): 0. Max coverage (-): 0

Region: chr14 16794949-16794985. Max. coverage (+): 0. Max coverage (-): 0

Region: chr14 16794986-16795022. Max. coverage (+): 0. Max coverage (-): 0

Region: chr14 16795023-16795059. Max. coverage (+): 0. Max coverage (-): 0

Region: chr14 16795060-16795095. Max. coverage (+): 0. Max coverage (-): 0

Region: chr14 16795096-16795132. Max. coverage (+): 0. Max coverage (-): 0

Region: chr14 16795133-16795169. Max. coverage (+): 0. Max coverage (-): 0

Region: chr14 16795170-16795205. Max. coverage (+): 7.96. Max coverage (-): 0

Region: chr14 16795206-16795242. Max. coverage (+): 0. Max coverage (-): 0

Region: chr14 16795243-16795279. Max. coverage (+): 7.89. Max coverage (-): 0

Region: chr14 16795280-16795315. Max. coverage (+): 0. Max coverage (-): 0

Region: chr14 16795316-16795352. Max. coverage (+): 3.17. Max coverage (-): 0

Region: chr14 16795353-16795389. Max. coverage (+): 3.17. Max coverage (-): 0

Region: chr14 16795390-16795425. Max. coverage (+): 0. Max coverage (-): 0

Region: chr14 16795426-16795462. Max. coverage (+): 0. Max coverage (-): 0

Region: chr14 16795463-16795499. Max. coverage (+): 0. Max coverage (-): 0

Region: chr14 16795500-16795536. Max. coverage (+): 0. Max coverage (-): 0

Region: chr14 16795537-16795572. Max. coverage (+): 0. Max coverage (-): 0

Region: chr14 16795573-16795609. Max. coverage (+): 0. Max coverage (-): 0

Region: chr14 16795610-16795646. Max. coverage (+): 0. Max coverage (-): 0

Region: chr14 16795647-16795682. Max. coverage (+): 0. Max coverage (-): 0

Region: chr14 16795683-16795719. Max. coverage (+): 0. Max coverage (-): 0

Region: chr14 16795720-16795756. Max. coverage (+): 28.79. Max coverage (-): 0

Region: chr14 16795757-16795792. Max. coverage (+): 10.22. Max coverage (-): 0

Region: chr14 16795793-16795829. Max. coverage (+): 6.89. Max coverage (-): 0

Region: chr14 16795830-16795866. Max. coverage (+): 0. Max coverage (-): 0

Region: chr14 16795867-16795902. Max. coverage (+): 0. Max coverage (-): 0

Region: chr14 16795903-16795939. Max. coverage (+): 0. Max coverage (-): 0

Region: chr14 16795940-16795976. Max. coverage (+): 0. Max coverage (-): 0

Region: chr14 16795977-16796012. Max. coverage (+): 0. Max coverage (-): 0

Region: chr14 16796013-16796049. Max. coverage (+): 0. Max coverage (-): 0

Region: chr14 16796050-16796086. Max. coverage (+): 0. Max coverage (-): 0

Region: chr14 16796087-16796123. Max. coverage (+): 0. Max coverage (-): 0

Region: chr14 16796124-16796159. Max. coverage (+): 0. Max coverage (-): 0

Region: chr14 16796160-16796196. Max. coverage (+): 2.69. Max coverage (-): 0

Region: chr14 16796197-16796233. Max. coverage (+): 0. Max coverage (-): 0

Region: chr14 16796234-16796269. Max. coverage (+): 0.48. Max coverage (-): 0

Region: chr14 16796270-16796306. Max. coverage (+): 1.86. Max coverage (-): 0

Region: chr14 16796307-16796343. Max. coverage (+): 0. Max coverage (-): 0

Region: chr14 16796344-16796379. Max. coverage (+): 1.24. Max coverage (-): 0

Region: chr14 16796380-16796416. Max. coverage (+): 9.6. Max coverage (-): 0

Region: chr14 16796417-16796453. Max. coverage (+): 0. Max coverage (-): 0

Region: chr14 16796454-16796489. Max. coverage (+): 0. Max coverage (-): 0

Region: chr14 16796490-16796526. Max. coverage (+): 0. Max coverage (-): 0

Region: chr14 16796527-16796563. Max. coverage (+): 0. Max coverage (-): 0

Region: chr14 16796564-16796600. Max. coverage (+): 11.59. Max coverage (-): 0

Region: chr14 16796601-16796636. Max. coverage (+): 15.37. Max coverage (-): 0

Region: chr14 16796637-16796673. Max. coverage (+): 14.03. Max coverage (-): 0

Region: chr14 16796674-16796710. Max. coverage (+): 2.41. Max coverage (-): 0

Region: chr14 16796711-16796746. Max. coverage (+): 4.95. Max coverage (-): 0

Region: chr14 16796747-16796783. Max. coverage (+): 0. Max coverage (-): 0

Region: chr14 16796784-16796820. Max. coverage (+): 0. Max coverage (-): 0

Region: chr14 16796821-16796856. Max. coverage (+): 0. Max coverage (-): 0

Region: chr14 16796857-16796893. Max. coverage (+): 0. Max coverage (-): 0

Region: chr14 16796894-16796930. Max. coverage (+): 0. Max coverage (-): 0

Region: chr14 16796931-16796966. Max. coverage (+): 0. Max coverage (-): 0

Region: chr14 16796967-16797003. Max. coverage (+): 4.94. Max coverage (-): 0

Region: chr14 16797004-16797040. Max. coverage (+): 0. Max coverage (-): 0

Region: chr14 16797041-16797076. Max. coverage (+): 6.87. Max coverage (-): 0

Region: chr14 16797077-16797113. Max. coverage (+): 11.91. Max coverage (-): 0

Region: chr14 16797114-16797150. Max. coverage (+): 3.35. Max coverage (-): 0

Region: chr14 16797151-16797187. Max. coverage (+): 6.27. Max coverage (-): 0

Region: chr14 16797188-16797223. Max. coverage (+): 14.4. Max coverage (-): 0

Region: chr14 16797224-16797260. Max. coverage (+): 5.49. Max coverage (-): 0

Region: chr14 16797261-16797297. Max. coverage (+): 0. Max coverage (-): 0

Region: chr14 16797298-16797333. Max. coverage (+): 0. Max coverage (-): 0

Region: chr14 16797334-16797370. Max. coverage (+): 0. Max coverage (-): 0

Region: chr14 16797371-16797407. Max. coverage (+): 8.16. Max coverage (-): 0

Region: chr14 16797408-16797443. Max. coverage (+): 8.16. Max coverage (-): 0

Region: chr14 16797444-16797480. Max. coverage (+): 4.29. Max coverage (-): 0

Region: chr14 16797481-16797517. Max. coverage (+): 0. Max coverage (-): 0

Region: chr14 16797518-16797553. Max. coverage (+): 0. Max coverage (-): 0

Region: chr14 16797554-16797590. Max. coverage (+): 0. Max coverage (-): 0

Region: chr14 16797591-16797627. Max. coverage (+): 0. Max coverage (-): 0

Region: chr14 16797628-16797664. Max. coverage (+): 0. Max coverage (-): 0

Region: chr14 16797665-16797700. Max. coverage (+): 0. Max coverage (-): 0

Region: chr14 16797701-16797737. Max. coverage (+): 0. Max coverage (-): 0

Region: chr14 16797738-16797774. Max. coverage (+): 0. Max coverage (-): 0

Region: chr14 16797775-16797810. Max. coverage (+): 11.87. Max coverage (-): 0

Region: chr14 16797811-16797847. Max. coverage (+): 9.12. Max coverage (-): 0

Region: chr14 16797848-16797884. Max. coverage (+): 0. Max coverage (-): 0

Region: chr14 16797885-16797920. Max. coverage (+): 0. Max coverage (-): 0

Region: chr14 16797921-16797957. Max. coverage (+): 0. Max coverage (-): 0

Region: chr14 16797958-16797994. Max. coverage (+): 0. Max coverage (-): 0

Region: chr14 16797995-16798030. Max. coverage (+): 0. Max coverage (-): 0

Region: chr14 16798031-16798067. Max. coverage (+): 0. Max coverage (-): 0

Region: chr14 16798068-16798104. Max. coverage (+): 0. Max coverage (-): 0

Region: chr14 16798105-16798140. Max. coverage (+): 0. Max coverage (-): 0

Region: chr14 16798141-16798177. Max. coverage (+): 0. Max coverage (-): 0

Region: chr14 16798178-16798214. Max. coverage (+): 0. Max coverage (-): 0

Region: chr14 16798215-16798251. Max. coverage (+): 0. Max coverage (-): 0

Region: chr14 16798252-16798287. Max. coverage (+): 0. Max coverage (-): 0

Region: chr14 16798288-16798324. Max. coverage (+): 0. Max coverage (-): 0

Region: chr14 16798325-16798361. Max. coverage (+): 0. Max coverage (-): 0

Region: chr14 16798362-16798397. Max. coverage (+): 0. Max coverage (-): 0

Region: chr14 16798398-16798434. Max. coverage (+): 0. Max coverage (-): 0

Region: chr14 16798435-16798471. Max. coverage (+): 0. Max coverage (-): 0

Region: chr14 16798472-16798507. Max. coverage (+): 0. Max coverage (-): 0

Region: chr14 16798508-16798544. Max. coverage (+): 0. Max coverage (-): 0

Region: chr14 16798545-16798581. Max. coverage (+): 0. Max coverage (-): 0

Region: chr14 16798582-16798617. Max. coverage (+): 0. Max coverage (-): 0

Region: chr14 16798618-16798654. Max. coverage (+): 0. Max coverage (-): 0

Region: chr14 16798655-16798691. Max. coverage (+): 4.89. Max coverage (-): 0

Region: chr14 16798692-16798728. Max. coverage (+): 0. Max coverage (-): 0

Region: chr14 16798729-16798764. Max. coverage (+): 0. Max coverage (-): 0

Region: chr14 16798765-16798801. Max. coverage (+): 0. Max coverage (-): 0

Region: chr14 16798802-16798838. Max. coverage (+): 0. Max coverage (-): 0

Region: chr14 16798839-16798874. Max. coverage (+): 0. Max coverage (-): 0

Region: chr14 16798875-16798911. Max. coverage (+): 0. Max coverage (-): 0

Region: chr14 16798912-16798948. Max. coverage (+): 0. Max coverage (-): 0

Region: chr14 16798949-16798984. Max. coverage (+): 0. Max coverage (-): 0

Region: chr14 16798985-16799021. Max. coverage (+): 0. Max coverage (-): 0

Region: chr14 16799022-16799058. Max. coverage (+): 0. Max coverage (-): 0

Region: chr14 16799059-16799094. Max. coverage (+): 0. Max coverage (-): 0

Region: chr14 16799095-16799131. Max. coverage (+): 0. Max coverage (-): 0

Region: chr14 16799132-16799168. Max. coverage (+): 0. Max coverage (-): 0

Region: chr14 16799169-16799205. Max. coverage (+): 3.73. Max coverage (-): 0

Region: chr14 16799206-16799241. Max. coverage (+): 17.64. Max coverage (-): 0

Region: chr14 16799242-16799278. Max. coverage (+): 10.59. Max coverage (-): 0

Region: chr14 16799279-16799315. Max. coverage (+): 0. Max coverage (-): 0

Region: chr14 16799316-16799351. Max. coverage (+): 0. Max coverage (-): 0

Region: chr14 16799352-16799388. Max. coverage (+): 0. Max coverage (-): 0

Region: chr14 16799389-16799425. Max. coverage (+): 3.33. Max coverage (-): 0

Region: chr14 16799426-16799461. Max. coverage (+): 0. Max coverage (-): 0

Region: chr14 16799462-16799498. Max. coverage (+): 4.52. Max coverage (-): 0

Region: chr14 16799499-16799535. Max. coverage (+): 4.52. Max coverage (-): 0

Region: chr14 16799536-16799571. Max. coverage (+): 0. Max coverage (-): 0

Region: chr14 16799572-16799608. Max. coverage (+): 0. Max coverage (-): 0

Region: chr14 16799609-16799645. Max. coverage (+): 0. Max coverage (-): 0

Region: chr14 16799646-16799681. Max. coverage (+): 0. Max coverage (-): 0

Region: chr14 16799682-16799718. Max. coverage (+): 0. Max coverage (-): 0

Region: chr14 16799719-16799755. Max. coverage (+): 0. Max coverage (-): 0

Region: chr14 16799756-16799792. Max. coverage (+): 0. Max coverage (-): 0

Region: chr14 16799793-16799828. Max. coverage (+): 0. Max coverage (-): 0

Region: chr14 16799829-16799865. Max. coverage (+): 0. Max coverage (-): 0

Region: chr14 16799866-16799902. Max. coverage (+): 0. Max coverage (-): 0

Region: chr14 16799903-16799938. Max. coverage (+): 3.79. Max coverage (-): 0

Region: chr14 16799939-16799975. Max. coverage (+): 3.81. Max coverage (-): 0

Region: chr14 16799976-16800012. Max. coverage (+): 3.81. Max coverage (-): 0

Region: chr14 16800013-16800048. Max. coverage (+): 0. Max coverage (-): 0

Region: chr14 16800049-16800085. Max. coverage (+): 0. Max coverage (-): 0

Region: chr14 16800086-16800122. Max. coverage (+): 0. Max coverage (-): 0

Region: chr14 16800123-16800158. Max. coverage (+): 0. Max coverage (-): 0

Region: chr14 16800159-16800195. Max. coverage (+): 0. Max coverage (-): 0

Region: chr14 16800196-16800232. Max. coverage (+): 0. Max coverage (-): 0

Region: chr14 16800233-16800269. Max. coverage (+): 0. Max coverage (-): 0

Region: chr14 16800270-16800305. Max. coverage (+): 0. Max coverage (-): 0

Region: chr14 16800306-16800342. Max. coverage (+): 0. Max coverage (-): 0

Region: chr14 16800343-16800379. Max. coverage (+): 0. Max coverage (-): 0

Region: chr14 16800380-16800415. Max. coverage (+): 0. Max coverage (-): 0

Region: chr14 16800416-16800452. Max. coverage (+): 0. Max coverage (-): 0

Region: chr14 16800453-16800489. Max. coverage (+): 0. Max coverage (-): 0

Region: chr14 16800490-16800525. Max. coverage (+): 0. Max coverage (-): 0

Region: chr14 16800526-16800562. Max. coverage (+): 0. Max coverage (-): 0

Region: chr14 16800563-16800599. Max. coverage (+): 0. Max coverage (-): 0

Region: chr14 16800600-16800635. Max. coverage (+): 0. Max coverage (-): 0

Region: chr14 16800636-16800672. Max. coverage (+): 0. Max coverage (-): 0

Region: chr14 16800673-16800709. Max. coverage (+): 0. Max coverage (-): 0

Region: chr14 16800710-16800745. Max. coverage (+): 0. Max coverage (-): 0

Region: chr14 16800746-16800782. Max. coverage (+): 0. Max coverage (-): 0

Region: chr14 16800783-16800819. Max. coverage (+): 0. Max coverage (-): 0

Region: chr14 16800820-16800856. Max. coverage (+): 0. Max coverage (-): 0

Region: chr14 16800857-16800892. Max. coverage (+): 0. Max coverage (-): 0

Region: chr14 16800893-16800929. Max. coverage (+): 0. Max coverage (-): 0

Region: chr14 16800930-16800966. Max. coverage (+): 0. Max coverage (-): 0

Region: chr14 16800967-16801002. Max. coverage (+): 0. Max coverage (-): 0

Region: chr14 16801003-16801039. Max. coverage (+): 0. Max coverage (-): 0

Region: chr14 16801040-16801076. Max. coverage (+): 0. Max coverage (-): 0

Region: chr14 16801077-16801112. Max. coverage (+): 0. Max coverage (-): 0

Region: chr14 16801113-16801149. Max. coverage (+): 0. Max coverage (-): 0

Region: chr14 16801150-16801186. Max. coverage (+): 0. Max coverage (-): 0

Region: chr14 16801187-16801222. Max. coverage (+): 0. Max coverage (-): 0

Region: chr14 16801223-16801259. Max. coverage (+): 0. Max coverage (-): 0

Region: chr14 16801260-16801296. Max. coverage (+): 0. Max coverage (-): 0

Region: chr14 16801297-16801333. Max. coverage (+): 0. Max coverage (-): 0

Region: chr14 16801334-16801369. Max. coverage (+): 0. Max coverage (-): 0

Region: chr14 16801370-16801406. Max. coverage (+): 0. Max coverage (-): 0

Region: chr14 16801407-16801443. Max. coverage (+): 0. Max coverage (-): 0

Region: chr14 16801444-16801479. Max. coverage (+): 0. Max coverage (-): 0

Region: chr14 16801480-16801516. Max. coverage (+): 0. Max coverage (-): 0

Region: chr14 16801517-16801553. Max. coverage (+): 0. Max coverage (-): 0

Region: chr14 16801554-16801589. Max. coverage (+): 0. Max coverage (-): 0

Region: chr14 16801590-16801626. Max. coverage (+): 0. Max coverage (-): 0

Region: chr14 16801627-16801663. Max. coverage (+): 0. Max coverage (-): 0

Region: chr14 16801664-16801699. Max. coverage (+): 0. Max coverage (-): 0

Region: chr14 16801700-16801736. Max. coverage (+): 0. Max coverage (-): 0

Region: chr14 16801737-16801773. Max. coverage (+): 0. Max coverage (-): 0

Region: chr14 16801774-16801809. Max. coverage (+): 0. Max coverage (-): 0

Region: chr14 16801810-16801846. Max. coverage (+): 0. Max coverage (-): 0

Region: chr14 16801847-16801883. Max. coverage (+): 0. Max coverage (-): 0

Region: chr14 16801884-16801920. Max. coverage (+): 0. Max coverage (-): 0

Region: chr14 16801921-16801956. Max. coverage (+): 0. Max coverage (-): 0

Region: chr14 16801957-16801993. Max. coverage (+): 0. Max coverage (-): 0

Region: chr14 16801994-16802030. Max. coverage (+): 0. Max coverage (-): 0

Region: chr14 16802031-16802066. Max. coverage (+): 0. Max coverage (-): 0

Region: chr14 16802067-16802103. Max. coverage (+): 0. Max coverage (-): 0

Region: chr14 16802104-16802140. Max. coverage (+): 0. Max coverage (-): 0

Region: chr14 16802141-16802176. Max. coverage (+): 0. Max coverage (-): 0

Region: chr14 16802177-16802213. Max. coverage (+): 0. Max coverage (-): 0

Region: chr14 16802214-16802250. Max. coverage (+): 0. Max coverage (-): 0

Region: chr14 16802251-16802286. Max. coverage (+): 0. Max coverage (-): 0

Region: chr14 16802287-16802323. Max. coverage (+): 0. Max coverage (-): 0

Region: chr14 16802324-16802360. Max. coverage (+): 0. Max coverage (-): 0

Region: chr14 16802361-16802397. Max. coverage (+): 0. Max coverage (-): 0

Region: chr14 16802398-16802433. Max. coverage (+): 0. Max coverage (-): 0

Region: chr14 16802434-16802470. Max. coverage (+): 12.39. Max coverage (-): 0

Region: chr14 16802471-16802507. Max. coverage (+): 56.99. Max coverage (-): 0

Region: chr14 16802508-16802543. Max. coverage (+): 20.68. Max coverage (-): 0

Region: chr14 16802544-16802580. Max. coverage (+): 0. Max coverage (-): 0

Region: chr14 16802581-16802617. Max. coverage (+): 0. Max coverage (-): 0

Region: chr14 16802618-16802653. Max. coverage (+): 0. Max coverage (-): 0

Region: chr14 16802654-16802690. Max. coverage (+): 0. Max coverage (-): 0

Region: chr14 16802691-16802727. Max. coverage (+): 6.93. Max coverage (-): 0

Region: chr14 16802728-16802763. Max. coverage (+): 20.48. Max coverage (-): 0

Region: chr14 16802764-16802800. Max. coverage (+): 3.08. Max coverage (-): 0

Region: chr14 16802801-16802837. Max. coverage (+): 0. Max coverage (-): 0

Region: chr14 16802838-16802874. Max. coverage (+): 0. Max coverage (-): 0

Region: chr14 16802875-16802910. Max. coverage (+): 0. Max coverage (-): 0

Region: chr14 16802911-16802947. Max. coverage (+): 0. Max coverage (-): 0

Region: chr14 16802948-16802984. Max. coverage (+): 0. Max coverage (-): 0

Region: chr14 16802985-16803020. Max. coverage (+): 0. Max coverage (-): 0

Region: chr14 16803021-16803057. Max. coverage (+): 0. Max coverage (-): 0

Region: chr14 16803058-16803094. Max. coverage (+): 0. Max coverage (-): 0

Region: chr14 16803095-16803130. Max. coverage (+): 0. Max coverage (-): 0

Region: chr14 16803131-16803167. Max. coverage (+): 0. Max coverage (-): 0

Region: chr14 16803168-16803204. Max. coverage (+): 0. Max coverage (-): 0

Region: chr14 16803205-16803240. Max. coverage (+): 0. Max coverage (-): 0

Region: chr14 16803241-16803277. Max. coverage (+): 0. Max coverage (-): 0

Region: chr14 16803278-16803314. Max. coverage (+): 0. Max coverage (-): 0

Region: chr14 16803315-16803350. Max. coverage (+): 0. Max coverage (-): 0

Region: chr14 16803351-16803387. Max. coverage (+): 0. Max coverage (-): 0

Region: chr14 16803388-16803424. Max. coverage (+): 0. Max coverage (-): 0

Region: chr14 16803425-16803461. Max. coverage (+): 0. Max coverage (-): 0

Region: chr14 16803462-16803497. Max. coverage (+): 0. Max coverage (-): 0

Region: chr14 16803498-16803534. Max. coverage (+): 0. Max coverage (-): 0

Region: chr14 16803535-16803571. Max. coverage (+): 0. Max coverage (-): 0

Region: chr14 16803572-16803607. Max. coverage (+): 0. Max coverage (-): 0

Region: chr14 16803608-16803644. Max. coverage (+): 0. Max coverage (-): 0

Region: chr14 16803645-16803681. Max. coverage (+): 0. Max coverage (-): 0

Region: chr14 16803682-16803717. Max. coverage (+): 0. Max coverage (-): 0

Region: chr14 16803718-16803754. Max. coverage (+): 0. Max coverage (-): 0

Region: chr14 16803755-16803791. Max. coverage (+): 0. Max coverage (-): 0

Region: chr14 16803792-16803827. Max. coverage (+): 0. Max coverage (-): 0

Region: chr14 16803828-16803864. Max. coverage (+): 0. Max coverage (-): 0

Region: chr14 16803865-16803901. Max. coverage (+): 0. Max coverage (-): 0

Region: chr14 16803902-16803938. Max. coverage (+): 0. Max coverage (-): 0

Region: chr14 16803939-16803974. Max. coverage (+): 20.2. Max coverage (-): 0

Region: chr14 16803975-16804011. Max. coverage (+): 0. Max coverage (-): 0

Region: chr14 16804012-16804048. Max. coverage (+): 4.88. Max coverage (-): 0

Region: chr14 16804049-16804084. Max. coverage (+): 0. Max coverage (-): 0

Region: chr14 16804085-16804121. Max. coverage (+): 36.04. Max coverage (-): 0

Region: chr14 16804122-16804158. Max. coverage (+): 36.04. Max coverage (-): 0

Region: chr14 16804159-16804194. Max. coverage (+): 0. Max coverage (-): 0

Region: chr14 16804195-16804231. Max. coverage (+): 6.13. Max coverage (-): 0

Region: chr14 16804232-16804268. Max. coverage (+): 6.13. Max coverage (-): 0

Region: chr14 16804269-16804304. Max. coverage (+): 0. Max coverage (-): 0

Region: chr14 16804305-16804341. Max. coverage (+): 10.89. Max coverage (-): 0

Region: chr14 16804342-16804378. Max. coverage (+): 7.49. Max coverage (-): 0

Region: chr14 16804379-16804414. Max. coverage (+): 0. Max coverage (-): 0

Region: chr14 16804415-16804451. Max. coverage (+): 0. Max coverage (-): 0

Region: chr14 16804452-16804488. Max. coverage (+): 0. Max coverage (-): 0

Region: chr14 16804489-16804525. Max. coverage (+): 0. Max coverage (-): 0

Region: chr14 16804526-16804561. Max. coverage (+): 0. Max coverage (-): 0

Region: chr14 16804562-16804598. Max. coverage (+): 0. Max coverage (-): 0

Region: chr14 16804599-16804635. Max. coverage (+): 0. Max coverage (-): 0

Region: chr14 16804636-16804671. Max. coverage (+): 9.89. Max coverage (-): 0

Region: chr14 16804672-16804708. Max. coverage (+): 68.26. Max coverage (-): 0

Region: chr14 16804709-16804745. Max. coverage (+): 18.38. Max coverage (-): 0

Region: chr14 16804746-16804781. Max. coverage (+): 18.38. Max coverage (-): 0

Region: chr14 16804782-16804818. Max. coverage (+): 26.3. Max coverage (-): 0

Region: chr14 16804819-16804855. Max. coverage (+): 11.78. Max coverage (-): 0

Region: chr14 16804856-16804891. Max. coverage (+): 0. Max coverage (-): 0

Region: chr14 16804892-16804928. Max. coverage (+): 0. Max coverage (-): 0

Region: chr14 16804929-16804965. Max. coverage (+): 0. Max coverage (-): 0

Region: chr14 16804966-16805002. Max. coverage (+): 0. Max coverage (-): 0

Region: chr14 16805003-16805038. Max. coverage (+): 0. Max coverage (-): 0

Region: chr14 16805039-16805075. Max. coverage (+): 15.12. Max coverage (-): 0

Region: chr14 16805076-16805112. Max. coverage (+): 15.12. Max coverage (-): 0

Region: chr14 16805113-16805148. Max. coverage (+): 0. Max coverage (-): 0

Region: chr14 16805149-16805185. Max. coverage (+): 36.04. Max coverage (-): 0

Region: chr14 16805186-16805222. Max. coverage (+): 25.37. Max coverage (-): 0

Region: chr14 16805223-16805258. Max. coverage (+): 28.02. Max coverage (-): 0

Region: chr14 16805259-16805295. Max. coverage (+): 0. Max coverage (-): 0

Region: chr14 16805296-16805332. Max. coverage (+): 30.99. Max coverage (-): 0

Region: chr14 16805333-16805368. Max. coverage (+): 12.77. Max coverage (-): 0

Region: chr14 16805369-16805405. Max. coverage (+): 0. Max coverage (-): 0

Region: chr14 16805406-16805442. Max. coverage (+): 6.09. Max coverage (-): 0

Region: chr14 16805443-16805478. Max. coverage (+): 3.17. Max coverage (-): 0

Region: chr14 16805479-16805515. Max. coverage (+): 3.17. Max coverage (-): 0

Region: chr14 16805516-16805552. Max. coverage (+): 18.46. Max coverage (-): 0

Region: chr14 16805553-16805589. Max. coverage (+): 10.53. Max coverage (-): 0

Region: chr14 16805590-16805625. Max. coverage (+): 1.95. Max coverage (-): 0

Region: chr14 16805626-16805662. Max. coverage (+): 20.93. Max coverage (-): 0

Region: chr14 16805663-16805699. Max. coverage (+): 3.15. Max coverage (-): 0

Region: chr14 16805700-16805735. Max. coverage (+): 72.63. Max coverage (-): 0

Region: chr14 16805736-16805772. Max. coverage (+): 11.61. Max coverage (-): 0

Region: chr14 16805773-16805809. Max. coverage (+): 10.67. Max coverage (-): 0

Region: chr14 16805810-16805845. Max. coverage (+): 5.95. Max coverage (-): 0

Region: chr14 16805846-16805882. Max. coverage (+): 0. Max coverage (-): 0

Region: chr14 16805883-16805919. Max. coverage (+): 0. Max coverage (-): 0

Region: chr14 16805920-16805955. Max. coverage (+): 0. Max coverage (-): 0

Region: chr14 16805956-16805992. Max. coverage (+): 0. Max coverage (-): 0

Region: chr14 16805993-16806029. Max. coverage (+): 0. Max coverage (-): 0

Region: chr14 16806030-16806066. Max. coverage (+): 0. Max coverage (-): 0

Region: chr14 16806067-16806102. Max. coverage (+): 0. Max coverage (-): 0

Region: chr14 16806103-16806139. Max. coverage (+): 0. Max coverage (-): 0

Region: chr14 16806140-16806176. Max. coverage (+): 8.94. Max coverage (-): 0

Region: chr14 16806177-16806212. Max. coverage (+): 10.23. Max coverage (-): 0

Region: chr14 16806213-16806249. Max. coverage (+): 4.54. Max coverage (-): 0

Region: chr14 16806250-16806286. Max. coverage (+): 0. Max coverage (-): 0

Region: chr14 16806287-16806322. Max. coverage (+): 0. Max coverage (-): 0

Region: chr14 16806323-16806359. Max. coverage (+): 0. Max coverage (-): 0

Region: chr14 16806360-16806396. Max. coverage (+): 18.02. Max coverage (-): 0

Region: chr14 16806397-16806432. Max. coverage (+): 1. Max coverage (-): 0

Region: chr14 16806433-16806469. Max. coverage (+): 41.15. Max coverage (-): 0

Region: chr14 16806470-16806506. Max. coverage (+): 23.9. Max coverage (-): 0

Region: chr14 16806507-16806543. Max. coverage (+): 23.9. Max coverage (-): 0

Region: chr14 16806544-16806579. Max. coverage (+): 10.76. Max coverage (-): 0

Region: chr14 16806580-16806616. Max. coverage (+): 0. Max coverage (-): 0

Region: chr14 16806617-16806653. Max. coverage (+): 0. Max coverage (-): 0

Region: chr14 16806654-16806689. Max. coverage (+): 0. Max coverage (-): 0

Region: chr14 16806690-16806726. Max. coverage (+): 0. Max coverage (-): 0

Region: chr14 16806727-16806763. Max. coverage (+): 0. Max coverage (-): 0

Region: chr14 16806764-16806799. Max. coverage (+): 2.05. Max coverage (-): 0

Region: chr14 16806800-16806836. Max. coverage (+): 0. Max coverage (-): 0

Region: chr14 16806837-16806873. Max. coverage (+): 0. Max coverage (-): 0

Region: chr14 16806874-16806909. Max. coverage (+): 0. Max coverage (-): 0

Region: chr14 16806910-16806946. Max. coverage (+): 3.81. Max coverage (-): 0

Region: chr14 16806947-16806983. Max. coverage (+): 3.81. Max coverage (-): 0

Region: chr14 16806984-16807019. Max. coverage (+): 0. Max coverage (-): 0

Region: chr14 16807020-16807056. Max. coverage (+): 0. Max coverage (-): 0

Region: chr14 16807057-16807093. Max. coverage (+): 0. Max coverage (-): 0

Region: chr14 16807094-16807130. Max. coverage (+): 0. Max coverage (-): 0

Region: chr14 16807131-16807166. Max. coverage (+): 0. Max coverage (-): 0

Region: chr14 16807167-16807203. Max. coverage (+): 0. Max coverage (-): 0

Region: chr14 16807204-16807240. Max. coverage (+): 0. Max coverage (-): 0

Region: chr14 16807241-16807276. Max. coverage (+): 0. Max coverage (-): 0

Region: chr14 16807277-16807313. Max. coverage (+): 0. Max coverage (-): 0

Region: chr14 16807314-16807350. Max. coverage (+): 0. Max coverage (-): 0

Region: chr14 16807351-16807386. Max. coverage (+): 0. Max coverage (-): 0

Region: chr14 16807387-16807423. Max. coverage (+): 0. Max coverage (-): 0

Region: chr14 16807424-16807460. Max. coverage (+): 0. Max coverage (-): 0

Region: chr14 16807461-16807496. Max. coverage (+): 0. Max coverage (-): 0

Region: chr14 16807497-16807533. Max. coverage (+): 0. Max coverage (-): 0

Region: chr14 16807534-16807570. Max. coverage (+): 0. Max coverage (-): 0

Region: chr14 16807571-16807607. Max. coverage (+): 4.83. Max coverage (-): 0

Region: chr14 16807608-16807643. Max. coverage (+): 0. Max coverage (-): 0

Region: chr14 16807644-16807680. Max. coverage (+): 10.41. Max coverage (-): 0

Region: chr14 16807681-16807717. Max. coverage (+): 0. Max coverage (-): 0

Region: chr14 16807718-16807753. Max. coverage (+): 0. Max coverage (-): 0

Region: chr14 16807754-16807790. Max. coverage (+): 0. Max coverage (-): 0

Region: chr14 16807791-16807827. Max. coverage (+): 0. Max coverage (-): 0

Region: chr14 16807828-16807863. Max. coverage (+): 4.93. Max coverage (-): 0

Region: chr14 16807864-16807900. Max. coverage (+): 0. Max coverage (-): 0

Region: chr14 16807901-16807937. Max. coverage (+): 0. Max coverage (-): 0

Region: chr14 16807938-16807973. Max. coverage (+): 0. Max coverage (-): 0

Region: chr14 16807974-16808010. Max. coverage (+): 2.68. Max coverage (-): 0

Region: chr14 16808011-16808047. Max. coverage (+): 0. Max coverage (-): 0

Region: chr14 16808048-16808083. Max. coverage (+): 0. Max coverage (-): 0

Region: chr14 16808084-16808120. Max. coverage (+): 3.35. Max coverage (-): 0

Region: chr14 16808121-16808157. Max. coverage (+): 0. Max coverage (-): 0

Region: chr14 16808158-16808194. Max. coverage (+): 0. Max coverage (-): 0

Region: chr14 16808195-16808230. Max. coverage (+): 0. Max coverage (-): 0

Region: chr14 16808231-16808267. Max. coverage (+): 0. Max coverage (-): 0

Region: chr14 16808268-16808304. Max. coverage (+): 0. Max coverage (-): 0

Region: chr14 16808305-16808340. Max. coverage (+): 0. Max coverage (-): 0

Region: chr14 16808341-16808377. Max. coverage (+): 0. Max coverage (-): 0

Region: chr14 16808378-16808414. Max. coverage (+): 0. Max coverage (-): 0

Region: chr14 16808415-16808450. Max. coverage (+): 0. Max coverage (-): 0

Region: chr14 16808451-16808487. Max. coverage (+): 5.35. Max coverage (-): 0

Region: chr14 16808488-16808524. Max. coverage (+): 0. Max coverage (-): 0

Region: chr14 16808525-16808560. Max. coverage (+): 0. Max coverage (-): 0

Region: chr14 16808561-16808597. Max. coverage (+): 0. Max coverage (-): 0

Region: chr14 16808598-16808634. Max. coverage (+): 0. Max coverage (-): 0

Region: chr14 16808635-16808671. Max. coverage (+): 0. Max coverage (-): 0

Region: chr14 16808672-16808707. Max. coverage (+): 0. Max coverage (-): 0

Region: chr14 16808708-16808744. Max. coverage (+): 7.2. Max coverage (-): 0

Region: chr14 16808745-16808781. Max. coverage (+): 3.4. Max coverage (-): 0

Region: chr14 16808782-16808817. Max. coverage (+): 16.15. Max coverage (-): 0

Region: chr14 16808818-16808854. Max. coverage (+): 27.46. Max coverage (-): 0

Region: chr14 16808855-16808891. Max. coverage (+): 27.46. Max coverage (-): 0

Region: chr14 16808892-16808927. Max. coverage (+): 6.38. Max coverage (-): 0

Region: chr14 16808928-16808964. Max. coverage (+): 1.64. Max coverage (-): 0

Region: chr14 16808965-16809001. Max. coverage (+): 1.64. Max coverage (-): 0

Region: chr14 16809002-16809037. Max. coverage (+): 24.09. Max coverage (-): 0

Region: chr14 16809038-16809074. Max. coverage (+): 0. Max coverage (-): 0

Region: chr14 16809075-16809111. Max. coverage (+): 6.86. Max coverage (-): 0

Region: chr14 16809112-16809147. Max. coverage (+): 6.86. Max coverage (-): 0

Region: chr14 16809148-16809184. Max. coverage (+): 12.92. Max coverage (-): 0

Region: chr14 16809185-16809221. Max. coverage (+): 12.92. Max coverage (-): 0

Region: chr14 16809222-16809258. Max. coverage (+): 0. Max coverage (-): 0

Region: chr14 16809259-16809294. Max. coverage (+): 2.43. Max coverage (-): 0

Region: chr14 16809295-16809331. Max. coverage (+): 0. Max coverage (-): 0

Region: chr14 16809332-16809368. Max. coverage (+): 0. Max coverage (-): 0

Region: chr14 16809369-16809404. Max. coverage (+): 0. Max coverage (-): 0

Region: chr14 16809405-16809441. Max. coverage (+): 23.33. Max coverage (-): 0

Region: chr14 16809442-16809478. Max. coverage (+): 0. Max coverage (-): 0

Region: chr14 16809479-16809514. Max. coverage (+): 0. Max coverage (-): 0

Region: chr14 16809515-16809551. Max. coverage (+): 0. Max coverage (-): 0

Region: chr14 16809552-16809588. Max. coverage (+): 0. Max coverage (-): 0

Region: chr14 16809589-16809624. Max. coverage (+): 0. Max coverage (-): 0

Region: chr14 16809625-16809661. Max. coverage (+): 1.33. Max coverage (-): 0

Region: chr14 16809662-. Max. coverage (+): 0. Max coverage (-): 0

RepeatMasker Color Code

**+**

100-98% Identity

<98-95% Identity

<95-90% Identity

<90-85% Identity

<85-80% Identity

<80-75% Identity

<75-70% Identity

<70% Identity

**-**

Gene Set Color Code

**+**

Gene

Pseudogene

**-**

Topology/Coverage Color Code

Coverage Plus Strand

Coverage Minus Strand

Mainstrand: Plus

Mainstrand: Minus

Complementary Strand

Flanking Region  
(if option -flank >0)

Gene Set Annotation  
  
RepeatMasker Annotation  

**1. L1MC5a**: 16791499-16791816 (-), Divergence to consensus: 46.6%  
**2. L3**: 16792035-16792152 (-), Divergence to consensus: 41.5%  
**3. Bov-tA2**: 16792227-16792400 (-), Divergence to consensus: 34.2%  
**4. Bov-tA2**: 16792444-16792576 (-), Divergence to consensus: 18.8%  
**5. BOV-A2**: 16792593-16792863 (-), Divergence to consensus: 5.2%  
**6. MIR**: 16793275-16793504 (+), Divergence to consensus: 41.4%  
**7. MIR**: 16793512-16793645 (+), Divergence to consensus: 43.8%  
**8. Bov-tA1**: 16793646-16793869 (-), Divergence to consensus: 13.8%  
**9. MIR**: 16793870-16793955 (+), Divergence to consensus: 43.8%  
**10. MamGypLTR1c**: 16793990-16794253 (-), Divergence to consensus: 48.9%  
**11. CHR-2\_BT**: 16794397-16794627 (+), Divergence to consensus: 33.4%  
**12. L2c**: 16794864-16794975 (-), Divergence to consensus: 39.7%  
**13. MER20**: 16794980-16795151 (+), Divergence to consensus: 18.6%  
**14. MIR**: 16795638-16795715 (-), Divergence to consensus: 28.2%  
**15. L2b**: 16795826-16796034 (+), Divergence to consensus: 42.3%  
**16. CHRL**: 16796433-16796588 (+), Divergence to consensus: 30.5%  
**17. Bov-tA1**: 16796691-16796936 (-), Divergence to consensus: 30.7%  
**18. L2b**: 16797321-16797356 (+), Divergence to consensus: 22.2%  
**19. L2b**: 16797507-16797771 (+), Divergence to consensus: 47.2%  
**20. MLT1F**: 16797860-16798411 (+), Divergence to consensus: 43.1%  
**21. Bov-tA2**: 16798449-16798655 (-), Divergence to consensus: 15.5%  
**22. L2a**: 16798714-16798897 (-), Divergence to consensus: 36.7%  
**23. MIRb**: 16798915-16799112 (-), Divergence to consensus: 36.9%  
**24. MIR**: 16799546-16799738 (-), Divergence to consensus: 47.9%  
**25. L2b**: 16799842-16799899 (-), Divergence to consensus: 25.9%  
**26. L2a**: 16800265-16800519 (-), Divergence to consensus: 45.8%  
**27. MIRc**: 16800555-16800655 (-), Divergence to consensus: 36.3%  
**28. L2b**: 16800779-16801342 (-), Divergence to consensus: 46.2%  
**29. L1-3\_BT**: 16801387-16801541 (-), Divergence to consensus: 7.2%  
**30. L1MD1**: 16801543-16801664 (-), Divergence to consensus: 27.2%  
**31. Bov-tA1**: 16801704-16801924 (-), Divergence to consensus: 14.4%  
**32. L1MD1**: 16801926-16802276 (-), Divergence to consensus: 24.1%  
**33. L2b**: 16802278-16802357 (-), Divergence to consensus: 32.6%  
**34. MIRb**: 16802571-16802689 (-), Divergence to consensus: 34.8%  
**35. Charlie1a**: 16802803-16803060 (+), Divergence to consensus: 23.1%  
**36. Charlie1**: 16803066-16803453 (+), Divergence to consensus: 32.6%  
**37. Bov-tA3**: 16803452-16803549 (-), Divergence to consensus: 13.4%  
**38. Charlie1**: 16803541-16803947 (+), Divergence to consensus: 33.2%  
**39. Charlie1**: 16804429-16804617 (+), Divergence to consensus: 31%  
**40. MIRc**: 16804839-16805045 (+), Divergence to consensus: 49.8%  
**41. CHR-2B**: 16805853-16806086 (-), Divergence to consensus: 49.1%  
**42. L2b**: 16806311-16806355 (-), Divergence to consensus: 17.8%  
**43. MIRb**: 16806647-16806682 (-), Divergence to consensus: 25%  
**44. MLT1J**: 16806687-16806751 (+), Divergence to consensus: 27.7%  
**45. MLT1J**: 16806817-16806889 (+), Divergence to consensus: 43.8%  
**46. MLT1A0**: 16807094-16807229 (-), Divergence to consensus: 25.8%  
**47. Bov-tA1**: 16807241-16807458 (-), Divergence to consensus: 17.5%  
**48. MLT1A0**: 16807461-16807584 (-), Divergence to consensus: 22.4%  
**49. MIRb**: 16808144-16808284 (-), Divergence to consensus: 36.6%  
**50. CHRL1\_BT**: 16808324-16808394 (+), Divergence to consensus: 20.3%  
**51. L4\_C\_Mam**: 16808516-16808581 (+), Divergence to consensus: 28.8%  
**52. AT\_rich**: 16809270-16809301 (+), Divergence to consensus: 50%

  
Transcription Factor Binding Sites  

**RFX4\_2** (Sequence: GTATCCAGG (-): 16797823)  
**RFX4\_1** (Sequence: CTTGGCAAC (+): 16802485)  
**SPZ1** (Sequence: CTCAAACCCT (-): 16794665)  
**RFX4\_2** (Sequence: CATAGATAC (+): 16792987)  
**SOX9** (Sequence: AACAATAA (-): 16805139)  
**SOX9** (Sequence: AACAATAG (-): 16805203)  
**SOX9** (Sequence: AACAATAA (-): 16809579)
