# Supplementary material for: Small RNA sequencing of cryopreserved semen from single bull revealed altered miRNAs and piRNAs expression between High- and Low-motile sperm populations
Source: BMC Genomics. 2017 Jan 4;18:14. doi: 10.1186/s12864-016-3394-7 (PMC5209821; doi:10.1186/s12864-016-3394-7)
Supplement: Additional file 4: — Details for each piRNA clusters found in Low Motile (LM) sperm fraction. Genes, repeats, transposable elements and transcription factors binding sites falling within the cluster regions were reported. (ZIP 1034 kb) [file 12864_2016_3394_MOESM4_ESM.zip › 14.html]

piRNA cluster 14


Predicted piRNA cluster no. 14     previous   next
  

Show proTRAC run info
Hide proTRAC run info

================================= proTRAC ====================================  
VERSION: 2.1                                    LAST MODIFIED: 06. October 2015  
  
Please cite:  
Rosenkranz D, Zischler H. proTRAC - a software for probabilistic piRNA cluster  
detection, visualization and analysis. 2012. BMC Bioinformatics 13:5.  
  
and (for proTRAC 2.0 and later):  
Rosenkranz D, Rudloff S, Bastuck K, Ketting RF, Zischler H. Tupaia small RNAs  
provide insights into function and evolution of RNAi-based transposon defense  
in mammals. 2015. RNA 21(5):911-922.  
  
Contact:  
David Rosenkranz  
Institute of Anthropology, small RNA group  
Johannes Gutenberg University Mainz  
email: rosenkranz@uni-mainz.de  
  
You can find the latest proTRAC version at:  
http://sourceforge.net/projects/protrac/files  
http://www.smallRNAgroup-mainz.de/software  
==============================================================================  
  
PARAMETERS:  
Map file: .............../storage/core/barbara/genhome/smallRNA/fertility/Sample\_not\_motile/pirna/Sample\_not\_motile\_26-33\_collapsed.fa.no-dust.map.weighted-10000-1000-b-0  
Genome file: ............/storage/core/barbara/genhome/smallRNA/fertility/Sample\_all/pirna/bt\_311\_chrY.fa  
RepeatMasker annotation: /storage/genomes/bt\_umd31/GCF\_000003055.6\_Bos\_taurus\_UMD\_3.1.1\_repeatMasker\_chr.out  
GeneSet:................./storage/core/barbara/genhome/smallRNA/fertility/Sample\_all/pirna/full.gtf  
  
Significant (p<=0.01) hit density will be calculated based  
on observed hit distribution.  
  
Sliding window size: ........................................ 5000 bp  
Sliding window increament: .................................. 1000 bp  
Normalize each hit by number of genomic hits: ............... 1 [0=no/1=yes]  
Normalize each hit by number of sequence reads: ............. 1 [0=no/1=yes]  
Normalize values (-> per million mapped reads): ............. 1 [0=no/1=yes]  
Min. fraction of hits with 1T(U) or 10A: .................... 0.75  
Alternatively: Min. fraction of hits with 1T(U) and 10A: .... 0.5  
Min. fraction of hits with typical piRNA length: ............ 0.75  
Typical piRNA length: ....................................... 26-33 nt  
Min. size of a piRNA cluster: ............................... 5000 bp.  
Min. number of hits (absolute): ............................. 0  
Min. number of hits (normalized): ........................... 0  
Min. fraction of hits on the mainstrand: .................... 0.75  
Top fraction of mapped sequences (in terms of read counts): . 1%  
Top fraction accounts for max. n% of sequence reads: ........ 90%  
Min. fraction of hits on each arm of a bidirectional cluster: 0.1  
Output image file for each cluster: ......................... 0 [0=no/1=yes]  
Output html file for each cluster: .......................... 1 [0=no/1=yes]  
Output a summary table: ..................................... 1 [0=no/1=yes]  
Output a FASTA file for each cluster (piRNA sequences): ..... 1 [0=no/1=yes]  
Output a FASTA file comprising cluster sequences: ........... 1 [0=no/1=yes]  
Search DNA motifs in clusters: .............................. 1 [0=no/1=yes]  
Output flanking sequences: +/- .............................. 0 bp  
Output ~.pTi file: .......................................... 1 [0=no/1=yes]  
==============================================================================  
  
  
Genome size (without gaps): ............ 2678902517 bp  
Gaps (N/X/-): .......................... 53837044 bp  
Mapped reads: .......................... 738059667487  
Non-identical sequences: ............... 277001  
Genomic hits: .......................... 533816  
Significant densitiy of mapped reads: .. 15118061 reads/kb

Show proTRAC cluster info
Hide proTRAC cluster info

|  |  |
| --- | --- |
| Location | chr15 |
| Coordinates | 30405928-30413553 |
| Size [bp] | 7626 |
| Sequence hit loci | 188 |
| Mapped reads (normalized) | 494828488 |
| Mapped reads (normalized) per kb | 64887029.6 |
| Normalized reads with 1T (1U) | 93.5% |
| Normalized reads with 10A | 25.3% |
| Normalized reads with length 26-33 nt | 100% |
| Normalized reads on the main strand(s) | 100% |
| Predicted directionality | mono:plus |

100%

0%

1T (1U)  
reads

10A reads

26-33 nt  
reads

reads on mainstrand

**Either the amount of reads with 1T (1U) OR 10A has to exceed 75% (set with option: -1Tor10A)  
Alternatively the amount of reads with 1T (1U) AND 10A has to exceed 50% (set with option: -1Tand10A)  
Minimum amount of reads with preferred size is 75% (set with option: -pisize)  
Minimum amount of reads on the main strand(s) is 75% (set with option: -clstrand)**

Show read coverage
Hide read coverage

WHAT DO I SEE HERE?  
This chart shows the location of mapped sequence reads within a predicted piRNA cluster. The color refers to the number of genomic hits produced by the sequence read in question. A dark red bar indicates that this sequence read produces many other hits elsewhere in the genome. Many adjacent red or yellow bars can indicate the presence of a multi-copy element such as transposons or rRNA genes. A dark green bar indicates that this sequence read maps uniquely to this locus.

1 hit

2-5 hits

6-10 hits

11-20 hits

21-50 hits

51-100 hits

> 100 hits

chr15

30405928

30413553

Gene Set

RepeatMasker

Mapped  
Reads

47.01

plus strand

minus strand

47.01

Region: chr15 16809663-30405935. Max. coverage (+): 0.14. Max coverage (-): 0

Region: chr15 30405936-30405950. Max. coverage (+): 0. Max coverage (-): 0

Region: chr15 30405951-30405966. Max. coverage (+): 14.61. Max coverage (-): 0

Region: chr15 30405967-30405981. Max. coverage (+): 7.47. Max coverage (-): 0

Region: chr15 30405982-30405996. Max. coverage (+): 0. Max coverage (-): 0

Region: chr15 30405997-30406011. Max. coverage (+): 0. Max coverage (-): 0

Region: chr15 30406012-30406027. Max. coverage (+): 0. Max coverage (-): 0

Region: chr15 30406028-30406042. Max. coverage (+): 0. Max coverage (-): 0

Region: chr15 30406043-30406057. Max. coverage (+): 0. Max coverage (-): 0

Region: chr15 30406058-30406072. Max. coverage (+): 0. Max coverage (-): 0

Region: chr15 30406073-30406088. Max. coverage (+): 0. Max coverage (-): 0

Region: chr15 30406089-30406103. Max. coverage (+): 0. Max coverage (-): 0

Region: chr15 30406104-30406118. Max. coverage (+): 0. Max coverage (-): 0

Region: chr15 30406119-30406133. Max. coverage (+): 0. Max coverage (-): 0

Region: chr15 30406134-30406149. Max. coverage (+): 0. Max coverage (-): 0

Region: chr15 30406150-30406164. Max. coverage (+): 0. Max coverage (-): 0

Region: chr15 30406165-30406179. Max. coverage (+): 0. Max coverage (-): 0

Region: chr15 30406180-30406194. Max. coverage (+): 0. Max coverage (-): 0

Region: chr15 30406195-30406210. Max. coverage (+): 0. Max coverage (-): 0

Region: chr15 30406211-30406225. Max. coverage (+): 0. Max coverage (-): 0

Region: chr15 30406226-30406240. Max. coverage (+): 0. Max coverage (-): 0

Region: chr15 30406241-30406255. Max. coverage (+): 0. Max coverage (-): 0

Region: chr15 30406256-30406271. Max. coverage (+): 0. Max coverage (-): 0

Region: chr15 30406272-30406286. Max. coverage (+): 0. Max coverage (-): 0

Region: chr15 30406287-30406301. Max. coverage (+): 0. Max coverage (-): 0

Region: chr15 30406302-30406316. Max. coverage (+): 0. Max coverage (-): 0

Region: chr15 30406317-30406332. Max. coverage (+): 0. Max coverage (-): 0

Region: chr15 30406333-30406347. Max. coverage (+): 0. Max coverage (-): 0

Region: chr15 30406348-30406362. Max. coverage (+): 0. Max coverage (-): 0

Region: chr15 30406363-30406377. Max. coverage (+): 0. Max coverage (-): 0

Region: chr15 30406378-30406393. Max. coverage (+): 0. Max coverage (-): 0

Region: chr15 30406394-30406408. Max. coverage (+): 0. Max coverage (-): 0

Region: chr15 30406409-30406423. Max. coverage (+): 0. Max coverage (-): 0

Region: chr15 30406424-30406438. Max. coverage (+): 0. Max coverage (-): 0

Region: chr15 30406439-30406454. Max. coverage (+): 0. Max coverage (-): 0

Region: chr15 30406455-30406469. Max. coverage (+): 0. Max coverage (-): 0

Region: chr15 30406470-30406484. Max. coverage (+): 0. Max coverage (-): 0

Region: chr15 30406485-30406499. Max. coverage (+): 0. Max coverage (-): 0

Region: chr15 30406500-30406515. Max. coverage (+): 0. Max coverage (-): 0

Region: chr15 30406516-30406530. Max. coverage (+): 0. Max coverage (-): 0

Region: chr15 30406531-30406545. Max. coverage (+): 0. Max coverage (-): 0

Region: chr15 30406546-30406560. Max. coverage (+): 0. Max coverage (-): 0

Region: chr15 30406561-30406576. Max. coverage (+): 0. Max coverage (-): 0

Region: chr15 30406577-30406591. Max. coverage (+): 0. Max coverage (-): 0

Region: chr15 30406592-30406606. Max. coverage (+): 0. Max coverage (-): 0

Region: chr15 30406607-30406621. Max. coverage (+): 0. Max coverage (-): 0

Region: chr15 30406622-30406637. Max. coverage (+): 0. Max coverage (-): 0

Region: chr15 30406638-30406652. Max. coverage (+): 0. Max coverage (-): 0

Region: chr15 30406653-30406667. Max. coverage (+): 0. Max coverage (-): 0

Region: chr15 30406668-30406682. Max. coverage (+): 0. Max coverage (-): 0

Region: chr15 30406683-30406698. Max. coverage (+): 0. Max coverage (-): 0

Region: chr15 30406699-30406713. Max. coverage (+): 0. Max coverage (-): 0

Region: chr15 30406714-30406728. Max. coverage (+): 0. Max coverage (-): 0

Region: chr15 30406729-30406743. Max. coverage (+): 0. Max coverage (-): 0

Region: chr15 30406744-30406759. Max. coverage (+): 0. Max coverage (-): 0

Region: chr15 30406760-30406774. Max. coverage (+): 0. Max coverage (-): 0

Region: chr15 30406775-30406789. Max. coverage (+): 0. Max coverage (-): 0

Region: chr15 30406790-30406804. Max. coverage (+): 0. Max coverage (-): 0

Region: chr15 30406805-30406820. Max. coverage (+): 0. Max coverage (-): 0

Region: chr15 30406821-30406835. Max. coverage (+): 0. Max coverage (-): 0

Region: chr15 30406836-30406850. Max. coverage (+): 0. Max coverage (-): 0

Region: chr15 30406851-30406865. Max. coverage (+): 6.43. Max coverage (-): 0

Region: chr15 30406866-30406881. Max. coverage (+): 22.25. Max coverage (-): 0

Region: chr15 30406882-30406896. Max. coverage (+): 0. Max coverage (-): 0

Region: chr15 30406897-30406911. Max. coverage (+): 0. Max coverage (-): 0

Region: chr15 30406912-30406927. Max. coverage (+): 0. Max coverage (-): 0

Region: chr15 30406928-30406942. Max. coverage (+): 0. Max coverage (-): 0

Region: chr15 30406943-30406957. Max. coverage (+): 0. Max coverage (-): 0

Region: chr15 30406958-30406972. Max. coverage (+): 0. Max coverage (-): 0

Region: chr15 30406973-30406988. Max. coverage (+): 0. Max coverage (-): 0

Region: chr15 30406989-30407003. Max. coverage (+): 0. Max coverage (-): 0

Region: chr15 30407004-30407018. Max. coverage (+): 0. Max coverage (-): 0

Region: chr15 30407019-30407033. Max. coverage (+): 0. Max coverage (-): 0

Region: chr15 30407034-30407049. Max. coverage (+): 0. Max coverage (-): 0

Region: chr15 30407050-30407064. Max. coverage (+): 0. Max coverage (-): 0

Region: chr15 30407065-30407079. Max. coverage (+): 0. Max coverage (-): 0

Region: chr15 30407080-30407094. Max. coverage (+): 0. Max coverage (-): 0

Region: chr15 30407095-30407110. Max. coverage (+): 0. Max coverage (-): 0

Region: chr15 30407111-30407125. Max. coverage (+): 0. Max coverage (-): 0

Region: chr15 30407126-30407140. Max. coverage (+): 0. Max coverage (-): 0

Region: chr15 30407141-30407155. Max. coverage (+): 0. Max coverage (-): 0

Region: chr15 30407156-30407171. Max. coverage (+): 0. Max coverage (-): 0

Region: chr15 30407172-30407186. Max. coverage (+): 0. Max coverage (-): 0

Region: chr15 30407187-30407201. Max. coverage (+): 0. Max coverage (-): 0

Region: chr15 30407202-30407216. Max. coverage (+): 0. Max coverage (-): 0

Region: chr15 30407217-30407232. Max. coverage (+): 0. Max coverage (-): 0

Region: chr15 30407233-30407247. Max. coverage (+): 0. Max coverage (-): 0

Region: chr15 30407248-30407262. Max. coverage (+): 0. Max coverage (-): 0

Region: chr15 30407263-30407277. Max. coverage (+): 0.74. Max coverage (-): 0

Region: chr15 30407278-30407293. Max. coverage (+): 0.74. Max coverage (-): 0

Region: chr15 30407294-30407308. Max. coverage (+): 0. Max coverage (-): 0

Region: chr15 30407309-30407323. Max. coverage (+): 0. Max coverage (-): 0

Region: chr15 30407324-30407338. Max. coverage (+): 0. Max coverage (-): 0

Region: chr15 30407339-30407354. Max. coverage (+): 0. Max coverage (-): 0

Region: chr15 30407355-30407369. Max. coverage (+): 0. Max coverage (-): 0

Region: chr15 30407370-30407384. Max. coverage (+): 0. Max coverage (-): 0

Region: chr15 30407385-30407399. Max. coverage (+): 0. Max coverage (-): 0

Region: chr15 30407400-30407415. Max. coverage (+): 0. Max coverage (-): 0

Region: chr15 30407416-30407430. Max. coverage (+): 0. Max coverage (-): 0

Region: chr15 30407431-30407445. Max. coverage (+): 0. Max coverage (-): 0

Region: chr15 30407446-30407460. Max. coverage (+): 0. Max coverage (-): 0

Region: chr15 30407461-30407476. Max. coverage (+): 1.93. Max coverage (-): 0

Region: chr15 30407477-30407491. Max. coverage (+): 3.59. Max coverage (-): 0

Region: chr15 30407492-30407506. Max. coverage (+): 3.59. Max coverage (-): 0

Region: chr15 30407507-30407521. Max. coverage (+): 0. Max coverage (-): 0

Region: chr15 30407522-30407537. Max. coverage (+): 0. Max coverage (-): 0

Region: chr15 30407538-30407552. Max. coverage (+): 0. Max coverage (-): 0

Region: chr15 30407553-30407567. Max. coverage (+): 2.5. Max coverage (-): 0

Region: chr15 30407568-30407582. Max. coverage (+): 0. Max coverage (-): 0

Region: chr15 30407583-30407598. Max. coverage (+): 0. Max coverage (-): 0

Region: chr15 30407599-30407613. Max. coverage (+): 0. Max coverage (-): 0

Region: chr15 30407614-30407628. Max. coverage (+): 7.51. Max coverage (-): 0

Region: chr15 30407629-30407643. Max. coverage (+): 0. Max coverage (-): 0

Region: chr15 30407644-30407659. Max. coverage (+): 0. Max coverage (-): 0

Region: chr15 30407660-30407674. Max. coverage (+): 7.37. Max coverage (-): 0

Region: chr15 30407675-30407689. Max. coverage (+): 13.3. Max coverage (-): 0

Region: chr15 30407690-30407704. Max. coverage (+): 0. Max coverage (-): 0

Region: chr15 30407705-30407720. Max. coverage (+): 0. Max coverage (-): 0

Region: chr15 30407721-30407735. Max. coverage (+): 0. Max coverage (-): 0

Region: chr15 30407736-30407750. Max. coverage (+): 0. Max coverage (-): 0

Region: chr15 30407751-30407765. Max. coverage (+): 0. Max coverage (-): 0

Region: chr15 30407766-30407781. Max. coverage (+): 3.69. Max coverage (-): 0

Region: chr15 30407782-30407796. Max. coverage (+): 3.69. Max coverage (-): 0

Region: chr15 30407797-30407811. Max. coverage (+): 0. Max coverage (-): 0

Region: chr15 30407812-30407826. Max. coverage (+): 0. Max coverage (-): 0

Region: chr15 30407827-30407842. Max. coverage (+): 5.02. Max coverage (-): 0

Region: chr15 30407843-30407857. Max. coverage (+): 0. Max coverage (-): 0

Region: chr15 30407858-30407872. Max. coverage (+): 5.27. Max coverage (-): 0

Region: chr15 30407873-30407887. Max. coverage (+): 5.27. Max coverage (-): 0

Region: chr15 30407888-30407903. Max. coverage (+): 9.21. Max coverage (-): 0

Region: chr15 30407904-30407918. Max. coverage (+): 3.69. Max coverage (-): 0

Region: chr15 30407919-30407933. Max. coverage (+): 0. Max coverage (-): 0

Region: chr15 30407934-30407948. Max. coverage (+): 2.3. Max coverage (-): 0

Region: chr15 30407949-30407964. Max. coverage (+): 3.14. Max coverage (-): 0

Region: chr15 30407965-30407979. Max. coverage (+): 9.43. Max coverage (-): 0

Region: chr15 30407980-30407994. Max. coverage (+): 0. Max coverage (-): 0

Region: chr15 30407995-30408009. Max. coverage (+): 4.24. Max coverage (-): 0

Region: chr15 30408010-30408025. Max. coverage (+): 0. Max coverage (-): 0

Region: chr15 30408026-30408040. Max. coverage (+): 0. Max coverage (-): 0

Region: chr15 30408041-30408055. Max. coverage (+): 0. Max coverage (-): 0

Region: chr15 30408056-30408070. Max. coverage (+): 0. Max coverage (-): 0

Region: chr15 30408071-30408086. Max. coverage (+): 0. Max coverage (-): 0

Region: chr15 30408087-30408101. Max. coverage (+): 0. Max coverage (-): 0

Region: chr15 30408102-30408116. Max. coverage (+): 0. Max coverage (-): 0

Region: chr15 30408117-30408131. Max. coverage (+): 0. Max coverage (-): 0

Region: chr15 30408132-30408147. Max. coverage (+): 0. Max coverage (-): 0

Region: chr15 30408148-30408162. Max. coverage (+): 20.45. Max coverage (-): 0

Region: chr15 30408163-30408177. Max. coverage (+): 13.35. Max coverage (-): 0

Region: chr15 30408178-30408192. Max. coverage (+): 4.34. Max coverage (-): 0

Region: chr15 30408193-30408208. Max. coverage (+): 0. Max coverage (-): 0

Region: chr15 30408209-30408223. Max. coverage (+): 0. Max coverage (-): 0

Region: chr15 30408224-30408238. Max. coverage (+): 2.11. Max coverage (-): 0

Region: chr15 30408239-30408253. Max. coverage (+): 10.62. Max coverage (-): 0

Region: chr15 30408254-30408269. Max. coverage (+): 22.22. Max coverage (-): 0

Region: chr15 30408270-30408284. Max. coverage (+): 3.91. Max coverage (-): 0

Region: chr15 30408285-30408299. Max. coverage (+): 0. Max coverage (-): 0

Region: chr15 30408300-30408314. Max. coverage (+): 0. Max coverage (-): 0

Region: chr15 30408315-30408330. Max. coverage (+): 0. Max coverage (-): 0

Region: chr15 30408331-30408345. Max. coverage (+): 0. Max coverage (-): 0

Region: chr15 30408346-30408360. Max. coverage (+): 0. Max coverage (-): 0

Region: chr15 30408361-30408375. Max. coverage (+): 0. Max coverage (-): 0

Region: chr15 30408376-30408391. Max. coverage (+): 6.96. Max coverage (-): 0

Region: chr15 30408392-30408406. Max. coverage (+): 6.96. Max coverage (-): 0

Region: chr15 30408407-30408421. Max. coverage (+): 0. Max coverage (-): 0

Region: chr15 30408422-30408436. Max. coverage (+): 0. Max coverage (-): 0

Region: chr15 30408437-30408452. Max. coverage (+): 0. Max coverage (-): 0

Region: chr15 30408453-30408467. Max. coverage (+): 0. Max coverage (-): 0

Region: chr15 30408468-30408482. Max. coverage (+): 9.73. Max coverage (-): 0

Region: chr15 30408483-30408497. Max. coverage (+): 12.94. Max coverage (-): 0

Region: chr15 30408498-30408513. Max. coverage (+): 6.04. Max coverage (-): 0

Region: chr15 30408514-30408528. Max. coverage (+): 6.04. Max coverage (-): 0

Region: chr15 30408529-30408543. Max. coverage (+): 2.15. Max coverage (-): 0

Region: chr15 30408544-30408558. Max. coverage (+): 0. Max coverage (-): 0

Region: chr15 30408559-30408574. Max. coverage (+): 0. Max coverage (-): 0

Region: chr15 30408575-30408589. Max. coverage (+): 0. Max coverage (-): 0

Region: chr15 30408590-30408604. Max. coverage (+): 6.06. Max coverage (-): 0

Region: chr15 30408605-30408619. Max. coverage (+): 6.06. Max coverage (-): 0

Region: chr15 30408620-30408635. Max. coverage (+): 0. Max coverage (-): 0

Region: chr15 30408636-30408650. Max. coverage (+): 5.08. Max coverage (-): 0

Region: chr15 30408651-30408665. Max. coverage (+): 0. Max coverage (-): 0

Region: chr15 30408666-30408680. Max. coverage (+): 3.53. Max coverage (-): 0

Region: chr15 30408681-30408696. Max. coverage (+): 9.14. Max coverage (-): 0

Region: chr15 30408697-30408711. Max. coverage (+): 0. Max coverage (-): 0

Region: chr15 30408712-30408726. Max. coverage (+): 3.83. Max coverage (-): 0

Region: chr15 30408727-30408741. Max. coverage (+): 3.83. Max coverage (-): 0

Region: chr15 30408742-30408757. Max. coverage (+): 5.07. Max coverage (-): 0

Region: chr15 30408758-30408772. Max. coverage (+): 9.11. Max coverage (-): 0

Region: chr15 30408773-30408787. Max. coverage (+): 3.15. Max coverage (-): 0

Region: chr15 30408788-30408803. Max. coverage (+): 3.15. Max coverage (-): 0

Region: chr15 30408804-30408818. Max. coverage (+): 0. Max coverage (-): 0

Region: chr15 30408819-30408833. Max. coverage (+): 0. Max coverage (-): 0

Region: chr15 30408834-30408848. Max. coverage (+): 11.64. Max coverage (-): 0

Region: chr15 30408849-30408864. Max. coverage (+): 11.64. Max coverage (-): 0

Region: chr15 30408865-30408879. Max. coverage (+): 0. Max coverage (-): 0

Region: chr15 30408880-30408894. Max. coverage (+): 0. Max coverage (-): 0

Region: chr15 30408895-30408909. Max. coverage (+): 0. Max coverage (-): 0

Region: chr15 30408910-30408925. Max. coverage (+): 5.5. Max coverage (-): 0

Region: chr15 30408926-30408940. Max. coverage (+): 0. Max coverage (-): 0

Region: chr15 30408941-30408955. Max. coverage (+): 0. Max coverage (-): 0

Region: chr15 30408956-30408970. Max. coverage (+): 0. Max coverage (-): 0

Region: chr15 30408971-30408986. Max. coverage (+): 0. Max coverage (-): 0

Region: chr15 30408987-30409001. Max. coverage (+): 0. Max coverage (-): 0

Region: chr15 30409002-30409016. Max. coverage (+): 0. Max coverage (-): 0

Region: chr15 30409017-30409031. Max. coverage (+): 0. Max coverage (-): 0

Region: chr15 30409032-30409047. Max. coverage (+): 0. Max coverage (-): 0

Region: chr15 30409048-30409062. Max. coverage (+): 0. Max coverage (-): 0

Region: chr15 30409063-30409077. Max. coverage (+): 0. Max coverage (-): 0

Region: chr15 30409078-30409092. Max. coverage (+): 0. Max coverage (-): 0

Region: chr15 30409093-30409108. Max. coverage (+): 3.9. Max coverage (-): 0

Region: chr15 30409109-30409123. Max. coverage (+): 0. Max coverage (-): 0

Region: chr15 30409124-30409138. Max. coverage (+): 0. Max coverage (-): 0

Region: chr15 30409139-30409153. Max. coverage (+): 0. Max coverage (-): 0

Region: chr15 30409154-30409169. Max. coverage (+): 0. Max coverage (-): 0

Region: chr15 30409170-30409184. Max. coverage (+): 0. Max coverage (-): 0

Region: chr15 30409185-30409199. Max. coverage (+): 0. Max coverage (-): 0

Region: chr15 30409200-30409214. Max. coverage (+): 0. Max coverage (-): 0

Region: chr15 30409215-30409230. Max. coverage (+): 0. Max coverage (-): 0

Region: chr15 30409231-30409245. Max. coverage (+): 9.14. Max coverage (-): 0

Region: chr15 30409246-30409260. Max. coverage (+): 0. Max coverage (-): 0

Region: chr15 30409261-30409275. Max. coverage (+): 0. Max coverage (-): 0

Region: chr15 30409276-30409291. Max. coverage (+): 0. Max coverage (-): 0

Region: chr15 30409292-30409306. Max. coverage (+): 5.79. Max coverage (-): 0

Region: chr15 30409307-30409321. Max. coverage (+): 10.06. Max coverage (-): 0

Region: chr15 30409322-30409336. Max. coverage (+): 4.01. Max coverage (-): 0

Region: chr15 30409337-30409352. Max. coverage (+): 0. Max coverage (-): 0

Region: chr15 30409353-30409367. Max. coverage (+): 1.54. Max coverage (-): 0

Region: chr15 30409368-30409382. Max. coverage (+): 0. Max coverage (-): 0

Region: chr15 30409383-30409397. Max. coverage (+): 0. Max coverage (-): 0

Region: chr15 30409398-30409413. Max. coverage (+): 0. Max coverage (-): 0

Region: chr15 30409414-30409428. Max. coverage (+): 0. Max coverage (-): 0

Region: chr15 30409429-30409443. Max. coverage (+): 0. Max coverage (-): 0

Region: chr15 30409444-30409458. Max. coverage (+): 0. Max coverage (-): 0

Region: chr15 30409459-30409474. Max. coverage (+): 0. Max coverage (-): 0

Region: chr15 30409475-30409489. Max. coverage (+): 0. Max coverage (-): 0

Region: chr15 30409490-30409504. Max. coverage (+): 0. Max coverage (-): 0

Region: chr15 30409505-30409519. Max. coverage (+): 0. Max coverage (-): 0

Region: chr15 30409520-30409535. Max. coverage (+): 0. Max coverage (-): 0

Region: chr15 30409536-30409550. Max. coverage (+): 0. Max coverage (-): 0

Region: chr15 30409551-30409565. Max. coverage (+): 0. Max coverage (-): 0

Region: chr15 30409566-30409580. Max. coverage (+): 12.3. Max coverage (-): 0

Region: chr15 30409581-30409596. Max. coverage (+): 12.3. Max coverage (-): 0

Region: chr15 30409597-30409611. Max. coverage (+): 0. Max coverage (-): 0

Region: chr15 30409612-30409626. Max. coverage (+): 13.61. Max coverage (-): 0

Region: chr15 30409627-30409641. Max. coverage (+): 9.59. Max coverage (-): 0

Region: chr15 30409642-30409657. Max. coverage (+): 0. Max coverage (-): 0

Region: chr15 30409658-30409672. Max. coverage (+): 0. Max coverage (-): 0

Region: chr15 30409673-30409687. Max. coverage (+): 0. Max coverage (-): 0

Region: chr15 30409688-30409702. Max. coverage (+): 0. Max coverage (-): 0

Region: chr15 30409703-30409718. Max. coverage (+): 28.8. Max coverage (-): 0

Region: chr15 30409719-30409733. Max. coverage (+): 0. Max coverage (-): 0

Region: chr15 30409734-30409748. Max. coverage (+): 0. Max coverage (-): 0

Region: chr15 30409749-30409763. Max. coverage (+): 0. Max coverage (-): 0

Region: chr15 30409764-30409779. Max. coverage (+): 8.95. Max coverage (-): 0

Region: chr15 30409780-30409794. Max. coverage (+): 15.26. Max coverage (-): 0

Region: chr15 30409795-30409809. Max. coverage (+): 9.84. Max coverage (-): 0

Region: chr15 30409810-30409824. Max. coverage (+): 0. Max coverage (-): 0

Region: chr15 30409825-30409840. Max. coverage (+): 0. Max coverage (-): 0

Region: chr15 30409841-30409855. Max. coverage (+): 0. Max coverage (-): 0

Region: chr15 30409856-30409870. Max. coverage (+): 4.92. Max coverage (-): 0

Region: chr15 30409871-30409885. Max. coverage (+): 5.97. Max coverage (-): 0

Region: chr15 30409886-30409901. Max. coverage (+): 5.97. Max coverage (-): 0

Region: chr15 30409902-30409916. Max. coverage (+): 6.38. Max coverage (-): 0

Region: chr15 30409917-30409931. Max. coverage (+): 0. Max coverage (-): 0

Region: chr15 30409932-30409946. Max. coverage (+): 2.18. Max coverage (-): 0

Region: chr15 30409947-30409962. Max. coverage (+): 2.18. Max coverage (-): 0

Region: chr15 30409963-30409977. Max. coverage (+): 0. Max coverage (-): 0

Region: chr15 30409978-30409992. Max. coverage (+): 0.23. Max coverage (-): 0

Region: chr15 30409993-30410007. Max. coverage (+): 0. Max coverage (-): 0

Region: chr15 30410008-30410023. Max. coverage (+): 0. Max coverage (-): 0

Region: chr15 30410024-30410038. Max. coverage (+): 0. Max coverage (-): 0

Region: chr15 30410039-30410053. Max. coverage (+): 0. Max coverage (-): 0

Region: chr15 30410054-30410068. Max. coverage (+): 0. Max coverage (-): 0

Region: chr15 30410069-30410084. Max. coverage (+): 0. Max coverage (-): 0

Region: chr15 30410085-30410099. Max. coverage (+): 0. Max coverage (-): 0

Region: chr15 30410100-30410114. Max. coverage (+): 4.36. Max coverage (-): 0

Region: chr15 30410115-30410129. Max. coverage (+): 0. Max coverage (-): 0

Region: chr15 30410130-30410145. Max. coverage (+): 4.67. Max coverage (-): 0

Region: chr15 30410146-30410160. Max. coverage (+): 0. Max coverage (-): 0

Region: chr15 30410161-30410175. Max. coverage (+): 0. Max coverage (-): 0

Region: chr15 30410176-30410190. Max. coverage (+): 0. Max coverage (-): 0

Region: chr15 30410191-30410206. Max. coverage (+): 0. Max coverage (-): 0

Region: chr15 30410207-30410221. Max. coverage (+): 3.1. Max coverage (-): 0

Region: chr15 30410222-30410236. Max. coverage (+): 3.1. Max coverage (-): 0

Region: chr15 30410237-30410251. Max. coverage (+): 1.14. Max coverage (-): 0

Region: chr15 30410252-30410267. Max. coverage (+): 0. Max coverage (-): 0

Region: chr15 30410268-30410282. Max. coverage (+): 0. Max coverage (-): 0

Region: chr15 30410283-30410297. Max. coverage (+): 0. Max coverage (-): 0

Region: chr15 30410298-30410312. Max. coverage (+): 0. Max coverage (-): 0

Region: chr15 30410313-30410328. Max. coverage (+): 0. Max coverage (-): 0

Region: chr15 30410329-30410343. Max. coverage (+): 0. Max coverage (-): 0

Region: chr15 30410344-30410358. Max. coverage (+): 0. Max coverage (-): 0

Region: chr15 30410359-30410373. Max. coverage (+): 1.25. Max coverage (-): 0

Region: chr15 30410374-30410389. Max. coverage (+): 0. Max coverage (-): 0

Region: chr15 30410390-30410404. Max. coverage (+): 0. Max coverage (-): 0

Region: chr15 30410405-30410419. Max. coverage (+): 0. Max coverage (-): 0

Region: chr15 30410420-30410434. Max. coverage (+): 5.14. Max coverage (-): 0

Region: chr15 30410435-30410450. Max. coverage (+): 2.03. Max coverage (-): 0

Region: chr15 30410451-30410465. Max. coverage (+): 0. Max coverage (-): 0

Region: chr15 30410466-30410480. Max. coverage (+): 0. Max coverage (-): 0

Region: chr15 30410481-30410495. Max. coverage (+): 0. Max coverage (-): 0

Region: chr15 30410496-30410511. Max. coverage (+): 0. Max coverage (-): 0

Region: chr15 30410512-30410526. Max. coverage (+): 0. Max coverage (-): 0

Region: chr15 30410527-30410541. Max. coverage (+): 0. Max coverage (-): 0

Region: chr15 30410542-30410556. Max. coverage (+): 0. Max coverage (-): 0

Region: chr15 30410557-30410572. Max. coverage (+): 0. Max coverage (-): 0

Region: chr15 30410573-30410587. Max. coverage (+): 0. Max coverage (-): 0

Region: chr15 30410588-30410602. Max. coverage (+): 0. Max coverage (-): 0

Region: chr15 30410603-30410617. Max. coverage (+): 0. Max coverage (-): 0

Region: chr15 30410618-30410633. Max. coverage (+): 0. Max coverage (-): 0

Region: chr15 30410634-30410648. Max. coverage (+): 0. Max coverage (-): 0

Region: chr15 30410649-30410663. Max. coverage (+): 0. Max coverage (-): 0

Region: chr15 30410664-30410678. Max. coverage (+): 0. Max coverage (-): 0

Region: chr15 30410679-30410694. Max. coverage (+): 0. Max coverage (-): 0

Region: chr15 30410695-30410709. Max. coverage (+): 0. Max coverage (-): 0

Region: chr15 30410710-30410724. Max. coverage (+): 0. Max coverage (-): 0

Region: chr15 30410725-30410740. Max. coverage (+): 0. Max coverage (-): 0

Region: chr15 30410741-30410755. Max. coverage (+): 0. Max coverage (-): 0

Region: chr15 30410756-30410770. Max. coverage (+): 4.1. Max coverage (-): 0

Region: chr15 30410771-30410785. Max. coverage (+): 4.1. Max coverage (-): 0

Region: chr15 30410786-30410801. Max. coverage (+): 0. Max coverage (-): 0

Region: chr15 30410802-30410816. Max. coverage (+): 0. Max coverage (-): 0

Region: chr15 30410817-30410831. Max. coverage (+): 0. Max coverage (-): 0

Region: chr15 30410832-30410846. Max. coverage (+): 0. Max coverage (-): 0

Region: chr15 30410847-30410862. Max. coverage (+): 0. Max coverage (-): 0

Region: chr15 30410863-30410877. Max. coverage (+): 0. Max coverage (-): 0

Region: chr15 30410878-30410892. Max. coverage (+): 0. Max coverage (-): 0

Region: chr15 30410893-30410907. Max. coverage (+): 0. Max coverage (-): 0

Region: chr15 30410908-30410923. Max. coverage (+): 0. Max coverage (-): 0

Region: chr15 30410924-30410938. Max. coverage (+): 0. Max coverage (-): 0

Region: chr15 30410939-30410953. Max. coverage (+): 0. Max coverage (-): 0

Region: chr15 30410954-30410968. Max. coverage (+): 0. Max coverage (-): 0

Region: chr15 30410969-30410984. Max. coverage (+): 0. Max coverage (-): 0

Region: chr15 30410985-30410999. Max. coverage (+): 0. Max coverage (-): 0

Region: chr15 30411000-30411014. Max. coverage (+): 0. Max coverage (-): 0

Region: chr15 30411015-30411029. Max. coverage (+): 0. Max coverage (-): 0

Region: chr15 30411030-30411045. Max. coverage (+): 0. Max coverage (-): 0

Region: chr15 30411046-30411060. Max. coverage (+): 0. Max coverage (-): 0

Region: chr15 30411061-30411075. Max. coverage (+): 4.57. Max coverage (-): 0

Region: chr15 30411076-30411090. Max. coverage (+): 4.57. Max coverage (-): 0

Region: chr15 30411091-30411106. Max. coverage (+): 0. Max coverage (-): 0

Region: chr15 30411107-30411121. Max. coverage (+): 0. Max coverage (-): 0

Region: chr15 30411122-30411136. Max. coverage (+): 0. Max coverage (-): 0

Region: chr15 30411137-30411151. Max. coverage (+): 0. Max coverage (-): 0

Region: chr15 30411152-30411167. Max. coverage (+): 0. Max coverage (-): 0

Region: chr15 30411168-30411182. Max. coverage (+): 0. Max coverage (-): 0

Region: chr15 30411183-30411197. Max. coverage (+): 0. Max coverage (-): 0

Region: chr15 30411198-30411212. Max. coverage (+): 0. Max coverage (-): 0

Region: chr15 30411213-30411228. Max. coverage (+): 0. Max coverage (-): 0

Region: chr15 30411229-30411243. Max. coverage (+): 0. Max coverage (-): 0

Region: chr15 30411244-30411258. Max. coverage (+): 2.22. Max coverage (-): 0

Region: chr15 30411259-30411273. Max. coverage (+): 2.22. Max coverage (-): 0

Region: chr15 30411274-30411289. Max. coverage (+): 7.95. Max coverage (-): 0

Region: chr15 30411290-30411304. Max. coverage (+): 6.18. Max coverage (-): 0

Region: chr15 30411305-30411319. Max. coverage (+): 13.69. Max coverage (-): 0

Region: chr15 30411320-30411334. Max. coverage (+): 13.69. Max coverage (-): 0

Region: chr15 30411335-30411350. Max. coverage (+): 0. Max coverage (-): 0

Region: chr15 30411351-30411365. Max. coverage (+): 11.39. Max coverage (-): 0

Region: chr15 30411366-30411380. Max. coverage (+): 5.28. Max coverage (-): 0

Region: chr15 30411381-30411395. Max. coverage (+): 4.28. Max coverage (-): 0

Region: chr15 30411396-30411411. Max. coverage (+): 0. Max coverage (-): 0

Region: chr15 30411412-30411426. Max. coverage (+): 0. Max coverage (-): 0

Region: chr15 30411427-30411441. Max. coverage (+): 0.79. Max coverage (-): 0

Region: chr15 30411442-30411456. Max. coverage (+): 3.48. Max coverage (-): 0

Region: chr15 30411457-30411472. Max. coverage (+): 0. Max coverage (-): 0

Region: chr15 30411473-30411487. Max. coverage (+): 0. Max coverage (-): 0

Region: chr15 30411488-30411502. Max. coverage (+): 0. Max coverage (-): 0

Region: chr15 30411503-30411517. Max. coverage (+): 0. Max coverage (-): 0

Region: chr15 30411518-30411533. Max. coverage (+): 2.41. Max coverage (-): 0

Region: chr15 30411534-30411548. Max. coverage (+): 0. Max coverage (-): 0

Region: chr15 30411549-30411563. Max. coverage (+): 0. Max coverage (-): 0

Region: chr15 30411564-30411578. Max. coverage (+): 0. Max coverage (-): 0

Region: chr15 30411579-30411594. Max. coverage (+): 0. Max coverage (-): 0

Region: chr15 30411595-30411609. Max. coverage (+): 4.63. Max coverage (-): 0

Region: chr15 30411610-30411624. Max. coverage (+): 0. Max coverage (-): 0

Region: chr15 30411625-30411639. Max. coverage (+): 0. Max coverage (-): 0

Region: chr15 30411640-30411655. Max. coverage (+): 5.48. Max coverage (-): 0

Region: chr15 30411656-30411670. Max. coverage (+): 0. Max coverage (-): 0

Region: chr15 30411671-30411685. Max. coverage (+): 0. Max coverage (-): 0

Region: chr15 30411686-30411700. Max. coverage (+): 0. Max coverage (-): 0

Region: chr15 30411701-30411716. Max. coverage (+): 10.95. Max coverage (-): 0

Region: chr15 30411717-30411731. Max. coverage (+): 10.95. Max coverage (-): 0

Region: chr15 30411732-30411746. Max. coverage (+): 0. Max coverage (-): 0

Region: chr15 30411747-30411761. Max. coverage (+): 0. Max coverage (-): 0

Region: chr15 30411762-30411777. Max. coverage (+): 5.97. Max coverage (-): 0

Region: chr15 30411778-30411792. Max. coverage (+): 4. Max coverage (-): 0

Region: chr15 30411793-30411807. Max. coverage (+): 3.15. Max coverage (-): 0

Region: chr15 30411808-30411822. Max. coverage (+): 0. Max coverage (-): 0

Region: chr15 30411823-30411838. Max. coverage (+): 0. Max coverage (-): 0

Region: chr15 30411839-30411853. Max. coverage (+): 0. Max coverage (-): 0

Region: chr15 30411854-30411868. Max. coverage (+): 0. Max coverage (-): 0

Region: chr15 30411869-30411883. Max. coverage (+): 0. Max coverage (-): 0

Region: chr15 30411884-30411899. Max. coverage (+): 1.13. Max coverage (-): 0

Region: chr15 30411900-30411914. Max. coverage (+): 1.13. Max coverage (-): 0

Region: chr15 30411915-30411929. Max. coverage (+): 0. Max coverage (-): 0

Region: chr15 30411930-30411944. Max. coverage (+): 0. Max coverage (-): 0

Region: chr15 30411945-30411960. Max. coverage (+): 0. Max coverage (-): 0

Region: chr15 30411961-30411975. Max. coverage (+): 0. Max coverage (-): 0

Region: chr15 30411976-30411990. Max. coverage (+): 0. Max coverage (-): 0

Region: chr15 30411991-30412005. Max. coverage (+): 0. Max coverage (-): 0

Region: chr15 30412006-30412021. Max. coverage (+): 0. Max coverage (-): 0

Region: chr15 30412022-30412036. Max. coverage (+): 0. Max coverage (-): 0

Region: chr15 30412037-30412051. Max. coverage (+): 0. Max coverage (-): 0

Region: chr15 30412052-30412066. Max. coverage (+): 0. Max coverage (-): 0

Region: chr15 30412067-30412082. Max. coverage (+): 0. Max coverage (-): 0

Region: chr15 30412083-30412097. Max. coverage (+): 0. Max coverage (-): 0

Region: chr15 30412098-30412112. Max. coverage (+): 0. Max coverage (-): 0

Region: chr15 30412113-30412127. Max. coverage (+): 0. Max coverage (-): 0

Region: chr15 30412128-30412143. Max. coverage (+): 12.15. Max coverage (-): 0

Region: chr15 30412144-30412158. Max. coverage (+): 47.01. Max coverage (-): 0

Region: chr15 30412159-30412173. Max. coverage (+): 0. Max coverage (-): 0

Region: chr15 30412174-30412188. Max. coverage (+): 0. Max coverage (-): 0

Region: chr15 30412189-30412204. Max. coverage (+): 11.14. Max coverage (-): 0

Region: chr15 30412205-30412219. Max. coverage (+): 0. Max coverage (-): 0

Region: chr15 30412220-30412234. Max. coverage (+): 4.4. Max coverage (-): 0

Region: chr15 30412235-30412249. Max. coverage (+): 5.55. Max coverage (-): 0

Region: chr15 30412250-30412265. Max. coverage (+): 3.3. Max coverage (-): 0

Region: chr15 30412266-30412280. Max. coverage (+): 21.04. Max coverage (-): 0

Region: chr15 30412281-30412295. Max. coverage (+): 0. Max coverage (-): 0

Region: chr15 30412296-30412310. Max. coverage (+): 11.87. Max coverage (-): 0

Region: chr15 30412311-30412326. Max. coverage (+): 8.04. Max coverage (-): 0

Region: chr15 30412327-30412341. Max. coverage (+): 0. Max coverage (-): 0

Region: chr15 30412342-30412356. Max. coverage (+): 0. Max coverage (-): 0

Region: chr15 30412357-30412371. Max. coverage (+): 0. Max coverage (-): 0

Region: chr15 30412372-30412387. Max. coverage (+): 0. Max coverage (-): 0

Region: chr15 30412388-30412402. Max. coverage (+): 0. Max coverage (-): 0

Region: chr15 30412403-30412417. Max. coverage (+): 0. Max coverage (-): 0

Region: chr15 30412418-30412432. Max. coverage (+): 0. Max coverage (-): 0

Region: chr15 30412433-30412448. Max. coverage (+): 0. Max coverage (-): 0

Region: chr15 30412449-30412463. Max. coverage (+): 0. Max coverage (-): 0

Region: chr15 30412464-30412478. Max. coverage (+): 0. Max coverage (-): 0

Region: chr15 30412479-30412493. Max. coverage (+): 0. Max coverage (-): 0

Region: chr15 30412494-30412509. Max. coverage (+): 0. Max coverage (-): 0

Region: chr15 30412510-30412524. Max. coverage (+): 0. Max coverage (-): 0

Region: chr15 30412525-30412539. Max. coverage (+): 0. Max coverage (-): 0

Region: chr15 30412540-30412554. Max. coverage (+): 0. Max coverage (-): 0

Region: chr15 30412555-30412570. Max. coverage (+): 0. Max coverage (-): 0

Region: chr15 30412571-30412585. Max. coverage (+): 5.4. Max coverage (-): 0

Region: chr15 30412586-30412600. Max. coverage (+): 0. Max coverage (-): 0

Region: chr15 30412601-30412616. Max. coverage (+): 0. Max coverage (-): 0

Region: chr15 30412617-30412631. Max. coverage (+): 0. Max coverage (-): 0

Region: chr15 30412632-30412646. Max. coverage (+): 4.45. Max coverage (-): 0

Region: chr15 30412647-30412661. Max. coverage (+): 4.45. Max coverage (-): 0

Region: chr15 30412662-30412677. Max. coverage (+): 0. Max coverage (-): 0

Region: chr15 30412678-30412692. Max. coverage (+): 0. Max coverage (-): 0

Region: chr15 30412693-30412707. Max. coverage (+): 0. Max coverage (-): 0

Region: chr15 30412708-30412722. Max. coverage (+): 0. Max coverage (-): 0

Region: chr15 30412723-30412738. Max. coverage (+): 0. Max coverage (-): 0

Region: chr15 30412739-30412753. Max. coverage (+): 0. Max coverage (-): 0

Region: chr15 30412754-30412768. Max. coverage (+): 5.99. Max coverage (-): 0

Region: chr15 30412769-30412783. Max. coverage (+): 5.99. Max coverage (-): 0

Region: chr15 30412784-30412799. Max. coverage (+): 0. Max coverage (-): 0

Region: chr15 30412800-30412814. Max. coverage (+): 0. Max coverage (-): 0

Region: chr15 30412815-30412829. Max. coverage (+): 0. Max coverage (-): 0

Region: chr15 30412830-30412844. Max. coverage (+): 4.04. Max coverage (-): 0

Region: chr15 30412845-30412860. Max. coverage (+): 4.94. Max coverage (-): 0

Region: chr15 30412861-30412875. Max. coverage (+): 0. Max coverage (-): 0

Region: chr15 30412876-30412890. Max. coverage (+): 0. Max coverage (-): 0

Region: chr15 30412891-30412905. Max. coverage (+): 0. Max coverage (-): 0

Region: chr15 30412906-30412921. Max. coverage (+): 0. Max coverage (-): 0

Region: chr15 30412922-30412936. Max. coverage (+): 0. Max coverage (-): 0

Region: chr15 30412937-30412951. Max. coverage (+): 0. Max coverage (-): 0

Region: chr15 30412952-30412966. Max. coverage (+): 0. Max coverage (-): 0

Region: chr15 30412967-30412982. Max. coverage (+): 12.91. Max coverage (-): 0

Region: chr15 30412983-30412997. Max. coverage (+): 12.91. Max coverage (-): 0

Region: chr15 30412998-30413012. Max. coverage (+): 0. Max coverage (-): 0

Region: chr15 30413013-30413027. Max. coverage (+): 0. Max coverage (-): 0

Region: chr15 30413028-30413043. Max. coverage (+): 5.88. Max coverage (-): 0

Region: chr15 30413044-30413058. Max. coverage (+): 0. Max coverage (-): 0

Region: chr15 30413059-30413073. Max. coverage (+): 0. Max coverage (-): 0

Region: chr15 30413074-30413088. Max. coverage (+): 0. Max coverage (-): 0

Region: chr15 30413089-30413104. Max. coverage (+): 0. Max coverage (-): 0

Region: chr15 30413105-30413119. Max. coverage (+): 0. Max coverage (-): 0

Region: chr15 30413120-30413134. Max. coverage (+): 0. Max coverage (-): 0

Region: chr15 30413135-30413149. Max. coverage (+): 0. Max coverage (-): 0

Region: chr15 30413150-30413165. Max. coverage (+): 0. Max coverage (-): 0

Region: chr15 30413166-30413180. Max. coverage (+): 2.73. Max coverage (-): 0

Region: chr15 30413181-30413195. Max. coverage (+): 0. Max coverage (-): 0

Region: chr15 30413196-30413210. Max. coverage (+): 0. Max coverage (-): 0

Region: chr15 30413211-30413226. Max. coverage (+): 0. Max coverage (-): 0

Region: chr15 30413227-30413241. Max. coverage (+): 0. Max coverage (-): 0

Region: chr15 30413242-30413256. Max. coverage (+): 0. Max coverage (-): 0

Region: chr15 30413257-30413271. Max. coverage (+): 0. Max coverage (-): 0

Region: chr15 30413272-30413287. Max. coverage (+): 0. Max coverage (-): 0

Region: chr15 30413288-30413302. Max. coverage (+): 0. Max coverage (-): 0

Region: chr15 30413303-30413317. Max. coverage (+): 0. Max coverage (-): 0

Region: chr15 30413318-30413332. Max. coverage (+): 0. Max coverage (-): 0

Region: chr15 30413333-30413348. Max. coverage (+): 0. Max coverage (-): 0

Region: chr15 30413349-30413363. Max. coverage (+): 0. Max coverage (-): 0

Region: chr15 30413364-30413378. Max. coverage (+): 0. Max coverage (-): 0

Region: chr15 30413379-30413393. Max. coverage (+): 0. Max coverage (-): 0

Region: chr15 30413394-30413409. Max. coverage (+): 0. Max coverage (-): 0

Region: chr15 30413410-30413424. Max. coverage (+): 1.23. Max coverage (-): 0

Region: chr15 30413425-30413439. Max. coverage (+): 1.23. Max coverage (-): 0

Region: chr15 30413440-30413454. Max. coverage (+): 0. Max coverage (-): 0

Region: chr15 30413455-30413470. Max. coverage (+): 0. Max coverage (-): 0

Region: chr15 30413471-30413485. Max. coverage (+): 0. Max coverage (-): 0

Region: chr15 30413486-30413500. Max. coverage (+): 0. Max coverage (-): 0

Region: chr15 30413501-30413515. Max. coverage (+): 0. Max coverage (-): 0

Region: chr15 30413516-30413531. Max. coverage (+): 5.83. Max coverage (-): 0

Region: chr15 30413532-30413546. Max. coverage (+): 5.83. Max coverage (-): 0

Region: chr15 30413547-. Max. coverage (+): 0. Max coverage (-): 0

RepeatMasker Color Code

**+**

100-98% Identity

<98-95% Identity

<95-90% Identity

<90-85% Identity

<85-80% Identity

<80-75% Identity

<75-70% Identity

<70% Identity

**-**

Gene Set Color Code

**+**

Gene

Pseudogene

**-**

Topology/Coverage Color Code

Coverage Plus Strand

Coverage Minus Strand

Mainstrand: Plus

Mainstrand: Minus

Complementary Strand

Flanking Region  
(if option -flank >0)

Gene Set Annotation  

**1. CBL (protein coding, ENSBTAG00000006817) Tr:00000008961 Ex:16**: 30405793-30406073 (+)

  
RepeatMasker Annotation  
  
Transcription Factor Binding Sites  

**RFX4\_1** (Sequence: GTTGCCAGG (-): 30411619)  
**RFX4\_2** (Sequence: CGTGGTTAC (+): 30407980)  
**Gata4** (Sequence: GTTATCT (+): 30409936)  
**Gata4** (Sequence: CTTATCT (+): 30411526)
