# Supplementary material for: Small RNA sequencing of cryopreserved semen from single bull revealed altered miRNAs and piRNAs expression between High- and Low-motile sperm populations
Source: BMC Genomics. 2017 Jan 4;18:14. doi: 10.1186/s12864-016-3394-7 (PMC5209821; doi:10.1186/s12864-016-3394-7)
Supplement: Additional file 4: — Details for each piRNA clusters found in Low Motile (LM) sperm fraction. Genes, repeats, transposable elements and transcription factors binding sites falling within the cluster regions were reported. (ZIP 1034 kb) [file 12864_2016_3394_MOESM4_ESM.zip › 15.html]

piRNA cluster 15


Predicted piRNA cluster no. 15     previous   next
  

Show proTRAC run info
Hide proTRAC run info

================================= proTRAC ====================================  
VERSION: 2.1                                    LAST MODIFIED: 06. October 2015  
  
Please cite:  
Rosenkranz D, Zischler H. proTRAC - a software for probabilistic piRNA cluster  
detection, visualization and analysis. 2012. BMC Bioinformatics 13:5.  
  
and (for proTRAC 2.0 and later):  
Rosenkranz D, Rudloff S, Bastuck K, Ketting RF, Zischler H. Tupaia small RNAs  
provide insights into function and evolution of RNAi-based transposon defense  
in mammals. 2015. RNA 21(5):911-922.  
  
Contact:  
David Rosenkranz  
Institute of Anthropology, small RNA group  
Johannes Gutenberg University Mainz  
email: rosenkranz@uni-mainz.de  
  
You can find the latest proTRAC version at:  
http://sourceforge.net/projects/protrac/files  
http://www.smallRNAgroup-mainz.de/software  
==============================================================================  
  
PARAMETERS:  
Map file: .............../storage/core/barbara/genhome/smallRNA/fertility/Sample\_not\_motile/pirna/Sample\_not\_motile\_26-33\_collapsed.fa.no-dust.map.weighted-10000-1000-b-0  
Genome file: ............/storage/core/barbara/genhome/smallRNA/fertility/Sample\_all/pirna/bt\_311\_chrY.fa  
RepeatMasker annotation: /storage/genomes/bt\_umd31/GCF\_000003055.6\_Bos\_taurus\_UMD\_3.1.1\_repeatMasker\_chr.out  
GeneSet:................./storage/core/barbara/genhome/smallRNA/fertility/Sample\_all/pirna/full.gtf  
  
Significant (p<=0.01) hit density will be calculated based  
on observed hit distribution.  
  
Sliding window size: ........................................ 5000 bp  
Sliding window increament: .................................. 1000 bp  
Normalize each hit by number of genomic hits: ............... 1 [0=no/1=yes]  
Normalize each hit by number of sequence reads: ............. 1 [0=no/1=yes]  
Normalize values (-> per million mapped reads): ............. 1 [0=no/1=yes]  
Min. fraction of hits with 1T(U) or 10A: .................... 0.75  
Alternatively: Min. fraction of hits with 1T(U) and 10A: .... 0.5  
Min. fraction of hits with typical piRNA length: ............ 0.75  
Typical piRNA length: ....................................... 26-33 nt  
Min. size of a piRNA cluster: ............................... 5000 bp.  
Min. number of hits (absolute): ............................. 0  
Min. number of hits (normalized): ........................... 0  
Min. fraction of hits on the mainstrand: .................... 0.75  
Top fraction of mapped sequences (in terms of read counts): . 1%  
Top fraction accounts for max. n% of sequence reads: ........ 90%  
Min. fraction of hits on each arm of a bidirectional cluster: 0.1  
Output image file for each cluster: ......................... 0 [0=no/1=yes]  
Output html file for each cluster: .......................... 1 [0=no/1=yes]  
Output a summary table: ..................................... 1 [0=no/1=yes]  
Output a FASTA file for each cluster (piRNA sequences): ..... 1 [0=no/1=yes]  
Output a FASTA file comprising cluster sequences: ........... 1 [0=no/1=yes]  
Search DNA motifs in clusters: .............................. 1 [0=no/1=yes]  
Output flanking sequences: +/- .............................. 0 bp  
Output ~.pTi file: .......................................... 1 [0=no/1=yes]  
==============================================================================  
  
  
Genome size (without gaps): ............ 2678902517 bp  
Gaps (N/X/-): .......................... 53837044 bp  
Mapped reads: .......................... 738059667487  
Non-identical sequences: ............... 277001  
Genomic hits: .......................... 533816  
Significant densitiy of mapped reads: .. 15118061 reads/kb

Show proTRAC cluster info
Hide proTRAC cluster info

|  |  |
| --- | --- |
| Location | chr15 |
| Coordinates | 55041118-55056635 |
| Size [bp] | 15518 |
| Sequence hit loci | 286 |
| Mapped reads (normalized) | 807809872.2 |
| Mapped reads (normalized) per kb | 52056313.5 |
| Normalized reads with 1T (1U) | 89.6% |
| Normalized reads with 10A | 26.1% |
| Normalized reads with length 26-33 nt | 100% |
| Normalized reads on the main strand(s) | 100% |
| Predicted directionality | mono:plus |

100%

0%

1T (1U)  
reads

10A reads

26-33 nt  
reads

reads on mainstrand

**Either the amount of reads with 1T (1U) OR 10A has to exceed 75% (set with option: -1Tor10A)  
Alternatively the amount of reads with 1T (1U) AND 10A has to exceed 50% (set with option: -1Tand10A)  
Minimum amount of reads with preferred size is 75% (set with option: -pisize)  
Minimum amount of reads on the main strand(s) is 75% (set with option: -clstrand)**

Show read coverage
Hide read coverage

WHAT DO I SEE HERE?  
This chart shows the location of mapped sequence reads within a predicted piRNA cluster. The color refers to the number of genomic hits produced by the sequence read in question. A dark red bar indicates that this sequence read produces many other hits elsewhere in the genome. Many adjacent red or yellow bars can indicate the presence of a multi-copy element such as transposons or rRNA genes. A dark green bar indicates that this sequence read maps uniquely to this locus.

1 hit

2-5 hits

6-10 hits

11-20 hits

21-50 hits

51-100 hits

> 100 hits

chr15

55041118

55056635

Gene Set

RepeatMasker

Mapped  
Reads

30.63

plus strand

minus strand

30.63

Region: chr15 30413548-55041133. Max. coverage (+): 7.1. Max coverage (-): 0

Region: chr15 55041134-55041164. Max. coverage (+): 0. Max coverage (-): 0

Region: chr15 55041165-55041195. Max. coverage (+): 0. Max coverage (-): 0

Region: chr15 55041196-55041226. Max. coverage (+): 0. Max coverage (-): 0

Region: chr15 55041227-55041257. Max. coverage (+): 0. Max coverage (-): 0

Region: chr15 55041258-55041288. Max. coverage (+): 0. Max coverage (-): 0

Region: chr15 55041289-55041319. Max. coverage (+): 0. Max coverage (-): 0

Region: chr15 55041320-55041350. Max. coverage (+): 0. Max coverage (-): 0

Region: chr15 55041351-55041381. Max. coverage (+): 0. Max coverage (-): 0

Region: chr15 55041382-55041412. Max. coverage (+): 5.68. Max coverage (-): 0

Region: chr15 55041413-55041443. Max. coverage (+): 1.84. Max coverage (-): 0

Region: chr15 55041444-55041474. Max. coverage (+): 0. Max coverage (-): 0

Region: chr15 55041475-55041505. Max. coverage (+): 4.95. Max coverage (-): 0

Region: chr15 55041506-55041536. Max. coverage (+): 0. Max coverage (-): 0

Region: chr15 55041537-55041568. Max. coverage (+): 0. Max coverage (-): 0

Region: chr15 55041569-55041599. Max. coverage (+): 0. Max coverage (-): 0

Region: chr15 55041600-55041630. Max. coverage (+): 0. Max coverage (-): 0

Region: chr15 55041631-55041661. Max. coverage (+): 2.14. Max coverage (-): 0

Region: chr15 55041662-55041692. Max. coverage (+): 0. Max coverage (-): 0

Region: chr15 55041693-55041723. Max. coverage (+): 0. Max coverage (-): 0

Region: chr15 55041724-55041754. Max. coverage (+): 0. Max coverage (-): 0

Region: chr15 55041755-55041785. Max. coverage (+): 0. Max coverage (-): 0

Region: chr15 55041786-55041816. Max. coverage (+): 0. Max coverage (-): 0

Region: chr15 55041817-55041847. Max. coverage (+): 0. Max coverage (-): 0

Region: chr15 55041848-55041878. Max. coverage (+): 0. Max coverage (-): 0

Region: chr15 55041879-55041909. Max. coverage (+): 3.29. Max coverage (-): 0

Region: chr15 55041910-55041940. Max. coverage (+): 0. Max coverage (-): 0

Region: chr15 55041941-55041971. Max. coverage (+): 0. Max coverage (-): 0

Region: chr15 55041972-55042002. Max. coverage (+): 0. Max coverage (-): 0

Region: chr15 55042003-55042033. Max. coverage (+): 5.92. Max coverage (-): 0

Region: chr15 55042034-55042064. Max. coverage (+): 0. Max coverage (-): 0

Region: chr15 55042065-55042095. Max. coverage (+): 7.47. Max coverage (-): 0

Region: chr15 55042096-55042126. Max. coverage (+): 3.08. Max coverage (-): 0

Region: chr15 55042127-55042157. Max. coverage (+): 3.08. Max coverage (-): 0

Region: chr15 55042158-55042188. Max. coverage (+): 0. Max coverage (-): 0

Region: chr15 55042189-55042219. Max. coverage (+): 0. Max coverage (-): 0

Region: chr15 55042220-55042250. Max. coverage (+): 0. Max coverage (-): 0

Region: chr15 55042251-55042281. Max. coverage (+): 6.5. Max coverage (-): 0

Region: chr15 55042282-55042312. Max. coverage (+): 1.7. Max coverage (-): 0

Region: chr15 55042313-55042343. Max. coverage (+): 4.27. Max coverage (-): 0

Region: chr15 55042344-55042374. Max. coverage (+): 0. Max coverage (-): 0

Region: chr15 55042375-55042405. Max. coverage (+): 0. Max coverage (-): 0

Region: chr15 55042406-55042437. Max. coverage (+): 0. Max coverage (-): 0

Region: chr15 55042438-55042468. Max. coverage (+): 0. Max coverage (-): 0

Region: chr15 55042469-55042499. Max. coverage (+): 0. Max coverage (-): 0

Region: chr15 55042500-55042530. Max. coverage (+): 0. Max coverage (-): 0

Region: chr15 55042531-55042561. Max. coverage (+): 3.98. Max coverage (-): 0

Region: chr15 55042562-55042592. Max. coverage (+): 6.86. Max coverage (-): 0

Region: chr15 55042593-55042623. Max. coverage (+): 0. Max coverage (-): 0

Region: chr15 55042624-55042654. Max. coverage (+): 0. Max coverage (-): 0

Region: chr15 55042655-55042685. Max. coverage (+): 0. Max coverage (-): 0

Region: chr15 55042686-55042716. Max. coverage (+): 0.61. Max coverage (-): 0

Region: chr15 55042717-55042747. Max. coverage (+): 10.25. Max coverage (-): 0

Region: chr15 55042748-55042778. Max. coverage (+): 8.29. Max coverage (-): 0

Region: chr15 55042779-55042809. Max. coverage (+): 8.29. Max coverage (-): 0

Region: chr15 55042810-55042840. Max. coverage (+): 11.38. Max coverage (-): 0

Region: chr15 55042841-55042871. Max. coverage (+): 2.82. Max coverage (-): 0

Region: chr15 55042872-55042902. Max. coverage (+): 16.45. Max coverage (-): 0

Region: chr15 55042903-55042933. Max. coverage (+): 3.24. Max coverage (-): 0

Region: chr15 55042934-55042964. Max. coverage (+): 21.41. Max coverage (-): 0

Region: chr15 55042965-55042995. Max. coverage (+): 15.34. Max coverage (-): 0

Region: chr15 55042996-55043026. Max. coverage (+): 0. Max coverage (-): 0

Region: chr15 55043027-55043057. Max. coverage (+): 0. Max coverage (-): 0

Region: chr15 55043058-55043088. Max. coverage (+): 0. Max coverage (-): 0

Region: chr15 55043089-55043119. Max. coverage (+): 0. Max coverage (-): 0

Region: chr15 55043120-55043150. Max. coverage (+): 4.3. Max coverage (-): 0

Region: chr15 55043151-55043181. Max. coverage (+): 15.66. Max coverage (-): 0

Region: chr15 55043182-55043212. Max. coverage (+): 6.82. Max coverage (-): 0

Region: chr15 55043213-55043243. Max. coverage (+): 18.25. Max coverage (-): 0

Region: chr15 55043244-55043275. Max. coverage (+): 11.29. Max coverage (-): 0

Region: chr15 55043276-55043306. Max. coverage (+): 14.05. Max coverage (-): 0

Region: chr15 55043307-55043337. Max. coverage (+): 28.87. Max coverage (-): 0

Region: chr15 55043338-55043368. Max. coverage (+): 4.09. Max coverage (-): 0

Region: chr15 55043369-55043399. Max. coverage (+): 7.79. Max coverage (-): 0

Region: chr15 55043400-55043430. Max. coverage (+): 4.43. Max coverage (-): 0

Region: chr15 55043431-55043461. Max. coverage (+): 9.45. Max coverage (-): 0

Region: chr15 55043462-55043492. Max. coverage (+): 22.2. Max coverage (-): 0

Region: chr15 55043493-55043523. Max. coverage (+): 29.85. Max coverage (-): 0

Region: chr15 55043524-55043554. Max. coverage (+): 14.32. Max coverage (-): 0

Region: chr15 55043555-55043585. Max. coverage (+): 21.07. Max coverage (-): 0

Region: chr15 55043586-55043616. Max. coverage (+): 7. Max coverage (-): 0

Region: chr15 55043617-55043647. Max. coverage (+): 0.35. Max coverage (-): 0

Region: chr15 55043648-55043678. Max. coverage (+): 0. Max coverage (-): 0

Region: chr15 55043679-55043709. Max. coverage (+): 0. Max coverage (-): 0

Region: chr15 55043710-55043740. Max. coverage (+): 4.19. Max coverage (-): 0

Region: chr15 55043741-55043771. Max. coverage (+): 0. Max coverage (-): 0

Region: chr15 55043772-55043802. Max. coverage (+): 0. Max coverage (-): 0

Region: chr15 55043803-55043833. Max. coverage (+): 0. Max coverage (-): 0

Region: chr15 55043834-55043864. Max. coverage (+): 2.89. Max coverage (-): 0

Region: chr15 55043865-55043895. Max. coverage (+): 0. Max coverage (-): 0

Region: chr15 55043896-55043926. Max. coverage (+): 3.51. Max coverage (-): 0

Region: chr15 55043927-55043957. Max. coverage (+): 9.17. Max coverage (-): 0

Region: chr15 55043958-55043988. Max. coverage (+): 6.87. Max coverage (-): 0

Region: chr15 55043989-55044019. Max. coverage (+): 6.87. Max coverage (-): 0

Region: chr15 55044020-55044050. Max. coverage (+): 0. Max coverage (-): 0

Region: chr15 55044051-55044081. Max. coverage (+): 0. Max coverage (-): 0

Region: chr15 55044082-55044112. Max. coverage (+): 0. Max coverage (-): 0

Region: chr15 55044113-55044144. Max. coverage (+): 4.09. Max coverage (-): 0

Region: chr15 55044145-55044175. Max. coverage (+): 5.94. Max coverage (-): 0

Region: chr15 55044176-55044206. Max. coverage (+): 5.94. Max coverage (-): 0

Region: chr15 55044207-55044237. Max. coverage (+): 7.65. Max coverage (-): 0

Region: chr15 55044238-55044268. Max. coverage (+): 0. Max coverage (-): 0

Region: chr15 55044269-55044299. Max. coverage (+): 0. Max coverage (-): 0

Region: chr15 55044300-55044330. Max. coverage (+): 0. Max coverage (-): 0

Region: chr15 55044331-55044361. Max. coverage (+): 0. Max coverage (-): 0

Region: chr15 55044362-55044392. Max. coverage (+): 0. Max coverage (-): 0

Region: chr15 55044393-55044423. Max. coverage (+): 10.31. Max coverage (-): 0

Region: chr15 55044424-55044454. Max. coverage (+): 5.15. Max coverage (-): 0

Region: chr15 55044455-55044485. Max. coverage (+): 2.35. Max coverage (-): 0

Region: chr15 55044486-55044516. Max. coverage (+): 0. Max coverage (-): 0

Region: chr15 55044517-55044547. Max. coverage (+): 0. Max coverage (-): 0

Region: chr15 55044548-55044578. Max. coverage (+): 0. Max coverage (-): 0

Region: chr15 55044579-55044609. Max. coverage (+): 0. Max coverage (-): 0

Region: chr15 55044610-55044640. Max. coverage (+): 0. Max coverage (-): 0

Region: chr15 55044641-55044671. Max. coverage (+): 0. Max coverage (-): 0

Region: chr15 55044672-55044702. Max. coverage (+): 0. Max coverage (-): 0

Region: chr15 55044703-55044733. Max. coverage (+): 9.71. Max coverage (-): 0

Region: chr15 55044734-55044764. Max. coverage (+): 0. Max coverage (-): 0

Region: chr15 55044765-55044795. Max. coverage (+): 1.18. Max coverage (-): 0

Region: chr15 55044796-55044826. Max. coverage (+): 6.94. Max coverage (-): 0

Region: chr15 55044827-55044857. Max. coverage (+): 0. Max coverage (-): 0

Region: chr15 55044858-55044888. Max. coverage (+): 0.9. Max coverage (-): 0

Region: chr15 55044889-55044919. Max. coverage (+): 0. Max coverage (-): 0

Region: chr15 55044920-55044950. Max. coverage (+): 0. Max coverage (-): 0

Region: chr15 55044951-55044981. Max. coverage (+): 6.88. Max coverage (-): 0

Region: chr15 55044982-55045013. Max. coverage (+): 0. Max coverage (-): 0

Region: chr15 55045014-55045044. Max. coverage (+): 0. Max coverage (-): 0

Region: chr15 55045045-55045075. Max. coverage (+): 0. Max coverage (-): 0

Region: chr15 55045076-55045106. Max. coverage (+): 0. Max coverage (-): 0

Region: chr15 55045107-55045137. Max. coverage (+): 0. Max coverage (-): 0

Region: chr15 55045138-55045168. Max. coverage (+): 0. Max coverage (-): 0

Region: chr15 55045169-55045199. Max. coverage (+): 0. Max coverage (-): 0

Region: chr15 55045200-55045230. Max. coverage (+): 0. Max coverage (-): 0

Region: chr15 55045231-55045261. Max. coverage (+): 0. Max coverage (-): 0

Region: chr15 55045262-55045292. Max. coverage (+): 0. Max coverage (-): 0

Region: chr15 55045293-55045323. Max. coverage (+): 0. Max coverage (-): 0

Region: chr15 55045324-55045354. Max. coverage (+): 0. Max coverage (-): 0

Region: chr15 55045355-55045385. Max. coverage (+): 0. Max coverage (-): 0

Region: chr15 55045386-55045416. Max. coverage (+): 16.94. Max coverage (-): 0

Region: chr15 55045417-55045447. Max. coverage (+): 0. Max coverage (-): 0

Region: chr15 55045448-55045478. Max. coverage (+): 0. Max coverage (-): 0

Region: chr15 55045479-55045509. Max. coverage (+): 2.25. Max coverage (-): 0

Region: chr15 55045510-55045540. Max. coverage (+): 2.25. Max coverage (-): 0

Region: chr15 55045541-55045571. Max. coverage (+): 0. Max coverage (-): 0

Region: chr15 55045572-55045602. Max. coverage (+): 10.23. Max coverage (-): 0

Region: chr15 55045603-55045633. Max. coverage (+): 0. Max coverage (-): 0

Region: chr15 55045634-55045664. Max. coverage (+): 0. Max coverage (-): 0

Region: chr15 55045665-55045695. Max. coverage (+): 0. Max coverage (-): 0

Region: chr15 55045696-55045726. Max. coverage (+): 0. Max coverage (-): 0

Region: chr15 55045727-55045757. Max. coverage (+): 0. Max coverage (-): 0

Region: chr15 55045758-55045788. Max. coverage (+): 5.9. Max coverage (-): 0

Region: chr15 55045789-55045819. Max. coverage (+): 8.43. Max coverage (-): 0

Region: chr15 55045820-55045850. Max. coverage (+): 0. Max coverage (-): 0

Region: chr15 55045851-55045882. Max. coverage (+): 0. Max coverage (-): 0

Region: chr15 55045883-55045913. Max. coverage (+): 0. Max coverage (-): 0

Region: chr15 55045914-55045944. Max. coverage (+): 0. Max coverage (-): 0

Region: chr15 55045945-55045975. Max. coverage (+): 0. Max coverage (-): 0

Region: chr15 55045976-55046006. Max. coverage (+): 0. Max coverage (-): 0

Region: chr15 55046007-55046037. Max. coverage (+): 0. Max coverage (-): 0

Region: chr15 55046038-55046068. Max. coverage (+): 2.31. Max coverage (-): 0

Region: chr15 55046069-55046099. Max. coverage (+): 11.02. Max coverage (-): 0

Region: chr15 55046100-55046130. Max. coverage (+): 5.79. Max coverage (-): 0

Region: chr15 55046131-55046161. Max. coverage (+): 5.79. Max coverage (-): 0

Region: chr15 55046162-55046192. Max. coverage (+): 0. Max coverage (-): 0

Region: chr15 55046193-55046223. Max. coverage (+): 0. Max coverage (-): 0

Region: chr15 55046224-55046254. Max. coverage (+): 7.01. Max coverage (-): 0

Region: chr15 55046255-55046285. Max. coverage (+): 0. Max coverage (-): 0

Region: chr15 55046286-55046316. Max. coverage (+): 0. Max coverage (-): 0

Region: chr15 55046317-55046347. Max. coverage (+): 30.63. Max coverage (-): 0

Region: chr15 55046348-55046378. Max. coverage (+): 6.64. Max coverage (-): 0

Region: chr15 55046379-55046409. Max. coverage (+): 5.33. Max coverage (-): 0

Region: chr15 55046410-55046440. Max. coverage (+): 0. Max coverage (-): 0

Region: chr15 55046441-55046471. Max. coverage (+): 0. Max coverage (-): 0

Region: chr15 55046472-55046502. Max. coverage (+): 0. Max coverage (-): 0

Region: chr15 55046503-55046533. Max. coverage (+): 4.34. Max coverage (-): 0

Region: chr15 55046534-55046564. Max. coverage (+): 0. Max coverage (-): 0

Region: chr15 55046565-55046595. Max. coverage (+): 6.29. Max coverage (-): 0

Region: chr15 55046596-55046626. Max. coverage (+): 6.29. Max coverage (-): 0

Region: chr15 55046627-55046657. Max. coverage (+): 0. Max coverage (-): 0

Region: chr15 55046658-55046688. Max. coverage (+): 0. Max coverage (-): 0

Region: chr15 55046689-55046719. Max. coverage (+): 0. Max coverage (-): 0

Region: chr15 55046720-55046751. Max. coverage (+): 0. Max coverage (-): 0

Region: chr15 55046752-55046782. Max. coverage (+): 5.92. Max coverage (-): 0

Region: chr15 55046783-55046813. Max. coverage (+): 0. Max coverage (-): 0

Region: chr15 55046814-55046844. Max. coverage (+): 4.07. Max coverage (-): 0

Region: chr15 55046845-55046875. Max. coverage (+): 2.81. Max coverage (-): 0

Region: chr15 55046876-55046906. Max. coverage (+): 0. Max coverage (-): 0

Region: chr15 55046907-55046937. Max. coverage (+): 0. Max coverage (-): 0

Region: chr15 55046938-55046968. Max. coverage (+): 0. Max coverage (-): 0

Region: chr15 55046969-55046999. Max. coverage (+): 0. Max coverage (-): 0

Region: chr15 55047000-55047030. Max. coverage (+): 0. Max coverage (-): 0

Region: chr15 55047031-55047061. Max. coverage (+): 0. Max coverage (-): 0

Region: chr15 55047062-55047092. Max. coverage (+): 11.34. Max coverage (-): 0

Region: chr15 55047093-55047123. Max. coverage (+): 11.34. Max coverage (-): 0

Region: chr15 55047124-55047154. Max. coverage (+): 0. Max coverage (-): 0

Region: chr15 55047155-55047185. Max. coverage (+): 2.06. Max coverage (-): 0

Region: chr15 55047186-55047216. Max. coverage (+): 2.06. Max coverage (-): 0

Region: chr15 55047217-55047247. Max. coverage (+): 0. Max coverage (-): 0

Region: chr15 55047248-55047278. Max. coverage (+): 0. Max coverage (-): 0

Region: chr15 55047279-55047309. Max. coverage (+): 0. Max coverage (-): 0

Region: chr15 55047310-55047340. Max. coverage (+): 0. Max coverage (-): 0

Region: chr15 55047341-55047371. Max. coverage (+): 0. Max coverage (-): 0

Region: chr15 55047372-55047402. Max. coverage (+): 0. Max coverage (-): 0

Region: chr15 55047403-55047433. Max. coverage (+): 0.79. Max coverage (-): 0

Region: chr15 55047434-55047464. Max. coverage (+): 0. Max coverage (-): 0

Region: chr15 55047465-55047495. Max. coverage (+): 0. Max coverage (-): 0

Region: chr15 55047496-55047526. Max. coverage (+): 0. Max coverage (-): 0

Region: chr15 55047527-55047557. Max. coverage (+): 0. Max coverage (-): 0

Region: chr15 55047558-55047589. Max. coverage (+): 0. Max coverage (-): 0

Region: chr15 55047590-55047620. Max. coverage (+): 3.9. Max coverage (-): 0

Region: chr15 55047621-55047651. Max. coverage (+): 5.04. Max coverage (-): 0

Region: chr15 55047652-55047682. Max. coverage (+): 0. Max coverage (-): 0

Region: chr15 55047683-55047713. Max. coverage (+): 0. Max coverage (-): 0

Region: chr15 55047714-55047744. Max. coverage (+): 5.14. Max coverage (-): 0

Region: chr15 55047745-55047775. Max. coverage (+): 0. Max coverage (-): 0

Region: chr15 55047776-55047806. Max. coverage (+): 0. Max coverage (-): 0

Region: chr15 55047807-55047837. Max. coverage (+): 0. Max coverage (-): 0

Region: chr15 55047838-55047868. Max. coverage (+): 0. Max coverage (-): 0

Region: chr15 55047869-55047899. Max. coverage (+): 2.4. Max coverage (-): 0

Region: chr15 55047900-55047930. Max. coverage (+): 2.4. Max coverage (-): 0

Region: chr15 55047931-55047961. Max. coverage (+): 0. Max coverage (-): 0

Region: chr15 55047962-55047992. Max. coverage (+): 0. Max coverage (-): 0

Region: chr15 55047993-55048023. Max. coverage (+): 0. Max coverage (-): 0

Region: chr15 55048024-55048054. Max. coverage (+): 0. Max coverage (-): 0

Region: chr15 55048055-55048085. Max. coverage (+): 0. Max coverage (-): 0

Region: chr15 55048086-55048116. Max. coverage (+): 0. Max coverage (-): 0

Region: chr15 55048117-55048147. Max. coverage (+): 0. Max coverage (-): 0

Region: chr15 55048148-55048178. Max. coverage (+): 0. Max coverage (-): 0

Region: chr15 55048179-55048209. Max. coverage (+): 9.37. Max coverage (-): 0

Region: chr15 55048210-55048240. Max. coverage (+): 13.65. Max coverage (-): 0

Region: chr15 55048241-55048271. Max. coverage (+): 0. Max coverage (-): 0

Region: chr15 55048272-55048302. Max. coverage (+): 0. Max coverage (-): 0

Region: chr15 55048303-55048333. Max. coverage (+): 0. Max coverage (-): 0

Region: chr15 55048334-55048364. Max. coverage (+): 4.76. Max coverage (-): 0

Region: chr15 55048365-55048395. Max. coverage (+): 13.56. Max coverage (-): 0

Region: chr15 55048396-55048426. Max. coverage (+): 2.53. Max coverage (-): 0

Region: chr15 55048427-55048458. Max. coverage (+): 0. Max coverage (-): 0

Region: chr15 55048459-55048489. Max. coverage (+): 1.34. Max coverage (-): 0

Region: chr15 55048490-55048520. Max. coverage (+): 1.34. Max coverage (-): 0

Region: chr15 55048521-55048551. Max. coverage (+): 0. Max coverage (-): 0

Region: chr15 55048552-55048582. Max. coverage (+): 0. Max coverage (-): 0

Region: chr15 55048583-55048613. Max. coverage (+): 0. Max coverage (-): 0

Region: chr15 55048614-55048644. Max. coverage (+): 6.75. Max coverage (-): 0

Region: chr15 55048645-55048675. Max. coverage (+): 6.12. Max coverage (-): 0

Region: chr15 55048676-55048706. Max. coverage (+): 0. Max coverage (-): 0

Region: chr15 55048707-55048737. Max. coverage (+): 0. Max coverage (-): 0

Region: chr15 55048738-55048768. Max. coverage (+): 0. Max coverage (-): 0

Region: chr15 55048769-55048799. Max. coverage (+): 0. Max coverage (-): 0

Region: chr15 55048800-55048830. Max. coverage (+): 0. Max coverage (-): 0

Region: chr15 55048831-55048861. Max. coverage (+): 0. Max coverage (-): 0

Region: chr15 55048862-55048892. Max. coverage (+): 0. Max coverage (-): 0

Region: chr15 55048893-55048923. Max. coverage (+): 0. Max coverage (-): 0

Region: chr15 55048924-55048954. Max. coverage (+): 0. Max coverage (-): 0

Region: chr15 55048955-55048985. Max. coverage (+): 0. Max coverage (-): 0

Region: chr15 55048986-55049016. Max. coverage (+): 2.93. Max coverage (-): 0

Region: chr15 55049017-55049047. Max. coverage (+): 2.93. Max coverage (-): 0

Region: chr15 55049048-55049078. Max. coverage (+): 0. Max coverage (-): 0

Region: chr15 55049079-55049109. Max. coverage (+): 0. Max coverage (-): 0

Region: chr15 55049110-55049140. Max. coverage (+): 0. Max coverage (-): 0

Region: chr15 55049141-55049171. Max. coverage (+): 0. Max coverage (-): 0

Region: chr15 55049172-55049202. Max. coverage (+): 0. Max coverage (-): 0

Region: chr15 55049203-55049233. Max. coverage (+): 0. Max coverage (-): 0

Region: chr15 55049234-55049264. Max. coverage (+): 0. Max coverage (-): 0

Region: chr15 55049265-55049295. Max. coverage (+): 0. Max coverage (-): 0

Region: chr15 55049296-55049327. Max. coverage (+): 0. Max coverage (-): 0

Region: chr15 55049328-55049358. Max. coverage (+): 0. Max coverage (-): 0

Region: chr15 55049359-55049389. Max. coverage (+): 0. Max coverage (-): 0

Region: chr15 55049390-55049420. Max. coverage (+): 0. Max coverage (-): 0

Region: chr15 55049421-55049451. Max. coverage (+): 0. Max coverage (-): 0

Region: chr15 55049452-55049482. Max. coverage (+): 0. Max coverage (-): 0

Region: chr15 55049483-55049513. Max. coverage (+): 0. Max coverage (-): 0

Region: chr15 55049514-55049544. Max. coverage (+): 0. Max coverage (-): 0

Region: chr15 55049545-55049575. Max. coverage (+): 0. Max coverage (-): 0

Region: chr15 55049576-55049606. Max. coverage (+): 0. Max coverage (-): 0

Region: chr15 55049607-55049637. Max. coverage (+): 0. Max coverage (-): 0

Region: chr15 55049638-55049668. Max. coverage (+): 0. Max coverage (-): 0

Region: chr15 55049669-55049699. Max. coverage (+): 0. Max coverage (-): 0

Region: chr15 55049700-55049730. Max. coverage (+): 0. Max coverage (-): 0

Region: chr15 55049731-55049761. Max. coverage (+): 0. Max coverage (-): 0

Region: chr15 55049762-55049792. Max. coverage (+): 0. Max coverage (-): 0

Region: chr15 55049793-55049823. Max. coverage (+): 0. Max coverage (-): 0

Region: chr15 55049824-55049854. Max. coverage (+): 0. Max coverage (-): 0

Region: chr15 55049855-55049885. Max. coverage (+): 0. Max coverage (-): 0

Region: chr15 55049886-55049916. Max. coverage (+): 0. Max coverage (-): 0

Region: chr15 55049917-55049947. Max. coverage (+): 0. Max coverage (-): 0

Region: chr15 55049948-55049978. Max. coverage (+): 0. Max coverage (-): 0

Region: chr15 55049979-55050009. Max. coverage (+): 0. Max coverage (-): 0

Region: chr15 55050010-55050040. Max. coverage (+): 0. Max coverage (-): 0

Region: chr15 55050041-55050071. Max. coverage (+): 0. Max coverage (-): 0

Region: chr15 55050072-55050102. Max. coverage (+): 0. Max coverage (-): 0

Region: chr15 55050103-55050133. Max. coverage (+): 0. Max coverage (-): 0

Region: chr15 55050134-55050164. Max. coverage (+): 0. Max coverage (-): 0

Region: chr15 55050165-55050196. Max. coverage (+): 0. Max coverage (-): 0

Region: chr15 55050197-55050227. Max. coverage (+): 0. Max coverage (-): 0

Region: chr15 55050228-55050258. Max. coverage (+): 0. Max coverage (-): 0

Region: chr15 55050259-55050289. Max. coverage (+): 0. Max coverage (-): 0

Region: chr15 55050290-55050320. Max. coverage (+): 0. Max coverage (-): 0

Region: chr15 55050321-55050351. Max. coverage (+): 0. Max coverage (-): 0

Region: chr15 55050352-55050382. Max. coverage (+): 0. Max coverage (-): 0

Region: chr15 55050383-55050413. Max. coverage (+): 0. Max coverage (-): 0

Region: chr15 55050414-55050444. Max. coverage (+): 0. Max coverage (-): 0

Region: chr15 55050445-55050475. Max. coverage (+): 0. Max coverage (-): 0

Region: chr15 55050476-55050506. Max. coverage (+): 0. Max coverage (-): 0

Region: chr15 55050507-55050537. Max. coverage (+): 0. Max coverage (-): 0

Region: chr15 55050538-55050568. Max. coverage (+): 0. Max coverage (-): 0

Region: chr15 55050569-55050599. Max. coverage (+): 0. Max coverage (-): 0

Region: chr15 55050600-55050630. Max. coverage (+): 0. Max coverage (-): 0

Region: chr15 55050631-55050661. Max. coverage (+): 0. Max coverage (-): 0

Region: chr15 55050662-55050692. Max. coverage (+): 0. Max coverage (-): 0

Region: chr15 55050693-55050723. Max. coverage (+): 4.98. Max coverage (-): 0

Region: chr15 55050724-55050754. Max. coverage (+): 2.63. Max coverage (-): 0

Region: chr15 55050755-55050785. Max. coverage (+): 0. Max coverage (-): 0

Region: chr15 55050786-55050816. Max. coverage (+): 0. Max coverage (-): 0

Region: chr15 55050817-55050847. Max. coverage (+): 0. Max coverage (-): 0

Region: chr15 55050848-55050878. Max. coverage (+): 1.95. Max coverage (-): 0

Region: chr15 55050879-55050909. Max. coverage (+): 0. Max coverage (-): 0

Region: chr15 55050910-55050940. Max. coverage (+): 0. Max coverage (-): 0

Region: chr15 55050941-55050971. Max. coverage (+): 0. Max coverage (-): 0

Region: chr15 55050972-55051002. Max. coverage (+): 0. Max coverage (-): 0

Region: chr15 55051003-55051034. Max. coverage (+): 0. Max coverage (-): 0

Region: chr15 55051035-55051065. Max. coverage (+): 0. Max coverage (-): 0

Region: chr15 55051066-55051096. Max. coverage (+): 0. Max coverage (-): 0

Region: chr15 55051097-55051127. Max. coverage (+): 0. Max coverage (-): 0

Region: chr15 55051128-55051158. Max. coverage (+): 0. Max coverage (-): 0

Region: chr15 55051159-55051189. Max. coverage (+): 4.14. Max coverage (-): 0

Region: chr15 55051190-55051220. Max. coverage (+): 0.73. Max coverage (-): 0

Region: chr15 55051221-55051251. Max. coverage (+): 6.4. Max coverage (-): 0

Region: chr15 55051252-55051282. Max. coverage (+): 0. Max coverage (-): 0

Region: chr15 55051283-55051313. Max. coverage (+): 9.07. Max coverage (-): 0

Region: chr15 55051314-55051344. Max. coverage (+): 15.37. Max coverage (-): 0

Region: chr15 55051345-55051375. Max. coverage (+): 0. Max coverage (-): 0

Region: chr15 55051376-55051406. Max. coverage (+): 0. Max coverage (-): 0

Region: chr15 55051407-55051437. Max. coverage (+): 1.7. Max coverage (-): 0

Region: chr15 55051438-55051468. Max. coverage (+): 0. Max coverage (-): 0

Region: chr15 55051469-55051499. Max. coverage (+): 21.08. Max coverage (-): 0

Region: chr15 55051500-55051530. Max. coverage (+): 9.83. Max coverage (-): 0

Region: chr15 55051531-55051561. Max. coverage (+): 1.05. Max coverage (-): 0

Region: chr15 55051562-55051592. Max. coverage (+): 4.15. Max coverage (-): 0

Region: chr15 55051593-55051623. Max. coverage (+): 4.15. Max coverage (-): 0

Region: chr15 55051624-55051654. Max. coverage (+): 0. Max coverage (-): 0

Region: chr15 55051655-55051685. Max. coverage (+): 0. Max coverage (-): 0

Region: chr15 55051686-55051716. Max. coverage (+): 0. Max coverage (-): 0

Region: chr15 55051717-55051747. Max. coverage (+): 0. Max coverage (-): 0

Region: chr15 55051748-55051778. Max. coverage (+): 0. Max coverage (-): 0

Region: chr15 55051779-55051809. Max. coverage (+): 0. Max coverage (-): 0

Region: chr15 55051810-55051840. Max. coverage (+): 0. Max coverage (-): 0

Region: chr15 55051841-55051871. Max. coverage (+): 0. Max coverage (-): 0

Region: chr15 55051872-55051903. Max. coverage (+): 0. Max coverage (-): 0

Region: chr15 55051904-55051934. Max. coverage (+): 0. Max coverage (-): 0

Region: chr15 55051935-55051965. Max. coverage (+): 0. Max coverage (-): 0

Region: chr15 55051966-55051996. Max. coverage (+): 0. Max coverage (-): 0

Region: chr15 55051997-55052027. Max. coverage (+): 0. Max coverage (-): 0

Region: chr15 55052028-55052058. Max. coverage (+): 4.4. Max coverage (-): 0

Region: chr15 55052059-55052089. Max. coverage (+): 7.93. Max coverage (-): 0

Region: chr15 55052090-55052120. Max. coverage (+): 0. Max coverage (-): 0

Region: chr15 55052121-55052151. Max. coverage (+): 20.28. Max coverage (-): 0

Region: chr15 55052152-55052182. Max. coverage (+): 11.67. Max coverage (-): 0

Region: chr15 55052183-55052213. Max. coverage (+): 4.32. Max coverage (-): 0

Region: chr15 55052214-55052244. Max. coverage (+): 5.31. Max coverage (-): 0

Region: chr15 55052245-55052275. Max. coverage (+): 0. Max coverage (-): 0

Region: chr15 55052276-55052306. Max. coverage (+): 0. Max coverage (-): 0

Region: chr15 55052307-55052337. Max. coverage (+): 0. Max coverage (-): 0

Region: chr15 55052338-55052368. Max. coverage (+): 4.37. Max coverage (-): 0

Region: chr15 55052369-55052399. Max. coverage (+): 0. Max coverage (-): 0

Region: chr15 55052400-55052430. Max. coverage (+): 0. Max coverage (-): 0

Region: chr15 55052431-55052461. Max. coverage (+): 14.46. Max coverage (-): 0

Region: chr15 55052462-55052492. Max. coverage (+): 0. Max coverage (-): 0

Region: chr15 55052493-55052523. Max. coverage (+): 15.83. Max coverage (-): 0

Region: chr15 55052524-55052554. Max. coverage (+): 15.83. Max coverage (-): 0

Region: chr15 55052555-55052585. Max. coverage (+): 6.91. Max coverage (-): 0

Region: chr15 55052586-55052616. Max. coverage (+): 9.09. Max coverage (-): 0

Region: chr15 55052617-55052647. Max. coverage (+): 14.28. Max coverage (-): 0

Region: chr15 55052648-55052678. Max. coverage (+): 5.54. Max coverage (-): 0

Region: chr15 55052679-55052709. Max. coverage (+): 0. Max coverage (-): 0

Region: chr15 55052710-55052740. Max. coverage (+): 0. Max coverage (-): 0

Region: chr15 55052741-55052772. Max. coverage (+): 0. Max coverage (-): 0

Region: chr15 55052773-55052803. Max. coverage (+): 0. Max coverage (-): 0

Region: chr15 55052804-55052834. Max. coverage (+): 4.72. Max coverage (-): 0

Region: chr15 55052835-55052865. Max. coverage (+): 0. Max coverage (-): 0

Region: chr15 55052866-55052896. Max. coverage (+): 9.99. Max coverage (-): 0

Region: chr15 55052897-55052927. Max. coverage (+): 9.99. Max coverage (-): 0

Region: chr15 55052928-55052958. Max. coverage (+): 23.12. Max coverage (-): 0

Region: chr15 55052959-55052989. Max. coverage (+): 0. Max coverage (-): 0

Region: chr15 55052990-55053020. Max. coverage (+): 9.1. Max coverage (-): 0

Region: chr15 55053021-55053051. Max. coverage (+): 8.64. Max coverage (-): 0

Region: chr15 55053052-55053082. Max. coverage (+): 0. Max coverage (-): 0

Region: chr15 55053083-55053113. Max. coverage (+): 0. Max coverage (-): 0

Region: chr15 55053114-55053144. Max. coverage (+): 0. Max coverage (-): 0

Region: chr15 55053145-55053175. Max. coverage (+): 10.39. Max coverage (-): 0

Region: chr15 55053176-55053206. Max. coverage (+): 3.72. Max coverage (-): 0

Region: chr15 55053207-55053237. Max. coverage (+): 17.59. Max coverage (-): 0

Region: chr15 55053238-55053268. Max. coverage (+): 0. Max coverage (-): 0

Region: chr15 55053269-55053299. Max. coverage (+): 0. Max coverage (-): 0

Region: chr15 55053300-55053330. Max. coverage (+): 0. Max coverage (-): 0

Region: chr15 55053331-55053361. Max. coverage (+): 0. Max coverage (-): 0

Region: chr15 55053362-55053392. Max. coverage (+): 0. Max coverage (-): 0

Region: chr15 55053393-55053423. Max. coverage (+): 0. Max coverage (-): 0

Region: chr15 55053424-55053454. Max. coverage (+): 0. Max coverage (-): 0

Region: chr15 55053455-55053485. Max. coverage (+): 3.87. Max coverage (-): 0

Region: chr15 55053486-55053516. Max. coverage (+): 0. Max coverage (-): 0

Region: chr15 55053517-55053547. Max. coverage (+): 2.27. Max coverage (-): 0

Region: chr15 55053548-55053578. Max. coverage (+): 7.61. Max coverage (-): 0

Region: chr15 55053579-55053609. Max. coverage (+): 0. Max coverage (-): 0

Region: chr15 55053610-55053641. Max. coverage (+): 14.4. Max coverage (-): 0

Region: chr15 55053642-55053672. Max. coverage (+): 0. Max coverage (-): 0

Region: chr15 55053673-55053703. Max. coverage (+): 0. Max coverage (-): 0

Region: chr15 55053704-55053734. Max. coverage (+): 0. Max coverage (-): 0

Region: chr15 55053735-55053765. Max. coverage (+): 0. Max coverage (-): 0

Region: chr15 55053766-55053796. Max. coverage (+): 0. Max coverage (-): 0

Region: chr15 55053797-55053827. Max. coverage (+): 0. Max coverage (-): 0

Region: chr15 55053828-55053858. Max. coverage (+): 0. Max coverage (-): 0

Region: chr15 55053859-55053889. Max. coverage (+): 0. Max coverage (-): 0

Region: chr15 55053890-55053920. Max. coverage (+): 0. Max coverage (-): 0

Region: chr15 55053921-55053951. Max. coverage (+): 0. Max coverage (-): 0

Region: chr15 55053952-55053982. Max. coverage (+): 0. Max coverage (-): 0

Region: chr15 55053983-55054013. Max. coverage (+): 0. Max coverage (-): 0

Region: chr15 55054014-55054044. Max. coverage (+): 0. Max coverage (-): 0

Region: chr15 55054045-55054075. Max. coverage (+): 7.38. Max coverage (-): 0

Region: chr15 55054076-55054106. Max. coverage (+): 0. Max coverage (-): 0

Region: chr15 55054107-55054137. Max. coverage (+): 0.27. Max coverage (-): 0

Region: chr15 55054138-55054168. Max. coverage (+): 4.13. Max coverage (-): 0

Region: chr15 55054169-55054199. Max. coverage (+): 4.13. Max coverage (-): 0

Region: chr15 55054200-55054230. Max. coverage (+): 5.95. Max coverage (-): 0

Region: chr15 55054231-55054261. Max. coverage (+): 0. Max coverage (-): 0

Region: chr15 55054262-55054292. Max. coverage (+): 0. Max coverage (-): 0

Region: chr15 55054293-55054323. Max. coverage (+): 0. Max coverage (-): 0

Region: chr15 55054324-55054354. Max. coverage (+): 0. Max coverage (-): 0

Region: chr15 55054355-55054385. Max. coverage (+): 3.83. Max coverage (-): 0

Region: chr15 55054386-55054416. Max. coverage (+): 0. Max coverage (-): 0

Region: chr15 55054417-55054447. Max. coverage (+): 0. Max coverage (-): 0

Region: chr15 55054448-55054478. Max. coverage (+): 6.78. Max coverage (-): 0

Region: chr15 55054479-55054510. Max. coverage (+): 0. Max coverage (-): 0

Region: chr15 55054511-55054541. Max. coverage (+): 0. Max coverage (-): 0

Region: chr15 55054542-55054572. Max. coverage (+): 0. Max coverage (-): 0

Region: chr15 55054573-55054603. Max. coverage (+): 0. Max coverage (-): 0

Region: chr15 55054604-55054634. Max. coverage (+): 0. Max coverage (-): 0

Region: chr15 55054635-55054665. Max. coverage (+): 0. Max coverage (-): 0

Region: chr15 55054666-55054696. Max. coverage (+): 4.17. Max coverage (-): 0

Region: chr15 55054697-55054727. Max. coverage (+): 3.71. Max coverage (-): 0

Region: chr15 55054728-55054758. Max. coverage (+): 0. Max coverage (-): 0

Region: chr15 55054759-55054789. Max. coverage (+): 0. Max coverage (-): 0

Region: chr15 55054790-55054820. Max. coverage (+): 0. Max coverage (-): 0

Region: chr15 55054821-55054851. Max. coverage (+): 0. Max coverage (-): 0

Region: chr15 55054852-55054882. Max. coverage (+): 0. Max coverage (-): 0

Region: chr15 55054883-55054913. Max. coverage (+): 0. Max coverage (-): 0

Region: chr15 55054914-55054944. Max. coverage (+): 0. Max coverage (-): 0

Region: chr15 55054945-55054975. Max. coverage (+): 0. Max coverage (-): 0

Region: chr15 55054976-55055006. Max. coverage (+): 0. Max coverage (-): 0

Region: chr15 55055007-55055037. Max. coverage (+): 0. Max coverage (-): 0

Region: chr15 55055038-55055068. Max. coverage (+): 0. Max coverage (-): 0

Region: chr15 55055069-55055099. Max. coverage (+): 0. Max coverage (-): 0

Region: chr15 55055100-55055130. Max. coverage (+): 0. Max coverage (-): 0

Region: chr15 55055131-55055161. Max. coverage (+): 0. Max coverage (-): 0

Region: chr15 55055162-55055192. Max. coverage (+): 0. Max coverage (-): 0

Region: chr15 55055193-55055223. Max. coverage (+): 12.27. Max coverage (-): 0

Region: chr15 55055224-55055254. Max. coverage (+): 6.12. Max coverage (-): 0

Region: chr15 55055255-55055285. Max. coverage (+): 11.43. Max coverage (-): 0

Region: chr15 55055286-55055316. Max. coverage (+): 0. Max coverage (-): 0

Region: chr15 55055317-55055348. Max. coverage (+): 0. Max coverage (-): 0

Region: chr15 55055349-55055379. Max. coverage (+): 0. Max coverage (-): 0

Region: chr15 55055380-55055410. Max. coverage (+): 0. Max coverage (-): 0

Region: chr15 55055411-55055441. Max. coverage (+): 0. Max coverage (-): 0

Region: chr15 55055442-55055472. Max. coverage (+): 0. Max coverage (-): 0

Region: chr15 55055473-55055503. Max. coverage (+): 0. Max coverage (-): 0

Region: chr15 55055504-55055534. Max. coverage (+): 2.93. Max coverage (-): 0

Region: chr15 55055535-55055565. Max. coverage (+): 2.93. Max coverage (-): 0

Region: chr15 55055566-55055596. Max. coverage (+): 0. Max coverage (-): 0

Region: chr15 55055597-55055627. Max. coverage (+): 0. Max coverage (-): 0

Region: chr15 55055628-55055658. Max. coverage (+): 0. Max coverage (-): 0

Region: chr15 55055659-55055689. Max. coverage (+): 0. Max coverage (-): 0

Region: chr15 55055690-55055720. Max. coverage (+): 0. Max coverage (-): 0

Region: chr15 55055721-55055751. Max. coverage (+): 0. Max coverage (-): 0

Region: chr15 55055752-55055782. Max. coverage (+): 0. Max coverage (-): 0

Region: chr15 55055783-55055813. Max. coverage (+): 0. Max coverage (-): 0

Region: chr15 55055814-55055844. Max. coverage (+): 2.83. Max coverage (-): 0

Region: chr15 55055845-55055875. Max. coverage (+): 2.83. Max coverage (-): 0

Region: chr15 55055876-55055906. Max. coverage (+): 0. Max coverage (-): 0

Region: chr15 55055907-55055937. Max. coverage (+): 0. Max coverage (-): 0

Region: chr15 55055938-55055968. Max. coverage (+): 0. Max coverage (-): 0

Region: chr15 55055969-55055999. Max. coverage (+): 0. Max coverage (-): 0

Region: chr15 55056000-55056030. Max. coverage (+): 0. Max coverage (-): 0

Region: chr15 55056031-55056061. Max. coverage (+): 0. Max coverage (-): 0

Region: chr15 55056062-55056092. Max. coverage (+): 0. Max coverage (-): 0

Region: chr15 55056093-55056123. Max. coverage (+): 0. Max coverage (-): 0

Region: chr15 55056124-55056154. Max. coverage (+): 0. Max coverage (-): 0

Region: chr15 55056155-55056185. Max. coverage (+): 0. Max coverage (-): 0

Region: chr15 55056186-55056217. Max. coverage (+): 0. Max coverage (-): 0

Region: chr15 55056218-55056248. Max. coverage (+): 0. Max coverage (-): 0

Region: chr15 55056249-55056279. Max. coverage (+): 0. Max coverage (-): 0

Region: chr15 55056280-55056310. Max. coverage (+): 0. Max coverage (-): 0

Region: chr15 55056311-55056341. Max. coverage (+): 0. Max coverage (-): 0

Region: chr15 55056342-55056372. Max. coverage (+): 0. Max coverage (-): 0

Region: chr15 55056373-55056403. Max. coverage (+): 0. Max coverage (-): 0

Region: chr15 55056404-55056434. Max. coverage (+): 0. Max coverage (-): 0

Region: chr15 55056435-55056465. Max. coverage (+): 0. Max coverage (-): 0

Region: chr15 55056466-55056496. Max. coverage (+): 0. Max coverage (-): 0

Region: chr15 55056497-55056527. Max. coverage (+): 0. Max coverage (-): 0

Region: chr15 55056528-55056558. Max. coverage (+): 0. Max coverage (-): 0

Region: chr15 55056559-55056589. Max. coverage (+): 0. Max coverage (-): 0

Region: chr15 55056590-55056620. Max. coverage (+): 7.17. Max coverage (-): 0

Region: chr15 55056621-. Max. coverage (+): 0. Max coverage (-): 0

RepeatMasker Color Code

**+**

100-98% Identity

<98-95% Identity

<95-90% Identity

<90-85% Identity

<85-80% Identity

<80-75% Identity

<75-70% Identity

<70% Identity

**-**

Gene Set Color Code

**+**

Gene

Pseudogene

**-**

Topology/Coverage Color Code

Coverage Plus Strand

Coverage Minus Strand

Mainstrand: Plus

Mainstrand: Minus

Complementary Strand

Flanking Region  
(if option -flank >0)

Gene Set Annotation  

**1. NEU3 (protein coding, ENSBTAG00000025931) Tr:00000036738 Ex:3**: 55041084-55043623 (+)

  
RepeatMasker Annotation  

**1. MIR3**: 55042356-55042515 (+), Divergence to consensus: 32.8%  
**2. L2c**: 55043020-55043063 (-), Divergence to consensus: 26.9%  
**3. Bov-tA2**: 55045183-55045390 (-), Divergence to consensus: 16.4%  
**4. L2c**: 55045741-55045807 (+), Divergence to consensus: 35.8%  
**5. L2b**: 55045845-55045957 (+), Divergence to consensus: 28.4%  
**6. MamRep1151**: 55047220-55047295 (-), Divergence to consensus: 30.3%  
**7. AT\_rich**: 55047718-55047739 (+), Divergence to consensus: 40.9%  
**8. MIRc**: 55047941-55048125 (+), Divergence to consensus: 39.7%  
**9. MIRc**: 55049215-55049312 (-), Divergence to consensus: 44.9%  
**10. BovB**: 55049446-55050184 (+), Divergence to consensus: 10.9%  
**11. ART2A**: 55050186-55050718 (+), Divergence to consensus: 15.3%  
**12. L2c**: 55051053-55051151 (+), Divergence to consensus: 34.3%  
**13. LTR18C\_BT**: 55051688-55051913 (+), Divergence to consensus: 6.2%  
**14. ERVL-B4-int**: 55053707-55054045 (+), Divergence to consensus: 34.8%  
**15. ART2A**: 55054746-55055229 (+), Divergence to consensus: 17%  
**16. LTR18C\_BT**: 55056019-55056290 (+), Divergence to consensus: 6.6%  
**17. MER91A**: 55056428-55056485 (+), Divergence to consensus: 34.5%

  
Transcription Factor Binding Sites  

**SPZ1** (Sequence: CTGAAACCCT (-): 55053629)  
**SOX9** (Sequence: AACAATGG (-): 55047212)  
**SOX9** (Sequence: CCATTGTT (+): 55048183)  
**Gata4** (Sequence: CTTATCT (+): 55042575)  
**Gata4** (Sequence: CTTATCT (+): 55045617)
