# Supplementary material for: Small RNA sequencing of cryopreserved semen from single bull revealed altered miRNAs and piRNAs expression between High- and Low-motile sperm populations
Source: BMC Genomics. 2017 Jan 4;18:14. doi: 10.1186/s12864-016-3394-7 (PMC5209821; doi:10.1186/s12864-016-3394-7)
Supplement: Additional file 4: — Details for each piRNA clusters found in Low Motile (LM) sperm fraction. Genes, repeats, transposable elements and transcription factors binding sites falling within the cluster regions were reported. (ZIP 1034 kb) [file 12864_2016_3394_MOESM4_ESM.zip › 16.html]

piRNA cluster 16


Predicted piRNA cluster no. 16     previous   next
  

Show proTRAC run info
Hide proTRAC run info

================================= proTRAC ====================================  
VERSION: 2.1                                    LAST MODIFIED: 06. October 2015  
  
Please cite:  
Rosenkranz D, Zischler H. proTRAC - a software for probabilistic piRNA cluster  
detection, visualization and analysis. 2012. BMC Bioinformatics 13:5.  
  
and (for proTRAC 2.0 and later):  
Rosenkranz D, Rudloff S, Bastuck K, Ketting RF, Zischler H. Tupaia small RNAs  
provide insights into function and evolution of RNAi-based transposon defense  
in mammals. 2015. RNA 21(5):911-922.  
  
Contact:  
David Rosenkranz  
Institute of Anthropology, small RNA group  
Johannes Gutenberg University Mainz  
email: rosenkranz@uni-mainz.de  
  
You can find the latest proTRAC version at:  
http://sourceforge.net/projects/protrac/files  
http://www.smallRNAgroup-mainz.de/software  
==============================================================================  
  
PARAMETERS:  
Map file: .............../storage/core/barbara/genhome/smallRNA/fertility/Sample\_not\_motile/pirna/Sample\_not\_motile\_26-33\_collapsed.fa.no-dust.map.weighted-10000-1000-b-0  
Genome file: ............/storage/core/barbara/genhome/smallRNA/fertility/Sample\_all/pirna/bt\_311\_chrY.fa  
RepeatMasker annotation: /storage/genomes/bt\_umd31/GCF\_000003055.6\_Bos\_taurus\_UMD\_3.1.1\_repeatMasker\_chr.out  
GeneSet:................./storage/core/barbara/genhome/smallRNA/fertility/Sample\_all/pirna/full.gtf  
  
Significant (p<=0.01) hit density will be calculated based  
on observed hit distribution.  
  
Sliding window size: ........................................ 5000 bp  
Sliding window increament: .................................. 1000 bp  
Normalize each hit by number of genomic hits: ............... 1 [0=no/1=yes]  
Normalize each hit by number of sequence reads: ............. 1 [0=no/1=yes]  
Normalize values (-> per million mapped reads): ............. 1 [0=no/1=yes]  
Min. fraction of hits with 1T(U) or 10A: .................... 0.75  
Alternatively: Min. fraction of hits with 1T(U) and 10A: .... 0.5  
Min. fraction of hits with typical piRNA length: ............ 0.75  
Typical piRNA length: ....................................... 26-33 nt  
Min. size of a piRNA cluster: ............................... 5000 bp.  
Min. number of hits (absolute): ............................. 0  
Min. number of hits (normalized): ........................... 0  
Min. fraction of hits on the mainstrand: .................... 0.75  
Top fraction of mapped sequences (in terms of read counts): . 1%  
Top fraction accounts for max. n% of sequence reads: ........ 90%  
Min. fraction of hits on each arm of a bidirectional cluster: 0.1  
Output image file for each cluster: ......................... 0 [0=no/1=yes]  
Output html file for each cluster: .......................... 1 [0=no/1=yes]  
Output a summary table: ..................................... 1 [0=no/1=yes]  
Output a FASTA file for each cluster (piRNA sequences): ..... 1 [0=no/1=yes]  
Output a FASTA file comprising cluster sequences: ........... 1 [0=no/1=yes]  
Search DNA motifs in clusters: .............................. 1 [0=no/1=yes]  
Output flanking sequences: +/- .............................. 0 bp  
Output ~.pTi file: .......................................... 1 [0=no/1=yes]  
==============================================================================  
  
  
Genome size (without gaps): ............ 2678902517 bp  
Gaps (N/X/-): .......................... 53837044 bp  
Mapped reads: .......................... 738059667487  
Non-identical sequences: ............... 277001  
Genomic hits: .......................... 533816  
Significant densitiy of mapped reads: .. 15118061 reads/kb

Show proTRAC cluster info
Hide proTRAC cluster info

|  |  |
| --- | --- |
| Location | chr15 |
| Coordinates | 76597133-76654524 |
| Size [bp] | 57392 |
| Sequence hit loci | 4919 |
| Mapped reads (normalized) | 13045788432 |
| Mapped reads (normalized) per kb | 227310225 |
| Normalized reads with 1T (1U) | 83.7% |
| Normalized reads with 10A | 31.9% |
| Normalized reads with length 26-33 nt | 100% |
| Normalized reads on the main strand(s) | 97.4% |
| Predicted directionality | mono:minus |

100%

0%

1T (1U)  
reads

10A reads

26-33 nt  
reads

reads on mainstrand

**Either the amount of reads with 1T (1U) OR 10A has to exceed 75% (set with option: -1Tor10A)  
Alternatively the amount of reads with 1T (1U) AND 10A has to exceed 50% (set with option: -1Tand10A)  
Minimum amount of reads with preferred size is 75% (set with option: -pisize)  
Minimum amount of reads on the main strand(s) is 75% (set with option: -clstrand)**

Show read coverage
Hide read coverage

WHAT DO I SEE HERE?  
This chart shows the location of mapped sequence reads within a predicted piRNA cluster. The color refers to the number of genomic hits produced by the sequence read in question. A dark red bar indicates that this sequence read produces many other hits elsewhere in the genome. Many adjacent red or yellow bars can indicate the presence of a multi-copy element such as transposons or rRNA genes. A dark green bar indicates that this sequence read maps uniquely to this locus.

1 hit

2-5 hits

6-10 hits

11-20 hits

21-50 hits

51-100 hits

> 100 hits

chr15

76597133

76654524

Gene Set

RepeatMasker

Mapped  
Reads

157.34

plus strand

minus strand

157.34

Region: chr15 55056622-76597190. Max. coverage (+): 0. Max coverage (-): 4.35

Region: chr15 76597191-76597305. Max. coverage (+): 0. Max coverage (-): 0

Region: chr15 76597306-76597419. Max. coverage (+): 0. Max coverage (-): 0

Region: chr15 76597420-76597534. Max. coverage (+): 0. Max coverage (-): 6.06

Region: chr15 76597535-76597649. Max. coverage (+): 0. Max coverage (-): 13.23

Region: chr15 76597650-76597764. Max. coverage (+): 0. Max coverage (-): 11.34

Region: chr15 76597765-76597879. Max. coverage (+): 0. Max coverage (-): 0

Region: chr15 76597880-76597993. Max. coverage (+): 0. Max coverage (-): 5.82

Region: chr15 76597994-76598108. Max. coverage (+): 0. Max coverage (-): 5.82

Region: chr15 76598109-76598223. Max. coverage (+): 0. Max coverage (-): 6.7

Region: chr15 76598224-76598338. Max. coverage (+): 0. Max coverage (-): 0

Region: chr15 76598339-76598453. Max. coverage (+): 0. Max coverage (-): 0

Region: chr15 76598454-76598567. Max. coverage (+): 0. Max coverage (-): 1.13

Region: chr15 76598568-76598682. Max. coverage (+): 0. Max coverage (-): 4.31

Region: chr15 76598683-76598797. Max. coverage (+): 0. Max coverage (-): 0

Region: chr15 76598798-76598912. Max. coverage (+): 0. Max coverage (-): 5.63

Region: chr15 76598913-76599026. Max. coverage (+): 0. Max coverage (-): 0

Region: chr15 76599027-76599141. Max. coverage (+): 0. Max coverage (-): 0

Region: chr15 76599142-76599256. Max. coverage (+): 0. Max coverage (-): 7.63

Region: chr15 76599257-76599371. Max. coverage (+): 0. Max coverage (-): 121.83

Region: chr15 76599372-76599486. Max. coverage (+): 0. Max coverage (-): 0

Region: chr15 76599487-76599600. Max. coverage (+): 0. Max coverage (-): 0

Region: chr15 76599601-76599715. Max. coverage (+): 0. Max coverage (-): 0

Region: chr15 76599716-76599830. Max. coverage (+): 0. Max coverage (-): 4.26

Region: chr15 76599831-76599945. Max. coverage (+): 0. Max coverage (-): 0

Region: chr15 76599946-76600059. Max. coverage (+): 0. Max coverage (-): 4.76

Region: chr15 76600060-76600174. Max. coverage (+): 0. Max coverage (-): 0

Region: chr15 76600175-76600289. Max. coverage (+): 0. Max coverage (-): 0

Region: chr15 76600290-76600404. Max. coverage (+): 0. Max coverage (-): 0

Region: chr15 76600405-76600519. Max. coverage (+): 0. Max coverage (-): 18.64

Region: chr15 76600520-76600633. Max. coverage (+): 0. Max coverage (-): 4.43

Region: chr15 76600634-76600748. Max. coverage (+): 0. Max coverage (-): 0

Region: chr15 76600749-76600863. Max. coverage (+): 0. Max coverage (-): 36.54

Region: chr15 76600864-76600978. Max. coverage (+): 0. Max coverage (-): 21.52

Region: chr15 76600979-76601093. Max. coverage (+): 0. Max coverage (-): 39.8

Region: chr15 76601094-76601207. Max. coverage (+): 0. Max coverage (-): 4.74

Region: chr15 76601208-76601322. Max. coverage (+): 0. Max coverage (-): 3.62

Region: chr15 76601323-76601437. Max. coverage (+): 0. Max coverage (-): 2.79

Region: chr15 76601438-76601552. Max. coverage (+): 0. Max coverage (-): 8.33

Region: chr15 76601553-76601666. Max. coverage (+): 0. Max coverage (-): 12.83

Region: chr15 76601667-76601781. Max. coverage (+): 0. Max coverage (-): 4.21

Region: chr15 76601782-76601896. Max. coverage (+): 0. Max coverage (-): 10.88

Region: chr15 76601897-76602011. Max. coverage (+): 0. Max coverage (-): 71.96

Region: chr15 76602012-76602126. Max. coverage (+): 0. Max coverage (-): 54.74

Region: chr15 76602127-76602240. Max. coverage (+): 0. Max coverage (-): 0

Region: chr15 76602241-76602355. Max. coverage (+): 0. Max coverage (-): 7.13

Region: chr15 76602356-76602470. Max. coverage (+): 0. Max coverage (-): 20.11

Region: chr15 76602471-76602585. Max. coverage (+): 0. Max coverage (-): 20.68

Region: chr15 76602586-76602700. Max. coverage (+): 0. Max coverage (-): 11.8

Region: chr15 76602701-76602814. Max. coverage (+): 0. Max coverage (-): 21.95

Region: chr15 76602815-76602929. Max. coverage (+): 0. Max coverage (-): 30.4

Region: chr15 76602930-76603044. Max. coverage (+): 0. Max coverage (-): 68.61

Region: chr15 76603045-76603159. Max. coverage (+): 0. Max coverage (-): 30.4

Region: chr15 76603160-76603273. Max. coverage (+): 0. Max coverage (-): 0

Region: chr15 76603274-76603388. Max. coverage (+): 0. Max coverage (-): 0

Region: chr15 76603389-76603503. Max. coverage (+): 0. Max coverage (-): 16.83

Region: chr15 76603504-76603618. Max. coverage (+): 0. Max coverage (-): 14.4

Region: chr15 76603619-76603733. Max. coverage (+): 0. Max coverage (-): 59.4

Region: chr15 76603734-76603847. Max. coverage (+): 0. Max coverage (-): 1.58

Region: chr15 76603848-76603962. Max. coverage (+): 0. Max coverage (-): 2.99

Region: chr15 76603963-76604077. Max. coverage (+): 0. Max coverage (-): 0

Region: chr15 76604078-76604192. Max. coverage (+): 0. Max coverage (-): 43.59

Region: chr15 76604193-76604306. Max. coverage (+): 0. Max coverage (-): 5.64

Region: chr15 76604307-76604421. Max. coverage (+): 0. Max coverage (-): 6.87

Region: chr15 76604422-76604536. Max. coverage (+): 0. Max coverage (-): 36.47

Region: chr15 76604537-76604651. Max. coverage (+): 0. Max coverage (-): 0

Region: chr15 76604652-76604766. Max. coverage (+): 0. Max coverage (-): 0

Region: chr15 76604767-76604880. Max. coverage (+): 0. Max coverage (-): 33

Region: chr15 76604881-76604995. Max. coverage (+): 0. Max coverage (-): 37.98

Region: chr15 76604996-76605110. Max. coverage (+): 0. Max coverage (-): 78.42

Region: chr15 76605111-76605225. Max. coverage (+): 0. Max coverage (-): 86.41

Region: chr15 76605226-76605340. Max. coverage (+): 0. Max coverage (-): 5.5

Region: chr15 76605341-76605454. Max. coverage (+): 0. Max coverage (-): 4.67

Region: chr15 76605455-76605569. Max. coverage (+): 0. Max coverage (-): 8.03

Region: chr15 76605570-76605684. Max. coverage (+): 0. Max coverage (-): 0

Region: chr15 76605685-76605799. Max. coverage (+): 0. Max coverage (-): 28.22

Region: chr15 76605800-76605913. Max. coverage (+): 0. Max coverage (-): 0

Region: chr15 76605914-76606028. Max. coverage (+): 0. Max coverage (-): 8.31

Region: chr15 76606029-76606143. Max. coverage (+): 0. Max coverage (-): 47.62

Region: chr15 76606144-76606258. Max. coverage (+): 0. Max coverage (-): 0

Region: chr15 76606259-76606373. Max. coverage (+): 0. Max coverage (-): 0

Region: chr15 76606374-76606487. Max. coverage (+): 0. Max coverage (-): 11.8

Region: chr15 76606488-76606602. Max. coverage (+): 0. Max coverage (-): 5.61

Region: chr15 76606603-76606717. Max. coverage (+): 0. Max coverage (-): 32.11

Region: chr15 76606718-76606832. Max. coverage (+): 0. Max coverage (-): 16.2

Region: chr15 76606833-76606947. Max. coverage (+): 0. Max coverage (-): 12.24

Region: chr15 76606948-76607061. Max. coverage (+): 0. Max coverage (-): 13.45

Region: chr15 76607062-76607176. Max. coverage (+): 0. Max coverage (-): 20.85

Region: chr15 76607177-76607291. Max. coverage (+): 0. Max coverage (-): 10.74

Region: chr15 76607292-76607406. Max. coverage (+): 0. Max coverage (-): 22.03

Region: chr15 76607407-76607520. Max. coverage (+): 0. Max coverage (-): 15.87

Region: chr15 76607521-76607635. Max. coverage (+): 0. Max coverage (-): 60.31

Region: chr15 76607636-76607750. Max. coverage (+): 0. Max coverage (-): 36.09

Region: chr15 76607751-76607865. Max. coverage (+): 0. Max coverage (-): 7.98

Region: chr15 76607866-76607980. Max. coverage (+): 0. Max coverage (-): 4.9

Region: chr15 76607981-76608094. Max. coverage (+): 0. Max coverage (-): 15.47

Region: chr15 76608095-76608209. Max. coverage (+): 0. Max coverage (-): 0.78

Region: chr15 76608210-76608324. Max. coverage (+): 4.09. Max coverage (-): 26.61

Region: chr15 76608325-76608439. Max. coverage (+): 0. Max coverage (-): 26.2

Region: chr15 76608440-76608554. Max. coverage (+): 0. Max coverage (-): 54.18

Region: chr15 76608555-76608668. Max. coverage (+): 0. Max coverage (-): 61.24

Region: chr15 76608669-76608783. Max. coverage (+): 0. Max coverage (-): 24.84

Region: chr15 76608784-76608898. Max. coverage (+): 0. Max coverage (-): 1.67

Region: chr15 76608899-76609013. Max. coverage (+): 0. Max coverage (-): 13.9

Region: chr15 76609014-76609127. Max. coverage (+): 0. Max coverage (-): 14.38

Region: chr15 76609128-76609242. Max. coverage (+): 0. Max coverage (-): 27.92

Region: chr15 76609243-76609357. Max. coverage (+): 0. Max coverage (-): 26.3

Region: chr15 76609358-76609472. Max. coverage (+): 0. Max coverage (-): 80.26

Region: chr15 76609473-76609587. Max. coverage (+): 0. Max coverage (-): 21.38

Region: chr15 76609588-76609701. Max. coverage (+): 0. Max coverage (-): 24.15

Region: chr15 76609702-76609816. Max. coverage (+): 0. Max coverage (-): 24.15

Region: chr15 76609817-76609931. Max. coverage (+): 0. Max coverage (-): 25.35

Region: chr15 76609932-76610046. Max. coverage (+): 0. Max coverage (-): 38.22

Region: chr15 76610047-76610160. Max. coverage (+): 0. Max coverage (-): 22.13

Region: chr15 76610161-76610275. Max. coverage (+): 0. Max coverage (-): 45.36

Region: chr15 76610276-76610390. Max. coverage (+): 0. Max coverage (-): 0

Region: chr15 76610391-76610505. Max. coverage (+): 0. Max coverage (-): 19.15

Region: chr15 76610506-76610620. Max. coverage (+): 0. Max coverage (-): 25.65

Region: chr15 76610621-76610734. Max. coverage (+): 0. Max coverage (-): 0

Region: chr15 76610735-76610849. Max. coverage (+): 0. Max coverage (-): 14.54

Region: chr15 76610850-76610964. Max. coverage (+): 0. Max coverage (-): 2.93

Region: chr15 76610965-76611079. Max. coverage (+): 0. Max coverage (-): 9.77

Region: chr15 76611080-76611194. Max. coverage (+): 0. Max coverage (-): 7.11

Region: chr15 76611195-76611308. Max. coverage (+): 0. Max coverage (-): 3.48

Region: chr15 76611309-76611423. Max. coverage (+): 0. Max coverage (-): 12.96

Region: chr15 76611424-76611538. Max. coverage (+): 0. Max coverage (-): 8.26

Region: chr15 76611539-76611653. Max. coverage (+): 0. Max coverage (-): 15.31

Region: chr15 76611654-76611767. Max. coverage (+): 0. Max coverage (-): 21.13

Region: chr15 76611768-76611882. Max. coverage (+): 0. Max coverage (-): 42.33

Region: chr15 76611883-76611997. Max. coverage (+): 0. Max coverage (-): 56.53

Region: chr15 76611998-76612112. Max. coverage (+): 0. Max coverage (-): 74.46

Region: chr15 76612113-76612227. Max. coverage (+): 0. Max coverage (-): 66.69

Region: chr15 76612228-76612341. Max. coverage (+): 0. Max coverage (-): 0

Region: chr15 76612342-76612456. Max. coverage (+): 0. Max coverage (-): 0

Region: chr15 76612457-76612571. Max. coverage (+): 0. Max coverage (-): 0

Region: chr15 76612572-76612686. Max. coverage (+): 0. Max coverage (-): 0

Region: chr15 76612687-76612801. Max. coverage (+): 0. Max coverage (-): 8.87

Region: chr15 76612802-76612915. Max. coverage (+): 0. Max coverage (-): 38.72

Region: chr15 76612916-76613030. Max. coverage (+): 0. Max coverage (-): 0

Region: chr15 76613031-76613145. Max. coverage (+): 0. Max coverage (-): 6.93

Region: chr15 76613146-76613260. Max. coverage (+): 0. Max coverage (-): 102.57

Region: chr15 76613261-76613374. Max. coverage (+): 0. Max coverage (-): 9.89

Region: chr15 76613375-76613489. Max. coverage (+): 0. Max coverage (-): 7.08

Region: chr15 76613490-76613604. Max. coverage (+): 0. Max coverage (-): 40.5

Region: chr15 76613605-76613719. Max. coverage (+): 0. Max coverage (-): 53.83

Region: chr15 76613720-76613834. Max. coverage (+): 0. Max coverage (-): 18.35

Region: chr15 76613835-76613948. Max. coverage (+): 0. Max coverage (-): 38.78

Region: chr15 76613949-76614063. Max. coverage (+): 0. Max coverage (-): 3.1

Region: chr15 76614064-76614178. Max. coverage (+): 0. Max coverage (-): 29.96

Region: chr15 76614179-76614293. Max. coverage (+): 0. Max coverage (-): 16.89

Region: chr15 76614294-76614407. Max. coverage (+): 0. Max coverage (-): 20.97

Region: chr15 76614408-76614522. Max. coverage (+): 0. Max coverage (-): 15.37

Region: chr15 76614523-76614637. Max. coverage (+): 0. Max coverage (-): 19.3

Region: chr15 76614638-76614752. Max. coverage (+): 0. Max coverage (-): 34.59

Region: chr15 76614753-76614867. Max. coverage (+): 0. Max coverage (-): 10.1

Region: chr15 76614868-76614981. Max. coverage (+): 0. Max coverage (-): 17.9

Region: chr15 76614982-76615096. Max. coverage (+): 0. Max coverage (-): 22.8

Region: chr15 76615097-76615211. Max. coverage (+): 0. Max coverage (-): 15.49

Region: chr15 76615212-76615326. Max. coverage (+): 0. Max coverage (-): 13.51

Region: chr15 76615327-76615441. Max. coverage (+): 0. Max coverage (-): 138

Region: chr15 76615442-76615555. Max. coverage (+): 0. Max coverage (-): 21

Region: chr15 76615556-76615670. Max. coverage (+): 0. Max coverage (-): 25.83

Region: chr15 76615671-76615785. Max. coverage (+): 0. Max coverage (-): 64.73

Region: chr15 76615786-76615900. Max. coverage (+): 0. Max coverage (-): 22.24

Region: chr15 76615901-76616014. Max. coverage (+): 0. Max coverage (-): 104.64

Region: chr15 76616015-76616129. Max. coverage (+): 0. Max coverage (-): 100.64

Region: chr15 76616130-76616244. Max. coverage (+): 0. Max coverage (-): 39.87

Region: chr15 76616245-76616359. Max. coverage (+): 0. Max coverage (-): 23.89

Region: chr15 76616360-76616474. Max. coverage (+): 0. Max coverage (-): 33.28

Region: chr15 76616475-76616588. Max. coverage (+): 0. Max coverage (-): 28.74

Region: chr15 76616589-76616703. Max. coverage (+): 0. Max coverage (-): 0

Region: chr15 76616704-76616818. Max. coverage (+): 0. Max coverage (-): 0

Region: chr15 76616819-76616933. Max. coverage (+): 0. Max coverage (-): 0

Region: chr15 76616934-76617048. Max. coverage (+): 0. Max coverage (-): 7.42

Region: chr15 76617049-76617162. Max. coverage (+): 0. Max coverage (-): 0

Region: chr15 76617163-76617277. Max. coverage (+): 0. Max coverage (-): 0

Region: chr15 76617278-76617392. Max. coverage (+): 0. Max coverage (-): 9.38

Region: chr15 76617393-76617507. Max. coverage (+): 0. Max coverage (-): 1.03

Region: chr15 76617508-76617621. Max. coverage (+): 0. Max coverage (-): 24.06

Region: chr15 76617622-76617736. Max. coverage (+): 0. Max coverage (-): 89.91

Region: chr15 76617737-76617851. Max. coverage (+): 0. Max coverage (-): 31.99

Region: chr15 76617852-76617966. Max. coverage (+): 0. Max coverage (-): 42.31

Region: chr15 76617967-76618081. Max. coverage (+): 0. Max coverage (-): 2.63

Region: chr15 76618082-76618195. Max. coverage (+): 0. Max coverage (-): 29.2

Region: chr15 76618196-76618310. Max. coverage (+): 0. Max coverage (-): 32.49

Region: chr15 76618311-76618425. Max. coverage (+): 0. Max coverage (-): 27.37

Region: chr15 76618426-76618540. Max. coverage (+): 0. Max coverage (-): 0

Region: chr15 76618541-76618654. Max. coverage (+): 0. Max coverage (-): 5.78

Region: chr15 76618655-76618769. Max. coverage (+): 0. Max coverage (-): 79.51

Region: chr15 76618770-76618884. Max. coverage (+): 0. Max coverage (-): 0.69

Region: chr15 76618885-76618999. Max. coverage (+): 0. Max coverage (-): 60.24

Region: chr15 76619000-76619114. Max. coverage (+): 0. Max coverage (-): 50.99

Region: chr15 76619115-76619228. Max. coverage (+): 0. Max coverage (-): 25.14

Region: chr15 76619229-76619343. Max. coverage (+): 0. Max coverage (-): 37.24

Region: chr15 76619344-76619458. Max. coverage (+): 0. Max coverage (-): 94.68

Region: chr15 76619459-76619573. Max. coverage (+): 0. Max coverage (-): 34.41

Region: chr15 76619574-76619688. Max. coverage (+): 0. Max coverage (-): 0

Region: chr15 76619689-76619802. Max. coverage (+): 0. Max coverage (-): 0

Region: chr15 76619803-76619917. Max. coverage (+): 0. Max coverage (-): 1.29

Region: chr15 76619918-76620032. Max. coverage (+): 0. Max coverage (-): 27.67

Region: chr15 76620033-76620147. Max. coverage (+): 0. Max coverage (-): 8.11

Region: chr15 76620148-76620261. Max. coverage (+): 0. Max coverage (-): 46.02

Region: chr15 76620262-76620376. Max. coverage (+): 0. Max coverage (-): 11.12

Region: chr15 76620377-76620491. Max. coverage (+): 0. Max coverage (-): 15.73

Region: chr15 76620492-76620606. Max. coverage (+): 0. Max coverage (-): 26.45

Region: chr15 76620607-76620721. Max. coverage (+): 0. Max coverage (-): 31.56

Region: chr15 76620722-76620835. Max. coverage (+): 0. Max coverage (-): 112.37

Region: chr15 76620836-76620950. Max. coverage (+): 0. Max coverage (-): 72.34

Region: chr15 76620951-76621065. Max. coverage (+): 0. Max coverage (-): 0

Region: chr15 76621066-76621180. Max. coverage (+): 0. Max coverage (-): 55.2

Region: chr15 76621181-76621295. Max. coverage (+): 0. Max coverage (-): 5.07

Region: chr15 76621296-76621409. Max. coverage (+): 0. Max coverage (-): 89.26

Region: chr15 76621410-76621524. Max. coverage (+): 0. Max coverage (-): 12.48

Region: chr15 76621525-76621639. Max. coverage (+): 0. Max coverage (-): 23.43

Region: chr15 76621640-76621754. Max. coverage (+): 0. Max coverage (-): 25.62

Region: chr15 76621755-76621868. Max. coverage (+): 0. Max coverage (-): 30.84

Region: chr15 76621869-76621983. Max. coverage (+): 0. Max coverage (-): 29.43

Region: chr15 76621984-76622098. Max. coverage (+): 0. Max coverage (-): 20.13

Region: chr15 76622099-76622213. Max. coverage (+): 4.42. Max coverage (-): 34.67

Region: chr15 76622214-76622328. Max. coverage (+): 0. Max coverage (-): 6.38

Region: chr15 76622329-76622442. Max. coverage (+): 0. Max coverage (-): 11.63

Region: chr15 76622443-76622557. Max. coverage (+): 0. Max coverage (-): 11.63

Region: chr15 76622558-76622672. Max. coverage (+): 0. Max coverage (-): 73.15

Region: chr15 76622673-76622787. Max. coverage (+): 0. Max coverage (-): 0

Region: chr15 76622788-76622902. Max. coverage (+): 0. Max coverage (-): 0

Region: chr15 76622903-76623016. Max. coverage (+): 0. Max coverage (-): 9.91

Region: chr15 76623017-76623131. Max. coverage (+): 0. Max coverage (-): 36.72

Region: chr15 76623132-76623246. Max. coverage (+): 0. Max coverage (-): 19.1

Region: chr15 76623247-76623361. Max. coverage (+): 0. Max coverage (-): 9.95

Region: chr15 76623362-76623475. Max. coverage (+): 0. Max coverage (-): 19.11

Region: chr15 76623476-76623590. Max. coverage (+): 0. Max coverage (-): 5.42

Region: chr15 76623591-76623705. Max. coverage (+): 2.42. Max coverage (-): 64.32

Region: chr15 76623706-76623820. Max. coverage (+): 0. Max coverage (-): 26.09

Region: chr15 76623821-76623935. Max. coverage (+): 0. Max coverage (-): 14.98

Region: chr15 76623936-76624049. Max. coverage (+): 0. Max coverage (-): 14.98

Region: chr15 76624050-76624164. Max. coverage (+): 0. Max coverage (-): 3.41

Region: chr15 76624165-76624279. Max. coverage (+): 4.57. Max coverage (-): 31.45

Region: chr15 76624280-76624394. Max. coverage (+): 0. Max coverage (-): 40.85

Region: chr15 76624395-76624508. Max. coverage (+): 0. Max coverage (-): 0

Region: chr15 76624509-76624623. Max. coverage (+): 0. Max coverage (-): 19.8

Region: chr15 76624624-76624738. Max. coverage (+): 10.71. Max coverage (-): 54.59

Region: chr15 76624739-76624853. Max. coverage (+): 0. Max coverage (-): 0

Region: chr15 76624854-76624968. Max. coverage (+): 0. Max coverage (-): 0

Region: chr15 76624969-76625082. Max. coverage (+): 0. Max coverage (-): 0

Region: chr15 76625083-76625197. Max. coverage (+): 0. Max coverage (-): 11.28

Region: chr15 76625198-76625312. Max. coverage (+): 0. Max coverage (-): 39.25

Region: chr15 76625313-76625427. Max. coverage (+): 0. Max coverage (-): 15.74

Region: chr15 76625428-76625542. Max. coverage (+): 0. Max coverage (-): 0

Region: chr15 76625543-76625656. Max. coverage (+): 0. Max coverage (-): 24.46

Region: chr15 76625657-76625771. Max. coverage (+): 0. Max coverage (-): 6.47

Region: chr15 76625772-76625886. Max. coverage (+): 0. Max coverage (-): 13.5

Region: chr15 76625887-76626001. Max. coverage (+): 0. Max coverage (-): 33.91

Region: chr15 76626002-76626115. Max. coverage (+): 0. Max coverage (-): 37.86

Region: chr15 76626116-76626230. Max. coverage (+): 0. Max coverage (-): 0

Region: chr15 76626231-76626345. Max. coverage (+): 0. Max coverage (-): 0

Region: chr15 76626346-76626460. Max. coverage (+): 0. Max coverage (-): 0

Region: chr15 76626461-76626575. Max. coverage (+): 0. Max coverage (-): 41.02

Region: chr15 76626576-76626689. Max. coverage (+): 0. Max coverage (-): 17.84

Region: chr15 76626690-76626804. Max. coverage (+): 0. Max coverage (-): 16.81

Region: chr15 76626805-76626919. Max. coverage (+): 0. Max coverage (-): 0.49

Region: chr15 76626920-76627034. Max. coverage (+): 0. Max coverage (-): 1.16

Region: chr15 76627035-76627149. Max. coverage (+): 0. Max coverage (-): 17.88

Region: chr15 76627150-76627263. Max. coverage (+): 0. Max coverage (-): 19.4

Region: chr15 76627264-76627378. Max. coverage (+): 0. Max coverage (-): 9.88

Region: chr15 76627379-76627493. Max. coverage (+): 0. Max coverage (-): 11.88

Region: chr15 76627494-76627608. Max. coverage (+): 0.63. Max coverage (-): 32.85

Region: chr15 76627609-76627722. Max. coverage (+): 0. Max coverage (-): 28.28

Region: chr15 76627723-76627837. Max. coverage (+): 0. Max coverage (-): 49.05

Region: chr15 76627838-76627952. Max. coverage (+): 0. Max coverage (-): 0

Region: chr15 76627953-76628067. Max. coverage (+): 0. Max coverage (-): 31.62

Region: chr15 76628068-76628182. Max. coverage (+): 0. Max coverage (-): 25.95

Region: chr15 76628183-76628296. Max. coverage (+): 0. Max coverage (-): 17.45

Region: chr15 76628297-76628411. Max. coverage (+): 0. Max coverage (-): 27.13

Region: chr15 76628412-76628526. Max. coverage (+): 0. Max coverage (-): 76.45

Region: chr15 76628527-76628641. Max. coverage (+): 4.18. Max coverage (-): 17.88

Region: chr15 76628642-76628755. Max. coverage (+): 0. Max coverage (-): 9.2

Region: chr15 76628756-76628870. Max. coverage (+): 0. Max coverage (-): 0

Region: chr15 76628871-76628985. Max. coverage (+): 0. Max coverage (-): 6.85

Region: chr15 76628986-76629100. Max. coverage (+): 0. Max coverage (-): 14.86

Region: chr15 76629101-76629215. Max. coverage (+): 1.63. Max coverage (-): 30.61

Region: chr15 76629216-76629329. Max. coverage (+): 0. Max coverage (-): 0

Region: chr15 76629330-76629444. Max. coverage (+): 0. Max coverage (-): 0

Region: chr15 76629445-76629559. Max. coverage (+): 0. Max coverage (-): 21.14

Region: chr15 76629560-76629674. Max. coverage (+): 0. Max coverage (-): 6.75

Region: chr15 76629675-76629789. Max. coverage (+): 0. Max coverage (-): 29.07

Region: chr15 76629790-76629903. Max. coverage (+): 0. Max coverage (-): 6.72

Region: chr15 76629904-76630018. Max. coverage (+): 0. Max coverage (-): 55.54

Region: chr15 76630019-76630133. Max. coverage (+): 0. Max coverage (-): 6.16

Region: chr15 76630134-76630248. Max. coverage (+): 0. Max coverage (-): 16.51

Region: chr15 76630249-76630362. Max. coverage (+): 0. Max coverage (-): 16.5

Region: chr15 76630363-76630477. Max. coverage (+): 0. Max coverage (-): 18.23

Region: chr15 76630478-76630592. Max. coverage (+): 0. Max coverage (-): 27.5

Region: chr15 76630593-76630707. Max. coverage (+): 0. Max coverage (-): 3.01

Region: chr15 76630708-76630822. Max. coverage (+): 0. Max coverage (-): 25.31

Region: chr15 76630823-76630936. Max. coverage (+): 0. Max coverage (-): 23.68

Region: chr15 76630937-76631051. Max. coverage (+): 0. Max coverage (-): 23.12

Region: chr15 76631052-76631166. Max. coverage (+): 0. Max coverage (-): 34.94

Region: chr15 76631167-76631281. Max. coverage (+): 0. Max coverage (-): 33.21

Region: chr15 76631282-76631396. Max. coverage (+): 0. Max coverage (-): 0

Region: chr15 76631397-76631510. Max. coverage (+): 0. Max coverage (-): 37.28

Region: chr15 76631511-76631625. Max. coverage (+): 0. Max coverage (-): 0

Region: chr15 76631626-76631740. Max. coverage (+): 0. Max coverage (-): 25.14

Region: chr15 76631741-76631855. Max. coverage (+): 0. Max coverage (-): 6.35

Region: chr15 76631856-76631969. Max. coverage (+): 0. Max coverage (-): 0

Region: chr15 76631970-76632084. Max. coverage (+): 0. Max coverage (-): 1.35

Region: chr15 76632085-76632199. Max. coverage (+): 0. Max coverage (-): 0

Region: chr15 76632200-76632314. Max. coverage (+): 0. Max coverage (-): 1.84

Region: chr15 76632315-76632429. Max. coverage (+): 0. Max coverage (-): 0

Region: chr15 76632430-76632543. Max. coverage (+): 0. Max coverage (-): 3.16

Region: chr15 76632544-76632658. Max. coverage (+): 0. Max coverage (-): 8.21

Region: chr15 76632659-76632773. Max. coverage (+): 0. Max coverage (-): 2.04

Region: chr15 76632774-76632888. Max. coverage (+): 0. Max coverage (-): 16.82

Region: chr15 76632889-76633002. Max. coverage (+): 0. Max coverage (-): 6.2

Region: chr15 76633003-76633117. Max. coverage (+): 0. Max coverage (-): 6.2

Region: chr15 76633118-76633232. Max. coverage (+): 0. Max coverage (-): 15.72

Region: chr15 76633233-76633347. Max. coverage (+): 0. Max coverage (-): 39.03

Region: chr15 76633348-76633462. Max. coverage (+): 0. Max coverage (-): 57.96

Region: chr15 76633463-76633576. Max. coverage (+): 0. Max coverage (-): 0

Region: chr15 76633577-76633691. Max. coverage (+): 0. Max coverage (-): 0

Region: chr15 76633692-76633806. Max. coverage (+): 0. Max coverage (-): 0

Region: chr15 76633807-76633921. Max. coverage (+): 0. Max coverage (-): 0

Region: chr15 76633922-76634036. Max. coverage (+): 0. Max coverage (-): 0

Region: chr15 76634037-76634150. Max. coverage (+): 0. Max coverage (-): 0

Region: chr15 76634151-76634265. Max. coverage (+): 0. Max coverage (-): 0

Region: chr15 76634266-76634380. Max. coverage (+): 0. Max coverage (-): 0

Region: chr15 76634381-76634495. Max. coverage (+): 0. Max coverage (-): 0

Region: chr15 76634496-76634609. Max. coverage (+): 0. Max coverage (-): 8.84

Region: chr15 76634610-76634724. Max. coverage (+): 0. Max coverage (-): 7.22

Region: chr15 76634725-76634839. Max. coverage (+): 0. Max coverage (-): 0

Region: chr15 76634840-76634954. Max. coverage (+): 0. Max coverage (-): 1.75

Region: chr15 76634955-76635069. Max. coverage (+): 0. Max coverage (-): 7.16

Region: chr15 76635070-76635183. Max. coverage (+): 0. Max coverage (-): 43.33

Region: chr15 76635184-76635298. Max. coverage (+): 0. Max coverage (-): 6.1

Region: chr15 76635299-76635413. Max. coverage (+): 0. Max coverage (-): 17.13

Region: chr15 76635414-76635528. Max. coverage (+): 0. Max coverage (-): 9.66

Region: chr15 76635529-76635643. Max. coverage (+): 3.97. Max coverage (-): 29.88

Region: chr15 76635644-76635757. Max. coverage (+): 0. Max coverage (-): 12.66

Region: chr15 76635758-76635872. Max. coverage (+): 0. Max coverage (-): 13.77

Region: chr15 76635873-76635987. Max. coverage (+): 0. Max coverage (-): 15.81

Region: chr15 76635988-76636102. Max. coverage (+): 0. Max coverage (-): 15.65

Region: chr15 76636103-76636216. Max. coverage (+): 5.98. Max coverage (-): 53.91

Region: chr15 76636217-76636331. Max. coverage (+): 0. Max coverage (-): 18.13

Region: chr15 76636332-76636446. Max. coverage (+): 0. Max coverage (-): 20.39

Region: chr15 76636447-76636561. Max. coverage (+): 0. Max coverage (-): 20.2

Region: chr15 76636562-76636676. Max. coverage (+): 0. Max coverage (-): 31.83

Region: chr15 76636677-76636790. Max. coverage (+): 0. Max coverage (-): 12.71

Region: chr15 76636791-76636905. Max. coverage (+): 4.74. Max coverage (-): 29.37

Region: chr15 76636906-76637020. Max. coverage (+): 0. Max coverage (-): 50.33

Region: chr15 76637021-76637135. Max. coverage (+): 6. Max coverage (-): 14.4

Region: chr15 76637136-76637250. Max. coverage (+): 0. Max coverage (-): 6.46

Region: chr15 76637251-76637364. Max. coverage (+): 0. Max coverage (-): 0

Region: chr15 76637365-76637479. Max. coverage (+): 0. Max coverage (-): 0

Region: chr15 76637480-76637594. Max. coverage (+): 0. Max coverage (-): 0

Region: chr15 76637595-76637709. Max. coverage (+): 0. Max coverage (-): 0

Region: chr15 76637710-76637823. Max. coverage (+): 0. Max coverage (-): 0

Region: chr15 76637824-76637938. Max. coverage (+): 0. Max coverage (-): 0

Region: chr15 76637939-76638053. Max. coverage (+): 0. Max coverage (-): 0

Region: chr15 76638054-76638168. Max. coverage (+): 0. Max coverage (-): 26.36

Region: chr15 76638169-76638283. Max. coverage (+): 0. Max coverage (-): 22.64

Region: chr15 76638284-76638397. Max. coverage (+): 0. Max coverage (-): 59.1

Region: chr15 76638398-76638512. Max. coverage (+): 0. Max coverage (-): 46.4

Region: chr15 76638513-76638627. Max. coverage (+): 0. Max coverage (-): 4.91

Region: chr15 76638628-76638742. Max. coverage (+): 0. Max coverage (-): 0

Region: chr15 76638743-76638856. Max. coverage (+): 0. Max coverage (-): 0

Region: chr15 76638857-76638971. Max. coverage (+): 0. Max coverage (-): 0

Region: chr15 76638972-76639086. Max. coverage (+): 0. Max coverage (-): 0

Region: chr15 76639087-76639201. Max. coverage (+): 0. Max coverage (-): 0

Region: chr15 76639202-76639316. Max. coverage (+): 0. Max coverage (-): 10.51

Region: chr15 76639317-76639430. Max. coverage (+): 0. Max coverage (-): 15.07

Region: chr15 76639431-76639545. Max. coverage (+): 0. Max coverage (-): 7.51

Region: chr15 76639546-76639660. Max. coverage (+): 0. Max coverage (-): 2.83

Region: chr15 76639661-76639775. Max. coverage (+): 0. Max coverage (-): 0

Region: chr15 76639776-76639890. Max. coverage (+): 0. Max coverage (-): 11.9

Region: chr15 76639891-76640004. Max. coverage (+): 0. Max coverage (-): 9.45

Region: chr15 76640005-76640119. Max. coverage (+): 0. Max coverage (-): 0

Region: chr15 76640120-76640234. Max. coverage (+): 0. Max coverage (-): 4.79

Region: chr15 76640235-76640349. Max. coverage (+): 0. Max coverage (-): 0

Region: chr15 76640350-76640463. Max. coverage (+): 0. Max coverage (-): 6.53

Region: chr15 76640464-76640578. Max. coverage (+): 0. Max coverage (-): 14.98

Region: chr15 76640579-76640693. Max. coverage (+): 0. Max coverage (-): 26.35

Region: chr15 76640694-76640808. Max. coverage (+): 0. Max coverage (-): 0

Region: chr15 76640809-76640923. Max. coverage (+): 0. Max coverage (-): 19.22

Region: chr15 76640924-76641037. Max. coverage (+): 0. Max coverage (-): 10.05

Region: chr15 76641038-76641152. Max. coverage (+): 0. Max coverage (-): 12.07

Region: chr15 76641153-76641267. Max. coverage (+): 0. Max coverage (-): 22.9

Region: chr15 76641268-76641382. Max. coverage (+): 0. Max coverage (-): 6.49

Region: chr15 76641383-76641497. Max. coverage (+): 0. Max coverage (-): 7.17

Region: chr15 76641498-76641611. Max. coverage (+): 0. Max coverage (-): 6.19

Region: chr15 76641612-76641726. Max. coverage (+): 0. Max coverage (-): 5.78

Region: chr15 76641727-76641841. Max. coverage (+): 0. Max coverage (-): 9.65

Region: chr15 76641842-76641956. Max. coverage (+): 0. Max coverage (-): 6.98

Region: chr15 76641957-76642070. Max. coverage (+): 0. Max coverage (-): 12.52

Region: chr15 76642071-76642185. Max. coverage (+): 0. Max coverage (-): 6.63

Region: chr15 76642186-76642300. Max. coverage (+): 0. Max coverage (-): 0

Region: chr15 76642301-76642415. Max. coverage (+): 0. Max coverage (-): 22.84

Region: chr15 76642416-76642530. Max. coverage (+): 0. Max coverage (-): 24.52

Region: chr15 76642531-76642644. Max. coverage (+): 0. Max coverage (-): 12.68

Region: chr15 76642645-76642759. Max. coverage (+): 0. Max coverage (-): 21.32

Region: chr15 76642760-76642874. Max. coverage (+): 0. Max coverage (-): 18.08

Region: chr15 76642875-76642989. Max. coverage (+): 0. Max coverage (-): 18.08

Region: chr15 76642990-76643103. Max. coverage (+): 0. Max coverage (-): 22.43

Region: chr15 76643104-76643218. Max. coverage (+): 0. Max coverage (-): 45.52

Region: chr15 76643219-76643333. Max. coverage (+): 0. Max coverage (-): 25.22

Region: chr15 76643334-76643448. Max. coverage (+): 0. Max coverage (-): 30.71

Region: chr15 76643449-76643563. Max. coverage (+): 0. Max coverage (-): 19.57

Region: chr15 76643564-76643677. Max. coverage (+): 0. Max coverage (-): 16.89

Region: chr15 76643678-76643792. Max. coverage (+): 0. Max coverage (-): 2.98

Region: chr15 76643793-76643907. Max. coverage (+): 0. Max coverage (-): 12.34

Region: chr15 76643908-76644022. Max. coverage (+): 0. Max coverage (-): 20.95

Region: chr15 76644023-76644137. Max. coverage (+): 0. Max coverage (-): 5.56

Region: chr15 76644138-76644251. Max. coverage (+): 0. Max coverage (-): 12.9

Region: chr15 76644252-76644366. Max. coverage (+): 0. Max coverage (-): 21.15

Region: chr15 76644367-76644481. Max. coverage (+): 0. Max coverage (-): 29.9

Region: chr15 76644482-76644596. Max. coverage (+): 0. Max coverage (-): 27.32

Region: chr15 76644597-76644710. Max. coverage (+): 0. Max coverage (-): 54.03

Region: chr15 76644711-76644825. Max. coverage (+): 0. Max coverage (-): 29.32

Region: chr15 76644826-76644940. Max. coverage (+): 0. Max coverage (-): 19.6

Region: chr15 76644941-76645055. Max. coverage (+): 0. Max coverage (-): 13.91

Region: chr15 76645056-76645170. Max. coverage (+): 0. Max coverage (-): 15.52

Region: chr15 76645171-76645284. Max. coverage (+): 0. Max coverage (-): 40.59

Region: chr15 76645285-76645399. Max. coverage (+): 0. Max coverage (-): 30.02

Region: chr15 76645400-76645514. Max. coverage (+): 0. Max coverage (-): 39.54

Region: chr15 76645515-76645629. Max. coverage (+): 0. Max coverage (-): 6.84

Region: chr15 76645630-76645744. Max. coverage (+): 0. Max coverage (-): 11.24

Region: chr15 76645745-76645858. Max. coverage (+): 0. Max coverage (-): 0

Region: chr15 76645859-76645973. Max. coverage (+): 0. Max coverage (-): 6.02

Region: chr15 76645974-76646088. Max. coverage (+): 0. Max coverage (-): 26.75

Region: chr15 76646089-76646203. Max. coverage (+): 0. Max coverage (-): 83.1

Region: chr15 76646204-76646317. Max. coverage (+): 0. Max coverage (-): 2.24

Region: chr15 76646318-76646432. Max. coverage (+): 0. Max coverage (-): 10.82

Region: chr15 76646433-76646547. Max. coverage (+): 0. Max coverage (-): 30.99

Region: chr15 76646548-76646662. Max. coverage (+): 0. Max coverage (-): 12.23

Region: chr15 76646663-76646777. Max. coverage (+): 0. Max coverage (-): 7.19

Region: chr15 76646778-76646891. Max. coverage (+): 0. Max coverage (-): 14.48

Region: chr15 76646892-76647006. Max. coverage (+): 0. Max coverage (-): 41.17

Region: chr15 76647007-76647121. Max. coverage (+): 0. Max coverage (-): 59.57

Region: chr15 76647122-76647236. Max. coverage (+): 0. Max coverage (-): 118.57

Region: chr15 76647237-76647350. Max. coverage (+): 0. Max coverage (-): 60.27

Region: chr15 76647351-76647465. Max. coverage (+): 0. Max coverage (-): 16.67

Region: chr15 76647466-76647580. Max. coverage (+): 0. Max coverage (-): 19.53

Region: chr15 76647581-76647695. Max. coverage (+): 0. Max coverage (-): 25.79

Region: chr15 76647696-76647810. Max. coverage (+): 0.53. Max coverage (-): 26.53

Region: chr15 76647811-76647924. Max. coverage (+): 3.3. Max coverage (-): 76.01

Region: chr15 76647925-76648039. Max. coverage (+): 0. Max coverage (-): 6.55

Region: chr15 76648040-76648154. Max. coverage (+): 0. Max coverage (-): 38.23

Region: chr15 76648155-76648269. Max. coverage (+): 0. Max coverage (-): 25.37

Region: chr15 76648270-76648384. Max. coverage (+): 0. Max coverage (-): 23.35

Region: chr15 76648385-76648498. Max. coverage (+): 0. Max coverage (-): 0

Region: chr15 76648499-76648613. Max. coverage (+): 0. Max coverage (-): 64.8

Region: chr15 76648614-76648728. Max. coverage (+): 0. Max coverage (-): 10.52

Region: chr15 76648729-76648843. Max. coverage (+): 0. Max coverage (-): 8.84

Region: chr15 76648844-76648957. Max. coverage (+): 0. Max coverage (-): 0

Region: chr15 76648958-76649072. Max. coverage (+): 0. Max coverage (-): 39.39

Region: chr15 76649073-76649187. Max. coverage (+): 0. Max coverage (-): 70.75

Region: chr15 76649188-76649302. Max. coverage (+): 0. Max coverage (-): 21.81

Region: chr15 76649303-76649417. Max. coverage (+): 0. Max coverage (-): 32.46

Region: chr15 76649418-76649531. Max. coverage (+): 0. Max coverage (-): 2.62

Region: chr15 76649532-76649646. Max. coverage (+): 0. Max coverage (-): 13.38

Region: chr15 76649647-76649761. Max. coverage (+): 0. Max coverage (-): 5.67

Region: chr15 76649762-76649876. Max. coverage (+): 38.95. Max coverage (-): 0.57

Region: chr15 76649877-76649991. Max. coverage (+): 5.1. Max coverage (-): 0

Region: chr15 76649992-76650105. Max. coverage (+): 0. Max coverage (-): 0

Region: chr15 76650106-76650220. Max. coverage (+): 0. Max coverage (-): 0

Region: chr15 76650221-76650335. Max. coverage (+): 8.13. Max coverage (-): 0

Region: chr15 76650336-76650450. Max. coverage (+): 0. Max coverage (-): 0

Region: chr15 76650451-76650564. Max. coverage (+): 6.91. Max coverage (-): 0

Region: chr15 76650565-76650679. Max. coverage (+): 0. Max coverage (-): 0

Region: chr15 76650680-76650794. Max. coverage (+): 0. Max coverage (-): 0

Region: chr15 76650795-76650909. Max. coverage (+): 0. Max coverage (-): 0

Region: chr15 76650910-76651024. Max. coverage (+): 0. Max coverage (-): 4.07

Region: chr15 76651025-76651138. Max. coverage (+): 0. Max coverage (-): 0

Region: chr15 76651139-76651253. Max. coverage (+): 0.16. Max coverage (-): 0

Region: chr15 76651254-76651368. Max. coverage (+): 0. Max coverage (-): 0

Region: chr15 76651369-76651483. Max. coverage (+): 0. Max coverage (-): 0

Region: chr15 76651484-76651598. Max. coverage (+): 68.13. Max coverage (-): 0

Region: chr15 76651599-76651712. Max. coverage (+): 3.1. Max coverage (-): 0

Region: chr15 76651713-76651827. Max. coverage (+): 157.34. Max coverage (-): 0

Region: chr15 76651828-76651942. Max. coverage (+): 0. Max coverage (-): 0

Region: chr15 76651943-76652057. Max. coverage (+): 26.28. Max coverage (-): 0

Region: chr15 76652058-76652171. Max. coverage (+): 0. Max coverage (-): 0

Region: chr15 76652172-76652286. Max. coverage (+): 0. Max coverage (-): 0

Region: chr15 76652287-76652401. Max. coverage (+): 0. Max coverage (-): 0

Region: chr15 76652402-76652516. Max. coverage (+): 0. Max coverage (-): 0

Region: chr15 76652517-76652631. Max. coverage (+): 0. Max coverage (-): 0

Region: chr15 76652632-76652745. Max. coverage (+): 0. Max coverage (-): 0

Region: chr15 76652746-76652860. Max. coverage (+): 0. Max coverage (-): 0

Region: chr15 76652861-76652975. Max. coverage (+): 0. Max coverage (-): 0

Region: chr15 76652976-76653090. Max. coverage (+): 0. Max coverage (-): 0

Region: chr15 76653091-76653204. Max. coverage (+): 0. Max coverage (-): 0

Region: chr15 76653205-76653319. Max. coverage (+): 0. Max coverage (-): 0

Region: chr15 76653320-76653434. Max. coverage (+): 0. Max coverage (-): 0

Region: chr15 76653435-76653549. Max. coverage (+): 0. Max coverage (-): 0

Region: chr15 76653550-76653664. Max. coverage (+): 6.09. Max coverage (-): 0

Region: chr15 76653665-76653778. Max. coverage (+): 5.22. Max coverage (-): 0

Region: chr15 76653779-76653893. Max. coverage (+): 0. Max coverage (-): 0

Region: chr15 76653894-76654008. Max. coverage (+): 0. Max coverage (-): 0

Region: chr15 76654009-76654123. Max. coverage (+): 5.71. Max coverage (-): 0

Region: chr15 76654124-76654238. Max. coverage (+): 0. Max coverage (-): 5.43

Region: chr15 76654239-76654352. Max. coverage (+): 0. Max coverage (-): 0

Region: chr15 76654353-76654467. Max. coverage (+): 0. Max coverage (-): 0

Region: chr15 76654468-. Max. coverage (+): 3.24. Max coverage (-): 0

RepeatMasker Color Code

**+**

100-98% Identity

<98-95% Identity

<95-90% Identity

<90-85% Identity

<85-80% Identity

<80-75% Identity

<75-70% Identity

<70% Identity

**-**

Gene Set Color Code

**+**

Gene

Pseudogene

**-**

Topology/Coverage Color Code

Coverage Plus Strand

Coverage Minus Strand

Mainstrand: Plus

Mainstrand: Minus

Complementary Strand

Flanking Region  
(if option -flank >0)

Gene Set Annotation  
  
RepeatMasker Annotation  

**1. MIR**: 76599311-76599442 (-), Divergence to consensus: 50%  
**2. L1ME3**: 76599450-76599647 (+), Divergence to consensus: 39.9%  
**3. L1ME4b**: 76599642-76600004 (+), Divergence to consensus: 47.1%  
**4. MIR**: 76600597-76600725 (+), Divergence to consensus: 30.6%  
**5. MIRc**: 76601214-76601271 (+), Divergence to consensus: 29.4%  
**6. CHRL**: 76602077-76602284 (+), Divergence to consensus: 25.3%  
**7. MIR**: 76603204-76603329 (+), Divergence to consensus: 32.8%  
**8. L2a**: 76604589-76604775 (+), Divergence to consensus: 40.7%  
**9. ART2A**: 76605179-76605315 (-), Divergence to consensus: 11.7%  
**10. MER103C**: 76605797-76606024 (+), Divergence to consensus: 46.4%  
**11. CHRL1\_BT**: 76606240-76606352 (-), Divergence to consensus: 29.3%  
**12. MIR3**: 76606516-76606667 (+), Divergence to consensus: 45.4%  
**13. AT\_rich**: 76608370-76608392 (+), Divergence to consensus: 43.5%  
**14. MER90a**: 76609904-76609983 (+), Divergence to consensus: 28.1%  
**15. MER110-int**: 76610191-76610505 (+), Divergence to consensus: 42.3%  
**16. Bov-tA3**: 76610552-76610760 (+), Divergence to consensus: 14.1%  
**17. AT\_rich**: 76611025-76611046 (+), Divergence to consensus: 45.5%  
**18. Bov-tA3**: 76612224-76612377 (-), Divergence to consensus: 24.3%  
**19. MER90a**: 76612393-76612769 (+), Divergence to consensus: 21.2%  
**20. BOV-A2**: 76612972-76613085 (-), Divergence to consensus: 7.9%  
**21. MIR3**: 76613931-76613970 (-), Divergence to consensus: 22.5%  
**22. MIR3**: 76615021-76615125 (+), Divergence to consensus: 37.2%  
**23. MIR3**: 76616563-76616655 (-), Divergence to consensus: 33.3%  
**24. Bov-tA1**: 76617043-76617269 (+), Divergence to consensus: 13.2%  
**25. MIRb**: 76617988-76618059 (+), Divergence to consensus: 19.4%  
**26. MER5A**: 76618435-76618549 (+), Divergence to consensus: 30.1%  
**27. MIR**: 76619592-76619824 (-), Divergence to consensus: 33.4%  
**28. L1ME4a**: 76621881-76622121 (+), Divergence to consensus: 48.4%  
**29. L1ME4a**: 76622187-76622287 (+), Divergence to consensus: 41.8%  
**30. A-rich**: 76622304-76622381 (+), Divergence to consensus: 33.8%  
**31. T-rich**: 76622868-76622931 (+), Divergence to consensus: 28.6%  
**32. MIRb**: 76624409-76624597 (+), Divergence to consensus: 37.1%  
**33. (TTCA)n**: 76624692-76624723 (+), Divergence to consensus: 9.4%  
**34. L2a**: 76624762-76625084 (-), Divergence to consensus: 48.4%  
**35. CHRL**: 76625260-76625413 (-), Divergence to consensus: 20%  
**36. MIR3**: 76625489-76625609 (-), Divergence to consensus: 41.6%  
**37. L2c**: 76626061-76626437 (-), Divergence to consensus: 50.1%  
**38. L2c**: 76626862-76626959 (-), Divergence to consensus: 36%  
**39. MIRb**: 76627821-76627945 (-), Divergence to consensus: 36.9%  
**40. MIRc**: 76628826-76628978 (+), Divergence to consensus: 37.2%  
**41. MIRb**: 76629223-76629445 (+), Divergence to consensus: 48.6%  
**42. A-rich**: 76630071-76630107 (+), Divergence to consensus: 18.9%  
**43. MIRb**: 76630255-76630393 (-), Divergence to consensus: 37.7%  
**44. L2a**: 76631297-76631394 (+), Divergence to consensus: 38.3%  
**45. MER41\_BT**: 76631493-76631605 (+), Divergence to consensus: 23%  
**46. L2a**: 76632128-76632250 (+), Divergence to consensus: 40.1%  
**47. L2a**: 76633466-76634521 (-), Divergence to consensus: 48.4%  
**48. L2b**: 76634699-76634774 (-), Divergence to consensus: 29%  
**49. MamGypLTR1a**: 76635941-76636049 (+), Divergence to consensus: 38.5%  
**50. L3**: 76637175-76637393 (+), Divergence to consensus: 40.3%  
**51. L3**: 76637410-76637662 (+), Divergence to consensus: 43.9%  
**52. L3**: 76637686-76638067 (+), Divergence to consensus: 43.1%  
**53. ART2A**: 76638549-76639033 (+), Divergence to consensus: 18.3%  
**54. L2c**: 76639058-76639096 (-), Divergence to consensus: 25.6%  
**55. Tigger7**: 76639228-76639566 (+), Divergence to consensus: 26.5%  
**56. L2c**: 76639607-76639750 (-), Divergence to consensus: 40.2%  
**57. Charlie18a**: 76639998-76640118 (+), Divergence to consensus: 31.6%  
**58. L2c**: 76641033-76641134 (-), Divergence to consensus: 38.4%  
**59. CHRL**: 76642127-76642288 (+), Divergence to consensus: 18.5%  
**60. CT-rich**: 76642530-76642574 (+), Divergence to consensus: 24.4%  
**61. Bov-tA2**: 76644874-76645023 (-), Divergence to consensus: 13.5%  
**62. MIR**: 76648870-76648933 (-), Divergence to consensus: 24%  
**63. L2a**: 76648886-76648960 (-), Divergence to consensus: 48.2%  
**64. MIR3**: 76650019-76650156 (+), Divergence to consensus: 41.9%  
**65. C-rich**: 76650738-76650814 (+), Divergence to consensus: 37.8%  
**66. Bov-tA2**: 76651040-76651244 (+), Divergence to consensus: 19.3%  
**67. L1\_BT**: 76651935-76652009 (-), Divergence to consensus: 13.4%  
**68. MIRc**: 76652764-76652844 (+), Divergence to consensus: 39.5%  
**69. L2a**: 76652914-76653216 (+), Divergence to consensus: 50.4%  
**70. MIRb**: 76653232-76653407 (-), Divergence to consensus: 57.3%

  
Transcription Factor Binding Sites  

**RFX4\_2** (Sequence: GTAACCATG (-): 76633360)  
**RFX4\_1** (Sequence: CTTGGCAAC (+): 76628792)  
**RFX4\_1** (Sequence: CTTGGCAAC (+): 76647746)  
**SPZ1** (Sequence: CTGTTACCCC (-): 76619880)  
**SPZ1** (Sequence: CTCAAACCCC (-): 76620842)  
**RFX4\_2** (Sequence: CCTAGATAC (+): 76599356)  
**Gata4** (Sequence: AGATAAG (-): 76603600)  
**Gata4** (Sequence: AGATAAG (-): 76615741)  
**Gata4** (Sequence: AGATAAC (-): 76630015)  
**Gata4** (Sequence: AGATAAC (-): 76637146)  
**Gata4** (Sequence: AGATAAC (-): 76644843)  
**SOX9** (Sequence: AACAATGG (-): 76599117)  
**SOX9** (Sequence: AACAATGA (-): 76600014)  
**SOX9** (Sequence: AACAATAG (-): 76605104)  
**SOX9** (Sequence: AACAATGA (-): 76623990)  
**SOX9** (Sequence: AACAATGA (-): 76625150)  
**SOX9** (Sequence: AACAATAA (-): 76635602)  
**SOX9** (Sequence: AACAATGG (-): 76649595)  
**SOX9** (Sequence: CCATTGTT (+): 76614018)  
**SOX9** (Sequence: CCATTGTT (+): 76648066)  
**SPZ1** (Sequence: AGGGTTTCAG (+): 76602941)  
**SPZ1** (Sequence: GGGGTAAGAG (+): 76641570)  
**Mybl1\_1** (Sequence: AACCGTTA (+): 76649621)  
**Gata4** (Sequence: GTTATCT (+): 76602434)  
**Gata4** (Sequence: CTTATCT (+): 76606419)  
**Gata4** (Sequence: GTTATCT (+): 76622187)  
**Gata4** (Sequence: CTTATCT (+): 76632401)  
**Gata4** (Sequence: CTTATCT (+): 76638370)  
**Gata4** (Sequence: GTTATCT (+): 76640520)  
**Gata4** (Sequence: CTTATCT (+): 76643108)  
**Gata4** (Sequence: GTTATCT (+): 76643567)  
**Gata4** (Sequence: CTTATCT (+): 76647901)  
**Gata4** (Sequence: CTTATCT (+): 76648710)
