# Supplementary material for: Small RNA sequencing of cryopreserved semen from single bull revealed altered miRNAs and piRNAs expression between High- and Low-motile sperm populations
Source: BMC Genomics. 2017 Jan 4;18:14. doi: 10.1186/s12864-016-3394-7 (PMC5209821; doi:10.1186/s12864-016-3394-7)
Supplement: Additional file 4: — Details for each piRNA clusters found in Low Motile (LM) sperm fraction. Genes, repeats, transposable elements and transcription factors binding sites falling within the cluster regions were reported. (ZIP 1034 kb) [file 12864_2016_3394_MOESM4_ESM.zip › 17.html]

piRNA cluster 17


Predicted piRNA cluster no. 17     previous   next
  

Show proTRAC run info
Hide proTRAC run info

================================= proTRAC ====================================  
VERSION: 2.1                                    LAST MODIFIED: 06. October 2015  
  
Please cite:  
Rosenkranz D, Zischler H. proTRAC - a software for probabilistic piRNA cluster  
detection, visualization and analysis. 2012. BMC Bioinformatics 13:5.  
  
and (for proTRAC 2.0 and later):  
Rosenkranz D, Rudloff S, Bastuck K, Ketting RF, Zischler H. Tupaia small RNAs  
provide insights into function and evolution of RNAi-based transposon defense  
in mammals. 2015. RNA 21(5):911-922.  
  
Contact:  
David Rosenkranz  
Institute of Anthropology, small RNA group  
Johannes Gutenberg University Mainz  
email: rosenkranz@uni-mainz.de  
  
You can find the latest proTRAC version at:  
http://sourceforge.net/projects/protrac/files  
http://www.smallRNAgroup-mainz.de/software  
==============================================================================  
  
PARAMETERS:  
Map file: .............../storage/core/barbara/genhome/smallRNA/fertility/Sample\_not\_motile/pirna/Sample\_not\_motile\_26-33\_collapsed.fa.no-dust.map.weighted-10000-1000-b-0  
Genome file: ............/storage/core/barbara/genhome/smallRNA/fertility/Sample\_all/pirna/bt\_311\_chrY.fa  
RepeatMasker annotation: /storage/genomes/bt\_umd31/GCF\_000003055.6\_Bos\_taurus\_UMD\_3.1.1\_repeatMasker\_chr.out  
GeneSet:................./storage/core/barbara/genhome/smallRNA/fertility/Sample\_all/pirna/full.gtf  
  
Significant (p<=0.01) hit density will be calculated based  
on observed hit distribution.  
  
Sliding window size: ........................................ 5000 bp  
Sliding window increament: .................................. 1000 bp  
Normalize each hit by number of genomic hits: ............... 1 [0=no/1=yes]  
Normalize each hit by number of sequence reads: ............. 1 [0=no/1=yes]  
Normalize values (-> per million mapped reads): ............. 1 [0=no/1=yes]  
Min. fraction of hits with 1T(U) or 10A: .................... 0.75  
Alternatively: Min. fraction of hits with 1T(U) and 10A: .... 0.5  
Min. fraction of hits with typical piRNA length: ............ 0.75  
Typical piRNA length: ....................................... 26-33 nt  
Min. size of a piRNA cluster: ............................... 5000 bp.  
Min. number of hits (absolute): ............................. 0  
Min. number of hits (normalized): ........................... 0  
Min. fraction of hits on the mainstrand: .................... 0.75  
Top fraction of mapped sequences (in terms of read counts): . 1%  
Top fraction accounts for max. n% of sequence reads: ........ 90%  
Min. fraction of hits on each arm of a bidirectional cluster: 0.1  
Output image file for each cluster: ......................... 0 [0=no/1=yes]  
Output html file for each cluster: .......................... 1 [0=no/1=yes]  
Output a summary table: ..................................... 1 [0=no/1=yes]  
Output a FASTA file for each cluster (piRNA sequences): ..... 1 [0=no/1=yes]  
Output a FASTA file comprising cluster sequences: ........... 1 [0=no/1=yes]  
Search DNA motifs in clusters: .............................. 1 [0=no/1=yes]  
Output flanking sequences: +/- .............................. 0 bp  
Output ~.pTi file: .......................................... 1 [0=no/1=yes]  
==============================================================================  
  
  
Genome size (without gaps): ............ 2678902517 bp  
Gaps (N/X/-): .......................... 53837044 bp  
Mapped reads: .......................... 738059667487  
Non-identical sequences: ............... 277001  
Genomic hits: .......................... 533816  
Significant densitiy of mapped reads: .. 15118061 reads/kb

Show proTRAC cluster info
Hide proTRAC cluster info

|  |  |
| --- | --- |
| Location | chr17 |
| Coordinates | 67143268-67162496 |
| Size [bp] | 19229 |
| Sequence hit loci | 1850 |
| Mapped reads (normalized) | 4793770634 |
| Mapped reads (normalized) per kb | 249299008.5 |
| Normalized reads with 1T (1U) | 80.2% |
| Normalized reads with 10A | 26.3% |
| Normalized reads with length 26-33 nt | 100% |
| Normalized reads on the main strand(s) | 99.2% |
| Predicted directionality | mono:minus |

100%

0%

1T (1U)  
reads

10A reads

26-33 nt  
reads

reads on mainstrand

**Either the amount of reads with 1T (1U) OR 10A has to exceed 75% (set with option: -1Tor10A)  
Alternatively the amount of reads with 1T (1U) AND 10A has to exceed 50% (set with option: -1Tand10A)  
Minimum amount of reads with preferred size is 75% (set with option: -pisize)  
Minimum amount of reads on the main strand(s) is 75% (set with option: -clstrand)**

Show read coverage
Hide read coverage

WHAT DO I SEE HERE?  
This chart shows the location of mapped sequence reads within a predicted piRNA cluster. The color refers to the number of genomic hits produced by the sequence read in question. A dark red bar indicates that this sequence read produces many other hits elsewhere in the genome. Many adjacent red or yellow bars can indicate the presence of a multi-copy element such as transposons or rRNA genes. A dark green bar indicates that this sequence read maps uniquely to this locus.

1 hit

2-5 hits

6-10 hits

11-20 hits

21-50 hits

51-100 hits

> 100 hits

chr17

67143268

67162496

Gene Set

RepeatMasker

Mapped  
Reads

161.6

plus strand

minus strand

161.6

Region: chr17 76654469-67143287. Max. coverage (+): 0. Max coverage (-): 3.07

Region: chr17 67143288-67143325. Max. coverage (+): 0. Max coverage (-): 0

Region: chr17 67143326-67143364. Max. coverage (+): 0. Max coverage (-): 0

Region: chr17 67143365-67143402. Max. coverage (+): 0. Max coverage (-): 0

Region: chr17 67143403-67143441. Max. coverage (+): 0. Max coverage (-): 0

Region: chr17 67143442-67143479. Max. coverage (+): 0. Max coverage (-): 0

Region: chr17 67143480-67143517. Max. coverage (+): 0. Max coverage (-): 0

Region: chr17 67143518-67143556. Max. coverage (+): 0. Max coverage (-): 0

Region: chr17 67143557-67143594. Max. coverage (+): 0. Max coverage (-): 0

Region: chr17 67143595-67143633. Max. coverage (+): 0. Max coverage (-): 0

Region: chr17 67143634-67143671. Max. coverage (+): 0. Max coverage (-): 0

Region: chr17 67143672-67143710. Max. coverage (+): 0. Max coverage (-): 0

Region: chr17 67143711-67143748. Max. coverage (+): 0. Max coverage (-): 0

Region: chr17 67143749-67143787. Max. coverage (+): 0. Max coverage (-): 0

Region: chr17 67143788-67143825. Max. coverage (+): 0. Max coverage (-): 5.33

Region: chr17 67143826-67143864. Max. coverage (+): 0. Max coverage (-): 5.33

Region: chr17 67143865-67143902. Max. coverage (+): 0. Max coverage (-): 4.48

Region: chr17 67143903-67143941. Max. coverage (+): 0. Max coverage (-): 50.27

Region: chr17 67143942-67143979. Max. coverage (+): 0. Max coverage (-): 43.6

Region: chr17 67143980-67144017. Max. coverage (+): 0. Max coverage (-): 17.63

Region: chr17 67144018-67144056. Max. coverage (+): 0. Max coverage (-): 11.28

Region: chr17 67144057-67144094. Max. coverage (+): 0. Max coverage (-): 0

Region: chr17 67144095-67144133. Max. coverage (+): 0. Max coverage (-): 0

Region: chr17 67144134-67144171. Max. coverage (+): 0. Max coverage (-): 2.36

Region: chr17 67144172-67144210. Max. coverage (+): 0. Max coverage (-): 4.09

Region: chr17 67144211-67144248. Max. coverage (+): 0. Max coverage (-): 0

Region: chr17 67144249-67144287. Max. coverage (+): 0. Max coverage (-): 0

Region: chr17 67144288-67144325. Max. coverage (+): 0. Max coverage (-): 0

Region: chr17 67144326-67144364. Max. coverage (+): 0. Max coverage (-): 0

Region: chr17 67144365-67144402. Max. coverage (+): 0. Max coverage (-): 0

Region: chr17 67144403-67144440. Max. coverage (+): 0. Max coverage (-): 0

Region: chr17 67144441-67144479. Max. coverage (+): 0. Max coverage (-): 0

Region: chr17 67144480-67144517. Max. coverage (+): 0. Max coverage (-): 0

Region: chr17 67144518-67144556. Max. coverage (+): 0. Max coverage (-): 0

Region: chr17 67144557-67144594. Max. coverage (+): 0. Max coverage (-): 0

Region: chr17 67144595-67144633. Max. coverage (+): 0. Max coverage (-): 0

Region: chr17 67144634-67144671. Max. coverage (+): 0. Max coverage (-): 6.37

Region: chr17 67144672-67144710. Max. coverage (+): 0. Max coverage (-): 17.01

Region: chr17 67144711-67144748. Max. coverage (+): 0. Max coverage (-): 13.13

Region: chr17 67144749-67144787. Max. coverage (+): 0. Max coverage (-): 4.48

Region: chr17 67144788-67144825. Max. coverage (+): 0. Max coverage (-): 0

Region: chr17 67144826-67144864. Max. coverage (+): 0. Max coverage (-): 6.43

Region: chr17 67144865-67144902. Max. coverage (+): 0. Max coverage (-): 6.43

Region: chr17 67144903-67144940. Max. coverage (+): 0. Max coverage (-): 32.88

Region: chr17 67144941-67144979. Max. coverage (+): 0. Max coverage (-): 5.26

Region: chr17 67144980-67145017. Max. coverage (+): 0. Max coverage (-): 12.1

Region: chr17 67145018-67145056. Max. coverage (+): 0. Max coverage (-): 5.68

Region: chr17 67145057-67145094. Max. coverage (+): 0. Max coverage (-): 1

Region: chr17 67145095-67145133. Max. coverage (+): 0. Max coverage (-): 0

Region: chr17 67145134-67145171. Max. coverage (+): 0. Max coverage (-): 0

Region: chr17 67145172-67145210. Max. coverage (+): 0. Max coverage (-): 0

Region: chr17 67145211-67145248. Max. coverage (+): 0. Max coverage (-): 0

Region: chr17 67145249-67145287. Max. coverage (+): 0. Max coverage (-): 0

Region: chr17 67145288-67145325. Max. coverage (+): 0. Max coverage (-): 0

Region: chr17 67145326-67145363. Max. coverage (+): 0. Max coverage (-): 0

Region: chr17 67145364-67145402. Max. coverage (+): 0. Max coverage (-): 0

Region: chr17 67145403-67145440. Max. coverage (+): 0. Max coverage (-): 0

Region: chr17 67145441-67145479. Max. coverage (+): 0. Max coverage (-): 11.49

Region: chr17 67145480-67145517. Max. coverage (+): 0. Max coverage (-): 0

Region: chr17 67145518-67145556. Max. coverage (+): 0. Max coverage (-): 0

Region: chr17 67145557-67145594. Max. coverage (+): 0. Max coverage (-): 0

Region: chr17 67145595-67145633. Max. coverage (+): 0. Max coverage (-): 0

Region: chr17 67145634-67145671. Max. coverage (+): 0. Max coverage (-): 0

Region: chr17 67145672-67145710. Max. coverage (+): 0. Max coverage (-): 0

Region: chr17 67145711-67145748. Max. coverage (+): 0. Max coverage (-): 0

Region: chr17 67145749-67145786. Max. coverage (+): 0. Max coverage (-): 0

Region: chr17 67145787-67145825. Max. coverage (+): 0. Max coverage (-): 0

Region: chr17 67145826-67145863. Max. coverage (+): 0. Max coverage (-): 0

Region: chr17 67145864-67145902. Max. coverage (+): 0. Max coverage (-): 0

Region: chr17 67145903-67145940. Max. coverage (+): 0. Max coverage (-): 0

Region: chr17 67145941-67145979. Max. coverage (+): 0. Max coverage (-): 0

Region: chr17 67145980-67146017. Max. coverage (+): 0. Max coverage (-): 0

Region: chr17 67146018-67146056. Max. coverage (+): 0. Max coverage (-): 0

Region: chr17 67146057-67146094. Max. coverage (+): 0. Max coverage (-): 1.15

Region: chr17 67146095-67146133. Max. coverage (+): 0. Max coverage (-): 0

Region: chr17 67146134-67146171. Max. coverage (+): 0. Max coverage (-): 0

Region: chr17 67146172-67146210. Max. coverage (+): 0. Max coverage (-): 0

Region: chr17 67146211-67146248. Max. coverage (+): 0. Max coverage (-): 0

Region: chr17 67146249-67146286. Max. coverage (+): 0. Max coverage (-): 0

Region: chr17 67146287-67146325. Max. coverage (+): 0. Max coverage (-): 0

Region: chr17 67146326-67146363. Max. coverage (+): 0. Max coverage (-): 1.87

Region: chr17 67146364-67146402. Max. coverage (+): 0. Max coverage (-): 0

Region: chr17 67146403-67146440. Max. coverage (+): 0. Max coverage (-): 0

Region: chr17 67146441-67146479. Max. coverage (+): 0. Max coverage (-): 0

Region: chr17 67146480-67146517. Max. coverage (+): 0. Max coverage (-): 0

Region: chr17 67146518-67146556. Max. coverage (+): 0. Max coverage (-): 0

Region: chr17 67146557-67146594. Max. coverage (+): 0. Max coverage (-): 0

Region: chr17 67146595-67146633. Max. coverage (+): 0. Max coverage (-): 0

Region: chr17 67146634-67146671. Max. coverage (+): 0. Max coverage (-): 0

Region: chr17 67146672-67146709. Max. coverage (+): 0. Max coverage (-): 0

Region: chr17 67146710-67146748. Max. coverage (+): 0. Max coverage (-): 0

Region: chr17 67146749-67146786. Max. coverage (+): 0. Max coverage (-): 0

Region: chr17 67146787-67146825. Max. coverage (+): 0. Max coverage (-): 0

Region: chr17 67146826-67146863. Max. coverage (+): 0. Max coverage (-): 2.11

Region: chr17 67146864-67146902. Max. coverage (+): 0. Max coverage (-): 3.31

Region: chr17 67146903-67146940. Max. coverage (+): 0. Max coverage (-): 3.99

Region: chr17 67146941-67146979. Max. coverage (+): 0. Max coverage (-): 7.88

Region: chr17 67146980-67147017. Max. coverage (+): 0. Max coverage (-): 0

Region: chr17 67147018-67147056. Max. coverage (+): 0. Max coverage (-): 5.21

Region: chr17 67147057-67147094. Max. coverage (+): 0. Max coverage (-): 0

Region: chr17 67147095-67147133. Max. coverage (+): 0. Max coverage (-): 0

Region: chr17 67147134-67147171. Max. coverage (+): 0. Max coverage (-): 20.9

Region: chr17 67147172-67147209. Max. coverage (+): 0. Max coverage (-): 1.02

Region: chr17 67147210-67147248. Max. coverage (+): 0. Max coverage (-): 0

Region: chr17 67147249-67147286. Max. coverage (+): 0. Max coverage (-): 0

Region: chr17 67147287-67147325. Max. coverage (+): 0. Max coverage (-): 0.32

Region: chr17 67147326-67147363. Max. coverage (+): 0. Max coverage (-): 1.88

Region: chr17 67147364-67147402. Max. coverage (+): 0. Max coverage (-): 0

Region: chr17 67147403-67147440. Max. coverage (+): 0. Max coverage (-): 0

Region: chr17 67147441-67147479. Max. coverage (+): 0. Max coverage (-): 0

Region: chr17 67147480-67147517. Max. coverage (+): 0. Max coverage (-): 0

Region: chr17 67147518-67147556. Max. coverage (+): 0. Max coverage (-): 2.54

Region: chr17 67147557-67147594. Max. coverage (+): 0. Max coverage (-): 0.08

Region: chr17 67147595-67147632. Max. coverage (+): 0. Max coverage (-): 6.33

Region: chr17 67147633-67147671. Max. coverage (+): 0. Max coverage (-): 5.44

Region: chr17 67147672-67147709. Max. coverage (+): 0. Max coverage (-): 5.63

Region: chr17 67147710-67147748. Max. coverage (+): 0. Max coverage (-): 0

Region: chr17 67147749-67147786. Max. coverage (+): 0. Max coverage (-): 0

Region: chr17 67147787-67147825. Max. coverage (+): 0. Max coverage (-): 0

Region: chr17 67147826-67147863. Max. coverage (+): 0. Max coverage (-): 0

Region: chr17 67147864-67147902. Max. coverage (+): 0. Max coverage (-): 0

Region: chr17 67147903-67147940. Max. coverage (+): 0. Max coverage (-): 0

Region: chr17 67147941-67147979. Max. coverage (+): 0. Max coverage (-): 0

Region: chr17 67147980-67148017. Max. coverage (+): 0. Max coverage (-): 12.2

Region: chr17 67148018-67148056. Max. coverage (+): 0. Max coverage (-): 0

Region: chr17 67148057-67148094. Max. coverage (+): 0. Max coverage (-): 0

Region: chr17 67148095-67148132. Max. coverage (+): 0. Max coverage (-): 0

Region: chr17 67148133-67148171. Max. coverage (+): 0. Max coverage (-): 0

Region: chr17 67148172-67148209. Max. coverage (+): 0. Max coverage (-): 0

Region: chr17 67148210-67148248. Max. coverage (+): 0. Max coverage (-): 0

Region: chr17 67148249-67148286. Max. coverage (+): 0. Max coverage (-): 7.85

Region: chr17 67148287-67148325. Max. coverage (+): 0. Max coverage (-): 7.85

Region: chr17 67148326-67148363. Max. coverage (+): 0. Max coverage (-): 0

Region: chr17 67148364-67148402. Max. coverage (+): 0. Max coverage (-): 0

Region: chr17 67148403-67148440. Max. coverage (+): 0. Max coverage (-): 0

Region: chr17 67148441-67148479. Max. coverage (+): 0. Max coverage (-): 0

Region: chr17 67148480-67148517. Max. coverage (+): 0. Max coverage (-): 0

Region: chr17 67148518-67148555. Max. coverage (+): 0. Max coverage (-): 0

Region: chr17 67148556-67148594. Max. coverage (+): 0. Max coverage (-): 0

Region: chr17 67148595-67148632. Max. coverage (+): 0. Max coverage (-): 0

Region: chr17 67148633-67148671. Max. coverage (+): 0. Max coverage (-): 0

Region: chr17 67148672-67148709. Max. coverage (+): 0. Max coverage (-): 0

Region: chr17 67148710-67148748. Max. coverage (+): 0. Max coverage (-): 0

Region: chr17 67148749-67148786. Max. coverage (+): 0. Max coverage (-): 0

Region: chr17 67148787-67148825. Max. coverage (+): 0. Max coverage (-): 0

Region: chr17 67148826-67148863. Max. coverage (+): 0. Max coverage (-): 0

Region: chr17 67148864-67148902. Max. coverage (+): 0. Max coverage (-): 0

Region: chr17 67148903-67148940. Max. coverage (+): 0. Max coverage (-): 0

Region: chr17 67148941-67148979. Max. coverage (+): 0. Max coverage (-): 0

Region: chr17 67148980-67149017. Max. coverage (+): 0. Max coverage (-): 0

Region: chr17 67149018-67149055. Max. coverage (+): 0. Max coverage (-): 0

Region: chr17 67149056-67149094. Max. coverage (+): 0. Max coverage (-): 0

Region: chr17 67149095-67149132. Max. coverage (+): 0. Max coverage (-): 0

Region: chr17 67149133-67149171. Max. coverage (+): 0. Max coverage (-): 0

Region: chr17 67149172-67149209. Max. coverage (+): 0. Max coverage (-): 0

Region: chr17 67149210-67149248. Max. coverage (+): 0. Max coverage (-): 0

Region: chr17 67149249-67149286. Max. coverage (+): 0. Max coverage (-): 0

Region: chr17 67149287-67149325. Max. coverage (+): 0. Max coverage (-): 0

Region: chr17 67149326-67149363. Max. coverage (+): 0. Max coverage (-): 0

Region: chr17 67149364-67149402. Max. coverage (+): 0. Max coverage (-): 0

Region: chr17 67149403-67149440. Max. coverage (+): 0. Max coverage (-): 0

Region: chr17 67149441-67149478. Max. coverage (+): 0. Max coverage (-): 0

Region: chr17 67149479-67149517. Max. coverage (+): 0. Max coverage (-): 4.63

Region: chr17 67149518-67149555. Max. coverage (+): 0. Max coverage (-): 0

Region: chr17 67149556-67149594. Max. coverage (+): 0. Max coverage (-): 5.54

Region: chr17 67149595-67149632. Max. coverage (+): 0. Max coverage (-): 0.13

Region: chr17 67149633-67149671. Max. coverage (+): 0. Max coverage (-): 4.59

Region: chr17 67149672-67149709. Max. coverage (+): 0. Max coverage (-): 6.68

Region: chr17 67149710-67149748. Max. coverage (+): 0. Max coverage (-): 7.3

Region: chr17 67149749-67149786. Max. coverage (+): 0. Max coverage (-): 0.89

Region: chr17 67149787-67149825. Max. coverage (+): 0. Max coverage (-): 46.69

Region: chr17 67149826-67149863. Max. coverage (+): 0. Max coverage (-): 74.22

Region: chr17 67149864-67149902. Max. coverage (+): 0. Max coverage (-): 4.35

Region: chr17 67149903-67149940. Max. coverage (+): 0. Max coverage (-): 0

Region: chr17 67149941-67149978. Max. coverage (+): 0. Max coverage (-): 0

Region: chr17 67149979-67150017. Max. coverage (+): 0. Max coverage (-): 19.12

Region: chr17 67150018-67150055. Max. coverage (+): 0. Max coverage (-): 20.31

Region: chr17 67150056-67150094. Max. coverage (+): 0. Max coverage (-): 0

Region: chr17 67150095-67150132. Max. coverage (+): 0. Max coverage (-): 40.64

Region: chr17 67150133-67150171. Max. coverage (+): 0. Max coverage (-): 29.89

Region: chr17 67150172-67150209. Max. coverage (+): 0. Max coverage (-): 11.01

Region: chr17 67150210-67150248. Max. coverage (+): 0. Max coverage (-): 0

Region: chr17 67150249-67150286. Max. coverage (+): 0. Max coverage (-): 0

Region: chr17 67150287-67150325. Max. coverage (+): 0. Max coverage (-): 0

Region: chr17 67150326-67150363. Max. coverage (+): 0. Max coverage (-): 0

Region: chr17 67150364-67150401. Max. coverage (+): 0. Max coverage (-): 0

Region: chr17 67150402-67150440. Max. coverage (+): 0. Max coverage (-): 31.76

Region: chr17 67150441-67150478. Max. coverage (+): 0. Max coverage (-): 3.44

Region: chr17 67150479-67150517. Max. coverage (+): 0. Max coverage (-): 12.81

Region: chr17 67150518-67150555. Max. coverage (+): 0. Max coverage (-): 9.78

Region: chr17 67150556-67150594. Max. coverage (+): 0. Max coverage (-): 21.73

Region: chr17 67150595-67150632. Max. coverage (+): 0. Max coverage (-): 31.3

Region: chr17 67150633-67150671. Max. coverage (+): 0. Max coverage (-): 8.96

Region: chr17 67150672-67150709. Max. coverage (+): 0. Max coverage (-): 12.87

Region: chr17 67150710-67150748. Max. coverage (+): 0. Max coverage (-): 0

Region: chr17 67150749-67150786. Max. coverage (+): 0. Max coverage (-): 0

Region: chr17 67150787-67150824. Max. coverage (+): 0. Max coverage (-): 0

Region: chr17 67150825-67150863. Max. coverage (+): 0. Max coverage (-): 35.96

Region: chr17 67150864-67150901. Max. coverage (+): 0. Max coverage (-): 41.75

Region: chr17 67150902-67150940. Max. coverage (+): 0. Max coverage (-): 161.6

Region: chr17 67150941-67150978. Max. coverage (+): 0. Max coverage (-): 94.53

Region: chr17 67150979-67151017. Max. coverage (+): 0. Max coverage (-): 87.58

Region: chr17 67151018-67151055. Max. coverage (+): 0. Max coverage (-): 88.52

Region: chr17 67151056-67151094. Max. coverage (+): 0. Max coverage (-): 14.88

Region: chr17 67151095-67151132. Max. coverage (+): 0. Max coverage (-): 32.32

Region: chr17 67151133-67151171. Max. coverage (+): 0. Max coverage (-): 43.89

Region: chr17 67151172-67151209. Max. coverage (+): 0. Max coverage (-): 36.27

Region: chr17 67151210-67151248. Max. coverage (+): 6.99. Max coverage (-): 127.79

Region: chr17 67151249-67151286. Max. coverage (+): 0. Max coverage (-): 54.54

Region: chr17 67151287-67151324. Max. coverage (+): 0. Max coverage (-): 30.94

Region: chr17 67151325-67151363. Max. coverage (+): 0. Max coverage (-): 27.98

Region: chr17 67151364-67151401. Max. coverage (+): 0. Max coverage (-): 2.8

Region: chr17 67151402-67151440. Max. coverage (+): 0. Max coverage (-): 0

Region: chr17 67151441-67151478. Max. coverage (+): 0. Max coverage (-): 34.03

Region: chr17 67151479-67151517. Max. coverage (+): 0. Max coverage (-): 18.71

Region: chr17 67151518-67151555. Max. coverage (+): 0. Max coverage (-): 22.12

Region: chr17 67151556-67151594. Max. coverage (+): 0. Max coverage (-): 43.12

Region: chr17 67151595-67151632. Max. coverage (+): 0. Max coverage (-): 4.61

Region: chr17 67151633-67151671. Max. coverage (+): 0. Max coverage (-): 23.7

Region: chr17 67151672-67151709. Max. coverage (+): 0. Max coverage (-): 9.9

Region: chr17 67151710-67151747. Max. coverage (+): 0. Max coverage (-): 13.86

Region: chr17 67151748-67151786. Max. coverage (+): 0. Max coverage (-): 0

Region: chr17 67151787-67151824. Max. coverage (+): 0. Max coverage (-): 4.48

Region: chr17 67151825-67151863. Max. coverage (+): 0. Max coverage (-): 14.87

Region: chr17 67151864-67151901. Max. coverage (+): 0. Max coverage (-): 49.15

Region: chr17 67151902-67151940. Max. coverage (+): 0. Max coverage (-): 1.56

Region: chr17 67151941-67151978. Max. coverage (+): 0. Max coverage (-): 81.93

Region: chr17 67151979-67152017. Max. coverage (+): 0. Max coverage (-): 12.15

Region: chr17 67152018-67152055. Max. coverage (+): 0. Max coverage (-): 0

Region: chr17 67152056-67152094. Max. coverage (+): 0. Max coverage (-): 33.01

Region: chr17 67152095-67152132. Max. coverage (+): 0. Max coverage (-): 29.05

Region: chr17 67152133-67152171. Max. coverage (+): 0. Max coverage (-): 54.39

Region: chr17 67152172-67152209. Max. coverage (+): 0. Max coverage (-): 20.66

Region: chr17 67152210-67152247. Max. coverage (+): 0. Max coverage (-): 0

Region: chr17 67152248-67152286. Max. coverage (+): 0. Max coverage (-): 3.6

Region: chr17 67152287-67152324. Max. coverage (+): 0. Max coverage (-): 0

Region: chr17 67152325-67152363. Max. coverage (+): 0. Max coverage (-): 0

Region: chr17 67152364-67152401. Max. coverage (+): 0. Max coverage (-): 1.01

Region: chr17 67152402-67152440. Max. coverage (+): 0. Max coverage (-): 10.96

Region: chr17 67152441-67152478. Max. coverage (+): 0. Max coverage (-): 12.25

Region: chr17 67152479-67152517. Max. coverage (+): 0. Max coverage (-): 18.14

Region: chr17 67152518-67152555. Max. coverage (+): 0. Max coverage (-): 11.14

Region: chr17 67152556-67152594. Max. coverage (+): 0. Max coverage (-): 11.14

Region: chr17 67152595-67152632. Max. coverage (+): 0. Max coverage (-): 16.84

Region: chr17 67152633-67152670. Max. coverage (+): 0. Max coverage (-): 16.86

Region: chr17 67152671-67152709. Max. coverage (+): 0. Max coverage (-): 15.53

Region: chr17 67152710-67152747. Max. coverage (+): 0. Max coverage (-): 38.49

Region: chr17 67152748-67152786. Max. coverage (+): 0. Max coverage (-): 0

Region: chr17 67152787-67152824. Max. coverage (+): 0. Max coverage (-): 0

Region: chr17 67152825-67152863. Max. coverage (+): 0. Max coverage (-): 0

Region: chr17 67152864-67152901. Max. coverage (+): 0. Max coverage (-): 50.22

Region: chr17 67152902-67152940. Max. coverage (+): 0. Max coverage (-): 38.39

Region: chr17 67152941-67152978. Max. coverage (+): 0. Max coverage (-): 39.7

Region: chr17 67152979-67153017. Max. coverage (+): 0. Max coverage (-): 17.69

Region: chr17 67153018-67153055. Max. coverage (+): 0. Max coverage (-): 15.55

Region: chr17 67153056-67153094. Max. coverage (+): 0. Max coverage (-): 9.69

Region: chr17 67153095-67153132. Max. coverage (+): 0. Max coverage (-): 0

Region: chr17 67153133-67153170. Max. coverage (+): 0. Max coverage (-): 27.07

Region: chr17 67153171-67153209. Max. coverage (+): 0. Max coverage (-): 27.07

Region: chr17 67153210-67153247. Max. coverage (+): 0. Max coverage (-): 35.09

Region: chr17 67153248-67153286. Max. coverage (+): 0. Max coverage (-): 33.96

Region: chr17 67153287-67153324. Max. coverage (+): 0. Max coverage (-): 33.96

Region: chr17 67153325-67153363. Max. coverage (+): 0. Max coverage (-): 0

Region: chr17 67153364-67153401. Max. coverage (+): 0. Max coverage (-): 0.03

Region: chr17 67153402-67153440. Max. coverage (+): 0. Max coverage (-): 0

Region: chr17 67153441-67153478. Max. coverage (+): 0. Max coverage (-): 24.54

Region: chr17 67153479-67153517. Max. coverage (+): 0. Max coverage (-): 6.01

Region: chr17 67153518-67153555. Max. coverage (+): 0. Max coverage (-): 25.05

Region: chr17 67153556-67153593. Max. coverage (+): 0. Max coverage (-): 31.25

Region: chr17 67153594-67153632. Max. coverage (+): 0. Max coverage (-): 5.74

Region: chr17 67153633-67153670. Max. coverage (+): 0. Max coverage (-): 0

Region: chr17 67153671-67153709. Max. coverage (+): 0. Max coverage (-): 16

Region: chr17 67153710-67153747. Max. coverage (+): 0. Max coverage (-): 17.53

Region: chr17 67153748-67153786. Max. coverage (+): 0. Max coverage (-): 73.53

Region: chr17 67153787-67153824. Max. coverage (+): 0. Max coverage (-): 5.87

Region: chr17 67153825-67153863. Max. coverage (+): 0. Max coverage (-): 45.69

Region: chr17 67153864-67153901. Max. coverage (+): 0. Max coverage (-): 2.54

Region: chr17 67153902-67153940. Max. coverage (+): 0. Max coverage (-): 52.73

Region: chr17 67153941-67153978. Max. coverage (+): 0. Max coverage (-): 4.92

Region: chr17 67153979-67154017. Max. coverage (+): 0. Max coverage (-): 17.01

Region: chr17 67154018-67154055. Max. coverage (+): 0. Max coverage (-): 6.03

Region: chr17 67154056-67154093. Max. coverage (+): 0. Max coverage (-): 29.54

Region: chr17 67154094-67154132. Max. coverage (+): 0. Max coverage (-): 61.95

Region: chr17 67154133-67154170. Max. coverage (+): 0. Max coverage (-): 20.93

Region: chr17 67154171-67154209. Max. coverage (+): 0. Max coverage (-): 31.03

Region: chr17 67154210-67154247. Max. coverage (+): 0. Max coverage (-): 11.49

Region: chr17 67154248-67154286. Max. coverage (+): 0. Max coverage (-): 34.18

Region: chr17 67154287-67154324. Max. coverage (+): 0. Max coverage (-): 27.29

Region: chr17 67154325-67154363. Max. coverage (+): 0. Max coverage (-): 16.54

Region: chr17 67154364-67154401. Max. coverage (+): 0. Max coverage (-): 7.55

Region: chr17 67154402-67154440. Max. coverage (+): 0. Max coverage (-): 5.55

Region: chr17 67154441-67154478. Max. coverage (+): 1.6. Max coverage (-): 31.27

Region: chr17 67154479-67154516. Max. coverage (+): 0. Max coverage (-): 3.98

Region: chr17 67154517-67154555. Max. coverage (+): 0. Max coverage (-): 4.56

Region: chr17 67154556-67154593. Max. coverage (+): 0. Max coverage (-): 8.54

Region: chr17 67154594-67154632. Max. coverage (+): 0. Max coverage (-): 7.62

Region: chr17 67154633-67154670. Max. coverage (+): 0. Max coverage (-): 8.23

Region: chr17 67154671-67154709. Max. coverage (+): 0. Max coverage (-): 1.54

Region: chr17 67154710-67154747. Max. coverage (+): 0. Max coverage (-): 0

Region: chr17 67154748-67154786. Max. coverage (+): 0. Max coverage (-): 0

Region: chr17 67154787-67154824. Max. coverage (+): 0. Max coverage (-): 0

Region: chr17 67154825-67154863. Max. coverage (+): 0. Max coverage (-): 0

Region: chr17 67154864-67154901. Max. coverage (+): 0. Max coverage (-): 0

Region: chr17 67154902-67154940. Max. coverage (+): 0. Max coverage (-): 0

Region: chr17 67154941-67154978. Max. coverage (+): 0. Max coverage (-): 0

Region: chr17 67154979-67155016. Max. coverage (+): 0. Max coverage (-): 0.8

Region: chr17 67155017-67155055. Max. coverage (+): 0. Max coverage (-): 4.58

Region: chr17 67155056-67155093. Max. coverage (+): 0. Max coverage (-): 5.75

Region: chr17 67155094-67155132. Max. coverage (+): 0. Max coverage (-): 0

Region: chr17 67155133-67155170. Max. coverage (+): 0. Max coverage (-): 0

Region: chr17 67155171-67155209. Max. coverage (+): 0. Max coverage (-): 37.44

Region: chr17 67155210-67155247. Max. coverage (+): 0. Max coverage (-): 2.18

Region: chr17 67155248-67155286. Max. coverage (+): 0. Max coverage (-): 0

Region: chr17 67155287-67155324. Max. coverage (+): 0. Max coverage (-): 0.67

Region: chr17 67155325-67155363. Max. coverage (+): 0. Max coverage (-): 4.87

Region: chr17 67155364-67155401. Max. coverage (+): 0. Max coverage (-): 3.85

Region: chr17 67155402-67155439. Max. coverage (+): 4.2. Max coverage (-): 15.69

Region: chr17 67155440-67155478. Max. coverage (+): 0. Max coverage (-): 17.88

Region: chr17 67155479-67155516. Max. coverage (+): 0. Max coverage (-): 24.51

Region: chr17 67155517-67155555. Max. coverage (+): 0. Max coverage (-): 3.02

Region: chr17 67155556-67155593. Max. coverage (+): 0. Max coverage (-): 8.64

Region: chr17 67155594-67155632. Max. coverage (+): 0. Max coverage (-): 0

Region: chr17 67155633-67155670. Max. coverage (+): 0. Max coverage (-): 0

Region: chr17 67155671-67155709. Max. coverage (+): 0. Max coverage (-): 6.38

Region: chr17 67155710-67155747. Max. coverage (+): 0. Max coverage (-): 17.1

Region: chr17 67155748-67155786. Max. coverage (+): 0. Max coverage (-): 6.38

Region: chr17 67155787-67155824. Max. coverage (+): 0. Max coverage (-): 6.38

Region: chr17 67155825-67155862. Max. coverage (+): 0. Max coverage (-): 13.64

Region: chr17 67155863-67155901. Max. coverage (+): 0. Max coverage (-): 13.64

Region: chr17 67155902-67155939. Max. coverage (+): 0. Max coverage (-): 0

Region: chr17 67155940-67155978. Max. coverage (+): 0. Max coverage (-): 0

Region: chr17 67155979-67156016. Max. coverage (+): 0. Max coverage (-): 0

Region: chr17 67156017-67156055. Max. coverage (+): 0. Max coverage (-): 4.6

Region: chr17 67156056-67156093. Max. coverage (+): 0. Max coverage (-): 8.67

Region: chr17 67156094-67156132. Max. coverage (+): 0. Max coverage (-): 0

Region: chr17 67156133-67156170. Max. coverage (+): 0. Max coverage (-): 25.41

Region: chr17 67156171-67156209. Max. coverage (+): 0. Max coverage (-): 62.27

Region: chr17 67156210-67156247. Max. coverage (+): 0. Max coverage (-): 10.73

Region: chr17 67156248-67156286. Max. coverage (+): 0. Max coverage (-): 37.85

Region: chr17 67156287-67156324. Max. coverage (+): 0. Max coverage (-): 50

Region: chr17 67156325-67156362. Max. coverage (+): 0. Max coverage (-): 26.44

Region: chr17 67156363-67156401. Max. coverage (+): 0. Max coverage (-): 11.22

Region: chr17 67156402-67156439. Max. coverage (+): 0. Max coverage (-): 17.16

Region: chr17 67156440-67156478. Max. coverage (+): 0. Max coverage (-): 0

Region: chr17 67156479-67156516. Max. coverage (+): 0. Max coverage (-): 0

Region: chr17 67156517-67156555. Max. coverage (+): 0. Max coverage (-): 0

Region: chr17 67156556-67156593. Max. coverage (+): 0. Max coverage (-): 0

Region: chr17 67156594-67156632. Max. coverage (+): 0. Max coverage (-): 0

Region: chr17 67156633-67156670. Max. coverage (+): 0. Max coverage (-): 0

Region: chr17 67156671-67156709. Max. coverage (+): 0. Max coverage (-): 0

Region: chr17 67156710-67156747. Max. coverage (+): 0. Max coverage (-): 62.75

Region: chr17 67156748-67156785. Max. coverage (+): 0. Max coverage (-): 0

Region: chr17 67156786-67156824. Max. coverage (+): 0. Max coverage (-): 5.33

Region: chr17 67156825-67156862. Max. coverage (+): 0. Max coverage (-): 5.33

Region: chr17 67156863-67156901. Max. coverage (+): 0. Max coverage (-): 10.11

Region: chr17 67156902-67156939. Max. coverage (+): 0. Max coverage (-): 0

Region: chr17 67156940-67156978. Max. coverage (+): 0. Max coverage (-): 14.52

Region: chr17 67156979-67157016. Max. coverage (+): 0. Max coverage (-): 0.85

Region: chr17 67157017-67157055. Max. coverage (+): 0. Max coverage (-): 13.52

Region: chr17 67157056-67157093. Max. coverage (+): 0. Max coverage (-): 12.03

Region: chr17 67157094-67157132. Max. coverage (+): 0. Max coverage (-): 56.6

Region: chr17 67157133-67157170. Max. coverage (+): 0. Max coverage (-): 33.27

Region: chr17 67157171-67157209. Max. coverage (+): 2.27. Max coverage (-): 10.07

Region: chr17 67157210-67157247. Max. coverage (+): 0. Max coverage (-): 7.27

Region: chr17 67157248-67157285. Max. coverage (+): 0. Max coverage (-): 7.27

Region: chr17 67157286-67157324. Max. coverage (+): 0. Max coverage (-): 5.09

Region: chr17 67157325-67157362. Max. coverage (+): 0. Max coverage (-): 0.84

Region: chr17 67157363-67157401. Max. coverage (+): 0. Max coverage (-): 10.77

Region: chr17 67157402-67157439. Max. coverage (+): 0. Max coverage (-): 0

Region: chr17 67157440-67157478. Max. coverage (+): 0. Max coverage (-): 8.58

Region: chr17 67157479-67157516. Max. coverage (+): 0. Max coverage (-): 48.32

Region: chr17 67157517-67157555. Max. coverage (+): 0. Max coverage (-): 0.37

Region: chr17 67157556-67157593. Max. coverage (+): 0. Max coverage (-): 42.79

Region: chr17 67157594-67157632. Max. coverage (+): 0. Max coverage (-): 50

Region: chr17 67157633-67157670. Max. coverage (+): 0. Max coverage (-): 45.35

Region: chr17 67157671-67157708. Max. coverage (+): 0. Max coverage (-): 30.38

Region: chr17 67157709-67157747. Max. coverage (+): 0. Max coverage (-): 60.75

Region: chr17 67157748-67157785. Max. coverage (+): 0. Max coverage (-): 103.8

Region: chr17 67157786-67157824. Max. coverage (+): 0. Max coverage (-): 0

Region: chr17 67157825-67157862. Max. coverage (+): 0. Max coverage (-): 0

Region: chr17 67157863-67157901. Max. coverage (+): 0. Max coverage (-): 0

Region: chr17 67157902-67157939. Max. coverage (+): 0. Max coverage (-): 0

Region: chr17 67157940-67157978. Max. coverage (+): 0. Max coverage (-): 0

Region: chr17 67157979-67158016. Max. coverage (+): 0. Max coverage (-): 1.52

Region: chr17 67158017-67158055. Max. coverage (+): 0. Max coverage (-): 3.35

Region: chr17 67158056-67158093. Max. coverage (+): 0. Max coverage (-): 0

Region: chr17 67158094-67158132. Max. coverage (+): 0. Max coverage (-): 11.63

Region: chr17 67158133-67158170. Max. coverage (+): 1.23. Max coverage (-): 12.45

Region: chr17 67158171-67158208. Max. coverage (+): 0. Max coverage (-): 0

Region: chr17 67158209-67158247. Max. coverage (+): 0. Max coverage (-): 26.18

Region: chr17 67158248-67158285. Max. coverage (+): 0. Max coverage (-): 4.9

Region: chr17 67158286-67158324. Max. coverage (+): 0. Max coverage (-): 2.85

Region: chr17 67158325-67158362. Max. coverage (+): 0. Max coverage (-): 4.86

Region: chr17 67158363-67158401. Max. coverage (+): 0. Max coverage (-): 17.05

Region: chr17 67158402-67158439. Max. coverage (+): 0. Max coverage (-): 8.01

Region: chr17 67158440-67158478. Max. coverage (+): 0. Max coverage (-): 0

Region: chr17 67158479-67158516. Max. coverage (+): 0. Max coverage (-): 0

Region: chr17 67158517-67158555. Max. coverage (+): 0. Max coverage (-): 33.7

Region: chr17 67158556-67158593. Max. coverage (+): 0. Max coverage (-): 5.54

Region: chr17 67158594-67158631. Max. coverage (+): 0. Max coverage (-): 8.88

Region: chr17 67158632-67158670. Max. coverage (+): 0. Max coverage (-): 8.2

Region: chr17 67158671-67158708. Max. coverage (+): 0. Max coverage (-): 16.69

Region: chr17 67158709-67158747. Max. coverage (+): 0. Max coverage (-): 33.77

Region: chr17 67158748-67158785. Max. coverage (+): 0. Max coverage (-): 36.29

Region: chr17 67158786-67158824. Max. coverage (+): 0. Max coverage (-): 7.44

Region: chr17 67158825-67158862. Max. coverage (+): 0. Max coverage (-): 1.7

Region: chr17 67158863-67158901. Max. coverage (+): 0. Max coverage (-): 12.8

Region: chr17 67158902-67158939. Max. coverage (+): 0. Max coverage (-): 38.86

Region: chr17 67158940-67158978. Max. coverage (+): 0. Max coverage (-): 11.18

Region: chr17 67158979-67159016. Max. coverage (+): 0. Max coverage (-): 0

Region: chr17 67159017-67159055. Max. coverage (+): 0. Max coverage (-): 0

Region: chr17 67159056-67159093. Max. coverage (+): 0. Max coverage (-): 0

Region: chr17 67159094-67159131. Max. coverage (+): 0. Max coverage (-): 0

Region: chr17 67159132-67159170. Max. coverage (+): 0. Max coverage (-): 0

Region: chr17 67159171-67159208. Max. coverage (+): 0. Max coverage (-): 0

Region: chr17 67159209-67159247. Max. coverage (+): 0. Max coverage (-): 0

Region: chr17 67159248-67159285. Max. coverage (+): 0. Max coverage (-): 0

Region: chr17 67159286-67159324. Max. coverage (+): 0. Max coverage (-): 0

Region: chr17 67159325-67159362. Max. coverage (+): 0. Max coverage (-): 0

Region: chr17 67159363-67159401. Max. coverage (+): 0. Max coverage (-): 0

Region: chr17 67159402-67159439. Max. coverage (+): 0. Max coverage (-): 0

Region: chr17 67159440-67159478. Max. coverage (+): 2.22. Max coverage (-): 0

Region: chr17 67159479-67159516. Max. coverage (+): 3.75. Max coverage (-): 8.68

Region: chr17 67159517-67159554. Max. coverage (+): 0. Max coverage (-): 31.45

Region: chr17 67159555-67159593. Max. coverage (+): 0. Max coverage (-): 31.45

Region: chr17 67159594-67159631. Max. coverage (+): 0. Max coverage (-): 33.47

Region: chr17 67159632-67159670. Max. coverage (+): 0. Max coverage (-): 20.02

Region: chr17 67159671-67159708. Max. coverage (+): 0. Max coverage (-): 11.59

Region: chr17 67159709-67159747. Max. coverage (+): 0. Max coverage (-): 0

Region: chr17 67159748-67159785. Max. coverage (+): 0. Max coverage (-): 28.04

Region: chr17 67159786-67159824. Max. coverage (+): 0. Max coverage (-): 43.92

Region: chr17 67159825-67159862. Max. coverage (+): 0. Max coverage (-): 0

Region: chr17 67159863-67159901. Max. coverage (+): 0. Max coverage (-): 0

Region: chr17 67159902-67159939. Max. coverage (+): 0. Max coverage (-): 0

Region: chr17 67159940-67159978. Max. coverage (+): 0. Max coverage (-): 0

Region: chr17 67159979-67160016. Max. coverage (+): 0. Max coverage (-): 0

Region: chr17 67160017-67160054. Max. coverage (+): 0. Max coverage (-): 1.17

Region: chr17 67160055-67160093. Max. coverage (+): 0. Max coverage (-): 69.11

Region: chr17 67160094-67160131. Max. coverage (+): 0. Max coverage (-): 0

Region: chr17 67160132-67160170. Max. coverage (+): 0. Max coverage (-): 0

Region: chr17 67160171-67160208. Max. coverage (+): 0. Max coverage (-): 0

Region: chr17 67160209-67160247. Max. coverage (+): 0. Max coverage (-): 0

Region: chr17 67160248-67160285. Max. coverage (+): 0. Max coverage (-): 0

Region: chr17 67160286-67160324. Max. coverage (+): 0. Max coverage (-): 0

Region: chr17 67160325-67160362. Max. coverage (+): 0. Max coverage (-): 47.07

Region: chr17 67160363-67160401. Max. coverage (+): 0. Max coverage (-): 55.03

Region: chr17 67160402-67160439. Max. coverage (+): 0. Max coverage (-): 16.17

Region: chr17 67160440-67160477. Max. coverage (+): 0. Max coverage (-): 2.82

Region: chr17 67160478-67160516. Max. coverage (+): 0. Max coverage (-): 0

Region: chr17 67160517-67160554. Max. coverage (+): 0. Max coverage (-): 0

Region: chr17 67160555-67160593. Max. coverage (+): 0. Max coverage (-): 30.45

Region: chr17 67160594-67160631. Max. coverage (+): 7.01. Max coverage (-): 29.22

Region: chr17 67160632-67160670. Max. coverage (+): 0. Max coverage (-): 42.66

Region: chr17 67160671-67160708. Max. coverage (+): 0. Max coverage (-): 77.93

Region: chr17 67160709-67160747. Max. coverage (+): 0. Max coverage (-): 37.09

Region: chr17 67160748-67160785. Max. coverage (+): 0. Max coverage (-): 6.05

Region: chr17 67160786-67160824. Max. coverage (+): 0. Max coverage (-): 5.68

Region: chr17 67160825-67160862. Max. coverage (+): 0. Max coverage (-): 9.17

Region: chr17 67160863-67160900. Max. coverage (+): 0. Max coverage (-): 5.72

Region: chr17 67160901-67160939. Max. coverage (+): 0. Max coverage (-): 0

Region: chr17 67160940-67160977. Max. coverage (+): 0. Max coverage (-): 25.09

Region: chr17 67160978-67161016. Max. coverage (+): 0. Max coverage (-): 8.39

Region: chr17 67161017-67161054. Max. coverage (+): 0. Max coverage (-): 1.61

Region: chr17 67161055-67161093. Max. coverage (+): 0. Max coverage (-): 2.12

Region: chr17 67161094-67161131. Max. coverage (+): 0. Max coverage (-): 3.17

Region: chr17 67161132-67161170. Max. coverage (+): 0. Max coverage (-): 5.95

Region: chr17 67161171-67161208. Max. coverage (+): 0. Max coverage (-): 2.16

Region: chr17 67161209-67161247. Max. coverage (+): 3.85. Max coverage (-): 0

Region: chr17 67161248-67161285. Max. coverage (+): 3.85. Max coverage (-): 0

Region: chr17 67161286-67161324. Max. coverage (+): 0. Max coverage (-): 0

Region: chr17 67161325-67161362. Max. coverage (+): 0. Max coverage (-): 0

Region: chr17 67161363-67161400. Max. coverage (+): 1.68. Max coverage (-): 0

Region: chr17 67161401-67161439. Max. coverage (+): 0. Max coverage (-): 5.58

Region: chr17 67161440-67161477. Max. coverage (+): 6.93. Max coverage (-): 9.56

Region: chr17 67161478-67161516. Max. coverage (+): 0. Max coverage (-): 6.58

Region: chr17 67161517-67161554. Max. coverage (+): 0. Max coverage (-): 6.55

Region: chr17 67161555-67161593. Max. coverage (+): 0. Max coverage (-): 0

Region: chr17 67161594-67161631. Max. coverage (+): 0. Max coverage (-): 0

Region: chr17 67161632-67161670. Max. coverage (+): 0. Max coverage (-): 0

Region: chr17 67161671-67161708. Max. coverage (+): 0. Max coverage (-): 0

Region: chr17 67161709-67161747. Max. coverage (+): 0. Max coverage (-): 5.75

Region: chr17 67161748-67161785. Max. coverage (+): 0. Max coverage (-): 0

Region: chr17 67161786-67161823. Max. coverage (+): 0.23. Max coverage (-): 0

Region: chr17 67161824-67161862. Max. coverage (+): 0. Max coverage (-): 1

Region: chr17 67161863-67161900. Max. coverage (+): 0. Max coverage (-): 0

Region: chr17 67161901-67161939. Max. coverage (+): 0. Max coverage (-): 0

Region: chr17 67161940-67161977. Max. coverage (+): 0. Max coverage (-): 0

Region: chr17 67161978-67162016. Max. coverage (+): 0. Max coverage (-): 0

Region: chr17 67162017-67162054. Max. coverage (+): 0. Max coverage (-): 0

Region: chr17 67162055-67162093. Max. coverage (+): 0. Max coverage (-): 0

Region: chr17 67162094-67162131. Max. coverage (+): 0. Max coverage (-): 0

Region: chr17 67162132-67162170. Max. coverage (+): 0. Max coverage (-): 0

Region: chr17 67162171-67162208. Max. coverage (+): 0. Max coverage (-): 0

Region: chr17 67162209-67162247. Max. coverage (+): 0. Max coverage (-): 6.3

Region: chr17 67162248-67162285. Max. coverage (+): 0. Max coverage (-): 6.3

Region: chr17 67162286-67162323. Max. coverage (+): 0. Max coverage (-): 0

Region: chr17 67162324-67162362. Max. coverage (+): 0. Max coverage (-): 0

Region: chr17 67162363-67162400. Max. coverage (+): 0. Max coverage (-): 0.88

Region: chr17 67162401-67162439. Max. coverage (+): 4.61. Max coverage (-): 0

Region: chr17 67162440-67162477. Max. coverage (+): 5.69. Max coverage (-): 0

Region: chr17 67162478-. Max. coverage (+): 0. Max coverage (-): 0

RepeatMasker Color Code

**+**

100-98% Identity

<98-95% Identity

<95-90% Identity

<90-85% Identity

<85-80% Identity

<80-75% Identity

<75-70% Identity

<70% Identity

**-**

Gene Set Color Code

**+**

Gene

Pseudogene

**-**

Topology/Coverage Color Code

Coverage Plus Strand

Coverage Minus Strand

Mainstrand: Plus

Mainstrand: Minus

Complementary Strand

Flanking Region  
(if option -flank >0)

Gene Set Annotation  
  
RepeatMasker Annotation  

**1. BTLTR1J3**: 67143352-67143749 (-), Divergence to consensus: 5.2%  
**2. MER33**: 67144303-67144578 (+), Divergence to consensus: 42.6%  
**3. CHR-2A**: 67145108-67145442 (-), Divergence to consensus: 34.4%  
**4. L1-2\_BT**: 67145662-67145957 (-), Divergence to consensus: 40.2%  
**5. Bov-tA2**: 67146378-67146562 (+), Divergence to consensus: 28.1%  
**6. BOV-A2**: 67147055-67147116 (+), Divergence to consensus: 3.2%  
**7. MLT1B**: 67147360-67147538 (+), Divergence to consensus: 22.5%  
**8. Bov-tA2**: 67147785-67147993 (-), Divergence to consensus: 17.6%  
**9. SINE2-2\_BT**: 67148150-67148278 (-), Divergence to consensus: 22.6%  
**10. AT\_rich**: 67148282-67148302 (+), Divergence to consensus: 42.9%  
**11. L1MB3**: 67148474-67149091 (+), Divergence to consensus: 29%  
**12. Bov-tA2**: 67149092-67149276 (+), Divergence to consensus: 20%  
**13. L1MB3**: 67149277-67149408 (+), Divergence to consensus: 32.2%  
**14. MIR**: 67150245-67150387 (-), Divergence to consensus: 42.7%  
**15. MER70C**: 67150729-67150803 (-), Divergence to consensus: 25.3%  
**16. (CACCC)n**: 67156449-67156543 (+), Divergence to consensus: 30.4%  
**17. ART2A**: 67157833-67157952 (+), Divergence to consensus: 20.8%  
**18. L1ME3A**: 67158994-67159133 (+), Divergence to consensus: 37.6%  
**19. L1M5**: 67159114-67159435 (+), Divergence to consensus: 37.2%  
**20. MIRc**: 67159837-67160023 (+), Divergence to consensus: 47.3%  
**21. MIR**: 67160113-67160350 (-), Divergence to consensus: 41.6%  
**22. MIRc**: 67161565-67161667 (+), Divergence to consensus: 32%  
**23. MIR**: 67161886-67162026 (-), Divergence to consensus: 41.7%

  
Transcription Factor Binding Sites  

**RFX4\_2** (Sequence: GTATCCAGG (-): 67161482)  
**Gata4** (Sequence: AGATAAC (-): 67147693)  
**Gata4** (Sequence: AGATAAG (-): 67156841)  
**Gata4** (Sequence: AGATAAC (-): 67157262)  
**SPZ1** (Sequence: AGGGTTTCAG (+): 67159798)  
**Gata4** (Sequence: CTTATCT (+): 67152923)
