# Supplementary material for: Small RNA sequencing of cryopreserved semen from single bull revealed altered miRNAs and piRNAs expression between High- and Low-motile sperm populations
Source: BMC Genomics. 2017 Jan 4;18:14. doi: 10.1186/s12864-016-3394-7 (PMC5209821; doi:10.1186/s12864-016-3394-7)
Supplement: Additional file 4: — Details for each piRNA clusters found in Low Motile (LM) sperm fraction. Genes, repeats, transposable elements and transcription factors binding sites falling within the cluster regions were reported. (ZIP 1034 kb) [file 12864_2016_3394_MOESM4_ESM.zip › 18.html]

piRNA cluster 18


Predicted piRNA cluster no. 18     previous   next
  

Show proTRAC run info
Hide proTRAC run info

================================= proTRAC ====================================  
VERSION: 2.1                                    LAST MODIFIED: 06. October 2015  
  
Please cite:  
Rosenkranz D, Zischler H. proTRAC - a software for probabilistic piRNA cluster  
detection, visualization and analysis. 2012. BMC Bioinformatics 13:5.  
  
and (for proTRAC 2.0 and later):  
Rosenkranz D, Rudloff S, Bastuck K, Ketting RF, Zischler H. Tupaia small RNAs  
provide insights into function and evolution of RNAi-based transposon defense  
in mammals. 2015. RNA 21(5):911-922.  
  
Contact:  
David Rosenkranz  
Institute of Anthropology, small RNA group  
Johannes Gutenberg University Mainz  
email: rosenkranz@uni-mainz.de  
  
You can find the latest proTRAC version at:  
http://sourceforge.net/projects/protrac/files  
http://www.smallRNAgroup-mainz.de/software  
==============================================================================  
  
PARAMETERS:  
Map file: .............../storage/core/barbara/genhome/smallRNA/fertility/Sample\_not\_motile/pirna/Sample\_not\_motile\_26-33\_collapsed.fa.no-dust.map.weighted-10000-1000-b-0  
Genome file: ............/storage/core/barbara/genhome/smallRNA/fertility/Sample\_all/pirna/bt\_311\_chrY.fa  
RepeatMasker annotation: /storage/genomes/bt\_umd31/GCF\_000003055.6\_Bos\_taurus\_UMD\_3.1.1\_repeatMasker\_chr.out  
GeneSet:................./storage/core/barbara/genhome/smallRNA/fertility/Sample\_all/pirna/full.gtf  
  
Significant (p<=0.01) hit density will be calculated based  
on observed hit distribution.  
  
Sliding window size: ........................................ 5000 bp  
Sliding window increament: .................................. 1000 bp  
Normalize each hit by number of genomic hits: ............... 1 [0=no/1=yes]  
Normalize each hit by number of sequence reads: ............. 1 [0=no/1=yes]  
Normalize values (-> per million mapped reads): ............. 1 [0=no/1=yes]  
Min. fraction of hits with 1T(U) or 10A: .................... 0.75  
Alternatively: Min. fraction of hits with 1T(U) and 10A: .... 0.5  
Min. fraction of hits with typical piRNA length: ............ 0.75  
Typical piRNA length: ....................................... 26-33 nt  
Min. size of a piRNA cluster: ............................... 5000 bp.  
Min. number of hits (absolute): ............................. 0  
Min. number of hits (normalized): ........................... 0  
Min. fraction of hits on the mainstrand: .................... 0.75  
Top fraction of mapped sequences (in terms of read counts): . 1%  
Top fraction accounts for max. n% of sequence reads: ........ 90%  
Min. fraction of hits on each arm of a bidirectional cluster: 0.1  
Output image file for each cluster: ......................... 0 [0=no/1=yes]  
Output html file for each cluster: .......................... 1 [0=no/1=yes]  
Output a summary table: ..................................... 1 [0=no/1=yes]  
Output a FASTA file for each cluster (piRNA sequences): ..... 1 [0=no/1=yes]  
Output a FASTA file comprising cluster sequences: ........... 1 [0=no/1=yes]  
Search DNA motifs in clusters: .............................. 1 [0=no/1=yes]  
Output flanking sequences: +/- .............................. 0 bp  
Output ~.pTi file: .......................................... 1 [0=no/1=yes]  
==============================================================================  
  
  
Genome size (without gaps): ............ 2678902517 bp  
Gaps (N/X/-): .......................... 53837044 bp  
Mapped reads: .......................... 738059667487  
Non-identical sequences: ............... 277001  
Genomic hits: .......................... 533816  
Significant densitiy of mapped reads: .. 15118061 reads/kb

Show proTRAC cluster info
Hide proTRAC cluster info

|  |  |
| --- | --- |
| Location | chr17 |
| Coordinates | 67199038-67226976 |
| Size [bp] | 27939 |
| Sequence hit loci | 1643 |
| Mapped reads (normalized) | 4134803925.3 |
| Mapped reads (normalized) per kb | 147993984.2 |
| Normalized reads with 1T (1U) | 80.3% |
| Normalized reads with 10A | 33.5% |
| Normalized reads with length 26-33 nt | 100% |
| Normalized reads on the main strand(s) | 97.4% |
| Predicted directionality | mono:minus |

100%

0%

1T (1U)  
reads

10A reads

26-33 nt  
reads

reads on mainstrand

**Either the amount of reads with 1T (1U) OR 10A has to exceed 75% (set with option: -1Tor10A)  
Alternatively the amount of reads with 1T (1U) AND 10A has to exceed 50% (set with option: -1Tand10A)  
Minimum amount of reads with preferred size is 75% (set with option: -pisize)  
Minimum amount of reads on the main strand(s) is 75% (set with option: -clstrand)**

Show read coverage
Hide read coverage

WHAT DO I SEE HERE?  
This chart shows the location of mapped sequence reads within a predicted piRNA cluster. The color refers to the number of genomic hits produced by the sequence read in question. A dark red bar indicates that this sequence read produces many other hits elsewhere in the genome. Many adjacent red or yellow bars can indicate the presence of a multi-copy element such as transposons or rRNA genes. A dark green bar indicates that this sequence read maps uniquely to this locus.

1 hit

2-5 hits

6-10 hits

11-20 hits

21-50 hits

51-100 hits

> 100 hits

chr17

67199038

67226976

Gene Set

RepeatMasker

Mapped  
Reads

151.83

plus strand

minus strand

151.83

Region: chr17 67162479-67199065. Max. coverage (+): 0. Max coverage (-): 13.59

Region: chr17 67199066-67199121. Max. coverage (+): 0. Max coverage (-): 0

Region: chr17 67199122-67199177. Max. coverage (+): 0. Max coverage (-): 0

Region: chr17 67199178-67199233. Max. coverage (+): 0. Max coverage (-): 0

Region: chr17 67199234-67199289. Max. coverage (+): 0. Max coverage (-): 72.54

Region: chr17 67199290-67199345. Max. coverage (+): 0. Max coverage (-): 0

Region: chr17 67199346-67199401. Max. coverage (+): 0. Max coverage (-): 0

Region: chr17 67199402-67199457. Max. coverage (+): 0. Max coverage (-): 0

Region: chr17 67199458-67199512. Max. coverage (+): 0. Max coverage (-): 0

Region: chr17 67199513-67199568. Max. coverage (+): 0. Max coverage (-): 0

Region: chr17 67199569-67199624. Max. coverage (+): 0. Max coverage (-): 0

Region: chr17 67199625-67199680. Max. coverage (+): 0. Max coverage (-): 0

Region: chr17 67199681-67199736. Max. coverage (+): 0. Max coverage (-): 0

Region: chr17 67199737-67199792. Max. coverage (+): 0. Max coverage (-): 0

Region: chr17 67199793-67199848. Max. coverage (+): 0. Max coverage (-): 0

Region: chr17 67199849-67199904. Max. coverage (+): 0. Max coverage (-): 0

Region: chr17 67199905-67199959. Max. coverage (+): 0. Max coverage (-): 0

Region: chr17 67199960-67200015. Max. coverage (+): 0. Max coverage (-): 0

Region: chr17 67200016-67200071. Max. coverage (+): 0. Max coverage (-): 0

Region: chr17 67200072-67200127. Max. coverage (+): 0. Max coverage (-): 0

Region: chr17 67200128-67200183. Max. coverage (+): 0. Max coverage (-): 0

Region: chr17 67200184-67200239. Max. coverage (+): 0. Max coverage (-): 0

Region: chr17 67200240-67200295. Max. coverage (+): 0. Max coverage (-): 0

Region: chr17 67200296-67200351. Max. coverage (+): 0. Max coverage (-): 0

Region: chr17 67200352-67200407. Max. coverage (+): 3.05. Max coverage (-): 8.03

Region: chr17 67200408-67200462. Max. coverage (+): 0. Max coverage (-): 0

Region: chr17 67200463-67200518. Max. coverage (+): 0. Max coverage (-): 0

Region: chr17 67200519-67200574. Max. coverage (+): 0. Max coverage (-): 5.69

Region: chr17 67200575-67200630. Max. coverage (+): 0. Max coverage (-): 11.12

Region: chr17 67200631-67200686. Max. coverage (+): 0. Max coverage (-): 29.98

Region: chr17 67200687-67200742. Max. coverage (+): 0. Max coverage (-): 0

Region: chr17 67200743-67200798. Max. coverage (+): 0. Max coverage (-): 0

Region: chr17 67200799-67200854. Max. coverage (+): 0. Max coverage (-): 0

Region: chr17 67200855-67200909. Max. coverage (+): 0. Max coverage (-): 11.7

Region: chr17 67200910-67200965. Max. coverage (+): 0. Max coverage (-): 11.7

Region: chr17 67200966-67201021. Max. coverage (+): 0. Max coverage (-): 0

Region: chr17 67201022-67201077. Max. coverage (+): 0. Max coverage (-): 0

Region: chr17 67201078-67201133. Max. coverage (+): 0. Max coverage (-): 0

Region: chr17 67201134-67201189. Max. coverage (+): 0. Max coverage (-): 0

Region: chr17 67201190-67201245. Max. coverage (+): 0. Max coverage (-): 0

Region: chr17 67201246-67201301. Max. coverage (+): 0. Max coverage (-): 0

Region: chr17 67201302-67201356. Max. coverage (+): 0. Max coverage (-): 0

Region: chr17 67201357-67201412. Max. coverage (+): 0. Max coverage (-): 0

Region: chr17 67201413-67201468. Max. coverage (+): 0. Max coverage (-): 0

Region: chr17 67201469-67201524. Max. coverage (+): 0. Max coverage (-): 0

Region: chr17 67201525-67201580. Max. coverage (+): 0. Max coverage (-): 0

Region: chr17 67201581-67201636. Max. coverage (+): 0. Max coverage (-): 0

Region: chr17 67201637-67201692. Max. coverage (+): 0. Max coverage (-): 20.04

Region: chr17 67201693-67201748. Max. coverage (+): 0. Max coverage (-): 4.21

Region: chr17 67201749-67201803. Max. coverage (+): 0. Max coverage (-): 8.4

Region: chr17 67201804-67201859. Max. coverage (+): 0. Max coverage (-): 5.28

Region: chr17 67201860-67201915. Max. coverage (+): 0. Max coverage (-): 18.78

Region: chr17 67201916-67201971. Max. coverage (+): 0. Max coverage (-): 31.77

Region: chr17 67201972-67202027. Max. coverage (+): 0. Max coverage (-): 13.79

Region: chr17 67202028-67202083. Max. coverage (+): 0. Max coverage (-): 13.79

Region: chr17 67202084-67202139. Max. coverage (+): 0. Max coverage (-): 7.67

Region: chr17 67202140-67202195. Max. coverage (+): 0. Max coverage (-): 0

Region: chr17 67202196-67202250. Max. coverage (+): 0. Max coverage (-): 25.69

Region: chr17 67202251-67202306. Max. coverage (+): 0. Max coverage (-): 0

Region: chr17 67202307-67202362. Max. coverage (+): 0. Max coverage (-): 22.64

Region: chr17 67202363-67202418. Max. coverage (+): 0. Max coverage (-): 72.44

Region: chr17 67202419-67202474. Max. coverage (+): 0. Max coverage (-): 6.17

Region: chr17 67202475-67202530. Max. coverage (+): 0. Max coverage (-): 14.93

Region: chr17 67202531-67202586. Max. coverage (+): 0. Max coverage (-): 19.72

Region: chr17 67202587-67202642. Max. coverage (+): 0. Max coverage (-): 6.86

Region: chr17 67202643-67202698. Max. coverage (+): 0. Max coverage (-): 25.31

Region: chr17 67202699-67202753. Max. coverage (+): 0. Max coverage (-): 9.3

Region: chr17 67202754-67202809. Max. coverage (+): 0. Max coverage (-): 26.88

Region: chr17 67202810-67202865. Max. coverage (+): 0. Max coverage (-): 2.98

Region: chr17 67202866-67202921. Max. coverage (+): 0. Max coverage (-): 7.06

Region: chr17 67202922-67202977. Max. coverage (+): 0. Max coverage (-): 54.03

Region: chr17 67202978-67203033. Max. coverage (+): 0. Max coverage (-): 21.96

Region: chr17 67203034-67203089. Max. coverage (+): 0. Max coverage (-): 40.58

Region: chr17 67203090-67203145. Max. coverage (+): 0. Max coverage (-): 106.67

Region: chr17 67203146-67203200. Max. coverage (+): 0. Max coverage (-): 18.49

Region: chr17 67203201-67203256. Max. coverage (+): 0. Max coverage (-): 33.47

Region: chr17 67203257-67203312. Max. coverage (+): 0. Max coverage (-): 35.73

Region: chr17 67203313-67203368. Max. coverage (+): 0. Max coverage (-): 13.41

Region: chr17 67203369-67203424. Max. coverage (+): 0. Max coverage (-): 27.9

Region: chr17 67203425-67203480. Max. coverage (+): 0. Max coverage (-): 18.16

Region: chr17 67203481-67203536. Max. coverage (+): 2.12. Max coverage (-): 14.1

Region: chr17 67203537-67203592. Max. coverage (+): 0. Max coverage (-): 2.11

Region: chr17 67203593-67203647. Max. coverage (+): 0. Max coverage (-): 82.91

Region: chr17 67203648-67203703. Max. coverage (+): 0. Max coverage (-): 27.35

Region: chr17 67203704-67203759. Max. coverage (+): 0. Max coverage (-): 5.54

Region: chr17 67203760-67203815. Max. coverage (+): 0. Max coverage (-): 48.81

Region: chr17 67203816-67203871. Max. coverage (+): 0. Max coverage (-): 48.81

Region: chr17 67203872-67203927. Max. coverage (+): 0. Max coverage (-): 34.86

Region: chr17 67203928-67203983. Max. coverage (+): 0. Max coverage (-): 5.1

Region: chr17 67203984-67204039. Max. coverage (+): 0. Max coverage (-): 0

Region: chr17 67204040-67204094. Max. coverage (+): 0. Max coverage (-): 0

Region: chr17 67204095-67204150. Max. coverage (+): 0. Max coverage (-): 0

Region: chr17 67204151-67204206. Max. coverage (+): 0. Max coverage (-): 62.79

Region: chr17 67204207-67204262. Max. coverage (+): 0. Max coverage (-): 34.69

Region: chr17 67204263-67204318. Max. coverage (+): 0. Max coverage (-): 41.57

Region: chr17 67204319-67204374. Max. coverage (+): 0. Max coverage (-): 26.51

Region: chr17 67204375-67204430. Max. coverage (+): 0. Max coverage (-): 6.17

Region: chr17 67204431-67204486. Max. coverage (+): 0. Max coverage (-): 37.32

Region: chr17 67204487-67204541. Max. coverage (+): 0. Max coverage (-): 91.38

Region: chr17 67204542-67204597. Max. coverage (+): 0. Max coverage (-): 17.91

Region: chr17 67204598-67204653. Max. coverage (+): 0. Max coverage (-): 6.48

Region: chr17 67204654-67204709. Max. coverage (+): 0. Max coverage (-): 6.1

Region: chr17 67204710-67204765. Max. coverage (+): 0. Max coverage (-): 0

Region: chr17 67204766-67204821. Max. coverage (+): 0. Max coverage (-): 6.78

Region: chr17 67204822-67204877. Max. coverage (+): 0. Max coverage (-): 0

Region: chr17 67204878-67204933. Max. coverage (+): 0. Max coverage (-): 0

Region: chr17 67204934-67204989. Max. coverage (+): 0. Max coverage (-): 0

Region: chr17 67204990-67205044. Max. coverage (+): 0. Max coverage (-): 0

Region: chr17 67205045-67205100. Max. coverage (+): 0. Max coverage (-): 8.2

Region: chr17 67205101-67205156. Max. coverage (+): 0. Max coverage (-): 0.66

Region: chr17 67205157-67205212. Max. coverage (+): 0. Max coverage (-): 4.74

Region: chr17 67205213-67205268. Max. coverage (+): 0. Max coverage (-): 0

Region: chr17 67205269-67205324. Max. coverage (+): 0. Max coverage (-): 1.58

Region: chr17 67205325-67205380. Max. coverage (+): 0. Max coverage (-): 30.87

Region: chr17 67205381-67205436. Max. coverage (+): 0. Max coverage (-): 0

Region: chr17 67205437-67205491. Max. coverage (+): 0. Max coverage (-): 22.68

Region: chr17 67205492-67205547. Max. coverage (+): 0. Max coverage (-): 0.3

Region: chr17 67205548-67205603. Max. coverage (+): 0. Max coverage (-): 0

Region: chr17 67205604-67205659. Max. coverage (+): 0. Max coverage (-): 0

Region: chr17 67205660-67205715. Max. coverage (+): 0. Max coverage (-): 19.71

Region: chr17 67205716-67205771. Max. coverage (+): 0. Max coverage (-): 0

Region: chr17 67205772-67205827. Max. coverage (+): 0. Max coverage (-): 7.03

Region: chr17 67205828-67205883. Max. coverage (+): 0. Max coverage (-): 1.23

Region: chr17 67205884-67205938. Max. coverage (+): 0. Max coverage (-): 0

Region: chr17 67205939-67205994. Max. coverage (+): 0. Max coverage (-): 0

Region: chr17 67205995-67206050. Max. coverage (+): 0. Max coverage (-): 0

Region: chr17 67206051-67206106. Max. coverage (+): 0. Max coverage (-): 0

Region: chr17 67206107-67206162. Max. coverage (+): 0. Max coverage (-): 0

Region: chr17 67206163-67206218. Max. coverage (+): 0. Max coverage (-): 5.15

Region: chr17 67206219-67206274. Max. coverage (+): 0. Max coverage (-): 7.05

Region: chr17 67206275-67206330. Max. coverage (+): 0. Max coverage (-): 49.3

Region: chr17 67206331-67206385. Max. coverage (+): 0. Max coverage (-): 22.16

Region: chr17 67206386-67206441. Max. coverage (+): 0. Max coverage (-): 27.23

Region: chr17 67206442-67206497. Max. coverage (+): 0. Max coverage (-): 12.98

Region: chr17 67206498-67206553. Max. coverage (+): 0. Max coverage (-): 0

Region: chr17 67206554-67206609. Max. coverage (+): 0. Max coverage (-): 0

Region: chr17 67206610-67206665. Max. coverage (+): 0. Max coverage (-): 0

Region: chr17 67206666-67206721. Max. coverage (+): 0. Max coverage (-): 0

Region: chr17 67206722-67206777. Max. coverage (+): 0. Max coverage (-): 17.06

Region: chr17 67206778-67206832. Max. coverage (+): 0. Max coverage (-): 68.64

Region: chr17 67206833-67206888. Max. coverage (+): 0. Max coverage (-): 17.16

Region: chr17 67206889-67206944. Max. coverage (+): 0. Max coverage (-): 12.18

Region: chr17 67206945-67207000. Max. coverage (+): 0. Max coverage (-): 19.64

Region: chr17 67207001-67207056. Max. coverage (+): 0. Max coverage (-): 36.78

Region: chr17 67207057-67207112. Max. coverage (+): 0. Max coverage (-): 18.92

Region: chr17 67207113-67207168. Max. coverage (+): 0. Max coverage (-): 96.45

Region: chr17 67207169-67207224. Max. coverage (+): 0. Max coverage (-): 80.1

Region: chr17 67207225-67207280. Max. coverage (+): 0. Max coverage (-): 0

Region: chr17 67207281-67207335. Max. coverage (+): 0. Max coverage (-): 0

Region: chr17 67207336-67207391. Max. coverage (+): 0. Max coverage (-): 6.19

Region: chr17 67207392-67207447. Max. coverage (+): 0. Max coverage (-): 0

Region: chr17 67207448-67207503. Max. coverage (+): 0. Max coverage (-): 9.08

Region: chr17 67207504-67207559. Max. coverage (+): 0. Max coverage (-): 0.82

Region: chr17 67207560-67207615. Max. coverage (+): 0. Max coverage (-): 43.66

Region: chr17 67207616-67207671. Max. coverage (+): 0. Max coverage (-): 71.22

Region: chr17 67207672-67207727. Max. coverage (+): 0. Max coverage (-): 15.57

Region: chr17 67207728-67207782. Max. coverage (+): 0. Max coverage (-): 8.25

Region: chr17 67207783-67207838. Max. coverage (+): 0. Max coverage (-): 7.63

Region: chr17 67207839-67207894. Max. coverage (+): 0. Max coverage (-): 8.93

Region: chr17 67207895-67207950. Max. coverage (+): 0. Max coverage (-): 33.35

Region: chr17 67207951-67208006. Max. coverage (+): 0. Max coverage (-): 1.53

Region: chr17 67208007-67208062. Max. coverage (+): 0. Max coverage (-): 1.53

Region: chr17 67208063-67208118. Max. coverage (+): 0. Max coverage (-): 2.99

Region: chr17 67208119-67208174. Max. coverage (+): 1.24. Max coverage (-): 12.57

Region: chr17 67208175-67208229. Max. coverage (+): 0. Max coverage (-): 40.01

Region: chr17 67208230-67208285. Max. coverage (+): 0. Max coverage (-): 5.38

Region: chr17 67208286-67208341. Max. coverage (+): 0. Max coverage (-): 4.95

Region: chr17 67208342-67208397. Max. coverage (+): 0. Max coverage (-): 4.9

Region: chr17 67208398-67208453. Max. coverage (+): 0. Max coverage (-): 17.22

Region: chr17 67208454-67208509. Max. coverage (+): 0. Max coverage (-): 35.21

Region: chr17 67208510-67208565. Max. coverage (+): 0. Max coverage (-): 113.87

Region: chr17 67208566-67208621. Max. coverage (+): 0. Max coverage (-): 23.8

Region: chr17 67208622-67208676. Max. coverage (+): 0. Max coverage (-): 16.85

Region: chr17 67208677-67208732. Max. coverage (+): 0. Max coverage (-): 7.68

Region: chr17 67208733-67208788. Max. coverage (+): 0. Max coverage (-): 0

Region: chr17 67208789-67208844. Max. coverage (+): 0. Max coverage (-): 0

Region: chr17 67208845-67208900. Max. coverage (+): 0. Max coverage (-): 0

Region: chr17 67208901-67208956. Max. coverage (+): 0. Max coverage (-): 4.4

Region: chr17 67208957-67209012. Max. coverage (+): 0. Max coverage (-): 7.24

Region: chr17 67209013-67209068. Max. coverage (+): 0. Max coverage (-): 2.91

Region: chr17 67209069-67209123. Max. coverage (+): 0. Max coverage (-): 0.5

Region: chr17 67209124-67209179. Max. coverage (+): 0. Max coverage (-): 9.58

Region: chr17 67209180-67209235. Max. coverage (+): 0. Max coverage (-): 0

Region: chr17 67209236-67209291. Max. coverage (+): 0. Max coverage (-): 0

Region: chr17 67209292-67209347. Max. coverage (+): 0. Max coverage (-): 0

Region: chr17 67209348-67209403. Max. coverage (+): 0. Max coverage (-): 0

Region: chr17 67209404-67209459. Max. coverage (+): 0. Max coverage (-): 0

Region: chr17 67209460-67209515. Max. coverage (+): 0. Max coverage (-): 0

Region: chr17 67209516-67209571. Max. coverage (+): 0. Max coverage (-): 0

Region: chr17 67209572-67209626. Max. coverage (+): 0. Max coverage (-): 0

Region: chr17 67209627-67209682. Max. coverage (+): 0. Max coverage (-): 0

Region: chr17 67209683-67209738. Max. coverage (+): 0. Max coverage (-): 0

Region: chr17 67209739-67209794. Max. coverage (+): 0. Max coverage (-): 0

Region: chr17 67209795-67209850. Max. coverage (+): 0. Max coverage (-): 0

Region: chr17 67209851-67209906. Max. coverage (+): 0. Max coverage (-): 0

Region: chr17 67209907-67209962. Max. coverage (+): 0. Max coverage (-): 0

Region: chr17 67209963-67210018. Max. coverage (+): 0. Max coverage (-): 0

Region: chr17 67210019-67210073. Max. coverage (+): 0. Max coverage (-): 0

Region: chr17 67210074-67210129. Max. coverage (+): 0. Max coverage (-): 0

Region: chr17 67210130-67210185. Max. coverage (+): 0. Max coverage (-): 3.41

Region: chr17 67210186-67210241. Max. coverage (+): 0. Max coverage (-): 9.64

Region: chr17 67210242-67210297. Max. coverage (+): 0. Max coverage (-): 0

Region: chr17 67210298-67210353. Max. coverage (+): 0. Max coverage (-): 0

Region: chr17 67210354-67210409. Max. coverage (+): 0. Max coverage (-): 0

Region: chr17 67210410-67210465. Max. coverage (+): 0. Max coverage (-): 0

Region: chr17 67210466-67210520. Max. coverage (+): 0. Max coverage (-): 0

Region: chr17 67210521-67210576. Max. coverage (+): 0. Max coverage (-): 0

Region: chr17 67210577-67210632. Max. coverage (+): 0. Max coverage (-): 3.94

Region: chr17 67210633-67210688. Max. coverage (+): 0. Max coverage (-): 3.94

Region: chr17 67210689-67210744. Max. coverage (+): 0. Max coverage (-): 3.78

Region: chr17 67210745-67210800. Max. coverage (+): 0. Max coverage (-): 2.04

Region: chr17 67210801-67210856. Max. coverage (+): 0. Max coverage (-): 1.15

Region: chr17 67210857-67210912. Max. coverage (+): 0. Max coverage (-): 30.77

Region: chr17 67210913-67210967. Max. coverage (+): 0. Max coverage (-): 0.63

Region: chr17 67210968-67211023. Max. coverage (+): 0. Max coverage (-): 9.15

Region: chr17 67211024-67211079. Max. coverage (+): 0. Max coverage (-): 9.15

Region: chr17 67211080-67211135. Max. coverage (+): 0. Max coverage (-): 2.22

Region: chr17 67211136-67211191. Max. coverage (+): 0. Max coverage (-): 1.13

Region: chr17 67211192-67211247. Max. coverage (+): 0. Max coverage (-): 46.12

Region: chr17 67211248-67211303. Max. coverage (+): 0. Max coverage (-): 5.59

Region: chr17 67211304-67211359. Max. coverage (+): 0. Max coverage (-): 20.11

Region: chr17 67211360-67211414. Max. coverage (+): 0. Max coverage (-): 38.03

Region: chr17 67211415-67211470. Max. coverage (+): 0. Max coverage (-): 0

Region: chr17 67211471-67211526. Max. coverage (+): 0. Max coverage (-): 0

Region: chr17 67211527-67211582. Max. coverage (+): 0. Max coverage (-): 1.61

Region: chr17 67211583-67211638. Max. coverage (+): 0. Max coverage (-): 17.15

Region: chr17 67211639-67211694. Max. coverage (+): 0. Max coverage (-): 81.87

Region: chr17 67211695-67211750. Max. coverage (+): 0. Max coverage (-): 0

Region: chr17 67211751-67211806. Max. coverage (+): 0. Max coverage (-): 0

Region: chr17 67211807-67211862. Max. coverage (+): 0. Max coverage (-): 0

Region: chr17 67211863-67211917. Max. coverage (+): 0. Max coverage (-): 0

Region: chr17 67211918-67211973. Max. coverage (+): 0. Max coverage (-): 18.17

Region: chr17 67211974-67212029. Max. coverage (+): 0. Max coverage (-): 46.64

Region: chr17 67212030-67212085. Max. coverage (+): 0. Max coverage (-): 25.42

Region: chr17 67212086-67212141. Max. coverage (+): 0. Max coverage (-): 4.7

Region: chr17 67212142-67212197. Max. coverage (+): 0. Max coverage (-): 0

Region: chr17 67212198-67212253. Max. coverage (+): 0. Max coverage (-): 0

Region: chr17 67212254-67212309. Max. coverage (+): 0. Max coverage (-): 0

Region: chr17 67212310-67212364. Max. coverage (+): 0. Max coverage (-): 0

Region: chr17 67212365-67212420. Max. coverage (+): 0. Max coverage (-): 6.14

Region: chr17 67212421-67212476. Max. coverage (+): 0. Max coverage (-): 23.42

Region: chr17 67212477-67212532. Max. coverage (+): 0. Max coverage (-): 23.42

Region: chr17 67212533-67212588. Max. coverage (+): 0. Max coverage (-): 151.83

Region: chr17 67212589-67212644. Max. coverage (+): 0. Max coverage (-): 133.27

Region: chr17 67212645-67212700. Max. coverage (+): 0. Max coverage (-): 40.38

Region: chr17 67212701-67212756. Max. coverage (+): 0. Max coverage (-): 7.91

Region: chr17 67212757-67212811. Max. coverage (+): 0. Max coverage (-): 6.76

Region: chr17 67212812-67212867. Max. coverage (+): 0. Max coverage (-): 3.8

Region: chr17 67212868-67212923. Max. coverage (+): 0. Max coverage (-): 10.86

Region: chr17 67212924-67212979. Max. coverage (+): 0. Max coverage (-): 6.9

Region: chr17 67212980-67213035. Max. coverage (+): 0. Max coverage (-): 2.71

Region: chr17 67213036-67213091. Max. coverage (+): 0. Max coverage (-): 10.14

Region: chr17 67213092-67213147. Max. coverage (+): 0. Max coverage (-): 36.89

Region: chr17 67213148-67213203. Max. coverage (+): 0. Max coverage (-): 16.44

Region: chr17 67213204-67213258. Max. coverage (+): 5.81. Max coverage (-): 5.36

Region: chr17 67213259-67213314. Max. coverage (+): 0. Max coverage (-): 21.43

Region: chr17 67213315-67213370. Max. coverage (+): 0. Max coverage (-): 5.75

Region: chr17 67213371-67213426. Max. coverage (+): 0. Max coverage (-): 5.75

Region: chr17 67213427-67213482. Max. coverage (+): 0. Max coverage (-): 54.51

Region: chr17 67213483-67213538. Max. coverage (+): 0. Max coverage (-): 1.72

Region: chr17 67213539-67213594. Max. coverage (+): 0. Max coverage (-): 0

Region: chr17 67213595-67213650. Max. coverage (+): 0. Max coverage (-): 0

Region: chr17 67213651-67213705. Max. coverage (+): 0. Max coverage (-): 0

Region: chr17 67213706-67213761. Max. coverage (+): 0.23. Max coverage (-): 13.73

Region: chr17 67213762-67213817. Max. coverage (+): 0.23. Max coverage (-): 49.3

Region: chr17 67213818-67213873. Max. coverage (+): 0. Max coverage (-): 0

Region: chr17 67213874-67213929. Max. coverage (+): 0. Max coverage (-): 0

Region: chr17 67213930-67213985. Max. coverage (+): 0. Max coverage (-): 0

Region: chr17 67213986-67214041. Max. coverage (+): 0. Max coverage (-): 0

Region: chr17 67214042-67214097. Max. coverage (+): 0. Max coverage (-): 0

Region: chr17 67214098-67214152. Max. coverage (+): 0. Max coverage (-): 0

Region: chr17 67214153-67214208. Max. coverage (+): 0. Max coverage (-): 0

Region: chr17 67214209-67214264. Max. coverage (+): 0. Max coverage (-): 0

Region: chr17 67214265-67214320. Max. coverage (+): 0. Max coverage (-): 0

Region: chr17 67214321-67214376. Max. coverage (+): 0. Max coverage (-): 8.47

Region: chr17 67214377-67214432. Max. coverage (+): 0. Max coverage (-): 2.69

Region: chr17 67214433-67214488. Max. coverage (+): 0. Max coverage (-): 1.82

Region: chr17 67214489-67214544. Max. coverage (+): 0. Max coverage (-): 8.65

Region: chr17 67214545-67214600. Max. coverage (+): 0. Max coverage (-): 29.52

Region: chr17 67214601-67214655. Max. coverage (+): 0. Max coverage (-): 10.91

Region: chr17 67214656-67214711. Max. coverage (+): 0. Max coverage (-): 15.66

Region: chr17 67214712-67214767. Max. coverage (+): 0. Max coverage (-): 3.93

Region: chr17 67214768-67214823. Max. coverage (+): 0. Max coverage (-): 3.96

Region: chr17 67214824-67214879. Max. coverage (+): 0. Max coverage (-): 6.45

Region: chr17 67214880-67214935. Max. coverage (+): 0. Max coverage (-): 24.29

Region: chr17 67214936-67214991. Max. coverage (+): 0. Max coverage (-): 1.11

Region: chr17 67214992-67215047. Max. coverage (+): 0. Max coverage (-): 5.78

Region: chr17 67215048-67215102. Max. coverage (+): 0. Max coverage (-): 4.12

Region: chr17 67215103-67215158. Max. coverage (+): 0. Max coverage (-): 1.5

Region: chr17 67215159-67215214. Max. coverage (+): 2.66. Max coverage (-): 0

Region: chr17 67215215-67215270. Max. coverage (+): 0. Max coverage (-): 0

Region: chr17 67215271-67215326. Max. coverage (+): 1.35. Max coverage (-): 0

Region: chr17 67215327-67215382. Max. coverage (+): 0. Max coverage (-): 0

Region: chr17 67215383-67215438. Max. coverage (+): 0. Max coverage (-): 0

Region: chr17 67215439-67215494. Max. coverage (+): 0. Max coverage (-): 0

Region: chr17 67215495-67215549. Max. coverage (+): 0. Max coverage (-): 0

Region: chr17 67215550-67215605. Max. coverage (+): 0. Max coverage (-): 0

Region: chr17 67215606-67215661. Max. coverage (+): 0. Max coverage (-): 0

Region: chr17 67215662-67215717. Max. coverage (+): 0. Max coverage (-): 0

Region: chr17 67215718-67215773. Max. coverage (+): 0. Max coverage (-): 0

Region: chr17 67215774-67215829. Max. coverage (+): 0. Max coverage (-): 0

Region: chr17 67215830-67215885. Max. coverage (+): 0. Max coverage (-): 0

Region: chr17 67215886-67215941. Max. coverage (+): 0. Max coverage (-): 0

Region: chr17 67215942-67215996. Max. coverage (+): 0. Max coverage (-): 0

Region: chr17 67215997-67216052. Max. coverage (+): 0. Max coverage (-): 0

Region: chr17 67216053-67216108. Max. coverage (+): 0. Max coverage (-): 0

Region: chr17 67216109-67216164. Max. coverage (+): 0. Max coverage (-): 0

Region: chr17 67216165-67216220. Max. coverage (+): 0. Max coverage (-): 0

Region: chr17 67216221-67216276. Max. coverage (+): 0. Max coverage (-): 0

Region: chr17 67216277-67216332. Max. coverage (+): 0. Max coverage (-): 0

Region: chr17 67216333-67216388. Max. coverage (+): 0. Max coverage (-): 0

Region: chr17 67216389-67216443. Max. coverage (+): 0. Max coverage (-): 0

Region: chr17 67216444-67216499. Max. coverage (+): 0. Max coverage (-): 0

Region: chr17 67216500-67216555. Max. coverage (+): 0. Max coverage (-): 0

Region: chr17 67216556-67216611. Max. coverage (+): 0. Max coverage (-): 0

Region: chr17 67216612-67216667. Max. coverage (+): 0. Max coverage (-): 0

Region: chr17 67216668-67216723. Max. coverage (+): 0. Max coverage (-): 0

Region: chr17 67216724-67216779. Max. coverage (+): 0. Max coverage (-): 0

Region: chr17 67216780-67216835. Max. coverage (+): 0. Max coverage (-): 0

Region: chr17 67216836-67216891. Max. coverage (+): 0. Max coverage (-): 0

Region: chr17 67216892-67216946. Max. coverage (+): 0. Max coverage (-): 0

Region: chr17 67216947-67217002. Max. coverage (+): 0. Max coverage (-): 0

Region: chr17 67217003-67217058. Max. coverage (+): 0. Max coverage (-): 0

Region: chr17 67217059-67217114. Max. coverage (+): 0. Max coverage (-): 0

Region: chr17 67217115-67217170. Max. coverage (+): 0. Max coverage (-): 0

Region: chr17 67217171-67217226. Max. coverage (+): 0. Max coverage (-): 0

Region: chr17 67217227-67217282. Max. coverage (+): 0. Max coverage (-): 0

Region: chr17 67217283-67217338. Max. coverage (+): 0. Max coverage (-): 0

Region: chr17 67217339-67217393. Max. coverage (+): 0. Max coverage (-): 0

Region: chr17 67217394-67217449. Max. coverage (+): 0. Max coverage (-): 0

Region: chr17 67217450-67217505. Max. coverage (+): 0. Max coverage (-): 0

Region: chr17 67217506-67217561. Max. coverage (+): 0. Max coverage (-): 0

Region: chr17 67217562-67217617. Max. coverage (+): 0. Max coverage (-): 0

Region: chr17 67217618-67217673. Max. coverage (+): 0. Max coverage (-): 0

Region: chr17 67217674-67217729. Max. coverage (+): 0. Max coverage (-): 0

Region: chr17 67217730-67217785. Max. coverage (+): 0. Max coverage (-): 0

Region: chr17 67217786-67217840. Max. coverage (+): 0. Max coverage (-): 0

Region: chr17 67217841-67217896. Max. coverage (+): 0. Max coverage (-): 0

Region: chr17 67217897-67217952. Max. coverage (+): 0. Max coverage (-): 0

Region: chr17 67217953-67218008. Max. coverage (+): 0. Max coverage (-): 0

Region: chr17 67218009-67218064. Max. coverage (+): 0. Max coverage (-): 0

Region: chr17 67218065-67218120. Max. coverage (+): 0. Max coverage (-): 0

Region: chr17 67218121-67218176. Max. coverage (+): 0. Max coverage (-): 0

Region: chr17 67218177-67218232. Max. coverage (+): 0. Max coverage (-): 0

Region: chr17 67218233-67218287. Max. coverage (+): 0. Max coverage (-): 0

Region: chr17 67218288-67218343. Max. coverage (+): 0. Max coverage (-): 0

Region: chr17 67218344-67218399. Max. coverage (+): 0. Max coverage (-): 0

Region: chr17 67218400-67218455. Max. coverage (+): 0. Max coverage (-): 0

Region: chr17 67218456-67218511. Max. coverage (+): 0. Max coverage (-): 0

Region: chr17 67218512-67218567. Max. coverage (+): 0. Max coverage (-): 0

Region: chr17 67218568-67218623. Max. coverage (+): 0. Max coverage (-): 0

Region: chr17 67218624-67218679. Max. coverage (+): 0. Max coverage (-): 0

Region: chr17 67218680-67218734. Max. coverage (+): 0. Max coverage (-): 0

Region: chr17 67218735-67218790. Max. coverage (+): 0. Max coverage (-): 0

Region: chr17 67218791-67218846. Max. coverage (+): 0. Max coverage (-): 0

Region: chr17 67218847-67218902. Max. coverage (+): 0. Max coverage (-): 0

Region: chr17 67218903-67218958. Max. coverage (+): 0. Max coverage (-): 0

Region: chr17 67218959-67219014. Max. coverage (+): 0. Max coverage (-): 0

Region: chr17 67219015-67219070. Max. coverage (+): 0. Max coverage (-): 0

Region: chr17 67219071-67219126. Max. coverage (+): 0. Max coverage (-): 0

Region: chr17 67219127-67219182. Max. coverage (+): 0. Max coverage (-): 0

Region: chr17 67219183-67219237. Max. coverage (+): 0. Max coverage (-): 0

Region: chr17 67219238-67219293. Max. coverage (+): 0. Max coverage (-): 0

Region: chr17 67219294-67219349. Max. coverage (+): 0. Max coverage (-): 0

Region: chr17 67219350-67219405. Max. coverage (+): 0. Max coverage (-): 0

Region: chr17 67219406-67219461. Max. coverage (+): 0. Max coverage (-): 0

Region: chr17 67219462-67219517. Max. coverage (+): 0. Max coverage (-): 0

Region: chr17 67219518-67219573. Max. coverage (+): 0. Max coverage (-): 0

Region: chr17 67219574-67219629. Max. coverage (+): 0. Max coverage (-): 0

Region: chr17 67219630-67219684. Max. coverage (+): 0. Max coverage (-): 0

Region: chr17 67219685-67219740. Max. coverage (+): 0. Max coverage (-): 0

Region: chr17 67219741-67219796. Max. coverage (+): 0. Max coverage (-): 0

Region: chr17 67219797-67219852. Max. coverage (+): 0. Max coverage (-): 0

Region: chr17 67219853-67219908. Max. coverage (+): 0. Max coverage (-): 0

Region: chr17 67219909-67219964. Max. coverage (+): 0. Max coverage (-): 0

Region: chr17 67219965-67220020. Max. coverage (+): 0. Max coverage (-): 0

Region: chr17 67220021-67220076. Max. coverage (+): 0. Max coverage (-): 0

Region: chr17 67220077-67220131. Max. coverage (+): 0. Max coverage (-): 0

Region: chr17 67220132-67220187. Max. coverage (+): 0. Max coverage (-): 0

Region: chr17 67220188-67220243. Max. coverage (+): 0. Max coverage (-): 0

Region: chr17 67220244-67220299. Max. coverage (+): 0. Max coverage (-): 0

Region: chr17 67220300-67220355. Max. coverage (+): 0. Max coverage (-): 0

Region: chr17 67220356-67220411. Max. coverage (+): 0. Max coverage (-): 0

Region: chr17 67220412-67220467. Max. coverage (+): 0. Max coverage (-): 0

Region: chr17 67220468-67220523. Max. coverage (+): 0. Max coverage (-): 0

Region: chr17 67220524-67220578. Max. coverage (+): 0. Max coverage (-): 0

Region: chr17 67220579-67220634. Max. coverage (+): 0. Max coverage (-): 0

Region: chr17 67220635-67220690. Max. coverage (+): 0. Max coverage (-): 0

Region: chr17 67220691-67220746. Max. coverage (+): 0. Max coverage (-): 0

Region: chr17 67220747-67220802. Max. coverage (+): 0. Max coverage (-): 0

Region: chr17 67220803-67220858. Max. coverage (+): 0. Max coverage (-): 0

Region: chr17 67220859-67220914. Max. coverage (+): 0. Max coverage (-): 0

Region: chr17 67220915-67220970. Max. coverage (+): 0. Max coverage (-): 0

Region: chr17 67220971-67221025. Max. coverage (+): 0. Max coverage (-): 0

Region: chr17 67221026-67221081. Max. coverage (+): 0. Max coverage (-): 0

Region: chr17 67221082-67221137. Max. coverage (+): 0. Max coverage (-): 0

Region: chr17 67221138-67221193. Max. coverage (+): 0. Max coverage (-): 0

Region: chr17 67221194-67221249. Max. coverage (+): 0. Max coverage (-): 0

Region: chr17 67221250-67221305. Max. coverage (+): 0. Max coverage (-): 0

Region: chr17 67221306-67221361. Max. coverage (+): 0. Max coverage (-): 0

Region: chr17 67221362-67221417. Max. coverage (+): 0. Max coverage (-): 0

Region: chr17 67221418-67221473. Max. coverage (+): 0. Max coverage (-): 0

Region: chr17 67221474-67221528. Max. coverage (+): 0. Max coverage (-): 0

Region: chr17 67221529-67221584. Max. coverage (+): 0. Max coverage (-): 0

Region: chr17 67221585-67221640. Max. coverage (+): 0. Max coverage (-): 0

Region: chr17 67221641-67221696. Max. coverage (+): 0. Max coverage (-): 0

Region: chr17 67221697-67221752. Max. coverage (+): 0. Max coverage (-): 0

Region: chr17 67221753-67221808. Max. coverage (+): 0. Max coverage (-): 0

Region: chr17 67221809-67221864. Max. coverage (+): 0. Max coverage (-): 0

Region: chr17 67221865-67221920. Max. coverage (+): 0. Max coverage (-): 0

Region: chr17 67221921-67221975. Max. coverage (+): 0. Max coverage (-): 0

Region: chr17 67221976-67222031. Max. coverage (+): 0. Max coverage (-): 0

Region: chr17 67222032-67222087. Max. coverage (+): 0. Max coverage (-): 0

Region: chr17 67222088-67222143. Max. coverage (+): 0. Max coverage (-): 0

Region: chr17 67222144-67222199. Max. coverage (+): 0. Max coverage (-): 0

Region: chr17 67222200-67222255. Max. coverage (+): 0. Max coverage (-): 0

Region: chr17 67222256-67222311. Max. coverage (+): 0. Max coverage (-): 0

Region: chr17 67222312-67222367. Max. coverage (+): 0. Max coverage (-): 0

Region: chr17 67222368-67222422. Max. coverage (+): 0. Max coverage (-): 0

Region: chr17 67222423-67222478. Max. coverage (+): 0. Max coverage (-): 0

Region: chr17 67222479-67222534. Max. coverage (+): 0. Max coverage (-): 0

Region: chr17 67222535-67222590. Max. coverage (+): 0. Max coverage (-): 0

Region: chr17 67222591-67222646. Max. coverage (+): 0. Max coverage (-): 0

Region: chr17 67222647-67222702. Max. coverage (+): 0. Max coverage (-): 0

Region: chr17 67222703-67222758. Max. coverage (+): 0. Max coverage (-): 0

Region: chr17 67222759-67222814. Max. coverage (+): 0. Max coverage (-): 0

Region: chr17 67222815-67222869. Max. coverage (+): 0. Max coverage (-): 0

Region: chr17 67222870-67222925. Max. coverage (+): 0. Max coverage (-): 0

Region: chr17 67222926-67222981. Max. coverage (+): 0. Max coverage (-): 0

Region: chr17 67222982-67223037. Max. coverage (+): 0. Max coverage (-): 0

Region: chr17 67223038-67223093. Max. coverage (+): 0. Max coverage (-): 0

Region: chr17 67223094-67223149. Max. coverage (+): 0. Max coverage (-): 0

Region: chr17 67223150-67223205. Max. coverage (+): 0. Max coverage (-): 0

Region: chr17 67223206-67223261. Max. coverage (+): 0. Max coverage (-): 0

Region: chr17 67223262-67223316. Max. coverage (+): 0. Max coverage (-): 0

Region: chr17 67223317-67223372. Max. coverage (+): 0. Max coverage (-): 0

Region: chr17 67223373-67223428. Max. coverage (+): 0. Max coverage (-): 0

Region: chr17 67223429-67223484. Max. coverage (+): 0. Max coverage (-): 0

Region: chr17 67223485-67223540. Max. coverage (+): 0. Max coverage (-): 0

Region: chr17 67223541-67223596. Max. coverage (+): 0. Max coverage (-): 0

Region: chr17 67223597-67223652. Max. coverage (+): 0. Max coverage (-): 0

Region: chr17 67223653-67223708. Max. coverage (+): 0. Max coverage (-): 0

Region: chr17 67223709-67223764. Max. coverage (+): 0. Max coverage (-): 0

Region: chr17 67223765-67223819. Max. coverage (+): 0. Max coverage (-): 0

Region: chr17 67223820-67223875. Max. coverage (+): 0. Max coverage (-): 0

Region: chr17 67223876-67223931. Max. coverage (+): 0. Max coverage (-): 0

Region: chr17 67223932-67223987. Max. coverage (+): 0. Max coverage (-): 0

Region: chr17 67223988-67224043. Max. coverage (+): 0. Max coverage (-): 0

Region: chr17 67224044-67224099. Max. coverage (+): 0. Max coverage (-): 0

Region: chr17 67224100-67224155. Max. coverage (+): 0. Max coverage (-): 0

Region: chr17 67224156-67224211. Max. coverage (+): 0. Max coverage (-): 0

Region: chr17 67224212-67224266. Max. coverage (+): 0. Max coverage (-): 0

Region: chr17 67224267-67224322. Max. coverage (+): 0. Max coverage (-): 0

Region: chr17 67224323-67224378. Max. coverage (+): 0. Max coverage (-): 0

Region: chr17 67224379-67224434. Max. coverage (+): 0. Max coverage (-): 0

Region: chr17 67224435-67224490. Max. coverage (+): 0. Max coverage (-): 0

Region: chr17 67224491-67224546. Max. coverage (+): 0. Max coverage (-): 0

Region: chr17 67224547-67224602. Max. coverage (+): 0. Max coverage (-): 0

Region: chr17 67224603-67224658. Max. coverage (+): 0. Max coverage (-): 0

Region: chr17 67224659-67224713. Max. coverage (+): 0. Max coverage (-): 0

Region: chr17 67224714-67224769. Max. coverage (+): 21.5. Max coverage (-): 0

Region: chr17 67224770-67224825. Max. coverage (+): 0.62. Max coverage (-): 0

Region: chr17 67224826-67224881. Max. coverage (+): 4.75. Max coverage (-): 0

Region: chr17 67224882-67224937. Max. coverage (+): 10.16. Max coverage (-): 0

Region: chr17 67224938-67224993. Max. coverage (+): 18.27. Max coverage (-): 0

Region: chr17 67224994-67225049. Max. coverage (+): 2.03. Max coverage (-): 0

Region: chr17 67225050-67225105. Max. coverage (+): 2.03. Max coverage (-): 0

Region: chr17 67225106-67225160. Max. coverage (+): 5.68. Max coverage (-): 0

Region: chr17 67225161-67225216. Max. coverage (+): 0. Max coverage (-): 0

Region: chr17 67225217-67225272. Max. coverage (+): 0.9. Max coverage (-): 0

Region: chr17 67225273-67225328. Max. coverage (+): 0.9. Max coverage (-): 0

Region: chr17 67225329-67225384. Max. coverage (+): 0. Max coverage (-): 0

Region: chr17 67225385-67225440. Max. coverage (+): 0. Max coverage (-): 0

Region: chr17 67225441-67225496. Max. coverage (+): 0. Max coverage (-): 0

Region: chr17 67225497-67225552. Max. coverage (+): 0. Max coverage (-): 0

Region: chr17 67225553-67225607. Max. coverage (+): 0. Max coverage (-): 0

Region: chr17 67225608-67225663. Max. coverage (+): 0. Max coverage (-): 0

Region: chr17 67225664-67225719. Max. coverage (+): 0. Max coverage (-): 0

Region: chr17 67225720-67225775. Max. coverage (+): 0. Max coverage (-): 0

Region: chr17 67225776-67225831. Max. coverage (+): 0. Max coverage (-): 0

Region: chr17 67225832-67225887. Max. coverage (+): 0. Max coverage (-): 0

Region: chr17 67225888-67225943. Max. coverage (+): 38.66. Max coverage (-): 0

Region: chr17 67225944-67225999. Max. coverage (+): 5.61. Max coverage (-): 0

Region: chr17 67226000-67226055. Max. coverage (+): 0. Max coverage (-): 0

Region: chr17 67226056-67226110. Max. coverage (+): 0. Max coverage (-): 0

Region: chr17 67226111-67226166. Max. coverage (+): 0. Max coverage (-): 0

Region: chr17 67226167-67226222. Max. coverage (+): 3.52. Max coverage (-): 0

Region: chr17 67226223-67226278. Max. coverage (+): 3.52. Max coverage (-): 0

Region: chr17 67226279-67226334. Max. coverage (+): 0. Max coverage (-): 0

Region: chr17 67226335-67226390. Max. coverage (+): 0. Max coverage (-): 0

Region: chr17 67226391-67226446. Max. coverage (+): 0. Max coverage (-): 0

Region: chr17 67226447-67226502. Max. coverage (+): 0. Max coverage (-): 0

Region: chr17 67226503-67226557. Max. coverage (+): 0. Max coverage (-): 0

Region: chr17 67226558-67226613. Max. coverage (+): 0. Max coverage (-): 0

Region: chr17 67226614-67226669. Max. coverage (+): 0. Max coverage (-): 0

Region: chr17 67226670-67226725. Max. coverage (+): 0. Max coverage (-): 0

Region: chr17 67226726-67226781. Max. coverage (+): 3.97. Max coverage (-): 0

Region: chr17 67226782-67226837. Max. coverage (+): 0. Max coverage (-): 0

Region: chr17 67226838-67226893. Max. coverage (+): 0. Max coverage (-): 0

Region: chr17 67226894-67226949. Max. coverage (+): 1.39. Max coverage (-): 0

Region: chr17 67226950-. Max. coverage (+): 1.39. Max coverage (-): 0

RepeatMasker Color Code

**+**

100-98% Identity

<98-95% Identity

<95-90% Identity

<90-85% Identity

<85-80% Identity

<80-75% Identity

<75-70% Identity

<70% Identity

**-**

Gene Set Color Code

**+**

Gene

Pseudogene

**-**

Topology/Coverage Color Code

Coverage Plus Strand

Coverage Minus Strand

Mainstrand: Plus

Mainstrand: Minus

Complementary Strand

Flanking Region  
(if option -flank >0)

Gene Set Annotation  

**1. PIWIL3 (protein coding, ENSBTAG00000015526) Tr:00000020633 Ex:16**: 67226882-67226985 (-)  
**2. PIWIL3 (protein coding, ENSBTAG00000015526) Tr:00000020633 Ex:17**: 67225872-67225942 (-)  
**3. PIWIL3 (protein coding, ENSBTAG00000015526) Tr:00000020633 Ex:18**: 67225285-67225438 (-)  
**4. PIWIL3 (protein coding, ENSBTAG00000015526) Tr:00000020633 Ex:19**: 67224435-67224487 (-)  
**5. PIWIL3 (protein coding, ENSBTAG00000015526) Tr:00000020633 Ex:20**: 67224314-67224367 (-)  
**6. PIWIL3 (protein coding, ENSBTAG00000015526) Tr:00000020633 Ex:21**: 67224135-67224138 (-)  
**7. PIWIL3 (protein coding, ENSBTAG00000015526) Tr:00000020633 Ex:22**: 67223379-67223512 (-)  
**8. PIWIL3 (protein coding, ENSBTAG00000015526) Tr:00000020633 Ex:23**: 67222565-67222701 (-)

  
RepeatMasker Annotation  

**1. MER21-int**: 67199073-67199215 (-), Divergence to consensus: 30.5%  
**2. MER21-int**: 67199287-67199462 (-), Divergence to consensus: 30%  
**3. MER21-int**: 67199477-67199839 (-), Divergence to consensus: 31.6%  
**4. MER21B**: 67199896-67199976 (-), Divergence to consensus: 26.5%  
**5. BOV-A2**: 67200003-67200273 (+), Divergence to consensus: 5.9%  
**6. LTR10B\_BT**: 67200439-67200513 (+), Divergence to consensus: 29.4%  
**7. MER21C**: 67200745-67200794 (-), Divergence to consensus: 24.5%  
**8. BOV-A2**: 67200799-67200862 (-), Divergence to consensus: 19.3%  
**9. MER21C\_BT**: 67200956-67201086 (-), Divergence to consensus: 41.9%  
**10. Bov-tA2**: 67201112-67201264 (+), Divergence to consensus: 16.2%  
**11. MER21C**: 67201271-67201538 (-), Divergence to consensus: 37.3%  
**12. L1MA10**: 67201544-67201630 (+), Divergence to consensus: 26.6%  
**13. (TA)n**: 67202176-67202202 (+), Divergence to consensus: 0%  
**14. MER3**: 67203950-67204121 (-), Divergence to consensus: 36.5%  
**15. MIR**: 67205540-67205660 (+), Divergence to consensus: 46.6%  
**16. MIR3**: 67206039-67206126 (-), Divergence to consensus: 36.4%  
**17. Bov-tA3**: 67208715-67208912 (-), Divergence to consensus: 15.2%  
**18. L1ME3A**: 67209193-67209303 (+), Divergence to consensus: 36.3%  
**19. L1MA9**: 67209306-67210128 (+), Divergence to consensus: 33.9%  
**20. L1M5**: 67210235-67210516 (+), Divergence to consensus: 33.1%  
**21. MIRc**: 67211412-67211582 (+), Divergence to consensus: 48%  
**22. Bov-tA2**: 67211728-67211833 (+), Divergence to consensus: 26.4%  
**23. MIR**: 67212111-67212340 (-), Divergence to consensus: 39.2%  
**24. MIRc**: 67213530-67213691 (+), Divergence to consensus: 44%  
**25. MIR**: 67213812-67213869 (-), Divergence to consensus: 50.4%  
**26. BOV-A2**: 67213870-67214128 (+), Divergence to consensus: 5.8%  
**27. (CAG)n**: 67214129-67214148 (+), Divergence to consensus: 0%  
**28. MIR**: 67214149-67214289 (-), Divergence to consensus: 50.4%  
**29. MIRc**: 67215483-67215609 (+), Divergence to consensus: 33.4%  
**30. L1M4**: 67215628-67216419 (-), Divergence to consensus: 32.7%  
**31. Bov-tA2**: 67216437-67216556 (+), Divergence to consensus: 16.7%  
**32. Bov-tA2**: 67216557-67216730 (+), Divergence to consensus: 23.3%  
**33. BTLTR1C**: 67216731-67217968 (-), Divergence to consensus: 37.3%  
**34. Bov-tA2**: 67217969-67218000 (+), Divergence to consensus: 27.4%  
**35. L1M4**: 67218043-67218209 (-), Divergence to consensus: 40.8%  
**36. SINE2-1\_BT**: 67218210-67218330 (-), Divergence to consensus: 25.6%  
**37. L1M4**: 67218331-67218476 (-), Divergence to consensus: 40.8%  
**38. Bov-tA2**: 67218498-67218615 (+), Divergence to consensus: 20.3%  
**39. Bov-tA2**: 67218616-67218796 (+), Divergence to consensus: 24.3%  
**40. Bov-tA2**: 67218813-67219024 (+), Divergence to consensus: 21.3%  
**41. L1M4**: 67219032-67219341 (-), Divergence to consensus: 44.3%  
**42. MLT1C**: 67219409-67219676 (+), Divergence to consensus: 33.8%  
**43. L1M4**: 67219680-67219860 (-), Divergence to consensus: 27.3%  
**44. L1\_BT**: 67219860-67219911 (+), Divergence to consensus: 17.3%  
**45. MLT1D**: 67220176-67220580 (+), Divergence to consensus: 40.3%  
**46. Bov-tA1**: 67220644-67220819 (+), Divergence to consensus: 21%  
**47. BOV-A2**: 67221005-67221123 (+), Divergence to consensus: 7.6%  
**48. L1-2\_BT**: 67222833-67223377 (+), Divergence to consensus: 33.5%  
**49. BOV-A2**: 67224597-67224718 (+), Divergence to consensus: 4.9%  
**50. MER20**: 67225590-67225772 (-), Divergence to consensus: 38%  
**51. MLT1E3**: 67226598-67226716 (+), Divergence to consensus: 38.9%

  
Transcription Factor Binding Sites  

**RFX4\_2** (Sequence: GTAACTAAG (-): 67205666)  
**RFX4\_2** (Sequence: GTATCCAGG (-): 67215417)  
**RFX4\_1** (Sequence: GTTGCCAAG (-): 67202406)  
**Gata4** (Sequence: AGATAAG (-): 67209159)  
**Gata4** (Sequence: GTTATCT (+): 67221478)  
**Gata4** (Sequence: CTTATCT (+): 67221851)  
**Gata4** (Sequence: GTTATCT (+): 67224526)
