# Supplementary material for: Small RNA sequencing of cryopreserved semen from single bull revealed altered miRNAs and piRNAs expression between High- and Low-motile sperm populations
Source: BMC Genomics. 2017 Jan 4;18:14. doi: 10.1186/s12864-016-3394-7 (PMC5209821; doi:10.1186/s12864-016-3394-7)
Supplement: Additional file 4: — Details for each piRNA clusters found in Low Motile (LM) sperm fraction. Genes, repeats, transposable elements and transcription factors binding sites falling within the cluster regions were reported. (ZIP 1034 kb) [file 12864_2016_3394_MOESM4_ESM.zip › 19.html]

piRNA cluster 19


Predicted piRNA cluster no. 19     previous   next
  

Show proTRAC run info
Hide proTRAC run info

================================= proTRAC ====================================  
VERSION: 2.1                                    LAST MODIFIED: 06. October 2015  
  
Please cite:  
Rosenkranz D, Zischler H. proTRAC - a software for probabilistic piRNA cluster  
detection, visualization and analysis. 2012. BMC Bioinformatics 13:5.  
  
and (for proTRAC 2.0 and later):  
Rosenkranz D, Rudloff S, Bastuck K, Ketting RF, Zischler H. Tupaia small RNAs  
provide insights into function and evolution of RNAi-based transposon defense  
in mammals. 2015. RNA 21(5):911-922.  
  
Contact:  
David Rosenkranz  
Institute of Anthropology, small RNA group  
Johannes Gutenberg University Mainz  
email: rosenkranz@uni-mainz.de  
  
You can find the latest proTRAC version at:  
http://sourceforge.net/projects/protrac/files  
http://www.smallRNAgroup-mainz.de/software  
==============================================================================  
  
PARAMETERS:  
Map file: .............../storage/core/barbara/genhome/smallRNA/fertility/Sample\_not\_motile/pirna/Sample\_not\_motile\_26-33\_collapsed.fa.no-dust.map.weighted-10000-1000-b-0  
Genome file: ............/storage/core/barbara/genhome/smallRNA/fertility/Sample\_all/pirna/bt\_311\_chrY.fa  
RepeatMasker annotation: /storage/genomes/bt\_umd31/GCF\_000003055.6\_Bos\_taurus\_UMD\_3.1.1\_repeatMasker\_chr.out  
GeneSet:................./storage/core/barbara/genhome/smallRNA/fertility/Sample\_all/pirna/full.gtf  
  
Significant (p<=0.01) hit density will be calculated based  
on observed hit distribution.  
  
Sliding window size: ........................................ 5000 bp  
Sliding window increament: .................................. 1000 bp  
Normalize each hit by number of genomic hits: ............... 1 [0=no/1=yes]  
Normalize each hit by number of sequence reads: ............. 1 [0=no/1=yes]  
Normalize values (-> per million mapped reads): ............. 1 [0=no/1=yes]  
Min. fraction of hits with 1T(U) or 10A: .................... 0.75  
Alternatively: Min. fraction of hits with 1T(U) and 10A: .... 0.5  
Min. fraction of hits with typical piRNA length: ............ 0.75  
Typical piRNA length: ....................................... 26-33 nt  
Min. size of a piRNA cluster: ............................... 5000 bp.  
Min. number of hits (absolute): ............................. 0  
Min. number of hits (normalized): ........................... 0  
Min. fraction of hits on the mainstrand: .................... 0.75  
Top fraction of mapped sequences (in terms of read counts): . 1%  
Top fraction accounts for max. n% of sequence reads: ........ 90%  
Min. fraction of hits on each arm of a bidirectional cluster: 0.1  
Output image file for each cluster: ......................... 0 [0=no/1=yes]  
Output html file for each cluster: .......................... 1 [0=no/1=yes]  
Output a summary table: ..................................... 1 [0=no/1=yes]  
Output a FASTA file for each cluster (piRNA sequences): ..... 1 [0=no/1=yes]  
Output a FASTA file comprising cluster sequences: ........... 1 [0=no/1=yes]  
Search DNA motifs in clusters: .............................. 1 [0=no/1=yes]  
Output flanking sequences: +/- .............................. 0 bp  
Output ~.pTi file: .......................................... 1 [0=no/1=yes]  
==============================================================================  
  
  
Genome size (without gaps): ............ 2678902517 bp  
Gaps (N/X/-): .......................... 53837044 bp  
Mapped reads: .......................... 738059667487  
Non-identical sequences: ............... 277001  
Genomic hits: .......................... 533816  
Significant densitiy of mapped reads: .. 15118061 reads/kb

Show proTRAC cluster info
Hide proTRAC cluster info

|  |  |
| --- | --- |
| Location | chr17 |
| Coordinates | 72232684-72240627 |
| Size [bp] | 7944 |
| Sequence hit loci | 50 |
| Mapped reads (normalized) | 137892922 |
| Mapped reads (normalized) per kb | 17358122.1 |
| Normalized reads with 1T (1U) | 82.4% |
| Normalized reads with 10A | 32.1% |
| Normalized reads with length 26-33 nt | 100% |
| Normalized reads on the main strand(s) | 100% |
| Predicted directionality | mono:plus |

100%

0%

1T (1U)  
reads

10A reads

26-33 nt  
reads

reads on mainstrand

**Either the amount of reads with 1T (1U) OR 10A has to exceed 75% (set with option: -1Tor10A)  
Alternatively the amount of reads with 1T (1U) AND 10A has to exceed 50% (set with option: -1Tand10A)  
Minimum amount of reads with preferred size is 75% (set with option: -pisize)  
Minimum amount of reads on the main strand(s) is 75% (set with option: -clstrand)**

Show read coverage
Hide read coverage

WHAT DO I SEE HERE?  
This chart shows the location of mapped sequence reads within a predicted piRNA cluster. The color refers to the number of genomic hits produced by the sequence read in question. A dark red bar indicates that this sequence read produces many other hits elsewhere in the genome. Many adjacent red or yellow bars can indicate the presence of a multi-copy element such as transposons or rRNA genes. A dark green bar indicates that this sequence read maps uniquely to this locus.

1 hit

2-5 hits

6-10 hits

11-20 hits

21-50 hits

51-100 hits

> 100 hits

chr17

72232684

72240627

Gene Set

RepeatMasker

Mapped  
Reads

13.13

plus strand

minus strand

13.13

Region: chr17 67226951-72232691. Max. coverage (+): 4.89. Max coverage (-): 0

Region: chr17 72232692-72232707. Max. coverage (+): 0. Max coverage (-): 0

Region: chr17 72232708-72232723. Max. coverage (+): 0. Max coverage (-): 0

Region: chr17 72232724-72232739. Max. coverage (+): 0. Max coverage (-): 0

Region: chr17 72232740-72232755. Max. coverage (+): 0. Max coverage (-): 0

Region: chr17 72232756-72232771. Max. coverage (+): 1.61. Max coverage (-): 0

Region: chr17 72232772-72232787. Max. coverage (+): 0. Max coverage (-): 0

Region: chr17 72232788-72232803. Max. coverage (+): 0. Max coverage (-): 0

Region: chr17 72232804-72232819. Max. coverage (+): 0. Max coverage (-): 0

Region: chr17 72232820-72232834. Max. coverage (+): 0. Max coverage (-): 0

Region: chr17 72232835-72232850. Max. coverage (+): 0. Max coverage (-): 0

Region: chr17 72232851-72232866. Max. coverage (+): 0. Max coverage (-): 0

Region: chr17 72232867-72232882. Max. coverage (+): 0. Max coverage (-): 0

Region: chr17 72232883-72232898. Max. coverage (+): 0. Max coverage (-): 0

Region: chr17 72232899-72232914. Max. coverage (+): 0. Max coverage (-): 0

Region: chr17 72232915-72232930. Max. coverage (+): 0. Max coverage (-): 0

Region: chr17 72232931-72232946. Max. coverage (+): 0. Max coverage (-): 0

Region: chr17 72232947-72232962. Max. coverage (+): 0. Max coverage (-): 0

Region: chr17 72232963-72232977. Max. coverage (+): 0. Max coverage (-): 0

Region: chr17 72232978-72232993. Max. coverage (+): 0. Max coverage (-): 0

Region: chr17 72232994-72233009. Max. coverage (+): 0. Max coverage (-): 0

Region: chr17 72233010-72233025. Max. coverage (+): 0. Max coverage (-): 0

Region: chr17 72233026-72233041. Max. coverage (+): 1.34. Max coverage (-): 0

Region: chr17 72233042-72233057. Max. coverage (+): 0. Max coverage (-): 0

Region: chr17 72233058-72233073. Max. coverage (+): 0. Max coverage (-): 0

Region: chr17 72233074-72233089. Max. coverage (+): 0. Max coverage (-): 0

Region: chr17 72233090-72233105. Max. coverage (+): 0. Max coverage (-): 0

Region: chr17 72233106-72233120. Max. coverage (+): 0. Max coverage (-): 0

Region: chr17 72233121-72233136. Max. coverage (+): 0. Max coverage (-): 0

Region: chr17 72233137-72233152. Max. coverage (+): 0. Max coverage (-): 0

Region: chr17 72233153-72233168. Max. coverage (+): 0. Max coverage (-): 0

Region: chr17 72233169-72233184. Max. coverage (+): 0. Max coverage (-): 0

Region: chr17 72233185-72233200. Max. coverage (+): 0. Max coverage (-): 0

Region: chr17 72233201-72233216. Max. coverage (+): 0. Max coverage (-): 0

Region: chr17 72233217-72233232. Max. coverage (+): 0. Max coverage (-): 0

Region: chr17 72233233-72233248. Max. coverage (+): 0. Max coverage (-): 0

Region: chr17 72233249-72233263. Max. coverage (+): 0. Max coverage (-): 0

Region: chr17 72233264-72233279. Max. coverage (+): 0. Max coverage (-): 0

Region: chr17 72233280-72233295. Max. coverage (+): 0. Max coverage (-): 0

Region: chr17 72233296-72233311. Max. coverage (+): 0. Max coverage (-): 0

Region: chr17 72233312-72233327. Max. coverage (+): 0. Max coverage (-): 0

Region: chr17 72233328-72233343. Max. coverage (+): 0. Max coverage (-): 0

Region: chr17 72233344-72233359. Max. coverage (+): 0. Max coverage (-): 0

Region: chr17 72233360-72233375. Max. coverage (+): 0. Max coverage (-): 0

Region: chr17 72233376-72233391. Max. coverage (+): 0. Max coverage (-): 0

Region: chr17 72233392-72233406. Max. coverage (+): 0. Max coverage (-): 0

Region: chr17 72233407-72233422. Max. coverage (+): 5.47. Max coverage (-): 0

Region: chr17 72233423-72233438. Max. coverage (+): 5.47. Max coverage (-): 0

Region: chr17 72233439-72233454. Max. coverage (+): 0. Max coverage (-): 0

Region: chr17 72233455-72233470. Max. coverage (+): 0. Max coverage (-): 0

Region: chr17 72233471-72233486. Max. coverage (+): 0. Max coverage (-): 0

Region: chr17 72233487-72233502. Max. coverage (+): 0. Max coverage (-): 0

Region: chr17 72233503-72233518. Max. coverage (+): 0. Max coverage (-): 0

Region: chr17 72233519-72233534. Max. coverage (+): 0. Max coverage (-): 0

Region: chr17 72233535-72233549. Max. coverage (+): 0. Max coverage (-): 0

Region: chr17 72233550-72233565. Max. coverage (+): 0. Max coverage (-): 0

Region: chr17 72233566-72233581. Max. coverage (+): 0. Max coverage (-): 0

Region: chr17 72233582-72233597. Max. coverage (+): 0. Max coverage (-): 0

Region: chr17 72233598-72233613. Max. coverage (+): 0. Max coverage (-): 0

Region: chr17 72233614-72233629. Max. coverage (+): 0. Max coverage (-): 0

Region: chr17 72233630-72233645. Max. coverage (+): 0. Max coverage (-): 0

Region: chr17 72233646-72233661. Max. coverage (+): 0. Max coverage (-): 0

Region: chr17 72233662-72233676. Max. coverage (+): 0. Max coverage (-): 0

Region: chr17 72233677-72233692. Max. coverage (+): 0. Max coverage (-): 0

Region: chr17 72233693-72233708. Max. coverage (+): 0. Max coverage (-): 0

Region: chr17 72233709-72233724. Max. coverage (+): 0. Max coverage (-): 0

Region: chr17 72233725-72233740. Max. coverage (+): 0. Max coverage (-): 0

Region: chr17 72233741-72233756. Max. coverage (+): 0. Max coverage (-): 0

Region: chr17 72233757-72233772. Max. coverage (+): 0. Max coverage (-): 0

Region: chr17 72233773-72233788. Max. coverage (+): 0. Max coverage (-): 0

Region: chr17 72233789-72233804. Max. coverage (+): 0. Max coverage (-): 0

Region: chr17 72233805-72233819. Max. coverage (+): 0. Max coverage (-): 0

Region: chr17 72233820-72233835. Max. coverage (+): 0. Max coverage (-): 0

Region: chr17 72233836-72233851. Max. coverage (+): 0. Max coverage (-): 0

Region: chr17 72233852-72233867. Max. coverage (+): 3.45. Max coverage (-): 0

Region: chr17 72233868-72233883. Max. coverage (+): 0. Max coverage (-): 0

Region: chr17 72233884-72233899. Max. coverage (+): 0. Max coverage (-): 0

Region: chr17 72233900-72233915. Max. coverage (+): 0. Max coverage (-): 0

Region: chr17 72233916-72233931. Max. coverage (+): 0. Max coverage (-): 0

Region: chr17 72233932-72233947. Max. coverage (+): 0. Max coverage (-): 0

Region: chr17 72233948-72233962. Max. coverage (+): 0. Max coverage (-): 0

Region: chr17 72233963-72233978. Max. coverage (+): 0. Max coverage (-): 0

Region: chr17 72233979-72233994. Max. coverage (+): 0. Max coverage (-): 0

Region: chr17 72233995-72234010. Max. coverage (+): 0. Max coverage (-): 0

Region: chr17 72234011-72234026. Max. coverage (+): 0. Max coverage (-): 0

Region: chr17 72234027-72234042. Max. coverage (+): 0. Max coverage (-): 0

Region: chr17 72234043-72234058. Max. coverage (+): 1.94. Max coverage (-): 0

Region: chr17 72234059-72234074. Max. coverage (+): 0. Max coverage (-): 0

Region: chr17 72234075-72234090. Max. coverage (+): 0. Max coverage (-): 0

Region: chr17 72234091-72234105. Max. coverage (+): 0. Max coverage (-): 0

Region: chr17 72234106-72234121. Max. coverage (+): 0. Max coverage (-): 0

Region: chr17 72234122-72234137. Max. coverage (+): 0. Max coverage (-): 0

Region: chr17 72234138-72234153. Max. coverage (+): 0. Max coverage (-): 0

Region: chr17 72234154-72234169. Max. coverage (+): 0. Max coverage (-): 0

Region: chr17 72234170-72234185. Max. coverage (+): 0. Max coverage (-): 0

Region: chr17 72234186-72234201. Max. coverage (+): 0. Max coverage (-): 0

Region: chr17 72234202-72234217. Max. coverage (+): 0. Max coverage (-): 0

Region: chr17 72234218-72234233. Max. coverage (+): 0. Max coverage (-): 0

Region: chr17 72234234-72234248. Max. coverage (+): 0. Max coverage (-): 0

Region: chr17 72234249-72234264. Max. coverage (+): 0. Max coverage (-): 0

Region: chr17 72234265-72234280. Max. coverage (+): 0. Max coverage (-): 0

Region: chr17 72234281-72234296. Max. coverage (+): 0. Max coverage (-): 0

Region: chr17 72234297-72234312. Max. coverage (+): 0. Max coverage (-): 0

Region: chr17 72234313-72234328. Max. coverage (+): 0. Max coverage (-): 0

Region: chr17 72234329-72234344. Max. coverage (+): 0. Max coverage (-): 0

Region: chr17 72234345-72234360. Max. coverage (+): 9.69. Max coverage (-): 0

Region: chr17 72234361-72234376. Max. coverage (+): 0. Max coverage (-): 0

Region: chr17 72234377-72234391. Max. coverage (+): 0. Max coverage (-): 0

Region: chr17 72234392-72234407. Max. coverage (+): 0. Max coverage (-): 0

Region: chr17 72234408-72234423. Max. coverage (+): 0. Max coverage (-): 0

Region: chr17 72234424-72234439. Max. coverage (+): 0. Max coverage (-): 0

Region: chr17 72234440-72234455. Max. coverage (+): 0. Max coverage (-): 0

Region: chr17 72234456-72234471. Max. coverage (+): 0. Max coverage (-): 0

Region: chr17 72234472-72234487. Max. coverage (+): 0. Max coverage (-): 0

Region: chr17 72234488-72234503. Max. coverage (+): 0. Max coverage (-): 0

Region: chr17 72234504-72234519. Max. coverage (+): 0. Max coverage (-): 0

Region: chr17 72234520-72234534. Max. coverage (+): 0. Max coverage (-): 0

Region: chr17 72234535-72234550. Max. coverage (+): 0. Max coverage (-): 0

Region: chr17 72234551-72234566. Max. coverage (+): 0. Max coverage (-): 0

Region: chr17 72234567-72234582. Max. coverage (+): 0. Max coverage (-): 0

Region: chr17 72234583-72234598. Max. coverage (+): 0. Max coverage (-): 0

Region: chr17 72234599-72234614. Max. coverage (+): 0. Max coverage (-): 0

Region: chr17 72234615-72234630. Max. coverage (+): 0. Max coverage (-): 0

Region: chr17 72234631-72234646. Max. coverage (+): 0. Max coverage (-): 0

Region: chr17 72234647-72234662. Max. coverage (+): 0. Max coverage (-): 0

Region: chr17 72234663-72234677. Max. coverage (+): 0. Max coverage (-): 0

Region: chr17 72234678-72234693. Max. coverage (+): 0. Max coverage (-): 0

Region: chr17 72234694-72234709. Max. coverage (+): 0. Max coverage (-): 0

Region: chr17 72234710-72234725. Max. coverage (+): 0. Max coverage (-): 0

Region: chr17 72234726-72234741. Max. coverage (+): 0. Max coverage (-): 0

Region: chr17 72234742-72234757. Max. coverage (+): 0. Max coverage (-): 0

Region: chr17 72234758-72234773. Max. coverage (+): 0. Max coverage (-): 0

Region: chr17 72234774-72234789. Max. coverage (+): 0. Max coverage (-): 0

Region: chr17 72234790-72234805. Max. coverage (+): 0. Max coverage (-): 0

Region: chr17 72234806-72234820. Max. coverage (+): 0. Max coverage (-): 0

Region: chr17 72234821-72234836. Max. coverage (+): 0. Max coverage (-): 0

Region: chr17 72234837-72234852. Max. coverage (+): 0. Max coverage (-): 0

Region: chr17 72234853-72234868. Max. coverage (+): 0. Max coverage (-): 0

Region: chr17 72234869-72234884. Max. coverage (+): 0. Max coverage (-): 0

Region: chr17 72234885-72234900. Max. coverage (+): 0. Max coverage (-): 0

Region: chr17 72234901-72234916. Max. coverage (+): 0. Max coverage (-): 0

Region: chr17 72234917-72234932. Max. coverage (+): 0. Max coverage (-): 0

Region: chr17 72234933-72234948. Max. coverage (+): 0. Max coverage (-): 0

Region: chr17 72234949-72234963. Max. coverage (+): 0. Max coverage (-): 0

Region: chr17 72234964-72234979. Max. coverage (+): 0. Max coverage (-): 0

Region: chr17 72234980-72234995. Max. coverage (+): 0. Max coverage (-): 0

Region: chr17 72234996-72235011. Max. coverage (+): 0. Max coverage (-): 0

Region: chr17 72235012-72235027. Max. coverage (+): 0. Max coverage (-): 0

Region: chr17 72235028-72235043. Max. coverage (+): 0. Max coverage (-): 0

Region: chr17 72235044-72235059. Max. coverage (+): 0. Max coverage (-): 0

Region: chr17 72235060-72235075. Max. coverage (+): 0. Max coverage (-): 0

Region: chr17 72235076-72235091. Max. coverage (+): 0. Max coverage (-): 0

Region: chr17 72235092-72235106. Max. coverage (+): 1.82. Max coverage (-): 0

Region: chr17 72235107-72235122. Max. coverage (+): 1.82. Max coverage (-): 0

Region: chr17 72235123-72235138. Max. coverage (+): 4.83. Max coverage (-): 0

Region: chr17 72235139-72235154. Max. coverage (+): 4.83. Max coverage (-): 0

Region: chr17 72235155-72235170. Max. coverage (+): 0. Max coverage (-): 0

Region: chr17 72235171-72235186. Max. coverage (+): 0. Max coverage (-): 0

Region: chr17 72235187-72235202. Max. coverage (+): 0. Max coverage (-): 0

Region: chr17 72235203-72235218. Max. coverage (+): 0. Max coverage (-): 0

Region: chr17 72235219-72235234. Max. coverage (+): 0. Max coverage (-): 0

Region: chr17 72235235-72235249. Max. coverage (+): 0. Max coverage (-): 0

Region: chr17 72235250-72235265. Max. coverage (+): 0. Max coverage (-): 0

Region: chr17 72235266-72235281. Max. coverage (+): 0. Max coverage (-): 0

Region: chr17 72235282-72235297. Max. coverage (+): 0. Max coverage (-): 0

Region: chr17 72235298-72235313. Max. coverage (+): 0. Max coverage (-): 0

Region: chr17 72235314-72235329. Max. coverage (+): 0. Max coverage (-): 0

Region: chr17 72235330-72235345. Max. coverage (+): 0. Max coverage (-): 0

Region: chr17 72235346-72235361. Max. coverage (+): 0. Max coverage (-): 0

Region: chr17 72235362-72235377. Max. coverage (+): 0. Max coverage (-): 0

Region: chr17 72235378-72235392. Max. coverage (+): 0. Max coverage (-): 0

Region: chr17 72235393-72235408. Max. coverage (+): 0. Max coverage (-): 0

Region: chr17 72235409-72235424. Max. coverage (+): 0. Max coverage (-): 0

Region: chr17 72235425-72235440. Max. coverage (+): 0. Max coverage (-): 0

Region: chr17 72235441-72235456. Max. coverage (+): 0. Max coverage (-): 0

Region: chr17 72235457-72235472. Max. coverage (+): 0. Max coverage (-): 0

Region: chr17 72235473-72235488. Max. coverage (+): 0. Max coverage (-): 0

Region: chr17 72235489-72235504. Max. coverage (+): 0. Max coverage (-): 0

Region: chr17 72235505-72235520. Max. coverage (+): 0. Max coverage (-): 0

Region: chr17 72235521-72235535. Max. coverage (+): 0. Max coverage (-): 0

Region: chr17 72235536-72235551. Max. coverage (+): 0. Max coverage (-): 0

Region: chr17 72235552-72235567. Max. coverage (+): 0. Max coverage (-): 0

Region: chr17 72235568-72235583. Max. coverage (+): 0. Max coverage (-): 0

Region: chr17 72235584-72235599. Max. coverage (+): 0. Max coverage (-): 0

Region: chr17 72235600-72235615. Max. coverage (+): 4. Max coverage (-): 0

Region: chr17 72235616-72235631. Max. coverage (+): 4. Max coverage (-): 0

Region: chr17 72235632-72235647. Max. coverage (+): 9.96. Max coverage (-): 0

Region: chr17 72235648-72235662. Max. coverage (+): 2.47. Max coverage (-): 0

Region: chr17 72235663-72235678. Max. coverage (+): 0. Max coverage (-): 0

Region: chr17 72235679-72235694. Max. coverage (+): 0. Max coverage (-): 0

Region: chr17 72235695-72235710. Max. coverage (+): 13.13. Max coverage (-): 0

Region: chr17 72235711-72235726. Max. coverage (+): 13.13. Max coverage (-): 0

Region: chr17 72235727-72235742. Max. coverage (+): 5.55. Max coverage (-): 0

Region: chr17 72235743-72235758. Max. coverage (+): 0. Max coverage (-): 0

Region: chr17 72235759-72235774. Max. coverage (+): 0. Max coverage (-): 0

Region: chr17 72235775-72235790. Max. coverage (+): 0. Max coverage (-): 0

Region: chr17 72235791-72235805. Max. coverage (+): 0. Max coverage (-): 0

Region: chr17 72235806-72235821. Max. coverage (+): 2.29. Max coverage (-): 0

Region: chr17 72235822-72235837. Max. coverage (+): 0. Max coverage (-): 0

Region: chr17 72235838-72235853. Max. coverage (+): 0. Max coverage (-): 0

Region: chr17 72235854-72235869. Max. coverage (+): 0. Max coverage (-): 0

Region: chr17 72235870-72235885. Max. coverage (+): 0. Max coverage (-): 0

Region: chr17 72235886-72235901. Max. coverage (+): 0. Max coverage (-): 0

Region: chr17 72235902-72235917. Max. coverage (+): 0. Max coverage (-): 0

Region: chr17 72235918-72235933. Max. coverage (+): 0. Max coverage (-): 0

Region: chr17 72235934-72235948. Max. coverage (+): 0. Max coverage (-): 0

Region: chr17 72235949-72235964. Max. coverage (+): 0. Max coverage (-): 0

Region: chr17 72235965-72235980. Max. coverage (+): 0. Max coverage (-): 0

Region: chr17 72235981-72235996. Max. coverage (+): 3.22. Max coverage (-): 0

Region: chr17 72235997-72236012. Max. coverage (+): 0. Max coverage (-): 0

Region: chr17 72236013-72236028. Max. coverage (+): 0. Max coverage (-): 0

Region: chr17 72236029-72236044. Max. coverage (+): 0. Max coverage (-): 0

Region: chr17 72236045-72236060. Max. coverage (+): 4.72. Max coverage (-): 0

Region: chr17 72236061-72236076. Max. coverage (+): 0. Max coverage (-): 0

Region: chr17 72236077-72236091. Max. coverage (+): 0. Max coverage (-): 0

Region: chr17 72236092-72236107. Max. coverage (+): 0. Max coverage (-): 0

Region: chr17 72236108-72236123. Max. coverage (+): 0. Max coverage (-): 0

Region: chr17 72236124-72236139. Max. coverage (+): 0. Max coverage (-): 0

Region: chr17 72236140-72236155. Max. coverage (+): 0. Max coverage (-): 0

Region: chr17 72236156-72236171. Max. coverage (+): 0. Max coverage (-): 0

Region: chr17 72236172-72236187. Max. coverage (+): 0. Max coverage (-): 0

Region: chr17 72236188-72236203. Max. coverage (+): 0. Max coverage (-): 0

Region: chr17 72236204-72236219. Max. coverage (+): 0. Max coverage (-): 0

Region: chr17 72236220-72236234. Max. coverage (+): 0. Max coverage (-): 0

Region: chr17 72236235-72236250. Max. coverage (+): 0. Max coverage (-): 0

Region: chr17 72236251-72236266. Max. coverage (+): 0. Max coverage (-): 0

Region: chr17 72236267-72236282. Max. coverage (+): 0. Max coverage (-): 0

Region: chr17 72236283-72236298. Max. coverage (+): 0. Max coverage (-): 0

Region: chr17 72236299-72236314. Max. coverage (+): 0. Max coverage (-): 0

Region: chr17 72236315-72236330. Max. coverage (+): 0. Max coverage (-): 0

Region: chr17 72236331-72236346. Max. coverage (+): 8.27. Max coverage (-): 0

Region: chr17 72236347-72236362. Max. coverage (+): 0. Max coverage (-): 0

Region: chr17 72236363-72236377. Max. coverage (+): 0. Max coverage (-): 0

Region: chr17 72236378-72236393. Max. coverage (+): 0. Max coverage (-): 0

Region: chr17 72236394-72236409. Max. coverage (+): 0. Max coverage (-): 0

Region: chr17 72236410-72236425. Max. coverage (+): 0. Max coverage (-): 0

Region: chr17 72236426-72236441. Max. coverage (+): 0. Max coverage (-): 0

Region: chr17 72236442-72236457. Max. coverage (+): 0. Max coverage (-): 0

Region: chr17 72236458-72236473. Max. coverage (+): 0. Max coverage (-): 0

Region: chr17 72236474-72236489. Max. coverage (+): 0. Max coverage (-): 0

Region: chr17 72236490-72236505. Max. coverage (+): 0. Max coverage (-): 0

Region: chr17 72236506-72236520. Max. coverage (+): 0. Max coverage (-): 0

Region: chr17 72236521-72236536. Max. coverage (+): 0. Max coverage (-): 0

Region: chr17 72236537-72236552. Max. coverage (+): 0. Max coverage (-): 0

Region: chr17 72236553-72236568. Max. coverage (+): 0. Max coverage (-): 0

Region: chr17 72236569-72236584. Max. coverage (+): 0. Max coverage (-): 0

Region: chr17 72236585-72236600. Max. coverage (+): 0. Max coverage (-): 0

Region: chr17 72236601-72236616. Max. coverage (+): 0. Max coverage (-): 0

Region: chr17 72236617-72236632. Max. coverage (+): 0. Max coverage (-): 0

Region: chr17 72236633-72236648. Max. coverage (+): 0. Max coverage (-): 0

Region: chr17 72236649-72236663. Max. coverage (+): 0. Max coverage (-): 0

Region: chr17 72236664-72236679. Max. coverage (+): 5.66. Max coverage (-): 0

Region: chr17 72236680-72236695. Max. coverage (+): 0. Max coverage (-): 0

Region: chr17 72236696-72236711. Max. coverage (+): 0. Max coverage (-): 0

Region: chr17 72236712-72236727. Max. coverage (+): 0. Max coverage (-): 0

Region: chr17 72236728-72236743. Max. coverage (+): 0. Max coverage (-): 0

Region: chr17 72236744-72236759. Max. coverage (+): 0. Max coverage (-): 0

Region: chr17 72236760-72236775. Max. coverage (+): 0. Max coverage (-): 0

Region: chr17 72236776-72236791. Max. coverage (+): 0. Max coverage (-): 0

Region: chr17 72236792-72236806. Max. coverage (+): 0. Max coverage (-): 0

Region: chr17 72236807-72236822. Max. coverage (+): 0. Max coverage (-): 0

Region: chr17 72236823-72236838. Max. coverage (+): 0. Max coverage (-): 0

Region: chr17 72236839-72236854. Max. coverage (+): 0. Max coverage (-): 0

Region: chr17 72236855-72236870. Max. coverage (+): 0. Max coverage (-): 0

Region: chr17 72236871-72236886. Max. coverage (+): 0. Max coverage (-): 0

Region: chr17 72236887-72236902. Max. coverage (+): 9.56. Max coverage (-): 0

Region: chr17 72236903-72236918. Max. coverage (+): 0. Max coverage (-): 0

Region: chr17 72236919-72236934. Max. coverage (+): 0. Max coverage (-): 0

Region: chr17 72236935-72236949. Max. coverage (+): 0. Max coverage (-): 0

Region: chr17 72236950-72236965. Max. coverage (+): 0. Max coverage (-): 0

Region: chr17 72236966-72236981. Max. coverage (+): 0. Max coverage (-): 0

Region: chr17 72236982-72236997. Max. coverage (+): 0. Max coverage (-): 0

Region: chr17 72236998-72237013. Max. coverage (+): 0. Max coverage (-): 0

Region: chr17 72237014-72237029. Max. coverage (+): 0. Max coverage (-): 0

Region: chr17 72237030-72237045. Max. coverage (+): 0. Max coverage (-): 0

Region: chr17 72237046-72237061. Max. coverage (+): 0. Max coverage (-): 0

Region: chr17 72237062-72237077. Max. coverage (+): 0. Max coverage (-): 0

Region: chr17 72237078-72237092. Max. coverage (+): 0.57. Max coverage (-): 0

Region: chr17 72237093-72237108. Max. coverage (+): 0. Max coverage (-): 0

Region: chr17 72237109-72237124. Max. coverage (+): 0. Max coverage (-): 0

Region: chr17 72237125-72237140. Max. coverage (+): 0. Max coverage (-): 0

Region: chr17 72237141-72237156. Max. coverage (+): 0. Max coverage (-): 0

Region: chr17 72237157-72237172. Max. coverage (+): 0. Max coverage (-): 0

Region: chr17 72237173-72237188. Max. coverage (+): 0. Max coverage (-): 0

Region: chr17 72237189-72237204. Max. coverage (+): 0. Max coverage (-): 0

Region: chr17 72237205-72237220. Max. coverage (+): 0. Max coverage (-): 0

Region: chr17 72237221-72237235. Max. coverage (+): 0. Max coverage (-): 0

Region: chr17 72237236-72237251. Max. coverage (+): 0. Max coverage (-): 0

Region: chr17 72237252-72237267. Max. coverage (+): 0. Max coverage (-): 0

Region: chr17 72237268-72237283. Max. coverage (+): 0. Max coverage (-): 0

Region: chr17 72237284-72237299. Max. coverage (+): 0. Max coverage (-): 0

Region: chr17 72237300-72237315. Max. coverage (+): 0. Max coverage (-): 0

Region: chr17 72237316-72237331. Max. coverage (+): 0. Max coverage (-): 0

Region: chr17 72237332-72237347. Max. coverage (+): 0. Max coverage (-): 0

Region: chr17 72237348-72237363. Max. coverage (+): 0. Max coverage (-): 0

Region: chr17 72237364-72237378. Max. coverage (+): 0. Max coverage (-): 0

Region: chr17 72237379-72237394. Max. coverage (+): 0. Max coverage (-): 0

Region: chr17 72237395-72237410. Max. coverage (+): 0. Max coverage (-): 0

Region: chr17 72237411-72237426. Max. coverage (+): 0. Max coverage (-): 0

Region: chr17 72237427-72237442. Max. coverage (+): 0. Max coverage (-): 0

Region: chr17 72237443-72237458. Max. coverage (+): 0. Max coverage (-): 0

Region: chr17 72237459-72237474. Max. coverage (+): 0. Max coverage (-): 0

Region: chr17 72237475-72237490. Max. coverage (+): 0. Max coverage (-): 0

Region: chr17 72237491-72237506. Max. coverage (+): 0. Max coverage (-): 0

Region: chr17 72237507-72237521. Max. coverage (+): 0. Max coverage (-): 0

Region: chr17 72237522-72237537. Max. coverage (+): 0. Max coverage (-): 0

Region: chr17 72237538-72237553. Max. coverage (+): 0. Max coverage (-): 0

Region: chr17 72237554-72237569. Max. coverage (+): 0. Max coverage (-): 0

Region: chr17 72237570-72237585. Max. coverage (+): 0. Max coverage (-): 0

Region: chr17 72237586-72237601. Max. coverage (+): 2.56. Max coverage (-): 0

Region: chr17 72237602-72237617. Max. coverage (+): 2.56. Max coverage (-): 0

Region: chr17 72237618-72237633. Max. coverage (+): 0. Max coverage (-): 0

Region: chr17 72237634-72237648. Max. coverage (+): 0. Max coverage (-): 0

Region: chr17 72237649-72237664. Max. coverage (+): 5.06. Max coverage (-): 0

Region: chr17 72237665-72237680. Max. coverage (+): 0. Max coverage (-): 0

Region: chr17 72237681-72237696. Max. coverage (+): 0. Max coverage (-): 0

Region: chr17 72237697-72237712. Max. coverage (+): 0. Max coverage (-): 0

Region: chr17 72237713-72237728. Max. coverage (+): 0. Max coverage (-): 0

Region: chr17 72237729-72237744. Max. coverage (+): 0. Max coverage (-): 0

Region: chr17 72237745-72237760. Max. coverage (+): 0. Max coverage (-): 0

Region: chr17 72237761-72237776. Max. coverage (+): 6.24. Max coverage (-): 0

Region: chr17 72237777-72237791. Max. coverage (+): 2.13. Max coverage (-): 0

Region: chr17 72237792-72237807. Max. coverage (+): 8.5. Max coverage (-): 0

Region: chr17 72237808-72237823. Max. coverage (+): 4.52. Max coverage (-): 0

Region: chr17 72237824-72237839. Max. coverage (+): 3.39. Max coverage (-): 0

Region: chr17 72237840-72237855. Max. coverage (+): 0. Max coverage (-): 0

Region: chr17 72237856-72237871. Max. coverage (+): 0. Max coverage (-): 0

Region: chr17 72237872-72237887. Max. coverage (+): 0. Max coverage (-): 0

Region: chr17 72237888-72237903. Max. coverage (+): 0. Max coverage (-): 0

Region: chr17 72237904-72237919. Max. coverage (+): 0. Max coverage (-): 0

Region: chr17 72237920-72237934. Max. coverage (+): 0. Max coverage (-): 0

Region: chr17 72237935-72237950. Max. coverage (+): 0. Max coverage (-): 0

Region: chr17 72237951-72237966. Max. coverage (+): 0. Max coverage (-): 0

Region: chr17 72237967-72237982. Max. coverage (+): 0. Max coverage (-): 0

Region: chr17 72237983-72237998. Max. coverage (+): 0. Max coverage (-): 0

Region: chr17 72237999-72238014. Max. coverage (+): 0. Max coverage (-): 0

Region: chr17 72238015-72238030. Max. coverage (+): 0. Max coverage (-): 0

Region: chr17 72238031-72238046. Max. coverage (+): 0. Max coverage (-): 0

Region: chr17 72238047-72238062. Max. coverage (+): 0. Max coverage (-): 0

Region: chr17 72238063-72238077. Max. coverage (+): 0. Max coverage (-): 0

Region: chr17 72238078-72238093. Max. coverage (+): 0. Max coverage (-): 0

Region: chr17 72238094-72238109. Max. coverage (+): 0. Max coverage (-): 0

Region: chr17 72238110-72238125. Max. coverage (+): 0. Max coverage (-): 0

Region: chr17 72238126-72238141. Max. coverage (+): 0. Max coverage (-): 0

Region: chr17 72238142-72238157. Max. coverage (+): 0. Max coverage (-): 0

Region: chr17 72238158-72238173. Max. coverage (+): 0. Max coverage (-): 0

Region: chr17 72238174-72238189. Max. coverage (+): 0. Max coverage (-): 0

Region: chr17 72238190-72238205. Max. coverage (+): 0. Max coverage (-): 0

Region: chr17 72238206-72238220. Max. coverage (+): 0. Max coverage (-): 0

Region: chr17 72238221-72238236. Max. coverage (+): 0. Max coverage (-): 0

Region: chr17 72238237-72238252. Max. coverage (+): 0. Max coverage (-): 0

Region: chr17 72238253-72238268. Max. coverage (+): 0. Max coverage (-): 0

Region: chr17 72238269-72238284. Max. coverage (+): 0. Max coverage (-): 0

Region: chr17 72238285-72238300. Max. coverage (+): 0. Max coverage (-): 0

Region: chr17 72238301-72238316. Max. coverage (+): 0. Max coverage (-): 0

Region: chr17 72238317-72238332. Max. coverage (+): 0. Max coverage (-): 0

Region: chr17 72238333-72238348. Max. coverage (+): 0. Max coverage (-): 0

Region: chr17 72238349-72238363. Max. coverage (+): 0. Max coverage (-): 0

Region: chr17 72238364-72238379. Max. coverage (+): 0. Max coverage (-): 0

Region: chr17 72238380-72238395. Max. coverage (+): 0. Max coverage (-): 0

Region: chr17 72238396-72238411. Max. coverage (+): 0. Max coverage (-): 0

Region: chr17 72238412-72238427. Max. coverage (+): 0. Max coverage (-): 0

Region: chr17 72238428-72238443. Max. coverage (+): 0. Max coverage (-): 0

Region: chr17 72238444-72238459. Max. coverage (+): 0. Max coverage (-): 0

Region: chr17 72238460-72238475. Max. coverage (+): 0. Max coverage (-): 0

Region: chr17 72238476-72238491. Max. coverage (+): 7.04. Max coverage (-): 0

Region: chr17 72238492-72238506. Max. coverage (+): 7.04. Max coverage (-): 0

Region: chr17 72238507-72238522. Max. coverage (+): 0. Max coverage (-): 0

Region: chr17 72238523-72238538. Max. coverage (+): 0. Max coverage (-): 0

Region: chr17 72238539-72238554. Max. coverage (+): 0. Max coverage (-): 0

Region: chr17 72238555-72238570. Max. coverage (+): 0. Max coverage (-): 0

Region: chr17 72238571-72238586. Max. coverage (+): 0. Max coverage (-): 0

Region: chr17 72238587-72238602. Max. coverage (+): 0. Max coverage (-): 0

Region: chr17 72238603-72238618. Max. coverage (+): 0. Max coverage (-): 0

Region: chr17 72238619-72238634. Max. coverage (+): 0. Max coverage (-): 0

Region: chr17 72238635-72238649. Max. coverage (+): 0. Max coverage (-): 0

Region: chr17 72238650-72238665. Max. coverage (+): 0. Max coverage (-): 0

Region: chr17 72238666-72238681. Max. coverage (+): 4.94. Max coverage (-): 0

Region: chr17 72238682-72238697. Max. coverage (+): 4.94. Max coverage (-): 0

Region: chr17 72238698-72238713. Max. coverage (+): 0. Max coverage (-): 0

Region: chr17 72238714-72238729. Max. coverage (+): 0. Max coverage (-): 0

Region: chr17 72238730-72238745. Max. coverage (+): 6.82. Max coverage (-): 0

Region: chr17 72238746-72238761. Max. coverage (+): 6.82. Max coverage (-): 0

Region: chr17 72238762-72238777. Max. coverage (+): 0. Max coverage (-): 0

Region: chr17 72238778-72238792. Max. coverage (+): 3.3. Max coverage (-): 0

Region: chr17 72238793-72238808. Max. coverage (+): 3.3. Max coverage (-): 0

Region: chr17 72238809-72238824. Max. coverage (+): 5.3. Max coverage (-): 0

Region: chr17 72238825-72238840. Max. coverage (+): 5.3. Max coverage (-): 0

Region: chr17 72238841-72238856. Max. coverage (+): 0. Max coverage (-): 0

Region: chr17 72238857-72238872. Max. coverage (+): 0. Max coverage (-): 0

Region: chr17 72238873-72238888. Max. coverage (+): 0. Max coverage (-): 0

Region: chr17 72238889-72238904. Max. coverage (+): 0. Max coverage (-): 0

Region: chr17 72238905-72238920. Max. coverage (+): 0. Max coverage (-): 0

Region: chr17 72238921-72238935. Max. coverage (+): 0. Max coverage (-): 0

Region: chr17 72238936-72238951. Max. coverage (+): 0. Max coverage (-): 0

Region: chr17 72238952-72238967. Max. coverage (+): 0. Max coverage (-): 0

Region: chr17 72238968-72238983. Max. coverage (+): 0. Max coverage (-): 0

Region: chr17 72238984-72238999. Max. coverage (+): 6.33. Max coverage (-): 0

Region: chr17 72239000-72239015. Max. coverage (+): 0. Max coverage (-): 0

Region: chr17 72239016-72239031. Max. coverage (+): 0. Max coverage (-): 0

Region: chr17 72239032-72239047. Max. coverage (+): 0. Max coverage (-): 0

Region: chr17 72239048-72239063. Max. coverage (+): 0. Max coverage (-): 0

Region: chr17 72239064-72239078. Max. coverage (+): 0. Max coverage (-): 0

Region: chr17 72239079-72239094. Max. coverage (+): 0. Max coverage (-): 0

Region: chr17 72239095-72239110. Max. coverage (+): 0. Max coverage (-): 0

Region: chr17 72239111-72239126. Max. coverage (+): 0. Max coverage (-): 0

Region: chr17 72239127-72239142. Max. coverage (+): 0. Max coverage (-): 0

Region: chr17 72239143-72239158. Max. coverage (+): 0. Max coverage (-): 0

Region: chr17 72239159-72239174. Max. coverage (+): 0. Max coverage (-): 0

Region: chr17 72239175-72239190. Max. coverage (+): 0. Max coverage (-): 0

Region: chr17 72239191-72239206. Max. coverage (+): 0. Max coverage (-): 0

Region: chr17 72239207-72239221. Max. coverage (+): 0. Max coverage (-): 0

Region: chr17 72239222-72239237. Max. coverage (+): 0. Max coverage (-): 0

Region: chr17 72239238-72239253. Max. coverage (+): 0. Max coverage (-): 0

Region: chr17 72239254-72239269. Max. coverage (+): 0. Max coverage (-): 0

Region: chr17 72239270-72239285. Max. coverage (+): 0. Max coverage (-): 0

Region: chr17 72239286-72239301. Max. coverage (+): 0. Max coverage (-): 0

Region: chr17 72239302-72239317. Max. coverage (+): 0. Max coverage (-): 0

Region: chr17 72239318-72239333. Max. coverage (+): 0. Max coverage (-): 0

Region: chr17 72239334-72239349. Max. coverage (+): 0. Max coverage (-): 0

Region: chr17 72239350-72239364. Max. coverage (+): 0. Max coverage (-): 0

Region: chr17 72239365-72239380. Max. coverage (+): 0. Max coverage (-): 0

Region: chr17 72239381-72239396. Max. coverage (+): 0. Max coverage (-): 0

Region: chr17 72239397-72239412. Max. coverage (+): 0. Max coverage (-): 0

Region: chr17 72239413-72239428. Max. coverage (+): 0. Max coverage (-): 0

Region: chr17 72239429-72239444. Max. coverage (+): 5.35. Max coverage (-): 0

Region: chr17 72239445-72239460. Max. coverage (+): 0. Max coverage (-): 0

Region: chr17 72239461-72239476. Max. coverage (+): 0. Max coverage (-): 0

Region: chr17 72239477-72239492. Max. coverage (+): 0. Max coverage (-): 0

Region: chr17 72239493-72239507. Max. coverage (+): 0. Max coverage (-): 0

Region: chr17 72239508-72239523. Max. coverage (+): 0. Max coverage (-): 0

Region: chr17 72239524-72239539. Max. coverage (+): 0. Max coverage (-): 0

Region: chr17 72239540-72239555. Max. coverage (+): 0. Max coverage (-): 0

Region: chr17 72239556-72239571. Max. coverage (+): 0. Max coverage (-): 0

Region: chr17 72239572-72239587. Max. coverage (+): 0. Max coverage (-): 0

Region: chr17 72239588-72239603. Max. coverage (+): 0. Max coverage (-): 0

Region: chr17 72239604-72239619. Max. coverage (+): 0. Max coverage (-): 0

Region: chr17 72239620-72239634. Max. coverage (+): 0. Max coverage (-): 0

Region: chr17 72239635-72239650. Max. coverage (+): 0. Max coverage (-): 0

Region: chr17 72239651-72239666. Max. coverage (+): 0. Max coverage (-): 0

Region: chr17 72239667-72239682. Max. coverage (+): 0. Max coverage (-): 0

Region: chr17 72239683-72239698. Max. coverage (+): 0. Max coverage (-): 0

Region: chr17 72239699-72239714. Max. coverage (+): 0. Max coverage (-): 0

Region: chr17 72239715-72239730. Max. coverage (+): 0. Max coverage (-): 0

Region: chr17 72239731-72239746. Max. coverage (+): 0. Max coverage (-): 0

Region: chr17 72239747-72239762. Max. coverage (+): 0. Max coverage (-): 0

Region: chr17 72239763-72239777. Max. coverage (+): 0. Max coverage (-): 0

Region: chr17 72239778-72239793. Max. coverage (+): 0. Max coverage (-): 0

Region: chr17 72239794-72239809. Max. coverage (+): 0. Max coverage (-): 0

Region: chr17 72239810-72239825. Max. coverage (+): 0. Max coverage (-): 0

Region: chr17 72239826-72239841. Max. coverage (+): 0. Max coverage (-): 0

Region: chr17 72239842-72239857. Max. coverage (+): 0. Max coverage (-): 0

Region: chr17 72239858-72239873. Max. coverage (+): 0. Max coverage (-): 0

Region: chr17 72239874-72239889. Max. coverage (+): 0. Max coverage (-): 0

Region: chr17 72239890-72239905. Max. coverage (+): 0. Max coverage (-): 0

Region: chr17 72239906-72239920. Max. coverage (+): 0. Max coverage (-): 0

Region: chr17 72239921-72239936. Max. coverage (+): 0. Max coverage (-): 0

Region: chr17 72239937-72239952. Max. coverage (+): 0. Max coverage (-): 0

Region: chr17 72239953-72239968. Max. coverage (+): 0. Max coverage (-): 0

Region: chr17 72239969-72239984. Max. coverage (+): 0. Max coverage (-): 0

Region: chr17 72239985-72240000. Max. coverage (+): 0. Max coverage (-): 0

Region: chr17 72240001-72240016. Max. coverage (+): 0. Max coverage (-): 0

Region: chr17 72240017-72240032. Max. coverage (+): 0. Max coverage (-): 0

Region: chr17 72240033-72240048. Max. coverage (+): 0. Max coverage (-): 0

Region: chr17 72240049-72240063. Max. coverage (+): 0.53. Max coverage (-): 0

Region: chr17 72240064-72240079. Max. coverage (+): 0.53. Max coverage (-): 0

Region: chr17 72240080-72240095. Max. coverage (+): 0. Max coverage (-): 0

Region: chr17 72240096-72240111. Max. coverage (+): 0. Max coverage (-): 0

Region: chr17 72240112-72240127. Max. coverage (+): 0. Max coverage (-): 0

Region: chr17 72240128-72240143. Max. coverage (+): 0. Max coverage (-): 0

Region: chr17 72240144-72240159. Max. coverage (+): 0. Max coverage (-): 0

Region: chr17 72240160-72240175. Max. coverage (+): 0. Max coverage (-): 0

Region: chr17 72240176-72240191. Max. coverage (+): 0. Max coverage (-): 0

Region: chr17 72240192-72240206. Max. coverage (+): 0. Max coverage (-): 0

Region: chr17 72240207-72240222. Max. coverage (+): 0. Max coverage (-): 0

Region: chr17 72240223-72240238. Max. coverage (+): 0. Max coverage (-): 0

Region: chr17 72240239-72240254. Max. coverage (+): 0. Max coverage (-): 0

Region: chr17 72240255-72240270. Max. coverage (+): 0. Max coverage (-): 0

Region: chr17 72240271-72240286. Max. coverage (+): 0. Max coverage (-): 0

Region: chr17 72240287-72240302. Max. coverage (+): 0. Max coverage (-): 0

Region: chr17 72240303-72240318. Max. coverage (+): 0. Max coverage (-): 0

Region: chr17 72240319-72240334. Max. coverage (+): 0. Max coverage (-): 0

Region: chr17 72240335-72240349. Max. coverage (+): 0. Max coverage (-): 0

Region: chr17 72240350-72240365. Max. coverage (+): 0. Max coverage (-): 0

Region: chr17 72240366-72240381. Max. coverage (+): 0. Max coverage (-): 0

Region: chr17 72240382-72240397. Max. coverage (+): 0. Max coverage (-): 0

Region: chr17 72240398-72240413. Max. coverage (+): 0. Max coverage (-): 0

Region: chr17 72240414-72240429. Max. coverage (+): 0. Max coverage (-): 0

Region: chr17 72240430-72240445. Max. coverage (+): 0. Max coverage (-): 0

Region: chr17 72240446-72240461. Max. coverage (+): 0. Max coverage (-): 0

Region: chr17 72240462-72240477. Max. coverage (+): 0. Max coverage (-): 0

Region: chr17 72240478-72240492. Max. coverage (+): 0. Max coverage (-): 0

Region: chr17 72240493-72240508. Max. coverage (+): 0. Max coverage (-): 0

Region: chr17 72240509-72240524. Max. coverage (+): 0. Max coverage (-): 0

Region: chr17 72240525-72240540. Max. coverage (+): 0. Max coverage (-): 0

Region: chr17 72240541-72240556. Max. coverage (+): 0. Max coverage (-): 0

Region: chr17 72240557-72240572. Max. coverage (+): 0. Max coverage (-): 0

Region: chr17 72240573-72240588. Max. coverage (+): 0. Max coverage (-): 0

Region: chr17 72240589-72240604. Max. coverage (+): 11.42. Max coverage (-): 0

Region: chr17 72240605-72240620. Max. coverage (+): 11.42. Max coverage (-): 0

Region: chr17 72240621-. Max. coverage (+): 0. Max coverage (-): 0

RepeatMasker Color Code

**+**

100-98% Identity

<98-95% Identity

<95-90% Identity

<90-85% Identity

<85-80% Identity

<80-75% Identity

<75-70% Identity

<70% Identity

**-**

Gene Set Color Code

**+**

Gene

Pseudogene

**-**

Topology/Coverage Color Code

Coverage Plus Strand

Coverage Minus Strand

Mainstrand: Plus

Mainstrand: Minus

Complementary Strand

Flanking Region  
(if option -flank >0)

Gene Set Annotation  

**1. PATZ1 (protein coding, ENSBTAG00000005478) Tr:00000029432 Ex:3**: 72238208-72238379 (-)

  
RepeatMasker Annotation  

**1. CHRL1\_BT**: 72233704-72233853 (-), Divergence to consensus: 31.4%  
**2. Bov-tA1**: 72234161-72234345 (-), Divergence to consensus: 22.3%  
**3. MIRc**: 72237211-72237437 (-), Divergence to consensus: 38.6%  
**4. Charlie15b**: 72239591-72239762 (+), Divergence to consensus: 43.3%  
**5. Tigger2a\_Art**: 72239763-72239997 (+), Divergence to consensus: 38%

  
Transcription Factor Binding Sites  

**SPZ1** (Sequence: CTGAAACCCT (-): 72236765)  
**SOX9** (Sequence: AACAATGA (-): 72233174)  
**SOX9** (Sequence: CTATTGTT (+): 72233908)
