# Supplementary material for: Small RNA sequencing of cryopreserved semen from single bull revealed altered miRNAs and piRNAs expression between High- and Low-motile sperm populations
Source: BMC Genomics. 2017 Jan 4;18:14. doi: 10.1186/s12864-016-3394-7 (PMC5209821; doi:10.1186/s12864-016-3394-7)
Supplement: Additional file 4: — Details for each piRNA clusters found in Low Motile (LM) sperm fraction. Genes, repeats, transposable elements and transcription factors binding sites falling within the cluster regions were reported. (ZIP 1034 kb) [file 12864_2016_3394_MOESM4_ESM.zip › 2.html]

piRNA cluster 2


Predicted piRNA cluster no. 2     previous   next
  

Show proTRAC run info
Hide proTRAC run info

================================= proTRAC ====================================  
VERSION: 2.1                                    LAST MODIFIED: 06. October 2015  
  
Please cite:  
Rosenkranz D, Zischler H. proTRAC - a software for probabilistic piRNA cluster  
detection, visualization and analysis. 2012. BMC Bioinformatics 13:5.  
  
and (for proTRAC 2.0 and later):  
Rosenkranz D, Rudloff S, Bastuck K, Ketting RF, Zischler H. Tupaia small RNAs  
provide insights into function and evolution of RNAi-based transposon defense  
in mammals. 2015. RNA 21(5):911-922.  
  
Contact:  
David Rosenkranz  
Institute of Anthropology, small RNA group  
Johannes Gutenberg University Mainz  
email: rosenkranz@uni-mainz.de  
  
You can find the latest proTRAC version at:  
http://sourceforge.net/projects/protrac/files  
http://www.smallRNAgroup-mainz.de/software  
==============================================================================  
  
PARAMETERS:  
Map file: .............../storage/core/barbara/genhome/smallRNA/fertility/Sample\_not\_motile/pirna/Sample\_not\_motile\_26-33\_collapsed.fa.no-dust.map.weighted-10000-1000-b-0  
Genome file: ............/storage/core/barbara/genhome/smallRNA/fertility/Sample\_all/pirna/bt\_311\_chrY.fa  
RepeatMasker annotation: /storage/genomes/bt\_umd31/GCF\_000003055.6\_Bos\_taurus\_UMD\_3.1.1\_repeatMasker\_chr.out  
GeneSet:................./storage/core/barbara/genhome/smallRNA/fertility/Sample\_all/pirna/full.gtf  
  
Significant (p<=0.01) hit density will be calculated based  
on observed hit distribution.  
  
Sliding window size: ........................................ 5000 bp  
Sliding window increament: .................................. 1000 bp  
Normalize each hit by number of genomic hits: ............... 1 [0=no/1=yes]  
Normalize each hit by number of sequence reads: ............. 1 [0=no/1=yes]  
Normalize values (-> per million mapped reads): ............. 1 [0=no/1=yes]  
Min. fraction of hits with 1T(U) or 10A: .................... 0.75  
Alternatively: Min. fraction of hits with 1T(U) and 10A: .... 0.5  
Min. fraction of hits with typical piRNA length: ............ 0.75  
Typical piRNA length: ....................................... 26-33 nt  
Min. size of a piRNA cluster: ............................... 5000 bp.  
Min. number of hits (absolute): ............................. 0  
Min. number of hits (normalized): ........................... 0  
Min. fraction of hits on the mainstrand: .................... 0.75  
Top fraction of mapped sequences (in terms of read counts): . 1%  
Top fraction accounts for max. n% of sequence reads: ........ 90%  
Min. fraction of hits on each arm of a bidirectional cluster: 0.1  
Output image file for each cluster: ......................... 0 [0=no/1=yes]  
Output html file for each cluster: .......................... 1 [0=no/1=yes]  
Output a summary table: ..................................... 1 [0=no/1=yes]  
Output a FASTA file for each cluster (piRNA sequences): ..... 1 [0=no/1=yes]  
Output a FASTA file comprising cluster sequences: ........... 1 [0=no/1=yes]  
Search DNA motifs in clusters: .............................. 1 [0=no/1=yes]  
Output flanking sequences: +/- .............................. 0 bp  
Output ~.pTi file: .......................................... 1 [0=no/1=yes]  
==============================================================================  
  
  
Genome size (without gaps): ............ 2678902517 bp  
Gaps (N/X/-): .......................... 53837044 bp  
Mapped reads: .......................... 738059667487  
Non-identical sequences: ............... 277001  
Genomic hits: .......................... 533816  
Significant densitiy of mapped reads: .. 15118061 reads/kb

Show proTRAC cluster info
Hide proTRAC cluster info

|  |  |
| --- | --- |
| Location | chr10 |
| Coordinates | 47899195-47913228 |
| Size [bp] | 14034 |
| Sequence hit loci | 1109 |
| Mapped reads (normalized) | 2942610028 |
| Mapped reads (normalized) per kb | 209677214.5 |
| Normalized reads with 1T (1U) | 85.4% |
| Normalized reads with 10A | 34.9% |
| Normalized reads with length 26-33 nt | 100% |
| Normalized reads on the main strand(s) | 100% |
| Predicted directionality | mono:minus |

100%

0%

1T (1U)  
reads

10A reads

26-33 nt  
reads

reads on mainstrand

**Either the amount of reads with 1T (1U) OR 10A has to exceed 75% (set with option: -1Tor10A)  
Alternatively the amount of reads with 1T (1U) AND 10A has to exceed 50% (set with option: -1Tand10A)  
Minimum amount of reads with preferred size is 75% (set with option: -pisize)  
Minimum amount of reads on the main strand(s) is 75% (set with option: -clstrand)**

Show read coverage
Hide read coverage

WHAT DO I SEE HERE?  
This chart shows the location of mapped sequence reads within a predicted piRNA cluster. The color refers to the number of genomic hits produced by the sequence read in question. A dark red bar indicates that this sequence read produces many other hits elsewhere in the genome. Many adjacent red or yellow bars can indicate the presence of a multi-copy element such as transposons or rRNA genes. A dark green bar indicates that this sequence read maps uniquely to this locus.

1 hit

2-5 hits

6-10 hits

11-20 hits

21-50 hits

51-100 hits

> 100 hits

chr10

47899195

47913228

Gene Set

RepeatMasker

Mapped  
Reads

126.45

plus strand

minus strand

126.45

Region: chr10 80736001-47899209. Max. coverage (+): 0. Max coverage (-): 4.15

Region: chr10 47899210-47899237. Max. coverage (+): 0. Max coverage (-): 0

Region: chr10 47899238-47899265. Max. coverage (+): 0. Max coverage (-): 0

Region: chr10 47899266-47899293. Max. coverage (+): 0. Max coverage (-): 0

Region: chr10 47899294-47899321. Max. coverage (+): 0. Max coverage (-): 0

Region: chr10 47899322-47899349. Max. coverage (+): 0. Max coverage (-): 0

Region: chr10 47899350-47899377. Max. coverage (+): 0. Max coverage (-): 0

Region: chr10 47899378-47899405. Max. coverage (+): 0. Max coverage (-): 0

Region: chr10 47899406-47899433. Max. coverage (+): 0. Max coverage (-): 0

Region: chr10 47899434-47899461. Max. coverage (+): 0. Max coverage (-): 0

Region: chr10 47899462-47899489. Max. coverage (+): 0. Max coverage (-): 0

Region: chr10 47899490-47899517. Max. coverage (+): 0. Max coverage (-): 0

Region: chr10 47899518-47899545. Max. coverage (+): 0. Max coverage (-): 0

Region: chr10 47899546-47899573. Max. coverage (+): 0. Max coverage (-): 0

Region: chr10 47899574-47899601. Max. coverage (+): 0. Max coverage (-): 0

Region: chr10 47899602-47899630. Max. coverage (+): 0. Max coverage (-): 0

Region: chr10 47899631-47899658. Max. coverage (+): 0. Max coverage (-): 0

Region: chr10 47899659-47899686. Max. coverage (+): 0. Max coverage (-): 0

Region: chr10 47899687-47899714. Max. coverage (+): 0. Max coverage (-): 0.96

Region: chr10 47899715-47899742. Max. coverage (+): 0. Max coverage (-): 0

Region: chr10 47899743-47899770. Max. coverage (+): 0. Max coverage (-): 0

Region: chr10 47899771-47899798. Max. coverage (+): 0. Max coverage (-): 0

Region: chr10 47899799-47899826. Max. coverage (+): 0. Max coverage (-): 0

Region: chr10 47899827-47899854. Max. coverage (+): 0. Max coverage (-): 0

Region: chr10 47899855-47899882. Max. coverage (+): 0. Max coverage (-): 0

Region: chr10 47899883-47899910. Max. coverage (+): 0. Max coverage (-): 4.54

Region: chr10 47899911-47899938. Max. coverage (+): 0. Max coverage (-): 4.54

Region: chr10 47899939-47899966. Max. coverage (+): 0. Max coverage (-): 6.04

Region: chr10 47899967-47899994. Max. coverage (+): 0. Max coverage (-): 0

Region: chr10 47899995-47900023. Max. coverage (+): 0. Max coverage (-): 14.67

Region: chr10 47900024-47900051. Max. coverage (+): 0. Max coverage (-): 3.33

Region: chr10 47900052-47900079. Max. coverage (+): 0. Max coverage (-): 0

Region: chr10 47900080-47900107. Max. coverage (+): 0. Max coverage (-): 0

Region: chr10 47900108-47900135. Max. coverage (+): 0. Max coverage (-): 0

Region: chr10 47900136-47900163. Max. coverage (+): 0. Max coverage (-): 0

Region: chr10 47900164-47900191. Max. coverage (+): 0. Max coverage (-): 0

Region: chr10 47900192-47900219. Max. coverage (+): 0. Max coverage (-): 0

Region: chr10 47900220-47900247. Max. coverage (+): 0. Max coverage (-): 0

Region: chr10 47900248-47900275. Max. coverage (+): 0. Max coverage (-): 0

Region: chr10 47900276-47900303. Max. coverage (+): 0. Max coverage (-): 0

Region: chr10 47900304-47900331. Max. coverage (+): 0. Max coverage (-): 0

Region: chr10 47900332-47900359. Max. coverage (+): 0. Max coverage (-): 0

Region: chr10 47900360-47900387. Max. coverage (+): 0. Max coverage (-): 0

Region: chr10 47900388-47900415. Max. coverage (+): 0. Max coverage (-): 0

Region: chr10 47900416-47900444. Max. coverage (+): 0. Max coverage (-): 0

Region: chr10 47900445-47900472. Max. coverage (+): 0. Max coverage (-): 0

Region: chr10 47900473-47900500. Max. coverage (+): 0. Max coverage (-): 0

Region: chr10 47900501-47900528. Max. coverage (+): 0. Max coverage (-): 0

Region: chr10 47900529-47900556. Max. coverage (+): 0. Max coverage (-): 0

Region: chr10 47900557-47900584. Max. coverage (+): 0. Max coverage (-): 0

Region: chr10 47900585-47900612. Max. coverage (+): 0. Max coverage (-): 0

Region: chr10 47900613-47900640. Max. coverage (+): 0. Max coverage (-): 0

Region: chr10 47900641-47900668. Max. coverage (+): 0. Max coverage (-): 0

Region: chr10 47900669-47900696. Max. coverage (+): 0. Max coverage (-): 0

Region: chr10 47900697-47900724. Max. coverage (+): 0. Max coverage (-): 0

Region: chr10 47900725-47900752. Max. coverage (+): 0. Max coverage (-): 0

Region: chr10 47900753-47900780. Max. coverage (+): 0. Max coverage (-): 0

Region: chr10 47900781-47900808. Max. coverage (+): 0. Max coverage (-): 0

Region: chr10 47900809-47900836. Max. coverage (+): 0. Max coverage (-): 0

Region: chr10 47900837-47900865. Max. coverage (+): 0. Max coverage (-): 0

Region: chr10 47900866-47900893. Max. coverage (+): 0. Max coverage (-): 0

Region: chr10 47900894-47900921. Max. coverage (+): 0. Max coverage (-): 0

Region: chr10 47900922-47900949. Max. coverage (+): 0. Max coverage (-): 0

Region: chr10 47900950-47900977. Max. coverage (+): 0. Max coverage (-): 0

Region: chr10 47900978-47901005. Max. coverage (+): 0. Max coverage (-): 0

Region: chr10 47901006-47901033. Max. coverage (+): 0. Max coverage (-): 0

Region: chr10 47901034-47901061. Max. coverage (+): 0. Max coverage (-): 0

Region: chr10 47901062-47901089. Max. coverage (+): 0. Max coverage (-): 0

Region: chr10 47901090-47901117. Max. coverage (+): 0. Max coverage (-): 0

Region: chr10 47901118-47901145. Max. coverage (+): 0. Max coverage (-): 0

Region: chr10 47901146-47901173. Max. coverage (+): 0. Max coverage (-): 0

Region: chr10 47901174-47901201. Max. coverage (+): 0. Max coverage (-): 0

Region: chr10 47901202-47901229. Max. coverage (+): 0. Max coverage (-): 0

Region: chr10 47901230-47901257. Max. coverage (+): 0. Max coverage (-): 0

Region: chr10 47901258-47901286. Max. coverage (+): 0. Max coverage (-): 0

Region: chr10 47901287-47901314. Max. coverage (+): 0. Max coverage (-): 0

Region: chr10 47901315-47901342. Max. coverage (+): 0. Max coverage (-): 0

Region: chr10 47901343-47901370. Max. coverage (+): 0. Max coverage (-): 0

Region: chr10 47901371-47901398. Max. coverage (+): 0. Max coverage (-): 0

Region: chr10 47901399-47901426. Max. coverage (+): 0. Max coverage (-): 0

Region: chr10 47901427-47901454. Max. coverage (+): 0. Max coverage (-): 0

Region: chr10 47901455-47901482. Max. coverage (+): 0. Max coverage (-): 0

Region: chr10 47901483-47901510. Max. coverage (+): 0. Max coverage (-): 0

Region: chr10 47901511-47901538. Max. coverage (+): 0. Max coverage (-): 0

Region: chr10 47901539-47901566. Max. coverage (+): 0. Max coverage (-): 0

Region: chr10 47901567-47901594. Max. coverage (+): 0. Max coverage (-): 0

Region: chr10 47901595-47901622. Max. coverage (+): 0. Max coverage (-): 0

Region: chr10 47901623-47901650. Max. coverage (+): 0. Max coverage (-): 0

Region: chr10 47901651-47901679. Max. coverage (+): 0. Max coverage (-): 0

Region: chr10 47901680-47901707. Max. coverage (+): 0. Max coverage (-): 0

Region: chr10 47901708-47901735. Max. coverage (+): 0. Max coverage (-): 0

Region: chr10 47901736-47901763. Max. coverage (+): 0. Max coverage (-): 9.42

Region: chr10 47901764-47901791. Max. coverage (+): 0. Max coverage (-): 0

Region: chr10 47901792-47901819. Max. coverage (+): 0. Max coverage (-): 0

Region: chr10 47901820-47901847. Max. coverage (+): 0. Max coverage (-): 0

Region: chr10 47901848-47901875. Max. coverage (+): 0. Max coverage (-): 0

Region: chr10 47901876-47901903. Max. coverage (+): 0. Max coverage (-): 0

Region: chr10 47901904-47901931. Max. coverage (+): 0. Max coverage (-): 0

Region: chr10 47901932-47901959. Max. coverage (+): 0. Max coverage (-): 0

Region: chr10 47901960-47901987. Max. coverage (+): 0. Max coverage (-): 0

Region: chr10 47901988-47902015. Max. coverage (+): 0. Max coverage (-): 0

Region: chr10 47902016-47902043. Max. coverage (+): 0. Max coverage (-): 0.94

Region: chr10 47902044-47902071. Max. coverage (+): 0. Max coverage (-): 4.46

Region: chr10 47902072-47902100. Max. coverage (+): 0. Max coverage (-): 3.45

Region: chr10 47902101-47902128. Max. coverage (+): 0. Max coverage (-): 9.33

Region: chr10 47902129-47902156. Max. coverage (+): 0. Max coverage (-): 9.93

Region: chr10 47902157-47902184. Max. coverage (+): 0. Max coverage (-): 3.12

Region: chr10 47902185-47902212. Max. coverage (+): 0. Max coverage (-): 2.03

Region: chr10 47902213-47902240. Max. coverage (+): 0. Max coverage (-): 1.35

Region: chr10 47902241-47902268. Max. coverage (+): 0. Max coverage (-): 6.18

Region: chr10 47902269-47902296. Max. coverage (+): 0. Max coverage (-): 2.87

Region: chr10 47902297-47902324. Max. coverage (+): 0. Max coverage (-): 0

Region: chr10 47902325-47902352. Max. coverage (+): 0. Max coverage (-): 0

Region: chr10 47902353-47902380. Max. coverage (+): 0. Max coverage (-): 0

Region: chr10 47902381-47902408. Max. coverage (+): 0. Max coverage (-): 91.47

Region: chr10 47902409-47902436. Max. coverage (+): 0. Max coverage (-): 1.68

Region: chr10 47902437-47902464. Max. coverage (+): 0. Max coverage (-): 0

Region: chr10 47902465-47902492. Max. coverage (+): 0. Max coverage (-): 0

Region: chr10 47902493-47902521. Max. coverage (+): 0. Max coverage (-): 3.99

Region: chr10 47902522-47902549. Max. coverage (+): 0. Max coverage (-): 0

Region: chr10 47902550-47902577. Max. coverage (+): 0. Max coverage (-): 25.91

Region: chr10 47902578-47902605. Max. coverage (+): 0. Max coverage (-): 2.15

Region: chr10 47902606-47902633. Max. coverage (+): 0. Max coverage (-): 2.15

Region: chr10 47902634-47902661. Max. coverage (+): 0. Max coverage (-): 126.45

Region: chr10 47902662-47902689. Max. coverage (+): 0. Max coverage (-): 50.75

Region: chr10 47902690-47902717. Max. coverage (+): 0. Max coverage (-): 3.2

Region: chr10 47902718-47902745. Max. coverage (+): 0. Max coverage (-): 6.35

Region: chr10 47902746-47902773. Max. coverage (+): 0. Max coverage (-): 14.33

Region: chr10 47902774-47902801. Max. coverage (+): 0. Max coverage (-): 9.99

Region: chr10 47902802-47902829. Max. coverage (+): 0. Max coverage (-): 23.9

Region: chr10 47902830-47902857. Max. coverage (+): 0. Max coverage (-): 18.52

Region: chr10 47902858-47902885. Max. coverage (+): 0. Max coverage (-): 18.35

Region: chr10 47902886-47902914. Max. coverage (+): 0. Max coverage (-): 58.71

Region: chr10 47902915-47902942. Max. coverage (+): 0. Max coverage (-): 66.94

Region: chr10 47902943-47902970. Max. coverage (+): 0. Max coverage (-): 30.38

Region: chr10 47902971-47902998. Max. coverage (+): 0. Max coverage (-): 0

Region: chr10 47902999-47903026. Max. coverage (+): 0. Max coverage (-): 9.18

Region: chr10 47903027-47903054. Max. coverage (+): 0. Max coverage (-): 4.35

Region: chr10 47903055-47903082. Max. coverage (+): 0. Max coverage (-): 50.85

Region: chr10 47903083-47903110. Max. coverage (+): 0. Max coverage (-): 38.34

Region: chr10 47903111-47903138. Max. coverage (+): 0. Max coverage (-): 3.74

Region: chr10 47903139-47903166. Max. coverage (+): 0. Max coverage (-): 10.41

Region: chr10 47903167-47903194. Max. coverage (+): 0. Max coverage (-): 21.9

Region: chr10 47903195-47903222. Max. coverage (+): 0. Max coverage (-): 6.17

Region: chr10 47903223-47903250. Max. coverage (+): 0. Max coverage (-): 12.58

Region: chr10 47903251-47903278. Max. coverage (+): 0. Max coverage (-): 25.44

Region: chr10 47903279-47903306. Max. coverage (+): 0. Max coverage (-): 34.49

Region: chr10 47903307-47903335. Max. coverage (+): 0. Max coverage (-): 16.47

Region: chr10 47903336-47903363. Max. coverage (+): 0. Max coverage (-): 5.36

Region: chr10 47903364-47903391. Max. coverage (+): 0. Max coverage (-): 17.49

Region: chr10 47903392-47903419. Max. coverage (+): 0. Max coverage (-): 17.49

Region: chr10 47903420-47903447. Max. coverage (+): 0. Max coverage (-): 0

Region: chr10 47903448-47903475. Max. coverage (+): 0. Max coverage (-): 0

Region: chr10 47903476-47903503. Max. coverage (+): 0. Max coverage (-): 0

Region: chr10 47903504-47903531. Max. coverage (+): 0. Max coverage (-): 0

Region: chr10 47903532-47903559. Max. coverage (+): 0. Max coverage (-): 0

Region: chr10 47903560-47903587. Max. coverage (+): 0. Max coverage (-): 0

Region: chr10 47903588-47903615. Max. coverage (+): 0. Max coverage (-): 0

Region: chr10 47903616-47903643. Max. coverage (+): 0. Max coverage (-): 0

Region: chr10 47903644-47903671. Max. coverage (+): 0. Max coverage (-): 0

Region: chr10 47903672-47903699. Max. coverage (+): 0. Max coverage (-): 0

Region: chr10 47903700-47903727. Max. coverage (+): 0. Max coverage (-): 0

Region: chr10 47903728-47903756. Max. coverage (+): 0. Max coverage (-): 0.19

Region: chr10 47903757-47903784. Max. coverage (+): 0. Max coverage (-): 0

Region: chr10 47903785-47903812. Max. coverage (+): 0. Max coverage (-): 5.37

Region: chr10 47903813-47903840. Max. coverage (+): 0. Max coverage (-): 7.27

Region: chr10 47903841-47903868. Max. coverage (+): 0. Max coverage (-): 18.92

Region: chr10 47903869-47903896. Max. coverage (+): 0. Max coverage (-): 0

Region: chr10 47903897-47903924. Max. coverage (+): 0. Max coverage (-): 1.47

Region: chr10 47903925-47903952. Max. coverage (+): 0. Max coverage (-): 7.35

Region: chr10 47903953-47903980. Max. coverage (+): 0. Max coverage (-): 7.13

Region: chr10 47903981-47904008. Max. coverage (+): 0. Max coverage (-): 14.6

Region: chr10 47904009-47904036. Max. coverage (+): 0. Max coverage (-): 5.26

Region: chr10 47904037-47904064. Max. coverage (+): 0. Max coverage (-): 10.38

Region: chr10 47904065-47904092. Max. coverage (+): 0. Max coverage (-): 0

Region: chr10 47904093-47904120. Max. coverage (+): 0. Max coverage (-): 12.09

Region: chr10 47904121-47904149. Max. coverage (+): 0. Max coverage (-): 0

Region: chr10 47904150-47904177. Max. coverage (+): 0. Max coverage (-): 0

Region: chr10 47904178-47904205. Max. coverage (+): 0. Max coverage (-): 8.09

Region: chr10 47904206-47904233. Max. coverage (+): 0. Max coverage (-): 10.57

Region: chr10 47904234-47904261. Max. coverage (+): 0. Max coverage (-): 10.57

Region: chr10 47904262-47904289. Max. coverage (+): 0. Max coverage (-): 3.88

Region: chr10 47904290-47904317. Max. coverage (+): 0. Max coverage (-): 6.79

Region: chr10 47904318-47904345. Max. coverage (+): 0. Max coverage (-): 6.71

Region: chr10 47904346-47904373. Max. coverage (+): 0. Max coverage (-): 6.08

Region: chr10 47904374-47904401. Max. coverage (+): 0. Max coverage (-): 7.18

Region: chr10 47904402-47904429. Max. coverage (+): 0. Max coverage (-): 15

Region: chr10 47904430-47904457. Max. coverage (+): 0. Max coverage (-): 4.58

Region: chr10 47904458-47904485. Max. coverage (+): 0. Max coverage (-): 0

Region: chr10 47904486-47904513. Max. coverage (+): 0. Max coverage (-): 0

Region: chr10 47904514-47904541. Max. coverage (+): 0. Max coverage (-): 0

Region: chr10 47904542-47904570. Max. coverage (+): 0. Max coverage (-): 0

Region: chr10 47904571-47904598. Max. coverage (+): 0. Max coverage (-): 0

Region: chr10 47904599-47904626. Max. coverage (+): 0. Max coverage (-): 0

Region: chr10 47904627-47904654. Max. coverage (+): 0. Max coverage (-): 0

Region: chr10 47904655-47904682. Max. coverage (+): 0. Max coverage (-): 0

Region: chr10 47904683-47904710. Max. coverage (+): 0. Max coverage (-): 0

Region: chr10 47904711-47904738. Max. coverage (+): 0. Max coverage (-): 7.88

Region: chr10 47904739-47904766. Max. coverage (+): 0. Max coverage (-): 4.81

Region: chr10 47904767-47904794. Max. coverage (+): 0. Max coverage (-): 0

Region: chr10 47904795-47904822. Max. coverage (+): 0. Max coverage (-): 0

Region: chr10 47904823-47904850. Max. coverage (+): 0. Max coverage (-): 0

Region: chr10 47904851-47904878. Max. coverage (+): 0. Max coverage (-): 0

Region: chr10 47904879-47904906. Max. coverage (+): 0. Max coverage (-): 0

Region: chr10 47904907-47904934. Max. coverage (+): 0. Max coverage (-): 3.93

Region: chr10 47904935-47904962. Max. coverage (+): 0. Max coverage (-): 14.51

Region: chr10 47904963-47904991. Max. coverage (+): 0. Max coverage (-): 0

Region: chr10 47904992-47905019. Max. coverage (+): 0. Max coverage (-): 0

Region: chr10 47905020-47905047. Max. coverage (+): 0. Max coverage (-): 0

Region: chr10 47905048-47905075. Max. coverage (+): 0. Max coverage (-): 0

Region: chr10 47905076-47905103. Max. coverage (+): 0. Max coverage (-): 2.24

Region: chr10 47905104-47905131. Max. coverage (+): 0. Max coverage (-): 2.24

Region: chr10 47905132-47905159. Max. coverage (+): 0. Max coverage (-): 0

Region: chr10 47905160-47905187. Max. coverage (+): 0. Max coverage (-): 0

Region: chr10 47905188-47905215. Max. coverage (+): 0. Max coverage (-): 6.61

Region: chr10 47905216-47905243. Max. coverage (+): 0. Max coverage (-): 20.46

Region: chr10 47905244-47905271. Max. coverage (+): 0. Max coverage (-): 0

Region: chr10 47905272-47905299. Max. coverage (+): 0. Max coverage (-): 0

Region: chr10 47905300-47905327. Max. coverage (+): 0. Max coverage (-): 3

Region: chr10 47905328-47905355. Max. coverage (+): 0. Max coverage (-): 6.34

Region: chr10 47905356-47905383. Max. coverage (+): 0. Max coverage (-): 53.43

Region: chr10 47905384-47905412. Max. coverage (+): 0. Max coverage (-): 7.59

Region: chr10 47905413-47905440. Max. coverage (+): 0. Max coverage (-): 3.69

Region: chr10 47905441-47905468. Max. coverage (+): 0. Max coverage (-): 16.82

Region: chr10 47905469-47905496. Max. coverage (+): 0. Max coverage (-): 0

Region: chr10 47905497-47905524. Max. coverage (+): 0. Max coverage (-): 20.31

Region: chr10 47905525-47905552. Max. coverage (+): 0. Max coverage (-): 21.75

Region: chr10 47905553-47905580. Max. coverage (+): 0. Max coverage (-): 0.73

Region: chr10 47905581-47905608. Max. coverage (+): 0. Max coverage (-): 61.83

Region: chr10 47905609-47905636. Max. coverage (+): 0. Max coverage (-): 58.71

Region: chr10 47905637-47905664. Max. coverage (+): 0. Max coverage (-): 0

Region: chr10 47905665-47905692. Max. coverage (+): 0. Max coverage (-): 0

Region: chr10 47905693-47905720. Max. coverage (+): 0. Max coverage (-): 20.58

Region: chr10 47905721-47905748. Max. coverage (+): 0. Max coverage (-): 20.58

Region: chr10 47905749-47905776. Max. coverage (+): 0. Max coverage (-): 57.93

Region: chr10 47905777-47905805. Max. coverage (+): 0. Max coverage (-): 16.63

Region: chr10 47905806-47905833. Max. coverage (+): 0. Max coverage (-): 8.4

Region: chr10 47905834-47905861. Max. coverage (+): 0. Max coverage (-): 6.6

Region: chr10 47905862-47905889. Max. coverage (+): 0. Max coverage (-): 0

Region: chr10 47905890-47905917. Max. coverage (+): 0. Max coverage (-): 24.34

Region: chr10 47905918-47905945. Max. coverage (+): 0. Max coverage (-): 15.61

Region: chr10 47905946-47905973. Max. coverage (+): 0. Max coverage (-): 0

Region: chr10 47905974-47906001. Max. coverage (+): 0. Max coverage (-): 3.75

Region: chr10 47906002-47906029. Max. coverage (+): 0. Max coverage (-): 5.22

Region: chr10 47906030-47906057. Max. coverage (+): 0. Max coverage (-): 0

Region: chr10 47906058-47906085. Max. coverage (+): 0. Max coverage (-): 14.43

Region: chr10 47906086-47906113. Max. coverage (+): 0. Max coverage (-): 2.37

Region: chr10 47906114-47906141. Max. coverage (+): 0. Max coverage (-): 2.37

Region: chr10 47906142-47906169. Max. coverage (+): 0. Max coverage (-): 0

Region: chr10 47906170-47906197. Max. coverage (+): 0. Max coverage (-): 0

Region: chr10 47906198-47906226. Max. coverage (+): 0. Max coverage (-): 0

Region: chr10 47906227-47906254. Max. coverage (+): 0. Max coverage (-): 0

Region: chr10 47906255-47906282. Max. coverage (+): 0. Max coverage (-): 0

Region: chr10 47906283-47906310. Max. coverage (+): 0. Max coverage (-): 0

Region: chr10 47906311-47906338. Max. coverage (+): 0. Max coverage (-): 0

Region: chr10 47906339-47906366. Max. coverage (+): 0. Max coverage (-): 53.06

Region: chr10 47906367-47906394. Max. coverage (+): 0. Max coverage (-): 64.67

Region: chr10 47906395-47906422. Max. coverage (+): 0. Max coverage (-): 0

Region: chr10 47906423-47906450. Max. coverage (+): 0. Max coverage (-): 0

Region: chr10 47906451-47906478. Max. coverage (+): 0. Max coverage (-): 0

Region: chr10 47906479-47906506. Max. coverage (+): 0. Max coverage (-): 0

Region: chr10 47906507-47906534. Max. coverage (+): 0. Max coverage (-): 0

Region: chr10 47906535-47906562. Max. coverage (+): 0. Max coverage (-): 11.69

Region: chr10 47906563-47906590. Max. coverage (+): 0. Max coverage (-): 11.69

Region: chr10 47906591-47906618. Max. coverage (+): 0. Max coverage (-): 9.74

Region: chr10 47906619-47906647. Max. coverage (+): 0. Max coverage (-): 0

Region: chr10 47906648-47906675. Max. coverage (+): 0. Max coverage (-): 7.64

Region: chr10 47906676-47906703. Max. coverage (+): 0. Max coverage (-): 7.64

Region: chr10 47906704-47906731. Max. coverage (+): 0. Max coverage (-): 2.99

Region: chr10 47906732-47906759. Max. coverage (+): 0. Max coverage (-): 13.73

Region: chr10 47906760-47906787. Max. coverage (+): 0. Max coverage (-): 0

Region: chr10 47906788-47906815. Max. coverage (+): 0. Max coverage (-): 0

Region: chr10 47906816-47906843. Max. coverage (+): 0. Max coverage (-): 0

Region: chr10 47906844-47906871. Max. coverage (+): 0. Max coverage (-): 0

Region: chr10 47906872-47906899. Max. coverage (+): 0. Max coverage (-): 6.49

Region: chr10 47906900-47906927. Max. coverage (+): 0. Max coverage (-): 26.69

Region: chr10 47906928-47906955. Max. coverage (+): 0. Max coverage (-): 16.23

Region: chr10 47906956-47906983. Max. coverage (+): 0. Max coverage (-): 0.18

Region: chr10 47906984-47907011. Max. coverage (+): 0. Max coverage (-): 6.1

Region: chr10 47907012-47907040. Max. coverage (+): 0. Max coverage (-): 23.73

Region: chr10 47907041-47907068. Max. coverage (+): 0. Max coverage (-): 15.65

Region: chr10 47907069-47907096. Max. coverage (+): 0. Max coverage (-): 0

Region: chr10 47907097-47907124. Max. coverage (+): 0. Max coverage (-): 7.73

Region: chr10 47907125-47907152. Max. coverage (+): 0. Max coverage (-): 7.73

Region: chr10 47907153-47907180. Max. coverage (+): 0. Max coverage (-): 4.3

Region: chr10 47907181-47907208. Max. coverage (+): 0. Max coverage (-): 7.43

Region: chr10 47907209-47907236. Max. coverage (+): 0. Max coverage (-): 7.03

Region: chr10 47907237-47907264. Max. coverage (+): 0. Max coverage (-): 28.14

Region: chr10 47907265-47907292. Max. coverage (+): 0. Max coverage (-): 28.14

Region: chr10 47907293-47907320. Max. coverage (+): 0. Max coverage (-): 31.66

Region: chr10 47907321-47907348. Max. coverage (+): 0. Max coverage (-): 24.28

Region: chr10 47907349-47907376. Max. coverage (+): 0. Max coverage (-): 31.22

Region: chr10 47907377-47907404. Max. coverage (+): 0. Max coverage (-): 0

Region: chr10 47907405-47907432. Max. coverage (+): 0. Max coverage (-): 13.47

Region: chr10 47907433-47907461. Max. coverage (+): 0. Max coverage (-): 20.97

Region: chr10 47907462-47907489. Max. coverage (+): 0. Max coverage (-): 6.85

Region: chr10 47907490-47907517. Max. coverage (+): 0. Max coverage (-): 0

Region: chr10 47907518-47907545. Max. coverage (+): 0. Max coverage (-): 0

Region: chr10 47907546-47907573. Max. coverage (+): 0. Max coverage (-): 0

Region: chr10 47907574-47907601. Max. coverage (+): 0. Max coverage (-): 3.51

Region: chr10 47907602-47907629. Max. coverage (+): 0. Max coverage (-): 3.11

Region: chr10 47907630-47907657. Max. coverage (+): 0. Max coverage (-): 10.66

Region: chr10 47907658-47907685. Max. coverage (+): 0. Max coverage (-): 7.87

Region: chr10 47907686-47907713. Max. coverage (+): 0. Max coverage (-): 6.25

Region: chr10 47907714-47907741. Max. coverage (+): 0. Max coverage (-): 15.32

Region: chr10 47907742-47907769. Max. coverage (+): 0. Max coverage (-): 53.89

Region: chr10 47907770-47907797. Max. coverage (+): 0. Max coverage (-): 0.45

Region: chr10 47907798-47907825. Max. coverage (+): 0. Max coverage (-): 4.85

Region: chr10 47907826-47907853. Max. coverage (+): 0. Max coverage (-): 18.07

Region: chr10 47907854-47907882. Max. coverage (+): 0. Max coverage (-): 18.23

Region: chr10 47907883-47907910. Max. coverage (+): 0. Max coverage (-): 0

Region: chr10 47907911-47907938. Max. coverage (+): 0. Max coverage (-): 12.05

Region: chr10 47907939-47907966. Max. coverage (+): 0. Max coverage (-): 34.35

Region: chr10 47907967-47907994. Max. coverage (+): 0. Max coverage (-): 7.71

Region: chr10 47907995-47908022. Max. coverage (+): 0. Max coverage (-): 0

Region: chr10 47908023-47908050. Max. coverage (+): 0. Max coverage (-): 51.76

Region: chr10 47908051-47908078. Max. coverage (+): 0. Max coverage (-): 0.37

Region: chr10 47908079-47908106. Max. coverage (+): 0. Max coverage (-): 0

Region: chr10 47908107-47908134. Max. coverage (+): 0. Max coverage (-): 10.52

Region: chr10 47908135-47908162. Max. coverage (+): 0. Max coverage (-): 11.06

Region: chr10 47908163-47908190. Max. coverage (+): 0. Max coverage (-): 0

Region: chr10 47908191-47908218. Max. coverage (+): 0. Max coverage (-): 6.92

Region: chr10 47908219-47908246. Max. coverage (+): 0. Max coverage (-): 17.66

Region: chr10 47908247-47908274. Max. coverage (+): 0. Max coverage (-): 17.66

Region: chr10 47908275-47908303. Max. coverage (+): 0. Max coverage (-): 45.56

Region: chr10 47908304-47908331. Max. coverage (+): 0. Max coverage (-): 64.63

Region: chr10 47908332-47908359. Max. coverage (+): 0. Max coverage (-): 9.56

Region: chr10 47908360-47908387. Max. coverage (+): 0. Max coverage (-): 22.46

Region: chr10 47908388-47908415. Max. coverage (+): 0. Max coverage (-): 19.12

Region: chr10 47908416-47908443. Max. coverage (+): 0. Max coverage (-): 12.86

Region: chr10 47908444-47908471. Max. coverage (+): 0. Max coverage (-): 30.3

Region: chr10 47908472-47908499. Max. coverage (+): 0. Max coverage (-): 49.6

Region: chr10 47908500-47908527. Max. coverage (+): 0. Max coverage (-): 27.13

Region: chr10 47908528-47908555. Max. coverage (+): 0. Max coverage (-): 17.04

Region: chr10 47908556-47908583. Max. coverage (+): 0. Max coverage (-): 0.59

Region: chr10 47908584-47908611. Max. coverage (+): 0. Max coverage (-): 59.59

Region: chr10 47908612-47908639. Max. coverage (+): 0. Max coverage (-): 80.6

Region: chr10 47908640-47908667. Max. coverage (+): 0. Max coverage (-): 84.44

Region: chr10 47908668-47908696. Max. coverage (+): 0. Max coverage (-): 108.44

Region: chr10 47908697-47908724. Max. coverage (+): 0. Max coverage (-): 89.21

Region: chr10 47908725-47908752. Max. coverage (+): 0. Max coverage (-): 59.4

Region: chr10 47908753-47908780. Max. coverage (+): 0. Max coverage (-): 5.98

Region: chr10 47908781-47908808. Max. coverage (+): 0. Max coverage (-): 5.98

Region: chr10 47908809-47908836. Max. coverage (+): 0. Max coverage (-): 61.2

Region: chr10 47908837-47908864. Max. coverage (+): 0. Max coverage (-): 0

Region: chr10 47908865-47908892. Max. coverage (+): 0. Max coverage (-): 47.84

Region: chr10 47908893-47908920. Max. coverage (+): 0. Max coverage (-): 0

Region: chr10 47908921-47908948. Max. coverage (+): 0. Max coverage (-): 0

Region: chr10 47908949-47908976. Max. coverage (+): 0. Max coverage (-): 0

Region: chr10 47908977-47909004. Max. coverage (+): 0. Max coverage (-): 11.85

Region: chr10 47909005-47909032. Max. coverage (+): 0. Max coverage (-): 5.32

Region: chr10 47909033-47909060. Max. coverage (+): 0. Max coverage (-): 3.97

Region: chr10 47909061-47909088. Max. coverage (+): 0. Max coverage (-): 0

Region: chr10 47909089-47909117. Max. coverage (+): 0. Max coverage (-): 2.76

Region: chr10 47909118-47909145. Max. coverage (+): 0. Max coverage (-): 0

Region: chr10 47909146-47909173. Max. coverage (+): 0. Max coverage (-): 0

Region: chr10 47909174-47909201. Max. coverage (+): 0. Max coverage (-): 40.3

Region: chr10 47909202-47909229. Max. coverage (+): 0. Max coverage (-): 18.2

Region: chr10 47909230-47909257. Max. coverage (+): 0. Max coverage (-): 4.13

Region: chr10 47909258-47909285. Max. coverage (+): 0. Max coverage (-): 12.95

Region: chr10 47909286-47909313. Max. coverage (+): 0. Max coverage (-): 12.95

Region: chr10 47909314-47909341. Max. coverage (+): 0. Max coverage (-): 0

Region: chr10 47909342-47909369. Max. coverage (+): 0. Max coverage (-): 0

Region: chr10 47909370-47909397. Max. coverage (+): 0. Max coverage (-): 0

Region: chr10 47909398-47909425. Max. coverage (+): 0. Max coverage (-): 0

Region: chr10 47909426-47909453. Max. coverage (+): 0. Max coverage (-): 0

Region: chr10 47909454-47909481. Max. coverage (+): 0. Max coverage (-): 0

Region: chr10 47909482-47909509. Max. coverage (+): 0. Max coverage (-): 0

Region: chr10 47909510-47909538. Max. coverage (+): 0. Max coverage (-): 0

Region: chr10 47909539-47909566. Max. coverage (+): 0. Max coverage (-): 0

Region: chr10 47909567-47909594. Max. coverage (+): 0. Max coverage (-): 0

Region: chr10 47909595-47909622. Max. coverage (+): 0. Max coverage (-): 0

Region: chr10 47909623-47909650. Max. coverage (+): 0. Max coverage (-): 0

Region: chr10 47909651-47909678. Max. coverage (+): 0. Max coverage (-): 0

Region: chr10 47909679-47909706. Max. coverage (+): 0. Max coverage (-): 0

Region: chr10 47909707-47909734. Max. coverage (+): 0. Max coverage (-): 0

Region: chr10 47909735-47909762. Max. coverage (+): 0. Max coverage (-): 0

Region: chr10 47909763-47909790. Max. coverage (+): 0. Max coverage (-): 7.72

Region: chr10 47909791-47909818. Max. coverage (+): 0. Max coverage (-): 7.72

Region: chr10 47909819-47909846. Max. coverage (+): 0. Max coverage (-): 3.76

Region: chr10 47909847-47909874. Max. coverage (+): 0. Max coverage (-): 2.85

Region: chr10 47909875-47909902. Max. coverage (+): 0. Max coverage (-): 0

Region: chr10 47909903-47909931. Max. coverage (+): 0. Max coverage (-): 0

Region: chr10 47909932-47909959. Max. coverage (+): 0. Max coverage (-): 0

Region: chr10 47909960-47909987. Max. coverage (+): 0. Max coverage (-): 0

Region: chr10 47909988-47910015. Max. coverage (+): 0. Max coverage (-): 0

Region: chr10 47910016-47910043. Max. coverage (+): 0. Max coverage (-): 0

Region: chr10 47910044-47910071. Max. coverage (+): 0. Max coverage (-): 0

Region: chr10 47910072-47910099. Max. coverage (+): 0. Max coverage (-): 0

Region: chr10 47910100-47910127. Max. coverage (+): 0. Max coverage (-): 0

Region: chr10 47910128-47910155. Max. coverage (+): 0. Max coverage (-): 0

Region: chr10 47910156-47910183. Max. coverage (+): 0. Max coverage (-): 0

Region: chr10 47910184-47910211. Max. coverage (+): 0. Max coverage (-): 0

Region: chr10 47910212-47910239. Max. coverage (+): 0. Max coverage (-): 8.49

Region: chr10 47910240-47910267. Max. coverage (+): 0. Max coverage (-): 8.49

Region: chr10 47910268-47910295. Max. coverage (+): 0. Max coverage (-): 0

Region: chr10 47910296-47910323. Max. coverage (+): 0. Max coverage (-): 4.89

Region: chr10 47910324-47910352. Max. coverage (+): 0. Max coverage (-): 0

Region: chr10 47910353-47910380. Max. coverage (+): 0. Max coverage (-): 1.31

Region: chr10 47910381-47910408. Max. coverage (+): 0. Max coverage (-): 0

Region: chr10 47910409-47910436. Max. coverage (+): 0. Max coverage (-): 0

Region: chr10 47910437-47910464. Max. coverage (+): 0. Max coverage (-): 0

Region: chr10 47910465-47910492. Max. coverage (+): 0. Max coverage (-): 0.4

Region: chr10 47910493-47910520. Max. coverage (+): 0. Max coverage (-): 21.7

Region: chr10 47910521-47910548. Max. coverage (+): 0. Max coverage (-): 0

Region: chr10 47910549-47910576. Max. coverage (+): 0. Max coverage (-): 0

Region: chr10 47910577-47910604. Max. coverage (+): 0. Max coverage (-): 0

Region: chr10 47910605-47910632. Max. coverage (+): 0. Max coverage (-): 1.87

Region: chr10 47910633-47910660. Max. coverage (+): 0. Max coverage (-): 5.19

Region: chr10 47910661-47910688. Max. coverage (+): 0. Max coverage (-): 56

Region: chr10 47910689-47910716. Max. coverage (+): 0. Max coverage (-): 65.37

Region: chr10 47910717-47910744. Max. coverage (+): 0. Max coverage (-): 7.14

Region: chr10 47910745-47910773. Max. coverage (+): 0. Max coverage (-): 7.59

Region: chr10 47910774-47910801. Max. coverage (+): 0. Max coverage (-): 0

Region: chr10 47910802-47910829. Max. coverage (+): 0. Max coverage (-): 0

Region: chr10 47910830-47910857. Max. coverage (+): 0. Max coverage (-): 0

Region: chr10 47910858-47910885. Max. coverage (+): 0. Max coverage (-): 3.84

Region: chr10 47910886-47910913. Max. coverage (+): 0. Max coverage (-): 6.43

Region: chr10 47910914-47910941. Max. coverage (+): 0. Max coverage (-): 6.13

Region: chr10 47910942-47910969. Max. coverage (+): 0. Max coverage (-): 0

Region: chr10 47910970-47910997. Max. coverage (+): 0. Max coverage (-): 52.7

Region: chr10 47910998-47911025. Max. coverage (+): 0. Max coverage (-): 0

Region: chr10 47911026-47911053. Max. coverage (+): 0. Max coverage (-): 0

Region: chr10 47911054-47911081. Max. coverage (+): 0. Max coverage (-): 0

Region: chr10 47911082-47911109. Max. coverage (+): 0. Max coverage (-): 2.82

Region: chr10 47911110-47911137. Max. coverage (+): 0. Max coverage (-): 0

Region: chr10 47911138-47911166. Max. coverage (+): 0. Max coverage (-): 0

Region: chr10 47911167-47911194. Max. coverage (+): 0. Max coverage (-): 0

Region: chr10 47911195-47911222. Max. coverage (+): 0. Max coverage (-): 6.25

Region: chr10 47911223-47911250. Max. coverage (+): 0. Max coverage (-): 12.62

Region: chr10 47911251-47911278. Max. coverage (+): 0. Max coverage (-): 2.31

Region: chr10 47911279-47911306. Max. coverage (+): 0. Max coverage (-): 0

Region: chr10 47911307-47911334. Max. coverage (+): 0. Max coverage (-): 14.23

Region: chr10 47911335-47911362. Max. coverage (+): 0. Max coverage (-): 4.87

Region: chr10 47911363-47911390. Max. coverage (+): 0. Max coverage (-): 9.29

Region: chr10 47911391-47911418. Max. coverage (+): 0. Max coverage (-): 6.04

Region: chr10 47911419-47911446. Max. coverage (+): 0. Max coverage (-): 0

Region: chr10 47911447-47911474. Max. coverage (+): 0. Max coverage (-): 14.4

Region: chr10 47911475-47911502. Max. coverage (+): 0. Max coverage (-): 6.7

Region: chr10 47911503-47911530. Max. coverage (+): 0. Max coverage (-): 0

Region: chr10 47911531-47911558. Max. coverage (+): 0. Max coverage (-): 1.72

Region: chr10 47911559-47911587. Max. coverage (+): 0. Max coverage (-): 5.98

Region: chr10 47911588-47911615. Max. coverage (+): 0. Max coverage (-): 5.98

Region: chr10 47911616-47911643. Max. coverage (+): 0. Max coverage (-): 0

Region: chr10 47911644-47911671. Max. coverage (+): 0. Max coverage (-): 0

Region: chr10 47911672-47911699. Max. coverage (+): 0. Max coverage (-): 0

Region: chr10 47911700-47911727. Max. coverage (+): 0. Max coverage (-): 0

Region: chr10 47911728-47911755. Max. coverage (+): 0. Max coverage (-): 0

Region: chr10 47911756-47911783. Max. coverage (+): 0. Max coverage (-): 11.68

Region: chr10 47911784-47911811. Max. coverage (+): 0. Max coverage (-): 0

Region: chr10 47911812-47911839. Max. coverage (+): 0. Max coverage (-): 0

Region: chr10 47911840-47911867. Max. coverage (+): 0. Max coverage (-): 3.34

Region: chr10 47911868-47911895. Max. coverage (+): 0. Max coverage (-): 0

Region: chr10 47911896-47911923. Max. coverage (+): 0. Max coverage (-): 0

Region: chr10 47911924-47911951. Max. coverage (+): 0. Max coverage (-): 0

Region: chr10 47911952-47911979. Max. coverage (+): 0. Max coverage (-): 0

Region: chr10 47911980-47912008. Max. coverage (+): 0. Max coverage (-): 0

Region: chr10 47912009-47912036. Max. coverage (+): 0. Max coverage (-): 0

Region: chr10 47912037-47912064. Max. coverage (+): 0. Max coverage (-): 10.18

Region: chr10 47912065-47912092. Max. coverage (+): 0. Max coverage (-): 10.18

Region: chr10 47912093-47912120. Max. coverage (+): 0. Max coverage (-): 13.92

Region: chr10 47912121-47912148. Max. coverage (+): 0. Max coverage (-): 0

Region: chr10 47912149-47912176. Max. coverage (+): 0. Max coverage (-): 6.53

Region: chr10 47912177-47912204. Max. coverage (+): 0. Max coverage (-): 0

Region: chr10 47912205-47912232. Max. coverage (+): 0. Max coverage (-): 0

Region: chr10 47912233-47912260. Max. coverage (+): 0. Max coverage (-): 8.43

Region: chr10 47912261-47912288. Max. coverage (+): 0. Max coverage (-): 14.49

Region: chr10 47912289-47912316. Max. coverage (+): 0. Max coverage (-): 14.49

Region: chr10 47912317-47912344. Max. coverage (+): 0. Max coverage (-): 5.66

Region: chr10 47912345-47912372. Max. coverage (+): 0. Max coverage (-): 0

Region: chr10 47912373-47912400. Max. coverage (+): 0. Max coverage (-): 0

Region: chr10 47912401-47912429. Max. coverage (+): 0. Max coverage (-): 6.51

Region: chr10 47912430-47912457. Max. coverage (+): 0. Max coverage (-): 0

Region: chr10 47912458-47912485. Max. coverage (+): 0. Max coverage (-): 0

Region: chr10 47912486-47912513. Max. coverage (+): 0. Max coverage (-): 0

Region: chr10 47912514-47912541. Max. coverage (+): 0. Max coverage (-): 0

Region: chr10 47912542-47912569. Max. coverage (+): 0. Max coverage (-): 0

Region: chr10 47912570-47912597. Max. coverage (+): 0. Max coverage (-): 6.9

Region: chr10 47912598-47912625. Max. coverage (+): 0. Max coverage (-): 9.9

Region: chr10 47912626-47912653. Max. coverage (+): 0. Max coverage (-): 12.45

Region: chr10 47912654-47912681. Max. coverage (+): 0. Max coverage (-): 12.45

Region: chr10 47912682-47912709. Max. coverage (+): 0. Max coverage (-): 0

Region: chr10 47912710-47912737. Max. coverage (+): 0. Max coverage (-): 6.99

Region: chr10 47912738-47912765. Max. coverage (+): 0. Max coverage (-): 6.99

Region: chr10 47912766-47912793. Max. coverage (+): 0. Max coverage (-): 11.69

Region: chr10 47912794-47912822. Max. coverage (+): 0. Max coverage (-): 4

Region: chr10 47912823-47912850. Max. coverage (+): 0. Max coverage (-): 3.65

Region: chr10 47912851-47912878. Max. coverage (+): 0. Max coverage (-): 5.3

Region: chr10 47912879-47912906. Max. coverage (+): 0. Max coverage (-): 6.68

Region: chr10 47912907-47912934. Max. coverage (+): 0. Max coverage (-): 1.81

Region: chr10 47912935-47912962. Max. coverage (+): 0. Max coverage (-): 3.64

Region: chr10 47912963-47912990. Max. coverage (+): 0. Max coverage (-): 19.14

Region: chr10 47912991-47913018. Max. coverage (+): 0. Max coverage (-): 19.14

Region: chr10 47913019-47913046. Max. coverage (+): 0. Max coverage (-): 0

Region: chr10 47913047-47913074. Max. coverage (+): 0. Max coverage (-): 4.49

Region: chr10 47913075-47913102. Max. coverage (+): 0. Max coverage (-): 7.88

Region: chr10 47913103-47913130. Max. coverage (+): 0. Max coverage (-): 0

Region: chr10 47913131-47913158. Max. coverage (+): 0. Max coverage (-): 6.24

Region: chr10 47913159-47913186. Max. coverage (+): 0. Max coverage (-): 26.53

Region: chr10 47913187-47913214. Max. coverage (+): 0. Max coverage (-): 2.47

Region: chr10 47913215-. Max. coverage (+): 0. Max coverage (-): 0

RepeatMasker Color Code

**+**

100-98% Identity

<98-95% Identity

<95-90% Identity

<90-85% Identity

<85-80% Identity

<80-75% Identity

<75-70% Identity

<70% Identity

**-**

Gene Set Color Code

**+**

Gene

Pseudogene

**-**

Topology/Coverage Color Code

Coverage Plus Strand

Coverage Minus Strand

Mainstrand: Plus

Mainstrand: Minus

Complementary Strand

Flanking Region  
(if option -flank >0)

Gene Set Annotation  
  
RepeatMasker Annotation  

**1. MIR3**: 47899410-47899530 (-), Divergence to consensus: 31.8%  
**2. Charlie4z**: 47899774-47899895 (-), Divergence to consensus: 37.4%  
**3. BTLTR1**: 47900092-47900123 (+), Divergence to consensus: 12.5%  
**4. BovB**: 47900124-47900384 (-), Divergence to consensus: 4.2%  
**5. BovB**: 47900377-47901218 (+), Divergence to consensus: 3%  
**6. ART2A**: 47901219-47901689 (+), Divergence to consensus: 19.1%  
**7. (AACTG)n**: 47901690-47901713 (+), Divergence to consensus: 4.2%  
**8. ART2A**: 47903422-47903721 (+), Divergence to consensus: 18.4%  
**9. CHR-2B**: 47904464-47904703 (-), Divergence to consensus: 47.8%  
**10. AT\_rich**: 47904721-47904746 (+), Divergence to consensus: 38.5%  
**11. AT\_rich**: 47904724-47904749 (+), Divergence to consensus: 69.2%  
**12. CHR-2B**: 47909351-47909591 (-), Divergence to consensus: 51.3%  
**13. AT\_rich**: 47911793-47911835 (+), Divergence to consensus: 72.1%  
**14. L2c**: 47911939-47912035 (+), Divergence to consensus: 46.4%

  
Transcription Factor Binding Sites  

**SPZ1** (Sequence: CTGAAACCCT (-): 47899754)  
**SPZ1** (Sequence: CTCTAACCCC (-): 47908460)  
**Gata4** (Sequence: AGATAAG (-): 47905236)  
**Mybl1\_1** (Sequence: AACCGTTA (+): 47911834)
