# Supplementary material for: Small RNA sequencing of cryopreserved semen from single bull revealed altered miRNAs and piRNAs expression between High- and Low-motile sperm populations
Source: BMC Genomics. 2017 Jan 4;18:14. doi: 10.1186/s12864-016-3394-7 (PMC5209821; doi:10.1186/s12864-016-3394-7)
Supplement: Additional file 4: — Details for each piRNA clusters found in Low Motile (LM) sperm fraction. Genes, repeats, transposable elements and transcription factors binding sites falling within the cluster regions were reported. (ZIP 1034 kb) [file 12864_2016_3394_MOESM4_ESM.zip › 20.html]

piRNA cluster 20


Predicted piRNA cluster no. 20     previous   next
  

Show proTRAC run info
Hide proTRAC run info

================================= proTRAC ====================================  
VERSION: 2.1                                    LAST MODIFIED: 06. October 2015  
  
Please cite:  
Rosenkranz D, Zischler H. proTRAC - a software for probabilistic piRNA cluster  
detection, visualization and analysis. 2012. BMC Bioinformatics 13:5.  
  
and (for proTRAC 2.0 and later):  
Rosenkranz D, Rudloff S, Bastuck K, Ketting RF, Zischler H. Tupaia small RNAs  
provide insights into function and evolution of RNAi-based transposon defense  
in mammals. 2015. RNA 21(5):911-922.  
  
Contact:  
David Rosenkranz  
Institute of Anthropology, small RNA group  
Johannes Gutenberg University Mainz  
email: rosenkranz@uni-mainz.de  
  
You can find the latest proTRAC version at:  
http://sourceforge.net/projects/protrac/files  
http://www.smallRNAgroup-mainz.de/software  
==============================================================================  
  
PARAMETERS:  
Map file: .............../storage/core/barbara/genhome/smallRNA/fertility/Sample\_not\_motile/pirna/Sample\_not\_motile\_26-33\_collapsed.fa.no-dust.map.weighted-10000-1000-b-0  
Genome file: ............/storage/core/barbara/genhome/smallRNA/fertility/Sample\_all/pirna/bt\_311\_chrY.fa  
RepeatMasker annotation: /storage/genomes/bt\_umd31/GCF\_000003055.6\_Bos\_taurus\_UMD\_3.1.1\_repeatMasker\_chr.out  
GeneSet:................./storage/core/barbara/genhome/smallRNA/fertility/Sample\_all/pirna/full.gtf  
  
Significant (p<=0.01) hit density will be calculated based  
on observed hit distribution.  
  
Sliding window size: ........................................ 5000 bp  
Sliding window increament: .................................. 1000 bp  
Normalize each hit by number of genomic hits: ............... 1 [0=no/1=yes]  
Normalize each hit by number of sequence reads: ............. 1 [0=no/1=yes]  
Normalize values (-> per million mapped reads): ............. 1 [0=no/1=yes]  
Min. fraction of hits with 1T(U) or 10A: .................... 0.75  
Alternatively: Min. fraction of hits with 1T(U) and 10A: .... 0.5  
Min. fraction of hits with typical piRNA length: ............ 0.75  
Typical piRNA length: ....................................... 26-33 nt  
Min. size of a piRNA cluster: ............................... 5000 bp.  
Min. number of hits (absolute): ............................. 0  
Min. number of hits (normalized): ........................... 0  
Min. fraction of hits on the mainstrand: .................... 0.75  
Top fraction of mapped sequences (in terms of read counts): . 1%  
Top fraction accounts for max. n% of sequence reads: ........ 90%  
Min. fraction of hits on each arm of a bidirectional cluster: 0.1  
Output image file for each cluster: ......................... 0 [0=no/1=yes]  
Output html file for each cluster: .......................... 1 [0=no/1=yes]  
Output a summary table: ..................................... 1 [0=no/1=yes]  
Output a FASTA file for each cluster (piRNA sequences): ..... 1 [0=no/1=yes]  
Output a FASTA file comprising cluster sequences: ........... 1 [0=no/1=yes]  
Search DNA motifs in clusters: .............................. 1 [0=no/1=yes]  
Output flanking sequences: +/- .............................. 0 bp  
Output ~.pTi file: .......................................... 1 [0=no/1=yes]  
==============================================================================  
  
  
Genome size (without gaps): ............ 2678902517 bp  
Gaps (N/X/-): .......................... 53837044 bp  
Mapped reads: .......................... 738059667487  
Non-identical sequences: ............... 277001  
Genomic hits: .......................... 533816  
Significant densitiy of mapped reads: .. 15118061 reads/kb

Show proTRAC cluster info
Hide proTRAC cluster info

|  |  |
| --- | --- |
| Location | chr17 |
| Coordinates | 73269266-73286813 |
| Size [bp] | 17548 |
| Sequence hit loci | 1471 |
| Mapped reads (normalized) | 3909486432 |
| Mapped reads (normalized) per kb | 222788148.6 |
| Normalized reads with 1T (1U) | 81.5% |
| Normalized reads with 10A | 30.6% |
| Normalized reads with length 26-33 nt | 100% |
| Normalized reads on the main strand(s) | 99.6% |
| Predicted directionality | mono:minus |

100%

0%

1T (1U)  
reads

10A reads

26-33 nt  
reads

reads on mainstrand

**Either the amount of reads with 1T (1U) OR 10A has to exceed 75% (set with option: -1Tor10A)  
Alternatively the amount of reads with 1T (1U) AND 10A has to exceed 50% (set with option: -1Tand10A)  
Minimum amount of reads with preferred size is 75% (set with option: -pisize)  
Minimum amount of reads on the main strand(s) is 75% (set with option: -clstrand)**

Show read coverage
Hide read coverage

WHAT DO I SEE HERE?  
This chart shows the location of mapped sequence reads within a predicted piRNA cluster. The color refers to the number of genomic hits produced by the sequence read in question. A dark red bar indicates that this sequence read produces many other hits elsewhere in the genome. Many adjacent red or yellow bars can indicate the presence of a multi-copy element such as transposons or rRNA genes. A dark green bar indicates that this sequence read maps uniquely to this locus.

1 hit

2-5 hits

6-10 hits

11-20 hits

21-50 hits

51-100 hits

> 100 hits

chr17

73269266

73286813

Gene Set

RepeatMasker

Mapped  
Reads

185.63

plus strand

minus strand

185.63

Region: chr17 72240622-73269283. Max. coverage (+): 0. Max coverage (-): 9.96

Region: chr17 73269284-73269318. Max. coverage (+): 0. Max coverage (-): 0

Region: chr17 73269319-73269353. Max. coverage (+): 0. Max coverage (-): 0

Region: chr17 73269354-73269388. Max. coverage (+): 0. Max coverage (-): 0

Region: chr17 73269389-73269423. Max. coverage (+): 0. Max coverage (-): 0

Region: chr17 73269424-73269459. Max. coverage (+): 0. Max coverage (-): 0

Region: chr17 73269460-73269494. Max. coverage (+): 0. Max coverage (-): 0

Region: chr17 73269495-73269529. Max. coverage (+): 0. Max coverage (-): 0

Region: chr17 73269530-73269564. Max. coverage (+): 0. Max coverage (-): 0.01

Region: chr17 73269565-73269599. Max. coverage (+): 0. Max coverage (-): 0

Region: chr17 73269600-73269634. Max. coverage (+): 0. Max coverage (-): 0

Region: chr17 73269635-73269669. Max. coverage (+): 0. Max coverage (-): 0

Region: chr17 73269670-73269704. Max. coverage (+): 0. Max coverage (-): 0

Region: chr17 73269705-73269739. Max. coverage (+): 0. Max coverage (-): 0

Region: chr17 73269740-73269774. Max. coverage (+): 0. Max coverage (-): 5.85

Region: chr17 73269775-73269809. Max. coverage (+): 0. Max coverage (-): 1.6

Region: chr17 73269810-73269845. Max. coverage (+): 0. Max coverage (-): 0

Region: chr17 73269846-73269880. Max. coverage (+): 0. Max coverage (-): 0

Region: chr17 73269881-73269915. Max. coverage (+): 0. Max coverage (-): 0

Region: chr17 73269916-73269950. Max. coverage (+): 0. Max coverage (-): 0

Region: chr17 73269951-73269985. Max. coverage (+): 0. Max coverage (-): 0

Region: chr17 73269986-73270020. Max. coverage (+): 0. Max coverage (-): 0

Region: chr17 73270021-73270055. Max. coverage (+): 0. Max coverage (-): 0

Region: chr17 73270056-73270090. Max. coverage (+): 0. Max coverage (-): 0

Region: chr17 73270091-73270125. Max. coverage (+): 0. Max coverage (-): 0

Region: chr17 73270126-73270160. Max. coverage (+): 0. Max coverage (-): 0

Region: chr17 73270161-73270196. Max. coverage (+): 0. Max coverage (-): 0

Region: chr17 73270197-73270231. Max. coverage (+): 0. Max coverage (-): 0

Region: chr17 73270232-73270266. Max. coverage (+): 0. Max coverage (-): 0

Region: chr17 73270267-73270301. Max. coverage (+): 0. Max coverage (-): 0

Region: chr17 73270302-73270336. Max. coverage (+): 0. Max coverage (-): 0

Region: chr17 73270337-73270371. Max. coverage (+): 0. Max coverage (-): 0.59

Region: chr17 73270372-73270406. Max. coverage (+): 0. Max coverage (-): 0

Region: chr17 73270407-73270441. Max. coverage (+): 0. Max coverage (-): 0

Region: chr17 73270442-73270476. Max. coverage (+): 0. Max coverage (-): 0

Region: chr17 73270477-73270511. Max. coverage (+): 0. Max coverage (-): 7.5

Region: chr17 73270512-73270547. Max. coverage (+): 0. Max coverage (-): 7.5

Region: chr17 73270548-73270582. Max. coverage (+): 0. Max coverage (-): 0

Region: chr17 73270583-73270617. Max. coverage (+): 0. Max coverage (-): 0

Region: chr17 73270618-73270652. Max. coverage (+): 0. Max coverage (-): 2.62

Region: chr17 73270653-73270687. Max. coverage (+): 0. Max coverage (-): 10.69

Region: chr17 73270688-73270722. Max. coverage (+): 0. Max coverage (-): 0

Region: chr17 73270723-73270757. Max. coverage (+): 0. Max coverage (-): 7.73

Region: chr17 73270758-73270792. Max. coverage (+): 0. Max coverage (-): 0

Region: chr17 73270793-73270827. Max. coverage (+): 0. Max coverage (-): 0

Region: chr17 73270828-73270862. Max. coverage (+): 0. Max coverage (-): 0

Region: chr17 73270863-73270897. Max. coverage (+): 0. Max coverage (-): 0

Region: chr17 73270898-73270933. Max. coverage (+): 0. Max coverage (-): 0

Region: chr17 73270934-73270968. Max. coverage (+): 0. Max coverage (-): 0

Region: chr17 73270969-73271003. Max. coverage (+): 0. Max coverage (-): 0

Region: chr17 73271004-73271038. Max. coverage (+): 0. Max coverage (-): 0

Region: chr17 73271039-73271073. Max. coverage (+): 0. Max coverage (-): 0

Region: chr17 73271074-73271108. Max. coverage (+): 0. Max coverage (-): 0

Region: chr17 73271109-73271143. Max. coverage (+): 0. Max coverage (-): 0

Region: chr17 73271144-73271178. Max. coverage (+): 0. Max coverage (-): 0

Region: chr17 73271179-73271213. Max. coverage (+): 0. Max coverage (-): 0

Region: chr17 73271214-73271248. Max. coverage (+): 0. Max coverage (-): 0

Region: chr17 73271249-73271284. Max. coverage (+): 0. Max coverage (-): 0

Region: chr17 73271285-73271319. Max. coverage (+): 0. Max coverage (-): 0

Region: chr17 73271320-73271354. Max. coverage (+): 0. Max coverage (-): 0

Region: chr17 73271355-73271389. Max. coverage (+): 0. Max coverage (-): 16.07

Region: chr17 73271390-73271424. Max. coverage (+): 0. Max coverage (-): 5.07

Region: chr17 73271425-73271459. Max. coverage (+): 0. Max coverage (-): 5.66

Region: chr17 73271460-73271494. Max. coverage (+): 0. Max coverage (-): 12.08

Region: chr17 73271495-73271529. Max. coverage (+): 0. Max coverage (-): 6.22

Region: chr17 73271530-73271564. Max. coverage (+): 0. Max coverage (-): 6.22

Region: chr17 73271565-73271599. Max. coverage (+): 0. Max coverage (-): 0

Region: chr17 73271600-73271634. Max. coverage (+): 0. Max coverage (-): 4.97

Region: chr17 73271635-73271670. Max. coverage (+): 0. Max coverage (-): 0

Region: chr17 73271671-73271705. Max. coverage (+): 0. Max coverage (-): 0

Region: chr17 73271706-73271740. Max. coverage (+): 0. Max coverage (-): 0

Region: chr17 73271741-73271775. Max. coverage (+): 0. Max coverage (-): 0

Region: chr17 73271776-73271810. Max. coverage (+): 0. Max coverage (-): 35.66

Region: chr17 73271811-73271845. Max. coverage (+): 0. Max coverage (-): 0.15

Region: chr17 73271846-73271880. Max. coverage (+): 0. Max coverage (-): 8.04

Region: chr17 73271881-73271915. Max. coverage (+): 0. Max coverage (-): 13.32

Region: chr17 73271916-73271950. Max. coverage (+): 0. Max coverage (-): 5.1

Region: chr17 73271951-73271985. Max. coverage (+): 0. Max coverage (-): 16.7

Region: chr17 73271986-73272021. Max. coverage (+): 0. Max coverage (-): 0

Region: chr17 73272022-73272056. Max. coverage (+): 0. Max coverage (-): 0

Region: chr17 73272057-73272091. Max. coverage (+): 0. Max coverage (-): 44.29

Region: chr17 73272092-73272126. Max. coverage (+): 0. Max coverage (-): 4.92

Region: chr17 73272127-73272161. Max. coverage (+): 0. Max coverage (-): 12.5

Region: chr17 73272162-73272196. Max. coverage (+): 0. Max coverage (-): 31.48

Region: chr17 73272197-73272231. Max. coverage (+): 0. Max coverage (-): 25.13

Region: chr17 73272232-73272266. Max. coverage (+): 0. Max coverage (-): 0

Region: chr17 73272267-73272301. Max. coverage (+): 0. Max coverage (-): 12.56

Region: chr17 73272302-73272336. Max. coverage (+): 0. Max coverage (-): 8.49

Region: chr17 73272337-73272371. Max. coverage (+): 0. Max coverage (-): 0

Region: chr17 73272372-73272407. Max. coverage (+): 0. Max coverage (-): 0

Region: chr17 73272408-73272442. Max. coverage (+): 0. Max coverage (-): 10.11

Region: chr17 73272443-73272477. Max. coverage (+): 0. Max coverage (-): 13.6

Region: chr17 73272478-73272512. Max. coverage (+): 0. Max coverage (-): 0

Region: chr17 73272513-73272547. Max. coverage (+): 0. Max coverage (-): 24.15

Region: chr17 73272548-73272582. Max. coverage (+): 0. Max coverage (-): 0

Region: chr17 73272583-73272617. Max. coverage (+): 0. Max coverage (-): 0

Region: chr17 73272618-73272652. Max. coverage (+): 0. Max coverage (-): 0

Region: chr17 73272653-73272687. Max. coverage (+): 0. Max coverage (-): 0

Region: chr17 73272688-73272722. Max. coverage (+): 0. Max coverage (-): 0

Region: chr17 73272723-73272758. Max. coverage (+): 0. Max coverage (-): 0

Region: chr17 73272759-73272793. Max. coverage (+): 0. Max coverage (-): 0

Region: chr17 73272794-73272828. Max. coverage (+): 0. Max coverage (-): 0

Region: chr17 73272829-73272863. Max. coverage (+): 0. Max coverage (-): 0

Region: chr17 73272864-73272898. Max. coverage (+): 0. Max coverage (-): 0

Region: chr17 73272899-73272933. Max. coverage (+): 0. Max coverage (-): 0

Region: chr17 73272934-73272968. Max. coverage (+): 0. Max coverage (-): 0

Region: chr17 73272969-73273003. Max. coverage (+): 0. Max coverage (-): 0

Region: chr17 73273004-73273038. Max. coverage (+): 0. Max coverage (-): 0

Region: chr17 73273039-73273073. Max. coverage (+): 0. Max coverage (-): 0

Region: chr17 73273074-73273109. Max. coverage (+): 0. Max coverage (-): 0

Region: chr17 73273110-73273144. Max. coverage (+): 0. Max coverage (-): 0

Region: chr17 73273145-73273179. Max. coverage (+): 0. Max coverage (-): 0

Region: chr17 73273180-73273214. Max. coverage (+): 0. Max coverage (-): 0

Region: chr17 73273215-73273249. Max. coverage (+): 0. Max coverage (-): 9.74

Region: chr17 73273250-73273284. Max. coverage (+): 0. Max coverage (-): 0

Region: chr17 73273285-73273319. Max. coverage (+): 0. Max coverage (-): 0

Region: chr17 73273320-73273354. Max. coverage (+): 0. Max coverage (-): 0

Region: chr17 73273355-73273389. Max. coverage (+): 0. Max coverage (-): 0

Region: chr17 73273390-73273424. Max. coverage (+): 0. Max coverage (-): 0.3

Region: chr17 73273425-73273459. Max. coverage (+): 0. Max coverage (-): 0

Region: chr17 73273460-73273495. Max. coverage (+): 0. Max coverage (-): 0

Region: chr17 73273496-73273530. Max. coverage (+): 0. Max coverage (-): 0

Region: chr17 73273531-73273565. Max. coverage (+): 0. Max coverage (-): 0

Region: chr17 73273566-73273600. Max. coverage (+): 0. Max coverage (-): 0

Region: chr17 73273601-73273635. Max. coverage (+): 0. Max coverage (-): 0

Region: chr17 73273636-73273670. Max. coverage (+): 0. Max coverage (-): 0

Region: chr17 73273671-73273705. Max. coverage (+): 0. Max coverage (-): 0

Region: chr17 73273706-73273740. Max. coverage (+): 0. Max coverage (-): 4.77

Region: chr17 73273741-73273775. Max. coverage (+): 0. Max coverage (-): 0

Region: chr17 73273776-73273810. Max. coverage (+): 0. Max coverage (-): 0

Region: chr17 73273811-73273846. Max. coverage (+): 0. Max coverage (-): 0

Region: chr17 73273847-73273881. Max. coverage (+): 0. Max coverage (-): 0

Region: chr17 73273882-73273916. Max. coverage (+): 0. Max coverage (-): 0

Region: chr17 73273917-73273951. Max. coverage (+): 0. Max coverage (-): 1.38

Region: chr17 73273952-73273986. Max. coverage (+): 0. Max coverage (-): 8.91

Region: chr17 73273987-73274021. Max. coverage (+): 0. Max coverage (-): 0.94

Region: chr17 73274022-73274056. Max. coverage (+): 0. Max coverage (-): 0

Region: chr17 73274057-73274091. Max. coverage (+): 0. Max coverage (-): 0

Region: chr17 73274092-73274126. Max. coverage (+): 0. Max coverage (-): 0

Region: chr17 73274127-73274161. Max. coverage (+): 0. Max coverage (-): 0

Region: chr17 73274162-73274196. Max. coverage (+): 0. Max coverage (-): 3.92

Region: chr17 73274197-73274232. Max. coverage (+): 0. Max coverage (-): 0

Region: chr17 73274233-73274267. Max. coverage (+): 0. Max coverage (-): 5.86

Region: chr17 73274268-73274302. Max. coverage (+): 0. Max coverage (-): 0

Region: chr17 73274303-73274337. Max. coverage (+): 5.34. Max coverage (-): 0

Region: chr17 73274338-73274372. Max. coverage (+): 0. Max coverage (-): 0

Region: chr17 73274373-73274407. Max. coverage (+): 0. Max coverage (-): 0

Region: chr17 73274408-73274442. Max. coverage (+): 0. Max coverage (-): 4.8

Region: chr17 73274443-73274477. Max. coverage (+): 0. Max coverage (-): 29.43

Region: chr17 73274478-73274512. Max. coverage (+): 0. Max coverage (-): 4.82

Region: chr17 73274513-73274547. Max. coverage (+): 0. Max coverage (-): 5.93

Region: chr17 73274548-73274583. Max. coverage (+): 0. Max coverage (-): 6.18

Region: chr17 73274584-73274618. Max. coverage (+): 0. Max coverage (-): 25.78

Region: chr17 73274619-73274653. Max. coverage (+): 0. Max coverage (-): 0

Region: chr17 73274654-73274688. Max. coverage (+): 0. Max coverage (-): 52.18

Region: chr17 73274689-73274723. Max. coverage (+): 0. Max coverage (-): 114.46

Region: chr17 73274724-73274758. Max. coverage (+): 0. Max coverage (-): 26.7

Region: chr17 73274759-73274793. Max. coverage (+): 0. Max coverage (-): 34.13

Region: chr17 73274794-73274828. Max. coverage (+): 0. Max coverage (-): 11.07

Region: chr17 73274829-73274863. Max. coverage (+): 0. Max coverage (-): 6.89

Region: chr17 73274864-73274898. Max. coverage (+): 0. Max coverage (-): 21.33

Region: chr17 73274899-73274934. Max. coverage (+): 0. Max coverage (-): 39.77

Region: chr17 73274935-73274969. Max. coverage (+): 0. Max coverage (-): 0

Region: chr17 73274970-73275004. Max. coverage (+): 0. Max coverage (-): 4.78

Region: chr17 73275005-73275039. Max. coverage (+): 0. Max coverage (-): 17.4

Region: chr17 73275040-73275074. Max. coverage (+): 0. Max coverage (-): 25.19

Region: chr17 73275075-73275109. Max. coverage (+): 0. Max coverage (-): 29.97

Region: chr17 73275110-73275144. Max. coverage (+): 0. Max coverage (-): 6.32

Region: chr17 73275145-73275179. Max. coverage (+): 1.95. Max coverage (-): 6.32

Region: chr17 73275180-73275214. Max. coverage (+): 0. Max coverage (-): 4.09

Region: chr17 73275215-73275249. Max. coverage (+): 0. Max coverage (-): 0

Region: chr17 73275250-73275284. Max. coverage (+): 0. Max coverage (-): 0

Region: chr17 73275285-73275320. Max. coverage (+): 0. Max coverage (-): 8.81

Region: chr17 73275321-73275355. Max. coverage (+): 0. Max coverage (-): 12.1

Region: chr17 73275356-73275390. Max. coverage (+): 0. Max coverage (-): 0

Region: chr17 73275391-73275425. Max. coverage (+): 0. Max coverage (-): 1.67

Region: chr17 73275426-73275460. Max. coverage (+): 0. Max coverage (-): 0

Region: chr17 73275461-73275495. Max. coverage (+): 0. Max coverage (-): 4.74

Region: chr17 73275496-73275530. Max. coverage (+): 0. Max coverage (-): 29.95

Region: chr17 73275531-73275565. Max. coverage (+): 0. Max coverage (-): 0

Region: chr17 73275566-73275600. Max. coverage (+): 0. Max coverage (-): 24.23

Region: chr17 73275601-73275635. Max. coverage (+): 0. Max coverage (-): 24.31

Region: chr17 73275636-73275671. Max. coverage (+): 0. Max coverage (-): 0

Region: chr17 73275672-73275706. Max. coverage (+): 0.51. Max coverage (-): 7.03

Region: chr17 73275707-73275741. Max. coverage (+): 0. Max coverage (-): 0

Region: chr17 73275742-73275776. Max. coverage (+): 0. Max coverage (-): 0

Region: chr17 73275777-73275811. Max. coverage (+): 0. Max coverage (-): 0

Region: chr17 73275812-73275846. Max. coverage (+): 0. Max coverage (-): 0

Region: chr17 73275847-73275881. Max. coverage (+): 0. Max coverage (-): 9.2

Region: chr17 73275882-73275916. Max. coverage (+): 0. Max coverage (-): 0

Region: chr17 73275917-73275951. Max. coverage (+): 0. Max coverage (-): 0

Region: chr17 73275952-73275986. Max. coverage (+): 0. Max coverage (-): 0

Region: chr17 73275987-73276021. Max. coverage (+): 0. Max coverage (-): 6.14

Region: chr17 73276022-73276057. Max. coverage (+): 0. Max coverage (-): 31.98

Region: chr17 73276058-73276092. Max. coverage (+): 0. Max coverage (-): 0

Region: chr17 73276093-73276127. Max. coverage (+): 0. Max coverage (-): 2.78

Region: chr17 73276128-73276162. Max. coverage (+): 5.72. Max coverage (-): 2.78

Region: chr17 73276163-73276197. Max. coverage (+): 0. Max coverage (-): 0

Region: chr17 73276198-73276232. Max. coverage (+): 0. Max coverage (-): 0

Region: chr17 73276233-73276267. Max. coverage (+): 0. Max coverage (-): 0

Region: chr17 73276268-73276302. Max. coverage (+): 0. Max coverage (-): 42.91

Region: chr17 73276303-73276337. Max. coverage (+): 0. Max coverage (-): 0

Region: chr17 73276338-73276372. Max. coverage (+): 0. Max coverage (-): 6.19

Region: chr17 73276373-73276408. Max. coverage (+): 0. Max coverage (-): 7.14

Region: chr17 73276409-73276443. Max. coverage (+): 0. Max coverage (-): 3.42

Region: chr17 73276444-73276478. Max. coverage (+): 0. Max coverage (-): 4.42

Region: chr17 73276479-73276513. Max. coverage (+): 0. Max coverage (-): 1.87

Region: chr17 73276514-73276548. Max. coverage (+): 0. Max coverage (-): 0

Region: chr17 73276549-73276583. Max. coverage (+): 0. Max coverage (-): 0

Region: chr17 73276584-73276618. Max. coverage (+): 0. Max coverage (-): 0

Region: chr17 73276619-73276653. Max. coverage (+): 0. Max coverage (-): 5.82

Region: chr17 73276654-73276688. Max. coverage (+): 0. Max coverage (-): 0

Region: chr17 73276689-73276723. Max. coverage (+): 0. Max coverage (-): 0

Region: chr17 73276724-73276758. Max. coverage (+): 0. Max coverage (-): 2.7

Region: chr17 73276759-73276794. Max. coverage (+): 0. Max coverage (-): 0

Region: chr17 73276795-73276829. Max. coverage (+): 0. Max coverage (-): 0

Region: chr17 73276830-73276864. Max. coverage (+): 0. Max coverage (-): 0

Region: chr17 73276865-73276899. Max. coverage (+): 0. Max coverage (-): 0

Region: chr17 73276900-73276934. Max. coverage (+): 0. Max coverage (-): 0

Region: chr17 73276935-73276969. Max. coverage (+): 0. Max coverage (-): 4.52

Region: chr17 73276970-73277004. Max. coverage (+): 0. Max coverage (-): 16.85

Region: chr17 73277005-73277039. Max. coverage (+): 0. Max coverage (-): 3.7

Region: chr17 73277040-73277074. Max. coverage (+): 0. Max coverage (-): 22.33

Region: chr17 73277075-73277109. Max. coverage (+): 0. Max coverage (-): 22.33

Region: chr17 73277110-73277145. Max. coverage (+): 0. Max coverage (-): 0.75

Region: chr17 73277146-73277180. Max. coverage (+): 0. Max coverage (-): 1.76

Region: chr17 73277181-73277215. Max. coverage (+): 0. Max coverage (-): 35.51

Region: chr17 73277216-73277250. Max. coverage (+): 0. Max coverage (-): 26.66

Region: chr17 73277251-73277285. Max. coverage (+): 0. Max coverage (-): 1.56

Region: chr17 73277286-73277320. Max. coverage (+): 0. Max coverage (-): 20.5

Region: chr17 73277321-73277355. Max. coverage (+): 0. Max coverage (-): 15.69

Region: chr17 73277356-73277390. Max. coverage (+): 0. Max coverage (-): 0

Region: chr17 73277391-73277425. Max. coverage (+): 0. Max coverage (-): 0

Region: chr17 73277426-73277460. Max. coverage (+): 0. Max coverage (-): 10.45

Region: chr17 73277461-73277496. Max. coverage (+): 0. Max coverage (-): 50.29

Region: chr17 73277497-73277531. Max. coverage (+): 0. Max coverage (-): 47

Region: chr17 73277532-73277566. Max. coverage (+): 0. Max coverage (-): 69.25

Region: chr17 73277567-73277601. Max. coverage (+): 0. Max coverage (-): 24.48

Region: chr17 73277602-73277636. Max. coverage (+): 0. Max coverage (-): 47.83

Region: chr17 73277637-73277671. Max. coverage (+): 0. Max coverage (-): 0

Region: chr17 73277672-73277706. Max. coverage (+): 0. Max coverage (-): 0

Region: chr17 73277707-73277741. Max. coverage (+): 0. Max coverage (-): 22.32

Region: chr17 73277742-73277776. Max. coverage (+): 0. Max coverage (-): 0

Region: chr17 73277777-73277811. Max. coverage (+): 0. Max coverage (-): 17.4

Region: chr17 73277812-73277846. Max. coverage (+): 0. Max coverage (-): 37.99

Region: chr17 73277847-73277882. Max. coverage (+): 0. Max coverage (-): 54.81

Region: chr17 73277883-73277917. Max. coverage (+): 0. Max coverage (-): 0

Region: chr17 73277918-73277952. Max. coverage (+): 0. Max coverage (-): 0

Region: chr17 73277953-73277987. Max. coverage (+): 0. Max coverage (-): 20.27

Region: chr17 73277988-73278022. Max. coverage (+): 0. Max coverage (-): 58.34

Region: chr17 73278023-73278057. Max. coverage (+): 0. Max coverage (-): 21.05

Region: chr17 73278058-73278092. Max. coverage (+): 0. Max coverage (-): 11.88

Region: chr17 73278093-73278127. Max. coverage (+): 0. Max coverage (-): 14.98

Region: chr17 73278128-73278162. Max. coverage (+): 0. Max coverage (-): 32.64

Region: chr17 73278163-73278197. Max. coverage (+): 0. Max coverage (-): 25.46

Region: chr17 73278198-73278233. Max. coverage (+): 0. Max coverage (-): 34.18

Region: chr17 73278234-73278268. Max. coverage (+): 0. Max coverage (-): 8.51

Region: chr17 73278269-73278303. Max. coverage (+): 0. Max coverage (-): 55.83

Region: chr17 73278304-73278338. Max. coverage (+): 0. Max coverage (-): 52.26

Region: chr17 73278339-73278373. Max. coverage (+): 0. Max coverage (-): 0.7

Region: chr17 73278374-73278408. Max. coverage (+): 0. Max coverage (-): 0

Region: chr17 73278409-73278443. Max. coverage (+): 0. Max coverage (-): 0

Region: chr17 73278444-73278478. Max. coverage (+): 0. Max coverage (-): 0

Region: chr17 73278479-73278513. Max. coverage (+): 0. Max coverage (-): 0

Region: chr17 73278514-73278548. Max. coverage (+): 0. Max coverage (-): 0

Region: chr17 73278549-73278583. Max. coverage (+): 0. Max coverage (-): 0

Region: chr17 73278584-73278619. Max. coverage (+): 0. Max coverage (-): 8.32

Region: chr17 73278620-73278654. Max. coverage (+): 0. Max coverage (-): 37.89

Region: chr17 73278655-73278689. Max. coverage (+): 0. Max coverage (-): 13.37

Region: chr17 73278690-73278724. Max. coverage (+): 0. Max coverage (-): 0

Region: chr17 73278725-73278759. Max. coverage (+): 0. Max coverage (-): 13.91

Region: chr17 73278760-73278794. Max. coverage (+): 0. Max coverage (-): 20.15

Region: chr17 73278795-73278829. Max. coverage (+): 0. Max coverage (-): 0

Region: chr17 73278830-73278864. Max. coverage (+): 0. Max coverage (-): 0

Region: chr17 73278865-73278899. Max. coverage (+): 0. Max coverage (-): 10.64

Region: chr17 73278900-73278934. Max. coverage (+): 0. Max coverage (-): 10.64

Region: chr17 73278935-73278970. Max. coverage (+): 0. Max coverage (-): 5.43

Region: chr17 73278971-73279005. Max. coverage (+): 0. Max coverage (-): 0.12

Region: chr17 73279006-73279040. Max. coverage (+): 0. Max coverage (-): 27.66

Region: chr17 73279041-73279075. Max. coverage (+): 0. Max coverage (-): 18.5

Region: chr17 73279076-73279110. Max. coverage (+): 0. Max coverage (-): 46.17

Region: chr17 73279111-73279145. Max. coverage (+): 0. Max coverage (-): 57.75

Region: chr17 73279146-73279180. Max. coverage (+): 0. Max coverage (-): 6.13

Region: chr17 73279181-73279215. Max. coverage (+): 0. Max coverage (-): 0

Region: chr17 73279216-73279250. Max. coverage (+): 0. Max coverage (-): 5.09

Region: chr17 73279251-73279285. Max. coverage (+): 2.32. Max coverage (-): 14.92

Region: chr17 73279286-73279321. Max. coverage (+): 2.32. Max coverage (-): 0

Region: chr17 73279322-73279356. Max. coverage (+): 0. Max coverage (-): 0

Region: chr17 73279357-73279391. Max. coverage (+): 0. Max coverage (-): 0

Region: chr17 73279392-73279426. Max. coverage (+): 0. Max coverage (-): 0

Region: chr17 73279427-73279461. Max. coverage (+): 0. Max coverage (-): 0

Region: chr17 73279462-73279496. Max. coverage (+): 0. Max coverage (-): 0

Region: chr17 73279497-73279531. Max. coverage (+): 0. Max coverage (-): 0

Region: chr17 73279532-73279566. Max. coverage (+): 0. Max coverage (-): 6.33

Region: chr17 73279567-73279601. Max. coverage (+): 0. Max coverage (-): 20.47

Region: chr17 73279602-73279636. Max. coverage (+): 0. Max coverage (-): 36.38

Region: chr17 73279637-73279671. Max. coverage (+): 0. Max coverage (-): 29.21

Region: chr17 73279672-73279707. Max. coverage (+): 0. Max coverage (-): 11.72

Region: chr17 73279708-73279742. Max. coverage (+): 0. Max coverage (-): 114.73

Region: chr17 73279743-73279777. Max. coverage (+): 0. Max coverage (-): 44.17

Region: chr17 73279778-73279812. Max. coverage (+): 0. Max coverage (-): 86

Region: chr17 73279813-73279847. Max. coverage (+): 0. Max coverage (-): 70.13

Region: chr17 73279848-73279882. Max. coverage (+): 0. Max coverage (-): 29.1

Region: chr17 73279883-73279917. Max. coverage (+): 0. Max coverage (-): 12.03

Region: chr17 73279918-73279952. Max. coverage (+): 0. Max coverage (-): 21.5

Region: chr17 73279953-73279987. Max. coverage (+): 0. Max coverage (-): 0

Region: chr17 73279988-73280022. Max. coverage (+): 0. Max coverage (-): 0.22

Region: chr17 73280023-73280058. Max. coverage (+): 0. Max coverage (-): 0

Region: chr17 73280059-73280093. Max. coverage (+): 0. Max coverage (-): 0

Region: chr17 73280094-73280128. Max. coverage (+): 0. Max coverage (-): 0

Region: chr17 73280129-73280163. Max. coverage (+): 0. Max coverage (-): 0

Region: chr17 73280164-73280198. Max. coverage (+): 0. Max coverage (-): 0

Region: chr17 73280199-73280233. Max. coverage (+): 0. Max coverage (-): 10.77

Region: chr17 73280234-73280268. Max. coverage (+): 0. Max coverage (-): 10.77

Region: chr17 73280269-73280303. Max. coverage (+): 0. Max coverage (-): 0

Region: chr17 73280304-73280338. Max. coverage (+): 0. Max coverage (-): 0

Region: chr17 73280339-73280373. Max. coverage (+): 0. Max coverage (-): 5.99

Region: chr17 73280374-73280408. Max. coverage (+): 0. Max coverage (-): 5.99

Region: chr17 73280409-73280444. Max. coverage (+): 0. Max coverage (-): 0

Region: chr17 73280445-73280479. Max. coverage (+): 0. Max coverage (-): 5.36

Region: chr17 73280480-73280514. Max. coverage (+): 0. Max coverage (-): 0

Region: chr17 73280515-73280549. Max. coverage (+): 0. Max coverage (-): 0

Region: chr17 73280550-73280584. Max. coverage (+): 0. Max coverage (-): 0

Region: chr17 73280585-73280619. Max. coverage (+): 0. Max coverage (-): 0

Region: chr17 73280620-73280654. Max. coverage (+): 0. Max coverage (-): 0

Region: chr17 73280655-73280689. Max. coverage (+): 0. Max coverage (-): 0

Region: chr17 73280690-73280724. Max. coverage (+): 0. Max coverage (-): 0

Region: chr17 73280725-73280759. Max. coverage (+): 0. Max coverage (-): 3.56

Region: chr17 73280760-73280795. Max. coverage (+): 0. Max coverage (-): 3.07

Region: chr17 73280796-73280830. Max. coverage (+): 0. Max coverage (-): 1.15

Region: chr17 73280831-73280865. Max. coverage (+): 0. Max coverage (-): 0

Region: chr17 73280866-73280900. Max. coverage (+): 0. Max coverage (-): 0

Region: chr17 73280901-73280935. Max. coverage (+): 0. Max coverage (-): 0

Region: chr17 73280936-73280970. Max. coverage (+): 0. Max coverage (-): 0

Region: chr17 73280971-73281005. Max. coverage (+): 0. Max coverage (-): 4.67

Region: chr17 73281006-73281040. Max. coverage (+): 0. Max coverage (-): 0

Region: chr17 73281041-73281075. Max. coverage (+): 0.66. Max coverage (-): 0

Region: chr17 73281076-73281110. Max. coverage (+): 0.66. Max coverage (-): 138.71

Region: chr17 73281111-73281145. Max. coverage (+): 0. Max coverage (-): 0.1

Region: chr17 73281146-73281181. Max. coverage (+): 0. Max coverage (-): 0

Region: chr17 73281182-73281216. Max. coverage (+): 0. Max coverage (-): 0

Region: chr17 73281217-73281251. Max. coverage (+): 0. Max coverage (-): 0.13

Region: chr17 73281252-73281286. Max. coverage (+): 0. Max coverage (-): 16

Region: chr17 73281287-73281321. Max. coverage (+): 0. Max coverage (-): 18.93

Region: chr17 73281322-73281356. Max. coverage (+): 0. Max coverage (-): 22.28

Region: chr17 73281357-73281391. Max. coverage (+): 0. Max coverage (-): 20.53

Region: chr17 73281392-73281426. Max. coverage (+): 0. Max coverage (-): 5.96

Region: chr17 73281427-73281461. Max. coverage (+): 0. Max coverage (-): 18.18

Region: chr17 73281462-73281496. Max. coverage (+): 0. Max coverage (-): 13.23

Region: chr17 73281497-73281532. Max. coverage (+): 0. Max coverage (-): 8.05

Region: chr17 73281533-73281567. Max. coverage (+): 0. Max coverage (-): 3.68

Region: chr17 73281568-73281602. Max. coverage (+): 0. Max coverage (-): 0

Region: chr17 73281603-73281637. Max. coverage (+): 0. Max coverage (-): 0

Region: chr17 73281638-73281672. Max. coverage (+): 0. Max coverage (-): 7.62

Region: chr17 73281673-73281707. Max. coverage (+): 0. Max coverage (-): 6.5

Region: chr17 73281708-73281742. Max. coverage (+): 0. Max coverage (-): 6.5

Region: chr17 73281743-73281777. Max. coverage (+): 0. Max coverage (-): 0

Region: chr17 73281778-73281812. Max. coverage (+): 0. Max coverage (-): 0

Region: chr17 73281813-73281847. Max. coverage (+): 0. Max coverage (-): 1.14

Region: chr17 73281848-73281883. Max. coverage (+): 0. Max coverage (-): 1.14

Region: chr17 73281884-73281918. Max. coverage (+): 0. Max coverage (-): 22.71

Region: chr17 73281919-73281953. Max. coverage (+): 0. Max coverage (-): 113.12

Region: chr17 73281954-73281988. Max. coverage (+): 0. Max coverage (-): 39.56

Region: chr17 73281989-73282023. Max. coverage (+): 0. Max coverage (-): 19.85

Region: chr17 73282024-73282058. Max. coverage (+): 0. Max coverage (-): 0

Region: chr17 73282059-73282093. Max. coverage (+): 0. Max coverage (-): 0

Region: chr17 73282094-73282128. Max. coverage (+): 0. Max coverage (-): 0

Region: chr17 73282129-73282163. Max. coverage (+): 0. Max coverage (-): 8.89

Region: chr17 73282164-73282198. Max. coverage (+): 0. Max coverage (-): 64.15

Region: chr17 73282199-73282233. Max. coverage (+): 0. Max coverage (-): 39.37

Region: chr17 73282234-73282269. Max. coverage (+): 0. Max coverage (-): 4.28

Region: chr17 73282270-73282304. Max. coverage (+): 0. Max coverage (-): 29.52

Region: chr17 73282305-73282339. Max. coverage (+): 0. Max coverage (-): 0

Region: chr17 73282340-73282374. Max. coverage (+): 0. Max coverage (-): 0

Region: chr17 73282375-73282409. Max. coverage (+): 0. Max coverage (-): 0

Region: chr17 73282410-73282444. Max. coverage (+): 0. Max coverage (-): 0

Region: chr17 73282445-73282479. Max. coverage (+): 0. Max coverage (-): 16.58

Region: chr17 73282480-73282514. Max. coverage (+): 0. Max coverage (-): 0

Region: chr17 73282515-73282549. Max. coverage (+): 0. Max coverage (-): 4.64

Region: chr17 73282550-73282584. Max. coverage (+): 0. Max coverage (-): 6.99

Region: chr17 73282585-73282620. Max. coverage (+): 0. Max coverage (-): 11.59

Region: chr17 73282621-73282655. Max. coverage (+): 0. Max coverage (-): 0

Region: chr17 73282656-73282690. Max. coverage (+): 0. Max coverage (-): 14.35

Region: chr17 73282691-73282725. Max. coverage (+): 0. Max coverage (-): 13.28

Region: chr17 73282726-73282760. Max. coverage (+): 0. Max coverage (-): 4.51

Region: chr17 73282761-73282795. Max. coverage (+): 0. Max coverage (-): 0

Region: chr17 73282796-73282830. Max. coverage (+): 0. Max coverage (-): 40.32

Region: chr17 73282831-73282865. Max. coverage (+): 0. Max coverage (-): 26.99

Region: chr17 73282866-73282900. Max. coverage (+): 0. Max coverage (-): 24.14

Region: chr17 73282901-73282935. Max. coverage (+): 0. Max coverage (-): 185.63

Region: chr17 73282936-73282970. Max. coverage (+): 0. Max coverage (-): 0

Region: chr17 73282971-73283006. Max. coverage (+): 0. Max coverage (-): 44.74

Region: chr17 73283007-73283041. Max. coverage (+): 0. Max coverage (-): 24.93

Region: chr17 73283042-73283076. Max. coverage (+): 0. Max coverage (-): 0

Region: chr17 73283077-73283111. Max. coverage (+): 0. Max coverage (-): 0

Region: chr17 73283112-73283146. Max. coverage (+): 0. Max coverage (-): 0

Region: chr17 73283147-73283181. Max. coverage (+): 0. Max coverage (-): 0

Region: chr17 73283182-73283216. Max. coverage (+): 0. Max coverage (-): 0

Region: chr17 73283217-73283251. Max. coverage (+): 0. Max coverage (-): 0

Region: chr17 73283252-73283286. Max. coverage (+): 0. Max coverage (-): 0

Region: chr17 73283287-73283321. Max. coverage (+): 0. Max coverage (-): 6.78

Region: chr17 73283322-73283357. Max. coverage (+): 0. Max coverage (-): 5.66

Region: chr17 73283358-73283392. Max. coverage (+): 0. Max coverage (-): 5.56

Region: chr17 73283393-73283427. Max. coverage (+): 3.75. Max coverage (-): 3.4

Region: chr17 73283428-73283462. Max. coverage (+): 0. Max coverage (-): 3.56

Region: chr17 73283463-73283497. Max. coverage (+): 0. Max coverage (-): 9.3

Region: chr17 73283498-73283532. Max. coverage (+): 0. Max coverage (-): 4.73

Region: chr17 73283533-73283567. Max. coverage (+): 0. Max coverage (-): 0

Region: chr17 73283568-73283602. Max. coverage (+): 0. Max coverage (-): 4.38

Region: chr17 73283603-73283637. Max. coverage (+): 0. Max coverage (-): 0

Region: chr17 73283638-73283672. Max. coverage (+): 0. Max coverage (-): 0

Region: chr17 73283673-73283708. Max. coverage (+): 0. Max coverage (-): 0

Region: chr17 73283709-73283743. Max. coverage (+): 0. Max coverage (-): 0

Region: chr17 73283744-73283778. Max. coverage (+): 0. Max coverage (-): 0

Region: chr17 73283779-73283813. Max. coverage (+): 0. Max coverage (-): 0

Region: chr17 73283814-73283848. Max. coverage (+): 0. Max coverage (-): 0

Region: chr17 73283849-73283883. Max. coverage (+): 0. Max coverage (-): 1.13

Region: chr17 73283884-73283918. Max. coverage (+): 0. Max coverage (-): 5.14

Region: chr17 73283919-73283953. Max. coverage (+): 0. Max coverage (-): 0

Region: chr17 73283954-73283988. Max. coverage (+): 0. Max coverage (-): 0

Region: chr17 73283989-73284023. Max. coverage (+): 0. Max coverage (-): 0

Region: chr17 73284024-73284058. Max. coverage (+): 0. Max coverage (-): 0

Region: chr17 73284059-73284094. Max. coverage (+): 0. Max coverage (-): 0

Region: chr17 73284095-73284129. Max. coverage (+): 0. Max coverage (-): 0

Region: chr17 73284130-73284164. Max. coverage (+): 0. Max coverage (-): 0

Region: chr17 73284165-73284199. Max. coverage (+): 0. Max coverage (-): 0

Region: chr17 73284200-73284234. Max. coverage (+): 0. Max coverage (-): 0

Region: chr17 73284235-73284269. Max. coverage (+): 0. Max coverage (-): 6.44

Region: chr17 73284270-73284304. Max. coverage (+): 0. Max coverage (-): 0

Region: chr17 73284305-73284339. Max. coverage (+): 0. Max coverage (-): 0

Region: chr17 73284340-73284374. Max. coverage (+): 0. Max coverage (-): 0

Region: chr17 73284375-73284409. Max. coverage (+): 0. Max coverage (-): 0

Region: chr17 73284410-73284445. Max. coverage (+): 0. Max coverage (-): 0

Region: chr17 73284446-73284480. Max. coverage (+): 0. Max coverage (-): 14.75

Region: chr17 73284481-73284515. Max. coverage (+): 0. Max coverage (-): 5.49

Region: chr17 73284516-73284550. Max. coverage (+): 0. Max coverage (-): 0

Region: chr17 73284551-73284585. Max. coverage (+): 0. Max coverage (-): 0

Region: chr17 73284586-73284620. Max. coverage (+): 0. Max coverage (-): 5.9

Region: chr17 73284621-73284655. Max. coverage (+): 0. Max coverage (-): 7.14

Region: chr17 73284656-73284690. Max. coverage (+): 0. Max coverage (-): 11.82

Region: chr17 73284691-73284725. Max. coverage (+): 0. Max coverage (-): 0

Region: chr17 73284726-73284760. Max. coverage (+): 0. Max coverage (-): 0

Region: chr17 73284761-73284795. Max. coverage (+): 0. Max coverage (-): 0

Region: chr17 73284796-73284831. Max. coverage (+): 0. Max coverage (-): 0

Region: chr17 73284832-73284866. Max. coverage (+): 0. Max coverage (-): 0

Region: chr17 73284867-73284901. Max. coverage (+): 0. Max coverage (-): 0

Region: chr17 73284902-73284936. Max. coverage (+): 0. Max coverage (-): 0

Region: chr17 73284937-73284971. Max. coverage (+): 0. Max coverage (-): 0

Region: chr17 73284972-73285006. Max. coverage (+): 0. Max coverage (-): 0

Region: chr17 73285007-73285041. Max. coverage (+): 0. Max coverage (-): 0

Region: chr17 73285042-73285076. Max. coverage (+): 0. Max coverage (-): 0

Region: chr17 73285077-73285111. Max. coverage (+): 0. Max coverage (-): 1.64

Region: chr17 73285112-73285146. Max. coverage (+): 0. Max coverage (-): 1.64

Region: chr17 73285147-73285182. Max. coverage (+): 0. Max coverage (-): 0

Region: chr17 73285183-73285217. Max. coverage (+): 0. Max coverage (-): 14.03

Region: chr17 73285218-73285252. Max. coverage (+): 0. Max coverage (-): 6

Region: chr17 73285253-73285287. Max. coverage (+): 0. Max coverage (-): 52.34

Region: chr17 73285288-73285322. Max. coverage (+): 0. Max coverage (-): 38.07

Region: chr17 73285323-73285357. Max. coverage (+): 0. Max coverage (-): 0

Region: chr17 73285358-73285392. Max. coverage (+): 0. Max coverage (-): 16.22

Region: chr17 73285393-73285427. Max. coverage (+): 0. Max coverage (-): 16.22

Region: chr17 73285428-73285462. Max. coverage (+): 0. Max coverage (-): 11.5

Region: chr17 73285463-73285497. Max. coverage (+): 0. Max coverage (-): 56.26

Region: chr17 73285498-73285532. Max. coverage (+): 0. Max coverage (-): 0

Region: chr17 73285533-73285568. Max. coverage (+): 0. Max coverage (-): 70.26

Region: chr17 73285569-73285603. Max. coverage (+): 0. Max coverage (-): 4.57

Region: chr17 73285604-73285638. Max. coverage (+): 0. Max coverage (-): 8.02

Region: chr17 73285639-73285673. Max. coverage (+): 0. Max coverage (-): 15.56

Region: chr17 73285674-73285708. Max. coverage (+): 0. Max coverage (-): 0

Region: chr17 73285709-73285743. Max. coverage (+): 0. Max coverage (-): 0

Region: chr17 73285744-73285778. Max. coverage (+): 0. Max coverage (-): 0

Region: chr17 73285779-73285813. Max. coverage (+): 0. Max coverage (-): 0

Region: chr17 73285814-73285848. Max. coverage (+): 0. Max coverage (-): 0

Region: chr17 73285849-73285883. Max. coverage (+): 0. Max coverage (-): 0

Region: chr17 73285884-73285919. Max. coverage (+): 0.42. Max coverage (-): 17.04

Region: chr17 73285920-73285954. Max. coverage (+): 0. Max coverage (-): 61.45

Region: chr17 73285955-73285989. Max. coverage (+): 0. Max coverage (-): 63.93

Region: chr17 73285990-73286024. Max. coverage (+): 0. Max coverage (-): 7

Region: chr17 73286025-73286059. Max. coverage (+): 0. Max coverage (-): 4.2

Region: chr17 73286060-73286094. Max. coverage (+): 0. Max coverage (-): 0

Region: chr17 73286095-73286129. Max. coverage (+): 0. Max coverage (-): 19.58

Region: chr17 73286130-73286164. Max. coverage (+): 0. Max coverage (-): 16.67

Region: chr17 73286165-73286199. Max. coverage (+): 0. Max coverage (-): 6.52

Region: chr17 73286200-73286234. Max. coverage (+): 0. Max coverage (-): 59.7

Region: chr17 73286235-73286270. Max. coverage (+): 0. Max coverage (-): 26.94

Region: chr17 73286271-73286305. Max. coverage (+): 0. Max coverage (-): 10.99

Region: chr17 73286306-73286340. Max. coverage (+): 0. Max coverage (-): 6.88

Region: chr17 73286341-73286375. Max. coverage (+): 0. Max coverage (-): 22.39

Region: chr17 73286376-73286410. Max. coverage (+): 0. Max coverage (-): 56.48

Region: chr17 73286411-73286445. Max. coverage (+): 0. Max coverage (-): 0

Region: chr17 73286446-73286480. Max. coverage (+): 0. Max coverage (-): 0

Region: chr17 73286481-73286515. Max. coverage (+): 0. Max coverage (-): 2.9

Region: chr17 73286516-73286550. Max. coverage (+): 0. Max coverage (-): 8.4

Region: chr17 73286551-73286585. Max. coverage (+): 0. Max coverage (-): 11.41

Region: chr17 73286586-73286620. Max. coverage (+): 0. Max coverage (-): 0

Region: chr17 73286621-73286656. Max. coverage (+): 0. Max coverage (-): 0

Region: chr17 73286657-73286691. Max. coverage (+): 0. Max coverage (-): 0

Region: chr17 73286692-73286726. Max. coverage (+): 0. Max coverage (-): 4.25

Region: chr17 73286727-73286761. Max. coverage (+): 0. Max coverage (-): 4.25

Region: chr17 73286762-73286796. Max. coverage (+): 0. Max coverage (-): 13.25

Region: chr17 73286797-. Max. coverage (+): 0. Max coverage (-): 0

RepeatMasker Color Code

**+**

100-98% Identity

<98-95% Identity

<95-90% Identity

<90-85% Identity

<85-80% Identity

<80-75% Identity

<75-70% Identity

<70% Identity

**-**

Gene Set Color Code

**+**

Gene

Pseudogene

**-**

Topology/Coverage Color Code

Coverage Plus Strand

Coverage Minus Strand

Mainstrand: Plus

Mainstrand: Minus

Complementary Strand

Flanking Region  
(if option -flank >0)

Gene Set Annotation  

**1. MIF (protein coding, ENSBTAG00000007375) Tr:00000009699 Ex:1**: 73273379-73273583 (+)  
**2. MIF (protein coding, ENSBTAG00000007375) Tr:00000009699 Ex:2**: 73273723-73273895 (+)  
**3. MIF (protein coding, ENSBTAG00000007375) Tr:00000009699 Ex:3**: 73273978-73274171 (+)

  
RepeatMasker Annotation  

**1. MIRb**: 73269812-73269935 (-), Divergence to consensus: 45.6%  
**2. MLT1E2**: 73270808-73271322 (-), Divergence to consensus: 31.5%  
**3. MIR**: 73271641-73271768 (-), Divergence to consensus: 43.5%  
**4. MLT1I**: 73271990-73272069 (-), Divergence to consensus: 35%  
**5. L2b**: 73272691-73272781 (-), Divergence to consensus: 40.3%  
**6. GC\_rich**: 73273304-73273393 (+), Divergence to consensus: 85.6%  
**7. GC\_rich**: 73273902-73273953 (+), Divergence to consensus: 75%  
**8. GC\_rich**: 73274091-73274117 (+), Divergence to consensus: 51.9%  
**9. GC\_rich**: 73274099-73274123 (+), Divergence to consensus: 52%  
**10. MIR3**: 73275749-73275794 (-), Divergence to consensus: 28.2%  
**11. MIR3**: 73276847-73276890 (+), Divergence to consensus: 18.6%  
**12. CHRL1\_BT**: 73278395-73278480 (+), Divergence to consensus: 25.6%  
**13. L1M5**: 73279335-73279542 (+), Divergence to consensus: 47.1%  
**14. L1M5**: 73279829-73280004 (+), Divergence to consensus: 45.2%  
**15. L1M5**: 73280056-73280119 (+), Divergence to consensus: 23.5%  
**16. GC\_rich**: 73282987-73283007 (+), Divergence to consensus: 47.6%  
**17. MIRb**: 73284016-73284191 (-), Divergence to consensus: 32.9%  
**18. C-rich**: 73284194-73284334 (+), Divergence to consensus: 37.6%  
**19. Arthur1B**: 73284720-73284898 (+), Divergence to consensus: 48.8%  
**20. Arthur1B**: 73284939-73285109 (+), Divergence to consensus: 30.6%  
**21. MIRb**: 73285682-73285774 (-), Divergence to consensus: 24.5%

  
Transcription Factor Binding Sites  

**RFX4\_1** (Sequence: GTTGCCAAG (-): 73286266)  
**RFX4\_2** (Sequence: CCTGGATAC (+): 73275387)  
**SOX9** (Sequence: TTATTGTT (+): 73270756)  
**SPZ1** (Sequence: GGGGTAAGAG (+): 73272321)
