# Supplementary material for: Small RNA sequencing of cryopreserved semen from single bull revealed altered miRNAs and piRNAs expression between High- and Low-motile sperm populations
Source: BMC Genomics. 2017 Jan 4;18:14. doi: 10.1186/s12864-016-3394-7 (PMC5209821; doi:10.1186/s12864-016-3394-7)
Supplement: Additional file 4: — Details for each piRNA clusters found in Low Motile (LM) sperm fraction. Genes, repeats, transposable elements and transcription factors binding sites falling within the cluster regions were reported. (ZIP 1034 kb) [file 12864_2016_3394_MOESM4_ESM.zip › 21.html]

piRNA cluster 21


Predicted piRNA cluster no. 21     previous   next
  

Show proTRAC run info
Hide proTRAC run info

================================= proTRAC ====================================  
VERSION: 2.1                                    LAST MODIFIED: 06. October 2015  
  
Please cite:  
Rosenkranz D, Zischler H. proTRAC - a software for probabilistic piRNA cluster  
detection, visualization and analysis. 2012. BMC Bioinformatics 13:5.  
  
and (for proTRAC 2.0 and later):  
Rosenkranz D, Rudloff S, Bastuck K, Ketting RF, Zischler H. Tupaia small RNAs  
provide insights into function and evolution of RNAi-based transposon defense  
in mammals. 2015. RNA 21(5):911-922.  
  
Contact:  
David Rosenkranz  
Institute of Anthropology, small RNA group  
Johannes Gutenberg University Mainz  
email: rosenkranz@uni-mainz.de  
  
You can find the latest proTRAC version at:  
http://sourceforge.net/projects/protrac/files  
http://www.smallRNAgroup-mainz.de/software  
==============================================================================  
  
PARAMETERS:  
Map file: .............../storage/core/barbara/genhome/smallRNA/fertility/Sample\_not\_motile/pirna/Sample\_not\_motile\_26-33\_collapsed.fa.no-dust.map.weighted-10000-1000-b-0  
Genome file: ............/storage/core/barbara/genhome/smallRNA/fertility/Sample\_all/pirna/bt\_311\_chrY.fa  
RepeatMasker annotation: /storage/genomes/bt\_umd31/GCF\_000003055.6\_Bos\_taurus\_UMD\_3.1.1\_repeatMasker\_chr.out  
GeneSet:................./storage/core/barbara/genhome/smallRNA/fertility/Sample\_all/pirna/full.gtf  
  
Significant (p<=0.01) hit density will be calculated based  
on observed hit distribution.  
  
Sliding window size: ........................................ 5000 bp  
Sliding window increament: .................................. 1000 bp  
Normalize each hit by number of genomic hits: ............... 1 [0=no/1=yes]  
Normalize each hit by number of sequence reads: ............. 1 [0=no/1=yes]  
Normalize values (-> per million mapped reads): ............. 1 [0=no/1=yes]  
Min. fraction of hits with 1T(U) or 10A: .................... 0.75  
Alternatively: Min. fraction of hits with 1T(U) and 10A: .... 0.5  
Min. fraction of hits with typical piRNA length: ............ 0.75  
Typical piRNA length: ....................................... 26-33 nt  
Min. size of a piRNA cluster: ............................... 5000 bp.  
Min. number of hits (absolute): ............................. 0  
Min. number of hits (normalized): ........................... 0  
Min. fraction of hits on the mainstrand: .................... 0.75  
Top fraction of mapped sequences (in terms of read counts): . 1%  
Top fraction accounts for max. n% of sequence reads: ........ 90%  
Min. fraction of hits on each arm of a bidirectional cluster: 0.1  
Output image file for each cluster: ......................... 0 [0=no/1=yes]  
Output html file for each cluster: .......................... 1 [0=no/1=yes]  
Output a summary table: ..................................... 1 [0=no/1=yes]  
Output a FASTA file for each cluster (piRNA sequences): ..... 1 [0=no/1=yes]  
Output a FASTA file comprising cluster sequences: ........... 1 [0=no/1=yes]  
Search DNA motifs in clusters: .............................. 1 [0=no/1=yes]  
Output flanking sequences: +/- .............................. 0 bp  
Output ~.pTi file: .......................................... 1 [0=no/1=yes]  
==============================================================================  
  
  
Genome size (without gaps): ............ 2678902517 bp  
Gaps (N/X/-): .......................... 53837044 bp  
Mapped reads: .......................... 738059667487  
Non-identical sequences: ............... 277001  
Genomic hits: .......................... 533816  
Significant densitiy of mapped reads: .. 15118061 reads/kb

Show proTRAC cluster info
Hide proTRAC cluster info

|  |  |
| --- | --- |
| Location | chr17 |
| Coordinates | 73758093-73773204 |
| Size [bp] | 15112 |
| Sequence hit loci | 105 |
| Mapped reads (normalized) | 273711399 |
| Mapped reads (normalized) per kb | 18112188.9 |
| Normalized reads with 1T (1U) | 86.9% |
| Normalized reads with 10A | 26% |
| Normalized reads with length 26-33 nt | 100% |
| Normalized reads on the main strand(s) | 99.8% |
| Predicted directionality | mono:minus |

100%

0%

1T (1U)  
reads

10A reads

26-33 nt  
reads

reads on mainstrand

**Either the amount of reads with 1T (1U) OR 10A has to exceed 75% (set with option: -1Tor10A)  
Alternatively the amount of reads with 1T (1U) AND 10A has to exceed 50% (set with option: -1Tand10A)  
Minimum amount of reads with preferred size is 75% (set with option: -pisize)  
Minimum amount of reads on the main strand(s) is 75% (set with option: -clstrand)**

Show read coverage
Hide read coverage

WHAT DO I SEE HERE?  
This chart shows the location of mapped sequence reads within a predicted piRNA cluster. The color refers to the number of genomic hits produced by the sequence read in question. A dark red bar indicates that this sequence read produces many other hits elsewhere in the genome. Many adjacent red or yellow bars can indicate the presence of a multi-copy element such as transposons or rRNA genes. A dark green bar indicates that this sequence read maps uniquely to this locus.

1 hit

2-5 hits

6-10 hits

11-20 hits

21-50 hits

51-100 hits

> 100 hits

chr17

73758093

73773204

Gene Set

RepeatMasker

Mapped  
Reads

23.24

plus strand

minus strand

23.24

Region: chr17 73286798-73758108. Max. coverage (+): 0. Max coverage (-): 12.99

Region: chr17 73758109-73758138. Max. coverage (+): 0. Max coverage (-): 0

Region: chr17 73758139-73758168. Max. coverage (+): 0. Max coverage (-): 0

Region: chr17 73758169-73758198. Max. coverage (+): 0. Max coverage (-): 6.24

Region: chr17 73758199-73758229. Max. coverage (+): 0. Max coverage (-): 0

Region: chr17 73758230-73758259. Max. coverage (+): 0. Max coverage (-): 0

Region: chr17 73758260-73758289. Max. coverage (+): 0. Max coverage (-): 0

Region: chr17 73758290-73758319. Max. coverage (+): 0. Max coverage (-): 0

Region: chr17 73758320-73758349. Max. coverage (+): 0. Max coverage (-): 0

Region: chr17 73758350-73758380. Max. coverage (+): 0. Max coverage (-): 2.62

Region: chr17 73758381-73758410. Max. coverage (+): 0. Max coverage (-): 0

Region: chr17 73758411-73758440. Max. coverage (+): 0. Max coverage (-): 0

Region: chr17 73758441-73758470. Max. coverage (+): 0. Max coverage (-): 0

Region: chr17 73758471-73758501. Max. coverage (+): 0. Max coverage (-): 0

Region: chr17 73758502-73758531. Max. coverage (+): 0. Max coverage (-): 0

Region: chr17 73758532-73758561. Max. coverage (+): 0. Max coverage (-): 0

Region: chr17 73758562-73758591. Max. coverage (+): 0. Max coverage (-): 0

Region: chr17 73758592-73758621. Max. coverage (+): 0. Max coverage (-): 0

Region: chr17 73758622-73758652. Max. coverage (+): 0. Max coverage (-): 0

Region: chr17 73758653-73758682. Max. coverage (+): 0. Max coverage (-): 4.75

Region: chr17 73758683-73758712. Max. coverage (+): 0. Max coverage (-): 0

Region: chr17 73758713-73758742. Max. coverage (+): 0. Max coverage (-): 0

Region: chr17 73758743-73758773. Max. coverage (+): 0. Max coverage (-): 0

Region: chr17 73758774-73758803. Max. coverage (+): 0. Max coverage (-): 0

Region: chr17 73758804-73758833. Max. coverage (+): 0. Max coverage (-): 0

Region: chr17 73758834-73758863. Max. coverage (+): 0. Max coverage (-): 0

Region: chr17 73758864-73758893. Max. coverage (+): 0. Max coverage (-): 0

Region: chr17 73758894-73758924. Max. coverage (+): 0. Max coverage (-): 0

Region: chr17 73758925-73758954. Max. coverage (+): 0. Max coverage (-): 0

Region: chr17 73758955-73758984. Max. coverage (+): 0. Max coverage (-): 0

Region: chr17 73758985-73759014. Max. coverage (+): 0. Max coverage (-): 0

Region: chr17 73759015-73759045. Max. coverage (+): 0. Max coverage (-): 0

Region: chr17 73759046-73759075. Max. coverage (+): 0. Max coverage (-): 0

Region: chr17 73759076-73759105. Max. coverage (+): 0. Max coverage (-): 0

Region: chr17 73759106-73759135. Max. coverage (+): 0. Max coverage (-): 0

Region: chr17 73759136-73759165. Max. coverage (+): 0. Max coverage (-): 0

Region: chr17 73759166-73759196. Max. coverage (+): 0. Max coverage (-): 0

Region: chr17 73759197-73759226. Max. coverage (+): 0. Max coverage (-): 0

Region: chr17 73759227-73759256. Max. coverage (+): 0. Max coverage (-): 0

Region: chr17 73759257-73759286. Max. coverage (+): 0. Max coverage (-): 0

Region: chr17 73759287-73759317. Max. coverage (+): 0. Max coverage (-): 0

Region: chr17 73759318-73759347. Max. coverage (+): 0. Max coverage (-): 0

Region: chr17 73759348-73759377. Max. coverage (+): 0. Max coverage (-): 0

Region: chr17 73759378-73759407. Max. coverage (+): 0. Max coverage (-): 0

Region: chr17 73759408-73759437. Max. coverage (+): 0. Max coverage (-): 0

Region: chr17 73759438-73759468. Max. coverage (+): 0. Max coverage (-): 0

Region: chr17 73759469-73759498. Max. coverage (+): 0. Max coverage (-): 0

Region: chr17 73759499-73759528. Max. coverage (+): 0. Max coverage (-): 0

Region: chr17 73759529-73759558. Max. coverage (+): 0. Max coverage (-): 0

Region: chr17 73759559-73759589. Max. coverage (+): 0. Max coverage (-): 0

Region: chr17 73759590-73759619. Max. coverage (+): 0. Max coverage (-): 0

Region: chr17 73759620-73759649. Max. coverage (+): 0. Max coverage (-): 0

Region: chr17 73759650-73759679. Max. coverage (+): 0. Max coverage (-): 0

Region: chr17 73759680-73759709. Max. coverage (+): 0. Max coverage (-): 4.73

Region: chr17 73759710-73759740. Max. coverage (+): 0. Max coverage (-): 0

Region: chr17 73759741-73759770. Max. coverage (+): 0. Max coverage (-): 0

Region: chr17 73759771-73759800. Max. coverage (+): 0. Max coverage (-): 0

Region: chr17 73759801-73759830. Max. coverage (+): 0. Max coverage (-): 0

Region: chr17 73759831-73759861. Max. coverage (+): 0. Max coverage (-): 0

Region: chr17 73759862-73759891. Max. coverage (+): 0. Max coverage (-): 0

Region: chr17 73759892-73759921. Max. coverage (+): 0. Max coverage (-): 0

Region: chr17 73759922-73759951. Max. coverage (+): 0. Max coverage (-): 0

Region: chr17 73759952-73759981. Max. coverage (+): 0. Max coverage (-): 0

Region: chr17 73759982-73760012. Max. coverage (+): 0. Max coverage (-): 0

Region: chr17 73760013-73760042. Max. coverage (+): 0. Max coverage (-): 0

Region: chr17 73760043-73760072. Max. coverage (+): 0. Max coverage (-): 0

Region: chr17 73760073-73760102. Max. coverage (+): 0. Max coverage (-): 0

Region: chr17 73760103-73760133. Max. coverage (+): 0. Max coverage (-): 0

Region: chr17 73760134-73760163. Max. coverage (+): 0. Max coverage (-): 0

Region: chr17 73760164-73760193. Max. coverage (+): 0. Max coverage (-): 0

Region: chr17 73760194-73760223. Max. coverage (+): 0. Max coverage (-): 0

Region: chr17 73760224-73760254. Max. coverage (+): 0. Max coverage (-): 0

Region: chr17 73760255-73760284. Max. coverage (+): 0. Max coverage (-): 0

Region: chr17 73760285-73760314. Max. coverage (+): 0. Max coverage (-): 0

Region: chr17 73760315-73760344. Max. coverage (+): 0. Max coverage (-): 0

Region: chr17 73760345-73760374. Max. coverage (+): 0. Max coverage (-): 0

Region: chr17 73760375-73760405. Max. coverage (+): 0. Max coverage (-): 0

Region: chr17 73760406-73760435. Max. coverage (+): 0. Max coverage (-): 0

Region: chr17 73760436-73760465. Max. coverage (+): 0. Max coverage (-): 0

Region: chr17 73760466-73760495. Max. coverage (+): 0. Max coverage (-): 0

Region: chr17 73760496-73760526. Max. coverage (+): 0. Max coverage (-): 0

Region: chr17 73760527-73760556. Max. coverage (+): 0. Max coverage (-): 0

Region: chr17 73760557-73760586. Max. coverage (+): 0. Max coverage (-): 0

Region: chr17 73760587-73760616. Max. coverage (+): 0. Max coverage (-): 0

Region: chr17 73760617-73760646. Max. coverage (+): 0. Max coverage (-): 0

Region: chr17 73760647-73760677. Max. coverage (+): 0. Max coverage (-): 0

Region: chr17 73760678-73760707. Max. coverage (+): 0. Max coverage (-): 0

Region: chr17 73760708-73760737. Max. coverage (+): 0. Max coverage (-): 0

Region: chr17 73760738-73760767. Max. coverage (+): 0. Max coverage (-): 0

Region: chr17 73760768-73760798. Max. coverage (+): 0. Max coverage (-): 0

Region: chr17 73760799-73760828. Max. coverage (+): 0. Max coverage (-): 0

Region: chr17 73760829-73760858. Max. coverage (+): 0. Max coverage (-): 0

Region: chr17 73760859-73760888. Max. coverage (+): 0. Max coverage (-): 0

Region: chr17 73760889-73760918. Max. coverage (+): 0. Max coverage (-): 10.37

Region: chr17 73760919-73760949. Max. coverage (+): 0. Max coverage (-): 1.44

Region: chr17 73760950-73760979. Max. coverage (+): 0. Max coverage (-): 0

Region: chr17 73760980-73761009. Max. coverage (+): 0. Max coverage (-): 0

Region: chr17 73761010-73761039. Max. coverage (+): 0. Max coverage (-): 0

Region: chr17 73761040-73761070. Max. coverage (+): 0. Max coverage (-): 0

Region: chr17 73761071-73761100. Max. coverage (+): 0. Max coverage (-): 0

Region: chr17 73761101-73761130. Max. coverage (+): 0. Max coverage (-): 0

Region: chr17 73761131-73761160. Max. coverage (+): 0. Max coverage (-): 0

Region: chr17 73761161-73761190. Max. coverage (+): 0. Max coverage (-): 0

Region: chr17 73761191-73761221. Max. coverage (+): 0. Max coverage (-): 8.57

Region: chr17 73761222-73761251. Max. coverage (+): 0. Max coverage (-): 0

Region: chr17 73761252-73761281. Max. coverage (+): 0. Max coverage (-): 5.29

Region: chr17 73761282-73761311. Max. coverage (+): 0. Max coverage (-): 0

Region: chr17 73761312-73761342. Max. coverage (+): 0. Max coverage (-): 0

Region: chr17 73761343-73761372. Max. coverage (+): 0. Max coverage (-): 0

Region: chr17 73761373-73761402. Max. coverage (+): 0. Max coverage (-): 0

Region: chr17 73761403-73761432. Max. coverage (+): 0. Max coverage (-): 0

Region: chr17 73761433-73761462. Max. coverage (+): 0. Max coverage (-): 0

Region: chr17 73761463-73761493. Max. coverage (+): 0. Max coverage (-): 0

Region: chr17 73761494-73761523. Max. coverage (+): 0. Max coverage (-): 6.08

Region: chr17 73761524-73761553. Max. coverage (+): 0. Max coverage (-): 0

Region: chr17 73761554-73761583. Max. coverage (+): 0. Max coverage (-): 16.12

Region: chr17 73761584-73761614. Max. coverage (+): 0. Max coverage (-): 4.59

Region: chr17 73761615-73761644. Max. coverage (+): 0. Max coverage (-): 1.56

Region: chr17 73761645-73761674. Max. coverage (+): 0. Max coverage (-): 0

Region: chr17 73761675-73761704. Max. coverage (+): 0. Max coverage (-): 0

Region: chr17 73761705-73761734. Max. coverage (+): 0. Max coverage (-): 0

Region: chr17 73761735-73761765. Max. coverage (+): 0. Max coverage (-): 0

Region: chr17 73761766-73761795. Max. coverage (+): 0. Max coverage (-): 0

Region: chr17 73761796-73761825. Max. coverage (+): 0. Max coverage (-): 0

Region: chr17 73761826-73761855. Max. coverage (+): 0. Max coverage (-): 0.61

Region: chr17 73761856-73761886. Max. coverage (+): 0. Max coverage (-): 0

Region: chr17 73761887-73761916. Max. coverage (+): 0. Max coverage (-): 0

Region: chr17 73761917-73761946. Max. coverage (+): 0. Max coverage (-): 0

Region: chr17 73761947-73761976. Max. coverage (+): 0. Max coverage (-): 0

Region: chr17 73761977-73762007. Max. coverage (+): 0. Max coverage (-): 0

Region: chr17 73762008-73762037. Max. coverage (+): 0. Max coverage (-): 0

Region: chr17 73762038-73762067. Max. coverage (+): 0. Max coverage (-): 0

Region: chr17 73762068-73762097. Max. coverage (+): 0. Max coverage (-): 0

Region: chr17 73762098-73762127. Max. coverage (+): 0. Max coverage (-): 0

Region: chr17 73762128-73762158. Max. coverage (+): 0. Max coverage (-): 9.57

Region: chr17 73762159-73762188. Max. coverage (+): 0. Max coverage (-): 1.2

Region: chr17 73762189-73762218. Max. coverage (+): 0. Max coverage (-): 0

Region: chr17 73762219-73762248. Max. coverage (+): 0. Max coverage (-): 0

Region: chr17 73762249-73762279. Max. coverage (+): 0. Max coverage (-): 0

Region: chr17 73762280-73762309. Max. coverage (+): 0. Max coverage (-): 0

Region: chr17 73762310-73762339. Max. coverage (+): 0. Max coverage (-): 0

Region: chr17 73762340-73762369. Max. coverage (+): 0. Max coverage (-): 0

Region: chr17 73762370-73762399. Max. coverage (+): 0. Max coverage (-): 0

Region: chr17 73762400-73762430. Max. coverage (+): 0. Max coverage (-): 0

Region: chr17 73762431-73762460. Max. coverage (+): 0. Max coverage (-): 0

Region: chr17 73762461-73762490. Max. coverage (+): 0. Max coverage (-): 0

Region: chr17 73762491-73762520. Max. coverage (+): 0. Max coverage (-): 0

Region: chr17 73762521-73762551. Max. coverage (+): 0. Max coverage (-): 0

Region: chr17 73762552-73762581. Max. coverage (+): 0. Max coverage (-): 0

Region: chr17 73762582-73762611. Max. coverage (+): 0. Max coverage (-): 0

Region: chr17 73762612-73762641. Max. coverage (+): 0. Max coverage (-): 0

Region: chr17 73762642-73762671. Max. coverage (+): 0. Max coverage (-): 0

Region: chr17 73762672-73762702. Max. coverage (+): 0. Max coverage (-): 0

Region: chr17 73762703-73762732. Max. coverage (+): 0. Max coverage (-): 0

Region: chr17 73762733-73762762. Max. coverage (+): 0. Max coverage (-): 5.7

Region: chr17 73762763-73762792. Max. coverage (+): 0. Max coverage (-): 13.59

Region: chr17 73762793-73762823. Max. coverage (+): 0. Max coverage (-): 16.97

Region: chr17 73762824-73762853. Max. coverage (+): 0. Max coverage (-): 0

Region: chr17 73762854-73762883. Max. coverage (+): 0. Max coverage (-): 0

Region: chr17 73762884-73762913. Max. coverage (+): 0. Max coverage (-): 0

Region: chr17 73762914-73762943. Max. coverage (+): 0. Max coverage (-): 0

Region: chr17 73762944-73762974. Max. coverage (+): 0. Max coverage (-): 0

Region: chr17 73762975-73763004. Max. coverage (+): 0. Max coverage (-): 0

Region: chr17 73763005-73763034. Max. coverage (+): 0. Max coverage (-): 0

Region: chr17 73763035-73763064. Max. coverage (+): 0. Max coverage (-): 0

Region: chr17 73763065-73763095. Max. coverage (+): 0. Max coverage (-): 0

Region: chr17 73763096-73763125. Max. coverage (+): 0. Max coverage (-): 0

Region: chr17 73763126-73763155. Max. coverage (+): 0. Max coverage (-): 0

Region: chr17 73763156-73763185. Max. coverage (+): 0. Max coverage (-): 0

Region: chr17 73763186-73763215. Max. coverage (+): 0. Max coverage (-): 10.38

Region: chr17 73763216-73763246. Max. coverage (+): 0. Max coverage (-): 10.38

Region: chr17 73763247-73763276. Max. coverage (+): 0. Max coverage (-): 20.52

Region: chr17 73763277-73763306. Max. coverage (+): 0. Max coverage (-): 0

Region: chr17 73763307-73763336. Max. coverage (+): 0. Max coverage (-): 0

Region: chr17 73763337-73763367. Max. coverage (+): 0. Max coverage (-): 0

Region: chr17 73763368-73763397. Max. coverage (+): 0. Max coverage (-): 0

Region: chr17 73763398-73763427. Max. coverage (+): 0. Max coverage (-): 0

Region: chr17 73763428-73763457. Max. coverage (+): 0. Max coverage (-): 0

Region: chr17 73763458-73763487. Max. coverage (+): 0. Max coverage (-): 0

Region: chr17 73763488-73763518. Max. coverage (+): 0. Max coverage (-): 0

Region: chr17 73763519-73763548. Max. coverage (+): 0. Max coverage (-): 0

Region: chr17 73763549-73763578. Max. coverage (+): 0. Max coverage (-): 0

Region: chr17 73763579-73763608. Max. coverage (+): 0. Max coverage (-): 0

Region: chr17 73763609-73763639. Max. coverage (+): 0. Max coverage (-): 0

Region: chr17 73763640-73763669. Max. coverage (+): 0. Max coverage (-): 0

Region: chr17 73763670-73763699. Max. coverage (+): 0. Max coverage (-): 0

Region: chr17 73763700-73763729. Max. coverage (+): 0. Max coverage (-): 0

Region: chr17 73763730-73763759. Max. coverage (+): 0. Max coverage (-): 0

Region: chr17 73763760-73763790. Max. coverage (+): 0. Max coverage (-): 0

Region: chr17 73763791-73763820. Max. coverage (+): 0. Max coverage (-): 0

Region: chr17 73763821-73763850. Max. coverage (+): 0. Max coverage (-): 0

Region: chr17 73763851-73763880. Max. coverage (+): 0. Max coverage (-): 0

Region: chr17 73763881-73763911. Max. coverage (+): 0. Max coverage (-): 0

Region: chr17 73763912-73763941. Max. coverage (+): 0. Max coverage (-): 0

Region: chr17 73763942-73763971. Max. coverage (+): 0. Max coverage (-): 0

Region: chr17 73763972-73764001. Max. coverage (+): 0. Max coverage (-): 0

Region: chr17 73764002-73764032. Max. coverage (+): 0. Max coverage (-): 0

Region: chr17 73764033-73764062. Max. coverage (+): 0. Max coverage (-): 0

Region: chr17 73764063-73764092. Max. coverage (+): 0. Max coverage (-): 0

Region: chr17 73764093-73764122. Max. coverage (+): 0. Max coverage (-): 0

Region: chr17 73764123-73764152. Max. coverage (+): 0. Max coverage (-): 0

Region: chr17 73764153-73764183. Max. coverage (+): 0. Max coverage (-): 0

Region: chr17 73764184-73764213. Max. coverage (+): 0. Max coverage (-): 0

Region: chr17 73764214-73764243. Max. coverage (+): 0. Max coverage (-): 0

Region: chr17 73764244-73764273. Max. coverage (+): 0. Max coverage (-): 0.86

Region: chr17 73764274-73764304. Max. coverage (+): 0. Max coverage (-): 0

Region: chr17 73764305-73764334. Max. coverage (+): 0. Max coverage (-): 0

Region: chr17 73764335-73764364. Max. coverage (+): 0. Max coverage (-): 0

Region: chr17 73764365-73764394. Max. coverage (+): 0. Max coverage (-): 0

Region: chr17 73764395-73764424. Max. coverage (+): 0. Max coverage (-): 0

Region: chr17 73764425-73764455. Max. coverage (+): 0. Max coverage (-): 1.27

Region: chr17 73764456-73764485. Max. coverage (+): 0. Max coverage (-): 0

Region: chr17 73764486-73764515. Max. coverage (+): 0. Max coverage (-): 0

Region: chr17 73764516-73764545. Max. coverage (+): 0. Max coverage (-): 0

Region: chr17 73764546-73764576. Max. coverage (+): 0. Max coverage (-): 0

Region: chr17 73764577-73764606. Max. coverage (+): 0. Max coverage (-): 0

Region: chr17 73764607-73764636. Max. coverage (+): 0. Max coverage (-): 0

Region: chr17 73764637-73764666. Max. coverage (+): 0. Max coverage (-): 0

Region: chr17 73764667-73764696. Max. coverage (+): 0. Max coverage (-): 0

Region: chr17 73764697-73764727. Max. coverage (+): 0. Max coverage (-): 0

Region: chr17 73764728-73764757. Max. coverage (+): 0. Max coverage (-): 0

Region: chr17 73764758-73764787. Max. coverage (+): 0. Max coverage (-): 0

Region: chr17 73764788-73764817. Max. coverage (+): 0. Max coverage (-): 0

Region: chr17 73764818-73764848. Max. coverage (+): 0. Max coverage (-): 0

Region: chr17 73764849-73764878. Max. coverage (+): 0. Max coverage (-): 0

Region: chr17 73764879-73764908. Max. coverage (+): 0. Max coverage (-): 0

Region: chr17 73764909-73764938. Max. coverage (+): 0. Max coverage (-): 0

Region: chr17 73764939-73764968. Max. coverage (+): 0. Max coverage (-): 0

Region: chr17 73764969-73764999. Max. coverage (+): 0. Max coverage (-): 0

Region: chr17 73765000-73765029. Max. coverage (+): 0. Max coverage (-): 0

Region: chr17 73765030-73765059. Max. coverage (+): 0. Max coverage (-): 0

Region: chr17 73765060-73765089. Max. coverage (+): 0. Max coverage (-): 0.2

Region: chr17 73765090-73765120. Max. coverage (+): 0. Max coverage (-): 0

Region: chr17 73765121-73765150. Max. coverage (+): 0. Max coverage (-): 0

Region: chr17 73765151-73765180. Max. coverage (+): 0. Max coverage (-): 0

Region: chr17 73765181-73765210. Max. coverage (+): 0. Max coverage (-): 0

Region: chr17 73765211-73765240. Max. coverage (+): 0. Max coverage (-): 0

Region: chr17 73765241-73765271. Max. coverage (+): 0. Max coverage (-): 0

Region: chr17 73765272-73765301. Max. coverage (+): 0. Max coverage (-): 0

Region: chr17 73765302-73765331. Max. coverage (+): 0. Max coverage (-): 0

Region: chr17 73765332-73765361. Max. coverage (+): 0. Max coverage (-): 0

Region: chr17 73765362-73765392. Max. coverage (+): 0. Max coverage (-): 0

Region: chr17 73765393-73765422. Max. coverage (+): 0. Max coverage (-): 0

Region: chr17 73765423-73765452. Max. coverage (+): 0. Max coverage (-): 0

Region: chr17 73765453-73765482. Max. coverage (+): 0. Max coverage (-): 0

Region: chr17 73765483-73765512. Max. coverage (+): 0. Max coverage (-): 0

Region: chr17 73765513-73765543. Max. coverage (+): 0. Max coverage (-): 0

Region: chr17 73765544-73765573. Max. coverage (+): 0. Max coverage (-): 6.23

Region: chr17 73765574-73765603. Max. coverage (+): 0. Max coverage (-): 0

Region: chr17 73765604-73765633. Max. coverage (+): 0. Max coverage (-): 0

Region: chr17 73765634-73765664. Max. coverage (+): 0. Max coverage (-): 0

Region: chr17 73765665-73765694. Max. coverage (+): 0. Max coverage (-): 0

Region: chr17 73765695-73765724. Max. coverage (+): 0. Max coverage (-): 0

Region: chr17 73765725-73765754. Max. coverage (+): 0. Max coverage (-): 0

Region: chr17 73765755-73765785. Max. coverage (+): 0. Max coverage (-): 0

Region: chr17 73765786-73765815. Max. coverage (+): 0. Max coverage (-): 4.48

Region: chr17 73765816-73765845. Max. coverage (+): 0. Max coverage (-): 4.48

Region: chr17 73765846-73765875. Max. coverage (+): 0. Max coverage (-): 0

Region: chr17 73765876-73765905. Max. coverage (+): 0. Max coverage (-): 7.06

Region: chr17 73765906-73765936. Max. coverage (+): 0. Max coverage (-): 1.03

Region: chr17 73765937-73765966. Max. coverage (+): 0. Max coverage (-): 2

Region: chr17 73765967-73765996. Max. coverage (+): 0. Max coverage (-): 0

Region: chr17 73765997-73766026. Max. coverage (+): 0. Max coverage (-): 6.59

Region: chr17 73766027-73766057. Max. coverage (+): 0. Max coverage (-): 6.87

Region: chr17 73766058-73766087. Max. coverage (+): 0. Max coverage (-): 1.82

Region: chr17 73766088-73766117. Max. coverage (+): 0. Max coverage (-): 9.21

Region: chr17 73766118-73766147. Max. coverage (+): 0. Max coverage (-): 0

Region: chr17 73766148-73766177. Max. coverage (+): 0. Max coverage (-): 0

Region: chr17 73766178-73766208. Max. coverage (+): 0. Max coverage (-): 0

Region: chr17 73766209-73766238. Max. coverage (+): 0. Max coverage (-): 0

Region: chr17 73766239-73766268. Max. coverage (+): 0. Max coverage (-): 0

Region: chr17 73766269-73766298. Max. coverage (+): 0. Max coverage (-): 0

Region: chr17 73766299-73766329. Max. coverage (+): 0. Max coverage (-): 0

Region: chr17 73766330-73766359. Max. coverage (+): 0. Max coverage (-): 0

Region: chr17 73766360-73766389. Max. coverage (+): 0. Max coverage (-): 0

Region: chr17 73766390-73766419. Max. coverage (+): 0. Max coverage (-): 0

Region: chr17 73766420-73766449. Max. coverage (+): 0. Max coverage (-): 0

Region: chr17 73766450-73766480. Max. coverage (+): 0. Max coverage (-): 0

Region: chr17 73766481-73766510. Max. coverage (+): 0. Max coverage (-): 0

Region: chr17 73766511-73766540. Max. coverage (+): 0. Max coverage (-): 0

Region: chr17 73766541-73766570. Max. coverage (+): 0. Max coverage (-): 0

Region: chr17 73766571-73766601. Max. coverage (+): 0. Max coverage (-): 0

Region: chr17 73766602-73766631. Max. coverage (+): 0. Max coverage (-): 0

Region: chr17 73766632-73766661. Max. coverage (+): 0. Max coverage (-): 0

Region: chr17 73766662-73766691. Max. coverage (+): 0. Max coverage (-): 0

Region: chr17 73766692-73766721. Max. coverage (+): 0. Max coverage (-): 0

Region: chr17 73766722-73766752. Max. coverage (+): 0. Max coverage (-): 0

Region: chr17 73766753-73766782. Max. coverage (+): 0. Max coverage (-): 0

Region: chr17 73766783-73766812. Max. coverage (+): 0. Max coverage (-): 0

Region: chr17 73766813-73766842. Max. coverage (+): 0. Max coverage (-): 0

Region: chr17 73766843-73766873. Max. coverage (+): 0. Max coverage (-): 0

Region: chr17 73766874-73766903. Max. coverage (+): 0. Max coverage (-): 0

Region: chr17 73766904-73766933. Max. coverage (+): 0. Max coverage (-): 0

Region: chr17 73766934-73766963. Max. coverage (+): 0. Max coverage (-): 0

Region: chr17 73766964-73766993. Max. coverage (+): 0. Max coverage (-): 0

Region: chr17 73766994-73767024. Max. coverage (+): 0. Max coverage (-): 0

Region: chr17 73767025-73767054. Max. coverage (+): 0. Max coverage (-): 0

Region: chr17 73767055-73767084. Max. coverage (+): 0. Max coverage (-): 0

Region: chr17 73767085-73767114. Max. coverage (+): 0. Max coverage (-): 0

Region: chr17 73767115-73767145. Max. coverage (+): 0. Max coverage (-): 0

Region: chr17 73767146-73767175. Max. coverage (+): 0. Max coverage (-): 0

Region: chr17 73767176-73767205. Max. coverage (+): 0. Max coverage (-): 0

Region: chr17 73767206-73767235. Max. coverage (+): 0. Max coverage (-): 0

Region: chr17 73767236-73767265. Max. coverage (+): 0. Max coverage (-): 0

Region: chr17 73767266-73767296. Max. coverage (+): 0. Max coverage (-): 0

Region: chr17 73767297-73767326. Max. coverage (+): 0. Max coverage (-): 0

Region: chr17 73767327-73767356. Max. coverage (+): 0. Max coverage (-): 0

Region: chr17 73767357-73767386. Max. coverage (+): 0. Max coverage (-): 0

Region: chr17 73767387-73767417. Max. coverage (+): 0. Max coverage (-): 0

Region: chr17 73767418-73767447. Max. coverage (+): 0. Max coverage (-): 0

Region: chr17 73767448-73767477. Max. coverage (+): 0. Max coverage (-): 0

Region: chr17 73767478-73767507. Max. coverage (+): 0. Max coverage (-): 0

Region: chr17 73767508-73767537. Max. coverage (+): 0. Max coverage (-): 0

Region: chr17 73767538-73767568. Max. coverage (+): 0. Max coverage (-): 0

Region: chr17 73767569-73767598. Max. coverage (+): 0. Max coverage (-): 0

Region: chr17 73767599-73767628. Max. coverage (+): 0. Max coverage (-): 2.87

Region: chr17 73767629-73767658. Max. coverage (+): 0. Max coverage (-): 0

Region: chr17 73767659-73767689. Max. coverage (+): 0. Max coverage (-): 9.68

Region: chr17 73767690-73767719. Max. coverage (+): 0. Max coverage (-): 0

Region: chr17 73767720-73767749. Max. coverage (+): 0. Max coverage (-): 0

Region: chr17 73767750-73767779. Max. coverage (+): 0. Max coverage (-): 0

Region: chr17 73767780-73767810. Max. coverage (+): 0. Max coverage (-): 0

Region: chr17 73767811-73767840. Max. coverage (+): 0. Max coverage (-): 0

Region: chr17 73767841-73767870. Max. coverage (+): 0. Max coverage (-): 0

Region: chr17 73767871-73767900. Max. coverage (+): 0. Max coverage (-): 0

Region: chr17 73767901-73767930. Max. coverage (+): 0. Max coverage (-): 0

Region: chr17 73767931-73767961. Max. coverage (+): 0. Max coverage (-): 0

Region: chr17 73767962-73767991. Max. coverage (+): 0. Max coverage (-): 0

Region: chr17 73767992-73768021. Max. coverage (+): 0. Max coverage (-): 0

Region: chr17 73768022-73768051. Max. coverage (+): 0. Max coverage (-): 2.4

Region: chr17 73768052-73768082. Max. coverage (+): 0. Max coverage (-): 0

Region: chr17 73768083-73768112. Max. coverage (+): 0. Max coverage (-): 0

Region: chr17 73768113-73768142. Max. coverage (+): 0. Max coverage (-): 3.43

Region: chr17 73768143-73768172. Max. coverage (+): 0. Max coverage (-): 0

Region: chr17 73768173-73768202. Max. coverage (+): 0. Max coverage (-): 0

Region: chr17 73768203-73768233. Max. coverage (+): 0. Max coverage (-): 0

Region: chr17 73768234-73768263. Max. coverage (+): 0. Max coverage (-): 0

Region: chr17 73768264-73768293. Max. coverage (+): 0. Max coverage (-): 0

Region: chr17 73768294-73768323. Max. coverage (+): 0. Max coverage (-): 0

Region: chr17 73768324-73768354. Max. coverage (+): 0. Max coverage (-): 0

Region: chr17 73768355-73768384. Max. coverage (+): 0. Max coverage (-): 0

Region: chr17 73768385-73768414. Max. coverage (+): 0. Max coverage (-): 0

Region: chr17 73768415-73768444. Max. coverage (+): 0. Max coverage (-): 0

Region: chr17 73768445-73768474. Max. coverage (+): 0. Max coverage (-): 0

Region: chr17 73768475-73768505. Max. coverage (+): 0. Max coverage (-): 0

Region: chr17 73768506-73768535. Max. coverage (+): 0. Max coverage (-): 0

Region: chr17 73768536-73768565. Max. coverage (+): 0. Max coverage (-): 0

Region: chr17 73768566-73768595. Max. coverage (+): 0. Max coverage (-): 0

Region: chr17 73768596-73768626. Max. coverage (+): 0. Max coverage (-): 0

Region: chr17 73768627-73768656. Max. coverage (+): 0. Max coverage (-): 0

Region: chr17 73768657-73768686. Max. coverage (+): 0. Max coverage (-): 0

Region: chr17 73768687-73768716. Max. coverage (+): 0. Max coverage (-): 5.77

Region: chr17 73768717-73768746. Max. coverage (+): 0. Max coverage (-): 5.77

Region: chr17 73768747-73768777. Max. coverage (+): 0. Max coverage (-): 7.23

Region: chr17 73768778-73768807. Max. coverage (+): 0. Max coverage (-): 0

Region: chr17 73768808-73768837. Max. coverage (+): 0. Max coverage (-): 0

Region: chr17 73768838-73768867. Max. coverage (+): 0. Max coverage (-): 0

Region: chr17 73768868-73768898. Max. coverage (+): 0. Max coverage (-): 0

Region: chr17 73768899-73768928. Max. coverage (+): 0. Max coverage (-): 0

Region: chr17 73768929-73768958. Max. coverage (+): 0. Max coverage (-): 0

Region: chr17 73768959-73768988. Max. coverage (+): 0. Max coverage (-): 0

Region: chr17 73768989-73769018. Max. coverage (+): 0. Max coverage (-): 0

Region: chr17 73769019-73769049. Max. coverage (+): 0. Max coverage (-): 0

Region: chr17 73769050-73769079. Max. coverage (+): 0. Max coverage (-): 0

Region: chr17 73769080-73769109. Max. coverage (+): 0. Max coverage (-): 0

Region: chr17 73769110-73769139. Max. coverage (+): 0. Max coverage (-): 0

Region: chr17 73769140-73769170. Max. coverage (+): 0. Max coverage (-): 0

Region: chr17 73769171-73769200. Max. coverage (+): 0.61. Max coverage (-): 6.23

Region: chr17 73769201-73769230. Max. coverage (+): 0. Max coverage (-): 0

Region: chr17 73769231-73769260. Max. coverage (+): 0. Max coverage (-): 0

Region: chr17 73769261-73769290. Max. coverage (+): 0. Max coverage (-): 0

Region: chr17 73769291-73769321. Max. coverage (+): 0. Max coverage (-): 0

Region: chr17 73769322-73769351. Max. coverage (+): 0. Max coverage (-): 0

Region: chr17 73769352-73769381. Max. coverage (+): 0. Max coverage (-): 0

Region: chr17 73769382-73769411. Max. coverage (+): 0. Max coverage (-): 0

Region: chr17 73769412-73769442. Max. coverage (+): 0. Max coverage (-): 0

Region: chr17 73769443-73769472. Max. coverage (+): 0. Max coverage (-): 0

Region: chr17 73769473-73769502. Max. coverage (+): 0. Max coverage (-): 0

Region: chr17 73769503-73769532. Max. coverage (+): 0. Max coverage (-): 0

Region: chr17 73769533-73769563. Max. coverage (+): 0. Max coverage (-): 0

Region: chr17 73769564-73769593. Max. coverage (+): 0. Max coverage (-): 0

Region: chr17 73769594-73769623. Max. coverage (+): 0. Max coverage (-): 0

Region: chr17 73769624-73769653. Max. coverage (+): 0. Max coverage (-): 0

Region: chr17 73769654-73769683. Max. coverage (+): 0. Max coverage (-): 23.24

Region: chr17 73769684-73769714. Max. coverage (+): 0. Max coverage (-): 0

Region: chr17 73769715-73769744. Max. coverage (+): 0. Max coverage (-): 0

Region: chr17 73769745-73769774. Max. coverage (+): 0. Max coverage (-): 0

Region: chr17 73769775-73769804. Max. coverage (+): 0. Max coverage (-): 3.33

Region: chr17 73769805-73769835. Max. coverage (+): 0. Max coverage (-): 0.82

Region: chr17 73769836-73769865. Max. coverage (+): 0. Max coverage (-): 0

Region: chr17 73769866-73769895. Max. coverage (+): 0. Max coverage (-): 0

Region: chr17 73769896-73769925. Max. coverage (+): 0. Max coverage (-): 0

Region: chr17 73769926-73769955. Max. coverage (+): 0. Max coverage (-): 0

Region: chr17 73769956-73769986. Max. coverage (+): 0. Max coverage (-): 0

Region: chr17 73769987-73770016. Max. coverage (+): 0. Max coverage (-): 0

Region: chr17 73770017-73770046. Max. coverage (+): 0. Max coverage (-): 0

Region: chr17 73770047-73770076. Max. coverage (+): 0. Max coverage (-): 0

Region: chr17 73770077-73770107. Max. coverage (+): 0. Max coverage (-): 0

Region: chr17 73770108-73770137. Max. coverage (+): 0. Max coverage (-): 10.15

Region: chr17 73770138-73770167. Max. coverage (+): 0. Max coverage (-): 0

Region: chr17 73770168-73770197. Max. coverage (+): 0. Max coverage (-): 0

Region: chr17 73770198-73770227. Max. coverage (+): 0. Max coverage (-): 0

Region: chr17 73770228-73770258. Max. coverage (+): 0. Max coverage (-): 0

Region: chr17 73770259-73770288. Max. coverage (+): 0. Max coverage (-): 0

Region: chr17 73770289-73770318. Max. coverage (+): 0. Max coverage (-): 0

Region: chr17 73770319-73770348. Max. coverage (+): 0. Max coverage (-): 0

Region: chr17 73770349-73770379. Max. coverage (+): 0. Max coverage (-): 0

Region: chr17 73770380-73770409. Max. coverage (+): 0. Max coverage (-): 0

Region: chr17 73770410-73770439. Max. coverage (+): 0. Max coverage (-): 0

Region: chr17 73770440-73770469. Max. coverage (+): 0. Max coverage (-): 0

Region: chr17 73770470-73770499. Max. coverage (+): 0. Max coverage (-): 0

Region: chr17 73770500-73770530. Max. coverage (+): 0. Max coverage (-): 0

Region: chr17 73770531-73770560. Max. coverage (+): 0. Max coverage (-): 0

Region: chr17 73770561-73770590. Max. coverage (+): 0. Max coverage (-): 0

Region: chr17 73770591-73770620. Max. coverage (+): 0. Max coverage (-): 0

Region: chr17 73770621-73770651. Max. coverage (+): 0. Max coverage (-): 0

Region: chr17 73770652-73770681. Max. coverage (+): 0. Max coverage (-): 0

Region: chr17 73770682-73770711. Max. coverage (+): 0. Max coverage (-): 0

Region: chr17 73770712-73770741. Max. coverage (+): 0. Max coverage (-): 0

Region: chr17 73770742-73770771. Max. coverage (+): 0. Max coverage (-): 0

Region: chr17 73770772-73770802. Max. coverage (+): 0. Max coverage (-): 0

Region: chr17 73770803-73770832. Max. coverage (+): 0. Max coverage (-): 0

Region: chr17 73770833-73770862. Max. coverage (+): 0. Max coverage (-): 12.88

Region: chr17 73770863-73770892. Max. coverage (+): 0. Max coverage (-): 0

Region: chr17 73770893-73770923. Max. coverage (+): 0. Max coverage (-): 0

Region: chr17 73770924-73770953. Max. coverage (+): 0. Max coverage (-): 0

Region: chr17 73770954-73770983. Max. coverage (+): 0. Max coverage (-): 0

Region: chr17 73770984-73771013. Max. coverage (+): 0. Max coverage (-): 0

Region: chr17 73771014-73771043. Max. coverage (+): 0. Max coverage (-): 0

Region: chr17 73771044-73771074. Max. coverage (+): 0. Max coverage (-): 0

Region: chr17 73771075-73771104. Max. coverage (+): 0. Max coverage (-): 0

Region: chr17 73771105-73771134. Max. coverage (+): 0. Max coverage (-): 0

Region: chr17 73771135-73771164. Max. coverage (+): 0. Max coverage (-): 0

Region: chr17 73771165-73771195. Max. coverage (+): 0. Max coverage (-): 0

Region: chr17 73771196-73771225. Max. coverage (+): 0. Max coverage (-): 0

Region: chr17 73771226-73771255. Max. coverage (+): 0. Max coverage (-): 0

Region: chr17 73771256-73771285. Max. coverage (+): 0. Max coverage (-): 0

Region: chr17 73771286-73771315. Max. coverage (+): 0. Max coverage (-): 6.93

Region: chr17 73771316-73771346. Max. coverage (+): 0. Max coverage (-): 0

Region: chr17 73771347-73771376. Max. coverage (+): 0. Max coverage (-): 0

Region: chr17 73771377-73771406. Max. coverage (+): 0. Max coverage (-): 0

Region: chr17 73771407-73771436. Max. coverage (+): 0. Max coverage (-): 0

Region: chr17 73771437-73771467. Max. coverage (+): 0. Max coverage (-): 0

Region: chr17 73771468-73771497. Max. coverage (+): 0. Max coverage (-): 0

Region: chr17 73771498-73771527. Max. coverage (+): 0. Max coverage (-): 2.55

Region: chr17 73771528-73771557. Max. coverage (+): 0. Max coverage (-): 0

Region: chr17 73771558-73771588. Max. coverage (+): 0. Max coverage (-): 0

Region: chr17 73771589-73771618. Max. coverage (+): 0. Max coverage (-): 2.96

Region: chr17 73771619-73771648. Max. coverage (+): 0. Max coverage (-): 2.96

Region: chr17 73771649-73771678. Max. coverage (+): 0. Max coverage (-): 0

Region: chr17 73771679-73771708. Max. coverage (+): 0. Max coverage (-): 0

Region: chr17 73771709-73771739. Max. coverage (+): 0. Max coverage (-): 0

Region: chr17 73771740-73771769. Max. coverage (+): 0. Max coverage (-): 0

Region: chr17 73771770-73771799. Max. coverage (+): 0. Max coverage (-): 0

Region: chr17 73771800-73771829. Max. coverage (+): 0. Max coverage (-): 0

Region: chr17 73771830-73771860. Max. coverage (+): 0. Max coverage (-): 0

Region: chr17 73771861-73771890. Max. coverage (+): 0. Max coverage (-): 0

Region: chr17 73771891-73771920. Max. coverage (+): 0. Max coverage (-): 0

Region: chr17 73771921-73771950. Max. coverage (+): 0. Max coverage (-): 0

Region: chr17 73771951-73771980. Max. coverage (+): 0. Max coverage (-): 0

Region: chr17 73771981-73772011. Max. coverage (+): 0. Max coverage (-): 0

Region: chr17 73772012-73772041. Max. coverage (+): 0. Max coverage (-): 0

Region: chr17 73772042-73772071. Max. coverage (+): 0. Max coverage (-): 0

Region: chr17 73772072-73772101. Max. coverage (+): 0. Max coverage (-): 0

Region: chr17 73772102-73772132. Max. coverage (+): 0. Max coverage (-): 0

Region: chr17 73772133-73772162. Max. coverage (+): 0. Max coverage (-): 0.16

Region: chr17 73772163-73772192. Max. coverage (+): 0. Max coverage (-): 6.32

Region: chr17 73772193-73772222. Max. coverage (+): 0. Max coverage (-): 8.68

Region: chr17 73772223-73772252. Max. coverage (+): 0. Max coverage (-): 0

Region: chr17 73772253-73772283. Max. coverage (+): 0. Max coverage (-): 0

Region: chr17 73772284-73772313. Max. coverage (+): 0. Max coverage (-): 0

Region: chr17 73772314-73772343. Max. coverage (+): 0. Max coverage (-): 0

Region: chr17 73772344-73772373. Max. coverage (+): 0. Max coverage (-): 0

Region: chr17 73772374-73772404. Max. coverage (+): 0. Max coverage (-): 0

Region: chr17 73772405-73772434. Max. coverage (+): 0. Max coverage (-): 4.07

Region: chr17 73772435-73772464. Max. coverage (+): 0. Max coverage (-): 6.92

Region: chr17 73772465-73772494. Max. coverage (+): 0. Max coverage (-): 0

Region: chr17 73772495-73772524. Max. coverage (+): 0. Max coverage (-): 9.21

Region: chr17 73772525-73772555. Max. coverage (+): 0. Max coverage (-): 1.04

Region: chr17 73772556-73772585. Max. coverage (+): 0. Max coverage (-): 1.04

Region: chr17 73772586-73772615. Max. coverage (+): 0. Max coverage (-): 0

Region: chr17 73772616-73772645. Max. coverage (+): 0. Max coverage (-): 0

Region: chr17 73772646-73772676. Max. coverage (+): 0. Max coverage (-): 2.09

Region: chr17 73772677-73772706. Max. coverage (+): 0. Max coverage (-): 5.83

Region: chr17 73772707-73772736. Max. coverage (+): 0. Max coverage (-): 5.83

Region: chr17 73772737-73772766. Max. coverage (+): 0. Max coverage (-): 0

Region: chr17 73772767-73772796. Max. coverage (+): 0. Max coverage (-): 0

Region: chr17 73772797-73772827. Max. coverage (+): 0. Max coverage (-): 0

Region: chr17 73772828-73772857. Max. coverage (+): 0. Max coverage (-): 6.62

Region: chr17 73772858-73772887. Max. coverage (+): 0. Max coverage (-): 6.62

Region: chr17 73772888-73772917. Max. coverage (+): 0. Max coverage (-): 0

Region: chr17 73772918-73772948. Max. coverage (+): 0. Max coverage (-): 0

Region: chr17 73772949-73772978. Max. coverage (+): 0. Max coverage (-): 0

Region: chr17 73772979-73773008. Max. coverage (+): 0. Max coverage (-): 0

Region: chr17 73773009-73773038. Max. coverage (+): 0. Max coverage (-): 0

Region: chr17 73773039-73773068. Max. coverage (+): 0. Max coverage (-): 3.4

Region: chr17 73773069-73773099. Max. coverage (+): 0. Max coverage (-): 0

Region: chr17 73773100-73773129. Max. coverage (+): 0. Max coverage (-): 0

Region: chr17 73773130-73773159. Max. coverage (+): 0. Max coverage (-): 0

Region: chr17 73773160-73773189. Max. coverage (+): 0. Max coverage (-): 3.71

Region: chr17 73773190-. Max. coverage (+): 0. Max coverage (-): 0

RepeatMasker Color Code

**+**

100-98% Identity

<98-95% Identity

<95-90% Identity

<90-85% Identity

<85-80% Identity

<80-75% Identity

<75-70% Identity

<70% Identity

**-**

Gene Set Color Code

**+**

Gene

Pseudogene

**-**

Topology/Coverage Color Code

Coverage Plus Strand

Coverage Minus Strand

Mainstrand: Plus

Mainstrand: Minus

Complementary Strand

Flanking Region  
(if option -flank >0)

Gene Set Annotation  
  
RepeatMasker Annotation  

**1. L2a**: 73759432-73759552 (-), Divergence to consensus: 38.6%  
**2. CHR-2\_BT**: 73759779-73759877 (+), Divergence to consensus: 19.2%  
**3. GC\_rich**: 73760267-73760288 (+), Divergence to consensus: 54.5%  
**4. L1MC4a**: 73760364-73760430 (-), Divergence to consensus: 27.6%  
**5. L1ME4c**: 73761666-73761734 (-), Divergence to consensus: 24.8%  
**6. L1MB3**: 73762465-73762707 (+), Divergence to consensus: 42.8%  
**7. SINE2-3\_BT**: 73763321-73763482 (+), Divergence to consensus: 36.5%  
**8. GC\_rich**: 73764944-73764965 (+), Divergence to consensus: 45.5%  
**9. GC\_rich**: 73765277-73765315 (+), Divergence to consensus: 69.2%  
**10. Bov-tA2**: 73766139-73766345 (-), Divergence to consensus: 12.8%  
**11. Bov-tA2**: 73766379-73766428 (+), Divergence to consensus: 22%  
**12. L1MB2**: 73766544-73766956 (+), Divergence to consensus: 45.6%  
**13. (TGGA)n**: 73767927-73767996 (+), Divergence to consensus: 12.9%  
**14. L1MC5a**: 73768164-73768207 (-), Divergence to consensus: 18.2%  
**15. Bov-tA2**: 73768793-73768969 (-), Divergence to consensus: 27.6%  
**16. Bov-tA2**: 73768970-73769090 (-), Divergence to consensus: 14.9%  
**17. MLT1E1**: 73769314-73769504 (+), Divergence to consensus: 36.6%

  
Transcription Factor Binding Sites  

**Gata4** (Sequence: AGATAAG (-): 73764094)
