# Supplementary material for: Small RNA sequencing of cryopreserved semen from single bull revealed altered miRNAs and piRNAs expression between High- and Low-motile sperm populations
Source: BMC Genomics. 2017 Jan 4;18:14. doi: 10.1186/s12864-016-3394-7 (PMC5209821; doi:10.1186/s12864-016-3394-7)
Supplement: Additional file 4: — Details for each piRNA clusters found in Low Motile (LM) sperm fraction. Genes, repeats, transposable elements and transcription factors binding sites falling within the cluster regions were reported. (ZIP 1034 kb) [file 12864_2016_3394_MOESM4_ESM.zip › 22.html]

piRNA cluster 22


Predicted piRNA cluster no. 22     previous   next
  

Show proTRAC run info
Hide proTRAC run info

================================= proTRAC ====================================  
VERSION: 2.1                                    LAST MODIFIED: 06. October 2015  
  
Please cite:  
Rosenkranz D, Zischler H. proTRAC - a software for probabilistic piRNA cluster  
detection, visualization and analysis. 2012. BMC Bioinformatics 13:5.  
  
and (for proTRAC 2.0 and later):  
Rosenkranz D, Rudloff S, Bastuck K, Ketting RF, Zischler H. Tupaia small RNAs  
provide insights into function and evolution of RNAi-based transposon defense  
in mammals. 2015. RNA 21(5):911-922.  
  
Contact:  
David Rosenkranz  
Institute of Anthropology, small RNA group  
Johannes Gutenberg University Mainz  
email: rosenkranz@uni-mainz.de  
  
You can find the latest proTRAC version at:  
http://sourceforge.net/projects/protrac/files  
http://www.smallRNAgroup-mainz.de/software  
==============================================================================  
  
PARAMETERS:  
Map file: .............../storage/core/barbara/genhome/smallRNA/fertility/Sample\_not\_motile/pirna/Sample\_not\_motile\_26-33\_collapsed.fa.no-dust.map.weighted-10000-1000-b-0  
Genome file: ............/storage/core/barbara/genhome/smallRNA/fertility/Sample\_all/pirna/bt\_311\_chrY.fa  
RepeatMasker annotation: /storage/genomes/bt\_umd31/GCF\_000003055.6\_Bos\_taurus\_UMD\_3.1.1\_repeatMasker\_chr.out  
GeneSet:................./storage/core/barbara/genhome/smallRNA/fertility/Sample\_all/pirna/full.gtf  
  
Significant (p<=0.01) hit density will be calculated based  
on observed hit distribution.  
  
Sliding window size: ........................................ 5000 bp  
Sliding window increament: .................................. 1000 bp  
Normalize each hit by number of genomic hits: ............... 1 [0=no/1=yes]  
Normalize each hit by number of sequence reads: ............. 1 [0=no/1=yes]  
Normalize values (-> per million mapped reads): ............. 1 [0=no/1=yes]  
Min. fraction of hits with 1T(U) or 10A: .................... 0.75  
Alternatively: Min. fraction of hits with 1T(U) and 10A: .... 0.5  
Min. fraction of hits with typical piRNA length: ............ 0.75  
Typical piRNA length: ....................................... 26-33 nt  
Min. size of a piRNA cluster: ............................... 5000 bp.  
Min. number of hits (absolute): ............................. 0  
Min. number of hits (normalized): ........................... 0  
Min. fraction of hits on the mainstrand: .................... 0.75  
Top fraction of mapped sequences (in terms of read counts): . 1%  
Top fraction accounts for max. n% of sequence reads: ........ 90%  
Min. fraction of hits on each arm of a bidirectional cluster: 0.1  
Output image file for each cluster: ......................... 0 [0=no/1=yes]  
Output html file for each cluster: .......................... 1 [0=no/1=yes]  
Output a summary table: ..................................... 1 [0=no/1=yes]  
Output a FASTA file for each cluster (piRNA sequences): ..... 1 [0=no/1=yes]  
Output a FASTA file comprising cluster sequences: ........... 1 [0=no/1=yes]  
Search DNA motifs in clusters: .............................. 1 [0=no/1=yes]  
Output flanking sequences: +/- .............................. 0 bp  
Output ~.pTi file: .......................................... 1 [0=no/1=yes]  
==============================================================================  
  
  
Genome size (without gaps): ............ 2678902517 bp  
Gaps (N/X/-): .......................... 53837044 bp  
Mapped reads: .......................... 738059667487  
Non-identical sequences: ............... 277001  
Genomic hits: .......................... 533816  
Significant densitiy of mapped reads: .. 15118061 reads/kb

Show proTRAC cluster info
Hide proTRAC cluster info

|  |  |
| --- | --- |
| Location | chr18 |
| Coordinates | 39793155-39800537 |
| Size [bp] | 7383 |
| Sequence hit loci | 729 |
| Mapped reads (normalized) | 1929692110 |
| Mapped reads (normalized) per kb | 261369647.8 |
| Normalized reads with 1T (1U) | 85.4% |
| Normalized reads with 10A | 25.4% |
| Normalized reads with length 26-33 nt | 100% |
| Normalized reads on the main strand(s) | 100% |
| Predicted directionality | mono:plus |

100%

0%

1T (1U)  
reads

10A reads

26-33 nt  
reads

reads on mainstrand

**Either the amount of reads with 1T (1U) OR 10A has to exceed 75% (set with option: -1Tor10A)  
Alternatively the amount of reads with 1T (1U) AND 10A has to exceed 50% (set with option: -1Tand10A)  
Minimum amount of reads with preferred size is 75% (set with option: -pisize)  
Minimum amount of reads on the main strand(s) is 75% (set with option: -clstrand)**

Show read coverage
Hide read coverage

WHAT DO I SEE HERE?  
This chart shows the location of mapped sequence reads within a predicted piRNA cluster. The color refers to the number of genomic hits produced by the sequence read in question. A dark red bar indicates that this sequence read produces many other hits elsewhere in the genome. Many adjacent red or yellow bars can indicate the presence of a multi-copy element such as transposons or rRNA genes. A dark green bar indicates that this sequence read maps uniquely to this locus.

1 hit

2-5 hits

6-10 hits

11-20 hits

21-50 hits

51-100 hits

> 100 hits

chr18

39793155

39800537

Gene Set

RepeatMasker

Mapped  
Reads

95.41

plus strand

minus strand

95.41

Region: chr18 73773191-39793162. Max. coverage (+): 7.29. Max coverage (-): 0

Region: chr18 39793163-39793177. Max. coverage (+): 5.38. Max coverage (-): 0

Region: chr18 39793178-39793191. Max. coverage (+): 0.85. Max coverage (-): 0

Region: chr18 39793192-39793206. Max. coverage (+): 3.28. Max coverage (-): 0

Region: chr18 39793207-39793221. Max. coverage (+): 0. Max coverage (-): 0

Region: chr18 39793222-39793236. Max. coverage (+): 13.11. Max coverage (-): 0

Region: chr18 39793237-39793250. Max. coverage (+): 0. Max coverage (-): 0

Region: chr18 39793251-39793265. Max. coverage (+): 0. Max coverage (-): 0

Region: chr18 39793266-39793280. Max. coverage (+): 0. Max coverage (-): 0

Region: chr18 39793281-39793295. Max. coverage (+): 0. Max coverage (-): 0

Region: chr18 39793296-39793310. Max. coverage (+): 0. Max coverage (-): 0

Region: chr18 39793311-39793324. Max. coverage (+): 5.39. Max coverage (-): 0

Region: chr18 39793325-39793339. Max. coverage (+): 5.39. Max coverage (-): 0

Region: chr18 39793340-39793354. Max. coverage (+): 0.99. Max coverage (-): 0

Region: chr18 39793355-39793369. Max. coverage (+): 5.92. Max coverage (-): 0

Region: chr18 39793370-39793383. Max. coverage (+): 0. Max coverage (-): 0

Region: chr18 39793384-39793398. Max. coverage (+): 1.22. Max coverage (-): 0

Region: chr18 39793399-39793413. Max. coverage (+): 11.14. Max coverage (-): 0

Region: chr18 39793414-39793428. Max. coverage (+): 2.47. Max coverage (-): 0

Region: chr18 39793429-39793442. Max. coverage (+): 0. Max coverage (-): 0

Region: chr18 39793443-39793457. Max. coverage (+): 0. Max coverage (-): 0

Region: chr18 39793458-39793472. Max. coverage (+): 0. Max coverage (-): 0

Region: chr18 39793473-39793487. Max. coverage (+): 0. Max coverage (-): 0

Region: chr18 39793488-39793502. Max. coverage (+): 0.22. Max coverage (-): 0

Region: chr18 39793503-39793516. Max. coverage (+): 4.24. Max coverage (-): 0

Region: chr18 39793517-39793531. Max. coverage (+): 4.24. Max coverage (-): 0

Region: chr18 39793532-39793546. Max. coverage (+): 16.53. Max coverage (-): 0

Region: chr18 39793547-39793561. Max. coverage (+): 0. Max coverage (-): 0

Region: chr18 39793562-39793575. Max. coverage (+): 0. Max coverage (-): 0

Region: chr18 39793576-39793590. Max. coverage (+): 0. Max coverage (-): 0

Region: chr18 39793591-39793605. Max. coverage (+): 0. Max coverage (-): 0

Region: chr18 39793606-39793620. Max. coverage (+): 0. Max coverage (-): 0

Region: chr18 39793621-39793634. Max. coverage (+): 0. Max coverage (-): 0

Region: chr18 39793635-39793649. Max. coverage (+): 3.26. Max coverage (-): 0

Region: chr18 39793650-39793664. Max. coverage (+): 6.02. Max coverage (-): 0

Region: chr18 39793665-39793679. Max. coverage (+): 0. Max coverage (-): 0

Region: chr18 39793680-39793693. Max. coverage (+): 0. Max coverage (-): 0

Region: chr18 39793694-39793708. Max. coverage (+): 0. Max coverage (-): 0

Region: chr18 39793709-39793723. Max. coverage (+): 0. Max coverage (-): 0

Region: chr18 39793724-39793738. Max. coverage (+): 0. Max coverage (-): 0

Region: chr18 39793739-39793753. Max. coverage (+): 0. Max coverage (-): 0

Region: chr18 39793754-39793767. Max. coverage (+): 0. Max coverage (-): 0

Region: chr18 39793768-39793782. Max. coverage (+): 0. Max coverage (-): 0

Region: chr18 39793783-39793797. Max. coverage (+): 0. Max coverage (-): 0

Region: chr18 39793798-39793812. Max. coverage (+): 0. Max coverage (-): 0

Region: chr18 39793813-39793826. Max. coverage (+): 0. Max coverage (-): 0

Region: chr18 39793827-39793841. Max. coverage (+): 2.05. Max coverage (-): 0

Region: chr18 39793842-39793856. Max. coverage (+): 0. Max coverage (-): 0

Region: chr18 39793857-39793871. Max. coverage (+): 0. Max coverage (-): 0

Region: chr18 39793872-39793885. Max. coverage (+): 0. Max coverage (-): 0

Region: chr18 39793886-39793900. Max. coverage (+): 0. Max coverage (-): 0

Region: chr18 39793901-39793915. Max. coverage (+): 0. Max coverage (-): 0

Region: chr18 39793916-39793930. Max. coverage (+): 0. Max coverage (-): 0

Region: chr18 39793931-39793944. Max. coverage (+): 0. Max coverage (-): 0

Region: chr18 39793945-39793959. Max. coverage (+): 0. Max coverage (-): 0

Region: chr18 39793960-39793974. Max. coverage (+): 0. Max coverage (-): 0

Region: chr18 39793975-39793989. Max. coverage (+): 0. Max coverage (-): 0

Region: chr18 39793990-39794004. Max. coverage (+): 0. Max coverage (-): 0

Region: chr18 39794005-39794018. Max. coverage (+): 0. Max coverage (-): 0

Region: chr18 39794019-39794033. Max. coverage (+): 0. Max coverage (-): 0

Region: chr18 39794034-39794048. Max. coverage (+): 0. Max coverage (-): 0

Region: chr18 39794049-39794063. Max. coverage (+): 0. Max coverage (-): 0

Region: chr18 39794064-39794077. Max. coverage (+): 0. Max coverage (-): 0

Region: chr18 39794078-39794092. Max. coverage (+): 0. Max coverage (-): 0

Region: chr18 39794093-39794107. Max. coverage (+): 0. Max coverage (-): 0

Region: chr18 39794108-39794122. Max. coverage (+): 0. Max coverage (-): 0

Region: chr18 39794123-39794136. Max. coverage (+): 0. Max coverage (-): 0

Region: chr18 39794137-39794151. Max. coverage (+): 0. Max coverage (-): 0

Region: chr18 39794152-39794166. Max. coverage (+): 0. Max coverage (-): 0

Region: chr18 39794167-39794181. Max. coverage (+): 0. Max coverage (-): 0

Region: chr18 39794182-39794196. Max. coverage (+): 0. Max coverage (-): 0

Region: chr18 39794197-39794210. Max. coverage (+): 0. Max coverage (-): 0

Region: chr18 39794211-39794225. Max. coverage (+): 23.49. Max coverage (-): 0

Region: chr18 39794226-39794240. Max. coverage (+): 23.49. Max coverage (-): 0

Region: chr18 39794241-39794255. Max. coverage (+): 0. Max coverage (-): 0

Region: chr18 39794256-39794269. Max. coverage (+): 0. Max coverage (-): 0

Region: chr18 39794270-39794284. Max. coverage (+): 0. Max coverage (-): 0

Region: chr18 39794285-39794299. Max. coverage (+): 0. Max coverage (-): 0

Region: chr18 39794300-39794314. Max. coverage (+): 0. Max coverage (-): 0

Region: chr18 39794315-39794328. Max. coverage (+): 0. Max coverage (-): 0

Region: chr18 39794329-39794343. Max. coverage (+): 15.08. Max coverage (-): 0

Region: chr18 39794344-39794358. Max. coverage (+): 0. Max coverage (-): 0

Region: chr18 39794359-39794373. Max. coverage (+): 0. Max coverage (-): 0

Region: chr18 39794374-39794387. Max. coverage (+): 0. Max coverage (-): 0

Region: chr18 39794388-39794402. Max. coverage (+): 0. Max coverage (-): 0

Region: chr18 39794403-39794417. Max. coverage (+): 0. Max coverage (-): 0

Region: chr18 39794418-39794432. Max. coverage (+): 0. Max coverage (-): 0

Region: chr18 39794433-39794447. Max. coverage (+): 6.91. Max coverage (-): 0

Region: chr18 39794448-39794461. Max. coverage (+): 2.6. Max coverage (-): 0

Region: chr18 39794462-39794476. Max. coverage (+): 0. Max coverage (-): 0

Region: chr18 39794477-39794491. Max. coverage (+): 0. Max coverage (-): 0

Region: chr18 39794492-39794506. Max. coverage (+): 0. Max coverage (-): 0

Region: chr18 39794507-39794520. Max. coverage (+): 0. Max coverage (-): 0

Region: chr18 39794521-39794535. Max. coverage (+): 0. Max coverage (-): 0

Region: chr18 39794536-39794550. Max. coverage (+): 0. Max coverage (-): 0

Region: chr18 39794551-39794565. Max. coverage (+): 0. Max coverage (-): 0

Region: chr18 39794566-39794579. Max. coverage (+): 0. Max coverage (-): 0

Region: chr18 39794580-39794594. Max. coverage (+): 0. Max coverage (-): 0

Region: chr18 39794595-39794609. Max. coverage (+): 0. Max coverage (-): 0

Region: chr18 39794610-39794624. Max. coverage (+): 0. Max coverage (-): 0

Region: chr18 39794625-39794638. Max. coverage (+): 0. Max coverage (-): 0

Region: chr18 39794639-39794653. Max. coverage (+): 0. Max coverage (-): 0

Region: chr18 39794654-39794668. Max. coverage (+): 0. Max coverage (-): 0

Region: chr18 39794669-39794683. Max. coverage (+): 0. Max coverage (-): 0

Region: chr18 39794684-39794698. Max. coverage (+): 0. Max coverage (-): 0

Region: chr18 39794699-39794712. Max. coverage (+): 0. Max coverage (-): 0

Region: chr18 39794713-39794727. Max. coverage (+): 0. Max coverage (-): 0

Region: chr18 39794728-39794742. Max. coverage (+): 0. Max coverage (-): 0

Region: chr18 39794743-39794757. Max. coverage (+): 5.47. Max coverage (-): 0

Region: chr18 39794758-39794771. Max. coverage (+): 49.81. Max coverage (-): 0

Region: chr18 39794772-39794786. Max. coverage (+): 0. Max coverage (-): 0

Region: chr18 39794787-39794801. Max. coverage (+): 1.08. Max coverage (-): 0

Region: chr18 39794802-39794816. Max. coverage (+): 1.08. Max coverage (-): 0

Region: chr18 39794817-39794830. Max. coverage (+): 0. Max coverage (-): 0

Region: chr18 39794831-39794845. Max. coverage (+): 29.74. Max coverage (-): 0

Region: chr18 39794846-39794860. Max. coverage (+): 17.94. Max coverage (-): 0

Region: chr18 39794861-39794875. Max. coverage (+): 11.24. Max coverage (-): 0

Region: chr18 39794876-39794890. Max. coverage (+): 14.91. Max coverage (-): 0

Region: chr18 39794891-39794904. Max. coverage (+): 16.52. Max coverage (-): 0

Region: chr18 39794905-39794919. Max. coverage (+): 16.52. Max coverage (-): 0

Region: chr18 39794920-39794934. Max. coverage (+): 3.05. Max coverage (-): 0

Region: chr18 39794935-39794949. Max. coverage (+): 20.49. Max coverage (-): 0

Region: chr18 39794950-39794963. Max. coverage (+): 18.15. Max coverage (-): 0

Region: chr18 39794964-39794978. Max. coverage (+): 3.24. Max coverage (-): 0

Region: chr18 39794979-39794993. Max. coverage (+): 8.54. Max coverage (-): 0

Region: chr18 39794994-39795008. Max. coverage (+): 11.15. Max coverage (-): 0

Region: chr18 39795009-39795022. Max. coverage (+): 0. Max coverage (-): 0

Region: chr18 39795023-39795037. Max. coverage (+): 26.14. Max coverage (-): 0

Region: chr18 39795038-39795052. Max. coverage (+): 11.55. Max coverage (-): 0

Region: chr18 39795053-39795067. Max. coverage (+): 8. Max coverage (-): 0

Region: chr18 39795068-39795081. Max. coverage (+): 3.74. Max coverage (-): 0

Region: chr18 39795082-39795096. Max. coverage (+): 0.32. Max coverage (-): 0

Region: chr18 39795097-39795111. Max. coverage (+): 5.66. Max coverage (-): 0

Region: chr18 39795112-39795126. Max. coverage (+): 1.67. Max coverage (-): 0

Region: chr18 39795127-39795141. Max. coverage (+): 1.67. Max coverage (-): 0

Region: chr18 39795142-39795155. Max. coverage (+): 0. Max coverage (-): 0

Region: chr18 39795156-39795170. Max. coverage (+): 0. Max coverage (-): 0

Region: chr18 39795171-39795185. Max. coverage (+): 4.17. Max coverage (-): 0

Region: chr18 39795186-39795200. Max. coverage (+): 6.96. Max coverage (-): 0

Region: chr18 39795201-39795214. Max. coverage (+): 2.56. Max coverage (-): 0

Region: chr18 39795215-39795229. Max. coverage (+): 5.45. Max coverage (-): 0

Region: chr18 39795230-39795244. Max. coverage (+): 4.62. Max coverage (-): 0

Region: chr18 39795245-39795259. Max. coverage (+): 0. Max coverage (-): 0

Region: chr18 39795260-39795273. Max. coverage (+): 7.32. Max coverage (-): 0

Region: chr18 39795274-39795288. Max. coverage (+): 7.32. Max coverage (-): 0

Region: chr18 39795289-39795303. Max. coverage (+): 6.98. Max coverage (-): 0

Region: chr18 39795304-39795318. Max. coverage (+): 6.98. Max coverage (-): 0

Region: chr18 39795319-39795332. Max. coverage (+): 0. Max coverage (-): 0

Region: chr18 39795333-39795347. Max. coverage (+): 7.28. Max coverage (-): 0

Region: chr18 39795348-39795362. Max. coverage (+): 11.59. Max coverage (-): 0

Region: chr18 39795363-39795377. Max. coverage (+): 10.04. Max coverage (-): 0

Region: chr18 39795378-39795392. Max. coverage (+): 1.08. Max coverage (-): 0

Region: chr18 39795393-39795406. Max. coverage (+): 24.04. Max coverage (-): 0

Region: chr18 39795407-39795421. Max. coverage (+): 0. Max coverage (-): 0

Region: chr18 39795422-39795436. Max. coverage (+): 0. Max coverage (-): 0

Region: chr18 39795437-39795451. Max. coverage (+): 1. Max coverage (-): 0

Region: chr18 39795452-39795465. Max. coverage (+): 33.23. Max coverage (-): 0

Region: chr18 39795466-39795480. Max. coverage (+): 0. Max coverage (-): 0

Region: chr18 39795481-39795495. Max. coverage (+): 0. Max coverage (-): 0

Region: chr18 39795496-39795510. Max. coverage (+): 0. Max coverage (-): 0

Region: chr18 39795511-39795524. Max. coverage (+): 0. Max coverage (-): 0

Region: chr18 39795525-39795539. Max. coverage (+): 6.9. Max coverage (-): 0

Region: chr18 39795540-39795554. Max. coverage (+): 2.64. Max coverage (-): 0

Region: chr18 39795555-39795569. Max. coverage (+): 0. Max coverage (-): 0

Region: chr18 39795570-39795584. Max. coverage (+): 0. Max coverage (-): 0

Region: chr18 39795585-39795598. Max. coverage (+): 0. Max coverage (-): 0

Region: chr18 39795599-39795613. Max. coverage (+): 0. Max coverage (-): 0

Region: chr18 39795614-39795628. Max. coverage (+): 6.57. Max coverage (-): 0

Region: chr18 39795629-39795643. Max. coverage (+): 6.57. Max coverage (-): 0

Region: chr18 39795644-39795657. Max. coverage (+): 9.72. Max coverage (-): 0

Region: chr18 39795658-39795672. Max. coverage (+): 4.38. Max coverage (-): 0

Region: chr18 39795673-39795687. Max. coverage (+): 0. Max coverage (-): 0

Region: chr18 39795688-39795702. Max. coverage (+): 0. Max coverage (-): 0

Region: chr18 39795703-39795716. Max. coverage (+): 0. Max coverage (-): 0

Region: chr18 39795717-39795731. Max. coverage (+): 0.03. Max coverage (-): 0

Region: chr18 39795732-39795746. Max. coverage (+): 0. Max coverage (-): 0

Region: chr18 39795747-39795761. Max. coverage (+): 0. Max coverage (-): 0

Region: chr18 39795762-39795775. Max. coverage (+): 0. Max coverage (-): 0

Region: chr18 39795776-39795790. Max. coverage (+): 0. Max coverage (-): 0

Region: chr18 39795791-39795805. Max. coverage (+): 26.29. Max coverage (-): 0

Region: chr18 39795806-39795820. Max. coverage (+): 0. Max coverage (-): 0

Region: chr18 39795821-39795835. Max. coverage (+): 0. Max coverage (-): 0

Region: chr18 39795836-39795849. Max. coverage (+): 0. Max coverage (-): 0

Region: chr18 39795850-39795864. Max. coverage (+): 14.81. Max coverage (-): 0

Region: chr18 39795865-39795879. Max. coverage (+): 14.81. Max coverage (-): 0

Region: chr18 39795880-39795894. Max. coverage (+): 13.85. Max coverage (-): 0

Region: chr18 39795895-39795908. Max. coverage (+): 13.85. Max coverage (-): 0

Region: chr18 39795909-39795923. Max. coverage (+): 0. Max coverage (-): 0

Region: chr18 39795924-39795938. Max. coverage (+): 6.59. Max coverage (-): 0

Region: chr18 39795939-39795953. Max. coverage (+): 6.59. Max coverage (-): 0

Region: chr18 39795954-39795967. Max. coverage (+): 4.22. Max coverage (-): 0

Region: chr18 39795968-39795982. Max. coverage (+): 6.77. Max coverage (-): 0

Region: chr18 39795983-39795997. Max. coverage (+): 0. Max coverage (-): 0

Region: chr18 39795998-39796012. Max. coverage (+): 20.93. Max coverage (-): 0

Region: chr18 39796013-39796026. Max. coverage (+): 37.58. Max coverage (-): 0

Region: chr18 39796027-39796041. Max. coverage (+): 32.97. Max coverage (-): 0

Region: chr18 39796042-39796056. Max. coverage (+): 29.16. Max coverage (-): 0

Region: chr18 39796057-39796071. Max. coverage (+): 33.84. Max coverage (-): 0

Region: chr18 39796072-39796086. Max. coverage (+): 4.93. Max coverage (-): 0

Region: chr18 39796087-39796100. Max. coverage (+): 0. Max coverage (-): 0

Region: chr18 39796101-39796115. Max. coverage (+): 0. Max coverage (-): 0

Region: chr18 39796116-39796130. Max. coverage (+): 3.33. Max coverage (-): 0

Region: chr18 39796131-39796145. Max. coverage (+): 6.32. Max coverage (-): 0

Region: chr18 39796146-39796159. Max. coverage (+): 16.8. Max coverage (-): 0

Region: chr18 39796160-39796174. Max. coverage (+): 95.41. Max coverage (-): 0

Region: chr18 39796175-39796189. Max. coverage (+): 20.68. Max coverage (-): 0

Region: chr18 39796190-39796204. Max. coverage (+): 6.14. Max coverage (-): 0

Region: chr18 39796205-39796218. Max. coverage (+): 0. Max coverage (-): 0

Region: chr18 39796219-39796233. Max. coverage (+): 0. Max coverage (-): 0

Region: chr18 39796234-39796248. Max. coverage (+): 0. Max coverage (-): 0

Region: chr18 39796249-39796263. Max. coverage (+): 0. Max coverage (-): 0

Region: chr18 39796264-39796278. Max. coverage (+): 16.73. Max coverage (-): 0

Region: chr18 39796279-39796292. Max. coverage (+): 43.13. Max coverage (-): 0

Region: chr18 39796293-39796307. Max. coverage (+): 6.7. Max coverage (-): 0

Region: chr18 39796308-39796322. Max. coverage (+): 6.7. Max coverage (-): 0

Region: chr18 39796323-39796337. Max. coverage (+): 0. Max coverage (-): 0

Region: chr18 39796338-39796351. Max. coverage (+): 0. Max coverage (-): 0

Region: chr18 39796352-39796366. Max. coverage (+): 5.87. Max coverage (-): 0

Region: chr18 39796367-39796381. Max. coverage (+): 12.16. Max coverage (-): 0

Region: chr18 39796382-39796396. Max. coverage (+): 25.56. Max coverage (-): 0

Region: chr18 39796397-39796410. Max. coverage (+): 25.56. Max coverage (-): 0

Region: chr18 39796411-39796425. Max. coverage (+): 14.63. Max coverage (-): 0

Region: chr18 39796426-39796440. Max. coverage (+): 0. Max coverage (-): 0

Region: chr18 39796441-39796455. Max. coverage (+): 93.14. Max coverage (-): 0

Region: chr18 39796456-39796469. Max. coverage (+): 90.33. Max coverage (-): 0

Region: chr18 39796470-39796484. Max. coverage (+): 0. Max coverage (-): 0

Region: chr18 39796485-39796499. Max. coverage (+): 0. Max coverage (-): 0

Region: chr18 39796500-39796514. Max. coverage (+): 0. Max coverage (-): 0

Region: chr18 39796515-39796529. Max. coverage (+): 0. Max coverage (-): 0

Region: chr18 39796530-39796543. Max. coverage (+): 0. Max coverage (-): 0

Region: chr18 39796544-39796558. Max. coverage (+): 0. Max coverage (-): 0

Region: chr18 39796559-39796573. Max. coverage (+): 7.56. Max coverage (-): 0

Region: chr18 39796574-39796588. Max. coverage (+): 0. Max coverage (-): 0

Region: chr18 39796589-39796602. Max. coverage (+): 0. Max coverage (-): 0

Region: chr18 39796603-39796617. Max. coverage (+): 5.41. Max coverage (-): 0

Region: chr18 39796618-39796632. Max. coverage (+): 5.41. Max coverage (-): 0

Region: chr18 39796633-39796647. Max. coverage (+): 18.77. Max coverage (-): 0

Region: chr18 39796648-39796661. Max. coverage (+): 20.94. Max coverage (-): 0

Region: chr18 39796662-39796676. Max. coverage (+): 15.77. Max coverage (-): 0

Region: chr18 39796677-39796691. Max. coverage (+): 18.96. Max coverage (-): 0

Region: chr18 39796692-39796706. Max. coverage (+): 0. Max coverage (-): 0

Region: chr18 39796707-39796720. Max. coverage (+): 5.96. Max coverage (-): 0

Region: chr18 39796721-39796735. Max. coverage (+): 5.96. Max coverage (-): 0

Region: chr18 39796736-39796750. Max. coverage (+): 0. Max coverage (-): 0

Region: chr18 39796751-39796765. Max. coverage (+): 0. Max coverage (-): 0

Region: chr18 39796766-39796780. Max. coverage (+): 0. Max coverage (-): 0

Region: chr18 39796781-39796794. Max. coverage (+): 0. Max coverage (-): 0

Region: chr18 39796795-39796809. Max. coverage (+): 6.07. Max coverage (-): 0

Region: chr18 39796810-39796824. Max. coverage (+): 19.71. Max coverage (-): 0

Region: chr18 39796825-39796839. Max. coverage (+): 16.51. Max coverage (-): 0

Region: chr18 39796840-39796853. Max. coverage (+): 0. Max coverage (-): 0

Region: chr18 39796854-39796868. Max. coverage (+): 0. Max coverage (-): 0

Region: chr18 39796869-39796883. Max. coverage (+): 2.83. Max coverage (-): 0

Region: chr18 39796884-39796898. Max. coverage (+): 3.5. Max coverage (-): 0

Region: chr18 39796899-39796912. Max. coverage (+): 2. Max coverage (-): 0

Region: chr18 39796913-39796927. Max. coverage (+): 0. Max coverage (-): 0

Region: chr18 39796928-39796942. Max. coverage (+): 0. Max coverage (-): 0

Region: chr18 39796943-39796957. Max. coverage (+): 0. Max coverage (-): 0

Region: chr18 39796958-39796972. Max. coverage (+): 4.21. Max coverage (-): 0

Region: chr18 39796973-39796986. Max. coverage (+): 0. Max coverage (-): 0

Region: chr18 39796987-39797001. Max. coverage (+): 0. Max coverage (-): 0

Region: chr18 39797002-39797016. Max. coverage (+): 0. Max coverage (-): 0

Region: chr18 39797017-39797031. Max. coverage (+): 0. Max coverage (-): 0

Region: chr18 39797032-39797045. Max. coverage (+): 0. Max coverage (-): 0

Region: chr18 39797046-39797060. Max. coverage (+): 10.53. Max coverage (-): 0

Region: chr18 39797061-39797075. Max. coverage (+): 14.76. Max coverage (-): 0

Region: chr18 39797076-39797090. Max. coverage (+): 9.65. Max coverage (-): 0

Region: chr18 39797091-39797104. Max. coverage (+): 0. Max coverage (-): 0

Region: chr18 39797105-39797119. Max. coverage (+): 11.65. Max coverage (-): 0

Region: chr18 39797120-39797134. Max. coverage (+): 32.32. Max coverage (-): 0

Region: chr18 39797135-39797149. Max. coverage (+): 42.14. Max coverage (-): 0

Region: chr18 39797150-39797163. Max. coverage (+): 6.17. Max coverage (-): 0

Region: chr18 39797164-39797178. Max. coverage (+): 6.17. Max coverage (-): 0

Region: chr18 39797179-39797193. Max. coverage (+): 5.67. Max coverage (-): 0

Region: chr18 39797194-39797208. Max. coverage (+): 5.52. Max coverage (-): 0

Region: chr18 39797209-39797223. Max. coverage (+): 5.52. Max coverage (-): 0

Region: chr18 39797224-39797237. Max. coverage (+): 25.13. Max coverage (-): 0

Region: chr18 39797238-39797252. Max. coverage (+): 15.14. Max coverage (-): 0

Region: chr18 39797253-39797267. Max. coverage (+): 0. Max coverage (-): 0

Region: chr18 39797268-39797282. Max. coverage (+): 0. Max coverage (-): 0

Region: chr18 39797283-39797296. Max. coverage (+): 27.83. Max coverage (-): 0

Region: chr18 39797297-39797311. Max. coverage (+): 16.8. Max coverage (-): 0

Region: chr18 39797312-39797326. Max. coverage (+): 0.28. Max coverage (-): 0

Region: chr18 39797327-39797341. Max. coverage (+): 10.02. Max coverage (-): 0

Region: chr18 39797342-39797355. Max. coverage (+): 8.85. Max coverage (-): 0

Region: chr18 39797356-39797370. Max. coverage (+): 10.78. Max coverage (-): 0

Region: chr18 39797371-39797385. Max. coverage (+): 0.56. Max coverage (-): 0

Region: chr18 39797386-39797400. Max. coverage (+): 0. Max coverage (-): 0

Region: chr18 39797401-39797414. Max. coverage (+): 15.82. Max coverage (-): 0

Region: chr18 39797415-39797429. Max. coverage (+): 0. Max coverage (-): 0

Region: chr18 39797430-39797444. Max. coverage (+): 0. Max coverage (-): 0

Region: chr18 39797445-39797459. Max. coverage (+): 8.3. Max coverage (-): 0

Region: chr18 39797460-39797474. Max. coverage (+): 14.11. Max coverage (-): 0

Region: chr18 39797475-39797488. Max. coverage (+): 0. Max coverage (-): 0

Region: chr18 39797489-39797503. Max. coverage (+): 0. Max coverage (-): 0

Region: chr18 39797504-39797518. Max. coverage (+): 0. Max coverage (-): 0

Region: chr18 39797519-39797533. Max. coverage (+): 10.47. Max coverage (-): 0

Region: chr18 39797534-39797547. Max. coverage (+): 10.47. Max coverage (-): 0

Region: chr18 39797548-39797562. Max. coverage (+): 21.01. Max coverage (-): 0

Region: chr18 39797563-39797577. Max. coverage (+): 29.93. Max coverage (-): 0

Region: chr18 39797578-39797592. Max. coverage (+): 11.5. Max coverage (-): 0

Region: chr18 39797593-39797606. Max. coverage (+): 0. Max coverage (-): 0

Region: chr18 39797607-39797621. Max. coverage (+): 20.83. Max coverage (-): 0

Region: chr18 39797622-39797636. Max. coverage (+): 27.19. Max coverage (-): 0

Region: chr18 39797637-39797651. Max. coverage (+): 26.38. Max coverage (-): 0

Region: chr18 39797652-39797666. Max. coverage (+): 0. Max coverage (-): 0

Region: chr18 39797667-39797680. Max. coverage (+): 0. Max coverage (-): 0

Region: chr18 39797681-39797695. Max. coverage (+): 16.1. Max coverage (-): 0

Region: chr18 39797696-39797710. Max. coverage (+): 26.8. Max coverage (-): 0

Region: chr18 39797711-39797725. Max. coverage (+): 6.47. Max coverage (-): 0

Region: chr18 39797726-39797739. Max. coverage (+): 9.05. Max coverage (-): 0

Region: chr18 39797740-39797754. Max. coverage (+): 4.59. Max coverage (-): 0

Region: chr18 39797755-39797769. Max. coverage (+): 0. Max coverage (-): 0

Region: chr18 39797770-39797784. Max. coverage (+): 5. Max coverage (-): 0

Region: chr18 39797785-39797798. Max. coverage (+): 10.83. Max coverage (-): 0

Region: chr18 39797799-39797813. Max. coverage (+): 15.15. Max coverage (-): 0

Region: chr18 39797814-39797828. Max. coverage (+): 24.18. Max coverage (-): 0

Region: chr18 39797829-39797843. Max. coverage (+): 25.05. Max coverage (-): 0

Region: chr18 39797844-39797857. Max. coverage (+): 30.88. Max coverage (-): 0

Region: chr18 39797858-39797872. Max. coverage (+): 11.19. Max coverage (-): 0

Region: chr18 39797873-39797887. Max. coverage (+): 15.94. Max coverage (-): 0

Region: chr18 39797888-39797902. Max. coverage (+): 4.63. Max coverage (-): 0

Region: chr18 39797903-39797917. Max. coverage (+): 1.33. Max coverage (-): 0

Region: chr18 39797918-39797931. Max. coverage (+): 1.33. Max coverage (-): 0

Region: chr18 39797932-39797946. Max. coverage (+): 0. Max coverage (-): 0

Region: chr18 39797947-39797961. Max. coverage (+): 0. Max coverage (-): 0

Region: chr18 39797962-39797976. Max. coverage (+): 0. Max coverage (-): 0

Region: chr18 39797977-39797990. Max. coverage (+): 0. Max coverage (-): 0

Region: chr18 39797991-39798005. Max. coverage (+): 0. Max coverage (-): 0

Region: chr18 39798006-39798020. Max. coverage (+): 10.17. Max coverage (-): 0

Region: chr18 39798021-39798035. Max. coverage (+): 51.78. Max coverage (-): 0

Region: chr18 39798036-39798049. Max. coverage (+): 18.61. Max coverage (-): 0

Region: chr18 39798050-39798064. Max. coverage (+): 0. Max coverage (-): 0

Region: chr18 39798065-39798079. Max. coverage (+): 8.12. Max coverage (-): 0

Region: chr18 39798080-39798094. Max. coverage (+): 43.18. Max coverage (-): 0

Region: chr18 39798095-39798108. Max. coverage (+): 3.04. Max coverage (-): 0

Region: chr18 39798109-39798123. Max. coverage (+): 0. Max coverage (-): 0

Region: chr18 39798124-39798138. Max. coverage (+): 3.7. Max coverage (-): 0

Region: chr18 39798139-39798153. Max. coverage (+): 20.44. Max coverage (-): 0

Region: chr18 39798154-39798168. Max. coverage (+): 10.39. Max coverage (-): 0

Region: chr18 39798169-39798182. Max. coverage (+): 13.3. Max coverage (-): 0

Region: chr18 39798183-39798197. Max. coverage (+): 33.94. Max coverage (-): 0

Region: chr18 39798198-39798212. Max. coverage (+): 19.75. Max coverage (-): 0

Region: chr18 39798213-39798227. Max. coverage (+): 0. Max coverage (-): 0

Region: chr18 39798228-39798241. Max. coverage (+): 5.99. Max coverage (-): 0

Region: chr18 39798242-39798256. Max. coverage (+): 50.65. Max coverage (-): 0

Region: chr18 39798257-39798271. Max. coverage (+): 0. Max coverage (-): 0

Region: chr18 39798272-39798286. Max. coverage (+): 0. Max coverage (-): 0

Region: chr18 39798287-39798300. Max. coverage (+): 11.9. Max coverage (-): 0

Region: chr18 39798301-39798315. Max. coverage (+): 17.89. Max coverage (-): 0

Region: chr18 39798316-39798330. Max. coverage (+): 17.64. Max coverage (-): 0

Region: chr18 39798331-39798345. Max. coverage (+): 0. Max coverage (-): 0

Region: chr18 39798346-39798360. Max. coverage (+): 17.04. Max coverage (-): 0

Region: chr18 39798361-39798374. Max. coverage (+): 2.44. Max coverage (-): 0

Region: chr18 39798375-39798389. Max. coverage (+): 0. Max coverage (-): 0

Region: chr18 39798390-39798404. Max. coverage (+): 26.28. Max coverage (-): 0

Region: chr18 39798405-39798419. Max. coverage (+): 22.74. Max coverage (-): 0

Region: chr18 39798420-39798433. Max. coverage (+): 0.65. Max coverage (-): 0

Region: chr18 39798434-39798448. Max. coverage (+): 0. Max coverage (-): 0

Region: chr18 39798449-39798463. Max. coverage (+): 0. Max coverage (-): 0

Region: chr18 39798464-39798478. Max. coverage (+): 0. Max coverage (-): 0

Region: chr18 39798479-39798492. Max. coverage (+): 12.23. Max coverage (-): 0

Region: chr18 39798493-39798507. Max. coverage (+): 12.23. Max coverage (-): 0

Region: chr18 39798508-39798522. Max. coverage (+): 3.08. Max coverage (-): 0

Region: chr18 39798523-39798537. Max. coverage (+): 3.08. Max coverage (-): 0

Region: chr18 39798538-39798551. Max. coverage (+): 0. Max coverage (-): 0

Region: chr18 39798552-39798566. Max. coverage (+): 6.54. Max coverage (-): 0

Region: chr18 39798567-39798581. Max. coverage (+): 0. Max coverage (-): 0

Region: chr18 39798582-39798596. Max. coverage (+): 14.7. Max coverage (-): 0

Region: chr18 39798597-39798611. Max. coverage (+): 11.52. Max coverage (-): 0

Region: chr18 39798612-39798625. Max. coverage (+): 3.84. Max coverage (-): 0

Region: chr18 39798626-39798640. Max. coverage (+): 5.02. Max coverage (-): 0

Region: chr18 39798641-39798655. Max. coverage (+): 0. Max coverage (-): 0

Region: chr18 39798656-39798670. Max. coverage (+): 15.3. Max coverage (-): 0

Region: chr18 39798671-39798684. Max. coverage (+): 0. Max coverage (-): 0

Region: chr18 39798685-39798699. Max. coverage (+): 0. Max coverage (-): 0

Region: chr18 39798700-39798714. Max. coverage (+): 0. Max coverage (-): 0

Region: chr18 39798715-39798729. Max. coverage (+): 0. Max coverage (-): 0

Region: chr18 39798730-39798743. Max. coverage (+): 0. Max coverage (-): 0

Region: chr18 39798744-39798758. Max. coverage (+): 0. Max coverage (-): 0

Region: chr18 39798759-39798773. Max. coverage (+): 0. Max coverage (-): 0

Region: chr18 39798774-39798788. Max. coverage (+): 0. Max coverage (-): 0

Region: chr18 39798789-39798802. Max. coverage (+): 0. Max coverage (-): 0

Region: chr18 39798803-39798817. Max. coverage (+): 0. Max coverage (-): 0

Region: chr18 39798818-39798832. Max. coverage (+): 0. Max coverage (-): 0

Region: chr18 39798833-39798847. Max. coverage (+): 0. Max coverage (-): 0

Region: chr18 39798848-39798862. Max. coverage (+): 0. Max coverage (-): 0

Region: chr18 39798863-39798876. Max. coverage (+): 0. Max coverage (-): 0

Region: chr18 39798877-39798891. Max. coverage (+): 0. Max coverage (-): 0

Region: chr18 39798892-39798906. Max. coverage (+): 5.83. Max coverage (-): 0

Region: chr18 39798907-39798921. Max. coverage (+): 5.83. Max coverage (-): 0

Region: chr18 39798922-39798935. Max. coverage (+): 0. Max coverage (-): 0

Region: chr18 39798936-39798950. Max. coverage (+): 2.06. Max coverage (-): 0

Region: chr18 39798951-39798965. Max. coverage (+): 0. Max coverage (-): 0

Region: chr18 39798966-39798980. Max. coverage (+): 0. Max coverage (-): 0

Region: chr18 39798981-39798994. Max. coverage (+): 0. Max coverage (-): 0

Region: chr18 39798995-39799009. Max. coverage (+): 0. Max coverage (-): 0

Region: chr18 39799010-39799024. Max. coverage (+): 24.8. Max coverage (-): 0

Region: chr18 39799025-39799039. Max. coverage (+): 33.74. Max coverage (-): 0

Region: chr18 39799040-39799054. Max. coverage (+): 33.74. Max coverage (-): 0

Region: chr18 39799055-39799068. Max. coverage (+): 11.02. Max coverage (-): 0

Region: chr18 39799069-39799083. Max. coverage (+): 7.11. Max coverage (-): 0

Region: chr18 39799084-39799098. Max. coverage (+): 0. Max coverage (-): 0

Region: chr18 39799099-39799113. Max. coverage (+): 0. Max coverage (-): 0

Region: chr18 39799114-39799127. Max. coverage (+): 0. Max coverage (-): 0

Region: chr18 39799128-39799142. Max. coverage (+): 0. Max coverage (-): 0

Region: chr18 39799143-39799157. Max. coverage (+): 0. Max coverage (-): 0

Region: chr18 39799158-39799172. Max. coverage (+): 50.07. Max coverage (-): 0

Region: chr18 39799173-39799186. Max. coverage (+): 50.07. Max coverage (-): 0

Region: chr18 39799187-39799201. Max. coverage (+): 6.94. Max coverage (-): 0

Region: chr18 39799202-39799216. Max. coverage (+): 0. Max coverage (-): 0

Region: chr18 39799217-39799231. Max. coverage (+): 0. Max coverage (-): 0

Region: chr18 39799232-39799245. Max. coverage (+): 35.85. Max coverage (-): 0

Region: chr18 39799246-39799260. Max. coverage (+): 35.85. Max coverage (-): 0

Region: chr18 39799261-39799275. Max. coverage (+): 10.67. Max coverage (-): 0

Region: chr18 39799276-39799290. Max. coverage (+): 5.06. Max coverage (-): 0

Region: chr18 39799291-39799305. Max. coverage (+): 2.81. Max coverage (-): 0

Region: chr18 39799306-39799319. Max. coverage (+): 8.61. Max coverage (-): 0

Region: chr18 39799320-39799334. Max. coverage (+): 30.48. Max coverage (-): 0

Region: chr18 39799335-39799349. Max. coverage (+): 33.66. Max coverage (-): 0

Region: chr18 39799350-39799364. Max. coverage (+): 0. Max coverage (-): 0

Region: chr18 39799365-39799378. Max. coverage (+): 0. Max coverage (-): 0

Region: chr18 39799379-39799393. Max. coverage (+): 3.33. Max coverage (-): 0

Region: chr18 39799394-39799408. Max. coverage (+): 10.79. Max coverage (-): 0

Region: chr18 39799409-39799423. Max. coverage (+): 12.55. Max coverage (-): 0

Region: chr18 39799424-39799437. Max. coverage (+): 9.58. Max coverage (-): 0

Region: chr18 39799438-39799452. Max. coverage (+): 25.75. Max coverage (-): 0

Region: chr18 39799453-39799467. Max. coverage (+): 25.75. Max coverage (-): 0

Region: chr18 39799468-39799482. Max. coverage (+): 6.4. Max coverage (-): 0

Region: chr18 39799483-39799496. Max. coverage (+): 6.4. Max coverage (-): 0

Region: chr18 39799497-39799511. Max. coverage (+): 0. Max coverage (-): 0

Region: chr18 39799512-39799526. Max. coverage (+): 0. Max coverage (-): 0

Region: chr18 39799527-39799541. Max. coverage (+): 0. Max coverage (-): 0

Region: chr18 39799542-39799556. Max. coverage (+): 0. Max coverage (-): 0

Region: chr18 39799557-39799570. Max. coverage (+): 3.52. Max coverage (-): 0

Region: chr18 39799571-39799585. Max. coverage (+): 15.98. Max coverage (-): 0

Region: chr18 39799586-39799600. Max. coverage (+): 0. Max coverage (-): 0

Region: chr18 39799601-39799615. Max. coverage (+): 0. Max coverage (-): 0

Region: chr18 39799616-39799629. Max. coverage (+): 0. Max coverage (-): 0

Region: chr18 39799630-39799644. Max. coverage (+): 4.9. Max coverage (-): 0

Region: chr18 39799645-39799659. Max. coverage (+): 6.48. Max coverage (-): 0

Region: chr18 39799660-39799674. Max. coverage (+): 0. Max coverage (-): 0

Region: chr18 39799675-39799688. Max. coverage (+): 0. Max coverage (-): 0

Region: chr18 39799689-39799703. Max. coverage (+): 17.34. Max coverage (-): 0

Region: chr18 39799704-39799718. Max. coverage (+): 5.86. Max coverage (-): 0

Region: chr18 39799719-39799733. Max. coverage (+): 0. Max coverage (-): 0

Region: chr18 39799734-39799748. Max. coverage (+): 14.11. Max coverage (-): 0

Region: chr18 39799749-39799762. Max. coverage (+): 14.11. Max coverage (-): 0

Region: chr18 39799763-39799777. Max. coverage (+): 0. Max coverage (-): 0

Region: chr18 39799778-39799792. Max. coverage (+): 0. Max coverage (-): 0

Region: chr18 39799793-39799807. Max. coverage (+): 0. Max coverage (-): 0

Region: chr18 39799808-39799821. Max. coverage (+): 0. Max coverage (-): 0

Region: chr18 39799822-39799836. Max. coverage (+): 0. Max coverage (-): 0

Region: chr18 39799837-39799851. Max. coverage (+): 3.24. Max coverage (-): 0

Region: chr18 39799852-39799866. Max. coverage (+): 0. Max coverage (-): 0

Region: chr18 39799867-39799880. Max. coverage (+): 15.69. Max coverage (-): 0

Region: chr18 39799881-39799895. Max. coverage (+): 0. Max coverage (-): 0

Region: chr18 39799896-39799910. Max. coverage (+): 0. Max coverage (-): 0

Region: chr18 39799911-39799925. Max. coverage (+): 0. Max coverage (-): 0

Region: chr18 39799926-39799939. Max. coverage (+): 0. Max coverage (-): 0

Region: chr18 39799940-39799954. Max. coverage (+): 0. Max coverage (-): 0

Region: chr18 39799955-39799969. Max. coverage (+): 0. Max coverage (-): 0

Region: chr18 39799970-39799984. Max. coverage (+): 0. Max coverage (-): 0

Region: chr18 39799985-39799999. Max. coverage (+): 0. Max coverage (-): 0

Region: chr18 39800000-39800013. Max. coverage (+): 0. Max coverage (-): 0

Region: chr18 39800014-39800028. Max. coverage (+): 0. Max coverage (-): 0

Region: chr18 39800029-39800043. Max. coverage (+): 0. Max coverage (-): 0

Region: chr18 39800044-39800058. Max. coverage (+): 0. Max coverage (-): 0

Region: chr18 39800059-39800072. Max. coverage (+): 0. Max coverage (-): 0

Region: chr18 39800073-39800087. Max. coverage (+): 0. Max coverage (-): 0

Region: chr18 39800088-39800102. Max. coverage (+): 0. Max coverage (-): 0

Region: chr18 39800103-39800117. Max. coverage (+): 0. Max coverage (-): 0

Region: chr18 39800118-39800131. Max. coverage (+): 0. Max coverage (-): 0

Region: chr18 39800132-39800146. Max. coverage (+): 0. Max coverage (-): 0

Region: chr18 39800147-39800161. Max. coverage (+): 0. Max coverage (-): 0

Region: chr18 39800162-39800176. Max. coverage (+): 0. Max coverage (-): 0

Region: chr18 39800177-39800190. Max. coverage (+): 0. Max coverage (-): 0

Region: chr18 39800191-39800205. Max. coverage (+): 0. Max coverage (-): 0

Region: chr18 39800206-39800220. Max. coverage (+): 0. Max coverage (-): 0

Region: chr18 39800221-39800235. Max. coverage (+): 0. Max coverage (-): 0

Region: chr18 39800236-39800250. Max. coverage (+): 0. Max coverage (-): 0

Region: chr18 39800251-39800264. Max. coverage (+): 0. Max coverage (-): 0

Region: chr18 39800265-39800279. Max. coverage (+): 0. Max coverage (-): 0

Region: chr18 39800280-39800294. Max. coverage (+): 0. Max coverage (-): 0

Region: chr18 39800295-39800309. Max. coverage (+): 0. Max coverage (-): 0

Region: chr18 39800310-39800323. Max. coverage (+): 1.13. Max coverage (-): 0

Region: chr18 39800324-39800338. Max. coverage (+): 19.29. Max coverage (-): 0

Region: chr18 39800339-39800353. Max. coverage (+): 0. Max coverage (-): 0

Region: chr18 39800354-39800368. Max. coverage (+): 3.14. Max coverage (-): 0

Region: chr18 39800369-39800382. Max. coverage (+): 0. Max coverage (-): 0

Region: chr18 39800383-39800397. Max. coverage (+): 0. Max coverage (-): 0

Region: chr18 39800398-39800412. Max. coverage (+): 0. Max coverage (-): 0

Region: chr18 39800413-39800427. Max. coverage (+): 2.45. Max coverage (-): 0

Region: chr18 39800428-39800442. Max. coverage (+): 2.45. Max coverage (-): 0

Region: chr18 39800443-39800456. Max. coverage (+): 0. Max coverage (-): 0

Region: chr18 39800457-39800471. Max. coverage (+): 0. Max coverage (-): 0

Region: chr18 39800472-39800486. Max. coverage (+): 0. Max coverage (-): 0

Region: chr18 39800487-39800501. Max. coverage (+): 3.28. Max coverage (-): 0

Region: chr18 39800502-39800515. Max. coverage (+): 3.28. Max coverage (-): 0

Region: chr18 39800516-39800530. Max. coverage (+): 0.51. Max coverage (-): 0

Region: chr18 39800531-. Max. coverage (+): 0. Max coverage (-): 0

RepeatMasker Color Code

**+**

100-98% Identity

<98-95% Identity

<95-90% Identity

<90-85% Identity

<85-80% Identity

<80-75% Identity

<75-70% Identity

<70% Identity

**-**

Gene Set Color Code

**+**

Gene

Pseudogene

**-**

Topology/Coverage Color Code

Coverage Plus Strand

Coverage Minus Strand

Mainstrand: Plus

Mainstrand: Minus

Complementary Strand

Flanking Region  
(if option -flank >0)

Gene Set Annotation  
  
RepeatMasker Annotation  

**1. MIR**: 39794006-39794167 (+), Divergence to consensus: 32.6%  
**2. AT\_rich**: 39794169-39794194 (+), Divergence to consensus: 61.5%  
**3. MER20**: 39794466-39794697 (-), Divergence to consensus: 28.8%  
**4. LTR16C**: 39797962-39798018 (-), Divergence to consensus: 19.3%  
**5. MER45A**: 39798701-39798859 (+), Divergence to consensus: 42.7%  
**6. Charlie1b**: 39800057-39800250 (-), Divergence to consensus: 28.3%  
**7. Charlie1b**: 39800223-39800275 (-), Divergence to consensus: 15.1%  
**8. LTR37-int**: 39800466-39800522 (+), Divergence to consensus: 17.6%

  
Transcription Factor Binding Sites  

**RFX4\_1** (Sequence: CCTGGCAAC (+): 39795910)  
**Gata4** (Sequence: AGATAAC (-): 39795059)  
**SOX9** (Sequence: TCATTGTT (+): 39796574)  
**SOX9** (Sequence: CTATTGTT (+): 39799355)  
**SPZ1** (Sequence: AGGGTTTCAG (+): 39796430)
