# Supplementary material for: Small RNA sequencing of cryopreserved semen from single bull revealed altered miRNAs and piRNAs expression between High- and Low-motile sperm populations
Source: BMC Genomics. 2017 Jan 4;18:14. doi: 10.1186/s12864-016-3394-7 (PMC5209821; doi:10.1186/s12864-016-3394-7)
Supplement: Additional file 4: — Details for each piRNA clusters found in Low Motile (LM) sperm fraction. Genes, repeats, transposable elements and transcription factors binding sites falling within the cluster regions were reported. (ZIP 1034 kb) [file 12864_2016_3394_MOESM4_ESM.zip › 23.html]

piRNA cluster 23


Predicted piRNA cluster no. 23     previous   next
  

Show proTRAC run info
Hide proTRAC run info

================================= proTRAC ====================================  
VERSION: 2.1                                    LAST MODIFIED: 06. October 2015  
  
Please cite:  
Rosenkranz D, Zischler H. proTRAC - a software for probabilistic piRNA cluster  
detection, visualization and analysis. 2012. BMC Bioinformatics 13:5.  
  
and (for proTRAC 2.0 and later):  
Rosenkranz D, Rudloff S, Bastuck K, Ketting RF, Zischler H. Tupaia small RNAs  
provide insights into function and evolution of RNAi-based transposon defense  
in mammals. 2015. RNA 21(5):911-922.  
  
Contact:  
David Rosenkranz  
Institute of Anthropology, small RNA group  
Johannes Gutenberg University Mainz  
email: rosenkranz@uni-mainz.de  
  
You can find the latest proTRAC version at:  
http://sourceforge.net/projects/protrac/files  
http://www.smallRNAgroup-mainz.de/software  
==============================================================================  
  
PARAMETERS:  
Map file: .............../storage/core/barbara/genhome/smallRNA/fertility/Sample\_not\_motile/pirna/Sample\_not\_motile\_26-33\_collapsed.fa.no-dust.map.weighted-10000-1000-b-0  
Genome file: ............/storage/core/barbara/genhome/smallRNA/fertility/Sample\_all/pirna/bt\_311\_chrY.fa  
RepeatMasker annotation: /storage/genomes/bt\_umd31/GCF\_000003055.6\_Bos\_taurus\_UMD\_3.1.1\_repeatMasker\_chr.out  
GeneSet:................./storage/core/barbara/genhome/smallRNA/fertility/Sample\_all/pirna/full.gtf  
  
Significant (p<=0.01) hit density will be calculated based  
on observed hit distribution.  
  
Sliding window size: ........................................ 5000 bp  
Sliding window increament: .................................. 1000 bp  
Normalize each hit by number of genomic hits: ............... 1 [0=no/1=yes]  
Normalize each hit by number of sequence reads: ............. 1 [0=no/1=yes]  
Normalize values (-> per million mapped reads): ............. 1 [0=no/1=yes]  
Min. fraction of hits with 1T(U) or 10A: .................... 0.75  
Alternatively: Min. fraction of hits with 1T(U) and 10A: .... 0.5  
Min. fraction of hits with typical piRNA length: ............ 0.75  
Typical piRNA length: ....................................... 26-33 nt  
Min. size of a piRNA cluster: ............................... 5000 bp.  
Min. number of hits (absolute): ............................. 0  
Min. number of hits (normalized): ........................... 0  
Min. fraction of hits on the mainstrand: .................... 0.75  
Top fraction of mapped sequences (in terms of read counts): . 1%  
Top fraction accounts for max. n% of sequence reads: ........ 90%  
Min. fraction of hits on each arm of a bidirectional cluster: 0.1  
Output image file for each cluster: ......................... 0 [0=no/1=yes]  
Output html file for each cluster: .......................... 1 [0=no/1=yes]  
Output a summary table: ..................................... 1 [0=no/1=yes]  
Output a FASTA file for each cluster (piRNA sequences): ..... 1 [0=no/1=yes]  
Output a FASTA file comprising cluster sequences: ........... 1 [0=no/1=yes]  
Search DNA motifs in clusters: .............................. 1 [0=no/1=yes]  
Output flanking sequences: +/- .............................. 0 bp  
Output ~.pTi file: .......................................... 1 [0=no/1=yes]  
==============================================================================  
  
  
Genome size (without gaps): ............ 2678902517 bp  
Gaps (N/X/-): .......................... 53837044 bp  
Mapped reads: .......................... 738059667487  
Non-identical sequences: ............... 277001  
Genomic hits: .......................... 533816  
Significant densitiy of mapped reads: .. 15118061 reads/kb

Show proTRAC cluster info
Hide proTRAC cluster info

|  |  |
| --- | --- |
| Location | chr18 |
| Coordinates | 45799158-45805752 |
| Size [bp] | 6595 |
| Sequence hit loci | 163 |
| Mapped reads (normalized) | 407111417 |
| Mapped reads (normalized) per kb | 61730313.4 |
| Normalized reads with 1T (1U) | 92.5% |
| Normalized reads with 10A | 38.8% |
| Normalized reads with length 26-33 nt | 100% |
| Normalized reads on the main strand(s) | 100% |
| Predicted directionality | mono:minus |

100%

0%

1T (1U)  
reads

10A reads

26-33 nt  
reads

reads on mainstrand

**Either the amount of reads with 1T (1U) OR 10A has to exceed 75% (set with option: -1Tor10A)  
Alternatively the amount of reads with 1T (1U) AND 10A has to exceed 50% (set with option: -1Tand10A)  
Minimum amount of reads with preferred size is 75% (set with option: -pisize)  
Minimum amount of reads on the main strand(s) is 75% (set with option: -clstrand)**

Show read coverage
Hide read coverage

WHAT DO I SEE HERE?  
This chart shows the location of mapped sequence reads within a predicted piRNA cluster. The color refers to the number of genomic hits produced by the sequence read in question. A dark red bar indicates that this sequence read produces many other hits elsewhere in the genome. Many adjacent red or yellow bars can indicate the presence of a multi-copy element such as transposons or rRNA genes. A dark green bar indicates that this sequence read maps uniquely to this locus.

1 hit

2-5 hits

6-10 hits

11-20 hits

21-50 hits

51-100 hits

> 100 hits

chr18

45799158

45805752

Gene Set

RepeatMasker

Mapped  
Reads

66.45

plus strand

minus strand

66.45

Region: chr18 39800532-45799164. Max. coverage (+): 0. Max coverage (-): 5.07

Region: chr18 45799165-45799177. Max. coverage (+): 0. Max coverage (-): 5.07

Region: chr18 45799178-45799190. Max. coverage (+): 0. Max coverage (-): 0

Region: chr18 45799191-45799204. Max. coverage (+): 0. Max coverage (-): 0

Region: chr18 45799205-45799217. Max. coverage (+): 0. Max coverage (-): 0

Region: chr18 45799218-45799230. Max. coverage (+): 0. Max coverage (-): 0

Region: chr18 45799231-45799243. Max. coverage (+): 0. Max coverage (-): 0

Region: chr18 45799244-45799256. Max. coverage (+): 0. Max coverage (-): 0

Region: chr18 45799257-45799270. Max. coverage (+): 0. Max coverage (-): 0

Region: chr18 45799271-45799283. Max. coverage (+): 0. Max coverage (-): 0

Region: chr18 45799284-45799296. Max. coverage (+): 0. Max coverage (-): 0

Region: chr18 45799297-45799309. Max. coverage (+): 0. Max coverage (-): 0

Region: chr18 45799310-45799322. Max. coverage (+): 0. Max coverage (-): 0

Region: chr18 45799323-45799336. Max. coverage (+): 0. Max coverage (-): 0

Region: chr18 45799337-45799349. Max. coverage (+): 0. Max coverage (-): 0

Region: chr18 45799350-45799362. Max. coverage (+): 0. Max coverage (-): 0

Region: chr18 45799363-45799375. Max. coverage (+): 0. Max coverage (-): 0

Region: chr18 45799376-45799388. Max. coverage (+): 0. Max coverage (-): 0

Region: chr18 45799389-45799402. Max. coverage (+): 0. Max coverage (-): 0

Region: chr18 45799403-45799415. Max. coverage (+): 0. Max coverage (-): 0

Region: chr18 45799416-45799428. Max. coverage (+): 0. Max coverage (-): 0

Region: chr18 45799429-45799441. Max. coverage (+): 0. Max coverage (-): 0

Region: chr18 45799442-45799454. Max. coverage (+): 0. Max coverage (-): 0

Region: chr18 45799455-45799467. Max. coverage (+): 0. Max coverage (-): 0

Region: chr18 45799468-45799481. Max. coverage (+): 0. Max coverage (-): 0

Region: chr18 45799482-45799494. Max. coverage (+): 0. Max coverage (-): 0

Region: chr18 45799495-45799507. Max. coverage (+): 0. Max coverage (-): 0

Region: chr18 45799508-45799520. Max. coverage (+): 0. Max coverage (-): 0

Region: chr18 45799521-45799533. Max. coverage (+): 0. Max coverage (-): 0

Region: chr18 45799534-45799547. Max. coverage (+): 0. Max coverage (-): 0

Region: chr18 45799548-45799560. Max. coverage (+): 0. Max coverage (-): 0

Region: chr18 45799561-45799573. Max. coverage (+): 0. Max coverage (-): 0

Region: chr18 45799574-45799586. Max. coverage (+): 0. Max coverage (-): 0

Region: chr18 45799587-45799599. Max. coverage (+): 0. Max coverage (-): 0

Region: chr18 45799600-45799613. Max. coverage (+): 0. Max coverage (-): 0

Region: chr18 45799614-45799626. Max. coverage (+): 0. Max coverage (-): 0

Region: chr18 45799627-45799639. Max. coverage (+): 0. Max coverage (-): 0

Region: chr18 45799640-45799652. Max. coverage (+): 0. Max coverage (-): 0

Region: chr18 45799653-45799665. Max. coverage (+): 0. Max coverage (-): 0

Region: chr18 45799666-45799679. Max. coverage (+): 0. Max coverage (-): 0

Region: chr18 45799680-45799692. Max. coverage (+): 0. Max coverage (-): 0

Region: chr18 45799693-45799705. Max. coverage (+): 0. Max coverage (-): 0

Region: chr18 45799706-45799718. Max. coverage (+): 0. Max coverage (-): 0

Region: chr18 45799719-45799731. Max. coverage (+): 0. Max coverage (-): 0

Region: chr18 45799732-45799744. Max. coverage (+): 0. Max coverage (-): 0

Region: chr18 45799745-45799758. Max. coverage (+): 0. Max coverage (-): 0

Region: chr18 45799759-45799771. Max. coverage (+): 0. Max coverage (-): 0

Region: chr18 45799772-45799784. Max. coverage (+): 0. Max coverage (-): 0

Region: chr18 45799785-45799797. Max. coverage (+): 0. Max coverage (-): 0

Region: chr18 45799798-45799810. Max. coverage (+): 0. Max coverage (-): 0

Region: chr18 45799811-45799824. Max. coverage (+): 0. Max coverage (-): 0

Region: chr18 45799825-45799837. Max. coverage (+): 0. Max coverage (-): 0

Region: chr18 45799838-45799850. Max. coverage (+): 0. Max coverage (-): 0

Region: chr18 45799851-45799863. Max. coverage (+): 0. Max coverage (-): 0

Region: chr18 45799864-45799876. Max. coverage (+): 0. Max coverage (-): 0

Region: chr18 45799877-45799890. Max. coverage (+): 0. Max coverage (-): 0

Region: chr18 45799891-45799903. Max. coverage (+): 0. Max coverage (-): 0

Region: chr18 45799904-45799916. Max. coverage (+): 0. Max coverage (-): 0

Region: chr18 45799917-45799929. Max. coverage (+): 0. Max coverage (-): 0

Region: chr18 45799930-45799942. Max. coverage (+): 0. Max coverage (-): 0

Region: chr18 45799943-45799955. Max. coverage (+): 0. Max coverage (-): 0

Region: chr18 45799956-45799969. Max. coverage (+): 0. Max coverage (-): 0

Region: chr18 45799970-45799982. Max. coverage (+): 0. Max coverage (-): 0

Region: chr18 45799983-45799995. Max. coverage (+): 0. Max coverage (-): 0

Region: chr18 45799996-45800008. Max. coverage (+): 0. Max coverage (-): 0

Region: chr18 45800009-45800021. Max. coverage (+): 0. Max coverage (-): 0

Region: chr18 45800022-45800035. Max. coverage (+): 0. Max coverage (-): 0

Region: chr18 45800036-45800048. Max. coverage (+): 0. Max coverage (-): 0

Region: chr18 45800049-45800061. Max. coverage (+): 0. Max coverage (-): 0

Region: chr18 45800062-45800074. Max. coverage (+): 0. Max coverage (-): 0

Region: chr18 45800075-45800087. Max. coverage (+): 0. Max coverage (-): 0

Region: chr18 45800088-45800101. Max. coverage (+): 0. Max coverage (-): 0

Region: chr18 45800102-45800114. Max. coverage (+): 0. Max coverage (-): 0

Region: chr18 45800115-45800127. Max. coverage (+): 0. Max coverage (-): 0

Region: chr18 45800128-45800140. Max. coverage (+): 0. Max coverage (-): 0

Region: chr18 45800141-45800153. Max. coverage (+): 0. Max coverage (-): 0

Region: chr18 45800154-45800167. Max. coverage (+): 0. Max coverage (-): 0

Region: chr18 45800168-45800180. Max. coverage (+): 0. Max coverage (-): 0

Region: chr18 45800181-45800193. Max. coverage (+): 0. Max coverage (-): 0

Region: chr18 45800194-45800206. Max. coverage (+): 0. Max coverage (-): 0

Region: chr18 45800207-45800219. Max. coverage (+): 0. Max coverage (-): 0

Region: chr18 45800220-45800232. Max. coverage (+): 0. Max coverage (-): 0

Region: chr18 45800233-45800246. Max. coverage (+): 0. Max coverage (-): 0

Region: chr18 45800247-45800259. Max. coverage (+): 0. Max coverage (-): 0

Region: chr18 45800260-45800272. Max. coverage (+): 0. Max coverage (-): 0

Region: chr18 45800273-45800285. Max. coverage (+): 0. Max coverage (-): 0

Region: chr18 45800286-45800298. Max. coverage (+): 0. Max coverage (-): 0

Region: chr18 45800299-45800312. Max. coverage (+): 0. Max coverage (-): 0

Region: chr18 45800313-45800325. Max. coverage (+): 0. Max coverage (-): 0

Region: chr18 45800326-45800338. Max. coverage (+): 0. Max coverage (-): 0

Region: chr18 45800339-45800351. Max. coverage (+): 0. Max coverage (-): 0

Region: chr18 45800352-45800364. Max. coverage (+): 0. Max coverage (-): 0

Region: chr18 45800365-45800378. Max. coverage (+): 0. Max coverage (-): 0

Region: chr18 45800379-45800391. Max. coverage (+): 0. Max coverage (-): 0

Region: chr18 45800392-45800404. Max. coverage (+): 0. Max coverage (-): 0

Region: chr18 45800405-45800417. Max. coverage (+): 0. Max coverage (-): 0

Region: chr18 45800418-45800430. Max. coverage (+): 0. Max coverage (-): 0

Region: chr18 45800431-45800444. Max. coverage (+): 0. Max coverage (-): 0

Region: chr18 45800445-45800457. Max. coverage (+): 0. Max coverage (-): 0

Region: chr18 45800458-45800470. Max. coverage (+): 0. Max coverage (-): 0

Region: chr18 45800471-45800483. Max. coverage (+): 0. Max coverage (-): 0

Region: chr18 45800484-45800496. Max. coverage (+): 0. Max coverage (-): 0

Region: chr18 45800497-45800509. Max. coverage (+): 0. Max coverage (-): 0

Region: chr18 45800510-45800523. Max. coverage (+): 0. Max coverage (-): 0

Region: chr18 45800524-45800536. Max. coverage (+): 0. Max coverage (-): 0

Region: chr18 45800537-45800549. Max. coverage (+): 0. Max coverage (-): 0

Region: chr18 45800550-45800562. Max. coverage (+): 0. Max coverage (-): 0

Region: chr18 45800563-45800575. Max. coverage (+): 0. Max coverage (-): 0

Region: chr18 45800576-45800589. Max. coverage (+): 0. Max coverage (-): 0

Region: chr18 45800590-45800602. Max. coverage (+): 0. Max coverage (-): 0

Region: chr18 45800603-45800615. Max. coverage (+): 0. Max coverage (-): 0

Region: chr18 45800616-45800628. Max. coverage (+): 0. Max coverage (-): 0

Region: chr18 45800629-45800641. Max. coverage (+): 0. Max coverage (-): 0

Region: chr18 45800642-45800655. Max. coverage (+): 0. Max coverage (-): 0

Region: chr18 45800656-45800668. Max. coverage (+): 0. Max coverage (-): 0

Region: chr18 45800669-45800681. Max. coverage (+): 0. Max coverage (-): 0

Region: chr18 45800682-45800694. Max. coverage (+): 0. Max coverage (-): 0

Region: chr18 45800695-45800707. Max. coverage (+): 0. Max coverage (-): 0

Region: chr18 45800708-45800721. Max. coverage (+): 0. Max coverage (-): 0

Region: chr18 45800722-45800734. Max. coverage (+): 0. Max coverage (-): 0

Region: chr18 45800735-45800747. Max. coverage (+): 0. Max coverage (-): 0

Region: chr18 45800748-45800760. Max. coverage (+): 0. Max coverage (-): 0

Region: chr18 45800761-45800773. Max. coverage (+): 0. Max coverage (-): 0

Region: chr18 45800774-45800786. Max. coverage (+): 0. Max coverage (-): 2.46

Region: chr18 45800787-45800800. Max. coverage (+): 0. Max coverage (-): 0

Region: chr18 45800801-45800813. Max. coverage (+): 0. Max coverage (-): 0

Region: chr18 45800814-45800826. Max. coverage (+): 0. Max coverage (-): 0

Region: chr18 45800827-45800839. Max. coverage (+): 0. Max coverage (-): 0

Region: chr18 45800840-45800852. Max. coverage (+): 0. Max coverage (-): 0

Region: chr18 45800853-45800866. Max. coverage (+): 0. Max coverage (-): 0

Region: chr18 45800867-45800879. Max. coverage (+): 0. Max coverage (-): 0

Region: chr18 45800880-45800892. Max. coverage (+): 0. Max coverage (-): 0

Region: chr18 45800893-45800905. Max. coverage (+): 0. Max coverage (-): 0

Region: chr18 45800906-45800918. Max. coverage (+): 0. Max coverage (-): 0

Region: chr18 45800919-45800932. Max. coverage (+): 0. Max coverage (-): 0

Region: chr18 45800933-45800945. Max. coverage (+): 0. Max coverage (-): 0

Region: chr18 45800946-45800958. Max. coverage (+): 0. Max coverage (-): 0

Region: chr18 45800959-45800971. Max. coverage (+): 0. Max coverage (-): 0

Region: chr18 45800972-45800984. Max. coverage (+): 0. Max coverage (-): 0

Region: chr18 45800985-45800998. Max. coverage (+): 0. Max coverage (-): 0

Region: chr18 45800999-45801011. Max. coverage (+): 0. Max coverage (-): 0

Region: chr18 45801012-45801024. Max. coverage (+): 0. Max coverage (-): 0

Region: chr18 45801025-45801037. Max. coverage (+): 0. Max coverage (-): 0

Region: chr18 45801038-45801050. Max. coverage (+): 0. Max coverage (-): 0

Region: chr18 45801051-45801063. Max. coverage (+): 0. Max coverage (-): 0

Region: chr18 45801064-45801077. Max. coverage (+): 0. Max coverage (-): 0

Region: chr18 45801078-45801090. Max. coverage (+): 0. Max coverage (-): 0

Region: chr18 45801091-45801103. Max. coverage (+): 0. Max coverage (-): 0

Region: chr18 45801104-45801116. Max. coverage (+): 0. Max coverage (-): 0

Region: chr18 45801117-45801129. Max. coverage (+): 0. Max coverage (-): 0

Region: chr18 45801130-45801143. Max. coverage (+): 0. Max coverage (-): 0

Region: chr18 45801144-45801156. Max. coverage (+): 0. Max coverage (-): 0

Region: chr18 45801157-45801169. Max. coverage (+): 0. Max coverage (-): 0

Region: chr18 45801170-45801182. Max. coverage (+): 0. Max coverage (-): 0

Region: chr18 45801183-45801195. Max. coverage (+): 0. Max coverage (-): 0

Region: chr18 45801196-45801209. Max. coverage (+): 0. Max coverage (-): 0

Region: chr18 45801210-45801222. Max. coverage (+): 0. Max coverage (-): 0

Region: chr18 45801223-45801235. Max. coverage (+): 0. Max coverage (-): 0

Region: chr18 45801236-45801248. Max. coverage (+): 0. Max coverage (-): 0

Region: chr18 45801249-45801261. Max. coverage (+): 0. Max coverage (-): 0

Region: chr18 45801262-45801274. Max. coverage (+): 0. Max coverage (-): 0

Region: chr18 45801275-45801288. Max. coverage (+): 0. Max coverage (-): 0

Region: chr18 45801289-45801301. Max. coverage (+): 0. Max coverage (-): 0

Region: chr18 45801302-45801314. Max. coverage (+): 0. Max coverage (-): 0

Region: chr18 45801315-45801327. Max. coverage (+): 0. Max coverage (-): 0

Region: chr18 45801328-45801340. Max. coverage (+): 0. Max coverage (-): 0

Region: chr18 45801341-45801354. Max. coverage (+): 0. Max coverage (-): 2.24

Region: chr18 45801355-45801367. Max. coverage (+): 0. Max coverage (-): 2.24

Region: chr18 45801368-45801380. Max. coverage (+): 0. Max coverage (-): 0

Region: chr18 45801381-45801393. Max. coverage (+): 0. Max coverage (-): 0

Region: chr18 45801394-45801406. Max. coverage (+): 0. Max coverage (-): 0.49

Region: chr18 45801407-45801420. Max. coverage (+): 0. Max coverage (-): 0

Region: chr18 45801421-45801433. Max. coverage (+): 0. Max coverage (-): 0

Region: chr18 45801434-45801446. Max. coverage (+): 0. Max coverage (-): 0

Region: chr18 45801447-45801459. Max. coverage (+): 0. Max coverage (-): 0

Region: chr18 45801460-45801472. Max. coverage (+): 0. Max coverage (-): 0

Region: chr18 45801473-45801486. Max. coverage (+): 0. Max coverage (-): 0

Region: chr18 45801487-45801499. Max. coverage (+): 0. Max coverage (-): 5.99

Region: chr18 45801500-45801512. Max. coverage (+): 0. Max coverage (-): 1.16

Region: chr18 45801513-45801525. Max. coverage (+): 0. Max coverage (-): 0

Region: chr18 45801526-45801538. Max. coverage (+): 0. Max coverage (-): 0

Region: chr18 45801539-45801551. Max. coverage (+): 0. Max coverage (-): 0

Region: chr18 45801552-45801565. Max. coverage (+): 0. Max coverage (-): 0

Region: chr18 45801566-45801578. Max. coverage (+): 0. Max coverage (-): 0

Region: chr18 45801579-45801591. Max. coverage (+): 0. Max coverage (-): 0

Region: chr18 45801592-45801604. Max. coverage (+): 0. Max coverage (-): 0

Region: chr18 45801605-45801617. Max. coverage (+): 0. Max coverage (-): 0

Region: chr18 45801618-45801631. Max. coverage (+): 0. Max coverage (-): 0

Region: chr18 45801632-45801644. Max. coverage (+): 0. Max coverage (-): 0

Region: chr18 45801645-45801657. Max. coverage (+): 0. Max coverage (-): 0

Region: chr18 45801658-45801670. Max. coverage (+): 0. Max coverage (-): 0

Region: chr18 45801671-45801683. Max. coverage (+): 0. Max coverage (-): 0

Region: chr18 45801684-45801697. Max. coverage (+): 0. Max coverage (-): 0

Region: chr18 45801698-45801710. Max. coverage (+): 0. Max coverage (-): 0

Region: chr18 45801711-45801723. Max. coverage (+): 0. Max coverage (-): 0

Region: chr18 45801724-45801736. Max. coverage (+): 0. Max coverage (-): 0

Region: chr18 45801737-45801749. Max. coverage (+): 0. Max coverage (-): 0

Region: chr18 45801750-45801763. Max. coverage (+): 0. Max coverage (-): 0

Region: chr18 45801764-45801776. Max. coverage (+): 0. Max coverage (-): 0

Region: chr18 45801777-45801789. Max. coverage (+): 0. Max coverage (-): 0

Region: chr18 45801790-45801802. Max. coverage (+): 0. Max coverage (-): 1.15

Region: chr18 45801803-45801815. Max. coverage (+): 0. Max coverage (-): 0

Region: chr18 45801816-45801828. Max. coverage (+): 0. Max coverage (-): 0

Region: chr18 45801829-45801842. Max. coverage (+): 0. Max coverage (-): 0

Region: chr18 45801843-45801855. Max. coverage (+): 0. Max coverage (-): 0

Region: chr18 45801856-45801868. Max. coverage (+): 0. Max coverage (-): 9.82

Region: chr18 45801869-45801881. Max. coverage (+): 0. Max coverage (-): 27.03

Region: chr18 45801882-45801894. Max. coverage (+): 0. Max coverage (-): 5.68

Region: chr18 45801895-45801908. Max. coverage (+): 0. Max coverage (-): 0

Region: chr18 45801909-45801921. Max. coverage (+): 0. Max coverage (-): 0

Region: chr18 45801922-45801934. Max. coverage (+): 0. Max coverage (-): 0

Region: chr18 45801935-45801947. Max. coverage (+): 0. Max coverage (-): 0

Region: chr18 45801948-45801960. Max. coverage (+): 0. Max coverage (-): 0

Region: chr18 45801961-45801974. Max. coverage (+): 0. Max coverage (-): 0

Region: chr18 45801975-45801987. Max. coverage (+): 0. Max coverage (-): 0

Region: chr18 45801988-45802000. Max. coverage (+): 0. Max coverage (-): 0

Region: chr18 45802001-45802013. Max. coverage (+): 0. Max coverage (-): 0

Region: chr18 45802014-45802026. Max. coverage (+): 0. Max coverage (-): 3.39

Region: chr18 45802027-45802040. Max. coverage (+): 0. Max coverage (-): 9.82

Region: chr18 45802041-45802053. Max. coverage (+): 0. Max coverage (-): 0

Region: chr18 45802054-45802066. Max. coverage (+): 0. Max coverage (-): 5.08

Region: chr18 45802067-45802079. Max. coverage (+): 0. Max coverage (-): 5.08

Region: chr18 45802080-45802092. Max. coverage (+): 0. Max coverage (-): 10.74

Region: chr18 45802093-45802105. Max. coverage (+): 0. Max coverage (-): 10.74

Region: chr18 45802106-45802119. Max. coverage (+): 0. Max coverage (-): 0

Region: chr18 45802120-45802132. Max. coverage (+): 0. Max coverage (-): 0

Region: chr18 45802133-45802145. Max. coverage (+): 0. Max coverage (-): 0

Region: chr18 45802146-45802158. Max. coverage (+): 0. Max coverage (-): 2.18

Region: chr18 45802159-45802171. Max. coverage (+): 0. Max coverage (-): 0

Region: chr18 45802172-45802185. Max. coverage (+): 0. Max coverage (-): 0

Region: chr18 45802186-45802198. Max. coverage (+): 0. Max coverage (-): 0

Region: chr18 45802199-45802211. Max. coverage (+): 0. Max coverage (-): 6.7

Region: chr18 45802212-45802224. Max. coverage (+): 0. Max coverage (-): 6.7

Region: chr18 45802225-45802237. Max. coverage (+): 0. Max coverage (-): 6.6

Region: chr18 45802238-45802251. Max. coverage (+): 0. Max coverage (-): 4.76

Region: chr18 45802252-45802264. Max. coverage (+): 0. Max coverage (-): 0

Region: chr18 45802265-45802277. Max. coverage (+): 0. Max coverage (-): 0

Region: chr18 45802278-45802290. Max. coverage (+): 0. Max coverage (-): 0

Region: chr18 45802291-45802303. Max. coverage (+): 0. Max coverage (-): 0

Region: chr18 45802304-45802317. Max. coverage (+): 0. Max coverage (-): 0

Region: chr18 45802318-45802330. Max. coverage (+): 0. Max coverage (-): 30.13

Region: chr18 45802331-45802343. Max. coverage (+): 0. Max coverage (-): 25.11

Region: chr18 45802344-45802356. Max. coverage (+): 0. Max coverage (-): 0

Region: chr18 45802357-45802369. Max. coverage (+): 0. Max coverage (-): 0

Region: chr18 45802370-45802382. Max. coverage (+): 0. Max coverage (-): 19.29

Region: chr18 45802383-45802396. Max. coverage (+): 0. Max coverage (-): 0

Region: chr18 45802397-45802409. Max. coverage (+): 0. Max coverage (-): 0

Region: chr18 45802410-45802422. Max. coverage (+): 0. Max coverage (-): 0

Region: chr18 45802423-45802435. Max. coverage (+): 0. Max coverage (-): 0

Region: chr18 45802436-45802448. Max. coverage (+): 0. Max coverage (-): 0

Region: chr18 45802449-45802462. Max. coverage (+): 0. Max coverage (-): 0

Region: chr18 45802463-45802475. Max. coverage (+): 0. Max coverage (-): 0

Region: chr18 45802476-45802488. Max. coverage (+): 0. Max coverage (-): 0.01

Region: chr18 45802489-45802501. Max. coverage (+): 0. Max coverage (-): 0

Region: chr18 45802502-45802514. Max. coverage (+): 0. Max coverage (-): 0.69

Region: chr18 45802515-45802528. Max. coverage (+): 0. Max coverage (-): 0.69

Region: chr18 45802529-45802541. Max. coverage (+): 0. Max coverage (-): 0

Region: chr18 45802542-45802554. Max. coverage (+): 0. Max coverage (-): 0

Region: chr18 45802555-45802567. Max. coverage (+): 0. Max coverage (-): 0

Region: chr18 45802568-45802580. Max. coverage (+): 0. Max coverage (-): 0.17

Region: chr18 45802581-45802593. Max. coverage (+): 0. Max coverage (-): 0.17

Region: chr18 45802594-45802607. Max. coverage (+): 0. Max coverage (-): 0

Region: chr18 45802608-45802620. Max. coverage (+): 0. Max coverage (-): 0

Region: chr18 45802621-45802633. Max. coverage (+): 0. Max coverage (-): 0

Region: chr18 45802634-45802646. Max. coverage (+): 0. Max coverage (-): 0

Region: chr18 45802647-45802659. Max. coverage (+): 0. Max coverage (-): 0

Region: chr18 45802660-45802673. Max. coverage (+): 0. Max coverage (-): 0

Region: chr18 45802674-45802686. Max. coverage (+): 0. Max coverage (-): 0

Region: chr18 45802687-45802699. Max. coverage (+): 0. Max coverage (-): 0

Region: chr18 45802700-45802712. Max. coverage (+): 0. Max coverage (-): 0

Region: chr18 45802713-45802725. Max. coverage (+): 0. Max coverage (-): 0

Region: chr18 45802726-45802739. Max. coverage (+): 0. Max coverage (-): 6.98

Region: chr18 45802740-45802752. Max. coverage (+): 0. Max coverage (-): 6.98

Region: chr18 45802753-45802765. Max. coverage (+): 0. Max coverage (-): 11.43

Region: chr18 45802766-45802778. Max. coverage (+): 0. Max coverage (-): 0

Region: chr18 45802779-45802791. Max. coverage (+): 0. Max coverage (-): 0

Region: chr18 45802792-45802805. Max. coverage (+): 0. Max coverage (-): 0

Region: chr18 45802806-45802818. Max. coverage (+): 0. Max coverage (-): 0

Region: chr18 45802819-45802831. Max. coverage (+): 0. Max coverage (-): 0

Region: chr18 45802832-45802844. Max. coverage (+): 0. Max coverage (-): 0

Region: chr18 45802845-45802857. Max. coverage (+): 0. Max coverage (-): 0

Region: chr18 45802858-45802870. Max. coverage (+): 0. Max coverage (-): 0

Region: chr18 45802871-45802884. Max. coverage (+): 0. Max coverage (-): 0

Region: chr18 45802885-45802897. Max. coverage (+): 0. Max coverage (-): 0

Region: chr18 45802898-45802910. Max. coverage (+): 0. Max coverage (-): 8.78

Region: chr18 45802911-45802923. Max. coverage (+): 0. Max coverage (-): 12.12

Region: chr18 45802924-45802936. Max. coverage (+): 0. Max coverage (-): 11.08

Region: chr18 45802937-45802950. Max. coverage (+): 0. Max coverage (-): 17.84

Region: chr18 45802951-45802963. Max. coverage (+): 0. Max coverage (-): 0

Region: chr18 45802964-45802976. Max. coverage (+): 0. Max coverage (-): 0.61

Region: chr18 45802977-45802989. Max. coverage (+): 0. Max coverage (-): 3.44

Region: chr18 45802990-45803002. Max. coverage (+): 0. Max coverage (-): 3.44

Region: chr18 45803003-45803016. Max. coverage (+): 0. Max coverage (-): 0

Region: chr18 45803017-45803029. Max. coverage (+): 0. Max coverage (-): 0

Region: chr18 45803030-45803042. Max. coverage (+): 0. Max coverage (-): 0

Region: chr18 45803043-45803055. Max. coverage (+): 0. Max coverage (-): 3.97

Region: chr18 45803056-45803068. Max. coverage (+): 0. Max coverage (-): 0

Region: chr18 45803069-45803082. Max. coverage (+): 0. Max coverage (-): 0

Region: chr18 45803083-45803095. Max. coverage (+): 0. Max coverage (-): 6.18

Region: chr18 45803096-45803108. Max. coverage (+): 0. Max coverage (-): 8.21

Region: chr18 45803109-45803121. Max. coverage (+): 0. Max coverage (-): 8.21

Region: chr18 45803122-45803134. Max. coverage (+): 0. Max coverage (-): 0

Region: chr18 45803135-45803147. Max. coverage (+): 0. Max coverage (-): 0

Region: chr18 45803148-45803161. Max. coverage (+): 0. Max coverage (-): 0

Region: chr18 45803162-45803174. Max. coverage (+): 0. Max coverage (-): 6.88

Region: chr18 45803175-45803187. Max. coverage (+): 0. Max coverage (-): 10.39

Region: chr18 45803188-45803200. Max. coverage (+): 0. Max coverage (-): 13.85

Region: chr18 45803201-45803213. Max. coverage (+): 0. Max coverage (-): 0

Region: chr18 45803214-45803227. Max. coverage (+): 0. Max coverage (-): 9.59

Region: chr18 45803228-45803240. Max. coverage (+): 0. Max coverage (-): 9.59

Region: chr18 45803241-45803253. Max. coverage (+): 0. Max coverage (-): 1.97

Region: chr18 45803254-45803266. Max. coverage (+): 0. Max coverage (-): 2.05

Region: chr18 45803267-45803279. Max. coverage (+): 0. Max coverage (-): 4.98

Region: chr18 45803280-45803293. Max. coverage (+): 0. Max coverage (-): 0

Region: chr18 45803294-45803306. Max. coverage (+): 0. Max coverage (-): 0

Region: chr18 45803307-45803319. Max. coverage (+): 0. Max coverage (-): 0.06

Region: chr18 45803320-45803332. Max. coverage (+): 0. Max coverage (-): 0.06

Region: chr18 45803333-45803345. Max. coverage (+): 0. Max coverage (-): 0

Region: chr18 45803346-45803359. Max. coverage (+): 0. Max coverage (-): 14.23

Region: chr18 45803360-45803372. Max. coverage (+): 0. Max coverage (-): 14.23

Region: chr18 45803373-45803385. Max. coverage (+): 0. Max coverage (-): 6.06

Region: chr18 45803386-45803398. Max. coverage (+): 0. Max coverage (-): 25.5

Region: chr18 45803399-45803411. Max. coverage (+): 0. Max coverage (-): 0

Region: chr18 45803412-45803424. Max. coverage (+): 0. Max coverage (-): 0

Region: chr18 45803425-45803438. Max. coverage (+): 0. Max coverage (-): 0

Region: chr18 45803439-45803451. Max. coverage (+): 0. Max coverage (-): 3.56

Region: chr18 45803452-45803464. Max. coverage (+): 0. Max coverage (-): 3.56

Region: chr18 45803465-45803477. Max. coverage (+): 0. Max coverage (-): 3.95

Region: chr18 45803478-45803490. Max. coverage (+): 0. Max coverage (-): 2.85

Region: chr18 45803491-45803504. Max. coverage (+): 0. Max coverage (-): 2.85

Region: chr18 45803505-45803517. Max. coverage (+): 0. Max coverage (-): 0

Region: chr18 45803518-45803530. Max. coverage (+): 0. Max coverage (-): 0

Region: chr18 45803531-45803543. Max. coverage (+): 0. Max coverage (-): 0

Region: chr18 45803544-45803556. Max. coverage (+): 0. Max coverage (-): 33.32

Region: chr18 45803557-45803570. Max. coverage (+): 0. Max coverage (-): 33.32

Region: chr18 45803571-45803583. Max. coverage (+): 0. Max coverage (-): 0

Region: chr18 45803584-45803596. Max. coverage (+): 0. Max coverage (-): 0

Region: chr18 45803597-45803609. Max. coverage (+): 0. Max coverage (-): 0

Region: chr18 45803610-45803622. Max. coverage (+): 0. Max coverage (-): 3.66

Region: chr18 45803623-45803636. Max. coverage (+): 0. Max coverage (-): 0

Region: chr18 45803637-45803649. Max. coverage (+): 0. Max coverage (-): 0

Region: chr18 45803650-45803662. Max. coverage (+): 0. Max coverage (-): 5.16

Region: chr18 45803663-45803675. Max. coverage (+): 0. Max coverage (-): 5.82

Region: chr18 45803676-45803688. Max. coverage (+): 0. Max coverage (-): 5.82

Region: chr18 45803689-45803701. Max. coverage (+): 0. Max coverage (-): 0

Region: chr18 45803702-45803715. Max. coverage (+): 0. Max coverage (-): 0

Region: chr18 45803716-45803728. Max. coverage (+): 0. Max coverage (-): 2.05

Region: chr18 45803729-45803741. Max. coverage (+): 0. Max coverage (-): 0

Region: chr18 45803742-45803754. Max. coverage (+): 0. Max coverage (-): 0

Region: chr18 45803755-45803767. Max. coverage (+): 0. Max coverage (-): 1.31

Region: chr18 45803768-45803781. Max. coverage (+): 0. Max coverage (-): 1.31

Region: chr18 45803782-45803794. Max. coverage (+): 0. Max coverage (-): 0

Region: chr18 45803795-45803807. Max. coverage (+): 0. Max coverage (-): 0

Region: chr18 45803808-45803820. Max. coverage (+): 0. Max coverage (-): 0

Region: chr18 45803821-45803833. Max. coverage (+): 0. Max coverage (-): 6.61

Region: chr18 45803834-45803847. Max. coverage (+): 0. Max coverage (-): 0.29

Region: chr18 45803848-45803860. Max. coverage (+): 0. Max coverage (-): 0

Region: chr18 45803861-45803873. Max. coverage (+): 0. Max coverage (-): 0

Region: chr18 45803874-45803886. Max. coverage (+): 0. Max coverage (-): 0

Region: chr18 45803887-45803899. Max. coverage (+): 0. Max coverage (-): 0

Region: chr18 45803900-45803912. Max. coverage (+): 0. Max coverage (-): 7.65

Region: chr18 45803913-45803926. Max. coverage (+): 0. Max coverage (-): 19.34

Region: chr18 45803927-45803939. Max. coverage (+): 0. Max coverage (-): 11.94

Region: chr18 45803940-45803952. Max. coverage (+): 0. Max coverage (-): 3.47

Region: chr18 45803953-45803965. Max. coverage (+): 0. Max coverage (-): 0

Region: chr18 45803966-45803978. Max. coverage (+): 0. Max coverage (-): 0

Region: chr18 45803979-45803992. Max. coverage (+): 0. Max coverage (-): 3.6

Region: chr18 45803993-45804005. Max. coverage (+): 0. Max coverage (-): 3.6

Region: chr18 45804006-45804018. Max. coverage (+): 0. Max coverage (-): 14.04

Region: chr18 45804019-45804031. Max. coverage (+): 0. Max coverage (-): 9.31

Region: chr18 45804032-45804044. Max. coverage (+): 0. Max coverage (-): 0.83

Region: chr18 45804045-45804058. Max. coverage (+): 0. Max coverage (-): 3.08

Region: chr18 45804059-45804071. Max. coverage (+): 0. Max coverage (-): 3.08

Region: chr18 45804072-45804084. Max. coverage (+): 0. Max coverage (-): 11.8

Region: chr18 45804085-45804097. Max. coverage (+): 0. Max coverage (-): 23.89

Region: chr18 45804098-45804110. Max. coverage (+): 0. Max coverage (-): 66.45

Region: chr18 45804111-45804124. Max. coverage (+): 0. Max coverage (-): 8.48

Region: chr18 45804125-45804137. Max. coverage (+): 0. Max coverage (-): 22.35

Region: chr18 45804138-45804150. Max. coverage (+): 0. Max coverage (-): 8.09

Region: chr18 45804151-45804163. Max. coverage (+): 0. Max coverage (-): 0

Region: chr18 45804164-45804176. Max. coverage (+): 0. Max coverage (-): 0

Region: chr18 45804177-45804189. Max. coverage (+): 0. Max coverage (-): 0

Region: chr18 45804190-45804203. Max. coverage (+): 0. Max coverage (-): 0

Region: chr18 45804204-45804216. Max. coverage (+): 0. Max coverage (-): 0

Region: chr18 45804217-45804229. Max. coverage (+): 0. Max coverage (-): 0

Region: chr18 45804230-45804242. Max. coverage (+): 0. Max coverage (-): 0

Region: chr18 45804243-45804255. Max. coverage (+): 0. Max coverage (-): 0

Region: chr18 45804256-45804269. Max. coverage (+): 0. Max coverage (-): 0

Region: chr18 45804270-45804282. Max. coverage (+): 0. Max coverage (-): 0

Region: chr18 45804283-45804295. Max. coverage (+): 0. Max coverage (-): 0

Region: chr18 45804296-45804308. Max. coverage (+): 0. Max coverage (-): 0

Region: chr18 45804309-45804321. Max. coverage (+): 0. Max coverage (-): 0

Region: chr18 45804322-45804335. Max. coverage (+): 0. Max coverage (-): 0

Region: chr18 45804336-45804348. Max. coverage (+): 0. Max coverage (-): 0

Region: chr18 45804349-45804361. Max. coverage (+): 0. Max coverage (-): 0

Region: chr18 45804362-45804374. Max. coverage (+): 0. Max coverage (-): 0

Region: chr18 45804375-45804387. Max. coverage (+): 0. Max coverage (-): 0

Region: chr18 45804388-45804401. Max. coverage (+): 0. Max coverage (-): 0

Region: chr18 45804402-45804414. Max. coverage (+): 0. Max coverage (-): 0

Region: chr18 45804415-45804427. Max. coverage (+): 0. Max coverage (-): 0

Region: chr18 45804428-45804440. Max. coverage (+): 0. Max coverage (-): 0

Region: chr18 45804441-45804453. Max. coverage (+): 0. Max coverage (-): 0

Region: chr18 45804454-45804466. Max. coverage (+): 0. Max coverage (-): 0

Region: chr18 45804467-45804480. Max. coverage (+): 0. Max coverage (-): 0

Region: chr18 45804481-45804493. Max. coverage (+): 0. Max coverage (-): 0

Region: chr18 45804494-45804506. Max. coverage (+): 0. Max coverage (-): 0

Region: chr18 45804507-45804519. Max. coverage (+): 0. Max coverage (-): 0

Region: chr18 45804520-45804532. Max. coverage (+): 0. Max coverage (-): 0

Region: chr18 45804533-45804546. Max. coverage (+): 0. Max coverage (-): 0

Region: chr18 45804547-45804559. Max. coverage (+): 0. Max coverage (-): 0

Region: chr18 45804560-45804572. Max. coverage (+): 0. Max coverage (-): 0

Region: chr18 45804573-45804585. Max. coverage (+): 0. Max coverage (-): 0

Region: chr18 45804586-45804598. Max. coverage (+): 0. Max coverage (-): 0

Region: chr18 45804599-45804612. Max. coverage (+): 0. Max coverage (-): 0

Region: chr18 45804613-45804625. Max. coverage (+): 0. Max coverage (-): 0

Region: chr18 45804626-45804638. Max. coverage (+): 0. Max coverage (-): 0

Region: chr18 45804639-45804651. Max. coverage (+): 0. Max coverage (-): 0

Region: chr18 45804652-45804664. Max. coverage (+): 0. Max coverage (-): 0

Region: chr18 45804665-45804678. Max. coverage (+): 0. Max coverage (-): 0

Region: chr18 45804679-45804691. Max. coverage (+): 0. Max coverage (-): 0

Region: chr18 45804692-45804704. Max. coverage (+): 0. Max coverage (-): 0

Region: chr18 45804705-45804717. Max. coverage (+): 0. Max coverage (-): 0

Region: chr18 45804718-45804730. Max. coverage (+): 0. Max coverage (-): 0

Region: chr18 45804731-45804743. Max. coverage (+): 0. Max coverage (-): 0

Region: chr18 45804744-45804757. Max. coverage (+): 0. Max coverage (-): 0

Region: chr18 45804758-45804770. Max. coverage (+): 0. Max coverage (-): 0

Region: chr18 45804771-45804783. Max. coverage (+): 0. Max coverage (-): 0

Region: chr18 45804784-45804796. Max. coverage (+): 0. Max coverage (-): 0

Region: chr18 45804797-45804809. Max. coverage (+): 0. Max coverage (-): 0

Region: chr18 45804810-45804823. Max. coverage (+): 0. Max coverage (-): 0

Region: chr18 45804824-45804836. Max. coverage (+): 0. Max coverage (-): 0

Region: chr18 45804837-45804849. Max. coverage (+): 0. Max coverage (-): 0

Region: chr18 45804850-45804862. Max. coverage (+): 0. Max coverage (-): 0

Region: chr18 45804863-45804875. Max. coverage (+): 0. Max coverage (-): 0

Region: chr18 45804876-45804889. Max. coverage (+): 0. Max coverage (-): 0

Region: chr18 45804890-45804902. Max. coverage (+): 0. Max coverage (-): 0

Region: chr18 45804903-45804915. Max. coverage (+): 0. Max coverage (-): 0

Region: chr18 45804916-45804928. Max. coverage (+): 0. Max coverage (-): 0

Region: chr18 45804929-45804941. Max. coverage (+): 0. Max coverage (-): 0

Region: chr18 45804942-45804955. Max. coverage (+): 0. Max coverage (-): 0

Region: chr18 45804956-45804968. Max. coverage (+): 0. Max coverage (-): 0

Region: chr18 45804969-45804981. Max. coverage (+): 0. Max coverage (-): 0

Region: chr18 45804982-45804994. Max. coverage (+): 0. Max coverage (-): 0

Region: chr18 45804995-45805007. Max. coverage (+): 0. Max coverage (-): 0

Region: chr18 45805008-45805020. Max. coverage (+): 0. Max coverage (-): 0

Region: chr18 45805021-45805034. Max. coverage (+): 0. Max coverage (-): 0

Region: chr18 45805035-45805047. Max. coverage (+): 0. Max coverage (-): 0

Region: chr18 45805048-45805060. Max. coverage (+): 0. Max coverage (-): 0

Region: chr18 45805061-45805073. Max. coverage (+): 0. Max coverage (-): 0

Region: chr18 45805074-45805086. Max. coverage (+): 0. Max coverage (-): 0

Region: chr18 45805087-45805100. Max. coverage (+): 0. Max coverage (-): 0

Region: chr18 45805101-45805113. Max. coverage (+): 0. Max coverage (-): 0

Region: chr18 45805114-45805126. Max. coverage (+): 0. Max coverage (-): 0

Region: chr18 45805127-45805139. Max. coverage (+): 0. Max coverage (-): 0

Region: chr18 45805140-45805152. Max. coverage (+): 0. Max coverage (-): 0

Region: chr18 45805153-45805166. Max. coverage (+): 0. Max coverage (-): 0

Region: chr18 45805167-45805179. Max. coverage (+): 0. Max coverage (-): 0

Region: chr18 45805180-45805192. Max. coverage (+): 0. Max coverage (-): 0

Region: chr18 45805193-45805205. Max. coverage (+): 0. Max coverage (-): 0

Region: chr18 45805206-45805218. Max. coverage (+): 0. Max coverage (-): 0

Region: chr18 45805219-45805231. Max. coverage (+): 0. Max coverage (-): 0

Region: chr18 45805232-45805245. Max. coverage (+): 0. Max coverage (-): 0

Region: chr18 45805246-45805258. Max. coverage (+): 0. Max coverage (-): 0

Region: chr18 45805259-45805271. Max. coverage (+): 0. Max coverage (-): 0

Region: chr18 45805272-45805284. Max. coverage (+): 0. Max coverage (-): 0

Region: chr18 45805285-45805297. Max. coverage (+): 0. Max coverage (-): 0

Region: chr18 45805298-45805311. Max. coverage (+): 0. Max coverage (-): 0

Region: chr18 45805312-45805324. Max. coverage (+): 0. Max coverage (-): 0

Region: chr18 45805325-45805337. Max. coverage (+): 0. Max coverage (-): 0

Region: chr18 45805338-45805350. Max. coverage (+): 0. Max coverage (-): 0

Region: chr18 45805351-45805363. Max. coverage (+): 0. Max coverage (-): 0

Region: chr18 45805364-45805377. Max. coverage (+): 0. Max coverage (-): 0

Region: chr18 45805378-45805390. Max. coverage (+): 0. Max coverage (-): 0

Region: chr18 45805391-45805403. Max. coverage (+): 0. Max coverage (-): 0

Region: chr18 45805404-45805416. Max. coverage (+): 0. Max coverage (-): 0

Region: chr18 45805417-45805429. Max. coverage (+): 0. Max coverage (-): 0

Region: chr18 45805430-45805443. Max. coverage (+): 0. Max coverage (-): 0

Region: chr18 45805444-45805456. Max. coverage (+): 0. Max coverage (-): 0

Region: chr18 45805457-45805469. Max. coverage (+): 0. Max coverage (-): 0

Region: chr18 45805470-45805482. Max. coverage (+): 0. Max coverage (-): 0

Region: chr18 45805483-45805495. Max. coverage (+): 0. Max coverage (-): 0

Region: chr18 45805496-45805508. Max. coverage (+): 0. Max coverage (-): 0

Region: chr18 45805509-45805522. Max. coverage (+): 0. Max coverage (-): 0

Region: chr18 45805523-45805535. Max. coverage (+): 0. Max coverage (-): 0

Region: chr18 45805536-45805548. Max. coverage (+): 0. Max coverage (-): 0

Region: chr18 45805549-45805561. Max. coverage (+): 0. Max coverage (-): 0

Region: chr18 45805562-45805574. Max. coverage (+): 0. Max coverage (-): 0

Region: chr18 45805575-45805588. Max. coverage (+): 0. Max coverage (-): 0

Region: chr18 45805589-45805601. Max. coverage (+): 0. Max coverage (-): 0

Region: chr18 45805602-45805614. Max. coverage (+): 0. Max coverage (-): 0

Region: chr18 45805615-45805627. Max. coverage (+): 0. Max coverage (-): 0

Region: chr18 45805628-45805640. Max. coverage (+): 0. Max coverage (-): 0

Region: chr18 45805641-45805654. Max. coverage (+): 0. Max coverage (-): 0

Region: chr18 45805655-45805667. Max. coverage (+): 0. Max coverage (-): 0

Region: chr18 45805668-45805680. Max. coverage (+): 0. Max coverage (-): 0

Region: chr18 45805681-45805693. Max. coverage (+): 0. Max coverage (-): 0

Region: chr18 45805694-45805706. Max. coverage (+): 0. Max coverage (-): 0

Region: chr18 45805707-45805720. Max. coverage (+): 0. Max coverage (-): 0

Region: chr18 45805721-45805733. Max. coverage (+): 0. Max coverage (-): 1.82

Region: chr18 45805734-45805746. Max. coverage (+): 0. Max coverage (-): 0

Region: chr18 45805747-. Max. coverage (+): 0. Max coverage (-): 0

RepeatMasker Color Code

**+**

100-98% Identity

<98-95% Identity

<95-90% Identity

<90-85% Identity

<85-80% Identity

<80-75% Identity

<75-70% Identity

<70% Identity

**-**

Gene Set Color Code

**+**

Gene

Pseudogene

**-**

Topology/Coverage Color Code

Coverage Plus Strand

Coverage Minus Strand

Mainstrand: Plus

Mainstrand: Minus

Complementary Strand

Flanking Region  
(if option -flank >0)

Gene Set Annotation  
  
RepeatMasker Annotation  

**1. L1M5**: 45799261-45799589 (+), Divergence to consensus: 38.5%  
**2. SINE2-2\_BT**: 45799590-45799704 (-), Divergence to consensus: 23.5%  
**3. L1M5**: 45799705-45800017 (+), Divergence to consensus: 36.7%  
**4. L1M5**: 45800019-45800486 (+), Divergence to consensus: 37.5%  
**5. MIRb**: 45800865-45801011 (+), Divergence to consensus: 44.4%  
**6. L1\_Art**: 45804437-45804534 (-), Divergence to consensus: 23.7%  
**7. Bov-tA2**: 45804535-45804578 (+), Divergence to consensus: 4.5%  
**8. L1ME1**: 45804732-45804894 (+), Divergence to consensus: 28.8%  
**9. Bov-tA2**: 45804899-45805088 (+), Divergence to consensus: 11.2%  
**10. MER58C**: 45805129-45805316 (-), Divergence to consensus: 39.3%  
**11. L1ME1**: 45805319-45805419 (+), Divergence to consensus: 24.8%  
**12. LTR87**: 45805426-45805718 (-), Divergence to consensus: 46.6%

  
Transcription Factor Binding Sites  

**Gata4** (Sequence: AGATAAC (-): 45800606)  
**Gata4** (Sequence: AGATAAC (-): 45805539)  
**Gata4** (Sequence: GTTATCT (+): 45804359)
