# Supplementary material for: Small RNA sequencing of cryopreserved semen from single bull revealed altered miRNAs and piRNAs expression between High- and Low-motile sperm populations
Source: BMC Genomics. 2017 Jan 4;18:14. doi: 10.1186/s12864-016-3394-7 (PMC5209821; doi:10.1186/s12864-016-3394-7)
Supplement: Additional file 4: — Details for each piRNA clusters found in Low Motile (LM) sperm fraction. Genes, repeats, transposable elements and transcription factors binding sites falling within the cluster regions were reported. (ZIP 1034 kb) [file 12864_2016_3394_MOESM4_ESM.zip › 24.html]

piRNA cluster 24


Predicted piRNA cluster no. 24     previous   next
  

Show proTRAC run info
Hide proTRAC run info

================================= proTRAC ====================================  
VERSION: 2.1                                    LAST MODIFIED: 06. October 2015  
  
Please cite:  
Rosenkranz D, Zischler H. proTRAC - a software for probabilistic piRNA cluster  
detection, visualization and analysis. 2012. BMC Bioinformatics 13:5.  
  
and (for proTRAC 2.0 and later):  
Rosenkranz D, Rudloff S, Bastuck K, Ketting RF, Zischler H. Tupaia small RNAs  
provide insights into function and evolution of RNAi-based transposon defense  
in mammals. 2015. RNA 21(5):911-922.  
  
Contact:  
David Rosenkranz  
Institute of Anthropology, small RNA group  
Johannes Gutenberg University Mainz  
email: rosenkranz@uni-mainz.de  
  
You can find the latest proTRAC version at:  
http://sourceforge.net/projects/protrac/files  
http://www.smallRNAgroup-mainz.de/software  
==============================================================================  
  
PARAMETERS:  
Map file: .............../storage/core/barbara/genhome/smallRNA/fertility/Sample\_not\_motile/pirna/Sample\_not\_motile\_26-33\_collapsed.fa.no-dust.map.weighted-10000-1000-b-0  
Genome file: ............/storage/core/barbara/genhome/smallRNA/fertility/Sample\_all/pirna/bt\_311\_chrY.fa  
RepeatMasker annotation: /storage/genomes/bt\_umd31/GCF\_000003055.6\_Bos\_taurus\_UMD\_3.1.1\_repeatMasker\_chr.out  
GeneSet:................./storage/core/barbara/genhome/smallRNA/fertility/Sample\_all/pirna/full.gtf  
  
Significant (p<=0.01) hit density will be calculated based  
on observed hit distribution.  
  
Sliding window size: ........................................ 5000 bp  
Sliding window increament: .................................. 1000 bp  
Normalize each hit by number of genomic hits: ............... 1 [0=no/1=yes]  
Normalize each hit by number of sequence reads: ............. 1 [0=no/1=yes]  
Normalize values (-> per million mapped reads): ............. 1 [0=no/1=yes]  
Min. fraction of hits with 1T(U) or 10A: .................... 0.75  
Alternatively: Min. fraction of hits with 1T(U) and 10A: .... 0.5  
Min. fraction of hits with typical piRNA length: ............ 0.75  
Typical piRNA length: ....................................... 26-33 nt  
Min. size of a piRNA cluster: ............................... 5000 bp.  
Min. number of hits (absolute): ............................. 0  
Min. number of hits (normalized): ........................... 0  
Min. fraction of hits on the mainstrand: .................... 0.75  
Top fraction of mapped sequences (in terms of read counts): . 1%  
Top fraction accounts for max. n% of sequence reads: ........ 90%  
Min. fraction of hits on each arm of a bidirectional cluster: 0.1  
Output image file for each cluster: ......................... 0 [0=no/1=yes]  
Output html file for each cluster: .......................... 1 [0=no/1=yes]  
Output a summary table: ..................................... 1 [0=no/1=yes]  
Output a FASTA file for each cluster (piRNA sequences): ..... 1 [0=no/1=yes]  
Output a FASTA file comprising cluster sequences: ........... 1 [0=no/1=yes]  
Search DNA motifs in clusters: .............................. 1 [0=no/1=yes]  
Output flanking sequences: +/- .............................. 0 bp  
Output ~.pTi file: .......................................... 1 [0=no/1=yes]  
==============================================================================  
  
  
Genome size (without gaps): ............ 2678902517 bp  
Gaps (N/X/-): .......................... 53837044 bp  
Mapped reads: .......................... 738059667487  
Non-identical sequences: ............... 277001  
Genomic hits: .......................... 533816  
Significant densitiy of mapped reads: .. 15118061 reads/kb

Show proTRAC cluster info
Hide proTRAC cluster info

|  |  |
| --- | --- |
| Location | chr19 |
| Coordinates | 57721915-57730880 |
| Size [bp] | 8966 |
| Sequence hit loci | 146 |
| Mapped reads (normalized) | 289692420.4 |
| Mapped reads (normalized) per kb | 32310107.1 |
| Normalized reads with 1T (1U) | 77.4% |
| Normalized reads with 10A | 37.7% |
| Normalized reads with length 26-33 nt | 100% |
| Normalized reads on the main strand(s) | 81.8% |
| Predicted directionality | mono:plus |

100%

0%

1T (1U)  
reads

10A reads

26-33 nt  
reads

reads on mainstrand

**Either the amount of reads with 1T (1U) OR 10A has to exceed 75% (set with option: -1Tor10A)  
Alternatively the amount of reads with 1T (1U) AND 10A has to exceed 50% (set with option: -1Tand10A)  
Minimum amount of reads with preferred size is 75% (set with option: -pisize)  
Minimum amount of reads on the main strand(s) is 75% (set with option: -clstrand)**

Show read coverage
Hide read coverage

WHAT DO I SEE HERE?  
This chart shows the location of mapped sequence reads within a predicted piRNA cluster. The color refers to the number of genomic hits produced by the sequence read in question. A dark red bar indicates that this sequence read produces many other hits elsewhere in the genome. Many adjacent red or yellow bars can indicate the presence of a multi-copy element such as transposons or rRNA genes. A dark green bar indicates that this sequence read maps uniquely to this locus.

1 hit

2-5 hits

6-10 hits

11-20 hits

21-50 hits

51-100 hits

> 100 hits

chr19

57721915

57730880

Gene Set

RepeatMasker

Mapped  
Reads

35.05

plus strand

minus strand

35.05

Region: chr19 45805748-57721923. Max. coverage (+): 0.47. Max coverage (-): 0

Region: chr19 57721924-57721941. Max. coverage (+): 0. Max coverage (-): 0

Region: chr19 57721942-57721959. Max. coverage (+): 0. Max coverage (-): 0

Region: chr19 57721960-57721977. Max. coverage (+): 0. Max coverage (-): 0

Region: chr19 57721978-57721995. Max. coverage (+): 0. Max coverage (-): 0

Region: chr19 57721996-57722013. Max. coverage (+): 0. Max coverage (-): 0

Region: chr19 57722014-57722031. Max. coverage (+): 0. Max coverage (-): 0

Region: chr19 57722032-57722049. Max. coverage (+): 0. Max coverage (-): 0

Region: chr19 57722050-57722067. Max. coverage (+): 0. Max coverage (-): 0

Region: chr19 57722068-57722085. Max. coverage (+): 1.33. Max coverage (-): 0

Region: chr19 57722086-57722103. Max. coverage (+): 1.33. Max coverage (-): 0

Region: chr19 57722104-57722121. Max. coverage (+): 0. Max coverage (-): 0

Region: chr19 57722122-57722139. Max. coverage (+): 0. Max coverage (-): 0

Region: chr19 57722140-57722157. Max. coverage (+): 0. Max coverage (-): 0

Region: chr19 57722158-57722175. Max. coverage (+): 7.34. Max coverage (-): 0

Region: chr19 57722176-57722192. Max. coverage (+): 0. Max coverage (-): 0

Region: chr19 57722193-57722210. Max. coverage (+): 0. Max coverage (-): 0

Region: chr19 57722211-57722228. Max. coverage (+): 0. Max coverage (-): 0

Region: chr19 57722229-57722246. Max. coverage (+): 0. Max coverage (-): 0

Region: chr19 57722247-57722264. Max. coverage (+): 0. Max coverage (-): 0

Region: chr19 57722265-57722282. Max. coverage (+): 0. Max coverage (-): 0

Region: chr19 57722283-57722300. Max. coverage (+): 0. Max coverage (-): 0

Region: chr19 57722301-57722318. Max. coverage (+): 0. Max coverage (-): 0

Region: chr19 57722319-57722336. Max. coverage (+): 0. Max coverage (-): 0

Region: chr19 57722337-57722354. Max. coverage (+): 0. Max coverage (-): 0

Region: chr19 57722355-57722372. Max. coverage (+): 0. Max coverage (-): 0

Region: chr19 57722373-57722390. Max. coverage (+): 0. Max coverage (-): 0

Region: chr19 57722391-57722408. Max. coverage (+): 0. Max coverage (-): 0

Region: chr19 57722409-57722426. Max. coverage (+): 0. Max coverage (-): 0

Region: chr19 57722427-57722443. Max. coverage (+): 0. Max coverage (-): 0

Region: chr19 57722444-57722461. Max. coverage (+): 0. Max coverage (-): 0

Region: chr19 57722462-57722479. Max. coverage (+): 0. Max coverage (-): 0

Region: chr19 57722480-57722497. Max. coverage (+): 0. Max coverage (-): 0

Region: chr19 57722498-57722515. Max. coverage (+): 0. Max coverage (-): 0

Region: chr19 57722516-57722533. Max. coverage (+): 0. Max coverage (-): 0

Region: chr19 57722534-57722551. Max. coverage (+): 0. Max coverage (-): 0

Region: chr19 57722552-57722569. Max. coverage (+): 0. Max coverage (-): 0

Region: chr19 57722570-57722587. Max. coverage (+): 0. Max coverage (-): 0

Region: chr19 57722588-57722605. Max. coverage (+): 0. Max coverage (-): 0

Region: chr19 57722606-57722623. Max. coverage (+): 0. Max coverage (-): 0

Region: chr19 57722624-57722641. Max. coverage (+): 0. Max coverage (-): 0

Region: chr19 57722642-57722659. Max. coverage (+): 0. Max coverage (-): 0

Region: chr19 57722660-57722677. Max. coverage (+): 0. Max coverage (-): 0

Region: chr19 57722678-57722695. Max. coverage (+): 0. Max coverage (-): 0

Region: chr19 57722696-57722712. Max. coverage (+): 0. Max coverage (-): 0

Region: chr19 57722713-57722730. Max. coverage (+): 0. Max coverage (-): 0

Region: chr19 57722731-57722748. Max. coverage (+): 0. Max coverage (-): 0

Region: chr19 57722749-57722766. Max. coverage (+): 0. Max coverage (-): 0

Region: chr19 57722767-57722784. Max. coverage (+): 0. Max coverage (-): 0

Region: chr19 57722785-57722802. Max. coverage (+): 0. Max coverage (-): 0

Region: chr19 57722803-57722820. Max. coverage (+): 0. Max coverage (-): 0

Region: chr19 57722821-57722838. Max. coverage (+): 0. Max coverage (-): 0

Region: chr19 57722839-57722856. Max. coverage (+): 0. Max coverage (-): 0

Region: chr19 57722857-57722874. Max. coverage (+): 0. Max coverage (-): 0

Region: chr19 57722875-57722892. Max. coverage (+): 0. Max coverage (-): 0

Region: chr19 57722893-57722910. Max. coverage (+): 11.71. Max coverage (-): 0

Region: chr19 57722911-57722928. Max. coverage (+): 19.51. Max coverage (-): 0

Region: chr19 57722929-57722946. Max. coverage (+): 0. Max coverage (-): 0

Region: chr19 57722947-57722964. Max. coverage (+): 2.78. Max coverage (-): 0.35

Region: chr19 57722965-57722981. Max. coverage (+): 2.78. Max coverage (-): 0

Region: chr19 57722982-57722999. Max. coverage (+): 0.35. Max coverage (-): 0

Region: chr19 57723000-57723017. Max. coverage (+): 0. Max coverage (-): 0

Region: chr19 57723018-57723035. Max. coverage (+): 0. Max coverage (-): 0

Region: chr19 57723036-57723053. Max. coverage (+): 0. Max coverage (-): 0

Region: chr19 57723054-57723071. Max. coverage (+): 4.3. Max coverage (-): 0

Region: chr19 57723072-57723089. Max. coverage (+): 4.3. Max coverage (-): 0

Region: chr19 57723090-57723107. Max. coverage (+): 0. Max coverage (-): 0

Region: chr19 57723108-57723125. Max. coverage (+): 0. Max coverage (-): 0

Region: chr19 57723126-57723143. Max. coverage (+): 0. Max coverage (-): 0

Region: chr19 57723144-57723161. Max. coverage (+): 0. Max coverage (-): 0

Region: chr19 57723162-57723179. Max. coverage (+): 0. Max coverage (-): 0

Region: chr19 57723180-57723197. Max. coverage (+): 0. Max coverage (-): 0

Region: chr19 57723198-57723215. Max. coverage (+): 0. Max coverage (-): 0

Region: chr19 57723216-57723233. Max. coverage (+): 0. Max coverage (-): 0

Region: chr19 57723234-57723250. Max. coverage (+): 0. Max coverage (-): 0

Region: chr19 57723251-57723268. Max. coverage (+): 0. Max coverage (-): 0

Region: chr19 57723269-57723286. Max. coverage (+): 0. Max coverage (-): 0

Region: chr19 57723287-57723304. Max. coverage (+): 0. Max coverage (-): 0

Region: chr19 57723305-57723322. Max. coverage (+): 0. Max coverage (-): 0

Region: chr19 57723323-57723340. Max. coverage (+): 0. Max coverage (-): 0

Region: chr19 57723341-57723358. Max. coverage (+): 0. Max coverage (-): 0

Region: chr19 57723359-57723376. Max. coverage (+): 0. Max coverage (-): 0

Region: chr19 57723377-57723394. Max. coverage (+): 0. Max coverage (-): 0

Region: chr19 57723395-57723412. Max. coverage (+): 0. Max coverage (-): 0

Region: chr19 57723413-57723430. Max. coverage (+): 0. Max coverage (-): 0

Region: chr19 57723431-57723448. Max. coverage (+): 0. Max coverage (-): 0

Region: chr19 57723449-57723466. Max. coverage (+): 0. Max coverage (-): 0

Region: chr19 57723467-57723484. Max. coverage (+): 0. Max coverage (-): 0

Region: chr19 57723485-57723501. Max. coverage (+): 0. Max coverage (-): 0

Region: chr19 57723502-57723519. Max. coverage (+): 0. Max coverage (-): 0

Region: chr19 57723520-57723537. Max. coverage (+): 0. Max coverage (-): 0

Region: chr19 57723538-57723555. Max. coverage (+): 0. Max coverage (-): 0

Region: chr19 57723556-57723573. Max. coverage (+): 1.62. Max coverage (-): 0

Region: chr19 57723574-57723591. Max. coverage (+): 15.48. Max coverage (-): 0

Region: chr19 57723592-57723609. Max. coverage (+): 15.48. Max coverage (-): 0

Region: chr19 57723610-57723627. Max. coverage (+): 0.47. Max coverage (-): 0

Region: chr19 57723628-57723645. Max. coverage (+): 7.06. Max coverage (-): 0

Region: chr19 57723646-57723663. Max. coverage (+): 0. Max coverage (-): 0

Region: chr19 57723664-57723681. Max. coverage (+): 0. Max coverage (-): 0

Region: chr19 57723682-57723699. Max. coverage (+): 0. Max coverage (-): 2.22

Region: chr19 57723700-57723717. Max. coverage (+): 0. Max coverage (-): 2.22

Region: chr19 57723718-57723735. Max. coverage (+): 0. Max coverage (-): 0

Region: chr19 57723736-57723753. Max. coverage (+): 0. Max coverage (-): 0

Region: chr19 57723754-57723770. Max. coverage (+): 0. Max coverage (-): 0

Region: chr19 57723771-57723788. Max. coverage (+): 0. Max coverage (-): 0

Region: chr19 57723789-57723806. Max. coverage (+): 0. Max coverage (-): 0

Region: chr19 57723807-57723824. Max. coverage (+): 0. Max coverage (-): 0

Region: chr19 57723825-57723842. Max. coverage (+): 0. Max coverage (-): 0

Region: chr19 57723843-57723860. Max. coverage (+): 0. Max coverage (-): 4.98

Region: chr19 57723861-57723878. Max. coverage (+): 0. Max coverage (-): 0

Region: chr19 57723879-57723896. Max. coverage (+): 0. Max coverage (-): 0

Region: chr19 57723897-57723914. Max. coverage (+): 0. Max coverage (-): 0

Region: chr19 57723915-57723932. Max. coverage (+): 0. Max coverage (-): 0

Region: chr19 57723933-57723950. Max. coverage (+): 0. Max coverage (-): 0

Region: chr19 57723951-57723968. Max. coverage (+): 0. Max coverage (-): 0

Region: chr19 57723969-57723986. Max. coverage (+): 0. Max coverage (-): 0

Region: chr19 57723987-57724004. Max. coverage (+): 0. Max coverage (-): 0

Region: chr19 57724005-57724022. Max. coverage (+): 0. Max coverage (-): 0

Region: chr19 57724023-57724039. Max. coverage (+): 0. Max coverage (-): 0

Region: chr19 57724040-57724057. Max. coverage (+): 0. Max coverage (-): 0

Region: chr19 57724058-57724075. Max. coverage (+): 0. Max coverage (-): 0

Region: chr19 57724076-57724093. Max. coverage (+): 0. Max coverage (-): 0

Region: chr19 57724094-57724111. Max. coverage (+): 0. Max coverage (-): 0

Region: chr19 57724112-57724129. Max. coverage (+): 0. Max coverage (-): 0

Region: chr19 57724130-57724147. Max. coverage (+): 0. Max coverage (-): 0

Region: chr19 57724148-57724165. Max. coverage (+): 0. Max coverage (-): 0

Region: chr19 57724166-57724183. Max. coverage (+): 0. Max coverage (-): 0

Region: chr19 57724184-57724201. Max. coverage (+): 0. Max coverage (-): 0

Region: chr19 57724202-57724219. Max. coverage (+): 0. Max coverage (-): 0

Region: chr19 57724220-57724237. Max. coverage (+): 0. Max coverage (-): 0

Region: chr19 57724238-57724255. Max. coverage (+): 0. Max coverage (-): 0

Region: chr19 57724256-57724273. Max. coverage (+): 0. Max coverage (-): 0

Region: chr19 57724274-57724290. Max. coverage (+): 0. Max coverage (-): 0

Region: chr19 57724291-57724308. Max. coverage (+): 0. Max coverage (-): 0

Region: chr19 57724309-57724326. Max. coverage (+): 0. Max coverage (-): 0

Region: chr19 57724327-57724344. Max. coverage (+): 0. Max coverage (-): 0

Region: chr19 57724345-57724362. Max. coverage (+): 0. Max coverage (-): 0

Region: chr19 57724363-57724380. Max. coverage (+): 15.74. Max coverage (-): 0

Region: chr19 57724381-57724398. Max. coverage (+): 26.22. Max coverage (-): 0

Region: chr19 57724399-57724416. Max. coverage (+): 0. Max coverage (-): 0

Region: chr19 57724417-57724434. Max. coverage (+): 0. Max coverage (-): 0.47

Region: chr19 57724435-57724452. Max. coverage (+): 3.74. Max coverage (-): 0

Region: chr19 57724453-57724470. Max. coverage (+): 0.47. Max coverage (-): 0

Region: chr19 57724471-57724488. Max. coverage (+): 0. Max coverage (-): 0

Region: chr19 57724489-57724506. Max. coverage (+): 0. Max coverage (-): 0

Region: chr19 57724507-57724524. Max. coverage (+): 0. Max coverage (-): 0

Region: chr19 57724525-57724542. Max. coverage (+): 5.77. Max coverage (-): 0

Region: chr19 57724543-57724559. Max. coverage (+): 5.77. Max coverage (-): 0

Region: chr19 57724560-57724577. Max. coverage (+): 0. Max coverage (-): 0

Region: chr19 57724578-57724595. Max. coverage (+): 0. Max coverage (-): 0

Region: chr19 57724596-57724613. Max. coverage (+): 0. Max coverage (-): 0

Region: chr19 57724614-57724631. Max. coverage (+): 0. Max coverage (-): 0

Region: chr19 57724632-57724649. Max. coverage (+): 0. Max coverage (-): 0

Region: chr19 57724650-57724667. Max. coverage (+): 0. Max coverage (-): 0

Region: chr19 57724668-57724685. Max. coverage (+): 0. Max coverage (-): 0

Region: chr19 57724686-57724703. Max. coverage (+): 0. Max coverage (-): 0

Region: chr19 57724704-57724721. Max. coverage (+): 0. Max coverage (-): 0

Region: chr19 57724722-57724739. Max. coverage (+): 0. Max coverage (-): 0

Region: chr19 57724740-57724757. Max. coverage (+): 0. Max coverage (-): 0

Region: chr19 57724758-57724775. Max. coverage (+): 0. Max coverage (-): 0

Region: chr19 57724776-57724793. Max. coverage (+): 0. Max coverage (-): 0

Region: chr19 57724794-57724811. Max. coverage (+): 0. Max coverage (-): 0

Region: chr19 57724812-57724828. Max. coverage (+): 0. Max coverage (-): 0

Region: chr19 57724829-57724846. Max. coverage (+): 0. Max coverage (-): 0

Region: chr19 57724847-57724864. Max. coverage (+): 0. Max coverage (-): 0

Region: chr19 57724865-57724882. Max. coverage (+): 0. Max coverage (-): 0

Region: chr19 57724883-57724900. Max. coverage (+): 0. Max coverage (-): 0

Region: chr19 57724901-57724918. Max. coverage (+): 0. Max coverage (-): 0

Region: chr19 57724919-57724936. Max. coverage (+): 0. Max coverage (-): 0

Region: chr19 57724937-57724954. Max. coverage (+): 0. Max coverage (-): 0

Region: chr19 57724955-57724972. Max. coverage (+): 0. Max coverage (-): 0

Region: chr19 57724973-57724990. Max. coverage (+): 0. Max coverage (-): 0

Region: chr19 57724991-57725008. Max. coverage (+): 0. Max coverage (-): 0

Region: chr19 57725009-57725026. Max. coverage (+): 0. Max coverage (-): 0

Region: chr19 57725027-57725044. Max. coverage (+): 0. Max coverage (-): 0

Region: chr19 57725045-57725062. Max. coverage (+): 0. Max coverage (-): 0

Region: chr19 57725063-57725079. Max. coverage (+): 0. Max coverage (-): 0

Region: chr19 57725080-57725097. Max. coverage (+): 0. Max coverage (-): 0

Region: chr19 57725098-57725115. Max. coverage (+): 0. Max coverage (-): 0

Region: chr19 57725116-57725133. Max. coverage (+): 0. Max coverage (-): 0

Region: chr19 57725134-57725151. Max. coverage (+): 0. Max coverage (-): 0

Region: chr19 57725152-57725169. Max. coverage (+): 0. Max coverage (-): 0

Region: chr19 57725170-57725187. Max. coverage (+): 0. Max coverage (-): 0

Region: chr19 57725188-57725205. Max. coverage (+): 0. Max coverage (-): 0

Region: chr19 57725206-57725223. Max. coverage (+): 0. Max coverage (-): 0

Region: chr19 57725224-57725241. Max. coverage (+): 0. Max coverage (-): 0

Region: chr19 57725242-57725259. Max. coverage (+): 0. Max coverage (-): 0

Region: chr19 57725260-57725277. Max. coverage (+): 0. Max coverage (-): 0

Region: chr19 57725278-57725295. Max. coverage (+): 0. Max coverage (-): 0

Region: chr19 57725296-57725313. Max. coverage (+): 0. Max coverage (-): 0

Region: chr19 57725314-57725331. Max. coverage (+): 0. Max coverage (-): 0

Region: chr19 57725332-57725348. Max. coverage (+): 0. Max coverage (-): 0

Region: chr19 57725349-57725366. Max. coverage (+): 0. Max coverage (-): 0

Region: chr19 57725367-57725384. Max. coverage (+): 0. Max coverage (-): 0

Region: chr19 57725385-57725402. Max. coverage (+): 0. Max coverage (-): 0

Region: chr19 57725403-57725420. Max. coverage (+): 0. Max coverage (-): 0

Region: chr19 57725421-57725438. Max. coverage (+): 0. Max coverage (-): 0

Region: chr19 57725439-57725456. Max. coverage (+): 0. Max coverage (-): 0

Region: chr19 57725457-57725474. Max. coverage (+): 0. Max coverage (-): 0

Region: chr19 57725475-57725492. Max. coverage (+): 0. Max coverage (-): 0

Region: chr19 57725493-57725510. Max. coverage (+): 1.96. Max coverage (-): 0

Region: chr19 57725511-57725528. Max. coverage (+): 18.8. Max coverage (-): 0

Region: chr19 57725529-57725546. Max. coverage (+): 18.8. Max coverage (-): 0

Region: chr19 57725547-57725564. Max. coverage (+): 0.58. Max coverage (-): 0

Region: chr19 57725565-57725582. Max. coverage (+): 2.44. Max coverage (-): 26.76

Region: chr19 57725583-57725600. Max. coverage (+): 0. Max coverage (-): 16.4

Region: chr19 57725601-57725617. Max. coverage (+): 0. Max coverage (-): 0

Region: chr19 57725618-57725635. Max. coverage (+): 0. Max coverage (-): 0

Region: chr19 57725636-57725653. Max. coverage (+): 0. Max coverage (-): 2.7

Region: chr19 57725654-57725671. Max. coverage (+): 0. Max coverage (-): 0

Region: chr19 57725672-57725689. Max. coverage (+): 0. Max coverage (-): 0

Region: chr19 57725690-57725707. Max. coverage (+): 0. Max coverage (-): 0

Region: chr19 57725708-57725725. Max. coverage (+): 0. Max coverage (-): 0

Region: chr19 57725726-57725743. Max. coverage (+): 0. Max coverage (-): 0

Region: chr19 57725744-57725761. Max. coverage (+): 0. Max coverage (-): 0

Region: chr19 57725762-57725779. Max. coverage (+): 0. Max coverage (-): 0

Region: chr19 57725780-57725797. Max. coverage (+): 0. Max coverage (-): 6.05

Region: chr19 57725798-57725815. Max. coverage (+): 4.13. Max coverage (-): 0

Region: chr19 57725816-57725833. Max. coverage (+): 3.05. Max coverage (-): 0

Region: chr19 57725834-57725851. Max. coverage (+): 0. Max coverage (-): 0

Region: chr19 57725852-57725869. Max. coverage (+): 4.2. Max coverage (-): 0

Region: chr19 57725870-57725886. Max. coverage (+): 0. Max coverage (-): 0

Region: chr19 57725887-57725904. Max. coverage (+): 0. Max coverage (-): 0

Region: chr19 57725905-57725922. Max. coverage (+): 2.61. Max coverage (-): 0

Region: chr19 57725923-57725940. Max. coverage (+): 0. Max coverage (-): 0

Region: chr19 57725941-57725958. Max. coverage (+): 0. Max coverage (-): 0

Region: chr19 57725959-57725976. Max. coverage (+): 0. Max coverage (-): 0

Region: chr19 57725977-57725994. Max. coverage (+): 0. Max coverage (-): 0

Region: chr19 57725995-57726012. Max. coverage (+): 8.42. Max coverage (-): 0

Region: chr19 57726013-57726030. Max. coverage (+): 0. Max coverage (-): 0

Region: chr19 57726031-57726048. Max. coverage (+): 0. Max coverage (-): 0

Region: chr19 57726049-57726066. Max. coverage (+): 0. Max coverage (-): 0

Region: chr19 57726067-57726084. Max. coverage (+): 0. Max coverage (-): 0

Region: chr19 57726085-57726102. Max. coverage (+): 0. Max coverage (-): 0

Region: chr19 57726103-57726120. Max. coverage (+): 0. Max coverage (-): 0

Region: chr19 57726121-57726137. Max. coverage (+): 0. Max coverage (-): 0

Region: chr19 57726138-57726155. Max. coverage (+): 0. Max coverage (-): 0

Region: chr19 57726156-57726173. Max. coverage (+): 0. Max coverage (-): 0

Region: chr19 57726174-57726191. Max. coverage (+): 0. Max coverage (-): 0

Region: chr19 57726192-57726209. Max. coverage (+): 0. Max coverage (-): 0

Region: chr19 57726210-57726227. Max. coverage (+): 0. Max coverage (-): 0

Region: chr19 57726228-57726245. Max. coverage (+): 0. Max coverage (-): 0

Region: chr19 57726246-57726263. Max. coverage (+): 9.36. Max coverage (-): 0

Region: chr19 57726264-57726281. Max. coverage (+): 0. Max coverage (-): 0

Region: chr19 57726282-57726299. Max. coverage (+): 0. Max coverage (-): 0

Region: chr19 57726300-57726317. Max. coverage (+): 1.19. Max coverage (-): 0

Region: chr19 57726318-57726335. Max. coverage (+): 3.75. Max coverage (-): 0

Region: chr19 57726336-57726353. Max. coverage (+): 0. Max coverage (-): 5.65

Region: chr19 57726354-57726371. Max. coverage (+): 0. Max coverage (-): 5.65

Region: chr19 57726372-57726389. Max. coverage (+): 0. Max coverage (-): 0

Region: chr19 57726390-57726406. Max. coverage (+): 0. Max coverage (-): 0

Region: chr19 57726407-57726424. Max. coverage (+): 0. Max coverage (-): 0

Region: chr19 57726425-57726442. Max. coverage (+): 0. Max coverage (-): 0

Region: chr19 57726443-57726460. Max. coverage (+): 0. Max coverage (-): 0

Region: chr19 57726461-57726478. Max. coverage (+): 1.12. Max coverage (-): 0

Region: chr19 57726479-57726496. Max. coverage (+): 12.33. Max coverage (-): 0

Region: chr19 57726497-57726514. Max. coverage (+): 8.38. Max coverage (-): 0

Region: chr19 57726515-57726532. Max. coverage (+): 0. Max coverage (-): 0

Region: chr19 57726533-57726550. Max. coverage (+): 0. Max coverage (-): 0

Region: chr19 57726551-57726568. Max. coverage (+): 0. Max coverage (-): 0

Region: chr19 57726569-57726586. Max. coverage (+): 0. Max coverage (-): 0.22

Region: chr19 57726587-57726604. Max. coverage (+): 3.52. Max coverage (-): 0.22

Region: chr19 57726605-57726622. Max. coverage (+): 0. Max coverage (-): 0

Region: chr19 57726623-57726640. Max. coverage (+): 0. Max coverage (-): 0

Region: chr19 57726641-57726658. Max. coverage (+): 0. Max coverage (-): 0

Region: chr19 57726659-57726675. Max. coverage (+): 0. Max coverage (-): 0

Region: chr19 57726676-57726693. Max. coverage (+): 0. Max coverage (-): 0

Region: chr19 57726694-57726711. Max. coverage (+): 2.42. Max coverage (-): 0

Region: chr19 57726712-57726729. Max. coverage (+): 3.89. Max coverage (-): 0

Region: chr19 57726730-57726747. Max. coverage (+): 0. Max coverage (-): 0

Region: chr19 57726748-57726765. Max. coverage (+): 0. Max coverage (-): 0

Region: chr19 57726766-57726783. Max. coverage (+): 0. Max coverage (-): 0

Region: chr19 57726784-57726801. Max. coverage (+): 0. Max coverage (-): 0

Region: chr19 57726802-57726819. Max. coverage (+): 0. Max coverage (-): 0

Region: chr19 57726820-57726837. Max. coverage (+): 0. Max coverage (-): 0

Region: chr19 57726838-57726855. Max. coverage (+): 0. Max coverage (-): 0

Region: chr19 57726856-57726873. Max. coverage (+): 0. Max coverage (-): 0

Region: chr19 57726874-57726891. Max. coverage (+): 0. Max coverage (-): 0

Region: chr19 57726892-57726909. Max. coverage (+): 4.25. Max coverage (-): 0

Region: chr19 57726910-57726926. Max. coverage (+): 6.84. Max coverage (-): 0

Region: chr19 57726927-57726944. Max. coverage (+): 6.84. Max coverage (-): 0

Region: chr19 57726945-57726962. Max. coverage (+): 0. Max coverage (-): 0

Region: chr19 57726963-57726980. Max. coverage (+): 0. Max coverage (-): 0

Region: chr19 57726981-57726998. Max. coverage (+): 0. Max coverage (-): 0

Region: chr19 57726999-57727016. Max. coverage (+): 0. Max coverage (-): 0

Region: chr19 57727017-57727034. Max. coverage (+): 6.36. Max coverage (-): 0

Region: chr19 57727035-57727052. Max. coverage (+): 6.36. Max coverage (-): 0

Region: chr19 57727053-57727070. Max. coverage (+): 0. Max coverage (-): 0

Region: chr19 57727071-57727088. Max. coverage (+): 0. Max coverage (-): 0

Region: chr19 57727089-57727106. Max. coverage (+): 0. Max coverage (-): 0

Region: chr19 57727107-57727124. Max. coverage (+): 35.05. Max coverage (-): 0

Region: chr19 57727125-57727142. Max. coverage (+): 35.05. Max coverage (-): 0

Region: chr19 57727143-57727160. Max. coverage (+): 0. Max coverage (-): 0

Region: chr19 57727161-57727178. Max. coverage (+): 0. Max coverage (-): 0

Region: chr19 57727179-57727195. Max. coverage (+): 0. Max coverage (-): 0

Region: chr19 57727196-57727213. Max. coverage (+): 0. Max coverage (-): 0

Region: chr19 57727214-57727231. Max. coverage (+): 0. Max coverage (-): 0

Region: chr19 57727232-57727249. Max. coverage (+): 0. Max coverage (-): 0

Region: chr19 57727250-57727267. Max. coverage (+): 0. Max coverage (-): 0

Region: chr19 57727268-57727285. Max. coverage (+): 0. Max coverage (-): 0

Region: chr19 57727286-57727303. Max. coverage (+): 0. Max coverage (-): 0

Region: chr19 57727304-57727321. Max. coverage (+): 0. Max coverage (-): 0

Region: chr19 57727322-57727339. Max. coverage (+): 0. Max coverage (-): 0

Region: chr19 57727340-57727357. Max. coverage (+): 0. Max coverage (-): 0

Region: chr19 57727358-57727375. Max. coverage (+): 0. Max coverage (-): 0

Region: chr19 57727376-57727393. Max. coverage (+): 0. Max coverage (-): 0

Region: chr19 57727394-57727411. Max. coverage (+): 0. Max coverage (-): 0

Region: chr19 57727412-57727429. Max. coverage (+): 0. Max coverage (-): 0

Region: chr19 57727430-57727447. Max. coverage (+): 0. Max coverage (-): 0

Region: chr19 57727448-57727464. Max. coverage (+): 0. Max coverage (-): 0

Region: chr19 57727465-57727482. Max. coverage (+): 0. Max coverage (-): 0

Region: chr19 57727483-57727500. Max. coverage (+): 0. Max coverage (-): 0

Region: chr19 57727501-57727518. Max. coverage (+): 0. Max coverage (-): 0

Region: chr19 57727519-57727536. Max. coverage (+): 0. Max coverage (-): 0

Region: chr19 57727537-57727554. Max. coverage (+): 0. Max coverage (-): 0

Region: chr19 57727555-57727572. Max. coverage (+): 0. Max coverage (-): 0

Region: chr19 57727573-57727590. Max. coverage (+): 7.37. Max coverage (-): 0

Region: chr19 57727591-57727608. Max. coverage (+): 3.71. Max coverage (-): 0

Region: chr19 57727609-57727626. Max. coverage (+): 0. Max coverage (-): 0

Region: chr19 57727627-57727644. Max. coverage (+): 4.52. Max coverage (-): 0

Region: chr19 57727645-57727662. Max. coverage (+): 0. Max coverage (-): 0

Region: chr19 57727663-57727680. Max. coverage (+): 0. Max coverage (-): 0

Region: chr19 57727681-57727698. Max. coverage (+): 2.11. Max coverage (-): 0

Region: chr19 57727699-57727716. Max. coverage (+): 0. Max coverage (-): 0

Region: chr19 57727717-57727733. Max. coverage (+): 0. Max coverage (-): 0

Region: chr19 57727734-57727751. Max. coverage (+): 0. Max coverage (-): 0

Region: chr19 57727752-57727769. Max. coverage (+): 0. Max coverage (-): 0

Region: chr19 57727770-57727787. Max. coverage (+): 0. Max coverage (-): 0

Region: chr19 57727788-57727805. Max. coverage (+): 6.89. Max coverage (-): 0

Region: chr19 57727806-57727823. Max. coverage (+): 0. Max coverage (-): 0

Region: chr19 57727824-57727841. Max. coverage (+): 0. Max coverage (-): 0

Region: chr19 57727842-57727859. Max. coverage (+): 0. Max coverage (-): 0

Region: chr19 57727860-57727877. Max. coverage (+): 0. Max coverage (-): 0

Region: chr19 57727878-57727895. Max. coverage (+): 0. Max coverage (-): 0

Region: chr19 57727896-57727913. Max. coverage (+): 0. Max coverage (-): 0

Region: chr19 57727914-57727931. Max. coverage (+): 0. Max coverage (-): 5.1

Region: chr19 57727932-57727949. Max. coverage (+): 0. Max coverage (-): 5.24

Region: chr19 57727950-57727967. Max. coverage (+): 0. Max coverage (-): 0

Region: chr19 57727968-57727984. Max. coverage (+): 0. Max coverage (-): 0

Region: chr19 57727985-57728002. Max. coverage (+): 0. Max coverage (-): 0

Region: chr19 57728003-57728020. Max. coverage (+): 12.56. Max coverage (-): 0

Region: chr19 57728021-57728038. Max. coverage (+): 0. Max coverage (-): 0

Region: chr19 57728039-57728056. Max. coverage (+): 0. Max coverage (-): 0

Region: chr19 57728057-57728074. Max. coverage (+): 0. Max coverage (-): 0

Region: chr19 57728075-57728092. Max. coverage (+): 0. Max coverage (-): 0

Region: chr19 57728093-57728110. Max. coverage (+): 0. Max coverage (-): 0

Region: chr19 57728111-57728128. Max. coverage (+): 0. Max coverage (-): 0

Region: chr19 57728129-57728146. Max. coverage (+): 0. Max coverage (-): 0

Region: chr19 57728147-57728164. Max. coverage (+): 0. Max coverage (-): 0

Region: chr19 57728165-57728182. Max. coverage (+): 0. Max coverage (-): 0

Region: chr19 57728183-57728200. Max. coverage (+): 0. Max coverage (-): 0

Region: chr19 57728201-57728218. Max. coverage (+): 0. Max coverage (-): 0

Region: chr19 57728219-57728236. Max. coverage (+): 0. Max coverage (-): 0

Region: chr19 57728237-57728253. Max. coverage (+): 0. Max coverage (-): 0

Region: chr19 57728254-57728271. Max. coverage (+): 0. Max coverage (-): 0

Region: chr19 57728272-57728289. Max. coverage (+): 0. Max coverage (-): 0

Region: chr19 57728290-57728307. Max. coverage (+): 0. Max coverage (-): 0

Region: chr19 57728308-57728325. Max. coverage (+): 16.78. Max coverage (-): 0

Region: chr19 57728326-57728343. Max. coverage (+): 30.68. Max coverage (-): 0

Region: chr19 57728344-57728361. Max. coverage (+): 0. Max coverage (-): 0

Region: chr19 57728362-57728379. Max. coverage (+): 0. Max coverage (-): 0

Region: chr19 57728380-57728397. Max. coverage (+): 0. Max coverage (-): 0

Region: chr19 57728398-57728415. Max. coverage (+): 0. Max coverage (-): 0

Region: chr19 57728416-57728433. Max. coverage (+): 0. Max coverage (-): 0

Region: chr19 57728434-57728451. Max. coverage (+): 0. Max coverage (-): 0

Region: chr19 57728452-57728469. Max. coverage (+): 0. Max coverage (-): 0

Region: chr19 57728470-57728487. Max. coverage (+): 0. Max coverage (-): 0

Region: chr19 57728488-57728505. Max. coverage (+): 0. Max coverage (-): 0

Region: chr19 57728506-57728522. Max. coverage (+): 0. Max coverage (-): 0

Region: chr19 57728523-57728540. Max. coverage (+): 0. Max coverage (-): 0

Region: chr19 57728541-57728558. Max. coverage (+): 0. Max coverage (-): 0

Region: chr19 57728559-57728576. Max. coverage (+): 0. Max coverage (-): 2.48

Region: chr19 57728577-57728594. Max. coverage (+): 0. Max coverage (-): 0

Region: chr19 57728595-57728612. Max. coverage (+): 0. Max coverage (-): 0

Region: chr19 57728613-57728630. Max. coverage (+): 0. Max coverage (-): 0

Region: chr19 57728631-57728648. Max. coverage (+): 0. Max coverage (-): 0

Region: chr19 57728649-57728666. Max. coverage (+): 1.45. Max coverage (-): 0

Region: chr19 57728667-57728684. Max. coverage (+): 1.45. Max coverage (-): 0

Region: chr19 57728685-57728702. Max. coverage (+): 0. Max coverage (-): 0

Region: chr19 57728703-57728720. Max. coverage (+): 0. Max coverage (-): 0

Region: chr19 57728721-57728738. Max. coverage (+): 0. Max coverage (-): 0

Region: chr19 57728739-57728756. Max. coverage (+): 0. Max coverage (-): 0

Region: chr19 57728757-57728773. Max. coverage (+): 0. Max coverage (-): 0

Region: chr19 57728774-57728791. Max. coverage (+): 0. Max coverage (-): 0

Region: chr19 57728792-57728809. Max. coverage (+): 0. Max coverage (-): 0

Region: chr19 57728810-57728827. Max. coverage (+): 0. Max coverage (-): 0

Region: chr19 57728828-57728845. Max. coverage (+): 0. Max coverage (-): 0

Region: chr19 57728846-57728863. Max. coverage (+): 0. Max coverage (-): 0

Region: chr19 57728864-57728881. Max. coverage (+): 11.25. Max coverage (-): 0

Region: chr19 57728882-57728899. Max. coverage (+): 0. Max coverage (-): 0

Region: chr19 57728900-57728917. Max. coverage (+): 0. Max coverage (-): 0

Region: chr19 57728918-57728935. Max. coverage (+): 0. Max coverage (-): 0

Region: chr19 57728936-57728953. Max. coverage (+): 0. Max coverage (-): 0

Region: chr19 57728954-57728971. Max. coverage (+): 1.77. Max coverage (-): 0

Region: chr19 57728972-57728989. Max. coverage (+): 0. Max coverage (-): 0

Region: chr19 57728990-57729007. Max. coverage (+): 0. Max coverage (-): 0

Region: chr19 57729008-57729025. Max. coverage (+): 0. Max coverage (-): 0

Region: chr19 57729026-57729042. Max. coverage (+): 0. Max coverage (-): 0

Region: chr19 57729043-57729060. Max. coverage (+): 0. Max coverage (-): 0

Region: chr19 57729061-57729078. Max. coverage (+): 0. Max coverage (-): 0

Region: chr19 57729079-57729096. Max. coverage (+): 0. Max coverage (-): 0

Region: chr19 57729097-57729114. Max. coverage (+): 0. Max coverage (-): 0

Region: chr19 57729115-57729132. Max. coverage (+): 0. Max coverage (-): 0

Region: chr19 57729133-57729150. Max. coverage (+): 0. Max coverage (-): 0

Region: chr19 57729151-57729168. Max. coverage (+): 0. Max coverage (-): 0

Region: chr19 57729169-57729186. Max. coverage (+): 0. Max coverage (-): 0

Region: chr19 57729187-57729204. Max. coverage (+): 0. Max coverage (-): 0

Region: chr19 57729205-57729222. Max. coverage (+): 0. Max coverage (-): 0

Region: chr19 57729223-57729240. Max. coverage (+): 0. Max coverage (-): 0

Region: chr19 57729241-57729258. Max. coverage (+): 0. Max coverage (-): 0

Region: chr19 57729259-57729276. Max. coverage (+): 0. Max coverage (-): 0

Region: chr19 57729277-57729294. Max. coverage (+): 0. Max coverage (-): 0

Region: chr19 57729295-57729311. Max. coverage (+): 0. Max coverage (-): 0

Region: chr19 57729312-57729329. Max. coverage (+): 0. Max coverage (-): 0

Region: chr19 57729330-57729347. Max. coverage (+): 0. Max coverage (-): 0

Region: chr19 57729348-57729365. Max. coverage (+): 0. Max coverage (-): 0

Region: chr19 57729366-57729383. Max. coverage (+): 0. Max coverage (-): 0

Region: chr19 57729384-57729401. Max. coverage (+): 0. Max coverage (-): 0

Region: chr19 57729402-57729419. Max. coverage (+): 0. Max coverage (-): 0

Region: chr19 57729420-57729437. Max. coverage (+): 0.13. Max coverage (-): 0

Region: chr19 57729438-57729455. Max. coverage (+): 0. Max coverage (-): 0

Region: chr19 57729456-57729473. Max. coverage (+): 0. Max coverage (-): 0

Region: chr19 57729474-57729491. Max. coverage (+): 0. Max coverage (-): 0

Region: chr19 57729492-57729509. Max. coverage (+): 0. Max coverage (-): 0

Region: chr19 57729510-57729527. Max. coverage (+): 0. Max coverage (-): 0

Region: chr19 57729528-57729545. Max. coverage (+): 0. Max coverage (-): 0

Region: chr19 57729546-57729562. Max. coverage (+): 0. Max coverage (-): 0

Region: chr19 57729563-57729580. Max. coverage (+): 0. Max coverage (-): 0

Region: chr19 57729581-57729598. Max. coverage (+): 0. Max coverage (-): 0

Region: chr19 57729599-57729616. Max. coverage (+): 0. Max coverage (-): 0

Region: chr19 57729617-57729634. Max. coverage (+): 0. Max coverage (-): 0

Region: chr19 57729635-57729652. Max. coverage (+): 0. Max coverage (-): 0

Region: chr19 57729653-57729670. Max. coverage (+): 0. Max coverage (-): 0

Region: chr19 57729671-57729688. Max. coverage (+): 0. Max coverage (-): 0

Region: chr19 57729689-57729706. Max. coverage (+): 0. Max coverage (-): 0

Region: chr19 57729707-57729724. Max. coverage (+): 0. Max coverage (-): 0

Region: chr19 57729725-57729742. Max. coverage (+): 0. Max coverage (-): 0

Region: chr19 57729743-57729760. Max. coverage (+): 0. Max coverage (-): 0

Region: chr19 57729761-57729778. Max. coverage (+): 0. Max coverage (-): 0

Region: chr19 57729779-57729796. Max. coverage (+): 0. Max coverage (-): 0

Region: chr19 57729797-57729814. Max. coverage (+): 0. Max coverage (-): 0

Region: chr19 57729815-57729831. Max. coverage (+): 0. Max coverage (-): 0

Region: chr19 57729832-57729849. Max. coverage (+): 0. Max coverage (-): 0

Region: chr19 57729850-57729867. Max. coverage (+): 0. Max coverage (-): 0

Region: chr19 57729868-57729885. Max. coverage (+): 0. Max coverage (-): 0

Region: chr19 57729886-57729903. Max. coverage (+): 0. Max coverage (-): 0

Region: chr19 57729904-57729921. Max. coverage (+): 0. Max coverage (-): 0

Region: chr19 57729922-57729939. Max. coverage (+): 0. Max coverage (-): 0

Region: chr19 57729940-57729957. Max. coverage (+): 0. Max coverage (-): 0

Region: chr19 57729958-57729975. Max. coverage (+): 0. Max coverage (-): 0

Region: chr19 57729976-57729993. Max. coverage (+): 0. Max coverage (-): 0

Region: chr19 57729994-57730011. Max. coverage (+): 0. Max coverage (-): 0

Region: chr19 57730012-57730029. Max. coverage (+): 0. Max coverage (-): 0

Region: chr19 57730030-57730047. Max. coverage (+): 0. Max coverage (-): 0

Region: chr19 57730048-57730065. Max. coverage (+): 0. Max coverage (-): 0

Region: chr19 57730066-57730083. Max. coverage (+): 0. Max coverage (-): 0

Region: chr19 57730084-57730100. Max. coverage (+): 0. Max coverage (-): 0

Region: chr19 57730101-57730118. Max. coverage (+): 0. Max coverage (-): 0

Region: chr19 57730119-57730136. Max. coverage (+): 0. Max coverage (-): 0

Region: chr19 57730137-57730154. Max. coverage (+): 0. Max coverage (-): 0

Region: chr19 57730155-57730172. Max. coverage (+): 0. Max coverage (-): 0

Region: chr19 57730173-57730190. Max. coverage (+): 0. Max coverage (-): 0

Region: chr19 57730191-57730208. Max. coverage (+): 0. Max coverage (-): 0

Region: chr19 57730209-57730226. Max. coverage (+): 0. Max coverage (-): 0

Region: chr19 57730227-57730244. Max. coverage (+): 0. Max coverage (-): 0

Region: chr19 57730245-57730262. Max. coverage (+): 0. Max coverage (-): 0

Region: chr19 57730263-57730280. Max. coverage (+): 0. Max coverage (-): 0

Region: chr19 57730281-57730298. Max. coverage (+): 0. Max coverage (-): 0

Region: chr19 57730299-57730316. Max. coverage (+): 0. Max coverage (-): 0

Region: chr19 57730317-57730334. Max. coverage (+): 0. Max coverage (-): 0

Region: chr19 57730335-57730352. Max. coverage (+): 0. Max coverage (-): 0

Region: chr19 57730353-57730369. Max. coverage (+): 0. Max coverage (-): 0

Region: chr19 57730370-57730387. Max. coverage (+): 0. Max coverage (-): 0

Region: chr19 57730388-57730405. Max. coverage (+): 0. Max coverage (-): 0

Region: chr19 57730406-57730423. Max. coverage (+): 0. Max coverage (-): 0

Region: chr19 57730424-57730441. Max. coverage (+): 0. Max coverage (-): 0

Region: chr19 57730442-57730459. Max. coverage (+): 0. Max coverage (-): 0

Region: chr19 57730460-57730477. Max. coverage (+): 0. Max coverage (-): 0

Region: chr19 57730478-57730495. Max. coverage (+): 0. Max coverage (-): 0

Region: chr19 57730496-57730513. Max. coverage (+): 0. Max coverage (-): 0

Region: chr19 57730514-57730531. Max. coverage (+): 0. Max coverage (-): 0

Region: chr19 57730532-57730549. Max. coverage (+): 0. Max coverage (-): 0

Region: chr19 57730550-57730567. Max. coverage (+): 0. Max coverage (-): 0

Region: chr19 57730568-57730585. Max. coverage (+): 0. Max coverage (-): 0

Region: chr19 57730586-57730603. Max. coverage (+): 0. Max coverage (-): 0

Region: chr19 57730604-57730620. Max. coverage (+): 0. Max coverage (-): 0

Region: chr19 57730621-57730638. Max. coverage (+): 0. Max coverage (-): 0

Region: chr19 57730639-57730656. Max. coverage (+): 0. Max coverage (-): 0

Region: chr19 57730657-57730674. Max. coverage (+): 0. Max coverage (-): 0

Region: chr19 57730675-57730692. Max. coverage (+): 0. Max coverage (-): 0

Region: chr19 57730693-57730710. Max. coverage (+): 0. Max coverage (-): 0

Region: chr19 57730711-57730728. Max. coverage (+): 0. Max coverage (-): 0

Region: chr19 57730729-57730746. Max. coverage (+): 0. Max coverage (-): 0

Region: chr19 57730747-57730764. Max. coverage (+): 0. Max coverage (-): 0

Region: chr19 57730765-57730782. Max. coverage (+): 0. Max coverage (-): 0

Region: chr19 57730783-57730800. Max. coverage (+): 0. Max coverage (-): 0

Region: chr19 57730801-57730818. Max. coverage (+): 0. Max coverage (-): 0

Region: chr19 57730819-57730836. Max. coverage (+): 0. Max coverage (-): 0

Region: chr19 57730837-57730854. Max. coverage (+): 0. Max coverage (-): 2.89

Region: chr19 57730855-57730872. Max. coverage (+): 0. Max coverage (-): 2.89

Region: chr19 57730873-. Max. coverage (+): 0. Max coverage (-): 0

RepeatMasker Color Code

**+**

100-98% Identity

<98-95% Identity

<95-90% Identity

<90-85% Identity

<85-80% Identity

<80-75% Identity

<75-70% Identity

<70% Identity

**-**

Gene Set Color Code

**+**

Gene

Pseudogene

**-**

Topology/Coverage Color Code

Coverage Plus Strand

Coverage Minus Strand

Mainstrand: Plus

Mainstrand: Minus

Complementary Strand

Flanking Region  
(if option -flank >0)

Gene Set Annotation  

**1. BTBD17 (protein coding, ENSBTAG00000030166) Tr:00000042549 Ex:3**: 57721368-57722442 (+)  
**2. KIF19 (protein coding, ENSBTAG00000004617) Tr:00000006057 Ex:12**: 57730681-57730879 (-)  
**3. KIF19 (protein coding, ENSBTAG00000004617) Tr:00000006057 Ex:13**: 57729314-57729584 (-)  
**4. KIF19 (protein coding, ENSBTAG00000004617) Tr:00000006057 Ex:14**: 57729100-57729221 (-)  
**5. KIF19 (protein coding, ENSBTAG00000004617) Tr:00000006057 Ex:15**: 57728498-57728634 (-)  
**6. KIF19 (protein coding, ENSBTAG00000004617) Tr:00000006057 Ex:16**: 57728148-57728253 (-)  
**7. KIF19 (protein coding, ENSBTAG00000004617) Tr:00000006057 Ex:17**: 57727919-57728006 (-)  
**8. KIF19 (protein coding, ENSBTAG00000004617) Tr:00000006057 Ex:18**: 57726861-57727255 (-)  
**9. KIF19 (protein coding, ENSBTAG00000004617) Tr:00000006057 Ex:19**: 57726500-57726662 (-)  
**10. KIF19 (protein coding, ENSBTAG00000004617) Tr:00000006057 Ex:20**: 57726115-57726245 (-)

  
RepeatMasker Annotation  

**1. Bov-tA1**: 57722707-57722900 (-), Divergence to consensus: 26.5%  
**2. Bov-tA1**: 57723347-57723555 (-), Divergence to consensus: 17.2%  
**3. Bov-tA1**: 57724167-57724371 (-), Divergence to consensus: 22.2%  
**4. Bov-tA1**: 57724818-57725024 (-), Divergence to consensus: 17.9%  
**5. Bov-tA1**: 57725286-57725494 (-), Divergence to consensus: 17.7%  
**6. MIRb**: 57727347-57727445 (+), Divergence to consensus: 43.5%  
**7. SINE2-2\_BT**: 57727452-57727573 (-), Divergence to consensus: 18%  
**8. Bov-tA3**: 57729817-57730021 (-), Divergence to consensus: 13.7%  
**9. MIRb**: 57730147-57730251 (-), Divergence to consensus: 35%  
**10. Bov-tA2**: 57730252-57730348 (+), Divergence to consensus: 13.9%  
**11. Bov-tA2**: 57730355-57730543 (-), Divergence to consensus: 20.6%

  
Transcription Factor Binding Sites  

**SOX9** (Sequence: TTATTGTT (+): 57723638)  
**SOX9** (Sequence: TTATTGTT (+): 57725577)
