# Supplementary material for: Small RNA sequencing of cryopreserved semen from single bull revealed altered miRNAs and piRNAs expression between High- and Low-motile sperm populations
Source: BMC Genomics. 2017 Jan 4;18:14. doi: 10.1186/s12864-016-3394-7 (PMC5209821; doi:10.1186/s12864-016-3394-7)
Supplement: Additional file 4: — Details for each piRNA clusters found in Low Motile (LM) sperm fraction. Genes, repeats, transposable elements and transcription factors binding sites falling within the cluster regions were reported. (ZIP 1034 kb) [file 12864_2016_3394_MOESM4_ESM.zip › 25.html]

piRNA cluster 25


Predicted piRNA cluster no. 25     previous   next
  

Show proTRAC run info
Hide proTRAC run info

================================= proTRAC ====================================  
VERSION: 2.1                                    LAST MODIFIED: 06. October 2015  
  
Please cite:  
Rosenkranz D, Zischler H. proTRAC - a software for probabilistic piRNA cluster  
detection, visualization and analysis. 2012. BMC Bioinformatics 13:5.  
  
and (for proTRAC 2.0 and later):  
Rosenkranz D, Rudloff S, Bastuck K, Ketting RF, Zischler H. Tupaia small RNAs  
provide insights into function and evolution of RNAi-based transposon defense  
in mammals. 2015. RNA 21(5):911-922.  
  
Contact:  
David Rosenkranz  
Institute of Anthropology, small RNA group  
Johannes Gutenberg University Mainz  
email: rosenkranz@uni-mainz.de  
  
You can find the latest proTRAC version at:  
http://sourceforge.net/projects/protrac/files  
http://www.smallRNAgroup-mainz.de/software  
==============================================================================  
  
PARAMETERS:  
Map file: .............../storage/core/barbara/genhome/smallRNA/fertility/Sample\_not\_motile/pirna/Sample\_not\_motile\_26-33\_collapsed.fa.no-dust.map.weighted-10000-1000-b-0  
Genome file: ............/storage/core/barbara/genhome/smallRNA/fertility/Sample\_all/pirna/bt\_311\_chrY.fa  
RepeatMasker annotation: /storage/genomes/bt\_umd31/GCF\_000003055.6\_Bos\_taurus\_UMD\_3.1.1\_repeatMasker\_chr.out  
GeneSet:................./storage/core/barbara/genhome/smallRNA/fertility/Sample\_all/pirna/full.gtf  
  
Significant (p<=0.01) hit density will be calculated based  
on observed hit distribution.  
  
Sliding window size: ........................................ 5000 bp  
Sliding window increament: .................................. 1000 bp  
Normalize each hit by number of genomic hits: ............... 1 [0=no/1=yes]  
Normalize each hit by number of sequence reads: ............. 1 [0=no/1=yes]  
Normalize values (-> per million mapped reads): ............. 1 [0=no/1=yes]  
Min. fraction of hits with 1T(U) or 10A: .................... 0.75  
Alternatively: Min. fraction of hits with 1T(U) and 10A: .... 0.5  
Min. fraction of hits with typical piRNA length: ............ 0.75  
Typical piRNA length: ....................................... 26-33 nt  
Min. size of a piRNA cluster: ............................... 5000 bp.  
Min. number of hits (absolute): ............................. 0  
Min. number of hits (normalized): ........................... 0  
Min. fraction of hits on the mainstrand: .................... 0.75  
Top fraction of mapped sequences (in terms of read counts): . 1%  
Top fraction accounts for max. n% of sequence reads: ........ 90%  
Min. fraction of hits on each arm of a bidirectional cluster: 0.1  
Output image file for each cluster: ......................... 0 [0=no/1=yes]  
Output html file for each cluster: .......................... 1 [0=no/1=yes]  
Output a summary table: ..................................... 1 [0=no/1=yes]  
Output a FASTA file for each cluster (piRNA sequences): ..... 1 [0=no/1=yes]  
Output a FASTA file comprising cluster sequences: ........... 1 [0=no/1=yes]  
Search DNA motifs in clusters: .............................. 1 [0=no/1=yes]  
Output flanking sequences: +/- .............................. 0 bp  
Output ~.pTi file: .......................................... 1 [0=no/1=yes]  
==============================================================================  
  
  
Genome size (without gaps): ............ 2678902517 bp  
Gaps (N/X/-): .......................... 53837044 bp  
Mapped reads: .......................... 738059667487  
Non-identical sequences: ............... 277001  
Genomic hits: .......................... 533816  
Significant densitiy of mapped reads: .. 15118061 reads/kb

Show proTRAC cluster info
Hide proTRAC cluster info

|  |  |
| --- | --- |
| Location | chr21 |
| Coordinates | 10316232-10341841 |
| Size [bp] | 25610 |
| Sequence hit loci | 1764 |
| Mapped reads (normalized) | 4022212920 |
| Mapped reads (normalized) per kb | 157056342.1 |
| Normalized reads with 1T (1U) | 81.7% |
| Normalized reads with 10A | 28.1% |
| Normalized reads with length 26-33 nt | 100% |
| Normalized reads on the main strand(s) | 99.6% |
| Predicted directionality | bi:minus-plus (split between 10321513 and 10321521) |

100%

0%

1T (1U)  
reads

10A reads

26-33 nt  
reads

reads on mainstrand

**Either the amount of reads with 1T (1U) OR 10A has to exceed 75% (set with option: -1Tor10A)  
Alternatively the amount of reads with 1T (1U) AND 10A has to exceed 50% (set with option: -1Tand10A)  
Minimum amount of reads with preferred size is 75% (set with option: -pisize)  
Minimum amount of reads on the main strand(s) is 75% (set with option: -clstrand)**

Show read coverage
Hide read coverage

WHAT DO I SEE HERE?  
This chart shows the location of mapped sequence reads within a predicted piRNA cluster. The color refers to the number of genomic hits produced by the sequence read in question. A dark red bar indicates that this sequence read produces many other hits elsewhere in the genome. Many adjacent red or yellow bars can indicate the presence of a multi-copy element such as transposons or rRNA genes. A dark green bar indicates that this sequence read maps uniquely to this locus.

1 hit

2-5 hits

6-10 hits

11-20 hits

21-50 hits

51-100 hits

> 100 hits

chr21

10316232

10341841

Gene Set

RepeatMasker

Mapped  
Reads

91.6

plus strand

minus strand

91.6

Region: chr21 57730874-10316257. Max. coverage (+): 0. Max coverage (-): 4.11

Region: chr21 10316258-10316308. Max. coverage (+): 0. Max coverage (-): 0

Region: chr21 10316309-10316360. Max. coverage (+): 0. Max coverage (-): 0

Region: chr21 10316361-10316411. Max. coverage (+): 0. Max coverage (-): 0

Region: chr21 10316412-10316462. Max. coverage (+): 0. Max coverage (-): 0

Region: chr21 10316463-10316513. Max. coverage (+): 0. Max coverage (-): 0

Region: chr21 10316514-10316564. Max. coverage (+): 0. Max coverage (-): 0

Region: chr21 10316565-10316616. Max. coverage (+): 0. Max coverage (-): 0

Region: chr21 10316617-10316667. Max. coverage (+): 0. Max coverage (-): 0

Region: chr21 10316668-10316718. Max. coverage (+): 0. Max coverage (-): 0

Region: chr21 10316719-10316769. Max. coverage (+): 0. Max coverage (-): 0

Region: chr21 10316770-10316821. Max. coverage (+): 0. Max coverage (-): 0

Region: chr21 10316822-10316872. Max. coverage (+): 0. Max coverage (-): 0

Region: chr21 10316873-10316923. Max. coverage (+): 0. Max coverage (-): 0

Region: chr21 10316924-10316974. Max. coverage (+): 0. Max coverage (-): 0

Region: chr21 10316975-10317025. Max. coverage (+): 0. Max coverage (-): 5.7

Region: chr21 10317026-10317077. Max. coverage (+): 0. Max coverage (-): 0

Region: chr21 10317078-10317128. Max. coverage (+): 0. Max coverage (-): 0

Region: chr21 10317129-10317179. Max. coverage (+): 0. Max coverage (-): 0

Region: chr21 10317180-10317230. Max. coverage (+): 0. Max coverage (-): 0

Region: chr21 10317231-10317282. Max. coverage (+): 0. Max coverage (-): 0

Region: chr21 10317283-10317333. Max. coverage (+): 0. Max coverage (-): 0

Region: chr21 10317334-10317384. Max. coverage (+): 0. Max coverage (-): 0

Region: chr21 10317385-10317435. Max. coverage (+): 0. Max coverage (-): 0

Region: chr21 10317436-10317486. Max. coverage (+): 0. Max coverage (-): 0

Region: chr21 10317487-10317538. Max. coverage (+): 0. Max coverage (-): 0

Region: chr21 10317539-10317589. Max. coverage (+): 0. Max coverage (-): 0

Region: chr21 10317590-10317640. Max. coverage (+): 0. Max coverage (-): 0

Region: chr21 10317641-10317691. Max. coverage (+): 0. Max coverage (-): 0

Region: chr21 10317692-10317742. Max. coverage (+): 0. Max coverage (-): 0

Region: chr21 10317743-10317794. Max. coverage (+): 0. Max coverage (-): 0

Region: chr21 10317795-10317845. Max. coverage (+): 0. Max coverage (-): 0

Region: chr21 10317846-10317896. Max. coverage (+): 0. Max coverage (-): 0

Region: chr21 10317897-10317947. Max. coverage (+): 0. Max coverage (-): 0

Region: chr21 10317948-10317999. Max. coverage (+): 0. Max coverage (-): 0

Region: chr21 10318000-10318050. Max. coverage (+): 0. Max coverage (-): 0

Region: chr21 10318051-10318101. Max. coverage (+): 0. Max coverage (-): 0

Region: chr21 10318102-10318152. Max. coverage (+): 0. Max coverage (-): 0

Region: chr21 10318153-10318203. Max. coverage (+): 0. Max coverage (-): 0

Region: chr21 10318204-10318255. Max. coverage (+): 0. Max coverage (-): 0

Region: chr21 10318256-10318306. Max. coverage (+): 0. Max coverage (-): 0

Region: chr21 10318307-10318357. Max. coverage (+): 0. Max coverage (-): 0

Region: chr21 10318358-10318408. Max. coverage (+): 0. Max coverage (-): 0

Region: chr21 10318409-10318460. Max. coverage (+): 0. Max coverage (-): 0

Region: chr21 10318461-10318511. Max. coverage (+): 0. Max coverage (-): 0

Region: chr21 10318512-10318562. Max. coverage (+): 0. Max coverage (-): 0

Region: chr21 10318563-10318613. Max. coverage (+): 0. Max coverage (-): 5.45

Region: chr21 10318614-10318664. Max. coverage (+): 0. Max coverage (-): 0

Region: chr21 10318665-10318716. Max. coverage (+): 0. Max coverage (-): 7.75

Region: chr21 10318717-10318767. Max. coverage (+): 0. Max coverage (-): 0

Region: chr21 10318768-10318818. Max. coverage (+): 0. Max coverage (-): 0

Region: chr21 10318819-10318869. Max. coverage (+): 0. Max coverage (-): 0

Region: chr21 10318870-10318921. Max. coverage (+): 0. Max coverage (-): 0

Region: chr21 10318922-10318972. Max. coverage (+): 0. Max coverage (-): 0

Region: chr21 10318973-10319023. Max. coverage (+): 0. Max coverage (-): 5.45

Region: chr21 10319024-10319074. Max. coverage (+): 0. Max coverage (-): 5.45

Region: chr21 10319075-10319125. Max. coverage (+): 0. Max coverage (-): 0

Region: chr21 10319126-10319177. Max. coverage (+): 0. Max coverage (-): 6.37

Region: chr21 10319178-10319228. Max. coverage (+): 0. Max coverage (-): 8.87

Region: chr21 10319229-10319279. Max. coverage (+): 0. Max coverage (-): 0

Region: chr21 10319280-10319330. Max. coverage (+): 0. Max coverage (-): 29.24

Region: chr21 10319331-10319382. Max. coverage (+): 0. Max coverage (-): 12.94

Region: chr21 10319383-10319433. Max. coverage (+): 0. Max coverage (-): 18.03

Region: chr21 10319434-10319484. Max. coverage (+): 0. Max coverage (-): 16.16

Region: chr21 10319485-10319535. Max. coverage (+): 0. Max coverage (-): 16.16

Region: chr21 10319536-10319586. Max. coverage (+): 0. Max coverage (-): 0

Region: chr21 10319587-10319638. Max. coverage (+): 0. Max coverage (-): 0.31

Region: chr21 10319639-10319689. Max. coverage (+): 4.6. Max coverage (-): 7.89

Region: chr21 10319690-10319740. Max. coverage (+): 0. Max coverage (-): 28.05

Region: chr21 10319741-10319791. Max. coverage (+): 0. Max coverage (-): 15.77

Region: chr21 10319792-10319843. Max. coverage (+): 1.04. Max coverage (-): 41.81

Region: chr21 10319844-10319894. Max. coverage (+): 0. Max coverage (-): 21.46

Region: chr21 10319895-10319945. Max. coverage (+): 0. Max coverage (-): 48.71

Region: chr21 10319946-10319996. Max. coverage (+): 0. Max coverage (-): 21

Region: chr21 10319997-10320047. Max. coverage (+): 0. Max coverage (-): 0

Region: chr21 10320048-10320099. Max. coverage (+): 0. Max coverage (-): 5.5

Region: chr21 10320100-10320150. Max. coverage (+): 0. Max coverage (-): 7.16

Region: chr21 10320151-10320201. Max. coverage (+): 0. Max coverage (-): 3.19

Region: chr21 10320202-10320252. Max. coverage (+): 0. Max coverage (-): 33.18

Region: chr21 10320253-10320303. Max. coverage (+): 0. Max coverage (-): 7.12

Region: chr21 10320304-10320355. Max. coverage (+): 0. Max coverage (-): 7.86

Region: chr21 10320356-10320406. Max. coverage (+): 0. Max coverage (-): 12.14

Region: chr21 10320407-10320457. Max. coverage (+): 0. Max coverage (-): 11.67

Region: chr21 10320458-10320508. Max. coverage (+): 0. Max coverage (-): 0

Region: chr21 10320509-10320560. Max. coverage (+): 0. Max coverage (-): 0

Region: chr21 10320561-10320611. Max. coverage (+): 0. Max coverage (-): 0

Region: chr21 10320612-10320662. Max. coverage (+): 0. Max coverage (-): 0

Region: chr21 10320663-10320713. Max. coverage (+): 0. Max coverage (-): 0

Region: chr21 10320714-10320764. Max. coverage (+): 0. Max coverage (-): 0

Region: chr21 10320765-10320816. Max. coverage (+): 0. Max coverage (-): 3

Region: chr21 10320817-10320867. Max. coverage (+): 0. Max coverage (-): 4

Region: chr21 10320868-10320918. Max. coverage (+): 0. Max coverage (-): 11.06

Region: chr21 10320919-10320969. Max. coverage (+): 0. Max coverage (-): 11.06

Region: chr21 10320970-10321021. Max. coverage (+): 0. Max coverage (-): 0

Region: chr21 10321022-10321072. Max. coverage (+): 0. Max coverage (-): 1.06

Region: chr21 10321073-10321123. Max. coverage (+): 0. Max coverage (-): 0

Region: chr21 10321124-10321174. Max. coverage (+): 0. Max coverage (-): 7.22

Region: chr21 10321175-10321225. Max. coverage (+): 0. Max coverage (-): 1.95

Region: chr21 10321226-10321277. Max. coverage (+): 1.35. Max coverage (-): 6.29

Region: chr21 10321278-10321328. Max. coverage (+): 0. Max coverage (-): 0

Region: chr21 10321329-10321379. Max. coverage (+): 0. Max coverage (-): 3.52

Region: chr21 10321380-10321430. Max. coverage (+): 0. Max coverage (-): 0

Region: chr21 10321431-10321482. Max. coverage (+): 5.64. Max coverage (-): 0

Region: chr21 10321483-10321533. Max. coverage (+): 5.33. Max coverage (-): 5.01

Region: chr21 10321534-10321584. Max. coverage (+): 0. Max coverage (-): 0

Region: chr21 10321585-10321635. Max. coverage (+): 2.19. Max coverage (-): 0

Region: chr21 10321636-10321686. Max. coverage (+): 8.2. Max coverage (-): 0

Region: chr21 10321687-10321738. Max. coverage (+): 5.6. Max coverage (-): 0

Region: chr21 10321739-10321789. Max. coverage (+): 0. Max coverage (-): 0

Region: chr21 10321790-10321840. Max. coverage (+): 0. Max coverage (-): 0

Region: chr21 10321841-10321891. Max. coverage (+): 0. Max coverage (-): 0

Region: chr21 10321892-10321943. Max. coverage (+): 0. Max coverage (-): 0

Region: chr21 10321944-10321994. Max. coverage (+): 0. Max coverage (-): 0

Region: chr21 10321995-10322045. Max. coverage (+): 0. Max coverage (-): 0

Region: chr21 10322046-10322096. Max. coverage (+): 0. Max coverage (-): 0

Region: chr21 10322097-10322147. Max. coverage (+): 0. Max coverage (-): 0

Region: chr21 10322148-10322199. Max. coverage (+): 0. Max coverage (-): 0

Region: chr21 10322200-10322250. Max. coverage (+): 19.98. Max coverage (-): 0

Region: chr21 10322251-10322301. Max. coverage (+): 14.04. Max coverage (-): 0

Region: chr21 10322302-10322352. Max. coverage (+): 0. Max coverage (-): 0

Region: chr21 10322353-10322404. Max. coverage (+): 0. Max coverage (-): 3.81

Region: chr21 10322405-10322455. Max. coverage (+): 24.49. Max coverage (-): 0

Region: chr21 10322456-10322506. Max. coverage (+): 0. Max coverage (-): 0

Region: chr21 10322507-10322557. Max. coverage (+): 0. Max coverage (-): 0

Region: chr21 10322558-10322608. Max. coverage (+): 14.48. Max coverage (-): 0

Region: chr21 10322609-10322660. Max. coverage (+): 17.71. Max coverage (-): 0

Region: chr21 10322661-10322711. Max. coverage (+): 41.8. Max coverage (-): 1.57

Region: chr21 10322712-10322762. Max. coverage (+): 10.01. Max coverage (-): 0

Region: chr21 10322763-10322813. Max. coverage (+): 0. Max coverage (-): 0

Region: chr21 10322814-10322864. Max. coverage (+): 0. Max coverage (-): 0

Region: chr21 10322865-10322916. Max. coverage (+): 32.45. Max coverage (-): 0

Region: chr21 10322917-10322967. Max. coverage (+): 18.92. Max coverage (-): 0

Region: chr21 10322968-10323018. Max. coverage (+): 0. Max coverage (-): 0

Region: chr21 10323019-10323069. Max. coverage (+): 0. Max coverage (-): 0

Region: chr21 10323070-10323121. Max. coverage (+): 0. Max coverage (-): 0

Region: chr21 10323122-10323172. Max. coverage (+): 26.03. Max coverage (-): 0

Region: chr21 10323173-10323223. Max. coverage (+): 26.03. Max coverage (-): 0

Region: chr21 10323224-10323274. Max. coverage (+): 24.06. Max coverage (-): 0

Region: chr21 10323275-10323325. Max. coverage (+): 24.76. Max coverage (-): 0

Region: chr21 10323326-10323377. Max. coverage (+): 11.55. Max coverage (-): 0

Region: chr21 10323378-10323428. Max. coverage (+): 6.33. Max coverage (-): 0

Region: chr21 10323429-10323479. Max. coverage (+): 15.51. Max coverage (-): 0

Region: chr21 10323480-10323530. Max. coverage (+): 46.37. Max coverage (-): 0

Region: chr21 10323531-10323582. Max. coverage (+): 5.44. Max coverage (-): 0

Region: chr21 10323583-10323633. Max. coverage (+): 0. Max coverage (-): 0

Region: chr21 10323634-10323684. Max. coverage (+): 0.26. Max coverage (-): 0

Region: chr21 10323685-10323735. Max. coverage (+): 37.95. Max coverage (-): 0

Region: chr21 10323736-10323786. Max. coverage (+): 19.68. Max coverage (-): 0

Region: chr21 10323787-10323838. Max. coverage (+): 23.75. Max coverage (-): 0

Region: chr21 10323839-10323889. Max. coverage (+): 20.42. Max coverage (-): 2.71

Region: chr21 10323890-10323940. Max. coverage (+): 34.25. Max coverage (-): 0

Region: chr21 10323941-10323991. Max. coverage (+): 56.38. Max coverage (-): 0

Region: chr21 10323992-10324043. Max. coverage (+): 46.02. Max coverage (-): 0

Region: chr21 10324044-10324094. Max. coverage (+): 17.11. Max coverage (-): 0

Region: chr21 10324095-10324145. Max. coverage (+): 67.65. Max coverage (-): 0

Region: chr21 10324146-10324196. Max. coverage (+): 23.39. Max coverage (-): 0

Region: chr21 10324197-10324247. Max. coverage (+): 0. Max coverage (-): 0

Region: chr21 10324248-10324299. Max. coverage (+): 0. Max coverage (-): 0

Region: chr21 10324300-10324350. Max. coverage (+): 14.89. Max coverage (-): 0

Region: chr21 10324351-10324401. Max. coverage (+): 7.93. Max coverage (-): 0

Region: chr21 10324402-10324452. Max. coverage (+): 3. Max coverage (-): 0

Region: chr21 10324453-10324504. Max. coverage (+): 43.73. Max coverage (-): 0

Region: chr21 10324505-10324555. Max. coverage (+): 29.49. Max coverage (-): 0

Region: chr21 10324556-10324606. Max. coverage (+): 14.42. Max coverage (-): 0

Region: chr21 10324607-10324657. Max. coverage (+): 28.22. Max coverage (-): 0

Region: chr21 10324658-10324708. Max. coverage (+): 25.82. Max coverage (-): 0

Region: chr21 10324709-10324760. Max. coverage (+): 35.96. Max coverage (-): 0

Region: chr21 10324761-10324811. Max. coverage (+): 15.7. Max coverage (-): 0

Region: chr21 10324812-10324862. Max. coverage (+): 4.2. Max coverage (-): 0

Region: chr21 10324863-10324913. Max. coverage (+): 67.67. Max coverage (-): 0

Region: chr21 10324914-10324965. Max. coverage (+): 33.56. Max coverage (-): 0

Region: chr21 10324966-10325016. Max. coverage (+): 11.97. Max coverage (-): 0

Region: chr21 10325017-10325067. Max. coverage (+): 19.77. Max coverage (-): 0

Region: chr21 10325068-10325118. Max. coverage (+): 55.1. Max coverage (-): 0

Region: chr21 10325119-10325169. Max. coverage (+): 12.97. Max coverage (-): 0

Region: chr21 10325170-10325221. Max. coverage (+): 15.85. Max coverage (-): 0

Region: chr21 10325222-10325272. Max. coverage (+): 15.85. Max coverage (-): 0

Region: chr21 10325273-10325323. Max. coverage (+): 0. Max coverage (-): 0

Region: chr21 10325324-10325374. Max. coverage (+): 16.12. Max coverage (-): 0

Region: chr21 10325375-10325425. Max. coverage (+): 16.7. Max coverage (-): 0

Region: chr21 10325426-10325477. Max. coverage (+): 9.13. Max coverage (-): 0

Region: chr21 10325478-10325528. Max. coverage (+): 16.63. Max coverage (-): 0

Region: chr21 10325529-10325579. Max. coverage (+): 1.96. Max coverage (-): 0

Region: chr21 10325580-10325630. Max. coverage (+): 18.78. Max coverage (-): 0

Region: chr21 10325631-10325682. Max. coverage (+): 0. Max coverage (-): 0

Region: chr21 10325683-10325733. Max. coverage (+): 0. Max coverage (-): 0

Region: chr21 10325734-10325784. Max. coverage (+): 0. Max coverage (-): 0

Region: chr21 10325785-10325835. Max. coverage (+): 0. Max coverage (-): 0

Region: chr21 10325836-10325886. Max. coverage (+): 16.79. Max coverage (-): 0

Region: chr21 10325887-10325938. Max. coverage (+): 34.53. Max coverage (-): 0

Region: chr21 10325939-10325989. Max. coverage (+): 0.54. Max coverage (-): 0

Region: chr21 10325990-10326040. Max. coverage (+): 15.56. Max coverage (-): 0

Region: chr21 10326041-10326091. Max. coverage (+): 23.31. Max coverage (-): 0

Region: chr21 10326092-10326143. Max. coverage (+): 13.2. Max coverage (-): 0

Region: chr21 10326144-10326194. Max. coverage (+): 12.45. Max coverage (-): 0

Region: chr21 10326195-10326245. Max. coverage (+): 0.4. Max coverage (-): 0

Region: chr21 10326246-10326296. Max. coverage (+): 2.35. Max coverage (-): 0

Region: chr21 10326297-10326347. Max. coverage (+): 12.6. Max coverage (-): 0

Region: chr21 10326348-10326399. Max. coverage (+): 12.6. Max coverage (-): 0

Region: chr21 10326400-10326450. Max. coverage (+): 0. Max coverage (-): 0

Region: chr21 10326451-10326501. Max. coverage (+): 0. Max coverage (-): 0

Region: chr21 10326502-10326552. Max. coverage (+): 0. Max coverage (-): 0

Region: chr21 10326553-10326604. Max. coverage (+): 0.51. Max coverage (-): 0

Region: chr21 10326605-10326655. Max. coverage (+): 0. Max coverage (-): 0

Region: chr21 10326656-10326706. Max. coverage (+): 0. Max coverage (-): 0

Region: chr21 10326707-10326757. Max. coverage (+): 3.9. Max coverage (-): 0

Region: chr21 10326758-10326808. Max. coverage (+): 3.9. Max coverage (-): 0

Region: chr21 10326809-10326860. Max. coverage (+): 8.92. Max coverage (-): 0

Region: chr21 10326861-10326911. Max. coverage (+): 6.88. Max coverage (-): 0

Region: chr21 10326912-10326962. Max. coverage (+): 2.5. Max coverage (-): 0

Region: chr21 10326963-10327013. Max. coverage (+): 34.37. Max coverage (-): 0

Region: chr21 10327014-10327065. Max. coverage (+): 16.02. Max coverage (-): 0

Region: chr21 10327066-10327116. Max. coverage (+): 38.63. Max coverage (-): 0

Region: chr21 10327117-10327167. Max. coverage (+): 6.62. Max coverage (-): 0

Region: chr21 10327168-10327218. Max. coverage (+): 0.83. Max coverage (-): 0

Region: chr21 10327219-10327269. Max. coverage (+): 10.97. Max coverage (-): 0

Region: chr21 10327270-10327321. Max. coverage (+): 7. Max coverage (-): 0

Region: chr21 10327322-10327372. Max. coverage (+): 12.31. Max coverage (-): 0

Region: chr21 10327373-10327423. Max. coverage (+): 18.9. Max coverage (-): 0

Region: chr21 10327424-10327474. Max. coverage (+): 9.16. Max coverage (-): 0

Region: chr21 10327475-10327526. Max. coverage (+): 17.97. Max coverage (-): 0

Region: chr21 10327527-10327577. Max. coverage (+): 11.85. Max coverage (-): 0

Region: chr21 10327578-10327628. Max. coverage (+): 0. Max coverage (-): 0

Region: chr21 10327629-10327679. Max. coverage (+): 13.46. Max coverage (-): 0

Region: chr21 10327680-10327730. Max. coverage (+): 6.36. Max coverage (-): 0

Region: chr21 10327731-10327782. Max. coverage (+): 0. Max coverage (-): 0

Region: chr21 10327783-10327833. Max. coverage (+): 0. Max coverage (-): 0

Region: chr21 10327834-10327884. Max. coverage (+): 2.72. Max coverage (-): 0

Region: chr21 10327885-10327935. Max. coverage (+): 0. Max coverage (-): 0

Region: chr21 10327936-10327986. Max. coverage (+): 4.69. Max coverage (-): 0

Region: chr21 10327987-10328038. Max. coverage (+): 19.63. Max coverage (-): 0

Region: chr21 10328039-10328089. Max. coverage (+): 0. Max coverage (-): 0

Region: chr21 10328090-10328140. Max. coverage (+): 0. Max coverage (-): 0

Region: chr21 10328141-10328191. Max. coverage (+): 0. Max coverage (-): 0

Region: chr21 10328192-10328243. Max. coverage (+): 0. Max coverage (-): 0

Region: chr21 10328244-10328294. Max. coverage (+): 0. Max coverage (-): 0

Region: chr21 10328295-10328345. Max. coverage (+): 0. Max coverage (-): 0

Region: chr21 10328346-10328396. Max. coverage (+): 6.77. Max coverage (-): 0

Region: chr21 10328397-10328447. Max. coverage (+): 49.23. Max coverage (-): 0

Region: chr21 10328448-10328499. Max. coverage (+): 0. Max coverage (-): 0

Region: chr21 10328500-10328550. Max. coverage (+): 0. Max coverage (-): 0

Region: chr21 10328551-10328601. Max. coverage (+): 0.46. Max coverage (-): 0

Region: chr21 10328602-10328652. Max. coverage (+): 9.02. Max coverage (-): 0

Region: chr21 10328653-10328704. Max. coverage (+): 5.98. Max coverage (-): 0

Region: chr21 10328705-10328755. Max. coverage (+): 8.89. Max coverage (-): 0

Region: chr21 10328756-10328806. Max. coverage (+): 0. Max coverage (-): 0

Region: chr21 10328807-10328857. Max. coverage (+): 6.64. Max coverage (-): 0

Region: chr21 10328858-10328908. Max. coverage (+): 7.93. Max coverage (-): 0

Region: chr21 10328909-10328960. Max. coverage (+): 32.16. Max coverage (-): 0

Region: chr21 10328961-10329011. Max. coverage (+): 4.7. Max coverage (-): 0

Region: chr21 10329012-10329062. Max. coverage (+): 4.7. Max coverage (-): 0

Region: chr21 10329063-10329113. Max. coverage (+): 5.1. Max coverage (-): 0

Region: chr21 10329114-10329165. Max. coverage (+): 0. Max coverage (-): 0

Region: chr21 10329166-10329216. Max. coverage (+): 6.59. Max coverage (-): 0

Region: chr21 10329217-10329267. Max. coverage (+): 16.97. Max coverage (-): 0

Region: chr21 10329268-10329318. Max. coverage (+): 5.14. Max coverage (-): 0

Region: chr21 10329319-10329369. Max. coverage (+): 1.27. Max coverage (-): 0

Region: chr21 10329370-10329421. Max. coverage (+): 0. Max coverage (-): 0

Region: chr21 10329422-10329472. Max. coverage (+): 0. Max coverage (-): 0

Region: chr21 10329473-10329523. Max. coverage (+): 0. Max coverage (-): 0

Region: chr21 10329524-10329574. Max. coverage (+): 7.68. Max coverage (-): 0

Region: chr21 10329575-10329626. Max. coverage (+): 5. Max coverage (-): 0

Region: chr21 10329627-10329677. Max. coverage (+): 0. Max coverage (-): 0

Region: chr21 10329678-10329728. Max. coverage (+): 0. Max coverage (-): 0

Region: chr21 10329729-10329779. Max. coverage (+): 0. Max coverage (-): 0

Region: chr21 10329780-10329830. Max. coverage (+): 0. Max coverage (-): 0

Region: chr21 10329831-10329882. Max. coverage (+): 24.64. Max coverage (-): 2.83

Region: chr21 10329883-10329933. Max. coverage (+): 0. Max coverage (-): 0

Region: chr21 10329934-10329984. Max. coverage (+): 0. Max coverage (-): 0

Region: chr21 10329985-10330035. Max. coverage (+): 0. Max coverage (-): 0

Region: chr21 10330036-10330087. Max. coverage (+): 3.95. Max coverage (-): 0

Region: chr21 10330088-10330138. Max. coverage (+): 8.14. Max coverage (-): 0

Region: chr21 10330139-10330189. Max. coverage (+): 0. Max coverage (-): 0

Region: chr21 10330190-10330240. Max. coverage (+): 0. Max coverage (-): 0

Region: chr21 10330241-10330291. Max. coverage (+): 0. Max coverage (-): 0

Region: chr21 10330292-10330343. Max. coverage (+): 2.31. Max coverage (-): 0

Region: chr21 10330344-10330394. Max. coverage (+): 0. Max coverage (-): 0

Region: chr21 10330395-10330445. Max. coverage (+): 0. Max coverage (-): 0

Region: chr21 10330446-10330496. Max. coverage (+): 0. Max coverage (-): 0

Region: chr21 10330497-10330547. Max. coverage (+): 0. Max coverage (-): 0

Region: chr21 10330548-10330599. Max. coverage (+): 0. Max coverage (-): 0

Region: chr21 10330600-10330650. Max. coverage (+): 0. Max coverage (-): 0

Region: chr21 10330651-10330701. Max. coverage (+): 0. Max coverage (-): 0

Region: chr21 10330702-10330752. Max. coverage (+): 0. Max coverage (-): 0

Region: chr21 10330753-10330804. Max. coverage (+): 0. Max coverage (-): 0

Region: chr21 10330805-10330855. Max. coverage (+): 0. Max coverage (-): 0

Region: chr21 10330856-10330906. Max. coverage (+): 24.34. Max coverage (-): 0

Region: chr21 10330907-10330957. Max. coverage (+): 5.96. Max coverage (-): 0

Region: chr21 10330958-10331008. Max. coverage (+): 5.94. Max coverage (-): 0

Region: chr21 10331009-10331060. Max. coverage (+): 0. Max coverage (-): 0

Region: chr21 10331061-10331111. Max. coverage (+): 0. Max coverage (-): 0

Region: chr21 10331112-10331162. Max. coverage (+): 0. Max coverage (-): 0

Region: chr21 10331163-10331213. Max. coverage (+): 0. Max coverage (-): 0

Region: chr21 10331214-10331265. Max. coverage (+): 0. Max coverage (-): 0

Region: chr21 10331266-10331316. Max. coverage (+): 0. Max coverage (-): 0

Region: chr21 10331317-10331367. Max. coverage (+): 0. Max coverage (-): 0

Region: chr21 10331368-10331418. Max. coverage (+): 0. Max coverage (-): 0

Region: chr21 10331419-10331469. Max. coverage (+): 0. Max coverage (-): 0

Region: chr21 10331470-10331521. Max. coverage (+): 0. Max coverage (-): 0

Region: chr21 10331522-10331572. Max. coverage (+): 0. Max coverage (-): 0

Region: chr21 10331573-10331623. Max. coverage (+): 0. Max coverage (-): 0

Region: chr21 10331624-10331674. Max. coverage (+): 0. Max coverage (-): 0

Region: chr21 10331675-10331726. Max. coverage (+): 0. Max coverage (-): 0

Region: chr21 10331727-10331777. Max. coverage (+): 0. Max coverage (-): 0

Region: chr21 10331778-10331828. Max. coverage (+): 4.77. Max coverage (-): 0

Region: chr21 10331829-10331879. Max. coverage (+): 0. Max coverage (-): 0

Region: chr21 10331880-10331930. Max. coverage (+): 0. Max coverage (-): 0

Region: chr21 10331931-10331982. Max. coverage (+): 0. Max coverage (-): 0

Region: chr21 10331983-10332033. Max. coverage (+): 0. Max coverage (-): 0

Region: chr21 10332034-10332084. Max. coverage (+): 0. Max coverage (-): 0

Region: chr21 10332085-10332135. Max. coverage (+): 0. Max coverage (-): 0

Region: chr21 10332136-10332187. Max. coverage (+): 0. Max coverage (-): 0

Region: chr21 10332188-10332238. Max. coverage (+): 0. Max coverage (-): 0

Region: chr21 10332239-10332289. Max. coverage (+): 0. Max coverage (-): 0

Region: chr21 10332290-10332340. Max. coverage (+): 0. Max coverage (-): 0

Region: chr21 10332341-10332391. Max. coverage (+): 0. Max coverage (-): 0

Region: chr21 10332392-10332443. Max. coverage (+): 0. Max coverage (-): 0

Region: chr21 10332444-10332494. Max. coverage (+): 69.65. Max coverage (-): 0

Region: chr21 10332495-10332545. Max. coverage (+): 28.34. Max coverage (-): 0

Region: chr21 10332546-10332596. Max. coverage (+): 11.7. Max coverage (-): 0

Region: chr21 10332597-10332648. Max. coverage (+): 41.42. Max coverage (-): 0

Region: chr21 10332649-10332699. Max. coverage (+): 20.17. Max coverage (-): 0

Region: chr21 10332700-10332750. Max. coverage (+): 6.56. Max coverage (-): 0

Region: chr21 10332751-10332801. Max. coverage (+): 9.02. Max coverage (-): 0

Region: chr21 10332802-10332852. Max. coverage (+): 0. Max coverage (-): 0

Region: chr21 10332853-10332904. Max. coverage (+): 3.79. Max coverage (-): 0

Region: chr21 10332905-10332955. Max. coverage (+): 3.35. Max coverage (-): 0

Region: chr21 10332956-10333006. Max. coverage (+): 0. Max coverage (-): 0

Region: chr21 10333007-10333057. Max. coverage (+): 3.97. Max coverage (-): 0

Region: chr21 10333058-10333108. Max. coverage (+): 9.43. Max coverage (-): 0

Region: chr21 10333109-10333160. Max. coverage (+): 28.03. Max coverage (-): 0

Region: chr21 10333161-10333211. Max. coverage (+): 3.89. Max coverage (-): 0

Region: chr21 10333212-10333262. Max. coverage (+): 10.61. Max coverage (-): 0

Region: chr21 10333263-10333313. Max. coverage (+): 16.14. Max coverage (-): 0

Region: chr21 10333314-10333365. Max. coverage (+): 0. Max coverage (-): 0

Region: chr21 10333366-10333416. Max. coverage (+): 0. Max coverage (-): 0

Region: chr21 10333417-10333467. Max. coverage (+): 0. Max coverage (-): 0

Region: chr21 10333468-10333518. Max. coverage (+): 0. Max coverage (-): 0

Region: chr21 10333519-10333569. Max. coverage (+): 6.27. Max coverage (-): 0

Region: chr21 10333570-10333621. Max. coverage (+): 4.52. Max coverage (-): 0

Region: chr21 10333622-10333672. Max. coverage (+): 11.28. Max coverage (-): 0

Region: chr21 10333673-10333723. Max. coverage (+): 10.66. Max coverage (-): 0

Region: chr21 10333724-10333774. Max. coverage (+): 20.97. Max coverage (-): 0

Region: chr21 10333775-10333826. Max. coverage (+): 5.96. Max coverage (-): 0

Region: chr21 10333827-10333877. Max. coverage (+): 5.96. Max coverage (-): 0

Region: chr21 10333878-10333928. Max. coverage (+): 0. Max coverage (-): 0

Region: chr21 10333929-10333979. Max. coverage (+): 0. Max coverage (-): 0

Region: chr21 10333980-10334030. Max. coverage (+): 34.76. Max coverage (-): 0

Region: chr21 10334031-10334082. Max. coverage (+): 76.84. Max coverage (-): 0

Region: chr21 10334083-10334133. Max. coverage (+): 26.23. Max coverage (-): 0

Region: chr21 10334134-10334184. Max. coverage (+): 14.46. Max coverage (-): 0

Region: chr21 10334185-10334235. Max. coverage (+): 6.6. Max coverage (-): 0

Region: chr21 10334236-10334287. Max. coverage (+): 67.74. Max coverage (-): 0

Region: chr21 10334288-10334338. Max. coverage (+): 28.15. Max coverage (-): 0

Region: chr21 10334339-10334389. Max. coverage (+): 11.52. Max coverage (-): 0

Region: chr21 10334390-10334440. Max. coverage (+): 6.8. Max coverage (-): 0

Region: chr21 10334441-10334491. Max. coverage (+): 4.28. Max coverage (-): 0

Region: chr21 10334492-10334543. Max. coverage (+): 0. Max coverage (-): 0

Region: chr21 10334544-10334594. Max. coverage (+): 5.09. Max coverage (-): 0

Region: chr21 10334595-10334645. Max. coverage (+): 21.72. Max coverage (-): 0

Region: chr21 10334646-10334696. Max. coverage (+): 15.88. Max coverage (-): 0

Region: chr21 10334697-10334748. Max. coverage (+): 0. Max coverage (-): 0

Region: chr21 10334749-10334799. Max. coverage (+): 0. Max coverage (-): 0

Region: chr21 10334800-10334850. Max. coverage (+): 7.16. Max coverage (-): 0

Region: chr21 10334851-10334901. Max. coverage (+): 7.16. Max coverage (-): 0

Region: chr21 10334902-10334952. Max. coverage (+): 6.29. Max coverage (-): 0

Region: chr21 10334953-10335004. Max. coverage (+): 4.6. Max coverage (-): 0

Region: chr21 10335005-10335055. Max. coverage (+): 0. Max coverage (-): 0

Region: chr21 10335056-10335106. Max. coverage (+): 0. Max coverage (-): 0

Region: chr21 10335107-10335157. Max. coverage (+): 0. Max coverage (-): 0

Region: chr21 10335158-10335209. Max. coverage (+): 0. Max coverage (-): 0

Region: chr21 10335210-10335260. Max. coverage (+): 0. Max coverage (-): 0

Region: chr21 10335261-10335311. Max. coverage (+): 0. Max coverage (-): 0

Region: chr21 10335312-10335362. Max. coverage (+): 0. Max coverage (-): 0

Region: chr21 10335363-10335413. Max. coverage (+): 0. Max coverage (-): 0

Region: chr21 10335414-10335465. Max. coverage (+): 0. Max coverage (-): 0

Region: chr21 10335466-10335516. Max. coverage (+): 0. Max coverage (-): 0

Region: chr21 10335517-10335567. Max. coverage (+): 5.24. Max coverage (-): 0

Region: chr21 10335568-10335618. Max. coverage (+): 0. Max coverage (-): 0

Region: chr21 10335619-10335669. Max. coverage (+): 0. Max coverage (-): 0

Region: chr21 10335670-10335721. Max. coverage (+): 0. Max coverage (-): 0

Region: chr21 10335722-10335772. Max. coverage (+): 0. Max coverage (-): 0

Region: chr21 10335773-10335823. Max. coverage (+): 0. Max coverage (-): 0

Region: chr21 10335824-10335874. Max. coverage (+): 0. Max coverage (-): 0

Region: chr21 10335875-10335926. Max. coverage (+): 0. Max coverage (-): 0

Region: chr21 10335927-10335977. Max. coverage (+): 8.53. Max coverage (-): 0

Region: chr21 10335978-10336028. Max. coverage (+): 44.75. Max coverage (-): 0

Region: chr21 10336029-10336079. Max. coverage (+): 64.77. Max coverage (-): 0

Region: chr21 10336080-10336130. Max. coverage (+): 18.27. Max coverage (-): 0

Region: chr21 10336131-10336182. Max. coverage (+): 24.02. Max coverage (-): 0

Region: chr21 10336183-10336233. Max. coverage (+): 20.52. Max coverage (-): 0

Region: chr21 10336234-10336284. Max. coverage (+): 7.29. Max coverage (-): 0

Region: chr21 10336285-10336335. Max. coverage (+): 0.57. Max coverage (-): 0

Region: chr21 10336336-10336387. Max. coverage (+): 11.56. Max coverage (-): 0

Region: chr21 10336388-10336438. Max. coverage (+): 2.96. Max coverage (-): 0

Region: chr21 10336439-10336489. Max. coverage (+): 1.58. Max coverage (-): 0

Region: chr21 10336490-10336540. Max. coverage (+): 8.66. Max coverage (-): 0

Region: chr21 10336541-10336591. Max. coverage (+): 32.14. Max coverage (-): 0

Region: chr21 10336592-10336643. Max. coverage (+): 16.13. Max coverage (-): 0

Region: chr21 10336644-10336694. Max. coverage (+): 25.11. Max coverage (-): 0

Region: chr21 10336695-10336745. Max. coverage (+): 2.96. Max coverage (-): 0

Region: chr21 10336746-10336796. Max. coverage (+): 91.6. Max coverage (-): 0

Region: chr21 10336797-10336848. Max. coverage (+): 17.09. Max coverage (-): 0

Region: chr21 10336849-10336899. Max. coverage (+): 11.54. Max coverage (-): 0

Region: chr21 10336900-10336950. Max. coverage (+): 0. Max coverage (-): 0

Region: chr21 10336951-10337001. Max. coverage (+): 0. Max coverage (-): 0

Region: chr21 10337002-10337052. Max. coverage (+): 7.4. Max coverage (-): 0

Region: chr21 10337053-10337104. Max. coverage (+): 0. Max coverage (-): 0

Region: chr21 10337105-10337155. Max. coverage (+): 1.59. Max coverage (-): 0

Region: chr21 10337156-10337206. Max. coverage (+): 6.47. Max coverage (-): 0

Region: chr21 10337207-10337257. Max. coverage (+): 13.95. Max coverage (-): 0

Region: chr21 10337258-10337309. Max. coverage (+): 10.09. Max coverage (-): 0

Region: chr21 10337310-10337360. Max. coverage (+): 7.08. Max coverage (-): 0

Region: chr21 10337361-10337411. Max. coverage (+): 7.08. Max coverage (-): 0

Region: chr21 10337412-10337462. Max. coverage (+): 12.62. Max coverage (-): 0

Region: chr21 10337463-10337513. Max. coverage (+): 1.1. Max coverage (-): 0

Region: chr21 10337514-10337565. Max. coverage (+): 12.54. Max coverage (-): 0

Region: chr21 10337566-10337616. Max. coverage (+): 0. Max coverage (-): 0

Region: chr21 10337617-10337667. Max. coverage (+): 0. Max coverage (-): 0

Region: chr21 10337668-10337718. Max. coverage (+): 0. Max coverage (-): 0

Region: chr21 10337719-10337770. Max. coverage (+): 9.74. Max coverage (-): 0

Region: chr21 10337771-10337821. Max. coverage (+): 0. Max coverage (-): 0

Region: chr21 10337822-10337872. Max. coverage (+): 13.93. Max coverage (-): 0

Region: chr21 10337873-10337923. Max. coverage (+): 10.32. Max coverage (-): 0

Region: chr21 10337924-10337974. Max. coverage (+): 18.33. Max coverage (-): 0

Region: chr21 10337975-10338026. Max. coverage (+): 11.01. Max coverage (-): 0

Region: chr21 10338027-10338077. Max. coverage (+): 16.4. Max coverage (-): 0

Region: chr21 10338078-10338128. Max. coverage (+): 0.51. Max coverage (-): 0

Region: chr21 10338129-10338179. Max. coverage (+): 10.32. Max coverage (-): 0

Region: chr21 10338180-10338230. Max. coverage (+): 1.68. Max coverage (-): 0

Region: chr21 10338231-10338282. Max. coverage (+): 5.67. Max coverage (-): 0

Region: chr21 10338283-10338333. Max. coverage (+): 1.78. Max coverage (-): 0

Region: chr21 10338334-10338384. Max. coverage (+): 28.7. Max coverage (-): 0

Region: chr21 10338385-10338435. Max. coverage (+): 24.52. Max coverage (-): 0

Region: chr21 10338436-10338487. Max. coverage (+): 13.21. Max coverage (-): 0

Region: chr21 10338488-10338538. Max. coverage (+): 49.56. Max coverage (-): 0

Region: chr21 10338539-10338589. Max. coverage (+): 13.33. Max coverage (-): 0

Region: chr21 10338590-10338640. Max. coverage (+): 10.31. Max coverage (-): 0

Region: chr21 10338641-10338691. Max. coverage (+): 0. Max coverage (-): 0

Region: chr21 10338692-10338743. Max. coverage (+): 0. Max coverage (-): 0

Region: chr21 10338744-10338794. Max. coverage (+): 1.92. Max coverage (-): 0

Region: chr21 10338795-10338845. Max. coverage (+): 0. Max coverage (-): 0

Region: chr21 10338846-10338896. Max. coverage (+): 9.05. Max coverage (-): 0

Region: chr21 10338897-10338948. Max. coverage (+): 0. Max coverage (-): 0

Region: chr21 10338949-10338999. Max. coverage (+): 17.9. Max coverage (-): 0

Region: chr21 10339000-10339050. Max. coverage (+): 17.9. Max coverage (-): 0

Region: chr21 10339051-10339101. Max. coverage (+): 0. Max coverage (-): 0

Region: chr21 10339102-10339152. Max. coverage (+): 5.95. Max coverage (-): 0

Region: chr21 10339153-10339204. Max. coverage (+): 17.31. Max coverage (-): 0

Region: chr21 10339205-10339255. Max. coverage (+): 0. Max coverage (-): 0

Region: chr21 10339256-10339306. Max. coverage (+): 2.69. Max coverage (-): 0

Region: chr21 10339307-10339357. Max. coverage (+): 4.23. Max coverage (-): 0

Region: chr21 10339358-10339409. Max. coverage (+): 0. Max coverage (-): 0

Region: chr21 10339410-10339460. Max. coverage (+): 0. Max coverage (-): 0

Region: chr21 10339461-10339511. Max. coverage (+): 3.61. Max coverage (-): 0

Region: chr21 10339512-10339562. Max. coverage (+): 11.66. Max coverage (-): 0

Region: chr21 10339563-10339613. Max. coverage (+): 17.71. Max coverage (-): 0

Region: chr21 10339614-10339665. Max. coverage (+): 15.28. Max coverage (-): 0

Region: chr21 10339666-10339716. Max. coverage (+): 65.41. Max coverage (-): 0

Region: chr21 10339717-10339767. Max. coverage (+): 5.34. Max coverage (-): 0

Region: chr21 10339768-10339818. Max. coverage (+): 5.34. Max coverage (-): 0

Region: chr21 10339819-10339870. Max. coverage (+): 0. Max coverage (-): 0

Region: chr21 10339871-10339921. Max. coverage (+): 24.26. Max coverage (-): 0

Region: chr21 10339922-10339972. Max. coverage (+): 35.59. Max coverage (-): 0

Region: chr21 10339973-10340023. Max. coverage (+): 4.44. Max coverage (-): 0

Region: chr21 10340024-10340074. Max. coverage (+): 8.67. Max coverage (-): 0

Region: chr21 10340075-10340126. Max. coverage (+): 22.45. Max coverage (-): 0

Region: chr21 10340127-10340177. Max. coverage (+): 17.54. Max coverage (-): 0

Region: chr21 10340178-10340228. Max. coverage (+): 17.54. Max coverage (-): 0

Region: chr21 10340229-10340279. Max. coverage (+): 38.78. Max coverage (-): 0

Region: chr21 10340280-10340331. Max. coverage (+): 11.94. Max coverage (-): 0

Region: chr21 10340332-10340382. Max. coverage (+): 7.58. Max coverage (-): 0

Region: chr21 10340383-10340433. Max. coverage (+): 0. Max coverage (-): 0

Region: chr21 10340434-10340484. Max. coverage (+): 0. Max coverage (-): 0

Region: chr21 10340485-10340535. Max. coverage (+): 11.09. Max coverage (-): 0

Region: chr21 10340536-10340587. Max. coverage (+): 11.64. Max coverage (-): 0

Region: chr21 10340588-10340638. Max. coverage (+): 12.42. Max coverage (-): 0

Region: chr21 10340639-10340689. Max. coverage (+): 23.95. Max coverage (-): 0

Region: chr21 10340690-10340740. Max. coverage (+): 18.31. Max coverage (-): 0

Region: chr21 10340741-10340791. Max. coverage (+): 6.97. Max coverage (-): 0

Region: chr21 10340792-10340843. Max. coverage (+): 0.41. Max coverage (-): 0

Region: chr21 10340844-10340894. Max. coverage (+): 0. Max coverage (-): 0

Region: chr21 10340895-10340945. Max. coverage (+): 0. Max coverage (-): 0

Region: chr21 10340946-10340996. Max. coverage (+): 0. Max coverage (-): 0

Region: chr21 10340997-10341048. Max. coverage (+): 0. Max coverage (-): 0

Region: chr21 10341049-10341099. Max. coverage (+): 6.86. Max coverage (-): 0

Region: chr21 10341100-10341150. Max. coverage (+): 1.71. Max coverage (-): 0

Region: chr21 10341151-10341201. Max. coverage (+): 5.69. Max coverage (-): 0

Region: chr21 10341202-10341252. Max. coverage (+): 0. Max coverage (-): 0

Region: chr21 10341253-10341304. Max. coverage (+): 0. Max coverage (-): 0

Region: chr21 10341305-10341355. Max. coverage (+): 0. Max coverage (-): 0

Region: chr21 10341356-10341406. Max. coverage (+): 0. Max coverage (-): 0

Region: chr21 10341407-10341457. Max. coverage (+): 0. Max coverage (-): 0

Region: chr21 10341458-10341509. Max. coverage (+): 0. Max coverage (-): 0

Region: chr21 10341510-10341560. Max. coverage (+): 0. Max coverage (-): 0

Region: chr21 10341561-10341611. Max. coverage (+): 0. Max coverage (-): 0

Region: chr21 10341612-10341662. Max. coverage (+): 0. Max coverage (-): 0

Region: chr21 10341663-10341713. Max. coverage (+): 0. Max coverage (-): 0

Region: chr21 10341714-10341765. Max. coverage (+): 0. Max coverage (-): 0

Region: chr21 10341766-10341816. Max. coverage (+): 16.98. Max coverage (-): 0

Region: chr21 10341817-. Max. coverage (+): 14.8. Max coverage (-): 0

RepeatMasker Color Code

**+**

100-98% Identity

<98-95% Identity

<95-90% Identity

<90-85% Identity

<85-80% Identity

<80-75% Identity

<75-70% Identity

<70% Identity

**-**

Gene Set Color Code

**+**

Gene

Pseudogene

**-**

Topology/Coverage Color Code

Coverage Plus Strand

Coverage Minus Strand

Mainstrand: Plus

Mainstrand: Minus

Complementary Strand

Flanking Region  
(if option -flank >0)

Gene Set Annotation  
  
RepeatMasker Annotation  

**1. L2b**: 10316687-10316752 (-), Divergence to consensus: 33.3%  
**2. MIRb**: 10316787-10317002 (-), Divergence to consensus: 51.6%  
**3. ALTR2B1\_BT**: 10317030-10317342 (-), Divergence to consensus: 20.6%  
**4. Bov-tA2**: 10317351-10317534 (+), Divergence to consensus: 17.9%  
**5. ALTR2B1\_BT**: 10317531-10317751 (-), Divergence to consensus: 15.8%  
**6. LTR39C\_BT**: 10317758-10317929 (-), Divergence to consensus: 24.6%  
**7. ART2A**: 10317929-10318352 (+), Divergence to consensus: 21.1%  
**8. Bov-tA2**: 10318817-10319003 (+), Divergence to consensus: 19.8%  
**9. L2b**: 10319274-10319372 (-), Divergence to consensus: 43.6%  
**10. MamTip2**: 10321003-10321137 (-), Divergence to consensus: 41.5%  
**11. L1\_BT**: 10321926-10322209 (+), Divergence to consensus: 11.6%  
**12. L2c**: 10323564-10323708 (-), Divergence to consensus: 38.8%  
**13. L2c**: 10325222-10325277 (-), Divergence to consensus: 32.5%  
**14. Bov-tA2**: 10325656-10325853 (-), Divergence to consensus: 16.1%  
**15. Bov-tA2**: 10328152-10328357 (-), Divergence to consensus: 15.1%  
**16. BOV-A2**: 10330562-10330833 (-), Divergence to consensus: 4.8%  
**17. (A)n**: 10331140-10331159 (+), Divergence to consensus: 0%  
**18. AT\_rich**: 10331195-10331225 (+), Divergence to consensus: 58.1%  
**19. L1\_BT**: 10331900-10332436 (+), Divergence to consensus: 17%  
**20. LTR78**: 10333313-10333503 (+), Divergence to consensus: 38.8%  
**21. L2c**: 10333882-10334018 (+), Divergence to consensus: 38.1%  
**22. MER2**: 10334708-10334827 (-), Divergence to consensus: 51.9%  
**23. MER41\_BT**: 10335026-10335370 (+), Divergence to consensus: 28.4%  
**24. MIR3**: 10335459-10335540 (-), Divergence to consensus: 41.5%  
**25. Bov-tA3**: 10335825-10335941 (+), Divergence to consensus: 17.5%  
**26. MER2**: 10337235-10337336 (-), Divergence to consensus: 35.3%  
**27. AT\_rich**: 10337518-10337545 (+), Divergence to consensus: 60.7%  
**28. Bov-tA3**: 10337598-10337713 (+), Divergence to consensus: 22.5%  
**29. MER2**: 10339003-10339104 (-), Divergence to consensus: 31.8%  
**30. Bov-tA3**: 10339350-10339460 (+), Divergence to consensus: 14.5%  
**31. MER2**: 10340714-10340817 (-), Divergence to consensus: 32.1%  
**32. Bov-tA3**: 10340878-10341075 (+), Divergence to consensus: 7.1%  
**33. MER41\_BT**: 10341221-10341803 (+), Divergence to consensus: 31.6%

  
Transcription Factor Binding Sites  

**RFX4\_2** (Sequence: GTATCCACG (-): 10337346)  
**SPZ1** (Sequence: CTCTAACCCC (-): 10327112)  
**SPZ1** (Sequence: CTCTTACCCT (-): 10337048)  
**RFX4\_2** (Sequence: CATGGATAC (+): 10334778)  
**RFX4\_2** (Sequence: CATAGATAC (+): 10337287)  
**RFX4\_2** (Sequence: CATGGATAC (+): 10339053)  
**RFX4\_2** (Sequence: CTTAGATAC (+): 10339665)  
**RFX4\_2** (Sequence: CATGGATAC (+): 10340766)  
**Gata4** (Sequence: AGATAAG (-): 10331070)  
**SOX9** (Sequence: AACAATGA (-): 10325445)
