# Supplementary material for: Small RNA sequencing of cryopreserved semen from single bull revealed altered miRNAs and piRNAs expression between High- and Low-motile sperm populations
Source: BMC Genomics. 2017 Jan 4;18:14. doi: 10.1186/s12864-016-3394-7 (PMC5209821; doi:10.1186/s12864-016-3394-7)
Supplement: Additional file 4: — Details for each piRNA clusters found in Low Motile (LM) sperm fraction. Genes, repeats, transposable elements and transcription factors binding sites falling within the cluster regions were reported. (ZIP 1034 kb) [file 12864_2016_3394_MOESM4_ESM.zip › 26.html]

piRNA cluster 26


Predicted piRNA cluster no. 26     previous   next
  

Show proTRAC run info
Hide proTRAC run info

================================= proTRAC ====================================  
VERSION: 2.1                                    LAST MODIFIED: 06. October 2015  
  
Please cite:  
Rosenkranz D, Zischler H. proTRAC - a software for probabilistic piRNA cluster  
detection, visualization and analysis. 2012. BMC Bioinformatics 13:5.  
  
and (for proTRAC 2.0 and later):  
Rosenkranz D, Rudloff S, Bastuck K, Ketting RF, Zischler H. Tupaia small RNAs  
provide insights into function and evolution of RNAi-based transposon defense  
in mammals. 2015. RNA 21(5):911-922.  
  
Contact:  
David Rosenkranz  
Institute of Anthropology, small RNA group  
Johannes Gutenberg University Mainz  
email: rosenkranz@uni-mainz.de  
  
You can find the latest proTRAC version at:  
http://sourceforge.net/projects/protrac/files  
http://www.smallRNAgroup-mainz.de/software  
==============================================================================  
  
PARAMETERS:  
Map file: .............../storage/core/barbara/genhome/smallRNA/fertility/Sample\_not\_motile/pirna/Sample\_not\_motile\_26-33\_collapsed.fa.no-dust.map.weighted-10000-1000-b-0  
Genome file: ............/storage/core/barbara/genhome/smallRNA/fertility/Sample\_all/pirna/bt\_311\_chrY.fa  
RepeatMasker annotation: /storage/genomes/bt\_umd31/GCF\_000003055.6\_Bos\_taurus\_UMD\_3.1.1\_repeatMasker\_chr.out  
GeneSet:................./storage/core/barbara/genhome/smallRNA/fertility/Sample\_all/pirna/full.gtf  
  
Significant (p<=0.01) hit density will be calculated based  
on observed hit distribution.  
  
Sliding window size: ........................................ 5000 bp  
Sliding window increament: .................................. 1000 bp  
Normalize each hit by number of genomic hits: ............... 1 [0=no/1=yes]  
Normalize each hit by number of sequence reads: ............. 1 [0=no/1=yes]  
Normalize values (-> per million mapped reads): ............. 1 [0=no/1=yes]  
Min. fraction of hits with 1T(U) or 10A: .................... 0.75  
Alternatively: Min. fraction of hits with 1T(U) and 10A: .... 0.5  
Min. fraction of hits with typical piRNA length: ............ 0.75  
Typical piRNA length: ....................................... 26-33 nt  
Min. size of a piRNA cluster: ............................... 5000 bp.  
Min. number of hits (absolute): ............................. 0  
Min. number of hits (normalized): ........................... 0  
Min. fraction of hits on the mainstrand: .................... 0.75  
Top fraction of mapped sequences (in terms of read counts): . 1%  
Top fraction accounts for max. n% of sequence reads: ........ 90%  
Min. fraction of hits on each arm of a bidirectional cluster: 0.1  
Output image file for each cluster: ......................... 0 [0=no/1=yes]  
Output html file for each cluster: .......................... 1 [0=no/1=yes]  
Output a summary table: ..................................... 1 [0=no/1=yes]  
Output a FASTA file for each cluster (piRNA sequences): ..... 1 [0=no/1=yes]  
Output a FASTA file comprising cluster sequences: ........... 1 [0=no/1=yes]  
Search DNA motifs in clusters: .............................. 1 [0=no/1=yes]  
Output flanking sequences: +/- .............................. 0 bp  
Output ~.pTi file: .......................................... 1 [0=no/1=yes]  
==============================================================================  
  
  
Genome size (without gaps): ............ 2678902517 bp  
Gaps (N/X/-): .......................... 53837044 bp  
Mapped reads: .......................... 738059667487  
Non-identical sequences: ............... 277001  
Genomic hits: .......................... 533816  
Significant densitiy of mapped reads: .. 15118061 reads/kb

Show proTRAC cluster info
Hide proTRAC cluster info

|  |  |
| --- | --- |
| Location | chr21 |
| Coordinates | 14005205-14030111 |
| Size [bp] | 24907 |
| Sequence hit loci | 2237 |
| Mapped reads (normalized) | 5948381353 |
| Mapped reads (normalized) per kb | 238823678.2 |
| Normalized reads with 1T (1U) | 81.8% |
| Normalized reads with 10A | 30.8% |
| Normalized reads with length 26-33 nt | 100% |
| Normalized reads on the main strand(s) | 99.4% |
| Predicted directionality | bi:minus-plus (split between 14012410 and 14012433) |

100%

0%

1T (1U)  
reads

10A reads

26-33 nt  
reads

reads on mainstrand

**Either the amount of reads with 1T (1U) OR 10A has to exceed 75% (set with option: -1Tor10A)  
Alternatively the amount of reads with 1T (1U) AND 10A has to exceed 50% (set with option: -1Tand10A)  
Minimum amount of reads with preferred size is 75% (set with option: -pisize)  
Minimum amount of reads on the main strand(s) is 75% (set with option: -clstrand)**

Show read coverage
Hide read coverage

WHAT DO I SEE HERE?  
This chart shows the location of mapped sequence reads within a predicted piRNA cluster. The color refers to the number of genomic hits produced by the sequence read in question. A dark red bar indicates that this sequence read produces many other hits elsewhere in the genome. Many adjacent red or yellow bars can indicate the presence of a multi-copy element such as transposons or rRNA genes. A dark green bar indicates that this sequence read maps uniquely to this locus.

1 hit

2-5 hits

6-10 hits

11-20 hits

21-50 hits

51-100 hits

> 100 hits

chr21

14005205

14030111

Gene Set

RepeatMasker

Mapped  
Reads

118.75

plus strand

minus strand

118.75

Region: chr21 10341818-14005229. Max. coverage (+): 0. Max coverage (-): 2.01

Region: chr21 14005230-14005279. Max. coverage (+): 0. Max coverage (-): 0

Region: chr21 14005280-14005329. Max. coverage (+): 0. Max coverage (-): 0

Region: chr21 14005330-14005379. Max. coverage (+): 0. Max coverage (-): 0

Region: chr21 14005380-14005429. Max. coverage (+): 0. Max coverage (-): 0

Region: chr21 14005430-14005478. Max. coverage (+): 0. Max coverage (-): 0

Region: chr21 14005479-14005528. Max. coverage (+): 0. Max coverage (-): 0

Region: chr21 14005529-14005578. Max. coverage (+): 0. Max coverage (-): 0

Region: chr21 14005579-14005628. Max. coverage (+): 0. Max coverage (-): 0

Region: chr21 14005629-14005678. Max. coverage (+): 0. Max coverage (-): 0

Region: chr21 14005679-14005728. Max. coverage (+): 0. Max coverage (-): 6.4

Region: chr21 14005729-14005777. Max. coverage (+): 0. Max coverage (-): 0

Region: chr21 14005778-14005827. Max. coverage (+): 0. Max coverage (-): 0

Region: chr21 14005828-14005877. Max. coverage (+): 0. Max coverage (-): 0

Region: chr21 14005878-14005927. Max. coverage (+): 0. Max coverage (-): 0

Region: chr21 14005928-14005977. Max. coverage (+): 0. Max coverage (-): 0

Region: chr21 14005978-14006026. Max. coverage (+): 0. Max coverage (-): 0

Region: chr21 14006027-14006076. Max. coverage (+): 0. Max coverage (-): 0

Region: chr21 14006077-14006126. Max. coverage (+): 0. Max coverage (-): 0

Region: chr21 14006127-14006176. Max. coverage (+): 0. Max coverage (-): 0

Region: chr21 14006177-14006226. Max. coverage (+): 0. Max coverage (-): 0

Region: chr21 14006227-14006276. Max. coverage (+): 0. Max coverage (-): 0

Region: chr21 14006277-14006325. Max. coverage (+): 0. Max coverage (-): 0

Region: chr21 14006326-14006375. Max. coverage (+): 0. Max coverage (-): 0

Region: chr21 14006376-14006425. Max. coverage (+): 0. Max coverage (-): 0

Region: chr21 14006426-14006475. Max. coverage (+): 0. Max coverage (-): 0

Region: chr21 14006476-14006525. Max. coverage (+): 0. Max coverage (-): 0

Region: chr21 14006526-14006574. Max. coverage (+): 0. Max coverage (-): 0

Region: chr21 14006575-14006624. Max. coverage (+): 0. Max coverage (-): 4.49

Region: chr21 14006625-14006674. Max. coverage (+): 0. Max coverage (-): 9.99

Region: chr21 14006675-14006724. Max. coverage (+): 0. Max coverage (-): 8.19

Region: chr21 14006725-14006774. Max. coverage (+): 0. Max coverage (-): 0

Region: chr21 14006775-14006823. Max. coverage (+): 0. Max coverage (-): 0

Region: chr21 14006824-14006873. Max. coverage (+): 0. Max coverage (-): 0

Region: chr21 14006874-14006923. Max. coverage (+): 0. Max coverage (-): 2.66

Region: chr21 14006924-14006973. Max. coverage (+): 0. Max coverage (-): 0

Region: chr21 14006974-14007023. Max. coverage (+): 0. Max coverage (-): 0

Region: chr21 14007024-14007073. Max. coverage (+): 0. Max coverage (-): 0

Region: chr21 14007074-14007122. Max. coverage (+): 0. Max coverage (-): 0

Region: chr21 14007123-14007172. Max. coverage (+): 0. Max coverage (-): 0

Region: chr21 14007173-14007222. Max. coverage (+): 0. Max coverage (-): 0

Region: chr21 14007223-14007272. Max. coverage (+): 0. Max coverage (-): 0

Region: chr21 14007273-14007322. Max. coverage (+): 0. Max coverage (-): 0

Region: chr21 14007323-14007371. Max. coverage (+): 0. Max coverage (-): 5.01

Region: chr21 14007372-14007421. Max. coverage (+): 0. Max coverage (-): 1.82

Region: chr21 14007422-14007471. Max. coverage (+): 0. Max coverage (-): 0

Region: chr21 14007472-14007521. Max. coverage (+): 0. Max coverage (-): 0

Region: chr21 14007522-14007571. Max. coverage (+): 0. Max coverage (-): 0

Region: chr21 14007572-14007620. Max. coverage (+): 0. Max coverage (-): 0

Region: chr21 14007621-14007670. Max. coverage (+): 0. Max coverage (-): 6.86

Region: chr21 14007671-14007720. Max. coverage (+): 0. Max coverage (-): 33.37

Region: chr21 14007721-14007770. Max. coverage (+): 0. Max coverage (-): 4.34

Region: chr21 14007771-14007820. Max. coverage (+): 0. Max coverage (-): 19.33

Region: chr21 14007821-14007870. Max. coverage (+): 0. Max coverage (-): 14.32

Region: chr21 14007871-14007919. Max. coverage (+): 0. Max coverage (-): 4.87

Region: chr21 14007920-14007969. Max. coverage (+): 0. Max coverage (-): 14.15

Region: chr21 14007970-14008019. Max. coverage (+): 0. Max coverage (-): 22.66

Region: chr21 14008020-14008069. Max. coverage (+): 0. Max coverage (-): 22.19

Region: chr21 14008070-14008119. Max. coverage (+): 0. Max coverage (-): 34.29

Region: chr21 14008120-14008168. Max. coverage (+): 0. Max coverage (-): 17.98

Region: chr21 14008169-14008218. Max. coverage (+): 0. Max coverage (-): 4.77

Region: chr21 14008219-14008268. Max. coverage (+): 0. Max coverage (-): 1.47

Region: chr21 14008269-14008318. Max. coverage (+): 0. Max coverage (-): 5.06

Region: chr21 14008319-14008368. Max. coverage (+): 0. Max coverage (-): 1.99

Region: chr21 14008369-14008418. Max. coverage (+): 0. Max coverage (-): 0

Region: chr21 14008419-14008467. Max. coverage (+): 0. Max coverage (-): 0

Region: chr21 14008468-14008517. Max. coverage (+): 0. Max coverage (-): 46.38

Region: chr21 14008518-14008567. Max. coverage (+): 0. Max coverage (-): 22.47

Region: chr21 14008568-14008617. Max. coverage (+): 0. Max coverage (-): 2.66

Region: chr21 14008618-14008667. Max. coverage (+): 0. Max coverage (-): 19.77

Region: chr21 14008668-14008716. Max. coverage (+): 0. Max coverage (-): 10.14

Region: chr21 14008717-14008766. Max. coverage (+): 0. Max coverage (-): 16.07

Region: chr21 14008767-14008816. Max. coverage (+): 0. Max coverage (-): 0

Region: chr21 14008817-14008866. Max. coverage (+): 0. Max coverage (-): 0

Region: chr21 14008867-14008916. Max. coverage (+): 0. Max coverage (-): 0

Region: chr21 14008917-14008965. Max. coverage (+): 0. Max coverage (-): 0

Region: chr21 14008966-14009015. Max. coverage (+): 0. Max coverage (-): 0

Region: chr21 14009016-14009065. Max. coverage (+): 0. Max coverage (-): 0

Region: chr21 14009066-14009115. Max. coverage (+): 0. Max coverage (-): 6.4

Region: chr21 14009116-14009165. Max. coverage (+): 0. Max coverage (-): 0

Region: chr21 14009166-14009215. Max. coverage (+): 0. Max coverage (-): 0

Region: chr21 14009216-14009264. Max. coverage (+): 0. Max coverage (-): 0

Region: chr21 14009265-14009314. Max. coverage (+): 0. Max coverage (-): 4.82

Region: chr21 14009315-14009364. Max. coverage (+): 0. Max coverage (-): 8.8

Region: chr21 14009365-14009414. Max. coverage (+): 0. Max coverage (-): 6.17

Region: chr21 14009415-14009464. Max. coverage (+): 0. Max coverage (-): 0

Region: chr21 14009465-14009513. Max. coverage (+): 0. Max coverage (-): 3.63

Region: chr21 14009514-14009563. Max. coverage (+): 0. Max coverage (-): 29.53

Region: chr21 14009564-14009613. Max. coverage (+): 0. Max coverage (-): 13.62

Region: chr21 14009614-14009663. Max. coverage (+): 0. Max coverage (-): 0

Region: chr21 14009664-14009713. Max. coverage (+): 0. Max coverage (-): 0

Region: chr21 14009714-14009762. Max. coverage (+): 0. Max coverage (-): 0

Region: chr21 14009763-14009812. Max. coverage (+): 0. Max coverage (-): 12.97

Region: chr21 14009813-14009862. Max. coverage (+): 0. Max coverage (-): 4.22

Region: chr21 14009863-14009912. Max. coverage (+): 0. Max coverage (-): 0

Region: chr21 14009913-14009962. Max. coverage (+): 0. Max coverage (-): 0

Region: chr21 14009963-14010012. Max. coverage (+): 0. Max coverage (-): 3.05

Region: chr21 14010013-14010061. Max. coverage (+): 0. Max coverage (-): 6.9

Region: chr21 14010062-14010111. Max. coverage (+): 0. Max coverage (-): 17.96

Region: chr21 14010112-14010161. Max. coverage (+): 0. Max coverage (-): 5.83

Region: chr21 14010162-14010211. Max. coverage (+): 0. Max coverage (-): 11.13

Region: chr21 14010212-14010261. Max. coverage (+): 0. Max coverage (-): 18.05

Region: chr21 14010262-14010310. Max. coverage (+): 0. Max coverage (-): 17.05

Region: chr21 14010311-14010360. Max. coverage (+): 0. Max coverage (-): 32.26

Region: chr21 14010361-14010410. Max. coverage (+): 0. Max coverage (-): 33.28

Region: chr21 14010411-14010460. Max. coverage (+): 0. Max coverage (-): 0

Region: chr21 14010461-14010510. Max. coverage (+): 0. Max coverage (-): 13.47

Region: chr21 14010511-14010560. Max. coverage (+): 0. Max coverage (-): 56.91

Region: chr21 14010561-14010609. Max. coverage (+): 0. Max coverage (-): 13.16

Region: chr21 14010610-14010659. Max. coverage (+): 0. Max coverage (-): 15.47

Region: chr21 14010660-14010709. Max. coverage (+): 0. Max coverage (-): 20.17

Region: chr21 14010710-14010759. Max. coverage (+): 0. Max coverage (-): 22.26

Region: chr21 14010760-14010809. Max. coverage (+): 0. Max coverage (-): 18.48

Region: chr21 14010810-14010858. Max. coverage (+): 0. Max coverage (-): 10.23

Region: chr21 14010859-14010908. Max. coverage (+): 0. Max coverage (-): 40.81

Region: chr21 14010909-14010958. Max. coverage (+): 0. Max coverage (-): 15.73

Region: chr21 14010959-14011008. Max. coverage (+): 0. Max coverage (-): 52

Region: chr21 14011009-14011058. Max. coverage (+): 0. Max coverage (-): 18.56

Region: chr21 14011059-14011107. Max. coverage (+): 0. Max coverage (-): 3.21

Region: chr21 14011108-14011157. Max. coverage (+): 0. Max coverage (-): 0

Region: chr21 14011158-14011207. Max. coverage (+): 0. Max coverage (-): 0

Region: chr21 14011208-14011257. Max. coverage (+): 0. Max coverage (-): 0

Region: chr21 14011258-14011307. Max. coverage (+): 0. Max coverage (-): 0

Region: chr21 14011308-14011357. Max. coverage (+): 0. Max coverage (-): 0

Region: chr21 14011358-14011406. Max. coverage (+): 0. Max coverage (-): 0

Region: chr21 14011407-14011456. Max. coverage (+): 0. Max coverage (-): 0

Region: chr21 14011457-14011506. Max. coverage (+): 0. Max coverage (-): 0

Region: chr21 14011507-14011556. Max. coverage (+): 0. Max coverage (-): 3.11

Region: chr21 14011557-14011606. Max. coverage (+): 0. Max coverage (-): 0.55

Region: chr21 14011607-14011655. Max. coverage (+): 0. Max coverage (-): 0

Region: chr21 14011656-14011705. Max. coverage (+): 0. Max coverage (-): 0

Region: chr21 14011706-14011755. Max. coverage (+): 0. Max coverage (-): 0

Region: chr21 14011756-14011805. Max. coverage (+): 0. Max coverage (-): 0

Region: chr21 14011806-14011855. Max. coverage (+): 0. Max coverage (-): 4.32

Region: chr21 14011856-14011904. Max. coverage (+): 6.13. Max coverage (-): 2.11

Region: chr21 14011905-14011954. Max. coverage (+): 0. Max coverage (-): 14.59

Region: chr21 14011955-14012004. Max. coverage (+): 0. Max coverage (-): 12.86

Region: chr21 14012005-14012054. Max. coverage (+): 0. Max coverage (-): 12.86

Region: chr21 14012055-14012104. Max. coverage (+): 0. Max coverage (-): 0

Region: chr21 14012105-14012154. Max. coverage (+): 5.62. Max coverage (-): 0

Region: chr21 14012155-14012203. Max. coverage (+): 0. Max coverage (-): 0

Region: chr21 14012204-14012253. Max. coverage (+): 0. Max coverage (-): 8.18

Region: chr21 14012254-14012303. Max. coverage (+): 0. Max coverage (-): 8.18

Region: chr21 14012304-14012353. Max. coverage (+): 9.1. Max coverage (-): 0

Region: chr21 14012354-14012403. Max. coverage (+): 6.71. Max coverage (-): 7.16

Region: chr21 14012404-14012452. Max. coverage (+): 5.95. Max coverage (-): 7.14

Region: chr21 14012453-14012502. Max. coverage (+): 0. Max coverage (-): 0

Region: chr21 14012503-14012552. Max. coverage (+): 0. Max coverage (-): 0

Region: chr21 14012553-14012602. Max. coverage (+): 0. Max coverage (-): 0

Region: chr21 14012603-14012652. Max. coverage (+): 0. Max coverage (-): 0

Region: chr21 14012653-14012702. Max. coverage (+): 0. Max coverage (-): 0

Region: chr21 14012703-14012751. Max. coverage (+): 4.75. Max coverage (-): 0

Region: chr21 14012752-14012801. Max. coverage (+): 3.96. Max coverage (-): 0

Region: chr21 14012802-14012851. Max. coverage (+): 3.96. Max coverage (-): 0

Region: chr21 14012852-14012901. Max. coverage (+): 3.92. Max coverage (-): 0

Region: chr21 14012902-14012951. Max. coverage (+): 6.13. Max coverage (-): 4.09

Region: chr21 14012952-14013000. Max. coverage (+): 8.27. Max coverage (-): 0

Region: chr21 14013001-14013050. Max. coverage (+): 0. Max coverage (-): 0

Region: chr21 14013051-14013100. Max. coverage (+): 0. Max coverage (-): 0

Region: chr21 14013101-14013150. Max. coverage (+): 0. Max coverage (-): 0

Region: chr21 14013151-14013200. Max. coverage (+): 0. Max coverage (-): 0

Region: chr21 14013201-14013249. Max. coverage (+): 0. Max coverage (-): 0

Region: chr21 14013250-14013299. Max. coverage (+): 0. Max coverage (-): 0

Region: chr21 14013300-14013349. Max. coverage (+): 0. Max coverage (-): 0

Region: chr21 14013350-14013399. Max. coverage (+): 0. Max coverage (-): 0

Region: chr21 14013400-14013449. Max. coverage (+): 0. Max coverage (-): 0

Region: chr21 14013450-14013499. Max. coverage (+): 23.31. Max coverage (-): 0

Region: chr21 14013500-14013548. Max. coverage (+): 22.14. Max coverage (-): 4.98

Region: chr21 14013549-14013598. Max. coverage (+): 71.92. Max coverage (-): 4.98

Region: chr21 14013599-14013648. Max. coverage (+): 18.98. Max coverage (-): 0

Region: chr21 14013649-14013698. Max. coverage (+): 116.55. Max coverage (-): 0

Region: chr21 14013699-14013748. Max. coverage (+): 7.21. Max coverage (-): 0

Region: chr21 14013749-14013797. Max. coverage (+): 25.7. Max coverage (-): 0

Region: chr21 14013798-14013847. Max. coverage (+): 6.37. Max coverage (-): 0

Region: chr21 14013848-14013897. Max. coverage (+): 0. Max coverage (-): 0

Region: chr21 14013898-14013947. Max. coverage (+): 0. Max coverage (-): 0

Region: chr21 14013948-14013997. Max. coverage (+): 0. Max coverage (-): 0

Region: chr21 14013998-14014046. Max. coverage (+): 9.2. Max coverage (-): 0

Region: chr21 14014047-14014096. Max. coverage (+): 12.29. Max coverage (-): 6.75

Region: chr21 14014097-14014146. Max. coverage (+): 5.54. Max coverage (-): 0

Region: chr21 14014147-14014196. Max. coverage (+): 5.54. Max coverage (-): 0

Region: chr21 14014197-14014246. Max. coverage (+): 22.49. Max coverage (-): 0

Region: chr21 14014247-14014296. Max. coverage (+): 22.49. Max coverage (-): 0

Region: chr21 14014297-14014345. Max. coverage (+): 78.89. Max coverage (-): 0

Region: chr21 14014346-14014395. Max. coverage (+): 18.21. Max coverage (-): 0

Region: chr21 14014396-14014445. Max. coverage (+): 31.95. Max coverage (-): 0

Region: chr21 14014446-14014495. Max. coverage (+): 74.52. Max coverage (-): 0

Region: chr21 14014496-14014545. Max. coverage (+): 44.73. Max coverage (-): 0

Region: chr21 14014546-14014594. Max. coverage (+): 18.2. Max coverage (-): 0

Region: chr21 14014595-14014644. Max. coverage (+): 1.88. Max coverage (-): 0

Region: chr21 14014645-14014694. Max. coverage (+): 17.98. Max coverage (-): 0

Region: chr21 14014695-14014744. Max. coverage (+): 13.32. Max coverage (-): 0

Region: chr21 14014745-14014794. Max. coverage (+): 33.79. Max coverage (-): 0

Region: chr21 14014795-14014844. Max. coverage (+): 28.38. Max coverage (-): 3.34

Region: chr21 14014845-14014893. Max. coverage (+): 32.14. Max coverage (-): 0

Region: chr21 14014894-14014943. Max. coverage (+): 0. Max coverage (-): 0

Region: chr21 14014944-14014993. Max. coverage (+): 3.06. Max coverage (-): 0

Region: chr21 14014994-14015043. Max. coverage (+): 23.65. Max coverage (-): 0

Region: chr21 14015044-14015093. Max. coverage (+): 24.15. Max coverage (-): 0

Region: chr21 14015094-14015142. Max. coverage (+): 49.24. Max coverage (-): 0

Region: chr21 14015143-14015192. Max. coverage (+): 2.6. Max coverage (-): 0

Region: chr21 14015193-14015242. Max. coverage (+): 0. Max coverage (-): 0

Region: chr21 14015243-14015292. Max. coverage (+): 17.83. Max coverage (-): 0

Region: chr21 14015293-14015342. Max. coverage (+): 5.62. Max coverage (-): 0

Region: chr21 14015343-14015391. Max. coverage (+): 78.8. Max coverage (-): 0

Region: chr21 14015392-14015441. Max. coverage (+): 0. Max coverage (-): 0

Region: chr21 14015442-14015491. Max. coverage (+): 0. Max coverage (-): 0

Region: chr21 14015492-14015541. Max. coverage (+): 0. Max coverage (-): 0

Region: chr21 14015542-14015591. Max. coverage (+): 13.78. Max coverage (-): 0

Region: chr21 14015592-14015641. Max. coverage (+): 21.35. Max coverage (-): 0

Region: chr21 14015642-14015690. Max. coverage (+): 60.15. Max coverage (-): 0

Region: chr21 14015691-14015740. Max. coverage (+): 0. Max coverage (-): 0

Region: chr21 14015741-14015790. Max. coverage (+): 0. Max coverage (-): 0

Region: chr21 14015791-14015840. Max. coverage (+): 0. Max coverage (-): 0

Region: chr21 14015841-14015890. Max. coverage (+): 18.91. Max coverage (-): 0

Region: chr21 14015891-14015939. Max. coverage (+): 6.47. Max coverage (-): 0

Region: chr21 14015940-14015989. Max. coverage (+): 3.94. Max coverage (-): 0

Region: chr21 14015990-14016039. Max. coverage (+): 24.84. Max coverage (-): 0

Region: chr21 14016040-14016089. Max. coverage (+): 27.35. Max coverage (-): 0

Region: chr21 14016090-14016139. Max. coverage (+): 44.94. Max coverage (-): 0

Region: chr21 14016140-14016188. Max. coverage (+): 42.08. Max coverage (-): 0

Region: chr21 14016189-14016238. Max. coverage (+): 64.87. Max coverage (-): 0

Region: chr21 14016239-14016288. Max. coverage (+): 0. Max coverage (-): 0

Region: chr21 14016289-14016338. Max. coverage (+): 14.36. Max coverage (-): 0

Region: chr21 14016339-14016388. Max. coverage (+): 8.46. Max coverage (-): 0

Region: chr21 14016389-14016438. Max. coverage (+): 35.66. Max coverage (-): 0

Region: chr21 14016439-14016487. Max. coverage (+): 23.58. Max coverage (-): 0

Region: chr21 14016488-14016537. Max. coverage (+): 39.2. Max coverage (-): 0

Region: chr21 14016538-14016587. Max. coverage (+): 77.82. Max coverage (-): 0

Region: chr21 14016588-14016637. Max. coverage (+): 16.09. Max coverage (-): 0

Region: chr21 14016638-14016687. Max. coverage (+): 42.19. Max coverage (-): 0

Region: chr21 14016688-14016736. Max. coverage (+): 22.46. Max coverage (-): 0

Region: chr21 14016737-14016786. Max. coverage (+): 35.4. Max coverage (-): 0

Region: chr21 14016787-14016836. Max. coverage (+): 1.37. Max coverage (-): 0

Region: chr21 14016837-14016886. Max. coverage (+): 16.65. Max coverage (-): 0

Region: chr21 14016887-14016936. Max. coverage (+): 4.55. Max coverage (-): 0

Region: chr21 14016937-14016986. Max. coverage (+): 11.06. Max coverage (-): 0

Region: chr21 14016987-14017035. Max. coverage (+): 6.55. Max coverage (-): 0

Region: chr21 14017036-14017085. Max. coverage (+): 10.3. Max coverage (-): 0

Region: chr21 14017086-14017135. Max. coverage (+): 16.95. Max coverage (-): 0

Region: chr21 14017136-14017185. Max. coverage (+): 16.23. Max coverage (-): 0

Region: chr21 14017186-14017235. Max. coverage (+): 56.21. Max coverage (-): 0

Region: chr21 14017236-14017284. Max. coverage (+): 57.76. Max coverage (-): 0

Region: chr21 14017285-14017334. Max. coverage (+): 27.21. Max coverage (-): 0

Region: chr21 14017335-14017384. Max. coverage (+): 8.01. Max coverage (-): 0

Region: chr21 14017385-14017434. Max. coverage (+): 3.79. Max coverage (-): 0

Region: chr21 14017435-14017484. Max. coverage (+): 24.61. Max coverage (-): 0

Region: chr21 14017485-14017533. Max. coverage (+): 39.89. Max coverage (-): 0

Region: chr21 14017534-14017583. Max. coverage (+): 19.93. Max coverage (-): 0

Region: chr21 14017584-14017633. Max. coverage (+): 30.17. Max coverage (-): 0

Region: chr21 14017634-14017683. Max. coverage (+): 30.92. Max coverage (-): 0

Region: chr21 14017684-14017733. Max. coverage (+): 29.11. Max coverage (-): 0

Region: chr21 14017734-14017783. Max. coverage (+): 31.27. Max coverage (-): 0

Region: chr21 14017784-14017832. Max. coverage (+): 59.7. Max coverage (-): 0

Region: chr21 14017833-14017882. Max. coverage (+): 0. Max coverage (-): 0

Region: chr21 14017883-14017932. Max. coverage (+): 13.45. Max coverage (-): 0

Region: chr21 14017933-14017982. Max. coverage (+): 6.73. Max coverage (-): 0

Region: chr21 14017983-14018032. Max. coverage (+): 7.76. Max coverage (-): 0

Region: chr21 14018033-14018081. Max. coverage (+): 18.75. Max coverage (-): 0

Region: chr21 14018082-14018131. Max. coverage (+): 16.06. Max coverage (-): 0

Region: chr21 14018132-14018181. Max. coverage (+): 0. Max coverage (-): 0

Region: chr21 14018182-14018231. Max. coverage (+): 0. Max coverage (-): 0

Region: chr21 14018232-14018281. Max. coverage (+): 59.63. Max coverage (-): 0

Region: chr21 14018282-14018330. Max. coverage (+): 31.5. Max coverage (-): 0

Region: chr21 14018331-14018380. Max. coverage (+): 14.45. Max coverage (-): 0

Region: chr21 14018381-14018430. Max. coverage (+): 0. Max coverage (-): 0

Region: chr21 14018431-14018480. Max. coverage (+): 6.99. Max coverage (-): 0

Region: chr21 14018481-14018530. Max. coverage (+): 19.14. Max coverage (-): 0

Region: chr21 14018531-14018580. Max. coverage (+): 40.51. Max coverage (-): 0

Region: chr21 14018581-14018629. Max. coverage (+): 14.9. Max coverage (-): 0

Region: chr21 14018630-14018679. Max. coverage (+): 55.7. Max coverage (-): 0

Region: chr21 14018680-14018729. Max. coverage (+): 88.85. Max coverage (-): 0

Region: chr21 14018730-14018779. Max. coverage (+): 44.43. Max coverage (-): 0

Region: chr21 14018780-14018829. Max. coverage (+): 50.35. Max coverage (-): 0

Region: chr21 14018830-14018878. Max. coverage (+): 19.08. Max coverage (-): 0

Region: chr21 14018879-14018928. Max. coverage (+): 11.24. Max coverage (-): 0

Region: chr21 14018929-14018978. Max. coverage (+): 8.46. Max coverage (-): 0

Region: chr21 14018979-14019028. Max. coverage (+): 14.35. Max coverage (-): 0

Region: chr21 14019029-14019078. Max. coverage (+): 20.72. Max coverage (-): 0

Region: chr21 14019079-14019128. Max. coverage (+): 15.65. Max coverage (-): 0

Region: chr21 14019129-14019177. Max. coverage (+): 15.17. Max coverage (-): 0

Region: chr21 14019178-14019227. Max. coverage (+): 14.89. Max coverage (-): 2.62

Region: chr21 14019228-14019277. Max. coverage (+): 2.13. Max coverage (-): 0

Region: chr21 14019278-14019327. Max. coverage (+): 22.69. Max coverage (-): 0

Region: chr21 14019328-14019377. Max. coverage (+): 11.37. Max coverage (-): 0

Region: chr21 14019378-14019426. Max. coverage (+): 4.43. Max coverage (-): 0

Region: chr21 14019427-14019476. Max. coverage (+): 0. Max coverage (-): 0

Region: chr21 14019477-14019526. Max. coverage (+): 13.16. Max coverage (-): 0

Region: chr21 14019527-14019576. Max. coverage (+): 4.54. Max coverage (-): 0

Region: chr21 14019577-14019626. Max. coverage (+): 11.28. Max coverage (-): 0

Region: chr21 14019627-14019675. Max. coverage (+): 35.67. Max coverage (-): 0

Region: chr21 14019676-14019725. Max. coverage (+): 29.24. Max coverage (-): 0

Region: chr21 14019726-14019775. Max. coverage (+): 5.51. Max coverage (-): 0

Region: chr21 14019776-14019825. Max. coverage (+): 14.22. Max coverage (-): 0

Region: chr21 14019826-14019875. Max. coverage (+): 60.12. Max coverage (-): 0

Region: chr21 14019876-14019925. Max. coverage (+): 47.56. Max coverage (-): 0

Region: chr21 14019926-14019974. Max. coverage (+): 42.97. Max coverage (-): 0

Region: chr21 14019975-14020024. Max. coverage (+): 0. Max coverage (-): 0

Region: chr21 14020025-14020074. Max. coverage (+): 8.3. Max coverage (-): 0

Region: chr21 14020075-14020124. Max. coverage (+): 8.3. Max coverage (-): 0

Region: chr21 14020125-14020174. Max. coverage (+): 24.17. Max coverage (-): 0.08

Region: chr21 14020175-14020223. Max. coverage (+): 25.06. Max coverage (-): 2.49

Region: chr21 14020224-14020273. Max. coverage (+): 27.52. Max coverage (-): 0

Region: chr21 14020274-14020323. Max. coverage (+): 35.06. Max coverage (-): 0

Region: chr21 14020324-14020373. Max. coverage (+): 8.3. Max coverage (-): 0

Region: chr21 14020374-14020423. Max. coverage (+): 36.53. Max coverage (-): 0

Region: chr21 14020424-14020472. Max. coverage (+): 4.89. Max coverage (-): 0

Region: chr21 14020473-14020522. Max. coverage (+): 66.21. Max coverage (-): 0

Region: chr21 14020523-14020572. Max. coverage (+): 3.01. Max coverage (-): 0

Region: chr21 14020573-14020622. Max. coverage (+): 8.86. Max coverage (-): 0

Region: chr21 14020623-14020672. Max. coverage (+): 30.79. Max coverage (-): 0

Region: chr21 14020673-14020722. Max. coverage (+): 21.91. Max coverage (-): 0

Region: chr21 14020723-14020771. Max. coverage (+): 20.35. Max coverage (-): 0

Region: chr21 14020772-14020821. Max. coverage (+): 50.2. Max coverage (-): 0

Region: chr21 14020822-14020871. Max. coverage (+): 6.93. Max coverage (-): 0

Region: chr21 14020872-14020921. Max. coverage (+): 3.14. Max coverage (-): 0

Region: chr21 14020922-14020971. Max. coverage (+): 23.49. Max coverage (-): 0

Region: chr21 14020972-14021020. Max. coverage (+): 24.48. Max coverage (-): 0

Region: chr21 14021021-14021070. Max. coverage (+): 24.49. Max coverage (-): 0

Region: chr21 14021071-14021120. Max. coverage (+): 63.38. Max coverage (-): 0

Region: chr21 14021121-14021170. Max. coverage (+): 55.45. Max coverage (-): 0

Region: chr21 14021171-14021220. Max. coverage (+): 9.39. Max coverage (-): 0

Region: chr21 14021221-14021270. Max. coverage (+): 0.54. Max coverage (-): 0

Region: chr21 14021271-14021319. Max. coverage (+): 0.3. Max coverage (-): 0

Region: chr21 14021320-14021369. Max. coverage (+): 0. Max coverage (-): 0

Region: chr21 14021370-14021419. Max. coverage (+): 0. Max coverage (-): 0

Region: chr21 14021420-14021469. Max. coverage (+): 0.39. Max coverage (-): 0

Region: chr21 14021470-14021519. Max. coverage (+): 0. Max coverage (-): 0

Region: chr21 14021520-14021568. Max. coverage (+): 15.75. Max coverage (-): 0

Region: chr21 14021569-14021618. Max. coverage (+): 15.75. Max coverage (-): 0

Region: chr21 14021619-14021668. Max. coverage (+): 37.05. Max coverage (-): 0

Region: chr21 14021669-14021718. Max. coverage (+): 5.7. Max coverage (-): 3.73

Region: chr21 14021719-14021768. Max. coverage (+): 0. Max coverage (-): 0

Region: chr21 14021769-14021817. Max. coverage (+): 6.19. Max coverage (-): 0

Region: chr21 14021818-14021867. Max. coverage (+): 19.46. Max coverage (-): 0

Region: chr21 14021868-14021917. Max. coverage (+): 87.35. Max coverage (-): 0

Region: chr21 14021918-14021967. Max. coverage (+): 17.39. Max coverage (-): 0

Region: chr21 14021968-14022017. Max. coverage (+): 3.96. Max coverage (-): 0

Region: chr21 14022018-14022067. Max. coverage (+): 0. Max coverage (-): 0

Region: chr21 14022068-14022116. Max. coverage (+): 0. Max coverage (-): 0

Region: chr21 14022117-14022166. Max. coverage (+): 0. Max coverage (-): 0

Region: chr21 14022167-14022216. Max. coverage (+): 84.61. Max coverage (-): 0

Region: chr21 14022217-14022266. Max. coverage (+): 67.44. Max coverage (-): 0

Region: chr21 14022267-14022316. Max. coverage (+): 118.75. Max coverage (-): 0

Region: chr21 14022317-14022365. Max. coverage (+): 9.95. Max coverage (-): 0

Region: chr21 14022366-14022415. Max. coverage (+): 21.64. Max coverage (-): 0

Region: chr21 14022416-14022465. Max. coverage (+): 31.02. Max coverage (-): 0

Region: chr21 14022466-14022515. Max. coverage (+): 36.11. Max coverage (-): 0

Region: chr21 14022516-14022565. Max. coverage (+): 68.25. Max coverage (-): 0

Region: chr21 14022566-14022614. Max. coverage (+): 27.64. Max coverage (-): 0

Region: chr21 14022615-14022664. Max. coverage (+): 0. Max coverage (-): 0

Region: chr21 14022665-14022714. Max. coverage (+): 9.12. Max coverage (-): 0

Region: chr21 14022715-14022764. Max. coverage (+): 28.57. Max coverage (-): 0

Region: chr21 14022765-14022814. Max. coverage (+): 7.88. Max coverage (-): 0

Region: chr21 14022815-14022864. Max. coverage (+): 3.87. Max coverage (-): 0

Region: chr21 14022865-14022913. Max. coverage (+): 6.96. Max coverage (-): 0

Region: chr21 14022914-14022963. Max. coverage (+): 6.96. Max coverage (-): 0

Region: chr21 14022964-14023013. Max. coverage (+): 13.59. Max coverage (-): 0

Region: chr21 14023014-14023063. Max. coverage (+): 7.2. Max coverage (-): 0

Region: chr21 14023064-14023113. Max. coverage (+): 5.09. Max coverage (-): 0

Region: chr21 14023114-14023162. Max. coverage (+): 12.94. Max coverage (-): 0

Region: chr21 14023163-14023212. Max. coverage (+): 32.85. Max coverage (-): 0

Region: chr21 14023213-14023262. Max. coverage (+): 39.42. Max coverage (-): 0

Region: chr21 14023263-14023312. Max. coverage (+): 8.62. Max coverage (-): 0

Region: chr21 14023313-14023362. Max. coverage (+): 109.42. Max coverage (-): 0

Region: chr21 14023363-14023412. Max. coverage (+): 28.96. Max coverage (-): 0

Region: chr21 14023413-14023461. Max. coverage (+): 15.97. Max coverage (-): 0

Region: chr21 14023462-14023511. Max. coverage (+): 70.57. Max coverage (-): 0

Region: chr21 14023512-14023561. Max. coverage (+): 46.04. Max coverage (-): 0

Region: chr21 14023562-14023611. Max. coverage (+): 7.44. Max coverage (-): 0

Region: chr21 14023612-14023661. Max. coverage (+): 29.73. Max coverage (-): 0

Region: chr21 14023662-14023710. Max. coverage (+): 16.26. Max coverage (-): 0

Region: chr21 14023711-14023760. Max. coverage (+): 4.59. Max coverage (-): 0

Region: chr21 14023761-14023810. Max. coverage (+): 22. Max coverage (-): 0

Region: chr21 14023811-14023860. Max. coverage (+): 64.01. Max coverage (-): 0

Region: chr21 14023861-14023910. Max. coverage (+): 19.83. Max coverage (-): 0

Region: chr21 14023911-14023959. Max. coverage (+): 34.54. Max coverage (-): 0

Region: chr21 14023960-14024009. Max. coverage (+): 19.28. Max coverage (-): 0

Region: chr21 14024010-14024059. Max. coverage (+): 28.51. Max coverage (-): 0

Region: chr21 14024060-14024109. Max. coverage (+): 20.42. Max coverage (-): 0

Region: chr21 14024110-14024159. Max. coverage (+): 14.39. Max coverage (-): 0

Region: chr21 14024160-14024209. Max. coverage (+): 0. Max coverage (-): 0

Region: chr21 14024210-14024258. Max. coverage (+): 0. Max coverage (-): 0

Region: chr21 14024259-14024308. Max. coverage (+): 6.7. Max coverage (-): 0

Region: chr21 14024309-14024358. Max. coverage (+): 3.82. Max coverage (-): 0

Region: chr21 14024359-14024408. Max. coverage (+): 0. Max coverage (-): 0

Region: chr21 14024409-14024458. Max. coverage (+): 5.26. Max coverage (-): 0

Region: chr21 14024459-14024507. Max. coverage (+): 0.75. Max coverage (-): 0

Region: chr21 14024508-14024557. Max. coverage (+): 0.75. Max coverage (-): 0

Region: chr21 14024558-14024607. Max. coverage (+): 2.96. Max coverage (-): 0

Region: chr21 14024608-14024657. Max. coverage (+): 5.63. Max coverage (-): 0

Region: chr21 14024658-14024707. Max. coverage (+): 0. Max coverage (-): 0

Region: chr21 14024708-14024756. Max. coverage (+): 17.03. Max coverage (-): 0

Region: chr21 14024757-14024806. Max. coverage (+): 0. Max coverage (-): 0

Region: chr21 14024807-14024856. Max. coverage (+): 0. Max coverage (-): 0

Region: chr21 14024857-14024906. Max. coverage (+): 7.7. Max coverage (-): 0

Region: chr21 14024907-14024956. Max. coverage (+): 4.49. Max coverage (-): 0

Region: chr21 14024957-14025006. Max. coverage (+): 9.42. Max coverage (-): 0

Region: chr21 14025007-14025055. Max. coverage (+): 0. Max coverage (-): 0

Region: chr21 14025056-14025105. Max. coverage (+): 0. Max coverage (-): 0

Region: chr21 14025106-14025155. Max. coverage (+): 0. Max coverage (-): 0

Region: chr21 14025156-14025205. Max. coverage (+): 0. Max coverage (-): 0

Region: chr21 14025206-14025255. Max. coverage (+): 0. Max coverage (-): 0

Region: chr21 14025256-14025304. Max. coverage (+): 0. Max coverage (-): 0

Region: chr21 14025305-14025354. Max. coverage (+): 0. Max coverage (-): 0

Region: chr21 14025355-14025404. Max. coverage (+): 5.94. Max coverage (-): 0

Region: chr21 14025405-14025454. Max. coverage (+): 1.17. Max coverage (-): 0

Region: chr21 14025455-14025504. Max. coverage (+): 0. Max coverage (-): 0

Region: chr21 14025505-14025554. Max. coverage (+): 1.52. Max coverage (-): 0

Region: chr21 14025555-14025603. Max. coverage (+): 0. Max coverage (-): 0

Region: chr21 14025604-14025653. Max. coverage (+): 0. Max coverage (-): 0

Region: chr21 14025654-14025703. Max. coverage (+): 0. Max coverage (-): 0

Region: chr21 14025704-14025753. Max. coverage (+): 6.15. Max coverage (-): 0

Region: chr21 14025754-14025803. Max. coverage (+): 0. Max coverage (-): 0

Region: chr21 14025804-14025852. Max. coverage (+): 0. Max coverage (-): 0

Region: chr21 14025853-14025902. Max. coverage (+): 0. Max coverage (-): 0

Region: chr21 14025903-14025952. Max. coverage (+): 0. Max coverage (-): 0

Region: chr21 14025953-14026002. Max. coverage (+): 6.96. Max coverage (-): 0

Region: chr21 14026003-14026052. Max. coverage (+): 0. Max coverage (-): 0

Region: chr21 14026053-14026101. Max. coverage (+): 0. Max coverage (-): 0

Region: chr21 14026102-14026151. Max. coverage (+): 0. Max coverage (-): 0

Region: chr21 14026152-14026201. Max. coverage (+): 0. Max coverage (-): 0

Region: chr21 14026202-14026251. Max. coverage (+): 12.89. Max coverage (-): 0

Region: chr21 14026252-14026301. Max. coverage (+): 10.51. Max coverage (-): 0

Region: chr21 14026302-14026351. Max. coverage (+): 9.81. Max coverage (-): 0

Region: chr21 14026352-14026400. Max. coverage (+): 3.84. Max coverage (-): 0

Region: chr21 14026401-14026450. Max. coverage (+): 6.03. Max coverage (-): 0

Region: chr21 14026451-14026500. Max. coverage (+): 3.59. Max coverage (-): 0

Region: chr21 14026501-14026550. Max. coverage (+): 0. Max coverage (-): 0

Region: chr21 14026551-14026600. Max. coverage (+): 0. Max coverage (-): 0

Region: chr21 14026601-14026649. Max. coverage (+): 0. Max coverage (-): 0

Region: chr21 14026650-14026699. Max. coverage (+): 4.4. Max coverage (-): 0

Region: chr21 14026700-14026749. Max. coverage (+): 0. Max coverage (-): 0

Region: chr21 14026750-14026799. Max. coverage (+): 0. Max coverage (-): 0

Region: chr21 14026800-14026849. Max. coverage (+): 0. Max coverage (-): 0

Region: chr21 14026850-14026898. Max. coverage (+): 0. Max coverage (-): 0

Region: chr21 14026899-14026948. Max. coverage (+): 0. Max coverage (-): 0

Region: chr21 14026949-14026998. Max. coverage (+): 0. Max coverage (-): 0

Region: chr21 14026999-14027048. Max. coverage (+): 0. Max coverage (-): 0

Region: chr21 14027049-14027098. Max. coverage (+): 0. Max coverage (-): 0

Region: chr21 14027099-14027148. Max. coverage (+): 0. Max coverage (-): 0

Region: chr21 14027149-14027197. Max. coverage (+): 4.55. Max coverage (-): 0

Region: chr21 14027198-14027247. Max. coverage (+): 6.39. Max coverage (-): 0

Region: chr21 14027248-14027297. Max. coverage (+): 11.44. Max coverage (-): 0

Region: chr21 14027298-14027347. Max. coverage (+): 0. Max coverage (-): 0

Region: chr21 14027348-14027397. Max. coverage (+): 0.43. Max coverage (-): 0

Region: chr21 14027398-14027446. Max. coverage (+): 0. Max coverage (-): 0

Region: chr21 14027447-14027496. Max. coverage (+): 0. Max coverage (-): 0

Region: chr21 14027497-14027546. Max. coverage (+): 0. Max coverage (-): 0

Region: chr21 14027547-14027596. Max. coverage (+): 0. Max coverage (-): 0

Region: chr21 14027597-14027646. Max. coverage (+): 0. Max coverage (-): 0

Region: chr21 14027647-14027696. Max. coverage (+): 0. Max coverage (-): 0

Region: chr21 14027697-14027745. Max. coverage (+): 0. Max coverage (-): 0

Region: chr21 14027746-14027795. Max. coverage (+): 3.75. Max coverage (-): 0

Region: chr21 14027796-14027845. Max. coverage (+): 0. Max coverage (-): 0

Region: chr21 14027846-14027895. Max. coverage (+): 11.27. Max coverage (-): 0

Region: chr21 14027896-14027945. Max. coverage (+): 2.28. Max coverage (-): 0

Region: chr21 14027946-14027994. Max. coverage (+): 2.09. Max coverage (-): 0

Region: chr21 14027995-14028044. Max. coverage (+): 1.84. Max coverage (-): 0

Region: chr21 14028045-14028094. Max. coverage (+): 0. Max coverage (-): 0

Region: chr21 14028095-14028144. Max. coverage (+): 23.59. Max coverage (-): 0

Region: chr21 14028145-14028194. Max. coverage (+): 6.32. Max coverage (-): 0

Region: chr21 14028195-14028243. Max. coverage (+): 16.71. Max coverage (-): 0

Region: chr21 14028244-14028293. Max. coverage (+): 0. Max coverage (-): 0

Region: chr21 14028294-14028343. Max. coverage (+): 0. Max coverage (-): 0

Region: chr21 14028344-14028393. Max. coverage (+): 0. Max coverage (-): 0

Region: chr21 14028394-14028443. Max. coverage (+): 0. Max coverage (-): 0

Region: chr21 14028444-14028493. Max. coverage (+): 0. Max coverage (-): 0

Region: chr21 14028494-14028542. Max. coverage (+): 0. Max coverage (-): 0

Region: chr21 14028543-14028592. Max. coverage (+): 0. Max coverage (-): 0

Region: chr21 14028593-14028642. Max. coverage (+): 0. Max coverage (-): 0

Region: chr21 14028643-14028692. Max. coverage (+): 1.41. Max coverage (-): 0

Region: chr21 14028693-14028742. Max. coverage (+): 0. Max coverage (-): 0

Region: chr21 14028743-14028791. Max. coverage (+): 0. Max coverage (-): 0

Region: chr21 14028792-14028841. Max. coverage (+): 0. Max coverage (-): 0

Region: chr21 14028842-14028891. Max. coverage (+): 0. Max coverage (-): 0

Region: chr21 14028892-14028941. Max. coverage (+): 0. Max coverage (-): 0

Region: chr21 14028942-14028991. Max. coverage (+): 0. Max coverage (-): 0

Region: chr21 14028992-14029040. Max. coverage (+): 0. Max coverage (-): 0

Region: chr21 14029041-14029090. Max. coverage (+): 0. Max coverage (-): 0

Region: chr21 14029091-14029140. Max. coverage (+): 0. Max coverage (-): 0

Region: chr21 14029141-14029190. Max. coverage (+): 0. Max coverage (-): 0

Region: chr21 14029191-14029240. Max. coverage (+): 0. Max coverage (-): 0

Region: chr21 14029241-14029290. Max. coverage (+): 0. Max coverage (-): 0

Region: chr21 14029291-14029339. Max. coverage (+): 0. Max coverage (-): 0

Region: chr21 14029340-14029389. Max. coverage (+): 0. Max coverage (-): 0

Region: chr21 14029390-14029439. Max. coverage (+): 0. Max coverage (-): 0

Region: chr21 14029440-14029489. Max. coverage (+): 0. Max coverage (-): 0

Region: chr21 14029490-14029539. Max. coverage (+): 0. Max coverage (-): 0

Region: chr21 14029540-14029588. Max. coverage (+): 0. Max coverage (-): 0

Region: chr21 14029589-14029638. Max. coverage (+): 0. Max coverage (-): 0

Region: chr21 14029639-14029688. Max. coverage (+): 0. Max coverage (-): 0

Region: chr21 14029689-14029738. Max. coverage (+): 0. Max coverage (-): 0

Region: chr21 14029739-14029788. Max. coverage (+): 6.63. Max coverage (-): 0

Region: chr21 14029789-14029838. Max. coverage (+): 0. Max coverage (-): 0

Region: chr21 14029839-14029887. Max. coverage (+): 0. Max coverage (-): 0

Region: chr21 14029888-14029937. Max. coverage (+): 0. Max coverage (-): 0

Region: chr21 14029938-14029987. Max. coverage (+): 0. Max coverage (-): 0

Region: chr21 14029988-14030037. Max. coverage (+): 0. Max coverage (-): 0

Region: chr21 14030038-14030087. Max. coverage (+): 6.72. Max coverage (-): 0

Region: chr21 14030088-. Max. coverage (+): 6.72. Max coverage (-): 0

RepeatMasker Color Code

**+**

100-98% Identity

<98-95% Identity

<95-90% Identity

<90-85% Identity

<85-80% Identity

<80-75% Identity

<75-70% Identity

<70% Identity

**-**

Gene Set Color Code

**+**

Gene

Pseudogene

**-**

Topology/Coverage Color Code

Coverage Plus Strand

Coverage Minus Strand

Mainstrand: Plus

Mainstrand: Minus

Complementary Strand

Flanking Region  
(if option -flank >0)

Gene Set Annotation  
  
RepeatMasker Annotation  

**1. BOV-A2**: 14005422-14005684 (-), Divergence to consensus: 6.8%  
**2. L1-2\_BT**: 14005953-14006181 (+), Divergence to consensus: 13.9%  
**3. Bov-tA2**: 14006186-14006405 (-), Divergence to consensus: 14.5%  
**4. (CTG)n**: 14008389-14008414 (+), Divergence to consensus: 0%  
**5. BOV-A2**: 14008415-14008449 (-), Divergence to consensus: 5.7%  
**6. MIRc**: 14008829-14008934 (-), Divergence to consensus: 30.2%  
**7. (T)n**: 14009158-14009177 (+), Divergence to consensus: 0%  
**8. L2b**: 14010996-14011065 (+), Divergence to consensus: 32.9%  
**9. L2b**: 14011241-14011518 (+), Divergence to consensus: 48.2%  
**10. L2b**: 14011584-14011707 (+), Divergence to consensus: 41.8%  
**11. (TCC)n**: 14012043-14012078 (+), Divergence to consensus: 0%  
**12. (TTCA)n**: 14012997-14013029 (+), Divergence to consensus: 6.1%  
**13. L2c**: 14013030-14013093 (-), Divergence to consensus: 31.6%  
**14. L2c**: 14013218-14013426 (-), Divergence to consensus: 47.3%  
**15. MIRc**: 14013832-14013983 (-), Divergence to consensus: 40.6%  
**16. L2c**: 14018136-14018262 (-), Divergence to consensus: 31.7%  
**17. G-rich**: 14018395-14018446 (+), Divergence to consensus: 21.1%  
**18. L2c**: 14020049-14020153 (-), Divergence to consensus: 33.5%  
**19. LTR48**: 14021699-14021742 (+), Divergence to consensus: 22.7%  
**20. (CA)n**: 14021750-14021782 (+), Divergence to consensus: 0%  
**21. L2c**: 14022004-14022173 (+), Divergence to consensus: 34.4%  
**22. MIRb**: 14023428-14023573 (+), Divergence to consensus: 46.6%  
**23. (TCCA)n**: 14023752-14023782 (+), Divergence to consensus: 16.1%  
**24. MER5A1**: 14025012-14025136 (+), Divergence to consensus: 29.7%  
**25. MIR**: 14025174-14025332 (-), Divergence to consensus: 28.2%  
**26. BOV-A2**: 14026080-14026205 (+), Divergence to consensus: 15.8%  
**27. Bov-tA2**: 14026909-14027097 (-), Divergence to consensus: 15%  
**28. AT\_rich**: 14027098-14027163 (+), Divergence to consensus: 84.8%  
**29. L2a**: 14027696-14027738 (+), Divergence to consensus: 20.9%  
**30. MIR**: 14028435-14028614 (+), Divergence to consensus: 37.3%  
**31. L1M4c**: 14028759-14028888 (-), Divergence to consensus: 25.4%  
**32. L1ME2**: 14028891-14029472 (+), Divergence to consensus: 30.6%  
**33. MER20**: 14029473-14029671 (+), Divergence to consensus: 34.9%  
**34. L1ME2**: 14029672-14029737 (+), Divergence to consensus: 36.8%

  
Transcription Factor Binding Sites  

**RFX4\_2** (Sequence: GTATCTAAG (-): 14018270)  
**RFX4\_2** (Sequence: GTAACCATG (-): 14019949)  
**RFX4\_2** (Sequence: GTATCCACG (-): 14022237)  
**RFX4\_1** (Sequence: CTTGGCAAC (+): 14019922)  
**Gata4** (Sequence: AGATAAG (-): 14007189)  
**Gata4** (Sequence: AGATAAG (-): 14008329)  
**Gata4** (Sequence: AGATAAC (-): 14023226)  
**SOX9** (Sequence: CCATTGTT (+): 14026865)  
**Gata4** (Sequence: CTTATCT (+): 14023028)  
**Gata4** (Sequence: GTTATCT (+): 14024343)
