# Supplementary material for: Small RNA sequencing of cryopreserved semen from single bull revealed altered miRNAs and piRNAs expression between High- and Low-motile sperm populations
Source: BMC Genomics. 2017 Jan 4;18:14. doi: 10.1186/s12864-016-3394-7 (PMC5209821; doi:10.1186/s12864-016-3394-7)
Supplement: Additional file 4: — Details for each piRNA clusters found in Low Motile (LM) sperm fraction. Genes, repeats, transposable elements and transcription factors binding sites falling within the cluster regions were reported. (ZIP 1034 kb) [file 12864_2016_3394_MOESM4_ESM.zip › 27.html]

piRNA cluster 27


Predicted piRNA cluster no. 27     previous   next
  

Show proTRAC run info
Hide proTRAC run info

================================= proTRAC ====================================  
VERSION: 2.1                                    LAST MODIFIED: 06. October 2015  
  
Please cite:  
Rosenkranz D, Zischler H. proTRAC - a software for probabilistic piRNA cluster  
detection, visualization and analysis. 2012. BMC Bioinformatics 13:5.  
  
and (for proTRAC 2.0 and later):  
Rosenkranz D, Rudloff S, Bastuck K, Ketting RF, Zischler H. Tupaia small RNAs  
provide insights into function and evolution of RNAi-based transposon defense  
in mammals. 2015. RNA 21(5):911-922.  
  
Contact:  
David Rosenkranz  
Institute of Anthropology, small RNA group  
Johannes Gutenberg University Mainz  
email: rosenkranz@uni-mainz.de  
  
You can find the latest proTRAC version at:  
http://sourceforge.net/projects/protrac/files  
http://www.smallRNAgroup-mainz.de/software  
==============================================================================  
  
PARAMETERS:  
Map file: .............../storage/core/barbara/genhome/smallRNA/fertility/Sample\_not\_motile/pirna/Sample\_not\_motile\_26-33\_collapsed.fa.no-dust.map.weighted-10000-1000-b-0  
Genome file: ............/storage/core/barbara/genhome/smallRNA/fertility/Sample\_all/pirna/bt\_311\_chrY.fa  
RepeatMasker annotation: /storage/genomes/bt\_umd31/GCF\_000003055.6\_Bos\_taurus\_UMD\_3.1.1\_repeatMasker\_chr.out  
GeneSet:................./storage/core/barbara/genhome/smallRNA/fertility/Sample\_all/pirna/full.gtf  
  
Significant (p<=0.01) hit density will be calculated based  
on observed hit distribution.  
  
Sliding window size: ........................................ 5000 bp  
Sliding window increament: .................................. 1000 bp  
Normalize each hit by number of genomic hits: ............... 1 [0=no/1=yes]  
Normalize each hit by number of sequence reads: ............. 1 [0=no/1=yes]  
Normalize values (-> per million mapped reads): ............. 1 [0=no/1=yes]  
Min. fraction of hits with 1T(U) or 10A: .................... 0.75  
Alternatively: Min. fraction of hits with 1T(U) and 10A: .... 0.5  
Min. fraction of hits with typical piRNA length: ............ 0.75  
Typical piRNA length: ....................................... 26-33 nt  
Min. size of a piRNA cluster: ............................... 5000 bp.  
Min. number of hits (absolute): ............................. 0  
Min. number of hits (normalized): ........................... 0  
Min. fraction of hits on the mainstrand: .................... 0.75  
Top fraction of mapped sequences (in terms of read counts): . 1%  
Top fraction accounts for max. n% of sequence reads: ........ 90%  
Min. fraction of hits on each arm of a bidirectional cluster: 0.1  
Output image file for each cluster: ......................... 0 [0=no/1=yes]  
Output html file for each cluster: .......................... 1 [0=no/1=yes]  
Output a summary table: ..................................... 1 [0=no/1=yes]  
Output a FASTA file for each cluster (piRNA sequences): ..... 1 [0=no/1=yes]  
Output a FASTA file comprising cluster sequences: ........... 1 [0=no/1=yes]  
Search DNA motifs in clusters: .............................. 1 [0=no/1=yes]  
Output flanking sequences: +/- .............................. 0 bp  
Output ~.pTi file: .......................................... 1 [0=no/1=yes]  
==============================================================================  
  
  
Genome size (without gaps): ............ 2678902517 bp  
Gaps (N/X/-): .......................... 53837044 bp  
Mapped reads: .......................... 738059667487  
Non-identical sequences: ............... 277001  
Genomic hits: .......................... 533816  
Significant densitiy of mapped reads: .. 15118061 reads/kb

Show proTRAC cluster info
Hide proTRAC cluster info

|  |  |
| --- | --- |
| Location | chr21 |
| Coordinates | 14729396-14782417 |
| Size [bp] | 53022 |
| Sequence hit loci | 4223 |
| Mapped reads (normalized) | 11103096475 |
| Mapped reads (normalized) per kb | 209405463.3 |
| Normalized reads with 1T (1U) | 79% |
| Normalized reads with 10A | 34% |
| Normalized reads with length 26-33 nt | 100% |
| Normalized reads on the main strand(s) | 98.4% |
| Predicted directionality | bi:minus-plus (split between 14764248 and 14764368) |

100%

0%

1T (1U)  
reads

10A reads

26-33 nt  
reads

reads on mainstrand

**Either the amount of reads with 1T (1U) OR 10A has to exceed 75% (set with option: -1Tor10A)  
Alternatively the amount of reads with 1T (1U) AND 10A has to exceed 50% (set with option: -1Tand10A)  
Minimum amount of reads with preferred size is 75% (set with option: -pisize)  
Minimum amount of reads on the main strand(s) is 75% (set with option: -clstrand)**

Show read coverage
Hide read coverage

WHAT DO I SEE HERE?  
This chart shows the location of mapped sequence reads within a predicted piRNA cluster. The color refers to the number of genomic hits produced by the sequence read in question. A dark red bar indicates that this sequence read produces many other hits elsewhere in the genome. Many adjacent red or yellow bars can indicate the presence of a multi-copy element such as transposons or rRNA genes. A dark green bar indicates that this sequence read maps uniquely to this locus.

1 hit

2-5 hits

6-10 hits

11-20 hits

21-50 hits

51-100 hits

> 100 hits

chr21

14729396

14782417

Gene Set

RepeatMasker

Mapped  
Reads

192.98

plus strand

minus strand

192.98

Region: chr21 14030089-14729449. Max. coverage (+): 0. Max coverage (-): 4.07

Region: chr21 14729450-14729555. Max. coverage (+): 0. Max coverage (-): 0

Region: chr21 14729556-14729661. Max. coverage (+): 0. Max coverage (-): 0

Region: chr21 14729662-14729767. Max. coverage (+): 0. Max coverage (-): 0

Region: chr21 14729768-14729873. Max. coverage (+): 0. Max coverage (-): 0

Region: chr21 14729874-14729979. Max. coverage (+): 0. Max coverage (-): 0

Region: chr21 14729980-14730085. Max. coverage (+): 0. Max coverage (-): 0

Region: chr21 14730086-14730191. Max. coverage (+): 0. Max coverage (-): 0

Region: chr21 14730192-14730297. Max. coverage (+): 0. Max coverage (-): 0

Region: chr21 14730298-14730403. Max. coverage (+): 0. Max coverage (-): 0

Region: chr21 14730404-14730509. Max. coverage (+): 0. Max coverage (-): 4.57

Region: chr21 14730510-14730615. Max. coverage (+): 0. Max coverage (-): 0

Region: chr21 14730616-14730721. Max. coverage (+): 0. Max coverage (-): 0

Region: chr21 14730722-14730827. Max. coverage (+): 0. Max coverage (-): 0

Region: chr21 14730828-14730933. Max. coverage (+): 0. Max coverage (-): 0

Region: chr21 14730934-14731039. Max. coverage (+): 0. Max coverage (-): 0

Region: chr21 14731040-14731145. Max. coverage (+): 0. Max coverage (-): 0

Region: chr21 14731146-14731251. Max. coverage (+): 0. Max coverage (-): 0

Region: chr21 14731252-14731357. Max. coverage (+): 0. Max coverage (-): 0

Region: chr21 14731358-14731463. Max. coverage (+): 0. Max coverage (-): 0

Region: chr21 14731464-14731569. Max. coverage (+): 0. Max coverage (-): 0

Region: chr21 14731570-14731675. Max. coverage (+): 0. Max coverage (-): 1.63

Region: chr21 14731676-14731781. Max. coverage (+): 0. Max coverage (-): 0

Region: chr21 14731782-14731888. Max. coverage (+): 0. Max coverage (-): 0

Region: chr21 14731889-14731994. Max. coverage (+): 0. Max coverage (-): 0

Region: chr21 14731995-14732100. Max. coverage (+): 0. Max coverage (-): 0

Region: chr21 14732101-14732206. Max. coverage (+): 0. Max coverage (-): 5.19

Region: chr21 14732207-14732312. Max. coverage (+): 0. Max coverage (-): 4.55

Region: chr21 14732313-14732418. Max. coverage (+): 0. Max coverage (-): 32.28

Region: chr21 14732419-14732524. Max. coverage (+): 0. Max coverage (-): 7.2

Region: chr21 14732525-14732630. Max. coverage (+): 0. Max coverage (-): 0

Region: chr21 14732631-14732736. Max. coverage (+): 0. Max coverage (-): 0

Region: chr21 14732737-14732842. Max. coverage (+): 0. Max coverage (-): 6.69

Region: chr21 14732843-14732948. Max. coverage (+): 0. Max coverage (-): 0

Region: chr21 14732949-14733054. Max. coverage (+): 0. Max coverage (-): 4.56

Region: chr21 14733055-14733160. Max. coverage (+): 0. Max coverage (-): 62.18

Region: chr21 14733161-14733266. Max. coverage (+): 0. Max coverage (-): 76.97

Region: chr21 14733267-14733372. Max. coverage (+): 0. Max coverage (-): 39.37

Region: chr21 14733373-14733478. Max. coverage (+): 0. Max coverage (-): 41.16

Region: chr21 14733479-14733584. Max. coverage (+): 0.08. Max coverage (-): 0

Region: chr21 14733585-14733690. Max. coverage (+): 0. Max coverage (-): 0

Region: chr21 14733691-14733796. Max. coverage (+): 0. Max coverage (-): 27.34

Region: chr21 14733797-14733902. Max. coverage (+): 0. Max coverage (-): 2.41

Region: chr21 14733903-14734008. Max. coverage (+): 0. Max coverage (-): 0

Region: chr21 14734009-14734114. Max. coverage (+): 0. Max coverage (-): 17.36

Region: chr21 14734115-14734221. Max. coverage (+): 0. Max coverage (-): 7.81

Region: chr21 14734222-14734327. Max. coverage (+): 0. Max coverage (-): 29.73

Region: chr21 14734328-14734433. Max. coverage (+): 0. Max coverage (-): 22.23

Region: chr21 14734434-14734539. Max. coverage (+): 0. Max coverage (-): 41.93

Region: chr21 14734540-14734645. Max. coverage (+): 0. Max coverage (-): 2.97

Region: chr21 14734646-14734751. Max. coverage (+): 0. Max coverage (-): 52.83

Region: chr21 14734752-14734857. Max. coverage (+): 0. Max coverage (-): 7.41

Region: chr21 14734858-14734963. Max. coverage (+): 0. Max coverage (-): 37.7

Region: chr21 14734964-14735069. Max. coverage (+): 0. Max coverage (-): 10.32

Region: chr21 14735070-14735175. Max. coverage (+): 0. Max coverage (-): 29.01

Region: chr21 14735176-14735281. Max. coverage (+): 0. Max coverage (-): 19.68

Region: chr21 14735282-14735387. Max. coverage (+): 0. Max coverage (-): 15.9

Region: chr21 14735388-14735493. Max. coverage (+): 0. Max coverage (-): 125.25

Region: chr21 14735494-14735599. Max. coverage (+): 0. Max coverage (-): 0

Region: chr21 14735600-14735705. Max. coverage (+): 0. Max coverage (-): 17.85

Region: chr21 14735706-14735811. Max. coverage (+): 0. Max coverage (-): 23.92

Region: chr21 14735812-14735917. Max. coverage (+): 0. Max coverage (-): 6.32

Region: chr21 14735918-14736023. Max. coverage (+): 0. Max coverage (-): 10.03

Region: chr21 14736024-14736129. Max. coverage (+): 0. Max coverage (-): 24.6

Region: chr21 14736130-14736235. Max. coverage (+): 0. Max coverage (-): 4.02

Region: chr21 14736236-14736341. Max. coverage (+): 0. Max coverage (-): 14.53

Region: chr21 14736342-14736447. Max. coverage (+): 0. Max coverage (-): 23.94

Region: chr21 14736448-14736553. Max. coverage (+): 0. Max coverage (-): 14.42

Region: chr21 14736554-14736660. Max. coverage (+): 0. Max coverage (-): 38.16

Region: chr21 14736661-14736766. Max. coverage (+): 0. Max coverage (-): 44.11

Region: chr21 14736767-14736872. Max. coverage (+): 0. Max coverage (-): 12.13

Region: chr21 14736873-14736978. Max. coverage (+): 0. Max coverage (-): 53.86

Region: chr21 14736979-14737084. Max. coverage (+): 0. Max coverage (-): 6.01

Region: chr21 14737085-14737190. Max. coverage (+): 0. Max coverage (-): 5.16

Region: chr21 14737191-14737296. Max. coverage (+): 0. Max coverage (-): 21.78

Region: chr21 14737297-14737402. Max. coverage (+): 0. Max coverage (-): 47.65

Region: chr21 14737403-14737508. Max. coverage (+): 0. Max coverage (-): 0

Region: chr21 14737509-14737614. Max. coverage (+): 0. Max coverage (-): 10.87

Region: chr21 14737615-14737720. Max. coverage (+): 2.75. Max coverage (-): 57.92

Region: chr21 14737721-14737826. Max. coverage (+): 0. Max coverage (-): 10.32

Region: chr21 14737827-14737932. Max. coverage (+): 0. Max coverage (-): 0

Region: chr21 14737933-14738038. Max. coverage (+): 0. Max coverage (-): 0

Region: chr21 14738039-14738144. Max. coverage (+): 0. Max coverage (-): 49.39

Region: chr21 14738145-14738250. Max. coverage (+): 0. Max coverage (-): 9.16

Region: chr21 14738251-14738356. Max. coverage (+): 0. Max coverage (-): 6.95

Region: chr21 14738357-14738462. Max. coverage (+): 0. Max coverage (-): 31.08

Region: chr21 14738463-14738568. Max. coverage (+): 0. Max coverage (-): 9.21

Region: chr21 14738569-14738674. Max. coverage (+): 0. Max coverage (-): 22.98

Region: chr21 14738675-14738780. Max. coverage (+): 0. Max coverage (-): 96.95

Region: chr21 14738781-14738886. Max. coverage (+): 0. Max coverage (-): 16.1

Region: chr21 14738887-14738992. Max. coverage (+): 0. Max coverage (-): 37.8

Region: chr21 14738993-14739099. Max. coverage (+): 0. Max coverage (-): 20.2

Region: chr21 14739100-14739205. Max. coverage (+): 0. Max coverage (-): 46.96

Region: chr21 14739206-14739311. Max. coverage (+): 0. Max coverage (-): 16.9

Region: chr21 14739312-14739417. Max. coverage (+): 0. Max coverage (-): 29.22

Region: chr21 14739418-14739523. Max. coverage (+): 0. Max coverage (-): 18.34

Region: chr21 14739524-14739629. Max. coverage (+): 0. Max coverage (-): 35.19

Region: chr21 14739630-14739735. Max. coverage (+): 0. Max coverage (-): 4.5

Region: chr21 14739736-14739841. Max. coverage (+): 0. Max coverage (-): 11.07

Region: chr21 14739842-14739947. Max. coverage (+): 0. Max coverage (-): 0

Region: chr21 14739948-14740053. Max. coverage (+): 0. Max coverage (-): 7.8

Region: chr21 14740054-14740159. Max. coverage (+): 0. Max coverage (-): 1.35

Region: chr21 14740160-14740265. Max. coverage (+): 0. Max coverage (-): 5.61

Region: chr21 14740266-14740371. Max. coverage (+): 0. Max coverage (-): 26.53

Region: chr21 14740372-14740477. Max. coverage (+): 0. Max coverage (-): 185.46

Region: chr21 14740478-14740583. Max. coverage (+): 0. Max coverage (-): 14.16

Region: chr21 14740584-14740689. Max. coverage (+): 0. Max coverage (-): 6.19

Region: chr21 14740690-14740795. Max. coverage (+): 0. Max coverage (-): 19.54

Region: chr21 14740796-14740901. Max. coverage (+): 0. Max coverage (-): 18.69

Region: chr21 14740902-14741007. Max. coverage (+): 0. Max coverage (-): 19.1

Region: chr21 14741008-14741113. Max. coverage (+): 1.89. Max coverage (-): 20.26

Region: chr21 14741114-14741219. Max. coverage (+): 0. Max coverage (-): 0

Region: chr21 14741220-14741325. Max. coverage (+): 0. Max coverage (-): 69.66

Region: chr21 14741326-14741431. Max. coverage (+): 0. Max coverage (-): 9.22

Region: chr21 14741432-14741538. Max. coverage (+): 0. Max coverage (-): 25.78

Region: chr21 14741539-14741644. Max. coverage (+): 0. Max coverage (-): 21.59

Region: chr21 14741645-14741750. Max. coverage (+): 0. Max coverage (-): 22.62

Region: chr21 14741751-14741856. Max. coverage (+): 0. Max coverage (-): 26.66

Region: chr21 14741857-14741962. Max. coverage (+): 0. Max coverage (-): 25.38

Region: chr21 14741963-14742068. Max. coverage (+): 0. Max coverage (-): 71.68

Region: chr21 14742069-14742174. Max. coverage (+): 0. Max coverage (-): 35.24

Region: chr21 14742175-14742280. Max. coverage (+): 0. Max coverage (-): 31.4

Region: chr21 14742281-14742386. Max. coverage (+): 0. Max coverage (-): 21.24

Region: chr21 14742387-14742492. Max. coverage (+): 0. Max coverage (-): 100.36

Region: chr21 14742493-14742598. Max. coverage (+): 0. Max coverage (-): 0

Region: chr21 14742599-14742704. Max. coverage (+): 0. Max coverage (-): 0

Region: chr21 14742705-14742810. Max. coverage (+): 0. Max coverage (-): 36.57

Region: chr21 14742811-14742916. Max. coverage (+): 0. Max coverage (-): 21.63

Region: chr21 14742917-14743022. Max. coverage (+): 0. Max coverage (-): 12.16

Region: chr21 14743023-14743128. Max. coverage (+): 0. Max coverage (-): 22.73

Region: chr21 14743129-14743234. Max. coverage (+): 0. Max coverage (-): 1.98

Region: chr21 14743235-14743340. Max. coverage (+): 0. Max coverage (-): 9.33

Region: chr21 14743341-14743446. Max. coverage (+): 0. Max coverage (-): 37.29

Region: chr21 14743447-14743552. Max. coverage (+): 0. Max coverage (-): 25.46

Region: chr21 14743553-14743658. Max. coverage (+): 0. Max coverage (-): 76.77

Region: chr21 14743659-14743764. Max. coverage (+): 0. Max coverage (-): 11.66

Region: chr21 14743765-14743871. Max. coverage (+): 0. Max coverage (-): 0

Region: chr21 14743872-14743977. Max. coverage (+): 0. Max coverage (-): 41.99

Region: chr21 14743978-14744083. Max. coverage (+): 0. Max coverage (-): 26.73

Region: chr21 14744084-14744189. Max. coverage (+): 0. Max coverage (-): 6.72

Region: chr21 14744190-14744295. Max. coverage (+): 0. Max coverage (-): 8.18

Region: chr21 14744296-14744401. Max. coverage (+): 0. Max coverage (-): 0

Region: chr21 14744402-14744507. Max. coverage (+): 0. Max coverage (-): 15.21

Region: chr21 14744508-14744613. Max. coverage (+): 0.91. Max coverage (-): 21.59

Region: chr21 14744614-14744719. Max. coverage (+): 0.91. Max coverage (-): 14.32

Region: chr21 14744720-14744825. Max. coverage (+): 0. Max coverage (-): 0

Region: chr21 14744826-14744931. Max. coverage (+): 0. Max coverage (-): 34.25

Region: chr21 14744932-14745037. Max. coverage (+): 0. Max coverage (-): 44.56

Region: chr21 14745038-14745143. Max. coverage (+): 0. Max coverage (-): 11.52

Region: chr21 14745144-14745249. Max. coverage (+): 0. Max coverage (-): 21.02

Region: chr21 14745250-14745355. Max. coverage (+): 0. Max coverage (-): 53.39

Region: chr21 14745356-14745461. Max. coverage (+): 0. Max coverage (-): 10.6

Region: chr21 14745462-14745567. Max. coverage (+): 0. Max coverage (-): 2.97

Region: chr21 14745568-14745673. Max. coverage (+): 0. Max coverage (-): 28.46

Region: chr21 14745674-14745779. Max. coverage (+): 0. Max coverage (-): 34.56

Region: chr21 14745780-14745885. Max. coverage (+): 0. Max coverage (-): 13.9

Region: chr21 14745886-14745991. Max. coverage (+): 0. Max coverage (-): 16.15

Region: chr21 14745992-14746097. Max. coverage (+): 0. Max coverage (-): 25.91

Region: chr21 14746098-14746203. Max. coverage (+): 0. Max coverage (-): 25.94

Region: chr21 14746204-14746310. Max. coverage (+): 0. Max coverage (-): 10.28

Region: chr21 14746311-14746416. Max. coverage (+): 0. Max coverage (-): 17.63

Region: chr21 14746417-14746522. Max. coverage (+): 0. Max coverage (-): 16.53

Region: chr21 14746523-14746628. Max. coverage (+): 0. Max coverage (-): 5.56

Region: chr21 14746629-14746734. Max. coverage (+): 0. Max coverage (-): 33.67

Region: chr21 14746735-14746840. Max. coverage (+): 0. Max coverage (-): 12.06

Region: chr21 14746841-14746946. Max. coverage (+): 0. Max coverage (-): 10.21

Region: chr21 14746947-14747052. Max. coverage (+): 0. Max coverage (-): 18.2

Region: chr21 14747053-14747158. Max. coverage (+): 0. Max coverage (-): 10.89

Region: chr21 14747159-14747264. Max. coverage (+): 0. Max coverage (-): 25.24

Region: chr21 14747265-14747370. Max. coverage (+): 0. Max coverage (-): 5.83

Region: chr21 14747371-14747476. Max. coverage (+): 0. Max coverage (-): 0

Region: chr21 14747477-14747582. Max. coverage (+): 0. Max coverage (-): 0

Region: chr21 14747583-14747688. Max. coverage (+): 0. Max coverage (-): 19.15

Region: chr21 14747689-14747794. Max. coverage (+): 0. Max coverage (-): 16.12

Region: chr21 14747795-14747900. Max. coverage (+): 0. Max coverage (-): 0

Region: chr21 14747901-14748006. Max. coverage (+): 0. Max coverage (-): 0

Region: chr21 14748007-14748112. Max. coverage (+): 0. Max coverage (-): 0

Region: chr21 14748113-14748218. Max. coverage (+): 0. Max coverage (-): 6.69

Region: chr21 14748219-14748324. Max. coverage (+): 0. Max coverage (-): 14.59

Region: chr21 14748325-14748430. Max. coverage (+): 0. Max coverage (-): 36.32

Region: chr21 14748431-14748536. Max. coverage (+): 0. Max coverage (-): 20.69

Region: chr21 14748537-14748642. Max. coverage (+): 0. Max coverage (-): 11.36

Region: chr21 14748643-14748749. Max. coverage (+): 0. Max coverage (-): 12.02

Region: chr21 14748750-14748855. Max. coverage (+): 0. Max coverage (-): 0

Region: chr21 14748856-14748961. Max. coverage (+): 0. Max coverage (-): 0

Region: chr21 14748962-14749067. Max. coverage (+): 0. Max coverage (-): 0

Region: chr21 14749068-14749173. Max. coverage (+): 0. Max coverage (-): 0

Region: chr21 14749174-14749279. Max. coverage (+): 0. Max coverage (-): 0

Region: chr21 14749280-14749385. Max. coverage (+): 0. Max coverage (-): 0

Region: chr21 14749386-14749491. Max. coverage (+): 0. Max coverage (-): 0

Region: chr21 14749492-14749597. Max. coverage (+): 0. Max coverage (-): 0

Region: chr21 14749598-14749703. Max. coverage (+): 0. Max coverage (-): 0

Region: chr21 14749704-14749809. Max. coverage (+): 0. Max coverage (-): 0

Region: chr21 14749810-14749915. Max. coverage (+): 0. Max coverage (-): 0

Region: chr21 14749916-14750021. Max. coverage (+): 0. Max coverage (-): 0

Region: chr21 14750022-14750127. Max. coverage (+): 0. Max coverage (-): 0

Region: chr21 14750128-14750233. Max. coverage (+): 0. Max coverage (-): 0.6

Region: chr21 14750234-14750339. Max. coverage (+): 0. Max coverage (-): 0

Region: chr21 14750340-14750445. Max. coverage (+): 0. Max coverage (-): 0

Region: chr21 14750446-14750551. Max. coverage (+): 0. Max coverage (-): 0

Region: chr21 14750552-14750657. Max. coverage (+): 0. Max coverage (-): 0

Region: chr21 14750658-14750763. Max. coverage (+): 0. Max coverage (-): 0

Region: chr21 14750764-14750869. Max. coverage (+): 0. Max coverage (-): 0

Region: chr21 14750870-14750975. Max. coverage (+): 0. Max coverage (-): 0

Region: chr21 14750976-14751081. Max. coverage (+): 0. Max coverage (-): 0

Region: chr21 14751082-14751188. Max. coverage (+): 0. Max coverage (-): 0

Region: chr21 14751189-14751294. Max. coverage (+): 0. Max coverage (-): 0

Region: chr21 14751295-14751400. Max. coverage (+): 0. Max coverage (-): 0

Region: chr21 14751401-14751506. Max. coverage (+): 0. Max coverage (-): 12.7

Region: chr21 14751507-14751612. Max. coverage (+): 0. Max coverage (-): 25.51

Region: chr21 14751613-14751718. Max. coverage (+): 0. Max coverage (-): 24.94

Region: chr21 14751719-14751824. Max. coverage (+): 0. Max coverage (-): 0

Region: chr21 14751825-14751930. Max. coverage (+): 0. Max coverage (-): 2.57

Region: chr21 14751931-14752036. Max. coverage (+): 0. Max coverage (-): 0

Region: chr21 14752037-14752142. Max. coverage (+): 0. Max coverage (-): 7.14

Region: chr21 14752143-14752248. Max. coverage (+): 0. Max coverage (-): 0

Region: chr21 14752249-14752354. Max. coverage (+): 0. Max coverage (-): 0

Region: chr21 14752355-14752460. Max. coverage (+): 0. Max coverage (-): 0

Region: chr21 14752461-14752566. Max. coverage (+): 0. Max coverage (-): 0

Region: chr21 14752567-14752672. Max. coverage (+): 0. Max coverage (-): 7.11

Region: chr21 14752673-14752778. Max. coverage (+): 0. Max coverage (-): 0

Region: chr21 14752779-14752884. Max. coverage (+): 0. Max coverage (-): 0

Region: chr21 14752885-14752990. Max. coverage (+): 0. Max coverage (-): 0

Region: chr21 14752991-14753096. Max. coverage (+): 0. Max coverage (-): 0

Region: chr21 14753097-14753202. Max. coverage (+): 0. Max coverage (-): 0

Region: chr21 14753203-14753308. Max. coverage (+): 0. Max coverage (-): 0

Region: chr21 14753309-14753414. Max. coverage (+): 0. Max coverage (-): 11.77

Region: chr21 14753415-14753521. Max. coverage (+): 0. Max coverage (-): 6.68

Region: chr21 14753522-14753627. Max. coverage (+): 0. Max coverage (-): 35.44

Region: chr21 14753628-14753733. Max. coverage (+): 0. Max coverage (-): 43.12

Region: chr21 14753734-14753839. Max. coverage (+): 0. Max coverage (-): 46.26

Region: chr21 14753840-14753945. Max. coverage (+): 0. Max coverage (-): 27.86

Region: chr21 14753946-14754051. Max. coverage (+): 0. Max coverage (-): 35.24

Region: chr21 14754052-14754157. Max. coverage (+): 0. Max coverage (-): 33.18

Region: chr21 14754158-14754263. Max. coverage (+): 0. Max coverage (-): 49.79

Region: chr21 14754264-14754369. Max. coverage (+): 0. Max coverage (-): 7.2

Region: chr21 14754370-14754475. Max. coverage (+): 0. Max coverage (-): 0

Region: chr21 14754476-14754581. Max. coverage (+): 0. Max coverage (-): 0.23

Region: chr21 14754582-14754687. Max. coverage (+): 0. Max coverage (-): 22.93

Region: chr21 14754688-14754793. Max. coverage (+): 0. Max coverage (-): 23.47

Region: chr21 14754794-14754899. Max. coverage (+): 0. Max coverage (-): 0

Region: chr21 14754900-14755005. Max. coverage (+): 0. Max coverage (-): 0

Region: chr21 14755006-14755111. Max. coverage (+): 0. Max coverage (-): 0

Region: chr21 14755112-14755217. Max. coverage (+): 0. Max coverage (-): 0

Region: chr21 14755218-14755323. Max. coverage (+): 0. Max coverage (-): 0

Region: chr21 14755324-14755429. Max. coverage (+): 0. Max coverage (-): 0

Region: chr21 14755430-14755535. Max. coverage (+): 0. Max coverage (-): 5.12

Region: chr21 14755536-14755641. Max. coverage (+): 0. Max coverage (-): 15.34

Region: chr21 14755642-14755747. Max. coverage (+): 1.19. Max coverage (-): 81.72

Region: chr21 14755748-14755853. Max. coverage (+): 0. Max coverage (-): 29.88

Region: chr21 14755854-14755960. Max. coverage (+): 0. Max coverage (-): 26.06

Region: chr21 14755961-14756066. Max. coverage (+): 0.89. Max coverage (-): 5.38

Region: chr21 14756067-14756172. Max. coverage (+): 0. Max coverage (-): 17.21

Region: chr21 14756173-14756278. Max. coverage (+): 0. Max coverage (-): 33.08

Region: chr21 14756279-14756384. Max. coverage (+): 0. Max coverage (-): 62.21

Region: chr21 14756385-14756490. Max. coverage (+): 0. Max coverage (-): 25.04

Region: chr21 14756491-14756596. Max. coverage (+): 0. Max coverage (-): 39.32

Region: chr21 14756597-14756702. Max. coverage (+): 0. Max coverage (-): 26.82

Region: chr21 14756703-14756808. Max. coverage (+): 6.17. Max coverage (-): 90.48

Region: chr21 14756809-14756914. Max. coverage (+): 0. Max coverage (-): 184.66

Region: chr21 14756915-14757020. Max. coverage (+): 1.7. Max coverage (-): 52.78

Region: chr21 14757021-14757126. Max. coverage (+): 0. Max coverage (-): 7.11

Region: chr21 14757127-14757232. Max. coverage (+): 0. Max coverage (-): 0

Region: chr21 14757233-14757338. Max. coverage (+): 0. Max coverage (-): 0

Region: chr21 14757339-14757444. Max. coverage (+): 0. Max coverage (-): 0

Region: chr21 14757445-14757550. Max. coverage (+): 0. Max coverage (-): 7.13

Region: chr21 14757551-14757656. Max. coverage (+): 0. Max coverage (-): 5.96

Region: chr21 14757657-14757762. Max. coverage (+): 0. Max coverage (-): 3.36

Region: chr21 14757763-14757868. Max. coverage (+): 0. Max coverage (-): 0

Region: chr21 14757869-14757974. Max. coverage (+): 0. Max coverage (-): 0

Region: chr21 14757975-14758080. Max. coverage (+): 0. Max coverage (-): 0

Region: chr21 14758081-14758186. Max. coverage (+): 0. Max coverage (-): 0

Region: chr21 14758187-14758292. Max. coverage (+): 0. Max coverage (-): 0

Region: chr21 14758293-14758399. Max. coverage (+): 0. Max coverage (-): 0

Region: chr21 14758400-14758505. Max. coverage (+): 0. Max coverage (-): 0

Region: chr21 14758506-14758611. Max. coverage (+): 0. Max coverage (-): 0

Region: chr21 14758612-14758717. Max. coverage (+): 0. Max coverage (-): 0

Region: chr21 14758718-14758823. Max. coverage (+): 0. Max coverage (-): 6.27

Region: chr21 14758824-14758929. Max. coverage (+): 0. Max coverage (-): 6.9

Region: chr21 14758930-14759035. Max. coverage (+): 0. Max coverage (-): 0

Region: chr21 14759036-14759141. Max. coverage (+): 0. Max coverage (-): 1.23

Region: chr21 14759142-14759247. Max. coverage (+): 0. Max coverage (-): 42.03

Region: chr21 14759248-14759353. Max. coverage (+): 0. Max coverage (-): 6.71

Region: chr21 14759354-14759459. Max. coverage (+): 0. Max coverage (-): 37.96

Region: chr21 14759460-14759565. Max. coverage (+): 0. Max coverage (-): 0

Region: chr21 14759566-14759671. Max. coverage (+): 0. Max coverage (-): 6.61

Region: chr21 14759672-14759777. Max. coverage (+): 0. Max coverage (-): 23.2

Region: chr21 14759778-14759883. Max. coverage (+): 0.39. Max coverage (-): 11.17

Region: chr21 14759884-14759989. Max. coverage (+): 0. Max coverage (-): 1.16

Region: chr21 14759990-14760095. Max. coverage (+): 0. Max coverage (-): 0

Region: chr21 14760096-14760201. Max. coverage (+): 0. Max coverage (-): 5.39

Region: chr21 14760202-14760307. Max. coverage (+): 0. Max coverage (-): 22

Region: chr21 14760308-14760413. Max. coverage (+): 0. Max coverage (-): 5.76

Region: chr21 14760414-14760519. Max. coverage (+): 0. Max coverage (-): 1.35

Region: chr21 14760520-14760625. Max. coverage (+): 0. Max coverage (-): 23.97

Region: chr21 14760626-14760732. Max. coverage (+): 0. Max coverage (-): 13.16

Region: chr21 14760733-14760838. Max. coverage (+): 0. Max coverage (-): 25.07

Region: chr21 14760839-14760944. Max. coverage (+): 6.17. Max coverage (-): 50.31

Region: chr21 14760945-14761050. Max. coverage (+): 6.97. Max coverage (-): 0

Region: chr21 14761051-14761156. Max. coverage (+): 0. Max coverage (-): 0

Region: chr21 14761157-14761262. Max. coverage (+): 0. Max coverage (-): 15.19

Region: chr21 14761263-14761368. Max. coverage (+): 0. Max coverage (-): 3.86

Region: chr21 14761369-14761474. Max. coverage (+): 2.68. Max coverage (-): 11.07

Region: chr21 14761475-14761580. Max. coverage (+): 0. Max coverage (-): 3.44

Region: chr21 14761581-14761686. Max. coverage (+): 0. Max coverage (-): 0

Region: chr21 14761687-14761792. Max. coverage (+): 5.17. Max coverage (-): 67.11

Region: chr21 14761793-14761898. Max. coverage (+): 5.08. Max coverage (-): 36.92

Region: chr21 14761899-14762004. Max. coverage (+): 3.51. Max coverage (-): 16.51

Region: chr21 14762005-14762110. Max. coverage (+): 0. Max coverage (-): 8.92

Region: chr21 14762111-14762216. Max. coverage (+): 0. Max coverage (-): 4.81

Region: chr21 14762217-14762322. Max. coverage (+): 0. Max coverage (-): 40.21

Region: chr21 14762323-14762428. Max. coverage (+): 2.61. Max coverage (-): 6.62

Region: chr21 14762429-14762534. Max. coverage (+): 0. Max coverage (-): 4.94

Region: chr21 14762535-14762640. Max. coverage (+): 22.16. Max coverage (-): 44.31

Region: chr21 14762641-14762746. Max. coverage (+): 0. Max coverage (-): 32.86

Region: chr21 14762747-14762852. Max. coverage (+): 0. Max coverage (-): 0

Region: chr21 14762853-14762958. Max. coverage (+): 0. Max coverage (-): 0

Region: chr21 14762959-14763064. Max. coverage (+): 0. Max coverage (-): 0

Region: chr21 14763065-14763171. Max. coverage (+): 3.87. Max coverage (-): 16.1

Region: chr21 14763172-14763277. Max. coverage (+): 0. Max coverage (-): 5.73

Region: chr21 14763278-14763383. Max. coverage (+): 0. Max coverage (-): 8.32

Region: chr21 14763384-14763489. Max. coverage (+): 0.03. Max coverage (-): 8.32

Region: chr21 14763490-14763595. Max. coverage (+): 0. Max coverage (-): 22.5

Region: chr21 14763596-14763701. Max. coverage (+): 0.54. Max coverage (-): 10.15

Region: chr21 14763702-14763807. Max. coverage (+): 0. Max coverage (-): 0

Region: chr21 14763808-14763913. Max. coverage (+): 0. Max coverage (-): 0

Region: chr21 14763914-14764019. Max. coverage (+): 0. Max coverage (-): 0

Region: chr21 14764020-14764125. Max. coverage (+): 0. Max coverage (-): 1.02

Region: chr21 14764126-14764231. Max. coverage (+): 6.17. Max coverage (-): 17.84

Region: chr21 14764232-14764337. Max. coverage (+): 0.98. Max coverage (-): 13.61

Region: chr21 14764338-14764443. Max. coverage (+): 14.44. Max coverage (-): 3.44

Region: chr21 14764444-14764549. Max. coverage (+): 0. Max coverage (-): 0

Region: chr21 14764550-14764655. Max. coverage (+): 12.73. Max coverage (-): 0.26

Region: chr21 14764656-14764761. Max. coverage (+): 8.53. Max coverage (-): 5.01

Region: chr21 14764762-14764867. Max. coverage (+): 42.83. Max coverage (-): 1.59

Region: chr21 14764868-14764973. Max. coverage (+): 5.91. Max coverage (-): 0

Region: chr21 14764974-14765079. Max. coverage (+): 11.26. Max coverage (-): 0

Region: chr21 14765080-14765185. Max. coverage (+): 6.01. Max coverage (-): 5.95

Region: chr21 14765186-14765291. Max. coverage (+): 0. Max coverage (-): 0

Region: chr21 14765292-14765397. Max. coverage (+): 2.59. Max coverage (-): 0

Region: chr21 14765398-14765503. Max. coverage (+): 7.17. Max coverage (-): 0

Region: chr21 14765504-14765610. Max. coverage (+): 17.5. Max coverage (-): 7.64

Region: chr21 14765611-14765716. Max. coverage (+): 0. Max coverage (-): 0

Region: chr21 14765717-14765822. Max. coverage (+): 13.91. Max coverage (-): 5.58

Region: chr21 14765823-14765928. Max. coverage (+): 10.94. Max coverage (-): 0

Region: chr21 14765929-14766034. Max. coverage (+): 0. Max coverage (-): 0

Region: chr21 14766035-14766140. Max. coverage (+): 61.11. Max coverage (-): 0

Region: chr21 14766141-14766246. Max. coverage (+): 6.19. Max coverage (-): 0

Region: chr21 14766247-14766352. Max. coverage (+): 8.59. Max coverage (-): 0

Region: chr21 14766353-14766458. Max. coverage (+): 0. Max coverage (-): 0

Region: chr21 14766459-14766564. Max. coverage (+): 29.92. Max coverage (-): 0

Region: chr21 14766565-14766670. Max. coverage (+): 0. Max coverage (-): 0

Region: chr21 14766671-14766776. Max. coverage (+): 0. Max coverage (-): 0

Region: chr21 14766777-14766882. Max. coverage (+): 0. Max coverage (-): 0

Region: chr21 14766883-14766988. Max. coverage (+): 5.57. Max coverage (-): 0

Region: chr21 14766989-14767094. Max. coverage (+): 6.4. Max coverage (-): 0

Region: chr21 14767095-14767200. Max. coverage (+): 12.11. Max coverage (-): 0

Region: chr21 14767201-14767306. Max. coverage (+): 0. Max coverage (-): 0

Region: chr21 14767307-14767412. Max. coverage (+): 73.75. Max coverage (-): 0

Region: chr21 14767413-14767518. Max. coverage (+): 82.02. Max coverage (-): 0

Region: chr21 14767519-14767624. Max. coverage (+): 0.73. Max coverage (-): 0

Region: chr21 14767625-14767730. Max. coverage (+): 8.2. Max coverage (-): 0

Region: chr21 14767731-14767836. Max. coverage (+): 0. Max coverage (-): 0

Region: chr21 14767837-14767942. Max. coverage (+): 0. Max coverage (-): 0

Region: chr21 14767943-14768049. Max. coverage (+): 0. Max coverage (-): 0

Region: chr21 14768050-14768155. Max. coverage (+): 93.75. Max coverage (-): 3.2

Region: chr21 14768156-14768261. Max. coverage (+): 17.31. Max coverage (-): 3.2

Region: chr21 14768262-14768367. Max. coverage (+): 141.97. Max coverage (-): 2.42

Region: chr21 14768368-14768473. Max. coverage (+): 117.56. Max coverage (-): 0.75

Region: chr21 14768474-14768579. Max. coverage (+): 7.62. Max coverage (-): 0

Region: chr21 14768580-14768685. Max. coverage (+): 6.96. Max coverage (-): 0

Region: chr21 14768686-14768791. Max. coverage (+): 44.79. Max coverage (-): 0

Region: chr21 14768792-14768897. Max. coverage (+): 17.09. Max coverage (-): 0

Region: chr21 14768898-14769003. Max. coverage (+): 17.8. Max coverage (-): 0

Region: chr21 14769004-14769109. Max. coverage (+): 4.14. Max coverage (-): 5.04

Region: chr21 14769110-14769215. Max. coverage (+): 27.41. Max coverage (-): 0

Region: chr21 14769216-14769321. Max. coverage (+): 18.47. Max coverage (-): 0

Region: chr21 14769322-14769427. Max. coverage (+): 25.6. Max coverage (-): 7.58

Region: chr21 14769428-14769533. Max. coverage (+): 2.65. Max coverage (-): 1.49

Region: chr21 14769534-14769639. Max. coverage (+): 0. Max coverage (-): 0

Region: chr21 14769640-14769745. Max. coverage (+): 0. Max coverage (-): 0

Region: chr21 14769746-14769851. Max. coverage (+): 0. Max coverage (-): 0

Region: chr21 14769852-14769957. Max. coverage (+): 1.08. Max coverage (-): 0

Region: chr21 14769958-14770063. Max. coverage (+): 111.98. Max coverage (-): 0

Region: chr21 14770064-14770169. Max. coverage (+): 105.06. Max coverage (-): 0.7

Region: chr21 14770170-14770275. Max. coverage (+): 16.67. Max coverage (-): 0

Region: chr21 14770276-14770382. Max. coverage (+): 12.67. Max coverage (-): 0

Region: chr21 14770383-14770488. Max. coverage (+): 64.13. Max coverage (-): 5.13

Region: chr21 14770489-14770594. Max. coverage (+): 18.43. Max coverage (-): 0

Region: chr21 14770595-14770700. Max. coverage (+): 18. Max coverage (-): 4.14

Region: chr21 14770701-14770806. Max. coverage (+): 11.45. Max coverage (-): 0

Region: chr21 14770807-14770912. Max. coverage (+): 19.51. Max coverage (-): 0

Region: chr21 14770913-14771018. Max. coverage (+): 14.37. Max coverage (-): 0

Region: chr21 14771019-14771124. Max. coverage (+): 32.96. Max coverage (-): 0

Region: chr21 14771125-14771230. Max. coverage (+): 17.03. Max coverage (-): 2.61

Region: chr21 14771231-14771336. Max. coverage (+): 31.16. Max coverage (-): 2.61

Region: chr21 14771337-14771442. Max. coverage (+): 44.96. Max coverage (-): 0

Region: chr21 14771443-14771548. Max. coverage (+): 0. Max coverage (-): 0

Region: chr21 14771549-14771654. Max. coverage (+): 0. Max coverage (-): 0

Region: chr21 14771655-14771760. Max. coverage (+): 0. Max coverage (-): 0

Region: chr21 14771761-14771866. Max. coverage (+): 6.97. Max coverage (-): 0

Region: chr21 14771867-14771972. Max. coverage (+): 6.09. Max coverage (-): 0

Region: chr21 14771973-14772078. Max. coverage (+): 64.7. Max coverage (-): 0

Region: chr21 14772079-14772184. Max. coverage (+): 7.99. Max coverage (-): 0

Region: chr21 14772185-14772290. Max. coverage (+): 11.92. Max coverage (-): 0

Region: chr21 14772291-14772396. Max. coverage (+): 4.61. Max coverage (-): 0

Region: chr21 14772397-14772502. Max. coverage (+): 3.81. Max coverage (-): 3.24

Region: chr21 14772503-14772608. Max. coverage (+): 25.34. Max coverage (-): 6.71

Region: chr21 14772609-14772714. Max. coverage (+): 14.54. Max coverage (-): 0

Region: chr21 14772715-14772821. Max. coverage (+): 0. Max coverage (-): 0

Region: chr21 14772822-14772927. Max. coverage (+): 0. Max coverage (-): 0

Region: chr21 14772928-14773033. Max. coverage (+): 0. Max coverage (-): 0

Region: chr21 14773034-14773139. Max. coverage (+): 0. Max coverage (-): 0

Region: chr21 14773140-14773245. Max. coverage (+): 17.61. Max coverage (-): 0

Region: chr21 14773246-14773351. Max. coverage (+): 65.22. Max coverage (-): 13.53

Region: chr21 14773352-14773457. Max. coverage (+): 9. Max coverage (-): 0

Region: chr21 14773458-14773563. Max. coverage (+): 21.6. Max coverage (-): 9.93

Region: chr21 14773564-14773669. Max. coverage (+): 51.85. Max coverage (-): 0

Region: chr21 14773670-14773775. Max. coverage (+): 14.53. Max coverage (-): 8.11

Region: chr21 14773776-14773881. Max. coverage (+): 4.19. Max coverage (-): 6.6

Region: chr21 14773882-14773987. Max. coverage (+): 66.62. Max coverage (-): 0

Region: chr21 14773988-14774093. Max. coverage (+): 4.77. Max coverage (-): 0

Region: chr21 14774094-14774199. Max. coverage (+): 23.87. Max coverage (-): 2.92

Region: chr21 14774200-14774305. Max. coverage (+): 5.6. Max coverage (-): 0

Region: chr21 14774306-14774411. Max. coverage (+): 15.48. Max coverage (-): 0

Region: chr21 14774412-14774517. Max. coverage (+): 17.45. Max coverage (-): 0

Region: chr21 14774518-14774623. Max. coverage (+): 39.22. Max coverage (-): 0

Region: chr21 14774624-14774729. Max. coverage (+): 30.21. Max coverage (-): 0

Region: chr21 14774730-14774835. Max. coverage (+): 14.1. Max coverage (-): 0.8

Region: chr21 14774836-14774941. Max. coverage (+): 13.91. Max coverage (-): 0

Region: chr21 14774942-14775047. Max. coverage (+): 18.38. Max coverage (-): 0

Region: chr21 14775048-14775153. Max. coverage (+): 13.49. Max coverage (-): 0

Region: chr21 14775154-14775260. Max. coverage (+): 70.35. Max coverage (-): 0

Region: chr21 14775261-14775366. Max. coverage (+): 1.73. Max coverage (-): 0

Region: chr21 14775367-14775472. Max. coverage (+): 7.8. Max coverage (-): 0

Region: chr21 14775473-14775578. Max. coverage (+): 0. Max coverage (-): 0

Region: chr21 14775579-14775684. Max. coverage (+): 2.02. Max coverage (-): 0

Region: chr21 14775685-14775790. Max. coverage (+): 3.37. Max coverage (-): 0

Region: chr21 14775791-14775896. Max. coverage (+): 6.61. Max coverage (-): 0

Region: chr21 14775897-14776002. Max. coverage (+): 0. Max coverage (-): 0

Region: chr21 14776003-14776108. Max. coverage (+): 0. Max coverage (-): 0

Region: chr21 14776109-14776214. Max. coverage (+): 5.54. Max coverage (-): 0

Region: chr21 14776215-14776320. Max. coverage (+): 0. Max coverage (-): 0

Region: chr21 14776321-14776426. Max. coverage (+): 0. Max coverage (-): 0

Region: chr21 14776427-14776532. Max. coverage (+): 61.24. Max coverage (-): 0

Region: chr21 14776533-14776638. Max. coverage (+): 56.99. Max coverage (-): 0

Region: chr21 14776639-14776744. Max. coverage (+): 16.25. Max coverage (-): 0

Region: chr21 14776745-14776850. Max. coverage (+): 11.15. Max coverage (-): 0

Region: chr21 14776851-14776956. Max. coverage (+): 138.58. Max coverage (-): 0

Region: chr21 14776957-14777062. Max. coverage (+): 11.99. Max coverage (-): 0

Region: chr21 14777063-14777168. Max. coverage (+): 52.99. Max coverage (-): 0

Region: chr21 14777169-14777274. Max. coverage (+): 192.98. Max coverage (-): 0

Region: chr21 14777275-14777380. Max. coverage (+): 14.66. Max coverage (-): 0

Region: chr21 14777381-14777486. Max. coverage (+): 7.05. Max coverage (-): 0

Region: chr21 14777487-14777592. Max. coverage (+): 61.89. Max coverage (-): 0

Region: chr21 14777593-14777699. Max. coverage (+): 22.78. Max coverage (-): 0

Region: chr21 14777700-14777805. Max. coverage (+): 6.67. Max coverage (-): 0

Region: chr21 14777806-14777911. Max. coverage (+): 14.63. Max coverage (-): 0

Region: chr21 14777912-14778017. Max. coverage (+): 67.9. Max coverage (-): 0

Region: chr21 14778018-14778123. Max. coverage (+): 119.03. Max coverage (-): 0

Region: chr21 14778124-14778229. Max. coverage (+): 23.84. Max coverage (-): 0

Region: chr21 14778230-14778335. Max. coverage (+): 22.17. Max coverage (-): 0

Region: chr21 14778336-14778441. Max. coverage (+): 51.49. Max coverage (-): 0

Region: chr21 14778442-14778547. Max. coverage (+): 30.38. Max coverage (-): 0

Region: chr21 14778548-14778653. Max. coverage (+): 16.49. Max coverage (-): 0

Region: chr21 14778654-14778759. Max. coverage (+): 21.34. Max coverage (-): 0

Region: chr21 14778760-14778865. Max. coverage (+): 63.47. Max coverage (-): 0

Region: chr21 14778866-14778971. Max. coverage (+): 4.12. Max coverage (-): 0

Region: chr21 14778972-14779077. Max. coverage (+): 18.83. Max coverage (-): 0

Region: chr21 14779078-14779183. Max. coverage (+): 15.72. Max coverage (-): 0

Region: chr21 14779184-14779289. Max. coverage (+): 14.28. Max coverage (-): 0

Region: chr21 14779290-14779395. Max. coverage (+): 9.41. Max coverage (-): 0

Region: chr21 14779396-14779501. Max. coverage (+): 0. Max coverage (-): 0

Region: chr21 14779502-14779607. Max. coverage (+): 0. Max coverage (-): 0

Region: chr21 14779608-14779713. Max. coverage (+): 0. Max coverage (-): 0

Region: chr21 14779714-14779819. Max. coverage (+): 2.67. Max coverage (-): 0

Region: chr21 14779820-14779925. Max. coverage (+): 0. Max coverage (-): 0

Region: chr21 14779926-14780032. Max. coverage (+): 0. Max coverage (-): 0

Region: chr21 14780033-14780138. Max. coverage (+): 0. Max coverage (-): 0

Region: chr21 14780139-14780244. Max. coverage (+): 6.32. Max coverage (-): 0

Region: chr21 14780245-14780350. Max. coverage (+): 0. Max coverage (-): 0

Region: chr21 14780351-14780456. Max. coverage (+): 0. Max coverage (-): 0

Region: chr21 14780457-14780562. Max. coverage (+): 0. Max coverage (-): 0

Region: chr21 14780563-14780668. Max. coverage (+): 0. Max coverage (-): 0

Region: chr21 14780669-14780774. Max. coverage (+): 0. Max coverage (-): 0

Region: chr21 14780775-14780880. Max. coverage (+): 0. Max coverage (-): 0

Region: chr21 14780881-14780986. Max. coverage (+): 92.98. Max coverage (-): 0

Region: chr21 14780987-14781092. Max. coverage (+): 17.22. Max coverage (-): 0

Region: chr21 14781093-14781198. Max. coverage (+): 17.23. Max coverage (-): 0

Region: chr21 14781199-14781304. Max. coverage (+): 9.7. Max coverage (-): 0

Region: chr21 14781305-14781410. Max. coverage (+): 18.29. Max coverage (-): 0

Region: chr21 14781411-14781516. Max. coverage (+): 4.35. Max coverage (-): 0

Region: chr21 14781517-14781622. Max. coverage (+): 0. Max coverage (-): 0

Region: chr21 14781623-14781728. Max. coverage (+): 0. Max coverage (-): 0

Region: chr21 14781729-14781834. Max. coverage (+): 14.64. Max coverage (-): 0

Region: chr21 14781835-14781940. Max. coverage (+): 23.35. Max coverage (-): 0

Region: chr21 14781941-14782046. Max. coverage (+): 15.86. Max coverage (-): 0

Region: chr21 14782047-14782152. Max. coverage (+): 8.98. Max coverage (-): 0

Region: chr21 14782153-14782258. Max. coverage (+): 0. Max coverage (-): 0

Region: chr21 14782259-14782364. Max. coverage (+): 10.94. Max coverage (-): 0

Region: chr21 14782365-. Max. coverage (+): 11.25. Max coverage (-): 0

RepeatMasker Color Code

**+**

100-98% Identity

<98-95% Identity

<95-90% Identity

<90-85% Identity

<85-80% Identity

<80-75% Identity

<75-70% Identity

<70% Identity

**-**

Gene Set Color Code

**+**

Gene

Pseudogene

**-**

Topology/Coverage Color Code

Coverage Plus Strand

Coverage Minus Strand

Mainstrand: Plus

Mainstrand: Minus

Complementary Strand

Flanking Region  
(if option -flank >0)

Gene Set Annotation  

**1. FAM174B (protein coding, ENSBTAG00000022989) Tr:00000031231 Ex:4**: 14729341-14729722 (+)

  
RepeatMasker Annotation  

**1. AT\_rich**: 14729618-14729639 (+), Divergence to consensus: 50%  
**2. (TG)n**: 14729949-14729990 (+), Divergence to consensus: 19.1%  
**3. Bov-tA2**: 14731699-14731904 (-), Divergence to consensus: 19.5%  
**4. L2b**: 14732197-14732249 (-), Divergence to consensus: 26.5%  
**5. (CA)n**: 14733617-14733656 (+), Divergence to consensus: 2.5%  
**6. MER5A1**: 14733945-14733988 (-), Divergence to consensus: 13.6%  
**7. GC\_rich**: 14734026-14734053 (+), Divergence to consensus: 53.6%  
**8. L1MEg**: 14737565-14738036 (-), Divergence to consensus: 47.5%  
**9. L2a**: 14739815-14739950 (-), Divergence to consensus: 40.6%  
**10. (TGGGGG)n**: 14740836-14740856 (+), Divergence to consensus: 0%  
**11. MLT1J1**: 14742504-14742757 (-), Divergence to consensus: 39.6%  
**12. ART2A**: 14742873-14742949 (+), Divergence to consensus: 26.3%  
**13. BOV-A2**: 14742884-14742950 (+), Divergence to consensus: 3%  
**14. (CAG)n**: 14742951-14742970 (+), Divergence to consensus: 0%  
**15. MER112**: 14745154-14745293 (+), Divergence to consensus: 43.6%  
**16. L1ME4a**: 14747394-14747535 (-), Divergence to consensus: 39.7%  
**17. LTR16B1**: 14747810-14748134 (-), Divergence to consensus: 48.9%  
**18. Bov-tA1**: 14748899-14749121 (-), Divergence to consensus: 22.4%  
**19. L1M5**: 14749224-14749846 (-), Divergence to consensus: 47.8%  
**20. Bov-tA2**: 14750457-14750629 (-), Divergence to consensus: 30.6%  
**21. Charlie21a**: 14750652-14751388 (+), Divergence to consensus: 44.6%  
**22. L4\_A\_Mam**: 14751671-14751847 (-), Divergence to consensus: 45%  
**23. L4\_A\_Mam**: 14752208-14752346 (-), Divergence to consensus: 38.8%  
**24. BovB**: 14752375-14752469 (+), Divergence to consensus: 19.4%  
**25. Bov-tA2**: 14752690-14752889 (-), Divergence to consensus: 16.7%  
**26. L4\_A\_Mam**: 14752902-14753283 (-), Divergence to consensus: 47%  
**27. AT\_rich**: 14753294-14753314 (+), Divergence to consensus: 42.9%  
**28. MamGypLTR3**: 14753811-14753983 (+), Divergence to consensus: 38.2%  
**29. BOV-A2**: 14754302-14754368 (+), Divergence to consensus: 8.3%  
**30. ART2A**: 14754369-14754437 (+), Divergence to consensus: 31.1%  
**31. (CAG)n**: 14754438-14754457 (+), Divergence to consensus: 0%  
**32. ART2A**: 14754458-14754486 (+), Divergence to consensus: 31.1%  
**33. BOV-A2**: 14757099-14757368 (+), Divergence to consensus: 8.2%  
**34. BovB**: 14757716-14758186 (+), Divergence to consensus: 10.9%  
**35. ART2A**: 14758188-14758704 (+), Divergence to consensus: 17.1%  
**36. (AACTG)n**: 14758705-14758724 (+), Divergence to consensus: 0%  
**37. L2c**: 14759996-14760140 (+), Divergence to consensus: 37.8%  
**38. MER112**: 14761536-14761635 (+), Divergence to consensus: 29.3%  
**39. L1-3\_BT**: 14762779-14763018 (+), Divergence to consensus: 19.4%  
**40. MER119**: 14763874-14763999 (+), Divergence to consensus: 28.6%  
**41. L2c**: 14766759-14766852 (+), Divergence to consensus: 43.9%  
**42. L1ME4a**: 14767211-14767339 (-), Divergence to consensus: 32.1%  
**43. L1ME4b**: 14767498-14767605 (-), Divergence to consensus: 28.9%  
**44. Bov-tA1**: 14767696-14767899 (-), Divergence to consensus: 14.7%  
**45. L1ME4a**: 14767905-14768045 (-), Divergence to consensus: 36.7%  
**46. MLT1C**: 14769465-14769892 (-), Divergence to consensus: 39%  
**47. AT\_rich**: 14769902-14769930 (+), Divergence to consensus: 65.5%  
**48. L4\_A\_Mam**: 14771472-14771774 (+), Divergence to consensus: 43.8%  
**49. L2c**: 14772335-14772456 (+), Divergence to consensus: 40.7%  
**50. MLT1D**: 14772743-14772946 (-), Divergence to consensus: 23.6%  
**51. MIRc**: 14773181-14773275 (-), Divergence to consensus: 41.8%  
**52. C-rich**: 14775478-14775513 (+), Divergence to consensus: 19.4%  
**53. MIR**: 14775952-14776202 (+), Divergence to consensus: 32.3%  
**54. Bov-tA2**: 14776237-14776429 (+), Divergence to consensus: 22%  
**55. AT\_rich**: 14776820-14776840 (+), Divergence to consensus: 66.7%  
**56. LTR107\_Mam**: 14777110-14777181 (-), Divergence to consensus: 23.6%  
**57. AT\_rich**: 14779045-14779065 (+), Divergence to consensus: 47.6%  
**58. AT\_rich**: 14779342-14779378 (+), Divergence to consensus: 73%  
**59. Bov-tA3**: 14779467-14779594 (+), Divergence to consensus: 15.1%  
**60. L1-2\_BT**: 14779606-14779737 (-), Divergence to consensus: 31.9%  
**61. AT\_rich**: 14779889-14779915 (+), Divergence to consensus: 51.9%  
**62. Bov-tA1**: 14780002-14780193 (-), Divergence to consensus: 21.9%  
**63. BOV-A2**: 14780273-14780350 (-), Divergence to consensus: 25.7%  
**64. ART2A**: 14780283-14780800 (-), Divergence to consensus: 15.2%  
**65. BovB**: 14780802-14780857 (-), Divergence to consensus: 3.6%  
**66. LTR16A2**: 14781521-14781764 (+), Divergence to consensus: 37.8%  
**67. Bov-tA2**: 14782123-14782329 (-), Divergence to consensus: 14.1%

  
Transcription Factor Binding Sites  

**RFX4\_2** (Sequence: GTATCCAGG (-): 14765698)  
**RFX4\_1** (Sequence: CATAGCAAC (+): 14742991)  
**RFX4\_1** (Sequence: CTTGGCAAC (+): 14763640)  
**RFX4\_2** (Sequence: CTTAGTTAC (+): 14734102)  
**RFX4\_2** (Sequence: CATAGTTAC (+): 14779231)  
**Gata4** (Sequence: AGATAAG (-): 14731412)  
**Gata4** (Sequence: AGATAAC (-): 14732134)  
**Gata4** (Sequence: AGATAAC (-): 14749937)  
**SOX9** (Sequence: AACAATAA (-): 14753627)  
**SOX9** (Sequence: AACAATAA (-): 14756114)  
**SOX9** (Sequence: TCATTGTT (+): 14753404)  
**SOX9** (Sequence: CCATTGTT (+): 14756266)  
**A-MYB** (Sequence: CCAACTGCCA (-): 14745171)  
**SPZ1** (Sequence: GGGGTATCAG (+): 14755766)  
**SPZ1** (Sequence: GGGGTTACAG (+): 14781135)  
**Gata4** (Sequence: CTTATCT (+): 14742106)  
**Gata4** (Sequence: CTTATCT (+): 14777300)
