# Supplementary material for: Small RNA sequencing of cryopreserved semen from single bull revealed altered miRNAs and piRNAs expression between High- and Low-motile sperm populations
Source: BMC Genomics. 2017 Jan 4;18:14. doi: 10.1186/s12864-016-3394-7 (PMC5209821; doi:10.1186/s12864-016-3394-7)
Supplement: Additional file 4: — Details for each piRNA clusters found in Low Motile (LM) sperm fraction. Genes, repeats, transposable elements and transcription factors binding sites falling within the cluster regions were reported. (ZIP 1034 kb) [file 12864_2016_3394_MOESM4_ESM.zip › 28.html]

piRNA cluster 28


Predicted piRNA cluster no. 28     previous   next
  

Show proTRAC run info
Hide proTRAC run info

================================= proTRAC ====================================  
VERSION: 2.1                                    LAST MODIFIED: 06. October 2015  
  
Please cite:  
Rosenkranz D, Zischler H. proTRAC - a software for probabilistic piRNA cluster  
detection, visualization and analysis. 2012. BMC Bioinformatics 13:5.  
  
and (for proTRAC 2.0 and later):  
Rosenkranz D, Rudloff S, Bastuck K, Ketting RF, Zischler H. Tupaia small RNAs  
provide insights into function and evolution of RNAi-based transposon defense  
in mammals. 2015. RNA 21(5):911-922.  
  
Contact:  
David Rosenkranz  
Institute of Anthropology, small RNA group  
Johannes Gutenberg University Mainz  
email: rosenkranz@uni-mainz.de  
  
You can find the latest proTRAC version at:  
http://sourceforge.net/projects/protrac/files  
http://www.smallRNAgroup-mainz.de/software  
==============================================================================  
  
PARAMETERS:  
Map file: .............../storage/core/barbara/genhome/smallRNA/fertility/Sample\_not\_motile/pirna/Sample\_not\_motile\_26-33\_collapsed.fa.no-dust.map.weighted-10000-1000-b-0  
Genome file: ............/storage/core/barbara/genhome/smallRNA/fertility/Sample\_all/pirna/bt\_311\_chrY.fa  
RepeatMasker annotation: /storage/genomes/bt\_umd31/GCF\_000003055.6\_Bos\_taurus\_UMD\_3.1.1\_repeatMasker\_chr.out  
GeneSet:................./storage/core/barbara/genhome/smallRNA/fertility/Sample\_all/pirna/full.gtf  
  
Significant (p<=0.01) hit density will be calculated based  
on observed hit distribution.  
  
Sliding window size: ........................................ 5000 bp  
Sliding window increament: .................................. 1000 bp  
Normalize each hit by number of genomic hits: ............... 1 [0=no/1=yes]  
Normalize each hit by number of sequence reads: ............. 1 [0=no/1=yes]  
Normalize values (-> per million mapped reads): ............. 1 [0=no/1=yes]  
Min. fraction of hits with 1T(U) or 10A: .................... 0.75  
Alternatively: Min. fraction of hits with 1T(U) and 10A: .... 0.5  
Min. fraction of hits with typical piRNA length: ............ 0.75  
Typical piRNA length: ....................................... 26-33 nt  
Min. size of a piRNA cluster: ............................... 5000 bp.  
Min. number of hits (absolute): ............................. 0  
Min. number of hits (normalized): ........................... 0  
Min. fraction of hits on the mainstrand: .................... 0.75  
Top fraction of mapped sequences (in terms of read counts): . 1%  
Top fraction accounts for max. n% of sequence reads: ........ 90%  
Min. fraction of hits on each arm of a bidirectional cluster: 0.1  
Output image file for each cluster: ......................... 0 [0=no/1=yes]  
Output html file for each cluster: .......................... 1 [0=no/1=yes]  
Output a summary table: ..................................... 1 [0=no/1=yes]  
Output a FASTA file for each cluster (piRNA sequences): ..... 1 [0=no/1=yes]  
Output a FASTA file comprising cluster sequences: ........... 1 [0=no/1=yes]  
Search DNA motifs in clusters: .............................. 1 [0=no/1=yes]  
Output flanking sequences: +/- .............................. 0 bp  
Output ~.pTi file: .......................................... 1 [0=no/1=yes]  
==============================================================================  
  
  
Genome size (without gaps): ............ 2678902517 bp  
Gaps (N/X/-): .......................... 53837044 bp  
Mapped reads: .......................... 738059667487  
Non-identical sequences: ............... 277001  
Genomic hits: .......................... 533816  
Significant densitiy of mapped reads: .. 15118061 reads/kb

Show proTRAC cluster info
Hide proTRAC cluster info

|  |  |
| --- | --- |
| Location | chr21 |
| Coordinates | 29761078-29769203 |
| Size [bp] | 8126 |
| Sequence hit loci | 82 |
| Mapped reads (normalized) | 221745579 |
| Mapped reads (normalized) per kb | 27288405 |
| Normalized reads with 1T (1U) | 87.6% |
| Normalized reads with 10A | 28.8% |
| Normalized reads with length 26-33 nt | 100% |
| Normalized reads on the main strand(s) | 100% |
| Predicted directionality | mono:plus |

100%

0%

1T (1U)  
reads

10A reads

26-33 nt  
reads

reads on mainstrand

**Either the amount of reads with 1T (1U) OR 10A has to exceed 75% (set with option: -1Tor10A)  
Alternatively the amount of reads with 1T (1U) AND 10A has to exceed 50% (set with option: -1Tand10A)  
Minimum amount of reads with preferred size is 75% (set with option: -pisize)  
Minimum amount of reads on the main strand(s) is 75% (set with option: -clstrand)**

Show read coverage
Hide read coverage

WHAT DO I SEE HERE?  
This chart shows the location of mapped sequence reads within a predicted piRNA cluster. The color refers to the number of genomic hits produced by the sequence read in question. A dark red bar indicates that this sequence read produces many other hits elsewhere in the genome. Many adjacent red or yellow bars can indicate the presence of a multi-copy element such as transposons or rRNA genes. A dark green bar indicates that this sequence read maps uniquely to this locus.

1 hit

2-5 hits

6-10 hits

11-20 hits

21-50 hits

51-100 hits

> 100 hits

chr21

29761078

29769203

Gene Set

RepeatMasker

Mapped  
Reads

20.86

plus strand

minus strand

20.86

Region: chr21 14782366-29761086. Max. coverage (+): 1.47. Max coverage (-): 0

Region: chr21 29761087-29761102. Max. coverage (+): 0. Max coverage (-): 0

Region: chr21 29761103-29761118. Max. coverage (+): 0. Max coverage (-): 0

Region: chr21 29761119-29761134. Max. coverage (+): 0. Max coverage (-): 0

Region: chr21 29761135-29761151. Max. coverage (+): 0. Max coverage (-): 0

Region: chr21 29761152-29761167. Max. coverage (+): 0. Max coverage (-): 0

Region: chr21 29761168-29761183. Max. coverage (+): 0. Max coverage (-): 0

Region: chr21 29761184-29761199. Max. coverage (+): 3.19. Max coverage (-): 0

Region: chr21 29761200-29761216. Max. coverage (+): 0. Max coverage (-): 0

Region: chr21 29761217-29761232. Max. coverage (+): 0. Max coverage (-): 0

Region: chr21 29761233-29761248. Max. coverage (+): 0. Max coverage (-): 0

Region: chr21 29761249-29761264. Max. coverage (+): 0. Max coverage (-): 0

Region: chr21 29761265-29761281. Max. coverage (+): 0. Max coverage (-): 0

Region: chr21 29761282-29761297. Max. coverage (+): 0. Max coverage (-): 0

Region: chr21 29761298-29761313. Max. coverage (+): 0. Max coverage (-): 0

Region: chr21 29761314-29761329. Max. coverage (+): 0. Max coverage (-): 0

Region: chr21 29761330-29761346. Max. coverage (+): 0. Max coverage (-): 0

Region: chr21 29761347-29761362. Max. coverage (+): 0. Max coverage (-): 0

Region: chr21 29761363-29761378. Max. coverage (+): 0. Max coverage (-): 0

Region: chr21 29761379-29761394. Max. coverage (+): 0. Max coverage (-): 0

Region: chr21 29761395-29761411. Max. coverage (+): 0. Max coverage (-): 0

Region: chr21 29761412-29761427. Max. coverage (+): 0. Max coverage (-): 0

Region: chr21 29761428-29761443. Max. coverage (+): 0. Max coverage (-): 0

Region: chr21 29761444-29761459. Max. coverage (+): 0. Max coverage (-): 0

Region: chr21 29761460-29761476. Max. coverage (+): 0. Max coverage (-): 0

Region: chr21 29761477-29761492. Max. coverage (+): 0. Max coverage (-): 0

Region: chr21 29761493-29761508. Max. coverage (+): 0. Max coverage (-): 0

Region: chr21 29761509-29761524. Max. coverage (+): 0. Max coverage (-): 0

Region: chr21 29761525-29761541. Max. coverage (+): 0. Max coverage (-): 0

Region: chr21 29761542-29761557. Max. coverage (+): 0. Max coverage (-): 0

Region: chr21 29761558-29761573. Max. coverage (+): 0. Max coverage (-): 0

Region: chr21 29761574-29761589. Max. coverage (+): 0. Max coverage (-): 0

Region: chr21 29761590-29761606. Max. coverage (+): 0. Max coverage (-): 0

Region: chr21 29761607-29761622. Max. coverage (+): 0. Max coverage (-): 0

Region: chr21 29761623-29761638. Max. coverage (+): 0. Max coverage (-): 0

Region: chr21 29761639-29761654. Max. coverage (+): 0. Max coverage (-): 0

Region: chr21 29761655-29761671. Max. coverage (+): 0. Max coverage (-): 0

Region: chr21 29761672-29761687. Max. coverage (+): 0. Max coverage (-): 0

Region: chr21 29761688-29761703. Max. coverage (+): 0. Max coverage (-): 0

Region: chr21 29761704-29761719. Max. coverage (+): 2.72. Max coverage (-): 0

Region: chr21 29761720-29761736. Max. coverage (+): 2.72. Max coverage (-): 0

Region: chr21 29761737-29761752. Max. coverage (+): 0. Max coverage (-): 0

Region: chr21 29761753-29761768. Max. coverage (+): 0. Max coverage (-): 0

Region: chr21 29761769-29761784. Max. coverage (+): 0. Max coverage (-): 0

Region: chr21 29761785-29761801. Max. coverage (+): 0. Max coverage (-): 0

Region: chr21 29761802-29761817. Max. coverage (+): 0. Max coverage (-): 0

Region: chr21 29761818-29761833. Max. coverage (+): 0. Max coverage (-): 0

Region: chr21 29761834-29761849. Max. coverage (+): 0. Max coverage (-): 0

Region: chr21 29761850-29761866. Max. coverage (+): 0. Max coverage (-): 0

Region: chr21 29761867-29761882. Max. coverage (+): 0. Max coverage (-): 0

Region: chr21 29761883-29761898. Max. coverage (+): 0. Max coverage (-): 0

Region: chr21 29761899-29761914. Max. coverage (+): 0. Max coverage (-): 0

Region: chr21 29761915-29761931. Max. coverage (+): 0. Max coverage (-): 0

Region: chr21 29761932-29761947. Max. coverage (+): 0. Max coverage (-): 0

Region: chr21 29761948-29761963. Max. coverage (+): 0. Max coverage (-): 0

Region: chr21 29761964-29761979. Max. coverage (+): 0. Max coverage (-): 0

Region: chr21 29761980-29761996. Max. coverage (+): 13.81. Max coverage (-): 0

Region: chr21 29761997-29762012. Max. coverage (+): 2.49. Max coverage (-): 0

Region: chr21 29762013-29762028. Max. coverage (+): 0.74. Max coverage (-): 0

Region: chr21 29762029-29762044. Max. coverage (+): 6.93. Max coverage (-): 0

Region: chr21 29762045-29762061. Max. coverage (+): 0. Max coverage (-): 0

Region: chr21 29762062-29762077. Max. coverage (+): 0. Max coverage (-): 0

Region: chr21 29762078-29762093. Max. coverage (+): 0. Max coverage (-): 0

Region: chr21 29762094-29762110. Max. coverage (+): 0. Max coverage (-): 0

Region: chr21 29762111-29762126. Max. coverage (+): 0. Max coverage (-): 0

Region: chr21 29762127-29762142. Max. coverage (+): 0. Max coverage (-): 0

Region: chr21 29762143-29762158. Max. coverage (+): 0. Max coverage (-): 0

Region: chr21 29762159-29762175. Max. coverage (+): 0. Max coverage (-): 0

Region: chr21 29762176-29762191. Max. coverage (+): 0. Max coverage (-): 0

Region: chr21 29762192-29762207. Max. coverage (+): 0. Max coverage (-): 0

Region: chr21 29762208-29762223. Max. coverage (+): 0. Max coverage (-): 0

Region: chr21 29762224-29762240. Max. coverage (+): 6.95. Max coverage (-): 0

Region: chr21 29762241-29762256. Max. coverage (+): 0. Max coverage (-): 0

Region: chr21 29762257-29762272. Max. coverage (+): 0. Max coverage (-): 0

Region: chr21 29762273-29762288. Max. coverage (+): 0. Max coverage (-): 0

Region: chr21 29762289-29762305. Max. coverage (+): 0. Max coverage (-): 0

Region: chr21 29762306-29762321. Max. coverage (+): 0. Max coverage (-): 0

Region: chr21 29762322-29762337. Max. coverage (+): 0. Max coverage (-): 0

Region: chr21 29762338-29762353. Max. coverage (+): 0. Max coverage (-): 0

Region: chr21 29762354-29762370. Max. coverage (+): 0. Max coverage (-): 0

Region: chr21 29762371-29762386. Max. coverage (+): 0. Max coverage (-): 0

Region: chr21 29762387-29762402. Max. coverage (+): 3.2. Max coverage (-): 0

Region: chr21 29762403-29762418. Max. coverage (+): 0. Max coverage (-): 0

Region: chr21 29762419-29762435. Max. coverage (+): 0. Max coverage (-): 0

Region: chr21 29762436-29762451. Max. coverage (+): 0. Max coverage (-): 0

Region: chr21 29762452-29762467. Max. coverage (+): 0. Max coverage (-): 0

Region: chr21 29762468-29762483. Max. coverage (+): 0. Max coverage (-): 0

Region: chr21 29762484-29762500. Max. coverage (+): 0. Max coverage (-): 0

Region: chr21 29762501-29762516. Max. coverage (+): 0. Max coverage (-): 0

Region: chr21 29762517-29762532. Max. coverage (+): 0. Max coverage (-): 0

Region: chr21 29762533-29762548. Max. coverage (+): 0. Max coverage (-): 0

Region: chr21 29762549-29762565. Max. coverage (+): 0. Max coverage (-): 0

Region: chr21 29762566-29762581. Max. coverage (+): 0. Max coverage (-): 0

Region: chr21 29762582-29762597. Max. coverage (+): 0. Max coverage (-): 0

Region: chr21 29762598-29762613. Max. coverage (+): 0. Max coverage (-): 0

Region: chr21 29762614-29762630. Max. coverage (+): 0. Max coverage (-): 0

Region: chr21 29762631-29762646. Max. coverage (+): 0. Max coverage (-): 0

Region: chr21 29762647-29762662. Max. coverage (+): 0. Max coverage (-): 0

Region: chr21 29762663-29762678. Max. coverage (+): 0. Max coverage (-): 0

Region: chr21 29762679-29762695. Max. coverage (+): 0. Max coverage (-): 0

Region: chr21 29762696-29762711. Max. coverage (+): 0. Max coverage (-): 0

Region: chr21 29762712-29762727. Max. coverage (+): 0. Max coverage (-): 0

Region: chr21 29762728-29762743. Max. coverage (+): 0. Max coverage (-): 0

Region: chr21 29762744-29762760. Max. coverage (+): 0. Max coverage (-): 0

Region: chr21 29762761-29762776. Max. coverage (+): 0. Max coverage (-): 0

Region: chr21 29762777-29762792. Max. coverage (+): 0. Max coverage (-): 0

Region: chr21 29762793-29762808. Max. coverage (+): 0. Max coverage (-): 0

Region: chr21 29762809-29762825. Max. coverage (+): 0. Max coverage (-): 0

Region: chr21 29762826-29762841. Max. coverage (+): 0. Max coverage (-): 0

Region: chr21 29762842-29762857. Max. coverage (+): 11. Max coverage (-): 0

Region: chr21 29762858-29762873. Max. coverage (+): 6.21. Max coverage (-): 0

Region: chr21 29762874-29762890. Max. coverage (+): 0. Max coverage (-): 0

Region: chr21 29762891-29762906. Max. coverage (+): 15.27. Max coverage (-): 0

Region: chr21 29762907-29762922. Max. coverage (+): 15.27. Max coverage (-): 0

Region: chr21 29762923-29762938. Max. coverage (+): 5.58. Max coverage (-): 0

Region: chr21 29762939-29762955. Max. coverage (+): 3.55. Max coverage (-): 0

Region: chr21 29762956-29762971. Max. coverage (+): 3.98. Max coverage (-): 0

Region: chr21 29762972-29762987. Max. coverage (+): 0. Max coverage (-): 0

Region: chr21 29762988-29763003. Max. coverage (+): 0. Max coverage (-): 0

Region: chr21 29763004-29763020. Max. coverage (+): 0. Max coverage (-): 0

Region: chr21 29763021-29763036. Max. coverage (+): 0. Max coverage (-): 0

Region: chr21 29763037-29763052. Max. coverage (+): 1.85. Max coverage (-): 0

Region: chr21 29763053-29763068. Max. coverage (+): 1.85. Max coverage (-): 0

Region: chr21 29763069-29763085. Max. coverage (+): 0. Max coverage (-): 0

Region: chr21 29763086-29763101. Max. coverage (+): 0. Max coverage (-): 0

Region: chr21 29763102-29763117. Max. coverage (+): 0. Max coverage (-): 0

Region: chr21 29763118-29763133. Max. coverage (+): 0. Max coverage (-): 0

Region: chr21 29763134-29763150. Max. coverage (+): 0. Max coverage (-): 0

Region: chr21 29763151-29763166. Max. coverage (+): 0. Max coverage (-): 0

Region: chr21 29763167-29763182. Max. coverage (+): 0. Max coverage (-): 0

Region: chr21 29763183-29763198. Max. coverage (+): 0. Max coverage (-): 0

Region: chr21 29763199-29763215. Max. coverage (+): 0. Max coverage (-): 0

Region: chr21 29763216-29763231. Max. coverage (+): 0. Max coverage (-): 0

Region: chr21 29763232-29763247. Max. coverage (+): 0. Max coverage (-): 0

Region: chr21 29763248-29763263. Max. coverage (+): 0. Max coverage (-): 0

Region: chr21 29763264-29763280. Max. coverage (+): 0. Max coverage (-): 0

Region: chr21 29763281-29763296. Max. coverage (+): 0. Max coverage (-): 0

Region: chr21 29763297-29763312. Max. coverage (+): 2.84. Max coverage (-): 0

Region: chr21 29763313-29763328. Max. coverage (+): 20.86. Max coverage (-): 0

Region: chr21 29763329-29763345. Max. coverage (+): 9.15. Max coverage (-): 0

Region: chr21 29763346-29763361. Max. coverage (+): 0. Max coverage (-): 0

Region: chr21 29763362-29763377. Max. coverage (+): 0. Max coverage (-): 0

Region: chr21 29763378-29763393. Max. coverage (+): 0. Max coverage (-): 0

Region: chr21 29763394-29763410. Max. coverage (+): 0. Max coverage (-): 0

Region: chr21 29763411-29763426. Max. coverage (+): 0. Max coverage (-): 0

Region: chr21 29763427-29763442. Max. coverage (+): 0. Max coverage (-): 0

Region: chr21 29763443-29763458. Max. coverage (+): 2.28. Max coverage (-): 0

Region: chr21 29763459-29763475. Max. coverage (+): 2.28. Max coverage (-): 0

Region: chr21 29763476-29763491. Max. coverage (+): 0. Max coverage (-): 0

Region: chr21 29763492-29763507. Max. coverage (+): 0. Max coverage (-): 0

Region: chr21 29763508-29763523. Max. coverage (+): 0. Max coverage (-): 0

Region: chr21 29763524-29763540. Max. coverage (+): 0. Max coverage (-): 0

Region: chr21 29763541-29763556. Max. coverage (+): 0. Max coverage (-): 0

Region: chr21 29763557-29763572. Max. coverage (+): 0. Max coverage (-): 0

Region: chr21 29763573-29763588. Max. coverage (+): 0. Max coverage (-): 0

Region: chr21 29763589-29763605. Max. coverage (+): 0. Max coverage (-): 0

Region: chr21 29763606-29763621. Max. coverage (+): 0. Max coverage (-): 0

Region: chr21 29763622-29763637. Max. coverage (+): 0. Max coverage (-): 0

Region: chr21 29763638-29763653. Max. coverage (+): 0. Max coverage (-): 0

Region: chr21 29763654-29763670. Max. coverage (+): 0. Max coverage (-): 0

Region: chr21 29763671-29763686. Max. coverage (+): 0. Max coverage (-): 0

Region: chr21 29763687-29763702. Max. coverage (+): 0. Max coverage (-): 0

Region: chr21 29763703-29763718. Max. coverage (+): 0. Max coverage (-): 0

Region: chr21 29763719-29763735. Max. coverage (+): 0. Max coverage (-): 0

Region: chr21 29763736-29763751. Max. coverage (+): 0. Max coverage (-): 0

Region: chr21 29763752-29763767. Max. coverage (+): 0. Max coverage (-): 0

Region: chr21 29763768-29763783. Max. coverage (+): 0. Max coverage (-): 0

Region: chr21 29763784-29763800. Max. coverage (+): 0. Max coverage (-): 0

Region: chr21 29763801-29763816. Max. coverage (+): 0. Max coverage (-): 0

Region: chr21 29763817-29763832. Max. coverage (+): 5.76. Max coverage (-): 0

Region: chr21 29763833-29763848. Max. coverage (+): 0. Max coverage (-): 0

Region: chr21 29763849-29763865. Max. coverage (+): 0. Max coverage (-): 0

Region: chr21 29763866-29763881. Max. coverage (+): 0. Max coverage (-): 0

Region: chr21 29763882-29763897. Max. coverage (+): 0. Max coverage (-): 0

Region: chr21 29763898-29763913. Max. coverage (+): 0. Max coverage (-): 0

Region: chr21 29763914-29763930. Max. coverage (+): 0. Max coverage (-): 0

Region: chr21 29763931-29763946. Max. coverage (+): 0. Max coverage (-): 0

Region: chr21 29763947-29763962. Max. coverage (+): 0. Max coverage (-): 0

Region: chr21 29763963-29763978. Max. coverage (+): 0. Max coverage (-): 0

Region: chr21 29763979-29763995. Max. coverage (+): 0. Max coverage (-): 0

Region: chr21 29763996-29764011. Max. coverage (+): 0. Max coverage (-): 0

Region: chr21 29764012-29764027. Max. coverage (+): 0. Max coverage (-): 0

Region: chr21 29764028-29764043. Max. coverage (+): 0. Max coverage (-): 0

Region: chr21 29764044-29764060. Max. coverage (+): 0. Max coverage (-): 0

Region: chr21 29764061-29764076. Max. coverage (+): 0. Max coverage (-): 0

Region: chr21 29764077-29764092. Max. coverage (+): 0. Max coverage (-): 0

Region: chr21 29764093-29764108. Max. coverage (+): 0. Max coverage (-): 0

Region: chr21 29764109-29764125. Max. coverage (+): 0. Max coverage (-): 0

Region: chr21 29764126-29764141. Max. coverage (+): 0. Max coverage (-): 0

Region: chr21 29764142-29764157. Max. coverage (+): 0. Max coverage (-): 0

Region: chr21 29764158-29764174. Max. coverage (+): 0. Max coverage (-): 0

Region: chr21 29764175-29764190. Max. coverage (+): 0. Max coverage (-): 0

Region: chr21 29764191-29764206. Max. coverage (+): 0. Max coverage (-): 0

Region: chr21 29764207-29764222. Max. coverage (+): 0. Max coverage (-): 0

Region: chr21 29764223-29764239. Max. coverage (+): 6.13. Max coverage (-): 0

Region: chr21 29764240-29764255. Max. coverage (+): 6.13. Max coverage (-): 0

Region: chr21 29764256-29764271. Max. coverage (+): 0. Max coverage (-): 0

Region: chr21 29764272-29764287. Max. coverage (+): 0. Max coverage (-): 0

Region: chr21 29764288-29764304. Max. coverage (+): 0. Max coverage (-): 0

Region: chr21 29764305-29764320. Max. coverage (+): 0. Max coverage (-): 0

Region: chr21 29764321-29764336. Max. coverage (+): 0. Max coverage (-): 0

Region: chr21 29764337-29764352. Max. coverage (+): 13.16. Max coverage (-): 0

Region: chr21 29764353-29764369. Max. coverage (+): 0.68. Max coverage (-): 0

Region: chr21 29764370-29764385. Max. coverage (+): 10.39. Max coverage (-): 0

Region: chr21 29764386-29764401. Max. coverage (+): 0. Max coverage (-): 0

Region: chr21 29764402-29764417. Max. coverage (+): 0. Max coverage (-): 0

Region: chr21 29764418-29764434. Max. coverage (+): 0. Max coverage (-): 0

Region: chr21 29764435-29764450. Max. coverage (+): 0. Max coverage (-): 0

Region: chr21 29764451-29764466. Max. coverage (+): 0. Max coverage (-): 0

Region: chr21 29764467-29764482. Max. coverage (+): 0. Max coverage (-): 0

Region: chr21 29764483-29764499. Max. coverage (+): 0. Max coverage (-): 0

Region: chr21 29764500-29764515. Max. coverage (+): 0. Max coverage (-): 0

Region: chr21 29764516-29764531. Max. coverage (+): 0. Max coverage (-): 0

Region: chr21 29764532-29764547. Max. coverage (+): 0. Max coverage (-): 0

Region: chr21 29764548-29764564. Max. coverage (+): 0. Max coverage (-): 0

Region: chr21 29764565-29764580. Max. coverage (+): 0. Max coverage (-): 0

Region: chr21 29764581-29764596. Max. coverage (+): 0. Max coverage (-): 0

Region: chr21 29764597-29764612. Max. coverage (+): 0. Max coverage (-): 0

Region: chr21 29764613-29764629. Max. coverage (+): 0. Max coverage (-): 0

Region: chr21 29764630-29764645. Max. coverage (+): 1.77. Max coverage (-): 0

Region: chr21 29764646-29764661. Max. coverage (+): 0. Max coverage (-): 0

Region: chr21 29764662-29764677. Max. coverage (+): 0. Max coverage (-): 0

Region: chr21 29764678-29764694. Max. coverage (+): 0. Max coverage (-): 0

Region: chr21 29764695-29764710. Max. coverage (+): 0. Max coverage (-): 0

Region: chr21 29764711-29764726. Max. coverage (+): 0. Max coverage (-): 0

Region: chr21 29764727-29764742. Max. coverage (+): 0. Max coverage (-): 0

Region: chr21 29764743-29764759. Max. coverage (+): 0. Max coverage (-): 0

Region: chr21 29764760-29764775. Max. coverage (+): 0. Max coverage (-): 0

Region: chr21 29764776-29764791. Max. coverage (+): 2.42. Max coverage (-): 0

Region: chr21 29764792-29764807. Max. coverage (+): 0. Max coverage (-): 0

Region: chr21 29764808-29764824. Max. coverage (+): 0. Max coverage (-): 0

Region: chr21 29764825-29764840. Max. coverage (+): 1.15. Max coverage (-): 0

Region: chr21 29764841-29764856. Max. coverage (+): 1.15. Max coverage (-): 0

Region: chr21 29764857-29764872. Max. coverage (+): 0. Max coverage (-): 0

Region: chr21 29764873-29764889. Max. coverage (+): 0. Max coverage (-): 0

Region: chr21 29764890-29764905. Max. coverage (+): 0. Max coverage (-): 0

Region: chr21 29764906-29764921. Max. coverage (+): 0. Max coverage (-): 0

Region: chr21 29764922-29764937. Max. coverage (+): 0. Max coverage (-): 0

Region: chr21 29764938-29764954. Max. coverage (+): 3.93. Max coverage (-): 0

Region: chr21 29764955-29764970. Max. coverage (+): 5.76. Max coverage (-): 0

Region: chr21 29764971-29764986. Max. coverage (+): 0. Max coverage (-): 0

Region: chr21 29764987-29765002. Max. coverage (+): 0. Max coverage (-): 0

Region: chr21 29765003-29765019. Max. coverage (+): 0. Max coverage (-): 0

Region: chr21 29765020-29765035. Max. coverage (+): 0. Max coverage (-): 0

Region: chr21 29765036-29765051. Max. coverage (+): 0. Max coverage (-): 0

Region: chr21 29765052-29765067. Max. coverage (+): 0. Max coverage (-): 0

Region: chr21 29765068-29765084. Max. coverage (+): 0. Max coverage (-): 0

Region: chr21 29765085-29765100. Max. coverage (+): 0. Max coverage (-): 0

Region: chr21 29765101-29765116. Max. coverage (+): 0. Max coverage (-): 0

Region: chr21 29765117-29765132. Max. coverage (+): 0. Max coverage (-): 0

Region: chr21 29765133-29765149. Max. coverage (+): 1.17. Max coverage (-): 0

Region: chr21 29765150-29765165. Max. coverage (+): 11.36. Max coverage (-): 0

Region: chr21 29765166-29765181. Max. coverage (+): 0. Max coverage (-): 0

Region: chr21 29765182-29765197. Max. coverage (+): 0. Max coverage (-): 0

Region: chr21 29765198-29765214. Max. coverage (+): 0. Max coverage (-): 0

Region: chr21 29765215-29765230. Max. coverage (+): 0. Max coverage (-): 0

Region: chr21 29765231-29765246. Max. coverage (+): 0. Max coverage (-): 0

Region: chr21 29765247-29765262. Max. coverage (+): 0. Max coverage (-): 0

Region: chr21 29765263-29765279. Max. coverage (+): 0. Max coverage (-): 0

Region: chr21 29765280-29765295. Max. coverage (+): 0. Max coverage (-): 0

Region: chr21 29765296-29765311. Max. coverage (+): 18.65. Max coverage (-): 0

Region: chr21 29765312-29765327. Max. coverage (+): 0. Max coverage (-): 0

Region: chr21 29765328-29765344. Max. coverage (+): 0. Max coverage (-): 0

Region: chr21 29765345-29765360. Max. coverage (+): 0. Max coverage (-): 0

Region: chr21 29765361-29765376. Max. coverage (+): 0. Max coverage (-): 0

Region: chr21 29765377-29765392. Max. coverage (+): 0. Max coverage (-): 0

Region: chr21 29765393-29765409. Max. coverage (+): 0. Max coverage (-): 0

Region: chr21 29765410-29765425. Max. coverage (+): 0. Max coverage (-): 0

Region: chr21 29765426-29765441. Max. coverage (+): 0. Max coverage (-): 0

Region: chr21 29765442-29765457. Max. coverage (+): 0. Max coverage (-): 0

Region: chr21 29765458-29765474. Max. coverage (+): 0. Max coverage (-): 0

Region: chr21 29765475-29765490. Max. coverage (+): 0. Max coverage (-): 0

Region: chr21 29765491-29765506. Max. coverage (+): 16.04. Max coverage (-): 0

Region: chr21 29765507-29765522. Max. coverage (+): 0. Max coverage (-): 0

Region: chr21 29765523-29765539. Max. coverage (+): 0. Max coverage (-): 0

Region: chr21 29765540-29765555. Max. coverage (+): 0. Max coverage (-): 0

Region: chr21 29765556-29765571. Max. coverage (+): 0. Max coverage (-): 0

Region: chr21 29765572-29765587. Max. coverage (+): 0. Max coverage (-): 0

Region: chr21 29765588-29765604. Max. coverage (+): 0. Max coverage (-): 0

Region: chr21 29765605-29765620. Max. coverage (+): 0. Max coverage (-): 0

Region: chr21 29765621-29765636. Max. coverage (+): 7.22. Max coverage (-): 0

Region: chr21 29765637-29765652. Max. coverage (+): 7.22. Max coverage (-): 0

Region: chr21 29765653-29765669. Max. coverage (+): 5.49. Max coverage (-): 0

Region: chr21 29765670-29765685. Max. coverage (+): 5.49. Max coverage (-): 0

Region: chr21 29765686-29765701. Max. coverage (+): 0. Max coverage (-): 0

Region: chr21 29765702-29765717. Max. coverage (+): 0. Max coverage (-): 0

Region: chr21 29765718-29765734. Max. coverage (+): 0. Max coverage (-): 0

Region: chr21 29765735-29765750. Max. coverage (+): 0. Max coverage (-): 0

Region: chr21 29765751-29765766. Max. coverage (+): 0. Max coverage (-): 0

Region: chr21 29765767-29765782. Max. coverage (+): 3.41. Max coverage (-): 0

Region: chr21 29765783-29765799. Max. coverage (+): 3.41. Max coverage (-): 0

Region: chr21 29765800-29765815. Max. coverage (+): 0. Max coverage (-): 0

Region: chr21 29765816-29765831. Max. coverage (+): 0. Max coverage (-): 0

Region: chr21 29765832-29765847. Max. coverage (+): 0. Max coverage (-): 0

Region: chr21 29765848-29765864. Max. coverage (+): 1.93. Max coverage (-): 0

Region: chr21 29765865-29765880. Max. coverage (+): 0. Max coverage (-): 0

Region: chr21 29765881-29765896. Max. coverage (+): 0. Max coverage (-): 0

Region: chr21 29765897-29765912. Max. coverage (+): 2.78. Max coverage (-): 0

Region: chr21 29765913-29765929. Max. coverage (+): 2.78. Max coverage (-): 0

Region: chr21 29765930-29765945. Max. coverage (+): 0. Max coverage (-): 0

Region: chr21 29765946-29765961. Max. coverage (+): 0. Max coverage (-): 0

Region: chr21 29765962-29765977. Max. coverage (+): 0. Max coverage (-): 0

Region: chr21 29765978-29765994. Max. coverage (+): 0. Max coverage (-): 0

Region: chr21 29765995-29766010. Max. coverage (+): 0. Max coverage (-): 0

Region: chr21 29766011-29766026. Max. coverage (+): 2.93. Max coverage (-): 0

Region: chr21 29766027-29766042. Max. coverage (+): 2.93. Max coverage (-): 0

Region: chr21 29766043-29766059. Max. coverage (+): 0. Max coverage (-): 0

Region: chr21 29766060-29766075. Max. coverage (+): 0. Max coverage (-): 0

Region: chr21 29766076-29766091. Max. coverage (+): 0. Max coverage (-): 0

Region: chr21 29766092-29766107. Max. coverage (+): 0. Max coverage (-): 0

Region: chr21 29766108-29766124. Max. coverage (+): 0. Max coverage (-): 0

Region: chr21 29766125-29766140. Max. coverage (+): 0. Max coverage (-): 0

Region: chr21 29766141-29766156. Max. coverage (+): 0. Max coverage (-): 0

Region: chr21 29766157-29766173. Max. coverage (+): 0. Max coverage (-): 0

Region: chr21 29766174-29766189. Max. coverage (+): 0. Max coverage (-): 0

Region: chr21 29766190-29766205. Max. coverage (+): 0. Max coverage (-): 0

Region: chr21 29766206-29766221. Max. coverage (+): 16.32. Max coverage (-): 0

Region: chr21 29766222-29766238. Max. coverage (+): 8.25. Max coverage (-): 0

Region: chr21 29766239-29766254. Max. coverage (+): 0. Max coverage (-): 0

Region: chr21 29766255-29766270. Max. coverage (+): 0. Max coverage (-): 0

Region: chr21 29766271-29766286. Max. coverage (+): 0. Max coverage (-): 0

Region: chr21 29766287-29766303. Max. coverage (+): 0. Max coverage (-): 0

Region: chr21 29766304-29766319. Max. coverage (+): 0. Max coverage (-): 0

Region: chr21 29766320-29766335. Max. coverage (+): 3.12. Max coverage (-): 0

Region: chr21 29766336-29766351. Max. coverage (+): 3.12. Max coverage (-): 0

Region: chr21 29766352-29766368. Max. coverage (+): 0. Max coverage (-): 0

Region: chr21 29766369-29766384. Max. coverage (+): 0. Max coverage (-): 0

Region: chr21 29766385-29766400. Max. coverage (+): 5.75. Max coverage (-): 0

Region: chr21 29766401-29766416. Max. coverage (+): 5.75. Max coverage (-): 0

Region: chr21 29766417-29766433. Max. coverage (+): 0. Max coverage (-): 0

Region: chr21 29766434-29766449. Max. coverage (+): 0. Max coverage (-): 0

Region: chr21 29766450-29766465. Max. coverage (+): 9.18. Max coverage (-): 0

Region: chr21 29766466-29766481. Max. coverage (+): 7.28. Max coverage (-): 0

Region: chr21 29766482-29766498. Max. coverage (+): 6.21. Max coverage (-): 0

Region: chr21 29766499-29766514. Max. coverage (+): 0. Max coverage (-): 0

Region: chr21 29766515-29766530. Max. coverage (+): 0. Max coverage (-): 0

Region: chr21 29766531-29766546. Max. coverage (+): 0. Max coverage (-): 0

Region: chr21 29766547-29766563. Max. coverage (+): 0. Max coverage (-): 0

Region: chr21 29766564-29766579. Max. coverage (+): 0. Max coverage (-): 0

Region: chr21 29766580-29766595. Max. coverage (+): 0. Max coverage (-): 0

Region: chr21 29766596-29766611. Max. coverage (+): 0. Max coverage (-): 0

Region: chr21 29766612-29766628. Max. coverage (+): 0. Max coverage (-): 0

Region: chr21 29766629-29766644. Max. coverage (+): 0. Max coverage (-): 0

Region: chr21 29766645-29766660. Max. coverage (+): 0. Max coverage (-): 0

Region: chr21 29766661-29766676. Max. coverage (+): 0. Max coverage (-): 0

Region: chr21 29766677-29766693. Max. coverage (+): 0. Max coverage (-): 0

Region: chr21 29766694-29766709. Max. coverage (+): 0. Max coverage (-): 0

Region: chr21 29766710-29766725. Max. coverage (+): 0. Max coverage (-): 0

Region: chr21 29766726-29766741. Max. coverage (+): 0. Max coverage (-): 0

Region: chr21 29766742-29766758. Max. coverage (+): 0. Max coverage (-): 0

Region: chr21 29766759-29766774. Max. coverage (+): 0. Max coverage (-): 0

Region: chr21 29766775-29766790. Max. coverage (+): 0. Max coverage (-): 0

Region: chr21 29766791-29766806. Max. coverage (+): 11.36. Max coverage (-): 0

Region: chr21 29766807-29766823. Max. coverage (+): 0. Max coverage (-): 0

Region: chr21 29766824-29766839. Max. coverage (+): 0. Max coverage (-): 0

Region: chr21 29766840-29766855. Max. coverage (+): 0. Max coverage (-): 0

Region: chr21 29766856-29766871. Max. coverage (+): 0. Max coverage (-): 0

Region: chr21 29766872-29766888. Max. coverage (+): 0. Max coverage (-): 0

Region: chr21 29766889-29766904. Max. coverage (+): 0. Max coverage (-): 0

Region: chr21 29766905-29766920. Max. coverage (+): 0. Max coverage (-): 0

Region: chr21 29766921-29766936. Max. coverage (+): 0. Max coverage (-): 0

Region: chr21 29766937-29766953. Max. coverage (+): 0. Max coverage (-): 0

Region: chr21 29766954-29766969. Max. coverage (+): 1.83. Max coverage (-): 0

Region: chr21 29766970-29766985. Max. coverage (+): 2.75. Max coverage (-): 0

Region: chr21 29766986-29767001. Max. coverage (+): 0. Max coverage (-): 0

Region: chr21 29767002-29767018. Max. coverage (+): 0. Max coverage (-): 0

Region: chr21 29767019-29767034. Max. coverage (+): 0. Max coverage (-): 0

Region: chr21 29767035-29767050. Max. coverage (+): 0. Max coverage (-): 0

Region: chr21 29767051-29767066. Max. coverage (+): 0. Max coverage (-): 0

Region: chr21 29767067-29767083. Max. coverage (+): 0. Max coverage (-): 0

Region: chr21 29767084-29767099. Max. coverage (+): 0. Max coverage (-): 0

Region: chr21 29767100-29767115. Max. coverage (+): 0. Max coverage (-): 0

Region: chr21 29767116-29767131. Max. coverage (+): 0. Max coverage (-): 0

Region: chr21 29767132-29767148. Max. coverage (+): 0. Max coverage (-): 0

Region: chr21 29767149-29767164. Max. coverage (+): 0. Max coverage (-): 0

Region: chr21 29767165-29767180. Max. coverage (+): 0. Max coverage (-): 0

Region: chr21 29767181-29767196. Max. coverage (+): 0. Max coverage (-): 0

Region: chr21 29767197-29767213. Max. coverage (+): 0. Max coverage (-): 0

Region: chr21 29767214-29767229. Max. coverage (+): 0. Max coverage (-): 0

Region: chr21 29767230-29767245. Max. coverage (+): 0. Max coverage (-): 0

Region: chr21 29767246-29767261. Max. coverage (+): 0. Max coverage (-): 0

Region: chr21 29767262-29767278. Max. coverage (+): 0. Max coverage (-): 0

Region: chr21 29767279-29767294. Max. coverage (+): 0. Max coverage (-): 0

Region: chr21 29767295-29767310. Max. coverage (+): 0. Max coverage (-): 0

Region: chr21 29767311-29767326. Max. coverage (+): 0. Max coverage (-): 0

Region: chr21 29767327-29767343. Max. coverage (+): 0. Max coverage (-): 0

Region: chr21 29767344-29767359. Max. coverage (+): 0. Max coverage (-): 0

Region: chr21 29767360-29767375. Max. coverage (+): 6.5. Max coverage (-): 0

Region: chr21 29767376-29767391. Max. coverage (+): 6.5. Max coverage (-): 0

Region: chr21 29767392-29767408. Max. coverage (+): 0. Max coverage (-): 0

Region: chr21 29767409-29767424. Max. coverage (+): 0. Max coverage (-): 0

Region: chr21 29767425-29767440. Max. coverage (+): 0. Max coverage (-): 0

Region: chr21 29767441-29767456. Max. coverage (+): 0. Max coverage (-): 0

Region: chr21 29767457-29767473. Max. coverage (+): 0. Max coverage (-): 0

Region: chr21 29767474-29767489. Max. coverage (+): 0. Max coverage (-): 0

Region: chr21 29767490-29767505. Max. coverage (+): 0. Max coverage (-): 0

Region: chr21 29767506-29767521. Max. coverage (+): 0. Max coverage (-): 0

Region: chr21 29767522-29767538. Max. coverage (+): 0. Max coverage (-): 0

Region: chr21 29767539-29767554. Max. coverage (+): 0. Max coverage (-): 0

Region: chr21 29767555-29767570. Max. coverage (+): 0. Max coverage (-): 0

Region: chr21 29767571-29767586. Max. coverage (+): 0. Max coverage (-): 0

Region: chr21 29767587-29767603. Max. coverage (+): 0. Max coverage (-): 0

Region: chr21 29767604-29767619. Max. coverage (+): 0. Max coverage (-): 0

Region: chr21 29767620-29767635. Max. coverage (+): 0. Max coverage (-): 0

Region: chr21 29767636-29767651. Max. coverage (+): 0. Max coverage (-): 0

Region: chr21 29767652-29767668. Max. coverage (+): 0. Max coverage (-): 0

Region: chr21 29767669-29767684. Max. coverage (+): 0. Max coverage (-): 0

Region: chr21 29767685-29767700. Max. coverage (+): 0. Max coverage (-): 0

Region: chr21 29767701-29767716. Max. coverage (+): 0. Max coverage (-): 0

Region: chr21 29767717-29767733. Max. coverage (+): 0. Max coverage (-): 0

Region: chr21 29767734-29767749. Max. coverage (+): 0. Max coverage (-): 0

Region: chr21 29767750-29767765. Max. coverage (+): 0. Max coverage (-): 0

Region: chr21 29767766-29767781. Max. coverage (+): 0. Max coverage (-): 0

Region: chr21 29767782-29767798. Max. coverage (+): 0. Max coverage (-): 0

Region: chr21 29767799-29767814. Max. coverage (+): 2.33. Max coverage (-): 0

Region: chr21 29767815-29767830. Max. coverage (+): 2.33. Max coverage (-): 0

Region: chr21 29767831-29767846. Max. coverage (+): 0. Max coverage (-): 0

Region: chr21 29767847-29767863. Max. coverage (+): 0. Max coverage (-): 0

Region: chr21 29767864-29767879. Max. coverage (+): 0. Max coverage (-): 0

Region: chr21 29767880-29767895. Max. coverage (+): 0. Max coverage (-): 0

Region: chr21 29767896-29767911. Max. coverage (+): 0. Max coverage (-): 0

Region: chr21 29767912-29767928. Max. coverage (+): 0. Max coverage (-): 0

Region: chr21 29767929-29767944. Max. coverage (+): 0. Max coverage (-): 0

Region: chr21 29767945-29767960. Max. coverage (+): 0. Max coverage (-): 0

Region: chr21 29767961-29767976. Max. coverage (+): 0. Max coverage (-): 0

Region: chr21 29767977-29767993. Max. coverage (+): 0. Max coverage (-): 0

Region: chr21 29767994-29768009. Max. coverage (+): 0. Max coverage (-): 0

Region: chr21 29768010-29768025. Max. coverage (+): 0. Max coverage (-): 0

Region: chr21 29768026-29768041. Max. coverage (+): 0. Max coverage (-): 0

Region: chr21 29768042-29768058. Max. coverage (+): 0. Max coverage (-): 0

Region: chr21 29768059-29768074. Max. coverage (+): 0. Max coverage (-): 0

Region: chr21 29768075-29768090. Max. coverage (+): 0. Max coverage (-): 0

Region: chr21 29768091-29768106. Max. coverage (+): 0. Max coverage (-): 0

Region: chr21 29768107-29768123. Max. coverage (+): 0. Max coverage (-): 0

Region: chr21 29768124-29768139. Max. coverage (+): 0. Max coverage (-): 0

Region: chr21 29768140-29768155. Max. coverage (+): 0. Max coverage (-): 0

Region: chr21 29768156-29768171. Max. coverage (+): 0. Max coverage (-): 0

Region: chr21 29768172-29768188. Max. coverage (+): 0. Max coverage (-): 0

Region: chr21 29768189-29768204. Max. coverage (+): 0. Max coverage (-): 0

Region: chr21 29768205-29768220. Max. coverage (+): 0. Max coverage (-): 0

Region: chr21 29768221-29768237. Max. coverage (+): 0. Max coverage (-): 0

Region: chr21 29768238-29768253. Max. coverage (+): 0. Max coverage (-): 0

Region: chr21 29768254-29768269. Max. coverage (+): 0. Max coverage (-): 0

Region: chr21 29768270-29768285. Max. coverage (+): 0. Max coverage (-): 0

Region: chr21 29768286-29768302. Max. coverage (+): 1.19. Max coverage (-): 0

Region: chr21 29768303-29768318. Max. coverage (+): 0. Max coverage (-): 0

Region: chr21 29768319-29768334. Max. coverage (+): 0. Max coverage (-): 0

Region: chr21 29768335-29768350. Max. coverage (+): 0. Max coverage (-): 0

Region: chr21 29768351-29768367. Max. coverage (+): 0. Max coverage (-): 0

Region: chr21 29768368-29768383. Max. coverage (+): 0. Max coverage (-): 0

Region: chr21 29768384-29768399. Max. coverage (+): 0. Max coverage (-): 0

Region: chr21 29768400-29768415. Max. coverage (+): 0. Max coverage (-): 0

Region: chr21 29768416-29768432. Max. coverage (+): 0. Max coverage (-): 0

Region: chr21 29768433-29768448. Max. coverage (+): 0. Max coverage (-): 0

Region: chr21 29768449-29768464. Max. coverage (+): 0. Max coverage (-): 0

Region: chr21 29768465-29768480. Max. coverage (+): 0. Max coverage (-): 0

Region: chr21 29768481-29768497. Max. coverage (+): 0. Max coverage (-): 0

Region: chr21 29768498-29768513. Max. coverage (+): 0. Max coverage (-): 0

Region: chr21 29768514-29768529. Max. coverage (+): 0. Max coverage (-): 0

Region: chr21 29768530-29768545. Max. coverage (+): 0. Max coverage (-): 0

Region: chr21 29768546-29768562. Max. coverage (+): 0. Max coverage (-): 0

Region: chr21 29768563-29768578. Max. coverage (+): 0. Max coverage (-): 0

Region: chr21 29768579-29768594. Max. coverage (+): 0. Max coverage (-): 0

Region: chr21 29768595-29768610. Max. coverage (+): 0. Max coverage (-): 0

Region: chr21 29768611-29768627. Max. coverage (+): 0. Max coverage (-): 0

Region: chr21 29768628-29768643. Max. coverage (+): 0. Max coverage (-): 0

Region: chr21 29768644-29768659. Max. coverage (+): 0. Max coverage (-): 0

Region: chr21 29768660-29768675. Max. coverage (+): 0. Max coverage (-): 0

Region: chr21 29768676-29768692. Max. coverage (+): 0. Max coverage (-): 0

Region: chr21 29768693-29768708. Max. coverage (+): 0. Max coverage (-): 0

Region: chr21 29768709-29768724. Max. coverage (+): 0. Max coverage (-): 0

Region: chr21 29768725-29768740. Max. coverage (+): 0. Max coverage (-): 0

Region: chr21 29768741-29768757. Max. coverage (+): 0. Max coverage (-): 0

Region: chr21 29768758-29768773. Max. coverage (+): 0. Max coverage (-): 0

Region: chr21 29768774-29768789. Max. coverage (+): 0. Max coverage (-): 0

Region: chr21 29768790-29768805. Max. coverage (+): 0. Max coverage (-): 0

Region: chr21 29768806-29768822. Max. coverage (+): 0. Max coverage (-): 0

Region: chr21 29768823-29768838. Max. coverage (+): 0. Max coverage (-): 0

Region: chr21 29768839-29768854. Max. coverage (+): 0. Max coverage (-): 0

Region: chr21 29768855-29768870. Max. coverage (+): 0. Max coverage (-): 0

Region: chr21 29768871-29768887. Max. coverage (+): 0. Max coverage (-): 0

Region: chr21 29768888-29768903. Max. coverage (+): 0. Max coverage (-): 0

Region: chr21 29768904-29768919. Max. coverage (+): 0. Max coverage (-): 0

Region: chr21 29768920-29768935. Max. coverage (+): 0. Max coverage (-): 0

Region: chr21 29768936-29768952. Max. coverage (+): 0. Max coverage (-): 0

Region: chr21 29768953-29768968. Max. coverage (+): 0. Max coverage (-): 0

Region: chr21 29768969-29768984. Max. coverage (+): 0. Max coverage (-): 0

Region: chr21 29768985-29769000. Max. coverage (+): 0. Max coverage (-): 0

Region: chr21 29769001-29769017. Max. coverage (+): 0. Max coverage (-): 0

Region: chr21 29769018-29769033. Max. coverage (+): 0. Max coverage (-): 0

Region: chr21 29769034-29769049. Max. coverage (+): 0. Max coverage (-): 0

Region: chr21 29769050-29769065. Max. coverage (+): 0. Max coverage (-): 0

Region: chr21 29769066-29769082. Max. coverage (+): 0. Max coverage (-): 0

Region: chr21 29769083-29769098. Max. coverage (+): 0. Max coverage (-): 0

Region: chr21 29769099-29769114. Max. coverage (+): 0. Max coverage (-): 0

Region: chr21 29769115-29769130. Max. coverage (+): 0. Max coverage (-): 0

Region: chr21 29769131-29769147. Max. coverage (+): 0. Max coverage (-): 0

Region: chr21 29769148-29769163. Max. coverage (+): 6.08. Max coverage (-): 0

Region: chr21 29769164-29769179. Max. coverage (+): 6.08. Max coverage (-): 0

Region: chr21 29769180-29769195. Max. coverage (+): 4.13. Max coverage (-): 0

Region: chr21 29769196-. Max. coverage (+): 0. Max coverage (-): 0

RepeatMasker Color Code

**+**

100-98% Identity

<98-95% Identity

<95-90% Identity

<90-85% Identity

<85-80% Identity

<80-75% Identity

<75-70% Identity

<70% Identity

**-**

Gene Set Color Code

**+**

Gene

Pseudogene

**-**

Topology/Coverage Color Code

Coverage Plus Strand

Coverage Minus Strand

Mainstrand: Plus

Mainstrand: Minus

Complementary Strand

Flanking Region  
(if option -flank >0)

Gene Set Annotation  

**1. (protein coding, ENSBTAG00000003957) Tr:00000005175 Ex:1**: 29766181-29766253 (-)  
**2. (protein coding, ENSBTAG00000003957) Tr:00000005175 Ex:2**: 29765462-29765730 (-)  
**3. (protein coding, ENSBTAG00000003957) Tr:00000005175 Ex:3**: 29764983-29765050 (-)  
**4. (protein coding, ENSBTAG00000003957) Tr:00000005175 Ex:4**: 29763705-29763898 (-)  
**5. (protein coding, ENSBTAG00000003957) Tr:00000005175 Ex:5**: 29762148-29762413 (-)

  
RepeatMasker Annotation  

**1. CHRL1\_BT**: 29761379-29761527 (-), Divergence to consensus: 23.5%  
**2. BOV-A2**: 29762591-29762793 (-), Divergence to consensus: 11.8%  
**3. AT\_rich**: 29766505-29766541 (+), Divergence to consensus: 70.3%  
**4. SINE2-2\_BT**: 29766565-29766677 (-), Divergence to consensus: 22.1%  
**5. L2a**: 29766835-29766916 (-), Divergence to consensus: 34.5%  
**6. MIRc**: 29767032-29767159 (-), Divergence to consensus: 44.5%  
**7. ERV1-2C-LTR\_BT**: 29768355-29768836 (+), Divergence to consensus: 18.9%  
**8. ERV1-2-I\_BT-int**: 29768873-29769533 (+), Divergence to consensus: 21.5%

  
Transcription Factor Binding Sites
