# Supplementary material for: Small RNA sequencing of cryopreserved semen from single bull revealed altered miRNAs and piRNAs expression between High- and Low-motile sperm populations
Source: BMC Genomics. 2017 Jan 4;18:14. doi: 10.1186/s12864-016-3394-7 (PMC5209821; doi:10.1186/s12864-016-3394-7)
Supplement: Additional file 4: — Details for each piRNA clusters found in Low Motile (LM) sperm fraction. Genes, repeats, transposable elements and transcription factors binding sites falling within the cluster regions were reported. (ZIP 1034 kb) [file 12864_2016_3394_MOESM4_ESM.zip › 29.html]

piRNA cluster 29


Predicted piRNA cluster no. 29     previous   next
  

Show proTRAC run info
Hide proTRAC run info

================================= proTRAC ====================================  
VERSION: 2.1                                    LAST MODIFIED: 06. October 2015  
  
Please cite:  
Rosenkranz D, Zischler H. proTRAC - a software for probabilistic piRNA cluster  
detection, visualization and analysis. 2012. BMC Bioinformatics 13:5.  
  
and (for proTRAC 2.0 and later):  
Rosenkranz D, Rudloff S, Bastuck K, Ketting RF, Zischler H. Tupaia small RNAs  
provide insights into function and evolution of RNAi-based transposon defense  
in mammals. 2015. RNA 21(5):911-922.  
  
Contact:  
David Rosenkranz  
Institute of Anthropology, small RNA group  
Johannes Gutenberg University Mainz  
email: rosenkranz@uni-mainz.de  
  
You can find the latest proTRAC version at:  
http://sourceforge.net/projects/protrac/files  
http://www.smallRNAgroup-mainz.de/software  
==============================================================================  
  
PARAMETERS:  
Map file: .............../storage/core/barbara/genhome/smallRNA/fertility/Sample\_not\_motile/pirna/Sample\_not\_motile\_26-33\_collapsed.fa.no-dust.map.weighted-10000-1000-b-0  
Genome file: ............/storage/core/barbara/genhome/smallRNA/fertility/Sample\_all/pirna/bt\_311\_chrY.fa  
RepeatMasker annotation: /storage/genomes/bt\_umd31/GCF\_000003055.6\_Bos\_taurus\_UMD\_3.1.1\_repeatMasker\_chr.out  
GeneSet:................./storage/core/barbara/genhome/smallRNA/fertility/Sample\_all/pirna/full.gtf  
  
Significant (p<=0.01) hit density will be calculated based  
on observed hit distribution.  
  
Sliding window size: ........................................ 5000 bp  
Sliding window increament: .................................. 1000 bp  
Normalize each hit by number of genomic hits: ............... 1 [0=no/1=yes]  
Normalize each hit by number of sequence reads: ............. 1 [0=no/1=yes]  
Normalize values (-> per million mapped reads): ............. 1 [0=no/1=yes]  
Min. fraction of hits with 1T(U) or 10A: .................... 0.75  
Alternatively: Min. fraction of hits with 1T(U) and 10A: .... 0.5  
Min. fraction of hits with typical piRNA length: ............ 0.75  
Typical piRNA length: ....................................... 26-33 nt  
Min. size of a piRNA cluster: ............................... 5000 bp.  
Min. number of hits (absolute): ............................. 0  
Min. number of hits (normalized): ........................... 0  
Min. fraction of hits on the mainstrand: .................... 0.75  
Top fraction of mapped sequences (in terms of read counts): . 1%  
Top fraction accounts for max. n% of sequence reads: ........ 90%  
Min. fraction of hits on each arm of a bidirectional cluster: 0.1  
Output image file for each cluster: ......................... 0 [0=no/1=yes]  
Output html file for each cluster: .......................... 1 [0=no/1=yes]  
Output a summary table: ..................................... 1 [0=no/1=yes]  
Output a FASTA file for each cluster (piRNA sequences): ..... 1 [0=no/1=yes]  
Output a FASTA file comprising cluster sequences: ........... 1 [0=no/1=yes]  
Search DNA motifs in clusters: .............................. 1 [0=no/1=yes]  
Output flanking sequences: +/- .............................. 0 bp  
Output ~.pTi file: .......................................... 1 [0=no/1=yes]  
==============================================================================  
  
  
Genome size (without gaps): ............ 2678902517 bp  
Gaps (N/X/-): .......................... 53837044 bp  
Mapped reads: .......................... 738059667487  
Non-identical sequences: ............... 277001  
Genomic hits: .......................... 533816  
Significant densitiy of mapped reads: .. 15118061 reads/kb

Show proTRAC cluster info
Hide proTRAC cluster info

|  |  |
| --- | --- |
| Location | chr22 |
| Coordinates | 51416353-51438984 |
| Size [bp] | 22632 |
| Sequence hit loci | 168 |
| Mapped reads (normalized) | 446114660 |
| Mapped reads (normalized) per kb | 19711676.4 |
| Normalized reads with 1T (1U) | 77% |
| Normalized reads with 10A | 32.1% |
| Normalized reads with length 26-33 nt | 100% |
| Normalized reads on the main strand(s) | 88.3% |
| Predicted directionality | mono:plus |

100%

0%

1T (1U)  
reads

10A reads

26-33 nt  
reads

reads on mainstrand

**Either the amount of reads with 1T (1U) OR 10A has to exceed 75% (set with option: -1Tor10A)  
Alternatively the amount of reads with 1T (1U) AND 10A has to exceed 50% (set with option: -1Tand10A)  
Minimum amount of reads with preferred size is 75% (set with option: -pisize)  
Minimum amount of reads on the main strand(s) is 75% (set with option: -clstrand)**

Show read coverage
Hide read coverage

WHAT DO I SEE HERE?  
This chart shows the location of mapped sequence reads within a predicted piRNA cluster. The color refers to the number of genomic hits produced by the sequence read in question. A dark red bar indicates that this sequence read produces many other hits elsewhere in the genome. Many adjacent red or yellow bars can indicate the presence of a multi-copy element such as transposons or rRNA genes. A dark green bar indicates that this sequence read maps uniquely to this locus.

1 hit

2-5 hits

6-10 hits

11-20 hits

21-50 hits

51-100 hits

> 100 hits

chr22

51416353

51438984

Gene Set

RepeatMasker

Mapped  
Reads

29.04

plus strand

minus strand

29.04

Region: chr22 29769197-51416375. Max. coverage (+): 0. Max coverage (-): 0.72

Region: chr22 51416376-51416420. Max. coverage (+): 1.51. Max coverage (-): 0

Region: chr22 51416421-51416466. Max. coverage (+): 0. Max coverage (-): 0

Region: chr22 51416467-51416511. Max. coverage (+): 0. Max coverage (-): 0

Region: chr22 51416512-51416556. Max. coverage (+): 4.31. Max coverage (-): 0

Region: chr22 51416557-51416601. Max. coverage (+): 7.06. Max coverage (-): 0

Region: chr22 51416602-51416647. Max. coverage (+): 7.06. Max coverage (-): 0

Region: chr22 51416648-51416692. Max. coverage (+): 0.76. Max coverage (-): 0

Region: chr22 51416693-51416737. Max. coverage (+): 0. Max coverage (-): 0

Region: chr22 51416738-51416783. Max. coverage (+): 0. Max coverage (-): 0

Region: chr22 51416784-51416828. Max. coverage (+): 0. Max coverage (-): 0

Region: chr22 51416829-51416873. Max. coverage (+): 0. Max coverage (-): 0

Region: chr22 51416874-51416918. Max. coverage (+): 0. Max coverage (-): 0

Region: chr22 51416919-51416964. Max. coverage (+): 0. Max coverage (-): 0

Region: chr22 51416965-51417009. Max. coverage (+): 0. Max coverage (-): 0

Region: chr22 51417010-51417054. Max. coverage (+): 0. Max coverage (-): 0

Region: chr22 51417055-51417099. Max. coverage (+): 0. Max coverage (-): 0

Region: chr22 51417100-51417145. Max. coverage (+): 0. Max coverage (-): 0

Region: chr22 51417146-51417190. Max. coverage (+): 0. Max coverage (-): 0

Region: chr22 51417191-51417235. Max. coverage (+): 0. Max coverage (-): 0

Region: chr22 51417236-51417280. Max. coverage (+): 0. Max coverage (-): 0

Region: chr22 51417281-51417326. Max. coverage (+): 0. Max coverage (-): 0

Region: chr22 51417327-51417371. Max. coverage (+): 0. Max coverage (-): 0

Region: chr22 51417372-51417416. Max. coverage (+): 0. Max coverage (-): 0

Region: chr22 51417417-51417461. Max. coverage (+): 0. Max coverage (-): 0

Region: chr22 51417462-51417507. Max. coverage (+): 0. Max coverage (-): 0

Region: chr22 51417508-51417552. Max. coverage (+): 0. Max coverage (-): 0

Region: chr22 51417553-51417597. Max. coverage (+): 0. Max coverage (-): 0

Region: chr22 51417598-51417643. Max. coverage (+): 0. Max coverage (-): 0

Region: chr22 51417644-51417688. Max. coverage (+): 0. Max coverage (-): 0

Region: chr22 51417689-51417733. Max. coverage (+): 0. Max coverage (-): 0

Region: chr22 51417734-51417778. Max. coverage (+): 0. Max coverage (-): 0

Region: chr22 51417779-51417824. Max. coverage (+): 0. Max coverage (-): 0

Region: chr22 51417825-51417869. Max. coverage (+): 0. Max coverage (-): 0

Region: chr22 51417870-51417914. Max. coverage (+): 1.71. Max coverage (-): 0

Region: chr22 51417915-51417959. Max. coverage (+): 3.8. Max coverage (-): 0

Region: chr22 51417960-51418005. Max. coverage (+): 4.58. Max coverage (-): 0

Region: chr22 51418006-51418050. Max. coverage (+): 0. Max coverage (-): 0

Region: chr22 51418051-51418095. Max. coverage (+): 8.01. Max coverage (-): 0

Region: chr22 51418096-51418140. Max. coverage (+): 0. Max coverage (-): 0

Region: chr22 51418141-51418186. Max. coverage (+): 6.18. Max coverage (-): 0

Region: chr22 51418187-51418231. Max. coverage (+): 6.18. Max coverage (-): 0

Region: chr22 51418232-51418276. Max. coverage (+): 9.91. Max coverage (-): 0

Region: chr22 51418277-51418321. Max. coverage (+): 3.9. Max coverage (-): 0

Region: chr22 51418322-51418367. Max. coverage (+): 0. Max coverage (-): 0

Region: chr22 51418368-51418412. Max. coverage (+): 4.14. Max coverage (-): 0

Region: chr22 51418413-51418457. Max. coverage (+): 29.04. Max coverage (-): 0

Region: chr22 51418458-51418503. Max. coverage (+): 1.73. Max coverage (-): 0

Region: chr22 51418504-51418548. Max. coverage (+): 1.73. Max coverage (-): 0

Region: chr22 51418549-51418593. Max. coverage (+): 0. Max coverage (-): 0

Region: chr22 51418594-51418638. Max. coverage (+): 0. Max coverage (-): 0

Region: chr22 51418639-51418684. Max. coverage (+): 0. Max coverage (-): 0

Region: chr22 51418685-51418729. Max. coverage (+): 0. Max coverage (-): 0

Region: chr22 51418730-51418774. Max. coverage (+): 0. Max coverage (-): 0

Region: chr22 51418775-51418819. Max. coverage (+): 0. Max coverage (-): 0

Region: chr22 51418820-51418865. Max. coverage (+): 0. Max coverage (-): 0

Region: chr22 51418866-51418910. Max. coverage (+): 6.7. Max coverage (-): 0

Region: chr22 51418911-51418955. Max. coverage (+): 9.6. Max coverage (-): 0

Region: chr22 51418956-51419000. Max. coverage (+): 0. Max coverage (-): 0

Region: chr22 51419001-51419046. Max. coverage (+): 0. Max coverage (-): 0

Region: chr22 51419047-51419091. Max. coverage (+): 0. Max coverage (-): 0

Region: chr22 51419092-51419136. Max. coverage (+): 0. Max coverage (-): 0

Region: chr22 51419137-51419181. Max. coverage (+): 0. Max coverage (-): 0

Region: chr22 51419182-51419227. Max. coverage (+): 0. Max coverage (-): 0

Region: chr22 51419228-51419272. Max. coverage (+): 0. Max coverage (-): 0

Region: chr22 51419273-51419317. Max. coverage (+): 0. Max coverage (-): 0

Region: chr22 51419318-51419363. Max. coverage (+): 0. Max coverage (-): 0

Region: chr22 51419364-51419408. Max. coverage (+): 12.17. Max coverage (-): 0

Region: chr22 51419409-51419453. Max. coverage (+): 0. Max coverage (-): 0

Region: chr22 51419454-51419498. Max. coverage (+): 0. Max coverage (-): 0

Region: chr22 51419499-51419544. Max. coverage (+): 0. Max coverage (-): 0

Region: chr22 51419545-51419589. Max. coverage (+): 0. Max coverage (-): 0

Region: chr22 51419590-51419634. Max. coverage (+): 0. Max coverage (-): 0

Region: chr22 51419635-51419679. Max. coverage (+): 11.34. Max coverage (-): 4.24

Region: chr22 51419680-51419725. Max. coverage (+): 0. Max coverage (-): 0

Region: chr22 51419726-51419770. Max. coverage (+): 0. Max coverage (-): 0

Region: chr22 51419771-51419815. Max. coverage (+): 0. Max coverage (-): 0

Region: chr22 51419816-51419860. Max. coverage (+): 6.27. Max coverage (-): 0

Region: chr22 51419861-51419906. Max. coverage (+): 0. Max coverage (-): 0

Region: chr22 51419907-51419951. Max. coverage (+): 0. Max coverage (-): 0

Region: chr22 51419952-51419996. Max. coverage (+): 1.59. Max coverage (-): 0

Region: chr22 51419997-51420042. Max. coverage (+): 0. Max coverage (-): 5.56

Region: chr22 51420043-51420087. Max. coverage (+): 0. Max coverage (-): 6.24

Region: chr22 51420088-51420132. Max. coverage (+): 5. Max coverage (-): 0

Region: chr22 51420133-51420177. Max. coverage (+): 0. Max coverage (-): 0

Region: chr22 51420178-51420223. Max. coverage (+): 0. Max coverage (-): 0

Region: chr22 51420224-51420268. Max. coverage (+): 0. Max coverage (-): 0

Region: chr22 51420269-51420313. Max. coverage (+): 0. Max coverage (-): 0

Region: chr22 51420314-51420358. Max. coverage (+): 0. Max coverage (-): 0

Region: chr22 51420359-51420404. Max. coverage (+): 0. Max coverage (-): 0

Region: chr22 51420405-51420449. Max. coverage (+): 0. Max coverage (-): 0

Region: chr22 51420450-51420494. Max. coverage (+): 0. Max coverage (-): 0

Region: chr22 51420495-51420539. Max. coverage (+): 0. Max coverage (-): 0

Region: chr22 51420540-51420585. Max. coverage (+): 0. Max coverage (-): 0

Region: chr22 51420586-51420630. Max. coverage (+): 0. Max coverage (-): 0

Region: chr22 51420631-51420675. Max. coverage (+): 0. Max coverage (-): 0

Region: chr22 51420676-51420720. Max. coverage (+): 12.09. Max coverage (-): 0

Region: chr22 51420721-51420766. Max. coverage (+): 4.09. Max coverage (-): 0

Region: chr22 51420767-51420811. Max. coverage (+): 0. Max coverage (-): 0

Region: chr22 51420812-51420856. Max. coverage (+): 0. Max coverage (-): 0

Region: chr22 51420857-51420902. Max. coverage (+): 0. Max coverage (-): 0

Region: chr22 51420903-51420947. Max. coverage (+): 0. Max coverage (-): 0

Region: chr22 51420948-51420992. Max. coverage (+): 0. Max coverage (-): 0

Region: chr22 51420993-51421037. Max. coverage (+): 0. Max coverage (-): 0

Region: chr22 51421038-51421083. Max. coverage (+): 0. Max coverage (-): 0

Region: chr22 51421084-51421128. Max. coverage (+): 5.45. Max coverage (-): 0

Region: chr22 51421129-51421173. Max. coverage (+): 4.6. Max coverage (-): 0

Region: chr22 51421174-51421218. Max. coverage (+): 0. Max coverage (-): 0

Region: chr22 51421219-51421264. Max. coverage (+): 1.52. Max coverage (-): 0

Region: chr22 51421265-51421309. Max. coverage (+): 0. Max coverage (-): 0

Region: chr22 51421310-51421354. Max. coverage (+): 0. Max coverage (-): 0

Region: chr22 51421355-51421399. Max. coverage (+): 0. Max coverage (-): 0

Region: chr22 51421400-51421445. Max. coverage (+): 0. Max coverage (-): 0

Region: chr22 51421446-51421490. Max. coverage (+): 0. Max coverage (-): 0

Region: chr22 51421491-51421535. Max. coverage (+): 0. Max coverage (-): 0

Region: chr22 51421536-51421580. Max. coverage (+): 0. Max coverage (-): 0

Region: chr22 51421581-51421626. Max. coverage (+): 0. Max coverage (-): 0

Region: chr22 51421627-51421671. Max. coverage (+): 0. Max coverage (-): 0

Region: chr22 51421672-51421716. Max. coverage (+): 0. Max coverage (-): 0

Region: chr22 51421717-51421762. Max. coverage (+): 0. Max coverage (-): 0

Region: chr22 51421763-51421807. Max. coverage (+): 0. Max coverage (-): 0

Region: chr22 51421808-51421852. Max. coverage (+): 0. Max coverage (-): 0

Region: chr22 51421853-51421897. Max. coverage (+): 0. Max coverage (-): 0

Region: chr22 51421898-51421943. Max. coverage (+): 0. Max coverage (-): 0

Region: chr22 51421944-51421988. Max. coverage (+): 0. Max coverage (-): 0

Region: chr22 51421989-51422033. Max. coverage (+): 0. Max coverage (-): 0

Region: chr22 51422034-51422078. Max. coverage (+): 0. Max coverage (-): 0

Region: chr22 51422079-51422124. Max. coverage (+): 0. Max coverage (-): 0

Region: chr22 51422125-51422169. Max. coverage (+): 0. Max coverage (-): 0

Region: chr22 51422170-51422214. Max. coverage (+): 0. Max coverage (-): 0

Region: chr22 51422215-51422259. Max. coverage (+): 0. Max coverage (-): 0

Region: chr22 51422260-51422305. Max. coverage (+): 5.49. Max coverage (-): 0

Region: chr22 51422306-51422350. Max. coverage (+): 5.36. Max coverage (-): 0

Region: chr22 51422351-51422395. Max. coverage (+): 0. Max coverage (-): 0

Region: chr22 51422396-51422441. Max. coverage (+): 0. Max coverage (-): 0

Region: chr22 51422442-51422486. Max. coverage (+): 12.61. Max coverage (-): 0

Region: chr22 51422487-51422531. Max. coverage (+): 5.5. Max coverage (-): 0

Region: chr22 51422532-51422576. Max. coverage (+): 5.5. Max coverage (-): 0

Region: chr22 51422577-51422622. Max. coverage (+): 0. Max coverage (-): 0

Region: chr22 51422623-51422667. Max. coverage (+): 10.63. Max coverage (-): 0

Region: chr22 51422668-51422712. Max. coverage (+): 10.63. Max coverage (-): 0

Region: chr22 51422713-51422757. Max. coverage (+): 1.19. Max coverage (-): 0

Region: chr22 51422758-51422803. Max. coverage (+): 1.19. Max coverage (-): 0

Region: chr22 51422804-51422848. Max. coverage (+): 0. Max coverage (-): 0

Region: chr22 51422849-51422893. Max. coverage (+): 2.09. Max coverage (-): 0

Region: chr22 51422894-51422938. Max. coverage (+): 6.05. Max coverage (-): 0

Region: chr22 51422939-51422984. Max. coverage (+): 6.05. Max coverage (-): 0

Region: chr22 51422985-51423029. Max. coverage (+): 0. Max coverage (-): 0

Region: chr22 51423030-51423074. Max. coverage (+): 0. Max coverage (-): 0

Region: chr22 51423075-51423119. Max. coverage (+): 1.89. Max coverage (-): 0

Region: chr22 51423120-51423165. Max. coverage (+): 0. Max coverage (-): 0

Region: chr22 51423166-51423210. Max. coverage (+): 0. Max coverage (-): 0

Region: chr22 51423211-51423255. Max. coverage (+): 4.47. Max coverage (-): 0

Region: chr22 51423256-51423301. Max. coverage (+): 0. Max coverage (-): 0

Region: chr22 51423302-51423346. Max. coverage (+): 0. Max coverage (-): 0

Region: chr22 51423347-51423391. Max. coverage (+): 4.92. Max coverage (-): 0

Region: chr22 51423392-51423436. Max. coverage (+): 0. Max coverage (-): 0

Region: chr22 51423437-51423482. Max. coverage (+): 0. Max coverage (-): 0

Region: chr22 51423483-51423527. Max. coverage (+): 0. Max coverage (-): 0

Region: chr22 51423528-51423572. Max. coverage (+): 0. Max coverage (-): 0

Region: chr22 51423573-51423617. Max. coverage (+): 0. Max coverage (-): 0

Region: chr22 51423618-51423663. Max. coverage (+): 0. Max coverage (-): 0

Region: chr22 51423664-51423708. Max. coverage (+): 0. Max coverage (-): 0

Region: chr22 51423709-51423753. Max. coverage (+): 0. Max coverage (-): 0

Region: chr22 51423754-51423798. Max. coverage (+): 0. Max coverage (-): 0

Region: chr22 51423799-51423844. Max. coverage (+): 0. Max coverage (-): 0

Region: chr22 51423845-51423889. Max. coverage (+): 0. Max coverage (-): 0

Region: chr22 51423890-51423934. Max. coverage (+): 0. Max coverage (-): 0

Region: chr22 51423935-51423979. Max. coverage (+): 0. Max coverage (-): 0

Region: chr22 51423980-51424025. Max. coverage (+): 0. Max coverage (-): 0

Region: chr22 51424026-51424070. Max. coverage (+): 0. Max coverage (-): 0

Region: chr22 51424071-51424115. Max. coverage (+): 0. Max coverage (-): 0

Region: chr22 51424116-51424161. Max. coverage (+): 0. Max coverage (-): 0

Region: chr22 51424162-51424206. Max. coverage (+): 0. Max coverage (-): 0

Region: chr22 51424207-51424251. Max. coverage (+): 0. Max coverage (-): 0

Region: chr22 51424252-51424296. Max. coverage (+): 0. Max coverage (-): 0

Region: chr22 51424297-51424342. Max. coverage (+): 0. Max coverage (-): 0

Region: chr22 51424343-51424387. Max. coverage (+): 0. Max coverage (-): 0

Region: chr22 51424388-51424432. Max. coverage (+): 0. Max coverage (-): 0

Region: chr22 51424433-51424477. Max. coverage (+): 0. Max coverage (-): 0

Region: chr22 51424478-51424523. Max. coverage (+): 0. Max coverage (-): 0

Region: chr22 51424524-51424568. Max. coverage (+): 0. Max coverage (-): 0

Region: chr22 51424569-51424613. Max. coverage (+): 0. Max coverage (-): 0

Region: chr22 51424614-51424658. Max. coverage (+): 0. Max coverage (-): 0

Region: chr22 51424659-51424704. Max. coverage (+): 0. Max coverage (-): 0

Region: chr22 51424705-51424749. Max. coverage (+): 0. Max coverage (-): 0

Region: chr22 51424750-51424794. Max. coverage (+): 0. Max coverage (-): 0

Region: chr22 51424795-51424839. Max. coverage (+): 0. Max coverage (-): 0

Region: chr22 51424840-51424885. Max. coverage (+): 0. Max coverage (-): 0

Region: chr22 51424886-51424930. Max. coverage (+): 0. Max coverage (-): 0

Region: chr22 51424931-51424975. Max. coverage (+): 0. Max coverage (-): 0

Region: chr22 51424976-51425021. Max. coverage (+): 0. Max coverage (-): 0

Region: chr22 51425022-51425066. Max. coverage (+): 0. Max coverage (-): 0

Region: chr22 51425067-51425111. Max. coverage (+): 0. Max coverage (-): 0

Region: chr22 51425112-51425156. Max. coverage (+): 0. Max coverage (-): 0

Region: chr22 51425157-51425202. Max. coverage (+): 0. Max coverage (-): 0

Region: chr22 51425203-51425247. Max. coverage (+): 0. Max coverage (-): 0

Region: chr22 51425248-51425292. Max. coverage (+): 0. Max coverage (-): 0

Region: chr22 51425293-51425337. Max. coverage (+): 0. Max coverage (-): 0

Region: chr22 51425338-51425383. Max. coverage (+): 0. Max coverage (-): 0

Region: chr22 51425384-51425428. Max. coverage (+): 0. Max coverage (-): 0

Region: chr22 51425429-51425473. Max. coverage (+): 0. Max coverage (-): 0

Region: chr22 51425474-51425518. Max. coverage (+): 0. Max coverage (-): 0

Region: chr22 51425519-51425564. Max. coverage (+): 0. Max coverage (-): 0

Region: chr22 51425565-51425609. Max. coverage (+): 0. Max coverage (-): 0

Region: chr22 51425610-51425654. Max. coverage (+): 10.1. Max coverage (-): 0

Region: chr22 51425655-51425700. Max. coverage (+): 0. Max coverage (-): 0

Region: chr22 51425701-51425745. Max. coverage (+): 0. Max coverage (-): 0

Region: chr22 51425746-51425790. Max. coverage (+): 1.37. Max coverage (-): 0

Region: chr22 51425791-51425835. Max. coverage (+): 0. Max coverage (-): 0

Region: chr22 51425836-51425881. Max. coverage (+): 0. Max coverage (-): 0

Region: chr22 51425882-51425926. Max. coverage (+): 0. Max coverage (-): 0

Region: chr22 51425927-51425971. Max. coverage (+): 0. Max coverage (-): 0

Region: chr22 51425972-51426016. Max. coverage (+): 0. Max coverage (-): 0

Region: chr22 51426017-51426062. Max. coverage (+): 0. Max coverage (-): 0

Region: chr22 51426063-51426107. Max. coverage (+): 1.62. Max coverage (-): 0

Region: chr22 51426108-51426152. Max. coverage (+): 0. Max coverage (-): 0

Region: chr22 51426153-51426197. Max. coverage (+): 0. Max coverage (-): 0

Region: chr22 51426198-51426243. Max. coverage (+): 0.97. Max coverage (-): 0

Region: chr22 51426244-51426288. Max. coverage (+): 0.97. Max coverage (-): 0

Region: chr22 51426289-51426333. Max. coverage (+): 0. Max coverage (-): 0

Region: chr22 51426334-51426378. Max. coverage (+): 5.15. Max coverage (-): 0

Region: chr22 51426379-51426424. Max. coverage (+): 0. Max coverage (-): 0

Region: chr22 51426425-51426469. Max. coverage (+): 0. Max coverage (-): 0

Region: chr22 51426470-51426514. Max. coverage (+): 0. Max coverage (-): 0

Region: chr22 51426515-51426560. Max. coverage (+): 0. Max coverage (-): 0

Region: chr22 51426561-51426605. Max. coverage (+): 0. Max coverage (-): 0

Region: chr22 51426606-51426650. Max. coverage (+): 0. Max coverage (-): 0

Region: chr22 51426651-51426695. Max. coverage (+): 0. Max coverage (-): 0

Region: chr22 51426696-51426741. Max. coverage (+): 0. Max coverage (-): 0

Region: chr22 51426742-51426786. Max. coverage (+): 0. Max coverage (-): 0

Region: chr22 51426787-51426831. Max. coverage (+): 2.81. Max coverage (-): 0

Region: chr22 51426832-51426876. Max. coverage (+): 0. Max coverage (-): 0

Region: chr22 51426877-51426922. Max. coverage (+): 4.11. Max coverage (-): 0

Region: chr22 51426923-51426967. Max. coverage (+): 0. Max coverage (-): 0

Region: chr22 51426968-51427012. Max. coverage (+): 0. Max coverage (-): 0

Region: chr22 51427013-51427057. Max. coverage (+): 0. Max coverage (-): 0

Region: chr22 51427058-51427103. Max. coverage (+): 0. Max coverage (-): 0

Region: chr22 51427104-51427148. Max. coverage (+): 0. Max coverage (-): 0

Region: chr22 51427149-51427193. Max. coverage (+): 0. Max coverage (-): 0

Region: chr22 51427194-51427238. Max. coverage (+): 0. Max coverage (-): 0

Region: chr22 51427239-51427284. Max. coverage (+): 0. Max coverage (-): 0

Region: chr22 51427285-51427329. Max. coverage (+): 0. Max coverage (-): 0

Region: chr22 51427330-51427374. Max. coverage (+): 0. Max coverage (-): 0

Region: chr22 51427375-51427420. Max. coverage (+): 0. Max coverage (-): 0

Region: chr22 51427421-51427465. Max. coverage (+): 3.9. Max coverage (-): 0

Region: chr22 51427466-51427510. Max. coverage (+): 0. Max coverage (-): 0

Region: chr22 51427511-51427555. Max. coverage (+): 0. Max coverage (-): 0

Region: chr22 51427556-51427601. Max. coverage (+): 0. Max coverage (-): 0

Region: chr22 51427602-51427646. Max. coverage (+): 0. Max coverage (-): 0

Region: chr22 51427647-51427691. Max. coverage (+): 0. Max coverage (-): 0

Region: chr22 51427692-51427736. Max. coverage (+): 0. Max coverage (-): 0

Region: chr22 51427737-51427782. Max. coverage (+): 0.42. Max coverage (-): 6.85

Region: chr22 51427783-51427827. Max. coverage (+): 0. Max coverage (-): 0.66

Region: chr22 51427828-51427872. Max. coverage (+): 1.66. Max coverage (-): 0

Region: chr22 51427873-51427917. Max. coverage (+): 0. Max coverage (-): 0

Region: chr22 51427918-51427963. Max. coverage (+): 0. Max coverage (-): 0

Region: chr22 51427964-51428008. Max. coverage (+): 0. Max coverage (-): 5.78

Region: chr22 51428009-51428053. Max. coverage (+): 0. Max coverage (-): 0

Region: chr22 51428054-51428099. Max. coverage (+): 0. Max coverage (-): 6.69

Region: chr22 51428100-51428144. Max. coverage (+): 0. Max coverage (-): 6.69

Region: chr22 51428145-51428189. Max. coverage (+): 0. Max coverage (-): 0

Region: chr22 51428190-51428234. Max. coverage (+): 0. Max coverage (-): 0

Region: chr22 51428235-51428280. Max. coverage (+): 0. Max coverage (-): 0

Region: chr22 51428281-51428325. Max. coverage (+): 0. Max coverage (-): 0

Region: chr22 51428326-51428370. Max. coverage (+): 0. Max coverage (-): 0

Region: chr22 51428371-51428415. Max. coverage (+): 0. Max coverage (-): 0

Region: chr22 51428416-51428461. Max. coverage (+): 0. Max coverage (-): 0

Region: chr22 51428462-51428506. Max. coverage (+): 0. Max coverage (-): 0

Region: chr22 51428507-51428551. Max. coverage (+): 0. Max coverage (-): 0

Region: chr22 51428552-51428596. Max. coverage (+): 7.05. Max coverage (-): 0

Region: chr22 51428597-51428642. Max. coverage (+): 0. Max coverage (-): 0

Region: chr22 51428643-51428687. Max. coverage (+): 0. Max coverage (-): 0

Region: chr22 51428688-51428732. Max. coverage (+): 0. Max coverage (-): 0

Region: chr22 51428733-51428777. Max. coverage (+): 0. Max coverage (-): 0

Region: chr22 51428778-51428823. Max. coverage (+): 0. Max coverage (-): 0

Region: chr22 51428824-51428868. Max. coverage (+): 0. Max coverage (-): 1.81

Region: chr22 51428869-51428913. Max. coverage (+): 0. Max coverage (-): 0

Region: chr22 51428914-51428959. Max. coverage (+): 0. Max coverage (-): 0

Region: chr22 51428960-51429004. Max. coverage (+): 0. Max coverage (-): 0

Region: chr22 51429005-51429049. Max. coverage (+): 0. Max coverage (-): 5.22

Region: chr22 51429050-51429094. Max. coverage (+): 0. Max coverage (-): 0

Region: chr22 51429095-51429140. Max. coverage (+): 0. Max coverage (-): 0

Region: chr22 51429141-51429185. Max. coverage (+): 6.65. Max coverage (-): 0

Region: chr22 51429186-51429230. Max. coverage (+): 0. Max coverage (-): 0

Region: chr22 51429231-51429275. Max. coverage (+): 0. Max coverage (-): 0

Region: chr22 51429276-51429321. Max. coverage (+): 0. Max coverage (-): 0

Region: chr22 51429322-51429366. Max. coverage (+): 0. Max coverage (-): 0

Region: chr22 51429367-51429411. Max. coverage (+): 0. Max coverage (-): 0

Region: chr22 51429412-51429456. Max. coverage (+): 0. Max coverage (-): 0

Region: chr22 51429457-51429502. Max. coverage (+): 8.55. Max coverage (-): 12.88

Region: chr22 51429503-51429547. Max. coverage (+): 7.19. Max coverage (-): 0

Region: chr22 51429548-51429592. Max. coverage (+): 0. Max coverage (-): 0

Region: chr22 51429593-51429637. Max. coverage (+): 0. Max coverage (-): 0

Region: chr22 51429638-51429683. Max. coverage (+): 0. Max coverage (-): 0

Region: chr22 51429684-51429728. Max. coverage (+): 0. Max coverage (-): 0

Region: chr22 51429729-51429773. Max. coverage (+): 0. Max coverage (-): 0

Region: chr22 51429774-51429819. Max. coverage (+): 0. Max coverage (-): 0

Region: chr22 51429820-51429864. Max. coverage (+): 0. Max coverage (-): 0

Region: chr22 51429865-51429909. Max. coverage (+): 0. Max coverage (-): 0

Region: chr22 51429910-51429954. Max. coverage (+): 3.44. Max coverage (-): 0

Region: chr22 51429955-51430000. Max. coverage (+): 0. Max coverage (-): 0

Region: chr22 51430001-51430045. Max. coverage (+): 0. Max coverage (-): 0

Region: chr22 51430046-51430090. Max. coverage (+): 8.3. Max coverage (-): 0

Region: chr22 51430091-51430135. Max. coverage (+): 0. Max coverage (-): 0

Region: chr22 51430136-51430181. Max. coverage (+): 0. Max coverage (-): 0

Region: chr22 51430182-51430226. Max. coverage (+): 0. Max coverage (-): 0

Region: chr22 51430227-51430271. Max. coverage (+): 0. Max coverage (-): 0

Region: chr22 51430272-51430316. Max. coverage (+): 0. Max coverage (-): 0

Region: chr22 51430317-51430362. Max. coverage (+): 3.5. Max coverage (-): 0

Region: chr22 51430363-51430407. Max. coverage (+): 3.25. Max coverage (-): 0

Region: chr22 51430408-51430452. Max. coverage (+): 0. Max coverage (-): 0

Region: chr22 51430453-51430497. Max. coverage (+): 0. Max coverage (-): 0

Region: chr22 51430498-51430543. Max. coverage (+): 0. Max coverage (-): 0

Region: chr22 51430544-51430588. Max. coverage (+): 0. Max coverage (-): 0

Region: chr22 51430589-51430633. Max. coverage (+): 0. Max coverage (-): 0

Region: chr22 51430634-51430679. Max. coverage (+): 0. Max coverage (-): 0

Region: chr22 51430680-51430724. Max. coverage (+): 0. Max coverage (-): 0

Region: chr22 51430725-51430769. Max. coverage (+): 0. Max coverage (-): 0

Region: chr22 51430770-51430814. Max. coverage (+): 0. Max coverage (-): 0

Region: chr22 51430815-51430860. Max. coverage (+): 0. Max coverage (-): 0

Region: chr22 51430861-51430905. Max. coverage (+): 0. Max coverage (-): 0

Region: chr22 51430906-51430950. Max. coverage (+): 0. Max coverage (-): 0

Region: chr22 51430951-51430995. Max. coverage (+): 0. Max coverage (-): 0

Region: chr22 51430996-51431041. Max. coverage (+): 0. Max coverage (-): 0

Region: chr22 51431042-51431086. Max. coverage (+): 0. Max coverage (-): 0

Region: chr22 51431087-51431131. Max. coverage (+): 18.14. Max coverage (-): 0

Region: chr22 51431132-51431176. Max. coverage (+): 0. Max coverage (-): 0

Region: chr22 51431177-51431222. Max. coverage (+): 0. Max coverage (-): 0

Region: chr22 51431223-51431267. Max. coverage (+): 22.85. Max coverage (-): 0

Region: chr22 51431268-51431312. Max. coverage (+): 16.04. Max coverage (-): 0

Region: chr22 51431313-51431358. Max. coverage (+): 0. Max coverage (-): 0

Region: chr22 51431359-51431403. Max. coverage (+): 0. Max coverage (-): 0

Region: chr22 51431404-51431448. Max. coverage (+): 0. Max coverage (-): 0

Region: chr22 51431449-51431493. Max. coverage (+): 0. Max coverage (-): 0

Region: chr22 51431494-51431539. Max. coverage (+): 0.89. Max coverage (-): 0

Region: chr22 51431540-51431584. Max. coverage (+): 0. Max coverage (-): 0

Region: chr22 51431585-51431629. Max. coverage (+): 6.2. Max coverage (-): 0

Region: chr22 51431630-51431674. Max. coverage (+): 0. Max coverage (-): 0

Region: chr22 51431675-51431720. Max. coverage (+): 1.23. Max coverage (-): 0

Region: chr22 51431721-51431765. Max. coverage (+): 0. Max coverage (-): 0

Region: chr22 51431766-51431810. Max. coverage (+): 0. Max coverage (-): 0

Region: chr22 51431811-51431855. Max. coverage (+): 0. Max coverage (-): 0

Region: chr22 51431856-51431901. Max. coverage (+): 0. Max coverage (-): 0

Region: chr22 51431902-51431946. Max. coverage (+): 0. Max coverage (-): 0

Region: chr22 51431947-51431991. Max. coverage (+): 0. Max coverage (-): 0

Region: chr22 51431992-51432036. Max. coverage (+): 0. Max coverage (-): 0

Region: chr22 51432037-51432082. Max. coverage (+): 0. Max coverage (-): 0

Region: chr22 51432083-51432127. Max. coverage (+): 0. Max coverage (-): 0

Region: chr22 51432128-51432172. Max. coverage (+): 0. Max coverage (-): 0

Region: chr22 51432173-51432218. Max. coverage (+): 0. Max coverage (-): 0

Region: chr22 51432219-51432263. Max. coverage (+): 0. Max coverage (-): 0

Region: chr22 51432264-51432308. Max. coverage (+): 0. Max coverage (-): 0

Region: chr22 51432309-51432353. Max. coverage (+): 0. Max coverage (-): 0

Region: chr22 51432354-51432399. Max. coverage (+): 0. Max coverage (-): 0

Region: chr22 51432400-51432444. Max. coverage (+): 0. Max coverage (-): 0

Region: chr22 51432445-51432489. Max. coverage (+): 0. Max coverage (-): 0

Region: chr22 51432490-51432534. Max. coverage (+): 2.55. Max coverage (-): 0

Region: chr22 51432535-51432580. Max. coverage (+): 13.43. Max coverage (-): 0

Region: chr22 51432581-51432625. Max. coverage (+): 0. Max coverage (-): 0

Region: chr22 51432626-51432670. Max. coverage (+): 0. Max coverage (-): 0

Region: chr22 51432671-51432715. Max. coverage (+): 0. Max coverage (-): 0

Region: chr22 51432716-51432761. Max. coverage (+): 0. Max coverage (-): 0

Region: chr22 51432762-51432806. Max. coverage (+): 2.22. Max coverage (-): 0

Region: chr22 51432807-51432851. Max. coverage (+): 0. Max coverage (-): 0

Region: chr22 51432852-51432896. Max. coverage (+): 0. Max coverage (-): 0

Region: chr22 51432897-51432942. Max. coverage (+): 0. Max coverage (-): 0

Region: chr22 51432943-51432987. Max. coverage (+): 6.67. Max coverage (-): 0

Region: chr22 51432988-51433032. Max. coverage (+): 0. Max coverage (-): 0

Region: chr22 51433033-51433078. Max. coverage (+): 4.57. Max coverage (-): 0

Region: chr22 51433079-51433123. Max. coverage (+): 0. Max coverage (-): 0

Region: chr22 51433124-51433168. Max. coverage (+): 0. Max coverage (-): 0

Region: chr22 51433169-51433213. Max. coverage (+): 0. Max coverage (-): 0

Region: chr22 51433214-51433259. Max. coverage (+): 0. Max coverage (-): 0

Region: chr22 51433260-51433304. Max. coverage (+): 0. Max coverage (-): 0

Region: chr22 51433305-51433349. Max. coverage (+): 0. Max coverage (-): 0

Region: chr22 51433350-51433394. Max. coverage (+): 0. Max coverage (-): 0

Region: chr22 51433395-51433440. Max. coverage (+): 0. Max coverage (-): 0

Region: chr22 51433441-51433485. Max. coverage (+): 0. Max coverage (-): 0

Region: chr22 51433486-51433530. Max. coverage (+): 0. Max coverage (-): 0

Region: chr22 51433531-51433575. Max. coverage (+): 0. Max coverage (-): 0

Region: chr22 51433576-51433621. Max. coverage (+): 0. Max coverage (-): 0

Region: chr22 51433622-51433666. Max. coverage (+): 0. Max coverage (-): 0

Region: chr22 51433667-51433711. Max. coverage (+): 0. Max coverage (-): 0

Region: chr22 51433712-51433757. Max. coverage (+): 0. Max coverage (-): 0

Region: chr22 51433758-51433802. Max. coverage (+): 0. Max coverage (-): 0

Region: chr22 51433803-51433847. Max. coverage (+): 0. Max coverage (-): 0

Region: chr22 51433848-51433892. Max. coverage (+): 0. Max coverage (-): 8.18

Region: chr22 51433893-51433938. Max. coverage (+): 0. Max coverage (-): 0

Region: chr22 51433939-51433983. Max. coverage (+): 0. Max coverage (-): 0

Region: chr22 51433984-51434028. Max. coverage (+): 5.49. Max coverage (-): 0

Region: chr22 51434029-51434073. Max. coverage (+): 6.73. Max coverage (-): 0

Region: chr22 51434074-51434119. Max. coverage (+): 0. Max coverage (-): 0

Region: chr22 51434120-51434164. Max. coverage (+): 0. Max coverage (-): 0

Region: chr22 51434165-51434209. Max. coverage (+): 0. Max coverage (-): 0

Region: chr22 51434210-51434254. Max. coverage (+): 5.96. Max coverage (-): 0

Region: chr22 51434255-51434300. Max. coverage (+): 0. Max coverage (-): 0

Region: chr22 51434301-51434345. Max. coverage (+): 0. Max coverage (-): 0

Region: chr22 51434346-51434390. Max. coverage (+): 0. Max coverage (-): 0

Region: chr22 51434391-51434435. Max. coverage (+): 0. Max coverage (-): 0

Region: chr22 51434436-51434481. Max. coverage (+): 0. Max coverage (-): 0

Region: chr22 51434482-51434526. Max. coverage (+): 0. Max coverage (-): 0

Region: chr22 51434527-51434571. Max. coverage (+): 5.17. Max coverage (-): 0

Region: chr22 51434572-51434617. Max. coverage (+): 0. Max coverage (-): 0

Region: chr22 51434618-51434662. Max. coverage (+): 6.34. Max coverage (-): 0

Region: chr22 51434663-51434707. Max. coverage (+): 6.34. Max coverage (-): 0

Region: chr22 51434708-51434752. Max. coverage (+): 0. Max coverage (-): 0

Region: chr22 51434753-51434798. Max. coverage (+): 8.15. Max coverage (-): 0

Region: chr22 51434799-51434843. Max. coverage (+): 0. Max coverage (-): 0

Region: chr22 51434844-51434888. Max. coverage (+): 16.44. Max coverage (-): 0

Region: chr22 51434889-51434933. Max. coverage (+): 0. Max coverage (-): 0

Region: chr22 51434934-51434979. Max. coverage (+): 5.49. Max coverage (-): 0

Region: chr22 51434980-51435024. Max. coverage (+): 0. Max coverage (-): 0

Region: chr22 51435025-51435069. Max. coverage (+): 0. Max coverage (-): 0

Region: chr22 51435070-51435114. Max. coverage (+): 0. Max coverage (-): 0

Region: chr22 51435115-51435160. Max. coverage (+): 0. Max coverage (-): 0

Region: chr22 51435161-51435205. Max. coverage (+): 5.93. Max coverage (-): 0

Region: chr22 51435206-51435250. Max. coverage (+): 0. Max coverage (-): 0

Region: chr22 51435251-51435295. Max. coverage (+): 0. Max coverage (-): 0

Region: chr22 51435296-51435341. Max. coverage (+): 0. Max coverage (-): 0

Region: chr22 51435342-51435386. Max. coverage (+): 5.37. Max coverage (-): 0

Region: chr22 51435387-51435431. Max. coverage (+): 7.15. Max coverage (-): 0

Region: chr22 51435432-51435477. Max. coverage (+): 0. Max coverage (-): 0

Region: chr22 51435478-51435522. Max. coverage (+): 0. Max coverage (-): 0

Region: chr22 51435523-51435567. Max. coverage (+): 0. Max coverage (-): 0

Region: chr22 51435568-51435612. Max. coverage (+): 0. Max coverage (-): 0

Region: chr22 51435613-51435658. Max. coverage (+): 0. Max coverage (-): 0

Region: chr22 51435659-51435703. Max. coverage (+): 0. Max coverage (-): 0

Region: chr22 51435704-51435748. Max. coverage (+): 0. Max coverage (-): 0

Region: chr22 51435749-51435793. Max. coverage (+): 0. Max coverage (-): 0

Region: chr22 51435794-51435839. Max. coverage (+): 0. Max coverage (-): 0

Region: chr22 51435840-51435884. Max. coverage (+): 0. Max coverage (-): 0

Region: chr22 51435885-51435929. Max. coverage (+): 0. Max coverage (-): 0

Region: chr22 51435930-51435974. Max. coverage (+): 0. Max coverage (-): 0

Region: chr22 51435975-51436020. Max. coverage (+): 0. Max coverage (-): 0

Region: chr22 51436021-51436065. Max. coverage (+): 0. Max coverage (-): 0

Region: chr22 51436066-51436110. Max. coverage (+): 0. Max coverage (-): 0

Region: chr22 51436111-51436155. Max. coverage (+): 0. Max coverage (-): 0

Region: chr22 51436156-51436201. Max. coverage (+): 0. Max coverage (-): 0

Region: chr22 51436202-51436246. Max. coverage (+): 0. Max coverage (-): 0

Region: chr22 51436247-51436291. Max. coverage (+): 0. Max coverage (-): 0

Region: chr22 51436292-51436337. Max. coverage (+): 0. Max coverage (-): 0

Region: chr22 51436338-51436382. Max. coverage (+): 5.85. Max coverage (-): 0

Region: chr22 51436383-51436427. Max. coverage (+): 0. Max coverage (-): 0

Region: chr22 51436428-51436472. Max. coverage (+): 0. Max coverage (-): 0

Region: chr22 51436473-51436518. Max. coverage (+): 0. Max coverage (-): 0

Region: chr22 51436519-51436563. Max. coverage (+): 0. Max coverage (-): 0

Region: chr22 51436564-51436608. Max. coverage (+): 0. Max coverage (-): 0

Region: chr22 51436609-51436653. Max. coverage (+): 0. Max coverage (-): 0

Region: chr22 51436654-51436699. Max. coverage (+): 0. Max coverage (-): 0

Region: chr22 51436700-51436744. Max. coverage (+): 0. Max coverage (-): 0

Region: chr22 51436745-51436789. Max. coverage (+): 0. Max coverage (-): 0

Region: chr22 51436790-51436834. Max. coverage (+): 0. Max coverage (-): 0

Region: chr22 51436835-51436880. Max. coverage (+): 0. Max coverage (-): 0

Region: chr22 51436881-51436925. Max. coverage (+): 9.6. Max coverage (-): 0

Region: chr22 51436926-51436970. Max. coverage (+): 9.6. Max coverage (-): 0

Region: chr22 51436971-51437016. Max. coverage (+): 2.79. Max coverage (-): 0

Region: chr22 51437017-51437061. Max. coverage (+): 15.61. Max coverage (-): 0

Region: chr22 51437062-51437106. Max. coverage (+): 0. Max coverage (-): 0

Region: chr22 51437107-51437151. Max. coverage (+): 0. Max coverage (-): 0

Region: chr22 51437152-51437197. Max. coverage (+): 0. Max coverage (-): 0

Region: chr22 51437198-51437242. Max. coverage (+): 0. Max coverage (-): 0

Region: chr22 51437243-51437287. Max. coverage (+): 0. Max coverage (-): 0

Region: chr22 51437288-51437332. Max. coverage (+): 0. Max coverage (-): 0

Region: chr22 51437333-51437378. Max. coverage (+): 3.94. Max coverage (-): 0

Region: chr22 51437379-51437423. Max. coverage (+): 0. Max coverage (-): 0

Region: chr22 51437424-51437468. Max. coverage (+): 2.41. Max coverage (-): 0

Region: chr22 51437469-51437513. Max. coverage (+): 2.41. Max coverage (-): 0

Region: chr22 51437514-51437559. Max. coverage (+): 7.1. Max coverage (-): 0

Region: chr22 51437560-51437604. Max. coverage (+): 7.1. Max coverage (-): 0

Region: chr22 51437605-51437649. Max. coverage (+): 0. Max coverage (-): 0

Region: chr22 51437650-51437694. Max. coverage (+): 0. Max coverage (-): 0

Region: chr22 51437695-51437740. Max. coverage (+): 0. Max coverage (-): 0

Region: chr22 51437741-51437785. Max. coverage (+): 0.33. Max coverage (-): 0

Region: chr22 51437786-51437830. Max. coverage (+): 0. Max coverage (-): 0

Region: chr22 51437831-51437876. Max. coverage (+): 0. Max coverage (-): 0

Region: chr22 51437877-51437921. Max. coverage (+): 0. Max coverage (-): 0

Region: chr22 51437922-51437966. Max. coverage (+): 0. Max coverage (-): 0

Region: chr22 51437967-51438011. Max. coverage (+): 0. Max coverage (-): 0

Region: chr22 51438012-51438057. Max. coverage (+): 0. Max coverage (-): 0

Region: chr22 51438058-51438102. Max. coverage (+): 0. Max coverage (-): 0

Region: chr22 51438103-51438147. Max. coverage (+): 0. Max coverage (-): 0

Region: chr22 51438148-51438192. Max. coverage (+): 0. Max coverage (-): 0

Region: chr22 51438193-51438238. Max. coverage (+): 0. Max coverage (-): 0

Region: chr22 51438239-51438283. Max. coverage (+): 0. Max coverage (-): 0

Region: chr22 51438284-51438328. Max. coverage (+): 0. Max coverage (-): 0

Region: chr22 51438329-51438373. Max. coverage (+): 0. Max coverage (-): 0

Region: chr22 51438374-51438419. Max. coverage (+): 0. Max coverage (-): 0

Region: chr22 51438420-51438464. Max. coverage (+): 0. Max coverage (-): 0

Region: chr22 51438465-51438509. Max. coverage (+): 0. Max coverage (-): 0

Region: chr22 51438510-51438554. Max. coverage (+): 0. Max coverage (-): 0

Region: chr22 51438555-51438600. Max. coverage (+): 0. Max coverage (-): 0

Region: chr22 51438601-51438645. Max. coverage (+): 0. Max coverage (-): 0

Region: chr22 51438646-51438690. Max. coverage (+): 0. Max coverage (-): 0

Region: chr22 51438691-51438736. Max. coverage (+): 0. Max coverage (-): 0

Region: chr22 51438737-51438781. Max. coverage (+): 0. Max coverage (-): 0

Region: chr22 51438782-51438826. Max. coverage (+): 0. Max coverage (-): 0

Region: chr22 51438827-51438871. Max. coverage (+): 0. Max coverage (-): 0

Region: chr22 51438872-51438917. Max. coverage (+): 0. Max coverage (-): 0

Region: chr22 51438918-51438962. Max. coverage (+): 3.18. Max coverage (-): 0

Region: chr22 51438963-. Max. coverage (+): 0. Max coverage (-): 0

RepeatMasker Color Code

**+**

100-98% Identity

<98-95% Identity

<95-90% Identity

<90-85% Identity

<85-80% Identity

<80-75% Identity

<75-70% Identity

<70% Identity

**-**

Gene Set Color Code

**+**

Gene

Pseudogene

**-**

Topology/Coverage Color Code

Coverage Plus Strand

Coverage Minus Strand

Mainstrand: Plus

Mainstrand: Minus

Complementary Strand

Flanking Region  
(if option -flank >0)

Gene Set Annotation  

**1. C3orf84 (protein coding, ENSBTAG00000000982) Tr:00000001300 Ex:1**: 51418820-51418935 (+)  
**2. C3orf84 (protein coding, ENSBTAG00000000982) Tr:00000001300 Ex:2**: 51420789-51420902 (+)  
**3. C3orf84 (protein coding, ENSBTAG00000000982) Tr:00000001300 Ex:3**: 51425932-51425988 (+)  
**4. C3orf84 (protein coding, ENSBTAG00000000982) Tr:00000001300 Ex:4**: 51426395-51426814 (+)  
**5. KLHDC8B (protein coding, ENSBTAG00000000981) Tr:00000001299 Ex:1**: 51432193-51432321 (-)  
**6. KLHDC8B (protein coding, ENSBTAG00000000981) Tr:00000001299 Ex:2**: 51430752-51431261 (-)  
**7. KLHDC8B (protein coding, ENSBTAG00000000981) Tr:00000001299 Ex:3**: 51429530-51429694 (-)  
**8. KLHDC8B (protein coding, ENSBTAG00000000981) Tr:00000001299 Ex:4**: 51428801-51428902 (-)  
**9. KLHDC8B (protein coding, ENSBTAG00000000981) Tr:00000001299 Ex:5**: 51427513-51428371 (-)  
**10. KLHDC8B (protein coding, ENSBTAG00000000981) Tr:00000001298 Ex:1**: 51432193-51432248 (-)  
**11. KLHDC8B (protein coding, ENSBTAG00000000981) Tr:00000001298 Ex:2**: 51430752-51431261 (-)  
**12. KLHDC8B (protein coding, ENSBTAG00000000981) Tr:00000001298 Ex:3**: 51429530-51429694 (-)  
**13. KLHDC8B (protein coding, ENSBTAG00000000981) Tr:00000001298 Ex:4**: 51429004-51429228 (-)  
**14. KLHDC8B (protein coding, ENSBTAG00000000981) Tr:00000001298 Ex:5**: 51428801-51428902 (-)  
**15. KLHDC8B (protein coding, ENSBTAG00000000981) Tr:00000001298 Ex:6**: 51427513-51428371 (-)  
**16. CCDC71 (protein coding, ENSBTAG00000013932) Tr:00000018512 Ex:1**: 51435769-51435809 (+)  
**17. CCDC71 (protein coding, ENSBTAG00000013932) Tr:00000018512 Ex:2**: 51437781-51439453 (+)

  
RepeatMasker Annotation  

**1. AT\_rich**: 51416976-51416996 (+), Divergence to consensus: 47.6%  
**2. L1MC1**: 51417140-51417383 (+), Divergence to consensus: 39%  
**3. L1ME1**: 51417414-51417763 (+), Divergence to consensus: 31.7%  
**4. L1ME3C**: 51417741-51417768 (-), Divergence to consensus: 44.1%  
**5. SINE2-1\_BT**: 51417769-51417887 (-), Divergence to consensus: 19.3%  
**6. L1ME3C**: 51417888-51418147 (-), Divergence to consensus: 44.1%  
**7. L2c**: 51419234-51419351 (+), Divergence to consensus: 40.3%  
**8. L2b**: 51420168-51420296 (+), Divergence to consensus: 36.4%  
**9. GA-rich**: 51420477-51420561 (+), Divergence to consensus: 22.2%  
**10. Bov-tA2**: 51421364-51421577 (+), Divergence to consensus: 18.3%  
**11. LTR33**: 51421877-51421957 (-), Divergence to consensus: 30.1%  
**12. L1MD2**: 51423564-51423761 (-), Divergence to consensus: 24.7%  
**13. SINE2-1\_BT**: 51423779-51423895 (+), Divergence to consensus: 18.8%  
**14. L1-2\_BT**: 51423900-51424081 (-), Divergence to consensus: 42.3%  
**15. AT\_rich**: 51424082-51424117 (+), Divergence to consensus: 61.1%  
**16. Bov-tA2**: 51424200-51424375 (-), Divergence to consensus: 27.8%  
**17. Bov-tA2**: 51424378-51424494 (-), Divergence to consensus: 16.2%  
**18. CHRL**: 51424500-51424636 (-), Divergence to consensus: 17.5%  
**19. L2a**: 51424649-51425611 (-), Divergence to consensus: 45.9%  
**20. MIRc**: 51427523-51427685 (-), Divergence to consensus: 25.2%  
**21. GC\_rich**: 51432192-51432220 (+), Divergence to consensus: 72.4%  
**22. LTR16C**: 51433252-51433552 (-), Divergence to consensus: 33.8%  
**23. CHR-2\_BT**: 51434326-51434533 (+), Divergence to consensus: 22.6%  
**24. L2c**: 51435562-51435634 (+), Divergence to consensus: 40.4%  
**25. ART2A**: 51436833-51436939 (-), Divergence to consensus: 27.1%  
**26. AmnSINE2**: 51437202-51437268 (+), Divergence to consensus: 35.8%

  
Transcription Factor Binding Sites  

**RFX4\_1** (Sequence: CCTGGCAAC (+): 51425833)  
**RFX4\_1** (Sequence: CATGGCAAC (+): 51430297)  
**SPZ1** (Sequence: CTGAAACCCT (-): 51435188)  
**Gata4** (Sequence: AGATAAG (-): 51419425)  
**SOX9** (Sequence: AACAATGA (-): 51419537)  
**SOX9** (Sequence: AACAATGG (-): 51426010)  
**Gata4** (Sequence: CTTATCT (+): 51416951)
