# Supplementary material for: Small RNA sequencing of cryopreserved semen from single bull revealed altered miRNAs and piRNAs expression between High- and Low-motile sperm populations
Source: BMC Genomics. 2017 Jan 4;18:14. doi: 10.1186/s12864-016-3394-7 (PMC5209821; doi:10.1186/s12864-016-3394-7)
Supplement: Additional file 4: — Details for each piRNA clusters found in Low Motile (LM) sperm fraction. Genes, repeats, transposable elements and transcription factors binding sites falling within the cluster regions were reported. (ZIP 1034 kb) [file 12864_2016_3394_MOESM4_ESM.zip › 3.html]

piRNA cluster 3


Predicted piRNA cluster no. 3     previous   next
  

Show proTRAC run info
Hide proTRAC run info

================================= proTRAC ====================================  
VERSION: 2.1                                    LAST MODIFIED: 06. October 2015  
  
Please cite:  
Rosenkranz D, Zischler H. proTRAC - a software for probabilistic piRNA cluster  
detection, visualization and analysis. 2012. BMC Bioinformatics 13:5.  
  
and (for proTRAC 2.0 and later):  
Rosenkranz D, Rudloff S, Bastuck K, Ketting RF, Zischler H. Tupaia small RNAs  
provide insights into function and evolution of RNAi-based transposon defense  
in mammals. 2015. RNA 21(5):911-922.  
  
Contact:  
David Rosenkranz  
Institute of Anthropology, small RNA group  
Johannes Gutenberg University Mainz  
email: rosenkranz@uni-mainz.de  
  
You can find the latest proTRAC version at:  
http://sourceforge.net/projects/protrac/files  
http://www.smallRNAgroup-mainz.de/software  
==============================================================================  
  
PARAMETERS:  
Map file: .............../storage/core/barbara/genhome/smallRNA/fertility/Sample\_not\_motile/pirna/Sample\_not\_motile\_26-33\_collapsed.fa.no-dust.map.weighted-10000-1000-b-0  
Genome file: ............/storage/core/barbara/genhome/smallRNA/fertility/Sample\_all/pirna/bt\_311\_chrY.fa  
RepeatMasker annotation: /storage/genomes/bt\_umd31/GCF\_000003055.6\_Bos\_taurus\_UMD\_3.1.1\_repeatMasker\_chr.out  
GeneSet:................./storage/core/barbara/genhome/smallRNA/fertility/Sample\_all/pirna/full.gtf  
  
Significant (p<=0.01) hit density will be calculated based  
on observed hit distribution.  
  
Sliding window size: ........................................ 5000 bp  
Sliding window increament: .................................. 1000 bp  
Normalize each hit by number of genomic hits: ............... 1 [0=no/1=yes]  
Normalize each hit by number of sequence reads: ............. 1 [0=no/1=yes]  
Normalize values (-> per million mapped reads): ............. 1 [0=no/1=yes]  
Min. fraction of hits with 1T(U) or 10A: .................... 0.75  
Alternatively: Min. fraction of hits with 1T(U) and 10A: .... 0.5  
Min. fraction of hits with typical piRNA length: ............ 0.75  
Typical piRNA length: ....................................... 26-33 nt  
Min. size of a piRNA cluster: ............................... 5000 bp.  
Min. number of hits (absolute): ............................. 0  
Min. number of hits (normalized): ........................... 0  
Min. fraction of hits on the mainstrand: .................... 0.75  
Top fraction of mapped sequences (in terms of read counts): . 1%  
Top fraction accounts for max. n% of sequence reads: ........ 90%  
Min. fraction of hits on each arm of a bidirectional cluster: 0.1  
Output image file for each cluster: ......................... 0 [0=no/1=yes]  
Output html file for each cluster: .......................... 1 [0=no/1=yes]  
Output a summary table: ..................................... 1 [0=no/1=yes]  
Output a FASTA file for each cluster (piRNA sequences): ..... 1 [0=no/1=yes]  
Output a FASTA file comprising cluster sequences: ........... 1 [0=no/1=yes]  
Search DNA motifs in clusters: .............................. 1 [0=no/1=yes]  
Output flanking sequences: +/- .............................. 0 bp  
Output ~.pTi file: .......................................... 1 [0=no/1=yes]  
==============================================================================  
  
  
Genome size (without gaps): ............ 2678902517 bp  
Gaps (N/X/-): .......................... 53837044 bp  
Mapped reads: .......................... 738059667487  
Non-identical sequences: ............... 277001  
Genomic hits: .......................... 533816  
Significant densitiy of mapped reads: .. 15118061 reads/kb

Show proTRAC cluster info
Hide proTRAC cluster info

|  |  |
| --- | --- |
| Location | chr10 |
| Coordinates | 47926396-47938304 |
| Size [bp] | 11909 |
| Sequence hit loci | 1028 |
| Mapped reads (normalized) | 2772073550 |
| Mapped reads (normalized) per kb | 232771311.6 |
| Normalized reads with 1T (1U) | 84.4% |
| Normalized reads with 10A | 34.9% |
| Normalized reads with length 26-33 nt | 100% |
| Normalized reads on the main strand(s) | 99.2% |
| Predicted directionality | bi:minus-plus (split between 47930512 and 47930604) |

100%

0%

1T (1U)  
reads

10A reads

26-33 nt  
reads

reads on mainstrand

**Either the amount of reads with 1T (1U) OR 10A has to exceed 75% (set with option: -1Tor10A)  
Alternatively the amount of reads with 1T (1U) AND 10A has to exceed 50% (set with option: -1Tand10A)  
Minimum amount of reads with preferred size is 75% (set with option: -pisize)  
Minimum amount of reads on the main strand(s) is 75% (set with option: -clstrand)**

Show read coverage
Hide read coverage

WHAT DO I SEE HERE?  
This chart shows the location of mapped sequence reads within a predicted piRNA cluster. The color refers to the number of genomic hits produced by the sequence read in question. A dark red bar indicates that this sequence read produces many other hits elsewhere in the genome. Many adjacent red or yellow bars can indicate the presence of a multi-copy element such as transposons or rRNA genes. A dark green bar indicates that this sequence read maps uniquely to this locus.

1 hit

2-5 hits

6-10 hits

11-20 hits

21-50 hits

51-100 hits

> 100 hits

chr10

47926396

47938304

Gene Set

RepeatMasker

Mapped  
Reads

146.81

plus strand

minus strand

146.81

Region: chr10 47913216-47926407. Max. coverage (+): 0. Max coverage (-): 11.64

Region: chr10 47926408-47926431. Max. coverage (+): 0. Max coverage (-): 0

Region: chr10 47926432-47926455. Max. coverage (+): 0. Max coverage (-): 0

Region: chr10 47926456-47926479. Max. coverage (+): 0. Max coverage (-): 0

Region: chr10 47926480-47926503. Max. coverage (+): 0. Max coverage (-): 0

Region: chr10 47926504-47926526. Max. coverage (+): 0. Max coverage (-): 5.42

Region: chr10 47926527-47926550. Max. coverage (+): 0. Max coverage (-): 0

Region: chr10 47926551-47926574. Max. coverage (+): 0. Max coverage (-): 0

Region: chr10 47926575-47926598. Max. coverage (+): 0. Max coverage (-): 6.16

Region: chr10 47926599-47926622. Max. coverage (+): 0. Max coverage (-): 0

Region: chr10 47926623-47926646. Max. coverage (+): 0. Max coverage (-): 0

Region: chr10 47926647-47926669. Max. coverage (+): 0. Max coverage (-): 0

Region: chr10 47926670-47926693. Max. coverage (+): 0. Max coverage (-): 0

Region: chr10 47926694-47926717. Max. coverage (+): 0. Max coverage (-): 0

Region: chr10 47926718-47926741. Max. coverage (+): 0. Max coverage (-): 0

Region: chr10 47926742-47926765. Max. coverage (+): 0. Max coverage (-): 0

Region: chr10 47926766-47926788. Max. coverage (+): 0. Max coverage (-): 0

Region: chr10 47926789-47926812. Max. coverage (+): 0. Max coverage (-): 0

Region: chr10 47926813-47926836. Max. coverage (+): 0. Max coverage (-): 0

Region: chr10 47926837-47926860. Max. coverage (+): 0. Max coverage (-): 0

Region: chr10 47926861-47926884. Max. coverage (+): 0. Max coverage (-): 0

Region: chr10 47926885-47926908. Max. coverage (+): 0. Max coverage (-): 0

Region: chr10 47926909-47926931. Max. coverage (+): 0. Max coverage (-): 0

Region: chr10 47926932-47926955. Max. coverage (+): 0. Max coverage (-): 0

Region: chr10 47926956-47926979. Max. coverage (+): 0. Max coverage (-): 0

Region: chr10 47926980-47927003. Max. coverage (+): 0. Max coverage (-): 0

Region: chr10 47927004-47927027. Max. coverage (+): 0. Max coverage (-): 0

Region: chr10 47927028-47927050. Max. coverage (+): 0. Max coverage (-): 4.07

Region: chr10 47927051-47927074. Max. coverage (+): 0. Max coverage (-): 4.67

Region: chr10 47927075-47927098. Max. coverage (+): 0. Max coverage (-): 4.67

Region: chr10 47927099-47927122. Max. coverage (+): 0. Max coverage (-): 6.11

Region: chr10 47927123-47927146. Max. coverage (+): 0. Max coverage (-): 0

Region: chr10 47927147-47927170. Max. coverage (+): 0. Max coverage (-): 0

Region: chr10 47927171-47927193. Max. coverage (+): 0. Max coverage (-): 0

Region: chr10 47927194-47927217. Max. coverage (+): 0. Max coverage (-): 0

Region: chr10 47927218-47927241. Max. coverage (+): 0. Max coverage (-): 0

Region: chr10 47927242-47927265. Max. coverage (+): 0. Max coverage (-): 0

Region: chr10 47927266-47927289. Max. coverage (+): 0. Max coverage (-): 0

Region: chr10 47927290-47927312. Max. coverage (+): 0. Max coverage (-): 0

Region: chr10 47927313-47927336. Max. coverage (+): 0. Max coverage (-): 0

Region: chr10 47927337-47927360. Max. coverage (+): 0. Max coverage (-): 0

Region: chr10 47927361-47927384. Max. coverage (+): 0. Max coverage (-): 0

Region: chr10 47927385-47927408. Max. coverage (+): 0. Max coverage (-): 0

Region: chr10 47927409-47927432. Max. coverage (+): 0. Max coverage (-): 0

Region: chr10 47927433-47927455. Max. coverage (+): 0. Max coverage (-): 0

Region: chr10 47927456-47927479. Max. coverage (+): 0. Max coverage (-): 0

Region: chr10 47927480-47927503. Max. coverage (+): 0. Max coverage (-): 0

Region: chr10 47927504-47927527. Max. coverage (+): 0. Max coverage (-): 0

Region: chr10 47927528-47927551. Max. coverage (+): 0. Max coverage (-): 0

Region: chr10 47927552-47927574. Max. coverage (+): 0. Max coverage (-): 0

Region: chr10 47927575-47927598. Max. coverage (+): 0. Max coverage (-): 0

Region: chr10 47927599-47927622. Max. coverage (+): 0. Max coverage (-): 0

Region: chr10 47927623-47927646. Max. coverage (+): 0. Max coverage (-): 0

Region: chr10 47927647-47927670. Max. coverage (+): 0. Max coverage (-): 0

Region: chr10 47927671-47927694. Max. coverage (+): 0. Max coverage (-): 0

Region: chr10 47927695-47927717. Max. coverage (+): 0. Max coverage (-): 0

Region: chr10 47927718-47927741. Max. coverage (+): 0. Max coverage (-): 0

Region: chr10 47927742-47927765. Max. coverage (+): 0. Max coverage (-): 0

Region: chr10 47927766-47927789. Max. coverage (+): 0. Max coverage (-): 0

Region: chr10 47927790-47927813. Max. coverage (+): 0. Max coverage (-): 0

Region: chr10 47927814-47927836. Max. coverage (+): 0. Max coverage (-): 0

Region: chr10 47927837-47927860. Max. coverage (+): 0. Max coverage (-): 0

Region: chr10 47927861-47927884. Max. coverage (+): 0. Max coverage (-): 0

Region: chr10 47927885-47927908. Max. coverage (+): 0. Max coverage (-): 0

Region: chr10 47927909-47927932. Max. coverage (+): 0. Max coverage (-): 0

Region: chr10 47927933-47927956. Max. coverage (+): 0. Max coverage (-): 0

Region: chr10 47927957-47927979. Max. coverage (+): 0. Max coverage (-): 0

Region: chr10 47927980-47928003. Max. coverage (+): 0. Max coverage (-): 0

Region: chr10 47928004-47928027. Max. coverage (+): 0. Max coverage (-): 0

Region: chr10 47928028-47928051. Max. coverage (+): 0. Max coverage (-): 0

Region: chr10 47928052-47928075. Max. coverage (+): 0. Max coverage (-): 0

Region: chr10 47928076-47928098. Max. coverage (+): 0. Max coverage (-): 0

Region: chr10 47928099-47928122. Max. coverage (+): 0. Max coverage (-): 0

Region: chr10 47928123-47928146. Max. coverage (+): 0. Max coverage (-): 0

Region: chr10 47928147-47928170. Max. coverage (+): 0. Max coverage (-): 0

Region: chr10 47928171-47928194. Max. coverage (+): 0. Max coverage (-): 0

Region: chr10 47928195-47928218. Max. coverage (+): 0. Max coverage (-): 0

Region: chr10 47928219-47928241. Max. coverage (+): 0. Max coverage (-): 0

Region: chr10 47928242-47928265. Max. coverage (+): 0. Max coverage (-): 0

Region: chr10 47928266-47928289. Max. coverage (+): 0. Max coverage (-): 0

Region: chr10 47928290-47928313. Max. coverage (+): 0. Max coverage (-): 0

Region: chr10 47928314-47928337. Max. coverage (+): 0. Max coverage (-): 0

Region: chr10 47928338-47928360. Max. coverage (+): 0. Max coverage (-): 0

Region: chr10 47928361-47928384. Max. coverage (+): 0. Max coverage (-): 0

Region: chr10 47928385-47928408. Max. coverage (+): 0. Max coverage (-): 0

Region: chr10 47928409-47928432. Max. coverage (+): 0. Max coverage (-): 0

Region: chr10 47928433-47928456. Max. coverage (+): 0. Max coverage (-): 0

Region: chr10 47928457-47928480. Max. coverage (+): 0. Max coverage (-): 0

Region: chr10 47928481-47928503. Max. coverage (+): 0. Max coverage (-): 0

Region: chr10 47928504-47928527. Max. coverage (+): 0. Max coverage (-): 0

Region: chr10 47928528-47928551. Max. coverage (+): 0. Max coverage (-): 3.84

Region: chr10 47928552-47928575. Max. coverage (+): 0. Max coverage (-): 0

Region: chr10 47928576-47928599. Max. coverage (+): 0. Max coverage (-): 13.56

Region: chr10 47928600-47928622. Max. coverage (+): 0. Max coverage (-): 1.39

Region: chr10 47928623-47928646. Max. coverage (+): 0. Max coverage (-): 0

Region: chr10 47928647-47928670. Max. coverage (+): 0. Max coverage (-): 0

Region: chr10 47928671-47928694. Max. coverage (+): 0. Max coverage (-): 0

Region: chr10 47928695-47928718. Max. coverage (+): 0. Max coverage (-): 5.41

Region: chr10 47928719-47928742. Max. coverage (+): 0. Max coverage (-): 20.1

Region: chr10 47928743-47928765. Max. coverage (+): 0. Max coverage (-): 0

Region: chr10 47928766-47928789. Max. coverage (+): 0. Max coverage (-): 0

Region: chr10 47928790-47928813. Max. coverage (+): 0. Max coverage (-): 0

Region: chr10 47928814-47928837. Max. coverage (+): 0. Max coverage (-): 4.04

Region: chr10 47928838-47928861. Max. coverage (+): 0. Max coverage (-): 13.78

Region: chr10 47928862-47928884. Max. coverage (+): 4.49. Max coverage (-): 3.8

Region: chr10 47928885-47928908. Max. coverage (+): 0. Max coverage (-): 3.8

Region: chr10 47928909-47928932. Max. coverage (+): 0. Max coverage (-): 0

Region: chr10 47928933-47928956. Max. coverage (+): 0. Max coverage (-): 83.69

Region: chr10 47928957-47928980. Max. coverage (+): 0. Max coverage (-): 3.72

Region: chr10 47928981-47929004. Max. coverage (+): 0. Max coverage (-): 7.66

Region: chr10 47929005-47929027. Max. coverage (+): 0. Max coverage (-): 6.85

Region: chr10 47929028-47929051. Max. coverage (+): 0. Max coverage (-): 3.52

Region: chr10 47929052-47929075. Max. coverage (+): 0. Max coverage (-): 5.98

Region: chr10 47929076-47929099. Max. coverage (+): 0. Max coverage (-): 19.53

Region: chr10 47929100-47929123. Max. coverage (+): 0. Max coverage (-): 0

Region: chr10 47929124-47929146. Max. coverage (+): 0. Max coverage (-): 0

Region: chr10 47929147-47929170. Max. coverage (+): 0. Max coverage (-): 0

Region: chr10 47929171-47929194. Max. coverage (+): 0. Max coverage (-): 0

Region: chr10 47929195-47929218. Max. coverage (+): 0. Max coverage (-): 0

Region: chr10 47929219-47929242. Max. coverage (+): 0. Max coverage (-): 0

Region: chr10 47929243-47929266. Max. coverage (+): 0. Max coverage (-): 0

Region: chr10 47929267-47929289. Max. coverage (+): 0. Max coverage (-): 11.27

Region: chr10 47929290-47929313. Max. coverage (+): 0. Max coverage (-): 14.96

Region: chr10 47929314-47929337. Max. coverage (+): 0. Max coverage (-): 2.54

Region: chr10 47929338-47929361. Max. coverage (+): 0. Max coverage (-): 0

Region: chr10 47929362-47929385. Max. coverage (+): 0. Max coverage (-): 2.93

Region: chr10 47929386-47929408. Max. coverage (+): 0. Max coverage (-): 5.44

Region: chr10 47929409-47929432. Max. coverage (+): 0. Max coverage (-): 8.79

Region: chr10 47929433-47929456. Max. coverage (+): 0. Max coverage (-): 54.13

Region: chr10 47929457-47929480. Max. coverage (+): 0. Max coverage (-): 48.33

Region: chr10 47929481-47929504. Max. coverage (+): 0. Max coverage (-): 0

Region: chr10 47929505-47929528. Max. coverage (+): 0. Max coverage (-): 0

Region: chr10 47929529-47929551. Max. coverage (+): 0. Max coverage (-): 0

Region: chr10 47929552-47929575. Max. coverage (+): 0. Max coverage (-): 6.04

Region: chr10 47929576-47929599. Max. coverage (+): 0. Max coverage (-): 9.06

Region: chr10 47929600-47929623. Max. coverage (+): 0. Max coverage (-): 7.65

Region: chr10 47929624-47929647. Max. coverage (+): 0. Max coverage (-): 19.16

Region: chr10 47929648-47929670. Max. coverage (+): 0. Max coverage (-): 23.74

Region: chr10 47929671-47929694. Max. coverage (+): 0. Max coverage (-): 3.95

Region: chr10 47929695-47929718. Max. coverage (+): 0. Max coverage (-): 101.51

Region: chr10 47929719-47929742. Max. coverage (+): 0. Max coverage (-): 1.55

Region: chr10 47929743-47929766. Max. coverage (+): 0. Max coverage (-): 0.76

Region: chr10 47929767-47929790. Max. coverage (+): 0. Max coverage (-): 14.93

Region: chr10 47929791-47929813. Max. coverage (+): 0. Max coverage (-): 6.21

Region: chr10 47929814-47929837. Max. coverage (+): 0. Max coverage (-): 7.2

Region: chr10 47929838-47929861. Max. coverage (+): 0. Max coverage (-): 2.45

Region: chr10 47929862-47929885. Max. coverage (+): 0. Max coverage (-): 0

Region: chr10 47929886-47929909. Max. coverage (+): 0. Max coverage (-): 12.58

Region: chr10 47929910-47929932. Max. coverage (+): 0. Max coverage (-): 6.15

Region: chr10 47929933-47929956. Max. coverage (+): 0. Max coverage (-): 5.43

Region: chr10 47929957-47929980. Max. coverage (+): 0. Max coverage (-): 5.43

Region: chr10 47929981-47930004. Max. coverage (+): 0. Max coverage (-): 0

Region: chr10 47930005-47930028. Max. coverage (+): 0. Max coverage (-): 0

Region: chr10 47930029-47930052. Max. coverage (+): 0. Max coverage (-): 0.75

Region: chr10 47930053-47930075. Max. coverage (+): 0. Max coverage (-): 4.41

Region: chr10 47930076-47930099. Max. coverage (+): 0. Max coverage (-): 6.8

Region: chr10 47930100-47930123. Max. coverage (+): 0. Max coverage (-): 2.29

Region: chr10 47930124-47930147. Max. coverage (+): 0. Max coverage (-): 2.29

Region: chr10 47930148-47930171. Max. coverage (+): 0. Max coverage (-): 10.92

Region: chr10 47930172-47930194. Max. coverage (+): 0. Max coverage (-): 0

Region: chr10 47930195-47930218. Max. coverage (+): 0. Max coverage (-): 1.02

Region: chr10 47930219-47930242. Max. coverage (+): 0. Max coverage (-): 0

Region: chr10 47930243-47930266. Max. coverage (+): 0. Max coverage (-): 4.99

Region: chr10 47930267-47930290. Max. coverage (+): 0. Max coverage (-): 17.2

Region: chr10 47930291-47930314. Max. coverage (+): 0. Max coverage (-): 0

Region: chr10 47930315-47930337. Max. coverage (+): 0. Max coverage (-): 0

Region: chr10 47930338-47930361. Max. coverage (+): 0. Max coverage (-): 0

Region: chr10 47930362-47930385. Max. coverage (+): 0. Max coverage (-): 0

Region: chr10 47930386-47930409. Max. coverage (+): 0. Max coverage (-): 7.2

Region: chr10 47930410-47930433. Max. coverage (+): 0. Max coverage (-): 0

Region: chr10 47930434-47930456. Max. coverage (+): 0. Max coverage (-): 4.78

Region: chr10 47930457-47930480. Max. coverage (+): 0. Max coverage (-): 5.78

Region: chr10 47930481-47930504. Max. coverage (+): 0. Max coverage (-): 6.68

Region: chr10 47930505-47930528. Max. coverage (+): 0. Max coverage (-): 6.68

Region: chr10 47930529-47930552. Max. coverage (+): 0. Max coverage (-): 0

Region: chr10 47930553-47930576. Max. coverage (+): 0. Max coverage (-): 0

Region: chr10 47930577-47930599. Max. coverage (+): 0. Max coverage (-): 0

Region: chr10 47930600-47930623. Max. coverage (+): 5.23. Max coverage (-): 1.61

Region: chr10 47930624-47930647. Max. coverage (+): 0. Max coverage (-): 0

Region: chr10 47930648-47930671. Max. coverage (+): 0. Max coverage (-): 0

Region: chr10 47930672-47930695. Max. coverage (+): 0. Max coverage (-): 0

Region: chr10 47930696-47930718. Max. coverage (+): 0. Max coverage (-): 0

Region: chr10 47930719-47930742. Max. coverage (+): 0. Max coverage (-): 0

Region: chr10 47930743-47930766. Max. coverage (+): 0. Max coverage (-): 0

Region: chr10 47930767-47930790. Max. coverage (+): 0. Max coverage (-): 0

Region: chr10 47930791-47930814. Max. coverage (+): 0. Max coverage (-): 0

Region: chr10 47930815-47930838. Max. coverage (+): 0. Max coverage (-): 0

Region: chr10 47930839-47930861. Max. coverage (+): 0. Max coverage (-): 0

Region: chr10 47930862-47930885. Max. coverage (+): 0. Max coverage (-): 0

Region: chr10 47930886-47930909. Max. coverage (+): 5.79. Max coverage (-): 0

Region: chr10 47930910-47930933. Max. coverage (+): 0. Max coverage (-): 0

Region: chr10 47930934-47930957. Max. coverage (+): 0. Max coverage (-): 0

Region: chr10 47930958-47930980. Max. coverage (+): 0. Max coverage (-): 0

Region: chr10 47930981-47931004. Max. coverage (+): 0. Max coverage (-): 0

Region: chr10 47931005-47931028. Max. coverage (+): 0. Max coverage (-): 0

Region: chr10 47931029-47931052. Max. coverage (+): 2.53. Max coverage (-): 0

Region: chr10 47931053-47931076. Max. coverage (+): 0. Max coverage (-): 0

Region: chr10 47931077-47931100. Max. coverage (+): 0. Max coverage (-): 0

Region: chr10 47931101-47931123. Max. coverage (+): 13.82. Max coverage (-): 0

Region: chr10 47931124-47931147. Max. coverage (+): 22.61. Max coverage (-): 0

Region: chr10 47931148-47931171. Max. coverage (+): 5.71. Max coverage (-): 0

Region: chr10 47931172-47931195. Max. coverage (+): 6.31. Max coverage (-): 0

Region: chr10 47931196-47931219. Max. coverage (+): 22.67. Max coverage (-): 0

Region: chr10 47931220-47931242. Max. coverage (+): 8.4. Max coverage (-): 0

Region: chr10 47931243-47931266. Max. coverage (+): 6.35. Max coverage (-): 0

Region: chr10 47931267-47931290. Max. coverage (+): 7.1. Max coverage (-): 0

Region: chr10 47931291-47931314. Max. coverage (+): 0. Max coverage (-): 2.93

Region: chr10 47931315-47931338. Max. coverage (+): 0. Max coverage (-): 0

Region: chr10 47931339-47931362. Max. coverage (+): 19.13. Max coverage (-): 0

Region: chr10 47931363-47931385. Max. coverage (+): 2.6. Max coverage (-): 0

Region: chr10 47931386-47931409. Max. coverage (+): 13.64. Max coverage (-): 0

Region: chr10 47931410-47931433. Max. coverage (+): 13.64. Max coverage (-): 0

Region: chr10 47931434-47931457. Max. coverage (+): 13.17. Max coverage (-): 0

Region: chr10 47931458-47931481. Max. coverage (+): 2.43. Max coverage (-): 0

Region: chr10 47931482-47931504. Max. coverage (+): 0. Max coverage (-): 0

Region: chr10 47931505-47931528. Max. coverage (+): 11.44. Max coverage (-): 0

Region: chr10 47931529-47931552. Max. coverage (+): 0. Max coverage (-): 0

Region: chr10 47931553-47931576. Max. coverage (+): 0. Max coverage (-): 0

Region: chr10 47931577-47931600. Max. coverage (+): 21.16. Max coverage (-): 0

Region: chr10 47931601-47931624. Max. coverage (+): 4.03. Max coverage (-): 0

Region: chr10 47931625-47931647. Max. coverage (+): 17.66. Max coverage (-): 0

Region: chr10 47931648-47931671. Max. coverage (+): 17.52. Max coverage (-): 0

Region: chr10 47931672-47931695. Max. coverage (+): 0. Max coverage (-): 0

Region: chr10 47931696-47931719. Max. coverage (+): 2.13. Max coverage (-): 0

Region: chr10 47931720-47931743. Max. coverage (+): 13.57. Max coverage (-): 0

Region: chr10 47931744-47931766. Max. coverage (+): 0. Max coverage (-): 0

Region: chr10 47931767-47931790. Max. coverage (+): 4.82. Max coverage (-): 0

Region: chr10 47931791-47931814. Max. coverage (+): 0. Max coverage (-): 0

Region: chr10 47931815-47931838. Max. coverage (+): 0. Max coverage (-): 0

Region: chr10 47931839-47931862. Max. coverage (+): 0. Max coverage (-): 5.37

Region: chr10 47931863-47931886. Max. coverage (+): 0. Max coverage (-): 0

Region: chr10 47931887-47931909. Max. coverage (+): 0. Max coverage (-): 0

Region: chr10 47931910-47931933. Max. coverage (+): 0. Max coverage (-): 0

Region: chr10 47931934-47931957. Max. coverage (+): 0. Max coverage (-): 0

Region: chr10 47931958-47931981. Max. coverage (+): 20.07. Max coverage (-): 0

Region: chr10 47931982-47932005. Max. coverage (+): 83.88. Max coverage (-): 0

Region: chr10 47932006-47932028. Max. coverage (+): 4.63. Max coverage (-): 0

Region: chr10 47932029-47932052. Max. coverage (+): 12.23. Max coverage (-): 0

Region: chr10 47932053-47932076. Max. coverage (+): 0. Max coverage (-): 0

Region: chr10 47932077-47932100. Max. coverage (+): 4.49. Max coverage (-): 0

Region: chr10 47932101-47932124. Max. coverage (+): 1.9. Max coverage (-): 0

Region: chr10 47932125-47932148. Max. coverage (+): 0. Max coverage (-): 0

Region: chr10 47932149-47932171. Max. coverage (+): 23.41. Max coverage (-): 0

Region: chr10 47932172-47932195. Max. coverage (+): 7.58. Max coverage (-): 3.54

Region: chr10 47932196-47932219. Max. coverage (+): 17.4. Max coverage (-): 0

Region: chr10 47932220-47932243. Max. coverage (+): 21.11. Max coverage (-): 6.05

Region: chr10 47932244-47932267. Max. coverage (+): 21.11. Max coverage (-): 0

Region: chr10 47932268-47932290. Max. coverage (+): 54.84. Max coverage (-): 0

Region: chr10 47932291-47932314. Max. coverage (+): 14.29. Max coverage (-): 0

Region: chr10 47932315-47932338. Max. coverage (+): 58.43. Max coverage (-): 0

Region: chr10 47932339-47932362. Max. coverage (+): 58.43. Max coverage (-): 0

Region: chr10 47932363-47932386. Max. coverage (+): 32.89. Max coverage (-): 0

Region: chr10 47932387-47932410. Max. coverage (+): 41.22. Max coverage (-): 0

Region: chr10 47932411-47932433. Max. coverage (+): 30.04. Max coverage (-): 0

Region: chr10 47932434-47932457. Max. coverage (+): 35.85. Max coverage (-): 0

Region: chr10 47932458-47932481. Max. coverage (+): 35.85. Max coverage (-): 0

Region: chr10 47932482-47932505. Max. coverage (+): 0. Max coverage (-): 0

Region: chr10 47932506-47932529. Max. coverage (+): 1.91. Max coverage (-): 0

Region: chr10 47932530-47932552. Max. coverage (+): 0. Max coverage (-): 0

Region: chr10 47932553-47932576. Max. coverage (+): 12.51. Max coverage (-): 0

Region: chr10 47932577-47932600. Max. coverage (+): 18.51. Max coverage (-): 0

Region: chr10 47932601-47932624. Max. coverage (+): 4.36. Max coverage (-): 0

Region: chr10 47932625-47932648. Max. coverage (+): 0. Max coverage (-): 0

Region: chr10 47932649-47932672. Max. coverage (+): 11.28. Max coverage (-): 0

Region: chr10 47932673-47932695. Max. coverage (+): 6.37. Max coverage (-): 0

Region: chr10 47932696-47932719. Max. coverage (+): 5.54. Max coverage (-): 0

Region: chr10 47932720-47932743. Max. coverage (+): 5.54. Max coverage (-): 0

Region: chr10 47932744-47932767. Max. coverage (+): 0. Max coverage (-): 0

Region: chr10 47932768-47932791. Max. coverage (+): 1.75. Max coverage (-): 0

Region: chr10 47932792-47932814. Max. coverage (+): 0. Max coverage (-): 0

Region: chr10 47932815-47932838. Max. coverage (+): 4.14. Max coverage (-): 0

Region: chr10 47932839-47932862. Max. coverage (+): 4.06. Max coverage (-): 0

Region: chr10 47932863-47932886. Max. coverage (+): 16.61. Max coverage (-): 0

Region: chr10 47932887-47932910. Max. coverage (+): 3.21. Max coverage (-): 0

Region: chr10 47932911-47932934. Max. coverage (+): 0. Max coverage (-): 0

Region: chr10 47932935-47932957. Max. coverage (+): 9.4. Max coverage (-): 0

Region: chr10 47932958-47932981. Max. coverage (+): 9.4. Max coverage (-): 0

Region: chr10 47932982-47933005. Max. coverage (+): 0. Max coverage (-): 0

Region: chr10 47933006-47933029. Max. coverage (+): 0. Max coverage (-): 0

Region: chr10 47933030-47933053. Max. coverage (+): 0. Max coverage (-): 0

Region: chr10 47933054-47933076. Max. coverage (+): 3.78. Max coverage (-): 0

Region: chr10 47933077-47933100. Max. coverage (+): 0. Max coverage (-): 0

Region: chr10 47933101-47933124. Max. coverage (+): 0. Max coverage (-): 0

Region: chr10 47933125-47933148. Max. coverage (+): 0. Max coverage (-): 0

Region: chr10 47933149-47933172. Max. coverage (+): 39.73. Max coverage (-): 0

Region: chr10 47933173-47933196. Max. coverage (+): 0. Max coverage (-): 0

Region: chr10 47933197-47933219. Max. coverage (+): 7.8. Max coverage (-): 0

Region: chr10 47933220-47933243. Max. coverage (+): 8.9. Max coverage (-): 0

Region: chr10 47933244-47933267. Max. coverage (+): 8.91. Max coverage (-): 0

Region: chr10 47933268-47933291. Max. coverage (+): 146.81. Max coverage (-): 0

Region: chr10 47933292-47933315. Max. coverage (+): 137.09. Max coverage (-): 0

Region: chr10 47933316-47933338. Max. coverage (+): 11.55. Max coverage (-): 0

Region: chr10 47933339-47933362. Max. coverage (+): 0. Max coverage (-): 0

Region: chr10 47933363-47933386. Max. coverage (+): 17.69. Max coverage (-): 0

Region: chr10 47933387-47933410. Max. coverage (+): 9.73. Max coverage (-): 0

Region: chr10 47933411-47933434. Max. coverage (+): 10.19. Max coverage (-): 0

Region: chr10 47933435-47933458. Max. coverage (+): 28.91. Max coverage (-): 0

Region: chr10 47933459-47933481. Max. coverage (+): 26.07. Max coverage (-): 0

Region: chr10 47933482-47933505. Max. coverage (+): 49.51. Max coverage (-): 0

Region: chr10 47933506-47933529. Max. coverage (+): 16.29. Max coverage (-): 0

Region: chr10 47933530-47933553. Max. coverage (+): 16.29. Max coverage (-): 0

Region: chr10 47933554-47933577. Max. coverage (+): 10.66. Max coverage (-): 0

Region: chr10 47933578-47933600. Max. coverage (+): 24.84. Max coverage (-): 0

Region: chr10 47933601-47933624. Max. coverage (+): 8.61. Max coverage (-): 0

Region: chr10 47933625-47933648. Max. coverage (+): 13.79. Max coverage (-): 0

Region: chr10 47933649-47933672. Max. coverage (+): 10.82. Max coverage (-): 0

Region: chr10 47933673-47933696. Max. coverage (+): 0. Max coverage (-): 0

Region: chr10 47933697-47933720. Max. coverage (+): 6.18. Max coverage (-): 0

Region: chr10 47933721-47933743. Max. coverage (+): 5.15. Max coverage (-): 0

Region: chr10 47933744-47933767. Max. coverage (+): 0. Max coverage (-): 0

Region: chr10 47933768-47933791. Max. coverage (+): 0. Max coverage (-): 0

Region: chr10 47933792-47933815. Max. coverage (+): 0. Max coverage (-): 0

Region: chr10 47933816-47933839. Max. coverage (+): 0. Max coverage (-): 0

Region: chr10 47933840-47933862. Max. coverage (+): 0. Max coverage (-): 0

Region: chr10 47933863-47933886. Max. coverage (+): 24.21. Max coverage (-): 0

Region: chr10 47933887-47933910. Max. coverage (+): 0. Max coverage (-): 0

Region: chr10 47933911-47933934. Max. coverage (+): 50.48. Max coverage (-): 0

Region: chr10 47933935-47933958. Max. coverage (+): 36.95. Max coverage (-): 0

Region: chr10 47933959-47933982. Max. coverage (+): 21.17. Max coverage (-): 0

Region: chr10 47933983-47934005. Max. coverage (+): 41.57. Max coverage (-): 0

Region: chr10 47934006-47934029. Max. coverage (+): 18.7. Max coverage (-): 0

Region: chr10 47934030-47934053. Max. coverage (+): 18.7. Max coverage (-): 0

Region: chr10 47934054-47934077. Max. coverage (+): 3.51. Max coverage (-): 0

Region: chr10 47934078-47934101. Max. coverage (+): 3.51. Max coverage (-): 0

Region: chr10 47934102-47934124. Max. coverage (+): 2.33. Max coverage (-): 0

Region: chr10 47934125-47934148. Max. coverage (+): 17.71. Max coverage (-): 0

Region: chr10 47934149-47934172. Max. coverage (+): 0.7. Max coverage (-): 0

Region: chr10 47934173-47934196. Max. coverage (+): 0. Max coverage (-): 0

Region: chr10 47934197-47934220. Max. coverage (+): 9.26. Max coverage (-): 0

Region: chr10 47934221-47934244. Max. coverage (+): 0. Max coverage (-): 0

Region: chr10 47934245-47934267. Max. coverage (+): 0. Max coverage (-): 0

Region: chr10 47934268-47934291. Max. coverage (+): 9.23. Max coverage (-): 0

Region: chr10 47934292-47934315. Max. coverage (+): 9.23. Max coverage (-): 0

Region: chr10 47934316-47934339. Max. coverage (+): 0. Max coverage (-): 0

Region: chr10 47934340-47934363. Max. coverage (+): 0. Max coverage (-): 0

Region: chr10 47934364-47934386. Max. coverage (+): 0. Max coverage (-): 0

Region: chr10 47934387-47934410. Max. coverage (+): 6.79. Max coverage (-): 0

Region: chr10 47934411-47934434. Max. coverage (+): 6.79. Max coverage (-): 0

Region: chr10 47934435-47934458. Max. coverage (+): 4.03. Max coverage (-): 0

Region: chr10 47934459-47934482. Max. coverage (+): 143.6. Max coverage (-): 0

Region: chr10 47934483-47934506. Max. coverage (+): 29.79. Max coverage (-): 0

Region: chr10 47934507-47934529. Max. coverage (+): 9.74. Max coverage (-): 0

Region: chr10 47934530-47934553. Max. coverage (+): 22.68. Max coverage (-): 0

Region: chr10 47934554-47934577. Max. coverage (+): 0. Max coverage (-): 0

Region: chr10 47934578-47934601. Max. coverage (+): 0. Max coverage (-): 0

Region: chr10 47934602-47934625. Max. coverage (+): 16.5. Max coverage (-): 0

Region: chr10 47934626-47934648. Max. coverage (+): 10.13. Max coverage (-): 0

Region: chr10 47934649-47934672. Max. coverage (+): 31.4. Max coverage (-): 0

Region: chr10 47934673-47934696. Max. coverage (+): 22.8. Max coverage (-): 0

Region: chr10 47934697-47934720. Max. coverage (+): 4. Max coverage (-): 0

Region: chr10 47934721-47934744. Max. coverage (+): 31.56. Max coverage (-): 0

Region: chr10 47934745-47934768. Max. coverage (+): 15.46. Max coverage (-): 0

Region: chr10 47934769-47934791. Max. coverage (+): 18.12. Max coverage (-): 0

Region: chr10 47934792-47934815. Max. coverage (+): 0. Max coverage (-): 0

Region: chr10 47934816-47934839. Max. coverage (+): 31.36. Max coverage (-): 0

Region: chr10 47934840-47934863. Max. coverage (+): 0. Max coverage (-): 0

Region: chr10 47934864-47934887. Max. coverage (+): 35.61. Max coverage (-): 0

Region: chr10 47934888-47934910. Max. coverage (+): 34.87. Max coverage (-): 0

Region: chr10 47934911-47934934. Max. coverage (+): 18.19. Max coverage (-): 0

Region: chr10 47934935-47934958. Max. coverage (+): 10.5. Max coverage (-): 0

Region: chr10 47934959-47934982. Max. coverage (+): 5.54. Max coverage (-): 0

Region: chr10 47934983-47935006. Max. coverage (+): 3.23. Max coverage (-): 0

Region: chr10 47935007-47935030. Max. coverage (+): 11.19. Max coverage (-): 0

Region: chr10 47935031-47935053. Max. coverage (+): 0. Max coverage (-): 0

Region: chr10 47935054-47935077. Max. coverage (+): 7.56. Max coverage (-): 0

Region: chr10 47935078-47935101. Max. coverage (+): 34.03. Max coverage (-): 0

Region: chr10 47935102-47935125. Max. coverage (+): 40.78. Max coverage (-): 0

Region: chr10 47935126-47935149. Max. coverage (+): 5.25. Max coverage (-): 0

Region: chr10 47935150-47935172. Max. coverage (+): 8. Max coverage (-): 0

Region: chr10 47935173-47935196. Max. coverage (+): 15.3. Max coverage (-): 0

Region: chr10 47935197-47935220. Max. coverage (+): 11.98. Max coverage (-): 0

Region: chr10 47935221-47935244. Max. coverage (+): 23.69. Max coverage (-): 0

Region: chr10 47935245-47935268. Max. coverage (+): 16.39. Max coverage (-): 5.61

Region: chr10 47935269-47935292. Max. coverage (+): 8.65. Max coverage (-): 0

Region: chr10 47935293-47935315. Max. coverage (+): 3.29. Max coverage (-): 0

Region: chr10 47935316-47935339. Max. coverage (+): 11.7. Max coverage (-): 0

Region: chr10 47935340-47935363. Max. coverage (+): 11.7. Max coverage (-): 0

Region: chr10 47935364-47935387. Max. coverage (+): 30.74. Max coverage (-): 0

Region: chr10 47935388-47935411. Max. coverage (+): 0.88. Max coverage (-): 0

Region: chr10 47935412-47935434. Max. coverage (+): 71.1. Max coverage (-): 0

Region: chr10 47935435-47935458. Max. coverage (+): 15.06. Max coverage (-): 0

Region: chr10 47935459-47935482. Max. coverage (+): 20.9. Max coverage (-): 0

Region: chr10 47935483-47935506. Max. coverage (+): 20.9. Max coverage (-): 0

Region: chr10 47935507-47935530. Max. coverage (+): 0. Max coverage (-): 0

Region: chr10 47935531-47935554. Max. coverage (+): 4.53. Max coverage (-): 0

Region: chr10 47935555-47935577. Max. coverage (+): 30.33. Max coverage (-): 0

Region: chr10 47935578-47935601. Max. coverage (+): 63.62. Max coverage (-): 0

Region: chr10 47935602-47935625. Max. coverage (+): 4.83. Max coverage (-): 0

Region: chr10 47935626-47935649. Max. coverage (+): 0. Max coverage (-): 0

Region: chr10 47935650-47935673. Max. coverage (+): 0. Max coverage (-): 0

Region: chr10 47935674-47935696. Max. coverage (+): 0. Max coverage (-): 0

Region: chr10 47935697-47935720. Max. coverage (+): 0. Max coverage (-): 0

Region: chr10 47935721-47935744. Max. coverage (+): 0. Max coverage (-): 0

Region: chr10 47935745-47935768. Max. coverage (+): 0. Max coverage (-): 0

Region: chr10 47935769-47935792. Max. coverage (+): 0. Max coverage (-): 0

Region: chr10 47935793-47935816. Max. coverage (+): 0. Max coverage (-): 0

Region: chr10 47935817-47935839. Max. coverage (+): 0. Max coverage (-): 0

Region: chr10 47935840-47935863. Max. coverage (+): 0. Max coverage (-): 0

Region: chr10 47935864-47935887. Max. coverage (+): 0. Max coverage (-): 0

Region: chr10 47935888-47935911. Max. coverage (+): 0. Max coverage (-): 0

Region: chr10 47935912-47935935. Max. coverage (+): 0. Max coverage (-): 0

Region: chr10 47935936-47935958. Max. coverage (+): 0. Max coverage (-): 0

Region: chr10 47935959-47935982. Max. coverage (+): 0. Max coverage (-): 0

Region: chr10 47935983-47936006. Max. coverage (+): 0. Max coverage (-): 0

Region: chr10 47936007-47936030. Max. coverage (+): 0. Max coverage (-): 0

Region: chr10 47936031-47936054. Max. coverage (+): 0. Max coverage (-): 0

Region: chr10 47936055-47936078. Max. coverage (+): 0. Max coverage (-): 0

Region: chr10 47936079-47936101. Max. coverage (+): 0. Max coverage (-): 0

Region: chr10 47936102-47936125. Max. coverage (+): 9.12. Max coverage (-): 0

Region: chr10 47936126-47936149. Max. coverage (+): 7.8. Max coverage (-): 0

Region: chr10 47936150-47936173. Max. coverage (+): 4.47. Max coverage (-): 0

Region: chr10 47936174-47936197. Max. coverage (+): 4.47. Max coverage (-): 0

Region: chr10 47936198-47936220. Max. coverage (+): 0. Max coverage (-): 0

Region: chr10 47936221-47936244. Max. coverage (+): 0. Max coverage (-): 0

Region: chr10 47936245-47936268. Max. coverage (+): 0. Max coverage (-): 0

Region: chr10 47936269-47936292. Max. coverage (+): 0. Max coverage (-): 0

Region: chr10 47936293-47936316. Max. coverage (+): 0. Max coverage (-): 0

Region: chr10 47936317-47936340. Max. coverage (+): 0. Max coverage (-): 0

Region: chr10 47936341-47936363. Max. coverage (+): 0. Max coverage (-): 0

Region: chr10 47936364-47936387. Max. coverage (+): 0. Max coverage (-): 0

Region: chr10 47936388-47936411. Max. coverage (+): 0. Max coverage (-): 0

Region: chr10 47936412-47936435. Max. coverage (+): 5.82. Max coverage (-): 0

Region: chr10 47936436-47936459. Max. coverage (+): 9.02. Max coverage (-): 0

Region: chr10 47936460-47936482. Max. coverage (+): 0. Max coverage (-): 0

Region: chr10 47936483-47936506. Max. coverage (+): 0. Max coverage (-): 0

Region: chr10 47936507-47936530. Max. coverage (+): 0. Max coverage (-): 0

Region: chr10 47936531-47936554. Max. coverage (+): 0. Max coverage (-): 0

Region: chr10 47936555-47936578. Max. coverage (+): 0. Max coverage (-): 0

Region: chr10 47936579-47936602. Max. coverage (+): 0. Max coverage (-): 0

Region: chr10 47936603-47936625. Max. coverage (+): 0. Max coverage (-): 0

Region: chr10 47936626-47936649. Max. coverage (+): 0. Max coverage (-): 0

Region: chr10 47936650-47936673. Max. coverage (+): 6.37. Max coverage (-): 0

Region: chr10 47936674-47936697. Max. coverage (+): 0. Max coverage (-): 0

Region: chr10 47936698-47936721. Max. coverage (+): 0. Max coverage (-): 0

Region: chr10 47936722-47936744. Max. coverage (+): 0. Max coverage (-): 0

Region: chr10 47936745-47936768. Max. coverage (+): 0. Max coverage (-): 0

Region: chr10 47936769-47936792. Max. coverage (+): 0. Max coverage (-): 0

Region: chr10 47936793-47936816. Max. coverage (+): 0. Max coverage (-): 0

Region: chr10 47936817-47936840. Max. coverage (+): 0. Max coverage (-): 0

Region: chr10 47936841-47936864. Max. coverage (+): 0. Max coverage (-): 0

Region: chr10 47936865-47936887. Max. coverage (+): 6.67. Max coverage (-): 0

Region: chr10 47936888-47936911. Max. coverage (+): 0. Max coverage (-): 0

Region: chr10 47936912-47936935. Max. coverage (+): 0. Max coverage (-): 0

Region: chr10 47936936-47936959. Max. coverage (+): 0. Max coverage (-): 0

Region: chr10 47936960-47936983. Max. coverage (+): 0. Max coverage (-): 0

Region: chr10 47936984-47937006. Max. coverage (+): 7.04. Max coverage (-): 0

Region: chr10 47937007-47937030. Max. coverage (+): 7.04. Max coverage (-): 0

Region: chr10 47937031-47937054. Max. coverage (+): 13.72. Max coverage (-): 0

Region: chr10 47937055-47937078. Max. coverage (+): 1.93. Max coverage (-): 0

Region: chr10 47937079-47937102. Max. coverage (+): 0. Max coverage (-): 0

Region: chr10 47937103-47937126. Max. coverage (+): 0. Max coverage (-): 0

Region: chr10 47937127-47937149. Max. coverage (+): 0. Max coverage (-): 0

Region: chr10 47937150-47937173. Max. coverage (+): 0. Max coverage (-): 0

Region: chr10 47937174-47937197. Max. coverage (+): 6.05. Max coverage (-): 0

Region: chr10 47937198-47937221. Max. coverage (+): 6.05. Max coverage (-): 0

Region: chr10 47937222-47937245. Max. coverage (+): 0. Max coverage (-): 0

Region: chr10 47937246-47937268. Max. coverage (+): 0. Max coverage (-): 0

Region: chr10 47937269-47937292. Max. coverage (+): 0. Max coverage (-): 0

Region: chr10 47937293-47937316. Max. coverage (+): 0. Max coverage (-): 0

Region: chr10 47937317-47937340. Max. coverage (+): 0. Max coverage (-): 0

Region: chr10 47937341-47937364. Max. coverage (+): 0. Max coverage (-): 0

Region: chr10 47937365-47937388. Max. coverage (+): 3.37. Max coverage (-): 0

Region: chr10 47937389-47937411. Max. coverage (+): 0. Max coverage (-): 0

Region: chr10 47937412-47937435. Max. coverage (+): 0. Max coverage (-): 0

Region: chr10 47937436-47937459. Max. coverage (+): 0. Max coverage (-): 0

Region: chr10 47937460-47937483. Max. coverage (+): 0. Max coverage (-): 0

Region: chr10 47937484-47937507. Max. coverage (+): 0. Max coverage (-): 0

Region: chr10 47937508-47937530. Max. coverage (+): 0. Max coverage (-): 0

Region: chr10 47937531-47937554. Max. coverage (+): 0. Max coverage (-): 0

Region: chr10 47937555-47937578. Max. coverage (+): 0. Max coverage (-): 0

Region: chr10 47937579-47937602. Max. coverage (+): 0. Max coverage (-): 0

Region: chr10 47937603-47937626. Max. coverage (+): 0. Max coverage (-): 0

Region: chr10 47937627-47937650. Max. coverage (+): 1.38. Max coverage (-): 0

Region: chr10 47937651-47937673. Max. coverage (+): 1.38. Max coverage (-): 0

Region: chr10 47937674-47937697. Max. coverage (+): 0. Max coverage (-): 0

Region: chr10 47937698-47937721. Max. coverage (+): 0. Max coverage (-): 0

Region: chr10 47937722-47937745. Max. coverage (+): 0. Max coverage (-): 0

Region: chr10 47937746-47937769. Max. coverage (+): 0. Max coverage (-): 0

Region: chr10 47937770-47937792. Max. coverage (+): 0. Max coverage (-): 0

Region: chr10 47937793-47937816. Max. coverage (+): 0. Max coverage (-): 0

Region: chr10 47937817-47937840. Max. coverage (+): 0. Max coverage (-): 0

Region: chr10 47937841-47937864. Max. coverage (+): 0. Max coverage (-): 0

Region: chr10 47937865-47937888. Max. coverage (+): 0. Max coverage (-): 0

Region: chr10 47937889-47937912. Max. coverage (+): 0. Max coverage (-): 0

Region: chr10 47937913-47937935. Max. coverage (+): 0. Max coverage (-): 0

Region: chr10 47937936-47937959. Max. coverage (+): 0. Max coverage (-): 0

Region: chr10 47937960-47937983. Max. coverage (+): 12.13. Max coverage (-): 0

Region: chr10 47937984-47938007. Max. coverage (+): 19.14. Max coverage (-): 0

Region: chr10 47938008-47938031. Max. coverage (+): 22.73. Max coverage (-): 0

Region: chr10 47938032-47938054. Max. coverage (+): 0. Max coverage (-): 0

Region: chr10 47938055-47938078. Max. coverage (+): 0. Max coverage (-): 0

Region: chr10 47938079-47938102. Max. coverage (+): 22.29. Max coverage (-): 0

Region: chr10 47938103-47938126. Max. coverage (+): 2.02. Max coverage (-): 0

Region: chr10 47938127-47938150. Max. coverage (+): 0. Max coverage (-): 0

Region: chr10 47938151-47938174. Max. coverage (+): 0. Max coverage (-): 0

Region: chr10 47938175-47938197. Max. coverage (+): 0. Max coverage (-): 0

Region: chr10 47938198-47938221. Max. coverage (+): 0. Max coverage (-): 0

Region: chr10 47938222-47938245. Max. coverage (+): 0. Max coverage (-): 0

Region: chr10 47938246-47938269. Max. coverage (+): 0. Max coverage (-): 0

Region: chr10 47938270-47938293. Max. coverage (+): 0.96. Max coverage (-): 0

Region: chr10 47938294-. Max. coverage (+): 0. Max coverage (-): 0

RepeatMasker Color Code

**+**

100-98% Identity

<98-95% Identity

<95-90% Identity

<90-85% Identity

<85-80% Identity

<80-75% Identity

<75-70% Identity

<70% Identity

**-**

Gene Set Color Code

**+**

Gene

Pseudogene

**-**

Topology/Coverage Color Code

Coverage Plus Strand

Coverage Minus Strand

Mainstrand: Plus

Mainstrand: Minus

Complementary Strand

Flanking Region  
(if option -flank >0)

Gene Set Annotation  
  
RepeatMasker Annotation  

**1. (TA)n**: 47926792-47926910 (+), Divergence to consensus: 31.3%  
**2. L1MEj**: 47927693-47928080 (-), Divergence to consensus: 40.6%  
**3. MIRb**: 47929138-47929286 (-), Divergence to consensus: 41.7%  
**4. GC\_rich**: 47930581-47930602 (+), Divergence to consensus: 36.4%  
**5. L2b**: 47933757-47933858 (-), Divergence to consensus: 37.4%  
**6. LTR5\_BT**: 47935638-47936062 (+), Divergence to consensus: 14.4%  
**7. MIRb**: 47936233-47936389 (+), Divergence to consensus: 28.3%  
**8. Charlie4z**: 47936546-47936643 (+), Divergence to consensus: 32.7%  
**9. MER94B**: 47936777-47936819 (-), Divergence to consensus: 27.8%  
**10. MIR3**: 47936905-47936979 (+), Divergence to consensus: 35.8%  
**11. L1MC2**: 47937560-47937624 (-), Divergence to consensus: 22.9%  
**12. L1M5**: 47937677-47937924 (-), Divergence to consensus: 32.3%

  
Transcription Factor Binding Sites  

**SOX9** (Sequence: AACAATAG (-): 47926684)  
**SOX9** (Sequence: AACAATAA (-): 47927485)  
**SOX9** (Sequence: AACAATAG (-): 47934668)  
**SOX9** (Sequence: AACAATGG (-): 47935150)  
**SOX9** (Sequence: CCATTGTT (+): 47929953)  
**Gata4** (Sequence: CTTATCT (+): 47927470)
