# Supplementary material for: Small RNA sequencing of cryopreserved semen from single bull revealed altered miRNAs and piRNAs expression between High- and Low-motile sperm populations
Source: BMC Genomics. 2017 Jan 4;18:14. doi: 10.1186/s12864-016-3394-7 (PMC5209821; doi:10.1186/s12864-016-3394-7)
Supplement: Additional file 4: — Details for each piRNA clusters found in Low Motile (LM) sperm fraction. Genes, repeats, transposable elements and transcription factors binding sites falling within the cluster regions were reported. (ZIP 1034 kb) [file 12864_2016_3394_MOESM4_ESM.zip › 30.html]

piRNA cluster 30


Predicted piRNA cluster no. 30     previous   next
  

Show proTRAC run info
Hide proTRAC run info

================================= proTRAC ====================================  
VERSION: 2.1                                    LAST MODIFIED: 06. October 2015  
  
Please cite:  
Rosenkranz D, Zischler H. proTRAC - a software for probabilistic piRNA cluster  
detection, visualization and analysis. 2012. BMC Bioinformatics 13:5.  
  
and (for proTRAC 2.0 and later):  
Rosenkranz D, Rudloff S, Bastuck K, Ketting RF, Zischler H. Tupaia small RNAs  
provide insights into function and evolution of RNAi-based transposon defense  
in mammals. 2015. RNA 21(5):911-922.  
  
Contact:  
David Rosenkranz  
Institute of Anthropology, small RNA group  
Johannes Gutenberg University Mainz  
email: rosenkranz@uni-mainz.de  
  
You can find the latest proTRAC version at:  
http://sourceforge.net/projects/protrac/files  
http://www.smallRNAgroup-mainz.de/software  
==============================================================================  
  
PARAMETERS:  
Map file: .............../storage/core/barbara/genhome/smallRNA/fertility/Sample\_not\_motile/pirna/Sample\_not\_motile\_26-33\_collapsed.fa.no-dust.map.weighted-10000-1000-b-0  
Genome file: ............/storage/core/barbara/genhome/smallRNA/fertility/Sample\_all/pirna/bt\_311\_chrY.fa  
RepeatMasker annotation: /storage/genomes/bt\_umd31/GCF\_000003055.6\_Bos\_taurus\_UMD\_3.1.1\_repeatMasker\_chr.out  
GeneSet:................./storage/core/barbara/genhome/smallRNA/fertility/Sample\_all/pirna/full.gtf  
  
Significant (p<=0.01) hit density will be calculated based  
on observed hit distribution.  
  
Sliding window size: ........................................ 5000 bp  
Sliding window increament: .................................. 1000 bp  
Normalize each hit by number of genomic hits: ............... 1 [0=no/1=yes]  
Normalize each hit by number of sequence reads: ............. 1 [0=no/1=yes]  
Normalize values (-> per million mapped reads): ............. 1 [0=no/1=yes]  
Min. fraction of hits with 1T(U) or 10A: .................... 0.75  
Alternatively: Min. fraction of hits with 1T(U) and 10A: .... 0.5  
Min. fraction of hits with typical piRNA length: ............ 0.75  
Typical piRNA length: ....................................... 26-33 nt  
Min. size of a piRNA cluster: ............................... 5000 bp.  
Min. number of hits (absolute): ............................. 0  
Min. number of hits (normalized): ........................... 0  
Min. fraction of hits on the mainstrand: .................... 0.75  
Top fraction of mapped sequences (in terms of read counts): . 1%  
Top fraction accounts for max. n% of sequence reads: ........ 90%  
Min. fraction of hits on each arm of a bidirectional cluster: 0.1  
Output image file for each cluster: ......................... 0 [0=no/1=yes]  
Output html file for each cluster: .......................... 1 [0=no/1=yes]  
Output a summary table: ..................................... 1 [0=no/1=yes]  
Output a FASTA file for each cluster (piRNA sequences): ..... 1 [0=no/1=yes]  
Output a FASTA file comprising cluster sequences: ........... 1 [0=no/1=yes]  
Search DNA motifs in clusters: .............................. 1 [0=no/1=yes]  
Output flanking sequences: +/- .............................. 0 bp  
Output ~.pTi file: .......................................... 1 [0=no/1=yes]  
==============================================================================  
  
  
Genome size (without gaps): ............ 2678902517 bp  
Gaps (N/X/-): .......................... 53837044 bp  
Mapped reads: .......................... 738059667487  
Non-identical sequences: ............... 277001  
Genomic hits: .......................... 533816  
Significant densitiy of mapped reads: .. 15118061 reads/kb

Show proTRAC cluster info
Hide proTRAC cluster info

|  |  |
| --- | --- |
| Location | chr22 |
| Coordinates | 59838230-59850052 |
| Size [bp] | 11823 |
| Sequence hit loci | 404 |
| Mapped reads (normalized) | 1082890588 |
| Mapped reads (normalized) per kb | 91591862.3 |
| Normalized reads with 1T (1U) | 80.4% |
| Normalized reads with 10A | 24.5% |
| Normalized reads with length 26-33 nt | 100% |
| Normalized reads on the main strand(s) | 99% |
| Predicted directionality | bi:minus-plus (split between 59846844 and 59847127) |

100%

0%

1T (1U)  
reads

10A reads

26-33 nt  
reads

reads on mainstrand

**Either the amount of reads with 1T (1U) OR 10A has to exceed 75% (set with option: -1Tor10A)  
Alternatively the amount of reads with 1T (1U) AND 10A has to exceed 50% (set with option: -1Tand10A)  
Minimum amount of reads with preferred size is 75% (set with option: -pisize)  
Minimum amount of reads on the main strand(s) is 75% (set with option: -clstrand)**

Show read coverage
Hide read coverage

WHAT DO I SEE HERE?  
This chart shows the location of mapped sequence reads within a predicted piRNA cluster. The color refers to the number of genomic hits produced by the sequence read in question. A dark red bar indicates that this sequence read produces many other hits elsewhere in the genome. Many adjacent red or yellow bars can indicate the presence of a multi-copy element such as transposons or rRNA genes. A dark green bar indicates that this sequence read maps uniquely to this locus.

1 hit

2-5 hits

6-10 hits

11-20 hits

21-50 hits

51-100 hits

> 100 hits

chr22

59838230

59850052

Gene Set

RepeatMasker

Mapped  
Reads

72.29

plus strand

minus strand

72.29

Region: chr22 51438964-59838241. Max. coverage (+): 0. Max coverage (-): 6.24

Region: chr22 59838242-59838265. Max. coverage (+): 0. Max coverage (-): 0

Region: chr22 59838266-59838289. Max. coverage (+): 0. Max coverage (-): 0

Region: chr22 59838290-59838312. Max. coverage (+): 0. Max coverage (-): 10.66

Region: chr22 59838313-59838336. Max. coverage (+): 0. Max coverage (-): 0

Region: chr22 59838337-59838360. Max. coverage (+): 0. Max coverage (-): 0

Region: chr22 59838361-59838383. Max. coverage (+): 0. Max coverage (-): 0

Region: chr22 59838384-59838407. Max. coverage (+): 0. Max coverage (-): 0

Region: chr22 59838408-59838430. Max. coverage (+): 0. Max coverage (-): 0

Region: chr22 59838431-59838454. Max. coverage (+): 0. Max coverage (-): 0

Region: chr22 59838455-59838478. Max. coverage (+): 0. Max coverage (-): 0

Region: chr22 59838479-59838501. Max. coverage (+): 0. Max coverage (-): 0

Region: chr22 59838502-59838525. Max. coverage (+): 0. Max coverage (-): 0

Region: chr22 59838526-59838549. Max. coverage (+): 0. Max coverage (-): 0

Region: chr22 59838550-59838572. Max. coverage (+): 0. Max coverage (-): 0

Region: chr22 59838573-59838596. Max. coverage (+): 0. Max coverage (-): 0

Region: chr22 59838597-59838620. Max. coverage (+): 0. Max coverage (-): 0.13

Region: chr22 59838621-59838643. Max. coverage (+): 0. Max coverage (-): 0

Region: chr22 59838644-59838667. Max. coverage (+): 0. Max coverage (-): 0

Region: chr22 59838668-59838691. Max. coverage (+): 0. Max coverage (-): 0

Region: chr22 59838692-59838714. Max. coverage (+): 0. Max coverage (-): 0

Region: chr22 59838715-59838738. Max. coverage (+): 0. Max coverage (-): 0

Region: chr22 59838739-59838762. Max. coverage (+): 0. Max coverage (-): 0

Region: chr22 59838763-59838785. Max. coverage (+): 0. Max coverage (-): 0

Region: chr22 59838786-59838809. Max. coverage (+): 0. Max coverage (-): 0

Region: chr22 59838810-59838832. Max. coverage (+): 0. Max coverage (-): 0

Region: chr22 59838833-59838856. Max. coverage (+): 0. Max coverage (-): 0

Region: chr22 59838857-59838880. Max. coverage (+): 0. Max coverage (-): 0

Region: chr22 59838881-59838903. Max. coverage (+): 0. Max coverage (-): 0

Region: chr22 59838904-59838927. Max. coverage (+): 0. Max coverage (-): 0

Region: chr22 59838928-59838951. Max. coverage (+): 0. Max coverage (-): 0

Region: chr22 59838952-59838974. Max. coverage (+): 0. Max coverage (-): 0

Region: chr22 59838975-59838998. Max. coverage (+): 0. Max coverage (-): 0

Region: chr22 59838999-59839022. Max. coverage (+): 0. Max coverage (-): 0

Region: chr22 59839023-59839045. Max. coverage (+): 0. Max coverage (-): 0

Region: chr22 59839046-59839069. Max. coverage (+): 0. Max coverage (-): 0

Region: chr22 59839070-59839093. Max. coverage (+): 0. Max coverage (-): 0

Region: chr22 59839094-59839116. Max. coverage (+): 0. Max coverage (-): 0

Region: chr22 59839117-59839140. Max. coverage (+): 0. Max coverage (-): 0

Region: chr22 59839141-59839164. Max. coverage (+): 0. Max coverage (-): 6.64

Region: chr22 59839165-59839187. Max. coverage (+): 0. Max coverage (-): 1.91

Region: chr22 59839188-59839211. Max. coverage (+): 0. Max coverage (-): 1.91

Region: chr22 59839212-59839234. Max. coverage (+): 0. Max coverage (-): 0.27

Region: chr22 59839235-59839258. Max. coverage (+): 0. Max coverage (-): 0

Region: chr22 59839259-59839282. Max. coverage (+): 0. Max coverage (-): 0

Region: chr22 59839283-59839305. Max. coverage (+): 0. Max coverage (-): 0

Region: chr22 59839306-59839329. Max. coverage (+): 0. Max coverage (-): 0

Region: chr22 59839330-59839353. Max. coverage (+): 0. Max coverage (-): 0

Region: chr22 59839354-59839376. Max. coverage (+): 0. Max coverage (-): 1.57

Region: chr22 59839377-59839400. Max. coverage (+): 0. Max coverage (-): 0

Region: chr22 59839401-59839424. Max. coverage (+): 0. Max coverage (-): 0

Region: chr22 59839425-59839447. Max. coverage (+): 0. Max coverage (-): 0

Region: chr22 59839448-59839471. Max. coverage (+): 0. Max coverage (-): 0

Region: chr22 59839472-59839495. Max. coverage (+): 0. Max coverage (-): 0

Region: chr22 59839496-59839518. Max. coverage (+): 0. Max coverage (-): 0

Region: chr22 59839519-59839542. Max. coverage (+): 0. Max coverage (-): 0

Region: chr22 59839543-59839565. Max. coverage (+): 0. Max coverage (-): 0

Region: chr22 59839566-59839589. Max. coverage (+): 0. Max coverage (-): 0

Region: chr22 59839590-59839613. Max. coverage (+): 0. Max coverage (-): 0

Region: chr22 59839614-59839636. Max. coverage (+): 0. Max coverage (-): 0

Region: chr22 59839637-59839660. Max. coverage (+): 0. Max coverage (-): 0

Region: chr22 59839661-59839684. Max. coverage (+): 0. Max coverage (-): 0

Region: chr22 59839685-59839707. Max. coverage (+): 0. Max coverage (-): 0

Region: chr22 59839708-59839731. Max. coverage (+): 0. Max coverage (-): 0

Region: chr22 59839732-59839755. Max. coverage (+): 0. Max coverage (-): 0

Region: chr22 59839756-59839778. Max. coverage (+): 0. Max coverage (-): 15.69

Region: chr22 59839779-59839802. Max. coverage (+): 0. Max coverage (-): 1.85

Region: chr22 59839803-59839826. Max. coverage (+): 0. Max coverage (-): 0

Region: chr22 59839827-59839849. Max. coverage (+): 0. Max coverage (-): 2.01

Region: chr22 59839850-59839873. Max. coverage (+): 0. Max coverage (-): 0

Region: chr22 59839874-59839897. Max. coverage (+): 0. Max coverage (-): 5.43

Region: chr22 59839898-59839920. Max. coverage (+): 0. Max coverage (-): 0

Region: chr22 59839921-59839944. Max. coverage (+): 0. Max coverage (-): 0

Region: chr22 59839945-59839967. Max. coverage (+): 0. Max coverage (-): 0

Region: chr22 59839968-59839991. Max. coverage (+): 0. Max coverage (-): 0

Region: chr22 59839992-59840015. Max. coverage (+): 0. Max coverage (-): 0

Region: chr22 59840016-59840038. Max. coverage (+): 0. Max coverage (-): 0

Region: chr22 59840039-59840062. Max. coverage (+): 0. Max coverage (-): 0

Region: chr22 59840063-59840086. Max. coverage (+): 0. Max coverage (-): 0

Region: chr22 59840087-59840109. Max. coverage (+): 0. Max coverage (-): 0

Region: chr22 59840110-59840133. Max. coverage (+): 0. Max coverage (-): 0

Region: chr22 59840134-59840157. Max. coverage (+): 0. Max coverage (-): 0

Region: chr22 59840158-59840180. Max. coverage (+): 0. Max coverage (-): 7.52

Region: chr22 59840181-59840204. Max. coverage (+): 0. Max coverage (-): 0

Region: chr22 59840205-59840228. Max. coverage (+): 4.94. Max coverage (-): 5.8

Region: chr22 59840229-59840251. Max. coverage (+): 0. Max coverage (-): 5.8

Region: chr22 59840252-59840275. Max. coverage (+): 0. Max coverage (-): 17.58

Region: chr22 59840276-59840299. Max. coverage (+): 0. Max coverage (-): 0

Region: chr22 59840300-59840322. Max. coverage (+): 0. Max coverage (-): 0

Region: chr22 59840323-59840346. Max. coverage (+): 0. Max coverage (-): 10.73

Region: chr22 59840347-59840369. Max. coverage (+): 0. Max coverage (-): 0.94

Region: chr22 59840370-59840393. Max. coverage (+): 0. Max coverage (-): 9.76

Region: chr22 59840394-59840417. Max. coverage (+): 0. Max coverage (-): 18.91

Region: chr22 59840418-59840440. Max. coverage (+): 0. Max coverage (-): 0

Region: chr22 59840441-59840464. Max. coverage (+): 0. Max coverage (-): 6.73

Region: chr22 59840465-59840488. Max. coverage (+): 0. Max coverage (-): 0

Region: chr22 59840489-59840511. Max. coverage (+): 0. Max coverage (-): 0

Region: chr22 59840512-59840535. Max. coverage (+): 0. Max coverage (-): 8.76

Region: chr22 59840536-59840559. Max. coverage (+): 0. Max coverage (-): 0

Region: chr22 59840560-59840582. Max. coverage (+): 0. Max coverage (-): 0

Region: chr22 59840583-59840606. Max. coverage (+): 0. Max coverage (-): 0

Region: chr22 59840607-59840630. Max. coverage (+): 0. Max coverage (-): 0

Region: chr22 59840631-59840653. Max. coverage (+): 0. Max coverage (-): 0

Region: chr22 59840654-59840677. Max. coverage (+): 0. Max coverage (-): 0

Region: chr22 59840678-59840701. Max. coverage (+): 0. Max coverage (-): 0

Region: chr22 59840702-59840724. Max. coverage (+): 0. Max coverage (-): 0

Region: chr22 59840725-59840748. Max. coverage (+): 0. Max coverage (-): 0

Region: chr22 59840749-59840771. Max. coverage (+): 0. Max coverage (-): 0

Region: chr22 59840772-59840795. Max. coverage (+): 0. Max coverage (-): 4.69

Region: chr22 59840796-59840819. Max. coverage (+): 0. Max coverage (-): 0

Region: chr22 59840820-59840842. Max. coverage (+): 0. Max coverage (-): 3.03

Region: chr22 59840843-59840866. Max. coverage (+): 0. Max coverage (-): 0

Region: chr22 59840867-59840890. Max. coverage (+): 0. Max coverage (-): 0

Region: chr22 59840891-59840913. Max. coverage (+): 0. Max coverage (-): 0

Region: chr22 59840914-59840937. Max. coverage (+): 0. Max coverage (-): 0

Region: chr22 59840938-59840961. Max. coverage (+): 0. Max coverage (-): 0

Region: chr22 59840962-59840984. Max. coverage (+): 0. Max coverage (-): 0

Region: chr22 59840985-59841008. Max. coverage (+): 0. Max coverage (-): 0

Region: chr22 59841009-59841032. Max. coverage (+): 0. Max coverage (-): 55.47

Region: chr22 59841033-59841055. Max. coverage (+): 0. Max coverage (-): 55.47

Region: chr22 59841056-59841079. Max. coverage (+): 0. Max coverage (-): 0

Region: chr22 59841080-59841102. Max. coverage (+): 0. Max coverage (-): 0

Region: chr22 59841103-59841126. Max. coverage (+): 0. Max coverage (-): 0

Region: chr22 59841127-59841150. Max. coverage (+): 0. Max coverage (-): 0

Region: chr22 59841151-59841173. Max. coverage (+): 0. Max coverage (-): 4.74

Region: chr22 59841174-59841197. Max. coverage (+): 0. Max coverage (-): 0

Region: chr22 59841198-59841221. Max. coverage (+): 0. Max coverage (-): 0

Region: chr22 59841222-59841244. Max. coverage (+): 0. Max coverage (-): 0

Region: chr22 59841245-59841268. Max. coverage (+): 0. Max coverage (-): 0

Region: chr22 59841269-59841292. Max. coverage (+): 0. Max coverage (-): 0

Region: chr22 59841293-59841315. Max. coverage (+): 0. Max coverage (-): 0

Region: chr22 59841316-59841339. Max. coverage (+): 0. Max coverage (-): 0

Region: chr22 59841340-59841363. Max. coverage (+): 0. Max coverage (-): 0

Region: chr22 59841364-59841386. Max. coverage (+): 0. Max coverage (-): 0

Region: chr22 59841387-59841410. Max. coverage (+): 0. Max coverage (-): 0

Region: chr22 59841411-59841434. Max. coverage (+): 0. Max coverage (-): 0

Region: chr22 59841435-59841457. Max. coverage (+): 0. Max coverage (-): 0

Region: chr22 59841458-59841481. Max. coverage (+): 0. Max coverage (-): 0

Region: chr22 59841482-59841504. Max. coverage (+): 0. Max coverage (-): 0

Region: chr22 59841505-59841528. Max. coverage (+): 0. Max coverage (-): 0

Region: chr22 59841529-59841552. Max. coverage (+): 0. Max coverage (-): 0

Region: chr22 59841553-59841575. Max. coverage (+): 0. Max coverage (-): 0.11

Region: chr22 59841576-59841599. Max. coverage (+): 0. Max coverage (-): 0.11

Region: chr22 59841600-59841623. Max. coverage (+): 0. Max coverage (-): 0

Region: chr22 59841624-59841646. Max. coverage (+): 0. Max coverage (-): 0

Region: chr22 59841647-59841670. Max. coverage (+): 0. Max coverage (-): 0

Region: chr22 59841671-59841694. Max. coverage (+): 0. Max coverage (-): 0

Region: chr22 59841695-59841717. Max. coverage (+): 0. Max coverage (-): 0

Region: chr22 59841718-59841741. Max. coverage (+): 0. Max coverage (-): 0

Region: chr22 59841742-59841765. Max. coverage (+): 0. Max coverage (-): 0

Region: chr22 59841766-59841788. Max. coverage (+): 0. Max coverage (-): 0

Region: chr22 59841789-59841812. Max. coverage (+): 0. Max coverage (-): 0

Region: chr22 59841813-59841836. Max. coverage (+): 0. Max coverage (-): 0

Region: chr22 59841837-59841859. Max. coverage (+): 0. Max coverage (-): 10.07

Region: chr22 59841860-59841883. Max. coverage (+): 0. Max coverage (-): 0

Region: chr22 59841884-59841906. Max. coverage (+): 0. Max coverage (-): 0

Region: chr22 59841907-59841930. Max. coverage (+): 0. Max coverage (-): 0

Region: chr22 59841931-59841954. Max. coverage (+): 0. Max coverage (-): 0

Region: chr22 59841955-59841977. Max. coverage (+): 0. Max coverage (-): 0

Region: chr22 59841978-59842001. Max. coverage (+): 0. Max coverage (-): 0

Region: chr22 59842002-59842025. Max. coverage (+): 0. Max coverage (-): 0

Region: chr22 59842026-59842048. Max. coverage (+): 0. Max coverage (-): 0

Region: chr22 59842049-59842072. Max. coverage (+): 0. Max coverage (-): 0

Region: chr22 59842073-59842096. Max. coverage (+): 0. Max coverage (-): 0

Region: chr22 59842097-59842119. Max. coverage (+): 0. Max coverage (-): 0

Region: chr22 59842120-59842143. Max. coverage (+): 0. Max coverage (-): 0

Region: chr22 59842144-59842167. Max. coverage (+): 0. Max coverage (-): 0

Region: chr22 59842168-59842190. Max. coverage (+): 0. Max coverage (-): 0

Region: chr22 59842191-59842214. Max. coverage (+): 0. Max coverage (-): 0

Region: chr22 59842215-59842237. Max. coverage (+): 0. Max coverage (-): 0

Region: chr22 59842238-59842261. Max. coverage (+): 0. Max coverage (-): 0

Region: chr22 59842262-59842285. Max. coverage (+): 0. Max coverage (-): 0

Region: chr22 59842286-59842308. Max. coverage (+): 0. Max coverage (-): 0

Region: chr22 59842309-59842332. Max. coverage (+): 0. Max coverage (-): 0

Region: chr22 59842333-59842356. Max. coverage (+): 0. Max coverage (-): 0

Region: chr22 59842357-59842379. Max. coverage (+): 0. Max coverage (-): 0

Region: chr22 59842380-59842403. Max. coverage (+): 0. Max coverage (-): 0

Region: chr22 59842404-59842427. Max. coverage (+): 0. Max coverage (-): 0

Region: chr22 59842428-59842450. Max. coverage (+): 0. Max coverage (-): 0

Region: chr22 59842451-59842474. Max. coverage (+): 0. Max coverage (-): 0

Region: chr22 59842475-59842498. Max. coverage (+): 0. Max coverage (-): 0

Region: chr22 59842499-59842521. Max. coverage (+): 0. Max coverage (-): 0

Region: chr22 59842522-59842545. Max. coverage (+): 0. Max coverage (-): 0

Region: chr22 59842546-59842569. Max. coverage (+): 0. Max coverage (-): 4.75

Region: chr22 59842570-59842592. Max. coverage (+): 0. Max coverage (-): 0

Region: chr22 59842593-59842616. Max. coverage (+): 0. Max coverage (-): 0

Region: chr22 59842617-59842639. Max. coverage (+): 0. Max coverage (-): 0.55

Region: chr22 59842640-59842663. Max. coverage (+): 0. Max coverage (-): 8.12

Region: chr22 59842664-59842687. Max. coverage (+): 0. Max coverage (-): 0

Region: chr22 59842688-59842710. Max. coverage (+): 0. Max coverage (-): 0

Region: chr22 59842711-59842734. Max. coverage (+): 0. Max coverage (-): 0

Region: chr22 59842735-59842758. Max. coverage (+): 0. Max coverage (-): 0

Region: chr22 59842759-59842781. Max. coverage (+): 0. Max coverage (-): 0

Region: chr22 59842782-59842805. Max. coverage (+): 0. Max coverage (-): 0

Region: chr22 59842806-59842829. Max. coverage (+): 0. Max coverage (-): 0

Region: chr22 59842830-59842852. Max. coverage (+): 0. Max coverage (-): 13.97

Region: chr22 59842853-59842876. Max. coverage (+): 0. Max coverage (-): 0

Region: chr22 59842877-59842900. Max. coverage (+): 0. Max coverage (-): 0

Region: chr22 59842901-59842923. Max. coverage (+): 0. Max coverage (-): 0

Region: chr22 59842924-59842947. Max. coverage (+): 0. Max coverage (-): 0

Region: chr22 59842948-59842971. Max. coverage (+): 0. Max coverage (-): 0

Region: chr22 59842972-59842994. Max. coverage (+): 0. Max coverage (-): 0

Region: chr22 59842995-59843018. Max. coverage (+): 0. Max coverage (-): 0

Region: chr22 59843019-59843041. Max. coverage (+): 0. Max coverage (-): 0

Region: chr22 59843042-59843065. Max. coverage (+): 0. Max coverage (-): 0

Region: chr22 59843066-59843089. Max. coverage (+): 0. Max coverage (-): 0

Region: chr22 59843090-59843112. Max. coverage (+): 0. Max coverage (-): 0

Region: chr22 59843113-59843136. Max. coverage (+): 0. Max coverage (-): 62.05

Region: chr22 59843137-59843160. Max. coverage (+): 0. Max coverage (-): 0

Region: chr22 59843161-59843183. Max. coverage (+): 0. Max coverage (-): 0

Region: chr22 59843184-59843207. Max. coverage (+): 0. Max coverage (-): 10.43

Region: chr22 59843208-59843231. Max. coverage (+): 0. Max coverage (-): 0

Region: chr22 59843232-59843254. Max. coverage (+): 0. Max coverage (-): 0

Region: chr22 59843255-59843278. Max. coverage (+): 0. Max coverage (-): 1.76

Region: chr22 59843279-59843302. Max. coverage (+): 0. Max coverage (-): 1.76

Region: chr22 59843303-59843325. Max. coverage (+): 0. Max coverage (-): 0

Region: chr22 59843326-59843349. Max. coverage (+): 0. Max coverage (-): 0

Region: chr22 59843350-59843373. Max. coverage (+): 0. Max coverage (-): 1.44

Region: chr22 59843374-59843396. Max. coverage (+): 0. Max coverage (-): 0

Region: chr22 59843397-59843420. Max. coverage (+): 0. Max coverage (-): 0

Region: chr22 59843421-59843443. Max. coverage (+): 0. Max coverage (-): 7.34

Region: chr22 59843444-59843467. Max. coverage (+): 0. Max coverage (-): 18.07

Region: chr22 59843468-59843491. Max. coverage (+): 6.63. Max coverage (-): 14.23

Region: chr22 59843492-59843514. Max. coverage (+): 6.63. Max coverage (-): 3.79

Region: chr22 59843515-59843538. Max. coverage (+): 0. Max coverage (-): 0

Region: chr22 59843539-59843562. Max. coverage (+): 0. Max coverage (-): 0

Region: chr22 59843563-59843585. Max. coverage (+): 0. Max coverage (-): 0

Region: chr22 59843586-59843609. Max. coverage (+): 0. Max coverage (-): 0

Region: chr22 59843610-59843633. Max. coverage (+): 0. Max coverage (-): 0

Region: chr22 59843634-59843656. Max. coverage (+): 0. Max coverage (-): 0

Region: chr22 59843657-59843680. Max. coverage (+): 0. Max coverage (-): 0

Region: chr22 59843681-59843704. Max. coverage (+): 0. Max coverage (-): 0

Region: chr22 59843705-59843727. Max. coverage (+): 0. Max coverage (-): 38.89

Region: chr22 59843728-59843751. Max. coverage (+): 0. Max coverage (-): 38.67

Region: chr22 59843752-59843774. Max. coverage (+): 0. Max coverage (-): 0

Region: chr22 59843775-59843798. Max. coverage (+): 0. Max coverage (-): 0

Region: chr22 59843799-59843822. Max. coverage (+): 0. Max coverage (-): 0

Region: chr22 59843823-59843845. Max. coverage (+): 0. Max coverage (-): 0

Region: chr22 59843846-59843869. Max. coverage (+): 0. Max coverage (-): 3.29

Region: chr22 59843870-59843893. Max. coverage (+): 0. Max coverage (-): 10.33

Region: chr22 59843894-59843916. Max. coverage (+): 0. Max coverage (-): 0

Region: chr22 59843917-59843940. Max. coverage (+): 0. Max coverage (-): 0

Region: chr22 59843941-59843964. Max. coverage (+): 0. Max coverage (-): 0

Region: chr22 59843965-59843987. Max. coverage (+): 0. Max coverage (-): 0

Region: chr22 59843988-59844011. Max. coverage (+): 0. Max coverage (-): 0

Region: chr22 59844012-59844035. Max. coverage (+): 0. Max coverage (-): 0

Region: chr22 59844036-59844058. Max. coverage (+): 0. Max coverage (-): 0

Region: chr22 59844059-59844082. Max. coverage (+): 0. Max coverage (-): 13.18

Region: chr22 59844083-59844106. Max. coverage (+): 0. Max coverage (-): 2.73

Region: chr22 59844107-59844129. Max. coverage (+): 0. Max coverage (-): 72.29

Region: chr22 59844130-59844153. Max. coverage (+): 0. Max coverage (-): 29.34

Region: chr22 59844154-59844176. Max. coverage (+): 0. Max coverage (-): 0

Region: chr22 59844177-59844200. Max. coverage (+): 0. Max coverage (-): 0

Region: chr22 59844201-59844224. Max. coverage (+): 0. Max coverage (-): 3.72

Region: chr22 59844225-59844247. Max. coverage (+): 0. Max coverage (-): 7.73

Region: chr22 59844248-59844271. Max. coverage (+): 0. Max coverage (-): 7.73

Region: chr22 59844272-59844295. Max. coverage (+): 0. Max coverage (-): 7.55

Region: chr22 59844296-59844318. Max. coverage (+): 0. Max coverage (-): 11.03

Region: chr22 59844319-59844342. Max. coverage (+): 0. Max coverage (-): 0

Region: chr22 59844343-59844366. Max. coverage (+): 2.92. Max coverage (-): 1.66

Region: chr22 59844367-59844389. Max. coverage (+): 0. Max coverage (-): 3.94

Region: chr22 59844390-59844413. Max. coverage (+): 0. Max coverage (-): 20.27

Region: chr22 59844414-59844437. Max. coverage (+): 0. Max coverage (-): 0

Region: chr22 59844438-59844460. Max. coverage (+): 0. Max coverage (-): 0

Region: chr22 59844461-59844484. Max. coverage (+): 0. Max coverage (-): 3.39

Region: chr22 59844485-59844508. Max. coverage (+): 0. Max coverage (-): 15.78

Region: chr22 59844509-59844531. Max. coverage (+): 0. Max coverage (-): 5.98

Region: chr22 59844532-59844555. Max. coverage (+): 0. Max coverage (-): 0

Region: chr22 59844556-59844578. Max. coverage (+): 0. Max coverage (-): 0

Region: chr22 59844579-59844602. Max. coverage (+): 0. Max coverage (-): 0

Region: chr22 59844603-59844626. Max. coverage (+): 0. Max coverage (-): 0

Region: chr22 59844627-59844649. Max. coverage (+): 0. Max coverage (-): 0

Region: chr22 59844650-59844673. Max. coverage (+): 0. Max coverage (-): 30.06

Region: chr22 59844674-59844697. Max. coverage (+): 0. Max coverage (-): 2.44

Region: chr22 59844698-59844720. Max. coverage (+): 0. Max coverage (-): 11.88

Region: chr22 59844721-59844744. Max. coverage (+): 0. Max coverage (-): 3.71

Region: chr22 59844745-59844768. Max. coverage (+): 0. Max coverage (-): 3.71

Region: chr22 59844769-59844791. Max. coverage (+): 0. Max coverage (-): 6.25

Region: chr22 59844792-59844815. Max. coverage (+): 0. Max coverage (-): 17.66

Region: chr22 59844816-59844839. Max. coverage (+): 0. Max coverage (-): 0

Region: chr22 59844840-59844862. Max. coverage (+): 0. Max coverage (-): 4.93

Region: chr22 59844863-59844886. Max. coverage (+): 0. Max coverage (-): 21.97

Region: chr22 59844887-59844909. Max. coverage (+): 0. Max coverage (-): 0

Region: chr22 59844910-59844933. Max. coverage (+): 0. Max coverage (-): 0

Region: chr22 59844934-59844957. Max. coverage (+): 0. Max coverage (-): 3.15

Region: chr22 59844958-59844980. Max. coverage (+): 0. Max coverage (-): 4.29

Region: chr22 59844981-59845004. Max. coverage (+): 0. Max coverage (-): 0

Region: chr22 59845005-59845028. Max. coverage (+): 0. Max coverage (-): 0

Region: chr22 59845029-59845051. Max. coverage (+): 0. Max coverage (-): 0

Region: chr22 59845052-59845075. Max. coverage (+): 0. Max coverage (-): 0

Region: chr22 59845076-59845099. Max. coverage (+): 0. Max coverage (-): 0

Region: chr22 59845100-59845122. Max. coverage (+): 0. Max coverage (-): 9.33

Region: chr22 59845123-59845146. Max. coverage (+): 0. Max coverage (-): 27.82

Region: chr22 59845147-59845170. Max. coverage (+): 0. Max coverage (-): 0

Region: chr22 59845171-59845193. Max. coverage (+): 0. Max coverage (-): 0

Region: chr22 59845194-59845217. Max. coverage (+): 0. Max coverage (-): 0

Region: chr22 59845218-59845241. Max. coverage (+): 0. Max coverage (-): 0

Region: chr22 59845242-59845264. Max. coverage (+): 0. Max coverage (-): 0

Region: chr22 59845265-59845288. Max. coverage (+): 0. Max coverage (-): 0.59

Region: chr22 59845289-59845311. Max. coverage (+): 0. Max coverage (-): 0

Region: chr22 59845312-59845335. Max. coverage (+): 0. Max coverage (-): 0

Region: chr22 59845336-59845359. Max. coverage (+): 0. Max coverage (-): 0

Region: chr22 59845360-59845382. Max. coverage (+): 0. Max coverage (-): 0

Region: chr22 59845383-59845406. Max. coverage (+): 0. Max coverage (-): 0

Region: chr22 59845407-59845430. Max. coverage (+): 0. Max coverage (-): 14.43

Region: chr22 59845431-59845453. Max. coverage (+): 0. Max coverage (-): 0

Region: chr22 59845454-59845477. Max. coverage (+): 0. Max coverage (-): 0

Region: chr22 59845478-59845501. Max. coverage (+): 0. Max coverage (-): 0

Region: chr22 59845502-59845524. Max. coverage (+): 0. Max coverage (-): 8.71

Region: chr22 59845525-59845548. Max. coverage (+): 0. Max coverage (-): 8.71

Region: chr22 59845549-59845572. Max. coverage (+): 0. Max coverage (-): 0

Region: chr22 59845573-59845595. Max. coverage (+): 0. Max coverage (-): 0

Region: chr22 59845596-59845619. Max. coverage (+): 0. Max coverage (-): 0

Region: chr22 59845620-59845643. Max. coverage (+): 0. Max coverage (-): 0

Region: chr22 59845644-59845666. Max. coverage (+): 0. Max coverage (-): 0

Region: chr22 59845667-59845690. Max. coverage (+): 0. Max coverage (-): 0

Region: chr22 59845691-59845713. Max. coverage (+): 0. Max coverage (-): 0

Region: chr22 59845714-59845737. Max. coverage (+): 0. Max coverage (-): 0

Region: chr22 59845738-59845761. Max. coverage (+): 0. Max coverage (-): 0

Region: chr22 59845762-59845784. Max. coverage (+): 0. Max coverage (-): 0

Region: chr22 59845785-59845808. Max. coverage (+): 0. Max coverage (-): 0

Region: chr22 59845809-59845832. Max. coverage (+): 0. Max coverage (-): 0

Region: chr22 59845833-59845855. Max. coverage (+): 0. Max coverage (-): 0

Region: chr22 59845856-59845879. Max. coverage (+): 0. Max coverage (-): 0

Region: chr22 59845880-59845903. Max. coverage (+): 0. Max coverage (-): 0

Region: chr22 59845904-59845926. Max. coverage (+): 0. Max coverage (-): 0

Region: chr22 59845927-59845950. Max. coverage (+): 0. Max coverage (-): 0

Region: chr22 59845951-59845974. Max. coverage (+): 0. Max coverage (-): 0

Region: chr22 59845975-59845997. Max. coverage (+): 0. Max coverage (-): 0

Region: chr22 59845998-59846021. Max. coverage (+): 0. Max coverage (-): 0

Region: chr22 59846022-59846045. Max. coverage (+): 0. Max coverage (-): 0

Region: chr22 59846046-59846068. Max. coverage (+): 0. Max coverage (-): 0

Region: chr22 59846069-59846092. Max. coverage (+): 0. Max coverage (-): 0

Region: chr22 59846093-59846115. Max. coverage (+): 0. Max coverage (-): 0

Region: chr22 59846116-59846139. Max. coverage (+): 0. Max coverage (-): 17.29

Region: chr22 59846140-59846163. Max. coverage (+): 0. Max coverage (-): 17.29

Region: chr22 59846164-59846186. Max. coverage (+): 0. Max coverage (-): 0.28

Region: chr22 59846187-59846210. Max. coverage (+): 0. Max coverage (-): 0

Region: chr22 59846211-59846234. Max. coverage (+): 0. Max coverage (-): 0

Region: chr22 59846235-59846257. Max. coverage (+): 0. Max coverage (-): 0

Region: chr22 59846258-59846281. Max. coverage (+): 0. Max coverage (-): 0

Region: chr22 59846282-59846305. Max. coverage (+): 0. Max coverage (-): 0

Region: chr22 59846306-59846328. Max. coverage (+): 0. Max coverage (-): 0

Region: chr22 59846329-59846352. Max. coverage (+): 0. Max coverage (-): 3.8

Region: chr22 59846353-59846376. Max. coverage (+): 0. Max coverage (-): 0

Region: chr22 59846377-59846399. Max. coverage (+): 0. Max coverage (-): 2.98

Region: chr22 59846400-59846423. Max. coverage (+): 0. Max coverage (-): 0

Region: chr22 59846424-59846446. Max. coverage (+): 0. Max coverage (-): 0

Region: chr22 59846447-59846470. Max. coverage (+): 0. Max coverage (-): 6.77

Region: chr22 59846471-59846494. Max. coverage (+): 0. Max coverage (-): 1.29

Region: chr22 59846495-59846517. Max. coverage (+): 0. Max coverage (-): 4.55

Region: chr22 59846518-59846541. Max. coverage (+): 0. Max coverage (-): 0

Region: chr22 59846542-59846565. Max. coverage (+): 0. Max coverage (-): 0

Region: chr22 59846566-59846588. Max. coverage (+): 0. Max coverage (-): 6.59

Region: chr22 59846589-59846612. Max. coverage (+): 0. Max coverage (-): 1.42

Region: chr22 59846613-59846636. Max. coverage (+): 0. Max coverage (-): 0

Region: chr22 59846637-59846659. Max. coverage (+): 0. Max coverage (-): 0

Region: chr22 59846660-59846683. Max. coverage (+): 0. Max coverage (-): 15.81

Region: chr22 59846684-59846707. Max. coverage (+): 0. Max coverage (-): 15.81

Region: chr22 59846708-59846730. Max. coverage (+): 0. Max coverage (-): 6.18

Region: chr22 59846731-59846754. Max. coverage (+): 0. Max coverage (-): 0

Region: chr22 59846755-59846778. Max. coverage (+): 0. Max coverage (-): 0

Region: chr22 59846779-59846801. Max. coverage (+): 0. Max coverage (-): 40.64

Region: chr22 59846802-59846825. Max. coverage (+): 0. Max coverage (-): 8.02

Region: chr22 59846826-59846848. Max. coverage (+): 0. Max coverage (-): 13.68

Region: chr22 59846849-59846872. Max. coverage (+): 0. Max coverage (-): 13.68

Region: chr22 59846873-59846896. Max. coverage (+): 0. Max coverage (-): 0

Region: chr22 59846897-59846919. Max. coverage (+): 0. Max coverage (-): 0

Region: chr22 59846920-59846943. Max. coverage (+): 0. Max coverage (-): 0

Region: chr22 59846944-59846967. Max. coverage (+): 0. Max coverage (-): 0

Region: chr22 59846968-59846990. Max. coverage (+): 0. Max coverage (-): 0

Region: chr22 59846991-59847014. Max. coverage (+): 0. Max coverage (-): 0

Region: chr22 59847015-59847038. Max. coverage (+): 0. Max coverage (-): 0

Region: chr22 59847039-59847061. Max. coverage (+): 0. Max coverage (-): 0

Region: chr22 59847062-59847085. Max. coverage (+): 0. Max coverage (-): 0

Region: chr22 59847086-59847109. Max. coverage (+): 0. Max coverage (-): 0

Region: chr22 59847110-59847132. Max. coverage (+): 6.04. Max coverage (-): 0

Region: chr22 59847133-59847156. Max. coverage (+): 6.04. Max coverage (-): 0

Region: chr22 59847157-59847180. Max. coverage (+): 0. Max coverage (-): 0

Region: chr22 59847181-59847203. Max. coverage (+): 0. Max coverage (-): 0

Region: chr22 59847204-59847227. Max. coverage (+): 0. Max coverage (-): 0

Region: chr22 59847228-59847250. Max. coverage (+): 0. Max coverage (-): 0

Region: chr22 59847251-59847274. Max. coverage (+): 0. Max coverage (-): 0

Region: chr22 59847275-59847298. Max. coverage (+): 0. Max coverage (-): 0

Region: chr22 59847299-59847321. Max. coverage (+): 0. Max coverage (-): 0

Region: chr22 59847322-59847345. Max. coverage (+): 0. Max coverage (-): 0

Region: chr22 59847346-59847369. Max. coverage (+): 0. Max coverage (-): 0

Region: chr22 59847370-59847392. Max. coverage (+): 0. Max coverage (-): 0

Region: chr22 59847393-59847416. Max. coverage (+): 0. Max coverage (-): 0

Region: chr22 59847417-59847440. Max. coverage (+): 0. Max coverage (-): 0

Region: chr22 59847441-59847463. Max. coverage (+): 0. Max coverage (-): 0

Region: chr22 59847464-59847487. Max. coverage (+): 0. Max coverage (-): 0

Region: chr22 59847488-59847511. Max. coverage (+): 0. Max coverage (-): 0

Region: chr22 59847512-59847534. Max. coverage (+): 0. Max coverage (-): 0

Region: chr22 59847535-59847558. Max. coverage (+): 0. Max coverage (-): 0

Region: chr22 59847559-59847581. Max. coverage (+): 0. Max coverage (-): 0

Region: chr22 59847582-59847605. Max. coverage (+): 0. Max coverage (-): 0

Region: chr22 59847606-59847629. Max. coverage (+): 0. Max coverage (-): 0

Region: chr22 59847630-59847652. Max. coverage (+): 0. Max coverage (-): 0

Region: chr22 59847653-59847676. Max. coverage (+): 0. Max coverage (-): 0

Region: chr22 59847677-59847700. Max. coverage (+): 0. Max coverage (-): 0

Region: chr22 59847701-59847723. Max. coverage (+): 0. Max coverage (-): 0

Region: chr22 59847724-59847747. Max. coverage (+): 0. Max coverage (-): 0

Region: chr22 59847748-59847771. Max. coverage (+): 0. Max coverage (-): 0

Region: chr22 59847772-59847794. Max. coverage (+): 0. Max coverage (-): 0

Region: chr22 59847795-59847818. Max. coverage (+): 0. Max coverage (-): 0

Region: chr22 59847819-59847842. Max. coverage (+): 0. Max coverage (-): 0

Region: chr22 59847843-59847865. Max. coverage (+): 0. Max coverage (-): 0

Region: chr22 59847866-59847889. Max. coverage (+): 0. Max coverage (-): 0

Region: chr22 59847890-59847913. Max. coverage (+): 0. Max coverage (-): 0

Region: chr22 59847914-59847936. Max. coverage (+): 0. Max coverage (-): 0

Region: chr22 59847937-59847960. Max. coverage (+): 0. Max coverage (-): 0

Region: chr22 59847961-59847983. Max. coverage (+): 0. Max coverage (-): 0

Region: chr22 59847984-59848007. Max. coverage (+): 8.69. Max coverage (-): 0

Region: chr22 59848008-59848031. Max. coverage (+): 7.59. Max coverage (-): 0

Region: chr22 59848032-59848054. Max. coverage (+): 0. Max coverage (-): 0

Region: chr22 59848055-59848078. Max. coverage (+): 11.08. Max coverage (-): 0

Region: chr22 59848079-59848102. Max. coverage (+): 0. Max coverage (-): 0

Region: chr22 59848103-59848125. Max. coverage (+): 0. Max coverage (-): 0

Region: chr22 59848126-59848149. Max. coverage (+): 0. Max coverage (-): 0

Region: chr22 59848150-59848173. Max. coverage (+): 14.67. Max coverage (-): 0

Region: chr22 59848174-59848196. Max. coverage (+): 8.7. Max coverage (-): 0

Region: chr22 59848197-59848220. Max. coverage (+): 8.7. Max coverage (-): 0

Region: chr22 59848221-59848244. Max. coverage (+): 0. Max coverage (-): 0

Region: chr22 59848245-59848267. Max. coverage (+): 0. Max coverage (-): 0

Region: chr22 59848268-59848291. Max. coverage (+): 0. Max coverage (-): 0

Region: chr22 59848292-59848315. Max. coverage (+): 0. Max coverage (-): 0

Region: chr22 59848316-59848338. Max. coverage (+): 0. Max coverage (-): 0

Region: chr22 59848339-59848362. Max. coverage (+): 0. Max coverage (-): 0

Region: chr22 59848363-59848385. Max. coverage (+): 0. Max coverage (-): 0

Region: chr22 59848386-59848409. Max. coverage (+): 0. Max coverage (-): 0

Region: chr22 59848410-59848433. Max. coverage (+): 19.69. Max coverage (-): 0

Region: chr22 59848434-59848456. Max. coverage (+): 0. Max coverage (-): 0

Region: chr22 59848457-59848480. Max. coverage (+): 5.33. Max coverage (-): 0

Region: chr22 59848481-59848504. Max. coverage (+): 5.33. Max coverage (-): 0

Region: chr22 59848505-59848527. Max. coverage (+): 0. Max coverage (-): 0

Region: chr22 59848528-59848551. Max. coverage (+): 5.61. Max coverage (-): 0

Region: chr22 59848552-59848575. Max. coverage (+): 0. Max coverage (-): 0

Region: chr22 59848576-59848598. Max. coverage (+): 4.64. Max coverage (-): 0

Region: chr22 59848599-59848622. Max. coverage (+): 2.15. Max coverage (-): 0

Region: chr22 59848623-59848646. Max. coverage (+): 0. Max coverage (-): 0

Region: chr22 59848647-59848669. Max. coverage (+): 1.53. Max coverage (-): 0

Region: chr22 59848670-59848693. Max. coverage (+): 0. Max coverage (-): 0

Region: chr22 59848694-59848717. Max. coverage (+): 35.78. Max coverage (-): 0

Region: chr22 59848718-59848740. Max. coverage (+): 35.78. Max coverage (-): 0

Region: chr22 59848741-59848764. Max. coverage (+): 0. Max coverage (-): 0

Region: chr22 59848765-59848787. Max. coverage (+): 0. Max coverage (-): 0

Region: chr22 59848788-59848811. Max. coverage (+): 0. Max coverage (-): 0

Region: chr22 59848812-59848835. Max. coverage (+): 14.38. Max coverage (-): 0

Region: chr22 59848836-59848858. Max. coverage (+): 0. Max coverage (-): 0

Region: chr22 59848859-59848882. Max. coverage (+): 0. Max coverage (-): 0

Region: chr22 59848883-59848906. Max. coverage (+): 0. Max coverage (-): 0

Region: chr22 59848907-59848929. Max. coverage (+): 0. Max coverage (-): 0

Region: chr22 59848930-59848953. Max. coverage (+): 0. Max coverage (-): 0

Region: chr22 59848954-59848977. Max. coverage (+): 0. Max coverage (-): 0

Region: chr22 59848978-59849000. Max. coverage (+): 0. Max coverage (-): 0

Region: chr22 59849001-59849024. Max. coverage (+): 0.53. Max coverage (-): 0

Region: chr22 59849025-59849048. Max. coverage (+): 9.31. Max coverage (-): 0

Region: chr22 59849049-59849071. Max. coverage (+): 16.14. Max coverage (-): 0

Region: chr22 59849072-59849095. Max. coverage (+): 0. Max coverage (-): 0

Region: chr22 59849096-59849118. Max. coverage (+): 0. Max coverage (-): 0

Region: chr22 59849119-59849142. Max. coverage (+): 0. Max coverage (-): 0

Region: chr22 59849143-59849166. Max. coverage (+): 0. Max coverage (-): 0

Region: chr22 59849167-59849189. Max. coverage (+): 0. Max coverage (-): 0

Region: chr22 59849190-59849213. Max. coverage (+): 0. Max coverage (-): 0

Region: chr22 59849214-59849237. Max. coverage (+): 4.34. Max coverage (-): 0

Region: chr22 59849238-59849260. Max. coverage (+): 40.63. Max coverage (-): 0

Region: chr22 59849261-59849284. Max. coverage (+): 0. Max coverage (-): 0

Region: chr22 59849285-59849308. Max. coverage (+): 22.17. Max coverage (-): 0

Region: chr22 59849309-59849331. Max. coverage (+): 0. Max coverage (-): 0

Region: chr22 59849332-59849355. Max. coverage (+): 0. Max coverage (-): 0

Region: chr22 59849356-59849379. Max. coverage (+): 21.3. Max coverage (-): 0

Region: chr22 59849380-59849402. Max. coverage (+): 8.5. Max coverage (-): 0

Region: chr22 59849403-59849426. Max. coverage (+): 57.59. Max coverage (-): 0

Region: chr22 59849427-59849450. Max. coverage (+): 57.59. Max coverage (-): 0

Region: chr22 59849451-59849473. Max. coverage (+): 0. Max coverage (-): 0

Region: chr22 59849474-59849497. Max. coverage (+): 47.26. Max coverage (-): 0

Region: chr22 59849498-59849520. Max. coverage (+): 12.15. Max coverage (-): 0

Region: chr22 59849521-59849544. Max. coverage (+): 0. Max coverage (-): 0

Region: chr22 59849545-59849568. Max. coverage (+): 0. Max coverage (-): 0

Region: chr22 59849569-59849591. Max. coverage (+): 0. Max coverage (-): 0

Region: chr22 59849592-59849615. Max. coverage (+): 0. Max coverage (-): 0

Region: chr22 59849616-59849639. Max. coverage (+): 18.39. Max coverage (-): 0

Region: chr22 59849640-59849662. Max. coverage (+): 6.1. Max coverage (-): 0

Region: chr22 59849663-59849686. Max. coverage (+): 0. Max coverage (-): 0

Region: chr22 59849687-59849710. Max. coverage (+): 0. Max coverage (-): 0

Region: chr22 59849711-59849733. Max. coverage (+): 0. Max coverage (-): 0

Region: chr22 59849734-59849757. Max. coverage (+): 0. Max coverage (-): 0

Region: chr22 59849758-59849781. Max. coverage (+): 0. Max coverage (-): 0

Region: chr22 59849782-59849804. Max. coverage (+): 0. Max coverage (-): 0

Region: chr22 59849805-59849828. Max. coverage (+): 0. Max coverage (-): 0

Region: chr22 59849829-59849852. Max. coverage (+): 0. Max coverage (-): 0

Region: chr22 59849853-59849875. Max. coverage (+): 0. Max coverage (-): 0

Region: chr22 59849876-59849899. Max. coverage (+): 5.36. Max coverage (-): 0

Region: chr22 59849900-59849922. Max. coverage (+): 0. Max coverage (-): 0

Region: chr22 59849923-59849946. Max. coverage (+): 0. Max coverage (-): 0

Region: chr22 59849947-59849970. Max. coverage (+): 0. Max coverage (-): 0

Region: chr22 59849971-59849993. Max. coverage (+): 0. Max coverage (-): 0

Region: chr22 59849994-59850017. Max. coverage (+): 0. Max coverage (-): 0

Region: chr22 59850018-59850041. Max. coverage (+): 5.58. Max coverage (-): 0

Region: chr22 59850042-. Max. coverage (+): 0. Max coverage (-): 0

RepeatMasker Color Code

**+**

100-98% Identity

<98-95% Identity

<95-90% Identity

<90-85% Identity

<85-80% Identity

<80-75% Identity

<75-70% Identity

<70% Identity

**-**

Gene Set Color Code

**+**

Gene

Pseudogene

**-**

Topology/Coverage Color Code

Coverage Plus Strand

Coverage Minus Strand

Mainstrand: Plus

Mainstrand: Minus

Complementary Strand

Flanking Region  
(if option -flank >0)

Gene Set Annotation  

**1. H1FX (protein coding, ENSBTAG00000040116) Tr:00000052022 Ex:1**: 59841218-59841862 (-)

  
RepeatMasker Annotation  

**1. CHR-2B**: 59838332-59838559 (-), Divergence to consensus: 42.1%  
**2. MLT1K**: 59838648-59839131 (-), Divergence to consensus: 42.6%  
**3. GC\_rich**: 59840747-59840768 (+), Divergence to consensus: 31.8%  
**4. GC\_rich**: 59841388-59841422 (+), Divergence to consensus: 65.7%  
**5. GC\_rich**: 59841459-59841493 (+), Divergence to consensus: 74.3%  
**6. GC\_rich**: 59841897-59841917 (+), Divergence to consensus: 66.7%  
**7. GC\_rich**: 59841931-59841971 (+), Divergence to consensus: 68.3%  
**8. (CTGGGG)n**: 59841985-59842088 (+), Divergence to consensus: 34.2%  
**9. L2a**: 59845170-59845227 (+), Divergence to consensus: 32.8%  
**10. GC\_rich**: 59846992-59847029 (+), Divergence to consensus: 60.5%  
**11. (CGGG)n**: 59847132-59847151 (+), Divergence to consensus: 0%  
**12. L2a**: 59847542-59847830 (-), Divergence to consensus: 50.6%  
**13. L2a**: 59847908-59847979 (-), Divergence to consensus: 33.9%  
**14. L2a**: 59849125-59849219 (-), Divergence to consensus: 17.1%

  
Transcription Factor Binding Sites  

**RFX4\_2** (Sequence: GTAACCAAG (-): 59840980)  
**RFX4\_2** (Sequence: GTATCTAAG (-): 59846163)  
**SPZ1** (Sequence: CTGTTACCCC (-): 59840109)  
**SOX9** (Sequence: AACAATAA (-): 59841057)  
**SOX9** (Sequence: AACAATGG (-): 59844396)
