# Supplementary material for: Small RNA sequencing of cryopreserved semen from single bull revealed altered miRNAs and piRNAs expression between High- and Low-motile sperm populations
Source: BMC Genomics. 2017 Jan 4;18:14. doi: 10.1186/s12864-016-3394-7 (PMC5209821; doi:10.1186/s12864-016-3394-7)
Supplement: Additional file 4: — Details for each piRNA clusters found in Low Motile (LM) sperm fraction. Genes, repeats, transposable elements and transcription factors binding sites falling within the cluster regions were reported. (ZIP 1034 kb) [file 12864_2016_3394_MOESM4_ESM.zip › 31.html]

piRNA cluster 31


Predicted piRNA cluster no. 31     previous   next
  

Show proTRAC run info
Hide proTRAC run info

================================= proTRAC ====================================  
VERSION: 2.1                                    LAST MODIFIED: 06. October 2015  
  
Please cite:  
Rosenkranz D, Zischler H. proTRAC - a software for probabilistic piRNA cluster  
detection, visualization and analysis. 2012. BMC Bioinformatics 13:5.  
  
and (for proTRAC 2.0 and later):  
Rosenkranz D, Rudloff S, Bastuck K, Ketting RF, Zischler H. Tupaia small RNAs  
provide insights into function and evolution of RNAi-based transposon defense  
in mammals. 2015. RNA 21(5):911-922.  
  
Contact:  
David Rosenkranz  
Institute of Anthropology, small RNA group  
Johannes Gutenberg University Mainz  
email: rosenkranz@uni-mainz.de  
  
You can find the latest proTRAC version at:  
http://sourceforge.net/projects/protrac/files  
http://www.smallRNAgroup-mainz.de/software  
==============================================================================  
  
PARAMETERS:  
Map file: .............../storage/core/barbara/genhome/smallRNA/fertility/Sample\_not\_motile/pirna/Sample\_not\_motile\_26-33\_collapsed.fa.no-dust.map.weighted-10000-1000-b-0  
Genome file: ............/storage/core/barbara/genhome/smallRNA/fertility/Sample\_all/pirna/bt\_311\_chrY.fa  
RepeatMasker annotation: /storage/genomes/bt\_umd31/GCF\_000003055.6\_Bos\_taurus\_UMD\_3.1.1\_repeatMasker\_chr.out  
GeneSet:................./storage/core/barbara/genhome/smallRNA/fertility/Sample\_all/pirna/full.gtf  
  
Significant (p<=0.01) hit density will be calculated based  
on observed hit distribution.  
  
Sliding window size: ........................................ 5000 bp  
Sliding window increament: .................................. 1000 bp  
Normalize each hit by number of genomic hits: ............... 1 [0=no/1=yes]  
Normalize each hit by number of sequence reads: ............. 1 [0=no/1=yes]  
Normalize values (-> per million mapped reads): ............. 1 [0=no/1=yes]  
Min. fraction of hits with 1T(U) or 10A: .................... 0.75  
Alternatively: Min. fraction of hits with 1T(U) and 10A: .... 0.5  
Min. fraction of hits with typical piRNA length: ............ 0.75  
Typical piRNA length: ....................................... 26-33 nt  
Min. size of a piRNA cluster: ............................... 5000 bp.  
Min. number of hits (absolute): ............................. 0  
Min. number of hits (normalized): ........................... 0  
Min. fraction of hits on the mainstrand: .................... 0.75  
Top fraction of mapped sequences (in terms of read counts): . 1%  
Top fraction accounts for max. n% of sequence reads: ........ 90%  
Min. fraction of hits on each arm of a bidirectional cluster: 0.1  
Output image file for each cluster: ......................... 0 [0=no/1=yes]  
Output html file for each cluster: .......................... 1 [0=no/1=yes]  
Output a summary table: ..................................... 1 [0=no/1=yes]  
Output a FASTA file for each cluster (piRNA sequences): ..... 1 [0=no/1=yes]  
Output a FASTA file comprising cluster sequences: ........... 1 [0=no/1=yes]  
Search DNA motifs in clusters: .............................. 1 [0=no/1=yes]  
Output flanking sequences: +/- .............................. 0 bp  
Output ~.pTi file: .......................................... 1 [0=no/1=yes]  
==============================================================================  
  
  
Genome size (without gaps): ............ 2678902517 bp  
Gaps (N/X/-): .......................... 53837044 bp  
Mapped reads: .......................... 738059667487  
Non-identical sequences: ............... 277001  
Genomic hits: .......................... 533816  
Significant densitiy of mapped reads: .. 15118061 reads/kb

Show proTRAC cluster info
Hide proTRAC cluster info

|  |  |
| --- | --- |
| Location | chr23 |
| Coordinates | 7924310-7985620 |
| Size [bp] | 61311 |
| Sequence hit loci | 5793 |
| Mapped reads (normalized) | 15416036719 |
| Mapped reads (normalized) per kb | 251439981.7 |
| Normalized reads with 1T (1U) | 82.1% |
| Normalized reads with 10A | 28.4% |
| Normalized reads with length 26-33 nt | 100% |
| Normalized reads on the main strand(s) | 88.8% |
| Predicted directionality | bi:minus-plus (split between 7953196 and 7953205) |

100%

0%

1T (1U)  
reads

10A reads

26-33 nt  
reads

reads on mainstrand

**Either the amount of reads with 1T (1U) OR 10A has to exceed 75% (set with option: -1Tor10A)  
Alternatively the amount of reads with 1T (1U) AND 10A has to exceed 50% (set with option: -1Tand10A)  
Minimum amount of reads with preferred size is 75% (set with option: -pisize)  
Minimum amount of reads on the main strand(s) is 75% (set with option: -clstrand)**

Show read coverage
Hide read coverage

WHAT DO I SEE HERE?  
This chart shows the location of mapped sequence reads within a predicted piRNA cluster. The color refers to the number of genomic hits produced by the sequence read in question. A dark red bar indicates that this sequence read produces many other hits elsewhere in the genome. Many adjacent red or yellow bars can indicate the presence of a multi-copy element such as transposons or rRNA genes. A dark green bar indicates that this sequence read maps uniquely to this locus.

1 hit

2-5 hits

6-10 hits

11-20 hits

21-50 hits

51-100 hits

> 100 hits

chr23

7924310

7985620

Gene Set

RepeatMasker

Mapped  
Reads

265.97

plus strand

minus strand

265.97

Region: chr23 59850043-7924371. Max. coverage (+): 0. Max coverage (-): 6.45

Region: chr23 7924372-7924493. Max. coverage (+): 0. Max coverage (-): 0

Region: chr23 7924494-7924616. Max. coverage (+): 0. Max coverage (-): 0

Region: chr23 7924617-7924739. Max. coverage (+): 0. Max coverage (-): 0

Region: chr23 7924740-7924861. Max. coverage (+): 0. Max coverage (-): 0

Region: chr23 7924862-7924984. Max. coverage (+): 0. Max coverage (-): 16.83

Region: chr23 7924985-7925107. Max. coverage (+): 0. Max coverage (-): 10.89

Region: chr23 7925108-7925229. Max. coverage (+): 0. Max coverage (-): 0

Region: chr23 7925230-7925352. Max. coverage (+): 0. Max coverage (-): 5.65

Region: chr23 7925353-7925474. Max. coverage (+): 0. Max coverage (-): 164.54

Region: chr23 7925475-7925597. Max. coverage (+): 0. Max coverage (-): 9.84

Region: chr23 7925598-7925720. Max. coverage (+): 0. Max coverage (-): 0

Region: chr23 7925721-7925842. Max. coverage (+): 0. Max coverage (-): 5.64

Region: chr23 7925843-7925965. Max. coverage (+): 0. Max coverage (-): 18.47

Region: chr23 7925966-7926088. Max. coverage (+): 0. Max coverage (-): 7.11

Region: chr23 7926089-7926210. Max. coverage (+): 0. Max coverage (-): 0

Region: chr23 7926211-7926333. Max. coverage (+): 0. Max coverage (-): 8.69

Region: chr23 7926334-7926455. Max. coverage (+): 0. Max coverage (-): 30.55

Region: chr23 7926456-7926578. Max. coverage (+): 0. Max coverage (-): 0

Region: chr23 7926579-7926701. Max. coverage (+): 0. Max coverage (-): 0

Region: chr23 7926702-7926823. Max. coverage (+): 0. Max coverage (-): 0

Region: chr23 7926824-7926946. Max. coverage (+): 0. Max coverage (-): 1.47

Region: chr23 7926947-7927068. Max. coverage (+): 0. Max coverage (-): 0

Region: chr23 7927069-7927191. Max. coverage (+): 0. Max coverage (-): 0

Region: chr23 7927192-7927314. Max. coverage (+): 0. Max coverage (-): 0

Region: chr23 7927315-7927436. Max. coverage (+): 0. Max coverage (-): 0

Region: chr23 7927437-7927559. Max. coverage (+): 0. Max coverage (-): 0

Region: chr23 7927560-7927682. Max. coverage (+): 0. Max coverage (-): 0

Region: chr23 7927683-7927804. Max. coverage (+): 0. Max coverage (-): 11.09

Region: chr23 7927805-7927927. Max. coverage (+): 0. Max coverage (-): 2.98

Region: chr23 7927928-7928049. Max. coverage (+): 0. Max coverage (-): 16.37

Region: chr23 7928050-7928172. Max. coverage (+): 0. Max coverage (-): 18.6

Region: chr23 7928173-7928295. Max. coverage (+): 0. Max coverage (-): 0

Region: chr23 7928296-7928417. Max. coverage (+): 0. Max coverage (-): 0

Region: chr23 7928418-7928540. Max. coverage (+): 0. Max coverage (-): 0

Region: chr23 7928541-7928663. Max. coverage (+): 0. Max coverage (-): 11.39

Region: chr23 7928664-7928785. Max. coverage (+): 0. Max coverage (-): 27.98

Region: chr23 7928786-7928908. Max. coverage (+): 0. Max coverage (-): 10.52

Region: chr23 7928909-7929030. Max. coverage (+): 0. Max coverage (-): 11.98

Region: chr23 7929031-7929153. Max. coverage (+): 0. Max coverage (-): 41.84

Region: chr23 7929154-7929276. Max. coverage (+): 0. Max coverage (-): 40.06

Region: chr23 7929277-7929398. Max. coverage (+): 0. Max coverage (-): 0

Region: chr23 7929399-7929521. Max. coverage (+): 0. Max coverage (-): 0

Region: chr23 7929522-7929644. Max. coverage (+): 0. Max coverage (-): 3.12

Region: chr23 7929645-7929766. Max. coverage (+): 0. Max coverage (-): 4.92

Region: chr23 7929767-7929889. Max. coverage (+): 0. Max coverage (-): 4.87

Region: chr23 7929890-7930011. Max. coverage (+): 0. Max coverage (-): 9.8

Region: chr23 7930012-7930134. Max. coverage (+): 0. Max coverage (-): 8.71

Region: chr23 7930135-7930257. Max. coverage (+): 0. Max coverage (-): 77.78

Region: chr23 7930258-7930379. Max. coverage (+): 0. Max coverage (-): 5.13

Region: chr23 7930380-7930502. Max. coverage (+): 0. Max coverage (-): 31.78

Region: chr23 7930503-7930625. Max. coverage (+): 0. Max coverage (-): 7.77

Region: chr23 7930626-7930747. Max. coverage (+): 0. Max coverage (-): 19.34

Region: chr23 7930748-7930870. Max. coverage (+): 0. Max coverage (-): 12.7

Region: chr23 7930871-7930992. Max. coverage (+): 0. Max coverage (-): 0

Region: chr23 7930993-7931115. Max. coverage (+): 0. Max coverage (-): 7.04

Region: chr23 7931116-7931238. Max. coverage (+): 0. Max coverage (-): 7.04

Region: chr23 7931239-7931360. Max. coverage (+): 0. Max coverage (-): 0

Region: chr23 7931361-7931483. Max. coverage (+): 0. Max coverage (-): 1.7

Region: chr23 7931484-7931606. Max. coverage (+): 0. Max coverage (-): 10.44

Region: chr23 7931607-7931728. Max. coverage (+): 0. Max coverage (-): 16.7

Region: chr23 7931729-7931851. Max. coverage (+): 0. Max coverage (-): 7

Region: chr23 7931852-7931973. Max. coverage (+): 0. Max coverage (-): 24.48

Region: chr23 7931974-7932096. Max. coverage (+): 0. Max coverage (-): 24.36

Region: chr23 7932097-7932219. Max. coverage (+): 0. Max coverage (-): 0

Region: chr23 7932220-7932341. Max. coverage (+): 0. Max coverage (-): 11.92

Region: chr23 7932342-7932464. Max. coverage (+): 0. Max coverage (-): 10.44

Region: chr23 7932465-7932586. Max. coverage (+): 0. Max coverage (-): 20.64

Region: chr23 7932587-7932709. Max. coverage (+): 0. Max coverage (-): 40.04

Region: chr23 7932710-7932832. Max. coverage (+): 0. Max coverage (-): 58.67

Region: chr23 7932833-7932954. Max. coverage (+): 0. Max coverage (-): 9.19

Region: chr23 7932955-7933077. Max. coverage (+): 0. Max coverage (-): 0

Region: chr23 7933078-7933200. Max. coverage (+): 0. Max coverage (-): 5.18

Region: chr23 7933201-7933322. Max. coverage (+): 0. Max coverage (-): 34.22

Region: chr23 7933323-7933445. Max. coverage (+): 0. Max coverage (-): 27.06

Region: chr23 7933446-7933567. Max. coverage (+): 0. Max coverage (-): 12.91

Region: chr23 7933568-7933690. Max. coverage (+): 0. Max coverage (-): 30.06

Region: chr23 7933691-7933813. Max. coverage (+): 0. Max coverage (-): 17.31

Region: chr23 7933814-7933935. Max. coverage (+): 0. Max coverage (-): 29.31

Region: chr23 7933936-7934058. Max. coverage (+): 0. Max coverage (-): 31.89

Region: chr23 7934059-7934181. Max. coverage (+): 4.47. Max coverage (-): 11.67

Region: chr23 7934182-7934303. Max. coverage (+): 0. Max coverage (-): 0

Region: chr23 7934304-7934426. Max. coverage (+): 0. Max coverage (-): 0.66

Region: chr23 7934427-7934548. Max. coverage (+): 0. Max coverage (-): 12.55

Region: chr23 7934549-7934671. Max. coverage (+): 0. Max coverage (-): 0

Region: chr23 7934672-7934794. Max. coverage (+): 0. Max coverage (-): 5.04

Region: chr23 7934795-7934916. Max. coverage (+): 0. Max coverage (-): 9.1

Region: chr23 7934917-7935039. Max. coverage (+): 0. Max coverage (-): 35.96

Region: chr23 7935040-7935162. Max. coverage (+): 0. Max coverage (-): 4.96

Region: chr23 7935163-7935284. Max. coverage (+): 0. Max coverage (-): 0

Region: chr23 7935285-7935407. Max. coverage (+): 0. Max coverage (-): 0

Region: chr23 7935408-7935529. Max. coverage (+): 0. Max coverage (-): 0

Region: chr23 7935530-7935652. Max. coverage (+): 19.46. Max coverage (-): 0

Region: chr23 7935653-7935775. Max. coverage (+): 36.22. Max coverage (-): 0

Region: chr23 7935776-7935897. Max. coverage (+): 35.35. Max coverage (-): 0

Region: chr23 7935898-7936020. Max. coverage (+): 26.62. Max coverage (-): 0

Region: chr23 7936021-7936143. Max. coverage (+): 6.62. Max coverage (-): 0

Region: chr23 7936144-7936265. Max. coverage (+): 265.97. Max coverage (-): 0

Region: chr23 7936266-7936388. Max. coverage (+): 6.78. Max coverage (-): 0

Region: chr23 7936389-7936510. Max. coverage (+): 50.11. Max coverage (-): 0

Region: chr23 7936511-7936633. Max. coverage (+): 15.19. Max coverage (-): 0

Region: chr23 7936634-7936756. Max. coverage (+): 0. Max coverage (-): 0

Region: chr23 7936757-7936878. Max. coverage (+): 30.65. Max coverage (-): 3.7

Region: chr23 7936879-7937001. Max. coverage (+): 5.95. Max coverage (-): 0

Region: chr23 7937002-7937123. Max. coverage (+): 26.74. Max coverage (-): 2.49

Region: chr23 7937124-7937246. Max. coverage (+): 51.39. Max coverage (-): 0

Region: chr23 7937247-7937369. Max. coverage (+): 9.88. Max coverage (-): 0

Region: chr23 7937370-7937491. Max. coverage (+): 10.28. Max coverage (-): 0

Region: chr23 7937492-7937614. Max. coverage (+): 59.17. Max coverage (-): 0

Region: chr23 7937615-7937737. Max. coverage (+): 161.17. Max coverage (-): 0

Region: chr23 7937738-7937859. Max. coverage (+): 6.54. Max coverage (-): 0

Region: chr23 7937860-7937982. Max. coverage (+): 0.82. Max coverage (-): 0

Region: chr23 7937983-7938104. Max. coverage (+): 7.02. Max coverage (-): 0

Region: chr23 7938105-7938227. Max. coverage (+): 3.53. Max coverage (-): 0

Region: chr23 7938228-7938350. Max. coverage (+): 27.11. Max coverage (-): 0

Region: chr23 7938351-7938472. Max. coverage (+): 43.97. Max coverage (-): 0

Region: chr23 7938473-7938595. Max. coverage (+): 37.96. Max coverage (-): 0

Region: chr23 7938596-7938718. Max. coverage (+): 51.8. Max coverage (-): 0

Region: chr23 7938719-7938840. Max. coverage (+): 12.83. Max coverage (-): 0

Region: chr23 7938841-7938963. Max. coverage (+): 46.21. Max coverage (-): 0

Region: chr23 7938964-7939085. Max. coverage (+): 29.78. Max coverage (-): 0

Region: chr23 7939086-7939208. Max. coverage (+): 38.93. Max coverage (-): 0

Region: chr23 7939209-7939331. Max. coverage (+): 79.24. Max coverage (-): 0

Region: chr23 7939332-7939453. Max. coverage (+): 6.96. Max coverage (-): 0

Region: chr23 7939454-7939576. Max. coverage (+): 29.87. Max coverage (-): 0

Region: chr23 7939577-7939699. Max. coverage (+): 0. Max coverage (-): 0

Region: chr23 7939700-7939821. Max. coverage (+): 17.62. Max coverage (-): 10.19

Region: chr23 7939822-7939944. Max. coverage (+): 31.63. Max coverage (-): 0

Region: chr23 7939945-7940066. Max. coverage (+): 46.57. Max coverage (-): 0

Region: chr23 7940067-7940189. Max. coverage (+): 11.23. Max coverage (-): 0

Region: chr23 7940190-7940312. Max. coverage (+): 48.13. Max coverage (-): 0

Region: chr23 7940313-7940434. Max. coverage (+): 4.47. Max coverage (-): 0

Region: chr23 7940435-7940557. Max. coverage (+): 0. Max coverage (-): 0

Region: chr23 7940558-7940680. Max. coverage (+): 0. Max coverage (-): 0

Region: chr23 7940681-7940802. Max. coverage (+): 0. Max coverage (-): 0

Region: chr23 7940803-7940925. Max. coverage (+): 0. Max coverage (-): 0

Region: chr23 7940926-7941047. Max. coverage (+): 0. Max coverage (-): 0

Region: chr23 7941048-7941170. Max. coverage (+): 0. Max coverage (-): 52.64

Region: chr23 7941171-7941293. Max. coverage (+): 5.57. Max coverage (-): 76.14

Region: chr23 7941294-7941415. Max. coverage (+): 0. Max coverage (-): 6.07

Region: chr23 7941416-7941538. Max. coverage (+): 0. Max coverage (-): 43.66

Region: chr23 7941539-7941661. Max. coverage (+): 0. Max coverage (-): 26.72

Region: chr23 7941662-7941783. Max. coverage (+): 0. Max coverage (-): 53.97

Region: chr23 7941784-7941906. Max. coverage (+): 0. Max coverage (-): 26.6

Region: chr23 7941907-7942028. Max. coverage (+): 0. Max coverage (-): 42.5

Region: chr23 7942029-7942151. Max. coverage (+): 0. Max coverage (-): 10.73

Region: chr23 7942152-7942274. Max. coverage (+): 0. Max coverage (-): 43.05

Region: chr23 7942275-7942396. Max. coverage (+): 0. Max coverage (-): 17.67

Region: chr23 7942397-7942519. Max. coverage (+): 0. Max coverage (-): 59.01

Region: chr23 7942520-7942641. Max. coverage (+): 4.05. Max coverage (-): 74.69

Region: chr23 7942642-7942764. Max. coverage (+): 0. Max coverage (-): 12.98

Region: chr23 7942765-7942887. Max. coverage (+): 0. Max coverage (-): 12.52

Region: chr23 7942888-7943009. Max. coverage (+): 0. Max coverage (-): 13.54

Region: chr23 7943010-7943132. Max. coverage (+): 0. Max coverage (-): 51.06

Region: chr23 7943133-7943255. Max. coverage (+): 0. Max coverage (-): 15.4

Region: chr23 7943256-7943377. Max. coverage (+): 0. Max coverage (-): 42.82

Region: chr23 7943378-7943500. Max. coverage (+): 0. Max coverage (-): 69.56

Region: chr23 7943501-7943622. Max. coverage (+): 0. Max coverage (-): 6.66

Region: chr23 7943623-7943745. Max. coverage (+): 5.1. Max coverage (-): 15.41

Region: chr23 7943746-7943868. Max. coverage (+): 0. Max coverage (-): 56.49

Region: chr23 7943869-7943990. Max. coverage (+): 0. Max coverage (-): 52.8

Region: chr23 7943991-7944113. Max. coverage (+): 0. Max coverage (-): 72.31

Region: chr23 7944114-7944236. Max. coverage (+): 0. Max coverage (-): 19.53

Region: chr23 7944237-7944358. Max. coverage (+): 0. Max coverage (-): 6.93

Region: chr23 7944359-7944481. Max. coverage (+): 0. Max coverage (-): 29.86

Region: chr23 7944482-7944603. Max. coverage (+): 0. Max coverage (-): 11.8

Region: chr23 7944604-7944726. Max. coverage (+): 0. Max coverage (-): 15.73

Region: chr23 7944727-7944849. Max. coverage (+): 0. Max coverage (-): 26.09

Region: chr23 7944850-7944971. Max. coverage (+): 0. Max coverage (-): 14.3

Region: chr23 7944972-7945094. Max. coverage (+): 0. Max coverage (-): 5.39

Region: chr23 7945095-7945217. Max. coverage (+): 0. Max coverage (-): 78.22

Region: chr23 7945218-7945339. Max. coverage (+): 0. Max coverage (-): 47.77

Region: chr23 7945340-7945462. Max. coverage (+): 0. Max coverage (-): 20.94

Region: chr23 7945463-7945584. Max. coverage (+): 0. Max coverage (-): 3.45

Region: chr23 7945585-7945707. Max. coverage (+): 0. Max coverage (-): 39.78

Region: chr23 7945708-7945830. Max. coverage (+): 5.02. Max coverage (-): 7.33

Region: chr23 7945831-7945952. Max. coverage (+): 0. Max coverage (-): 7.13

Region: chr23 7945953-7946075. Max. coverage (+): 0. Max coverage (-): 0

Region: chr23 7946076-7946198. Max. coverage (+): 0.83. Max coverage (-): 21.51

Region: chr23 7946199-7946320. Max. coverage (+): 3.45. Max coverage (-): 7.33

Region: chr23 7946321-7946443. Max. coverage (+): 0. Max coverage (-): 82.93

Region: chr23 7946444-7946565. Max. coverage (+): 0. Max coverage (-): 27.77

Region: chr23 7946566-7946688. Max. coverage (+): 0. Max coverage (-): 19.22

Region: chr23 7946689-7946811. Max. coverage (+): 0. Max coverage (-): 149.97

Region: chr23 7946812-7946933. Max. coverage (+): 0. Max coverage (-): 0.1

Region: chr23 7946934-7947056. Max. coverage (+): 0. Max coverage (-): 7.09

Region: chr23 7947057-7947179. Max. coverage (+): 0. Max coverage (-): 21.32

Region: chr23 7947180-7947301. Max. coverage (+): 0. Max coverage (-): 8.3

Region: chr23 7947302-7947424. Max. coverage (+): 0. Max coverage (-): 47.9

Region: chr23 7947425-7947546. Max. coverage (+): 0. Max coverage (-): 52.06

Region: chr23 7947547-7947669. Max. coverage (+): 0. Max coverage (-): 27.71

Region: chr23 7947670-7947792. Max. coverage (+): 0. Max coverage (-): 21.69

Region: chr23 7947793-7947914. Max. coverage (+): 0. Max coverage (-): 20.79

Region: chr23 7947915-7948037. Max. coverage (+): 0. Max coverage (-): 0

Region: chr23 7948038-7948159. Max. coverage (+): 0. Max coverage (-): 0

Region: chr23 7948160-7948282. Max. coverage (+): 0. Max coverage (-): 0

Region: chr23 7948283-7948405. Max. coverage (+): 0. Max coverage (-): 0

Region: chr23 7948406-7948527. Max. coverage (+): 0. Max coverage (-): 0

Region: chr23 7948528-7948650. Max. coverage (+): 0. Max coverage (-): 32.48

Region: chr23 7948651-7948773. Max. coverage (+): 0. Max coverage (-): 5.38

Region: chr23 7948774-7948895. Max. coverage (+): 0. Max coverage (-): 35.3

Region: chr23 7948896-7949018. Max. coverage (+): 0. Max coverage (-): 1.06

Region: chr23 7949019-7949140. Max. coverage (+): 0. Max coverage (-): 0

Region: chr23 7949141-7949263. Max. coverage (+): 0. Max coverage (-): 11.52

Region: chr23 7949264-7949386. Max. coverage (+): 0. Max coverage (-): 13.31

Region: chr23 7949387-7949508. Max. coverage (+): 0. Max coverage (-): 18.02

Region: chr23 7949509-7949631. Max. coverage (+): 0. Max coverage (-): 66.53

Region: chr23 7949632-7949754. Max. coverage (+): 0. Max coverage (-): 36.8

Region: chr23 7949755-7949876. Max. coverage (+): 0. Max coverage (-): 21.61

Region: chr23 7949877-7949999. Max. coverage (+): 0. Max coverage (-): 9.59

Region: chr23 7950000-7950121. Max. coverage (+): 0. Max coverage (-): 45.78

Region: chr23 7950122-7950244. Max. coverage (+): 0. Max coverage (-): 75.65

Region: chr23 7950245-7950367. Max. coverage (+): 0. Max coverage (-): 33.38

Region: chr23 7950368-7950489. Max. coverage (+): 0. Max coverage (-): 0

Region: chr23 7950490-7950612. Max. coverage (+): 0. Max coverage (-): 10.29

Region: chr23 7950613-7950735. Max. coverage (+): 0. Max coverage (-): 10.23

Region: chr23 7950736-7950857. Max. coverage (+): 0. Max coverage (-): 16.34

Region: chr23 7950858-7950980. Max. coverage (+): 0. Max coverage (-): 4.68

Region: chr23 7950981-7951102. Max. coverage (+): 0. Max coverage (-): 8.88

Region: chr23 7951103-7951225. Max. coverage (+): 0. Max coverage (-): 17.01

Region: chr23 7951226-7951348. Max. coverage (+): 0. Max coverage (-): 80.78

Region: chr23 7951349-7951470. Max. coverage (+): 0. Max coverage (-): 9.86

Region: chr23 7951471-7951593. Max. coverage (+): 0.62. Max coverage (-): 6.5

Region: chr23 7951594-7951716. Max. coverage (+): 0. Max coverage (-): 24.43

Region: chr23 7951717-7951838. Max. coverage (+): 0. Max coverage (-): 7.48

Region: chr23 7951839-7951961. Max. coverage (+): 0. Max coverage (-): 0.2

Region: chr23 7951962-7952083. Max. coverage (+): 0. Max coverage (-): 0

Region: chr23 7952084-7952206. Max. coverage (+): 0. Max coverage (-): 0

Region: chr23 7952207-7952329. Max. coverage (+): 0. Max coverage (-): 0

Region: chr23 7952330-7952451. Max. coverage (+): 0. Max coverage (-): 18.83

Region: chr23 7952452-7952574. Max. coverage (+): 3.69. Max coverage (-): 37.77

Region: chr23 7952575-7952696. Max. coverage (+): 3.59. Max coverage (-): 49.7

Region: chr23 7952697-7952819. Max. coverage (+): 0. Max coverage (-): 1.56

Region: chr23 7952820-7952942. Max. coverage (+): 0. Max coverage (-): 0

Region: chr23 7952943-7953064. Max. coverage (+): 0. Max coverage (-): 0

Region: chr23 7953065-7953187. Max. coverage (+): 1.02. Max coverage (-): 2.01

Region: chr23 7953188-7953310. Max. coverage (+): 9.71. Max coverage (-): 2.36

Region: chr23 7953311-7953432. Max. coverage (+): 0. Max coverage (-): 0

Region: chr23 7953433-7953555. Max. coverage (+): 0. Max coverage (-): 0

Region: chr23 7953556-7953677. Max. coverage (+): 8.01. Max coverage (-): 0

Region: chr23 7953678-7953800. Max. coverage (+): 94.27. Max coverage (-): 0.67

Region: chr23 7953801-7953923. Max. coverage (+): 46.38. Max coverage (-): 0

Region: chr23 7953924-7954045. Max. coverage (+): 15.15. Max coverage (-): 0

Region: chr23 7954046-7954168. Max. coverage (+): 35.21. Max coverage (-): 0

Region: chr23 7954169-7954291. Max. coverage (+): 4.41. Max coverage (-): 0

Region: chr23 7954292-7954413. Max. coverage (+): 6.36. Max coverage (-): 0

Region: chr23 7954414-7954536. Max. coverage (+): 22.39. Max coverage (-): 0

Region: chr23 7954537-7954658. Max. coverage (+): 0. Max coverage (-): 0

Region: chr23 7954659-7954781. Max. coverage (+): 43.19. Max coverage (-): 0

Region: chr23 7954782-7954904. Max. coverage (+): 74.2. Max coverage (-): 0

Region: chr23 7954905-7955026. Max. coverage (+): 94.35. Max coverage (-): 0

Region: chr23 7955027-7955149. Max. coverage (+): 46.91. Max coverage (-): 7.06

Region: chr23 7955150-7955272. Max. coverage (+): 41.28. Max coverage (-): 3.7

Region: chr23 7955273-7955394. Max. coverage (+): 36.19. Max coverage (-): 0

Region: chr23 7955395-7955517. Max. coverage (+): 47.28. Max coverage (-): 0

Region: chr23 7955518-7955639. Max. coverage (+): 18.46. Max coverage (-): 0

Region: chr23 7955640-7955762. Max. coverage (+): 35.04. Max coverage (-): 0

Region: chr23 7955763-7955885. Max. coverage (+): 82.7. Max coverage (-): 0

Region: chr23 7955886-7956007. Max. coverage (+): 10.09. Max coverage (-): 0

Region: chr23 7956008-7956130. Max. coverage (+): 9.22. Max coverage (-): 0

Region: chr23 7956131-7956253. Max. coverage (+): 5.49. Max coverage (-): 0

Region: chr23 7956254-7956375. Max. coverage (+): 46.72. Max coverage (-): 0

Region: chr23 7956376-7956498. Max. coverage (+): 15.28. Max coverage (-): 0

Region: chr23 7956499-7956620. Max. coverage (+): 200.39. Max coverage (-): 0.99

Region: chr23 7956621-7956743. Max. coverage (+): 65.99. Max coverage (-): 5.9

Region: chr23 7956744-7956866. Max. coverage (+): 108.73. Max coverage (-): 0

Region: chr23 7956867-7956988. Max. coverage (+): 53.86. Max coverage (-): 0

Region: chr23 7956989-7957111. Max. coverage (+): 29.38. Max coverage (-): 0

Region: chr23 7957112-7957234. Max. coverage (+): 23.69. Max coverage (-): 0

Region: chr23 7957235-7957356. Max. coverage (+): 42.05. Max coverage (-): 0

Region: chr23 7957357-7957479. Max. coverage (+): 31.64. Max coverage (-): 0

Region: chr23 7957480-7957601. Max. coverage (+): 46.82. Max coverage (-): 0

Region: chr23 7957602-7957724. Max. coverage (+): 17.48. Max coverage (-): 0

Region: chr23 7957725-7957847. Max. coverage (+): 25.19. Max coverage (-): 0

Region: chr23 7957848-7957969. Max. coverage (+): 33.99. Max coverage (-): 0

Region: chr23 7957970-7958092. Max. coverage (+): 90.01. Max coverage (-): 0

Region: chr23 7958093-7958214. Max. coverage (+): 17.06. Max coverage (-): 0

Region: chr23 7958215-7958337. Max. coverage (+): 42.14. Max coverage (-): 0

Region: chr23 7958338-7958460. Max. coverage (+): 32.88. Max coverage (-): 0

Region: chr23 7958461-7958582. Max. coverage (+): 42.74. Max coverage (-): 0

Region: chr23 7958583-7958705. Max. coverage (+): 36.86. Max coverage (-): 0

Region: chr23 7958706-7958828. Max. coverage (+): 55.73. Max coverage (-): 0

Region: chr23 7958829-7958950. Max. coverage (+): 69.63. Max coverage (-): 0

Region: chr23 7958951-7959073. Max. coverage (+): 182.82. Max coverage (-): 0

Region: chr23 7959074-7959195. Max. coverage (+): 56.73. Max coverage (-): 1.39

Region: chr23 7959196-7959318. Max. coverage (+): 31.25. Max coverage (-): 0

Region: chr23 7959319-7959441. Max. coverage (+): 14.75. Max coverage (-): 0

Region: chr23 7959442-7959563. Max. coverage (+): 22.82. Max coverage (-): 3.53

Region: chr23 7959564-7959686. Max. coverage (+): 35.26. Max coverage (-): 0

Region: chr23 7959687-7959809. Max. coverage (+): 6.97. Max coverage (-): 0

Region: chr23 7959810-7959931. Max. coverage (+): 56.84. Max coverage (-): 0

Region: chr23 7959932-7960054. Max. coverage (+): 10.68. Max coverage (-): 0

Region: chr23 7960055-7960176. Max. coverage (+): 29.59. Max coverage (-): 0

Region: chr23 7960177-7960299. Max. coverage (+): 0. Max coverage (-): 0

Region: chr23 7960300-7960422. Max. coverage (+): 0. Max coverage (-): 0

Region: chr23 7960423-7960544. Max. coverage (+): 0. Max coverage (-): 0

Region: chr23 7960545-7960667. Max. coverage (+): 73.12. Max coverage (-): 0

Region: chr23 7960668-7960790. Max. coverage (+): 171.74. Max coverage (-): 0

Region: chr23 7960791-7960912. Max. coverage (+): 21.99. Max coverage (-): 0

Region: chr23 7960913-7961035. Max. coverage (+): 10.14. Max coverage (-): 0

Region: chr23 7961036-7961157. Max. coverage (+): 35.7. Max coverage (-): 0

Region: chr23 7961158-7961280. Max. coverage (+): 59.39. Max coverage (-): 0

Region: chr23 7961281-7961403. Max. coverage (+): 33.56. Max coverage (-): 0

Region: chr23 7961404-7961525. Max. coverage (+): 0.43. Max coverage (-): 0

Region: chr23 7961526-7961648. Max. coverage (+): 6.46. Max coverage (-): 0

Region: chr23 7961649-7961771. Max. coverage (+): 23.15. Max coverage (-): 0

Region: chr23 7961772-7961893. Max. coverage (+): 6.29. Max coverage (-): 0

Region: chr23 7961894-7962016. Max. coverage (+): 6.75. Max coverage (-): 0

Region: chr23 7962017-7962138. Max. coverage (+): 69.16. Max coverage (-): 0

Region: chr23 7962139-7962261. Max. coverage (+): 6.53. Max coverage (-): 0

Region: chr23 7962262-7962384. Max. coverage (+): 0. Max coverage (-): 0

Region: chr23 7962385-7962506. Max. coverage (+): 0. Max coverage (-): 0

Region: chr23 7962507-7962629. Max. coverage (+): 0. Max coverage (-): 0

Region: chr23 7962630-7962751. Max. coverage (+): 34.17. Max coverage (-): 0

Region: chr23 7962752-7962874. Max. coverage (+): 35.28. Max coverage (-): 13.02

Region: chr23 7962875-7962997. Max. coverage (+): 9.83. Max coverage (-): 13.02

Region: chr23 7962998-7963119. Max. coverage (+): 29.76. Max coverage (-): 0

Region: chr23 7963120-7963242. Max. coverage (+): 33.12. Max coverage (-): 0

Region: chr23 7963243-7963365. Max. coverage (+): 0. Max coverage (-): 0

Region: chr23 7963366-7963487. Max. coverage (+): 15.13. Max coverage (-): 0

Region: chr23 7963488-7963610. Max. coverage (+): 6.48. Max coverage (-): 0

Region: chr23 7963611-7963732. Max. coverage (+): 11.62. Max coverage (-): 5.63

Region: chr23 7963733-7963855. Max. coverage (+): 14.56. Max coverage (-): 0

Region: chr23 7963856-7963978. Max. coverage (+): 6.84. Max coverage (-): 2.25

Region: chr23 7963979-7964100. Max. coverage (+): 14.77. Max coverage (-): 0

Region: chr23 7964101-7964223. Max. coverage (+): 14.77. Max coverage (-): 0

Region: chr23 7964224-7964346. Max. coverage (+): 16.49. Max coverage (-): 0

Region: chr23 7964347-7964468. Max. coverage (+): 26.32. Max coverage (-): 0

Region: chr23 7964469-7964591. Max. coverage (+): 6.57. Max coverage (-): 5.67

Region: chr23 7964592-7964713. Max. coverage (+): 17.27. Max coverage (-): 0

Region: chr23 7964714-7964836. Max. coverage (+): 0. Max coverage (-): 0

Region: chr23 7964837-7964959. Max. coverage (+): 23.97. Max coverage (-): 0

Region: chr23 7964960-7965081. Max. coverage (+): 58.61. Max coverage (-): 0

Region: chr23 7965082-7965204. Max. coverage (+): 24.36. Max coverage (-): 0

Region: chr23 7965205-7965327. Max. coverage (+): 28.34. Max coverage (-): 0

Region: chr23 7965328-7965449. Max. coverage (+): 26.51. Max coverage (-): 0

Region: chr23 7965450-7965572. Max. coverage (+): 53.15. Max coverage (-): 0

Region: chr23 7965573-7965694. Max. coverage (+): 6.86. Max coverage (-): 0

Region: chr23 7965695-7965817. Max. coverage (+): 20.79. Max coverage (-): 1.11

Region: chr23 7965818-7965940. Max. coverage (+): 5.59. Max coverage (-): 0

Region: chr23 7965941-7966062. Max. coverage (+): 4.69. Max coverage (-): 0

Region: chr23 7966063-7966185. Max. coverage (+): 13.71. Max coverage (-): 0

Region: chr23 7966186-7966308. Max. coverage (+): 0. Max coverage (-): 0

Region: chr23 7966309-7966430. Max. coverage (+): 97.53. Max coverage (-): 4.39

Region: chr23 7966431-7966553. Max. coverage (+): 26.65. Max coverage (-): 0

Region: chr23 7966554-7966675. Max. coverage (+): 5.01. Max coverage (-): 0

Region: chr23 7966676-7966798. Max. coverage (+): 3.31. Max coverage (-): 0.67

Region: chr23 7966799-7966921. Max. coverage (+): 5.17. Max coverage (-): 0

Region: chr23 7966922-7967043. Max. coverage (+): 0. Max coverage (-): 0

Region: chr23 7967044-7967166. Max. coverage (+): 5.9. Max coverage (-): 0

Region: chr23 7967167-7967289. Max. coverage (+): 8.45. Max coverage (-): 0

Region: chr23 7967290-7967411. Max. coverage (+): 0. Max coverage (-): 0

Region: chr23 7967412-7967534. Max. coverage (+): 13.51. Max coverage (-): 0

Region: chr23 7967535-7967656. Max. coverage (+): 0. Max coverage (-): 4.33

Region: chr23 7967657-7967779. Max. coverage (+): 5.05. Max coverage (-): 0

Region: chr23 7967780-7967902. Max. coverage (+): 10.27. Max coverage (-): 4.1

Region: chr23 7967903-7968024. Max. coverage (+): 0. Max coverage (-): 0

Region: chr23 7968025-7968147. Max. coverage (+): 53.06. Max coverage (-): 2.93

Region: chr23 7968148-7968269. Max. coverage (+): 44.62. Max coverage (-): 7.19

Region: chr23 7968270-7968392. Max. coverage (+): 11.95. Max coverage (-): 7.22

Region: chr23 7968393-7968515. Max. coverage (+): 36.74. Max coverage (-): 5.99

Region: chr23 7968516-7968637. Max. coverage (+): 77.25. Max coverage (-): 4.35

Region: chr23 7968638-7968760. Max. coverage (+): 0. Max coverage (-): 0

Region: chr23 7968761-7968883. Max. coverage (+): 0. Max coverage (-): 0

Region: chr23 7968884-7969005. Max. coverage (+): 5.66. Max coverage (-): 0.45

Region: chr23 7969006-7969128. Max. coverage (+): 167.94. Max coverage (-): 3.28

Region: chr23 7969129-7969250. Max. coverage (+): 22.74. Max coverage (-): 5.9

Region: chr23 7969251-7969373. Max. coverage (+): 136.77. Max coverage (-): 0

Region: chr23 7969374-7969496. Max. coverage (+): 47.31. Max coverage (-): 0

Region: chr23 7969497-7969618. Max. coverage (+): 115.52. Max coverage (-): 0

Region: chr23 7969619-7969741. Max. coverage (+): 75.05. Max coverage (-): 0

Region: chr23 7969742-7969864. Max. coverage (+): 50.17. Max coverage (-): 5.39

Region: chr23 7969865-7969986. Max. coverage (+): 60.16. Max coverage (-): 1.72

Region: chr23 7969987-7970109. Max. coverage (+): 27.51. Max coverage (-): 0

Region: chr23 7970110-7970231. Max. coverage (+): 0. Max coverage (-): 0

Region: chr23 7970232-7970354. Max. coverage (+): 5.59. Max coverage (-): 0

Region: chr23 7970355-7970477. Max. coverage (+): 25.29. Max coverage (-): 0

Region: chr23 7970478-7970599. Max. coverage (+): 86.89. Max coverage (-): 0

Region: chr23 7970600-7970722. Max. coverage (+): 30.69. Max coverage (-): 0

Region: chr23 7970723-7970845. Max. coverage (+): 0. Max coverage (-): 0

Region: chr23 7970846-7970967. Max. coverage (+): 21.23. Max coverage (-): 0

Region: chr23 7970968-7971090. Max. coverage (+): 47.04. Max coverage (-): 0

Region: chr23 7971091-7971212. Max. coverage (+): 16.42. Max coverage (-): 0

Region: chr23 7971213-7971335. Max. coverage (+): 21.62. Max coverage (-): 0

Region: chr23 7971336-7971458. Max. coverage (+): 5.86. Max coverage (-): 0

Region: chr23 7971459-7971580. Max. coverage (+): 5.86. Max coverage (-): 0

Region: chr23 7971581-7971703. Max. coverage (+): 0. Max coverage (-): 0

Region: chr23 7971704-7971826. Max. coverage (+): 0. Max coverage (-): 0

Region: chr23 7971827-7971948. Max. coverage (+): 16.16. Max coverage (-): 0

Region: chr23 7971949-7972071. Max. coverage (+): 40.9. Max coverage (-): 0

Region: chr23 7972072-7972193. Max. coverage (+): 23.44. Max coverage (-): 0

Region: chr23 7972194-7972316. Max. coverage (+): 17.79. Max coverage (-): 0

Region: chr23 7972317-7972439. Max. coverage (+): 20.42. Max coverage (-): 0

Region: chr23 7972440-7972561. Max. coverage (+): 37.58. Max coverage (-): 0

Region: chr23 7972562-7972684. Max. coverage (+): 21.71. Max coverage (-): 0

Region: chr23 7972685-7972807. Max. coverage (+): 60.18. Max coverage (-): 0

Region: chr23 7972808-7972929. Max. coverage (+): 59.38. Max coverage (-): 0

Region: chr23 7972930-7973052. Max. coverage (+): 30.83. Max coverage (-): 0

Region: chr23 7973053-7973174. Max. coverage (+): 5.96. Max coverage (-): 0

Region: chr23 7973175-7973297. Max. coverage (+): 5.02. Max coverage (-): 0

Region: chr23 7973298-7973420. Max. coverage (+): 0.83. Max coverage (-): 0

Region: chr23 7973421-7973542. Max. coverage (+): 0.83. Max coverage (-): 0

Region: chr23 7973543-7973665. Max. coverage (+): 0. Max coverage (-): 0

Region: chr23 7973666-7973787. Max. coverage (+): 33.24. Max coverage (-): 0

Region: chr23 7973788-7973910. Max. coverage (+): 20.94. Max coverage (-): 0

Region: chr23 7973911-7974033. Max. coverage (+): 1.4. Max coverage (-): 0

Region: chr23 7974034-7974155. Max. coverage (+): 15.86. Max coverage (-): 0

Region: chr23 7974156-7974278. Max. coverage (+): 6.5. Max coverage (-): 0

Region: chr23 7974279-7974401. Max. coverage (+): 15.68. Max coverage (-): 0

Region: chr23 7974402-7974523. Max. coverage (+): 0. Max coverage (-): 0

Region: chr23 7974524-7974646. Max. coverage (+): 11.57. Max coverage (-): 0

Region: chr23 7974647-7974768. Max. coverage (+): 11.9. Max coverage (-): 0

Region: chr23 7974769-7974891. Max. coverage (+): 0. Max coverage (-): 0

Region: chr23 7974892-7975014. Max. coverage (+): 0. Max coverage (-): 0

Region: chr23 7975015-7975136. Max. coverage (+): 42.65. Max coverage (-): 0

Region: chr23 7975137-7975259. Max. coverage (+): 18.76. Max coverage (-): 0

Region: chr23 7975260-7975382. Max. coverage (+): 27.27. Max coverage (-): 0

Region: chr23 7975383-7975504. Max. coverage (+): 25.78. Max coverage (-): 6.9

Region: chr23 7975505-7975627. Max. coverage (+): 0.55. Max coverage (-): 0

Region: chr23 7975628-7975749. Max. coverage (+): 81.47. Max coverage (-): 0

Region: chr23 7975750-7975872. Max. coverage (+): 8.48. Max coverage (-): 0

Region: chr23 7975873-7975995. Max. coverage (+): 37.08. Max coverage (-): 0

Region: chr23 7975996-7976117. Max. coverage (+): 21.47. Max coverage (-): 0

Region: chr23 7976118-7976240. Max. coverage (+): 24.48. Max coverage (-): 0

Region: chr23 7976241-7976363. Max. coverage (+): 1.99. Max coverage (-): 0

Region: chr23 7976364-7976485. Max. coverage (+): 0. Max coverage (-): 0

Region: chr23 7976486-7976608. Max. coverage (+): 28.67. Max coverage (-): 0

Region: chr23 7976609-7976730. Max. coverage (+): 0. Max coverage (-): 0

Region: chr23 7976731-7976853. Max. coverage (+): 18.25. Max coverage (-): 0

Region: chr23 7976854-7976976. Max. coverage (+): 44.37. Max coverage (-): 0

Region: chr23 7976977-7977098. Max. coverage (+): 0. Max coverage (-): 0

Region: chr23 7977099-7977221. Max. coverage (+): 22.01. Max coverage (-): 0

Region: chr23 7977222-7977344. Max. coverage (+): 22.29. Max coverage (-): 0

Region: chr23 7977345-7977466. Max. coverage (+): 3.7. Max coverage (-): 0

Region: chr23 7977467-7977589. Max. coverage (+): 6.41. Max coverage (-): 5.25

Region: chr23 7977590-7977711. Max. coverage (+): 3.65. Max coverage (-): 0

Region: chr23 7977712-7977834. Max. coverage (+): 5.01. Max coverage (-): 0

Region: chr23 7977835-7977957. Max. coverage (+): 6.94. Max coverage (-): 0

Region: chr23 7977958-7978079. Max. coverage (+): 0. Max coverage (-): 0

Region: chr23 7978080-7978202. Max. coverage (+): 8.8. Max coverage (-): 0

Region: chr23 7978203-7978324. Max. coverage (+): 15.39. Max coverage (-): 0

Region: chr23 7978325-7978447. Max. coverage (+): 3.45. Max coverage (-): 0

Region: chr23 7978448-7978570. Max. coverage (+): 27.83. Max coverage (-): 0

Region: chr23 7978571-7978692. Max. coverage (+): 0. Max coverage (-): 0

Region: chr23 7978693-7978815. Max. coverage (+): 62.54. Max coverage (-): 0

Region: chr23 7978816-7978938. Max. coverage (+): 12.73. Max coverage (-): 0

Region: chr23 7978939-7979060. Max. coverage (+): 0. Max coverage (-): 0

Region: chr23 7979061-7979183. Max. coverage (+): 19.83. Max coverage (-): 0

Region: chr23 7979184-7979305. Max. coverage (+): 28.67. Max coverage (-): 0

Region: chr23 7979306-7979428. Max. coverage (+): 0. Max coverage (-): 0

Region: chr23 7979429-7979551. Max. coverage (+): 0. Max coverage (-): 0

Region: chr23 7979552-7979673. Max. coverage (+): 0. Max coverage (-): 0

Region: chr23 7979674-7979796. Max. coverage (+): 19.41. Max coverage (-): 0

Region: chr23 7979797-7979919. Max. coverage (+): 0. Max coverage (-): 0

Region: chr23 7979920-7980041. Max. coverage (+): 0. Max coverage (-): 0

Region: chr23 7980042-7980164. Max. coverage (+): 14.54. Max coverage (-): 0

Region: chr23 7980165-7980286. Max. coverage (+): 45.73. Max coverage (-): 0

Region: chr23 7980287-7980409. Max. coverage (+): 0. Max coverage (-): 0

Region: chr23 7980410-7980532. Max. coverage (+): 1.41. Max coverage (-): 0

Region: chr23 7980533-7980654. Max. coverage (+): 19.08. Max coverage (-): 0

Region: chr23 7980655-7980777. Max. coverage (+): 15.63. Max coverage (-): 0

Region: chr23 7980778-7980900. Max. coverage (+): 18.36. Max coverage (-): 0

Region: chr23 7980901-7981022. Max. coverage (+): 34.27. Max coverage (-): 0

Region: chr23 7981023-7981145. Max. coverage (+): 2.9. Max coverage (-): 0

Region: chr23 7981146-7981267. Max. coverage (+): 0. Max coverage (-): 0

Region: chr23 7981268-7981390. Max. coverage (+): 0. Max coverage (-): 0

Region: chr23 7981391-7981513. Max. coverage (+): 0. Max coverage (-): 0

Region: chr23 7981514-7981635. Max. coverage (+): 0. Max coverage (-): 0

Region: chr23 7981636-7981758. Max. coverage (+): 0. Max coverage (-): 0

Region: chr23 7981759-7981881. Max. coverage (+): 0. Max coverage (-): 0

Region: chr23 7981882-7982003. Max. coverage (+): 0. Max coverage (-): 0

Region: chr23 7982004-7982126. Max. coverage (+): 0. Max coverage (-): 0

Region: chr23 7982127-7982248. Max. coverage (+): 0. Max coverage (-): 0

Region: chr23 7982249-7982371. Max. coverage (+): 23.14. Max coverage (-): 0

Region: chr23 7982372-7982494. Max. coverage (+): 6.35. Max coverage (-): 0

Region: chr23 7982495-7982616. Max. coverage (+): 0. Max coverage (-): 0

Region: chr23 7982617-7982739. Max. coverage (+): 4.89. Max coverage (-): 0

Region: chr23 7982740-7982862. Max. coverage (+): 5.77. Max coverage (-): 0

Region: chr23 7982863-7982984. Max. coverage (+): 0. Max coverage (-): 0

Region: chr23 7982985-7983107. Max. coverage (+): 1.91. Max coverage (-): 0

Region: chr23 7983108-7983229. Max. coverage (+): 2.38. Max coverage (-): 0

Region: chr23 7983230-7983352. Max. coverage (+): 0. Max coverage (-): 0

Region: chr23 7983353-7983475. Max. coverage (+): 0.58. Max coverage (-): 0

Region: chr23 7983476-7983597. Max. coverage (+): 3.15. Max coverage (-): 0

Region: chr23 7983598-7983720. Max. coverage (+): 0. Max coverage (-): 0

Region: chr23 7983721-7983842. Max. coverage (+): 0. Max coverage (-): 0

Region: chr23 7983843-7983965. Max. coverage (+): 0. Max coverage (-): 0

Region: chr23 7983966-7984088. Max. coverage (+): 0. Max coverage (-): 0

Region: chr23 7984089-7984210. Max. coverage (+): 0. Max coverage (-): 0

Region: chr23 7984211-7984333. Max. coverage (+): 0. Max coverage (-): 0

Region: chr23 7984334-7984456. Max. coverage (+): 5.9. Max coverage (-): 0

Region: chr23 7984457-7984578. Max. coverage (+): 4.91. Max coverage (-): 0

Region: chr23 7984579-7984701. Max. coverage (+): 10.07. Max coverage (-): 0

Region: chr23 7984702-7984823. Max. coverage (+): 4.41. Max coverage (-): 0

Region: chr23 7984824-7984946. Max. coverage (+): 3.96. Max coverage (-): 0

Region: chr23 7984947-7985069. Max. coverage (+): 0. Max coverage (-): 0

Region: chr23 7985070-7985191. Max. coverage (+): 0. Max coverage (-): 0

Region: chr23 7985192-7985314. Max. coverage (+): 5.05. Max coverage (-): 0

Region: chr23 7985315-7985437. Max. coverage (+): 0. Max coverage (-): 0

Region: chr23 7985438-7985559. Max. coverage (+): 0. Max coverage (-): 0

Region: chr23 7985560-. Max. coverage (+): 10.3. Max coverage (-): 0

RepeatMasker Color Code

**+**

100-98% Identity

<98-95% Identity

<95-90% Identity

<90-85% Identity

<85-80% Identity

<80-75% Identity

<75-70% Identity

<70% Identity

**-**

Gene Set Color Code

**+**

Gene

Pseudogene

**-**

Topology/Coverage Color Code

Coverage Plus Strand

Coverage Minus Strand

Mainstrand: Plus

Mainstrand: Minus

Complementary Strand

Flanking Region  
(if option -flank >0)

Gene Set Annotation  
  
RepeatMasker Annotation  

**1. CHRL**: 7925535-7925710 (-), Divergence to consensus: 28.2%  
**2. MER63A**: 7926066-7926263 (-), Divergence to consensus: 37.4%  
**3. MamGypLTR1a**: 7926533-7926633 (-), Divergence to consensus: 34.4%  
**4. MIRb**: 7926759-7926814 (+), Divergence to consensus: 25%  
**5. L2a**: 7926920-7927372 (+), Divergence to consensus: 45%  
**6. L2a**: 7927511-7927710 (+), Divergence to consensus: 48.3%  
**7. MER102b**: 7928221-7928482 (+), Divergence to consensus: 46.7%  
**8. MIR3**: 7928985-7929037 (-), Divergence to consensus: 26.4%  
**9. (TG)n**: 7930542-7930595 (+), Divergence to consensus: 22.2%  
**10. (TG)n**: 7930841-7930885 (+), Divergence to consensus: 17.8%  
**11. Charlie9**: 7930891-7930957 (-), Divergence to consensus: 25.7%  
**12. C-rich**: 7931246-7931346 (+), Divergence to consensus: 33.8%  
**13. MLT1N2**: 7932275-7932483 (-), Divergence to consensus: 46.2%  
**14. MamRep605**: 7932972-7933053 (+), Divergence to consensus: 26.9%  
**15. (TGGA)n**: 7933464-7933504 (+), Divergence to consensus: 7.3%  
**16. Bov-tA1**: 7934118-7934319 (+), Divergence to consensus: 23.3%  
**17. MIRc**: 7938202-7938305 (+), Divergence to consensus: 32.7%  
**18. MIR3**: 7939588-7939723 (-), Divergence to consensus: 37.7%  
**19. MIR3**: 7942711-7942860 (-), Divergence to consensus: 50.4%  
**20. L2a**: 7944179-7944288 (+), Divergence to consensus: 33.9%  
**21. MIRc**: 7945922-7946004 (-), Divergence to consensus: 38.5%  
**22. MIR3**: 7946003-7946116 (-), Divergence to consensus: 43%  
**23. MIRc**: 7946774-7946872 (+), Divergence to consensus: 30.6%  
**24. GC\_rich**: 7947248-7947268 (+), Divergence to consensus: 23.8%  
**25. MIR3**: 7947647-7947773 (+), Divergence to consensus: 37.8%  
**26. BOV-A2**: 7947880-7948140 (+), Divergence to consensus: 4.6%  
**27. (CAG)n**: 7948141-7948166 (+), Divergence to consensus: 0%  
**28. Bov-tA2**: 7950383-7950567 (+), Divergence to consensus: 17.3%  
**29. (CGGGG)n**: 7952748-7952955 (+), Divergence to consensus: 29.8%  
**30. BOV-A2**: 7953353-7953599 (-), Divergence to consensus: 6.1%  
**31. LTR75**: 7954483-7954682 (-), Divergence to consensus: 25.1%  
**32. L2c**: 7960188-7960636 (+), Divergence to consensus: 47.2%  
**33. Bov-tA2**: 7962300-7962487 (-), Divergence to consensus: 27.7%  
**34. Bov-tA2**: 7962488-7962605 (-), Divergence to consensus: 17.8%  
**35. L2a**: 7962654-7962720 (-), Divergence to consensus: 16.4%  
**36. L1ME3A**: 7967217-7967640 (+), Divergence to consensus: 37.1%  
**37. Zaphod2**: 7967997-7968045 (+), Divergence to consensus: 12.6%  
**38. CR1\_Mam**: 7968661-7968986 (-), Divergence to consensus: 44.7%  
**39. L2a**: 7971531-7971846 (+), Divergence to consensus: 43.4%  
**40. AT\_rich**: 7973462-7973490 (+), Divergence to consensus: 75.9%  
**41. CHRL**: 7973491-7973653 (-), Divergence to consensus: 22.7%  
**42. BOV-A2**: 7974724-7974995 (+), Divergence to consensus: 5.5%  
**43. (GGGTG)n**: 7978071-7978102 (+), Divergence to consensus: 12.5%  
**44. BOV-A2**: 7978324-7978375 (+), Divergence to consensus: 11.6%  
**45. L2b**: 7978951-7979117 (-), Divergence to consensus: 48.8%  
**46. ERV1-1B-LTR\_BT**: 7979242-7979445 (+), Divergence to consensus: 49.6%  
**47. 5S**: 7979444-7979493 (-), Divergence to consensus: 18%  
**48. ERV1-1B-LTR\_BT**: 7979493-7979742 (+), Divergence to consensus: 39%  
**49. ART2A**: 7981320-7981868 (-), Divergence to consensus: 10.6%  
**50. BovB**: 7981869-7982271 (-), Divergence to consensus: 4.4%  
**51. MER63A**: 7983437-7983604 (-), Divergence to consensus: 51.3%  
**52. (CA)n**: 7985135-7985176 (+), Divergence to consensus: 0%

  
Transcription Factor Binding Sites  

**RFX4\_1** (Sequence: GTTGCCAAG (-): 7931850)  
**RFX4\_1** (Sequence: GTTGCCAAG (-): 7974262)  
**RFX4\_1** (Sequence: GTTGCCAGG (-): 7982892)  
**RFX4\_1** (Sequence: CCTGGCAAC (+): 7947225)  
**SPZ1** (Sequence: CTCAAACCCC (-): 7951102)  
**RFX4\_2** (Sequence: CATGGATAC (+): 7967864)  
**RFX4\_2** (Sequence: CATGGATAC (+): 7985003)  
**Gata4** (Sequence: AGATAAG (-): 7947813)  
**Gata4** (Sequence: AGATAAC (-): 7954983)  
**Gata4** (Sequence: AGATAAG (-): 7955814)  
**Gata4** (Sequence: AGATAAG (-): 7985600)  
**SOX9** (Sequence: AACAATAG (-): 7968156)  
**A-MYB** (Sequence: TGACAGTTGG (+): 7925135)  
**Gata4** (Sequence: CTTATCT (+): 7932289)  
**Gata4** (Sequence: GTTATCT (+): 7955073)  
**Gata4** (Sequence: CTTATCT (+): 7965222)  
**Gata4** (Sequence: CTTATCT (+): 7973044)
