# Supplementary material for: Small RNA sequencing of cryopreserved semen from single bull revealed altered miRNAs and piRNAs expression between High- and Low-motile sperm populations
Source: BMC Genomics. 2017 Jan 4;18:14. doi: 10.1186/s12864-016-3394-7 (PMC5209821; doi:10.1186/s12864-016-3394-7)
Supplement: Additional file 4: — Details for each piRNA clusters found in Low Motile (LM) sperm fraction. Genes, repeats, transposable elements and transcription factors binding sites falling within the cluster regions were reported. (ZIP 1034 kb) [file 12864_2016_3394_MOESM4_ESM.zip › 32.html]

piRNA cluster 32


Predicted piRNA cluster no. 32     previous   next
  

Show proTRAC run info
Hide proTRAC run info

================================= proTRAC ====================================  
VERSION: 2.1                                    LAST MODIFIED: 06. October 2015  
  
Please cite:  
Rosenkranz D, Zischler H. proTRAC - a software for probabilistic piRNA cluster  
detection, visualization and analysis. 2012. BMC Bioinformatics 13:5.  
  
and (for proTRAC 2.0 and later):  
Rosenkranz D, Rudloff S, Bastuck K, Ketting RF, Zischler H. Tupaia small RNAs  
provide insights into function and evolution of RNAi-based transposon defense  
in mammals. 2015. RNA 21(5):911-922.  
  
Contact:  
David Rosenkranz  
Institute of Anthropology, small RNA group  
Johannes Gutenberg University Mainz  
email: rosenkranz@uni-mainz.de  
  
You can find the latest proTRAC version at:  
http://sourceforge.net/projects/protrac/files  
http://www.smallRNAgroup-mainz.de/software  
==============================================================================  
  
PARAMETERS:  
Map file: .............../storage/core/barbara/genhome/smallRNA/fertility/Sample\_not\_motile/pirna/Sample\_not\_motile\_26-33\_collapsed.fa.no-dust.map.weighted-10000-1000-b-0  
Genome file: ............/storage/core/barbara/genhome/smallRNA/fertility/Sample\_all/pirna/bt\_311\_chrY.fa  
RepeatMasker annotation: /storage/genomes/bt\_umd31/GCF\_000003055.6\_Bos\_taurus\_UMD\_3.1.1\_repeatMasker\_chr.out  
GeneSet:................./storage/core/barbara/genhome/smallRNA/fertility/Sample\_all/pirna/full.gtf  
  
Significant (p<=0.01) hit density will be calculated based  
on observed hit distribution.  
  
Sliding window size: ........................................ 5000 bp  
Sliding window increament: .................................. 1000 bp  
Normalize each hit by number of genomic hits: ............... 1 [0=no/1=yes]  
Normalize each hit by number of sequence reads: ............. 1 [0=no/1=yes]  
Normalize values (-> per million mapped reads): ............. 1 [0=no/1=yes]  
Min. fraction of hits with 1T(U) or 10A: .................... 0.75  
Alternatively: Min. fraction of hits with 1T(U) and 10A: .... 0.5  
Min. fraction of hits with typical piRNA length: ............ 0.75  
Typical piRNA length: ....................................... 26-33 nt  
Min. size of a piRNA cluster: ............................... 5000 bp.  
Min. number of hits (absolute): ............................. 0  
Min. number of hits (normalized): ........................... 0  
Min. fraction of hits on the mainstrand: .................... 0.75  
Top fraction of mapped sequences (in terms of read counts): . 1%  
Top fraction accounts for max. n% of sequence reads: ........ 90%  
Min. fraction of hits on each arm of a bidirectional cluster: 0.1  
Output image file for each cluster: ......................... 0 [0=no/1=yes]  
Output html file for each cluster: .......................... 1 [0=no/1=yes]  
Output a summary table: ..................................... 1 [0=no/1=yes]  
Output a FASTA file for each cluster (piRNA sequences): ..... 1 [0=no/1=yes]  
Output a FASTA file comprising cluster sequences: ........... 1 [0=no/1=yes]  
Search DNA motifs in clusters: .............................. 1 [0=no/1=yes]  
Output flanking sequences: +/- .............................. 0 bp  
Output ~.pTi file: .......................................... 1 [0=no/1=yes]  
==============================================================================  
  
  
Genome size (without gaps): ............ 2678902517 bp  
Gaps (N/X/-): .......................... 53837044 bp  
Mapped reads: .......................... 738059667487  
Non-identical sequences: ............... 277001  
Genomic hits: .......................... 533816  
Significant densitiy of mapped reads: .. 15118061 reads/kb

Show proTRAC cluster info
Hide proTRAC cluster info

|  |  |
| --- | --- |
| Location | chr23 |
| Coordinates | 47783733-47788743 |
| Size [bp] | 5011 |
| Sequence hit loci | 459 |
| Mapped reads (normalized) | 1244091427 |
| Mapped reads (normalized) per kb | 248272086.8 |
| Normalized reads with 1T (1U) | 86.1% |
| Normalized reads with 10A | 31.1% |
| Normalized reads with length 26-33 nt | 100% |
| Normalized reads on the main strand(s) | 100% |
| Predicted directionality | mono:minus |

100%

0%

1T (1U)  
reads

10A reads

26-33 nt  
reads

reads on mainstrand

**Either the amount of reads with 1T (1U) OR 10A has to exceed 75% (set with option: -1Tor10A)  
Alternatively the amount of reads with 1T (1U) AND 10A has to exceed 50% (set with option: -1Tand10A)  
Minimum amount of reads with preferred size is 75% (set with option: -pisize)  
Minimum amount of reads on the main strand(s) is 75% (set with option: -clstrand)**

Show read coverage
Hide read coverage

WHAT DO I SEE HERE?  
This chart shows the location of mapped sequence reads within a predicted piRNA cluster. The color refers to the number of genomic hits produced by the sequence read in question. A dark red bar indicates that this sequence read produces many other hits elsewhere in the genome. Many adjacent red or yellow bars can indicate the presence of a multi-copy element such as transposons or rRNA genes. A dark green bar indicates that this sequence read maps uniquely to this locus.

1 hit

2-5 hits

6-10 hits

11-20 hits

21-50 hits

51-100 hits

> 100 hits

chr23

47783733

47788743

Gene Set

RepeatMasker

Mapped  
Reads

112.85

plus strand

minus strand

112.85

Region: chr23 7985561-47783738. Max. coverage (+): 0. Max coverage (-): 7.18

Region: chr23 47783739-47783748. Max. coverage (+): 0. Max coverage (-): 7.18

Region: chr23 47783749-47783758. Max. coverage (+): 0. Max coverage (-): 0

Region: chr23 47783759-47783768. Max. coverage (+): 0. Max coverage (-): 0

Region: chr23 47783769-47783778. Max. coverage (+): 0. Max coverage (-): 0

Region: chr23 47783779-47783788. Max. coverage (+): 0. Max coverage (-): 5.31

Region: chr23 47783789-47783798. Max. coverage (+): 0. Max coverage (-): 7.25

Region: chr23 47783799-47783808. Max. coverage (+): 0. Max coverage (-): 4.46

Region: chr23 47783809-47783818. Max. coverage (+): 0. Max coverage (-): 0

Region: chr23 47783819-47783828. Max. coverage (+): 0. Max coverage (-): 0

Region: chr23 47783829-47783838. Max. coverage (+): 0. Max coverage (-): 0

Region: chr23 47783839-47783848. Max. coverage (+): 0. Max coverage (-): 0

Region: chr23 47783849-47783858. Max. coverage (+): 0. Max coverage (-): 0

Region: chr23 47783859-47783868. Max. coverage (+): 0. Max coverage (-): 7.15

Region: chr23 47783869-47783878. Max. coverage (+): 0. Max coverage (-): 0

Region: chr23 47783879-47783888. Max. coverage (+): 0. Max coverage (-): 0

Region: chr23 47783889-47783898. Max. coverage (+): 0. Max coverage (-): 4.34

Region: chr23 47783899-47783908. Max. coverage (+): 0. Max coverage (-): 4.34

Region: chr23 47783909-47783918. Max. coverage (+): 0. Max coverage (-): 0

Region: chr23 47783919-47783928. Max. coverage (+): 0. Max coverage (-): 21.02

Region: chr23 47783929-47783938. Max. coverage (+): 0. Max coverage (-): 18.74

Region: chr23 47783939-47783948. Max. coverage (+): 0. Max coverage (-): 0

Region: chr23 47783949-47783958. Max. coverage (+): 0. Max coverage (-): 37.47

Region: chr23 47783959-47783968. Max. coverage (+): 0. Max coverage (-): 77.3

Region: chr23 47783969-47783978. Max. coverage (+): 0. Max coverage (-): 5.75

Region: chr23 47783979-47783988. Max. coverage (+): 0. Max coverage (-): 0

Region: chr23 47783989-47783998. Max. coverage (+): 0. Max coverage (-): 0

Region: chr23 47783999-47784008. Max. coverage (+): 0. Max coverage (-): 0

Region: chr23 47784009-47784018. Max. coverage (+): 0. Max coverage (-): 11.69

Region: chr23 47784019-47784028. Max. coverage (+): 0. Max coverage (-): 11.69

Region: chr23 47784029-47784038. Max. coverage (+): 0. Max coverage (-): 0

Region: chr23 47784039-47784048. Max. coverage (+): 0. Max coverage (-): 0

Region: chr23 47784049-47784058. Max. coverage (+): 0. Max coverage (-): 73.95

Region: chr23 47784059-47784068. Max. coverage (+): 0. Max coverage (-): 65.03

Region: chr23 47784069-47784078. Max. coverage (+): 0. Max coverage (-): 21.94

Region: chr23 47784079-47784088. Max. coverage (+): 0. Max coverage (-): 0

Region: chr23 47784089-47784098. Max. coverage (+): 0. Max coverage (-): 0

Region: chr23 47784099-47784108. Max. coverage (+): 0. Max coverage (-): 0

Region: chr23 47784109-47784118. Max. coverage (+): 0. Max coverage (-): 0

Region: chr23 47784119-47784128. Max. coverage (+): 0. Max coverage (-): 0

Region: chr23 47784129-47784138. Max. coverage (+): 0. Max coverage (-): 4.1

Region: chr23 47784139-47784148. Max. coverage (+): 0. Max coverage (-): 9.06

Region: chr23 47784149-47784158. Max. coverage (+): 0. Max coverage (-): 13.13

Region: chr23 47784159-47784168. Max. coverage (+): 0. Max coverage (-): 0

Region: chr23 47784169-47784178. Max. coverage (+): 0. Max coverage (-): 0

Region: chr23 47784179-47784189. Max. coverage (+): 0. Max coverage (-): 8.44

Region: chr23 47784190-47784199. Max. coverage (+): 0. Max coverage (-): 6.41

Region: chr23 47784200-47784209. Max. coverage (+): 0. Max coverage (-): 6.41

Region: chr23 47784210-47784219. Max. coverage (+): 0. Max coverage (-): 0

Region: chr23 47784220-47784229. Max. coverage (+): 0. Max coverage (-): 0

Region: chr23 47784230-47784239. Max. coverage (+): 0. Max coverage (-): 0

Region: chr23 47784240-47784249. Max. coverage (+): 0. Max coverage (-): 0

Region: chr23 47784250-47784259. Max. coverage (+): 0. Max coverage (-): 0

Region: chr23 47784260-47784269. Max. coverage (+): 0. Max coverage (-): 0

Region: chr23 47784270-47784279. Max. coverage (+): 0. Max coverage (-): 0

Region: chr23 47784280-47784289. Max. coverage (+): 0. Max coverage (-): 0

Region: chr23 47784290-47784299. Max. coverage (+): 0. Max coverage (-): 0

Region: chr23 47784300-47784309. Max. coverage (+): 0. Max coverage (-): 0

Region: chr23 47784310-47784319. Max. coverage (+): 0. Max coverage (-): 0

Region: chr23 47784320-47784329. Max. coverage (+): 0. Max coverage (-): 0

Region: chr23 47784330-47784339. Max. coverage (+): 0. Max coverage (-): 0

Region: chr23 47784340-47784349. Max. coverage (+): 0. Max coverage (-): 0

Region: chr23 47784350-47784359. Max. coverage (+): 0. Max coverage (-): 0

Region: chr23 47784360-47784369. Max. coverage (+): 0. Max coverage (-): 0

Region: chr23 47784370-47784379. Max. coverage (+): 0. Max coverage (-): 0

Region: chr23 47784380-47784389. Max. coverage (+): 0. Max coverage (-): 0

Region: chr23 47784390-47784399. Max. coverage (+): 0. Max coverage (-): 0

Region: chr23 47784400-47784409. Max. coverage (+): 0. Max coverage (-): 0

Region: chr23 47784410-47784419. Max. coverage (+): 0. Max coverage (-): 0

Region: chr23 47784420-47784429. Max. coverage (+): 0. Max coverage (-): 0

Region: chr23 47784430-47784439. Max. coverage (+): 0. Max coverage (-): 0

Region: chr23 47784440-47784449. Max. coverage (+): 0. Max coverage (-): 0

Region: chr23 47784450-47784459. Max. coverage (+): 0. Max coverage (-): 0

Region: chr23 47784460-47784469. Max. coverage (+): 0. Max coverage (-): 0

Region: chr23 47784470-47784479. Max. coverage (+): 0. Max coverage (-): 0

Region: chr23 47784480-47784489. Max. coverage (+): 0. Max coverage (-): 0

Region: chr23 47784490-47784499. Max. coverage (+): 0. Max coverage (-): 0

Region: chr23 47784500-47784509. Max. coverage (+): 0. Max coverage (-): 0

Region: chr23 47784510-47784519. Max. coverage (+): 0. Max coverage (-): 0

Region: chr23 47784520-47784529. Max. coverage (+): 0. Max coverage (-): 0

Region: chr23 47784530-47784539. Max. coverage (+): 0. Max coverage (-): 0

Region: chr23 47784540-47784549. Max. coverage (+): 0. Max coverage (-): 0

Region: chr23 47784550-47784559. Max. coverage (+): 0. Max coverage (-): 0

Region: chr23 47784560-47784569. Max. coverage (+): 0. Max coverage (-): 12.51

Region: chr23 47784570-47784579. Max. coverage (+): 0. Max coverage (-): 7.19

Region: chr23 47784580-47784589. Max. coverage (+): 0. Max coverage (-): 0

Region: chr23 47784590-47784599. Max. coverage (+): 0. Max coverage (-): 6.05

Region: chr23 47784600-47784609. Max. coverage (+): 0. Max coverage (-): 10.47

Region: chr23 47784610-47784619. Max. coverage (+): 0. Max coverage (-): 2.85

Region: chr23 47784620-47784629. Max. coverage (+): 0. Max coverage (-): 0

Region: chr23 47784630-47784639. Max. coverage (+): 0. Max coverage (-): 20.3

Region: chr23 47784640-47784650. Max. coverage (+): 0. Max coverage (-): 23.91

Region: chr23 47784651-47784660. Max. coverage (+): 0. Max coverage (-): 5.84

Region: chr23 47784661-47784670. Max. coverage (+): 0. Max coverage (-): 0

Region: chr23 47784671-47784680. Max. coverage (+): 0. Max coverage (-): 0

Region: chr23 47784681-47784690. Max. coverage (+): 0. Max coverage (-): 0

Region: chr23 47784691-47784700. Max. coverage (+): 0. Max coverage (-): 0

Region: chr23 47784701-47784710. Max. coverage (+): 0. Max coverage (-): 0

Region: chr23 47784711-47784720. Max. coverage (+): 0. Max coverage (-): 0

Region: chr23 47784721-47784730. Max. coverage (+): 0. Max coverage (-): 0

Region: chr23 47784731-47784740. Max. coverage (+): 0. Max coverage (-): 0

Region: chr23 47784741-47784750. Max. coverage (+): 0. Max coverage (-): 0

Region: chr23 47784751-47784760. Max. coverage (+): 0. Max coverage (-): 0

Region: chr23 47784761-47784770. Max. coverage (+): 0. Max coverage (-): 0

Region: chr23 47784771-47784780. Max. coverage (+): 0. Max coverage (-): 0

Region: chr23 47784781-47784790. Max. coverage (+): 0. Max coverage (-): 0

Region: chr23 47784791-47784800. Max. coverage (+): 0. Max coverage (-): 0

Region: chr23 47784801-47784810. Max. coverage (+): 0. Max coverage (-): 0

Region: chr23 47784811-47784820. Max. coverage (+): 0. Max coverage (-): 0

Region: chr23 47784821-47784830. Max. coverage (+): 0. Max coverage (-): 0

Region: chr23 47784831-47784840. Max. coverage (+): 0. Max coverage (-): 0

Region: chr23 47784841-47784850. Max. coverage (+): 0. Max coverage (-): 0

Region: chr23 47784851-47784860. Max. coverage (+): 0. Max coverage (-): 0

Region: chr23 47784861-47784870. Max. coverage (+): 0. Max coverage (-): 0

Region: chr23 47784871-47784880. Max. coverage (+): 0. Max coverage (-): 0

Region: chr23 47784881-47784890. Max. coverage (+): 0. Max coverage (-): 0

Region: chr23 47784891-47784900. Max. coverage (+): 0. Max coverage (-): 4.48

Region: chr23 47784901-47784910. Max. coverage (+): 0. Max coverage (-): 4.48

Region: chr23 47784911-47784920. Max. coverage (+): 0. Max coverage (-): 0

Region: chr23 47784921-47784930. Max. coverage (+): 0. Max coverage (-): 0

Region: chr23 47784931-47784940. Max. coverage (+): 0. Max coverage (-): 0

Region: chr23 47784941-47784950. Max. coverage (+): 0. Max coverage (-): 0

Region: chr23 47784951-47784960. Max. coverage (+): 0. Max coverage (-): 0

Region: chr23 47784961-47784970. Max. coverage (+): 0. Max coverage (-): 0

Region: chr23 47784971-47784980. Max. coverage (+): 0. Max coverage (-): 0

Region: chr23 47784981-47784990. Max. coverage (+): 0. Max coverage (-): 0

Region: chr23 47784991-47785000. Max. coverage (+): 0. Max coverage (-): 0

Region: chr23 47785001-47785010. Max. coverage (+): 0. Max coverage (-): 0

Region: chr23 47785011-47785020. Max. coverage (+): 0. Max coverage (-): 0

Region: chr23 47785021-47785030. Max. coverage (+): 0. Max coverage (-): 0

Region: chr23 47785031-47785040. Max. coverage (+): 0. Max coverage (-): 0

Region: chr23 47785041-47785050. Max. coverage (+): 0. Max coverage (-): 0

Region: chr23 47785051-47785060. Max. coverage (+): 0. Max coverage (-): 0

Region: chr23 47785061-47785070. Max. coverage (+): 0. Max coverage (-): 0

Region: chr23 47785071-47785080. Max. coverage (+): 0. Max coverage (-): 0

Region: chr23 47785081-47785090. Max. coverage (+): 0. Max coverage (-): 0

Region: chr23 47785091-47785101. Max. coverage (+): 0. Max coverage (-): 0

Region: chr23 47785102-47785111. Max. coverage (+): 0. Max coverage (-): 0

Region: chr23 47785112-47785121. Max. coverage (+): 0. Max coverage (-): 0

Region: chr23 47785122-47785131. Max. coverage (+): 0. Max coverage (-): 0

Region: chr23 47785132-47785141. Max. coverage (+): 0. Max coverage (-): 0

Region: chr23 47785142-47785151. Max. coverage (+): 0. Max coverage (-): 0

Region: chr23 47785152-47785161. Max. coverage (+): 0. Max coverage (-): 0

Region: chr23 47785162-47785171. Max. coverage (+): 0. Max coverage (-): 0.65

Region: chr23 47785172-47785181. Max. coverage (+): 0. Max coverage (-): 0.65

Region: chr23 47785182-47785191. Max. coverage (+): 0. Max coverage (-): 0

Region: chr23 47785192-47785201. Max. coverage (+): 0. Max coverage (-): 0

Region: chr23 47785202-47785211. Max. coverage (+): 0. Max coverage (-): 0

Region: chr23 47785212-47785221. Max. coverage (+): 0. Max coverage (-): 0

Region: chr23 47785222-47785231. Max. coverage (+): 0. Max coverage (-): 0

Region: chr23 47785232-47785241. Max. coverage (+): 0. Max coverage (-): 0

Region: chr23 47785242-47785251. Max. coverage (+): 0. Max coverage (-): 4.87

Region: chr23 47785252-47785261. Max. coverage (+): 0. Max coverage (-): 24.01

Region: chr23 47785262-47785271. Max. coverage (+): 0. Max coverage (-): 29.41

Region: chr23 47785272-47785281. Max. coverage (+): 0. Max coverage (-): 0.51

Region: chr23 47785282-47785291. Max. coverage (+): 0. Max coverage (-): 0.51

Region: chr23 47785292-47785301. Max. coverage (+): 0. Max coverage (-): 1.53

Region: chr23 47785302-47785311. Max. coverage (+): 0. Max coverage (-): 8.26

Region: chr23 47785312-47785321. Max. coverage (+): 0. Max coverage (-): 8.26

Region: chr23 47785322-47785331. Max. coverage (+): 0. Max coverage (-): 7.42

Region: chr23 47785332-47785341. Max. coverage (+): 0. Max coverage (-): 0

Region: chr23 47785342-47785351. Max. coverage (+): 0. Max coverage (-): 0

Region: chr23 47785352-47785361. Max. coverage (+): 0. Max coverage (-): 0

Region: chr23 47785362-47785371. Max. coverage (+): 0. Max coverage (-): 0

Region: chr23 47785372-47785381. Max. coverage (+): 0. Max coverage (-): 0

Region: chr23 47785382-47785391. Max. coverage (+): 0. Max coverage (-): 0

Region: chr23 47785392-47785401. Max. coverage (+): 0. Max coverage (-): 0

Region: chr23 47785402-47785411. Max. coverage (+): 0. Max coverage (-): 5.74

Region: chr23 47785412-47785421. Max. coverage (+): 0. Max coverage (-): 5.74

Region: chr23 47785422-47785431. Max. coverage (+): 0. Max coverage (-): 0

Region: chr23 47785432-47785441. Max. coverage (+): 0. Max coverage (-): 0

Region: chr23 47785442-47785451. Max. coverage (+): 0. Max coverage (-): 0

Region: chr23 47785452-47785461. Max. coverage (+): 0. Max coverage (-): 0

Region: chr23 47785462-47785471. Max. coverage (+): 0. Max coverage (-): 7.18

Region: chr23 47785472-47785481. Max. coverage (+): 0. Max coverage (-): 7.18

Region: chr23 47785482-47785491. Max. coverage (+): 0. Max coverage (-): 0

Region: chr23 47785492-47785501. Max. coverage (+): 0. Max coverage (-): 0

Region: chr23 47785502-47785511. Max. coverage (+): 0. Max coverage (-): 0

Region: chr23 47785512-47785521. Max. coverage (+): 0. Max coverage (-): 37.06

Region: chr23 47785522-47785531. Max. coverage (+): 0. Max coverage (-): 37.06

Region: chr23 47785532-47785541. Max. coverage (+): 0. Max coverage (-): 5.93

Region: chr23 47785542-47785551. Max. coverage (+): 0. Max coverage (-): 8.21

Region: chr23 47785552-47785562. Max. coverage (+): 0. Max coverage (-): 22.3

Region: chr23 47785563-47785572. Max. coverage (+): 0. Max coverage (-): 22.3

Region: chr23 47785573-47785582. Max. coverage (+): 0. Max coverage (-): 0

Region: chr23 47785583-47785592. Max. coverage (+): 0. Max coverage (-): 0

Region: chr23 47785593-47785602. Max. coverage (+): 0. Max coverage (-): 0

Region: chr23 47785603-47785612. Max. coverage (+): 0. Max coverage (-): 11.26

Region: chr23 47785613-47785622. Max. coverage (+): 0. Max coverage (-): 0

Region: chr23 47785623-47785632. Max. coverage (+): 0. Max coverage (-): 0

Region: chr23 47785633-47785642. Max. coverage (+): 0. Max coverage (-): 0

Region: chr23 47785643-47785652. Max. coverage (+): 0. Max coverage (-): 0

Region: chr23 47785653-47785662. Max. coverage (+): 0. Max coverage (-): 2.14

Region: chr23 47785663-47785672. Max. coverage (+): 0. Max coverage (-): 5.77

Region: chr23 47785673-47785682. Max. coverage (+): 0. Max coverage (-): 5.77

Region: chr23 47785683-47785692. Max. coverage (+): 0. Max coverage (-): 0

Region: chr23 47785693-47785702. Max. coverage (+): 0. Max coverage (-): 0

Region: chr23 47785703-47785712. Max. coverage (+): 0. Max coverage (-): 0

Region: chr23 47785713-47785722. Max. coverage (+): 0. Max coverage (-): 0

Region: chr23 47785723-47785732. Max. coverage (+): 0. Max coverage (-): 0

Region: chr23 47785733-47785742. Max. coverage (+): 0. Max coverage (-): 6.06

Region: chr23 47785743-47785752. Max. coverage (+): 0. Max coverage (-): 30.61

Region: chr23 47785753-47785762. Max. coverage (+): 0. Max coverage (-): 14.92

Region: chr23 47785763-47785772. Max. coverage (+): 0. Max coverage (-): 30.25

Region: chr23 47785773-47785782. Max. coverage (+): 0. Max coverage (-): 5.96

Region: chr23 47785783-47785792. Max. coverage (+): 0. Max coverage (-): 3.88

Region: chr23 47785793-47785802. Max. coverage (+): 0. Max coverage (-): 3.88

Region: chr23 47785803-47785812. Max. coverage (+): 0. Max coverage (-): 14

Region: chr23 47785813-47785822. Max. coverage (+): 0. Max coverage (-): 14

Region: chr23 47785823-47785832. Max. coverage (+): 0. Max coverage (-): 12.03

Region: chr23 47785833-47785842. Max. coverage (+): 0. Max coverage (-): 1.03

Region: chr23 47785843-47785852. Max. coverage (+): 0. Max coverage (-): 0

Region: chr23 47785853-47785862. Max. coverage (+): 0. Max coverage (-): 0

Region: chr23 47785863-47785872. Max. coverage (+): 0. Max coverage (-): 0

Region: chr23 47785873-47785882. Max. coverage (+): 0. Max coverage (-): 0

Region: chr23 47785883-47785892. Max. coverage (+): 0. Max coverage (-): 0

Region: chr23 47785893-47785902. Max. coverage (+): 0. Max coverage (-): 0

Region: chr23 47785903-47785912. Max. coverage (+): 0. Max coverage (-): 0

Region: chr23 47785913-47785922. Max. coverage (+): 0. Max coverage (-): 0

Region: chr23 47785923-47785932. Max. coverage (+): 0. Max coverage (-): 0

Region: chr23 47785933-47785942. Max. coverage (+): 0. Max coverage (-): 6.72

Region: chr23 47785943-47785952. Max. coverage (+): 0. Max coverage (-): 0

Region: chr23 47785953-47785962. Max. coverage (+): 0. Max coverage (-): 0

Region: chr23 47785963-47785972. Max. coverage (+): 0. Max coverage (-): 0.11

Region: chr23 47785973-47785982. Max. coverage (+): 0. Max coverage (-): 3.5

Region: chr23 47785983-47785992. Max. coverage (+): 0. Max coverage (-): 6.68

Region: chr23 47785993-47786002. Max. coverage (+): 0. Max coverage (-): 13.76

Region: chr23 47786003-47786013. Max. coverage (+): 0. Max coverage (-): 11.87

Region: chr23 47786014-47786023. Max. coverage (+): 0. Max coverage (-): 13.63

Region: chr23 47786024-47786033. Max. coverage (+): 0. Max coverage (-): 0

Region: chr23 47786034-47786043. Max. coverage (+): 0. Max coverage (-): 3.4

Region: chr23 47786044-47786053. Max. coverage (+): 0. Max coverage (-): 24.24

Region: chr23 47786054-47786063. Max. coverage (+): 0. Max coverage (-): 24.24

Region: chr23 47786064-47786073. Max. coverage (+): 0. Max coverage (-): 36.77

Region: chr23 47786074-47786083. Max. coverage (+): 0. Max coverage (-): 33.6

Region: chr23 47786084-47786093. Max. coverage (+): 0. Max coverage (-): 0

Region: chr23 47786094-47786103. Max. coverage (+): 0. Max coverage (-): 0

Region: chr23 47786104-47786113. Max. coverage (+): 0. Max coverage (-): 0

Region: chr23 47786114-47786123. Max. coverage (+): 0. Max coverage (-): 0

Region: chr23 47786124-47786133. Max. coverage (+): 0. Max coverage (-): 0

Region: chr23 47786134-47786143. Max. coverage (+): 0. Max coverage (-): 3.24

Region: chr23 47786144-47786153. Max. coverage (+): 0. Max coverage (-): 14.62

Region: chr23 47786154-47786163. Max. coverage (+): 0. Max coverage (-): 7.67

Region: chr23 47786164-47786173. Max. coverage (+): 0. Max coverage (-): 6.61

Region: chr23 47786174-47786183. Max. coverage (+): 0. Max coverage (-): 5.5

Region: chr23 47786184-47786193. Max. coverage (+): 0. Max coverage (-): 0

Region: chr23 47786194-47786203. Max. coverage (+): 0. Max coverage (-): 0

Region: chr23 47786204-47786213. Max. coverage (+): 0. Max coverage (-): 1.65

Region: chr23 47786214-47786223. Max. coverage (+): 0. Max coverage (-): 3.27

Region: chr23 47786224-47786233. Max. coverage (+): 0. Max coverage (-): 3.27

Region: chr23 47786234-47786243. Max. coverage (+): 0. Max coverage (-): 9.57

Region: chr23 47786244-47786253. Max. coverage (+): 0. Max coverage (-): 23.99

Region: chr23 47786254-47786263. Max. coverage (+): 0. Max coverage (-): 19.77

Region: chr23 47786264-47786273. Max. coverage (+): 0. Max coverage (-): 14.24

Region: chr23 47786274-47786283. Max. coverage (+): 0. Max coverage (-): 14.24

Region: chr23 47786284-47786293. Max. coverage (+): 0. Max coverage (-): 3.18

Region: chr23 47786294-47786303. Max. coverage (+): 0. Max coverage (-): 1.62

Region: chr23 47786304-47786313. Max. coverage (+): 0. Max coverage (-): 2.56

Region: chr23 47786314-47786323. Max. coverage (+): 0. Max coverage (-): 2.97

Region: chr23 47786324-47786333. Max. coverage (+): 0. Max coverage (-): 5.75

Region: chr23 47786334-47786343. Max. coverage (+): 0. Max coverage (-): 4.42

Region: chr23 47786344-47786353. Max. coverage (+): 0. Max coverage (-): 5.24

Region: chr23 47786354-47786363. Max. coverage (+): 0. Max coverage (-): 29.27

Region: chr23 47786364-47786373. Max. coverage (+): 0. Max coverage (-): 40.35

Region: chr23 47786374-47786383. Max. coverage (+): 0. Max coverage (-): 11.49

Region: chr23 47786384-47786393. Max. coverage (+): 0. Max coverage (-): 7.18

Region: chr23 47786394-47786403. Max. coverage (+): 0. Max coverage (-): 14.08

Region: chr23 47786404-47786413. Max. coverage (+): 0. Max coverage (-): 8.99

Region: chr23 47786414-47786423. Max. coverage (+): 0. Max coverage (-): 4.18

Region: chr23 47786424-47786433. Max. coverage (+): 0. Max coverage (-): 4.61

Region: chr23 47786434-47786443. Max. coverage (+): 0. Max coverage (-): 4.61

Region: chr23 47786444-47786453. Max. coverage (+): 0. Max coverage (-): 0

Region: chr23 47786454-47786463. Max. coverage (+): 0. Max coverage (-): 0

Region: chr23 47786464-47786474. Max. coverage (+): 0. Max coverage (-): 0

Region: chr23 47786475-47786484. Max. coverage (+): 0. Max coverage (-): 6.23

Region: chr23 47786485-47786494. Max. coverage (+): 0. Max coverage (-): 6.23

Region: chr23 47786495-47786504. Max. coverage (+): 0. Max coverage (-): 22.87

Region: chr23 47786505-47786514. Max. coverage (+): 0. Max coverage (-): 110.54

Region: chr23 47786515-47786524. Max. coverage (+): 0. Max coverage (-): 106.08

Region: chr23 47786525-47786534. Max. coverage (+): 0. Max coverage (-): 12.38

Region: chr23 47786535-47786544. Max. coverage (+): 0. Max coverage (-): 5.97

Region: chr23 47786545-47786554. Max. coverage (+): 0. Max coverage (-): 12.89

Region: chr23 47786555-47786564. Max. coverage (+): 0. Max coverage (-): 9.35

Region: chr23 47786565-47786574. Max. coverage (+): 0. Max coverage (-): 6.17

Region: chr23 47786575-47786584. Max. coverage (+): 0. Max coverage (-): 0

Region: chr23 47786585-47786594. Max. coverage (+): 0. Max coverage (-): 7.19

Region: chr23 47786595-47786604. Max. coverage (+): 0. Max coverage (-): 5.54

Region: chr23 47786605-47786614. Max. coverage (+): 0. Max coverage (-): 0

Region: chr23 47786615-47786624. Max. coverage (+): 0. Max coverage (-): 0

Region: chr23 47786625-47786634. Max. coverage (+): 0. Max coverage (-): 0

Region: chr23 47786635-47786644. Max. coverage (+): 0. Max coverage (-): 0

Region: chr23 47786645-47786654. Max. coverage (+): 0. Max coverage (-): 0

Region: chr23 47786655-47786664. Max. coverage (+): 0. Max coverage (-): 0

Region: chr23 47786665-47786674. Max. coverage (+): 0. Max coverage (-): 0

Region: chr23 47786675-47786684. Max. coverage (+): 0. Max coverage (-): 3.28

Region: chr23 47786685-47786694. Max. coverage (+): 0. Max coverage (-): 3.28

Region: chr23 47786695-47786704. Max. coverage (+): 0. Max coverage (-): 2.55

Region: chr23 47786705-47786714. Max. coverage (+): 0. Max coverage (-): 2.55

Region: chr23 47786715-47786724. Max. coverage (+): 0. Max coverage (-): 0

Region: chr23 47786725-47786734. Max. coverage (+): 0. Max coverage (-): 0

Region: chr23 47786735-47786744. Max. coverage (+): 0. Max coverage (-): 4.23

Region: chr23 47786745-47786754. Max. coverage (+): 0. Max coverage (-): 4.23

Region: chr23 47786755-47786764. Max. coverage (+): 0. Max coverage (-): 18.9

Region: chr23 47786765-47786774. Max. coverage (+): 0. Max coverage (-): 20.37

Region: chr23 47786775-47786784. Max. coverage (+): 0. Max coverage (-): 0

Region: chr23 47786785-47786794. Max. coverage (+): 0. Max coverage (-): 0

Region: chr23 47786795-47786804. Max. coverage (+): 0. Max coverage (-): 0

Region: chr23 47786805-47786814. Max. coverage (+): 0. Max coverage (-): 0

Region: chr23 47786815-47786824. Max. coverage (+): 0. Max coverage (-): 0

Region: chr23 47786825-47786834. Max. coverage (+): 0. Max coverage (-): 0

Region: chr23 47786835-47786844. Max. coverage (+): 0. Max coverage (-): 0

Region: chr23 47786845-47786854. Max. coverage (+): 0. Max coverage (-): 92.85

Region: chr23 47786855-47786864. Max. coverage (+): 0. Max coverage (-): 112.85

Region: chr23 47786865-47786874. Max. coverage (+): 0. Max coverage (-): 3.74

Region: chr23 47786875-47786884. Max. coverage (+): 0. Max coverage (-): 0

Region: chr23 47786885-47786894. Max. coverage (+): 0. Max coverage (-): 0

Region: chr23 47786895-47786904. Max. coverage (+): 0. Max coverage (-): 4.4

Region: chr23 47786905-47786914. Max. coverage (+): 0. Max coverage (-): 4.4

Region: chr23 47786915-47786925. Max. coverage (+): 0. Max coverage (-): 3.42

Region: chr23 47786926-47786935. Max. coverage (+): 0. Max coverage (-): 3.42

Region: chr23 47786936-47786945. Max. coverage (+): 0. Max coverage (-): 11.61

Region: chr23 47786946-47786955. Max. coverage (+): 0. Max coverage (-): 8

Region: chr23 47786956-47786965. Max. coverage (+): 0. Max coverage (-): 33.17

Region: chr23 47786966-47786975. Max. coverage (+): 0. Max coverage (-): 28.68

Region: chr23 47786976-47786985. Max. coverage (+): 0. Max coverage (-): 13.21

Region: chr23 47786986-47786995. Max. coverage (+): 0. Max coverage (-): 3.33

Region: chr23 47786996-47787005. Max. coverage (+): 0. Max coverage (-): 8.69

Region: chr23 47787006-47787015. Max. coverage (+): 0. Max coverage (-): 16.35

Region: chr23 47787016-47787025. Max. coverage (+): 0. Max coverage (-): 0

Region: chr23 47787026-47787035. Max. coverage (+): 0. Max coverage (-): 0

Region: chr23 47787036-47787045. Max. coverage (+): 0. Max coverage (-): 12.69

Region: chr23 47787046-47787055. Max. coverage (+): 0. Max coverage (-): 34.97

Region: chr23 47787056-47787065. Max. coverage (+): 0. Max coverage (-): 17.65

Region: chr23 47787066-47787075. Max. coverage (+): 0. Max coverage (-): 0

Region: chr23 47787076-47787085. Max. coverage (+): 0. Max coverage (-): 0

Region: chr23 47787086-47787095. Max. coverage (+): 0. Max coverage (-): 1.03

Region: chr23 47787096-47787105. Max. coverage (+): 0. Max coverage (-): 1.03

Region: chr23 47787106-47787115. Max. coverage (+): 0. Max coverage (-): 0

Region: chr23 47787116-47787125. Max. coverage (+): 0. Max coverage (-): 5.22

Region: chr23 47787126-47787135. Max. coverage (+): 0. Max coverage (-): 10.57

Region: chr23 47787136-47787145. Max. coverage (+): 0. Max coverage (-): 0

Region: chr23 47787146-47787155. Max. coverage (+): 0. Max coverage (-): 26.89

Region: chr23 47787156-47787165. Max. coverage (+): 0. Max coverage (-): 26.89

Region: chr23 47787166-47787175. Max. coverage (+): 0. Max coverage (-): 2.67

Region: chr23 47787176-47787185. Max. coverage (+): 0. Max coverage (-): 6.97

Region: chr23 47787186-47787195. Max. coverage (+): 0. Max coverage (-): 6.97

Region: chr23 47787196-47787205. Max. coverage (+): 0. Max coverage (-): 5.13

Region: chr23 47787206-47787215. Max. coverage (+): 0. Max coverage (-): 0

Region: chr23 47787216-47787225. Max. coverage (+): 0. Max coverage (-): 1.63

Region: chr23 47787226-47787235. Max. coverage (+): 0. Max coverage (-): 1.63

Region: chr23 47787236-47787245. Max. coverage (+): 0. Max coverage (-): 0

Region: chr23 47787246-47787255. Max. coverage (+): 0. Max coverage (-): 0

Region: chr23 47787256-47787265. Max. coverage (+): 0. Max coverage (-): 0

Region: chr23 47787266-47787275. Max. coverage (+): 0. Max coverage (-): 0

Region: chr23 47787276-47787285. Max. coverage (+): 0. Max coverage (-): 0

Region: chr23 47787286-47787295. Max. coverage (+): 0. Max coverage (-): 0

Region: chr23 47787296-47787305. Max. coverage (+): 0. Max coverage (-): 0

Region: chr23 47787306-47787315. Max. coverage (+): 0. Max coverage (-): 0

Region: chr23 47787316-47787325. Max. coverage (+): 0. Max coverage (-): 11.21

Region: chr23 47787326-47787335. Max. coverage (+): 0. Max coverage (-): 6.66

Region: chr23 47787336-47787345. Max. coverage (+): 0. Max coverage (-): 0.79

Region: chr23 47787346-47787355. Max. coverage (+): 0. Max coverage (-): 6.45

Region: chr23 47787356-47787365. Max. coverage (+): 0. Max coverage (-): 5.65

Region: chr23 47787366-47787375. Max. coverage (+): 0. Max coverage (-): 0

Region: chr23 47787376-47787386. Max. coverage (+): 0. Max coverage (-): 0

Region: chr23 47787387-47787396. Max. coverage (+): 0. Max coverage (-): 0

Region: chr23 47787397-47787406. Max. coverage (+): 0. Max coverage (-): 0

Region: chr23 47787407-47787416. Max. coverage (+): 0. Max coverage (-): 0

Region: chr23 47787417-47787426. Max. coverage (+): 0. Max coverage (-): 0

Region: chr23 47787427-47787436. Max. coverage (+): 0. Max coverage (-): 0

Region: chr23 47787437-47787446. Max. coverage (+): 0. Max coverage (-): 0

Region: chr23 47787447-47787456. Max. coverage (+): 0. Max coverage (-): 0

Region: chr23 47787457-47787466. Max. coverage (+): 0. Max coverage (-): 1.62

Region: chr23 47787467-47787476. Max. coverage (+): 0. Max coverage (-): 8.88

Region: chr23 47787477-47787486. Max. coverage (+): 0. Max coverage (-): 10.16

Region: chr23 47787487-47787496. Max. coverage (+): 0. Max coverage (-): 10.16

Region: chr23 47787497-47787506. Max. coverage (+): 0. Max coverage (-): 0

Region: chr23 47787507-47787516. Max. coverage (+): 0. Max coverage (-): 2.91

Region: chr23 47787517-47787526. Max. coverage (+): 0. Max coverage (-): 12.07

Region: chr23 47787527-47787536. Max. coverage (+): 0. Max coverage (-): 10.59

Region: chr23 47787537-47787546. Max. coverage (+): 0. Max coverage (-): 7.66

Region: chr23 47787547-47787556. Max. coverage (+): 0. Max coverage (-): 49.74

Region: chr23 47787557-47787566. Max. coverage (+): 0. Max coverage (-): 48.07

Region: chr23 47787567-47787576. Max. coverage (+): 0. Max coverage (-): 0.88

Region: chr23 47787577-47787586. Max. coverage (+): 0. Max coverage (-): 0

Region: chr23 47787587-47787596. Max. coverage (+): 0. Max coverage (-): 15.36

Region: chr23 47787597-47787606. Max. coverage (+): 0. Max coverage (-): 4.84

Region: chr23 47787607-47787616. Max. coverage (+): 0. Max coverage (-): 0

Region: chr23 47787617-47787626. Max. coverage (+): 0. Max coverage (-): 0

Region: chr23 47787627-47787636. Max. coverage (+): 0. Max coverage (-): 0.7

Region: chr23 47787637-47787646. Max. coverage (+): 0. Max coverage (-): 23.96

Region: chr23 47787647-47787656. Max. coverage (+): 0. Max coverage (-): 5.47

Region: chr23 47787657-47787666. Max. coverage (+): 0. Max coverage (-): 5.47

Region: chr23 47787667-47787676. Max. coverage (+): 0. Max coverage (-): 30.14

Region: chr23 47787677-47787686. Max. coverage (+): 0. Max coverage (-): 30.14

Region: chr23 47787687-47787696. Max. coverage (+): 0. Max coverage (-): 31.91

Region: chr23 47787697-47787706. Max. coverage (+): 0. Max coverage (-): 31.91

Region: chr23 47787707-47787716. Max. coverage (+): 0. Max coverage (-): 0

Region: chr23 47787717-47787726. Max. coverage (+): 0. Max coverage (-): 0

Region: chr23 47787727-47787736. Max. coverage (+): 0. Max coverage (-): 0

Region: chr23 47787737-47787746. Max. coverage (+): 0. Max coverage (-): 0

Region: chr23 47787747-47787756. Max. coverage (+): 0. Max coverage (-): 0

Region: chr23 47787757-47787766. Max. coverage (+): 0. Max coverage (-): 0

Region: chr23 47787767-47787776. Max. coverage (+): 0. Max coverage (-): 0

Region: chr23 47787777-47787786. Max. coverage (+): 0. Max coverage (-): 0

Region: chr23 47787787-47787796. Max. coverage (+): 0. Max coverage (-): 0

Region: chr23 47787797-47787806. Max. coverage (+): 0. Max coverage (-): 0

Region: chr23 47787807-47787816. Max. coverage (+): 0. Max coverage (-): 0

Region: chr23 47787817-47787826. Max. coverage (+): 0. Max coverage (-): 0

Region: chr23 47787827-47787837. Max. coverage (+): 0. Max coverage (-): 0

Region: chr23 47787838-47787847. Max. coverage (+): 0. Max coverage (-): 0

Region: chr23 47787848-47787857. Max. coverage (+): 0. Max coverage (-): 0

Region: chr23 47787858-47787867. Max. coverage (+): 0. Max coverage (-): 0

Region: chr23 47787868-47787877. Max. coverage (+): 0. Max coverage (-): 0

Region: chr23 47787878-47787887. Max. coverage (+): 0. Max coverage (-): 0

Region: chr23 47787888-47787897. Max. coverage (+): 0. Max coverage (-): 0

Region: chr23 47787898-47787907. Max. coverage (+): 0. Max coverage (-): 0

Region: chr23 47787908-47787917. Max. coverage (+): 0. Max coverage (-): 0

Region: chr23 47787918-47787927. Max. coverage (+): 0. Max coverage (-): 0

Region: chr23 47787928-47787937. Max. coverage (+): 0. Max coverage (-): 0

Region: chr23 47787938-47787947. Max. coverage (+): 0. Max coverage (-): 0

Region: chr23 47787948-47787957. Max. coverage (+): 0. Max coverage (-): 0

Region: chr23 47787958-47787967. Max. coverage (+): 0. Max coverage (-): 0

Region: chr23 47787968-47787977. Max. coverage (+): 0. Max coverage (-): 0

Region: chr23 47787978-47787987. Max. coverage (+): 0. Max coverage (-): 0

Region: chr23 47787988-47787997. Max. coverage (+): 0. Max coverage (-): 0

Region: chr23 47787998-47788007. Max. coverage (+): 0. Max coverage (-): 0

Region: chr23 47788008-47788017. Max. coverage (+): 0. Max coverage (-): 0

Region: chr23 47788018-47788027. Max. coverage (+): 0. Max coverage (-): 0

Region: chr23 47788028-47788037. Max. coverage (+): 0. Max coverage (-): 0

Region: chr23 47788038-47788047. Max. coverage (+): 0. Max coverage (-): 0

Region: chr23 47788048-47788057. Max. coverage (+): 0. Max coverage (-): 0

Region: chr23 47788058-47788067. Max. coverage (+): 0. Max coverage (-): 0

Region: chr23 47788068-47788077. Max. coverage (+): 0. Max coverage (-): 0

Region: chr23 47788078-47788087. Max. coverage (+): 0. Max coverage (-): 0

Region: chr23 47788088-47788097. Max. coverage (+): 0. Max coverage (-): 0

Region: chr23 47788098-47788107. Max. coverage (+): 0. Max coverage (-): 0

Region: chr23 47788108-47788117. Max. coverage (+): 0. Max coverage (-): 0

Region: chr23 47788118-47788127. Max. coverage (+): 0. Max coverage (-): 0

Region: chr23 47788128-47788137. Max. coverage (+): 0. Max coverage (-): 0

Region: chr23 47788138-47788147. Max. coverage (+): 0. Max coverage (-): 0

Region: chr23 47788148-47788157. Max. coverage (+): 0. Max coverage (-): 0

Region: chr23 47788158-47788167. Max. coverage (+): 0. Max coverage (-): 0

Region: chr23 47788168-47788177. Max. coverage (+): 0. Max coverage (-): 0

Region: chr23 47788178-47788187. Max. coverage (+): 0. Max coverage (-): 0

Region: chr23 47788188-47788197. Max. coverage (+): 0. Max coverage (-): 0

Region: chr23 47788198-47788207. Max. coverage (+): 0. Max coverage (-): 0

Region: chr23 47788208-47788217. Max. coverage (+): 0. Max coverage (-): 0

Region: chr23 47788218-47788227. Max. coverage (+): 0. Max coverage (-): 10.76

Region: chr23 47788228-47788237. Max. coverage (+): 0. Max coverage (-): 10.76

Region: chr23 47788238-47788247. Max. coverage (+): 0. Max coverage (-): 0

Region: chr23 47788248-47788257. Max. coverage (+): 0. Max coverage (-): 0

Region: chr23 47788258-47788267. Max. coverage (+): 0. Max coverage (-): 0

Region: chr23 47788268-47788277. Max. coverage (+): 0. Max coverage (-): 0

Region: chr23 47788278-47788287. Max. coverage (+): 0. Max coverage (-): 0

Region: chr23 47788288-47788298. Max. coverage (+): 0. Max coverage (-): 0

Region: chr23 47788299-47788308. Max. coverage (+): 0. Max coverage (-): 0

Region: chr23 47788309-47788318. Max. coverage (+): 0. Max coverage (-): 0

Region: chr23 47788319-47788328. Max. coverage (+): 0. Max coverage (-): 0

Region: chr23 47788329-47788338. Max. coverage (+): 0. Max coverage (-): 5.04

Region: chr23 47788339-47788348. Max. coverage (+): 0. Max coverage (-): 12.19

Region: chr23 47788349-47788358. Max. coverage (+): 0. Max coverage (-): 5.39

Region: chr23 47788359-47788368. Max. coverage (+): 0. Max coverage (-): 18.93

Region: chr23 47788369-47788378. Max. coverage (+): 0. Max coverage (-): 16.22

Region: chr23 47788379-47788388. Max. coverage (+): 0. Max coverage (-): 0

Region: chr23 47788389-47788398. Max. coverage (+): 0. Max coverage (-): 0

Region: chr23 47788399-47788408. Max. coverage (+): 0. Max coverage (-): 0

Region: chr23 47788409-47788418. Max. coverage (+): 0. Max coverage (-): 0

Region: chr23 47788419-47788428. Max. coverage (+): 0. Max coverage (-): 0

Region: chr23 47788429-47788438. Max. coverage (+): 0. Max coverage (-): 0

Region: chr23 47788439-47788448. Max. coverage (+): 0. Max coverage (-): 0

Region: chr23 47788449-47788458. Max. coverage (+): 0. Max coverage (-): 0

Region: chr23 47788459-47788468. Max. coverage (+): 0. Max coverage (-): 0

Region: chr23 47788469-47788478. Max. coverage (+): 0. Max coverage (-): 0

Region: chr23 47788479-47788488. Max. coverage (+): 0. Max coverage (-): 0

Region: chr23 47788489-47788498. Max. coverage (+): 0. Max coverage (-): 0

Region: chr23 47788499-47788508. Max. coverage (+): 0. Max coverage (-): 0

Region: chr23 47788509-47788518. Max. coverage (+): 0. Max coverage (-): 0

Region: chr23 47788519-47788528. Max. coverage (+): 0. Max coverage (-): 0

Region: chr23 47788529-47788538. Max. coverage (+): 0. Max coverage (-): 0

Region: chr23 47788539-47788548. Max. coverage (+): 0. Max coverage (-): 0

Region: chr23 47788549-47788558. Max. coverage (+): 0. Max coverage (-): 0

Region: chr23 47788559-47788568. Max. coverage (+): 0. Max coverage (-): 0

Region: chr23 47788569-47788578. Max. coverage (+): 0. Max coverage (-): 0

Region: chr23 47788579-47788588. Max. coverage (+): 0. Max coverage (-): 0

Region: chr23 47788589-47788598. Max. coverage (+): 0. Max coverage (-): 0

Region: chr23 47788599-47788608. Max. coverage (+): 0. Max coverage (-): 0

Region: chr23 47788609-47788618. Max. coverage (+): 0. Max coverage (-): 0

Region: chr23 47788619-47788628. Max. coverage (+): 0. Max coverage (-): 0

Region: chr23 47788629-47788638. Max. coverage (+): 0. Max coverage (-): 0

Region: chr23 47788639-47788648. Max. coverage (+): 0. Max coverage (-): 0

Region: chr23 47788649-47788658. Max. coverage (+): 0. Max coverage (-): 0

Region: chr23 47788659-47788668. Max. coverage (+): 0. Max coverage (-): 0

Region: chr23 47788669-47788678. Max. coverage (+): 0. Max coverage (-): 6.73

Region: chr23 47788679-47788688. Max. coverage (+): 0. Max coverage (-): 6.73

Region: chr23 47788689-47788698. Max. coverage (+): 0. Max coverage (-): 0

Region: chr23 47788699-47788708. Max. coverage (+): 0. Max coverage (-): 0

Region: chr23 47788709-47788718. Max. coverage (+): 0. Max coverage (-): 6.84

Region: chr23 47788719-47788728. Max. coverage (+): 0. Max coverage (-): 6.84

Region: chr23 47788729-47788738. Max. coverage (+): 0. Max coverage (-): 0

Region: chr23 47788739-. Max. coverage (+): 0. Max coverage (-): 0

RepeatMasker Color Code

**+**

100-98% Identity

<98-95% Identity

<95-90% Identity

<90-85% Identity

<85-80% Identity

<80-75% Identity

<75-70% Identity

<70% Identity

**-**

Gene Set Color Code

**+**

Gene

Pseudogene

**-**

Topology/Coverage Color Code

Coverage Plus Strand

Coverage Minus Strand

Mainstrand: Plus

Mainstrand: Minus

Complementary Strand

Flanking Region  
(if option -flank >0)

Gene Set Annotation  
  
RepeatMasker Annotation  

**1. L1MC5a**: 47784258-47784654 (-), Divergence to consensus: 50.8%  
**2. L1MC5a**: 47784698-47784875 (-), Divergence to consensus: 41.8%  
**3. L2a**: 47785036-47785173 (+), Divergence to consensus: 38.6%  
**4. L2a**: 47787738-47787988 (+), Divergence to consensus: 42.7%  
**5. L2a**: 47788001-47788221 (+), Divergence to consensus: 38.2%  
**6. L2a**: 47788268-47788327 (+), Divergence to consensus: 33.3%  
**7. BOV-A2**: 47788404-47788672 (+), Divergence to consensus: 4.8%

  
Transcription Factor Binding Sites
