# Supplementary material for: Small RNA sequencing of cryopreserved semen from single bull revealed altered miRNAs and piRNAs expression between High- and Low-motile sperm populations
Source: BMC Genomics. 2017 Jan 4;18:14. doi: 10.1186/s12864-016-3394-7 (PMC5209821; doi:10.1186/s12864-016-3394-7)
Supplement: Additional file 4: — Details for each piRNA clusters found in Low Motile (LM) sperm fraction. Genes, repeats, transposable elements and transcription factors binding sites falling within the cluster regions were reported. (ZIP 1034 kb) [file 12864_2016_3394_MOESM4_ESM.zip › 33.html]

piRNA cluster 33


Predicted piRNA cluster no. 33     previous   next
  

Show proTRAC run info
Hide proTRAC run info

================================= proTRAC ====================================  
VERSION: 2.1                                    LAST MODIFIED: 06. October 2015  
  
Please cite:  
Rosenkranz D, Zischler H. proTRAC - a software for probabilistic piRNA cluster  
detection, visualization and analysis. 2012. BMC Bioinformatics 13:5.  
  
and (for proTRAC 2.0 and later):  
Rosenkranz D, Rudloff S, Bastuck K, Ketting RF, Zischler H. Tupaia small RNAs  
provide insights into function and evolution of RNAi-based transposon defense  
in mammals. 2015. RNA 21(5):911-922.  
  
Contact:  
David Rosenkranz  
Institute of Anthropology, small RNA group  
Johannes Gutenberg University Mainz  
email: rosenkranz@uni-mainz.de  
  
You can find the latest proTRAC version at:  
http://sourceforge.net/projects/protrac/files  
http://www.smallRNAgroup-mainz.de/software  
==============================================================================  
  
PARAMETERS:  
Map file: .............../storage/core/barbara/genhome/smallRNA/fertility/Sample\_not\_motile/pirna/Sample\_not\_motile\_26-33\_collapsed.fa.no-dust.map.weighted-10000-1000-b-0  
Genome file: ............/storage/core/barbara/genhome/smallRNA/fertility/Sample\_all/pirna/bt\_311\_chrY.fa  
RepeatMasker annotation: /storage/genomes/bt\_umd31/GCF\_000003055.6\_Bos\_taurus\_UMD\_3.1.1\_repeatMasker\_chr.out  
GeneSet:................./storage/core/barbara/genhome/smallRNA/fertility/Sample\_all/pirna/full.gtf  
  
Significant (p<=0.01) hit density will be calculated based  
on observed hit distribution.  
  
Sliding window size: ........................................ 5000 bp  
Sliding window increament: .................................. 1000 bp  
Normalize each hit by number of genomic hits: ............... 1 [0=no/1=yes]  
Normalize each hit by number of sequence reads: ............. 1 [0=no/1=yes]  
Normalize values (-> per million mapped reads): ............. 1 [0=no/1=yes]  
Min. fraction of hits with 1T(U) or 10A: .................... 0.75  
Alternatively: Min. fraction of hits with 1T(U) and 10A: .... 0.5  
Min. fraction of hits with typical piRNA length: ............ 0.75  
Typical piRNA length: ....................................... 26-33 nt  
Min. size of a piRNA cluster: ............................... 5000 bp.  
Min. number of hits (absolute): ............................. 0  
Min. number of hits (normalized): ........................... 0  
Min. fraction of hits on the mainstrand: .................... 0.75  
Top fraction of mapped sequences (in terms of read counts): . 1%  
Top fraction accounts for max. n% of sequence reads: ........ 90%  
Min. fraction of hits on each arm of a bidirectional cluster: 0.1  
Output image file for each cluster: ......................... 0 [0=no/1=yes]  
Output html file for each cluster: .......................... 1 [0=no/1=yes]  
Output a summary table: ..................................... 1 [0=no/1=yes]  
Output a FASTA file for each cluster (piRNA sequences): ..... 1 [0=no/1=yes]  
Output a FASTA file comprising cluster sequences: ........... 1 [0=no/1=yes]  
Search DNA motifs in clusters: .............................. 1 [0=no/1=yes]  
Output flanking sequences: +/- .............................. 0 bp  
Output ~.pTi file: .......................................... 1 [0=no/1=yes]  
==============================================================================  
  
  
Genome size (without gaps): ............ 2678902517 bp  
Gaps (N/X/-): .......................... 53837044 bp  
Mapped reads: .......................... 738059667487  
Non-identical sequences: ............... 277001  
Genomic hits: .......................... 533816  
Significant densitiy of mapped reads: .. 15118061 reads/kb

Show proTRAC cluster info
Hide proTRAC cluster info

|  |  |
| --- | --- |
| Location | chr24 |
| Coordinates | 43014207-43024557 |
| Size [bp] | 10351 |
| Sequence hit loci | 332 |
| Mapped reads (normalized) | 871727550 |
| Mapped reads (normalized) per kb | 84216747.2 |
| Normalized reads with 1T (1U) | 89.7% |
| Normalized reads with 10A | 32.7% |
| Normalized reads with length 26-33 nt | 100% |
| Normalized reads on the main strand(s) | 100% |
| Predicted directionality | mono:minus |

100%

0%

1T (1U)  
reads

10A reads

26-33 nt  
reads

reads on mainstrand

**Either the amount of reads with 1T (1U) OR 10A has to exceed 75% (set with option: -1Tor10A)  
Alternatively the amount of reads with 1T (1U) AND 10A has to exceed 50% (set with option: -1Tand10A)  
Minimum amount of reads with preferred size is 75% (set with option: -pisize)  
Minimum amount of reads on the main strand(s) is 75% (set with option: -clstrand)**

Show read coverage
Hide read coverage

WHAT DO I SEE HERE?  
This chart shows the location of mapped sequence reads within a predicted piRNA cluster. The color refers to the number of genomic hits produced by the sequence read in question. A dark red bar indicates that this sequence read produces many other hits elsewhere in the genome. Many adjacent red or yellow bars can indicate the presence of a multi-copy element such as transposons or rRNA genes. A dark green bar indicates that this sequence read maps uniquely to this locus.

1 hit

2-5 hits

6-10 hits

11-20 hits

21-50 hits

51-100 hits

> 100 hits

chr24

43014207

43024557

Gene Set

RepeatMasker

Mapped  
Reads

85.14

plus strand

minus strand

85.14

Region: chr24 47788740-43014217. Max. coverage (+): 0. Max coverage (-): 3.37

Region: chr24 43014218-43014238. Max. coverage (+): 0. Max coverage (-): 0

Region: chr24 43014239-43014258. Max. coverage (+): 0. Max coverage (-): 0

Region: chr24 43014259-43014279. Max. coverage (+): 0. Max coverage (-): 0

Region: chr24 43014280-43014300. Max. coverage (+): 0. Max coverage (-): 0

Region: chr24 43014301-43014320. Max. coverage (+): 0. Max coverage (-): 0

Region: chr24 43014321-43014341. Max. coverage (+): 0. Max coverage (-): 0

Region: chr24 43014342-43014362. Max. coverage (+): 0. Max coverage (-): 0

Region: chr24 43014363-43014382. Max. coverage (+): 0. Max coverage (-): 7.19

Region: chr24 43014383-43014403. Max. coverage (+): 0. Max coverage (-): 1

Region: chr24 43014404-43014424. Max. coverage (+): 0. Max coverage (-): 3.04

Region: chr24 43014425-43014445. Max. coverage (+): 0. Max coverage (-): 6.45

Region: chr24 43014446-43014465. Max. coverage (+): 0. Max coverage (-): 0

Region: chr24 43014466-43014486. Max. coverage (+): 0. Max coverage (-): 0

Region: chr24 43014487-43014507. Max. coverage (+): 0. Max coverage (-): 1.9

Region: chr24 43014508-43014527. Max. coverage (+): 0. Max coverage (-): 0.62

Region: chr24 43014528-43014548. Max. coverage (+): 0. Max coverage (-): 0

Region: chr24 43014549-43014569. Max. coverage (+): 0. Max coverage (-): 0

Region: chr24 43014570-43014589. Max. coverage (+): 0. Max coverage (-): 0

Region: chr24 43014590-43014610. Max. coverage (+): 0. Max coverage (-): 0

Region: chr24 43014611-43014631. Max. coverage (+): 0. Max coverage (-): 0

Region: chr24 43014632-43014652. Max. coverage (+): 0. Max coverage (-): 0

Region: chr24 43014653-43014672. Max. coverage (+): 0. Max coverage (-): 0

Region: chr24 43014673-43014693. Max. coverage (+): 0. Max coverage (-): 0

Region: chr24 43014694-43014714. Max. coverage (+): 0. Max coverage (-): 0

Region: chr24 43014715-43014734. Max. coverage (+): 0. Max coverage (-): 0

Region: chr24 43014735-43014755. Max. coverage (+): 0. Max coverage (-): 0

Region: chr24 43014756-43014776. Max. coverage (+): 0. Max coverage (-): 0

Region: chr24 43014777-43014797. Max. coverage (+): 0. Max coverage (-): 0

Region: chr24 43014798-43014817. Max. coverage (+): 0. Max coverage (-): 0

Region: chr24 43014818-43014838. Max. coverage (+): 0. Max coverage (-): 0

Region: chr24 43014839-43014859. Max. coverage (+): 0. Max coverage (-): 0

Region: chr24 43014860-43014879. Max. coverage (+): 0. Max coverage (-): 0

Region: chr24 43014880-43014900. Max. coverage (+): 0. Max coverage (-): 0

Region: chr24 43014901-43014921. Max. coverage (+): 0. Max coverage (-): 0

Region: chr24 43014922-43014941. Max. coverage (+): 0. Max coverage (-): 0

Region: chr24 43014942-43014962. Max. coverage (+): 0. Max coverage (-): 0

Region: chr24 43014963-43014983. Max. coverage (+): 0. Max coverage (-): 0

Region: chr24 43014984-43015004. Max. coverage (+): 0. Max coverage (-): 0

Region: chr24 43015005-43015024. Max. coverage (+): 0. Max coverage (-): 0

Region: chr24 43015025-43015045. Max. coverage (+): 0. Max coverage (-): 0

Region: chr24 43015046-43015066. Max. coverage (+): 0. Max coverage (-): 0

Region: chr24 43015067-43015086. Max. coverage (+): 0. Max coverage (-): 0

Region: chr24 43015087-43015107. Max. coverage (+): 0. Max coverage (-): 0

Region: chr24 43015108-43015128. Max. coverage (+): 0. Max coverage (-): 0

Region: chr24 43015129-43015148. Max. coverage (+): 0. Max coverage (-): 0

Region: chr24 43015149-43015169. Max. coverage (+): 0. Max coverage (-): 0

Region: chr24 43015170-43015190. Max. coverage (+): 0. Max coverage (-): 0

Region: chr24 43015191-43015211. Max. coverage (+): 0. Max coverage (-): 0

Region: chr24 43015212-43015231. Max. coverage (+): 0. Max coverage (-): 0

Region: chr24 43015232-43015252. Max. coverage (+): 0. Max coverage (-): 3.91

Region: chr24 43015253-43015273. Max. coverage (+): 0. Max coverage (-): 0

Region: chr24 43015274-43015293. Max. coverage (+): 0. Max coverage (-): 0

Region: chr24 43015294-43015314. Max. coverage (+): 0. Max coverage (-): 0

Region: chr24 43015315-43015335. Max. coverage (+): 0. Max coverage (-): 0

Region: chr24 43015336-43015355. Max. coverage (+): 0. Max coverage (-): 0

Region: chr24 43015356-43015376. Max. coverage (+): 0. Max coverage (-): 0

Region: chr24 43015377-43015397. Max. coverage (+): 0. Max coverage (-): 0

Region: chr24 43015398-43015418. Max. coverage (+): 0. Max coverage (-): 0

Region: chr24 43015419-43015438. Max. coverage (+): 0. Max coverage (-): 0

Region: chr24 43015439-43015459. Max. coverage (+): 0. Max coverage (-): 0

Region: chr24 43015460-43015480. Max. coverage (+): 0. Max coverage (-): 0

Region: chr24 43015481-43015500. Max. coverage (+): 0. Max coverage (-): 0

Region: chr24 43015501-43015521. Max. coverage (+): 0. Max coverage (-): 2.91

Region: chr24 43015522-43015542. Max. coverage (+): 0. Max coverage (-): 6.67

Region: chr24 43015543-43015562. Max. coverage (+): 0. Max coverage (-): 6.67

Region: chr24 43015563-43015583. Max. coverage (+): 0. Max coverage (-): 0

Region: chr24 43015584-43015604. Max. coverage (+): 0. Max coverage (-): 8.61

Region: chr24 43015605-43015625. Max. coverage (+): 0. Max coverage (-): 13.46

Region: chr24 43015626-43015645. Max. coverage (+): 0. Max coverage (-): 27.58

Region: chr24 43015646-43015666. Max. coverage (+): 0. Max coverage (-): 0

Region: chr24 43015667-43015687. Max. coverage (+): 0. Max coverage (-): 0

Region: chr24 43015688-43015707. Max. coverage (+): 0. Max coverage (-): 0

Region: chr24 43015708-43015728. Max. coverage (+): 0. Max coverage (-): 0

Region: chr24 43015729-43015749. Max. coverage (+): 0. Max coverage (-): 0

Region: chr24 43015750-43015770. Max. coverage (+): 0. Max coverage (-): 0

Region: chr24 43015771-43015790. Max. coverage (+): 0. Max coverage (-): 0

Region: chr24 43015791-43015811. Max. coverage (+): 0. Max coverage (-): 0

Region: chr24 43015812-43015832. Max. coverage (+): 0. Max coverage (-): 0

Region: chr24 43015833-43015852. Max. coverage (+): 0. Max coverage (-): 0

Region: chr24 43015853-43015873. Max. coverage (+): 0. Max coverage (-): 0

Region: chr24 43015874-43015894. Max. coverage (+): 0. Max coverage (-): 0

Region: chr24 43015895-43015914. Max. coverage (+): 0. Max coverage (-): 0

Region: chr24 43015915-43015935. Max. coverage (+): 0. Max coverage (-): 0

Region: chr24 43015936-43015956. Max. coverage (+): 0. Max coverage (-): 0

Region: chr24 43015957-43015977. Max. coverage (+): 0. Max coverage (-): 0

Region: chr24 43015978-43015997. Max. coverage (+): 0. Max coverage (-): 0

Region: chr24 43015998-43016018. Max. coverage (+): 0. Max coverage (-): 6.11

Region: chr24 43016019-43016039. Max. coverage (+): 0. Max coverage (-): 11.48

Region: chr24 43016040-43016059. Max. coverage (+): 0. Max coverage (-): 11.38

Region: chr24 43016060-43016080. Max. coverage (+): 0. Max coverage (-): 6.45

Region: chr24 43016081-43016101. Max. coverage (+): 0. Max coverage (-): 2.34

Region: chr24 43016102-43016121. Max. coverage (+): 0. Max coverage (-): 0

Region: chr24 43016122-43016142. Max. coverage (+): 0. Max coverage (-): 16.26

Region: chr24 43016143-43016163. Max. coverage (+): 0. Max coverage (-): 10.96

Region: chr24 43016164-43016184. Max. coverage (+): 0. Max coverage (-): 0

Region: chr24 43016185-43016204. Max. coverage (+): 0. Max coverage (-): 0

Region: chr24 43016205-43016225. Max. coverage (+): 0. Max coverage (-): 5.35

Region: chr24 43016226-43016246. Max. coverage (+): 0. Max coverage (-): 5.35

Region: chr24 43016247-43016266. Max. coverage (+): 0. Max coverage (-): 3.94

Region: chr24 43016267-43016287. Max. coverage (+): 0. Max coverage (-): 0

Region: chr24 43016288-43016308. Max. coverage (+): 0. Max coverage (-): 3.84

Region: chr24 43016309-43016328. Max. coverage (+): 0. Max coverage (-): 4.53

Region: chr24 43016329-43016349. Max. coverage (+): 0. Max coverage (-): 0

Region: chr24 43016350-43016370. Max. coverage (+): 0. Max coverage (-): 0

Region: chr24 43016371-43016391. Max. coverage (+): 0. Max coverage (-): 0

Region: chr24 43016392-43016411. Max. coverage (+): 0. Max coverage (-): 11.79

Region: chr24 43016412-43016432. Max. coverage (+): 0. Max coverage (-): 11.79

Region: chr24 43016433-43016453. Max. coverage (+): 0. Max coverage (-): 42.33

Region: chr24 43016454-43016473. Max. coverage (+): 0. Max coverage (-): 0

Region: chr24 43016474-43016494. Max. coverage (+): 0. Max coverage (-): 0

Region: chr24 43016495-43016515. Max. coverage (+): 0. Max coverage (-): 0

Region: chr24 43016516-43016535. Max. coverage (+): 0. Max coverage (-): 27.02

Region: chr24 43016536-43016556. Max. coverage (+): 0. Max coverage (-): 19.62

Region: chr24 43016557-43016577. Max. coverage (+): 0. Max coverage (-): 0

Region: chr24 43016578-43016598. Max. coverage (+): 0. Max coverage (-): 0

Region: chr24 43016599-43016618. Max. coverage (+): 0. Max coverage (-): 0

Region: chr24 43016619-43016639. Max. coverage (+): 0. Max coverage (-): 0

Region: chr24 43016640-43016660. Max. coverage (+): 0. Max coverage (-): 0

Region: chr24 43016661-43016680. Max. coverage (+): 0. Max coverage (-): 0

Region: chr24 43016681-43016701. Max. coverage (+): 0. Max coverage (-): 0

Region: chr24 43016702-43016722. Max. coverage (+): 0. Max coverage (-): 0

Region: chr24 43016723-43016742. Max. coverage (+): 0. Max coverage (-): 0

Region: chr24 43016743-43016763. Max. coverage (+): 0. Max coverage (-): 0

Region: chr24 43016764-43016784. Max. coverage (+): 0. Max coverage (-): 0

Region: chr24 43016785-43016805. Max. coverage (+): 0. Max coverage (-): 0

Region: chr24 43016806-43016825. Max. coverage (+): 0. Max coverage (-): 0

Region: chr24 43016826-43016846. Max. coverage (+): 0. Max coverage (-): 0

Region: chr24 43016847-43016867. Max. coverage (+): 0. Max coverage (-): 0

Region: chr24 43016868-43016887. Max. coverage (+): 0. Max coverage (-): 0

Region: chr24 43016888-43016908. Max. coverage (+): 0. Max coverage (-): 0

Region: chr24 43016909-43016929. Max. coverage (+): 0. Max coverage (-): 0

Region: chr24 43016930-43016950. Max. coverage (+): 0. Max coverage (-): 0

Region: chr24 43016951-43016970. Max. coverage (+): 0. Max coverage (-): 0

Region: chr24 43016971-43016991. Max. coverage (+): 0. Max coverage (-): 0

Region: chr24 43016992-43017012. Max. coverage (+): 0. Max coverage (-): 1.27

Region: chr24 43017013-43017032. Max. coverage (+): 0. Max coverage (-): 1.27

Region: chr24 43017033-43017053. Max. coverage (+): 0. Max coverage (-): 0

Region: chr24 43017054-43017074. Max. coverage (+): 0. Max coverage (-): 0.04

Region: chr24 43017075-43017094. Max. coverage (+): 0. Max coverage (-): 3.18

Region: chr24 43017095-43017115. Max. coverage (+): 0. Max coverage (-): 0

Region: chr24 43017116-43017136. Max. coverage (+): 0. Max coverage (-): 5.39

Region: chr24 43017137-43017157. Max. coverage (+): 0. Max coverage (-): 0.36

Region: chr24 43017158-43017177. Max. coverage (+): 0. Max coverage (-): 0.36

Region: chr24 43017178-43017198. Max. coverage (+): 0. Max coverage (-): 0

Region: chr24 43017199-43017219. Max. coverage (+): 0. Max coverage (-): 0

Region: chr24 43017220-43017239. Max. coverage (+): 0. Max coverage (-): 0

Region: chr24 43017240-43017260. Max. coverage (+): 0. Max coverage (-): 0

Region: chr24 43017261-43017281. Max. coverage (+): 0. Max coverage (-): 0

Region: chr24 43017282-43017301. Max. coverage (+): 0. Max coverage (-): 0

Region: chr24 43017302-43017322. Max. coverage (+): 0. Max coverage (-): 0

Region: chr24 43017323-43017343. Max. coverage (+): 0. Max coverage (-): 0

Region: chr24 43017344-43017364. Max. coverage (+): 0. Max coverage (-): 0

Region: chr24 43017365-43017384. Max. coverage (+): 0. Max coverage (-): 0

Region: chr24 43017385-43017405. Max. coverage (+): 0. Max coverage (-): 0

Region: chr24 43017406-43017426. Max. coverage (+): 0. Max coverage (-): 14.28

Region: chr24 43017427-43017446. Max. coverage (+): 0. Max coverage (-): 6.69

Region: chr24 43017447-43017467. Max. coverage (+): 0. Max coverage (-): 0

Region: chr24 43017468-43017488. Max. coverage (+): 0. Max coverage (-): 0

Region: chr24 43017489-43017508. Max. coverage (+): 0. Max coverage (-): 0

Region: chr24 43017509-43017529. Max. coverage (+): 0. Max coverage (-): 0

Region: chr24 43017530-43017550. Max. coverage (+): 0. Max coverage (-): 0

Region: chr24 43017551-43017571. Max. coverage (+): 0. Max coverage (-): 0

Region: chr24 43017572-43017591. Max. coverage (+): 0. Max coverage (-): 0

Region: chr24 43017592-43017612. Max. coverage (+): 0. Max coverage (-): 0

Region: chr24 43017613-43017633. Max. coverage (+): 0. Max coverage (-): 0

Region: chr24 43017634-43017653. Max. coverage (+): 0. Max coverage (-): 0

Region: chr24 43017654-43017674. Max. coverage (+): 0. Max coverage (-): 0

Region: chr24 43017675-43017695. Max. coverage (+): 0. Max coverage (-): 0

Region: chr24 43017696-43017715. Max. coverage (+): 0. Max coverage (-): 0

Region: chr24 43017716-43017736. Max. coverage (+): 0. Max coverage (-): 0

Region: chr24 43017737-43017757. Max. coverage (+): 0. Max coverage (-): 1.03

Region: chr24 43017758-43017778. Max. coverage (+): 0. Max coverage (-): 0

Region: chr24 43017779-43017798. Max. coverage (+): 0. Max coverage (-): 0

Region: chr24 43017799-43017819. Max. coverage (+): 0. Max coverage (-): 0

Region: chr24 43017820-43017840. Max. coverage (+): 0. Max coverage (-): 0

Region: chr24 43017841-43017860. Max. coverage (+): 0. Max coverage (-): 0

Region: chr24 43017861-43017881. Max. coverage (+): 0. Max coverage (-): 0

Region: chr24 43017882-43017902. Max. coverage (+): 0. Max coverage (-): 6.62

Region: chr24 43017903-43017923. Max. coverage (+): 0. Max coverage (-): 17.4

Region: chr24 43017924-43017943. Max. coverage (+): 0. Max coverage (-): 5.77

Region: chr24 43017944-43017964. Max. coverage (+): 0. Max coverage (-): 5.77

Region: chr24 43017965-43017985. Max. coverage (+): 0. Max coverage (-): 0

Region: chr24 43017986-43018005. Max. coverage (+): 0. Max coverage (-): 0

Region: chr24 43018006-43018026. Max. coverage (+): 0. Max coverage (-): 0

Region: chr24 43018027-43018047. Max. coverage (+): 0. Max coverage (-): 0

Region: chr24 43018048-43018067. Max. coverage (+): 0. Max coverage (-): 0

Region: chr24 43018068-43018088. Max. coverage (+): 0. Max coverage (-): 0

Region: chr24 43018089-43018109. Max. coverage (+): 0. Max coverage (-): 0

Region: chr24 43018110-43018130. Max. coverage (+): 0. Max coverage (-): 0

Region: chr24 43018131-43018150. Max. coverage (+): 0. Max coverage (-): 0

Region: chr24 43018151-43018171. Max. coverage (+): 0. Max coverage (-): 0

Region: chr24 43018172-43018192. Max. coverage (+): 0. Max coverage (-): 0

Region: chr24 43018193-43018212. Max. coverage (+): 0. Max coverage (-): 0

Region: chr24 43018213-43018233. Max. coverage (+): 0. Max coverage (-): 0

Region: chr24 43018234-43018254. Max. coverage (+): 0. Max coverage (-): 0

Region: chr24 43018255-43018274. Max. coverage (+): 0. Max coverage (-): 0

Region: chr24 43018275-43018295. Max. coverage (+): 0. Max coverage (-): 0

Region: chr24 43018296-43018316. Max. coverage (+): 0. Max coverage (-): 0

Region: chr24 43018317-43018337. Max. coverage (+): 0. Max coverage (-): 0

Region: chr24 43018338-43018357. Max. coverage (+): 0. Max coverage (-): 0

Region: chr24 43018358-43018378. Max. coverage (+): 0. Max coverage (-): 0

Region: chr24 43018379-43018399. Max. coverage (+): 0. Max coverage (-): 0

Region: chr24 43018400-43018419. Max. coverage (+): 0. Max coverage (-): 0

Region: chr24 43018420-43018440. Max. coverage (+): 0. Max coverage (-): 0

Region: chr24 43018441-43018461. Max. coverage (+): 0. Max coverage (-): 0

Region: chr24 43018462-43018481. Max. coverage (+): 0. Max coverage (-): 0

Region: chr24 43018482-43018502. Max. coverage (+): 0. Max coverage (-): 0

Region: chr24 43018503-43018523. Max. coverage (+): 0. Max coverage (-): 3.11

Region: chr24 43018524-43018544. Max. coverage (+): 0. Max coverage (-): 0

Region: chr24 43018545-43018564. Max. coverage (+): 0. Max coverage (-): 0

Region: chr24 43018565-43018585. Max. coverage (+): 0. Max coverage (-): 0

Region: chr24 43018586-43018606. Max. coverage (+): 0. Max coverage (-): 0

Region: chr24 43018607-43018626. Max. coverage (+): 0. Max coverage (-): 0

Region: chr24 43018627-43018647. Max. coverage (+): 0. Max coverage (-): 0

Region: chr24 43018648-43018668. Max. coverage (+): 0. Max coverage (-): 0

Region: chr24 43018669-43018688. Max. coverage (+): 0. Max coverage (-): 0

Region: chr24 43018689-43018709. Max. coverage (+): 0. Max coverage (-): 0

Region: chr24 43018710-43018730. Max. coverage (+): 0. Max coverage (-): 0

Region: chr24 43018731-43018751. Max. coverage (+): 0. Max coverage (-): 0

Region: chr24 43018752-43018771. Max. coverage (+): 0. Max coverage (-): 0

Region: chr24 43018772-43018792. Max. coverage (+): 0. Max coverage (-): 5.61

Region: chr24 43018793-43018813. Max. coverage (+): 0. Max coverage (-): 5.61

Region: chr24 43018814-43018833. Max. coverage (+): 0. Max coverage (-): 0

Region: chr24 43018834-43018854. Max. coverage (+): 0. Max coverage (-): 0

Region: chr24 43018855-43018875. Max. coverage (+): 0. Max coverage (-): 0

Region: chr24 43018876-43018896. Max. coverage (+): 0. Max coverage (-): 0

Region: chr24 43018897-43018916. Max. coverage (+): 0. Max coverage (-): 0

Region: chr24 43018917-43018937. Max. coverage (+): 0. Max coverage (-): 0

Region: chr24 43018938-43018958. Max. coverage (+): 0. Max coverage (-): 0

Region: chr24 43018959-43018978. Max. coverage (+): 0. Max coverage (-): 0

Region: chr24 43018979-43018999. Max. coverage (+): 0. Max coverage (-): 0

Region: chr24 43019000-43019020. Max. coverage (+): 0. Max coverage (-): 0

Region: chr24 43019021-43019040. Max. coverage (+): 0. Max coverage (-): 0

Region: chr24 43019041-43019061. Max. coverage (+): 0. Max coverage (-): 6.91

Region: chr24 43019062-43019082. Max. coverage (+): 0. Max coverage (-): 0

Region: chr24 43019083-43019103. Max. coverage (+): 0. Max coverage (-): 0

Region: chr24 43019104-43019123. Max. coverage (+): 0. Max coverage (-): 0

Region: chr24 43019124-43019144. Max. coverage (+): 0. Max coverage (-): 0

Region: chr24 43019145-43019165. Max. coverage (+): 0. Max coverage (-): 0

Region: chr24 43019166-43019185. Max. coverage (+): 0. Max coverage (-): 0

Region: chr24 43019186-43019206. Max. coverage (+): 0. Max coverage (-): 6.98

Region: chr24 43019207-43019227. Max. coverage (+): 0. Max coverage (-): 0

Region: chr24 43019228-43019247. Max. coverage (+): 0. Max coverage (-): 12

Region: chr24 43019248-43019268. Max. coverage (+): 0. Max coverage (-): 12

Region: chr24 43019269-43019289. Max. coverage (+): 0. Max coverage (-): 0

Region: chr24 43019290-43019310. Max. coverage (+): 0. Max coverage (-): 0

Region: chr24 43019311-43019330. Max. coverage (+): 0. Max coverage (-): 0

Region: chr24 43019331-43019351. Max. coverage (+): 0. Max coverage (-): 0

Region: chr24 43019352-43019372. Max. coverage (+): 0. Max coverage (-): 0

Region: chr24 43019373-43019392. Max. coverage (+): 0. Max coverage (-): 0

Region: chr24 43019393-43019413. Max. coverage (+): 0. Max coverage (-): 0

Region: chr24 43019414-43019434. Max. coverage (+): 0. Max coverage (-): 0

Region: chr24 43019435-43019454. Max. coverage (+): 0. Max coverage (-): 0

Region: chr24 43019455-43019475. Max. coverage (+): 0. Max coverage (-): 5.67

Region: chr24 43019476-43019496. Max. coverage (+): 0. Max coverage (-): 0

Region: chr24 43019497-43019517. Max. coverage (+): 0. Max coverage (-): 0

Region: chr24 43019518-43019537. Max. coverage (+): 0. Max coverage (-): 0

Region: chr24 43019538-43019558. Max. coverage (+): 0. Max coverage (-): 0

Region: chr24 43019559-43019579. Max. coverage (+): 0. Max coverage (-): 0

Region: chr24 43019580-43019599. Max. coverage (+): 0. Max coverage (-): 0

Region: chr24 43019600-43019620. Max. coverage (+): 0. Max coverage (-): 0

Region: chr24 43019621-43019641. Max. coverage (+): 0. Max coverage (-): 0

Region: chr24 43019642-43019661. Max. coverage (+): 0. Max coverage (-): 0

Region: chr24 43019662-43019682. Max. coverage (+): 0. Max coverage (-): 0

Region: chr24 43019683-43019703. Max. coverage (+): 0. Max coverage (-): 0

Region: chr24 43019704-43019724. Max. coverage (+): 0. Max coverage (-): 4.12

Region: chr24 43019725-43019744. Max. coverage (+): 0. Max coverage (-): 0

Region: chr24 43019745-43019765. Max. coverage (+): 0. Max coverage (-): 0

Region: chr24 43019766-43019786. Max. coverage (+): 0. Max coverage (-): 0

Region: chr24 43019787-43019806. Max. coverage (+): 0. Max coverage (-): 7.12

Region: chr24 43019807-43019827. Max. coverage (+): 0. Max coverage (-): 10.58

Region: chr24 43019828-43019848. Max. coverage (+): 0. Max coverage (-): 2.57

Region: chr24 43019849-43019868. Max. coverage (+): 0. Max coverage (-): 6.41

Region: chr24 43019869-43019889. Max. coverage (+): 0. Max coverage (-): 6.32

Region: chr24 43019890-43019910. Max. coverage (+): 0. Max coverage (-): 12.09

Region: chr24 43019911-43019931. Max. coverage (+): 0. Max coverage (-): 12.92

Region: chr24 43019932-43019951. Max. coverage (+): 0. Max coverage (-): 0

Region: chr24 43019952-43019972. Max. coverage (+): 0. Max coverage (-): 0

Region: chr24 43019973-43019993. Max. coverage (+): 0. Max coverage (-): 0

Region: chr24 43019994-43020013. Max. coverage (+): 0. Max coverage (-): 5.23

Region: chr24 43020014-43020034. Max. coverage (+): 0. Max coverage (-): 0

Region: chr24 43020035-43020055. Max. coverage (+): 0. Max coverage (-): 0

Region: chr24 43020056-43020076. Max. coverage (+): 0. Max coverage (-): 5.54

Region: chr24 43020077-43020096. Max. coverage (+): 0. Max coverage (-): 1.28

Region: chr24 43020097-43020117. Max. coverage (+): 0. Max coverage (-): 0

Region: chr24 43020118-43020138. Max. coverage (+): 0. Max coverage (-): 11.44

Region: chr24 43020139-43020158. Max. coverage (+): 0. Max coverage (-): 2.95

Region: chr24 43020159-43020179. Max. coverage (+): 0. Max coverage (-): 5.41

Region: chr24 43020180-43020200. Max. coverage (+): 0. Max coverage (-): 0

Region: chr24 43020201-43020220. Max. coverage (+): 0. Max coverage (-): 0

Region: chr24 43020221-43020241. Max. coverage (+): 0. Max coverage (-): 0

Region: chr24 43020242-43020262. Max. coverage (+): 0. Max coverage (-): 7.89

Region: chr24 43020263-43020283. Max. coverage (+): 0. Max coverage (-): 10.32

Region: chr24 43020284-43020303. Max. coverage (+): 0. Max coverage (-): 4.84

Region: chr24 43020304-43020324. Max. coverage (+): 0. Max coverage (-): 15.97

Region: chr24 43020325-43020345. Max. coverage (+): 0. Max coverage (-): 5.47

Region: chr24 43020346-43020365. Max. coverage (+): 0. Max coverage (-): 0

Region: chr24 43020366-43020386. Max. coverage (+): 0. Max coverage (-): 0

Region: chr24 43020387-43020407. Max. coverage (+): 0. Max coverage (-): 10.59

Region: chr24 43020408-43020427. Max. coverage (+): 0. Max coverage (-): 9.64

Region: chr24 43020428-43020448. Max. coverage (+): 0. Max coverage (-): 1.03

Region: chr24 43020449-43020469. Max. coverage (+): 0. Max coverage (-): 16.89

Region: chr24 43020470-43020490. Max. coverage (+): 0. Max coverage (-): 85.14

Region: chr24 43020491-43020510. Max. coverage (+): 0. Max coverage (-): 72.97

Region: chr24 43020511-43020531. Max. coverage (+): 0. Max coverage (-): 15.63

Region: chr24 43020532-43020552. Max. coverage (+): 0. Max coverage (-): 9.84

Region: chr24 43020553-43020572. Max. coverage (+): 0. Max coverage (-): 0

Region: chr24 43020573-43020593. Max. coverage (+): 0. Max coverage (-): 24.62

Region: chr24 43020594-43020614. Max. coverage (+): 0. Max coverage (-): 0

Region: chr24 43020615-43020634. Max. coverage (+): 0. Max coverage (-): 0

Region: chr24 43020635-43020655. Max. coverage (+): 0. Max coverage (-): 30.07

Region: chr24 43020656-43020676. Max. coverage (+): 0. Max coverage (-): 25.51

Region: chr24 43020677-43020697. Max. coverage (+): 0. Max coverage (-): 0

Region: chr24 43020698-43020717. Max. coverage (+): 0. Max coverage (-): 0

Region: chr24 43020718-43020738. Max. coverage (+): 0. Max coverage (-): 0

Region: chr24 43020739-43020759. Max. coverage (+): 0. Max coverage (-): 0.57

Region: chr24 43020760-43020779. Max. coverage (+): 0. Max coverage (-): 0

Region: chr24 43020780-43020800. Max. coverage (+): 0. Max coverage (-): 0

Region: chr24 43020801-43020821. Max. coverage (+): 0. Max coverage (-): 0

Region: chr24 43020822-43020841. Max. coverage (+): 0. Max coverage (-): 0

Region: chr24 43020842-43020862. Max. coverage (+): 0. Max coverage (-): 0

Region: chr24 43020863-43020883. Max. coverage (+): 0. Max coverage (-): 0

Region: chr24 43020884-43020904. Max. coverage (+): 0. Max coverage (-): 34.98

Region: chr24 43020905-43020924. Max. coverage (+): 0. Max coverage (-): 12.5

Region: chr24 43020925-43020945. Max. coverage (+): 0. Max coverage (-): 0

Region: chr24 43020946-43020966. Max. coverage (+): 0. Max coverage (-): 0

Region: chr24 43020967-43020986. Max. coverage (+): 0. Max coverage (-): 0

Region: chr24 43020987-43021007. Max. coverage (+): 0. Max coverage (-): 12.3

Region: chr24 43021008-43021028. Max. coverage (+): 0. Max coverage (-): 6.33

Region: chr24 43021029-43021049. Max. coverage (+): 0. Max coverage (-): 6.33

Region: chr24 43021050-43021069. Max. coverage (+): 0. Max coverage (-): 0

Region: chr24 43021070-43021090. Max. coverage (+): 0. Max coverage (-): 3.76

Region: chr24 43021091-43021111. Max. coverage (+): 0. Max coverage (-): 6.91

Region: chr24 43021112-43021131. Max. coverage (+): 0. Max coverage (-): 6.91

Region: chr24 43021132-43021152. Max. coverage (+): 0. Max coverage (-): 79.7

Region: chr24 43021153-43021173. Max. coverage (+): 0. Max coverage (-): 3.78

Region: chr24 43021174-43021193. Max. coverage (+): 0. Max coverage (-): 0

Region: chr24 43021194-43021214. Max. coverage (+): 0. Max coverage (-): 0

Region: chr24 43021215-43021235. Max. coverage (+): 0. Max coverage (-): 5.09

Region: chr24 43021236-43021256. Max. coverage (+): 0. Max coverage (-): 9.56

Region: chr24 43021257-43021276. Max. coverage (+): 0. Max coverage (-): 0

Region: chr24 43021277-43021297. Max. coverage (+): 0. Max coverage (-): 9.5

Region: chr24 43021298-43021318. Max. coverage (+): 0. Max coverage (-): 9.5

Region: chr24 43021319-43021338. Max. coverage (+): 0. Max coverage (-): 0

Region: chr24 43021339-43021359. Max. coverage (+): 0. Max coverage (-): 0

Region: chr24 43021360-43021380. Max. coverage (+): 0. Max coverage (-): 0

Region: chr24 43021381-43021400. Max. coverage (+): 0. Max coverage (-): 0

Region: chr24 43021401-43021421. Max. coverage (+): 0. Max coverage (-): 4.47

Region: chr24 43021422-43021442. Max. coverage (+): 0. Max coverage (-): 4.47

Region: chr24 43021443-43021463. Max. coverage (+): 0. Max coverage (-): 0

Region: chr24 43021464-43021483. Max. coverage (+): 0. Max coverage (-): 52.15

Region: chr24 43021484-43021504. Max. coverage (+): 0. Max coverage (-): 0.5

Region: chr24 43021505-43021525. Max. coverage (+): 0. Max coverage (-): 0

Region: chr24 43021526-43021545. Max. coverage (+): 0. Max coverage (-): 0

Region: chr24 43021546-43021566. Max. coverage (+): 0. Max coverage (-): 0

Region: chr24 43021567-43021587. Max. coverage (+): 0. Max coverage (-): 0

Region: chr24 43021588-43021607. Max. coverage (+): 0. Max coverage (-): 0

Region: chr24 43021608-43021628. Max. coverage (+): 0. Max coverage (-): 0

Region: chr24 43021629-43021649. Max. coverage (+): 0. Max coverage (-): 0

Region: chr24 43021650-43021670. Max. coverage (+): 0. Max coverage (-): 0

Region: chr24 43021671-43021690. Max. coverage (+): 0. Max coverage (-): 0

Region: chr24 43021691-43021711. Max. coverage (+): 0. Max coverage (-): 0

Region: chr24 43021712-43021732. Max. coverage (+): 0. Max coverage (-): 0

Region: chr24 43021733-43021752. Max. coverage (+): 0. Max coverage (-): 0

Region: chr24 43021753-43021773. Max. coverage (+): 0. Max coverage (-): 0

Region: chr24 43021774-43021794. Max. coverage (+): 0. Max coverage (-): 0

Region: chr24 43021795-43021814. Max. coverage (+): 0. Max coverage (-): 0

Region: chr24 43021815-43021835. Max. coverage (+): 0. Max coverage (-): 0

Region: chr24 43021836-43021856. Max. coverage (+): 0. Max coverage (-): 0

Region: chr24 43021857-43021877. Max. coverage (+): 0. Max coverage (-): 0

Region: chr24 43021878-43021897. Max. coverage (+): 0. Max coverage (-): 0

Region: chr24 43021898-43021918. Max. coverage (+): 0. Max coverage (-): 0

Region: chr24 43021919-43021939. Max. coverage (+): 0. Max coverage (-): 0

Region: chr24 43021940-43021959. Max. coverage (+): 0. Max coverage (-): 0

Region: chr24 43021960-43021980. Max. coverage (+): 0. Max coverage (-): 0

Region: chr24 43021981-43022001. Max. coverage (+): 0. Max coverage (-): 0

Region: chr24 43022002-43022022. Max. coverage (+): 0. Max coverage (-): 0

Region: chr24 43022023-43022042. Max. coverage (+): 0. Max coverage (-): 0

Region: chr24 43022043-43022063. Max. coverage (+): 0. Max coverage (-): 0

Region: chr24 43022064-43022084. Max. coverage (+): 0. Max coverage (-): 0

Region: chr24 43022085-43022104. Max. coverage (+): 0. Max coverage (-): 0

Region: chr24 43022105-43022125. Max. coverage (+): 0. Max coverage (-): 0

Region: chr24 43022126-43022146. Max. coverage (+): 0. Max coverage (-): 0

Region: chr24 43022147-43022166. Max. coverage (+): 0. Max coverage (-): 0

Region: chr24 43022167-43022187. Max. coverage (+): 0. Max coverage (-): 0

Region: chr24 43022188-43022208. Max. coverage (+): 0. Max coverage (-): 0

Region: chr24 43022209-43022229. Max. coverage (+): 0. Max coverage (-): 0

Region: chr24 43022230-43022249. Max. coverage (+): 0. Max coverage (-): 0

Region: chr24 43022250-43022270. Max. coverage (+): 0. Max coverage (-): 0

Region: chr24 43022271-43022291. Max. coverage (+): 0. Max coverage (-): 0

Region: chr24 43022292-43022311. Max. coverage (+): 0. Max coverage (-): 0

Region: chr24 43022312-43022332. Max. coverage (+): 0. Max coverage (-): 0

Region: chr24 43022333-43022353. Max. coverage (+): 0. Max coverage (-): 0

Region: chr24 43022354-43022373. Max. coverage (+): 0. Max coverage (-): 0

Region: chr24 43022374-43022394. Max. coverage (+): 0. Max coverage (-): 0

Region: chr24 43022395-43022415. Max. coverage (+): 0. Max coverage (-): 0

Region: chr24 43022416-43022436. Max. coverage (+): 0. Max coverage (-): 0

Region: chr24 43022437-43022456. Max. coverage (+): 0. Max coverage (-): 0

Region: chr24 43022457-43022477. Max. coverage (+): 0. Max coverage (-): 23.76

Region: chr24 43022478-43022498. Max. coverage (+): 0. Max coverage (-): 8.69

Region: chr24 43022499-43022518. Max. coverage (+): 0. Max coverage (-): 17.63

Region: chr24 43022519-43022539. Max. coverage (+): 0. Max coverage (-): 10.6

Region: chr24 43022540-43022560. Max. coverage (+): 0. Max coverage (-): 0

Region: chr24 43022561-43022580. Max. coverage (+): 0. Max coverage (-): 0

Region: chr24 43022581-43022601. Max. coverage (+): 0. Max coverage (-): 0

Region: chr24 43022602-43022622. Max. coverage (+): 0. Max coverage (-): 0

Region: chr24 43022623-43022643. Max. coverage (+): 0. Max coverage (-): 0

Region: chr24 43022644-43022663. Max. coverage (+): 0. Max coverage (-): 3.46

Region: chr24 43022664-43022684. Max. coverage (+): 0. Max coverage (-): 3.46

Region: chr24 43022685-43022705. Max. coverage (+): 0. Max coverage (-): 0

Region: chr24 43022706-43022725. Max. coverage (+): 0. Max coverage (-): 0

Region: chr24 43022726-43022746. Max. coverage (+): 0. Max coverage (-): 0

Region: chr24 43022747-43022767. Max. coverage (+): 0. Max coverage (-): 7.6

Region: chr24 43022768-43022787. Max. coverage (+): 0. Max coverage (-): 7.6

Region: chr24 43022788-43022808. Max. coverage (+): 0. Max coverage (-): 0

Region: chr24 43022809-43022829. Max. coverage (+): 0. Max coverage (-): 8.28

Region: chr24 43022830-43022850. Max. coverage (+): 0. Max coverage (-): 0

Region: chr24 43022851-43022870. Max. coverage (+): 0. Max coverage (-): 0

Region: chr24 43022871-43022891. Max. coverage (+): 0. Max coverage (-): 0

Region: chr24 43022892-43022912. Max. coverage (+): 0. Max coverage (-): 0

Region: chr24 43022913-43022932. Max. coverage (+): 0. Max coverage (-): 0

Region: chr24 43022933-43022953. Max. coverage (+): 0. Max coverage (-): 0

Region: chr24 43022954-43022974. Max. coverage (+): 0. Max coverage (-): 0

Region: chr24 43022975-43022994. Max. coverage (+): 0. Max coverage (-): 3.82

Region: chr24 43022995-43023015. Max. coverage (+): 0. Max coverage (-): 11.92

Region: chr24 43023016-43023036. Max. coverage (+): 0. Max coverage (-): 4.43

Region: chr24 43023037-43023057. Max. coverage (+): 0. Max coverage (-): 0

Region: chr24 43023058-43023077. Max. coverage (+): 0. Max coverage (-): 0

Region: chr24 43023078-43023098. Max. coverage (+): 0. Max coverage (-): 11.73

Region: chr24 43023099-43023119. Max. coverage (+): 0. Max coverage (-): 0

Region: chr24 43023120-43023139. Max. coverage (+): 0. Max coverage (-): 0

Region: chr24 43023140-43023160. Max. coverage (+): 0. Max coverage (-): 8.13

Region: chr24 43023161-43023181. Max. coverage (+): 0. Max coverage (-): 0

Region: chr24 43023182-43023202. Max. coverage (+): 0. Max coverage (-): 3.76

Region: chr24 43023203-43023222. Max. coverage (+): 0. Max coverage (-): 0

Region: chr24 43023223-43023243. Max. coverage (+): 0. Max coverage (-): 0

Region: chr24 43023244-43023264. Max. coverage (+): 0. Max coverage (-): 0

Region: chr24 43023265-43023284. Max. coverage (+): 0. Max coverage (-): 0

Region: chr24 43023285-43023305. Max. coverage (+): 0. Max coverage (-): 0

Region: chr24 43023306-43023326. Max. coverage (+): 0. Max coverage (-): 0

Region: chr24 43023327-43023346. Max. coverage (+): 0. Max coverage (-): 0

Region: chr24 43023347-43023367. Max. coverage (+): 0. Max coverage (-): 0

Region: chr24 43023368-43023388. Max. coverage (+): 0. Max coverage (-): 12.17

Region: chr24 43023389-43023409. Max. coverage (+): 0. Max coverage (-): 5.29

Region: chr24 43023410-43023429. Max. coverage (+): 0. Max coverage (-): 0

Region: chr24 43023430-43023450. Max. coverage (+): 0. Max coverage (-): 0

Region: chr24 43023451-43023471. Max. coverage (+): 0. Max coverage (-): 0

Region: chr24 43023472-43023491. Max. coverage (+): 0. Max coverage (-): 0

Region: chr24 43023492-43023512. Max. coverage (+): 0. Max coverage (-): 0

Region: chr24 43023513-43023533. Max. coverage (+): 0. Max coverage (-): 0

Region: chr24 43023534-43023553. Max. coverage (+): 0. Max coverage (-): 0

Region: chr24 43023554-43023574. Max. coverage (+): 0. Max coverage (-): 0

Region: chr24 43023575-43023595. Max. coverage (+): 0. Max coverage (-): 0

Region: chr24 43023596-43023616. Max. coverage (+): 0. Max coverage (-): 0

Region: chr24 43023617-43023636. Max. coverage (+): 0. Max coverage (-): 0

Region: chr24 43023637-43023657. Max. coverage (+): 0. Max coverage (-): 0

Region: chr24 43023658-43023678. Max. coverage (+): 0. Max coverage (-): 0

Region: chr24 43023679-43023698. Max. coverage (+): 0. Max coverage (-): 0

Region: chr24 43023699-43023719. Max. coverage (+): 0. Max coverage (-): 4.44

Region: chr24 43023720-43023740. Max. coverage (+): 0. Max coverage (-): 0

Region: chr24 43023741-43023760. Max. coverage (+): 0. Max coverage (-): 6.79

Region: chr24 43023761-43023781. Max. coverage (+): 0. Max coverage (-): 6.79

Region: chr24 43023782-43023802. Max. coverage (+): 0. Max coverage (-): 6.27

Region: chr24 43023803-43023823. Max. coverage (+): 0. Max coverage (-): 1.21

Region: chr24 43023824-43023843. Max. coverage (+): 0. Max coverage (-): 0

Region: chr24 43023844-43023864. Max. coverage (+): 0. Max coverage (-): 0

Region: chr24 43023865-43023885. Max. coverage (+): 0. Max coverage (-): 0

Region: chr24 43023886-43023905. Max. coverage (+): 0. Max coverage (-): 0

Region: chr24 43023906-43023926. Max. coverage (+): 0. Max coverage (-): 0

Region: chr24 43023927-43023947. Max. coverage (+): 0. Max coverage (-): 0

Region: chr24 43023948-43023967. Max. coverage (+): 0. Max coverage (-): 0

Region: chr24 43023968-43023988. Max. coverage (+): 0. Max coverage (-): 0

Region: chr24 43023989-43024009. Max. coverage (+): 0. Max coverage (-): 0

Region: chr24 43024010-43024030. Max. coverage (+): 0. Max coverage (-): 0

Region: chr24 43024031-43024050. Max. coverage (+): 0. Max coverage (-): 0

Region: chr24 43024051-43024071. Max. coverage (+): 0. Max coverage (-): 0

Region: chr24 43024072-43024092. Max. coverage (+): 0. Max coverage (-): 0

Region: chr24 43024093-43024112. Max. coverage (+): 0. Max coverage (-): 0

Region: chr24 43024113-43024133. Max. coverage (+): 0. Max coverage (-): 0

Region: chr24 43024134-43024154. Max. coverage (+): 0. Max coverage (-): 0

Region: chr24 43024155-43024175. Max. coverage (+): 0. Max coverage (-): 0

Region: chr24 43024176-43024195. Max. coverage (+): 0. Max coverage (-): 0

Region: chr24 43024196-43024216. Max. coverage (+): 0. Max coverage (-): 0

Region: chr24 43024217-43024237. Max. coverage (+): 0. Max coverage (-): 0

Region: chr24 43024238-43024257. Max. coverage (+): 0. Max coverage (-): 0

Region: chr24 43024258-43024278. Max. coverage (+): 0. Max coverage (-): 0

Region: chr24 43024279-43024299. Max. coverage (+): 0. Max coverage (-): 0

Region: chr24 43024300-43024319. Max. coverage (+): 0. Max coverage (-): 0

Region: chr24 43024320-43024340. Max. coverage (+): 0. Max coverage (-): 0

Region: chr24 43024341-43024361. Max. coverage (+): 0. Max coverage (-): 0

Region: chr24 43024362-43024382. Max. coverage (+): 0. Max coverage (-): 0

Region: chr24 43024383-43024402. Max. coverage (+): 0. Max coverage (-): 0

Region: chr24 43024403-43024423. Max. coverage (+): 0. Max coverage (-): 0

Region: chr24 43024424-43024444. Max. coverage (+): 0. Max coverage (-): 0

Region: chr24 43024445-43024464. Max. coverage (+): 0. Max coverage (-): 0

Region: chr24 43024465-43024485. Max. coverage (+): 0. Max coverage (-): 0

Region: chr24 43024486-43024506. Max. coverage (+): 0. Max coverage (-): 0

Region: chr24 43024507-43024526. Max. coverage (+): 0. Max coverage (-): 2.42

Region: chr24 43024527-43024547. Max. coverage (+): 0. Max coverage (-): 2.42

Region: chr24 43024548-. Max. coverage (+): 0. Max coverage (-): 0

RepeatMasker Color Code

**+**

100-98% Identity

<98-95% Identity

<95-90% Identity

<90-85% Identity

<85-80% Identity

<80-75% Identity

<75-70% Identity

<70% Identity

**-**

Gene Set Color Code

**+**

Gene

Pseudogene

**-**

Topology/Coverage Color Code

Coverage Plus Strand

Coverage Minus Strand

Mainstrand: Plus

Mainstrand: Minus

Complementary Strand

Flanking Region  
(if option -flank >0)

Gene Set Annotation  
  
RepeatMasker Annotation  

**1. LTR39B\_BT**: 43014626-43015234 (-), Divergence to consensus: 26.7%  
**2. L2b**: 43015665-43015952 (+), Divergence to consensus: 42.9%  
**3. L2b**: 43016357-43016471 (+), Divergence to consensus: 40.7%  
**4. L2b**: 43016587-43016958 (+), Divergence to consensus: 47.7%  
**5. AT\_rich**: 43017564-43017600 (+), Divergence to consensus: 64.9%  
**6. L1MB1**: 43017974-43018176 (-), Divergence to consensus: 28.6%  
**7. L1MA9**: 43018176-43018274 (+), Divergence to consensus: 22.4%  
**8. Bov-tA1**: 43018277-43018491 (+), Divergence to consensus: 16.8%  
**9. Bov-tA2**: 43019298-43019394 (-), Divergence to consensus: 21.6%  
**10. MIRb**: 43020797-43020856 (-), Divergence to consensus: 36.6%  
**11. Bov-tA2**: 43021510-43021622 (+), Divergence to consensus: 18.6%  
**12. Tigger19a**: 43022236-43022340 (-), Divergence to consensus: 44.4%  
**13. L1-3\_BT**: 43023428-43023684 (+), Divergence to consensus: 21.1%  
**14. Bov-tA2**: 43023886-43024086 (+), Divergence to consensus: 14.5%

  
Transcription Factor Binding Sites  

**SOX9** (Sequence: AACAATAA (-): 43024185)  
**Gata4** (Sequence: GTTATCT (+): 43017650)  
**Gata4** (Sequence: GTTATCT (+): 43018561)  
**Gata4** (Sequence: GTTATCT (+): 43020519)  
**Gata4** (Sequence: CTTATCT (+): 43023194)
