# Supplementary material for: Small RNA sequencing of cryopreserved semen from single bull revealed altered miRNAs and piRNAs expression between High- and Low-motile sperm populations
Source: BMC Genomics. 2017 Jan 4;18:14. doi: 10.1186/s12864-016-3394-7 (PMC5209821; doi:10.1186/s12864-016-3394-7)
Supplement: Additional file 4: — Details for each piRNA clusters found in Low Motile (LM) sperm fraction. Genes, repeats, transposable elements and transcription factors binding sites falling within the cluster regions were reported. (ZIP 1034 kb) [file 12864_2016_3394_MOESM4_ESM.zip › 34.html]

piRNA cluster 34


Predicted piRNA cluster no. 34     previous   next
  

Show proTRAC run info
Hide proTRAC run info

================================= proTRAC ====================================  
VERSION: 2.1                                    LAST MODIFIED: 06. October 2015  
  
Please cite:  
Rosenkranz D, Zischler H. proTRAC - a software for probabilistic piRNA cluster  
detection, visualization and analysis. 2012. BMC Bioinformatics 13:5.  
  
and (for proTRAC 2.0 and later):  
Rosenkranz D, Rudloff S, Bastuck K, Ketting RF, Zischler H. Tupaia small RNAs  
provide insights into function and evolution of RNAi-based transposon defense  
in mammals. 2015. RNA 21(5):911-922.  
  
Contact:  
David Rosenkranz  
Institute of Anthropology, small RNA group  
Johannes Gutenberg University Mainz  
email: rosenkranz@uni-mainz.de  
  
You can find the latest proTRAC version at:  
http://sourceforge.net/projects/protrac/files  
http://www.smallRNAgroup-mainz.de/software  
==============================================================================  
  
PARAMETERS:  
Map file: .............../storage/core/barbara/genhome/smallRNA/fertility/Sample\_not\_motile/pirna/Sample\_not\_motile\_26-33\_collapsed.fa.no-dust.map.weighted-10000-1000-b-0  
Genome file: ............/storage/core/barbara/genhome/smallRNA/fertility/Sample\_all/pirna/bt\_311\_chrY.fa  
RepeatMasker annotation: /storage/genomes/bt\_umd31/GCF\_000003055.6\_Bos\_taurus\_UMD\_3.1.1\_repeatMasker\_chr.out  
GeneSet:................./storage/core/barbara/genhome/smallRNA/fertility/Sample\_all/pirna/full.gtf  
  
Significant (p<=0.01) hit density will be calculated based  
on observed hit distribution.  
  
Sliding window size: ........................................ 5000 bp  
Sliding window increament: .................................. 1000 bp  
Normalize each hit by number of genomic hits: ............... 1 [0=no/1=yes]  
Normalize each hit by number of sequence reads: ............. 1 [0=no/1=yes]  
Normalize values (-> per million mapped reads): ............. 1 [0=no/1=yes]  
Min. fraction of hits with 1T(U) or 10A: .................... 0.75  
Alternatively: Min. fraction of hits with 1T(U) and 10A: .... 0.5  
Min. fraction of hits with typical piRNA length: ............ 0.75  
Typical piRNA length: ....................................... 26-33 nt  
Min. size of a piRNA cluster: ............................... 5000 bp.  
Min. number of hits (absolute): ............................. 0  
Min. number of hits (normalized): ........................... 0  
Min. fraction of hits on the mainstrand: .................... 0.75  
Top fraction of mapped sequences (in terms of read counts): . 1%  
Top fraction accounts for max. n% of sequence reads: ........ 90%  
Min. fraction of hits on each arm of a bidirectional cluster: 0.1  
Output image file for each cluster: ......................... 0 [0=no/1=yes]  
Output html file for each cluster: .......................... 1 [0=no/1=yes]  
Output a summary table: ..................................... 1 [0=no/1=yes]  
Output a FASTA file for each cluster (piRNA sequences): ..... 1 [0=no/1=yes]  
Output a FASTA file comprising cluster sequences: ........... 1 [0=no/1=yes]  
Search DNA motifs in clusters: .............................. 1 [0=no/1=yes]  
Output flanking sequences: +/- .............................. 0 bp  
Output ~.pTi file: .......................................... 1 [0=no/1=yes]  
==============================================================================  
  
  
Genome size (without gaps): ............ 2678902517 bp  
Gaps (N/X/-): .......................... 53837044 bp  
Mapped reads: .......................... 738059667487  
Non-identical sequences: ............... 277001  
Genomic hits: .......................... 533816  
Significant densitiy of mapped reads: .. 15118061 reads/kb

Show proTRAC cluster info
Hide proTRAC cluster info

|  |  |
| --- | --- |
| Location | chr24 |
| Coordinates | 43040852-43049351 |
| Size [bp] | 8500 |
| Sequence hit loci | 88 |
| Mapped reads (normalized) | 204597356 |
| Mapped reads (normalized) per kb | 24070277.2 |
| Normalized reads with 1T (1U) | 97.6% |
| Normalized reads with 10A | 37.9% |
| Normalized reads with length 26-33 nt | 100% |
| Normalized reads on the main strand(s) | 100% |
| Predicted directionality | mono:minus |

100%

0%

1T (1U)  
reads

10A reads

26-33 nt  
reads

reads on mainstrand

**Either the amount of reads with 1T (1U) OR 10A has to exceed 75% (set with option: -1Tor10A)  
Alternatively the amount of reads with 1T (1U) AND 10A has to exceed 50% (set with option: -1Tand10A)  
Minimum amount of reads with preferred size is 75% (set with option: -pisize)  
Minimum amount of reads on the main strand(s) is 75% (set with option: -clstrand)**

Show read coverage
Hide read coverage

WHAT DO I SEE HERE?  
This chart shows the location of mapped sequence reads within a predicted piRNA cluster. The color refers to the number of genomic hits produced by the sequence read in question. A dark red bar indicates that this sequence read produces many other hits elsewhere in the genome. Many adjacent red or yellow bars can indicate the presence of a multi-copy element such as transposons or rRNA genes. A dark green bar indicates that this sequence read maps uniquely to this locus.

1 hit

2-5 hits

6-10 hits

11-20 hits

21-50 hits

51-100 hits

> 100 hits

chr24

43040852

43049351

Gene Set

RepeatMasker

Mapped  
Reads

19

plus strand

minus strand

19

Region: chr24 43024549-43040860. Max. coverage (+): 0. Max coverage (-): 7.56

Region: chr24 43040861-43040877. Max. coverage (+): 0. Max coverage (-): 2.74

Region: chr24 43040878-43040894. Max. coverage (+): 0. Max coverage (-): 0

Region: chr24 43040895-43040911. Max. coverage (+): 0. Max coverage (-): 0

Region: chr24 43040912-43040928. Max. coverage (+): 0. Max coverage (-): 0

Region: chr24 43040929-43040945. Max. coverage (+): 0. Max coverage (-): 0

Region: chr24 43040946-43040962. Max. coverage (+): 0. Max coverage (-): 0

Region: chr24 43040963-43040979. Max. coverage (+): 0. Max coverage (-): 0

Region: chr24 43040980-43040996. Max. coverage (+): 0. Max coverage (-): 0

Region: chr24 43040997-43041013. Max. coverage (+): 0. Max coverage (-): 0

Region: chr24 43041014-43041030. Max. coverage (+): 0. Max coverage (-): 0

Region: chr24 43041031-43041047. Max. coverage (+): 0. Max coverage (-): 0

Region: chr24 43041048-43041064. Max. coverage (+): 0. Max coverage (-): 6.73

Region: chr24 43041065-43041081. Max. coverage (+): 0. Max coverage (-): 8.16

Region: chr24 43041082-43041098. Max. coverage (+): 0. Max coverage (-): 0

Region: chr24 43041099-43041115. Max. coverage (+): 0. Max coverage (-): 0

Region: chr24 43041116-43041132. Max. coverage (+): 0. Max coverage (-): 5.85

Region: chr24 43041133-43041149. Max. coverage (+): 0. Max coverage (-): 5.85

Region: chr24 43041150-43041166. Max. coverage (+): 0. Max coverage (-): 0

Region: chr24 43041167-43041183. Max. coverage (+): 0. Max coverage (-): 0

Region: chr24 43041184-43041200. Max. coverage (+): 0. Max coverage (-): 0

Region: chr24 43041201-43041217. Max. coverage (+): 0. Max coverage (-): 0

Region: chr24 43041218-43041234. Max. coverage (+): 0. Max coverage (-): 0

Region: chr24 43041235-43041251. Max. coverage (+): 0. Max coverage (-): 0

Region: chr24 43041252-43041268. Max. coverage (+): 0. Max coverage (-): 0

Region: chr24 43041269-43041285. Max. coverage (+): 0. Max coverage (-): 0

Region: chr24 43041286-43041302. Max. coverage (+): 0. Max coverage (-): 0

Region: chr24 43041303-43041319. Max. coverage (+): 0. Max coverage (-): 0

Region: chr24 43041320-43041336. Max. coverage (+): 0. Max coverage (-): 0

Region: chr24 43041337-43041353. Max. coverage (+): 0. Max coverage (-): 0

Region: chr24 43041354-43041370. Max. coverage (+): 0. Max coverage (-): 0

Region: chr24 43041371-43041387. Max. coverage (+): 0. Max coverage (-): 0

Region: chr24 43041388-43041404. Max. coverage (+): 0. Max coverage (-): 0

Region: chr24 43041405-43041421. Max. coverage (+): 0. Max coverage (-): 0

Region: chr24 43041422-43041438. Max. coverage (+): 0. Max coverage (-): 0

Region: chr24 43041439-43041455. Max. coverage (+): 0. Max coverage (-): 0

Region: chr24 43041456-43041472. Max. coverage (+): 0. Max coverage (-): 0

Region: chr24 43041473-43041489. Max. coverage (+): 0. Max coverage (-): 0

Region: chr24 43041490-43041506. Max. coverage (+): 0. Max coverage (-): 0

Region: chr24 43041507-43041523. Max. coverage (+): 0. Max coverage (-): 0

Region: chr24 43041524-43041540. Max. coverage (+): 0. Max coverage (-): 0

Region: chr24 43041541-43041557. Max. coverage (+): 0. Max coverage (-): 0

Region: chr24 43041558-43041574. Max. coverage (+): 0. Max coverage (-): 3.34

Region: chr24 43041575-43041591. Max. coverage (+): 0. Max coverage (-): 4.72

Region: chr24 43041592-43041608. Max. coverage (+): 0. Max coverage (-): 10.51

Region: chr24 43041609-43041625. Max. coverage (+): 0. Max coverage (-): 11.22

Region: chr24 43041626-43041642. Max. coverage (+): 0. Max coverage (-): 0

Region: chr24 43041643-43041659. Max. coverage (+): 0. Max coverage (-): 0

Region: chr24 43041660-43041676. Max. coverage (+): 0. Max coverage (-): 18.93

Region: chr24 43041677-43041693. Max. coverage (+): 0. Max coverage (-): 18.93

Region: chr24 43041694-43041710. Max. coverage (+): 0. Max coverage (-): 0

Region: chr24 43041711-43041727. Max. coverage (+): 0. Max coverage (-): 1.29

Region: chr24 43041728-43041744. Max. coverage (+): 0. Max coverage (-): 0

Region: chr24 43041745-43041761. Max. coverage (+): 0. Max coverage (-): 0

Region: chr24 43041762-43041778. Max. coverage (+): 0. Max coverage (-): 0

Region: chr24 43041779-43041795. Max. coverage (+): 0. Max coverage (-): 4.78

Region: chr24 43041796-43041812. Max. coverage (+): 0. Max coverage (-): 0

Region: chr24 43041813-43041829. Max. coverage (+): 0. Max coverage (-): 0

Region: chr24 43041830-43041846. Max. coverage (+): 0. Max coverage (-): 0

Region: chr24 43041847-43041863. Max. coverage (+): 0. Max coverage (-): 0

Region: chr24 43041864-43041880. Max. coverage (+): 0. Max coverage (-): 0

Region: chr24 43041881-43041897. Max. coverage (+): 0. Max coverage (-): 0

Region: chr24 43041898-43041914. Max. coverage (+): 0. Max coverage (-): 13.16

Region: chr24 43041915-43041931. Max. coverage (+): 0. Max coverage (-): 8.35

Region: chr24 43041932-43041948. Max. coverage (+): 0. Max coverage (-): 0

Region: chr24 43041949-43041965. Max. coverage (+): 0. Max coverage (-): 6.09

Region: chr24 43041966-43041982. Max. coverage (+): 0. Max coverage (-): 0

Region: chr24 43041983-43041999. Max. coverage (+): 0. Max coverage (-): 0

Region: chr24 43042000-43042016. Max. coverage (+): 0. Max coverage (-): 0

Region: chr24 43042017-43042033. Max. coverage (+): 0. Max coverage (-): 5.13

Region: chr24 43042034-43042050. Max. coverage (+): 0. Max coverage (-): 5.22

Region: chr24 43042051-43042067. Max. coverage (+): 0. Max coverage (-): 0.63

Region: chr24 43042068-43042084. Max. coverage (+): 0. Max coverage (-): 0

Region: chr24 43042085-43042101. Max. coverage (+): 0. Max coverage (-): 0

Region: chr24 43042102-43042118. Max. coverage (+): 0. Max coverage (-): 0

Region: chr24 43042119-43042135. Max. coverage (+): 0. Max coverage (-): 0

Region: chr24 43042136-43042152. Max. coverage (+): 0. Max coverage (-): 0

Region: chr24 43042153-43042169. Max. coverage (+): 0. Max coverage (-): 0

Region: chr24 43042170-43042186. Max. coverage (+): 0. Max coverage (-): 0

Region: chr24 43042187-43042203. Max. coverage (+): 0. Max coverage (-): 0

Region: chr24 43042204-43042220. Max. coverage (+): 0. Max coverage (-): 0

Region: chr24 43042221-43042237. Max. coverage (+): 0. Max coverage (-): 0

Region: chr24 43042238-43042254. Max. coverage (+): 0. Max coverage (-): 0

Region: chr24 43042255-43042271. Max. coverage (+): 0. Max coverage (-): 0

Region: chr24 43042272-43042288. Max. coverage (+): 0. Max coverage (-): 0

Region: chr24 43042289-43042305. Max. coverage (+): 0. Max coverage (-): 0

Region: chr24 43042306-43042322. Max. coverage (+): 0. Max coverage (-): 0

Region: chr24 43042323-43042339. Max. coverage (+): 0. Max coverage (-): 0

Region: chr24 43042340-43042356. Max. coverage (+): 0. Max coverage (-): 0

Region: chr24 43042357-43042373. Max. coverage (+): 0. Max coverage (-): 0

Region: chr24 43042374-43042390. Max. coverage (+): 0. Max coverage (-): 0

Region: chr24 43042391-43042407. Max. coverage (+): 0. Max coverage (-): 0

Region: chr24 43042408-43042424. Max. coverage (+): 0. Max coverage (-): 0

Region: chr24 43042425-43042441. Max. coverage (+): 0. Max coverage (-): 0

Region: chr24 43042442-43042458. Max. coverage (+): 0. Max coverage (-): 0

Region: chr24 43042459-43042475. Max. coverage (+): 0. Max coverage (-): 0

Region: chr24 43042476-43042492. Max. coverage (+): 0. Max coverage (-): 0

Region: chr24 43042493-43042509. Max. coverage (+): 0. Max coverage (-): 0

Region: chr24 43042510-43042526. Max. coverage (+): 0. Max coverage (-): 0

Region: chr24 43042527-43042543. Max. coverage (+): 0. Max coverage (-): 0

Region: chr24 43042544-43042560. Max. coverage (+): 0. Max coverage (-): 0

Region: chr24 43042561-43042577. Max. coverage (+): 0. Max coverage (-): 0

Region: chr24 43042578-43042594. Max. coverage (+): 0. Max coverage (-): 0

Region: chr24 43042595-43042611. Max. coverage (+): 0. Max coverage (-): 0

Region: chr24 43042612-43042628. Max. coverage (+): 0. Max coverage (-): 0

Region: chr24 43042629-43042645. Max. coverage (+): 0. Max coverage (-): 0

Region: chr24 43042646-43042662. Max. coverage (+): 0. Max coverage (-): 0

Region: chr24 43042663-43042679. Max. coverage (+): 0. Max coverage (-): 0

Region: chr24 43042680-43042696. Max. coverage (+): 0. Max coverage (-): 0

Region: chr24 43042697-43042713. Max. coverage (+): 0. Max coverage (-): 0

Region: chr24 43042714-43042730. Max. coverage (+): 0. Max coverage (-): 0

Region: chr24 43042731-43042747. Max. coverage (+): 0. Max coverage (-): 0

Region: chr24 43042748-43042764. Max. coverage (+): 0. Max coverage (-): 0

Region: chr24 43042765-43042781. Max. coverage (+): 0. Max coverage (-): 0

Region: chr24 43042782-43042798. Max. coverage (+): 0. Max coverage (-): 0

Region: chr24 43042799-43042815. Max. coverage (+): 0. Max coverage (-): 0

Region: chr24 43042816-43042832. Max. coverage (+): 0. Max coverage (-): 0

Region: chr24 43042833-43042849. Max. coverage (+): 0. Max coverage (-): 0

Region: chr24 43042850-43042866. Max. coverage (+): 0. Max coverage (-): 0

Region: chr24 43042867-43042883. Max. coverage (+): 0. Max coverage (-): 0

Region: chr24 43042884-43042900. Max. coverage (+): 0. Max coverage (-): 0

Region: chr24 43042901-43042917. Max. coverage (+): 0. Max coverage (-): 0

Region: chr24 43042918-43042934. Max. coverage (+): 0. Max coverage (-): 0

Region: chr24 43042935-43042951. Max. coverage (+): 0. Max coverage (-): 0

Region: chr24 43042952-43042968. Max. coverage (+): 0. Max coverage (-): 0

Region: chr24 43042969-43042985. Max. coverage (+): 0. Max coverage (-): 0

Region: chr24 43042986-43043002. Max. coverage (+): 0. Max coverage (-): 0

Region: chr24 43043003-43043019. Max. coverage (+): 0. Max coverage (-): 0

Region: chr24 43043020-43043036. Max. coverage (+): 0. Max coverage (-): 0

Region: chr24 43043037-43043053. Max. coverage (+): 0. Max coverage (-): 0

Region: chr24 43043054-43043070. Max. coverage (+): 0. Max coverage (-): 0

Region: chr24 43043071-43043087. Max. coverage (+): 0. Max coverage (-): 0

Region: chr24 43043088-43043104. Max. coverage (+): 0. Max coverage (-): 0

Region: chr24 43043105-43043121. Max. coverage (+): 0. Max coverage (-): 0

Region: chr24 43043122-43043138. Max. coverage (+): 0. Max coverage (-): 0

Region: chr24 43043139-43043155. Max. coverage (+): 0. Max coverage (-): 0

Region: chr24 43043156-43043172. Max. coverage (+): 0. Max coverage (-): 0

Region: chr24 43043173-43043189. Max. coverage (+): 0. Max coverage (-): 0

Region: chr24 43043190-43043206. Max. coverage (+): 0. Max coverage (-): 0

Region: chr24 43043207-43043223. Max. coverage (+): 0. Max coverage (-): 0

Region: chr24 43043224-43043240. Max. coverage (+): 0. Max coverage (-): 0

Region: chr24 43043241-43043257. Max. coverage (+): 0. Max coverage (-): 0

Region: chr24 43043258-43043274. Max. coverage (+): 0. Max coverage (-): 0

Region: chr24 43043275-43043291. Max. coverage (+): 0. Max coverage (-): 0

Region: chr24 43043292-43043308. Max. coverage (+): 0. Max coverage (-): 0

Region: chr24 43043309-43043325. Max. coverage (+): 0. Max coverage (-): 0

Region: chr24 43043326-43043342. Max. coverage (+): 0. Max coverage (-): 0

Region: chr24 43043343-43043359. Max. coverage (+): 0. Max coverage (-): 0

Region: chr24 43043360-43043376. Max. coverage (+): 0. Max coverage (-): 0

Region: chr24 43043377-43043393. Max. coverage (+): 0. Max coverage (-): 0

Region: chr24 43043394-43043410. Max. coverage (+): 0. Max coverage (-): 0

Region: chr24 43043411-43043427. Max. coverage (+): 0. Max coverage (-): 0

Region: chr24 43043428-43043444. Max. coverage (+): 0. Max coverage (-): 0

Region: chr24 43043445-43043461. Max. coverage (+): 0. Max coverage (-): 0

Region: chr24 43043462-43043478. Max. coverage (+): 0. Max coverage (-): 0

Region: chr24 43043479-43043495. Max. coverage (+): 0. Max coverage (-): 0

Region: chr24 43043496-43043512. Max. coverage (+): 0. Max coverage (-): 0

Region: chr24 43043513-43043529. Max. coverage (+): 0. Max coverage (-): 0

Region: chr24 43043530-43043546. Max. coverage (+): 0. Max coverage (-): 0

Region: chr24 43043547-43043563. Max. coverage (+): 0. Max coverage (-): 0

Region: chr24 43043564-43043580. Max. coverage (+): 0. Max coverage (-): 0

Region: chr24 43043581-43043597. Max. coverage (+): 0. Max coverage (-): 0

Region: chr24 43043598-43043614. Max. coverage (+): 0. Max coverage (-): 0

Region: chr24 43043615-43043631. Max. coverage (+): 0. Max coverage (-): 0

Region: chr24 43043632-43043648. Max. coverage (+): 0. Max coverage (-): 0

Region: chr24 43043649-43043665. Max. coverage (+): 0. Max coverage (-): 0

Region: chr24 43043666-43043682. Max. coverage (+): 0. Max coverage (-): 0

Region: chr24 43043683-43043699. Max. coverage (+): 0. Max coverage (-): 0

Region: chr24 43043700-43043716. Max. coverage (+): 0. Max coverage (-): 0

Region: chr24 43043717-43043733. Max. coverage (+): 0. Max coverage (-): 0

Region: chr24 43043734-43043750. Max. coverage (+): 0. Max coverage (-): 0

Region: chr24 43043751-43043767. Max. coverage (+): 0. Max coverage (-): 0

Region: chr24 43043768-43043784. Max. coverage (+): 0. Max coverage (-): 0

Region: chr24 43043785-43043801. Max. coverage (+): 0. Max coverage (-): 0

Region: chr24 43043802-43043818. Max. coverage (+): 0. Max coverage (-): 0

Region: chr24 43043819-43043835. Max. coverage (+): 0. Max coverage (-): 0

Region: chr24 43043836-43043852. Max. coverage (+): 0. Max coverage (-): 0

Region: chr24 43043853-43043869. Max. coverage (+): 0. Max coverage (-): 0

Region: chr24 43043870-43043886. Max. coverage (+): 0. Max coverage (-): 0

Region: chr24 43043887-43043903. Max. coverage (+): 0. Max coverage (-): 0

Region: chr24 43043904-43043920. Max. coverage (+): 0. Max coverage (-): 0

Region: chr24 43043921-43043937. Max. coverage (+): 0. Max coverage (-): 0

Region: chr24 43043938-43043954. Max. coverage (+): 0. Max coverage (-): 0

Region: chr24 43043955-43043971. Max. coverage (+): 0. Max coverage (-): 0

Region: chr24 43043972-43043988. Max. coverage (+): 0. Max coverage (-): 0

Region: chr24 43043989-43044005. Max. coverage (+): 0. Max coverage (-): 0

Region: chr24 43044006-43044022. Max. coverage (+): 0. Max coverage (-): 0

Region: chr24 43044023-43044039. Max. coverage (+): 0. Max coverage (-): 0

Region: chr24 43044040-43044056. Max. coverage (+): 0. Max coverage (-): 0

Region: chr24 43044057-43044073. Max. coverage (+): 0. Max coverage (-): 0

Region: chr24 43044074-43044090. Max. coverage (+): 0. Max coverage (-): 0

Region: chr24 43044091-43044107. Max. coverage (+): 0. Max coverage (-): 0

Region: chr24 43044108-43044124. Max. coverage (+): 0. Max coverage (-): 0

Region: chr24 43044125-43044141. Max. coverage (+): 0. Max coverage (-): 0

Region: chr24 43044142-43044158. Max. coverage (+): 0. Max coverage (-): 0

Region: chr24 43044159-43044175. Max. coverage (+): 0. Max coverage (-): 0

Region: chr24 43044176-43044192. Max. coverage (+): 0. Max coverage (-): 0

Region: chr24 43044193-43044209. Max. coverage (+): 0. Max coverage (-): 0

Region: chr24 43044210-43044226. Max. coverage (+): 0. Max coverage (-): 0

Region: chr24 43044227-43044243. Max. coverage (+): 0. Max coverage (-): 0

Region: chr24 43044244-43044260. Max. coverage (+): 0. Max coverage (-): 0

Region: chr24 43044261-43044277. Max. coverage (+): 0. Max coverage (-): 0

Region: chr24 43044278-43044294. Max. coverage (+): 0. Max coverage (-): 0

Region: chr24 43044295-43044311. Max. coverage (+): 0. Max coverage (-): 0

Region: chr24 43044312-43044328. Max. coverage (+): 0. Max coverage (-): 0

Region: chr24 43044329-43044345. Max. coverage (+): 0. Max coverage (-): 0

Region: chr24 43044346-43044362. Max. coverage (+): 0. Max coverage (-): 0

Region: chr24 43044363-43044379. Max. coverage (+): 0. Max coverage (-): 0

Region: chr24 43044380-43044396. Max. coverage (+): 0. Max coverage (-): 0

Region: chr24 43044397-43044413. Max. coverage (+): 0. Max coverage (-): 0

Region: chr24 43044414-43044430. Max. coverage (+): 0. Max coverage (-): 0

Region: chr24 43044431-43044447. Max. coverage (+): 0. Max coverage (-): 0

Region: chr24 43044448-43044464. Max. coverage (+): 0. Max coverage (-): 0

Region: chr24 43044465-43044481. Max. coverage (+): 0. Max coverage (-): 0

Region: chr24 43044482-43044498. Max. coverage (+): 0. Max coverage (-): 0

Region: chr24 43044499-43044515. Max. coverage (+): 0. Max coverage (-): 0

Region: chr24 43044516-43044532. Max. coverage (+): 0. Max coverage (-): 0

Region: chr24 43044533-43044549. Max. coverage (+): 0. Max coverage (-): 0

Region: chr24 43044550-43044566. Max. coverage (+): 0. Max coverage (-): 0

Region: chr24 43044567-43044583. Max. coverage (+): 0. Max coverage (-): 0

Region: chr24 43044584-43044600. Max. coverage (+): 0. Max coverage (-): 0

Region: chr24 43044601-43044617. Max. coverage (+): 0. Max coverage (-): 0

Region: chr24 43044618-43044634. Max. coverage (+): 0. Max coverage (-): 0

Region: chr24 43044635-43044651. Max. coverage (+): 0. Max coverage (-): 0

Region: chr24 43044652-43044668. Max. coverage (+): 0. Max coverage (-): 0

Region: chr24 43044669-43044685. Max. coverage (+): 0. Max coverage (-): 0

Region: chr24 43044686-43044702. Max. coverage (+): 0. Max coverage (-): 0

Region: chr24 43044703-43044719. Max. coverage (+): 0. Max coverage (-): 0

Region: chr24 43044720-43044736. Max. coverage (+): 0. Max coverage (-): 0

Region: chr24 43044737-43044753. Max. coverage (+): 0. Max coverage (-): 0

Region: chr24 43044754-43044770. Max. coverage (+): 0. Max coverage (-): 0

Region: chr24 43044771-43044787. Max. coverage (+): 0. Max coverage (-): 0

Region: chr24 43044788-43044804. Max. coverage (+): 0. Max coverage (-): 0

Region: chr24 43044805-43044821. Max. coverage (+): 0. Max coverage (-): 0

Region: chr24 43044822-43044838. Max. coverage (+): 0. Max coverage (-): 0

Region: chr24 43044839-43044855. Max. coverage (+): 0. Max coverage (-): 0

Region: chr24 43044856-43044872. Max. coverage (+): 0. Max coverage (-): 0

Region: chr24 43044873-43044889. Max. coverage (+): 0. Max coverage (-): 0

Region: chr24 43044890-43044906. Max. coverage (+): 0. Max coverage (-): 0

Region: chr24 43044907-43044923. Max. coverage (+): 0. Max coverage (-): 0

Region: chr24 43044924-43044940. Max. coverage (+): 0. Max coverage (-): 0

Region: chr24 43044941-43044957. Max. coverage (+): 0. Max coverage (-): 0

Region: chr24 43044958-43044974. Max. coverage (+): 0. Max coverage (-): 0

Region: chr24 43044975-43044991. Max. coverage (+): 0. Max coverage (-): 0

Region: chr24 43044992-43045008. Max. coverage (+): 0. Max coverage (-): 0

Region: chr24 43045009-43045025. Max. coverage (+): 0. Max coverage (-): 0

Region: chr24 43045026-43045042. Max. coverage (+): 0. Max coverage (-): 0

Region: chr24 43045043-43045059. Max. coverage (+): 0. Max coverage (-): 0

Region: chr24 43045060-43045076. Max. coverage (+): 0. Max coverage (-): 0

Region: chr24 43045077-43045093. Max. coverage (+): 0. Max coverage (-): 0

Region: chr24 43045094-43045110. Max. coverage (+): 0. Max coverage (-): 0

Region: chr24 43045111-43045127. Max. coverage (+): 0. Max coverage (-): 0

Region: chr24 43045128-43045144. Max. coverage (+): 0. Max coverage (-): 0

Region: chr24 43045145-43045161. Max. coverage (+): 0. Max coverage (-): 0

Region: chr24 43045162-43045178. Max. coverage (+): 0. Max coverage (-): 0

Region: chr24 43045179-43045195. Max. coverage (+): 0. Max coverage (-): 0

Region: chr24 43045196-43045212. Max. coverage (+): 0. Max coverage (-): 0

Region: chr24 43045213-43045229. Max. coverage (+): 0. Max coverage (-): 0

Region: chr24 43045230-43045246. Max. coverage (+): 0. Max coverage (-): 0

Region: chr24 43045247-43045263. Max. coverage (+): 0. Max coverage (-): 0

Region: chr24 43045264-43045280. Max. coverage (+): 0. Max coverage (-): 0

Region: chr24 43045281-43045297. Max. coverage (+): 0. Max coverage (-): 0

Region: chr24 43045298-43045314. Max. coverage (+): 0. Max coverage (-): 0

Region: chr24 43045315-43045331. Max. coverage (+): 0. Max coverage (-): 0

Region: chr24 43045332-43045348. Max. coverage (+): 0. Max coverage (-): 0

Region: chr24 43045349-43045365. Max. coverage (+): 0. Max coverage (-): 0

Region: chr24 43045366-43045382. Max. coverage (+): 0. Max coverage (-): 0

Region: chr24 43045383-43045399. Max. coverage (+): 0. Max coverage (-): 0

Region: chr24 43045400-43045416. Max. coverage (+): 0. Max coverage (-): 0

Region: chr24 43045417-43045433. Max. coverage (+): 0. Max coverage (-): 0

Region: chr24 43045434-43045450. Max. coverage (+): 0. Max coverage (-): 0

Region: chr24 43045451-43045467. Max. coverage (+): 0. Max coverage (-): 0

Region: chr24 43045468-43045484. Max. coverage (+): 0. Max coverage (-): 0

Region: chr24 43045485-43045501. Max. coverage (+): 0. Max coverage (-): 0

Region: chr24 43045502-43045518. Max. coverage (+): 0. Max coverage (-): 0

Region: chr24 43045519-43045535. Max. coverage (+): 0. Max coverage (-): 0

Region: chr24 43045536-43045552. Max. coverage (+): 0. Max coverage (-): 0

Region: chr24 43045553-43045569. Max. coverage (+): 0. Max coverage (-): 0

Region: chr24 43045570-43045586. Max. coverage (+): 0. Max coverage (-): 0

Region: chr24 43045587-43045603. Max. coverage (+): 0. Max coverage (-): 0

Region: chr24 43045604-43045620. Max. coverage (+): 0. Max coverage (-): 0

Region: chr24 43045621-43045637. Max. coverage (+): 0. Max coverage (-): 0

Region: chr24 43045638-43045654. Max. coverage (+): 0. Max coverage (-): 0

Region: chr24 43045655-43045671. Max. coverage (+): 0. Max coverage (-): 0

Region: chr24 43045672-43045688. Max. coverage (+): 0. Max coverage (-): 0

Region: chr24 43045689-43045705. Max. coverage (+): 0. Max coverage (-): 0

Region: chr24 43045706-43045722. Max. coverage (+): 0. Max coverage (-): 0

Region: chr24 43045723-43045739. Max. coverage (+): 0. Max coverage (-): 0

Region: chr24 43045740-43045756. Max. coverage (+): 0. Max coverage (-): 0

Region: chr24 43045757-43045773. Max. coverage (+): 0. Max coverage (-): 0

Region: chr24 43045774-43045790. Max. coverage (+): 0. Max coverage (-): 0

Region: chr24 43045791-43045807. Max. coverage (+): 0. Max coverage (-): 0

Region: chr24 43045808-43045824. Max. coverage (+): 0. Max coverage (-): 0

Region: chr24 43045825-43045841. Max. coverage (+): 0. Max coverage (-): 0

Region: chr24 43045842-43045858. Max. coverage (+): 0. Max coverage (-): 0

Region: chr24 43045859-43045875. Max. coverage (+): 0. Max coverage (-): 0

Region: chr24 43045876-43045892. Max. coverage (+): 0. Max coverage (-): 11.19

Region: chr24 43045893-43045909. Max. coverage (+): 0. Max coverage (-): 0

Region: chr24 43045910-43045926. Max. coverage (+): 0. Max coverage (-): 0

Region: chr24 43045927-43045943. Max. coverage (+): 0. Max coverage (-): 0

Region: chr24 43045944-43045960. Max. coverage (+): 0. Max coverage (-): 0

Region: chr24 43045961-43045977. Max. coverage (+): 0. Max coverage (-): 0

Region: chr24 43045978-43045994. Max. coverage (+): 0. Max coverage (-): 0

Region: chr24 43045995-43046011. Max. coverage (+): 0. Max coverage (-): 0

Region: chr24 43046012-43046028. Max. coverage (+): 0. Max coverage (-): 0

Region: chr24 43046029-43046045. Max. coverage (+): 0. Max coverage (-): 0

Region: chr24 43046046-43046062. Max. coverage (+): 0. Max coverage (-): 0

Region: chr24 43046063-43046079. Max. coverage (+): 0. Max coverage (-): 0.85

Region: chr24 43046080-43046096. Max. coverage (+): 0. Max coverage (-): 0

Region: chr24 43046097-43046113. Max. coverage (+): 0. Max coverage (-): 0.05

Region: chr24 43046114-43046130. Max. coverage (+): 0. Max coverage (-): 0

Region: chr24 43046131-43046147. Max. coverage (+): 0. Max coverage (-): 0

Region: chr24 43046148-43046164. Max. coverage (+): 0. Max coverage (-): 0

Region: chr24 43046165-43046181. Max. coverage (+): 0. Max coverage (-): 0

Region: chr24 43046182-43046198. Max. coverage (+): 0. Max coverage (-): 0

Region: chr24 43046199-43046215. Max. coverage (+): 0. Max coverage (-): 0

Region: chr24 43046216-43046232. Max. coverage (+): 0. Max coverage (-): 0

Region: chr24 43046233-43046249. Max. coverage (+): 0. Max coverage (-): 0

Region: chr24 43046250-43046266. Max. coverage (+): 0. Max coverage (-): 0

Region: chr24 43046267-43046283. Max. coverage (+): 0. Max coverage (-): 0

Region: chr24 43046284-43046300. Max. coverage (+): 0. Max coverage (-): 0

Region: chr24 43046301-43046317. Max. coverage (+): 0. Max coverage (-): 0

Region: chr24 43046318-43046334. Max. coverage (+): 0. Max coverage (-): 0

Region: chr24 43046335-43046351. Max. coverage (+): 0. Max coverage (-): 0

Region: chr24 43046352-43046368. Max. coverage (+): 0. Max coverage (-): 0

Region: chr24 43046369-43046385. Max. coverage (+): 0. Max coverage (-): 0

Region: chr24 43046386-43046402. Max. coverage (+): 0. Max coverage (-): 0

Region: chr24 43046403-43046419. Max. coverage (+): 0. Max coverage (-): 0

Region: chr24 43046420-43046436. Max. coverage (+): 0. Max coverage (-): 0

Region: chr24 43046437-43046453. Max. coverage (+): 0. Max coverage (-): 0

Region: chr24 43046454-43046470. Max. coverage (+): 0. Max coverage (-): 0

Region: chr24 43046471-43046487. Max. coverage (+): 0. Max coverage (-): 0

Region: chr24 43046488-43046504. Max. coverage (+): 0. Max coverage (-): 0

Region: chr24 43046505-43046521. Max. coverage (+): 0. Max coverage (-): 0

Region: chr24 43046522-43046538. Max. coverage (+): 0. Max coverage (-): 0

Region: chr24 43046539-43046555. Max. coverage (+): 0. Max coverage (-): 0

Region: chr24 43046556-43046572. Max. coverage (+): 0. Max coverage (-): 0

Region: chr24 43046573-43046589. Max. coverage (+): 0. Max coverage (-): 0

Region: chr24 43046590-43046606. Max. coverage (+): 0. Max coverage (-): 0

Region: chr24 43046607-43046623. Max. coverage (+): 0. Max coverage (-): 0

Region: chr24 43046624-43046640. Max. coverage (+): 0. Max coverage (-): 0

Region: chr24 43046641-43046657. Max. coverage (+): 0. Max coverage (-): 0

Region: chr24 43046658-43046674. Max. coverage (+): 0. Max coverage (-): 0

Region: chr24 43046675-43046691. Max. coverage (+): 0. Max coverage (-): 0

Region: chr24 43046692-43046708. Max. coverage (+): 0. Max coverage (-): 0

Region: chr24 43046709-43046725. Max. coverage (+): 0. Max coverage (-): 0

Region: chr24 43046726-43046742. Max. coverage (+): 0. Max coverage (-): 0

Region: chr24 43046743-43046759. Max. coverage (+): 0. Max coverage (-): 0

Region: chr24 43046760-43046776. Max. coverage (+): 0. Max coverage (-): 0

Region: chr24 43046777-43046793. Max. coverage (+): 0. Max coverage (-): 0

Region: chr24 43046794-43046810. Max. coverage (+): 0. Max coverage (-): 0

Region: chr24 43046811-43046827. Max. coverage (+): 0. Max coverage (-): 0

Region: chr24 43046828-43046844. Max. coverage (+): 0. Max coverage (-): 0

Region: chr24 43046845-43046861. Max. coverage (+): 0. Max coverage (-): 0

Region: chr24 43046862-43046878. Max. coverage (+): 0. Max coverage (-): 0

Region: chr24 43046879-43046895. Max. coverage (+): 0. Max coverage (-): 0

Region: chr24 43046896-43046912. Max. coverage (+): 0. Max coverage (-): 0

Region: chr24 43046913-43046929. Max. coverage (+): 0. Max coverage (-): 0

Region: chr24 43046930-43046946. Max. coverage (+): 0. Max coverage (-): 0

Region: chr24 43046947-43046963. Max. coverage (+): 0. Max coverage (-): 0

Region: chr24 43046964-43046980. Max. coverage (+): 0. Max coverage (-): 0

Region: chr24 43046981-43046997. Max. coverage (+): 0. Max coverage (-): 0

Region: chr24 43046998-43047014. Max. coverage (+): 0. Max coverage (-): 0

Region: chr24 43047015-43047031. Max. coverage (+): 0. Max coverage (-): 0

Region: chr24 43047032-43047048. Max. coverage (+): 0. Max coverage (-): 0

Region: chr24 43047049-43047065. Max. coverage (+): 0. Max coverage (-): 0

Region: chr24 43047066-43047082. Max. coverage (+): 0. Max coverage (-): 0

Region: chr24 43047083-43047099. Max. coverage (+): 0. Max coverage (-): 0

Region: chr24 43047100-43047116. Max. coverage (+): 0. Max coverage (-): 0

Region: chr24 43047117-43047133. Max. coverage (+): 0. Max coverage (-): 0

Region: chr24 43047134-43047150. Max. coverage (+): 0. Max coverage (-): 0

Region: chr24 43047151-43047167. Max. coverage (+): 0. Max coverage (-): 0

Region: chr24 43047168-43047184. Max. coverage (+): 0. Max coverage (-): 0.19

Region: chr24 43047185-43047201. Max. coverage (+): 0. Max coverage (-): 0

Region: chr24 43047202-43047218. Max. coverage (+): 0. Max coverage (-): 0

Region: chr24 43047219-43047235. Max. coverage (+): 0. Max coverage (-): 0

Region: chr24 43047236-43047252. Max. coverage (+): 0. Max coverage (-): 0

Region: chr24 43047253-43047269. Max. coverage (+): 0. Max coverage (-): 0

Region: chr24 43047270-43047286. Max. coverage (+): 0. Max coverage (-): 1.02

Region: chr24 43047287-43047303. Max. coverage (+): 0. Max coverage (-): 0

Region: chr24 43047304-43047320. Max. coverage (+): 0. Max coverage (-): 0

Region: chr24 43047321-43047337. Max. coverage (+): 0. Max coverage (-): 0

Region: chr24 43047338-43047354. Max. coverage (+): 0. Max coverage (-): 0

Region: chr24 43047355-43047371. Max. coverage (+): 0. Max coverage (-): 0

Region: chr24 43047372-43047388. Max. coverage (+): 0. Max coverage (-): 0

Region: chr24 43047389-43047405. Max. coverage (+): 0. Max coverage (-): 0

Region: chr24 43047406-43047422. Max. coverage (+): 0. Max coverage (-): 0

Region: chr24 43047423-43047439. Max. coverage (+): 0. Max coverage (-): 0

Region: chr24 43047440-43047456. Max. coverage (+): 0. Max coverage (-): 0

Region: chr24 43047457-43047473. Max. coverage (+): 0. Max coverage (-): 0

Region: chr24 43047474-43047490. Max. coverage (+): 0. Max coverage (-): 0

Region: chr24 43047491-43047507. Max. coverage (+): 0. Max coverage (-): 1.7

Region: chr24 43047508-43047524. Max. coverage (+): 0. Max coverage (-): 0

Region: chr24 43047525-43047541. Max. coverage (+): 0. Max coverage (-): 4.15

Region: chr24 43047542-43047558. Max. coverage (+): 0. Max coverage (-): 4.15

Region: chr24 43047559-43047575. Max. coverage (+): 0. Max coverage (-): 0

Region: chr24 43047576-43047592. Max. coverage (+): 0. Max coverage (-): 0

Region: chr24 43047593-43047609. Max. coverage (+): 0. Max coverage (-): 0.26

Region: chr24 43047610-43047626. Max. coverage (+): 0. Max coverage (-): 0.26

Region: chr24 43047627-43047643. Max. coverage (+): 0. Max coverage (-): 0

Region: chr24 43047644-43047660. Max. coverage (+): 0. Max coverage (-): 0

Region: chr24 43047661-43047677. Max. coverage (+): 0. Max coverage (-): 7.27

Region: chr24 43047678-43047694. Max. coverage (+): 0. Max coverage (-): 6.04

Region: chr24 43047695-43047711. Max. coverage (+): 0. Max coverage (-): 6.04

Region: chr24 43047712-43047728. Max. coverage (+): 0. Max coverage (-): 0

Region: chr24 43047729-43047745. Max. coverage (+): 0. Max coverage (-): 0

Region: chr24 43047746-43047762. Max. coverage (+): 0. Max coverage (-): 0

Region: chr24 43047763-43047779. Max. coverage (+): 0. Max coverage (-): 0

Region: chr24 43047780-43047796. Max. coverage (+): 0. Max coverage (-): 3.08

Region: chr24 43047797-43047813. Max. coverage (+): 0. Max coverage (-): 0

Region: chr24 43047814-43047830. Max. coverage (+): 0. Max coverage (-): 0

Region: chr24 43047831-43047847. Max. coverage (+): 0. Max coverage (-): 0

Region: chr24 43047848-43047864. Max. coverage (+): 0. Max coverage (-): 0

Region: chr24 43047865-43047881. Max. coverage (+): 0. Max coverage (-): 0

Region: chr24 43047882-43047898. Max. coverage (+): 0. Max coverage (-): 0

Region: chr24 43047899-43047915. Max. coverage (+): 0. Max coverage (-): 0

Region: chr24 43047916-43047932. Max. coverage (+): 0. Max coverage (-): 0

Region: chr24 43047933-43047949. Max. coverage (+): 0. Max coverage (-): 6.71

Region: chr24 43047950-43047966. Max. coverage (+): 0. Max coverage (-): 0

Region: chr24 43047967-43047983. Max. coverage (+): 0. Max coverage (-): 0

Region: chr24 43047984-43048000. Max. coverage (+): 0. Max coverage (-): 0

Region: chr24 43048001-43048017. Max. coverage (+): 0. Max coverage (-): 11

Region: chr24 43048018-43048034. Max. coverage (+): 0. Max coverage (-): 11

Region: chr24 43048035-43048051. Max. coverage (+): 0. Max coverage (-): 0

Region: chr24 43048052-43048068. Max. coverage (+): 0. Max coverage (-): 0

Region: chr24 43048069-43048085. Max. coverage (+): 0. Max coverage (-): 0

Region: chr24 43048086-43048102. Max. coverage (+): 0. Max coverage (-): 0

Region: chr24 43048103-43048119. Max. coverage (+): 0. Max coverage (-): 0

Region: chr24 43048120-43048136. Max. coverage (+): 0. Max coverage (-): 0

Region: chr24 43048137-43048153. Max. coverage (+): 0. Max coverage (-): 0

Region: chr24 43048154-43048170. Max. coverage (+): 0. Max coverage (-): 0

Region: chr24 43048171-43048187. Max. coverage (+): 0. Max coverage (-): 0

Region: chr24 43048188-43048204. Max. coverage (+): 0. Max coverage (-): 0

Region: chr24 43048205-43048221. Max. coverage (+): 0. Max coverage (-): 0

Region: chr24 43048222-43048238. Max. coverage (+): 0. Max coverage (-): 11.18

Region: chr24 43048239-43048255. Max. coverage (+): 0. Max coverage (-): 19

Region: chr24 43048256-43048272. Max. coverage (+): 0. Max coverage (-): 0

Region: chr24 43048273-43048289. Max. coverage (+): 0. Max coverage (-): 0

Region: chr24 43048290-43048306. Max. coverage (+): 0. Max coverage (-): 0

Region: chr24 43048307-43048323. Max. coverage (+): 0. Max coverage (-): 0

Region: chr24 43048324-43048340. Max. coverage (+): 0. Max coverage (-): 0

Region: chr24 43048341-43048357. Max. coverage (+): 0. Max coverage (-): 0

Region: chr24 43048358-43048374. Max. coverage (+): 0. Max coverage (-): 0

Region: chr24 43048375-43048391. Max. coverage (+): 0. Max coverage (-): 0

Region: chr24 43048392-43048408. Max. coverage (+): 0. Max coverage (-): 0

Region: chr24 43048409-43048425. Max. coverage (+): 0. Max coverage (-): 0

Region: chr24 43048426-43048442. Max. coverage (+): 0. Max coverage (-): 0

Region: chr24 43048443-43048459. Max. coverage (+): 0. Max coverage (-): 0

Region: chr24 43048460-43048476. Max. coverage (+): 0. Max coverage (-): 0

Region: chr24 43048477-43048493. Max. coverage (+): 0. Max coverage (-): 0.93

Region: chr24 43048494-43048510. Max. coverage (+): 0. Max coverage (-): 2.53

Region: chr24 43048511-43048527. Max. coverage (+): 0. Max coverage (-): 0

Region: chr24 43048528-43048544. Max. coverage (+): 0. Max coverage (-): 0

Region: chr24 43048545-43048561. Max. coverage (+): 0. Max coverage (-): 0

Region: chr24 43048562-43048578. Max. coverage (+): 0. Max coverage (-): 0

Region: chr24 43048579-43048595. Max. coverage (+): 0. Max coverage (-): 0

Region: chr24 43048596-43048612. Max. coverage (+): 0. Max coverage (-): 0

Region: chr24 43048613-43048629. Max. coverage (+): 0. Max coverage (-): 0

Region: chr24 43048630-43048646. Max. coverage (+): 0. Max coverage (-): 0

Region: chr24 43048647-43048663. Max. coverage (+): 0. Max coverage (-): 0

Region: chr24 43048664-43048680. Max. coverage (+): 0. Max coverage (-): 0

Region: chr24 43048681-43048697. Max. coverage (+): 0. Max coverage (-): 0

Region: chr24 43048698-43048714. Max. coverage (+): 0. Max coverage (-): 0

Region: chr24 43048715-43048731. Max. coverage (+): 0. Max coverage (-): 0

Region: chr24 43048732-43048748. Max. coverage (+): 0. Max coverage (-): 0

Region: chr24 43048749-43048765. Max. coverage (+): 0. Max coverage (-): 0

Region: chr24 43048766-43048782. Max. coverage (+): 0. Max coverage (-): 0

Region: chr24 43048783-43048799. Max. coverage (+): 0. Max coverage (-): 0

Region: chr24 43048800-43048816. Max. coverage (+): 0. Max coverage (-): 0

Region: chr24 43048817-43048833. Max. coverage (+): 0. Max coverage (-): 0

Region: chr24 43048834-43048850. Max. coverage (+): 0. Max coverage (-): 0

Region: chr24 43048851-43048867. Max. coverage (+): 0. Max coverage (-): 0

Region: chr24 43048868-43048884. Max. coverage (+): 0. Max coverage (-): 0

Region: chr24 43048885-43048901. Max. coverage (+): 0. Max coverage (-): 0

Region: chr24 43048902-43048918. Max. coverage (+): 0. Max coverage (-): 0

Region: chr24 43048919-43048935. Max. coverage (+): 0. Max coverage (-): 3.05

Region: chr24 43048936-43048952. Max. coverage (+): 0. Max coverage (-): 0.36

Region: chr24 43048953-43048969. Max. coverage (+): 0. Max coverage (-): 4.89

Region: chr24 43048970-43048986. Max. coverage (+): 0. Max coverage (-): 0

Region: chr24 43048987-43049003. Max. coverage (+): 0. Max coverage (-): 2.01

Region: chr24 43049004-43049020. Max. coverage (+): 0. Max coverage (-): 14.17

Region: chr24 43049021-43049037. Max. coverage (+): 0. Max coverage (-): 8.7

Region: chr24 43049038-43049054. Max. coverage (+): 0. Max coverage (-): 0

Region: chr24 43049055-43049071. Max. coverage (+): 0. Max coverage (-): 0

Region: chr24 43049072-43049088. Max. coverage (+): 0. Max coverage (-): 0

Region: chr24 43049089-43049105. Max. coverage (+): 0. Max coverage (-): 0

Region: chr24 43049106-43049122. Max. coverage (+): 0. Max coverage (-): 0

Region: chr24 43049123-43049139. Max. coverage (+): 0. Max coverage (-): 0

Region: chr24 43049140-43049156. Max. coverage (+): 0. Max coverage (-): 17.55

Region: chr24 43049157-43049173. Max. coverage (+): 0. Max coverage (-): 0

Region: chr24 43049174-43049190. Max. coverage (+): 0. Max coverage (-): 0

Region: chr24 43049191-43049207. Max. coverage (+): 0. Max coverage (-): 5.38

Region: chr24 43049208-43049224. Max. coverage (+): 0. Max coverage (-): 6.15

Region: chr24 43049225-43049241. Max. coverage (+): 0. Max coverage (-): 15.34

Region: chr24 43049242-43049258. Max. coverage (+): 0. Max coverage (-): 15.34

Region: chr24 43049259-43049275. Max. coverage (+): 0. Max coverage (-): 6.15

Region: chr24 43049276-43049292. Max. coverage (+): 0. Max coverage (-): 9.52

Region: chr24 43049293-43049309. Max. coverage (+): 0. Max coverage (-): 0

Region: chr24 43049310-43049326. Max. coverage (+): 0. Max coverage (-): 6.35

Region: chr24 43049327-43049343. Max. coverage (+): 0. Max coverage (-): 5.88

Region: chr24 43049344-. Max. coverage (+): 0. Max coverage (-): 0

RepeatMasker Color Code

**+**

100-98% Identity

<98-95% Identity

<95-90% Identity

<90-85% Identity

<85-80% Identity

<80-75% Identity

<75-70% Identity

<70% Identity

**-**

Gene Set Color Code

**+**

Gene

Pseudogene

**-**

Topology/Coverage Color Code

Coverage Plus Strand

Coverage Minus Strand

Mainstrand: Plus

Mainstrand: Minus

Complementary Strand

Flanking Region  
(if option -flank >0)

Gene Set Annotation  
  
RepeatMasker Annotation  

**1. MLT1D**: 43041117-43041506 (+), Divergence to consensus: 47.9%  
**2. 5S**: 43041852-43041893 (-), Divergence to consensus: 19.1%  
**3. BovB**: 43042105-43042826 (+), Divergence to consensus: 9.3%  
**4. BTLTR1**: 43042827-43042897 (-), Divergence to consensus: 12.7%  
**5. BovB**: 43042898-43043750 (+), Divergence to consensus: 5.5%  
**6. ART2A**: 43043751-43044252 (+), Divergence to consensus: 15.2%  
**7. (AACTG)n**: 43044253-43044282 (+), Divergence to consensus: 6.7%  
**8. LTR10A\_BT**: 43044283-43044348 (-), Divergence to consensus: 23.4%  
**9. Bov-tA1**: 43044367-43044563 (-), Divergence to consensus: 14.2%  
**10. LTR10A\_BT**: 43044569-43044770 (-), Divergence to consensus: 26.1%  
**11. L1\_Art**: 43044775-43044869 (+), Divergence to consensus: 23.1%  
**12. LTR10A\_BT**: 43044897-43045016 (-), Divergence to consensus: 16.7%  
**13. Bov-tA1**: 43045030-43045224 (-), Divergence to consensus: 28.2%  
**14. LTR10A\_BT**: 43045225-43045516 (-), Divergence to consensus: 18.4%  
**15. AT\_rich**: 43046818-43046838 (+), Divergence to consensus: 52.4%  
**16. Charlie13a**: 43048057-43048239 (+), Divergence to consensus: 40.7%  
**17. Bov-tA2**: 43048275-43048480 (+), Divergence to consensus: 12.6%  
**18. Charlie13a**: 43048575-43048859 (+), Divergence to consensus: 48.2%

  
Transcription Factor Binding Sites  

**RFX4\_1** (Sequence: GTTGCCAGG (-): 43048591)  
**SOX9** (Sequence: AACAATGA (-): 43048549)  
**SOX9** (Sequence: CCATTGTT (+): 43041524)  
**Gata4** (Sequence: CTTATCT (+): 43047438)  
**Gata4** (Sequence: GTTATCT (+): 43048703)  
**Gata4** (Sequence: CTTATCT (+): 43049288)
