# Supplementary material for: Small RNA sequencing of cryopreserved semen from single bull revealed altered miRNAs and piRNAs expression between High- and Low-motile sperm populations
Source: BMC Genomics. 2017 Jan 4;18:14. doi: 10.1186/s12864-016-3394-7 (PMC5209821; doi:10.1186/s12864-016-3394-7)
Supplement: Additional file 4: — Details for each piRNA clusters found in Low Motile (LM) sperm fraction. Genes, repeats, transposable elements and transcription factors binding sites falling within the cluster regions were reported. (ZIP 1034 kb) [file 12864_2016_3394_MOESM4_ESM.zip › 35.html]

piRNA cluster 35


Predicted piRNA cluster no. 35     previous   next
  

Show proTRAC run info
Hide proTRAC run info

================================= proTRAC ====================================  
VERSION: 2.1                                    LAST MODIFIED: 06. October 2015  
  
Please cite:  
Rosenkranz D, Zischler H. proTRAC - a software for probabilistic piRNA cluster  
detection, visualization and analysis. 2012. BMC Bioinformatics 13:5.  
  
and (for proTRAC 2.0 and later):  
Rosenkranz D, Rudloff S, Bastuck K, Ketting RF, Zischler H. Tupaia small RNAs  
provide insights into function and evolution of RNAi-based transposon defense  
in mammals. 2015. RNA 21(5):911-922.  
  
Contact:  
David Rosenkranz  
Institute of Anthropology, small RNA group  
Johannes Gutenberg University Mainz  
email: rosenkranz@uni-mainz.de  
  
You can find the latest proTRAC version at:  
http://sourceforge.net/projects/protrac/files  
http://www.smallRNAgroup-mainz.de/software  
==============================================================================  
  
PARAMETERS:  
Map file: .............../storage/core/barbara/genhome/smallRNA/fertility/Sample\_not\_motile/pirna/Sample\_not\_motile\_26-33\_collapsed.fa.no-dust.map.weighted-10000-1000-b-0  
Genome file: ............/storage/core/barbara/genhome/smallRNA/fertility/Sample\_all/pirna/bt\_311\_chrY.fa  
RepeatMasker annotation: /storage/genomes/bt\_umd31/GCF\_000003055.6\_Bos\_taurus\_UMD\_3.1.1\_repeatMasker\_chr.out  
GeneSet:................./storage/core/barbara/genhome/smallRNA/fertility/Sample\_all/pirna/full.gtf  
  
Significant (p<=0.01) hit density will be calculated based  
on observed hit distribution.  
  
Sliding window size: ........................................ 5000 bp  
Sliding window increament: .................................. 1000 bp  
Normalize each hit by number of genomic hits: ............... 1 [0=no/1=yes]  
Normalize each hit by number of sequence reads: ............. 1 [0=no/1=yes]  
Normalize values (-> per million mapped reads): ............. 1 [0=no/1=yes]  
Min. fraction of hits with 1T(U) or 10A: .................... 0.75  
Alternatively: Min. fraction of hits with 1T(U) and 10A: .... 0.5  
Min. fraction of hits with typical piRNA length: ............ 0.75  
Typical piRNA length: ....................................... 26-33 nt  
Min. size of a piRNA cluster: ............................... 5000 bp.  
Min. number of hits (absolute): ............................. 0  
Min. number of hits (normalized): ........................... 0  
Min. fraction of hits on the mainstrand: .................... 0.75  
Top fraction of mapped sequences (in terms of read counts): . 1%  
Top fraction accounts for max. n% of sequence reads: ........ 90%  
Min. fraction of hits on each arm of a bidirectional cluster: 0.1  
Output image file for each cluster: ......................... 0 [0=no/1=yes]  
Output html file for each cluster: .......................... 1 [0=no/1=yes]  
Output a summary table: ..................................... 1 [0=no/1=yes]  
Output a FASTA file for each cluster (piRNA sequences): ..... 1 [0=no/1=yes]  
Output a FASTA file comprising cluster sequences: ........... 1 [0=no/1=yes]  
Search DNA motifs in clusters: .............................. 1 [0=no/1=yes]  
Output flanking sequences: +/- .............................. 0 bp  
Output ~.pTi file: .......................................... 1 [0=no/1=yes]  
==============================================================================  
  
  
Genome size (without gaps): ............ 2678902517 bp  
Gaps (N/X/-): .......................... 53837044 bp  
Mapped reads: .......................... 738059667487  
Non-identical sequences: ............... 277001  
Genomic hits: .......................... 533816  
Significant densitiy of mapped reads: .. 15118061 reads/kb

Show proTRAC cluster info
Hide proTRAC cluster info

|  |  |
| --- | --- |
| Location | chr24 |
| Coordinates | 43053380-43074695 |
| Size [bp] | 21316 |
| Sequence hit loci | 1520 |
| Mapped reads (normalized) | 3895296549 |
| Mapped reads (normalized) per kb | 182740502.4 |
| Normalized reads with 1T (1U) | 80.9% |
| Normalized reads with 10A | 29.6% |
| Normalized reads with length 26-33 nt | 100% |
| Normalized reads on the main strand(s) | 92% |
| Predicted directionality | mono:minus |

100%

0%

1T (1U)  
reads

10A reads

26-33 nt  
reads

reads on mainstrand

**Either the amount of reads with 1T (1U) OR 10A has to exceed 75% (set with option: -1Tor10A)  
Alternatively the amount of reads with 1T (1U) AND 10A has to exceed 50% (set with option: -1Tand10A)  
Minimum amount of reads with preferred size is 75% (set with option: -pisize)  
Minimum amount of reads on the main strand(s) is 75% (set with option: -clstrand)**

Show read coverage
Hide read coverage

WHAT DO I SEE HERE?  
This chart shows the location of mapped sequence reads within a predicted piRNA cluster. The color refers to the number of genomic hits produced by the sequence read in question. A dark red bar indicates that this sequence read produces many other hits elsewhere in the genome. Many adjacent red or yellow bars can indicate the presence of a multi-copy element such as transposons or rRNA genes. A dark green bar indicates that this sequence read maps uniquely to this locus.

1 hit

2-5 hits

6-10 hits

11-20 hits

21-50 hits

51-100 hits

> 100 hits

chr24

43053380

43074695

Gene Set

RepeatMasker

Mapped  
Reads

91.3

plus strand

minus strand

91.3

Region: chr24 43049345-43053401. Max. coverage (+): 0. Max coverage (-): 14.49

Region: chr24 43053402-43053443. Max. coverage (+): 0. Max coverage (-): 0

Region: chr24 43053444-43053486. Max. coverage (+): 0. Max coverage (-): 0

Region: chr24 43053487-43053529. Max. coverage (+): 0. Max coverage (-): 48.69

Region: chr24 43053530-43053571. Max. coverage (+): 0. Max coverage (-): 19.6

Region: chr24 43053572-43053614. Max. coverage (+): 0. Max coverage (-): 29.96

Region: chr24 43053615-43053657. Max. coverage (+): 0. Max coverage (-): 0

Region: chr24 43053658-43053699. Max. coverage (+): 0. Max coverage (-): 0

Region: chr24 43053700-43053742. Max. coverage (+): 0. Max coverage (-): 15.88

Region: chr24 43053743-43053785. Max. coverage (+): 0. Max coverage (-): 10.9

Region: chr24 43053786-43053827. Max. coverage (+): 0. Max coverage (-): 10.81

Region: chr24 43053828-43053870. Max. coverage (+): 0. Max coverage (-): 0

Region: chr24 43053871-43053912. Max. coverage (+): 0. Max coverage (-): 0

Region: chr24 43053913-43053955. Max. coverage (+): 0. Max coverage (-): 3.15

Region: chr24 43053956-43053998. Max. coverage (+): 0. Max coverage (-): 9.57

Region: chr24 43053999-43054040. Max. coverage (+): 0. Max coverage (-): 3.8

Region: chr24 43054041-43054083. Max. coverage (+): 0. Max coverage (-): 12.04

Region: chr24 43054084-43054126. Max. coverage (+): 0. Max coverage (-): 31.03

Region: chr24 43054127-43054168. Max. coverage (+): 0. Max coverage (-): 57.52

Region: chr24 43054169-43054211. Max. coverage (+): 0. Max coverage (-): 12.76

Region: chr24 43054212-43054253. Max. coverage (+): 0. Max coverage (-): 0

Region: chr24 43054254-43054296. Max. coverage (+): 0. Max coverage (-): 32.24

Region: chr24 43054297-43054339. Max. coverage (+): 0. Max coverage (-): 3.69

Region: chr24 43054340-43054381. Max. coverage (+): 0. Max coverage (-): 6.26

Region: chr24 43054382-43054424. Max. coverage (+): 0. Max coverage (-): 1.07

Region: chr24 43054425-43054467. Max. coverage (+): 0. Max coverage (-): 26.85

Region: chr24 43054468-43054509. Max. coverage (+): 0. Max coverage (-): 26.85

Region: chr24 43054510-43054552. Max. coverage (+): 3.46. Max coverage (-): 17.04

Region: chr24 43054553-43054595. Max. coverage (+): 0. Max coverage (-): 40.96

Region: chr24 43054596-43054637. Max. coverage (+): 0. Max coverage (-): 20.65

Region: chr24 43054638-43054680. Max. coverage (+): 0. Max coverage (-): 7.41

Region: chr24 43054681-43054722. Max. coverage (+): 0. Max coverage (-): 3.12

Region: chr24 43054723-43054765. Max. coverage (+): 0. Max coverage (-): 0

Region: chr24 43054766-43054808. Max. coverage (+): 0. Max coverage (-): 0

Region: chr24 43054809-43054850. Max. coverage (+): 0. Max coverage (-): 0

Region: chr24 43054851-43054893. Max. coverage (+): 0. Max coverage (-): 0

Region: chr24 43054894-43054936. Max. coverage (+): 0. Max coverage (-): 28.98

Region: chr24 43054937-43054978. Max. coverage (+): 0. Max coverage (-): 39.93

Region: chr24 43054979-43055021. Max. coverage (+): 0. Max coverage (-): 4.1

Region: chr24 43055022-43055063. Max. coverage (+): 0. Max coverage (-): 0

Region: chr24 43055064-43055106. Max. coverage (+): 0. Max coverage (-): 27.25

Region: chr24 43055107-43055149. Max. coverage (+): 0. Max coverage (-): 0

Region: chr24 43055150-43055191. Max. coverage (+): 0. Max coverage (-): 0

Region: chr24 43055192-43055234. Max. coverage (+): 0. Max coverage (-): 0

Region: chr24 43055235-43055277. Max. coverage (+): 0. Max coverage (-): 0

Region: chr24 43055278-43055319. Max. coverage (+): 0. Max coverage (-): 0

Region: chr24 43055320-43055362. Max. coverage (+): 0. Max coverage (-): 80.93

Region: chr24 43055363-43055405. Max. coverage (+): 0. Max coverage (-): 56.6

Region: chr24 43055406-43055447. Max. coverage (+): 0. Max coverage (-): 6.3

Region: chr24 43055448-43055490. Max. coverage (+): 0. Max coverage (-): 10.8

Region: chr24 43055491-43055532. Max. coverage (+): 0. Max coverage (-): 0

Region: chr24 43055533-43055575. Max. coverage (+): 0. Max coverage (-): 0

Region: chr24 43055576-43055618. Max. coverage (+): 4.65. Max coverage (-): 13.43

Region: chr24 43055619-43055660. Max. coverage (+): 4.65. Max coverage (-): 71.56

Region: chr24 43055661-43055703. Max. coverage (+): 0. Max coverage (-): 0

Region: chr24 43055704-43055746. Max. coverage (+): 0. Max coverage (-): 0

Region: chr24 43055747-43055788. Max. coverage (+): 0. Max coverage (-): 0

Region: chr24 43055789-43055831. Max. coverage (+): 0. Max coverage (-): 0

Region: chr24 43055832-43055873. Max. coverage (+): 0. Max coverage (-): 36.96

Region: chr24 43055874-43055916. Max. coverage (+): 0. Max coverage (-): 17.55

Region: chr24 43055917-43055959. Max. coverage (+): 0. Max coverage (-): 36.08

Region: chr24 43055960-43056001. Max. coverage (+): 0. Max coverage (-): 21.87

Region: chr24 43056002-43056044. Max. coverage (+): 0. Max coverage (-): 7.37

Region: chr24 43056045-43056087. Max. coverage (+): 0. Max coverage (-): 1.19

Region: chr24 43056088-43056129. Max. coverage (+): 0. Max coverage (-): 15.64

Region: chr24 43056130-43056172. Max. coverage (+): 0. Max coverage (-): 6.04

Region: chr24 43056173-43056215. Max. coverage (+): 0. Max coverage (-): 0

Region: chr24 43056216-43056257. Max. coverage (+): 0. Max coverage (-): 0

Region: chr24 43056258-43056300. Max. coverage (+): 0. Max coverage (-): 0

Region: chr24 43056301-43056342. Max. coverage (+): 0. Max coverage (-): 0

Region: chr24 43056343-43056385. Max. coverage (+): 0. Max coverage (-): 0

Region: chr24 43056386-43056428. Max. coverage (+): 0. Max coverage (-): 4.77

Region: chr24 43056429-43056470. Max. coverage (+): 0. Max coverage (-): 15.22

Region: chr24 43056471-43056513. Max. coverage (+): 0. Max coverage (-): 22.98

Region: chr24 43056514-43056556. Max. coverage (+): 0. Max coverage (-): 86.38

Region: chr24 43056557-43056598. Max. coverage (+): 0. Max coverage (-): 1.8

Region: chr24 43056599-43056641. Max. coverage (+): 0. Max coverage (-): 0

Region: chr24 43056642-43056683. Max. coverage (+): 0. Max coverage (-): 0

Region: chr24 43056684-43056726. Max. coverage (+): 0. Max coverage (-): 0

Region: chr24 43056727-43056769. Max. coverage (+): 0. Max coverage (-): 0

Region: chr24 43056770-43056811. Max. coverage (+): 0. Max coverage (-): 0

Region: chr24 43056812-43056854. Max. coverage (+): 0. Max coverage (-): 0

Region: chr24 43056855-43056897. Max. coverage (+): 0. Max coverage (-): 0

Region: chr24 43056898-43056939. Max. coverage (+): 0. Max coverage (-): 2.27

Region: chr24 43056940-43056982. Max. coverage (+): 0. Max coverage (-): 1.88

Region: chr24 43056983-43057025. Max. coverage (+): 0. Max coverage (-): 1.45

Region: chr24 43057026-43057067. Max. coverage (+): 0. Max coverage (-): 0

Region: chr24 43057068-43057110. Max. coverage (+): 0. Max coverage (-): 4.97

Region: chr24 43057111-43057152. Max. coverage (+): 0. Max coverage (-): 4.56

Region: chr24 43057153-43057195. Max. coverage (+): 0. Max coverage (-): 2.55

Region: chr24 43057196-43057238. Max. coverage (+): 0. Max coverage (-): 0

Region: chr24 43057239-43057280. Max. coverage (+): 0. Max coverage (-): 12.32

Region: chr24 43057281-43057323. Max. coverage (+): 0. Max coverage (-): 6.58

Region: chr24 43057324-43057366. Max. coverage (+): 0. Max coverage (-): 5.74

Region: chr24 43057367-43057408. Max. coverage (+): 0. Max coverage (-): 10.85

Region: chr24 43057409-43057451. Max. coverage (+): 0. Max coverage (-): 2.81

Region: chr24 43057452-43057493. Max. coverage (+): 0. Max coverage (-): 2.81

Region: chr24 43057494-43057536. Max. coverage (+): 0. Max coverage (-): 28.75

Region: chr24 43057537-43057579. Max. coverage (+): 0. Max coverage (-): 4.44

Region: chr24 43057580-43057621. Max. coverage (+): 0. Max coverage (-): 77.78

Region: chr24 43057622-43057664. Max. coverage (+): 0. Max coverage (-): 47.64

Region: chr24 43057665-43057707. Max. coverage (+): 0. Max coverage (-): 33.26

Region: chr24 43057708-43057749. Max. coverage (+): 0. Max coverage (-): 25.88

Region: chr24 43057750-43057792. Max. coverage (+): 0. Max coverage (-): 7.79

Region: chr24 43057793-43057835. Max. coverage (+): 0. Max coverage (-): 10.17

Region: chr24 43057836-43057877. Max. coverage (+): 0. Max coverage (-): 60.51

Region: chr24 43057878-43057920. Max. coverage (+): 0. Max coverage (-): 19.67

Region: chr24 43057921-43057962. Max. coverage (+): 0. Max coverage (-): 0

Region: chr24 43057963-43058005. Max. coverage (+): 0. Max coverage (-): 0

Region: chr24 43058006-43058048. Max. coverage (+): 0. Max coverage (-): 14.63

Region: chr24 43058049-43058090. Max. coverage (+): 0. Max coverage (-): 0

Region: chr24 43058091-43058133. Max. coverage (+): 0. Max coverage (-): 10.07

Region: chr24 43058134-43058176. Max. coverage (+): 0. Max coverage (-): 0

Region: chr24 43058177-43058218. Max. coverage (+): 0. Max coverage (-): 0

Region: chr24 43058219-43058261. Max. coverage (+): 0. Max coverage (-): 1.64

Region: chr24 43058262-43058303. Max. coverage (+): 0. Max coverage (-): 0

Region: chr24 43058304-43058346. Max. coverage (+): 0. Max coverage (-): 0

Region: chr24 43058347-43058389. Max. coverage (+): 0. Max coverage (-): 3

Region: chr24 43058390-43058431. Max. coverage (+): 0. Max coverage (-): 0.41

Region: chr24 43058432-43058474. Max. coverage (+): 0. Max coverage (-): 0.41

Region: chr24 43058475-43058517. Max. coverage (+): 0. Max coverage (-): 6.59

Region: chr24 43058518-43058559. Max. coverage (+): 0. Max coverage (-): 10.65

Region: chr24 43058560-43058602. Max. coverage (+): 0. Max coverage (-): 0

Region: chr24 43058603-43058645. Max. coverage (+): 0. Max coverage (-): 0

Region: chr24 43058646-43058687. Max. coverage (+): 0. Max coverage (-): 0

Region: chr24 43058688-43058730. Max. coverage (+): 0. Max coverage (-): 0

Region: chr24 43058731-43058772. Max. coverage (+): 0. Max coverage (-): 0

Region: chr24 43058773-43058815. Max. coverage (+): 0. Max coverage (-): 0.58

Region: chr24 43058816-43058858. Max. coverage (+): 0. Max coverage (-): 0

Region: chr24 43058859-43058900. Max. coverage (+): 0. Max coverage (-): 0

Region: chr24 43058901-43058943. Max. coverage (+): 0. Max coverage (-): 2.42

Region: chr24 43058944-43058986. Max. coverage (+): 0. Max coverage (-): 0

Region: chr24 43058987-43059028. Max. coverage (+): 0. Max coverage (-): 2.42

Region: chr24 43059029-43059071. Max. coverage (+): 0. Max coverage (-): 0

Region: chr24 43059072-43059114. Max. coverage (+): 0. Max coverage (-): 3.76

Region: chr24 43059115-43059156. Max. coverage (+): 0. Max coverage (-): 0

Region: chr24 43059157-43059199. Max. coverage (+): 0. Max coverage (-): 16.44

Region: chr24 43059200-43059241. Max. coverage (+): 0. Max coverage (-): 18.66

Region: chr24 43059242-43059284. Max. coverage (+): 0. Max coverage (-): 0

Region: chr24 43059285-43059327. Max. coverage (+): 0. Max coverage (-): 0

Region: chr24 43059328-43059369. Max. coverage (+): 0. Max coverage (-): 0

Region: chr24 43059370-43059412. Max. coverage (+): 0. Max coverage (-): 0

Region: chr24 43059413-43059455. Max. coverage (+): 0. Max coverage (-): 7.52

Region: chr24 43059456-43059497. Max. coverage (+): 0. Max coverage (-): 33.46

Region: chr24 43059498-43059540. Max. coverage (+): 0. Max coverage (-): 33.04

Region: chr24 43059541-43059582. Max. coverage (+): 0. Max coverage (-): 0

Region: chr24 43059583-43059625. Max. coverage (+): 0. Max coverage (-): 7.98

Region: chr24 43059626-43059668. Max. coverage (+): 0. Max coverage (-): 0

Region: chr24 43059669-43059710. Max. coverage (+): 0. Max coverage (-): 22.48

Region: chr24 43059711-43059753. Max. coverage (+): 0. Max coverage (-): 0

Region: chr24 43059754-43059796. Max. coverage (+): 0. Max coverage (-): 0

Region: chr24 43059797-43059838. Max. coverage (+): 0. Max coverage (-): 0

Region: chr24 43059839-43059881. Max. coverage (+): 0. Max coverage (-): 11.16

Region: chr24 43059882-43059924. Max. coverage (+): 0. Max coverage (-): 0

Region: chr24 43059925-43059966. Max. coverage (+): 0. Max coverage (-): 23.12

Region: chr24 43059967-43060009. Max. coverage (+): 0. Max coverage (-): 47.97

Region: chr24 43060010-43060051. Max. coverage (+): 0. Max coverage (-): 5.64

Region: chr24 43060052-43060094. Max. coverage (+): 0. Max coverage (-): 16.53

Region: chr24 43060095-43060137. Max. coverage (+): 0. Max coverage (-): 0

Region: chr24 43060138-43060179. Max. coverage (+): 8.22. Max coverage (-): 0

Region: chr24 43060180-43060222. Max. coverage (+): 0. Max coverage (-): 16.67

Region: chr24 43060223-43060265. Max. coverage (+): 1.86. Max coverage (-): 12.75

Region: chr24 43060266-43060307. Max. coverage (+): 0. Max coverage (-): 10.56

Region: chr24 43060308-43060350. Max. coverage (+): 0. Max coverage (-): 14.77

Region: chr24 43060351-43060392. Max. coverage (+): 0. Max coverage (-): 0

Region: chr24 43060393-43060435. Max. coverage (+): 0. Max coverage (-): 0

Region: chr24 43060436-43060478. Max. coverage (+): 4.9. Max coverage (-): 0

Region: chr24 43060479-43060520. Max. coverage (+): 0. Max coverage (-): 6.12

Region: chr24 43060521-43060563. Max. coverage (+): 0. Max coverage (-): 7.37

Region: chr24 43060564-43060606. Max. coverage (+): 0. Max coverage (-): 64.7

Region: chr24 43060607-43060648. Max. coverage (+): 0. Max coverage (-): 2.24

Region: chr24 43060649-43060691. Max. coverage (+): 0. Max coverage (-): 4.79

Region: chr24 43060692-43060734. Max. coverage (+): 0. Max coverage (-): 0.66

Region: chr24 43060735-43060776. Max. coverage (+): 0. Max coverage (-): 23.36

Region: chr24 43060777-43060819. Max. coverage (+): 1.93. Max coverage (-): 32.17

Region: chr24 43060820-43060861. Max. coverage (+): 0. Max coverage (-): 28.45

Region: chr24 43060862-43060904. Max. coverage (+): 0. Max coverage (-): 22.59

Region: chr24 43060905-43060947. Max. coverage (+): 0. Max coverage (-): 28.61

Region: chr24 43060948-43060989. Max. coverage (+): 3.85. Max coverage (-): 13.87

Region: chr24 43060990-43061032. Max. coverage (+): 0. Max coverage (-): 0

Region: chr24 43061033-43061075. Max. coverage (+): 0. Max coverage (-): 0

Region: chr24 43061076-43061117. Max. coverage (+): 0. Max coverage (-): 6.93

Region: chr24 43061118-43061160. Max. coverage (+): 0. Max coverage (-): 64.59

Region: chr24 43061161-43061202. Max. coverage (+): 0. Max coverage (-): 46.99

Region: chr24 43061203-43061245. Max. coverage (+): 0. Max coverage (-): 24.19

Region: chr24 43061246-43061288. Max. coverage (+): 0. Max coverage (-): 23.24

Region: chr24 43061289-43061330. Max. coverage (+): 0. Max coverage (-): 20.5

Region: chr24 43061331-43061373. Max. coverage (+): 0. Max coverage (-): 14.61

Region: chr24 43061374-43061416. Max. coverage (+): 0. Max coverage (-): 0

Region: chr24 43061417-43061458. Max. coverage (+): 0. Max coverage (-): 23.2

Region: chr24 43061459-43061501. Max. coverage (+): 0. Max coverage (-): 14.84

Region: chr24 43061502-43061544. Max. coverage (+): 0. Max coverage (-): 12.18

Region: chr24 43061545-43061586. Max. coverage (+): 0. Max coverage (-): 15.36

Region: chr24 43061587-43061629. Max. coverage (+): 0. Max coverage (-): 17.74

Region: chr24 43061630-43061671. Max. coverage (+): 0. Max coverage (-): 9.94

Region: chr24 43061672-43061714. Max. coverage (+): 0. Max coverage (-): 14.52

Region: chr24 43061715-43061757. Max. coverage (+): 0. Max coverage (-): 6

Region: chr24 43061758-43061799. Max. coverage (+): 0. Max coverage (-): 0

Region: chr24 43061800-43061842. Max. coverage (+): 0. Max coverage (-): 17.7

Region: chr24 43061843-43061885. Max. coverage (+): 0. Max coverage (-): 0

Region: chr24 43061886-43061927. Max. coverage (+): 0. Max coverage (-): 0

Region: chr24 43061928-43061970. Max. coverage (+): 0. Max coverage (-): 0

Region: chr24 43061971-43062012. Max. coverage (+): 0. Max coverage (-): 0

Region: chr24 43062013-43062055. Max. coverage (+): 0. Max coverage (-): 6.32

Region: chr24 43062056-43062098. Max. coverage (+): 0.14. Max coverage (-): 21.62

Region: chr24 43062099-43062140. Max. coverage (+): 0. Max coverage (-): 21.08

Region: chr24 43062141-43062183. Max. coverage (+): 0. Max coverage (-): 19.44

Region: chr24 43062184-43062226. Max. coverage (+): 0. Max coverage (-): 3.9

Region: chr24 43062227-43062268. Max. coverage (+): 0. Max coverage (-): 7.99

Region: chr24 43062269-43062311. Max. coverage (+): 0. Max coverage (-): 2.43

Region: chr24 43062312-43062354. Max. coverage (+): 0. Max coverage (-): 0

Region: chr24 43062355-43062396. Max. coverage (+): 0. Max coverage (-): 0

Region: chr24 43062397-43062439. Max. coverage (+): 0. Max coverage (-): 4.4

Region: chr24 43062440-43062481. Max. coverage (+): 0. Max coverage (-): 0

Region: chr24 43062482-43062524. Max. coverage (+): 0. Max coverage (-): 0

Region: chr24 43062525-43062567. Max. coverage (+): 0. Max coverage (-): 20.45

Region: chr24 43062568-43062609. Max. coverage (+): 0. Max coverage (-): 7.35

Region: chr24 43062610-43062652. Max. coverage (+): 0. Max coverage (-): 10.99

Region: chr24 43062653-43062695. Max. coverage (+): 0. Max coverage (-): 3.23

Region: chr24 43062696-43062737. Max. coverage (+): 0. Max coverage (-): 3.1

Region: chr24 43062738-43062780. Max. coverage (+): 0. Max coverage (-): 23.73

Region: chr24 43062781-43062822. Max. coverage (+): 0. Max coverage (-): 13.34

Region: chr24 43062823-43062865. Max. coverage (+): 0. Max coverage (-): 1.14

Region: chr24 43062866-43062908. Max. coverage (+): 0. Max coverage (-): 5.14

Region: chr24 43062909-43062950. Max. coverage (+): 0. Max coverage (-): 24.85

Region: chr24 43062951-43062993. Max. coverage (+): 0. Max coverage (-): 37.71

Region: chr24 43062994-43063036. Max. coverage (+): 0. Max coverage (-): 0

Region: chr24 43063037-43063078. Max. coverage (+): 0. Max coverage (-): 0

Region: chr24 43063079-43063121. Max. coverage (+): 0. Max coverage (-): 0

Region: chr24 43063122-43063164. Max. coverage (+): 0. Max coverage (-): 0

Region: chr24 43063165-43063206. Max. coverage (+): 0. Max coverage (-): 0

Region: chr24 43063207-43063249. Max. coverage (+): 0. Max coverage (-): 0

Region: chr24 43063250-43063291. Max. coverage (+): 0. Max coverage (-): 4.84

Region: chr24 43063292-43063334. Max. coverage (+): 0. Max coverage (-): 0

Region: chr24 43063335-43063377. Max. coverage (+): 0. Max coverage (-): 0

Region: chr24 43063378-43063419. Max. coverage (+): 0. Max coverage (-): 0

Region: chr24 43063420-43063462. Max. coverage (+): 0. Max coverage (-): 0

Region: chr24 43063463-43063505. Max. coverage (+): 0. Max coverage (-): 11.28

Region: chr24 43063506-43063547. Max. coverage (+): 0. Max coverage (-): 12.73

Region: chr24 43063548-43063590. Max. coverage (+): 0. Max coverage (-): 0

Region: chr24 43063591-43063632. Max. coverage (+): 5.46. Max coverage (-): 0

Region: chr24 43063633-43063675. Max. coverage (+): 0. Max coverage (-): 0

Region: chr24 43063676-43063718. Max. coverage (+): 0. Max coverage (-): 6.71

Region: chr24 43063719-43063760. Max. coverage (+): 0. Max coverage (-): 0

Region: chr24 43063761-43063803. Max. coverage (+): 0. Max coverage (-): 10.97

Region: chr24 43063804-43063846. Max. coverage (+): 0. Max coverage (-): 0

Region: chr24 43063847-43063888. Max. coverage (+): 0. Max coverage (-): 0

Region: chr24 43063889-43063931. Max. coverage (+): 0. Max coverage (-): 5.7

Region: chr24 43063932-43063974. Max. coverage (+): 0. Max coverage (-): 43.61

Region: chr24 43063975-43064016. Max. coverage (+): 0. Max coverage (-): 0.4

Region: chr24 43064017-43064059. Max. coverage (+): 0. Max coverage (-): 0

Region: chr24 43064060-43064101. Max. coverage (+): 0. Max coverage (-): 0

Region: chr24 43064102-43064144. Max. coverage (+): 0. Max coverage (-): 2.92

Region: chr24 43064145-43064187. Max. coverage (+): 0. Max coverage (-): 0.19

Region: chr24 43064188-43064229. Max. coverage (+): 0. Max coverage (-): 25.88

Region: chr24 43064230-43064272. Max. coverage (+): 0. Max coverage (-): 91.3

Region: chr24 43064273-43064315. Max. coverage (+): 0. Max coverage (-): 0

Region: chr24 43064316-43064357. Max. coverage (+): 0. Max coverage (-): 0

Region: chr24 43064358-43064400. Max. coverage (+): 0. Max coverage (-): 4.44

Region: chr24 43064401-43064443. Max. coverage (+): 0. Max coverage (-): 7.93

Region: chr24 43064444-43064485. Max. coverage (+): 0. Max coverage (-): 1.5

Region: chr24 43064486-43064528. Max. coverage (+): 0. Max coverage (-): 0

Region: chr24 43064529-43064570. Max. coverage (+): 0. Max coverage (-): 0

Region: chr24 43064571-43064613. Max. coverage (+): 0. Max coverage (-): 0

Region: chr24 43064614-43064656. Max. coverage (+): 0. Max coverage (-): 0

Region: chr24 43064657-43064698. Max. coverage (+): 0. Max coverage (-): 0

Region: chr24 43064699-43064741. Max. coverage (+): 0. Max coverage (-): 3.01

Region: chr24 43064742-43064784. Max. coverage (+): 0. Max coverage (-): 2.01

Region: chr24 43064785-43064826. Max. coverage (+): 0. Max coverage (-): 15.18

Region: chr24 43064827-43064869. Max. coverage (+): 0. Max coverage (-): 16.63

Region: chr24 43064870-43064911. Max. coverage (+): 0. Max coverage (-): 0

Region: chr24 43064912-43064954. Max. coverage (+): 0. Max coverage (-): 0

Region: chr24 43064955-43064997. Max. coverage (+): 0. Max coverage (-): 1.5

Region: chr24 43064998-43065039. Max. coverage (+): 0. Max coverage (-): 0

Region: chr24 43065040-43065082. Max. coverage (+): 0. Max coverage (-): 19.1

Region: chr24 43065083-43065125. Max. coverage (+): 0. Max coverage (-): 0

Region: chr24 43065126-43065167. Max. coverage (+): 0. Max coverage (-): 6.95

Region: chr24 43065168-43065210. Max. coverage (+): 0. Max coverage (-): 20.14

Region: chr24 43065211-43065253. Max. coverage (+): 0. Max coverage (-): 8.65

Region: chr24 43065254-43065295. Max. coverage (+): 0. Max coverage (-): 8.65

Region: chr24 43065296-43065338. Max. coverage (+): 0. Max coverage (-): 4.44

Region: chr24 43065339-43065380. Max. coverage (+): 0. Max coverage (-): 29.68

Region: chr24 43065381-43065423. Max. coverage (+): 0. Max coverage (-): 17.51

Region: chr24 43065424-43065466. Max. coverage (+): 0. Max coverage (-): 0

Region: chr24 43065467-43065508. Max. coverage (+): 0. Max coverage (-): 0

Region: chr24 43065509-43065551. Max. coverage (+): 0. Max coverage (-): 18.64

Region: chr24 43065552-43065594. Max. coverage (+): 0. Max coverage (-): 27.73

Region: chr24 43065595-43065636. Max. coverage (+): 0. Max coverage (-): 27.56

Region: chr24 43065637-43065679. Max. coverage (+): 0. Max coverage (-): 13.93

Region: chr24 43065680-43065721. Max. coverage (+): 0. Max coverage (-): 14.33

Region: chr24 43065722-43065764. Max. coverage (+): 0. Max coverage (-): 3.33

Region: chr24 43065765-43065807. Max. coverage (+): 0. Max coverage (-): 10.99

Region: chr24 43065808-43065849. Max. coverage (+): 0. Max coverage (-): 0

Region: chr24 43065850-43065892. Max. coverage (+): 0. Max coverage (-): 0

Region: chr24 43065893-43065935. Max. coverage (+): 0. Max coverage (-): 0

Region: chr24 43065936-43065977. Max. coverage (+): 0. Max coverage (-): 0

Region: chr24 43065978-43066020. Max. coverage (+): 0. Max coverage (-): 0

Region: chr24 43066021-43066063. Max. coverage (+): 0. Max coverage (-): 0

Region: chr24 43066064-43066105. Max. coverage (+): 0. Max coverage (-): 0

Region: chr24 43066106-43066148. Max. coverage (+): 0. Max coverage (-): 0

Region: chr24 43066149-43066190. Max. coverage (+): 0. Max coverage (-): 0

Region: chr24 43066191-43066233. Max. coverage (+): 0. Max coverage (-): 3.35

Region: chr24 43066234-43066276. Max. coverage (+): 0. Max coverage (-): 3.35

Region: chr24 43066277-43066318. Max. coverage (+): 0. Max coverage (-): 0

Region: chr24 43066319-43066361. Max. coverage (+): 0. Max coverage (-): 0

Region: chr24 43066362-43066404. Max. coverage (+): 0. Max coverage (-): 3.5

Region: chr24 43066405-43066446. Max. coverage (+): 0. Max coverage (-): 0

Region: chr24 43066447-43066489. Max. coverage (+): 0. Max coverage (-): 0

Region: chr24 43066490-43066531. Max. coverage (+): 0. Max coverage (-): 0

Region: chr24 43066532-43066574. Max. coverage (+): 0. Max coverage (-): 0

Region: chr24 43066575-43066617. Max. coverage (+): 0. Max coverage (-): 0

Region: chr24 43066618-43066659. Max. coverage (+): 0. Max coverage (-): 0

Region: chr24 43066660-43066702. Max. coverage (+): 0. Max coverage (-): 0

Region: chr24 43066703-43066745. Max. coverage (+): 0. Max coverage (-): 0

Region: chr24 43066746-43066787. Max. coverage (+): 0. Max coverage (-): 0

Region: chr24 43066788-43066830. Max. coverage (+): 0. Max coverage (-): 0

Region: chr24 43066831-43066873. Max. coverage (+): 2.64. Max coverage (-): 5.74

Region: chr24 43066874-43066915. Max. coverage (+): 5.24. Max coverage (-): 6.28

Region: chr24 43066916-43066958. Max. coverage (+): 0. Max coverage (-): 13.65

Region: chr24 43066959-43067000. Max. coverage (+): 0. Max coverage (-): 15.89

Region: chr24 43067001-43067043. Max. coverage (+): 0. Max coverage (-): 0

Region: chr24 43067044-43067086. Max. coverage (+): 0. Max coverage (-): 0

Region: chr24 43067087-43067128. Max. coverage (+): 0. Max coverage (-): 0

Region: chr24 43067129-43067171. Max. coverage (+): 0. Max coverage (-): 0

Region: chr24 43067172-43067214. Max. coverage (+): 0. Max coverage (-): 0

Region: chr24 43067215-43067256. Max. coverage (+): 0. Max coverage (-): 0

Region: chr24 43067257-43067299. Max. coverage (+): 0. Max coverage (-): 16.35

Region: chr24 43067300-43067341. Max. coverage (+): 0. Max coverage (-): 4.25

Region: chr24 43067342-43067384. Max. coverage (+): 0. Max coverage (-): 3.27

Region: chr24 43067385-43067427. Max. coverage (+): 0. Max coverage (-): 0

Region: chr24 43067428-43067469. Max. coverage (+): 0. Max coverage (-): 17.93

Region: chr24 43067470-43067512. Max. coverage (+): 5.44. Max coverage (-): 4.44

Region: chr24 43067513-43067555. Max. coverage (+): 0. Max coverage (-): 8.45

Region: chr24 43067556-43067597. Max. coverage (+): 0. Max coverage (-): 0

Region: chr24 43067598-43067640. Max. coverage (+): 7.45. Max coverage (-): 0.55

Region: chr24 43067641-43067683. Max. coverage (+): 0. Max coverage (-): 5

Region: chr24 43067684-43067725. Max. coverage (+): 0. Max coverage (-): 0

Region: chr24 43067726-43067768. Max. coverage (+): 0. Max coverage (-): 0

Region: chr24 43067769-43067810. Max. coverage (+): 0. Max coverage (-): 3.47

Region: chr24 43067811-43067853. Max. coverage (+): 0. Max coverage (-): 22.34

Region: chr24 43067854-43067896. Max. coverage (+): 0. Max coverage (-): 48.52

Region: chr24 43067897-43067938. Max. coverage (+): 0. Max coverage (-): 35.91

Region: chr24 43067939-43067981. Max. coverage (+): 0. Max coverage (-): 37.24

Region: chr24 43067982-43068024. Max. coverage (+): 0. Max coverage (-): 0

Region: chr24 43068025-43068066. Max. coverage (+): 0. Max coverage (-): 6.2

Region: chr24 43068067-43068109. Max. coverage (+): 0. Max coverage (-): 10.62

Region: chr24 43068110-43068151. Max. coverage (+): 0. Max coverage (-): 4.04

Region: chr24 43068152-43068194. Max. coverage (+): 0. Max coverage (-): 3.54

Region: chr24 43068195-43068237. Max. coverage (+): 0. Max coverage (-): 25.91

Region: chr24 43068238-43068279. Max. coverage (+): 0. Max coverage (-): 17.26

Region: chr24 43068280-43068322. Max. coverage (+): 0. Max coverage (-): 6.63

Region: chr24 43068323-43068365. Max. coverage (+): 3.68. Max coverage (-): 0

Region: chr24 43068366-43068407. Max. coverage (+): 0. Max coverage (-): 6.01

Region: chr24 43068408-43068450. Max. coverage (+): 0. Max coverage (-): 5.53

Region: chr24 43068451-43068493. Max. coverage (+): 0. Max coverage (-): 4.07

Region: chr24 43068494-43068535. Max. coverage (+): 0. Max coverage (-): 0.65

Region: chr24 43068536-43068578. Max. coverage (+): 0. Max coverage (-): 0

Region: chr24 43068579-43068620. Max. coverage (+): 0. Max coverage (-): 4.07

Region: chr24 43068621-43068663. Max. coverage (+): 0. Max coverage (-): 5.39

Region: chr24 43068664-43068706. Max. coverage (+): 3.28. Max coverage (-): 3.97

Region: chr24 43068707-43068748. Max. coverage (+): 0. Max coverage (-): 10.34

Region: chr24 43068749-43068791. Max. coverage (+): 0. Max coverage (-): 59.41

Region: chr24 43068792-43068834. Max. coverage (+): 0. Max coverage (-): 9.52

Region: chr24 43068835-43068876. Max. coverage (+): 0. Max coverage (-): 6.32

Region: chr24 43068877-43068919. Max. coverage (+): 0. Max coverage (-): 7.53

Region: chr24 43068920-43068961. Max. coverage (+): 0. Max coverage (-): 0

Region: chr24 43068962-43069004. Max. coverage (+): 0. Max coverage (-): 10.5

Region: chr24 43069005-43069047. Max. coverage (+): 0. Max coverage (-): 67.29

Region: chr24 43069048-43069089. Max. coverage (+): 0. Max coverage (-): 72.88

Region: chr24 43069090-43069132. Max. coverage (+): 0. Max coverage (-): 0

Region: chr24 43069133-43069175. Max. coverage (+): 0. Max coverage (-): 0

Region: chr24 43069176-43069217. Max. coverage (+): 0. Max coverage (-): 26.73

Region: chr24 43069218-43069260. Max. coverage (+): 0. Max coverage (-): 30.5

Region: chr24 43069261-43069303. Max. coverage (+): 0. Max coverage (-): 1.98

Region: chr24 43069304-43069345. Max. coverage (+): 0. Max coverage (-): 25.68

Region: chr24 43069346-43069388. Max. coverage (+): 0. Max coverage (-): 5.94

Region: chr24 43069389-43069430. Max. coverage (+): 0. Max coverage (-): 18.98

Region: chr24 43069431-43069473. Max. coverage (+): 0. Max coverage (-): 0

Region: chr24 43069474-43069516. Max. coverage (+): 6.04. Max coverage (-): 28.72

Region: chr24 43069517-43069558. Max. coverage (+): 0. Max coverage (-): 5.98

Region: chr24 43069559-43069601. Max. coverage (+): 0. Max coverage (-): 17.34

Region: chr24 43069602-43069644. Max. coverage (+): 3.13. Max coverage (-): 0

Region: chr24 43069645-43069686. Max. coverage (+): 2.08. Max coverage (-): 13.73

Region: chr24 43069687-43069729. Max. coverage (+): 0. Max coverage (-): 16.98

Region: chr24 43069730-43069772. Max. coverage (+): 0. Max coverage (-): 0

Region: chr24 43069773-43069814. Max. coverage (+): 0. Max coverage (-): 0

Region: chr24 43069815-43069857. Max. coverage (+): 0. Max coverage (-): 0

Region: chr24 43069858-43069899. Max. coverage (+): 0. Max coverage (-): 0

Region: chr24 43069900-43069942. Max. coverage (+): 0. Max coverage (-): 5.21

Region: chr24 43069943-43069985. Max. coverage (+): 0. Max coverage (-): 0

Region: chr24 43069986-43070027. Max. coverage (+): 0. Max coverage (-): 0

Region: chr24 43070028-43070070. Max. coverage (+): 0. Max coverage (-): 6.22

Region: chr24 43070071-43070113. Max. coverage (+): 0. Max coverage (-): 6.22

Region: chr24 43070114-43070155. Max. coverage (+): 0. Max coverage (-): 1.37

Region: chr24 43070156-43070198. Max. coverage (+): 0. Max coverage (-): 5.22

Region: chr24 43070199-43070240. Max. coverage (+): 4.81. Max coverage (-): 5.22

Region: chr24 43070241-43070283. Max. coverage (+): 5.64. Max coverage (-): 6.72

Region: chr24 43070284-43070326. Max. coverage (+): 0. Max coverage (-): 0

Region: chr24 43070327-43070368. Max. coverage (+): 0. Max coverage (-): 3.05

Region: chr24 43070369-43070411. Max. coverage (+): 3.29. Max coverage (-): 0

Region: chr24 43070412-43070454. Max. coverage (+): 3.29. Max coverage (-): 0

Region: chr24 43070455-43070496. Max. coverage (+): 4.46. Max coverage (-): 0

Region: chr24 43070497-43070539. Max. coverage (+): 0. Max coverage (-): 0

Region: chr24 43070540-43070582. Max. coverage (+): 0. Max coverage (-): 1.56

Region: chr24 43070583-43070624. Max. coverage (+): 3.2. Max coverage (-): 3.73

Region: chr24 43070625-43070667. Max. coverage (+): 0. Max coverage (-): 3.73

Region: chr24 43070668-43070709. Max. coverage (+): 6.04. Max coverage (-): 0.19

Region: chr24 43070710-43070752. Max. coverage (+): 4.04. Max coverage (-): 0

Region: chr24 43070753-43070795. Max. coverage (+): 0. Max coverage (-): 0

Region: chr24 43070796-43070837. Max. coverage (+): 0. Max coverage (-): 1.57

Region: chr24 43070838-43070880. Max. coverage (+): 13.58. Max coverage (-): 2.17

Region: chr24 43070881-43070923. Max. coverage (+): 0. Max coverage (-): 0

Region: chr24 43070924-43070965. Max. coverage (+): 0. Max coverage (-): 0

Region: chr24 43070966-43071008. Max. coverage (+): 11.06. Max coverage (-): 0

Region: chr24 43071009-43071050. Max. coverage (+): 23.13. Max coverage (-): 10.99

Region: chr24 43071051-43071093. Max. coverage (+): 0. Max coverage (-): 0

Region: chr24 43071094-43071136. Max. coverage (+): 0. Max coverage (-): 0

Region: chr24 43071137-43071178. Max. coverage (+): 26.77. Max coverage (-): 5.17

Region: chr24 43071179-43071221. Max. coverage (+): 12.41. Max coverage (-): 0

Region: chr24 43071222-43071264. Max. coverage (+): 0. Max coverage (-): 0

Region: chr24 43071265-43071306. Max. coverage (+): 0. Max coverage (-): 0

Region: chr24 43071307-43071349. Max. coverage (+): 0. Max coverage (-): 0

Region: chr24 43071350-43071392. Max. coverage (+): 0. Max coverage (-): 0

Region: chr24 43071393-43071434. Max. coverage (+): 0. Max coverage (-): 0

Region: chr24 43071435-43071477. Max. coverage (+): 11.79. Max coverage (-): 0

Region: chr24 43071478-43071519. Max. coverage (+): 18.57. Max coverage (-): 0

Region: chr24 43071520-43071562. Max. coverage (+): 0. Max coverage (-): 0

Region: chr24 43071563-43071605. Max. coverage (+): 0. Max coverage (-): 0

Region: chr24 43071606-43071647. Max. coverage (+): 0. Max coverage (-): 0

Region: chr24 43071648-43071690. Max. coverage (+): 0. Max coverage (-): 0

Region: chr24 43071691-43071733. Max. coverage (+): 21.81. Max coverage (-): 0

Region: chr24 43071734-43071775. Max. coverage (+): 34.26. Max coverage (-): 0

Region: chr24 43071776-43071818. Max. coverage (+): 11.64. Max coverage (-): 0

Region: chr24 43071819-43071860. Max. coverage (+): 0. Max coverage (-): 0

Region: chr24 43071861-43071903. Max. coverage (+): 0. Max coverage (-): 0

Region: chr24 43071904-43071946. Max. coverage (+): 4.17. Max coverage (-): 0

Region: chr24 43071947-43071988. Max. coverage (+): 0. Max coverage (-): 2.06

Region: chr24 43071989-43072031. Max. coverage (+): 0. Max coverage (-): 0

Region: chr24 43072032-43072074. Max. coverage (+): 0. Max coverage (-): 0

Region: chr24 43072075-43072116. Max. coverage (+): 0. Max coverage (-): 0

Region: chr24 43072117-43072159. Max. coverage (+): 0. Max coverage (-): 0

Region: chr24 43072160-43072202. Max. coverage (+): 0. Max coverage (-): 0

Region: chr24 43072203-43072244. Max. coverage (+): 0. Max coverage (-): 0

Region: chr24 43072245-43072287. Max. coverage (+): 5.68. Max coverage (-): 0

Region: chr24 43072288-43072329. Max. coverage (+): 1.57. Max coverage (-): 0

Region: chr24 43072330-43072372. Max. coverage (+): 0. Max coverage (-): 0

Region: chr24 43072373-43072415. Max. coverage (+): 0. Max coverage (-): 0

Region: chr24 43072416-43072457. Max. coverage (+): 9.27. Max coverage (-): 0

Region: chr24 43072458-43072500. Max. coverage (+): 0. Max coverage (-): 0

Region: chr24 43072501-43072543. Max. coverage (+): 0. Max coverage (-): 0

Region: chr24 43072544-43072585. Max. coverage (+): 0. Max coverage (-): 0

Region: chr24 43072586-43072628. Max. coverage (+): 0. Max coverage (-): 0

Region: chr24 43072629-43072670. Max. coverage (+): 0. Max coverage (-): 6.54

Region: chr24 43072671-43072713. Max. coverage (+): 0. Max coverage (-): 0

Region: chr24 43072714-43072756. Max. coverage (+): 0. Max coverage (-): 0

Region: chr24 43072757-43072798. Max. coverage (+): 0. Max coverage (-): 0

Region: chr24 43072799-43072841. Max. coverage (+): 0. Max coverage (-): 0

Region: chr24 43072842-43072884. Max. coverage (+): 0. Max coverage (-): 0

Region: chr24 43072885-43072926. Max. coverage (+): 5.25. Max coverage (-): 0

Region: chr24 43072927-43072969. Max. coverage (+): 3.94. Max coverage (-): 0

Region: chr24 43072970-43073012. Max. coverage (+): 0.04. Max coverage (-): 0

Region: chr24 43073013-43073054. Max. coverage (+): 0. Max coverage (-): 0

Region: chr24 43073055-43073097. Max. coverage (+): 13.92. Max coverage (-): 0

Region: chr24 43073098-43073139. Max. coverage (+): 0. Max coverage (-): 0

Region: chr24 43073140-43073182. Max. coverage (+): 2.25. Max coverage (-): 0

Region: chr24 43073183-43073225. Max. coverage (+): 3.41. Max coverage (-): 0

Region: chr24 43073226-43073267. Max. coverage (+): 0. Max coverage (-): 0

Region: chr24 43073268-43073310. Max. coverage (+): 20.91. Max coverage (-): 0

Region: chr24 43073311-43073353. Max. coverage (+): 21. Max coverage (-): 0

Region: chr24 43073354-43073395. Max. coverage (+): 0. Max coverage (-): 0

Region: chr24 43073396-43073438. Max. coverage (+): 1.55. Max coverage (-): 0

Region: chr24 43073439-43073480. Max. coverage (+): 2.64. Max coverage (-): 0

Region: chr24 43073481-43073523. Max. coverage (+): 0. Max coverage (-): 0

Region: chr24 43073524-43073566. Max. coverage (+): 0. Max coverage (-): 0

Region: chr24 43073567-43073608. Max. coverage (+): 0. Max coverage (-): 0

Region: chr24 43073609-43073651. Max. coverage (+): 0. Max coverage (-): 0

Region: chr24 43073652-43073694. Max. coverage (+): 0. Max coverage (-): 0

Region: chr24 43073695-43073736. Max. coverage (+): 0. Max coverage (-): 0

Region: chr24 43073737-43073779. Max. coverage (+): 0. Max coverage (-): 0

Region: chr24 43073780-43073822. Max. coverage (+): 0. Max coverage (-): 0

Region: chr24 43073823-43073864. Max. coverage (+): 0. Max coverage (-): 0

Region: chr24 43073865-43073907. Max. coverage (+): 0. Max coverage (-): 0

Region: chr24 43073908-43073949. Max. coverage (+): 0. Max coverage (-): 0

Region: chr24 43073950-43073992. Max. coverage (+): 0. Max coverage (-): 0

Region: chr24 43073993-43074035. Max. coverage (+): 0. Max coverage (-): 0

Region: chr24 43074036-43074077. Max. coverage (+): 0. Max coverage (-): 0

Region: chr24 43074078-43074120. Max. coverage (+): 0. Max coverage (-): 0

Region: chr24 43074121-43074163. Max. coverage (+): 0. Max coverage (-): 0

Region: chr24 43074164-43074205. Max. coverage (+): 3.88. Max coverage (-): 0

Region: chr24 43074206-43074248. Max. coverage (+): 0. Max coverage (-): 0

Region: chr24 43074249-43074290. Max. coverage (+): 1.44. Max coverage (-): 0

Region: chr24 43074291-43074333. Max. coverage (+): 0. Max coverage (-): 0

Region: chr24 43074334-43074376. Max. coverage (+): 3.7. Max coverage (-): 0

Region: chr24 43074377-43074418. Max. coverage (+): 0. Max coverage (-): 0

Region: chr24 43074419-43074461. Max. coverage (+): 0. Max coverage (-): 0

Region: chr24 43074462-43074504. Max. coverage (+): 0. Max coverage (-): 0

Region: chr24 43074505-43074546. Max. coverage (+): 2.04. Max coverage (-): 0

Region: chr24 43074547-43074589. Max. coverage (+): 0. Max coverage (-): 0

Region: chr24 43074590-43074632. Max. coverage (+): 0. Max coverage (-): 0

Region: chr24 43074633-43074674. Max. coverage (+): 5.27. Max coverage (-): 0

Region: chr24 43074675-. Max. coverage (+): 0. Max coverage (-): 0

RepeatMasker Color Code

**+**

100-98% Identity

<98-95% Identity

<95-90% Identity

<90-85% Identity

<85-80% Identity

<80-75% Identity

<75-70% Identity

<70% Identity

**-**

Gene Set Color Code

**+**

Gene

Pseudogene

**-**

Topology/Coverage Color Code

Coverage Plus Strand

Coverage Minus Strand

Mainstrand: Plus

Mainstrand: Minus

Complementary Strand

Flanking Region  
(if option -flank >0)

Gene Set Annotation  
  
RepeatMasker Annotation  

**1. L1M5**: 43054729-43055089 (+), Divergence to consensus: 44.2%  
**2. MamRep38**: 43055155-43055239 (-), Divergence to consensus: 24.1%  
**3. MLT1F**: 43055659-43055889 (+), Divergence to consensus: 36.9%  
**4. MLT1F-int**: 43056202-43056402 (+), Divergence to consensus: 40.2%  
**5. L1\_BT**: 43056583-43056744 (+), Divergence to consensus: 17.3%  
**6. MLT1F-int**: 43056760-43056913 (+), Divergence to consensus: 42%  
**7. MIR3**: 43058808-43058865 (-), Divergence to consensus: 24.1%  
**8. L1M3**: 43059250-43059397 (-), Divergence to consensus: 20.4%  
**9. (TCCCC)n**: 43059791-43059819 (+), Divergence to consensus: 6.9%  
**10. MIRc**: 43063027-43063082 (-), Divergence to consensus: 32.1%  
**11. ART2A**: 43065904-43066205 (+), Divergence to consensus: 18.7%  
**12. Bov-tA2**: 43066418-43066599 (+), Divergence to consensus: 24.7%  
**13. (CACTT)n**: 43066601-43066628 (+), Divergence to consensus: 0%  
**14. MLT1K**: 43067026-43067250 (+), Divergence to consensus: 43%  
**15. LTR16A2**: 43072672-43072774 (+), Divergence to consensus: 28.1%  
**16. SINE2-3\_BT**: 43072815-43072874 (+), Divergence to consensus: 26.7%  
**17. MER63B**: 43073025-43073135 (-), Divergence to consensus: 32.7%  
**18. L2b**: 43073489-43073771 (-), Divergence to consensus: 41.2%  
**19. L2b**: 43073813-43074136 (-), Divergence to consensus: 48.9%

  
Transcription Factor Binding Sites  

**RFX4\_2** (Sequence: GTAACCATG (-): 43057874)  
**RFX4\_2** (Sequence: CTTGGTTAC (+): 43065675)  
**Gata4** (Sequence: AGATAAG (-): 43064756)  
**SOX9** (Sequence: AACAATGG (-): 43057333)  
**SOX9** (Sequence: CCATTGTT (+): 43063980)  
**A-MYB** (Sequence: CCAACTGCCT (-): 43070409)
