# Supplementary material for: Small RNA sequencing of cryopreserved semen from single bull revealed altered miRNAs and piRNAs expression between High- and Low-motile sperm populations
Source: BMC Genomics. 2017 Jan 4;18:14. doi: 10.1186/s12864-016-3394-7 (PMC5209821; doi:10.1186/s12864-016-3394-7)
Supplement: Additional file 4: — Details for each piRNA clusters found in Low Motile (LM) sperm fraction. Genes, repeats, transposable elements and transcription factors binding sites falling within the cluster regions were reported. (ZIP 1034 kb) [file 12864_2016_3394_MOESM4_ESM.zip › 36.html]

piRNA cluster 36


Predicted piRNA cluster no. 36     previous   next
  

Show proTRAC run info
Hide proTRAC run info

================================= proTRAC ====================================  
VERSION: 2.1                                    LAST MODIFIED: 06. October 2015  
  
Please cite:  
Rosenkranz D, Zischler H. proTRAC - a software for probabilistic piRNA cluster  
detection, visualization and analysis. 2012. BMC Bioinformatics 13:5.  
  
and (for proTRAC 2.0 and later):  
Rosenkranz D, Rudloff S, Bastuck K, Ketting RF, Zischler H. Tupaia small RNAs  
provide insights into function and evolution of RNAi-based transposon defense  
in mammals. 2015. RNA 21(5):911-922.  
  
Contact:  
David Rosenkranz  
Institute of Anthropology, small RNA group  
Johannes Gutenberg University Mainz  
email: rosenkranz@uni-mainz.de  
  
You can find the latest proTRAC version at:  
http://sourceforge.net/projects/protrac/files  
http://www.smallRNAgroup-mainz.de/software  
==============================================================================  
  
PARAMETERS:  
Map file: .............../storage/core/barbara/genhome/smallRNA/fertility/Sample\_not\_motile/pirna/Sample\_not\_motile\_26-33\_collapsed.fa.no-dust.map.weighted-10000-1000-b-0  
Genome file: ............/storage/core/barbara/genhome/smallRNA/fertility/Sample\_all/pirna/bt\_311\_chrY.fa  
RepeatMasker annotation: /storage/genomes/bt\_umd31/GCF\_000003055.6\_Bos\_taurus\_UMD\_3.1.1\_repeatMasker\_chr.out  
GeneSet:................./storage/core/barbara/genhome/smallRNA/fertility/Sample\_all/pirna/full.gtf  
  
Significant (p<=0.01) hit density will be calculated based  
on observed hit distribution.  
  
Sliding window size: ........................................ 5000 bp  
Sliding window increament: .................................. 1000 bp  
Normalize each hit by number of genomic hits: ............... 1 [0=no/1=yes]  
Normalize each hit by number of sequence reads: ............. 1 [0=no/1=yes]  
Normalize values (-> per million mapped reads): ............. 1 [0=no/1=yes]  
Min. fraction of hits with 1T(U) or 10A: .................... 0.75  
Alternatively: Min. fraction of hits with 1T(U) and 10A: .... 0.5  
Min. fraction of hits with typical piRNA length: ............ 0.75  
Typical piRNA length: ....................................... 26-33 nt  
Min. size of a piRNA cluster: ............................... 5000 bp.  
Min. number of hits (absolute): ............................. 0  
Min. number of hits (normalized): ........................... 0  
Min. fraction of hits on the mainstrand: .................... 0.75  
Top fraction of mapped sequences (in terms of read counts): . 1%  
Top fraction accounts for max. n% of sequence reads: ........ 90%  
Min. fraction of hits on each arm of a bidirectional cluster: 0.1  
Output image file for each cluster: ......................... 0 [0=no/1=yes]  
Output html file for each cluster: .......................... 1 [0=no/1=yes]  
Output a summary table: ..................................... 1 [0=no/1=yes]  
Output a FASTA file for each cluster (piRNA sequences): ..... 1 [0=no/1=yes]  
Output a FASTA file comprising cluster sequences: ........... 1 [0=no/1=yes]  
Search DNA motifs in clusters: .............................. 1 [0=no/1=yes]  
Output flanking sequences: +/- .............................. 0 bp  
Output ~.pTi file: .......................................... 1 [0=no/1=yes]  
==============================================================================  
  
  
Genome size (without gaps): ............ 2678902517 bp  
Gaps (N/X/-): .......................... 53837044 bp  
Mapped reads: .......................... 738059667487  
Non-identical sequences: ............... 277001  
Genomic hits: .......................... 533816  
Significant densitiy of mapped reads: .. 15118061 reads/kb

Show proTRAC cluster info
Hide proTRAC cluster info

|  |  |
| --- | --- |
| Location | chr25 |
| Coordinates | 29348932-29354852 |
| Size [bp] | 5921 |
| Sequence hit loci | 56 |
| Mapped reads (normalized) | 132540808 |
| Mapped reads (normalized) per kb | 22384868.8 |
| Normalized reads with 1T (1U) | 86.3% |
| Normalized reads with 10A | 28.4% |
| Normalized reads with length 26-33 nt | 100% |
| Normalized reads on the main strand(s) | 97.7% |
| Predicted directionality | mono:plus |

100%

0%

1T (1U)  
reads

10A reads

26-33 nt  
reads

reads on mainstrand

**Either the amount of reads with 1T (1U) OR 10A has to exceed 75% (set with option: -1Tor10A)  
Alternatively the amount of reads with 1T (1U) AND 10A has to exceed 50% (set with option: -1Tand10A)  
Minimum amount of reads with preferred size is 75% (set with option: -pisize)  
Minimum amount of reads on the main strand(s) is 75% (set with option: -clstrand)**

Show read coverage
Hide read coverage

WHAT DO I SEE HERE?  
This chart shows the location of mapped sequence reads within a predicted piRNA cluster. The color refers to the number of genomic hits produced by the sequence read in question. A dark red bar indicates that this sequence read produces many other hits elsewhere in the genome. Many adjacent red or yellow bars can indicate the presence of a multi-copy element such as transposons or rRNA genes. A dark green bar indicates that this sequence read maps uniquely to this locus.

1 hit

2-5 hits

6-10 hits

11-20 hits

21-50 hits

51-100 hits

> 100 hits

chr25

29348932

29354852

Gene Set

RepeatMasker

Mapped  
Reads

27.55

plus strand

minus strand

27.55

Region: chr25 43074676-29348937. Max. coverage (+): 1.1. Max coverage (-): 0

Region: chr25 29348938-29348949. Max. coverage (+): 1.1. Max coverage (-): 0

Region: chr25 29348950-29348961. Max. coverage (+): 0. Max coverage (-): 0

Region: chr25 29348962-29348973. Max. coverage (+): 0. Max coverage (-): 0

Region: chr25 29348974-29348985. Max. coverage (+): 0. Max coverage (-): 0

Region: chr25 29348986-29348997. Max. coverage (+): 0. Max coverage (-): 0

Region: chr25 29348998-29349008. Max. coverage (+): 0. Max coverage (-): 0

Region: chr25 29349009-29349020. Max. coverage (+): 0. Max coverage (-): 0

Region: chr25 29349021-29349032. Max. coverage (+): 0. Max coverage (-): 0

Region: chr25 29349033-29349044. Max. coverage (+): 0. Max coverage (-): 0

Region: chr25 29349045-29349056. Max. coverage (+): 0. Max coverage (-): 0

Region: chr25 29349057-29349068. Max. coverage (+): 0. Max coverage (-): 0

Region: chr25 29349069-29349080. Max. coverage (+): 0. Max coverage (-): 0

Region: chr25 29349081-29349091. Max. coverage (+): 0. Max coverage (-): 0

Region: chr25 29349092-29349103. Max. coverage (+): 0. Max coverage (-): 0

Region: chr25 29349104-29349115. Max. coverage (+): 0. Max coverage (-): 0

Region: chr25 29349116-29349127. Max. coverage (+): 0. Max coverage (-): 0

Region: chr25 29349128-29349139. Max. coverage (+): 0. Max coverage (-): 0

Region: chr25 29349140-29349151. Max. coverage (+): 0. Max coverage (-): 0

Region: chr25 29349152-29349162. Max. coverage (+): 0. Max coverage (-): 0

Region: chr25 29349163-29349174. Max. coverage (+): 0. Max coverage (-): 0

Region: chr25 29349175-29349186. Max. coverage (+): 0. Max coverage (-): 0

Region: chr25 29349187-29349198. Max. coverage (+): 0. Max coverage (-): 0

Region: chr25 29349199-29349210. Max. coverage (+): 0. Max coverage (-): 0

Region: chr25 29349211-29349222. Max. coverage (+): 0. Max coverage (-): 0

Region: chr25 29349223-29349233. Max. coverage (+): 0. Max coverage (-): 0

Region: chr25 29349234-29349245. Max. coverage (+): 0. Max coverage (-): 0

Region: chr25 29349246-29349257. Max. coverage (+): 0. Max coverage (-): 0

Region: chr25 29349258-29349269. Max. coverage (+): 0. Max coverage (-): 0

Region: chr25 29349270-29349281. Max. coverage (+): 0. Max coverage (-): 0

Region: chr25 29349282-29349293. Max. coverage (+): 3.47. Max coverage (-): 0

Region: chr25 29349294-29349305. Max. coverage (+): 0. Max coverage (-): 0

Region: chr25 29349306-29349316. Max. coverage (+): 0. Max coverage (-): 0

Region: chr25 29349317-29349328. Max. coverage (+): 0. Max coverage (-): 0

Region: chr25 29349329-29349340. Max. coverage (+): 0. Max coverage (-): 0

Region: chr25 29349341-29349352. Max. coverage (+): 0. Max coverage (-): 0

Region: chr25 29349353-29349364. Max. coverage (+): 0. Max coverage (-): 0

Region: chr25 29349365-29349376. Max. coverage (+): 0. Max coverage (-): 0

Region: chr25 29349377-29349387. Max. coverage (+): 0. Max coverage (-): 0

Region: chr25 29349388-29349399. Max. coverage (+): 0. Max coverage (-): 0

Region: chr25 29349400-29349411. Max. coverage (+): 0. Max coverage (-): 0

Region: chr25 29349412-29349423. Max. coverage (+): 0. Max coverage (-): 0

Region: chr25 29349424-29349435. Max. coverage (+): 0. Max coverage (-): 0

Region: chr25 29349436-29349447. Max. coverage (+): 0. Max coverage (-): 0

Region: chr25 29349448-29349458. Max. coverage (+): 0. Max coverage (-): 0

Region: chr25 29349459-29349470. Max. coverage (+): 0. Max coverage (-): 0

Region: chr25 29349471-29349482. Max. coverage (+): 0. Max coverage (-): 0

Region: chr25 29349483-29349494. Max. coverage (+): 0. Max coverage (-): 0

Region: chr25 29349495-29349506. Max. coverage (+): 0. Max coverage (-): 0

Region: chr25 29349507-29349518. Max. coverage (+): 0. Max coverage (-): 0

Region: chr25 29349519-29349530. Max. coverage (+): 0. Max coverage (-): 0

Region: chr25 29349531-29349541. Max. coverage (+): 0. Max coverage (-): 0

Region: chr25 29349542-29349553. Max. coverage (+): 0. Max coverage (-): 0

Region: chr25 29349554-29349565. Max. coverage (+): 0. Max coverage (-): 0

Region: chr25 29349566-29349577. Max. coverage (+): 0. Max coverage (-): 0

Region: chr25 29349578-29349589. Max. coverage (+): 0. Max coverage (-): 0

Region: chr25 29349590-29349601. Max. coverage (+): 0. Max coverage (-): 0

Region: chr25 29349602-29349612. Max. coverage (+): 0. Max coverage (-): 0

Region: chr25 29349613-29349624. Max. coverage (+): 0. Max coverage (-): 0

Region: chr25 29349625-29349636. Max. coverage (+): 0. Max coverage (-): 0

Region: chr25 29349637-29349648. Max. coverage (+): 0. Max coverage (-): 0

Region: chr25 29349649-29349660. Max. coverage (+): 0. Max coverage (-): 0

Region: chr25 29349661-29349672. Max. coverage (+): 0. Max coverage (-): 0

Region: chr25 29349673-29349683. Max. coverage (+): 0. Max coverage (-): 0

Region: chr25 29349684-29349695. Max. coverage (+): 0. Max coverage (-): 0

Region: chr25 29349696-29349707. Max. coverage (+): 0. Max coverage (-): 0

Region: chr25 29349708-29349719. Max. coverage (+): 0. Max coverage (-): 0

Region: chr25 29349720-29349731. Max. coverage (+): 0. Max coverage (-): 0

Region: chr25 29349732-29349743. Max. coverage (+): 0. Max coverage (-): 0

Region: chr25 29349744-29349755. Max. coverage (+): 0. Max coverage (-): 0

Region: chr25 29349756-29349766. Max. coverage (+): 0. Max coverage (-): 0

Region: chr25 29349767-29349778. Max. coverage (+): 0. Max coverage (-): 0

Region: chr25 29349779-29349790. Max. coverage (+): 0. Max coverage (-): 0

Region: chr25 29349791-29349802. Max. coverage (+): 12. Max coverage (-): 0

Region: chr25 29349803-29349814. Max. coverage (+): 0. Max coverage (-): 0

Region: chr25 29349815-29349826. Max. coverage (+): 0. Max coverage (-): 0

Region: chr25 29349827-29349837. Max. coverage (+): 0. Max coverage (-): 0

Region: chr25 29349838-29349849. Max. coverage (+): 0. Max coverage (-): 0

Region: chr25 29349850-29349861. Max. coverage (+): 0. Max coverage (-): 0

Region: chr25 29349862-29349873. Max. coverage (+): 0. Max coverage (-): 0

Region: chr25 29349874-29349885. Max. coverage (+): 3.39. Max coverage (-): 0

Region: chr25 29349886-29349897. Max. coverage (+): 3.39. Max coverage (-): 0

Region: chr25 29349898-29349908. Max. coverage (+): 2.41. Max coverage (-): 0

Region: chr25 29349909-29349920. Max. coverage (+): 2.41. Max coverage (-): 0

Region: chr25 29349921-29349932. Max. coverage (+): 0. Max coverage (-): 0

Region: chr25 29349933-29349944. Max. coverage (+): 0. Max coverage (-): 0

Region: chr25 29349945-29349956. Max. coverage (+): 0. Max coverage (-): 0

Region: chr25 29349957-29349968. Max. coverage (+): 0. Max coverage (-): 0

Region: chr25 29349969-29349980. Max. coverage (+): 0. Max coverage (-): 0

Region: chr25 29349981-29349991. Max. coverage (+): 0. Max coverage (-): 0

Region: chr25 29349992-29350003. Max. coverage (+): 0. Max coverage (-): 0

Region: chr25 29350004-29350015. Max. coverage (+): 0. Max coverage (-): 0

Region: chr25 29350016-29350027. Max. coverage (+): 0. Max coverage (-): 0

Region: chr25 29350028-29350039. Max. coverage (+): 0. Max coverage (-): 0

Region: chr25 29350040-29350051. Max. coverage (+): 0. Max coverage (-): 0

Region: chr25 29350052-29350062. Max. coverage (+): 0. Max coverage (-): 0

Region: chr25 29350063-29350074. Max. coverage (+): 0. Max coverage (-): 0

Region: chr25 29350075-29350086. Max. coverage (+): 0. Max coverage (-): 0

Region: chr25 29350087-29350098. Max. coverage (+): 0. Max coverage (-): 0

Region: chr25 29350099-29350110. Max. coverage (+): 0. Max coverage (-): 0

Region: chr25 29350111-29350122. Max. coverage (+): 0. Max coverage (-): 0

Region: chr25 29350123-29350133. Max. coverage (+): 0. Max coverage (-): 0

Region: chr25 29350134-29350145. Max. coverage (+): 0. Max coverage (-): 0

Region: chr25 29350146-29350157. Max. coverage (+): 0. Max coverage (-): 0

Region: chr25 29350158-29350169. Max. coverage (+): 0. Max coverage (-): 0

Region: chr25 29350170-29350181. Max. coverage (+): 0. Max coverage (-): 0

Region: chr25 29350182-29350193. Max. coverage (+): 0. Max coverage (-): 0

Region: chr25 29350194-29350205. Max. coverage (+): 4.92. Max coverage (-): 0

Region: chr25 29350206-29350216. Max. coverage (+): 0. Max coverage (-): 0

Region: chr25 29350217-29350228. Max. coverage (+): 0. Max coverage (-): 0

Region: chr25 29350229-29350240. Max. coverage (+): 0. Max coverage (-): 0

Region: chr25 29350241-29350252. Max. coverage (+): 0. Max coverage (-): 0

Region: chr25 29350253-29350264. Max. coverage (+): 3.36. Max coverage (-): 0

Region: chr25 29350265-29350276. Max. coverage (+): 0. Max coverage (-): 0

Region: chr25 29350277-29350287. Max. coverage (+): 0. Max coverage (-): 0

Region: chr25 29350288-29350299. Max. coverage (+): 0. Max coverage (-): 0

Region: chr25 29350300-29350311. Max. coverage (+): 0. Max coverage (-): 0

Region: chr25 29350312-29350323. Max. coverage (+): 0. Max coverage (-): 0

Region: chr25 29350324-29350335. Max. coverage (+): 0. Max coverage (-): 0

Region: chr25 29350336-29350347. Max. coverage (+): 0. Max coverage (-): 0

Region: chr25 29350348-29350358. Max. coverage (+): 0. Max coverage (-): 0

Region: chr25 29350359-29350370. Max. coverage (+): 0. Max coverage (-): 0

Region: chr25 29350371-29350382. Max. coverage (+): 0. Max coverage (-): 0

Region: chr25 29350383-29350394. Max. coverage (+): 5.36. Max coverage (-): 0

Region: chr25 29350395-29350406. Max. coverage (+): 0. Max coverage (-): 0

Region: chr25 29350407-29350418. Max. coverage (+): 0. Max coverage (-): 0

Region: chr25 29350419-29350430. Max. coverage (+): 0. Max coverage (-): 0

Region: chr25 29350431-29350441. Max. coverage (+): 0. Max coverage (-): 0

Region: chr25 29350442-29350453. Max. coverage (+): 0. Max coverage (-): 0

Region: chr25 29350454-29350465. Max. coverage (+): 0. Max coverage (-): 0

Region: chr25 29350466-29350477. Max. coverage (+): 0. Max coverage (-): 0

Region: chr25 29350478-29350489. Max. coverage (+): 0. Max coverage (-): 0

Region: chr25 29350490-29350501. Max. coverage (+): 4.85. Max coverage (-): 0

Region: chr25 29350502-29350512. Max. coverage (+): 4.85. Max coverage (-): 0

Region: chr25 29350513-29350524. Max. coverage (+): 5.11. Max coverage (-): 0

Region: chr25 29350525-29350536. Max. coverage (+): 5.11. Max coverage (-): 0

Region: chr25 29350537-29350548. Max. coverage (+): 0. Max coverage (-): 0

Region: chr25 29350549-29350560. Max. coverage (+): 0. Max coverage (-): 0

Region: chr25 29350561-29350572. Max. coverage (+): 0. Max coverage (-): 0

Region: chr25 29350573-29350583. Max. coverage (+): 0. Max coverage (-): 0

Region: chr25 29350584-29350595. Max. coverage (+): 0. Max coverage (-): 0

Region: chr25 29350596-29350607. Max. coverage (+): 0. Max coverage (-): 0

Region: chr25 29350608-29350619. Max. coverage (+): 0. Max coverage (-): 0

Region: chr25 29350620-29350631. Max. coverage (+): 0. Max coverage (-): 0

Region: chr25 29350632-29350643. Max. coverage (+): 0. Max coverage (-): 0

Region: chr25 29350644-29350655. Max. coverage (+): 0. Max coverage (-): 0

Region: chr25 29350656-29350666. Max. coverage (+): 0. Max coverage (-): 0

Region: chr25 29350667-29350678. Max. coverage (+): 0. Max coverage (-): 0

Region: chr25 29350679-29350690. Max. coverage (+): 0. Max coverage (-): 0

Region: chr25 29350691-29350702. Max. coverage (+): 0. Max coverage (-): 0

Region: chr25 29350703-29350714. Max. coverage (+): 0. Max coverage (-): 0

Region: chr25 29350715-29350726. Max. coverage (+): 12.57. Max coverage (-): 0

Region: chr25 29350727-29350737. Max. coverage (+): 12.57. Max coverage (-): 0

Region: chr25 29350738-29350749. Max. coverage (+): 0. Max coverage (-): 0

Region: chr25 29350750-29350761. Max. coverage (+): 0. Max coverage (-): 0

Region: chr25 29350762-29350773. Max. coverage (+): 0. Max coverage (-): 0

Region: chr25 29350774-29350785. Max. coverage (+): 0. Max coverage (-): 0

Region: chr25 29350786-29350797. Max. coverage (+): 0. Max coverage (-): 0

Region: chr25 29350798-29350808. Max. coverage (+): 0. Max coverage (-): 0

Region: chr25 29350809-29350820. Max. coverage (+): 0. Max coverage (-): 0

Region: chr25 29350821-29350832. Max. coverage (+): 0. Max coverage (-): 0

Region: chr25 29350833-29350844. Max. coverage (+): 0. Max coverage (-): 0

Region: chr25 29350845-29350856. Max. coverage (+): 0. Max coverage (-): 0

Region: chr25 29350857-29350868. Max. coverage (+): 0. Max coverage (-): 0

Region: chr25 29350869-29350880. Max. coverage (+): 0. Max coverage (-): 0

Region: chr25 29350881-29350891. Max. coverage (+): 0. Max coverage (-): 0

Region: chr25 29350892-29350903. Max. coverage (+): 0. Max coverage (-): 0

Region: chr25 29350904-29350915. Max. coverage (+): 0. Max coverage (-): 0

Region: chr25 29350916-29350927. Max. coverage (+): 0. Max coverage (-): 0

Region: chr25 29350928-29350939. Max. coverage (+): 0. Max coverage (-): 0

Region: chr25 29350940-29350951. Max. coverage (+): 0. Max coverage (-): 0

Region: chr25 29350952-29350962. Max. coverage (+): 0. Max coverage (-): 0

Region: chr25 29350963-29350974. Max. coverage (+): 0. Max coverage (-): 0

Region: chr25 29350975-29350986. Max. coverage (+): 0. Max coverage (-): 0

Region: chr25 29350987-29350998. Max. coverage (+): 0. Max coverage (-): 0

Region: chr25 29350999-29351010. Max. coverage (+): 0. Max coverage (-): 0

Region: chr25 29351011-29351022. Max. coverage (+): 6.12. Max coverage (-): 0

Region: chr25 29351023-29351033. Max. coverage (+): 7.67. Max coverage (-): 0

Region: chr25 29351034-29351045. Max. coverage (+): 0. Max coverage (-): 0

Region: chr25 29351046-29351057. Max. coverage (+): 0. Max coverage (-): 0

Region: chr25 29351058-29351069. Max. coverage (+): 0.33. Max coverage (-): 0

Region: chr25 29351070-29351081. Max. coverage (+): 0. Max coverage (-): 0

Region: chr25 29351082-29351093. Max. coverage (+): 0. Max coverage (-): 0

Region: chr25 29351094-29351105. Max. coverage (+): 0. Max coverage (-): 0

Region: chr25 29351106-29351116. Max. coverage (+): 0. Max coverage (-): 0

Region: chr25 29351117-29351128. Max. coverage (+): 0. Max coverage (-): 0

Region: chr25 29351129-29351140. Max. coverage (+): 0. Max coverage (-): 0

Region: chr25 29351141-29351152. Max. coverage (+): 1.79. Max coverage (-): 0

Region: chr25 29351153-29351164. Max. coverage (+): 1.79. Max coverage (-): 0

Region: chr25 29351165-29351176. Max. coverage (+): 0. Max coverage (-): 0

Region: chr25 29351177-29351187. Max. coverage (+): 0. Max coverage (-): 0

Region: chr25 29351188-29351199. Max. coverage (+): 0. Max coverage (-): 0

Region: chr25 29351200-29351211. Max. coverage (+): 0. Max coverage (-): 0

Region: chr25 29351212-29351223. Max. coverage (+): 0. Max coverage (-): 0

Region: chr25 29351224-29351235. Max. coverage (+): 0. Max coverage (-): 0

Region: chr25 29351236-29351247. Max. coverage (+): 0. Max coverage (-): 0

Region: chr25 29351248-29351258. Max. coverage (+): 0. Max coverage (-): 0

Region: chr25 29351259-29351270. Max. coverage (+): 0. Max coverage (-): 0

Region: chr25 29351271-29351282. Max. coverage (+): 0. Max coverage (-): 0

Region: chr25 29351283-29351294. Max. coverage (+): 4.47. Max coverage (-): 0

Region: chr25 29351295-29351306. Max. coverage (+): 4.47. Max coverage (-): 0

Region: chr25 29351307-29351318. Max. coverage (+): 0. Max coverage (-): 0

Region: chr25 29351319-29351330. Max. coverage (+): 0. Max coverage (-): 0

Region: chr25 29351331-29351341. Max. coverage (+): 0. Max coverage (-): 0

Region: chr25 29351342-29351353. Max. coverage (+): 0. Max coverage (-): 0

Region: chr25 29351354-29351365. Max. coverage (+): 0. Max coverage (-): 0

Region: chr25 29351366-29351377. Max. coverage (+): 0. Max coverage (-): 0

Region: chr25 29351378-29351389. Max. coverage (+): 0. Max coverage (-): 0

Region: chr25 29351390-29351401. Max. coverage (+): 0. Max coverage (-): 0

Region: chr25 29351402-29351412. Max. coverage (+): 0. Max coverage (-): 0

Region: chr25 29351413-29351424. Max. coverage (+): 0. Max coverage (-): 0

Region: chr25 29351425-29351436. Max. coverage (+): 0. Max coverage (-): 0

Region: chr25 29351437-29351448. Max. coverage (+): 0. Max coverage (-): 0

Region: chr25 29351449-29351460. Max. coverage (+): 0. Max coverage (-): 0

Region: chr25 29351461-29351472. Max. coverage (+): 0. Max coverage (-): 0

Region: chr25 29351473-29351483. Max. coverage (+): 0. Max coverage (-): 0

Region: chr25 29351484-29351495. Max. coverage (+): 0. Max coverage (-): 0

Region: chr25 29351496-29351507. Max. coverage (+): 0. Max coverage (-): 0

Region: chr25 29351508-29351519. Max. coverage (+): 0. Max coverage (-): 0

Region: chr25 29351520-29351531. Max. coverage (+): 0. Max coverage (-): 0

Region: chr25 29351532-29351543. Max. coverage (+): 0. Max coverage (-): 0

Region: chr25 29351544-29351555. Max. coverage (+): 0. Max coverage (-): 0

Region: chr25 29351556-29351566. Max. coverage (+): 0. Max coverage (-): 0

Region: chr25 29351567-29351578. Max. coverage (+): 0. Max coverage (-): 0

Region: chr25 29351579-29351590. Max. coverage (+): 0. Max coverage (-): 0

Region: chr25 29351591-29351602. Max. coverage (+): 0. Max coverage (-): 0

Region: chr25 29351603-29351614. Max. coverage (+): 3.94. Max coverage (-): 0

Region: chr25 29351615-29351626. Max. coverage (+): 3.94. Max coverage (-): 0

Region: chr25 29351627-29351637. Max. coverage (+): 0. Max coverage (-): 0

Region: chr25 29351638-29351649. Max. coverage (+): 0. Max coverage (-): 0

Region: chr25 29351650-29351661. Max. coverage (+): 0. Max coverage (-): 0

Region: chr25 29351662-29351673. Max. coverage (+): 0. Max coverage (-): 0

Region: chr25 29351674-29351685. Max. coverage (+): 0. Max coverage (-): 0

Region: chr25 29351686-29351697. Max. coverage (+): 0. Max coverage (-): 0

Region: chr25 29351698-29351708. Max. coverage (+): 0. Max coverage (-): 0

Region: chr25 29351709-29351720. Max. coverage (+): 0. Max coverage (-): 0

Region: chr25 29351721-29351732. Max. coverage (+): 0. Max coverage (-): 0

Region: chr25 29351733-29351744. Max. coverage (+): 0. Max coverage (-): 0

Region: chr25 29351745-29351756. Max. coverage (+): 0. Max coverage (-): 0

Region: chr25 29351757-29351768. Max. coverage (+): 0. Max coverage (-): 0

Region: chr25 29351769-29351780. Max. coverage (+): 0. Max coverage (-): 0

Region: chr25 29351781-29351791. Max. coverage (+): 0. Max coverage (-): 0

Region: chr25 29351792-29351803. Max. coverage (+): 0. Max coverage (-): 0

Region: chr25 29351804-29351815. Max. coverage (+): 0. Max coverage (-): 0

Region: chr25 29351816-29351827. Max. coverage (+): 0. Max coverage (-): 0

Region: chr25 29351828-29351839. Max. coverage (+): 0. Max coverage (-): 0

Region: chr25 29351840-29351851. Max. coverage (+): 0. Max coverage (-): 0

Region: chr25 29351852-29351862. Max. coverage (+): 0. Max coverage (-): 0

Region: chr25 29351863-29351874. Max. coverage (+): 0. Max coverage (-): 0

Region: chr25 29351875-29351886. Max. coverage (+): 0. Max coverage (-): 0

Region: chr25 29351887-29351898. Max. coverage (+): 0. Max coverage (-): 0

Region: chr25 29351899-29351910. Max. coverage (+): 0. Max coverage (-): 0

Region: chr25 29351911-29351922. Max. coverage (+): 0. Max coverage (-): 0

Region: chr25 29351923-29351933. Max. coverage (+): 0. Max coverage (-): 0

Region: chr25 29351934-29351945. Max. coverage (+): 0. Max coverage (-): 0

Region: chr25 29351946-29351957. Max. coverage (+): 0. Max coverage (-): 0

Region: chr25 29351958-29351969. Max. coverage (+): 0. Max coverage (-): 0

Region: chr25 29351970-29351981. Max. coverage (+): 0. Max coverage (-): 0

Region: chr25 29351982-29351993. Max. coverage (+): 5.24. Max coverage (-): 0

Region: chr25 29351994-29352004. Max. coverage (+): 5.24. Max coverage (-): 0

Region: chr25 29352005-29352016. Max. coverage (+): 0. Max coverage (-): 0

Region: chr25 29352017-29352028. Max. coverage (+): 0. Max coverage (-): 0

Region: chr25 29352029-29352040. Max. coverage (+): 0. Max coverage (-): 0

Region: chr25 29352041-29352052. Max. coverage (+): 0. Max coverage (-): 0

Region: chr25 29352053-29352064. Max. coverage (+): 0. Max coverage (-): 0

Region: chr25 29352065-29352076. Max. coverage (+): 0. Max coverage (-): 0

Region: chr25 29352077-29352087. Max. coverage (+): 0. Max coverage (-): 0

Region: chr25 29352088-29352099. Max. coverage (+): 0. Max coverage (-): 0

Region: chr25 29352100-29352111. Max. coverage (+): 0. Max coverage (-): 0

Region: chr25 29352112-29352123. Max. coverage (+): 0. Max coverage (-): 0

Region: chr25 29352124-29352135. Max. coverage (+): 0. Max coverage (-): 0

Region: chr25 29352136-29352147. Max. coverage (+): 0. Max coverage (-): 0

Region: chr25 29352148-29352158. Max. coverage (+): 0. Max coverage (-): 0

Region: chr25 29352159-29352170. Max. coverage (+): 0. Max coverage (-): 0

Region: chr25 29352171-29352182. Max. coverage (+): 0. Max coverage (-): 0

Region: chr25 29352183-29352194. Max. coverage (+): 0. Max coverage (-): 0

Region: chr25 29352195-29352206. Max. coverage (+): 6.43. Max coverage (-): 0

Region: chr25 29352207-29352218. Max. coverage (+): 6.43. Max coverage (-): 0

Region: chr25 29352219-29352229. Max. coverage (+): 0. Max coverage (-): 0

Region: chr25 29352230-29352241. Max. coverage (+): 0. Max coverage (-): 0

Region: chr25 29352242-29352253. Max. coverage (+): 0. Max coverage (-): 0

Region: chr25 29352254-29352265. Max. coverage (+): 0. Max coverage (-): 0

Region: chr25 29352266-29352277. Max. coverage (+): 0. Max coverage (-): 0

Region: chr25 29352278-29352289. Max. coverage (+): 0. Max coverage (-): 0

Region: chr25 29352290-29352301. Max. coverage (+): 0. Max coverage (-): 0

Region: chr25 29352302-29352312. Max. coverage (+): 0. Max coverage (-): 4.06

Region: chr25 29352313-29352324. Max. coverage (+): 0. Max coverage (-): 0

Region: chr25 29352325-29352336. Max. coverage (+): 0. Max coverage (-): 0

Region: chr25 29352337-29352348. Max. coverage (+): 2.09. Max coverage (-): 0

Region: chr25 29352349-29352360. Max. coverage (+): 2.09. Max coverage (-): 0

Region: chr25 29352361-29352372. Max. coverage (+): 0. Max coverage (-): 0

Region: chr25 29352373-29352383. Max. coverage (+): 0. Max coverage (-): 0

Region: chr25 29352384-29352395. Max. coverage (+): 0. Max coverage (-): 0

Region: chr25 29352396-29352407. Max. coverage (+): 0. Max coverage (-): 0

Region: chr25 29352408-29352419. Max. coverage (+): 8.06. Max coverage (-): 0

Region: chr25 29352420-29352431. Max. coverage (+): 3.6. Max coverage (-): 0

Region: chr25 29352432-29352443. Max. coverage (+): 0. Max coverage (-): 0

Region: chr25 29352444-29352454. Max. coverage (+): 0. Max coverage (-): 0

Region: chr25 29352455-29352466. Max. coverage (+): 0. Max coverage (-): 0

Region: chr25 29352467-29352478. Max. coverage (+): 0. Max coverage (-): 0

Region: chr25 29352479-29352490. Max. coverage (+): 1.88. Max coverage (-): 0

Region: chr25 29352491-29352502. Max. coverage (+): 0. Max coverage (-): 0

Region: chr25 29352503-29352514. Max. coverage (+): 0. Max coverage (-): 0

Region: chr25 29352515-29352526. Max. coverage (+): 0. Max coverage (-): 0

Region: chr25 29352527-29352537. Max. coverage (+): 0. Max coverage (-): 0

Region: chr25 29352538-29352549. Max. coverage (+): 0. Max coverage (-): 0

Region: chr25 29352550-29352561. Max. coverage (+): 0. Max coverage (-): 0

Region: chr25 29352562-29352573. Max. coverage (+): 0. Max coverage (-): 0

Region: chr25 29352574-29352585. Max. coverage (+): 0. Max coverage (-): 0

Region: chr25 29352586-29352597. Max. coverage (+): 2.43. Max coverage (-): 0

Region: chr25 29352598-29352608. Max. coverage (+): 0. Max coverage (-): 0

Region: chr25 29352609-29352620. Max. coverage (+): 0. Max coverage (-): 0

Region: chr25 29352621-29352632. Max. coverage (+): 0. Max coverage (-): 0

Region: chr25 29352633-29352644. Max. coverage (+): 0. Max coverage (-): 0

Region: chr25 29352645-29352656. Max. coverage (+): 0. Max coverage (-): 0

Region: chr25 29352657-29352668. Max. coverage (+): 0. Max coverage (-): 0

Region: chr25 29352669-29352679. Max. coverage (+): 0. Max coverage (-): 0

Region: chr25 29352680-29352691. Max. coverage (+): 0. Max coverage (-): 0

Region: chr25 29352692-29352703. Max. coverage (+): 0. Max coverage (-): 0

Region: chr25 29352704-29352715. Max. coverage (+): 0. Max coverage (-): 0

Region: chr25 29352716-29352727. Max. coverage (+): 1.55. Max coverage (-): 0

Region: chr25 29352728-29352739. Max. coverage (+): 1.55. Max coverage (-): 0

Region: chr25 29352740-29352751. Max. coverage (+): 0. Max coverage (-): 0

Region: chr25 29352752-29352762. Max. coverage (+): 0. Max coverage (-): 0

Region: chr25 29352763-29352774. Max. coverage (+): 0. Max coverage (-): 0

Region: chr25 29352775-29352786. Max. coverage (+): 0. Max coverage (-): 0

Region: chr25 29352787-29352798. Max. coverage (+): 0. Max coverage (-): 0

Region: chr25 29352799-29352810. Max. coverage (+): 0. Max coverage (-): 0

Region: chr25 29352811-29352822. Max. coverage (+): 3.56. Max coverage (-): 0

Region: chr25 29352823-29352833. Max. coverage (+): 3.56. Max coverage (-): 0

Region: chr25 29352834-29352845. Max. coverage (+): 0. Max coverage (-): 0

Region: chr25 29352846-29352857. Max. coverage (+): 0. Max coverage (-): 0

Region: chr25 29352858-29352869. Max. coverage (+): 0. Max coverage (-): 0

Region: chr25 29352870-29352881. Max. coverage (+): 0. Max coverage (-): 0

Region: chr25 29352882-29352893. Max. coverage (+): 0. Max coverage (-): 0

Region: chr25 29352894-29352904. Max. coverage (+): 0. Max coverage (-): 0

Region: chr25 29352905-29352916. Max. coverage (+): 0. Max coverage (-): 0

Region: chr25 29352917-29352928. Max. coverage (+): 0. Max coverage (-): 0

Region: chr25 29352929-29352940. Max. coverage (+): 1.66. Max coverage (-): 0

Region: chr25 29352941-29352952. Max. coverage (+): 1.66. Max coverage (-): 0

Region: chr25 29352953-29352964. Max. coverage (+): 0. Max coverage (-): 0

Region: chr25 29352965-29352976. Max. coverage (+): 0. Max coverage (-): 0

Region: chr25 29352977-29352987. Max. coverage (+): 0. Max coverage (-): 0

Region: chr25 29352988-29352999. Max. coverage (+): 0. Max coverage (-): 0

Region: chr25 29353000-29353011. Max. coverage (+): 0. Max coverage (-): 0

Region: chr25 29353012-29353023. Max. coverage (+): 0. Max coverage (-): 0

Region: chr25 29353024-29353035. Max. coverage (+): 0.33. Max coverage (-): 0

Region: chr25 29353036-29353047. Max. coverage (+): 7.38. Max coverage (-): 0

Region: chr25 29353048-29353058. Max. coverage (+): 0. Max coverage (-): 0

Region: chr25 29353059-29353070. Max. coverage (+): 0. Max coverage (-): 0

Region: chr25 29353071-29353082. Max. coverage (+): 0. Max coverage (-): 0

Region: chr25 29353083-29353094. Max. coverage (+): 0. Max coverage (-): 0

Region: chr25 29353095-29353106. Max. coverage (+): 0. Max coverage (-): 0

Region: chr25 29353107-29353118. Max. coverage (+): 0. Max coverage (-): 0

Region: chr25 29353119-29353129. Max. coverage (+): 0. Max coverage (-): 0

Region: chr25 29353130-29353141. Max. coverage (+): 0. Max coverage (-): 0

Region: chr25 29353142-29353153. Max. coverage (+): 0. Max coverage (-): 0

Region: chr25 29353154-29353165. Max. coverage (+): 0. Max coverage (-): 0

Region: chr25 29353166-29353177. Max. coverage (+): 0. Max coverage (-): 0

Region: chr25 29353178-29353189. Max. coverage (+): 0. Max coverage (-): 0

Region: chr25 29353190-29353201. Max. coverage (+): 0. Max coverage (-): 0

Region: chr25 29353202-29353212. Max. coverage (+): 0. Max coverage (-): 0

Region: chr25 29353213-29353224. Max. coverage (+): 0. Max coverage (-): 0

Region: chr25 29353225-29353236. Max. coverage (+): 0. Max coverage (-): 0

Region: chr25 29353237-29353248. Max. coverage (+): 0. Max coverage (-): 0

Region: chr25 29353249-29353260. Max. coverage (+): 11.31. Max coverage (-): 0

Region: chr25 29353261-29353272. Max. coverage (+): 27.55. Max coverage (-): 0

Region: chr25 29353273-29353283. Max. coverage (+): 0. Max coverage (-): 0

Region: chr25 29353284-29353295. Max. coverage (+): 0. Max coverage (-): 0

Region: chr25 29353296-29353307. Max. coverage (+): 0. Max coverage (-): 0

Region: chr25 29353308-29353319. Max. coverage (+): 0. Max coverage (-): 0

Region: chr25 29353320-29353331. Max. coverage (+): 0. Max coverage (-): 0

Region: chr25 29353332-29353343. Max. coverage (+): 0. Max coverage (-): 0

Region: chr25 29353344-29353354. Max. coverage (+): 0. Max coverage (-): 0

Region: chr25 29353355-29353366. Max. coverage (+): 0. Max coverage (-): 0

Region: chr25 29353367-29353378. Max. coverage (+): 0. Max coverage (-): 0

Region: chr25 29353379-29353390. Max. coverage (+): 0. Max coverage (-): 0

Region: chr25 29353391-29353402. Max. coverage (+): 0. Max coverage (-): 0

Region: chr25 29353403-29353414. Max. coverage (+): 0. Max coverage (-): 0

Region: chr25 29353415-29353426. Max. coverage (+): 0. Max coverage (-): 0

Region: chr25 29353427-29353437. Max. coverage (+): 0. Max coverage (-): 0

Region: chr25 29353438-29353449. Max. coverage (+): 0. Max coverage (-): 0

Region: chr25 29353450-29353461. Max. coverage (+): 0. Max coverage (-): 0

Region: chr25 29353462-29353473. Max. coverage (+): 0. Max coverage (-): 0

Region: chr25 29353474-29353485. Max. coverage (+): 0. Max coverage (-): 0

Region: chr25 29353486-29353497. Max. coverage (+): 0. Max coverage (-): 0

Region: chr25 29353498-29353508. Max. coverage (+): 0. Max coverage (-): 0

Region: chr25 29353509-29353520. Max. coverage (+): 0. Max coverage (-): 0

Region: chr25 29353521-29353532. Max. coverage (+): 0. Max coverage (-): 0

Region: chr25 29353533-29353544. Max. coverage (+): 0. Max coverage (-): 0

Region: chr25 29353545-29353556. Max. coverage (+): 0. Max coverage (-): 0

Region: chr25 29353557-29353568. Max. coverage (+): 0. Max coverage (-): 0

Region: chr25 29353569-29353579. Max. coverage (+): 0. Max coverage (-): 0

Region: chr25 29353580-29353591. Max. coverage (+): 0. Max coverage (-): 0

Region: chr25 29353592-29353603. Max. coverage (+): 0. Max coverage (-): 0

Region: chr25 29353604-29353615. Max. coverage (+): 0. Max coverage (-): 0

Region: chr25 29353616-29353627. Max. coverage (+): 0. Max coverage (-): 0

Region: chr25 29353628-29353639. Max. coverage (+): 0. Max coverage (-): 0

Region: chr25 29353640-29353651. Max. coverage (+): 0. Max coverage (-): 0

Region: chr25 29353652-29353662. Max. coverage (+): 0. Max coverage (-): 0

Region: chr25 29353663-29353674. Max. coverage (+): 0. Max coverage (-): 0

Region: chr25 29353675-29353686. Max. coverage (+): 0. Max coverage (-): 0

Region: chr25 29353687-29353698. Max. coverage (+): 0. Max coverage (-): 0

Region: chr25 29353699-29353710. Max. coverage (+): 0.92. Max coverage (-): 0

Region: chr25 29353711-29353722. Max. coverage (+): 0. Max coverage (-): 0

Region: chr25 29353723-29353733. Max. coverage (+): 0. Max coverage (-): 0

Region: chr25 29353734-29353745. Max. coverage (+): 0. Max coverage (-): 0

Region: chr25 29353746-29353757. Max. coverage (+): 4.47. Max coverage (-): 0

Region: chr25 29353758-29353769. Max. coverage (+): 4.47. Max coverage (-): 0

Region: chr25 29353770-29353781. Max. coverage (+): 0. Max coverage (-): 0

Region: chr25 29353782-29353793. Max. coverage (+): 0. Max coverage (-): 0

Region: chr25 29353794-29353804. Max. coverage (+): 0. Max coverage (-): 0

Region: chr25 29353805-29353816. Max. coverage (+): 10.45. Max coverage (-): 0

Region: chr25 29353817-29353828. Max. coverage (+): 0. Max coverage (-): 0

Region: chr25 29353829-29353840. Max. coverage (+): 0. Max coverage (-): 0

Region: chr25 29353841-29353852. Max. coverage (+): 0. Max coverage (-): 0

Region: chr25 29353853-29353864. Max. coverage (+): 0. Max coverage (-): 0

Region: chr25 29353865-29353876. Max. coverage (+): 0. Max coverage (-): 0

Region: chr25 29353877-29353887. Max. coverage (+): 0. Max coverage (-): 0

Region: chr25 29353888-29353899. Max. coverage (+): 0. Max coverage (-): 0

Region: chr25 29353900-29353911. Max. coverage (+): 0. Max coverage (-): 0

Region: chr25 29353912-29353923. Max. coverage (+): 1.32. Max coverage (-): 0

Region: chr25 29353924-29353935. Max. coverage (+): 0. Max coverage (-): 0

Region: chr25 29353936-29353947. Max. coverage (+): 0. Max coverage (-): 0

Region: chr25 29353948-29353958. Max. coverage (+): 0. Max coverage (-): 0

Region: chr25 29353959-29353970. Max. coverage (+): 0. Max coverage (-): 0

Region: chr25 29353971-29353982. Max. coverage (+): 4.2. Max coverage (-): 0

Region: chr25 29353983-29353994. Max. coverage (+): 0. Max coverage (-): 0

Region: chr25 29353995-29354006. Max. coverage (+): 0. Max coverage (-): 0

Region: chr25 29354007-29354018. Max. coverage (+): 0. Max coverage (-): 0

Region: chr25 29354019-29354029. Max. coverage (+): 0. Max coverage (-): 0

Region: chr25 29354030-29354041. Max. coverage (+): 0. Max coverage (-): 0

Region: chr25 29354042-29354053. Max. coverage (+): 0. Max coverage (-): 0

Region: chr25 29354054-29354065. Max. coverage (+): 0. Max coverage (-): 0

Region: chr25 29354066-29354077. Max. coverage (+): 0. Max coverage (-): 0

Region: chr25 29354078-29354089. Max. coverage (+): 0. Max coverage (-): 0

Region: chr25 29354090-29354101. Max. coverage (+): 0. Max coverage (-): 0

Region: chr25 29354102-29354112. Max. coverage (+): 0. Max coverage (-): 0

Region: chr25 29354113-29354124. Max. coverage (+): 0. Max coverage (-): 0

Region: chr25 29354125-29354136. Max. coverage (+): 0. Max coverage (-): 0

Region: chr25 29354137-29354148. Max. coverage (+): 0. Max coverage (-): 0

Region: chr25 29354149-29354160. Max. coverage (+): 0. Max coverage (-): 0

Region: chr25 29354161-29354172. Max. coverage (+): 0. Max coverage (-): 0

Region: chr25 29354173-29354183. Max. coverage (+): 0. Max coverage (-): 0

Region: chr25 29354184-29354195. Max. coverage (+): 0. Max coverage (-): 0

Region: chr25 29354196-29354207. Max. coverage (+): 0. Max coverage (-): 0

Region: chr25 29354208-29354219. Max. coverage (+): 0. Max coverage (-): 0

Region: chr25 29354220-29354231. Max. coverage (+): 0. Max coverage (-): 0

Region: chr25 29354232-29354243. Max. coverage (+): 0. Max coverage (-): 0

Region: chr25 29354244-29354254. Max. coverage (+): 0. Max coverage (-): 0

Region: chr25 29354255-29354266. Max. coverage (+): 0. Max coverage (-): 0

Region: chr25 29354267-29354278. Max. coverage (+): 0. Max coverage (-): 0

Region: chr25 29354279-29354290. Max. coverage (+): 0. Max coverage (-): 0

Region: chr25 29354291-29354302. Max. coverage (+): 0. Max coverage (-): 0

Region: chr25 29354303-29354314. Max. coverage (+): 0. Max coverage (-): 0

Region: chr25 29354315-29354326. Max. coverage (+): 0. Max coverage (-): 0

Region: chr25 29354327-29354337. Max. coverage (+): 0. Max coverage (-): 0

Region: chr25 29354338-29354349. Max. coverage (+): 0. Max coverage (-): 0

Region: chr25 29354350-29354361. Max. coverage (+): 0. Max coverage (-): 0

Region: chr25 29354362-29354373. Max. coverage (+): 0. Max coverage (-): 0

Region: chr25 29354374-29354385. Max. coverage (+): 0. Max coverage (-): 0

Region: chr25 29354386-29354397. Max. coverage (+): 0. Max coverage (-): 0

Region: chr25 29354398-29354408. Max. coverage (+): 0. Max coverage (-): 0

Region: chr25 29354409-29354420. Max. coverage (+): 0. Max coverage (-): 0

Region: chr25 29354421-29354432. Max. coverage (+): 0. Max coverage (-): 0

Region: chr25 29354433-29354444. Max. coverage (+): 0.88. Max coverage (-): 0

Region: chr25 29354445-29354456. Max. coverage (+): 0. Max coverage (-): 0

Region: chr25 29354457-29354468. Max. coverage (+): 0. Max coverage (-): 0

Region: chr25 29354469-29354479. Max. coverage (+): 0. Max coverage (-): 0

Region: chr25 29354480-29354491. Max. coverage (+): 0. Max coverage (-): 0

Region: chr25 29354492-29354503. Max. coverage (+): 0. Max coverage (-): 0

Region: chr25 29354504-29354515. Max. coverage (+): 0. Max coverage (-): 0

Region: chr25 29354516-29354527. Max. coverage (+): 0. Max coverage (-): 0

Region: chr25 29354528-29354539. Max. coverage (+): 0. Max coverage (-): 0

Region: chr25 29354540-29354551. Max. coverage (+): 0. Max coverage (-): 0

Region: chr25 29354552-29354562. Max. coverage (+): 0. Max coverage (-): 0

Region: chr25 29354563-29354574. Max. coverage (+): 0. Max coverage (-): 0

Region: chr25 29354575-29354586. Max. coverage (+): 0. Max coverage (-): 0

Region: chr25 29354587-29354598. Max. coverage (+): 0. Max coverage (-): 0

Region: chr25 29354599-29354610. Max. coverage (+): 0. Max coverage (-): 0

Region: chr25 29354611-29354622. Max. coverage (+): 0. Max coverage (-): 0

Region: chr25 29354623-29354633. Max. coverage (+): 0. Max coverage (-): 0

Region: chr25 29354634-29354645. Max. coverage (+): 0. Max coverage (-): 0

Region: chr25 29354646-29354657. Max. coverage (+): 0. Max coverage (-): 0

Region: chr25 29354658-29354669. Max. coverage (+): 0. Max coverage (-): 0

Region: chr25 29354670-29354681. Max. coverage (+): 0. Max coverage (-): 0

Region: chr25 29354682-29354693. Max. coverage (+): 0. Max coverage (-): 0

Region: chr25 29354694-29354704. Max. coverage (+): 0. Max coverage (-): 0

Region: chr25 29354705-29354716. Max. coverage (+): 0. Max coverage (-): 0

Region: chr25 29354717-29354728. Max. coverage (+): 0. Max coverage (-): 0

Region: chr25 29354729-29354740. Max. coverage (+): 0. Max coverage (-): 0

Region: chr25 29354741-29354752. Max. coverage (+): 0. Max coverage (-): 0

Region: chr25 29354753-29354764. Max. coverage (+): 0. Max coverage (-): 0

Region: chr25 29354765-29354776. Max. coverage (+): 0. Max coverage (-): 0

Region: chr25 29354777-29354787. Max. coverage (+): 0. Max coverage (-): 0

Region: chr25 29354788-29354799. Max. coverage (+): 0. Max coverage (-): 0

Region: chr25 29354800-29354811. Max. coverage (+): 0. Max coverage (-): 0

Region: chr25 29354812-29354823. Max. coverage (+): 2.86. Max coverage (-): 0

Region: chr25 29354824-29354835. Max. coverage (+): 2.86. Max coverage (-): 0

Region: chr25 29354836-29354847. Max. coverage (+): 0. Max coverage (-): 0

Region: chr25 29354848-. Max. coverage (+): 0. Max coverage (-): 0

RepeatMasker Color Code

**+**

100-98% Identity

<98-95% Identity

<95-90% Identity

<90-85% Identity

<85-80% Identity

<80-75% Identity

<75-70% Identity

<70% Identity

**-**

Gene Set Color Code

**+**

Gene

Pseudogene

**-**

Topology/Coverage Color Code

Coverage Plus Strand

Coverage Minus Strand

Mainstrand: Plus

Mainstrand: Minus

Complementary Strand

Flanking Region  
(if option -flank >0)

Gene Set Annotation  

**1. CALN1 (protein coding, ENSBTAG00000043969) Tr:00000061255 Ex:6**: 29348906-29349030 (+)

  
RepeatMasker Annotation  

**1. (CCCCCA)n**: 29349432-29349459 (+), Divergence to consensus: 7.1%  
**2. AT\_rich**: 29352254-29352274 (+), Divergence to consensus: 28.6%  
**3. L3b**: 29353365-29353450 (+), Divergence to consensus: 38%  
**4. MIR3**: 29354644-29354764 (+), Divergence to consensus: 45.9%

  
Transcription Factor Binding Sites  

**RFX4\_1** (Sequence: GTTGCTAAG (-): 29351578)  
**RFX4\_1** (Sequence: GTTGCCATG (-): 29352279)  
**Gata4** (Sequence: AGATAAC (-): 29353913)  
**SOX9** (Sequence: TTATTGTT (+): 29354199)  
**SPZ1** (Sequence: AGGGTTTGAG (+): 29350808)  
**SPZ1** (Sequence: GGGGTATGAG (+): 29351195)
