# Supplementary material for: Small RNA sequencing of cryopreserved semen from single bull revealed altered miRNAs and piRNAs expression between High- and Low-motile sperm populations
Source: BMC Genomics. 2017 Jan 4;18:14. doi: 10.1186/s12864-016-3394-7 (PMC5209821; doi:10.1186/s12864-016-3394-7)
Supplement: Additional file 4: — Details for each piRNA clusters found in Low Motile (LM) sperm fraction. Genes, repeats, transposable elements and transcription factors binding sites falling within the cluster regions were reported. (ZIP 1034 kb) [file 12864_2016_3394_MOESM4_ESM.zip › 37.html]

piRNA cluster 37


Predicted piRNA cluster no. 37     previous   next
  

Show proTRAC run info
Hide proTRAC run info

================================= proTRAC ====================================  
VERSION: 2.1                                    LAST MODIFIED: 06. October 2015  
  
Please cite:  
Rosenkranz D, Zischler H. proTRAC - a software for probabilistic piRNA cluster  
detection, visualization and analysis. 2012. BMC Bioinformatics 13:5.  
  
and (for proTRAC 2.0 and later):  
Rosenkranz D, Rudloff S, Bastuck K, Ketting RF, Zischler H. Tupaia small RNAs  
provide insights into function and evolution of RNAi-based transposon defense  
in mammals. 2015. RNA 21(5):911-922.  
  
Contact:  
David Rosenkranz  
Institute of Anthropology, small RNA group  
Johannes Gutenberg University Mainz  
email: rosenkranz@uni-mainz.de  
  
You can find the latest proTRAC version at:  
http://sourceforge.net/projects/protrac/files  
http://www.smallRNAgroup-mainz.de/software  
==============================================================================  
  
PARAMETERS:  
Map file: .............../storage/core/barbara/genhome/smallRNA/fertility/Sample\_not\_motile/pirna/Sample\_not\_motile\_26-33\_collapsed.fa.no-dust.map.weighted-10000-1000-b-0  
Genome file: ............/storage/core/barbara/genhome/smallRNA/fertility/Sample\_all/pirna/bt\_311\_chrY.fa  
RepeatMasker annotation: /storage/genomes/bt\_umd31/GCF\_000003055.6\_Bos\_taurus\_UMD\_3.1.1\_repeatMasker\_chr.out  
GeneSet:................./storage/core/barbara/genhome/smallRNA/fertility/Sample\_all/pirna/full.gtf  
  
Significant (p<=0.01) hit density will be calculated based  
on observed hit distribution.  
  
Sliding window size: ........................................ 5000 bp  
Sliding window increament: .................................. 1000 bp  
Normalize each hit by number of genomic hits: ............... 1 [0=no/1=yes]  
Normalize each hit by number of sequence reads: ............. 1 [0=no/1=yes]  
Normalize values (-> per million mapped reads): ............. 1 [0=no/1=yes]  
Min. fraction of hits with 1T(U) or 10A: .................... 0.75  
Alternatively: Min. fraction of hits with 1T(U) and 10A: .... 0.5  
Min. fraction of hits with typical piRNA length: ............ 0.75  
Typical piRNA length: ....................................... 26-33 nt  
Min. size of a piRNA cluster: ............................... 5000 bp.  
Min. number of hits (absolute): ............................. 0  
Min. number of hits (normalized): ........................... 0  
Min. fraction of hits on the mainstrand: .................... 0.75  
Top fraction of mapped sequences (in terms of read counts): . 1%  
Top fraction accounts for max. n% of sequence reads: ........ 90%  
Min. fraction of hits on each arm of a bidirectional cluster: 0.1  
Output image file for each cluster: ......................... 0 [0=no/1=yes]  
Output html file for each cluster: .......................... 1 [0=no/1=yes]  
Output a summary table: ..................................... 1 [0=no/1=yes]  
Output a FASTA file for each cluster (piRNA sequences): ..... 1 [0=no/1=yes]  
Output a FASTA file comprising cluster sequences: ........... 1 [0=no/1=yes]  
Search DNA motifs in clusters: .............................. 1 [0=no/1=yes]  
Output flanking sequences: +/- .............................. 0 bp  
Output ~.pTi file: .......................................... 1 [0=no/1=yes]  
==============================================================================  
  
  
Genome size (without gaps): ............ 2678902517 bp  
Gaps (N/X/-): .......................... 53837044 bp  
Mapped reads: .......................... 738059667487  
Non-identical sequences: ............... 277001  
Genomic hits: .......................... 533816  
Significant densitiy of mapped reads: .. 15118061 reads/kb

Show proTRAC cluster info
Hide proTRAC cluster info

|  |  |
| --- | --- |
| Location | chr28 |
| Coordinates | 1795898-1801531 |
| Size [bp] | 5634 |
| Sequence hit loci | 248 |
| Mapped reads (normalized) | 304263274.7 |
| Mapped reads (normalized) per kb | 54004841.1 |
| Normalized reads with 1T (1U) | 92.9% |
| Normalized reads with 10A | 34.5% |
| Normalized reads with length 26-33 nt | 100% |
| Normalized reads on the main strand(s) | 100% |
| Predicted directionality | mono:plus |

100%

0%

1T (1U)  
reads

10A reads

26-33 nt  
reads

reads on mainstrand

**Either the amount of reads with 1T (1U) OR 10A has to exceed 75% (set with option: -1Tor10A)  
Alternatively the amount of reads with 1T (1U) AND 10A has to exceed 50% (set with option: -1Tand10A)  
Minimum amount of reads with preferred size is 75% (set with option: -pisize)  
Minimum amount of reads on the main strand(s) is 75% (set with option: -clstrand)**

Show read coverage
Hide read coverage

WHAT DO I SEE HERE?  
This chart shows the location of mapped sequence reads within a predicted piRNA cluster. The color refers to the number of genomic hits produced by the sequence read in question. A dark red bar indicates that this sequence read produces many other hits elsewhere in the genome. Many adjacent red or yellow bars can indicate the presence of a multi-copy element such as transposons or rRNA genes. A dark green bar indicates that this sequence read maps uniquely to this locus.

1 hit

2-5 hits

6-10 hits

11-20 hits

21-50 hits

51-100 hits

> 100 hits

chr28

1795898

1801531

Gene Set

RepeatMasker

Mapped  
Reads

41.75

plus strand

minus strand

41.75

Region: chr28 29354849-1795903. Max. coverage (+): 3.55. Max coverage (-): 0

Region: chr28 1795904-1795914. Max. coverage (+): 3.55. Max coverage (-): 0

Region: chr28 1795915-1795926. Max. coverage (+): 0. Max coverage (-): 0

Region: chr28 1795927-1795937. Max. coverage (+): 0. Max coverage (-): 0

Region: chr28 1795938-1795948. Max. coverage (+): 0. Max coverage (-): 0

Region: chr28 1795949-1795959. Max. coverage (+): 0. Max coverage (-): 0

Region: chr28 1795960-1795971. Max. coverage (+): 0. Max coverage (-): 0

Region: chr28 1795972-1795982. Max. coverage (+): 0. Max coverage (-): 0

Region: chr28 1795983-1795993. Max. coverage (+): 0. Max coverage (-): 0

Region: chr28 1795994-1796005. Max. coverage (+): 0. Max coverage (-): 0

Region: chr28 1796006-1796016. Max. coverage (+): 0. Max coverage (-): 0

Region: chr28 1796017-1796027. Max. coverage (+): 0. Max coverage (-): 0

Region: chr28 1796028-1796038. Max. coverage (+): 0. Max coverage (-): 0

Region: chr28 1796039-1796050. Max. coverage (+): 0. Max coverage (-): 0

Region: chr28 1796051-1796061. Max. coverage (+): 0. Max coverage (-): 0

Region: chr28 1796062-1796072. Max. coverage (+): 0.19. Max coverage (-): 0

Region: chr28 1796073-1796083. Max. coverage (+): 0. Max coverage (-): 0

Region: chr28 1796084-1796095. Max. coverage (+): 0. Max coverage (-): 0

Region: chr28 1796096-1796106. Max. coverage (+): 0. Max coverage (-): 0

Region: chr28 1796107-1796117. Max. coverage (+): 0. Max coverage (-): 0

Region: chr28 1796118-1796128. Max. coverage (+): 0. Max coverage (-): 0

Region: chr28 1796129-1796140. Max. coverage (+): 0. Max coverage (-): 0

Region: chr28 1796141-1796151. Max. coverage (+): 0. Max coverage (-): 0

Region: chr28 1796152-1796162. Max. coverage (+): 0. Max coverage (-): 0

Region: chr28 1796163-1796174. Max. coverage (+): 0. Max coverage (-): 0

Region: chr28 1796175-1796185. Max. coverage (+): 0. Max coverage (-): 0

Region: chr28 1796186-1796196. Max. coverage (+): 0. Max coverage (-): 0

Region: chr28 1796197-1796207. Max. coverage (+): 0. Max coverage (-): 0

Region: chr28 1796208-1796219. Max. coverage (+): 0. Max coverage (-): 0

Region: chr28 1796220-1796230. Max. coverage (+): 0. Max coverage (-): 0

Region: chr28 1796231-1796241. Max. coverage (+): 0. Max coverage (-): 0

Region: chr28 1796242-1796252. Max. coverage (+): 0. Max coverage (-): 0

Region: chr28 1796253-1796264. Max. coverage (+): 0. Max coverage (-): 0

Region: chr28 1796265-1796275. Max. coverage (+): 0. Max coverage (-): 0

Region: chr28 1796276-1796286. Max. coverage (+): 0. Max coverage (-): 0

Region: chr28 1796287-1796298. Max. coverage (+): 0. Max coverage (-): 0

Region: chr28 1796299-1796309. Max. coverage (+): 0. Max coverage (-): 0

Region: chr28 1796310-1796320. Max. coverage (+): 0. Max coverage (-): 0

Region: chr28 1796321-1796331. Max. coverage (+): 0. Max coverage (-): 0

Region: chr28 1796332-1796343. Max. coverage (+): 0. Max coverage (-): 0

Region: chr28 1796344-1796354. Max. coverage (+): 0. Max coverage (-): 0

Region: chr28 1796355-1796365. Max. coverage (+): 5.61. Max coverage (-): 0

Region: chr28 1796366-1796376. Max. coverage (+): 9.92. Max coverage (-): 0

Region: chr28 1796377-1796388. Max. coverage (+): 0. Max coverage (-): 0

Region: chr28 1796389-1796399. Max. coverage (+): 0. Max coverage (-): 0

Region: chr28 1796400-1796410. Max. coverage (+): 0. Max coverage (-): 0

Region: chr28 1796411-1796421. Max. coverage (+): 0. Max coverage (-): 0

Region: chr28 1796422-1796433. Max. coverage (+): 0. Max coverage (-): 0

Region: chr28 1796434-1796444. Max. coverage (+): 0. Max coverage (-): 0

Region: chr28 1796445-1796455. Max. coverage (+): 0. Max coverage (-): 0

Region: chr28 1796456-1796467. Max. coverage (+): 0. Max coverage (-): 0

Region: chr28 1796468-1796478. Max. coverage (+): 0. Max coverage (-): 0

Region: chr28 1796479-1796489. Max. coverage (+): 0. Max coverage (-): 0

Region: chr28 1796490-1796500. Max. coverage (+): 0. Max coverage (-): 0

Region: chr28 1796501-1796512. Max. coverage (+): 0. Max coverage (-): 0

Region: chr28 1796513-1796523. Max. coverage (+): 0. Max coverage (-): 0

Region: chr28 1796524-1796534. Max. coverage (+): 0. Max coverage (-): 0

Region: chr28 1796535-1796545. Max. coverage (+): 0. Max coverage (-): 0

Region: chr28 1796546-1796557. Max. coverage (+): 0. Max coverage (-): 0

Region: chr28 1796558-1796568. Max. coverage (+): 0. Max coverage (-): 0

Region: chr28 1796569-1796579. Max. coverage (+): 0. Max coverage (-): 0

Region: chr28 1796580-1796590. Max. coverage (+): 0. Max coverage (-): 0

Region: chr28 1796591-1796602. Max. coverage (+): 0. Max coverage (-): 0

Region: chr28 1796603-1796613. Max. coverage (+): 0. Max coverage (-): 0

Region: chr28 1796614-1796624. Max. coverage (+): 0. Max coverage (-): 0

Region: chr28 1796625-1796636. Max. coverage (+): 0. Max coverage (-): 0

Region: chr28 1796637-1796647. Max. coverage (+): 0. Max coverage (-): 0

Region: chr28 1796648-1796658. Max. coverage (+): 0. Max coverage (-): 0

Region: chr28 1796659-1796669. Max. coverage (+): 0. Max coverage (-): 0

Region: chr28 1796670-1796681. Max. coverage (+): 1.2. Max coverage (-): 0

Region: chr28 1796682-1796692. Max. coverage (+): 0. Max coverage (-): 0

Region: chr28 1796693-1796703. Max. coverage (+): 0. Max coverage (-): 0

Region: chr28 1796704-1796714. Max. coverage (+): 0. Max coverage (-): 0

Region: chr28 1796715-1796726. Max. coverage (+): 0. Max coverage (-): 0

Region: chr28 1796727-1796737. Max. coverage (+): 0. Max coverage (-): 0

Region: chr28 1796738-1796748. Max. coverage (+): 0. Max coverage (-): 0

Region: chr28 1796749-1796760. Max. coverage (+): 0. Max coverage (-): 0

Region: chr28 1796761-1796771. Max. coverage (+): 0. Max coverage (-): 0

Region: chr28 1796772-1796782. Max. coverage (+): 0. Max coverage (-): 0

Region: chr28 1796783-1796793. Max. coverage (+): 0. Max coverage (-): 0

Region: chr28 1796794-1796805. Max. coverage (+): 0. Max coverage (-): 0

Region: chr28 1796806-1796816. Max. coverage (+): 0. Max coverage (-): 0

Region: chr28 1796817-1796827. Max. coverage (+): 0. Max coverage (-): 0

Region: chr28 1796828-1796838. Max. coverage (+): 0. Max coverage (-): 0

Region: chr28 1796839-1796850. Max. coverage (+): 0. Max coverage (-): 0

Region: chr28 1796851-1796861. Max. coverage (+): 0. Max coverage (-): 0

Region: chr28 1796862-1796872. Max. coverage (+): 0. Max coverage (-): 0

Region: chr28 1796873-1796883. Max. coverage (+): 0. Max coverage (-): 0

Region: chr28 1796884-1796895. Max. coverage (+): 0. Max coverage (-): 0

Region: chr28 1796896-1796906. Max. coverage (+): 0. Max coverage (-): 0

Region: chr28 1796907-1796917. Max. coverage (+): 0. Max coverage (-): 0

Region: chr28 1796918-1796929. Max. coverage (+): 0. Max coverage (-): 0

Region: chr28 1796930-1796940. Max. coverage (+): 0. Max coverage (-): 0

Region: chr28 1796941-1796951. Max. coverage (+): 0. Max coverage (-): 0

Region: chr28 1796952-1796962. Max. coverage (+): 0. Max coverage (-): 0

Region: chr28 1796963-1796974. Max. coverage (+): 0. Max coverage (-): 0

Region: chr28 1796975-1796985. Max. coverage (+): 0. Max coverage (-): 0

Region: chr28 1796986-1796996. Max. coverage (+): 0. Max coverage (-): 0

Region: chr28 1796997-1797007. Max. coverage (+): 0. Max coverage (-): 0

Region: chr28 1797008-1797019. Max. coverage (+): 0. Max coverage (-): 0

Region: chr28 1797020-1797030. Max. coverage (+): 0. Max coverage (-): 0

Region: chr28 1797031-1797041. Max. coverage (+): 0. Max coverage (-): 0

Region: chr28 1797042-1797052. Max. coverage (+): 0. Max coverage (-): 0

Region: chr28 1797053-1797064. Max. coverage (+): 0. Max coverage (-): 0

Region: chr28 1797065-1797075. Max. coverage (+): 0. Max coverage (-): 0

Region: chr28 1797076-1797086. Max. coverage (+): 0. Max coverage (-): 0

Region: chr28 1797087-1797098. Max. coverage (+): 0. Max coverage (-): 0

Region: chr28 1797099-1797109. Max. coverage (+): 0. Max coverage (-): 0

Region: chr28 1797110-1797120. Max. coverage (+): 0. Max coverage (-): 0

Region: chr28 1797121-1797131. Max. coverage (+): 0. Max coverage (-): 0

Region: chr28 1797132-1797143. Max. coverage (+): 0. Max coverage (-): 0

Region: chr28 1797144-1797154. Max. coverage (+): 0. Max coverage (-): 0

Region: chr28 1797155-1797165. Max. coverage (+): 0. Max coverage (-): 0

Region: chr28 1797166-1797176. Max. coverage (+): 0. Max coverage (-): 0

Region: chr28 1797177-1797188. Max. coverage (+): 0. Max coverage (-): 0

Region: chr28 1797189-1797199. Max. coverage (+): 0. Max coverage (-): 0

Region: chr28 1797200-1797210. Max. coverage (+): 0. Max coverage (-): 0

Region: chr28 1797211-1797221. Max. coverage (+): 0. Max coverage (-): 0

Region: chr28 1797222-1797233. Max. coverage (+): 0. Max coverage (-): 0

Region: chr28 1797234-1797244. Max. coverage (+): 0. Max coverage (-): 0

Region: chr28 1797245-1797255. Max. coverage (+): 0. Max coverage (-): 0

Region: chr28 1797256-1797267. Max. coverage (+): 0. Max coverage (-): 0

Region: chr28 1797268-1797278. Max. coverage (+): 0. Max coverage (-): 0

Region: chr28 1797279-1797289. Max. coverage (+): 0. Max coverage (-): 0

Region: chr28 1797290-1797300. Max. coverage (+): 0. Max coverage (-): 0

Region: chr28 1797301-1797312. Max. coverage (+): 0. Max coverage (-): 0

Region: chr28 1797313-1797323. Max. coverage (+): 0. Max coverage (-): 0

Region: chr28 1797324-1797334. Max. coverage (+): 0. Max coverage (-): 0

Region: chr28 1797335-1797345. Max. coverage (+): 0. Max coverage (-): 0

Region: chr28 1797346-1797357. Max. coverage (+): 0. Max coverage (-): 0

Region: chr28 1797358-1797368. Max. coverage (+): 0. Max coverage (-): 0

Region: chr28 1797369-1797379. Max. coverage (+): 0. Max coverage (-): 0

Region: chr28 1797380-1797391. Max. coverage (+): 0. Max coverage (-): 0

Region: chr28 1797392-1797402. Max. coverage (+): 0. Max coverage (-): 0

Region: chr28 1797403-1797413. Max. coverage (+): 0. Max coverage (-): 0

Region: chr28 1797414-1797424. Max. coverage (+): 0. Max coverage (-): 0

Region: chr28 1797425-1797436. Max. coverage (+): 0. Max coverage (-): 0

Region: chr28 1797437-1797447. Max. coverage (+): 0. Max coverage (-): 0

Region: chr28 1797448-1797458. Max. coverage (+): 0. Max coverage (-): 0

Region: chr28 1797459-1797469. Max. coverage (+): 0. Max coverage (-): 0

Region: chr28 1797470-1797481. Max. coverage (+): 0. Max coverage (-): 0

Region: chr28 1797482-1797492. Max. coverage (+): 0. Max coverage (-): 0

Region: chr28 1797493-1797503. Max. coverage (+): 0. Max coverage (-): 0

Region: chr28 1797504-1797514. Max. coverage (+): 0. Max coverage (-): 0

Region: chr28 1797515-1797526. Max. coverage (+): 0. Max coverage (-): 0

Region: chr28 1797527-1797537. Max. coverage (+): 0. Max coverage (-): 0

Region: chr28 1797538-1797548. Max. coverage (+): 0. Max coverage (-): 0

Region: chr28 1797549-1797560. Max. coverage (+): 0. Max coverage (-): 0

Region: chr28 1797561-1797571. Max. coverage (+): 0. Max coverage (-): 0

Region: chr28 1797572-1797582. Max. coverage (+): 0. Max coverage (-): 0

Region: chr28 1797583-1797593. Max. coverage (+): 0. Max coverage (-): 0

Region: chr28 1797594-1797605. Max. coverage (+): 0. Max coverage (-): 0

Region: chr28 1797606-1797616. Max. coverage (+): 0. Max coverage (-): 0

Region: chr28 1797617-1797627. Max. coverage (+): 0. Max coverage (-): 0

Region: chr28 1797628-1797638. Max. coverage (+): 0. Max coverage (-): 0

Region: chr28 1797639-1797650. Max. coverage (+): 0. Max coverage (-): 0

Region: chr28 1797651-1797661. Max. coverage (+): 0. Max coverage (-): 0

Region: chr28 1797662-1797672. Max. coverage (+): 0. Max coverage (-): 0

Region: chr28 1797673-1797683. Max. coverage (+): 0. Max coverage (-): 0

Region: chr28 1797684-1797695. Max. coverage (+): 0. Max coverage (-): 0

Region: chr28 1797696-1797706. Max. coverage (+): 0. Max coverage (-): 0

Region: chr28 1797707-1797717. Max. coverage (+): 0. Max coverage (-): 0

Region: chr28 1797718-1797729. Max. coverage (+): 0. Max coverage (-): 0

Region: chr28 1797730-1797740. Max. coverage (+): 0. Max coverage (-): 0

Region: chr28 1797741-1797751. Max. coverage (+): 0. Max coverage (-): 0

Region: chr28 1797752-1797762. Max. coverage (+): 0. Max coverage (-): 0

Region: chr28 1797763-1797774. Max. coverage (+): 5.31. Max coverage (-): 0

Region: chr28 1797775-1797785. Max. coverage (+): 5.31. Max coverage (-): 0

Region: chr28 1797786-1797796. Max. coverage (+): 0. Max coverage (-): 0

Region: chr28 1797797-1797807. Max. coverage (+): 0. Max coverage (-): 0

Region: chr28 1797808-1797819. Max. coverage (+): 0. Max coverage (-): 0

Region: chr28 1797820-1797830. Max. coverage (+): 0. Max coverage (-): 0

Region: chr28 1797831-1797841. Max. coverage (+): 0. Max coverage (-): 0

Region: chr28 1797842-1797852. Max. coverage (+): 0. Max coverage (-): 0

Region: chr28 1797853-1797864. Max. coverage (+): 0. Max coverage (-): 0

Region: chr28 1797865-1797875. Max. coverage (+): 0. Max coverage (-): 0

Region: chr28 1797876-1797886. Max. coverage (+): 0. Max coverage (-): 0

Region: chr28 1797887-1797898. Max. coverage (+): 0. Max coverage (-): 0

Region: chr28 1797899-1797909. Max. coverage (+): 0. Max coverage (-): 0

Region: chr28 1797910-1797920. Max. coverage (+): 0. Max coverage (-): 0

Region: chr28 1797921-1797931. Max. coverage (+): 0. Max coverage (-): 0

Region: chr28 1797932-1797943. Max. coverage (+): 0. Max coverage (-): 0

Region: chr28 1797944-1797954. Max. coverage (+): 0. Max coverage (-): 0

Region: chr28 1797955-1797965. Max. coverage (+): 0. Max coverage (-): 0

Region: chr28 1797966-1797976. Max. coverage (+): 0. Max coverage (-): 0

Region: chr28 1797977-1797988. Max. coverage (+): 0. Max coverage (-): 0

Region: chr28 1797989-1797999. Max. coverage (+): 0. Max coverage (-): 0

Region: chr28 1798000-1798010. Max. coverage (+): 0. Max coverage (-): 0

Region: chr28 1798011-1798022. Max. coverage (+): 0. Max coverage (-): 0

Region: chr28 1798023-1798033. Max. coverage (+): 0. Max coverage (-): 0

Region: chr28 1798034-1798044. Max. coverage (+): 0. Max coverage (-): 0

Region: chr28 1798045-1798055. Max. coverage (+): 0. Max coverage (-): 0

Region: chr28 1798056-1798067. Max. coverage (+): 0. Max coverage (-): 0

Region: chr28 1798068-1798078. Max. coverage (+): 0. Max coverage (-): 0

Region: chr28 1798079-1798089. Max. coverage (+): 0. Max coverage (-): 0

Region: chr28 1798090-1798100. Max. coverage (+): 0. Max coverage (-): 0

Region: chr28 1798101-1798112. Max. coverage (+): 0. Max coverage (-): 0

Region: chr28 1798113-1798123. Max. coverage (+): 0. Max coverage (-): 0

Region: chr28 1798124-1798134. Max. coverage (+): 0. Max coverage (-): 0

Region: chr28 1798135-1798145. Max. coverage (+): 0. Max coverage (-): 0

Region: chr28 1798146-1798157. Max. coverage (+): 0. Max coverage (-): 0

Region: chr28 1798158-1798168. Max. coverage (+): 0. Max coverage (-): 0

Region: chr28 1798169-1798179. Max. coverage (+): 0. Max coverage (-): 0

Region: chr28 1798180-1798191. Max. coverage (+): 0. Max coverage (-): 0

Region: chr28 1798192-1798202. Max. coverage (+): 0. Max coverage (-): 0

Region: chr28 1798203-1798213. Max. coverage (+): 0. Max coverage (-): 0

Region: chr28 1798214-1798224. Max. coverage (+): 0. Max coverage (-): 0

Region: chr28 1798225-1798236. Max. coverage (+): 0. Max coverage (-): 0

Region: chr28 1798237-1798247. Max. coverage (+): 0. Max coverage (-): 0

Region: chr28 1798248-1798258. Max. coverage (+): 0. Max coverage (-): 0

Region: chr28 1798259-1798269. Max. coverage (+): 0. Max coverage (-): 0

Region: chr28 1798270-1798281. Max. coverage (+): 0. Max coverage (-): 0

Region: chr28 1798282-1798292. Max. coverage (+): 0. Max coverage (-): 0

Region: chr28 1798293-1798303. Max. coverage (+): 0. Max coverage (-): 0

Region: chr28 1798304-1798314. Max. coverage (+): 0. Max coverage (-): 0

Region: chr28 1798315-1798326. Max. coverage (+): 0. Max coverage (-): 0

Region: chr28 1798327-1798337. Max. coverage (+): 0. Max coverage (-): 0

Region: chr28 1798338-1798348. Max. coverage (+): 0. Max coverage (-): 0

Region: chr28 1798349-1798360. Max. coverage (+): 0. Max coverage (-): 0

Region: chr28 1798361-1798371. Max. coverage (+): 0. Max coverage (-): 0

Region: chr28 1798372-1798382. Max. coverage (+): 0. Max coverage (-): 0

Region: chr28 1798383-1798393. Max. coverage (+): 0. Max coverage (-): 0

Region: chr28 1798394-1798405. Max. coverage (+): 0. Max coverage (-): 0

Region: chr28 1798406-1798416. Max. coverage (+): 0. Max coverage (-): 0

Region: chr28 1798417-1798427. Max. coverage (+): 0. Max coverage (-): 0

Region: chr28 1798428-1798438. Max. coverage (+): 0. Max coverage (-): 0

Region: chr28 1798439-1798450. Max. coverage (+): 0. Max coverage (-): 0

Region: chr28 1798451-1798461. Max. coverage (+): 0. Max coverage (-): 0

Region: chr28 1798462-1798472. Max. coverage (+): 0. Max coverage (-): 0

Region: chr28 1798473-1798484. Max. coverage (+): 0. Max coverage (-): 0

Region: chr28 1798485-1798495. Max. coverage (+): 0. Max coverage (-): 0

Region: chr28 1798496-1798506. Max. coverage (+): 0. Max coverage (-): 0

Region: chr28 1798507-1798517. Max. coverage (+): 0. Max coverage (-): 0

Region: chr28 1798518-1798529. Max. coverage (+): 0. Max coverage (-): 0

Region: chr28 1798530-1798540. Max. coverage (+): 0. Max coverage (-): 0

Region: chr28 1798541-1798551. Max. coverage (+): 0. Max coverage (-): 0

Region: chr28 1798552-1798562. Max. coverage (+): 0. Max coverage (-): 0

Region: chr28 1798563-1798574. Max. coverage (+): 0. Max coverage (-): 0

Region: chr28 1798575-1798585. Max. coverage (+): 0. Max coverage (-): 0

Region: chr28 1798586-1798596. Max. coverage (+): 0. Max coverage (-): 0

Region: chr28 1798597-1798607. Max. coverage (+): 0. Max coverage (-): 0

Region: chr28 1798608-1798619. Max. coverage (+): 0. Max coverage (-): 0

Region: chr28 1798620-1798630. Max. coverage (+): 0. Max coverage (-): 0

Region: chr28 1798631-1798641. Max. coverage (+): 0. Max coverage (-): 0

Region: chr28 1798642-1798653. Max. coverage (+): 0. Max coverage (-): 0

Region: chr28 1798654-1798664. Max. coverage (+): 0. Max coverage (-): 0

Region: chr28 1798665-1798675. Max. coverage (+): 1.27. Max coverage (-): 0

Region: chr28 1798676-1798686. Max. coverage (+): 1.27. Max coverage (-): 0

Region: chr28 1798687-1798698. Max. coverage (+): 0. Max coverage (-): 0

Region: chr28 1798699-1798709. Max. coverage (+): 0. Max coverage (-): 0

Region: chr28 1798710-1798720. Max. coverage (+): 0. Max coverage (-): 0

Region: chr28 1798721-1798731. Max. coverage (+): 0. Max coverage (-): 0

Region: chr28 1798732-1798743. Max. coverage (+): 0. Max coverage (-): 0

Region: chr28 1798744-1798754. Max. coverage (+): 5.79. Max coverage (-): 0

Region: chr28 1798755-1798765. Max. coverage (+): 8.01. Max coverage (-): 0

Region: chr28 1798766-1798776. Max. coverage (+): 0. Max coverage (-): 0

Region: chr28 1798777-1798788. Max. coverage (+): 0. Max coverage (-): 0

Region: chr28 1798789-1798799. Max. coverage (+): 0. Max coverage (-): 0

Region: chr28 1798800-1798810. Max. coverage (+): 0. Max coverage (-): 0

Region: chr28 1798811-1798822. Max. coverage (+): 0. Max coverage (-): 0

Region: chr28 1798823-1798833. Max. coverage (+): 0. Max coverage (-): 0

Region: chr28 1798834-1798844. Max. coverage (+): 0. Max coverage (-): 0

Region: chr28 1798845-1798855. Max. coverage (+): 7.14. Max coverage (-): 0

Region: chr28 1798856-1798867. Max. coverage (+): 0. Max coverage (-): 0

Region: chr28 1798868-1798878. Max. coverage (+): 0. Max coverage (-): 0

Region: chr28 1798879-1798889. Max. coverage (+): 0. Max coverage (-): 0

Region: chr28 1798890-1798900. Max. coverage (+): 11.62. Max coverage (-): 0

Region: chr28 1798901-1798912. Max. coverage (+): 0. Max coverage (-): 0

Region: chr28 1798913-1798923. Max. coverage (+): 0. Max coverage (-): 0

Region: chr28 1798924-1798934. Max. coverage (+): 0. Max coverage (-): 0

Region: chr28 1798935-1798945. Max. coverage (+): 0. Max coverage (-): 0

Region: chr28 1798946-1798957. Max. coverage (+): 0. Max coverage (-): 0

Region: chr28 1798958-1798968. Max. coverage (+): 0. Max coverage (-): 0

Region: chr28 1798969-1798979. Max. coverage (+): 0. Max coverage (-): 0

Region: chr28 1798980-1798991. Max. coverage (+): 0. Max coverage (-): 0

Region: chr28 1798992-1799002. Max. coverage (+): 0. Max coverage (-): 0

Region: chr28 1799003-1799013. Max. coverage (+): 0. Max coverage (-): 0

Region: chr28 1799014-1799024. Max. coverage (+): 0. Max coverage (-): 0

Region: chr28 1799025-1799036. Max. coverage (+): 0. Max coverage (-): 0

Region: chr28 1799037-1799047. Max. coverage (+): 0. Max coverage (-): 0

Region: chr28 1799048-1799058. Max. coverage (+): 0. Max coverage (-): 0

Region: chr28 1799059-1799069. Max. coverage (+): 0. Max coverage (-): 0

Region: chr28 1799070-1799081. Max. coverage (+): 0. Max coverage (-): 0

Region: chr28 1799082-1799092. Max. coverage (+): 0. Max coverage (-): 0

Region: chr28 1799093-1799103. Max. coverage (+): 0. Max coverage (-): 0

Region: chr28 1799104-1799115. Max. coverage (+): 0. Max coverage (-): 0

Region: chr28 1799116-1799126. Max. coverage (+): 0. Max coverage (-): 0

Region: chr28 1799127-1799137. Max. coverage (+): 0. Max coverage (-): 0

Region: chr28 1799138-1799148. Max. coverage (+): 0. Max coverage (-): 0

Region: chr28 1799149-1799160. Max. coverage (+): 0. Max coverage (-): 0

Region: chr28 1799161-1799171. Max. coverage (+): 0. Max coverage (-): 0

Region: chr28 1799172-1799182. Max. coverage (+): 0. Max coverage (-): 0

Region: chr28 1799183-1799193. Max. coverage (+): 0. Max coverage (-): 0

Region: chr28 1799194-1799205. Max. coverage (+): 0. Max coverage (-): 0

Region: chr28 1799206-1799216. Max. coverage (+): 0. Max coverage (-): 0

Region: chr28 1799217-1799227. Max. coverage (+): 0. Max coverage (-): 0

Region: chr28 1799228-1799238. Max. coverage (+): 0. Max coverage (-): 0

Region: chr28 1799239-1799250. Max. coverage (+): 0. Max coverage (-): 0

Region: chr28 1799251-1799261. Max. coverage (+): 0. Max coverage (-): 0

Region: chr28 1799262-1799272. Max. coverage (+): 0. Max coverage (-): 0

Region: chr28 1799273-1799284. Max. coverage (+): 0. Max coverage (-): 0

Region: chr28 1799285-1799295. Max. coverage (+): 0. Max coverage (-): 0

Region: chr28 1799296-1799306. Max. coverage (+): 0. Max coverage (-): 0

Region: chr28 1799307-1799317. Max. coverage (+): 0. Max coverage (-): 0

Region: chr28 1799318-1799329. Max. coverage (+): 0. Max coverage (-): 0

Region: chr28 1799330-1799340. Max. coverage (+): 0. Max coverage (-): 0

Region: chr28 1799341-1799351. Max. coverage (+): 0. Max coverage (-): 0

Region: chr28 1799352-1799362. Max. coverage (+): 0. Max coverage (-): 0

Region: chr28 1799363-1799374. Max. coverage (+): 0. Max coverage (-): 0

Region: chr28 1799375-1799385. Max. coverage (+): 0. Max coverage (-): 0

Region: chr28 1799386-1799396. Max. coverage (+): 0. Max coverage (-): 0

Region: chr28 1799397-1799407. Max. coverage (+): 0. Max coverage (-): 0

Region: chr28 1799408-1799419. Max. coverage (+): 0. Max coverage (-): 0

Region: chr28 1799420-1799430. Max. coverage (+): 4.57. Max coverage (-): 0

Region: chr28 1799431-1799441. Max. coverage (+): 6.55. Max coverage (-): 0

Region: chr28 1799442-1799453. Max. coverage (+): 0. Max coverage (-): 0

Region: chr28 1799454-1799464. Max. coverage (+): 0. Max coverage (-): 0

Region: chr28 1799465-1799475. Max. coverage (+): 0. Max coverage (-): 0

Region: chr28 1799476-1799486. Max. coverage (+): 2.69. Max coverage (-): 0

Region: chr28 1799487-1799498. Max. coverage (+): 0.4. Max coverage (-): 0

Region: chr28 1799499-1799509. Max. coverage (+): 0. Max coverage (-): 0

Region: chr28 1799510-1799520. Max. coverage (+): 6.75. Max coverage (-): 0

Region: chr28 1799521-1799531. Max. coverage (+): 0. Max coverage (-): 0

Region: chr28 1799532-1799543. Max. coverage (+): 0. Max coverage (-): 0

Region: chr28 1799544-1799554. Max. coverage (+): 0. Max coverage (-): 0

Region: chr28 1799555-1799565. Max. coverage (+): 0. Max coverage (-): 0

Region: chr28 1799566-1799577. Max. coverage (+): 0. Max coverage (-): 0

Region: chr28 1799578-1799588. Max. coverage (+): 4.46. Max coverage (-): 0

Region: chr28 1799589-1799599. Max. coverage (+): 4.47. Max coverage (-): 0

Region: chr28 1799600-1799610. Max. coverage (+): 3.02. Max coverage (-): 0

Region: chr28 1799611-1799622. Max. coverage (+): 21.91. Max coverage (-): 0

Region: chr28 1799623-1799633. Max. coverage (+): 18.43. Max coverage (-): 0

Region: chr28 1799634-1799644. Max. coverage (+): 8.41. Max coverage (-): 0

Region: chr28 1799645-1799655. Max. coverage (+): 1.45. Max coverage (-): 0

Region: chr28 1799656-1799667. Max. coverage (+): 0. Max coverage (-): 0

Region: chr28 1799668-1799678. Max. coverage (+): 0. Max coverage (-): 0

Region: chr28 1799679-1799689. Max. coverage (+): 0. Max coverage (-): 0

Region: chr28 1799690-1799700. Max. coverage (+): 0. Max coverage (-): 0

Region: chr28 1799701-1799712. Max. coverage (+): 0.6. Max coverage (-): 0

Region: chr28 1799713-1799723. Max. coverage (+): 2.41. Max coverage (-): 0

Region: chr28 1799724-1799734. Max. coverage (+): 4.71. Max coverage (-): 0

Region: chr28 1799735-1799746. Max. coverage (+): 3.6. Max coverage (-): 0

Region: chr28 1799747-1799757. Max. coverage (+): 1.09. Max coverage (-): 0

Region: chr28 1799758-1799768. Max. coverage (+): 0.69. Max coverage (-): 0

Region: chr28 1799769-1799779. Max. coverage (+): 0. Max coverage (-): 0

Region: chr28 1799780-1799791. Max. coverage (+): 0. Max coverage (-): 0

Region: chr28 1799792-1799802. Max. coverage (+): 0. Max coverage (-): 0

Region: chr28 1799803-1799813. Max. coverage (+): 0. Max coverage (-): 0

Region: chr28 1799814-1799824. Max. coverage (+): 0. Max coverage (-): 0

Region: chr28 1799825-1799836. Max. coverage (+): 0. Max coverage (-): 0

Region: chr28 1799837-1799847. Max. coverage (+): 0. Max coverage (-): 0

Region: chr28 1799848-1799858. Max. coverage (+): 0. Max coverage (-): 0

Region: chr28 1799859-1799869. Max. coverage (+): 0. Max coverage (-): 0

Region: chr28 1799870-1799881. Max. coverage (+): 0. Max coverage (-): 0

Region: chr28 1799882-1799892. Max. coverage (+): 1.4. Max coverage (-): 0

Region: chr28 1799893-1799903. Max. coverage (+): 10.01. Max coverage (-): 0

Region: chr28 1799904-1799915. Max. coverage (+): 10.01. Max coverage (-): 0

Region: chr28 1799916-1799926. Max. coverage (+): 0. Max coverage (-): 0

Region: chr28 1799927-1799937. Max. coverage (+): 1.47. Max coverage (-): 0

Region: chr28 1799938-1799948. Max. coverage (+): 32.65. Max coverage (-): 0

Region: chr28 1799949-1799960. Max. coverage (+): 25.11. Max coverage (-): 0

Region: chr28 1799961-1799971. Max. coverage (+): 0. Max coverage (-): 0

Region: chr28 1799972-1799982. Max. coverage (+): 2.39. Max coverage (-): 0

Region: chr28 1799983-1799993. Max. coverage (+): 1.62. Max coverage (-): 0

Region: chr28 1799994-1800005. Max. coverage (+): 0. Max coverage (-): 0

Region: chr28 1800006-1800016. Max. coverage (+): 0. Max coverage (-): 0

Region: chr28 1800017-1800027. Max. coverage (+): 0. Max coverage (-): 0

Region: chr28 1800028-1800038. Max. coverage (+): 0. Max coverage (-): 0

Region: chr28 1800039-1800050. Max. coverage (+): 6.28. Max coverage (-): 0

Region: chr28 1800051-1800061. Max. coverage (+): 6.28. Max coverage (-): 0

Region: chr28 1800062-1800072. Max. coverage (+): 41.75. Max coverage (-): 0

Region: chr28 1800073-1800084. Max. coverage (+): 6.75. Max coverage (-): 0

Region: chr28 1800085-1800095. Max. coverage (+): 0. Max coverage (-): 0

Region: chr28 1800096-1800106. Max. coverage (+): 0. Max coverage (-): 0

Region: chr28 1800107-1800117. Max. coverage (+): 2.4. Max coverage (-): 0

Region: chr28 1800118-1800129. Max. coverage (+): 37.94. Max coverage (-): 0

Region: chr28 1800130-1800140. Max. coverage (+): 7.21. Max coverage (-): 0

Region: chr28 1800141-1800151. Max. coverage (+): 0. Max coverage (-): 0

Region: chr28 1800152-1800162. Max. coverage (+): 0. Max coverage (-): 0

Region: chr28 1800163-1800174. Max. coverage (+): 0.31. Max coverage (-): 0

Region: chr28 1800175-1800185. Max. coverage (+): 0. Max coverage (-): 0

Region: chr28 1800186-1800196. Max. coverage (+): 0. Max coverage (-): 0

Region: chr28 1800197-1800208. Max. coverage (+): 0. Max coverage (-): 0

Region: chr28 1800209-1800219. Max. coverage (+): 0. Max coverage (-): 0

Region: chr28 1800220-1800230. Max. coverage (+): 0. Max coverage (-): 0

Region: chr28 1800231-1800241. Max. coverage (+): 0. Max coverage (-): 0

Region: chr28 1800242-1800253. Max. coverage (+): 0. Max coverage (-): 0

Region: chr28 1800254-1800264. Max. coverage (+): 4.37. Max coverage (-): 0

Region: chr28 1800265-1800275. Max. coverage (+): 0. Max coverage (-): 0

Region: chr28 1800276-1800286. Max. coverage (+): 0. Max coverage (-): 0

Region: chr28 1800287-1800298. Max. coverage (+): 0. Max coverage (-): 0

Region: chr28 1800299-1800309. Max. coverage (+): 0. Max coverage (-): 0

Region: chr28 1800310-1800320. Max. coverage (+): 0. Max coverage (-): 0

Region: chr28 1800321-1800331. Max. coverage (+): 0. Max coverage (-): 0

Region: chr28 1800332-1800343. Max. coverage (+): 0. Max coverage (-): 0

Region: chr28 1800344-1800354. Max. coverage (+): 0. Max coverage (-): 0

Region: chr28 1800355-1800365. Max. coverage (+): 0. Max coverage (-): 0

Region: chr28 1800366-1800377. Max. coverage (+): 0. Max coverage (-): 0

Region: chr28 1800378-1800388. Max. coverage (+): 0. Max coverage (-): 0

Region: chr28 1800389-1800399. Max. coverage (+): 8.3. Max coverage (-): 0

Region: chr28 1800400-1800410. Max. coverage (+): 4.03. Max coverage (-): 0

Region: chr28 1800411-1800422. Max. coverage (+): 0. Max coverage (-): 0

Region: chr28 1800423-1800433. Max. coverage (+): 0. Max coverage (-): 0

Region: chr28 1800434-1800444. Max. coverage (+): 17.34. Max coverage (-): 0

Region: chr28 1800445-1800455. Max. coverage (+): 17.34. Max coverage (-): 0

Region: chr28 1800456-1800467. Max. coverage (+): 2.89. Max coverage (-): 0

Region: chr28 1800468-1800478. Max. coverage (+): 1.53. Max coverage (-): 0

Region: chr28 1800479-1800489. Max. coverage (+): 1.53. Max coverage (-): 0

Region: chr28 1800490-1800500. Max. coverage (+): 0. Max coverage (-): 0

Region: chr28 1800501-1800512. Max. coverage (+): 0. Max coverage (-): 0

Region: chr28 1800513-1800523. Max. coverage (+): 0. Max coverage (-): 0

Region: chr28 1800524-1800534. Max. coverage (+): 1.45. Max coverage (-): 0

Region: chr28 1800535-1800546. Max. coverage (+): 1.45. Max coverage (-): 0

Region: chr28 1800547-1800557. Max. coverage (+): 0. Max coverage (-): 0

Region: chr28 1800558-1800568. Max. coverage (+): 13.76. Max coverage (-): 0

Region: chr28 1800569-1800579. Max. coverage (+): 0. Max coverage (-): 0

Region: chr28 1800580-1800591. Max. coverage (+): 0. Max coverage (-): 0

Region: chr28 1800592-1800602. Max. coverage (+): 0. Max coverage (-): 0

Region: chr28 1800603-1800613. Max. coverage (+): 7.29. Max coverage (-): 0

Region: chr28 1800614-1800624. Max. coverage (+): 0. Max coverage (-): 0

Region: chr28 1800625-1800636. Max. coverage (+): 0. Max coverage (-): 0

Region: chr28 1800637-1800647. Max. coverage (+): 0. Max coverage (-): 0

Region: chr28 1800648-1800658. Max. coverage (+): 0. Max coverage (-): 0

Region: chr28 1800659-1800669. Max. coverage (+): 0. Max coverage (-): 0

Region: chr28 1800670-1800681. Max. coverage (+): 0. Max coverage (-): 0

Region: chr28 1800682-1800692. Max. coverage (+): 0. Max coverage (-): 0

Region: chr28 1800693-1800703. Max. coverage (+): 0. Max coverage (-): 0

Region: chr28 1800704-1800715. Max. coverage (+): 0. Max coverage (-): 0

Region: chr28 1800716-1800726. Max. coverage (+): 1.95. Max coverage (-): 0

Region: chr28 1800727-1800737. Max. coverage (+): 1.95. Max coverage (-): 0

Region: chr28 1800738-1800748. Max. coverage (+): 0. Max coverage (-): 0

Region: chr28 1800749-1800760. Max. coverage (+): 7.04. Max coverage (-): 0

Region: chr28 1800761-1800771. Max. coverage (+): 11.57. Max coverage (-): 0

Region: chr28 1800772-1800782. Max. coverage (+): 0. Max coverage (-): 0

Region: chr28 1800783-1800793. Max. coverage (+): 0. Max coverage (-): 0

Region: chr28 1800794-1800805. Max. coverage (+): 0. Max coverage (-): 0

Region: chr28 1800806-1800816. Max. coverage (+): 5.58. Max coverage (-): 0

Region: chr28 1800817-1800827. Max. coverage (+): 4.04. Max coverage (-): 0

Region: chr28 1800828-1800839. Max. coverage (+): 0. Max coverage (-): 0

Region: chr28 1800840-1800850. Max. coverage (+): 0. Max coverage (-): 0

Region: chr28 1800851-1800861. Max. coverage (+): 0. Max coverage (-): 0

Region: chr28 1800862-1800872. Max. coverage (+): 2.89. Max coverage (-): 0

Region: chr28 1800873-1800884. Max. coverage (+): 2.89. Max coverage (-): 0

Region: chr28 1800885-1800895. Max. coverage (+): 7.23. Max coverage (-): 0

Region: chr28 1800896-1800906. Max. coverage (+): 0.99. Max coverage (-): 0

Region: chr28 1800907-1800917. Max. coverage (+): 0.48. Max coverage (-): 0

Region: chr28 1800918-1800929. Max. coverage (+): 0. Max coverage (-): 0

Region: chr28 1800930-1800940. Max. coverage (+): 4.84. Max coverage (-): 0

Region: chr28 1800941-1800951. Max. coverage (+): 4.84. Max coverage (-): 0

Region: chr28 1800952-1800962. Max. coverage (+): 0. Max coverage (-): 0

Region: chr28 1800963-1800974. Max. coverage (+): 0.82. Max coverage (-): 0

Region: chr28 1800975-1800985. Max. coverage (+): 5.3. Max coverage (-): 0

Region: chr28 1800986-1800996. Max. coverage (+): 5.02. Max coverage (-): 0

Region: chr28 1800997-1801008. Max. coverage (+): 4.55. Max coverage (-): 0

Region: chr28 1801009-1801019. Max. coverage (+): 0.33. Max coverage (-): 0

Region: chr28 1801020-1801030. Max. coverage (+): 0.03. Max coverage (-): 0

Region: chr28 1801031-1801041. Max. coverage (+): 0.03. Max coverage (-): 0

Region: chr28 1801042-1801053. Max. coverage (+): 3.78. Max coverage (-): 0

Region: chr28 1801054-1801064. Max. coverage (+): 0. Max coverage (-): 0

Region: chr28 1801065-1801075. Max. coverage (+): 0. Max coverage (-): 0

Region: chr28 1801076-1801086. Max. coverage (+): 0. Max coverage (-): 0

Region: chr28 1801087-1801098. Max. coverage (+): 0. Max coverage (-): 0

Region: chr28 1801099-1801109. Max. coverage (+): 0. Max coverage (-): 0

Region: chr28 1801110-1801120. Max. coverage (+): 0. Max coverage (-): 0

Region: chr28 1801121-1801131. Max. coverage (+): 0. Max coverage (-): 0

Region: chr28 1801132-1801143. Max. coverage (+): 0. Max coverage (-): 0

Region: chr28 1801144-1801154. Max. coverage (+): 0. Max coverage (-): 0

Region: chr28 1801155-1801165. Max. coverage (+): 0. Max coverage (-): 0

Region: chr28 1801166-1801177. Max. coverage (+): 0. Max coverage (-): 0

Region: chr28 1801178-1801188. Max. coverage (+): 0. Max coverage (-): 0

Region: chr28 1801189-1801199. Max. coverage (+): 0. Max coverage (-): 0

Region: chr28 1801200-1801210. Max. coverage (+): 0. Max coverage (-): 0

Region: chr28 1801211-1801222. Max. coverage (+): 0. Max coverage (-): 0

Region: chr28 1801223-1801233. Max. coverage (+): 0. Max coverage (-): 0

Region: chr28 1801234-1801244. Max. coverage (+): 0. Max coverage (-): 0

Region: chr28 1801245-1801255. Max. coverage (+): 3.15. Max coverage (-): 0

Region: chr28 1801256-1801267. Max. coverage (+): 7.04. Max coverage (-): 0

Region: chr28 1801268-1801278. Max. coverage (+): 0. Max coverage (-): 0

Region: chr28 1801279-1801289. Max. coverage (+): 2.87. Max coverage (-): 0

Region: chr28 1801290-1801301. Max. coverage (+): 4.91. Max coverage (-): 0

Region: chr28 1801302-1801312. Max. coverage (+): 0. Max coverage (-): 0

Region: chr28 1801313-1801323. Max. coverage (+): 0. Max coverage (-): 0

Region: chr28 1801324-1801334. Max. coverage (+): 3.5. Max coverage (-): 0

Region: chr28 1801335-1801346. Max. coverage (+): 3.5. Max coverage (-): 0

Region: chr28 1801347-1801357. Max. coverage (+): 1.41. Max coverage (-): 0

Region: chr28 1801358-1801368. Max. coverage (+): 0. Max coverage (-): 0

Region: chr28 1801369-1801379. Max. coverage (+): 0.71. Max coverage (-): 0

Region: chr28 1801380-1801391. Max. coverage (+): 0.71. Max coverage (-): 0

Region: chr28 1801392-1801402. Max. coverage (+): 0. Max coverage (-): 0

Region: chr28 1801403-1801413. Max. coverage (+): 0. Max coverage (-): 0

Region: chr28 1801414-1801424. Max. coverage (+): 1.9. Max coverage (-): 0

Region: chr28 1801425-1801436. Max. coverage (+): 1.9. Max coverage (-): 0

Region: chr28 1801437-1801447. Max. coverage (+): 0.43. Max coverage (-): 0

Region: chr28 1801448-1801458. Max. coverage (+): 0. Max coverage (-): 0

Region: chr28 1801459-1801470. Max. coverage (+): 0. Max coverage (-): 0

Region: chr28 1801471-1801481. Max. coverage (+): 0. Max coverage (-): 0

Region: chr28 1801482-1801492. Max. coverage (+): 0. Max coverage (-): 0

Region: chr28 1801493-1801503. Max. coverage (+): 0. Max coverage (-): 0

Region: chr28 1801504-1801515. Max. coverage (+): 1.04. Max coverage (-): 0

Region: chr28 1801516-1801526. Max. coverage (+): 0. Max coverage (-): 0

Region: chr28 1801527-. Max. coverage (+): 0. Max coverage (-): 0

RepeatMasker Color Code

**+**

100-98% Identity

<98-95% Identity

<95-90% Identity

<90-85% Identity

<85-80% Identity

<80-75% Identity

<75-70% Identity

<70% Identity

**-**

Gene Set Color Code

**+**

Gene

Pseudogene

**-**

Topology/Coverage Color Code

Coverage Plus Strand

Coverage Minus Strand

Mainstrand: Plus

Mainstrand: Minus

Complementary Strand

Flanking Region  
(if option -flank >0)

Gene Set Annotation  
  
RepeatMasker Annotation  

**1. L2c**: 1796613-1796653 (-), Divergence to consensus: 21.9%  
**2. AT\_rich**: 1796802-1796825 (+), Divergence to consensus: 50%  
**3. SINE2-1\_BT**: 1796841-1796947 (-), Divergence to consensus: 27.1%  
**4. MER74A**: 1796973-1797287 (+), Divergence to consensus: 38.6%  
**5. Bov-tA3**: 1799194-1799396 (+), Divergence to consensus: 11.3%

  
Transcription Factor Binding Sites
