# Supplementary material for: Small RNA sequencing of cryopreserved semen from single bull revealed altered miRNAs and piRNAs expression between High- and Low-motile sperm populations
Source: BMC Genomics. 2017 Jan 4;18:14. doi: 10.1186/s12864-016-3394-7 (PMC5209821; doi:10.1186/s12864-016-3394-7)
Supplement: Additional file 4: — Details for each piRNA clusters found in Low Motile (LM) sperm fraction. Genes, repeats, transposable elements and transcription factors binding sites falling within the cluster regions were reported. (ZIP 1034 kb) [file 12864_2016_3394_MOESM4_ESM.zip › 38.html]

piRNA cluster 38


Predicted piRNA cluster no. 38     previous   next
  

Show proTRAC run info
Hide proTRAC run info

================================= proTRAC ====================================  
VERSION: 2.1                                    LAST MODIFIED: 06. October 2015  
  
Please cite:  
Rosenkranz D, Zischler H. proTRAC - a software for probabilistic piRNA cluster  
detection, visualization and analysis. 2012. BMC Bioinformatics 13:5.  
  
and (for proTRAC 2.0 and later):  
Rosenkranz D, Rudloff S, Bastuck K, Ketting RF, Zischler H. Tupaia small RNAs  
provide insights into function and evolution of RNAi-based transposon defense  
in mammals. 2015. RNA 21(5):911-922.  
  
Contact:  
David Rosenkranz  
Institute of Anthropology, small RNA group  
Johannes Gutenberg University Mainz  
email: rosenkranz@uni-mainz.de  
  
You can find the latest proTRAC version at:  
http://sourceforge.net/projects/protrac/files  
http://www.smallRNAgroup-mainz.de/software  
==============================================================================  
  
PARAMETERS:  
Map file: .............../storage/core/barbara/genhome/smallRNA/fertility/Sample\_not\_motile/pirna/Sample\_not\_motile\_26-33\_collapsed.fa.no-dust.map.weighted-10000-1000-b-0  
Genome file: ............/storage/core/barbara/genhome/smallRNA/fertility/Sample\_all/pirna/bt\_311\_chrY.fa  
RepeatMasker annotation: /storage/genomes/bt\_umd31/GCF\_000003055.6\_Bos\_taurus\_UMD\_3.1.1\_repeatMasker\_chr.out  
GeneSet:................./storage/core/barbara/genhome/smallRNA/fertility/Sample\_all/pirna/full.gtf  
  
Significant (p<=0.01) hit density will be calculated based  
on observed hit distribution.  
  
Sliding window size: ........................................ 5000 bp  
Sliding window increament: .................................. 1000 bp  
Normalize each hit by number of genomic hits: ............... 1 [0=no/1=yes]  
Normalize each hit by number of sequence reads: ............. 1 [0=no/1=yes]  
Normalize values (-> per million mapped reads): ............. 1 [0=no/1=yes]  
Min. fraction of hits with 1T(U) or 10A: .................... 0.75  
Alternatively: Min. fraction of hits with 1T(U) and 10A: .... 0.5  
Min. fraction of hits with typical piRNA length: ............ 0.75  
Typical piRNA length: ....................................... 26-33 nt  
Min. size of a piRNA cluster: ............................... 5000 bp.  
Min. number of hits (absolute): ............................. 0  
Min. number of hits (normalized): ........................... 0  
Min. fraction of hits on the mainstrand: .................... 0.75  
Top fraction of mapped sequences (in terms of read counts): . 1%  
Top fraction accounts for max. n% of sequence reads: ........ 90%  
Min. fraction of hits on each arm of a bidirectional cluster: 0.1  
Output image file for each cluster: ......................... 0 [0=no/1=yes]  
Output html file for each cluster: .......................... 1 [0=no/1=yes]  
Output a summary table: ..................................... 1 [0=no/1=yes]  
Output a FASTA file for each cluster (piRNA sequences): ..... 1 [0=no/1=yes]  
Output a FASTA file comprising cluster sequences: ........... 1 [0=no/1=yes]  
Search DNA motifs in clusters: .............................. 1 [0=no/1=yes]  
Output flanking sequences: +/- .............................. 0 bp  
Output ~.pTi file: .......................................... 1 [0=no/1=yes]  
==============================================================================  
  
  
Genome size (without gaps): ............ 2678902517 bp  
Gaps (N/X/-): .......................... 53837044 bp  
Mapped reads: .......................... 738059667487  
Non-identical sequences: ............... 277001  
Genomic hits: .......................... 533816  
Significant densitiy of mapped reads: .. 15118061 reads/kb

Show proTRAC cluster info
Hide proTRAC cluster info

|  |  |
| --- | --- |
| Location | chr28 |
| Coordinates | 25302730-25325908 |
| Size [bp] | 23179 |
| Sequence hit loci | 895 |
| Mapped reads (normalized) | 2357808557 |
| Mapped reads (normalized) per kb | 101721754.9 |
| Normalized reads with 1T (1U) | 86.7% |
| Normalized reads with 10A | 36.9% |
| Normalized reads with length 26-33 nt | 100% |
| Normalized reads on the main strand(s) | 100% |
| Predicted directionality | mono:minus |

100%

0%

1T (1U)  
reads

10A reads

26-33 nt  
reads

reads on mainstrand

**Either the amount of reads with 1T (1U) OR 10A has to exceed 75% (set with option: -1Tor10A)  
Alternatively the amount of reads with 1T (1U) AND 10A has to exceed 50% (set with option: -1Tand10A)  
Minimum amount of reads with preferred size is 75% (set with option: -pisize)  
Minimum amount of reads on the main strand(s) is 75% (set with option: -clstrand)**

Show read coverage
Hide read coverage

WHAT DO I SEE HERE?  
This chart shows the location of mapped sequence reads within a predicted piRNA cluster. The color refers to the number of genomic hits produced by the sequence read in question. A dark red bar indicates that this sequence read produces many other hits elsewhere in the genome. Many adjacent red or yellow bars can indicate the presence of a multi-copy element such as transposons or rRNA genes. A dark green bar indicates that this sequence read maps uniquely to this locus.

1 hit

2-5 hits

6-10 hits

11-20 hits

21-50 hits

51-100 hits

> 100 hits

chr28

25302730

25325908

Gene Set

RepeatMasker

Mapped  
Reads

213.45

plus strand

minus strand

213.45

Region: chr28 1801528-25302753. Max. coverage (+): 0. Max coverage (-): 5.33

Region: chr28 25302754-25302799. Max. coverage (+): 0. Max coverage (-): 3.94

Region: chr28 25302800-25302845. Max. coverage (+): 0. Max coverage (-): 8.27

Region: chr28 25302846-25302892. Max. coverage (+): 0. Max coverage (-): 6.25

Region: chr28 25302893-25302938. Max. coverage (+): 0. Max coverage (-): 0

Region: chr28 25302939-25302984. Max. coverage (+): 0. Max coverage (-): 0

Region: chr28 25302985-25303031. Max. coverage (+): 0. Max coverage (-): 0

Region: chr28 25303032-25303077. Max. coverage (+): 0. Max coverage (-): 0

Region: chr28 25303078-25303124. Max. coverage (+): 0. Max coverage (-): 0

Region: chr28 25303125-25303170. Max. coverage (+): 0. Max coverage (-): 0

Region: chr28 25303171-25303216. Max. coverage (+): 0. Max coverage (-): 0

Region: chr28 25303217-25303263. Max. coverage (+): 0. Max coverage (-): 0

Region: chr28 25303264-25303309. Max. coverage (+): 0. Max coverage (-): 0

Region: chr28 25303310-25303355. Max. coverage (+): 0. Max coverage (-): 0

Region: chr28 25303356-25303402. Max. coverage (+): 0. Max coverage (-): 0

Region: chr28 25303403-25303448. Max. coverage (+): 0. Max coverage (-): 0

Region: chr28 25303449-25303494. Max. coverage (+): 0. Max coverage (-): 0

Region: chr28 25303495-25303541. Max. coverage (+): 0. Max coverage (-): 0

Region: chr28 25303542-25303587. Max. coverage (+): 0. Max coverage (-): 0.47

Region: chr28 25303588-25303633. Max. coverage (+): 0. Max coverage (-): 0

Region: chr28 25303634-25303680. Max. coverage (+): 0. Max coverage (-): 0

Region: chr28 25303681-25303726. Max. coverage (+): 0. Max coverage (-): 17.38

Region: chr28 25303727-25303773. Max. coverage (+): 0. Max coverage (-): 18.8

Region: chr28 25303774-25303819. Max. coverage (+): 0. Max coverage (-): 5.72

Region: chr28 25303820-25303865. Max. coverage (+): 0. Max coverage (-): 0

Region: chr28 25303866-25303912. Max. coverage (+): 0. Max coverage (-): 0

Region: chr28 25303913-25303958. Max. coverage (+): 0. Max coverage (-): 2.89

Region: chr28 25303959-25304004. Max. coverage (+): 0. Max coverage (-): 0

Region: chr28 25304005-25304051. Max. coverage (+): 0. Max coverage (-): 0

Region: chr28 25304052-25304097. Max. coverage (+): 0. Max coverage (-): 0

Region: chr28 25304098-25304143. Max. coverage (+): 0. Max coverage (-): 0

Region: chr28 25304144-25304190. Max. coverage (+): 0. Max coverage (-): 0

Region: chr28 25304191-25304236. Max. coverage (+): 0. Max coverage (-): 0

Region: chr28 25304237-25304282. Max. coverage (+): 0. Max coverage (-): 0

Region: chr28 25304283-25304329. Max. coverage (+): 0. Max coverage (-): 0

Region: chr28 25304330-25304375. Max. coverage (+): 0. Max coverage (-): 0

Region: chr28 25304376-25304422. Max. coverage (+): 0. Max coverage (-): 0

Region: chr28 25304423-25304468. Max. coverage (+): 0. Max coverage (-): 0

Region: chr28 25304469-25304514. Max. coverage (+): 0. Max coverage (-): 0

Region: chr28 25304515-25304561. Max. coverage (+): 0. Max coverage (-): 0

Region: chr28 25304562-25304607. Max. coverage (+): 0. Max coverage (-): 0

Region: chr28 25304608-25304653. Max. coverage (+): 0. Max coverage (-): 0

Region: chr28 25304654-25304700. Max. coverage (+): 0. Max coverage (-): 5.65

Region: chr28 25304701-25304746. Max. coverage (+): 0. Max coverage (-): 11.92

Region: chr28 25304747-25304792. Max. coverage (+): 0. Max coverage (-): 0

Region: chr28 25304793-25304839. Max. coverage (+): 0. Max coverage (-): 0

Region: chr28 25304840-25304885. Max. coverage (+): 0. Max coverage (-): 0

Region: chr28 25304886-25304932. Max. coverage (+): 0. Max coverage (-): 0

Region: chr28 25304933-25304978. Max. coverage (+): 0. Max coverage (-): 0

Region: chr28 25304979-25305024. Max. coverage (+): 0. Max coverage (-): 0

Region: chr28 25305025-25305071. Max. coverage (+): 0. Max coverage (-): 0

Region: chr28 25305072-25305117. Max. coverage (+): 0. Max coverage (-): 0

Region: chr28 25305118-25305163. Max. coverage (+): 0. Max coverage (-): 0

Region: chr28 25305164-25305210. Max. coverage (+): 0. Max coverage (-): 0

Region: chr28 25305211-25305256. Max. coverage (+): 0. Max coverage (-): 0

Region: chr28 25305257-25305302. Max. coverage (+): 0. Max coverage (-): 0

Region: chr28 25305303-25305349. Max. coverage (+): 0. Max coverage (-): 0

Region: chr28 25305350-25305395. Max. coverage (+): 0. Max coverage (-): 0

Region: chr28 25305396-25305441. Max. coverage (+): 0. Max coverage (-): 0

Region: chr28 25305442-25305488. Max. coverage (+): 0. Max coverage (-): 0

Region: chr28 25305489-25305534. Max. coverage (+): 0. Max coverage (-): 12.77

Region: chr28 25305535-25305581. Max. coverage (+): 0. Max coverage (-): 9.22

Region: chr28 25305582-25305627. Max. coverage (+): 0. Max coverage (-): 14.52

Region: chr28 25305628-25305673. Max. coverage (+): 0. Max coverage (-): 12.29

Region: chr28 25305674-25305720. Max. coverage (+): 0. Max coverage (-): 8.85

Region: chr28 25305721-25305766. Max. coverage (+): 0. Max coverage (-): 0

Region: chr28 25305767-25305812. Max. coverage (+): 0. Max coverage (-): 0

Region: chr28 25305813-25305859. Max. coverage (+): 0. Max coverage (-): 0

Region: chr28 25305860-25305905. Max. coverage (+): 0. Max coverage (-): 0

Region: chr28 25305906-25305951. Max. coverage (+): 0. Max coverage (-): 0

Region: chr28 25305952-25305998. Max. coverage (+): 0. Max coverage (-): 0

Region: chr28 25305999-25306044. Max. coverage (+): 0. Max coverage (-): 0

Region: chr28 25306045-25306090. Max. coverage (+): 0. Max coverage (-): 0

Region: chr28 25306091-25306137. Max. coverage (+): 0. Max coverage (-): 0

Region: chr28 25306138-25306183. Max. coverage (+): 0. Max coverage (-): 0

Region: chr28 25306184-25306230. Max. coverage (+): 0. Max coverage (-): 0

Region: chr28 25306231-25306276. Max. coverage (+): 0. Max coverage (-): 0

Region: chr28 25306277-25306322. Max. coverage (+): 0. Max coverage (-): 0

Region: chr28 25306323-25306369. Max. coverage (+): 0. Max coverage (-): 0

Region: chr28 25306370-25306415. Max. coverage (+): 0. Max coverage (-): 17.14

Region: chr28 25306416-25306461. Max. coverage (+): 0. Max coverage (-): 0

Region: chr28 25306462-25306508. Max. coverage (+): 0. Max coverage (-): 0

Region: chr28 25306509-25306554. Max. coverage (+): 0. Max coverage (-): 0

Region: chr28 25306555-25306600. Max. coverage (+): 0. Max coverage (-): 0

Region: chr28 25306601-25306647. Max. coverage (+): 0. Max coverage (-): 0

Region: chr28 25306648-25306693. Max. coverage (+): 0. Max coverage (-): 0

Region: chr28 25306694-25306739. Max. coverage (+): 0. Max coverage (-): 0

Region: chr28 25306740-25306786. Max. coverage (+): 0. Max coverage (-): 0

Region: chr28 25306787-25306832. Max. coverage (+): 0. Max coverage (-): 8.05

Region: chr28 25306833-25306879. Max. coverage (+): 0. Max coverage (-): 0

Region: chr28 25306880-25306925. Max. coverage (+): 0. Max coverage (-): 1.65

Region: chr28 25306926-25306971. Max. coverage (+): 0. Max coverage (-): 21.17

Region: chr28 25306972-25307018. Max. coverage (+): 0. Max coverage (-): 213.45

Region: chr28 25307019-25307064. Max. coverage (+): 0. Max coverage (-): 21.95

Region: chr28 25307065-25307110. Max. coverage (+): 0. Max coverage (-): 44.06

Region: chr28 25307111-25307157. Max. coverage (+): 0. Max coverage (-): 3.37

Region: chr28 25307158-25307203. Max. coverage (+): 0. Max coverage (-): 61.9

Region: chr28 25307204-25307249. Max. coverage (+): 0. Max coverage (-): 10.98

Region: chr28 25307250-25307296. Max. coverage (+): 0. Max coverage (-): 46.3

Region: chr28 25307297-25307342. Max. coverage (+): 0. Max coverage (-): 26.57

Region: chr28 25307343-25307388. Max. coverage (+): 0. Max coverage (-): 0

Region: chr28 25307389-25307435. Max. coverage (+): 0. Max coverage (-): 0

Region: chr28 25307436-25307481. Max. coverage (+): 0. Max coverage (-): 0

Region: chr28 25307482-25307528. Max. coverage (+): 0. Max coverage (-): 0

Region: chr28 25307529-25307574. Max. coverage (+): 0. Max coverage (-): 0

Region: chr28 25307575-25307620. Max. coverage (+): 0. Max coverage (-): 0

Region: chr28 25307621-25307667. Max. coverage (+): 0. Max coverage (-): 0

Region: chr28 25307668-25307713. Max. coverage (+): 0. Max coverage (-): 0

Region: chr28 25307714-25307759. Max. coverage (+): 0. Max coverage (-): 0

Region: chr28 25307760-25307806. Max. coverage (+): 0. Max coverage (-): 0

Region: chr28 25307807-25307852. Max. coverage (+): 0. Max coverage (-): 0

Region: chr28 25307853-25307898. Max. coverage (+): 0. Max coverage (-): 0

Region: chr28 25307899-25307945. Max. coverage (+): 0. Max coverage (-): 4.25

Region: chr28 25307946-25307991. Max. coverage (+): 0. Max coverage (-): 4.25

Region: chr28 25307992-25308037. Max. coverage (+): 0. Max coverage (-): 0

Region: chr28 25308038-25308084. Max. coverage (+): 0. Max coverage (-): 0.58

Region: chr28 25308085-25308130. Max. coverage (+): 0. Max coverage (-): 0

Region: chr28 25308131-25308177. Max. coverage (+): 0. Max coverage (-): 6.87

Region: chr28 25308178-25308223. Max. coverage (+): 0. Max coverage (-): 0

Region: chr28 25308224-25308269. Max. coverage (+): 0. Max coverage (-): 4.32

Region: chr28 25308270-25308316. Max. coverage (+): 0. Max coverage (-): 4.32

Region: chr28 25308317-25308362. Max. coverage (+): 0. Max coverage (-): 0

Region: chr28 25308363-25308408. Max. coverage (+): 0. Max coverage (-): 0

Region: chr28 25308409-25308455. Max. coverage (+): 0. Max coverage (-): 0

Region: chr28 25308456-25308501. Max. coverage (+): 0. Max coverage (-): 0

Region: chr28 25308502-25308547. Max. coverage (+): 0. Max coverage (-): 0

Region: chr28 25308548-25308594. Max. coverage (+): 0. Max coverage (-): 4.24

Region: chr28 25308595-25308640. Max. coverage (+): 0. Max coverage (-): 19.69

Region: chr28 25308641-25308687. Max. coverage (+): 0. Max coverage (-): 0

Region: chr28 25308688-25308733. Max. coverage (+): 0. Max coverage (-): 7.29

Region: chr28 25308734-25308779. Max. coverage (+): 0. Max coverage (-): 7.29

Region: chr28 25308780-25308826. Max. coverage (+): 0. Max coverage (-): 6.76

Region: chr28 25308827-25308872. Max. coverage (+): 0. Max coverage (-): 6.76

Region: chr28 25308873-25308918. Max. coverage (+): 0. Max coverage (-): 5.9

Region: chr28 25308919-25308965. Max. coverage (+): 0. Max coverage (-): 0

Region: chr28 25308966-25309011. Max. coverage (+): 0. Max coverage (-): 6.82

Region: chr28 25309012-25309057. Max. coverage (+): 0. Max coverage (-): 8.19

Region: chr28 25309058-25309104. Max. coverage (+): 0. Max coverage (-): 9.45

Region: chr28 25309105-25309150. Max. coverage (+): 0. Max coverage (-): 0

Region: chr28 25309151-25309196. Max. coverage (+): 0. Max coverage (-): 7.37

Region: chr28 25309197-25309243. Max. coverage (+): 0. Max coverage (-): 0

Region: chr28 25309244-25309289. Max. coverage (+): 0. Max coverage (-): 0

Region: chr28 25309290-25309336. Max. coverage (+): 0. Max coverage (-): 0

Region: chr28 25309337-25309382. Max. coverage (+): 0. Max coverage (-): 0

Region: chr28 25309383-25309428. Max. coverage (+): 0. Max coverage (-): 0

Region: chr28 25309429-25309475. Max. coverage (+): 0. Max coverage (-): 0

Region: chr28 25309476-25309521. Max. coverage (+): 0. Max coverage (-): 0

Region: chr28 25309522-25309567. Max. coverage (+): 0. Max coverage (-): 6.53

Region: chr28 25309568-25309614. Max. coverage (+): 0. Max coverage (-): 0

Region: chr28 25309615-25309660. Max. coverage (+): 0. Max coverage (-): 0

Region: chr28 25309661-25309706. Max. coverage (+): 0. Max coverage (-): 0

Region: chr28 25309707-25309753. Max. coverage (+): 0. Max coverage (-): 0

Region: chr28 25309754-25309799. Max. coverage (+): 0. Max coverage (-): 2.79

Region: chr28 25309800-25309845. Max. coverage (+): 0. Max coverage (-): 7.2

Region: chr28 25309846-25309892. Max. coverage (+): 0. Max coverage (-): 2.85

Region: chr28 25309893-25309938. Max. coverage (+): 0. Max coverage (-): 0

Region: chr28 25309939-25309985. Max. coverage (+): 0. Max coverage (-): 0

Region: chr28 25309986-25310031. Max. coverage (+): 0. Max coverage (-): 0

Region: chr28 25310032-25310077. Max. coverage (+): 0. Max coverage (-): 0

Region: chr28 25310078-25310124. Max. coverage (+): 0. Max coverage (-): 0

Region: chr28 25310125-25310170. Max. coverage (+): 0. Max coverage (-): 0

Region: chr28 25310171-25310216. Max. coverage (+): 0. Max coverage (-): 0

Region: chr28 25310217-25310263. Max. coverage (+): 0. Max coverage (-): 0

Region: chr28 25310264-25310309. Max. coverage (+): 0. Max coverage (-): 0

Region: chr28 25310310-25310355. Max. coverage (+): 0. Max coverage (-): 0

Region: chr28 25310356-25310402. Max. coverage (+): 0. Max coverage (-): 0

Region: chr28 25310403-25310448. Max. coverage (+): 0. Max coverage (-): 0

Region: chr28 25310449-25310494. Max. coverage (+): 0. Max coverage (-): 0

Region: chr28 25310495-25310541. Max. coverage (+): 0. Max coverage (-): 1.31

Region: chr28 25310542-25310587. Max. coverage (+): 0. Max coverage (-): 16.03

Region: chr28 25310588-25310634. Max. coverage (+): 0. Max coverage (-): 7.55

Region: chr28 25310635-25310680. Max. coverage (+): 0. Max coverage (-): 0

Region: chr28 25310681-25310726. Max. coverage (+): 0. Max coverage (-): 0

Region: chr28 25310727-25310773. Max. coverage (+): 0. Max coverage (-): 0

Region: chr28 25310774-25310819. Max. coverage (+): 0. Max coverage (-): 0

Region: chr28 25310820-25310865. Max. coverage (+): 0. Max coverage (-): 0

Region: chr28 25310866-25310912. Max. coverage (+): 0. Max coverage (-): 12.3

Region: chr28 25310913-25310958. Max. coverage (+): 0. Max coverage (-): 0

Region: chr28 25310959-25311004. Max. coverage (+): 0. Max coverage (-): 0

Region: chr28 25311005-25311051. Max. coverage (+): 0. Max coverage (-): 0

Region: chr28 25311052-25311097. Max. coverage (+): 0. Max coverage (-): 7.81

Region: chr28 25311098-25311143. Max. coverage (+): 0. Max coverage (-): 3.02

Region: chr28 25311144-25311190. Max. coverage (+): 0. Max coverage (-): 0

Region: chr28 25311191-25311236. Max. coverage (+): 0. Max coverage (-): 0

Region: chr28 25311237-25311283. Max. coverage (+): 0. Max coverage (-): 1.26

Region: chr28 25311284-25311329. Max. coverage (+): 0. Max coverage (-): 16

Region: chr28 25311330-25311375. Max. coverage (+): 0. Max coverage (-): 0

Region: chr28 25311376-25311422. Max. coverage (+): 0. Max coverage (-): 0

Region: chr28 25311423-25311468. Max. coverage (+): 0. Max coverage (-): 0

Region: chr28 25311469-25311514. Max. coverage (+): 0. Max coverage (-): 0

Region: chr28 25311515-25311561. Max. coverage (+): 0. Max coverage (-): 5.79

Region: chr28 25311562-25311607. Max. coverage (+): 0. Max coverage (-): 6.62

Region: chr28 25311608-25311653. Max. coverage (+): 0. Max coverage (-): 19.59

Region: chr28 25311654-25311700. Max. coverage (+): 0. Max coverage (-): 19.59

Region: chr28 25311701-25311746. Max. coverage (+): 0. Max coverage (-): 5.93

Region: chr28 25311747-25311792. Max. coverage (+): 0. Max coverage (-): 21.82

Region: chr28 25311793-25311839. Max. coverage (+): 0. Max coverage (-): 44.54

Region: chr28 25311840-25311885. Max. coverage (+): 0. Max coverage (-): 0

Region: chr28 25311886-25311932. Max. coverage (+): 0. Max coverage (-): 0

Region: chr28 25311933-25311978. Max. coverage (+): 0. Max coverage (-): 1.3

Region: chr28 25311979-25312024. Max. coverage (+): 0. Max coverage (-): 0

Region: chr28 25312025-25312071. Max. coverage (+): 0. Max coverage (-): 0

Region: chr28 25312072-25312117. Max. coverage (+): 0. Max coverage (-): 0

Region: chr28 25312118-25312163. Max. coverage (+): 0. Max coverage (-): 0

Region: chr28 25312164-25312210. Max. coverage (+): 0. Max coverage (-): 0

Region: chr28 25312211-25312256. Max. coverage (+): 0. Max coverage (-): 15.27

Region: chr28 25312257-25312302. Max. coverage (+): 0. Max coverage (-): 2.51

Region: chr28 25312303-25312349. Max. coverage (+): 0. Max coverage (-): 19.98

Region: chr28 25312350-25312395. Max. coverage (+): 0. Max coverage (-): 4.82

Region: chr28 25312396-25312442. Max. coverage (+): 0. Max coverage (-): 0

Region: chr28 25312443-25312488. Max. coverage (+): 0. Max coverage (-): 1.26

Region: chr28 25312489-25312534. Max. coverage (+): 0. Max coverage (-): 0

Region: chr28 25312535-25312581. Max. coverage (+): 0. Max coverage (-): 0

Region: chr28 25312582-25312627. Max. coverage (+): 0. Max coverage (-): 0

Region: chr28 25312628-25312673. Max. coverage (+): 0. Max coverage (-): 0

Region: chr28 25312674-25312720. Max. coverage (+): 0. Max coverage (-): 0

Region: chr28 25312721-25312766. Max. coverage (+): 0. Max coverage (-): 0

Region: chr28 25312767-25312812. Max. coverage (+): 0. Max coverage (-): 0

Region: chr28 25312813-25312859. Max. coverage (+): 0. Max coverage (-): 0

Region: chr28 25312860-25312905. Max. coverage (+): 0. Max coverage (-): 0

Region: chr28 25312906-25312951. Max. coverage (+): 0. Max coverage (-): 0

Region: chr28 25312952-25312998. Max. coverage (+): 0. Max coverage (-): 5.86

Region: chr28 25312999-25313044. Max. coverage (+): 0. Max coverage (-): 5.04

Region: chr28 25313045-25313091. Max. coverage (+): 0. Max coverage (-): 0

Region: chr28 25313092-25313137. Max. coverage (+): 0. Max coverage (-): 4.42

Region: chr28 25313138-25313183. Max. coverage (+): 0. Max coverage (-): 11.08

Region: chr28 25313184-25313230. Max. coverage (+): 0. Max coverage (-): 15.32

Region: chr28 25313231-25313276. Max. coverage (+): 0. Max coverage (-): 0.77

Region: chr28 25313277-25313322. Max. coverage (+): 0. Max coverage (-): 18.53

Region: chr28 25313323-25313369. Max. coverage (+): 0. Max coverage (-): 9.75

Region: chr28 25313370-25313415. Max. coverage (+): 0. Max coverage (-): 10.37

Region: chr28 25313416-25313461. Max. coverage (+): 0. Max coverage (-): 11.07

Region: chr28 25313462-25313508. Max. coverage (+): 0. Max coverage (-): 5.5

Region: chr28 25313509-25313554. Max. coverage (+): 0. Max coverage (-): 11.89

Region: chr28 25313555-25313600. Max. coverage (+): 0. Max coverage (-): 7.09

Region: chr28 25313601-25313647. Max. coverage (+): 0. Max coverage (-): 29.95

Region: chr28 25313648-25313693. Max. coverage (+): 0. Max coverage (-): 39.07

Region: chr28 25313694-25313740. Max. coverage (+): 0. Max coverage (-): 18.96

Region: chr28 25313741-25313786. Max. coverage (+): 0. Max coverage (-): 5.4

Region: chr28 25313787-25313832. Max. coverage (+): 0. Max coverage (-): 17.6

Region: chr28 25313833-25313879. Max. coverage (+): 0. Max coverage (-): 55.8

Region: chr28 25313880-25313925. Max. coverage (+): 0. Max coverage (-): 16.25

Region: chr28 25313926-25313971. Max. coverage (+): 0. Max coverage (-): 6.08

Region: chr28 25313972-25314018. Max. coverage (+): 0. Max coverage (-): 3.61

Region: chr28 25314019-25314064. Max. coverage (+): 0. Max coverage (-): 9.32

Region: chr28 25314065-25314110. Max. coverage (+): 0. Max coverage (-): 5.65

Region: chr28 25314111-25314157. Max. coverage (+): 0. Max coverage (-): 18.61

Region: chr28 25314158-25314203. Max. coverage (+): 0. Max coverage (-): 5.45

Region: chr28 25314204-25314249. Max. coverage (+): 0. Max coverage (-): 0

Region: chr28 25314250-25314296. Max. coverage (+): 0. Max coverage (-): 0

Region: chr28 25314297-25314342. Max. coverage (+): 0. Max coverage (-): 8.44

Region: chr28 25314343-25314389. Max. coverage (+): 0. Max coverage (-): 6.08

Region: chr28 25314390-25314435. Max. coverage (+): 0. Max coverage (-): 3.66

Region: chr28 25314436-25314481. Max. coverage (+): 0. Max coverage (-): 6.12

Region: chr28 25314482-25314528. Max. coverage (+): 0. Max coverage (-): 0

Region: chr28 25314529-25314574. Max. coverage (+): 0. Max coverage (-): 3.52

Region: chr28 25314575-25314620. Max. coverage (+): 0. Max coverage (-): 2.11

Region: chr28 25314621-25314667. Max. coverage (+): 0. Max coverage (-): 0

Region: chr28 25314668-25314713. Max. coverage (+): 0. Max coverage (-): 4.75

Region: chr28 25314714-25314759. Max. coverage (+): 0. Max coverage (-): 4.41

Region: chr28 25314760-25314806. Max. coverage (+): 0. Max coverage (-): 4.66

Region: chr28 25314807-25314852. Max. coverage (+): 0. Max coverage (-): 6.31

Region: chr28 25314853-25314898. Max. coverage (+): 0. Max coverage (-): 0

Region: chr28 25314899-25314945. Max. coverage (+): 0. Max coverage (-): 20.57

Region: chr28 25314946-25314991. Max. coverage (+): 0. Max coverage (-): 5.11

Region: chr28 25314992-25315038. Max. coverage (+): 0. Max coverage (-): 16.9

Region: chr28 25315039-25315084. Max. coverage (+): 0. Max coverage (-): 5.53

Region: chr28 25315085-25315130. Max. coverage (+): 0. Max coverage (-): 0.14

Region: chr28 25315131-25315177. Max. coverage (+): 0. Max coverage (-): 0

Region: chr28 25315178-25315223. Max. coverage (+): 0. Max coverage (-): 17.25

Region: chr28 25315224-25315269. Max. coverage (+): 0. Max coverage (-): 16.54

Region: chr28 25315270-25315316. Max. coverage (+): 0. Max coverage (-): 53.28

Region: chr28 25315317-25315362. Max. coverage (+): 0. Max coverage (-): 28.26

Region: chr28 25315363-25315408. Max. coverage (+): 0. Max coverage (-): 1.46

Region: chr28 25315409-25315455. Max. coverage (+): 0. Max coverage (-): 8.95

Region: chr28 25315456-25315501. Max. coverage (+): 0. Max coverage (-): 6.47

Region: chr28 25315502-25315547. Max. coverage (+): 0. Max coverage (-): 5.63

Region: chr28 25315548-25315594. Max. coverage (+): 0. Max coverage (-): 0

Region: chr28 25315595-25315640. Max. coverage (+): 0. Max coverage (-): 29.34

Region: chr28 25315641-25315687. Max. coverage (+): 0. Max coverage (-): 23.71

Region: chr28 25315688-25315733. Max. coverage (+): 0. Max coverage (-): 12.73

Region: chr28 25315734-25315779. Max. coverage (+): 0. Max coverage (-): 9.11

Region: chr28 25315780-25315826. Max. coverage (+): 0. Max coverage (-): 5.13

Region: chr28 25315827-25315872. Max. coverage (+): 0. Max coverage (-): 19

Region: chr28 25315873-25315918. Max. coverage (+): 0. Max coverage (-): 32.61

Region: chr28 25315919-25315965. Max. coverage (+): 0. Max coverage (-): 6.75

Region: chr28 25315966-25316011. Max. coverage (+): 0. Max coverage (-): 15.31

Region: chr28 25316012-25316057. Max. coverage (+): 0. Max coverage (-): 97.7

Region: chr28 25316058-25316104. Max. coverage (+): 0. Max coverage (-): 16.07

Region: chr28 25316105-25316150. Max. coverage (+): 0. Max coverage (-): 2.97

Region: chr28 25316151-25316196. Max. coverage (+): 0. Max coverage (-): 6.04

Region: chr28 25316197-25316243. Max. coverage (+): 0. Max coverage (-): 8.6

Region: chr28 25316244-25316289. Max. coverage (+): 0. Max coverage (-): 4.16

Region: chr28 25316290-25316336. Max. coverage (+): 0. Max coverage (-): 3.26

Region: chr28 25316337-25316382. Max. coverage (+): 0. Max coverage (-): 4.33

Region: chr28 25316383-25316428. Max. coverage (+): 0. Max coverage (-): 0

Region: chr28 25316429-25316475. Max. coverage (+): 0. Max coverage (-): 7.14

Region: chr28 25316476-25316521. Max. coverage (+): 0. Max coverage (-): 10.58

Region: chr28 25316522-25316567. Max. coverage (+): 0. Max coverage (-): 10.62

Region: chr28 25316568-25316614. Max. coverage (+): 0. Max coverage (-): 0

Region: chr28 25316615-25316660. Max. coverage (+): 0. Max coverage (-): 24.07

Region: chr28 25316661-25316706. Max. coverage (+): 0. Max coverage (-): 4.64

Region: chr28 25316707-25316753. Max. coverage (+): 0. Max coverage (-): 4.64

Region: chr28 25316754-25316799. Max. coverage (+): 0. Max coverage (-): 37.95

Region: chr28 25316800-25316846. Max. coverage (+): 0. Max coverage (-): 34.01

Region: chr28 25316847-25316892. Max. coverage (+): 0. Max coverage (-): 33.51

Region: chr28 25316893-25316938. Max. coverage (+): 0. Max coverage (-): 42.16

Region: chr28 25316939-25316985. Max. coverage (+): 0. Max coverage (-): 41.27

Region: chr28 25316986-25317031. Max. coverage (+): 0. Max coverage (-): 40.58

Region: chr28 25317032-25317077. Max. coverage (+): 0. Max coverage (-): 0

Region: chr28 25317078-25317124. Max. coverage (+): 0. Max coverage (-): 15.2

Region: chr28 25317125-25317170. Max. coverage (+): 0. Max coverage (-): 0

Region: chr28 25317171-25317216. Max. coverage (+): 0. Max coverage (-): 39.42

Region: chr28 25317217-25317263. Max. coverage (+): 0. Max coverage (-): 22.7

Region: chr28 25317264-25317309. Max. coverage (+): 0. Max coverage (-): 0

Region: chr28 25317310-25317355. Max. coverage (+): 0. Max coverage (-): 0

Region: chr28 25317356-25317402. Max. coverage (+): 0. Max coverage (-): 7.01

Region: chr28 25317403-25317448. Max. coverage (+): 0. Max coverage (-): 0

Region: chr28 25317449-25317495. Max. coverage (+): 0. Max coverage (-): 13.29

Region: chr28 25317496-25317541. Max. coverage (+): 0. Max coverage (-): 9.26

Region: chr28 25317542-25317587. Max. coverage (+): 0. Max coverage (-): 2.8

Region: chr28 25317588-25317634. Max. coverage (+): 0. Max coverage (-): 8.64

Region: chr28 25317635-25317680. Max. coverage (+): 0. Max coverage (-): 11.67

Region: chr28 25317681-25317726. Max. coverage (+): 0. Max coverage (-): 20.54

Region: chr28 25317727-25317773. Max. coverage (+): 0. Max coverage (-): 61.21

Region: chr28 25317774-25317819. Max. coverage (+): 0. Max coverage (-): 6.29

Region: chr28 25317820-25317865. Max. coverage (+): 0. Max coverage (-): 1.62

Region: chr28 25317866-25317912. Max. coverage (+): 0. Max coverage (-): 0

Region: chr28 25317913-25317958. Max. coverage (+): 0. Max coverage (-): 0

Region: chr28 25317959-25318004. Max. coverage (+): 0. Max coverage (-): 0

Region: chr28 25318005-25318051. Max. coverage (+): 0. Max coverage (-): 0

Region: chr28 25318052-25318097. Max. coverage (+): 0. Max coverage (-): 12.41

Region: chr28 25318098-25318144. Max. coverage (+): 0. Max coverage (-): 5.12

Region: chr28 25318145-25318190. Max. coverage (+): 0. Max coverage (-): 0.95

Region: chr28 25318191-25318236. Max. coverage (+): 0. Max coverage (-): 0.95

Region: chr28 25318237-25318283. Max. coverage (+): 0. Max coverage (-): 0

Region: chr28 25318284-25318329. Max. coverage (+): 0. Max coverage (-): 4.09

Region: chr28 25318330-25318375. Max. coverage (+): 0. Max coverage (-): 0.46

Region: chr28 25318376-25318422. Max. coverage (+): 0. Max coverage (-): 12.48

Region: chr28 25318423-25318468. Max. coverage (+): 0. Max coverage (-): 3.63

Region: chr28 25318469-25318514. Max. coverage (+): 0. Max coverage (-): 8.21

Region: chr28 25318515-25318561. Max. coverage (+): 0. Max coverage (-): 2.68

Region: chr28 25318562-25318607. Max. coverage (+): 0. Max coverage (-): 6.82

Region: chr28 25318608-25318653. Max. coverage (+): 0. Max coverage (-): 1.77

Region: chr28 25318654-25318700. Max. coverage (+): 0. Max coverage (-): 0

Region: chr28 25318701-25318746. Max. coverage (+): 0. Max coverage (-): 0

Region: chr28 25318747-25318793. Max. coverage (+): 0. Max coverage (-): 0

Region: chr28 25318794-25318839. Max. coverage (+): 0. Max coverage (-): 0

Region: chr28 25318840-25318885. Max. coverage (+): 0. Max coverage (-): 0

Region: chr28 25318886-25318932. Max. coverage (+): 0. Max coverage (-): 3.29

Region: chr28 25318933-25318978. Max. coverage (+): 0. Max coverage (-): 3.29

Region: chr28 25318979-25319024. Max. coverage (+): 0. Max coverage (-): 3.58

Region: chr28 25319025-25319071. Max. coverage (+): 0. Max coverage (-): 0

Region: chr28 25319072-25319117. Max. coverage (+): 0. Max coverage (-): 0

Region: chr28 25319118-25319163. Max. coverage (+): 0. Max coverage (-): 0

Region: chr28 25319164-25319210. Max. coverage (+): 0. Max coverage (-): 0

Region: chr28 25319211-25319256. Max. coverage (+): 0. Max coverage (-): 0

Region: chr28 25319257-25319302. Max. coverage (+): 0. Max coverage (-): 13.43

Region: chr28 25319303-25319349. Max. coverage (+): 0. Max coverage (-): 0.56

Region: chr28 25319350-25319395. Max. coverage (+): 0. Max coverage (-): 0

Region: chr28 25319396-25319442. Max. coverage (+): 0. Max coverage (-): 0

Region: chr28 25319443-25319488. Max. coverage (+): 0. Max coverage (-): 0

Region: chr28 25319489-25319534. Max. coverage (+): 0. Max coverage (-): 0

Region: chr28 25319535-25319581. Max. coverage (+): 0. Max coverage (-): 0

Region: chr28 25319582-25319627. Max. coverage (+): 0. Max coverage (-): 0

Region: chr28 25319628-25319673. Max. coverage (+): 0. Max coverage (-): 0

Region: chr28 25319674-25319720. Max. coverage (+): 0. Max coverage (-): 0

Region: chr28 25319721-25319766. Max. coverage (+): 0. Max coverage (-): 0

Region: chr28 25319767-25319812. Max. coverage (+): 0. Max coverage (-): 0

Region: chr28 25319813-25319859. Max. coverage (+): 0. Max coverage (-): 0

Region: chr28 25319860-25319905. Max. coverage (+): 0. Max coverage (-): 0

Region: chr28 25319906-25319951. Max. coverage (+): 0. Max coverage (-): 0

Region: chr28 25319952-25319998. Max. coverage (+): 0. Max coverage (-): 0

Region: chr28 25319999-25320044. Max. coverage (+): 0. Max coverage (-): 0

Region: chr28 25320045-25320091. Max. coverage (+): 0. Max coverage (-): 0

Region: chr28 25320092-25320137. Max. coverage (+): 0. Max coverage (-): 0

Region: chr28 25320138-25320183. Max. coverage (+): 0. Max coverage (-): 0

Region: chr28 25320184-25320230. Max. coverage (+): 0. Max coverage (-): 0

Region: chr28 25320231-25320276. Max. coverage (+): 0. Max coverage (-): 0

Region: chr28 25320277-25320322. Max. coverage (+): 0. Max coverage (-): 0

Region: chr28 25320323-25320369. Max. coverage (+): 0. Max coverage (-): 0

Region: chr28 25320370-25320415. Max. coverage (+): 0. Max coverage (-): 0

Region: chr28 25320416-25320461. Max. coverage (+): 0. Max coverage (-): 0

Region: chr28 25320462-25320508. Max. coverage (+): 0. Max coverage (-): 0

Region: chr28 25320509-25320554. Max. coverage (+): 0. Max coverage (-): 0

Region: chr28 25320555-25320601. Max. coverage (+): 0. Max coverage (-): 0

Region: chr28 25320602-25320647. Max. coverage (+): 0. Max coverage (-): 0

Region: chr28 25320648-25320693. Max. coverage (+): 0. Max coverage (-): 0

Region: chr28 25320694-25320740. Max. coverage (+): 0. Max coverage (-): 0

Region: chr28 25320741-25320786. Max. coverage (+): 0. Max coverage (-): 0

Region: chr28 25320787-25320832. Max. coverage (+): 0. Max coverage (-): 0

Region: chr28 25320833-25320879. Max. coverage (+): 0. Max coverage (-): 0

Region: chr28 25320880-25320925. Max. coverage (+): 0. Max coverage (-): 0

Region: chr28 25320926-25320971. Max. coverage (+): 0. Max coverage (-): 0.81

Region: chr28 25320972-25321018. Max. coverage (+): 0. Max coverage (-): 0.81

Region: chr28 25321019-25321064. Max. coverage (+): 0. Max coverage (-): 0

Region: chr28 25321065-25321110. Max. coverage (+): 0. Max coverage (-): 0

Region: chr28 25321111-25321157. Max. coverage (+): 0. Max coverage (-): 0

Region: chr28 25321158-25321203. Max. coverage (+): 0. Max coverage (-): 0

Region: chr28 25321204-25321250. Max. coverage (+): 0. Max coverage (-): 0

Region: chr28 25321251-25321296. Max. coverage (+): 0. Max coverage (-): 0

Region: chr28 25321297-25321342. Max. coverage (+): 0. Max coverage (-): 0

Region: chr28 25321343-25321389. Max. coverage (+): 0. Max coverage (-): 0

Region: chr28 25321390-25321435. Max. coverage (+): 0. Max coverage (-): 0

Region: chr28 25321436-25321481. Max. coverage (+): 0. Max coverage (-): 0

Region: chr28 25321482-25321528. Max. coverage (+): 0. Max coverage (-): 0

Region: chr28 25321529-25321574. Max. coverage (+): 0. Max coverage (-): 0

Region: chr28 25321575-25321620. Max. coverage (+): 0. Max coverage (-): 0

Region: chr28 25321621-25321667. Max. coverage (+): 0. Max coverage (-): 3.45

Region: chr28 25321668-25321713. Max. coverage (+): 0. Max coverage (-): 0

Region: chr28 25321714-25321759. Max. coverage (+): 0. Max coverage (-): 0

Region: chr28 25321760-25321806. Max. coverage (+): 0. Max coverage (-): 0

Region: chr28 25321807-25321852. Max. coverage (+): 0. Max coverage (-): 0.08

Region: chr28 25321853-25321899. Max. coverage (+): 0. Max coverage (-): 2.55

Region: chr28 25321900-25321945. Max. coverage (+): 0. Max coverage (-): 0

Region: chr28 25321946-25321991. Max. coverage (+): 0. Max coverage (-): 0

Region: chr28 25321992-25322038. Max. coverage (+): 0. Max coverage (-): 0

Region: chr28 25322039-25322084. Max. coverage (+): 0. Max coverage (-): 0

Region: chr28 25322085-25322130. Max. coverage (+): 0. Max coverage (-): 0

Region: chr28 25322131-25322177. Max. coverage (+): 0. Max coverage (-): 0

Region: chr28 25322178-25322223. Max. coverage (+): 0. Max coverage (-): 0

Region: chr28 25322224-25322269. Max. coverage (+): 0. Max coverage (-): 0

Region: chr28 25322270-25322316. Max. coverage (+): 0. Max coverage (-): 1.12

Region: chr28 25322317-25322362. Max. coverage (+): 0. Max coverage (-): 0

Region: chr28 25322363-25322408. Max. coverage (+): 0. Max coverage (-): 0

Region: chr28 25322409-25322455. Max. coverage (+): 0. Max coverage (-): 0

Region: chr28 25322456-25322501. Max. coverage (+): 0. Max coverage (-): 0

Region: chr28 25322502-25322548. Max. coverage (+): 0. Max coverage (-): 0

Region: chr28 25322549-25322594. Max. coverage (+): 0. Max coverage (-): 0

Region: chr28 25322595-25322640. Max. coverage (+): 0. Max coverage (-): 0

Region: chr28 25322641-25322687. Max. coverage (+): 0. Max coverage (-): 0

Region: chr28 25322688-25322733. Max. coverage (+): 0. Max coverage (-): 0

Region: chr28 25322734-25322779. Max. coverage (+): 0. Max coverage (-): 0

Region: chr28 25322780-25322826. Max. coverage (+): 0. Max coverage (-): 0

Region: chr28 25322827-25322872. Max. coverage (+): 0. Max coverage (-): 0

Region: chr28 25322873-25322918. Max. coverage (+): 0. Max coverage (-): 0

Region: chr28 25322919-25322965. Max. coverage (+): 0. Max coverage (-): 0

Region: chr28 25322966-25323011. Max. coverage (+): 0. Max coverage (-): 0

Region: chr28 25323012-25323057. Max. coverage (+): 0. Max coverage (-): 0

Region: chr28 25323058-25323104. Max. coverage (+): 0. Max coverage (-): 0

Region: chr28 25323105-25323150. Max. coverage (+): 0. Max coverage (-): 5

Region: chr28 25323151-25323197. Max. coverage (+): 0. Max coverage (-): 0

Region: chr28 25323198-25323243. Max. coverage (+): 0. Max coverage (-): 0

Region: chr28 25323244-25323289. Max. coverage (+): 0. Max coverage (-): 0

Region: chr28 25323290-25323336. Max. coverage (+): 0. Max coverage (-): 10.72

Region: chr28 25323337-25323382. Max. coverage (+): 0. Max coverage (-): 10.72

Region: chr28 25323383-25323428. Max. coverage (+): 0. Max coverage (-): 0

Region: chr28 25323429-25323475. Max. coverage (+): 0. Max coverage (-): 0

Region: chr28 25323476-25323521. Max. coverage (+): 0. Max coverage (-): 3.88

Region: chr28 25323522-25323567. Max. coverage (+): 0. Max coverage (-): 0

Region: chr28 25323568-25323614. Max. coverage (+): 0. Max coverage (-): 0

Region: chr28 25323615-25323660. Max. coverage (+): 0. Max coverage (-): 0

Region: chr28 25323661-25323706. Max. coverage (+): 0. Max coverage (-): 0.4

Region: chr28 25323707-25323753. Max. coverage (+): 0. Max coverage (-): 0.4

Region: chr28 25323754-25323799. Max. coverage (+): 0. Max coverage (-): 6.15

Region: chr28 25323800-25323846. Max. coverage (+): 0. Max coverage (-): 0

Region: chr28 25323847-25323892. Max. coverage (+): 0. Max coverage (-): 0

Region: chr28 25323893-25323938. Max. coverage (+): 0. Max coverage (-): 0

Region: chr28 25323939-25323985. Max. coverage (+): 0. Max coverage (-): 0

Region: chr28 25323986-25324031. Max. coverage (+): 0. Max coverage (-): 0

Region: chr28 25324032-25324077. Max. coverage (+): 0. Max coverage (-): 0

Region: chr28 25324078-25324124. Max. coverage (+): 0. Max coverage (-): 0

Region: chr28 25324125-25324170. Max. coverage (+): 0. Max coverage (-): 0

Region: chr28 25324171-25324216. Max. coverage (+): 0. Max coverage (-): 0

Region: chr28 25324217-25324263. Max. coverage (+): 0. Max coverage (-): 0

Region: chr28 25324264-25324309. Max. coverage (+): 0. Max coverage (-): 0

Region: chr28 25324310-25324356. Max. coverage (+): 0. Max coverage (-): 0

Region: chr28 25324357-25324402. Max. coverage (+): 0. Max coverage (-): 5.4

Region: chr28 25324403-25324448. Max. coverage (+): 0. Max coverage (-): 0

Region: chr28 25324449-25324495. Max. coverage (+): 0. Max coverage (-): 0

Region: chr28 25324496-25324541. Max. coverage (+): 0. Max coverage (-): 3.28

Region: chr28 25324542-25324587. Max. coverage (+): 0. Max coverage (-): 0

Region: chr28 25324588-25324634. Max. coverage (+): 0. Max coverage (-): 0

Region: chr28 25324635-25324680. Max. coverage (+): 0. Max coverage (-): 0

Region: chr28 25324681-25324726. Max. coverage (+): 0. Max coverage (-): 6.83

Region: chr28 25324727-25324773. Max. coverage (+): 0. Max coverage (-): 8.34

Region: chr28 25324774-25324819. Max. coverage (+): 0. Max coverage (-): 6.85

Region: chr28 25324820-25324865. Max. coverage (+): 0. Max coverage (-): 28.02

Region: chr28 25324866-25324912. Max. coverage (+): 0. Max coverage (-): 6.27

Region: chr28 25324913-25324958. Max. coverage (+): 0. Max coverage (-): 6.85

Region: chr28 25324959-25325005. Max. coverage (+): 0. Max coverage (-): 5.81

Region: chr28 25325006-25325051. Max. coverage (+): 0. Max coverage (-): 0

Region: chr28 25325052-25325097. Max. coverage (+): 0. Max coverage (-): 0.8

Region: chr28 25325098-25325144. Max. coverage (+): 0. Max coverage (-): 0

Region: chr28 25325145-25325190. Max. coverage (+): 0. Max coverage (-): 0

Region: chr28 25325191-25325236. Max. coverage (+): 0. Max coverage (-): 4.86

Region: chr28 25325237-25325283. Max. coverage (+): 0. Max coverage (-): 9.81

Region: chr28 25325284-25325329. Max. coverage (+): 0. Max coverage (-): 10.68

Region: chr28 25325330-25325375. Max. coverage (+): 0. Max coverage (-): 7.2

Region: chr28 25325376-25325422. Max. coverage (+): 0. Max coverage (-): 0

Region: chr28 25325423-25325468. Max. coverage (+): 0. Max coverage (-): 0

Region: chr28 25325469-25325514. Max. coverage (+): 0. Max coverage (-): 0

Region: chr28 25325515-25325561. Max. coverage (+): 0. Max coverage (-): 3.81

Region: chr28 25325562-25325607. Max. coverage (+): 0. Max coverage (-): 2.77

Region: chr28 25325608-25325654. Max. coverage (+): 0. Max coverage (-): 0

Region: chr28 25325655-25325700. Max. coverage (+): 0. Max coverage (-): 0

Region: chr28 25325701-25325746. Max. coverage (+): 0. Max coverage (-): 0

Region: chr28 25325747-25325793. Max. coverage (+): 0. Max coverage (-): 0

Region: chr28 25325794-25325839. Max. coverage (+): 0. Max coverage (-): 0

Region: chr28 25325840-25325885. Max. coverage (+): 0. Max coverage (-): 15.57

Region: chr28 25325886-. Max. coverage (+): 0. Max coverage (-): 15.57

RepeatMasker Color Code

**+**

100-98% Identity

<98-95% Identity

<95-90% Identity

<90-85% Identity

<85-80% Identity

<80-75% Identity

<75-70% Identity

<70% Identity

**-**

Gene Set Color Code

**+**

Gene

Pseudogene

**-**

Topology/Coverage Color Code

Coverage Plus Strand

Coverage Minus Strand

Mainstrand: Plus

Mainstrand: Minus

Complementary Strand

Flanking Region  
(if option -flank >0)

Gene Set Annotation  

**1. STOX1 (protein coding, ENSBTAG00000019028) Tr:00000025324 Ex:2**: 25320940-25321092 (+)  
**2. STOX1 (protein coding, ENSBTAG00000019028) Tr:00000025324 Ex:3**: 25323161-25325504 (+)

  
RepeatMasker Annotation  

**1. HERVL40-int**: 25302913-25303126 (-), Divergence to consensus: 40%  
**2. Bov-tA2**: 25303144-25303345 (+), Divergence to consensus: 15.4%  
**3. Bov-tA1**: 25303367-25303552 (+), Divergence to consensus: 12.4%  
**4. MER68-int**: 25303997-25304875 (-), Divergence to consensus: 46.7%  
**5. MER41\_BT**: 25304876-25305442 (-), Divergence to consensus: 29.4%  
**6. MER68-int**: 25305443-25306389 (-), Divergence to consensus: 53.8%  
**7. MER68-int**: 25306482-25306757 (-), Divergence to consensus: 36.6%  
**8. LTR68**: 25307367-25307842 (+), Divergence to consensus: 39.9%  
**9. MLT1L**: 25310066-25310316 (-), Divergence to consensus: 39.1%  
**10. Bov-tA3**: 25310650-25310890 (+), Divergence to consensus: 12.9%  
**11. MIRc**: 25311147-25311247 (-), Divergence to consensus: 38.7%  
**12. L2c**: 25311365-25311535 (+), Divergence to consensus: 31.6%  
**13. MER103C**: 25311854-25312050 (+), Divergence to consensus: 50.4%  
**14. MIR3**: 25312051-25312170 (-), Divergence to consensus: 39.6%  
**15. MLT1E2**: 25312532-25313033 (+), Divergence to consensus: 27%  
**16. AT\_rich**: 25315574-25315597 (+), Divergence to consensus: 54.2%  
**17. MER5A1**: 25317868-25318034 (+), Divergence to consensus: 32.1%  
**18. MER20**: 25318641-25318825 (+), Divergence to consensus: 34.5%  
**19. MER81**: 25318828-25318907 (+), Divergence to consensus: 23.7%  
**20. SINE2-1\_BT**: 25319049-25319151 (-), Divergence to consensus: 50.2%  
**21. AT\_rich**: 25319169-25319189 (+), Divergence to consensus: 23.8%  
**22. SINE2-3\_BT**: 25319199-25319253 (-), Divergence to consensus: 26.7%  
**23. BovB**: 25319336-25319976 (+), Divergence to consensus: 12.4%  
**24. ART2A**: 25319978-25320509 (+), Divergence to consensus: 20.8%  
**25. CHR-2B**: 25321209-25321508 (-), Divergence to consensus: 25.2%  
**26. SINE2-2\_BT**: 25321706-25321824 (-), Divergence to consensus: 29.6%  
**27. Bov-tA1**: 25321962-25322185 (+), Divergence to consensus: 20.5%  
**28. Bov-tA1**: 25322398-25322620 (+), Divergence to consensus: 20.5%

  
Transcription Factor Binding Sites  

**RFX4\_1** (Sequence: CTTAGCAAC (+): 25316186)  
**Gata4** (Sequence: AGATAAC (-): 25313619)  
**Gata4** (Sequence: AGATAAC (-): 25323735)  
**SOX9** (Sequence: TTATTGTT (+): 25315196)  
**SOX9** (Sequence: TCATTGTT (+): 25315557)  
**Gata4** (Sequence: CTTATCT (+): 25309701)  
**Gata4** (Sequence: CTTATCT (+): 25320583)  
**Gata4** (Sequence: GTTATCT (+): 25325570)
