# Supplementary material for: Small RNA sequencing of cryopreserved semen from single bull revealed altered miRNAs and piRNAs expression between High- and Low-motile sperm populations
Source: BMC Genomics. 2017 Jan 4;18:14. doi: 10.1186/s12864-016-3394-7 (PMC5209821; doi:10.1186/s12864-016-3394-7)
Supplement: Additional file 4: — Details for each piRNA clusters found in Low Motile (LM) sperm fraction. Genes, repeats, transposable elements and transcription factors binding sites falling within the cluster regions were reported. (ZIP 1034 kb) [file 12864_2016_3394_MOESM4_ESM.zip › 39.html]

piRNA cluster 39


Predicted piRNA cluster no. 39     previous   next
  

Show proTRAC run info
Hide proTRAC run info

================================= proTRAC ====================================  
VERSION: 2.1                                    LAST MODIFIED: 06. October 2015  
  
Please cite:  
Rosenkranz D, Zischler H. proTRAC - a software for probabilistic piRNA cluster  
detection, visualization and analysis. 2012. BMC Bioinformatics 13:5.  
  
and (for proTRAC 2.0 and later):  
Rosenkranz D, Rudloff S, Bastuck K, Ketting RF, Zischler H. Tupaia small RNAs  
provide insights into function and evolution of RNAi-based transposon defense  
in mammals. 2015. RNA 21(5):911-922.  
  
Contact:  
David Rosenkranz  
Institute of Anthropology, small RNA group  
Johannes Gutenberg University Mainz  
email: rosenkranz@uni-mainz.de  
  
You can find the latest proTRAC version at:  
http://sourceforge.net/projects/protrac/files  
http://www.smallRNAgroup-mainz.de/software  
==============================================================================  
  
PARAMETERS:  
Map file: .............../storage/core/barbara/genhome/smallRNA/fertility/Sample\_not\_motile/pirna/Sample\_not\_motile\_26-33\_collapsed.fa.no-dust.map.weighted-10000-1000-b-0  
Genome file: ............/storage/core/barbara/genhome/smallRNA/fertility/Sample\_all/pirna/bt\_311\_chrY.fa  
RepeatMasker annotation: /storage/genomes/bt\_umd31/GCF\_000003055.6\_Bos\_taurus\_UMD\_3.1.1\_repeatMasker\_chr.out  
GeneSet:................./storage/core/barbara/genhome/smallRNA/fertility/Sample\_all/pirna/full.gtf  
  
Significant (p<=0.01) hit density will be calculated based  
on observed hit distribution.  
  
Sliding window size: ........................................ 5000 bp  
Sliding window increament: .................................. 1000 bp  
Normalize each hit by number of genomic hits: ............... 1 [0=no/1=yes]  
Normalize each hit by number of sequence reads: ............. 1 [0=no/1=yes]  
Normalize values (-> per million mapped reads): ............. 1 [0=no/1=yes]  
Min. fraction of hits with 1T(U) or 10A: .................... 0.75  
Alternatively: Min. fraction of hits with 1T(U) and 10A: .... 0.5  
Min. fraction of hits with typical piRNA length: ............ 0.75  
Typical piRNA length: ....................................... 26-33 nt  
Min. size of a piRNA cluster: ............................... 5000 bp.  
Min. number of hits (absolute): ............................. 0  
Min. number of hits (normalized): ........................... 0  
Min. fraction of hits on the mainstrand: .................... 0.75  
Top fraction of mapped sequences (in terms of read counts): . 1%  
Top fraction accounts for max. n% of sequence reads: ........ 90%  
Min. fraction of hits on each arm of a bidirectional cluster: 0.1  
Output image file for each cluster: ......................... 0 [0=no/1=yes]  
Output html file for each cluster: .......................... 1 [0=no/1=yes]  
Output a summary table: ..................................... 1 [0=no/1=yes]  
Output a FASTA file for each cluster (piRNA sequences): ..... 1 [0=no/1=yes]  
Output a FASTA file comprising cluster sequences: ........... 1 [0=no/1=yes]  
Search DNA motifs in clusters: .............................. 1 [0=no/1=yes]  
Output flanking sequences: +/- .............................. 0 bp  
Output ~.pTi file: .......................................... 1 [0=no/1=yes]  
==============================================================================  
  
  
Genome size (without gaps): ............ 2678902517 bp  
Gaps (N/X/-): .......................... 53837044 bp  
Mapped reads: .......................... 738059667487  
Non-identical sequences: ............... 277001  
Genomic hits: .......................... 533816  
Significant densitiy of mapped reads: .. 15118061 reads/kb

Show proTRAC cluster info
Hide proTRAC cluster info

|  |  |
| --- | --- |
| Location | chr28 |
| Coordinates | 33690966-33725536 |
| Size [bp] | 34571 |
| Sequence hit loci | 2138 |
| Mapped reads (normalized) | 5584432318 |
| Mapped reads (normalized) per kb | 161535168.7 |
| Normalized reads with 1T (1U) | 83.4% |
| Normalized reads with 10A | 30.9% |
| Normalized reads with length 26-33 nt | 100% |
| Normalized reads on the main strand(s) | 98.5% |
| Predicted directionality | mono:plus |

100%

0%

1T (1U)  
reads

10A reads

26-33 nt  
reads

reads on mainstrand

**Either the amount of reads with 1T (1U) OR 10A has to exceed 75% (set with option: -1Tor10A)  
Alternatively the amount of reads with 1T (1U) AND 10A has to exceed 50% (set with option: -1Tand10A)  
Minimum amount of reads with preferred size is 75% (set with option: -pisize)  
Minimum amount of reads on the main strand(s) is 75% (set with option: -clstrand)**

Show read coverage
Hide read coverage

WHAT DO I SEE HERE?  
This chart shows the location of mapped sequence reads within a predicted piRNA cluster. The color refers to the number of genomic hits produced by the sequence read in question. A dark red bar indicates that this sequence read produces many other hits elsewhere in the genome. Many adjacent red or yellow bars can indicate the presence of a multi-copy element such as transposons or rRNA genes. A dark green bar indicates that this sequence read maps uniquely to this locus.

1 hit

2-5 hits

6-10 hits

11-20 hits

21-50 hits

51-100 hits

> 100 hits

chr28

33690966

33725536

Gene Set

RepeatMasker

Mapped  
Reads

164.38

plus strand

minus strand

164.38

Region: chr28 25325887-33691000. Max. coverage (+): 1.06. Max coverage (-): 7.03

Region: chr28 33691001-33691069. Max. coverage (+): 0. Max coverage (-): 7.03

Region: chr28 33691070-33691138. Max. coverage (+): 0. Max coverage (-): 0

Region: chr28 33691139-33691207. Max. coverage (+): 0. Max coverage (-): 0

Region: chr28 33691208-33691277. Max. coverage (+): 0. Max coverage (-): 0

Region: chr28 33691278-33691346. Max. coverage (+): 0. Max coverage (-): 0

Region: chr28 33691347-33691415. Max. coverage (+): 0. Max coverage (-): 0

Region: chr28 33691416-33691484. Max. coverage (+): 8.53. Max coverage (-): 0

Region: chr28 33691485-33691553. Max. coverage (+): 8.46. Max coverage (-): 0

Region: chr28 33691554-33691622. Max. coverage (+): 13.59. Max coverage (-): 0

Region: chr28 33691623-33691691. Max. coverage (+): 19.26. Max coverage (-): 0.85

Region: chr28 33691692-33691761. Max. coverage (+): 3.8. Max coverage (-): 8.38

Region: chr28 33691762-33691830. Max. coverage (+): 27.46. Max coverage (-): 8.38

Region: chr28 33691831-33691899. Max. coverage (+): 23.55. Max coverage (-): 0

Region: chr28 33691900-33691968. Max. coverage (+): 13.31. Max coverage (-): 0

Region: chr28 33691969-33692037. Max. coverage (+): 7.18. Max coverage (-): 0

Region: chr28 33692038-33692106. Max. coverage (+): 0. Max coverage (-): 0

Region: chr28 33692107-33692175. Max. coverage (+): 6.97. Max coverage (-): 0

Region: chr28 33692176-33692245. Max. coverage (+): 20.7. Max coverage (-): 0

Region: chr28 33692246-33692314. Max. coverage (+): 18.21. Max coverage (-): 6.69

Region: chr28 33692315-33692383. Max. coverage (+): 18. Max coverage (-): 0

Region: chr28 33692384-33692452. Max. coverage (+): 6.33. Max coverage (-): 0

Region: chr28 33692453-33692521. Max. coverage (+): 6.32. Max coverage (-): 5.84

Region: chr28 33692522-33692590. Max. coverage (+): 29.21. Max coverage (-): 0

Region: chr28 33692591-33692659. Max. coverage (+): 21.76. Max coverage (-): 0

Region: chr28 33692660-33692729. Max. coverage (+): 11.46. Max coverage (-): 0

Region: chr28 33692730-33692798. Max. coverage (+): 8.99. Max coverage (-): 0

Region: chr28 33692799-33692867. Max. coverage (+): 0. Max coverage (-): 0

Region: chr28 33692868-33692936. Max. coverage (+): 4.22. Max coverage (-): 0

Region: chr28 33692937-33693005. Max. coverage (+): 10.74. Max coverage (-): 0

Region: chr28 33693006-33693074. Max. coverage (+): 3.42. Max coverage (-): 0

Region: chr28 33693075-33693143. Max. coverage (+): 17.97. Max coverage (-): 0

Region: chr28 33693144-33693213. Max. coverage (+): 8.28. Max coverage (-): 0

Region: chr28 33693214-33693282. Max. coverage (+): 68.47. Max coverage (-): 6.09

Region: chr28 33693283-33693351. Max. coverage (+): 43.18. Max coverage (-): 0

Region: chr28 33693352-33693420. Max. coverage (+): 43.18. Max coverage (-): 0

Region: chr28 33693421-33693489. Max. coverage (+): 21.78. Max coverage (-): 6.63

Region: chr28 33693490-33693558. Max. coverage (+): 35.22. Max coverage (-): 4.26

Region: chr28 33693559-33693627. Max. coverage (+): 0. Max coverage (-): 0

Region: chr28 33693628-33693697. Max. coverage (+): 6.31. Max coverage (-): 0

Region: chr28 33693698-33693766. Max. coverage (+): 8.41. Max coverage (-): 0

Region: chr28 33693767-33693835. Max. coverage (+): 16.98. Max coverage (-): 0

Region: chr28 33693836-33693904. Max. coverage (+): 13.69. Max coverage (-): 0

Region: chr28 33693905-33693973. Max. coverage (+): 36.84. Max coverage (-): 0

Region: chr28 33693974-33694042. Max. coverage (+): 0. Max coverage (-): 0

Region: chr28 33694043-33694111. Max. coverage (+): 4.59. Max coverage (-): 0

Region: chr28 33694112-33694181. Max. coverage (+): 9.04. Max coverage (-): 11.48

Region: chr28 33694182-33694250. Max. coverage (+): 20.78. Max coverage (-): 8.38

Region: chr28 33694251-33694319. Max. coverage (+): 18.55. Max coverage (-): 0

Region: chr28 33694320-33694388. Max. coverage (+): 0. Max coverage (-): 0

Region: chr28 33694389-33694457. Max. coverage (+): 0. Max coverage (-): 0

Region: chr28 33694458-33694526. Max. coverage (+): 0. Max coverage (-): 0

Region: chr28 33694527-33694595. Max. coverage (+): 20.18. Max coverage (-): 0

Region: chr28 33694596-33694665. Max. coverage (+): 5.79. Max coverage (-): 0

Region: chr28 33694666-33694734. Max. coverage (+): 3.5. Max coverage (-): 0

Region: chr28 33694735-33694803. Max. coverage (+): 2.18. Max coverage (-): 5.51

Region: chr28 33694804-33694872. Max. coverage (+): 53.92. Max coverage (-): 0

Region: chr28 33694873-33694941. Max. coverage (+): 17.51. Max coverage (-): 0

Region: chr28 33694942-33695010. Max. coverage (+): 47.12. Max coverage (-): 0

Region: chr28 33695011-33695079. Max. coverage (+): 47.12. Max coverage (-): 0

Region: chr28 33695080-33695149. Max. coverage (+): 26.2. Max coverage (-): 0

Region: chr28 33695150-33695218. Max. coverage (+): 20.59. Max coverage (-): 0

Region: chr28 33695219-33695287. Max. coverage (+): 13.12. Max coverage (-): 0

Region: chr28 33695288-33695356. Max. coverage (+): 6.3. Max coverage (-): 0

Region: chr28 33695357-33695425. Max. coverage (+): 55.22. Max coverage (-): 0

Region: chr28 33695426-33695494. Max. coverage (+): 34.78. Max coverage (-): 0

Region: chr28 33695495-33695563. Max. coverage (+): 12.25. Max coverage (-): 0

Region: chr28 33695564-33695633. Max. coverage (+): 28.35. Max coverage (-): 0

Region: chr28 33695634-33695702. Max. coverage (+): 19.08. Max coverage (-): 0

Region: chr28 33695703-33695771. Max. coverage (+): 6.02. Max coverage (-): 0

Region: chr28 33695772-33695840. Max. coverage (+): 30.5. Max coverage (-): 0

Region: chr28 33695841-33695909. Max. coverage (+): 67.95. Max coverage (-): 0

Region: chr28 33695910-33695978. Max. coverage (+): 69.97. Max coverage (-): 0

Region: chr28 33695979-33696047. Max. coverage (+): 1.63. Max coverage (-): 0

Region: chr28 33696048-33696117. Max. coverage (+): 0. Max coverage (-): 0

Region: chr28 33696118-33696186. Max. coverage (+): 0. Max coverage (-): 0

Region: chr28 33696187-33696255. Max. coverage (+): 0. Max coverage (-): 0

Region: chr28 33696256-33696324. Max. coverage (+): 0. Max coverage (-): 0

Region: chr28 33696325-33696393. Max. coverage (+): 11.51. Max coverage (-): 0

Region: chr28 33696394-33696462. Max. coverage (+): 0. Max coverage (-): 0

Region: chr28 33696463-33696531. Max. coverage (+): 0. Max coverage (-): 0

Region: chr28 33696532-33696601. Max. coverage (+): 0. Max coverage (-): 0

Region: chr28 33696602-33696670. Max. coverage (+): 19.26. Max coverage (-): 0

Region: chr28 33696671-33696739. Max. coverage (+): 36.99. Max coverage (-): 0

Region: chr28 33696740-33696808. Max. coverage (+): 24. Max coverage (-): 0

Region: chr28 33696809-33696877. Max. coverage (+): 0. Max coverage (-): 0

Region: chr28 33696878-33696946. Max. coverage (+): 4.34. Max coverage (-): 0

Region: chr28 33696947-33697015. Max. coverage (+): 5.48. Max coverage (-): 0

Region: chr28 33697016-33697085. Max. coverage (+): 0.33. Max coverage (-): 0

Region: chr28 33697086-33697154. Max. coverage (+): 0. Max coverage (-): 0

Region: chr28 33697155-33697223. Max. coverage (+): 1.46. Max coverage (-): 0

Region: chr28 33697224-33697292. Max. coverage (+): 70.39. Max coverage (-): 1.02

Region: chr28 33697293-33697361. Max. coverage (+): 5.81. Max coverage (-): 0

Region: chr28 33697362-33697430. Max. coverage (+): 0. Max coverage (-): 0

Region: chr28 33697431-33697499. Max. coverage (+): 6.2. Max coverage (-): 0

Region: chr28 33697500-33697569. Max. coverage (+): 12.54. Max coverage (-): 0

Region: chr28 33697570-33697638. Max. coverage (+): 0. Max coverage (-): 0

Region: chr28 33697639-33697707. Max. coverage (+): 23.73. Max coverage (-): 0

Region: chr28 33697708-33697776. Max. coverage (+): 0. Max coverage (-): 0

Region: chr28 33697777-33697845. Max. coverage (+): 0. Max coverage (-): 0

Region: chr28 33697846-33697914. Max. coverage (+): 3.9. Max coverage (-): 0

Region: chr28 33697915-33697983. Max. coverage (+): 41.27. Max coverage (-): 0

Region: chr28 33697984-33698053. Max. coverage (+): 61.55. Max coverage (-): 0

Region: chr28 33698054-33698122. Max. coverage (+): 71.68. Max coverage (-): 0

Region: chr28 33698123-33698191. Max. coverage (+): 24.68. Max coverage (-): 0

Region: chr28 33698192-33698260. Max. coverage (+): 14.01. Max coverage (-): 0

Region: chr28 33698261-33698329. Max. coverage (+): 30.35. Max coverage (-): 3.23

Region: chr28 33698330-33698398. Max. coverage (+): 22.64. Max coverage (-): 3.23

Region: chr28 33698399-33698467. Max. coverage (+): 56.56. Max coverage (-): 0

Region: chr28 33698468-33698537. Max. coverage (+): 11.57. Max coverage (-): 0

Region: chr28 33698538-33698606. Max. coverage (+): 18.74. Max coverage (-): 0

Region: chr28 33698607-33698675. Max. coverage (+): 16.41. Max coverage (-): 0

Region: chr28 33698676-33698744. Max. coverage (+): 0. Max coverage (-): 0

Region: chr28 33698745-33698813. Max. coverage (+): 8.49. Max coverage (-): 0

Region: chr28 33698814-33698882. Max. coverage (+): 4.68. Max coverage (-): 0

Region: chr28 33698883-33698951. Max. coverage (+): 4.68. Max coverage (-): 0

Region: chr28 33698952-33699021. Max. coverage (+): 2.91. Max coverage (-): 0

Region: chr28 33699022-33699090. Max. coverage (+): 0. Max coverage (-): 0

Region: chr28 33699091-33699159. Max. coverage (+): 6.86. Max coverage (-): 0

Region: chr28 33699160-33699228. Max. coverage (+): 14.75. Max coverage (-): 0

Region: chr28 33699229-33699297. Max. coverage (+): 9.27. Max coverage (-): 0

Region: chr28 33699298-33699366. Max. coverage (+): 20.28. Max coverage (-): 0

Region: chr28 33699367-33699435. Max. coverage (+): 44.74. Max coverage (-): 0

Region: chr28 33699436-33699505. Max. coverage (+): 0. Max coverage (-): 0

Region: chr28 33699506-33699574. Max. coverage (+): 0. Max coverage (-): 0

Region: chr28 33699575-33699643. Max. coverage (+): 0. Max coverage (-): 0

Region: chr28 33699644-33699712. Max. coverage (+): 0. Max coverage (-): 0

Region: chr28 33699713-33699781. Max. coverage (+): 0. Max coverage (-): 0

Region: chr28 33699782-33699850. Max. coverage (+): 0. Max coverage (-): 0

Region: chr28 33699851-33699919. Max. coverage (+): 0. Max coverage (-): 0

Region: chr28 33699920-33699989. Max. coverage (+): 0. Max coverage (-): 0

Region: chr28 33699990-33700058. Max. coverage (+): 0. Max coverage (-): 0

Region: chr28 33700059-33700127. Max. coverage (+): 0. Max coverage (-): 0

Region: chr28 33700128-33700196. Max. coverage (+): 0. Max coverage (-): 0

Region: chr28 33700197-33700265. Max. coverage (+): 0. Max coverage (-): 0

Region: chr28 33700266-33700334. Max. coverage (+): 0. Max coverage (-): 0

Region: chr28 33700335-33700403. Max. coverage (+): 0. Max coverage (-): 0

Region: chr28 33700404-33700473. Max. coverage (+): 0. Max coverage (-): 0

Region: chr28 33700474-33700542. Max. coverage (+): 0. Max coverage (-): 0

Region: chr28 33700543-33700611. Max. coverage (+): 0. Max coverage (-): 0

Region: chr28 33700612-33700680. Max. coverage (+): 0. Max coverage (-): 0

Region: chr28 33700681-33700749. Max. coverage (+): 0. Max coverage (-): 0

Region: chr28 33700750-33700818. Max. coverage (+): 0. Max coverage (-): 0

Region: chr28 33700819-33700887. Max. coverage (+): 4.17. Max coverage (-): 0

Region: chr28 33700888-33700957. Max. coverage (+): 0. Max coverage (-): 0

Region: chr28 33700958-33701026. Max. coverage (+): 0. Max coverage (-): 0

Region: chr28 33701027-33701095. Max. coverage (+): 0. Max coverage (-): 0

Region: chr28 33701096-33701164. Max. coverage (+): 6.18. Max coverage (-): 0

Region: chr28 33701165-33701233. Max. coverage (+): 3.77. Max coverage (-): 0

Region: chr28 33701234-33701302. Max. coverage (+): 0. Max coverage (-): 0

Region: chr28 33701303-33701371. Max. coverage (+): 5.5. Max coverage (-): 0

Region: chr28 33701372-33701441. Max. coverage (+): 0. Max coverage (-): 0

Region: chr28 33701442-33701510. Max. coverage (+): 0. Max coverage (-): 0

Region: chr28 33701511-33701579. Max. coverage (+): 0. Max coverage (-): 0

Region: chr28 33701580-33701648. Max. coverage (+): 0. Max coverage (-): 0

Region: chr28 33701649-33701717. Max. coverage (+): 0. Max coverage (-): 0

Region: chr28 33701718-33701786. Max. coverage (+): 0. Max coverage (-): 0

Region: chr28 33701787-33701855. Max. coverage (+): 0. Max coverage (-): 0

Region: chr28 33701856-33701925. Max. coverage (+): 0. Max coverage (-): 0

Region: chr28 33701926-33701994. Max. coverage (+): 0. Max coverage (-): 0

Region: chr28 33701995-33702063. Max. coverage (+): 0. Max coverage (-): 0

Region: chr28 33702064-33702132. Max. coverage (+): 0. Max coverage (-): 0

Region: chr28 33702133-33702201. Max. coverage (+): 0. Max coverage (-): 0

Region: chr28 33702202-33702270. Max. coverage (+): 0. Max coverage (-): 0

Region: chr28 33702271-33702339. Max. coverage (+): 0. Max coverage (-): 0

Region: chr28 33702340-33702409. Max. coverage (+): 0. Max coverage (-): 0

Region: chr28 33702410-33702478. Max. coverage (+): 0. Max coverage (-): 0

Region: chr28 33702479-33702547. Max. coverage (+): 0. Max coverage (-): 0

Region: chr28 33702548-33702616. Max. coverage (+): 0. Max coverage (-): 0

Region: chr28 33702617-33702685. Max. coverage (+): 0. Max coverage (-): 0

Region: chr28 33702686-33702754. Max. coverage (+): 0. Max coverage (-): 0

Region: chr28 33702755-33702823. Max. coverage (+): 0. Max coverage (-): 0

Region: chr28 33702824-33702892. Max. coverage (+): 0. Max coverage (-): 0

Region: chr28 33702893-33702962. Max. coverage (+): 0. Max coverage (-): 0

Region: chr28 33702963-33703031. Max. coverage (+): 0. Max coverage (-): 0

Region: chr28 33703032-33703100. Max. coverage (+): 0. Max coverage (-): 0

Region: chr28 33703101-33703169. Max. coverage (+): 0. Max coverage (-): 0

Region: chr28 33703170-33703238. Max. coverage (+): 0. Max coverage (-): 0

Region: chr28 33703239-33703307. Max. coverage (+): 0. Max coverage (-): 0

Region: chr28 33703308-33703376. Max. coverage (+): 0. Max coverage (-): 0

Region: chr28 33703377-33703446. Max. coverage (+): 0. Max coverage (-): 0

Region: chr28 33703447-33703515. Max. coverage (+): 0. Max coverage (-): 0

Region: chr28 33703516-33703584. Max. coverage (+): 0. Max coverage (-): 0

Region: chr28 33703585-33703653. Max. coverage (+): 0. Max coverage (-): 0

Region: chr28 33703654-33703722. Max. coverage (+): 0. Max coverage (-): 0

Region: chr28 33703723-33703791. Max. coverage (+): 10.86. Max coverage (-): 0

Region: chr28 33703792-33703860. Max. coverage (+): 1.78. Max coverage (-): 0

Region: chr28 33703861-33703930. Max. coverage (+): 10.11. Max coverage (-): 0

Region: chr28 33703931-33703999. Max. coverage (+): 0. Max coverage (-): 0

Region: chr28 33704000-33704068. Max. coverage (+): 0. Max coverage (-): 0

Region: chr28 33704069-33704137. Max. coverage (+): 0. Max coverage (-): 0

Region: chr28 33704138-33704206. Max. coverage (+): 0. Max coverage (-): 0

Region: chr28 33704207-33704275. Max. coverage (+): 0. Max coverage (-): 0

Region: chr28 33704276-33704344. Max. coverage (+): 0. Max coverage (-): 0

Region: chr28 33704345-33704414. Max. coverage (+): 4.31. Max coverage (-): 0

Region: chr28 33704415-33704483. Max. coverage (+): 33.83. Max coverage (-): 0

Region: chr28 33704484-33704552. Max. coverage (+): 21.28. Max coverage (-): 0

Region: chr28 33704553-33704621. Max. coverage (+): 19.91. Max coverage (-): 0

Region: chr28 33704622-33704690. Max. coverage (+): 12.01. Max coverage (-): 0

Region: chr28 33704691-33704759. Max. coverage (+): 10.35. Max coverage (-): 0

Region: chr28 33704760-33704828. Max. coverage (+): 11.44. Max coverage (-): 0

Region: chr28 33704829-33704898. Max. coverage (+): 9.41. Max coverage (-): 0

Region: chr28 33704899-33704967. Max. coverage (+): 24.67. Max coverage (-): 3.49

Region: chr28 33704968-33705036. Max. coverage (+): 24.67. Max coverage (-): 23.01

Region: chr28 33705037-33705105. Max. coverage (+): 1.25. Max coverage (-): 0

Region: chr28 33705106-33705174. Max. coverage (+): 45.54. Max coverage (-): 0

Region: chr28 33705175-33705243. Max. coverage (+): 8.71. Max coverage (-): 0

Region: chr28 33705244-33705312. Max. coverage (+): 61.39. Max coverage (-): 0

Region: chr28 33705313-33705382. Max. coverage (+): 76.86. Max coverage (-): 0

Region: chr28 33705383-33705451. Max. coverage (+): 31.61. Max coverage (-): 0

Region: chr28 33705452-33705520. Max. coverage (+): 10.47. Max coverage (-): 0

Region: chr28 33705521-33705589. Max. coverage (+): 16.46. Max coverage (-): 5.12

Region: chr28 33705590-33705658. Max. coverage (+): 25.42. Max coverage (-): 0

Region: chr28 33705659-33705727. Max. coverage (+): 0.92. Max coverage (-): 0

Region: chr28 33705728-33705796. Max. coverage (+): 7.69. Max coverage (-): 0

Region: chr28 33705797-33705866. Max. coverage (+): 25.97. Max coverage (-): 0

Region: chr28 33705867-33705935. Max. coverage (+): 14.47. Max coverage (-): 0

Region: chr28 33705936-33706004. Max. coverage (+): 35.88. Max coverage (-): 0

Region: chr28 33706005-33706073. Max. coverage (+): 0. Max coverage (-): 0

Region: chr28 33706074-33706142. Max. coverage (+): 0. Max coverage (-): 0

Region: chr28 33706143-33706211. Max. coverage (+): 14.73. Max coverage (-): 0

Region: chr28 33706212-33706280. Max. coverage (+): 11.32. Max coverage (-): 0

Region: chr28 33706281-33706350. Max. coverage (+): 22.58. Max coverage (-): 0

Region: chr28 33706351-33706419. Max. coverage (+): 164.38. Max coverage (-): 0

Region: chr28 33706420-33706488. Max. coverage (+): 20.34. Max coverage (-): 3.09

Region: chr28 33706489-33706557. Max. coverage (+): 3.69. Max coverage (-): 3.09

Region: chr28 33706558-33706626. Max. coverage (+): 0. Max coverage (-): 0

Region: chr28 33706627-33706695. Max. coverage (+): 21.11. Max coverage (-): 0

Region: chr28 33706696-33706764. Max. coverage (+): 69.15. Max coverage (-): 0

Region: chr28 33706765-33706834. Max. coverage (+): 40.41. Max coverage (-): 0

Region: chr28 33706835-33706903. Max. coverage (+): 7.4. Max coverage (-): 0

Region: chr28 33706904-33706972. Max. coverage (+): 9.33. Max coverage (-): 3.21

Region: chr28 33706973-33707041. Max. coverage (+): 37.78. Max coverage (-): 0

Region: chr28 33707042-33707110. Max. coverage (+): 0. Max coverage (-): 0

Region: chr28 33707111-33707179. Max. coverage (+): 37.14. Max coverage (-): 0

Region: chr28 33707180-33707248. Max. coverage (+): 7.59. Max coverage (-): 8.46

Region: chr28 33707249-33707318. Max. coverage (+): 19.24. Max coverage (-): 0

Region: chr28 33707319-33707387. Max. coverage (+): 36.66. Max coverage (-): 0

Region: chr28 33707388-33707456. Max. coverage (+): 47.28. Max coverage (-): 0

Region: chr28 33707457-33707525. Max. coverage (+): 6.37. Max coverage (-): 0

Region: chr28 33707526-33707594. Max. coverage (+): 9.09. Max coverage (-): 0

Region: chr28 33707595-33707663. Max. coverage (+): 9.13. Max coverage (-): 0

Region: chr28 33707664-33707732. Max. coverage (+): 2.53. Max coverage (-): 0

Region: chr28 33707733-33707802. Max. coverage (+): 0. Max coverage (-): 0

Region: chr28 33707803-33707871. Max. coverage (+): 0. Max coverage (-): 0

Region: chr28 33707872-33707940. Max. coverage (+): 0. Max coverage (-): 0

Region: chr28 33707941-33708009. Max. coverage (+): 30.22. Max coverage (-): 0

Region: chr28 33708010-33708078. Max. coverage (+): 47.88. Max coverage (-): 0

Region: chr28 33708079-33708147. Max. coverage (+): 34.09. Max coverage (-): 0

Region: chr28 33708148-33708216. Max. coverage (+): 4.41. Max coverage (-): 0

Region: chr28 33708217-33708286. Max. coverage (+): 10.8. Max coverage (-): 0

Region: chr28 33708287-33708355. Max. coverage (+): 10.8. Max coverage (-): 0

Region: chr28 33708356-33708424. Max. coverage (+): 16.63. Max coverage (-): 0

Region: chr28 33708425-33708493. Max. coverage (+): 22.08. Max coverage (-): 0

Region: chr28 33708494-33708562. Max. coverage (+): 35.7. Max coverage (-): 0

Region: chr28 33708563-33708631. Max. coverage (+): 78.53. Max coverage (-): 0

Region: chr28 33708632-33708700. Max. coverage (+): 51.96. Max coverage (-): 0

Region: chr28 33708701-33708770. Max. coverage (+): 0. Max coverage (-): 0

Region: chr28 33708771-33708839. Max. coverage (+): 0. Max coverage (-): 0

Region: chr28 33708840-33708908. Max. coverage (+): 23.86. Max coverage (-): 0

Region: chr28 33708909-33708977. Max. coverage (+): 6.66. Max coverage (-): 0

Region: chr28 33708978-33709046. Max. coverage (+): 0. Max coverage (-): 0

Region: chr28 33709047-33709115. Max. coverage (+): 0. Max coverage (-): 0

Region: chr28 33709116-33709184. Max. coverage (+): 0. Max coverage (-): 0

Region: chr28 33709185-33709254. Max. coverage (+): 0. Max coverage (-): 0

Region: chr28 33709255-33709323. Max. coverage (+): 0. Max coverage (-): 0

Region: chr28 33709324-33709392. Max. coverage (+): 0. Max coverage (-): 0

Region: chr28 33709393-33709461. Max. coverage (+): 0. Max coverage (-): 0

Region: chr28 33709462-33709530. Max. coverage (+): 4.31. Max coverage (-): 0

Region: chr28 33709531-33709599. Max. coverage (+): 3.39. Max coverage (-): 0

Region: chr28 33709600-33709668. Max. coverage (+): 6.9. Max coverage (-): 0

Region: chr28 33709669-33709738. Max. coverage (+): 6.9. Max coverage (-): 0

Region: chr28 33709739-33709807. Max. coverage (+): 0. Max coverage (-): 0

Region: chr28 33709808-33709876. Max. coverage (+): 0. Max coverage (-): 0

Region: chr28 33709877-33709945. Max. coverage (+): 7.33. Max coverage (-): 0

Region: chr28 33709946-33710014. Max. coverage (+): 9.99. Max coverage (-): 0

Region: chr28 33710015-33710083. Max. coverage (+): 23.62. Max coverage (-): 0

Region: chr28 33710084-33710152. Max. coverage (+): 9.11. Max coverage (-): 0

Region: chr28 33710153-33710222. Max. coverage (+): 0. Max coverage (-): 0

Region: chr28 33710223-33710291. Max. coverage (+): 14.03. Max coverage (-): 0

Region: chr28 33710292-33710360. Max. coverage (+): 9.47. Max coverage (-): 0

Region: chr28 33710361-33710429. Max. coverage (+): 3.6. Max coverage (-): 0

Region: chr28 33710430-33710498. Max. coverage (+): 12.79. Max coverage (-): 0

Region: chr28 33710499-33710567. Max. coverage (+): 6.66. Max coverage (-): 0

Region: chr28 33710568-33710636. Max. coverage (+): 7.44. Max coverage (-): 0

Region: chr28 33710637-33710706. Max. coverage (+): 10.98. Max coverage (-): 0

Region: chr28 33710707-33710775. Max. coverage (+): 0. Max coverage (-): 0

Region: chr28 33710776-33710844. Max. coverage (+): 3.93. Max coverage (-): 0

Region: chr28 33710845-33710913. Max. coverage (+): 2.44. Max coverage (-): 0

Region: chr28 33710914-33710982. Max. coverage (+): 0. Max coverage (-): 0

Region: chr28 33710983-33711051. Max. coverage (+): 0. Max coverage (-): 0

Region: chr28 33711052-33711120. Max. coverage (+): 0. Max coverage (-): 0

Region: chr28 33711121-33711190. Max. coverage (+): 0. Max coverage (-): 0

Region: chr28 33711191-33711259. Max. coverage (+): 0. Max coverage (-): 0

Region: chr28 33711260-33711328. Max. coverage (+): 0. Max coverage (-): 0

Region: chr28 33711329-33711397. Max. coverage (+): 0. Max coverage (-): 0

Region: chr28 33711398-33711466. Max. coverage (+): 0. Max coverage (-): 0

Region: chr28 33711467-33711535. Max. coverage (+): 12.28. Max coverage (-): 0

Region: chr28 33711536-33711604. Max. coverage (+): 26.74. Max coverage (-): 0

Region: chr28 33711605-33711674. Max. coverage (+): 15.58. Max coverage (-): 0

Region: chr28 33711675-33711743. Max. coverage (+): 41.35. Max coverage (-): 0

Region: chr28 33711744-33711812. Max. coverage (+): 82.72. Max coverage (-): 0

Region: chr28 33711813-33711881. Max. coverage (+): 8.56. Max coverage (-): 0

Region: chr28 33711882-33711950. Max. coverage (+): 12.82. Max coverage (-): 0

Region: chr28 33711951-33712019. Max. coverage (+): 33.67. Max coverage (-): 0

Region: chr28 33712020-33712088. Max. coverage (+): 74.92. Max coverage (-): 0

Region: chr28 33712089-33712158. Max. coverage (+): 23.53. Max coverage (-): 0

Region: chr28 33712159-33712227. Max. coverage (+): 20.75. Max coverage (-): 0

Region: chr28 33712228-33712296. Max. coverage (+): 28.02. Max coverage (-): 0

Region: chr28 33712297-33712365. Max. coverage (+): 34.51. Max coverage (-): 0

Region: chr28 33712366-33712434. Max. coverage (+): 97.03. Max coverage (-): 0

Region: chr28 33712435-33712503. Max. coverage (+): 27.53. Max coverage (-): 0

Region: chr28 33712504-33712572. Max. coverage (+): 50.62. Max coverage (-): 0

Region: chr28 33712573-33712642. Max. coverage (+): 23.71. Max coverage (-): 0

Region: chr28 33712643-33712711. Max. coverage (+): 13.23. Max coverage (-): 0

Region: chr28 33712712-33712780. Max. coverage (+): 24.68. Max coverage (-): 0

Region: chr28 33712781-33712849. Max. coverage (+): 11.95. Max coverage (-): 0

Region: chr28 33712850-33712918. Max. coverage (+): 13.17. Max coverage (-): 0

Region: chr28 33712919-33712987. Max. coverage (+): 36.67. Max coverage (-): 0

Region: chr28 33712988-33713056. Max. coverage (+): 13.11. Max coverage (-): 0

Region: chr28 33713057-33713126. Max. coverage (+): 5.81. Max coverage (-): 0

Region: chr28 33713127-33713195. Max. coverage (+): 15.77. Max coverage (-): 0

Region: chr28 33713196-33713264. Max. coverage (+): 6.17. Max coverage (-): 0

Region: chr28 33713265-33713333. Max. coverage (+): 9.82. Max coverage (-): 0

Region: chr28 33713334-33713402. Max. coverage (+): 34.29. Max coverage (-): 0

Region: chr28 33713403-33713471. Max. coverage (+): 11.8. Max coverage (-): 0

Region: chr28 33713472-33713540. Max. coverage (+): 14.72. Max coverage (-): 0

Region: chr28 33713541-33713610. Max. coverage (+): 6.56. Max coverage (-): 0

Region: chr28 33713611-33713679. Max. coverage (+): 10.25. Max coverage (-): 0

Region: chr28 33713680-33713748. Max. coverage (+): 9.28. Max coverage (-): 0

Region: chr28 33713749-33713817. Max. coverage (+): 24.39. Max coverage (-): 0

Region: chr28 33713818-33713886. Max. coverage (+): 14.41. Max coverage (-): 0

Region: chr28 33713887-33713955. Max. coverage (+): 12.89. Max coverage (-): 0

Region: chr28 33713956-33714024. Max. coverage (+): 5.32. Max coverage (-): 0

Region: chr28 33714025-33714093. Max. coverage (+): 11.75. Max coverage (-): 0

Region: chr28 33714094-33714163. Max. coverage (+): 11.75. Max coverage (-): 0

Region: chr28 33714164-33714232. Max. coverage (+): 0. Max coverage (-): 0

Region: chr28 33714233-33714301. Max. coverage (+): 0. Max coverage (-): 0

Region: chr28 33714302-33714370. Max. coverage (+): 0. Max coverage (-): 0

Region: chr28 33714371-33714439. Max. coverage (+): 6.06. Max coverage (-): 0

Region: chr28 33714440-33714508. Max. coverage (+): 164.09. Max coverage (-): 0

Region: chr28 33714509-33714577. Max. coverage (+): 20.63. Max coverage (-): 0

Region: chr28 33714578-33714647. Max. coverage (+): 13.02. Max coverage (-): 0

Region: chr28 33714648-33714716. Max. coverage (+): 9.64. Max coverage (-): 0

Region: chr28 33714717-33714785. Max. coverage (+): 30.41. Max coverage (-): 0

Region: chr28 33714786-33714854. Max. coverage (+): 90.42. Max coverage (-): 0

Region: chr28 33714855-33714923. Max. coverage (+): 10.7. Max coverage (-): 0

Region: chr28 33714924-33714992. Max. coverage (+): 8.74. Max coverage (-): 0

Region: chr28 33714993-33715061. Max. coverage (+): 6.2. Max coverage (-): 0

Region: chr28 33715062-33715131. Max. coverage (+): 0. Max coverage (-): 0

Region: chr28 33715132-33715200. Max. coverage (+): 1.46. Max coverage (-): 0

Region: chr28 33715201-33715269. Max. coverage (+): 9.34. Max coverage (-): 0

Region: chr28 33715270-33715338. Max. coverage (+): 7.09. Max coverage (-): 0

Region: chr28 33715339-33715407. Max. coverage (+): 0. Max coverage (-): 0

Region: chr28 33715408-33715476. Max. coverage (+): 0. Max coverage (-): 0

Region: chr28 33715477-33715545. Max. coverage (+): 6.98. Max coverage (-): 0

Region: chr28 33715546-33715615. Max. coverage (+): 4.8. Max coverage (-): 0

Region: chr28 33715616-33715684. Max. coverage (+): 0. Max coverage (-): 0

Region: chr28 33715685-33715753. Max. coverage (+): 4.75. Max coverage (-): 0

Region: chr28 33715754-33715822. Max. coverage (+): 0. Max coverage (-): 0

Region: chr28 33715823-33715891. Max. coverage (+): 0. Max coverage (-): 0

Region: chr28 33715892-33715960. Max. coverage (+): 0. Max coverage (-): 0

Region: chr28 33715961-33716029. Max. coverage (+): 0. Max coverage (-): 0

Region: chr28 33716030-33716099. Max. coverage (+): 0. Max coverage (-): 0

Region: chr28 33716100-33716168. Max. coverage (+): 0. Max coverage (-): 0

Region: chr28 33716169-33716237. Max. coverage (+): 0. Max coverage (-): 0

Region: chr28 33716238-33716306. Max. coverage (+): 0. Max coverage (-): 0

Region: chr28 33716307-33716375. Max. coverage (+): 0. Max coverage (-): 0

Region: chr28 33716376-33716444. Max. coverage (+): 0. Max coverage (-): 0

Region: chr28 33716445-33716513. Max. coverage (+): 0. Max coverage (-): 0

Region: chr28 33716514-33716583. Max. coverage (+): 0. Max coverage (-): 0

Region: chr28 33716584-33716652. Max. coverage (+): 0. Max coverage (-): 0

Region: chr28 33716653-33716721. Max. coverage (+): 0. Max coverage (-): 0

Region: chr28 33716722-33716790. Max. coverage (+): 0. Max coverage (-): 0

Region: chr28 33716791-33716859. Max. coverage (+): 1.75. Max coverage (-): 0

Region: chr28 33716860-33716928. Max. coverage (+): 0. Max coverage (-): 0

Region: chr28 33716929-33716997. Max. coverage (+): 0. Max coverage (-): 0

Region: chr28 33716998-33717067. Max. coverage (+): 4.37. Max coverage (-): 0

Region: chr28 33717068-33717136. Max. coverage (+): 1.1. Max coverage (-): 0

Region: chr28 33717137-33717205. Max. coverage (+): 0. Max coverage (-): 0

Region: chr28 33717206-33717274. Max. coverage (+): 0. Max coverage (-): 0

Region: chr28 33717275-33717343. Max. coverage (+): 0. Max coverage (-): 0

Region: chr28 33717344-33717412. Max. coverage (+): 0. Max coverage (-): 0

Region: chr28 33717413-33717481. Max. coverage (+): 0. Max coverage (-): 0

Region: chr28 33717482-33717551. Max. coverage (+): 0. Max coverage (-): 0

Region: chr28 33717552-33717620. Max. coverage (+): 0. Max coverage (-): 0

Region: chr28 33717621-33717689. Max. coverage (+): 3.68. Max coverage (-): 0

Region: chr28 33717690-33717758. Max. coverage (+): 19.07. Max coverage (-): 0

Region: chr28 33717759-33717827. Max. coverage (+): 13.8. Max coverage (-): 0

Region: chr28 33717828-33717896. Max. coverage (+): 0. Max coverage (-): 0

Region: chr28 33717897-33717965. Max. coverage (+): 0.76. Max coverage (-): 0

Region: chr28 33717966-33718035. Max. coverage (+): 0. Max coverage (-): 0

Region: chr28 33718036-33718104. Max. coverage (+): 0. Max coverage (-): 0

Region: chr28 33718105-33718173. Max. coverage (+): 0. Max coverage (-): 0

Region: chr28 33718174-33718242. Max. coverage (+): 1.38. Max coverage (-): 0

Region: chr28 33718243-33718311. Max. coverage (+): 0. Max coverage (-): 0

Region: chr28 33718312-33718380. Max. coverage (+): 1.53. Max coverage (-): 0

Region: chr28 33718381-33718449. Max. coverage (+): 0. Max coverage (-): 0

Region: chr28 33718450-33718519. Max. coverage (+): 0. Max coverage (-): 0

Region: chr28 33718520-33718588. Max. coverage (+): 7.37. Max coverage (-): 0

Region: chr28 33718589-33718657. Max. coverage (+): 4.59. Max coverage (-): 0

Region: chr28 33718658-33718726. Max. coverage (+): 0. Max coverage (-): 0

Region: chr28 33718727-33718795. Max. coverage (+): 0. Max coverage (-): 0

Region: chr28 33718796-33718864. Max. coverage (+): 0. Max coverage (-): 0

Region: chr28 33718865-33718933. Max. coverage (+): 8.26. Max coverage (-): 0

Region: chr28 33718934-33719003. Max. coverage (+): 3.79. Max coverage (-): 0

Region: chr28 33719004-33719072. Max. coverage (+): 4.04. Max coverage (-): 0

Region: chr28 33719073-33719141. Max. coverage (+): 0. Max coverage (-): 0

Region: chr28 33719142-33719210. Max. coverage (+): 4.17. Max coverage (-): 0

Region: chr28 33719211-33719279. Max. coverage (+): 0. Max coverage (-): 0

Region: chr28 33719280-33719348. Max. coverage (+): 0. Max coverage (-): 0

Region: chr28 33719349-33719417. Max. coverage (+): 7.97. Max coverage (-): 0

Region: chr28 33719418-33719487. Max. coverage (+): 7.97. Max coverage (-): 0

Region: chr28 33719488-33719556. Max. coverage (+): 0. Max coverage (-): 0

Region: chr28 33719557-33719625. Max. coverage (+): 15.16. Max coverage (-): 0

Region: chr28 33719626-33719694. Max. coverage (+): 0. Max coverage (-): 0

Region: chr28 33719695-33719763. Max. coverage (+): 0. Max coverage (-): 0

Region: chr28 33719764-33719832. Max. coverage (+): 0. Max coverage (-): 0

Region: chr28 33719833-33719901. Max. coverage (+): 6.87. Max coverage (-): 0

Region: chr28 33719902-33719971. Max. coverage (+): 0. Max coverage (-): 0

Region: chr28 33719972-33720040. Max. coverage (+): 0. Max coverage (-): 0

Region: chr28 33720041-33720109. Max. coverage (+): 0. Max coverage (-): 0

Region: chr28 33720110-33720178. Max. coverage (+): 3.79. Max coverage (-): 0

Region: chr28 33720179-33720247. Max. coverage (+): 1.38. Max coverage (-): 0

Region: chr28 33720248-33720316. Max. coverage (+): 1.38. Max coverage (-): 0

Region: chr28 33720317-33720385. Max. coverage (+): 0. Max coverage (-): 0

Region: chr28 33720386-33720455. Max. coverage (+): 2.89. Max coverage (-): 0

Region: chr28 33720456-33720524. Max. coverage (+): 0. Max coverage (-): 0

Region: chr28 33720525-33720593. Max. coverage (+): 0. Max coverage (-): 0

Region: chr28 33720594-33720662. Max. coverage (+): 0. Max coverage (-): 0

Region: chr28 33720663-33720731. Max. coverage (+): 0. Max coverage (-): 0

Region: chr28 33720732-33720800. Max. coverage (+): 0. Max coverage (-): 0

Region: chr28 33720801-33720869. Max. coverage (+): 0. Max coverage (-): 0

Region: chr28 33720870-33720939. Max. coverage (+): 0. Max coverage (-): 0

Region: chr28 33720940-33721008. Max. coverage (+): 3.76. Max coverage (-): 0

Region: chr28 33721009-33721077. Max. coverage (+): 0. Max coverage (-): 0

Region: chr28 33721078-33721146. Max. coverage (+): 0. Max coverage (-): 0

Region: chr28 33721147-33721215. Max. coverage (+): 0. Max coverage (-): 0

Region: chr28 33721216-33721284. Max. coverage (+): 0. Max coverage (-): 0

Region: chr28 33721285-33721353. Max. coverage (+): 0. Max coverage (-): 0

Region: chr28 33721354-33721423. Max. coverage (+): 0. Max coverage (-): 0

Region: chr28 33721424-33721492. Max. coverage (+): 0. Max coverage (-): 0

Region: chr28 33721493-33721561. Max. coverage (+): 0. Max coverage (-): 0

Region: chr28 33721562-33721630. Max. coverage (+): 6.34. Max coverage (-): 0

Region: chr28 33721631-33721699. Max. coverage (+): 0. Max coverage (-): 0

Region: chr28 33721700-33721768. Max. coverage (+): 0. Max coverage (-): 0

Region: chr28 33721769-33721837. Max. coverage (+): 2.79. Max coverage (-): 0

Region: chr28 33721838-33721907. Max. coverage (+): 0. Max coverage (-): 0

Region: chr28 33721908-33721976. Max. coverage (+): 0. Max coverage (-): 0

Region: chr28 33721977-33722045. Max. coverage (+): 0. Max coverage (-): 0

Region: chr28 33722046-33722114. Max. coverage (+): 0. Max coverage (-): 0

Region: chr28 33722115-33722183. Max. coverage (+): 0. Max coverage (-): 0

Region: chr28 33722184-33722252. Max. coverage (+): 0. Max coverage (-): 0

Region: chr28 33722253-33722321. Max. coverage (+): 0.6. Max coverage (-): 0

Region: chr28 33722322-33722391. Max. coverage (+): 0. Max coverage (-): 0

Region: chr28 33722392-33722460. Max. coverage (+): 0. Max coverage (-): 0

Region: chr28 33722461-33722529. Max. coverage (+): 0. Max coverage (-): 0

Region: chr28 33722530-33722598. Max. coverage (+): 0. Max coverage (-): 0

Region: chr28 33722599-33722667. Max. coverage (+): 17.94. Max coverage (-): 0

Region: chr28 33722668-33722736. Max. coverage (+): 10.1. Max coverage (-): 0

Region: chr28 33722737-33722805. Max. coverage (+): 0. Max coverage (-): 0

Region: chr28 33722806-33722875. Max. coverage (+): 1.65. Max coverage (-): 0

Region: chr28 33722876-33722944. Max. coverage (+): 1.65. Max coverage (-): 0

Region: chr28 33722945-33723013. Max. coverage (+): 0. Max coverage (-): 0

Region: chr28 33723014-33723082. Max. coverage (+): 0. Max coverage (-): 0

Region: chr28 33723083-33723151. Max. coverage (+): 0. Max coverage (-): 0

Region: chr28 33723152-33723220. Max. coverage (+): 0. Max coverage (-): 0

Region: chr28 33723221-33723289. Max. coverage (+): 0. Max coverage (-): 0

Region: chr28 33723290-33723359. Max. coverage (+): 0. Max coverage (-): 0

Region: chr28 33723360-33723428. Max. coverage (+): 10.36. Max coverage (-): 0

Region: chr28 33723429-33723497. Max. coverage (+): 0.69. Max coverage (-): 0

Region: chr28 33723498-33723566. Max. coverage (+): 0. Max coverage (-): 0

Region: chr28 33723567-33723635. Max. coverage (+): 6.2. Max coverage (-): 0

Region: chr28 33723636-33723704. Max. coverage (+): 6.2. Max coverage (-): 0

Region: chr28 33723705-33723773. Max. coverage (+): 0. Max coverage (-): 0

Region: chr28 33723774-33723843. Max. coverage (+): 0. Max coverage (-): 0

Region: chr28 33723844-33723912. Max. coverage (+): 1.56. Max coverage (-): 0

Region: chr28 33723913-33723981. Max. coverage (+): 1.56. Max coverage (-): 0

Region: chr28 33723982-33724050. Max. coverage (+): 0. Max coverage (-): 0

Region: chr28 33724051-33724119. Max. coverage (+): 10.56. Max coverage (-): 0

Region: chr28 33724120-33724188. Max. coverage (+): 5.3. Max coverage (-): 0

Region: chr28 33724189-33724257. Max. coverage (+): 6.18. Max coverage (-): 0

Region: chr28 33724258-33724327. Max. coverage (+): 0. Max coverage (-): 0

Region: chr28 33724328-33724396. Max. coverage (+): 9.21. Max coverage (-): 0

Region: chr28 33724397-33724465. Max. coverage (+): 6.49. Max coverage (-): 0

Region: chr28 33724466-33724534. Max. coverage (+): 0. Max coverage (-): 0

Region: chr28 33724535-33724603. Max. coverage (+): 0. Max coverage (-): 0

Region: chr28 33724604-33724672. Max. coverage (+): 0. Max coverage (-): 0

Region: chr28 33724673-33724741. Max. coverage (+): 0. Max coverage (-): 0

Region: chr28 33724742-33724811. Max. coverage (+): 3.85. Max coverage (-): 0

Region: chr28 33724812-33724880. Max. coverage (+): 2.71. Max coverage (-): 0

Region: chr28 33724881-33724949. Max. coverage (+): 5.49. Max coverage (-): 0

Region: chr28 33724950-33725018. Max. coverage (+): 0. Max coverage (-): 0

Region: chr28 33725019-33725087. Max. coverage (+): 0. Max coverage (-): 0

Region: chr28 33725088-33725156. Max. coverage (+): 0. Max coverage (-): 0

Region: chr28 33725157-33725225. Max. coverage (+): 0. Max coverage (-): 0

Region: chr28 33725226-33725295. Max. coverage (+): 0. Max coverage (-): 0

Region: chr28 33725296-33725364. Max. coverage (+): 0. Max coverage (-): 0

Region: chr28 33725365-33725433. Max. coverage (+): 4.62. Max coverage (-): 0

Region: chr28 33725434-33725502. Max. coverage (+): 4.62. Max coverage (-): 0

Region: chr28 33725503-. Max. coverage (+): 5.3. Max coverage (-): 0

RepeatMasker Color Code

**+**

100-98% Identity

<98-95% Identity

<95-90% Identity

<90-85% Identity

<85-80% Identity

<80-75% Identity

<75-70% Identity

<70% Identity

**-**

Gene Set Color Code

**+**

Gene

Pseudogene

**-**

Topology/Coverage Color Code

Coverage Plus Strand

Coverage Minus Strand

Mainstrand: Plus

Mainstrand: Minus

Complementary Strand

Flanking Region  
(if option -flank >0)

Gene Set Annotation  

**1. DLG5 (protein coding, ENSBTAG00000013187) Tr:00000017554 Ex:28**: 33724499-33724642 (-)  
**2. DLG5 (protein coding, ENSBTAG00000013187) Tr:00000017554 Ex:29**: 33724131-33724258 (-)  
**3. DLG5 (protein coding, ENSBTAG00000013187) Tr:00000017554 Ex:30**: 33723633-33723742 (-)  
**4. DLG5 (protein coding, ENSBTAG00000013187) Tr:00000017554 Ex:31**: 33722861-33722970 (-)  
**5. DLG5 (protein coding, ENSBTAG00000013187) Tr:00000017554 Ex:32**: 33719674-33721398 (-)

  
RepeatMasker Annotation  

**1. BOV-A2**: 33691282-33691410 (+), Divergence to consensus: 8.5%  
**2. L4\_C\_Mam**: 33691990-33692131 (-), Divergence to consensus: 35.6%  
**3. Arthur1A**: 33693070-33693221 (-), Divergence to consensus: 37.6%  
**4. (TTCA)n**: 33693935-33693965 (+), Divergence to consensus: 6.5%  
**5. G-rich**: 33695677-33695769 (+), Divergence to consensus: 20.1%  
**6. AT\_rich**: 33695959-33695987 (+), Divergence to consensus: 79.3%  
**7. L1MEd**: 33696006-33696398 (+), Divergence to consensus: 43.4%  
**8. L1MEd**: 33696418-33696647 (+), Divergence to consensus: 40.3%  
**9. L2c**: 33697080-33697133 (+), Divergence to consensus: 33.5%  
**10. L2c**: 33699039-33699136 (+), Divergence to consensus: 28.5%  
**11. BOV-A2**: 33699494-33699572 (+), Divergence to consensus: 11.4%  
**12. BTLTR1C**: 33699573-33700780 (-), Divergence to consensus: 30.7%  
**13. Tigger15a**: 33700795-33700898 (-), Divergence to consensus: 43%  
**14. L1ME3G**: 33701353-33701566 (+), Divergence to consensus: 47.4%  
**15. L1MCc**: 33701552-33702657 (+), Divergence to consensus: 38.9%  
**16. LTR13B\_BT**: 33702752-33703028 (+), Divergence to consensus: 19.3%  
**17. L1MB5**: 33703050-33703365 (+), Divergence to consensus: 27.5%  
**18. CHRL**: 33703366-33703516 (+), Divergence to consensus: 37.7%  
**19. L1MB5**: 33703517-33703760 (+), Divergence to consensus: 27.5%  
**20. L1MCc**: 33703948-33704098 (+), Divergence to consensus: 28.4%  
**21. MER110**: 33704144-33704372 (+), Divergence to consensus: 40.3%  
**22. MIR3**: 33705231-33705287 (-), Divergence to consensus: 31.6%  
**23. L3**: 33706073-33706159 (-), Divergence to consensus: 39.1%  
**24. MamGypLTR3a**: 33706538-33706652 (-), Divergence to consensus: 39.9%  
**25. MamGypLTR3a**: 33706895-33706960 (-), Divergence to consensus: 31.8%  
**26. MIR**: 33707773-33707952 (+), Divergence to consensus: 35%  
**27. LTR16B1**: 33709018-33709465 (+), Divergence to consensus: 34.5%  
**28. L1ME4a**: 33709500-33709647 (-), Divergence to consensus: 37.2%  
**29. MLT1N2**: 33709701-33709852 (+), Divergence to consensus: 36.4%  
**30. MIR**: 33709933-33710021 (-), Divergence to consensus: 40.2%  
**31. MIRb**: 33710179-33710249 (+), Divergence to consensus: 32.7%  
**32. MER89**: 33711101-33711470 (+), Divergence to consensus: 21.7%  
**33. BOV-A2**: 33714161-33714432 (-), Divergence to consensus: 4.1%  
**34. MLT2C1**: 33715759-33715967 (+), Divergence to consensus: 45.2%  
**35. MLT2C1**: 33716076-33716179 (+), Divergence to consensus: 46.8%  
**36. Bov-tA2**: 33716180-33716369 (+), Divergence to consensus: 20%  
**37. MLT2C1**: 33716370-33716402 (+), Divergence to consensus: 46.8%  
**38. HAL1**: 33716582-33716755 (+), Divergence to consensus: 36.7%  
**39. HAL1**: 33716885-33716998 (+), Divergence to consensus: 30.9%  
**40. HAL1**: 33717086-33717238 (+), Divergence to consensus: 47%  
**41. ART2A**: 33717322-33717630 (+), Divergence to consensus: 21%  
**42. L1ME3D**: 33718129-33718340 (+), Divergence to consensus: 48.7%  
**43. MER5C**: 33722042-33722235 (+), Divergence to consensus: 30.1%  
**44. MER81**: 33724786-33724848 (+), Divergence to consensus: 20.6%  
**45. MLT1J**: 33725051-33725314 (+), Divergence to consensus: 43.1%

  
Transcription Factor Binding Sites  

**RFX4\_1** (Sequence: GTTGCCAGG (-): 33691230)  
**RFX4\_1** (Sequence: GTTGCTAGG (-): 33692553)  
**RFX4\_1** (Sequence: CCTGGCAAC (+): 33718603)  
**RFX4\_2** (Sequence: CCTGGTTAC (+): 33719431)  
**Gata4** (Sequence: AGATAAC (-): 33694539)  
**Gata4** (Sequence: AGATAAC (-): 33696922)  
**Gata4** (Sequence: AGATAAG (-): 33714447)  
**SOX9** (Sequence: AACAATAG (-): 33693135)  
**SOX9** (Sequence: AACAATGA (-): 33701499)  
**SOX9** (Sequence: AACAATAA (-): 33715680)  
**SOX9** (Sequence: AACAATAA (-): 33717290)  
**A-MYB** (Sequence: CCAACTGTCA (-): 33694548)  
**Gata4** (Sequence: CTTATCT (+): 33696965)
