# Supplementary material for: Small RNA sequencing of cryopreserved semen from single bull revealed altered miRNAs and piRNAs expression between High- and Low-motile sperm populations
Source: BMC Genomics. 2017 Jan 4;18:14. doi: 10.1186/s12864-016-3394-7 (PMC5209821; doi:10.1186/s12864-016-3394-7)
Supplement: Additional file 4: — Details for each piRNA clusters found in Low Motile (LM) sperm fraction. Genes, repeats, transposable elements and transcription factors binding sites falling within the cluster regions were reported. (ZIP 1034 kb) [file 12864_2016_3394_MOESM4_ESM.zip › 4.html]

piRNA cluster 4


Predicted piRNA cluster no. 4     previous   next
  

Show proTRAC run info
Hide proTRAC run info

================================= proTRAC ====================================  
VERSION: 2.1                                    LAST MODIFIED: 06. October 2015  
  
Please cite:  
Rosenkranz D, Zischler H. proTRAC - a software for probabilistic piRNA cluster  
detection, visualization and analysis. 2012. BMC Bioinformatics 13:5.  
  
and (for proTRAC 2.0 and later):  
Rosenkranz D, Rudloff S, Bastuck K, Ketting RF, Zischler H. Tupaia small RNAs  
provide insights into function and evolution of RNAi-based transposon defense  
in mammals. 2015. RNA 21(5):911-922.  
  
Contact:  
David Rosenkranz  
Institute of Anthropology, small RNA group  
Johannes Gutenberg University Mainz  
email: rosenkranz@uni-mainz.de  
  
You can find the latest proTRAC version at:  
http://sourceforge.net/projects/protrac/files  
http://www.smallRNAgroup-mainz.de/software  
==============================================================================  
  
PARAMETERS:  
Map file: .............../storage/core/barbara/genhome/smallRNA/fertility/Sample\_not\_motile/pirna/Sample\_not\_motile\_26-33\_collapsed.fa.no-dust.map.weighted-10000-1000-b-0  
Genome file: ............/storage/core/barbara/genhome/smallRNA/fertility/Sample\_all/pirna/bt\_311\_chrY.fa  
RepeatMasker annotation: /storage/genomes/bt\_umd31/GCF\_000003055.6\_Bos\_taurus\_UMD\_3.1.1\_repeatMasker\_chr.out  
GeneSet:................./storage/core/barbara/genhome/smallRNA/fertility/Sample\_all/pirna/full.gtf  
  
Significant (p<=0.01) hit density will be calculated based  
on observed hit distribution.  
  
Sliding window size: ........................................ 5000 bp  
Sliding window increament: .................................. 1000 bp  
Normalize each hit by number of genomic hits: ............... 1 [0=no/1=yes]  
Normalize each hit by number of sequence reads: ............. 1 [0=no/1=yes]  
Normalize values (-> per million mapped reads): ............. 1 [0=no/1=yes]  
Min. fraction of hits with 1T(U) or 10A: .................... 0.75  
Alternatively: Min. fraction of hits with 1T(U) and 10A: .... 0.5  
Min. fraction of hits with typical piRNA length: ............ 0.75  
Typical piRNA length: ....................................... 26-33 nt  
Min. size of a piRNA cluster: ............................... 5000 bp.  
Min. number of hits (absolute): ............................. 0  
Min. number of hits (normalized): ........................... 0  
Min. fraction of hits on the mainstrand: .................... 0.75  
Top fraction of mapped sequences (in terms of read counts): . 1%  
Top fraction accounts for max. n% of sequence reads: ........ 90%  
Min. fraction of hits on each arm of a bidirectional cluster: 0.1  
Output image file for each cluster: ......................... 0 [0=no/1=yes]  
Output html file for each cluster: .......................... 1 [0=no/1=yes]  
Output a summary table: ..................................... 1 [0=no/1=yes]  
Output a FASTA file for each cluster (piRNA sequences): ..... 1 [0=no/1=yes]  
Output a FASTA file comprising cluster sequences: ........... 1 [0=no/1=yes]  
Search DNA motifs in clusters: .............................. 1 [0=no/1=yes]  
Output flanking sequences: +/- .............................. 0 bp  
Output ~.pTi file: .......................................... 1 [0=no/1=yes]  
==============================================================================  
  
  
Genome size (without gaps): ............ 2678902517 bp  
Gaps (N/X/-): .......................... 53837044 bp  
Mapped reads: .......................... 738059667487  
Non-identical sequences: ............... 277001  
Genomic hits: .......................... 533816  
Significant densitiy of mapped reads: .. 15118061 reads/kb

Show proTRAC cluster info
Hide proTRAC cluster info

|  |  |
| --- | --- |
| Location | chr10 |
| Coordinates | 59179390-59224895 |
| Size [bp] | 45506 |
| Sequence hit loci | 1532 |
| Mapped reads (normalized) | 4228234501 |
| Mapped reads (normalized) per kb | 92915978.1 |
| Normalized reads with 1T (1U) | 85.6% |
| Normalized reads with 10A | 31.5% |
| Normalized reads with length 26-33 nt | 100% |
| Normalized reads on the main strand(s) | 99.2% |
| Predicted directionality | mono:plus |

100%

0%

1T (1U)  
reads

10A reads

26-33 nt  
reads

reads on mainstrand

**Either the amount of reads with 1T (1U) OR 10A has to exceed 75% (set with option: -1Tor10A)  
Alternatively the amount of reads with 1T (1U) AND 10A has to exceed 50% (set with option: -1Tand10A)  
Minimum amount of reads with preferred size is 75% (set with option: -pisize)  
Minimum amount of reads on the main strand(s) is 75% (set with option: -clstrand)**

Show read coverage
Hide read coverage

WHAT DO I SEE HERE?  
This chart shows the location of mapped sequence reads within a predicted piRNA cluster. The color refers to the number of genomic hits produced by the sequence read in question. A dark red bar indicates that this sequence read produces many other hits elsewhere in the genome. Many adjacent red or yellow bars can indicate the presence of a multi-copy element such as transposons or rRNA genes. A dark green bar indicates that this sequence read maps uniquely to this locus.

1 hit

2-5 hits

6-10 hits

11-20 hits

21-50 hits

51-100 hits

> 100 hits

chr10

59179390

59224895

Gene Set

RepeatMasker

Mapped  
Reads

141.52

plus strand

minus strand

141.52

Region: chr10 47938295-59179435. Max. coverage (+): 0. Max coverage (-): 0.4

Region: chr10 59179436-59179526. Max. coverage (+): 0. Max coverage (-): 0

Region: chr10 59179527-59179617. Max. coverage (+): 0. Max coverage (-): 0

Region: chr10 59179618-59179708. Max. coverage (+): 0. Max coverage (-): 0

Region: chr10 59179709-59179799. Max. coverage (+): 0. Max coverage (-): 0

Region: chr10 59179800-59179890. Max. coverage (+): 0. Max coverage (-): 0

Region: chr10 59179891-59179981. Max. coverage (+): 0. Max coverage (-): 0

Region: chr10 59179982-59180072. Max. coverage (+): 0. Max coverage (-): 0

Region: chr10 59180073-59180163. Max. coverage (+): 0. Max coverage (-): 0

Region: chr10 59180164-59180254. Max. coverage (+): 0. Max coverage (-): 0

Region: chr10 59180255-59180345. Max. coverage (+): 0. Max coverage (-): 0

Region: chr10 59180346-59180436. Max. coverage (+): 0. Max coverage (-): 0

Region: chr10 59180437-59180527. Max. coverage (+): 0. Max coverage (-): 0

Region: chr10 59180528-59180618. Max. coverage (+): 0. Max coverage (-): 8.5

Region: chr10 59180619-59180709. Max. coverage (+): 0. Max coverage (-): 0

Region: chr10 59180710-59180800. Max. coverage (+): 0. Max coverage (-): 0

Region: chr10 59180801-59180891. Max. coverage (+): 0. Max coverage (-): 0

Region: chr10 59180892-59180982. Max. coverage (+): 0. Max coverage (-): 0

Region: chr10 59180983-59181073. Max. coverage (+): 0. Max coverage (-): 0

Region: chr10 59181074-59181164. Max. coverage (+): 0. Max coverage (-): 0

Region: chr10 59181165-59181255. Max. coverage (+): 0. Max coverage (-): 0

Region: chr10 59181256-59181346. Max. coverage (+): 0. Max coverage (-): 0

Region: chr10 59181347-59181437. Max. coverage (+): 0. Max coverage (-): 0

Region: chr10 59181438-59181528. Max. coverage (+): 0. Max coverage (-): 3.62

Region: chr10 59181529-59181619. Max. coverage (+): 0. Max coverage (-): 0

Region: chr10 59181620-59181710. Max. coverage (+): 0. Max coverage (-): 0

Region: chr10 59181711-59181801. Max. coverage (+): 0. Max coverage (-): 0

Region: chr10 59181802-59181892. Max. coverage (+): 0. Max coverage (-): 4.58

Region: chr10 59181893-59181983. Max. coverage (+): 0. Max coverage (-): 0

Region: chr10 59181984-59182074. Max. coverage (+): 0. Max coverage (-): 0

Region: chr10 59182075-59182165. Max. coverage (+): 0. Max coverage (-): 0

Region: chr10 59182166-59182256. Max. coverage (+): 0. Max coverage (-): 0

Region: chr10 59182257-59182347. Max. coverage (+): 0. Max coverage (-): 0

Region: chr10 59182348-59182438. Max. coverage (+): 0. Max coverage (-): 0

Region: chr10 59182439-59182529. Max. coverage (+): 0. Max coverage (-): 0

Region: chr10 59182530-59182620. Max. coverage (+): 0. Max coverage (-): 0

Region: chr10 59182621-59182711. Max. coverage (+): 0. Max coverage (-): 0

Region: chr10 59182712-59182802. Max. coverage (+): 0. Max coverage (-): 0

Region: chr10 59182803-59182893. Max. coverage (+): 0. Max coverage (-): 0

Region: chr10 59182894-59182984. Max. coverage (+): 38.66. Max coverage (-): 0

Region: chr10 59182985-59183075. Max. coverage (+): 16.87. Max coverage (-): 0

Region: chr10 59183076-59183166. Max. coverage (+): 2.2. Max coverage (-): 0

Region: chr10 59183167-59183258. Max. coverage (+): 3.18. Max coverage (-): 0

Region: chr10 59183259-59183349. Max. coverage (+): 10.8. Max coverage (-): 0

Region: chr10 59183350-59183440. Max. coverage (+): 2.03. Max coverage (-): 0

Region: chr10 59183441-59183531. Max. coverage (+): 13.19. Max coverage (-): 0

Region: chr10 59183532-59183622. Max. coverage (+): 6.91. Max coverage (-): 0

Region: chr10 59183623-59183713. Max. coverage (+): 22.81. Max coverage (-): 0

Region: chr10 59183714-59183804. Max. coverage (+): 15.24. Max coverage (-): 0

Region: chr10 59183805-59183895. Max. coverage (+): 9.7. Max coverage (-): 0

Region: chr10 59183896-59183986. Max. coverage (+): 2.23. Max coverage (-): 0

Region: chr10 59183987-59184077. Max. coverage (+): 3.31. Max coverage (-): 0

Region: chr10 59184078-59184168. Max. coverage (+): 3.31. Max coverage (-): 0

Region: chr10 59184169-59184259. Max. coverage (+): 5.99. Max coverage (-): 0

Region: chr10 59184260-59184350. Max. coverage (+): 2.2. Max coverage (-): 0

Region: chr10 59184351-59184441. Max. coverage (+): 2.91. Max coverage (-): 0

Region: chr10 59184442-59184532. Max. coverage (+): 9.82. Max coverage (-): 0

Region: chr10 59184533-59184623. Max. coverage (+): 9.82. Max coverage (-): 0

Region: chr10 59184624-59184714. Max. coverage (+): 4.48. Max coverage (-): 0

Region: chr10 59184715-59184805. Max. coverage (+): 0. Max coverage (-): 0

Region: chr10 59184806-59184896. Max. coverage (+): 0. Max coverage (-): 0

Region: chr10 59184897-59184987. Max. coverage (+): 10.14. Max coverage (-): 0

Region: chr10 59184988-59185078. Max. coverage (+): 141.52. Max coverage (-): 0

Region: chr10 59185079-59185169. Max. coverage (+): 45.36. Max coverage (-): 0

Region: chr10 59185170-59185260. Max. coverage (+): 46.68. Max coverage (-): 0

Region: chr10 59185261-59185351. Max. coverage (+): 8.81. Max coverage (-): 0

Region: chr10 59185352-59185442. Max. coverage (+): 3.91. Max coverage (-): 0

Region: chr10 59185443-59185533. Max. coverage (+): 9.11. Max coverage (-): 0

Region: chr10 59185534-59185624. Max. coverage (+): 0. Max coverage (-): 0

Region: chr10 59185625-59185715. Max. coverage (+): 11.93. Max coverage (-): 0

Region: chr10 59185716-59185806. Max. coverage (+): 5.7. Max coverage (-): 0

Region: chr10 59185807-59185897. Max. coverage (+): 6.48. Max coverage (-): 0

Region: chr10 59185898-59185988. Max. coverage (+): 17.42. Max coverage (-): 0

Region: chr10 59185989-59186079. Max. coverage (+): 18.92. Max coverage (-): 0

Region: chr10 59186080-59186170. Max. coverage (+): 16.08. Max coverage (-): 0

Region: chr10 59186171-59186261. Max. coverage (+): 7.66. Max coverage (-): 0

Region: chr10 59186262-59186352. Max. coverage (+): 0. Max coverage (-): 0

Region: chr10 59186353-59186443. Max. coverage (+): 0. Max coverage (-): 0

Region: chr10 59186444-59186534. Max. coverage (+): 9.07. Max coverage (-): 0

Region: chr10 59186535-59186625. Max. coverage (+): 14.35. Max coverage (-): 2.12

Region: chr10 59186626-59186716. Max. coverage (+): 28.04. Max coverage (-): 0

Region: chr10 59186717-59186807. Max. coverage (+): 16.56. Max coverage (-): 0

Region: chr10 59186808-59186898. Max. coverage (+): 0. Max coverage (-): 0

Region: chr10 59186899-59186989. Max. coverage (+): 0. Max coverage (-): 0

Region: chr10 59186990-59187080. Max. coverage (+): 6.8. Max coverage (-): 0

Region: chr10 59187081-59187171. Max. coverage (+): 31.82. Max coverage (-): 0

Region: chr10 59187172-59187262. Max. coverage (+): 3.7. Max coverage (-): 0

Region: chr10 59187263-59187353. Max. coverage (+): 5.7. Max coverage (-): 0

Region: chr10 59187354-59187444. Max. coverage (+): 6.28. Max coverage (-): 0

Region: chr10 59187445-59187535. Max. coverage (+): 0.47. Max coverage (-): 3.05

Region: chr10 59187536-59187626. Max. coverage (+): 0. Max coverage (-): 0

Region: chr10 59187627-59187717. Max. coverage (+): 10.74. Max coverage (-): 0

Region: chr10 59187718-59187808. Max. coverage (+): 40.02. Max coverage (-): 0

Region: chr10 59187809-59187899. Max. coverage (+): 13.4. Max coverage (-): 0

Region: chr10 59187900-59187990. Max. coverage (+): 11.64. Max coverage (-): 0

Region: chr10 59187991-59188081. Max. coverage (+): 0. Max coverage (-): 0

Region: chr10 59188082-59188172. Max. coverage (+): 22.69. Max coverage (-): 0

Region: chr10 59188173-59188263. Max. coverage (+): 5.09. Max coverage (-): 0

Region: chr10 59188264-59188354. Max. coverage (+): 14.52. Max coverage (-): 0

Region: chr10 59188355-59188445. Max. coverage (+): 63.62. Max coverage (-): 0

Region: chr10 59188446-59188536. Max. coverage (+): 26.85. Max coverage (-): 0

Region: chr10 59188537-59188627. Max. coverage (+): 10.48. Max coverage (-): 0

Region: chr10 59188628-59188718. Max. coverage (+): 45.24. Max coverage (-): 0

Region: chr10 59188719-59188809. Max. coverage (+): 16.34. Max coverage (-): 0

Region: chr10 59188810-59188900. Max. coverage (+): 0. Max coverage (-): 0

Region: chr10 59188901-59188991. Max. coverage (+): 0. Max coverage (-): 0

Region: chr10 59188992-59189082. Max. coverage (+): 0. Max coverage (-): 0

Region: chr10 59189083-59189173. Max. coverage (+): 31.81. Max coverage (-): 0

Region: chr10 59189174-59189264. Max. coverage (+): 8.68. Max coverage (-): 0

Region: chr10 59189265-59189355. Max. coverage (+): 18.88. Max coverage (-): 0

Region: chr10 59189356-59189446. Max. coverage (+): 15.21. Max coverage (-): 0

Region: chr10 59189447-59189537. Max. coverage (+): 37.39. Max coverage (-): 0

Region: chr10 59189538-59189628. Max. coverage (+): 32.86. Max coverage (-): 0

Region: chr10 59189629-59189719. Max. coverage (+): 11.11. Max coverage (-): 0

Region: chr10 59189720-59189810. Max. coverage (+): 35.63. Max coverage (-): 0

Region: chr10 59189811-59189901. Max. coverage (+): 28.6. Max coverage (-): 0

Region: chr10 59189902-59189992. Max. coverage (+): 35.55. Max coverage (-): 0

Region: chr10 59189993-59190083. Max. coverage (+): 11.72. Max coverage (-): 0

Region: chr10 59190084-59190174. Max. coverage (+): 26.9. Max coverage (-): 0

Region: chr10 59190175-59190265. Max. coverage (+): 44.41. Max coverage (-): 0

Region: chr10 59190266-59190356. Max. coverage (+): 17.65. Max coverage (-): 0

Region: chr10 59190357-59190447. Max. coverage (+): 7.17. Max coverage (-): 0

Region: chr10 59190448-59190538. Max. coverage (+): 22.63. Max coverage (-): 0

Region: chr10 59190539-59190629. Max. coverage (+): 26.07. Max coverage (-): 0

Region: chr10 59190630-59190720. Max. coverage (+): 26.74. Max coverage (-): 0

Region: chr10 59190721-59190812. Max. coverage (+): 0. Max coverage (-): 0

Region: chr10 59190813-59190903. Max. coverage (+): 22.64. Max coverage (-): 0

Region: chr10 59190904-59190994. Max. coverage (+): 0. Max coverage (-): 0

Region: chr10 59190995-59191085. Max. coverage (+): 4.15. Max coverage (-): 0

Region: chr10 59191086-59191176. Max. coverage (+): 0. Max coverage (-): 0

Region: chr10 59191177-59191267. Max. coverage (+): 53.11. Max coverage (-): 0

Region: chr10 59191268-59191358. Max. coverage (+): 13.74. Max coverage (-): 0

Region: chr10 59191359-59191449. Max. coverage (+): 2.32. Max coverage (-): 0

Region: chr10 59191450-59191540. Max. coverage (+): 12.95. Max coverage (-): 0

Region: chr10 59191541-59191631. Max. coverage (+): 19.99. Max coverage (-): 0

Region: chr10 59191632-59191722. Max. coverage (+): 0. Max coverage (-): 0

Region: chr10 59191723-59191813. Max. coverage (+): 0. Max coverage (-): 0

Region: chr10 59191814-59191904. Max. coverage (+): 12.1. Max coverage (-): 0

Region: chr10 59191905-59191995. Max. coverage (+): 40.87. Max coverage (-): 0

Region: chr10 59191996-59192086. Max. coverage (+): 6.3. Max coverage (-): 0

Region: chr10 59192087-59192177. Max. coverage (+): 6.6. Max coverage (-): 0

Region: chr10 59192178-59192268. Max. coverage (+): 0. Max coverage (-): 0

Region: chr10 59192269-59192359. Max. coverage (+): 0. Max coverage (-): 0

Region: chr10 59192360-59192450. Max. coverage (+): 0.8. Max coverage (-): 0

Region: chr10 59192451-59192541. Max. coverage (+): 0. Max coverage (-): 0

Region: chr10 59192542-59192632. Max. coverage (+): 11.37. Max coverage (-): 0

Region: chr10 59192633-59192723. Max. coverage (+): 0.14. Max coverage (-): 0

Region: chr10 59192724-59192814. Max. coverage (+): 1.48. Max coverage (-): 0

Region: chr10 59192815-59192905. Max. coverage (+): 0. Max coverage (-): 0

Region: chr10 59192906-59192996. Max. coverage (+): 1.24. Max coverage (-): 0

Region: chr10 59192997-59193087. Max. coverage (+): 5.59. Max coverage (-): 0

Region: chr10 59193088-59193178. Max. coverage (+): 0.59. Max coverage (-): 0

Region: chr10 59193179-59193269. Max. coverage (+): 2.15. Max coverage (-): 0

Region: chr10 59193270-59193360. Max. coverage (+): 6.48. Max coverage (-): 0

Region: chr10 59193361-59193451. Max. coverage (+): 6.48. Max coverage (-): 0

Region: chr10 59193452-59193542. Max. coverage (+): 3.43. Max coverage (-): 0

Region: chr10 59193543-59193633. Max. coverage (+): 4.92. Max coverage (-): 0

Region: chr10 59193634-59193724. Max. coverage (+): 0. Max coverage (-): 0

Region: chr10 59193725-59193815. Max. coverage (+): 65.26. Max coverage (-): 0

Region: chr10 59193816-59193906. Max. coverage (+): 65.26. Max coverage (-): 0

Region: chr10 59193907-59193997. Max. coverage (+): 4.48. Max coverage (-): 0

Region: chr10 59193998-59194088. Max. coverage (+): 34.65. Max coverage (-): 4.4

Region: chr10 59194089-59194179. Max. coverage (+): 22.87. Max coverage (-): 0

Region: chr10 59194180-59194270. Max. coverage (+): 20.05. Max coverage (-): 0

Region: chr10 59194271-59194361. Max. coverage (+): 6.82. Max coverage (-): 0

Region: chr10 59194362-59194452. Max. coverage (+): 3.36. Max coverage (-): 0

Region: chr10 59194453-59194543. Max. coverage (+): 21.19. Max coverage (-): 0

Region: chr10 59194544-59194634. Max. coverage (+): 0. Max coverage (-): 0

Region: chr10 59194635-59194725. Max. coverage (+): 8.43. Max coverage (-): 0

Region: chr10 59194726-59194816. Max. coverage (+): 47.33. Max coverage (-): 0

Region: chr10 59194817-59194907. Max. coverage (+): 6.81. Max coverage (-): 0

Region: chr10 59194908-59194998. Max. coverage (+): 4.56. Max coverage (-): 0

Region: chr10 59194999-59195089. Max. coverage (+): 18.01. Max coverage (-): 0

Region: chr10 59195090-59195180. Max. coverage (+): 0. Max coverage (-): 0

Region: chr10 59195181-59195271. Max. coverage (+): 9.25. Max coverage (-): 0

Region: chr10 59195272-59195362. Max. coverage (+): 27.73. Max coverage (-): 0

Region: chr10 59195363-59195453. Max. coverage (+): 0. Max coverage (-): 0

Region: chr10 59195454-59195544. Max. coverage (+): 36.68. Max coverage (-): 0

Region: chr10 59195545-59195635. Max. coverage (+): 19.38. Max coverage (-): 0

Region: chr10 59195636-59195726. Max. coverage (+): 0. Max coverage (-): 0

Region: chr10 59195727-59195817. Max. coverage (+): 0. Max coverage (-): 0

Region: chr10 59195818-59195908. Max. coverage (+): 0. Max coverage (-): 0

Region: chr10 59195909-59195999. Max. coverage (+): 42.85. Max coverage (-): 0

Region: chr10 59196000-59196090. Max. coverage (+): 18.38. Max coverage (-): 0

Region: chr10 59196091-59196181. Max. coverage (+): 19.02. Max coverage (-): 0

Region: chr10 59196182-59196272. Max. coverage (+): 18.16. Max coverage (-): 0

Region: chr10 59196273-59196363. Max. coverage (+): 19.88. Max coverage (-): 0

Region: chr10 59196364-59196454. Max. coverage (+): 5.71. Max coverage (-): 0

Region: chr10 59196455-59196545. Max. coverage (+): 60.2. Max coverage (-): 0

Region: chr10 59196546-59196636. Max. coverage (+): 0. Max coverage (-): 0

Region: chr10 59196637-59196727. Max. coverage (+): 0. Max coverage (-): 0

Region: chr10 59196728-59196818. Max. coverage (+): 20.82. Max coverage (-): 0

Region: chr10 59196819-59196909. Max. coverage (+): 28.86. Max coverage (-): 0

Region: chr10 59196910-59197000. Max. coverage (+): 10.63. Max coverage (-): 0

Region: chr10 59197001-59197091. Max. coverage (+): 13.57. Max coverage (-): 0

Region: chr10 59197092-59197182. Max. coverage (+): 7.12. Max coverage (-): 0

Region: chr10 59197183-59197273. Max. coverage (+): 6.2. Max coverage (-): 0

Region: chr10 59197274-59197364. Max. coverage (+): 24.31. Max coverage (-): 0

Region: chr10 59197365-59197455. Max. coverage (+): 12.51. Max coverage (-): 0

Region: chr10 59197456-59197546. Max. coverage (+): 3.83. Max coverage (-): 0

Region: chr10 59197547-59197637. Max. coverage (+): 13.81. Max coverage (-): 0

Region: chr10 59197638-59197728. Max. coverage (+): 0. Max coverage (-): 0

Region: chr10 59197729-59197819. Max. coverage (+): 0. Max coverage (-): 0

Region: chr10 59197820-59197910. Max. coverage (+): 0. Max coverage (-): 0

Region: chr10 59197911-59198001. Max. coverage (+): 9.22. Max coverage (-): 0

Region: chr10 59198002-59198092. Max. coverage (+): 65.73. Max coverage (-): 0

Region: chr10 59198093-59198183. Max. coverage (+): 43.37. Max coverage (-): 0

Region: chr10 59198184-59198274. Max. coverage (+): 41.54. Max coverage (-): 0

Region: chr10 59198275-59198366. Max. coverage (+): 47.26. Max coverage (-): 0

Region: chr10 59198367-59198457. Max. coverage (+): 10.17. Max coverage (-): 0

Region: chr10 59198458-59198548. Max. coverage (+): 5.63. Max coverage (-): 0

Region: chr10 59198549-59198639. Max. coverage (+): 27.8. Max coverage (-): 0

Region: chr10 59198640-59198730. Max. coverage (+): 37.52. Max coverage (-): 0

Region: chr10 59198731-59198821. Max. coverage (+): 36.4. Max coverage (-): 0

Region: chr10 59198822-59198912. Max. coverage (+): 0.66. Max coverage (-): 0

Region: chr10 59198913-59199003. Max. coverage (+): 18.84. Max coverage (-): 0

Region: chr10 59199004-59199094. Max. coverage (+): 13.05. Max coverage (-): 0

Region: chr10 59199095-59199185. Max. coverage (+): 16.08. Max coverage (-): 0

Region: chr10 59199186-59199276. Max. coverage (+): 27.14. Max coverage (-): 0

Region: chr10 59199277-59199367. Max. coverage (+): 19.83. Max coverage (-): 0

Region: chr10 59199368-59199458. Max. coverage (+): 10.03. Max coverage (-): 0

Region: chr10 59199459-59199549. Max. coverage (+): 5.51. Max coverage (-): 0

Region: chr10 59199550-59199640. Max. coverage (+): 20.46. Max coverage (-): 0

Region: chr10 59199641-59199731. Max. coverage (+): 0. Max coverage (-): 0

Region: chr10 59199732-59199822. Max. coverage (+): 2.74. Max coverage (-): 1.33

Region: chr10 59199823-59199913. Max. coverage (+): 3.48. Max coverage (-): 0

Region: chr10 59199914-59200004. Max. coverage (+): 1.12. Max coverage (-): 0

Region: chr10 59200005-59200095. Max. coverage (+): 5.15. Max coverage (-): 0

Region: chr10 59200096-59200186. Max. coverage (+): 5.15. Max coverage (-): 0

Region: chr10 59200187-59200277. Max. coverage (+): 7.02. Max coverage (-): 0

Region: chr10 59200278-59200368. Max. coverage (+): 29.15. Max coverage (-): 0

Region: chr10 59200369-59200459. Max. coverage (+): 5.6. Max coverage (-): 0

Region: chr10 59200460-59200550. Max. coverage (+): 0. Max coverage (-): 0

Region: chr10 59200551-59200641. Max. coverage (+): 12.72. Max coverage (-): 0

Region: chr10 59200642-59200732. Max. coverage (+): 6.31. Max coverage (-): 0

Region: chr10 59200733-59200823. Max. coverage (+): 6.86. Max coverage (-): 0

Region: chr10 59200824-59200914. Max. coverage (+): 4.85. Max coverage (-): 1.28

Region: chr10 59200915-59201005. Max. coverage (+): 11.07. Max coverage (-): 0

Region: chr10 59201006-59201096. Max. coverage (+): 19.48. Max coverage (-): 0

Region: chr10 59201097-59201187. Max. coverage (+): 0. Max coverage (-): 0

Region: chr10 59201188-59201278. Max. coverage (+): 0. Max coverage (-): 0

Region: chr10 59201279-59201369. Max. coverage (+): 20.5. Max coverage (-): 0

Region: chr10 59201370-59201460. Max. coverage (+): 0. Max coverage (-): 0

Region: chr10 59201461-59201551. Max. coverage (+): 0. Max coverage (-): 0

Region: chr10 59201552-59201642. Max. coverage (+): 0. Max coverage (-): 0

Region: chr10 59201643-59201733. Max. coverage (+): 0. Max coverage (-): 0

Region: chr10 59201734-59201824. Max. coverage (+): 0. Max coverage (-): 0

Region: chr10 59201825-59201915. Max. coverage (+): 0. Max coverage (-): 0

Region: chr10 59201916-59202006. Max. coverage (+): 0. Max coverage (-): 0

Region: chr10 59202007-59202097. Max. coverage (+): 0. Max coverage (-): 0.36

Region: chr10 59202098-59202188. Max. coverage (+): 12.09. Max coverage (-): 0

Region: chr10 59202189-59202279. Max. coverage (+): 3.91. Max coverage (-): 0

Region: chr10 59202280-59202370. Max. coverage (+): 0. Max coverage (-): 0

Region: chr10 59202371-59202461. Max. coverage (+): 0. Max coverage (-): 0

Region: chr10 59202462-59202552. Max. coverage (+): 0. Max coverage (-): 0

Region: chr10 59202553-59202643. Max. coverage (+): 0. Max coverage (-): 0

Region: chr10 59202644-59202734. Max. coverage (+): 0. Max coverage (-): 0

Region: chr10 59202735-59202825. Max. coverage (+): 2.47. Max coverage (-): 0

Region: chr10 59202826-59202916. Max. coverage (+): 0.2. Max coverage (-): 0

Region: chr10 59202917-59203007. Max. coverage (+): 0. Max coverage (-): 0

Region: chr10 59203008-59203098. Max. coverage (+): 0. Max coverage (-): 0

Region: chr10 59203099-59203189. Max. coverage (+): 6.4. Max coverage (-): 0

Region: chr10 59203190-59203280. Max. coverage (+): 6.7. Max coverage (-): 0

Region: chr10 59203281-59203371. Max. coverage (+): 4.77. Max coverage (-): 0

Region: chr10 59203372-59203462. Max. coverage (+): 1.23. Max coverage (-): 0

Region: chr10 59203463-59203553. Max. coverage (+): 0. Max coverage (-): 0

Region: chr10 59203554-59203644. Max. coverage (+): 0. Max coverage (-): 0

Region: chr10 59203645-59203735. Max. coverage (+): 0. Max coverage (-): 0

Region: chr10 59203736-59203826. Max. coverage (+): 1.9. Max coverage (-): 0

Region: chr10 59203827-59203917. Max. coverage (+): 7.2. Max coverage (-): 0

Region: chr10 59203918-59204008. Max. coverage (+): 7.64. Max coverage (-): 0

Region: chr10 59204009-59204099. Max. coverage (+): 2.36. Max coverage (-): 0

Region: chr10 59204100-59204190. Max. coverage (+): 9.23. Max coverage (-): 0

Region: chr10 59204191-59204281. Max. coverage (+): 4.32. Max coverage (-): 0

Region: chr10 59204282-59204372. Max. coverage (+): 12.63. Max coverage (-): 0

Region: chr10 59204373-59204463. Max. coverage (+): 0. Max coverage (-): 0

Region: chr10 59204464-59204554. Max. coverage (+): 3.65. Max coverage (-): 0

Region: chr10 59204555-59204645. Max. coverage (+): 0. Max coverage (-): 0

Region: chr10 59204646-59204736. Max. coverage (+): 0. Max coverage (-): 0

Region: chr10 59204737-59204827. Max. coverage (+): 0. Max coverage (-): 0

Region: chr10 59204828-59204918. Max. coverage (+): 7.16. Max coverage (-): 0

Region: chr10 59204919-59205009. Max. coverage (+): 28.28. Max coverage (-): 0

Region: chr10 59205010-59205100. Max. coverage (+): 9.5. Max coverage (-): 0

Region: chr10 59205101-59205191. Max. coverage (+): 25.06. Max coverage (-): 0

Region: chr10 59205192-59205282. Max. coverage (+): 0. Max coverage (-): 0

Region: chr10 59205283-59205373. Max. coverage (+): 19.88. Max coverage (-): 0

Region: chr10 59205374-59205464. Max. coverage (+): 18.74. Max coverage (-): 0

Region: chr10 59205465-59205555. Max. coverage (+): 5.27. Max coverage (-): 0

Region: chr10 59205556-59205646. Max. coverage (+): 23.63. Max coverage (-): 0

Region: chr10 59205647-59205737. Max. coverage (+): 23.63. Max coverage (-): 0

Region: chr10 59205738-59205828. Max. coverage (+): 33.8. Max coverage (-): 0

Region: chr10 59205829-59205919. Max. coverage (+): 12.49. Max coverage (-): 0

Region: chr10 59205920-59206011. Max. coverage (+): 3.33. Max coverage (-): 0

Region: chr10 59206012-59206102. Max. coverage (+): 9.31. Max coverage (-): 0

Region: chr10 59206103-59206193. Max. coverage (+): 28.62. Max coverage (-): 0

Region: chr10 59206194-59206284. Max. coverage (+): 2.35. Max coverage (-): 0

Region: chr10 59206285-59206375. Max. coverage (+): 2.35. Max coverage (-): 0

Region: chr10 59206376-59206466. Max. coverage (+): 0. Max coverage (-): 0

Region: chr10 59206467-59206557. Max. coverage (+): 0. Max coverage (-): 0

Region: chr10 59206558-59206648. Max. coverage (+): 6.34. Max coverage (-): 0

Region: chr10 59206649-59206739. Max. coverage (+): 5.05. Max coverage (-): 0

Region: chr10 59206740-59206830. Max. coverage (+): 3.6. Max coverage (-): 0

Region: chr10 59206831-59206921. Max. coverage (+): 3.43. Max coverage (-): 0

Region: chr10 59206922-59207012. Max. coverage (+): 0. Max coverage (-): 0

Region: chr10 59207013-59207103. Max. coverage (+): 0. Max coverage (-): 0

Region: chr10 59207104-59207194. Max. coverage (+): 0. Max coverage (-): 0

Region: chr10 59207195-59207285. Max. coverage (+): 0. Max coverage (-): 0

Region: chr10 59207286-59207376. Max. coverage (+): 0. Max coverage (-): 0

Region: chr10 59207377-59207467. Max. coverage (+): 3.83. Max coverage (-): 0

Region: chr10 59207468-59207558. Max. coverage (+): 0. Max coverage (-): 0

Region: chr10 59207559-59207649. Max. coverage (+): 0. Max coverage (-): 0

Region: chr10 59207650-59207740. Max. coverage (+): 0. Max coverage (-): 0

Region: chr10 59207741-59207831. Max. coverage (+): 0. Max coverage (-): 0

Region: chr10 59207832-59207922. Max. coverage (+): 0. Max coverage (-): 0

Region: chr10 59207923-59208013. Max. coverage (+): 0. Max coverage (-): 0

Region: chr10 59208014-59208104. Max. coverage (+): 0. Max coverage (-): 0

Region: chr10 59208105-59208195. Max. coverage (+): 0. Max coverage (-): 0

Region: chr10 59208196-59208286. Max. coverage (+): 0. Max coverage (-): 0

Region: chr10 59208287-59208377. Max. coverage (+): 0. Max coverage (-): 0

Region: chr10 59208378-59208468. Max. coverage (+): 0. Max coverage (-): 0

Region: chr10 59208469-59208559. Max. coverage (+): 0. Max coverage (-): 0

Region: chr10 59208560-59208650. Max. coverage (+): 0. Max coverage (-): 0

Region: chr10 59208651-59208741. Max. coverage (+): 0. Max coverage (-): 0

Region: chr10 59208742-59208832. Max. coverage (+): 0. Max coverage (-): 0

Region: chr10 59208833-59208923. Max. coverage (+): 0. Max coverage (-): 0

Region: chr10 59208924-59209014. Max. coverage (+): 0. Max coverage (-): 0

Region: chr10 59209015-59209105. Max. coverage (+): 0. Max coverage (-): 0

Region: chr10 59209106-59209196. Max. coverage (+): 0. Max coverage (-): 0

Region: chr10 59209197-59209287. Max. coverage (+): 0. Max coverage (-): 0

Region: chr10 59209288-59209378. Max. coverage (+): 0. Max coverage (-): 0

Region: chr10 59209379-59209469. Max. coverage (+): 0. Max coverage (-): 0

Region: chr10 59209470-59209560. Max. coverage (+): 0. Max coverage (-): 0

Region: chr10 59209561-59209651. Max. coverage (+): 0. Max coverage (-): 0

Region: chr10 59209652-59209742. Max. coverage (+): 0. Max coverage (-): 0

Region: chr10 59209743-59209833. Max. coverage (+): 0. Max coverage (-): 0

Region: chr10 59209834-59209924. Max. coverage (+): 0. Max coverage (-): 0

Region: chr10 59209925-59210015. Max. coverage (+): 0. Max coverage (-): 0

Region: chr10 59210016-59210106. Max. coverage (+): 0. Max coverage (-): 0

Region: chr10 59210107-59210197. Max. coverage (+): 0. Max coverage (-): 0

Region: chr10 59210198-59210288. Max. coverage (+): 0. Max coverage (-): 0

Region: chr10 59210289-59210379. Max. coverage (+): 0. Max coverage (-): 0

Region: chr10 59210380-59210470. Max. coverage (+): 0. Max coverage (-): 0

Region: chr10 59210471-59210561. Max. coverage (+): 0. Max coverage (-): 0

Region: chr10 59210562-59210652. Max. coverage (+): 0. Max coverage (-): 0

Region: chr10 59210653-59210743. Max. coverage (+): 0. Max coverage (-): 0

Region: chr10 59210744-59210834. Max. coverage (+): 0. Max coverage (-): 0

Region: chr10 59210835-59210925. Max. coverage (+): 0. Max coverage (-): 0

Region: chr10 59210926-59211016. Max. coverage (+): 0. Max coverage (-): 0

Region: chr10 59211017-59211107. Max. coverage (+): 0. Max coverage (-): 0

Region: chr10 59211108-59211198. Max. coverage (+): 0. Max coverage (-): 0

Region: chr10 59211199-59211289. Max. coverage (+): 0. Max coverage (-): 0

Region: chr10 59211290-59211380. Max. coverage (+): 0. Max coverage (-): 0

Region: chr10 59211381-59211471. Max. coverage (+): 0. Max coverage (-): 0

Region: chr10 59211472-59211562. Max. coverage (+): 0. Max coverage (-): 0

Region: chr10 59211563-59211653. Max. coverage (+): 0. Max coverage (-): 0

Region: chr10 59211654-59211744. Max. coverage (+): 0. Max coverage (-): 0

Region: chr10 59211745-59211835. Max. coverage (+): 0. Max coverage (-): 0

Region: chr10 59211836-59211926. Max. coverage (+): 0. Max coverage (-): 0

Region: chr10 59211927-59212017. Max. coverage (+): 0. Max coverage (-): 0

Region: chr10 59212018-59212108. Max. coverage (+): 0. Max coverage (-): 0

Region: chr10 59212109-59212199. Max. coverage (+): 0. Max coverage (-): 0

Region: chr10 59212200-59212290. Max. coverage (+): 0. Max coverage (-): 0

Region: chr10 59212291-59212381. Max. coverage (+): 0. Max coverage (-): 0

Region: chr10 59212382-59212472. Max. coverage (+): 5.62. Max coverage (-): 0

Region: chr10 59212473-59212563. Max. coverage (+): 19.24. Max coverage (-): 0

Region: chr10 59212564-59212654. Max. coverage (+): 28.76. Max coverage (-): 0

Region: chr10 59212655-59212745. Max. coverage (+): 3.72. Max coverage (-): 0

Region: chr10 59212746-59212836. Max. coverage (+): 0. Max coverage (-): 0

Region: chr10 59212837-59212927. Max. coverage (+): 0. Max coverage (-): 0

Region: chr10 59212928-59213018. Max. coverage (+): 9.75. Max coverage (-): 0

Region: chr10 59213019-59213109. Max. coverage (+): 10.58. Max coverage (-): 0

Region: chr10 59213110-59213200. Max. coverage (+): 0. Max coverage (-): 0

Region: chr10 59213201-59213291. Max. coverage (+): 8.58. Max coverage (-): 0

Region: chr10 59213292-59213382. Max. coverage (+): 1.34. Max coverage (-): 0

Region: chr10 59213383-59213473. Max. coverage (+): 15.72. Max coverage (-): 0.47

Region: chr10 59213474-59213565. Max. coverage (+): 0. Max coverage (-): 0

Region: chr10 59213566-59213656. Max. coverage (+): 19.83. Max coverage (-): 1.89

Region: chr10 59213657-59213747. Max. coverage (+): 5.74. Max coverage (-): 3.71

Region: chr10 59213748-59213838. Max. coverage (+): 4.21. Max coverage (-): 6.59

Region: chr10 59213839-59213929. Max. coverage (+): 6.54. Max coverage (-): 0

Region: chr10 59213930-59214020. Max. coverage (+): 9.84. Max coverage (-): 0

Region: chr10 59214021-59214111. Max. coverage (+): 0. Max coverage (-): 0

Region: chr10 59214112-59214202. Max. coverage (+): 6.21. Max coverage (-): 0

Region: chr10 59214203-59214293. Max. coverage (+): 23.08. Max coverage (-): 0

Region: chr10 59214294-59214384. Max. coverage (+): 0. Max coverage (-): 0

Region: chr10 59214385-59214475. Max. coverage (+): 10.17. Max coverage (-): 0

Region: chr10 59214476-59214566. Max. coverage (+): 14.4. Max coverage (-): 0

Region: chr10 59214567-59214657. Max. coverage (+): 0. Max coverage (-): 0

Region: chr10 59214658-59214748. Max. coverage (+): 0. Max coverage (-): 0

Region: chr10 59214749-59214839. Max. coverage (+): 0. Max coverage (-): 0

Region: chr10 59214840-59214930. Max. coverage (+): 0. Max coverage (-): 0

Region: chr10 59214931-59215021. Max. coverage (+): 0. Max coverage (-): 0

Region: chr10 59215022-59215112. Max. coverage (+): 0. Max coverage (-): 0

Region: chr10 59215113-59215203. Max. coverage (+): 0. Max coverage (-): 0

Region: chr10 59215204-59215294. Max. coverage (+): 0. Max coverage (-): 0

Region: chr10 59215295-59215385. Max. coverage (+): 0. Max coverage (-): 0

Region: chr10 59215386-59215476. Max. coverage (+): 0. Max coverage (-): 0

Region: chr10 59215477-59215567. Max. coverage (+): 0. Max coverage (-): 0

Region: chr10 59215568-59215658. Max. coverage (+): 3.31. Max coverage (-): 0

Region: chr10 59215659-59215749. Max. coverage (+): 0. Max coverage (-): 0

Region: chr10 59215750-59215840. Max. coverage (+): 11.27. Max coverage (-): 0

Region: chr10 59215841-59215931. Max. coverage (+): 5.01. Max coverage (-): 0

Region: chr10 59215932-59216022. Max. coverage (+): 5.31. Max coverage (-): 0

Region: chr10 59216023-59216113. Max. coverage (+): 0. Max coverage (-): 0

Region: chr10 59216114-59216204. Max. coverage (+): 0. Max coverage (-): 0

Region: chr10 59216205-59216295. Max. coverage (+): 0. Max coverage (-): 0

Region: chr10 59216296-59216386. Max. coverage (+): 0. Max coverage (-): 0

Region: chr10 59216387-59216477. Max. coverage (+): 0. Max coverage (-): 0

Region: chr10 59216478-59216568. Max. coverage (+): 0. Max coverage (-): 0

Region: chr10 59216569-59216659. Max. coverage (+): 0. Max coverage (-): 0

Region: chr10 59216660-59216750. Max. coverage (+): 0. Max coverage (-): 0

Region: chr10 59216751-59216841. Max. coverage (+): 0. Max coverage (-): 0

Region: chr10 59216842-59216932. Max. coverage (+): 0. Max coverage (-): 0

Region: chr10 59216933-59217023. Max. coverage (+): 0.36. Max coverage (-): 0

Region: chr10 59217024-59217114. Max. coverage (+): 0. Max coverage (-): 0

Region: chr10 59217115-59217205. Max. coverage (+): 0. Max coverage (-): 0

Region: chr10 59217206-59217296. Max. coverage (+): 9.23. Max coverage (-): 0

Region: chr10 59217297-59217387. Max. coverage (+): 6.41. Max coverage (-): 0

Region: chr10 59217388-59217478. Max. coverage (+): 0. Max coverage (-): 0

Region: chr10 59217479-59217569. Max. coverage (+): 0. Max coverage (-): 0

Region: chr10 59217570-59217660. Max. coverage (+): 0. Max coverage (-): 0

Region: chr10 59217661-59217751. Max. coverage (+): 0. Max coverage (-): 0

Region: chr10 59217752-59217842. Max. coverage (+): 0. Max coverage (-): 0

Region: chr10 59217843-59217933. Max. coverage (+): 0. Max coverage (-): 0

Region: chr10 59217934-59218024. Max. coverage (+): 0. Max coverage (-): 0

Region: chr10 59218025-59218115. Max. coverage (+): 0. Max coverage (-): 0

Region: chr10 59218116-59218206. Max. coverage (+): 6.83. Max coverage (-): 0

Region: chr10 59218207-59218297. Max. coverage (+): 0. Max coverage (-): 0

Region: chr10 59218298-59218388. Max. coverage (+): 0. Max coverage (-): 0

Region: chr10 59218389-59218479. Max. coverage (+): 0. Max coverage (-): 0

Region: chr10 59218480-59218570. Max. coverage (+): 0. Max coverage (-): 0

Region: chr10 59218571-59218661. Max. coverage (+): 0. Max coverage (-): 0

Region: chr10 59218662-59218752. Max. coverage (+): 0. Max coverage (-): 0

Region: chr10 59218753-59218843. Max. coverage (+): 4.97. Max coverage (-): 0

Region: chr10 59218844-59218934. Max. coverage (+): 0. Max coverage (-): 0

Region: chr10 59218935-59219025. Max. coverage (+): 9.91. Max coverage (-): 0

Region: chr10 59219026-59219116. Max. coverage (+): 0. Max coverage (-): 0

Region: chr10 59219117-59219207. Max. coverage (+): 0. Max coverage (-): 0

Region: chr10 59219208-59219298. Max. coverage (+): 6.27. Max coverage (-): 0

Region: chr10 59219299-59219389. Max. coverage (+): 0. Max coverage (-): 0

Region: chr10 59219390-59219480. Max. coverage (+): 6.18. Max coverage (-): 0

Region: chr10 59219481-59219571. Max. coverage (+): 5.85. Max coverage (-): 0

Region: chr10 59219572-59219662. Max. coverage (+): 0. Max coverage (-): 0

Region: chr10 59219663-59219753. Max. coverage (+): 0. Max coverage (-): 0

Region: chr10 59219754-59219844. Max. coverage (+): 0. Max coverage (-): 0

Region: chr10 59219845-59219935. Max. coverage (+): 5.06. Max coverage (-): 0

Region: chr10 59219936-59220026. Max. coverage (+): 31.59. Max coverage (-): 0

Region: chr10 59220027-59220117. Max. coverage (+): 6.75. Max coverage (-): 0

Region: chr10 59220118-59220208. Max. coverage (+): 2.87. Max coverage (-): 0

Region: chr10 59220209-59220299. Max. coverage (+): 0. Max coverage (-): 0

Region: chr10 59220300-59220390. Max. coverage (+): 2.58. Max coverage (-): 0

Region: chr10 59220391-59220481. Max. coverage (+): 0. Max coverage (-): 0

Region: chr10 59220482-59220572. Max. coverage (+): 0. Max coverage (-): 0

Region: chr10 59220573-59220663. Max. coverage (+): 10.89. Max coverage (-): 0

Region: chr10 59220664-59220754. Max. coverage (+): 4.06. Max coverage (-): 0

Region: chr10 59220755-59220845. Max. coverage (+): 0. Max coverage (-): 0

Region: chr10 59220846-59220936. Max. coverage (+): 0. Max coverage (-): 0

Region: chr10 59220937-59221027. Max. coverage (+): 0. Max coverage (-): 0

Region: chr10 59221028-59221119. Max. coverage (+): 0. Max coverage (-): 0

Region: chr10 59221120-59221210. Max. coverage (+): 0. Max coverage (-): 0

Region: chr10 59221211-59221301. Max. coverage (+): 6.18. Max coverage (-): 0

Region: chr10 59221302-59221392. Max. coverage (+): 17.11. Max coverage (-): 0

Region: chr10 59221393-59221483. Max. coverage (+): 0. Max coverage (-): 0

Region: chr10 59221484-59221574. Max. coverage (+): 8.04. Max coverage (-): 0

Region: chr10 59221575-59221665. Max. coverage (+): 0.53. Max coverage (-): 0

Region: chr10 59221666-59221756. Max. coverage (+): 5.89. Max coverage (-): 0

Region: chr10 59221757-59221847. Max. coverage (+): 3.4. Max coverage (-): 0

Region: chr10 59221848-59221938. Max. coverage (+): 16.72. Max coverage (-): 0

Region: chr10 59221939-59222029. Max. coverage (+): 0. Max coverage (-): 0

Region: chr10 59222030-59222120. Max. coverage (+): 0. Max coverage (-): 0

Region: chr10 59222121-59222211. Max. coverage (+): 0. Max coverage (-): 0

Region: chr10 59222212-59222302. Max. coverage (+): 0. Max coverage (-): 0

Region: chr10 59222303-59222393. Max. coverage (+): 0. Max coverage (-): 0

Region: chr10 59222394-59222484. Max. coverage (+): 0. Max coverage (-): 0

Region: chr10 59222485-59222575. Max. coverage (+): 0. Max coverage (-): 0

Region: chr10 59222576-59222666. Max. coverage (+): 0. Max coverage (-): 0

Region: chr10 59222667-59222757. Max. coverage (+): 5.27. Max coverage (-): 0

Region: chr10 59222758-59222848. Max. coverage (+): 0. Max coverage (-): 0

Region: chr10 59222849-59222939. Max. coverage (+): 0. Max coverage (-): 0

Region: chr10 59222940-59223030. Max. coverage (+): 0. Max coverage (-): 0

Region: chr10 59223031-59223121. Max. coverage (+): 0. Max coverage (-): 0

Region: chr10 59223122-59223212. Max. coverage (+): 0. Max coverage (-): 0

Region: chr10 59223213-59223303. Max. coverage (+): 0. Max coverage (-): 0

Region: chr10 59223304-59223394. Max. coverage (+): 3.25. Max coverage (-): 0

Region: chr10 59223395-59223485. Max. coverage (+): 0. Max coverage (-): 0

Region: chr10 59223486-59223576. Max. coverage (+): 0. Max coverage (-): 0

Region: chr10 59223577-59223667. Max. coverage (+): 0. Max coverage (-): 0

Region: chr10 59223668-59223758. Max. coverage (+): 0. Max coverage (-): 0

Region: chr10 59223759-59223849. Max. coverage (+): 0. Max coverage (-): 0

Region: chr10 59223850-59223940. Max. coverage (+): 0. Max coverage (-): 0

Region: chr10 59223941-59224031. Max. coverage (+): 0. Max coverage (-): 0

Region: chr10 59224032-59224122. Max. coverage (+): 0. Max coverage (-): 0

Region: chr10 59224123-59224213. Max. coverage (+): 0. Max coverage (-): 0

Region: chr10 59224214-59224304. Max. coverage (+): 0. Max coverage (-): 0

Region: chr10 59224305-59224395. Max. coverage (+): 0. Max coverage (-): 0

Region: chr10 59224396-59224486. Max. coverage (+): 0. Max coverage (-): 0

Region: chr10 59224487-59224577. Max. coverage (+): 4.76. Max coverage (-): 0

Region: chr10 59224578-59224668. Max. coverage (+): 0. Max coverage (-): 0

Region: chr10 59224669-59224759. Max. coverage (+): 0. Max coverage (-): 0

Region: chr10 59224760-59224850. Max. coverage (+): 0. Max coverage (-): 0

Region: chr10 59224851-. Max. coverage (+): 0.68. Max coverage (-): 0

RepeatMasker Color Code

**+**

100-98% Identity

<98-95% Identity

<95-90% Identity

<90-85% Identity

<85-80% Identity

<80-75% Identity

<75-70% Identity

<70% Identity

**-**

Gene Set Color Code

**+**

Gene

Pseudogene

**-**

Topology/Coverage Color Code

Coverage Plus Strand

Coverage Minus Strand

Mainstrand: Plus

Mainstrand: Minus

Complementary Strand

Flanking Region  
(if option -flank >0)

Gene Set Annotation  
  
RepeatMasker Annotation  

**1. MLT1E1**: 59179463-59179971 (-), Divergence to consensus: 31.4%  
**2. Bov-tA2**: 59179972-59180083 (+), Divergence to consensus: 10%  
**3. L1ME4a**: 59180901-59180980 (-), Divergence to consensus: 35%  
**4. MamTip2**: 59182273-59182377 (+), Divergence to consensus: 40.6%  
**5. L2c**: 59182564-59182882 (-), Divergence to consensus: 49.6%  
**6. L2b**: 59185481-59185601 (+), Divergence to consensus: 39.5%  
**7. MIR3**: 59186817-59187011 (-), Divergence to consensus: 41.1%  
**8. L2c**: 59187538-59187610 (+), Divergence to consensus: 33.2%  
**9. Charlie18a**: 59188809-59189029 (-), Divergence to consensus: 41.7%  
**10. AT\_rich**: 59190374-59190396 (+), Divergence to consensus: 21.7%  
**11. MER91A**: 59191083-59191193 (-), Divergence to consensus: 46.8%  
**12. MIRb**: 59192234-59192342 (+), Divergence to consensus: 42.2%  
**13. L3**: 59192730-59192763 (-), Divergence to consensus: 15.6%  
**14. MIR**: 59193728-59193778 (-), Divergence to consensus: 11.8%  
**15. L2c**: 59193736-59193809 (-), Divergence to consensus: 32.4%  
**16. (TG)n**: 59194985-59195030 (+), Divergence to consensus: 0%  
**17. L2c**: 59195893-59195962 (-), Divergence to consensus: 33.2%  
**18. (A)n**: 59200739-59200772 (+), Divergence to consensus: 14.7%  
**19. (TA)n**: 59201248-59201290 (+), Divergence to consensus: 2.3%  
**20. MamTip2**: 59201558-59201595 (+), Divergence to consensus: 16.2%  
**21. MER94B**: 59203458-59203516 (-), Divergence to consensus: 30.5%  
**22. AT\_rich**: 59203690-59203712 (+), Divergence to consensus: 39.1%  
**23. MIRb**: 59204676-59204875 (-), Divergence to consensus: 42.3%  
**24. MIRc**: 59206311-59206459 (-), Divergence to consensus: 35.3%  
**25. MIRc**: 59206490-59206524 (-), Divergence to consensus: 20%  
**26. MIRb**: 59207024-59207151 (-), Divergence to consensus: 41.7%  
**27. L2c**: 59207192-59207306 (-), Divergence to consensus: 34.6%  
**28. MIRb**: 59208817-59208983 (+), Divergence to consensus: 42%  
**29. AT\_rich**: 59209221-59209243 (+), Divergence to consensus: 60.9%  
**30. L1ME1**: 59209255-59209662 (+), Divergence to consensus: 31.8%  
**31. LTR16B1**: 59209669-59210112 (-), Divergence to consensus: 36%  
**32. CHRL**: 59210118-59210301 (-), Divergence to consensus: 29.7%  
**33. L1ME1**: 59210319-59210971 (+), Divergence to consensus: 35.2%  
**34. SINE2-1\_BT**: 59210972-59211089 (+), Divergence to consensus: 28%  
**35. L1ME1**: 59211090-59211146 (+), Divergence to consensus: 35.2%  
**36. ART2A**: 59211304-59211808 (-), Divergence to consensus: 9.6%  
**37. L1ME1**: 59211809-59212461 (+), Divergence to consensus: 43.1%  
**38. MER45C**: 59212782-59212962 (+), Divergence to consensus: 31%  
**39. MER45C**: 59213073-59213231 (+), Divergence to consensus: 43.4%  
**40. MIR3**: 59214085-59214171 (+), Divergence to consensus: 38%  
**41. MER102b**: 59214590-59214765 (-), Divergence to consensus: 38.8%  
**42. BOV-A2**: 59215279-59215550 (-), Divergence to consensus: 4.8%  
**43. L2**: 59216187-59216647 (+), Divergence to consensus: 46.2%  
**44. (TAGA)n**: 59216648-59216668 (+), Divergence to consensus: 0%  
**45. L2**: 59216669-59216858 (+), Divergence to consensus: 51.7%  
**46. CR1\_Mam**: 59217920-59218151 (-), Divergence to consensus: 39.6%  
**47. AT\_rich**: 59218483-59218517 (+), Divergence to consensus: 65.7%  
**48. MIRc**: 59218551-59218755 (-), Divergence to consensus: 42.8%  
**49. MER91B**: 59219191-59219246 (+), Divergence to consensus: 33.9%  
**50. MIR**: 59219651-59219878 (+), Divergence to consensus: 37.9%  
**51. MIRb**: 59220474-59220585 (+), Divergence to consensus: 43.3%  
**52. MER94**: 59220686-59220797 (-), Divergence to consensus: 32.2%  
**53. MIRb**: 59220893-59221089 (-), Divergence to consensus: 42.1%  
**54. L2c**: 59222447-59222567 (+), Divergence to consensus: 44.4%  
**55. MIRc**: 59222932-59223107 (-), Divergence to consensus: 46.3%  
**56. L1MEd**: 59223763-59224114 (+), Divergence to consensus: 49.9%  
**57. L1MEd**: 59224565-59224805 (+), Divergence to consensus: 45.9%  
**58. MARNA**: 59224848-59225029 (-), Divergence to consensus: 43.3%

  
Transcription Factor Binding Sites  

**Mybl1\_1** (Sequence: TAACGGTT (-): 59181159)  
**Mybl1\_1** (Sequence: TAACGGTT (-): 59196938)  
**RFX4\_2** (Sequence: GTAACCATG (-): 59213579)  
**RFX4\_1** (Sequence: GTTGCCAAG (-): 59195076)  
**RFX4\_1** (Sequence: CTTAGCAAC (+): 59202299)  
**RFX4\_2** (Sequence: CGTGGTTAC (+): 59197438)  
**Gata4** (Sequence: AGATAAG (-): 59183862)  
**Gata4** (Sequence: AGATAAG (-): 59212475)  
**Gata4** (Sequence: AGATAAG (-): 59214285)  
**Gata4** (Sequence: AGATAAC (-): 59220076)  
**SOX9** (Sequence: AACAATAA (-): 59181291)  
**SOX9** (Sequence: AACAATGA (-): 59183154)  
**SOX9** (Sequence: AACAATGA (-): 59204111)  
**SOX9** (Sequence: AACAATAA (-): 59207638)  
**SOX9** (Sequence: AACAATGG (-): 59207959)  
**SOX9** (Sequence: TCATTGTT (+): 59182493)  
**SOX9** (Sequence: CTATTGTT (+): 59184581)  
**SOX9** (Sequence: TTATTGTT (+): 59220875)  
**A-MYB** (Sequence: CCAACTGCCA (-): 59200978)  
**A-MYB** (Sequence: TGGCAGTTGG (+): 59199634)  
**SPZ1** (Sequence: AGGGTTTGAG (+): 59185855)  
**SPZ1** (Sequence: AGGGTTAGAG (+): 59207427)  
**Mybl1\_1** (Sequence: AACCGTTA (+): 59193599)  
**Gata4** (Sequence: CTTATCT (+): 59185624)  
**Gata4** (Sequence: GTTATCT (+): 59189535)  
**Gata4** (Sequence: GTTATCT (+): 59192668)  
**Gata4** (Sequence: CTTATCT (+): 59199404)  
**Gata4** (Sequence: CTTATCT (+): 59203382)
