# Supplementary material for: Small RNA sequencing of cryopreserved semen from single bull revealed altered miRNAs and piRNAs expression between High- and Low-motile sperm populations
Source: BMC Genomics. 2017 Jan 4;18:14. doi: 10.1186/s12864-016-3394-7 (PMC5209821; doi:10.1186/s12864-016-3394-7)
Supplement: Additional file 4: — Details for each piRNA clusters found in Low Motile (LM) sperm fraction. Genes, repeats, transposable elements and transcription factors binding sites falling within the cluster regions were reported. (ZIP 1034 kb) [file 12864_2016_3394_MOESM4_ESM.zip › 40.html]

piRNA cluster 40


Predicted piRNA cluster no. 40     previous   next
  

Show proTRAC run info
Hide proTRAC run info

================================= proTRAC ====================================  
VERSION: 2.1                                    LAST MODIFIED: 06. October 2015  
  
Please cite:  
Rosenkranz D, Zischler H. proTRAC - a software for probabilistic piRNA cluster  
detection, visualization and analysis. 2012. BMC Bioinformatics 13:5.  
  
and (for proTRAC 2.0 and later):  
Rosenkranz D, Rudloff S, Bastuck K, Ketting RF, Zischler H. Tupaia small RNAs  
provide insights into function and evolution of RNAi-based transposon defense  
in mammals. 2015. RNA 21(5):911-922.  
  
Contact:  
David Rosenkranz  
Institute of Anthropology, small RNA group  
Johannes Gutenberg University Mainz  
email: rosenkranz@uni-mainz.de  
  
You can find the latest proTRAC version at:  
http://sourceforge.net/projects/protrac/files  
http://www.smallRNAgroup-mainz.de/software  
==============================================================================  
  
PARAMETERS:  
Map file: .............../storage/core/barbara/genhome/smallRNA/fertility/Sample\_not\_motile/pirna/Sample\_not\_motile\_26-33\_collapsed.fa.no-dust.map.weighted-10000-1000-b-0  
Genome file: ............/storage/core/barbara/genhome/smallRNA/fertility/Sample\_all/pirna/bt\_311\_chrY.fa  
RepeatMasker annotation: /storage/genomes/bt\_umd31/GCF\_000003055.6\_Bos\_taurus\_UMD\_3.1.1\_repeatMasker\_chr.out  
GeneSet:................./storage/core/barbara/genhome/smallRNA/fertility/Sample\_all/pirna/full.gtf  
  
Significant (p<=0.01) hit density will be calculated based  
on observed hit distribution.  
  
Sliding window size: ........................................ 5000 bp  
Sliding window increament: .................................. 1000 bp  
Normalize each hit by number of genomic hits: ............... 1 [0=no/1=yes]  
Normalize each hit by number of sequence reads: ............. 1 [0=no/1=yes]  
Normalize values (-> per million mapped reads): ............. 1 [0=no/1=yes]  
Min. fraction of hits with 1T(U) or 10A: .................... 0.75  
Alternatively: Min. fraction of hits with 1T(U) and 10A: .... 0.5  
Min. fraction of hits with typical piRNA length: ............ 0.75  
Typical piRNA length: ....................................... 26-33 nt  
Min. size of a piRNA cluster: ............................... 5000 bp.  
Min. number of hits (absolute): ............................. 0  
Min. number of hits (normalized): ........................... 0  
Min. fraction of hits on the mainstrand: .................... 0.75  
Top fraction of mapped sequences (in terms of read counts): . 1%  
Top fraction accounts for max. n% of sequence reads: ........ 90%  
Min. fraction of hits on each arm of a bidirectional cluster: 0.1  
Output image file for each cluster: ......................... 0 [0=no/1=yes]  
Output html file for each cluster: .......................... 1 [0=no/1=yes]  
Output a summary table: ..................................... 1 [0=no/1=yes]  
Output a FASTA file for each cluster (piRNA sequences): ..... 1 [0=no/1=yes]  
Output a FASTA file comprising cluster sequences: ........... 1 [0=no/1=yes]  
Search DNA motifs in clusters: .............................. 1 [0=no/1=yes]  
Output flanking sequences: +/- .............................. 0 bp  
Output ~.pTi file: .......................................... 1 [0=no/1=yes]  
==============================================================================  
  
  
Genome size (without gaps): ............ 2678902517 bp  
Gaps (N/X/-): .......................... 53837044 bp  
Mapped reads: .......................... 738059667487  
Non-identical sequences: ............... 277001  
Genomic hits: .......................... 533816  
Significant densitiy of mapped reads: .. 15118061 reads/kb

Show proTRAC cluster info
Hide proTRAC cluster info

|  |  |
| --- | --- |
| Location | chr3 |
| Coordinates | 94040986-94049155 |
| Size [bp] | 8170 |
| Sequence hit loci | 203 |
| Mapped reads (normalized) | 577089622 |
| Mapped reads (normalized) per kb | 70635204.7 |
| Normalized reads with 1T (1U) | 80.8% |
| Normalized reads with 10A | 21.3% |
| Normalized reads with length 26-33 nt | 100% |
| Normalized reads on the main strand(s) | 99.3% |
| Predicted directionality | mono:minus |

100%

0%

1T (1U)  
reads

10A reads

26-33 nt  
reads

reads on mainstrand

**Either the amount of reads with 1T (1U) OR 10A has to exceed 75% (set with option: -1Tor10A)  
Alternatively the amount of reads with 1T (1U) AND 10A has to exceed 50% (set with option: -1Tand10A)  
Minimum amount of reads with preferred size is 75% (set with option: -pisize)  
Minimum amount of reads on the main strand(s) is 75% (set with option: -clstrand)**

Show read coverage
Hide read coverage

WHAT DO I SEE HERE?  
This chart shows the location of mapped sequence reads within a predicted piRNA cluster. The color refers to the number of genomic hits produced by the sequence read in question. A dark red bar indicates that this sequence read produces many other hits elsewhere in the genome. Many adjacent red or yellow bars can indicate the presence of a multi-copy element such as transposons or rRNA genes. A dark green bar indicates that this sequence read maps uniquely to this locus.

1 hit

2-5 hits

6-10 hits

11-20 hits

21-50 hits

51-100 hits

> 100 hits

chr3

94040986

94049155

Gene Set

RepeatMasker

Mapped  
Reads

78.13

plus strand

minus strand

78.13

Region: chr3 33725504-94040994. Max. coverage (+): 0. Max coverage (-): 5.51

Region: chr3 94040995-94041010. Max. coverage (+): 0. Max coverage (-): 0

Region: chr3 94041011-94041026. Max. coverage (+): 0. Max coverage (-): 0

Region: chr3 94041027-94041043. Max. coverage (+): 0. Max coverage (-): 0

Region: chr3 94041044-94041059. Max. coverage (+): 0. Max coverage (-): 0

Region: chr3 94041060-94041075. Max. coverage (+): 0. Max coverage (-): 0

Region: chr3 94041076-94041092. Max. coverage (+): 0. Max coverage (-): 4.87

Region: chr3 94041093-94041108. Max. coverage (+): 0. Max coverage (-): 4.87

Region: chr3 94041109-94041124. Max. coverage (+): 0. Max coverage (-): 0

Region: chr3 94041125-94041141. Max. coverage (+): 0. Max coverage (-): 0

Region: chr3 94041142-94041157. Max. coverage (+): 0. Max coverage (-): 0

Region: chr3 94041158-94041173. Max. coverage (+): 0. Max coverage (-): 0

Region: chr3 94041174-94041190. Max. coverage (+): 0. Max coverage (-): 0

Region: chr3 94041191-94041206. Max. coverage (+): 0. Max coverage (-): 0

Region: chr3 94041207-94041222. Max. coverage (+): 0. Max coverage (-): 0

Region: chr3 94041223-94041239. Max. coverage (+): 0. Max coverage (-): 0

Region: chr3 94041240-94041255. Max. coverage (+): 0. Max coverage (-): 0

Region: chr3 94041256-94041271. Max. coverage (+): 0. Max coverage (-): 0

Region: chr3 94041272-94041288. Max. coverage (+): 0. Max coverage (-): 0

Region: chr3 94041289-94041304. Max. coverage (+): 0. Max coverage (-): 0

Region: chr3 94041305-94041320. Max. coverage (+): 0. Max coverage (-): 0

Region: chr3 94041321-94041337. Max. coverage (+): 0. Max coverage (-): 13.44

Region: chr3 94041338-94041353. Max. coverage (+): 0. Max coverage (-): 0

Region: chr3 94041354-94041369. Max. coverage (+): 0. Max coverage (-): 0

Region: chr3 94041370-94041386. Max. coverage (+): 0. Max coverage (-): 0

Region: chr3 94041387-94041402. Max. coverage (+): 0. Max coverage (-): 0

Region: chr3 94041403-94041419. Max. coverage (+): 0. Max coverage (-): 0

Region: chr3 94041420-94041435. Max. coverage (+): 0. Max coverage (-): 0

Region: chr3 94041436-94041451. Max. coverage (+): 0. Max coverage (-): 0

Region: chr3 94041452-94041468. Max. coverage (+): 0. Max coverage (-): 0

Region: chr3 94041469-94041484. Max. coverage (+): 0. Max coverage (-): 0

Region: chr3 94041485-94041500. Max. coverage (+): 0. Max coverage (-): 0

Region: chr3 94041501-94041517. Max. coverage (+): 0. Max coverage (-): 0

Region: chr3 94041518-94041533. Max. coverage (+): 0. Max coverage (-): 0

Region: chr3 94041534-94041549. Max. coverage (+): 0. Max coverage (-): 0

Region: chr3 94041550-94041566. Max. coverage (+): 0. Max coverage (-): 1.98

Region: chr3 94041567-94041582. Max. coverage (+): 0. Max coverage (-): 1.98

Region: chr3 94041583-94041598. Max. coverage (+): 0. Max coverage (-): 2.14

Region: chr3 94041599-94041615. Max. coverage (+): 0. Max coverage (-): 2.14

Region: chr3 94041616-94041631. Max. coverage (+): 0. Max coverage (-): 0.59

Region: chr3 94041632-94041647. Max. coverage (+): 0. Max coverage (-): 0.59

Region: chr3 94041648-94041664. Max. coverage (+): 0. Max coverage (-): 0

Region: chr3 94041665-94041680. Max. coverage (+): 0. Max coverage (-): 0

Region: chr3 94041681-94041696. Max. coverage (+): 0. Max coverage (-): 0

Region: chr3 94041697-94041713. Max. coverage (+): 0. Max coverage (-): 0

Region: chr3 94041714-94041729. Max. coverage (+): 0. Max coverage (-): 0

Region: chr3 94041730-94041745. Max. coverage (+): 0. Max coverage (-): 0

Region: chr3 94041746-94041762. Max. coverage (+): 0. Max coverage (-): 0

Region: chr3 94041763-94041778. Max. coverage (+): 0. Max coverage (-): 0

Region: chr3 94041779-94041794. Max. coverage (+): 0. Max coverage (-): 0

Region: chr3 94041795-94041811. Max. coverage (+): 0. Max coverage (-): 0

Region: chr3 94041812-94041827. Max. coverage (+): 0. Max coverage (-): 0

Region: chr3 94041828-94041843. Max. coverage (+): 0. Max coverage (-): 0

Region: chr3 94041844-94041860. Max. coverage (+): 0. Max coverage (-): 0

Region: chr3 94041861-94041876. Max. coverage (+): 0. Max coverage (-): 0

Region: chr3 94041877-94041892. Max. coverage (+): 0. Max coverage (-): 0

Region: chr3 94041893-94041909. Max. coverage (+): 0. Max coverage (-): 0

Region: chr3 94041910-94041925. Max. coverage (+): 0. Max coverage (-): 0

Region: chr3 94041926-94041941. Max. coverage (+): 0. Max coverage (-): 0

Region: chr3 94041942-94041958. Max. coverage (+): 0. Max coverage (-): 0

Region: chr3 94041959-94041974. Max. coverage (+): 0. Max coverage (-): 2.58

Region: chr3 94041975-94041990. Max. coverage (+): 0. Max coverage (-): 2.58

Region: chr3 94041991-94042007. Max. coverage (+): 0. Max coverage (-): 0

Region: chr3 94042008-94042023. Max. coverage (+): 0. Max coverage (-): 0

Region: chr3 94042024-94042039. Max. coverage (+): 0. Max coverage (-): 0

Region: chr3 94042040-94042056. Max. coverage (+): 0. Max coverage (-): 0

Region: chr3 94042057-94042072. Max. coverage (+): 0. Max coverage (-): 0

Region: chr3 94042073-94042088. Max. coverage (+): 0. Max coverage (-): 0

Region: chr3 94042089-94042105. Max. coverage (+): 0. Max coverage (-): 0

Region: chr3 94042106-94042121. Max. coverage (+): 0. Max coverage (-): 0

Region: chr3 94042122-94042137. Max. coverage (+): 0. Max coverage (-): 0

Region: chr3 94042138-94042154. Max. coverage (+): 0. Max coverage (-): 0

Region: chr3 94042155-94042170. Max. coverage (+): 0. Max coverage (-): 0

Region: chr3 94042171-94042186. Max. coverage (+): 0. Max coverage (-): 0

Region: chr3 94042187-94042203. Max. coverage (+): 0. Max coverage (-): 0

Region: chr3 94042204-94042219. Max. coverage (+): 0. Max coverage (-): 0

Region: chr3 94042220-94042236. Max. coverage (+): 0. Max coverage (-): 0

Region: chr3 94042237-94042252. Max. coverage (+): 0. Max coverage (-): 0

Region: chr3 94042253-94042268. Max. coverage (+): 0. Max coverage (-): 0

Region: chr3 94042269-94042285. Max. coverage (+): 0. Max coverage (-): 0

Region: chr3 94042286-94042301. Max. coverage (+): 0. Max coverage (-): 0

Region: chr3 94042302-94042317. Max. coverage (+): 0. Max coverage (-): 6.98

Region: chr3 94042318-94042334. Max. coverage (+): 0. Max coverage (-): 0

Region: chr3 94042335-94042350. Max. coverage (+): 0. Max coverage (-): 2.7

Region: chr3 94042351-94042366. Max. coverage (+): 0. Max coverage (-): 2.7

Region: chr3 94042367-94042383. Max. coverage (+): 0. Max coverage (-): 0

Region: chr3 94042384-94042399. Max. coverage (+): 0. Max coverage (-): 2.06

Region: chr3 94042400-94042415. Max. coverage (+): 0. Max coverage (-): 0

Region: chr3 94042416-94042432. Max. coverage (+): 0. Max coverage (-): 0

Region: chr3 94042433-94042448. Max. coverage (+): 0. Max coverage (-): 0

Region: chr3 94042449-94042464. Max. coverage (+): 0. Max coverage (-): 0

Region: chr3 94042465-94042481. Max. coverage (+): 0. Max coverage (-): 0

Region: chr3 94042482-94042497. Max. coverage (+): 0. Max coverage (-): 0

Region: chr3 94042498-94042513. Max. coverage (+): 0. Max coverage (-): 0

Region: chr3 94042514-94042530. Max. coverage (+): 0. Max coverage (-): 0

Region: chr3 94042531-94042546. Max. coverage (+): 0. Max coverage (-): 0

Region: chr3 94042547-94042562. Max. coverage (+): 0. Max coverage (-): 0

Region: chr3 94042563-94042579. Max. coverage (+): 0. Max coverage (-): 0

Region: chr3 94042580-94042595. Max. coverage (+): 0. Max coverage (-): 0

Region: chr3 94042596-94042611. Max. coverage (+): 0. Max coverage (-): 0

Region: chr3 94042612-94042628. Max. coverage (+): 0. Max coverage (-): 0

Region: chr3 94042629-94042644. Max. coverage (+): 0. Max coverage (-): 0

Region: chr3 94042645-94042660. Max. coverage (+): 0. Max coverage (-): 0

Region: chr3 94042661-94042677. Max. coverage (+): 0. Max coverage (-): 0

Region: chr3 94042678-94042693. Max. coverage (+): 0. Max coverage (-): 0

Region: chr3 94042694-94042709. Max. coverage (+): 0. Max coverage (-): 0

Region: chr3 94042710-94042726. Max. coverage (+): 0. Max coverage (-): 0

Region: chr3 94042727-94042742. Max. coverage (+): 0. Max coverage (-): 0

Region: chr3 94042743-94042758. Max. coverage (+): 0. Max coverage (-): 0

Region: chr3 94042759-94042775. Max. coverage (+): 0. Max coverage (-): 0

Region: chr3 94042776-94042791. Max. coverage (+): 0. Max coverage (-): 0

Region: chr3 94042792-94042807. Max. coverage (+): 0. Max coverage (-): 0

Region: chr3 94042808-94042824. Max. coverage (+): 0. Max coverage (-): 0

Region: chr3 94042825-94042840. Max. coverage (+): 0. Max coverage (-): 0

Region: chr3 94042841-94042856. Max. coverage (+): 0. Max coverage (-): 0

Region: chr3 94042857-94042873. Max. coverage (+): 0. Max coverage (-): 0

Region: chr3 94042874-94042889. Max. coverage (+): 0. Max coverage (-): 0

Region: chr3 94042890-94042905. Max. coverage (+): 0. Max coverage (-): 0

Region: chr3 94042906-94042922. Max. coverage (+): 0. Max coverage (-): 0

Region: chr3 94042923-94042938. Max. coverage (+): 0. Max coverage (-): 0

Region: chr3 94042939-94042954. Max. coverage (+): 0. Max coverage (-): 0

Region: chr3 94042955-94042971. Max. coverage (+): 0. Max coverage (-): 0

Region: chr3 94042972-94042987. Max. coverage (+): 0. Max coverage (-): 0

Region: chr3 94042988-94043003. Max. coverage (+): 0. Max coverage (-): 0

Region: chr3 94043004-94043020. Max. coverage (+): 0. Max coverage (-): 0

Region: chr3 94043021-94043036. Max. coverage (+): 0. Max coverage (-): 0

Region: chr3 94043037-94043053. Max. coverage (+): 0. Max coverage (-): 0

Region: chr3 94043054-94043069. Max. coverage (+): 0. Max coverage (-): 0

Region: chr3 94043070-94043085. Max. coverage (+): 0. Max coverage (-): 0

Region: chr3 94043086-94043102. Max. coverage (+): 0. Max coverage (-): 0

Region: chr3 94043103-94043118. Max. coverage (+): 0. Max coverage (-): 0

Region: chr3 94043119-94043134. Max. coverage (+): 0. Max coverage (-): 0

Region: chr3 94043135-94043151. Max. coverage (+): 0. Max coverage (-): 0

Region: chr3 94043152-94043167. Max. coverage (+): 0. Max coverage (-): 0

Region: chr3 94043168-94043183. Max. coverage (+): 0. Max coverage (-): 0

Region: chr3 94043184-94043200. Max. coverage (+): 0. Max coverage (-): 0

Region: chr3 94043201-94043216. Max. coverage (+): 0. Max coverage (-): 0

Region: chr3 94043217-94043232. Max. coverage (+): 0. Max coverage (-): 0

Region: chr3 94043233-94043249. Max. coverage (+): 0. Max coverage (-): 0

Region: chr3 94043250-94043265. Max. coverage (+): 0. Max coverage (-): 0

Region: chr3 94043266-94043281. Max. coverage (+): 0. Max coverage (-): 0

Region: chr3 94043282-94043298. Max. coverage (+): 0. Max coverage (-): 0

Region: chr3 94043299-94043314. Max. coverage (+): 0. Max coverage (-): 0

Region: chr3 94043315-94043330. Max. coverage (+): 0. Max coverage (-): 0

Region: chr3 94043331-94043347. Max. coverage (+): 0. Max coverage (-): 0

Region: chr3 94043348-94043363. Max. coverage (+): 0. Max coverage (-): 0

Region: chr3 94043364-94043379. Max. coverage (+): 0. Max coverage (-): 0

Region: chr3 94043380-94043396. Max. coverage (+): 0. Max coverage (-): 0

Region: chr3 94043397-94043412. Max. coverage (+): 0. Max coverage (-): 0

Region: chr3 94043413-94043428. Max. coverage (+): 0. Max coverage (-): 0

Region: chr3 94043429-94043445. Max. coverage (+): 0. Max coverage (-): 0

Region: chr3 94043446-94043461. Max. coverage (+): 0. Max coverage (-): 0

Region: chr3 94043462-94043477. Max. coverage (+): 0. Max coverage (-): 0

Region: chr3 94043478-94043494. Max. coverage (+): 0. Max coverage (-): 0

Region: chr3 94043495-94043510. Max. coverage (+): 0. Max coverage (-): 0

Region: chr3 94043511-94043526. Max. coverage (+): 0. Max coverage (-): 0

Region: chr3 94043527-94043543. Max. coverage (+): 0. Max coverage (-): 0

Region: chr3 94043544-94043559. Max. coverage (+): 0. Max coverage (-): 0

Region: chr3 94043560-94043575. Max. coverage (+): 0. Max coverage (-): 0

Region: chr3 94043576-94043592. Max. coverage (+): 0. Max coverage (-): 0

Region: chr3 94043593-94043608. Max. coverage (+): 0. Max coverage (-): 0

Region: chr3 94043609-94043624. Max. coverage (+): 0. Max coverage (-): 0

Region: chr3 94043625-94043641. Max. coverage (+): 0. Max coverage (-): 0

Region: chr3 94043642-94043657. Max. coverage (+): 0. Max coverage (-): 0

Region: chr3 94043658-94043673. Max. coverage (+): 0. Max coverage (-): 0

Region: chr3 94043674-94043690. Max. coverage (+): 0. Max coverage (-): 0

Region: chr3 94043691-94043706. Max. coverage (+): 0. Max coverage (-): 5.83

Region: chr3 94043707-94043722. Max. coverage (+): 0. Max coverage (-): 5.83

Region: chr3 94043723-94043739. Max. coverage (+): 0. Max coverage (-): 2.29

Region: chr3 94043740-94043755. Max. coverage (+): 0. Max coverage (-): 2.29

Region: chr3 94043756-94043771. Max. coverage (+): 0. Max coverage (-): 0

Region: chr3 94043772-94043788. Max. coverage (+): 0. Max coverage (-): 9.64

Region: chr3 94043789-94043804. Max. coverage (+): 0. Max coverage (-): 0

Region: chr3 94043805-94043820. Max. coverage (+): 0. Max coverage (-): 0

Region: chr3 94043821-94043837. Max. coverage (+): 0. Max coverage (-): 0

Region: chr3 94043838-94043853. Max. coverage (+): 0. Max coverage (-): 0

Region: chr3 94043854-94043870. Max. coverage (+): 0. Max coverage (-): 0

Region: chr3 94043871-94043886. Max. coverage (+): 0. Max coverage (-): 0

Region: chr3 94043887-94043902. Max. coverage (+): 0. Max coverage (-): 0

Region: chr3 94043903-94043919. Max. coverage (+): 0. Max coverage (-): 0

Region: chr3 94043920-94043935. Max. coverage (+): 0. Max coverage (-): 4.77

Region: chr3 94043936-94043951. Max. coverage (+): 0. Max coverage (-): 4.77

Region: chr3 94043952-94043968. Max. coverage (+): 0. Max coverage (-): 0

Region: chr3 94043969-94043984. Max. coverage (+): 0. Max coverage (-): 4.9

Region: chr3 94043985-94044000. Max. coverage (+): 0. Max coverage (-): 13.2

Region: chr3 94044001-94044017. Max. coverage (+): 0. Max coverage (-): 17.45

Region: chr3 94044018-94044033. Max. coverage (+): 0. Max coverage (-): 6.51

Region: chr3 94044034-94044049. Max. coverage (+): 0. Max coverage (-): 6.51

Region: chr3 94044050-94044066. Max. coverage (+): 0. Max coverage (-): 0

Region: chr3 94044067-94044082. Max. coverage (+): 0. Max coverage (-): 0

Region: chr3 94044083-94044098. Max. coverage (+): 0. Max coverage (-): 5.13

Region: chr3 94044099-94044115. Max. coverage (+): 0. Max coverage (-): 6.62

Region: chr3 94044116-94044131. Max. coverage (+): 0. Max coverage (-): 0

Region: chr3 94044132-94044147. Max. coverage (+): 0. Max coverage (-): 0

Region: chr3 94044148-94044164. Max. coverage (+): 0. Max coverage (-): 0

Region: chr3 94044165-94044180. Max. coverage (+): 0. Max coverage (-): 5.12

Region: chr3 94044181-94044196. Max. coverage (+): 0. Max coverage (-): 0

Region: chr3 94044197-94044213. Max. coverage (+): 0. Max coverage (-): 27.22

Region: chr3 94044214-94044229. Max. coverage (+): 0. Max coverage (-): 0

Region: chr3 94044230-94044245. Max. coverage (+): 0. Max coverage (-): 0

Region: chr3 94044246-94044262. Max. coverage (+): 0. Max coverage (-): 0

Region: chr3 94044263-94044278. Max. coverage (+): 0. Max coverage (-): 0

Region: chr3 94044279-94044294. Max. coverage (+): 0. Max coverage (-): 0

Region: chr3 94044295-94044311. Max. coverage (+): 0. Max coverage (-): 0

Region: chr3 94044312-94044327. Max. coverage (+): 0. Max coverage (-): 0

Region: chr3 94044328-94044343. Max. coverage (+): 0. Max coverage (-): 0

Region: chr3 94044344-94044360. Max. coverage (+): 0. Max coverage (-): 0

Region: chr3 94044361-94044376. Max. coverage (+): 0. Max coverage (-): 0

Region: chr3 94044377-94044392. Max. coverage (+): 0. Max coverage (-): 0

Region: chr3 94044393-94044409. Max. coverage (+): 0. Max coverage (-): 0

Region: chr3 94044410-94044425. Max. coverage (+): 0. Max coverage (-): 0

Region: chr3 94044426-94044441. Max. coverage (+): 0. Max coverage (-): 0

Region: chr3 94044442-94044458. Max. coverage (+): 0. Max coverage (-): 9.76

Region: chr3 94044459-94044474. Max. coverage (+): 0. Max coverage (-): 0

Region: chr3 94044475-94044490. Max. coverage (+): 0. Max coverage (-): 0

Region: chr3 94044491-94044507. Max. coverage (+): 0. Max coverage (-): 0

Region: chr3 94044508-94044523. Max. coverage (+): 0. Max coverage (-): 0

Region: chr3 94044524-94044539. Max. coverage (+): 0. Max coverage (-): 0

Region: chr3 94044540-94044556. Max. coverage (+): 0. Max coverage (-): 0

Region: chr3 94044557-94044572. Max. coverage (+): 0. Max coverage (-): 0

Region: chr3 94044573-94044588. Max. coverage (+): 0. Max coverage (-): 4.91

Region: chr3 94044589-94044605. Max. coverage (+): 0. Max coverage (-): 4.91

Region: chr3 94044606-94044621. Max. coverage (+): 0. Max coverage (-): 0

Region: chr3 94044622-94044637. Max. coverage (+): 0. Max coverage (-): 0

Region: chr3 94044638-94044654. Max. coverage (+): 0. Max coverage (-): 6.17

Region: chr3 94044655-94044670. Max. coverage (+): 0. Max coverage (-): 6.17

Region: chr3 94044671-94044687. Max. coverage (+): 0. Max coverage (-): 2.33

Region: chr3 94044688-94044703. Max. coverage (+): 0. Max coverage (-): 0

Region: chr3 94044704-94044719. Max. coverage (+): 0. Max coverage (-): 2.67

Region: chr3 94044720-94044736. Max. coverage (+): 0. Max coverage (-): 78.13

Region: chr3 94044737-94044752. Max. coverage (+): 0. Max coverage (-): 5.13

Region: chr3 94044753-94044768. Max. coverage (+): 0. Max coverage (-): 0

Region: chr3 94044769-94044785. Max. coverage (+): 0. Max coverage (-): 0

Region: chr3 94044786-94044801. Max. coverage (+): 0. Max coverage (-): 0

Region: chr3 94044802-94044817. Max. coverage (+): 0. Max coverage (-): 0

Region: chr3 94044818-94044834. Max. coverage (+): 0. Max coverage (-): 0

Region: chr3 94044835-94044850. Max. coverage (+): 0. Max coverage (-): 0

Region: chr3 94044851-94044866. Max. coverage (+): 0. Max coverage (-): 0

Region: chr3 94044867-94044883. Max. coverage (+): 0. Max coverage (-): 0

Region: chr3 94044884-94044899. Max. coverage (+): 0. Max coverage (-): 0

Region: chr3 94044900-94044915. Max. coverage (+): 0. Max coverage (-): 0

Region: chr3 94044916-94044932. Max. coverage (+): 0. Max coverage (-): 0

Region: chr3 94044933-94044948. Max. coverage (+): 0. Max coverage (-): 0

Region: chr3 94044949-94044964. Max. coverage (+): 0. Max coverage (-): 0

Region: chr3 94044965-94044981. Max. coverage (+): 0. Max coverage (-): 0

Region: chr3 94044982-94044997. Max. coverage (+): 0. Max coverage (-): 0

Region: chr3 94044998-94045013. Max. coverage (+): 0. Max coverage (-): 0

Region: chr3 94045014-94045030. Max. coverage (+): 0. Max coverage (-): 0

Region: chr3 94045031-94045046. Max. coverage (+): 0. Max coverage (-): 0

Region: chr3 94045047-94045062. Max. coverage (+): 0. Max coverage (-): 0

Region: chr3 94045063-94045079. Max. coverage (+): 0. Max coverage (-): 0

Region: chr3 94045080-94045095. Max. coverage (+): 0. Max coverage (-): 0

Region: chr3 94045096-94045111. Max. coverage (+): 0. Max coverage (-): 0

Region: chr3 94045112-94045128. Max. coverage (+): 0. Max coverage (-): 0

Region: chr3 94045129-94045144. Max. coverage (+): 0. Max coverage (-): 0

Region: chr3 94045145-94045160. Max. coverage (+): 0. Max coverage (-): 6.86

Region: chr3 94045161-94045177. Max. coverage (+): 0. Max coverage (-): 0

Region: chr3 94045178-94045193. Max. coverage (+): 0. Max coverage (-): 0

Region: chr3 94045194-94045209. Max. coverage (+): 0. Max coverage (-): 0

Region: chr3 94045210-94045226. Max. coverage (+): 0. Max coverage (-): 0

Region: chr3 94045227-94045242. Max. coverage (+): 0. Max coverage (-): 0

Region: chr3 94045243-94045258. Max. coverage (+): 0. Max coverage (-): 0

Region: chr3 94045259-94045275. Max. coverage (+): 0. Max coverage (-): 0

Region: chr3 94045276-94045291. Max. coverage (+): 0. Max coverage (-): 7.95

Region: chr3 94045292-94045307. Max. coverage (+): 0. Max coverage (-): 2.4

Region: chr3 94045308-94045324. Max. coverage (+): 0. Max coverage (-): 2.4

Region: chr3 94045325-94045340. Max. coverage (+): 0. Max coverage (-): 0

Region: chr3 94045341-94045356. Max. coverage (+): 0. Max coverage (-): 3.22

Region: chr3 94045357-94045373. Max. coverage (+): 0. Max coverage (-): 3.09

Region: chr3 94045374-94045389. Max. coverage (+): 0. Max coverage (-): 0

Region: chr3 94045390-94045405. Max. coverage (+): 0. Max coverage (-): 0

Region: chr3 94045406-94045422. Max. coverage (+): 0. Max coverage (-): 0

Region: chr3 94045423-94045438. Max. coverage (+): 0. Max coverage (-): 0

Region: chr3 94045439-94045454. Max. coverage (+): 0. Max coverage (-): 0

Region: chr3 94045455-94045471. Max. coverage (+): 0. Max coverage (-): 0.41

Region: chr3 94045472-94045487. Max. coverage (+): 0. Max coverage (-): 4.8

Region: chr3 94045488-94045504. Max. coverage (+): 0. Max coverage (-): 0

Region: chr3 94045505-94045520. Max. coverage (+): 0. Max coverage (-): 2.7

Region: chr3 94045521-94045536. Max. coverage (+): 5.81. Max coverage (-): 0

Region: chr3 94045537-94045553. Max. coverage (+): 0. Max coverage (-): 28.68

Region: chr3 94045554-94045569. Max. coverage (+): 0. Max coverage (-): 0

Region: chr3 94045570-94045585. Max. coverage (+): 0. Max coverage (-): 0

Region: chr3 94045586-94045602. Max. coverage (+): 0. Max coverage (-): 0

Region: chr3 94045603-94045618. Max. coverage (+): 0. Max coverage (-): 0

Region: chr3 94045619-94045634. Max. coverage (+): 0. Max coverage (-): 5.53

Region: chr3 94045635-94045651. Max. coverage (+): 0. Max coverage (-): 19.1

Region: chr3 94045652-94045667. Max. coverage (+): 0. Max coverage (-): 19.1

Region: chr3 94045668-94045683. Max. coverage (+): 0. Max coverage (-): 0

Region: chr3 94045684-94045700. Max. coverage (+): 0. Max coverage (-): 7.99

Region: chr3 94045701-94045716. Max. coverage (+): 0. Max coverage (-): 0

Region: chr3 94045717-94045732. Max. coverage (+): 0. Max coverage (-): 1.31

Region: chr3 94045733-94045749. Max. coverage (+): 0. Max coverage (-): 20.76

Region: chr3 94045750-94045765. Max. coverage (+): 0. Max coverage (-): 27.63

Region: chr3 94045766-94045781. Max. coverage (+): 0. Max coverage (-): 0

Region: chr3 94045782-94045798. Max. coverage (+): 0. Max coverage (-): 0

Region: chr3 94045799-94045814. Max. coverage (+): 0. Max coverage (-): 16.9

Region: chr3 94045815-94045830. Max. coverage (+): 0. Max coverage (-): 16.9

Region: chr3 94045831-94045847. Max. coverage (+): 0. Max coverage (-): 0

Region: chr3 94045848-94045863. Max. coverage (+): 0. Max coverage (-): 0

Region: chr3 94045864-94045879. Max. coverage (+): 0. Max coverage (-): 0

Region: chr3 94045880-94045896. Max. coverage (+): 0. Max coverage (-): 0

Region: chr3 94045897-94045912. Max. coverage (+): 0. Max coverage (-): 10.41

Region: chr3 94045913-94045928. Max. coverage (+): 0. Max coverage (-): 35.99

Region: chr3 94045929-94045945. Max. coverage (+): 0. Max coverage (-): 39.78

Region: chr3 94045946-94045961. Max. coverage (+): 0. Max coverage (-): 0

Region: chr3 94045962-94045977. Max. coverage (+): 0. Max coverage (-): 0

Region: chr3 94045978-94045994. Max. coverage (+): 0. Max coverage (-): 10.56

Region: chr3 94045995-94046010. Max. coverage (+): 0. Max coverage (-): 2.58

Region: chr3 94046011-94046026. Max. coverage (+): 0. Max coverage (-): 6.72

Region: chr3 94046027-94046043. Max. coverage (+): 0. Max coverage (-): 4.33

Region: chr3 94046044-94046059. Max. coverage (+): 0. Max coverage (-): 0

Region: chr3 94046060-94046075. Max. coverage (+): 0. Max coverage (-): 0

Region: chr3 94046076-94046092. Max. coverage (+): 0. Max coverage (-): 0

Region: chr3 94046093-94046108. Max. coverage (+): 0. Max coverage (-): 0

Region: chr3 94046109-94046124. Max. coverage (+): 0. Max coverage (-): 0

Region: chr3 94046125-94046141. Max. coverage (+): 0. Max coverage (-): 0

Region: chr3 94046142-94046157. Max. coverage (+): 0. Max coverage (-): 0

Region: chr3 94046158-94046173. Max. coverage (+): 0. Max coverage (-): 0

Region: chr3 94046174-94046190. Max. coverage (+): 0. Max coverage (-): 0

Region: chr3 94046191-94046206. Max. coverage (+): 0. Max coverage (-): 0

Region: chr3 94046207-94046222. Max. coverage (+): 0. Max coverage (-): 0

Region: chr3 94046223-94046239. Max. coverage (+): 0. Max coverage (-): 10.64

Region: chr3 94046240-94046255. Max. coverage (+): 0. Max coverage (-): 10.64

Region: chr3 94046256-94046271. Max. coverage (+): 0. Max coverage (-): 0

Region: chr3 94046272-94046288. Max. coverage (+): 0. Max coverage (-): 0

Region: chr3 94046289-94046304. Max. coverage (+): 0. Max coverage (-): 0

Region: chr3 94046305-94046321. Max. coverage (+): 0. Max coverage (-): 0

Region: chr3 94046322-94046337. Max. coverage (+): 0. Max coverage (-): 0

Region: chr3 94046338-94046353. Max. coverage (+): 0. Max coverage (-): 0

Region: chr3 94046354-94046370. Max. coverage (+): 0. Max coverage (-): 0

Region: chr3 94046371-94046386. Max. coverage (+): 0. Max coverage (-): 2.62

Region: chr3 94046387-94046402. Max. coverage (+): 0. Max coverage (-): 0

Region: chr3 94046403-94046419. Max. coverage (+): 0. Max coverage (-): 4.51

Region: chr3 94046420-94046435. Max. coverage (+): 0. Max coverage (-): 0

Region: chr3 94046436-94046451. Max. coverage (+): 0. Max coverage (-): 0

Region: chr3 94046452-94046468. Max. coverage (+): 0. Max coverage (-): 0

Region: chr3 94046469-94046484. Max. coverage (+): 0. Max coverage (-): 0

Region: chr3 94046485-94046500. Max. coverage (+): 0. Max coverage (-): 0

Region: chr3 94046501-94046517. Max. coverage (+): 0. Max coverage (-): 0

Region: chr3 94046518-94046533. Max. coverage (+): 0. Max coverage (-): 0

Region: chr3 94046534-94046549. Max. coverage (+): 0. Max coverage (-): 0

Region: chr3 94046550-94046566. Max. coverage (+): 0. Max coverage (-): 0

Region: chr3 94046567-94046582. Max. coverage (+): 0. Max coverage (-): 0

Region: chr3 94046583-94046598. Max. coverage (+): 0. Max coverage (-): 2.83

Region: chr3 94046599-94046615. Max. coverage (+): 0. Max coverage (-): 0

Region: chr3 94046616-94046631. Max. coverage (+): 0. Max coverage (-): 0

Region: chr3 94046632-94046647. Max. coverage (+): 0. Max coverage (-): 0

Region: chr3 94046648-94046664. Max. coverage (+): 0. Max coverage (-): 0

Region: chr3 94046665-94046680. Max. coverage (+): 0. Max coverage (-): 0

Region: chr3 94046681-94046696. Max. coverage (+): 0. Max coverage (-): 0

Region: chr3 94046697-94046713. Max. coverage (+): 0. Max coverage (-): 0

Region: chr3 94046714-94046729. Max. coverage (+): 0. Max coverage (-): 18.56

Region: chr3 94046730-94046745. Max. coverage (+): 0. Max coverage (-): 8.1

Region: chr3 94046746-94046762. Max. coverage (+): 0. Max coverage (-): 13.41

Region: chr3 94046763-94046778. Max. coverage (+): 0. Max coverage (-): 2.51

Region: chr3 94046779-94046794. Max. coverage (+): 0. Max coverage (-): 0

Region: chr3 94046795-94046811. Max. coverage (+): 0. Max coverage (-): 6.99

Region: chr3 94046812-94046827. Max. coverage (+): 0. Max coverage (-): 0

Region: chr3 94046828-94046843. Max. coverage (+): 0. Max coverage (-): 0

Region: chr3 94046844-94046860. Max. coverage (+): 0. Max coverage (-): 0

Region: chr3 94046861-94046876. Max. coverage (+): 0. Max coverage (-): 0

Region: chr3 94046877-94046892. Max. coverage (+): 0. Max coverage (-): 0

Region: chr3 94046893-94046909. Max. coverage (+): 0. Max coverage (-): 0

Region: chr3 94046910-94046925. Max. coverage (+): 0. Max coverage (-): 0

Region: chr3 94046926-94046941. Max. coverage (+): 0. Max coverage (-): 0

Region: chr3 94046942-94046958. Max. coverage (+): 0. Max coverage (-): 0

Region: chr3 94046959-94046974. Max. coverage (+): 0. Max coverage (-): 0

Region: chr3 94046975-94046990. Max. coverage (+): 0. Max coverage (-): 0

Region: chr3 94046991-94047007. Max. coverage (+): 0. Max coverage (-): 0

Region: chr3 94047008-94047023. Max. coverage (+): 0. Max coverage (-): 0

Region: chr3 94047024-94047039. Max. coverage (+): 0. Max coverage (-): 0

Region: chr3 94047040-94047056. Max. coverage (+): 0. Max coverage (-): 0

Region: chr3 94047057-94047072. Max. coverage (+): 0. Max coverage (-): 0

Region: chr3 94047073-94047088. Max. coverage (+): 0. Max coverage (-): 0

Region: chr3 94047089-94047105. Max. coverage (+): 0. Max coverage (-): 7.12

Region: chr3 94047106-94047121. Max. coverage (+): 0. Max coverage (-): 4.06

Region: chr3 94047122-94047138. Max. coverage (+): 0. Max coverage (-): 4.06

Region: chr3 94047139-94047154. Max. coverage (+): 0. Max coverage (-): 1.81

Region: chr3 94047155-94047170. Max. coverage (+): 0. Max coverage (-): 0

Region: chr3 94047171-94047187. Max. coverage (+): 0. Max coverage (-): 0

Region: chr3 94047188-94047203. Max. coverage (+): 0. Max coverage (-): 4.15

Region: chr3 94047204-94047219. Max. coverage (+): 0. Max coverage (-): 7.03

Region: chr3 94047220-94047236. Max. coverage (+): 0. Max coverage (-): 0

Region: chr3 94047237-94047252. Max. coverage (+): 0. Max coverage (-): 0

Region: chr3 94047253-94047268. Max. coverage (+): 0. Max coverage (-): 0

Region: chr3 94047269-94047285. Max. coverage (+): 0. Max coverage (-): 1.48

Region: chr3 94047286-94047301. Max. coverage (+): 0. Max coverage (-): 1.48

Region: chr3 94047302-94047317. Max. coverage (+): 0. Max coverage (-): 32.28

Region: chr3 94047318-94047334. Max. coverage (+): 0. Max coverage (-): 29.8

Region: chr3 94047335-94047350. Max. coverage (+): 0. Max coverage (-): 20.29

Region: chr3 94047351-94047366. Max. coverage (+): 0. Max coverage (-): 0

Region: chr3 94047367-94047383. Max. coverage (+): 0. Max coverage (-): 0

Region: chr3 94047384-94047399. Max. coverage (+): 0. Max coverage (-): 0

Region: chr3 94047400-94047415. Max. coverage (+): 0. Max coverage (-): 0

Region: chr3 94047416-94047432. Max. coverage (+): 0. Max coverage (-): 2.35

Region: chr3 94047433-94047448. Max. coverage (+): 0. Max coverage (-): 0

Region: chr3 94047449-94047464. Max. coverage (+): 0. Max coverage (-): 17.3

Region: chr3 94047465-94047481. Max. coverage (+): 0. Max coverage (-): 17.3

Region: chr3 94047482-94047497. Max. coverage (+): 0. Max coverage (-): 0

Region: chr3 94047498-94047513. Max. coverage (+): 0. Max coverage (-): 5.87

Region: chr3 94047514-94047530. Max. coverage (+): 0. Max coverage (-): 21.33

Region: chr3 94047531-94047546. Max. coverage (+): 0. Max coverage (-): 21.33

Region: chr3 94047547-94047562. Max. coverage (+): 0. Max coverage (-): 2.28

Region: chr3 94047563-94047579. Max. coverage (+): 0. Max coverage (-): 13.55

Region: chr3 94047580-94047595. Max. coverage (+): 0. Max coverage (-): 0

Region: chr3 94047596-94047611. Max. coverage (+): 0. Max coverage (-): 0

Region: chr3 94047612-94047628. Max. coverage (+): 0. Max coverage (-): 0

Region: chr3 94047629-94047644. Max. coverage (+): 0. Max coverage (-): 0

Region: chr3 94047645-94047660. Max. coverage (+): 0. Max coverage (-): 0

Region: chr3 94047661-94047677. Max. coverage (+): 0. Max coverage (-): 0

Region: chr3 94047678-94047693. Max. coverage (+): 0. Max coverage (-): 0

Region: chr3 94047694-94047709. Max. coverage (+): 0. Max coverage (-): 0

Region: chr3 94047710-94047726. Max. coverage (+): 0. Max coverage (-): 0

Region: chr3 94047727-94047742. Max. coverage (+): 0. Max coverage (-): 0

Region: chr3 94047743-94047758. Max. coverage (+): 0. Max coverage (-): 0

Region: chr3 94047759-94047775. Max. coverage (+): 0. Max coverage (-): 0

Region: chr3 94047776-94047791. Max. coverage (+): 0. Max coverage (-): 0

Region: chr3 94047792-94047807. Max. coverage (+): 0. Max coverage (-): 0

Region: chr3 94047808-94047824. Max. coverage (+): 0. Max coverage (-): 0

Region: chr3 94047825-94047840. Max. coverage (+): 0. Max coverage (-): 0

Region: chr3 94047841-94047856. Max. coverage (+): 0. Max coverage (-): 0

Region: chr3 94047857-94047873. Max. coverage (+): 0. Max coverage (-): 0

Region: chr3 94047874-94047889. Max. coverage (+): 0. Max coverage (-): 9.35

Region: chr3 94047890-94047905. Max. coverage (+): 0. Max coverage (-): 9.35

Region: chr3 94047906-94047922. Max. coverage (+): 0. Max coverage (-): 0

Region: chr3 94047923-94047938. Max. coverage (+): 0. Max coverage (-): 0

Region: chr3 94047939-94047955. Max. coverage (+): 0. Max coverage (-): 0

Region: chr3 94047956-94047971. Max. coverage (+): 0. Max coverage (-): 0

Region: chr3 94047972-94047987. Max. coverage (+): 0. Max coverage (-): 0

Region: chr3 94047988-94048004. Max. coverage (+): 0. Max coverage (-): 0

Region: chr3 94048005-94048020. Max. coverage (+): 0. Max coverage (-): 0

Region: chr3 94048021-94048036. Max. coverage (+): 0. Max coverage (-): 0

Region: chr3 94048037-94048053. Max. coverage (+): 0. Max coverage (-): 0

Region: chr3 94048054-94048069. Max. coverage (+): 0. Max coverage (-): 0

Region: chr3 94048070-94048085. Max. coverage (+): 0. Max coverage (-): 0

Region: chr3 94048086-94048102. Max. coverage (+): 0. Max coverage (-): 0

Region: chr3 94048103-94048118. Max. coverage (+): 0. Max coverage (-): 0

Region: chr3 94048119-94048134. Max. coverage (+): 0. Max coverage (-): 2.76

Region: chr3 94048135-94048151. Max. coverage (+): 0. Max coverage (-): 2.76

Region: chr3 94048152-94048167. Max. coverage (+): 0. Max coverage (-): 0

Region: chr3 94048168-94048183. Max. coverage (+): 0. Max coverage (-): 0

Region: chr3 94048184-94048200. Max. coverage (+): 0. Max coverage (-): 0

Region: chr3 94048201-94048216. Max. coverage (+): 0. Max coverage (-): 0

Region: chr3 94048217-94048232. Max. coverage (+): 0. Max coverage (-): 0

Region: chr3 94048233-94048249. Max. coverage (+): 0. Max coverage (-): 0

Region: chr3 94048250-94048265. Max. coverage (+): 0. Max coverage (-): 0

Region: chr3 94048266-94048281. Max. coverage (+): 0. Max coverage (-): 0

Region: chr3 94048282-94048298. Max. coverage (+): 0. Max coverage (-): 0

Region: chr3 94048299-94048314. Max. coverage (+): 0. Max coverage (-): 0

Region: chr3 94048315-94048330. Max. coverage (+): 0. Max coverage (-): 0

Region: chr3 94048331-94048347. Max. coverage (+): 0. Max coverage (-): 0

Region: chr3 94048348-94048363. Max. coverage (+): 0. Max coverage (-): 0

Region: chr3 94048364-94048379. Max. coverage (+): 0. Max coverage (-): 0.44

Region: chr3 94048380-94048396. Max. coverage (+): 0. Max coverage (-): 0

Region: chr3 94048397-94048412. Max. coverage (+): 0. Max coverage (-): 2.52

Region: chr3 94048413-94048428. Max. coverage (+): 0. Max coverage (-): 2.52

Region: chr3 94048429-94048445. Max. coverage (+): 0. Max coverage (-): 0

Region: chr3 94048446-94048461. Max. coverage (+): 0. Max coverage (-): 0

Region: chr3 94048462-94048477. Max. coverage (+): 0. Max coverage (-): 0

Region: chr3 94048478-94048494. Max. coverage (+): 0. Max coverage (-): 0

Region: chr3 94048495-94048510. Max. coverage (+): 0. Max coverage (-): 0

Region: chr3 94048511-94048526. Max. coverage (+): 0. Max coverage (-): 0

Region: chr3 94048527-94048543. Max. coverage (+): 0. Max coverage (-): 0

Region: chr3 94048544-94048559. Max. coverage (+): 0. Max coverage (-): 0

Region: chr3 94048560-94048575. Max. coverage (+): 0. Max coverage (-): 0

Region: chr3 94048576-94048592. Max. coverage (+): 0. Max coverage (-): 0

Region: chr3 94048593-94048608. Max. coverage (+): 0. Max coverage (-): 0

Region: chr3 94048609-94048624. Max. coverage (+): 0. Max coverage (-): 1.15

Region: chr3 94048625-94048641. Max. coverage (+): 0. Max coverage (-): 1.15

Region: chr3 94048642-94048657. Max. coverage (+): 0. Max coverage (-): 0

Region: chr3 94048658-94048673. Max. coverage (+): 0. Max coverage (-): 0

Region: chr3 94048674-94048690. Max. coverage (+): 0. Max coverage (-): 0

Region: chr3 94048691-94048706. Max. coverage (+): 0. Max coverage (-): 0

Region: chr3 94048707-94048722. Max. coverage (+): 0. Max coverage (-): 0

Region: chr3 94048723-94048739. Max. coverage (+): 0. Max coverage (-): 0

Region: chr3 94048740-94048755. Max. coverage (+): 0. Max coverage (-): 0

Region: chr3 94048756-94048772. Max. coverage (+): 0. Max coverage (-): 0

Region: chr3 94048773-94048788. Max. coverage (+): 0. Max coverage (-): 0

Region: chr3 94048789-94048804. Max. coverage (+): 0. Max coverage (-): 0

Region: chr3 94048805-94048821. Max. coverage (+): 0. Max coverage (-): 5.43

Region: chr3 94048822-94048837. Max. coverage (+): 0. Max coverage (-): 5.43

Region: chr3 94048838-94048853. Max. coverage (+): 0. Max coverage (-): 0

Region: chr3 94048854-94048870. Max. coverage (+): 0. Max coverage (-): 0

Region: chr3 94048871-94048886. Max. coverage (+): 0. Max coverage (-): 0

Region: chr3 94048887-94048902. Max. coverage (+): 0. Max coverage (-): 0

Region: chr3 94048903-94048919. Max. coverage (+): 0. Max coverage (-): 0

Region: chr3 94048920-94048935. Max. coverage (+): 0. Max coverage (-): 0

Region: chr3 94048936-94048951. Max. coverage (+): 0. Max coverage (-): 0

Region: chr3 94048952-94048968. Max. coverage (+): 0. Max coverage (-): 0

Region: chr3 94048969-94048984. Max. coverage (+): 0. Max coverage (-): 0

Region: chr3 94048985-94049000. Max. coverage (+): 0. Max coverage (-): 0

Region: chr3 94049001-94049017. Max. coverage (+): 0. Max coverage (-): 0

Region: chr3 94049018-94049033. Max. coverage (+): 0. Max coverage (-): 4.61

Region: chr3 94049034-94049049. Max. coverage (+): 0. Max coverage (-): 0

Region: chr3 94049050-94049066. Max. coverage (+): 0. Max coverage (-): 0

Region: chr3 94049067-94049082. Max. coverage (+): 0. Max coverage (-): 4.46

Region: chr3 94049083-94049098. Max. coverage (+): 0. Max coverage (-): 0

Region: chr3 94049099-94049115. Max. coverage (+): 0. Max coverage (-): 0

Region: chr3 94049116-94049131. Max. coverage (+): 0. Max coverage (-): 5.16

Region: chr3 94049132-94049147. Max. coverage (+): 0. Max coverage (-): 5.16

Region: chr3 94049148-. Max. coverage (+): 0. Max coverage (-): 0

RepeatMasker Color Code

**+**

100-98% Identity

<98-95% Identity

<95-90% Identity

<90-85% Identity

<85-80% Identity

<80-75% Identity

<75-70% Identity

<70% Identity

**-**

Gene Set Color Code

**+**

Gene

Pseudogene

**-**

Topology/Coverage Color Code

Coverage Plus Strand

Coverage Minus Strand

Mainstrand: Plus

Mainstrand: Minus

Complementary Strand

Flanking Region  
(if option -flank >0)

Gene Set Annotation  

**1. (protein coding, ENSBTAG00000002910) Tr:00000003779 Ex:8**: 94043983-94044033 (+)  
**2. (protein coding, ENSBTAG00000002910) Tr:00000003779 Ex:9**: 94045451-94045498 (+)  
**3. (protein coding, ENSBTAG00000002910) Tr:00000003779 Ex:10**: 94046306-94046956 (+)

  
RepeatMasker Annotation  

**1. Tigger2a\_Art**: 94041673-94041864 (+), Divergence to consensus: 39.1%  
**2. MER81**: 94042034-94042115 (+), Divergence to consensus: 37.8%  
**3. L1ME4b**: 94042146-94042245 (-), Divergence to consensus: 35%  
**4. L1MC4a**: 94042448-94042987 (-), Divergence to consensus: 43.5%  
**5. (TA)n**: 94043007-94043062 (+), Divergence to consensus: 3.6%  
**6. MIRb**: 94043426-94043477 (-), Divergence to consensus: 26.9%  
**7. MIR3**: 94043543-94043649 (+), Divergence to consensus: 40%  
**8. MIR3**: 94044247-94044379 (-), Divergence to consensus: 42.6%  
**9. L2c**: 94044506-94044546 (-), Divergence to consensus: 24.4%  
**10. MIR**: 94044793-94044940 (+), Divergence to consensus: 33.1%  
**11. MIR3**: 94046486-94046532 (+), Divergence to consensus: 23.4%  
**12. MIRc**: 94046987-94047054 (+), Divergence to consensus: 28.4%  
**13. Bov-tA3**: 94047607-94047810 (-), Divergence to consensus: 11.3%  
**14. AT\_rich**: 94048699-94048722 (+), Divergence to consensus: 54.2%

  
Transcription Factor Binding Sites  

**RFX4\_1** (Sequence: GTTGCCAGG (-): 94045021)  
**SOX9** (Sequence: AACAATAA (-): 94046670)  
**Gata4** (Sequence: CTTATCT (+): 94047591)
