# Supplementary material for: Small RNA sequencing of cryopreserved semen from single bull revealed altered miRNAs and piRNAs expression between High- and Low-motile sperm populations
Source: BMC Genomics. 2017 Jan 4;18:14. doi: 10.1186/s12864-016-3394-7 (PMC5209821; doi:10.1186/s12864-016-3394-7)
Supplement: Additional file 4: — Details for each piRNA clusters found in Low Motile (LM) sperm fraction. Genes, repeats, transposable elements and transcription factors binding sites falling within the cluster regions were reported. (ZIP 1034 kb) [file 12864_2016_3394_MOESM4_ESM.zip › 41.html]

piRNA cluster 41


Predicted piRNA cluster no. 41     previous   next
  

Show proTRAC run info
Hide proTRAC run info

================================= proTRAC ====================================  
VERSION: 2.1                                    LAST MODIFIED: 06. October 2015  
  
Please cite:  
Rosenkranz D, Zischler H. proTRAC - a software for probabilistic piRNA cluster  
detection, visualization and analysis. 2012. BMC Bioinformatics 13:5.  
  
and (for proTRAC 2.0 and later):  
Rosenkranz D, Rudloff S, Bastuck K, Ketting RF, Zischler H. Tupaia small RNAs  
provide insights into function and evolution of RNAi-based transposon defense  
in mammals. 2015. RNA 21(5):911-922.  
  
Contact:  
David Rosenkranz  
Institute of Anthropology, small RNA group  
Johannes Gutenberg University Mainz  
email: rosenkranz@uni-mainz.de  
  
You can find the latest proTRAC version at:  
http://sourceforge.net/projects/protrac/files  
http://www.smallRNAgroup-mainz.de/software  
==============================================================================  
  
PARAMETERS:  
Map file: .............../storage/core/barbara/genhome/smallRNA/fertility/Sample\_not\_motile/pirna/Sample\_not\_motile\_26-33\_collapsed.fa.no-dust.map.weighted-10000-1000-b-0  
Genome file: ............/storage/core/barbara/genhome/smallRNA/fertility/Sample\_all/pirna/bt\_311\_chrY.fa  
RepeatMasker annotation: /storage/genomes/bt\_umd31/GCF\_000003055.6\_Bos\_taurus\_UMD\_3.1.1\_repeatMasker\_chr.out  
GeneSet:................./storage/core/barbara/genhome/smallRNA/fertility/Sample\_all/pirna/full.gtf  
  
Significant (p<=0.01) hit density will be calculated based  
on observed hit distribution.  
  
Sliding window size: ........................................ 5000 bp  
Sliding window increament: .................................. 1000 bp  
Normalize each hit by number of genomic hits: ............... 1 [0=no/1=yes]  
Normalize each hit by number of sequence reads: ............. 1 [0=no/1=yes]  
Normalize values (-> per million mapped reads): ............. 1 [0=no/1=yes]  
Min. fraction of hits with 1T(U) or 10A: .................... 0.75  
Alternatively: Min. fraction of hits with 1T(U) and 10A: .... 0.5  
Min. fraction of hits with typical piRNA length: ............ 0.75  
Typical piRNA length: ....................................... 26-33 nt  
Min. size of a piRNA cluster: ............................... 5000 bp.  
Min. number of hits (absolute): ............................. 0  
Min. number of hits (normalized): ........................... 0  
Min. fraction of hits on the mainstrand: .................... 0.75  
Top fraction of mapped sequences (in terms of read counts): . 1%  
Top fraction accounts for max. n% of sequence reads: ........ 90%  
Min. fraction of hits on each arm of a bidirectional cluster: 0.1  
Output image file for each cluster: ......................... 0 [0=no/1=yes]  
Output html file for each cluster: .......................... 1 [0=no/1=yes]  
Output a summary table: ..................................... 1 [0=no/1=yes]  
Output a FASTA file for each cluster (piRNA sequences): ..... 1 [0=no/1=yes]  
Output a FASTA file comprising cluster sequences: ........... 1 [0=no/1=yes]  
Search DNA motifs in clusters: .............................. 1 [0=no/1=yes]  
Output flanking sequences: +/- .............................. 0 bp  
Output ~.pTi file: .......................................... 1 [0=no/1=yes]  
==============================================================================  
  
  
Genome size (without gaps): ............ 2678902517 bp  
Gaps (N/X/-): .......................... 53837044 bp  
Mapped reads: .......................... 738059667487  
Non-identical sequences: ............... 277001  
Genomic hits: .......................... 533816  
Significant densitiy of mapped reads: .. 15118061 reads/kb

Show proTRAC cluster info
Hide proTRAC cluster info

|  |  |
| --- | --- |
| Location | chr3 |
| Coordinates | 118175302-118180942 |
| Size [bp] | 5641 |
| Sequence hit loci | 157 |
| Mapped reads (normalized) | 465274038 |
| Mapped reads (normalized) per kb | 82480772.6 |
| Normalized reads with 1T (1U) | 93.6% |
| Normalized reads with 10A | 25.8% |
| Normalized reads with length 26-33 nt | 100% |
| Normalized reads on the main strand(s) | 98% |
| Predicted directionality | mono:minus |

100%

0%

1T (1U)  
reads

10A reads

26-33 nt  
reads

reads on mainstrand

**Either the amount of reads with 1T (1U) OR 10A has to exceed 75% (set with option: -1Tor10A)  
Alternatively the amount of reads with 1T (1U) AND 10A has to exceed 50% (set with option: -1Tand10A)  
Minimum amount of reads with preferred size is 75% (set with option: -pisize)  
Minimum amount of reads on the main strand(s) is 75% (set with option: -clstrand)**

Show read coverage
Hide read coverage

WHAT DO I SEE HERE?  
This chart shows the location of mapped sequence reads within a predicted piRNA cluster. The color refers to the number of genomic hits produced by the sequence read in question. A dark red bar indicates that this sequence read produces many other hits elsewhere in the genome. Many adjacent red or yellow bars can indicate the presence of a multi-copy element such as transposons or rRNA genes. A dark green bar indicates that this sequence read maps uniquely to this locus.

1 hit

2-5 hits

6-10 hits

11-20 hits

21-50 hits

51-100 hits

> 100 hits

chr3

118175302

118180942

Gene Set

RepeatMasker

Mapped  
Reads

139.02

plus strand

minus strand

139.02

Region: chr3 94049149-118175307. Max. coverage (+): 0. Max coverage (-): 5.02

Region: chr3 118175308-118175318. Max. coverage (+): 0. Max coverage (-): 5.02

Region: chr3 118175319-118175330. Max. coverage (+): 0. Max coverage (-): 0

Region: chr3 118175331-118175341. Max. coverage (+): 0. Max coverage (-): 0

Region: chr3 118175342-118175352. Max. coverage (+): 0. Max coverage (-): 0

Region: chr3 118175353-118175364. Max. coverage (+): 0. Max coverage (-): 0

Region: chr3 118175365-118175375. Max. coverage (+): 0. Max coverage (-): 0

Region: chr3 118175376-118175386. Max. coverage (+): 0. Max coverage (-): 0

Region: chr3 118175387-118175397. Max. coverage (+): 0. Max coverage (-): 3.56

Region: chr3 118175398-118175409. Max. coverage (+): 0. Max coverage (-): 3.56

Region: chr3 118175410-118175420. Max. coverage (+): 0. Max coverage (-): 0

Region: chr3 118175421-118175431. Max. coverage (+): 0. Max coverage (-): 1.16

Region: chr3 118175432-118175443. Max. coverage (+): 0. Max coverage (-): 1.16

Region: chr3 118175444-118175454. Max. coverage (+): 0. Max coverage (-): 0

Region: chr3 118175455-118175465. Max. coverage (+): 0. Max coverage (-): 5.99

Region: chr3 118175466-118175476. Max. coverage (+): 0. Max coverage (-): 0

Region: chr3 118175477-118175488. Max. coverage (+): 0. Max coverage (-): 0

Region: chr3 118175489-118175499. Max. coverage (+): 0. Max coverage (-): 0

Region: chr3 118175500-118175510. Max. coverage (+): 0. Max coverage (-): 0

Region: chr3 118175511-118175521. Max. coverage (+): 0. Max coverage (-): 0

Region: chr3 118175522-118175533. Max. coverage (+): 0. Max coverage (-): 0

Region: chr3 118175534-118175544. Max. coverage (+): 0. Max coverage (-): 0

Region: chr3 118175545-118175555. Max. coverage (+): 0. Max coverage (-): 0

Region: chr3 118175556-118175567. Max. coverage (+): 0. Max coverage (-): 0

Region: chr3 118175568-118175578. Max. coverage (+): 0. Max coverage (-): 0

Region: chr3 118175579-118175589. Max. coverage (+): 0. Max coverage (-): 0

Region: chr3 118175590-118175600. Max. coverage (+): 0. Max coverage (-): 0

Region: chr3 118175601-118175612. Max. coverage (+): 0. Max coverage (-): 0

Region: chr3 118175613-118175623. Max. coverage (+): 0. Max coverage (-): 0

Region: chr3 118175624-118175634. Max. coverage (+): 0. Max coverage (-): 0

Region: chr3 118175635-118175646. Max. coverage (+): 0. Max coverage (-): 0

Region: chr3 118175647-118175657. Max. coverage (+): 0. Max coverage (-): 0

Region: chr3 118175658-118175668. Max. coverage (+): 0. Max coverage (-): 0

Region: chr3 118175669-118175679. Max. coverage (+): 0. Max coverage (-): 0

Region: chr3 118175680-118175691. Max. coverage (+): 0. Max coverage (-): 0

Region: chr3 118175692-118175702. Max. coverage (+): 0. Max coverage (-): 1.02

Region: chr3 118175703-118175713. Max. coverage (+): 0. Max coverage (-): 67.23

Region: chr3 118175714-118175725. Max. coverage (+): 0. Max coverage (-): 20.12

Region: chr3 118175726-118175736. Max. coverage (+): 0. Max coverage (-): 20.69

Region: chr3 118175737-118175747. Max. coverage (+): 0. Max coverage (-): 4.61

Region: chr3 118175748-118175758. Max. coverage (+): 0. Max coverage (-): 6.39

Region: chr3 118175759-118175770. Max. coverage (+): 0. Max coverage (-): 0

Region: chr3 118175771-118175781. Max. coverage (+): 0. Max coverage (-): 0

Region: chr3 118175782-118175792. Max. coverage (+): 0. Max coverage (-): 0

Region: chr3 118175793-118175804. Max. coverage (+): 0. Max coverage (-): 0

Region: chr3 118175805-118175815. Max. coverage (+): 0. Max coverage (-): 0

Region: chr3 118175816-118175826. Max. coverage (+): 0. Max coverage (-): 0

Region: chr3 118175827-118175837. Max. coverage (+): 0. Max coverage (-): 0

Region: chr3 118175838-118175849. Max. coverage (+): 0. Max coverage (-): 0

Region: chr3 118175850-118175860. Max. coverage (+): 0. Max coverage (-): 0

Region: chr3 118175861-118175871. Max. coverage (+): 0. Max coverage (-): 0

Region: chr3 118175872-118175883. Max. coverage (+): 0. Max coverage (-): 0

Region: chr3 118175884-118175894. Max. coverage (+): 0. Max coverage (-): 0

Region: chr3 118175895-118175905. Max. coverage (+): 0. Max coverage (-): 0

Region: chr3 118175906-118175916. Max. coverage (+): 0. Max coverage (-): 0

Region: chr3 118175917-118175928. Max. coverage (+): 0. Max coverage (-): 0

Region: chr3 118175929-118175939. Max. coverage (+): 0. Max coverage (-): 0

Region: chr3 118175940-118175950. Max. coverage (+): 0. Max coverage (-): 0

Region: chr3 118175951-118175961. Max. coverage (+): 0. Max coverage (-): 12.94

Region: chr3 118175962-118175973. Max. coverage (+): 0. Max coverage (-): 9.29

Region: chr3 118175974-118175984. Max. coverage (+): 0. Max coverage (-): 0

Region: chr3 118175985-118175995. Max. coverage (+): 0. Max coverage (-): 0

Region: chr3 118175996-118176007. Max. coverage (+): 0. Max coverage (-): 0

Region: chr3 118176008-118176018. Max. coverage (+): 0. Max coverage (-): 0

Region: chr3 118176019-118176029. Max. coverage (+): 0. Max coverage (-): 0

Region: chr3 118176030-118176040. Max. coverage (+): 0. Max coverage (-): 0

Region: chr3 118176041-118176052. Max. coverage (+): 0. Max coverage (-): 0

Region: chr3 118176053-118176063. Max. coverage (+): 0. Max coverage (-): 6.39

Region: chr3 118176064-118176074. Max. coverage (+): 0. Max coverage (-): 8.06

Region: chr3 118176075-118176086. Max. coverage (+): 0. Max coverage (-): 10.82

Region: chr3 118176087-118176097. Max. coverage (+): 0. Max coverage (-): 0

Region: chr3 118176098-118176108. Max. coverage (+): 0. Max coverage (-): 0

Region: chr3 118176109-118176119. Max. coverage (+): 0. Max coverage (-): 0

Region: chr3 118176120-118176131. Max. coverage (+): 0. Max coverage (-): 5.31

Region: chr3 118176132-118176142. Max. coverage (+): 0. Max coverage (-): 5.31

Region: chr3 118176143-118176153. Max. coverage (+): 0. Max coverage (-): 0

Region: chr3 118176154-118176165. Max. coverage (+): 0. Max coverage (-): 0

Region: chr3 118176166-118176176. Max. coverage (+): 0. Max coverage (-): 0

Region: chr3 118176177-118176187. Max. coverage (+): 0. Max coverage (-): 0

Region: chr3 118176188-118176198. Max. coverage (+): 0. Max coverage (-): 0

Region: chr3 118176199-118176210. Max. coverage (+): 0. Max coverage (-): 0

Region: chr3 118176211-118176221. Max. coverage (+): 0. Max coverage (-): 18.08

Region: chr3 118176222-118176232. Max. coverage (+): 0. Max coverage (-): 18.08

Region: chr3 118176233-118176244. Max. coverage (+): 0. Max coverage (-): 11.22

Region: chr3 118176245-118176255. Max. coverage (+): 0. Max coverage (-): 42.84

Region: chr3 118176256-118176266. Max. coverage (+): 0. Max coverage (-): 70.6

Region: chr3 118176267-118176277. Max. coverage (+): 0. Max coverage (-): 26.23

Region: chr3 118176278-118176289. Max. coverage (+): 0. Max coverage (-): 0

Region: chr3 118176290-118176300. Max. coverage (+): 0. Max coverage (-): 0

Region: chr3 118176301-118176311. Max. coverage (+): 0. Max coverage (-): 5.62

Region: chr3 118176312-118176323. Max. coverage (+): 0. Max coverage (-): 0

Region: chr3 118176324-118176334. Max. coverage (+): 0. Max coverage (-): 0

Region: chr3 118176335-118176345. Max. coverage (+): 0. Max coverage (-): 0

Region: chr3 118176346-118176356. Max. coverage (+): 0. Max coverage (-): 0

Region: chr3 118176357-118176368. Max. coverage (+): 0. Max coverage (-): 0

Region: chr3 118176369-118176379. Max. coverage (+): 0. Max coverage (-): 0

Region: chr3 118176380-118176390. Max. coverage (+): 0. Max coverage (-): 0

Region: chr3 118176391-118176401. Max. coverage (+): 0. Max coverage (-): 0

Region: chr3 118176402-118176413. Max. coverage (+): 0. Max coverage (-): 0

Region: chr3 118176414-118176424. Max. coverage (+): 0. Max coverage (-): 12.76

Region: chr3 118176425-118176435. Max. coverage (+): 0. Max coverage (-): 12.76

Region: chr3 118176436-118176447. Max. coverage (+): 0. Max coverage (-): 0

Region: chr3 118176448-118176458. Max. coverage (+): 0. Max coverage (-): 0

Region: chr3 118176459-118176469. Max. coverage (+): 0. Max coverage (-): 0

Region: chr3 118176470-118176480. Max. coverage (+): 0. Max coverage (-): 0

Region: chr3 118176481-118176492. Max. coverage (+): 0. Max coverage (-): 0

Region: chr3 118176493-118176503. Max. coverage (+): 0. Max coverage (-): 0

Region: chr3 118176504-118176514. Max. coverage (+): 0. Max coverage (-): 0

Region: chr3 118176515-118176526. Max. coverage (+): 0. Max coverage (-): 0

Region: chr3 118176527-118176537. Max. coverage (+): 0. Max coverage (-): 0

Region: chr3 118176538-118176548. Max. coverage (+): 0. Max coverage (-): 0

Region: chr3 118176549-118176559. Max. coverage (+): 0. Max coverage (-): 0

Region: chr3 118176560-118176571. Max. coverage (+): 0. Max coverage (-): 0

Region: chr3 118176572-118176582. Max. coverage (+): 0. Max coverage (-): 0

Region: chr3 118176583-118176593. Max. coverage (+): 0. Max coverage (-): 0

Region: chr3 118176594-118176605. Max. coverage (+): 0. Max coverage (-): 0

Region: chr3 118176606-118176616. Max. coverage (+): 0. Max coverage (-): 0

Region: chr3 118176617-118176627. Max. coverage (+): 0. Max coverage (-): 0

Region: chr3 118176628-118176638. Max. coverage (+): 0. Max coverage (-): 0

Region: chr3 118176639-118176650. Max. coverage (+): 0. Max coverage (-): 0

Region: chr3 118176651-118176661. Max. coverage (+): 0. Max coverage (-): 0

Region: chr3 118176662-118176672. Max. coverage (+): 0. Max coverage (-): 0

Region: chr3 118176673-118176684. Max. coverage (+): 0. Max coverage (-): 0

Region: chr3 118176685-118176695. Max. coverage (+): 0. Max coverage (-): 0

Region: chr3 118176696-118176706. Max. coverage (+): 0. Max coverage (-): 0

Region: chr3 118176707-118176717. Max. coverage (+): 0. Max coverage (-): 0

Region: chr3 118176718-118176729. Max. coverage (+): 0. Max coverage (-): 0

Region: chr3 118176730-118176740. Max. coverage (+): 0. Max coverage (-): 0

Region: chr3 118176741-118176751. Max. coverage (+): 0. Max coverage (-): 0

Region: chr3 118176752-118176763. Max. coverage (+): 0. Max coverage (-): 0

Region: chr3 118176764-118176774. Max. coverage (+): 0. Max coverage (-): 0

Region: chr3 118176775-118176785. Max. coverage (+): 0. Max coverage (-): 2.43

Region: chr3 118176786-118176796. Max. coverage (+): 0. Max coverage (-): 2.43

Region: chr3 118176797-118176808. Max. coverage (+): 0. Max coverage (-): 0

Region: chr3 118176809-118176819. Max. coverage (+): 0. Max coverage (-): 0

Region: chr3 118176820-118176830. Max. coverage (+): 0. Max coverage (-): 0

Region: chr3 118176831-118176841. Max. coverage (+): 0. Max coverage (-): 0

Region: chr3 118176842-118176853. Max. coverage (+): 0. Max coverage (-): 0

Region: chr3 118176854-118176864. Max. coverage (+): 0. Max coverage (-): 0

Region: chr3 118176865-118176875. Max. coverage (+): 0. Max coverage (-): 0

Region: chr3 118176876-118176887. Max. coverage (+): 0. Max coverage (-): 0

Region: chr3 118176888-118176898. Max. coverage (+): 0. Max coverage (-): 0

Region: chr3 118176899-118176909. Max. coverage (+): 0. Max coverage (-): 0

Region: chr3 118176910-118176920. Max. coverage (+): 0. Max coverage (-): 0

Region: chr3 118176921-118176932. Max. coverage (+): 0. Max coverage (-): 0

Region: chr3 118176933-118176943. Max. coverage (+): 0. Max coverage (-): 0

Region: chr3 118176944-118176954. Max. coverage (+): 0. Max coverage (-): 0

Region: chr3 118176955-118176966. Max. coverage (+): 0. Max coverage (-): 0

Region: chr3 118176967-118176977. Max. coverage (+): 0. Max coverage (-): 0

Region: chr3 118176978-118176988. Max. coverage (+): 0. Max coverage (-): 0

Region: chr3 118176989-118176999. Max. coverage (+): 0. Max coverage (-): 0

Region: chr3 118177000-118177011. Max. coverage (+): 0. Max coverage (-): 0

Region: chr3 118177012-118177022. Max. coverage (+): 0. Max coverage (-): 0

Region: chr3 118177023-118177033. Max. coverage (+): 0. Max coverage (-): 0

Region: chr3 118177034-118177045. Max. coverage (+): 0. Max coverage (-): 0

Region: chr3 118177046-118177056. Max. coverage (+): 0. Max coverage (-): 0

Region: chr3 118177057-118177067. Max. coverage (+): 0. Max coverage (-): 0

Region: chr3 118177068-118177078. Max. coverage (+): 0. Max coverage (-): 0

Region: chr3 118177079-118177090. Max. coverage (+): 0. Max coverage (-): 0

Region: chr3 118177091-118177101. Max. coverage (+): 0. Max coverage (-): 0

Region: chr3 118177102-118177112. Max. coverage (+): 0. Max coverage (-): 0

Region: chr3 118177113-118177124. Max. coverage (+): 0. Max coverage (-): 0

Region: chr3 118177125-118177135. Max. coverage (+): 0. Max coverage (-): 0

Region: chr3 118177136-118177146. Max. coverage (+): 0. Max coverage (-): 0

Region: chr3 118177147-118177157. Max. coverage (+): 0. Max coverage (-): 0

Region: chr3 118177158-118177169. Max. coverage (+): 0. Max coverage (-): 0

Region: chr3 118177170-118177180. Max. coverage (+): 0. Max coverage (-): 0

Region: chr3 118177181-118177191. Max. coverage (+): 0. Max coverage (-): 0

Region: chr3 118177192-118177203. Max. coverage (+): 0. Max coverage (-): 0

Region: chr3 118177204-118177214. Max. coverage (+): 0. Max coverage (-): 0

Region: chr3 118177215-118177225. Max. coverage (+): 0. Max coverage (-): 0

Region: chr3 118177226-118177236. Max. coverage (+): 0. Max coverage (-): 0

Region: chr3 118177237-118177248. Max. coverage (+): 0. Max coverage (-): 0

Region: chr3 118177249-118177259. Max. coverage (+): 0. Max coverage (-): 0

Region: chr3 118177260-118177270. Max. coverage (+): 0. Max coverage (-): 0

Region: chr3 118177271-118177281. Max. coverage (+): 0. Max coverage (-): 0

Region: chr3 118177282-118177293. Max. coverage (+): 0. Max coverage (-): 0

Region: chr3 118177294-118177304. Max. coverage (+): 0. Max coverage (-): 0

Region: chr3 118177305-118177315. Max. coverage (+): 0. Max coverage (-): 0

Region: chr3 118177316-118177327. Max. coverage (+): 0. Max coverage (-): 0

Region: chr3 118177328-118177338. Max. coverage (+): 0. Max coverage (-): 0

Region: chr3 118177339-118177349. Max. coverage (+): 0. Max coverage (-): 0

Region: chr3 118177350-118177360. Max. coverage (+): 0. Max coverage (-): 0

Region: chr3 118177361-118177372. Max. coverage (+): 0. Max coverage (-): 0

Region: chr3 118177373-118177383. Max. coverage (+): 0. Max coverage (-): 0

Region: chr3 118177384-118177394. Max. coverage (+): 0. Max coverage (-): 0

Region: chr3 118177395-118177406. Max. coverage (+): 0. Max coverage (-): 0

Region: chr3 118177407-118177417. Max. coverage (+): 0. Max coverage (-): 0

Region: chr3 118177418-118177428. Max. coverage (+): 0. Max coverage (-): 0

Region: chr3 118177429-118177439. Max. coverage (+): 0. Max coverage (-): 0

Region: chr3 118177440-118177451. Max. coverage (+): 0. Max coverage (-): 0

Region: chr3 118177452-118177462. Max. coverage (+): 0. Max coverage (-): 0

Region: chr3 118177463-118177473. Max. coverage (+): 0. Max coverage (-): 0

Region: chr3 118177474-118177485. Max. coverage (+): 0. Max coverage (-): 0

Region: chr3 118177486-118177496. Max. coverage (+): 0. Max coverage (-): 0

Region: chr3 118177497-118177507. Max. coverage (+): 0. Max coverage (-): 0

Region: chr3 118177508-118177518. Max. coverage (+): 0. Max coverage (-): 0

Region: chr3 118177519-118177530. Max. coverage (+): 0. Max coverage (-): 0

Region: chr3 118177531-118177541. Max. coverage (+): 0. Max coverage (-): 0

Region: chr3 118177542-118177552. Max. coverage (+): 0. Max coverage (-): 0

Region: chr3 118177553-118177564. Max. coverage (+): 0. Max coverage (-): 0

Region: chr3 118177565-118177575. Max. coverage (+): 0. Max coverage (-): 0

Region: chr3 118177576-118177586. Max. coverage (+): 0. Max coverage (-): 0

Region: chr3 118177587-118177597. Max. coverage (+): 0. Max coverage (-): 0

Region: chr3 118177598-118177609. Max. coverage (+): 0. Max coverage (-): 0

Region: chr3 118177610-118177620. Max. coverage (+): 0. Max coverage (-): 0

Region: chr3 118177621-118177631. Max. coverage (+): 0. Max coverage (-): 0

Region: chr3 118177632-118177643. Max. coverage (+): 0. Max coverage (-): 0

Region: chr3 118177644-118177654. Max. coverage (+): 0. Max coverage (-): 0

Region: chr3 118177655-118177665. Max. coverage (+): 0. Max coverage (-): 0

Region: chr3 118177666-118177676. Max. coverage (+): 0. Max coverage (-): 0

Region: chr3 118177677-118177688. Max. coverage (+): 0. Max coverage (-): 0

Region: chr3 118177689-118177699. Max. coverage (+): 0. Max coverage (-): 0

Region: chr3 118177700-118177710. Max. coverage (+): 0. Max coverage (-): 0

Region: chr3 118177711-118177721. Max. coverage (+): 0. Max coverage (-): 0

Region: chr3 118177722-118177733. Max. coverage (+): 0. Max coverage (-): 0

Region: chr3 118177734-118177744. Max. coverage (+): 0. Max coverage (-): 0

Region: chr3 118177745-118177755. Max. coverage (+): 0. Max coverage (-): 0

Region: chr3 118177756-118177767. Max. coverage (+): 0. Max coverage (-): 0

Region: chr3 118177768-118177778. Max. coverage (+): 0. Max coverage (-): 0

Region: chr3 118177779-118177789. Max. coverage (+): 0. Max coverage (-): 0

Region: chr3 118177790-118177800. Max. coverage (+): 0. Max coverage (-): 6.51

Region: chr3 118177801-118177812. Max. coverage (+): 0. Max coverage (-): 6.51

Region: chr3 118177813-118177823. Max. coverage (+): 0. Max coverage (-): 0

Region: chr3 118177824-118177834. Max. coverage (+): 0. Max coverage (-): 0

Region: chr3 118177835-118177846. Max. coverage (+): 0. Max coverage (-): 0

Region: chr3 118177847-118177857. Max. coverage (+): 0. Max coverage (-): 0

Region: chr3 118177858-118177868. Max. coverage (+): 0. Max coverage (-): 0

Region: chr3 118177869-118177879. Max. coverage (+): 0. Max coverage (-): 0

Region: chr3 118177880-118177891. Max. coverage (+): 0. Max coverage (-): 0

Region: chr3 118177892-118177902. Max. coverage (+): 0. Max coverage (-): 0

Region: chr3 118177903-118177913. Max. coverage (+): 0. Max coverage (-): 0

Region: chr3 118177914-118177925. Max. coverage (+): 0. Max coverage (-): 0

Region: chr3 118177926-118177936. Max. coverage (+): 0. Max coverage (-): 0

Region: chr3 118177937-118177947. Max. coverage (+): 0. Max coverage (-): 0

Region: chr3 118177948-118177958. Max. coverage (+): 0. Max coverage (-): 0

Region: chr3 118177959-118177970. Max. coverage (+): 0. Max coverage (-): 0

Region: chr3 118177971-118177981. Max. coverage (+): 0. Max coverage (-): 0

Region: chr3 118177982-118177992. Max. coverage (+): 0. Max coverage (-): 0

Region: chr3 118177993-118178004. Max. coverage (+): 0. Max coverage (-): 0

Region: chr3 118178005-118178015. Max. coverage (+): 0. Max coverage (-): 0

Region: chr3 118178016-118178026. Max. coverage (+): 0. Max coverage (-): 0

Region: chr3 118178027-118178037. Max. coverage (+): 0. Max coverage (-): 0

Region: chr3 118178038-118178049. Max. coverage (+): 0. Max coverage (-): 0

Region: chr3 118178050-118178060. Max. coverage (+): 0. Max coverage (-): 0

Region: chr3 118178061-118178071. Max. coverage (+): 0. Max coverage (-): 0

Region: chr3 118178072-118178083. Max. coverage (+): 0. Max coverage (-): 0

Region: chr3 118178084-118178094. Max. coverage (+): 0. Max coverage (-): 0

Region: chr3 118178095-118178105. Max. coverage (+): 0. Max coverage (-): 0

Region: chr3 118178106-118178116. Max. coverage (+): 0. Max coverage (-): 0

Region: chr3 118178117-118178128. Max. coverage (+): 0. Max coverage (-): 0

Region: chr3 118178129-118178139. Max. coverage (+): 0. Max coverage (-): 0

Region: chr3 118178140-118178150. Max. coverage (+): 0. Max coverage (-): 0

Region: chr3 118178151-118178161. Max. coverage (+): 0. Max coverage (-): 0

Region: chr3 118178162-118178173. Max. coverage (+): 0. Max coverage (-): 0

Region: chr3 118178174-118178184. Max. coverage (+): 0. Max coverage (-): 0

Region: chr3 118178185-118178195. Max. coverage (+): 0. Max coverage (-): 0

Region: chr3 118178196-118178207. Max. coverage (+): 0. Max coverage (-): 0

Region: chr3 118178208-118178218. Max. coverage (+): 0. Max coverage (-): 0

Region: chr3 118178219-118178229. Max. coverage (+): 0. Max coverage (-): 0

Region: chr3 118178230-118178240. Max. coverage (+): 0. Max coverage (-): 0

Region: chr3 118178241-118178252. Max. coverage (+): 0. Max coverage (-): 0

Region: chr3 118178253-118178263. Max. coverage (+): 0. Max coverage (-): 0

Region: chr3 118178264-118178274. Max. coverage (+): 0. Max coverage (-): 0

Region: chr3 118178275-118178286. Max. coverage (+): 0. Max coverage (-): 0

Region: chr3 118178287-118178297. Max. coverage (+): 0. Max coverage (-): 0

Region: chr3 118178298-118178308. Max. coverage (+): 0. Max coverage (-): 0

Region: chr3 118178309-118178319. Max. coverage (+): 0. Max coverage (-): 0

Region: chr3 118178320-118178331. Max. coverage (+): 0. Max coverage (-): 0

Region: chr3 118178332-118178342. Max. coverage (+): 0. Max coverage (-): 0

Region: chr3 118178343-118178353. Max. coverage (+): 0. Max coverage (-): 0

Region: chr3 118178354-118178365. Max. coverage (+): 0. Max coverage (-): 0

Region: chr3 118178366-118178376. Max. coverage (+): 0. Max coverage (-): 0

Region: chr3 118178377-118178387. Max. coverage (+): 0. Max coverage (-): 0

Region: chr3 118178388-118178398. Max. coverage (+): 0. Max coverage (-): 0

Region: chr3 118178399-118178410. Max. coverage (+): 0. Max coverage (-): 0

Region: chr3 118178411-118178421. Max. coverage (+): 0. Max coverage (-): 0

Region: chr3 118178422-118178432. Max. coverage (+): 0. Max coverage (-): 0

Region: chr3 118178433-118178444. Max. coverage (+): 0. Max coverage (-): 0

Region: chr3 118178445-118178455. Max. coverage (+): 0. Max coverage (-): 0

Region: chr3 118178456-118178466. Max. coverage (+): 0. Max coverage (-): 0

Region: chr3 118178467-118178477. Max. coverage (+): 0. Max coverage (-): 0

Region: chr3 118178478-118178489. Max. coverage (+): 0. Max coverage (-): 0

Region: chr3 118178490-118178500. Max. coverage (+): 0. Max coverage (-): 0

Region: chr3 118178501-118178511. Max. coverage (+): 0. Max coverage (-): 0

Region: chr3 118178512-118178523. Max. coverage (+): 0. Max coverage (-): 0

Region: chr3 118178524-118178534. Max. coverage (+): 0. Max coverage (-): 0

Region: chr3 118178535-118178545. Max. coverage (+): 0. Max coverage (-): 0

Region: chr3 118178546-118178556. Max. coverage (+): 0. Max coverage (-): 0

Region: chr3 118178557-118178568. Max. coverage (+): 0. Max coverage (-): 0

Region: chr3 118178569-118178579. Max. coverage (+): 0. Max coverage (-): 0

Region: chr3 118178580-118178590. Max. coverage (+): 0. Max coverage (-): 0

Region: chr3 118178591-118178601. Max. coverage (+): 0. Max coverage (-): 0

Region: chr3 118178602-118178613. Max. coverage (+): 0. Max coverage (-): 0

Region: chr3 118178614-118178624. Max. coverage (+): 0. Max coverage (-): 0

Region: chr3 118178625-118178635. Max. coverage (+): 0. Max coverage (-): 0

Region: chr3 118178636-118178647. Max. coverage (+): 0. Max coverage (-): 0

Region: chr3 118178648-118178658. Max. coverage (+): 0. Max coverage (-): 0

Region: chr3 118178659-118178669. Max. coverage (+): 0. Max coverage (-): 0

Region: chr3 118178670-118178680. Max. coverage (+): 0. Max coverage (-): 0

Region: chr3 118178681-118178692. Max. coverage (+): 0. Max coverage (-): 0

Region: chr3 118178693-118178703. Max. coverage (+): 0. Max coverage (-): 0

Region: chr3 118178704-118178714. Max. coverage (+): 0. Max coverage (-): 0

Region: chr3 118178715-118178726. Max. coverage (+): 0. Max coverage (-): 0

Region: chr3 118178727-118178737. Max. coverage (+): 0. Max coverage (-): 0

Region: chr3 118178738-118178748. Max. coverage (+): 0. Max coverage (-): 0

Region: chr3 118178749-118178759. Max. coverage (+): 0. Max coverage (-): 0

Region: chr3 118178760-118178771. Max. coverage (+): 0. Max coverage (-): 0

Region: chr3 118178772-118178782. Max. coverage (+): 0. Max coverage (-): 0

Region: chr3 118178783-118178793. Max. coverage (+): 0. Max coverage (-): 0

Region: chr3 118178794-118178805. Max. coverage (+): 0. Max coverage (-): 0

Region: chr3 118178806-118178816. Max. coverage (+): 0. Max coverage (-): 0

Region: chr3 118178817-118178827. Max. coverage (+): 0. Max coverage (-): 0

Region: chr3 118178828-118178838. Max. coverage (+): 0. Max coverage (-): 0

Region: chr3 118178839-118178850. Max. coverage (+): 0. Max coverage (-): 0

Region: chr3 118178851-118178861. Max. coverage (+): 0. Max coverage (-): 0

Region: chr3 118178862-118178872. Max. coverage (+): 0. Max coverage (-): 0

Region: chr3 118178873-118178884. Max. coverage (+): 0. Max coverage (-): 0

Region: chr3 118178885-118178895. Max. coverage (+): 0. Max coverage (-): 0

Region: chr3 118178896-118178906. Max. coverage (+): 0. Max coverage (-): 0

Region: chr3 118178907-118178917. Max. coverage (+): 0. Max coverage (-): 0

Region: chr3 118178918-118178929. Max. coverage (+): 0. Max coverage (-): 0

Region: chr3 118178930-118178940. Max. coverage (+): 0. Max coverage (-): 0

Region: chr3 118178941-118178951. Max. coverage (+): 0. Max coverage (-): 0

Region: chr3 118178952-118178963. Max. coverage (+): 0. Max coverage (-): 0

Region: chr3 118178964-118178974. Max. coverage (+): 0. Max coverage (-): 0

Region: chr3 118178975-118178985. Max. coverage (+): 0. Max coverage (-): 0

Region: chr3 118178986-118178996. Max. coverage (+): 0. Max coverage (-): 0

Region: chr3 118178997-118179008. Max. coverage (+): 0. Max coverage (-): 0

Region: chr3 118179009-118179019. Max. coverage (+): 0. Max coverage (-): 0

Region: chr3 118179020-118179030. Max. coverage (+): 0. Max coverage (-): 0

Region: chr3 118179031-118179041. Max. coverage (+): 0. Max coverage (-): 0

Region: chr3 118179042-118179053. Max. coverage (+): 0. Max coverage (-): 0

Region: chr3 118179054-118179064. Max. coverage (+): 0. Max coverage (-): 0

Region: chr3 118179065-118179075. Max. coverage (+): 0. Max coverage (-): 0

Region: chr3 118179076-118179087. Max. coverage (+): 0. Max coverage (-): 0

Region: chr3 118179088-118179098. Max. coverage (+): 0. Max coverage (-): 0

Region: chr3 118179099-118179109. Max. coverage (+): 0. Max coverage (-): 0

Region: chr3 118179110-118179120. Max. coverage (+): 0. Max coverage (-): 0

Region: chr3 118179121-118179132. Max. coverage (+): 0. Max coverage (-): 0

Region: chr3 118179133-118179143. Max. coverage (+): 0. Max coverage (-): 0

Region: chr3 118179144-118179154. Max. coverage (+): 0. Max coverage (-): 0

Region: chr3 118179155-118179166. Max. coverage (+): 0. Max coverage (-): 0

Region: chr3 118179167-118179177. Max. coverage (+): 0. Max coverage (-): 0

Region: chr3 118179178-118179188. Max. coverage (+): 0. Max coverage (-): 0

Region: chr3 118179189-118179199. Max. coverage (+): 0. Max coverage (-): 0

Region: chr3 118179200-118179211. Max. coverage (+): 0. Max coverage (-): 0

Region: chr3 118179212-118179222. Max. coverage (+): 0. Max coverage (-): 0

Region: chr3 118179223-118179233. Max. coverage (+): 0. Max coverage (-): 0

Region: chr3 118179234-118179245. Max. coverage (+): 0. Max coverage (-): 0

Region: chr3 118179246-118179256. Max. coverage (+): 0. Max coverage (-): 0

Region: chr3 118179257-118179267. Max. coverage (+): 0. Max coverage (-): 0

Region: chr3 118179268-118179278. Max. coverage (+): 0. Max coverage (-): 0

Region: chr3 118179279-118179290. Max. coverage (+): 0. Max coverage (-): 0

Region: chr3 118179291-118179301. Max. coverage (+): 0. Max coverage (-): 0

Region: chr3 118179302-118179312. Max. coverage (+): 0. Max coverage (-): 0

Region: chr3 118179313-118179324. Max. coverage (+): 0. Max coverage (-): 0

Region: chr3 118179325-118179335. Max. coverage (+): 0. Max coverage (-): 0

Region: chr3 118179336-118179346. Max. coverage (+): 0. Max coverage (-): 0

Region: chr3 118179347-118179357. Max. coverage (+): 0. Max coverage (-): 0

Region: chr3 118179358-118179369. Max. coverage (+): 0. Max coverage (-): 0

Region: chr3 118179370-118179380. Max. coverage (+): 0. Max coverage (-): 0

Region: chr3 118179381-118179391. Max. coverage (+): 0. Max coverage (-): 0

Region: chr3 118179392-118179403. Max. coverage (+): 0. Max coverage (-): 0

Region: chr3 118179404-118179414. Max. coverage (+): 0. Max coverage (-): 0

Region: chr3 118179415-118179425. Max. coverage (+): 0. Max coverage (-): 0

Region: chr3 118179426-118179436. Max. coverage (+): 0. Max coverage (-): 0

Region: chr3 118179437-118179448. Max. coverage (+): 0. Max coverage (-): 0

Region: chr3 118179449-118179459. Max. coverage (+): 0. Max coverage (-): 0

Region: chr3 118179460-118179470. Max. coverage (+): 0. Max coverage (-): 0

Region: chr3 118179471-118179481. Max. coverage (+): 0. Max coverage (-): 0

Region: chr3 118179482-118179493. Max. coverage (+): 0. Max coverage (-): 0

Region: chr3 118179494-118179504. Max. coverage (+): 0. Max coverage (-): 0

Region: chr3 118179505-118179515. Max. coverage (+): 0. Max coverage (-): 0

Region: chr3 118179516-118179527. Max. coverage (+): 0. Max coverage (-): 0

Region: chr3 118179528-118179538. Max. coverage (+): 0. Max coverage (-): 2.42

Region: chr3 118179539-118179549. Max. coverage (+): 0. Max coverage (-): 47.59

Region: chr3 118179550-118179560. Max. coverage (+): 9.9. Max coverage (-): 8.76

Region: chr3 118179561-118179572. Max. coverage (+): 9.9. Max coverage (-): 6.1

Region: chr3 118179573-118179583. Max. coverage (+): 0. Max coverage (-): 56.56

Region: chr3 118179584-118179594. Max. coverage (+): 0. Max coverage (-): 56.56

Region: chr3 118179595-118179606. Max. coverage (+): 0. Max coverage (-): 0

Region: chr3 118179607-118179617. Max. coverage (+): 0. Max coverage (-): 0

Region: chr3 118179618-118179628. Max. coverage (+): 0. Max coverage (-): 0

Region: chr3 118179629-118179639. Max. coverage (+): 0. Max coverage (-): 0

Region: chr3 118179640-118179651. Max. coverage (+): 0. Max coverage (-): 0

Region: chr3 118179652-118179662. Max. coverage (+): 0. Max coverage (-): 0

Region: chr3 118179663-118179673. Max. coverage (+): 0. Max coverage (-): 0

Region: chr3 118179674-118179685. Max. coverage (+): 0. Max coverage (-): 0

Region: chr3 118179686-118179696. Max. coverage (+): 0. Max coverage (-): 0

Region: chr3 118179697-118179707. Max. coverage (+): 0. Max coverage (-): 0

Region: chr3 118179708-118179718. Max. coverage (+): 0. Max coverage (-): 0

Region: chr3 118179719-118179730. Max. coverage (+): 0. Max coverage (-): 0

Region: chr3 118179731-118179741. Max. coverage (+): 0. Max coverage (-): 139.02

Region: chr3 118179742-118179752. Max. coverage (+): 0. Max coverage (-): 139.02

Region: chr3 118179753-118179764. Max. coverage (+): 0. Max coverage (-): 0.37

Region: chr3 118179765-118179775. Max. coverage (+): 0. Max coverage (-): 16.46

Region: chr3 118179776-118179786. Max. coverage (+): 0. Max coverage (-): 40.49

Region: chr3 118179787-118179797. Max. coverage (+): 0. Max coverage (-): 8.02

Region: chr3 118179798-118179809. Max. coverage (+): 0. Max coverage (-): 0

Region: chr3 118179810-118179820. Max. coverage (+): 0. Max coverage (-): 0

Region: chr3 118179821-118179831. Max. coverage (+): 0. Max coverage (-): 0

Region: chr3 118179832-118179843. Max. coverage (+): 0. Max coverage (-): 0

Region: chr3 118179844-118179854. Max. coverage (+): 0. Max coverage (-): 0

Region: chr3 118179855-118179865. Max. coverage (+): 0. Max coverage (-): 0

Region: chr3 118179866-118179876. Max. coverage (+): 0. Max coverage (-): 0

Region: chr3 118179877-118179888. Max. coverage (+): 0. Max coverage (-): 0

Region: chr3 118179889-118179899. Max. coverage (+): 0. Max coverage (-): 0

Region: chr3 118179900-118179910. Max. coverage (+): 0. Max coverage (-): 0

Region: chr3 118179911-118179921. Max. coverage (+): 0. Max coverage (-): 0

Region: chr3 118179922-118179933. Max. coverage (+): 0. Max coverage (-): 0

Region: chr3 118179934-118179944. Max. coverage (+): 0. Max coverage (-): 0

Region: chr3 118179945-118179955. Max. coverage (+): 0. Max coverage (-): 0

Region: chr3 118179956-118179967. Max. coverage (+): 0. Max coverage (-): 0

Region: chr3 118179968-118179978. Max. coverage (+): 0. Max coverage (-): 0

Region: chr3 118179979-118179989. Max. coverage (+): 0. Max coverage (-): 0

Region: chr3 118179990-118180000. Max. coverage (+): 0. Max coverage (-): 0

Region: chr3 118180001-118180012. Max. coverage (+): 0. Max coverage (-): 0

Region: chr3 118180013-118180023. Max. coverage (+): 0. Max coverage (-): 0

Region: chr3 118180024-118180034. Max. coverage (+): 0. Max coverage (-): 0

Region: chr3 118180035-118180046. Max. coverage (+): 0. Max coverage (-): 0

Region: chr3 118180047-118180057. Max. coverage (+): 0. Max coverage (-): 0

Region: chr3 118180058-118180068. Max. coverage (+): 0. Max coverage (-): 0

Region: chr3 118180069-118180079. Max. coverage (+): 0. Max coverage (-): 0

Region: chr3 118180080-118180091. Max. coverage (+): 0. Max coverage (-): 0

Region: chr3 118180092-118180102. Max. coverage (+): 0. Max coverage (-): 0

Region: chr3 118180103-118180113. Max. coverage (+): 0. Max coverage (-): 0

Region: chr3 118180114-118180125. Max. coverage (+): 0. Max coverage (-): 0

Region: chr3 118180126-118180136. Max. coverage (+): 0. Max coverage (-): 0

Region: chr3 118180137-118180147. Max. coverage (+): 0. Max coverage (-): 0

Region: chr3 118180148-118180158. Max. coverage (+): 0. Max coverage (-): 0

Region: chr3 118180159-118180170. Max. coverage (+): 0. Max coverage (-): 0

Region: chr3 118180171-118180181. Max. coverage (+): 0. Max coverage (-): 0

Region: chr3 118180182-118180192. Max. coverage (+): 0. Max coverage (-): 0

Region: chr3 118180193-118180204. Max. coverage (+): 0. Max coverage (-): 0

Region: chr3 118180205-118180215. Max. coverage (+): 0. Max coverage (-): 0

Region: chr3 118180216-118180226. Max. coverage (+): 0. Max coverage (-): 0

Region: chr3 118180227-118180237. Max. coverage (+): 0. Max coverage (-): 0

Region: chr3 118180238-118180249. Max. coverage (+): 0. Max coverage (-): 0

Region: chr3 118180250-118180260. Max. coverage (+): 0. Max coverage (-): 0

Region: chr3 118180261-118180271. Max. coverage (+): 0. Max coverage (-): 0

Region: chr3 118180272-118180283. Max. coverage (+): 0. Max coverage (-): 0

Region: chr3 118180284-118180294. Max. coverage (+): 0. Max coverage (-): 0

Region: chr3 118180295-118180305. Max. coverage (+): 0. Max coverage (-): 0

Region: chr3 118180306-118180316. Max. coverage (+): 0. Max coverage (-): 0

Region: chr3 118180317-118180328. Max. coverage (+): 0. Max coverage (-): 0

Region: chr3 118180329-118180339. Max. coverage (+): 0. Max coverage (-): 0

Region: chr3 118180340-118180350. Max. coverage (+): 0. Max coverage (-): 0

Region: chr3 118180351-118180361. Max. coverage (+): 0. Max coverage (-): 0

Region: chr3 118180362-118180373. Max. coverage (+): 0. Max coverage (-): 0

Region: chr3 118180374-118180384. Max. coverage (+): 0. Max coverage (-): 0

Region: chr3 118180385-118180395. Max. coverage (+): 0. Max coverage (-): 0

Region: chr3 118180396-118180407. Max. coverage (+): 0. Max coverage (-): 0

Region: chr3 118180408-118180418. Max. coverage (+): 0. Max coverage (-): 0

Region: chr3 118180419-118180429. Max. coverage (+): 0. Max coverage (-): 0

Region: chr3 118180430-118180440. Max. coverage (+): 0. Max coverage (-): 0

Region: chr3 118180441-118180452. Max. coverage (+): 0. Max coverage (-): 0

Region: chr3 118180453-118180463. Max. coverage (+): 0. Max coverage (-): 0

Region: chr3 118180464-118180474. Max. coverage (+): 0. Max coverage (-): 0

Region: chr3 118180475-118180486. Max. coverage (+): 0. Max coverage (-): 0

Region: chr3 118180487-118180497. Max. coverage (+): 0. Max coverage (-): 0

Region: chr3 118180498-118180508. Max. coverage (+): 0. Max coverage (-): 0

Region: chr3 118180509-118180519. Max. coverage (+): 0. Max coverage (-): 0

Region: chr3 118180520-118180531. Max. coverage (+): 0. Max coverage (-): 0

Region: chr3 118180532-118180542. Max. coverage (+): 0. Max coverage (-): 0

Region: chr3 118180543-118180553. Max. coverage (+): 0. Max coverage (-): 0

Region: chr3 118180554-118180565. Max. coverage (+): 0. Max coverage (-): 0

Region: chr3 118180566-118180576. Max. coverage (+): 0. Max coverage (-): 0

Region: chr3 118180577-118180587. Max. coverage (+): 0. Max coverage (-): 0

Region: chr3 118180588-118180598. Max. coverage (+): 0. Max coverage (-): 0

Region: chr3 118180599-118180610. Max. coverage (+): 0. Max coverage (-): 0

Region: chr3 118180611-118180621. Max. coverage (+): 0. Max coverage (-): 0

Region: chr3 118180622-118180632. Max. coverage (+): 0. Max coverage (-): 0

Region: chr3 118180633-118180644. Max. coverage (+): 0. Max coverage (-): 0

Region: chr3 118180645-118180655. Max. coverage (+): 0. Max coverage (-): 0

Region: chr3 118180656-118180666. Max. coverage (+): 0. Max coverage (-): 0

Region: chr3 118180667-118180677. Max. coverage (+): 0. Max coverage (-): 0

Region: chr3 118180678-118180689. Max. coverage (+): 0. Max coverage (-): 0

Region: chr3 118180690-118180700. Max. coverage (+): 0. Max coverage (-): 0

Region: chr3 118180701-118180711. Max. coverage (+): 0. Max coverage (-): 0

Region: chr3 118180712-118180723. Max. coverage (+): 0. Max coverage (-): 0

Region: chr3 118180724-118180734. Max. coverage (+): 0. Max coverage (-): 0

Region: chr3 118180735-118180745. Max. coverage (+): 0. Max coverage (-): 0

Region: chr3 118180746-118180756. Max. coverage (+): 0. Max coverage (-): 0

Region: chr3 118180757-118180768. Max. coverage (+): 0. Max coverage (-): 0

Region: chr3 118180769-118180779. Max. coverage (+): 0. Max coverage (-): 0

Region: chr3 118180780-118180790. Max. coverage (+): 0. Max coverage (-): 0

Region: chr3 118180791-118180801. Max. coverage (+): 0. Max coverage (-): 0

Region: chr3 118180802-118180813. Max. coverage (+): 0. Max coverage (-): 0

Region: chr3 118180814-118180824. Max. coverage (+): 0. Max coverage (-): 0

Region: chr3 118180825-118180835. Max. coverage (+): 0. Max coverage (-): 0

Region: chr3 118180836-118180847. Max. coverage (+): 0. Max coverage (-): 0

Region: chr3 118180848-118180858. Max. coverage (+): 0. Max coverage (-): 0

Region: chr3 118180859-118180869. Max. coverage (+): 0. Max coverage (-): 0

Region: chr3 118180870-118180880. Max. coverage (+): 0. Max coverage (-): 0

Region: chr3 118180881-118180892. Max. coverage (+): 0. Max coverage (-): 0

Region: chr3 118180893-118180903. Max. coverage (+): 0. Max coverage (-): 0

Region: chr3 118180904-118180914. Max. coverage (+): 0. Max coverage (-): 0

Region: chr3 118180915-118180926. Max. coverage (+): 2.84. Max coverage (-): 0

Region: chr3 118180927-118180937. Max. coverage (+): 0. Max coverage (-): 0

Region: chr3 118180938-. Max. coverage (+): 0. Max coverage (-): 0

RepeatMasker Color Code

**+**

100-98% Identity

<98-95% Identity

<95-90% Identity

<90-85% Identity

<85-80% Identity

<80-75% Identity

<75-70% Identity

<70% Identity

**-**

Gene Set Color Code

**+**

Gene

Pseudogene

**-**

Topology/Coverage Color Code

Coverage Plus Strand

Coverage Minus Strand

Mainstrand: Plus

Mainstrand: Minus

Complementary Strand

Flanking Region  
(if option -flank >0)

Gene Set Annotation  
  
RepeatMasker Annotation  

**1. L1ME2z**: 118175037-118175423 (+), Divergence to consensus: 38%  
**2. LTR75**: 118175946-118176006 (-), Divergence to consensus: 24.9%  
**3. MLT1F2**: 118176502-118176754 (+), Divergence to consensus: 45.4%  
**4. Bov-tA3**: 118178165-118178376 (+), Divergence to consensus: 11.3%  
**5. L2a**: 118178455-118178707 (+), Divergence to consensus: 48.5%  
**6. L2**: 118180480-118180561 (-), Divergence to consensus: 41.4%  
**7. BOV-A2**: 118180608-118180877 (-), Divergence to consensus: 4.8%

  
Transcription Factor Binding Sites  

**SPZ1** (Sequence: CTCAAACCCC (-): 118177188)  
**A-MYB** (Sequence: CCAACTGCCA (-): 118176888)
