# Supplementary material for: Small RNA sequencing of cryopreserved semen from single bull revealed altered miRNAs and piRNAs expression between High- and Low-motile sperm populations
Source: BMC Genomics. 2017 Jan 4;18:14. doi: 10.1186/s12864-016-3394-7 (PMC5209821; doi:10.1186/s12864-016-3394-7)
Supplement: Additional file 4: — Details for each piRNA clusters found in Low Motile (LM) sperm fraction. Genes, repeats, transposable elements and transcription factors binding sites falling within the cluster regions were reported. (ZIP 1034 kb) [file 12864_2016_3394_MOESM4_ESM.zip › 42.html]

piRNA cluster 42


Predicted piRNA cluster no. 42     previous   next
  

Show proTRAC run info
Hide proTRAC run info

================================= proTRAC ====================================  
VERSION: 2.1                                    LAST MODIFIED: 06. October 2015  
  
Please cite:  
Rosenkranz D, Zischler H. proTRAC - a software for probabilistic piRNA cluster  
detection, visualization and analysis. 2012. BMC Bioinformatics 13:5.  
  
and (for proTRAC 2.0 and later):  
Rosenkranz D, Rudloff S, Bastuck K, Ketting RF, Zischler H. Tupaia small RNAs  
provide insights into function and evolution of RNAi-based transposon defense  
in mammals. 2015. RNA 21(5):911-922.  
  
Contact:  
David Rosenkranz  
Institute of Anthropology, small RNA group  
Johannes Gutenberg University Mainz  
email: rosenkranz@uni-mainz.de  
  
You can find the latest proTRAC version at:  
http://sourceforge.net/projects/protrac/files  
http://www.smallRNAgroup-mainz.de/software  
==============================================================================  
  
PARAMETERS:  
Map file: .............../storage/core/barbara/genhome/smallRNA/fertility/Sample\_not\_motile/pirna/Sample\_not\_motile\_26-33\_collapsed.fa.no-dust.map.weighted-10000-1000-b-0  
Genome file: ............/storage/core/barbara/genhome/smallRNA/fertility/Sample\_all/pirna/bt\_311\_chrY.fa  
RepeatMasker annotation: /storage/genomes/bt\_umd31/GCF\_000003055.6\_Bos\_taurus\_UMD\_3.1.1\_repeatMasker\_chr.out  
GeneSet:................./storage/core/barbara/genhome/smallRNA/fertility/Sample\_all/pirna/full.gtf  
  
Significant (p<=0.01) hit density will be calculated based  
on observed hit distribution.  
  
Sliding window size: ........................................ 5000 bp  
Sliding window increament: .................................. 1000 bp  
Normalize each hit by number of genomic hits: ............... 1 [0=no/1=yes]  
Normalize each hit by number of sequence reads: ............. 1 [0=no/1=yes]  
Normalize values (-> per million mapped reads): ............. 1 [0=no/1=yes]  
Min. fraction of hits with 1T(U) or 10A: .................... 0.75  
Alternatively: Min. fraction of hits with 1T(U) and 10A: .... 0.5  
Min. fraction of hits with typical piRNA length: ............ 0.75  
Typical piRNA length: ....................................... 26-33 nt  
Min. size of a piRNA cluster: ............................... 5000 bp.  
Min. number of hits (absolute): ............................. 0  
Min. number of hits (normalized): ........................... 0  
Min. fraction of hits on the mainstrand: .................... 0.75  
Top fraction of mapped sequences (in terms of read counts): . 1%  
Top fraction accounts for max. n% of sequence reads: ........ 90%  
Min. fraction of hits on each arm of a bidirectional cluster: 0.1  
Output image file for each cluster: ......................... 0 [0=no/1=yes]  
Output html file for each cluster: .......................... 1 [0=no/1=yes]  
Output a summary table: ..................................... 1 [0=no/1=yes]  
Output a FASTA file for each cluster (piRNA sequences): ..... 1 [0=no/1=yes]  
Output a FASTA file comprising cluster sequences: ........... 1 [0=no/1=yes]  
Search DNA motifs in clusters: .............................. 1 [0=no/1=yes]  
Output flanking sequences: +/- .............................. 0 bp  
Output ~.pTi file: .......................................... 1 [0=no/1=yes]  
==============================================================================  
  
  
Genome size (without gaps): ............ 2678902517 bp  
Gaps (N/X/-): .......................... 53837044 bp  
Mapped reads: .......................... 738059667487  
Non-identical sequences: ............... 277001  
Genomic hits: .......................... 533816  
Significant densitiy of mapped reads: .. 15118061 reads/kb

Show proTRAC cluster info
Hide proTRAC cluster info

|  |  |
| --- | --- |
| Location | chr3 |
| Coordinates | 118372298-118377376 |
| Size [bp] | 5079 |
| Sequence hit loci | 69 |
| Mapped reads (normalized) | 179793395 |
| Mapped reads (normalized) per kb | 35399369 |
| Normalized reads with 1T (1U) | 84.3% |
| Normalized reads with 10A | 35.2% |
| Normalized reads with length 26-33 nt | 100% |
| Normalized reads on the main strand(s) | 100% |
| Predicted directionality | mono:plus |

100%

0%

1T (1U)  
reads

10A reads

26-33 nt  
reads

reads on mainstrand

**Either the amount of reads with 1T (1U) OR 10A has to exceed 75% (set with option: -1Tor10A)  
Alternatively the amount of reads with 1T (1U) AND 10A has to exceed 50% (set with option: -1Tand10A)  
Minimum amount of reads with preferred size is 75% (set with option: -pisize)  
Minimum amount of reads on the main strand(s) is 75% (set with option: -clstrand)**

Show read coverage
Hide read coverage

WHAT DO I SEE HERE?  
This chart shows the location of mapped sequence reads within a predicted piRNA cluster. The color refers to the number of genomic hits produced by the sequence read in question. A dark red bar indicates that this sequence read produces many other hits elsewhere in the genome. Many adjacent red or yellow bars can indicate the presence of a multi-copy element such as transposons or rRNA genes. A dark green bar indicates that this sequence read maps uniquely to this locus.

1 hit

2-5 hits

6-10 hits

11-20 hits

21-50 hits

51-100 hits

> 100 hits

chr3

118372298

118377376

Gene Set

RepeatMasker

Mapped  
Reads

27.61

plus strand

minus strand

27.61

Region: chr3 118180939-118372303. Max. coverage (+): 27.61. Max coverage (-): 0

Region: chr3 118372304-118372313. Max. coverage (+): 27.61. Max coverage (-): 0

Region: chr3 118372314-118372323. Max. coverage (+): 3.16. Max coverage (-): 0

Region: chr3 118372324-118372333. Max. coverage (+): 0. Max coverage (-): 0

Region: chr3 118372334-118372343. Max. coverage (+): 0. Max coverage (-): 0

Region: chr3 118372344-118372353. Max. coverage (+): 0. Max coverage (-): 0

Region: chr3 118372354-118372364. Max. coverage (+): 7.7. Max coverage (-): 0

Region: chr3 118372365-118372374. Max. coverage (+): 7.7. Max coverage (-): 0

Region: chr3 118372375-118372384. Max. coverage (+): 0. Max coverage (-): 0

Region: chr3 118372385-118372394. Max. coverage (+): 0. Max coverage (-): 0

Region: chr3 118372395-118372404. Max. coverage (+): 0. Max coverage (-): 0

Region: chr3 118372405-118372414. Max. coverage (+): 0. Max coverage (-): 0

Region: chr3 118372415-118372424. Max. coverage (+): 0. Max coverage (-): 0

Region: chr3 118372425-118372435. Max. coverage (+): 0. Max coverage (-): 0

Region: chr3 118372436-118372445. Max. coverage (+): 0. Max coverage (-): 0

Region: chr3 118372446-118372455. Max. coverage (+): 3.2. Max coverage (-): 0

Region: chr3 118372456-118372465. Max. coverage (+): 1.71. Max coverage (-): 0

Region: chr3 118372466-118372475. Max. coverage (+): 1.71. Max coverage (-): 0

Region: chr3 118372476-118372485. Max. coverage (+): 0. Max coverage (-): 0

Region: chr3 118372486-118372496. Max. coverage (+): 0. Max coverage (-): 0

Region: chr3 118372497-118372506. Max. coverage (+): 0. Max coverage (-): 0

Region: chr3 118372507-118372516. Max. coverage (+): 0. Max coverage (-): 0

Region: chr3 118372517-118372526. Max. coverage (+): 0. Max coverage (-): 0

Region: chr3 118372527-118372536. Max. coverage (+): 0. Max coverage (-): 0

Region: chr3 118372537-118372546. Max. coverage (+): 0. Max coverage (-): 0

Region: chr3 118372547-118372557. Max. coverage (+): 0. Max coverage (-): 0

Region: chr3 118372558-118372567. Max. coverage (+): 7.21. Max coverage (-): 0

Region: chr3 118372568-118372577. Max. coverage (+): 7.21. Max coverage (-): 0

Region: chr3 118372578-118372587. Max. coverage (+): 0. Max coverage (-): 0

Region: chr3 118372588-118372597. Max. coverage (+): 0. Max coverage (-): 0

Region: chr3 118372598-118372607. Max. coverage (+): 0. Max coverage (-): 0

Region: chr3 118372608-118372617. Max. coverage (+): 0. Max coverage (-): 0

Region: chr3 118372618-118372628. Max. coverage (+): 8.58. Max coverage (-): 0

Region: chr3 118372629-118372638. Max. coverage (+): 14.81. Max coverage (-): 0

Region: chr3 118372639-118372648. Max. coverage (+): 0. Max coverage (-): 0

Region: chr3 118372649-118372658. Max. coverage (+): 0. Max coverage (-): 0

Region: chr3 118372659-118372668. Max. coverage (+): 0. Max coverage (-): 0

Region: chr3 118372669-118372678. Max. coverage (+): 0. Max coverage (-): 0

Region: chr3 118372679-118372689. Max. coverage (+): 0. Max coverage (-): 0

Region: chr3 118372690-118372699. Max. coverage (+): 0. Max coverage (-): 0

Region: chr3 118372700-118372709. Max. coverage (+): 0. Max coverage (-): 0

Region: chr3 118372710-118372719. Max. coverage (+): 0. Max coverage (-): 0

Region: chr3 118372720-118372729. Max. coverage (+): 14.8. Max coverage (-): 0

Region: chr3 118372730-118372739. Max. coverage (+): 14.8. Max coverage (-): 0

Region: chr3 118372740-118372750. Max. coverage (+): 0. Max coverage (-): 0

Region: chr3 118372751-118372760. Max. coverage (+): 3.21. Max coverage (-): 0

Region: chr3 118372761-118372770. Max. coverage (+): 3.21. Max coverage (-): 0

Region: chr3 118372771-118372780. Max. coverage (+): 0. Max coverage (-): 0

Region: chr3 118372781-118372790. Max. coverage (+): 0. Max coverage (-): 0

Region: chr3 118372791-118372800. Max. coverage (+): 0. Max coverage (-): 0

Region: chr3 118372801-118372810. Max. coverage (+): 0. Max coverage (-): 0

Region: chr3 118372811-118372821. Max. coverage (+): 0. Max coverage (-): 0

Region: chr3 118372822-118372831. Max. coverage (+): 0. Max coverage (-): 0

Region: chr3 118372832-118372841. Max. coverage (+): 0. Max coverage (-): 0

Region: chr3 118372842-118372851. Max. coverage (+): 0. Max coverage (-): 0

Region: chr3 118372852-118372861. Max. coverage (+): 0. Max coverage (-): 0

Region: chr3 118372862-118372871. Max. coverage (+): 0. Max coverage (-): 0

Region: chr3 118372872-118372882. Max. coverage (+): 0. Max coverage (-): 0

Region: chr3 118372883-118372892. Max. coverage (+): 0. Max coverage (-): 0

Region: chr3 118372893-118372902. Max. coverage (+): 0. Max coverage (-): 0

Region: chr3 118372903-118372912. Max. coverage (+): 0. Max coverage (-): 0

Region: chr3 118372913-118372922. Max. coverage (+): 0. Max coverage (-): 0

Region: chr3 118372923-118372932. Max. coverage (+): 0. Max coverage (-): 0

Region: chr3 118372933-118372943. Max. coverage (+): 0. Max coverage (-): 0

Region: chr3 118372944-118372953. Max. coverage (+): 0. Max coverage (-): 0

Region: chr3 118372954-118372963. Max. coverage (+): 0. Max coverage (-): 0

Region: chr3 118372964-118372973. Max. coverage (+): 4.77. Max coverage (-): 0

Region: chr3 118372974-118372983. Max. coverage (+): 4.77. Max coverage (-): 0

Region: chr3 118372984-118372993. Max. coverage (+): 2.3. Max coverage (-): 0

Region: chr3 118372994-118373003. Max. coverage (+): 0. Max coverage (-): 0

Region: chr3 118373004-118373014. Max. coverage (+): 0. Max coverage (-): 0

Region: chr3 118373015-118373024. Max. coverage (+): 0. Max coverage (-): 0

Region: chr3 118373025-118373034. Max. coverage (+): 0. Max coverage (-): 0

Region: chr3 118373035-118373044. Max. coverage (+): 0. Max coverage (-): 0

Region: chr3 118373045-118373054. Max. coverage (+): 14.69. Max coverage (-): 0

Region: chr3 118373055-118373064. Max. coverage (+): 14.69. Max coverage (-): 0

Region: chr3 118373065-118373075. Max. coverage (+): 0. Max coverage (-): 0

Region: chr3 118373076-118373085. Max. coverage (+): 0. Max coverage (-): 0

Region: chr3 118373086-118373095. Max. coverage (+): 0. Max coverage (-): 0

Region: chr3 118373096-118373105. Max. coverage (+): 9.07. Max coverage (-): 0

Region: chr3 118373106-118373115. Max. coverage (+): 9.07. Max coverage (-): 0

Region: chr3 118373116-118373125. Max. coverage (+): 0. Max coverage (-): 0

Region: chr3 118373126-118373136. Max. coverage (+): 0. Max coverage (-): 0

Region: chr3 118373137-118373146. Max. coverage (+): 0. Max coverage (-): 0

Region: chr3 118373147-118373156. Max. coverage (+): 1.81. Max coverage (-): 0

Region: chr3 118373157-118373166. Max. coverage (+): 0. Max coverage (-): 0

Region: chr3 118373167-118373176. Max. coverage (+): 7.6. Max coverage (-): 0

Region: chr3 118373177-118373186. Max. coverage (+): 7.6. Max coverage (-): 0

Region: chr3 118373187-118373196. Max. coverage (+): 0. Max coverage (-): 0

Region: chr3 118373197-118373207. Max. coverage (+): 1.56. Max coverage (-): 0

Region: chr3 118373208-118373217. Max. coverage (+): 1.56. Max coverage (-): 0

Region: chr3 118373218-118373227. Max. coverage (+): 0. Max coverage (-): 0

Region: chr3 118373228-118373237. Max. coverage (+): 0. Max coverage (-): 0

Region: chr3 118373238-118373247. Max. coverage (+): 0. Max coverage (-): 0

Region: chr3 118373248-118373257. Max. coverage (+): 11.76. Max coverage (-): 0

Region: chr3 118373258-118373268. Max. coverage (+): 11.76. Max coverage (-): 0

Region: chr3 118373269-118373278. Max. coverage (+): 8.62. Max coverage (-): 0

Region: chr3 118373279-118373288. Max. coverage (+): 8.62. Max coverage (-): 0

Region: chr3 118373289-118373298. Max. coverage (+): 0. Max coverage (-): 0

Region: chr3 118373299-118373308. Max. coverage (+): 0.31. Max coverage (-): 0

Region: chr3 118373309-118373318. Max. coverage (+): 0. Max coverage (-): 0

Region: chr3 118373319-118373329. Max. coverage (+): 0. Max coverage (-): 0

Region: chr3 118373330-118373339. Max. coverage (+): 0. Max coverage (-): 0

Region: chr3 118373340-118373349. Max. coverage (+): 0. Max coverage (-): 0

Region: chr3 118373350-118373359. Max. coverage (+): 0. Max coverage (-): 0

Region: chr3 118373360-118373369. Max. coverage (+): 0. Max coverage (-): 0

Region: chr3 118373370-118373379. Max. coverage (+): 0. Max coverage (-): 0

Region: chr3 118373380-118373389. Max. coverage (+): 0. Max coverage (-): 0

Region: chr3 118373390-118373400. Max. coverage (+): 0. Max coverage (-): 0

Region: chr3 118373401-118373410. Max. coverage (+): 0. Max coverage (-): 0

Region: chr3 118373411-118373420. Max. coverage (+): 0. Max coverage (-): 0

Region: chr3 118373421-118373430. Max. coverage (+): 0. Max coverage (-): 0

Region: chr3 118373431-118373440. Max. coverage (+): 0. Max coverage (-): 0

Region: chr3 118373441-118373450. Max. coverage (+): 0. Max coverage (-): 0

Region: chr3 118373451-118373461. Max. coverage (+): 0. Max coverage (-): 0

Region: chr3 118373462-118373471. Max. coverage (+): 3.17. Max coverage (-): 0

Region: chr3 118373472-118373481. Max. coverage (+): 3.17. Max coverage (-): 0

Region: chr3 118373482-118373491. Max. coverage (+): 0. Max coverage (-): 0

Region: chr3 118373492-118373501. Max. coverage (+): 0. Max coverage (-): 0

Region: chr3 118373502-118373511. Max. coverage (+): 0. Max coverage (-): 0

Region: chr3 118373512-118373522. Max. coverage (+): 7.44. Max coverage (-): 0

Region: chr3 118373523-118373532. Max. coverage (+): 7.44. Max coverage (-): 0

Region: chr3 118373533-118373542. Max. coverage (+): 0. Max coverage (-): 0

Region: chr3 118373543-118373552. Max. coverage (+): 0. Max coverage (-): 0

Region: chr3 118373553-118373562. Max. coverage (+): 0. Max coverage (-): 0

Region: chr3 118373563-118373572. Max. coverage (+): 0. Max coverage (-): 0

Region: chr3 118373573-118373582. Max. coverage (+): 0. Max coverage (-): 0

Region: chr3 118373583-118373593. Max. coverage (+): 0. Max coverage (-): 0

Region: chr3 118373594-118373603. Max. coverage (+): 0. Max coverage (-): 0

Region: chr3 118373604-118373613. Max. coverage (+): 0. Max coverage (-): 0

Region: chr3 118373614-118373623. Max. coverage (+): 0. Max coverage (-): 0

Region: chr3 118373624-118373633. Max. coverage (+): 0. Max coverage (-): 0

Region: chr3 118373634-118373643. Max. coverage (+): 0. Max coverage (-): 0

Region: chr3 118373644-118373654. Max. coverage (+): 0. Max coverage (-): 0

Region: chr3 118373655-118373664. Max. coverage (+): 0. Max coverage (-): 0

Region: chr3 118373665-118373674. Max. coverage (+): 0. Max coverage (-): 0

Region: chr3 118373675-118373684. Max. coverage (+): 0. Max coverage (-): 0

Region: chr3 118373685-118373694. Max. coverage (+): 0. Max coverage (-): 0

Region: chr3 118373695-118373704. Max. coverage (+): 0. Max coverage (-): 0

Region: chr3 118373705-118373715. Max. coverage (+): 3.26. Max coverage (-): 0

Region: chr3 118373716-118373725. Max. coverage (+): 3.26. Max coverage (-): 0

Region: chr3 118373726-118373735. Max. coverage (+): 0. Max coverage (-): 0

Region: chr3 118373736-118373745. Max. coverage (+): 0. Max coverage (-): 0

Region: chr3 118373746-118373755. Max. coverage (+): 0. Max coverage (-): 0

Region: chr3 118373756-118373765. Max. coverage (+): 0. Max coverage (-): 0

Region: chr3 118373766-118373775. Max. coverage (+): 4.51. Max coverage (-): 0

Region: chr3 118373776-118373786. Max. coverage (+): 4.51. Max coverage (-): 0

Region: chr3 118373787-118373796. Max. coverage (+): 0. Max coverage (-): 0

Region: chr3 118373797-118373806. Max. coverage (+): 0. Max coverage (-): 0

Region: chr3 118373807-118373816. Max. coverage (+): 0. Max coverage (-): 0

Region: chr3 118373817-118373826. Max. coverage (+): 0. Max coverage (-): 0

Region: chr3 118373827-118373836. Max. coverage (+): 0. Max coverage (-): 0

Region: chr3 118373837-118373847. Max. coverage (+): 0. Max coverage (-): 0

Region: chr3 118373848-118373857. Max. coverage (+): 0. Max coverage (-): 0

Region: chr3 118373858-118373867. Max. coverage (+): 0. Max coverage (-): 0

Region: chr3 118373868-118373877. Max. coverage (+): 0. Max coverage (-): 0

Region: chr3 118373878-118373887. Max. coverage (+): 0. Max coverage (-): 0

Region: chr3 118373888-118373897. Max. coverage (+): 0. Max coverage (-): 0

Region: chr3 118373898-118373908. Max. coverage (+): 0. Max coverage (-): 0

Region: chr3 118373909-118373918. Max. coverage (+): 0. Max coverage (-): 0

Region: chr3 118373919-118373928. Max. coverage (+): 0. Max coverage (-): 0

Region: chr3 118373929-118373938. Max. coverage (+): 0. Max coverage (-): 0

Region: chr3 118373939-118373948. Max. coverage (+): 0. Max coverage (-): 0

Region: chr3 118373949-118373958. Max. coverage (+): 0. Max coverage (-): 0

Region: chr3 118373959-118373968. Max. coverage (+): 0. Max coverage (-): 0

Region: chr3 118373969-118373979. Max. coverage (+): 0. Max coverage (-): 0

Region: chr3 118373980-118373989. Max. coverage (+): 0. Max coverage (-): 0

Region: chr3 118373990-118373999. Max. coverage (+): 16.85. Max coverage (-): 0

Region: chr3 118374000-118374009. Max. coverage (+): 16.85. Max coverage (-): 0

Region: chr3 118374010-118374019. Max. coverage (+): 0. Max coverage (-): 0

Region: chr3 118374020-118374029. Max. coverage (+): 0. Max coverage (-): 0

Region: chr3 118374030-118374040. Max. coverage (+): 0. Max coverage (-): 0

Region: chr3 118374041-118374050. Max. coverage (+): 0. Max coverage (-): 0

Region: chr3 118374051-118374060. Max. coverage (+): 0. Max coverage (-): 0

Region: chr3 118374061-118374070. Max. coverage (+): 0. Max coverage (-): 0

Region: chr3 118374071-118374080. Max. coverage (+): 0. Max coverage (-): 0

Region: chr3 118374081-118374090. Max. coverage (+): 0. Max coverage (-): 0

Region: chr3 118374091-118374101. Max. coverage (+): 0. Max coverage (-): 0

Region: chr3 118374102-118374111. Max. coverage (+): 0. Max coverage (-): 0

Region: chr3 118374112-118374121. Max. coverage (+): 0. Max coverage (-): 0

Region: chr3 118374122-118374131. Max. coverage (+): 0. Max coverage (-): 0

Region: chr3 118374132-118374141. Max. coverage (+): 0. Max coverage (-): 0

Region: chr3 118374142-118374151. Max. coverage (+): 0. Max coverage (-): 0

Region: chr3 118374152-118374161. Max. coverage (+): 0. Max coverage (-): 0

Region: chr3 118374162-118374172. Max. coverage (+): 0. Max coverage (-): 0

Region: chr3 118374173-118374182. Max. coverage (+): 0. Max coverage (-): 0

Region: chr3 118374183-118374192. Max. coverage (+): 0. Max coverage (-): 0

Region: chr3 118374193-118374202. Max. coverage (+): 0. Max coverage (-): 0

Region: chr3 118374203-118374212. Max. coverage (+): 0. Max coverage (-): 0

Region: chr3 118374213-118374222. Max. coverage (+): 0. Max coverage (-): 0

Region: chr3 118374223-118374233. Max. coverage (+): 0. Max coverage (-): 0

Region: chr3 118374234-118374243. Max. coverage (+): 0. Max coverage (-): 0

Region: chr3 118374244-118374253. Max. coverage (+): 0. Max coverage (-): 0

Region: chr3 118374254-118374263. Max. coverage (+): 0. Max coverage (-): 0

Region: chr3 118374264-118374273. Max. coverage (+): 0. Max coverage (-): 0

Region: chr3 118374274-118374283. Max. coverage (+): 0. Max coverage (-): 0

Region: chr3 118374284-118374294. Max. coverage (+): 0. Max coverage (-): 0

Region: chr3 118374295-118374304. Max. coverage (+): 0. Max coverage (-): 0

Region: chr3 118374305-118374314. Max. coverage (+): 0. Max coverage (-): 0

Region: chr3 118374315-118374324. Max. coverage (+): 0. Max coverage (-): 0

Region: chr3 118374325-118374334. Max. coverage (+): 0. Max coverage (-): 0

Region: chr3 118374335-118374344. Max. coverage (+): 0. Max coverage (-): 0

Region: chr3 118374345-118374354. Max. coverage (+): 0. Max coverage (-): 0

Region: chr3 118374355-118374365. Max. coverage (+): 0. Max coverage (-): 0

Region: chr3 118374366-118374375. Max. coverage (+): 0. Max coverage (-): 0

Region: chr3 118374376-118374385. Max. coverage (+): 0. Max coverage (-): 0

Region: chr3 118374386-118374395. Max. coverage (+): 0. Max coverage (-): 0

Region: chr3 118374396-118374405. Max. coverage (+): 0. Max coverage (-): 0

Region: chr3 118374406-118374415. Max. coverage (+): 0. Max coverage (-): 0

Region: chr3 118374416-118374426. Max. coverage (+): 0. Max coverage (-): 0

Region: chr3 118374427-118374436. Max. coverage (+): 0. Max coverage (-): 0

Region: chr3 118374437-118374446. Max. coverage (+): 0. Max coverage (-): 0

Region: chr3 118374447-118374456. Max. coverage (+): 0. Max coverage (-): 0

Region: chr3 118374457-118374466. Max. coverage (+): 0. Max coverage (-): 0

Region: chr3 118374467-118374476. Max. coverage (+): 0. Max coverage (-): 0

Region: chr3 118374477-118374487. Max. coverage (+): 0. Max coverage (-): 0

Region: chr3 118374488-118374497. Max. coverage (+): 0. Max coverage (-): 0

Region: chr3 118374498-118374507. Max. coverage (+): 0. Max coverage (-): 0

Region: chr3 118374508-118374517. Max. coverage (+): 0. Max coverage (-): 0

Region: chr3 118374518-118374527. Max. coverage (+): 0. Max coverage (-): 0

Region: chr3 118374528-118374537. Max. coverage (+): 0. Max coverage (-): 0

Region: chr3 118374538-118374547. Max. coverage (+): 0. Max coverage (-): 0

Region: chr3 118374548-118374558. Max. coverage (+): 0. Max coverage (-): 0

Region: chr3 118374559-118374568. Max. coverage (+): 0. Max coverage (-): 0

Region: chr3 118374569-118374578. Max. coverage (+): 0. Max coverage (-): 0

Region: chr3 118374579-118374588. Max. coverage (+): 0. Max coverage (-): 0

Region: chr3 118374589-118374598. Max. coverage (+): 6.84. Max coverage (-): 0

Region: chr3 118374599-118374608. Max. coverage (+): 6.84. Max coverage (-): 0

Region: chr3 118374609-118374619. Max. coverage (+): 0. Max coverage (-): 0

Region: chr3 118374620-118374629. Max. coverage (+): 0. Max coverage (-): 0

Region: chr3 118374630-118374639. Max. coverage (+): 0. Max coverage (-): 0

Region: chr3 118374640-118374649. Max. coverage (+): 0. Max coverage (-): 0

Region: chr3 118374650-118374659. Max. coverage (+): 0. Max coverage (-): 0

Region: chr3 118374660-118374669. Max. coverage (+): 0. Max coverage (-): 0

Region: chr3 118374670-118374680. Max. coverage (+): 0. Max coverage (-): 0

Region: chr3 118374681-118374690. Max. coverage (+): 0. Max coverage (-): 0

Region: chr3 118374691-118374700. Max. coverage (+): 0. Max coverage (-): 0

Region: chr3 118374701-118374710. Max. coverage (+): 0. Max coverage (-): 0

Region: chr3 118374711-118374720. Max. coverage (+): 0.12. Max coverage (-): 0

Region: chr3 118374721-118374730. Max. coverage (+): 0.12. Max coverage (-): 0

Region: chr3 118374731-118374740. Max. coverage (+): 0. Max coverage (-): 0

Region: chr3 118374741-118374751. Max. coverage (+): 0. Max coverage (-): 0

Region: chr3 118374752-118374761. Max. coverage (+): 0. Max coverage (-): 0

Region: chr3 118374762-118374771. Max. coverage (+): 0. Max coverage (-): 0

Region: chr3 118374772-118374781. Max. coverage (+): 0. Max coverage (-): 0

Region: chr3 118374782-118374791. Max. coverage (+): 0. Max coverage (-): 0

Region: chr3 118374792-118374801. Max. coverage (+): 0. Max coverage (-): 0

Region: chr3 118374802-118374812. Max. coverage (+): 5.61. Max coverage (-): 0

Region: chr3 118374813-118374822. Max. coverage (+): 5.61. Max coverage (-): 0

Region: chr3 118374823-118374832. Max. coverage (+): 0. Max coverage (-): 0

Region: chr3 118374833-118374842. Max. coverage (+): 10.18. Max coverage (-): 0

Region: chr3 118374843-118374852. Max. coverage (+): 4.08. Max coverage (-): 0

Region: chr3 118374853-118374862. Max. coverage (+): 0. Max coverage (-): 0

Region: chr3 118374863-118374873. Max. coverage (+): 0. Max coverage (-): 0

Region: chr3 118374874-118374883. Max. coverage (+): 0. Max coverage (-): 0

Region: chr3 118374884-118374893. Max. coverage (+): 0. Max coverage (-): 0

Region: chr3 118374894-118374903. Max. coverage (+): 0. Max coverage (-): 0

Region: chr3 118374904-118374913. Max. coverage (+): 0. Max coverage (-): 0

Region: chr3 118374914-118374923. Max. coverage (+): 0. Max coverage (-): 0

Region: chr3 118374924-118374934. Max. coverage (+): 3.21. Max coverage (-): 0

Region: chr3 118374935-118374944. Max. coverage (+): 0. Max coverage (-): 0

Region: chr3 118374945-118374954. Max. coverage (+): 0. Max coverage (-): 0

Region: chr3 118374955-118374964. Max. coverage (+): 0. Max coverage (-): 0

Region: chr3 118374965-118374974. Max. coverage (+): 0. Max coverage (-): 0

Region: chr3 118374975-118374984. Max. coverage (+): 0. Max coverage (-): 0

Region: chr3 118374985-118374994. Max. coverage (+): 0. Max coverage (-): 0

Region: chr3 118374995-118375005. Max. coverage (+): 0. Max coverage (-): 0

Region: chr3 118375006-118375015. Max. coverage (+): 0. Max coverage (-): 0

Region: chr3 118375016-118375025. Max. coverage (+): 0. Max coverage (-): 0

Region: chr3 118375026-118375035. Max. coverage (+): 0. Max coverage (-): 0

Region: chr3 118375036-118375045. Max. coverage (+): 0. Max coverage (-): 0

Region: chr3 118375046-118375055. Max. coverage (+): 0. Max coverage (-): 0

Region: chr3 118375056-118375066. Max. coverage (+): 0. Max coverage (-): 0

Region: chr3 118375067-118375076. Max. coverage (+): 0. Max coverage (-): 0

Region: chr3 118375077-118375086. Max. coverage (+): 0. Max coverage (-): 0

Region: chr3 118375087-118375096. Max. coverage (+): 0. Max coverage (-): 0

Region: chr3 118375097-118375106. Max. coverage (+): 0. Max coverage (-): 0

Region: chr3 118375107-118375116. Max. coverage (+): 0. Max coverage (-): 0

Region: chr3 118375117-118375127. Max. coverage (+): 0. Max coverage (-): 0

Region: chr3 118375128-118375137. Max. coverage (+): 0. Max coverage (-): 0

Region: chr3 118375138-118375147. Max. coverage (+): 0. Max coverage (-): 0

Region: chr3 118375148-118375157. Max. coverage (+): 0. Max coverage (-): 0

Region: chr3 118375158-118375167. Max. coverage (+): 0. Max coverage (-): 0

Region: chr3 118375168-118375177. Max. coverage (+): 0. Max coverage (-): 0

Region: chr3 118375178-118375187. Max. coverage (+): 0. Max coverage (-): 0

Region: chr3 118375188-118375198. Max. coverage (+): 0. Max coverage (-): 0

Region: chr3 118375199-118375208. Max. coverage (+): 0. Max coverage (-): 0

Region: chr3 118375209-118375218. Max. coverage (+): 0. Max coverage (-): 0

Region: chr3 118375219-118375228. Max. coverage (+): 0. Max coverage (-): 0

Region: chr3 118375229-118375238. Max. coverage (+): 0. Max coverage (-): 0

Region: chr3 118375239-118375248. Max. coverage (+): 0. Max coverage (-): 0

Region: chr3 118375249-118375259. Max. coverage (+): 0. Max coverage (-): 0

Region: chr3 118375260-118375269. Max. coverage (+): 0. Max coverage (-): 0

Region: chr3 118375270-118375279. Max. coverage (+): 0. Max coverage (-): 0

Region: chr3 118375280-118375289. Max. coverage (+): 2.77. Max coverage (-): 0

Region: chr3 118375290-118375299. Max. coverage (+): 2.77. Max coverage (-): 0

Region: chr3 118375300-118375309. Max. coverage (+): 0. Max coverage (-): 0

Region: chr3 118375310-118375320. Max. coverage (+): 0. Max coverage (-): 0

Region: chr3 118375321-118375330. Max. coverage (+): 0. Max coverage (-): 0

Region: chr3 118375331-118375340. Max. coverage (+): 0. Max coverage (-): 0

Region: chr3 118375341-118375350. Max. coverage (+): 0. Max coverage (-): 0

Region: chr3 118375351-118375360. Max. coverage (+): 0. Max coverage (-): 0

Region: chr3 118375361-118375370. Max. coverage (+): 0. Max coverage (-): 0

Region: chr3 118375371-118375380. Max. coverage (+): 0. Max coverage (-): 0

Region: chr3 118375381-118375391. Max. coverage (+): 0. Max coverage (-): 0

Region: chr3 118375392-118375401. Max. coverage (+): 0. Max coverage (-): 0

Region: chr3 118375402-118375411. Max. coverage (+): 14.11. Max coverage (-): 0

Region: chr3 118375412-118375421. Max. coverage (+): 14.11. Max coverage (-): 0

Region: chr3 118375422-118375431. Max. coverage (+): 0. Max coverage (-): 0

Region: chr3 118375432-118375441. Max. coverage (+): 0. Max coverage (-): 0

Region: chr3 118375442-118375452. Max. coverage (+): 0. Max coverage (-): 0

Region: chr3 118375453-118375462. Max. coverage (+): 0. Max coverage (-): 0

Region: chr3 118375463-118375472. Max. coverage (+): 0. Max coverage (-): 0

Region: chr3 118375473-118375482. Max. coverage (+): 0. Max coverage (-): 0

Region: chr3 118375483-118375492. Max. coverage (+): 0. Max coverage (-): 0

Region: chr3 118375493-118375502. Max. coverage (+): 0. Max coverage (-): 0

Region: chr3 118375503-118375513. Max. coverage (+): 0. Max coverage (-): 0

Region: chr3 118375514-118375523. Max. coverage (+): 0. Max coverage (-): 0

Region: chr3 118375524-118375533. Max. coverage (+): 0. Max coverage (-): 0

Region: chr3 118375534-118375543. Max. coverage (+): 0. Max coverage (-): 0

Region: chr3 118375544-118375553. Max. coverage (+): 0. Max coverage (-): 0

Region: chr3 118375554-118375563. Max. coverage (+): 0. Max coverage (-): 0

Region: chr3 118375564-118375573. Max. coverage (+): 0. Max coverage (-): 0

Region: chr3 118375574-118375584. Max. coverage (+): 0. Max coverage (-): 0

Region: chr3 118375585-118375594. Max. coverage (+): 0. Max coverage (-): 0

Region: chr3 118375595-118375604. Max. coverage (+): 0. Max coverage (-): 0

Region: chr3 118375605-118375614. Max. coverage (+): 0. Max coverage (-): 0

Region: chr3 118375615-118375624. Max. coverage (+): 0. Max coverage (-): 0

Region: chr3 118375625-118375634. Max. coverage (+): 0. Max coverage (-): 0

Region: chr3 118375635-118375645. Max. coverage (+): 0. Max coverage (-): 0

Region: chr3 118375646-118375655. Max. coverage (+): 0. Max coverage (-): 0

Region: chr3 118375656-118375665. Max. coverage (+): 0. Max coverage (-): 0

Region: chr3 118375666-118375675. Max. coverage (+): 0. Max coverage (-): 0

Region: chr3 118375676-118375685. Max. coverage (+): 0. Max coverage (-): 0

Region: chr3 118375686-118375695. Max. coverage (+): 0. Max coverage (-): 0

Region: chr3 118375696-118375706. Max. coverage (+): 0. Max coverage (-): 0

Region: chr3 118375707-118375716. Max. coverage (+): 0. Max coverage (-): 0

Region: chr3 118375717-118375726. Max. coverage (+): 0. Max coverage (-): 0

Region: chr3 118375727-118375736. Max. coverage (+): 0. Max coverage (-): 0

Region: chr3 118375737-118375746. Max. coverage (+): 0. Max coverage (-): 0

Region: chr3 118375747-118375756. Max. coverage (+): 0. Max coverage (-): 0

Region: chr3 118375757-118375766. Max. coverage (+): 0. Max coverage (-): 0

Region: chr3 118375767-118375777. Max. coverage (+): 0. Max coverage (-): 0

Region: chr3 118375778-118375787. Max. coverage (+): 0. Max coverage (-): 0

Region: chr3 118375788-118375797. Max. coverage (+): 0. Max coverage (-): 0

Region: chr3 118375798-118375807. Max. coverage (+): 0. Max coverage (-): 0

Region: chr3 118375808-118375817. Max. coverage (+): 0. Max coverage (-): 0

Region: chr3 118375818-118375827. Max. coverage (+): 0. Max coverage (-): 0

Region: chr3 118375828-118375838. Max. coverage (+): 0. Max coverage (-): 0

Region: chr3 118375839-118375848. Max. coverage (+): 0. Max coverage (-): 0

Region: chr3 118375849-118375858. Max. coverage (+): 0. Max coverage (-): 0

Region: chr3 118375859-118375868. Max. coverage (+): 0. Max coverage (-): 0

Region: chr3 118375869-118375878. Max. coverage (+): 0. Max coverage (-): 0

Region: chr3 118375879-118375888. Max. coverage (+): 0. Max coverage (-): 0

Region: chr3 118375889-118375899. Max. coverage (+): 0. Max coverage (-): 0

Region: chr3 118375900-118375909. Max. coverage (+): 0. Max coverage (-): 0

Region: chr3 118375910-118375919. Max. coverage (+): 0. Max coverage (-): 0

Region: chr3 118375920-118375929. Max. coverage (+): 0. Max coverage (-): 0

Region: chr3 118375930-118375939. Max. coverage (+): 0. Max coverage (-): 0

Region: chr3 118375940-118375949. Max. coverage (+): 0. Max coverage (-): 0

Region: chr3 118375950-118375959. Max. coverage (+): 0. Max coverage (-): 0

Region: chr3 118375960-118375970. Max. coverage (+): 0. Max coverage (-): 0

Region: chr3 118375971-118375980. Max. coverage (+): 0. Max coverage (-): 0

Region: chr3 118375981-118375990. Max. coverage (+): 0. Max coverage (-): 0

Region: chr3 118375991-118376000. Max. coverage (+): 0. Max coverage (-): 0

Region: chr3 118376001-118376010. Max. coverage (+): 0. Max coverage (-): 0

Region: chr3 118376011-118376020. Max. coverage (+): 0. Max coverage (-): 0

Region: chr3 118376021-118376031. Max. coverage (+): 0. Max coverage (-): 0

Region: chr3 118376032-118376041. Max. coverage (+): 0. Max coverage (-): 0

Region: chr3 118376042-118376051. Max. coverage (+): 0. Max coverage (-): 0

Region: chr3 118376052-118376061. Max. coverage (+): 0. Max coverage (-): 0

Region: chr3 118376062-118376071. Max. coverage (+): 0. Max coverage (-): 0

Region: chr3 118376072-118376081. Max. coverage (+): 0. Max coverage (-): 0

Region: chr3 118376082-118376092. Max. coverage (+): 0. Max coverage (-): 0

Region: chr3 118376093-118376102. Max. coverage (+): 0. Max coverage (-): 0

Region: chr3 118376103-118376112. Max. coverage (+): 0. Max coverage (-): 0

Region: chr3 118376113-118376122. Max. coverage (+): 0. Max coverage (-): 0

Region: chr3 118376123-118376132. Max. coverage (+): 0. Max coverage (-): 0

Region: chr3 118376133-118376142. Max. coverage (+): 0. Max coverage (-): 0

Region: chr3 118376143-118376152. Max. coverage (+): 0. Max coverage (-): 0

Region: chr3 118376153-118376163. Max. coverage (+): 0. Max coverage (-): 0

Region: chr3 118376164-118376173. Max. coverage (+): 0. Max coverage (-): 0

Region: chr3 118376174-118376183. Max. coverage (+): 2.89. Max coverage (-): 0

Region: chr3 118376184-118376193. Max. coverage (+): 2.89. Max coverage (-): 0

Region: chr3 118376194-118376203. Max. coverage (+): 0. Max coverage (-): 0

Region: chr3 118376204-118376213. Max. coverage (+): 0. Max coverage (-): 0

Region: chr3 118376214-118376224. Max. coverage (+): 0. Max coverage (-): 0

Region: chr3 118376225-118376234. Max. coverage (+): 0. Max coverage (-): 0

Region: chr3 118376235-118376244. Max. coverage (+): 0. Max coverage (-): 0

Region: chr3 118376245-118376254. Max. coverage (+): 0. Max coverage (-): 0

Region: chr3 118376255-118376264. Max. coverage (+): 0. Max coverage (-): 0

Region: chr3 118376265-118376274. Max. coverage (+): 0. Max coverage (-): 0

Region: chr3 118376275-118376285. Max. coverage (+): 0. Max coverage (-): 0

Region: chr3 118376286-118376295. Max. coverage (+): 0. Max coverage (-): 0

Region: chr3 118376296-118376305. Max. coverage (+): 0. Max coverage (-): 0

Region: chr3 118376306-118376315. Max. coverage (+): 0. Max coverage (-): 0

Region: chr3 118376316-118376325. Max. coverage (+): 0. Max coverage (-): 0

Region: chr3 118376326-118376335. Max. coverage (+): 0. Max coverage (-): 0

Region: chr3 118376336-118376345. Max. coverage (+): 0. Max coverage (-): 0

Region: chr3 118376346-118376356. Max. coverage (+): 0. Max coverage (-): 0

Region: chr3 118376357-118376366. Max. coverage (+): 0. Max coverage (-): 0

Region: chr3 118376367-118376376. Max. coverage (+): 0. Max coverage (-): 0

Region: chr3 118376377-118376386. Max. coverage (+): 0. Max coverage (-): 0

Region: chr3 118376387-118376396. Max. coverage (+): 0. Max coverage (-): 0

Region: chr3 118376397-118376406. Max. coverage (+): 0. Max coverage (-): 0

Region: chr3 118376407-118376417. Max. coverage (+): 0. Max coverage (-): 0

Region: chr3 118376418-118376427. Max. coverage (+): 0. Max coverage (-): 0

Region: chr3 118376428-118376437. Max. coverage (+): 0. Max coverage (-): 0

Region: chr3 118376438-118376447. Max. coverage (+): 0. Max coverage (-): 0

Region: chr3 118376448-118376457. Max. coverage (+): 0. Max coverage (-): 0

Region: chr3 118376458-118376467. Max. coverage (+): 0. Max coverage (-): 0

Region: chr3 118376468-118376478. Max. coverage (+): 0. Max coverage (-): 0

Region: chr3 118376479-118376488. Max. coverage (+): 0. Max coverage (-): 0

Region: chr3 118376489-118376498. Max. coverage (+): 0. Max coverage (-): 0

Region: chr3 118376499-118376508. Max. coverage (+): 0. Max coverage (-): 0

Region: chr3 118376509-118376518. Max. coverage (+): 0. Max coverage (-): 0

Region: chr3 118376519-118376528. Max. coverage (+): 0. Max coverage (-): 0

Region: chr3 118376529-118376538. Max. coverage (+): 0. Max coverage (-): 0

Region: chr3 118376539-118376549. Max. coverage (+): 0. Max coverage (-): 0

Region: chr3 118376550-118376559. Max. coverage (+): 0. Max coverage (-): 0

Region: chr3 118376560-118376569. Max. coverage (+): 0. Max coverage (-): 0

Region: chr3 118376570-118376579. Max. coverage (+): 0. Max coverage (-): 0

Region: chr3 118376580-118376589. Max. coverage (+): 0. Max coverage (-): 0

Region: chr3 118376590-118376599. Max. coverage (+): 0. Max coverage (-): 0

Region: chr3 118376600-118376610. Max. coverage (+): 0. Max coverage (-): 0

Region: chr3 118376611-118376620. Max. coverage (+): 0. Max coverage (-): 0

Region: chr3 118376621-118376630. Max. coverage (+): 0. Max coverage (-): 0

Region: chr3 118376631-118376640. Max. coverage (+): 0. Max coverage (-): 0

Region: chr3 118376641-118376650. Max. coverage (+): 0. Max coverage (-): 0

Region: chr3 118376651-118376660. Max. coverage (+): 0. Max coverage (-): 0

Region: chr3 118376661-118376671. Max. coverage (+): 0. Max coverage (-): 0

Region: chr3 118376672-118376681. Max. coverage (+): 0. Max coverage (-): 0

Region: chr3 118376682-118376691. Max. coverage (+): 0. Max coverage (-): 0

Region: chr3 118376692-118376701. Max. coverage (+): 0. Max coverage (-): 0

Region: chr3 118376702-118376711. Max. coverage (+): 5.2. Max coverage (-): 0

Region: chr3 118376712-118376721. Max. coverage (+): 0. Max coverage (-): 0

Region: chr3 118376722-118376731. Max. coverage (+): 0. Max coverage (-): 0

Region: chr3 118376732-118376742. Max. coverage (+): 0. Max coverage (-): 0

Region: chr3 118376743-118376752. Max. coverage (+): 0. Max coverage (-): 0

Region: chr3 118376753-118376762. Max. coverage (+): 0. Max coverage (-): 0

Region: chr3 118376763-118376772. Max. coverage (+): 0. Max coverage (-): 0

Region: chr3 118376773-118376782. Max. coverage (+): 0. Max coverage (-): 0

Region: chr3 118376783-118376792. Max. coverage (+): 0. Max coverage (-): 0

Region: chr3 118376793-118376803. Max. coverage (+): 0. Max coverage (-): 0

Region: chr3 118376804-118376813. Max. coverage (+): 0. Max coverage (-): 0

Region: chr3 118376814-118376823. Max. coverage (+): 0. Max coverage (-): 0

Region: chr3 118376824-118376833. Max. coverage (+): 0. Max coverage (-): 0

Region: chr3 118376834-118376843. Max. coverage (+): 0. Max coverage (-): 0

Region: chr3 118376844-118376853. Max. coverage (+): 0. Max coverage (-): 0

Region: chr3 118376854-118376864. Max. coverage (+): 0. Max coverage (-): 0

Region: chr3 118376865-118376874. Max. coverage (+): 0. Max coverage (-): 0

Region: chr3 118376875-118376884. Max. coverage (+): 0. Max coverage (-): 0

Region: chr3 118376885-118376894. Max. coverage (+): 0. Max coverage (-): 0

Region: chr3 118376895-118376904. Max. coverage (+): 0. Max coverage (-): 0

Region: chr3 118376905-118376914. Max. coverage (+): 0. Max coverage (-): 0

Region: chr3 118376915-118376924. Max. coverage (+): 0. Max coverage (-): 0

Region: chr3 118376925-118376935. Max. coverage (+): 0. Max coverage (-): 0

Region: chr3 118376936-118376945. Max. coverage (+): 0. Max coverage (-): 0

Region: chr3 118376946-118376955. Max. coverage (+): 0. Max coverage (-): 0

Region: chr3 118376956-118376965. Max. coverage (+): 0. Max coverage (-): 0

Region: chr3 118376966-118376975. Max. coverage (+): 0. Max coverage (-): 0

Region: chr3 118376976-118376985. Max. coverage (+): 0. Max coverage (-): 0

Region: chr3 118376986-118376996. Max. coverage (+): 0. Max coverage (-): 0

Region: chr3 118376997-118377006. Max. coverage (+): 0. Max coverage (-): 0

Region: chr3 118377007-118377016. Max. coverage (+): 0. Max coverage (-): 0

Region: chr3 118377017-118377026. Max. coverage (+): 0. Max coverage (-): 0

Region: chr3 118377027-118377036. Max. coverage (+): 0. Max coverage (-): 0

Region: chr3 118377037-118377046. Max. coverage (+): 0. Max coverage (-): 0

Region: chr3 118377047-118377057. Max. coverage (+): 0. Max coverage (-): 0

Region: chr3 118377058-118377067. Max. coverage (+): 0. Max coverage (-): 0

Region: chr3 118377068-118377077. Max. coverage (+): 0. Max coverage (-): 0

Region: chr3 118377078-118377087. Max. coverage (+): 0. Max coverage (-): 0

Region: chr3 118377088-118377097. Max. coverage (+): 7.08. Max coverage (-): 0

Region: chr3 118377098-118377107. Max. coverage (+): 7.08. Max coverage (-): 0

Region: chr3 118377108-118377117. Max. coverage (+): 0. Max coverage (-): 0

Region: chr3 118377118-118377128. Max. coverage (+): 0. Max coverage (-): 0

Region: chr3 118377129-118377138. Max. coverage (+): 0. Max coverage (-): 0

Region: chr3 118377139-118377148. Max. coverage (+): 0. Max coverage (-): 0

Region: chr3 118377149-118377158. Max. coverage (+): 0. Max coverage (-): 0

Region: chr3 118377159-118377168. Max. coverage (+): 0. Max coverage (-): 0

Region: chr3 118377169-118377178. Max. coverage (+): 0. Max coverage (-): 0

Region: chr3 118377179-118377189. Max. coverage (+): 0. Max coverage (-): 0

Region: chr3 118377190-118377199. Max. coverage (+): 0. Max coverage (-): 0

Region: chr3 118377200-118377209. Max. coverage (+): 0. Max coverage (-): 0

Region: chr3 118377210-118377219. Max. coverage (+): 0. Max coverage (-): 0

Region: chr3 118377220-118377229. Max. coverage (+): 0. Max coverage (-): 0

Region: chr3 118377230-118377239. Max. coverage (+): 0. Max coverage (-): 0

Region: chr3 118377240-118377250. Max. coverage (+): 0. Max coverage (-): 0

Region: chr3 118377251-118377260. Max. coverage (+): 0. Max coverage (-): 0

Region: chr3 118377261-118377270. Max. coverage (+): 0. Max coverage (-): 0

Region: chr3 118377271-118377280. Max. coverage (+): 0. Max coverage (-): 0

Region: chr3 118377281-118377290. Max. coverage (+): 0. Max coverage (-): 0

Region: chr3 118377291-118377300. Max. coverage (+): 0. Max coverage (-): 0

Region: chr3 118377301-118377310. Max. coverage (+): 0. Max coverage (-): 0

Region: chr3 118377311-118377321. Max. coverage (+): 0. Max coverage (-): 0

Region: chr3 118377322-118377331. Max. coverage (+): 0. Max coverage (-): 0

Region: chr3 118377332-118377341. Max. coverage (+): 0. Max coverage (-): 0

Region: chr3 118377342-118377351. Max. coverage (+): 4.43. Max coverage (-): 0

Region: chr3 118377352-118377361. Max. coverage (+): 4.43. Max coverage (-): 0

Region: chr3 118377362-118377371. Max. coverage (+): 0. Max coverage (-): 0

Region: chr3 118377372-. Max. coverage (+): 0. Max coverage (-): 0

RepeatMasker Color Code

**+**

100-98% Identity

<98-95% Identity

<95-90% Identity

<90-85% Identity

<85-80% Identity

<80-75% Identity

<75-70% Identity

<70% Identity

**-**

Gene Set Color Code

**+**

Gene

Pseudogene

**-**

Topology/Coverage Color Code

Coverage Plus Strand

Coverage Minus Strand

Mainstrand: Plus

Mainstrand: Minus

Complementary Strand

Flanking Region  
(if option -flank >0)

Gene Set Annotation  

**1. ASB1 (protein coding, ENSBTAG00000003376) Tr:00000004378 Ex:5**: 118372187-118372314 (+)

  
RepeatMasker Annotation  

**1. (CA)n**: 118372806-118372833 (+), Divergence to consensus: 10.7%  
**2. L4\_A\_Mam**: 118375357-118375400 (-), Divergence to consensus: 13.9%  
**3. MIRc**: 118375701-118375763 (+), Divergence to consensus: 30.2%  
**4. Charlie18a**: 118375973-118376166 (+), Divergence to consensus: 26.2%

  
Transcription Factor Binding Sites  

**RFX4\_2** (Sequence: GTATCCAGG (-): 118373476)
