# Supplementary material for: Small RNA sequencing of cryopreserved semen from single bull revealed altered miRNAs and piRNAs expression between High- and Low-motile sperm populations
Source: BMC Genomics. 2017 Jan 4;18:14. doi: 10.1186/s12864-016-3394-7 (PMC5209821; doi:10.1186/s12864-016-3394-7)
Supplement: Additional file 4: — Details for each piRNA clusters found in Low Motile (LM) sperm fraction. Genes, repeats, transposable elements and transcription factors binding sites falling within the cluster regions were reported. (ZIP 1034 kb) [file 12864_2016_3394_MOESM4_ESM.zip › 43.html]

piRNA cluster 43


Predicted piRNA cluster no. 43     previous   next
  

Show proTRAC run info
Hide proTRAC run info

================================= proTRAC ====================================  
VERSION: 2.1                                    LAST MODIFIED: 06. October 2015  
  
Please cite:  
Rosenkranz D, Zischler H. proTRAC - a software for probabilistic piRNA cluster  
detection, visualization and analysis. 2012. BMC Bioinformatics 13:5.  
  
and (for proTRAC 2.0 and later):  
Rosenkranz D, Rudloff S, Bastuck K, Ketting RF, Zischler H. Tupaia small RNAs  
provide insights into function and evolution of RNAi-based transposon defense  
in mammals. 2015. RNA 21(5):911-922.  
  
Contact:  
David Rosenkranz  
Institute of Anthropology, small RNA group  
Johannes Gutenberg University Mainz  
email: rosenkranz@uni-mainz.de  
  
You can find the latest proTRAC version at:  
http://sourceforge.net/projects/protrac/files  
http://www.smallRNAgroup-mainz.de/software  
==============================================================================  
  
PARAMETERS:  
Map file: .............../storage/core/barbara/genhome/smallRNA/fertility/Sample\_not\_motile/pirna/Sample\_not\_motile\_26-33\_collapsed.fa.no-dust.map.weighted-10000-1000-b-0  
Genome file: ............/storage/core/barbara/genhome/smallRNA/fertility/Sample\_all/pirna/bt\_311\_chrY.fa  
RepeatMasker annotation: /storage/genomes/bt\_umd31/GCF\_000003055.6\_Bos\_taurus\_UMD\_3.1.1\_repeatMasker\_chr.out  
GeneSet:................./storage/core/barbara/genhome/smallRNA/fertility/Sample\_all/pirna/full.gtf  
  
Significant (p<=0.01) hit density will be calculated based  
on observed hit distribution.  
  
Sliding window size: ........................................ 5000 bp  
Sliding window increament: .................................. 1000 bp  
Normalize each hit by number of genomic hits: ............... 1 [0=no/1=yes]  
Normalize each hit by number of sequence reads: ............. 1 [0=no/1=yes]  
Normalize values (-> per million mapped reads): ............. 1 [0=no/1=yes]  
Min. fraction of hits with 1T(U) or 10A: .................... 0.75  
Alternatively: Min. fraction of hits with 1T(U) and 10A: .... 0.5  
Min. fraction of hits with typical piRNA length: ............ 0.75  
Typical piRNA length: ....................................... 26-33 nt  
Min. size of a piRNA cluster: ............................... 5000 bp.  
Min. number of hits (absolute): ............................. 0  
Min. number of hits (normalized): ........................... 0  
Min. fraction of hits on the mainstrand: .................... 0.75  
Top fraction of mapped sequences (in terms of read counts): . 1%  
Top fraction accounts for max. n% of sequence reads: ........ 90%  
Min. fraction of hits on each arm of a bidirectional cluster: 0.1  
Output image file for each cluster: ......................... 0 [0=no/1=yes]  
Output html file for each cluster: .......................... 1 [0=no/1=yes]  
Output a summary table: ..................................... 1 [0=no/1=yes]  
Output a FASTA file for each cluster (piRNA sequences): ..... 1 [0=no/1=yes]  
Output a FASTA file comprising cluster sequences: ........... 1 [0=no/1=yes]  
Search DNA motifs in clusters: .............................. 1 [0=no/1=yes]  
Output flanking sequences: +/- .............................. 0 bp  
Output ~.pTi file: .......................................... 1 [0=no/1=yes]  
==============================================================================  
  
  
Genome size (without gaps): ............ 2678902517 bp  
Gaps (N/X/-): .......................... 53837044 bp  
Mapped reads: .......................... 738059667487  
Non-identical sequences: ............... 277001  
Genomic hits: .......................... 533816  
Significant densitiy of mapped reads: .. 15118061 reads/kb

Show proTRAC cluster info
Hide proTRAC cluster info

|  |  |
| --- | --- |
| Location | chr5 |
| Coordinates | 56763981-56770839 |
| Size [bp] | 6859 |
| Sequence hit loci | 107 |
| Mapped reads (normalized) | 301846453 |
| Mapped reads (normalized) per kb | 44007355.7 |
| Normalized reads with 1T (1U) | 89.9% |
| Normalized reads with 10A | 36.9% |
| Normalized reads with length 26-33 nt | 100% |
| Normalized reads on the main strand(s) | 99% |
| Predicted directionality | mono:plus |

100%

0%

1T (1U)  
reads

10A reads

26-33 nt  
reads

reads on mainstrand

**Either the amount of reads with 1T (1U) OR 10A has to exceed 75% (set with option: -1Tor10A)  
Alternatively the amount of reads with 1T (1U) AND 10A has to exceed 50% (set with option: -1Tand10A)  
Minimum amount of reads with preferred size is 75% (set with option: -pisize)  
Minimum amount of reads on the main strand(s) is 75% (set with option: -clstrand)**

Show read coverage
Hide read coverage

WHAT DO I SEE HERE?  
This chart shows the location of mapped sequence reads within a predicted piRNA cluster. The color refers to the number of genomic hits produced by the sequence read in question. A dark red bar indicates that this sequence read produces many other hits elsewhere in the genome. Many adjacent red or yellow bars can indicate the presence of a multi-copy element such as transposons or rRNA genes. A dark green bar indicates that this sequence read maps uniquely to this locus.

1 hit

2-5 hits

6-10 hits

11-20 hits

21-50 hits

51-100 hits

> 100 hits

chr5

56763981

56770839

Gene Set

RepeatMasker

Mapped  
Reads

38.53

plus strand

minus strand

38.53

Region: chr5 118377373-56763987. Max. coverage (+): 5.33. Max coverage (-): 0

Region: chr5 56763988-56764001. Max. coverage (+): 5.33. Max coverage (-): 0

Region: chr5 56764002-56764015. Max. coverage (+): 0. Max coverage (-): 0

Region: chr5 56764016-56764029. Max. coverage (+): 0. Max coverage (-): 0

Region: chr5 56764030-56764042. Max. coverage (+): 0. Max coverage (-): 0

Region: chr5 56764043-56764056. Max. coverage (+): 0. Max coverage (-): 0

Region: chr5 56764057-56764070. Max. coverage (+): 0. Max coverage (-): 0

Region: chr5 56764071-56764083. Max. coverage (+): 0. Max coverage (-): 0

Region: chr5 56764084-56764097. Max. coverage (+): 0. Max coverage (-): 0

Region: chr5 56764098-56764111. Max. coverage (+): 0. Max coverage (-): 0

Region: chr5 56764112-56764125. Max. coverage (+): 0. Max coverage (-): 0

Region: chr5 56764126-56764138. Max. coverage (+): 0. Max coverage (-): 0

Region: chr5 56764139-56764152. Max. coverage (+): 0. Max coverage (-): 0

Region: chr5 56764153-56764166. Max. coverage (+): 0. Max coverage (-): 0

Region: chr5 56764167-56764179. Max. coverage (+): 0. Max coverage (-): 0

Region: chr5 56764180-56764193. Max. coverage (+): 0. Max coverage (-): 0

Region: chr5 56764194-56764207. Max. coverage (+): 0. Max coverage (-): 0

Region: chr5 56764208-56764221. Max. coverage (+): 0. Max coverage (-): 0

Region: chr5 56764222-56764234. Max. coverage (+): 0. Max coverage (-): 0

Region: chr5 56764235-56764248. Max. coverage (+): 0. Max coverage (-): 0

Region: chr5 56764249-56764262. Max. coverage (+): 0. Max coverage (-): 0

Region: chr5 56764263-56764275. Max. coverage (+): 0. Max coverage (-): 0

Region: chr5 56764276-56764289. Max. coverage (+): 0. Max coverage (-): 0

Region: chr5 56764290-56764303. Max. coverage (+): 0. Max coverage (-): 0

Region: chr5 56764304-56764317. Max. coverage (+): 0. Max coverage (-): 0

Region: chr5 56764318-56764330. Max. coverage (+): 0. Max coverage (-): 0

Region: chr5 56764331-56764344. Max. coverage (+): 0. Max coverage (-): 0

Region: chr5 56764345-56764358. Max. coverage (+): 0. Max coverage (-): 0

Region: chr5 56764359-56764371. Max. coverage (+): 0. Max coverage (-): 0

Region: chr5 56764372-56764385. Max. coverage (+): 0. Max coverage (-): 0

Region: chr5 56764386-56764399. Max. coverage (+): 0. Max coverage (-): 0

Region: chr5 56764400-56764413. Max. coverage (+): 0. Max coverage (-): 0

Region: chr5 56764414-56764426. Max. coverage (+): 0. Max coverage (-): 0

Region: chr5 56764427-56764440. Max. coverage (+): 0. Max coverage (-): 0

Region: chr5 56764441-56764454. Max. coverage (+): 0. Max coverage (-): 0

Region: chr5 56764455-56764467. Max. coverage (+): 0. Max coverage (-): 0

Region: chr5 56764468-56764481. Max. coverage (+): 0. Max coverage (-): 0

Region: chr5 56764482-56764495. Max. coverage (+): 0. Max coverage (-): 0

Region: chr5 56764496-56764509. Max. coverage (+): 3.57. Max coverage (-): 0

Region: chr5 56764510-56764522. Max. coverage (+): 3.57. Max coverage (-): 0

Region: chr5 56764523-56764536. Max. coverage (+): 0. Max coverage (-): 0

Region: chr5 56764537-56764550. Max. coverage (+): 0. Max coverage (-): 0

Region: chr5 56764551-56764564. Max. coverage (+): 0. Max coverage (-): 0

Region: chr5 56764565-56764577. Max. coverage (+): 0. Max coverage (-): 0

Region: chr5 56764578-56764591. Max. coverage (+): 0. Max coverage (-): 0

Region: chr5 56764592-56764605. Max. coverage (+): 0. Max coverage (-): 0

Region: chr5 56764606-56764618. Max. coverage (+): 0. Max coverage (-): 0

Region: chr5 56764619-56764632. Max. coverage (+): 0. Max coverage (-): 0

Region: chr5 56764633-56764646. Max. coverage (+): 0. Max coverage (-): 0

Region: chr5 56764647-56764660. Max. coverage (+): 0. Max coverage (-): 0

Region: chr5 56764661-56764673. Max. coverage (+): 0. Max coverage (-): 0

Region: chr5 56764674-56764687. Max. coverage (+): 0. Max coverage (-): 0

Region: chr5 56764688-56764701. Max. coverage (+): 0. Max coverage (-): 0

Region: chr5 56764702-56764714. Max. coverage (+): 0. Max coverage (-): 0

Region: chr5 56764715-56764728. Max. coverage (+): 0. Max coverage (-): 0

Region: chr5 56764729-56764742. Max. coverage (+): 0. Max coverage (-): 0

Region: chr5 56764743-56764756. Max. coverage (+): 0. Max coverage (-): 0

Region: chr5 56764757-56764769. Max. coverage (+): 0. Max coverage (-): 0

Region: chr5 56764770-56764783. Max. coverage (+): 0. Max coverage (-): 0

Region: chr5 56764784-56764797. Max. coverage (+): 0. Max coverage (-): 0

Region: chr5 56764798-56764810. Max. coverage (+): 0. Max coverage (-): 0

Region: chr5 56764811-56764824. Max. coverage (+): 0. Max coverage (-): 0

Region: chr5 56764825-56764838. Max. coverage (+): 0. Max coverage (-): 0

Region: chr5 56764839-56764852. Max. coverage (+): 0. Max coverage (-): 0

Region: chr5 56764853-56764865. Max. coverage (+): 0. Max coverage (-): 0

Region: chr5 56764866-56764879. Max. coverage (+): 0. Max coverage (-): 0

Region: chr5 56764880-56764893. Max. coverage (+): 4.35. Max coverage (-): 0

Region: chr5 56764894-56764906. Max. coverage (+): 0. Max coverage (-): 0

Region: chr5 56764907-56764920. Max. coverage (+): 0. Max coverage (-): 0

Region: chr5 56764921-56764934. Max. coverage (+): 0. Max coverage (-): 0

Region: chr5 56764935-56764948. Max. coverage (+): 0. Max coverage (-): 0

Region: chr5 56764949-56764961. Max. coverage (+): 0. Max coverage (-): 0

Region: chr5 56764962-56764975. Max. coverage (+): 0. Max coverage (-): 0

Region: chr5 56764976-56764989. Max. coverage (+): 0. Max coverage (-): 0

Region: chr5 56764990-56765002. Max. coverage (+): 0. Max coverage (-): 0

Region: chr5 56765003-56765016. Max. coverage (+): 0. Max coverage (-): 0

Region: chr5 56765017-56765030. Max. coverage (+): 0. Max coverage (-): 0

Region: chr5 56765031-56765044. Max. coverage (+): 0. Max coverage (-): 0

Region: chr5 56765045-56765057. Max. coverage (+): 0. Max coverage (-): 0

Region: chr5 56765058-56765071. Max. coverage (+): 0. Max coverage (-): 0

Region: chr5 56765072-56765085. Max. coverage (+): 0. Max coverage (-): 0

Region: chr5 56765086-56765099. Max. coverage (+): 0. Max coverage (-): 0

Region: chr5 56765100-56765112. Max. coverage (+): 0. Max coverage (-): 0

Region: chr5 56765113-56765126. Max. coverage (+): 0. Max coverage (-): 0

Region: chr5 56765127-56765140. Max. coverage (+): 0. Max coverage (-): 0

Region: chr5 56765141-56765153. Max. coverage (+): 0. Max coverage (-): 0

Region: chr5 56765154-56765167. Max. coverage (+): 0. Max coverage (-): 0

Region: chr5 56765168-56765181. Max. coverage (+): 0. Max coverage (-): 0

Region: chr5 56765182-56765195. Max. coverage (+): 0. Max coverage (-): 0

Region: chr5 56765196-56765208. Max. coverage (+): 0. Max coverage (-): 0

Region: chr5 56765209-56765222. Max. coverage (+): 0. Max coverage (-): 0

Region: chr5 56765223-56765236. Max. coverage (+): 0. Max coverage (-): 0

Region: chr5 56765237-56765249. Max. coverage (+): 0. Max coverage (-): 0

Region: chr5 56765250-56765263. Max. coverage (+): 0. Max coverage (-): 0

Region: chr5 56765264-56765277. Max. coverage (+): 0. Max coverage (-): 0

Region: chr5 56765278-56765291. Max. coverage (+): 0. Max coverage (-): 0

Region: chr5 56765292-56765304. Max. coverage (+): 0. Max coverage (-): 0

Region: chr5 56765305-56765318. Max. coverage (+): 0. Max coverage (-): 0

Region: chr5 56765319-56765332. Max. coverage (+): 0. Max coverage (-): 0

Region: chr5 56765333-56765345. Max. coverage (+): 0. Max coverage (-): 0

Region: chr5 56765346-56765359. Max. coverage (+): 0. Max coverage (-): 0

Region: chr5 56765360-56765373. Max. coverage (+): 0. Max coverage (-): 0

Region: chr5 56765374-56765387. Max. coverage (+): 0. Max coverage (-): 0

Region: chr5 56765388-56765400. Max. coverage (+): 0. Max coverage (-): 0

Region: chr5 56765401-56765414. Max. coverage (+): 0. Max coverage (-): 0

Region: chr5 56765415-56765428. Max. coverage (+): 2.36. Max coverage (-): 0

Region: chr5 56765429-56765441. Max. coverage (+): 0. Max coverage (-): 0

Region: chr5 56765442-56765455. Max. coverage (+): 0. Max coverage (-): 0

Region: chr5 56765456-56765469. Max. coverage (+): 0. Max coverage (-): 0

Region: chr5 56765470-56765483. Max. coverage (+): 0. Max coverage (-): 0

Region: chr5 56765484-56765496. Max. coverage (+): 0. Max coverage (-): 0

Region: chr5 56765497-56765510. Max. coverage (+): 0. Max coverage (-): 0

Region: chr5 56765511-56765524. Max. coverage (+): 0. Max coverage (-): 0

Region: chr5 56765525-56765537. Max. coverage (+): 0. Max coverage (-): 0

Region: chr5 56765538-56765551. Max. coverage (+): 0. Max coverage (-): 0

Region: chr5 56765552-56765565. Max. coverage (+): 0. Max coverage (-): 0

Region: chr5 56765566-56765579. Max. coverage (+): 0. Max coverage (-): 0

Region: chr5 56765580-56765592. Max. coverage (+): 0. Max coverage (-): 0

Region: chr5 56765593-56765606. Max. coverage (+): 0. Max coverage (-): 0

Region: chr5 56765607-56765620. Max. coverage (+): 0. Max coverage (-): 0

Region: chr5 56765621-56765634. Max. coverage (+): 0. Max coverage (-): 0

Region: chr5 56765635-56765647. Max. coverage (+): 0. Max coverage (-): 0

Region: chr5 56765648-56765661. Max. coverage (+): 0. Max coverage (-): 0

Region: chr5 56765662-56765675. Max. coverage (+): 0. Max coverage (-): 0

Region: chr5 56765676-56765688. Max. coverage (+): 0. Max coverage (-): 0

Region: chr5 56765689-56765702. Max. coverage (+): 0. Max coverage (-): 0

Region: chr5 56765703-56765716. Max. coverage (+): 2.35. Max coverage (-): 0

Region: chr5 56765717-56765730. Max. coverage (+): 2.35. Max coverage (-): 0

Region: chr5 56765731-56765743. Max. coverage (+): 0. Max coverage (-): 0

Region: chr5 56765744-56765757. Max. coverage (+): 0. Max coverage (-): 0

Region: chr5 56765758-56765771. Max. coverage (+): 0. Max coverage (-): 0

Region: chr5 56765772-56765784. Max. coverage (+): 0. Max coverage (-): 0

Region: chr5 56765785-56765798. Max. coverage (+): 0. Max coverage (-): 0

Region: chr5 56765799-56765812. Max. coverage (+): 0. Max coverage (-): 0

Region: chr5 56765813-56765826. Max. coverage (+): 0. Max coverage (-): 0

Region: chr5 56765827-56765839. Max. coverage (+): 0. Max coverage (-): 0

Region: chr5 56765840-56765853. Max. coverage (+): 0. Max coverage (-): 0

Region: chr5 56765854-56765867. Max. coverage (+): 11.88. Max coverage (-): 0

Region: chr5 56765868-56765880. Max. coverage (+): 0. Max coverage (-): 0

Region: chr5 56765881-56765894. Max. coverage (+): 0. Max coverage (-): 0

Region: chr5 56765895-56765908. Max. coverage (+): 0. Max coverage (-): 0

Region: chr5 56765909-56765922. Max. coverage (+): 0. Max coverage (-): 0

Region: chr5 56765923-56765935. Max. coverage (+): 0. Max coverage (-): 0

Region: chr5 56765936-56765949. Max. coverage (+): 0. Max coverage (-): 0

Region: chr5 56765950-56765963. Max. coverage (+): 0. Max coverage (-): 0

Region: chr5 56765964-56765976. Max. coverage (+): 0. Max coverage (-): 0

Region: chr5 56765977-56765990. Max. coverage (+): 0. Max coverage (-): 0

Region: chr5 56765991-56766004. Max. coverage (+): 1.84. Max coverage (-): 0

Region: chr5 56766005-56766018. Max. coverage (+): 0. Max coverage (-): 0

Region: chr5 56766019-56766031. Max. coverage (+): 0. Max coverage (-): 0

Region: chr5 56766032-56766045. Max. coverage (+): 0. Max coverage (-): 0

Region: chr5 56766046-56766059. Max. coverage (+): 0. Max coverage (-): 0

Region: chr5 56766060-56766072. Max. coverage (+): 0. Max coverage (-): 0

Region: chr5 56766073-56766086. Max. coverage (+): 0. Max coverage (-): 0

Region: chr5 56766087-56766100. Max. coverage (+): 0. Max coverage (-): 0

Region: chr5 56766101-56766114. Max. coverage (+): 0. Max coverage (-): 0

Region: chr5 56766115-56766127. Max. coverage (+): 0. Max coverage (-): 0

Region: chr5 56766128-56766141. Max. coverage (+): 0. Max coverage (-): 0

Region: chr5 56766142-56766155. Max. coverage (+): 4.3. Max coverage (-): 0

Region: chr5 56766156-56766169. Max. coverage (+): 4.3. Max coverage (-): 0

Region: chr5 56766170-56766182. Max. coverage (+): 0. Max coverage (-): 0

Region: chr5 56766183-56766196. Max. coverage (+): 0. Max coverage (-): 0

Region: chr5 56766197-56766210. Max. coverage (+): 0. Max coverage (-): 0

Region: chr5 56766211-56766223. Max. coverage (+): 0. Max coverage (-): 0

Region: chr5 56766224-56766237. Max. coverage (+): 0. Max coverage (-): 0

Region: chr5 56766238-56766251. Max. coverage (+): 0. Max coverage (-): 0

Region: chr5 56766252-56766265. Max. coverage (+): 0. Max coverage (-): 0

Region: chr5 56766266-56766278. Max. coverage (+): 0. Max coverage (-): 0

Region: chr5 56766279-56766292. Max. coverage (+): 0. Max coverage (-): 0

Region: chr5 56766293-56766306. Max. coverage (+): 3.14. Max coverage (-): 0

Region: chr5 56766307-56766319. Max. coverage (+): 0. Max coverage (-): 0

Region: chr5 56766320-56766333. Max. coverage (+): 0. Max coverage (-): 0

Region: chr5 56766334-56766347. Max. coverage (+): 0. Max coverage (-): 0

Region: chr5 56766348-56766361. Max. coverage (+): 0. Max coverage (-): 0

Region: chr5 56766362-56766374. Max. coverage (+): 0. Max coverage (-): 0

Region: chr5 56766375-56766388. Max. coverage (+): 0. Max coverage (-): 0

Region: chr5 56766389-56766402. Max. coverage (+): 0. Max coverage (-): 0

Region: chr5 56766403-56766415. Max. coverage (+): 0. Max coverage (-): 0

Region: chr5 56766416-56766429. Max. coverage (+): 0. Max coverage (-): 0

Region: chr5 56766430-56766443. Max. coverage (+): 0. Max coverage (-): 0

Region: chr5 56766444-56766457. Max. coverage (+): 0. Max coverage (-): 0

Region: chr5 56766458-56766470. Max. coverage (+): 0. Max coverage (-): 0

Region: chr5 56766471-56766484. Max. coverage (+): 0. Max coverage (-): 0

Region: chr5 56766485-56766498. Max. coverage (+): 0. Max coverage (-): 0

Region: chr5 56766499-56766511. Max. coverage (+): 0. Max coverage (-): 0

Region: chr5 56766512-56766525. Max. coverage (+): 0. Max coverage (-): 0

Region: chr5 56766526-56766539. Max. coverage (+): 0. Max coverage (-): 0

Region: chr5 56766540-56766553. Max. coverage (+): 5.94. Max coverage (-): 0

Region: chr5 56766554-56766566. Max. coverage (+): 0. Max coverage (-): 0

Region: chr5 56766567-56766580. Max. coverage (+): 0. Max coverage (-): 0

Region: chr5 56766581-56766594. Max. coverage (+): 0. Max coverage (-): 0

Region: chr5 56766595-56766607. Max. coverage (+): 0. Max coverage (-): 0

Region: chr5 56766608-56766621. Max. coverage (+): 0. Max coverage (-): 0

Region: chr5 56766622-56766635. Max. coverage (+): 0. Max coverage (-): 0

Region: chr5 56766636-56766649. Max. coverage (+): 0. Max coverage (-): 0

Region: chr5 56766650-56766662. Max. coverage (+): 0. Max coverage (-): 0

Region: chr5 56766663-56766676. Max. coverage (+): 1.7. Max coverage (-): 0

Region: chr5 56766677-56766690. Max. coverage (+): 8.72. Max coverage (-): 0

Region: chr5 56766691-56766704. Max. coverage (+): 0. Max coverage (-): 0

Region: chr5 56766705-56766717. Max. coverage (+): 1.72. Max coverage (-): 0

Region: chr5 56766718-56766731. Max. coverage (+): 1.72. Max coverage (-): 0

Region: chr5 56766732-56766745. Max. coverage (+): 0. Max coverage (-): 0

Region: chr5 56766746-56766758. Max. coverage (+): 0. Max coverage (-): 0

Region: chr5 56766759-56766772. Max. coverage (+): 0. Max coverage (-): 0

Region: chr5 56766773-56766786. Max. coverage (+): 7.14. Max coverage (-): 0

Region: chr5 56766787-56766800. Max. coverage (+): 0. Max coverage (-): 0

Region: chr5 56766801-56766813. Max. coverage (+): 0. Max coverage (-): 0

Region: chr5 56766814-56766827. Max. coverage (+): 0. Max coverage (-): 0

Region: chr5 56766828-56766841. Max. coverage (+): 0. Max coverage (-): 0

Region: chr5 56766842-56766854. Max. coverage (+): 0. Max coverage (-): 0

Region: chr5 56766855-56766868. Max. coverage (+): 0. Max coverage (-): 0

Region: chr5 56766869-56766882. Max. coverage (+): 0. Max coverage (-): 0

Region: chr5 56766883-56766896. Max. coverage (+): 0. Max coverage (-): 0

Region: chr5 56766897-56766909. Max. coverage (+): 0. Max coverage (-): 0

Region: chr5 56766910-56766923. Max. coverage (+): 0. Max coverage (-): 0

Region: chr5 56766924-56766937. Max. coverage (+): 14.42. Max coverage (-): 0

Region: chr5 56766938-56766950. Max. coverage (+): 6.13. Max coverage (-): 0

Region: chr5 56766951-56766964. Max. coverage (+): 0. Max coverage (-): 0

Region: chr5 56766965-56766978. Max. coverage (+): 0. Max coverage (-): 0

Region: chr5 56766979-56766992. Max. coverage (+): 0. Max coverage (-): 0

Region: chr5 56766993-56767005. Max. coverage (+): 0. Max coverage (-): 0

Region: chr5 56767006-56767019. Max. coverage (+): 0. Max coverage (-): 0

Region: chr5 56767020-56767033. Max. coverage (+): 0. Max coverage (-): 0

Region: chr5 56767034-56767046. Max. coverage (+): 0. Max coverage (-): 0

Region: chr5 56767047-56767060. Max. coverage (+): 0. Max coverage (-): 0

Region: chr5 56767061-56767074. Max. coverage (+): 0. Max coverage (-): 0

Region: chr5 56767075-56767088. Max. coverage (+): 0. Max coverage (-): 0

Region: chr5 56767089-56767101. Max. coverage (+): 0. Max coverage (-): 0

Region: chr5 56767102-56767115. Max. coverage (+): 0. Max coverage (-): 0

Region: chr5 56767116-56767129. Max. coverage (+): 0. Max coverage (-): 0

Region: chr5 56767130-56767142. Max. coverage (+): 0. Max coverage (-): 0

Region: chr5 56767143-56767156. Max. coverage (+): 11.49. Max coverage (-): 0

Region: chr5 56767157-56767170. Max. coverage (+): 11.49. Max coverage (-): 0

Region: chr5 56767171-56767184. Max. coverage (+): 0. Max coverage (-): 0

Region: chr5 56767185-56767197. Max. coverage (+): 0. Max coverage (-): 0

Region: chr5 56767198-56767211. Max. coverage (+): 0. Max coverage (-): 0

Region: chr5 56767212-56767225. Max. coverage (+): 8.09. Max coverage (-): 0

Region: chr5 56767226-56767239. Max. coverage (+): 0. Max coverage (-): 0

Region: chr5 56767240-56767252. Max. coverage (+): 0. Max coverage (-): 0

Region: chr5 56767253-56767266. Max. coverage (+): 0. Max coverage (-): 0

Region: chr5 56767267-56767280. Max. coverage (+): 0. Max coverage (-): 0

Region: chr5 56767281-56767293. Max. coverage (+): 0. Max coverage (-): 0

Region: chr5 56767294-56767307. Max. coverage (+): 0. Max coverage (-): 0

Region: chr5 56767308-56767321. Max. coverage (+): 0. Max coverage (-): 0

Region: chr5 56767322-56767335. Max. coverage (+): 0. Max coverage (-): 4.03

Region: chr5 56767336-56767348. Max. coverage (+): 0. Max coverage (-): 0

Region: chr5 56767349-56767362. Max. coverage (+): 0. Max coverage (-): 0

Region: chr5 56767363-56767376. Max. coverage (+): 0. Max coverage (-): 0

Region: chr5 56767377-56767389. Max. coverage (+): 0. Max coverage (-): 0

Region: chr5 56767390-56767403. Max. coverage (+): 0. Max coverage (-): 0

Region: chr5 56767404-56767417. Max. coverage (+): 0. Max coverage (-): 0

Region: chr5 56767418-56767431. Max. coverage (+): 0. Max coverage (-): 0

Region: chr5 56767432-56767444. Max. coverage (+): 0. Max coverage (-): 0

Region: chr5 56767445-56767458. Max. coverage (+): 0. Max coverage (-): 0

Region: chr5 56767459-56767472. Max. coverage (+): 0. Max coverage (-): 0

Region: chr5 56767473-56767485. Max. coverage (+): 0. Max coverage (-): 0

Region: chr5 56767486-56767499. Max. coverage (+): 0. Max coverage (-): 0

Region: chr5 56767500-56767513. Max. coverage (+): 0. Max coverage (-): 0

Region: chr5 56767514-56767527. Max. coverage (+): 3.37. Max coverage (-): 0

Region: chr5 56767528-56767540. Max. coverage (+): 3.37. Max coverage (-): 0

Region: chr5 56767541-56767554. Max. coverage (+): 0. Max coverage (-): 0

Region: chr5 56767555-56767568. Max. coverage (+): 0. Max coverage (-): 0

Region: chr5 56767569-56767581. Max. coverage (+): 5.68. Max coverage (-): 0

Region: chr5 56767582-56767595. Max. coverage (+): 8.27. Max coverage (-): 0

Region: chr5 56767596-56767609. Max. coverage (+): 0. Max coverage (-): 0

Region: chr5 56767610-56767623. Max. coverage (+): 0. Max coverage (-): 0

Region: chr5 56767624-56767636. Max. coverage (+): 0. Max coverage (-): 0

Region: chr5 56767637-56767650. Max. coverage (+): 0. Max coverage (-): 0

Region: chr5 56767651-56767664. Max. coverage (+): 0. Max coverage (-): 0

Region: chr5 56767665-56767678. Max. coverage (+): 0. Max coverage (-): 0

Region: chr5 56767679-56767691. Max. coverage (+): 0. Max coverage (-): 0

Region: chr5 56767692-56767705. Max. coverage (+): 0. Max coverage (-): 0

Region: chr5 56767706-56767719. Max. coverage (+): 0. Max coverage (-): 0

Region: chr5 56767720-56767732. Max. coverage (+): 0. Max coverage (-): 0

Region: chr5 56767733-56767746. Max. coverage (+): 0. Max coverage (-): 0

Region: chr5 56767747-56767760. Max. coverage (+): 0. Max coverage (-): 0

Region: chr5 56767761-56767774. Max. coverage (+): 0. Max coverage (-): 0

Region: chr5 56767775-56767787. Max. coverage (+): 0. Max coverage (-): 0

Region: chr5 56767788-56767801. Max. coverage (+): 0. Max coverage (-): 0

Region: chr5 56767802-56767815. Max. coverage (+): 2.23. Max coverage (-): 0

Region: chr5 56767816-56767828. Max. coverage (+): 2.23. Max coverage (-): 0

Region: chr5 56767829-56767842. Max. coverage (+): 0. Max coverage (-): 0

Region: chr5 56767843-56767856. Max. coverage (+): 0. Max coverage (-): 0

Region: chr5 56767857-56767870. Max. coverage (+): 12. Max coverage (-): 0

Region: chr5 56767871-56767883. Max. coverage (+): 12. Max coverage (-): 0

Region: chr5 56767884-56767897. Max. coverage (+): 9.4. Max coverage (-): 0

Region: chr5 56767898-56767911. Max. coverage (+): 9.4. Max coverage (-): 0

Region: chr5 56767912-56767924. Max. coverage (+): 0. Max coverage (-): 0

Region: chr5 56767925-56767938. Max. coverage (+): 0. Max coverage (-): 0

Region: chr5 56767939-56767952. Max. coverage (+): 0. Max coverage (-): 0

Region: chr5 56767953-56767966. Max. coverage (+): 5.11. Max coverage (-): 0

Region: chr5 56767967-56767979. Max. coverage (+): 5.11. Max coverage (-): 0

Region: chr5 56767980-56767993. Max. coverage (+): 0. Max coverage (-): 0

Region: chr5 56767994-56768007. Max. coverage (+): 0. Max coverage (-): 0

Region: chr5 56768008-56768020. Max. coverage (+): 7. Max coverage (-): 0

Region: chr5 56768021-56768034. Max. coverage (+): 7. Max coverage (-): 0

Region: chr5 56768035-56768048. Max. coverage (+): 0. Max coverage (-): 0

Region: chr5 56768049-56768062. Max. coverage (+): 0. Max coverage (-): 0

Region: chr5 56768063-56768075. Max. coverage (+): 8.83. Max coverage (-): 0

Region: chr5 56768076-56768089. Max. coverage (+): 30.84. Max coverage (-): 0

Region: chr5 56768090-56768103. Max. coverage (+): 6.06. Max coverage (-): 0

Region: chr5 56768104-56768116. Max. coverage (+): 2.19. Max coverage (-): 0

Region: chr5 56768117-56768130. Max. coverage (+): 0. Max coverage (-): 0

Region: chr5 56768131-56768144. Max. coverage (+): 0. Max coverage (-): 0

Region: chr5 56768145-56768158. Max. coverage (+): 0. Max coverage (-): 0

Region: chr5 56768159-56768171. Max. coverage (+): 0. Max coverage (-): 0

Region: chr5 56768172-56768185. Max. coverage (+): 0. Max coverage (-): 0

Region: chr5 56768186-56768199. Max. coverage (+): 0. Max coverage (-): 0

Region: chr5 56768200-56768213. Max. coverage (+): 0. Max coverage (-): 0

Region: chr5 56768214-56768226. Max. coverage (+): 0. Max coverage (-): 0

Region: chr5 56768227-56768240. Max. coverage (+): 4.88. Max coverage (-): 0

Region: chr5 56768241-56768254. Max. coverage (+): 5.94. Max coverage (-): 0

Region: chr5 56768255-56768267. Max. coverage (+): 0. Max coverage (-): 0

Region: chr5 56768268-56768281. Max. coverage (+): 0. Max coverage (-): 0

Region: chr5 56768282-56768295. Max. coverage (+): 5. Max coverage (-): 0

Region: chr5 56768296-56768309. Max. coverage (+): 3.08. Max coverage (-): 0

Region: chr5 56768310-56768322. Max. coverage (+): 0. Max coverage (-): 0

Region: chr5 56768323-56768336. Max. coverage (+): 0. Max coverage (-): 0

Region: chr5 56768337-56768350. Max. coverage (+): 0. Max coverage (-): 0

Region: chr5 56768351-56768363. Max. coverage (+): 0. Max coverage (-): 0

Region: chr5 56768364-56768377. Max. coverage (+): 0. Max coverage (-): 0

Region: chr5 56768378-56768391. Max. coverage (+): 12.33. Max coverage (-): 0

Region: chr5 56768392-56768405. Max. coverage (+): 0. Max coverage (-): 0

Region: chr5 56768406-56768418. Max. coverage (+): 0. Max coverage (-): 0

Region: chr5 56768419-56768432. Max. coverage (+): 4.51. Max coverage (-): 0

Region: chr5 56768433-56768446. Max. coverage (+): 16.55. Max coverage (-): 0

Region: chr5 56768447-56768459. Max. coverage (+): 16.55. Max coverage (-): 0

Region: chr5 56768460-56768473. Max. coverage (+): 0. Max coverage (-): 0

Region: chr5 56768474-56768487. Max. coverage (+): 12.24. Max coverage (-): 0

Region: chr5 56768488-56768501. Max. coverage (+): 0. Max coverage (-): 0

Region: chr5 56768502-56768514. Max. coverage (+): 0. Max coverage (-): 0

Region: chr5 56768515-56768528. Max. coverage (+): 0. Max coverage (-): 0

Region: chr5 56768529-56768542. Max. coverage (+): 0. Max coverage (-): 0

Region: chr5 56768543-56768555. Max. coverage (+): 0. Max coverage (-): 0

Region: chr5 56768556-56768569. Max. coverage (+): 6.33. Max coverage (-): 0

Region: chr5 56768570-56768583. Max. coverage (+): 6.33. Max coverage (-): 0

Region: chr5 56768584-56768597. Max. coverage (+): 0. Max coverage (-): 0

Region: chr5 56768598-56768610. Max. coverage (+): 0. Max coverage (-): 0

Region: chr5 56768611-56768624. Max. coverage (+): 0. Max coverage (-): 0

Region: chr5 56768625-56768638. Max. coverage (+): 0. Max coverage (-): 0

Region: chr5 56768639-56768651. Max. coverage (+): 4.18. Max coverage (-): 0

Region: chr5 56768652-56768665. Max. coverage (+): 4.18. Max coverage (-): 0

Region: chr5 56768666-56768679. Max. coverage (+): 2.9. Max coverage (-): 0

Region: chr5 56768680-56768693. Max. coverage (+): 0. Max coverage (-): 0

Region: chr5 56768694-56768706. Max. coverage (+): 0. Max coverage (-): 0

Region: chr5 56768707-56768720. Max. coverage (+): 0. Max coverage (-): 0

Region: chr5 56768721-56768734. Max. coverage (+): 0. Max coverage (-): 0

Region: chr5 56768735-56768748. Max. coverage (+): 0. Max coverage (-): 0

Region: chr5 56768749-56768761. Max. coverage (+): 0. Max coverage (-): 0

Region: chr5 56768762-56768775. Max. coverage (+): 0. Max coverage (-): 0

Region: chr5 56768776-56768789. Max. coverage (+): 0. Max coverage (-): 0

Region: chr5 56768790-56768802. Max. coverage (+): 0. Max coverage (-): 0

Region: chr5 56768803-56768816. Max. coverage (+): 0. Max coverage (-): 0

Region: chr5 56768817-56768830. Max. coverage (+): 0. Max coverage (-): 0

Region: chr5 56768831-56768844. Max. coverage (+): 0. Max coverage (-): 0

Region: chr5 56768845-56768857. Max. coverage (+): 0. Max coverage (-): 0

Region: chr5 56768858-56768871. Max. coverage (+): 22.47. Max coverage (-): 0

Region: chr5 56768872-56768885. Max. coverage (+): 0. Max coverage (-): 0

Region: chr5 56768886-56768898. Max. coverage (+): 1.72. Max coverage (-): 0

Region: chr5 56768899-56768912. Max. coverage (+): 1.72. Max coverage (-): 0

Region: chr5 56768913-56768926. Max. coverage (+): 1.95. Max coverage (-): 0

Region: chr5 56768927-56768940. Max. coverage (+): 3.23. Max coverage (-): 0

Region: chr5 56768941-56768953. Max. coverage (+): 6.25. Max coverage (-): 0

Region: chr5 56768954-56768967. Max. coverage (+): 0. Max coverage (-): 0

Region: chr5 56768968-56768981. Max. coverage (+): 0. Max coverage (-): 0

Region: chr5 56768982-56768994. Max. coverage (+): 13.57. Max coverage (-): 0

Region: chr5 56768995-56769008. Max. coverage (+): 19.65. Max coverage (-): 0

Region: chr5 56769009-56769022. Max. coverage (+): 38.53. Max coverage (-): 0

Region: chr5 56769023-56769036. Max. coverage (+): 4.45. Max coverage (-): 0

Region: chr5 56769037-56769049. Max. coverage (+): 0. Max coverage (-): 0

Region: chr5 56769050-56769063. Max. coverage (+): 0. Max coverage (-): 0

Region: chr5 56769064-56769077. Max. coverage (+): 0. Max coverage (-): 0

Region: chr5 56769078-56769090. Max. coverage (+): 0. Max coverage (-): 0

Region: chr5 56769091-56769104. Max. coverage (+): 0. Max coverage (-): 0

Region: chr5 56769105-56769118. Max. coverage (+): 0. Max coverage (-): 0

Region: chr5 56769119-56769132. Max. coverage (+): 0. Max coverage (-): 0

Region: chr5 56769133-56769145. Max. coverage (+): 0. Max coverage (-): 0

Region: chr5 56769146-56769159. Max. coverage (+): 13.93. Max coverage (-): 0

Region: chr5 56769160-56769173. Max. coverage (+): 0. Max coverage (-): 0

Region: chr5 56769174-56769186. Max. coverage (+): 0. Max coverage (-): 0

Region: chr5 56769187-56769200. Max. coverage (+): 0. Max coverage (-): 0

Region: chr5 56769201-56769214. Max. coverage (+): 7.15. Max coverage (-): 0

Region: chr5 56769215-56769228. Max. coverage (+): 7.15. Max coverage (-): 0

Region: chr5 56769229-56769241. Max. coverage (+): 0. Max coverage (-): 0

Region: chr5 56769242-56769255. Max. coverage (+): 0. Max coverage (-): 0

Region: chr5 56769256-56769269. Max. coverage (+): 0. Max coverage (-): 0

Region: chr5 56769270-56769283. Max. coverage (+): 0. Max coverage (-): 0

Region: chr5 56769284-56769296. Max. coverage (+): 0. Max coverage (-): 0

Region: chr5 56769297-56769310. Max. coverage (+): 0. Max coverage (-): 0

Region: chr5 56769311-56769324. Max. coverage (+): 0. Max coverage (-): 0

Region: chr5 56769325-56769337. Max. coverage (+): 0. Max coverage (-): 0

Region: chr5 56769338-56769351. Max. coverage (+): 0. Max coverage (-): 0

Region: chr5 56769352-56769365. Max. coverage (+): 0. Max coverage (-): 0

Region: chr5 56769366-56769379. Max. coverage (+): 14.29. Max coverage (-): 0

Region: chr5 56769380-56769392. Max. coverage (+): 7.47. Max coverage (-): 0

Region: chr5 56769393-56769406. Max. coverage (+): 0. Max coverage (-): 0

Region: chr5 56769407-56769420. Max. coverage (+): 2.28. Max coverage (-): 0

Region: chr5 56769421-56769433. Max. coverage (+): 0. Max coverage (-): 0

Region: chr5 56769434-56769447. Max. coverage (+): 0. Max coverage (-): 0

Region: chr5 56769448-56769461. Max. coverage (+): 0. Max coverage (-): 0

Region: chr5 56769462-56769475. Max. coverage (+): 0. Max coverage (-): 0

Region: chr5 56769476-56769488. Max. coverage (+): 0. Max coverage (-): 0

Region: chr5 56769489-56769502. Max. coverage (+): 0. Max coverage (-): 0

Region: chr5 56769503-56769516. Max. coverage (+): 0. Max coverage (-): 0

Region: chr5 56769517-56769529. Max. coverage (+): 0. Max coverage (-): 0

Region: chr5 56769530-56769543. Max. coverage (+): 0. Max coverage (-): 0

Region: chr5 56769544-56769557. Max. coverage (+): 0. Max coverage (-): 0

Region: chr5 56769558-56769571. Max. coverage (+): 0. Max coverage (-): 0

Region: chr5 56769572-56769584. Max. coverage (+): 0. Max coverage (-): 0

Region: chr5 56769585-56769598. Max. coverage (+): 0. Max coverage (-): 0

Region: chr5 56769599-56769612. Max. coverage (+): 0. Max coverage (-): 0

Region: chr5 56769613-56769625. Max. coverage (+): 0. Max coverage (-): 0

Region: chr5 56769626-56769639. Max. coverage (+): 0. Max coverage (-): 0

Region: chr5 56769640-56769653. Max. coverage (+): 0. Max coverage (-): 0

Region: chr5 56769654-56769667. Max. coverage (+): 0. Max coverage (-): 0

Region: chr5 56769668-56769680. Max. coverage (+): 0. Max coverage (-): 0

Region: chr5 56769681-56769694. Max. coverage (+): 0. Max coverage (-): 0

Region: chr5 56769695-56769708. Max. coverage (+): 0. Max coverage (-): 0

Region: chr5 56769709-56769721. Max. coverage (+): 0. Max coverage (-): 0

Region: chr5 56769722-56769735. Max. coverage (+): 0. Max coverage (-): 0

Region: chr5 56769736-56769749. Max. coverage (+): 0. Max coverage (-): 0

Region: chr5 56769750-56769763. Max. coverage (+): 0. Max coverage (-): 0

Region: chr5 56769764-56769776. Max. coverage (+): 0. Max coverage (-): 0

Region: chr5 56769777-56769790. Max. coverage (+): 0. Max coverage (-): 0

Region: chr5 56769791-56769804. Max. coverage (+): 0. Max coverage (-): 0

Region: chr5 56769805-56769818. Max. coverage (+): 0. Max coverage (-): 0

Region: chr5 56769819-56769831. Max. coverage (+): 0. Max coverage (-): 0

Region: chr5 56769832-56769845. Max. coverage (+): 0. Max coverage (-): 0

Region: chr5 56769846-56769859. Max. coverage (+): 0. Max coverage (-): 0

Region: chr5 56769860-56769872. Max. coverage (+): 0. Max coverage (-): 0

Region: chr5 56769873-56769886. Max. coverage (+): 0. Max coverage (-): 0

Region: chr5 56769887-56769900. Max. coverage (+): 0. Max coverage (-): 0

Region: chr5 56769901-56769914. Max. coverage (+): 0. Max coverage (-): 0

Region: chr5 56769915-56769927. Max. coverage (+): 0. Max coverage (-): 0

Region: chr5 56769928-56769941. Max. coverage (+): 0. Max coverage (-): 0

Region: chr5 56769942-56769955. Max. coverage (+): 0. Max coverage (-): 0

Region: chr5 56769956-56769968. Max. coverage (+): 0. Max coverage (-): 0

Region: chr5 56769969-56769982. Max. coverage (+): 0. Max coverage (-): 0

Region: chr5 56769983-56769996. Max. coverage (+): 0. Max coverage (-): 0

Region: chr5 56769997-56770010. Max. coverage (+): 0. Max coverage (-): 0

Region: chr5 56770011-56770023. Max. coverage (+): 0. Max coverage (-): 0

Region: chr5 56770024-56770037. Max. coverage (+): 0. Max coverage (-): 0

Region: chr5 56770038-56770051. Max. coverage (+): 0. Max coverage (-): 0

Region: chr5 56770052-56770064. Max. coverage (+): 0. Max coverage (-): 0

Region: chr5 56770065-56770078. Max. coverage (+): 0. Max coverage (-): 0

Region: chr5 56770079-56770092. Max. coverage (+): 0. Max coverage (-): 0

Region: chr5 56770093-56770106. Max. coverage (+): 0. Max coverage (-): 0

Region: chr5 56770107-56770119. Max. coverage (+): 0. Max coverage (-): 0

Region: chr5 56770120-56770133. Max. coverage (+): 0. Max coverage (-): 0

Region: chr5 56770134-56770147. Max. coverage (+): 0. Max coverage (-): 0

Region: chr5 56770148-56770160. Max. coverage (+): 0. Max coverage (-): 0

Region: chr5 56770161-56770174. Max. coverage (+): 0. Max coverage (-): 0

Region: chr5 56770175-56770188. Max. coverage (+): 0. Max coverage (-): 0

Region: chr5 56770189-56770202. Max. coverage (+): 0. Max coverage (-): 0

Region: chr5 56770203-56770215. Max. coverage (+): 0. Max coverage (-): 0

Region: chr5 56770216-56770229. Max. coverage (+): 0. Max coverage (-): 0

Region: chr5 56770230-56770243. Max. coverage (+): 0. Max coverage (-): 0

Region: chr5 56770244-56770256. Max. coverage (+): 0. Max coverage (-): 0

Region: chr5 56770257-56770270. Max. coverage (+): 0. Max coverage (-): 0

Region: chr5 56770271-56770284. Max. coverage (+): 0. Max coverage (-): 0

Region: chr5 56770285-56770298. Max. coverage (+): 0. Max coverage (-): 0

Region: chr5 56770299-56770311. Max. coverage (+): 0. Max coverage (-): 0

Region: chr5 56770312-56770325. Max. coverage (+): 0. Max coverage (-): 0

Region: chr5 56770326-56770339. Max. coverage (+): 0. Max coverage (-): 0

Region: chr5 56770340-56770353. Max. coverage (+): 0. Max coverage (-): 0

Region: chr5 56770354-56770366. Max. coverage (+): 0. Max coverage (-): 0

Region: chr5 56770367-56770380. Max. coverage (+): 0. Max coverage (-): 0

Region: chr5 56770381-56770394. Max. coverage (+): 0. Max coverage (-): 0

Region: chr5 56770395-56770407. Max. coverage (+): 0. Max coverage (-): 0

Region: chr5 56770408-56770421. Max. coverage (+): 0. Max coverage (-): 0

Region: chr5 56770422-56770435. Max. coverage (+): 0. Max coverage (-): 0

Region: chr5 56770436-56770449. Max. coverage (+): 0. Max coverage (-): 0

Region: chr5 56770450-56770462. Max. coverage (+): 0. Max coverage (-): 0

Region: chr5 56770463-56770476. Max. coverage (+): 0. Max coverage (-): 0

Region: chr5 56770477-56770490. Max. coverage (+): 0. Max coverage (-): 0

Region: chr5 56770491-56770503. Max. coverage (+): 0. Max coverage (-): 0

Region: chr5 56770504-56770517. Max. coverage (+): 0. Max coverage (-): 0

Region: chr5 56770518-56770531. Max. coverage (+): 0. Max coverage (-): 0

Region: chr5 56770532-56770545. Max. coverage (+): 0. Max coverage (-): 0

Region: chr5 56770546-56770558. Max. coverage (+): 0. Max coverage (-): 0

Region: chr5 56770559-56770572. Max. coverage (+): 0. Max coverage (-): 0

Region: chr5 56770573-56770586. Max. coverage (+): 0. Max coverage (-): 0

Region: chr5 56770587-56770599. Max. coverage (+): 0. Max coverage (-): 0

Region: chr5 56770600-56770613. Max. coverage (+): 0. Max coverage (-): 0

Region: chr5 56770614-56770627. Max. coverage (+): 0. Max coverage (-): 0

Region: chr5 56770628-56770641. Max. coverage (+): 0. Max coverage (-): 0

Region: chr5 56770642-56770654. Max. coverage (+): 0. Max coverage (-): 0

Region: chr5 56770655-56770668. Max. coverage (+): 0. Max coverage (-): 0

Region: chr5 56770669-56770682. Max. coverage (+): 0. Max coverage (-): 0

Region: chr5 56770683-56770695. Max. coverage (+): 0. Max coverage (-): 0

Region: chr5 56770696-56770709. Max. coverage (+): 0. Max coverage (-): 0

Region: chr5 56770710-56770723. Max. coverage (+): 0. Max coverage (-): 0

Region: chr5 56770724-56770737. Max. coverage (+): 0. Max coverage (-): 0

Region: chr5 56770738-56770750. Max. coverage (+): 0. Max coverage (-): 0

Region: chr5 56770751-56770764. Max. coverage (+): 0. Max coverage (-): 0

Region: chr5 56770765-56770778. Max. coverage (+): 0. Max coverage (-): 0

Region: chr5 56770779-56770791. Max. coverage (+): 0. Max coverage (-): 0

Region: chr5 56770792-56770805. Max. coverage (+): 0. Max coverage (-): 0

Region: chr5 56770806-56770819. Max. coverage (+): 4.02. Max coverage (-): 0

Region: chr5 56770820-56770833. Max. coverage (+): 4.02. Max coverage (-): 0

Region: chr5 56770834-. Max. coverage (+): 0. Max coverage (-): 0

RepeatMasker Color Code

**+**

100-98% Identity

<98-95% Identity

<95-90% Identity

<90-85% Identity

<85-80% Identity

<80-75% Identity

<75-70% Identity

<70% Identity

**-**

Gene Set Color Code

**+**

Gene

Pseudogene

**-**

Topology/Coverage Color Code

Coverage Plus Strand

Coverage Minus Strand

Mainstrand: Plus

Mainstrand: Minus

Complementary Strand

Flanking Region  
(if option -flank >0)

Gene Set Annotation  

**1. ZBTB39 (protein coding, ENSBTAG00000038498) Tr:00000056411 Ex:1**: 56764459-56766582 (+)

  
RepeatMasker Annotation  

**1. AT\_rich**: 56768041-56768061 (+), Divergence to consensus: 38.1%  
**2. MER45A**: 56769484-56769554 (-), Divergence to consensus: 29.5%  
**3. Bov-tA2**: 56769597-56769805 (-), Divergence to consensus: 10.6%

  
Transcription Factor Binding Sites  

**SOX9** (Sequence: CCATTGTT (+): 56770645)  
**A-MYB** (Sequence: AGGCAGTTGG (+): 56766855)
