# Supplementary material for: Small RNA sequencing of cryopreserved semen from single bull revealed altered miRNAs and piRNAs expression between High- and Low-motile sperm populations
Source: BMC Genomics. 2017 Jan 4;18:14. doi: 10.1186/s12864-016-3394-7 (PMC5209821; doi:10.1186/s12864-016-3394-7)
Supplement: Additional file 4: — Details for each piRNA clusters found in Low Motile (LM) sperm fraction. Genes, repeats, transposable elements and transcription factors binding sites falling within the cluster regions were reported. (ZIP 1034 kb) [file 12864_2016_3394_MOESM4_ESM.zip › 44.html]

piRNA cluster 44


Predicted piRNA cluster no. 44     previous   next
  

Show proTRAC run info
Hide proTRAC run info

================================= proTRAC ====================================  
VERSION: 2.1                                    LAST MODIFIED: 06. October 2015  
  
Please cite:  
Rosenkranz D, Zischler H. proTRAC - a software for probabilistic piRNA cluster  
detection, visualization and analysis. 2012. BMC Bioinformatics 13:5.  
  
and (for proTRAC 2.0 and later):  
Rosenkranz D, Rudloff S, Bastuck K, Ketting RF, Zischler H. Tupaia small RNAs  
provide insights into function and evolution of RNAi-based transposon defense  
in mammals. 2015. RNA 21(5):911-922.  
  
Contact:  
David Rosenkranz  
Institute of Anthropology, small RNA group  
Johannes Gutenberg University Mainz  
email: rosenkranz@uni-mainz.de  
  
You can find the latest proTRAC version at:  
http://sourceforge.net/projects/protrac/files  
http://www.smallRNAgroup-mainz.de/software  
==============================================================================  
  
PARAMETERS:  
Map file: .............../storage/core/barbara/genhome/smallRNA/fertility/Sample\_not\_motile/pirna/Sample\_not\_motile\_26-33\_collapsed.fa.no-dust.map.weighted-10000-1000-b-0  
Genome file: ............/storage/core/barbara/genhome/smallRNA/fertility/Sample\_all/pirna/bt\_311\_chrY.fa  
RepeatMasker annotation: /storage/genomes/bt\_umd31/GCF\_000003055.6\_Bos\_taurus\_UMD\_3.1.1\_repeatMasker\_chr.out  
GeneSet:................./storage/core/barbara/genhome/smallRNA/fertility/Sample\_all/pirna/full.gtf  
  
Significant (p<=0.01) hit density will be calculated based  
on observed hit distribution.  
  
Sliding window size: ........................................ 5000 bp  
Sliding window increament: .................................. 1000 bp  
Normalize each hit by number of genomic hits: ............... 1 [0=no/1=yes]  
Normalize each hit by number of sequence reads: ............. 1 [0=no/1=yes]  
Normalize values (-> per million mapped reads): ............. 1 [0=no/1=yes]  
Min. fraction of hits with 1T(U) or 10A: .................... 0.75  
Alternatively: Min. fraction of hits with 1T(U) and 10A: .... 0.5  
Min. fraction of hits with typical piRNA length: ............ 0.75  
Typical piRNA length: ....................................... 26-33 nt  
Min. size of a piRNA cluster: ............................... 5000 bp.  
Min. number of hits (absolute): ............................. 0  
Min. number of hits (normalized): ........................... 0  
Min. fraction of hits on the mainstrand: .................... 0.75  
Top fraction of mapped sequences (in terms of read counts): . 1%  
Top fraction accounts for max. n% of sequence reads: ........ 90%  
Min. fraction of hits on each arm of a bidirectional cluster: 0.1  
Output image file for each cluster: ......................... 0 [0=no/1=yes]  
Output html file for each cluster: .......................... 1 [0=no/1=yes]  
Output a summary table: ..................................... 1 [0=no/1=yes]  
Output a FASTA file for each cluster (piRNA sequences): ..... 1 [0=no/1=yes]  
Output a FASTA file comprising cluster sequences: ........... 1 [0=no/1=yes]  
Search DNA motifs in clusters: .............................. 1 [0=no/1=yes]  
Output flanking sequences: +/- .............................. 0 bp  
Output ~.pTi file: .......................................... 1 [0=no/1=yes]  
==============================================================================  
  
  
Genome size (without gaps): ............ 2678902517 bp  
Gaps (N/X/-): .......................... 53837044 bp  
Mapped reads: .......................... 738059667487  
Non-identical sequences: ............... 277001  
Genomic hits: .......................... 533816  
Significant densitiy of mapped reads: .. 15118061 reads/kb

Show proTRAC cluster info
Hide proTRAC cluster info

|  |  |
| --- | --- |
| Location | chr5 |
| Coordinates | 76154278-76173336 |
| Size [bp] | 19059 |
| Sequence hit loci | 1138 |
| Mapped reads (normalized) | 2853269084.8 |
| Mapped reads (normalized) per kb | 149707176.9 |
| Normalized reads with 1T (1U) | 83.1% |
| Normalized reads with 10A | 26.6% |
| Normalized reads with length 26-33 nt | 100% |
| Normalized reads on the main strand(s) | 99.8% |
| Predicted directionality | mono:minus |

100%

0%

1T (1U)  
reads

10A reads

26-33 nt  
reads

reads on mainstrand

**Either the amount of reads with 1T (1U) OR 10A has to exceed 75% (set with option: -1Tor10A)  
Alternatively the amount of reads with 1T (1U) AND 10A has to exceed 50% (set with option: -1Tand10A)  
Minimum amount of reads with preferred size is 75% (set with option: -pisize)  
Minimum amount of reads on the main strand(s) is 75% (set with option: -clstrand)**

Show read coverage
Hide read coverage

WHAT DO I SEE HERE?  
This chart shows the location of mapped sequence reads within a predicted piRNA cluster. The color refers to the number of genomic hits produced by the sequence read in question. A dark red bar indicates that this sequence read produces many other hits elsewhere in the genome. Many adjacent red or yellow bars can indicate the presence of a multi-copy element such as transposons or rRNA genes. A dark green bar indicates that this sequence read maps uniquely to this locus.

1 hit

2-5 hits

6-10 hits

11-20 hits

21-50 hits

51-100 hits

> 100 hits

chr5

76154278

76173336

Gene Set

RepeatMasker

Mapped  
Reads

107.32

plus strand

minus strand

107.32

Region: chr5 56770835-76154297. Max. coverage (+): 0. Max coverage (-): 3.48

Region: chr5 76154298-76154335. Max. coverage (+): 0. Max coverage (-): 0

Region: chr5 76154336-76154373. Max. coverage (+): 0. Max coverage (-): 4.34

Region: chr5 76154374-76154411. Max. coverage (+): 0. Max coverage (-): 0.5

Region: chr5 76154412-76154449. Max. coverage (+): 0. Max coverage (-): 0.5

Region: chr5 76154450-76154487. Max. coverage (+): 0. Max coverage (-): 0

Region: chr5 76154488-76154525. Max. coverage (+): 0. Max coverage (-): 0

Region: chr5 76154526-76154563. Max. coverage (+): 0. Max coverage (-): 0

Region: chr5 76154564-76154602. Max. coverage (+): 0. Max coverage (-): 0

Region: chr5 76154603-76154640. Max. coverage (+): 0. Max coverage (-): 0

Region: chr5 76154641-76154678. Max. coverage (+): 0. Max coverage (-): 0

Region: chr5 76154679-76154716. Max. coverage (+): 0. Max coverage (-): 0

Region: chr5 76154717-76154754. Max. coverage (+): 0. Max coverage (-): 0

Region: chr5 76154755-76154792. Max. coverage (+): 0. Max coverage (-): 0

Region: chr5 76154793-76154830. Max. coverage (+): 0. Max coverage (-): 0

Region: chr5 76154831-76154868. Max. coverage (+): 0. Max coverage (-): 0

Region: chr5 76154869-76154906. Max. coverage (+): 0. Max coverage (-): 0

Region: chr5 76154907-76154945. Max. coverage (+): 0. Max coverage (-): 0

Region: chr5 76154946-76154983. Max. coverage (+): 0. Max coverage (-): 0

Region: chr5 76154984-76155021. Max. coverage (+): 0. Max coverage (-): 0

Region: chr5 76155022-76155059. Max. coverage (+): 0. Max coverage (-): 0

Region: chr5 76155060-76155097. Max. coverage (+): 0. Max coverage (-): 0

Region: chr5 76155098-76155135. Max. coverage (+): 0. Max coverage (-): 0

Region: chr5 76155136-76155173. Max. coverage (+): 0. Max coverage (-): 0

Region: chr5 76155174-76155211. Max. coverage (+): 0. Max coverage (-): 0

Region: chr5 76155212-76155250. Max. coverage (+): 0. Max coverage (-): 0

Region: chr5 76155251-76155288. Max. coverage (+): 0. Max coverage (-): 0

Region: chr5 76155289-76155326. Max. coverage (+): 0. Max coverage (-): 0

Region: chr5 76155327-76155364. Max. coverage (+): 0. Max coverage (-): 0

Region: chr5 76155365-76155402. Max. coverage (+): 0. Max coverage (-): 0

Region: chr5 76155403-76155440. Max. coverage (+): 0. Max coverage (-): 0

Region: chr5 76155441-76155478. Max. coverage (+): 0. Max coverage (-): 0

Region: chr5 76155479-76155516. Max. coverage (+): 0. Max coverage (-): 0

Region: chr5 76155517-76155554. Max. coverage (+): 0. Max coverage (-): 0

Region: chr5 76155555-76155593. Max. coverage (+): 0. Max coverage (-): 0

Region: chr5 76155594-76155631. Max. coverage (+): 0. Max coverage (-): 0

Region: chr5 76155632-76155669. Max. coverage (+): 0. Max coverage (-): 0

Region: chr5 76155670-76155707. Max. coverage (+): 0. Max coverage (-): 0

Region: chr5 76155708-76155745. Max. coverage (+): 0. Max coverage (-): 0

Region: chr5 76155746-76155783. Max. coverage (+): 0. Max coverage (-): 0

Region: chr5 76155784-76155821. Max. coverage (+): 0. Max coverage (-): 0

Region: chr5 76155822-76155859. Max. coverage (+): 0. Max coverage (-): 0

Region: chr5 76155860-76155898. Max. coverage (+): 0. Max coverage (-): 7.66

Region: chr5 76155899-76155936. Max. coverage (+): 0. Max coverage (-): 3

Region: chr5 76155937-76155974. Max. coverage (+): 0. Max coverage (-): 3.97

Region: chr5 76155975-76156012. Max. coverage (+): 0. Max coverage (-): 6.98

Region: chr5 76156013-76156050. Max. coverage (+): 0. Max coverage (-): 0

Region: chr5 76156051-76156088. Max. coverage (+): 0. Max coverage (-): 6.55

Region: chr5 76156089-76156126. Max. coverage (+): 0. Max coverage (-): 6.13

Region: chr5 76156127-76156164. Max. coverage (+): 0. Max coverage (-): 0

Region: chr5 76156165-76156202. Max. coverage (+): 0. Max coverage (-): 0

Region: chr5 76156203-76156241. Max. coverage (+): 0. Max coverage (-): 0

Region: chr5 76156242-76156279. Max. coverage (+): 0. Max coverage (-): 8.79

Region: chr5 76156280-76156317. Max. coverage (+): 0. Max coverage (-): 12.34

Region: chr5 76156318-76156355. Max. coverage (+): 0. Max coverage (-): 0

Region: chr5 76156356-76156393. Max. coverage (+): 0. Max coverage (-): 0

Region: chr5 76156394-76156431. Max. coverage (+): 0. Max coverage (-): 0

Region: chr5 76156432-76156469. Max. coverage (+): 0. Max coverage (-): 0

Region: chr5 76156470-76156507. Max. coverage (+): 0. Max coverage (-): 0

Region: chr5 76156508-76156546. Max. coverage (+): 0. Max coverage (-): 0

Region: chr5 76156547-76156584. Max. coverage (+): 0. Max coverage (-): 0

Region: chr5 76156585-76156622. Max. coverage (+): 0. Max coverage (-): 6.39

Region: chr5 76156623-76156660. Max. coverage (+): 0. Max coverage (-): 0

Region: chr5 76156661-76156698. Max. coverage (+): 0. Max coverage (-): 0

Region: chr5 76156699-76156736. Max. coverage (+): 0. Max coverage (-): 0

Region: chr5 76156737-76156774. Max. coverage (+): 0. Max coverage (-): 0

Region: chr5 76156775-76156812. Max. coverage (+): 0. Max coverage (-): 0

Region: chr5 76156813-76156850. Max. coverage (+): 0. Max coverage (-): 0

Region: chr5 76156851-76156889. Max. coverage (+): 0. Max coverage (-): 0

Region: chr5 76156890-76156927. Max. coverage (+): 0. Max coverage (-): 0

Region: chr5 76156928-76156965. Max. coverage (+): 0. Max coverage (-): 0

Region: chr5 76156966-76157003. Max. coverage (+): 0. Max coverage (-): 0

Region: chr5 76157004-76157041. Max. coverage (+): 0. Max coverage (-): 1.05

Region: chr5 76157042-76157079. Max. coverage (+): 0. Max coverage (-): 2.02

Region: chr5 76157080-76157117. Max. coverage (+): 0. Max coverage (-): 10.86

Region: chr5 76157118-76157155. Max. coverage (+): 0. Max coverage (-): 15.1

Region: chr5 76157156-76157194. Max. coverage (+): 0. Max coverage (-): 0

Region: chr5 76157195-76157232. Max. coverage (+): 0. Max coverage (-): 5.52

Region: chr5 76157233-76157270. Max. coverage (+): 0. Max coverage (-): 3.43

Region: chr5 76157271-76157308. Max. coverage (+): 0. Max coverage (-): 10.92

Region: chr5 76157309-76157346. Max. coverage (+): 0. Max coverage (-): 15.34

Region: chr5 76157347-76157384. Max. coverage (+): 0. Max coverage (-): 2.97

Region: chr5 76157385-76157422. Max. coverage (+): 0. Max coverage (-): 0

Region: chr5 76157423-76157460. Max. coverage (+): 0. Max coverage (-): 0

Region: chr5 76157461-76157498. Max. coverage (+): 0. Max coverage (-): 13.24

Region: chr5 76157499-76157537. Max. coverage (+): 0. Max coverage (-): 11.29

Region: chr5 76157538-76157575. Max. coverage (+): 0. Max coverage (-): 0

Region: chr5 76157576-76157613. Max. coverage (+): 0. Max coverage (-): 10.55

Region: chr5 76157614-76157651. Max. coverage (+): 0. Max coverage (-): 0

Region: chr5 76157652-76157689. Max. coverage (+): 0. Max coverage (-): 6.57

Region: chr5 76157690-76157727. Max. coverage (+): 0. Max coverage (-): 11.68

Region: chr5 76157728-76157765. Max. coverage (+): 0. Max coverage (-): 6.55

Region: chr5 76157766-76157803. Max. coverage (+): 0. Max coverage (-): 0

Region: chr5 76157804-76157842. Max. coverage (+): 0. Max coverage (-): 4.38

Region: chr5 76157843-76157880. Max. coverage (+): 0. Max coverage (-): 25.85

Region: chr5 76157881-76157918. Max. coverage (+): 0. Max coverage (-): 12.11

Region: chr5 76157919-76157956. Max. coverage (+): 0. Max coverage (-): 19.62

Region: chr5 76157957-76157994. Max. coverage (+): 0. Max coverage (-): 2.71

Region: chr5 76157995-76158032. Max. coverage (+): 0. Max coverage (-): 0

Region: chr5 76158033-76158070. Max. coverage (+): 0. Max coverage (-): 0

Region: chr5 76158071-76158108. Max. coverage (+): 0. Max coverage (-): 0

Region: chr5 76158109-76158146. Max. coverage (+): 0. Max coverage (-): 0

Region: chr5 76158147-76158185. Max. coverage (+): 0. Max coverage (-): 0

Region: chr5 76158186-76158223. Max. coverage (+): 0. Max coverage (-): 0

Region: chr5 76158224-76158261. Max. coverage (+): 0. Max coverage (-): 0

Region: chr5 76158262-76158299. Max. coverage (+): 0. Max coverage (-): 0

Region: chr5 76158300-76158337. Max. coverage (+): 0. Max coverage (-): 0

Region: chr5 76158338-76158375. Max. coverage (+): 0. Max coverage (-): 0

Region: chr5 76158376-76158413. Max. coverage (+): 0. Max coverage (-): 0

Region: chr5 76158414-76158451. Max. coverage (+): 0. Max coverage (-): 0

Region: chr5 76158452-76158490. Max. coverage (+): 0. Max coverage (-): 0

Region: chr5 76158491-76158528. Max. coverage (+): 0. Max coverage (-): 0

Region: chr5 76158529-76158566. Max. coverage (+): 0. Max coverage (-): 0

Region: chr5 76158567-76158604. Max. coverage (+): 0. Max coverage (-): 0

Region: chr5 76158605-76158642. Max. coverage (+): 0. Max coverage (-): 0

Region: chr5 76158643-76158680. Max. coverage (+): 0. Max coverage (-): 6.4

Region: chr5 76158681-76158718. Max. coverage (+): 0. Max coverage (-): 12.82

Region: chr5 76158719-76158756. Max. coverage (+): 0. Max coverage (-): 12.82

Region: chr5 76158757-76158794. Max. coverage (+): 0. Max coverage (-): 16.4

Region: chr5 76158795-76158833. Max. coverage (+): 0. Max coverage (-): 8.25

Region: chr5 76158834-76158871. Max. coverage (+): 0. Max coverage (-): 0

Region: chr5 76158872-76158909. Max. coverage (+): 0. Max coverage (-): 4.96

Region: chr5 76158910-76158947. Max. coverage (+): 0. Max coverage (-): 6.87

Region: chr5 76158948-76158985. Max. coverage (+): 0. Max coverage (-): 14.65

Region: chr5 76158986-76159023. Max. coverage (+): 0. Max coverage (-): 16.68

Region: chr5 76159024-76159061. Max. coverage (+): 0. Max coverage (-): 0

Region: chr5 76159062-76159099. Max. coverage (+): 0. Max coverage (-): 0

Region: chr5 76159100-76159138. Max. coverage (+): 0. Max coverage (-): 0

Region: chr5 76159139-76159176. Max. coverage (+): 0. Max coverage (-): 0

Region: chr5 76159177-76159214. Max. coverage (+): 0. Max coverage (-): 0

Region: chr5 76159215-76159252. Max. coverage (+): 0. Max coverage (-): 0

Region: chr5 76159253-76159290. Max. coverage (+): 0. Max coverage (-): 0

Region: chr5 76159291-76159328. Max. coverage (+): 0. Max coverage (-): 0

Region: chr5 76159329-76159366. Max. coverage (+): 0. Max coverage (-): 0

Region: chr5 76159367-76159404. Max. coverage (+): 0. Max coverage (-): 0

Region: chr5 76159405-76159442. Max. coverage (+): 0. Max coverage (-): 6.15

Region: chr5 76159443-76159481. Max. coverage (+): 0. Max coverage (-): 6.15

Region: chr5 76159482-76159519. Max. coverage (+): 0. Max coverage (-): 0

Region: chr5 76159520-76159557. Max. coverage (+): 0. Max coverage (-): 0

Region: chr5 76159558-76159595. Max. coverage (+): 0. Max coverage (-): 0

Region: chr5 76159596-76159633. Max. coverage (+): 0. Max coverage (-): 0

Region: chr5 76159634-76159671. Max. coverage (+): 0. Max coverage (-): 0

Region: chr5 76159672-76159709. Max. coverage (+): 0. Max coverage (-): 0.22

Region: chr5 76159710-76159747. Max. coverage (+): 0. Max coverage (-): 43.67

Region: chr5 76159748-76159786. Max. coverage (+): 0. Max coverage (-): 19.88

Region: chr5 76159787-76159824. Max. coverage (+): 0. Max coverage (-): 22.05

Region: chr5 76159825-76159862. Max. coverage (+): 0. Max coverage (-): 6.09

Region: chr5 76159863-76159900. Max. coverage (+): 0. Max coverage (-): 3.49

Region: chr5 76159901-76159938. Max. coverage (+): 0. Max coverage (-): 1.44

Region: chr5 76159939-76159976. Max. coverage (+): 0. Max coverage (-): 8.94

Region: chr5 76159977-76160014. Max. coverage (+): 0. Max coverage (-): 19.47

Region: chr5 76160015-76160052. Max. coverage (+): 0. Max coverage (-): 20.94

Region: chr5 76160053-76160090. Max. coverage (+): 0. Max coverage (-): 6.91

Region: chr5 76160091-76160129. Max. coverage (+): 0. Max coverage (-): 0

Region: chr5 76160130-76160167. Max. coverage (+): 0. Max coverage (-): 1.85

Region: chr5 76160168-76160205. Max. coverage (+): 0. Max coverage (-): 0

Region: chr5 76160206-76160243. Max. coverage (+): 0. Max coverage (-): 1.58

Region: chr5 76160244-76160281. Max. coverage (+): 0. Max coverage (-): 20.36

Region: chr5 76160282-76160319. Max. coverage (+): 0. Max coverage (-): 0

Region: chr5 76160320-76160357. Max. coverage (+): 0. Max coverage (-): 15.65

Region: chr5 76160358-76160395. Max. coverage (+): 0. Max coverage (-): 1.9

Region: chr5 76160396-76160434. Max. coverage (+): 0. Max coverage (-): 26.82

Region: chr5 76160435-76160472. Max. coverage (+): 0. Max coverage (-): 29.05

Region: chr5 76160473-76160510. Max. coverage (+): 0. Max coverage (-): 25.83

Region: chr5 76160511-76160548. Max. coverage (+): 0. Max coverage (-): 20.56

Region: chr5 76160549-76160586. Max. coverage (+): 0. Max coverage (-): 7.82

Region: chr5 76160587-76160624. Max. coverage (+): 0. Max coverage (-): 24.26

Region: chr5 76160625-76160662. Max. coverage (+): 0. Max coverage (-): 0

Region: chr5 76160663-76160700. Max. coverage (+): 0. Max coverage (-): 0

Region: chr5 76160701-76160739. Max. coverage (+): 0. Max coverage (-): 0

Region: chr5 76160740-76160777. Max. coverage (+): 0. Max coverage (-): 0

Region: chr5 76160778-76160815. Max. coverage (+): 0. Max coverage (-): 0

Region: chr5 76160816-76160853. Max. coverage (+): 0. Max coverage (-): 0

Region: chr5 76160854-76160891. Max. coverage (+): 0. Max coverage (-): 0

Region: chr5 76160892-76160929. Max. coverage (+): 0.03. Max coverage (-): 1.1

Region: chr5 76160930-76160967. Max. coverage (+): 0. Max coverage (-): 3.81

Region: chr5 76160968-76161005. Max. coverage (+): 0. Max coverage (-): 0

Region: chr5 76161006-76161043. Max. coverage (+): 0. Max coverage (-): 0.05

Region: chr5 76161044-76161082. Max. coverage (+): 0. Max coverage (-): 0

Region: chr5 76161083-76161120. Max. coverage (+): 0. Max coverage (-): 0

Region: chr5 76161121-76161158. Max. coverage (+): 0. Max coverage (-): 0.09

Region: chr5 76161159-76161196. Max. coverage (+): 0. Max coverage (-): 0

Region: chr5 76161197-76161234. Max. coverage (+): 0. Max coverage (-): 0

Region: chr5 76161235-76161272. Max. coverage (+): 0. Max coverage (-): 0

Region: chr5 76161273-76161310. Max. coverage (+): 0. Max coverage (-): 0

Region: chr5 76161311-76161348. Max. coverage (+): 0. Max coverage (-): 0

Region: chr5 76161349-76161387. Max. coverage (+): 0. Max coverage (-): 0

Region: chr5 76161388-76161425. Max. coverage (+): 0. Max coverage (-): 0

Region: chr5 76161426-76161463. Max. coverage (+): 0. Max coverage (-): 0

Region: chr5 76161464-76161501. Max. coverage (+): 0. Max coverage (-): 0

Region: chr5 76161502-76161539. Max. coverage (+): 0. Max coverage (-): 3.34

Region: chr5 76161540-76161577. Max. coverage (+): 0. Max coverage (-): 7.78

Region: chr5 76161578-76161615. Max. coverage (+): 0. Max coverage (-): 2.87

Region: chr5 76161616-76161653. Max. coverage (+): 0. Max coverage (-): 2.57

Region: chr5 76161654-76161691. Max. coverage (+): 0. Max coverage (-): 0.17

Region: chr5 76161692-76161730. Max. coverage (+): 0. Max coverage (-): 9.47

Region: chr5 76161731-76161768. Max. coverage (+): 0. Max coverage (-): 0

Region: chr5 76161769-76161806. Max. coverage (+): 0. Max coverage (-): 0

Region: chr5 76161807-76161844. Max. coverage (+): 0. Max coverage (-): 0

Region: chr5 76161845-76161882. Max. coverage (+): 0. Max coverage (-): 0

Region: chr5 76161883-76161920. Max. coverage (+): 0. Max coverage (-): 6.33

Region: chr5 76161921-76161958. Max. coverage (+): 0. Max coverage (-): 0

Region: chr5 76161959-76161996. Max. coverage (+): 0. Max coverage (-): 2.05

Region: chr5 76161997-76162035. Max. coverage (+): 0. Max coverage (-): 0.78

Region: chr5 76162036-76162073. Max. coverage (+): 0. Max coverage (-): 0

Region: chr5 76162074-76162111. Max. coverage (+): 0. Max coverage (-): 0

Region: chr5 76162112-76162149. Max. coverage (+): 0. Max coverage (-): 3.1

Region: chr5 76162150-76162187. Max. coverage (+): 0. Max coverage (-): 5.34

Region: chr5 76162188-76162225. Max. coverage (+): 0. Max coverage (-): 7.88

Region: chr5 76162226-76162263. Max. coverage (+): 0. Max coverage (-): 3.67

Region: chr5 76162264-76162301. Max. coverage (+): 0. Max coverage (-): 21.51

Region: chr5 76162302-76162339. Max. coverage (+): 0. Max coverage (-): 0

Region: chr5 76162340-76162378. Max. coverage (+): 0. Max coverage (-): 0

Region: chr5 76162379-76162416. Max. coverage (+): 0. Max coverage (-): 0

Region: chr5 76162417-76162454. Max. coverage (+): 0. Max coverage (-): 0

Region: chr5 76162455-76162492. Max. coverage (+): 0. Max coverage (-): 0

Region: chr5 76162493-76162530. Max. coverage (+): 0. Max coverage (-): 0

Region: chr5 76162531-76162568. Max. coverage (+): 0. Max coverage (-): 0

Region: chr5 76162569-76162606. Max. coverage (+): 0. Max coverage (-): 0

Region: chr5 76162607-76162644. Max. coverage (+): 0. Max coverage (-): 0

Region: chr5 76162645-76162683. Max. coverage (+): 0. Max coverage (-): 0

Region: chr5 76162684-76162721. Max. coverage (+): 0. Max coverage (-): 0

Region: chr5 76162722-76162759. Max. coverage (+): 0. Max coverage (-): 0

Region: chr5 76162760-76162797. Max. coverage (+): 0. Max coverage (-): 0

Region: chr5 76162798-76162835. Max. coverage (+): 0. Max coverage (-): 0

Region: chr5 76162836-76162873. Max. coverage (+): 0. Max coverage (-): 0

Region: chr5 76162874-76162911. Max. coverage (+): 0. Max coverage (-): 0

Region: chr5 76162912-76162949. Max. coverage (+): 0. Max coverage (-): 0

Region: chr5 76162950-76162987. Max. coverage (+): 0. Max coverage (-): 0

Region: chr5 76162988-76163026. Max. coverage (+): 0. Max coverage (-): 0

Region: chr5 76163027-76163064. Max. coverage (+): 0. Max coverage (-): 6.88

Region: chr5 76163065-76163102. Max. coverage (+): 0. Max coverage (-): 7.64

Region: chr5 76163103-76163140. Max. coverage (+): 0. Max coverage (-): 2.09

Region: chr5 76163141-76163178. Max. coverage (+): 0. Max coverage (-): 59.49

Region: chr5 76163179-76163216. Max. coverage (+): 0. Max coverage (-): 5.6

Region: chr5 76163217-76163254. Max. coverage (+): 0. Max coverage (-): 0

Region: chr5 76163255-76163292. Max. coverage (+): 0. Max coverage (-): 3.11

Region: chr5 76163293-76163331. Max. coverage (+): 0. Max coverage (-): 1.77

Region: chr5 76163332-76163369. Max. coverage (+): 0. Max coverage (-): 15.15

Region: chr5 76163370-76163407. Max. coverage (+): 0. Max coverage (-): 0

Region: chr5 76163408-76163445. Max. coverage (+): 0. Max coverage (-): 23.63

Region: chr5 76163446-76163483. Max. coverage (+): 0. Max coverage (-): 12.22

Region: chr5 76163484-76163521. Max. coverage (+): 0. Max coverage (-): 0

Region: chr5 76163522-76163559. Max. coverage (+): 0. Max coverage (-): 1.58

Region: chr5 76163560-76163597. Max. coverage (+): 0. Max coverage (-): 0

Region: chr5 76163598-76163635. Max. coverage (+): 0. Max coverage (-): 6.4

Region: chr5 76163636-76163674. Max. coverage (+): 0. Max coverage (-): 7.17

Region: chr5 76163675-76163712. Max. coverage (+): 0. Max coverage (-): 0

Region: chr5 76163713-76163750. Max. coverage (+): 0. Max coverage (-): 3.96

Region: chr5 76163751-76163788. Max. coverage (+): 0. Max coverage (-): 22.86

Region: chr5 76163789-76163826. Max. coverage (+): 0. Max coverage (-): 2.55

Region: chr5 76163827-76163864. Max. coverage (+): 0. Max coverage (-): 10.7

Region: chr5 76163865-76163902. Max. coverage (+): 0. Max coverage (-): 12.5

Region: chr5 76163903-76163940. Max. coverage (+): 0. Max coverage (-): 29.1

Region: chr5 76163941-76163979. Max. coverage (+): 0. Max coverage (-): 50.52

Region: chr5 76163980-76164017. Max. coverage (+): 0. Max coverage (-): 0

Region: chr5 76164018-76164055. Max. coverage (+): 0. Max coverage (-): 0

Region: chr5 76164056-76164093. Max. coverage (+): 0. Max coverage (-): 0

Region: chr5 76164094-76164131. Max. coverage (+): 0. Max coverage (-): 0

Region: chr5 76164132-76164169. Max. coverage (+): 0. Max coverage (-): 4.68

Region: chr5 76164170-76164207. Max. coverage (+): 0. Max coverage (-): 23.08

Region: chr5 76164208-76164245. Max. coverage (+): 0. Max coverage (-): 0

Region: chr5 76164246-76164283. Max. coverage (+): 0. Max coverage (-): 4.75

Region: chr5 76164284-76164322. Max. coverage (+): 0. Max coverage (-): 19.33

Region: chr5 76164323-76164360. Max. coverage (+): 0. Max coverage (-): 40.21

Region: chr5 76164361-76164398. Max. coverage (+): 0. Max coverage (-): 30.8

Region: chr5 76164399-76164436. Max. coverage (+): 0. Max coverage (-): 10.88

Region: chr5 76164437-76164474. Max. coverage (+): 0. Max coverage (-): 23.66

Region: chr5 76164475-76164512. Max. coverage (+): 0. Max coverage (-): 23.76

Region: chr5 76164513-76164550. Max. coverage (+): 0. Max coverage (-): 4.31

Region: chr5 76164551-76164588. Max. coverage (+): 0. Max coverage (-): 0

Region: chr5 76164589-76164627. Max. coverage (+): 0. Max coverage (-): 0

Region: chr5 76164628-76164665. Max. coverage (+): 0. Max coverage (-): 0

Region: chr5 76164666-76164703. Max. coverage (+): 0. Max coverage (-): 0

Region: chr5 76164704-76164741. Max. coverage (+): 0. Max coverage (-): 0

Region: chr5 76164742-76164779. Max. coverage (+): 0. Max coverage (-): 0

Region: chr5 76164780-76164817. Max. coverage (+): 0. Max coverage (-): 0

Region: chr5 76164818-76164855. Max. coverage (+): 0. Max coverage (-): 0

Region: chr5 76164856-76164893. Max. coverage (+): 0. Max coverage (-): 0

Region: chr5 76164894-76164931. Max. coverage (+): 0. Max coverage (-): 5.86

Region: chr5 76164932-76164970. Max. coverage (+): 0. Max coverage (-): 5.86

Region: chr5 76164971-76165008. Max. coverage (+): 0. Max coverage (-): 0

Region: chr5 76165009-76165046. Max. coverage (+): 0. Max coverage (-): 0

Region: chr5 76165047-76165084. Max. coverage (+): 0. Max coverage (-): 0.73

Region: chr5 76165085-76165122. Max. coverage (+): 0. Max coverage (-): 5.18

Region: chr5 76165123-76165160. Max. coverage (+): 0. Max coverage (-): 0.73

Region: chr5 76165161-76165198. Max. coverage (+): 0. Max coverage (-): 1.01

Region: chr5 76165199-76165236. Max. coverage (+): 0. Max coverage (-): 0

Region: chr5 76165237-76165275. Max. coverage (+): 0. Max coverage (-): 0

Region: chr5 76165276-76165313. Max. coverage (+): 0. Max coverage (-): 6.99

Region: chr5 76165314-76165351. Max. coverage (+): 0. Max coverage (-): 6.06

Region: chr5 76165352-76165389. Max. coverage (+): 0. Max coverage (-): 23.13

Region: chr5 76165390-76165427. Max. coverage (+): 0. Max coverage (-): 13.96

Region: chr5 76165428-76165465. Max. coverage (+): 0. Max coverage (-): 0

Region: chr5 76165466-76165503. Max. coverage (+): 0. Max coverage (-): 6.64

Region: chr5 76165504-76165541. Max. coverage (+): 0. Max coverage (-): 12.64

Region: chr5 76165542-76165579. Max. coverage (+): 0. Max coverage (-): 11.09

Region: chr5 76165580-76165618. Max. coverage (+): 0. Max coverage (-): 52.55

Region: chr5 76165619-76165656. Max. coverage (+): 0. Max coverage (-): 5.85

Region: chr5 76165657-76165694. Max. coverage (+): 0. Max coverage (-): 4.03

Region: chr5 76165695-76165732. Max. coverage (+): 0. Max coverage (-): 4.03

Region: chr5 76165733-76165770. Max. coverage (+): 0. Max coverage (-): 0

Region: chr5 76165771-76165808. Max. coverage (+): 0. Max coverage (-): 0

Region: chr5 76165809-76165846. Max. coverage (+): 0. Max coverage (-): 23.9

Region: chr5 76165847-76165884. Max. coverage (+): 0. Max coverage (-): 78.94

Region: chr5 76165885-76165923. Max. coverage (+): 0. Max coverage (-): 47.61

Region: chr5 76165924-76165961. Max. coverage (+): 0. Max coverage (-): 6.59

Region: chr5 76165962-76165999. Max. coverage (+): 0. Max coverage (-): 0.55

Region: chr5 76166000-76166037. Max. coverage (+): 0. Max coverage (-): 14.08

Region: chr5 76166038-76166075. Max. coverage (+): 0. Max coverage (-): 4.6

Region: chr5 76166076-76166113. Max. coverage (+): 0. Max coverage (-): 4.03

Region: chr5 76166114-76166151. Max. coverage (+): 0. Max coverage (-): 12.12

Region: chr5 76166152-76166189. Max. coverage (+): 0. Max coverage (-): 6.38

Region: chr5 76166190-76166227. Max. coverage (+): 0. Max coverage (-): 2.49

Region: chr5 76166228-76166266. Max. coverage (+): 0. Max coverage (-): 11.27

Region: chr5 76166267-76166304. Max. coverage (+): 0. Max coverage (-): 1.58

Region: chr5 76166305-76166342. Max. coverage (+): 0. Max coverage (-): 7

Region: chr5 76166343-76166380. Max. coverage (+): 0. Max coverage (-): 0.13

Region: chr5 76166381-76166418. Max. coverage (+): 0. Max coverage (-): 16.53

Region: chr5 76166419-76166456. Max. coverage (+): 0. Max coverage (-): 27.63

Region: chr5 76166457-76166494. Max. coverage (+): 0. Max coverage (-): 95.76

Region: chr5 76166495-76166532. Max. coverage (+): 0. Max coverage (-): 23.55

Region: chr5 76166533-76166571. Max. coverage (+): 0. Max coverage (-): 12.47

Region: chr5 76166572-76166609. Max. coverage (+): 0. Max coverage (-): 9.85

Region: chr5 76166610-76166647. Max. coverage (+): 0. Max coverage (-): 20.85

Region: chr5 76166648-76166685. Max. coverage (+): 0. Max coverage (-): 0

Region: chr5 76166686-76166723. Max. coverage (+): 0. Max coverage (-): 0

Region: chr5 76166724-76166761. Max. coverage (+): 0. Max coverage (-): 0

Region: chr5 76166762-76166799. Max. coverage (+): 0. Max coverage (-): 0

Region: chr5 76166800-76166837. Max. coverage (+): 0. Max coverage (-): 0

Region: chr5 76166838-76166875. Max. coverage (+): 0. Max coverage (-): 0

Region: chr5 76166876-76166914. Max. coverage (+): 0. Max coverage (-): 25.92

Region: chr5 76166915-76166952. Max. coverage (+): 0. Max coverage (-): 7.84

Region: chr5 76166953-76166990. Max. coverage (+): 0. Max coverage (-): 6.66

Region: chr5 76166991-76167028. Max. coverage (+): 0. Max coverage (-): 3.69

Region: chr5 76167029-76167066. Max. coverage (+): 0. Max coverage (-): 0

Region: chr5 76167067-76167104. Max. coverage (+): 0. Max coverage (-): 36.27

Region: chr5 76167105-76167142. Max. coverage (+): 0. Max coverage (-): 29.85

Region: chr5 76167143-76167180. Max. coverage (+): 0. Max coverage (-): 24.19

Region: chr5 76167181-76167219. Max. coverage (+): 0. Max coverage (-): 11.93

Region: chr5 76167220-76167257. Max. coverage (+): 0. Max coverage (-): 1.23

Region: chr5 76167258-76167295. Max. coverage (+): 0. Max coverage (-): 0

Region: chr5 76167296-76167333. Max. coverage (+): 0. Max coverage (-): 0

Region: chr5 76167334-76167371. Max. coverage (+): 0. Max coverage (-): 7.67

Region: chr5 76167372-76167409. Max. coverage (+): 0. Max coverage (-): 0

Region: chr5 76167410-76167447. Max. coverage (+): 0. Max coverage (-): 2.48

Region: chr5 76167448-76167485. Max. coverage (+): 0. Max coverage (-): 0.95

Region: chr5 76167486-76167524. Max. coverage (+): 0. Max coverage (-): 0

Region: chr5 76167525-76167562. Max. coverage (+): 0. Max coverage (-): 3.25

Region: chr5 76167563-76167600. Max. coverage (+): 0. Max coverage (-): 3.75

Region: chr5 76167601-76167638. Max. coverage (+): 0. Max coverage (-): 7.69

Region: chr5 76167639-76167676. Max. coverage (+): 0. Max coverage (-): 12.92

Region: chr5 76167677-76167714. Max. coverage (+): 0. Max coverage (-): 3.72

Region: chr5 76167715-76167752. Max. coverage (+): 0. Max coverage (-): 26.04

Region: chr5 76167753-76167790. Max. coverage (+): 0. Max coverage (-): 0

Region: chr5 76167791-76167828. Max. coverage (+): 0. Max coverage (-): 21.61

Region: chr5 76167829-76167867. Max. coverage (+): 0. Max coverage (-): 71.27

Region: chr5 76167868-76167905. Max. coverage (+): 0. Max coverage (-): 10.94

Region: chr5 76167906-76167943. Max. coverage (+): 0. Max coverage (-): 16.04

Region: chr5 76167944-76167981. Max. coverage (+): 0. Max coverage (-): 20.85

Region: chr5 76167982-76168019. Max. coverage (+): 0. Max coverage (-): 0

Region: chr5 76168020-76168057. Max. coverage (+): 0. Max coverage (-): 0

Region: chr5 76168058-76168095. Max. coverage (+): 0. Max coverage (-): 55.66

Region: chr5 76168096-76168133. Max. coverage (+): 0. Max coverage (-): 58.34

Region: chr5 76168134-76168172. Max. coverage (+): 0. Max coverage (-): 8.38

Region: chr5 76168173-76168210. Max. coverage (+): 0. Max coverage (-): 0.18

Region: chr5 76168211-76168248. Max. coverage (+): 0. Max coverage (-): 0

Region: chr5 76168249-76168286. Max. coverage (+): 0. Max coverage (-): 0

Region: chr5 76168287-76168324. Max. coverage (+): 0. Max coverage (-): 0

Region: chr5 76168325-76168362. Max. coverage (+): 0. Max coverage (-): 0

Region: chr5 76168363-76168400. Max. coverage (+): 0. Max coverage (-): 0

Region: chr5 76168401-76168438. Max. coverage (+): 0. Max coverage (-): 0.72

Region: chr5 76168439-76168476. Max. coverage (+): 0. Max coverage (-): 0

Region: chr5 76168477-76168515. Max. coverage (+): 0. Max coverage (-): 13.7

Region: chr5 76168516-76168553. Max. coverage (+): 0. Max coverage (-): 15.6

Region: chr5 76168554-76168591. Max. coverage (+): 0. Max coverage (-): 24.73

Region: chr5 76168592-76168629. Max. coverage (+): 0. Max coverage (-): 23.47

Region: chr5 76168630-76168667. Max. coverage (+): 0. Max coverage (-): 0

Region: chr5 76168668-76168705. Max. coverage (+): 0. Max coverage (-): 0

Region: chr5 76168706-76168743. Max. coverage (+): 0. Max coverage (-): 13.87

Region: chr5 76168744-76168781. Max. coverage (+): 0. Max coverage (-): 18.76

Region: chr5 76168782-76168820. Max. coverage (+): 0. Max coverage (-): 6.79

Region: chr5 76168821-76168858. Max. coverage (+): 0. Max coverage (-): 0

Region: chr5 76168859-76168896. Max. coverage (+): 0. Max coverage (-): 0

Region: chr5 76168897-76168934. Max. coverage (+): 0. Max coverage (-): 4.82

Region: chr5 76168935-76168972. Max. coverage (+): 0. Max coverage (-): 0

Region: chr5 76168973-76169010. Max. coverage (+): 0. Max coverage (-): 0

Region: chr5 76169011-76169048. Max. coverage (+): 0. Max coverage (-): 36.62

Region: chr5 76169049-76169086. Max. coverage (+): 0. Max coverage (-): 27.52

Region: chr5 76169087-76169124. Max. coverage (+): 0. Max coverage (-): 3.67

Region: chr5 76169125-76169163. Max. coverage (+): 0. Max coverage (-): 30.53

Region: chr5 76169164-76169201. Max. coverage (+): 0. Max coverage (-): 0

Region: chr5 76169202-76169239. Max. coverage (+): 0. Max coverage (-): 0

Region: chr5 76169240-76169277. Max. coverage (+): 0. Max coverage (-): 0

Region: chr5 76169278-76169315. Max. coverage (+): 0. Max coverage (-): 0.45

Region: chr5 76169316-76169353. Max. coverage (+): 0. Max coverage (-): 0

Region: chr5 76169354-76169391. Max. coverage (+): 0. Max coverage (-): 11.12

Region: chr5 76169392-76169429. Max. coverage (+): 0. Max coverage (-): 10.49

Region: chr5 76169430-76169468. Max. coverage (+): 0. Max coverage (-): 5.78

Region: chr5 76169469-76169506. Max. coverage (+): 0. Max coverage (-): 40.05

Region: chr5 76169507-76169544. Max. coverage (+): 0. Max coverage (-): 0

Region: chr5 76169545-76169582. Max. coverage (+): 0. Max coverage (-): 107.32

Region: chr5 76169583-76169620. Max. coverage (+): 0. Max coverage (-): 6.44

Region: chr5 76169621-76169658. Max. coverage (+): 0. Max coverage (-): 4.31

Region: chr5 76169659-76169696. Max. coverage (+): 0. Max coverage (-): 4.31

Region: chr5 76169697-76169734. Max. coverage (+): 0. Max coverage (-): 3.93

Region: chr5 76169735-76169772. Max. coverage (+): 0. Max coverage (-): 3.93

Region: chr5 76169773-76169811. Max. coverage (+): 0. Max coverage (-): 5.85

Region: chr5 76169812-76169849. Max. coverage (+): 0. Max coverage (-): 0

Region: chr5 76169850-76169887. Max. coverage (+): 0. Max coverage (-): 0

Region: chr5 76169888-76169925. Max. coverage (+): 0. Max coverage (-): 9.25

Region: chr5 76169926-76169963. Max. coverage (+): 0. Max coverage (-): 0

Region: chr5 76169964-76170001. Max. coverage (+): 0. Max coverage (-): 0

Region: chr5 76170002-76170039. Max. coverage (+): 0. Max coverage (-): 0

Region: chr5 76170040-76170077. Max. coverage (+): 0. Max coverage (-): 6.52

Region: chr5 76170078-76170116. Max. coverage (+): 0. Max coverage (-): 12.16

Region: chr5 76170117-76170154. Max. coverage (+): 0. Max coverage (-): 15.36

Region: chr5 76170155-76170192. Max. coverage (+): 0. Max coverage (-): 3.15

Region: chr5 76170193-76170230. Max. coverage (+): 0. Max coverage (-): 12.48

Region: chr5 76170231-76170268. Max. coverage (+): 0. Max coverage (-): 23.9

Region: chr5 76170269-76170306. Max. coverage (+): 0. Max coverage (-): 1.13

Region: chr5 76170307-76170344. Max. coverage (+): 3.48. Max coverage (-): 4.32

Region: chr5 76170345-76170382. Max. coverage (+): 0. Max coverage (-): 37.54

Region: chr5 76170383-76170420. Max. coverage (+): 0. Max coverage (-): 37.54

Region: chr5 76170421-76170459. Max. coverage (+): 0. Max coverage (-): 7.9

Region: chr5 76170460-76170497. Max. coverage (+): 0. Max coverage (-): 17.35

Region: chr5 76170498-76170535. Max. coverage (+): 0. Max coverage (-): 0

Region: chr5 76170536-76170573. Max. coverage (+): 0. Max coverage (-): 13.67

Region: chr5 76170574-76170611. Max. coverage (+): 0. Max coverage (-): 6.36

Region: chr5 76170612-76170649. Max. coverage (+): 1.27. Max coverage (-): 5.87

Region: chr5 76170650-76170687. Max. coverage (+): 0. Max coverage (-): 0

Region: chr5 76170688-76170725. Max. coverage (+): 0. Max coverage (-): 37.27

Region: chr5 76170726-76170764. Max. coverage (+): 0. Max coverage (-): 0

Region: chr5 76170765-76170802. Max. coverage (+): 0. Max coverage (-): 9.04

Region: chr5 76170803-76170840. Max. coverage (+): 0. Max coverage (-): 0

Region: chr5 76170841-76170878. Max. coverage (+): 0. Max coverage (-): 0

Region: chr5 76170879-76170916. Max. coverage (+): 0. Max coverage (-): 8.8

Region: chr5 76170917-76170954. Max. coverage (+): 0. Max coverage (-): 0

Region: chr5 76170955-76170992. Max. coverage (+): 0. Max coverage (-): 0

Region: chr5 76170993-76171030. Max. coverage (+): 0. Max coverage (-): 0

Region: chr5 76171031-76171068. Max. coverage (+): 0. Max coverage (-): 0

Region: chr5 76171069-76171107. Max. coverage (+): 0. Max coverage (-): 15.87

Region: chr5 76171108-76171145. Max. coverage (+): 0. Max coverage (-): 0

Region: chr5 76171146-76171183. Max. coverage (+): 0. Max coverage (-): 36.65

Region: chr5 76171184-76171221. Max. coverage (+): 0. Max coverage (-): 56.56

Region: chr5 76171222-76171259. Max. coverage (+): 0. Max coverage (-): 0

Region: chr5 76171260-76171297. Max. coverage (+): 0. Max coverage (-): 25.45

Region: chr5 76171298-76171335. Max. coverage (+): 0. Max coverage (-): 19.36

Region: chr5 76171336-76171373. Max. coverage (+): 0. Max coverage (-): 13.5

Region: chr5 76171374-76171412. Max. coverage (+): 0. Max coverage (-): 16.85

Region: chr5 76171413-76171450. Max. coverage (+): 0. Max coverage (-): 0

Region: chr5 76171451-76171488. Max. coverage (+): 0. Max coverage (-): 32.49

Region: chr5 76171489-76171526. Max. coverage (+): 0. Max coverage (-): 26.82

Region: chr5 76171527-76171564. Max. coverage (+): 0. Max coverage (-): 28.95

Region: chr5 76171565-76171602. Max. coverage (+): 0. Max coverage (-): 13.4

Region: chr5 76171603-76171640. Max. coverage (+): 0. Max coverage (-): 1.82

Region: chr5 76171641-76171678. Max. coverage (+): 0. Max coverage (-): 10.56

Region: chr5 76171679-76171716. Max. coverage (+): 0. Max coverage (-): 6.91

Region: chr5 76171717-76171755. Max. coverage (+): 0. Max coverage (-): 9.52

Region: chr5 76171756-76171793. Max. coverage (+): 0. Max coverage (-): 0

Region: chr5 76171794-76171831. Max. coverage (+): 0. Max coverage (-): 15.02

Region: chr5 76171832-76171869. Max. coverage (+): 0. Max coverage (-): 15.02

Region: chr5 76171870-76171907. Max. coverage (+): 0. Max coverage (-): 0

Region: chr5 76171908-76171945. Max. coverage (+): 0. Max coverage (-): 7.01

Region: chr5 76171946-76171983. Max. coverage (+): 0. Max coverage (-): 0

Region: chr5 76171984-76172021. Max. coverage (+): 0. Max coverage (-): 0

Region: chr5 76172022-76172060. Max. coverage (+): 0. Max coverage (-): 0

Region: chr5 76172061-76172098. Max. coverage (+): 0. Max coverage (-): 0

Region: chr5 76172099-76172136. Max. coverage (+): 0. Max coverage (-): 0

Region: chr5 76172137-76172174. Max. coverage (+): 0. Max coverage (-): 0

Region: chr5 76172175-76172212. Max. coverage (+): 0. Max coverage (-): 0

Region: chr5 76172213-76172250. Max. coverage (+): 0. Max coverage (-): 0

Region: chr5 76172251-76172288. Max. coverage (+): 0. Max coverage (-): 0.51

Region: chr5 76172289-76172326. Max. coverage (+): 0. Max coverage (-): 0

Region: chr5 76172327-76172364. Max. coverage (+): 0. Max coverage (-): 0

Region: chr5 76172365-76172403. Max. coverage (+): 0. Max coverage (-): 0

Region: chr5 76172404-76172441. Max. coverage (+): 0. Max coverage (-): 0

Region: chr5 76172442-76172479. Max. coverage (+): 0. Max coverage (-): 0

Region: chr5 76172480-76172517. Max. coverage (+): 2.74. Max coverage (-): 0

Region: chr5 76172518-76172555. Max. coverage (+): 0. Max coverage (-): 5.82

Region: chr5 76172556-76172593. Max. coverage (+): 0. Max coverage (-): 0

Region: chr5 76172594-76172631. Max. coverage (+): 0. Max coverage (-): 0

Region: chr5 76172632-76172669. Max. coverage (+): 0. Max coverage (-): 0

Region: chr5 76172670-76172708. Max. coverage (+): 0. Max coverage (-): 0

Region: chr5 76172709-76172746. Max. coverage (+): 0. Max coverage (-): 0

Region: chr5 76172747-76172784. Max. coverage (+): 0. Max coverage (-): 5.18

Region: chr5 76172785-76172822. Max. coverage (+): 0. Max coverage (-): 0

Region: chr5 76172823-76172860. Max. coverage (+): 0. Max coverage (-): 16.34

Region: chr5 76172861-76172898. Max. coverage (+): 0. Max coverage (-): 0

Region: chr5 76172899-76172936. Max. coverage (+): 0. Max coverage (-): 0

Region: chr5 76172937-76172974. Max. coverage (+): 0. Max coverage (-): 0

Region: chr5 76172975-76173012. Max. coverage (+): 0. Max coverage (-): 0

Region: chr5 76173013-76173051. Max. coverage (+): 0. Max coverage (-): 0

Region: chr5 76173052-76173089. Max. coverage (+): 0. Max coverage (-): 0

Region: chr5 76173090-76173127. Max. coverage (+): 0. Max coverage (-): 0

Region: chr5 76173128-76173165. Max. coverage (+): 0. Max coverage (-): 0

Region: chr5 76173166-76173203. Max. coverage (+): 0. Max coverage (-): 0

Region: chr5 76173204-76173241. Max. coverage (+): 0. Max coverage (-): 6.09

Region: chr5 76173242-76173279. Max. coverage (+): 0. Max coverage (-): 5.68

Region: chr5 76173280-76173317. Max. coverage (+): 0. Max coverage (-): 10.03

Region: chr5 76173318-. Max. coverage (+): 0. Max coverage (-): 0

RepeatMasker Color Code

**+**

100-98% Identity

<98-95% Identity

<95-90% Identity

<90-85% Identity

<85-80% Identity

<80-75% Identity

<75-70% Identity

<70% Identity

**-**

Gene Set Color Code

**+**

Gene

Pseudogene

**-**

Topology/Coverage Color Code

Coverage Plus Strand

Coverage Minus Strand

Mainstrand: Plus

Mainstrand: Minus

Complementary Strand

Flanking Region  
(if option -flank >0)

Gene Set Annotation  
  
RepeatMasker Annotation  

**1. Plat\_L3**: 76154480-76154545 (-), Divergence to consensus: 24.5%  
**2. L3**: 76154550-76154782 (-), Divergence to consensus: 46%  
**3. MLT1L**: 76155132-76155284 (-), Divergence to consensus: 38.8%  
**4. X7B\_LINE**: 76155287-76155359 (+), Divergence to consensus: 38.4%  
**5. L2c**: 76155373-76155424 (-), Divergence to consensus: 32.7%  
**6. L2c**: 76155472-76155789 (-), Divergence to consensus: 43.9%  
**7. L2c**: 76155803-76155871 (-), Divergence to consensus: 23.2%  
**8. L3**: 76156307-76156350 (-), Divergence to consensus: 29.6%  
**9. MIR**: 76156379-76156513 (-), Divergence to consensus: 39.5%  
**10. MIRb**: 76156693-76156868 (+), Divergence to consensus: 49.5%  
**11. C-rich**: 76157421-76157464 (+), Divergence to consensus: 22%  
**12. MER115**: 76158059-76158633 (-), Divergence to consensus: 45.2%  
**13. MIRb**: 76159044-76159208 (+), Divergence to consensus: 41.2%  
**14. LTR67B**: 76159257-76159407 (+), Divergence to consensus: 38.4%  
**15. LTR67B**: 76159526-76159710 (+), Divergence to consensus: 39.5%  
**16. Bov-tA2**: 76160658-76160719 (-), Divergence to consensus: 17.7%  
**17. BovB**: 76160720-76160873 (-), Divergence to consensus: 19.9%  
**18. ART2A**: 76161187-76161513 (+), Divergence to consensus: 17.9%  
**19. MER41\_BT**: 76162340-76162380 (-), Divergence to consensus: 14.6%  
**20. MER41\_BT**: 76162367-76162939 (-), Divergence to consensus: 25.7%  
**21. Bov-tA3**: 76164553-76164777 (+), Divergence to consensus: 13.5%  
**22. MamRep1151**: 76166342-76166962 (+), Divergence to consensus: 44.7%  
**23. MIRb**: 76170924-76170987 (+), Divergence to consensus: 41.7%  
**24. MIRb**: 76172881-76173007 (+), Divergence to consensus: 40.2%

  
Transcription Factor Binding Sites  

**RFX4\_1** (Sequence: GTTGCTAGG (-): 76157073)  
**RFX4\_1** (Sequence: GTTGCCAAG (-): 76164539)  
**RFX4\_1** (Sequence: GTTGCCATG (-): 76172115)  
**RFX4\_1** (Sequence: CCTGGCAAC (+): 76160130)  
**SOX9** (Sequence: AACAATGA (-): 76160489)  
**SOX9** (Sequence: TTATTGTT (+): 76157251)  
**A-MYB** (Sequence: CCAACTGCCA (-): 76172097)
