# Supplementary material for: Small RNA sequencing of cryopreserved semen from single bull revealed altered miRNAs and piRNAs expression between High- and Low-motile sperm populations
Source: BMC Genomics. 2017 Jan 4;18:14. doi: 10.1186/s12864-016-3394-7 (PMC5209821; doi:10.1186/s12864-016-3394-7)
Supplement: Additional file 4: — Details for each piRNA clusters found in Low Motile (LM) sperm fraction. Genes, repeats, transposable elements and transcription factors binding sites falling within the cluster regions were reported. (ZIP 1034 kb) [file 12864_2016_3394_MOESM4_ESM.zip › 45.html]

piRNA cluster 45


Predicted piRNA cluster no. 45     previous   next
  

Show proTRAC run info
Hide proTRAC run info

================================= proTRAC ====================================  
VERSION: 2.1                                    LAST MODIFIED: 06. October 2015  
  
Please cite:  
Rosenkranz D, Zischler H. proTRAC - a software for probabilistic piRNA cluster  
detection, visualization and analysis. 2012. BMC Bioinformatics 13:5.  
  
and (for proTRAC 2.0 and later):  
Rosenkranz D, Rudloff S, Bastuck K, Ketting RF, Zischler H. Tupaia small RNAs  
provide insights into function and evolution of RNAi-based transposon defense  
in mammals. 2015. RNA 21(5):911-922.  
  
Contact:  
David Rosenkranz  
Institute of Anthropology, small RNA group  
Johannes Gutenberg University Mainz  
email: rosenkranz@uni-mainz.de  
  
You can find the latest proTRAC version at:  
http://sourceforge.net/projects/protrac/files  
http://www.smallRNAgroup-mainz.de/software  
==============================================================================  
  
PARAMETERS:  
Map file: .............../storage/core/barbara/genhome/smallRNA/fertility/Sample\_not\_motile/pirna/Sample\_not\_motile\_26-33\_collapsed.fa.no-dust.map.weighted-10000-1000-b-0  
Genome file: ............/storage/core/barbara/genhome/smallRNA/fertility/Sample\_all/pirna/bt\_311\_chrY.fa  
RepeatMasker annotation: /storage/genomes/bt\_umd31/GCF\_000003055.6\_Bos\_taurus\_UMD\_3.1.1\_repeatMasker\_chr.out  
GeneSet:................./storage/core/barbara/genhome/smallRNA/fertility/Sample\_all/pirna/full.gtf  
  
Significant (p<=0.01) hit density will be calculated based  
on observed hit distribution.  
  
Sliding window size: ........................................ 5000 bp  
Sliding window increament: .................................. 1000 bp  
Normalize each hit by number of genomic hits: ............... 1 [0=no/1=yes]  
Normalize each hit by number of sequence reads: ............. 1 [0=no/1=yes]  
Normalize values (-> per million mapped reads): ............. 1 [0=no/1=yes]  
Min. fraction of hits with 1T(U) or 10A: .................... 0.75  
Alternatively: Min. fraction of hits with 1T(U) and 10A: .... 0.5  
Min. fraction of hits with typical piRNA length: ............ 0.75  
Typical piRNA length: ....................................... 26-33 nt  
Min. size of a piRNA cluster: ............................... 5000 bp.  
Min. number of hits (absolute): ............................. 0  
Min. number of hits (normalized): ........................... 0  
Min. fraction of hits on the mainstrand: .................... 0.75  
Top fraction of mapped sequences (in terms of read counts): . 1%  
Top fraction accounts for max. n% of sequence reads: ........ 90%  
Min. fraction of hits on each arm of a bidirectional cluster: 0.1  
Output image file for each cluster: ......................... 0 [0=no/1=yes]  
Output html file for each cluster: .......................... 1 [0=no/1=yes]  
Output a summary table: ..................................... 1 [0=no/1=yes]  
Output a FASTA file for each cluster (piRNA sequences): ..... 1 [0=no/1=yes]  
Output a FASTA file comprising cluster sequences: ........... 1 [0=no/1=yes]  
Search DNA motifs in clusters: .............................. 1 [0=no/1=yes]  
Output flanking sequences: +/- .............................. 0 bp  
Output ~.pTi file: .......................................... 1 [0=no/1=yes]  
==============================================================================  
  
  
Genome size (without gaps): ............ 2678902517 bp  
Gaps (N/X/-): .......................... 53837044 bp  
Mapped reads: .......................... 738059667487  
Non-identical sequences: ............... 277001  
Genomic hits: .......................... 533816  
Significant densitiy of mapped reads: .. 15118061 reads/kb

Show proTRAC cluster info
Hide proTRAC cluster info

|  |  |
| --- | --- |
| Location | chr5 |
| Coordinates | 106887683-106930718 |
| Size [bp] | 43036 |
| Sequence hit loci | 3624 |
| Mapped reads (normalized) | 9611564054 |
| Mapped reads (normalized) per kb | 223337765 |
| Normalized reads with 1T (1U) | 82.6% |
| Normalized reads with 10A | 32.3% |
| Normalized reads with length 26-33 nt | 100% |
| Normalized reads on the main strand(s) | 98.5% |
| Predicted directionality | bi:minus-plus (split between 106907514 and 106907676) |

100%

0%

1T (1U)  
reads

10A reads

26-33 nt  
reads

reads on mainstrand

**Either the amount of reads with 1T (1U) OR 10A has to exceed 75% (set with option: -1Tor10A)  
Alternatively the amount of reads with 1T (1U) AND 10A has to exceed 50% (set with option: -1Tand10A)  
Minimum amount of reads with preferred size is 75% (set with option: -pisize)  
Minimum amount of reads on the main strand(s) is 75% (set with option: -clstrand)**

Show read coverage
Hide read coverage

WHAT DO I SEE HERE?  
This chart shows the location of mapped sequence reads within a predicted piRNA cluster. The color refers to the number of genomic hits produced by the sequence read in question. A dark red bar indicates that this sequence read produces many other hits elsewhere in the genome. Many adjacent red or yellow bars can indicate the presence of a multi-copy element such as transposons or rRNA genes. A dark green bar indicates that this sequence read maps uniquely to this locus.

1 hit

2-5 hits

6-10 hits

11-20 hits

21-50 hits

51-100 hits

> 100 hits

chr5

106887683

106930718

Gene Set

RepeatMasker

Mapped  
Reads

165.85

plus strand

minus strand

165.85

Region: chr5 76173319-106887726. Max. coverage (+): 0. Max coverage (-): 5.53

Region: chr5 106887727-106887812. Max. coverage (+): 0. Max coverage (-): 10.21

Region: chr5 106887813-106887898. Max. coverage (+): 0. Max coverage (-): 0

Region: chr5 106887899-106887984. Max. coverage (+): 0. Max coverage (-): 0

Region: chr5 106887985-106888070. Max. coverage (+): 0. Max coverage (-): 0

Region: chr5 106888071-106888156. Max. coverage (+): 0. Max coverage (-): 0

Region: chr5 106888157-106888242. Max. coverage (+): 0. Max coverage (-): 5.24

Region: chr5 106888243-106888328. Max. coverage (+): 0. Max coverage (-): 0

Region: chr5 106888329-106888414. Max. coverage (+): 0. Max coverage (-): 0

Region: chr5 106888415-106888500. Max. coverage (+): 0. Max coverage (-): 0

Region: chr5 106888501-106888586. Max. coverage (+): 0. Max coverage (-): 0

Region: chr5 106888587-106888672. Max. coverage (+): 0. Max coverage (-): 0

Region: chr5 106888673-106888758. Max. coverage (+): 0. Max coverage (-): 7

Region: chr5 106888759-106888844. Max. coverage (+): 0. Max coverage (-): 15.42

Region: chr5 106888845-106888931. Max. coverage (+): 0. Max coverage (-): 0

Region: chr5 106888932-106889017. Max. coverage (+): 0. Max coverage (-): 0

Region: chr5 106889018-106889103. Max. coverage (+): 0. Max coverage (-): 4.47

Region: chr5 106889104-106889189. Max. coverage (+): 0. Max coverage (-): 7.03

Region: chr5 106889190-106889275. Max. coverage (+): 0. Max coverage (-): 14.37

Region: chr5 106889276-106889361. Max. coverage (+): 0. Max coverage (-): 8.6

Region: chr5 106889362-106889447. Max. coverage (+): 0. Max coverage (-): 0

Region: chr5 106889448-106889533. Max. coverage (+): 0. Max coverage (-): 0

Region: chr5 106889534-106889619. Max. coverage (+): 0. Max coverage (-): 0

Region: chr5 106889620-106889705. Max. coverage (+): 0. Max coverage (-): 0

Region: chr5 106889706-106889791. Max. coverage (+): 0. Max coverage (-): 0

Region: chr5 106889792-106889877. Max. coverage (+): 0. Max coverage (-): 0

Region: chr5 106889878-106889963. Max. coverage (+): 0. Max coverage (-): 0

Region: chr5 106889964-106890049. Max. coverage (+): 0. Max coverage (-): 0

Region: chr5 106890050-106890136. Max. coverage (+): 0. Max coverage (-): 0

Region: chr5 106890137-106890222. Max. coverage (+): 0. Max coverage (-): 0

Region: chr5 106890223-106890308. Max. coverage (+): 0. Max coverage (-): 0

Region: chr5 106890309-106890394. Max. coverage (+): 0. Max coverage (-): 0

Region: chr5 106890395-106890480. Max. coverage (+): 0. Max coverage (-): 0

Region: chr5 106890481-106890566. Max. coverage (+): 0. Max coverage (-): 0

Region: chr5 106890567-106890652. Max. coverage (+): 0. Max coverage (-): 0

Region: chr5 106890653-106890738. Max. coverage (+): 0. Max coverage (-): 0

Region: chr5 106890739-106890824. Max. coverage (+): 0. Max coverage (-): 0

Region: chr5 106890825-106890910. Max. coverage (+): 0. Max coverage (-): 0

Region: chr5 106890911-106890996. Max. coverage (+): 0. Max coverage (-): 15.05

Region: chr5 106890997-106891082. Max. coverage (+): 0. Max coverage (-): 0

Region: chr5 106891083-106891168. Max. coverage (+): 0. Max coverage (-): 3.42

Region: chr5 106891169-106891254. Max. coverage (+): 0. Max coverage (-): 0

Region: chr5 106891255-106891341. Max. coverage (+): 0. Max coverage (-): 4.33

Region: chr5 106891342-106891427. Max. coverage (+): 0. Max coverage (-): 0

Region: chr5 106891428-106891513. Max. coverage (+): 0. Max coverage (-): 0

Region: chr5 106891514-106891599. Max. coverage (+): 0. Max coverage (-): 1.71

Region: chr5 106891600-106891685. Max. coverage (+): 0. Max coverage (-): 0

Region: chr5 106891686-106891771. Max. coverage (+): 0. Max coverage (-): 0

Region: chr5 106891772-106891857. Max. coverage (+): 0. Max coverage (-): 8.8

Region: chr5 106891858-106891943. Max. coverage (+): 0. Max coverage (-): 5.15

Region: chr5 106891944-106892029. Max. coverage (+): 0. Max coverage (-): 6.8

Region: chr5 106892030-106892115. Max. coverage (+): 0. Max coverage (-): 6.8

Region: chr5 106892116-106892201. Max. coverage (+): 0. Max coverage (-): 6.24

Region: chr5 106892202-106892287. Max. coverage (+): 0. Max coverage (-): 0

Region: chr5 106892288-106892373. Max. coverage (+): 0. Max coverage (-): 8.47

Region: chr5 106892374-106892459. Max. coverage (+): 0. Max coverage (-): 8.1

Region: chr5 106892460-106892546. Max. coverage (+): 0. Max coverage (-): 18.38

Region: chr5 106892547-106892632. Max. coverage (+): 0. Max coverage (-): 0

Region: chr5 106892633-106892718. Max. coverage (+): 0. Max coverage (-): 0

Region: chr5 106892719-106892804. Max. coverage (+): 0. Max coverage (-): 0

Region: chr5 106892805-106892890. Max. coverage (+): 0. Max coverage (-): 50.83

Region: chr5 106892891-106892976. Max. coverage (+): 0. Max coverage (-): 0

Region: chr5 106892977-106893062. Max. coverage (+): 0. Max coverage (-): 3.61

Region: chr5 106893063-106893148. Max. coverage (+): 0. Max coverage (-): 0

Region: chr5 106893149-106893234. Max. coverage (+): 0. Max coverage (-): 8.56

Region: chr5 106893235-106893320. Max. coverage (+): 0. Max coverage (-): 22.16

Region: chr5 106893321-106893406. Max. coverage (+): 0. Max coverage (-): 18.15

Region: chr5 106893407-106893492. Max. coverage (+): 0. Max coverage (-): 0

Region: chr5 106893493-106893578. Max. coverage (+): 0. Max coverage (-): 12.6

Region: chr5 106893579-106893665. Max. coverage (+): 0. Max coverage (-): 36.94

Region: chr5 106893666-106893751. Max. coverage (+): 0. Max coverage (-): 34.71

Region: chr5 106893752-106893837. Max. coverage (+): 0. Max coverage (-): 0

Region: chr5 106893838-106893923. Max. coverage (+): 0. Max coverage (-): 0.71

Region: chr5 106893924-106894009. Max. coverage (+): 0. Max coverage (-): 0

Region: chr5 106894010-106894095. Max. coverage (+): 0. Max coverage (-): 27.14

Region: chr5 106894096-106894181. Max. coverage (+): 0. Max coverage (-): 10.07

Region: chr5 106894182-106894267. Max. coverage (+): 0. Max coverage (-): 13.54

Region: chr5 106894268-106894353. Max. coverage (+): 0. Max coverage (-): 1.84

Region: chr5 106894354-106894439. Max. coverage (+): 0. Max coverage (-): 2.95

Region: chr5 106894440-106894525. Max. coverage (+): 0. Max coverage (-): 0

Region: chr5 106894526-106894611. Max. coverage (+): 0. Max coverage (-): 13.13

Region: chr5 106894612-106894697. Max. coverage (+): 0. Max coverage (-): 0.17

Region: chr5 106894698-106894783. Max. coverage (+): 0. Max coverage (-): 15.03

Region: chr5 106894784-106894870. Max. coverage (+): 0. Max coverage (-): 15.03

Region: chr5 106894871-106894956. Max. coverage (+): 0. Max coverage (-): 14.57

Region: chr5 106894957-106895042. Max. coverage (+): 0. Max coverage (-): 25.39

Region: chr5 106895043-106895128. Max. coverage (+): 0. Max coverage (-): 18.35

Region: chr5 106895129-106895214. Max. coverage (+): 0. Max coverage (-): 23.58

Region: chr5 106895215-106895300. Max. coverage (+): 0. Max coverage (-): 59.42

Region: chr5 106895301-106895386. Max. coverage (+): 0. Max coverage (-): 20.71

Region: chr5 106895387-106895472. Max. coverage (+): 0. Max coverage (-): 46.58

Region: chr5 106895473-106895558. Max. coverage (+): 0. Max coverage (-): 9.2

Region: chr5 106895559-106895644. Max. coverage (+): 0. Max coverage (-): 9.88

Region: chr5 106895645-106895730. Max. coverage (+): 0. Max coverage (-): 0

Region: chr5 106895731-106895816. Max. coverage (+): 0. Max coverage (-): 0

Region: chr5 106895817-106895902. Max. coverage (+): 0. Max coverage (-): 0

Region: chr5 106895903-106895988. Max. coverage (+): 0. Max coverage (-): 5.83

Region: chr5 106895989-106896075. Max. coverage (+): 0. Max coverage (-): 14.87

Region: chr5 106896076-106896161. Max. coverage (+): 0. Max coverage (-): 17.96

Region: chr5 106896162-106896247. Max. coverage (+): 0. Max coverage (-): 13.72

Region: chr5 106896248-106896333. Max. coverage (+): 0. Max coverage (-): 27.29

Region: chr5 106896334-106896419. Max. coverage (+): 0. Max coverage (-): 15.1

Region: chr5 106896420-106896505. Max. coverage (+): 0. Max coverage (-): 6.92

Region: chr5 106896506-106896591. Max. coverage (+): 0. Max coverage (-): 5.83

Region: chr5 106896592-106896677. Max. coverage (+): 0. Max coverage (-): 0

Region: chr5 106896678-106896763. Max. coverage (+): 0. Max coverage (-): 10.23

Region: chr5 106896764-106896849. Max. coverage (+): 0. Max coverage (-): 0

Region: chr5 106896850-106896935. Max. coverage (+): 0. Max coverage (-): 33.06

Region: chr5 106896936-106897021. Max. coverage (+): 0. Max coverage (-): 39.51

Region: chr5 106897022-106897107. Max. coverage (+): 0. Max coverage (-): 0

Region: chr5 106897108-106897193. Max. coverage (+): 0. Max coverage (-): 13.71

Region: chr5 106897194-106897280. Max. coverage (+): 0. Max coverage (-): 13.71

Region: chr5 106897281-106897366. Max. coverage (+): 0. Max coverage (-): 0

Region: chr5 106897367-106897452. Max. coverage (+): 0. Max coverage (-): 0

Region: chr5 106897453-106897538. Max. coverage (+): 0. Max coverage (-): 0.32

Region: chr5 106897539-106897624. Max. coverage (+): 0. Max coverage (-): 3.19

Region: chr5 106897625-106897710. Max. coverage (+): 0. Max coverage (-): 3.19

Region: chr5 106897711-106897796. Max. coverage (+): 0. Max coverage (-): 9.4

Region: chr5 106897797-106897882. Max. coverage (+): 0. Max coverage (-): 0

Region: chr5 106897883-106897968. Max. coverage (+): 0. Max coverage (-): 32.27

Region: chr5 106897969-106898054. Max. coverage (+): 0. Max coverage (-): 31.24

Region: chr5 106898055-106898140. Max. coverage (+): 0. Max coverage (-): 41.47

Region: chr5 106898141-106898226. Max. coverage (+): 0. Max coverage (-): 55.92

Region: chr5 106898227-106898312. Max. coverage (+): 0. Max coverage (-): 22.01

Region: chr5 106898313-106898398. Max. coverage (+): 0. Max coverage (-): 47.83

Region: chr5 106898399-106898485. Max. coverage (+): 0. Max coverage (-): 45.89

Region: chr5 106898486-106898571. Max. coverage (+): 0. Max coverage (-): 71.85

Region: chr5 106898572-106898657. Max. coverage (+): 0. Max coverage (-): 21.54

Region: chr5 106898658-106898743. Max. coverage (+): 0. Max coverage (-): 57.34

Region: chr5 106898744-106898829. Max. coverage (+): 0.52. Max coverage (-): 3.63

Region: chr5 106898830-106898915. Max. coverage (+): 0. Max coverage (-): 0

Region: chr5 106898916-106899001. Max. coverage (+): 0. Max coverage (-): 0

Region: chr5 106899002-106899087. Max. coverage (+): 0. Max coverage (-): 0

Region: chr5 106899088-106899173. Max. coverage (+): 0. Max coverage (-): 18.03

Region: chr5 106899174-106899259. Max. coverage (+): 0. Max coverage (-): 1.32

Region: chr5 106899260-106899345. Max. coverage (+): 0. Max coverage (-): 14.03

Region: chr5 106899346-106899431. Max. coverage (+): 0. Max coverage (-): 0.79

Region: chr5 106899432-106899517. Max. coverage (+): 0. Max coverage (-): 11.81

Region: chr5 106899518-106899603. Max. coverage (+): 0. Max coverage (-): 26.75

Region: chr5 106899604-106899690. Max. coverage (+): 0. Max coverage (-): 7.16

Region: chr5 106899691-106899776. Max. coverage (+): 0. Max coverage (-): 28.38

Region: chr5 106899777-106899862. Max. coverage (+): 5.46. Max coverage (-): 45.86

Region: chr5 106899863-106899948. Max. coverage (+): 3.68. Max coverage (-): 96.15

Region: chr5 106899949-106900034. Max. coverage (+): 0. Max coverage (-): 21.15

Region: chr5 106900035-106900120. Max. coverage (+): 0.91. Max coverage (-): 41.91

Region: chr5 106900121-106900206. Max. coverage (+): 0. Max coverage (-): 82.47

Region: chr5 106900207-106900292. Max. coverage (+): 0. Max coverage (-): 46.63

Region: chr5 106900293-106900378. Max. coverage (+): 0. Max coverage (-): 10.82

Region: chr5 106900379-106900464. Max. coverage (+): 0. Max coverage (-): 0

Region: chr5 106900465-106900550. Max. coverage (+): 0. Max coverage (-): 6.59

Region: chr5 106900551-106900636. Max. coverage (+): 0. Max coverage (-): 0

Region: chr5 106900637-106900722. Max. coverage (+): 0. Max coverage (-): 3.59

Region: chr5 106900723-106900808. Max. coverage (+): 0. Max coverage (-): 71.51

Region: chr5 106900809-106900895. Max. coverage (+): 0. Max coverage (-): 63.59

Region: chr5 106900896-106900981. Max. coverage (+): 0. Max coverage (-): 6.55

Region: chr5 106900982-106901067. Max. coverage (+): 0. Max coverage (-): 25.66

Region: chr5 106901068-106901153. Max. coverage (+): 0. Max coverage (-): 67.61

Region: chr5 106901154-106901239. Max. coverage (+): 0. Max coverage (-): 36.36

Region: chr5 106901240-106901325. Max. coverage (+): 0. Max coverage (-): 97.32

Region: chr5 106901326-106901411. Max. coverage (+): 0. Max coverage (-): 0

Region: chr5 106901412-106901497. Max. coverage (+): 0. Max coverage (-): 0

Region: chr5 106901498-106901583. Max. coverage (+): 0. Max coverage (-): 2.14

Region: chr5 106901584-106901669. Max. coverage (+): 0. Max coverage (-): 10.22

Region: chr5 106901670-106901755. Max. coverage (+): 0. Max coverage (-): 20.22

Region: chr5 106901756-106901841. Max. coverage (+): 0. Max coverage (-): 20.39

Region: chr5 106901842-106901927. Max. coverage (+): 0. Max coverage (-): 0

Region: chr5 106901928-106902013. Max. coverage (+): 0. Max coverage (-): 0

Region: chr5 106902014-106902100. Max. coverage (+): 0. Max coverage (-): 0

Region: chr5 106902101-106902186. Max. coverage (+): 0. Max coverage (-): 0

Region: chr5 106902187-106902272. Max. coverage (+): 0. Max coverage (-): 1.85

Region: chr5 106902273-106902358. Max. coverage (+): 0. Max coverage (-): 6.3

Region: chr5 106902359-106902444. Max. coverage (+): 5.64. Max coverage (-): 92.23

Region: chr5 106902445-106902530. Max. coverage (+): 6.36. Max coverage (-): 5.35

Region: chr5 106902531-106902616. Max. coverage (+): 6.36. Max coverage (-): 1.47

Region: chr5 106902617-106902702. Max. coverage (+): 0. Max coverage (-): 0

Region: chr5 106902703-106902788. Max. coverage (+): 0. Max coverage (-): 3.74

Region: chr5 106902789-106902874. Max. coverage (+): 0. Max coverage (-): 6.92

Region: chr5 106902875-106902960. Max. coverage (+): 0. Max coverage (-): 84.87

Region: chr5 106902961-106903046. Max. coverage (+): 0. Max coverage (-): 0.59

Region: chr5 106903047-106903132. Max. coverage (+): 0. Max coverage (-): 7.09

Region: chr5 106903133-106903218. Max. coverage (+): 0. Max coverage (-): 0

Region: chr5 106903219-106903305. Max. coverage (+): 0. Max coverage (-): 0

Region: chr5 106903306-106903391. Max. coverage (+): 0. Max coverage (-): 4.24

Region: chr5 106903392-106903477. Max. coverage (+): 0. Max coverage (-): 11.14

Region: chr5 106903478-106903563. Max. coverage (+): 0. Max coverage (-): 11.14

Region: chr5 106903564-106903649. Max. coverage (+): 0. Max coverage (-): 19.61

Region: chr5 106903650-106903735. Max. coverage (+): 0. Max coverage (-): 36.61

Region: chr5 106903736-106903821. Max. coverage (+): 6.55. Max coverage (-): 0

Region: chr5 106903822-106903907. Max. coverage (+): 0. Max coverage (-): 15.58

Region: chr5 106903908-106903993. Max. coverage (+): 0. Max coverage (-): 20.03

Region: chr5 106903994-106904079. Max. coverage (+): 22.64. Max coverage (-): 5.42

Region: chr5 106904080-106904165. Max. coverage (+): 0. Max coverage (-): 0

Region: chr5 106904166-106904251. Max. coverage (+): 0. Max coverage (-): 0

Region: chr5 106904252-106904337. Max. coverage (+): 0. Max coverage (-): 8.63

Region: chr5 106904338-106904424. Max. coverage (+): 0. Max coverage (-): 40.89

Region: chr5 106904425-106904510. Max. coverage (+): 4.51. Max coverage (-): 11.67

Region: chr5 106904511-106904596. Max. coverage (+): 0. Max coverage (-): 76.67

Region: chr5 106904597-106904682. Max. coverage (+): 0. Max coverage (-): 15.34

Region: chr5 106904683-106904768. Max. coverage (+): 0. Max coverage (-): 6.05

Region: chr5 106904769-106904854. Max. coverage (+): 0. Max coverage (-): 0.46

Region: chr5 106904855-106904940. Max. coverage (+): 0. Max coverage (-): 23.06

Region: chr5 106904941-106905026. Max. coverage (+): 0. Max coverage (-): 40.78

Region: chr5 106905027-106905112. Max. coverage (+): 0. Max coverage (-): 24.13

Region: chr5 106905113-106905198. Max. coverage (+): 5.97. Max coverage (-): 35.37

Region: chr5 106905199-106905284. Max. coverage (+): 4.18. Max coverage (-): 64.21

Region: chr5 106905285-106905370. Max. coverage (+): 0. Max coverage (-): 6.85

Region: chr5 106905371-106905456. Max. coverage (+): 0. Max coverage (-): 0

Region: chr5 106905457-106905542. Max. coverage (+): 8.01. Max coverage (-): 140.76

Region: chr5 106905543-106905629. Max. coverage (+): 0. Max coverage (-): 13.83

Region: chr5 106905630-106905715. Max. coverage (+): 5.47. Max coverage (-): 28.11

Region: chr5 106905716-106905801. Max. coverage (+): 3.33. Max coverage (-): 7.21

Region: chr5 106905802-106905887. Max. coverage (+): 0. Max coverage (-): 165.85

Region: chr5 106905888-106905973. Max. coverage (+): 0. Max coverage (-): 38.94

Region: chr5 106905974-106906059. Max. coverage (+): 0. Max coverage (-): 0

Region: chr5 106906060-106906145. Max. coverage (+): 0. Max coverage (-): 4.58

Region: chr5 106906146-106906231. Max. coverage (+): 0. Max coverage (-): 4.23

Region: chr5 106906232-106906317. Max. coverage (+): 0. Max coverage (-): 18.64

Region: chr5 106906318-106906403. Max. coverage (+): 0. Max coverage (-): 5.51

Region: chr5 106906404-106906489. Max. coverage (+): 3.75. Max coverage (-): 27.25

Region: chr5 106906490-106906575. Max. coverage (+): 0. Max coverage (-): 44.31

Region: chr5 106906576-106906661. Max. coverage (+): 0. Max coverage (-): 4.66

Region: chr5 106906662-106906747. Max. coverage (+): 6.31. Max coverage (-): 9.96

Region: chr5 106906748-106906834. Max. coverage (+): 0. Max coverage (-): 19.12

Region: chr5 106906835-106906920. Max. coverage (+): 0. Max coverage (-): 0

Region: chr5 106906921-106907006. Max. coverage (+): 0. Max coverage (-): 6.84

Region: chr5 106907007-106907092. Max. coverage (+): 0. Max coverage (-): 7.95

Region: chr5 106907093-106907178. Max. coverage (+): 0. Max coverage (-): 0

Region: chr5 106907179-106907264. Max. coverage (+): 0. Max coverage (-): 7.9

Region: chr5 106907265-106907350. Max. coverage (+): 0. Max coverage (-): 30.01

Region: chr5 106907351-106907436. Max. coverage (+): 1.57. Max coverage (-): 14.69

Region: chr5 106907437-106907522. Max. coverage (+): 0. Max coverage (-): 18.87

Region: chr5 106907523-106907608. Max. coverage (+): 0. Max coverage (-): 0

Region: chr5 106907609-106907694. Max. coverage (+): 6.54. Max coverage (-): 6.09

Region: chr5 106907695-106907780. Max. coverage (+): 8.99. Max coverage (-): 0

Region: chr5 106907781-106907866. Max. coverage (+): 5.05. Max coverage (-): 0

Region: chr5 106907867-106907952. Max. coverage (+): 0. Max coverage (-): 0

Region: chr5 106907953-106908039. Max. coverage (+): 2.49. Max coverage (-): 3.49

Region: chr5 106908040-106908125. Max. coverage (+): 0. Max coverage (-): 3.49

Region: chr5 106908126-106908211. Max. coverage (+): 8.11. Max coverage (-): 0

Region: chr5 106908212-106908297. Max. coverage (+): 19.92. Max coverage (-): 0

Region: chr5 106908298-106908383. Max. coverage (+): 6.21. Max coverage (-): 6

Region: chr5 106908384-106908469. Max. coverage (+): 21.64. Max coverage (-): 0

Region: chr5 106908470-106908555. Max. coverage (+): 58.12. Max coverage (-): 0

Region: chr5 106908556-106908641. Max. coverage (+): 53.7. Max coverage (-): 2.21

Region: chr5 106908642-106908727. Max. coverage (+): 6.28. Max coverage (-): 0

Region: chr5 106908728-106908813. Max. coverage (+): 0. Max coverage (-): 0

Region: chr5 106908814-106908899. Max. coverage (+): 51.18. Max coverage (-): 0

Region: chr5 106908900-106908985. Max. coverage (+): 8.3. Max coverage (-): 0

Region: chr5 106908986-106909071. Max. coverage (+): 13.99. Max coverage (-): 4.43

Region: chr5 106909072-106909157. Max. coverage (+): 25.87. Max coverage (-): 0

Region: chr5 106909158-106909244. Max. coverage (+): 34.72. Max coverage (-): 0

Region: chr5 106909245-106909330. Max. coverage (+): 16.53. Max coverage (-): 0

Region: chr5 106909331-106909416. Max. coverage (+): 10.14. Max coverage (-): 0

Region: chr5 106909417-106909502. Max. coverage (+): 18.86. Max coverage (-): 0

Region: chr5 106909503-106909588. Max. coverage (+): 19.07. Max coverage (-): 0

Region: chr5 106909589-106909674. Max. coverage (+): 28.07. Max coverage (-): 0

Region: chr5 106909675-106909760. Max. coverage (+): 33.76. Max coverage (-): 0

Region: chr5 106909761-106909846. Max. coverage (+): 64.69. Max coverage (-): 0

Region: chr5 106909847-106909932. Max. coverage (+): 29.12. Max coverage (-): 0.61

Region: chr5 106909933-106910018. Max. coverage (+): 53.09. Max coverage (-): 0.61

Region: chr5 106910019-106910104. Max. coverage (+): 127.82. Max coverage (-): 0

Region: chr5 106910105-106910190. Max. coverage (+): 33.53. Max coverage (-): 0

Region: chr5 106910191-106910276. Max. coverage (+): 44.86. Max coverage (-): 0

Region: chr5 106910277-106910362. Max. coverage (+): 8.38. Max coverage (-): 0

Region: chr5 106910363-106910449. Max. coverage (+): 0. Max coverage (-): 0

Region: chr5 106910450-106910535. Max. coverage (+): 0. Max coverage (-): 0

Region: chr5 106910536-106910621. Max. coverage (+): 13.81. Max coverage (-): 0

Region: chr5 106910622-106910707. Max. coverage (+): 32.25. Max coverage (-): 0

Region: chr5 106910708-106910793. Max. coverage (+): 0. Max coverage (-): 0

Region: chr5 106910794-106910879. Max. coverage (+): 91.58. Max coverage (-): 6.53

Region: chr5 106910880-106910965. Max. coverage (+): 54.51. Max coverage (-): 3.62

Region: chr5 106910966-106911051. Max. coverage (+): 29.52. Max coverage (-): 4.13

Region: chr5 106911052-106911137. Max. coverage (+): 51.64. Max coverage (-): 0

Region: chr5 106911138-106911223. Max. coverage (+): 31.92. Max coverage (-): 0

Region: chr5 106911224-106911309. Max. coverage (+): 13.97. Max coverage (-): 0

Region: chr5 106911310-106911395. Max. coverage (+): 28.07. Max coverage (-): 0

Region: chr5 106911396-106911481. Max. coverage (+): 34.94. Max coverage (-): 1.78

Region: chr5 106911482-106911567. Max. coverage (+): 18.86. Max coverage (-): 0

Region: chr5 106911568-106911654. Max. coverage (+): 29.11. Max coverage (-): 0

Region: chr5 106911655-106911740. Max. coverage (+): 55.99. Max coverage (-): 0

Region: chr5 106911741-106911826. Max. coverage (+): 42. Max coverage (-): 0.79

Region: chr5 106911827-106911912. Max. coverage (+): 16.12. Max coverage (-): 1.74

Region: chr5 106911913-106911998. Max. coverage (+): 27.72. Max coverage (-): 1.74

Region: chr5 106911999-106912084. Max. coverage (+): 72.66. Max coverage (-): 0

Region: chr5 106912085-106912170. Max. coverage (+): 65.86. Max coverage (-): 0

Region: chr5 106912171-106912256. Max. coverage (+): 115.97. Max coverage (-): 0

Region: chr5 106912257-106912342. Max. coverage (+): 99.11. Max coverage (-): 0

Region: chr5 106912343-106912428. Max. coverage (+): 34.57. Max coverage (-): 0

Region: chr5 106912429-106912514. Max. coverage (+): 36.43. Max coverage (-): 0

Region: chr5 106912515-106912600. Max. coverage (+): 59.5. Max coverage (-): 0

Region: chr5 106912601-106912686. Max. coverage (+): 21.26. Max coverage (-): 0

Region: chr5 106912687-106912772. Max. coverage (+): 16.62. Max coverage (-): 0

Region: chr5 106912773-106912859. Max. coverage (+): 34.58. Max coverage (-): 0

Region: chr5 106912860-106912945. Max. coverage (+): 9.06. Max coverage (-): 0

Region: chr5 106912946-106913031. Max. coverage (+): 12.25. Max coverage (-): 0

Region: chr5 106913032-106913117. Max. coverage (+): 11.44. Max coverage (-): 0

Region: chr5 106913118-106913203. Max. coverage (+): 62.08. Max coverage (-): 0

Region: chr5 106913204-106913289. Max. coverage (+): 0. Max coverage (-): 0

Region: chr5 106913290-106913375. Max. coverage (+): 12.7. Max coverage (-): 0

Region: chr5 106913376-106913461. Max. coverage (+): 4.74. Max coverage (-): 0.87

Region: chr5 106913462-106913547. Max. coverage (+): 4.23. Max coverage (-): 0

Region: chr5 106913548-106913633. Max. coverage (+): 40. Max coverage (-): 0

Region: chr5 106913634-106913719. Max. coverage (+): 71.61. Max coverage (-): 2.73

Region: chr5 106913720-106913805. Max. coverage (+): 9.49. Max coverage (-): 7.47

Region: chr5 106913806-106913891. Max. coverage (+): 0. Max coverage (-): 0

Region: chr5 106913892-106913977. Max. coverage (+): 0. Max coverage (-): 0

Region: chr5 106913978-106914064. Max. coverage (+): 0. Max coverage (-): 0

Region: chr5 106914065-106914150. Max. coverage (+): 19.76. Max coverage (-): 0

Region: chr5 106914151-106914236. Max. coverage (+): 32.44. Max coverage (-): 0.69

Region: chr5 106914237-106914322. Max. coverage (+): 0. Max coverage (-): 0

Region: chr5 106914323-106914408. Max. coverage (+): 24.92. Max coverage (-): 0

Region: chr5 106914409-106914494. Max. coverage (+): 10.55. Max coverage (-): 0

Region: chr5 106914495-106914580. Max. coverage (+): 47.94. Max coverage (-): 3.66

Region: chr5 106914581-106914666. Max. coverage (+): 62.94. Max coverage (-): 1.91

Region: chr5 106914667-106914752. Max. coverage (+): 7.12. Max coverage (-): 0

Region: chr5 106914753-106914838. Max. coverage (+): 0. Max coverage (-): 0

Region: chr5 106914839-106914924. Max. coverage (+): 0. Max coverage (-): 0

Region: chr5 106914925-106915010. Max. coverage (+): 20.14. Max coverage (-): 0

Region: chr5 106915011-106915096. Max. coverage (+): 26.56. Max coverage (-): 0

Region: chr5 106915097-106915183. Max. coverage (+): 161.2. Max coverage (-): 0

Region: chr5 106915184-106915269. Max. coverage (+): 38.08. Max coverage (-): 5.26

Region: chr5 106915270-106915355. Max. coverage (+): 19.95. Max coverage (-): 0

Region: chr5 106915356-106915441. Max. coverage (+): 84.18. Max coverage (-): 0

Region: chr5 106915442-106915527. Max. coverage (+): 14.7. Max coverage (-): 0

Region: chr5 106915528-106915613. Max. coverage (+): 47.52. Max coverage (-): 0

Region: chr5 106915614-106915699. Max. coverage (+): 54.32. Max coverage (-): 0

Region: chr5 106915700-106915785. Max. coverage (+): 19.81. Max coverage (-): 0

Region: chr5 106915786-106915871. Max. coverage (+): 4.48. Max coverage (-): 0

Region: chr5 106915872-106915957. Max. coverage (+): 4.19. Max coverage (-): 0

Region: chr5 106915958-106916043. Max. coverage (+): 33.37. Max coverage (-): 0

Region: chr5 106916044-106916129. Max. coverage (+): 16.8. Max coverage (-): 0

Region: chr5 106916130-106916215. Max. coverage (+): 49.45. Max coverage (-): 0

Region: chr5 106916216-106916301. Max. coverage (+): 0.12. Max coverage (-): 0

Region: chr5 106916302-106916388. Max. coverage (+): 18.79. Max coverage (-): 0

Region: chr5 106916389-106916474. Max. coverage (+): 24.28. Max coverage (-): 0

Region: chr5 106916475-106916560. Max. coverage (+): 28.34. Max coverage (-): 0

Region: chr5 106916561-106916646. Max. coverage (+): 7.07. Max coverage (-): 0

Region: chr5 106916647-106916732. Max. coverage (+): 57.12. Max coverage (-): 4.3

Region: chr5 106916733-106916818. Max. coverage (+): 34.8. Max coverage (-): 0

Region: chr5 106916819-106916904. Max. coverage (+): 6.81. Max coverage (-): 0

Region: chr5 106916905-106916990. Max. coverage (+): 17.48. Max coverage (-): 0

Region: chr5 106916991-106917076. Max. coverage (+): 19.16. Max coverage (-): 0

Region: chr5 106917077-106917162. Max. coverage (+): 1.51. Max coverage (-): 0

Region: chr5 106917163-106917248. Max. coverage (+): 54.26. Max coverage (-): 0

Region: chr5 106917249-106917334. Max. coverage (+): 18.58. Max coverage (-): 0

Region: chr5 106917335-106917420. Max. coverage (+): 15.71. Max coverage (-): 0

Region: chr5 106917421-106917506. Max. coverage (+): 11. Max coverage (-): 0

Region: chr5 106917507-106917593. Max. coverage (+): 41.9. Max coverage (-): 4.94

Region: chr5 106917594-106917679. Max. coverage (+): 67.64. Max coverage (-): 0

Region: chr5 106917680-106917765. Max. coverage (+): 0. Max coverage (-): 0

Region: chr5 106917766-106917851. Max. coverage (+): 0. Max coverage (-): 0

Region: chr5 106917852-106917937. Max. coverage (+): 20.4. Max coverage (-): 0

Region: chr5 106917938-106918023. Max. coverage (+): 0. Max coverage (-): 0

Region: chr5 106918024-106918109. Max. coverage (+): 8.5. Max coverage (-): 0

Region: chr5 106918110-106918195. Max. coverage (+): 4.74. Max coverage (-): 0

Region: chr5 106918196-106918281. Max. coverage (+): 0. Max coverage (-): 0

Region: chr5 106918282-106918367. Max. coverage (+): 6.34. Max coverage (-): 0

Region: chr5 106918368-106918453. Max. coverage (+): 3.39. Max coverage (-): 4.95

Region: chr5 106918454-106918539. Max. coverage (+): 0.79. Max coverage (-): 11.29

Region: chr5 106918540-106918625. Max. coverage (+): 0. Max coverage (-): 0

Region: chr5 106918626-106918711. Max. coverage (+): 24.09. Max coverage (-): 0

Region: chr5 106918712-106918798. Max. coverage (+): 22.44. Max coverage (-): 0

Region: chr5 106918799-106918884. Max. coverage (+): 0.49. Max coverage (-): 0

Region: chr5 106918885-106918970. Max. coverage (+): 1.14. Max coverage (-): 0

Region: chr5 106918971-106919056. Max. coverage (+): 18.44. Max coverage (-): 0

Region: chr5 106919057-106919142. Max. coverage (+): 90.11. Max coverage (-): 0

Region: chr5 106919143-106919228. Max. coverage (+): 42.99. Max coverage (-): 0

Region: chr5 106919229-106919314. Max. coverage (+): 110.96. Max coverage (-): 0

Region: chr5 106919315-106919400. Max. coverage (+): 27.68. Max coverage (-): 0

Region: chr5 106919401-106919486. Max. coverage (+): 0. Max coverage (-): 0

Region: chr5 106919487-106919572. Max. coverage (+): 0. Max coverage (-): 0

Region: chr5 106919573-106919658. Max. coverage (+): 16.22. Max coverage (-): 0

Region: chr5 106919659-106919744. Max. coverage (+): 12.41. Max coverage (-): 0

Region: chr5 106919745-106919830. Max. coverage (+): 47.66. Max coverage (-): 0

Region: chr5 106919831-106919916. Max. coverage (+): 11.53. Max coverage (-): 0

Region: chr5 106919917-106920003. Max. coverage (+): 24.43. Max coverage (-): 0

Region: chr5 106920004-106920089. Max. coverage (+): 2.29. Max coverage (-): 0

Region: chr5 106920090-106920175. Max. coverage (+): 5.02. Max coverage (-): 0

Region: chr5 106920176-106920261. Max. coverage (+): 0. Max coverage (-): 0

Region: chr5 106920262-106920347. Max. coverage (+): 0. Max coverage (-): 0

Region: chr5 106920348-106920433. Max. coverage (+): 2.48. Max coverage (-): 0

Region: chr5 106920434-106920519. Max. coverage (+): 9.55. Max coverage (-): 0

Region: chr5 106920520-106920605. Max. coverage (+): 8.46. Max coverage (-): 0

Region: chr5 106920606-106920691. Max. coverage (+): 8.25. Max coverage (-): 0

Region: chr5 106920692-106920777. Max. coverage (+): 6.94. Max coverage (-): 0

Region: chr5 106920778-106920863. Max. coverage (+): 64.08. Max coverage (-): 0

Region: chr5 106920864-106920949. Max. coverage (+): 6.68. Max coverage (-): 0

Region: chr5 106920950-106921035. Max. coverage (+): 67.94. Max coverage (-): 0

Region: chr5 106921036-106921121. Max. coverage (+): 0. Max coverage (-): 0

Region: chr5 106921122-106921208. Max. coverage (+): 1.75. Max coverage (-): 0

Region: chr5 106921209-106921294. Max. coverage (+): 1.72. Max coverage (-): 0

Region: chr5 106921295-106921380. Max. coverage (+): 7.71. Max coverage (-): 0

Region: chr5 106921381-106921466. Max. coverage (+): 113.72. Max coverage (-): 0

Region: chr5 106921467-106921552. Max. coverage (+): 1.27. Max coverage (-): 0

Region: chr5 106921553-106921638. Max. coverage (+): 7.02. Max coverage (-): 0

Region: chr5 106921639-106921724. Max. coverage (+): 5.08. Max coverage (-): 0

Region: chr5 106921725-106921810. Max. coverage (+): 12.39. Max coverage (-): 0

Region: chr5 106921811-106921896. Max. coverage (+): 18.48. Max coverage (-): 0

Region: chr5 106921897-106921982. Max. coverage (+): 8.05. Max coverage (-): 0

Region: chr5 106921983-106922068. Max. coverage (+): 8.05. Max coverage (-): 0

Region: chr5 106922069-106922154. Max. coverage (+): 30.45. Max coverage (-): 0

Region: chr5 106922155-106922240. Max. coverage (+): 51.27. Max coverage (-): 0

Region: chr5 106922241-106922326. Max. coverage (+): 3. Max coverage (-): 0

Region: chr5 106922327-106922413. Max. coverage (+): 4.64. Max coverage (-): 0

Region: chr5 106922414-106922499. Max. coverage (+): 0. Max coverage (-): 0

Region: chr5 106922500-106922585. Max. coverage (+): 0. Max coverage (-): 0

Region: chr5 106922586-106922671. Max. coverage (+): 0. Max coverage (-): 0

Region: chr5 106922672-106922757. Max. coverage (+): 0. Max coverage (-): 0

Region: chr5 106922758-106922843. Max. coverage (+): 0. Max coverage (-): 0

Region: chr5 106922844-106922929. Max. coverage (+): 0. Max coverage (-): 0

Region: chr5 106922930-106923015. Max. coverage (+): 0. Max coverage (-): 0

Region: chr5 106923016-106923101. Max. coverage (+): 0. Max coverage (-): 0

Region: chr5 106923102-106923187. Max. coverage (+): 0. Max coverage (-): 0

Region: chr5 106923188-106923273. Max. coverage (+): 0. Max coverage (-): 0

Region: chr5 106923274-106923359. Max. coverage (+): 0. Max coverage (-): 0

Region: chr5 106923360-106923445. Max. coverage (+): 0. Max coverage (-): 0

Region: chr5 106923446-106923531. Max. coverage (+): 0. Max coverage (-): 0

Region: chr5 106923532-106923618. Max. coverage (+): 0. Max coverage (-): 0

Region: chr5 106923619-106923704. Max. coverage (+): 0. Max coverage (-): 0

Region: chr5 106923705-106923790. Max. coverage (+): 0. Max coverage (-): 0

Region: chr5 106923791-106923876. Max. coverage (+): 0. Max coverage (-): 0

Region: chr5 106923877-106923962. Max. coverage (+): 6.65. Max coverage (-): 0

Region: chr5 106923963-106924048. Max. coverage (+): 59.17. Max coverage (-): 0

Region: chr5 106924049-106924134. Max. coverage (+): 27.98. Max coverage (-): 0

Region: chr5 106924135-106924220. Max. coverage (+): 46. Max coverage (-): 0

Region: chr5 106924221-106924306. Max. coverage (+): 7.16. Max coverage (-): 0

Region: chr5 106924307-106924392. Max. coverage (+): 16.03. Max coverage (-): 0

Region: chr5 106924393-106924478. Max. coverage (+): 5.07. Max coverage (-): 0

Region: chr5 106924479-106924564. Max. coverage (+): 4.47. Max coverage (-): 0

Region: chr5 106924565-106924650. Max. coverage (+): 6.91. Max coverage (-): 0

Region: chr5 106924651-106924736. Max. coverage (+): 6.91. Max coverage (-): 0

Region: chr5 106924737-106924823. Max. coverage (+): 0. Max coverage (-): 0

Region: chr5 106924824-106924909. Max. coverage (+): 0. Max coverage (-): 0

Region: chr5 106924910-106924995. Max. coverage (+): 0. Max coverage (-): 0

Region: chr5 106924996-106925081. Max. coverage (+): 0. Max coverage (-): 0

Region: chr5 106925082-106925167. Max. coverage (+): 0. Max coverage (-): 0

Region: chr5 106925168-106925253. Max. coverage (+): 0. Max coverage (-): 0

Region: chr5 106925254-106925339. Max. coverage (+): 0. Max coverage (-): 0

Region: chr5 106925340-106925425. Max. coverage (+): 0. Max coverage (-): 0

Region: chr5 106925426-106925511. Max. coverage (+): 0. Max coverage (-): 0

Region: chr5 106925512-106925597. Max. coverage (+): 0. Max coverage (-): 0

Region: chr5 106925598-106925683. Max. coverage (+): 0. Max coverage (-): 0

Region: chr5 106925684-106925769. Max. coverage (+): 0. Max coverage (-): 0

Region: chr5 106925770-106925855. Max. coverage (+): 0. Max coverage (-): 0

Region: chr5 106925856-106925942. Max. coverage (+): 0. Max coverage (-): 0

Region: chr5 106925943-106926028. Max. coverage (+): 0. Max coverage (-): 0

Region: chr5 106926029-106926114. Max. coverage (+): 0. Max coverage (-): 0

Region: chr5 106926115-106926200. Max. coverage (+): 0. Max coverage (-): 0

Region: chr5 106926201-106926286. Max. coverage (+): 7.01. Max coverage (-): 0

Region: chr5 106926287-106926372. Max. coverage (+): 10.3. Max coverage (-): 0

Region: chr5 106926373-106926458. Max. coverage (+): 0. Max coverage (-): 0

Region: chr5 106926459-106926544. Max. coverage (+): 0. Max coverage (-): 0

Region: chr5 106926545-106926630. Max. coverage (+): 0. Max coverage (-): 0

Region: chr5 106926631-106926716. Max. coverage (+): 0. Max coverage (-): 0

Region: chr5 106926717-106926802. Max. coverage (+): 12.58. Max coverage (-): 0

Region: chr5 106926803-106926888. Max. coverage (+): 18.79. Max coverage (-): 0

Region: chr5 106926889-106926974. Max. coverage (+): 14.58. Max coverage (-): 0

Region: chr5 106926975-106927060. Max. coverage (+): 0.18. Max coverage (-): 0

Region: chr5 106927061-106927147. Max. coverage (+): 8.29. Max coverage (-): 0

Region: chr5 106927148-106927233. Max. coverage (+): 0. Max coverage (-): 0

Region: chr5 106927234-106927319. Max. coverage (+): 0. Max coverage (-): 0

Region: chr5 106927320-106927405. Max. coverage (+): 0. Max coverage (-): 0

Region: chr5 106927406-106927491. Max. coverage (+): 0. Max coverage (-): 0

Region: chr5 106927492-106927577. Max. coverage (+): 5.77. Max coverage (-): 0

Region: chr5 106927578-106927663. Max. coverage (+): 5.77. Max coverage (-): 0

Region: chr5 106927664-106927749. Max. coverage (+): 18.85. Max coverage (-): 0

Region: chr5 106927750-106927835. Max. coverage (+): 2.76. Max coverage (-): 0

Region: chr5 106927836-106927921. Max. coverage (+): 0. Max coverage (-): 0

Region: chr5 106927922-106928007. Max. coverage (+): 5.98. Max coverage (-): 0

Region: chr5 106928008-106928093. Max. coverage (+): 0. Max coverage (-): 0

Region: chr5 106928094-106928179. Max. coverage (+): 3.08. Max coverage (-): 0

Region: chr5 106928180-106928265. Max. coverage (+): 0. Max coverage (-): 0

Region: chr5 106928266-106928352. Max. coverage (+): 0. Max coverage (-): 0

Region: chr5 106928353-106928438. Max. coverage (+): 0. Max coverage (-): 0

Region: chr5 106928439-106928524. Max. coverage (+): 0. Max coverage (-): 0

Region: chr5 106928525-106928610. Max. coverage (+): 0.79. Max coverage (-): 0

Region: chr5 106928611-106928696. Max. coverage (+): 0. Max coverage (-): 0

Region: chr5 106928697-106928782. Max. coverage (+): 0. Max coverage (-): 0

Region: chr5 106928783-106928868. Max. coverage (+): 0. Max coverage (-): 0

Region: chr5 106928869-106928954. Max. coverage (+): 0. Max coverage (-): 0

Region: chr5 106928955-106929040. Max. coverage (+): 0. Max coverage (-): 0

Region: chr5 106929041-106929126. Max. coverage (+): 0. Max coverage (-): 0

Region: chr5 106929127-106929212. Max. coverage (+): 0. Max coverage (-): 0

Region: chr5 106929213-106929298. Max. coverage (+): 0. Max coverage (-): 0

Region: chr5 106929299-106929384. Max. coverage (+): 0. Max coverage (-): 0

Region: chr5 106929385-106929470. Max. coverage (+): 0. Max coverage (-): 0

Region: chr5 106929471-106929557. Max. coverage (+): 18.75. Max coverage (-): 0

Region: chr5 106929558-106929643. Max. coverage (+): 0. Max coverage (-): 0

Region: chr5 106929644-106929729. Max. coverage (+): 8.88. Max coverage (-): 0

Region: chr5 106929730-106929815. Max. coverage (+): 10.31. Max coverage (-): 0

Region: chr5 106929816-106929901. Max. coverage (+): 0. Max coverage (-): 0

Region: chr5 106929902-106929987. Max. coverage (+): 0. Max coverage (-): 0

Region: chr5 106929988-106930073. Max. coverage (+): 0. Max coverage (-): 0

Region: chr5 106930074-106930159. Max. coverage (+): 0. Max coverage (-): 0

Region: chr5 106930160-106930245. Max. coverage (+): 0. Max coverage (-): 0

Region: chr5 106930246-106930331. Max. coverage (+): 1.79. Max coverage (-): 0

Region: chr5 106930332-106930417. Max. coverage (+): 1.79. Max coverage (-): 0

Region: chr5 106930418-106930503. Max. coverage (+): 0. Max coverage (-): 0

Region: chr5 106930504-106930589. Max. coverage (+): 0. Max coverage (-): 0

Region: chr5 106930590-106930675. Max. coverage (+): 0. Max coverage (-): 0

Region: chr5 106930676-. Max. coverage (+): 1.91. Max coverage (-): 0

RepeatMasker Color Code

**+**

100-98% Identity

<98-95% Identity

<95-90% Identity

<90-85% Identity

<85-80% Identity

<80-75% Identity

<75-70% Identity

<70% Identity

**-**

Gene Set Color Code

**+**

Gene

Pseudogene

**-**

Topology/Coverage Color Code

Coverage Plus Strand

Coverage Minus Strand

Mainstrand: Plus

Mainstrand: Minus

Complementary Strand

Flanking Region  
(if option -flank >0)

Gene Set Annotation  
  
RepeatMasker Annotation  

**1. MIRb**: 106888298-106888450 (+), Divergence to consensus: 49.8%  
**2. MIR3**: 106888510-106888700 (+), Divergence to consensus: 35.3%  
**3. MIR**: 106889717-106889855 (-), Divergence to consensus: 42.6%  
**4. MIRb**: 106889871-106889970 (+), Divergence to consensus: 38%  
**5. L1-2\_BT**: 106889973-106890863 (+), Divergence to consensus: 28.4%  
**6. LTR88a**: 106891932-106892146 (-), Divergence to consensus: 44.7%  
**7. Bov-tA1**: 106892560-106892764 (-), Divergence to consensus: 19.6%  
**8. MamTip2**: 106892918-106893007 (-), Divergence to consensus: 31.4%  
**9. MIR3**: 106893094-106893161 (+), Divergence to consensus: 35.8%  
**10. A-rich**: 106893505-106893554 (+), Divergence to consensus: 24%  
**11. Bov-tA2**: 106893704-106893879 (+), Divergence to consensus: 23.9%  
**12. (T)n**: 106894388-106894408 (+), Divergence to consensus: 0%  
**13. MIR3**: 106894451-106894508 (+), Divergence to consensus: 29.8%  
**14. HAL1ME**: 106895688-106896029 (-), Divergence to consensus: 45%  
**15. HAL1ME**: 106896909-106897137 (-), Divergence to consensus: 43.9%  
**16. L2a**: 106897138-106897177 (+), Divergence to consensus: 20%  
**17. BOV-A2**: 106897225-106897488 (+), Divergence to consensus: 9.1%  
**18. L2a**: 106898870-106898986 (+), Divergence to consensus: 42.1%  
**19. (TGAA)n**: 106898987-106899028 (+), Divergence to consensus: 7.1%  
**20. L2c**: 106901327-106901422 (-), Divergence to consensus: 33.3%  
**21. MIRb**: 106901872-106901968 (-), Divergence to consensus: 32%  
**22. MIRb**: 106902560-106902674 (+), Divergence to consensus: 20.8%  
**23. L1ME4a**: 106904060-106904162 (-), Divergence to consensus: 28.1%  
**24. Bov-tA3**: 106905976-106906135 (+), Divergence to consensus: 13.1%  
**25. L2b**: 106906794-106906841 (-), Divergence to consensus: 27.1%  
**26. MIR**: 106906851-106906979 (-), Divergence to consensus: 33.4%  
**27. MIRb**: 106908728-106908827 (+), Divergence to consensus: 52%  
**28. MIR3**: 106910358-106910547 (+), Divergence to consensus: 33.3%  
**29. MIRc**: 106910682-106910801 (+), Divergence to consensus: 31.7%  
**30. (CAGA)n**: 106913528-106913554 (+), Divergence to consensus: 7.4%  
**31. MER90a**: 106913853-106914116 (-), Divergence to consensus: 46.8%  
**32. MER90a**: 106914152-106914373 (-), Divergence to consensus: 42.9%  
**33. L1MB7**: 106914708-106914985 (+), Divergence to consensus: 41.8%  
**34. MIRb**: 106917663-106917855 (+), Divergence to consensus: 38.1%  
**35. ART2A**: 106922342-106922372 (-), Divergence to consensus: 13.6%  
**36. (CAGTT)n**: 106922373-106922392 (+), Divergence to consensus: 0%  
**37. ART2A**: 106922393-106922903 (-), Divergence to consensus: 13.6%  
**38. BovB**: 106922904-106923752 (-), Divergence to consensus: 3.7%  
**39. BTLTR1**: 106923753-106923823 (+), Divergence to consensus: 7%  
**40. BovB**: 106923838-106923927 (-), Divergence to consensus: 18.2%  
**41. MIR3**: 106924653-106924823 (+), Divergence to consensus: 32.9%  
**42. Charlie1a**: 106924943-106925047 (+), Divergence to consensus: 34.3%  
**43. BOV-A2**: 106925337-106925418 (-), Divergence to consensus: 13.4%  
**44. Charlie1a**: 106925537-106926270 (+), Divergence to consensus: 36.1%  
**45. MER20**: 106926350-106926503 (+), Divergence to consensus: 41.6%  
**46. Charlie1**: 106926504-106926610 (+), Divergence to consensus: 43%  
**47. (TA)n**: 106926611-106926639 (+), Divergence to consensus: 3.5%  
**48. Charlie1**: 106926640-106926773 (+), Divergence to consensus: 43%  
**49. L2c**: 106926991-106927368 (-), Divergence to consensus: 42.6%  
**50. ART2A**: 106928968-106929403 (-), Divergence to consensus: 17.3%  
**51. MIR**: 106929604-106929670 (-), Divergence to consensus: 28.4%

  
Transcription Factor Binding Sites  

**RFX4\_2** (Sequence: GTATCCAGG (-): 106889185)  
**RFX4\_2** (Sequence: GTATCCAAG (-): 106896707)  
**RFX4\_2** (Sequence: GTATCTAGG (-): 106907010)  
**RFX4\_2** (Sequence: GTAACCAGG (-): 106928535)  
**RFX4\_1** (Sequence: GTTGCCAAG (-): 106905234)  
**RFX4\_1** (Sequence: GTTGCCAAG (-): 106908660)  
**RFX4\_1** (Sequence: CTTGGCAAC (+): 106906451)  
**RFX4\_2** (Sequence: CCTGGTTAC (+): 106909127)  
**Gata4** (Sequence: AGATAAG (-): 106908050)  
**Gata4** (Sequence: AGATAAC (-): 106908868)  
**Gata4** (Sequence: AGATAAG (-): 106911421)  
**SOX9** (Sequence: AACAATAA (-): 106887869)  
**SOX9** (Sequence: CTATTGTT (+): 106897624)  
**A-MYB** (Sequence: TGACAGTTGG (+): 106917109)  
**SPZ1** (Sequence: GGGGTAACAG (+): 106918197)  
**SPZ1** (Sequence: AGGGTATCAG (+): 106926942)  
**Gata4** (Sequence: GTTATCT (+): 106894423)  
**Gata4** (Sequence: CTTATCT (+): 106914485)
