# Supplementary material for: Small RNA sequencing of cryopreserved semen from single bull revealed altered miRNAs and piRNAs expression between High- and Low-motile sperm populations
Source: BMC Genomics. 2017 Jan 4;18:14. doi: 10.1186/s12864-016-3394-7 (PMC5209821; doi:10.1186/s12864-016-3394-7)
Supplement: Additional file 4: — Details for each piRNA clusters found in Low Motile (LM) sperm fraction. Genes, repeats, transposable elements and transcription factors binding sites falling within the cluster regions were reported. (ZIP 1034 kb) [file 12864_2016_3394_MOESM4_ESM.zip › 46.html]

piRNA cluster 46


Predicted piRNA cluster no. 46     previous   next
  

Show proTRAC run info
Hide proTRAC run info

================================= proTRAC ====================================  
VERSION: 2.1                                    LAST MODIFIED: 06. October 2015  
  
Please cite:  
Rosenkranz D, Zischler H. proTRAC - a software for probabilistic piRNA cluster  
detection, visualization and analysis. 2012. BMC Bioinformatics 13:5.  
  
and (for proTRAC 2.0 and later):  
Rosenkranz D, Rudloff S, Bastuck K, Ketting RF, Zischler H. Tupaia small RNAs  
provide insights into function and evolution of RNAi-based transposon defense  
in mammals. 2015. RNA 21(5):911-922.  
  
Contact:  
David Rosenkranz  
Institute of Anthropology, small RNA group  
Johannes Gutenberg University Mainz  
email: rosenkranz@uni-mainz.de  
  
You can find the latest proTRAC version at:  
http://sourceforge.net/projects/protrac/files  
http://www.smallRNAgroup-mainz.de/software  
==============================================================================  
  
PARAMETERS:  
Map file: .............../storage/core/barbara/genhome/smallRNA/fertility/Sample\_not\_motile/pirna/Sample\_not\_motile\_26-33\_collapsed.fa.no-dust.map.weighted-10000-1000-b-0  
Genome file: ............/storage/core/barbara/genhome/smallRNA/fertility/Sample\_all/pirna/bt\_311\_chrY.fa  
RepeatMasker annotation: /storage/genomes/bt\_umd31/GCF\_000003055.6\_Bos\_taurus\_UMD\_3.1.1\_repeatMasker\_chr.out  
GeneSet:................./storage/core/barbara/genhome/smallRNA/fertility/Sample\_all/pirna/full.gtf  
  
Significant (p<=0.01) hit density will be calculated based  
on observed hit distribution.  
  
Sliding window size: ........................................ 5000 bp  
Sliding window increament: .................................. 1000 bp  
Normalize each hit by number of genomic hits: ............... 1 [0=no/1=yes]  
Normalize each hit by number of sequence reads: ............. 1 [0=no/1=yes]  
Normalize values (-> per million mapped reads): ............. 1 [0=no/1=yes]  
Min. fraction of hits with 1T(U) or 10A: .................... 0.75  
Alternatively: Min. fraction of hits with 1T(U) and 10A: .... 0.5  
Min. fraction of hits with typical piRNA length: ............ 0.75  
Typical piRNA length: ....................................... 26-33 nt  
Min. size of a piRNA cluster: ............................... 5000 bp.  
Min. number of hits (absolute): ............................. 0  
Min. number of hits (normalized): ........................... 0  
Min. fraction of hits on the mainstrand: .................... 0.75  
Top fraction of mapped sequences (in terms of read counts): . 1%  
Top fraction accounts for max. n% of sequence reads: ........ 90%  
Min. fraction of hits on each arm of a bidirectional cluster: 0.1  
Output image file for each cluster: ......................... 0 [0=no/1=yes]  
Output html file for each cluster: .......................... 1 [0=no/1=yes]  
Output a summary table: ..................................... 1 [0=no/1=yes]  
Output a FASTA file for each cluster (piRNA sequences): ..... 1 [0=no/1=yes]  
Output a FASTA file comprising cluster sequences: ........... 1 [0=no/1=yes]  
Search DNA motifs in clusters: .............................. 1 [0=no/1=yes]  
Output flanking sequences: +/- .............................. 0 bp  
Output ~.pTi file: .......................................... 1 [0=no/1=yes]  
==============================================================================  
  
  
Genome size (without gaps): ............ 2678902517 bp  
Gaps (N/X/-): .......................... 53837044 bp  
Mapped reads: .......................... 738059667487  
Non-identical sequences: ............... 277001  
Genomic hits: .......................... 533816  
Significant densitiy of mapped reads: .. 15118061 reads/kb

Show proTRAC cluster info
Hide proTRAC cluster info

|  |  |
| --- | --- |
| Location | chr7 |
| Coordinates | 15471449-15482110 |
| Size [bp] | 10662 |
| Sequence hit loci | 167 |
| Mapped reads (normalized) | 471911835 |
| Mapped reads (normalized) per kb | 44261098.8 |
| Normalized reads with 1T (1U) | 89.7% |
| Normalized reads with 10A | 24.7% |
| Normalized reads with length 26-33 nt | 100% |
| Normalized reads on the main strand(s) | 100% |
| Predicted directionality | mono:minus |

100%

0%

1T (1U)  
reads

10A reads

26-33 nt  
reads

reads on mainstrand

**Either the amount of reads with 1T (1U) OR 10A has to exceed 75% (set with option: -1Tor10A)  
Alternatively the amount of reads with 1T (1U) AND 10A has to exceed 50% (set with option: -1Tand10A)  
Minimum amount of reads with preferred size is 75% (set with option: -pisize)  
Minimum amount of reads on the main strand(s) is 75% (set with option: -clstrand)**

Show read coverage
Hide read coverage

WHAT DO I SEE HERE?  
This chart shows the location of mapped sequence reads within a predicted piRNA cluster. The color refers to the number of genomic hits produced by the sequence read in question. A dark red bar indicates that this sequence read produces many other hits elsewhere in the genome. Many adjacent red or yellow bars can indicate the presence of a multi-copy element such as transposons or rRNA genes. A dark green bar indicates that this sequence read maps uniquely to this locus.

1 hit

2-5 hits

6-10 hits

11-20 hits

21-50 hits

51-100 hits

> 100 hits

chr7

15471449

15482110

Gene Set

RepeatMasker

Mapped  
Reads

33.27

plus strand

minus strand

33.27

Region: chr7 106930677-15471459. Max. coverage (+): 0. Max coverage (-): 3.52

Region: chr7 15471460-15471480. Max. coverage (+): 0. Max coverage (-): 0

Region: chr7 15471481-15471502. Max. coverage (+): 0. Max coverage (-): 0

Region: chr7 15471503-15471523. Max. coverage (+): 0. Max coverage (-): 0

Region: chr7 15471524-15471544. Max. coverage (+): 0. Max coverage (-): 0

Region: chr7 15471545-15471566. Max. coverage (+): 0. Max coverage (-): 0

Region: chr7 15471567-15471587. Max. coverage (+): 0. Max coverage (-): 0

Region: chr7 15471588-15471608. Max. coverage (+): 0. Max coverage (-): 0

Region: chr7 15471609-15471630. Max. coverage (+): 0. Max coverage (-): 0

Region: chr7 15471631-15471651. Max. coverage (+): 0. Max coverage (-): 0

Region: chr7 15471652-15471672. Max. coverage (+): 0. Max coverage (-): 0

Region: chr7 15471673-15471694. Max. coverage (+): 0. Max coverage (-): 0

Region: chr7 15471695-15471715. Max. coverage (+): 0. Max coverage (-): 0

Region: chr7 15471716-15471736. Max. coverage (+): 0. Max coverage (-): 0

Region: chr7 15471737-15471758. Max. coverage (+): 0. Max coverage (-): 0

Region: chr7 15471759-15471779. Max. coverage (+): 0. Max coverage (-): 0

Region: chr7 15471780-15471800. Max. coverage (+): 0. Max coverage (-): 0

Region: chr7 15471801-15471822. Max. coverage (+): 0. Max coverage (-): 0

Region: chr7 15471823-15471843. Max. coverage (+): 0. Max coverage (-): 0

Region: chr7 15471844-15471864. Max. coverage (+): 0. Max coverage (-): 0

Region: chr7 15471865-15471886. Max. coverage (+): 0. Max coverage (-): 0

Region: chr7 15471887-15471907. Max. coverage (+): 0. Max coverage (-): 0

Region: chr7 15471908-15471928. Max. coverage (+): 0. Max coverage (-): 0

Region: chr7 15471929-15471950. Max. coverage (+): 0. Max coverage (-): 0

Region: chr7 15471951-15471971. Max. coverage (+): 0. Max coverage (-): 0

Region: chr7 15471972-15471992. Max. coverage (+): 0. Max coverage (-): 0

Region: chr7 15471993-15472014. Max. coverage (+): 0. Max coverage (-): 0

Region: chr7 15472015-15472035. Max. coverage (+): 0. Max coverage (-): 0

Region: chr7 15472036-15472056. Max. coverage (+): 0. Max coverage (-): 0

Region: chr7 15472057-15472078. Max. coverage (+): 0. Max coverage (-): 0

Region: chr7 15472079-15472099. Max. coverage (+): 0. Max coverage (-): 0

Region: chr7 15472100-15472120. Max. coverage (+): 0. Max coverage (-): 0

Region: chr7 15472121-15472142. Max. coverage (+): 0. Max coverage (-): 0

Region: chr7 15472143-15472163. Max. coverage (+): 0. Max coverage (-): 0

Region: chr7 15472164-15472184. Max. coverage (+): 0. Max coverage (-): 0

Region: chr7 15472185-15472206. Max. coverage (+): 0. Max coverage (-): 0

Region: chr7 15472207-15472227. Max. coverage (+): 0. Max coverage (-): 0

Region: chr7 15472228-15472248. Max. coverage (+): 0. Max coverage (-): 0

Region: chr7 15472249-15472269. Max. coverage (+): 0. Max coverage (-): 0

Region: chr7 15472270-15472291. Max. coverage (+): 0. Max coverage (-): 0

Region: chr7 15472292-15472312. Max. coverage (+): 0. Max coverage (-): 0

Region: chr7 15472313-15472333. Max. coverage (+): 0. Max coverage (-): 0

Region: chr7 15472334-15472355. Max. coverage (+): 0. Max coverage (-): 0

Region: chr7 15472356-15472376. Max. coverage (+): 0. Max coverage (-): 0

Region: chr7 15472377-15472397. Max. coverage (+): 0. Max coverage (-): 0

Region: chr7 15472398-15472419. Max. coverage (+): 0. Max coverage (-): 0

Region: chr7 15472420-15472440. Max. coverage (+): 0. Max coverage (-): 0.7

Region: chr7 15472441-15472461. Max. coverage (+): 0. Max coverage (-): 0.7

Region: chr7 15472462-15472483. Max. coverage (+): 0. Max coverage (-): 7.6

Region: chr7 15472484-15472504. Max. coverage (+): 0. Max coverage (-): 3.27

Region: chr7 15472505-15472525. Max. coverage (+): 0. Max coverage (-): 0

Region: chr7 15472526-15472547. Max. coverage (+): 0. Max coverage (-): 0

Region: chr7 15472548-15472568. Max. coverage (+): 0. Max coverage (-): 0

Region: chr7 15472569-15472589. Max. coverage (+): 0. Max coverage (-): 0

Region: chr7 15472590-15472611. Max. coverage (+): 0. Max coverage (-): 0

Region: chr7 15472612-15472632. Max. coverage (+): 0. Max coverage (-): 0

Region: chr7 15472633-15472653. Max. coverage (+): 0. Max coverage (-): 0

Region: chr7 15472654-15472675. Max. coverage (+): 0. Max coverage (-): 0

Region: chr7 15472676-15472696. Max. coverage (+): 0. Max coverage (-): 0

Region: chr7 15472697-15472717. Max. coverage (+): 0. Max coverage (-): 0

Region: chr7 15472718-15472739. Max. coverage (+): 0. Max coverage (-): 0

Region: chr7 15472740-15472760. Max. coverage (+): 0. Max coverage (-): 0

Region: chr7 15472761-15472781. Max. coverage (+): 0. Max coverage (-): 0

Region: chr7 15472782-15472803. Max. coverage (+): 0. Max coverage (-): 0

Region: chr7 15472804-15472824. Max. coverage (+): 0. Max coverage (-): 0

Region: chr7 15472825-15472845. Max. coverage (+): 0. Max coverage (-): 0

Region: chr7 15472846-15472867. Max. coverage (+): 0. Max coverage (-): 0

Region: chr7 15472868-15472888. Max. coverage (+): 0. Max coverage (-): 0

Region: chr7 15472889-15472909. Max. coverage (+): 0. Max coverage (-): 0

Region: chr7 15472910-15472931. Max. coverage (+): 0. Max coverage (-): 0

Region: chr7 15472932-15472952. Max. coverage (+): 0. Max coverage (-): 2.6

Region: chr7 15472953-15472973. Max. coverage (+): 0. Max coverage (-): 2.6

Region: chr7 15472974-15472994. Max. coverage (+): 0. Max coverage (-): 0

Region: chr7 15472995-15473016. Max. coverage (+): 0. Max coverage (-): 15.66

Region: chr7 15473017-15473037. Max. coverage (+): 0. Max coverage (-): 7.97

Region: chr7 15473038-15473058. Max. coverage (+): 0. Max coverage (-): 0

Region: chr7 15473059-15473080. Max. coverage (+): 0. Max coverage (-): 0

Region: chr7 15473081-15473101. Max. coverage (+): 0. Max coverage (-): 0

Region: chr7 15473102-15473122. Max. coverage (+): 0. Max coverage (-): 0

Region: chr7 15473123-15473144. Max. coverage (+): 0. Max coverage (-): 0

Region: chr7 15473145-15473165. Max. coverage (+): 0. Max coverage (-): 0

Region: chr7 15473166-15473186. Max. coverage (+): 0. Max coverage (-): 0

Region: chr7 15473187-15473208. Max. coverage (+): 0. Max coverage (-): 0

Region: chr7 15473209-15473229. Max. coverage (+): 0. Max coverage (-): 0

Region: chr7 15473230-15473250. Max. coverage (+): 0. Max coverage (-): 0

Region: chr7 15473251-15473272. Max. coverage (+): 0. Max coverage (-): 0

Region: chr7 15473273-15473293. Max. coverage (+): 0. Max coverage (-): 0

Region: chr7 15473294-15473314. Max. coverage (+): 0. Max coverage (-): 0

Region: chr7 15473315-15473336. Max. coverage (+): 0. Max coverage (-): 0

Region: chr7 15473337-15473357. Max. coverage (+): 0. Max coverage (-): 0

Region: chr7 15473358-15473378. Max. coverage (+): 0. Max coverage (-): 0

Region: chr7 15473379-15473400. Max. coverage (+): 0. Max coverage (-): 0

Region: chr7 15473401-15473421. Max. coverage (+): 0. Max coverage (-): 0

Region: chr7 15473422-15473442. Max. coverage (+): 0. Max coverage (-): 0

Region: chr7 15473443-15473464. Max. coverage (+): 0. Max coverage (-): 0

Region: chr7 15473465-15473485. Max. coverage (+): 0. Max coverage (-): 0

Region: chr7 15473486-15473506. Max. coverage (+): 0. Max coverage (-): 0

Region: chr7 15473507-15473528. Max. coverage (+): 0. Max coverage (-): 0

Region: chr7 15473529-15473549. Max. coverage (+): 0. Max coverage (-): 0

Region: chr7 15473550-15473570. Max. coverage (+): 0. Max coverage (-): 0

Region: chr7 15473571-15473592. Max. coverage (+): 0. Max coverage (-): 0

Region: chr7 15473593-15473613. Max. coverage (+): 0. Max coverage (-): 0

Region: chr7 15473614-15473634. Max. coverage (+): 0. Max coverage (-): 1.58

Region: chr7 15473635-15473656. Max. coverage (+): 0. Max coverage (-): 0

Region: chr7 15473657-15473677. Max. coverage (+): 0. Max coverage (-): 3.02

Region: chr7 15473678-15473698. Max. coverage (+): 0. Max coverage (-): 33

Region: chr7 15473699-15473720. Max. coverage (+): 0. Max coverage (-): 0

Region: chr7 15473721-15473741. Max. coverage (+): 0. Max coverage (-): 0

Region: chr7 15473742-15473762. Max. coverage (+): 0. Max coverage (-): 0

Region: chr7 15473763-15473783. Max. coverage (+): 0. Max coverage (-): 0

Region: chr7 15473784-15473805. Max. coverage (+): 0. Max coverage (-): 0

Region: chr7 15473806-15473826. Max. coverage (+): 0. Max coverage (-): 0

Region: chr7 15473827-15473847. Max. coverage (+): 0. Max coverage (-): 0

Region: chr7 15473848-15473869. Max. coverage (+): 0. Max coverage (-): 0

Region: chr7 15473870-15473890. Max. coverage (+): 0. Max coverage (-): 0

Region: chr7 15473891-15473911. Max. coverage (+): 0. Max coverage (-): 0

Region: chr7 15473912-15473933. Max. coverage (+): 0. Max coverage (-): 0

Region: chr7 15473934-15473954. Max. coverage (+): 0. Max coverage (-): 0

Region: chr7 15473955-15473975. Max. coverage (+): 0. Max coverage (-): 0

Region: chr7 15473976-15473997. Max. coverage (+): 0. Max coverage (-): 0

Region: chr7 15473998-15474018. Max. coverage (+): 0. Max coverage (-): 6.95

Region: chr7 15474019-15474039. Max. coverage (+): 0. Max coverage (-): 6.34

Region: chr7 15474040-15474061. Max. coverage (+): 0. Max coverage (-): 9.79

Region: chr7 15474062-15474082. Max. coverage (+): 0. Max coverage (-): 0

Region: chr7 15474083-15474103. Max. coverage (+): 0. Max coverage (-): 0

Region: chr7 15474104-15474125. Max. coverage (+): 0. Max coverage (-): 0

Region: chr7 15474126-15474146. Max. coverage (+): 0. Max coverage (-): 0

Region: chr7 15474147-15474167. Max. coverage (+): 0. Max coverage (-): 0

Region: chr7 15474168-15474189. Max. coverage (+): 0. Max coverage (-): 0

Region: chr7 15474190-15474210. Max. coverage (+): 0. Max coverage (-): 0

Region: chr7 15474211-15474231. Max. coverage (+): 0. Max coverage (-): 0

Region: chr7 15474232-15474253. Max. coverage (+): 0. Max coverage (-): 0

Region: chr7 15474254-15474274. Max. coverage (+): 0. Max coverage (-): 0

Region: chr7 15474275-15474295. Max. coverage (+): 0. Max coverage (-): 0

Region: chr7 15474296-15474317. Max. coverage (+): 0. Max coverage (-): 0

Region: chr7 15474318-15474338. Max. coverage (+): 0. Max coverage (-): 0

Region: chr7 15474339-15474359. Max. coverage (+): 0. Max coverage (-): 0

Region: chr7 15474360-15474381. Max. coverage (+): 0. Max coverage (-): 0

Region: chr7 15474382-15474402. Max. coverage (+): 0. Max coverage (-): 0

Region: chr7 15474403-15474423. Max. coverage (+): 0. Max coverage (-): 0

Region: chr7 15474424-15474445. Max. coverage (+): 0. Max coverage (-): 0

Region: chr7 15474446-15474466. Max. coverage (+): 0. Max coverage (-): 0

Region: chr7 15474467-15474487. Max. coverage (+): 0. Max coverage (-): 0

Region: chr7 15474488-15474508. Max. coverage (+): 0. Max coverage (-): 0

Region: chr7 15474509-15474530. Max. coverage (+): 0. Max coverage (-): 0

Region: chr7 15474531-15474551. Max. coverage (+): 0. Max coverage (-): 0

Region: chr7 15474552-15474572. Max. coverage (+): 0. Max coverage (-): 0

Region: chr7 15474573-15474594. Max. coverage (+): 0. Max coverage (-): 0

Region: chr7 15474595-15474615. Max. coverage (+): 0. Max coverage (-): 0

Region: chr7 15474616-15474636. Max. coverage (+): 0. Max coverage (-): 0

Region: chr7 15474637-15474658. Max. coverage (+): 0. Max coverage (-): 6.71

Region: chr7 15474659-15474679. Max. coverage (+): 0. Max coverage (-): 0

Region: chr7 15474680-15474700. Max. coverage (+): 0. Max coverage (-): 20.19

Region: chr7 15474701-15474722. Max. coverage (+): 0. Max coverage (-): 0

Region: chr7 15474723-15474743. Max. coverage (+): 0. Max coverage (-): 2.38

Region: chr7 15474744-15474764. Max. coverage (+): 0. Max coverage (-): 18.82

Region: chr7 15474765-15474786. Max. coverage (+): 0. Max coverage (-): 18.82

Region: chr7 15474787-15474807. Max. coverage (+): 0. Max coverage (-): 0

Region: chr7 15474808-15474828. Max. coverage (+): 0. Max coverage (-): 0

Region: chr7 15474829-15474850. Max. coverage (+): 0. Max coverage (-): 0

Region: chr7 15474851-15474871. Max. coverage (+): 0. Max coverage (-): 11.68

Region: chr7 15474872-15474892. Max. coverage (+): 0. Max coverage (-): 20.93

Region: chr7 15474893-15474914. Max. coverage (+): 0. Max coverage (-): 7.54

Region: chr7 15474915-15474935. Max. coverage (+): 0. Max coverage (-): 0

Region: chr7 15474936-15474956. Max. coverage (+): 0. Max coverage (-): 0

Region: chr7 15474957-15474978. Max. coverage (+): 0. Max coverage (-): 0

Region: chr7 15474979-15474999. Max. coverage (+): 0. Max coverage (-): 0

Region: chr7 15475000-15475020. Max. coverage (+): 0. Max coverage (-): 0

Region: chr7 15475021-15475042. Max. coverage (+): 0. Max coverage (-): 0

Region: chr7 15475043-15475063. Max. coverage (+): 0. Max coverage (-): 0

Region: chr7 15475064-15475084. Max. coverage (+): 0. Max coverage (-): 0

Region: chr7 15475085-15475106. Max. coverage (+): 0. Max coverage (-): 0

Region: chr7 15475107-15475127. Max. coverage (+): 0. Max coverage (-): 0

Region: chr7 15475128-15475148. Max. coverage (+): 0. Max coverage (-): 0

Region: chr7 15475149-15475170. Max. coverage (+): 0. Max coverage (-): 9.85

Region: chr7 15475171-15475191. Max. coverage (+): 0. Max coverage (-): 0

Region: chr7 15475192-15475212. Max. coverage (+): 0. Max coverage (-): 0

Region: chr7 15475213-15475234. Max. coverage (+): 0. Max coverage (-): 0

Region: chr7 15475235-15475255. Max. coverage (+): 0. Max coverage (-): 0

Region: chr7 15475256-15475276. Max. coverage (+): 0. Max coverage (-): 0

Region: chr7 15475277-15475297. Max. coverage (+): 0. Max coverage (-): 0

Region: chr7 15475298-15475319. Max. coverage (+): 0. Max coverage (-): 0

Region: chr7 15475320-15475340. Max. coverage (+): 0. Max coverage (-): 0

Region: chr7 15475341-15475361. Max. coverage (+): 0. Max coverage (-): 0

Region: chr7 15475362-15475383. Max. coverage (+): 0. Max coverage (-): 0

Region: chr7 15475384-15475404. Max. coverage (+): 0. Max coverage (-): 0

Region: chr7 15475405-15475425. Max. coverage (+): 0. Max coverage (-): 0

Region: chr7 15475426-15475447. Max. coverage (+): 0. Max coverage (-): 0

Region: chr7 15475448-15475468. Max. coverage (+): 0. Max coverage (-): 0

Region: chr7 15475469-15475489. Max. coverage (+): 0. Max coverage (-): 0

Region: chr7 15475490-15475511. Max. coverage (+): 0. Max coverage (-): 0

Region: chr7 15475512-15475532. Max. coverage (+): 0. Max coverage (-): 0

Region: chr7 15475533-15475553. Max. coverage (+): 0. Max coverage (-): 0

Region: chr7 15475554-15475575. Max. coverage (+): 0. Max coverage (-): 0

Region: chr7 15475576-15475596. Max. coverage (+): 0. Max coverage (-): 0

Region: chr7 15475597-15475617. Max. coverage (+): 0. Max coverage (-): 0

Region: chr7 15475618-15475639. Max. coverage (+): 0. Max coverage (-): 0

Region: chr7 15475640-15475660. Max. coverage (+): 0. Max coverage (-): 5.38

Region: chr7 15475661-15475681. Max. coverage (+): 0. Max coverage (-): 3.92

Region: chr7 15475682-15475703. Max. coverage (+): 0. Max coverage (-): 3.92

Region: chr7 15475704-15475724. Max. coverage (+): 0. Max coverage (-): 0

Region: chr7 15475725-15475745. Max. coverage (+): 0. Max coverage (-): 0

Region: chr7 15475746-15475767. Max. coverage (+): 0. Max coverage (-): 0

Region: chr7 15475768-15475788. Max. coverage (+): 0. Max coverage (-): 0

Region: chr7 15475789-15475809. Max. coverage (+): 0. Max coverage (-): 0

Region: chr7 15475810-15475831. Max. coverage (+): 0. Max coverage (-): 0

Region: chr7 15475832-15475852. Max. coverage (+): 0. Max coverage (-): 0

Region: chr7 15475853-15475873. Max. coverage (+): 0. Max coverage (-): 0

Region: chr7 15475874-15475895. Max. coverage (+): 0. Max coverage (-): 0

Region: chr7 15475896-15475916. Max. coverage (+): 0. Max coverage (-): 0

Region: chr7 15475917-15475937. Max. coverage (+): 0. Max coverage (-): 0

Region: chr7 15475938-15475959. Max. coverage (+): 0. Max coverage (-): 4.9

Region: chr7 15475960-15475980. Max. coverage (+): 0. Max coverage (-): 0

Region: chr7 15475981-15476001. Max. coverage (+): 0. Max coverage (-): 0

Region: chr7 15476002-15476022. Max. coverage (+): 0. Max coverage (-): 0

Region: chr7 15476023-15476044. Max. coverage (+): 0. Max coverage (-): 0

Region: chr7 15476045-15476065. Max. coverage (+): 0. Max coverage (-): 0

Region: chr7 15476066-15476086. Max. coverage (+): 0. Max coverage (-): 0

Region: chr7 15476087-15476108. Max. coverage (+): 0. Max coverage (-): 0

Region: chr7 15476109-15476129. Max. coverage (+): 0. Max coverage (-): 6.13

Region: chr7 15476130-15476150. Max. coverage (+): 0. Max coverage (-): 23.6

Region: chr7 15476151-15476172. Max. coverage (+): 0. Max coverage (-): 26.14

Region: chr7 15476173-15476193. Max. coverage (+): 0. Max coverage (-): 21.22

Region: chr7 15476194-15476214. Max. coverage (+): 0. Max coverage (-): 4.68

Region: chr7 15476215-15476236. Max. coverage (+): 0. Max coverage (-): 0

Region: chr7 15476237-15476257. Max. coverage (+): 0. Max coverage (-): 0

Region: chr7 15476258-15476278. Max. coverage (+): 0. Max coverage (-): 0

Region: chr7 15476279-15476300. Max. coverage (+): 0. Max coverage (-): 0

Region: chr7 15476301-15476321. Max. coverage (+): 0. Max coverage (-): 0

Region: chr7 15476322-15476342. Max. coverage (+): 0. Max coverage (-): 0

Region: chr7 15476343-15476364. Max. coverage (+): 0. Max coverage (-): 0

Region: chr7 15476365-15476385. Max. coverage (+): 0. Max coverage (-): 0

Region: chr7 15476386-15476406. Max. coverage (+): 0. Max coverage (-): 0

Region: chr7 15476407-15476428. Max. coverage (+): 0. Max coverage (-): 0

Region: chr7 15476429-15476449. Max. coverage (+): 0. Max coverage (-): 0

Region: chr7 15476450-15476470. Max. coverage (+): 0. Max coverage (-): 0

Region: chr7 15476471-15476492. Max. coverage (+): 0. Max coverage (-): 0

Region: chr7 15476493-15476513. Max. coverage (+): 0. Max coverage (-): 0

Region: chr7 15476514-15476534. Max. coverage (+): 0. Max coverage (-): 0

Region: chr7 15476535-15476556. Max. coverage (+): 0. Max coverage (-): 0

Region: chr7 15476557-15476577. Max. coverage (+): 0. Max coverage (-): 0

Region: chr7 15476578-15476598. Max. coverage (+): 0. Max coverage (-): 0

Region: chr7 15476599-15476620. Max. coverage (+): 0. Max coverage (-): 0

Region: chr7 15476621-15476641. Max. coverage (+): 0. Max coverage (-): 0

Region: chr7 15476642-15476662. Max. coverage (+): 0. Max coverage (-): 0

Region: chr7 15476663-15476684. Max. coverage (+): 0. Max coverage (-): 0

Region: chr7 15476685-15476705. Max. coverage (+): 0. Max coverage (-): 0

Region: chr7 15476706-15476726. Max. coverage (+): 0. Max coverage (-): 3.1

Region: chr7 15476727-15476748. Max. coverage (+): 0. Max coverage (-): 0

Region: chr7 15476749-15476769. Max. coverage (+): 0. Max coverage (-): 4.29

Region: chr7 15476770-15476790. Max. coverage (+): 0. Max coverage (-): 5.73

Region: chr7 15476791-15476811. Max. coverage (+): 0. Max coverage (-): 0

Region: chr7 15476812-15476833. Max. coverage (+): 0. Max coverage (-): 0

Region: chr7 15476834-15476854. Max. coverage (+): 0. Max coverage (-): 5.44

Region: chr7 15476855-15476875. Max. coverage (+): 0. Max coverage (-): 1.88

Region: chr7 15476876-15476897. Max. coverage (+): 0. Max coverage (-): 10.79

Region: chr7 15476898-15476918. Max. coverage (+): 0. Max coverage (-): 4.47

Region: chr7 15476919-15476939. Max. coverage (+): 0. Max coverage (-): 3.85

Region: chr7 15476940-15476961. Max. coverage (+): 0. Max coverage (-): 13.29

Region: chr7 15476962-15476982. Max. coverage (+): 0. Max coverage (-): 7.11

Region: chr7 15476983-15477003. Max. coverage (+): 0. Max coverage (-): 7.11

Region: chr7 15477004-15477025. Max. coverage (+): 0. Max coverage (-): 27.69

Region: chr7 15477026-15477046. Max. coverage (+): 0. Max coverage (-): 0

Region: chr7 15477047-15477067. Max. coverage (+): 0. Max coverage (-): 3.08

Region: chr7 15477068-15477089. Max. coverage (+): 0. Max coverage (-): 11.95

Region: chr7 15477090-15477110. Max. coverage (+): 0. Max coverage (-): 33.27

Region: chr7 15477111-15477131. Max. coverage (+): 0. Max coverage (-): 9.29

Region: chr7 15477132-15477153. Max. coverage (+): 0. Max coverage (-): 3.3

Region: chr7 15477154-15477174. Max. coverage (+): 0. Max coverage (-): 20.97

Region: chr7 15477175-15477195. Max. coverage (+): 0. Max coverage (-): 0

Region: chr7 15477196-15477217. Max. coverage (+): 0. Max coverage (-): 0

Region: chr7 15477218-15477238. Max. coverage (+): 0. Max coverage (-): 0

Region: chr7 15477239-15477259. Max. coverage (+): 0. Max coverage (-): 0

Region: chr7 15477260-15477281. Max. coverage (+): 0. Max coverage (-): 0

Region: chr7 15477282-15477302. Max. coverage (+): 0. Max coverage (-): 0

Region: chr7 15477303-15477323. Max. coverage (+): 0. Max coverage (-): 0

Region: chr7 15477324-15477345. Max. coverage (+): 0. Max coverage (-): 0

Region: chr7 15477346-15477366. Max. coverage (+): 0. Max coverage (-): 0

Region: chr7 15477367-15477387. Max. coverage (+): 0. Max coverage (-): 0

Region: chr7 15477388-15477409. Max. coverage (+): 0. Max coverage (-): 0

Region: chr7 15477410-15477430. Max. coverage (+): 0. Max coverage (-): 0

Region: chr7 15477431-15477451. Max. coverage (+): 0. Max coverage (-): 0

Region: chr7 15477452-15477473. Max. coverage (+): 0. Max coverage (-): 0

Region: chr7 15477474-15477494. Max. coverage (+): 0. Max coverage (-): 0

Region: chr7 15477495-15477515. Max. coverage (+): 0. Max coverage (-): 0

Region: chr7 15477516-15477537. Max. coverage (+): 0. Max coverage (-): 0

Region: chr7 15477538-15477558. Max. coverage (+): 0. Max coverage (-): 0

Region: chr7 15477559-15477579. Max. coverage (+): 0. Max coverage (-): 0

Region: chr7 15477580-15477600. Max. coverage (+): 0. Max coverage (-): 0

Region: chr7 15477601-15477622. Max. coverage (+): 0. Max coverage (-): 0

Region: chr7 15477623-15477643. Max. coverage (+): 0. Max coverage (-): 0

Region: chr7 15477644-15477664. Max. coverage (+): 0. Max coverage (-): 0

Region: chr7 15477665-15477686. Max. coverage (+): 0. Max coverage (-): 0

Region: chr7 15477687-15477707. Max. coverage (+): 0. Max coverage (-): 0

Region: chr7 15477708-15477728. Max. coverage (+): 0. Max coverage (-): 0

Region: chr7 15477729-15477750. Max. coverage (+): 0. Max coverage (-): 0

Region: chr7 15477751-15477771. Max. coverage (+): 0. Max coverage (-): 0

Region: chr7 15477772-15477792. Max. coverage (+): 0. Max coverage (-): 0

Region: chr7 15477793-15477814. Max. coverage (+): 0. Max coverage (-): 0

Region: chr7 15477815-15477835. Max. coverage (+): 0. Max coverage (-): 0

Region: chr7 15477836-15477856. Max. coverage (+): 0. Max coverage (-): 0

Region: chr7 15477857-15477878. Max. coverage (+): 0. Max coverage (-): 0

Region: chr7 15477879-15477899. Max. coverage (+): 0. Max coverage (-): 0

Region: chr7 15477900-15477920. Max. coverage (+): 0. Max coverage (-): 0

Region: chr7 15477921-15477942. Max. coverage (+): 0. Max coverage (-): 0

Region: chr7 15477943-15477963. Max. coverage (+): 0. Max coverage (-): 0

Region: chr7 15477964-15477984. Max. coverage (+): 0. Max coverage (-): 0

Region: chr7 15477985-15478006. Max. coverage (+): 0. Max coverage (-): 0

Region: chr7 15478007-15478027. Max. coverage (+): 0. Max coverage (-): 0

Region: chr7 15478028-15478048. Max. coverage (+): 0. Max coverage (-): 0

Region: chr7 15478049-15478070. Max. coverage (+): 0. Max coverage (-): 0

Region: chr7 15478071-15478091. Max. coverage (+): 0. Max coverage (-): 0

Region: chr7 15478092-15478112. Max. coverage (+): 0. Max coverage (-): 6.56

Region: chr7 15478113-15478134. Max. coverage (+): 0. Max coverage (-): 6.56

Region: chr7 15478135-15478155. Max. coverage (+): 0. Max coverage (-): 0

Region: chr7 15478156-15478176. Max. coverage (+): 0. Max coverage (-): 0

Region: chr7 15478177-15478198. Max. coverage (+): 0. Max coverage (-): 0

Region: chr7 15478199-15478219. Max. coverage (+): 0. Max coverage (-): 0

Region: chr7 15478220-15478240. Max. coverage (+): 0. Max coverage (-): 0

Region: chr7 15478241-15478262. Max. coverage (+): 0. Max coverage (-): 0

Region: chr7 15478263-15478283. Max. coverage (+): 0. Max coverage (-): 0

Region: chr7 15478284-15478304. Max. coverage (+): 0. Max coverage (-): 0

Region: chr7 15478305-15478325. Max. coverage (+): 0. Max coverage (-): 6.89

Region: chr7 15478326-15478347. Max. coverage (+): 0. Max coverage (-): 0

Region: chr7 15478348-15478368. Max. coverage (+): 0. Max coverage (-): 0

Region: chr7 15478369-15478389. Max. coverage (+): 0. Max coverage (-): 0

Region: chr7 15478390-15478411. Max. coverage (+): 0. Max coverage (-): 0

Region: chr7 15478412-15478432. Max. coverage (+): 0. Max coverage (-): 0

Region: chr7 15478433-15478453. Max. coverage (+): 0. Max coverage (-): 0

Region: chr7 15478454-15478475. Max. coverage (+): 0. Max coverage (-): 0

Region: chr7 15478476-15478496. Max. coverage (+): 0. Max coverage (-): 0

Region: chr7 15478497-15478517. Max. coverage (+): 0. Max coverage (-): 0

Region: chr7 15478518-15478539. Max. coverage (+): 0. Max coverage (-): 0

Region: chr7 15478540-15478560. Max. coverage (+): 0. Max coverage (-): 0

Region: chr7 15478561-15478581. Max. coverage (+): 0. Max coverage (-): 0

Region: chr7 15478582-15478603. Max. coverage (+): 0. Max coverage (-): 0

Region: chr7 15478604-15478624. Max. coverage (+): 0. Max coverage (-): 0

Region: chr7 15478625-15478645. Max. coverage (+): 0. Max coverage (-): 6.77

Region: chr7 15478646-15478667. Max. coverage (+): 0. Max coverage (-): 9.07

Region: chr7 15478668-15478688. Max. coverage (+): 0. Max coverage (-): 0

Region: chr7 15478689-15478709. Max. coverage (+): 0. Max coverage (-): 0

Region: chr7 15478710-15478731. Max. coverage (+): 0. Max coverage (-): 0

Region: chr7 15478732-15478752. Max. coverage (+): 0. Max coverage (-): 0

Region: chr7 15478753-15478773. Max. coverage (+): 0. Max coverage (-): 0

Region: chr7 15478774-15478795. Max. coverage (+): 0. Max coverage (-): 0

Region: chr7 15478796-15478816. Max. coverage (+): 0. Max coverage (-): 0

Region: chr7 15478817-15478837. Max. coverage (+): 0. Max coverage (-): 0

Region: chr7 15478838-15478859. Max. coverage (+): 0. Max coverage (-): 0

Region: chr7 15478860-15478880. Max. coverage (+): 0. Max coverage (-): 0

Region: chr7 15478881-15478901. Max. coverage (+): 0. Max coverage (-): 0

Region: chr7 15478902-15478923. Max. coverage (+): 0. Max coverage (-): 0

Region: chr7 15478924-15478944. Max. coverage (+): 0. Max coverage (-): 8.36

Region: chr7 15478945-15478965. Max. coverage (+): 0. Max coverage (-): 8.36

Region: chr7 15478966-15478987. Max. coverage (+): 0. Max coverage (-): 0

Region: chr7 15478988-15479008. Max. coverage (+): 0. Max coverage (-): 0

Region: chr7 15479009-15479029. Max. coverage (+): 0. Max coverage (-): 0

Region: chr7 15479030-15479051. Max. coverage (+): 0. Max coverage (-): 0

Region: chr7 15479052-15479072. Max. coverage (+): 0. Max coverage (-): 0

Region: chr7 15479073-15479093. Max. coverage (+): 0. Max coverage (-): 0

Region: chr7 15479094-15479114. Max. coverage (+): 0. Max coverage (-): 0

Region: chr7 15479115-15479136. Max. coverage (+): 0. Max coverage (-): 0

Region: chr7 15479137-15479157. Max. coverage (+): 0. Max coverage (-): 0

Region: chr7 15479158-15479178. Max. coverage (+): 0. Max coverage (-): 0

Region: chr7 15479179-15479200. Max. coverage (+): 0. Max coverage (-): 0

Region: chr7 15479201-15479221. Max. coverage (+): 0. Max coverage (-): 0

Region: chr7 15479222-15479242. Max. coverage (+): 0. Max coverage (-): 0

Region: chr7 15479243-15479264. Max. coverage (+): 0. Max coverage (-): 2.17

Region: chr7 15479265-15479285. Max. coverage (+): 0. Max coverage (-): 0

Region: chr7 15479286-15479306. Max. coverage (+): 0. Max coverage (-): 0

Region: chr7 15479307-15479328. Max. coverage (+): 0. Max coverage (-): 0

Region: chr7 15479329-15479349. Max. coverage (+): 0. Max coverage (-): 0

Region: chr7 15479350-15479370. Max. coverage (+): 0. Max coverage (-): 0

Region: chr7 15479371-15479392. Max. coverage (+): 0. Max coverage (-): 0

Region: chr7 15479393-15479413. Max. coverage (+): 0. Max coverage (-): 0

Region: chr7 15479414-15479434. Max. coverage (+): 0. Max coverage (-): 0

Region: chr7 15479435-15479456. Max. coverage (+): 0. Max coverage (-): 0

Region: chr7 15479457-15479477. Max. coverage (+): 0. Max coverage (-): 0

Region: chr7 15479478-15479498. Max. coverage (+): 0. Max coverage (-): 0

Region: chr7 15479499-15479520. Max. coverage (+): 0. Max coverage (-): 0

Region: chr7 15479521-15479541. Max. coverage (+): 0. Max coverage (-): 0

Region: chr7 15479542-15479562. Max. coverage (+): 0. Max coverage (-): 6.64

Region: chr7 15479563-15479584. Max. coverage (+): 0. Max coverage (-): 0

Region: chr7 15479585-15479605. Max. coverage (+): 0. Max coverage (-): 2.14

Region: chr7 15479606-15479626. Max. coverage (+): 0. Max coverage (-): 21.99

Region: chr7 15479627-15479648. Max. coverage (+): 0. Max coverage (-): 13.66

Region: chr7 15479649-15479669. Max. coverage (+): 0. Max coverage (-): 4.31

Region: chr7 15479670-15479690. Max. coverage (+): 0. Max coverage (-): 0

Region: chr7 15479691-15479712. Max. coverage (+): 0. Max coverage (-): 2.41

Region: chr7 15479713-15479733. Max. coverage (+): 0. Max coverage (-): 0

Region: chr7 15479734-15479754. Max. coverage (+): 0. Max coverage (-): 0

Region: chr7 15479755-15479776. Max. coverage (+): 0. Max coverage (-): 0

Region: chr7 15479777-15479797. Max. coverage (+): 0. Max coverage (-): 0

Region: chr7 15479798-15479818. Max. coverage (+): 0. Max coverage (-): 0

Region: chr7 15479819-15479839. Max. coverage (+): 0. Max coverage (-): 0

Region: chr7 15479840-15479861. Max. coverage (+): 0. Max coverage (-): 0

Region: chr7 15479862-15479882. Max. coverage (+): 0. Max coverage (-): 0

Region: chr7 15479883-15479903. Max. coverage (+): 0. Max coverage (-): 0

Region: chr7 15479904-15479925. Max. coverage (+): 0. Max coverage (-): 0

Region: chr7 15479926-15479946. Max. coverage (+): 0. Max coverage (-): 0

Region: chr7 15479947-15479967. Max. coverage (+): 0. Max coverage (-): 0

Region: chr7 15479968-15479989. Max. coverage (+): 0. Max coverage (-): 0

Region: chr7 15479990-15480010. Max. coverage (+): 0. Max coverage (-): 0

Region: chr7 15480011-15480031. Max. coverage (+): 0. Max coverage (-): 0

Region: chr7 15480032-15480053. Max. coverage (+): 0. Max coverage (-): 0

Region: chr7 15480054-15480074. Max. coverage (+): 0. Max coverage (-): 5.52

Region: chr7 15480075-15480095. Max. coverage (+): 0. Max coverage (-): 5.52

Region: chr7 15480096-15480117. Max. coverage (+): 0. Max coverage (-): 5.12

Region: chr7 15480118-15480138. Max. coverage (+): 0. Max coverage (-): 5.12

Region: chr7 15480139-15480159. Max. coverage (+): 0. Max coverage (-): 0

Region: chr7 15480160-15480181. Max. coverage (+): 0. Max coverage (-): 0

Region: chr7 15480182-15480202. Max. coverage (+): 0. Max coverage (-): 0

Region: chr7 15480203-15480223. Max. coverage (+): 0. Max coverage (-): 0

Region: chr7 15480224-15480245. Max. coverage (+): 0. Max coverage (-): 0

Region: chr7 15480246-15480266. Max. coverage (+): 0. Max coverage (-): 0

Region: chr7 15480267-15480287. Max. coverage (+): 0. Max coverage (-): 0

Region: chr7 15480288-15480309. Max. coverage (+): 0. Max coverage (-): 0

Region: chr7 15480310-15480330. Max. coverage (+): 0. Max coverage (-): 0

Region: chr7 15480331-15480351. Max. coverage (+): 0. Max coverage (-): 0

Region: chr7 15480352-15480373. Max. coverage (+): 0. Max coverage (-): 0

Region: chr7 15480374-15480394. Max. coverage (+): 0. Max coverage (-): 0

Region: chr7 15480395-15480415. Max. coverage (+): 0. Max coverage (-): 0

Region: chr7 15480416-15480437. Max. coverage (+): 0. Max coverage (-): 0

Region: chr7 15480438-15480458. Max. coverage (+): 0. Max coverage (-): 0

Region: chr7 15480459-15480479. Max. coverage (+): 0. Max coverage (-): 0

Region: chr7 15480480-15480501. Max. coverage (+): 0. Max coverage (-): 0

Region: chr7 15480502-15480522. Max. coverage (+): 0. Max coverage (-): 0

Region: chr7 15480523-15480543. Max. coverage (+): 0. Max coverage (-): 0

Region: chr7 15480544-15480565. Max. coverage (+): 0. Max coverage (-): 0

Region: chr7 15480566-15480586. Max. coverage (+): 0. Max coverage (-): 0

Region: chr7 15480587-15480607. Max. coverage (+): 0. Max coverage (-): 0

Region: chr7 15480608-15480628. Max. coverage (+): 0. Max coverage (-): 6.96

Region: chr7 15480629-15480650. Max. coverage (+): 0. Max coverage (-): 0

Region: chr7 15480651-15480671. Max. coverage (+): 0. Max coverage (-): 0

Region: chr7 15480672-15480692. Max. coverage (+): 0. Max coverage (-): 0

Region: chr7 15480693-15480714. Max. coverage (+): 0. Max coverage (-): 0

Region: chr7 15480715-15480735. Max. coverage (+): 0. Max coverage (-): 6.05

Region: chr7 15480736-15480756. Max. coverage (+): 0. Max coverage (-): 12.69

Region: chr7 15480757-15480778. Max. coverage (+): 0. Max coverage (-): 0

Region: chr7 15480779-15480799. Max. coverage (+): 0. Max coverage (-): 0

Region: chr7 15480800-15480820. Max. coverage (+): 0. Max coverage (-): 0

Region: chr7 15480821-15480842. Max. coverage (+): 0. Max coverage (-): 0

Region: chr7 15480843-15480863. Max. coverage (+): 0. Max coverage (-): 0

Region: chr7 15480864-15480884. Max. coverage (+): 0. Max coverage (-): 0

Region: chr7 15480885-15480906. Max. coverage (+): 0. Max coverage (-): 0

Region: chr7 15480907-15480927. Max. coverage (+): 0. Max coverage (-): 4.45

Region: chr7 15480928-15480948. Max. coverage (+): 0. Max coverage (-): 0

Region: chr7 15480949-15480970. Max. coverage (+): 0. Max coverage (-): 0

Region: chr7 15480971-15480991. Max. coverage (+): 0. Max coverage (-): 2.48

Region: chr7 15480992-15481012. Max. coverage (+): 0. Max coverage (-): 0

Region: chr7 15481013-15481034. Max. coverage (+): 0. Max coverage (-): 0

Region: chr7 15481035-15481055. Max. coverage (+): 0. Max coverage (-): 4.65

Region: chr7 15481056-15481076. Max. coverage (+): 0. Max coverage (-): 0

Region: chr7 15481077-15481098. Max. coverage (+): 0. Max coverage (-): 0

Region: chr7 15481099-15481119. Max. coverage (+): 0. Max coverage (-): 0

Region: chr7 15481120-15481140. Max. coverage (+): 0. Max coverage (-): 0

Region: chr7 15481141-15481162. Max. coverage (+): 0. Max coverage (-): 0

Region: chr7 15481163-15481183. Max. coverage (+): 0. Max coverage (-): 0

Region: chr7 15481184-15481204. Max. coverage (+): 0. Max coverage (-): 0

Region: chr7 15481205-15481226. Max. coverage (+): 0. Max coverage (-): 0

Region: chr7 15481227-15481247. Max. coverage (+): 0. Max coverage (-): 0

Region: chr7 15481248-15481268. Max. coverage (+): 0. Max coverage (-): 0

Region: chr7 15481269-15481290. Max. coverage (+): 0. Max coverage (-): 0

Region: chr7 15481291-15481311. Max. coverage (+): 0. Max coverage (-): 0

Region: chr7 15481312-15481332. Max. coverage (+): 0. Max coverage (-): 0

Region: chr7 15481333-15481353. Max. coverage (+): 0. Max coverage (-): 0

Region: chr7 15481354-15481375. Max. coverage (+): 0. Max coverage (-): 0

Region: chr7 15481376-15481396. Max. coverage (+): 0. Max coverage (-): 0

Region: chr7 15481397-15481417. Max. coverage (+): 0. Max coverage (-): 0

Region: chr7 15481418-15481439. Max. coverage (+): 0. Max coverage (-): 0

Region: chr7 15481440-15481460. Max. coverage (+): 0. Max coverage (-): 0

Region: chr7 15481461-15481481. Max. coverage (+): 0. Max coverage (-): 0

Region: chr7 15481482-15481503. Max. coverage (+): 0. Max coverage (-): 0

Region: chr7 15481504-15481524. Max. coverage (+): 0. Max coverage (-): 0

Region: chr7 15481525-15481545. Max. coverage (+): 0. Max coverage (-): 0

Region: chr7 15481546-15481567. Max. coverage (+): 0. Max coverage (-): 0

Region: chr7 15481568-15481588. Max. coverage (+): 0. Max coverage (-): 0

Region: chr7 15481589-15481609. Max. coverage (+): 0. Max coverage (-): 0

Region: chr7 15481610-15481631. Max. coverage (+): 0. Max coverage (-): 0

Region: chr7 15481632-15481652. Max. coverage (+): 0. Max coverage (-): 0

Region: chr7 15481653-15481673. Max. coverage (+): 0. Max coverage (-): 0

Region: chr7 15481674-15481695. Max. coverage (+): 0. Max coverage (-): 0

Region: chr7 15481696-15481716. Max. coverage (+): 0. Max coverage (-): 0

Region: chr7 15481717-15481737. Max. coverage (+): 0. Max coverage (-): 0

Region: chr7 15481738-15481759. Max. coverage (+): 0. Max coverage (-): 0

Region: chr7 15481760-15481780. Max. coverage (+): 0. Max coverage (-): 0

Region: chr7 15481781-15481801. Max. coverage (+): 0. Max coverage (-): 0

Region: chr7 15481802-15481823. Max. coverage (+): 0. Max coverage (-): 0

Region: chr7 15481824-15481844. Max. coverage (+): 0. Max coverage (-): 0

Region: chr7 15481845-15481865. Max. coverage (+): 0. Max coverage (-): 0

Region: chr7 15481866-15481887. Max. coverage (+): 0. Max coverage (-): 0

Region: chr7 15481888-15481908. Max. coverage (+): 0. Max coverage (-): 0

Region: chr7 15481909-15481929. Max. coverage (+): 0. Max coverage (-): 0

Region: chr7 15481930-15481951. Max. coverage (+): 0. Max coverage (-): 0

Region: chr7 15481952-15481972. Max. coverage (+): 0. Max coverage (-): 0

Region: chr7 15481973-15481993. Max. coverage (+): 0. Max coverage (-): 0

Region: chr7 15481994-15482015. Max. coverage (+): 0. Max coverage (-): 0

Region: chr7 15482016-15482036. Max. coverage (+): 0. Max coverage (-): 0

Region: chr7 15482037-15482057. Max. coverage (+): 0. Max coverage (-): 0

Region: chr7 15482058-15482079. Max. coverage (+): 0. Max coverage (-): 0

Region: chr7 15482080-15482100. Max. coverage (+): 0. Max coverage (-): 12.02

Region: chr7 15482101-. Max. coverage (+): 0. Max coverage (-): 0

RepeatMasker Color Code

**+**

100-98% Identity

<98-95% Identity

<95-90% Identity

<90-85% Identity

<85-80% Identity

<80-75% Identity

<75-70% Identity

<70% Identity

**-**

Gene Set Color Code

**+**

Gene

Pseudogene

**-**

Topology/Coverage Color Code

Coverage Plus Strand

Coverage Minus Strand

Mainstrand: Plus

Mainstrand: Minus

Complementary Strand

Flanking Region  
(if option -flank >0)

Gene Set Annotation  

**1. (protein coding, ENSBTAG00000039523) Tr:00000054432 Ex:5**: 15471211-15472604 (+)

  
RepeatMasker Annotation  

**1. BovB**: 15471490-15471890 (+), Divergence to consensus: 6%  
**2. ART2A**: 15471891-15472417 (+), Divergence to consensus: 7.8%  
**3. L2**: 15472608-15472715 (+), Divergence to consensus: 32.4%  
**4. LTR16B**: 15472754-15473038 (-), Divergence to consensus: 44.5%  
**5. CHR-2\_BT**: 15473088-15473304 (+), Divergence to consensus: 21.4%  
**6. CHR-2A**: 15473717-15473989 (+), Divergence to consensus: 34.8%  
**7. L1M5**: 15474131-15474456 (+), Divergence to consensus: 40.7%  
**8. L1-2\_BT**: 15474475-15474564 (+), Divergence to consensus: 30%  
**9. SINE2-1\_BT**: 15474936-15475051 (-), Divergence to consensus: 25.8%  
**10. AT\_rich**: 15475057-15475095 (+), Divergence to consensus: 79.5%  
**11. LTR40a**: 15475218-15475648 (+), Divergence to consensus: 42.3%  
**12. Bov-tA2**: 15475737-15475924 (+), Divergence to consensus: 12.9%  
**13. L1M5**: 15475999-15476106 (+), Divergence to consensus: 41.7%  
**14. L2a**: 15476251-15476335 (-), Divergence to consensus: 25.3%  
**15. L1M5**: 15476336-15476715 (+), Divergence to consensus: 38.2%  
**16. L1MB7**: 15477212-15477293 (-), Divergence to consensus: 27.1%  
**17. Bov-tA3**: 15477295-15477458 (+), Divergence to consensus: 9.8%  
**18. L1MB7**: 15477482-15477720 (-), Divergence to consensus: 34.8%  
**19. SINE2-2\_BT**: 15477721-15477843 (+), Divergence to consensus: 30.4%  
**20. L1MB7**: 15477844-15478015 (-), Divergence to consensus: 34.8%  
**21. AT\_rich**: 15478138-15478162 (+), Divergence to consensus: 44%  
**22. AT\_rich**: 15478140-15478165 (+), Divergence to consensus: 69.2%  
**23. SINE2-1\_BT**: 15478167-15478279 (-), Divergence to consensus: 17.7%  
**24. MER58A**: 15478437-15478624 (+), Divergence to consensus: 27.2%  
**25. CHRL1\_BT**: 15478688-15478839 (+), Divergence to consensus: 22.3%  
**26. SINE2-1\_BT**: 15479105-15479218 (-), Divergence to consensus: 27.5%  
**27. L1ME2**: 15479317-15479439 (+), Divergence to consensus: 23.6%  
**28. LTR65**: 15479830-15480070 (-), Divergence to consensus: 29.7%  
**29. Bov-tA2**: 15480144-15480337 (+), Divergence to consensus: 20.2%  
**30. L1M5**: 15480346-15480466 (+), Divergence to consensus: 35.5%  
**31. Bov-tA2**: 15481489-15481690 (-), Divergence to consensus: 13.4%

  
Transcription Factor Binding Sites  

**Gata4** (Sequence: AGATAAC (-): 15473601)  
**SOX9** (Sequence: AACAATGG (-): 15479690)  
**SOX9** (Sequence: CTATTGTT (+): 15478842)  
**Gata4** (Sequence: GTTATCT (+): 15472725)  
**Gata4** (Sequence: CTTATCT (+): 15481915)
