# Supplementary material for: Small RNA sequencing of cryopreserved semen from single bull revealed altered miRNAs and piRNAs expression between High- and Low-motile sperm populations
Source: BMC Genomics. 2017 Jan 4;18:14. doi: 10.1186/s12864-016-3394-7 (PMC5209821; doi:10.1186/s12864-016-3394-7)
Supplement: Additional file 4: — Details for each piRNA clusters found in Low Motile (LM) sperm fraction. Genes, repeats, transposable elements and transcription factors binding sites falling within the cluster regions were reported. (ZIP 1034 kb) [file 12864_2016_3394_MOESM4_ESM.zip › 47.html]

piRNA cluster 47


Predicted piRNA cluster no. 47     previous   next
  

Show proTRAC run info
Hide proTRAC run info

================================= proTRAC ====================================  
VERSION: 2.1                                    LAST MODIFIED: 06. October 2015  
  
Please cite:  
Rosenkranz D, Zischler H. proTRAC - a software for probabilistic piRNA cluster  
detection, visualization and analysis. 2012. BMC Bioinformatics 13:5.  
  
and (for proTRAC 2.0 and later):  
Rosenkranz D, Rudloff S, Bastuck K, Ketting RF, Zischler H. Tupaia small RNAs  
provide insights into function and evolution of RNAi-based transposon defense  
in mammals. 2015. RNA 21(5):911-922.  
  
Contact:  
David Rosenkranz  
Institute of Anthropology, small RNA group  
Johannes Gutenberg University Mainz  
email: rosenkranz@uni-mainz.de  
  
You can find the latest proTRAC version at:  
http://sourceforge.net/projects/protrac/files  
http://www.smallRNAgroup-mainz.de/software  
==============================================================================  
  
PARAMETERS:  
Map file: .............../storage/core/barbara/genhome/smallRNA/fertility/Sample\_not\_motile/pirna/Sample\_not\_motile\_26-33\_collapsed.fa.no-dust.map.weighted-10000-1000-b-0  
Genome file: ............/storage/core/barbara/genhome/smallRNA/fertility/Sample\_all/pirna/bt\_311\_chrY.fa  
RepeatMasker annotation: /storage/genomes/bt\_umd31/GCF\_000003055.6\_Bos\_taurus\_UMD\_3.1.1\_repeatMasker\_chr.out  
GeneSet:................./storage/core/barbara/genhome/smallRNA/fertility/Sample\_all/pirna/full.gtf  
  
Significant (p<=0.01) hit density will be calculated based  
on observed hit distribution.  
  
Sliding window size: ........................................ 5000 bp  
Sliding window increament: .................................. 1000 bp  
Normalize each hit by number of genomic hits: ............... 1 [0=no/1=yes]  
Normalize each hit by number of sequence reads: ............. 1 [0=no/1=yes]  
Normalize values (-> per million mapped reads): ............. 1 [0=no/1=yes]  
Min. fraction of hits with 1T(U) or 10A: .................... 0.75  
Alternatively: Min. fraction of hits with 1T(U) and 10A: .... 0.5  
Min. fraction of hits with typical piRNA length: ............ 0.75  
Typical piRNA length: ....................................... 26-33 nt  
Min. size of a piRNA cluster: ............................... 5000 bp.  
Min. number of hits (absolute): ............................. 0  
Min. number of hits (normalized): ........................... 0  
Min. fraction of hits on the mainstrand: .................... 0.75  
Top fraction of mapped sequences (in terms of read counts): . 1%  
Top fraction accounts for max. n% of sequence reads: ........ 90%  
Min. fraction of hits on each arm of a bidirectional cluster: 0.1  
Output image file for each cluster: ......................... 0 [0=no/1=yes]  
Output html file for each cluster: .......................... 1 [0=no/1=yes]  
Output a summary table: ..................................... 1 [0=no/1=yes]  
Output a FASTA file for each cluster (piRNA sequences): ..... 1 [0=no/1=yes]  
Output a FASTA file comprising cluster sequences: ........... 1 [0=no/1=yes]  
Search DNA motifs in clusters: .............................. 1 [0=no/1=yes]  
Output flanking sequences: +/- .............................. 0 bp  
Output ~.pTi file: .......................................... 1 [0=no/1=yes]  
==============================================================================  
  
  
Genome size (without gaps): ............ 2678902517 bp  
Gaps (N/X/-): .......................... 53837044 bp  
Mapped reads: .......................... 738059667487  
Non-identical sequences: ............... 277001  
Genomic hits: .......................... 533816  
Significant densitiy of mapped reads: .. 15118061 reads/kb

Show proTRAC cluster info
Hide proTRAC cluster info

|  |  |
| --- | --- |
| Location | chr7 |
| Coordinates | 16605431-16616557 |
| Size [bp] | 11127 |
| Sequence hit loci | 602 |
| Mapped reads (normalized) | 1477326130 |
| Mapped reads (normalized) per kb | 132769491.3 |
| Normalized reads with 1T (1U) | 78.9% |
| Normalized reads with 10A | 27.3% |
| Normalized reads with length 26-33 nt | 100% |
| Normalized reads on the main strand(s) | 96.6% |
| Predicted directionality | bi:minus-plus (split between 16610483 and 16610680) |

100%

0%

1T (1U)  
reads

10A reads

26-33 nt  
reads

reads on mainstrand

**Either the amount of reads with 1T (1U) OR 10A has to exceed 75% (set with option: -1Tor10A)  
Alternatively the amount of reads with 1T (1U) AND 10A has to exceed 50% (set with option: -1Tand10A)  
Minimum amount of reads with preferred size is 75% (set with option: -pisize)  
Minimum amount of reads on the main strand(s) is 75% (set with option: -clstrand)**

Show read coverage
Hide read coverage

WHAT DO I SEE HERE?  
This chart shows the location of mapped sequence reads within a predicted piRNA cluster. The color refers to the number of genomic hits produced by the sequence read in question. A dark red bar indicates that this sequence read produces many other hits elsewhere in the genome. Many adjacent red or yellow bars can indicate the presence of a multi-copy element such as transposons or rRNA genes. A dark green bar indicates that this sequence read maps uniquely to this locus.

1 hit

2-5 hits

6-10 hits

11-20 hits

21-50 hits

51-100 hits

> 100 hits

chr7

16605431

16616557

Gene Set

RepeatMasker

Mapped  
Reads

133.56

plus strand

minus strand

133.56

Region: chr7 15482102-16605442. Max. coverage (+): 0. Max coverage (-): 11.53

Region: chr7 16605443-16605464. Max. coverage (+): 0. Max coverage (-): 0

Region: chr7 16605465-16605486. Max. coverage (+): 0. Max coverage (-): 0

Region: chr7 16605487-16605508. Max. coverage (+): 0. Max coverage (-): 0

Region: chr7 16605509-16605531. Max. coverage (+): 0. Max coverage (-): 0

Region: chr7 16605532-16605553. Max. coverage (+): 0. Max coverage (-): 32.94

Region: chr7 16605554-16605575. Max. coverage (+): 0. Max coverage (-): 13.7

Region: chr7 16605576-16605597. Max. coverage (+): 0. Max coverage (-): 0

Region: chr7 16605598-16605620. Max. coverage (+): 0. Max coverage (-): 0

Region: chr7 16605621-16605642. Max. coverage (+): 0. Max coverage (-): 0

Region: chr7 16605643-16605664. Max. coverage (+): 0. Max coverage (-): 0

Region: chr7 16605665-16605686. Max. coverage (+): 0. Max coverage (-): 0

Region: chr7 16605687-16605709. Max. coverage (+): 0. Max coverage (-): 0

Region: chr7 16605710-16605731. Max. coverage (+): 0. Max coverage (-): 43.05

Region: chr7 16605732-16605753. Max. coverage (+): 0. Max coverage (-): 0

Region: chr7 16605754-16605775. Max. coverage (+): 0. Max coverage (-): 3.53

Region: chr7 16605776-16605798. Max. coverage (+): 0. Max coverage (-): 0

Region: chr7 16605799-16605820. Max. coverage (+): 0. Max coverage (-): 0

Region: chr7 16605821-16605842. Max. coverage (+): 0. Max coverage (-): 0

Region: chr7 16605843-16605864. Max. coverage (+): 0. Max coverage (-): 0

Region: chr7 16605865-16605887. Max. coverage (+): 0. Max coverage (-): 0

Region: chr7 16605888-16605909. Max. coverage (+): 0. Max coverage (-): 0

Region: chr7 16605910-16605931. Max. coverage (+): 0. Max coverage (-): 0

Region: chr7 16605932-16605953. Max. coverage (+): 0. Max coverage (-): 0

Region: chr7 16605954-16605976. Max. coverage (+): 0. Max coverage (-): 0

Region: chr7 16605977-16605998. Max. coverage (+): 0. Max coverage (-): 0

Region: chr7 16605999-16606020. Max. coverage (+): 0. Max coverage (-): 0

Region: chr7 16606021-16606042. Max. coverage (+): 0. Max coverage (-): 0

Region: chr7 16606043-16606065. Max. coverage (+): 0. Max coverage (-): 0

Region: chr7 16606066-16606087. Max. coverage (+): 0. Max coverage (-): 0

Region: chr7 16606088-16606109. Max. coverage (+): 0. Max coverage (-): 5.94

Region: chr7 16606110-16606132. Max. coverage (+): 0. Max coverage (-): 0

Region: chr7 16606133-16606154. Max. coverage (+): 0. Max coverage (-): 0

Region: chr7 16606155-16606176. Max. coverage (+): 0. Max coverage (-): 0

Region: chr7 16606177-16606198. Max. coverage (+): 0. Max coverage (-): 0

Region: chr7 16606199-16606221. Max. coverage (+): 0. Max coverage (-): 0

Region: chr7 16606222-16606243. Max. coverage (+): 0. Max coverage (-): 0

Region: chr7 16606244-16606265. Max. coverage (+): 0. Max coverage (-): 9.16

Region: chr7 16606266-16606287. Max. coverage (+): 0. Max coverage (-): 0

Region: chr7 16606288-16606310. Max. coverage (+): 0. Max coverage (-): 0

Region: chr7 16606311-16606332. Max. coverage (+): 0. Max coverage (-): 0

Region: chr7 16606333-16606354. Max. coverage (+): 0. Max coverage (-): 0

Region: chr7 16606355-16606376. Max. coverage (+): 0. Max coverage (-): 0

Region: chr7 16606377-16606399. Max. coverage (+): 0. Max coverage (-): 0

Region: chr7 16606400-16606421. Max. coverage (+): 0. Max coverage (-): 2.64

Region: chr7 16606422-16606443. Max. coverage (+): 0. Max coverage (-): 2.64

Region: chr7 16606444-16606465. Max. coverage (+): 0. Max coverage (-): 0

Region: chr7 16606466-16606488. Max. coverage (+): 0. Max coverage (-): 0

Region: chr7 16606489-16606510. Max. coverage (+): 0. Max coverage (-): 0

Region: chr7 16606511-16606532. Max. coverage (+): 0. Max coverage (-): 0

Region: chr7 16606533-16606554. Max. coverage (+): 0. Max coverage (-): 0

Region: chr7 16606555-16606577. Max. coverage (+): 0. Max coverage (-): 0

Region: chr7 16606578-16606599. Max. coverage (+): 0. Max coverage (-): 0

Region: chr7 16606600-16606621. Max. coverage (+): 0. Max coverage (-): 0

Region: chr7 16606622-16606643. Max. coverage (+): 0. Max coverage (-): 0

Region: chr7 16606644-16606666. Max. coverage (+): 0. Max coverage (-): 0

Region: chr7 16606667-16606688. Max. coverage (+): 0. Max coverage (-): 0

Region: chr7 16606689-16606710. Max. coverage (+): 0. Max coverage (-): 0

Region: chr7 16606711-16606732. Max. coverage (+): 0. Max coverage (-): 0

Region: chr7 16606733-16606755. Max. coverage (+): 0. Max coverage (-): 0

Region: chr7 16606756-16606777. Max. coverage (+): 0. Max coverage (-): 0

Region: chr7 16606778-16606799. Max. coverage (+): 0. Max coverage (-): 0

Region: chr7 16606800-16606821. Max. coverage (+): 0. Max coverage (-): 0

Region: chr7 16606822-16606844. Max. coverage (+): 0. Max coverage (-): 0

Region: chr7 16606845-16606866. Max. coverage (+): 0. Max coverage (-): 0

Region: chr7 16606867-16606888. Max. coverage (+): 0. Max coverage (-): 0

Region: chr7 16606889-16606910. Max. coverage (+): 0. Max coverage (-): 0

Region: chr7 16606911-16606933. Max. coverage (+): 0. Max coverage (-): 0

Region: chr7 16606934-16606955. Max. coverage (+): 0. Max coverage (-): 0

Region: chr7 16606956-16606977. Max. coverage (+): 0. Max coverage (-): 0

Region: chr7 16606978-16606999. Max. coverage (+): 0. Max coverage (-): 0

Region: chr7 16607000-16607022. Max. coverage (+): 0. Max coverage (-): 0

Region: chr7 16607023-16607044. Max. coverage (+): 0. Max coverage (-): 0

Region: chr7 16607045-16607066. Max. coverage (+): 0. Max coverage (-): 0

Region: chr7 16607067-16607088. Max. coverage (+): 0. Max coverage (-): 0

Region: chr7 16607089-16607111. Max. coverage (+): 0. Max coverage (-): 0

Region: chr7 16607112-16607133. Max. coverage (+): 0. Max coverage (-): 0

Region: chr7 16607134-16607155. Max. coverage (+): 0. Max coverage (-): 0

Region: chr7 16607156-16607177. Max. coverage (+): 0. Max coverage (-): 0

Region: chr7 16607178-16607200. Max. coverage (+): 0. Max coverage (-): 0

Region: chr7 16607201-16607222. Max. coverage (+): 0. Max coverage (-): 0

Region: chr7 16607223-16607244. Max. coverage (+): 0. Max coverage (-): 0

Region: chr7 16607245-16607266. Max. coverage (+): 0. Max coverage (-): 0

Region: chr7 16607267-16607289. Max. coverage (+): 0. Max coverage (-): 0

Region: chr7 16607290-16607311. Max. coverage (+): 0. Max coverage (-): 0

Region: chr7 16607312-16607333. Max. coverage (+): 0. Max coverage (-): 0

Region: chr7 16607334-16607355. Max. coverage (+): 0. Max coverage (-): 0

Region: chr7 16607356-16607378. Max. coverage (+): 0. Max coverage (-): 0

Region: chr7 16607379-16607400. Max. coverage (+): 0.19. Max coverage (-): 0

Region: chr7 16607401-16607422. Max. coverage (+): 0.19. Max coverage (-): 0

Region: chr7 16607423-16607444. Max. coverage (+): 0. Max coverage (-): 0

Region: chr7 16607445-16607467. Max. coverage (+): 0. Max coverage (-): 0

Region: chr7 16607468-16607489. Max. coverage (+): 0. Max coverage (-): 0

Region: chr7 16607490-16607511. Max. coverage (+): 0. Max coverage (-): 0

Region: chr7 16607512-16607534. Max. coverage (+): 0. Max coverage (-): 0

Region: chr7 16607535-16607556. Max. coverage (+): 0. Max coverage (-): 0

Region: chr7 16607557-16607578. Max. coverage (+): 0. Max coverage (-): 0

Region: chr7 16607579-16607600. Max. coverage (+): 0. Max coverage (-): 0

Region: chr7 16607601-16607623. Max. coverage (+): 0. Max coverage (-): 0

Region: chr7 16607624-16607645. Max. coverage (+): 0. Max coverage (-): 0

Region: chr7 16607646-16607667. Max. coverage (+): 0. Max coverage (-): 0

Region: chr7 16607668-16607689. Max. coverage (+): 0. Max coverage (-): 0

Region: chr7 16607690-16607712. Max. coverage (+): 0. Max coverage (-): 0

Region: chr7 16607713-16607734. Max. coverage (+): 0. Max coverage (-): 0

Region: chr7 16607735-16607756. Max. coverage (+): 0. Max coverage (-): 0

Region: chr7 16607757-16607778. Max. coverage (+): 0. Max coverage (-): 0

Region: chr7 16607779-16607801. Max. coverage (+): 9.71. Max coverage (-): 0

Region: chr7 16607802-16607823. Max. coverage (+): 9.71. Max coverage (-): 0

Region: chr7 16607824-16607845. Max. coverage (+): 1.09. Max coverage (-): 0

Region: chr7 16607846-16607867. Max. coverage (+): 0. Max coverage (-): 0

Region: chr7 16607868-16607890. Max. coverage (+): 0.83. Max coverage (-): 0

Region: chr7 16607891-16607912. Max. coverage (+): 0. Max coverage (-): 0

Region: chr7 16607913-16607934. Max. coverage (+): 0. Max coverage (-): 0

Region: chr7 16607935-16607956. Max. coverage (+): 1.93. Max coverage (-): 0

Region: chr7 16607957-16607979. Max. coverage (+): 1.93. Max coverage (-): 0

Region: chr7 16607980-16608001. Max. coverage (+): 0. Max coverage (-): 0

Region: chr7 16608002-16608023. Max. coverage (+): 0. Max coverage (-): 0

Region: chr7 16608024-16608045. Max. coverage (+): 0. Max coverage (-): 0

Region: chr7 16608046-16608068. Max. coverage (+): 7.2. Max coverage (-): 1.94

Region: chr7 16608069-16608090. Max. coverage (+): 9.99. Max coverage (-): 1.75

Region: chr7 16608091-16608112. Max. coverage (+): 1.12. Max coverage (-): 0

Region: chr7 16608113-16608134. Max. coverage (+): 0.33. Max coverage (-): 0

Region: chr7 16608135-16608157. Max. coverage (+): 0. Max coverage (-): 0

Region: chr7 16608158-16608179. Max. coverage (+): 0. Max coverage (-): 0

Region: chr7 16608180-16608201. Max. coverage (+): 0. Max coverage (-): 0

Region: chr7 16608202-16608223. Max. coverage (+): 0. Max coverage (-): 0

Region: chr7 16608224-16608246. Max. coverage (+): 0. Max coverage (-): 0

Region: chr7 16608247-16608268. Max. coverage (+): 0. Max coverage (-): 0

Region: chr7 16608269-16608290. Max. coverage (+): 0. Max coverage (-): 0

Region: chr7 16608291-16608312. Max. coverage (+): 0. Max coverage (-): 0

Region: chr7 16608313-16608335. Max. coverage (+): 0. Max coverage (-): 0

Region: chr7 16608336-16608357. Max. coverage (+): 0. Max coverage (-): 0

Region: chr7 16608358-16608379. Max. coverage (+): 0. Max coverage (-): 0

Region: chr7 16608380-16608401. Max. coverage (+): 0. Max coverage (-): 0

Region: chr7 16608402-16608424. Max. coverage (+): 0. Max coverage (-): 0

Region: chr7 16608425-16608446. Max. coverage (+): 0. Max coverage (-): 0

Region: chr7 16608447-16608468. Max. coverage (+): 0. Max coverage (-): 0

Region: chr7 16608469-16608490. Max. coverage (+): 0. Max coverage (-): 0

Region: chr7 16608491-16608513. Max. coverage (+): 0. Max coverage (-): 0

Region: chr7 16608514-16608535. Max. coverage (+): 0. Max coverage (-): 0

Region: chr7 16608536-16608557. Max. coverage (+): 2.39. Max coverage (-): 0

Region: chr7 16608558-16608579. Max. coverage (+): 0. Max coverage (-): 0

Region: chr7 16608580-16608602. Max. coverage (+): 0. Max coverage (-): 0

Region: chr7 16608603-16608624. Max. coverage (+): 0. Max coverage (-): 0

Region: chr7 16608625-16608646. Max. coverage (+): 0. Max coverage (-): 0

Region: chr7 16608647-16608668. Max. coverage (+): 0. Max coverage (-): 0

Region: chr7 16608669-16608691. Max. coverage (+): 0. Max coverage (-): 0

Region: chr7 16608692-16608713. Max. coverage (+): 0. Max coverage (-): 0

Region: chr7 16608714-16608735. Max. coverage (+): 0. Max coverage (-): 0

Region: chr7 16608736-16608757. Max. coverage (+): 0. Max coverage (-): 0

Region: chr7 16608758-16608780. Max. coverage (+): 0. Max coverage (-): 40.43

Region: chr7 16608781-16608802. Max. coverage (+): 0. Max coverage (-): 0

Region: chr7 16608803-16608824. Max. coverage (+): 0. Max coverage (-): 11.33

Region: chr7 16608825-16608846. Max. coverage (+): 0. Max coverage (-): 0

Region: chr7 16608847-16608869. Max. coverage (+): 0. Max coverage (-): 5.06

Region: chr7 16608870-16608891. Max. coverage (+): 0. Max coverage (-): 5.06

Region: chr7 16608892-16608913. Max. coverage (+): 0. Max coverage (-): 0

Region: chr7 16608914-16608936. Max. coverage (+): 0. Max coverage (-): 0

Region: chr7 16608937-16608958. Max. coverage (+): 0. Max coverage (-): 0

Region: chr7 16608959-16608980. Max. coverage (+): 0. Max coverage (-): 0

Region: chr7 16608981-16609002. Max. coverage (+): 0. Max coverage (-): 0

Region: chr7 16609003-16609025. Max. coverage (+): 0. Max coverage (-): 47.09

Region: chr7 16609026-16609047. Max. coverage (+): 0. Max coverage (-): 15.7

Region: chr7 16609048-16609069. Max. coverage (+): 0. Max coverage (-): 0

Region: chr7 16609070-16609091. Max. coverage (+): 0. Max coverage (-): 0

Region: chr7 16609092-16609114. Max. coverage (+): 0. Max coverage (-): 0

Region: chr7 16609115-16609136. Max. coverage (+): 0. Max coverage (-): 0

Region: chr7 16609137-16609158. Max. coverage (+): 0. Max coverage (-): 0

Region: chr7 16609159-16609180. Max. coverage (+): 0. Max coverage (-): 0

Region: chr7 16609181-16609203. Max. coverage (+): 0. Max coverage (-): 0

Region: chr7 16609204-16609225. Max. coverage (+): 0. Max coverage (-): 0

Region: chr7 16609226-16609247. Max. coverage (+): 0. Max coverage (-): 0

Region: chr7 16609248-16609269. Max. coverage (+): 0. Max coverage (-): 0

Region: chr7 16609270-16609292. Max. coverage (+): 0. Max coverage (-): 0

Region: chr7 16609293-16609314. Max. coverage (+): 0. Max coverage (-): 0

Region: chr7 16609315-16609336. Max. coverage (+): 0. Max coverage (-): 0

Region: chr7 16609337-16609358. Max. coverage (+): 0. Max coverage (-): 0

Region: chr7 16609359-16609381. Max. coverage (+): 0. Max coverage (-): 0

Region: chr7 16609382-16609403. Max. coverage (+): 0. Max coverage (-): 0

Region: chr7 16609404-16609425. Max. coverage (+): 0. Max coverage (-): 0

Region: chr7 16609426-16609447. Max. coverage (+): 0. Max coverage (-): 0

Region: chr7 16609448-16609470. Max. coverage (+): 0. Max coverage (-): 0

Region: chr7 16609471-16609492. Max. coverage (+): 0. Max coverage (-): 0

Region: chr7 16609493-16609514. Max. coverage (+): 0. Max coverage (-): 7.06

Region: chr7 16609515-16609536. Max. coverage (+): 0. Max coverage (-): 7.06

Region: chr7 16609537-16609559. Max. coverage (+): 0. Max coverage (-): 2.53

Region: chr7 16609560-16609581. Max. coverage (+): 0. Max coverage (-): 39.55

Region: chr7 16609582-16609603. Max. coverage (+): 0. Max coverage (-): 4.79

Region: chr7 16609604-16609625. Max. coverage (+): 0. Max coverage (-): 0

Region: chr7 16609626-16609648. Max. coverage (+): 0. Max coverage (-): 0

Region: chr7 16609649-16609670. Max. coverage (+): 0. Max coverage (-): 0

Region: chr7 16609671-16609692. Max. coverage (+): 0. Max coverage (-): 0

Region: chr7 16609693-16609714. Max. coverage (+): 0. Max coverage (-): 0

Region: chr7 16609715-16609737. Max. coverage (+): 0. Max coverage (-): 0

Region: chr7 16609738-16609759. Max. coverage (+): 0. Max coverage (-): 0

Region: chr7 16609760-16609781. Max. coverage (+): 0. Max coverage (-): 0

Region: chr7 16609782-16609803. Max. coverage (+): 0. Max coverage (-): 0

Region: chr7 16609804-16609826. Max. coverage (+): 0. Max coverage (-): 0

Region: chr7 16609827-16609848. Max. coverage (+): 0. Max coverage (-): 0

Region: chr7 16609849-16609870. Max. coverage (+): 0. Max coverage (-): 0

Region: chr7 16609871-16609892. Max. coverage (+): 0. Max coverage (-): 0

Region: chr7 16609893-16609915. Max. coverage (+): 0. Max coverage (-): 0

Region: chr7 16609916-16609937. Max. coverage (+): 0. Max coverage (-): 0

Region: chr7 16609938-16609959. Max. coverage (+): 0. Max coverage (-): 0

Region: chr7 16609960-16609981. Max. coverage (+): 0. Max coverage (-): 0

Region: chr7 16609982-16610004. Max. coverage (+): 0. Max coverage (-): 0

Region: chr7 16610005-16610026. Max. coverage (+): 0. Max coverage (-): 0

Region: chr7 16610027-16610048. Max. coverage (+): 0. Max coverage (-): 0

Region: chr7 16610049-16610070. Max. coverage (+): 0. Max coverage (-): 0

Region: chr7 16610071-16610093. Max. coverage (+): 0. Max coverage (-): 0

Region: chr7 16610094-16610115. Max. coverage (+): 0. Max coverage (-): 0

Region: chr7 16610116-16610137. Max. coverage (+): 0. Max coverage (-): 0

Region: chr7 16610138-16610159. Max. coverage (+): 0. Max coverage (-): 0

Region: chr7 16610160-16610182. Max. coverage (+): 0. Max coverage (-): 0

Region: chr7 16610183-16610204. Max. coverage (+): 0. Max coverage (-): 0

Region: chr7 16610205-16610226. Max. coverage (+): 0. Max coverage (-): 0

Region: chr7 16610227-16610248. Max. coverage (+): 0. Max coverage (-): 2.08

Region: chr7 16610249-16610271. Max. coverage (+): 0. Max coverage (-): 0

Region: chr7 16610272-16610293. Max. coverage (+): 0. Max coverage (-): 0

Region: chr7 16610294-16610315. Max. coverage (+): 0. Max coverage (-): 0

Region: chr7 16610316-16610338. Max. coverage (+): 0. Max coverage (-): 0

Region: chr7 16610339-16610360. Max. coverage (+): 0. Max coverage (-): 0

Region: chr7 16610361-16610382. Max. coverage (+): 0. Max coverage (-): 0

Region: chr7 16610383-16610404. Max. coverage (+): 0. Max coverage (-): 0

Region: chr7 16610405-16610427. Max. coverage (+): 0. Max coverage (-): 0

Region: chr7 16610428-16610449. Max. coverage (+): 0. Max coverage (-): 0

Region: chr7 16610450-16610471. Max. coverage (+): 0. Max coverage (-): 0

Region: chr7 16610472-16610493. Max. coverage (+): 0. Max coverage (-): 0.4

Region: chr7 16610494-16610516. Max. coverage (+): 0. Max coverage (-): 0

Region: chr7 16610517-16610538. Max. coverage (+): 0. Max coverage (-): 0

Region: chr7 16610539-16610560. Max. coverage (+): 0. Max coverage (-): 0

Region: chr7 16610561-16610582. Max. coverage (+): 0. Max coverage (-): 0

Region: chr7 16610583-16610605. Max. coverage (+): 0. Max coverage (-): 0

Region: chr7 16610606-16610627. Max. coverage (+): 0. Max coverage (-): 0

Region: chr7 16610628-16610649. Max. coverage (+): 0. Max coverage (-): 0

Region: chr7 16610650-16610671. Max. coverage (+): 0. Max coverage (-): 0

Region: chr7 16610672-16610694. Max. coverage (+): 6.5. Max coverage (-): 0

Region: chr7 16610695-16610716. Max. coverage (+): 8.8. Max coverage (-): 1.28

Region: chr7 16610717-16610738. Max. coverage (+): 3.55. Max coverage (-): 1.28

Region: chr7 16610739-16610760. Max. coverage (+): 10.46. Max coverage (-): 2.79

Region: chr7 16610761-16610783. Max. coverage (+): 5.23. Max coverage (-): 0

Region: chr7 16610784-16610805. Max. coverage (+): 5.23. Max coverage (-): 0

Region: chr7 16610806-16610827. Max. coverage (+): 0. Max coverage (-): 0

Region: chr7 16610828-16610849. Max. coverage (+): 6.72. Max coverage (-): 0

Region: chr7 16610850-16610872. Max. coverage (+): 0. Max coverage (-): 0

Region: chr7 16610873-16610894. Max. coverage (+): 0. Max coverage (-): 0

Region: chr7 16610895-16610916. Max. coverage (+): 8.77. Max coverage (-): 0

Region: chr7 16610917-16610938. Max. coverage (+): 45.72. Max coverage (-): 0

Region: chr7 16610939-16610961. Max. coverage (+): 0. Max coverage (-): 0

Region: chr7 16610962-16610983. Max. coverage (+): 17.1. Max coverage (-): 0

Region: chr7 16610984-16611005. Max. coverage (+): 4.89. Max coverage (-): 0

Region: chr7 16611006-16611027. Max. coverage (+): 0. Max coverage (-): 0

Region: chr7 16611028-16611050. Max. coverage (+): 0. Max coverage (-): 0

Region: chr7 16611051-16611072. Max. coverage (+): 0. Max coverage (-): 0

Region: chr7 16611073-16611094. Max. coverage (+): 0. Max coverage (-): 0

Region: chr7 16611095-16611116. Max. coverage (+): 0. Max coverage (-): 0

Region: chr7 16611117-16611139. Max. coverage (+): 0. Max coverage (-): 0

Region: chr7 16611140-16611161. Max. coverage (+): 0. Max coverage (-): 0

Region: chr7 16611162-16611183. Max. coverage (+): 0. Max coverage (-): 0

Region: chr7 16611184-16611205. Max. coverage (+): 0. Max coverage (-): 0

Region: chr7 16611206-16611228. Max. coverage (+): 0. Max coverage (-): 0

Region: chr7 16611229-16611250. Max. coverage (+): 0. Max coverage (-): 0

Region: chr7 16611251-16611272. Max. coverage (+): 6.02. Max coverage (-): 0

Region: chr7 16611273-16611294. Max. coverage (+): 0. Max coverage (-): 0

Region: chr7 16611295-16611317. Max. coverage (+): 0. Max coverage (-): 0

Region: chr7 16611318-16611339. Max. coverage (+): 0. Max coverage (-): 0

Region: chr7 16611340-16611361. Max. coverage (+): 0. Max coverage (-): 0

Region: chr7 16611362-16611383. Max. coverage (+): 0. Max coverage (-): 0

Region: chr7 16611384-16611406. Max. coverage (+): 0. Max coverage (-): 0

Region: chr7 16611407-16611428. Max. coverage (+): 0. Max coverage (-): 0

Region: chr7 16611429-16611450. Max. coverage (+): 0. Max coverage (-): 0

Region: chr7 16611451-16611472. Max. coverage (+): 0. Max coverage (-): 0

Region: chr7 16611473-16611495. Max. coverage (+): 0. Max coverage (-): 0

Region: chr7 16611496-16611517. Max. coverage (+): 0. Max coverage (-): 0

Region: chr7 16611518-16611539. Max. coverage (+): 0. Max coverage (-): 0

Region: chr7 16611540-16611561. Max. coverage (+): 0. Max coverage (-): 0

Region: chr7 16611562-16611584. Max. coverage (+): 0. Max coverage (-): 0

Region: chr7 16611585-16611606. Max. coverage (+): 16.79. Max coverage (-): 0

Region: chr7 16611607-16611628. Max. coverage (+): 0. Max coverage (-): 0

Region: chr7 16611629-16611650. Max. coverage (+): 0. Max coverage (-): 0

Region: chr7 16611651-16611673. Max. coverage (+): 0.96. Max coverage (-): 0

Region: chr7 16611674-16611695. Max. coverage (+): 34.2. Max coverage (-): 0

Region: chr7 16611696-16611717. Max. coverage (+): 0. Max coverage (-): 0

Region: chr7 16611718-16611740. Max. coverage (+): 0. Max coverage (-): 0

Region: chr7 16611741-16611762. Max. coverage (+): 0. Max coverage (-): 0

Region: chr7 16611763-16611784. Max. coverage (+): 133.56. Max coverage (-): 0

Region: chr7 16611785-16611806. Max. coverage (+): 0. Max coverage (-): 0

Region: chr7 16611807-16611829. Max. coverage (+): 0. Max coverage (-): 0

Region: chr7 16611830-16611851. Max. coverage (+): 13.2. Max coverage (-): 0

Region: chr7 16611852-16611873. Max. coverage (+): 0.96. Max coverage (-): 0

Region: chr7 16611874-16611895. Max. coverage (+): 19.58. Max coverage (-): 0

Region: chr7 16611896-16611918. Max. coverage (+): 17.34. Max coverage (-): 0

Region: chr7 16611919-16611940. Max. coverage (+): 25.96. Max coverage (-): 0

Region: chr7 16611941-16611962. Max. coverage (+): 0. Max coverage (-): 0

Region: chr7 16611963-16611984. Max. coverage (+): 15.6. Max coverage (-): 0

Region: chr7 16611985-16612007. Max. coverage (+): 0. Max coverage (-): 0

Region: chr7 16612008-16612029. Max. coverage (+): 54.62. Max coverage (-): 0

Region: chr7 16612030-16612051. Max. coverage (+): 0. Max coverage (-): 0

Region: chr7 16612052-16612073. Max. coverage (+): 0. Max coverage (-): 0

Region: chr7 16612074-16612096. Max. coverage (+): 0. Max coverage (-): 0

Region: chr7 16612097-16612118. Max. coverage (+): 0. Max coverage (-): 0

Region: chr7 16612119-16612140. Max. coverage (+): 0. Max coverage (-): 0

Region: chr7 16612141-16612162. Max. coverage (+): 6.6. Max coverage (-): 0

Region: chr7 16612163-16612185. Max. coverage (+): 0. Max coverage (-): 0

Region: chr7 16612186-16612207. Max. coverage (+): 50.63. Max coverage (-): 0

Region: chr7 16612208-16612229. Max. coverage (+): 10.29. Max coverage (-): 0

Region: chr7 16612230-16612251. Max. coverage (+): 0. Max coverage (-): 0

Region: chr7 16612252-16612274. Max. coverage (+): 0. Max coverage (-): 0

Region: chr7 16612275-16612296. Max. coverage (+): 0. Max coverage (-): 0

Region: chr7 16612297-16612318. Max. coverage (+): 0. Max coverage (-): 0

Region: chr7 16612319-16612340. Max. coverage (+): 0. Max coverage (-): 0

Region: chr7 16612341-16612363. Max. coverage (+): 0. Max coverage (-): 0

Region: chr7 16612364-16612385. Max. coverage (+): 0. Max coverage (-): 0

Region: chr7 16612386-16612407. Max. coverage (+): 0. Max coverage (-): 0

Region: chr7 16612408-16612429. Max. coverage (+): 0. Max coverage (-): 0

Region: chr7 16612430-16612452. Max. coverage (+): 6.72. Max coverage (-): 0

Region: chr7 16612453-16612474. Max. coverage (+): 6.72. Max coverage (-): 0

Region: chr7 16612475-16612496. Max. coverage (+): 4.5. Max coverage (-): 0

Region: chr7 16612497-16612518. Max. coverage (+): 0. Max coverage (-): 0

Region: chr7 16612519-16612541. Max. coverage (+): 0. Max coverage (-): 0

Region: chr7 16612542-16612563. Max. coverage (+): 0. Max coverage (-): 0

Region: chr7 16612564-16612585. Max. coverage (+): 0. Max coverage (-): 0

Region: chr7 16612586-16612607. Max. coverage (+): 15.41. Max coverage (-): 0

Region: chr7 16612608-16612630. Max. coverage (+): 15.41. Max coverage (-): 0

Region: chr7 16612631-16612652. Max. coverage (+): 0. Max coverage (-): 0

Region: chr7 16612653-16612674. Max. coverage (+): 0. Max coverage (-): 0

Region: chr7 16612675-16612696. Max. coverage (+): 0. Max coverage (-): 0

Region: chr7 16612697-16612719. Max. coverage (+): 0. Max coverage (-): 0

Region: chr7 16612720-16612741. Max. coverage (+): 0. Max coverage (-): 0

Region: chr7 16612742-16612763. Max. coverage (+): 0. Max coverage (-): 0

Region: chr7 16612764-16612785. Max. coverage (+): 0. Max coverage (-): 0

Region: chr7 16612786-16612808. Max. coverage (+): 0.45. Max coverage (-): 0

Region: chr7 16612809-16612830. Max. coverage (+): 0. Max coverage (-): 0

Region: chr7 16612831-16612852. Max. coverage (+): 0. Max coverage (-): 0

Region: chr7 16612853-16612874. Max. coverage (+): 0. Max coverage (-): 0

Region: chr7 16612875-16612897. Max. coverage (+): 0. Max coverage (-): 0

Region: chr7 16612898-16612919. Max. coverage (+): 0. Max coverage (-): 0

Region: chr7 16612920-16612941. Max. coverage (+): 0. Max coverage (-): 0

Region: chr7 16612942-16612963. Max. coverage (+): 0. Max coverage (-): 0

Region: chr7 16612964-16612986. Max. coverage (+): 0. Max coverage (-): 0

Region: chr7 16612987-16613008. Max. coverage (+): 0. Max coverage (-): 0

Region: chr7 16613009-16613030. Max. coverage (+): 0. Max coverage (-): 0

Region: chr7 16613031-16613052. Max. coverage (+): 0. Max coverage (-): 0

Region: chr7 16613053-16613075. Max. coverage (+): 0. Max coverage (-): 0

Region: chr7 16613076-16613097. Max. coverage (+): 0. Max coverage (-): 0

Region: chr7 16613098-16613119. Max. coverage (+): 0.18. Max coverage (-): 0

Region: chr7 16613120-16613142. Max. coverage (+): 0. Max coverage (-): 0

Region: chr7 16613143-16613164. Max. coverage (+): 0. Max coverage (-): 0

Region: chr7 16613165-16613186. Max. coverage (+): 35.47. Max coverage (-): 0

Region: chr7 16613187-16613208. Max. coverage (+): 25.9. Max coverage (-): 0

Region: chr7 16613209-16613231. Max. coverage (+): 23.06. Max coverage (-): 0

Region: chr7 16613232-16613253. Max. coverage (+): 0. Max coverage (-): 0

Region: chr7 16613254-16613275. Max. coverage (+): 0. Max coverage (-): 0

Region: chr7 16613276-16613297. Max. coverage (+): 1.97. Max coverage (-): 0

Region: chr7 16613298-16613320. Max. coverage (+): 0. Max coverage (-): 0

Region: chr7 16613321-16613342. Max. coverage (+): 0. Max coverage (-): 0

Region: chr7 16613343-16613364. Max. coverage (+): 4.58. Max coverage (-): 0

Region: chr7 16613365-16613386. Max. coverage (+): 4.58. Max coverage (-): 0

Region: chr7 16613387-16613409. Max. coverage (+): 0. Max coverage (-): 0

Region: chr7 16613410-16613431. Max. coverage (+): 0. Max coverage (-): 0

Region: chr7 16613432-16613453. Max. coverage (+): 0. Max coverage (-): 4.61

Region: chr7 16613454-16613475. Max. coverage (+): 17.09. Max coverage (-): 4.61

Region: chr7 16613476-16613498. Max. coverage (+): 23.73. Max coverage (-): 1.32

Region: chr7 16613499-16613520. Max. coverage (+): 2.65. Max coverage (-): 0

Region: chr7 16613521-16613542. Max. coverage (+): 0.79. Max coverage (-): 0

Region: chr7 16613543-16613564. Max. coverage (+): 15.39. Max coverage (-): 0

Region: chr7 16613565-16613587. Max. coverage (+): 15.39. Max coverage (-): 0

Region: chr7 16613588-16613609. Max. coverage (+): 0. Max coverage (-): 0

Region: chr7 16613610-16613631. Max. coverage (+): 0. Max coverage (-): 0

Region: chr7 16613632-16613653. Max. coverage (+): 0. Max coverage (-): 0

Region: chr7 16613654-16613676. Max. coverage (+): 0. Max coverage (-): 0

Region: chr7 16613677-16613698. Max. coverage (+): 0. Max coverage (-): 0

Region: chr7 16613699-16613720. Max. coverage (+): 0. Max coverage (-): 0

Region: chr7 16613721-16613742. Max. coverage (+): 0. Max coverage (-): 0

Region: chr7 16613743-16613765. Max. coverage (+): 0. Max coverage (-): 0

Region: chr7 16613766-16613787. Max. coverage (+): 0. Max coverage (-): 0

Region: chr7 16613788-16613809. Max. coverage (+): 0. Max coverage (-): 0

Region: chr7 16613810-16613831. Max. coverage (+): 0. Max coverage (-): 0

Region: chr7 16613832-16613854. Max. coverage (+): 0. Max coverage (-): 0

Region: chr7 16613855-16613876. Max. coverage (+): 0. Max coverage (-): 0

Region: chr7 16613877-16613898. Max. coverage (+): 0. Max coverage (-): 0

Region: chr7 16613899-16613920. Max. coverage (+): 0. Max coverage (-): 0

Region: chr7 16613921-16613943. Max. coverage (+): 0. Max coverage (-): 0

Region: chr7 16613944-16613965. Max. coverage (+): 4.01. Max coverage (-): 0

Region: chr7 16613966-16613987. Max. coverage (+): 0. Max coverage (-): 0

Region: chr7 16613988-16614009. Max. coverage (+): 0. Max coverage (-): 0

Region: chr7 16614010-16614032. Max. coverage (+): 0. Max coverage (-): 0

Region: chr7 16614033-16614054. Max. coverage (+): 0. Max coverage (-): 0

Region: chr7 16614055-16614076. Max. coverage (+): 0. Max coverage (-): 0

Region: chr7 16614077-16614098. Max. coverage (+): 0. Max coverage (-): 0

Region: chr7 16614099-16614121. Max. coverage (+): 0. Max coverage (-): 0

Region: chr7 16614122-16614143. Max. coverage (+): 0. Max coverage (-): 0

Region: chr7 16614144-16614165. Max. coverage (+): 0. Max coverage (-): 0

Region: chr7 16614166-16614187. Max. coverage (+): 3.53. Max coverage (-): 0

Region: chr7 16614188-16614210. Max. coverage (+): 54.03. Max coverage (-): 0

Region: chr7 16614211-16614232. Max. coverage (+): 0. Max coverage (-): 0

Region: chr7 16614233-16614254. Max. coverage (+): 34.74. Max coverage (-): 0

Region: chr7 16614255-16614276. Max. coverage (+): 50.5. Max coverage (-): 0

Region: chr7 16614277-16614299. Max. coverage (+): 0. Max coverage (-): 0

Region: chr7 16614300-16614321. Max. coverage (+): 0. Max coverage (-): 0

Region: chr7 16614322-16614343. Max. coverage (+): 0. Max coverage (-): 0

Region: chr7 16614344-16614365. Max. coverage (+): 1.03. Max coverage (-): 0

Region: chr7 16614366-16614388. Max. coverage (+): 0. Max coverage (-): 0

Region: chr7 16614389-16614410. Max. coverage (+): 89.21. Max coverage (-): 0

Region: chr7 16614411-16614432. Max. coverage (+): 25.7. Max coverage (-): 0

Region: chr7 16614433-16614454. Max. coverage (+): 0. Max coverage (-): 0

Region: chr7 16614455-16614477. Max. coverage (+): 24.7. Max coverage (-): 0

Region: chr7 16614478-16614499. Max. coverage (+): 16.18. Max coverage (-): 0

Region: chr7 16614500-16614521. Max. coverage (+): 18.75. Max coverage (-): 0

Region: chr7 16614522-16614544. Max. coverage (+): 0. Max coverage (-): 0

Region: chr7 16614545-16614566. Max. coverage (+): 0. Max coverage (-): 0

Region: chr7 16614567-16614588. Max. coverage (+): 14.84. Max coverage (-): 0

Region: chr7 16614589-16614610. Max. coverage (+): 0. Max coverage (-): 0

Region: chr7 16614611-16614633. Max. coverage (+): 0. Max coverage (-): 9.02

Region: chr7 16614634-16614655. Max. coverage (+): 7.51. Max coverage (-): 9.02

Region: chr7 16614656-16614677. Max. coverage (+): 6.2. Max coverage (-): 0

Region: chr7 16614678-16614699. Max. coverage (+): 24.97. Max coverage (-): 0

Region: chr7 16614700-16614722. Max. coverage (+): 26.06. Max coverage (-): 0

Region: chr7 16614723-16614744. Max. coverage (+): 25.63. Max coverage (-): 0

Region: chr7 16614745-16614766. Max. coverage (+): 0. Max coverage (-): 0

Region: chr7 16614767-16614788. Max. coverage (+): 0. Max coverage (-): 0

Region: chr7 16614789-16614811. Max. coverage (+): 11.86. Max coverage (-): 0

Region: chr7 16614812-16614833. Max. coverage (+): 81.85. Max coverage (-): 0

Region: chr7 16614834-16614855. Max. coverage (+): 0. Max coverage (-): 0

Region: chr7 16614856-16614877. Max. coverage (+): 9.39. Max coverage (-): 0

Region: chr7 16614878-16614900. Max. coverage (+): 6.56. Max coverage (-): 0

Region: chr7 16614901-16614922. Max. coverage (+): 6.56. Max coverage (-): 0

Region: chr7 16614923-16614944. Max. coverage (+): 0. Max coverage (-): 0

Region: chr7 16614945-16614966. Max. coverage (+): 0. Max coverage (-): 0

Region: chr7 16614967-16614989. Max. coverage (+): 2.92. Max coverage (-): 0

Region: chr7 16614990-16615011. Max. coverage (+): 4.29. Max coverage (-): 0

Region: chr7 16615012-16615033. Max. coverage (+): 8.03. Max coverage (-): 0

Region: chr7 16615034-16615055. Max. coverage (+): 8.03. Max coverage (-): 0

Region: chr7 16615056-16615078. Max. coverage (+): 7.53. Max coverage (-): 0

Region: chr7 16615079-16615100. Max. coverage (+): 56.12. Max coverage (-): 0

Region: chr7 16615101-16615122. Max. coverage (+): 66.36. Max coverage (-): 0

Region: chr7 16615123-16615144. Max. coverage (+): 36.79. Max coverage (-): 0

Region: chr7 16615145-16615167. Max. coverage (+): 1.1. Max coverage (-): 0

Region: chr7 16615168-16615189. Max. coverage (+): 44.28. Max coverage (-): 0

Region: chr7 16615190-16615211. Max. coverage (+): 24.61. Max coverage (-): 0

Region: chr7 16615212-16615233. Max. coverage (+): 1.07. Max coverage (-): 0

Region: chr7 16615234-16615256. Max. coverage (+): 0. Max coverage (-): 0

Region: chr7 16615257-16615278. Max. coverage (+): 6.55. Max coverage (-): 0

Region: chr7 16615279-16615300. Max. coverage (+): 12.13. Max coverage (-): 0

Region: chr7 16615301-16615322. Max. coverage (+): 10.62. Max coverage (-): 0

Region: chr7 16615323-16615345. Max. coverage (+): 30.24. Max coverage (-): 0

Region: chr7 16615346-16615367. Max. coverage (+): 0. Max coverage (-): 0

Region: chr7 16615368-16615389. Max. coverage (+): 7.35. Max coverage (-): 0

Region: chr7 16615390-16615411. Max. coverage (+): 0. Max coverage (-): 0

Region: chr7 16615412-16615434. Max. coverage (+): 0. Max coverage (-): 0

Region: chr7 16615435-16615456. Max. coverage (+): 0. Max coverage (-): 0

Region: chr7 16615457-16615478. Max. coverage (+): 0. Max coverage (-): 0

Region: chr7 16615479-16615500. Max. coverage (+): 0. Max coverage (-): 0

Region: chr7 16615501-16615523. Max. coverage (+): 5.72. Max coverage (-): 0

Region: chr7 16615524-16615545. Max. coverage (+): 0. Max coverage (-): 0

Region: chr7 16615546-16615567. Max. coverage (+): 24.67. Max coverage (-): 0

Region: chr7 16615568-16615589. Max. coverage (+): 0. Max coverage (-): 0

Region: chr7 16615590-16615612. Max. coverage (+): 0. Max coverage (-): 0

Region: chr7 16615613-16615634. Max. coverage (+): 0. Max coverage (-): 0

Region: chr7 16615635-16615656. Max. coverage (+): 0. Max coverage (-): 0

Region: chr7 16615657-16615678. Max. coverage (+): 0. Max coverage (-): 0

Region: chr7 16615679-16615701. Max. coverage (+): 0. Max coverage (-): 0

Region: chr7 16615702-16615723. Max. coverage (+): 0. Max coverage (-): 0

Region: chr7 16615724-16615745. Max. coverage (+): 0. Max coverage (-): 0

Region: chr7 16615746-16615767. Max. coverage (+): 0. Max coverage (-): 0

Region: chr7 16615768-16615790. Max. coverage (+): 0. Max coverage (-): 0

Region: chr7 16615791-16615812. Max. coverage (+): 0. Max coverage (-): 0

Region: chr7 16615813-16615834. Max. coverage (+): 0. Max coverage (-): 0

Region: chr7 16615835-16615856. Max. coverage (+): 0. Max coverage (-): 0

Region: chr7 16615857-16615879. Max. coverage (+): 0. Max coverage (-): 0

Region: chr7 16615880-16615901. Max. coverage (+): 0. Max coverage (-): 0

Region: chr7 16615902-16615923. Max. coverage (+): 0. Max coverage (-): 0

Region: chr7 16615924-16615946. Max. coverage (+): 0. Max coverage (-): 0

Region: chr7 16615947-16615968. Max. coverage (+): 0. Max coverage (-): 0

Region: chr7 16615969-16615990. Max. coverage (+): 0. Max coverage (-): 0

Region: chr7 16615991-16616012. Max. coverage (+): 0. Max coverage (-): 0

Region: chr7 16616013-16616035. Max. coverage (+): 0. Max coverage (-): 0

Region: chr7 16616036-16616057. Max. coverage (+): 0. Max coverage (-): 0

Region: chr7 16616058-16616079. Max. coverage (+): 0. Max coverage (-): 0

Region: chr7 16616080-16616101. Max. coverage (+): 0. Max coverage (-): 0

Region: chr7 16616102-16616124. Max. coverage (+): 0. Max coverage (-): 0

Region: chr7 16616125-16616146. Max. coverage (+): 0. Max coverage (-): 0

Region: chr7 16616147-16616168. Max. coverage (+): 0. Max coverage (-): 0

Region: chr7 16616169-16616190. Max. coverage (+): 0. Max coverage (-): 0

Region: chr7 16616191-16616213. Max. coverage (+): 0. Max coverage (-): 0

Region: chr7 16616214-16616235. Max. coverage (+): 0. Max coverage (-): 0

Region: chr7 16616236-16616257. Max. coverage (+): 0. Max coverage (-): 0

Region: chr7 16616258-16616279. Max. coverage (+): 0. Max coverage (-): 0

Region: chr7 16616280-16616302. Max. coverage (+): 0. Max coverage (-): 0

Region: chr7 16616303-16616324. Max. coverage (+): 0. Max coverage (-): 0

Region: chr7 16616325-16616346. Max. coverage (+): 0. Max coverage (-): 0

Region: chr7 16616347-16616368. Max. coverage (+): 0. Max coverage (-): 0

Region: chr7 16616369-16616391. Max. coverage (+): 0. Max coverage (-): 0

Region: chr7 16616392-16616413. Max. coverage (+): 0. Max coverage (-): 0

Region: chr7 16616414-16616435. Max. coverage (+): 0. Max coverage (-): 0

Region: chr7 16616436-16616457. Max. coverage (+): 0. Max coverage (-): 0

Region: chr7 16616458-16616480. Max. coverage (+): 0. Max coverage (-): 0

Region: chr7 16616481-16616502. Max. coverage (+): 0. Max coverage (-): 0

Region: chr7 16616503-16616524. Max. coverage (+): 0. Max coverage (-): 0

Region: chr7 16616525-16616546. Max. coverage (+): 5.55. Max coverage (-): 0

Region: chr7 16616547-. Max. coverage (+): 0. Max coverage (-): 0

RepeatMasker Color Code

**+**

100-98% Identity

<98-95% Identity

<95-90% Identity

<90-85% Identity

<85-80% Identity

<80-75% Identity

<75-70% Identity

<70% Identity

**-**

Gene Set Color Code

**+**

Gene

Pseudogene

**-**

Topology/Coverage Color Code

Coverage Plus Strand

Coverage Minus Strand

Mainstrand: Plus

Mainstrand: Minus

Complementary Strand

Flanking Region  
(if option -flank >0)

Gene Set Annotation  

**1. (protein coding, ENSBTAG00000032364) Tr:00000045920 Ex:1**: 16615321-16615398 (-)  
**2. (protein coding, ENSBTAG00000032364) Tr:00000045920 Ex:2**: 16614477-16614559 (-)  
**3. (protein coding, ENSBTAG00000032364) Tr:00000045920 Ex:3**: 16612153-16612192 (-)  
**4. (protein coding, ENSBTAG00000032364) Tr:00000045920 Ex:4**: 16611845-16611925 (-)  
**5. (protein coding, ENSBTAG00000032364) Tr:00000045920 Ex:5**: 16611469-16611576 (-)  
**6. (protein coding, ENSBTAG00000032364) Tr:00000045920 Ex:6**: 16610680-16610789 (-)  
**7. (protein coding, ENSBTAG00000032364) Tr:00000045920 Ex:7**: 16608954-16609083 (-)  
**8. (protein coding, ENSBTAG00000032364) Tr:00000045920 Ex:8**: 16608766-16608808 (-)  
**9. (protein coding, ENSBTAG00000032364) Tr:00000045920 Ex:9**: 16608042-16608133 (-)  
**10. (protein coding, ENSBTAG00000032364) Tr:00000045920 Ex:10**: 16607764-16607820 (-)  
**11. (protein coding, ENSBTAG00000032364) Tr:00000045920 Ex:11**: 16607194-16607298 (-)

  
RepeatMasker Annotation  

**1. SINE2-1\_BT**: 16609650-16609765 (-), Divergence to consensus: 24.2%  
**2. BOV-A2**: 16609815-16610058 (+), Divergence to consensus: 14.8%  
**3. SINE2-2\_BT**: 16610069-16610185 (+), Divergence to consensus: 23.9%  
**4. GC\_rich**: 16610870-16610897 (+), Divergence to consensus: 82.1%  
**5. (CGGGG)n**: 16611189-16611241 (+), Divergence to consensus: 24.9%  
**6. L1M3**: 16615584-16615810 (+), Divergence to consensus: 43.4%  
**7. L1MB8**: 16615814-16616073 (+), Divergence to consensus: 34.7%

  
Transcription Factor Binding Sites  

**RFX4\_2** (Sequence: CATGGATAC (+): 16611556)
