# Supplementary material for: Small RNA sequencing of cryopreserved semen from single bull revealed altered miRNAs and piRNAs expression between High- and Low-motile sperm populations
Source: BMC Genomics. 2017 Jan 4;18:14. doi: 10.1186/s12864-016-3394-7 (PMC5209821; doi:10.1186/s12864-016-3394-7)
Supplement: Additional file 4: — Details for each piRNA clusters found in Low Motile (LM) sperm fraction. Genes, repeats, transposable elements and transcription factors binding sites falling within the cluster regions were reported. (ZIP 1034 kb) [file 12864_2016_3394_MOESM4_ESM.zip › 48.html]

piRNA cluster 48


Predicted piRNA cluster no. 48     previous   next
  

Show proTRAC run info
Hide proTRAC run info

================================= proTRAC ====================================  
VERSION: 2.1                                    LAST MODIFIED: 06. October 2015  
  
Please cite:  
Rosenkranz D, Zischler H. proTRAC - a software for probabilistic piRNA cluster  
detection, visualization and analysis. 2012. BMC Bioinformatics 13:5.  
  
and (for proTRAC 2.0 and later):  
Rosenkranz D, Rudloff S, Bastuck K, Ketting RF, Zischler H. Tupaia small RNAs  
provide insights into function and evolution of RNAi-based transposon defense  
in mammals. 2015. RNA 21(5):911-922.  
  
Contact:  
David Rosenkranz  
Institute of Anthropology, small RNA group  
Johannes Gutenberg University Mainz  
email: rosenkranz@uni-mainz.de  
  
You can find the latest proTRAC version at:  
http://sourceforge.net/projects/protrac/files  
http://www.smallRNAgroup-mainz.de/software  
==============================================================================  
  
PARAMETERS:  
Map file: .............../storage/core/barbara/genhome/smallRNA/fertility/Sample\_not\_motile/pirna/Sample\_not\_motile\_26-33\_collapsed.fa.no-dust.map.weighted-10000-1000-b-0  
Genome file: ............/storage/core/barbara/genhome/smallRNA/fertility/Sample\_all/pirna/bt\_311\_chrY.fa  
RepeatMasker annotation: /storage/genomes/bt\_umd31/GCF\_000003055.6\_Bos\_taurus\_UMD\_3.1.1\_repeatMasker\_chr.out  
GeneSet:................./storage/core/barbara/genhome/smallRNA/fertility/Sample\_all/pirna/full.gtf  
  
Significant (p<=0.01) hit density will be calculated based  
on observed hit distribution.  
  
Sliding window size: ........................................ 5000 bp  
Sliding window increament: .................................. 1000 bp  
Normalize each hit by number of genomic hits: ............... 1 [0=no/1=yes]  
Normalize each hit by number of sequence reads: ............. 1 [0=no/1=yes]  
Normalize values (-> per million mapped reads): ............. 1 [0=no/1=yes]  
Min. fraction of hits with 1T(U) or 10A: .................... 0.75  
Alternatively: Min. fraction of hits with 1T(U) and 10A: .... 0.5  
Min. fraction of hits with typical piRNA length: ............ 0.75  
Typical piRNA length: ....................................... 26-33 nt  
Min. size of a piRNA cluster: ............................... 5000 bp.  
Min. number of hits (absolute): ............................. 0  
Min. number of hits (normalized): ........................... 0  
Min. fraction of hits on the mainstrand: .................... 0.75  
Top fraction of mapped sequences (in terms of read counts): . 1%  
Top fraction accounts for max. n% of sequence reads: ........ 90%  
Min. fraction of hits on each arm of a bidirectional cluster: 0.1  
Output image file for each cluster: ......................... 0 [0=no/1=yes]  
Output html file for each cluster: .......................... 1 [0=no/1=yes]  
Output a summary table: ..................................... 1 [0=no/1=yes]  
Output a FASTA file for each cluster (piRNA sequences): ..... 1 [0=no/1=yes]  
Output a FASTA file comprising cluster sequences: ........... 1 [0=no/1=yes]  
Search DNA motifs in clusters: .............................. 1 [0=no/1=yes]  
Output flanking sequences: +/- .............................. 0 bp  
Output ~.pTi file: .......................................... 1 [0=no/1=yes]  
==============================================================================  
  
  
Genome size (without gaps): ............ 2678902517 bp  
Gaps (N/X/-): .......................... 53837044 bp  
Mapped reads: .......................... 738059667487  
Non-identical sequences: ............... 277001  
Genomic hits: .......................... 533816  
Significant densitiy of mapped reads: .. 15118061 reads/kb

Show proTRAC cluster info
Hide proTRAC cluster info

|  |  |
| --- | --- |
| Location | chr8 |
| Coordinates | 17577034-17611473 |
| Size [bp] | 34440 |
| Sequence hit loci | 3982 |
| Mapped reads (normalized) | 10565463417 |
| Mapped reads (normalized) per kb | 306778844.9 |
| Normalized reads with 1T (1U) | 81.3% |
| Normalized reads with 10A | 30.9% |
| Normalized reads with length 26-33 nt | 100% |
| Normalized reads on the main strand(s) | 94.4% |
| Predicted directionality | mono:plus |

100%

0%

1T (1U)  
reads

10A reads

26-33 nt  
reads

reads on mainstrand

**Either the amount of reads with 1T (1U) OR 10A has to exceed 75% (set with option: -1Tor10A)  
Alternatively the amount of reads with 1T (1U) AND 10A has to exceed 50% (set with option: -1Tand10A)  
Minimum amount of reads with preferred size is 75% (set with option: -pisize)  
Minimum amount of reads on the main strand(s) is 75% (set with option: -clstrand)**

Show read coverage
Hide read coverage

WHAT DO I SEE HERE?  
This chart shows the location of mapped sequence reads within a predicted piRNA cluster. The color refers to the number of genomic hits produced by the sequence read in question. A dark red bar indicates that this sequence read produces many other hits elsewhere in the genome. Many adjacent red or yellow bars can indicate the presence of a multi-copy element such as transposons or rRNA genes. A dark green bar indicates that this sequence read maps uniquely to this locus.

1 hit

2-5 hits

6-10 hits

11-20 hits

21-50 hits

51-100 hits

> 100 hits

chr8

17577034

17611473

Gene Set

RepeatMasker

Mapped  
Reads

506.88

plus strand

minus strand

506.88

Region: chr8 16616548-17577068. Max. coverage (+): 1.07. Max coverage (-): 0

Region: chr8 17577069-17577137. Max. coverage (+): 4.86. Max coverage (-): 0

Region: chr8 17577138-17577206. Max. coverage (+): 0. Max coverage (-): 1.6

Region: chr8 17577207-17577275. Max. coverage (+): 0. Max coverage (-): 0

Region: chr8 17577276-17577343. Max. coverage (+): 0. Max coverage (-): 0

Region: chr8 17577344-17577412. Max. coverage (+): 0. Max coverage (-): 0

Region: chr8 17577413-17577481. Max. coverage (+): 0. Max coverage (-): 0

Region: chr8 17577482-17577550. Max. coverage (+): 6.17. Max coverage (-): 0

Region: chr8 17577551-17577619. Max. coverage (+): 0. Max coverage (-): 0

Region: chr8 17577620-17577688. Max. coverage (+): 0. Max coverage (-): 0

Region: chr8 17577689-17577757. Max. coverage (+): 0. Max coverage (-): 0

Region: chr8 17577758-17577826. Max. coverage (+): 0. Max coverage (-): 0

Region: chr8 17577827-17577894. Max. coverage (+): 0. Max coverage (-): 0

Region: chr8 17577895-17577963. Max. coverage (+): 0. Max coverage (-): 0

Region: chr8 17577964-17578032. Max. coverage (+): 0. Max coverage (-): 0

Region: chr8 17578033-17578101. Max. coverage (+): 0. Max coverage (-): 0

Region: chr8 17578102-17578170. Max. coverage (+): 0. Max coverage (-): 0

Region: chr8 17578171-17578239. Max. coverage (+): 0. Max coverage (-): 0

Region: chr8 17578240-17578308. Max. coverage (+): 0. Max coverage (-): 0

Region: chr8 17578309-17578377. Max. coverage (+): 7.61. Max coverage (-): 0

Region: chr8 17578378-17578446. Max. coverage (+): 6.01. Max coverage (-): 0.3

Region: chr8 17578447-17578514. Max. coverage (+): 8.48. Max coverage (-): 0

Region: chr8 17578515-17578583. Max. coverage (+): 1.09. Max coverage (-): 0

Region: chr8 17578584-17578652. Max. coverage (+): 9.6. Max coverage (-): 0

Region: chr8 17578653-17578721. Max. coverage (+): 22.6. Max coverage (-): 0

Region: chr8 17578722-17578790. Max. coverage (+): 13.34. Max coverage (-): 0

Region: chr8 17578791-17578859. Max. coverage (+): 11.78. Max coverage (-): 0

Region: chr8 17578860-17578928. Max. coverage (+): 2.09. Max coverage (-): 0

Region: chr8 17578929-17578997. Max. coverage (+): 5.67. Max coverage (-): 0

Region: chr8 17578998-17579065. Max. coverage (+): 43.85. Max coverage (-): 0

Region: chr8 17579066-17579134. Max. coverage (+): 0. Max coverage (-): 0

Region: chr8 17579135-17579203. Max. coverage (+): 0. Max coverage (-): 0

Region: chr8 17579204-17579272. Max. coverage (+): 0. Max coverage (-): 0

Region: chr8 17579273-17579341. Max. coverage (+): 25.11. Max coverage (-): 0

Region: chr8 17579342-17579410. Max. coverage (+): 38. Max coverage (-): 12.95

Region: chr8 17579411-17579479. Max. coverage (+): 3.79. Max coverage (-): 3.02

Region: chr8 17579480-17579548. Max. coverage (+): 50.9. Max coverage (-): 0

Region: chr8 17579549-17579616. Max. coverage (+): 4.51. Max coverage (-): 0

Region: chr8 17579617-17579685. Max. coverage (+): 46.86. Max coverage (-): 0

Region: chr8 17579686-17579754. Max. coverage (+): 34.64. Max coverage (-): 0

Region: chr8 17579755-17579823. Max. coverage (+): 0. Max coverage (-): 0

Region: chr8 17579824-17579892. Max. coverage (+): 48.97. Max coverage (-): 0

Region: chr8 17579893-17579961. Max. coverage (+): 0. Max coverage (-): 0

Region: chr8 17579962-17580030. Max. coverage (+): 11.51. Max coverage (-): 0

Region: chr8 17580031-17580099. Max. coverage (+): 14.64. Max coverage (-): 0

Region: chr8 17580100-17580168. Max. coverage (+): 11.68. Max coverage (-): 0

Region: chr8 17580169-17580236. Max. coverage (+): 1.37. Max coverage (-): 0

Region: chr8 17580237-17580305. Max. coverage (+): 7.02. Max coverage (-): 0

Region: chr8 17580306-17580374. Max. coverage (+): 5.58. Max coverage (-): 1.04

Region: chr8 17580375-17580443. Max. coverage (+): 9.77. Max coverage (-): 0

Region: chr8 17580444-17580512. Max. coverage (+): 4.05. Max coverage (-): 0

Region: chr8 17580513-17580581. Max. coverage (+): 15.43. Max coverage (-): 0

Region: chr8 17580582-17580650. Max. coverage (+): 3.12. Max coverage (-): 0

Region: chr8 17580651-17580719. Max. coverage (+): 6.69. Max coverage (-): 0

Region: chr8 17580720-17580787. Max. coverage (+): 10.83. Max coverage (-): 0

Region: chr8 17580788-17580856. Max. coverage (+): 21.11. Max coverage (-): 0

Region: chr8 17580857-17580925. Max. coverage (+): 55. Max coverage (-): 0

Region: chr8 17580926-17580994. Max. coverage (+): 57.19. Max coverage (-): 0

Region: chr8 17580995-17581063. Max. coverage (+): 67.89. Max coverage (-): 0

Region: chr8 17581064-17581132. Max. coverage (+): 26.54. Max coverage (-): 0

Region: chr8 17581133-17581201. Max. coverage (+): 14.93. Max coverage (-): 0

Region: chr8 17581202-17581270. Max. coverage (+): 25.45. Max coverage (-): 0

Region: chr8 17581271-17581338. Max. coverage (+): 0. Max coverage (-): 0

Region: chr8 17581339-17581407. Max. coverage (+): 0. Max coverage (-): 0

Region: chr8 17581408-17581476. Max. coverage (+): 5.53. Max coverage (-): 0

Region: chr8 17581477-17581545. Max. coverage (+): 43.25. Max coverage (-): 0

Region: chr8 17581546-17581614. Max. coverage (+): 19.83. Max coverage (-): 0

Region: chr8 17581615-17581683. Max. coverage (+): 15.47. Max coverage (-): 0

Region: chr8 17581684-17581752. Max. coverage (+): 15.15. Max coverage (-): 0

Region: chr8 17581753-17581821. Max. coverage (+): 15.45. Max coverage (-): 5

Region: chr8 17581822-17581890. Max. coverage (+): 2.22. Max coverage (-): 4.52

Region: chr8 17581891-17581958. Max. coverage (+): 6.55. Max coverage (-): 0

Region: chr8 17581959-17582027. Max. coverage (+): 32.22. Max coverage (-): 0

Region: chr8 17582028-17582096. Max. coverage (+): 12.65. Max coverage (-): 0

Region: chr8 17582097-17582165. Max. coverage (+): 38.45. Max coverage (-): 0

Region: chr8 17582166-17582234. Max. coverage (+): 23.71. Max coverage (-): 0

Region: chr8 17582235-17582303. Max. coverage (+): 24.36. Max coverage (-): 0

Region: chr8 17582304-17582372. Max. coverage (+): 8.18. Max coverage (-): 0

Region: chr8 17582373-17582441. Max. coverage (+): 14.48. Max coverage (-): 0

Region: chr8 17582442-17582509. Max. coverage (+): 29.42. Max coverage (-): 0

Region: chr8 17582510-17582578. Max. coverage (+): 3.63. Max coverage (-): 0

Region: chr8 17582579-17582647. Max. coverage (+): 19.15. Max coverage (-): 0

Region: chr8 17582648-17582716. Max. coverage (+): 10.29. Max coverage (-): 0

Region: chr8 17582717-17582785. Max. coverage (+): 32.19. Max coverage (-): 0

Region: chr8 17582786-17582854. Max. coverage (+): 8.37. Max coverage (-): 0

Region: chr8 17582855-17582923. Max. coverage (+): 5.19. Max coverage (-): 0

Region: chr8 17582924-17582992. Max. coverage (+): 6.43. Max coverage (-): 0

Region: chr8 17582993-17583060. Max. coverage (+): 0. Max coverage (-): 0

Region: chr8 17583061-17583129. Max. coverage (+): 5.01. Max coverage (-): 0

Region: chr8 17583130-17583198. Max. coverage (+): 1.09. Max coverage (-): 0

Region: chr8 17583199-17583267. Max. coverage (+): 6.28. Max coverage (-): 0

Region: chr8 17583268-17583336. Max. coverage (+): 0. Max coverage (-): 0

Region: chr8 17583337-17583405. Max. coverage (+): 0. Max coverage (-): 0

Region: chr8 17583406-17583474. Max. coverage (+): 0. Max coverage (-): 0

Region: chr8 17583475-17583543. Max. coverage (+): 50.49. Max coverage (-): 0

Region: chr8 17583544-17583612. Max. coverage (+): 29.01. Max coverage (-): 0

Region: chr8 17583613-17583680. Max. coverage (+): 11.63. Max coverage (-): 0

Region: chr8 17583681-17583749. Max. coverage (+): 23.29. Max coverage (-): 4.89

Region: chr8 17583750-17583818. Max. coverage (+): 0. Max coverage (-): 0

Region: chr8 17583819-17583887. Max. coverage (+): 16.75. Max coverage (-): 0

Region: chr8 17583888-17583956. Max. coverage (+): 29.55. Max coverage (-): 0

Region: chr8 17583957-17584025. Max. coverage (+): 40.36. Max coverage (-): 0

Region: chr8 17584026-17584094. Max. coverage (+): 16.68. Max coverage (-): 0

Region: chr8 17584095-17584163. Max. coverage (+): 55.93. Max coverage (-): 0

Region: chr8 17584164-17584231. Max. coverage (+): 51.7. Max coverage (-): 0

Region: chr8 17584232-17584300. Max. coverage (+): 13.92. Max coverage (-): 0

Region: chr8 17584301-17584369. Max. coverage (+): 12.08. Max coverage (-): 0.98

Region: chr8 17584370-17584438. Max. coverage (+): 5.87. Max coverage (-): 0.98

Region: chr8 17584439-17584507. Max. coverage (+): 7.13. Max coverage (-): 0

Region: chr8 17584508-17584576. Max. coverage (+): 14.38. Max coverage (-): 0

Region: chr8 17584577-17584645. Max. coverage (+): 6.22. Max coverage (-): 0

Region: chr8 17584646-17584714. Max. coverage (+): 0. Max coverage (-): 0

Region: chr8 17584715-17584782. Max. coverage (+): 1.55. Max coverage (-): 23.14

Region: chr8 17584783-17584851. Max. coverage (+): 6.39. Max coverage (-): 23.14

Region: chr8 17584852-17584920. Max. coverage (+): 19.78. Max coverage (-): 0

Region: chr8 17584921-17584989. Max. coverage (+): 30.01. Max coverage (-): 0

Region: chr8 17584990-17585058. Max. coverage (+): 92.92. Max coverage (-): 6.94

Region: chr8 17585059-17585127. Max. coverage (+): 52.57. Max coverage (-): 0

Region: chr8 17585128-17585196. Max. coverage (+): 1.65. Max coverage (-): 0

Region: chr8 17585197-17585265. Max. coverage (+): 21.74. Max coverage (-): 0

Region: chr8 17585266-17585334. Max. coverage (+): 90.09. Max coverage (-): 0

Region: chr8 17585335-17585402. Max. coverage (+): 88.39. Max coverage (-): 3.02

Region: chr8 17585403-17585471. Max. coverage (+): 61.88. Max coverage (-): 0

Region: chr8 17585472-17585540. Max. coverage (+): 19.37. Max coverage (-): 0

Region: chr8 17585541-17585609. Max. coverage (+): 22.72. Max coverage (-): 0

Region: chr8 17585610-17585678. Max. coverage (+): 8.33. Max coverage (-): 0

Region: chr8 17585679-17585747. Max. coverage (+): 41.02. Max coverage (-): 0

Region: chr8 17585748-17585816. Max. coverage (+): 35.21. Max coverage (-): 0

Region: chr8 17585817-17585885. Max. coverage (+): 152.67. Max coverage (-): 0

Region: chr8 17585886-17585953. Max. coverage (+): 49.5. Max coverage (-): 0

Region: chr8 17585954-17586022. Max. coverage (+): 506.88. Max coverage (-): 5.71

Region: chr8 17586023-17586091. Max. coverage (+): 62.39. Max coverage (-): 6.44

Region: chr8 17586092-17586160. Max. coverage (+): 43.7. Max coverage (-): 4.83

Region: chr8 17586161-17586229. Max. coverage (+): 42.65. Max coverage (-): 4.77

Region: chr8 17586230-17586298. Max. coverage (+): 58.26. Max coverage (-): 0

Region: chr8 17586299-17586367. Max. coverage (+): 34.71. Max coverage (-): 0

Region: chr8 17586368-17586436. Max. coverage (+): 10.09. Max coverage (-): 0

Region: chr8 17586437-17586504. Max. coverage (+): 17.28. Max coverage (-): 0

Region: chr8 17586505-17586573. Max. coverage (+): 118.28. Max coverage (-): 0

Region: chr8 17586574-17586642. Max. coverage (+): 23.98. Max coverage (-): 0

Region: chr8 17586643-17586711. Max. coverage (+): 16.39. Max coverage (-): 0

Region: chr8 17586712-17586780. Max. coverage (+): 10.9. Max coverage (-): 0

Region: chr8 17586781-17586849. Max. coverage (+): 126.63. Max coverage (-): 0

Region: chr8 17586850-17586918. Max. coverage (+): 19.21. Max coverage (-): 0

Region: chr8 17586919-17586987. Max. coverage (+): 24.82. Max coverage (-): 1.78

Region: chr8 17586988-17587056. Max. coverage (+): 29.58. Max coverage (-): 2.34

Region: chr8 17587057-17587124. Max. coverage (+): 13.97. Max coverage (-): 9.71

Region: chr8 17587125-17587193. Max. coverage (+): 44.13. Max coverage (-): 13.6

Region: chr8 17587194-17587262. Max. coverage (+): 24.04. Max coverage (-): 6.72

Region: chr8 17587263-17587331. Max. coverage (+): 80.53. Max coverage (-): 27.36

Region: chr8 17587332-17587400. Max. coverage (+): 80.53. Max coverage (-): 6.62

Region: chr8 17587401-17587469. Max. coverage (+): 20.7. Max coverage (-): 1.06

Region: chr8 17587470-17587538. Max. coverage (+): 34.56. Max coverage (-): 20.2

Region: chr8 17587539-17587607. Max. coverage (+): 10.95. Max coverage (-): 18.97

Region: chr8 17587608-17587675. Max. coverage (+): 34.48. Max coverage (-): 13.26

Region: chr8 17587676-17587744. Max. coverage (+): 5.72. Max coverage (-): 35.93

Region: chr8 17587745-17587813. Max. coverage (+): 5.47. Max coverage (-): 0

Region: chr8 17587814-17587882. Max. coverage (+): 0. Max coverage (-): 0

Region: chr8 17587883-17587951. Max. coverage (+): 10.21. Max coverage (-): 0

Region: chr8 17587952-17588020. Max. coverage (+): 26.23. Max coverage (-): 0

Region: chr8 17588021-17588089. Max. coverage (+): 116.93. Max coverage (-): 6.85

Region: chr8 17588090-17588158. Max. coverage (+): 5.44. Max coverage (-): 0

Region: chr8 17588159-17588226. Max. coverage (+): 11.86. Max coverage (-): 6.35

Region: chr8 17588227-17588295. Max. coverage (+): 41.12. Max coverage (-): 17.7

Region: chr8 17588296-17588364. Max. coverage (+): 15.4. Max coverage (-): 0

Region: chr8 17588365-17588433. Max. coverage (+): 35.97. Max coverage (-): 6.01

Region: chr8 17588434-17588502. Max. coverage (+): 0. Max coverage (-): 0

Region: chr8 17588503-17588571. Max. coverage (+): 0. Max coverage (-): 0

Region: chr8 17588572-17588640. Max. coverage (+): 2.21. Max coverage (-): 5.76

Region: chr8 17588641-17588709. Max. coverage (+): 7.64. Max coverage (-): 5.76

Region: chr8 17588710-17588778. Max. coverage (+): 5.58. Max coverage (-): 2.26

Region: chr8 17588779-17588846. Max. coverage (+): 0. Max coverage (-): 2.26

Region: chr8 17588847-17588915. Max. coverage (+): 1.8. Max coverage (-): 0

Region: chr8 17588916-17588984. Max. coverage (+): 10.01. Max coverage (-): 0

Region: chr8 17588985-17589053. Max. coverage (+): 41.51. Max coverage (-): 0

Region: chr8 17589054-17589122. Max. coverage (+): 24.89. Max coverage (-): 6.37

Region: chr8 17589123-17589191. Max. coverage (+): 9.41. Max coverage (-): 6.37

Region: chr8 17589192-17589260. Max. coverage (+): 38.48. Max coverage (-): 5.51

Region: chr8 17589261-17589329. Max. coverage (+): 99.84. Max coverage (-): 7.86

Region: chr8 17589330-17589397. Max. coverage (+): 61.3. Max coverage (-): 0

Region: chr8 17589398-17589466. Max. coverage (+): 80.67. Max coverage (-): 0

Region: chr8 17589467-17589535. Max. coverage (+): 58.92. Max coverage (-): 0

Region: chr8 17589536-17589604. Max. coverage (+): 66.59. Max coverage (-): 0

Region: chr8 17589605-17589673. Max. coverage (+): 49.32. Max coverage (-): 0

Region: chr8 17589674-17589742. Max. coverage (+): 58.02. Max coverage (-): 0

Region: chr8 17589743-17589811. Max. coverage (+): 62.34. Max coverage (-): 0

Region: chr8 17589812-17589880. Max. coverage (+): 56.94. Max coverage (-): 0

Region: chr8 17589881-17589948. Max. coverage (+): 14.21. Max coverage (-): 0

Region: chr8 17589949-17590017. Max. coverage (+): 11.12. Max coverage (-): 0

Region: chr8 17590018-17590086. Max. coverage (+): 15.86. Max coverage (-): 0

Region: chr8 17590087-17590155. Max. coverage (+): 21.94. Max coverage (-): 0

Region: chr8 17590156-17590224. Max. coverage (+): 71.13. Max coverage (-): 0

Region: chr8 17590225-17590293. Max. coverage (+): 71.13. Max coverage (-): 0.43

Region: chr8 17590294-17590362. Max. coverage (+): 38.58. Max coverage (-): 12.02

Region: chr8 17590363-17590431. Max. coverage (+): 14.35. Max coverage (-): 0.98

Region: chr8 17590432-17590500. Max. coverage (+): 0. Max coverage (-): 2.2

Region: chr8 17590501-17590568. Max. coverage (+): 7.01. Max coverage (-): 0.26

Region: chr8 17590569-17590637. Max. coverage (+): 6.94. Max coverage (-): 5.43

Region: chr8 17590638-17590706. Max. coverage (+): 11.95. Max coverage (-): 0.22

Region: chr8 17590707-17590775. Max. coverage (+): 17.94. Max coverage (-): 0

Region: chr8 17590776-17590844. Max. coverage (+): 2.82. Max coverage (-): 3.33

Region: chr8 17590845-17590913. Max. coverage (+): 65.68. Max coverage (-): 0

Region: chr8 17590914-17590982. Max. coverage (+): 11.6. Max coverage (-): 3.84

Region: chr8 17590983-17591051. Max. coverage (+): 8.66. Max coverage (-): 7.07

Region: chr8 17591052-17591119. Max. coverage (+): 0. Max coverage (-): 0

Region: chr8 17591120-17591188. Max. coverage (+): 9.83. Max coverage (-): 2.29

Region: chr8 17591189-17591257. Max. coverage (+): 0.34. Max coverage (-): 0.5

Region: chr8 17591258-17591326. Max. coverage (+): 0. Max coverage (-): 3.79

Region: chr8 17591327-17591395. Max. coverage (+): 49.12. Max coverage (-): 5.63

Region: chr8 17591396-17591464. Max. coverage (+): 24.36. Max coverage (-): 0

Region: chr8 17591465-17591533. Max. coverage (+): 41.58. Max coverage (-): 1.02

Region: chr8 17591534-17591602. Max. coverage (+): 22.23. Max coverage (-): 18.25

Region: chr8 17591603-17591670. Max. coverage (+): 14.75. Max coverage (-): 17.42

Region: chr8 17591671-17591739. Max. coverage (+): 21.84. Max coverage (-): 0

Region: chr8 17591740-17591808. Max. coverage (+): 22.95. Max coverage (-): 3.84

Region: chr8 17591809-17591877. Max. coverage (+): 15.54. Max coverage (-): 5.82

Region: chr8 17591878-17591946. Max. coverage (+): 2.68. Max coverage (-): 0

Region: chr8 17591947-17592015. Max. coverage (+): 2.68. Max coverage (-): 0

Region: chr8 17592016-17592084. Max. coverage (+): 4.21. Max coverage (-): 0

Region: chr8 17592085-17592153. Max. coverage (+): 13.9. Max coverage (-): 6.45

Region: chr8 17592154-17592222. Max. coverage (+): 20.83. Max coverage (-): 0

Region: chr8 17592223-17592290. Max. coverage (+): 34.44. Max coverage (-): 13.49

Region: chr8 17592291-17592359. Max. coverage (+): 119.4. Max coverage (-): 6.81

Region: chr8 17592360-17592428. Max. coverage (+): 76.37. Max coverage (-): 17.32

Region: chr8 17592429-17592497. Max. coverage (+): 76.37. Max coverage (-): 56.58

Region: chr8 17592498-17592566. Max. coverage (+): 52.03. Max coverage (-): 3.51

Region: chr8 17592567-17592635. Max. coverage (+): 31.16. Max coverage (-): 3.7

Region: chr8 17592636-17592704. Max. coverage (+): 38.02. Max coverage (-): 17.22

Region: chr8 17592705-17592773. Max. coverage (+): 86.36. Max coverage (-): 7.62

Region: chr8 17592774-17592841. Max. coverage (+): 15.8. Max coverage (-): 37.96

Region: chr8 17592842-17592910. Max. coverage (+): 6.72. Max coverage (-): 7

Region: chr8 17592911-17592979. Max. coverage (+): 39.1. Max coverage (-): 4.71

Region: chr8 17592980-17593048. Max. coverage (+): 42.99. Max coverage (-): 5.38

Region: chr8 17593049-17593117. Max. coverage (+): 36.04. Max coverage (-): 0

Region: chr8 17593118-17593186. Max. coverage (+): 11.79. Max coverage (-): 0

Region: chr8 17593187-17593255. Max. coverage (+): 69.43. Max coverage (-): 0

Region: chr8 17593256-17593324. Max. coverage (+): 21.12. Max coverage (-): 0

Region: chr8 17593325-17593392. Max. coverage (+): 10.88. Max coverage (-): 0

Region: chr8 17593393-17593461. Max. coverage (+): 3.89. Max coverage (-): 0

Region: chr8 17593462-17593530. Max. coverage (+): 3.85. Max coverage (-): 0

Region: chr8 17593531-17593599. Max. coverage (+): 3.73. Max coverage (-): 0

Region: chr8 17593600-17593668. Max. coverage (+): 7.2. Max coverage (-): 0

Region: chr8 17593669-17593737. Max. coverage (+): 16.35. Max coverage (-): 0

Region: chr8 17593738-17593806. Max. coverage (+): 3.65. Max coverage (-): 0

Region: chr8 17593807-17593875. Max. coverage (+): 6.69. Max coverage (-): 0

Region: chr8 17593876-17593944. Max. coverage (+): 9.77. Max coverage (-): 0

Region: chr8 17593945-17594012. Max. coverage (+): 25.74. Max coverage (-): 0

Region: chr8 17594013-17594081. Max. coverage (+): 0. Max coverage (-): 0

Region: chr8 17594082-17594150. Max. coverage (+): 0. Max coverage (-): 0

Region: chr8 17594151-17594219. Max. coverage (+): 0. Max coverage (-): 0

Region: chr8 17594220-17594288. Max. coverage (+): 7.42. Max coverage (-): 0

Region: chr8 17594289-17594357. Max. coverage (+): 24.16. Max coverage (-): 0

Region: chr8 17594358-17594426. Max. coverage (+): 16.2. Max coverage (-): 0

Region: chr8 17594427-17594495. Max. coverage (+): 2.64. Max coverage (-): 0

Region: chr8 17594496-17594563. Max. coverage (+): 4.8. Max coverage (-): 0

Region: chr8 17594564-17594632. Max. coverage (+): 2.97. Max coverage (-): 0

Region: chr8 17594633-17594701. Max. coverage (+): 6.75. Max coverage (-): 0

Region: chr8 17594702-17594770. Max. coverage (+): 0. Max coverage (-): 0

Region: chr8 17594771-17594839. Max. coverage (+): 14.94. Max coverage (-): 0

Region: chr8 17594840-17594908. Max. coverage (+): 45.94. Max coverage (-): 0

Region: chr8 17594909-17594977. Max. coverage (+): 21.6. Max coverage (-): 0

Region: chr8 17594978-17595046. Max. coverage (+): 96.72. Max coverage (-): 0

Region: chr8 17595047-17595114. Max. coverage (+): 67.69. Max coverage (-): 0

Region: chr8 17595115-17595183. Max. coverage (+): 45.47. Max coverage (-): 0

Region: chr8 17595184-17595252. Max. coverage (+): 5.91. Max coverage (-): 0

Region: chr8 17595253-17595321. Max. coverage (+): 45.76. Max coverage (-): 0

Region: chr8 17595322-17595390. Max. coverage (+): 4.13. Max coverage (-): 0

Region: chr8 17595391-17595459. Max. coverage (+): 9.6. Max coverage (-): 0

Region: chr8 17595460-17595528. Max. coverage (+): 12.09. Max coverage (-): 0

Region: chr8 17595529-17595597. Max. coverage (+): 6.24. Max coverage (-): 0

Region: chr8 17595598-17595666. Max. coverage (+): 13.23. Max coverage (-): 0

Region: chr8 17595667-17595734. Max. coverage (+): 4.61. Max coverage (-): 0

Region: chr8 17595735-17595803. Max. coverage (+): 0. Max coverage (-): 0

Region: chr8 17595804-17595872. Max. coverage (+): 0.07. Max coverage (-): 0

Region: chr8 17595873-17595941. Max. coverage (+): 0.07. Max coverage (-): 0

Region: chr8 17595942-17596010. Max. coverage (+): 0. Max coverage (-): 0

Region: chr8 17596011-17596079. Max. coverage (+): 0. Max coverage (-): 0

Region: chr8 17596080-17596148. Max. coverage (+): 0. Max coverage (-): 0

Region: chr8 17596149-17596217. Max. coverage (+): 0. Max coverage (-): 0

Region: chr8 17596218-17596285. Max. coverage (+): 7.13. Max coverage (-): 0

Region: chr8 17596286-17596354. Max. coverage (+): 11.12. Max coverage (-): 0

Region: chr8 17596355-17596423. Max. coverage (+): 0. Max coverage (-): 0

Region: chr8 17596424-17596492. Max. coverage (+): 0. Max coverage (-): 0

Region: chr8 17596493-17596561. Max. coverage (+): 0. Max coverage (-): 0

Region: chr8 17596562-17596630. Max. coverage (+): 0. Max coverage (-): 0

Region: chr8 17596631-17596699. Max. coverage (+): 0. Max coverage (-): 0

Region: chr8 17596700-17596768. Max. coverage (+): 0. Max coverage (-): 0

Region: chr8 17596769-17596836. Max. coverage (+): 0. Max coverage (-): 0

Region: chr8 17596837-17596905. Max. coverage (+): 0. Max coverage (-): 0

Region: chr8 17596906-17596974. Max. coverage (+): 1.05. Max coverage (-): 0

Region: chr8 17596975-17597043. Max. coverage (+): 25.03. Max coverage (-): 0

Region: chr8 17597044-17597112. Max. coverage (+): 25.09. Max coverage (-): 0

Region: chr8 17597113-17597181. Max. coverage (+): 33.28. Max coverage (-): 0

Region: chr8 17597182-17597250. Max. coverage (+): 33.42. Max coverage (-): 0

Region: chr8 17597251-17597319. Max. coverage (+): 26.66. Max coverage (-): 0

Region: chr8 17597320-17597388. Max. coverage (+): 41.18. Max coverage (-): 0

Region: chr8 17597389-17597456. Max. coverage (+): 22.93. Max coverage (-): 0

Region: chr8 17597457-17597525. Max. coverage (+): 52.93. Max coverage (-): 0

Region: chr8 17597526-17597594. Max. coverage (+): 6.3. Max coverage (-): 0

Region: chr8 17597595-17597663. Max. coverage (+): 45.23. Max coverage (-): 0

Region: chr8 17597664-17597732. Max. coverage (+): 20.35. Max coverage (-): 0

Region: chr8 17597733-17597801. Max. coverage (+): 34.67. Max coverage (-): 0

Region: chr8 17597802-17597870. Max. coverage (+): 42.78. Max coverage (-): 0

Region: chr8 17597871-17597939. Max. coverage (+): 33.17. Max coverage (-): 0

Region: chr8 17597940-17598007. Max. coverage (+): 33.17. Max coverage (-): 0

Region: chr8 17598008-17598076. Max. coverage (+): 12.15. Max coverage (-): 0

Region: chr8 17598077-17598145. Max. coverage (+): 53.94. Max coverage (-): 2.09

Region: chr8 17598146-17598214. Max. coverage (+): 53.94. Max coverage (-): 0

Region: chr8 17598215-17598283. Max. coverage (+): 17.53. Max coverage (-): 0

Region: chr8 17598284-17598352. Max. coverage (+): 20.03. Max coverage (-): 0

Region: chr8 17598353-17598421. Max. coverage (+): 23.61. Max coverage (-): 0

Region: chr8 17598422-17598490. Max. coverage (+): 55.89. Max coverage (-): 0

Region: chr8 17598491-17598558. Max. coverage (+): 28.72. Max coverage (-): 0

Region: chr8 17598559-17598627. Max. coverage (+): 17.84. Max coverage (-): 0

Region: chr8 17598628-17598696. Max. coverage (+): 12.49. Max coverage (-): 0

Region: chr8 17598697-17598765. Max. coverage (+): 9.01. Max coverage (-): 0

Region: chr8 17598766-17598834. Max. coverage (+): 30.99. Max coverage (-): 0

Region: chr8 17598835-17598903. Max. coverage (+): 24.7. Max coverage (-): 0

Region: chr8 17598904-17598972. Max. coverage (+): 21.68. Max coverage (-): 0

Region: chr8 17598973-17599041. Max. coverage (+): 17.45. Max coverage (-): 0

Region: chr8 17599042-17599110. Max. coverage (+): 15.61. Max coverage (-): 0

Region: chr8 17599111-17599178. Max. coverage (+): 8.88. Max coverage (-): 0

Region: chr8 17599179-17599247. Max. coverage (+): 9.26. Max coverage (-): 0

Region: chr8 17599248-17599316. Max. coverage (+): 0. Max coverage (-): 0

Region: chr8 17599317-17599385. Max. coverage (+): 49.51. Max coverage (-): 0

Region: chr8 17599386-17599454. Max. coverage (+): 13.41. Max coverage (-): 0

Region: chr8 17599455-17599523. Max. coverage (+): 15.89. Max coverage (-): 0

Region: chr8 17599524-17599592. Max. coverage (+): 13.26. Max coverage (-): 0

Region: chr8 17599593-17599661. Max. coverage (+): 15.2. Max coverage (-): 0

Region: chr8 17599662-17599729. Max. coverage (+): 2.89. Max coverage (-): 0

Region: chr8 17599730-17599798. Max. coverage (+): 56.2. Max coverage (-): 0

Region: chr8 17599799-17599867. Max. coverage (+): 96.5. Max coverage (-): 0

Region: chr8 17599868-17599936. Max. coverage (+): 38.32. Max coverage (-): 0

Region: chr8 17599937-17600005. Max. coverage (+): 18.81. Max coverage (-): 0

Region: chr8 17600006-17600074. Max. coverage (+): 25.57. Max coverage (-): 0

Region: chr8 17600075-17600143. Max. coverage (+): 8.14. Max coverage (-): 0

Region: chr8 17600144-17600212. Max. coverage (+): 10.86. Max coverage (-): 0

Region: chr8 17600213-17600280. Max. coverage (+): 10.53. Max coverage (-): 0

Region: chr8 17600281-17600349. Max. coverage (+): 14.92. Max coverage (-): 0

Region: chr8 17600350-17600418. Max. coverage (+): 3.02. Max coverage (-): 0

Region: chr8 17600419-17600487. Max. coverage (+): 0. Max coverage (-): 0

Region: chr8 17600488-17600556. Max. coverage (+): 0. Max coverage (-): 0

Region: chr8 17600557-17600625. Max. coverage (+): 8.08. Max coverage (-): 0

Region: chr8 17600626-17600694. Max. coverage (+): 0. Max coverage (-): 0

Region: chr8 17600695-17600763. Max. coverage (+): 13.49. Max coverage (-): 0

Region: chr8 17600764-17600832. Max. coverage (+): 39.22. Max coverage (-): 0

Region: chr8 17600833-17600900. Max. coverage (+): 9.25. Max coverage (-): 0

Region: chr8 17600901-17600969. Max. coverage (+): 3.61. Max coverage (-): 0

Region: chr8 17600970-17601038. Max. coverage (+): 1.1. Max coverage (-): 0

Region: chr8 17601039-17601107. Max. coverage (+): 18.86. Max coverage (-): 0

Region: chr8 17601108-17601176. Max. coverage (+): 13.01. Max coverage (-): 0

Region: chr8 17601177-17601245. Max. coverage (+): 13.01. Max coverage (-): 0

Region: chr8 17601246-17601314. Max. coverage (+): 2.27. Max coverage (-): 0

Region: chr8 17601315-17601383. Max. coverage (+): 52.73. Max coverage (-): 0

Region: chr8 17601384-17601451. Max. coverage (+): 31.54. Max coverage (-): 0

Region: chr8 17601452-17601520. Max. coverage (+): 12.19. Max coverage (-): 0

Region: chr8 17601521-17601589. Max. coverage (+): 5.88. Max coverage (-): 0

Region: chr8 17601590-17601658. Max. coverage (+): 6.26. Max coverage (-): 0

Region: chr8 17601659-17601727. Max. coverage (+): 1.94. Max coverage (-): 0

Region: chr8 17601728-17601796. Max. coverage (+): 0. Max coverage (-): 0

Region: chr8 17601797-17601865. Max. coverage (+): 0.85. Max coverage (-): 0

Region: chr8 17601866-17601934. Max. coverage (+): 6.87. Max coverage (-): 0

Region: chr8 17601935-17602002. Max. coverage (+): 15.31. Max coverage (-): 0

Region: chr8 17602003-17602071. Max. coverage (+): 4.02. Max coverage (-): 0

Region: chr8 17602072-17602140. Max. coverage (+): 17.74. Max coverage (-): 0

Region: chr8 17602141-17602209. Max. coverage (+): 5.27. Max coverage (-): 0

Region: chr8 17602210-17602278. Max. coverage (+): 0. Max coverage (-): 0

Region: chr8 17602279-17602347. Max. coverage (+): 0. Max coverage (-): 0

Region: chr8 17602348-17602416. Max. coverage (+): 0. Max coverage (-): 0

Region: chr8 17602417-17602485. Max. coverage (+): 0. Max coverage (-): 0

Region: chr8 17602486-17602554. Max. coverage (+): 0. Max coverage (-): 0

Region: chr8 17602555-17602622. Max. coverage (+): 0. Max coverage (-): 0

Region: chr8 17602623-17602691. Max. coverage (+): 11.08. Max coverage (-): 0

Region: chr8 17602692-17602760. Max. coverage (+): 6.48. Max coverage (-): 0

Region: chr8 17602761-17602829. Max. coverage (+): 0. Max coverage (-): 0

Region: chr8 17602830-17602898. Max. coverage (+): 0. Max coverage (-): 0

Region: chr8 17602899-17602967. Max. coverage (+): 0. Max coverage (-): 0

Region: chr8 17602968-17603036. Max. coverage (+): 0. Max coverage (-): 0

Region: chr8 17603037-17603105. Max. coverage (+): 3.02. Max coverage (-): 0

Region: chr8 17603106-17603173. Max. coverage (+): 7.1. Max coverage (-): 0

Region: chr8 17603174-17603242. Max. coverage (+): 4.58. Max coverage (-): 0

Region: chr8 17603243-17603311. Max. coverage (+): 0. Max coverage (-): 0

Region: chr8 17603312-17603380. Max. coverage (+): 5.11. Max coverage (-): 0

Region: chr8 17603381-17603449. Max. coverage (+): 0. Max coverage (-): 0

Region: chr8 17603450-17603518. Max. coverage (+): 0. Max coverage (-): 0

Region: chr8 17603519-17603587. Max. coverage (+): 0. Max coverage (-): 0

Region: chr8 17603588-17603656. Max. coverage (+): 7.24. Max coverage (-): 0

Region: chr8 17603657-17603724. Max. coverage (+): 3.97. Max coverage (-): 0

Region: chr8 17603725-17603793. Max. coverage (+): 0. Max coverage (-): 0

Region: chr8 17603794-17603862. Max. coverage (+): 0. Max coverage (-): 0

Region: chr8 17603863-17603931. Max. coverage (+): 0. Max coverage (-): 0

Region: chr8 17603932-17604000. Max. coverage (+): 0. Max coverage (-): 0

Region: chr8 17604001-17604069. Max. coverage (+): 0. Max coverage (-): 0

Region: chr8 17604070-17604138. Max. coverage (+): 5.03. Max coverage (-): 0

Region: chr8 17604139-17604207. Max. coverage (+): 0. Max coverage (-): 0

Region: chr8 17604208-17604276. Max. coverage (+): 0. Max coverage (-): 0

Region: chr8 17604277-17604344. Max. coverage (+): 0. Max coverage (-): 0

Region: chr8 17604345-17604413. Max. coverage (+): 10.99. Max coverage (-): 0

Region: chr8 17604414-17604482. Max. coverage (+): 0. Max coverage (-): 0

Region: chr8 17604483-17604551. Max. coverage (+): 0. Max coverage (-): 0

Region: chr8 17604552-17604620. Max. coverage (+): 0. Max coverage (-): 0

Region: chr8 17604621-17604689. Max. coverage (+): 0. Max coverage (-): 0

Region: chr8 17604690-17604758. Max. coverage (+): 0. Max coverage (-): 0

Region: chr8 17604759-17604827. Max. coverage (+): 0. Max coverage (-): 0

Region: chr8 17604828-17604895. Max. coverage (+): 0. Max coverage (-): 0

Region: chr8 17604896-17604964. Max. coverage (+): 0. Max coverage (-): 0

Region: chr8 17604965-17605033. Max. coverage (+): 0. Max coverage (-): 0

Region: chr8 17605034-17605102. Max. coverage (+): 0. Max coverage (-): 0

Region: chr8 17605103-17605171. Max. coverage (+): 0. Max coverage (-): 0

Region: chr8 17605172-17605240. Max. coverage (+): 0. Max coverage (-): 0

Region: chr8 17605241-17605309. Max. coverage (+): 0. Max coverage (-): 0

Region: chr8 17605310-17605378. Max. coverage (+): 0. Max coverage (-): 0

Region: chr8 17605379-17605446. Max. coverage (+): 0. Max coverage (-): 0

Region: chr8 17605447-17605515. Max. coverage (+): 0. Max coverage (-): 0

Region: chr8 17605516-17605584. Max. coverage (+): 0. Max coverage (-): 0

Region: chr8 17605585-17605653. Max. coverage (+): 0. Max coverage (-): 0

Region: chr8 17605654-17605722. Max. coverage (+): 0. Max coverage (-): 0

Region: chr8 17605723-17605791. Max. coverage (+): 0. Max coverage (-): 0

Region: chr8 17605792-17605860. Max. coverage (+): 0. Max coverage (-): 0

Region: chr8 17605861-17605929. Max. coverage (+): 0. Max coverage (-): 0

Region: chr8 17605930-17605998. Max. coverage (+): 0. Max coverage (-): 0

Region: chr8 17605999-17606066. Max. coverage (+): 0. Max coverage (-): 0

Region: chr8 17606067-17606135. Max. coverage (+): 0. Max coverage (-): 0

Region: chr8 17606136-17606204. Max. coverage (+): 0. Max coverage (-): 0

Region: chr8 17606205-17606273. Max. coverage (+): 0. Max coverage (-): 0

Region: chr8 17606274-17606342. Max. coverage (+): 0. Max coverage (-): 0

Region: chr8 17606343-17606411. Max. coverage (+): 0. Max coverage (-): 0

Region: chr8 17606412-17606480. Max. coverage (+): 0. Max coverage (-): 0

Region: chr8 17606481-17606549. Max. coverage (+): 0. Max coverage (-): 0

Region: chr8 17606550-17606617. Max. coverage (+): 0. Max coverage (-): 0

Region: chr8 17606618-17606686. Max. coverage (+): 0. Max coverage (-): 0

Region: chr8 17606687-17606755. Max. coverage (+): 0. Max coverage (-): 0

Region: chr8 17606756-17606824. Max. coverage (+): 0. Max coverage (-): 0

Region: chr8 17606825-17606893. Max. coverage (+): 0. Max coverage (-): 0

Region: chr8 17606894-17606962. Max. coverage (+): 0. Max coverage (-): 0

Region: chr8 17606963-17607031. Max. coverage (+): 0. Max coverage (-): 0

Region: chr8 17607032-17607100. Max. coverage (+): 0. Max coverage (-): 0

Region: chr8 17607101-17607168. Max. coverage (+): 0. Max coverage (-): 0

Region: chr8 17607169-17607237. Max. coverage (+): 0. Max coverage (-): 0

Region: chr8 17607238-17607306. Max. coverage (+): 0. Max coverage (-): 0

Region: chr8 17607307-17607375. Max. coverage (+): 0. Max coverage (-): 0

Region: chr8 17607376-17607444. Max. coverage (+): 0. Max coverage (-): 0

Region: chr8 17607445-17607513. Max. coverage (+): 0. Max coverage (-): 0

Region: chr8 17607514-17607582. Max. coverage (+): 0. Max coverage (-): 0

Region: chr8 17607583-17607651. Max. coverage (+): 0. Max coverage (-): 0

Region: chr8 17607652-17607720. Max. coverage (+): 0. Max coverage (-): 0

Region: chr8 17607721-17607788. Max. coverage (+): 0. Max coverage (-): 0

Region: chr8 17607789-17607857. Max. coverage (+): 0. Max coverage (-): 0

Region: chr8 17607858-17607926. Max. coverage (+): 0. Max coverage (-): 0

Region: chr8 17607927-17607995. Max. coverage (+): 0. Max coverage (-): 0

Region: chr8 17607996-17608064. Max. coverage (+): 0. Max coverage (-): 0

Region: chr8 17608065-17608133. Max. coverage (+): 0. Max coverage (-): 0

Region: chr8 17608134-17608202. Max. coverage (+): 0. Max coverage (-): 0

Region: chr8 17608203-17608271. Max. coverage (+): 0. Max coverage (-): 0

Region: chr8 17608272-17608339. Max. coverage (+): 0. Max coverage (-): 0

Region: chr8 17608340-17608408. Max. coverage (+): 0. Max coverage (-): 0

Region: chr8 17608409-17608477. Max. coverage (+): 0. Max coverage (-): 0

Region: chr8 17608478-17608546. Max. coverage (+): 5.82. Max coverage (-): 0

Region: chr8 17608547-17608615. Max. coverage (+): 0. Max coverage (-): 0

Region: chr8 17608616-17608684. Max. coverage (+): 0. Max coverage (-): 0

Region: chr8 17608685-17608753. Max. coverage (+): 0. Max coverage (-): 0

Region: chr8 17608754-17608822. Max. coverage (+): 0. Max coverage (-): 0

Region: chr8 17608823-17608890. Max. coverage (+): 0. Max coverage (-): 0

Region: chr8 17608891-17608959. Max. coverage (+): 0. Max coverage (-): 0

Region: chr8 17608960-17609028. Max. coverage (+): 0. Max coverage (-): 0

Region: chr8 17609029-17609097. Max. coverage (+): 5.68. Max coverage (-): 0

Region: chr8 17609098-17609166. Max. coverage (+): 0. Max coverage (-): 0

Region: chr8 17609167-17609235. Max. coverage (+): 0. Max coverage (-): 0

Region: chr8 17609236-17609304. Max. coverage (+): 0. Max coverage (-): 0

Region: chr8 17609305-17609373. Max. coverage (+): 0. Max coverage (-): 0

Region: chr8 17609374-17609442. Max. coverage (+): 0. Max coverage (-): 0

Region: chr8 17609443-17609510. Max. coverage (+): 0. Max coverage (-): 0

Region: chr8 17609511-17609579. Max. coverage (+): 0. Max coverage (-): 0

Region: chr8 17609580-17609648. Max. coverage (+): 0. Max coverage (-): 0

Region: chr8 17609649-17609717. Max. coverage (+): 0. Max coverage (-): 0

Region: chr8 17609718-17609786. Max. coverage (+): 0. Max coverage (-): 0

Region: chr8 17609787-17609855. Max. coverage (+): 0. Max coverage (-): 0

Region: chr8 17609856-17609924. Max. coverage (+): 0. Max coverage (-): 0

Region: chr8 17609925-17609993. Max. coverage (+): 0. Max coverage (-): 0

Region: chr8 17609994-17610061. Max. coverage (+): 22.21. Max coverage (-): 0

Region: chr8 17610062-17610130. Max. coverage (+): 15.8. Max coverage (-): 0

Region: chr8 17610131-17610199. Max. coverage (+): 0. Max coverage (-): 0

Region: chr8 17610200-17610268. Max. coverage (+): 0. Max coverage (-): 0

Region: chr8 17610269-17610337. Max. coverage (+): 56.49. Max coverage (-): 0

Region: chr8 17610338-17610406. Max. coverage (+): 22.3. Max coverage (-): 0

Region: chr8 17610407-17610475. Max. coverage (+): 9.76. Max coverage (-): 0

Region: chr8 17610476-17610544. Max. coverage (+): 24.97. Max coverage (-): 0

Region: chr8 17610545-17610612. Max. coverage (+): 23.49. Max coverage (-): 0

Region: chr8 17610613-17610681. Max. coverage (+): 13.88. Max coverage (-): 0

Region: chr8 17610682-17610750. Max. coverage (+): 15.23. Max coverage (-): 0

Region: chr8 17610751-17610819. Max. coverage (+): 8.51. Max coverage (-): 0

Region: chr8 17610820-17610888. Max. coverage (+): 0. Max coverage (-): 0

Region: chr8 17610889-17610957. Max. coverage (+): 0. Max coverage (-): 0

Region: chr8 17610958-17611026. Max. coverage (+): 7.25. Max coverage (-): 0

Region: chr8 17611027-17611095. Max. coverage (+): 13.22. Max coverage (-): 0

Region: chr8 17611096-17611164. Max. coverage (+): 10.44. Max coverage (-): 0

Region: chr8 17611165-17611232. Max. coverage (+): 9.24. Max coverage (-): 0

Region: chr8 17611233-17611301. Max. coverage (+): 13.55. Max coverage (-): 0

Region: chr8 17611302-17611370. Max. coverage (+): 6.05. Max coverage (-): 0

Region: chr8 17611371-17611439. Max. coverage (+): 0. Max coverage (-): 0

Region: chr8 17611440-. Max. coverage (+): 17.64. Max coverage (-): 0

RepeatMasker Color Code

**+**

100-98% Identity

<98-95% Identity

<95-90% Identity

<90-85% Identity

<85-80% Identity

<80-75% Identity

<75-70% Identity

<70% Identity

**-**

Gene Set Color Code

**+**

Gene

Pseudogene

**-**

Topology/Coverage Color Code

Coverage Plus Strand

Coverage Minus Strand

Mainstrand: Plus

Mainstrand: Minus

Complementary Strand

Flanking Region  
(if option -flank >0)

Gene Set Annotation  
  
RepeatMasker Annotation  

**1. L1ME4a**: 17577730-17578253 (-), Divergence to consensus: 41.7%  
**2. L2c**: 17579075-17579133 (+), Divergence to consensus: 30.5%  
**3. MIRb**: 17579196-17579331 (-), Divergence to consensus: 33%  
**4. L2a**: 17581334-17581446 (+), Divergence to consensus: 38.5%  
**5. (TG)n**: 17582375-17582406 (+), Divergence to consensus: 6.2%  
**6. ART2A**: 17583268-17583517 (+), Divergence to consensus: 14.5%  
**7. Bov-tA3**: 17594040-17594252 (+), Divergence to consensus: 13.8%  
**8. Bov-tA3**: 17594660-17594772 (+), Divergence to consensus: 7.1%  
**9. L2c**: 17595532-17595612 (+), Divergence to consensus: 37.5%  
**10. BOV-A2**: 17596407-17596647 (-), Divergence to consensus: 15.4%  
**11. L1\_BT**: 17596648-17596965 (+), Divergence to consensus: 20.5%  
**12. Bov-tA2**: 17596996-17597030 (-), Divergence to consensus: 2.9%  
**13. MIR**: 17599298-17599392 (+), Divergence to consensus: 28.1%  
**14. L1ME4c**: 17600319-17600551 (-), Divergence to consensus: 43.4%  
**15. AT\_rich**: 17600966-17601005 (+), Divergence to consensus: 70%  
**16. MIRb**: 17602370-17602610 (-), Divergence to consensus: 40.5%  
**17. MIRb**: 17602980-17603069 (-), Divergence to consensus: 37.8%  
**18. MIRc**: 17604003-17604096 (-), Divergence to consensus: 37.2%  
**19. Bov-tA3**: 17604147-17604342 (-), Divergence to consensus: 18.3%  
**20. L1ME2**: 17604422-17604557 (+), Divergence to consensus: 30.8%  
**21. L1MEc**: 17604590-17605107 (+), Divergence to consensus: 43.6%  
**22. Bov-tA2**: 17605111-17605311 (-), Divergence to consensus: 38.5%  
**23. ART2A**: 17605335-17605855 (-), Divergence to consensus: 20.1%  
**24. BovB**: 17605856-17606691 (-), Divergence to consensus: 10.8%  
**25. BTLTR1**: 17606693-17606763 (+), Divergence to consensus: 17.5%  
**26. BovB**: 17606765-17607525 (-), Divergence to consensus: 11.9%  
**27. L1MEc**: 17607521-17607762 (+), Divergence to consensus: 29.8%  
**28. L1ME3**: 17607762-17607930 (+), Divergence to consensus: 43.4%  
**29. Bov-tA2**: 17607948-17608155 (-), Divergence to consensus: 18.6%  
**30. BOV-A2**: 17608167-17608431 (-), Divergence to consensus: 5.7%  
**31. BovB**: 17609333-17609462 (+), Divergence to consensus: 4.6%  
**32. ART2A**: 17609463-17609979 (+), Divergence to consensus: 9.7%  
**33. (AACTG)n**: 17609980-17609999 (+), Divergence to consensus: 0%  
**34. BOV-A2**: 17610145-17610273 (-), Divergence to consensus: 7.8%  
**35. MLT1H**: 17610820-17610878 (+), Divergence to consensus: 20.3%  
**36. MLT1H**: 17610891-17611015 (+), Divergence to consensus: 35.5%

  
Transcription Factor Binding Sites  

**RFX4\_2** (Sequence: CCTGGATAC (+): 17597290)  
**RFX4\_2** (Sequence: CATGGATAC (+): 17597300)  
**Gata4** (Sequence: AGATAAG (-): 17594841)  
**Gata4** (Sequence: AGATAAG (-): 17600114)  
**SOX9** (Sequence: AACAATGA (-): 17580126)  
**SOX9** (Sequence: AACAATAA (-): 17581264)  
**SOX9** (Sequence: AACAATGA (-): 17592501)  
**SOX9** (Sequence: AACAATGA (-): 17593454)  
**SOX9** (Sequence: AACAATAA (-): 17593477)  
**SOX9** (Sequence: AACAATGA (-): 17610561)  
**SOX9** (Sequence: TTATTGTT (+): 17584659)  
**SOX9** (Sequence: TTATTGTT (+): 17590137)  
**SOX9** (Sequence: CCATTGTT (+): 17600388)  
**SOX9** (Sequence: CTATTGTT (+): 17608985)  
**Gata4** (Sequence: GTTATCT (+): 17577568)  
**Gata4** (Sequence: CTTATCT (+): 17592792)  
**Gata4** (Sequence: GTTATCT (+): 17595751)  
**Gata4** (Sequence: GTTATCT (+): 17598558)  
**Gata4** (Sequence: CTTATCT (+): 17603517)  
**Gata4** (Sequence: GTTATCT (+): 17611382)
