# Supplementary material for: Small RNA sequencing of cryopreserved semen from single bull revealed altered miRNAs and piRNAs expression between High- and Low-motile sperm populations
Source: BMC Genomics. 2017 Jan 4;18:14. doi: 10.1186/s12864-016-3394-7 (PMC5209821; doi:10.1186/s12864-016-3394-7)
Supplement: Additional file 4: — Details for each piRNA clusters found in Low Motile (LM) sperm fraction. Genes, repeats, transposable elements and transcription factors binding sites falling within the cluster regions were reported. (ZIP 1034 kb) [file 12864_2016_3394_MOESM4_ESM.zip › 49.html]

piRNA cluster 49


Predicted piRNA cluster no. 49     previous   next
  

Show proTRAC run info
Hide proTRAC run info

================================= proTRAC ====================================  
VERSION: 2.1                                    LAST MODIFIED: 06. October 2015  
  
Please cite:  
Rosenkranz D, Zischler H. proTRAC - a software for probabilistic piRNA cluster  
detection, visualization and analysis. 2012. BMC Bioinformatics 13:5.  
  
and (for proTRAC 2.0 and later):  
Rosenkranz D, Rudloff S, Bastuck K, Ketting RF, Zischler H. Tupaia small RNAs  
provide insights into function and evolution of RNAi-based transposon defense  
in mammals. 2015. RNA 21(5):911-922.  
  
Contact:  
David Rosenkranz  
Institute of Anthropology, small RNA group  
Johannes Gutenberg University Mainz  
email: rosenkranz@uni-mainz.de  
  
You can find the latest proTRAC version at:  
http://sourceforge.net/projects/protrac/files  
http://www.smallRNAgroup-mainz.de/software  
==============================================================================  
  
PARAMETERS:  
Map file: .............../storage/core/barbara/genhome/smallRNA/fertility/Sample\_not\_motile/pirna/Sample\_not\_motile\_26-33\_collapsed.fa.no-dust.map.weighted-10000-1000-b-0  
Genome file: ............/storage/core/barbara/genhome/smallRNA/fertility/Sample\_all/pirna/bt\_311\_chrY.fa  
RepeatMasker annotation: /storage/genomes/bt\_umd31/GCF\_000003055.6\_Bos\_taurus\_UMD\_3.1.1\_repeatMasker\_chr.out  
GeneSet:................./storage/core/barbara/genhome/smallRNA/fertility/Sample\_all/pirna/full.gtf  
  
Significant (p<=0.01) hit density will be calculated based  
on observed hit distribution.  
  
Sliding window size: ........................................ 5000 bp  
Sliding window increament: .................................. 1000 bp  
Normalize each hit by number of genomic hits: ............... 1 [0=no/1=yes]  
Normalize each hit by number of sequence reads: ............. 1 [0=no/1=yes]  
Normalize values (-> per million mapped reads): ............. 1 [0=no/1=yes]  
Min. fraction of hits with 1T(U) or 10A: .................... 0.75  
Alternatively: Min. fraction of hits with 1T(U) and 10A: .... 0.5  
Min. fraction of hits with typical piRNA length: ............ 0.75  
Typical piRNA length: ....................................... 26-33 nt  
Min. size of a piRNA cluster: ............................... 5000 bp.  
Min. number of hits (absolute): ............................. 0  
Min. number of hits (normalized): ........................... 0  
Min. fraction of hits on the mainstrand: .................... 0.75  
Top fraction of mapped sequences (in terms of read counts): . 1%  
Top fraction accounts for max. n% of sequence reads: ........ 90%  
Min. fraction of hits on each arm of a bidirectional cluster: 0.1  
Output image file for each cluster: ......................... 0 [0=no/1=yes]  
Output html file for each cluster: .......................... 1 [0=no/1=yes]  
Output a summary table: ..................................... 1 [0=no/1=yes]  
Output a FASTA file for each cluster (piRNA sequences): ..... 1 [0=no/1=yes]  
Output a FASTA file comprising cluster sequences: ........... 1 [0=no/1=yes]  
Search DNA motifs in clusters: .............................. 1 [0=no/1=yes]  
Output flanking sequences: +/- .............................. 0 bp  
Output ~.pTi file: .......................................... 1 [0=no/1=yes]  
==============================================================================  
  
  
Genome size (without gaps): ............ 2678902517 bp  
Gaps (N/X/-): .......................... 53837044 bp  
Mapped reads: .......................... 738059667487  
Non-identical sequences: ............... 277001  
Genomic hits: .......................... 533816  
Significant densitiy of mapped reads: .. 15118061 reads/kb

Show proTRAC cluster info
Hide proTRAC cluster info

|  |  |
| --- | --- |
| Location | chr8 |
| Coordinates | 87062543-87069683 |
| Size [bp] | 7141 |
| Sequence hit loci | 122 |
| Mapped reads (normalized) | 327794637 |
| Mapped reads (normalized) per kb | 45903184 |
| Normalized reads with 1T (1U) | 84.5% |
| Normalized reads with 10A | 39.2% |
| Normalized reads with length 26-33 nt | 100% |
| Normalized reads on the main strand(s) | 97.3% |
| Predicted directionality | mono:plus |

100%

0%

1T (1U)  
reads

10A reads

26-33 nt  
reads

reads on mainstrand

**Either the amount of reads with 1T (1U) OR 10A has to exceed 75% (set with option: -1Tor10A)  
Alternatively the amount of reads with 1T (1U) AND 10A has to exceed 50% (set with option: -1Tand10A)  
Minimum amount of reads with preferred size is 75% (set with option: -pisize)  
Minimum amount of reads on the main strand(s) is 75% (set with option: -clstrand)**

Show read coverage
Hide read coverage

WHAT DO I SEE HERE?  
This chart shows the location of mapped sequence reads within a predicted piRNA cluster. The color refers to the number of genomic hits produced by the sequence read in question. A dark red bar indicates that this sequence read produces many other hits elsewhere in the genome. Many adjacent red or yellow bars can indicate the presence of a multi-copy element such as transposons or rRNA genes. A dark green bar indicates that this sequence read maps uniquely to this locus.

1 hit

2-5 hits

6-10 hits

11-20 hits

21-50 hits

51-100 hits

> 100 hits

chr8

87062543

87069683

Gene Set

RepeatMasker

Mapped  
Reads

42.42

plus strand

minus strand

42.42

Region: chr8 17611441-87062550. Max. coverage (+): 6.02. Max coverage (-): 0

Region: chr8 87062551-87062564. Max. coverage (+): 0. Max coverage (-): 0

Region: chr8 87062565-87062578. Max. coverage (+): 0. Max coverage (-): 0

Region: chr8 87062579-87062592. Max. coverage (+): 0. Max coverage (-): 0

Region: chr8 87062593-87062607. Max. coverage (+): 3.27. Max coverage (-): 0

Region: chr8 87062608-87062621. Max. coverage (+): 0. Max coverage (-): 0

Region: chr8 87062622-87062635. Max. coverage (+): 0. Max coverage (-): 0

Region: chr8 87062636-87062650. Max. coverage (+): 0. Max coverage (-): 0

Region: chr8 87062651-87062664. Max. coverage (+): 0. Max coverage (-): 0

Region: chr8 87062665-87062678. Max. coverage (+): 0. Max coverage (-): 0

Region: chr8 87062679-87062692. Max. coverage (+): 0. Max coverage (-): 0

Region: chr8 87062693-87062707. Max. coverage (+): 0. Max coverage (-): 0

Region: chr8 87062708-87062721. Max. coverage (+): 0. Max coverage (-): 0

Region: chr8 87062722-87062735. Max. coverage (+): 0. Max coverage (-): 0

Region: chr8 87062736-87062750. Max. coverage (+): 0. Max coverage (-): 0

Region: chr8 87062751-87062764. Max. coverage (+): 0. Max coverage (-): 0

Region: chr8 87062765-87062778. Max. coverage (+): 0. Max coverage (-): 0

Region: chr8 87062779-87062792. Max. coverage (+): 0. Max coverage (-): 0

Region: chr8 87062793-87062807. Max. coverage (+): 0. Max coverage (-): 0

Region: chr8 87062808-87062821. Max. coverage (+): 0. Max coverage (-): 0

Region: chr8 87062822-87062835. Max. coverage (+): 0. Max coverage (-): 0

Region: chr8 87062836-87062850. Max. coverage (+): 0. Max coverage (-): 0

Region: chr8 87062851-87062864. Max. coverage (+): 0. Max coverage (-): 0

Region: chr8 87062865-87062878. Max. coverage (+): 0. Max coverage (-): 0

Region: chr8 87062879-87062892. Max. coverage (+): 0. Max coverage (-): 0

Region: chr8 87062893-87062907. Max. coverage (+): 0. Max coverage (-): 0

Region: chr8 87062908-87062921. Max. coverage (+): 0. Max coverage (-): 0

Region: chr8 87062922-87062935. Max. coverage (+): 0. Max coverage (-): 0

Region: chr8 87062936-87062950. Max. coverage (+): 0. Max coverage (-): 0

Region: chr8 87062951-87062964. Max. coverage (+): 0. Max coverage (-): 0

Region: chr8 87062965-87062978. Max. coverage (+): 0. Max coverage (-): 0

Region: chr8 87062979-87062992. Max. coverage (+): 0. Max coverage (-): 0

Region: chr8 87062993-87063007. Max. coverage (+): 0. Max coverage (-): 0

Region: chr8 87063008-87063021. Max. coverage (+): 5.92. Max coverage (-): 0

Region: chr8 87063022-87063035. Max. coverage (+): 0. Max coverage (-): 0

Region: chr8 87063036-87063050. Max. coverage (+): 0. Max coverage (-): 0

Region: chr8 87063051-87063064. Max. coverage (+): 0. Max coverage (-): 0

Region: chr8 87063065-87063078. Max. coverage (+): 0. Max coverage (-): 0

Region: chr8 87063079-87063092. Max. coverage (+): 0. Max coverage (-): 0

Region: chr8 87063093-87063107. Max. coverage (+): 0. Max coverage (-): 0

Region: chr8 87063108-87063121. Max. coverage (+): 0. Max coverage (-): 0

Region: chr8 87063122-87063135. Max. coverage (+): 0. Max coverage (-): 0

Region: chr8 87063136-87063149. Max. coverage (+): 0. Max coverage (-): 0

Region: chr8 87063150-87063164. Max. coverage (+): 0. Max coverage (-): 0

Region: chr8 87063165-87063178. Max. coverage (+): 0. Max coverage (-): 0

Region: chr8 87063179-87063192. Max. coverage (+): 0. Max coverage (-): 0

Region: chr8 87063193-87063207. Max. coverage (+): 0. Max coverage (-): 0

Region: chr8 87063208-87063221. Max. coverage (+): 0. Max coverage (-): 0

Region: chr8 87063222-87063235. Max. coverage (+): 0. Max coverage (-): 0

Region: chr8 87063236-87063249. Max. coverage (+): 0. Max coverage (-): 0

Region: chr8 87063250-87063264. Max. coverage (+): 0. Max coverage (-): 0

Region: chr8 87063265-87063278. Max. coverage (+): 0. Max coverage (-): 0

Region: chr8 87063279-87063292. Max. coverage (+): 0. Max coverage (-): 0

Region: chr8 87063293-87063307. Max. coverage (+): 0. Max coverage (-): 0

Region: chr8 87063308-87063321. Max. coverage (+): 0. Max coverage (-): 0

Region: chr8 87063322-87063335. Max. coverage (+): 0. Max coverage (-): 0

Region: chr8 87063336-87063349. Max. coverage (+): 0. Max coverage (-): 0

Region: chr8 87063350-87063364. Max. coverage (+): 6.29. Max coverage (-): 0

Region: chr8 87063365-87063378. Max. coverage (+): 0. Max coverage (-): 0

Region: chr8 87063379-87063392. Max. coverage (+): 0. Max coverage (-): 0

Region: chr8 87063393-87063407. Max. coverage (+): 4.07. Max coverage (-): 0

Region: chr8 87063408-87063421. Max. coverage (+): 4.07. Max coverage (-): 0

Region: chr8 87063422-87063435. Max. coverage (+): 0. Max coverage (-): 0

Region: chr8 87063436-87063449. Max. coverage (+): 0. Max coverage (-): 0

Region: chr8 87063450-87063464. Max. coverage (+): 0. Max coverage (-): 0

Region: chr8 87063465-87063478. Max. coverage (+): 0. Max coverage (-): 0

Region: chr8 87063479-87063492. Max. coverage (+): 0. Max coverage (-): 0

Region: chr8 87063493-87063507. Max. coverage (+): 0. Max coverage (-): 0

Region: chr8 87063508-87063521. Max. coverage (+): 0.56. Max coverage (-): 0

Region: chr8 87063522-87063535. Max. coverage (+): 0. Max coverage (-): 0

Region: chr8 87063536-87063549. Max. coverage (+): 0. Max coverage (-): 0

Region: chr8 87063550-87063564. Max. coverage (+): 0. Max coverage (-): 0

Region: chr8 87063565-87063578. Max. coverage (+): 0. Max coverage (-): 0

Region: chr8 87063579-87063592. Max. coverage (+): 3.3. Max coverage (-): 0

Region: chr8 87063593-87063607. Max. coverage (+): 0. Max coverage (-): 0

Region: chr8 87063608-87063621. Max. coverage (+): 0. Max coverage (-): 0

Region: chr8 87063622-87063635. Max. coverage (+): 0. Max coverage (-): 0

Region: chr8 87063636-87063649. Max. coverage (+): 0. Max coverage (-): 0

Region: chr8 87063650-87063664. Max. coverage (+): 0. Max coverage (-): 0

Region: chr8 87063665-87063678. Max. coverage (+): 0. Max coverage (-): 0

Region: chr8 87063679-87063692. Max. coverage (+): 0. Max coverage (-): 0

Region: chr8 87063693-87063706. Max. coverage (+): 0. Max coverage (-): 0

Region: chr8 87063707-87063721. Max. coverage (+): 0. Max coverage (-): 0

Region: chr8 87063722-87063735. Max. coverage (+): 0. Max coverage (-): 0

Region: chr8 87063736-87063749. Max. coverage (+): 0. Max coverage (-): 0

Region: chr8 87063750-87063764. Max. coverage (+): 0. Max coverage (-): 0

Region: chr8 87063765-87063778. Max. coverage (+): 0. Max coverage (-): 0

Region: chr8 87063779-87063792. Max. coverage (+): 0. Max coverage (-): 0

Region: chr8 87063793-87063806. Max. coverage (+): 0. Max coverage (-): 0

Region: chr8 87063807-87063821. Max. coverage (+): 0. Max coverage (-): 0

Region: chr8 87063822-87063835. Max. coverage (+): 0. Max coverage (-): 0

Region: chr8 87063836-87063849. Max. coverage (+): 0. Max coverage (-): 0

Region: chr8 87063850-87063864. Max. coverage (+): 10.99. Max coverage (-): 0

Region: chr8 87063865-87063878. Max. coverage (+): 10.99. Max coverage (-): 0

Region: chr8 87063879-87063892. Max. coverage (+): 0. Max coverage (-): 0

Region: chr8 87063893-87063906. Max. coverage (+): 0. Max coverage (-): 0

Region: chr8 87063907-87063921. Max. coverage (+): 0. Max coverage (-): 0

Region: chr8 87063922-87063935. Max. coverage (+): 0. Max coverage (-): 0

Region: chr8 87063936-87063949. Max. coverage (+): 0. Max coverage (-): 0

Region: chr8 87063950-87063964. Max. coverage (+): 0. Max coverage (-): 0

Region: chr8 87063965-87063978. Max. coverage (+): 0. Max coverage (-): 0

Region: chr8 87063979-87063992. Max. coverage (+): 0. Max coverage (-): 0

Region: chr8 87063993-87064006. Max. coverage (+): 7.6. Max coverage (-): 0

Region: chr8 87064007-87064021. Max. coverage (+): 4.67. Max coverage (-): 0

Region: chr8 87064022-87064035. Max. coverage (+): 4.67. Max coverage (-): 0

Region: chr8 87064036-87064049. Max. coverage (+): 0. Max coverage (-): 0

Region: chr8 87064050-87064064. Max. coverage (+): 8.03. Max coverage (-): 0

Region: chr8 87064065-87064078. Max. coverage (+): 8.03. Max coverage (-): 0

Region: chr8 87064079-87064092. Max. coverage (+): 0. Max coverage (-): 0

Region: chr8 87064093-87064106. Max. coverage (+): 4.81. Max coverage (-): 0

Region: chr8 87064107-87064121. Max. coverage (+): 0. Max coverage (-): 0

Region: chr8 87064122-87064135. Max. coverage (+): 0. Max coverage (-): 0

Region: chr8 87064136-87064149. Max. coverage (+): 0. Max coverage (-): 0

Region: chr8 87064150-87064164. Max. coverage (+): 0. Max coverage (-): 0

Region: chr8 87064165-87064178. Max. coverage (+): 0. Max coverage (-): 0

Region: chr8 87064179-87064192. Max. coverage (+): 0. Max coverage (-): 0

Region: chr8 87064193-87064206. Max. coverage (+): 0. Max coverage (-): 0

Region: chr8 87064207-87064221. Max. coverage (+): 0. Max coverage (-): 0

Region: chr8 87064222-87064235. Max. coverage (+): 0. Max coverage (-): 0

Region: chr8 87064236-87064249. Max. coverage (+): 0. Max coverage (-): 0

Region: chr8 87064250-87064263. Max. coverage (+): 0. Max coverage (-): 0

Region: chr8 87064264-87064278. Max. coverage (+): 0. Max coverage (-): 0

Region: chr8 87064279-87064292. Max. coverage (+): 0. Max coverage (-): 0

Region: chr8 87064293-87064306. Max. coverage (+): 0. Max coverage (-): 0

Region: chr8 87064307-87064321. Max. coverage (+): 0. Max coverage (-): 0

Region: chr8 87064322-87064335. Max. coverage (+): 0. Max coverage (-): 0

Region: chr8 87064336-87064349. Max. coverage (+): 0. Max coverage (-): 0

Region: chr8 87064350-87064363. Max. coverage (+): 0. Max coverage (-): 0

Region: chr8 87064364-87064378. Max. coverage (+): 0. Max coverage (-): 0

Region: chr8 87064379-87064392. Max. coverage (+): 0. Max coverage (-): 0

Region: chr8 87064393-87064406. Max. coverage (+): 0. Max coverage (-): 0

Region: chr8 87064407-87064421. Max. coverage (+): 0. Max coverage (-): 0

Region: chr8 87064422-87064435. Max. coverage (+): 0. Max coverage (-): 0

Region: chr8 87064436-87064449. Max. coverage (+): 0. Max coverage (-): 0

Region: chr8 87064450-87064463. Max. coverage (+): 0. Max coverage (-): 0

Region: chr8 87064464-87064478. Max. coverage (+): 0. Max coverage (-): 0

Region: chr8 87064479-87064492. Max. coverage (+): 0. Max coverage (-): 0

Region: chr8 87064493-87064506. Max. coverage (+): 0. Max coverage (-): 0

Region: chr8 87064507-87064521. Max. coverage (+): 0. Max coverage (-): 0

Region: chr8 87064522-87064535. Max. coverage (+): 0. Max coverage (-): 0

Region: chr8 87064536-87064549. Max. coverage (+): 0. Max coverage (-): 0

Region: chr8 87064550-87064563. Max. coverage (+): 0. Max coverage (-): 0

Region: chr8 87064564-87064578. Max. coverage (+): 0. Max coverage (-): 0

Region: chr8 87064579-87064592. Max. coverage (+): 0. Max coverage (-): 0

Region: chr8 87064593-87064606. Max. coverage (+): 0. Max coverage (-): 0

Region: chr8 87064607-87064621. Max. coverage (+): 0. Max coverage (-): 0

Region: chr8 87064622-87064635. Max. coverage (+): 8.07. Max coverage (-): 0

Region: chr8 87064636-87064649. Max. coverage (+): 0. Max coverage (-): 0

Region: chr8 87064650-87064663. Max. coverage (+): 0. Max coverage (-): 0

Region: chr8 87064664-87064678. Max. coverage (+): 0. Max coverage (-): 0

Region: chr8 87064679-87064692. Max. coverage (+): 0. Max coverage (-): 0

Region: chr8 87064693-87064706. Max. coverage (+): 0. Max coverage (-): 0

Region: chr8 87064707-87064721. Max. coverage (+): 0. Max coverage (-): 0

Region: chr8 87064722-87064735. Max. coverage (+): 0. Max coverage (-): 0

Region: chr8 87064736-87064749. Max. coverage (+): 0. Max coverage (-): 0

Region: chr8 87064750-87064763. Max. coverage (+): 0. Max coverage (-): 0

Region: chr8 87064764-87064778. Max. coverage (+): 0. Max coverage (-): 0

Region: chr8 87064779-87064792. Max. coverage (+): 0. Max coverage (-): 0

Region: chr8 87064793-87064806. Max. coverage (+): 0. Max coverage (-): 0

Region: chr8 87064807-87064820. Max. coverage (+): 0. Max coverage (-): 0

Region: chr8 87064821-87064835. Max. coverage (+): 0. Max coverage (-): 0

Region: chr8 87064836-87064849. Max. coverage (+): 4.26. Max coverage (-): 0

Region: chr8 87064850-87064863. Max. coverage (+): 0. Max coverage (-): 0

Region: chr8 87064864-87064878. Max. coverage (+): 0. Max coverage (-): 0

Region: chr8 87064879-87064892. Max. coverage (+): 0. Max coverage (-): 0

Region: chr8 87064893-87064906. Max. coverage (+): 0. Max coverage (-): 0

Region: chr8 87064907-87064920. Max. coverage (+): 0. Max coverage (-): 0

Region: chr8 87064921-87064935. Max. coverage (+): 1.47. Max coverage (-): 0

Region: chr8 87064936-87064949. Max. coverage (+): 0. Max coverage (-): 0

Region: chr8 87064950-87064963. Max. coverage (+): 0. Max coverage (-): 0

Region: chr8 87064964-87064978. Max. coverage (+): 4.1. Max coverage (-): 0

Region: chr8 87064979-87064992. Max. coverage (+): 6.66. Max coverage (-): 0

Region: chr8 87064993-87065006. Max. coverage (+): 6.66. Max coverage (-): 0

Region: chr8 87065007-87065020. Max. coverage (+): 0. Max coverage (-): 0

Region: chr8 87065021-87065035. Max. coverage (+): 0. Max coverage (-): 0

Region: chr8 87065036-87065049. Max. coverage (+): 0. Max coverage (-): 0

Region: chr8 87065050-87065063. Max. coverage (+): 0. Max coverage (-): 0

Region: chr8 87065064-87065078. Max. coverage (+): 0. Max coverage (-): 0

Region: chr8 87065079-87065092. Max. coverage (+): 0. Max coverage (-): 0

Region: chr8 87065093-87065106. Max. coverage (+): 0. Max coverage (-): 0

Region: chr8 87065107-87065120. Max. coverage (+): 0. Max coverage (-): 0

Region: chr8 87065121-87065135. Max. coverage (+): 0. Max coverage (-): 0

Region: chr8 87065136-87065149. Max. coverage (+): 0. Max coverage (-): 0

Region: chr8 87065150-87065163. Max. coverage (+): 6.46. Max coverage (-): 0

Region: chr8 87065164-87065178. Max. coverage (+): 6.46. Max coverage (-): 0

Region: chr8 87065179-87065192. Max. coverage (+): 0.55. Max coverage (-): 0

Region: chr8 87065193-87065206. Max. coverage (+): 0. Max coverage (-): 0

Region: chr8 87065207-87065220. Max. coverage (+): 0. Max coverage (-): 0

Region: chr8 87065221-87065235. Max. coverage (+): 0. Max coverage (-): 0

Region: chr8 87065236-87065249. Max. coverage (+): 0. Max coverage (-): 0

Region: chr8 87065250-87065263. Max. coverage (+): 0. Max coverage (-): 0

Region: chr8 87065264-87065278. Max. coverage (+): 0. Max coverage (-): 0

Region: chr8 87065279-87065292. Max. coverage (+): 0.19. Max coverage (-): 0

Region: chr8 87065293-87065306. Max. coverage (+): 0. Max coverage (-): 0

Region: chr8 87065307-87065320. Max. coverage (+): 2.41. Max coverage (-): 0

Region: chr8 87065321-87065335. Max. coverage (+): 0. Max coverage (-): 0

Region: chr8 87065336-87065349. Max. coverage (+): 0. Max coverage (-): 0

Region: chr8 87065350-87065363. Max. coverage (+): 0. Max coverage (-): 0

Region: chr8 87065364-87065377. Max. coverage (+): 0. Max coverage (-): 0

Region: chr8 87065378-87065392. Max. coverage (+): 16.03. Max coverage (-): 0

Region: chr8 87065393-87065406. Max. coverage (+): 0. Max coverage (-): 0

Region: chr8 87065407-87065420. Max. coverage (+): 0. Max coverage (-): 0

Region: chr8 87065421-87065435. Max. coverage (+): 0. Max coverage (-): 0

Region: chr8 87065436-87065449. Max. coverage (+): 0. Max coverage (-): 0

Region: chr8 87065450-87065463. Max. coverage (+): 0. Max coverage (-): 0

Region: chr8 87065464-87065477. Max. coverage (+): 0. Max coverage (-): 0

Region: chr8 87065478-87065492. Max. coverage (+): 0. Max coverage (-): 0

Region: chr8 87065493-87065506. Max. coverage (+): 0. Max coverage (-): 0

Region: chr8 87065507-87065520. Max. coverage (+): 0. Max coverage (-): 0

Region: chr8 87065521-87065535. Max. coverage (+): 0. Max coverage (-): 0

Region: chr8 87065536-87065549. Max. coverage (+): 0. Max coverage (-): 0

Region: chr8 87065550-87065563. Max. coverage (+): 0. Max coverage (-): 0

Region: chr8 87065564-87065577. Max. coverage (+): 0. Max coverage (-): 0

Region: chr8 87065578-87065592. Max. coverage (+): 0. Max coverage (-): 0

Region: chr8 87065593-87065606. Max. coverage (+): 0. Max coverage (-): 0

Region: chr8 87065607-87065620. Max. coverage (+): 0. Max coverage (-): 0

Region: chr8 87065621-87065635. Max. coverage (+): 0. Max coverage (-): 0

Region: chr8 87065636-87065649. Max. coverage (+): 0. Max coverage (-): 0

Region: chr8 87065650-87065663. Max. coverage (+): 0. Max coverage (-): 0

Region: chr8 87065664-87065677. Max. coverage (+): 0. Max coverage (-): 0

Region: chr8 87065678-87065692. Max. coverage (+): 0. Max coverage (-): 0

Region: chr8 87065693-87065706. Max. coverage (+): 6.36. Max coverage (-): 0

Region: chr8 87065707-87065720. Max. coverage (+): 6.36. Max coverage (-): 0

Region: chr8 87065721-87065735. Max. coverage (+): 0. Max coverage (-): 0

Region: chr8 87065736-87065749. Max. coverage (+): 0. Max coverage (-): 0

Region: chr8 87065750-87065763. Max. coverage (+): 0. Max coverage (-): 0

Region: chr8 87065764-87065777. Max. coverage (+): 0. Max coverage (-): 0

Region: chr8 87065778-87065792. Max. coverage (+): 0. Max coverage (-): 0

Region: chr8 87065793-87065806. Max. coverage (+): 0. Max coverage (-): 0

Region: chr8 87065807-87065820. Max. coverage (+): 0. Max coverage (-): 0

Region: chr8 87065821-87065835. Max. coverage (+): 0. Max coverage (-): 0

Region: chr8 87065836-87065849. Max. coverage (+): 0. Max coverage (-): 0

Region: chr8 87065850-87065863. Max. coverage (+): 0. Max coverage (-): 0

Region: chr8 87065864-87065877. Max. coverage (+): 0. Max coverage (-): 0

Region: chr8 87065878-87065892. Max. coverage (+): 0. Max coverage (-): 0

Region: chr8 87065893-87065906. Max. coverage (+): 3.73. Max coverage (-): 0

Region: chr8 87065907-87065920. Max. coverage (+): 10.48. Max coverage (-): 0

Region: chr8 87065921-87065934. Max. coverage (+): 0. Max coverage (-): 0

Region: chr8 87065935-87065949. Max. coverage (+): 14.42. Max coverage (-): 0

Region: chr8 87065950-87065963. Max. coverage (+): 0. Max coverage (-): 0

Region: chr8 87065964-87065977. Max. coverage (+): 3.1. Max coverage (-): 0

Region: chr8 87065978-87065992. Max. coverage (+): 3.1. Max coverage (-): 0

Region: chr8 87065993-87066006. Max. coverage (+): 0. Max coverage (-): 0

Region: chr8 87066007-87066020. Max. coverage (+): 0. Max coverage (-): 0

Region: chr8 87066021-87066034. Max. coverage (+): 0. Max coverage (-): 0

Region: chr8 87066035-87066049. Max. coverage (+): 0. Max coverage (-): 0

Region: chr8 87066050-87066063. Max. coverage (+): 4.46. Max coverage (-): 0

Region: chr8 87066064-87066077. Max. coverage (+): 0. Max coverage (-): 0

Region: chr8 87066078-87066092. Max. coverage (+): 4.53. Max coverage (-): 0

Region: chr8 87066093-87066106. Max. coverage (+): 0. Max coverage (-): 0

Region: chr8 87066107-87066120. Max. coverage (+): 0. Max coverage (-): 0

Region: chr8 87066121-87066134. Max. coverage (+): 0. Max coverage (-): 0

Region: chr8 87066135-87066149. Max. coverage (+): 0. Max coverage (-): 0

Region: chr8 87066150-87066163. Max. coverage (+): 0. Max coverage (-): 0

Region: chr8 87066164-87066177. Max. coverage (+): 0.43. Max coverage (-): 0

Region: chr8 87066178-87066192. Max. coverage (+): 0. Max coverage (-): 0

Region: chr8 87066193-87066206. Max. coverage (+): 0. Max coverage (-): 0

Region: chr8 87066207-87066220. Max. coverage (+): 0. Max coverage (-): 0

Region: chr8 87066221-87066234. Max. coverage (+): 2.65. Max coverage (-): 0

Region: chr8 87066235-87066249. Max. coverage (+): 1.55. Max coverage (-): 0

Region: chr8 87066250-87066263. Max. coverage (+): 7.5. Max coverage (-): 0

Region: chr8 87066264-87066277. Max. coverage (+): 7.5. Max coverage (-): 0

Region: chr8 87066278-87066292. Max. coverage (+): 3.7. Max coverage (-): 0

Region: chr8 87066293-87066306. Max. coverage (+): 0. Max coverage (-): 0

Region: chr8 87066307-87066320. Max. coverage (+): 0. Max coverage (-): 0

Region: chr8 87066321-87066334. Max. coverage (+): 0. Max coverage (-): 0

Region: chr8 87066335-87066349. Max. coverage (+): 0. Max coverage (-): 0

Region: chr8 87066350-87066363. Max. coverage (+): 0. Max coverage (-): 0

Region: chr8 87066364-87066377. Max. coverage (+): 13.22. Max coverage (-): 0

Region: chr8 87066378-87066391. Max. coverage (+): 0. Max coverage (-): 0

Region: chr8 87066392-87066406. Max. coverage (+): 14.84. Max coverage (-): 0

Region: chr8 87066407-87066420. Max. coverage (+): 4.87. Max coverage (-): 0

Region: chr8 87066421-87066434. Max. coverage (+): 4.87. Max coverage (-): 0

Region: chr8 87066435-87066449. Max. coverage (+): 0. Max coverage (-): 0

Region: chr8 87066450-87066463. Max. coverage (+): 0. Max coverage (-): 0

Region: chr8 87066464-87066477. Max. coverage (+): 0. Max coverage (-): 0

Region: chr8 87066478-87066491. Max. coverage (+): 0. Max coverage (-): 0

Region: chr8 87066492-87066506. Max. coverage (+): 0. Max coverage (-): 0

Region: chr8 87066507-87066520. Max. coverage (+): 0. Max coverage (-): 0

Region: chr8 87066521-87066534. Max. coverage (+): 0. Max coverage (-): 0

Region: chr8 87066535-87066549. Max. coverage (+): 0. Max coverage (-): 0

Region: chr8 87066550-87066563. Max. coverage (+): 10.26. Max coverage (-): 0

Region: chr8 87066564-87066577. Max. coverage (+): 10.26. Max coverage (-): 0

Region: chr8 87066578-87066591. Max. coverage (+): 0. Max coverage (-): 0

Region: chr8 87066592-87066606. Max. coverage (+): 0. Max coverage (-): 0

Region: chr8 87066607-87066620. Max. coverage (+): 0. Max coverage (-): 0

Region: chr8 87066621-87066634. Max. coverage (+): 0. Max coverage (-): 0

Region: chr8 87066635-87066649. Max. coverage (+): 3.23. Max coverage (-): 0

Region: chr8 87066650-87066663. Max. coverage (+): 0. Max coverage (-): 0

Region: chr8 87066664-87066677. Max. coverage (+): 0. Max coverage (-): 0

Region: chr8 87066678-87066691. Max. coverage (+): 0. Max coverage (-): 0

Region: chr8 87066692-87066706. Max. coverage (+): 0. Max coverage (-): 0

Region: chr8 87066707-87066720. Max. coverage (+): 0. Max coverage (-): 0

Region: chr8 87066721-87066734. Max. coverage (+): 0. Max coverage (-): 0

Region: chr8 87066735-87066749. Max. coverage (+): 0. Max coverage (-): 0

Region: chr8 87066750-87066763. Max. coverage (+): 0. Max coverage (-): 0

Region: chr8 87066764-87066777. Max. coverage (+): 0. Max coverage (-): 0

Region: chr8 87066778-87066791. Max. coverage (+): 0. Max coverage (-): 0

Region: chr8 87066792-87066806. Max. coverage (+): 0. Max coverage (-): 0

Region: chr8 87066807-87066820. Max. coverage (+): 1.8. Max coverage (-): 0

Region: chr8 87066821-87066834. Max. coverage (+): 1.8. Max coverage (-): 0

Region: chr8 87066835-87066849. Max. coverage (+): 0. Max coverage (-): 0

Region: chr8 87066850-87066863. Max. coverage (+): 0. Max coverage (-): 0

Region: chr8 87066864-87066877. Max. coverage (+): 0. Max coverage (-): 0

Region: chr8 87066878-87066891. Max. coverage (+): 0. Max coverage (-): 0

Region: chr8 87066892-87066906. Max. coverage (+): 0. Max coverage (-): 0

Region: chr8 87066907-87066920. Max. coverage (+): 0. Max coverage (-): 0

Region: chr8 87066921-87066934. Max. coverage (+): 0.71. Max coverage (-): 0

Region: chr8 87066935-87066948. Max. coverage (+): 0.71. Max coverage (-): 0

Region: chr8 87066949-87066963. Max. coverage (+): 0. Max coverage (-): 0

Region: chr8 87066964-87066977. Max. coverage (+): 0. Max coverage (-): 0

Region: chr8 87066978-87066991. Max. coverage (+): 0. Max coverage (-): 0

Region: chr8 87066992-87067006. Max. coverage (+): 0. Max coverage (-): 0

Region: chr8 87067007-87067020. Max. coverage (+): 0. Max coverage (-): 0

Region: chr8 87067021-87067034. Max. coverage (+): 0. Max coverage (-): 0

Region: chr8 87067035-87067048. Max. coverage (+): 0. Max coverage (-): 0

Region: chr8 87067049-87067063. Max. coverage (+): 0. Max coverage (-): 0

Region: chr8 87067064-87067077. Max. coverage (+): 0. Max coverage (-): 0

Region: chr8 87067078-87067091. Max. coverage (+): 0. Max coverage (-): 0

Region: chr8 87067092-87067106. Max. coverage (+): 0. Max coverage (-): 0

Region: chr8 87067107-87067120. Max. coverage (+): 0. Max coverage (-): 0

Region: chr8 87067121-87067134. Max. coverage (+): 0. Max coverage (-): 0

Region: chr8 87067135-87067148. Max. coverage (+): 0. Max coverage (-): 0

Region: chr8 87067149-87067163. Max. coverage (+): 0. Max coverage (-): 0

Region: chr8 87067164-87067177. Max. coverage (+): 0. Max coverage (-): 0

Region: chr8 87067178-87067191. Max. coverage (+): 0. Max coverage (-): 0

Region: chr8 87067192-87067206. Max. coverage (+): 0. Max coverage (-): 0

Region: chr8 87067207-87067220. Max. coverage (+): 0. Max coverage (-): 0

Region: chr8 87067221-87067234. Max. coverage (+): 0. Max coverage (-): 0

Region: chr8 87067235-87067248. Max. coverage (+): 0. Max coverage (-): 0

Region: chr8 87067249-87067263. Max. coverage (+): 0. Max coverage (-): 0

Region: chr8 87067264-87067277. Max. coverage (+): 3.31. Max coverage (-): 0

Region: chr8 87067278-87067291. Max. coverage (+): 3.31. Max coverage (-): 0

Region: chr8 87067292-87067306. Max. coverage (+): 0. Max coverage (-): 0

Region: chr8 87067307-87067320. Max. coverage (+): 0. Max coverage (-): 0

Region: chr8 87067321-87067334. Max. coverage (+): 0. Max coverage (-): 0

Region: chr8 87067335-87067348. Max. coverage (+): 0. Max coverage (-): 0

Region: chr8 87067349-87067363. Max. coverage (+): 0. Max coverage (-): 0

Region: chr8 87067364-87067377. Max. coverage (+): 0. Max coverage (-): 0

Region: chr8 87067378-87067391. Max. coverage (+): 0. Max coverage (-): 0

Region: chr8 87067392-87067406. Max. coverage (+): 0. Max coverage (-): 0

Region: chr8 87067407-87067420. Max. coverage (+): 6.64. Max coverage (-): 0

Region: chr8 87067421-87067434. Max. coverage (+): 6.64. Max coverage (-): 0

Region: chr8 87067435-87067448. Max. coverage (+): 0. Max coverage (-): 0

Region: chr8 87067449-87067463. Max. coverage (+): 4.84. Max coverage (-): 0

Region: chr8 87067464-87067477. Max. coverage (+): 4.84. Max coverage (-): 0

Region: chr8 87067478-87067491. Max. coverage (+): 2.59. Max coverage (-): 0

Region: chr8 87067492-87067505. Max. coverage (+): 0. Max coverage (-): 0

Region: chr8 87067506-87067520. Max. coverage (+): 0. Max coverage (-): 0

Region: chr8 87067521-87067534. Max. coverage (+): 0. Max coverage (-): 0

Region: chr8 87067535-87067548. Max. coverage (+): 0. Max coverage (-): 0

Region: chr8 87067549-87067563. Max. coverage (+): 5.51. Max coverage (-): 0

Region: chr8 87067564-87067577. Max. coverage (+): 0. Max coverage (-): 0

Region: chr8 87067578-87067591. Max. coverage (+): 0. Max coverage (-): 0

Region: chr8 87067592-87067605. Max. coverage (+): 0. Max coverage (-): 0

Region: chr8 87067606-87067620. Max. coverage (+): 0. Max coverage (-): 0

Region: chr8 87067621-87067634. Max. coverage (+): 0. Max coverage (-): 0

Region: chr8 87067635-87067648. Max. coverage (+): 0. Max coverage (-): 0

Region: chr8 87067649-87067663. Max. coverage (+): 42.42. Max coverage (-): 0

Region: chr8 87067664-87067677. Max. coverage (+): 42.42. Max coverage (-): 0

Region: chr8 87067678-87067691. Max. coverage (+): 0. Max coverage (-): 0

Region: chr8 87067692-87067705. Max. coverage (+): 5.98. Max coverage (-): 0

Region: chr8 87067706-87067720. Max. coverage (+): 0. Max coverage (-): 0

Region: chr8 87067721-87067734. Max. coverage (+): 0. Max coverage (-): 0

Region: chr8 87067735-87067748. Max. coverage (+): 13.2. Max coverage (-): 0

Region: chr8 87067749-87067763. Max. coverage (+): 13.2. Max coverage (-): 0

Region: chr8 87067764-87067777. Max. coverage (+): 4.36. Max coverage (-): 0

Region: chr8 87067778-87067791. Max. coverage (+): 0. Max coverage (-): 0

Region: chr8 87067792-87067805. Max. coverage (+): 0. Max coverage (-): 0

Region: chr8 87067806-87067820. Max. coverage (+): 0. Max coverage (-): 0

Region: chr8 87067821-87067834. Max. coverage (+): 13.41. Max coverage (-): 0

Region: chr8 87067835-87067848. Max. coverage (+): 6.35. Max coverage (-): 0

Region: chr8 87067849-87067863. Max. coverage (+): 0. Max coverage (-): 0

Region: chr8 87067864-87067877. Max. coverage (+): 0. Max coverage (-): 0

Region: chr8 87067878-87067891. Max. coverage (+): 0. Max coverage (-): 0

Region: chr8 87067892-87067905. Max. coverage (+): 0. Max coverage (-): 0

Region: chr8 87067906-87067920. Max. coverage (+): 0. Max coverage (-): 0

Region: chr8 87067921-87067934. Max. coverage (+): 0. Max coverage (-): 0

Region: chr8 87067935-87067948. Max. coverage (+): 0. Max coverage (-): 0

Region: chr8 87067949-87067963. Max. coverage (+): 0. Max coverage (-): 0

Region: chr8 87067964-87067977. Max. coverage (+): 0. Max coverage (-): 0

Region: chr8 87067978-87067991. Max. coverage (+): 0. Max coverage (-): 0

Region: chr8 87067992-87068005. Max. coverage (+): 0. Max coverage (-): 0

Region: chr8 87068006-87068020. Max. coverage (+): 0. Max coverage (-): 0

Region: chr8 87068021-87068034. Max. coverage (+): 0. Max coverage (-): 0

Region: chr8 87068035-87068048. Max. coverage (+): 0. Max coverage (-): 0

Region: chr8 87068049-87068062. Max. coverage (+): 0. Max coverage (-): 0

Region: chr8 87068063-87068077. Max. coverage (+): 0. Max coverage (-): 0

Region: chr8 87068078-87068091. Max. coverage (+): 0. Max coverage (-): 0

Region: chr8 87068092-87068105. Max. coverage (+): 0. Max coverage (-): 0

Region: chr8 87068106-87068120. Max. coverage (+): 0. Max coverage (-): 0

Region: chr8 87068121-87068134. Max. coverage (+): 0. Max coverage (-): 0

Region: chr8 87068135-87068148. Max. coverage (+): 0. Max coverage (-): 0

Region: chr8 87068149-87068162. Max. coverage (+): 0. Max coverage (-): 0

Region: chr8 87068163-87068177. Max. coverage (+): 0. Max coverage (-): 0

Region: chr8 87068178-87068191. Max. coverage (+): 0. Max coverage (-): 0

Region: chr8 87068192-87068205. Max. coverage (+): 0.53. Max coverage (-): 0

Region: chr8 87068206-87068220. Max. coverage (+): 0. Max coverage (-): 0

Region: chr8 87068221-87068234. Max. coverage (+): 0. Max coverage (-): 0

Region: chr8 87068235-87068248. Max. coverage (+): 0. Max coverage (-): 0

Region: chr8 87068249-87068262. Max. coverage (+): 4.18. Max coverage (-): 0

Region: chr8 87068263-87068277. Max. coverage (+): 5.56. Max coverage (-): 0

Region: chr8 87068278-87068291. Max. coverage (+): 5.56. Max coverage (-): 0

Region: chr8 87068292-87068305. Max. coverage (+): 0. Max coverage (-): 6.21

Region: chr8 87068306-87068320. Max. coverage (+): 0. Max coverage (-): 6.21

Region: chr8 87068321-87068334. Max. coverage (+): 0. Max coverage (-): 0

Region: chr8 87068335-87068348. Max. coverage (+): 0. Max coverage (-): 0

Region: chr8 87068349-87068362. Max. coverage (+): 0. Max coverage (-): 0

Region: chr8 87068363-87068377. Max. coverage (+): 0. Max coverage (-): 0

Region: chr8 87068378-87068391. Max. coverage (+): 13.21. Max coverage (-): 0

Region: chr8 87068392-87068405. Max. coverage (+): 0. Max coverage (-): 0

Region: chr8 87068406-87068420. Max. coverage (+): 0. Max coverage (-): 0

Region: chr8 87068421-87068434. Max. coverage (+): 0. Max coverage (-): 0

Region: chr8 87068435-87068448. Max. coverage (+): 0. Max coverage (-): 0

Region: chr8 87068449-87068462. Max. coverage (+): 0. Max coverage (-): 0

Region: chr8 87068463-87068477. Max. coverage (+): 0. Max coverage (-): 0

Region: chr8 87068478-87068491. Max. coverage (+): 0. Max coverage (-): 0

Region: chr8 87068492-87068505. Max. coverage (+): 0. Max coverage (-): 0

Region: chr8 87068506-87068520. Max. coverage (+): 8.3. Max coverage (-): 0

Region: chr8 87068521-87068534. Max. coverage (+): 8.3. Max coverage (-): 0

Region: chr8 87068535-87068548. Max. coverage (+): 0. Max coverage (-): 0

Region: chr8 87068549-87068562. Max. coverage (+): 0. Max coverage (-): 0

Region: chr8 87068563-87068577. Max. coverage (+): 0. Max coverage (-): 0

Region: chr8 87068578-87068591. Max. coverage (+): 0. Max coverage (-): 0

Region: chr8 87068592-87068605. Max. coverage (+): 0. Max coverage (-): 0

Region: chr8 87068606-87068619. Max. coverage (+): 0. Max coverage (-): 0

Region: chr8 87068620-87068634. Max. coverage (+): 0. Max coverage (-): 0

Region: chr8 87068635-87068648. Max. coverage (+): 0. Max coverage (-): 0

Region: chr8 87068649-87068662. Max. coverage (+): 0. Max coverage (-): 0

Region: chr8 87068663-87068677. Max. coverage (+): 5.67. Max coverage (-): 2.46

Region: chr8 87068678-87068691. Max. coverage (+): 5.67. Max coverage (-): 0

Region: chr8 87068692-87068705. Max. coverage (+): 0. Max coverage (-): 0

Region: chr8 87068706-87068719. Max. coverage (+): 0. Max coverage (-): 0

Region: chr8 87068720-87068734. Max. coverage (+): 0. Max coverage (-): 0

Region: chr8 87068735-87068748. Max. coverage (+): 0. Max coverage (-): 0

Region: chr8 87068749-87068762. Max. coverage (+): 0. Max coverage (-): 0

Region: chr8 87068763-87068777. Max. coverage (+): 0. Max coverage (-): 0

Region: chr8 87068778-87068791. Max. coverage (+): 0. Max coverage (-): 0

Region: chr8 87068792-87068805. Max. coverage (+): 0. Max coverage (-): 0

Region: chr8 87068806-87068819. Max. coverage (+): 0. Max coverage (-): 0

Region: chr8 87068820-87068834. Max. coverage (+): 0. Max coverage (-): 0

Region: chr8 87068835-87068848. Max. coverage (+): 0. Max coverage (-): 0

Region: chr8 87068849-87068862. Max. coverage (+): 0. Max coverage (-): 0

Region: chr8 87068863-87068877. Max. coverage (+): 0. Max coverage (-): 0

Region: chr8 87068878-87068891. Max. coverage (+): 0. Max coverage (-): 0

Region: chr8 87068892-87068905. Max. coverage (+): 0. Max coverage (-): 0

Region: chr8 87068906-87068919. Max. coverage (+): 0. Max coverage (-): 0

Region: chr8 87068920-87068934. Max. coverage (+): 0. Max coverage (-): 0

Region: chr8 87068935-87068948. Max. coverage (+): 0. Max coverage (-): 0

Region: chr8 87068949-87068962. Max. coverage (+): 0. Max coverage (-): 0

Region: chr8 87068963-87068977. Max. coverage (+): 0. Max coverage (-): 0

Region: chr8 87068978-87068991. Max. coverage (+): 0. Max coverage (-): 0

Region: chr8 87068992-87069005. Max. coverage (+): 0. Max coverage (-): 0

Region: chr8 87069006-87069019. Max. coverage (+): 0. Max coverage (-): 0

Region: chr8 87069020-87069034. Max. coverage (+): 0. Max coverage (-): 0

Region: chr8 87069035-87069048. Max. coverage (+): 0. Max coverage (-): 0

Region: chr8 87069049-87069062. Max. coverage (+): 4.03. Max coverage (-): 0

Region: chr8 87069063-87069077. Max. coverage (+): 0. Max coverage (-): 3.43

Region: chr8 87069078-87069091. Max. coverage (+): 0. Max coverage (-): 3.43

Region: chr8 87069092-87069105. Max. coverage (+): 0. Max coverage (-): 0

Region: chr8 87069106-87069119. Max. coverage (+): 0. Max coverage (-): 0

Region: chr8 87069120-87069134. Max. coverage (+): 0. Max coverage (-): 0

Region: chr8 87069135-87069148. Max. coverage (+): 0. Max coverage (-): 0

Region: chr8 87069149-87069162. Max. coverage (+): 0. Max coverage (-): 0

Region: chr8 87069163-87069176. Max. coverage (+): 0. Max coverage (-): 0

Region: chr8 87069177-87069191. Max. coverage (+): 2.17. Max coverage (-): 0

Region: chr8 87069192-87069205. Max. coverage (+): 2.17. Max coverage (-): 0

Region: chr8 87069206-87069219. Max. coverage (+): 0. Max coverage (-): 0

Region: chr8 87069220-87069234. Max. coverage (+): 0. Max coverage (-): 0

Region: chr8 87069235-87069248. Max. coverage (+): 0. Max coverage (-): 0

Region: chr8 87069249-87069262. Max. coverage (+): 0. Max coverage (-): 0

Region: chr8 87069263-87069276. Max. coverage (+): 0. Max coverage (-): 0

Region: chr8 87069277-87069291. Max. coverage (+): 0. Max coverage (-): 0

Region: chr8 87069292-87069305. Max. coverage (+): 0. Max coverage (-): 0

Region: chr8 87069306-87069319. Max. coverage (+): 0. Max coverage (-): 0

Region: chr8 87069320-87069334. Max. coverage (+): 0. Max coverage (-): 0

Region: chr8 87069335-87069348. Max. coverage (+): 0. Max coverage (-): 0

Region: chr8 87069349-87069362. Max. coverage (+): 40.98. Max coverage (-): 0

Region: chr8 87069363-87069376. Max. coverage (+): 40.98. Max coverage (-): 0

Region: chr8 87069377-87069391. Max. coverage (+): 0. Max coverage (-): 0

Region: chr8 87069392-87069405. Max. coverage (+): 0. Max coverage (-): 0

Region: chr8 87069406-87069419. Max. coverage (+): 0. Max coverage (-): 0

Region: chr8 87069420-87069434. Max. coverage (+): 0. Max coverage (-): 0

Region: chr8 87069435-87069448. Max. coverage (+): 0. Max coverage (-): 0

Region: chr8 87069449-87069462. Max. coverage (+): 0. Max coverage (-): 0

Region: chr8 87069463-87069476. Max. coverage (+): 0. Max coverage (-): 0

Region: chr8 87069477-87069491. Max. coverage (+): 0. Max coverage (-): 0

Region: chr8 87069492-87069505. Max. coverage (+): 0. Max coverage (-): 0

Region: chr8 87069506-87069519. Max. coverage (+): 0. Max coverage (-): 0

Region: chr8 87069520-87069534. Max. coverage (+): 0. Max coverage (-): 0

Region: chr8 87069535-87069548. Max. coverage (+): 0. Max coverage (-): 0

Region: chr8 87069549-87069562. Max. coverage (+): 0. Max coverage (-): 0

Region: chr8 87069563-87069576. Max. coverage (+): 0. Max coverage (-): 0

Region: chr8 87069577-87069591. Max. coverage (+): 0. Max coverage (-): 0

Region: chr8 87069592-87069605. Max. coverage (+): 0. Max coverage (-): 0

Region: chr8 87069606-87069619. Max. coverage (+): 0. Max coverage (-): 0

Region: chr8 87069620-87069634. Max. coverage (+): 0. Max coverage (-): 0

Region: chr8 87069635-87069648. Max. coverage (+): 7.89. Max coverage (-): 0

Region: chr8 87069649-87069662. Max. coverage (+): 13.44. Max coverage (-): 0

Region: chr8 87069663-87069676. Max. coverage (+): 0. Max coverage (-): 0

Region: chr8 87069677-. Max. coverage (+): 0. Max coverage (-): 0

RepeatMasker Color Code

**+**

100-98% Identity

<98-95% Identity

<95-90% Identity

<90-85% Identity

<85-80% Identity

<80-75% Identity

<75-70% Identity

<70% Identity

**-**

Gene Set Color Code

**+**

Gene

Pseudogene

**-**

Topology/Coverage Color Code

Coverage Plus Strand

Coverage Minus Strand

Mainstrand: Plus

Mainstrand: Minus

Complementary Strand

Flanking Region  
(if option -flank >0)

Gene Set Annotation  
  
RepeatMasker Annotation  
  
Transcription Factor Binding Sites  

**RFX4\_2** (Sequence: CCTGGATAC (+): 87068861)  
**SOX9** (Sequence: CCATTGTT (+): 87065433)  
**SPZ1** (Sequence: GGGGTAAGAG (+): 87068156)
