# Supplementary material for: Small RNA sequencing of cryopreserved semen from single bull revealed altered miRNAs and piRNAs expression between High- and Low-motile sperm populations
Source: BMC Genomics. 2017 Jan 4;18:14. doi: 10.1186/s12864-016-3394-7 (PMC5209821; doi:10.1186/s12864-016-3394-7)
Supplement: Additional file 4: — Details for each piRNA clusters found in Low Motile (LM) sperm fraction. Genes, repeats, transposable elements and transcription factors binding sites falling within the cluster regions were reported. (ZIP 1034 kb) [file 12864_2016_3394_MOESM4_ESM.zip › 5.html]

piRNA cluster 5


Predicted piRNA cluster no. 5     previous   next
  

Show proTRAC run info
Hide proTRAC run info

================================= proTRAC ====================================  
VERSION: 2.1                                    LAST MODIFIED: 06. October 2015  
  
Please cite:  
Rosenkranz D, Zischler H. proTRAC - a software for probabilistic piRNA cluster  
detection, visualization and analysis. 2012. BMC Bioinformatics 13:5.  
  
and (for proTRAC 2.0 and later):  
Rosenkranz D, Rudloff S, Bastuck K, Ketting RF, Zischler H. Tupaia small RNAs  
provide insights into function and evolution of RNAi-based transposon defense  
in mammals. 2015. RNA 21(5):911-922.  
  
Contact:  
David Rosenkranz  
Institute of Anthropology, small RNA group  
Johannes Gutenberg University Mainz  
email: rosenkranz@uni-mainz.de  
  
You can find the latest proTRAC version at:  
http://sourceforge.net/projects/protrac/files  
http://www.smallRNAgroup-mainz.de/software  
==============================================================================  
  
PARAMETERS:  
Map file: .............../storage/core/barbara/genhome/smallRNA/fertility/Sample\_not\_motile/pirna/Sample\_not\_motile\_26-33\_collapsed.fa.no-dust.map.weighted-10000-1000-b-0  
Genome file: ............/storage/core/barbara/genhome/smallRNA/fertility/Sample\_all/pirna/bt\_311\_chrY.fa  
RepeatMasker annotation: /storage/genomes/bt\_umd31/GCF\_000003055.6\_Bos\_taurus\_UMD\_3.1.1\_repeatMasker\_chr.out  
GeneSet:................./storage/core/barbara/genhome/smallRNA/fertility/Sample\_all/pirna/full.gtf  
  
Significant (p<=0.01) hit density will be calculated based  
on observed hit distribution.  
  
Sliding window size: ........................................ 5000 bp  
Sliding window increament: .................................. 1000 bp  
Normalize each hit by number of genomic hits: ............... 1 [0=no/1=yes]  
Normalize each hit by number of sequence reads: ............. 1 [0=no/1=yes]  
Normalize values (-> per million mapped reads): ............. 1 [0=no/1=yes]  
Min. fraction of hits with 1T(U) or 10A: .................... 0.75  
Alternatively: Min. fraction of hits with 1T(U) and 10A: .... 0.5  
Min. fraction of hits with typical piRNA length: ............ 0.75  
Typical piRNA length: ....................................... 26-33 nt  
Min. size of a piRNA cluster: ............................... 5000 bp.  
Min. number of hits (absolute): ............................. 0  
Min. number of hits (normalized): ........................... 0  
Min. fraction of hits on the mainstrand: .................... 0.75  
Top fraction of mapped sequences (in terms of read counts): . 1%  
Top fraction accounts for max. n% of sequence reads: ........ 90%  
Min. fraction of hits on each arm of a bidirectional cluster: 0.1  
Output image file for each cluster: ......................... 0 [0=no/1=yes]  
Output html file for each cluster: .......................... 1 [0=no/1=yes]  
Output a summary table: ..................................... 1 [0=no/1=yes]  
Output a FASTA file for each cluster (piRNA sequences): ..... 1 [0=no/1=yes]  
Output a FASTA file comprising cluster sequences: ........... 1 [0=no/1=yes]  
Search DNA motifs in clusters: .............................. 1 [0=no/1=yes]  
Output flanking sequences: +/- .............................. 0 bp  
Output ~.pTi file: .......................................... 1 [0=no/1=yes]  
==============================================================================  
  
  
Genome size (without gaps): ............ 2678902517 bp  
Gaps (N/X/-): .......................... 53837044 bp  
Mapped reads: .......................... 738059667487  
Non-identical sequences: ............... 277001  
Genomic hits: .......................... 533816  
Significant densitiy of mapped reads: .. 15118061 reads/kb

Show proTRAC cluster info
Hide proTRAC cluster info

|  |  |
| --- | --- |
| Location | chr10 |
| Coordinates | 100904597-100939947 |
| Size [bp] | 35351 |
| Sequence hit loci | 3606 |
| Mapped reads (normalized) | 9525802622 |
| Mapped reads (normalized) per kb | 269463455.7 |
| Normalized reads with 1T (1U) | 82.8% |
| Normalized reads with 10A | 29.5% |
| Normalized reads with length 26-33 nt | 100% |
| Normalized reads on the main strand(s) | 98.9% |
| Predicted directionality | bi:minus-plus (split between 100933440 and 100933652) |

100%

0%

1T (1U)  
reads

10A reads

26-33 nt  
reads

reads on mainstrand

**Either the amount of reads with 1T (1U) OR 10A has to exceed 75% (set with option: -1Tor10A)  
Alternatively the amount of reads with 1T (1U) AND 10A has to exceed 50% (set with option: -1Tand10A)  
Minimum amount of reads with preferred size is 75% (set with option: -pisize)  
Minimum amount of reads on the main strand(s) is 75% (set with option: -clstrand)**

Show read coverage
Hide read coverage

WHAT DO I SEE HERE?  
This chart shows the location of mapped sequence reads within a predicted piRNA cluster. The color refers to the number of genomic hits produced by the sequence read in question. A dark red bar indicates that this sequence read produces many other hits elsewhere in the genome. Many adjacent red or yellow bars can indicate the presence of a multi-copy element such as transposons or rRNA genes. A dark green bar indicates that this sequence read maps uniquely to this locus.

1 hit

2-5 hits

6-10 hits

11-20 hits

21-50 hits

51-100 hits

> 100 hits

chr10

100904597

100939947

Gene Set

RepeatMasker

Mapped  
Reads

190.47

plus strand

minus strand

190.47

Region: chr10 59224852-100904632. Max. coverage (+): 0. Max coverage (-): 4.65

Region: chr10 100904633-100904703. Max. coverage (+): 0. Max coverage (-): 0

Region: chr10 100904704-100904773. Max. coverage (+): 0. Max coverage (-): 0

Region: chr10 100904774-100904844. Max. coverage (+): 0. Max coverage (-): 0

Region: chr10 100904845-100904915. Max. coverage (+): 0. Max coverage (-): 0

Region: chr10 100904916-100904985. Max. coverage (+): 0. Max coverage (-): 0

Region: chr10 100904986-100905056. Max. coverage (+): 0. Max coverage (-): 0

Region: chr10 100905057-100905127. Max. coverage (+): 0. Max coverage (-): 0

Region: chr10 100905128-100905197. Max. coverage (+): 0. Max coverage (-): 0

Region: chr10 100905198-100905268. Max. coverage (+): 0. Max coverage (-): 0

Region: chr10 100905269-100905339. Max. coverage (+): 0. Max coverage (-): 0

Region: chr10 100905340-100905410. Max. coverage (+): 0. Max coverage (-): 0

Region: chr10 100905411-100905480. Max. coverage (+): 0. Max coverage (-): 0

Region: chr10 100905481-100905551. Max. coverage (+): 0. Max coverage (-): 0

Region: chr10 100905552-100905622. Max. coverage (+): 0. Max coverage (-): 0

Region: chr10 100905623-100905692. Max. coverage (+): 0. Max coverage (-): 0

Region: chr10 100905693-100905763. Max. coverage (+): 0. Max coverage (-): 0

Region: chr10 100905764-100905834. Max. coverage (+): 0. Max coverage (-): 10.78

Region: chr10 100905835-100905904. Max. coverage (+): 0. Max coverage (-): 2.3

Region: chr10 100905905-100905975. Max. coverage (+): 0. Max coverage (-): 0

Region: chr10 100905976-100906046. Max. coverage (+): 0. Max coverage (-): 0

Region: chr10 100906047-100906117. Max. coverage (+): 0. Max coverage (-): 0

Region: chr10 100906118-100906187. Max. coverage (+): 0. Max coverage (-): 0

Region: chr10 100906188-100906258. Max. coverage (+): 0. Max coverage (-): 3.76

Region: chr10 100906259-100906329. Max. coverage (+): 0. Max coverage (-): 4.75

Region: chr10 100906330-100906399. Max. coverage (+): 0. Max coverage (-): 0

Region: chr10 100906400-100906470. Max. coverage (+): 0. Max coverage (-): 0

Region: chr10 100906471-100906541. Max. coverage (+): 0. Max coverage (-): 0

Region: chr10 100906542-100906612. Max. coverage (+): 0. Max coverage (-): 1.3

Region: chr10 100906613-100906682. Max. coverage (+): 0. Max coverage (-): 0

Region: chr10 100906683-100906753. Max. coverage (+): 0. Max coverage (-): 0

Region: chr10 100906754-100906824. Max. coverage (+): 0. Max coverage (-): 0

Region: chr10 100906825-100906894. Max. coverage (+): 0. Max coverage (-): 0

Region: chr10 100906895-100906965. Max. coverage (+): 0. Max coverage (-): 16.45

Region: chr10 100906966-100907036. Max. coverage (+): 0. Max coverage (-): 3.88

Region: chr10 100907037-100907106. Max. coverage (+): 0. Max coverage (-): 6.95

Region: chr10 100907107-100907177. Max. coverage (+): 0. Max coverage (-): 14.79

Region: chr10 100907178-100907248. Max. coverage (+): 0. Max coverage (-): 7.37

Region: chr10 100907249-100907319. Max. coverage (+): 0. Max coverage (-): 0

Region: chr10 100907320-100907389. Max. coverage (+): 0. Max coverage (-): 0

Region: chr10 100907390-100907460. Max. coverage (+): 0. Max coverage (-): 0

Region: chr10 100907461-100907531. Max. coverage (+): 0. Max coverage (-): 0

Region: chr10 100907532-100907601. Max. coverage (+): 0. Max coverage (-): 0

Region: chr10 100907602-100907672. Max. coverage (+): 0. Max coverage (-): 0

Region: chr10 100907673-100907743. Max. coverage (+): 0. Max coverage (-): 0

Region: chr10 100907744-100907813. Max. coverage (+): 0. Max coverage (-): 0

Region: chr10 100907814-100907884. Max. coverage (+): 0. Max coverage (-): 0

Region: chr10 100907885-100907955. Max. coverage (+): 0. Max coverage (-): 0

Region: chr10 100907956-100908026. Max. coverage (+): 0. Max coverage (-): 0

Region: chr10 100908027-100908096. Max. coverage (+): 0. Max coverage (-): 0

Region: chr10 100908097-100908167. Max. coverage (+): 0. Max coverage (-): 5.7

Region: chr10 100908168-100908238. Max. coverage (+): 0. Max coverage (-): 0

Region: chr10 100908239-100908308. Max. coverage (+): 0. Max coverage (-): 0

Region: chr10 100908309-100908379. Max. coverage (+): 0. Max coverage (-): 2.31

Region: chr10 100908380-100908450. Max. coverage (+): 0. Max coverage (-): 3.14

Region: chr10 100908451-100908520. Max. coverage (+): 0. Max coverage (-): 8.95

Region: chr10 100908521-100908591. Max. coverage (+): 0. Max coverage (-): 10.86

Region: chr10 100908592-100908662. Max. coverage (+): 0. Max coverage (-): 0

Region: chr10 100908663-100908733. Max. coverage (+): 0. Max coverage (-): 10.81

Region: chr10 100908734-100908803. Max. coverage (+): 0. Max coverage (-): 6.56

Region: chr10 100908804-100908874. Max. coverage (+): 0. Max coverage (-): 4.42

Region: chr10 100908875-100908945. Max. coverage (+): 0. Max coverage (-): 14.16

Region: chr10 100908946-100909015. Max. coverage (+): 0. Max coverage (-): 14.16

Region: chr10 100909016-100909086. Max. coverage (+): 0. Max coverage (-): 8.85

Region: chr10 100909087-100909157. Max. coverage (+): 0. Max coverage (-): 0

Region: chr10 100909158-100909227. Max. coverage (+): 0. Max coverage (-): 0

Region: chr10 100909228-100909298. Max. coverage (+): 0. Max coverage (-): 0

Region: chr10 100909299-100909369. Max. coverage (+): 0. Max coverage (-): 0

Region: chr10 100909370-100909440. Max. coverage (+): 0. Max coverage (-): 0

Region: chr10 100909441-100909510. Max. coverage (+): 0. Max coverage (-): 0

Region: chr10 100909511-100909581. Max. coverage (+): 0. Max coverage (-): 0

Region: chr10 100909582-100909652. Max. coverage (+): 0. Max coverage (-): 0

Region: chr10 100909653-100909722. Max. coverage (+): 0. Max coverage (-): 3.94

Region: chr10 100909723-100909793. Max. coverage (+): 0. Max coverage (-): 0

Region: chr10 100909794-100909864. Max. coverage (+): 0. Max coverage (-): 12.39

Region: chr10 100909865-100909935. Max. coverage (+): 0. Max coverage (-): 8.71

Region: chr10 100909936-100910005. Max. coverage (+): 0. Max coverage (-): 13.42

Region: chr10 100910006-100910076. Max. coverage (+): 0. Max coverage (-): 31.61

Region: chr10 100910077-100910147. Max. coverage (+): 0. Max coverage (-): 13.68

Region: chr10 100910148-100910217. Max. coverage (+): 0. Max coverage (-): 2.97

Region: chr10 100910218-100910288. Max. coverage (+): 0. Max coverage (-): 0

Region: chr10 100910289-100910359. Max. coverage (+): 0. Max coverage (-): 0

Region: chr10 100910360-100910429. Max. coverage (+): 0. Max coverage (-): 0

Region: chr10 100910430-100910500. Max. coverage (+): 0. Max coverage (-): 2.65

Region: chr10 100910501-100910571. Max. coverage (+): 0. Max coverage (-): 35.4

Region: chr10 100910572-100910642. Max. coverage (+): 0. Max coverage (-): 15.76

Region: chr10 100910643-100910712. Max. coverage (+): 0. Max coverage (-): 15.76

Region: chr10 100910713-100910783. Max. coverage (+): 0. Max coverage (-): 19.78

Region: chr10 100910784-100910854. Max. coverage (+): 0. Max coverage (-): 61.48

Region: chr10 100910855-100910924. Max. coverage (+): 0. Max coverage (-): 26.21

Region: chr10 100910925-100910995. Max. coverage (+): 0. Max coverage (-): 28.8

Region: chr10 100910996-100911066. Max. coverage (+): 0. Max coverage (-): 58.67

Region: chr10 100911067-100911136. Max. coverage (+): 0. Max coverage (-): 28.92

Region: chr10 100911137-100911207. Max. coverage (+): 0. Max coverage (-): 44.25

Region: chr10 100911208-100911278. Max. coverage (+): 0. Max coverage (-): 44.25

Region: chr10 100911279-100911349. Max. coverage (+): 0. Max coverage (-): 42.87

Region: chr10 100911350-100911419. Max. coverage (+): 0. Max coverage (-): 17.06

Region: chr10 100911420-100911490. Max. coverage (+): 0. Max coverage (-): 20.52

Region: chr10 100911491-100911561. Max. coverage (+): 0. Max coverage (-): 11.43

Region: chr10 100911562-100911631. Max. coverage (+): 0. Max coverage (-): 19.22

Region: chr10 100911632-100911702. Max. coverage (+): 0. Max coverage (-): 30.98

Region: chr10 100911703-100911773. Max. coverage (+): 0. Max coverage (-): 40.89

Region: chr10 100911774-100911843. Max. coverage (+): 0. Max coverage (-): 38.74

Region: chr10 100911844-100911914. Max. coverage (+): 0. Max coverage (-): 56.73

Region: chr10 100911915-100911985. Max. coverage (+): 0. Max coverage (-): 4.75

Region: chr10 100911986-100912056. Max. coverage (+): 0. Max coverage (-): 0

Region: chr10 100912057-100912126. Max. coverage (+): 0. Max coverage (-): 0

Region: chr10 100912127-100912197. Max. coverage (+): 0. Max coverage (-): 0

Region: chr10 100912198-100912268. Max. coverage (+): 0. Max coverage (-): 49.31

Region: chr10 100912269-100912338. Max. coverage (+): 0. Max coverage (-): 11.7

Region: chr10 100912339-100912409. Max. coverage (+): 0. Max coverage (-): 5.24

Region: chr10 100912410-100912480. Max. coverage (+): 0. Max coverage (-): 69.19

Region: chr10 100912481-100912550. Max. coverage (+): 0. Max coverage (-): 0

Region: chr10 100912551-100912621. Max. coverage (+): 0. Max coverage (-): 0

Region: chr10 100912622-100912692. Max. coverage (+): 0. Max coverage (-): 9.11

Region: chr10 100912693-100912763. Max. coverage (+): 0. Max coverage (-): 0

Region: chr10 100912764-100912833. Max. coverage (+): 0. Max coverage (-): 33.54

Region: chr10 100912834-100912904. Max. coverage (+): 0. Max coverage (-): 51.93

Region: chr10 100912905-100912975. Max. coverage (+): 0. Max coverage (-): 14.65

Region: chr10 100912976-100913045. Max. coverage (+): 0. Max coverage (-): 3.29

Region: chr10 100913046-100913116. Max. coverage (+): 0. Max coverage (-): 10.58

Region: chr10 100913117-100913187. Max. coverage (+): 11.3. Max coverage (-): 16.76

Region: chr10 100913188-100913257. Max. coverage (+): 0. Max coverage (-): 57.04

Region: chr10 100913258-100913328. Max. coverage (+): 0. Max coverage (-): 98.74

Region: chr10 100913329-100913399. Max. coverage (+): 6.79. Max coverage (-): 11.24

Region: chr10 100913400-100913470. Max. coverage (+): 0. Max coverage (-): 11.37

Region: chr10 100913471-100913540. Max. coverage (+): 0. Max coverage (-): 9.61

Region: chr10 100913541-100913611. Max. coverage (+): 0. Max coverage (-): 14.02

Region: chr10 100913612-100913682. Max. coverage (+): 0. Max coverage (-): 5.68

Region: chr10 100913683-100913752. Max. coverage (+): 0. Max coverage (-): 27.21

Region: chr10 100913753-100913823. Max. coverage (+): 0. Max coverage (-): 29.78

Region: chr10 100913824-100913894. Max. coverage (+): 0. Max coverage (-): 21.48

Region: chr10 100913895-100913965. Max. coverage (+): 0. Max coverage (-): 33.93

Region: chr10 100913966-100914035. Max. coverage (+): 1.86. Max coverage (-): 12.72

Region: chr10 100914036-100914106. Max. coverage (+): 0. Max coverage (-): 12.88

Region: chr10 100914107-100914177. Max. coverage (+): 0. Max coverage (-): 25.22

Region: chr10 100914178-100914247. Max. coverage (+): 2.42. Max coverage (-): 19.36

Region: chr10 100914248-100914318. Max. coverage (+): 0. Max coverage (-): 15.95

Region: chr10 100914319-100914389. Max. coverage (+): 7.18. Max coverage (-): 13.97

Region: chr10 100914390-100914459. Max. coverage (+): 0. Max coverage (-): 49.11

Region: chr10 100914460-100914530. Max. coverage (+): 0. Max coverage (-): 15.27

Region: chr10 100914531-100914601. Max. coverage (+): 0. Max coverage (-): 12.09

Region: chr10 100914602-100914672. Max. coverage (+): 0. Max coverage (-): 23.31

Region: chr10 100914673-100914742. Max. coverage (+): 0. Max coverage (-): 12.18

Region: chr10 100914743-100914813. Max. coverage (+): 0. Max coverage (-): 55.59

Region: chr10 100914814-100914884. Max. coverage (+): 0. Max coverage (-): 5.51

Region: chr10 100914885-100914954. Max. coverage (+): 0. Max coverage (-): 15.47

Region: chr10 100914955-100915025. Max. coverage (+): 0. Max coverage (-): 4.31

Region: chr10 100915026-100915096. Max. coverage (+): 0. Max coverage (-): 3.55

Region: chr10 100915097-100915166. Max. coverage (+): 0. Max coverage (-): 0

Region: chr10 100915167-100915237. Max. coverage (+): 0. Max coverage (-): 4.25

Region: chr10 100915238-100915308. Max. coverage (+): 0. Max coverage (-): 5.48

Region: chr10 100915309-100915379. Max. coverage (+): 5.78. Max coverage (-): 111.31

Region: chr10 100915380-100915449. Max. coverage (+): 5.78. Max coverage (-): 18.29

Region: chr10 100915450-100915520. Max. coverage (+): 0. Max coverage (-): 20.92

Region: chr10 100915521-100915591. Max. coverage (+): 0. Max coverage (-): 4.13

Region: chr10 100915592-100915661. Max. coverage (+): 0. Max coverage (-): 38.12

Region: chr10 100915662-100915732. Max. coverage (+): 0. Max coverage (-): 33.18

Region: chr10 100915733-100915803. Max. coverage (+): 0. Max coverage (-): 59.11

Region: chr10 100915804-100915873. Max. coverage (+): 0.63. Max coverage (-): 44.91

Region: chr10 100915874-100915944. Max. coverage (+): 4.59. Max coverage (-): 12.29

Region: chr10 100915945-100916015. Max. coverage (+): 4.59. Max coverage (-): 0

Region: chr10 100916016-100916086. Max. coverage (+): 0. Max coverage (-): 4.49

Region: chr10 100916087-100916156. Max. coverage (+): 0. Max coverage (-): 20.77

Region: chr10 100916157-100916227. Max. coverage (+): 0. Max coverage (-): 7.38

Region: chr10 100916228-100916298. Max. coverage (+): 0. Max coverage (-): 44.54

Region: chr10 100916299-100916368. Max. coverage (+): 0. Max coverage (-): 43.01

Region: chr10 100916369-100916439. Max. coverage (+): 0. Max coverage (-): 24.65

Region: chr10 100916440-100916510. Max. coverage (+): 0. Max coverage (-): 34.64

Region: chr10 100916511-100916580. Max. coverage (+): 0. Max coverage (-): 0

Region: chr10 100916581-100916651. Max. coverage (+): 0. Max coverage (-): 0

Region: chr10 100916652-100916722. Max. coverage (+): 0. Max coverage (-): 0

Region: chr10 100916723-100916793. Max. coverage (+): 0. Max coverage (-): 13.98

Region: chr10 100916794-100916863. Max. coverage (+): 0. Max coverage (-): 3.16

Region: chr10 100916864-100916934. Max. coverage (+): 0. Max coverage (-): 8.9

Region: chr10 100916935-100917005. Max. coverage (+): 0. Max coverage (-): 4.43

Region: chr10 100917006-100917075. Max. coverage (+): 6.6. Max coverage (-): 47.65

Region: chr10 100917076-100917146. Max. coverage (+): 0. Max coverage (-): 14.15

Region: chr10 100917147-100917217. Max. coverage (+): 0. Max coverage (-): 4.3

Region: chr10 100917218-100917288. Max. coverage (+): 0. Max coverage (-): 21.87

Region: chr10 100917289-100917358. Max. coverage (+): 0. Max coverage (-): 21.87

Region: chr10 100917359-100917429. Max. coverage (+): 0. Max coverage (-): 8.91

Region: chr10 100917430-100917500. Max. coverage (+): 3.45. Max coverage (-): 20.71

Region: chr10 100917501-100917570. Max. coverage (+): 0. Max coverage (-): 13.07

Region: chr10 100917571-100917641. Max. coverage (+): 0. Max coverage (-): 14.34

Region: chr10 100917642-100917712. Max. coverage (+): 0. Max coverage (-): 47.7

Region: chr10 100917713-100917782. Max. coverage (+): 0. Max coverage (-): 54.28

Region: chr10 100917783-100917853. Max. coverage (+): 0. Max coverage (-): 26.43

Region: chr10 100917854-100917924. Max. coverage (+): 0. Max coverage (-): 1.23

Region: chr10 100917925-100917995. Max. coverage (+): 0. Max coverage (-): 3.2

Region: chr10 100917996-100918065. Max. coverage (+): 0. Max coverage (-): 3.49

Region: chr10 100918066-100918136. Max. coverage (+): 0. Max coverage (-): 0

Region: chr10 100918137-100918207. Max. coverage (+): 0. Max coverage (-): 4.69

Region: chr10 100918208-100918277. Max. coverage (+): 0. Max coverage (-): 0

Region: chr10 100918278-100918348. Max. coverage (+): 0. Max coverage (-): 13.14

Region: chr10 100918349-100918419. Max. coverage (+): 0. Max coverage (-): 28.12

Region: chr10 100918420-100918489. Max. coverage (+): 0. Max coverage (-): 1.13

Region: chr10 100918490-100918560. Max. coverage (+): 0. Max coverage (-): 14.77

Region: chr10 100918561-100918631. Max. coverage (+): 1.22. Max coverage (-): 34.87

Region: chr10 100918632-100918702. Max. coverage (+): 0. Max coverage (-): 40.24

Region: chr10 100918703-100918772. Max. coverage (+): 0. Max coverage (-): 28.57

Region: chr10 100918773-100918843. Max. coverage (+): 0. Max coverage (-): 27.38

Region: chr10 100918844-100918914. Max. coverage (+): 0. Max coverage (-): 7.38

Region: chr10 100918915-100918984. Max. coverage (+): 0. Max coverage (-): 6.8

Region: chr10 100918985-100919055. Max. coverage (+): 0. Max coverage (-): 0.46

Region: chr10 100919056-100919126. Max. coverage (+): 0. Max coverage (-): 0

Region: chr10 100919127-100919196. Max. coverage (+): 0. Max coverage (-): 0

Region: chr10 100919197-100919267. Max. coverage (+): 0. Max coverage (-): 31.36

Region: chr10 100919268-100919338. Max. coverage (+): 0. Max coverage (-): 34.03

Region: chr10 100919339-100919409. Max. coverage (+): 0. Max coverage (-): 0.3

Region: chr10 100919410-100919479. Max. coverage (+): 0. Max coverage (-): 0

Region: chr10 100919480-100919550. Max. coverage (+): 0. Max coverage (-): 7.12

Region: chr10 100919551-100919621. Max. coverage (+): 0. Max coverage (-): 54.37

Region: chr10 100919622-100919691. Max. coverage (+): 0. Max coverage (-): 0

Region: chr10 100919692-100919762. Max. coverage (+): 0. Max coverage (-): 6.65

Region: chr10 100919763-100919833. Max. coverage (+): 0. Max coverage (-): 0

Region: chr10 100919834-100919903. Max. coverage (+): 0. Max coverage (-): 0

Region: chr10 100919904-100919974. Max. coverage (+): 0. Max coverage (-): 7.05

Region: chr10 100919975-100920045. Max. coverage (+): 0. Max coverage (-): 21.7

Region: chr10 100920046-100920116. Max. coverage (+): 0. Max coverage (-): 9.06

Region: chr10 100920117-100920186. Max. coverage (+): 0. Max coverage (-): 17

Region: chr10 100920187-100920257. Max. coverage (+): 0. Max coverage (-): 32.66

Region: chr10 100920258-100920328. Max. coverage (+): 0. Max coverage (-): 19.97

Region: chr10 100920329-100920398. Max. coverage (+): 0. Max coverage (-): 18.25

Region: chr10 100920399-100920469. Max. coverage (+): 0. Max coverage (-): 21.87

Region: chr10 100920470-100920540. Max. coverage (+): 0. Max coverage (-): 19.93

Region: chr10 100920541-100920611. Max. coverage (+): 0. Max coverage (-): 5.68

Region: chr10 100920612-100920681. Max. coverage (+): 0. Max coverage (-): 46.86

Region: chr10 100920682-100920752. Max. coverage (+): 0. Max coverage (-): 34.04

Region: chr10 100920753-100920823. Max. coverage (+): 0. Max coverage (-): 17.35

Region: chr10 100920824-100920893. Max. coverage (+): 0. Max coverage (-): 74.5

Region: chr10 100920894-100920964. Max. coverage (+): 1.29. Max coverage (-): 24.38

Region: chr10 100920965-100921035. Max. coverage (+): 0. Max coverage (-): 64.26

Region: chr10 100921036-100921105. Max. coverage (+): 0. Max coverage (-): 8.29

Region: chr10 100921106-100921176. Max. coverage (+): 0. Max coverage (-): 14.37

Region: chr10 100921177-100921247. Max. coverage (+): 6.37. Max coverage (-): 34.78

Region: chr10 100921248-100921318. Max. coverage (+): 6.37. Max coverage (-): 21.85

Region: chr10 100921319-100921388. Max. coverage (+): 0. Max coverage (-): 26.51

Region: chr10 100921389-100921459. Max. coverage (+): 0. Max coverage (-): 11.44

Region: chr10 100921460-100921530. Max. coverage (+): 0. Max coverage (-): 3.16

Region: chr10 100921531-100921600. Max. coverage (+): 0. Max coverage (-): 46.39

Region: chr10 100921601-100921671. Max. coverage (+): 0. Max coverage (-): 58.84

Region: chr10 100921672-100921742. Max. coverage (+): 0. Max coverage (-): 13.79

Region: chr10 100921743-100921812. Max. coverage (+): 0. Max coverage (-): 0

Region: chr10 100921813-100921883. Max. coverage (+): 0. Max coverage (-): 0

Region: chr10 100921884-100921954. Max. coverage (+): 0. Max coverage (-): 0

Region: chr10 100921955-100922025. Max. coverage (+): 0. Max coverage (-): 0

Region: chr10 100922026-100922095. Max. coverage (+): 0. Max coverage (-): 1.01

Region: chr10 100922096-100922166. Max. coverage (+): 0. Max coverage (-): 17

Region: chr10 100922167-100922237. Max. coverage (+): 0. Max coverage (-): 4.49

Region: chr10 100922238-100922307. Max. coverage (+): 0. Max coverage (-): 0

Region: chr10 100922308-100922378. Max. coverage (+): 0. Max coverage (-): 0

Region: chr10 100922379-100922449. Max. coverage (+): 0. Max coverage (-): 27.19

Region: chr10 100922450-100922519. Max. coverage (+): 0. Max coverage (-): 27.19

Region: chr10 100922520-100922590. Max. coverage (+): 0. Max coverage (-): 0.74

Region: chr10 100922591-100922661. Max. coverage (+): 0. Max coverage (-): 2.6

Region: chr10 100922662-100922732. Max. coverage (+): 0. Max coverage (-): 3.35

Region: chr10 100922733-100922802. Max. coverage (+): 0. Max coverage (-): 9.42

Region: chr10 100922803-100922873. Max. coverage (+): 0. Max coverage (-): 22.97

Region: chr10 100922874-100922944. Max. coverage (+): 0. Max coverage (-): 21.28

Region: chr10 100922945-100923014. Max. coverage (+): 0. Max coverage (-): 29.36

Region: chr10 100923015-100923085. Max. coverage (+): 0. Max coverage (-): 0

Region: chr10 100923086-100923156. Max. coverage (+): 0. Max coverage (-): 0

Region: chr10 100923157-100923226. Max. coverage (+): 0. Max coverage (-): 0

Region: chr10 100923227-100923297. Max. coverage (+): 0. Max coverage (-): 0

Region: chr10 100923298-100923368. Max. coverage (+): 0. Max coverage (-): 3.08

Region: chr10 100923369-100923439. Max. coverage (+): 0. Max coverage (-): 7.94

Region: chr10 100923440-100923509. Max. coverage (+): 0. Max coverage (-): 0

Region: chr10 100923510-100923580. Max. coverage (+): 0. Max coverage (-): 8.19

Region: chr10 100923581-100923651. Max. coverage (+): 0. Max coverage (-): 18.77

Region: chr10 100923652-100923721. Max. coverage (+): 0. Max coverage (-): 22.6

Region: chr10 100923722-100923792. Max. coverage (+): 0. Max coverage (-): 40.04

Region: chr10 100923793-100923863. Max. coverage (+): 0. Max coverage (-): 13.95

Region: chr10 100923864-100923933. Max. coverage (+): 0. Max coverage (-): 0

Region: chr10 100923934-100924004. Max. coverage (+): 0. Max coverage (-): 0

Region: chr10 100924005-100924075. Max. coverage (+): 0. Max coverage (-): 0

Region: chr10 100924076-100924146. Max. coverage (+): 0. Max coverage (-): 0

Region: chr10 100924147-100924216. Max. coverage (+): 0. Max coverage (-): 5.15

Region: chr10 100924217-100924287. Max. coverage (+): 0. Max coverage (-): 6.54

Region: chr10 100924288-100924358. Max. coverage (+): 0. Max coverage (-): 0

Region: chr10 100924359-100924428. Max. coverage (+): 0. Max coverage (-): 15.13

Region: chr10 100924429-100924499. Max. coverage (+): 0. Max coverage (-): 10.34

Region: chr10 100924500-100924570. Max. coverage (+): 0. Max coverage (-): 4.54

Region: chr10 100924571-100924641. Max. coverage (+): 0. Max coverage (-): 22.18

Region: chr10 100924642-100924711. Max. coverage (+): 0. Max coverage (-): 11.04

Region: chr10 100924712-100924782. Max. coverage (+): 0. Max coverage (-): 29.62

Region: chr10 100924783-100924853. Max. coverage (+): 0. Max coverage (-): 0

Region: chr10 100924854-100924923. Max. coverage (+): 0. Max coverage (-): 54.71

Region: chr10 100924924-100924994. Max. coverage (+): 0. Max coverage (-): 44.86

Region: chr10 100924995-100925065. Max. coverage (+): 0. Max coverage (-): 122.11

Region: chr10 100925066-100925135. Max. coverage (+): 0. Max coverage (-): 6

Region: chr10 100925136-100925206. Max. coverage (+): 0. Max coverage (-): 77.13

Region: chr10 100925207-100925277. Max. coverage (+): 0. Max coverage (-): 98.79

Region: chr10 100925278-100925348. Max. coverage (+): 0. Max coverage (-): 10.9

Region: chr10 100925349-100925418. Max. coverage (+): 0. Max coverage (-): 10.25

Region: chr10 100925419-100925489. Max. coverage (+): 0. Max coverage (-): 0

Region: chr10 100925490-100925560. Max. coverage (+): 0. Max coverage (-): 2.86

Region: chr10 100925561-100925630. Max. coverage (+): 0. Max coverage (-): 4.18

Region: chr10 100925631-100925701. Max. coverage (+): 0. Max coverage (-): 11.38

Region: chr10 100925702-100925772. Max. coverage (+): 0. Max coverage (-): 14.14

Region: chr10 100925773-100925842. Max. coverage (+): 6.38. Max coverage (-): 21.49

Region: chr10 100925843-100925913. Max. coverage (+): 0. Max coverage (-): 5.76

Region: chr10 100925914-100925984. Max. coverage (+): 0. Max coverage (-): 0

Region: chr10 100925985-100926055. Max. coverage (+): 0. Max coverage (-): 26.34

Region: chr10 100926056-100926125. Max. coverage (+): 0. Max coverage (-): 17.33

Region: chr10 100926126-100926196. Max. coverage (+): 0. Max coverage (-): 18.19

Region: chr10 100926197-100926267. Max. coverage (+): 0. Max coverage (-): 5.14

Region: chr10 100926268-100926337. Max. coverage (+): 0. Max coverage (-): 18.21

Region: chr10 100926338-100926408. Max. coverage (+): 0. Max coverage (-): 6.71

Region: chr10 100926409-100926479. Max. coverage (+): 0. Max coverage (-): 7.16

Region: chr10 100926480-100926549. Max. coverage (+): 0. Max coverage (-): 10.83

Region: chr10 100926550-100926620. Max. coverage (+): 0. Max coverage (-): 17.75

Region: chr10 100926621-100926691. Max. coverage (+): 0. Max coverage (-): 66.86

Region: chr10 100926692-100926762. Max. coverage (+): 0. Max coverage (-): 52.02

Region: chr10 100926763-100926832. Max. coverage (+): 0. Max coverage (-): 21.26

Region: chr10 100926833-100926903. Max. coverage (+): 0. Max coverage (-): 16.63

Region: chr10 100926904-100926974. Max. coverage (+): 0. Max coverage (-): 11.8

Region: chr10 100926975-100927044. Max. coverage (+): 0. Max coverage (-): 37.43

Region: chr10 100927045-100927115. Max. coverage (+): 0. Max coverage (-): 28.33

Region: chr10 100927116-100927186. Max. coverage (+): 0. Max coverage (-): 33.42

Region: chr10 100927187-100927256. Max. coverage (+): 0. Max coverage (-): 82.55

Region: chr10 100927257-100927327. Max. coverage (+): 0. Max coverage (-): 6.2

Region: chr10 100927328-100927398. Max. coverage (+): 0. Max coverage (-): 0

Region: chr10 100927399-100927469. Max. coverage (+): 0. Max coverage (-): 0

Region: chr10 100927470-100927539. Max. coverage (+): 0. Max coverage (-): 8.94

Region: chr10 100927540-100927610. Max. coverage (+): 0. Max coverage (-): 7.18

Region: chr10 100927611-100927681. Max. coverage (+): 0. Max coverage (-): 5.9

Region: chr10 100927682-100927751. Max. coverage (+): 0. Max coverage (-): 13.61

Region: chr10 100927752-100927822. Max. coverage (+): 0. Max coverage (-): 22.31

Region: chr10 100927823-100927893. Max. coverage (+): 0. Max coverage (-): 9.66

Region: chr10 100927894-100927964. Max. coverage (+): 0. Max coverage (-): 9.66

Region: chr10 100927965-100928034. Max. coverage (+): 0. Max coverage (-): 3.38

Region: chr10 100928035-100928105. Max. coverage (+): 0. Max coverage (-): 0.29

Region: chr10 100928106-100928176. Max. coverage (+): 0. Max coverage (-): 28.03

Region: chr10 100928177-100928246. Max. coverage (+): 0. Max coverage (-): 7.64

Region: chr10 100928247-100928317. Max. coverage (+): 0. Max coverage (-): 41.27

Region: chr10 100928318-100928388. Max. coverage (+): 0. Max coverage (-): 190.47

Region: chr10 100928389-100928458. Max. coverage (+): 0. Max coverage (-): 50.44

Region: chr10 100928459-100928529. Max. coverage (+): 0. Max coverage (-): 0

Region: chr10 100928530-100928600. Max. coverage (+): 0. Max coverage (-): 0

Region: chr10 100928601-100928671. Max. coverage (+): 0. Max coverage (-): 0

Region: chr10 100928672-100928741. Max. coverage (+): 0. Max coverage (-): 0

Region: chr10 100928742-100928812. Max. coverage (+): 0. Max coverage (-): 0

Region: chr10 100928813-100928883. Max. coverage (+): 0. Max coverage (-): 0

Region: chr10 100928884-100928953. Max. coverage (+): 0. Max coverage (-): 0

Region: chr10 100928954-100929024. Max. coverage (+): 0. Max coverage (-): 0

Region: chr10 100929025-100929095. Max. coverage (+): 0. Max coverage (-): 5.45

Region: chr10 100929096-100929165. Max. coverage (+): 0. Max coverage (-): 5.04

Region: chr10 100929166-100929236. Max. coverage (+): 0. Max coverage (-): 10.34

Region: chr10 100929237-100929307. Max. coverage (+): 0. Max coverage (-): 5.67

Region: chr10 100929308-100929378. Max. coverage (+): 0. Max coverage (-): 1.04

Region: chr10 100929379-100929448. Max. coverage (+): 0. Max coverage (-): 9.44

Region: chr10 100929449-100929519. Max. coverage (+): 0. Max coverage (-): 16.26

Region: chr10 100929520-100929590. Max. coverage (+): 0. Max coverage (-): 11.93

Region: chr10 100929591-100929660. Max. coverage (+): 0. Max coverage (-): 16.97

Region: chr10 100929661-100929731. Max. coverage (+): 0. Max coverage (-): 21.48

Region: chr10 100929732-100929802. Max. coverage (+): 0. Max coverage (-): 64.33

Region: chr10 100929803-100929872. Max. coverage (+): 0. Max coverage (-): 76.29

Region: chr10 100929873-100929943. Max. coverage (+): 0. Max coverage (-): 105.17

Region: chr10 100929944-100930014. Max. coverage (+): 0. Max coverage (-): 54.69

Region: chr10 100930015-100930085. Max. coverage (+): 0. Max coverage (-): 22.31

Region: chr10 100930086-100930155. Max. coverage (+): 0. Max coverage (-): 15.43

Region: chr10 100930156-100930226. Max. coverage (+): 0. Max coverage (-): 16.44

Region: chr10 100930227-100930297. Max. coverage (+): 0. Max coverage (-): 19.38

Region: chr10 100930298-100930367. Max. coverage (+): 0. Max coverage (-): 5.84

Region: chr10 100930368-100930438. Max. coverage (+): 0. Max coverage (-): 12.83

Region: chr10 100930439-100930509. Max. coverage (+): 0. Max coverage (-): 52.25

Region: chr10 100930510-100930579. Max. coverage (+): 0. Max coverage (-): 44.39

Region: chr10 100930580-100930650. Max. coverage (+): 0. Max coverage (-): 1.68

Region: chr10 100930651-100930721. Max. coverage (+): 0. Max coverage (-): 98.75

Region: chr10 100930722-100930792. Max. coverage (+): 0. Max coverage (-): 13.27

Region: chr10 100930793-100930862. Max. coverage (+): 0. Max coverage (-): 13.27

Region: chr10 100930863-100930933. Max. coverage (+): 0. Max coverage (-): 40.3

Region: chr10 100930934-100931004. Max. coverage (+): 0. Max coverage (-): 7.63

Region: chr10 100931005-100931074. Max. coverage (+): 0. Max coverage (-): 0

Region: chr10 100931075-100931145. Max. coverage (+): 0. Max coverage (-): 2.88

Region: chr10 100931146-100931216. Max. coverage (+): 2.78. Max coverage (-): 15.06

Region: chr10 100931217-100931287. Max. coverage (+): 0. Max coverage (-): 14.84

Region: chr10 100931288-100931357. Max. coverage (+): 0. Max coverage (-): 46.59

Region: chr10 100931358-100931428. Max. coverage (+): 0. Max coverage (-): 3.88

Region: chr10 100931429-100931499. Max. coverage (+): 0. Max coverage (-): 6.72

Region: chr10 100931500-100931569. Max. coverage (+): 0. Max coverage (-): 65.49

Region: chr10 100931570-100931640. Max. coverage (+): 0. Max coverage (-): 17.06

Region: chr10 100931641-100931711. Max. coverage (+): 8.98. Max coverage (-): 51.71

Region: chr10 100931712-100931781. Max. coverage (+): 0.18. Max coverage (-): 42.15

Region: chr10 100931782-100931852. Max. coverage (+): 4. Max coverage (-): 164.05

Region: chr10 100931853-100931923. Max. coverage (+): 0. Max coverage (-): 0

Region: chr10 100931924-100931994. Max. coverage (+): 0. Max coverage (-): 0

Region: chr10 100931995-100932064. Max. coverage (+): 0. Max coverage (-): 0

Region: chr10 100932065-100932135. Max. coverage (+): 0. Max coverage (-): 0.92

Region: chr10 100932136-100932206. Max. coverage (+): 0. Max coverage (-): 0

Region: chr10 100932207-100932276. Max. coverage (+): 0. Max coverage (-): 0

Region: chr10 100932277-100932347. Max. coverage (+): 0. Max coverage (-): 7.15

Region: chr10 100932348-100932418. Max. coverage (+): 0. Max coverage (-): 1.48

Region: chr10 100932419-100932488. Max. coverage (+): 0. Max coverage (-): 5.17

Region: chr10 100932489-100932559. Max. coverage (+): 0. Max coverage (-): 12.48

Region: chr10 100932560-100932630. Max. coverage (+): 0. Max coverage (-): 7.61

Region: chr10 100932631-100932701. Max. coverage (+): 0. Max coverage (-): 13.56

Region: chr10 100932702-100932771. Max. coverage (+): 0. Max coverage (-): 28.09

Region: chr10 100932772-100932842. Max. coverage (+): 0. Max coverage (-): 5.71

Region: chr10 100932843-100932913. Max. coverage (+): 0. Max coverage (-): 11.7

Region: chr10 100932914-100932983. Max. coverage (+): 0. Max coverage (-): 12.79

Region: chr10 100932984-100933054. Max. coverage (+): 0. Max coverage (-): 12.62

Region: chr10 100933055-100933125. Max. coverage (+): 0. Max coverage (-): 33.21

Region: chr10 100933126-100933195. Max. coverage (+): 7.67. Max coverage (-): 31.19

Region: chr10 100933196-100933266. Max. coverage (+): 0. Max coverage (-): 8.52

Region: chr10 100933267-100933337. Max. coverage (+): 0. Max coverage (-): 0

Region: chr10 100933338-100933408. Max. coverage (+): 0. Max coverage (-): 8.66

Region: chr10 100933409-100933478. Max. coverage (+): 0. Max coverage (-): 8.52

Region: chr10 100933479-100933549. Max. coverage (+): 0. Max coverage (-): 0

Region: chr10 100933550-100933620. Max. coverage (+): 0. Max coverage (-): 0

Region: chr10 100933621-100933690. Max. coverage (+): 7.53. Max coverage (-): 0

Region: chr10 100933691-100933761. Max. coverage (+): 53.77. Max coverage (-): 0

Region: chr10 100933762-100933832. Max. coverage (+): 18.22. Max coverage (-): 6.63

Region: chr10 100933833-100933902. Max. coverage (+): 31.06. Max coverage (-): 6.63

Region: chr10 100933903-100933973. Max. coverage (+): 0. Max coverage (-): 0

Region: chr10 100933974-100934044. Max. coverage (+): 0. Max coverage (-): 0

Region: chr10 100934045-100934115. Max. coverage (+): 0. Max coverage (-): 0

Region: chr10 100934116-100934185. Max. coverage (+): 3.12. Max coverage (-): 0

Region: chr10 100934186-100934256. Max. coverage (+): 29.89. Max coverage (-): 0

Region: chr10 100934257-100934327. Max. coverage (+): 28.95. Max coverage (-): 0

Region: chr10 100934328-100934397. Max. coverage (+): 4.78. Max coverage (-): 0.76

Region: chr10 100934398-100934468. Max. coverage (+): 11.53. Max coverage (-): 0

Region: chr10 100934469-100934539. Max. coverage (+): 11.53. Max coverage (-): 0

Region: chr10 100934540-100934609. Max. coverage (+): 52.59. Max coverage (-): 0

Region: chr10 100934610-100934680. Max. coverage (+): 17.28. Max coverage (-): 0

Region: chr10 100934681-100934751. Max. coverage (+): 0.52. Max coverage (-): 0

Region: chr10 100934752-100934822. Max. coverage (+): 5.83. Max coverage (-): 0

Region: chr10 100934823-100934892. Max. coverage (+): 10.04. Max coverage (-): 0

Region: chr10 100934893-100934963. Max. coverage (+): 26.47. Max coverage (-): 3.22

Region: chr10 100934964-100935034. Max. coverage (+): 6.73. Max coverage (-): 1.57

Region: chr10 100935035-100935104. Max. coverage (+): 23.6. Max coverage (-): 0

Region: chr10 100935105-100935175. Max. coverage (+): 7.16. Max coverage (-): 0

Region: chr10 100935176-100935246. Max. coverage (+): 5.57. Max coverage (-): 0

Region: chr10 100935247-100935317. Max. coverage (+): 47.33. Max coverage (-): 0

Region: chr10 100935318-100935387. Max. coverage (+): 0. Max coverage (-): 0

Region: chr10 100935388-100935458. Max. coverage (+): 0. Max coverage (-): 0

Region: chr10 100935459-100935529. Max. coverage (+): 0. Max coverage (-): 0

Region: chr10 100935530-100935599. Max. coverage (+): 0. Max coverage (-): 0

Region: chr10 100935600-100935670. Max. coverage (+): 4.87. Max coverage (-): 0

Region: chr10 100935671-100935741. Max. coverage (+): 42.63. Max coverage (-): 0

Region: chr10 100935742-100935811. Max. coverage (+): 84.49. Max coverage (-): 0

Region: chr10 100935812-100935882. Max. coverage (+): 6.6. Max coverage (-): 0

Region: chr10 100935883-100935953. Max. coverage (+): 37.33. Max coverage (-): 0

Region: chr10 100935954-100936024. Max. coverage (+): 12.37. Max coverage (-): 0

Region: chr10 100936025-100936094. Max. coverage (+): 13.11. Max coverage (-): 0

Region: chr10 100936095-100936165. Max. coverage (+): 0. Max coverage (-): 0

Region: chr10 100936166-100936236. Max. coverage (+): 0.83. Max coverage (-): 0

Region: chr10 100936237-100936306. Max. coverage (+): 0. Max coverage (-): 0

Region: chr10 100936307-100936377. Max. coverage (+): 13.69. Max coverage (-): 0

Region: chr10 100936378-100936448. Max. coverage (+): 8.38. Max coverage (-): 0

Region: chr10 100936449-100936518. Max. coverage (+): 33.1. Max coverage (-): 0

Region: chr10 100936519-100936589. Max. coverage (+): 70.89. Max coverage (-): 0

Region: chr10 100936590-100936660. Max. coverage (+): 57.73. Max coverage (-): 0

Region: chr10 100936661-100936731. Max. coverage (+): 41.82. Max coverage (-): 0

Region: chr10 100936732-100936801. Max. coverage (+): 28.23. Max coverage (-): 0

Region: chr10 100936802-100936872. Max. coverage (+): 64.24. Max coverage (-): 0

Region: chr10 100936873-100936943. Max. coverage (+): 0. Max coverage (-): 0

Region: chr10 100936944-100937013. Max. coverage (+): 12.15. Max coverage (-): 0

Region: chr10 100937014-100937084. Max. coverage (+): 49.51. Max coverage (-): 0

Region: chr10 100937085-100937155. Max. coverage (+): 0. Max coverage (-): 0

Region: chr10 100937156-100937225. Max. coverage (+): 7.12. Max coverage (-): 0

Region: chr10 100937226-100937296. Max. coverage (+): 51.47. Max coverage (-): 0

Region: chr10 100937297-100937367. Max. coverage (+): 34.69. Max coverage (-): 0

Region: chr10 100937368-100937438. Max. coverage (+): 23.24. Max coverage (-): 0

Region: chr10 100937439-100937508. Max. coverage (+): 34.53. Max coverage (-): 3.83

Region: chr10 100937509-100937579. Max. coverage (+): 20.28. Max coverage (-): 0

Region: chr10 100937580-100937650. Max. coverage (+): 4.93. Max coverage (-): 0

Region: chr10 100937651-100937720. Max. coverage (+): 56.12. Max coverage (-): 0

Region: chr10 100937721-100937791. Max. coverage (+): 8.66. Max coverage (-): 0

Region: chr10 100937792-100937862. Max. coverage (+): 8.88. Max coverage (-): 0

Region: chr10 100937863-100937932. Max. coverage (+): 0. Max coverage (-): 0

Region: chr10 100937933-100938003. Max. coverage (+): 27.51. Max coverage (-): 0

Region: chr10 100938004-100938074. Max. coverage (+): 22.68. Max coverage (-): 0

Region: chr10 100938075-100938145. Max. coverage (+): 48.01. Max coverage (-): 0

Region: chr10 100938146-100938215. Max. coverage (+): 39.14. Max coverage (-): 0

Region: chr10 100938216-100938286. Max. coverage (+): 6.82. Max coverage (-): 0

Region: chr10 100938287-100938357. Max. coverage (+): 26.77. Max coverage (-): 0

Region: chr10 100938358-100938427. Max. coverage (+): 34.62. Max coverage (-): 0

Region: chr10 100938428-100938498. Max. coverage (+): 19.26. Max coverage (-): 0

Region: chr10 100938499-100938569. Max. coverage (+): 23.99. Max coverage (-): 0

Region: chr10 100938570-100938640. Max. coverage (+): 23.45. Max coverage (-): 0

Region: chr10 100938641-100938710. Max. coverage (+): 46.41. Max coverage (-): 0

Region: chr10 100938711-100938781. Max. coverage (+): 49.19. Max coverage (-): 0

Region: chr10 100938782-100938852. Max. coverage (+): 6.7. Max coverage (-): 0

Region: chr10 100938853-100938922. Max. coverage (+): 18.17. Max coverage (-): 0

Region: chr10 100938923-100938993. Max. coverage (+): 7.05. Max coverage (-): 0

Region: chr10 100938994-100939064. Max. coverage (+): 47.21. Max coverage (-): 0

Region: chr10 100939065-100939134. Max. coverage (+): 0. Max coverage (-): 5.7

Region: chr10 100939135-100939205. Max. coverage (+): 15.77. Max coverage (-): 3.07

Region: chr10 100939206-100939276. Max. coverage (+): 15.77. Max coverage (-): 0

Region: chr10 100939277-100939347. Max. coverage (+): 11.83. Max coverage (-): 0

Region: chr10 100939348-100939417. Max. coverage (+): 14.27. Max coverage (-): 0

Region: chr10 100939418-100939488. Max. coverage (+): 10.25. Max coverage (-): 0

Region: chr10 100939489-100939559. Max. coverage (+): 10.02. Max coverage (-): 0

Region: chr10 100939560-100939629. Max. coverage (+): 29.56. Max coverage (-): 3.06

Region: chr10 100939630-100939700. Max. coverage (+): 33.43. Max coverage (-): 10.15

Region: chr10 100939701-100939771. Max. coverage (+): 32.57. Max coverage (-): 0

Region: chr10 100939772-100939841. Max. coverage (+): 18.19. Max coverage (-): 0

Region: chr10 100939842-100939912. Max. coverage (+): 3.98. Max coverage (-): 0

Region: chr10 100939913-. Max. coverage (+): 1.63. Max coverage (-): 0

RepeatMasker Color Code

**+**

100-98% Identity

<98-95% Identity

<95-90% Identity

<90-85% Identity

<85-80% Identity

<80-75% Identity

<75-70% Identity

<70% Identity

**-**

Gene Set Color Code

**+**

Gene

Pseudogene

**-**

Topology/Coverage Color Code

Coverage Plus Strand

Coverage Minus Strand

Mainstrand: Plus

Mainstrand: Minus

Complementary Strand

Flanking Region  
(if option -flank >0)

Gene Set Annotation  
  
RepeatMasker Annotation  

**1. HAL1**: 100904597-100904932 (-), Divergence to consensus: 49.7%  
**2. HAL1**: 100904983-100905459 (-), Divergence to consensus: 44.5%  
**3. MER115**: 100905546-100905753 (+), Divergence to consensus: 40.7%  
**4. Zaphod**: 100905863-100906007 (+), Divergence to consensus: 31.9%  
**5. HAL1**: 100906015-100906058 (-), Divergence to consensus: 22.7%  
**6. HAL1**: 100906058-100906109 (-), Divergence to consensus: 23.1%  
**7. (TTTTG)n**: 100906334-100906357 (+), Divergence to consensus: 4.2%  
**8. L1\_BT**: 100907359-100907485 (+), Divergence to consensus: 4.7%  
**9. BTLTR1J**: 100907486-100907556 (-), Divergence to consensus: 28.2%  
**10. L1\_BT**: 100907557-100907971 (+), Divergence to consensus: 18.7%  
**11. AT\_rich**: 100907972-100907993 (+), Divergence to consensus: 50%  
**12. LSU-rRNA\_Hsa**: 100909090-100909358 (+), Divergence to consensus: 38.3%  
**13. LSU-rRNA\_Hsa**: 100909469-100909665 (+), Divergence to consensus: 42%  
**14. LTR78B**: 100909844-100910137 (-), Divergence to consensus: 46.9%  
**15. LTR78B**: 100910311-100910463 (-), Divergence to consensus: 40.5%  
**16. LTR78B**: 100910885-100910964 (-), Divergence to consensus: 35%  
**17. MamRep1894**: 100912514-100912592 (+), Divergence to consensus: 20.2%  
**18. MER94B**: 100916564-100916610 (+), Divergence to consensus: 21.3%  
**19. Bov-tA2**: 100921784-100921973 (-), Divergence to consensus: 27.5%  
**20. MIRb**: 100923010-100923220 (+), Divergence to consensus: 31.7%  
**21. L2b**: 100923866-100924023 (-), Divergence to consensus: 46.1%  
**22. (A)n**: 100924161-100924187 (+), Divergence to consensus: 11.1%  
**23. Bov-tA2**: 100925399-100925536 (+), Divergence to consensus: 13%  
**24. BOV-A2**: 100927300-100927462 (+), Divergence to consensus: 5.5%  
**25. Bov-tA2**: 100928490-100928669 (+), Divergence to consensus: 16.1%  
**26. MER5A1**: 100928791-100928939 (-), Divergence to consensus: 36.4%  
**27. AT\_rich**: 100930324-100930348 (+), Divergence to consensus: 32%  
**28. MIR**: 100931886-100932107 (-), Divergence to consensus: 39%  
**29. L2b**: 100933982-100934084 (+), Divergence to consensus: 40%  
**30. MIR3**: 100934088-100934132 (+), Divergence to consensus: 24.4%  
**31. GA-rich**: 100934728-100934801 (+), Divergence to consensus: 22%  
**32. L2c**: 100935305-100935640 (+), Divergence to consensus: 46.6%  
**33. L3**: 100936053-100936159 (+), Divergence to consensus: 42.8%  
**34. G-rich**: 100937115-100937166 (+), Divergence to consensus: 23.1%  
**35. GC\_rich**: 100939546-100939567 (+), Divergence to consensus: 50%  
**36. GC\_rich**: 100939548-100939585 (+), Divergence to consensus: 65.8%

  
Transcription Factor Binding Sites  

**RFX4\_2** (Sequence: GTATCTAAG (-): 100907130)  
**RFX4\_2** (Sequence: GTATCTAAG (-): 100930933)  
**RFX4\_2** (Sequence: GTAACCATG (-): 100938759)  
**RFX4\_1** (Sequence: GTTGCTATG (-): 100908054)  
**RFX4\_1** (Sequence: GTTGCCACG (-): 100912973)  
**RFX4\_1** (Sequence: GTTGCCAAG (-): 100917775)  
**SPZ1** (Sequence: CTGAAACCCT (-): 100908736)  
**SPZ1** (Sequence: CTGATACCCT (-): 100914108)  
**RFX4\_2** (Sequence: CTTGGATAC (+): 100923398)  
**Gata4** (Sequence: AGATAAC (-): 100910564)  
**Gata4** (Sequence: AGATAAG (-): 100911433)  
**Gata4** (Sequence: AGATAAG (-): 100929022)  
**Gata4** (Sequence: AGATAAG (-): 100938434)  
**SOX9** (Sequence: TTATTGTT (+): 100927146)  
**Gata4** (Sequence: GTTATCT (+): 100906553)  
**Gata4** (Sequence: CTTATCT (+): 100923460)  
**Gata4** (Sequence: CTTATCT (+): 100924957)  
**Gata4** (Sequence: CTTATCT (+): 100932396)
