# Supplementary material for: Small RNA sequencing of cryopreserved semen from single bull revealed altered miRNAs and piRNAs expression between High- and Low-motile sperm populations
Source: BMC Genomics. 2017 Jan 4;18:14. doi: 10.1186/s12864-016-3394-7 (PMC5209821; doi:10.1186/s12864-016-3394-7)
Supplement: Additional file 4: — Details for each piRNA clusters found in Low Motile (LM) sperm fraction. Genes, repeats, transposable elements and transcription factors binding sites falling within the cluster regions were reported. (ZIP 1034 kb) [file 12864_2016_3394_MOESM4_ESM.zip › 50.html]

piRNA cluster 50


Predicted piRNA cluster no. 50     previous   next
  

Show proTRAC run info
Hide proTRAC run info

================================= proTRAC ====================================  
VERSION: 2.1                                    LAST MODIFIED: 06. October 2015  
  
Please cite:  
Rosenkranz D, Zischler H. proTRAC - a software for probabilistic piRNA cluster  
detection, visualization and analysis. 2012. BMC Bioinformatics 13:5.  
  
and (for proTRAC 2.0 and later):  
Rosenkranz D, Rudloff S, Bastuck K, Ketting RF, Zischler H. Tupaia small RNAs  
provide insights into function and evolution of RNAi-based transposon defense  
in mammals. 2015. RNA 21(5):911-922.  
  
Contact:  
David Rosenkranz  
Institute of Anthropology, small RNA group  
Johannes Gutenberg University Mainz  
email: rosenkranz@uni-mainz.de  
  
You can find the latest proTRAC version at:  
http://sourceforge.net/projects/protrac/files  
http://www.smallRNAgroup-mainz.de/software  
==============================================================================  
  
PARAMETERS:  
Map file: .............../storage/core/barbara/genhome/smallRNA/fertility/Sample\_not\_motile/pirna/Sample\_not\_motile\_26-33\_collapsed.fa.no-dust.map.weighted-10000-1000-b-0  
Genome file: ............/storage/core/barbara/genhome/smallRNA/fertility/Sample\_all/pirna/bt\_311\_chrY.fa  
RepeatMasker annotation: /storage/genomes/bt\_umd31/GCF\_000003055.6\_Bos\_taurus\_UMD\_3.1.1\_repeatMasker\_chr.out  
GeneSet:................./storage/core/barbara/genhome/smallRNA/fertility/Sample\_all/pirna/full.gtf  
  
Significant (p<=0.01) hit density will be calculated based  
on observed hit distribution.  
  
Sliding window size: ........................................ 5000 bp  
Sliding window increament: .................................. 1000 bp  
Normalize each hit by number of genomic hits: ............... 1 [0=no/1=yes]  
Normalize each hit by number of sequence reads: ............. 1 [0=no/1=yes]  
Normalize values (-> per million mapped reads): ............. 1 [0=no/1=yes]  
Min. fraction of hits with 1T(U) or 10A: .................... 0.75  
Alternatively: Min. fraction of hits with 1T(U) and 10A: .... 0.5  
Min. fraction of hits with typical piRNA length: ............ 0.75  
Typical piRNA length: ....................................... 26-33 nt  
Min. size of a piRNA cluster: ............................... 5000 bp.  
Min. number of hits (absolute): ............................. 0  
Min. number of hits (normalized): ........................... 0  
Min. fraction of hits on the mainstrand: .................... 0.75  
Top fraction of mapped sequences (in terms of read counts): . 1%  
Top fraction accounts for max. n% of sequence reads: ........ 90%  
Min. fraction of hits on each arm of a bidirectional cluster: 0.1  
Output image file for each cluster: ......................... 0 [0=no/1=yes]  
Output html file for each cluster: .......................... 1 [0=no/1=yes]  
Output a summary table: ..................................... 1 [0=no/1=yes]  
Output a FASTA file for each cluster (piRNA sequences): ..... 1 [0=no/1=yes]  
Output a FASTA file comprising cluster sequences: ........... 1 [0=no/1=yes]  
Search DNA motifs in clusters: .............................. 1 [0=no/1=yes]  
Output flanking sequences: +/- .............................. 0 bp  
Output ~.pTi file: .......................................... 1 [0=no/1=yes]  
==============================================================================  
  
  
Genome size (without gaps): ............ 2678902517 bp  
Gaps (N/X/-): .......................... 53837044 bp  
Mapped reads: .......................... 738059667487  
Non-identical sequences: ............... 277001  
Genomic hits: .......................... 533816  
Significant densitiy of mapped reads: .. 15118061 reads/kb

Show proTRAC cluster info
Hide proTRAC cluster info

|  |  |
| --- | --- |
| Location | chr8 |
| Coordinates | 100787494-100793012 |
| Size [bp] | 5519 |
| Sequence hit loci | 141 |
| Mapped reads (normalized) | 356593621.9 |
| Mapped reads (normalized) per kb | 64611998.9 |
| Normalized reads with 1T (1U) | 87.1% |
| Normalized reads with 10A | 39.3% |
| Normalized reads with length 26-33 nt | 100% |
| Normalized reads on the main strand(s) | 100% |
| Predicted directionality | mono:minus |

100%

0%

1T (1U)  
reads

10A reads

26-33 nt  
reads

reads on mainstrand

**Either the amount of reads with 1T (1U) OR 10A has to exceed 75% (set with option: -1Tor10A)  
Alternatively the amount of reads with 1T (1U) AND 10A has to exceed 50% (set with option: -1Tand10A)  
Minimum amount of reads with preferred size is 75% (set with option: -pisize)  
Minimum amount of reads on the main strand(s) is 75% (set with option: -clstrand)**

Show read coverage
Hide read coverage

WHAT DO I SEE HERE?  
This chart shows the location of mapped sequence reads within a predicted piRNA cluster. The color refers to the number of genomic hits produced by the sequence read in question. A dark red bar indicates that this sequence read produces many other hits elsewhere in the genome. Many adjacent red or yellow bars can indicate the presence of a multi-copy element such as transposons or rRNA genes. A dark green bar indicates that this sequence read maps uniquely to this locus.

1 hit

2-5 hits

6-10 hits

11-20 hits

21-50 hits

51-100 hits

> 100 hits

chr8

100787494

100793012

Gene Set

RepeatMasker

Mapped  
Reads

43.31

plus strand

minus strand

43.31

Region: chr8 87069678-100787499. Max. coverage (+): 0. Max coverage (-): 2.36

Region: chr8 100787500-100787510. Max. coverage (+): 0. Max coverage (-): 2.36

Region: chr8 100787511-100787521. Max. coverage (+): 0. Max coverage (-): 0

Region: chr8 100787522-100787532. Max. coverage (+): 0. Max coverage (-): 0

Region: chr8 100787533-100787543. Max. coverage (+): 0. Max coverage (-): 0

Region: chr8 100787544-100787554. Max. coverage (+): 0. Max coverage (-): 0

Region: chr8 100787555-100787565. Max. coverage (+): 0. Max coverage (-): 0

Region: chr8 100787566-100787576. Max. coverage (+): 0. Max coverage (-): 0

Region: chr8 100787577-100787587. Max. coverage (+): 0. Max coverage (-): 0

Region: chr8 100787588-100787598. Max. coverage (+): 0. Max coverage (-): 0

Region: chr8 100787599-100787609. Max. coverage (+): 0. Max coverage (-): 0

Region: chr8 100787610-100787620. Max. coverage (+): 0. Max coverage (-): 0

Region: chr8 100787621-100787631. Max. coverage (+): 0. Max coverage (-): 0

Region: chr8 100787632-100787643. Max. coverage (+): 0. Max coverage (-): 0

Region: chr8 100787644-100787654. Max. coverage (+): 0. Max coverage (-): 0

Region: chr8 100787655-100787665. Max. coverage (+): 0. Max coverage (-): 0

Region: chr8 100787666-100787676. Max. coverage (+): 0. Max coverage (-): 0

Region: chr8 100787677-100787687. Max. coverage (+): 0. Max coverage (-): 0

Region: chr8 100787688-100787698. Max. coverage (+): 0. Max coverage (-): 0

Region: chr8 100787699-100787709. Max. coverage (+): 0. Max coverage (-): 0

Region: chr8 100787710-100787720. Max. coverage (+): 0. Max coverage (-): 0

Region: chr8 100787721-100787731. Max. coverage (+): 0. Max coverage (-): 0

Region: chr8 100787732-100787742. Max. coverage (+): 0. Max coverage (-): 0

Region: chr8 100787743-100787753. Max. coverage (+): 0. Max coverage (-): 0

Region: chr8 100787754-100787764. Max. coverage (+): 0. Max coverage (-): 0

Region: chr8 100787765-100787775. Max. coverage (+): 0. Max coverage (-): 0

Region: chr8 100787776-100787786. Max. coverage (+): 0. Max coverage (-): 0

Region: chr8 100787787-100787797. Max. coverage (+): 0. Max coverage (-): 0

Region: chr8 100787798-100787808. Max. coverage (+): 0. Max coverage (-): 0

Region: chr8 100787809-100787819. Max. coverage (+): 0. Max coverage (-): 0

Region: chr8 100787820-100787830. Max. coverage (+): 0. Max coverage (-): 0

Region: chr8 100787831-100787841. Max. coverage (+): 0. Max coverage (-): 0

Region: chr8 100787842-100787852. Max. coverage (+): 0. Max coverage (-): 0

Region: chr8 100787853-100787863. Max. coverage (+): 0. Max coverage (-): 3.14

Region: chr8 100787864-100787874. Max. coverage (+): 0. Max coverage (-): 3.14

Region: chr8 100787875-100787885. Max. coverage (+): 0. Max coverage (-): 0

Region: chr8 100787886-100787896. Max. coverage (+): 0. Max coverage (-): 0

Region: chr8 100787897-100787907. Max. coverage (+): 0. Max coverage (-): 0

Region: chr8 100787908-100787918. Max. coverage (+): 0. Max coverage (-): 0

Region: chr8 100787919-100787930. Max. coverage (+): 0. Max coverage (-): 0

Region: chr8 100787931-100787941. Max. coverage (+): 0. Max coverage (-): 0

Region: chr8 100787942-100787952. Max. coverage (+): 0. Max coverage (-): 0

Region: chr8 100787953-100787963. Max. coverage (+): 0. Max coverage (-): 0

Region: chr8 100787964-100787974. Max. coverage (+): 0. Max coverage (-): 0

Region: chr8 100787975-100787985. Max. coverage (+): 0. Max coverage (-): 0

Region: chr8 100787986-100787996. Max. coverage (+): 0. Max coverage (-): 0

Region: chr8 100787997-100788007. Max. coverage (+): 0. Max coverage (-): 0

Region: chr8 100788008-100788018. Max. coverage (+): 0. Max coverage (-): 0

Region: chr8 100788019-100788029. Max. coverage (+): 0. Max coverage (-): 0

Region: chr8 100788030-100788040. Max. coverage (+): 0. Max coverage (-): 0

Region: chr8 100788041-100788051. Max. coverage (+): 0. Max coverage (-): 0

Region: chr8 100788052-100788062. Max. coverage (+): 0. Max coverage (-): 0

Region: chr8 100788063-100788073. Max. coverage (+): 0. Max coverage (-): 0

Region: chr8 100788074-100788084. Max. coverage (+): 0. Max coverage (-): 0

Region: chr8 100788085-100788095. Max. coverage (+): 0. Max coverage (-): 0

Region: chr8 100788096-100788106. Max. coverage (+): 0. Max coverage (-): 0

Region: chr8 100788107-100788117. Max. coverage (+): 0. Max coverage (-): 0

Region: chr8 100788118-100788128. Max. coverage (+): 0. Max coverage (-): 0

Region: chr8 100788129-100788139. Max. coverage (+): 0. Max coverage (-): 0

Region: chr8 100788140-100788150. Max. coverage (+): 0. Max coverage (-): 0

Region: chr8 100788151-100788161. Max. coverage (+): 0. Max coverage (-): 0

Region: chr8 100788162-100788172. Max. coverage (+): 0. Max coverage (-): 0

Region: chr8 100788173-100788183. Max. coverage (+): 0. Max coverage (-): 0

Region: chr8 100788184-100788194. Max. coverage (+): 0. Max coverage (-): 0

Region: chr8 100788195-100788205. Max. coverage (+): 0. Max coverage (-): 0

Region: chr8 100788206-100788216. Max. coverage (+): 0. Max coverage (-): 0

Region: chr8 100788217-100788228. Max. coverage (+): 0. Max coverage (-): 0

Region: chr8 100788229-100788239. Max. coverage (+): 0. Max coverage (-): 0

Region: chr8 100788240-100788250. Max. coverage (+): 0. Max coverage (-): 5.35

Region: chr8 100788251-100788261. Max. coverage (+): 0. Max coverage (-): 5.35

Region: chr8 100788262-100788272. Max. coverage (+): 0. Max coverage (-): 0

Region: chr8 100788273-100788283. Max. coverage (+): 0. Max coverage (-): 0

Region: chr8 100788284-100788294. Max. coverage (+): 0. Max coverage (-): 0

Region: chr8 100788295-100788305. Max. coverage (+): 0. Max coverage (-): 0

Region: chr8 100788306-100788316. Max. coverage (+): 0. Max coverage (-): 0

Region: chr8 100788317-100788327. Max. coverage (+): 0. Max coverage (-): 0

Region: chr8 100788328-100788338. Max. coverage (+): 0. Max coverage (-): 0

Region: chr8 100788339-100788349. Max. coverage (+): 0. Max coverage (-): 0

Region: chr8 100788350-100788360. Max. coverage (+): 0. Max coverage (-): 0

Region: chr8 100788361-100788371. Max. coverage (+): 0. Max coverage (-): 0

Region: chr8 100788372-100788382. Max. coverage (+): 0. Max coverage (-): 0

Region: chr8 100788383-100788393. Max. coverage (+): 0. Max coverage (-): 0

Region: chr8 100788394-100788404. Max. coverage (+): 0. Max coverage (-): 0

Region: chr8 100788405-100788415. Max. coverage (+): 0. Max coverage (-): 0

Region: chr8 100788416-100788426. Max. coverage (+): 0. Max coverage (-): 0

Region: chr8 100788427-100788437. Max. coverage (+): 0. Max coverage (-): 0

Region: chr8 100788438-100788448. Max. coverage (+): 0. Max coverage (-): 0

Region: chr8 100788449-100788459. Max. coverage (+): 0. Max coverage (-): 0

Region: chr8 100788460-100788470. Max. coverage (+): 0. Max coverage (-): 6.15

Region: chr8 100788471-100788481. Max. coverage (+): 0. Max coverage (-): 0

Region: chr8 100788482-100788492. Max. coverage (+): 0. Max coverage (-): 0

Region: chr8 100788493-100788503. Max. coverage (+): 0. Max coverage (-): 0

Region: chr8 100788504-100788515. Max. coverage (+): 0. Max coverage (-): 0

Region: chr8 100788516-100788526. Max. coverage (+): 0. Max coverage (-): 0

Region: chr8 100788527-100788537. Max. coverage (+): 0. Max coverage (-): 0

Region: chr8 100788538-100788548. Max. coverage (+): 0. Max coverage (-): 0

Region: chr8 100788549-100788559. Max. coverage (+): 0. Max coverage (-): 0

Region: chr8 100788560-100788570. Max. coverage (+): 0. Max coverage (-): 0

Region: chr8 100788571-100788581. Max. coverage (+): 0. Max coverage (-): 0

Region: chr8 100788582-100788592. Max. coverage (+): 0. Max coverage (-): 0

Region: chr8 100788593-100788603. Max. coverage (+): 0. Max coverage (-): 0

Region: chr8 100788604-100788614. Max. coverage (+): 0. Max coverage (-): 0

Region: chr8 100788615-100788625. Max. coverage (+): 0. Max coverage (-): 0

Region: chr8 100788626-100788636. Max. coverage (+): 0. Max coverage (-): 0

Region: chr8 100788637-100788647. Max. coverage (+): 0. Max coverage (-): 0

Region: chr8 100788648-100788658. Max. coverage (+): 0. Max coverage (-): 0

Region: chr8 100788659-100788669. Max. coverage (+): 0. Max coverage (-): 0

Region: chr8 100788670-100788680. Max. coverage (+): 0. Max coverage (-): 0

Region: chr8 100788681-100788691. Max. coverage (+): 0. Max coverage (-): 0

Region: chr8 100788692-100788702. Max. coverage (+): 0. Max coverage (-): 0

Region: chr8 100788703-100788713. Max. coverage (+): 0. Max coverage (-): 0

Region: chr8 100788714-100788724. Max. coverage (+): 0. Max coverage (-): 0

Region: chr8 100788725-100788735. Max. coverage (+): 0. Max coverage (-): 0

Region: chr8 100788736-100788746. Max. coverage (+): 0. Max coverage (-): 0

Region: chr8 100788747-100788757. Max. coverage (+): 0. Max coverage (-): 0

Region: chr8 100788758-100788768. Max. coverage (+): 0. Max coverage (-): 0

Region: chr8 100788769-100788779. Max. coverage (+): 0. Max coverage (-): 0

Region: chr8 100788780-100788790. Max. coverage (+): 0. Max coverage (-): 0

Region: chr8 100788791-100788802. Max. coverage (+): 0. Max coverage (-): 0

Region: chr8 100788803-100788813. Max. coverage (+): 0. Max coverage (-): 0

Region: chr8 100788814-100788824. Max. coverage (+): 0. Max coverage (-): 0

Region: chr8 100788825-100788835. Max. coverage (+): 0. Max coverage (-): 0

Region: chr8 100788836-100788846. Max. coverage (+): 0. Max coverage (-): 0

Region: chr8 100788847-100788857. Max. coverage (+): 0. Max coverage (-): 0

Region: chr8 100788858-100788868. Max. coverage (+): 0. Max coverage (-): 0

Region: chr8 100788869-100788879. Max. coverage (+): 0. Max coverage (-): 0

Region: chr8 100788880-100788890. Max. coverage (+): 0. Max coverage (-): 0

Region: chr8 100788891-100788901. Max. coverage (+): 0. Max coverage (-): 0

Region: chr8 100788902-100788912. Max. coverage (+): 0. Max coverage (-): 0

Region: chr8 100788913-100788923. Max. coverage (+): 0. Max coverage (-): 0

Region: chr8 100788924-100788934. Max. coverage (+): 0. Max coverage (-): 0

Region: chr8 100788935-100788945. Max. coverage (+): 0. Max coverage (-): 0

Region: chr8 100788946-100788956. Max. coverage (+): 0. Max coverage (-): 0

Region: chr8 100788957-100788967. Max. coverage (+): 0. Max coverage (-): 0

Region: chr8 100788968-100788978. Max. coverage (+): 0. Max coverage (-): 0

Region: chr8 100788979-100788989. Max. coverage (+): 0. Max coverage (-): 0

Region: chr8 100788990-100789000. Max. coverage (+): 0. Max coverage (-): 0

Region: chr8 100789001-100789011. Max. coverage (+): 0. Max coverage (-): 0

Region: chr8 100789012-100789022. Max. coverage (+): 0. Max coverage (-): 0

Region: chr8 100789023-100789033. Max. coverage (+): 0. Max coverage (-): 0

Region: chr8 100789034-100789044. Max. coverage (+): 0. Max coverage (-): 0

Region: chr8 100789045-100789055. Max. coverage (+): 0. Max coverage (-): 0

Region: chr8 100789056-100789066. Max. coverage (+): 0. Max coverage (-): 0

Region: chr8 100789067-100789077. Max. coverage (+): 0. Max coverage (-): 0

Region: chr8 100789078-100789088. Max. coverage (+): 0. Max coverage (-): 0

Region: chr8 100789089-100789100. Max. coverage (+): 0. Max coverage (-): 0

Region: chr8 100789101-100789111. Max. coverage (+): 0. Max coverage (-): 0

Region: chr8 100789112-100789122. Max. coverage (+): 0. Max coverage (-): 0

Region: chr8 100789123-100789133. Max. coverage (+): 0. Max coverage (-): 0

Region: chr8 100789134-100789144. Max. coverage (+): 0. Max coverage (-): 0

Region: chr8 100789145-100789155. Max. coverage (+): 0. Max coverage (-): 0

Region: chr8 100789156-100789166. Max. coverage (+): 0. Max coverage (-): 0

Region: chr8 100789167-100789177. Max. coverage (+): 0. Max coverage (-): 0

Region: chr8 100789178-100789188. Max. coverage (+): 0. Max coverage (-): 0

Region: chr8 100789189-100789199. Max. coverage (+): 0. Max coverage (-): 0

Region: chr8 100789200-100789210. Max. coverage (+): 0. Max coverage (-): 10.98

Region: chr8 100789211-100789221. Max. coverage (+): 0. Max coverage (-): 0

Region: chr8 100789222-100789232. Max. coverage (+): 0. Max coverage (-): 0

Region: chr8 100789233-100789243. Max. coverage (+): 0. Max coverage (-): 0

Region: chr8 100789244-100789254. Max. coverage (+): 0. Max coverage (-): 0

Region: chr8 100789255-100789265. Max. coverage (+): 0. Max coverage (-): 0

Region: chr8 100789266-100789276. Max. coverage (+): 0. Max coverage (-): 0

Region: chr8 100789277-100789287. Max. coverage (+): 0. Max coverage (-): 6.72

Region: chr8 100789288-100789298. Max. coverage (+): 0. Max coverage (-): 6.72

Region: chr8 100789299-100789309. Max. coverage (+): 0. Max coverage (-): 0

Region: chr8 100789310-100789320. Max. coverage (+): 0. Max coverage (-): 0

Region: chr8 100789321-100789331. Max. coverage (+): 0. Max coverage (-): 0

Region: chr8 100789332-100789342. Max. coverage (+): 0. Max coverage (-): 0

Region: chr8 100789343-100789353. Max. coverage (+): 0. Max coverage (-): 0

Region: chr8 100789354-100789364. Max. coverage (+): 0. Max coverage (-): 0

Region: chr8 100789365-100789375. Max. coverage (+): 0. Max coverage (-): 0

Region: chr8 100789376-100789387. Max. coverage (+): 0. Max coverage (-): 0

Region: chr8 100789388-100789398. Max. coverage (+): 0. Max coverage (-): 0

Region: chr8 100789399-100789409. Max. coverage (+): 0. Max coverage (-): 0

Region: chr8 100789410-100789420. Max. coverage (+): 0. Max coverage (-): 0

Region: chr8 100789421-100789431. Max. coverage (+): 0. Max coverage (-): 0

Region: chr8 100789432-100789442. Max. coverage (+): 0. Max coverage (-): 0

Region: chr8 100789443-100789453. Max. coverage (+): 0. Max coverage (-): 0

Region: chr8 100789454-100789464. Max. coverage (+): 0. Max coverage (-): 0

Region: chr8 100789465-100789475. Max. coverage (+): 0. Max coverage (-): 0

Region: chr8 100789476-100789486. Max. coverage (+): 0. Max coverage (-): 0

Region: chr8 100789487-100789497. Max. coverage (+): 0. Max coverage (-): 0

Region: chr8 100789498-100789508. Max. coverage (+): 0. Max coverage (-): 0

Region: chr8 100789509-100789519. Max. coverage (+): 0. Max coverage (-): 0

Region: chr8 100789520-100789530. Max. coverage (+): 0. Max coverage (-): 0

Region: chr8 100789531-100789541. Max. coverage (+): 0. Max coverage (-): 0

Region: chr8 100789542-100789552. Max. coverage (+): 0. Max coverage (-): 0

Region: chr8 100789553-100789563. Max. coverage (+): 0. Max coverage (-): 0

Region: chr8 100789564-100789574. Max. coverage (+): 0. Max coverage (-): 0

Region: chr8 100789575-100789585. Max. coverage (+): 0. Max coverage (-): 0

Region: chr8 100789586-100789596. Max. coverage (+): 0. Max coverage (-): 0

Region: chr8 100789597-100789607. Max. coverage (+): 0. Max coverage (-): 0

Region: chr8 100789608-100789618. Max. coverage (+): 0. Max coverage (-): 0

Region: chr8 100789619-100789629. Max. coverage (+): 0. Max coverage (-): 0

Region: chr8 100789630-100789640. Max. coverage (+): 0. Max coverage (-): 0

Region: chr8 100789641-100789651. Max. coverage (+): 0. Max coverage (-): 0

Region: chr8 100789652-100789662. Max. coverage (+): 0. Max coverage (-): 0

Region: chr8 100789663-100789674. Max. coverage (+): 0. Max coverage (-): 0

Region: chr8 100789675-100789685. Max. coverage (+): 0. Max coverage (-): 0

Region: chr8 100789686-100789696. Max. coverage (+): 0. Max coverage (-): 0

Region: chr8 100789697-100789707. Max. coverage (+): 0. Max coverage (-): 0

Region: chr8 100789708-100789718. Max. coverage (+): 0. Max coverage (-): 0

Region: chr8 100789719-100789729. Max. coverage (+): 0. Max coverage (-): 2.28

Region: chr8 100789730-100789740. Max. coverage (+): 0. Max coverage (-): 2.28

Region: chr8 100789741-100789751. Max. coverage (+): 0. Max coverage (-): 0

Region: chr8 100789752-100789762. Max. coverage (+): 0. Max coverage (-): 0

Region: chr8 100789763-100789773. Max. coverage (+): 0. Max coverage (-): 0

Region: chr8 100789774-100789784. Max. coverage (+): 0. Max coverage (-): 0

Region: chr8 100789785-100789795. Max. coverage (+): 0. Max coverage (-): 0

Region: chr8 100789796-100789806. Max. coverage (+): 0. Max coverage (-): 0

Region: chr8 100789807-100789817. Max. coverage (+): 0. Max coverage (-): 0

Region: chr8 100789818-100789828. Max. coverage (+): 0. Max coverage (-): 0

Region: chr8 100789829-100789839. Max. coverage (+): 0. Max coverage (-): 0

Region: chr8 100789840-100789850. Max. coverage (+): 0. Max coverage (-): 0

Region: chr8 100789851-100789861. Max. coverage (+): 0. Max coverage (-): 0

Region: chr8 100789862-100789872. Max. coverage (+): 0. Max coverage (-): 0

Region: chr8 100789873-100789883. Max. coverage (+): 0. Max coverage (-): 0

Region: chr8 100789884-100789894. Max. coverage (+): 0. Max coverage (-): 0

Region: chr8 100789895-100789905. Max. coverage (+): 0. Max coverage (-): 0

Region: chr8 100789906-100789916. Max. coverage (+): 0. Max coverage (-): 0

Region: chr8 100789917-100789927. Max. coverage (+): 0. Max coverage (-): 0

Region: chr8 100789928-100789938. Max. coverage (+): 0. Max coverage (-): 0

Region: chr8 100789939-100789949. Max. coverage (+): 0. Max coverage (-): 0

Region: chr8 100789950-100789960. Max. coverage (+): 0. Max coverage (-): 0

Region: chr8 100789961-100789972. Max. coverage (+): 0. Max coverage (-): 0

Region: chr8 100789973-100789983. Max. coverage (+): 0. Max coverage (-): 0

Region: chr8 100789984-100789994. Max. coverage (+): 0. Max coverage (-): 0

Region: chr8 100789995-100790005. Max. coverage (+): 0. Max coverage (-): 0

Region: chr8 100790006-100790016. Max. coverage (+): 0. Max coverage (-): 0

Region: chr8 100790017-100790027. Max. coverage (+): 0. Max coverage (-): 0

Region: chr8 100790028-100790038. Max. coverage (+): 0. Max coverage (-): 0

Region: chr8 100790039-100790049. Max. coverage (+): 0. Max coverage (-): 0

Region: chr8 100790050-100790060. Max. coverage (+): 0. Max coverage (-): 0

Region: chr8 100790061-100790071. Max. coverage (+): 0. Max coverage (-): 0

Region: chr8 100790072-100790082. Max. coverage (+): 0. Max coverage (-): 0

Region: chr8 100790083-100790093. Max. coverage (+): 0. Max coverage (-): 0

Region: chr8 100790094-100790104. Max. coverage (+): 0. Max coverage (-): 0

Region: chr8 100790105-100790115. Max. coverage (+): 0. Max coverage (-): 1.17

Region: chr8 100790116-100790126. Max. coverage (+): 0. Max coverage (-): 0

Region: chr8 100790127-100790137. Max. coverage (+): 0. Max coverage (-): 6.24

Region: chr8 100790138-100790148. Max. coverage (+): 0. Max coverage (-): 0

Region: chr8 100790149-100790159. Max. coverage (+): 0. Max coverage (-): 0

Region: chr8 100790160-100790170. Max. coverage (+): 0. Max coverage (-): 0

Region: chr8 100790171-100790181. Max. coverage (+): 0. Max coverage (-): 0

Region: chr8 100790182-100790192. Max. coverage (+): 0. Max coverage (-): 0

Region: chr8 100790193-100790203. Max. coverage (+): 0. Max coverage (-): 0

Region: chr8 100790204-100790214. Max. coverage (+): 0. Max coverage (-): 3.16

Region: chr8 100790215-100790225. Max. coverage (+): 0. Max coverage (-): 3.16

Region: chr8 100790226-100790236. Max. coverage (+): 0. Max coverage (-): 0

Region: chr8 100790237-100790247. Max. coverage (+): 0. Max coverage (-): 0

Region: chr8 100790248-100790259. Max. coverage (+): 0. Max coverage (-): 6.46

Region: chr8 100790260-100790270. Max. coverage (+): 0. Max coverage (-): 8.39

Region: chr8 100790271-100790281. Max. coverage (+): 0. Max coverage (-): 6.82

Region: chr8 100790282-100790292. Max. coverage (+): 0. Max coverage (-): 0

Region: chr8 100790293-100790303. Max. coverage (+): 0. Max coverage (-): 0

Region: chr8 100790304-100790314. Max. coverage (+): 0. Max coverage (-): 0

Region: chr8 100790315-100790325. Max. coverage (+): 0. Max coverage (-): 0

Region: chr8 100790326-100790336. Max. coverage (+): 0. Max coverage (-): 0

Region: chr8 100790337-100790347. Max. coverage (+): 0. Max coverage (-): 14.43

Region: chr8 100790348-100790358. Max. coverage (+): 0. Max coverage (-): 7.66

Region: chr8 100790359-100790369. Max. coverage (+): 0. Max coverage (-): 0

Region: chr8 100790370-100790380. Max. coverage (+): 0. Max coverage (-): 0

Region: chr8 100790381-100790391. Max. coverage (+): 0. Max coverage (-): 0

Region: chr8 100790392-100790402. Max. coverage (+): 0. Max coverage (-): 0

Region: chr8 100790403-100790413. Max. coverage (+): 0. Max coverage (-): 0

Region: chr8 100790414-100790424. Max. coverage (+): 0. Max coverage (-): 0

Region: chr8 100790425-100790435. Max. coverage (+): 0. Max coverage (-): 0

Region: chr8 100790436-100790446. Max. coverage (+): 0. Max coverage (-): 0

Region: chr8 100790447-100790457. Max. coverage (+): 0. Max coverage (-): 0

Region: chr8 100790458-100790468. Max. coverage (+): 0. Max coverage (-): 0

Region: chr8 100790469-100790479. Max. coverage (+): 0. Max coverage (-): 0

Region: chr8 100790480-100790490. Max. coverage (+): 0. Max coverage (-): 0

Region: chr8 100790491-100790501. Max. coverage (+): 0. Max coverage (-): 0

Region: chr8 100790502-100790512. Max. coverage (+): 0. Max coverage (-): 0

Region: chr8 100790513-100790523. Max. coverage (+): 0. Max coverage (-): 0

Region: chr8 100790524-100790534. Max. coverage (+): 0. Max coverage (-): 0

Region: chr8 100790535-100790546. Max. coverage (+): 0. Max coverage (-): 0

Region: chr8 100790547-100790557. Max. coverage (+): 0. Max coverage (-): 0

Region: chr8 100790558-100790568. Max. coverage (+): 0. Max coverage (-): 0

Region: chr8 100790569-100790579. Max. coverage (+): 0. Max coverage (-): 0

Region: chr8 100790580-100790590. Max. coverage (+): 0. Max coverage (-): 0

Region: chr8 100790591-100790601. Max. coverage (+): 0. Max coverage (-): 0

Region: chr8 100790602-100790612. Max. coverage (+): 0. Max coverage (-): 0

Region: chr8 100790613-100790623. Max. coverage (+): 0. Max coverage (-): 0

Region: chr8 100790624-100790634. Max. coverage (+): 0. Max coverage (-): 0

Region: chr8 100790635-100790645. Max. coverage (+): 0. Max coverage (-): 0

Region: chr8 100790646-100790656. Max. coverage (+): 0. Max coverage (-): 0

Region: chr8 100790657-100790667. Max. coverage (+): 0. Max coverage (-): 0

Region: chr8 100790668-100790678. Max. coverage (+): 0. Max coverage (-): 0

Region: chr8 100790679-100790689. Max. coverage (+): 0. Max coverage (-): 0

Region: chr8 100790690-100790700. Max. coverage (+): 0. Max coverage (-): 0

Region: chr8 100790701-100790711. Max. coverage (+): 0. Max coverage (-): 0

Region: chr8 100790712-100790722. Max. coverage (+): 0. Max coverage (-): 0

Region: chr8 100790723-100790733. Max. coverage (+): 0. Max coverage (-): 0

Region: chr8 100790734-100790744. Max. coverage (+): 0. Max coverage (-): 0

Region: chr8 100790745-100790755. Max. coverage (+): 0. Max coverage (-): 0

Region: chr8 100790756-100790766. Max. coverage (+): 0. Max coverage (-): 0

Region: chr8 100790767-100790777. Max. coverage (+): 0. Max coverage (-): 0

Region: chr8 100790778-100790788. Max. coverage (+): 0. Max coverage (-): 0

Region: chr8 100790789-100790799. Max. coverage (+): 0. Max coverage (-): 0

Region: chr8 100790800-100790810. Max. coverage (+): 0. Max coverage (-): 0

Region: chr8 100790811-100790821. Max. coverage (+): 0. Max coverage (-): 0

Region: chr8 100790822-100790832. Max. coverage (+): 0. Max coverage (-): 0

Region: chr8 100790833-100790844. Max. coverage (+): 0. Max coverage (-): 0

Region: chr8 100790845-100790855. Max. coverage (+): 0. Max coverage (-): 0

Region: chr8 100790856-100790866. Max. coverage (+): 0. Max coverage (-): 0

Region: chr8 100790867-100790877. Max. coverage (+): 0. Max coverage (-): 0

Region: chr8 100790878-100790888. Max. coverage (+): 0. Max coverage (-): 0

Region: chr8 100790889-100790899. Max. coverage (+): 0. Max coverage (-): 0

Region: chr8 100790900-100790910. Max. coverage (+): 0. Max coverage (-): 0

Region: chr8 100790911-100790921. Max. coverage (+): 0. Max coverage (-): 0

Region: chr8 100790922-100790932. Max. coverage (+): 0. Max coverage (-): 0

Region: chr8 100790933-100790943. Max. coverage (+): 0. Max coverage (-): 0

Region: chr8 100790944-100790954. Max. coverage (+): 0. Max coverage (-): 0

Region: chr8 100790955-100790965. Max. coverage (+): 0. Max coverage (-): 0

Region: chr8 100790966-100790976. Max. coverage (+): 0. Max coverage (-): 0

Region: chr8 100790977-100790987. Max. coverage (+): 0. Max coverage (-): 0

Region: chr8 100790988-100790998. Max. coverage (+): 0. Max coverage (-): 0

Region: chr8 100790999-100791009. Max. coverage (+): 0. Max coverage (-): 0

Region: chr8 100791010-100791020. Max. coverage (+): 0. Max coverage (-): 0

Region: chr8 100791021-100791031. Max. coverage (+): 0. Max coverage (-): 0

Region: chr8 100791032-100791042. Max. coverage (+): 0. Max coverage (-): 0

Region: chr8 100791043-100791053. Max. coverage (+): 0. Max coverage (-): 0

Region: chr8 100791054-100791064. Max. coverage (+): 0. Max coverage (-): 0

Region: chr8 100791065-100791075. Max. coverage (+): 0. Max coverage (-): 0

Region: chr8 100791076-100791086. Max. coverage (+): 0. Max coverage (-): 0

Region: chr8 100791087-100791097. Max. coverage (+): 0. Max coverage (-): 0

Region: chr8 100791098-100791108. Max. coverage (+): 0. Max coverage (-): 0

Region: chr8 100791109-100791119. Max. coverage (+): 0. Max coverage (-): 0

Region: chr8 100791120-100791131. Max. coverage (+): 0. Max coverage (-): 4.77

Region: chr8 100791132-100791142. Max. coverage (+): 0. Max coverage (-): 4.77

Region: chr8 100791143-100791153. Max. coverage (+): 0. Max coverage (-): 0

Region: chr8 100791154-100791164. Max. coverage (+): 0. Max coverage (-): 0

Region: chr8 100791165-100791175. Max. coverage (+): 0. Max coverage (-): 0

Region: chr8 100791176-100791186. Max. coverage (+): 0. Max coverage (-): 0

Region: chr8 100791187-100791197. Max. coverage (+): 0. Max coverage (-): 0

Region: chr8 100791198-100791208. Max. coverage (+): 0. Max coverage (-): 0

Region: chr8 100791209-100791219. Max. coverage (+): 0. Max coverage (-): 0

Region: chr8 100791220-100791230. Max. coverage (+): 0. Max coverage (-): 0

Region: chr8 100791231-100791241. Max. coverage (+): 0. Max coverage (-): 0

Region: chr8 100791242-100791252. Max. coverage (+): 0. Max coverage (-): 0

Region: chr8 100791253-100791263. Max. coverage (+): 0. Max coverage (-): 0

Region: chr8 100791264-100791274. Max. coverage (+): 0. Max coverage (-): 0

Region: chr8 100791275-100791285. Max. coverage (+): 0. Max coverage (-): 6.72

Region: chr8 100791286-100791296. Max. coverage (+): 0. Max coverage (-): 6.72

Region: chr8 100791297-100791307. Max. coverage (+): 0. Max coverage (-): 0

Region: chr8 100791308-100791318. Max. coverage (+): 0. Max coverage (-): 0

Region: chr8 100791319-100791329. Max. coverage (+): 0. Max coverage (-): 0

Region: chr8 100791330-100791340. Max. coverage (+): 0. Max coverage (-): 0

Region: chr8 100791341-100791351. Max. coverage (+): 0. Max coverage (-): 0

Region: chr8 100791352-100791362. Max. coverage (+): 0. Max coverage (-): 0

Region: chr8 100791363-100791373. Max. coverage (+): 0. Max coverage (-): 0

Region: chr8 100791374-100791384. Max. coverage (+): 0. Max coverage (-): 0

Region: chr8 100791385-100791395. Max. coverage (+): 0. Max coverage (-): 0

Region: chr8 100791396-100791406. Max. coverage (+): 0. Max coverage (-): 0

Region: chr8 100791407-100791418. Max. coverage (+): 0. Max coverage (-): 5.11

Region: chr8 100791419-100791429. Max. coverage (+): 0. Max coverage (-): 0

Region: chr8 100791430-100791440. Max. coverage (+): 0. Max coverage (-): 0

Region: chr8 100791441-100791451. Max. coverage (+): 0. Max coverage (-): 0

Region: chr8 100791452-100791462. Max. coverage (+): 0. Max coverage (-): 0

Region: chr8 100791463-100791473. Max. coverage (+): 0. Max coverage (-): 0

Region: chr8 100791474-100791484. Max. coverage (+): 0. Max coverage (-): 0

Region: chr8 100791485-100791495. Max. coverage (+): 0. Max coverage (-): 0

Region: chr8 100791496-100791506. Max. coverage (+): 0. Max coverage (-): 0

Region: chr8 100791507-100791517. Max. coverage (+): 0. Max coverage (-): 0.26

Region: chr8 100791518-100791528. Max. coverage (+): 0. Max coverage (-): 0.26

Region: chr8 100791529-100791539. Max. coverage (+): 0. Max coverage (-): 0

Region: chr8 100791540-100791550. Max. coverage (+): 0. Max coverage (-): 8.14

Region: chr8 100791551-100791561. Max. coverage (+): 0. Max coverage (-): 8.14

Region: chr8 100791562-100791572. Max. coverage (+): 0. Max coverage (-): 0

Region: chr8 100791573-100791583. Max. coverage (+): 0. Max coverage (-): 0

Region: chr8 100791584-100791594. Max. coverage (+): 0. Max coverage (-): 0

Region: chr8 100791595-100791605. Max. coverage (+): 0. Max coverage (-): 0

Region: chr8 100791606-100791616. Max. coverage (+): 0. Max coverage (-): 0

Region: chr8 100791617-100791627. Max. coverage (+): 0. Max coverage (-): 0

Region: chr8 100791628-100791638. Max. coverage (+): 0. Max coverage (-): 0

Region: chr8 100791639-100791649. Max. coverage (+): 0. Max coverage (-): 0

Region: chr8 100791650-100791660. Max. coverage (+): 0. Max coverage (-): 0

Region: chr8 100791661-100791671. Max. coverage (+): 0. Max coverage (-): 2.41

Region: chr8 100791672-100791682. Max. coverage (+): 0. Max coverage (-): 0

Region: chr8 100791683-100791693. Max. coverage (+): 0. Max coverage (-): 2.12

Region: chr8 100791694-100791704. Max. coverage (+): 0. Max coverage (-): 8.2

Region: chr8 100791705-100791716. Max. coverage (+): 0. Max coverage (-): 8.2

Region: chr8 100791717-100791727. Max. coverage (+): 0. Max coverage (-): 13.14

Region: chr8 100791728-100791738. Max. coverage (+): 0. Max coverage (-): 3.83

Region: chr8 100791739-100791749. Max. coverage (+): 0. Max coverage (-): 0

Region: chr8 100791750-100791760. Max. coverage (+): 0. Max coverage (-): 10.18

Region: chr8 100791761-100791771. Max. coverage (+): 0. Max coverage (-): 10.18

Region: chr8 100791772-100791782. Max. coverage (+): 0. Max coverage (-): 0

Region: chr8 100791783-100791793. Max. coverage (+): 0. Max coverage (-): 0

Region: chr8 100791794-100791804. Max. coverage (+): 0. Max coverage (-): 0

Region: chr8 100791805-100791815. Max. coverage (+): 0. Max coverage (-): 12.1

Region: chr8 100791816-100791826. Max. coverage (+): 0. Max coverage (-): 7.88

Region: chr8 100791827-100791837. Max. coverage (+): 0. Max coverage (-): 0

Region: chr8 100791838-100791848. Max. coverage (+): 0. Max coverage (-): 0

Region: chr8 100791849-100791859. Max. coverage (+): 0. Max coverage (-): 2.13

Region: chr8 100791860-100791870. Max. coverage (+): 0. Max coverage (-): 2.13

Region: chr8 100791871-100791881. Max. coverage (+): 0. Max coverage (-): 4.52

Region: chr8 100791882-100791892. Max. coverage (+): 0. Max coverage (-): 0.27

Region: chr8 100791893-100791903. Max. coverage (+): 0. Max coverage (-): 5.87

Region: chr8 100791904-100791914. Max. coverage (+): 0. Max coverage (-): 13.06

Region: chr8 100791915-100791925. Max. coverage (+): 0. Max coverage (-): 7.18

Region: chr8 100791926-100791936. Max. coverage (+): 0. Max coverage (-): 2.33

Region: chr8 100791937-100791947. Max. coverage (+): 0. Max coverage (-): 6.93

Region: chr8 100791948-100791958. Max. coverage (+): 0. Max coverage (-): 11.17

Region: chr8 100791959-100791969. Max. coverage (+): 0. Max coverage (-): 0.76

Region: chr8 100791970-100791980. Max. coverage (+): 0. Max coverage (-): 0.41

Region: chr8 100791981-100791991. Max. coverage (+): 0. Max coverage (-): 8.31

Region: chr8 100791992-100792003. Max. coverage (+): 0. Max coverage (-): 6.89

Region: chr8 100792004-100792014. Max. coverage (+): 0. Max coverage (-): 5.82

Region: chr8 100792015-100792025. Max. coverage (+): 0. Max coverage (-): 5.82

Region: chr8 100792026-100792036. Max. coverage (+): 0. Max coverage (-): 0

Region: chr8 100792037-100792047. Max. coverage (+): 0. Max coverage (-): 0

Region: chr8 100792048-100792058. Max. coverage (+): 0. Max coverage (-): 3.12

Region: chr8 100792059-100792069. Max. coverage (+): 0. Max coverage (-): 3.12

Region: chr8 100792070-100792080. Max. coverage (+): 0. Max coverage (-): 0

Region: chr8 100792081-100792091. Max. coverage (+): 0. Max coverage (-): 0

Region: chr8 100792092-100792102. Max. coverage (+): 0. Max coverage (-): 0.84

Region: chr8 100792103-100792113. Max. coverage (+): 0. Max coverage (-): 0

Region: chr8 100792114-100792124. Max. coverage (+): 0. Max coverage (-): 24.33

Region: chr8 100792125-100792135. Max. coverage (+): 0. Max coverage (-): 31.75

Region: chr8 100792136-100792146. Max. coverage (+): 0. Max coverage (-): 5.5

Region: chr8 100792147-100792157. Max. coverage (+): 0. Max coverage (-): 0

Region: chr8 100792158-100792168. Max. coverage (+): 0. Max coverage (-): 0

Region: chr8 100792169-100792179. Max. coverage (+): 0. Max coverage (-): 0

Region: chr8 100792180-100792190. Max. coverage (+): 0. Max coverage (-): 0

Region: chr8 100792191-100792201. Max. coverage (+): 0. Max coverage (-): 3.74

Region: chr8 100792202-100792212. Max. coverage (+): 0. Max coverage (-): 12.19

Region: chr8 100792213-100792223. Max. coverage (+): 0. Max coverage (-): 8.45

Region: chr8 100792224-100792234. Max. coverage (+): 0. Max coverage (-): 0

Region: chr8 100792235-100792245. Max. coverage (+): 0. Max coverage (-): 25.82

Region: chr8 100792246-100792256. Max. coverage (+): 0. Max coverage (-): 25.82

Region: chr8 100792257-100792267. Max. coverage (+): 0. Max coverage (-): 23.27

Region: chr8 100792268-100792278. Max. coverage (+): 0. Max coverage (-): 0

Region: chr8 100792279-100792290. Max. coverage (+): 0. Max coverage (-): 0

Region: chr8 100792291-100792301. Max. coverage (+): 0. Max coverage (-): 0

Region: chr8 100792302-100792312. Max. coverage (+): 0. Max coverage (-): 0

Region: chr8 100792313-100792323. Max. coverage (+): 0. Max coverage (-): 0

Region: chr8 100792324-100792334. Max. coverage (+): 0. Max coverage (-): 0

Region: chr8 100792335-100792345. Max. coverage (+): 0. Max coverage (-): 0

Region: chr8 100792346-100792356. Max. coverage (+): 0. Max coverage (-): 3.79

Region: chr8 100792357-100792367. Max. coverage (+): 0. Max coverage (-): 14.67

Region: chr8 100792368-100792378. Max. coverage (+): 0. Max coverage (-): 14.67

Region: chr8 100792379-100792389. Max. coverage (+): 0. Max coverage (-): 13.38

Region: chr8 100792390-100792400. Max. coverage (+): 0. Max coverage (-): 8.91

Region: chr8 100792401-100792411. Max. coverage (+): 0. Max coverage (-): 12.43

Region: chr8 100792412-100792422. Max. coverage (+): 0. Max coverage (-): 5.38

Region: chr8 100792423-100792433. Max. coverage (+): 0. Max coverage (-): 5.38

Region: chr8 100792434-100792444. Max. coverage (+): 0. Max coverage (-): 0

Region: chr8 100792445-100792455. Max. coverage (+): 0. Max coverage (-): 0

Region: chr8 100792456-100792466. Max. coverage (+): 0. Max coverage (-): 0

Region: chr8 100792467-100792477. Max. coverage (+): 0. Max coverage (-): 0

Region: chr8 100792478-100792488. Max. coverage (+): 0. Max coverage (-): 0

Region: chr8 100792489-100792499. Max. coverage (+): 0. Max coverage (-): 0

Region: chr8 100792500-100792510. Max. coverage (+): 0. Max coverage (-): 0

Region: chr8 100792511-100792521. Max. coverage (+): 0. Max coverage (-): 0

Region: chr8 100792522-100792532. Max. coverage (+): 0. Max coverage (-): 2.15

Region: chr8 100792533-100792543. Max. coverage (+): 0. Max coverage (-): 3.16

Region: chr8 100792544-100792554. Max. coverage (+): 0. Max coverage (-): 1.01

Region: chr8 100792555-100792565. Max. coverage (+): 0. Max coverage (-): 0

Region: chr8 100792566-100792576. Max. coverage (+): 0. Max coverage (-): 0

Region: chr8 100792577-100792588. Max. coverage (+): 0. Max coverage (-): 0

Region: chr8 100792589-100792599. Max. coverage (+): 0. Max coverage (-): 2.01

Region: chr8 100792600-100792610. Max. coverage (+): 0. Max coverage (-): 18.62

Region: chr8 100792611-100792621. Max. coverage (+): 0. Max coverage (-): 18.62

Region: chr8 100792622-100792632. Max. coverage (+): 0. Max coverage (-): 6.46

Region: chr8 100792633-100792643. Max. coverage (+): 0. Max coverage (-): 0

Region: chr8 100792644-100792654. Max. coverage (+): 0. Max coverage (-): 0

Region: chr8 100792655-100792665. Max. coverage (+): 0. Max coverage (-): 0

Region: chr8 100792666-100792676. Max. coverage (+): 0. Max coverage (-): 6

Region: chr8 100792677-100792687. Max. coverage (+): 0. Max coverage (-): 6.7

Region: chr8 100792688-100792698. Max. coverage (+): 0. Max coverage (-): 6.7

Region: chr8 100792699-100792709. Max. coverage (+): 0. Max coverage (-): 0

Region: chr8 100792710-100792720. Max. coverage (+): 0. Max coverage (-): 0

Region: chr8 100792721-100792731. Max. coverage (+): 0. Max coverage (-): 0

Region: chr8 100792732-100792742. Max. coverage (+): 0. Max coverage (-): 0

Region: chr8 100792743-100792753. Max. coverage (+): 0. Max coverage (-): 5.99

Region: chr8 100792754-100792764. Max. coverage (+): 0. Max coverage (-): 5.99

Region: chr8 100792765-100792775. Max. coverage (+): 0. Max coverage (-): 0

Region: chr8 100792776-100792786. Max. coverage (+): 0. Max coverage (-): 0

Region: chr8 100792787-100792797. Max. coverage (+): 0. Max coverage (-): 0

Region: chr8 100792798-100792808. Max. coverage (+): 0. Max coverage (-): 0

Region: chr8 100792809-100792819. Max. coverage (+): 0. Max coverage (-): 6.29

Region: chr8 100792820-100792830. Max. coverage (+): 0. Max coverage (-): 43.31

Region: chr8 100792831-100792841. Max. coverage (+): 0. Max coverage (-): 41.15

Region: chr8 100792842-100792852. Max. coverage (+): 0. Max coverage (-): 0

Region: chr8 100792853-100792863. Max. coverage (+): 0. Max coverage (-): 0

Region: chr8 100792864-100792875. Max. coverage (+): 0. Max coverage (-): 0

Region: chr8 100792876-100792886. Max. coverage (+): 0. Max coverage (-): 0

Region: chr8 100792887-100792897. Max. coverage (+): 0. Max coverage (-): 0

Region: chr8 100792898-100792908. Max. coverage (+): 0. Max coverage (-): 0

Region: chr8 100792909-100792919. Max. coverage (+): 0. Max coverage (-): 0

Region: chr8 100792920-100792930. Max. coverage (+): 0. Max coverage (-): 0

Region: chr8 100792931-100792941. Max. coverage (+): 0. Max coverage (-): 3.01

Region: chr8 100792942-100792952. Max. coverage (+): 0. Max coverage (-): 3.01

Region: chr8 100792953-100792963. Max. coverage (+): 0. Max coverage (-): 0

Region: chr8 100792964-100792974. Max. coverage (+): 0. Max coverage (-): 8.14

Region: chr8 100792975-100792985. Max. coverage (+): 0. Max coverage (-): 8.14

Region: chr8 100792986-100792996. Max. coverage (+): 0. Max coverage (-): 0.73

Region: chr8 100792997-100793007. Max. coverage (+): 0. Max coverage (-): 0

Region: chr8 100793008-. Max. coverage (+): 0. Max coverage (-): 0

RepeatMasker Color Code

**+**

100-98% Identity

<98-95% Identity

<95-90% Identity

<90-85% Identity

<85-80% Identity

<80-75% Identity

<75-70% Identity

<70% Identity

**-**

Gene Set Color Code

**+**

Gene

Pseudogene

**-**

Topology/Coverage Color Code

Coverage Plus Strand

Coverage Minus Strand

Mainstrand: Plus

Mainstrand: Minus

Complementary Strand

Flanking Region  
(if option -flank >0)

Gene Set Annotation  
  
RepeatMasker Annotation  

**1. AT\_rich**: 100787756-100787806 (+), Divergence to consensus: 80.4%  
**2. MIR**: 100788857-100789011 (-), Divergence to consensus: 47.9%  
**3. MIRc**: 100789040-100789137 (-), Divergence to consensus: 45.6%  
**4. L2a**: 100789352-100789637 (+), Divergence to consensus: 45.1%  
**5. L1MC4a**: 100789816-100790370 (-), Divergence to consensus: 53.6%  
**6. LTR68**: 100790374-100790531 (-), Divergence to consensus: 24.7%  
**7. (CAGTT)n**: 100790532-100790574 (+), Divergence to consensus: 9.4%  
**8. ART2A**: 100790575-100790777 (-), Divergence to consensus: 10.9%  
**9. ART2A**: 100790752-100790826 (-), Divergence to consensus: 22.6%  
**10. LTR68**: 100790819-100791226 (-), Divergence to consensus: 35.2%  
**11. L1MC4a**: 100791448-100791545 (-), Divergence to consensus: 25.2%

  
Transcription Factor Binding Sites  

**SOX9** (Sequence: AACAATAG (-): 100788682)  
**SOX9** (Sequence: AACAATAA (-): 100788839)  
**SOX9** (Sequence: CCATTGTT (+): 100787680)  
**SOX9** (Sequence: TCATTGTT (+): 100789937)
