# Supplementary material for: Small RNA sequencing of cryopreserved semen from single bull revealed altered miRNAs and piRNAs expression between High- and Low-motile sperm populations
Source: BMC Genomics. 2017 Jan 4;18:14. doi: 10.1186/s12864-016-3394-7 (PMC5209821; doi:10.1186/s12864-016-3394-7)
Supplement: Additional file 4: — Details for each piRNA clusters found in Low Motile (LM) sperm fraction. Genes, repeats, transposable elements and transcription factors binding sites falling within the cluster regions were reported. (ZIP 1034 kb) [file 12864_2016_3394_MOESM4_ESM.zip › 51.html]

piRNA cluster 51


Predicted piRNA cluster no. 51     previous   next
  

Show proTRAC run info
Hide proTRAC run info

================================= proTRAC ====================================  
VERSION: 2.1                                    LAST MODIFIED: 06. October 2015  
  
Please cite:  
Rosenkranz D, Zischler H. proTRAC - a software for probabilistic piRNA cluster  
detection, visualization and analysis. 2012. BMC Bioinformatics 13:5.  
  
and (for proTRAC 2.0 and later):  
Rosenkranz D, Rudloff S, Bastuck K, Ketting RF, Zischler H. Tupaia small RNAs  
provide insights into function and evolution of RNAi-based transposon defense  
in mammals. 2015. RNA 21(5):911-922.  
  
Contact:  
David Rosenkranz  
Institute of Anthropology, small RNA group  
Johannes Gutenberg University Mainz  
email: rosenkranz@uni-mainz.de  
  
You can find the latest proTRAC version at:  
http://sourceforge.net/projects/protrac/files  
http://www.smallRNAgroup-mainz.de/software  
==============================================================================  
  
PARAMETERS:  
Map file: .............../storage/core/barbara/genhome/smallRNA/fertility/Sample\_not\_motile/pirna/Sample\_not\_motile\_26-33\_collapsed.fa.no-dust.map.weighted-10000-1000-b-0  
Genome file: ............/storage/core/barbara/genhome/smallRNA/fertility/Sample\_all/pirna/bt\_311\_chrY.fa  
RepeatMasker annotation: /storage/genomes/bt\_umd31/GCF\_000003055.6\_Bos\_taurus\_UMD\_3.1.1\_repeatMasker\_chr.out  
GeneSet:................./storage/core/barbara/genhome/smallRNA/fertility/Sample\_all/pirna/full.gtf  
  
Significant (p<=0.01) hit density will be calculated based  
on observed hit distribution.  
  
Sliding window size: ........................................ 5000 bp  
Sliding window increament: .................................. 1000 bp  
Normalize each hit by number of genomic hits: ............... 1 [0=no/1=yes]  
Normalize each hit by number of sequence reads: ............. 1 [0=no/1=yes]  
Normalize values (-> per million mapped reads): ............. 1 [0=no/1=yes]  
Min. fraction of hits with 1T(U) or 10A: .................... 0.75  
Alternatively: Min. fraction of hits with 1T(U) and 10A: .... 0.5  
Min. fraction of hits with typical piRNA length: ............ 0.75  
Typical piRNA length: ....................................... 26-33 nt  
Min. size of a piRNA cluster: ............................... 5000 bp.  
Min. number of hits (absolute): ............................. 0  
Min. number of hits (normalized): ........................... 0  
Min. fraction of hits on the mainstrand: .................... 0.75  
Top fraction of mapped sequences (in terms of read counts): . 1%  
Top fraction accounts for max. n% of sequence reads: ........ 90%  
Min. fraction of hits on each arm of a bidirectional cluster: 0.1  
Output image file for each cluster: ......................... 0 [0=no/1=yes]  
Output html file for each cluster: .......................... 1 [0=no/1=yes]  
Output a summary table: ..................................... 1 [0=no/1=yes]  
Output a FASTA file for each cluster (piRNA sequences): ..... 1 [0=no/1=yes]  
Output a FASTA file comprising cluster sequences: ........... 1 [0=no/1=yes]  
Search DNA motifs in clusters: .............................. 1 [0=no/1=yes]  
Output flanking sequences: +/- .............................. 0 bp  
Output ~.pTi file: .......................................... 1 [0=no/1=yes]  
==============================================================================  
  
  
Genome size (without gaps): ............ 2678902517 bp  
Gaps (N/X/-): .......................... 53837044 bp  
Mapped reads: .......................... 738059667487  
Non-identical sequences: ............... 277001  
Genomic hits: .......................... 533816  
Significant densitiy of mapped reads: .. 15118061 reads/kb

Show proTRAC cluster info
Hide proTRAC cluster info

|  |  |
| --- | --- |
| Location | chr8 |
| Coordinates | 104027800-104035391 |
| Size [bp] | 7592 |
| Sequence hit loci | 759 |
| Mapped reads (normalized) | 1259684799 |
| Mapped reads (normalized) per kb | 165922655.3 |
| Normalized reads with 1T (1U) | 81% |
| Normalized reads with 10A | 27.3% |
| Normalized reads with length 26-33 nt | 100% |
| Normalized reads on the main strand(s) | 100% |
| Predicted directionality | bi:minus-plus (split between 104031395 and 104031574) |

100%

0%

1T (1U)  
reads

10A reads

26-33 nt  
reads

reads on mainstrand

**Either the amount of reads with 1T (1U) OR 10A has to exceed 75% (set with option: -1Tor10A)  
Alternatively the amount of reads with 1T (1U) AND 10A has to exceed 50% (set with option: -1Tand10A)  
Minimum amount of reads with preferred size is 75% (set with option: -pisize)  
Minimum amount of reads on the main strand(s) is 75% (set with option: -clstrand)**

Show read coverage
Hide read coverage

WHAT DO I SEE HERE?  
This chart shows the location of mapped sequence reads within a predicted piRNA cluster. The color refers to the number of genomic hits produced by the sequence read in question. A dark red bar indicates that this sequence read produces many other hits elsewhere in the genome. Many adjacent red or yellow bars can indicate the presence of a multi-copy element such as transposons or rRNA genes. A dark green bar indicates that this sequence read maps uniquely to this locus.

1 hit

2-5 hits

6-10 hits

11-20 hits

21-50 hits

51-100 hits

> 100 hits

chr8

104027800

104035391

Gene Set

RepeatMasker

Mapped  
Reads

182.09

plus strand

minus strand

182.09

Region: chr8 100793009-104027807. Max. coverage (+): 0. Max coverage (-): 6.3

Region: chr8 104027808-104027822. Max. coverage (+): 0. Max coverage (-): 14.94

Region: chr8 104027823-104027837. Max. coverage (+): 0. Max coverage (-): 14.94

Region: chr8 104027838-104027853. Max. coverage (+): 0. Max coverage (-): 0

Region: chr8 104027854-104027868. Max. coverage (+): 0. Max coverage (-): 0

Region: chr8 104027869-104027883. Max. coverage (+): 0. Max coverage (-): 0

Region: chr8 104027884-104027898. Max. coverage (+): 0. Max coverage (-): 0

Region: chr8 104027899-104027913. Max. coverage (+): 0. Max coverage (-): 0

Region: chr8 104027914-104027929. Max. coverage (+): 0. Max coverage (-): 0

Region: chr8 104027930-104027944. Max. coverage (+): 0. Max coverage (-): 0

Region: chr8 104027945-104027959. Max. coverage (+): 0. Max coverage (-): 0

Region: chr8 104027960-104027974. Max. coverage (+): 0. Max coverage (-): 0

Region: chr8 104027975-104027989. Max. coverage (+): 0. Max coverage (-): 0

Region: chr8 104027990-104028004. Max. coverage (+): 0. Max coverage (-): 0

Region: chr8 104028005-104028020. Max. coverage (+): 0. Max coverage (-): 0

Region: chr8 104028021-104028035. Max. coverage (+): 0. Max coverage (-): 0

Region: chr8 104028036-104028050. Max. coverage (+): 0. Max coverage (-): 0

Region: chr8 104028051-104028065. Max. coverage (+): 0. Max coverage (-): 0

Region: chr8 104028066-104028080. Max. coverage (+): 0. Max coverage (-): 0

Region: chr8 104028081-104028096. Max. coverage (+): 0. Max coverage (-): 0

Region: chr8 104028097-104028111. Max. coverage (+): 0. Max coverage (-): 0

Region: chr8 104028112-104028126. Max. coverage (+): 0. Max coverage (-): 0

Region: chr8 104028127-104028141. Max. coverage (+): 0. Max coverage (-): 0

Region: chr8 104028142-104028156. Max. coverage (+): 0. Max coverage (-): 0

Region: chr8 104028157-104028172. Max. coverage (+): 0. Max coverage (-): 0

Region: chr8 104028173-104028187. Max. coverage (+): 0. Max coverage (-): 0

Region: chr8 104028188-104028202. Max. coverage (+): 0. Max coverage (-): 0

Region: chr8 104028203-104028217. Max. coverage (+): 0. Max coverage (-): 1.56

Region: chr8 104028218-104028232. Max. coverage (+): 0. Max coverage (-): 1.56

Region: chr8 104028233-104028247. Max. coverage (+): 0. Max coverage (-): 0

Region: chr8 104028248-104028263. Max. coverage (+): 0. Max coverage (-): 33.84

Region: chr8 104028264-104028278. Max. coverage (+): 0. Max coverage (-): 4.02

Region: chr8 104028279-104028293. Max. coverage (+): 0. Max coverage (-): 0

Region: chr8 104028294-104028308. Max. coverage (+): 0. Max coverage (-): 0

Region: chr8 104028309-104028323. Max. coverage (+): 0. Max coverage (-): 0

Region: chr8 104028324-104028339. Max. coverage (+): 0. Max coverage (-): 14.55

Region: chr8 104028340-104028354. Max. coverage (+): 0. Max coverage (-): 19.58

Region: chr8 104028355-104028369. Max. coverage (+): 0. Max coverage (-): 0

Region: chr8 104028370-104028384. Max. coverage (+): 0. Max coverage (-): 0

Region: chr8 104028385-104028399. Max. coverage (+): 0. Max coverage (-): 5.92

Region: chr8 104028400-104028414. Max. coverage (+): 0. Max coverage (-): 5.92

Region: chr8 104028415-104028430. Max. coverage (+): 0. Max coverage (-): 7.64

Region: chr8 104028431-104028445. Max. coverage (+): 0. Max coverage (-): 5.67

Region: chr8 104028446-104028460. Max. coverage (+): 0. Max coverage (-): 2.9

Region: chr8 104028461-104028475. Max. coverage (+): 0. Max coverage (-): 0

Region: chr8 104028476-104028490. Max. coverage (+): 0. Max coverage (-): 0

Region: chr8 104028491-104028506. Max. coverage (+): 0. Max coverage (-): 2.82

Region: chr8 104028507-104028521. Max. coverage (+): 0. Max coverage (-): 2.82

Region: chr8 104028522-104028536. Max. coverage (+): 0. Max coverage (-): 0.22

Region: chr8 104028537-104028551. Max. coverage (+): 0. Max coverage (-): 0

Region: chr8 104028552-104028566. Max. coverage (+): 0. Max coverage (-): 0

Region: chr8 104028567-104028581. Max. coverage (+): 0. Max coverage (-): 0

Region: chr8 104028582-104028597. Max. coverage (+): 0. Max coverage (-): 0

Region: chr8 104028598-104028612. Max. coverage (+): 0. Max coverage (-): 0

Region: chr8 104028613-104028627. Max. coverage (+): 0. Max coverage (-): 0

Region: chr8 104028628-104028642. Max. coverage (+): 0. Max coverage (-): 0

Region: chr8 104028643-104028657. Max. coverage (+): 0. Max coverage (-): 0

Region: chr8 104028658-104028673. Max. coverage (+): 0. Max coverage (-): 0

Region: chr8 104028674-104028688. Max. coverage (+): 0. Max coverage (-): 0

Region: chr8 104028689-104028703. Max. coverage (+): 0. Max coverage (-): 0

Region: chr8 104028704-104028718. Max. coverage (+): 0. Max coverage (-): 0

Region: chr8 104028719-104028733. Max. coverage (+): 0. Max coverage (-): 0

Region: chr8 104028734-104028748. Max. coverage (+): 0. Max coverage (-): 0

Region: chr8 104028749-104028764. Max. coverage (+): 0. Max coverage (-): 0

Region: chr8 104028765-104028779. Max. coverage (+): 0. Max coverage (-): 43.11

Region: chr8 104028780-104028794. Max. coverage (+): 0. Max coverage (-): 3.35

Region: chr8 104028795-104028809. Max. coverage (+): 0. Max coverage (-): 20.95

Region: chr8 104028810-104028824. Max. coverage (+): 0. Max coverage (-): 0

Region: chr8 104028825-104028840. Max. coverage (+): 0. Max coverage (-): 0.11

Region: chr8 104028841-104028855. Max. coverage (+): 0. Max coverage (-): 3.88

Region: chr8 104028856-104028870. Max. coverage (+): 0. Max coverage (-): 2.52

Region: chr8 104028871-104028885. Max. coverage (+): 0. Max coverage (-): 0

Region: chr8 104028886-104028900. Max. coverage (+): 0. Max coverage (-): 0

Region: chr8 104028901-104028916. Max. coverage (+): 0. Max coverage (-): 0

Region: chr8 104028917-104028931. Max. coverage (+): 0. Max coverage (-): 21.8

Region: chr8 104028932-104028946. Max. coverage (+): 0. Max coverage (-): 10.8

Region: chr8 104028947-104028961. Max. coverage (+): 0. Max coverage (-): 1.02

Region: chr8 104028962-104028976. Max. coverage (+): 0. Max coverage (-): 41.49

Region: chr8 104028977-104028991. Max. coverage (+): 0. Max coverage (-): 15.83

Region: chr8 104028992-104029007. Max. coverage (+): 0. Max coverage (-): 0

Region: chr8 104029008-104029022. Max. coverage (+): 0. Max coverage (-): 0

Region: chr8 104029023-104029037. Max. coverage (+): 0. Max coverage (-): 0

Region: chr8 104029038-104029052. Max. coverage (+): 0. Max coverage (-): 0

Region: chr8 104029053-104029067. Max. coverage (+): 0. Max coverage (-): 0

Region: chr8 104029068-104029083. Max. coverage (+): 0. Max coverage (-): 0

Region: chr8 104029084-104029098. Max. coverage (+): 0. Max coverage (-): 0

Region: chr8 104029099-104029113. Max. coverage (+): 0. Max coverage (-): 0

Region: chr8 104029114-104029128. Max. coverage (+): 0. Max coverage (-): 4.03

Region: chr8 104029129-104029143. Max. coverage (+): 0. Max coverage (-): 7.17

Region: chr8 104029144-104029158. Max. coverage (+): 0. Max coverage (-): 7.17

Region: chr8 104029159-104029174. Max. coverage (+): 0. Max coverage (-): 0

Region: chr8 104029175-104029189. Max. coverage (+): 0. Max coverage (-): 13.78

Region: chr8 104029190-104029204. Max. coverage (+): 0. Max coverage (-): 13.78

Region: chr8 104029205-104029219. Max. coverage (+): 0. Max coverage (-): 0

Region: chr8 104029220-104029234. Max. coverage (+): 0. Max coverage (-): 4.92

Region: chr8 104029235-104029250. Max. coverage (+): 0. Max coverage (-): 0

Region: chr8 104029251-104029265. Max. coverage (+): 0. Max coverage (-): 9.73

Region: chr8 104029266-104029280. Max. coverage (+): 0. Max coverage (-): 3.1

Region: chr8 104029281-104029295. Max. coverage (+): 0. Max coverage (-): 0

Region: chr8 104029296-104029310. Max. coverage (+): 0. Max coverage (-): 0

Region: chr8 104029311-104029325. Max. coverage (+): 0. Max coverage (-): 1.31

Region: chr8 104029326-104029341. Max. coverage (+): 0. Max coverage (-): 0

Region: chr8 104029342-104029356. Max. coverage (+): 0. Max coverage (-): 0

Region: chr8 104029357-104029371. Max. coverage (+): 0. Max coverage (-): 0

Region: chr8 104029372-104029386. Max. coverage (+): 0. Max coverage (-): 0

Region: chr8 104029387-104029401. Max. coverage (+): 0. Max coverage (-): 0

Region: chr8 104029402-104029417. Max. coverage (+): 0. Max coverage (-): 0

Region: chr8 104029418-104029432. Max. coverage (+): 0. Max coverage (-): 0

Region: chr8 104029433-104029447. Max. coverage (+): 0. Max coverage (-): 0.25

Region: chr8 104029448-104029462. Max. coverage (+): 0. Max coverage (-): 0.25

Region: chr8 104029463-104029477. Max. coverage (+): 0. Max coverage (-): 0.21

Region: chr8 104029478-104029493. Max. coverage (+): 0. Max coverage (-): 0.21

Region: chr8 104029494-104029508. Max. coverage (+): 0. Max coverage (-): 0

Region: chr8 104029509-104029523. Max. coverage (+): 0. Max coverage (-): 0

Region: chr8 104029524-104029538. Max. coverage (+): 0. Max coverage (-): 0

Region: chr8 104029539-104029553. Max. coverage (+): 0. Max coverage (-): 0

Region: chr8 104029554-104029568. Max. coverage (+): 0. Max coverage (-): 15.02

Region: chr8 104029569-104029584. Max. coverage (+): 0. Max coverage (-): 6.46

Region: chr8 104029585-104029599. Max. coverage (+): 0. Max coverage (-): 0

Region: chr8 104029600-104029614. Max. coverage (+): 0. Max coverage (-): 6.71

Region: chr8 104029615-104029629. Max. coverage (+): 0. Max coverage (-): 6.71

Region: chr8 104029630-104029644. Max. coverage (+): 0. Max coverage (-): 4.6

Region: chr8 104029645-104029660. Max. coverage (+): 0. Max coverage (-): 0

Region: chr8 104029661-104029675. Max. coverage (+): 0. Max coverage (-): 0

Region: chr8 104029676-104029690. Max. coverage (+): 0. Max coverage (-): 0

Region: chr8 104029691-104029705. Max. coverage (+): 0. Max coverage (-): 0

Region: chr8 104029706-104029720. Max. coverage (+): 0. Max coverage (-): 6.26

Region: chr8 104029721-104029735. Max. coverage (+): 0. Max coverage (-): 6.26

Region: chr8 104029736-104029751. Max. coverage (+): 0. Max coverage (-): 11.93

Region: chr8 104029752-104029766. Max. coverage (+): 0. Max coverage (-): 11.05

Region: chr8 104029767-104029781. Max. coverage (+): 0. Max coverage (-): 0

Region: chr8 104029782-104029796. Max. coverage (+): 0. Max coverage (-): 0

Region: chr8 104029797-104029811. Max. coverage (+): 0. Max coverage (-): 16.64

Region: chr8 104029812-104029827. Max. coverage (+): 0. Max coverage (-): 10.78

Region: chr8 104029828-104029842. Max. coverage (+): 0. Max coverage (-): 42.74

Region: chr8 104029843-104029857. Max. coverage (+): 0. Max coverage (-): 32.24

Region: chr8 104029858-104029872. Max. coverage (+): 0. Max coverage (-): 0

Region: chr8 104029873-104029887. Max. coverage (+): 0. Max coverage (-): 0

Region: chr8 104029888-104029902. Max. coverage (+): 0. Max coverage (-): 2.96

Region: chr8 104029903-104029918. Max. coverage (+): 0. Max coverage (-): 13.31

Region: chr8 104029919-104029933. Max. coverage (+): 0. Max coverage (-): 13.4

Region: chr8 104029934-104029948. Max. coverage (+): 0. Max coverage (-): 37.87

Region: chr8 104029949-104029963. Max. coverage (+): 0. Max coverage (-): 0

Region: chr8 104029964-104029978. Max. coverage (+): 0. Max coverage (-): 0

Region: chr8 104029979-104029994. Max. coverage (+): 0. Max coverage (-): 2.36

Region: chr8 104029995-104030009. Max. coverage (+): 0. Max coverage (-): 2.71

Region: chr8 104030010-104030024. Max. coverage (+): 0. Max coverage (-): 0

Region: chr8 104030025-104030039. Max. coverage (+): 0. Max coverage (-): 0

Region: chr8 104030040-104030054. Max. coverage (+): 0. Max coverage (-): 0

Region: chr8 104030055-104030070. Max. coverage (+): 0. Max coverage (-): 0

Region: chr8 104030071-104030085. Max. coverage (+): 0. Max coverage (-): 0

Region: chr8 104030086-104030100. Max. coverage (+): 0. Max coverage (-): 0

Region: chr8 104030101-104030115. Max. coverage (+): 0. Max coverage (-): 1.55

Region: chr8 104030116-104030130. Max. coverage (+): 0. Max coverage (-): 0

Region: chr8 104030131-104030145. Max. coverage (+): 0. Max coverage (-): 0

Region: chr8 104030146-104030161. Max. coverage (+): 0. Max coverage (-): 0

Region: chr8 104030162-104030176. Max. coverage (+): 0. Max coverage (-): 0

Region: chr8 104030177-104030191. Max. coverage (+): 0. Max coverage (-): 0

Region: chr8 104030192-104030206. Max. coverage (+): 0. Max coverage (-): 14.65

Region: chr8 104030207-104030221. Max. coverage (+): 0. Max coverage (-): 12.01

Region: chr8 104030222-104030237. Max. coverage (+): 0. Max coverage (-): 0.39

Region: chr8 104030238-104030252. Max. coverage (+): 0. Max coverage (-): 5.57

Region: chr8 104030253-104030267. Max. coverage (+): 0. Max coverage (-): 12.13

Region: chr8 104030268-104030282. Max. coverage (+): 0. Max coverage (-): 0.59

Region: chr8 104030283-104030297. Max. coverage (+): 0. Max coverage (-): 0

Region: chr8 104030298-104030312. Max. coverage (+): 0. Max coverage (-): 16.39

Region: chr8 104030313-104030328. Max. coverage (+): 0. Max coverage (-): 16.39

Region: chr8 104030329-104030343. Max. coverage (+): 0. Max coverage (-): 0.13

Region: chr8 104030344-104030358. Max. coverage (+): 0. Max coverage (-): 0

Region: chr8 104030359-104030373. Max. coverage (+): 0. Max coverage (-): 0

Region: chr8 104030374-104030388. Max. coverage (+): 0. Max coverage (-): 0

Region: chr8 104030389-104030404. Max. coverage (+): 0. Max coverage (-): 0

Region: chr8 104030405-104030419. Max. coverage (+): 0. Max coverage (-): 3.14

Region: chr8 104030420-104030434. Max. coverage (+): 0. Max coverage (-): 0

Region: chr8 104030435-104030449. Max. coverage (+): 0. Max coverage (-): 0

Region: chr8 104030450-104030464. Max. coverage (+): 0. Max coverage (-): 0

Region: chr8 104030465-104030479. Max. coverage (+): 0. Max coverage (-): 0

Region: chr8 104030480-104030495. Max. coverage (+): 0. Max coverage (-): 0

Region: chr8 104030496-104030510. Max. coverage (+): 0. Max coverage (-): 4.06

Region: chr8 104030511-104030525. Max. coverage (+): 0. Max coverage (-): 8.12

Region: chr8 104030526-104030540. Max. coverage (+): 0. Max coverage (-): 5.78

Region: chr8 104030541-104030555. Max. coverage (+): 0. Max coverage (-): 0

Region: chr8 104030556-104030571. Max. coverage (+): 0. Max coverage (-): 6.69

Region: chr8 104030572-104030586. Max. coverage (+): 0. Max coverage (-): 6.69

Region: chr8 104030587-104030601. Max. coverage (+): 0. Max coverage (-): 0

Region: chr8 104030602-104030616. Max. coverage (+): 0. Max coverage (-): 0

Region: chr8 104030617-104030631. Max. coverage (+): 0. Max coverage (-): 0

Region: chr8 104030632-104030646. Max. coverage (+): 0. Max coverage (-): 0

Region: chr8 104030647-104030662. Max. coverage (+): 0. Max coverage (-): 0

Region: chr8 104030663-104030677. Max. coverage (+): 0. Max coverage (-): 0

Region: chr8 104030678-104030692. Max. coverage (+): 0. Max coverage (-): 0

Region: chr8 104030693-104030707. Max. coverage (+): 0. Max coverage (-): 12.71

Region: chr8 104030708-104030722. Max. coverage (+): 0. Max coverage (-): 37.07

Region: chr8 104030723-104030738. Max. coverage (+): 0. Max coverage (-): 0

Region: chr8 104030739-104030753. Max. coverage (+): 0. Max coverage (-): 4.14

Region: chr8 104030754-104030768. Max. coverage (+): 0. Max coverage (-): 5.82

Region: chr8 104030769-104030783. Max. coverage (+): 0. Max coverage (-): 1.97

Region: chr8 104030784-104030798. Max. coverage (+): 0. Max coverage (-): 1.15

Region: chr8 104030799-104030814. Max. coverage (+): 0. Max coverage (-): 5.6

Region: chr8 104030815-104030829. Max. coverage (+): 0. Max coverage (-): 0

Region: chr8 104030830-104030844. Max. coverage (+): 0. Max coverage (-): 2.37

Region: chr8 104030845-104030859. Max. coverage (+): 0. Max coverage (-): 2.37

Region: chr8 104030860-104030874. Max. coverage (+): 0. Max coverage (-): 0

Region: chr8 104030875-104030889. Max. coverage (+): 0. Max coverage (-): 0

Region: chr8 104030890-104030905. Max. coverage (+): 0. Max coverage (-): 0

Region: chr8 104030906-104030920. Max. coverage (+): 0. Max coverage (-): 10.67

Region: chr8 104030921-104030935. Max. coverage (+): 0. Max coverage (-): 16.87

Region: chr8 104030936-104030950. Max. coverage (+): 0. Max coverage (-): 0

Region: chr8 104030951-104030965. Max. coverage (+): 0. Max coverage (-): 0

Region: chr8 104030966-104030981. Max. coverage (+): 0. Max coverage (-): 10.06

Region: chr8 104030982-104030996. Max. coverage (+): 0. Max coverage (-): 17

Region: chr8 104030997-104031011. Max. coverage (+): 0. Max coverage (-): 20.66

Region: chr8 104031012-104031026. Max. coverage (+): 0. Max coverage (-): 22.68

Region: chr8 104031027-104031041. Max. coverage (+): 0. Max coverage (-): 2.19

Region: chr8 104031042-104031056. Max. coverage (+): 0. Max coverage (-): 2.19

Region: chr8 104031057-104031072. Max. coverage (+): 0. Max coverage (-): 10.06

Region: chr8 104031073-104031087. Max. coverage (+): 0. Max coverage (-): 13.15

Region: chr8 104031088-104031102. Max. coverage (+): 0. Max coverage (-): 0

Region: chr8 104031103-104031117. Max. coverage (+): 0. Max coverage (-): 5.63

Region: chr8 104031118-104031132. Max. coverage (+): 0. Max coverage (-): 9.94

Region: chr8 104031133-104031148. Max. coverage (+): 0. Max coverage (-): 1.06

Region: chr8 104031149-104031163. Max. coverage (+): 0. Max coverage (-): 3.17

Region: chr8 104031164-104031178. Max. coverage (+): 0. Max coverage (-): 15.11

Region: chr8 104031179-104031193. Max. coverage (+): 0. Max coverage (-): 19.29

Region: chr8 104031194-104031208. Max. coverage (+): 0. Max coverage (-): 27.13

Region: chr8 104031209-104031223. Max. coverage (+): 0. Max coverage (-): 0

Region: chr8 104031224-104031239. Max. coverage (+): 0. Max coverage (-): 0

Region: chr8 104031240-104031254. Max. coverage (+): 0. Max coverage (-): 0

Region: chr8 104031255-104031269. Max. coverage (+): 0. Max coverage (-): 0

Region: chr8 104031270-104031284. Max. coverage (+): 0. Max coverage (-): 4.78

Region: chr8 104031285-104031299. Max. coverage (+): 0. Max coverage (-): 9.78

Region: chr8 104031300-104031315. Max. coverage (+): 0. Max coverage (-): 2.66

Region: chr8 104031316-104031330. Max. coverage (+): 0. Max coverage (-): 0

Region: chr8 104031331-104031345. Max. coverage (+): 0. Max coverage (-): 6.34

Region: chr8 104031346-104031360. Max. coverage (+): 0. Max coverage (-): 34.63

Region: chr8 104031361-104031375. Max. coverage (+): 0. Max coverage (-): 23.68

Region: chr8 104031376-104031391. Max. coverage (+): 0. Max coverage (-): 11.26

Region: chr8 104031392-104031406. Max. coverage (+): 0. Max coverage (-): 15.01

Region: chr8 104031407-104031421. Max. coverage (+): 0. Max coverage (-): 0

Region: chr8 104031422-104031436. Max. coverage (+): 0. Max coverage (-): 0

Region: chr8 104031437-104031451. Max. coverage (+): 0. Max coverage (-): 0

Region: chr8 104031452-104031466. Max. coverage (+): 0. Max coverage (-): 0

Region: chr8 104031467-104031482. Max. coverage (+): 0. Max coverage (-): 0

Region: chr8 104031483-104031497. Max. coverage (+): 0. Max coverage (-): 0

Region: chr8 104031498-104031512. Max. coverage (+): 0. Max coverage (-): 0

Region: chr8 104031513-104031527. Max. coverage (+): 0. Max coverage (-): 0

Region: chr8 104031528-104031542. Max. coverage (+): 0. Max coverage (-): 0

Region: chr8 104031543-104031558. Max. coverage (+): 0. Max coverage (-): 0

Region: chr8 104031559-104031573. Max. coverage (+): 0. Max coverage (-): 0

Region: chr8 104031574-104031588. Max. coverage (+): 18.04. Max coverage (-): 0

Region: chr8 104031589-104031603. Max. coverage (+): 9.46. Max coverage (-): 0

Region: chr8 104031604-104031618. Max. coverage (+): 30.86. Max coverage (-): 0

Region: chr8 104031619-104031633. Max. coverage (+): 38.13. Max coverage (-): 0

Region: chr8 104031634-104031649. Max. coverage (+): 6.34. Max coverage (-): 0

Region: chr8 104031650-104031664. Max. coverage (+): 0.31. Max coverage (-): 0

Region: chr8 104031665-104031679. Max. coverage (+): 2.66. Max coverage (-): 0

Region: chr8 104031680-104031694. Max. coverage (+): 5.01. Max coverage (-): 0

Region: chr8 104031695-104031709. Max. coverage (+): 0. Max coverage (-): 0

Region: chr8 104031710-104031725. Max. coverage (+): 0. Max coverage (-): 0

Region: chr8 104031726-104031740. Max. coverage (+): 0. Max coverage (-): 0

Region: chr8 104031741-104031755. Max. coverage (+): 0. Max coverage (-): 0

Region: chr8 104031756-104031770. Max. coverage (+): 0. Max coverage (-): 0

Region: chr8 104031771-104031785. Max. coverage (+): 14.92. Max coverage (-): 0

Region: chr8 104031786-104031800. Max. coverage (+): 13.59. Max coverage (-): 0

Region: chr8 104031801-104031816. Max. coverage (+): 13.59. Max coverage (-): 0

Region: chr8 104031817-104031831. Max. coverage (+): 1.91. Max coverage (-): 0

Region: chr8 104031832-104031846. Max. coverage (+): 0.95. Max coverage (-): 0

Region: chr8 104031847-104031861. Max. coverage (+): 9.12. Max coverage (-): 0

Region: chr8 104031862-104031876. Max. coverage (+): 5.06. Max coverage (-): 0

Region: chr8 104031877-104031892. Max. coverage (+): 0. Max coverage (-): 0

Region: chr8 104031893-104031907. Max. coverage (+): 14.95. Max coverage (-): 0

Region: chr8 104031908-104031922. Max. coverage (+): 9.04. Max coverage (-): 0

Region: chr8 104031923-104031937. Max. coverage (+): 1.97. Max coverage (-): 0

Region: chr8 104031938-104031952. Max. coverage (+): 1.97. Max coverage (-): 0

Region: chr8 104031953-104031968. Max. coverage (+): 20.4. Max coverage (-): 0

Region: chr8 104031969-104031983. Max. coverage (+): 9.01. Max coverage (-): 0

Region: chr8 104031984-104031998. Max. coverage (+): 15.29. Max coverage (-): 0

Region: chr8 104031999-104032013. Max. coverage (+): 9.05. Max coverage (-): 0

Region: chr8 104032014-104032028. Max. coverage (+): 0. Max coverage (-): 0

Region: chr8 104032029-104032043. Max. coverage (+): 0. Max coverage (-): 0

Region: chr8 104032044-104032059. Max. coverage (+): 19.67. Max coverage (-): 0

Region: chr8 104032060-104032074. Max. coverage (+): 16.4. Max coverage (-): 0

Region: chr8 104032075-104032089. Max. coverage (+): 0. Max coverage (-): 0

Region: chr8 104032090-104032104. Max. coverage (+): 0. Max coverage (-): 0

Region: chr8 104032105-104032119. Max. coverage (+): 0. Max coverage (-): 0

Region: chr8 104032120-104032135. Max. coverage (+): 2.13. Max coverage (-): 0

Region: chr8 104032136-104032150. Max. coverage (+): 2.13. Max coverage (-): 0

Region: chr8 104032151-104032165. Max. coverage (+): 0. Max coverage (-): 0

Region: chr8 104032166-104032180. Max. coverage (+): 5.03. Max coverage (-): 0

Region: chr8 104032181-104032195. Max. coverage (+): 1.04. Max coverage (-): 0

Region: chr8 104032196-104032210. Max. coverage (+): 1.77. Max coverage (-): 0

Region: chr8 104032211-104032226. Max. coverage (+): 5.23. Max coverage (-): 0

Region: chr8 104032227-104032241. Max. coverage (+): 3.72. Max coverage (-): 0

Region: chr8 104032242-104032256. Max. coverage (+): 0. Max coverage (-): 0

Region: chr8 104032257-104032271. Max. coverage (+): 96.25. Max coverage (-): 0

Region: chr8 104032272-104032286. Max. coverage (+): 182.09. Max coverage (-): 0

Region: chr8 104032287-104032302. Max. coverage (+): 14.52. Max coverage (-): 0

Region: chr8 104032303-104032317. Max. coverage (+): 0. Max coverage (-): 0

Region: chr8 104032318-104032332. Max. coverage (+): 0. Max coverage (-): 0

Region: chr8 104032333-104032347. Max. coverage (+): 0. Max coverage (-): 0

Region: chr8 104032348-104032362. Max. coverage (+): 0. Max coverage (-): 0

Region: chr8 104032363-104032377. Max. coverage (+): 0. Max coverage (-): 0

Region: chr8 104032378-104032393. Max. coverage (+): 0. Max coverage (-): 0

Region: chr8 104032394-104032408. Max. coverage (+): 5.22. Max coverage (-): 0

Region: chr8 104032409-104032423. Max. coverage (+): 5.22. Max coverage (-): 0

Region: chr8 104032424-104032438. Max. coverage (+): 0. Max coverage (-): 0

Region: chr8 104032439-104032453. Max. coverage (+): 2.22. Max coverage (-): 0

Region: chr8 104032454-104032469. Max. coverage (+): 6.34. Max coverage (-): 0

Region: chr8 104032470-104032484. Max. coverage (+): 3.17. Max coverage (-): 0

Region: chr8 104032485-104032499. Max. coverage (+): 0. Max coverage (-): 0

Region: chr8 104032500-104032514. Max. coverage (+): 7.1. Max coverage (-): 0

Region: chr8 104032515-104032529. Max. coverage (+): 0. Max coverage (-): 0

Region: chr8 104032530-104032544. Max. coverage (+): 0. Max coverage (-): 0

Region: chr8 104032545-104032560. Max. coverage (+): 0. Max coverage (-): 0

Region: chr8 104032561-104032575. Max. coverage (+): 2.45. Max coverage (-): 0

Region: chr8 104032576-104032590. Max. coverage (+): 0. Max coverage (-): 0

Region: chr8 104032591-104032605. Max. coverage (+): 0. Max coverage (-): 0

Region: chr8 104032606-104032620. Max. coverage (+): 0. Max coverage (-): 0

Region: chr8 104032621-104032636. Max. coverage (+): 0. Max coverage (-): 0

Region: chr8 104032637-104032651. Max. coverage (+): 0.1. Max coverage (-): 0

Region: chr8 104032652-104032666. Max. coverage (+): 12.8. Max coverage (-): 0

Region: chr8 104032667-104032681. Max. coverage (+): 4.49. Max coverage (-): 0

Region: chr8 104032682-104032696. Max. coverage (+): 0. Max coverage (-): 0

Region: chr8 104032697-104032712. Max. coverage (+): 0.46. Max coverage (-): 0

Region: chr8 104032713-104032727. Max. coverage (+): 9.25. Max coverage (-): 0

Region: chr8 104032728-104032742. Max. coverage (+): 2.08. Max coverage (-): 0

Region: chr8 104032743-104032757. Max. coverage (+): 0.29. Max coverage (-): 0

Region: chr8 104032758-104032772. Max. coverage (+): 7.4. Max coverage (-): 0

Region: chr8 104032773-104032787. Max. coverage (+): 10.87. Max coverage (-): 0

Region: chr8 104032788-104032803. Max. coverage (+): 0. Max coverage (-): 0

Region: chr8 104032804-104032818. Max. coverage (+): 0. Max coverage (-): 0

Region: chr8 104032819-104032833. Max. coverage (+): 6.52. Max coverage (-): 0

Region: chr8 104032834-104032848. Max. coverage (+): 6.52. Max coverage (-): 0

Region: chr8 104032849-104032863. Max. coverage (+): 0. Max coverage (-): 0

Region: chr8 104032864-104032879. Max. coverage (+): 1.15. Max coverage (-): 0

Region: chr8 104032880-104032894. Max. coverage (+): 0. Max coverage (-): 0

Region: chr8 104032895-104032909. Max. coverage (+): 0. Max coverage (-): 0

Region: chr8 104032910-104032924. Max. coverage (+): 0. Max coverage (-): 0

Region: chr8 104032925-104032939. Max. coverage (+): 0. Max coverage (-): 0

Region: chr8 104032940-104032954. Max. coverage (+): 0. Max coverage (-): 0

Region: chr8 104032955-104032970. Max. coverage (+): 1.35. Max coverage (-): 0

Region: chr8 104032971-104032985. Max. coverage (+): 1.75. Max coverage (-): 0

Region: chr8 104032986-104033000. Max. coverage (+): 1.09. Max coverage (-): 0

Region: chr8 104033001-104033015. Max. coverage (+): 0. Max coverage (-): 0

Region: chr8 104033016-104033030. Max. coverage (+): 0. Max coverage (-): 0

Region: chr8 104033031-104033046. Max. coverage (+): 31.71. Max coverage (-): 0

Region: chr8 104033047-104033061. Max. coverage (+): 3.15. Max coverage (-): 0

Region: chr8 104033062-104033076. Max. coverage (+): 9.88. Max coverage (-): 0

Region: chr8 104033077-104033091. Max. coverage (+): 2.19. Max coverage (-): 0

Region: chr8 104033092-104033106. Max. coverage (+): 0. Max coverage (-): 0

Region: chr8 104033107-104033121. Max. coverage (+): 0. Max coverage (-): 0

Region: chr8 104033122-104033137. Max. coverage (+): 26.76. Max coverage (-): 0

Region: chr8 104033138-104033152. Max. coverage (+): 30.94. Max coverage (-): 0

Region: chr8 104033153-104033167. Max. coverage (+): 11.89. Max coverage (-): 0

Region: chr8 104033168-104033182. Max. coverage (+): 13.21. Max coverage (-): 0

Region: chr8 104033183-104033197. Max. coverage (+): 0. Max coverage (-): 0

Region: chr8 104033198-104033213. Max. coverage (+): 2.27. Max coverage (-): 0

Region: chr8 104033214-104033228. Max. coverage (+): 8.2. Max coverage (-): 0

Region: chr8 104033229-104033243. Max. coverage (+): 8.85. Max coverage (-): 0

Region: chr8 104033244-104033258. Max. coverage (+): 4.64. Max coverage (-): 0

Region: chr8 104033259-104033273. Max. coverage (+): 4.64. Max coverage (-): 0

Region: chr8 104033274-104033289. Max. coverage (+): 0. Max coverage (-): 0

Region: chr8 104033290-104033304. Max. coverage (+): 0. Max coverage (-): 0

Region: chr8 104033305-104033319. Max. coverage (+): 0. Max coverage (-): 0

Region: chr8 104033320-104033334. Max. coverage (+): 0. Max coverage (-): 0

Region: chr8 104033335-104033349. Max. coverage (+): 0. Max coverage (-): 0

Region: chr8 104033350-104033364. Max. coverage (+): 0. Max coverage (-): 0

Region: chr8 104033365-104033380. Max. coverage (+): 0. Max coverage (-): 0

Region: chr8 104033381-104033395. Max. coverage (+): 8.53. Max coverage (-): 0

Region: chr8 104033396-104033410. Max. coverage (+): 11.86. Max coverage (-): 0

Region: chr8 104033411-104033425. Max. coverage (+): 11.12. Max coverage (-): 0

Region: chr8 104033426-104033440. Max. coverage (+): 0. Max coverage (-): 0

Region: chr8 104033441-104033456. Max. coverage (+): 0. Max coverage (-): 0

Region: chr8 104033457-104033471. Max. coverage (+): 6.88. Max coverage (-): 0

Region: chr8 104033472-104033486. Max. coverage (+): 6.88. Max coverage (-): 0

Region: chr8 104033487-104033501. Max. coverage (+): 0. Max coverage (-): 0

Region: chr8 104033502-104033516. Max. coverage (+): 0.11. Max coverage (-): 0

Region: chr8 104033517-104033531. Max. coverage (+): 0.19. Max coverage (-): 0

Region: chr8 104033532-104033547. Max. coverage (+): 0. Max coverage (-): 0

Region: chr8 104033548-104033562. Max. coverage (+): 0. Max coverage (-): 0

Region: chr8 104033563-104033577. Max. coverage (+): 0. Max coverage (-): 0

Region: chr8 104033578-104033592. Max. coverage (+): 0. Max coverage (-): 0

Region: chr8 104033593-104033607. Max. coverage (+): 0. Max coverage (-): 0

Region: chr8 104033608-104033623. Max. coverage (+): 0. Max coverage (-): 0

Region: chr8 104033624-104033638. Max. coverage (+): 0. Max coverage (-): 0

Region: chr8 104033639-104033653. Max. coverage (+): 0. Max coverage (-): 0

Region: chr8 104033654-104033668. Max. coverage (+): 0. Max coverage (-): 0

Region: chr8 104033669-104033683. Max. coverage (+): 0. Max coverage (-): 0

Region: chr8 104033684-104033698. Max. coverage (+): 0. Max coverage (-): 0

Region: chr8 104033699-104033714. Max. coverage (+): 7.2. Max coverage (-): 0

Region: chr8 104033715-104033729. Max. coverage (+): 7.2. Max coverage (-): 0

Region: chr8 104033730-104033744. Max. coverage (+): 0.96. Max coverage (-): 0

Region: chr8 104033745-104033759. Max. coverage (+): 0.96. Max coverage (-): 0

Region: chr8 104033760-104033774. Max. coverage (+): 0. Max coverage (-): 0

Region: chr8 104033775-104033790. Max. coverage (+): 11.2. Max coverage (-): 0

Region: chr8 104033791-104033805. Max. coverage (+): 1.21. Max coverage (-): 0

Region: chr8 104033806-104033820. Max. coverage (+): 0. Max coverage (-): 0

Region: chr8 104033821-104033835. Max. coverage (+): 8.24. Max coverage (-): 0

Region: chr8 104033836-104033850. Max. coverage (+): 3.05. Max coverage (-): 0

Region: chr8 104033851-104033866. Max. coverage (+): 2.97. Max coverage (-): 0

Region: chr8 104033867-104033881. Max. coverage (+): 0. Max coverage (-): 0

Region: chr8 104033882-104033896. Max. coverage (+): 0. Max coverage (-): 0

Region: chr8 104033897-104033911. Max. coverage (+): 0. Max coverage (-): 0

Region: chr8 104033912-104033926. Max. coverage (+): 0. Max coverage (-): 0

Region: chr8 104033927-104033941. Max. coverage (+): 0. Max coverage (-): 0

Region: chr8 104033942-104033957. Max. coverage (+): 0. Max coverage (-): 0

Region: chr8 104033958-104033972. Max. coverage (+): 0. Max coverage (-): 0

Region: chr8 104033973-104033987. Max. coverage (+): 0. Max coverage (-): 0

Region: chr8 104033988-104034002. Max. coverage (+): 30.31. Max coverage (-): 0

Region: chr8 104034003-104034017. Max. coverage (+): 33.22. Max coverage (-): 0

Region: chr8 104034018-104034033. Max. coverage (+): 5.08. Max coverage (-): 0

Region: chr8 104034034-104034048. Max. coverage (+): 13.42. Max coverage (-): 0

Region: chr8 104034049-104034063. Max. coverage (+): 10.52. Max coverage (-): 0

Region: chr8 104034064-104034078. Max. coverage (+): 0. Max coverage (-): 0

Region: chr8 104034079-104034093. Max. coverage (+): 0. Max coverage (-): 0

Region: chr8 104034094-104034108. Max. coverage (+): 1.86. Max coverage (-): 0

Region: chr8 104034109-104034124. Max. coverage (+): 2.87. Max coverage (-): 0

Region: chr8 104034125-104034139. Max. coverage (+): 2.87. Max coverage (-): 0

Region: chr8 104034140-104034154. Max. coverage (+): 0. Max coverage (-): 0

Region: chr8 104034155-104034169. Max. coverage (+): 3.75. Max coverage (-): 0

Region: chr8 104034170-104034184. Max. coverage (+): 0. Max coverage (-): 0

Region: chr8 104034185-104034200. Max. coverage (+): 19.82. Max coverage (-): 0

Region: chr8 104034201-104034215. Max. coverage (+): 4.12. Max coverage (-): 0

Region: chr8 104034216-104034230. Max. coverage (+): 0. Max coverage (-): 0

Region: chr8 104034231-104034245. Max. coverage (+): 0. Max coverage (-): 0

Region: chr8 104034246-104034260. Max. coverage (+): 0. Max coverage (-): 0

Region: chr8 104034261-104034275. Max. coverage (+): 0. Max coverage (-): 0

Region: chr8 104034276-104034291. Max. coverage (+): 0. Max coverage (-): 0

Region: chr8 104034292-104034306. Max. coverage (+): 0. Max coverage (-): 0

Region: chr8 104034307-104034321. Max. coverage (+): 0. Max coverage (-): 0

Region: chr8 104034322-104034336. Max. coverage (+): 0. Max coverage (-): 0

Region: chr8 104034337-104034351. Max. coverage (+): 0. Max coverage (-): 0

Region: chr8 104034352-104034367. Max. coverage (+): 0. Max coverage (-): 0

Region: chr8 104034368-104034382. Max. coverage (+): 0. Max coverage (-): 0

Region: chr8 104034383-104034397. Max. coverage (+): 0. Max coverage (-): 0

Region: chr8 104034398-104034412. Max. coverage (+): 0. Max coverage (-): 0

Region: chr8 104034413-104034427. Max. coverage (+): 0. Max coverage (-): 0

Region: chr8 104034428-104034442. Max. coverage (+): 0. Max coverage (-): 0

Region: chr8 104034443-104034458. Max. coverage (+): 0.15. Max coverage (-): 0

Region: chr8 104034459-104034473. Max. coverage (+): 1.98. Max coverage (-): 0

Region: chr8 104034474-104034488. Max. coverage (+): 0. Max coverage (-): 0

Region: chr8 104034489-104034503. Max. coverage (+): 0. Max coverage (-): 0

Region: chr8 104034504-104034518. Max. coverage (+): 0. Max coverage (-): 0

Region: chr8 104034519-104034534. Max. coverage (+): 6.02. Max coverage (-): 0

Region: chr8 104034535-104034549. Max. coverage (+): 3.98. Max coverage (-): 0

Region: chr8 104034550-104034564. Max. coverage (+): 0. Max coverage (-): 0

Region: chr8 104034565-104034579. Max. coverage (+): 0. Max coverage (-): 0

Region: chr8 104034580-104034594. Max. coverage (+): 0. Max coverage (-): 0

Region: chr8 104034595-104034610. Max. coverage (+): 0. Max coverage (-): 0

Region: chr8 104034611-104034625. Max. coverage (+): 1.44. Max coverage (-): 0

Region: chr8 104034626-104034640. Max. coverage (+): 0. Max coverage (-): 0

Region: chr8 104034641-104034655. Max. coverage (+): 0. Max coverage (-): 0

Region: chr8 104034656-104034670. Max. coverage (+): 0. Max coverage (-): 0

Region: chr8 104034671-104034685. Max. coverage (+): 0. Max coverage (-): 0

Region: chr8 104034686-104034701. Max. coverage (+): 0. Max coverage (-): 0

Region: chr8 104034702-104034716. Max. coverage (+): 23.76. Max coverage (-): 0

Region: chr8 104034717-104034731. Max. coverage (+): 0. Max coverage (-): 0

Region: chr8 104034732-104034746. Max. coverage (+): 13.92. Max coverage (-): 0

Region: chr8 104034747-104034761. Max. coverage (+): 13.92. Max coverage (-): 0

Region: chr8 104034762-104034777. Max. coverage (+): 0. Max coverage (-): 0

Region: chr8 104034778-104034792. Max. coverage (+): 0. Max coverage (-): 0

Region: chr8 104034793-104034807. Max. coverage (+): 0. Max coverage (-): 0

Region: chr8 104034808-104034822. Max. coverage (+): 0. Max coverage (-): 0

Region: chr8 104034823-104034837. Max. coverage (+): 0. Max coverage (-): 0

Region: chr8 104034838-104034852. Max. coverage (+): 0. Max coverage (-): 0

Region: chr8 104034853-104034868. Max. coverage (+): 0. Max coverage (-): 0

Region: chr8 104034869-104034883. Max. coverage (+): 0. Max coverage (-): 0

Region: chr8 104034884-104034898. Max. coverage (+): 0. Max coverage (-): 0

Region: chr8 104034899-104034913. Max. coverage (+): 0. Max coverage (-): 0

Region: chr8 104034914-104034928. Max. coverage (+): 0. Max coverage (-): 0

Region: chr8 104034929-104034944. Max. coverage (+): 0. Max coverage (-): 0

Region: chr8 104034945-104034959. Max. coverage (+): 0. Max coverage (-): 0

Region: chr8 104034960-104034974. Max. coverage (+): 0. Max coverage (-): 0

Region: chr8 104034975-104034989. Max. coverage (+): 0. Max coverage (-): 0

Region: chr8 104034990-104035004. Max. coverage (+): 0. Max coverage (-): 0

Region: chr8 104035005-104035019. Max. coverage (+): 0. Max coverage (-): 0

Region: chr8 104035020-104035035. Max. coverage (+): 0. Max coverage (-): 0

Region: chr8 104035036-104035050. Max. coverage (+): 0. Max coverage (-): 0

Region: chr8 104035051-104035065. Max. coverage (+): 0. Max coverage (-): 0

Region: chr8 104035066-104035080. Max. coverage (+): 0. Max coverage (-): 0

Region: chr8 104035081-104035095. Max. coverage (+): 0. Max coverage (-): 0

Region: chr8 104035096-104035111. Max. coverage (+): 0. Max coverage (-): 0

Region: chr8 104035112-104035126. Max. coverage (+): 0. Max coverage (-): 0

Region: chr8 104035127-104035141. Max. coverage (+): 0. Max coverage (-): 0

Region: chr8 104035142-104035156. Max. coverage (+): 0. Max coverage (-): 0

Region: chr8 104035157-104035171. Max. coverage (+): 0. Max coverage (-): 0

Region: chr8 104035172-104035187. Max. coverage (+): 0. Max coverage (-): 0

Region: chr8 104035188-104035202. Max. coverage (+): 0. Max coverage (-): 0

Region: chr8 104035203-104035217. Max. coverage (+): 0. Max coverage (-): 0

Region: chr8 104035218-104035232. Max. coverage (+): 0. Max coverage (-): 0

Region: chr8 104035233-104035247. Max. coverage (+): 0. Max coverage (-): 0

Region: chr8 104035248-104035262. Max. coverage (+): 0. Max coverage (-): 0

Region: chr8 104035263-104035278. Max. coverage (+): 5.57. Max coverage (-): 0

Region: chr8 104035279-104035293. Max. coverage (+): 5.57. Max coverage (-): 0

Region: chr8 104035294-104035308. Max. coverage (+): 0. Max coverage (-): 0

Region: chr8 104035309-104035323. Max. coverage (+): 0. Max coverage (-): 0

Region: chr8 104035324-104035338. Max. coverage (+): 0. Max coverage (-): 0

Region: chr8 104035339-104035354. Max. coverage (+): 1.3. Max coverage (-): 0

Region: chr8 104035355-104035369. Max. coverage (+): 1.94. Max coverage (-): 0

Region: chr8 104035370-104035384. Max. coverage (+): 1.94. Max coverage (-): 0

Region: chr8 104035385-. Max. coverage (+): 0. Max coverage (-): 0

RepeatMasker Color Code

**+**

100-98% Identity

<98-95% Identity

<95-90% Identity

<90-85% Identity

<85-80% Identity

<80-75% Identity

<75-70% Identity

<70% Identity

**-**

Gene Set Color Code

**+**

Gene

Pseudogene

**-**

Topology/Coverage Color Code

Coverage Plus Strand

Coverage Minus Strand

Mainstrand: Plus

Mainstrand: Minus

Complementary Strand

Flanking Region  
(if option -flank >0)

Gene Set Annotation  
  
RepeatMasker Annotation  

**1. MIRc**: 104030653-104030703 (+), Divergence to consensus: 29%  
**2. MIRb**: 104035174-104035252 (-), Divergence to consensus: 33.2%

  
Transcription Factor Binding Sites  

**SOX9** (Sequence: AACAATGA (-): 104031676)  
**SOX9** (Sequence: TCATTGTT (+): 104031312)  
**SOX9** (Sequence: CCATTGTT (+): 104035258)
