# Supplementary material for: Small RNA sequencing of cryopreserved semen from single bull revealed altered miRNAs and piRNAs expression between High- and Low-motile sperm populations
Source: BMC Genomics. 2017 Jan 4;18:14. doi: 10.1186/s12864-016-3394-7 (PMC5209821; doi:10.1186/s12864-016-3394-7)
Supplement: Additional file 4: — Details for each piRNA clusters found in Low Motile (LM) sperm fraction. Genes, repeats, transposable elements and transcription factors binding sites falling within the cluster regions were reported. (ZIP 1034 kb) [file 12864_2016_3394_MOESM4_ESM.zip › 6.html]

piRNA cluster 6


Predicted piRNA cluster no. 6     previous   next
  

Show proTRAC run info
Hide proTRAC run info

================================= proTRAC ====================================  
VERSION: 2.1                                    LAST MODIFIED: 06. October 2015  
  
Please cite:  
Rosenkranz D, Zischler H. proTRAC - a software for probabilistic piRNA cluster  
detection, visualization and analysis. 2012. BMC Bioinformatics 13:5.  
  
and (for proTRAC 2.0 and later):  
Rosenkranz D, Rudloff S, Bastuck K, Ketting RF, Zischler H. Tupaia small RNAs  
provide insights into function and evolution of RNAi-based transposon defense  
in mammals. 2015. RNA 21(5):911-922.  
  
Contact:  
David Rosenkranz  
Institute of Anthropology, small RNA group  
Johannes Gutenberg University Mainz  
email: rosenkranz@uni-mainz.de  
  
You can find the latest proTRAC version at:  
http://sourceforge.net/projects/protrac/files  
http://www.smallRNAgroup-mainz.de/software  
==============================================================================  
  
PARAMETERS:  
Map file: .............../storage/core/barbara/genhome/smallRNA/fertility/Sample\_not\_motile/pirna/Sample\_not\_motile\_26-33\_collapsed.fa.no-dust.map.weighted-10000-1000-b-0  
Genome file: ............/storage/core/barbara/genhome/smallRNA/fertility/Sample\_all/pirna/bt\_311\_chrY.fa  
RepeatMasker annotation: /storage/genomes/bt\_umd31/GCF\_000003055.6\_Bos\_taurus\_UMD\_3.1.1\_repeatMasker\_chr.out  
GeneSet:................./storage/core/barbara/genhome/smallRNA/fertility/Sample\_all/pirna/full.gtf  
  
Significant (p<=0.01) hit density will be calculated based  
on observed hit distribution.  
  
Sliding window size: ........................................ 5000 bp  
Sliding window increament: .................................. 1000 bp  
Normalize each hit by number of genomic hits: ............... 1 [0=no/1=yes]  
Normalize each hit by number of sequence reads: ............. 1 [0=no/1=yes]  
Normalize values (-> per million mapped reads): ............. 1 [0=no/1=yes]  
Min. fraction of hits with 1T(U) or 10A: .................... 0.75  
Alternatively: Min. fraction of hits with 1T(U) and 10A: .... 0.5  
Min. fraction of hits with typical piRNA length: ............ 0.75  
Typical piRNA length: ....................................... 26-33 nt  
Min. size of a piRNA cluster: ............................... 5000 bp.  
Min. number of hits (absolute): ............................. 0  
Min. number of hits (normalized): ........................... 0  
Min. fraction of hits on the mainstrand: .................... 0.75  
Top fraction of mapped sequences (in terms of read counts): . 1%  
Top fraction accounts for max. n% of sequence reads: ........ 90%  
Min. fraction of hits on each arm of a bidirectional cluster: 0.1  
Output image file for each cluster: ......................... 0 [0=no/1=yes]  
Output html file for each cluster: .......................... 1 [0=no/1=yes]  
Output a summary table: ..................................... 1 [0=no/1=yes]  
Output a FASTA file for each cluster (piRNA sequences): ..... 1 [0=no/1=yes]  
Output a FASTA file comprising cluster sequences: ........... 1 [0=no/1=yes]  
Search DNA motifs in clusters: .............................. 1 [0=no/1=yes]  
Output flanking sequences: +/- .............................. 0 bp  
Output ~.pTi file: .......................................... 1 [0=no/1=yes]  
==============================================================================  
  
  
Genome size (without gaps): ............ 2678902517 bp  
Gaps (N/X/-): .......................... 53837044 bp  
Mapped reads: .......................... 738059667487  
Non-identical sequences: ............... 277001  
Genomic hits: .......................... 533816  
Significant densitiy of mapped reads: .. 15118061 reads/kb

Show proTRAC cluster info
Hide proTRAC cluster info

|  |  |
| --- | --- |
| Location | chr10 |
| Coordinates | 100967850-100989247 |
| Size [bp] | 21398 |
| Sequence hit loci | 348 |
| Mapped reads (normalized) | 929225250 |
| Mapped reads (normalized) per kb | 43425799.1 |
| Normalized reads with 1T (1U) | 83.7% |
| Normalized reads with 10A | 32.4% |
| Normalized reads with length 26-33 nt | 100% |
| Normalized reads on the main strand(s) | 99.6% |
| Predicted directionality | mono:plus |

100%

0%

1T (1U)  
reads

10A reads

26-33 nt  
reads

reads on mainstrand

**Either the amount of reads with 1T (1U) OR 10A has to exceed 75% (set with option: -1Tor10A)  
Alternatively the amount of reads with 1T (1U) AND 10A has to exceed 50% (set with option: -1Tand10A)  
Minimum amount of reads with preferred size is 75% (set with option: -pisize)  
Minimum amount of reads on the main strand(s) is 75% (set with option: -clstrand)**

Show read coverage
Hide read coverage

WHAT DO I SEE HERE?  
This chart shows the location of mapped sequence reads within a predicted piRNA cluster. The color refers to the number of genomic hits produced by the sequence read in question. A dark red bar indicates that this sequence read produces many other hits elsewhere in the genome. Many adjacent red or yellow bars can indicate the presence of a multi-copy element such as transposons or rRNA genes. A dark green bar indicates that this sequence read maps uniquely to this locus.

1 hit

2-5 hits

6-10 hits

11-20 hits

21-50 hits

51-100 hits

> 100 hits

chr10

100967850

100989247

Gene Set

RepeatMasker

Mapped  
Reads

52.23

plus strand

minus strand

52.23

Region: chr10 100939914-100967871. Max. coverage (+): 4.72. Max coverage (-): 0

Region: chr10 100967872-100967914. Max. coverage (+): 0. Max coverage (-): 0

Region: chr10 100967915-100967956. Max. coverage (+): 0. Max coverage (-): 0

Region: chr10 100967957-100967999. Max. coverage (+): 2.6. Max coverage (-): 0

Region: chr10 100968000-100968042. Max. coverage (+): 4.72. Max coverage (-): 0

Region: chr10 100968043-100968085. Max. coverage (+): 0. Max coverage (-): 0

Region: chr10 100968086-100968128. Max. coverage (+): 0. Max coverage (-): 0

Region: chr10 100968129-100968170. Max. coverage (+): 0. Max coverage (-): 0

Region: chr10 100968171-100968213. Max. coverage (+): 0. Max coverage (-): 0

Region: chr10 100968214-100968256. Max. coverage (+): 0. Max coverage (-): 0

Region: chr10 100968257-100968299. Max. coverage (+): 0. Max coverage (-): 0

Region: chr10 100968300-100968342. Max. coverage (+): 0. Max coverage (-): 0

Region: chr10 100968343-100968384. Max. coverage (+): 0. Max coverage (-): 0

Region: chr10 100968385-100968427. Max. coverage (+): 0. Max coverage (-): 0

Region: chr10 100968428-100968470. Max. coverage (+): 0. Max coverage (-): 0

Region: chr10 100968471-100968513. Max. coverage (+): 0. Max coverage (-): 0

Region: chr10 100968514-100968556. Max. coverage (+): 0. Max coverage (-): 0

Region: chr10 100968557-100968598. Max. coverage (+): 0. Max coverage (-): 0

Region: chr10 100968599-100968641. Max. coverage (+): 0. Max coverage (-): 0

Region: chr10 100968642-100968684. Max. coverage (+): 0. Max coverage (-): 0

Region: chr10 100968685-100968727. Max. coverage (+): 0. Max coverage (-): 0

Region: chr10 100968728-100968770. Max. coverage (+): 0. Max coverage (-): 0.43

Region: chr10 100968771-100968812. Max. coverage (+): 11.68. Max coverage (-): 0.43

Region: chr10 100968813-100968855. Max. coverage (+): 4.22. Max coverage (-): 0

Region: chr10 100968856-100968898. Max. coverage (+): 4.03. Max coverage (-): 0

Region: chr10 100968899-100968941. Max. coverage (+): 0. Max coverage (-): 0

Region: chr10 100968942-100968984. Max. coverage (+): 23.83. Max coverage (-): 4.34

Region: chr10 100968985-100969026. Max. coverage (+): 4.56. Max coverage (-): 0

Region: chr10 100969027-100969069. Max. coverage (+): 0. Max coverage (-): 0

Region: chr10 100969070-100969112. Max. coverage (+): 0. Max coverage (-): 0

Region: chr10 100969113-100969155. Max. coverage (+): 0. Max coverage (-): 0

Region: chr10 100969156-100969198. Max. coverage (+): 0. Max coverage (-): 0

Region: chr10 100969199-100969240. Max. coverage (+): 6.38. Max coverage (-): 0

Region: chr10 100969241-100969283. Max. coverage (+): 0. Max coverage (-): 0

Region: chr10 100969284-100969326. Max. coverage (+): 4.6. Max coverage (-): 0

Region: chr10 100969327-100969369. Max. coverage (+): 25.29. Max coverage (-): 0

Region: chr10 100969370-100969412. Max. coverage (+): 5.49. Max coverage (-): 0

Region: chr10 100969413-100969454. Max. coverage (+): 0. Max coverage (-): 0

Region: chr10 100969455-100969497. Max. coverage (+): 0. Max coverage (-): 0

Region: chr10 100969498-100969540. Max. coverage (+): 0. Max coverage (-): 0

Region: chr10 100969541-100969583. Max. coverage (+): 32.57. Max coverage (-): 0

Region: chr10 100969584-100969626. Max. coverage (+): 2.61. Max coverage (-): 0

Region: chr10 100969627-100969668. Max. coverage (+): 0. Max coverage (-): 0

Region: chr10 100969669-100969711. Max. coverage (+): 0. Max coverage (-): 0

Region: chr10 100969712-100969754. Max. coverage (+): 1.81. Max coverage (-): 0

Region: chr10 100969755-100969797. Max. coverage (+): 6.87. Max coverage (-): 0

Region: chr10 100969798-100969840. Max. coverage (+): 12.52. Max coverage (-): 0

Region: chr10 100969841-100969882. Max. coverage (+): 0. Max coverage (-): 0

Region: chr10 100969883-100969925. Max. coverage (+): 0. Max coverage (-): 0

Region: chr10 100969926-100969968. Max. coverage (+): 17.38. Max coverage (-): 0

Region: chr10 100969969-100970011. Max. coverage (+): 3.89. Max coverage (-): 0

Region: chr10 100970012-100970053. Max. coverage (+): 0. Max coverage (-): 0

Region: chr10 100970054-100970096. Max. coverage (+): 0. Max coverage (-): 0

Region: chr10 100970097-100970139. Max. coverage (+): 0. Max coverage (-): 0

Region: chr10 100970140-100970182. Max. coverage (+): 13.33. Max coverage (-): 0

Region: chr10 100970183-100970225. Max. coverage (+): 18.84. Max coverage (-): 0

Region: chr10 100970226-100970267. Max. coverage (+): 4.49. Max coverage (-): 0

Region: chr10 100970268-100970310. Max. coverage (+): 12.52. Max coverage (-): 0

Region: chr10 100970311-100970353. Max. coverage (+): 17.27. Max coverage (-): 0

Region: chr10 100970354-100970396. Max. coverage (+): 0. Max coverage (-): 0

Region: chr10 100970397-100970439. Max. coverage (+): 12.39. Max coverage (-): 0

Region: chr10 100970440-100970481. Max. coverage (+): 6.65. Max coverage (-): 0

Region: chr10 100970482-100970524. Max. coverage (+): 21.05. Max coverage (-): 0

Region: chr10 100970525-100970567. Max. coverage (+): 11.26. Max coverage (-): 0

Region: chr10 100970568-100970610. Max. coverage (+): 0. Max coverage (-): 0

Region: chr10 100970611-100970653. Max. coverage (+): 0. Max coverage (-): 0

Region: chr10 100970654-100970695. Max. coverage (+): 0. Max coverage (-): 0

Region: chr10 100970696-100970738. Max. coverage (+): 52.23. Max coverage (-): 0

Region: chr10 100970739-100970781. Max. coverage (+): 12.75. Max coverage (-): 0

Region: chr10 100970782-100970824. Max. coverage (+): 0. Max coverage (-): 0

Region: chr10 100970825-100970867. Max. coverage (+): 0. Max coverage (-): 0

Region: chr10 100970868-100970909. Max. coverage (+): 0. Max coverage (-): 0

Region: chr10 100970910-100970952. Max. coverage (+): 0. Max coverage (-): 0

Region: chr10 100970953-100970995. Max. coverage (+): 0. Max coverage (-): 0

Region: chr10 100970996-100971038. Max. coverage (+): 0. Max coverage (-): 0

Region: chr10 100971039-100971081. Max. coverage (+): 0. Max coverage (-): 0

Region: chr10 100971082-100971123. Max. coverage (+): 0. Max coverage (-): 0

Region: chr10 100971124-100971166. Max. coverage (+): 0. Max coverage (-): 0

Region: chr10 100971167-100971209. Max. coverage (+): 0. Max coverage (-): 0

Region: chr10 100971210-100971252. Max. coverage (+): 0. Max coverage (-): 0

Region: chr10 100971253-100971295. Max. coverage (+): 0. Max coverage (-): 0

Region: chr10 100971296-100971337. Max. coverage (+): 2.28. Max coverage (-): 0

Region: chr10 100971338-100971380. Max. coverage (+): 2.3. Max coverage (-): 0

Region: chr10 100971381-100971423. Max. coverage (+): 40.49. Max coverage (-): 0

Region: chr10 100971424-100971466. Max. coverage (+): 0.19. Max coverage (-): 0

Region: chr10 100971467-100971509. Max. coverage (+): 0.56. Max coverage (-): 0

Region: chr10 100971510-100971551. Max. coverage (+): 11.86. Max coverage (-): 0

Region: chr10 100971552-100971594. Max. coverage (+): 6.76. Max coverage (-): 0

Region: chr10 100971595-100971637. Max. coverage (+): 6.76. Max coverage (-): 0

Region: chr10 100971638-100971680. Max. coverage (+): 0. Max coverage (-): 0

Region: chr10 100971681-100971723. Max. coverage (+): 8.41. Max coverage (-): 0

Region: chr10 100971724-100971765. Max. coverage (+): 4.39. Max coverage (-): 0

Region: chr10 100971766-100971808. Max. coverage (+): 0. Max coverage (-): 0

Region: chr10 100971809-100971851. Max. coverage (+): 25.6. Max coverage (-): 0

Region: chr10 100971852-100971894. Max. coverage (+): 1.86. Max coverage (-): 0

Region: chr10 100971895-100971937. Max. coverage (+): 17.85. Max coverage (-): 0

Region: chr10 100971938-100971979. Max. coverage (+): 2.46. Max coverage (-): 0

Region: chr10 100971980-100972022. Max. coverage (+): 0. Max coverage (-): 0

Region: chr10 100972023-100972065. Max. coverage (+): 10. Max coverage (-): 0

Region: chr10 100972066-100972108. Max. coverage (+): 0. Max coverage (-): 0

Region: chr10 100972109-100972150. Max. coverage (+): 3.83. Max coverage (-): 0

Region: chr10 100972151-100972193. Max. coverage (+): 0. Max coverage (-): 0

Region: chr10 100972194-100972236. Max. coverage (+): 0. Max coverage (-): 0

Region: chr10 100972237-100972279. Max. coverage (+): 0. Max coverage (-): 0

Region: chr10 100972280-100972322. Max. coverage (+): 0. Max coverage (-): 0

Region: chr10 100972323-100972364. Max. coverage (+): 14.78. Max coverage (-): 0

Region: chr10 100972365-100972407. Max. coverage (+): 3.03. Max coverage (-): 0

Region: chr10 100972408-100972450. Max. coverage (+): 0. Max coverage (-): 0

Region: chr10 100972451-100972493. Max. coverage (+): 0. Max coverage (-): 0

Region: chr10 100972494-100972536. Max. coverage (+): 0. Max coverage (-): 0

Region: chr10 100972537-100972578. Max. coverage (+): 0. Max coverage (-): 0

Region: chr10 100972579-100972621. Max. coverage (+): 0. Max coverage (-): 0

Region: chr10 100972622-100972664. Max. coverage (+): 0. Max coverage (-): 0

Region: chr10 100972665-100972707. Max. coverage (+): 0. Max coverage (-): 0

Region: chr10 100972708-100972750. Max. coverage (+): 0. Max coverage (-): 0

Region: chr10 100972751-100972792. Max. coverage (+): 0. Max coverage (-): 0

Region: chr10 100972793-100972835. Max. coverage (+): 0. Max coverage (-): 0

Region: chr10 100972836-100972878. Max. coverage (+): 0. Max coverage (-): 0

Region: chr10 100972879-100972921. Max. coverage (+): 0. Max coverage (-): 0

Region: chr10 100972922-100972964. Max. coverage (+): 19.02. Max coverage (-): 0

Region: chr10 100972965-100973006. Max. coverage (+): 0. Max coverage (-): 0

Region: chr10 100973007-100973049. Max. coverage (+): 0. Max coverage (-): 0

Region: chr10 100973050-100973092. Max. coverage (+): 0. Max coverage (-): 0

Region: chr10 100973093-100973135. Max. coverage (+): 0. Max coverage (-): 0

Region: chr10 100973136-100973178. Max. coverage (+): 0. Max coverage (-): 0

Region: chr10 100973179-100973220. Max. coverage (+): 0. Max coverage (-): 0

Region: chr10 100973221-100973263. Max. coverage (+): 0. Max coverage (-): 0

Region: chr10 100973264-100973306. Max. coverage (+): 0. Max coverage (-): 0

Region: chr10 100973307-100973349. Max. coverage (+): 0. Max coverage (-): 0

Region: chr10 100973350-100973392. Max. coverage (+): 0. Max coverage (-): 0

Region: chr10 100973393-100973434. Max. coverage (+): 0. Max coverage (-): 0

Region: chr10 100973435-100973477. Max. coverage (+): 0. Max coverage (-): 0

Region: chr10 100973478-100973520. Max. coverage (+): 12.77. Max coverage (-): 0

Region: chr10 100973521-100973563. Max. coverage (+): 3.99. Max coverage (-): 0

Region: chr10 100973564-100973606. Max. coverage (+): 0. Max coverage (-): 0

Region: chr10 100973607-100973648. Max. coverage (+): 21.91. Max coverage (-): 0

Region: chr10 100973649-100973691. Max. coverage (+): 1.46. Max coverage (-): 0

Region: chr10 100973692-100973734. Max. coverage (+): 0. Max coverage (-): 0

Region: chr10 100973735-100973777. Max. coverage (+): 11.25. Max coverage (-): 0

Region: chr10 100973778-100973820. Max. coverage (+): 11.25. Max coverage (-): 0

Region: chr10 100973821-100973862. Max. coverage (+): 0. Max coverage (-): 0

Region: chr10 100973863-100973905. Max. coverage (+): 2.47. Max coverage (-): 0

Region: chr10 100973906-100973948. Max. coverage (+): 0. Max coverage (-): 0

Region: chr10 100973949-100973991. Max. coverage (+): 24.28. Max coverage (-): 0

Region: chr10 100973992-100974034. Max. coverage (+): 0. Max coverage (-): 0

Region: chr10 100974035-100974076. Max. coverage (+): 0. Max coverage (-): 0

Region: chr10 100974077-100974119. Max. coverage (+): 0. Max coverage (-): 0

Region: chr10 100974120-100974162. Max. coverage (+): 0. Max coverage (-): 0

Region: chr10 100974163-100974205. Max. coverage (+): 4.41. Max coverage (-): 0

Region: chr10 100974206-100974248. Max. coverage (+): 0. Max coverage (-): 0

Region: chr10 100974249-100974290. Max. coverage (+): 0. Max coverage (-): 0

Region: chr10 100974291-100974333. Max. coverage (+): 0. Max coverage (-): 0

Region: chr10 100974334-100974376. Max. coverage (+): 0. Max coverage (-): 0

Region: chr10 100974377-100974419. Max. coverage (+): 0. Max coverage (-): 0

Region: chr10 100974420-100974461. Max. coverage (+): 0. Max coverage (-): 0

Region: chr10 100974462-100974504. Max. coverage (+): 0. Max coverage (-): 0

Region: chr10 100974505-100974547. Max. coverage (+): 0. Max coverage (-): 0

Region: chr10 100974548-100974590. Max. coverage (+): 0. Max coverage (-): 0

Region: chr10 100974591-100974633. Max. coverage (+): 0. Max coverage (-): 0

Region: chr10 100974634-100974675. Max. coverage (+): 0. Max coverage (-): 0

Region: chr10 100974676-100974718. Max. coverage (+): 0. Max coverage (-): 0

Region: chr10 100974719-100974761. Max. coverage (+): 0. Max coverage (-): 0

Region: chr10 100974762-100974804. Max. coverage (+): 0. Max coverage (-): 0

Region: chr10 100974805-100974847. Max. coverage (+): 2.87. Max coverage (-): 0

Region: chr10 100974848-100974889. Max. coverage (+): 0. Max coverage (-): 0

Region: chr10 100974890-100974932. Max. coverage (+): 6.71. Max coverage (-): 0

Region: chr10 100974933-100974975. Max. coverage (+): 0. Max coverage (-): 0

Region: chr10 100974976-100975018. Max. coverage (+): 12.76. Max coverage (-): 0

Region: chr10 100975019-100975061. Max. coverage (+): 12.76. Max coverage (-): 0

Region: chr10 100975062-100975103. Max. coverage (+): 9.56. Max coverage (-): 0

Region: chr10 100975104-100975146. Max. coverage (+): 0. Max coverage (-): 0

Region: chr10 100975147-100975189. Max. coverage (+): 13.9. Max coverage (-): 0

Region: chr10 100975190-100975232. Max. coverage (+): 0. Max coverage (-): 0

Region: chr10 100975233-100975275. Max. coverage (+): 0. Max coverage (-): 0

Region: chr10 100975276-100975317. Max. coverage (+): 0. Max coverage (-): 0

Region: chr10 100975318-100975360. Max. coverage (+): 0. Max coverage (-): 0

Region: chr10 100975361-100975403. Max. coverage (+): 0. Max coverage (-): 0

Region: chr10 100975404-100975446. Max. coverage (+): 0. Max coverage (-): 0

Region: chr10 100975447-100975489. Max. coverage (+): 0. Max coverage (-): 0

Region: chr10 100975490-100975531. Max. coverage (+): 0. Max coverage (-): 0

Region: chr10 100975532-100975574. Max. coverage (+): 0. Max coverage (-): 0

Region: chr10 100975575-100975617. Max. coverage (+): 0. Max coverage (-): 0

Region: chr10 100975618-100975660. Max. coverage (+): 0. Max coverage (-): 0

Region: chr10 100975661-100975703. Max. coverage (+): 0. Max coverage (-): 0

Region: chr10 100975704-100975745. Max. coverage (+): 0. Max coverage (-): 0

Region: chr10 100975746-100975788. Max. coverage (+): 6.49. Max coverage (-): 0

Region: chr10 100975789-100975831. Max. coverage (+): 6.49. Max coverage (-): 0

Region: chr10 100975832-100975874. Max. coverage (+): 0. Max coverage (-): 0

Region: chr10 100975875-100975917. Max. coverage (+): 0. Max coverage (-): 0

Region: chr10 100975918-100975959. Max. coverage (+): 9.86. Max coverage (-): 0

Region: chr10 100975960-100976002. Max. coverage (+): 0. Max coverage (-): 0

Region: chr10 100976003-100976045. Max. coverage (+): 0. Max coverage (-): 0

Region: chr10 100976046-100976088. Max. coverage (+): 0. Max coverage (-): 0

Region: chr10 100976089-100976131. Max. coverage (+): 0. Max coverage (-): 0

Region: chr10 100976132-100976173. Max. coverage (+): 0. Max coverage (-): 0

Region: chr10 100976174-100976216. Max. coverage (+): 0. Max coverage (-): 0

Region: chr10 100976217-100976259. Max. coverage (+): 0. Max coverage (-): 0

Region: chr10 100976260-100976302. Max. coverage (+): 0. Max coverage (-): 0

Region: chr10 100976303-100976345. Max. coverage (+): 2.83. Max coverage (-): 0

Region: chr10 100976346-100976387. Max. coverage (+): 2.83. Max coverage (-): 0

Region: chr10 100976388-100976430. Max. coverage (+): 0. Max coverage (-): 0

Region: chr10 100976431-100976473. Max. coverage (+): 0. Max coverage (-): 0

Region: chr10 100976474-100976516. Max. coverage (+): 6.45. Max coverage (-): 0

Region: chr10 100976517-100976558. Max. coverage (+): 0. Max coverage (-): 0

Region: chr10 100976559-100976601. Max. coverage (+): 0. Max coverage (-): 0

Region: chr10 100976602-100976644. Max. coverage (+): 0. Max coverage (-): 0

Region: chr10 100976645-100976687. Max. coverage (+): 0. Max coverage (-): 0

Region: chr10 100976688-100976730. Max. coverage (+): 0. Max coverage (-): 0

Region: chr10 100976731-100976772. Max. coverage (+): 0. Max coverage (-): 0

Region: chr10 100976773-100976815. Max. coverage (+): 0. Max coverage (-): 0

Region: chr10 100976816-100976858. Max. coverage (+): 0. Max coverage (-): 0

Region: chr10 100976859-100976901. Max. coverage (+): 0. Max coverage (-): 0

Region: chr10 100976902-100976944. Max. coverage (+): 0. Max coverage (-): 0

Region: chr10 100976945-100976986. Max. coverage (+): 0. Max coverage (-): 0

Region: chr10 100976987-100977029. Max. coverage (+): 0. Max coverage (-): 0

Region: chr10 100977030-100977072. Max. coverage (+): 12.64. Max coverage (-): 0

Region: chr10 100977073-100977115. Max. coverage (+): 0. Max coverage (-): 0

Region: chr10 100977116-100977158. Max. coverage (+): 0. Max coverage (-): 0

Region: chr10 100977159-100977200. Max. coverage (+): 0. Max coverage (-): 0

Region: chr10 100977201-100977243. Max. coverage (+): 4.93. Max coverage (-): 0

Region: chr10 100977244-100977286. Max. coverage (+): 0. Max coverage (-): 0

Region: chr10 100977287-100977329. Max. coverage (+): 0. Max coverage (-): 0

Region: chr10 100977330-100977372. Max. coverage (+): 0. Max coverage (-): 0

Region: chr10 100977373-100977414. Max. coverage (+): 3.53. Max coverage (-): 0

Region: chr10 100977415-100977457. Max. coverage (+): 0. Max coverage (-): 0

Region: chr10 100977458-100977500. Max. coverage (+): 0. Max coverage (-): 0

Region: chr10 100977501-100977543. Max. coverage (+): 0. Max coverage (-): 0

Region: chr10 100977544-100977586. Max. coverage (+): 0. Max coverage (-): 0

Region: chr10 100977587-100977628. Max. coverage (+): 0. Max coverage (-): 0

Region: chr10 100977629-100977671. Max. coverage (+): 0. Max coverage (-): 0

Region: chr10 100977672-100977714. Max. coverage (+): 0. Max coverage (-): 0

Region: chr10 100977715-100977757. Max. coverage (+): 0. Max coverage (-): 0

Region: chr10 100977758-100977800. Max. coverage (+): 0. Max coverage (-): 0

Region: chr10 100977801-100977842. Max. coverage (+): 0. Max coverage (-): 0

Region: chr10 100977843-100977885. Max. coverage (+): 0. Max coverage (-): 0

Region: chr10 100977886-100977928. Max. coverage (+): 0. Max coverage (-): 0

Region: chr10 100977929-100977971. Max. coverage (+): 0. Max coverage (-): 0

Region: chr10 100977972-100978014. Max. coverage (+): 0. Max coverage (-): 0

Region: chr10 100978015-100978056. Max. coverage (+): 7.69. Max coverage (-): 0

Region: chr10 100978057-100978099. Max. coverage (+): 0. Max coverage (-): 0

Region: chr10 100978100-100978142. Max. coverage (+): 0. Max coverage (-): 0

Region: chr10 100978143-100978185. Max. coverage (+): 1.24. Max coverage (-): 0

Region: chr10 100978186-100978228. Max. coverage (+): 1.24. Max coverage (-): 0

Region: chr10 100978229-100978270. Max. coverage (+): 0. Max coverage (-): 0

Region: chr10 100978271-100978313. Max. coverage (+): 0. Max coverage (-): 0

Region: chr10 100978314-100978356. Max. coverage (+): 3.46. Max coverage (-): 0

Region: chr10 100978357-100978399. Max. coverage (+): 7.42. Max coverage (-): 0

Region: chr10 100978400-100978442. Max. coverage (+): 0. Max coverage (-): 0

Region: chr10 100978443-100978484. Max. coverage (+): 0. Max coverage (-): 0

Region: chr10 100978485-100978527. Max. coverage (+): 0. Max coverage (-): 0

Region: chr10 100978528-100978570. Max. coverage (+): 0. Max coverage (-): 0

Region: chr10 100978571-100978613. Max. coverage (+): 0. Max coverage (-): 0

Region: chr10 100978614-100978655. Max. coverage (+): 0. Max coverage (-): 0

Region: chr10 100978656-100978698. Max. coverage (+): 0. Max coverage (-): 0

Region: chr10 100978699-100978741. Max. coverage (+): 0. Max coverage (-): 0

Region: chr10 100978742-100978784. Max. coverage (+): 0. Max coverage (-): 0

Region: chr10 100978785-100978827. Max. coverage (+): 18.46. Max coverage (-): 0

Region: chr10 100978828-100978869. Max. coverage (+): 0. Max coverage (-): 0

Region: chr10 100978870-100978912. Max. coverage (+): 1.61. Max coverage (-): 0

Region: chr10 100978913-100978955. Max. coverage (+): 0. Max coverage (-): 0

Region: chr10 100978956-100978998. Max. coverage (+): 0. Max coverage (-): 0

Region: chr10 100978999-100979041. Max. coverage (+): 0. Max coverage (-): 0

Region: chr10 100979042-100979083. Max. coverage (+): 0. Max coverage (-): 0

Region: chr10 100979084-100979126. Max. coverage (+): 0. Max coverage (-): 0

Region: chr10 100979127-100979169. Max. coverage (+): 13.6. Max coverage (-): 0

Region: chr10 100979170-100979212. Max. coverage (+): 22.39. Max coverage (-): 0

Region: chr10 100979213-100979255. Max. coverage (+): 3.24. Max coverage (-): 0

Region: chr10 100979256-100979297. Max. coverage (+): 3.71. Max coverage (-): 0

Region: chr10 100979298-100979340. Max. coverage (+): 0. Max coverage (-): 0

Region: chr10 100979341-100979383. Max. coverage (+): 0. Max coverage (-): 0

Region: chr10 100979384-100979426. Max. coverage (+): 4.7. Max coverage (-): 0

Region: chr10 100979427-100979469. Max. coverage (+): 0. Max coverage (-): 0

Region: chr10 100979470-100979511. Max. coverage (+): 0. Max coverage (-): 0

Region: chr10 100979512-100979554. Max. coverage (+): 0. Max coverage (-): 0

Region: chr10 100979555-100979597. Max. coverage (+): 0.96. Max coverage (-): 0

Region: chr10 100979598-100979640. Max. coverage (+): 6.05. Max coverage (-): 0

Region: chr10 100979641-100979683. Max. coverage (+): 0. Max coverage (-): 0

Region: chr10 100979684-100979725. Max. coverage (+): 0. Max coverage (-): 0

Region: chr10 100979726-100979768. Max. coverage (+): 0. Max coverage (-): 0

Region: chr10 100979769-100979811. Max. coverage (+): 1.88. Max coverage (-): 0

Region: chr10 100979812-100979854. Max. coverage (+): 3.04. Max coverage (-): 0

Region: chr10 100979855-100979897. Max. coverage (+): 0. Max coverage (-): 0

Region: chr10 100979898-100979939. Max. coverage (+): 0. Max coverage (-): 0

Region: chr10 100979940-100979982. Max. coverage (+): 0. Max coverage (-): 0

Region: chr10 100979983-100980025. Max. coverage (+): 0. Max coverage (-): 0

Region: chr10 100980026-100980068. Max. coverage (+): 0. Max coverage (-): 0

Region: chr10 100980069-100980111. Max. coverage (+): 0. Max coverage (-): 0

Region: chr10 100980112-100980153. Max. coverage (+): 5.46. Max coverage (-): 0

Region: chr10 100980154-100980196. Max. coverage (+): 0. Max coverage (-): 0

Region: chr10 100980197-100980239. Max. coverage (+): 0. Max coverage (-): 0

Region: chr10 100980240-100980282. Max. coverage (+): 0. Max coverage (-): 0

Region: chr10 100980283-100980325. Max. coverage (+): 0. Max coverage (-): 0

Region: chr10 100980326-100980367. Max. coverage (+): 5.15. Max coverage (-): 0

Region: chr10 100980368-100980410. Max. coverage (+): 5.79. Max coverage (-): 0

Region: chr10 100980411-100980453. Max. coverage (+): 0. Max coverage (-): 0

Region: chr10 100980454-100980496. Max. coverage (+): 5.57. Max coverage (-): 0

Region: chr10 100980497-100980539. Max. coverage (+): 0. Max coverage (-): 0

Region: chr10 100980540-100980581. Max. coverage (+): 0. Max coverage (-): 0

Region: chr10 100980582-100980624. Max. coverage (+): 7.26. Max coverage (-): 0

Region: chr10 100980625-100980667. Max. coverage (+): 7.26. Max coverage (-): 0

Region: chr10 100980668-100980710. Max. coverage (+): 0. Max coverage (-): 0

Region: chr10 100980711-100980752. Max. coverage (+): 2.29. Max coverage (-): 0

Region: chr10 100980753-100980795. Max. coverage (+): 3.09. Max coverage (-): 0

Region: chr10 100980796-100980838. Max. coverage (+): 6.57. Max coverage (-): 0

Region: chr10 100980839-100980881. Max. coverage (+): 6.57. Max coverage (-): 0

Region: chr10 100980882-100980924. Max. coverage (+): 0. Max coverage (-): 0

Region: chr10 100980925-100980966. Max. coverage (+): 0. Max coverage (-): 0

Region: chr10 100980967-100981009. Max. coverage (+): 6.41. Max coverage (-): 0

Region: chr10 100981010-100981052. Max. coverage (+): 0. Max coverage (-): 0

Region: chr10 100981053-100981095. Max. coverage (+): 0. Max coverage (-): 0

Region: chr10 100981096-100981138. Max. coverage (+): 0. Max coverage (-): 0

Region: chr10 100981139-100981180. Max. coverage (+): 2.2. Max coverage (-): 0

Region: chr10 100981181-100981223. Max. coverage (+): 5.73. Max coverage (-): 0

Region: chr10 100981224-100981266. Max. coverage (+): 5.73. Max coverage (-): 0

Region: chr10 100981267-100981309. Max. coverage (+): 0. Max coverage (-): 0

Region: chr10 100981310-100981352. Max. coverage (+): 1.72. Max coverage (-): 0

Region: chr10 100981353-100981394. Max. coverage (+): 5.56. Max coverage (-): 0

Region: chr10 100981395-100981437. Max. coverage (+): 0. Max coverage (-): 0

Region: chr10 100981438-100981480. Max. coverage (+): 0. Max coverage (-): 0

Region: chr10 100981481-100981523. Max. coverage (+): 0. Max coverage (-): 0

Region: chr10 100981524-100981566. Max. coverage (+): 0. Max coverage (-): 0

Region: chr10 100981567-100981608. Max. coverage (+): 1.65. Max coverage (-): 0

Region: chr10 100981609-100981651. Max. coverage (+): 9.94. Max coverage (-): 0

Region: chr10 100981652-100981694. Max. coverage (+): 0. Max coverage (-): 0

Region: chr10 100981695-100981737. Max. coverage (+): 0. Max coverage (-): 0

Region: chr10 100981738-100981780. Max. coverage (+): 0. Max coverage (-): 0

Region: chr10 100981781-100981822. Max. coverage (+): 0. Max coverage (-): 0

Region: chr10 100981823-100981865. Max. coverage (+): 0. Max coverage (-): 0

Region: chr10 100981866-100981908. Max. coverage (+): 0. Max coverage (-): 0

Region: chr10 100981909-100981951. Max. coverage (+): 0. Max coverage (-): 0

Region: chr10 100981952-100981994. Max. coverage (+): 0. Max coverage (-): 0

Region: chr10 100981995-100982036. Max. coverage (+): 0. Max coverage (-): 0

Region: chr10 100982037-100982079. Max. coverage (+): 0. Max coverage (-): 0

Region: chr10 100982080-100982122. Max. coverage (+): 0. Max coverage (-): 0

Region: chr10 100982123-100982165. Max. coverage (+): 0. Max coverage (-): 0

Region: chr10 100982166-100982208. Max. coverage (+): 0. Max coverage (-): 0

Region: chr10 100982209-100982250. Max. coverage (+): 0. Max coverage (-): 0

Region: chr10 100982251-100982293. Max. coverage (+): 0. Max coverage (-): 0

Region: chr10 100982294-100982336. Max. coverage (+): 0. Max coverage (-): 0

Region: chr10 100982337-100982379. Max. coverage (+): 0. Max coverage (-): 0

Region: chr10 100982380-100982422. Max. coverage (+): 0. Max coverage (-): 0

Region: chr10 100982423-100982464. Max. coverage (+): 0. Max coverage (-): 0

Region: chr10 100982465-100982507. Max. coverage (+): 0. Max coverage (-): 0

Region: chr10 100982508-100982550. Max. coverage (+): 0. Max coverage (-): 0

Region: chr10 100982551-100982593. Max. coverage (+): 0. Max coverage (-): 0

Region: chr10 100982594-100982636. Max. coverage (+): 0. Max coverage (-): 0

Region: chr10 100982637-100982678. Max. coverage (+): 0. Max coverage (-): 0

Region: chr10 100982679-100982721. Max. coverage (+): 2.94. Max coverage (-): 0

Region: chr10 100982722-100982764. Max. coverage (+): 0. Max coverage (-): 0

Region: chr10 100982765-100982807. Max. coverage (+): 0. Max coverage (-): 0

Region: chr10 100982808-100982849. Max. coverage (+): 0. Max coverage (-): 0

Region: chr10 100982850-100982892. Max. coverage (+): 0. Max coverage (-): 0

Region: chr10 100982893-100982935. Max. coverage (+): 0. Max coverage (-): 0

Region: chr10 100982936-100982978. Max. coverage (+): 2.58. Max coverage (-): 0

Region: chr10 100982979-100983021. Max. coverage (+): 1.88. Max coverage (-): 0

Region: chr10 100983022-100983063. Max. coverage (+): 0. Max coverage (-): 0

Region: chr10 100983064-100983106. Max. coverage (+): 0. Max coverage (-): 0

Region: chr10 100983107-100983149. Max. coverage (+): 16.43. Max coverage (-): 0

Region: chr10 100983150-100983192. Max. coverage (+): 0. Max coverage (-): 0

Region: chr10 100983193-100983235. Max. coverage (+): 2.37. Max coverage (-): 0

Region: chr10 100983236-100983277. Max. coverage (+): 0. Max coverage (-): 0

Region: chr10 100983278-100983320. Max. coverage (+): 0. Max coverage (-): 0

Region: chr10 100983321-100983363. Max. coverage (+): 0. Max coverage (-): 0

Region: chr10 100983364-100983406. Max. coverage (+): 2.16. Max coverage (-): 0

Region: chr10 100983407-100983449. Max. coverage (+): 0. Max coverage (-): 0

Region: chr10 100983450-100983491. Max. coverage (+): 0. Max coverage (-): 0

Region: chr10 100983492-100983534. Max. coverage (+): 0. Max coverage (-): 0

Region: chr10 100983535-100983577. Max. coverage (+): 0. Max coverage (-): 0

Region: chr10 100983578-100983620. Max. coverage (+): 0. Max coverage (-): 0

Region: chr10 100983621-100983663. Max. coverage (+): 0. Max coverage (-): 0

Region: chr10 100983664-100983705. Max. coverage (+): 0. Max coverage (-): 0

Region: chr10 100983706-100983748. Max. coverage (+): 0. Max coverage (-): 0

Region: chr10 100983749-100983791. Max. coverage (+): 0. Max coverage (-): 0

Region: chr10 100983792-100983834. Max. coverage (+): 0. Max coverage (-): 0

Region: chr10 100983835-100983877. Max. coverage (+): 0. Max coverage (-): 0

Region: chr10 100983878-100983919. Max. coverage (+): 0. Max coverage (-): 0

Region: chr10 100983920-100983962. Max. coverage (+): 0. Max coverage (-): 0

Region: chr10 100983963-100984005. Max. coverage (+): 0. Max coverage (-): 0

Region: chr10 100984006-100984048. Max. coverage (+): 0. Max coverage (-): 0

Region: chr10 100984049-100984091. Max. coverage (+): 0. Max coverage (-): 0

Region: chr10 100984092-100984133. Max. coverage (+): 0. Max coverage (-): 0

Region: chr10 100984134-100984176. Max. coverage (+): 0. Max coverage (-): 0

Region: chr10 100984177-100984219. Max. coverage (+): 38.3. Max coverage (-): 0

Region: chr10 100984220-100984262. Max. coverage (+): 2.74. Max coverage (-): 0

Region: chr10 100984263-100984305. Max. coverage (+): 2.55. Max coverage (-): 0

Region: chr10 100984306-100984347. Max. coverage (+): 0. Max coverage (-): 0

Region: chr10 100984348-100984390. Max. coverage (+): 0. Max coverage (-): 0

Region: chr10 100984391-100984433. Max. coverage (+): 0. Max coverage (-): 0

Region: chr10 100984434-100984476. Max. coverage (+): 0. Max coverage (-): 0

Region: chr10 100984477-100984519. Max. coverage (+): 0. Max coverage (-): 0

Region: chr10 100984520-100984561. Max. coverage (+): 0. Max coverage (-): 0

Region: chr10 100984562-100984604. Max. coverage (+): 0. Max coverage (-): 0

Region: chr10 100984605-100984647. Max. coverage (+): 0. Max coverage (-): 0

Region: chr10 100984648-100984690. Max. coverage (+): 0. Max coverage (-): 0

Region: chr10 100984691-100984733. Max. coverage (+): 0. Max coverage (-): 0

Region: chr10 100984734-100984775. Max. coverage (+): 0. Max coverage (-): 0

Region: chr10 100984776-100984818. Max. coverage (+): 5.43. Max coverage (-): 0

Region: chr10 100984819-100984861. Max. coverage (+): 31.16. Max coverage (-): 0

Region: chr10 100984862-100984904. Max. coverage (+): 8.34. Max coverage (-): 0

Region: chr10 100984905-100984947. Max. coverage (+): 0. Max coverage (-): 0

Region: chr10 100984948-100984989. Max. coverage (+): 0. Max coverage (-): 0

Region: chr10 100984990-100985032. Max. coverage (+): 0.02. Max coverage (-): 0

Region: chr10 100985033-100985075. Max. coverage (+): 15.32. Max coverage (-): 0

Region: chr10 100985076-100985118. Max. coverage (+): 5.76. Max coverage (-): 0

Region: chr10 100985119-100985160. Max. coverage (+): 21.44. Max coverage (-): 0

Region: chr10 100985161-100985203. Max. coverage (+): 0. Max coverage (-): 0

Region: chr10 100985204-100985246. Max. coverage (+): 0. Max coverage (-): 0

Region: chr10 100985247-100985289. Max. coverage (+): 0. Max coverage (-): 0

Region: chr10 100985290-100985332. Max. coverage (+): 7.01. Max coverage (-): 0

Region: chr10 100985333-100985374. Max. coverage (+): 11.88. Max coverage (-): 0

Region: chr10 100985375-100985417. Max. coverage (+): 0. Max coverage (-): 0

Region: chr10 100985418-100985460. Max. coverage (+): 0. Max coverage (-): 0

Region: chr10 100985461-100985503. Max. coverage (+): 0. Max coverage (-): 0

Region: chr10 100985504-100985546. Max. coverage (+): 0. Max coverage (-): 0

Region: chr10 100985547-100985588. Max. coverage (+): 3.08. Max coverage (-): 0

Region: chr10 100985589-100985631. Max. coverage (+): 6.68. Max coverage (-): 0

Region: chr10 100985632-100985674. Max. coverage (+): 6.68. Max coverage (-): 0

Region: chr10 100985675-100985717. Max. coverage (+): 0. Max coverage (-): 0

Region: chr10 100985718-100985760. Max. coverage (+): 0. Max coverage (-): 0

Region: chr10 100985761-100985802. Max. coverage (+): 0. Max coverage (-): 0

Region: chr10 100985803-100985845. Max. coverage (+): 0. Max coverage (-): 0

Region: chr10 100985846-100985888. Max. coverage (+): 0. Max coverage (-): 0

Region: chr10 100985889-100985931. Max. coverage (+): 0. Max coverage (-): 0

Region: chr10 100985932-100985974. Max. coverage (+): 0. Max coverage (-): 0

Region: chr10 100985975-100986016. Max. coverage (+): 0. Max coverage (-): 0

Region: chr10 100986017-100986059. Max. coverage (+): 0. Max coverage (-): 0

Region: chr10 100986060-100986102. Max. coverage (+): 0. Max coverage (-): 0

Region: chr10 100986103-100986145. Max. coverage (+): 0. Max coverage (-): 0

Region: chr10 100986146-100986188. Max. coverage (+): 0. Max coverage (-): 0

Region: chr10 100986189-100986230. Max. coverage (+): 0. Max coverage (-): 0

Region: chr10 100986231-100986273. Max. coverage (+): 0. Max coverage (-): 0

Region: chr10 100986274-100986316. Max. coverage (+): 0. Max coverage (-): 0

Region: chr10 100986317-100986359. Max. coverage (+): 0. Max coverage (-): 0

Region: chr10 100986360-100986402. Max. coverage (+): 0.04. Max coverage (-): 0

Region: chr10 100986403-100986444. Max. coverage (+): 0. Max coverage (-): 0

Region: chr10 100986445-100986487. Max. coverage (+): 0. Max coverage (-): 0

Region: chr10 100986488-100986530. Max. coverage (+): 0. Max coverage (-): 0

Region: chr10 100986531-100986573. Max. coverage (+): 0. Max coverage (-): 0

Region: chr10 100986574-100986616. Max. coverage (+): 0. Max coverage (-): 0

Region: chr10 100986617-100986658. Max. coverage (+): 0. Max coverage (-): 0

Region: chr10 100986659-100986701. Max. coverage (+): 0. Max coverage (-): 0

Region: chr10 100986702-100986744. Max. coverage (+): 3.17. Max coverage (-): 0

Region: chr10 100986745-100986787. Max. coverage (+): 5.6. Max coverage (-): 0

Region: chr10 100986788-100986830. Max. coverage (+): 0. Max coverage (-): 0

Region: chr10 100986831-100986872. Max. coverage (+): 0. Max coverage (-): 0

Region: chr10 100986873-100986915. Max. coverage (+): 0. Max coverage (-): 0

Region: chr10 100986916-100986958. Max. coverage (+): 0. Max coverage (-): 0

Region: chr10 100986959-100987001. Max. coverage (+): 0. Max coverage (-): 0

Region: chr10 100987002-100987044. Max. coverage (+): 0. Max coverage (-): 0

Region: chr10 100987045-100987086. Max. coverage (+): 6.05. Max coverage (-): 0

Region: chr10 100987087-100987129. Max. coverage (+): 0. Max coverage (-): 0

Region: chr10 100987130-100987172. Max. coverage (+): 0. Max coverage (-): 0

Region: chr10 100987173-100987215. Max. coverage (+): 0. Max coverage (-): 0

Region: chr10 100987216-100987257. Max. coverage (+): 0. Max coverage (-): 0

Region: chr10 100987258-100987300. Max. coverage (+): 0. Max coverage (-): 0

Region: chr10 100987301-100987343. Max. coverage (+): 0. Max coverage (-): 0

Region: chr10 100987344-100987386. Max. coverage (+): 0. Max coverage (-): 0

Region: chr10 100987387-100987429. Max. coverage (+): 0. Max coverage (-): 0

Region: chr10 100987430-100987471. Max. coverage (+): 0. Max coverage (-): 0

Region: chr10 100987472-100987514. Max. coverage (+): 0. Max coverage (-): 0

Region: chr10 100987515-100987557. Max. coverage (+): 0. Max coverage (-): 0

Region: chr10 100987558-100987600. Max. coverage (+): 0. Max coverage (-): 0

Region: chr10 100987601-100987643. Max. coverage (+): 0. Max coverage (-): 0

Region: chr10 100987644-100987685. Max. coverage (+): 0. Max coverage (-): 0

Region: chr10 100987686-100987728. Max. coverage (+): 0. Max coverage (-): 0

Region: chr10 100987729-100987771. Max. coverage (+): 3.66. Max coverage (-): 0

Region: chr10 100987772-100987814. Max. coverage (+): 3.26. Max coverage (-): 0

Region: chr10 100987815-100987857. Max. coverage (+): 0. Max coverage (-): 0

Region: chr10 100987858-100987899. Max. coverage (+): 0. Max coverage (-): 0

Region: chr10 100987900-100987942. Max. coverage (+): 0. Max coverage (-): 0

Region: chr10 100987943-100987985. Max. coverage (+): 0. Max coverage (-): 0

Region: chr10 100987986-100988028. Max. coverage (+): 0. Max coverage (-): 0

Region: chr10 100988029-100988071. Max. coverage (+): 0. Max coverage (-): 0

Region: chr10 100988072-100988113. Max. coverage (+): 0. Max coverage (-): 0

Region: chr10 100988114-100988156. Max. coverage (+): 0. Max coverage (-): 0

Region: chr10 100988157-100988199. Max. coverage (+): 4.57. Max coverage (-): 0

Region: chr10 100988200-100988242. Max. coverage (+): 0. Max coverage (-): 0

Region: chr10 100988243-100988285. Max. coverage (+): 0. Max coverage (-): 0

Region: chr10 100988286-100988327. Max. coverage (+): 7.69. Max coverage (-): 0

Region: chr10 100988328-100988370. Max. coverage (+): 0. Max coverage (-): 0

Region: chr10 100988371-100988413. Max. coverage (+): 0. Max coverage (-): 0

Region: chr10 100988414-100988456. Max. coverage (+): 0. Max coverage (-): 0

Region: chr10 100988457-100988499. Max. coverage (+): 5.66. Max coverage (-): 0

Region: chr10 100988500-100988541. Max. coverage (+): 0. Max coverage (-): 0

Region: chr10 100988542-100988584. Max. coverage (+): 0. Max coverage (-): 0

Region: chr10 100988585-100988627. Max. coverage (+): 0. Max coverage (-): 0

Region: chr10 100988628-100988670. Max. coverage (+): 0. Max coverage (-): 0

Region: chr10 100988671-100988713. Max. coverage (+): 0. Max coverage (-): 0

Region: chr10 100988714-100988755. Max. coverage (+): 3.67. Max coverage (-): 0

Region: chr10 100988756-100988798. Max. coverage (+): 0. Max coverage (-): 0

Region: chr10 100988799-100988841. Max. coverage (+): 0. Max coverage (-): 0

Region: chr10 100988842-100988884. Max. coverage (+): 0. Max coverage (-): 0

Region: chr10 100988885-100988927. Max. coverage (+): 0. Max coverage (-): 0

Region: chr10 100988928-100988969. Max. coverage (+): 0. Max coverage (-): 0

Region: chr10 100988970-100989012. Max. coverage (+): 0. Max coverage (-): 0

Region: chr10 100989013-100989055. Max. coverage (+): 0. Max coverage (-): 0

Region: chr10 100989056-100989098. Max. coverage (+): 0. Max coverage (-): 0

Region: chr10 100989099-100989141. Max. coverage (+): 0. Max coverage (-): 0

Region: chr10 100989142-100989183. Max. coverage (+): 0. Max coverage (-): 0

Region: chr10 100989184-100989226. Max. coverage (+): 3.04. Max coverage (-): 0

Region: chr10 100989227-. Max. coverage (+): 3.04. Max coverage (-): 0

RepeatMasker Color Code

**+**

100-98% Identity

<98-95% Identity

<95-90% Identity

<90-85% Identity

<85-80% Identity

<80-75% Identity

<75-70% Identity

<70% Identity

**-**

Gene Set Color Code

**+**

Gene

Pseudogene

**-**

Topology/Coverage Color Code

Coverage Plus Strand

Coverage Minus Strand

Mainstrand: Plus

Mainstrand: Minus

Complementary Strand

Flanking Region  
(if option -flank >0)

Gene Set Annotation  

**1. KCNK10 (protein coding, ENSBTAG00000019355) Tr:00000025779 Ex:4**: 100988552-100988738 (-)  
**2. KCNK10 (protein coding, ENSBTAG00000019355) Tr:00000025779 Ex:5**: 100984235-100984377 (-)  
**3. KCNK10 (protein coding, ENSBTAG00000019355) Tr:00000025779 Ex:6**: 100981353-100981970 (-)

  
RepeatMasker Annotation  

**1. MIR**: 100968311-100968497 (+), Divergence to consensus: 37.1%  
**2. L2c**: 100970875-100970973 (+), Divergence to consensus: 32.6%  
**3. Bov-tA3**: 100971033-100971227 (-), Divergence to consensus: 10.2%  
**4. AT\_rich**: 100971767-100971790 (+), Divergence to consensus: 50%  
**5. AT\_rich**: 100971769-100971792 (+), Divergence to consensus: 50%  
**6. L2c**: 100972188-100972319 (-), Divergence to consensus: 42.5%  
**7. Bov-tA1**: 100972720-100972933 (-), Divergence to consensus: 21.5%  
**8. Charlie1b**: 100972969-100973096 (-), Divergence to consensus: 35.2%  
**9. MER117**: 100973102-100973237 (+), Divergence to consensus: 39.2%  
**10. MIRb**: 100974467-100974599 (+), Divergence to consensus: 46%  
**11. Bov-tA2**: 100974609-100974797 (+), Divergence to consensus: 19.1%  
**12. ART2A**: 100974751-100974804 (+), Divergence to consensus: 27.8%  
**13. AT\_rich**: 100976323-100976343 (+), Divergence to consensus: 42.9%  
**14. MIR3**: 100977044-100977182 (-), Divergence to consensus: 44.8%  
**15. Bov-tA1**: 100977709-100977916 (-), Divergence to consensus: 22.2%  
**16. MIRb**: 100978562-100978787 (+), Divergence to consensus: 39.5%  
**17. Bov-tA2**: 100978970-100979154 (-), Divergence to consensus: 13%  
**18. GC\_rich**: 100981406-100981443 (+), Divergence to consensus: 76.3%  
**19. C-rich**: 100982027-100982191 (+), Divergence to consensus: 33%  
**20. T-rich**: 100983263-100983348 (+), Divergence to consensus: 30.2%  
**21. (CTG)n**: 100983910-100983934 (+), Divergence to consensus: 0%  
**22. BOV-A2**: 100983935-100984195 (-), Divergence to consensus: 5.5%  
**23. Bov-tA2**: 100984623-100984807 (-), Divergence to consensus: 26.5%  
**24. MIR3**: 100985225-100985306 (-), Divergence to consensus: 31%  
**25. L1-2\_BT**: 100986537-100986659 (+), Divergence to consensus: 38.2%  
**26. Charlie24**: 100988830-100988912 (-), Divergence to consensus: 22.2%

  
Transcription Factor Binding Sites  

**RFX4\_2** (Sequence: GTAACCAAG (-): 100985441)  
**RFX4\_1** (Sequence: GTTGCCATG (-): 100972539)  
**RFX4\_1** (Sequence: GTTGCCAGG (-): 100979705)  
**Gata4** (Sequence: AGATAAC (-): 100971605)  
**SOX9** (Sequence: AACAATGG (-): 100977392)  
**Gata4** (Sequence: GTTATCT (+): 100967910)  
**Gata4** (Sequence: GTTATCT (+): 100968059)  
**Gata4** (Sequence: CTTATCT (+): 100983326)
