# Supplementary material for: Small RNA sequencing of cryopreserved semen from single bull revealed altered miRNAs and piRNAs expression between High- and Low-motile sperm populations
Source: BMC Genomics. 2017 Jan 4;18:14. doi: 10.1186/s12864-016-3394-7 (PMC5209821; doi:10.1186/s12864-016-3394-7)
Supplement: Additional file 4: — Details for each piRNA clusters found in Low Motile (LM) sperm fraction. Genes, repeats, transposable elements and transcription factors binding sites falling within the cluster regions were reported. (ZIP 1034 kb) [file 12864_2016_3394_MOESM4_ESM.zip › 7.html]

piRNA cluster 7


Predicted piRNA cluster no. 7     previous   next
  

Show proTRAC run info
Hide proTRAC run info

================================= proTRAC ====================================  
VERSION: 2.1                                    LAST MODIFIED: 06. October 2015  
  
Please cite:  
Rosenkranz D, Zischler H. proTRAC - a software for probabilistic piRNA cluster  
detection, visualization and analysis. 2012. BMC Bioinformatics 13:5.  
  
and (for proTRAC 2.0 and later):  
Rosenkranz D, Rudloff S, Bastuck K, Ketting RF, Zischler H. Tupaia small RNAs  
provide insights into function and evolution of RNAi-based transposon defense  
in mammals. 2015. RNA 21(5):911-922.  
  
Contact:  
David Rosenkranz  
Institute of Anthropology, small RNA group  
Johannes Gutenberg University Mainz  
email: rosenkranz@uni-mainz.de  
  
You can find the latest proTRAC version at:  
http://sourceforge.net/projects/protrac/files  
http://www.smallRNAgroup-mainz.de/software  
==============================================================================  
  
PARAMETERS:  
Map file: .............../storage/core/barbara/genhome/smallRNA/fertility/Sample\_not\_motile/pirna/Sample\_not\_motile\_26-33\_collapsed.fa.no-dust.map.weighted-10000-1000-b-0  
Genome file: ............/storage/core/barbara/genhome/smallRNA/fertility/Sample\_all/pirna/bt\_311\_chrY.fa  
RepeatMasker annotation: /storage/genomes/bt\_umd31/GCF\_000003055.6\_Bos\_taurus\_UMD\_3.1.1\_repeatMasker\_chr.out  
GeneSet:................./storage/core/barbara/genhome/smallRNA/fertility/Sample\_all/pirna/full.gtf  
  
Significant (p<=0.01) hit density will be calculated based  
on observed hit distribution.  
  
Sliding window size: ........................................ 5000 bp  
Sliding window increament: .................................. 1000 bp  
Normalize each hit by number of genomic hits: ............... 1 [0=no/1=yes]  
Normalize each hit by number of sequence reads: ............. 1 [0=no/1=yes]  
Normalize values (-> per million mapped reads): ............. 1 [0=no/1=yes]  
Min. fraction of hits with 1T(U) or 10A: .................... 0.75  
Alternatively: Min. fraction of hits with 1T(U) and 10A: .... 0.5  
Min. fraction of hits with typical piRNA length: ............ 0.75  
Typical piRNA length: ....................................... 26-33 nt  
Min. size of a piRNA cluster: ............................... 5000 bp.  
Min. number of hits (absolute): ............................. 0  
Min. number of hits (normalized): ........................... 0  
Min. fraction of hits on the mainstrand: .................... 0.75  
Top fraction of mapped sequences (in terms of read counts): . 1%  
Top fraction accounts for max. n% of sequence reads: ........ 90%  
Min. fraction of hits on each arm of a bidirectional cluster: 0.1  
Output image file for each cluster: ......................... 0 [0=no/1=yes]  
Output html file for each cluster: .......................... 1 [0=no/1=yes]  
Output a summary table: ..................................... 1 [0=no/1=yes]  
Output a FASTA file for each cluster (piRNA sequences): ..... 1 [0=no/1=yes]  
Output a FASTA file comprising cluster sequences: ........... 1 [0=no/1=yes]  
Search DNA motifs in clusters: .............................. 1 [0=no/1=yes]  
Output flanking sequences: +/- .............................. 0 bp  
Output ~.pTi file: .......................................... 1 [0=no/1=yes]  
==============================================================================  
  
  
Genome size (without gaps): ............ 2678902517 bp  
Gaps (N/X/-): .......................... 53837044 bp  
Mapped reads: .......................... 738059667487  
Non-identical sequences: ............... 277001  
Genomic hits: .......................... 533816  
Significant densitiy of mapped reads: .. 15118061 reads/kb

Show proTRAC cluster info
Hide proTRAC cluster info

|  |  |
| --- | --- |
| Location | chr11 |
| Coordinates | 97604985-97610362 |
| Size [bp] | 5378 |
| Sequence hit loci | 67 |
| Mapped reads (normalized) | 182772358 |
| Mapped reads (normalized) per kb | 33985191.1 |
| Normalized reads with 1T (1U) | 92.6% |
| Normalized reads with 10A | 22.1% |
| Normalized reads with length 26-33 nt | 100% |
| Normalized reads on the main strand(s) | 100% |
| Predicted directionality | mono:plus |

100%

0%

1T (1U)  
reads

10A reads

26-33 nt  
reads

reads on mainstrand

**Either the amount of reads with 1T (1U) OR 10A has to exceed 75% (set with option: -1Tor10A)  
Alternatively the amount of reads with 1T (1U) AND 10A has to exceed 50% (set with option: -1Tand10A)  
Minimum amount of reads with preferred size is 75% (set with option: -pisize)  
Minimum amount of reads on the main strand(s) is 75% (set with option: -clstrand)**

Show read coverage
Hide read coverage

WHAT DO I SEE HERE?  
This chart shows the location of mapped sequence reads within a predicted piRNA cluster. The color refers to the number of genomic hits produced by the sequence read in question. A dark red bar indicates that this sequence read produces many other hits elsewhere in the genome. Many adjacent red or yellow bars can indicate the presence of a multi-copy element such as transposons or rRNA genes. A dark green bar indicates that this sequence read maps uniquely to this locus.

1 hit

2-5 hits

6-10 hits

11-20 hits

21-50 hits

51-100 hits

> 100 hits

chr11

97604985

97610362

Gene Set

RepeatMasker

Mapped  
Reads

35.91

plus strand

minus strand

35.91

Region: chr11 100989228-97604990. Max. coverage (+): 6.99. Max coverage (-): 0

Region: chr11 97604991-97605001. Max. coverage (+): 6.99. Max coverage (-): 0

Region: chr11 97605002-97605011. Max. coverage (+): 0. Max coverage (-): 0

Region: chr11 97605012-97605022. Max. coverage (+): 0. Max coverage (-): 0

Region: chr11 97605023-97605033. Max. coverage (+): 0. Max coverage (-): 0

Region: chr11 97605034-97605044. Max. coverage (+): 0. Max coverage (-): 0

Region: chr11 97605045-97605054. Max. coverage (+): 0. Max coverage (-): 0

Region: chr11 97605055-97605065. Max. coverage (+): 0. Max coverage (-): 0

Region: chr11 97605066-97605076. Max. coverage (+): 0. Max coverage (-): 0

Region: chr11 97605077-97605087. Max. coverage (+): 0. Max coverage (-): 0

Region: chr11 97605088-97605097. Max. coverage (+): 0. Max coverage (-): 0

Region: chr11 97605098-97605108. Max. coverage (+): 0. Max coverage (-): 0

Region: chr11 97605109-97605119. Max. coverage (+): 0. Max coverage (-): 0

Region: chr11 97605120-97605130. Max. coverage (+): 0. Max coverage (-): 0

Region: chr11 97605131-97605140. Max. coverage (+): 0. Max coverage (-): 0

Region: chr11 97605141-97605151. Max. coverage (+): 0. Max coverage (-): 0

Region: chr11 97605152-97605162. Max. coverage (+): 0. Max coverage (-): 0

Region: chr11 97605163-97605173. Max. coverage (+): 0. Max coverage (-): 0

Region: chr11 97605174-97605183. Max. coverage (+): 0. Max coverage (-): 0

Region: chr11 97605184-97605194. Max. coverage (+): 0. Max coverage (-): 0

Region: chr11 97605195-97605205. Max. coverage (+): 0. Max coverage (-): 0

Region: chr11 97605206-97605216. Max. coverage (+): 0. Max coverage (-): 0

Region: chr11 97605217-97605227. Max. coverage (+): 0. Max coverage (-): 0

Region: chr11 97605228-97605237. Max. coverage (+): 0. Max coverage (-): 0

Region: chr11 97605238-97605248. Max. coverage (+): 0. Max coverage (-): 0

Region: chr11 97605249-97605259. Max. coverage (+): 0. Max coverage (-): 0

Region: chr11 97605260-97605270. Max. coverage (+): 0. Max coverage (-): 0

Region: chr11 97605271-97605280. Max. coverage (+): 0. Max coverage (-): 0

Region: chr11 97605281-97605291. Max. coverage (+): 0. Max coverage (-): 0

Region: chr11 97605292-97605302. Max. coverage (+): 0. Max coverage (-): 0

Region: chr11 97605303-97605313. Max. coverage (+): 0. Max coverage (-): 0

Region: chr11 97605314-97605323. Max. coverage (+): 0. Max coverage (-): 0

Region: chr11 97605324-97605334. Max. coverage (+): 0. Max coverage (-): 0

Region: chr11 97605335-97605345. Max. coverage (+): 0. Max coverage (-): 0

Region: chr11 97605346-97605356. Max. coverage (+): 0. Max coverage (-): 0

Region: chr11 97605357-97605366. Max. coverage (+): 0. Max coverage (-): 0

Region: chr11 97605367-97605377. Max. coverage (+): 0. Max coverage (-): 0

Region: chr11 97605378-97605388. Max. coverage (+): 0. Max coverage (-): 0

Region: chr11 97605389-97605399. Max. coverage (+): 0.36. Max coverage (-): 0

Region: chr11 97605400-97605409. Max. coverage (+): 0. Max coverage (-): 0

Region: chr11 97605410-97605420. Max. coverage (+): 0. Max coverage (-): 0

Region: chr11 97605421-97605431. Max. coverage (+): 0. Max coverage (-): 0

Region: chr11 97605432-97605442. Max. coverage (+): 0. Max coverage (-): 0

Region: chr11 97605443-97605452. Max. coverage (+): 0. Max coverage (-): 0

Region: chr11 97605453-97605463. Max. coverage (+): 0. Max coverage (-): 0

Region: chr11 97605464-97605474. Max. coverage (+): 0. Max coverage (-): 0

Region: chr11 97605475-97605485. Max. coverage (+): 0. Max coverage (-): 0

Region: chr11 97605486-97605495. Max. coverage (+): 0. Max coverage (-): 0

Region: chr11 97605496-97605506. Max. coverage (+): 14.28. Max coverage (-): 0

Region: chr11 97605507-97605517. Max. coverage (+): 0. Max coverage (-): 0

Region: chr11 97605518-97605528. Max. coverage (+): 0. Max coverage (-): 0

Region: chr11 97605529-97605538. Max. coverage (+): 9.85. Max coverage (-): 0

Region: chr11 97605539-97605549. Max. coverage (+): 9.85. Max coverage (-): 0

Region: chr11 97605550-97605560. Max. coverage (+): 5.32. Max coverage (-): 0

Region: chr11 97605561-97605571. Max. coverage (+): 5.32. Max coverage (-): 0

Region: chr11 97605572-97605581. Max. coverage (+): 0.92. Max coverage (-): 0

Region: chr11 97605582-97605592. Max. coverage (+): 0.92. Max coverage (-): 0

Region: chr11 97605593-97605603. Max. coverage (+): 0. Max coverage (-): 0

Region: chr11 97605604-97605614. Max. coverage (+): 0. Max coverage (-): 0

Region: chr11 97605615-97605624. Max. coverage (+): 0. Max coverage (-): 0

Region: chr11 97605625-97605635. Max. coverage (+): 0. Max coverage (-): 0

Region: chr11 97605636-97605646. Max. coverage (+): 0. Max coverage (-): 0

Region: chr11 97605647-97605657. Max. coverage (+): 0. Max coverage (-): 0

Region: chr11 97605658-97605668. Max. coverage (+): 0. Max coverage (-): 0

Region: chr11 97605669-97605678. Max. coverage (+): 0. Max coverage (-): 0

Region: chr11 97605679-97605689. Max. coverage (+): 0. Max coverage (-): 0

Region: chr11 97605690-97605700. Max. coverage (+): 0. Max coverage (-): 0

Region: chr11 97605701-97605711. Max. coverage (+): 0. Max coverage (-): 0

Region: chr11 97605712-97605721. Max. coverage (+): 0. Max coverage (-): 0

Region: chr11 97605722-97605732. Max. coverage (+): 0. Max coverage (-): 0

Region: chr11 97605733-97605743. Max. coverage (+): 0. Max coverage (-): 0

Region: chr11 97605744-97605754. Max. coverage (+): 0. Max coverage (-): 0

Region: chr11 97605755-97605764. Max. coverage (+): 0. Max coverage (-): 0

Region: chr11 97605765-97605775. Max. coverage (+): 0. Max coverage (-): 0

Region: chr11 97605776-97605786. Max. coverage (+): 0. Max coverage (-): 0

Region: chr11 97605787-97605797. Max. coverage (+): 0. Max coverage (-): 0

Region: chr11 97605798-97605807. Max. coverage (+): 0. Max coverage (-): 0

Region: chr11 97605808-97605818. Max. coverage (+): 31.93. Max coverage (-): 0

Region: chr11 97605819-97605829. Max. coverage (+): 0. Max coverage (-): 0

Region: chr11 97605830-97605840. Max. coverage (+): 0. Max coverage (-): 0

Region: chr11 97605841-97605850. Max. coverage (+): 0. Max coverage (-): 0

Region: chr11 97605851-97605861. Max. coverage (+): 0. Max coverage (-): 0

Region: chr11 97605862-97605872. Max. coverage (+): 0. Max coverage (-): 0

Region: chr11 97605873-97605883. Max. coverage (+): 0. Max coverage (-): 0

Region: chr11 97605884-97605893. Max. coverage (+): 4.24. Max coverage (-): 0

Region: chr11 97605894-97605904. Max. coverage (+): 4.24. Max coverage (-): 0

Region: chr11 97605905-97605915. Max. coverage (+): 0. Max coverage (-): 0

Region: chr11 97605916-97605926. Max. coverage (+): 0. Max coverage (-): 0

Region: chr11 97605927-97605936. Max. coverage (+): 0. Max coverage (-): 0

Region: chr11 97605937-97605947. Max. coverage (+): 0. Max coverage (-): 0

Region: chr11 97605948-97605958. Max. coverage (+): 0. Max coverage (-): 0

Region: chr11 97605959-97605969. Max. coverage (+): 0. Max coverage (-): 0

Region: chr11 97605970-97605979. Max. coverage (+): 0. Max coverage (-): 0

Region: chr11 97605980-97605990. Max. coverage (+): 0. Max coverage (-): 0

Region: chr11 97605991-97606001. Max. coverage (+): 0. Max coverage (-): 0

Region: chr11 97606002-97606012. Max. coverage (+): 0. Max coverage (-): 0

Region: chr11 97606013-97606022. Max. coverage (+): 0. Max coverage (-): 0

Region: chr11 97606023-97606033. Max. coverage (+): 0. Max coverage (-): 0

Region: chr11 97606034-97606044. Max. coverage (+): 0. Max coverage (-): 0

Region: chr11 97606045-97606055. Max. coverage (+): 0. Max coverage (-): 0

Region: chr11 97606056-97606065. Max. coverage (+): 0. Max coverage (-): 0

Region: chr11 97606066-97606076. Max. coverage (+): 0. Max coverage (-): 0

Region: chr11 97606077-97606087. Max. coverage (+): 0. Max coverage (-): 0

Region: chr11 97606088-97606098. Max. coverage (+): 0. Max coverage (-): 0

Region: chr11 97606099-97606109. Max. coverage (+): 0. Max coverage (-): 0

Region: chr11 97606110-97606119. Max. coverage (+): 0. Max coverage (-): 0

Region: chr11 97606120-97606130. Max. coverage (+): 0. Max coverage (-): 0

Region: chr11 97606131-97606141. Max. coverage (+): 0. Max coverage (-): 0

Region: chr11 97606142-97606152. Max. coverage (+): 0. Max coverage (-): 0

Region: chr11 97606153-97606162. Max. coverage (+): 0. Max coverage (-): 0

Region: chr11 97606163-97606173. Max. coverage (+): 0. Max coverage (-): 0

Region: chr11 97606174-97606184. Max. coverage (+): 0. Max coverage (-): 0

Region: chr11 97606185-97606195. Max. coverage (+): 7.23. Max coverage (-): 0

Region: chr11 97606196-97606205. Max. coverage (+): 0. Max coverage (-): 0

Region: chr11 97606206-97606216. Max. coverage (+): 0. Max coverage (-): 0

Region: chr11 97606217-97606227. Max. coverage (+): 0. Max coverage (-): 0

Region: chr11 97606228-97606238. Max. coverage (+): 0. Max coverage (-): 0

Region: chr11 97606239-97606248. Max. coverage (+): 0. Max coverage (-): 0

Region: chr11 97606249-97606259. Max. coverage (+): 0. Max coverage (-): 0

Region: chr11 97606260-97606270. Max. coverage (+): 0. Max coverage (-): 0

Region: chr11 97606271-97606281. Max. coverage (+): 0. Max coverage (-): 0

Region: chr11 97606282-97606291. Max. coverage (+): 0. Max coverage (-): 0

Region: chr11 97606292-97606302. Max. coverage (+): 0. Max coverage (-): 0

Region: chr11 97606303-97606313. Max. coverage (+): 0. Max coverage (-): 0

Region: chr11 97606314-97606324. Max. coverage (+): 0. Max coverage (-): 0

Region: chr11 97606325-97606334. Max. coverage (+): 0. Max coverage (-): 0

Region: chr11 97606335-97606345. Max. coverage (+): 0. Max coverage (-): 0

Region: chr11 97606346-97606356. Max. coverage (+): 0. Max coverage (-): 0

Region: chr11 97606357-97606367. Max. coverage (+): 0. Max coverage (-): 0

Region: chr11 97606368-97606377. Max. coverage (+): 0. Max coverage (-): 0

Region: chr11 97606378-97606388. Max. coverage (+): 0. Max coverage (-): 0

Region: chr11 97606389-97606399. Max. coverage (+): 0. Max coverage (-): 0

Region: chr11 97606400-97606410. Max. coverage (+): 0. Max coverage (-): 0

Region: chr11 97606411-97606420. Max. coverage (+): 7.2. Max coverage (-): 0

Region: chr11 97606421-97606431. Max. coverage (+): 0. Max coverage (-): 0

Region: chr11 97606432-97606442. Max. coverage (+): 0. Max coverage (-): 0

Region: chr11 97606443-97606453. Max. coverage (+): 0. Max coverage (-): 0

Region: chr11 97606454-97606463. Max. coverage (+): 0. Max coverage (-): 0

Region: chr11 97606464-97606474. Max. coverage (+): 0. Max coverage (-): 0

Region: chr11 97606475-97606485. Max. coverage (+): 0. Max coverage (-): 0

Region: chr11 97606486-97606496. Max. coverage (+): 0. Max coverage (-): 0

Region: chr11 97606497-97606506. Max. coverage (+): 0. Max coverage (-): 0

Region: chr11 97606507-97606517. Max. coverage (+): 0. Max coverage (-): 0

Region: chr11 97606518-97606528. Max. coverage (+): 0. Max coverage (-): 0

Region: chr11 97606529-97606539. Max. coverage (+): 0. Max coverage (-): 0

Region: chr11 97606540-97606549. Max. coverage (+): 0. Max coverage (-): 0

Region: chr11 97606550-97606560. Max. coverage (+): 0. Max coverage (-): 0

Region: chr11 97606561-97606571. Max. coverage (+): 0. Max coverage (-): 0

Region: chr11 97606572-97606582. Max. coverage (+): 0. Max coverage (-): 0

Region: chr11 97606583-97606593. Max. coverage (+): 0. Max coverage (-): 0

Region: chr11 97606594-97606603. Max. coverage (+): 0. Max coverage (-): 0

Region: chr11 97606604-97606614. Max. coverage (+): 0. Max coverage (-): 0

Region: chr11 97606615-97606625. Max. coverage (+): 2.1. Max coverage (-): 0

Region: chr11 97606626-97606636. Max. coverage (+): 0. Max coverage (-): 0

Region: chr11 97606637-97606646. Max. coverage (+): 0. Max coverage (-): 0

Region: chr11 97606647-97606657. Max. coverage (+): 0. Max coverage (-): 0

Region: chr11 97606658-97606668. Max. coverage (+): 0.63. Max coverage (-): 0

Region: chr11 97606669-97606679. Max. coverage (+): 0. Max coverage (-): 0

Region: chr11 97606680-97606689. Max. coverage (+): 0. Max coverage (-): 0

Region: chr11 97606690-97606700. Max. coverage (+): 0. Max coverage (-): 0

Region: chr11 97606701-97606711. Max. coverage (+): 0. Max coverage (-): 0

Region: chr11 97606712-97606722. Max. coverage (+): 0. Max coverage (-): 0

Region: chr11 97606723-97606732. Max. coverage (+): 0. Max coverage (-): 0

Region: chr11 97606733-97606743. Max. coverage (+): 0. Max coverage (-): 0

Region: chr11 97606744-97606754. Max. coverage (+): 4.98. Max coverage (-): 0

Region: chr11 97606755-97606765. Max. coverage (+): 0. Max coverage (-): 0

Region: chr11 97606766-97606775. Max. coverage (+): 0. Max coverage (-): 0

Region: chr11 97606776-97606786. Max. coverage (+): 0. Max coverage (-): 0

Region: chr11 97606787-97606797. Max. coverage (+): 3.67. Max coverage (-): 0

Region: chr11 97606798-97606808. Max. coverage (+): 3.67. Max coverage (-): 0

Region: chr11 97606809-97606818. Max. coverage (+): 0. Max coverage (-): 0

Region: chr11 97606819-97606829. Max. coverage (+): 0. Max coverage (-): 0

Region: chr11 97606830-97606840. Max. coverage (+): 8.63. Max coverage (-): 0

Region: chr11 97606841-97606851. Max. coverage (+): 8.63. Max coverage (-): 0

Region: chr11 97606852-97606861. Max. coverage (+): 0. Max coverage (-): 0

Region: chr11 97606862-97606872. Max. coverage (+): 0. Max coverage (-): 0

Region: chr11 97606873-97606883. Max. coverage (+): 5.23. Max coverage (-): 0

Region: chr11 97606884-97606894. Max. coverage (+): 5.23. Max coverage (-): 0

Region: chr11 97606895-97606904. Max. coverage (+): 0. Max coverage (-): 0

Region: chr11 97606905-97606915. Max. coverage (+): 0. Max coverage (-): 0

Region: chr11 97606916-97606926. Max. coverage (+): 0. Max coverage (-): 0

Region: chr11 97606927-97606937. Max. coverage (+): 0. Max coverage (-): 0

Region: chr11 97606938-97606947. Max. coverage (+): 0. Max coverage (-): 0

Region: chr11 97606948-97606958. Max. coverage (+): 0. Max coverage (-): 0

Region: chr11 97606959-97606969. Max. coverage (+): 0. Max coverage (-): 0

Region: chr11 97606970-97606980. Max. coverage (+): 0.68. Max coverage (-): 0

Region: chr11 97606981-97606990. Max. coverage (+): 0. Max coverage (-): 0

Region: chr11 97606991-97607001. Max. coverage (+): 0. Max coverage (-): 0

Region: chr11 97607002-97607012. Max. coverage (+): 19.06. Max coverage (-): 0

Region: chr11 97607013-97607023. Max. coverage (+): 19.06. Max coverage (-): 0

Region: chr11 97607024-97607034. Max. coverage (+): 0. Max coverage (-): 0

Region: chr11 97607035-97607044. Max. coverage (+): 0. Max coverage (-): 0

Region: chr11 97607045-97607055. Max. coverage (+): 0. Max coverage (-): 0

Region: chr11 97607056-97607066. Max. coverage (+): 0. Max coverage (-): 0

Region: chr11 97607067-97607077. Max. coverage (+): 0. Max coverage (-): 0

Region: chr11 97607078-97607087. Max. coverage (+): 0. Max coverage (-): 0

Region: chr11 97607088-97607098. Max. coverage (+): 0. Max coverage (-): 0

Region: chr11 97607099-97607109. Max. coverage (+): 2.26. Max coverage (-): 0

Region: chr11 97607110-97607120. Max. coverage (+): 0. Max coverage (-): 0

Region: chr11 97607121-97607130. Max. coverage (+): 0.09. Max coverage (-): 0

Region: chr11 97607131-97607141. Max. coverage (+): 0.09. Max coverage (-): 0

Region: chr11 97607142-97607152. Max. coverage (+): 0. Max coverage (-): 0

Region: chr11 97607153-97607163. Max. coverage (+): 0. Max coverage (-): 0

Region: chr11 97607164-97607173. Max. coverage (+): 0. Max coverage (-): 0

Region: chr11 97607174-97607184. Max. coverage (+): 0. Max coverage (-): 0

Region: chr11 97607185-97607195. Max. coverage (+): 0. Max coverage (-): 0

Region: chr11 97607196-97607206. Max. coverage (+): 0. Max coverage (-): 0

Region: chr11 97607207-97607216. Max. coverage (+): 0. Max coverage (-): 0

Region: chr11 97607217-97607227. Max. coverage (+): 0. Max coverage (-): 0

Region: chr11 97607228-97607238. Max. coverage (+): 0. Max coverage (-): 0

Region: chr11 97607239-97607249. Max. coverage (+): 0. Max coverage (-): 0

Region: chr11 97607250-97607259. Max. coverage (+): 0. Max coverage (-): 0

Region: chr11 97607260-97607270. Max. coverage (+): 0. Max coverage (-): 0

Region: chr11 97607271-97607281. Max. coverage (+): 0. Max coverage (-): 0

Region: chr11 97607282-97607292. Max. coverage (+): 0. Max coverage (-): 0

Region: chr11 97607293-97607302. Max. coverage (+): 0. Max coverage (-): 0

Region: chr11 97607303-97607313. Max. coverage (+): 0. Max coverage (-): 0

Region: chr11 97607314-97607324. Max. coverage (+): 0. Max coverage (-): 0

Region: chr11 97607325-97607335. Max. coverage (+): 0. Max coverage (-): 0

Region: chr11 97607336-97607345. Max. coverage (+): 0. Max coverage (-): 0

Region: chr11 97607346-97607356. Max. coverage (+): 0. Max coverage (-): 0

Region: chr11 97607357-97607367. Max. coverage (+): 0. Max coverage (-): 0

Region: chr11 97607368-97607378. Max. coverage (+): 0. Max coverage (-): 0

Region: chr11 97607379-97607388. Max. coverage (+): 0. Max coverage (-): 0

Region: chr11 97607389-97607399. Max. coverage (+): 0. Max coverage (-): 0

Region: chr11 97607400-97607410. Max. coverage (+): 0. Max coverage (-): 0

Region: chr11 97607411-97607421. Max. coverage (+): 0. Max coverage (-): 0

Region: chr11 97607422-97607431. Max. coverage (+): 0. Max coverage (-): 0

Region: chr11 97607432-97607442. Max. coverage (+): 0. Max coverage (-): 0

Region: chr11 97607443-97607453. Max. coverage (+): 7.75. Max coverage (-): 0

Region: chr11 97607454-97607464. Max. coverage (+): 7.75. Max coverage (-): 0

Region: chr11 97607465-97607475. Max. coverage (+): 5.28. Max coverage (-): 0

Region: chr11 97607476-97607485. Max. coverage (+): 5.28. Max coverage (-): 0

Region: chr11 97607486-97607496. Max. coverage (+): 0.06. Max coverage (-): 0

Region: chr11 97607497-97607507. Max. coverage (+): 0. Max coverage (-): 0

Region: chr11 97607508-97607518. Max. coverage (+): 0. Max coverage (-): 0

Region: chr11 97607519-97607528. Max. coverage (+): 0. Max coverage (-): 0

Region: chr11 97607529-97607539. Max. coverage (+): 0. Max coverage (-): 0

Region: chr11 97607540-97607550. Max. coverage (+): 0. Max coverage (-): 0

Region: chr11 97607551-97607561. Max. coverage (+): 6.91. Max coverage (-): 0

Region: chr11 97607562-97607571. Max. coverage (+): 9.52. Max coverage (-): 0

Region: chr11 97607572-97607582. Max. coverage (+): 0. Max coverage (-): 0

Region: chr11 97607583-97607593. Max. coverage (+): 0. Max coverage (-): 0

Region: chr11 97607594-97607604. Max. coverage (+): 0. Max coverage (-): 0

Region: chr11 97607605-97607614. Max. coverage (+): 5.9. Max coverage (-): 0

Region: chr11 97607615-97607625. Max. coverage (+): 5.9. Max coverage (-): 0

Region: chr11 97607626-97607636. Max. coverage (+): 0. Max coverage (-): 0

Region: chr11 97607637-97607647. Max. coverage (+): 0. Max coverage (-): 0

Region: chr11 97607648-97607657. Max. coverage (+): 0. Max coverage (-): 0

Region: chr11 97607658-97607668. Max. coverage (+): 0. Max coverage (-): 0

Region: chr11 97607669-97607679. Max. coverage (+): 4.74. Max coverage (-): 0

Region: chr11 97607680-97607690. Max. coverage (+): 4.74. Max coverage (-): 0

Region: chr11 97607691-97607700. Max. coverage (+): 0. Max coverage (-): 0

Region: chr11 97607701-97607711. Max. coverage (+): 0. Max coverage (-): 0

Region: chr11 97607712-97607722. Max. coverage (+): 0. Max coverage (-): 0

Region: chr11 97607723-97607733. Max. coverage (+): 0. Max coverage (-): 0

Region: chr11 97607734-97607743. Max. coverage (+): 5.22. Max coverage (-): 0

Region: chr11 97607744-97607754. Max. coverage (+): 5.22. Max coverage (-): 0

Region: chr11 97607755-97607765. Max. coverage (+): 0. Max coverage (-): 0

Region: chr11 97607766-97607776. Max. coverage (+): 0. Max coverage (-): 0

Region: chr11 97607777-97607786. Max. coverage (+): 0. Max coverage (-): 0

Region: chr11 97607787-97607797. Max. coverage (+): 0. Max coverage (-): 0

Region: chr11 97607798-97607808. Max. coverage (+): 5.2. Max coverage (-): 0

Region: chr11 97607809-97607819. Max. coverage (+): 5.2. Max coverage (-): 0

Region: chr11 97607820-97607829. Max. coverage (+): 0. Max coverage (-): 0

Region: chr11 97607830-97607840. Max. coverage (+): 0. Max coverage (-): 0

Region: chr11 97607841-97607851. Max. coverage (+): 0. Max coverage (-): 0

Region: chr11 97607852-97607862. Max. coverage (+): 16.53. Max coverage (-): 0

Region: chr11 97607863-97607872. Max. coverage (+): 35.91. Max coverage (-): 0

Region: chr11 97607873-97607883. Max. coverage (+): 0. Max coverage (-): 0

Region: chr11 97607884-97607894. Max. coverage (+): 0. Max coverage (-): 0

Region: chr11 97607895-97607905. Max. coverage (+): 0. Max coverage (-): 0

Region: chr11 97607906-97607916. Max. coverage (+): 0. Max coverage (-): 0

Region: chr11 97607917-97607926. Max. coverage (+): 0. Max coverage (-): 0

Region: chr11 97607927-97607937. Max. coverage (+): 0. Max coverage (-): 0

Region: chr11 97607938-97607948. Max. coverage (+): 0. Max coverage (-): 0

Region: chr11 97607949-97607959. Max. coverage (+): 0. Max coverage (-): 0

Region: chr11 97607960-97607969. Max. coverage (+): 0. Max coverage (-): 0

Region: chr11 97607970-97607980. Max. coverage (+): 0. Max coverage (-): 0

Region: chr11 97607981-97607991. Max. coverage (+): 0. Max coverage (-): 0

Region: chr11 97607992-97608002. Max. coverage (+): 0. Max coverage (-): 0

Region: chr11 97608003-97608012. Max. coverage (+): 0. Max coverage (-): 0

Region: chr11 97608013-97608023. Max. coverage (+): 0. Max coverage (-): 0

Region: chr11 97608024-97608034. Max. coverage (+): 0. Max coverage (-): 0

Region: chr11 97608035-97608045. Max. coverage (+): 0. Max coverage (-): 0

Region: chr11 97608046-97608055. Max. coverage (+): 0. Max coverage (-): 0

Region: chr11 97608056-97608066. Max. coverage (+): 0. Max coverage (-): 0

Region: chr11 97608067-97608077. Max. coverage (+): 0. Max coverage (-): 0

Region: chr11 97608078-97608088. Max. coverage (+): 0. Max coverage (-): 0

Region: chr11 97608089-97608098. Max. coverage (+): 0. Max coverage (-): 0

Region: chr11 97608099-97608109. Max. coverage (+): 0. Max coverage (-): 0

Region: chr11 97608110-97608120. Max. coverage (+): 0. Max coverage (-): 0

Region: chr11 97608121-97608131. Max. coverage (+): 0. Max coverage (-): 0

Region: chr11 97608132-97608141. Max. coverage (+): 0. Max coverage (-): 0

Region: chr11 97608142-97608152. Max. coverage (+): 0. Max coverage (-): 0

Region: chr11 97608153-97608163. Max. coverage (+): 0. Max coverage (-): 0

Region: chr11 97608164-97608174. Max. coverage (+): 0. Max coverage (-): 0

Region: chr11 97608175-97608184. Max. coverage (+): 0. Max coverage (-): 0

Region: chr11 97608185-97608195. Max. coverage (+): 0. Max coverage (-): 0

Region: chr11 97608196-97608206. Max. coverage (+): 0. Max coverage (-): 0

Region: chr11 97608207-97608217. Max. coverage (+): 0. Max coverage (-): 0

Region: chr11 97608218-97608227. Max. coverage (+): 0. Max coverage (-): 0

Region: chr11 97608228-97608238. Max. coverage (+): 0.78. Max coverage (-): 0

Region: chr11 97608239-97608249. Max. coverage (+): 0.78. Max coverage (-): 0

Region: chr11 97608250-97608260. Max. coverage (+): 0. Max coverage (-): 0

Region: chr11 97608261-97608270. Max. coverage (+): 0. Max coverage (-): 0

Region: chr11 97608271-97608281. Max. coverage (+): 0. Max coverage (-): 0

Region: chr11 97608282-97608292. Max. coverage (+): 0. Max coverage (-): 0

Region: chr11 97608293-97608303. Max. coverage (+): 0. Max coverage (-): 0

Region: chr11 97608304-97608313. Max. coverage (+): 0. Max coverage (-): 0

Region: chr11 97608314-97608324. Max. coverage (+): 0. Max coverage (-): 0

Region: chr11 97608325-97608335. Max. coverage (+): 0. Max coverage (-): 0

Region: chr11 97608336-97608346. Max. coverage (+): 0. Max coverage (-): 0

Region: chr11 97608347-97608357. Max. coverage (+): 0. Max coverage (-): 0

Region: chr11 97608358-97608367. Max. coverage (+): 0. Max coverage (-): 0

Region: chr11 97608368-97608378. Max. coverage (+): 0. Max coverage (-): 0

Region: chr11 97608379-97608389. Max. coverage (+): 0. Max coverage (-): 0

Region: chr11 97608390-97608400. Max. coverage (+): 0. Max coverage (-): 0

Region: chr11 97608401-97608410. Max. coverage (+): 0. Max coverage (-): 0

Region: chr11 97608411-97608421. Max. coverage (+): 0.04. Max coverage (-): 0

Region: chr11 97608422-97608432. Max. coverage (+): 0.04. Max coverage (-): 0

Region: chr11 97608433-97608443. Max. coverage (+): 0. Max coverage (-): 0

Region: chr11 97608444-97608453. Max. coverage (+): 0. Max coverage (-): 0

Region: chr11 97608454-97608464. Max. coverage (+): 0. Max coverage (-): 0

Region: chr11 97608465-97608475. Max. coverage (+): 0. Max coverage (-): 0

Region: chr11 97608476-97608486. Max. coverage (+): 0. Max coverage (-): 0

Region: chr11 97608487-97608496. Max. coverage (+): 0. Max coverage (-): 0

Region: chr11 97608497-97608507. Max. coverage (+): 0. Max coverage (-): 0

Region: chr11 97608508-97608518. Max. coverage (+): 0. Max coverage (-): 0

Region: chr11 97608519-97608529. Max. coverage (+): 0. Max coverage (-): 0

Region: chr11 97608530-97608539. Max. coverage (+): 0. Max coverage (-): 0

Region: chr11 97608540-97608550. Max. coverage (+): 0. Max coverage (-): 0

Region: chr11 97608551-97608561. Max. coverage (+): 0. Max coverage (-): 0

Region: chr11 97608562-97608572. Max. coverage (+): 0. Max coverage (-): 0

Region: chr11 97608573-97608582. Max. coverage (+): 0. Max coverage (-): 0

Region: chr11 97608583-97608593. Max. coverage (+): 0. Max coverage (-): 0

Region: chr11 97608594-97608604. Max. coverage (+): 0. Max coverage (-): 0

Region: chr11 97608605-97608615. Max. coverage (+): 0. Max coverage (-): 0

Region: chr11 97608616-97608625. Max. coverage (+): 0. Max coverage (-): 0

Region: chr11 97608626-97608636. Max. coverage (+): 0. Max coverage (-): 0

Region: chr11 97608637-97608647. Max. coverage (+): 0. Max coverage (-): 0

Region: chr11 97608648-97608658. Max. coverage (+): 0. Max coverage (-): 0

Region: chr11 97608659-97608668. Max. coverage (+): 0. Max coverage (-): 0

Region: chr11 97608669-97608679. Max. coverage (+): 0. Max coverage (-): 0

Region: chr11 97608680-97608690. Max. coverage (+): 0. Max coverage (-): 0

Region: chr11 97608691-97608701. Max. coverage (+): 0. Max coverage (-): 0

Region: chr11 97608702-97608711. Max. coverage (+): 6.1. Max coverage (-): 0

Region: chr11 97608712-97608722. Max. coverage (+): 6.1. Max coverage (-): 0

Region: chr11 97608723-97608733. Max. coverage (+): 0. Max coverage (-): 0

Region: chr11 97608734-97608744. Max. coverage (+): 0.25. Max coverage (-): 0

Region: chr11 97608745-97608754. Max. coverage (+): 0. Max coverage (-): 0

Region: chr11 97608755-97608765. Max. coverage (+): 0. Max coverage (-): 0

Region: chr11 97608766-97608776. Max. coverage (+): 0. Max coverage (-): 0

Region: chr11 97608777-97608787. Max. coverage (+): 0. Max coverage (-): 0

Region: chr11 97608788-97608798. Max. coverage (+): 0. Max coverage (-): 0

Region: chr11 97608799-97608808. Max. coverage (+): 0. Max coverage (-): 0

Region: chr11 97608809-97608819. Max. coverage (+): 0. Max coverage (-): 0

Region: chr11 97608820-97608830. Max. coverage (+): 0. Max coverage (-): 0

Region: chr11 97608831-97608841. Max. coverage (+): 0. Max coverage (-): 0

Region: chr11 97608842-97608851. Max. coverage (+): 0. Max coverage (-): 0

Region: chr11 97608852-97608862. Max. coverage (+): 0. Max coverage (-): 0

Region: chr11 97608863-97608873. Max. coverage (+): 0. Max coverage (-): 0

Region: chr11 97608874-97608884. Max. coverage (+): 0. Max coverage (-): 0

Region: chr11 97608885-97608894. Max. coverage (+): 6.74. Max coverage (-): 0

Region: chr11 97608895-97608905. Max. coverage (+): 6.74. Max coverage (-): 0

Region: chr11 97608906-97608916. Max. coverage (+): 0. Max coverage (-): 0

Region: chr11 97608917-97608927. Max. coverage (+): 0. Max coverage (-): 0

Region: chr11 97608928-97608937. Max. coverage (+): 0. Max coverage (-): 0

Region: chr11 97608938-97608948. Max. coverage (+): 0. Max coverage (-): 0

Region: chr11 97608949-97608959. Max. coverage (+): 0. Max coverage (-): 0

Region: chr11 97608960-97608970. Max. coverage (+): 0. Max coverage (-): 0

Region: chr11 97608971-97608980. Max. coverage (+): 0. Max coverage (-): 0

Region: chr11 97608981-97608991. Max. coverage (+): 0. Max coverage (-): 0

Region: chr11 97608992-97609002. Max. coverage (+): 0. Max coverage (-): 0

Region: chr11 97609003-97609013. Max. coverage (+): 5.27. Max coverage (-): 0

Region: chr11 97609014-97609023. Max. coverage (+): 0. Max coverage (-): 0

Region: chr11 97609024-97609034. Max. coverage (+): 0. Max coverage (-): 0

Region: chr11 97609035-97609045. Max. coverage (+): 2.33. Max coverage (-): 0

Region: chr11 97609046-97609056. Max. coverage (+): 2.33. Max coverage (-): 0

Region: chr11 97609057-97609066. Max. coverage (+): 0. Max coverage (-): 0

Region: chr11 97609067-97609077. Max. coverage (+): 0. Max coverage (-): 0

Region: chr11 97609078-97609088. Max. coverage (+): 0. Max coverage (-): 0

Region: chr11 97609089-97609099. Max. coverage (+): 0. Max coverage (-): 0

Region: chr11 97609100-97609109. Max. coverage (+): 0. Max coverage (-): 0

Region: chr11 97609110-97609120. Max. coverage (+): 0. Max coverage (-): 0

Region: chr11 97609121-97609131. Max. coverage (+): 0. Max coverage (-): 0

Region: chr11 97609132-97609142. Max. coverage (+): 0. Max coverage (-): 0

Region: chr11 97609143-97609152. Max. coverage (+): 0. Max coverage (-): 0

Region: chr11 97609153-97609163. Max. coverage (+): 0. Max coverage (-): 0

Region: chr11 97609164-97609174. Max. coverage (+): 0. Max coverage (-): 0

Region: chr11 97609175-97609185. Max. coverage (+): 0. Max coverage (-): 0

Region: chr11 97609186-97609195. Max. coverage (+): 0. Max coverage (-): 0

Region: chr11 97609196-97609206. Max. coverage (+): 0. Max coverage (-): 0

Region: chr11 97609207-97609217. Max. coverage (+): 1.94. Max coverage (-): 0

Region: chr11 97609218-97609228. Max. coverage (+): 1.94. Max coverage (-): 0

Region: chr11 97609229-97609238. Max. coverage (+): 0. Max coverage (-): 0

Region: chr11 97609239-97609249. Max. coverage (+): 0. Max coverage (-): 0

Region: chr11 97609250-97609260. Max. coverage (+): 0. Max coverage (-): 0

Region: chr11 97609261-97609271. Max. coverage (+): 0. Max coverage (-): 0

Region: chr11 97609272-97609282. Max. coverage (+): 0. Max coverage (-): 0

Region: chr11 97609283-97609292. Max. coverage (+): 0. Max coverage (-): 0

Region: chr11 97609293-97609303. Max. coverage (+): 0. Max coverage (-): 0

Region: chr11 97609304-97609314. Max. coverage (+): 0. Max coverage (-): 0

Region: chr11 97609315-97609325. Max. coverage (+): 0. Max coverage (-): 0

Region: chr11 97609326-97609335. Max. coverage (+): 0. Max coverage (-): 0

Region: chr11 97609336-97609346. Max. coverage (+): 0. Max coverage (-): 0

Region: chr11 97609347-97609357. Max. coverage (+): 0. Max coverage (-): 0

Region: chr11 97609358-97609368. Max. coverage (+): 0. Max coverage (-): 0

Region: chr11 97609369-97609378. Max. coverage (+): 0. Max coverage (-): 0

Region: chr11 97609379-97609389. Max. coverage (+): 0. Max coverage (-): 0

Region: chr11 97609390-97609400. Max. coverage (+): 0. Max coverage (-): 0

Region: chr11 97609401-97609411. Max. coverage (+): 0. Max coverage (-): 0

Region: chr11 97609412-97609421. Max. coverage (+): 0. Max coverage (-): 0

Region: chr11 97609422-97609432. Max. coverage (+): 0. Max coverage (-): 0

Region: chr11 97609433-97609443. Max. coverage (+): 0. Max coverage (-): 0

Region: chr11 97609444-97609454. Max. coverage (+): 0. Max coverage (-): 0

Region: chr11 97609455-97609464. Max. coverage (+): 0. Max coverage (-): 0

Region: chr11 97609465-97609475. Max. coverage (+): 0. Max coverage (-): 0

Region: chr11 97609476-97609486. Max. coverage (+): 0. Max coverage (-): 0

Region: chr11 97609487-97609497. Max. coverage (+): 0. Max coverage (-): 0

Region: chr11 97609498-97609507. Max. coverage (+): 0. Max coverage (-): 0

Region: chr11 97609508-97609518. Max. coverage (+): 0. Max coverage (-): 0

Region: chr11 97609519-97609529. Max. coverage (+): 0. Max coverage (-): 0

Region: chr11 97609530-97609540. Max. coverage (+): 0. Max coverage (-): 0

Region: chr11 97609541-97609550. Max. coverage (+): 0. Max coverage (-): 0

Region: chr11 97609551-97609561. Max. coverage (+): 0. Max coverage (-): 0

Region: chr11 97609562-97609572. Max. coverage (+): 0. Max coverage (-): 0

Region: chr11 97609573-97609583. Max. coverage (+): 0. Max coverage (-): 0

Region: chr11 97609584-97609593. Max. coverage (+): 0. Max coverage (-): 0

Region: chr11 97609594-97609604. Max. coverage (+): 0. Max coverage (-): 0

Region: chr11 97609605-97609615. Max. coverage (+): 0. Max coverage (-): 0

Region: chr11 97609616-97609626. Max. coverage (+): 0. Max coverage (-): 0

Region: chr11 97609627-97609636. Max. coverage (+): 0. Max coverage (-): 0

Region: chr11 97609637-97609647. Max. coverage (+): 0. Max coverage (-): 0

Region: chr11 97609648-97609658. Max. coverage (+): 0. Max coverage (-): 0

Region: chr11 97609659-97609669. Max. coverage (+): 0. Max coverage (-): 0

Region: chr11 97609670-97609679. Max. coverage (+): 0. Max coverage (-): 0

Region: chr11 97609680-97609690. Max. coverage (+): 0. Max coverage (-): 0

Region: chr11 97609691-97609701. Max. coverage (+): 0. Max coverage (-): 0

Region: chr11 97609702-97609712. Max. coverage (+): 0. Max coverage (-): 0

Region: chr11 97609713-97609723. Max. coverage (+): 0. Max coverage (-): 0

Region: chr11 97609724-97609733. Max. coverage (+): 0. Max coverage (-): 0

Region: chr11 97609734-97609744. Max. coverage (+): 0. Max coverage (-): 0

Region: chr11 97609745-97609755. Max. coverage (+): 0. Max coverage (-): 0

Region: chr11 97609756-97609766. Max. coverage (+): 0. Max coverage (-): 0

Region: chr11 97609767-97609776. Max. coverage (+): 0. Max coverage (-): 0

Region: chr11 97609777-97609787. Max. coverage (+): 0. Max coverage (-): 0

Region: chr11 97609788-97609798. Max. coverage (+): 0. Max coverage (-): 0

Region: chr11 97609799-97609809. Max. coverage (+): 0. Max coverage (-): 0

Region: chr11 97609810-97609819. Max. coverage (+): 0. Max coverage (-): 0

Region: chr11 97609820-97609830. Max. coverage (+): 0. Max coverage (-): 0

Region: chr11 97609831-97609841. Max. coverage (+): 0. Max coverage (-): 0

Region: chr11 97609842-97609852. Max. coverage (+): 0. Max coverage (-): 0

Region: chr11 97609853-97609862. Max. coverage (+): 0. Max coverage (-): 0

Region: chr11 97609863-97609873. Max. coverage (+): 0. Max coverage (-): 0

Region: chr11 97609874-97609884. Max. coverage (+): 0. Max coverage (-): 0

Region: chr11 97609885-97609895. Max. coverage (+): 0. Max coverage (-): 0

Region: chr11 97609896-97609905. Max. coverage (+): 0. Max coverage (-): 0

Region: chr11 97609906-97609916. Max. coverage (+): 0. Max coverage (-): 0

Region: chr11 97609917-97609927. Max. coverage (+): 0. Max coverage (-): 0

Region: chr11 97609928-97609938. Max. coverage (+): 0. Max coverage (-): 0

Region: chr11 97609939-97609948. Max. coverage (+): 0. Max coverage (-): 0

Region: chr11 97609949-97609959. Max. coverage (+): 0. Max coverage (-): 0

Region: chr11 97609960-97609970. Max. coverage (+): 0. Max coverage (-): 0

Region: chr11 97609971-97609981. Max. coverage (+): 0. Max coverage (-): 0

Region: chr11 97609982-97609991. Max. coverage (+): 0. Max coverage (-): 0

Region: chr11 97609992-97610002. Max. coverage (+): 0. Max coverage (-): 0

Region: chr11 97610003-97610013. Max. coverage (+): 0. Max coverage (-): 0

Region: chr11 97610014-97610024. Max. coverage (+): 0. Max coverage (-): 0

Region: chr11 97610025-97610034. Max. coverage (+): 0. Max coverage (-): 0

Region: chr11 97610035-97610045. Max. coverage (+): 0. Max coverage (-): 0

Region: chr11 97610046-97610056. Max. coverage (+): 0. Max coverage (-): 0

Region: chr11 97610057-97610067. Max. coverage (+): 0. Max coverage (-): 0

Region: chr11 97610068-97610077. Max. coverage (+): 0. Max coverage (-): 0

Region: chr11 97610078-97610088. Max. coverage (+): 0. Max coverage (-): 0

Region: chr11 97610089-97610099. Max. coverage (+): 0. Max coverage (-): 0

Region: chr11 97610100-97610110. Max. coverage (+): 0. Max coverage (-): 0

Region: chr11 97610111-97610120. Max. coverage (+): 0. Max coverage (-): 0

Region: chr11 97610121-97610131. Max. coverage (+): 0. Max coverage (-): 0

Region: chr11 97610132-97610142. Max. coverage (+): 0. Max coverage (-): 0

Region: chr11 97610143-97610153. Max. coverage (+): 0. Max coverage (-): 0

Region: chr11 97610154-97610164. Max. coverage (+): 0. Max coverage (-): 0

Region: chr11 97610165-97610174. Max. coverage (+): 0. Max coverage (-): 0

Region: chr11 97610175-97610185. Max. coverage (+): 0. Max coverage (-): 0

Region: chr11 97610186-97610196. Max. coverage (+): 0. Max coverage (-): 0

Region: chr11 97610197-97610207. Max. coverage (+): 0. Max coverage (-): 0

Region: chr11 97610208-97610217. Max. coverage (+): 0. Max coverage (-): 0

Region: chr11 97610218-97610228. Max. coverage (+): 0. Max coverage (-): 0

Region: chr11 97610229-97610239. Max. coverage (+): 0. Max coverage (-): 0

Region: chr11 97610240-97610250. Max. coverage (+): 0. Max coverage (-): 0

Region: chr11 97610251-97610260. Max. coverage (+): 0. Max coverage (-): 0

Region: chr11 97610261-97610271. Max. coverage (+): 0. Max coverage (-): 0

Region: chr11 97610272-97610282. Max. coverage (+): 0. Max coverage (-): 0

Region: chr11 97610283-97610293. Max. coverage (+): 0. Max coverage (-): 0

Region: chr11 97610294-97610303. Max. coverage (+): 0. Max coverage (-): 0

Region: chr11 97610304-97610314. Max. coverage (+): 0. Max coverage (-): 0

Region: chr11 97610315-97610325. Max. coverage (+): 0. Max coverage (-): 0

Region: chr11 97610326-97610336. Max. coverage (+): 6.34. Max coverage (-): 0

Region: chr11 97610337-97610346. Max. coverage (+): 6.34. Max coverage (-): 0

Region: chr11 97610347-97610357. Max. coverage (+): 0. Max coverage (-): 0

Region: chr11 97610358-. Max. coverage (+): 0. Max coverage (-): 0

RepeatMasker Color Code

**+**

100-98% Identity

<98-95% Identity

<95-90% Identity

<90-85% Identity

<85-80% Identity

<80-75% Identity

<75-70% Identity

<70% Identity

**-**

Gene Set Color Code

**+**

Gene

Pseudogene

**-**

Topology/Coverage Color Code

Coverage Plus Strand

Coverage Minus Strand

Mainstrand: Plus

Mainstrand: Minus

Complementary Strand

Flanking Region  
(if option -flank >0)

Gene Set Annotation  

**1. ZBTB43 (protein coding, ENSBTAG00000003438) Tr:00000004465 Ex:2**: 97605347-97608142 (+)

  
RepeatMasker Annotation  

**1. AT\_rich**: 97608595-97608634 (+), Divergence to consensus: 65%  
**2. GC\_rich**: 97610100-97610122 (+), Divergence to consensus: 47.8%

  
Transcription Factor Binding Sites  

**SOX9** (Sequence: CCATTGTT (+): 97605475)  
**A-MYB** (Sequence: CCAACTGTCA (-): 97607858)
