# Supplementary material for: Small RNA sequencing of cryopreserved semen from single bull revealed altered miRNAs and piRNAs expression between High- and Low-motile sperm populations
Source: BMC Genomics. 2017 Jan 4;18:14. doi: 10.1186/s12864-016-3394-7 (PMC5209821; doi:10.1186/s12864-016-3394-7)
Supplement: Additional file 4: — Details for each piRNA clusters found in Low Motile (LM) sperm fraction. Genes, repeats, transposable elements and transcription factors binding sites falling within the cluster regions were reported. (ZIP 1034 kb) [file 12864_2016_3394_MOESM4_ESM.zip › 8.html]

piRNA cluster 8


Predicted piRNA cluster no. 8     previous   next
  

Show proTRAC run info
Hide proTRAC run info

================================= proTRAC ====================================  
VERSION: 2.1                                    LAST MODIFIED: 06. October 2015  
  
Please cite:  
Rosenkranz D, Zischler H. proTRAC - a software for probabilistic piRNA cluster  
detection, visualization and analysis. 2012. BMC Bioinformatics 13:5.  
  
and (for proTRAC 2.0 and later):  
Rosenkranz D, Rudloff S, Bastuck K, Ketting RF, Zischler H. Tupaia small RNAs  
provide insights into function and evolution of RNAi-based transposon defense  
in mammals. 2015. RNA 21(5):911-922.  
  
Contact:  
David Rosenkranz  
Institute of Anthropology, small RNA group  
Johannes Gutenberg University Mainz  
email: rosenkranz@uni-mainz.de  
  
You can find the latest proTRAC version at:  
http://sourceforge.net/projects/protrac/files  
http://www.smallRNAgroup-mainz.de/software  
==============================================================================  
  
PARAMETERS:  
Map file: .............../storage/core/barbara/genhome/smallRNA/fertility/Sample\_not\_motile/pirna/Sample\_not\_motile\_26-33\_collapsed.fa.no-dust.map.weighted-10000-1000-b-0  
Genome file: ............/storage/core/barbara/genhome/smallRNA/fertility/Sample\_all/pirna/bt\_311\_chrY.fa  
RepeatMasker annotation: /storage/genomes/bt\_umd31/GCF\_000003055.6\_Bos\_taurus\_UMD\_3.1.1\_repeatMasker\_chr.out  
GeneSet:................./storage/core/barbara/genhome/smallRNA/fertility/Sample\_all/pirna/full.gtf  
  
Significant (p<=0.01) hit density will be calculated based  
on observed hit distribution.  
  
Sliding window size: ........................................ 5000 bp  
Sliding window increament: .................................. 1000 bp  
Normalize each hit by number of genomic hits: ............... 1 [0=no/1=yes]  
Normalize each hit by number of sequence reads: ............. 1 [0=no/1=yes]  
Normalize values (-> per million mapped reads): ............. 1 [0=no/1=yes]  
Min. fraction of hits with 1T(U) or 10A: .................... 0.75  
Alternatively: Min. fraction of hits with 1T(U) and 10A: .... 0.5  
Min. fraction of hits with typical piRNA length: ............ 0.75  
Typical piRNA length: ....................................... 26-33 nt  
Min. size of a piRNA cluster: ............................... 5000 bp.  
Min. number of hits (absolute): ............................. 0  
Min. number of hits (normalized): ........................... 0  
Min. fraction of hits on the mainstrand: .................... 0.75  
Top fraction of mapped sequences (in terms of read counts): . 1%  
Top fraction accounts for max. n% of sequence reads: ........ 90%  
Min. fraction of hits on each arm of a bidirectional cluster: 0.1  
Output image file for each cluster: ......................... 0 [0=no/1=yes]  
Output html file for each cluster: .......................... 1 [0=no/1=yes]  
Output a summary table: ..................................... 1 [0=no/1=yes]  
Output a FASTA file for each cluster (piRNA sequences): ..... 1 [0=no/1=yes]  
Output a FASTA file comprising cluster sequences: ........... 1 [0=no/1=yes]  
Search DNA motifs in clusters: .............................. 1 [0=no/1=yes]  
Output flanking sequences: +/- .............................. 0 bp  
Output ~.pTi file: .......................................... 1 [0=no/1=yes]  
==============================================================================  
  
  
Genome size (without gaps): ............ 2678902517 bp  
Gaps (N/X/-): .......................... 53837044 bp  
Mapped reads: .......................... 738059667487  
Non-identical sequences: ............... 277001  
Genomic hits: .......................... 533816  
Significant densitiy of mapped reads: .. 15118061 reads/kb

Show proTRAC cluster info
Hide proTRAC cluster info

|  |  |
| --- | --- |
| Location | chr11 |
| Coordinates | 100484444-100490325 |
| Size [bp] | 5882 |
| Sequence hit loci | 158 |
| Mapped reads (normalized) | 464568911.1 |
| Mapped reads (normalized) per kb | 78981453.8 |
| Normalized reads with 1T (1U) | 78.1% |
| Normalized reads with 10A | 23% |
| Normalized reads with length 26-33 nt | 100% |
| Normalized reads on the main strand(s) | 87.8% |
| Predicted directionality | mono:plus |

100%

0%

1T (1U)  
reads

10A reads

26-33 nt  
reads

reads on mainstrand

**Either the amount of reads with 1T (1U) OR 10A has to exceed 75% (set with option: -1Tor10A)  
Alternatively the amount of reads with 1T (1U) AND 10A has to exceed 50% (set with option: -1Tand10A)  
Minimum amount of reads with preferred size is 75% (set with option: -pisize)  
Minimum amount of reads on the main strand(s) is 75% (set with option: -clstrand)**

Show read coverage
Hide read coverage

WHAT DO I SEE HERE?  
This chart shows the location of mapped sequence reads within a predicted piRNA cluster. The color refers to the number of genomic hits produced by the sequence read in question. A dark red bar indicates that this sequence read produces many other hits elsewhere in the genome. Many adjacent red or yellow bars can indicate the presence of a multi-copy element such as transposons or rRNA genes. A dark green bar indicates that this sequence read maps uniquely to this locus.

1 hit

2-5 hits

6-10 hits

11-20 hits

21-50 hits

51-100 hits

> 100 hits

chr11

100484444

100490325

Gene Set

RepeatMasker

Mapped  
Reads

52.18

plus strand

minus strand

52.18

Region: chr11 97610359-100484449. Max. coverage (+): 0.07. Max coverage (-): 0

Region: chr11 100484450-100484461. Max. coverage (+): 13.99. Max coverage (-): 0

Region: chr11 100484462-100484473. Max. coverage (+): 6.95. Max coverage (-): 0

Region: chr11 100484474-100484485. Max. coverage (+): 0. Max coverage (-): 0

Region: chr11 100484486-100484496. Max. coverage (+): 0. Max coverage (-): 0

Region: chr11 100484497-100484508. Max. coverage (+): 7.12. Max coverage (-): 0

Region: chr11 100484509-100484520. Max. coverage (+): 7.12. Max coverage (-): 0

Region: chr11 100484521-100484532. Max. coverage (+): 0. Max coverage (-): 0

Region: chr11 100484533-100484543. Max. coverage (+): 0. Max coverage (-): 0

Region: chr11 100484544-100484555. Max. coverage (+): 0. Max coverage (-): 0

Region: chr11 100484556-100484567. Max. coverage (+): 10.51. Max coverage (-): 0

Region: chr11 100484568-100484579. Max. coverage (+): 10.51. Max coverage (-): 0

Region: chr11 100484580-100484591. Max. coverage (+): 0. Max coverage (-): 0

Region: chr11 100484592-100484602. Max. coverage (+): 0. Max coverage (-): 0

Region: chr11 100484603-100484614. Max. coverage (+): 0. Max coverage (-): 0

Region: chr11 100484615-100484626. Max. coverage (+): 0. Max coverage (-): 0

Region: chr11 100484627-100484638. Max. coverage (+): 0. Max coverage (-): 0

Region: chr11 100484639-100484649. Max. coverage (+): 0. Max coverage (-): 0

Region: chr11 100484650-100484661. Max. coverage (+): 3.66. Max coverage (-): 0

Region: chr11 100484662-100484673. Max. coverage (+): 24.56. Max coverage (-): 0

Region: chr11 100484674-100484685. Max. coverage (+): 7.86. Max coverage (-): 0

Region: chr11 100484686-100484696. Max. coverage (+): 0. Max coverage (-): 0

Region: chr11 100484697-100484708. Max. coverage (+): 0. Max coverage (-): 0

Region: chr11 100484709-100484720. Max. coverage (+): 0. Max coverage (-): 0

Region: chr11 100484721-100484732. Max. coverage (+): 0. Max coverage (-): 0

Region: chr11 100484733-100484743. Max. coverage (+): 0. Max coverage (-): 0

Region: chr11 100484744-100484755. Max. coverage (+): 0. Max coverage (-): 0

Region: chr11 100484756-100484767. Max. coverage (+): 0. Max coverage (-): 0

Region: chr11 100484768-100484779. Max. coverage (+): 2.87. Max coverage (-): 0

Region: chr11 100484780-100484791. Max. coverage (+): 2.87. Max coverage (-): 0

Region: chr11 100484792-100484802. Max. coverage (+): 0. Max coverage (-): 0

Region: chr11 100484803-100484814. Max. coverage (+): 0. Max coverage (-): 0

Region: chr11 100484815-100484826. Max. coverage (+): 0. Max coverage (-): 0

Region: chr11 100484827-100484838. Max. coverage (+): 0. Max coverage (-): 0

Region: chr11 100484839-100484849. Max. coverage (+): 0. Max coverage (-): 0

Region: chr11 100484850-100484861. Max. coverage (+): 0. Max coverage (-): 0

Region: chr11 100484862-100484873. Max. coverage (+): 0. Max coverage (-): 0

Region: chr11 100484874-100484885. Max. coverage (+): 0. Max coverage (-): 0

Region: chr11 100484886-100484896. Max. coverage (+): 0. Max coverage (-): 0

Region: chr11 100484897-100484908. Max. coverage (+): 0. Max coverage (-): 0

Region: chr11 100484909-100484920. Max. coverage (+): 0. Max coverage (-): 0

Region: chr11 100484921-100484932. Max. coverage (+): 0. Max coverage (-): 0

Region: chr11 100484933-100484943. Max. coverage (+): 0. Max coverage (-): 0

Region: chr11 100484944-100484955. Max. coverage (+): 0. Max coverage (-): 0

Region: chr11 100484956-100484967. Max. coverage (+): 0. Max coverage (-): 0

Region: chr11 100484968-100484979. Max. coverage (+): 0. Max coverage (-): 0

Region: chr11 100484980-100484991. Max. coverage (+): 0. Max coverage (-): 0

Region: chr11 100484992-100485002. Max. coverage (+): 0. Max coverage (-): 0

Region: chr11 100485003-100485014. Max. coverage (+): 8.13. Max coverage (-): 0

Region: chr11 100485015-100485026. Max. coverage (+): 1.1. Max coverage (-): 0

Region: chr11 100485027-100485038. Max. coverage (+): 0. Max coverage (-): 0

Region: chr11 100485039-100485049. Max. coverage (+): 0. Max coverage (-): 0

Region: chr11 100485050-100485061. Max. coverage (+): 0. Max coverage (-): 0

Region: chr11 100485062-100485073. Max. coverage (+): 0. Max coverage (-): 0

Region: chr11 100485074-100485085. Max. coverage (+): 13.44. Max coverage (-): 0

Region: chr11 100485086-100485096. Max. coverage (+): 13.44. Max coverage (-): 0

Region: chr11 100485097-100485108. Max. coverage (+): 0. Max coverage (-): 0

Region: chr11 100485109-100485120. Max. coverage (+): 0. Max coverage (-): 0

Region: chr11 100485121-100485132. Max. coverage (+): 0. Max coverage (-): 0

Region: chr11 100485133-100485143. Max. coverage (+): 0. Max coverage (-): 0

Region: chr11 100485144-100485155. Max. coverage (+): 0. Max coverage (-): 0

Region: chr11 100485156-100485167. Max. coverage (+): 0. Max coverage (-): 0

Region: chr11 100485168-100485179. Max. coverage (+): 0. Max coverage (-): 0

Region: chr11 100485180-100485191. Max. coverage (+): 0. Max coverage (-): 0

Region: chr11 100485192-100485202. Max. coverage (+): 1.19. Max coverage (-): 0

Region: chr11 100485203-100485214. Max. coverage (+): 19.65. Max coverage (-): 0

Region: chr11 100485215-100485226. Max. coverage (+): 0. Max coverage (-): 0

Region: chr11 100485227-100485238. Max. coverage (+): 0. Max coverage (-): 0

Region: chr11 100485239-100485249. Max. coverage (+): 0. Max coverage (-): 0

Region: chr11 100485250-100485261. Max. coverage (+): 0. Max coverage (-): 0

Region: chr11 100485262-100485273. Max. coverage (+): 0. Max coverage (-): 0

Region: chr11 100485274-100485285. Max. coverage (+): 0. Max coverage (-): 0

Region: chr11 100485286-100485296. Max. coverage (+): 0. Max coverage (-): 0

Region: chr11 100485297-100485308. Max. coverage (+): 0. Max coverage (-): 0

Region: chr11 100485309-100485320. Max. coverage (+): 0. Max coverage (-): 0

Region: chr11 100485321-100485332. Max. coverage (+): 0. Max coverage (-): 0

Region: chr11 100485333-100485343. Max. coverage (+): 0. Max coverage (-): 0

Region: chr11 100485344-100485355. Max. coverage (+): 0. Max coverage (-): 0

Region: chr11 100485356-100485367. Max. coverage (+): 0. Max coverage (-): 0

Region: chr11 100485368-100485379. Max. coverage (+): 0. Max coverage (-): 0

Region: chr11 100485380-100485391. Max. coverage (+): 0. Max coverage (-): 0

Region: chr11 100485392-100485402. Max. coverage (+): 0. Max coverage (-): 0

Region: chr11 100485403-100485414. Max. coverage (+): 0. Max coverage (-): 0

Region: chr11 100485415-100485426. Max. coverage (+): 0. Max coverage (-): 0

Region: chr11 100485427-100485438. Max. coverage (+): 0. Max coverage (-): 0

Region: chr11 100485439-100485449. Max. coverage (+): 0. Max coverage (-): 0

Region: chr11 100485450-100485461. Max. coverage (+): 0. Max coverage (-): 0

Region: chr11 100485462-100485473. Max. coverage (+): 24.79. Max coverage (-): 0

Region: chr11 100485474-100485485. Max. coverage (+): 24.79. Max coverage (-): 0

Region: chr11 100485486-100485496. Max. coverage (+): 0. Max coverage (-): 0

Region: chr11 100485497-100485508. Max. coverage (+): 0. Max coverage (-): 0

Region: chr11 100485509-100485520. Max. coverage (+): 0. Max coverage (-): 0

Region: chr11 100485521-100485532. Max. coverage (+): 0. Max coverage (-): 0

Region: chr11 100485533-100485543. Max. coverage (+): 0. Max coverage (-): 0

Region: chr11 100485544-100485555. Max. coverage (+): 0. Max coverage (-): 0

Region: chr11 100485556-100485567. Max. coverage (+): 0. Max coverage (-): 0

Region: chr11 100485568-100485579. Max. coverage (+): 0. Max coverage (-): 0

Region: chr11 100485580-100485590. Max. coverage (+): 0. Max coverage (-): 0

Region: chr11 100485591-100485602. Max. coverage (+): 12.89. Max coverage (-): 0

Region: chr11 100485603-100485614. Max. coverage (+): 6.81. Max coverage (-): 0

Region: chr11 100485615-100485626. Max. coverage (+): 0. Max coverage (-): 0

Region: chr11 100485627-100485638. Max. coverage (+): 0. Max coverage (-): 0

Region: chr11 100485639-100485649. Max. coverage (+): 0. Max coverage (-): 0

Region: chr11 100485650-100485661. Max. coverage (+): 0. Max coverage (-): 0

Region: chr11 100485662-100485673. Max. coverage (+): 0. Max coverage (-): 0

Region: chr11 100485674-100485685. Max. coverage (+): 0. Max coverage (-): 0

Region: chr11 100485686-100485696. Max. coverage (+): 0. Max coverage (-): 0

Region: chr11 100485697-100485708. Max. coverage (+): 0. Max coverage (-): 0

Region: chr11 100485709-100485720. Max. coverage (+): 0. Max coverage (-): 0

Region: chr11 100485721-100485732. Max. coverage (+): 9.26. Max coverage (-): 0

Region: chr11 100485733-100485743. Max. coverage (+): 3.32. Max coverage (-): 0

Region: chr11 100485744-100485755. Max. coverage (+): 0. Max coverage (-): 0

Region: chr11 100485756-100485767. Max. coverage (+): 0. Max coverage (-): 0

Region: chr11 100485768-100485779. Max. coverage (+): 0. Max coverage (-): 0

Region: chr11 100485780-100485790. Max. coverage (+): 0. Max coverage (-): 0

Region: chr11 100485791-100485802. Max. coverage (+): 10.64. Max coverage (-): 0

Region: chr11 100485803-100485814. Max. coverage (+): 10.64. Max coverage (-): 0

Region: chr11 100485815-100485826. Max. coverage (+): 0. Max coverage (-): 0

Region: chr11 100485827-100485838. Max. coverage (+): 0. Max coverage (-): 0

Region: chr11 100485839-100485849. Max. coverage (+): 0. Max coverage (-): 0

Region: chr11 100485850-100485861. Max. coverage (+): 0. Max coverage (-): 0

Region: chr11 100485862-100485873. Max. coverage (+): 35.74. Max coverage (-): 0

Region: chr11 100485874-100485885. Max. coverage (+): 17.72. Max coverage (-): 0

Region: chr11 100485886-100485896. Max. coverage (+): 0. Max coverage (-): 0

Region: chr11 100485897-100485908. Max. coverage (+): 0. Max coverage (-): 0

Region: chr11 100485909-100485920. Max. coverage (+): 0. Max coverage (-): 0

Region: chr11 100485921-100485932. Max. coverage (+): 5.61. Max coverage (-): 0

Region: chr11 100485933-100485943. Max. coverage (+): 5.61. Max coverage (-): 0

Region: chr11 100485944-100485955. Max. coverage (+): 0. Max coverage (-): 0

Region: chr11 100485956-100485967. Max. coverage (+): 0. Max coverage (-): 0

Region: chr11 100485968-100485979. Max. coverage (+): 0. Max coverage (-): 0

Region: chr11 100485980-100485990. Max. coverage (+): 0. Max coverage (-): 0

Region: chr11 100485991-100486002. Max. coverage (+): 0. Max coverage (-): 0

Region: chr11 100486003-100486014. Max. coverage (+): 0. Max coverage (-): 0

Region: chr11 100486015-100486026. Max. coverage (+): 0. Max coverage (-): 0

Region: chr11 100486027-100486038. Max. coverage (+): 0. Max coverage (-): 0

Region: chr11 100486039-100486049. Max. coverage (+): 0. Max coverage (-): 0

Region: chr11 100486050-100486061. Max. coverage (+): 0. Max coverage (-): 0

Region: chr11 100486062-100486073. Max. coverage (+): 0. Max coverage (-): 0

Region: chr11 100486074-100486085. Max. coverage (+): 0. Max coverage (-): 0

Region: chr11 100486086-100486096. Max. coverage (+): 0. Max coverage (-): 0

Region: chr11 100486097-100486108. Max. coverage (+): 0. Max coverage (-): 0

Region: chr11 100486109-100486120. Max. coverage (+): 0. Max coverage (-): 0

Region: chr11 100486121-100486132. Max. coverage (+): 0. Max coverage (-): 0

Region: chr11 100486133-100486143. Max. coverage (+): 2.58. Max coverage (-): 0

Region: chr11 100486144-100486155. Max. coverage (+): 2.58. Max coverage (-): 0

Region: chr11 100486156-100486167. Max. coverage (+): 20.15. Max coverage (-): 0

Region: chr11 100486168-100486179. Max. coverage (+): 10.85. Max coverage (-): 0

Region: chr11 100486180-100486190. Max. coverage (+): 17.68. Max coverage (-): 0

Region: chr11 100486191-100486202. Max. coverage (+): 0. Max coverage (-): 0

Region: chr11 100486203-100486214. Max. coverage (+): 0. Max coverage (-): 0

Region: chr11 100486215-100486226. Max. coverage (+): 0. Max coverage (-): 0

Region: chr11 100486227-100486238. Max. coverage (+): 5.39. Max coverage (-): 0

Region: chr11 100486239-100486249. Max. coverage (+): 10.48. Max coverage (-): 0

Region: chr11 100486250-100486261. Max. coverage (+): 0. Max coverage (-): 0

Region: chr11 100486262-100486273. Max. coverage (+): 0. Max coverage (-): 0

Region: chr11 100486274-100486285. Max. coverage (+): 0. Max coverage (-): 0

Region: chr11 100486286-100486296. Max. coverage (+): 0. Max coverage (-): 0

Region: chr11 100486297-100486308. Max. coverage (+): 0.61. Max coverage (-): 0

Region: chr11 100486309-100486320. Max. coverage (+): 0.61. Max coverage (-): 0

Region: chr11 100486321-100486332. Max. coverage (+): 0. Max coverage (-): 0

Region: chr11 100486333-100486343. Max. coverage (+): 0. Max coverage (-): 0

Region: chr11 100486344-100486355. Max. coverage (+): 4.31. Max coverage (-): 0

Region: chr11 100486356-100486367. Max. coverage (+): 4.31. Max coverage (-): 0

Region: chr11 100486368-100486379. Max. coverage (+): 0. Max coverage (-): 0

Region: chr11 100486380-100486390. Max. coverage (+): 0. Max coverage (-): 0

Region: chr11 100486391-100486402. Max. coverage (+): 0. Max coverage (-): 0

Region: chr11 100486403-100486414. Max. coverage (+): 0. Max coverage (-): 0

Region: chr11 100486415-100486426. Max. coverage (+): 0. Max coverage (-): 0

Region: chr11 100486427-100486437. Max. coverage (+): 0. Max coverage (-): 0

Region: chr11 100486438-100486449. Max. coverage (+): 0. Max coverage (-): 0

Region: chr11 100486450-100486461. Max. coverage (+): 0. Max coverage (-): 0

Region: chr11 100486462-100486473. Max. coverage (+): 0. Max coverage (-): 0

Region: chr11 100486474-100486485. Max. coverage (+): 0. Max coverage (-): 0

Region: chr11 100486486-100486496. Max. coverage (+): 0. Max coverage (-): 0

Region: chr11 100486497-100486508. Max. coverage (+): 0. Max coverage (-): 0

Region: chr11 100486509-100486520. Max. coverage (+): 0. Max coverage (-): 0

Region: chr11 100486521-100486532. Max. coverage (+): 0. Max coverage (-): 0

Region: chr11 100486533-100486543. Max. coverage (+): 0. Max coverage (-): 0

Region: chr11 100486544-100486555. Max. coverage (+): 0. Max coverage (-): 0

Region: chr11 100486556-100486567. Max. coverage (+): 0. Max coverage (-): 0

Region: chr11 100486568-100486579. Max. coverage (+): 0. Max coverage (-): 0

Region: chr11 100486580-100486590. Max. coverage (+): 0. Max coverage (-): 0

Region: chr11 100486591-100486602. Max. coverage (+): 0. Max coverage (-): 0

Region: chr11 100486603-100486614. Max. coverage (+): 7.65. Max coverage (-): 0

Region: chr11 100486615-100486626. Max. coverage (+): 0. Max coverage (-): 0

Region: chr11 100486627-100486637. Max. coverage (+): 0. Max coverage (-): 0

Region: chr11 100486638-100486649. Max. coverage (+): 0. Max coverage (-): 0

Region: chr11 100486650-100486661. Max. coverage (+): 0. Max coverage (-): 0

Region: chr11 100486662-100486673. Max. coverage (+): 0. Max coverage (-): 0

Region: chr11 100486674-100486685. Max. coverage (+): 3.98. Max coverage (-): 0

Region: chr11 100486686-100486696. Max. coverage (+): 4.28. Max coverage (-): 0

Region: chr11 100486697-100486708. Max. coverage (+): 4.28. Max coverage (-): 0

Region: chr11 100486709-100486720. Max. coverage (+): 3.49. Max coverage (-): 0

Region: chr11 100486721-100486732. Max. coverage (+): 7. Max coverage (-): 0

Region: chr11 100486733-100486743. Max. coverage (+): 0. Max coverage (-): 0

Region: chr11 100486744-100486755. Max. coverage (+): 0. Max coverage (-): 0

Region: chr11 100486756-100486767. Max. coverage (+): 0. Max coverage (-): 0

Region: chr11 100486768-100486779. Max. coverage (+): 0. Max coverage (-): 0

Region: chr11 100486780-100486790. Max. coverage (+): 0. Max coverage (-): 0

Region: chr11 100486791-100486802. Max. coverage (+): 0. Max coverage (-): 0

Region: chr11 100486803-100486814. Max. coverage (+): 0. Max coverage (-): 0

Region: chr11 100486815-100486826. Max. coverage (+): 0. Max coverage (-): 0

Region: chr11 100486827-100486837. Max. coverage (+): 0. Max coverage (-): 0

Region: chr11 100486838-100486849. Max. coverage (+): 0. Max coverage (-): 0

Region: chr11 100486850-100486861. Max. coverage (+): 0. Max coverage (-): 0

Region: chr11 100486862-100486873. Max. coverage (+): 6.08. Max coverage (-): 0

Region: chr11 100486874-100486885. Max. coverage (+): 6.08. Max coverage (-): 0

Region: chr11 100486886-100486896. Max. coverage (+): 0. Max coverage (-): 0

Region: chr11 100486897-100486908. Max. coverage (+): 0. Max coverage (-): 0

Region: chr11 100486909-100486920. Max. coverage (+): 0. Max coverage (-): 0

Region: chr11 100486921-100486932. Max. coverage (+): 0. Max coverage (-): 0

Region: chr11 100486933-100486943. Max. coverage (+): 0. Max coverage (-): 0

Region: chr11 100486944-100486955. Max. coverage (+): 0. Max coverage (-): 0

Region: chr11 100486956-100486967. Max. coverage (+): 0. Max coverage (-): 0

Region: chr11 100486968-100486979. Max. coverage (+): 0. Max coverage (-): 0

Region: chr11 100486980-100486990. Max. coverage (+): 0. Max coverage (-): 0

Region: chr11 100486991-100487002. Max. coverage (+): 0. Max coverage (-): 0

Region: chr11 100487003-100487014. Max. coverage (+): 0. Max coverage (-): 0

Region: chr11 100487015-100487026. Max. coverage (+): 0. Max coverage (-): 0

Region: chr11 100487027-100487037. Max. coverage (+): 0. Max coverage (-): 0

Region: chr11 100487038-100487049. Max. coverage (+): 0. Max coverage (-): 0

Region: chr11 100487050-100487061. Max. coverage (+): 14.08. Max coverage (-): 0

Region: chr11 100487062-100487073. Max. coverage (+): 14.08. Max coverage (-): 0

Region: chr11 100487074-100487085. Max. coverage (+): 0. Max coverage (-): 0

Region: chr11 100487086-100487096. Max. coverage (+): 4.67. Max coverage (-): 0

Region: chr11 100487097-100487108. Max. coverage (+): 0. Max coverage (-): 0

Region: chr11 100487109-100487120. Max. coverage (+): 5.55. Max coverage (-): 0

Region: chr11 100487121-100487132. Max. coverage (+): 5.55. Max coverage (-): 0

Region: chr11 100487133-100487143. Max. coverage (+): 0. Max coverage (-): 0

Region: chr11 100487144-100487155. Max. coverage (+): 0. Max coverage (-): 0

Region: chr11 100487156-100487167. Max. coverage (+): 0. Max coverage (-): 0

Region: chr11 100487168-100487179. Max. coverage (+): 0. Max coverage (-): 0

Region: chr11 100487180-100487190. Max. coverage (+): 0. Max coverage (-): 0

Region: chr11 100487191-100487202. Max. coverage (+): 0. Max coverage (-): 0

Region: chr11 100487203-100487214. Max. coverage (+): 0. Max coverage (-): 0

Region: chr11 100487215-100487226. Max. coverage (+): 0. Max coverage (-): 0

Region: chr11 100487227-100487237. Max. coverage (+): 0.22. Max coverage (-): 0

Region: chr11 100487238-100487249. Max. coverage (+): 0. Max coverage (-): 0

Region: chr11 100487250-100487261. Max. coverage (+): 0. Max coverage (-): 0

Region: chr11 100487262-100487273. Max. coverage (+): 0. Max coverage (-): 0

Region: chr11 100487274-100487285. Max. coverage (+): 0. Max coverage (-): 0

Region: chr11 100487286-100487296. Max. coverage (+): 0. Max coverage (-): 0

Region: chr11 100487297-100487308. Max. coverage (+): 0. Max coverage (-): 0

Region: chr11 100487309-100487320. Max. coverage (+): 0. Max coverage (-): 0

Region: chr11 100487321-100487332. Max. coverage (+): 0. Max coverage (-): 0

Region: chr11 100487333-100487343. Max. coverage (+): 0. Max coverage (-): 0

Region: chr11 100487344-100487355. Max. coverage (+): 0. Max coverage (-): 0

Region: chr11 100487356-100487367. Max. coverage (+): 3.46. Max coverage (-): 0

Region: chr11 100487368-100487379. Max. coverage (+): 0. Max coverage (-): 0

Region: chr11 100487380-100487390. Max. coverage (+): 5.59. Max coverage (-): 0

Region: chr11 100487391-100487402. Max. coverage (+): 0. Max coverage (-): 0

Region: chr11 100487403-100487414. Max. coverage (+): 0. Max coverage (-): 0

Region: chr11 100487415-100487426. Max. coverage (+): 0. Max coverage (-): 0

Region: chr11 100487427-100487437. Max. coverage (+): 0. Max coverage (-): 0

Region: chr11 100487438-100487449. Max. coverage (+): 0. Max coverage (-): 0

Region: chr11 100487450-100487461. Max. coverage (+): 0. Max coverage (-): 0

Region: chr11 100487462-100487473. Max. coverage (+): 0. Max coverage (-): 0

Region: chr11 100487474-100487484. Max. coverage (+): 0. Max coverage (-): 0

Region: chr11 100487485-100487496. Max. coverage (+): 0. Max coverage (-): 0

Region: chr11 100487497-100487508. Max. coverage (+): 0. Max coverage (-): 0

Region: chr11 100487509-100487520. Max. coverage (+): 0. Max coverage (-): 0

Region: chr11 100487521-100487532. Max. coverage (+): 0. Max coverage (-): 0

Region: chr11 100487533-100487543. Max. coverage (+): 0. Max coverage (-): 0

Region: chr11 100487544-100487555. Max. coverage (+): 0. Max coverage (-): 0

Region: chr11 100487556-100487567. Max. coverage (+): 0. Max coverage (-): 0

Region: chr11 100487568-100487579. Max. coverage (+): 0. Max coverage (-): 0

Region: chr11 100487580-100487590. Max. coverage (+): 0. Max coverage (-): 0

Region: chr11 100487591-100487602. Max. coverage (+): 0. Max coverage (-): 0

Region: chr11 100487603-100487614. Max. coverage (+): 0. Max coverage (-): 0

Region: chr11 100487615-100487626. Max. coverage (+): 0. Max coverage (-): 0

Region: chr11 100487627-100487637. Max. coverage (+): 0. Max coverage (-): 0

Region: chr11 100487638-100487649. Max. coverage (+): 0. Max coverage (-): 0

Region: chr11 100487650-100487661. Max. coverage (+): 0. Max coverage (-): 1.68

Region: chr11 100487662-100487673. Max. coverage (+): 0. Max coverage (-): 1.68

Region: chr11 100487674-100487684. Max. coverage (+): 0. Max coverage (-): 0

Region: chr11 100487685-100487696. Max. coverage (+): 0. Max coverage (-): 0

Region: chr11 100487697-100487708. Max. coverage (+): 0. Max coverage (-): 0

Region: chr11 100487709-100487720. Max. coverage (+): 0. Max coverage (-): 0

Region: chr11 100487721-100487732. Max. coverage (+): 0. Max coverage (-): 0

Region: chr11 100487733-100487743. Max. coverage (+): 0. Max coverage (-): 0.39

Region: chr11 100487744-100487755. Max. coverage (+): 4.35. Max coverage (-): 0.39

Region: chr11 100487756-100487767. Max. coverage (+): 4.35. Max coverage (-): 4.94

Region: chr11 100487768-100487779. Max. coverage (+): 0. Max coverage (-): 0

Region: chr11 100487780-100487790. Max. coverage (+): 6.01. Max coverage (-): 0

Region: chr11 100487791-100487802. Max. coverage (+): 0. Max coverage (-): 0

Region: chr11 100487803-100487814. Max. coverage (+): 0. Max coverage (-): 0

Region: chr11 100487815-100487826. Max. coverage (+): 0. Max coverage (-): 0

Region: chr11 100487827-100487837. Max. coverage (+): 0. Max coverage (-): 0

Region: chr11 100487838-100487849. Max. coverage (+): 0. Max coverage (-): 0

Region: chr11 100487850-100487861. Max. coverage (+): 0. Max coverage (-): 0

Region: chr11 100487862-100487873. Max. coverage (+): 0. Max coverage (-): 0

Region: chr11 100487874-100487884. Max. coverage (+): 1.11. Max coverage (-): 0

Region: chr11 100487885-100487896. Max. coverage (+): 1.11. Max coverage (-): 0

Region: chr11 100487897-100487908. Max. coverage (+): 0. Max coverage (-): 0

Region: chr11 100487909-100487920. Max. coverage (+): 0. Max coverage (-): 26.92

Region: chr11 100487921-100487932. Max. coverage (+): 0. Max coverage (-): 11.45

Region: chr11 100487933-100487943. Max. coverage (+): 7.21. Max coverage (-): 1.93

Region: chr11 100487944-100487955. Max. coverage (+): 7.21. Max coverage (-): 4.11

Region: chr11 100487956-100487967. Max. coverage (+): 0. Max coverage (-): 4.11

Region: chr11 100487968-100487979. Max. coverage (+): 0. Max coverage (-): 0

Region: chr11 100487980-100487990. Max. coverage (+): 0. Max coverage (-): 0

Region: chr11 100487991-100488002. Max. coverage (+): 0. Max coverage (-): 0

Region: chr11 100488003-100488014. Max. coverage (+): 0. Max coverage (-): 0

Region: chr11 100488015-100488026. Max. coverage (+): 0. Max coverage (-): 0

Region: chr11 100488027-100488037. Max. coverage (+): 0. Max coverage (-): 0

Region: chr11 100488038-100488049. Max. coverage (+): 0. Max coverage (-): 0

Region: chr11 100488050-100488061. Max. coverage (+): 0. Max coverage (-): 2.31

Region: chr11 100488062-100488073. Max. coverage (+): 0. Max coverage (-): 2.31

Region: chr11 100488074-100488084. Max. coverage (+): 0. Max coverage (-): 0

Region: chr11 100488085-100488096. Max. coverage (+): 0. Max coverage (-): 0

Region: chr11 100488097-100488108. Max. coverage (+): 0. Max coverage (-): 0

Region: chr11 100488109-100488120. Max. coverage (+): 0. Max coverage (-): 0

Region: chr11 100488121-100488132. Max. coverage (+): 0. Max coverage (-): 0

Region: chr11 100488133-100488143. Max. coverage (+): 0. Max coverage (-): 2.16

Region: chr11 100488144-100488155. Max. coverage (+): 0. Max coverage (-): 2.16

Region: chr11 100488156-100488167. Max. coverage (+): 0. Max coverage (-): 5.9

Region: chr11 100488168-100488179. Max. coverage (+): 0. Max coverage (-): 0

Region: chr11 100488180-100488190. Max. coverage (+): 0. Max coverage (-): 0

Region: chr11 100488191-100488202. Max. coverage (+): 2.37. Max coverage (-): 3.54

Region: chr11 100488203-100488214. Max. coverage (+): 4.71. Max coverage (-): 4.44

Region: chr11 100488215-100488226. Max. coverage (+): 4.71. Max coverage (-): 0

Region: chr11 100488227-100488237. Max. coverage (+): 0. Max coverage (-): 1.19

Region: chr11 100488238-100488249. Max. coverage (+): 0. Max coverage (-): 5.73

Region: chr11 100488250-100488261. Max. coverage (+): 0. Max coverage (-): 0

Region: chr11 100488262-100488273. Max. coverage (+): 0. Max coverage (-): 0

Region: chr11 100488274-100488284. Max. coverage (+): 0. Max coverage (-): 0

Region: chr11 100488285-100488296. Max. coverage (+): 0. Max coverage (-): 0

Region: chr11 100488297-100488308. Max. coverage (+): 0. Max coverage (-): 2.63

Region: chr11 100488309-100488320. Max. coverage (+): 0. Max coverage (-): 2.63

Region: chr11 100488321-100488332. Max. coverage (+): 4.5. Max coverage (-): 0

Region: chr11 100488333-100488343. Max. coverage (+): 0. Max coverage (-): 0

Region: chr11 100488344-100488355. Max. coverage (+): 5.28. Max coverage (-): 0

Region: chr11 100488356-100488367. Max. coverage (+): 5.28. Max coverage (-): 0

Region: chr11 100488368-100488379. Max. coverage (+): 0. Max coverage (-): 3.61

Region: chr11 100488380-100488390. Max. coverage (+): 2.39. Max coverage (-): 3.61

Region: chr11 100488391-100488402. Max. coverage (+): 2.39. Max coverage (-): 0

Region: chr11 100488403-100488414. Max. coverage (+): 0. Max coverage (-): 6.54

Region: chr11 100488415-100488426. Max. coverage (+): 5.17. Max coverage (-): 0

Region: chr11 100488427-100488437. Max. coverage (+): 0. Max coverage (-): 0

Region: chr11 100488438-100488449. Max. coverage (+): 0. Max coverage (-): 0

Region: chr11 100488450-100488461. Max. coverage (+): 0. Max coverage (-): 0

Region: chr11 100488462-100488473. Max. coverage (+): 0. Max coverage (-): 0

Region: chr11 100488474-100488484. Max. coverage (+): 4.3. Max coverage (-): 0

Region: chr11 100488485-100488496. Max. coverage (+): 4.3. Max coverage (-): 0

Region: chr11 100488497-100488508. Max. coverage (+): 0. Max coverage (-): 0

Region: chr11 100488509-100488520. Max. coverage (+): 0. Max coverage (-): 0

Region: chr11 100488521-100488531. Max. coverage (+): 0. Max coverage (-): 0

Region: chr11 100488532-100488543. Max. coverage (+): 0. Max coverage (-): 0

Region: chr11 100488544-100488555. Max. coverage (+): 0. Max coverage (-): 0

Region: chr11 100488556-100488567. Max. coverage (+): 0. Max coverage (-): 0

Region: chr11 100488568-100488579. Max. coverage (+): 0. Max coverage (-): 0

Region: chr11 100488580-100488590. Max. coverage (+): 0. Max coverage (-): 0

Region: chr11 100488591-100488602. Max. coverage (+): 8.06. Max coverage (-): 0

Region: chr11 100488603-100488614. Max. coverage (+): 9.81. Max coverage (-): 0

Region: chr11 100488615-100488626. Max. coverage (+): 5.55. Max coverage (-): 0

Region: chr11 100488627-100488637. Max. coverage (+): 0. Max coverage (-): 0

Region: chr11 100488638-100488649. Max. coverage (+): 0. Max coverage (-): 0

Region: chr11 100488650-100488661. Max. coverage (+): 0. Max coverage (-): 0

Region: chr11 100488662-100488673. Max. coverage (+): 0. Max coverage (-): 0

Region: chr11 100488674-100488684. Max. coverage (+): 0. Max coverage (-): 0

Region: chr11 100488685-100488696. Max. coverage (+): 2.54. Max coverage (-): 0

Region: chr11 100488697-100488708. Max. coverage (+): 0. Max coverage (-): 0

Region: chr11 100488709-100488720. Max. coverage (+): 0. Max coverage (-): 0

Region: chr11 100488721-100488731. Max. coverage (+): 0. Max coverage (-): 0

Region: chr11 100488732-100488743. Max. coverage (+): 0. Max coverage (-): 0

Region: chr11 100488744-100488755. Max. coverage (+): 4.3. Max coverage (-): 0

Region: chr11 100488756-100488767. Max. coverage (+): 4.3. Max coverage (-): 0

Region: chr11 100488768-100488779. Max. coverage (+): 0. Max coverage (-): 0

Region: chr11 100488780-100488790. Max. coverage (+): 0. Max coverage (-): 0

Region: chr11 100488791-100488802. Max. coverage (+): 0. Max coverage (-): 0

Region: chr11 100488803-100488814. Max. coverage (+): 0. Max coverage (-): 0

Region: chr11 100488815-100488826. Max. coverage (+): 0. Max coverage (-): 0

Region: chr11 100488827-100488837. Max. coverage (+): 0. Max coverage (-): 0

Region: chr11 100488838-100488849. Max. coverage (+): 0. Max coverage (-): 0

Region: chr11 100488850-100488861. Max. coverage (+): 0. Max coverage (-): 0

Region: chr11 100488862-100488873. Max. coverage (+): 0. Max coverage (-): 0

Region: chr11 100488874-100488884. Max. coverage (+): 0. Max coverage (-): 0

Region: chr11 100488885-100488896. Max. coverage (+): 0. Max coverage (-): 0

Region: chr11 100488897-100488908. Max. coverage (+): 0. Max coverage (-): 0

Region: chr11 100488909-100488920. Max. coverage (+): 0. Max coverage (-): 0

Region: chr11 100488921-100488931. Max. coverage (+): 0. Max coverage (-): 0

Region: chr11 100488932-100488943. Max. coverage (+): 0. Max coverage (-): 0

Region: chr11 100488944-100488955. Max. coverage (+): 0. Max coverage (-): 0

Region: chr11 100488956-100488967. Max. coverage (+): 0. Max coverage (-): 0

Region: chr11 100488968-100488979. Max. coverage (+): 0. Max coverage (-): 0

Region: chr11 100488980-100488990. Max. coverage (+): 0. Max coverage (-): 0

Region: chr11 100488991-100489002. Max. coverage (+): 0. Max coverage (-): 0

Region: chr11 100489003-100489014. Max. coverage (+): 0. Max coverage (-): 0

Region: chr11 100489015-100489026. Max. coverage (+): 0. Max coverage (-): 0

Region: chr11 100489027-100489037. Max. coverage (+): 0. Max coverage (-): 0

Region: chr11 100489038-100489049. Max. coverage (+): 0. Max coverage (-): 0

Region: chr11 100489050-100489061. Max. coverage (+): 0. Max coverage (-): 0

Region: chr11 100489062-100489073. Max. coverage (+): 0. Max coverage (-): 0

Region: chr11 100489074-100489084. Max. coverage (+): 0. Max coverage (-): 0

Region: chr11 100489085-100489096. Max. coverage (+): 0. Max coverage (-): 0

Region: chr11 100489097-100489108. Max. coverage (+): 0. Max coverage (-): 0

Region: chr11 100489109-100489120. Max. coverage (+): 0. Max coverage (-): 0

Region: chr11 100489121-100489131. Max. coverage (+): 0. Max coverage (-): 0

Region: chr11 100489132-100489143. Max. coverage (+): 0. Max coverage (-): 0

Region: chr11 100489144-100489155. Max. coverage (+): 0. Max coverage (-): 0

Region: chr11 100489156-100489167. Max. coverage (+): 0. Max coverage (-): 0

Region: chr11 100489168-100489179. Max. coverage (+): 0. Max coverage (-): 0

Region: chr11 100489180-100489190. Max. coverage (+): 0. Max coverage (-): 0

Region: chr11 100489191-100489202. Max. coverage (+): 14.46. Max coverage (-): 0

Region: chr11 100489203-100489214. Max. coverage (+): 14.46. Max coverage (-): 0

Region: chr11 100489215-100489226. Max. coverage (+): 0. Max coverage (-): 0

Region: chr11 100489227-100489237. Max. coverage (+): 0. Max coverage (-): 0

Region: chr11 100489238-100489249. Max. coverage (+): 0. Max coverage (-): 0

Region: chr11 100489250-100489261. Max. coverage (+): 0. Max coverage (-): 0

Region: chr11 100489262-100489273. Max. coverage (+): 7.19. Max coverage (-): 0

Region: chr11 100489274-100489284. Max. coverage (+): 12.48. Max coverage (-): 0

Region: chr11 100489285-100489296. Max. coverage (+): 10.81. Max coverage (-): 0

Region: chr11 100489297-100489308. Max. coverage (+): 10.81. Max coverage (-): 0

Region: chr11 100489309-100489320. Max. coverage (+): 0. Max coverage (-): 0

Region: chr11 100489321-100489331. Max. coverage (+): 0. Max coverage (-): 0

Region: chr11 100489332-100489343. Max. coverage (+): 0. Max coverage (-): 0

Region: chr11 100489344-100489355. Max. coverage (+): 12.97. Max coverage (-): 0

Region: chr11 100489356-100489367. Max. coverage (+): 16.59. Max coverage (-): 0

Region: chr11 100489368-100489378. Max. coverage (+): 6.07. Max coverage (-): 0

Region: chr11 100489379-100489390. Max. coverage (+): 6.07. Max coverage (-): 0

Region: chr11 100489391-100489402. Max. coverage (+): 0. Max coverage (-): 0

Region: chr11 100489403-100489414. Max. coverage (+): 0. Max coverage (-): 0

Region: chr11 100489415-100489426. Max. coverage (+): 0. Max coverage (-): 0

Region: chr11 100489427-100489437. Max. coverage (+): 0. Max coverage (-): 0

Region: chr11 100489438-100489449. Max. coverage (+): 0. Max coverage (-): 0

Region: chr11 100489450-100489461. Max. coverage (+): 0. Max coverage (-): 0

Region: chr11 100489462-100489473. Max. coverage (+): 0. Max coverage (-): 0

Region: chr11 100489474-100489484. Max. coverage (+): 0. Max coverage (-): 0

Region: chr11 100489485-100489496. Max. coverage (+): 0. Max coverage (-): 0

Region: chr11 100489497-100489508. Max. coverage (+): 0. Max coverage (-): 0

Region: chr11 100489509-100489520. Max. coverage (+): 0. Max coverage (-): 0

Region: chr11 100489521-100489531. Max. coverage (+): 0. Max coverage (-): 0

Region: chr11 100489532-100489543. Max. coverage (+): 0. Max coverage (-): 0

Region: chr11 100489544-100489555. Max. coverage (+): 0. Max coverage (-): 0

Region: chr11 100489556-100489567. Max. coverage (+): 0. Max coverage (-): 0

Region: chr11 100489568-100489578. Max. coverage (+): 0. Max coverage (-): 0

Region: chr11 100489579-100489590. Max. coverage (+): 6.83. Max coverage (-): 0

Region: chr11 100489591-100489602. Max. coverage (+): 0. Max coverage (-): 0

Region: chr11 100489603-100489614. Max. coverage (+): 6.93. Max coverage (-): 0

Region: chr11 100489615-100489626. Max. coverage (+): 30.26. Max coverage (-): 0

Region: chr11 100489627-100489637. Max. coverage (+): 52.18. Max coverage (-): 0

Region: chr11 100489638-100489649. Max. coverage (+): 21.8. Max coverage (-): 0

Region: chr11 100489650-100489661. Max. coverage (+): 0. Max coverage (-): 0

Region: chr11 100489662-100489673. Max. coverage (+): 0. Max coverage (-): 0

Region: chr11 100489674-100489684. Max. coverage (+): 7.05. Max coverage (-): 0

Region: chr11 100489685-100489696. Max. coverage (+): 7.05. Max coverage (-): 0

Region: chr11 100489697-100489708. Max. coverage (+): 6.91. Max coverage (-): 0

Region: chr11 100489709-100489720. Max. coverage (+): 0. Max coverage (-): 0

Region: chr11 100489721-100489731. Max. coverage (+): 0. Max coverage (-): 0

Region: chr11 100489732-100489743. Max. coverage (+): 0. Max coverage (-): 0

Region: chr11 100489744-100489755. Max. coverage (+): 0. Max coverage (-): 0

Region: chr11 100489756-100489767. Max. coverage (+): 0. Max coverage (-): 0

Region: chr11 100489768-100489778. Max. coverage (+): 0. Max coverage (-): 0

Region: chr11 100489779-100489790. Max. coverage (+): 0. Max coverage (-): 0

Region: chr11 100489791-100489802. Max. coverage (+): 0. Max coverage (-): 0

Region: chr11 100489803-100489814. Max. coverage (+): 0. Max coverage (-): 0

Region: chr11 100489815-100489826. Max. coverage (+): 0. Max coverage (-): 0

Region: chr11 100489827-100489837. Max. coverage (+): 0. Max coverage (-): 0

Region: chr11 100489838-100489849. Max. coverage (+): 0. Max coverage (-): 0

Region: chr11 100489850-100489861. Max. coverage (+): 0. Max coverage (-): 0

Region: chr11 100489862-100489873. Max. coverage (+): 0. Max coverage (-): 0

Region: chr11 100489874-100489884. Max. coverage (+): 0. Max coverage (-): 0

Region: chr11 100489885-100489896. Max. coverage (+): 0. Max coverage (-): 0

Region: chr11 100489897-100489908. Max. coverage (+): 0. Max coverage (-): 0

Region: chr11 100489909-100489920. Max. coverage (+): 0. Max coverage (-): 0

Region: chr11 100489921-100489931. Max. coverage (+): 0. Max coverage (-): 0

Region: chr11 100489932-100489943. Max. coverage (+): 0. Max coverage (-): 0

Region: chr11 100489944-100489955. Max. coverage (+): 0. Max coverage (-): 0

Region: chr11 100489956-100489967. Max. coverage (+): 0. Max coverage (-): 0

Region: chr11 100489968-100489978. Max. coverage (+): 0. Max coverage (-): 0

Region: chr11 100489979-100489990. Max. coverage (+): 0. Max coverage (-): 0

Region: chr11 100489991-100490002. Max. coverage (+): 0. Max coverage (-): 0

Region: chr11 100490003-100490014. Max. coverage (+): 0. Max coverage (-): 0

Region: chr11 100490015-100490026. Max. coverage (+): 0. Max coverage (-): 0

Region: chr11 100490027-100490037. Max. coverage (+): 0. Max coverage (-): 0

Region: chr11 100490038-100490049. Max. coverage (+): 0. Max coverage (-): 0

Region: chr11 100490050-100490061. Max. coverage (+): 0. Max coverage (-): 0

Region: chr11 100490062-100490073. Max. coverage (+): 0. Max coverage (-): 0

Region: chr11 100490074-100490084. Max. coverage (+): 0. Max coverage (-): 0

Region: chr11 100490085-100490096. Max. coverage (+): 5.1. Max coverage (-): 0

Region: chr11 100490097-100490108. Max. coverage (+): 5.1. Max coverage (-): 0

Region: chr11 100490109-100490120. Max. coverage (+): 0. Max coverage (-): 0

Region: chr11 100490121-100490131. Max. coverage (+): 0. Max coverage (-): 0

Region: chr11 100490132-100490143. Max. coverage (+): 0. Max coverage (-): 0

Region: chr11 100490144-100490155. Max. coverage (+): 0. Max coverage (-): 0

Region: chr11 100490156-100490167. Max. coverage (+): 0. Max coverage (-): 0

Region: chr11 100490168-100490178. Max. coverage (+): 0. Max coverage (-): 0

Region: chr11 100490179-100490190. Max. coverage (+): 0. Max coverage (-): 0

Region: chr11 100490191-100490202. Max. coverage (+): 0. Max coverage (-): 0

Region: chr11 100490203-100490214. Max. coverage (+): 0. Max coverage (-): 0

Region: chr11 100490215-100490226. Max. coverage (+): 0. Max coverage (-): 0

Region: chr11 100490227-100490237. Max. coverage (+): 0. Max coverage (-): 0

Region: chr11 100490238-100490249. Max. coverage (+): 0. Max coverage (-): 0

Region: chr11 100490250-100490261. Max. coverage (+): 0. Max coverage (-): 0

Region: chr11 100490262-100490273. Max. coverage (+): 0. Max coverage (-): 0

Region: chr11 100490274-100490284. Max. coverage (+): 0. Max coverage (-): 0

Region: chr11 100490285-100490296. Max. coverage (+): 0. Max coverage (-): 0

Region: chr11 100490297-100490308. Max. coverage (+): 4.07. Max coverage (-): 0

Region: chr11 100490309-100490320. Max. coverage (+): 0. Max coverage (-): 0

Region: chr11 100490321-. Max. coverage (+): 0. Max coverage (-): 0

RepeatMasker Color Code

**+**

100-98% Identity

<98-95% Identity

<95-90% Identity

<90-85% Identity

<85-80% Identity

<80-75% Identity

<75-70% Identity

<70% Identity

**-**

Gene Set Color Code

**+**

Gene

Pseudogene

**-**

Topology/Coverage Color Code

Coverage Plus Strand

Coverage Minus Strand

Mainstrand: Plus

Mainstrand: Minus

Complementary Strand

Flanking Region  
(if option -flank >0)

Gene Set Annotation  

**1. GPR107 (protein coding, ENSBTAG00000004204) Tr:00000005512 Ex:18**: 100484432-100484528 (+)

  
RepeatMasker Annotation  

**1. LTR11B\_BT**: 100488819-100488993 (-), Divergence to consensus: 38.9%  
**2. Bov-tA2**: 100489006-100489119 (-), Divergence to consensus: 11.4%

  
Transcription Factor Binding Sites  

**SOX9** (Sequence: TCATTGTT (+): 100487839)  
**A-MYB** (Sequence: CCAACTGCCA (-): 100488534)
