# Supplementary material for: Small RNA sequencing of cryopreserved semen from single bull revealed altered miRNAs and piRNAs expression between High- and Low-motile sperm populations
Source: BMC Genomics. 2017 Jan 4;18:14. doi: 10.1186/s12864-016-3394-7 (PMC5209821; doi:10.1186/s12864-016-3394-7)
Supplement: Additional file 4: — Details for each piRNA clusters found in Low Motile (LM) sperm fraction. Genes, repeats, transposable elements and transcription factors binding sites falling within the cluster regions were reported. (ZIP 1034 kb) [file 12864_2016_3394_MOESM4_ESM.zip › 9.html]

piRNA cluster 9


Predicted piRNA cluster no. 9     previous   next
  

Show proTRAC run info
Hide proTRAC run info

================================= proTRAC ====================================  
VERSION: 2.1                                    LAST MODIFIED: 06. October 2015  
  
Please cite:  
Rosenkranz D, Zischler H. proTRAC - a software for probabilistic piRNA cluster  
detection, visualization and analysis. 2012. BMC Bioinformatics 13:5.  
  
and (for proTRAC 2.0 and later):  
Rosenkranz D, Rudloff S, Bastuck K, Ketting RF, Zischler H. Tupaia small RNAs  
provide insights into function and evolution of RNAi-based transposon defense  
in mammals. 2015. RNA 21(5):911-922.  
  
Contact:  
David Rosenkranz  
Institute of Anthropology, small RNA group  
Johannes Gutenberg University Mainz  
email: rosenkranz@uni-mainz.de  
  
You can find the latest proTRAC version at:  
http://sourceforge.net/projects/protrac/files  
http://www.smallRNAgroup-mainz.de/software  
==============================================================================  
  
PARAMETERS:  
Map file: .............../storage/core/barbara/genhome/smallRNA/fertility/Sample\_not\_motile/pirna/Sample\_not\_motile\_26-33\_collapsed.fa.no-dust.map.weighted-10000-1000-b-0  
Genome file: ............/storage/core/barbara/genhome/smallRNA/fertility/Sample\_all/pirna/bt\_311\_chrY.fa  
RepeatMasker annotation: /storage/genomes/bt\_umd31/GCF\_000003055.6\_Bos\_taurus\_UMD\_3.1.1\_repeatMasker\_chr.out  
GeneSet:................./storage/core/barbara/genhome/smallRNA/fertility/Sample\_all/pirna/full.gtf  
  
Significant (p<=0.01) hit density will be calculated based  
on observed hit distribution.  
  
Sliding window size: ........................................ 5000 bp  
Sliding window increament: .................................. 1000 bp  
Normalize each hit by number of genomic hits: ............... 1 [0=no/1=yes]  
Normalize each hit by number of sequence reads: ............. 1 [0=no/1=yes]  
Normalize values (-> per million mapped reads): ............. 1 [0=no/1=yes]  
Min. fraction of hits with 1T(U) or 10A: .................... 0.75  
Alternatively: Min. fraction of hits with 1T(U) and 10A: .... 0.5  
Min. fraction of hits with typical piRNA length: ............ 0.75  
Typical piRNA length: ....................................... 26-33 nt  
Min. size of a piRNA cluster: ............................... 5000 bp.  
Min. number of hits (absolute): ............................. 0  
Min. number of hits (normalized): ........................... 0  
Min. fraction of hits on the mainstrand: .................... 0.75  
Top fraction of mapped sequences (in terms of read counts): . 1%  
Top fraction accounts for max. n% of sequence reads: ........ 90%  
Min. fraction of hits on each arm of a bidirectional cluster: 0.1  
Output image file for each cluster: ......................... 0 [0=no/1=yes]  
Output html file for each cluster: .......................... 1 [0=no/1=yes]  
Output a summary table: ..................................... 1 [0=no/1=yes]  
Output a FASTA file for each cluster (piRNA sequences): ..... 1 [0=no/1=yes]  
Output a FASTA file comprising cluster sequences: ........... 1 [0=no/1=yes]  
Search DNA motifs in clusters: .............................. 1 [0=no/1=yes]  
Output flanking sequences: +/- .............................. 0 bp  
Output ~.pTi file: .......................................... 1 [0=no/1=yes]  
==============================================================================  
  
  
Genome size (without gaps): ............ 2678902517 bp  
Gaps (N/X/-): .......................... 53837044 bp  
Mapped reads: .......................... 738059667487  
Non-identical sequences: ............... 277001  
Genomic hits: .......................... 533816  
Significant densitiy of mapped reads: .. 15118061 reads/kb

Show proTRAC cluster info
Hide proTRAC cluster info

|  |  |
| --- | --- |
| Location | chr12 |
| Coordinates | 29589027-29637424 |
| Size [bp] | 48398 |
| Sequence hit loci | 2495 |
| Mapped reads (normalized) | 6601700364.5 |
| Mapped reads (normalized) per kb | 136404404.4 |
| Normalized reads with 1T (1U) | 84% |
| Normalized reads with 10A | 29.9% |
| Normalized reads with length 26-33 nt | 100% |
| Normalized reads on the main strand(s) | 99.3% |
| Predicted directionality | mono:plus |

100%

0%

1T (1U)  
reads

10A reads

26-33 nt  
reads

reads on mainstrand

**Either the amount of reads with 1T (1U) OR 10A has to exceed 75% (set with option: -1Tor10A)  
Alternatively the amount of reads with 1T (1U) AND 10A has to exceed 50% (set with option: -1Tand10A)  
Minimum amount of reads with preferred size is 75% (set with option: -pisize)  
Minimum amount of reads on the main strand(s) is 75% (set with option: -clstrand)**

Show read coverage
Hide read coverage

WHAT DO I SEE HERE?  
This chart shows the location of mapped sequence reads within a predicted piRNA cluster. The color refers to the number of genomic hits produced by the sequence read in question. A dark red bar indicates that this sequence read produces many other hits elsewhere in the genome. Many adjacent red or yellow bars can indicate the presence of a multi-copy element such as transposons or rRNA genes. A dark green bar indicates that this sequence read maps uniquely to this locus.

1 hit

2-5 hits

6-10 hits

11-20 hits

21-50 hits

51-100 hits

> 100 hits

chr12

29589027

29637424

Gene Set

RepeatMasker

Mapped  
Reads

149.4

plus strand

minus strand

149.4

Region: chr12 100490322-29589075. Max. coverage (+): 0. Max coverage (-): 4.52

Region: chr12 29589076-29589172. Max. coverage (+): 0. Max coverage (-): 0

Region: chr12 29589173-29589268. Max. coverage (+): 0. Max coverage (-): 0

Region: chr12 29589269-29589365. Max. coverage (+): 2.62. Max coverage (-): 0

Region: chr12 29589366-29589462. Max. coverage (+): 23.93. Max coverage (-): 0

Region: chr12 29589463-29589559. Max. coverage (+): 5.21. Max coverage (-): 0

Region: chr12 29589560-29589656. Max. coverage (+): 0. Max coverage (-): 0

Region: chr12 29589657-29589752. Max. coverage (+): 9.92. Max coverage (-): 0

Region: chr12 29589753-29589849. Max. coverage (+): 20.33. Max coverage (-): 0

Region: chr12 29589850-29589946. Max. coverage (+): 12.72. Max coverage (-): 0

Region: chr12 29589947-29590043. Max. coverage (+): 23.12. Max coverage (-): 0

Region: chr12 29590044-29590140. Max. coverage (+): 49.63. Max coverage (-): 0

Region: chr12 29590141-29590236. Max. coverage (+): 0.73. Max coverage (-): 0

Region: chr12 29590237-29590333. Max. coverage (+): 23.81. Max coverage (-): 0

Region: chr12 29590334-29590430. Max. coverage (+): 22.57. Max coverage (-): 0

Region: chr12 29590431-29590527. Max. coverage (+): 2.44. Max coverage (-): 0

Region: chr12 29590528-29590624. Max. coverage (+): 0. Max coverage (-): 0

Region: chr12 29590625-29590720. Max. coverage (+): 15.76. Max coverage (-): 0

Region: chr12 29590721-29590817. Max. coverage (+): 9.35. Max coverage (-): 0

Region: chr12 29590818-29590914. Max. coverage (+): 51.35. Max coverage (-): 0

Region: chr12 29590915-29591011. Max. coverage (+): 12.36. Max coverage (-): 0

Region: chr12 29591012-29591108. Max. coverage (+): 4.82. Max coverage (-): 0

Region: chr12 29591109-29591204. Max. coverage (+): 5.53. Max coverage (-): 0

Region: chr12 29591205-29591301. Max. coverage (+): 5.99. Max coverage (-): 0

Region: chr12 29591302-29591398. Max. coverage (+): 0.33. Max coverage (-): 0

Region: chr12 29591399-29591495. Max. coverage (+): 6.17. Max coverage (-): 0

Region: chr12 29591496-29591592. Max. coverage (+): 0. Max coverage (-): 0

Region: chr12 29591593-29591688. Max. coverage (+): 36.75. Max coverage (-): 0

Region: chr12 29591689-29591785. Max. coverage (+): 23.12. Max coverage (-): 0

Region: chr12 29591786-29591882. Max. coverage (+): 0. Max coverage (-): 0

Region: chr12 29591883-29591979. Max. coverage (+): 0. Max coverage (-): 0

Region: chr12 29591980-29592076. Max. coverage (+): 6.97. Max coverage (-): 0

Region: chr12 29592077-29592172. Max. coverage (+): 8.3. Max coverage (-): 0

Region: chr12 29592173-29592269. Max. coverage (+): 0. Max coverage (-): 0

Region: chr12 29592270-29592366. Max. coverage (+): 0. Max coverage (-): 0

Region: chr12 29592367-29592463. Max. coverage (+): 0. Max coverage (-): 0

Region: chr12 29592464-29592560. Max. coverage (+): 7.2. Max coverage (-): 0

Region: chr12 29592561-29592656. Max. coverage (+): 1.62. Max coverage (-): 0

Region: chr12 29592657-29592753. Max. coverage (+): 0. Max coverage (-): 0

Region: chr12 29592754-29592850. Max. coverage (+): 0. Max coverage (-): 0

Region: chr12 29592851-29592947. Max. coverage (+): 0. Max coverage (-): 0

Region: chr12 29592948-29593044. Max. coverage (+): 0. Max coverage (-): 0

Region: chr12 29593045-29593140. Max. coverage (+): 0. Max coverage (-): 0

Region: chr12 29593141-29593237. Max. coverage (+): 0. Max coverage (-): 0

Region: chr12 29593238-29593334. Max. coverage (+): 0. Max coverage (-): 0

Region: chr12 29593335-29593431. Max. coverage (+): 5.26. Max coverage (-): 0

Region: chr12 29593432-29593528. Max. coverage (+): 2.48. Max coverage (-): 0

Region: chr12 29593529-29593624. Max. coverage (+): 10.16. Max coverage (-): 0

Region: chr12 29593625-29593721. Max. coverage (+): 4.85. Max coverage (-): 0

Region: chr12 29593722-29593818. Max. coverage (+): 1.45. Max coverage (-): 0

Region: chr12 29593819-29593915. Max. coverage (+): 10.75. Max coverage (-): 0

Region: chr12 29593916-29594011. Max. coverage (+): 4.64. Max coverage (-): 0

Region: chr12 29594012-29594108. Max. coverage (+): 9.3. Max coverage (-): 0

Region: chr12 29594109-29594205. Max. coverage (+): 0.96. Max coverage (-): 0

Region: chr12 29594206-29594302. Max. coverage (+): 23.7. Max coverage (-): 0

Region: chr12 29594303-29594399. Max. coverage (+): 6.49. Max coverage (-): 0

Region: chr12 29594400-29594495. Max. coverage (+): 32.32. Max coverage (-): 0

Region: chr12 29594496-29594592. Max. coverage (+): 0. Max coverage (-): 0

Region: chr12 29594593-29594689. Max. coverage (+): 3.25. Max coverage (-): 0

Region: chr12 29594690-29594786. Max. coverage (+): 7.09. Max coverage (-): 0

Region: chr12 29594787-29594883. Max. coverage (+): 10.26. Max coverage (-): 5.39

Region: chr12 29594884-29594979. Max. coverage (+): 0. Max coverage (-): 5.39

Region: chr12 29594980-29595076. Max. coverage (+): 8.21. Max coverage (-): 0

Region: chr12 29595077-29595173. Max. coverage (+): 0. Max coverage (-): 0

Region: chr12 29595174-29595270. Max. coverage (+): 0. Max coverage (-): 0

Region: chr12 29595271-29595367. Max. coverage (+): 16.41. Max coverage (-): 0

Region: chr12 29595368-29595463. Max. coverage (+): 99.3. Max coverage (-): 0

Region: chr12 29595464-29595560. Max. coverage (+): 75.75. Max coverage (-): 0

Region: chr12 29595561-29595657. Max. coverage (+): 62.4. Max coverage (-): 0

Region: chr12 29595658-29595754. Max. coverage (+): 0. Max coverage (-): 0

Region: chr12 29595755-29595851. Max. coverage (+): 0. Max coverage (-): 0

Region: chr12 29595852-29595947. Max. coverage (+): 10.65. Max coverage (-): 0

Region: chr12 29595948-29596044. Max. coverage (+): 16.79. Max coverage (-): 0

Region: chr12 29596045-29596141. Max. coverage (+): 54.35. Max coverage (-): 0

Region: chr12 29596142-29596238. Max. coverage (+): 54.36. Max coverage (-): 0

Region: chr12 29596239-29596335. Max. coverage (+): 29.18. Max coverage (-): 0

Region: chr12 29596336-29596431. Max. coverage (+): 42.5. Max coverage (-): 0

Region: chr12 29596432-29596528. Max. coverage (+): 8.85. Max coverage (-): 0

Region: chr12 29596529-29596625. Max. coverage (+): 49.5. Max coverage (-): 0

Region: chr12 29596626-29596722. Max. coverage (+): 46.41. Max coverage (-): 0

Region: chr12 29596723-29596819. Max. coverage (+): 23.27. Max coverage (-): 0

Region: chr12 29596820-29596915. Max. coverage (+): 89.78. Max coverage (-): 0

Region: chr12 29596916-29597012. Max. coverage (+): 63.05. Max coverage (-): 0

Region: chr12 29597013-29597109. Max. coverage (+): 12.24. Max coverage (-): 0

Region: chr12 29597110-29597206. Max. coverage (+): 7.81. Max coverage (-): 0

Region: chr12 29597207-29597303. Max. coverage (+): 2.4. Max coverage (-): 0

Region: chr12 29597304-29597399. Max. coverage (+): 0. Max coverage (-): 0

Region: chr12 29597400-29597496. Max. coverage (+): 0. Max coverage (-): 0

Region: chr12 29597497-29597593. Max. coverage (+): 6.15. Max coverage (-): 0

Region: chr12 29597594-29597690. Max. coverage (+): 0. Max coverage (-): 0

Region: chr12 29597691-29597787. Max. coverage (+): 0. Max coverage (-): 0

Region: chr12 29597788-29597883. Max. coverage (+): 7.18. Max coverage (-): 5.2

Region: chr12 29597884-29597980. Max. coverage (+): 11.93. Max coverage (-): 0

Region: chr12 29597981-29598077. Max. coverage (+): 3.07. Max coverage (-): 0

Region: chr12 29598078-29598174. Max. coverage (+): 120.26. Max coverage (-): 0

Region: chr12 29598175-29598271. Max. coverage (+): 6.02. Max coverage (-): 14.18

Region: chr12 29598272-29598367. Max. coverage (+): 39.27. Max coverage (-): 0

Region: chr12 29598368-29598464. Max. coverage (+): 12.47. Max coverage (-): 0

Region: chr12 29598465-29598561. Max. coverage (+): 0. Max coverage (-): 0

Region: chr12 29598562-29598658. Max. coverage (+): 22.22. Max coverage (-): 0

Region: chr12 29598659-29598754. Max. coverage (+): 4.3. Max coverage (-): 0

Region: chr12 29598755-29598851. Max. coverage (+): 16.78. Max coverage (-): 0

Region: chr12 29598852-29598948. Max. coverage (+): 23. Max coverage (-): 0

Region: chr12 29598949-29599045. Max. coverage (+): 68.77. Max coverage (-): 0

Region: chr12 29599046-29599142. Max. coverage (+): 59.9. Max coverage (-): 0

Region: chr12 29599143-29599238. Max. coverage (+): 104.72. Max coverage (-): 0

Region: chr12 29599239-29599335. Max. coverage (+): 44.82. Max coverage (-): 0

Region: chr12 29599336-29599432. Max. coverage (+): 32.37. Max coverage (-): 0

Region: chr12 29599433-29599529. Max. coverage (+): 6.81. Max coverage (-): 0

Region: chr12 29599530-29599626. Max. coverage (+): 33.67. Max coverage (-): 0

Region: chr12 29599627-29599722. Max. coverage (+): 18.19. Max coverage (-): 0

Region: chr12 29599723-29599819. Max. coverage (+): 29.55. Max coverage (-): 0

Region: chr12 29599820-29599916. Max. coverage (+): 11.98. Max coverage (-): 0

Region: chr12 29599917-29600013. Max. coverage (+): 13.74. Max coverage (-): 0

Region: chr12 29600014-29600110. Max. coverage (+): 8.8. Max coverage (-): 0

Region: chr12 29600111-29600206. Max. coverage (+): 20.47. Max coverage (-): 0

Region: chr12 29600207-29600303. Max. coverage (+): 18.4. Max coverage (-): 0

Region: chr12 29600304-29600400. Max. coverage (+): 0. Max coverage (-): 0

Region: chr12 29600401-29600497. Max. coverage (+): 115.9. Max coverage (-): 0

Region: chr12 29600498-29600594. Max. coverage (+): 50.48. Max coverage (-): 0

Region: chr12 29600595-29600690. Max. coverage (+): 24.48. Max coverage (-): 0

Region: chr12 29600691-29600787. Max. coverage (+): 37.81. Max coverage (-): 0

Region: chr12 29600788-29600884. Max. coverage (+): 6.46. Max coverage (-): 0

Region: chr12 29600885-29600981. Max. coverage (+): 6.16. Max coverage (-): 0

Region: chr12 29600982-29601078. Max. coverage (+): 16.9. Max coverage (-): 0

Region: chr12 29601079-29601174. Max. coverage (+): 13.88. Max coverage (-): 0

Region: chr12 29601175-29601271. Max. coverage (+): 13.88. Max coverage (-): 0

Region: chr12 29601272-29601368. Max. coverage (+): 2.27. Max coverage (-): 0

Region: chr12 29601369-29601465. Max. coverage (+): 2.27. Max coverage (-): 0

Region: chr12 29601466-29601562. Max. coverage (+): 1.91. Max coverage (-): 0

Region: chr12 29601563-29601658. Max. coverage (+): 6.97. Max coverage (-): 0

Region: chr12 29601659-29601755. Max. coverage (+): 0. Max coverage (-): 0

Region: chr12 29601756-29601852. Max. coverage (+): 0. Max coverage (-): 0

Region: chr12 29601853-29601949. Max. coverage (+): 0. Max coverage (-): 0

Region: chr12 29601950-29602046. Max. coverage (+): 0. Max coverage (-): 0

Region: chr12 29602047-29602142. Max. coverage (+): 0. Max coverage (-): 0

Region: chr12 29602143-29602239. Max. coverage (+): 0. Max coverage (-): 0

Region: chr12 29602240-29602336. Max. coverage (+): 0. Max coverage (-): 0

Region: chr12 29602337-29602433. Max. coverage (+): 0. Max coverage (-): 0

Region: chr12 29602434-29602530. Max. coverage (+): 0. Max coverage (-): 0

Region: chr12 29602531-29602626. Max. coverage (+): 3.73. Max coverage (-): 0

Region: chr12 29602627-29602723. Max. coverage (+): 3.73. Max coverage (-): 0

Region: chr12 29602724-29602820. Max. coverage (+): 10.42. Max coverage (-): 0

Region: chr12 29602821-29602917. Max. coverage (+): 0. Max coverage (-): 0

Region: chr12 29602918-29603014. Max. coverage (+): 0. Max coverage (-): 0

Region: chr12 29603015-29603110. Max. coverage (+): 0. Max coverage (-): 0

Region: chr12 29603111-29603207. Max. coverage (+): 0. Max coverage (-): 0

Region: chr12 29603208-29603304. Max. coverage (+): 4.28. Max coverage (-): 0

Region: chr12 29603305-29603401. Max. coverage (+): 6.82. Max coverage (-): 0

Region: chr12 29603402-29603498. Max. coverage (+): 0. Max coverage (-): 0

Region: chr12 29603499-29603594. Max. coverage (+): 3.83. Max coverage (-): 0

Region: chr12 29603595-29603691. Max. coverage (+): 1.22. Max coverage (-): 0

Region: chr12 29603692-29603788. Max. coverage (+): 0. Max coverage (-): 0

Region: chr12 29603789-29603885. Max. coverage (+): 17.19. Max coverage (-): 0

Region: chr12 29603886-29603981. Max. coverage (+): 0. Max coverage (-): 0

Region: chr12 29603982-29604078. Max. coverage (+): 0. Max coverage (-): 0

Region: chr12 29604079-29604175. Max. coverage (+): 0. Max coverage (-): 0

Region: chr12 29604176-29604272. Max. coverage (+): 0. Max coverage (-): 0

Region: chr12 29604273-29604369. Max. coverage (+): 0. Max coverage (-): 0

Region: chr12 29604370-29604465. Max. coverage (+): 6.48. Max coverage (-): 0

Region: chr12 29604466-29604562. Max. coverage (+): 0. Max coverage (-): 0

Region: chr12 29604563-29604659. Max. coverage (+): 0. Max coverage (-): 0

Region: chr12 29604660-29604756. Max. coverage (+): 0. Max coverage (-): 0

Region: chr12 29604757-29604853. Max. coverage (+): 0. Max coverage (-): 0

Region: chr12 29604854-29604949. Max. coverage (+): 0. Max coverage (-): 0

Region: chr12 29604950-29605046. Max. coverage (+): 0. Max coverage (-): 0

Region: chr12 29605047-29605143. Max. coverage (+): 0. Max coverage (-): 0

Region: chr12 29605144-29605240. Max. coverage (+): 0. Max coverage (-): 0

Region: chr12 29605241-29605337. Max. coverage (+): 0. Max coverage (-): 0

Region: chr12 29605338-29605433. Max. coverage (+): 0. Max coverage (-): 0

Region: chr12 29605434-29605530. Max. coverage (+): 0. Max coverage (-): 0

Region: chr12 29605531-29605627. Max. coverage (+): 0. Max coverage (-): 0

Region: chr12 29605628-29605724. Max. coverage (+): 0. Max coverage (-): 0

Region: chr12 29605725-29605821. Max. coverage (+): 0. Max coverage (-): 0

Region: chr12 29605822-29605917. Max. coverage (+): 0. Max coverage (-): 0

Region: chr12 29605918-29606014. Max. coverage (+): 0. Max coverage (-): 0

Region: chr12 29606015-29606111. Max. coverage (+): 0. Max coverage (-): 0

Region: chr12 29606112-29606208. Max. coverage (+): 0. Max coverage (-): 0

Region: chr12 29606209-29606305. Max. coverage (+): 0. Max coverage (-): 0

Region: chr12 29606306-29606401. Max. coverage (+): 0. Max coverage (-): 0

Region: chr12 29606402-29606498. Max. coverage (+): 0. Max coverage (-): 0

Region: chr12 29606499-29606595. Max. coverage (+): 0. Max coverage (-): 0

Region: chr12 29606596-29606692. Max. coverage (+): 0. Max coverage (-): 0

Region: chr12 29606693-29606789. Max. coverage (+): 14.66. Max coverage (-): 0

Region: chr12 29606790-29606885. Max. coverage (+): 0. Max coverage (-): 0

Region: chr12 29606886-29606982. Max. coverage (+): 0. Max coverage (-): 0

Region: chr12 29606983-29607079. Max. coverage (+): 2.44. Max coverage (-): 0

Region: chr12 29607080-29607176. Max. coverage (+): 0. Max coverage (-): 0

Region: chr12 29607177-29607273. Max. coverage (+): 0. Max coverage (-): 5.74

Region: chr12 29607274-29607369. Max. coverage (+): 32.46. Max coverage (-): 0

Region: chr12 29607370-29607466. Max. coverage (+): 23.78. Max coverage (-): 0

Region: chr12 29607467-29607563. Max. coverage (+): 7. Max coverage (-): 0

Region: chr12 29607564-29607660. Max. coverage (+): 0. Max coverage (-): 0

Region: chr12 29607661-29607757. Max. coverage (+): 4.26. Max coverage (-): 0

Region: chr12 29607758-29607853. Max. coverage (+): 0. Max coverage (-): 0

Region: chr12 29607854-29607950. Max. coverage (+): 0. Max coverage (-): 0

Region: chr12 29607951-29608047. Max. coverage (+): 0. Max coverage (-): 0

Region: chr12 29608048-29608144. Max. coverage (+): 0. Max coverage (-): 0

Region: chr12 29608145-29608241. Max. coverage (+): 0. Max coverage (-): 0

Region: chr12 29608242-29608337. Max. coverage (+): 0. Max coverage (-): 0

Region: chr12 29608338-29608434. Max. coverage (+): 0. Max coverage (-): 0

Region: chr12 29608435-29608531. Max. coverage (+): 0. Max coverage (-): 0

Region: chr12 29608532-29608628. Max. coverage (+): 0. Max coverage (-): 0

Region: chr12 29608629-29608724. Max. coverage (+): 0. Max coverage (-): 0

Region: chr12 29608725-29608821. Max. coverage (+): 4.49. Max coverage (-): 0

Region: chr12 29608822-29608918. Max. coverage (+): 0. Max coverage (-): 0

Region: chr12 29608919-29609015. Max. coverage (+): 0. Max coverage (-): 0

Region: chr12 29609016-29609112. Max. coverage (+): 0. Max coverage (-): 0

Region: chr12 29609113-29609208. Max. coverage (+): 0. Max coverage (-): 0

Region: chr12 29609209-29609305. Max. coverage (+): 3.36. Max coverage (-): 0

Region: chr12 29609306-29609402. Max. coverage (+): 0. Max coverage (-): 0

Region: chr12 29609403-29609499. Max. coverage (+): 4.24. Max coverage (-): 0

Region: chr12 29609500-29609596. Max. coverage (+): 0. Max coverage (-): 0

Region: chr12 29609597-29609692. Max. coverage (+): 0. Max coverage (-): 0

Region: chr12 29609693-29609789. Max. coverage (+): 0. Max coverage (-): 0

Region: chr12 29609790-29609886. Max. coverage (+): 0. Max coverage (-): 0

Region: chr12 29609887-29609983. Max. coverage (+): 0. Max coverage (-): 0

Region: chr12 29609984-29610080. Max. coverage (+): 0. Max coverage (-): 0

Region: chr12 29610081-29610176. Max. coverage (+): 2.28. Max coverage (-): 0

Region: chr12 29610177-29610273. Max. coverage (+): 4.17. Max coverage (-): 0

Region: chr12 29610274-29610370. Max. coverage (+): 0. Max coverage (-): 0

Region: chr12 29610371-29610467. Max. coverage (+): 0. Max coverage (-): 0

Region: chr12 29610468-29610564. Max. coverage (+): 6.9. Max coverage (-): 0

Region: chr12 29610565-29610660. Max. coverage (+): 6.9. Max coverage (-): 0

Region: chr12 29610661-29610757. Max. coverage (+): 6.1. Max coverage (-): 0

Region: chr12 29610758-29610854. Max. coverage (+): 21. Max coverage (-): 0

Region: chr12 29610855-29610951. Max. coverage (+): 49.94. Max coverage (-): 0

Region: chr12 29610952-29611048. Max. coverage (+): 43.86. Max coverage (-): 0

Region: chr12 29611049-29611144. Max. coverage (+): 70.33. Max coverage (-): 0

Region: chr12 29611145-29611241. Max. coverage (+): 54.82. Max coverage (-): 0

Region: chr12 29611242-29611338. Max. coverage (+): 2.55. Max coverage (-): 0

Region: chr12 29611339-29611435. Max. coverage (+): 38.01. Max coverage (-): 0

Region: chr12 29611436-29611532. Max. coverage (+): 42.68. Max coverage (-): 0

Region: chr12 29611533-29611628. Max. coverage (+): 13.2. Max coverage (-): 0

Region: chr12 29611629-29611725. Max. coverage (+): 84.07. Max coverage (-): 0

Region: chr12 29611726-29611822. Max. coverage (+): 38.83. Max coverage (-): 0

Region: chr12 29611823-29611919. Max. coverage (+): 16.91. Max coverage (-): 0

Region: chr12 29611920-29612016. Max. coverage (+): 12.76. Max coverage (-): 0

Region: chr12 29612017-29612112. Max. coverage (+): 45.41. Max coverage (-): 0

Region: chr12 29612113-29612209. Max. coverage (+): 31.56. Max coverage (-): 0

Region: chr12 29612210-29612306. Max. coverage (+): 29.29. Max coverage (-): 0

Region: chr12 29612307-29612403. Max. coverage (+): 7.73. Max coverage (-): 0

Region: chr12 29612404-29612500. Max. coverage (+): 60.07. Max coverage (-): 0

Region: chr12 29612501-29612596. Max. coverage (+): 6.22. Max coverage (-): 12.37

Region: chr12 29612597-29612693. Max. coverage (+): 25.66. Max coverage (-): 0

Region: chr12 29612694-29612790. Max. coverage (+): 27.83. Max coverage (-): 0

Region: chr12 29612791-29612887. Max. coverage (+): 9.86. Max coverage (-): 0

Region: chr12 29612888-29612984. Max. coverage (+): 0. Max coverage (-): 0

Region: chr12 29612985-29613080. Max. coverage (+): 0. Max coverage (-): 0

Region: chr12 29613081-29613177. Max. coverage (+): 0. Max coverage (-): 0

Region: chr12 29613178-29613274. Max. coverage (+): 0. Max coverage (-): 0

Region: chr12 29613275-29613371. Max. coverage (+): 0. Max coverage (-): 0

Region: chr12 29613372-29613467. Max. coverage (+): 14.42. Max coverage (-): 0

Region: chr12 29613468-29613564. Max. coverage (+): 0. Max coverage (-): 0

Region: chr12 29613565-29613661. Max. coverage (+): 28.4. Max coverage (-): 0

Region: chr12 29613662-29613758. Max. coverage (+): 0. Max coverage (-): 0

Region: chr12 29613759-29613855. Max. coverage (+): 0. Max coverage (-): 0

Region: chr12 29613856-29613951. Max. coverage (+): 0. Max coverage (-): 0

Region: chr12 29613952-29614048. Max. coverage (+): 24.45. Max coverage (-): 0

Region: chr12 29614049-29614145. Max. coverage (+): 17.8. Max coverage (-): 0

Region: chr12 29614146-29614242. Max. coverage (+): 11.2. Max coverage (-): 0

Region: chr12 29614243-29614339. Max. coverage (+): 27.68. Max coverage (-): 0

Region: chr12 29614340-29614435. Max. coverage (+): 26.14. Max coverage (-): 0

Region: chr12 29614436-29614532. Max. coverage (+): 0. Max coverage (-): 0

Region: chr12 29614533-29614629. Max. coverage (+): 22.79. Max coverage (-): 0

Region: chr12 29614630-29614726. Max. coverage (+): 12.83. Max coverage (-): 0

Region: chr12 29614727-29614823. Max. coverage (+): 6.17. Max coverage (-): 0

Region: chr12 29614824-29614919. Max. coverage (+): 0.33. Max coverage (-): 0

Region: chr12 29614920-29615016. Max. coverage (+): 5.43. Max coverage (-): 0

Region: chr12 29615017-29615113. Max. coverage (+): 4.37. Max coverage (-): 0

Region: chr12 29615114-29615210. Max. coverage (+): 0. Max coverage (-): 0

Region: chr12 29615211-29615307. Max. coverage (+): 0. Max coverage (-): 0

Region: chr12 29615308-29615403. Max. coverage (+): 0. Max coverage (-): 0

Region: chr12 29615404-29615500. Max. coverage (+): 0. Max coverage (-): 0

Region: chr12 29615501-29615597. Max. coverage (+): 20.82. Max coverage (-): 0

Region: chr12 29615598-29615694. Max. coverage (+): 65.31. Max coverage (-): 0

Region: chr12 29615695-29615791. Max. coverage (+): 107.95. Max coverage (-): 0

Region: chr12 29615792-29615887. Max. coverage (+): 12.49. Max coverage (-): 0

Region: chr12 29615888-29615984. Max. coverage (+): 15. Max coverage (-): 0

Region: chr12 29615985-29616081. Max. coverage (+): 3.46. Max coverage (-): 0

Region: chr12 29616082-29616178. Max. coverage (+): 0. Max coverage (-): 0

Region: chr12 29616179-29616275. Max. coverage (+): 20.91. Max coverage (-): 0

Region: chr12 29616276-29616371. Max. coverage (+): 64.17. Max coverage (-): 0

Region: chr12 29616372-29616468. Max. coverage (+): 0. Max coverage (-): 0

Region: chr12 29616469-29616565. Max. coverage (+): 0. Max coverage (-): 0

Region: chr12 29616566-29616662. Max. coverage (+): 0. Max coverage (-): 0

Region: chr12 29616663-29616759. Max. coverage (+): 0. Max coverage (-): 0

Region: chr12 29616760-29616855. Max. coverage (+): 9.03. Max coverage (-): 0

Region: chr12 29616856-29616952. Max. coverage (+): 36.72. Max coverage (-): 0

Region: chr12 29616953-29617049. Max. coverage (+): 14.15. Max coverage (-): 0

Region: chr12 29617050-29617146. Max. coverage (+): 13.47. Max coverage (-): 0

Region: chr12 29617147-29617243. Max. coverage (+): 4.96. Max coverage (-): 0

Region: chr12 29617244-29617339. Max. coverage (+): 0. Max coverage (-): 0

Region: chr12 29617340-29617436. Max. coverage (+): 6.8. Max coverage (-): 0

Region: chr12 29617437-29617533. Max. coverage (+): 1.1. Max coverage (-): 0

Region: chr12 29617534-29617630. Max. coverage (+): 9.81. Max coverage (-): 0

Region: chr12 29617631-29617727. Max. coverage (+): 0. Max coverage (-): 0

Region: chr12 29617728-29617823. Max. coverage (+): 0. Max coverage (-): 0

Region: chr12 29617824-29617920. Max. coverage (+): 0. Max coverage (-): 0

Region: chr12 29617921-29618017. Max. coverage (+): 0. Max coverage (-): 0

Region: chr12 29618018-29618114. Max. coverage (+): 0. Max coverage (-): 0

Region: chr12 29618115-29618210. Max. coverage (+): 0. Max coverage (-): 0

Region: chr12 29618211-29618307. Max. coverage (+): 0. Max coverage (-): 0

Region: chr12 29618308-29618404. Max. coverage (+): 0. Max coverage (-): 0

Region: chr12 29618405-29618501. Max. coverage (+): 0. Max coverage (-): 0

Region: chr12 29618502-29618598. Max. coverage (+): 0. Max coverage (-): 0

Region: chr12 29618599-29618694. Max. coverage (+): 0. Max coverage (-): 0

Region: chr12 29618695-29618791. Max. coverage (+): 0. Max coverage (-): 0

Region: chr12 29618792-29618888. Max. coverage (+): 0. Max coverage (-): 0

Region: chr12 29618889-29618985. Max. coverage (+): 0. Max coverage (-): 0

Region: chr12 29618986-29619082. Max. coverage (+): 49.07. Max coverage (-): 0

Region: chr12 29619083-29619178. Max. coverage (+): 46.59. Max coverage (-): 0

Region: chr12 29619179-29619275. Max. coverage (+): 19.94. Max coverage (-): 0

Region: chr12 29619276-29619372. Max. coverage (+): 0. Max coverage (-): 0

Region: chr12 29619373-29619469. Max. coverage (+): 0. Max coverage (-): 0

Region: chr12 29619470-29619566. Max. coverage (+): 0. Max coverage (-): 0

Region: chr12 29619567-29619662. Max. coverage (+): 0. Max coverage (-): 0

Region: chr12 29619663-29619759. Max. coverage (+): 0. Max coverage (-): 0

Region: chr12 29619760-29619856. Max. coverage (+): 32.86. Max coverage (-): 0

Region: chr12 29619857-29619953. Max. coverage (+): 8.73. Max coverage (-): 0

Region: chr12 29619954-29620050. Max. coverage (+): 3.97. Max coverage (-): 0

Region: chr12 29620051-29620146. Max. coverage (+): 20.47. Max coverage (-): 0

Region: chr12 29620147-29620243. Max. coverage (+): 1.8. Max coverage (-): 0

Region: chr12 29620244-29620340. Max. coverage (+): 5.16. Max coverage (-): 0

Region: chr12 29620341-29620437. Max. coverage (+): 0. Max coverage (-): 0

Region: chr12 29620438-29620534. Max. coverage (+): 0. Max coverage (-): 0

Region: chr12 29620535-29620630. Max. coverage (+): 15.35. Max coverage (-): 0

Region: chr12 29620631-29620727. Max. coverage (+): 40.46. Max coverage (-): 0

Region: chr12 29620728-29620824. Max. coverage (+): 13.8. Max coverage (-): 0

Region: chr12 29620825-29620921. Max. coverage (+): 8.14. Max coverage (-): 0

Region: chr12 29620922-29621018. Max. coverage (+): 6.06. Max coverage (-): 0

Region: chr12 29621019-29621114. Max. coverage (+): 6.4. Max coverage (-): 0

Region: chr12 29621115-29621211. Max. coverage (+): 7.44. Max coverage (-): 0

Region: chr12 29621212-29621308. Max. coverage (+): 16.46. Max coverage (-): 0

Region: chr12 29621309-29621405. Max. coverage (+): 4.02. Max coverage (-): 0

Region: chr12 29621406-29621502. Max. coverage (+): 0. Max coverage (-): 0

Region: chr12 29621503-29621598. Max. coverage (+): 0. Max coverage (-): 0

Region: chr12 29621599-29621695. Max. coverage (+): 68.86. Max coverage (-): 0

Region: chr12 29621696-29621792. Max. coverage (+): 73.6. Max coverage (-): 0

Region: chr12 29621793-29621889. Max. coverage (+): 28.11. Max coverage (-): 0

Region: chr12 29621890-29621986. Max. coverage (+): 0. Max coverage (-): 0

Region: chr12 29621987-29622082. Max. coverage (+): 0. Max coverage (-): 0

Region: chr12 29622083-29622179. Max. coverage (+): 14.04. Max coverage (-): 0

Region: chr12 29622180-29622276. Max. coverage (+): 19.21. Max coverage (-): 0

Region: chr12 29622277-29622373. Max. coverage (+): 1.39. Max coverage (-): 0

Region: chr12 29622374-29622470. Max. coverage (+): 21.28. Max coverage (-): 0

Region: chr12 29622471-29622566. Max. coverage (+): 149.4. Max coverage (-): 0

Region: chr12 29622567-29622663. Max. coverage (+): 25. Max coverage (-): 0

Region: chr12 29622664-29622760. Max. coverage (+): 6.12. Max coverage (-): 0

Region: chr12 29622761-29622857. Max. coverage (+): 54.52. Max coverage (-): 0

Region: chr12 29622858-29622953. Max. coverage (+): 54.3. Max coverage (-): 0

Region: chr12 29622954-29623050. Max. coverage (+): 0. Max coverage (-): 0

Region: chr12 29623051-29623147. Max. coverage (+): 10.87. Max coverage (-): 0

Region: chr12 29623148-29623244. Max. coverage (+): 31.23. Max coverage (-): 0

Region: chr12 29623245-29623341. Max. coverage (+): 0. Max coverage (-): 0

Region: chr12 29623342-29623437. Max. coverage (+): 0. Max coverage (-): 0

Region: chr12 29623438-29623534. Max. coverage (+): 0. Max coverage (-): 0

Region: chr12 29623535-29623631. Max. coverage (+): 0. Max coverage (-): 0

Region: chr12 29623632-29623728. Max. coverage (+): 0. Max coverage (-): 0

Region: chr12 29623729-29623825. Max. coverage (+): 0. Max coverage (-): 0

Region: chr12 29623826-29623921. Max. coverage (+): 0. Max coverage (-): 0

Region: chr12 29623922-29624018. Max. coverage (+): 0. Max coverage (-): 0

Region: chr12 29624019-29624115. Max. coverage (+): 0. Max coverage (-): 0

Region: chr12 29624116-29624212. Max. coverage (+): 0. Max coverage (-): 0

Region: chr12 29624213-29624309. Max. coverage (+): 0. Max coverage (-): 0

Region: chr12 29624310-29624405. Max. coverage (+): 0. Max coverage (-): 0

Region: chr12 29624406-29624502. Max. coverage (+): 0. Max coverage (-): 0

Region: chr12 29624503-29624599. Max. coverage (+): 0. Max coverage (-): 0

Region: chr12 29624600-29624696. Max. coverage (+): 0. Max coverage (-): 0

Region: chr12 29624697-29624793. Max. coverage (+): 0. Max coverage (-): 0

Region: chr12 29624794-29624889. Max. coverage (+): 0. Max coverage (-): 0

Region: chr12 29624890-29624986. Max. coverage (+): 0. Max coverage (-): 0

Region: chr12 29624987-29625083. Max. coverage (+): 0. Max coverage (-): 0

Region: chr12 29625084-29625180. Max. coverage (+): 0. Max coverage (-): 0

Region: chr12 29625181-29625277. Max. coverage (+): 0. Max coverage (-): 0

Region: chr12 29625278-29625373. Max. coverage (+): 0. Max coverage (-): 0

Region: chr12 29625374-29625470. Max. coverage (+): 0. Max coverage (-): 0

Region: chr12 29625471-29625567. Max. coverage (+): 0. Max coverage (-): 0

Region: chr12 29625568-29625664. Max. coverage (+): 0. Max coverage (-): 0

Region: chr12 29625665-29625761. Max. coverage (+): 0. Max coverage (-): 0

Region: chr12 29625762-29625857. Max. coverage (+): 0. Max coverage (-): 0

Region: chr12 29625858-29625954. Max. coverage (+): 0. Max coverage (-): 0

Region: chr12 29625955-29626051. Max. coverage (+): 0. Max coverage (-): 0

Region: chr12 29626052-29626148. Max. coverage (+): 0. Max coverage (-): 0

Region: chr12 29626149-29626245. Max. coverage (+): 0. Max coverage (-): 0

Region: chr12 29626246-29626341. Max. coverage (+): 10.69. Max coverage (-): 0

Region: chr12 29626342-29626438. Max. coverage (+): 8.4. Max coverage (-): 0

Region: chr12 29626439-29626535. Max. coverage (+): 0. Max coverage (-): 0

Region: chr12 29626536-29626632. Max. coverage (+): 0. Max coverage (-): 0

Region: chr12 29626633-29626729. Max. coverage (+): 0. Max coverage (-): 0

Region: chr12 29626730-29626825. Max. coverage (+): 0. Max coverage (-): 0

Region: chr12 29626826-29626922. Max. coverage (+): 77.66. Max coverage (-): 0

Region: chr12 29626923-29627019. Max. coverage (+): 19.89. Max coverage (-): 0

Region: chr12 29627020-29627116. Max. coverage (+): 6.65. Max coverage (-): 0

Region: chr12 29627117-29627213. Max. coverage (+): 25.68. Max coverage (-): 0

Region: chr12 29627214-29627309. Max. coverage (+): 5.61. Max coverage (-): 0

Region: chr12 29627310-29627406. Max. coverage (+): 0. Max coverage (-): 0

Region: chr12 29627407-29627503. Max. coverage (+): 0. Max coverage (-): 0

Region: chr12 29627504-29627600. Max. coverage (+): 17.72. Max coverage (-): 0

Region: chr12 29627601-29627697. Max. coverage (+): 19.34. Max coverage (-): 0

Region: chr12 29627698-29627793. Max. coverage (+): 4.66. Max coverage (-): 0

Region: chr12 29627794-29627890. Max. coverage (+): 12.82. Max coverage (-): 0

Region: chr12 29627891-29627987. Max. coverage (+): 26.39. Max coverage (-): 0

Region: chr12 29627988-29628084. Max. coverage (+): 0. Max coverage (-): 0

Region: chr12 29628085-29628180. Max. coverage (+): 0. Max coverage (-): 0

Region: chr12 29628181-29628277. Max. coverage (+): 0. Max coverage (-): 0

Region: chr12 29628278-29628374. Max. coverage (+): 0. Max coverage (-): 0

Region: chr12 29628375-29628471. Max. coverage (+): 2.98. Max coverage (-): 0

Region: chr12 29628472-29628568. Max. coverage (+): 5.49. Max coverage (-): 0

Region: chr12 29628569-29628664. Max. coverage (+): 4.9. Max coverage (-): 0

Region: chr12 29628665-29628761. Max. coverage (+): 0. Max coverage (-): 0

Region: chr12 29628762-29628858. Max. coverage (+): 0. Max coverage (-): 0

Region: chr12 29628859-29628955. Max. coverage (+): 0. Max coverage (-): 0

Region: chr12 29628956-29629052. Max. coverage (+): 0. Max coverage (-): 0

Region: chr12 29629053-29629148. Max. coverage (+): 0. Max coverage (-): 0

Region: chr12 29629149-29629245. Max. coverage (+): 0. Max coverage (-): 0

Region: chr12 29629246-29629342. Max. coverage (+): 6.12. Max coverage (-): 0

Region: chr12 29629343-29629439. Max. coverage (+): 9.92. Max coverage (-): 0

Region: chr12 29629440-29629536. Max. coverage (+): 13.21. Max coverage (-): 0

Region: chr12 29629537-29629632. Max. coverage (+): 26.47. Max coverage (-): 0

Region: chr12 29629633-29629729. Max. coverage (+): 0. Max coverage (-): 0

Region: chr12 29629730-29629826. Max. coverage (+): 0. Max coverage (-): 0

Region: chr12 29629827-29629923. Max. coverage (+): 0. Max coverage (-): 0

Region: chr12 29629924-29630020. Max. coverage (+): 0. Max coverage (-): 0

Region: chr12 29630021-29630116. Max. coverage (+): 0. Max coverage (-): 0

Region: chr12 29630117-29630213. Max. coverage (+): 0. Max coverage (-): 0

Region: chr12 29630214-29630310. Max. coverage (+): 0. Max coverage (-): 0

Region: chr12 29630311-29630407. Max. coverage (+): 0. Max coverage (-): 0

Region: chr12 29630408-29630504. Max. coverage (+): 0. Max coverage (-): 0

Region: chr12 29630505-29630600. Max. coverage (+): 0. Max coverage (-): 0

Region: chr12 29630601-29630697. Max. coverage (+): 0. Max coverage (-): 0

Region: chr12 29630698-29630794. Max. coverage (+): 13.48. Max coverage (-): 0

Region: chr12 29630795-29630891. Max. coverage (+): 6.01. Max coverage (-): 0

Region: chr12 29630892-29630988. Max. coverage (+): 0. Max coverage (-): 0

Region: chr12 29630989-29631084. Max. coverage (+): 3.86. Max coverage (-): 0

Region: chr12 29631085-29631181. Max. coverage (+): 51.99. Max coverage (-): 6.96

Region: chr12 29631182-29631278. Max. coverage (+): 0. Max coverage (-): 0

Region: chr12 29631279-29631375. Max. coverage (+): 6.23. Max coverage (-): 0

Region: chr12 29631376-29631472. Max. coverage (+): 8.58. Max coverage (-): 0

Region: chr12 29631473-29631568. Max. coverage (+): 4.37. Max coverage (-): 0

Region: chr12 29631569-29631665. Max. coverage (+): 33.86. Max coverage (-): 0

Region: chr12 29631666-29631762. Max. coverage (+): 13.51. Max coverage (-): 0

Region: chr12 29631763-29631859. Max. coverage (+): 6.54. Max coverage (-): 0

Region: chr12 29631860-29631956. Max. coverage (+): 0. Max coverage (-): 0

Region: chr12 29631957-29632052. Max. coverage (+): 0. Max coverage (-): 0

Region: chr12 29632053-29632149. Max. coverage (+): 10.54. Max coverage (-): 0

Region: chr12 29632150-29632246. Max. coverage (+): 10.54. Max coverage (-): 0

Region: chr12 29632247-29632343. Max. coverage (+): 0. Max coverage (-): 0

Region: chr12 29632344-29632440. Max. coverage (+): 0. Max coverage (-): 0

Region: chr12 29632441-29632536. Max. coverage (+): 0. Max coverage (-): 0

Region: chr12 29632537-29632633. Max. coverage (+): 0. Max coverage (-): 0

Region: chr12 29632634-29632730. Max. coverage (+): 0. Max coverage (-): 0

Region: chr12 29632731-29632827. Max. coverage (+): 0. Max coverage (-): 0

Region: chr12 29632828-29632923. Max. coverage (+): 0. Max coverage (-): 0

Region: chr12 29632924-29633020. Max. coverage (+): 2. Max coverage (-): 0

Region: chr12 29633021-29633117. Max. coverage (+): 0. Max coverage (-): 0

Region: chr12 29633118-29633214. Max. coverage (+): 0. Max coverage (-): 0

Region: chr12 29633215-29633311. Max. coverage (+): 0. Max coverage (-): 0

Region: chr12 29633312-29633407. Max. coverage (+): 0. Max coverage (-): 0

Region: chr12 29633408-29633504. Max. coverage (+): 0. Max coverage (-): 6.41

Region: chr12 29633505-29633601. Max. coverage (+): 23.17. Max coverage (-): 0

Region: chr12 29633602-29633698. Max. coverage (+): 0. Max coverage (-): 0

Region: chr12 29633699-29633795. Max. coverage (+): 32.56. Max coverage (-): 1.15

Region: chr12 29633796-29633891. Max. coverage (+): 13.3. Max coverage (-): 0

Region: chr12 29633892-29633988. Max. coverage (+): 2.61. Max coverage (-): 0

Region: chr12 29633989-29634085. Max. coverage (+): 0. Max coverage (-): 0

Region: chr12 29634086-29634182. Max. coverage (+): 0. Max coverage (-): 0

Region: chr12 29634183-29634279. Max. coverage (+): 0. Max coverage (-): 0

Region: chr12 29634280-29634375. Max. coverage (+): 0. Max coverage (-): 0

Region: chr12 29634376-29634472. Max. coverage (+): 0.36. Max coverage (-): 0

Region: chr12 29634473-29634569. Max. coverage (+): 3.8. Max coverage (-): 0

Region: chr12 29634570-29634666. Max. coverage (+): 0. Max coverage (-): 0

Region: chr12 29634667-29634763. Max. coverage (+): 7.52. Max coverage (-): 0

Region: chr12 29634764-29634859. Max. coverage (+): 7.36. Max coverage (-): 0

Region: chr12 29634860-29634956. Max. coverage (+): 11.9. Max coverage (-): 0

Region: chr12 29634957-29635053. Max. coverage (+): 5.13. Max coverage (-): 0

Region: chr12 29635054-29635150. Max. coverage (+): 4.87. Max coverage (-): 0

Region: chr12 29635151-29635247. Max. coverage (+): 0. Max coverage (-): 0

Region: chr12 29635248-29635343. Max. coverage (+): 22.09. Max coverage (-): 0

Region: chr12 29635344-29635440. Max. coverage (+): 0. Max coverage (-): 0

Region: chr12 29635441-29635537. Max. coverage (+): 15.9. Max coverage (-): 0

Region: chr12 29635538-29635634. Max. coverage (+): 18.98. Max coverage (-): 0

Region: chr12 29635635-29635731. Max. coverage (+): 16.52. Max coverage (-): 0

Region: chr12 29635732-29635827. Max. coverage (+): 5.36. Max coverage (-): 0

Region: chr12 29635828-29635924. Max. coverage (+): 0. Max coverage (-): 0

Region: chr12 29635925-29636021. Max. coverage (+): 0. Max coverage (-): 0

Region: chr12 29636022-29636118. Max. coverage (+): 0. Max coverage (-): 0

Region: chr12 29636119-29636215. Max. coverage (+): 0. Max coverage (-): 0

Region: chr12 29636216-29636311. Max. coverage (+): 0. Max coverage (-): 0

Region: chr12 29636312-29636408. Max. coverage (+): 0. Max coverage (-): 0

Region: chr12 29636409-29636505. Max. coverage (+): 0. Max coverage (-): 0

Region: chr12 29636506-29636602. Max. coverage (+): 0. Max coverage (-): 0

Region: chr12 29636603-29636699. Max. coverage (+): 0. Max coverage (-): 0

Region: chr12 29636700-29636795. Max. coverage (+): 0. Max coverage (-): 0

Region: chr12 29636796-29636892. Max. coverage (+): 0. Max coverage (-): 0

Region: chr12 29636893-29636989. Max. coverage (+): 4.65. Max coverage (-): 0

Region: chr12 29636990-29637086. Max. coverage (+): 0. Max coverage (-): 0

Region: chr12 29637087-29637183. Max. coverage (+): 0. Max coverage (-): 0

Region: chr12 29637184-29637279. Max. coverage (+): 0. Max coverage (-): 0

Region: chr12 29637280-29637376. Max. coverage (+): 0. Max coverage (-): 0

Region: chr12 29637377-. Max. coverage (+): 4.05. Max coverage (-): 0

RepeatMasker Color Code

**+**

100-98% Identity

<98-95% Identity

<95-90% Identity

<90-85% Identity

<85-80% Identity

<80-75% Identity

<75-70% Identity

<70% Identity

**-**

Gene Set Color Code

**+**

Gene

Pseudogene

**-**

Topology/Coverage Color Code

Coverage Plus Strand

Coverage Minus Strand

Mainstrand: Plus

Mainstrand: Minus

Complementary Strand

Flanking Region  
(if option -flank >0)

Gene Set Annotation  
  
RepeatMasker Annotation  

**1. AT\_rich**: 29591399-29591426 (+), Divergence to consensus: 42.9%  
**2. L2**: 29593323-29593623 (+), Divergence to consensus: 45.6%  
**3. BOV-A2**: 29595205-29595321 (-), Divergence to consensus: 5.2%  
**4. L1ME3B**: 29595732-29595847 (+), Divergence to consensus: 34.5%  
**5. MIRb**: 29595973-29596043 (-), Divergence to consensus: 35.2%  
**6. Charlie16a**: 29597433-29597494 (+), Divergence to consensus: 27.6%  
**7. MIR**: 29597704-29597806 (+), Divergence to consensus: 30%  
**8. L2**: 29600239-29600500 (-), Divergence to consensus: 43.6%  
**9. L1\_Art**: 29602038-29602414 (-), Divergence to consensus: 34.7%  
**10. AT\_rich**: 29603464-29603506 (+), Divergence to consensus: 74.4%  
**11. L2b**: 29604402-29604454 (-), Divergence to consensus: 26.4%  
**12. L2c**: 29605051-29605122 (-), Divergence to consensus: 36.3%  
**13. MIR**: 29605128-29605265 (+), Divergence to consensus: 36.8%  
**14. BTLTR1C**: 29605280-29606538 (-), Divergence to consensus: 5.4%  
**15. G-rich**: 29606569-29606659 (+), Divergence to consensus: 32.2%  
**16. MIRb**: 29606678-29606753 (+), Divergence to consensus: 38.6%  
**17. MIR**: 29606818-29607015 (-), Divergence to consensus: 36.6%  
**18. L1MC5a**: 29607860-29608341 (-), Divergence to consensus: 42.2%  
**19. L1MC5a**: 29608393-29608641 (+), Divergence to consensus: 37.4%  
**20. (CCA)n**: 29608839-29608861 (+), Divergence to consensus: 4.3%  
**21. UCON2**: 29609779-29609906 (-), Divergence to consensus: 37.5%  
**22. MamGypLTR1a**: 29612503-29612545 (+), Divergence to consensus: 18.6%  
**23. MLT2F**: 29612835-29613389 (-), Divergence to consensus: 54.1%  
**24. Bov-tA2**: 29613494-29613623 (-), Divergence to consensus: 17.7%  
**25. MER102c**: 29613692-29613761 (-), Divergence to consensus: 31.4%  
**26. MER102b**: 29613796-29613967 (-), Divergence to consensus: 34.6%  
**27. L2b**: 29614502-29614592 (+), Divergence to consensus: 46%  
**28. BOV-A2**: 29615281-29615529 (-), Divergence to consensus: 10.4%  
**29. MIRb**: 29616025-29616055 (-), Divergence to consensus: 12.9%  
**30. L2**: 29616102-29616201 (-), Divergence to consensus: 39%  
**31. MLT1J2**: 29616397-29616791 (-), Divergence to consensus: 42.6%  
**32. MER102c**: 29617211-29617321 (-), Divergence to consensus: 28.4%  
**33. ERV2-1C-LTR\_BT**: 29617669-29618930 (-), Divergence to consensus: 21.3%  
**34. BTLTR1F**: 29618931-29618986 (-), Divergence to consensus: 8.9%  
**35. L1MC4**: 29619284-29619778 (+), Divergence to consensus: 35.2%  
**36. BOV-A2**: 29620282-29620359 (-), Divergence to consensus: 24.4%  
**37. ART2A**: 29620291-29620576 (-), Divergence to consensus: 18.2%  
**38. L1ME3**: 29621373-29621616 (-), Divergence to consensus: 37.4%  
**39. MER58A**: 29621737-29621800 (-), Divergence to consensus: 25.5%  
**40. MER58A**: 29621801-29621870 (-), Divergence to consensus: 25.7%  
**41. L1ME3**: 29621873-29622091 (-), Divergence to consensus: 36.2%  
**42. MLT1F2**: 29622293-29622489 (-), Divergence to consensus: 39.6%  
**43. L1ME3**: 29622718-29622922 (-), Divergence to consensus: 46.8%  
**44. MER20**: 29622924-29623067 (-), Divergence to consensus: 34.4%  
**45. L1ME3**: 29623208-29624074 (-), Divergence to consensus: 38.5%  
**46. L1ME3**: 29624069-29624332 (-), Divergence to consensus: 34.2%  
**47. SINE2-2\_BT**: 29624333-29624439 (-), Divergence to consensus: 31.8%  
**48. L1ME3**: 29624440-29624644 (-), Divergence to consensus: 34.2%  
**49. L1ME3**: 29624672-29625650 (-), Divergence to consensus: 42.7%  
**50. ART2A**: 29625685-29625806 (-), Divergence to consensus: 18%  
**51. L1ME3**: 29625824-29626260 (-), Divergence to consensus: 42.7%  
**52. L1ME3**: 29626479-29626603 (-), Divergence to consensus: 46.4%  
**53. ART2A**: 29626640-29626651 (-), Divergence to consensus: 28.1%  
**54. (CTG)n**: 29626652-29626677 (+), Divergence to consensus: 0%  
**55. ART2A**: 29626678-29626744 (-), Divergence to consensus: 28.1%  
**56. BOV-A2**: 29626745-29626853 (-), Divergence to consensus: 5.1%  
**57. MLT1L**: 29627282-29627501 (+), Divergence to consensus: 44.2%  
**58. MLT1A**: 29628042-29628431 (-), Divergence to consensus: 34.5%  
**59. FordPrefect**: 29628665-29629116 (+), Divergence to consensus: 34.2%  
**60. FordPrefect**: 29629116-29629301 (+), Divergence to consensus: 31.2%  
**61. MIRb**: 29629886-29630009 (+), Divergence to consensus: 37.6%  
**62. L1\_BT**: 29630082-29630783 (-), Divergence to consensus: 17.6%  
**63. LTR41B**: 29631165-29631360 (+), Divergence to consensus: 27.2%  
**64. MIR**: 29631807-29632014 (-), Divergence to consensus: 47.9%  
**65. L2**: 29632172-29632947 (+), Divergence to consensus: 47.8%  
**66. MER58C**: 29633038-29633168 (-), Divergence to consensus: 31.4%  
**67. (CAGTT)n**: 29633169-29633189 (+), Divergence to consensus: 0%  
**68. ART2A**: 29633190-29633493 (-), Divergence to consensus: 16.1%  
**69. MER58C**: 29633613-29633690 (-), Divergence to consensus: 29.9%  
**70. MLT1I**: 29633691-29633834 (-), Divergence to consensus: 36.2%  
**71. LTR40a**: 29633991-29634401 (+), Divergence to consensus: 34.5%  
**72. tRNA-Gly-GGG**: 29635874-29635909 (-), Divergence to consensus: 5.6%  
**73. CHR-2A**: 29636581-29636898 (-), Divergence to consensus: 22.5%  
**74. Charlie22a**: 29637211-29637383 (+), Divergence to consensus: 35.3%

  
Transcription Factor Binding Sites  

**RFX4\_2** (Sequence: GTAACTAAG (-): 29602838)  
**RFX4\_1** (Sequence: GTTGCCATG (-): 29597368)  
**RFX4\_1** (Sequence: CTTGGCAAC (+): 29611581)  
**SPZ1** (Sequence: CTCATACCCT (-): 29615076)  
**RFX4\_2** (Sequence: CATGGATAC (+): 29594476)  
**RFX4\_2** (Sequence: CCTGGATAC (+): 29627649)  
**Gata4** (Sequence: AGATAAG (-): 29604722)  
**Gata4** (Sequence: AGATAAC (-): 29629485)  
**SOX9** (Sequence: AACAATGA (-): 29629686)  
**SOX9** (Sequence: CCATTGTT (+): 29602421)  
**SOX9** (Sequence: TTATTGTT (+): 29612147)  
**SOX9** (Sequence: CCATTGTT (+): 29623101)  
**SOX9** (Sequence: TCATTGTT (+): 29635498)  
**A-MYB** (Sequence: CCAACTGTCT (-): 29623077)  
**SPZ1** (Sequence: AGGGTTACAG (+): 29603177)  
**SPZ1** (Sequence: GGGGTTAGAG (+): 29622679)  
**Mybl1\_1** (Sequence: AACCGTTA (+): 29621170)  
**Gata4** (Sequence: CTTATCT (+): 29593447)  
**Gata4** (Sequence: GTTATCT (+): 29612152)
